# Common microRNA–mRNA interactions exist among distinct porcine iPSC lines independent of their metastable pluripotent states

**DOI:** 10.1038/cddis.2017.426

**Published:** 2017-08-31

**Authors:** Shiqiang Zhang, Youlong Xie, Hongxia Cao, Huayan Wang

**Affiliations:** 1 College of Veterinary Medicine, Shaanxi Center of Stem Cells Engineering & Technology, Northwest A&F University, Yangling, Shaanxi 712100, China

## Abstract

Previous evidences have proved that porcine-induced pluripotent stem cells (piPSCs) could be induced to distinctive metastable pluripotent states. This raises the issue of whether there is a common transcriptomic profile existing among the piPSC lines at distinctive state. In this study, we performed conjoint analysis of small RNA-seq and mRNA-seq for three piPSC lines which represent LIF dependence, FGF2 dependence and LFB2i dependence, respectively. Interestingly, we found there are 16 common microRNAs which potentially target 13 common mRNAs among the three piPSC lines. Dual-luciferase reporter assay validated that miR-370, one of the 16 common microRNAs, could directly target the 3′UTR of *LIN28A*. When the differentiation occurred, miR-370 could be activated in piPSCs and switched off the expression of *LIN28A*. Ectopic expression of miR-370 in piPSCs could reduce *LIN28A* expression, decrease the alkaline phosphatase activity, slow down the proliferation, and further cause the downregulation of downstream pluripotent genes (*OCT4*, *SOX2*, *NANOG*, *SALL4* and *ESRRB*) and upregulation of differentiation relevant genes (*SOX9, JARID2* and *JMJD4*). Moreover, these phenotypes caused by miR-370 could be rescued by overexpressing *LIN28A.* Collectively, our findings suggest that a set of common miRNA–mRNA interactions exist among the distinct piPSC lines, which orchestrate the self-renewal and differentiation of piPSCs independent of their metastable pluripotent states.

MicroRNAs (miRNAs) are a group of small noncoding RNAs that consist of about 20–21 nucleotides and function in post-transcriptional regulation of gene expression. Several miRNA clusters, including miR-290–295/miR-371-373, miR-302 and miR-17-92b, are defined as the embryonic stem cell cycle-specific (ESCC) miRNAs.^1^ Recent evidences have showed that these ESCC miRNAs have an important role in maintaining pluripotency and cellular reprogramming.^2^ The miR-302/367 cluster was reported to regulate both cell cycle and apoptosis in human embryonic stem cells (hESCs).^3^ In addition, overexpression of miR-302a, miR-302b and miR-200c could improve the efficiency of porcine-induced pluripotent stem cells (piPSCs) induction.^4^

To further study the post-transcriptional regulation mechanism of global miRNAs on pluripotency, small RNA sequencing has been used extensively to reveal transcription profiles. The differentially expressed miRNAs have been documented between human-induced pluripotent stem cells (hiPSCs) and hESCs,^5^ in which miR-371-3 are highly expressed in hESCs *versus* hiPSCs. In contrast, miR-181a, miR-199b-3p and miR-214 are hiPSC-specific miRNAs, which are lowly expressed in hESCs. In addition, comparison of global miRNA profiles also showed differences between naive state and primed state on mice.^6^

Although many attempts have been tried to get piPSCs similar to mouse naïve pluripotent state, it is still challenging to achieve authentic piPSCs that can form germline-transmission-chimera with high efficiency. However, distinctive piPSC lines have been made, which featured by LIF dependence,^7^ FGF2 dependence^8^ and LFB2i dependence.^9^ The LIF-dependent piPSCs were generated by transducing four Yamanaka factors into porcine embryonic fibroblasts (PEFs) and propagated in the presence of leukemia inhibitor factor (LIF) and forskolin.^7^ By contrast, the FGF2-dependent piPSCs were induced by ectopically expressing four Yamanaka factors plus *LIN28* and *c-MYC* into porcine mesenchymal stem cells and maintained in the presence of fibroblast growth factor 2 (FGF2).^8^ Our previous study showed that LFB2i-dependent piPSCs could be made by overexpressing four Yamanaka factors into PEFs and maintained with addition of three growth factors (LIF, FGF2 and BMP4) and two inhibitors (2i: CHIR99021 and SB431542).^9^ Although these three types of piPSC lines were generated under diverse cellular context and by different reprogramming strategies, they represent different metastable pluripotent states. Recently, the miRNA profiles of piPSCs derived from culture conditions of both hESCs and mouse embryonic stem cells (mESCs) were investigated and showed the significant differences between the two culture conditions.^10^ However, it is unclear whether there is a common miRNA profile existing among the distinct piPSC lines.

In this study, we performed conjoint analysis of small RNA-seq and mRNA-seq for three metastable piPSC lines with dependence of distinctive signaling pathways. Interestingly, we found there were 16 common miRNAs that potentially target 13 common mRNAs among the three piPSC lines. MiR-370, one of the 16 miRNAs, was demonstrated to target the 3′UTR of *LIN28A*. Ectopic expression of miR-370 could reduce the expression level of *LIN28A* in piPSCs. Even more, miR-370 could be activated and switched off the expression of *LIN28A* upon piPSCs differentiation. We also found that ectopic expression of miR-370 in piPSCs could decrease the alkaline phosphatase activity, slow down the cellular proliferation, and further cause the downregulation of downstream pluripotent genes (*OCT4*, *SOX2*, *NANOG*, *SALL4* and *ESRRB*) and upregulation of differentiation relevant genes (*SOX9, JARID2* and *JMJD4*). Of note, these defects caused by miR-370 could be rescued by overexpressing *LIN28A.*Our study uncovered that a set of common miRNA–mRNA interactions exist among distinct piPSC lines independent of their metastable pluripotent states. The orchestration of miRNA–mRNA interactions is supposed to regulate the self-renewal and differentiation of piPSCs.

## Results

### Global miRNA expression pattern of distinct piPSC lines

**Figure 1:**
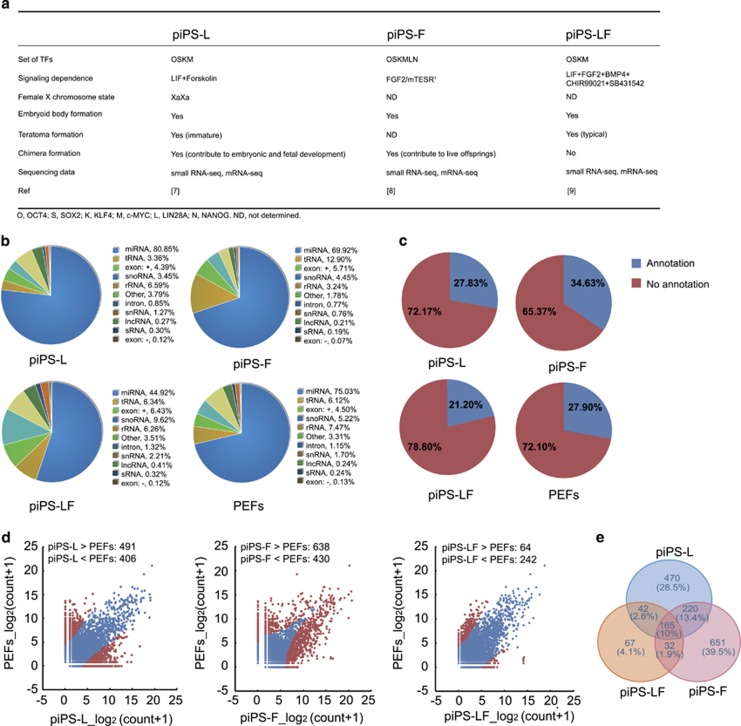
MicroRNA expression profiles of distinct piPSC lines. (**a**) Summary of cellular context about LIF-dependent piPSCs (piPS-L), FGF2-dependent piPSCs (piPS-F) and LFB2i-dependent piPSCs (piPS-LF). (**b**) Pie charts of small RNA-seq showing the percentage of small RNAs components from three piPSC lines and PEFs. (**c**) Pie charts of the percentage of miRNAs with and without annotation. (**d**) Scatter plots showing the differentially expressed miRNAs between PEFs and piPSC lines. (**e**) Venn analysis of differentially expressed miRNAs from piPS-L, piPS-F and piPS-LF

To determine whether there is a common miRNA profile existing in different types of piPSCs, three piPSC lines that represent LIF dependence, FGF2 dependence and LFB2i dependence were selected for small RNA-seq analysis. The reprogramming context of the three piPSC lines and sequencing information is summarized in Figure 1a. The small RNA-seq of PEFs was used as a somatic cell control. Small RNA-seq showed the number of miRNAs and its proportion in total small RNAs for LIF-dependent piPSCs (piPS-L), FGF2-dependent piPSCs (piPS-F), LFB2i-dependent piPSCs (piPS-LF) and PEFs is 11144 (80.85%), 6838 (69.92%), 10146 (44.92%) and 7661 (75.03%), respectively (Figure 1b). To annotate the acquired pig miRNAs, Blast analysis was performed based on pig miRBase as well as other animal species’ miRbases, including human, mouse, cattle and rat.^11^ Results showed that only 21.2 to 34.6% of porcine miRNAs was annotated. In contrast, most sequenced miRNAs (~78%) have no annotation due to the limited information of pig miRBase (Figure 1c). Besides, we compared the differentially expressed miRNAs between each line of piPSCs and PEFs. The number of differentially expressed miRNAs (FDR<0.05) in piPS-L, piPS-F and piPS-LF is 897, 1068 and 306, respectively (Figure 1d; Supplementary Table 1). Then, we carried out Venn analysis of the differentially expressed miRNAs and revealed that 470 miRNAs were specific for piPS-L, 651 for piPS-F, and 67 for piPS-LF. We also found that 220 miRNAs were shared by piPS-L and piPS-F, 32 for piPS-F and piPS-LF, and 42 for piPS-L and piPS-LF. Importantly, there were 165 miRNAs which were commonly expressed among the three piPSC lines (Figure 1e; Supplementary Table 2). Collectively, the result of small RNA-seq revealed that a common miRNA profile existed among distinct piPSC lines, although piPSC lines differed in their signaling dependence and original context.

### Analysis of common miRNAs revealed by small RNA-seq

**Figure 2:**
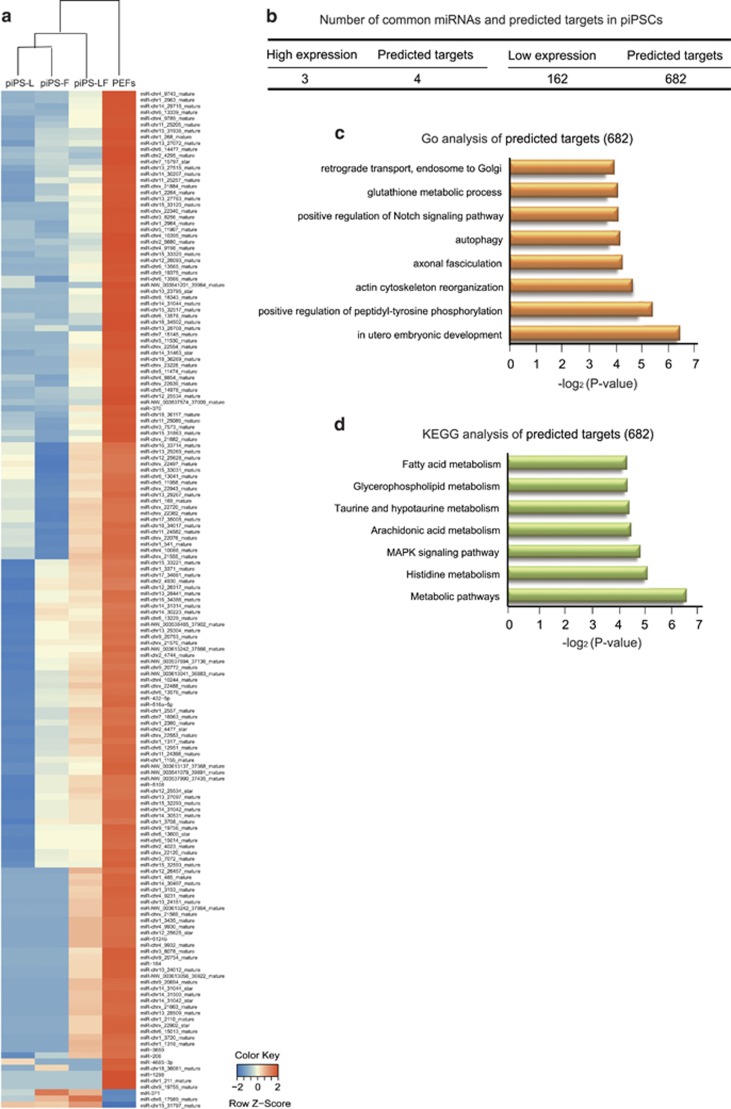
Analysis of common miRNAs revealed by small RNA-seq. (**a**) Heatmap of the 165 differentially expressed miRNAs shared by piPS-L, piPS-F and piPS-LF (*P*<0.05). (**b**) Statistical table showing the number of commonly high- and low-expression miRNAs and their predicted target mRNAs. (**c**) GO analysis of the predicted target mRNAs with commonly high expression in piPS-L, piPS-F and piPS-LF. (**d**) KEGG analysis of predicted target mRNAs with commonly high expression in piPS-L, piPS-F and piPS-LF

The heatmap of 165 common miRNAs showed that piPSC lines were clustered relatively closer *versus* PEFs. Three miRNAs (miR-371, chr8_17989_mature and chr15_31797_mature) were highly expressed in piPSC lines, but lowly expressed in PEFs. By contrast, the other 162 miRNAs showed the lower expression level in piPSC lines *versus* PEFs (Figure 2a). The three high-expressed miRNAs in piPSCs were predicted to target four mRNAs, while 162 lowly expressed miRNAs in piPSCs were predicted to target 682 mRNAs (Figure 2b; Supplementary Table 3). We then performed GO and KEGG analysis of the predicted 682 mRNAs. The enriched biological processes of GO analysis (*P*<0.05) included in utero embryonic development, positive regulation of peptidyl-tyrosine phosphorylation, actin cytoskeleton reorganization, axonal fasciculation, autophagy, positive regulation of Notch signaling pathway, glutathione metabolic process and retrograde transport, and endosome to Golgi (Figure 2c; Supplementary Table 4). The significantly enriched KEGG pathways (*P*<0.05) included metabolic pathways, histidine metabolism, MAPK signaling pathway, arachidonic acid metabolism, taurine and hypotaurine metabolism, glycerophospholipid metabolism and fatty acid metabolism (Figure 2d; Supplementary Table 4). These results indicated that a set of miRNAs were commonly expressed with low level in piPSC lines and they were supposed to target on genes involved in many important biological processes and pathways.

### Global mRNA expression pattern of distinct piPSC lines

**Figure 3:**
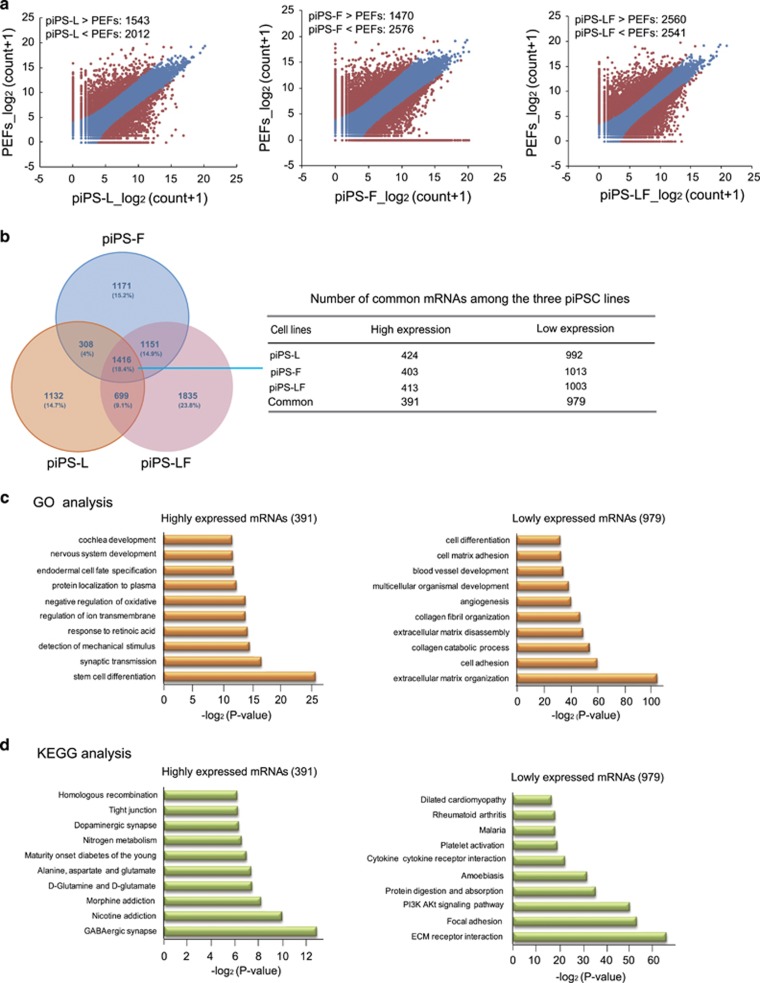
mRNA expression profiles of distinct piPSC lines. (**a**) Scatter plots showing the differentially expressed mRNAs between piPSC lines and PEFs (*P*<0.05). (**b**) Venn diagram of RNA-seq data showing the commonly high- and low-expression mRNAs in piPS-L, piPS-F and piPS-LF. (**c**) GO analysis of the mRNAs with commonly high- and low expression in piPS-L, piPS-F and piPS-LF. (**d**) KEGG analysis of the mRNAs commonly high- and low-expressed in piPS-L, piPS-F and piPS-LF

To explore the global mRNA expression pattern of distinct piPSC lines, we analyzed the mRNA-seq data of piPS-L, piPS-F and piPS-LF. The scatter plots showed that a significant number of differentially expressed transcripts existed between each type of piPSC line *versus* PEFs (Figure 3a; Supplementary Table 5). Venn analysis of differentially expressed transcripts uncovered 1416 common mRNAs that are shared by distinct piPSC lines independent of their metastable pluripotent states. Meanwhile, the Venn analysis also revealed 1132 mRNAs which are expressed exclusively in piPS-L, 1171 in piPS-F and 1835 in piPS-LF (Figure 3b,Supplementary Table 6). We then focused on the common mRNAs that were divisible into two categories: 391 highly expressed mRNAs and 979 lowly expressed mRNAs (Figure 3b; Supplementary Table 7). GO analysis and KEGG analysis were performed for these common mRNAs (Figures 3c and d; Supplementary Table 8). The common high-expression mRNAs are enriched in typical GO terms and pathways of stem cells, such as ‘stem cell differentiation’ and ‘tight junction’. In contrast, the common low-expression mRNAs are mostly enriched in GO terms and pathways relevant to extracellular matrix. This observation uncovered fundamental transcriptome differences between piPSCs and somatic cells.

### Conjoint analysis of small RNA-seq and mRNA-seq for distinct piPSC lines

**Figure 4:**
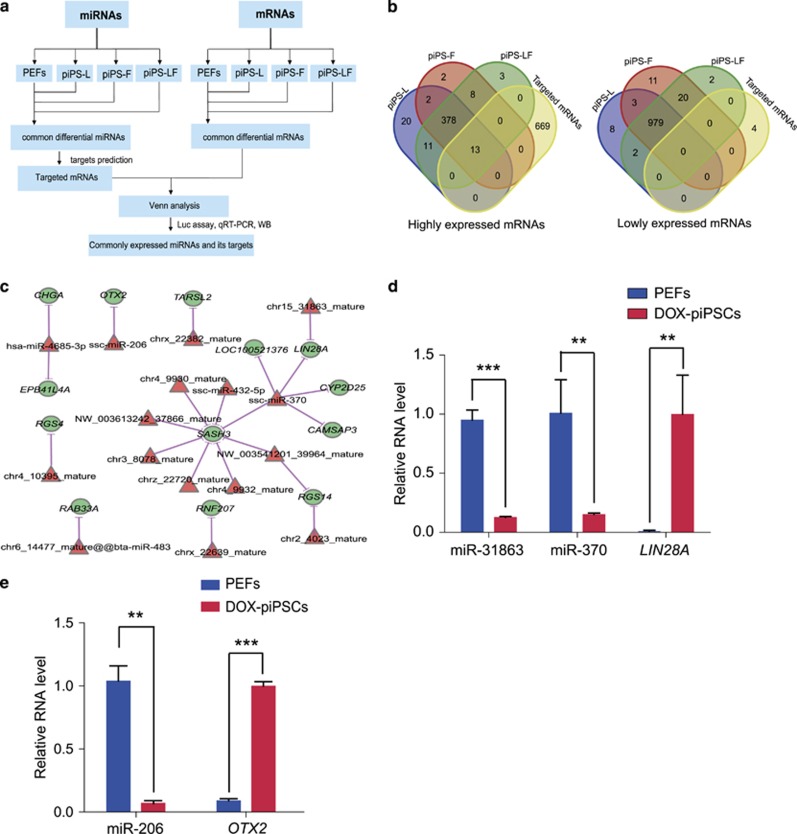
Conjoint analysis of small RNA-seq and mRNA-seq for distinct piPSC lines. (**a**) Flow diagram showing the conjoint analysis procedure of small RNA-seq and mRNA-seq data. (**b**) Venn analysis between the predicted miRNA target mRNAs and the differentially expressed mRNAs with the same expression trend in piPS-L, piPS-F and piPS-LF. (**c**) The network showing the common miRNAs and their targets generated by conjoint analysis of small RNA-seq and mRNA-seq. (**d**) qRT-PCR validation of the expression levels of two miRNAs (miR-31863 and miR-370) with their target *LIN28A* in Dox-piPSCs. (**e**) qRT-PCR showing the expression level of miR-206 and its target *OTX2* in Dox-piPSCs. **P<0.01, ***P<0.001

To increase the accuracy of predicted targets from the common miRNAs for distinct piPSC lines, conjoint analysis of small RNA-seq and mRNA-seq was performed and validated by wet-lab experiments (Figure 4a). The analysis was performed by aligning 682 mRNAs that were targeted by the 162 lowly expressed miRNAs in piPSCs (Figure 2b) with 391 mRNAs that were highly expressed in all three piPSC lines (Figure 3b), and vice versa. The Venn diagram revealed that 13 mRNAs with high-expression level are common among the distinct piPSC lines (Figure 4b; Supplementary Table 9). These common mRNAs include *CHGA, EPB414A, OTX2, TARSL2, LOC100521376, LIN28A, CYP2D25, CAMSAP3, SASH3, RGS4, RNF207* and *RAB33A*. Interestingly, two well-known pluripotent genes *OTX2* and *LIN28A* were found among the list. The interaction network of the common miRNAs and these common 13 mRNAs was constructed (Figure 4c). Interestingly, miR-206 was found to target *OTX2* while miR-370 and chr15_31863_mature (miR-31863) were found to target *LIN28A*. The negative correlation of miR-370 and miR-31863 with LIN28A was confirmed in another cell line of piPSCs (Dox-piPSCs) described in this lab^12^ and PEFs by qRT-PCR, showing that expression level of miR-370 and miR-31863 were high and *LIN28A* was low in PEFs, conversely the expression level of miR-370 and miR-31863 were low and *LIN28A* was high in piPSCs (Figure 4d). The negative correlation of miR-206 with *OTX2* was also validated (Figure 4e). Collectively, conjoint analysis of small RNA-seq and mRNA-seq indicated a set of common miRNA–mRNA interactions existed among the distinct piPSC lines independent of their signaling dependence.

### MiR-370 targets the 3′UTR of *LIN28A* mRNA

**Figure 5:**
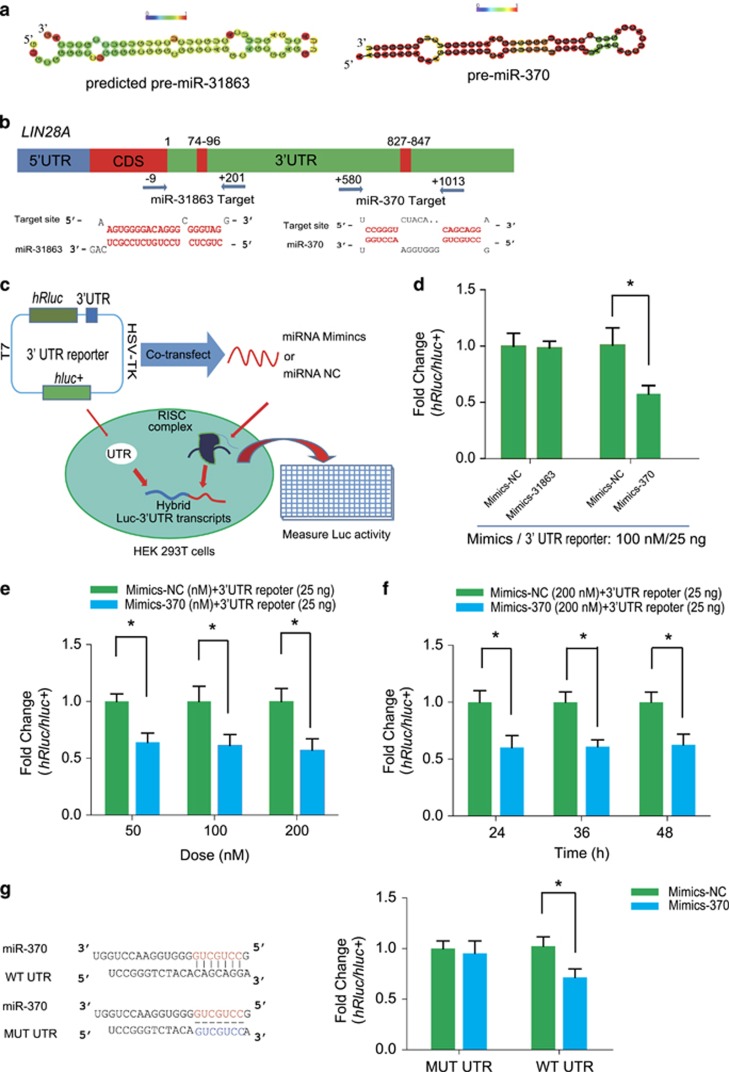
MiR-370 directly targets the 3′UTR of *LIN28A*. (**a**) Secondary structure of miR-31863 and miR-370 predicted by RNAFold. Scale, pseudo base-paring probabilities for each base pair. (**b**) Diagram showing the potential positions (+74~ +96 and +827~ +847) at 3′UTR of *LIN28A* targeted by miR-31863 and miR-370, respectively. (**c**) Diagram showing the procedure of cotransfecting dual-luciferase reporter plasmids with miRNA mimics into HEK 293T cells and measuring its relative luciferase activity. (**d**) Comparison of the relative luciferase activity by cotransfecting Mimcs-31863 and Mimics-370 with their corresponding *LIN28A* 3′UTR reporter plasmids. (**e**) Luciferase assay showing the dose-dependent repression activity of mimics-370 on its reporter plasmid that contains the 3′UTR (+580~+1013) of *LIN28A*. (**f**) The timecourse of mimics-370 with repression activity on the 3’UTR (+580~+1013) of *LIN28A*. (**g**) Mutation of the motif (CAGCAGG) in 3′UTR of *LIN28A* abolishes the repression effect by miR-370. Three independent experiments were performed in triplicates for each luciferase assay. *P<0.05, **P<0.01

The following study focused on the interaction of miR-370 and miR-31863 with *LIN28A* in piPSCs. Because miR-31863 is a novel miRNA found in pig, its secondary structure was predicted by using RNAFold and we found pre-miR-31863 could form a stem loop structure similar to pre-miR-370 (Figure 5a). The analysis showed that *LIN28A* 3′UTR retains two potential sites for miRNA targeting. One is for miR-31863 and the other one is for miR-370 (Figure 5b). To determine the actual interaction, a reporter vector that contains the targeted sites in 3′UTR of *LIN28A* (−9~201 bp or +580~+1013 bp) and a mimics of miR-31863 or miR-370 were cotransfected into HEK 293T cells (Figure 5c). Results showed that mimics of miR-370 (mimics-370) significantly downregulated luciferase activity, but mimics-31863 had no effect (Figure 5d). Then we focused on mimics-370 and examined its dose-dependent effect on *LIN28A* 3′UTR reporter. As the concentration of mimics-370 increased, the luciferase activity decreased gradually with dose-dependent course (Figure 5e). We also investigated the downregulation effect of luciferase activity by mimics-370 over the timecourse. However, no significant difference was observed during the treatment time from 24 h to 48 h (Figure 5f). To determine the specificity of mimics-370 targeting on *LIN28A*, we constructed a reporter vector that contained the mutated sequence of 3′UTR of *LIN28A* (MUT-UTR) (Figure 5g). The cotransfection result showed that the MUT-UTR completely abolished the mimics-370 effect when compared with WT-UTR (Figure 5g). These results suggest that miR-370 can directly target the 3′UTR of *LIN28A* mRNA.

### MiR-370 represses *LIN28A* expression

**Figure 6:**
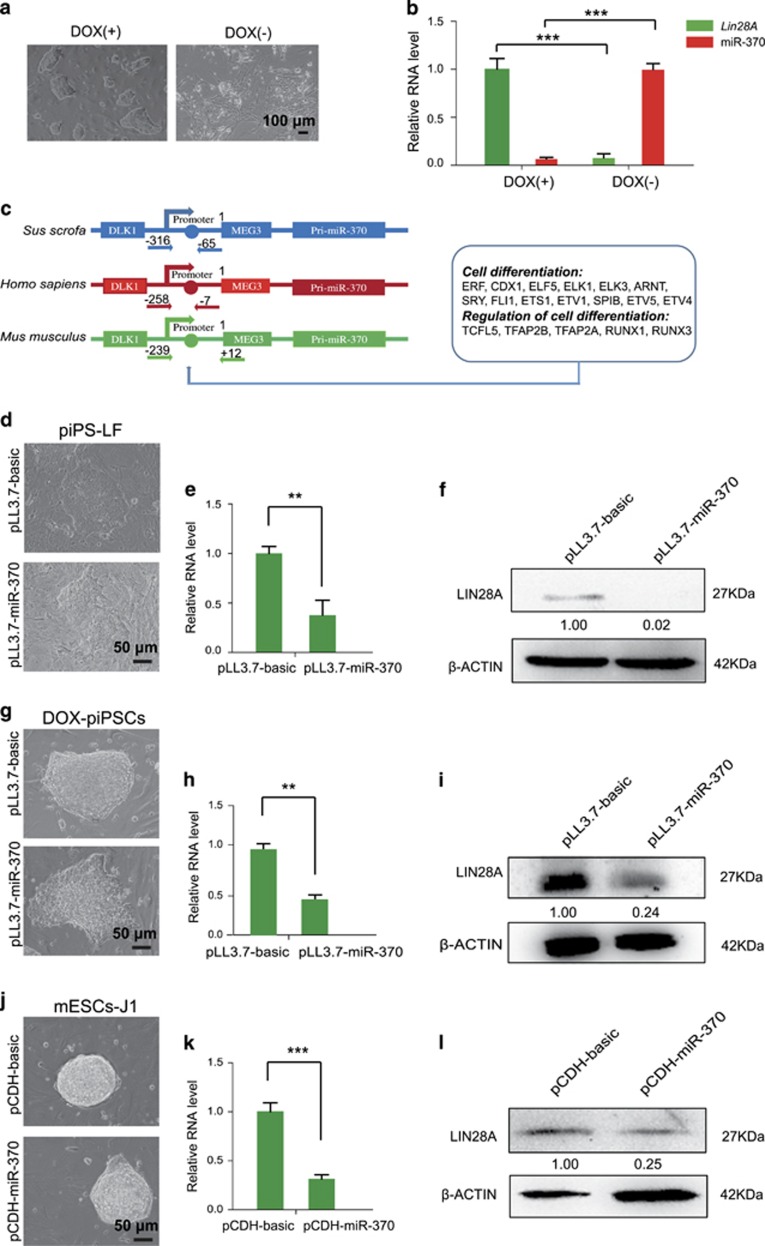
MiR-370 represses the expression level of *LIN28A*. (**a**) Images showing the morphology of Dox-piPSCs at undifferentiated (DOX+) and differentiated (DOX−) state. Scale bar, 100 *μ*m. (**b**) qRT-PCR analysis showing the expression level changes of *LIN28A* and miR-370 in Dox-piPSCs at undifferentiated and differentiated state. (**c**) Comparative analysis of Pri-miR-370 promoter of *Sus scrofa* (pig), *homo sapiens* (human) and *Mus musculus* (mouse).The conservative promoter region is predicted to be bound by 18 transcription factors (*ERF, CDX1, ELF5, ELK1, ELK3, ARNT, SRY, FLI1, ETS1, ETV1, SPIB, ETV5, ETV4, TCFL5, TFAP2B, TFAP2A, RUNX1* and *RUNX3*). (**d**,**g**,**j**) Colony images of piPS-LF, Dox-piPSCs and mESCs J1 transduced with vector control lentivirus and miR-370 lentivirus. Scale bar, 50 *μ*m. (**e**,**h**,**k**) qRT-PCR results showing the downregulation of *LIN28A* mRNA level in piPS-LF, Dox-piPSCs and mESCs J1 transduced with miR-370 lentivirus. The counterpart cells transduced with vector control lentivirus are used as control. **P<0.01, ***P<0.001. (**f**,**i**,**l**) Western blotting results showing the decreased LIN28A protein (27 kDa) level in piPS-LF, Dox-piPSCs and mESCs J1 transduced with miR-370 lentivirus. The counterpart cells transduced with vector control lentivirus are used as control. *β*-Actin (42 kDa) is used as internal control. Relative quantification of LIN28A after normalization with *β*-actin was also determined

To validate the regulation of *LIN28A* expression by miR-370, we investigated expression changes of miR-370 and *LIN28A* in Dox-piPSCs, a tetracycline operator (TetO)-inducible piPSCs line reported in this laboratory previously.^12^ The Dox-piPSCs were maintained in the pluripotent state with an addition of doxycycline, but started to differentiate soon after withdrawal of doxycycline (Figure 6a). In the differentiated Dox-piPSCs, miR-370 was significantly increased and the expression of *LIN28A* was significantly decreased (Figure 6b). This finding intrigued us to investigate which transcription factors may be potentially involved in activating miR-370 when the Dox-piPSCs initiated differentiation. We analyzed the promoter of Pri-miR-370 in the DLK1-MEG3 locus^13^ and found that the genomic DNA sequences of Pri-miR-370 promoter region were highly conserved among pig, human and mouse (Figure 6c). We further predicted transcription factor binding sites in the Pri-miR-370 promoter of pig, human and mouse, respectively, by searching JASPAR database. There were 216, 251 and 263 transcription factors that may potentially bind the Pri-miR-370 promoter of pig, human and mouse, respectively (Supplementary Figure 1a; Supplementary Table 10). Venn analysis uncovered that there were 204 transcription factors conserved among the three species for potentially regulating Pri-miR-370 expression (Supplementary Figure 1a; Supplementary Table 11). GO analysis of the 204 conserved transcription factors revealed that a total of 18 transcription factors were enriched for cell differentiation and regulation of cell differentiation (Figure 6c; Supplementary Figure 1b; Supplementary Table 12). Together these 18 transcription factors are suggested to potentially activate miR-370 when the piPSCs initiate differentiation.

To validate the repression of *LIN28A* expression by miR-370, we next transduced the piPS-LF with miR-370 lentiviruses. The piPS-LF colonies with miR-370 overexpression became morphologically incompact even cultured under LFB2i condition^9^ (Figure 6d). The endogenous *LIN28A* was significantly reduced on both the mRNA level and protein level in piPS-LF upon overexpressing miR-370 (Figures 6e and f). To test whether the interaction between *LIN28A* and miR-370 exists universally in other piPSCs, we further investigated the effects of miR-370 on Dox-piPSCs. The Dox-piPSCs transduced with miR-370 lentiviruses showed similar changes which were observed in piPS-LF (Figures 6g–i). Then we extended our investigation of interaction between Lin28a and miR-370 in mESCs line J1. The 3′UTR of mouse Lin28a mRNA also contains a targeting site for miR-370 (Supplementary Figure 2). So we asked whether overexpression of miR-370 in mESCs could also lead to similar phenotype as observed in piPSCs. Unexpectedly, the mESCs with miR-370 overexpression showed no obvious morphology change (Figure 6j), although the expression of Lin28a was significantly decreased (Figures 6k and l). Together the results above indicate that miR-370 can conservatively repress LIN28A expression in diverse pluripotent stem cells instead of a species-specific manner.

### MiR-370 affects self-renewal and differentiation of piPSCs by *LIN28A* dependence

**Figure 7:**
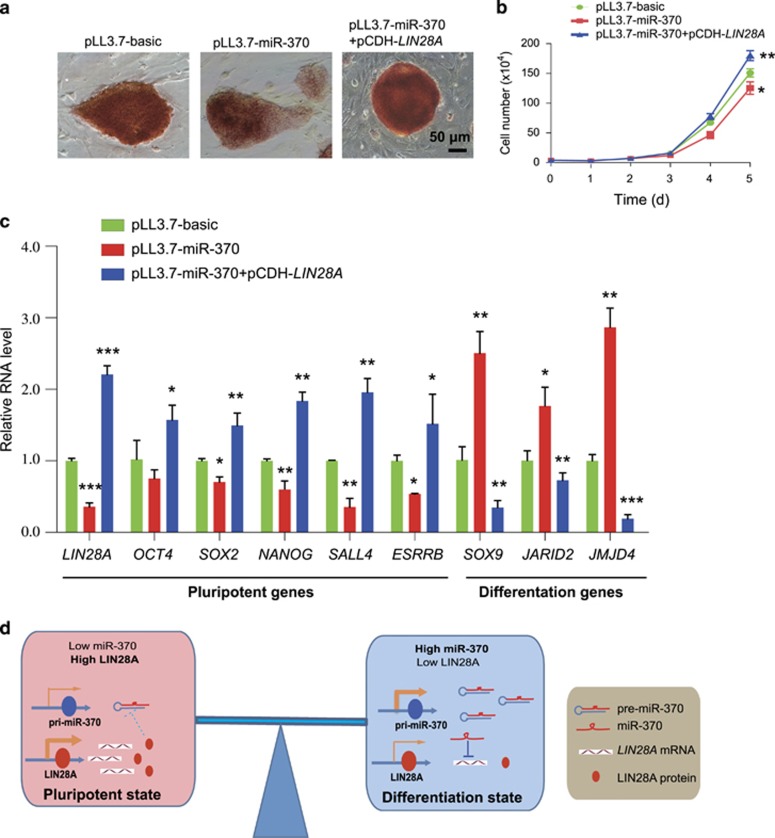
MiR-370 influences the self-renewal and differentiation of piPSCs by *LIN28A* dependence. (**a**) Alkaline phosphatase (AP) staining of Dox-piPSCs transduced with pLL3.7-basic lentivirus (vector control), miR-370 lentivirus, and miR-370+*LIN28A* lentivirus, respectively. Scale bar, 50 *μ*m. (**b**) Growth curve of Dox-piPSCs transduced with pLL3.7-basic lentivirus(vector control), miR-370 lentivirus, and miR-370+*LIN28A* lentivirus, respectively. *P<0.05, **P<0.01. (**c**) The expression level changes of pluripotent genes (*LIN28A*, *OCT4*, *SOX2*, *NANOG*, *SALL4* and *ESRRB*) and differentiation genes (*SOX9, JARID2* and *JMJD4*) in Dox-piPSCs transduced with pLL3.7-basic lentivirus(vector control), miR-370 lentivirus, and miR-370+*LIN28A* lentivirus, respectively. *P<0.05, **P<0.01, ***P<0.001. (**d**) The working model of interaction between miR-370 and LIN28A in piPSCs. In the pluripotent state, *LIN28A* is highly expressed while miR-370 is lowly expressed. LIN28A protein is speculated to inhibit the maturation of miR-370 precursors. When the differentiation initiates, miR-370 is highly activated and inhibits *LIN28A* expression at post-transcriptional level, which further results in reduced expression of LIN28A proteins

To further explore the effect of miR-370 on self-renewal and differentiation of piPSCs, we firstly investigated the impact of miR-370 on alkaline phosphatase (AP) activity. Interestingly, we found that miR-370 overexpression decreased the AP activity in Dox-piPSCs. However, *LIN28A* overexpression rescued the weakened AP activity in the Dox-piPSCs caused by miR-370 (Figure 7a).

Then we examined the impact of miR-370 on cellular proliferation. We noted that miR-370 overexpression slowed down the proliferation rate of Dox-piPSCs. Similarly, this proliferation defect caused by miR-370 could be rescued by *LIN28A* overexpression in Dox-piPSCs (Figure 7b).

Finally, we observed the impact of miR-370 on the expression of pluripotency and differentiation relevant genes. We found that miR-370 overexpression reduced the expression level of pluripotent genes (*LIN28A, OCT4, SOX2, NANOG, SALL4* and *ESRRB*) in Dox-piPSCs. By contrast, miR-370 overexpression in Dox-piPSCs upregulated the expression level of differentiation relevant genes (*SOX9, JARID2* and *JMJD4*) (Figure 7c). Of note, *LIN28A* overexpression could rescue the downregulation of pluripotent genes and upregulation of differentiation genes caused by miR-370 in Dox-piPSCs (Figure 7c). We also investigated the impact of miR-370 on the expression of pluripotent genes in piPS-LF and mESCs. Similar phenotype was observed although the expression change amplitudes of pluripotent genes were not exactly same as that observed in Dox-piPSCs (Supplementary Figure 3).

Taken together, these results indicate that miR-370 can regulate the self-renewal and differentiation of piPSCs by *LIN28A* dependence.

## Discussion

In this study, we proved that a set of common microRNA–mRNA interactions exists among distinct piPSC lines independent of their metastable pluripotent states. Furthermore, we validated the interaction of miR-370 with *LIN28A*, and demonstrated that miR-370 can target the 3′UTR of *LIN28A* and reduce the *LIN28A* expression in piPSCs. We propose the working model of interaction between miR-370 and LIN28A in piPSCs (Figure 7d). In the pluripotent state, *LIN28A* is highly expressed while miR-370 is lowly expressed. LIN28A protein is speculated to inhibit the maturation of miR-370 precursor.^14^ When the differentiation initiates, miR-370 is highly activated and inhibits *LIN28A* expression at post-transcriptional level, which further leads to less LIN28A proteins.

MiRNAs represent an important mechanism of post-transcriptional regulation and are highly conserved between invertebrates and vertebrates.^15^ We revealed there are 165 common miRNAs that are differentially expressed between distinct piPSC lines and somatic cells. Interestingly, only three out of the 165 common miRNAs are highly expressed in piPSCs and others are highly expressed in somatic cells. The three miRNAs include miR-371 and other two predicted miRNAs (chr8_17989_mature and chr15_31797_mature). This unique miRNA signature of piPSCs presented an obvious contrast against the specific miRNA signature of mouse and human PSCs. Substantial evidence has shown that miRNA clusters miR-302, miR-290 and miR-371 are expressed exclusively in both mouse and human pluripotent stem cells (PSCs).^16^ Taken the findings together, miR-371 seems to be more conserved than miR-302 and miR-290 in PSC across diverse species. Recent evidence suggested that miR-371 could promote pluripotency by regulating glycolytic metabolism via the Mbd2-Myc signaling pathway in mouse and human ESCs.^17^ Thus, it will be interesting to investigate if the analogous role of miR-371 exists in piPSCs in the future.

We also uncovered that there are 1416 common mRNAs differentially expressed between distinct piPSC lines and somatic cells. Out of the 1416 common mRNAs, many genes are commonly expressed in somatic cells with high levels and enriched in biological processes of extracellular matrix organization and cell adhesion. This feature of gene expression pattern reflects the fundamental differences on extracellular matrix between PSC and somatic cells. Extracellular matrix organization and cell adhesion of somatic cells are regarded as barriers to achieve fully reprogramming.^18^

Moreover, conjoint analysis of small RNA-seq and mRNA-seq was applied and revealed that sixteen miRNAs were commonly expressed lower in piPSC lines than that in somatic cells, and these miRNAs potentially targeted thirteen common mRNAs. These miRNA–mRNA interactions are common in distinct piPSC lines independent of their metastable pluripotent states. Interestingly, interactions of miR-206 on *OTX2* and miR-370 on *LIN28A* were found among the common interactions. Otx2, once as a transcription factor involved in brain development,^19^ was found recently to antagonize naïve pluripotency and promote the naïve-to-primed transition.^20^ We previously demonstrated that overexpression of *OTX2* decreased the percentage of alkaline phosphatase-positive piPSC colonies and downregulated the expressions of *NANOG* and *OCT4*.^21^ The common interaction of miR-206 on *OTX2* found in this study suggests the potential use of miR-206 to induce piPSCs with naïve pluripotency by downregulating *OTX2*.

LIN28A, as an RNA-binding protein, has important roles in tumorigenesis, embryonic development and pluripotency regulation.^22^ Overexpression of LIN28A and LIN28B could increase iPSCs derivation efficiency, while double knockout of LIN28A/B reduced the reprogramming efficiency and caused mouse iPSCs trapped in a more naïve state.^23^ In addition, mESCs at naïve state showed the low expression of Lin28a, whereas mouse epiblast stem cells at primed state express Lin28a with high level.^24^ Lin28a was further demonstrated to facilitate the naive-to-primed transition in mouse PSCs.^23^ All these evidences indicate that LIN28A can regulate PSCs exiting from naïve pluripotency as a key factor. Our findings showed that *LIN28A* was conservatively expressed in distinct piPSC lines, which indicated the primed pluripotent feature of these cell lines and might explain the limited chimera potential for these piPSCs.^7, 8, 9^ Moreover, we demonstrated that miR-370 could target on the 3′UTR of *LIN28A* and reduce the expression of *LIN28A* in piPSCs. Therefore, ectopic expression of miR-370 may logically push piPSCs to acquire naive pluripotency. Unexpectedly, we found ectopic expression of miR-370 could not make piPSCs acquire naïve pluripotency. In contrast, miR-370 overexpression led to the downregulation of classical pluripotent genes (including naïve pluripotency gene *NANOG*) in piPSCs. Taken these findings together, LIN28A seems to have different roles in PSC across species. Additionally, we also demonstrated that miR-370 was activated during the process of piPSCs differentiation and decreased the expression level of *LIN28A*, which suggests the role of miR-370 as an orchestrator for regulating self-renewal and differentiation of piPSCs. The similar interaction of miR-370 on *LIN28A* was once reported in human hepatocellular carcinoma and demonstrated that LIN28A protein could blocked the biogenesis of miR-370 by an inverse feedback.^14^ It will be interesting to investigate this inverse feedback effect of LIN28A on miR-370 in piPSCs (Figure 7d) in the future study.

Collectively, a set of common microRNA–mRNA interactions was uncovered among distinct piPSC lines independent of their metastable pluripotent states, which will provide insights into the mechanism of reprogramming and pluripotency regulation on pig.

## Materials and Methods

### Cell culture

The LIF-dependent piPSCs (piPS-L) were from Dr. Hanazono’s laboratory, and were cultured with LIF-containing medium on collagen I coated plate.^7^ The FGF2-dependent piPSCs (piPS-F) were from Dr. West’s laboratory, and were cultured with FGF2-containing mTeSR TM 1 medium on Matrigel-coated plate.^8^ The LFB2i-dependent piPSCs (piPS-LF) were generated by our laboratory, which were cultured on MEF feeders with medium containing LIF, FGF2, BMP4 and small molecules.^9^ DOX-piPSCs were generated by tetracycline operator (TetO)-inducible system and reported in this laboratory previously.^12^ The DOX-piPSCs are routinely maintained on MEF feeders and cultured in DMEM medium containing 15% FBS (HyClone, Logan, UT, USA), 1 mM l-glutamine (Invitrogen, Carlsbad, USA), 0.1 mM NEAA (Invitrogen), 0.1 mM *β*-mercaptoethanol (Invitrogen), 10 ng/ml human LIF (Millipore, Bedford, MA, USA), 10 ng/ml human FGF2 (PeproTech, Rocky Hill, USA), 3 *μ*M CHIR99021 (Selleck Chemicals, Houston, USA), 2 *μ*M SB431542 (Selleck Chemicals) and 4 *μ*g/ml doxycycline (Sigma-Aldrich, St. Louis, USA). Porcine embryonic fibroblasts (PEFs) derived from a 35-day-old fetal pig were cultured with medium consisting of DMEM (HyClone) supplemented with 15% FBS (HyClone), 0.1 mM NEAA (Invitrogen), 1 mM l-glutamine (Invitrogen) and 0.1 mM *β*-mercaptoethanol (Invitrogen). HEK 293T cells were cultured in DMEM medium with 10% FBS (HyClone). J1 mESCs were routinely cultured on MEF feeders with DMEM medium containing 15% FBS (HyClone), 0.1 mM NEAA (Invitrogen), 1 mM l-glutamine (Invitrogen), 0.1 mM *β*-mercaptoethanol (Invitrogen) and 1000 U/ml mouse LIF (Millipore).

### Small RNA sequencing

For small RNA sequencing, the RNA lysates of piPS-L and piPS-F was provided by Dr. Hanazono’s lab and Dr. West’s lab, respectively. The RNA lysates of piPS-LF was prepared by our lab. Three microgram RNA per sample was used as input material for the small RNA library. Sequencing libraries were generated using NEBNext Multiplex Small RNA Library Prep Set for Illumina (New England Biolabs, Ipswich, MA, USA) following manufacturer’s recommendations and index codes were added to attribute sequences to each sample. Briefly, NEB 3′ SR Adaptor was ligated to 3′ ends of miRNA, small interfering RNA (siRNA) and piwi-interacting RNA (piRNA) directly and specifically. After the 3′ ligation reaction, the SR RT Primer hybridized to the excess of 3′ SR Adaptor (that remained free after the 3′ ligation reaction) and transformed the single-stranded DNA adaptor into a double-stranded DNA molecule. The 5'end adapter was ligated to 5′ ends of miRNAs, siRNA and piRNA. Then first-strand cDNA was synthesized using M-MuLV Reverse Transcriptase (RNase H^–^). PCR amplification was performed using LongAmp Taq 2X Master Mix, SR Primer for illumina and index (X) primer. PCR products were purified on 8% polyacrylamide gel (100 V, 80 min). DNA fragments corresponding to 140–160 bp (the length of small noncoding RNA plus the 3′ and 5′ adaptors) were recovered and dissolved in 8 *μ*l elution buffer. The library quality was assessed on the Agilent Bioanalyzer 2100 system using DNA High Sensitivity Chips. The clustering of the index-coded samples was performed on a cBot Cluster Generation System using TruSeq SR Cluster Kit v3-cBot-HS (Illumia, San Diego, USA) according to the manufacturer’s instructions. After cluster generation, the library preparations were sequenced on an Illumina Hiseq 2500/2000 platform and 50 bp single-end reads were generated. Small RNA-seq data of piPS-L, piPS-F, piPS-LF and PEFs were deposited in EMBL-EBI database (http://www.ebi.ac.uk/) under accession number E-MTAB-2631.

### mRNA sequencing

For mRNA sequencing, a total amount of 3 *μ*g RNA per sample was used as input material for the RNA sample preparations. Sequencing libraries were generated using NEBNext® Ultra™ RNA Library Prep Kit for Illumina (New England Biolabs) following manufacturer’s recommendations and index codes were added to attribute sequences to each sample. Briefly, mRNA was purified from total RNA using poly-T oligo-attached magnetic beads. Fragmentation was carried out using divalent cations under elevated temperature in NEBNext First-Strand Synthesis Reaction Buffer (5X). First-strand cDNA was synthesized using random hexamer primer and M-MuLV Reverse Transcriptase (RNase H). Second strand cDNA synthesis was subsequently performed using DNA polymerase I and RNase H. Remaining overhangs were converted into blunt ends via exonuclease/polymerase activities. After adenylation of 3′ ends of DNA fragments, NEBNext Adaptor with the hairpin loop structure were ligated to prepare for hybridization. In order to select cDNA fragments of preferentially 150–200 bp in length, the library fragments were purified with AMPure XP system (Beckman Coulter, Beverly, USA). Then 3 *μ*l USER Enzyme (New England Biolabs) was used with size-selected, adaptor-ligated cDNA at 37 °C for 15 min followed by 95 °C 5 min before PCR. Then PCR was performed with Phusion High-Fidelity DNA polymerase, Universal PCR primers and Index (X) Primer. At last, PCR products were purified using AMPure XP system and library quality was assessed on the Agilent Bioanalyzer 2100 system. The clustering of the index-coded samples was performed on a cBot Cluster Generation System using TruSeq PE Cluster Kit v3-cBot-HS (Illumia) according to the manufacturer’s instructions. After cluster generation, the library preparations were sequenced on Illumina Hiseq 2000 platform and 100 bp paired-end reads were generated. The sequenced data have been deposited with the European Bioinformatics Institute (www.ebi.ac.uk/). The mRNA-seq data of piPS-L, piPS-LF and PEFs were deposited in EMBL-EBI database (http://www.ebi.ac.uk/) under accession number E-MTAB-2634. mRNA-seq data of piPS-F were downloaded from GEO under the accession number GSM881389.

### Data analysis

After removal of adaptors, low quality tags, and contaminants from the sequenced tags, clean reads were annotated. Index of the reference genome was built using Bowtie v2.0.6.^25^ Paired-end clean reads were aligned to the reference genome using TopHat v2.0.9.^26^ Normalization was performed before further analysis. Hierarchical clustering was done by using gplots package. The miRNA profiles were clustered using average linkage clustering with Euclidean distances, treating samples independently each other. Differentially expressed miRNAs between any two samples were calculated by EBSeq.^27^ Target genes of miRNAs were predicted using RNAhybrid.^28^ Gene Ontology (GO) analysis and Kyoto Encyclopedia of Genes and Genomes (KEGG) pathway analysis of differentially expressed genes were performed using the Database for Annotation and Visualization and Integrated Discovery (DAVID);^29, 30^ a hypergeometric test with the Benjamini and Hochberg false discovery rate (FDR) was performed using the default parameters to adjust the *P*-value.

Secondary structure of miRNA was predicted by RNAFold.^31^ The promoter region of miR-370 was predicted by the Web Promoter Scan Service (http://www-bimas.cit.nih.gov/molbio/proscan/). The potential transcription factors binding to the miR-370 promoter was predicted by the JASPAR.^32^

### Gene cloning and vector constructs

Genomic DNA (gDNA) was isolated from PEFs and its concentration and purity was examined using Nanodrop (Thermo Scientific, San Jose, CA, USA). The 3′UTR of pig LIN28A were amplified by PCR using pig gDNA and the mutated UTR was amplified by Overlap PCR. The cloned fragments were cut with XhoI and NotI, and ligated into pSICHECK2 vector (Promega, Madison, USA). The synthesized pre-mir-370 (80 bp) was cloned into pLL3.7 basic (Addgene, Cambridge, MA, USA) with *Hpa*I and *Xho*I. The CDS of porcine *LIN28A* and mouse Lin28a were amplified by PCR using mRNA isolated from DOX-piPSCs and J1. The cloned fragments were cut by EcoRI and BamHI, and ligated into pCD513B-1 vector (System Biosciences, Mountain View, CA, USA). The synthesized mouse pri-miR-370 (537 bp) fragment was also cloned into pCD513B-1 vector. All the primers used for vector construction are listed in Supplementary Table 13.

### Cell transfection and luciferase assay

All miRNA mimics were ordered from GenePharma. Cells were cotransfected by miRNA mimics reporter plasmids using X-tremeGENE siRNA Transfection Reagent (Roche, Mannheim, Germany). Cells were harvested 36 h after transfection and lysed for 15 min at room temperature. The firefly and renilla luciferase activities were analyzed using Dual-luciferase reporter assay system (Promega, Wisconsin, USA) on a Hamamatsu BHP9504 Luminometer (Hamamatsu, Peking, China). Firefly luciferase activity was normalized to the renilla luciferase activity.

### Lentivirus package and infection

HEK 293T cells were seeded in a 6-well plate the day before transfection. After 24 h culture, cells were transfected with lentivirus vector (pLL3.7-miR-370, pCDH-miR-370, pCDH-LIN28A (pig), or pCDH-Lin28a (mouse)) and its package plasmids (pSPAX2, pVSVG) using Lipofectamine 2000 (Invitrogen), according to the manufacturer’s instruction. At 48 h after transfection, the supernatant with lentivirus particles was collected and used for infecting Dox-piPSCs, piPS-LF or J1 in the presence of 4 ug/ml polybrene (Sigma-Aldrich). Two rounds of infection were conducted. The infected piPSCs colonies with GFP or puromycin resistance were picked up and expanded for further analysis.

### Quantitative RT-PCR

Total RNAs from Dox-piPSCs were reverse transcribed to cDNAs using Revert Aid First-Strand cDNA Synthesis Kit (Thermo Scientific) and Mir-X™ miRNA First-Strand Synthesis and SYBR qRT-PCR (Clontech, Palo Alto, CA, USA) according to the manufacturer’s instruction. For the quantitative RT-PCR, reactions were performed in triplicate using SYBR Green PCR Master Mix (TransGen Biotech, Peking, China), and detected with the CFX96 real-time PCR system (Bio-Rad, Hercules, USA). Relative expression levels of target genes and deferentially expressed miRNAs were normalized to either U6 (for miRNAs) or *β*-Actin expression (for mRNAs). The relative expression levels were calculated using 2^−ΔΔCt^.

### Western blotting

The cells were lysed with ice-cold RIPA buffer (50 mM Tris–HCl, pH 7.5, 150 mM NaCl, 1% NP-40, 0.25% sodium deoxycholate, 1 mM EDTA) supplemented with 1 mM protease inhibitor PMSF (Sigma-Aldrich). Protein concentration was measured using BCA protein assay kit (Pierce, Rockford, IL, USA) according to the manufacturer’s instructions. Protein samples were mixed with 4 × loading buffer (200 mM Tris–HCl, pH 6.8, 8% SDS, 0.2% bromophenol blue, 40% glycerine, 8% *β*-mercaptoethanol), heated at 95 °C for 5 min, and subjected to 10% SDS-PAGE. After electrophoresis, proteins were transferred to PVDF membrane (Millipore) by semidry electrophoretic transfer for 45 min at 15 V. The membrane was blocked with blocking buffer (5% dried nonfat milk with TBS-T buffer containing 20 mM Tris–HCl, pH 7.6, 137 mM NaCl, 0.1% Tween 20) for 1 h at 37 °C, and then incubated with the primary anti-LIN28A antibody (1:1000 dilution, BBI Life Sciences, Shanghai, China) and anti-*β*-actin (1:2000 dilution, Santa Cruz Biotechnology, Santa Cruz, CA, USA) overnight at 4 °C, respectively. After washing three times with TBS-T buffer, the membrane was incubated with the HRP-conjugated secondary antibody (1:3000 dilution, Sungene Biotech, Tianjin, China) for 1 h at 37 °C. After washing three times in TBS-T for 5 min at room temperature, the membrane was incubated in the enhanced chemiluminescent substrate (Pierce) for 1 min and detected with a Chemiluminescent Imaging System (Tanon, Shanghai, China).

### Alkaline phosphatase staining

The alkaline phosphatase (AP) activity of piPSCs was determined by AST Fast Red TR (Sigma-Aldrich) and a-Naphthol AS-MX Phosphate (Sigma-Aldrich) according to the manufacturer’s instruction. Briefly, cells were washed twice with ice-cold PBS, fixed with 4% paraformaldehyde (pH 7.4) for 15 min at room temperature, and followed by washing three times with ice-cold PBS. Then the cells were then incubated at room temperature with dye solution containing AST Fast Red TR (1.0 mg/ml), a-Naphthol AS-MX (0.4 mg/ml) in 0.1 M Tris Buffer. After 10–20 min incubation, the cells were washed three times with PBS and the images were documented with a Nikon microscope.

### Cell growth curve

Growth curve of Dox-piPSCs was determined by cell counting for five days. The cells were plated with 4 × 10^4^ cells/well in 12-well plate and harvested for cell counting every 24 h. The cells in triplicate wells were counted and averaged for each treatment at every time point.

### Statistics analysis

Calculated data are presented as mean±S.D. Student *t*-test was used to determine the differences between two groups and one-factor ANOVA with Bonferroni *post hoc* test was used to determine the differences among three groups in this study. All statistical analyses were done with GraphPad Prism 5.0 (GraphPad, La Jolla, CA, USA).
