# Supplementary figures and images for: Common microRNA–mRNA interactions exist among distinct porcine iPSC lines independent of their metastable pluripotent states
Source: Cell Death Dis. 2017 Aug 31;8(8):e3027–. doi: 10.1038/cddis.2017.426 (PMC5596602; doi:10.1038/cddis.2017.426)

a

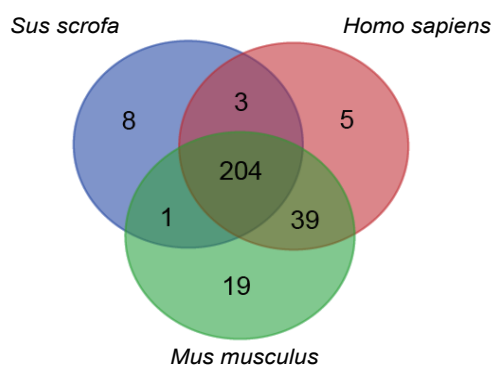

b

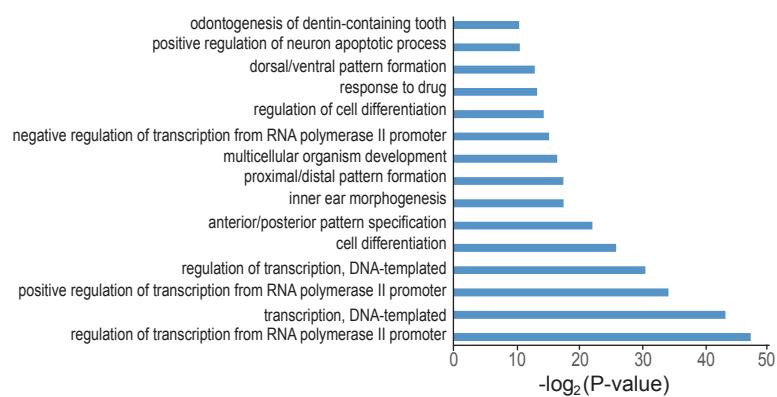

Fig.S1

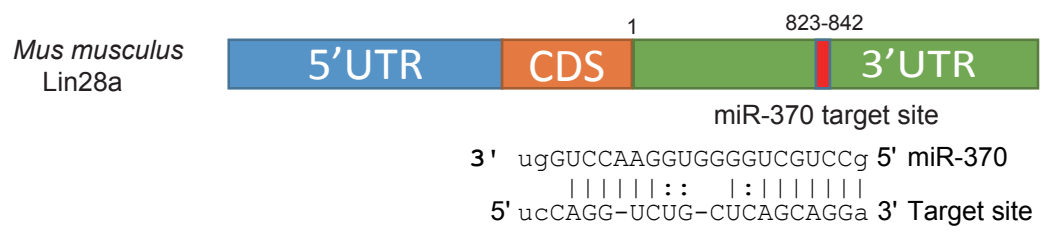

**Fig.S2**

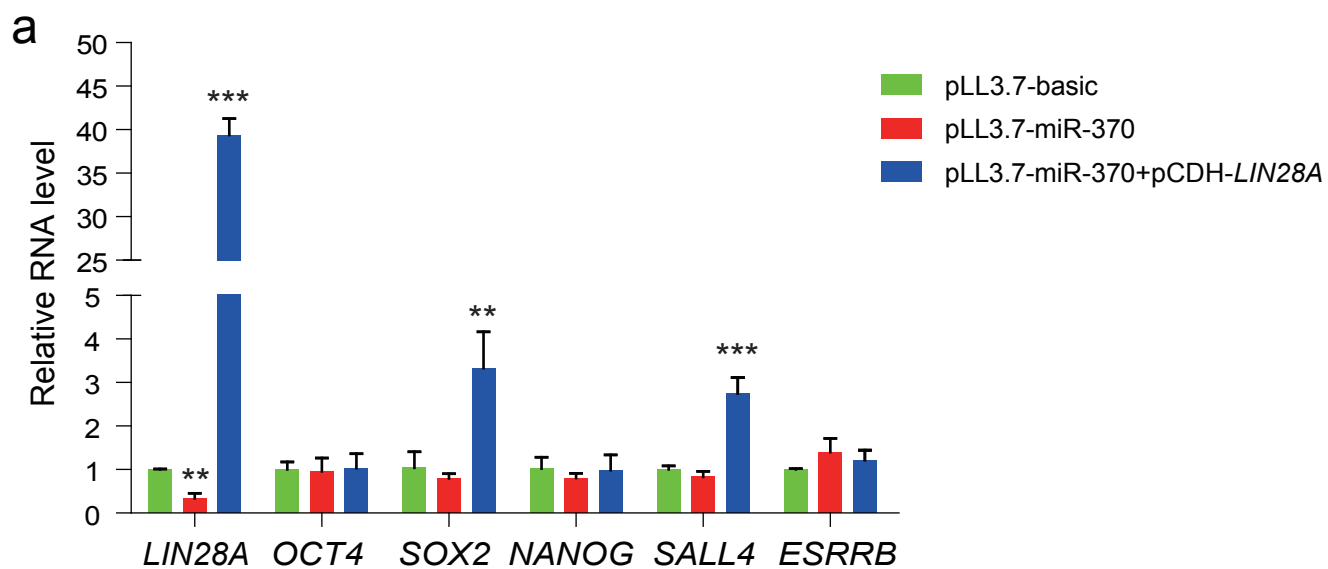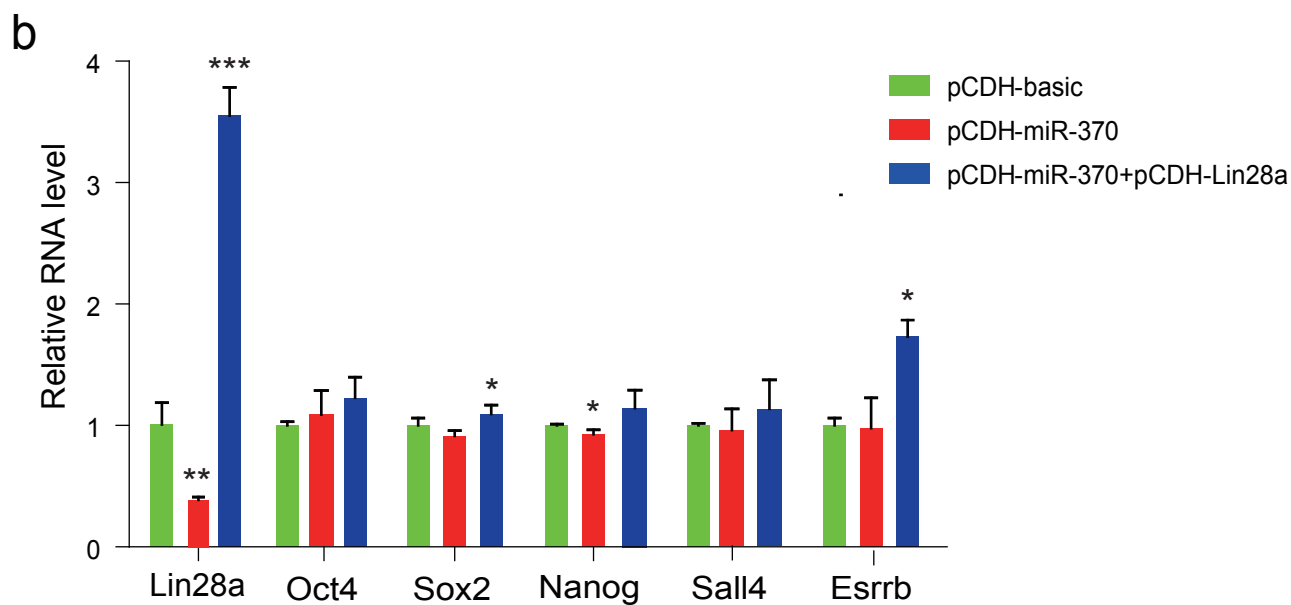

**Fig.S3**

Supplement: Supplementary Figure 1 [file cddis2017426x1.pdf]
