# Supplementary material for: Common microRNA–mRNA interactions exist among distinct porcine iPSC lines independent of their metastable pluripotent states
Source: Cell Death Dis. 2017 Aug 31;8(8):e3027–. doi: 10.1038/cddis.2017.426 (PMC5596602; doi:10.1038/cddis.2017.426)
Supplement: Supplementary Table 1 [file cddis2017426x2.pdf]

piPS-L Vs PEFs

AccID	piPS-L	PEF	piPS-L_log2(count+1)	PEF_log2(count+1)	PEF_log2(count+1)<0.0	Log2FC	FDR
rno-miR-351-5p	27370.37073	0.805927	14.74038006	0.852739935	0.852739935	15.05161	4.63E-08
rno-miR-298-5p	14916.10522	0.805927	13.86467998	0.852739935	0.852739935	14.17586	1.65E-07
mmu-miR-5099	6044.455867	0	12.56163542	0	0	20	3.23E-07
chr6_12933_star@@hsa-miR-520e	29877.34238	4.029637	14.86681249	2.330454351	2.330454351	12.85611	4.26E-07
rno-miR-872-3p	4087.664021	0	11.9974138	0	0	20	7.32E-07
ssc-miR-371-5p	103988.2814	16.92448	16.66607531	4.163859073	4.163859073	12.58502	8.75E-07
chr8_18357_star	2904.562526	0	11.50460179	0	0	20	1.49E-06
chr6_12933_mature	2181.376962	0	11.0916846	0	0	20	2.72E-06
hsa-miR-3659	0	13122.92	0	13.67991072	13.67991072	-20	3.02E-06
chr4_10088_mature	0	9929.832	0	13.27769889	13.27769889	-20	4.67E-06
chr2_4320_mature	2915.308225	0.805927	11.50992749	0.852739935	0.852739935	11.82071	4.99E-06
chr13_28872_mature	6854.681595	3.22371	12.74308439	2.078510713	2.078510713	11.05415	5.99E-06
chr4_9150_mature@@rno-miR-298-5	1419.50688	0	10.4721901	0	0	20	6.66E-06
chr4_9149_mature@@rno-miR-298-5	1391.568062	0	10.44353213	0	0	20	6.94E-06
chr1_3439_mature@@rno-miR-872-5	6064.872695	3.22371	12.56649951	2.078510713	2.078510713	10.87754	7.74E-06
NW_003538989_38416_mature	2325.369332	0.805927	11.18386444	0.852739935	0.852739935	11.49452	7.99E-06
chr18_36117_mature	1.074569932	13979.62	1.05281229	13.77114047	13.77114047	-13.6673	8E-06
ssc-miR-126-5p	10080.54053	5.641492	13.29942849	2.731507408	2.731507408	10.80321	8.33E-06
ssc-miR-206	1.074569932	11796.36	1.05281229	13.52617644	13.52617644	-13.4223	1.04E-05
chr1_3720_mature	0	5863.928	0	12.51789771	12.51789771	-20	1.07E-05
chrx_21555_mature	0	5591.525	0	12.44928399	12.44928399	-20	1.15E-05
mmu-miR-883a-3p	1090.688481	0	10.09234552	0	0	20	1.15E-05
chr15_32852_star	1884.79566	0.805927	10.88095764	0.852739935	0.852739935	11.19147	1.24E-05
chr5_11699_mature	971.4112184	0	9.925422727	0	0	20	1.47E-05
chr1_2086_mature	959.5909491	0	9.907778405	0	0	20	1.51E-05
chr7_16402_mature	3175.354149	2.417782	11.63315606	1.773060525	1.773060525	10.35902	1.79E-05
chr8_17989_mature@@bta-miR-302b	2325.369332	1.611855	11.18386444	1.38507475	1.38507475	10.49452	1.82E-05
chr16_34017_mature	0	3926.479	0	11.93938767	11.93938767	-20	2E-05
bta-miR-379	0	3912.778	0	11.93434612	11.93434612	-20	2.01E-05
chr6_12932_star@@bta-miR-292	1486.130216	0.805927	10.53831526	0.852739935	0.852739935	10.84862	2.03E-05
rno-miR-322-3p	804.8528789	0	9.654372666	0	0	20	2.17E-05
chr8_17983_mature@@bta-miR-302b	2088.963947	1.611855	11.02926234	1.38507475	1.38507475	10.33985	2.28E-05
rno-miR-3586-5p	1200.294614	0.805927	10.2303743	0.852739935	0.852739935	10.54045	3.17E-05
rno-miR-182	1188.474345	0.805927	10.21610844	0.852739935	0.852739935	10.52617	3.24E-05
chr2_4263_mature	1143.342407	0.805927	10.16030308	0.852739935	0.852739935	10.47032	3.51E-05
NW_003613310_38408_mature	638.2945395	0	9.320336961	0	0	20	3.51E-05
mmu-miR-344d-3p	620.0268507	0	9.278511836	0	0	20	3.73E-05
NW_003541079_39891_mature	2.149139864	7881.165	1.654957834	12.94437615	12.94437615	-11.8404	4.02E-05
NW_003538989_38422_mature	2056.72685	2.417782	11.00683577	1.773060525	1.773060525	9.73245	4.42E-05
chr1_1318_mature	0	2350.89	0	11.1996051	11.1996051	-20	4.47E-05
ssc-miR-153	999.3500366	0.805927	9.966289192	0.852739935	0.852739935	10.27612	4.64E-05
chr1_319_mature	949.9198197	0.805927	9.89317989	0.852739935	0.852739935	10.20294	5.15E-05
rno-miR-350	524.3901267	0	9.037245279	0	0	20	5.29E-05
chr1_485_mature	0	1921.331	0	10.90864108	10.90864108	-20	6.13E-05
NW_003537574_37009_mature	3.223709795	8313.142	2.078510713	13.02135161	13.02135161	-11.3325	6.34E-05
chr4_10011_mature	476.0344798	0	8.897949737	0	0	20	6.46E-05
ssc-miR-455-5p	0	1852.021	0	10.85566373	10.85566373	-20	6.5E-05
chr18_36269_mature	1.074569932	3564.617	1.05281229	11.79993607	11.79993607	-11.6958	6.81E-05
mmu-miR-467a-5p	456.692221	0	8.838233962	0	0	20	7.04E-05
chr11_24857_mature	0	1671.494	0	10.70778491	10.70778491	-20	7.63E-05
chr11_24582_mature	0	1636.033	0	10.67686744	10.67686744	-20	7.89E-05
chr7_15777_star	431.9771126	0	8.758146955	0	0	20	7.9E-05
chr9_19733_mature	428.7534028	0	8.747365253	0	0	20	8.02E-05
rno-miR-151-3p	1108.95617	1.611855	10.11628699	1.38507475	1.38507475	9.426265	8.5E-05
ssc-miR-1249	1028.363425	1.611855	10.00753671	1.38507475	1.38507475	9.317413	9.94E-05
chr8_18597_mature	363.204637	0	8.50860548	0	0	20	0.000113
chr8_18598_mature	352.4459376	0	8.465398812	0	0	20	0.00012
chr15_33570_mature	615.7285709	0.805927	9.268491873	0.852739935	0.852739935	9.577429	0.000127
chr2_4298_mature	1162.684666	2.417782	10.18448446	1.773060525	1.773060525	8.90956	0.000144
chrx_22076_mature	0	1105.732	0	10.11209079	10.11209079	-20	0.000146
chr7_15145_mature	1.074569932	2137.32	1.05281229	11.06226178	11.06226178	-10.9578	0.000152
chr2_4477_star	1.074569932	2107.5	1.05281229	11.0420015	11.0420015	-10.9376	0.000155
rno-miR-674-3p	1120.776439	2.417782	10.13156947	1.773060525	1.773060525	8.856598	0.000156
mmu-miR-669a-3p	309.4761404	0	8.278338593	0	0	20	0.000157
NW_003613137_37368_mature	1.074569932	1938.256	1.05281229	10.92128719	10.92128719	-10.8168	0.000177
chr1_3153_mature	0	967.1129	0	9.91903155	9.91903155	-20	0.00018
rno-miR-3068-3p	289.0593117	0	8.180204124	0	0	20	0.000181
chr5_11530_mature	1.074569932	1885.064	1.05281229	10.88116315	10.88116315	-10.7766	0.000185
chrx_22553_mature	1.074569932	1729.52	1.05281229	10.75699015	10.75699015	-10.6524	0.000211
mmu-miR-467d-5p	267.567913	0	8.06914314	0	0	20	0.000212
chr8_18153_mature	467.4379203	0.805927	8.871714057	0.852739935	0.852739935	9.179909	0.000224
NW_003538125_37558_mature	467.4379203	0.805927	8.871714057	0.852739935	0.852739935	9.179909	0.000224
chr12_25534_mature	3.223709795	3712.908	2.078510713	11.85872227	11.85872227	-10.1696	0.000224
mmu-miR-669a-5p	260.0459235	0	8.028159819	0	0	20	0.000225
chrx_22554_mature	1.074569932	1641.674	1.05281229	10.68183067	10.68183067	-10.5772	0.000229
chr6_12627_mature	1176.654075	3.22371	10.20170011	2.078510713	2.078510713	8.511753	0.000234
chr5_11364_mature	251.449364	0	7.979850233	0	0	20	0.000241
chr6_12983_mature@@rno-miR-200	249.3002242	0	7.967515773	0	0	20	0.000246
chr1_541_mature	0	772.8844	0	9.595974312	9.595974312	-20	0.000255
chr14_31463_star	2.149139864	2371.844	1.654957834	11.21240183	11.21240183	-10.108	0.000263
chr16_33920_mature	2508.046221	7.253347	11.29292333	3.044979305	3.044979305	8.433701	0.000264
chr1_1317_mature	2.149139864	2365.397	1.654957834	11.20847645	11.20847645	-10.1041	0.000264
chrx_21552_mature	1105.73246	3.22371	10.11209079	2.078510713	2.078510713	8.422065	0.000266
mmu-miR-690	238.5545249	0	7.904210254	0	0	20	0.000269
chr16_33919_mature	2479.032833	7.253347	11.2761435	3.044979305	3.044979305	8.416915	0.00027
mmu-miR-1198-5p	234.2562451	0	7.878089211	0	0	20	0.000279
chr18_36371_mature	1077.793642	3.22371	10.07520321	2.078510713	2.078510713	8.385143	0.000281
rno-miR-296-3p	229.9579654	0	7.851486493	0	0	20	0.00029
chr4_8839_mature	225.6596857	0	7.824384002	0	0	20	0.000301
NW_003538989_38414_mature	224.5851158	0	7.817528071	0	0	20	0.000304
chr2_6234_mature	221.361406	0	7.796762599	0	0	20	0.000314
chr4_9231_mature	0	660.8605	0	9.37038338	9.37038338	-20	0.000325
rno-miR-339-3p	215.9885563	0	7.761475149	0	0	20	0.00033
chr13_28643_mature	211.6902766	0	7.73261027	0	0	20	0.000344
rno-miR-1843-5p	964.9637988	3.22371	9.915825312	2.078510713	2.078510713	8.225609	0.000353
chr6_15010_mature	208.4665668	0	7.710576182	0	0	20	0.000355
chr9_20972_mature@@hsa-miR-488	548.0306652	1.611855	9.100742921	1.38507475	1.38507475	8.409391	0.000365
chr10_23883_mature	205.242857	0	7.688200344	0	0	20	0.000366
chr6_14978_mature	4.298279727	3488.86	2.405524014	11.76895342	11.76895342	-9.66478	0.000381
chr5_10827_mature	723.1855641	2.417782	9.500215608	1.773060525	1.773060525	8.224538	0.000385
rno-miR-466b-2-3p	191.2734479	0	7.587015736	0	0	20	0.000423
chr1_1915_mature	191.2734479	0	7.587015736	0	0	20	0.000423
chr6_12951_mature	1.074569932	1104.121	1.05281229	10.10998811	10.10998811	-10.0049	0.000426
chr14_30497_mature	0	550.4484	0	9.107082211	9.107082211	-20	0.000433
chr4_8854_mature	1.074569932	1067.048	1.05281229	10.06076069	10.06076069	-9.95565	0.000449
chr16_34772_mature	0	535.1358	0	9.066454733	9.066454733	-20	0.000452
chr10_24300_mature	183.7514583	0	7.529441942	0	0	20	0.000459
chr12_26282_star	327.7438292	0.805927	8.360820004	0.852739935	0.852739935	8.667703	0.000464
chr16_33714_mature	0	520.6291	0	9.026880633	9.026880633	-20	0.000472
chrx_22639_mature	1.074569932	1014.663	1.05281229	9.98820559	9.98820559	-9.88303	0.000486
chr1_3819_mature	1.074569932	1007.409	1.05281229	9.977865629	9.977865629	-9.87267	0.000491
chr13_28482_mature	177.3040388	0	7.478195571	0	0	20	0.000494
chr3_7573_mature	2.149139864	1579.618	1.654957834	10.62627284	10.62627284	-9.5216	0.000497
chrx_21838_star@@rno-miR-188-3p	174.080329	0	7.45187319	0	0	20	0.000513
bta-miR-302c	800.5545992	3.22371	9.646656985	2.078510713	2.078510713	7.956134	0.000518
chr5_11562_mature@@mmu-miR-30	454.5430812	1.611855	8.831443687	1.38507475	1.38507475	8.139551	0.000536
chr1_2557_mature	1.074569932	949.3825	1.05281229	9.892364514	9.892364514	-9.78709	0.000539
chr7_15832_mature	1991.178084	8.059274	10.9601309	3.179395517	3.179395517	7.948757	0.00054
chr4_10241_star	2433.900896	9.671129	11.24964734	3.415640968	3.415640968	7.97537	0.000547
mmu-miR-467e-5p	167.6329094	0	7.397742301	0	0	20	0.000554
chr7_16963_mature	1.074569932	930.0403	1.05281229	9.862699769	9.862699769	-9.75739	0.000556
ssc-miR-370	4.298279727	2733.706	2.405524014	11.41716998	11.41716998	-9.31288	0.000558
chr13_29269_mature	0	461.7964	0	8.85423392	8.85423392	-20	0.000569
mmu-miR-3082-3p	163.3346296	0	7.360492716	0	0	20	0.000584
mmu-miR-467c-5p	162.2600597	0	7.35102808	0	0	20	0.000592
bta-miR-877	290.1338816	0.805927	8.185538937	0.852739935	0.852739935	8.491853	0.000596
chr13_27061_mature	161.1854898	0	7.341500942	0	0	20	0.0006
mmu-miR-351-5p	159.0363499	0	7.322255819	0	0	20	0.000616
ssc-miR-145-5p	23.6405385	19900.77	4.62296188	14.28060887	14.28060887	-9.71735	0.000626
chr13_29267_mature	0	430.3653	0	8.752766176	8.752766176	-20	0.000635
chr11_24398_mature	1.074569932	849.4475	1.05281229	9.73207842	9.73207842	-9.62662	0.000641
chr15_32372_mature	155.8126401	0	7.292898044	0	0	20	0.000642
chr8_17778_mature	413.7094235	1.611855	8.695957021	1.38507475	1.38507475	8.003752	0.00065
ssc-miR-296-5p	877.9236343	4.029637	9.779594011	2.330454351	2.330454351	7.767302	0.000657
chr12_25526_mature	0	419.8882	0	8.717293256	8.717293256	-20	0.000659
chr3_8430_mature	153.6635003	0	7.272988959	0	0	20	0.000661
chr18_35940_mature	153.6635003	0	7.272988959	0	0	20	0.000661
chr18_35938_mature	149.3652205	0	7.2323271	0	0	20	0.0007
NW_003613237_37813_mature	147.2160807	0	7.211558171	0	0	20	0.000721
chr12_26457_mature	0	390.8748	0	8.614249038	8.614249038	-20	0.000737
chr16_34293_mature	1.074569932	774.4963	1.05281229	9.598976048	9.598976048	-9.49336	0.00074
chr1_169_mature	0	386.8452	0	8.599337045	8.599337045	-20	0.000749
chr3_8429_mature	143.9923709	0	7.179833181	0	0	20	0.000754
chr1_468_star	512.5698575	2.417782	9.00441672	1.773060525	1.773060525	7.72792	0.00078
chr12_25628_mature	0	371.5326	0	8.541222691	8.541222691	-20	0.000797
chr1_3926_mature	139.6940911	0	7.136417929	0	0	20	0.000802
chr6_14625_mature@@rno-miR-298-	791.9580398	4.029637	9.631100716	2.330454351	2.330454351	7.61863	0.000812
chrx_22943_mature	0	362.6674	0	8.506475604	8.506475604	-20	0.000828
chr16_34839_mature	492.1530288	2.417782	8.945891584	1.773060525	1.773060525	7.669279	0.000848
chr2_6101_mature	358.9063572	1.611855	8.491477775	1.38507475	1.38507475	7.798742	0.000869
chr14_30693_mature	0	348.9666	0	8.451073371	8.451073371	-20	0.000879
chr10_24151_mature	0	346.5488	0	8.441071766	8.441071766	-20	0.000888
chr15_33120_mature	1.074569932	686.6502	1.05281229	9.42553103	9.42553103	-9.31967	0.000892
chrx_22070_mature	235.3308151	0.805927	7.884663944	0.852739935	0.852739935	8.189825	0.000913
ssc-miR-150	342.7878083	1.611855	8.425374573	1.38507475	1.38507475	7.73245	0.000954
chr4_9327_mature	339.5640985	1.611855	8.411782547	1.38507475	1.38507475	7.718818	0.000973
chr1_3820_mature	2.149139864	1015.469	1.654957834	9.989349913	9.989349913	-8.88417	0.000988
chr18_35948_star@@rno-miR-96-3p	125.724682	0	6.985553734	0	0	20	0.000993
chr5_11958_mature	0	321.5651	0	8.33344633	8.33344633	-20	0.000998
chr15_32458_mature	335.2658187	1.611855	8.393458327	1.38507475	1.38507475	7.70044	0.000998
ssc-miR-192	4378.872472	20.14819	12.09667315	4.402462031	4.402462031	7.763766	0.001003
chr1_1155_mature	2.149139864	1000.156	1.654957834	9.967451025	9.967451025	-8.86225	0.001012
chr4_10395_mature	2.149139864	984.0374	1.654957834	9.944034714	9.944034714	-8.83881	0.001038
chrx_23228_mature	2.149139864	982.4256	1.654957834	9.941672042	9.941672042	-8.83644	0.00104
chr9_20478_mature	121.4264023	0	6.935770911	0	0	20	0.001065
chrx_22340_mature	1.074569932	610.0871	1.05281229	9.255234166	9.255234166	-9.14911	0.001072
ssc-miR-145-3p	2.149139864	955.83	1.654957834	9.902118745	9.902118745	-8.79685	0.001086
rno-miR-466c-5p	119.2772624	0	6.910220127	0	0	20	0.001104
mmu-miR-3470b	119.2772624	0	6.910220127	0	0	20	0.001104
chrx_22720_mature	0	299.805	0	8.23268479	8.23268479	-20	0.001112
ssc-miR-362	1.074569932	588.327	1.05281229	9.202924647	9.202924647	-9.09672	0.001134
rno-miR-466b-1-3p	210.6157066	0.805927	7.725302902	0.852739935	0.852739935	8.029747	0.001144
chr1_2360_mature	2.149139864	922.7869	1.654957834	9.851416323	9.851416323	-8.74609	0.001147
mmu-miR-1948-3p	116.0535526	0	6.871024911	0	0	20	0.001167
chr4_8769_mature	659.7859381	4.029637	9.368039176	2.330454351	2.330454351	7.355204	0.001179
chr13_27180_mature	206.3174269	0.805927	7.695697583	0.852739935	0.852739935	8	0.001193
chr16_34762_mature	0	283.6865	0	8.153230078	8.153230078	-20	0.001212
rno-miR-674-5p	199.8700073	0.805927	7.650118356	0.852739935	0.852739935	7.954196	0.001272
rno-miR-6215	110.680703	0	6.803236118	0	0	20	0.001283
ssc-miR-486	5661.908971	27.40153	12.46732762	4.827896911	4.827896911	7.690888	0.00131
chr13_28441_mature	5.372849659	1987.417	2.671938627	10.95740469	10.95740469	-8.53099	0.001318
NW_003613242_37864_mature	0	266.762	0	8.064807344	8.064807344	-20	0.001333
chr3_8078_mature	0	265.9561	0	8.060458479	8.060458479	-20	0.001339
ssc-miR-378	19.34225877	9122.293	4.346407978	13.1553389	13.1553389	-8.8815	0.001392
chrx_22424_star	182.6768884	0.805927	7.521026297	0.852739935	0.852739935	7.824428	0.001525
mmu-miR-1196-5p	182.6768884	0.805927	7.521026297	0.852739935	0.852739935	7.824428	0.001525
chr12_26049_mature	101.0095736	0	6.672560745	0	0	20	0.001542
chr8_18345_mature	0	241.7782	0	7.923495278	7.923495278	-20	0.001551
chr11_25089_mature	3.223709795	1072.689	2.078510713	10.06836104	10.06836104	-8.37829	0.001552
hsa-miR-516a-5p	1.074569932	473.8853	1.05281229	8.89143541	8.89143541	-8.78463	0.001586
bta-miR-449c	98.86043373	0	6.641841268	0	0	20	0.00161
chrx_22153_mature	98.86043373	0	6.641841268	0	0	20	0.00161
chr1_2964_mature	1.074569932	463.4083	1.05281229	8.859249894	8.859249894	-8.75238	0.001642
chr14_31530_mature	260.0459235	1.611855	8.028159819	1.38507475	1.38507475	7.333901	0.001671
chr5_11275_mature	647.9656689	4.835565	9.34199835	2.544872269	2.544872269	7.066089	0.001765
chr6_14899_mature	640.4436794	4.835565	9.325178788	2.544872269	2.544872269	7.049244	0.001808
chr1_3446_mature	166.5583394	0.805927	7.388519682	0.852739935	0.852739935	7.691162	0.001836
chr5_11967_mature	1.074569932	429.5593	1.05281229	8.750068242	8.750068242	-8.64295	0.001846
chr2_4519_mature	335.2658187	2.417782	8.393458327	1.773060525	1.773060525	7.115477	0.00185
chr5_11474_mature	2.149139864	676.9791	1.654957834	9.405096899	9.405096899	-8.29921	0.001854
chr3_8256_mature	1.074569932	417.4704	1.05281229	8.708981833	8.708981833	-8.60177	0.001929
mmu-miR-125b-2-3p	90.26387427	0	6.511971994	0	0	20	0.00193
rno-miR-3102	90.26387427	0	6.511971994	0	0	20	0.00193
chr1_211_mature	0	209.5411	0	7.717958332	7.717958332	-20	0.001935
chr1_589_mature	0	207.9293	0	7.706870893	7.706870893	-20	0.001958
ssc-miR-429	89.18930434	0	6.494884448	0	0	20	0.001977
chr16_34240_mature	156.88721	0.805927	7.302750497	0.852739935	0.852739935	7.604862	0.00207
chr16_34386_mature	5.372849659	1477.265	2.671938627	10.52968921	10.52968921	-8.10303	0.002089
chr18_36372_mature	1029.437895	8.059274	10.00904198	3.179395517	3.179395517	6.996991	0.002089
chr4_10244_mature	1.074569932	394.0985	1.05281229	8.626068641	8.626068641	-8.51865	0.002109
chr6_13041_mature	0	195.8404	0	7.620882324	7.620882324	-20	0.002147
chr15_33031_mature	0	195.8404	0	7.620882324	7.620882324	-20	0.002147
rno-miR-24-2-5p	84.89102461	0	6.424435476	0	0	20	0.002181
mmu-miR-350-5p	84.89102461	0	6.424435476	0	0	20	0.002181
NW_003613041_36883_mature	1.074569932	384.4274	1.05281229	8.5903153	8.5903153	-8.48281	0.002191
hsa-miR-6087	1593.587209	12.08891	10.63896729	3.710273245	3.710273245	7.04245	0.002228
rno-miR-503-3p	83.81645468	0	6.406272275	0	0	20	0.002237
chr1_986_star	481.4073295	4.029637	8.914108017	2.330454351	2.330454351	6.900464	0.002237
mmu-miR-339-3p	150.4397905	0.805927	7.24260051	0.852739935	0.852739935	7.544321	0.002252
chrx_22905_mature	223.5105458	1.611855	7.810639403	1.38507475	1.38507475	7.115477	0.002266
chr6_13566_mature	1.074569932	375.5622	1.05281229	8.556744343	8.556744343	-8.44915	0.002271
chr13_28748_mature	222.4359759	1.611855	7.803717686	1.38507475	1.38507475	7.108524	0.002288
chr8_17233_mature	82.74188475	0	6.387877484	0	0	20	0.002295
chrx_22120_mature	1.074569932	365.8911	1.05281229	8.519207948	8.519207948	-8.41151	0.002365
chr14_30387_mature	377.1740461	3.22371	8.562906545	2.078510713	2.078510713	6.870365	0.002403
chr15_32593_mature	1.074569932	359.4436	1.05281229	8.493629892	8.493629892	-8.38586	0.00243
NW_003613237_37814_mature	143.9923709	0.805927	7.179833181	0.852739935	0.852739935	7.481127	0.002457
chrx_22497_mature	0	178.9159	0	7.491178829	7.491178829	-20	0.002467
ssc-miR-7	69.84704557	37072.66	6.146635787	15.17810703	15.17810703	-9.05194	0.002474
bta-miR-33b	79.51817495	0	6.331242567	0	0	20	0.002483
chr9_20036_mature	0	178.11	0	7.484701805	7.484701805	-20	0.002484
rno-miR-344a-3p	78.44360502	0	6.311859186	0	0	20	0.002551
rno-miR-3560	77.36903509	0	6.292211829	0	0	20	0.002621
chr1_666_mature	137.5449513	0.805927	7.114210328	0.852739935	0.852739935	7.415037	0.002692
mmu-miR-5097	13593.30964	70.92162	13.73071527	6.168353522	6.168353522	7.582456	0.002724
chrx_22196_star	0	167.6329	0	7.397742301	7.397742301	-20	0.002726
chr2_6477_star	135.3958114	0.805927	7.091655531	0.852739935	0.852739935	7.392317	0.002778
chrx_22713_mature	601.7591618	5.641492	9.235437864	2.731507408	2.731507408	6.736966	0.002828
chrx_21682_mature	2.149139864	510.958	1.654957834	8.999881656	8.999881656	-7.8933	0.002863
chr8_17991_mature@@bta-miR-367	198.7954374	1.611855	7.642379827	1.38507475	1.38507475	6.946419	0.002865
chr15_32917_mature	1186.325205	10.47706	10.21349942	3.520680821	3.520680821	6.823122	0.002883
mmu-miR-466b-3p	132.1721016	0.805927	7.057148072	0.852739935	0.852739935	7.357552	0.002914
chr4_9236_mature	1.074569932	319.1473	1.05281229	8.322591896	8.322591896	-8.21432	0.002918
hsa-miR-762	196.6462975	1.611855	7.626777119	1.38507475	1.38507475	6.930737	0.002928
rno-miR-702-3p	73.07075536	0	6.210832145	0	0	20	0.002934
chrx_21569_mature	0	159.5736	0	7.327091221	7.327091221	-20	0.00294
chr2_5680_mature	3.223709795	701.1569	2.078510713	9.455649593	9.455649593	-7.76487	0.002996
mmu-miR-1935	130.0229618	0.805927	7.033675856	0.852739935	0.852739935	7.333901	0.003011
ssc-miR-187	262.1950634	2.417782	8.039988619	1.773060525	1.773060525	6.760812	0.003033
chr15_31863_mature	3.223709795	685.8443	2.078510713	9.423839197	9.423839197	-7.73302	0.0031
mmu-miR-5121	70.9216155	0	6.168353522	0	0	20	0.003111
chr11_25111_mature	70.9216155	0	6.168353522	0	0	20	0.003111
chr6_14250_mature	127.8738219	0.805927	7.009815429	0.852739935	0.852739935	7.309855	0.003112
chr13_29417_mature	190.1988779	1.611855	7.578930247	1.38507475	1.38507475	6.882643	0.003129
chr6_12650_mature	2.149139864	477.915	1.654957834	8.903625744	8.903625744	-7.79685	0.003173
rno-miR-871-3p	69.84704557	0	6.146635787	0	0	20	0.003206
rno-miR-501-3p	69.84704557	0	6.146635787	0	0	20	0.003206
mmu-miR-5117-3p	252.523934	2.417782	7.985978141	1.773060525	1.773060525	6.706592	0.00327
chr6_13339_mature	3.223709795	656.0249	2.078510713	9.359804332	9.359804332	-7.66888	0.003319
chrx_22488_mature	1.074569932	290.9398	1.05281229	8.189527141	8.189527141	-8.08082	0.003364
chr9_20754_mature	0	145.8729	0	7.198424102	7.198424102	-20	0.003372
mmu-miR-700-5p	67.6979057	0	6.102194213	0	0	20	0.003409
bta-miR-1246	5.372849659	1074.301	2.671938627	10.07052523	10.07052523	-7.6435	0.003418
chr12_25546_mature	0	142.6492	0	7.166405731	7.166405731	-20	0.003489

piPS-F Vs PEFs

AccID	piPS-F	PEF	piPS-F_log2(count+1)	PEF_log2(count+1)	PEF_log2(count+1)<0.0	Log2FC	FDR
chr11_25345_mature	251.449364	0	7.979850233	0	0	20	0
ssc-miR-545-3p	694.172176	20.14819	9.44122653	4.402462031	4.402462031	5.106572	0
chr6_13158_mature	3232.30635	22.56597	11.65879449	4.558633073	4.558633073	7.162271	0
chr2_5822_mature	167.632909	3.22371	7.397742301	2.078510713	2.078510713	5.70044	0
chrx_21682_mature	12.8948392	510.958	3.796477232	8.999881656	8.999881656	-5.30834	0
chr12_25587_mature	696.321316	109.6061	9.445679773	6.789287574	6.789287574	2.667425	0
chr7_15145_mature	2.14913986	2137.32	1.654957834	11.06226178	11.06226178	-9.95783	0
chr13_27072_mature	4.29827973	728.5584	2.405524014	9.510879685	9.510879685	-7.40514	0
chr9_19418_mature@@bta-miR-2452	10.7456993	436.8127	3.554060706	8.77416992	8.77416992	-5.34518	0
chr2_4241_mature	182.676888	0.805927	7.521026297	0.852739935	0.852739935	7.824428	0
chr3_8450_mature	15.043979	282.8805	4.003960082	8.149140118	8.149140118	-4.23293	0
chr11_25258_mature	4.29827973	394.0985	2.405524014	8.626068641	8.626068641	-6.51865	0
chr8_18024_mature	318.0727	5.641492	8.317741365	2.731507408	2.731507408	5.817136	0
chr15_33264_mature	3023.83979	22.56597	11.56264302	4.558633073	4.558633073	7.066089	0
chr11_25350_mature	2456.46686	333.654	11.26295625	8.386526292	8.386526292	2.88016	0
chr15_32759_mature	189.124308	3.22371	7.570799187	2.078510713	2.078510713	5.874469	0
chr2_5068_star	2772.39042	106.3824	11.43743501	6.746614057	6.746614057	4.703799	0
ssc-miR-30c-5p	16638.6408	1500.637	14.02233667	10.5523203	10.5523203	3.470891	0
chr2_6335_mature	1672.03081	9704.978	10.7082483	13.24465793	13.24465793	-2.53712	0
chr1_2125_mature	502.898728	67.69791	8.976990005	6.102194213	6.102194213	2.893085	0
hsa-miR-4792	11197.0187	205.5115	13.45095587	7.690078309	7.690078309	5.767752	0
chr1_2964_mature	4.29827973	463.4083	2.405524014	8.859249894	8.859249894	-6.75238	0
chr3_7304_star@@mmu-miR-216b-3p	3053.92775	8.865202	11.57692254	3.302348583	3.302348583	8.428297	0
chr14_29999_mature	5970.31054	244.196	12.54383188	7.937791734	7.937791734	4.611694	0
ssc-miR-143-3p	382.546896	647476.5	8.583259175	19.30447047	19.30447047	-10.725	0
chrx_22340_mature	2.14913986	610.0871	1.654957834	9.255234166	9.255234166	-8.14911	0
chr3_6703_star	760.795512	104.7706	9.573259978	6.72479443	6.72479443	2.860275	0
chr5_10765_star	1749.39985	855.089	10.7734688	9.741617018	9.741617018	1.032714	0
ssc-miR-143-5p	10.7456993	2311.4	3.554060706	11.17517521	11.17517521	-7.74886	0
chr6_13566_mature	15.043979	375.5622	4.003960082	8.556744343	8.556744343	-4.64179	0
hsa-miR-4705	507.197008	29.81932	8.989244071	4.94576292	4.94576292	4.088227	0
ssc-miR-542-3p	38.6845175	6947.095	5.310504361	12.76240168	12.76240168	-7.48851	0
chr4_9198_mature	6.44741959	257.8968	2.896740642	8.016233232	8.016233232	-5.32193	0
chr14_31467_mature	638.29454	35.46081	9.320336961	5.188274618	5.188274618	4.169925	0
chr12_25628_mature	8.59655945	371.5326	3.262517265	8.541222691	8.541222691	-5.43359	0
chr3_8257_mature@@bta-miR-2898	2993.75183	656.8309	11.54822074	9.361572903	9.361572903	2.188361	0
chr6_12438_mature	120403.412	10329.57	16.87752873	13.33463253	13.33463253	3.543024	0
chr4_9773_mature	221.361406	0.805927	7.796762599	0.852739935	0.852739935	8.101538	0
hsa-miR-3607-3p	1087.46477	166.827	10.088079	7.390830869	7.390830869	2.704544	0
chrx_22076_mature	2.14913986	1105.732	1.654957834	10.11209079	10.11209079	-9.00703	0
bta-miR-2889	421.231413	24.17782	8.721890106	4.654081667	4.654081667	4.122857	0
bta-miR-2887	550.179805	83.01053	9.106379219	6.392498216	6.392498216	2.728537	0
hsa-miR-3607-5p	281.537322	5.641492	8.142297645	2.731507408	2.731507408	5.641106	0
chr8_18026_mature	930.577561	8.865202	9.863532079	3.302348583	3.302348583	6.713829	0
bta-miR-2898	990.753477	274.0153	9.95383774	8.103368244	8.103368244	1.85427	0
ssc-miR-935	264.344203	9.671129	8.051721221	3.415640968	3.415640968	4.77259	0
chr2_5456_mature	5162.23395	517.4054	12.33405925	9.017936998	9.017936998	3.318628	0
chr7_15245_mature	2037.38459	39.49044	10.99321056	5.339509593	5.339509593	5.689071	0
chr18_36617_mature	438.424532	62.05641	8.779471605	5.978571212	5.978571212	2.820676	0
chr7_15287_mature	109066.699	8365.527	16.73486438	13.03041315	13.03041315	3.70461	0
ssc-miR-92a	1684551.7	14602.6	20.68393413	13.83403638	13.83403638	6.849996	0
chr12_25947_star	156.88721	2.417782	7.302750497	1.773060525	1.773060525	6.0199	0
chr6_12967_mature	578.118623	27.40153	9.177715082	4.827896911	4.827896911	4.399037	0
chr16_34772_mature	8.59655945	535.1358	3.262517265	9.066454733	9.066454733	-5.96	0
chr13_28823_star	1375.44951	100.7409	10.42673598	6.668756392	6.668756392	3.771181	0
chr3_6901_star	597.460882	10.47706	9.225113139	3.520680821	3.520680821	5.833539	0
ssc-miR-99a	292.283021	7699.025	8.196149744	12.9106474	12.9106474	-4.71924	0
ssc-miR-99b	49872.9397	11746.39	15.60599855	13.52005295	13.52005295	2.086039	0
chr5_10912_star	412.634854	5.641492	8.692213945	2.731507408	2.731507408	6.192645	0
chr1_541_mature	2.14913986	772.8844	1.654957834	9.595974312	9.595974312	-8.49035	0
chr7_16183_mature	6737.55347	394.0985	12.71822321	8.626068641	8.626068641	4.095597	0
chr3_7145_mature	1087.46477	184.5574	10.088079	7.535721616	7.535721616	2.558827	0
chr2_4834_mature	17.1931189	452.9312	4.185320985	8.826329926	8.826329926	-4.71939	0
chr5_12402_mature	496.451309	27.40153	8.958411509	4.827896911	4.827896911	4.179324	0
chr16_34017_mature	2.14913986	3926.479	1.654957834	11.93938767	11.93938767	-10.8353	0
ssc-miR-151-5p	76582.4499	734.1999	16.22474503	9.521992772	9.521992772	6.704697	0
chr6_12838_mature	33582.4595	2204.212	15.03546323	11.10670136	11.10670136	3.929373	0
chr10_23656_mature	339.564098	4.029637	8.411782547	2.330454351	2.330454351	6.39689	0
chr3_7983_star	1186.3252	29.81932	10.21349942	4.94576292	4.94576292	5.314109	0
chr3_8533_star	1689.22393	9.671129	10.72299868	3.415640968	3.415640968	7.448461	0
chr7_16724_mature	35828.3107	58.02678	15.12885267	5.883297648	5.883297648	9.270166	0
chr15_33053_mature	883.296484	11.28298	9.788386342	3.618589217	3.618589217	6.290677	0
chr4_10149_star	281.537322	20.14819	8.142297645	4.402462031	4.402462031	3.804604	0
ssc-miR-196a	17.1931189	338.4895	4.185320985	8.407223265	8.407223265	-4.29921	0
ssc-miR-196b	444.871952	50.77343	8.800485637	5.694139975	5.694139975	3.131245	0
chr16_34762_mature	4.29827973	283.6865	2.405524014	8.153230078	8.153230078	-6.04439	0
ssc-miR-151-3p	62737.6909	19804.86	15.93706781	14.27363981	14.27363981	1.663478	0
ssc-let-7d-3p	9501.34734	342.5192	13.21406823	8.424246783	8.424246783	4.793875	0
chr1_3820_mature	4.29827973	1015.469	2.405524014	9.989349913	9.989349913	-7.88417	0
chr2_6283_mature	236.405385	24.98375	7.891208849	4.699537803	4.699537803	3.242201	0
chr13_28221_mature	236.405385	0.805927	7.891208849	0.852739935	0.852739935	8.196397	0
hsa-miR-1291	1250.7994	145.0669	10.28978768	7.190485881	7.190485881	3.10806	0
chr7_15783_mature	264.344203	15.31262	8.051721221	4.027916745	4.027916745	4.109624	0
chr7_15101_mature	6471.06013	225.6597	12.6600093	7.824384002	7.824384002	4.841781	0
chr3_8533_mature	719.961854	15.31262	9.493779119	4.027916745	4.027916745	5.555127	0
chr7_15333_mature	8544.9801	236.9427	13.06103024	7.894470201	7.894470201	5.172467	0
chrx_21884_mature	2.14913986	253.0612	1.654957834	7.989032362	7.989032362	-6.87958	0
ssc-miR-9-1	5845.66043	255.479	12.51339709	8.002696903	8.002696903	4.516089	0
ssc-miR-9-2	6039.08302	227.2715	12.56035266	7.834607195	7.834607195	4.731841	0
chr4_9437_mature	2138.39416	31.43117	11.0629866	5.019309189	5.019309189	6.088188	0
ssc-miR-374b-3p	593.162602	11.28298	9.214713992	3.618589217	3.618589217	5.716207	0
chr13_29269_mature	4.29827973	461.7964	2.405524014	8.85423392	8.85423392	-6.74735	0
ssc-miR-423-3p	36963.0565	11045.24	15.17383547	13.43126719	13.43126719	1.74266	0
bta-let-7b	487.854749	78.17496	8.933262058	6.306972375	6.306972375	2.641673	0
ssc-miR-21	1168614.14	2238509	20.15636846	21.09410709	21.09410709	-0.93774	0
chr1_1444_mature	2.14913986	271.5976	1.654957834	8.090628787	8.090628787	-6.98157	0
chr6_13171_mature	221.361406	12.08891	7.796762599	3.710273245	3.710273245	4.194647	0
ssc-miR-31	12.8948392	601.2219	3.796477232	9.234151307	9.234151307	-5.54303	0
ssc-miR-32	851.059386	132.978	9.734810175	7.065852624	7.065852624	2.678072	0
ssc-miR-423-5p	13111.9023	6627.141	13.67869942	12.69438867	12.69438867	0.984418	0
ssc-miR-16	193293.639	2383.933	17.5604421	11.21973326	11.21973326	6.341306	0
chr14_29777_mature	4734.55512	1721.461	12.20931784	10.75025563	10.75025563	1.459595	0
ssc-miR-874	1590.3635	117.6654	10.6360477	6.890755622	6.890755622	3.756594	0
ssc-miR-499-5p	670.531637	36.26674	9.39131156	5.21981653	5.21981653	4.208587	0
chr3_7600_mature	1742.95243	61.25049	10.76814497	5.960013198	5.960013198	4.830668	0
ssc-miR-22-5p	1431.32715	141.0373	10.48414533	7.150126067	7.150126067	3.343205	0
ssc-miR-374b-5p	891.893043	333.654	9.80234356	8.386526292	8.386526292	1.418518	0
chr6_12802_mature	466.36335	12.08891	8.868400795	3.710273245	3.710273245	5.269698	0
ssc-miR-95	257.896784	2.417782	8.016233232	1.773060525	1.773060525	6.736966	0
chr4_10088_mature	2.14913986	9929.832	1.654957834	13.27769889	13.27769889	-12.1738	0
ssc-miR-22-3p	550998.627	21109.66	19.07169182	14.3656839	14.3656839	4.706074	0
ssc-miR-98	76169.815	1075.107	16.21695071	10.07160611	10.07160611	6.146667	0
ssc-miR-30e-3p	3696.52057	692.2917	11.85234246	9.437318634	9.437318634	2.416716	0
ssc-miR-10a-5p	19675.3755	59396.05	14.26417687	15.85810359	15.85810359	-1.59398	0
chr11_24857_mature	2.14913986	1671.494	1.654957834	10.70778491	10.70778491	-9.60316	0
NW_003613380_38805_mature	640.443679	50.77343	9.325178788	5.694139975	5.694139975	3.656926	0
bta-miR-193b	78512.3775	9638.086	16.26065087	13.23468069	13.23468069	3.026101	0
chr3_8127_star	406.187434	62.86234	8.66954923	5.996893534	5.996893534	2.691878	0
chr9_19936_mature	726.409274	146.6788	9.506623509	7.206318883	7.206318883	2.308122	0
ssc-miR-30e-5p	214.913986	8322.007	7.75431289	13.02288911	13.02288911	-5.2751	0
ssc-miR-181b	2413.48407	5820.408	11.23749923	12.50715243	12.50715243	-1.27	0
ssc-miR-181c	5265.39267	58.8327	12.36259938	5.902862353	5.902862353	6.483779	0
chr17_35020_mature	287.984742	38.68452	8.174849511	5.310504361	5.310504361	2.896164	0
ssc-miR-133a-3p	8347.25923	412.6349	13.02725968	8.692213945	8.692213945	4.338365	0
chr1_2963_mature	6.44741959	476.3031	2.896740642	8.898761964	8.898761964	-6.20701	0
mmu-miR-1900	3268.84173	502.8987	11.67500509	8.976990005	8.976990005	2.70044	0
chr13_27132_mature	629.69798	51.57936	9.300805502	5.716424587	5.716424587	3.609794	0
chr1_3195_mature@@bta-miR-2887	3159.2356	243.3901	11.6258164	7.933041972	7.933041972	3.698233	0
chrx_21844_mature@@hsa-miR-660-5	10434.074	230.4953	13.34915322	7.854838784	7.854838784	5.500422	0
ssc-miR-1306-5p	4425.07898	311.8939	12.11181348	8.289529827	8.289529827	3.826576	0
chrx_22664_mature	937.024981	4.029637	9.873482533	2.330454351	2.330454351	7.861294	0
ssc-let-7i	212218.965	79993.13	17.69520086	16.28760661	16.28760661	1.407605	0
ssc-let-7g	99756.6251	16362.74	16.6061395	13.99821534	13.99821534	2.607998	0
chr3_7304_mature@@bta-miR-216b	5458.81525	26.59561	12.41463642	4.786366653	4.786366653	7.681256	0
chr12_25666_mature	14485.2027	166.827	13.82239185	7.390830869	7.390830869	6.440083	0
ssc-let-7f	315534.566	20199.77	18.2674431	14.30212235	14.30212235	3.965388	0
chr4_9327_mature	152.58893	1.611855	7.26293043	1.38507475	1.38507475	6.564785	0
ssc-let-7e	22069.5173	3867.646	14.42983282	11.91761294	11.91761294	2.512527	0
ssc-let-7c	23511.5901	11989.78	14.52114585	13.54963821	13.54963821	0.971567	0
ssc-let-7a	270157.627	15987.18	18.04344722	13.96471835	13.96471835	4.078814	0
chr15_33456_mature	728.558414	114.4417	9.510879685	6.851020612	6.851020612	2.670432	0
chr13_28475_star	292.283021	5.641492	8.196149744	2.731507408	2.731507408	5.695145	0
mmu-miR-1983	3702.96799	1081.555	11.85485591	10.08022412	10.08022412	1.775576	0
NW_003539217_38656_mature	303.028721	4.835565	8.248063807	2.544872269	2.544872269	5.969626	0
chr9_20930_mature	2720.81107	282.8805	11.41035121	8.149140118	8.149140118	3.265772	0
chr15_33627_mature	992.902617	31.43117	9.956960693	5.019309189	5.019309189	4.981384	0
chr5_11530_mature	2.14913986	1885.064	1.654957834	10.88116315	10.88116315	-9.77664	0
chr14_31463_star	2.14913986	2371.844	1.654957834	11.21240183	11.21240183	-10.108	0
ssc-miR-4334-3p	1706.41705	182.9455	10.73759978	7.523134815	7.523134815	3.221484	0
chrx_22074_mature	292.283021	31.43117	8.196149744	5.019309189	5.019309189	3.217098	0
chr1_169_mature	2.14913986	386.8452	1.654957834	8.599337045	8.599337045	-7.49185	0
chr2_4615_mature	664.084218	16.92448	9.377393227	4.163859073	4.163859073	5.294183	0
chr16_34600_star	8306.42557	2051.891	13.02018575	11.00344151	11.00344151	2.017273	0
chr17_35449_mature	68198.6553	10954.97	16.05747683	13.41942984	13.41942984	2.638158	0
chr7_16464_mature	8252.69708	242.5842	13.01082477	7.928276522	7.928276522	5.088308	0
ssc-miR-758	58.0267763	392.4867	5.883297648	8.620170944	8.620170944	-2.75785	0
ssc-miR-744	16961.0118	2297.699	14.05001967	11.16660195	11.16660195	2.88396	0
ssc-miR-2320-3p	1399.09005	224.0478	10.45130391	7.814087849	7.814087849	2.64261	0
NW_003541078_39887_star	924.130141	174.0803	9.853512519	7.45187319	7.45187319	2.408343	0
chr8_17690_mature	324.520119	1112.18	8.346602905	10.12047102	10.12047102	-1.77701	0
ssc-miR-2320-5p	23337.5098	1463.564	14.51042482	10.51625577	10.51625577	3.995093	0
chr15_33547_mature	726.409274	3136.67	9.506623509	11.61547774	11.61547774	-2.11038	0
ssc-miR-708-5p	40.8336574	784.9733	5.38659223	9.618336558	9.618336558	-4.26481	0
ssc-miR-708-3p	77.3690351	1212.115	6.292211829	10.24450047	10.24450047	-3.96963	0
chr5_11960_mature	1287.33478	462.6024	10.33129182	8.856744087	8.856744087	1.476543	0
chr9_19375_mature	4.29827973	406.9934	2.405524014	8.672401869	8.672401869	-6.5651	0
chr1_468_star	8330.06611	2.417782	13.02428541	1.773060525	1.773060525	11.75043	0
chr4_10395_mature	6.44741959	984.0374	2.896740642	9.944034714	9.944034714	-7.25385	0
chr6_12448_star	2993.75183	66.89198	11.54822074	6.085169218	6.085169218	5.483978	0
ssc-miR-296-5p	984.306058	4.029637	9.944428117	2.330454351	2.330454351	7.932313	0
chr3_7145_star	775.839491	79.78682	9.601472732	6.336047992	6.336047992	3.281536	0
ssc-miR-628	1429.17801	15.31262	10.48197901	4.027916745	4.027916745	6.544321	0
chr18_36269_mature	2.14913986	3564.617	1.654957834	11.79993607	11.79993607	-10.6958	0
ssc-miR-652	206.317427	2.417782	7.695697583	1.773060525	1.773060525	6.415037	0
NW_003537879_37346_mature	143.992371	1.611855	7.179833181	1.38507475	1.38507475	6.481127	0
mmu-miR-6243	249.300224	939.7114	7.967515773	9.877608386	9.877608386	-1.91433	0
mmu-miR-5115	15869.2488	1928.584	13.95403712	10.91407442	10.91407442	3.04062	0
NW_003541078_39887_mature	7653.08705	39.49044	12.90201459	5.339509593	5.339509593	7.598394	0
mmu-miR-6236	2198.57008	62.86234	11.10300585	5.996893534	5.996893534	5.128226	0
mmu-miR-5105	5577.01795	641.5182	12.44553686	9.327593621	9.327593621	3.119932	0
mmu-miR-5109	1972.91039	7493.513	10.94684079	12.8716191	12.8716191	-1.92532	0
chr6_14159_mature	1265.84338	186.1692	10.30702246	7.548199552	7.548199552	2.765412	0
bta-miR-4286	6511.89379	510.958	12.66908298	8.999881656	8.999881656	3.671801	0
ssc-miR-361-5p	2490.8531	174.8863	11.28300331	7.458498946	7.458498946	3.832151	0
chr8_18750_mature	700.619596	10.47706	9.454545231	3.520680821	3.520680821	6.063326	0
chr13_26691_mature	831.717127	274.0153	9.701682687	8.103368244	8.103368244	1.601836	0
chr9_19153_mature	603.908302	7.253347	9.24057265	3.044979305	3.044979305	6.379539	0
chr4_10240_mature	17.1931189	599.61	4.185320985	9.230284738	9.230284738	-5.12412	0
chr7_16615_mature	19.3422588	290.1339	4.346407978	8.185538937	8.185538937	-3.90689	0
chr18_36516_mature	1672.03081	171.6625	10.7082483	7.431811362	7.431811362	3.283954	0
chr9_20193_mature	249.300224	29.81932	7.967515773	4.94576292	4.94576292	3.063565	0
chr6_14032_mature	8.59655945	1699.701	3.262517265	10.7319138	10.7319138	-7.62731	0
chr16_34601_mature	19.3422588	1191.967	4.346407978	10.22033805	10.22033805	-5.94544	0
chr5_11474_mature	4.29827973	676.9791	2.405524014	9.405096899	9.405096899	-7.29921	0
ssc-miR-615	19.3422588	456.1549	4.346407978	8.836539387	8.836539387	-4.5597	0
chr2_5281_mature	124.650112	920.3691	6.973268148	9.847635478	9.847635478	-2.88433	0
chr6_13339_mature	6.44741959	656.0249	2.896740642	9.359804332	9.359804332	-6.66888	0
chr13_28222_mature	287.984742	3.22371	8.174849511	2.078510713	2.078510713	6.481127	0
chrx_22663_mature	816.673148	15.31262	9.675380454	4.027916745	4.027916745	5.736966	0
chr11_24694_mature	1020.84144	8.865202	9.996955627	3.302348583	3.302348583	6.84739	0
chr11_25089_mature	17.1931189	1072.689	4.185320985	10.06836104	10.06836104	-5.96326	0
mmu-miR-5097	31104.5012	70.92162	14.92488213	6.168353522	6.168353522	8.776682	0
chr8_17417_mature	361.055497	12.08891	8.500067045	3.710273245	3.710273245	4.900464	0
chr6_14220_star	2978.70785	207.9293	11.54095517	7.706870893	7.706870893	3.840522	0
chr5_12267_mature	12.8948392	437.6186	3.796477232	8.776823197	8.776823197	-5.08481	0
ssc-miR-582	354.608078	59.63863	8.474144285	5.922165283	5.922165283	2.571906	0
chr7_15660_mature	326.669259	20.14819	8.356096521	4.402462031	4.402462031	4.019109	0
chr3_7739_mature	638.29454	35.46081	9.320336961	5.188274618	5.188274618	4.169925	0
chr4_10241_star	3032.43635	9.671129	11.56673732	3.415640968	3.415640968	8.292577	0
chr6_13306_mature	436.275392	26.59561	8.772398353	4.786366653	4.786366653	4.035979	0
chr2_5731_mature	303.028721	46.74379	8.248063807	5.577241251	5.577241251	2.696608	0
chr8_18872_star	563.074644	46.74379	9.139742278	5.577241251	5.577241251	3.59048	0
chr7_15769_mature	15.043979	523.8528	4.003960082	9.035769166	9.035769166	-5.1219	0
chr5_11650_mature	735.005833	74.95125	9.52357339	6.247001856	6.247001856	3.293731	0
ssc-miR-504	836.015407	221.63	9.709110369	7.798504517	7.798504517	1.915376	0
ssc-miR-505	2664.93343	642.3242	11.38042504	9.329402097	9.329402097	2.052726	0
ssc-miR-503	4.29827973	332.0421	2.405524014	8.379560789	8.379560789	-6.27146	0
ssc-miR-500	8587.9629	1333.81	13.06826822	10.38241861	10.38241861	2.686763	0
chr9_20919_mature	15950.9161	426.3356	13.9614421	8.739225767	8.739225767	5.225506	0
chr9_19140_mature	2086.81481	69.30976	11.02777803	6.135653077	6.135653077	4.9121	0
chr13_28716_mature	52742.0414	149.9025	15.68669315	7.237472949	7.237472949	8.458785	0
chr8_18343_mature	2.14913986	238.5545	1.654957834	7.904210254	7.904210254	-6.79442	0
bta-miR-1291	2551.02902	158.7677	11.31742902	7.319832027	7.319832027	4.00609	0
chr6_13122_mature@@mmu-miR-5097	7257.64532	175.6922	12.82548461	7.465094412	7.465094412	5.36838	0
chr2_5134_mature	853.208526	40.29637	9.738444487	5.367943153	5.367943153	4.404177	0
bta-miR-1246	12.8948392	1074.301	3.796477232	10.07052523	10.07052523	-6.38046	0
chr12_26006_mature@@mmu-miR-744	739.304113	14.50669	9.531974234	3.954819241	3.954819241	5.671377	0
chr17_35504_mature	154.73807	3.22371	7.282977844	2.078510713	2.078510713	5.584963	0
ssc-miR-425-5p	12993.6996	6349.902	13.66563566	12.63274588	12.63274588	1.033006	0
chr7_17017_star	399.740015	49.9675	8.646522763	5.671505736	5.671505736	3	0
NW_003541084_39898_mature	3240.90291	980.8137	11.66262517	9.939305495	9.939305495	1.724345	0
ssc-miR-425-3p	32.237098	309.4761	5.054722516	8.278338593	8.278338593	-3.26303	0
ssc-miR-30a-5p	844.611966	16701.23	9.723851982	14.02775351	14.02775351	-4.30552	0
chr3_6615_mature	702.768735	18.53633	9.458957614	4.288087668	4.288087668	5.244622	0
ssc-miR-497	638.29454	9.671129	9.320336961	3.415640968	3.415640968	6.044394	0
ssc-miR-486	29978.352	27.40153	14.87168158	4.827896911	4.827896911	10.09545	0
NW_003612993_36688_mature	4.29827973	444.066	2.405524014	8.797875562	8.797875562	-6.69087	0
chr12_26134_mature	167.632909	4.029637	7.397742301	2.330454351	2.330454351	5.378512	0
chr3_6620_mature	242.852805	5.641492	7.929866755	2.731507408	2.731507408	5.427862	0
NW_003540278_39378_star	94.562154	826.0756	6.578367468	9.691875458	9.691875458	-3.12694	0
chr3_6707_mature@@mmu-miR-590-3	5645.79042	14.50669	12.46321537	3.954819241	3.954819241	8.604313	0
ssc-miR-27b-5p	5800.52849	608.4752	12.50221733	9.251423764	9.251423764	3.252914	0
chr1_412_star	8779.23634	62.86234	13.10004406	5.996893534	5.996893534	7.125756	0
chr16_34245_mature	1220.71144	22.56597	10.25468786	4.558633073	4.558633073	5.75743	0
chr2_6439_mature	801.629169	35.46081	9.648589777	5.188274618	5.188274618	4.498638	0
ssc-miR-27b-3p	1541784.34	53677.99	20.55617049	15.71206995	15.71206995	4.844126	0
bta-miR-677	214.913986	1580.424	7.75431289	10.62700826	10.62700826	-2.87848	0
chr10_23743_mature	260.045924	18.53633	8.028159819	4.288087668	4.288087668	3.810339	0
chr6_13671_mature	3995.25101	450.5134	11.96443149	8.818625135	8.818625135	3.148644	0
ssc-miR-451	167.632909	2.417782	7.397742301	1.773060525	1.773060525	6.115477	0
ssc-miR-452	8.59655945	1299.155	3.262517265	10.34446796	10.34446796	-7.2396	0
chr16_34601_star	365.353777	20.14819	8.517093679	4.402462031	4.402462031	4.180572	0
chr18_35948_star@@rno-miR-96-3p	245.001944	0	7.942525909	0	0	20	0
chr4_8854_mature	8.59655945	1067.048	3.262517265	10.06076069	10.06076069	-6.95565	0
bta-miR-154c	68.7724756	759.1837	6.124586119	9.570204199	9.570204199	-3.46455	0
chr9_20434_mature@@rno-miR-34b-3	294.432161	13.70077	8.206683079	3.877819487	3.877819487	4.425607	0
chr7_17049_mature	279.388182	4397.946	8.131281734	12.1029422	12.1029422	-3.97649	0
chr18_35822_mature@@mmu-miR-511	1592.51264	246.6138	10.63799475	7.951947907	7.951947907	2.690979	0
chr15_31863_mature	27.9388182	685.8443	4.854934103	9.423839197	9.423839197	-4.61754	0
chr5_10827_mature	92576.3488	2.417782	16.49837163	1.773060525	1.773060525	15.22467	0
chr11_25205_mature	4.29827973	362.6674	2.405524014	8.506475604	8.506475604	-6.39874	0
chrx_21555_mature	2.14913986	5591.525	1.654957834	12.44928399	12.44928399	-11.3453	0
chrx_22210_mature	341.713238	1359.6	8.420858111	10.41002686	10.41002686	-1.99232	0
chrx_22424_star	464.214211	0.805927	8.861751356	0.852739935	0.852739935	9.169925	0

piPS-LF Vs PEFs

AccID	piPS-LF	PEF	piPS-LF_log2(count+1	PEF_log2(count+1	PEF_log2(count+1)<0.0	Log2FC	FDR
NW_003541079_39891_mature	4.835565	7881.165	2.544872269	12.94437615	12.94437615	-10.6705	6.92E-06
NW_003537574_37009_mature	5.37285	8313.142	2.671938627	13.02135161	13.02135161	-10.5955	7.91E-06
NW_003538989_38416_mature	17292.52	0.805927	14.07794365	0.852739935	0.852739935	14.38914	1.03E-05
NW_003613137_37368_mature	1.611855	1938.256	1.38507475	10.92128719	10.92128719	-10.2318	1.89E-05
chr8_17989_mature@@bta-miR-30	17222.67	1.611855	14.07210493	1.38507475	1.38507475	13.3833	1.95E-05
chr8_17983_mature@@bta-miR-30	16849.26	1.611855	14.04048293	1.38507475	1.38507475	13.35168	2.01E-05
chr18_36117_mature	13.43212	13979.62	3.851211749	13.77114047	13.77114047	-10.0234	2.75E-05
NW_003538989_38422_mature	16620.37	2.417782	14.02075195	1.773060525	1.773060525	12.74698	3.37E-05
bta-miR-302c	19119.29	3.22371	14.22281645	2.078510713	2.078510713	12.53402	4.08E-05
chr12_25534_mature	5.37285	3712.908	2.671938627	11.85872227	11.85872227	-9.43265	5.19E-05
chr6_14978_mature	5.910135	3488.86	2.788713818	11.76895342	11.76895342	-9.20535	7.62E-05
chr1_485_mature	4.29828	1921.331	2.405524014	10.90864108	10.90864108	-8.80413	0.00014
chr1_3153_mature	2.14914	967.1129	1.654957834	9.91903155	9.91903155	-8.81378	0.000159
chrx_22554_mature	4.29828	1641.674	2.405524014	10.68183067	10.68183067	-8.57719	0.000202
chr1_1155_mature	2.686425	1000.156	1.882222339	9.967451025	9.967451025	-8.54032	0.000227
chr15_32593_mature	0.537285	359.4436	0.620384622	8.493629892	8.493629892	-9.38586	0.00024
chr1_211_mature	0	209.5411	0	7.717958332	7.717958332	-20	0.000299
chr12_26311_mature	1.611855	584.2974	1.38507475	9.193026061	9.193026061	-8.50184	0.000307
chr2_4295_mature	1.611855	576.2381	1.38507475	9.173022781	9.173022781	-8.4818	0.000317
chr5_12267_mature	1.07457	437.6186	1.05281229	8.776823197	8.776823197	-8.66977	0.000326
chr13_28441_mature	6.44742	1987.417	2.896740642	10.95740469	10.95740469	-8.26796	0.000351
chr13_28708_mature	0.537285	294.9694	0.620384622	8.20930444	8.20930444	-9.10066	0.000378
chr4_9231_mature	2.14914	660.8605	1.654957834	9.37038338	9.37038338	-8.26444	0.000383
chr5_11530_mature	6.44742	1885.064	2.896740642	10.88116315	10.88116315	-8.19168	0.000397
chr6_14477_mature@@bta-miR-48	3.22371	888.938	2.078510713	9.797560981	9.797560981	-8.10722	0.000437
chr7_15145_mature	7.52199	2137.32	3.091190278	11.06226178	11.06226178	-8.15047	0.000442
chr3_8078_mature	0.537285	265.9561	0.620384622	8.060458479	8.060458479	-8.95128	0.00048
chrx_22120_mature	1.07457	365.8911	1.05281229	8.519207948	8.519207948	-8.41151	0.000492
chr2_5680_mature	2.686425	701.1569	1.882222339	9.455649593	9.455649593	-8.02791	0.000516
chr16_34017_mature	13.96941	3926.479	3.90394537	11.93938767	11.93938767	-8.13482	0.00059
bta-miR-154c	3.22371	759.1837	2.078510713	9.570204199	9.570204199	-7.87958	0.000629
chr1_3720_mature	19.87954	5863.928	4.384018281	12.51789771	12.51789771	-8.20444	0.000645
chr4_10395_mature	4.29828	984.0374	2.405524014	9.944034714	9.944034714	-7.83881	0.00066
chrx_22553_mature	7.52199	1729.52	3.091190278	10.75699015	10.75699015	-7.84504	0.000721
chr2_4477_star	9.133844	2107.5	3.34110968	11.0420015	11.0420015	-7.85009	0.000766
hsa-miR-4685-3p	0	134.5899	0	7.083105736	7.083105736	-20	0.000822
hsa-miR-516a-5p	2.14914	473.8853	1.654957834	8.89143541	8.89143541	-7.78463	0.000823
chr6_12951_mature	5.37285	1104.121	2.671938627	10.10998811	10.10998811	-7.68299	0.000863
NW_003541201_39964_mature	1.611855	365.8911	1.38507475	8.519207948	8.519207948	-7.82655	0.000899
chr9_19755_mature	0	128.1425	0	7.012819653	7.012819653	-20	0.00092
chr4_10088_mature	33.84895	9929.832	5.123043401	13.27769889	13.27769889	-8.19651	0.000927
chr15_31936_mature	4.835565	938.9055	2.544872269	9.876371869	9.876371869	-7.60115	0.000971
chr6_13229_mature	3.760995	724.5288	2.251263041	9.502889027	9.502889027	-7.58978	0.000982
chr13_29267_mature	2.14914	430.3653	1.654957834	8.752766176	8.752766176	-7.64566	0.001026
chrx_22340_mature	3.22371	610.0871	2.078510713	9.255234166	9.255234166	-7.56415	0.001039
chr3_7072_mature	1.07457	261.9264	1.05281229	8.038515312	8.038515312	-7.92926	0.001056
chrx_22076_mature	5.910135	1105.732	2.788713818	10.11209079	10.11209079	-7.5476	0.001089
NW_003613242_37866_mature	1.07457	257.0909	1.05281229	8.011735219	8.011735219	-7.90238	0.001102
chr11_24398_mature	4.835565	849.4475	2.544872269	9.73207842	9.73207842	-7.4567	0.001221
chr14_30497_mature	3.22371	550.4484	2.078510713	9.107082211	9.107082211	-7.41574	0.001315
chr18_36269_mature	17.19312	3564.617	4.185320985	11.79993607	11.79993607	-7.69577	0.001337
chr16_34386_mature	8.596559	1477.265	3.262517265	10.52968921	10.52968921	-7.42495	0.001474
chr1_3824_star	0	103.9646	0	6.713759604	6.713759604	-20	0.001476
chr18_36061_mature	0	103.1587	0	6.702639724	6.702639724	-20	0.001503
chr15_33120_mature	4.29828	686.6502	2.405524014	9.42553103	9.42553103	-7.31967	0.001506
chr1_541_mature	4.835565	772.8844	2.544872269	9.595974312	9.595974312	-7.32042	0.001515
chr9_19756_mature	0.537285	157.9618	0.620384622	7.312536122	7.312536122	-8.19967	0.001572
chr14_30223_mature	2.14914	344.131	1.654957834	8.43100034	8.43100034	-7.32305	0.001707
chr13_27515_mature	3.760995	560.1196	2.251263041	9.132164438	9.132164438	-7.21848	0.001769
chr9_20754_mature	0.537285	145.8729	0.620384622	7.198424102	7.198424102	-8.08481	0.001882
chr1_268_mature	4.835565	684.2324	2.544872269	9.420449567	9.420449567	-7.14466	0.002001
bta-miR-2429	0	90.26387	0	6.511971994	6.511971994	-20	0.002029
mmu-miR-5099	332.0421	0	8.379560789	0	0	20	0.002057
chr6_13578_mature	1.611855	252.2553	1.38507475	7.984448603	7.984448603	-7.29002	0.002094
chr4_8854_mature	7.52199	1067.048	3.091190278	10.06076069	10.06076069	-7.14829	0.002183
chr10_24151_mature	2.686425	346.5488	1.882222339	8.441071766	8.441071766	-7.01123	0.00258
hsa-miR-3659	59.10135	13122.92	5.909325402	13.67991072	13.67991072	-7.79468	0.002645
chr6_13600_star	0.537285	123.3069	0.620384622	6.957762565	6.957762565	-7.84235	0.002745
chrx_22639_mature	8.059274	1014.663	3.179395517	9.98820559	9.98820559	-6.97613	0.00293
ssc-miR-708-5p	6.44742	784.9733	2.896740642	9.618336558	9.618336558	-6.92778	0.00296
NW_003613242_37864_mature	2.14914	266.762	1.654957834	8.064807344	8.064807344	-6.95565	0.003035
ssc-miR-432-5p	2.14914	260.3146	1.654957834	8.029643734	8.029643734	-6.92035	0.003207
chr16_34502_mature	1.07457	159.5736	1.05281229	7.327091221	7.327091221	-7.21432	0.003235
chr14_31492_mature	0.537285	113.6358	0.620384622	6.840913474	6.840913474	-7.72451	0.003293
chrx_22943_mature	3.22371	362.6674	2.078510713	8.506475604	8.506475604	-6.81378	0.003385
chr14_31463_star	17.19312	2371.844	4.185320985	11.21240183	11.21240183	-7.10803	0.003401
chr3_8256_mature	3.760995	417.4704	2.251263041	8.708981833	8.708981833	-6.79442	0.003441
chr11_25257_mature	3.760995	416.6645	2.251263041	8.706200684	8.706200684	-6.79163	0.003456
chr5_11980_mature	1.611855	197.4522	1.38507475	7.632647927	7.632647927	-6.93664	0.003632
chr12_26422_mature	0	69.30976	0	6.135653077	6.135653077	-20	0.003654
chr1_2018_mature	3.760995	406.1874	2.251263041	8.66954923	8.66954923	-6.75489	0.00366
chr2_4744_mature	2.686425	294.1635	1.882222339	8.20537061	8.20537061	-6.77479	0.00373
NW_003537694_37136_mature	3.22371	346.5488	2.078510713	8.441071766	8.441071766	-6.74819	0.00375
chrx_21570_mature	1.611855	193.4226	1.38507475	7.603052029	7.603052029	-6.90689	0.003803
chr6_12932_star@@bta-miR-292	385.7706	0.805927	8.595334344	0.852739935	0.852739935	8.902878	0.00384
chr8_18343_mature	2.14914	238.5545	1.654957834	7.904210254	7.904210254	-6.79442	0.003899
chr17_34881_mature	5.910135	630.2353	2.788713818	9.302033996	9.302033996	-6.73656	0.003917
hsa-miR-184	0.537285	104.7706	0.620384622	6.72479443	6.72479443	-7.60733	0.003945
rno-miR-300-3p	214.914	0	7.75431289	0	0	20	0.004022
ssc-miR-371-5p	7189.41	16.92448	12.81185835	4.163859073	4.163859073	8.730618	0.004144
chr5_11967_mature	4.29828	429.5593	2.405524014	8.750068242	8.750068242	-6.64295	0.004356
bta-miR-302a	199.3327	0	7.64625428	0	0	20	0.004515
chr11_25099_mature	1.611855	176.4981	1.38507475	7.471659863	7.471659863	-6.77479	0.004663
chr16_33714_mature	5.37285	520.6291	2.671938627	9.026880633	9.026880633	-6.59842	0.004774
chr1_2964_mature	4.835565	463.4083	2.544872269	8.859249894	8.859249894	-6.58246	0.004826
NW_003538989_38414_mature	188.587	0	7.566716407	0	0	20	0.004915
chr9_20753_mature	1.611855	170.0507	1.38507475	7.418280128	7.418280128	-6.7211	0.005064
chr4_10244_mature	4.29828	394.0985	2.405524014	8.626068641	8.626068641	-6.51865	0.005282
chr10_24012_mature	0.537285	91.0698	0.620384622	6.524656134	6.524656134	-7.40514	0.005374
chr14_30282_mature	4.29828	390.8748	2.405524014	8.614249038	8.614249038	-6.5068	0.00538
NW_003538465_37902_mature	2.14914	205.5115	1.654957834	7.690078309	7.690078309	-6.57932	0.005434
chr7_15797_star	2.686425	241.7782	1.882222339	7.923495278	7.923495278	-6.49185	0.005776
NW_003538989_38416_star	169.2448	0	7.41146662	0	0	20	0.0058
chrx_21569_mature	1.611855	159.5736	1.38507475	7.327091221	7.327091221	-6.62936	0.005828
bta-miR-2899	0	55.60899	0	5.82295938	5.82295938	-20	0.005925
chr6_13041_mature	2.14914	195.8404	1.654957834	7.620882324	7.620882324	-6.50978	0.006046
NW_003613056_36922_mature	0.537285	86.23424	0.620384622	6.446822559	6.446822559	-7.32643	0.006057
hsa-miR-1298	0	54.80307	0	5.802272499	5.802272499	-20	0.006116
chr5_11958_mature	3.760995	321.5651	2.251263041	8.33344633	8.33344633	-6.41785	0.00617
chr13_29269_mature	5.37285	461.7964	2.671938627	8.85423392	8.85423392	-6.42543	0.006242
chr11_24582_mature	16.11855	1636.033	4.097488514	10.67686744	10.67686744	-6.66534	0.006575
chr11_25258_mature	4.835565	394.0985	2.544872269	8.626068641	8.626068641	-6.34873	0.006927
ssc-miR-206	75.75718	11796.36	6.262229808	13.52617644	13.52617644	-7.28274	0.007207
NW_003613103_37207_mature	0	50.77343	0	5.694139975	5.694139975	-20	0.007218
chr18_36523_mature	0.537285	78.98089	0.620384622	6.32158343	6.32158343	-7.19967	0.007336
chr14_31314_mature	3.760995	297.3872	2.251263041	8.221041977	8.221041977	-6.30508	0.007337
chr16_33806_mature@@bta-miR-3	239.6291	0.805927	7.910667281	0.852739935	0.852739935	8.215937	0.00796
chr15_32293_mature	1.611855	137.0077	1.38507475	7.108604601	7.108604601	-6.40939	0.008144
ssc-miR-455-3p	6.44742	497.2572	2.896740642	8.960746947	8.960746947	-6.26913	0.008248
mmu-miR-6370	0	47.54972	0	5.601391055	5.601391055	-20	0.008314
rno-miR-434-5p	133.2467	0	7.068742509	0	0	20	0.008347
chr14_31042_mature	1.611855	135.3958	1.38507475	7.091655531	7.091655531	-6.39232	0.008357
chr13_27072_mature	9.133844	728.5584	3.34110968	9.510879685	9.510879685	-6.31768	0.008571
chr14_30531_mature	1.611855	133.784	1.38507475	7.074504971	7.074504971	-6.37504	0.008578
chr1_1318_mature	23.64054	2350.89	4.62296188	11.1996051	11.1996051	-6.6358	0.008676
chr7_16963_mature	11.28298	930.0403	3.618589217	9.862699769	9.862699769	-6.36507	0.008724
ssc-miR-2366	1.07457	101.5469	1.05281229	6.680139486	6.680139486	-6.56224	0.00876
chr13_29304_mature	2.14914	164.4092	1.654957834	7.369895665	7.369895665	-6.25739	0.008878
chrx_23196_mature	127.8738	0	7.009815429	0	0	20	0.008885
bta-miR-483	1.07457	100.7409	1.05281229	6.668756392	6.668756392	-6.55075	0.008912
chr1_169_mature	5.37285	386.8452	2.671938627	8.599337045	8.599337045	-6.16993	0.009244
chr6_13576_mature	3.760995	265.9561	2.251263041	8.060458479	8.060458479	-6.14393	0.009382
chr2_4586_mature	320.7591	1.611855	8.329837252	1.38507475	1.38507475	7.636625	0.009436
chr1_1317_mature	24.71511	2365.397	4.684544332	11.20847645	11.20847645	-6.58055	0.00973
chr15_31863_mature	9.133844	685.8443	3.34110968	9.423839197	9.423839197	-6.23051	0.009802
rno-miR-541-5p	312.1626	1.611855	8.290767956	1.38507475	1.38507475	7.597432	0.009833
chr1_282_mature	206.3174	0.805927	7.695697583	0.852739935	0.852739935	8	0.00999
chr4_9198_mature	3.760995	257.8968	2.251263041	8.016233232	8.016233232	-6.09954	0.010036
chr5_11474_mature	9.133844	676.9791	3.34110968	9.405096899	9.405096899	-6.21175	0.010088
chr3_7573_mature	18.26769	1579.618	4.268111626	10.62627284	10.62627284	-6.43414	0.010127
chr9_19375_mature	5.910135	406.9934	2.788713818	8.672401869	8.672401869	-6.10567	0.010381
chrx_21555_mature	48.89293	5591.525	5.640763545	12.44928399	12.44928399	-6.83747	0.010457
chr17_34903_mature	0.537285	66.89198	0.620384622	6.085169218	6.085169218	-6.96	0.010498
hsa-miR-302a-5p	294.4322	1.611855	8.206683079	1.38507475	1.38507475	7.51307	0.010745
chr6_14032_mature	19.87954	1699.701	4.384018281	10.7319138	10.7319138	-6.41785	0.010924
chr1_2360_mature	12.35755	922.7869	3.739583968	9.851416323	9.851416323	-6.22253	0.011349
chrx_22902_star	0.537285	64.4742	0.620384622	6.032854532	6.032854532	-6.90689	0.011356
chr9_20880_mature	1.611855	116.8595	1.38507475	6.880923997	6.880923997	-6.17991	0.011496
chr9_20403_mature	277.7763	1.611855	8.122964248	1.38507475	1.38507475	7.429058	0.011734
NW_003613041_36883_mature	5.910135	384.4274	2.788713818	8.5903153	8.5903153	-6.02338	0.011765
chr6_12933_mature	104.2333	0	6.717447265	0	0	20	0.012096
chr14_30207_mature	5.37285	339.2955	2.671938627	8.410644077	8.410644077	-5.98071	0.012325
chr6_13566_mature	5.910135	375.5622	2.788713818	8.556744343	8.556744343	-5.98972	0.012381
chr14_29460_mature	1.611855	111.218	1.38507475	6.810160141	6.810160141	-6.10852	0.012784
chr8_18274_mature	1.07457	84.62238	1.05281229	6.419916069	6.419916069	-6.29921	0.012962
chr14_31044_mature	2.14914	137.8136	1.654957834	7.117005045	7.117005045	-6.00282	0.013008
chr5_11533_mature	2.686425	165.2151	1.882222339	7.376907876	7.376907876	-5.94251	0.013281
chr6_15013_mature	0.537285	59.63863	0.620384622	5.922165283	5.922165283	-6.79442	0.013401
hsa-miR-4474-5p	0.537285	59.63863	0.620384622	5.922165283	5.922165283	-6.79442	0.013401
chr4_9743_mature	8.596559	552.0603	3.262517265	9.111292982	9.111292982	-6.00492	0.013516
chr9_19709_star	95.63672	0	6.594499642	0	0	20	0.013763
chr6_15014_mature	0.537285	58.8327	0.620384622	5.902862353	5.902862353	-6.77479	0.013792
chrx_23228_mature	13.96941	982.4256	3.90394537	9.941672042	9.941672042	-6.13601	0.013792
chr4_8920_mature	243.3901	1.611855	7.933041972	1.38507475	1.38507475	7.238405	0.014318
ssc-miR-370	31.69981	2733.706	5.03121048	11.41716998	11.41716998	-6.43024	0.014491
chr10_23795_star	3.22371	186.9752	2.078510713	7.554398282	7.554398282	-5.85798	0.014591
chr2_4023_mature	0.537285	57.22085	0.620384622	5.863463969	5.863463969	-6.73471	0.014623
chr8_18995_mature	0	36.26674	0	5.21981653	5.21981653	-20	0.014758
chr1_3371_mature	8.596559	529.4943	3.262517265	9.051193532	9.051193532	-5.94471	0.014807
chr15_33320_mature	4.835565	277.239	2.544872269	8.120181062	8.120181062	-5.8413	0.014976
chr2_6447_mature	10.20841	642.3242	3.48651029	9.329402097	9.329402097	-5.97547	0.015146
chr1_3052_star	1.07457	77.36904	1.05281229	6.292211829	6.292211829	-6.16993	0.015673
chr14_31500_mature	1.07457	77.36904	1.05281229	6.292211829	6.292211829	-6.16993	0.015673
chr15_31797_mature	87.57745	0	6.468867551	0	0	20	0.015696
NW_003539007_38445_mature	228.8834	1.611855	7.844758453	1.38507475	1.38507475	7.149747	0.0157
chr15_32517_mature	2.14914	125.7247	1.654957834	6.985553734	6.985553734	-5.87036	0.015826
chr1_2963_mature	8.059274	476.3031	3.179395517	8.898761964	8.898761964	-5.88509	0.015829
chrx_22228_mature	224.5851	1.611855	7.817528071	1.38507475	1.38507475	7.122397	0.016152
chr14_30429_mature	3.760995	205.5115	2.251263041	7.690078309	7.690078309	-5.77196	0.016407
chr6_13339_mature	10.7457	656.0249	3.554060706	9.359804332	9.359804332	-5.93192	0.016547
chr7_16788_star	83.27917	0	6.397104196	0	0	20	0.016917
chr8_17991_mature@@bta-miR-36	216.5258	1.611855	7.765042988	1.38507475	1.38507475	7.069674	0.017059
chr12_26356_star	1.611855	96.71129	1.38507475	6.610453419	6.610453419	-5.90689	0.017203
chr1_2110_mature	0.537285	52.38528	0.620384622	5.738370208	5.738370208	-6.60733	0.017589
chr1_2557_mature	15.04398	949.3825	4.003960082	9.892364514	9.892364514	-5.97973	0.018199
chr16_34017_star	1.07457	70.92162	1.05281229	6.168353522	6.168353522	-6.04439	0.018809
chr14_31499_mature	1.07457	69.30976	1.05281229	6.135653077	6.135653077	-6.01123	0.019731
chrx_23205_mature	0.537285	49.16157	0.620384622	5.648510724	5.648510724	-6.5157	0.020062
chr14_31042_star	1.07457	68.50383	1.05281229	6.11902064	6.11902064	-5.99435	0.020216
chr9_20694_mature	1.611855	89.45795	1.38507475	6.499175346	6.499175346	-5.79442	0.020257
chr4_10238_mature	73.60804	0	6.22125921	0	0	20	0.020308
ssc-miR-615	8.596559	456.1549	3.262517265	8.836539387	8.836539387	-5.72962	0.020452
chr1_2112_mature	0.537285	48.35565	0.620384622	5.625143252	5.625143252	-6.49185	0.020757
chr12_26093_mature	3.760995	182.1396	2.251263041	7.516799994	7.516799994	-5.59779	0.021209
NW_003537990_37435_mature	2.14914	108.8002	1.654957834	6.778736946	6.778736946	-5.66178	0.021462
chr7_16615_mature	5.910135	290.1339	2.788713818	8.185538937	8.185538937	-5.61738	0.021604
chr8_17417_mature	1543.082	12.08891	10.59253405	3.710273245	3.710273245	6.995987	0.021745
chr9_20601_mature	1.07457	66.08605	1.05281229	6.067940913	6.067940913	-5.94251	0.021776
chr10_23437_mature	1.611855	86.23424	1.38507475	6.446822559	6.446822559	-5.74147	0.021864
chrx_22497_mature	3.760995	178.9159	2.251263041	7.491178829	7.491178829	-5.57202	0.022022
chr7_16567_mature	0	29.81932	0	4.94576292	4.94576292	-20	0.02208
NW_003539626_38995_mature	10.7457	571.4026	3.554060706	9.160886317	9.160886317	-5.73268	0.022315
chr12_25628_mature	7.52199	371.5326	3.091190278	8.541222691	8.541222691	-5.62623	0.022763
chr9_19579_mature	2.14914	105.5765	1.654957834	6.735745493	6.735745493	-5.61839	0.02285
chr12_26317_mature	7.52199	369.9207	3.091190278	8.534966969	8.534966969	-5.61996	0.022975
chr3_8179_mature	0.537285	45.93786	0.620384622	5.552680304	5.552680304	-6.41785	0.023056
chr17_35005_mature	2.686425	127.3365	1.882222339	7.003788148	7.003788148	-5.56682	0.023069
mmu-miR-5108	2.14914	104.7706	1.654957834	6.72479443	6.72479443	-5.60733	0.023217
mmu-miR-193b-3p	0	29.01339	0	4.907534286	4.907534286	-20	0.023339
hsa-miR-5702	0	29.01339	0	4.907534286	4.907534286	-20	0.023339
rno-miR-181a-2-3p	0	29.01339	0	4.907534286	4.907534286	-20	0.023339
chr2_4930_mature	10.7457	559.3136	3.554060706	9.130090828	9.130090828	-5.70183	0.023363
chr3_6868_star	0.537285	45.13194	0.620384622	5.527693969	5.527693969	-6.39232	0.023903
chr3_7304_star@@mmu-miR-216b	963.3519	8.865202	9.913415949	3.302348583	3.302348583	6.763766	0.024094
chr15_33031_mature	4.29828	195.8404	2.405524014	7.620882324	7.620882324	-5.50978	0.024186
chr9_20220_mature	850.5221	8.059274	9.733900165	3.179395517	3.179395517	6.721555	0.024272
chr16_34398_mature	2.14914	102.3528	1.654957834	6.691433469	6.691433469	-5.57365	0.024368
chr4_9789_mature	10.20841	514.9876	3.48651029	9.011192697	9.011192697	-5.65671	0.024424
chr9_20772_mature	3.760995	170.0507	2.251263041	7.418280128	7.418280128	-5.49871	0.024501
chrx_22720_mature	6.44742	299.805	2.896740642	8.23268479	8.23268479	-5.53916	0.024744
chr12_26098_mature	110.6807	0.805927	6.803236118	0.852739935	0.852739935	7.101538	0.025292
chr7_16002_mature	1.07457	61.25049	1.05281229	5.960013198	5.960013198	-5.83289	0.02545
chr14_31496_mature	62.32506	0	5.984704544	0	0	20	0.025917
chr9_19418_mature@@bta-miR-24	9.133844	436.8127	3.34110968	8.77416992	8.77416992	-5.57965	0.026139
chr4_9234_star	1.07457	60.44456	1.05281229	5.941213351	5.941213351	-5.81378	0.026147
rno-miR-6317	107.9943	0.805927	6.76810859	0.852739935	0.852739935	7.066089	0.026217
chr10_24172_mature	2.14914	98.32315	1.654957834	6.634058094	6.634058094	-5.5157	0.026472
chr9_19423_mature	106.9197	0.805927	6.753814543	0.852739935	0.852739935	7.051662	0.026603
chr1_3708_mature	1.611855	78.17496	1.38507475	6.306972375	6.306972375	-5.59991	0.026761
chrx_22559_mature	1.07457	59.63863	1.05281229	5.922165283	5.922165283	-5.79442	0.02687
chr6_14094_mature	2.14914	97.51722	1.654957834	6.622304032	6.622304032	-5.50383	0.026924
chr13_27783_mature	3.22371	139.4254	2.078510713	7.133660602	7.133660602	-5.43463	0.027109
chr5_11058_mature	217.6004	2.417782	7.772152305	1.773060525	1.773060525	6.491853	0.027167
ssc-miR-758	8.596559	392.4867	3.262517265	8.620170944	8.620170944	-5.51274	0.028173
chr12_26457_mature	8.596559	390.8748	3.262517265	8.614249038	8.614249038	-5.5068	0.028419
chrx_21884_mature	5.910135	253.0612	2.788713818	7.989032362	7.989032362	-5.42015	0.028823
chr5_11434_star	0.537285	41.1023	0.620384622	5.395827139	5.395827139	-6.25739	0.02887
chr14_29715_mature	8.596559	381.2037	3.262517265	8.578197871	8.578197871	-5.47066	0.029958
chr14_29516_mature	0.537285	40.29637	0.620384622	5.367943153	5.367943153	-6.22882	0.030036
chr6_13565_mature	7.52199	325.5947	3.091190278	8.351357522	8.351357522	-5.43582	0.030087
chr7_15769_mature	11.28298	523.8528	3.618589217	9.035769166	9.035769166	-5.53694	0.030467
chr3_8428_mature	2.14914	91.0698	1.654957834	6.524656134	6.524656134	-5.40514	0.03096
hsa-miR-1248	0	24.98375	0	4.699537803	4.699537803	-20	0.031441
chr11_24459_mature	0	24.98375	0	4.699537803	4.699537803	-20	0.031441
NW_003613367_38732_mature	0	24.98375	0	4.699537803	4.699537803	-20	0.031441
chr8_18354_mature	142.3805	1.611855	7.163705179	1.38507475	1.38507475	6.464886	0.031637
chr12_25693_mature	800.0173	8.865202	9.645689617	3.302348583	3.302348583	6.495734	0.031738
chr9_20219_mature	893.5049	9.671129	9.804945572	3.415640968	3.415640968	6.529647	0.031767
chrx_22488_mature	6.984705	290.9398	2.997239027	8.189527141	8.189527141	-5.38038	0.031867
chr1_3478_mature	94.02487	0.805927	6.570233227	0.852739935	0.852739935	6.866249	0.032059
chr1_2264_mature	4.835565	193.4226	2.544872269	7.603052029	7.603052029	-5.32193	0.032064
chrx_21682_mature	11.28298	510.958	3.618589217	8.999881656	8.999881656	-5.50098	0.032111
mmu-miR-5124b	2.686425	107.9943	1.882222339	6.76810859	6.76810859	-5.32912	0.032389
rno-miR-1188-5p	92.41301	0.805927	6.545551653	0.852739935	0.852739935	6.841302	0.032868
chr2_4844_mature	52.65393	0	5.745611853	0	0	20	0.033071
chr14_31044_star	1.611855	70.11569	1.38507475	6.152095947	6.152095947	-5.44294	0.033358
chr14_30497_star	6.984705	284.4924	2.997239027	8.157308477	8.157308477	-5.34805	0.033387
chr1_3069_mature	137.0077	1.611855	7.108604601	1.38507475	1.38507475	6.409391	0.033448
chr18_36189_mature	2.14914	87.04016	1.654957834	6.460089935	6.460089935	-5.33985	0.033927
chrx_22382_mature	3.22371	124.1128	2.078510713	6.967085898	6.967085898	-5.26679	0.034412
rno-miR-434-3p	51.04207	0	5.701606491	0	0	20	0.034576
chr6_14094_star	1.611855	67.69791	1.38507475	6.102194213	6.102194213	-5.39232	0.035781
hsa-miR-548ak	0	23.3719	0	4.607146585	4.607146585	-20	0.035812
chr7_16939_star	85.96559	0.805927	6.442372848	0.852739935	0.852739935	6.736966	0.036465

mmu-miR-1965	66.62333577	0	6.079449279	0	0	20	0.003517
chr17_34881_mature	3.223709795	630.2353	2.078510713	9.302033996	9.302033996	-7.61102	0.003531
NW_003612993_36688_mature	2.149139864	444.066	1.654957834	8.797875562	8.797875562	-7.69087	0.003552
NW_003537574_37016_mature	119.2772624	0.805927	6.910220127	0.852739935	0.852739935	7.209453	0.003571
chr12_26075_star	65.54876584	0	6.056340006	0	0	20	0.003631
chr1_3435_mature	0	138.6195	0	7.125356859	7.125356859	-20	0.003645
chr6_12448_star	10816.62093	66.89198	13.40109563	6.085169218	6.085169218	7.337201	0.003718
chr1_1444_mature	1.074569932	271.5976	1.05281229	8.090628787	8.090628787	-7.98157	0.003737
chr6_13600_mature	64.47419591	0	6.032854532	0	0	20	0.00375
mmu-miR-712-5p	64.47419591	0	6.032854532	0	0	20	0.00375
hsa-miR-4685-3p	0	134.5899	0	7.083105736	7.083105736	-20	0.003813
chr3_8310_star	0	134.5899	0	7.083105736	7.083105736	-20	0.003813
chr16_34839_star	298.730441	3.22371	8.227521803	2.078510713	2.078510713	6.533979	0.003835
chr6_13576_mature	1.074569932	265.9561	1.05281229	8.060458479	8.060458479	-7.95128	0.003859
mmu-miR-450b-5p	63.39962598	0	6.008980404	0	0	20	0.003875
hsa-miR-4524a-3p	113.9044128	0.805927	6.844290394	0.852739935	0.852739935	7.142958	0.003911
chr3_7072_mature	1.074569932	261.9264	1.05281229	8.038515312	8.038515312	-7.92926	0.003951
chr14_30236_mature	362.130067	4.029637	8.504342579	2.330454351	2.330454351	6.489714	0.003964
NW_003613522_39530_mature	168.7074793	1.611855	7.406906338	1.38507475	1.38507475	6.709658	0.00397
ssc-miR-432-5p	1.074569932	260.3146	1.05281229	8.029643734	8.029643734	-7.92035	0.003988
chr9_19375_mature	2.149139864	406.9934	1.654957834	8.672401869	8.672401869	-7.5651	0.00406
hsa-miR-4524b-5p	111.7552729	0.805927	6.817051091	0.852739935	0.852739935	7.115477	0.00406
chr13_27312_mature	1.074569932	257.0909	1.05281229	8.011735219	8.011735219	-7.90238	0.004065
chr3_8533_star	908.0115924	9.671129	9.828154883	3.415640968	3.415640968	6.552883	0.0041
chr9_19755_mature	0	128.1425	0	7.012819653	7.012819653	-20	0.004109
mmu-miR-669o-5p	61.25048611	0	5.960013198	0	0	20	0.004145
chr17_35005_mature	0	127.3365	0	7.003788148	7.003788148	-20	0.004148
ssc-miR-195	731.7821236	8.059274	9.517240499	3.179395517	3.179395517	6.50462	0.004166
chrx_21977_mature@@rno-miR-384-	164.4091996	1.611855	7.369895665	1.38507475	1.38507475	6.672425	0.004178
chr6_13578_mature	1.074569932	252.2553	1.05281229	7.984448603	7.984448603	-7.87498	0.004184
chr16_34380_mature	60.17591618	0	5.934891897	0	0	20	0.00429
chr17_35010_mature	60.17591618	0	5.934891897	0	0	20	0.00429
chrx_22382_mature	0	124.1128	0	6.967085898	6.967085898	-20	0.004313
hsa-miR-3202	0	123.3069	0	6.957762565	6.957762565	-20	0.004356
mmu-miR-669c-5p	107.4569932	0.805927	6.760979269	0.852739935	0.852739935	7.058894	0.004386
chr15_32108_mature	3.223709795	545.6129	2.078510713	9.094375652	9.094375652	-7.40301	0.004406
NW_003540835_39730_mature	0	121.695	0	6.938933175	6.938933175	-20	0.004444
ssc-miR-182	456053.9265	1181.49	18.79884807	10.20761183	10.20761183	8.592454	0.004473
chr10_23799_mature	2.149139864	381.2037	1.654957834	8.578197871	8.578197871	-7.47066	0.004488
chr2_6113_mature	0	120.0832	0	6.919854778	6.919854778	-20	0.004535
chr8_18343_mature	1.074569932	238.5545	1.05281229	7.904210254	7.904210254	-7.79442	0.004557
chr1_3371_mature	3.223709795	529.4943	2.078510713	9.051193532	9.051193532	-7.35975	0.004613
chr15_33531_mature	0	116.8595	0	6.880923997	6.880923997	-20	0.004726
chr9_20880_mature	0	116.8595	0	6.880923997	6.880923997	-20	0.004726
mmu-miR-466g	103.1587135	0.805927	6.702639724	0.852739935	0.852739935	7	0.004751
ssc-miR-18b	1096.06133	12.08891	10.09942847	3.710273245	3.710273245	6.5025	0.004755
chr5_12116_mature@@bta-miR-200(	56.95220639	0	5.856791684	0	0	20	0.004773
NW_003541201_39964_mature	2.149139864	365.8911	1.654957834	8.519207948	8.519207948	-7.41151	0.004779
chr3_8552_mature	2033.086311	20.14819	10.99016518	4.402462031	4.402462031	6.656878	0.004812
chr17_35521_mature	0	114.4417	0	6.851020612	6.851020612	-20	0.004878
ssc-miR-181c	7996.949433	58.8327	12.96541444	5.902862353	5.902862353	7.086688	0.004884
chr15_31749_star	101.0095736	0.805927	6.672560745	0.852739935	0.852739935	6.969626	0.00495
chr15_32711_mature	55.87763645	0	5.82978961	0	0	20	0.004953
chr13_27434_mature	55.87763645	0	5.82978961	0	0	20	0.004953
ssc-miR-1	8.596559455	1441.804	3.262517265	10.49465982	10.49465982	-7.3899	0.004954
chr15_33221_mature	3.223709795	502.0928	2.078510713	8.974680734	8.974680734	-7.28309	0.005004
chr13_28070_mature	0	112.0239	0	6.820484263	6.820484263	-20	0.005039
chr3_6707_mature@@mmu-miR-590	1320.646446	14.50669	10.36812058	3.954819241	3.954819241	6.508382	0.005079
chr7_16724_mature	7662.758184	58.02678	12.90383632	5.883297648	5.883297648	7.045001	0.005135
mmu-miR-669l-5p	54.80306652	0	5.802272499	0	0	20	0.005142
chr13_28475_star	444.8719518	5.641492	8.800485637	2.731507408	2.731507408	6.30117	0.005181
NW_003537694_37136_mature	2.149139864	346.5488	1.654957834	8.441071766	8.441071766	-7.33316	0.005192
chr4_9930_mature	0	108.8002	0	6.778736946	6.778736946	-20	0.005266
chrx_22335_mature	146.1415107	1.611855	7.201060499	1.38507475	1.38507475	6.5025	0.005268
mmu-miR-5124b	0	107.9943	0	6.76810859	6.76810859	-20	0.005326
NW_003538870_38317_mature	53.72849659	0	5.774220321	0	0	20	0.005342
chr18_35974_mature@@bta-miR-592	53.72849659	0	5.774220321	0	0	20	0.005342
NW_003538374_37783_mature	53.72849659	0	5.774220321	0	0	20	0.005342
hsa-miR-4760-3p	53.72849659	0	5.774220321	0	0	20	0.005342
chr3_6615_mature	1718.237321	18.53633	10.74755299	4.288087668	4.288087668	6.53443	0.005481
chr12_26608_mature	193.4225877	2.417782	7.603052029	1.773060525	1.773060525	6.321928	0.005546
mmu-miR-677-5p	52.65392666	0	5.745611853	0	0	20	0.005554
hsa-miR-184	0	104.7706	0	6.72479443	6.72479443	-20	0.005576
hsa-miR-210	192.3480178	2.417782	7.595056164	1.773060525	1.773060525	6.313891	0.005607
chr1_3229_mature	0	103.9646	0	6.713759604	6.713759604	-20	0.005641
chr1_3824_star	0	103.9646	0	6.713759604	6.713759604	-20	0.005641
NW_003613107_37244_mature	0	103.9646	0	6.713759604	6.713759604	-20	0.005641
chr6_13949_star	140.7686611	1.611855	7.14739484	1.38507475	1.38507475	6.448461	0.005669
chr13_27316_mature	1.074569932	206.3174	1.05281229	7.695697583	7.695697583	-7.58496	0.005683
chr10_23656_mature	301.9541508	4.029637	8.242955662	2.330454351	2.330454351	6.227536	0.005687
chr14_30616_star	556.6272247	7.253347	9.123157188	3.044979305	3.044979305	6.261921	0.005692
chr6_13565_mature	2.149139864	325.5947	1.654957834	8.351357522	8.351357522	-7.24317	0.005709
chr13_27664_mature	1.074569932	204.7056	1.05281229	7.684437063	7.684437063	-7.57365	0.005751
hsa-miR-1287	51.57935673	0	5.716424587	0	0	20	0.005778
ssc-miR-497	756.497232	9.671129	9.565096807	3.415640968	3.415640968	6.289507	0.005912
chr4_9932_mature	0	99.935	0	6.657282769	6.657282769	-20	0.005988
ssc-miR-545-5p	236.405385	3.22371	7.891208849	2.078510713	2.078510713	6.196397	0.006096
NW_003537589_37025_mature	1.074569932	196.6463	1.05281229	7.626777119	7.626777119	-7.5157	0.006112
NW_003613497_39427_mature	0	98.32315	0	6.634058094	6.634058094	-20	0.006136
chr9_20605_mature	0	98.32315	0	6.634058094	6.634058094	-20	0.006136
chr10_24172_mature	0	98.32315	0	6.634058094	6.634058094	-20	0.006136
chr4_9116_mature	1.074569932	195.8404	1.05281229	7.620882324	7.620882324	-7.50978	0.00615
chr9_19559_mature	90.26387427	0.805927	6.511971994	0.852739935	0.852739935	6.807355	0.00616
chr1_1386_mature	134.3212415	1.611855	7.080244508	1.38507475	1.38507475	6.380822	0.006212
chr13_29417_star	49.43021686	0	5.656216524	0	0	20	0.006269
mmu-miR-344b-3p	181.6023185	2.417782	7.512561273	1.773060525	1.773060525	6.230954	0.006276
chr12_26356_star	0	96.71129	0	6.610453419	6.610453419	-20	0.006291
NW_003538101_37534_mature	0	95.90537	0	6.598504656	6.598504656	-20	0.006371
chr5_11238_mature	462.0650707	6.44742	8.855071127	2.896740642	2.896740642	6.16323	0.006377
chr13_27515_mature	4.298279727	560.1196	2.405524014	9.132164438	9.132164438	-7.02583	0.006485
chr10_23795_star	1.074569932	186.9752	1.05281229	7.554398282	7.554398282	-7.44294	0.006597
chr16_34525_mature@@bta-miR-449	87.04016448	0.805927	6.460089935	0.852739935	0.852739935	6.754888	0.006609
chr4_10006_mature	0	93.48758	0	6.562052863	6.562052863	-20	0.00662
chr15_33053_mature	855.3576657	11.28298	9.742069667	3.618589217	3.618589217	6.244307	0.006631
chr15_33346_mature	0	91.87573	0	6.537229727	6.537229727	-20	0.006795
chr12_25625_star	0	91.87573	0	6.537229727	6.537229727	-20	0.006795
chr17_35756_mature	47.281077	0	5.593385953	0	0	20	0.006824
chr13_27058_mature	47.281077	0	5.593385953	0	0	20	0.006824
chr1_2742_star	47.281077	0	5.593385953	0	0	20	0.006824
chr10_24012_mature	0	91.0698	0	6.524656134	6.524656134	-20	0.006886
chr3_8118_star	1.074569932	178.9159	1.05281229	7.491178829	7.491178829	-7.37938	0.007051
chr9_20694_mature	0	89.45795	0	6.499175346	6.499175346	-20	0.007073
chr8_17373_mature	0	89.45795	0	6.499175346	6.499175346	-20	0.007073
rno-miR-3473	83.81645468	0.805927	6.406272275	0.852739935	0.852739935	6.70044	0.007108
NW_003613233_37802_mature	83.81645468	0.805927	6.406272275	0.852739935	0.852739935	6.70044	0.007108
mmu-miR-669p-5p	46.20650707	0	5.560913833	0	0	20	0.007129
chr16_34021_mature	46.20650707	0	5.560913833	0	0	20	0.007129
hsa-miR-3663-3p	46.20650707	0	5.560913833	0	0	20	0.007129
hsa-miR-3687	124.6501121	1.611855	6.973268148	1.38507475	1.38507475	6.273018	0.007183
chr4_9789_mature	4.298279727	514.9876	2.405524014	9.011192697	9.011192697	-6.90463	0.00737
mmu-miR-532-5p	45.13193714	0	5.527693969	0	0	20	0.007454
mmu-miR-539-5p	45.13193714	0	5.527693969	0	0	20	0.007454
NW_003613056_36922_mature	0	86.23424	0	6.446822559	6.446822559	-20	0.007472
chr10_23437_mature	0	86.23424	0	6.446822559	6.446822559	-20	0.007472
chr1_2437_mature	0	86.23424	0	6.446822559	6.446822559	-20	0.007472
chr12_26455_mature	3.223709795	385.2333	2.078510713	8.593328822	8.593328822	-6.90087	0.007491
chr15_31936_mature	7.521989523	938.9055	3.091190278	9.876371869	9.876371869	-6.96372	0.007507
chr14_29715_mature	3.223709795	381.2037	2.078510713	8.578197871	8.578197871	-6.8857	0.007612
chr15_33121_mature	80.59274489	0.805927	6.35036897	0.852739935	0.852739935	6.643856	0.007664
chr10_23786_mature	0	84.62238	0	6.419916069	6.419916069	-20	0.007686
ssc-miR-146b	35437.16721	214.3767	15.11301637	7.750718383	7.750718383	7.368971	0.007753
chr5_11528_mature	0	83.81645	0	6.406272275	6.406272275	-20	0.007797
chr6_13705_mature	0	83.81645	0	6.406272275	6.406272275	-20	0.007797
mmu-miR-467b-5p	44.0573672	0	5.493691111	0	0	20	0.007802
chr3_7600_mature	6661.259007	61.25049	12.70179573	5.960013198	5.960013198	6.76493	0.007879
chr1_1337_mature	118.2026925	1.611855	6.897273013	1.38507475	1.38507475	6.196397	0.00796
chr5_11672_mature	117.1281226	1.611855	6.884208655	1.38507475	1.38507475	6.183222	0.008101
NW_003613242_37866_mature	2.149139864	257.0909	1.654957834	8.011735219	8.011735219	-6.90238	0.008168
rno-miR-879-5p	42.98279727	0	5.458867456	0	0	20	0.008174
chr8_17595_mature	157.96178	2.417782	7.312536122	1.773060525	1.773060525	6.029747	0.008234
chr16_34601_mature	9.671129386	1191.967	3.415640968	10.22033805	10.22033805	-6.94544	0.008244
chr1_3926_star	77.36903509	0.805927	6.292211829	0.852739935	0.852739935	6.584963	0.008287
chr1_2963_mature	4.298279727	476.3031	2.405524014	8.898761964	8.898761964	-6.79198	0.008297
chr16_34502_mature	1.074569932	159.5736	1.05281229	7.327091221	7.327091221	-7.21432	0.008376
chr9_19904_mature	2.149139864	251.4494	1.654957834	7.979850233	7.979850233	-6.87036	0.008445
chr1_1185_mature	155.8126401	2.417782	7.292898044	1.773060525	1.773060525	6.009984	0.008455
chr9_19756_mature	1.074569932	157.9618	1.05281229	7.312536122	7.312536122	-7.19967	0.008505
chr18_36523_mature	0	78.98089	0	6.32158343	6.32158343	-20	0.008521
mmu-miR-466c-3p	41.90822734	0	5.423182395	0	0	20	0.008574
chr3_7706_mature	41.90822734	0	5.423182395	0	0	20	0.008574
mmu-miR-1981-3p	41.90822734	0	5.423182395	0	0	20	0.008574
chr6_12641_mature	41.90822734	0	5.423182395	0	0	20	0.008574
mmu-miR-3470a	41.90822734	0	5.423182395	0	0	20	0.008574
chr8_18374_mature	41.90822734	0	5.423182395	0	0	20	0.008574
chr9_19519_mature	0	78.17496	0	6.306972375	6.306972375	-20	0.008652
chr13_28627_star	153.6635003	2.417782	7.272988959	1.773060525	1.773060525	5.989946	0.008685
chr1_3052_star	0	77.36904	0	6.292211829	6.292211829	-20	0.008786
chr4_9333_star	0	77.36904	0	6.292211829	6.292211829	-20	0.008786
chr14_31500_mature	0	77.36904	0	6.292211829	6.292211829	-20	0.008786
chr15_33015_mature	618.9522807	9.671129	9.276013362	3.415640968	3.415640968	6	0.008802
chr13_28222_star	152.5889303	2.417782	7.26293043	1.773060525	1.773060525	5.979822	0.008803
bta-miR-2285t	1.074569932	153.9321	1.05281229	7.275492671	7.275492671	-7.16239	0.008841
chr4_9639_mature	40.83365741	0	5.38659223	0	0	20	0.009002
chr13_29078_mature	40.83365741	0	5.38659223	0	0	20	0.009002
mmu-miR-664-3p	40.83365741	0	5.38659223	0	0	20	0.009002
mmu-miR-1195	40.83365741	0	5.38659223	0	0	20	0.009002
chr5_11626_mature	786.5851901	12.08891	9.621292173	3.710273245	3.710273245	6.023847	0.009214
chr13_28489_mature	0	74.14533	0	6.231611453	6.231611453	-20	0.00936
chr4_9743_mature	5.372849659	552.0603	2.671938627	9.111292982	9.111292982	-6.68299	0.009462
chr1_3877_mature	39.75908748	0	5.349049847	0	0	20	0.009463
chr15_31808_mature	0	73.3394	0	6.216055097	6.216055097	-20	0.009513
chr14_31044_star	0	70.11569	0	6.152095947	6.152095947	-20	0.010169
ssc-miR-504	2.149139864	221.63	1.654957834	7.798504517	7.798504517	-6.68825	0.01021
chr1_1112_mature	140.7686611	2.417782	7.14739484	1.773060525	1.773060525	5.863498	0.01028
chr14_31499_mature	0	69.30976	0	6.135653077	6.135653077	-20	0.010344
chr18_36239_mature	0	69.30976	0	6.135653077	6.135653077	-20	0.010344
chr12_26422_mature	0	69.30976	0	6.135653077	6.135653077	-20	0.010344
chr9_20130_mature	68.77247564	0.805927	6.124586119	0.852739935	0.852739935	6.415037	0.010363
chr6_14477_mature@@bta-miR-483	8.596559455	888.938	3.262517265	9.797560981	9.797560981	-6.69218	0.01037
chr14_31044_mature	1.074569932	137.8136	1.05281229	7.117005045	7.117005045	-7.00282	0.01043
mmu-miR-5130	37.60994761	0	5.270900692	0	0	20	0.010495
hsa-miR-5095	37.60994761	0	5.270900692	0	0	20	0.010495
bta-miR-2317	37.60994761	0	5.270900692	0	0	20	0.010495
chr1_3945_mature	37.60994761	0	5.270900692	0	0	20	0.010495
chr15_32293_mature	1.074569932	137.0077	1.05281229	7.108604601	7.108604601	-6.99435	0.010522
chr14_31042_star	0	68.50383	0	6.11902064	6.11902064	-20	0.010525
chr13_28617_mature@@mmu-miR-6	67.6979057	0.805927	6.102194213	0.852739935	0.852739935	6.392317	0.010675
chrx_21977_star@@rno-miR-384-5p	67.6979057	0.805927	6.102194213	0.852739935	0.852739935	6.392317	0.010675
mmu-miR-1947-5p	67.6979057	0.805927	6.102194213	0.852739935	0.852739935	6.392317	0.010675
chr14_31042_mature	1.074569932	135.3958	1.05281229	7.091655531	7.091655531	-6.97728	0.010709
chr6_14094_star	0	67.69791	0	6.102194213	6.102194213	-20	0.01071
chr15_33264_mature	1572.09581	22.56597	10.61939083	4.558633073	4.558633073	6.122397	0.010711
chr1_3507_mature	0	66.89198	0	6.085169218	6.085169218	-20	0.010901
chr14_30531_mature	1.074569932	133.784	1.05281229	7.074504971	7.074504971	-6.96	0.010901
chr14_29507_mature	1.074569932	132.978	1.05281229	7.065852624	7.065852624	-6.95128	0.011
chr13_28708_mature	3.223709795	294.9694	2.078510713	8.20930444	8.20930444	-6.5157	0.011205
chr2_4744_mature	3.223709795	294.1635	2.078510713	8.20537061	8.20537061	-6.51175	0.011251
chr16_33764_mature	0	65.28012	0	6.050504383	6.050504383	-20	0.0113
chr5_12092_mature	1.074569932	130.5602	1.05281229	7.039579809	7.039579809	-6.92481	0.011304
chr13_28221_mature	65.54876584	0.805927	6.056340006	0.852739935	0.852739935	6.345775	0.011343
NW_003538465_37902_mature	2.149139864	205.5115	1.654957834	7.690078309	7.690078309	-6.57932	0.01143
chrx_22279_mature	0	64.4742	0	6.032854532	6.032854532	-20	0.011509
chrx_22902_star	0	64.4742	0	6.032854532	6.032854532	-20	0.011509
chrx_22973_mature	96.71129386	1.611855	6.610453419	1.38507475	1.38507475	5.906891	0.011678
NW_003538989_38416_star	35.46080775	0	5.188274618	0	0	20	0.011703
chr7_16877_mature	35.46080775	0	5.188274618	0	0	20	0.011703
chr15_31807_mature	0	63.66827	0	6.014986077	6.014986077	-20	0.011724
chr10_23842_mature	4.298279727	377.98	2.405524014	8.565977804	8.565977804	-6.45841	0.011759
chr15_32517_mature	1.074569932	125.7247	1.05281229	6.985553734	6.985553734	-6.87036	0.011955
chr2_4295_mature	6.447419591	576.2381	2.896740642	9.173022781	9.173022781	-6.4818	0.012028
chr1_268_mature	7.521989523	684.2324	3.091190278	9.420449567	9.420449567	-6.50723	0.01213
NW_003540828_39718_mature	0	62.05641	0	5.978571212	5.978571212	-20	0.012174
chr14_30912_mature	0	62.05641	0	5.978571212	5.978571212	-20	0.012174
chr15_33320_mature	3.223709795	277.239	2.078510713	8.120181062	8.120181062	-6.42626	0.012294
chr6_13600_star	1.074569932	123.3069	1.05281229	6.957762565	6.957762565	-6.84235	0.012304
NW_003537425_36910_star	34.38623782	0	5.145116481	0	0	20	0.012384
chr7_15205_mature	34.38623782	0	5.145116481	0	0	20	0.012384
chr16_34526_mature@@bta-miR-449	93.48758407	1.611855	6.562052863	1.38507475	1.38507475	5.857981	0.012449
chrx_22499_mature	0	60.44456	0	5.941213351	5.941213351	-20	0.012653
chr1_3508_mature	0	60.44456	0	5.941213351	5.941213351	-20	0.012653
rno-miR-101b-3p	281.5373221	5.641492	8.142297645	2.731507408	2.731507408	5.641106	0.012699
chr14_29632_star	92.41301414	1.611855	6.545551653	1.38507475	1.38507475	5.841302	0.012723
chr14_31142_mature	125.724682	2.417782	6.985553734	1.773060525	1.773060525	5.70044	0.012749
chr6_15013_mature	0	59.63863	0	5.922165283	5.922165283	-20	0.012905
chrx_22559_mature	0	59.63863	0	5.922165283	5.922165283	-20	0.012905
chr3_6620_mature	278.3136123	5.641492	8.125742075	2.731507408	2.731507408	5.624491	0.012983
chr15_31797_mature	33.31166789	0	5.100627352	0	0	20	0.013126
chr13_29302_mature	235.3308151	4.835565	7.884663944	2.544872269	2.544872269	5.604862	0.013165
chr4_9707_mature	1.074569932	116.0536	1.05281229	6.871024911	6.871024911	-6.75489	0.013457
chr5_12329_mature	271.8661928	5.641492	8.09204985	2.731507408	2.731507408	5.590676	0.013581
NW_003537105_36696_mature	1520.516454	24.17782	10.57129422	4.654081667	4.654081667	5.974733	0.013595
chr12_26093_mature	2.149139864	182.1396	1.654957834	7.516799994	7.516799994	-6.40514	0.013678
chr4_9198_mature	3.223709795	257.8968	2.078510713	8.016233232	8.016233232	-6.32193	0.013693
chr10_23626_mature	0	57.22085	0	5.863463969	5.863463969	-20	0.013709
chr7_15363_mature	59.10134625	0.805927	5.909325402	0.852739935	0.852739935	6.196397	0.013759
chr5_11567_mature	6.447419591	525.4647	2.896740642	9.040192981	9.040192981	-6.34873	0.013815
chr14_30207_mature	4.298279727	339.2955	2.405524014	8.410644077	8.410644077	-6.30264	0.01382
ssc-miR-935	491.0784588	9.671129	8.942744552	3.415640968	3.415640968	5.666125	0.013836
mmu-miR-5620-5p	32.23709795	0	5.054722516	0	0	20	0.013935
mmu-miR-1981-5p	32.23709795	0	5.054722516	0	0	20	0.013935
chr1_1283_mature	0	56.41492	0	5.843353819	5.843353819	-20	0.013996
chr17_35122_mature	2.149139864	178.9159	1.654957834	7.491178829	7.491178829	-6.37938	0.014044
chr11_24694_mature	440.573672	8.865202	8.786510347	3.302348583	3.302348583	5.635086	0.014047
chrx_21884_mature	3.223709795	253.0612	2.078510713	7.989030362	7.989030362	-6.29462	0.014084
chr9_20889_mature	1.074569932	112.0239	1.05281229	6.820484263	6.820484263	-6.7039	0.014175
chr11_25309_mature	0	55.60899	0	5.82295938	5.82295938	-20	0.014292
chr16_33868_mature	436.2753923	8.865202	8.772398353	3.302348583	3.302348583	5.620942	0.014316
hsa-miR-4750-5p	347.086088	7.253347	8.443300345	3.044979305	3.044979305	5.580503	0.014357
chr17_35678_mature	1411.98489	23.3719	10.46453032	4.607146585	4.607146585	5.916806	0.014451
chr8_17577_mature	21.49139864	2302.535	4.491301473	11.16963363	11.16963363	-6.74332	0.014463
chr14_29940_mature	150.4397905	3.22371	7.24260051	2.078510713	2.078510713	5.544321	0.014587
hsa-miR-1298	0	54.80307	0	5.802272499	5.802272499	-20	0.014599
chrx_22747_mature	301.9541508	6.44742	8.242955662	2.896740642	2.896740642	5.549464	0.014622
chr5_11313_star@@bta-miR-141	56.95220639	0.805927	5.856791684	0.852739935	0.852739935	6.142958	0.014734
NW_003537990_37435_mature	1.074569932	108.8002	1.05281229	6.778736946	6.778736946	-6.66178	0.014797
mmu-miR-3109-3p	31.16252802	0	5.007308904	0	0	20	0.014819
chr14_31110_mature	31.16252802	0	5.007308904	0	0	20	0.014819
rno-miR-322-5p	31.16252802	0	5.007308904	0	0	20	0.014819
chr7_16155_mature	31.16252802	0	5.007308904	0	0	20	0.014819
chr9_20370_star	31.16252802	0	5.007308904	0	0	20	0.014819
bta-miR-211	31.16252802	0	5.007308904	0	0	20	0.014819
chr16_34124_mature	31.16252802	0	5.007308904	0	0	20	0.014819
chr14_30043_mature	31.16252802	0	5.007308904	0	0	20	0.014819
mmu-miR-700-3p	84.89102461	1.611855	6.424435476	1.38507475	1.38507475	5.718818	0.014914
chrx_21664_mature	0	53.99714	0	5.781284667	5.781284667	-20	0.014917

mmu-miR-6412	651.189379	12.08891	9.349147136	3.710273245	3.710273245	5.751321	0
chr8_17418_mature	477.10905	21.76004	8.901195903	4.508431259	4.508431259	4.454566	0
chr5_11238_mature	262.195063	6.44742	8.039988619	2.896740642	2.896740642	5.345775	0
chr14_31430_mature	6.44741959	406.9934	2.896740642	8.672401869	8.672401869	-5.98014	0
chr15_32108_mature	4.29827973	545.6129	2.405524014	9.094375652	9.094375652	-6.98797	0
chr7_15797_star	6.44741959	241.7782	2.896740642	7.923495278	7.923495278	-5.22882	0
chr13_29267_mature	2.14913986	430.3653	1.654957834	8.752766176	8.752766176	-7.64566	0
chr6_12933_star@@hsa-miR-520e	371.801196	4.029637	8.54226268	2.330454351	2.330454351	6.527738	0
chr7_15273_mature	2048.13029	199.87	11.000796	7.650118356	7.650118356	3.357174	0
chr2_4295_mature	6.44741959	576.2381	2.896740642	9.173022781	9.173022781	-6.4818	0
chr9_20853_mature@@mmu-miR-6243	197.720867	2113.142	7.634599566	11.04585641	11.04585641	-3.41785	0
chr11_953_mature	952.06896	83.81645	9.896436794	6.406272275	6.406272275	3.505761	0
chr15_33243_star	277.239042	9.671129	8.120181062	3.415640968	3.415640968	4.841302	0
chr17_35470_mature	184.826028	9.671129	7.537808781	3.415640968	3.415640968	4.25634	0
chr1_3220_star	563.074644	23.3719	9.139742278	4.607146585	4.607146585	4.59048	0
ssc-miR-34a	32929.121	1682.777	15.00712019	10.71748495	10.71748495	4.290449	0
chr7_15358_mature	5052.62782	50.77343	12.3031037	5.694139975	5.694139975	6.636816	0
chr16_33741_star	272.940763	19.34226	8.097720146	4.346407978	4.346407978	3.81876	0
ssc-miR-342	2931.42677	378.7859	11.51787937	8.569042538	8.569042538	2.952148	0
chrx_21868_mature	2134.09588	318.3413	11.06008515	8.318955528	8.318955528	2.744979	0
ssc-miR-146a-5p	210.615707	13622.59	7.725302902	13.73381948	13.73381948	-6.01524	0
ssc-miR-340	10820.9192	1312.856	13.40166876	10.35959124	10.35959124	3.043043	0
ssc-miR-378	107.456993	9122.293	6.760979269	13.1553389	13.1553389	-6.40757	0
chr13_28352_mature	3010.94495	971.1426	11.55647969	9.925024107	9.925024107	1.632461	0
ssc-miR-370	6.44741959	2733.706	2.896740642	11.41716998	11.41716998	-8.72792	0
ssc-miR-34c	5622.14988	416.6645	12.45716279	8.706200684	8.706200684	3.754164	0
ssc-miR-363	575.969483	1648.122	9.172351205	10.6874821	10.6874821	-1.51676	0
ssc-miR-30d	992.902617	19309.22	9.956960693	14.23707666	14.23707666	-4.28149	0
ssc-miR-324	1083.16649	152.3203	10.08237061	7.260404801	7.260404801	2.830075	0
ssc-miR-320	6825.66821	11150.01	12.73696592	13.44488628	13.44488628	-0.708	0
ssc-miR-335	70.9216155	418.2763	6.168353522	8.711757631	8.711757631	-2.56016	0
ssc-miR-339	2776.6887	361.8614	11.43966921	8.503274883	8.503274883	2.939856	0
ssc-miR-221-5p	27.9388182	3056.883	4.854934103	11.5783174	11.5783174	-6.77365	0
ssc-miR-301	973.560358	38.68452	9.92860773	5.310504361	5.310504361	4.653442	0
chr15_33572_star	5108.50546	661.6664	12.31896795	9.372139038	9.372139038	2.948725	0
chr14_31443_mature	10.7456993	477.915	3.554060706	8.903625744	8.903625744	-5.47492	0
chr1_986_star	7979.75631	4.029637	12.96230976	2.330454351	2.330454351	10.95148	0
chr7_17173_mature	262.195063	5.641492	8.039988619	2.731507408	2.731507408	5.53842	0
ssc-miR-221-3p	15007.4437	31697.13	13.87348676	14.95210996	14.95210996	-1.07867	0
chr12_25534_mature	4.29827973	3712.908	2.405524014	11.85872227	11.85872227	-9.75457	0
chr2_4519_mature	373.950336	2.417782	8.550555707	1.773060525	1.773060525	7.273018	0
chr15_32917_mature	2224.35976	10.47706	11.11982287	3.520680821	3.520680821	7.730013	0
chr13_28708_mature	2.14913986	294.9694	1.654957834	8.20930444	8.20930444	-7.10066	0
chr13_28872_mature	22675.5747	3.22371	14.46891512	2.078510713	2.078510713	12.78013	0
chrx_22749_mature	434.126252	10.47706	8.765290251	3.520680821	3.520680821	5.372809	0
chr1_2879_mature	186.975168	4.835565	7.554398282	2.544872269	2.544872269	5.273018	0
chr12_25526_mature	12.8948392	419.8882	3.796477232	8.717293256	8.717293256	-5.02514	0
bta-miR-2904	5572.71967	381.2037	12.44442473	8.578197871	8.578197871	3.869748	0
chr2_5068_mature	3806.1267	16.92448	11.89448687	4.163859073	4.163859073	7.813069	0
chr4_10236_mature	330.967539	4977.408	8.374898366	12.28146873	12.28146873	-3.91063	0
chr13_29302_mature	165.48377	4.835565	7.379237726	2.544872269	2.544872269	5.096862	0
chr12_25501_mature	2.14913986	230.4953	1.654957834	7.854838784	7.854838784	-6.74483	0
chr13_27180_mature	765.093791	0.805927	9.581377219	0.852739935	0.852739935	9.890771	0
chr7_15331_mature	8555.7258	119.2773	13.06284314	6.910220127	6.910220127	6.164499	0
chr1_268_mature	4.29827973	684.2324	2.405524014	9.420449567	9.420449567	-7.31458	0
chr8_18458_star	116.053553	496.4513	6.871024911	8.958411509	8.958411509	-2.09686	0
chr5_11967_mature	4.29827973	429.5593	2.405524014	8.750068242	8.750068242	-6.64295	0
NW_003540278_39378_mature	1038.03455	32.2371	10.02102792	5.054722516	5.054722516	5.008989	0
chr4_8816_mature	8.59655945	239.3605	3.262517265	7.909055732	7.909055732	-4.79928	0
chr10_23371_mature	20365.2493	798.6741	14.3138927	9.643268355	9.643268355	4.672359	0
chr13_29176_star	339.564098	0	8.411782547	0	0	20	0
chr13_28627_mature	324.520119	2.417782	8.346602905	1.773060525	1.773060525	7.06848	0
chr13_27228_mature	801.629169	82.2046	9.648589777	6.378591382	6.378591382	3.285644	0
chr14_29715_mature	8.59655945	381.2037	3.262517265	8.578197871	8.578197871	-5.47066	0
NW_003537574_37009_mature	8.59655945	8313.142	3.262517265	13.02135161	13.02135161	-9.91742	0
NW_003537647_37094_star	391.143455	8.865202	8.615237712	3.302348583	3.302348583	5.463401	0
ssc-miR-92b-3p	60988.2911	744.677	15.89626833	9.542406961	9.542406961	6.355774	0
ssc-miR-29b	161.18549	2412.141	7.341500942	11.23669641	11.23669641	-3.90352	0
ssc-miR-29a	21.4913986	8630.677	4.491301473	13.07542517	13.07542517	-8.64957	0
chr8_18908_mature	2944.32161	75.75718	11.52420946	6.262229808	6.262229808	5.280409	0
hsa-miR-3676-5p	2538.13418	88.65202	11.31012092	6.486264174	6.486264174	4.839471	0
chr18_36618_mature	498.600448	58.8327	8.964630961	5.902862353	5.902862353	3.083194	0
chr1_3220_mature	69155.0225	14904.82	16.07756728	13.86358834	13.86358834	2.214055	0
ssc-miR-27a	106126.676	2232.419	16.6954414	11.12503824	11.12503824	5.571036	0
ssc-miR-26a	107575.196	20739.74	16.71499935	14.34017954	14.34017954	2.374876	0
ssc-miR-145-5p	6.44741959	19900.77	2.896740642	14.28060887	14.28060887	-11.5918	0
chr1_2148_mature	1392.64263	54.80307	10.44464495	5.802272499	5.802272499	4.667425	0
ssc-miR-23b	60410.1724	6179.852	15.88252777	12.59358993	12.59358993	3.289147	0
ssc-miR-23a	35645.6338	8160.821	15.12147822	12.99467542	12.99467542	2.126939	0
chr9_20339_mature@@mmu-miR-3096	12267.2903	24.17782	13.58264659	4.654081667	4.654081667	8.986917	0
chr1_782_mature	492.153029	45.13194	8.945891584	5.527693969	5.527693969	3.446886	0
chr4_8769_mature	199.870007	4.029637	7.650118356	2.330454351	2.330454351	5.632268	0
ssc-miR-222	13629.845	20983.93	13.73458738	14.35706624	14.35706624	-0.62252	0
ssc-miR-20a	46629.8876	2634.577	15.50899827	11.36390303	11.36390303	4.145612	0
chr16_33919_mature	5069.82094	7.253347	12.30800362	3.044979305	3.044979305	9.449072	0
chr18_35948_mature@@rno-miR-96-5p	6503.29723	87.04016	12.66717747	6.460089935	6.460089935	6.223346	0
ssc-miR-1296-5p	1987.95437	174.8863	10.95779447	7.458498946	7.458498946	3.506796	0
bta-miR-1434-5p	395.441735	15.31262	8.630965038	4.027916745	4.027916745	4.690672	0
chr7_15331_star	980.077778	86.23424	9.938120765	6.446822559	6.446822559	3.506461	0
chr6_14978_mature	4.29827973	3488.86	2.405524014	11.76895342	11.76895342	-9.66478	0
chr13_27210_mature	1237.90456	5.641492	10.27484934	2.731507408	2.731507408	7.777608	0
NW_003613116_37270_mature	315.92356	57.22085	8.307991103	5.863463969	5.863463969	2.464963	0
chr2_4155_mature@@hsa-miR-5701	7571.41974	901.8328	12.88653867	9.818315047	9.818315047	3.069632	0
chr2_5678_star	10685.5234	124.1128	13.38350496	6.967085898	6.967085898	6.427862	0
ssc-miR-30c-1-3p	421.231413	74.14533	8.721890106	6.231611453	6.231611453	2.506185	0
chr17_35129_star	891.893043	240.1664	9.80234356	7.91388499	7.91388499	1.892837	0
chr6_13451_star	107.456993	584.2974	6.760979269	9.193026061	9.193026061	-2.44294	0
chr8_18872_mature	1225.00972	144.261	10.2597547	7.182503739	7.182503739	3.08604	0
chr8_18593_mature	423.380553	62.05641	8.729214737	5.978571212	5.978571212	2.770303	0
chr8_17577_mature	163.33463	2302.535	7.360492716	11.16963363	11.16963363	-3.81732	0
chr6_13565_mature	4.29827973	325.5947	2.405524014	8.351357522	8.351357522	-6.24317	0
chr12_25849_mature	195.571728	8.865202	7.618912027	3.302348583	3.302348583	4.463401	0
chr15_31873_mature	447.021092	25.78968	8.807422842	4.743605355	4.743605355	4.115477	0
chrx_22266_mature	68574.7548	11104.07	16.06541098	13.43893066	13.43893066	2.626589	0
chr3_8256_mature	2.14913986	417.4704	1.654957834	8.708981833	8.708981833	-7.60177	0
chr4_10147_star	333.116679	13.70077	8.384208193	3.877819487	3.877819487	4.603699	0
ssc-miR-125a	38263.2861	2722.423	15.22371086	11.41120532	11.41120532	3.812998	0
ssc-miR-125b	81819.9037	30049.81	16.32018185	14.8751163	14.8751163	1.445096	0
ssc-miR-1249	2149.13986	1.611855	11.07021479	1.38507475	1.38507475	10.38082	0
chr5_12399_mature	15622.0977	940.5173	13.93139291	9.878843844	9.878843844	4.05399	0
bta-miR-147	4130.64682	46.74379	12.01250122	5.577241251	5.577241251	6.465449	0
chr7_15295_mature	971.411218	157.9618	9.925422727	7.312536122	7.312536122	2.620507	0
ssc-miR-129b	747.900673	1630.391	9.548630575	10.67188709	10.67188709	-1.1243	0
ssc-miR-129a	722.110994	101.5469	9.498073301	6.680139486	6.680139486	2.830075	0
chr1_2976_mature	105393.819	5434.369	16.68544442	12.40816221	12.40816221	4.277534	0
ssc-miR-345-3p	128.948392	586.7152	7.02179497	9.198973359	9.198973359	-2.18587	0
chr7_15205_mature	202.019147	0	7.665471987	0	0	20	0
mmu-miR-5117-5p	184.826028	2.417782	7.537808781	1.773060525	1.773060525	6.25634	0
ssc-miR-345-5p	1375.44951	352.9962	10.42673598	8.467590156	8.467590156	1.962179	0
chr14_30079_mature	384.696036	16.92448	8.591320507	4.163859073	4.163859073	4.506536	0
chr3_7724_mature	30236.2487	2997.244	14.88403926	11.54990217	11.54990217	3.334571	0
NW_003537370_36873_mature	9965.56155	1218.562	13.28288015	10.25214775	10.25214775	3.031771	0
chr15_32760_mature	202.019147	4.835565	7.665471987	2.544872269	2.544872269	5.384664	0
chr6_14477_mature@@bta-miR-483	8.59655945	888.938	3.262517265	9.797560981	9.797560981	-6.69218	0
chr16_33741_mature	279.388182	8.059274	8.131281734	3.179395517	3.179395517	5.115477	0
chr11_24694_star	206.317427	5.641492	7.695697583	2.731507408	2.731507408	5.192645	0
chr16_33714_mature	4.29827973	520.6291	2.405524014	9.026880633	9.026880633	-6.92035	0
ssc-miR-15a	7599.35856	329.6243	12.89185177	8.369049069	8.369049069	4.526983	0
ssc-miR-15b	11560.2233	212.7648	13.49700644	7.739880812	7.739880812	5.763766	0
chr7_15333_star	651.189379	20.14819	9.349147136	4.402462031	4.402462031	5.014355	0
mmu-miR-5615-5p	238.554525	1.611855	7.904210254	1.38507475	1.38507475	7.209453	0
chr11_24779_mature	4169.33134	47.54972	12.0259463	5.601391055	5.601391055	6.454235	0
ssc-miR-149	4186.52445	320.7591	12.0318819	8.329837252	8.329837252	3.706191	0
ssc-miR-150	189.124308	1.611855	7.570799187	1.38507475	1.38507475	6.874469	0
ssc-miR-152	870.401645	42144.36	9.767194026	15.36308633	15.36308633	-5.59751	0
NW_003541078_39885_star	782.28691	180.5277	9.613397039	7.504046287	7.504046287	2.115477	0
ssc-miR-671-3p	1577.46866	186.9752	10.6243099	7.554398282	7.554398282	3.076693	0
ssc-miR-10b	1021266.96	34525.13	19.96193003	15.07540084	15.07540084	4.88657	0
chr3_6707_star@@mmu-miR-590-5p	257.896784	1.611855	8.016233232	1.38507475	1.38507475	7.321928	0
chr15_33627_star	98.8604337	885.7143	6.641841268	9.792325477	9.792325477	-3.16338	0
ssc-miR-128	12245.7989	2152.632	13.58011709	11.07255618	11.07255618	2.508113	0
ssc-miR-127	649.040239	11950.29	9.344385216	13.54487899	13.54487899	-4.20259	0
chr5_11958_mature	2.14913986	321.5651	1.654957834	8.33344633	8.33344633	-7.22521	0
ssc-miR-138	6342.11174	92.68166	12.63097504	6.549694683	6.549694683	6.096536	0
ssc-miR-103	95445.4505	11249.94	16.54240383	13.45775808	13.45775808	3.084759	0
ssc-miR-101	71233.2408	22860.13	16.12028326	14.48060923	14.48060923	1.639717	0
ssc-miR-100	26230.252	102701.8	14.67899905	16.64811532	16.64811532	-1.96916	0
ssc-miR-107	11500.0474	420.6941	13.48947763	8.720053123	8.720053123	4.772724	0
mmu-miR-5615-3p	206.317427	1.611855	7.695697583	1.38507475	1.38507475	7	0
chr7_15706_mature	606.057442	8.865202	9.245689225	3.302348583	3.302348583	6.095157	0
chr4_9743_mature	8.59655945	552.0603	3.262517265	9.111292982	9.111292982	-6.00492	0
rno-miR-3074	692.023036	160.3796	9.436759498	7.334314072	7.334314072	2.10933	0
chr1_1437_mature	6419.48077	30.62524	12.64846562	4.983004663	4.983004663	7.711592	0
chr2_5426_mature	8695.41989	296.5813	13.08620588	8.217140066	8.217140066	4.873756	0
ssc-miR-19b	7526.2878	2019.654	12.87791442	10.98060673	10.98060673	1.89783	0
ssc-miR-19a	2606.90665	187.7811	11.34867652	7.560570491	7.560570491	3.795215	0
chr14_31373_mature	1607.55662	114.4417	10.651551	6.851020612	6.851020612	3.812185	0
chrx_22639_mature	4.29827973	1014.663	2.405524014	9.98820559	9.98820559	-7.88303	0
chr16_33754_mature	3475.15916	186.1692	11.76327842	7.548199552	7.548199552	4.222392	0
ssc-miR-182	1030542.65	1181.49	19.97497419	10.20761183	10.20761183	9.768582	0
ssc-miR-185	2088.96395	10970.28	11.02926234	13.42144481	13.42144481	-2.39274	0
ssc-miR-186	114020.466	3437.281	16.79894593	11.74747156	11.74747156	5.051881	0
ssc-miR-18a	35987.347	347.3547	15.13524222	8.444413344	8.444413344	6.694936	0
ssc-miR-192	2757.34645	20.14819	11.42958755	4.402462031	4.402462031	7.096487	0
ssc-miR-195	623.25056	8.059274	9.285981401	3.179395517	3.179395517	6.273018	0
chr4_9236_mature	4.29827973	319.1473	2.405524014	8.322591896	8.322591896	-6.21432	0
chr14_30000_mature	5993.95108	249.8375	12.54953227	7.970609288	7.970609288	4.584445	0
chr8_18458_mature@@mmu-miR-6243	240.703665	1860.886	7.917095537	10.8625494	10.8625494	-2.95066	0
bta-miR-211	156.88721	0	7.302750497	0	0	20	0
chr6_12598_mature	3159.2356	31.43117	11.6258164	5.019309189	5.019309189	6.651236	0
rno-miR-92a-3p	754.348092	7.253347	9.560997834	3.044979305	3.044979305	6.70044	0
ssc-miR-210	4667.93178	1474.041	12.18887679	10.52653964	10.52653964	1.663007	0
ssc-miR-216	1575.31952	12.08891	10.62234428	3.710273245	3.710273245	7.025816	0
ssc-miR-217	1048.78025	58.8327	10.03587165	5.902862353	5.902862353	4.15595	0
ssc-miR-214	40.8336574	9044.924	5.38659223	13.14305212	13.14305212	-7.79121	0
ssc-miR-205	1972.91039	4.029637	10.94684079	2.330454351	2.330454351	8.93546	0
ssc-miR-9	5845.66043	253.8671	12.51339709	7.993601605	7.993601605	4.52522	0
ssc-miR-7	700.619596	37072.66	9.454545231	15.17810703	15.17810703	-5.72558	0
ssc-miR-1	8.59655945	1441.804	3.262517265	10.49465982	10.49465982	-7.3899	0
chrx_22554_mature	2.14913986	1641.674	1.654957834	10.68183067	10.68183067	-9.57719	0
chr4_9141_mature	382.546896	22.56597	8.583259175	4.558633073	4.558633073	4.083416	0
chr5_11626_mature	2899.18968	12.08891	11.50193154	3.710273245	3.710273245	7.905822	0
chr5_11953_mature	141.843231	1200.832	7.158288862	10.2310194	10.2310194	-3.08166	0
chr12_26213_mature	35815.4158	250.6434	15.12833335	7.97523716	7.97523716	7.1588	0
chr6_14878_mature	926.279281	274.8213	9.85686011	8.107589853	8.107589853	1.752954	0
chr2_6533_mature	40.8336574	731.7821	5.38659223	9.517240499	9.517240499	-4.16358	0
chr11_25257_mature	2.14913986	416.6645	1.654957834	8.706200684	8.706200684	-7.59898	0
chr7_17188_mature	202.019147	8.865202	7.665471987	3.302348583	3.302348583	4.510195	0
chr1_3117_mature@@mmu-miR-1957a	1154.08811	73.3394	10.17378719	6.216055097	6.216055097	3.976021	0
bta-miR-106b	12914.1814	1622.332	13.65678029	10.66474233	10.66474233	2.992815	0
chr18_36117_mature	6.44741959	13979.62	2.896740642	13.77114047	13.77114047	-11.0423	0
chr7_16102_mature	752.198952	3.22371	9.556887183	2.078510713	2.078510713	7.866249	0
chr8_18128_mature	1805.27749	72.53347	10.81880393	6.200329169	6.200329169	4.63743	0
ssc-miR-365-3p	28665.2275	2520.941	14.80706444	11.30031884	11.30031884	3.507267	0
ssc-miR-4331	1800.97191	365.0851	10.81536665	8.516035381	8.516035381	2.302477	0
ssc-miR-4332	3247.35033	60.44456	11.66549152	5.941213351	5.941213351	5.747507	0
bta-miR-410	1003.64832	44.32601	9.97247485	5.502267253	5.502267253	4.500957	0
chr13_29407_mature	1708.56619	29.81932	10.73941457	4.94576292	4.94576292	5.840395	0
chr14_29589_mature	4.29827973	679.3968	2.405524014	9.410232629	9.410232629	-7.30435	0
chr2_5437_mature	55033.0245	215.9886	15.74803621	7.761475149	7.761475149	7.993199	0
chr18_36226_mature	674.829917	103.1587	9.400516406	6.702639724	6.702639724	2.709658	0
ssc-miR-331-5p	595.311742	53.19121	9.219922936	5.759986999	5.759986999	3.484386	0
rno-miR-203b-3p	1050.92939	58.8327	10.03882216	5.902862353	5.902862353	4.158904	0
chrx_22073_star	844.611966	124.918	9.723851867	6.976349367	6.976349367	2.757299	0
bta-miR-33a	771.541211	18.53633	9.593468085	4.288087668	4.288087668	5.379316	0
bta-miR-33b	444.871952	0	8.800485637	0	0	20	0
chr1_788_star	380.397756	2.417782	8.575152545	1.773060525	1.773060525	7.297681	0
ssc-miR-331-3p	4882.84577	1010.633	12.25380193	9.982470319	9.982470319	2.272463	0
chr11_24582_mature	4.29827973	1636.033	2.405524014	10.67686744	10.67686744	-8.57223	0
chr14_31622_mature	1435.62451	153.1262	10.48846824	7.26796846	7.26796846	3.228886	0
rno-miR-203b-5p	2125.49933	640.7123	11.05426468	9.325782875	9.325782875	1.730053	0
chr13_27580_mature	3743.80164	27.40153	11.87067359	4.827896911	4.827896911	7.094104	0
chr15_33320_mature	4.29827973	277.239	2.405524014	8.120181062	8.120181062	-6.01123	0
chr9_20767_mature	2273.78998	116.0536	11.15151764	6.871024911	6.871024911	4.292236	0
chr1_2018_mature	2.14913986	406.1874	1.654957834	8.66954923	8.66954923	-7.56224	0
chr13_29352_mature	186.975168	4.029637	7.554398282	2.330454351	2.330454351	5.536053	0
chr5_10971_star	204.168287	6.44742	7.68066394	2.896740642	2.896740642	4.984893	0
bta-miR-6529	37852.8004	6316.859	15.20815053	12.6252201	12.6252201	2.583121	0
NW_003537687_37130_mature	1553.82812	21.76004	10.60253939	4.508431259	4.508431259	6.158002	0
chr13_27072_star	202.019147	5.641492	7.665471987	2.731507408	2.731507408	5.162211	0
chr18_36406_mature	760.795512	52.38528	9.573259978	5.738370208	5.738370208	3.860275	0
chr10_23799_star	4.29827973	381.2037	2.405524014	8.578197871	8.578197871	-6.47066	0
chr14_30575_mature	1401.23919	134.5899	10.45351675	7.083105736	7.083105736	3.380061	0
chr13_28054_mature	2275.93912	1166.983	11.15288	10.18980349	10.18980349	0.963678	0
bta-miR-25	114452.445	8425.166	16.80440134	13.04066055	13.04066055	3.763899	0
chr3_6912_mature	1431.32715	11960.77	10.48414533	13.54614317	13.54614317	-3.06288	0
chr2_5680_mature	4.29827973	701.1569	2.405524014	9.455649593	9.455649593	-7.34983	0
chr7_17170_mature	6804.17681	397.3222	12.73241693	8.637792196	8.637792196	4.098039	0
ssc-miR-28-3p	14427.1759	17500.71	13.8166013	14.09520864	14.09520864	-0.27862	0
chr3_8144_mature	2372.65041	369.9207	11.21289176	8.534966969	8.534966969	2.681212	0
ssc-miR-148a-5p	1392.64263	4224.672	10.44464495	12.04496497	12.04496497	-1.60101	0
chr14_30414_mature	52864.5424	31351.38	15.69004006	14.93628751	14.93628751	0.753771	0
chr3_6770_mature	171.931189	4.029637	7.43405428	2.330454351	2.330454351	5.415037	0
ssc-miR-148a-3p	22989.3491	99491.74	14.48874075	16.60230369	16.60230369	-2.11361	0
chrx_22747_mature	350.309798	6.44742	8.456600003	2.896740642	2.896740642	5.763766	0
ssc-miR-532-5p	12774.4873	5248.2	13.64109071	12.35788173	12.35788173	1.283371	0
chrx_21506_mature	1085.31563	2153.438	10.08522763	11.07309596	11.07309596	-0.98853	0
chrx_22720_mature	2.14913986	299.805	1.654957834	8.23268479	8.23268479	-7.12412	0
bta-miR-93	36576.2113	2620.07	15.15865747	11.35594024	11.35594024	3.803228	0
ssc-miR-532-3p	1598.96006	132.1721	10.64382017	7.057148072	7.057148072	3.596644	0
chr14_30038_mature	2563.92386	97.51722	11.32470028	6.622304032	6.622304032	4.716553	0
ssc-miR-17-3p	5527.58773	116.8595	12.43269528	6.880923997	6.880923997	5.563803	0
chr12_26608_mature	330.967539	2.417782	8.374898366	1.773060525	1.773060525	7.096862	0
chr13_26633_star	745.751533	87.84609	9.544484483	6.473236413	6.473236413	3.085645	0
chr3_7983_mature	5641.49214	214.3767	12.46211679	7.750718383	7.750718383	4.717857	0
chr1_2073_mature@@bta-miR-2478	5551.22827	310.2821	12.43885117	8.282078659	8.282078659	4.161155	0
ssc-miR-17-5p	63737.0409	4266.58	15.95986706	12.05920245	12.05920245	3.90098	0
chr9_20768_mature	2336.11503	105.5765	11.19051303	6.735745493	6.735745493	4.467751	0
chr18_36225_mature	545.881525	120.0832	9.095084516	6.919854778	6.919854778	2.184554	0
chrx_22424_mature@@bta-miR-2478	5289.0332	278.8509	12.36906106	8.128154563	8.128154563	4.245438	0
chr15_32130_mature	7074.96843	12920.63	12.7887119	13.65750032	13.65750032	-0.86888	0
chr1_3118_mature@@mmu-miR-1957a	1166.98295	61.25049	10.18980349	5.960013198	5.960013198	4.251918	0
chr2_5067_mature	3672.88003	4.835565	11.8430888	2.544872269	2.544872269	9.569012	0
chr17_35678_mature	451.319371	23.3719	8.821197973	4.607146585	4.607146585	4.271302	0
chr13_26691_star	335.265819	29.01339	8.393458327	4.907534286	4.907534286	3.530515	0
NW_003541124_39922_mature	21.4913986	415.0526	4.491301473	8.70062225	8.70062225	-4.27146	0
chr12_26075_star	365.353777	0	8.517093679	0	0	20	0
chr15_33256_mature	490.003889	83.01053	8.939590641	6.392498216	6.392498216	2.561427	0
chrx_22543_star	212.764847	11.28298	7.739880812	3.618589217	3.618589217	4.237039	0
chr14_30207_mature	2.14913986	339.2955	1.654957834	8.410644077	8.410644077	-7.30264	0
chr14_31374_mature	1631.19176	107.1884	10.67259962	6.757401354	6.757401354	3.927711	0
chrx_21651_mature	1437.77457	568.9848	10.49062485	9.154779583	9.154779583	1.337376	0
chr2_5435_mature	55538.0724	221.63	15.76121546	7.798504517	7.798504517	7.96918	0
chr6_14390_mature	275.089903	4.029637	8.108994315	2.330454351	2.330454351	6.093109	0
chr4_10371_mature@@bta-miR-2478	6443.12131	245.0019	12.65376794	7.942525909	7.942525909	4.716895	0

chr1_3435_mature	3.760995	138.6195	2.251263041	7.125356859	7.125356859	-5.20387	0.037314
chr8_19026_mature	3.760995	137.8136	2.251263041	7.117005045	7.117005045	-5.19546	0.037757
chr4_9932_mature	2.686425	99.935	1.882222339	6.657282769	6.657282769	-5.21723	0.037876
chrx_21663_mature	1.07457	49.9675	1.05281229	5.671505736	5.671505736	-5.53916	0.038266
chr5_10910_mature	124.6501	1.611855	6.973268148	1.38507475	1.38507475	6.273018	0.03832
bta-miR-103	0	22.56597	0	4.558633073	4.558633073	-20	0.038323
chr13_27097_mature	1.611855	65.28012	1.38507475	6.050504383	6.050504383	-5.33985	0.038459
chr11_25205_mature	9.133844	362.6674	3.34110968	8.506475604	8.506475604	-5.31128	0.038599
chr4_9530_mature	2.14914	81.39867	1.654957834	6.364549187	6.364549187	-5.24317	0.038795
chr8_17677_mature	46.74379	0	5.577241251	0	0	20	0.039188
chr11_25089_mature	22.02868	1072.689	4.525360039	10.06836104	10.06836104	-5.60571	0.039638
bta-miR-2308	271.3289	4.029637	8.089206323	2.330454351	2.330454351	6.073249	0.040184
chrx_22145_mature	119.2773	1.611855	6.910220127	1.38507475	1.38507475	6.209453	0.040808
NW_003539597_38976_star	78.44361	0.805927	6.311859186	0.852739935	0.852739935	6.604862	0.041542
rno-miR-341	162.2601	2.417782	7.35102808	1.773060525	1.773060525	6.06848	0.041559
chr11_24729_mature	44.59465	0	5.510792714	0	0	20	0.041881
mmu-miR-1931	0.537285	33.84895	0.620384622	5.123043401	5.123043401	-5.97728	0.042275
chr3_7655_mature	0.537285	33.84895	0.620384622	5.123043401	5.123043401	-5.97728	0.042275
chr13_28643_star	44.05737	0	5.493691111	0	0	20	0.042602
chr3_7082_mature	44.05737	0	5.493691111	0	0	20	0.042602
chr1_3436_mature	3.22371	110.4121	2.078510713	6.799761604	6.799761604	-5.09803	0.043521
chr2_6092_mature	7.52199	270.7916	3.091190278	8.08635718	8.08635718	-5.16993	0.044013
chr8_18586_mature	42.9828	0	5.458867456	0	0	20	0.044105
chr12_25625_star	2.686425	91.87573	1.882222339	6.537229727	6.537229727	-5.09592	0.044761
chr4_9930_mature	3.22371	108.8002	2.078510713	6.778736946	6.778736946	-5.07682	0.044808
chr15_33605_mature	1.07457	45.93786	1.05281229	5.552680304	5.552680304	-5.41785	0.045068
chr11_24465_mature	1.07457	45.93786	1.05281229	5.552680304	5.552680304	-5.41785	0.045068
chr15_31817_mature	73.60804	0.805927	6.22125921	0.852739935	0.852739935	6.51307	0.045443
chr17_35576_mature	420.6941	6.44742	8.720053123	2.896740642	2.896740642	6.027906	0.045707
chr14_30496_mature	1.611855	59.63863	1.38507475	5.922165283	5.922165283	-5.20945	0.045906
chr17_35575_mature	416.3958	6.44742	8.705272441	2.896740642	2.896740642	6.01309	0.046385
chr4_10161_mature	41.37094	0	5.405003311	0	0	20	0.046527
chr13_28509_mature	1.07457	45.13194	1.05281229	5.527693969	5.527693969	-5.39232	0.046629
chr15_33221_mature	12.89484	502.0928	3.796477232	8.974680734	8.974680734	-5.28309	0.046853
chr4_9258_mature	1.611855	58.8327	1.38507475	5.902862353	5.902862353	-5.18982	0.047131
chr12_25534_star	2.686425	89.45795	1.882222339	6.499175346	6.499175346	-5.05745	0.047166
NW_003539007_38454_mature	0	20.14819	0	4.402462031	4.402462031	-20	0.047523
chr11_25362_mature	0	20.14819	0	4.402462031	4.402462031	-20	0.047523
chr1_3525_mature	40.29637	0	5.367943153	0	0	20	0.048264
mmu-miR-467a-5p	40.29637	0	5.367943153	0	0	20	0.048264
chr1_3523_mature	40.29637	0	5.367943153	0	0	20	0.048264
bta-miR-433	40.29637	0	5.367943153	0	0	20	0.048264
chr1_2437_mature	2.686425	86.23424	1.882222339	6.446822559		-5.0045	0.050663
chr12_25415_mature	183.7515	3.22371	7.529441942	2.078510713		5.83289	0.050747
chrx_23204_mature@@hsa-miR-50	38.68452	0	5.310504361	0		20	0.051074
chr11_24439_mature	67.69791	0.805927	6.102194213	0.852739935		6.392317	0.051083
NW_003613132_37331_mature	0.537285	30.62524	0.620384622	4.983004663		-5.83289	0.051135
ssc-miR-133b	3.22371	101.5469	2.078510713	6.680139486		-4.97728	0.051307
chr13_27058_mature	38.14723	0	5.290838418	0		20	0.05207
chr15_33531_mature	3.760995	116.8595	2.251263041	6.880923997		-4.95752	0.052403
chr11_25309_mature	1.611855	55.60899	1.38507475	5.82295938		-5.10852	0.052515
chr14_29507_mature	4.29828	132.978	2.405524014	7.065852624		-4.95128	0.053139
chr18_35895_mature	0.537285	29.81932	0.620384622	4.94576292		-5.79442	0.05375
rno-miR-3543	37.07266	0	5.250683565	0		20	0.054159
NW_003537574_37016_star	2.14914	68.50383	1.654957834	6.11902064		-4.99435	0.054313
hsa-miR-4426	173.0058	3.22371	7.442991245	2.078510713		5.745954	0.055228
chrx_21983_mature	36.53538	0	5.230179094	0		20	0.055255
chrx_22576_star	0	18.53633	0	4.288087668		-20	0.05546
hsa-miR-548w	0	18.53633	0	4.288087668		-20	0.05546
chr1_3507_mature	2.14914	66.89198	1.654957834	6.085169218		-4.96	0.056835
chr4_9234_mature	94.02487	1.611855	6.570233227	1.38507475		5.866249	0.056994
chr14_30455_mature	35.46081	0	5.188274618	0		20	0.057558
chr15_33182_mature	1.07457	40.29637	1.05281229	5.367943153		-5.22882	0.057782
chr3_6737_mature	303.566	5.641492	8.250611114	2.731507408		5.74979	0.058133
chr4_10313_mature	61.25049	0.805927	5.960013198	0.852739935		6.247928	0.05865
NW_003541128_39926_mature	1.611855	52.38528	1.38507475	5.738370208		-5.02237	0.058795
chr3_7264_mature	458.3041	8.059274	8.843305776	3.179395517		5.829511	0.059656
chrx_22416_mature	2.686425	78.98089	1.882222339	6.32158343		-4.87774	0.059971
chr6_15024_mature	34.38624	0	5.145116481	0		20	0.060021
chr6_14824_star	60.17592	0.805927	5.934891897	0.852739935		6.222392	0.060087
chr8_17418_mature	1720.924	21.76004	10.74980554	4.508431259		6.305358	0.060841
ssc-miR-10a-3p	5.37285	157.1559	2.671938627	7.305203133		-4.87036	0.061031
chr10_24271_mature	245.5392	4.835565	7.945673417	2.544872269		5.666125	0.061228
chrx_22543_star	693.6349	11.28298	9.440111068	3.618589217		5.941956	0.061297
rno-miR-182	59.10135	0.805927	5.909325402	0.852739935		6.196397	0.061582
mmu-miR-1943-5p	1.07457	38.68452	1.05281229	5.310504361		-5.16993	0.062324
chr9_20262_mature	1.611855	50.77343	1.38507475	5.694139975		-4.97728	0.06233
chr13_27087_mature	620.5641	10.47706	9.279759452	3.520680821		5.888275	0.0625
chr5_12321_mature	33.31167	0	5.100627352	0		20	0.062658
chr8_17983_star@@mmu-miR-302	33.31167	0	5.100627352	0		20	0.062658
chr4_9788_mature	0.537285	27.40153	0.620384622	4.827896911		-5.67243	0.062811
NW_003538331_37733_mature	87.57745	1.611855	6.468867551	1.38507475		5.763766	0.06284
chr9_20604_mature	2.686425	76.56311	1.882222339	6.277298702		-4.83289	0.063601
chr15_32108_mature	15.58126	545.6129	4.051482084	9.094375652		-5.12999	0.064011
chr17_35416_mature	194.4972	4.029637	7.611003822	2.330454351		5.592955	0.064311
chr10_23553_mature	118.2027	2.417782	6.897273013	1.773060525		5.611435	0.064766
chr8_18345_mature	8.059274	241.7782	3.179395517	7.923495278		-4.90689	0.064767
chr16_33835_mature	1.07457	37.87859	1.05281229	5.280903997		-5.13955	0.064782
chr5_11533_star	1.07457	37.87859	1.05281229	5.280903997		-5.13955	0.064782
chr16_33778_mature	32.2371	0	5.054722516	0		20	0.065488
chr3_8310_star	4.835565	134.5899	2.544872269	7.083105736		-4.79874	0.066022
chr14_30666_mature	6.984705	199.87	2.997239027	7.650118356		-4.83872	0.067813
chr2_5388_mature	1.611855	48.35565	1.38507475	5.625143252		-4.90689	0.068208
chr1_3508_mature	2.14914	60.44456	1.654957834	5.941213351		-4.81378	0.068721
chr12_25704_mature	112.8298	2.417782	6.830735029	1.773060525		5.544321	0.069009
chr2_5753_mature	0.537285	25.78968	0.620384622	4.743605355		-5.58496	0.070067
NW_003539007_38442_mature	1.07457	36.26674	1.05281229	5.21981653		-5.07682	0.070116
hsa-miR-4529-5p	30.62524	0	4.983004663	0		20	0.070138
chr2_5761_mature	1.611855	47.54972	1.38507475	5.601391055		-4.88264	0.070337
NW_003613103_37208_mature	1.611855	47.54972	1.38507475	5.601391055		-4.88264	0.070337
NW_003539097_38524_star	1.611855	47.54972	1.38507475	5.601391055		-4.88264	0.070337
chr5_11382_mature	0	16.11855	0	4.097488514		-20	0.071265
chr7_16991_star	30.08796	0	4.958283956	0		20	0.071807
chrx_22392_mature	1.611855	46.74379	1.38507475	5.577241251		-4.85798	0.072559
chr9_20605_mature	3.760995	98.32315	2.251263041	6.634058094		-4.70834	0.072878
chr7_15776_mature	2.686425	70.92162	1.882222339	6.168353522		-4.72247	0.07334
chr13_28323_mature	51.57936	0.805927	5.716424587	0.852739935		6	0.073971
chr13_28924_mature	51.57936	0.805927	5.716424587	0.852739935		6	0.073971
mmu-miR-3067-5p	0.537285	24.98375	0.620384622	4.699537803		-5.53916	0.074134
NW_003539074_38507_mature	106.3824	2.417782	6.746614057	1.773060525		5.459432	0.074724
chr1_1734_mature	4.29828	111.218	2.405524014	6.810160141		-4.69349	0.074796
hsa-miR-598	138.0822	3.22371	7.119794359	2.078510713		5.420662	0.075309
mmu-miR-1981-5p	29.01339	0	4.907534286	0		20	0.075344
NW_003537125_36714_mature	1.07457	34.65488	1.05281229	5.156027656		-5.01123	0.076074
NW_003613157_37452_mature	1.07457	34.65488	1.05281229	5.156027656		-5.01123	0.076074
NW_003538101_37534_mature	3.760995	95.90537	2.251263041	6.598504656		-4.67243	0.076328
chr18_36239_mature	2.686425	69.30976	1.882222339	6.135653077		-4.6893	0.076498
bta-miR-2435	2.686425	69.30976	1.882222339	6.135653077		-4.6893	0.076498
chr4_9937_mature	49.9675	0.805927	5.671505736	0.852739935		5.954196	0.077152
chr2_4396_mature	0	15.31262	0	4.027916745		-20	0.07792
chr14_30693_mature	11.82027	348.9666	3.680354657	8.451073371		-4.88375	0.078037
chr14_30669_mature	49.43022	0.805927	5.656216524	0.852739935		5.938599	0.078261
chr16_34304_mature	0.537285	24.17782	0.620384622	4.654081667		-5.49185	0.078533
chr6_14848_mature	3.760995	94.29351	2.251263041	6.57430608		-4.64797	0.078754
mmu-miR-3081-3p	27.93882	0	4.854934103	0		20	0.079171
chr6_12983_mature@@rno-miR-20	27.93882	0	4.854934103	0		20	0.079171
chr3_7304_mature@@bta-miR-216	1900.914	26.59561	10.89323646	4.786366653		6.159362	0.079311
chr16_34306_mature	1.07457	33.84895	1.05281229	5.123043401		-4.97728	0.079317
chr14_29906_mature	202.5564	4.835565	7.669284999	2.544872269		5.388496	0.079739
NW_003540835_39730_mature	4.835565	121.695	2.544872269	6.938933175		-4.65344	0.07981
chr17_35401_mature	132.1721	3.22371	7.057148072	2.078510713		5.357552	0.079874
ssc-miR-574	25.78968	933.264	4.743605355	9.867686445		-5.17742	0.080146
chr14_31575_mature	100.4723	2.417782	6.664941981	1.773060525		5.376969	0.080664
NW_003613290_38305_mature	27.40153	0	4.827896911	0		20	0.081203
chr17_35756_mature	27.40153	0	4.827896911	0		20	0.081203
chr10_23298_mature	8.596559	231.3012	3.262517265	7.859852659		-4.74987	0.081687
NW_003537589_37025_mature	7.52199	196.6463	3.091190278	7.626777119		-4.70834	0.082396
NW_003613519_39525_star	1.611855	43.52008	1.38507475	5.476384353		-4.75489	0.082481
NW_003613447_39176_mature	3.760995	91.87573	2.251263041	6.537229727		-4.6105	0.082595
chr7_15777_mature	1.07457	33.04303	1.05281229	5.08928735		-4.94251	0.082752
chr7_15474_mature	1.07457	33.04303	1.05281229	5.08928735		-4.94251	0.082752
chr1_495_mature	47.28108	0.805927	5.593385953	0.852739935		5.874469	0.082966
chr11_24562_mature	0.537285	23.3719	0.620384622	4.607146585		-5.44294	0.083299
chr8_17722_mature	26.86425	0	4.800343329	0		20	0.08332
chr1_3824_mature	4.29828	104.7706	2.405524014	6.72479443		-4.60733	0.08353
NW_003541201_39964_star	2.14914	53.99714	1.654957834	5.781284667		-4.65105	0.084328
chr5_11694_mature	69.84705	1.611855	6.146635787	1.38507475		5.437405	0.085101
chr8_18293_mature	1.611855	42.71415	1.38507475	5.4500286		-4.72792	0.08525
chr16_33764_mature	2.686425	65.28012	1.882222339	6.050504383		-4.60288	0.085256
chr13_27735_star	0	14.50669	0	3.954819241		-20	0.085463
chr6_13558_mature	26.32696	0	4.772253246	0		20	0.085527
chrx_22644_mature	95.63672	2.417782	6.594499642	1.773060525		5.305808	0.08611
chr13_29352_mature	156.3499	4.029637	7.297832681	2.330454351		5.277985	0.086488
chr11_24466_mature	2.14914	53.19121	1.654957834	5.759986999		-4.62936	0.086611
chr6_13322_mature	68.23519	1.611855	6.113433608	1.38507475		5.403722	0.087742
chr1_468_mature	25.25239	0	4.714377052	0		20	0.090234
chr1_2947_mature	1.07457	31.43117	1.05281229	5.019309189		-4.87036	0.09026
mmu-miR-1934-5p	1.07457	31.43117	1.05281229	5.019309189		-4.87036	0.09026
chr14_30452_star	1.07457	31.43117	1.05281229	5.019309189		-4.87036	0.09026
chr14_30427_mature	6.984705	171.6625	2.997239027	7.431811362		-4.61923	0.090373
chr3_8648_mature	1.611855	41.1023	1.38507475	5.395827139		-4.67243	0.091182
chr13_28489_mature	3.22371	74.14533	2.078510713	6.231611453		-4.52356	0.092291
chr15_31782_mature	65.54877	1.611855	6.056340006	1.38507475		5.345775	0.092445
ssc-miR-133a-3p	14.50669	412.6349	3.954819241	8.692123945		-4.83007	0.09248
ssc-miR-652	90.26387	2.417782	6.511971994	1.773060525		5.222392	0.092886
NW_003540828_39718_mature	2.686425	62.05641	1.882222339	5.978571212		-4.52982	0.093279
chr17_35504_mature	117.1281	3.22371	6.884208655	2.078510713		5.183222	0.093732
chr18_36042_mature	0	13.70077	0	3.877819487		-20	0.094046
NW_003538201_37611_mature	0	13.70077	0	3.877819487		-20	0.094046
chr5_11381_mature	0	13.70077	0	3.877819487		-20	0.094046
chr18_35964_mature	1.611855	40.29637	1.38507475	5.367943153		-4.64386	0.094359
chr14_30679_star	1.07457	30.62524	1.05281229	4.983004663		-4.83289	0.094365
rno-miR-3068-3p	24.17782	0	4.654081667	0		20	0.095371
chr15_32249_mature	42.44551	0.805927	5.441135257	0.852739935		5.718818	0.09538
chr12_26455_mature	13.96941	385.2333	3.90394537	8.593328822		-4.78539	0.096221
hsa-miR-4531	4.29828	96.71129	2.405524014	6.610453419		-4.49185	0.096534
chr2_4766_mature	41.90823	0.805927	5.423182395	0.852739935		5.70044	0.096941
chr2_6121_mature	41.90823	0.805927	5.423182395	0.852739935		5.70044	0.096941
chr17_35590_mature	5.910135	136.2017	2.788713818	7.100154956		-4.52641	0.097321
chr5_11575_mature	2.686425	60.44456	1.882222339	5.941213351		-4.49185	0.097679
chr5_12251_mature	111.7553	3.22371	6.817051091	2.078510713		5.115477	0.099637
chr9_20219_star	61.78777	1.611855	5.972411693	1.38507475		5.260528	0.099741
chr2_5943_mature	40.83366	0.805927	5.38659223	0.852739935		5.662965	0.10019
chr6_13244_mature	0.537285	20.95411	0.620384622	4.456419386		-5.2854	0.100211
ssc-miR-143-5p	55.87764	2311.4	5.82978961	11.17517521		-5.37035	0.101815
chr14_30374_mature	83.27917	2.417782	6.397104196	1.773060525		5.106199	0.103059
chr15_33101_mature	39.75909	0.805927	5.349049847	0.852739935		5.624491	0.103616
hsa-miR-3615	0	12.89484	0	3.796477232		-20	0.103852
mmu-miR-5106	0	12.89484	0	3.796477232		-20	0.103852
NW_003538201_37612_mature	0	12.89484	0	3.796477232		-20	0.103852
chr15_32429_mature	82.74188	2.417782	6.387877484	1.773060525		5.096862	0.103915
chrx_23193_mature	22.56597	0	4.558633073	0		20	0.104006
chr7_15151_star	1.611855	37.87859	1.38507475	5.280903997		-4.55459	0.104846
chr6_14946_mature	1.611855	37.87859	1.38507475	5.280903997		-4.55459	0.104846
chr5_11550_star	1.611855	37.87859	1.38507475	5.280903997		-4.55459	0.104846
chr15_33346_mature	4.29828	91.87573	2.405524014	6.537229727		-4.41785	0.105687
chr4_9707_mature	5.37285	116.0536	2.671938627	6.871024911		-4.43296	0.107098
chr1_986_mature	22.02868	0	4.525360039	0		20	0.107165
chr6_13334_mature	22.02868	0	4.525360039	0		20	0.107165
chr13_26768_star	2.686425	57.22085	1.882222339	5.863463969		-4.41278	0.107362
chr7_16555_mature	3.760995	78.98089	2.251263041	6.32158343		-4.39232	0.108089
chr17_35462_mature	3.760995	78.98089	2.251263041	6.32158343		-4.39232	0.108089
chr6_12830_mature	1.07457	28.20746	1.05281229	4.868265031		-4.71425	0.108312
NW_003613282_38228_mature	1.07457	28.20746	1.05281229	4.868265031		-4.71425	0.108312
chr2_5583_mature	4.29828	90.26387	2.405524014	6.511971994		-4.39232	0.108998
chr2_4893_mature	837.09	17.7304	9.710961329	4.227310103		5.561085	0.110446
chr8_17569_mature	21.4914	0	4.491301473	0		20	0.11048
chr16_33726_mature	21.4914	0	4.491301473	0		20	0.11048
chr2_5373_mature	21.4914	0	4.491301473	0		20	0.11048
chr5_11866_mature	21.4914	0	4.491301473	0		20	0.11048
chr4_9116_mature	8.596559	195.8404	3.262517265	7.620882324		-4.50978	0.110997
chr4_8813_mature	37.60995	0.805927	5.270900692	0.852739935		5.544321	0.111057
ssc-miR-150	56.41492	1.611855	5.843353819	1.38507475		5.129283	0.111882
chr8_17419_mature	185.3633	5.641492	7.541974074	2.731507408		5.038135	0.113056
chr8_17417_star	249.3002	7.253347	7.967515773	3.044979305		5.103093	0.113944
chr7_15205_mature	20.95411	0	4.456419386	0		20	0.113961
chr1_3536_mature	2.14914	45.13194	1.654957834	5.527693969		-4.39232	0.114861
chr5_12354_star	2.14914	45.13194	1.654957834	5.527693969		-4.39232	0.114861
chr14_30349_mature	36.53538	0.805927	5.230179094	0.852739935		5.5025	0.115101
rno-miR-758-3p	0	12.08891	0	3.710273245		-20	0.115104
chr11_24684_mature	0	12.08891	0	3.710273245		-20	0.115104
hsa-miR-100-5p	0	12.08891	0	3.710273245		-20	0.115104
chr5_12427_mature	0	12.08891	0	3.710273245		-20	0.115104
NW_003539007_38433_mature	1.611855	35.46081	1.38507475	5.188274618		-4.45943	0.116971
rno-miR-351-5p	35.99809	0.805927	5.209378995	0.852739935		5.481127	0.117212
chrx_22279_mature	3.22371	64.4742	2.078510713	6.032854532		-4.32193	0.117545
NW_003539588_38971_star	20.41683	0	4.420672964	0		20	0.117621
chr4_10240_mature	21.4914	599.61	4.491301473	9.230284738		-4.80219	0.118337
chrx_21664_mature	2.686425	53.99714	1.882222339	5.781284667		-4.32912	0.118386
chr6_13694_mature	35.46081	0.805927	5.188274618	0.852739935		5.459432	0.119385
chr1_3820_mature	32.2371	1015.469	5.054722516	9.989349913		-4.97728	0.120034
NW_003613335_38593_mature	1.611855	34.65488	1.38507475	5.156027656		-4.42626	0.12143
hsa-miR-5694	95.63672	3.22371	6.594499642	2.078510713		4.890771	0.121439
chr13_29080_mature	19.87954	0	4.384018281	0		20	0.121472
hsa-miR-514b-3p	19.87954	0	4.384018281	0		20	0.121472
hsa-miR-802	19.87954	0	4.384018281	0		20	0.121472
chr13_28676_mature	19.87954	0	4.384018281	0		20	0.121472
chr6_13376_mature	4.29828	84.62238	2.405524014	6.419916069		-4.29921	0.121752
chr6_12650_mature	18.26769	477.915	4.268111626	8.903625744		-4.70939	0.121772
chr1_1174_mature	0.537285	18.53633	0.620384622	4.288087668		-5.10852	0.122147
chr8_17641_mature	0.537285	18.53633	0.620384622	4.288087668		-5.10852	0.122147
chr13_27663_mature	10.20841	226.4656	3.48651029	7.829504654		-4.47146	0.12424
chr2_5754_mature	1.07457	25.78968	1.05281229	4.743605355		-4.58496	0.125192
chr9_20857_mature	3.22371	62.05641	2.078510713	5.978571212		-4.26679	0.125286
chr13_28716_star	3.22371	62.05641	2.078510713	5.978571212		-4.26679	0.125286
chr14_31358_mature	19.34226	0	4.346407978	0		20	0.125527
rno-miR-3544	19.34226	0	4.346407978	0		20	0.125527
NW_003538780_38224_mature	19.34226	0	4.346407978	0		20	0.125527
chr9_21075_mature	19.34226	0	4.346407978	0		20	0.125527
chr13_28675_mature	19.34226	0	4.346407978	0		20	0.125527
chr15_32263_mature	19.34226	0	4.346407978	0		20	0.125527
chr3_7381_mature	19.34226	0	4.346407978	0		20	0.125527
chr18_36331_mature	19.34226	0	4.346407978	0		20	0.125527
NW_003539007_38439_mature	1.611855	33.84895	1.38507475	5.123043401		-4.39232	0.12612
chr1_3819_mature	32.77438	1007.409	5.077857506	9.977865629		-4.94194	0.126897
chr8_17317_mature	70.38433	2.417782	6.15753552	1.773060525		4.863498	0.127213
mmu-miR-695	2.14914	41.90823	1.654957834	5.423182395		-4.2854	0.129658
NW_003613552_39654_mature	18.80497	0	4.307790888	0		20	0.129802
NW_003613265_38082_mature	18.80497	0	4.307790888	0		20	0.129802
chrx_23194_mature	18.80497	0	4.307790888	0		20	0.129802
chr15_32787_mature	18.80497	0	4.307790888	0		20	0.129802
chr15_33619_mature	18.80497	0	4.307790888	0		20	0.129802
chr1_915_star	49.9675	1.611855	5.671505736	1.38507475		4.954196	0.129859
chr5_11961_mature	288.522	8.865202	8.177529302	3.302348583		5.024384	0.130651
chr14_30452_mature	68.77248	2.417782	6.124586119	1.773060525		4.830075	0.130858

chr8_18026_mature	426.6042629	8.865202	8.740132426	3.302348583	3.302348583	5.588601	0.014948	
chr13_28171_mature	1.074569932	107.9943	1.05281229	6.76810859	6.76810859	-6.65105	0.014959	
hsa-miR-4750-3p	5514.69289	67.69791	12.42932642	6.102194213	6.102194213	6.348026	0.014996	
ssc-miR-194a	338.4895285	7.253347	8.407223265	3.044979305	3.044979305	5.544321	0.015065	
chr9_20772_mature	2.149139864	170.0507	1.654957834	7.418280128	7.418280128	-6.30606	0.015139	
chr9_20753_mature	2.149139864	170.0507	1.654957834	7.418280128	7.418280128	-6.30606	0.015139	
chr9_20339_mature@@mmu-miR-30	1434.550859	24.17782	10.48738873	4.654081667	4.654081667	5.890771	0.015242	
chr1_3819_star	0	53.19121	0	5.759986999	5.759986999	-20	0.015246	
chr3_7813_mature	55.87763645	0.805927	5.82978961	0.852739935	0.852739935	6.115477	0.01526	
hsa-miR-4791	83.81645468	1.611855	6.406272275	1.38507475	1.38507475	5.70044	0.015272	
chr6_14288_mature	180.5277485	4.029637	7.504046287	2.330454351	2.330454351	5.485427	0.015407	
chr9_19579_mature	1.074569932	105.5765	1.05281229	6.735745493	6.735745493	-6.61839	0.015464	
chr1_2110_mature	0	52.38528	0	5.738370208	5.738370208	-20	0.015588	
NW_003541128_39926_mature	0	52.38528	0	5.738370208	5.738370208	-20	0.015588	
chr7_16094_mature	0	52.38528	0	5.738370208	5.738370208	-20	0.015588	
mmu-miR-5108	1.074569932	104.7706	1.05281229	6.72479443	6.72479443	-6.60733	0.015638	
chr10_23743_mature	1009.021166	18.53633	9.980169811	4.288087668	4.288087668	5.766457	0.015759	
chr13_27448_mature	30.08795809	0	4.958283956	0	0	20	0.015789	
chr7_16991_mature	30.08795809	0	4.958283956	0	0	20	0.015789	
hsa-miR-4264	30.08795809	0	4.958283956	0	0	20	0.015789	
chr5_12332_star	30.08795809	0	4.958283956	0	0	20	0.015789	
chr7_16061_star	30.08795809	0	4.958283956	0	0	20	0.015789	
bta-miR-301a	54.80306652	0.805927	5.802272499	0.852739935	0.852739935	6.087463	0.015814	
NW_003539115_38548_star	54.80306652	0.805927	5.802272499	0.852739935	0.852739935	6.087463	0.015814	
chr13_29304_mature	2.149139864	164.4092	1.654957834	7.369895665	7.369895665	-6.25739	0.015911	
chr18_36061_mature	1.074569932	103.1587	1.05281229	6.702639724	6.702639724	-6.58496	0.015997	
chr10_23298_mature	3.223709795	231.3012	2.078510713	7.859852659	7.859852659	-6.16491	0.01609	
chr12_25501_mature	3.223709795	230.4953	2.078510713	7.854838784	7.854838784	-6.15987	0.016173	
chr16_34398_mature	1.074569932	102.3528	1.05281229	6.691433469	6.691433469	-6.57365	0.016181	
chr11_25154_mature	1.074569932	102.3528	1.05281229	6.691433469	6.691433469	-6.57365	0.016181	
chr1_1437_mature	1881.571951	30.62524	10.87848929	4.983004663	4.983004663	5.941073	0.016203	
mmu-miR-3096b-5p	636.1453996	12.89484	9.31547883	3.796477232	3.796477232	5.624491	0.016233	
ssc-miR-382	2.149139864	161.9914	1.654957834	7.348652187	7.348652187	-6.23601	0.016261	
chr9_20262_mature	0	50.77343	0	5.694139975	5.694139975	-20	0.016309	
mmu-miR-669d-5p	53.72849659	0.805927	5.774220321	0.852739935	0.852739935	6.058894	0.016398	

[illegible]

chr9_19205_mature	66.62333577	2.417782	6.079449279	1.773060525	1.773060525	4.784271	0.040253
chr9_19683_mature	85.96559455	3.22371	6.442372848	2.078510713	2.078510713	4.736966	0.040267
chr14_31118_mature	2.149139864	85.42831	1.654957834	6.433432039	6.433432039	-5.31288	0.040514
chr12_26014_mature	48.35564693	3535.604	5.625143252	11.78814886	11.78814886	-6.19213	0.040515
chrx_22749_mature	304.1032907	10.47706	8.25315393	3.520680821	3.520680821	4.859256	0.040533
mmu-miR-5615-3p	48.35564693	1.611855	5.625143252	1.38507475	1.38507475	4.906891	0.040629
chr9_20434_mature@@rno-miR-34b	420.1568433	13.70077	8.718213798	3.877819487	3.877819487	4.938599	0.04064
chr6_13376_mature	2.149139864	84.62238	1.654957834	6.419916069	6.419916069	-5.29921	0.041045
chr8_18274_mature	2.149139864	84.62238	1.654957834	6.419916069	6.419916069	-5.29921	0.041045
rno-miR-345-5p	17.19311891	0	4.185320985	0	0	20	0.04111
chr13_28152_star	17.19311891	0	4.185320985	0	0	20	0.04111
chr2_4630_mature	17.19311891	0	4.185320985	0	0	20	0.04111
chr13_27867_mature	17.19311891	0	4.185320985	0	0	20	0.04111
chr6_14288_star	17.19311891	0	4.185320985	0	0	20	0.04111
chr4_10618_star	17.19311891	0	4.185320985	0	0	20	0.04111
ssc-miR-196b-3p	17.19311891	0	4.185320985	0	0	20	0.04111
chr14_31314_mature	7.521989523	297.3872	3.091190278	8.221041977	8.221041977	-5.30508	0.041143
rno-miR-203b-5p	13.96940911	640.7123	3.90394537	9.325782875	9.325782875	-5.51933	0.041291
chr11_24779_mature	2044.90658	47.54972	10.99852456	5.601391055	5.601391055	5.426454	0.041585
chr1_1290_mature	1.074569932	52.38528	1.05281229	5.738370208	5.738370208	-5.60733	0.041676
hsa-miR-5707	1.074569932	52.38528	1.05281229	5.738370208	5.738370208	-5.60733	0.041676
rno-miR-466b-5p	1.074569932	52.38528	1.05281229	5.738370208	5.738370208	-5.60733	0.041676
ssc-miR-487b	0	25.78968	0	4.743605355	4.743605355	-20	0.041862
chr6_12811_star	0	25.78968	0	4.743605355	4.743605355	-20	0.041862
chr8_18357_mature	0	25.78968	0	4.743605355	4.743605355	-20	0.041862
chr2_5753_mature	0	25.78968	0	4.743605355	4.743605355	-20	0.041862
chr2_5754_mature	0	25.78968	0	4.743605355	4.743605355	-20	0.041862
hsa-miR-649	0	25.78968	0	4.743605355	4.743605355	-20	0.041862
chr14_30223_mature	8.596559455	344.131	3.262517265	8.43100034	8.43100034	-5.32305	0.041953
chr6_13411_mature	6.447419591	245.0019	2.896740642	7.942525909	7.942525909	-5.24793	0.041983
chr16_34464_mature	47.281077	1.611855	5.593385953	1.38507475	1.38507475	4.874469	0.042191
chr4_8813_mature	31.16252802	0.805927	5.007308904	0.852739935	0.852739935	5.273018	0.042394
chr16_34334_mature	31.16252802	0.805927	5.007308904	0.852739935	0.852739935	5.273018	0.042394
chr7_16992_mature	31.16252802	0.805927	5.007308904	0.852739935	0.852739935	5.273018	0.042394
chr13_26947_mature	64.47419591	2.417782	6.032854532	1.773060525	1.773060525	4.736966	0.042563
chr7_15797_star	6.447419591	241.7782	2.896740642	7.923495278	7.923495278	-5.22882	0.04277
mmu-miR-6236	2903.487956	62.86234	11.50406813	5.996893534	5.996893534	5.529447	0.043081
chr7_15783_mature	465.2887805	15.31262	8.865079907	4.027916745	4.027916745	4.925333	0.043326
NW_003613103_37207_mature	1.074569932	50.77343	1.05281229	5.694139975	5.694139975	-5.56224	0.04347
chr15_32760_mature	122.5009722	4.835565	6.948378589	2.544872269	2.544872269	4.662965	0.043602
hsa-miR-1248	0	24.98375	0	4.699537803	4.699537803	-20	0.043657
NW_003613367_38732_mature	0	24.98375	0	4.699537803	4.699537803	-20	0.043657
chr9_19915_mature	0	24.98375	0	4.699537803	4.699537803	-20	0.043657
chr3_8555_mature	0	24.98375	0	4.699537803	4.699537803	-20	0.043657
mmu-miR-222-3p	0	24.98375	0	4.699537803	4.699537803	-20	0.043657
chr3_6707_star@@mmu-miR-590-5p	46.20650707	1.611855	5.560913833	1.38507475	1.38507475	4.841302	0.043843
chr14_29589_star	46.20650707	1.611855	5.560913833	1.38507475	1.38507475	4.841302	0.043843
rno-miR-598-3p	318.0726998	11.28298	8.317741365	3.618589217	3.618589217	4.817136	0.043849
chrx_22864_mature	1.074569932	49.9675	1.05281229	5.671505736	5.671505736	-5.53916	0.044415
chr8_19054_mature	1.074569932	49.9675	1.05281229	5.671505736	5.671505736	-5.53916	0.044415
mmu-miR-466c-5p	30.08795809	0.805927	4.958283956	0.852739935	0.852739935	5.222392	0.044899
mmu-miR-3066-5p	30.08795809	0.805927	4.958283956	0.852739935	0.852739935	5.222392	0.044899
bta-miR-2478	30.08795809	0.805927	4.958283956	0.852739935	0.852739935	5.222392	0.044899
chr2_6291_star	30.08795809	0.805927	4.958283956	0.852739935	0.852739935	5.222392	0.044899
chr17_35401_mature	80.59274489	3.22371	6.35036897	2.078510713	2.078510713	4.643856	0.044972
chr1_1609_mature	99.93500366	4.029637	6.657282769	2.330454351	2.330454351	4.632268	0.044972
chr11_25215_mature	340.6386684	12.08891	8.416327465	3.710273245	3.710273245	4.816486	0.04503
rno-miR-760-5p	16.11854898	0	4.097488514	0	0	20	0.045554
rno-miR-300-3p	16.11854898	0	4.097488514	0	0	20	0.045554
mmu-miR-5123	16.11854898	0	4.097488514	0	0	20	0.045554
chrx_21884_star	16.11854898	0	4.097488514	0	0	20	0.045554
chr4_8812_mature	16.11854898	0	4.097488514	0	0	20	0.045554
mmu-miR-466n-5p	16.11854898	0	4.097488514	0	0	20	0.045554
chr1_3765_mature	16.11854898	0	4.097488514	0	0	20	0.045554
chr6_13566_star	16.11854898	0	4.097488514	0	0	20	0.045554
rno-miR-881-3p	16.11854898	0	4.097488514	0	0	20	0.045554
hsa-miR-4697-5p	16.11854898	0	4.097488514	0	0	20	0.045554
NW_003539279_38690_mature	0	24.17782	0	4.654081667	4.654081667	-20	0.04558
chr10_23377_mature	0	24.17782	0	4.654081667	4.654081667	-20	0.04558
chr6_13311_mature	0	24.17782	0	4.654081667	4.654081667	-20	0.04558
hsa-miR-4665-3p	45.13193714	1.611855	5.527693969	1.38507475	1.38507475	4.807355	0.04559
chr1_3708_mature	2.149139864	78.17496	1.654957834	6.306972375	6.306972375	-5.18488	0.045726
chr1_3436_mature	3.223709795	110.4121	2.078510713	6.799761604	6.799761604	-5.09803	0.046262
chr2_5388_mature	1.074569932	48.35565	1.05281229	5.625143252	5.625143252	-5.49185	0.046406
chr14_30524_star	306.2524306	11.28298	8.263280613	3.618589217	3.618589217	4.762501	0.046909
chr9_20604_mature	2.149139864	76.56311	1.654957834	6.277298702	6.277298702	-5.15482	0.047032
bta-miR-2887	4011.369555	83.01053	11.97023877	6.392498216	6.392498216	5.594657	0.047055
chr6_12988_mature@@mmu-miR-65	44.0573672	1.611855	5.493691111	1.38507475	1.38507475	4.77259	0.047439
mmu-miR-6370	1.074569932	47.54972	1.05281229	5.601391055	5.601391055	-5.46761	0.047457
NW_003539097_38524_star	1.074569932	47.54972	1.05281229	5.601391055	5.601391055	-5.46761	0.047457
chr9_20212_star	29.01338816	0.805927	4.907534286	0.852739935	0.852739935	5.169925	0.047624
hsa-miR-548ak	0	23.3719	0	4.607146585	4.607146585	-20	0.047644
chr11_24562_mature	0	23.3719	0	4.607146585	4.607146585	-20	0.047644
chr17_35097_mature	7.521989523	265.9561	3.091190278	8.060458479	8.060458479	-5.14393	0.048106
chr7_17178_star	77.36903509	3.22371	6.292211829	2.078510713	2.078510713	4.584963	0.048186
chr11_25193_mature	1.074569932	46.74379	1.05281229	5.577241251	5.577241251	-5.44294	0.048547
ssc-miR-451	59.10134625	2.417782	5.909325402	1.773060525	1.773060525	4.611435	0.049249
hsa-miR-5000-3p	42.98279727	1.611855	5.458867456	1.38507475	1.38507475	4.736966	0.049399
chr1_330_mature	42.98279727	1.611855	5.458867456	1.38507475	1.38507475	4.736966	0.049399
chr9_20255_mature	42.98279727	1.611855	5.458867456	1.38507475	1.38507475	4.736966	0.049399
chr1_3087_star	94.562154	4.029637	6.578367468	2.330454351	2.330454351	4.552541	0.04941
chr2_5067_mature	113.9044128	4.835565	6.844290394	2.544872269	2.544872269	4.557995	0.04941
chr13_27783_mature	4.298279727	139.4254	2.405524014	7.133660602	7.133660602	-5.01959	0.049627
ssc-miR-133a-5p	1.074569932	45.93786	1.05281229	5.552680304	5.552680304	-5.41785	0.049677
chr13_28621_mature	1.074569932	45.93786	1.05281229	5.552680304	5.552680304	-5.41785	0.049677
chr13_29181_mature	1.074569932	45.93786	1.05281229	5.552680304	5.552680304	-5.41785	0.049677
chr11_24465_mature	1.074569932	45.93786	1.05281229	5.552680304	5.552680304	-5.41785	0.049677
chr3_8509_mature	0	22.56597	0	4.558633073	4.558633073	-20	0.049864
chr11_24872_mature	0	22.56597	0	4.558633073	4.558633073	-20	0.049864
chr18_35896_mature	0	22.56597	0	4.558633073	4.558633073	-20	0.049864
bta-miR-103	0	22.56597	0	4.558633073	4.558633073	-20	0.049864
chr5_11025_mature	0	22.56597	0	4.558633073	4.558633073	-20	0.049864
chr4_10705_mature	0	22.56597	0	4.558633073	4.558633073	-20	0.049864
ssc-miR-582	2497.300522	59.63863	11.28673131	5.922165283		5.387978	0.050103
chr8_19026_mature	4.298279727	137.8136	2.405524014	7.117005045		-5.00282	0.05042
chr17_35034_mature	27.93881823	0.805927	4.854934103	0.852739935		5.115477	0.050595
chr1_1283_star	27.93881823	0.805927	4.854934103	0.852739935		5.115477	0.050595
chr8_18472_star	27.93881823	0.805927	4.854934103	0.852739935		5.115477	0.050595
chr5_10912_star	132.1721016	5.641492	7.057148072	2.731507408		4.550197	0.050696
rno-miR-298-3p	15.04397905	0	4.003960082	0		20	0.050732
chr2_4844_mature	15.04397905	0	4.003960082	0		20	0.050732
chr8_17918_mature	15.04397905	0	4.003960082	0		20	0.050732
chr15_31855_star	15.04397905	0	4.003960082	0		20	0.050732
chr15_32628_star	15.04397905	0	4.003960082	0		20	0.050732
chr16_34021_star	15.04397905	0	4.003960082	0		20	0.050732
mmu-miR-1843b-3p	15.04397905	0	4.003960082	0		20	0.050732
mmu-miR-5709	15.04397905	0	4.003960082	0		20	0.050732
NW_003613109_37260_mature	15.04397905	0	4.003960082	0		20	0.050732
hsa-miR-6089	15.04397905	0	4.003960082	0		20	0.050732
chr1_3536_mature	1.074569932	45.13194	1.05281229	5.527693969		-5.39232	0.050851
bta-miR-2487	1247.575691	35.46081	10.28606757	5.188274618		5.136758	0.050865
ssc-miR-374b-3p	292.2830215	11.28298	8.196149744	3.618589217		4.695145	0.05094
bta-miR-200a	41.90822734	1.611855	5.423182395	1.38507475		4.70044	0.051478
chr10_23668_mature	1.074569932	44.32601	1.05281229	5.502267253		-5.36632	0.052071
chr16_34017_star	2.149139864	70.92162	1.654957834	6.168353522		-5.04439	0.052114
chr1_989_mature	0	21.76004	0	4.508431259		-20	0.052256
chr15_33603_mature	0	21.76004	0	4.508431259		-20	0.052256
chr9_19418_mature@@bta-miR-245	11.82026925	436.8127	3.680354657	8.77416992		-5.20768	0.05235
chrx_21673_mature	4.298279727	133.784	2.405524014	7.074504971		-4.96	0.052493
chr7_15100_mature	40.83365741	1.611855	5.38659223	1.38507475		4.662965	0.053685
rno-miR-351-3p	26.8642483	0.805927	4.800343329	0.852739935		5.058894	0.053842
chr12_26311_mature	15.04397905	584.2974	4.003960082	9.193026061		-5.27944	0.053932
chr1_1181_mature	107.4569932	4.835565	6.760979269	2.544872269		4.473931	0.05453
chr8_18293_mature	1.074569932	42.71415	1.05281229	5.4500286		-5.31288	0.054656
chr1_577_mature	0	20.95411	0	4.456419386		-20	0.05484
chr4_10283_mature	0	20.95411	0	4.456419386		-20	0.05484
NW_003537639_37085_mature	0	20.95411	0	4.456419386		-20	0.05484
chr8_18998_mature	0	20.95411	0	4.456419386		-20	0.05484
mmu-miR-6413	434.1262525	16.11855	8.765290251	4.097488514		4.751321	0.054981
ssc-miR-34c	32246.76908	416.6645	14.97691174	8.706200684		6.274125	0.055174
hsa-miR-3607-5p	125.724682	5.641492	6.985553734	2.731507408		4.478047	0.055191
hsa-miR-4787-5p	602.8337318	20.95411	9.238007542	4.456419386		4.846455	0.055326
chr3_8405_mature	2.149139864	67.69791	1.654957834	6.102194213		-4.97728	0.05543
chr15_32567_star@@rno-miR-375-5	70.9216155	3.22371	6.168353522	2.078510713		4.459432	0.055692
rno-let-7b-3p	54.80306652	2.417782	5.802272499	1.773060525		4.5025	0.055749
chr1_771_mature	3.223709795	95.90537	2.078510713	6.598504656		-4.89482	0.055946
mmu-miR-695	1.074569932	41.90823	1.05281229	5.423182395		-5.2854	0.056027
chr13_29225_mature	1.074569932	41.90823	1.05281229	5.423182395		-5.2854	0.056027
chr9_20972_star@@rno-miR-488-5p	39.75908748	1.611855	5.349049847	1.38507475		4.624491	0.056032
ssc-miR-138	4221.985262	92.68166	12.0440475	6.549694683		5.509494	0.056309
chr15_33603_star	13.96940911	0	3.90394537	0		20	0.056811
chr18_36468_star	13.96940911	0	3.90394537	0		20	0.056811
hsa-miR-1284	13.96940911	0	3.90394537	0		20	0.056811
chr7_16788_star	13.96940911	0	3.90394537	0		20	0.056811
chr8_17337_mature	13.96940911	0	3.90394537	0		20	0.056811
mmu-miR-344d-3-5p	13.96940911	0	3.90394537	0		20	0.056811
chr6_15065_mature	13.96940911	0	3.90394537	0		20	0.056811
chr8_18502_mature	13.96940911	0	3.90394537	0		20	0.056811
rno-miR-743b-3p	13.96940911	0	3.90394537	0		20	0.056811
hsa-miR-4645-5p	13.96940911	0	3.90394537	0		20	0.056811
chr14_30479_mature	13.96940911	0	3.90394537	0		20	0.056811
rno-miR-501-5p	13.96940911	0	3.90394537	0		20	0.056811
chr16_33778_mature	13.96940911	0	3.90394537	0		20	0.056811
chrx_22063_mature	13.96940911	0	3.90394537	0		20	0.056811
mmu-miR-709	13.96940911	0	3.90394537	0		20	0.056811
mmu-miR-714	13.96940911	0	3.90394537	0		20	0.056811
mmu-miR-466h-5p	13.96940911	0	3.90394537	0		20	0.056811
hsa-miR-1908	13.96940911	0	3.90394537	0		20	0.056811
chr13_28222_mature	69.84704557	3.22371	6.146635787	2.078510713		4.437405	0.057105
chr9_20601_mature	2.149139864	66.08605	1.654957834	6.067940913		-4.94251	0.057217
chr11_24821_mature	25.78967836	0.805927	4.743605355	0.852739935		5	0.057398
chr2_5976_star	25.78967836	0.805927	4.743605355	0.852739935		5	0.057398
chr15_32065_star	25.78967836	0.805927	4.743605355	0.852739935		5	0.057398
chr12_26515_mature	25.78967836	0.805927	4.743605355	0.852739935		5	0.057398
chr6_12737_mature	25.78967836	0.805927	4.743605355	0.852739935		5	0.057398
chr5_11434_star	1.074569932	41.1023	1.05281229	5.395827139		-5.25739	0.057454
chr6_12549_mature	1.074569932	41.1023	1.05281229	5.395827139		-5.25739	0.057454
chr4_9787_mature	1.074569932	41.1023	1.05281229	5.395827139		-5.25739	0.057454
chr17_34938_mature	1.074569932	41.1023	1.05281229	5.395827139		-5.25739	0.057454
mmu-miR-669m-5p	53.72849659	2.417782	5.774220321	1.773060525		4.473931	0.057566
chr3_7233_mature	0	20.14819	0	4.402462031		-20	0.057636
chr14_30104_mature	0	20.14819	0	4.402462031		-20	0.057636
chr15_32699_mature	0	20.14819	0	4.402462031		-20	0.057636
chr13_28917_mature	0	20.14819	0	4.402462031		-20	0.057636
ssc-miR-490-3p	0	20.14819	0	4.402462031		-20	0.057636
chr9_20608_mature	2.149139864	65.28012	1.654957834	6.050504383		-4.92481	0.058146
chr2_4893_mature	471.7362001	17.7304	8.884891534	4.227310103		4.733683	0.05866
ssc-miR-15b	12600.40702	212.7648	13.62129721	7.739880812		5.888067	0.05881
chr13_29259_star	1.074569932	40.29637	1.05281229	5.367943153		-5.22882	0.058941
NW_003541078_39887_mature	1317.422736	39.49044	10.36459731	5.339509593		5.060071	0.059546
chr4_8769_star	466.3633504	17.7304	8.868400795	4.227310103		4.717157	0.059851
chr2_4381_mature	1.074569932	39.49044	1.05281229	5.339509593		-5.19967	0.060492
chr8_18294_mature	1.074569932	39.49044	1.05281229	5.339509593		-5.19967	0.060492
chr1_3640_mature	0	19.34226	0	4.346407978		-20	0.06067
chr1_273_mature	0	19.34226	0	4.346407978		-20	0.06067
chr13_28840_star	0	19.34226	0	4.346407978		-20	0.06067
hsa-miR-4647	0	19.34226	0	4.346407978		-20	0.06067
chr15_31685_mature	2.149139864	62.86234	1.654957834	5.996893534		-4.87036	0.061083
chr9_20088_mature	6.447419591	186.1692	2.896740642	7.548199552		-4.85175	0.061239
chr4_8669_mature	24.71510843	0.805927	4.684544332	0.852739935		4.938599	0.061302
mmu-miR-881-3p	24.71510843	0.805927	4.684544332	0.852739935		4.938599	0.061302
NW_003537647_37094_star	196.6462975	8.865202	7.626777119	3.302348583		4.471306	0.061489
ssc-miR-301	1258.32139	38.68452	10.2984308	5.310504361		5.0236	0.06159
chr8_17244_mature	82.74188475	4.029637	6.387877484	2.330454351		4.359896	0.061691
chr3_8165_star	82.74188475	4.029637	6.387877484	2.330454351		4.359896	0.061691
chr8_17231_mature	1.074569932	38.68452	1.05281229	5.310504361		-5.16993	0.062108
chr9_20857_mature	2.149139864	62.05641	1.654957834	5.978571212		-4.85175	0.062116
chr1_1729_mature	13.96940911	477.915	3.90394537	8.903625744		-5.09641	0.06229
mmu-miR-3096a-5p	306.2524306	12.89484	8.263280613	3.796477232		4.569856	0.062328
chr14_31584_mature	98.86043373	4.835565	6.641841268	2.544872269		4.353637	0.062631
rno-miR-92a-3p	153.6635003	7.253347	7.272988959	3.044979305		4.404984	0.062998
chr7_16002_mature	2.149139864	61.25049	1.654957834	5.960013198		-4.83289	0.063176
chr1_1256_mature	2.149139864	61.25049	1.654957834	5.960013198		-4.83289	0.063176
chr16_34498_star	1.074569932	37.87859	1.05281229	5.280903997		-5.13955	0.063796
mmu-miR-6406	1.074569932	37.87859	1.05281229	5.280903997		-5.13955	0.063796
chr5_11550_star	1.074569932	37.87859	1.05281229	5.280903997		-5.13955	0.063796
chr1_1174_mature	0	18.53633	0	4.288087668		-20	0.063971
chrx_21532_mature	0	18.53633	0	4.288087668		-20	0.063971
bta-miR-658	0	18.53633	0	4.288087668		-20	0.063971
hsa-miR-548w	0	18.53633	0	4.288087668		-20	0.063971
rno-miR-337-3p	12.89483918	0	3.796477232	0		20	0.064002
chr2_4263_star	12.89483918	0	3.796477232	0		20	0.064002
mmu-miR-3081-3p	12.89483918	0	3.796477232	0		20	0.064002
chr17_35808_star	12.89483918	0	3.796477232	0		20	0.064002
chr5_11364_star	36.53537768	1.611855	5.230179094	1.38507475		4.5025	0.064028
chr9_20110_mature	36.53537768	1.611855	5.230179094	1.38507475		4.5025	0.064028
chr5_11575_mature	2.149139864	60.44456	1.654957834	5.941213351		-4.81378	0.064267
bta-miR-147	1577.46866	46.74379	10.6243099	5.577241251		5.076693	0.064276
chr2_6521_mature	80.59274489	4.029637	6.35036897	2.330454351		4.321928	0.064388
chr5_11374_mature	80.59274489	4.029637	6.35036897	2.330454351		4.321928	0.064388
chr7_15098_mature	664.0842179	24.17782	9.377393227	4.654081667		4.77961	0.064519
chr5_11762_mature	210.6157066	9.671129	7.725302902	3.415640968		4.444785	0.065158
chr14_31492_mature	4.298279727	113.6358	2.405524014	6.840913474		-4.72451	0.065277
chr14_30496_mature	2.149139864	59.63863	1.654957834	5.922165283		-4.79442	0.065387
hsa-miR-4474-5p	2.149139864	59.63863	1.654957834	5.922165283		-4.79442	0.065387
chr3_8310_mature	1.074569932	37.07266	1.05281229	5.250683565		-5.10852	0.065558
chr13_28825_mature	1.074569932	37.07266	1.05281229	5.250683565		-5.10852	0.065558
chrx_22122_star	1.074569932	37.07266	1.05281229	5.250683565		-5.10852	0.065558
chr13_27664_star	23.6405385	0.805927	4.62296188	0.852739935		4.874469	0.0656
chr14_30038_star	23.6405385	0.805927	4.62296188	0.852739935		4.874469	0.0656
chr11_25099_mature	6.447419591	176.4981	2.896740642	7.471659863		-4.77479	0.065769
chr7_17190_star	95.63672393	4.835565	6.594499642	2.544872269		4.305808	0.066118
chr3_7552_mature	4.298279727	112.0239	2.405524014	6.820484263		-4.7039	0.066512
chr2_5437_mature	12006.16985	215.9886	13.55160852	7.761475149		5.796677	0.066841
chr1_246_star	148.2906506	7.253347	7.221980009	3.044979305		4.353637	0.066843
ssc-miR-3613	207.3919968	9.671129	7.703156063	3.415640968		4.422532	0.066872
chr1_861_mature	1.074569932	36.26674	1.05281229	5.21981653		-5.07682	0.0674
chr1_2018_mature	12.89483918	406.1874	3.796477232	8.66954923		-4.97728	0.067475
NW_003541078_39885_mature	1261.5451	40.29637	10.30211921	5.367943153		4.968398	0.067493
bta-miR-7	0	17.7304	0	4.227310103		-20	0.067571
hsa-miR-1276	0	17.7304	0	4.227310103		-20	0.067571
chrx_21531_mature	0	17.7304	0	4.227310103		-20	0.067571
chr3_7220_star	0	17.7304	0	4.227310103		-20	0.067571
chr6_12645_mature	0	17.7304	0	4.227310103		-20	0.067571
chr9_19233_mature	0	17.7304	0	4.227310103		-20	0.067571
rno-miR-370-5p	0	17.7304	0	4.227310103		-20	0.067571
chr3_7237_star	0	17.7304	0	4.227310103		-20	0.067571
chr5_11626_star	0	17.7304	0	4.227310103		-20	0.067571
chr17_34997_mature	0	17.7304	0	4.227310103		-20	0.067571
chr1_1781_mature	0	17.7304	0	4.227310103		-20	0.067571
chr7_16556_mature	3.223709795	82.2046	2.078510713	6.378591382		-4.67243	0.068496
chr12_26006_mature@@mmu-miR-7	335.2658187	14.50669	8.393458327	3.954819241		4.530515	0.068519
chr15_32589_mature	725.334704	26.59561	9.504490703	4.786366653		4.769387	0.068617
NW_003539948_39194_mature	430.9025427	17.7304	8.754562	4.227310103		4.603064	0.068661
chr13_26768_star	2.149139864	57.22085	1.654957834	5.863463969		-4.73471	0.068946

chr14_31521_mature	161.18549	18.53633	7.341500942	4.288087668	4.288087668	3.120294	5.96E-07
chr8_18502_mature	90.2638743	0	6.511971994	0	0	20	6.25E-07
chr9_20605_star	337.414959	99.12908	8.40264953	6.645717165	6.645717165	1.767144	6.82E-07
chr4_10247_mature	313.77442	87.04016	8.298174496	6.460089935	6.460089935	1.849975	8.69E-07
chr6_14444_mature	2.14913986	147.4847	1.654957834	7.214170697	7.214170697	-6.10066	1.08E-06
ssc-miR-191	4375.64876	5628.597	12.09561089	12.45881601	12.45881601	-0.36328	1.08E-06
ssc-miR-1306-3p	515.793567	1001.768	9.013444302	9.969771887	9.969771887	-0.95768	1.57E-06
chr4_10445_mature	141.843231	12.89484	7.158288862	3.796477232	3.796477232	3.459432	1.79E-06
chr18_36328_mature	229.957965	47.54972	7.851486493	5.601391055	5.601391055	2.273861	2.47E-06
chr16_34309_mature	105.307853	3.22371	6.732104367	2.078510713	2.078510713	5.029747	2.6E-06
chr12_25570_star	195.571728	33.04303	7.618912027	5.08928735	5.08928735	2.56528	3.35E-06
chr2_6291_star	92.4130141	0.805927	6.545551653	0.852739935	0.852739935	6.841302	3.51E-06
chr13_29223_mature	66.6233358	302.2228	6.079449279	8.244234395	8.244234395	-2.18151	3.76E-06
chr8_18246_mature	96.7112939	1.611855	6.610453419	1.38507475	1.38507475	5.906891	3.88E-06
chr6_14250_star	96.7112939	1.611855	6.610453419	1.38507475	1.38507475	5.906891	3.88E-06
chr14_30369_mature	1033.73627	564.1492	10.0150474	9.142488017	9.142488017	0.873719	4.88E-06
chr2_6521_mature	107.456993	4.029637	6.760979269	2.330454351	2.330454351	4.736966	5.72E-06
chr17_35617_mature	113.904413	5.641492	6.844290394	2.731507408	2.731507408	4.335603	6.41E-06
hsa-miR-4634	113.904413	5.641492	6.844290394	2.731507408	2.731507408	4.335603	6.41E-06
chr12_25546_mature	2.14913986	142.6492	1.654957834	7.166405731	7.166405731	-6.05257	6.67E-06
mmu-miR-6240	1240.0537	719.6932	10.27734983	9.493241447	9.493241447	0.784949	7.42E-06
chr15_31797_mature	85.9655945	0	6.442372848	0	0	20	7.75E-06
chr2_5678_mature	909.086162	475.4972	9.829859329	8.896323911	8.896323911	0.93498	7.77E-06
chr11_25149_mature	10.7456993	170.0507	3.554060706	7.418280128	7.418280128	-3.98413	1.05E-05
chr9_20220_star	109.606133	4.835565	6.789287574	2.544872269	2.544872269	4.5025	1.12E-05
chr2_6095_mature	98.8604337	2.417782	6.641841268	1.773060525	1.773060525	5.353637	1.14E-05
chr9_20088_mature	17.1931189	186.1692	4.185320985	7.548199552	7.548199552	-3.43671	2.12E-05
chr13_27783_mature	2.14913986	139.4254	1.654957834	7.133660602	7.133660602	-6.01959	2.24E-05
chr11_25373_mature	83.8164547	0	6.406272275	0	0	20	2.72E-05
bta-miR-2285t	6.44741959	153.9321	2.896740642	7.275492671	7.275492671	-4.57743	2.83E-05
chr6_14437_mature	2.14913986	138.6195	1.654957834	7.125356859	7.125356859	-6.01123	3.04E-05
chr8_19026_mature	2.14913986	137.8136	1.654957834	7.117005045	7.117005045	-6.00282	4.11E-05
chr14_31044_mature	2.14913986	137.8136	1.654957834	7.117005045	7.117005045	-6.00282	4.11E-05
chr6_14250_mature	88.1147344	0.805927	6.477592084	0.852739935	0.852739935	6.77259	4.17E-05
hsa-miR-3615	135.395811	12.89484	7.091655531	3.796477232	3.796477232	3.392317	4.27E-05
chr9_20255_mature	92.4130141	1.611855	6.545551653	1.38507475	1.38507475	5.841302	4.43E-05
chr12_25449_mature	124.650112	9.671129	6.973268148	3.415640968	3.415640968	3.688056	4.94E-05
ssc-miR-664-5p	255.747644	63.66827	8.004207226	6.014986077	6.014986077	2.006075	5.89E-05
chr4_9318_mature	238.554525	55.60899	7.904210254	5.82295938	5.82295938	2.100929	7.3E-05
chr2_5750_star	146.141511	16.92448	7.201060499	4.163859073	4.163859073	3.110183	8.02E-05
chr6_13566_star	81.6673148	0	6.369245121	0	0	20	9.57E-05
chr7_15749_mature	152.58893	19.34226	7.26293043	4.346407978	4.346407978	2.979822	9.57E-05
NW_003540770_39680_mature	206.317427	41.1023	7.695697583	5.395827139	5.395827139	2.327575	9.73E-05
chr2_5556_mature	113.904413	377.174	6.844290394	8.562906545	8.562906545	-1.72741	0.000124
chr1_3195_star	354.608078	117.6654	8.474144285	6.890755622	6.890755622	1.591535	0.00013
hsa-miR-4685-3p	2.14913986	134.5899	1.654957834	7.083105736	7.083105736	-5.96867	0.000138
chr3_8310_star	2.14913986	134.5899	1.654957834	7.083105736	7.083105736	-5.96867	0.000138
chr8_17989_mature@@bta-miR-302b	90.2638743	1.611855	6.511971994	1.38507475	1.38507475	5.807355	0.00015
chrx_21673_mature	2.14913986	133.784	1.654957834	7.074504971	7.074504971	-5.96	0.000187
chr13_28400_mature	206.317427	527.0766	7.695697583	9.044603272	9.044603272	-1.35315	0.000206
chr8_18403_mature	449.170232	881.6846	8.814326848	9.785754265	9.785754265	-0.973	0.00021
chr14_30429_mature	27.9388182	205.5115	4.854934103	7.690078309	7.690078309	-2.87888	0.000271
chr9_20679_mature	96.7112939	3.22371	6.610453419	2.078510713	2.078510713	4.906891	0.000298
chr7_15245_star	103.158713	4.835565	6.702639724	2.544872269	2.544872269	4.415037	0.000359
ssc-miR-30b-3p	193.422588	37.07266	7.603052029	5.250683565	5.250683565	2.383329	0.000436
chr3_6737_mature	105.307853	5.641492	6.732104367	2.731507408	2.731507408	4.222392	0.000618
chr5_12092_mature	2.14913986	130.5602	1.654957834	7.039579809	7.039579809	-5.92481	0.000627
chr2_5113_mature	15.043979	170.0507	4.003960082	7.418280128	7.418280128	-3.49871	0.000753
chr5_12341_mature	21.4913986	186.1692	4.491301473	7.548199552	7.548199552	-3.11478	0.000921
mmu-miR-532-5p	77.3690351	0	6.292211829	0	0	20	0.001174
bta-miR-454	171.931189	29.01339	7.43405428	4.907534286	4.907534286	2.567041	0.001197
ssc-miR-664-3p	133.246672	14.50669	7.068742509	3.954819241	3.954819241	3.199309	0.00128
chr3_7692_mature	96.7112939	4.029637	6.610453419	2.330454351	2.330454351	4.584963	0.001958
chr17_35005_mature	2.14913986	127.3365	1.654957834	7.003788148	7.003788148	-5.88874	0.002095
bta-miR-1248	124.650112	12.08891	6.973268148	3.710273245	3.710273245	3.366128	0.002387
chr17_35401_mature	92.4130141	3.22371	6.545551653	2.078510713	2.078510713	4.841302	0.003129
bta-miR-2424	167.632909	28.20746	7.397742301	4.868265031	4.868265031	2.571157	0.003221
hsa-miR-3659	0	13122.92	0	13.67991072	13.67991072	-20	0.003243
chr7_16829_mature	367.502917	131.3662	8.525532228	7.048390682	7.048390682	1.484162	0.003262
chr12_25999_mature	4.29827973	133.784	2.405524014	7.074504971	7.074504971	-4.96	0.003302
ssc-miR-206	0	11796.36	0	13.52617644	13.52617644	-20	0.003413
chr15_32517_mature	2.14913986	125.7247	1.654957834	6.985553734	6.985553734	-5.87036	0.003823
chr6_12502_mature	75.2198952	0	6.25209572	0	0	20	0.004095
NW_003541079_39891_mature	0	7881.165	0	12.94437615	12.94437615	-20	0.004142
chr14_30864_mature	88.1147344	2.417782	6.477592084	1.773060525	1.773060525	5.187627	0.004511
chr1_3720_mature	0	5863.928	0	12.51789771	12.51789771	-20	0.004773
hsa-miR-4485	79.518175	0.805927	6.331242567	0.852739935	0.852739935	6.624491	0.005727
bta-miR-379	0	3912.778	0	11.93434612	11.93434612	-20	0.005793
chr12_25530_mature	4.29827973	132.1721	2.405524014	7.057148072	7.057148072	-4.94251	0.005962
mmu-let-7j	109.606133	8.059274	6.789287574	3.179395517	3.179395517	3.765535	0.006257
chrx_22382_mature	2.14913986	124.1128	1.654957834	6.967085898	6.967085898	-5.85175	0.006965
chr1_1317_mature	0	2365.397	0	11.20847645	11.20847645	-20	0.007368
chr1_1318_mature	0	2350.89	0	11.1996051	11.1996051	-20	0.00739
chr2_4477_star	0	2107.5	0	11.0420015	11.0420015	-20	0.007786
chr13_28441_mature	0	1987.417	0	10.95740469	10.95740469	-20	0.008007
NW_003613137_37368_mature	0	1938.256	0	10.92128719	10.92128719	-20	0.008103
chr1_485_mature	0	1921.331	0	10.90864108	10.90864108	-20	0.008137
ssc-miR-455-5p	0	1852.021	0	10.85566373	10.85566373	-20	0.008281
chrx_22553_mature	0	1729.52	0	10.75699015	10.75699015	-20	0.008556
chr6_13928_star	103.158713	6.44742	6.702639724	2.896740642	2.896740642	4	0.00917
chr16_34386_mature	0	1477.265	0	10.52968921	10.52968921	-20	0.009223
hsa-miR-3202	2.14913986	123.3069	1.654957834	6.957762565	6.957762565	-5.84235	0.009392
chr6_12951_mature	0	1104.121	0	10.10998811	10.10998811	-20	0.010593
chr6_12841_mature	96.7112939	4.835565	6.610453419	2.544872269	2.544872269	4.321928	0.011082
chr1_1155_mature	0	1000.156	0	9.967451025	9.967451025	-20	0.011103
chr1_3153_mature	0	967.1129	0	9.91903155	9.91903155	-20	0.011281
ssc-miR-145-3p	0	955.83	0	9.902118745	9.902118745	-20	0.011344
chr1_2557_mature	0	949.3825	0	9.892364514	9.892364514	-20	0.011381
chr7_16963_mature	0	930.0403	0	9.862699769	9.862699769	-20	0.011493
chr1_2360_mature	0	922.7869	0	9.851416323	9.851416323	-20	0.011536
chr11_24398_mature	0	849.4475	0	9.73207842	9.73207842	-20	0.011998
chr6_14961_mature	2.14913986	122.501	1.654957834	6.948378589	6.948378589	-5.83289	0.012651
chr5_10730_mature	141.843231	19.34226	7.158288862	4.346407978	4.346407978	2.874469	0.012795
chr6_13229_mature	0	724.5288	0	9.502889027	9.502889027	-20	0.012937
chr4_9231_mature	0	660.8605	0	9.37038338	9.37038338	-20	0.013512
chr2_6447_mature	0	642.3242	0	9.329402097	9.329402097	-20	0.013695
chr17_34881_mature	0	630.2353	0	9.302033996	9.302033996	-20	0.013819
chr1_392_mature	73.0707554	0	6.210832145	0	0	20	0.014164
ssc-miR-362	0	588.327	0	9.202924647	9.202924647	-20	0.014276
chr12_26311_mature	0	584.2974	0	9.193026061	9.193026061	-20	0.014322
NW_003539626_38995_mature	0	571.4026	0	9.160886317	9.160886317	-20	0.014474
chr2_4930_mature	0	559.3136	0	9.130090828	9.130090828	-20	0.014621
chr14_30497_mature	0	550.4484	0	9.107082211	9.107082211	-20	0.014731
chr1_3371_mature	0	529.4943	0	9.051193532	9.051193532	-20	0.015004
chr15_33221_mature	0	502.0928	0	8.974680734	8.974680734	-20	0.015384
hsa-miR-516a-5p	0	473.8853	0	8.89143541	8.89143541	-20	0.015809
chr4_10244_mature	0	394.0985	0	8.626068641	8.626068641	-20	0.017241
chr14_30282_mature	0	390.8748	0	8.614249038	8.614249038	-20	0.017308
chr12_26457_mature	0	390.8748	0	8.614249038	8.614249038	-20	0.017308
NW_003613041_36883_mature	0	384.4274	0	8.5903153	8.5903153	-20	0.017444
chr17_35590_mature	6.44741959	136.2017	2.896740642	7.100154956	7.100154956	-4.40088	0.017593
chr12_26317_mature	0	369.9207	0	8.534966969	8.534966969	-20	0.017762
chrx_22120_mature	0	365.8911	0	8.519207948	8.519207948	-20	0.017853
chr15_32593_mature	0	359.4436	0	8.493629892	8.493629892	-20	0.018003
NW_003537694_37136_mature	0	346.5488	0	8.441071766	8.441071766	-20	0.018314
chr10_24151_mature	0	346.5488	0	8.441071766	8.441071766	-20	0.018314
chr14_30223_mature	0	344.131	0	8.43100034	8.43100034	-20	0.018374
chr3_7963_mature	107.456993	8.059274	6.760979269	3.179395517	3.179395517	3.736966	0.018413
chr11_24705_mature	494.302169	214.3767	8.952165127	7.750718383	7.750718383	1.205245	0.018501
chr14_29632_star	81.6673148	1.611855	6.369245121	1.38507475	1.38507475	5.662965	0.018636
chr14_31314_mature	0	297.3872	0	8.221041977	8.221041977	-20	0.019673
chr2_4744_mature	0	294.1635	0	8.20537061	8.20537061	-20	0.019774
chrx_22488_mature	0	290.9398	0	8.189527141	8.189527141	-20	0.019876
chr2_6092_mature	0	270.7916	0	8.08635718	8.08635718	-20	0.020553
NW_003613242_37864_mature	0	266.762	0	8.064807344	8.064807344	-20	0.020697
chr3_8078_mature	0	265.9561	0	8.060458479	8.060458479	-20	0.020726
chr6_13576_mature	0	265.9561	0	8.060458479	8.060458479	-20	0.020726
chr3_7072_mature	0	261.9264	0	8.038515312	8.038515312	-20	0.020874
ssc-miR-432-5p	0	260.3146	0	8.029643734	8.029643734	-20	0.020935
chr13_27312_mature	0	257.0909	0	8.011735219	8.011735219	-20	0.021056
NW_003613242_37866_mature	0	257.0909	0	8.011735219	8.011735219	-20	0.021056
chr10_23953_mature	0	255.479	0	8.002696903	8.002696903	-20	0.021118
chr6_13411_mature	0	245.0019	0	7.942525909	7.942525909	-20	0.021534
chr8_18345_mature	0	241.7782	0	7.923495278	7.923495278	-20	0.021667
rno-miR-143-3p	0	233.719	0	7.874790575	7.874790575	-20	0.022012
chr13_27663_mature	0	226.4656	0	7.829504654	7.829504654	-20	0.022337
chr1_211_mature	0	209.5411	0	7.717958332	7.717958332	-20	0.023157
NW_003538465_37902_mature	0	205.5115	0	7.690078309	7.690078309	-20	0.023366
NW_003537589_37025_mature	0	196.6463	0	7.626777119	7.626777119	-20	0.023848
chr4_9116_mature	0	195.8404	0	7.620882324	7.620882324	-20	0.023894
chrx_21570_mature	0	193.4226	0	7.603052029	7.603052029	-20	0.024031
chr17_35122_mature	0	178.9159	0	7.491178829	7.491178829	-20	0.024913
chr9_20036_mature	0	178.11	0	7.484701805	7.484701805	-20	0.024965
chr9_20772_mature	0	170.0507	0	7.418280128	7.418280128	-20	0.025504
chr9_20753_mature	0	170.0507	0	7.418280128	7.418280128	-20	0.025504
chr5_11533_mature	0	165.2151	0	7.376907876	7.376907876	-20	0.025846
chr13_29304_mature	0	164.4092	0	7.369895665	7.369895665	-20	0.025904
ssc-miR-382	0	161.9914	0	7.348652187	7.348652187	-20	0.026081
chrx_21569_mature	0	159.5736	0	7.327091221	7.327091221	-20	0.026262
chr9_19756_mature	0	157.9618	0	7.312536122	7.312536122	-20	0.026385
ssc-miR-10a-3p	0	157.1559	0	7.305203133	7.305203133	-20	0.026448
chr9_20754_mature	0	145.8729	0	7.198424102	7.198424102	-20	0.027368
chr1_3435_mature	0	138.6195	0	7.125356859	7.125356859	-20	0.028016
chr15_32293_mature	0	137.0077	0	7.108604601	7.108604601	-20	0.028166
chr14_31042_mature	0	135.3958	0	7.091655531	7.091655531	-20	0.028319
chr14_30531_mature	0	133.784	0	7.074504971	7.074504971	-20	0.028475
chr14_29507_mature	0	132.978	0	7.065852624	7.065852624	-20	0.028554
chr1_412_mature	0	130.5602	0	7.039579809	7.039579809	-20	0.028794
chr9_19755_mature	0	128.1425	0	7.012819653	7.012819653	-20	0.029041
chr13_27508_mature	0	124.9188	0	6.976349367	6.976349367	-20	0.029381
chr17_35522_mature	0	123.3069	0	6.957762565	6.957762565	-20	0.029556
chr6_13600_star	0	123.3069	0	6.957762565	6.957762565	-20	0.029556
chr13_28070_mature	0	112.0239	0	6.820484263	6.820484263	-20	0.030875
chr1_1734_mature	0	111.218	0	6.810160141	6.810160141	-20	0.030977
NW_003537990_37435_mature	0	108.8002	0	6.778736946	6.778736946	-20	0.031288
chr4_9930_mature	0	108.8002	0	6.778736946	6.778736946	-20	0.031288
mmu-miR-5124b	0	107.9943	0	6.76810859	6.76810859	-20	0.031393
chr16_34510_star	0	107.9943	0	6.76810859	6.76810859	-20	0.031393
hsa-miR-184	0	104.7706	0	6.72479443	6.72479443	-20	0.031828
mmu-miR-5108	0	104.7706	0	6.72479443	6.72479443	-20	0.031828
NW_003613107_37244_mature	0	103.9646	0	6.713759604	6.713759604	-20	0.031939
chr18_36061_mature	0	103.1587	0	6.702639724	6.702639724	-20	0.032052
NW_003613497_39428_mature	0	101.5469	0	6.680139486	6.680139486	-20	0.032281
bta-miR-483	0	100.7409	0	6.668756392	6.668756392	-20	0.032398
chr4_9932_mature	0	99.935	0	6.657282769	6.657282769	-20	0.032516
NW_003538101_37534_mature	0	95.90537	0	6.598504656	6.598504656	-20	0.033126
chr4_10006_mature	0	93.48758	0	6.562052863	6.562052863	-20	0.033509
NW_003613447_39176_mature	0	91.87573	0	6.537229727	6.537229727	-20	0.033773
chr6_13666_mature	0	91.87573	0	6.537229727	6.537229727	-20	0.033773
chr2_5590_mature	0	91.87573	0	6.537229727	6.537229727	-20	0.033773
chr12_25625_star	0	91.87573	0	6.537229727	6.537229727	-20	0.033773
chr10_24012_mature	0	91.0698	0	6.524656134	6.524656134	-20	0.033907
chr3_8428_mature	0	91.0698	0	6.524656134	6.524656134	-20	0.033907
chrx_22188_mature@@bta-miR-1298	0	91.0698	0	6.524656134	6.524656134	-20	0.033907
chr7_16961_mature	0	90.26387	0	6.511971994	6.511971994	-20	0.034043
chr2_5583_mature	0	90.26387	0	6.511971994	6.511971994	-20	0.034043
chr9_20694_mature	0	89.45795	0	6.499175346	6.499175346	-20	0.034181
chr12_25534_star	0	89.45795	0	6.499175346	6.499175346	-20	0.034181
chr8_17373_mature	0	89.45795	0	6.499175346	6.499175346	-20	0.034181
NW_003613056_36922_mature	0	86.23424	0	6.446822559	6.446822559	-20	0.034749
chr5_11528_mature	0	83.81645	0	6.406272275	6.406272275	-20	0.035195
chr1_478_mature	0	83.81645	0	6.406272275	6.406272275	-20	0.035195
chr7_16556_mature	0	82.2046	0	6.378591382	6.378591382	-20	0.035503
chr7_16555_mature	0	78.98089	0	6.32158343	6.32158343	-20	0.036144
chrx_22416_mature	0	78.98089	0	6.32158343	6.32158343	-20	0.036144
chr12_25822_mature	0	78.17496	0	6.306972375	6.306972375	-20	0.03631
chr1_3708_mature	0	78.17496	0	6.306972375	6.306972375	-20	0.03631
chr4_9333_star	0	77.36904	0	6.292211829	6.292211829	-20	0.036479
chr14_31500_mature	0	77.36904	0	6.292211829	6.292211829	-20	0.036479
chr5_12108_mature	0	77.36904	0	6.292211829	6.292211829	-20	0.036479
chr9_20604_mature	0	76.56311	0	6.277298702	6.277298702	-20	0.036649
ssc-miR-542-5p	0	74.95125	0	6.247001856	6.247001856	-20	0.036999
chr5_12375_mature	0	74.95125	0	6.247001856	6.247001856	-20	0.036999
chr13_28489_mature	0	74.14533	0	6.231611453	6.231611453	-20	0.037177
chr7_15776_mature	0	70.92162	0	6.168353522	6.168353522	-20	0.03792
chr14_31044_star	0	70.11569	0	6.152095947	6.152095947	-20	0.038113
chr18_36239_mature	0	69.30976	0	6.135653077	6.135653077	-20	0.038309
chr14_31042_star	0	68.50383	0	6.11902064	6.11902064	-20	0.038508
chr1_3507_mature	0	66.89198	0	6.085169218	6.085169218	-20	0.038916
chr13_27097_mature	0	65.28012	0	6.050504383	6.050504383	-20	0.039338
chrx_22902_star	0	64.4742	0	6.032854532	6.032854532	-20	0.039555
chr7_16781_star	0	62.05641	0	5.978571212	5.978571212	-20	0.040227
chr9_20857_mature	0	62.05641	0	5.978571212	5.978571212	-20	0.040227
chr13_28716_star	0	62.05641	0	5.978571212	5.978571212	-20	0.040227
NW_003540828_39718_mature	0	62.05641	0	5.978571212	5.978571212	-20	0.040227
chrx_22499_mature	0	60.44456	0	5.941213351	5.941213351	-20	0.040696
chr1_3508_mature	0	60.44456	0	5.941213351	5.941213351	-20	0.040696
chr6_15013_mature	0	59.63863	0	5.922165283	5.922165283	-20	0.040937
hsa-miR-4474-5p	0	59.63863	0	5.922165283	5.922165283	-20	0.040937
NW_003539103_38526_mature	0	58.8327	0	5.902862353	5.902862353	-20	0.041182
chr6_15014_mature	0	58.8327	0	5.902862353	5.902862353	-20	0.041182
chr4_9258_mature	0	58.8327	0	5.902862353	5.902862353	-20	0.041182
chrx_22845_star	0	58.02678	0	5.883297648	5.883297648	-20	0.041432
chr3_8352_mature	0	58.02678	0	5.883297648	5.883297648	-20	0.041432
chr2_4023_mature	0	57.22085	0	5.863463969	5.863463969	-20	0.041687
chr10_23626_mature	0	57.22085	0	5.863463969	5.863463969	-20	0.041687
chr13_26768_star	0	57.22085	0	5.863463969	5.863463969	-20	0.041687
chr1_1283_mature	0	56.41492	0	5.843353819	5.843353819	-20	0.041946
hsa-miR-1298	0	54.80307	0	5.802272499	5.802272499	-20	0.042481
chr6_13320_mature	0	53.19121	0	5.759986999	5.759986999	-20	0.043038
chr11_24466_mature	0	53.19121	0	5.759986999	5.759986999	-20	0.043038
chr1_2110_mature	0	52.38528	0	5.738370208	5.738370208	-20	0.043325
chr1_1290_mature	0	52.38528	0	5.738370208	5.738370208	-20	0.043325
hsa-miR-5707	0	52.38528	0	5.738370208	5.738370208	-20	0.043325
chrx_22864_mature	0	49.9675	0	5.671505736	5.671505736	-20	0.044224
chrx_21663_mature	0	49.9675	0	5.671505736	5.671505736	-20	0.044224
chrx_23205_mature	0	49.16157	0	5.648510724	5.648510724	-20	0.044537
chr2_5388_mature	0	48.35565	0	5.625143252	5.625143252	-20	0.044857
chr2_5761_mature	0	47.54972	0	5.601391055	5.601391055	-20	0.045184
NW_003613103_37208_mature	0	47.54972	0	5.601391055	5.601391055	-20	0.045184
NW_003539097_38524_star	0	47.54972	0	5.601391055	5.601391055	-20	0.045184
chr11_25193_mature	0	46.74379	0	5.577241251	5.577241251	-20	0.045519
ssc-miR-133a-5p	0	45.93786	0	5.552680304	5.552680304	-20	0.045861
chr13_28023_star	0	45.93786	0	5.552680304	5.552680304	-20	0.045861
chr13_28509_mature	0	45.13194	0	5.527693969	5.527693969	-20	0.046212
chr11_25311_mature	0	45.13194	0	5.527693969	5.527693969	-20	0.046212
chr9_20541_star	0	44.32601	0	5.502267253	5.502267253	-20	0.046571
chr16_34336_mature	0	43.52008	0	5.476384353	5.476384353	-20	0.046939
chr7_15130_mature	0	43.52008	0	5.476384353	5.476384353	-20	0.046939
chr14_31463_mature	0	42.71415	0	5.4500286	5.4500286	-20	0.047317
chr15_31872_mature	0	42.71415	0	5.4500286	5.4500286	-20	0.047317
chr15_31871_mature	0	42.71415	0	5.4500286	5.4500286	-20	0.047317
chr18_35994_mature	0	42.71415	0	5.4500286	5.4500286	-20	0.047317
chr13_28245_mature	0	42.71415	0	5.4500286	5.4500286	-20	0.047317
mmu-miR-695	0	41.90823	0	5.423182395	5.423182395	-20	0.047704

chr3_8450_mature	16.11855	282.8805	4.097488514	8.149140118		-4.1334	0.223132
chrx_22969_mature	121.4264	6.44742	6.935770911	2.896740642		4.235216	0.22491
chr16_34380_mature	11.28298	0	3.618589217	0		20	0.225502
chr14_30518_mature	11.28298	0	3.618589217	0		20	0.225502
chr1_1486_mature	11.28298	0	3.618589217	0		20	0.225502
NW_003541081_39894_mature	11.28298	0	3.618589217	0		20	0.225502
chr16_33778_star	11.28298	0	3.618589217	0		20	0.225502
chr4_8920_star	11.28298	0	3.618589217	0		20	0.225502
chr16_34458_mature	1.611855	22.56597	1.38507475	4.558633073		-3.80735	0.225999
chr14_30079_mature	1.07457	16.92448	1.05281229	4.163859073		-3.97728	0.227247
chr2_4343_mature	1.07457	16.92448	1.05281229	4.163859073		-3.97728	0.227247
ssc-miR-100	1237.367	102701.8	10.27422354	16.64811532		-6.37504	0.227256
chr2_6308_star	30.08796	1.611855	4.958283956	1.38507475		4.222392	0.227429
chr15_33667_mature	5.37285	71.72754	2.671938627	6.184429932		-3.73877	0.228833
chr15_31807_mature	4.835565	63.66827	2.544872269	6.014986077		-3.71882	0.229004
NW_003539080_38513_mature	2.686425	34.65488	1.882222339	5.156027656		-3.6893	0.229097
ssc-miR-139-3p	2.14914	28.20746	1.654957834	4.868265031		-3.71425	0.229893
chr3_8556_mature	2.14914	28.20746	1.654957834	4.868265031		-3.71425	0.229893
chr15_31782_star	2.14914	28.20746	1.654957834	4.868265031		-3.71425	0.229893
chr6_13302_mature	84.35374	4.835565	6.415382459	2.544872269		4.124696	0.230281
mmu-miR-5117-3p	41.37094	2.417782	5.405003311	1.773060525		4.096862	0.230452
chr6_12549_mature	3.22371	41.1023	2.078510713	5.395827139		-3.67243	0.231292
chr9_20972_star@@rno-miR-488-5	29.55067	1.611855	4.933132262	1.38507475		4.196397	0.231515
chr13_28432_star	29.55067	1.611855	4.933132262	1.38507475		4.196397	0.231515
chr1_1780_mature	29.55067	1.611855	4.933132262	1.38507475		4.196397	0.231515
chr4_10385_star	19.34226	0.805927	4.346407978	0.852739935		4.584963	0.232483
chr9_20618_mature@@rno-miR-13	0	7.253347	0	3.044979305		-20	0.232728
chr16_34772_star	0	7.253347	0	3.044979305		-20	0.232728
hsa-miR-5004-5p	0	7.253347	0	3.044979305		-20	0.232728
chr6_14945_star	0	7.253347	0	3.044979305		-20	0.232728
mmu-miR-6387	0	7.253347	0	3.044979305		-20	0.232728
bta-miR-2393	0	7.253347	0	3.044979305		-20	0.232728
bta-miR-208a	0	7.253347	0	3.044979305		-20	0.232728
chr1_917_star	40.83366	2.417782	5.38659223	1.773060525		4.078003	0.233478
chr6_13355_mature	29.01339	1.611855	4.907534286	1.38507475		4.169925	0.235713
chr6_12747_star	10.7457	0	3.554060706	0		20	0.236298
chr2_4178_star	10.7457	0	3.554060706	0		20	0.236298
chr9_20720_mature	10.7457	0	3.554060706	0		20	0.236298
chr16_34699_mature	10.7457	0	3.554060706	0		20	0.236298
chr12_26332_star	10.7457	0	3.554060706	0		20	0.236298
chr8_17991_star@@hsa-miR-367-	10.7457	0	3.554060706	0		20	0.236298
chr16_33810_star	10.7457	0	3.554060706	0		20	0.236298
chr4_9149_star	10.7457	0	3.554060706	0		20	0.236298
chr11_24910_mature	10.7457	0	3.554060706	0		20	0.236298
chr1_2401_mature	10.7457	0	3.554060706	0		20	0.236298
chr13_27643_star	10.7457	0	3.554060706	0		20	0.236298
chr13_28432_mature	10.7457	0	3.554060706	0		20	0.236298
chr3_7424_star	10.7457	0	3.554060706	0		20	0.236298
chr8_17482_mature	10.7457	0	3.554060706	0		20	0.236298
bta-let-7b	5.910135	78.17496	2.788713818	6.306972375		-3.72544	0.236301
NW_003538449_37886_mature	1.611855	21.76004	1.38507475	4.508431259		-3.75489	0.236502
chr17_34998_mature	1.611855	21.76004	1.38507475	4.508431259		-3.75489	0.236502
chr15_33603_mature	1.611855	21.76004	1.38507475	4.508431259		-3.75489	0.236502
chr1_3135_mature	1.611855	21.76004	1.38507475	4.508431259		-3.75489	0.236502
chr6_14945_mature	2.14914	27.40153	1.654957834	4.827896911		-3.67243	0.23846
chr1_1669_mature	2.14914	27.40153	1.654957834	4.827896911		-3.67243	0.23846
NW_003537574_37016_mature	18.80497	0.805927	4.307790888	0.852739935		4.544321	0.238851
chr4_8825_mature	18.80497	0.805927	4.307790888	0.852739935		4.544321	0.238851
hsa-miR-4485	18.80497	0.805927	4.307790888	0.852739935		4.544321	0.238851
chr3_7552_mature	8.059274	112.0239	3.179395517	6.820484263		-3.79701	0.239527
chr12_26195_mature	39.75909	2.417782	5.349049847	1.773060525		4.039528	0.239713
chr8_18702_mature	4.29828	53.99714	2.405524014	5.781284667		-3.65105	0.240014
rno-miR-329-3p	28.4761	1.611855	4.881473904	1.38507475		4.142958	0.240028
chr2_4356_mature	28.4761	1.611855	4.881473904	1.38507475		4.142958	0.240028
chrx_23076_mature	1.07457	16.11855	1.05281229	4.097488514		-3.90689	0.241137
chr11_25154_mature	7.52199	102.3528	3.091190278	6.691433469		-3.76629	0.241682
chr13_27641_mature	0.537285	11.28298	0.620384622	3.618589217		-4.39232	0.241724
chr4_10065_star	0.537285	11.28298	0.620384622	3.618589217		-4.39232	0.241724
chr14_30277_star	0.537285	11.28298	0.620384622	3.618589217		-4.39232	0.241724
rno-miR-741-5p	0.537285	11.28298	0.620384622	3.618589217		-4.39232	0.241724
hsa-miR-3157-3p	0.537285	11.28298	0.620384622	3.618589217		-4.39232	0.241724
chr4_9980_star	0.537285	11.28298	0.620384622	3.618589217		-4.39232	0.241724
mmu-miR-705	0.537285	11.28298	0.620384622	3.618589217		-4.39232	0.241724
hsa-miR-5699	0.537285	11.28298	0.620384622	3.618589217		-4.39232	0.241724
chr16_33739_mature	0.537285	11.28298	0.620384622	3.618589217		-4.39232	0.241724
chr11_24694_star	96.17401	5.641492	6.602498583	2.731507408		4.091498	0.242255
chr3_6707_mature@@mmu-miR-59	330.4303	14.50669	8.372561492	3.954819241		4.509555	0.242296
chr15_32289_mature	27.93882	1.611855	4.854934103	1.38507475		4.115477	0.244463
chr14_30332_mature	18.26769	0.805927	4.268111626	0.852739935		4.5025	0.245487
chr13_28023_star	3.760995	45.93786	2.251263041	5.552680304		-3.6105	0.245922
chr7_15778_mature	2.14914	26.59561	1.654957834	4.786366653		-3.62936	0.247419
chr1_291_mature	2.14914	26.59561	1.654957834	4.786366653		-3.62936	0.247419
chr11_24871_mature	1.611855	20.95411	1.38507475	4.456419386		-3.70044	0.247607
chr7_15929_star	10.20841	0	3.48651029	0		20	0.247905
chr6_12901_star@@hsa-miR-1233	10.20841	0	3.48651029	0		20	0.247905
chr3_7785_star	10.20841	0	3.48651029	0		20	0.247905
chr7_16724_star	10.20841	0	3.48651029	0		20	0.247905
bta-miR-154a	10.20841	0	3.48651029	0		20	0.247905
mmu-miR-6389	10.20841	0	3.48651029	0		20	0.247905
chr4_10208_mature	10.20841	0	3.48651029	0		20	0.247905
chr13_28533_mature	10.20841	0	3.48651029	0		20	0.247905
chr14_31486_mature	10.20841	0	3.48651029	0		20	0.247905
chr1_2742_star	10.20841	0	3.48651029	0		20	0.247905
bta-miR-489	50.50479	3.22371	5.686634616	2.078510713		3.969626	0.248292
chr10_23395_mature	27.40153	1.611855	4.827896911	1.38507475		4.087463	0.249023
chr1_3752_mature	3.22371	38.68452	2.078510713	5.310504361		-3.58496	0.249969
chr16_34391_mature	6.44742	83.01053	2.896740642	6.392498216		-3.6865	0.250027
chr6_12988_star	49.9675	3.22371	5.671505736	2.078510713		3.954196	0.250895
chr6_14477_star	2.686425	32.2371	1.882222339	5.054722516		-3.58496	0.251037
chr18_35961_star	3.760995	45.13194	2.251263041	5.527693969		-3.58496	0.251525
chr4_10265_mature	183.2142	9.671129	7.525240256	3.415640968		4.243703	0.251808
chr11_25086_mature	17.7304	0.805927	4.227310103	0.852739935		4.459432	0.252406
hsa-miR-3924	17.7304	0.805927	4.227310103	0.852739935		4.459432	0.252406
chr5_12405_mature	17.7304	0.805927	4.227310103	0.852739935		4.459432	0.252406
NW_003537531_36983_mature	17.7304	0.805927	4.227310103	0.852739935		4.459432	0.252406
bta-miR-2285n	5.910135	74.14533	2.788713818	6.231611453		-3.64909	0.253612
chr9_19609_mature@@rno-miR-13	1.07457	15.31262	1.05281229	4.027916745		-3.83289	0.256073
chr17_35074_mature	48.89293	3.22371	5.640763545	2.078510713		3.922832	0.256224
ssc-miR-187	37.07266	2.417782	5.250683565	1.773060525		3.938599	0.256446
chr16_34498_star	3.22371	37.87859	2.078510713	5.280903997		-3.55459	0.256597
chr1_384_mature	3.22371	37.87859	2.078510713	5.280903997		-3.55459	0.256597
chr2_5658_mature	3.22371	37.87859	2.078510713	5.280903997		-3.55459	0.256597
rno-miR-664-2-5p	2.14914	25.78968	1.654957834	4.743605355		-3.58496	0.256787
hsa-miR-649	2.14914	25.78968	1.654957834	4.743605355		-3.58496	0.256787
chr8_17373_mature	6.984705	89.45795	2.997239027	6.499175346		-3.67894	0.256931
chr15_31808_mature	5.910135	73.3394	2.788713818	6.216055097		-3.63333	0.257257
hsa-miR-591	3.760995	44.32601	2.251263041	5.502267253		-3.55897	0.257284
chr2_5207_mature	140.7687	8.059274	7.14739484	3.179395517		4.126532	0.25824
chr18_35894_mature	26.32696	1.611855	4.772253246	1.38507475		4.029747	0.258536
chr17_35289_mature	26.32696	1.611855	4.772253246	1.38507475		4.029747	0.258536
chr5_11550_mature	26.32696	1.611855	4.772253246	1.38507475		4.029747	0.258536
chr5_12388_mature	1.611855	20.14819	1.38507475	4.402462031		-3.64386	0.259346
chr5_11928_mature	17.19312	0.805927	4.185320985	0.852739935		4.415037	0.259623
chr15_32446_mature	17.19312	0.805927	4.185320985	0.852739935		4.415037	0.259623
chr7_15380_mature	4.29828	50.77343	2.405524014	5.694139975		-3.56224	0.259764
chr12_26534_star	104.7706	3207.591	6.72479443	11.6477243		-4.93618	0.26019
chr16_34452_mature	105.3079	6.44742	6.732104367	2.896740642		4.029747	0.26036
chr15_33139_star	9.671129	0	3.415640968	0		20	0.260402
hsa-miR-4752	9.671129	0	3.415640968	0		20	0.260402
chr6_13582_star	9.671129	0	3.415640968	0		20	0.260402
chr6_13983_mature	9.671129	0	3.415640968	0		20	0.260402
chr3_8026_mature	9.671129	0	3.415640968	0		20	0.260402
chr2_5367_mature	9.671129	0	3.415640968	0		20	0.260402
bta-miR-20b	9.671129	0	3.415640968	0		20	0.260402
chr5_11609_mature	9.671129	0	3.415640968	0		20	0.260402
chr1_2345_mature	9.671129	0	3.415640968	0		20	0.260402
chr2_5756_star	9.671129	0	3.415640968	0		20	0.260402
chr3_7756_star	9.671129	0	3.415640968	0		20	0.260402
chr16_34606_mature	9.671129	0	3.415640968	0		20	0.260402
NW_003539920_39188_mature	47.81836	3.22371	5.609351983	2.078510713		3.890771	0.261726
hsa-miR-1243	0.537285	10.47706	0.620384622	3.520680821		-4.2854	0.262955
chr4_10614_mature	0.537285	10.47706	0.620384622	3.520680821		-4.2854	0.262955
rno-miR-206-3p	0.537285	10.47706	0.620384622	3.520680821		-4.2854	0.262955
chr13_28748_mature	25.78968	1.611855	4.743605355	1.38507475		4	0.263498
hsa-miR-1258	0	6.44742	0	2.896740642		-20	0.26572
hsa-miR-216a-3p	0	6.44742	0	2.896740642		-20	0.26572
chr1_480_mature	0	6.44742	0	2.896740642		-20	0.26572
hsa-miR-224-3p	0	6.44742	0	2.896740642		-20	0.26572
hsa-miR-520c-5p	0	6.44742	0	2.896740642		-20	0.26572
chr1_3435_star	0	6.44742	0	2.896740642		-20	0.26572
mmu-miR-222-3p	2.14914	24.98375	1.654957834	4.699537803		-3.53916	0.266581
chr13_27663_star	16.65583	0.805927	4.142073061	0.852739935		4.369234	0.267154
NW_003540392_39464_mature	16.65583	0.805927	4.142073061	0.852739935		4.369234	0.267154
chr4_9980_mature	16.65583	0.805927	4.142073061	0.852739935		4.369234	0.267154
chr11_25386_mature	59.10135	4.029637	5.909325402	2.330454351		3.874469	0.267665
chrx_23005_mature	3.760995	42.71415	2.251263041	5.4500286		-3.50553	0.269279
chr2_4675_mature	46.20651	3.22371	5.560913833	2.078510713		3.841302	0.270314
chr9_19519_mature	6.44742	78.17496	2.896740642	6.306972375		-3.59991	0.270358
chr15_32917_mature	189.6616	10.47706	7.574870445	3.520680821		4.178122	0.2711
chr14_31221_mature	34.92352	2.417782	5.166856928	1.773060525		3.852443	0.271126
mmu-miR-344e-5p	1.611855	19.34226	1.38507475	4.346407978		-3.58496	0.271755
chr1_3640_mature	1.611855	19.34226	1.38507475	4.346407978		-3.58496	0.271755
chr9_19866_mature	1.611855	19.34226	1.38507475	4.346407978		-3.58496	0.271755
chr16_34498_mature	1.611855	19.34226	1.38507475	4.346407978		-3.58496	0.271755
chr16_34566_mature	1.611855	19.34226	1.38507475	4.346407978		-3.58496	0.271755
chr1_1173_mature	1.07457	14.50669	1.05281229	3.954819241		-3.75489	0.272132
chr9_19208_mature	1.07457	14.50669	1.05281229	3.954819241		-3.75489	0.272132
chr13_27644_mature	1.07457	14.50669	1.05281229	3.954819241		-3.75489	0.272132
chr13_27078_mature	24.71511	1.611855	4.684544332	1.38507475		3.938599	0.273857
chr14_30379_mature	24.71511	1.611855	4.684544332	1.38507475		3.938599	0.273857
chr14_30481_mature	24.71511	1.611855	4.684544332	1.38507475		3.938599	0.273857
chr13_27066_star	9.133844	0	3.34110968	0		20	0.273878
chr14_29727_star	9.133844	0	3.34110968	0		20	0.273878
chr10_23618_star	9.133844	0	3.34110968	0		20	0.273878
NW_003613280_38221_mature	9.133844	0	3.34110968	0		20	0.273878
chr1_1422_star	9.133844	0	3.34110968	0		20	0.273878
chr8_18418_mature	9.133844	0	3.34110968	0		20	0.273878
chr6_15010_mature	9.133844	0	3.34110968	0		20	0.273878
chr9_20919_star	9.133844	0	3.34110968	0		20	0.273878
chr8_18481_mature	9.133844	0	3.34110968	0		20	0.273878
chr15_33696_star	9.133844	0	3.34110968	0		20	0.273878
chr2_5371_star	9.133844	0	3.34110968	0		20	0.273878
chr18_36022_mature	9.133844	0	3.34110968	0		20	0.273878
chr9_19911_mature	9.133844	0	3.34110968	0		20	0.273878
chr2_5533_mature	9.133844	0	3.34110968	0		20	0.273878
chr4_10638_mature	9.133844	0	3.34110968	0		20	0.273878
hsa-miR-4473	9.133844	0	3.34110968	0		20	0.273878
chr9_21068_mature	9.133844	0	3.34110968	0		20	0.273878
chr11_24876_star	9.133844	0	3.34110968	0		20	0.273878
chr11_25207_mature	57.48949	4.029637	5.870105538	2.330454351		3.834576	0.274741
chr16_33929_mature	34.38624	2.417782	5.145116481	1.773060525		3.830075	0.274992
chr17_34922_mature	16.11855	0.805927	4.097488514	0.852739935		4.321928	0.275015
chr1_2519_mature	16.11855	0.805927	4.097488514	0.852739935		4.321928	0.275015
chr15_32921_mature	2.686425	29.81932	1.882222339	4.94576292		-3.47249	0.275549
chr14_30214_mature	2.686425	29.81932	1.882222339	4.94576292		-3.47249	0.275549
chr5_11052_mature	291.7457	14.50669	8.193504349	3.954819241		4.329921	0.27588
bta-miR-199c	2.14914	24.17782	1.654957834	4.654081667		-3.49185	0.276819
chr1_526_mature	24.17782	1.611855	4.654081667	1.38507475		3.906891	0.279264
chr9_20370_mature	24.17782	1.611855	4.654081667	1.38507475		3.906891	0.279264
chr1_2937_star	24.17782	1.611855	4.654081667	1.38507475		3.906891	0.279264
chr5_12291_mature	8.059274	99.12908	3.179395517	6.645717165		-3.62059	0.281798
chrx_22188_mature@@bta-miR-12	7.52199	91.0698	3.091190278	6.524656134		-3.59779	0.281883
chr6_12675_mature	33.31167	2.417782	5.100627352	1.773060525		3.784271	0.282972
chr13_29007_mature	15.58126	0.805927	4.051482084	0.852739935		4.273018	0.283226
hsa-miR-643	15.58126	0.805927	4.051482084	0.852739935		4.273018	0.283226
chr17_35528_mature	15.58126	0.805927	4.051482084	0.852739935		4.273018	0.283226
chr1_886_mature	15.58126	0.805927	4.051482084	0.852739935		4.273018	0.283226
chr6_13983_star	5.910135	67.69791	2.788713818	6.102194213		-3.51785	0.284594
chr3_8405_mature	5.910135	67.69791	2.788713818	6.102194213		-3.51785	0.284594
chr1_2381_mature	23.64054	1.611855	4.62296188	1.38507475		3.874469	0.284831
chr14_29793_mature	3.22371	34.65488	2.078510713	5.156027656		-3.42626	0.28528
NW_003538989_38422_star	43.52008	3.22371	5.476384353	2.078510713		3.754888	0.285588
ssc-miR-935	0.537285	9.671129	0.620384622	3.415640968		-4.16993	0.286463
chr7_15714_star	0.537285	9.671129	0.620384622	3.415640968		-4.16993	0.286463
hsa-miR-934	0.537285	9.671129	0.620384622	3.415640968		-4.16993	0.286463
hsa-miR-3611	2.14914	23.3719	1.654957834	4.607146585		-3.44294	0.287518
chr1_3819_star	4.835565	53.19121	2.544872269	5.759986999		-3.45943	0.287985
NW_003613192_37588_mature	8.596559	0	3.262517265	0		20	0.288431
chr11_25154_star	8.596559	0	3.262517265	0		20	0.288431
chr5_11345_mature	8.596559	0	3.262517265	0		20	0.288431
chr5_12116_mature@@bta-miR-20	8.596559	0	3.262517265	0		20	0.288431
chr16_33868_star	8.596559	0	3.262517265	0		20	0.288431
chr2_4579_mature	8.596559	0	3.262517265	0		20	0.288431
chrx_23194_star@@mmu-miR-201-	8.596559	0	3.262517265	0		20	0.288431
chr18_35974_mature@@bta-miR-5	8.596559	0	3.262517265	0		20	0.288431
chr16_34520_mature	8.596559	0	3.262517265	0		20	0.288431
chr15_31697_star	8.596559	0	3.262517265	0		20	0.288431
rno-miR-216a-3p	8.596559	0	3.262517265	0		20	0.288431
chr9_20138_mature	8.596559	0	3.262517265	0		20	0.288431
mmu-miR-101c	8.596559	0	3.262517265	0		20	0.288431
chr15_31965_star	8.596559	0	3.262517265	0		20	0.288431
chr8_17814_star	8.596559	0	3.262517265	0		20	0.288431
NW_003537297_36825_mature	8.596559	0	3.262517265	0		20	0.288431
chr5_11037_star	8.596559	0	3.262517265	0		20	0.288431
chr7_16877_mature	8.596559	0	3.262517265	0		20	0.288431
chr9_21017_star	8.596559	0	3.262517265	0		20	0.288431
chr13_29259_star	3.760995	40.29637	2.251263041	5.367943153		-3.42146	0.288529
chr9_19614_mature	3.760995	40.29637	2.251263041	5.367943153		-3.42146	0.288529
chr14_29727_mature	3.760995	40.29637	2.251263041	5.367943153		-3.42146	0.288529
chr17_35115_star	1.07457	13.70077	1.05281229	3.877819487		-3.67243	0.289389
hsa-miR-766-5p	1.07457	13.70077	1.05281229	3.877819487		-3.67243	0.289389
chr11_25110_star	1.07457	13.70077	1.05281229	3.877819487		-3.67243	0.289389
chrx_22916_star	1.07457	13.70077	1.05281229	3.877819487		-3.67243	0.289389
chr1_169_star	1.07457	13.70077	1.05281229	3.877819487		-3.67243	0.289389
chr3_6786_star	23.10325	1.611855	4.591155994	1.38507475		3.841302	0.290564
chr1_79_mature	23.10325	1.611855	4.591155994	1.38507475		3.841302	0.290564
bta-miR-3596	8.059274	96.71129	3.179395517	6.610453419		-3.58496	0.290682
chr10_23953_mature	17.19312	255.479	4.185320985	8.002696903		-3.8933	0.29106
chr17_35073_mature	32.2371	2.417782	5.054722516	1.773060525		3.736966	0.291298
mmu-miR-302a-3p	15.04398	0.805927	4.003960082	0.852739935		4.222392	0.291805
chr8_17571_mature	15.04398	0.805927	4.003960082	0.852739935		4.222392	0.291805
chr7_16536_mature	3.22371	33.84895	2.078510713	5.123043401		-3.39232	0.293024
ssc-miR-424-3p	4.835565	52.38528	2.544872269	5.738370208		-3.43741	0.293226
chr7_16094_mature	4.835565	52.38528	2.544872269	5.738370208		-3.43741	0.293226
chr1_990_mature	2.686425	28.20746	1.882222339	4.868265031		-3.39232	0.293452
chr6_14448_mature	5.37285	58.8327	2.671938627	5.902862353		-3.45286	0.294785
chrx_23221_mature	7.52199	87.84609	3.091190278	6.473236413		-3.54579	0.294794
NW_003538662_38098_mature	31.69981	2.417782	5.03121048	1.773060525		3.712718	0.295597
chr16_33741_mature	122.501	8.059274	6.948378589	3.179395517		3.925999	0.295731
chr13_27213_mature	22.56597	1.611855	4.558633073	1.38507475		3.807355	0.296467
chr4_9998_mature	22.56597	1.611855	4.558633073	1.38507475		3.807355	0.296467
chr9_20608_mature	5.910135	65.28012	2.788713818	6.050504383		-3.46538	0.297343
chr8_18750_mature	171.9312	10.47706	7.43405428	3.520680821		4.036526	0.298376
chr18_35896_mature	2.14914	22.56597	1.654957834	4.558633073		-3.39232	0.298696
chr3_7220_star	1.611855	17.7304	1.38507475	4.227310103		-3.45943	0.298713

chr6_13570_mature	1.074569932	35.46081	1.05281229	5.188274618		-5.04439	0.069327	NW_003613164_37468_mature	0	41.90823	0	5.423182395	5.423182395	-20	0.047704	rno-miR-370-5p	1.611855	17.7304	1.38507475	4.227310103		-3.45943	0.298713
chr17_35007_mature	1.074569932	35.46081	1.05281229	5.188274618		-5.04439	0.069327	chr6_13901_star	70.9216155	0	6.168353522	0	0	20	0.047732	chr14_30212_mature	136.4704	8.865202	7.102977006	3.302348583		3.944291	0.30047
chr6_14848_star	34.38623782	1.611855	5.145116481	1.38507475		4.415037	0.070297	chr5_11434_star	0	41.1023	0	5.395827139	5.395827139	-20	0.048101	chr14_30645_mature	14.50669	0.805927	3.954819241	0.852739935		4.169925	0.300772
chr4_10081_mature	34.38623782	1.611855	5.145116481	1.38507475		4.415037	0.070297	chr15_33182_mature	0	40.29637	0	5.367943153	5.367943153	-20	0.048509	NW_003538623_38054_star	14.50669	0.805927	3.954819241	0.852739935		4.169925	0.300772
ssc-miR-155-3p	34.38623782	1.611855	5.145116481	1.38507475		4.415037	0.070297	chr14_29516_mature	0	40.29637	0	5.367943153	5.367943153	-20	0.048509	bta-miR-664	14.50669	0.805927	3.954819241	0.852739935		4.169925	0.300772
bta-miR-2331-3p	34.38623782	1.611855	5.145116481	1.38507475		4.415037	0.070297	chr16_33835_mature	0	37.87859	0	5.280903997	5.280903997	-20	0.0498	chr5_11160_mature	14.50669	0.805927	3.954819241	0.852739935		4.169925	0.300772
chr4_9226_star	22.56596857	0.805927	4.558633073	0.852739935		4.807355	0.070344	chr16_34498_star	0	37.87859	0	5.280903997	5.280903997	-20	0.0498	chr1_916_mature	14.50669	0.805927	3.954819241	0.852739935		4.169925	0.300772
chr6_12739_mature	22.56596857	0.805927	4.558633073	0.852739935		4.807355	0.070344	mmu-miR-6406	0	37.87859	0	5.280903997	5.280903997	-20	0.0498	chr7_16933_mature	14.50669	0.805927	3.954819241	0.852739935		4.169925	0.300772
chr2_5482_star	22.56596857	0.805927	4.558633073	0.852739935		4.807355	0.070344	hsa-miR-526a	0	37.87859	0	5.280903997	5.280903997	-20	0.0498	bta-miR-378c	14.50669	0.805927	3.954819241	0.852739935		4.169925	0.300772
chr2_4081_mature	22.56596857	0.805927	4.558633073	0.852739935		4.807355	0.070344	chrx_22122_star	0	37.07266	0	5.250683565		-20	0.050255	NW_003539018_38471_mature	22.02868	1.611855	4.525360039	1.38507475		3.77259	0.302547
chr12_25952_mature	22.56596857	0.805927	4.558633073	0.852739935		4.807355	0.070344	NW_003613200_37636_mature	0	37.07266	0	5.250683565		-20	0.050255	chr15_32789_mature	22.02868	1.611855	4.525360039	1.38507475		3.77259	0.302547
chr15_32840_mature	22.56596857	0.805927	4.558633073	0.852739935		4.807355	0.070344	chr9_19519_star	0	36.26674	0	5.21981653		-20	0.050723	chr13_29005_mature	22.02868	1.611855	4.525360039	1.38507475		3.77259	0.302547
chr17_35808_mature	22.56596857	0.805927	4.558633073	0.852739935		4.807355	0.070344	chr9_19846_mature	0	36.26674	0	5.21981653		-20	0.050723	mmu-miR-344d-2-5p	22.02868	1.611855	4.525360039	1.38507475		3.77259	0.302547
chr18_36365_mature	47.281077	2.417782	5.593385953	1.773060525		4.289507	0.070488	chr5_11922_star	0	36.26674	0	5.21981653		-20	0.050723	chr7_17092_mature	8.059274	0	3.179395517	0		20	0.304171
chr2_5435_mature	12064.19662	221.63	13.55856381	7.798504517		5.766435	0.070652	chr5_11657_star	0	35.46081	0	5.188274618		-20	0.051206	chr18_35998_star	8.059274	0	3.179395517	0		20	0.304171
ssc-miR-425-3p	10.74569932	309.4761	3.554060706	8.278338593		-4.848	0.071069	chr2_4828_mature	0	35.46081	0	5.188274618		-20	0.051206	NW_003613483_39359_mature	8.059274	0	3.179395517	0		20	0.304171
NW_003537125_36714_mature	1.074569932	34.65488	1.05281229	5.156027656		-5.01123	0.071344	NW_003537125_36714_mature	0	34.65488	0	5.156027656		-20	0.051703	chrx_21525_mature	8.059274	0	3.179395517	0		20	0.304171
ssc-miR-29c	1.074569932	34.65488	1.05281229	5.156027656		-5.01123	0.071344	chr7_15959_mature	0	34.65488	0	5.156027656		-20	0.051703	hsa-miR-378e	8.059274	0	3.179395517	0		20	0.304171
NW_003539597_38976_mature	0	16.92448	0	4.163859073		-20	0.07151	ssc-miR-29c	0	34.65488	0	5.156027656		-20	0.051703	bta-miR-1256	8.059274	0	3.179395517	0		20	0.304171
chr7_15658_star	0	16.92448	0	4.163859073		-20	0.07151	NW_003539080_38513_mature	0	34.65488	0	5.156027656		-20	0.051703	chr17_35456_mature	8.059274	0	3.179395517	0		20	0.304171
chr2_4818_star	0	16.92448	0	4.163859073		-20	0.07151	chr7_15755_mature	0	33.84895	0	5.123043401		-20	0.052215	chr14_31128_mature	8.059274	0	3.179395517	0		20	0.304171
mmu-miR-6411	0	16.92448	0	4.163859073		-20	0.07151	chr11_24943_star	0	33.84895	0	5.123043401		-20	0.052215	chr2_4701_mature	8.059274	0	3.179395517	0		20	0.304171
chr14_30079_mature	0	16.92448	0	4.163859073		-20	0.07151	chr7_15777_mature	0	33.04303	0	5.08928735		-20	0.052744	chr2_4820_star	8.059274	0	3.179395517	0		20	0.304171
chr8_18198_mature	0	16.92448	0	4.163859073		-20	0.07151	chr9_19808_mature	0	33.04303	0	5.08928735		-20	0.052744	NW_003539513_38910_star	8.059274	0	3.179395517	0		20	0.304171
mmu-miR-26b-5p	0	16.92448	0	4.163859073		-20	0.07151	chr6_12618_mature	0	32.2371	0	5.054722516		-20	0.053289	chr13_27815_star	8.059274	0	3.179395517	0		20	0.304171
chr7_16555_mature	3.223709795	78.98089	2.078510713	6.32158343		-4.61471	0.072113	chr13_28842_star	0	32.2371	0	5.054722516		-20	0.053289	NW_003613342_38628_mature	8.059274	0	3.179395517	0		20	0.304171
chr4_9934_star	11.82026925	0	3.680354657	0		20	0.072584	chr16_34111_mature	0	32.2371	0	5.054722516		-20	0.053289	chr1_3554_mature	8.059274	0	3.179395517	0		20	0.304171
chr2_6500_mature	11.82026925	0	3.680354657	0		20	0.072584	chr9_20193_star	0	31.43117	0	5.019309189		-20	0.053853	chr1_2086_mature	8.059274	0	3.179395517	0		20	0.304171
chr3_7917_star	11.82026925	0	3.680354657	0		20	0.072584	chrx_22054_mature	0	31.43117	0	5.019309189		-20	0.053853	chr7_15085_star	8.059274	0	3.179395517	0		20	0.304171
mmu-miR-466d-5p	11.82026925	0	3.680354657	0		20	0.072584	chr14_30452_star	0	31.43117	0	5.019309189		-20	0.053853	bta-miR-487a	0	5.641492	0	2.731507408		-20	0.304599
chr7_16193_mature	11.82026925	0	3.680354657	0		20	0.072584	chr1_3642_mature	0	31.43117	0	5.019309189		-20	0.053853	rno-miR-222-5p	0	5.641492	0	2.731507408		-20	0.304599
mmu-miR-466n-3p	11.82026925	0	3.680354657	0		20	0.072584	chr1_826_mature	0	31.43117	0	5.019309189		-20	0.053853	bta-miR-181a	0	5.641492	0	2.731507408		-20	0.304599
mmu-miR-486-3p	11.82026925	0	3.680354657	0		20	0.072584	chr5_11222_mature	6.44741959	132.978	2.896740642	7.065852624		-4.36632	0.054294	hsa-miR-3672	0	5.641492	0	2.731507408		-20	0.304599
mmu-miR-297a-5p	11.82026925	0	3.680354657	0		20	0.072584	NW_003613132_37331_mature	0	30.62524	0	4.983004663		-20	0.054436	chr16_33720_mature	0	5.641492	0	2.731507408		-20	0.304599
mmu-miR-133a-5p	11.82026925	0	3.680354657	0		20	0.072584	chr9_20736_star	0	29.81932	0	4.94576292		-20	0.05504	chr12_26240_star	0	5.641492	0	2.731507408		-20	0.304599
chr9_21068_mature	11.82026925	0	3.680354657	0		20	0.072584	chr18_35895_mature	0	29.81932	0	4.94576292		-20	0.05504	NW_003613264_38074_star	0	5.641492	0	2.731507408		-20	0.304599
hsa-miR-1910	11.82026925	0	3.680354657	0		20	0.072584	chr13_28506_mature	0	29.81932	0	4.94576292		-20	0.05504	bta-miR-631	0	5.641492	0	2.731507408		-20	0.304599
chr7_15165_star	46.20650707	2.417782	5.560913833	1.773060525		4.25634	0.073038	chr7_16567_mature	0	29.81932	0	4.94576292		-20	0.05504	hsa-miR-4423-3p	0	5.641492	0	2.731507408		-20	0.304599
chr12_25822_mature	3.223709795	78.17496	2.078510713	6.306972375		-4.59991	0.073066	chr5_11241_mature	0	29.01339	0	4.907534286		-20	0.055664	bta-miR-323	0	5.641492	0	2.731507408		-20	0.304599
chr11_25364_mature	3.223709795	78.17496	2.078510713	6.306972375		-4.59991	0.073066	mmu-miR-193b-3p	0	29.01339	0	4.907534286		-20	0.055664	chr5_11238_star	0	5.641492	0	2.731507408		-20	0.304599
chr13_29016_mature	3.223709795	78.17496	2.078510713	6.306972375		-4.59991	0.073066	hsa-miR-5702	0	29.01339	0	4.907534286		-20	0.055664	NW_003538681_38110_mature	0	5.641492	0	2.731507408		-20	0.304599
chr6_14220_mature	1.074569932	33.84895	1.05281229	5.123043401		-4.97728	0.073456	NW_003537641_37089_mature	0	29.01339	0	4.907534286		-20	0.055664	chr10_24012_star	0	5.641492	0	2.731507408		-20	0.304599
chr3_7259_mature	1.074569932	33.84895	1.05281229	5.123043401		-4.97728	0.073456	chr1_990_mature	0	28.20746	0	4.868265031		-20	0.056312	chr6_12434_mature	0	5.641492	0	2.731507408		-20	0.304599
chr11_24943_star	1.074569932	33.84895	1.05281229	5.123043401		-4.97728	0.073456	NW_003539453_38866_mature	0	28.20746	0	4.868265031		-20	0.056312	chr9_20036_mature	13.43212	178.11	3.851211749	7.484701805		-3.72901	0.305234
ssc-miR-142-5p	74.1453253	4.029637	6.231611453	2.330454351		4.201634	0.073571	chr8_18274_star	0	28.20746	0	4.868265031		-20	0.056312	chr17_34936_mature	4.29828	44.32601	2.405524014	5.502267253		-3.36632	0.305352
chr15_32346_mature	33.31166789	1.611855	5.100627352	1.38507475		4.369234	0.073765	NW_003539146_38590_mature	0	28.20746	0	4.868265031		-20	0.056312	chr10_23668_mature	4.29828	44.32601	2.405524014	5.502267253		-3.36632	0.305352
chr7_16084_mature	89.18930434	4.835565	6.494884448	2.544872269		4.205114	0.073977	chr3_8556_mature	0	28.20746	0	4.868265031		-20	0.056312	chr12_26197_mature	40.29637	3.22371	5.367943153	2.078510713		3.643856	0.305654
chr5_12108_mature	3.223709795	77.36904	2.078510713	6.292211829		-4.58496	0.074039	NW_003613282_38228_mature	0	28.20746	0	4.868265031		-20	0.056312	ssc-miR-196b-5p	5.910135	63.66827	2.788713818	6.014986077		-3.42931	0.306208
chr5_10752_star	21.49139864	0.805927	4.491301473	0.852739935		4.736966	0.075593	chr5_12195_star	0	27.40153	0	4.827896911		-20	0.056984	chr11_24609_mature	75.2199	5.641492	6.25209572	2.731507408		3.736966	0.306613
mmu-miR-5128	21.49139864	0.805927	4.491301473	0.852739935		4.736966	0.075593	chr13_27155_star	0	27.40153	0	4.827896911		-20	0.056984	chr12_25511_mature	9.133844	107.9943	3.34110968	6.76810859		-3.56359	0.307451
chr8_17750_mature	21.49139864	0.805927	4.491301473	0.852739935		4.736966	0.075593	chr1_1669_mature	0	27.40153	0	4.827896911		-20	0.056984	chr2_4395_mature	1.07457	12.89484	1.05281229	3.796477232		-3.58496	0.30792
hsa-miR-2277-3p	21.49139864	0.805927	4.491301473	0.852739935		4.736966	0.075593	chr6_13274_mature	0	26.59561	0	4.786366653		-20	0.057682	chr13_28512_star	1.07457	12.89484	1.05281229	3.796477232		-3.58496	0.30792
chr10_23335_star	21.49139864	0.805927	4.491301473	0.852739935		4.736966	0.075593	chr15_32650_mature	0	26.59561	0	4.786366653		-20	0.057682	chr6_12875_mature	1.07457	12.89484	1.05281229	3.796477232		-3.58496	0.30792
chr11_24466_mature	2.149139864	53.19121	1.654957834	5.759986999		-4.62936	0.07561	chr7_15778_mature	0	26.59561	0	4.786366653		-20	0.057682	chr15_33696_mature	21.4914	1.611855	4.491301473	1.38507475		3.736966	0.308811
chr4_9640_mature	45.13193714	2.417782	5.527693969	1.773060525		4.222392	0.075721	chr7_16275_mature	0	26.59561	0	4.786366653		-20	0.057682	chr9_20818_mature	21.4914	1.611855	4.491301473	1.38507475		3.736966	0.308811
chr13_27245_star	0	16.11855	0	4.097488514		-20	0.075831	chr12_25999_star	0	26.59561	0	4.786366653		-20	0.057682	chr7_16298_mature	30.08796	2.417782	4.958283956	1.773060525		3.63743	0.309063
chr2_5595_star	0	16.11855	0	4.097488514		-20	0.075831	ssc-miR-487b	0	25.78968	0	4.743605355		-20	0.058407	chr13_29206_mature	3.22371	32.2371	2.078510713	5.054722516		-3.32193	0.309243
chrx_23076_mature	0	16.11855	0	4.097488514		-20	0.075831	chr6_12811_star	0	25.78968	0	4.743605355		-20	0.058407	NW_003538347_37746_star	3.760995	37.87859	2.251263041	5.280903997		-3.3322	0.309383
chr2_4860_mature	0	16.11855	0	4.097488514		-20	0.075831	rno-miR-664-2-5p	0	25.78968	0	4.743605355		-20	0.058407	chr6_14372_mature	5.37285	56.41492	2.671938627	5.843353819		-3.39232	0.309516
chr5_11357_star	0	16.11855	0	4.097488514		-20	0.075831	chr2_5753_mature	0	25.78968	0	4.743605355		-20	0.058407	chrx_22108_mature	12.89484	166.827	3.796477232	7.390830869		-3.69349	0.309995
chr8_18467_mature	0	16.11855	0	4.097488514		-20	0.075831	chr2_5754_mature	0	25.78968	0	4.743605355		-20	0.058407	NW_003540346_39431_mature	13.96941	0.805927	3.90394537	0.852739935		4.115477	0.310149
chr14_29634_star	0	16.11855	0	4.097488514		-20	0.075831	chr3_8285_mature	0	25.78968	0	4.743605355		-20	0.058407	chr16_34502_star	13.96941	0.805927	3.90394537	0.852739935		4.115477	0.310149
chr1_182_mature	0	16.11855	0	4.097488514		-20	0.075831	hsa-miR-1248	0	24.98375	0	4.699537803		-20	0.059162	chr3_8118_mature	13.96941	0.805927	3.90394537	0.852739935		4.115477	0.310149
chr9_20257_star	0	16.11855	0	4.097488514		-20	0.075831	mmu-miR-3067-5p	0	24.98375	0	4.699537803		-20	0.059162	NW_003537656_37100_mature	13.96941	0.805927	3.90394537	0.852739935		4.115477	0.310149
rno-miR-1843-3p	58.02677632	3.22371	5.883297648	2.078510713		4.169925	0.076721	NW_003613367_38732_mature	0	24.98375	0	4.699537803		-20	0.059162	chr14_30124_mature	13.96941	0.805927	3.90394537	0.852739935		4.115477	0.310149
chr13_27994_mature	249.3002242	12.08891	7.967515773	3.710273245		4.366128	0.077178	chr13_27184_mature	0	24.98375	0	4.699537803		-20	0.059162	chr6_14069_mature	13.96941	0.805927	3.90394537	0.852739935		4.115477	0.310149
rno-let-7c-5p	32.23709795	1.611855	5.054722516	1.38507475		4.321928	0.077482	chr15_31776_mature	0	24.98375	0	4.699537803		-20	0.059162	ssc-miR-145-3p	47.28108	955.83	5.593385953	9.902118745		-4.33742	0.310315
chr14_31020_mature	4.298279727	99.12908	2.405524014	6.645717165		-4.52748	0.077916	chr3_8555_mature	0	24.98375	0	4.699537803		-20	0.059162	chr14_31062_star	2.14914	21.76004	1.654957834	4.508431259		-3.33985	0.310368
chrx_23051_mature	1.074569932	32.2371	1.05281229	5.054722516		-4.90689	0.077994	mmu-miR-3097-5p	0	24.98375	0	4.699537803		-20	0.059162	chr13_26730_mature	0.537285	8.865202	0.620384622	3.302348583		-4.04439	0.312465
chr6_12618_mature	1.074569932	32.2371	1.05281229	5.054722516		-4.90689	0.077994	mmu-miR-222-3p	0	24.98375	0	4.699537803		-20	0.059162	hsa-miR-145-5p	0.537285	8.865202	0.620384622	3.302348583		-4.04439	0.312465
chr16_33909_mature	1.074569932	32.2371	1.05281229	5.054722516		-4.90689	0.077994	chr12_26328_mature	0	24.98375	0	4.699537803		-20	0.059162	chr11_24683_mature	0.537285	8.865202	0.620384622	3.302348583		-4.04439	0.312465
chr5_11589_star	44.0573672	2.417782	5.493691111	1.773060525		4.187627	0.078548	hsa-miR-5000-3p	79.518175	1.611855	6.331242567	1.38507475		5.624491	0.059606	bta-miR-6524	0.537285	8.865202	0.620384622	3.302348583		-4.04439	0.312465
chr14_31648_mature	4.298279727	96.71129	2.405524014	6.610453419		-4.49185	0.080404	chr5_11673_mature	79.518175	1.611855	6.331242567	1.38507475		5.624491	0.059606	chrx_22414_star	0.537285	8.865202	0.620384622	3.302348583		-4.04439	0.312465
chrx_21675_mature	0	15.31262	0	4.027916745		-20	0.080589	chr6_13311_mature	0	24.17782	0	4.654081667		-20	0.059948	hsa-miR-607	0.537285	8.865202	0.620384622	3.302348583		-4.04439	0.312465
chr14_31643_mature	0	15.31262	0	4.027916745		-20	0.080589	chr16_34304_mature	0	24.17782	0	4.654081667		-20	0.059948	rno-miR-34a-5p	0.537285	8.865202	0.620384622	3.302348583		-4.04439	0.312465
chr12_25675_mature	0	15.31262	0	4.027916745		-20	0.080589	hsa-miR-3611	0	23.3719	0	4.607146585		-20	0.060768	chr15_31984_mature	61.25049	4.835565	5.960013198	2.544872269		3.662965	0.312692
mmu-miR-1899	0	15.31262	0	4.027916745		-20	0.080589	NW_003537216_36773_star	0	23.3719	0	4.607146585		-20	0.060768	chr12_25999_star	2.686425	26.59561	1.882222339	4.786366653		-3.30743	0.312695
chr7_17161_star	0	15.31262	0	4.027916745		-20	0.080589	chr13_28990_mature	0	23.3719	0	4.607146585		-20	0.060768	chr14_29552_mature	1.611855	16.92448	1.38507475	4.163859073		-3.39232	0.313327
bta-miR-2367-3p	0	15.31262	0	4.027916745		-20	0.080589	chr11_24562_mature	0	23.3719	0	4.607146585		-20	0.060768	NW_003537425_36910_mature	1.611855	16.92448	1.38507475	4.163859073		-3.39232	0.313327
chr9_19811_mature	0	15.31262	0	4.027916745		-20	0.080589	chr11_24872_mature	0	22.56597	0	4.558633073		-20	0.061625	chr13_28063_mature	20.95411	1.611855	4.456419386	1.38507475		3.70044	0.315264
chr6_13188_star	0	15.31262	0	4.027916745		-20	0.080589	bta-miR-103	0	22.56597	0	4.558633073		-20	0.061625	chrx_22054_mature	3.22371	31.43117	2.078510713	5.019309189		-3.2854	0.317729
chrx_22191_star	0	15.31262	0	4.027916745		-20	0.080589	NW_003613199_37634_mature	0	22.56597	0	4.558633073		-20	0.061625	chr6_13188_mature	3.22371	31.43117	2.078510713	5.019309189		-3.2854	0.317729
mmu-miR-302a-5p	0	15.31262	0	4.027916745		-20	0.080589	chr15_31936_star	0	22.56597	0	4.558633073		-20	0.061625	chr14_31463_mature	4.29828	42.71415	2.405524014	5.4500286		-3.31288	0.318141
chr8_17912_mature	0	15.31262	0	4.027916745		-20	0.080589	chr5_11025_mature	0	22.56597	0	4.558633073		-20	0.061625	chrx_23015_mature	71.99619	5.641492	6.18974917	2.731507408		3.673772	0.318717
rno-miR-632	20.4168287	0.805927	4.420672964	0.852739935		4.662965	0.081419	chr1_989_mature	0	21.76004	0	4.508431259		-20	0.06252	chr6_14013_mature	15.04398	199.0641	4.003960082	7.644318354		-3.72597	0.319894
chr15_31929_mature	20.4168287	0.805927	4.420672964	0.852739935		4.662965	0.081419	NW_003538449_37886_mature	0	21.76004	0	4.508431259		-20	0.06252	chr8_17814_mature	13.43212	0.805927	3.851211749	0.852739935		4.058894	0.319957
chr1_1793_mature	20.4168287	0.805927	4.420672964	0.852739935		4.662965	0.081419	chr14_31062_star	0	21.76004	0	4.508431259		-20	0.06252	chr1_3657_mature	13.43212	0.805927	3.851211749	0.852739935		4.058894	0.319957
chr2_4586_mature	31.16252802	1.611855	5.007308904	1.38507475		4.273018	0.081471	chr17_34998_mature	0	21.76004	0	4.508431259		-20	0.06252	chr16_34245_star	13.43212	0.805927	3.851211749	0.852739935		4.058894	0.319957
chr14_31255_mature	31.16252802	1.611855	5.007308904	1.38507475		4.273018	0.081471	chr13_27363_mature	0	21.76004	0	4.508431259		-20	0.06252	chr15_31965_mature	13.43212	0.805927	3.851211749	0.852739935		4.058894	0.319957
chr5_11694_mature	31.16252802	1.611855	5.007308904	1.38507475		4.273018	0.081471	bta-miR-301a	75.2198952	0.805927	6.25209572	0.852739935		6.544321	0.062803	chr1_3113_mature	13.43212	0.805927	3.851211749	0.852739935		4.058894	0.319957
chr5_11653_mature	42.98279727	2.417782	5.458867456	1.773060525		4.152003	0.081526	chr1_577_mature	0	20.95411	0	4.456419386		-20	0.063458	chr15_33620_mature	13.43212	0.805927	3.851211749	0.852739935		4.058894	0.319957
chr3_8356_mature	42.98279727	2.417782	5.458867456	1.773060525		4.152003	0.081526	chr13_28542_mature	0	20.95411	0	4.456419386		-20	0.063458	chr11_25035_mature	13.43212	0.805927	3.851211749	0.852739935		4.058894	0.319957
NW_003539696_39032_mature	10.74569932	278.8509	3.554060706	8.128514563		-4.69766	0.081601	chr14_30089_mature	0	20.95411	0	4.456419386		-20	0.063458	chr8_17989_star@@mmu-miR-302	176.2295	11.28298	7.469474698	3.618589217		3.965235	0.320429
chr6_14032_mature	40.83365741	1699.701	5.38659223	10.7319138		-5.37938	0.081626	chr12_26051_star	0	20.95411	0	4.456419386		-20	0.063458	NW_003539513_38910_mature	7.52199	0	3.091190278	0		20	0.321219
chr3_7766_mature	2.149139864	49.9675	1.654957834	5.671505736		-4.53916	0.081719	chr14_29918_mature	0	20.95411	0	4.456419386		-20	0.063458	rno-miR-666-5p	7.52199	0	3.091190278	0		20	0.321219
chrx_22247_mature	3579.392443	95.90537	11.80590201	6.598504656		5.221959	0.082636	chr3_7233_mature	0	20.14819	0	4.402462031		-20	0.064442	chr12_25838_mature	7.52199	0	3.091190278	0		20	0.321219
chr13_28104_mature	10.74569932	0	3.554060706	0		20	0.082922	chr14_30104_mature	0	20.14819	0	4.402462031		-20	0.064442	chr14_30041_mature	7.52199	0	3.091190278	0		20	0.321219
chr12_25406_star	10.74569932	0	3.554060706	0		20	0.082922	chr18_36561_mature	0	20.14819	0	4.402462031		-20	0.064442	chr4_8749_star	7.52199	0	3.091190278	0		20	0.321219
chr11_24729_mature	10.74569932	0	3.554060706	0		20	0.082922	chr13_28917_mature	0	20.14819	0	4.402462031		-20	0.064442	chr5_11163_star	7.52199	0	3.091190278	0		20	0.321219
ssc-miR-208b	10.74569932	0	3.554060706	0		20	0.082922	mmu-miR-344e-5p	0	19.34226	0	4.346407978		-20	0.065476	chr8_19000_mature	7.52199	0	3.091190278	0		20	0.321219
hsa-miR-3960	10.74569932	0	3.554060706	0		20	0.082922	chr1_3640_mature	0	19.34226	0	4.346407978		-20	0.065476	chr5_11282_star	7.52199	0	3.091190278	0		20	0.321219
chr10_23364_mature	10.74569932	0	3.554060706	0		20	0.082922	chr8_17643_mature	0	19.34226	0	4.346407978		-20	0.065476	chr9_20434_star@@rno-miR-34b-5	7.52199	0	3.091190278	0		20	0.321219
hsa-miR-15b-5p	10.74569932	0	3.554060706	0		20	0.082922	chr3_7756_mature	0	19.34226	0	4.346407978		-20	0.065476	mmu-miR-654-5p	7.52199	0	3.091190278	0		20	0.321219
chr6_13601_star	10.74569932	0	3.554060706	0		20	0.082922	NW_003539453_38861_mature	0	19.34226	0	4.346407978		-20	0.065476	chr5_11515_mature	7.52199	0	3.091190278	0		20	0.321219
chr1_788_mature	10.74569932	0	3.554060706	0		20	0.082922	NW_003540824_39708_mature	0	19.34226	0	4.346407978		-20	0.065476	mmu-miR-3095-5p	7.52199	0	3.091190278	0		20	0.321219
chr12_26448_star	10.74569932	0	3.554060706	0		20	0.082922	bta-miR-26b	0	19.34226	0	4.346407978		-20	0.065476	bta-miR-2356	7.52199	0	3.091190278	0		20	0.321219
chr3_7521_mature	10.74569932	0	3.554060706	0		20	0.082922	chr6_14141_star	0	19.34226	0	4.346407978		-20	0.065476	NW_003541202_39972_mature	7.52199	0	3.091190278	0		20	0.321219
chr2_4586_star	10.74569932	0	3.554060706	0		20	0.082922	hsa-miR-4647	0	19.34226	0	4.346407978		-20	0.065476	ssc-miR-124a	20.41683	1.611855	4.420672964	1.38507475		3.662965	0.321914
chr15_33361_mature	10.74569932	0	3.554060706	0		20	0.082922	chr2_6005_mature	0	19.34226	0	4.346407978		-20	0.065476	chr4_10618_mature	20.41683	1.611855	4.420672964	1.38507475		3.662965	0.321914
chr2_5867_star	10.74569932	0	3.554060706	0		20	0.082922	chr16_34566_mature	0	19.34226	0	4.346407978		-20	0.065476	chr1_1780_star	20.41683	1.611855	4.420672964	1.38507475		3.662965	0.321914
NW_003538922_38350_mature	10.74569932	0	3.554060706	0		20	0.082922	chr1_1174_mature	0	18.53633	0	4.288087668		-20	0.066563	hsa-miR-4484	20.41683	1.611855	4.420672964	1.38507475		3.662965	0.321914
NW_003613359_38685_mature	10.74569932	0	3.554060706	0		20	0.082922	bta-miR-6120-3p	0	18.53633	0	4.288087668		-20	0.066563	chr4_10283_mature	2.14914	20.95411	1.654957834	4.456419386		-3.2854	0.322549
hsa-miR-4632-3p	10.74569932	0	3.554060706	0		20	0.082922	chr1_3589_mature	0	18.53633	0	4.288087668		-20	0.066563	chr7_15913_star	2.14914	20.95411	1.654957834	4.456419386		-3.2854	0.322549
chr11_25373_mature	10.74569932	0	3.554060706	0		20	0.082922	chr8_17485_mature	0	18.53633	0	4.288087668		-20	0.066563	ssc-miR-487b	2.686425	25.78968	1.882222339	4.743605355		-3.26303	0.322842
mmu-miR-3107-3p	10.74569932	0	3.554060706	0		20	0.082922	ssc-miR-450c-3p	0	18.53633	0	4.288087668		-20	0.066563	rno-miR-598-3p	174.6176	11.28298	7.45629374	3.618589217		3.951978	0.32313
chr11_24937_star	10.74569932	0	3.554060706	0		20	0.082922	chr8_17641_mature	0	18.53633	0	4.288087668		-20	0.066563	mmu-miR-5112	4.29828	41.90823	2.405524014	5.423182395		-3.2854	0.324758
hsa-miR-1268a	10.74569932	0	3.554060706	0		20	0.082922	chr1_217_mature	0	18.53633	0	4.288087668		-20	0.066563	chr13_27664_mature	15.58126	204.7056	4.051482084	7.684437063		-3.71567	0.327248
chr15_31760_star	10.74569932	0	3.554060706	0		20	0.082922	chr18_36348_mature	10.7456993	145.0669	3.554060706	7.190485881		-3.75489	0.067679	chr6_12802_mature	1.07457	12.08891	1.05281229	3.710273245		-3.49185	0.327795
chr2_4629_mature	10.74569932	0	3.554060706	0		20	0.082922	bta-miR-7	0	17.7304	0	4.227310103		-20	0.06771	rno-miR-349	1.07457	12.08891	1.05281229	3.710273245		-3.49185	0.327795
chr16_33806_star	1.074569932	30.62524	1.05281229	4.983004663		-4.83289	0.082998	NW_003538764_38206_mature	0	17.7304	0	4.227310103		-20	0.06771	rno-miR-31b	1.07457	12.08891	1.05281229	3.710273245		-3.49185	0.327795
NW_003539012_38462_mature	2.149139864	49.16157	1.654957834	5.648510724		-4.5157	0.083369	hsa-miR-1276	0	17.7304	0	4.227310103		-20	0.06771	chr4_9226_mature	1.07457	12.08891	1.05281229	3.710273245		-3.49185	0.327795
chr7_16043_mature	41.90822734	2.417782	5.423182395	1.773060525		4.115477	0.084668	chrx_21531_mature	0	17.7304	0	4.227310103		-20	0.06771	chr8_17371_star	1.611855	16.11855	1.38507475	4.097488514		-3.32193	0.328738
ssc-miR-374a-3p	6.447419591	144.261	2.896740642	7.182503739		-4.48382	0.085556	hsa-miR-374a-3p	0	17.7304	0	4.227310103		-20	0.06771	chr12_25647_mature	1.611855	16.11855	1.38507475	4.097488514		-3.32193	0.328738
NW_003539007_38427_mature	1.074569932	29.81932	1.05281229	4.94576292		-4.79442	0.085696	chr10_24135_mature	0	17.7304	0	4.227310103		-20	0.06771	chr8_17278_star	19.87954	1.611855	4.384018281	1.38507475		3.624491	0.328768
chr14_30214_mature	1.074569932	29.81932	1.05281229	4.94576292		-4.79442	0.085696	hsa-miR-4772-5p	0	17.7304	0	4.227310103		-20	0.06771	chr1_777_mature	19.87954	1.611855	4.384018281	1.38507475		3.624491	0.328768
NW_003537603_37043_mature	30.08795809	1.611855	4.958283956	1.38507475		4.222392	0.085758	chr5_11626_star	0	17.7304	0	4.227310103		-20	0.06771	bta-miR-138	19.87954	1.611855	4.384018281	1.38507475		3.624491	0.328768
chr16_33810_mature	30.08795809	1.611855	4.958283956	1.38507475		4.222392	0.085758	chrx_22097_mature	0	16.92448	0	4.163859073		-20	0.068921	chr9_19744_mature	19.87954	1.611855	4.384018281	1.38507475		3.624491	0.328768
hsa-miR-4449	30.08795809	1.611855	4.958283956	1.38507475		4.222392	0.085758	mmu-miR-6411	0	16.92448	0	4.163859073		-20	0.068921	chr1_1435_mature	8.059274	87.04016	3.179395517	6.460089935		-3.43296	0.329591
chr2_5321_star	4.298279727	91.87573	2.405524014	6.537229727		-4.41785	0.085777	bta-miR-2322-5p	0	16.92448	0	4.163859073		-20	0.068921	NW_003539506_38905_star	46.74379	4.029637	5.577241251	2.330454351		3.536053	0.329805
chr8_19000_star	0	14.50669	0	3.954819241		-20	0.085845	chr8_18198_mature	0	16.92448	0	4.163859073		-20	0.068921	chr7_15658_mature@@bta-miR-23	12.89484	0.805927	3.796477232	0.852739935		4	0.33022
chr9_20430_mature	0	14.50669	0	3.954819241		-20	0.085845	chr14_29552_mature	0	16.92448	0	4.163859073		-20	0.068921	chr11_25358_star	12.89484	0.805927	3.796477232	0.852739935		4	0.33022
chr1_1173_mature	0	14.50669	0	3.954819241		-20	0.085845	chr5_11269_mature	0	16.92448	0	4.163859073		-20	0.068921	rno-miR-298-5p	12.89484	0.805927	3.796477232	0.852739935		4	0.33022
chr3_8626_star	0	14.50669	0	3.954819241		-20	0.085845	chrx_23076_star	0	16.92448	0	4.163859073		-20	0.068921	chr14_30903_mature	12.89484	0.805927	3.796477232	0.852739935		4	0.33022
chr6_12877_mature	0	14.50669	0	3.954819241		-20	0.085845	chr5_11634_mature	0	16.92448	0	4.163859073		-20	0.068921	chr1_2507_mature	12.89484	0.805927	3.796477232	0.852739935		4	0.33022
chr10_23277_mature	0	14.50669	0	3.954819241		-20	0.085845	mmu-miR-26b-5p	0	16.92448	0	4.163859073		-20	0.068921	chr15_32587_mature	12.89484	0.805927	3.796477232	0.852739935		4	0.33022
hsa-miR-3920	0	14.50669	0	3.954819241		-20	0.085845	chr2_5595_star	0	16.11855	0	4.097488514		-20	0.070203	NW_003540830_39720_mature	12.89484	0.805927	3.796477232	0.852739935		4	0.33022
chrx_21688_mature	0	14.50669	0	3.954819241		-20	0.085845	chr6_13361_star	0	16.11855	0	4.097488514		-20	0.070203	chr1_3142_star	4.835565	46.74379	2.544872269	5.577241251		-3.27302	0.333088
NW_003538933_38357_mature	0	14.50669	0	3.954819241		-20	0.085845	chr13_26618_star	0	16.11855	0	4.097488514		-20	0.070203	chr9_19738_mature	2.686425	24.98375	1.882222339	4.699537803		-3.21723	0.333349
chr13_27489_mature	0	14.50669	0	3.954819241		-20	0.085845	chr8_17371_star	0	16.11855	0	4.097488514		-20	0.070203	chr3_8555_mature	2.686425	24.98375	1.882222339	4.699537803		-3.21723	0.333349
chr13_27644_mature	0	14.50669	0	3.954819241		-20	0.085845	chr5_11382_mature	0	16.11855	0	4.097488514		-20	0.070203	chr18_36191_mature	27.40153	2.417782	4.827896911	1.773060525		3.5025	0.333558
chr4_9231_star	0	14.50669	0	3.954819241		-20	0.085845	chr2_4860_mature	0	16.11855	0	4.097488514		-20	0.070203	hsa-miR-4443	5.910135	58.8327	2.788713818	5.902862353		-3.31536	0.334638
chr3_8626_mature	0	14.50669	0	3.954819241		-20	0.085845	chr1_742_mature	0	16.11855	0	4.097488514		-20	0.070203	chr3_7091_mature	2.14914	20.14819	1.654957834	4.402462031		-3.22882	0.335255
chr3_6901_star	195.5717276	10.47706	7.618912027	3.520680821		4.222392	0.086287	chr8_18467_mature	0	16.11855	0	4.097488514		-20	0.070203	chr1_2110_star	2.14914	20.14819	1.654957834	4.402462031		-3.22882	0.335255
chrx_22188_mature@@bta-miR-129	4.298279727	91.0698	2.405524014	6.524656134		-4.40514	0.086729	chr1_182_mature	0	16.11855	0	4.097488514		-20	0.070203	NW_003539007_38456_mature	2.14914	20.14819	1.654957834	4.402462031		-3.22882	0.335255
chr11_25149_mature	7.521989523	170.0507	3.091190278	7.418280128		-4.49871	0.087497	chr1_1984_star	0	16.11855	0	4.097488514		-20	0.070203	chr1_3253_mature	120.8891	8.865202	6.929425513	3.302348583		3.769387	0.335308
chr14_30666_mature	8.596559455	199.87	3.262517265	7.650118356		-4.53916	0.087557	chrx_21675_mature	0	15.31262	0	4.027916745		-20	0.071564	chr14_31062_mature	3.22371	29.81932	2.078510713	4.94576292		-3.20945	0.335478
chr2_5583_mature	4.298279727	90.26387	2.405524014	6.511971994		-4.39232	0.087697	chr14_31643_mature	0	15.31262	0	4.027916745		-20	0.071564	NW_003538401_37828_mature	35.99809	3.22371	5.209378995	2.078510713		3.481127	0.335746
chr11_25274_mature	19.34225877	0.805927	4.346407978	0.852739935		4.584963	0.087906	chr12_25675_mature	0	15.31262	0	4.027916745		-20	0.071564	chr4_9997_mature	19.34226	1.611855	4.346407978	1.38507475		3.584963	0.335831
mmu-miR-224-5p	19.34225877	0.805927	4.346407978	0.852739935		4.584963	0.087906	bta-miR-2367-3p	0	15.31262	0	4.027916745		-20	0.071564	chr13_29016_mature	7.52199	78.17496	3.091190278	6.306972375		-3.37752	0.337773
chr4_9373_mature	19.34225877	0.805927	4.346407978	0.852739935		4.584963	0.087906	chr9_19811_mature	0	15.31262	0	4.027916745		-20	0.071564	chrx_21606_mature	26.86425	2.417782	4.800343329	1.773060525		3.473931	0.338789
mmu-miR-363-5p	19.34225877	0.805927	4.346407978	0.852739935		4.584963	0.087906	chr11_24821_star	0	15.31262	0	4.027916745		-20	0.071564	chr6_14159_star	26.86425	2.417782	4.800343329	1.773060525		3.473931	0.338789
chrx_23149_mature	19.34225877	0.805927	4.346407978	0.852739935		4.584963	0.087906	chr2_4396_mature	0	15.31262	0	4.027916745		-20	0.071564	chr6_14411_mature	26.86425	2.417782	4.800343329	1.773060525		3.473931	0.338789
chr15_31817_mature	19.34225877	0.805927	4.346407978	0.852739935		4.584963	0.087906	chr18_36468_mature	0	15.31262	0	4.027916745		-20	0.071564	chr13_29181_mature	4.835565	45.93786	2.544872269	5.552680304		-3.24793	0.339257
chr14_31430_mature	15.04397905	406.9934	4.003960082	8.672401869		-4.75775	0.08811	chr5_11139_star	0	15.31262	0	4.027916745		-20	0.071564	chr6_14371_mature	4.835565	45.93786	2.544872269	5.552680304		-3.24793	0.339257
chr5_11241_mature	1.074569932	29.01339	1.05281229	4.907534286		-4.75489	0.088536	chr1_2367_mature@@mmu-miR-6243	0	15.31262	0	4.027916745		-20	0.071564	chr13_28104_mature	6.984705	0	2.997239027	0		20	0.33971
mmu-miR-193b-3p	1.074569932	29.01339	1.05281229	4.907534286		-4.75489	0.088536	chrx_21533_star	0	15.31262	0	4.027916745		-20	0.071564	chr6_14178_star	6.984705	0	2.997239027	0		20	0.33971
hsa-miR-2681-3p	1.074569932	29.01339	1.05281229	4.907534286		-4.75489	0.088536	chr8_17912_mature	0	15.31262	0	4.027916745		-20	0.071564	chr13_28423_mature	6.984705	0	2.997239027	0		20	0.33971
chr2_5550_mature	1.074569932	29.01339	1.05281229	4.907534286		-4.75489	0.088536	chr8_19000_star	0	14.50669	0	3.954819241		-20	0.073012	chr13_29304_star	6.984705	0	2.997239027	0		20	0.33971
chr15_33643_mature	1.074569932	29.01339	1.05281229	4.907534286		-4.75489	0.088536	chr1_1173_mature	0	14.50669	0	3.954819241		-20	0.073012	chr6_13898_mature	6.984705	0	2.997239027	0		20	0.33971
rno-miR-181a-2-3p	1.074569932	29.01339	1.05281229	4.907534286		-4.75489	0.088536	chr6_12877_mature	0	14.50669	0	3.954819241		-20	0.073012	mmu-miR-1967	6.984705	0	2.997239027	0		20	0.33971
chr4_10054_mature	3.223709795	66.89198	2.078510713	6.085169218		-4.37504	0.088839	chr9_19208_mature	0	14.50669	0	3.954819241		-20	0.073012	chr11_25079_mature	6.984705	0	2.997239027	0		20	0.33971
chr5_11980_mature	8.596559455	197.4522	3.262517265	7.632647927		-4.5216	0.088936	chr10_23277_mature	0	14.50669	0	3.954819241		-20	0.073012	mmu-miR-669a-5p	6.984705	0	2.997239027	0		20	0.33971
chrx_22210_mature	36.53537768	1359.6	5.230179094	10.41002686		-5.21774	0.089334	hsa-miR-3920	0	14.50669	0	3.954819241		-20	0.073012	rno-miR-759	6.984705	0	2.997239027	0		20	0.33971
chr13_29048_star	3.223709795	66.08605	2.078510713	6.067940913		-4.35755	0.090171	NW_003538933_38357_mature	0	14.50669	0	3.954819241		-20	0.073012	chr16_34371_mature	6.984705	0	2.997239027	0		20	0.33971
chr13_27245_mature	29.01338816	1.611855	4.907534286	1.38507475		4.169925	0.090372	hsa-miR-3646	0	14.50669	0	3.954819241		-20	0.073012	rno-miR-3560	6.984705	0	2.997239027	0		20	0.33971
chr3_8179_mature	2.149139864	45.93786	1.654957834	5.552680304		-4.41785	0.090537	chr7_16674_star	0	14.50669	0	3.954819241		-20	0.073012	chr4_8783_mature	6.984705	0	2.997239027	0		20	0.33971
chrx_21502_mature	4.298279727	87.84609	2.405524014	6.473236413		-4.35315	0.090707	chr10_23328_mature	0	14.50669	0	3.954819241		-20	0.073012	chr4_9373_star	6.984705	0	2.997239027	0		20	0.33971
chrx_23221_mature	4.298279727	87.84609	2.405524014	6.473236413		-4.35315	0.090707	chr13_27644_mature	0	14.50669	0	3.954819241		-20	0.073012	chr14_30563_star	6.984705	0	2.997239027	0		20	0.33971
chr2_6447_mature	21.49139864	642.3242	4.491301473	9.329402097		-4.90147	0.090916	chr13_28860_mature	0	14.50669	0	3.954819241		-20	0.073012	chr1_577_star	6.984705	0	2.997239027	0		20	0.33971
hsa-miR-492	1.074569932	28.20746	1.05281229	4.868265031		-4.71425	0.09153	bta-miR-2350	0	14.50669	0	3.954819241		-20	0.073012	bta-miR-2440	6.984705	0	2.997239027	0		20	0.33971
chr9_20062_star	1.074569932	28.20746	1.05281229	4.868265031		-4.71425	0.09153	chr4_9231_star	0	14.50669	0	3.954819241		-20	0.073012	chr7_16512_mature	6.984705	0	2.997239027	0		20	0.33971
ssc-miR-139-3p	1.074569932	28.20746	1.05281229	4.868265031		-4.71425	0.09153	chr11_24560_mature	0	13.70077	0	3.877819487		-20	0.074557	chr7_16725_mature	6.984705	0	2.997239027	0		20	0.33971
chr11_24876_mature	1.074569932	28.20746	1.05281229	4.868265031		-4.71425	0.09153	chrx_23209_star	0	13.70077	0	3.877819487		-20	0.074557	mmu-miR-3470b	6.984705	0	2.997239027	0		20	0.33971
NW_003539146_38590_mature	1.074569932	28.20746	1.05281229	4.868265031		-4.71425	0.09153	chr10_23892_mature	0	13.70077	0	3.877819487		-20	0.074557	chr1_3945_mature	6.984705	0	2.997239027	0		20	0.33971
NW_003613282_38228_mature	1.074569932	28.20746	1.05281229	4.868265031		-4.71425	0.09153	chr7_16662_mature	0	13.70077	0	3.877819487		-20	0.074557	mmu-miR-3089-5p	6.984705	0	2.997239027	0		20	0.33971
chr6_13601_mature	3.223709795	65.28012	2.078510713	6.050504383		-4.33985	0.091534	chr5_11381_mature	0	13.70077	0	3.877819487		-20	0.074557	chr5_11407_star	6.984705	0	2.997239027	0		20	0.33971
hsa-miR-766-5p	0	13.70077	0	3.877819487		-20	0.091672	chr1_169_star	0	13.70077	0	3.877819487		-20	0.074557	chr11_25205_star	12.35755	0.805927	3.739583968	0.852739935		3.938599	0.340962
NW_003538201_37611_mature	0	13.70077	0	3.877819487		-20	0.091672	chr1_3559_mature	0	13.70077	0	3.877819487		-20	0.074557	chr5_11099_mature	0.537285	8.059274	0.620384622	3.179395517		-3.90689	0.341171
chr13_28822_mature	0	13.70077	0	3.877819487		-20	0.091672	chr2_4280_star	0	13.70077	0	3.877819487		-20	0.074557	chr13_27566_star	0.537285	8.059274	0.620384622	3.179395517		-3.90689	0.341171
chr10_23892_mature	0	13.70077	0	3.877819487		-20	0.091672	chr18_36505_mature	0	13.70077	0	3.877819487		-20	0.074557	bta-miR-100	0.537285	8.059274	0.620384622	3.179395517		-3.90689	0.341171
NW_003538807_38238_star	0	13.70077	0	3.877819487		-20	0.091672	chr13_27643_mature	0	13.70077	0	3.877819487		-20	0.074557	ssc-miR-137	0.537285	8.059274	0.620384622	3.179395517		-3.90689	0.341171
chr7_16662_mature	0	13.70077	0	3.877819487		-20	0.091672	rno-miR-363-3p	0	12.89484	0	3.796477232		-20	0.07621	chr14_31645_mature	0.537285	8.059274	0.620384622	3.179395517		-3.90689	0.341171
chr5_11381_mature	0	13.70077	0	3.877819487		-20	0.091672	NW_003613233_37800_star	0	12.89484	0	3.796477232		-20	0.07621	hsa-miR-4442	0.537285	8.059274	0.620384622	3.179395517		-3.90689	0.341171
chr13_27643_mature	0	13.70077	0	3.877819487		-20	0.091672	chr13_27642_mature	0	12.89484	0	3.796477232		-20	0.07621	chr4_9430_star	0.537285	8.059274	0.620384622	3.179395517		-3.90689	0.341171
chr7_15701_mature	0	13.70077	0	3.877819487		-20	0.091672	chr1_924_mature	0	12.89484	0	3.796477232		-20	0.07621	chr8_17483_star	0.537285	8.059274	0.620384622	3.179395517		-3.90689	0.341171
chr17_35590_mature	6.447419591	136.2017	2.896740642	7.100154956		-4.40088	0.092017	chr2_4395_mature	0	12.89484	0	3.796477232		-20	0.07621	chr1_816_mature	18.80497	1.611855	4.307790888	1.38507475		3.544321	0.343112
chr11_25311_mature	2.149139864	45.13194	1.654957834	5.527693969		-4.39232	0.092483	chr8_17637_mature	0	12.89484	0	3.796477232		-20	0.07621	chr15_31781_mature	54.80307	4.835565	5.802272499	2.544872269		3.5025	0.343466
chr13_26844_mature	90.26387427	5.641492	6.511971994	2.731507408		4	0.094068	hsa-miR-4694-3p	0	12.89484	0	3.796477232		-20	0.07621	chr6_14340_mature	90.26387	7.253347	6.511971994	3.044979305		3.63743	0.343837
chr2_4549_mature	76.29446516	4.835565	6.272293205	2.544872269		3.979822	0.09424	rno-miR-741-3p	0	12.89484	0	3.796477232		-20	0.07621	chr2_4363_mature	34.92352	3.22371	5.166856928	2.078510713		3.437405	0.343929
chr5_12195_star	1.074569932	27.40153	1.05281229	4.827896911		-4.67243	0.094688	chr3_8153_mature	0	12.89484	0	3.796477232		-20	0.07621	chr14_29426_mature	26.32696	2.417782	4.772253246	1.773060525		3.444785	0.344136
chr15_33559_mature	18.26768884	0.805927	4.268111626	0.852739935		4.5025	0.095148	NW_003538201_37612_mature	0	12.89484	0	3.796477232		-20	0.07621	chr13_27562_mature	3.22371	29.01339	2.078510713	4.907534286		-3.16993	0.344752
chr10_23377_star	18.26768884	0.805927	4.268111626	0.852739935		4.5025	0.095148	chr14_30107_mature	0	12.89484	0	3.796477232		-20	0.07621	chr13_28371_star	3.22371	29.01339	2.078510713	4.907534286		-3.16993	0.344752
chr5_11163_mature	18.26768884	0.805927	4.268111626	0.852739935		4.5025	0.095148	chr1_3743_mature	0	12.89484	0	3.796477232		-20	0.07621	chr11_24821_mature	1.611855	15.31262	1.38507475	4.027916745		-3.24793	0.344974
chr14_30669_mature	18.26768884	0.805927	4.268111626	0.852739935		4.5025	0.095148	chr1_1055_mature	0	12.89484	0	3.796477232		-20	0.07621	chr15_32961_star	1.611855	15.31262	1.38507475	4.027916745		-3.24793	0.344974
rno-miR-196c-3p	18.26768884	0.805927	4.268111626	0.852739935		4.5025	0.095148	chr9_20736_mature	0	12.89484	0	3.796477232		-20	0.07621	chr3_8052_mature	54.26578	4.835565	5.788314591	2.544872269		3.488286	0.346223
chr17_34922_mature	18.26768884	0.805927	4.268111626	0.852739935		4.5025	0.095148	hsa-miR-5680	0	12.89484	0	3.796477232		-20	0.07621	ssc-miR-98	54.80307	1075.107	5.802272499	10.07160611		-4.29408	0.34798
mmu-miR-3060-3p	18.26768884	0.805927	4.268111626	0.852739935		4.5025	0.095148	chr13_27898_mature	0	12.89484	0	3.796477232		-20	0.07621	chr7_17051_mature	34.38624	3.22371	5.145116481	2.078510713		3.415037	0.348126
chr2_4423_mature	18.26768884	0.805927	4.268111626	0.852739935		4.5025	0.095148	chr5_11119_star	0	12.08891	0	3.710273245		-20	0.077985	chr1_2014_mature	1.07457	11.28298	1.05281229	3.618589217		-3.39232	0.349077
mmu-miR-1843b-5p	18.26768884	0.805927	4.268111626	0.852739935		4.5025	0.095148	bta-miR-2299-5p	0	12.08891	0	3.710273245		-20	0.077985	chr2_4824_mature	1.07457	11.28298	1.05281229	3.618589217		-3.39232	0.349077
mmu-miR-6538	18.26768884	0.805927	4.268111626	0.852739935		4.5025	0.095148	chr6_13504_mature	0	12.08891	0	3.710273245		-20	0.077985	chr13_29218_mature	1.07457	11.28298	1.05281229	3.618589217		-3.39232	0.349077
chr5_11673_star	38.68451755	2.417782	5.310504361	1.773060525		4	0.095188	chr5_11615_star	0	12.08891	0	3.710273245		-20	0.077985	chr4_9726_mature	1.07457	11.28298	1.05281229	3.618589217		-3.39232	0.349077
chr15_32429_mature	38.68451755	2.417782	5.310504361	1.773060525		4	0.095188	chr16_34524_star	0	12.08891	0	3.710273245		-20	0.077985	chr9_19555_mature	25.78968	2.417782	4.743605355	1.773060525		3.415037	0.349603
hsa-miR-4709-5p	27.93881823	1.611855	4.854934103	1.38507475		4.115477	0.095343	rno-miR-349	0	12.08891	0	3.710273245		-20	0.077985	ssc-miR-31	35.99809	601.2219	5.209378995	9.234513071		-4.06191	0.349674
mmu-miR-669e-5p	27.93881823	1.611855	4.854934103	1.38507475		4.115477	0.095343	rno-miR-758-3p	0	12.08891	0	3.710273245		-20	0.077985	ssc-let-7d-5p	87.57745	2043.832	6.468867551	10.99776661		-4.54457	0.349957
mmu-miR-3084-5p	9.671129386	0	3.415640968	0		20	0.095506	chr11_24519_star	0	12.08891	0	3.710273245		-20	0.077985	hsa-miR-103b	0	4.835565	0	2.544872269		-20	0.350342
hsa-miR-4751	9.671129386	0	3.415640968	0		20	0.095506	chr11_24684_mature	0	12.08891	0	3.710273245		-20	0.077985	NW_003539571_38947_mature	0	4.835565	0	2.544872269		-20	0.350342
chr5_10730_star	9.671129386	0	3.415640968	0		20	0.095506	NW_003540824_39708_star	0	12.08891	0	3.710273245		-20	0.077985	chr1_2751_star	0	4.835565	0	2.544872269		-20	0.350342
NW_003613280_38221_mature	9.671129386	0	3.415640968	0		20	0.095506	chr8_18439_mature	0	12.08891	0	3.710273245		-20	0.077985	NW_003613116_37270_star	0	4.835565	0	2.544872269		-20	0.350342
chr8_17367_star	9.671129386	0	3.415640968	0		20	0.095506	chr4_9226_mature	0	12.08891	0	3.710273245		-20	0.077985	chrx_21721_mature	0	4.835565	0	2.544872269		-20	0.350342
chr5_11345_mature	9.671129386	0	3.415640968	0		20	0.095506	rno-miR-31a-5p	0	12.08891	0	3.710273245		-20	0.077985	chr6_13027_star	0	4.835565	0	2.544872269		-20	0.350342
chr13_27913_star	9.671129386	0	3.415640968	0		20	0.095506	chr5_10912_mature	0	12.08891	0	3.710273245		-20	0.077985	chr11_24796_mature	18.26769	1.611855	4.268111626	1.38507475		3.5025	0.350618
chr15_33031_star	9.671129386	0	3.415640968	0		20	0.095506	hsa-miR-302b-5p	0	12.08891	0	3.710273245		-20	0.077985	chr1_3918_star	18.26769	1.611855	4.268111626	1.38507475		3.5025	0.350618
bta-miR-763	9.671129386	0	3.415640968	0		20	0.095506	chr14_31622_star	141.843231	20.95411	7.158288862	4.456419386		2.758992	0.079616	chr2_5588_star	128.9484	9.671129	7.02179497	3.415640968		3.736966	0.350731
chr1_2420_star	9.671129386	0	3.415640968	0		20	0.095506	chr1_251_mature	0	11.28298	0	3.618589217		-20	0.079898	chr16_33936_mature	4.835565	44.32601	2.544872269	5.502267253		-3.1964	0.351965
chr8_17991_star@@hsa-miR-367-5p	9.671129386	0	3.415640968	0		20	0.095506	chr13_27641_mature	0	11.28298	0	3.618589217		-20	0.079898	chr14_31373_star	11.82027	0.805927	3.680354657	0.852739935		3.874469	0.352208
mmu-miR-344c-3p	9.671129386	0	3.415640968	0		20	0.095506	chr7_15081_mature	0	11.28298	0	3.618589217		-20	0.079898	chr10_23795_mature	11.82027	0.805927	3.680354657	0.852739935		3.874469	0.352208
rno-miR-3075	9.671129386	0	3.415640968	0		20	0.095506	chr1_2668_mature	0	11.28298	0	3.618589217		-20	0.079898	chr8_17231_mature	4.29828	38.68452	2.405524014	5.310504361		-3.16993	0.352761
hsa-miR-4434	9.671129386	0	3.415640968	0		20	0.095506	NW_003613097_37151_mature	0	11.28298	0	3.618589217		-20	0.079898	chr9_19757_mature	3.22371	28.20746	2.078510713	4.868265031		-3.12928	0.354296
mmu-miR-3110-5p	9.671129386	0	3.415640968	0		20	0.095506	rno-miR-741-5p	0	11.28298	0	3.618589217		-20	0.079898	bta-miR-2898	20.41683	274.0153	4.420672964	8.103368244		-3.74643	0.355898
mmu-miR-704	9.671129386	0	3.415640968	0		20	0.095506	chr7_16939_mature	0	11.28298	0	3.618589217		-20	0.079898	chr14_29589_mature	39.75909	679.3968	5.349049847	9.410232629		-4.0949	0.355909
chr7_15085_star	9.671129386	0	3.415640968	0		20	0.095506	chr9_20735_mature	0	11.28298	0	3.618589217		-20	0.079898	chr9_19808_mature	3.760995	33.04303	2.251263041	5.08928735		-3.13516	0.356285
chr3_6792_mature	3.223709795	62.05641	2.078510713	5.978571212		-4.26679	0.097331	hsa-miR-3157-3p	0	11.28298	0	3.618589217		-20	0.079898	chr6_13334_star	17.7304	1.611855	4.227310103	1.38507475		3.459432	0.358355
chr11_24694_star	88.11473441	5.641492	6.477592084	2.731507408		3.965235	0.097568	chr8_17599_mature	0	11.28298	0	3.618589217		-20	0.079898	chr4_8831_mature	17.7304	1.611855	4.227310103	1.38507475		3.459432	0.358355
chr5_10971_star	102.0841435	6.44742	6.687678623	2.896740642		3.984893	0.097862	chr4_9980_star	0	11.28298	0	3.618589217		-20	0.079898	chrx_22916_mature	17.7304	1.611855	4.227310103	1.38507475		3.459432	0.358355
chr6_13274_mature	1.074569932	26.59561	1.05281229	4.786366653		-4.62936	0.098023	NW_003538201_37614_mature	0	11.28298	0	3.618589217		-20	0.079898	NW_003538989_38414_star	17.7304	1.611855	4.227310103	1.38507475		3.459432	0.358355
chr15_32650_mature	1.074569932	26.59561	1.05281229	4.786366653		-4.62936	0.098023	chr1_330_star	0	11.28298	0	3.618589217		-20	0.079898	chr4_9788_star	6.44742	0	2.896740642	0		20	0.359791
chr1_291_mature	1.074569932	26.59561	1.05281229	4.786366653		-4.62936	0.098023	chr14_29882_mature	0	11.28298	0	3.618589217		-20	0.079898	chr5_12300_star	6.44742	0	2.896740642	0		20	0.359791
chr14_30313_mature	1.074569932	26.59561	1.05281229	4.786366653		-4.62936	0.098023	bta-miR-2352	0	11.28298	0	3.618589217		-20	0.079898	chr7_16110_star	6.44742	0	2.896740642	0		20	0.359791
chr12_25666_mature	6852.532455	166.827	12.74263206	7.390830869		5.360213	0.098144	mmu-miR-705	0	11.28298	0	3.618589217		-20	0.079898	chr6_14470_mature	6.44742	0	2.896740642	0		20	0.359791
rno-miR-363-3p	0	12.89484	0	3.796477232		-20	0.09816	chr10_23305_mature	0	11.28298	0	3.618589217		-20	0.079898	chrx_23098_mature	6.44742	0	2.896740642	0		20	0.359791
chr13_27642_mature	0	12.89484	0	3.796477232		-20	0.09816	hsa-miR-5699	0	11.28298	0	3.618589217		-20	0.079898	NW_003537262_36795_mature	6.44742	0	2.896740642	0		20	0.359791
chr1_924_mature	0	12.89484	0	3.796477232		-20	0.09816	chr16_33739_mature	0	11.28298	0	3.618589217		-20	0.079898	chr15_32720_mature	6.44742	0	2.896740642	0		20	0.359791
chr2_4395_mature	0	12.89484	0	3.796477232		-20	0.09816	chr4_10714_mature	0	11.28298	0	3.618589217		-20	0.079898	chr10_24203_mature	6.44742	0	2.896740642	0		20	0.359791
chr8_17637_mature	0	12.89484	0	3.796477232		-20	0.09816	chr2_5807_mature	0	10.47706	0	3.520680821		-20	0.081968	ssc-miR-429	6.44742	0	2.896740642	0		20	0.359791
chr14_30107_mature	0	12.89484	0	3.796477232		-20	0.09816	chr14_30903_star	0	10.47706	0	3.520680821		-20	0.081968	chr1_2930_star	6.44742	0	2.896740642	0		20	0.359791
chr9_20571_mature	0	12.89484	0	3.796477232		-20	0.09816	hsa-miR-1243	0	10.47706	0	3.520680821		-20	0.081968	bta-miR-2285r	6.44742	0	2.896740642	0		20	0.359791
chr1_1055_mature	0	12.89484	0	3.796477232		-20	0.09816	chr14_31216_mature	0	10.47706	0	3.520680821		-20	0.081968	bta-miR-146a	6.44742	0	2.896740642	0		20	0.359791
chr13_28512_mature	0	12.89484	0	3.796477232		-20	0.09816	chr15_32006_mature	0	10.47706	0	3.520680821		-20	0.081968	chr3_8495_star	6.44742	0	2.896740642	0		20	0.359791
chrx_21678_mature	0	12.89484	0	3.796477232		-20	0.09816	chr4_10614_mature	0	10.47706	0	3.520680821		-20	0.081968	chr1_1416_star	6.44742	0	2.896740642	0		20	0.359791
bta-miR-2340	0	12.89484	0	3.796477232		-20	0.09816	chr17_35802_mature	0	10.47706	0	3.520680821		-20	0.081968	chr4_8756_mature	6.44742	0	2.896740642	0		20	0.359791
hsa-miR-5680	0	12.89484	0	3.796477232		-20	0.09816	chr5_11689_mature	0	10.47706	0	3.520680821		-20	0.081968	chr10_23778_star	6.44742	0	2.896740642	0		20	0.359791
chr13_27898_mature	0	12.89484	0	3.796477232		-20	0.09816	chr5_12267_star	0	10.47706	0	3.520680821		-20	0.081968	hsa-miR-4313	6.44742	0	2.896740642	0		20	0.359791
chr13_28245_mature	2.149139864	42.71415	1.654957834	5.4500286		-4.31288	0.098739	rno-miR-206-3p	0	10.47706	0	3.520680821		-20	0.081968	mmu-miR-690	6.44742	0	2.896740642	0		20	0.359791
chrx_21844_mature@@hsa-miR-660	10531.8599	230.4953	13.36260959	7.854838784		5.513879	0.099441	NW_003539061_38502_mature	0	10.47706	0	3.520680821		-20	0.081968	chr5_12251_star	6.44742	0	2.896740642	0		20	0.359791
chr8_17417_mature	212.7648465	12.08891	7.739880812	3.710273245		4.137504	0.099734	chr13_28744_mature	0	10.47706	0	3.520680821		-20	0.081968	hsa-miR-4504	6.44742	0	2.896740642	0		20	0.359791
chr5_11615_mature	447.0210916	21.76004	8.807422842	4.508431259		4.36059	0.100186	hsa-miR-637	0	10.47706	0	3.520680821		-20	0.081968	chr16_34510_star	10.20841	107.9943	3.48651029	6.76810859		-3.40312	0.361865
chr4_9234_star	3.223709795	60.44456	2.078510713	5.941213351		-4.22882	0.100451	chr4_9657_mature	0	10.47706	0	3.520680821		-20	0.081968	chr8_19000_star	1.611855	14.50669	1.38507475	3.954819241		-3.16993	0.362055
chr13_27323_mature	26.8642483	1.611855	4.800343329	1.38507475		4.058894	0.100708	chr1_3135_star	0	10.47706	0	3.520680821		-20	0.081968	chr6_12877_mature	1.611855	14.50669	1.38507475	3.954819241		-3.16993	0.362055
chr14_29851_mature	48.35564693	3.22371	5.625143252	2.078510713		3.906891	0.100751	bta-miR-380-5p	0	10.47706	0	3.520680821		-20	0.081968	chr12_25510_star	1.611855	14.50669	1.38507475	3.954819241		-3.16993	0.362055
chr11_24870_star	48.35564693	3.22371	5.625143252	2.078510713		3.906891	0.100751	chr6_13529_star	0	9.671129	0	3.415640968		-20	0.084218	chr6_14661_mature	2.14914	18.53633	1.654957834	4.288087668		-3.10852	0.362287
NW_003613164_37468_mature	2.149139864	41.90823	1.654957834	5.423182395		-4.2854	0.100975	NW_003538681_38109_mature	0	9.671129	0	3.415640968		-20	0.084218	chr1_3121_mature	2.14914	18.53633	1.654957834	4.288087668		-3.10852	0.362287
chr6_13308_star	338.4895285	17.7304	8.407223265	4.227310103		4.254814	0.102576	chr1_2964_star	0	9.671129	0	3.415640968		-20	0.084218	chr6_14638_mature	41.37094	4.029637	5.405003311	2.330454351		3.359896	0.363457
chr4_8977_mature	5.372849659	100.7409	2.671938627	6.668756392		-4.22882	0.103257	chr15_33015_mature	0	9.671129	0	3.415640968		-20	0.084218	chr5_11238_mature	72.53347	6.44742	6.200329169	2.896740642		3.491853	0.363657
hsa-miR-572	17.19311891	0.805927	4.185320985	0.852739935		4.415037	0.103262	chr13_29021_mature	0	9.671129	0	3.415640968		-20	0.084218	chr13_27489_mature	217.0631	14.50669	7.768602025	3.954819241		3.903324	0.36368
chr3_7917_mature	17.19311891	0.805927	4.185320985	0.852739935		4.415037	0.103262	chr7_15714_star	0	9.671129	0	3.415640968		-20	0.084218	chr11_25383_star	11.28298	0.805927	3.618589217	0.852739935		3.807355	0.363985
chr13_26857_star	17.19311891	0.805927	4.185320985	0.852739935		4.415037	0.103262	chr2_5658_star	0	9.671129	0	3.415640968		-20	0.084218	chrx_22845_mature	11.28298	0.805927	3.618589217	0.852739935		3.807355	0.363985
chr8_17927_star	17.19311891	0.805927	4.185320985	0.852739935		4.415037	0.103262	chr2_4016_mature	0	9.671129	0	3.415640968		-20	0.084218	chr1_3232_star	11.28298	0.805927	3.618589217	0.852739935		3.807355	0.363985
chr8_17371_mature	17.19311891	0.805927	4.185320985	0.852739935		4.415037	0.103262	chr7_17037_mature	0	9.671129	0	3.415640968		-20	0.084218	NW_003540770_39675_star	11.28298	0.805927	3.618589217	0.852739935		3.807355	0.363985
chrx_21844_star@@hsa-miR-660-3p	17.19311891	0.805927	4.185320985	0.852739935		4.415037	0.103262	chr7_16043_star	0	9.671129	0	3.415640968		-20	0.084218	chr16_34188_mature	11.28298	0.805927	3.618589217	0.852739935		3.807355	0.363985
chr12_25987_mature	17.19311891	0.805927	4.185320985	0.852739935		4.415037	0.103262	chr15_33605_star	0	9.671129	0	3.415640968		-20	0.084218	chr11_24676_star	11.28298	0.805927	3.618589217	0.852739935		3.807355	0.363985
bta-miR-2303	17.19311891	0.805927	4.185320985	0.852739935		4.415037	0.103262	chr6_14717_mature	0	9.671129	0	3.415640968		-20	0.084218	chrx_21533_mature	11.28298	0.805927	3.618589217	0.852739935		3.807355	0.363985
chr11_25258_mature	16.11854898	394.0985	4.097488514	8.626068641		-4.61176	0.103468	hsa-miR-2052	0	9.671129	0	3.415640968		-20	0.084218	rno-miR-770-3p	11.28298	0.805927	3.618589217	0.852739935		3.807355	0.363985
ssc-miR-4332	1691.373073	60.44456	10.72483192	5.941213351		4.806439	0.103527	chr16_33846_mature	0	9.671129	0	3.415640968		-20	0.084218	chr16_34111_mature	3.760995	32.2371	2.251263041	5.054722516		-3.09954	0.364811
NW_003539103_38526_mature	3.223709795	58.8327	2.078510713	5.902862353		-4.18982	0.103733	chr8_19043_mature	0	9.671129	0	3.415640968		-20	0.084218	chr17_35521_mature	10.7457	114.4417	3.554060706	6.851020612		-3.41278	0.365141
chr15_32589_star	47.281077	3.22371	5.593385953	2.078510713		3.874469	0.104053	chr2_4820_mature	0	9.671129	0	3.415640968		-20	0.084218	chr4_10437_mature	4.835565	42.71415	2.544872269	5.4500286		-3.14296	0.365176

chr10_23498_mature	111.7552729	7.253347	6.817051091	3.044979305		3.945552	0.104648	chr13_27981_mature	0	9.671129	0	3.415640968		-20	0.084218	chr7_16704_mature	17.19312	1.611855	4.185320985	1.38507475		3.415037	0.366333
chr6_14961_mature	6.447419591	122.501	2.896740642	6.948378589		-4.24793	0.104947	chr13_28533_star	0	9.671129	0	3.415640968		-20	0.084218	chr1_2672_mature	17.19312	1.611855	4.185320985	1.38507475		3.415037	0.366333
chr6_13311_star	1.074569932	24.98375	1.05281229	4.699537803		-4.53916	0.10528	chr2_4213_star	0	9.671129	0	3.415640968		-20	0.084218	NW_003538795_38232_star	17.19312	1.611855	4.185320985	1.38507475		3.415037	0.366333
chr9_19738_mature	1.074569932	24.98375	1.05281229	4.699537803		-4.53916	0.10528	hsa-miR-934	0	9.671129	0	3.415640968		-20	0.084218	chr11_25156_star	17.19312	1.611855	4.185320985	1.38507475		3.415037	0.366333
chrx_22000_mature	1.074569932	24.98375	1.05281229	4.699537803		-4.53916	0.10528	chr10_24076_mature	0	9.671129	0	3.415640968		-20	0.084218	chr11_24795_mature	17.19312	1.611855	4.185320985	1.38507475		3.415037	0.366333
chr11_24459_mature	1.074569932	24.98375	1.05281229	4.699537803		-4.53916	0.10528	chr2_5051_star	0	8.865202	0	3.302348583		-20	0.086676	chr1_766_mature	17.19312	1.611855	4.185320985	1.38507475		3.415037	0.366333
mmu-miR-3067-5p	1.074569932	24.98375	1.05281229	4.699537803		-4.53916	0.10528	chr2_4097_star	0	8.865202	0	3.302348583		-20	0.086676	chr6_14513_mature	24.17782	2.417782	4.654081667	1.773060525		3.321928	0.366752
NW_003613447_39176_star	1.074569932	24.98375	1.05281229	4.699537803		-4.53916	0.10528	chr10_23786_star	0	8.865202	0	3.302348583		-20	0.086676	chr13_28696_star	2.686425	22.56597	1.882222339	4.558633073		-3.07039	0.367105
chr5_12291_mature	5.372849659	99.12908	2.671938627	6.645717165		-4.20555	0.105297	chr16_34295_star	0	8.865202	0	3.302348583		-20	0.086676	NW_003613199_37634_mature	2.686425	22.56597	1.882222339	4.558633073		-3.07039	0.367105
chr1_3094_mature	0	12.08891	0	3.710273245		-20	0.105414	rno-miR-200b-3p	0	8.865202	0	3.302348583		-20	0.086676	chr5_11025_mature	2.686425	22.56597	1.882222339	4.558633073		-3.07039	0.367105
chr5_12128_star	0	12.08891	0	3.710273245		-20	0.105414	hsa-miR-563	0	8.865202	0	3.302348583		-20	0.086676	chr13_28673_mature	9.133844	91.87573	3.34110968	6.537229727		-3.33039	0.369279
bta-miR-2299-5p	0	12.08891	0	3.710273245		-20	0.105414	chr12_26249_star	0	8.865202	0	3.302348583		-20	0.086676	hsa-miR-637	1.07457	10.47706	1.05281229	3.520680821		-3.2854	0.371818
chr6_13504_mature	0	12.08891	0	3.710273245		-20	0.105414	chr13_26730_mature	0	8.865202	0	3.302348583		-20	0.086676	hsa-miR-1260b	1.07457	10.47706	1.05281229	3.520680821		-3.2854	0.371818
chr5_11615_star	0	12.08891	0	3.710273245		-20	0.105414	chr2_5550_star	0	8.865202	0	3.302348583		-20	0.086676	chr9_21017_mature	23.64054	2.417782	4.62296188	1.773060525		3.289507	0.372726
rno-miR-349	0	12.08891	0	3.710273245		-20	0.105414	chr14_30596_star	0	8.865202	0	3.302348583		-20	0.086676	chr4_9687_mature	0.537285	7.253347	0.620384622	3.044979305		-3.75489	0.372767
rno-miR-758-3p	0	12.08891	0	3.710273245		-20	0.105414	chrx_22056_mature	0	8.865202	0	3.302348583		-20	0.086676	mmu-miR-712-3p	0.537285	7.253347	0.620384622	3.044979305		-3.75489	0.372767
NW_003541007_39797_mature	0	12.08891	0	3.710273245		-20	0.105414	hsa-miR-145-5p	0	8.865202	0	3.302348583		-20	0.086676	hsa-miR-518f-3p	0.537285	7.253347	0.620384622	3.044979305		-3.75489	0.372767
chr16_33920_star	0	12.08891	0	3.710273245		-20	0.105414	chr9_20563_star	0	8.865202	0	3.302348583		-20	0.086676	chr5_12382_mature	0.537285	7.253347	0.620384622	3.044979305		-3.75489	0.372767
hsa-miR-100-5p	0	12.08891	0	3.710273245		-20	0.105414	chr11_24683_mature	0	8.865202	0	3.302348583		-20	0.086676	bta-miR-497	0.537285	7.253347	0.620384622	3.044979305		-3.75489	0.372767
hsa-miR-618	0	12.08891	0	3.710273245		-20	0.105414	chr16_33936_star	0	8.865202	0	3.302348583		-20	0.086676	chr13_27082_mature	0.537285	7.253347	0.620384622	3.044979305		-3.75489	0.372767
rno-miR-31a-5p	0	12.08891	0	3.710273245		-20	0.105414	chr15_31790_star	0	8.865202	0	3.302348583		-20	0.086676	chr17_34997_star	0.537285	7.253347	0.620384622	3.044979305		-3.75489	0.372767
hsa-miR-302b-5p	0	12.08891	0	3.710273245		-20	0.105414	bta-miR-761	0	8.865202	0	3.302348583		-20	0.086676	NW_003540202_39340_star	0.537285	7.253347	0.620384622	3.044979305		-3.75489	0.372767
chr18_35964_mature	2.149139864	40.29637	1.654957834	5.367943153		-4.22882	0.105695	chr8_19044_mature	0	8.865202	0	3.302348583		-20	0.086676	chr10_23904_mature	3.22371	26.59561	2.078510713	4.786366653		-3.04439	0.37421
NW_003537687_37130_star	25.78967836	1.611855	4.743605355	1.38507475		4	0.106506	chr6_14961_star	0	8.865202	0	3.302348583		-20	0.086676	NW_003540202_39340_mature	3.22371	26.59561	2.078510713	4.786366653		-3.04439	0.37421
mmu-miR-3057-5p	58.02677632	4.029637	5.883297648	2.330454351		3.847997	0.106546	chr10_23799_star	0	8.865202	0	3.302348583		-20	0.086676	chr7_16275_mature	3.22371	26.59561	2.078510713	4.786366653		-3.04439	0.37421
chrx_22277_mature	3.223709795	57.22085	2.078510713	5.863463969		-4.14975	0.107189	bta-miR-6524	0	8.865202	0	3.302348583		-20	0.086676	hsa-miR-509-5p	16.65583	1.611855	4.142073061	1.38507475		3.369234	0.374557
chr14_30497_star	12.89483918	284.4924	3.796477232	8.157308477		-4.46352	0.107565	chr6_12645_star	0	8.865202	0	3.302348583		-20	0.086676	chr13_27323_mature	16.65583	1.611855	4.142073061	1.38507475		3.369234	0.374557
chr2_4773_mature	35.46080775	2.417782	5.188274618	1.773060525		3.874469	0.107643	chr10_23275_mature	0	8.865202	0	3.302348583		-20	0.086676	chr13_27765_mature	16.65583	1.611855	4.142073061	1.38507475		3.369234	0.374557
chr7_15769_mature	20.4168287	523.8528	4.420672964	9.035769166		-4.68133	0.108211	chr1_1729_star	0	8.865202	0	3.302348583		-20	0.086676	ssc-miR-216	161.7228	12.08891	7.346272375	3.710273245		3.741767	0.375187
chr6_14372_mature	3.223709795	56.41492	2.078510713	5.843353819		-4.12928	0.108985	bta-miR-3432	0	8.865202	0	3.302348583		-20	0.086676	chr9_19519_star	4.29828	36.26674	2.405524014	5.21981653		-3.07682	0.375424
chr9_20315_mature	1.074569932	24.17782	1.05281229	4.654081667		-4.49185	0.109233	rno-miR-34a-5p	0	8.865202	0	3.302348583		-20	0.086676	ssc-miR-195	92.9503	8.059274	6.55382585	3.179395517		3.527738	0.37561
chr16_33881_mature	1.074569932	24.17782	1.05281229	4.654081667		-4.49185	0.109233	chr13_27796_star	0	8.865202	0	3.302348583		-20	0.086676	chr17_35440_mature	10.7457	0.805927	3.554060706	0.852739935		3.736966	0.376318
chrx_22644_star	1.074569932	24.17782	1.05281229	4.654081667		-4.49185	0.109233	chr16_33720_star	0	8.865202	0	3.302348583		-20	0.086676	rno-miR-3586-5p	10.7457	0.805927	3.554060706	0.852739935		3.736966	0.376318
hsa-miR-4273	1.074569932	24.17782	1.05281229	4.654081667		-4.49185	0.109233	NW_003613454_39203_mature	0	8.865202	0	3.302348583		-20	0.086676	chr16_33881_star	10.7457	0.805927	3.554060706	0.852739935		3.736966	0.376318
chr2_4612_mature	81.66731482	5.641492	6.369245121	2.731507408		3.85561	0.109234	chr1_2968_mature	0	8.865202	0	3.302348583		-20	0.086676	chr5_11163_mature	10.7457	0.805927	3.554060706	0.852739935		3.736966	0.376318
NW_003539151_38597_mature	973.5603582	41.1023	9.92860773	5.395827139		4.565979	0.110131	chr10_23707_mature	0	8.865202	0	3.302348583		-20	0.086676	chr6_13529_mature	10.7457	0.805927	3.554060706	0.852739935		3.736966	0.376318
chr7_15151_mature	2.149139864	38.68452	1.654957834	5.310504361		-4.16993	0.110774	ssc-miR-374a-3p	10.7456993	144.261	3.554060706	7.182503739		-3.74685	0.087833	chr15_32610_mature	10.7457	0.805927	3.554060706	0.852739935		3.736966	0.376318
chr5_11229_mature	107.4569932	7.253347	6.760979269	3.044979305		3.888969	0.110969	chr1_2437_star	0	8.059274	0	3.179395517		-20	0.089379	chrx_21531_mature	2.14914	17.7304	1.654957834	4.227310103		-3.04439	0.37663
chr1_2829_mature	8.596559455	0	3.262517265	0		20	0.110994	chr1_814_star	0	8.059274	0	3.179395517		-20	0.089379	chr6_12645_mature	2.14914	17.7304	1.654957834	4.227310103		-3.04439	0.37663
NW_003613192_37588_mature	8.596559455	0	3.262517265	0		20	0.110994	rno-miR-145-5p	0	8.059274	0	3.179395517		-20	0.089379	chr15_32058_mature	2.14914	17.7304	1.654957834	4.227310103		-3.04439	0.37663
chr17_35489_mature	8.596559455	0	3.262517265	0		20	0.110994	chr12_25510_mature	0	8.059274	0	3.179395517		-20	0.089379	chr9_19233_mature	2.14914	17.7304	1.654957834	4.227310103		-3.04439	0.37663
chr6_14386_mature	8.596559455	0	3.262517265	0		20	0.110994	chr14_30180_mature	0	8.059274	0	3.179395517		-20	0.089379	chr14_30331_mature	23.10325	2.417782	4.591155994	1.773060525		3.25634	0.378835
chr14_31358_mature	8.596559455	0	3.262517265	0		20	0.110994	chr10_23893_star	0	8.059274	0	3.179395517		-20	0.089379	chr9_20900_mature	2.686425	21.76004	1.882222339	4.508431259		-3.01792	0.379118
mmu-miR-1954	8.596559455	0	3.262517265	0		20	0.110994	rno-miR-222-3p	0	8.059274	0	3.179395517		-20	0.089379	chr13_27363_mature	2.686425	21.76004	1.882222339	4.508431259		-3.01792	0.379118
chr14_30807_mature	8.596559455	0	3.262517265	0		20	0.110994	chr4_9452_star	0	8.059274	0	3.179395517		-20	0.089379	chr6_12752_mature	1.611855	13.70077	1.38507475	3.877819487		-3.08746	0.379996
chr2_5420_star	8.596559455	0	3.262517265	0		20	0.110994	NW_003613497_39430_mature	0	8.059274	0	3.179395517		-20	0.089379	chr1_3231_star	1.611855	13.70077	1.38507475	3.877819487		-3.08746	0.379996
rno-miR-342-5p	8.596559455	0	3.262517265	0		20	0.110994	chr5_11099_mature	0	8.059274	0	3.179395517		-20	0.089379	chr2_4280_star	1.611855	13.70077	1.38507475	3.877819487		-3.08746	0.379996
mmu-miR-3062-5p	8.596559455	0	3.262517265	0		20	0.110994	chr16_34510_mature	0	8.059274	0	3.179395517		-20	0.089379	chr18_36505_mature	1.611855	13.70077	1.38507475	3.877819487		-3.08746	0.379996
rno-miR-743a-5p	8.596559455	0	3.262517265	0		20	0.110994	chr1_877_mature	0	8.059274	0	3.179395517		-20	0.089379	chr11_25144_mature	79.51817	7.253347	6.331242567	3.044979305		3.454566	0.380925
chr7_17045_star	8.596559455	0	3.262517265	0		20	0.110994	chr4_10537_mature	0	8.059274	0	3.179395517		-20	0.089379	rno-miR-380-3p	5.910135	0	2.788713818	0		20	0.381625
bta-miR-450b	8.596559455	0	3.262517265	0		20	0.110994	bta-miR-2284j	0	8.059274	0	3.179395517		-20	0.089379	hsa-miR-1256	5.910135	0	2.788713818	0		20	0.381625
chr2_4579_mature	8.596559455	0	3.262517265	0		20	0.110994	hsa-miR-582-5p	0	8.059274	0	3.179395517		-20	0.089379	hsa-miR-548aq-3p	5.910135	0	2.788713818	0		20	0.381625
chr1_607_mature	8.596559455	0	3.262517265	0		20	0.110994	bta-miR-100	0	8.059274	0	3.179395517		-20	0.089379	chr14_31505_mature	5.910135	0	2.788713818	0		20	0.381625
NW_003538465_37902_star	8.596559455	0	3.262517265	0		20	0.110994	chr9_20710_mature	0	8.059274	0	3.179395517		-20	0.089379	rno-miR-322-3p	5.910135	0	2.788713818	0		20	0.381625
hsa-miR-339-5p	8.596559455	0	3.262517265	0		20	0.110994	NW_003539115_38548_mature	0	8.059274	0	3.179395517		-20	0.089379	chr2_6082_star	5.910135	0	2.788713818	0		20	0.381625
chrx_21839_mature	8.596559455	0	3.262517265	0		20	0.110994	chr14_31645_mature	0	8.059274	0	3.179395517		-20	0.089379	chr4_9149_mature@@rno-miR-298	5.910135	0	2.788713818	0		20	0.381625
rno-miR-297	8.596559455	0	3.262517265	0		20	0.110994	chr2_6581_mature	0	8.059274	0	3.179395517		-20	0.089379	chr7_15714_mature	5.910135	0	2.788713818	0		20	0.381625
chr12_25921_mature	8.596559455	0	3.262517265	0		20	0.110994	chr5_11482_star	0	8.059274	0	3.179395517		-20	0.089379	chr12_25609_star	5.910135	0	2.788713818	0		20	0.381625
chr5_11515_mature	8.596559455	0	3.262517265	0		20	0.110994	chr16_34138_mature	0	8.059274	0	3.179395517		-20	0.089379	chr8_17361_mature	5.910135	0	2.788713818	0		20	0.381625
mmu-miR-1946b	8.596559455	0	3.262517265	0		20	0.110994	chrx_23221_star	0	8.059274	0	3.179395517		-20	0.089379	chr5_12150_star	5.910135	0	2.788713818	0		20	0.381625
NW_003613251_37928_star	8.596559455	0	3.262517265	0		20	0.110994	chr12_26098_star	0	8.059274	0	3.179395517		-20	0.089379	chr15_33120_star	5.910135	0	2.788713818	0		20	0.381625
chr10_23462_mature	8.596559455	0	3.262517265	0		20	0.110994	chr17_35178_star	0	8.059274	0	3.179395517		-20	0.089379	chr12_25838_star	5.910135	0	2.788713818	0		20	0.381625
rno-miR-296-5p	8.596559455	0	3.262517265	0		20	0.110994	mmu-miR-294-3p	0	8.059274	0	3.179395517		-20	0.089379	rno-miR-466b-2-3p	5.910135	0	2.788713818	0		20	0.381625
chr13_29380_star	8.596559455	0	3.262517265	0		20	0.110994	chr4_9430_star	0	8.059274	0	3.179395517		-20	0.089379	mmu-miR-6339	5.910135	0	2.788713818	0		20	0.381625
chr8_17814_star	8.596559455	0	3.262517265	0		20	0.110994	chr6_14759_mature	0	8.059274	0	3.179395517		-20	0.089379	chrx_22793_star	5.910135	0	2.788713818	0		20	0.381625
chr9_20403_star	8.596559455	0	3.262517265	0		20	0.110994	chr1_2966_mature	0	8.059274	0	3.179395517		-20	0.089379	chr4_9185_star	5.910135	0	2.788713818	0		20	0.381625
chrx_22202_mature	8.596559455	0	3.262517265	0		20	0.110994	chr9_19423_star	0	8.059274	0	3.179395517		-20	0.089379	chr1_3090_mature	5.910135	0	2.788713818	0		20	0.381625
chr12_25405_star	8.596559455	0	3.262517265	0		20	0.110994	chr12_25520_mature	0	8.059274	0	3.179395517		-20	0.089379	bta-miR-2284h-3p	5.910135	0	2.788713818	0		20	0.381625
chr12_25396_star	8.596559455	0	3.262517265	0		20	0.110994	chr15_31849_mature	0	7.253347	0	3.044979305		-20	0.092368	bta-miR-211	5.910135	0	2.788713818	0		20	0.381625
mmu-miR-3107-5p	8.596559455	0	3.262517265	0		20	0.110994	chr9_20618_mature@@rno-miR-135b-	0	7.253347	0	3.044979305		-20	0.092368	hsa-miR-3134	5.910135	0	2.788713818	0		20	0.381625
chr8_17482_mature	8.596559455	0	3.262517265	0		20	0.110994	chr16_34772_star	0	7.253347	0	3.044979305		-20	0.092368	rno-miR-195-5p	5.910135	0	2.788713818	0		20	0.381625
chr16_34606_mature	8.596559455	0	3.262517265	0		20	0.110994	hsa-miR-1252	0	7.253347	0	3.044979305		-20	0.092368	mmu-miR-361-3p	5.910135	0	2.788713818	0		20	0.381625
bta-miR-2285n	4.298279727	74.14533	2.405524014	6.231611453		-4.10852	0.11136	hsa-miR-581	0	7.253347	0	3.044979305		-20	0.092368	rno-miR-455-3p	5.910135	0	2.788713818	0		20	0.381625
chr8_18024_mature	80.59274489	5.641492	6.35036897	2.731507408		3.836501	0.111366	chr1_1317_star	0	7.253347	0	3.044979305		-20	0.092368	chr7_15713_mature	5.910135	0	2.788713818	0		20	0.381625
chr7_16110_mature	93.48758407	6.44742	6.562052863	2.896740642		3.857981	0.111617	chr2_5556_star	0	7.253347	0	3.044979305		-20	0.092368	chr18_36328_star	5.910135	0	2.788713818	0		20	0.381625
chr10_23656_star	34.38623782	2.417782	5.145116481	1.773060525		3.830075	0.112301	hsa-miR-5004-5p	0	7.253347	0	3.044979305		-20	0.092368	chr5_11369_star	5.910135	0	2.788713818	0		20	0.381625
chr2_4773_star	16.11854898	0.805927	4.097488514	0.852739935		4.321928	0.11238	NW_003613217_37697_star	0	7.253347	0	3.044979305		-20	0.092368	bta-miR-2287	5.910135	0	2.788713818	0		20	0.381625
chrx_22329_mature	16.11854898	0.805927	4.097488514	0.852739935		4.321928	0.11238	chr16_34837_mature	0	7.253347	0	3.044979305		-20	0.092368	chr8_17657_mature	5.910135	0	2.788713818	0		20	0.381625
mmu-let-7g-3p	16.11854898	0.805927	4.097488514	0.852739935		4.321928	0.11238	chr11_25109_star	0	7.253347	0	3.044979305		-20	0.092368	mmu-miR-466k	5.910135	0	2.788713818	0		20	0.381625
chr10_23348_star	16.11854898	0.805927	4.097488514	0.852739935		4.321928	0.11238	chr6_14945_star	0	7.253347	0	3.044979305		-20	0.092368	chr4_8979_star	5.910135	0	2.788713818	0		20	0.381625
bta-miR-2316	16.11854898	0.805927	4.097488514	0.852739935		4.321928	0.11238	mmu-miR-712-3p	0	7.253347	0	3.044979305		-20	0.092368	NW_003539513_38909_mature	5.910135	0	2.788713818	0		20	0.381625
chr12_26044_mature	16.11854898	0.805927	4.097488514	0.852739935		4.321928	0.11238	chr7_16117_mature	0	7.253347	0	3.044979305		-20	0.092368	chr15_33182_star	5.910135	0	2.788713818	0		20	0.381625
chr3_7100_mature	16.11854898	0.805927	4.097488514	0.852739935		4.321928	0.11238	chr7_16529_mature	0	7.253347	0	3.044979305		-20	0.092368	chr17_35278_star	5.910135	0	2.788713818	0		20	0.381625
chr7_16274_mature	650.1148088	30.62524	9.346768141	4.983004663		4.407901	0.112646	chr5_11229_mature	0	7.253347	0	3.044979305		-20	0.092368	hsa-miR-3942-5p	5.910135	0	2.788713818	0		20	0.381625
ssc-miR-204	229.9579654	13.70077	7.851486493	3.877819487		4.069042	0.112683	mmu-miR-6420	0	7.253347	0	3.044979305		-20	0.092368	chr18_36298_star	5.910135	0	2.788713818	0		20	0.381625
chr1_1793_star	24.71510843	1.611855	4.684544332	1.38507475		3.938599	0.112781	chr16_34016_mature	0	7.253347	0	3.044979305		-20	0.092368	rno-miR-141-5p	5.910135	0	2.788713818	0		20	0.381625
chr7_16063_star	24.71510843	1.611855	4.684544332	1.38507475		3.938599	0.112781	mmu-miR-6387	0	7.253347	0	3.044979305		-20	0.092368	hsa-miR-3940-3p	5.910135	0	2.788713818	0		20	0.381625
chr6_14437_mature	7.521989523	138.6195	3.091190278	7.125356859		-4.20387	0.112859	bta-miR-2285g	0	7.253347	0	3.044979305		-20	0.092368	chr2_6125_mature	38.68452	4.029637	5.310504361	2.330454351		3.263034	0.382102
chr8_17809_mature	1.074569932	23.3719	1.05281229	4.607146585		-4.44294	0.113426	chr1_495_star	0	7.253347	0	3.044979305		-20	0.092368	chr2_6509_star	16.11855	1.611855	4.097488514	1.38507475		3.321928	0.383036
bta-miR-409a	1.074569932	23.3719	1.05281229	4.607146585		-4.44294	0.113426	hsa-miR-518f-3p	0	7.253347	0	3.044979305		-20	0.092368	chrx_22089_mature	16.11855	1.611855	4.097488514	1.38507475		3.321928	0.383036
chr13_28990_mature	1.074569932	23.3719	1.05281229	4.607146585		-4.44294	0.113426	chr11_25333_star	0	7.253347	0	3.044979305		-20	0.092368	chr17_35500_star	16.11855	1.611855	4.097488514	1.38507475		3.321928	0.383036
chr10_24200_mature	1.074569932	23.3719	1.05281229	4.607146585		-4.44294	0.113426	chr16_34708_star	0	7.253347	0	3.044979305		-20	0.092368	chr7_15747_mature	16.11855	1.611855	4.097488514	1.38507475		3.321928	0.383036
hsa-miR-526a	2.149139864	37.87859	1.654957834	5.280903997		-4.13955	0.113459	chr5_12382_mature	0	7.253347	0	3.044979305		-20	0.092368	chr14_31116_mature	16.11855	1.611855	4.097488514	1.38507475		3.321928	0.383036
chr13_27641_mature	0	11.28298	0	3.618589217		-20	0.113563	chr15_33029_star	0	7.253347	0	3.044979305		-20	0.092368	chr2_4828_mature	4.29828	35.46081	2.405524014	5.188274618		-3.04439	0.383302
chr1_2014_mature	0	11.28298	0	3.618589217		-20	0.113563	chrx_23209_mature	0	7.253347	0	3.044979305		-20	0.092368	chr16_34849_mature	142.9178	11.28298	7.169101237	3.618589217		3.662965	0.383639
chr4_10065_star	0	11.28298	0	3.618589217		-20	0.113563	chr3_8179_star	0	7.253347	0	3.044979305		-20	0.092368	chr2_6348_mature	67.69791	6.44742	6.102194213	2.896740642		3.392317	0.383715
rno-miR-741-5p	0	11.28298	0	3.618589217		-20	0.113563	chr1_3743_star	0	7.253347	0	3.044979305		-20	0.092368	chr6_14709_mature	67.16062	6.44742	6.090866569	2.896740642		3.380822	0.386037
hsa-miR-3157-3p	0	11.28298	0	3.618589217		-20	0.113563	chr1_2345_star	0	7.253347	0	3.044979305		-20	0.092368	ssc-miR-27b-5p	38.68452	608.4752	5.310504361	9.251423764		-3.97537	0.388708
chr13_29218_mature	0	11.28298	0	3.618589217		-20	0.113563	chr9_19819_mature	0	7.253347	0	3.044979305		-20	0.092368	chr13_27641_star	10.20841	0.805927	3.48651029	0.852739935		3.662965	0.389235
chr2_4238_mature	0	11.28298	0	3.618589217		-20	0.113563	bta-miR-497	0	7.253347	0	3.044979305		-20	0.092368	chr2_6343_mature	10.20841	0.805927	3.48651029	0.852739935		3.662965	0.389235
mmu-miR-705	0	11.28298	0	3.618589217		-20	0.113563	chr2_6087_star	0	7.253347	0	3.044979305		-20	0.092368	chr9_19892_mature	10.20841	0.805927	3.48651029	0.852739935		3.662965	0.389235
hsa-miR-5699	0	11.28298	0	3.618589217		-20	0.113563	NW_003541128_39926_star	0	7.253347	0	3.044979305		-20	0.092368	chr5_11678_mature	29.55067	3.22371	4.933132262	2.078510713		3.196397	0.389324
chr3_8533_mature	263.2696333	15.31262	8.045866847	4.027916745		4.103748	0.113578	chr1_943_mature	0	7.253347	0	3.044979305		-20	0.092368	chr17_34904_mature	5.37285	45.13194	2.671938627	5.527693969		-3.07039	0.389779
mmu-miR-5103	44.0573672	3.22371	5.493691111	2.078510713		3.77259	0.114912	chr8_17373_star	0	7.253347	0	3.044979305		-20	0.092368	chr11_25311_mature	5.37285	45.13194	2.671938627	5.527693969		-3.07039	0.389779
hsa-miR-5100	44.0573672	3.22371	5.493691111	2.078510713		3.77259	0.114912	NW_003540202_39340_star	0	7.253347	0	3.044979305		-20	0.092368	ssc-miR-323	11.28298	114.4417	3.618589217	6.851020612		-3.34239	0.390475
chr1_3355_mature	44.0573672	3.22371	5.493691111	2.078510713		3.77259	0.114912	hsa-miR-4329	0	7.253347	0	3.044979305		-20	0.092368	NW_003538623_38054_mature	4.29828	34.65488	2.405524014	5.156027656		-3.01123	0.391342
chr13_28842_mature	44.0573672	3.22371	5.493691111	2.078510713		3.77259	0.114912	chr1_2375_mature	0	7.253347	0	3.044979305		-20	0.092368	chr1_3039_star	4.29828	34.65488	2.405524014	5.156027656		-3.01123	0.391342
ssc-miR-16	228232.2061	2383.933	17.80014918	11.21973326		6.581015	0.115538	chr1_1197_star	0	7.253347	0	3.044979305		-20	0.092368	hsa-miR-6723-5p	22.02868	2.417782	4.525360039	1.773060525		3.187627	0.391467
ssc-miR-218-3p	54.80306652	4.029637	5.802272499	2.330454351		3.765535	0.115565	bta-miR-208a	0	7.253347	0	3.044979305		-20	0.092368	mmu-miR-6411	2.14914	16.92448	1.654957834	4.163859073		-2.97728	0.391531
chr9_19555_mature	33.31166789	2.417782	5.100627352	1.773060525		3.784271	0.117244	chr15_33531_mature	2.14913986	116.8595	1.654957834	6.880923997		-5.76487	0.095189	chr13_26740_mature	46.20651	4.835565	5.560913833	2.544872269		3.25634	0.39158
chr1_3261_mature	9.671129386	182.9455	3.415640968	7.523134815		-4.24159	0.117511	chr1_1833_mature	2.14913986	116.8595	1.654957834	6.880923997		-5.76487	0.095189	chr12_25530_star	3.760995	29.81932	2.251263041	4.94576292		-2.98706	0.391639
chr6_13459_mature	103.1587135	7.253347	6.702639724	3.044979305		3.830075	0.117838	chr17_35020_star	0	6.44742	0	2.896740642		-20	0.095702	rno-miR-151-3p	15.58126	1.611855	4.051482084	1.38507475		3.273018	0.391778
NW_003613231_37780_star	1.074569932	22.56597	1.05281229	4.558633073		-4.39232	0.117879	NW_003541072_39884_mature	0	6.44742	0	2.896740642		-20	0.095702	hsa-miR-639	15.58126	1.611855	4.051482084	1.38507475		3.273018	0.391778
NW_003613199_37634_mature	1.074569932	22.56597	1.05281229	4.558633073		-4.39232	0.117879	chr13_27068_mature	0	6.44742	0	2.896740642		-20	0.095702	NW_003539018_38471_star	15.58126	1.611855	4.051482084	1.38507475		3.273018	0.391778
chr15_31936_star	1.074569932	22.56597	1.05281229	4.558633073		-4.39232	0.117879	chr5_10855_star	0	6.44742	0	2.896740642		-20	0.095702	chr13_29079_mature	15.58126	1.611855	4.051482084	1.38507475		3.273018	0.391778
chr16_34458_mature	1.074569932	22.56597	1.05281229	4.558633073		-4.39232	0.117879	hsa-miR-3935	0	6.44742	0	2.896740642		-20	0.095702	chr2_6577_mature	65.54877	6.44742	6.056340006	2.896740642		3.345775	0.393121
chr1_2426_star	3.223709795	52.38528	2.078510713	5.738370208		-4.02237	0.118725	chr2_4320_star	0	6.44742	0	2.896740642		-20	0.095702	chr2_4280_mature	8.596559	79.78682	3.262517265	6.336047992		-3.21432	0.393971
chr14_30822_star	42.98279727	3.22371	5.458867456	2.078510713		3.736966	0.118882	chr14_31524_mature	0	6.44742	0	2.896740642		-20	0.095702	chr4_9318_mature	6.44742	55.60899	2.896740642	5.82295938		-3.10852	0.3941
chr3_8247_mature	2.149139864	36.26674	1.654957834	5.21981653		-4.07682	0.119148	chr6_14709_mature	0	6.44742	0	2.896740642		-20	0.095702	chr1_1416_mature	5.910135	49.9675	2.788713818	5.671505736		-3.07973	0.394387
chr5_11922_star	2.149139864	36.26674	1.654957834	5.21981653		-4.07682	0.119148	hsa-miR-216a-3p	0	6.44742	0	2.896740642		-20	0.095702	chrx_22122_mature	3.22371	24.98375	2.078510713	4.699537803		-2.9542	0.395236
chr12_25930_mature	23.6405385	1.611855	4.62296188	1.38507475		3.874469	0.119582	chr2_6442_mature	0	6.44742	0	2.896740642		-20	0.095702	chr16_33846_mature	1.07457	9.671129	1.05281229	3.415640968		-3.16993	0.396048
chr12_25511_mature	6.447419591	107.9943	2.896740642	6.76810859		-4.06609	0.122081	chr14_29554_star	0	6.44742	0	2.896740642		-20	0.095702	chr8_19043_mature	1.07457	9.671129	1.05281229	3.415640968		-3.16993	0.396048
chr5_11657_star	2.149139864	35.46081	1.654957834	5.188274618		-4.04439	0.122163	chr8_18597_star	0	6.44742	0	2.896740642		-20	0.095702	chr10_24076_mature	1.07457	9.671129	1.05281229	3.415640968		-3.16993	0.396048
chr3_6770_mature	52.65392666	4.029637	5.745611853	2.330454351		3.707819	0.122177	chr13_27234_mature	0	6.44742	0	2.896740642		-20	0.095702	chr14_29856_mature	21.4914	2.417782	4.491301473	1.773060525		3.152003	0.397996
chr12_26594_mature	32.23709795	2.417782	5.054722516	1.773060525		3.736966	0.122494	chr1_2963_star	0	6.44742	0	2.896740642		-20	0.095702	chr13_28627_star	21.4914	2.417782	4.491301473	1.773060525		3.152003	0.397996
chr1_3877_star	32.23709795	2.417782	5.054722516	1.773060525		3.736966	0.122494	hsa-miR-526b-5p	0	6.44742	0	2.896740642		-20	0.095702	chr9_20275_mature	45.13194	4.835565	5.527693969	2.544872269		3.222392	0.398235
NW_003538449_37886_mature	1.074569932	21.76004	1.05281229	4.508431259		-4.33985	0.122614	hsa-miR-3160-3p	0	6.44742	0	2.896740642		-20	0.095702	chr10_23437_star	1.611855	12.89484	1.38507475	3.796477232		-3	0.398804
chr18_36565_mature	1.074569932	21.76004	1.05281229	4.508431259		-4.33985	0.122614	chr2_5590_star	0	6.44742	0	2.896740642		-20	0.095702	hsa-miR-4694-3p	1.611855	12.89484	1.38507475	3.796477232		-3	0.398804
chr14_31062_star	1.074569932	21.76004	1.05281229	4.508431259		-4.33985	0.122614	bta-miR-2285p	0	6.44742	0	2.896740642		-20	0.095702	chr14_30107_mature	1.611855	12.89484	1.38507475	3.796477232		-3	0.398804
chr17_34998_mature	1.074569932	21.76004	1.05281229	4.508431259		-4.33985	0.122614	hsa-miR-224-3p	0	6.44742	0	2.896740642		-20	0.095702	chr9_20889_mature	11.28298	112.0239	3.618589217	6.820484263		-3.31159	0.399035
bta-miR-2427	1.074569932	21.76004	1.05281229	4.508431259		-4.33985	0.122614	mmu-miR-3967	0	6.44742	0	2.896740642		-20	0.095702	ssc-miR-2320-5p	74.14533	1463.564	6.231611453	10.51625577		-4.30299	0.399134
chr15_32591_star	1.074569932	21.76004	1.05281229	4.508431259		-4.33985	0.122614	hsa-miR-520c-5p	0	6.44742	0	2.896740642		-20	0.095702	chr13_27948_mature	54.26578	5.641492	5.788314591	2.731507408		3.265894	0.399194
chr1_3135_mature	1.074569932	21.76004	1.05281229	4.508431259		-4.33985	0.122614	chr8_18290_mature	0	6.44742	0	2.896740642		-20	0.095702	chr2_6203_star	15.04398	1.611855	4.003960082	1.38507475		3.222392	0.400789
ssc-miR-2483	1.074569932	21.76004	1.05281229	4.508431259		-4.33985	0.122614	chr1_3435_star	0	6.44742	0	2.896740642		-20	0.095702	chr7_16134_mature	15.04398	1.611855	4.003960082	1.38507475		3.222392	0.400789
chr4_9639_star	15.04397905	0.805927	4.003960082	0.852739935		4.222392	0.122661	chr4_8847_star	0	6.44742	0	2.896740642		-20	0.095702	chr9_19409_mature	96.71129	8.865202	6.610453419	3.302348583		3.447459	0.401907
ssc-miR-190b	15.04397905	0.805927	4.003960082	0.852739935		4.222392	0.122661	chr7_16628_mature	0	6.44742	0	2.896740642		-20	0.095702	chr6_14308_mature	35.99809	4.029637	5.209378995	2.330454351		3.159199	0.402076
hsa-miR-3934-5p	15.04397905	0.805927	4.003960082	0.852739935		4.222392	0.122661	bta-miR-378	0	6.44742	0	2.896740642		-20	0.095702	mmu-miR-669c-5p	9.671129	0.805927	3.415640968	0.852739935		3.584963	0.402762
ssc-miR-325	15.04397905	0.805927	4.003960082	0.852739935		4.222392	0.122661	chr14_30768_star	0	6.44742	0	2.896740642		-20	0.095702	chr14_29799_mature	9.671129	0.805927	3.415640968	0.852739935		3.584963	0.402762
ssc-miR-144	15.04397905	0.805927	4.003960082	0.852739935		4.222392	0.122661	hsa-miR-4288	0	6.44742	0	2.896740642		-20	0.095702	chr2_4699_mature	9.671129	0.805927	3.415640968	0.852739935		3.584963	0.402762
chr17_35646_mature	15.04397905	0.805927	4.003960082	0.852739935		4.222392	0.122661	chr14_29946_mature	0	6.44742	0	2.896740642		-20	0.095702	chr9_20772_star	9.671129	0.805927	3.415640968	0.852739935		3.584963	0.402762
NW_003539221_38657_mature	15.04397905	0.805927	4.003960082	0.852739935		4.222392	0.122661	NW_003539115_38544_star	0	6.44742	0	2.896740642		-20	0.095702	chr13_28101_star	9.671129	0.805927	3.415640968	0.852739935		3.584963	0.402762
hsa-miR-4656	15.04397905	0.805927	4.003960082	0.852739935		4.222392	0.122661	NW_003613206_37650_mature	0	6.44742	0	2.896740642		-20	0.095702	chr17_35181_mature@@bta-miR-6	9.671129	0.805927	3.415640968	0.852739935		3.584963	0.402762
chr7_16194_mature	15.04397905	0.805927	4.003960082	0.852739935		4.222392	0.122661	chr16_34837_star	0	6.44742	0	2.896740642		-20	0.095702	chr1_3489_mature	9.671129	0.805927	3.415640968	0.852739935		3.584963	0.402762
chr3_7219_mature	0	10.47706	0	3.520680821		-20	0.122761	chr7_15961_star	0	6.44742	0	2.896740642		-20	0.095702	chr17_35576_star	9.671129	0.805927	3.415640968	0.852739935		3.584963	0.402762
hsa-miR-1243	0	10.47706	0	3.520680821		-20	0.122761	chr3_8070_mature	0	6.44742	0	2.896740642		-20	0.095702	chr13_27007_star	9.671129	0.805927	3.415640968	0.852739935		3.584963	0.402762
chrx_21983_star	0	10.47706	0	3.520680821		-20	0.122761	hsa-miR-1273e	0	6.44742	0	2.896740642		-20	0.095702	chr13_28621_star	9.671129	0.805927	3.415640968	0.852739935		3.584963	0.402762
chr15_32006_mature	0	10.47706	0	3.520680821		-20	0.122761	chr6_12531_mature	0	6.44742	0	2.896740642		-20	0.095702	chr12_25587_star	5.37285	43.52008	2.671938627	5.476384353		-3.01792	0.402882
chr5_11689_mature	0	10.47706	0	3.520680821		-20	0.122761	chr15_31672_mature	0	5.641492	0	2.731507408		-20	0.099452	hsa-miR-1290	0	4.029637	0	2.330454351		-20	0.403901
rno-miR-206-3p	0	10.47706	0	3.520680821		-20	0.122761	chr12_26266_mature	0	5.641492	0	2.731507408		-20	0.099452	hsa-miR-5092	0	4.029637	0	2.330454351		-20	0.403901
hsa-miR-637	0	10.47706	0	3.520680821		-20	0.122761	NW_003539074_38507_star	0	5.641492	0	2.731507408		-20	0.099452	chr8_17864_mature	0	4.029637	0	2.330454351		-20	0.403901
chr1_3135_star	0	10.47706	0	3.520680821		-20	0.122761	chr9_19622_mature	0	5.641492	0	2.731507408		-20	0.099452	chr4_10620_mature	0	4.029637	0	2.330454351		-20	0.403901
chr5_11270_mature	0	10.47706	0	3.520680821		-20	0.122761	rno-miR-33-3p	0	5.641492	0	2.731507408		-20	0.099452	chr18_36486_mature	0	4.029637	0	2.330454351		-20	0.403901
chr13_27786_mature	5.372849659	87.04016	2.671938627	6.460089935		-4.01792	0.122868	chr5_12211_star	0	5.641492	0	2.731507408		-20	0.099452	bta-miR-195	0	4.029637	0	2.330454351		-20	0.403901
chr18_36189_mature	5.372849659	87.04016	2.671938627	6.460089935		-4.01792	0.122868	chr13_26861_mature	0	5.641492	0	2.731507408		-20	0.099452	chr13_29025_star	0	4.029637	0	2.330454351		-20	0.403901
chr6_14013_mature	10.74569932	199.0641	3.554060706	7.644318354		-4.2114	0.124799	chr7_17039_mature	0	5.641492	0	2.731507408		-20	0.099452	mmu-miR-449a-5p	0	4.029637	0	2.330454351		-20	0.403901
NW_003538623_38054_mature	2.149139864	34.65488	1.654957834	5.156027656		-4.01123	0.1253	bta-miR-487a	0	5.641492	0	2.731507408		-20	0.099452	chr6_14288_mature	0	4.029637	0	2.330454351		-20	0.403901
chr8_18995_star	74.1453253	5.641492	6.231611453	2.731507408		3.716207	0.12546	hsa-miR-518d-5p	0	5.641492	0	2.731507408		-20	0.099452	mmu-miR-143-3p	0	4.029637	0	2.330454351		-20	0.403901
chr1_2751_mature	5.372849659	84.62238	2.671938627	6.419916069		-3.97728	0.126938	chr5_10971_mature	0	5.641492	0	2.731507408		-20	0.099452	hsa-miR-658	0	4.029637	0	2.330454351		-20	0.403901
chr9_20219_star	22.56596857	1.611855	4.558633073	1.38507475		3.807355	0.126963	rno-miR-222-5p	0	5.641492	0	2.731507408		-20	0.099452	rno-miR-582-5p	0	4.029637	0	2.330454351		-20	0.403901
chr15_32200_mature	22.56596857	1.611855	4.558633073	1.38507475		3.807355	0.126963	NW_003537206_36767_star	0	5.641492	0	2.731507408		-20	0.099452	mmu-miR-684	0	4.029637	0	2.330454351		-20	0.403901
chr13_26704_mature	22.56596857	1.611855	4.558633073	1.38507475		3.807355	0.126963	chrx_23015_mature	0	5.641492	0	2.731507408		-20	0.099452	chr3_8078_star	0	4.029637	0	2.330454351		-20	0.403901
chr2_5822_mature	40.83365741	3.22371	5.38659223	2.078510713		3.662965	0.127412	bta-miR-181a	0	5.641492	0	2.731507408		-20	0.099452	chr6_12992_star	0	4.029637	0	2.330454351		-20	0.403901
chr15_32017_mature	1.074569932	20.95411	1.05281229	4.456419386		-4.2854	0.127656	mmu-miR-5107-3p	0	5.641492	0	2.731507408		-20	0.099452	chr16_33995_mature	2.686425	20.14819	1.882222339	4.402462031		-2.90689	0.404297
chr16_34112_mature	1.074569932	20.95411	1.05281229	4.456419386		-4.2854	0.127656	chrx_23196_star	0	5.641492	0	2.731507408		-20	0.099452	chr5_12034_mature	27.93882	3.22371	4.854934103	2.078510713		3.115477	0.40453
chr7_16272_mature	1.074569932	20.95411	1.05281229	4.456419386		-4.2854	0.127656	NW_003613290_38304_star	0	5.641492	0	2.731507408		-20	0.099452	chr7_16043_mature	20.95411	2.417782	4.456419386	1.773060525		3.115477	0.40467
chrx_23008_mature	1.074569932	20.95411	1.05281229	4.456419386		-4.2854	0.127656	NW_003613031_36829_star	0	5.641492	0	2.731507408		-20	0.099452	chr1_3915_mature	20.95411	2.417782	4.456419386	1.773060525		3.115477	0.40467
bta-miR-574	1.074569932	20.95411	1.05281229	4.456419386		-4.2854	0.127656	hsa-miR-4433-5p	0	5.641492	0	2.731507408		-20	0.099452	chr2_6281_star	20.95411	2.417782	4.456419386	1.773060525		3.115477	0.40467
chr12_26051_star	1.074569932	20.95411	1.05281229	4.456419386		-4.2854	0.127656	chrx_21936_star	0	5.641492	0	2.731507408		-20	0.099452	chr10_23828_mature	20.95411	2.417782	4.456419386	1.773060525		3.115477	0.40467
chr6_13244_mature	1.074569932	20.95411	1.05281229	4.456419386		-4.2854	0.127656	chr10_23487_mature	0	5.641492	0	2.731507408		-20	0.099452	chr3_7627_mature	20.95411	2.417782	4.456419386	1.773060525		3.115477	0.40467
chr11_24871_mature	1.074569932	20.95411	1.05281229	4.456419386		-4.2854	0.127656	chr9_19579_star	0	5.641492	0	2.731507408		-20	0.099452	rno-miR-101b-3p	53.19121	5.641492	5.759986999	2.731507408		3.237039	0.404941
hsa-miR-517-5p	1.074569932	20.95411	1.05281229	4.456419386		-4.2854	0.127656	chr2_5656_mature	0	5.641492	0	2.731507408		-20	0.099452	ssc-miR-125b	664.6215	30049.81	9.37855823	14.8751163		-5.49868	0.405009
chr2_6135_mature	1.074569932	20.95411	1.05281229	4.456419386		-4.2854	0.127656	chr2_5587_mature	0	5.641492	0	2.731507408		-20	0.099452	chr1_478_mature	9.133844	83.81645	3.34110968	6.406272275		-3.19794	0.405019
chr3_7373_mature	31.16252802	2.417782	5.007308904	1.773060525		3.688056	0.128074	NW_003613264_38074_star	0	5.641492	0	2.731507408		-20	0.099452	chr18_36577_star	5.37285	0	2.671938627	0		20	0.405387
chr1_188_mature	31.16252802	2.417782	5.007308904	1.773060525		3.688056	0.128074	chr3_8428_star	0	5.641492	0	2.731507408		-20	0.099452	chr18_35896_star	5.37285	0	2.671938627	0		20	0.405387
mmu-miR-1931	2.149139864	33.84895	1.654957834	5.123043401		-3.97728	0.128565	mmu-miR-6540-3p	0	5.641492	0	2.731507408		-20	0.099452	chr3_6736_star	5.37285	0	2.671938627	0		20	0.405387
chr13_26618_mature	2.149139864	33.84895	1.654957834	5.123043401		-3.97728	0.128565	chr6_14536_star	0	5.641492	0	2.731507408		-20	0.099452	chr17_34920_mature	5.37285	0	2.671938627	0		20	0.405387
ssc-miR-143-5p	64.47419591	2311.4	6.032854532	11.17517521		-5.1639	0.128946	chr7_16945_mature	0	5.641492	0	2.731507408		-20	0.099452	rno-miR-134-5p	5.37285	0	2.671938627	0		20	0.405387
chr7_17049_mature	101.0095736	4397.946	6.672560745	12.1029422		-5.44427	0.129854	rno-miR-532-5p	0	5.641492	0	2.731507408		-20	0.099452	mmu-miR-146a-3p	5.37285	0	2.671938627	0		20	0.405387
chr13_27066_star	7.521989523	0	3.091190278	0		20	0.130285	chr8_18995_star	0	5.641492	0	2.731507408		-20	0.099452	chr17_35177_mature@@bta-miR-6	5.37285	0	2.671938627	0		20	0.405387
chr15_33671_mature	7.521989523	0	3.091190278	0		20	0.130285	rno-miR-3559-5p	0	5.641492	0	2.731507408		-20	0.099452	chr1_2128_star	5.37285	0	2.671938627	0		20	0.405387
chr12_25546_star	7.521989523	0	3.091190278	0		20	0.130285	chr9_19744_star	0	5.641492	0	2.731507408		-20	0.099452	mmu-miR-1912-5p	5.37285	0	2.671938627	0		20	0.405387
hsa-miR-1247-5p	7.521989523	0	3.091190278	0		20	0.130285	chr14_31047_mature	0	5.641492	0	2.731507408		-20	0.099452	hsa-miR-7-5p	5.37285	0	2.671938627	0		20	0.405387
chr12_25838_mature	7.521989523	0	3.091190278	0		20	0.130285	chr11_25110_mature	0	5.641492	0	2.731507408		-20	0.099452	mmu-miR-677-5p	5.37285	0	2.671938627	0		20	0.405387
chr16_34657_star	7.521989523	0	3.091190278	0		20	0.130285	chr2_4728_star	0	5.641492	0	2.731507408		-20	0.099452	NW_003613109_37254_star	5.37285	0	2.671938627	0		20	0.405387
rno-miR-759	7.521989523	0	3.091190278	0		20	0.130285	bta-miR-323	0	5.641492	0	2.731507408		-20	0.099452	chr12_26057_star	5.37285	0	2.671938627	0		20	0.405387
rno-miR-434-3p	7.521989523	0	3.091190278	0		20	0.130285	chr1_182_star	0	5.641492	0	2.731507408		-20	0.099452	rno-miR-6314	5.37285	0	2.671938627	0		20	0.405387
rno-miR-483-3p	7.521989523	0	3.091190278	0		20	0.130285	chr1_886_star	0	5.641492	0	2.731507408		-20	0.099452	chr1_2947_star	5.37285	0	2.671938627	0		20	0.405387
chr1_3489_star	7.521989523	0	3.091190278	0		20	0.130285	chr2_5816_star	0	5.641492	0	2.731507408		-20	0.099452	rno-miR-188-5p	5.37285	0	2.671938627	0		20	0.405387
chrx_23039_mature	7.521989523	0	3.091190278	0		20	0.130285	chr8_18125_mature	0	5.641492	0	2.731507408		-20	0.099452	chr2_6208_star	5.37285	0	2.671938627	0		20	0.405387
bta-miR-503-3p	7.521989523	0	3.091190278	0		20	0.130285	chr3_7580_star	0	5.641492	0	2.731507408		-20	0.099452	chr18_36156_star	5.37285	0	2.671938627	0		20	0.405387
rno-miR-6320	7.521989523	0	3.091190278	0		20	0.130285	chr1_816_star	0	5.641492	0	2.731507408		-20	0.099452	chr15_32591_mature	5.37285	0	2.671938627	0		20	0.405387
chr6_13131_mature	7.521989523	0	3.091190278	0		20	0.130285	rno-miR-101b-3p	0	5.641492	0	2.731507408		-20	0.099452	chr2_4071_star	5.37285	0	2.671938627	0		20	0.405387
chr14_29583_mature	7.521989523	0	3.091190278	0		20	0.130285	hsa-miR-4666b	0	5.641492	0	2.731507408		-20	0.099452	chr17_35707_mature	5.37285	0	2.671938627	0		20	0.405387
NW_003613109_37259_mature	7.521989523	0	3.091190278	0		20	0.130285	chr5_11238_star	0	5.641492	0	2.731507408		-20	0.099452	NW_003538922_38349_mature	5.37285	0	2.671938627	0		20	0.405387
chr14_31102_star	7.521989523	0	3.091190278	0		20	0.130285	chr5_11382_star	0	5.641492	0	2.731507408		-20	0.099452	chr2_4006_mature	5.37285	0	2.671938627	0		20	0.405387
chr15_33369_mature	7.521989523	0	3.091190278	0		20	0.130285	NW_003538681_38110_mature	0	5.641492	0	2.731507408		-20	0.099452	chr9_19896_mature	5.37285	0	2.671938627	0		20	0.405387
mmu-miR-3092-3p	7.521989523	0	3.091190278	0		20	0.130285	chr10_24012_star	0	5.641492	0	2.731507408		-20	0.099452	hsa-miR-3682-5p	5.37285	0	2.671938627	0		20	0.405387
chr1_3090_mature	7.521989523	0	3.091190278	0		20	0.130285	hsa-miR-3691-3p	0	5.641492	0	2.731507408		-20	0.099452	chr6_13396_mature	5.37285	0	2.671938627	0		20	0.405387
mmu-miR-466a-3p	7.521989523	0	3.091190278	0		20	0.130285	chr6_12619_mature	0	5.641492	0	2.731507408		-20	0.099452	chr1_384_star	5.37285	0	2.671938627	0		20	0.405387
chr17_35169_mature	7.521989523	0	3.091190278	0		20	0.130285	chr13_27764_star	0	5.641492	0	2.731507408		-20	0.099452	chr8_17973_star	5.37285	0	2.671938627	0		20	0.405387
mmu-miR-196a-1-3p	7.521989523	0	3.091190278	0		20	0.130285	chrx_21675_star	0	5.641492	0	2.731507408		-20	0.099452	bta-miR-34c	5.37285	0	2.671938627	0		20	0.405387
rno-miR-201-5p	7.521989523	0	3.091190278	0		20	0.130285	hsa-miR-891b	0	5.641492	0	2.731507408		-20	0.099452	hsa-miR-4264	5.37285	0	2.671938627	0		20	0.405387
chr2_5373_mature	7.521989523	0	3.091190278	0		20	0.130285	chr7_16992_star	0	5.641492	0	2.731507408		-20	0.099452	hsa-miR-4434	5.37285	0	2.671938627	0		20	0.405387
mmu-miR-466a-5p	7.521989523	0	3.091190278	0		20	0.130285	chr11_24683_star	0	5.641492	0	2.731507408		-20	0.099452	chr1_3342_star	5.37285	0	2.671938627	0		20	0.405387
chr3_7067_mature	7.521989523	0	3.091190278	0		20	0.130285	chr5_11980_star	0	5.641492	0	2.731507408		-20	0.099452	hsa-miR-619	5.37285	0	2.671938627	0		20	0.405387
mmu-miR-3091-5p	7.521989523	0	3.091190278	0		20	0.130285	chr2_5406_mature	0	5.641492	0	2.731507408		-20	0.099452	mmu-miR-467e-5p	5.37285	0	2.671938627	0		20	0.405387
chr6_12911_star	7.521989523	0	3.091190278	0		20	0.130285	chr1_918_star	0	4.835565	0	2.544872269		-20	0.103716	chr4_9118_mature	5.37285	0	2.671938627	0		20	0.405387
hsa-miR-4664-5p	7.521989523	0	3.091190278	0		20	0.130285	chr14_31492_star	0	4.835565	0	2.544872269		-20	0.103716	bta-miR-2379	5.37285	0	2.671938627	0		20	0.405387
mmu-miR-101c	7.521989523	0	3.091190278	0		20	0.130285	chr4_10320_star	0	4.835565	0	2.544872269		-20	0.103716	mmu-miR-3474	5.37285	0	2.671938627	0		20	0.405387
chr5_10979_star	7.521989523	0	3.091190278	0		20	0.130285	chr7_15648_mature	0	4.835565	0	2.544872269		-20	0.103716	chr9_19114_star	5.37285	0	2.671938627	0		20	0.405387
NW_003539513_38909_mature	7.521989523	0	3.091190278	0		20	0.130285	NW_003537624_37070_mature	0	4.835565	0	2.544872269		-20	0.103716	rno-miR-376b-3p	5.37285	0	2.671938627	0		20	0.405387
mmu-miR-503-3p	7.521989523	0	3.091190278	0		20	0.130285	NW_003613114_37265_star	0	4.835565	0	2.544872269		-20	0.103716	chr4_8848_star	5.37285	0	2.671938627	0		20	0.405387
chr4_9868_mature	7.521989523	0	3.091190278	0		20	0.130285	rno-miR-3585-5p	0	4.835565	0	2.544872269		-20	0.103716	hsa-miR-3691-5p	5.37285	0	2.671938627	0		20	0.405387
chrx_21544_star	7.521989523	0	3.091190278	0		20	0.130285	chr1_1197_mature	0	4.835565	0	2.544872269		-20	0.103716	hsa-miR-4760-3p	5.37285	0	2.671938627	0		20	0.405387
chr3_7118_mature	7.521989523	0	3.091190278	0		20	0.130285	hsa-miR-1293	0	4.835565	0	2.544872269		-20	0.103716	chr14_30043_mature	5.37285	0	2.671938627	0		20	0.405387
mmu-miR-669e-3p	7.521989523	0	3.091190278	0		20	0.130285	hsa-miR-1289	0	4.835565	0	2.544872269		-20	0.103716	chr4_8977_star	5.37285	0	2.671938627	0		20	0.405387
hsa-miR-940	7.521989523	0	3.091190278	0		20	0.130285	rno-miR-503-5p	0	4.835565	0	2.544872269		-20	0.103716	chr2_6398_mature	5.37285	0	2.671938627	0		20	0.405387
chrx_22200_mature	7.521989523	0	3.091190278	0		20	0.130285	chr2_6403_star	0	4.835565	0	2.544872269		-20	0.103716	rno-miR-3551-3p	5.37285	0	2.671938627	0		20	0.405387
chrx_21735_mature	83.81645468	6.44742	6.406272275	2.896740642		3.70044	0.130543	chr5_11515_star	0	4.835565	0	2.544872269		-20	0.103716	chr11_25077_star	5.37285	0	2.671938627	0		20	0.405387
chr4_8749_mature	6.447419591	101.5469	2.896740642	6.680139486		-3.97728	0.131157	NW_003613246_37882_star	0	4.835565	0	2.544872269		-20	0.103716	chr2_4155_star	5.37285	0	2.671938627	0		20	0.405387
bta-miR-33a	304.1032907	18.53633	8.25315393	4.288087668		4.036134	0.132461	chr5_12346_mature	0	4.835565	0	2.544872269		-20	0.103716	NW_003539279_38690_mature	3.22371	24.17782	2.078510713	4.654081667		-2.90689	0.406168
chr6_13911_mature	1.074569932	20.14819	1.05281229	4.402462031		-4.22882	0.133031	hsa-miR-548av-3p	0	4.835565	0	2.544872269		-20	0.103716	hsa-miR-4273	3.22371	24.17782	2.078510713	4.654081667		-2.90689	0.406168
chr5_12388_mature	1.074569932	20.14819	1.05281229	4.402462031		-4.22882	0.133031	NW_003613157_37452_star	0	4.835565	0	2.544872269		-20	0.103716	NW_003539467_38882_star	35.46081	4.029637	5.188274618	2.330454351		3.137504	0.406238
chr18_36561_mature	1.074569932	20.14819	1.05281229	4.402462031		-4.22882	0.133031	NW_003540835_39730_star	0	4.835565	0	2.544872269		-20	0.103716	chr11_24782_mature	35.46081	4.029637	5.188274618	2.330454351		3.137504	0.406238
chr14_29928_mature	1.074569932	20.14819	1.05281229	4.402462031		-4.22882	0.133031	hsa-miR-509-3p	0	4.835565	0	2.544872269		-20	0.103716	hsa-miR-1973	35.46081	4.029637	5.188274618	2.330454351		3.137504	0.406238
chr6_13529_star	0	9.671129	0	3.415640968		-20	0.133201	chr7_17037_star	0	4.835565	0	2.544872269		-20	0.103716	chr2_5595_star	2.14914	16.11855	1.654957834	4.097488514		-2.90689	0.406989
NW_003538681_38109_mature	0	9.671129	0	3.415640968		-20	0.133201	chr4_10650_star	0	4.835565	0	2.544872269		-20	0.103716	chr13_26618_star	2.14914	16.11855	1.654957834	4.097488514		-2.90689	0.406989
hsa-miR-1246	0	9.671129	0	3.415640968		-20	0.133201	chr7_16829_star	0	4.835565	0	2.544872269		-20	0.103716	chr2_4860_mature	2.14914	16.11855	1.654957834	4.097488514		-2.90689	0.406989
rno-miR-672-3p	0	9.671129	0	3.415640968		-20	0.133201	chr15_31984_mature	0	4.835565	0	2.544872269		-20	0.103716	chr2_6442_mature	0.537285	6.44742	0.620384622	2.896740642		-3.58496	0.407388
chr1_2009_mature	0	9.671129	0	3.415640968		-20	0.133201	chr15_31781_mature	0	4.835565	0	2.544872269		-20	0.103716	bta-miR-2285p	0.537285	6.44742	0.620384622	2.896740642		-3.58496	0.407388
chr13_29021_mature	0	9.671129	0	3.415640968		-20	0.133201	NW_003537641_37087_star	0	4.835565	0	2.544872269		-20	0.103716	chr13_29380_mature	0.537285	6.44742	0.620384622	2.896740642		-3.58496	0.407388
chr13_26614_mature	0	9.671129	0	3.415640968		-20	0.133201	chr1_1318_star	0	4.835565	0	2.544872269		-20	0.103716	chr16_34295_mature	0.537285	6.44742	0.620384622	2.896740642		-3.58496	0.407388
hsa-miR-2052	0	9.671129	0	3.415640968		-20	0.133201	chr11_25099_star	0	4.835565	0	2.544872269		-20	0.103716	chr16_34309_mature	27.40153	3.22371	4.827896911	2.078510713		3.087463	0.409774
chr16_33846_mature	0	9.671129	0	3.415640968		-20	0.133201	chr17_35550_star	0	4.835565	0	2.544872269		-20	0.103716	chr9_20685_mature	14.50669	1.611855	3.954819241	1.38507475		3.169925	0.410077
chr2_4213_star	0	9.671129	0	3.415640968		-20	0.133201	bta-miR-2284c	0	4.835565	0	2.544872269		-20	0.103716	chr9_20697_mature	14.50669	1.611855	3.954819241	1.38507475		3.169925	0.410077
hsa-miR-934	0	9.671129	0	3.415640968		-20	0.133201	bta-miR-2284b	0	4.835565	0	2.544872269		-20	0.103716	chr9_19974_mature	7.52199	64.4742	3.091190278	6.032854532		-3.09954	0.410611
chr10_24076_mature	0	9.671129	0	3.415640968		-20	0.133201	chr11_24964_mature	0	4.835565	0	2.544872269		-20	0.103716	chr3_8352_mature	6.984705	58.02678	2.997239027	5.883297648		-3.05445	0.41518
chr17_35547_mature	161.1854898	11.28298	7.341500942	3.618589217		3.836501	0.133477	chr9_20299_mature	0	4.835565	0	2.544872269		-20	0.103716	chr6_12618_mature	4.29828	32.2371	2.405524014	5.054722516		-2.90689	0.416437
mmu-miR-6539	133.2466715	9.671129	7.068742509	3.415640968		3.784271	0.133631	chr9_20604_star	0	4.835565	0	2.544872269		-20	0.103716	chr3_8074_mature	51.04207	5.641492	5.701606491	2.731507408		3.177538	0.416772
ssc-miR-92b-3p	43520.08224	744.677	15.40942681	9.542406961		5.868923	0.133835	hsa-miR-103b	0	4.835565	0	2.544872269		-20	0.103716	chrx_23064_star	9.133844	0.805927	3.34110968	0.852739935		3.5025	0.416928
chr13_28297_mature@@rno-miR-26	30.08795809	2.417782	4.958283956	1.773060525		3.63743	0.134009	mmu-miR-466p-5p	0	4.835565	0	2.544872269		-20	0.103716	chr4_9773_mature	9.133844	0.805927	3.34110968	0.852739935		3.5025	0.416928
NW_003539422_38814_mature	30.08795809	2.417782	4.958283956	1.773060525		3.63743	0.134009	chr14_29882_star	0	4.835565	0	2.544872269		-20	0.103716	chr5_11725_star	9.133844	0.805927	3.34110968	0.852739935		3.5025	0.416928
chr4_8951_mature	30.08795809	2.417782	4.958283956	1.773060525		3.63743	0.134009	chr14_29906_star	0	4.835565	0	2.544872269		-20	0.103716	chr18_36427_mature@@bta-miR-5	9.133844	0.805927	3.34110968	0.852739935		3.5025	0.416928
chr3_7250_star	13.96940911	0.805927	3.90394537	0.852739935		4.115477	0.134291	chr13_29352_star	0	4.835565	0	2.544872269		-20	0.103716	NW_003538331_37733_star	9.133844	0.805927	3.34110968	0.852739935		3.5025	0.416928
rno-miR-465-3p	13.96940911	0.805927	3.90394537	0.852739935		4.115477	0.134291	chr6_14638_star	0	4.835565	0	2.544872269		-20	0.103716	NW_003613233_37800_mature	9.133844	0.805927	3.34110968	0.852739935		3.5025	0.416928
chr2_5943_mature	13.96940911	0.805927	3.90394537	0.852739935		4.115477	0.134291	hsa-miR-5010-3p	0	4.835565	0	2.544872269		-20	0.103716	chr17_34966_star	9.133844	0.805927	3.34110968	0.852739935		3.5025	0.416928
chr15_32200_star	13.96940911	0.805927	3.90394537	0.852739935		4.115477	0.134291	chr4_10620_star	0	4.835565	0	2.544872269		-20	0.103716	chr13_27664_star	9.133844	0.805927	3.34110968	0.852739935		3.5025	0.416928
chr9_20720_star	13.96940911	0.805927	3.90394537	0.852739935		4.115477	0.134291	mmu-miR-3970	0	4.835565	0	2.544872269		-20	0.103716	chr6_12860_mature	9.133844	0.805927	3.34110968	0.852739935		3.5025	0.416928
chr11_25156_mature	13.96940911	0.805927	3.90394537	0.852739935		4.115477	0.134291	NW_003538795_38232_mature	0	4.835565	0	2.544872269		-20	0.103716	chr4_10320_mature	9.133844	0.805927	3.34110968	0.852739935		3.5025	0.416928
chr4_10320_mature	13.96940911	0.805927	3.90394537	0.852739935		4.115477	0.134291	chr7_17051_star	0	4.835565	0	2.544872269		-20	0.103716	hsa-miR-4760-5p	9.133844	0.805927	3.34110968	0.852739935		3.5025	0.416928
chr2_4571_star	13.96940911	0.805927	3.90394537	0.852739935		4.115477	0.134291	chr4_8989_star	0	4.835565	0	2.544872269		-20	0.103716	hsa-miR-4518	9.133844	0.805927	3.34110968	0.852739935		3.5025	0.416928
chr6_12919_star	13.96940911	0.805927	3.90394537	0.852739935		4.115477	0.134291	chr9_19153_star	0	4.835565	0	2.544872269		-20	0.103716	chr1_2742_mature	9.133844	0.805927	3.34110968	0.852739935		3.5025	0.416928
chr1_3478_mature	13.96940911	0.805927	3.90394537	0.852739935		4.115477	0.134291	chr4_9349_mature	0	4.835565	0	2.544872269		-20	0.103716	chr13_28840_star	2.686425	19.34226	1.882222339	4.346407978		-2.848	0.417459
chr18_36628_star	13.96940911	0.805927	3.90394537	0.852739935		4.115477	0.134291	chr14_30973_mature	0	4.835565	0	2.544872269		-20	0.103716	chr4_10393_mature	2.686425	19.34226	1.882222339	4.346407978		-2.848	0.417459
chr1_2420_mature	13.96940911	0.805927	3.90394537	0.852739935		4.115477	0.134291	NW_003539571_38947_mature	0	4.835565	0	2.544872269		-20	0.103716	chr5_10730_mature	2.686425	19.34226	1.882222339	4.346407978		-2.848	0.417459
chr13_28023_mature	13.96940911	0.805927	3.90394537	0.852739935		4.115477	0.134291	chr6_14899_mature	0	4.835565	0	2.544872269		-20	0.103716	chr12_26402_mature	19.87954	2.417782	4.384018281	1.773060525		3.039528	0.418466
chr1_3142_star	3.223709795	46.74379	2.078510713	5.577241251		-3.85798	0.134854	chr15_31883_mature	0	4.835565	0	2.544872269		-20	0.103716	NW_003539879_39158_mature	19.87954	2.417782	4.384018281	1.773060525		3.039528	0.418466
chrx_22392_mature	3.223709795	46.74379	2.078510713	5.577241251		-3.85798	0.134854	chr11_24516_mature	0	4.835565	0	2.544872269		-20	0.103716	chr5_11119_star	1.611855	12.08891	1.38507475	3.710273245		-2.90689	0.418475
chr13_27960_mature	3.223709795	46.74379	2.078510713	5.577241251		-3.85798	0.134854	mmu-miR-3079-5p	0	4.835565	0	2.544872269		-20	0.103716	chr6_12657_mature	1.611855	12.08891	1.38507475	3.710273245		-2.90689	0.418475
chr8_19035_star	3.223709795	46.74379	2.078510713	5.577241251		-3.85798	0.134854	chr15_33146_star	0	4.835565	0	2.544872269		-20	0.103716	chr14_30700_mature	114.979	10.47706	6.857719579	3.520680821		3.456065	0.418923
chr1_408_mature	21.49139864	1.611855	4.491301473	1.38507475		3.736966	0.134986	chr6_12619_star	0	4.835565	0	2.544872269		-20	0.103716	chr5_11962_star	13.96941	1.611855	3.90394537	1.38507475		3.115477	0.419648
mmu-miR-1191	21.49139864	1.611855	4.491301473	1.38507475		3.736966	0.134986	chr11_25333_mature	0	4.835565	0	2.544872269		-20	0.103716	chr5_11045_mature	13.96941	1.611855	3.90394537	1.38507475		3.115477	0.419648
chr5_11045_mature	21.49139864	1.611855	4.491301473	1.38507475		3.736966	0.134986	chr8_18077_mature	0	4.835565	0	2.544872269		-20	0.103716	bta-miR-30e-5p	13.96941	1.611855	3.90394537	1.38507475		3.115477	0.419648
chr13_28864_mature	21.49139864	1.611855	4.491301473	1.38507475		3.736966	0.134986	chr5_11313_mature@@hsa-miR-141-5p	0	4.835565	0	2.544872269		-20	0.103716	chr1_814_mature	26.32696	3.22371	4.772253246	2.078510713		3.029747	0.420531
chr13_29206_mature	2.149139864	32.2371	1.654957834	5.054722516		-3.90689	0.135508	chr8_17514_mature	0	4.835565	0	2.544872269		-20	0.103716	mmu-miR-466f-3p	1.07457	8.865202	1.05281229	3.302348583		-3.04439	0.421773

chr6_12841_mature	59.10134625	4.835565	5.909325402	2.544872269		3.611435	0.135741
chr13_27210_mature	69.84704557	5.641492	6.146635787	2.731507408		3.63005	0.136261
chr7_16781_star	4.298279727	62.05641	2.405524014	5.978571212		-3.85175	0.136537
chr15_32728_mature	38.68451755	3.22371	5.310504361	2.078510713		3.584963	0.136806
chr6_14967_mature	48.35564693	4.029637	5.625143252	2.330454351		3.584963	0.137059
chr17_35462_mature	5.372849659	78.98089	2.671938627	6.32158343		-3.87774	0.137301
chr6_14371_mature	3.223709795	45.93786	2.078510713	5.552680304		-3.83289	0.137437
chr13_28713_mature	200.9445773	13.70077	7.657815596	3.877819487		3.874469	0.137992
chr15_32726_mature	104.2332834	8.059274	6.717447265	3.179395517		3.693022	0.138607
mmu-miR-344e-5p	1.074569932	19.34226	1.05281229	4.346407978		-4.16993	0.138768
NW_003539453_38861_mature	1.074569932	19.34226	1.05281229	4.346407978		-4.16993	0.138768
chr9_19866_mature	1.074569932	19.34226	1.05281229	4.346407978		-4.16993	0.138768
NW_003540824_39708_mature	1.074569932	19.34226	1.05281229	4.346407978		-4.16993	0.138768
chr14_30846_mature	1.074569932	19.34226	1.05281229	4.346407978		-4.16993	0.138768
chr2_6005_mature	1.074569932	19.34226	1.05281229	4.346407978		-4.16993	0.138768
chr16_34566_mature	1.074569932	19.34226	1.05281229	4.346407978		-4.16993	0.138768
chr12_26240_mature	156.88721	11.28298	7.302750497	3.618589217		3.797507	0.138791
chr7_15120_mature	569.5220639	30.62524	9.156138871	4.983004663		4.216959	0.138812
chr9_20193_star	2.149139864	31.43117	1.654957834	5.019309189		-3.87036	0.139201
chr18_35961_star	3.223709795	45.13194	2.078510713	5.527693969		-3.80735	0.140097
chr5_12354_star	3.223709795	45.13194	2.078510713	5.527693969		-3.80735	0.140097
ssc-miR-95	29.01338816	2.417782	4.907534286	1.773060525		3.584963	0.140327
chr6_14411_mature	29.01338816	2.417782	4.907534286	1.773060525		3.584963	0.140327
chr13_27068_mature	79.51817495	6.44742	6.331242567	2.896740642		3.624491	0.140393
chr12_26596_star	37.60994761	3.22371	5.270900692	2.078510713		3.544321	0.141861
chr14_30679_star	2.149139864	30.62524	1.654957834	4.983004663		-3.83289	0.143054
NW_003613057_36932_star	2.149139864	30.62524	1.654957834	4.983004663		-3.83289	0.143054
chr2_6509_star	20.4168287	1.611855	4.420672964	1.38507475		3.662965	0.143718
hsa-miR-302a-5p	20.4168287	1.611855	4.420672964	1.38507475		3.662965	0.143718
chr2_5369_star	20.4168287	1.611855	4.420672964	1.38507475		3.662965	0.143718
chr11_24906_star	20.4168287	1.611855	4.420672964	1.38507475		3.662965	0.143718
chr15_32637_mature	20.4168287	1.611855	4.420672964	1.38507475		3.662965	0.143718
ssc-miR-217	4.298279727	58.8327	2.405524014	5.902862353		-3.77479	0.144776
chr4_9258_mature	4.298279727	58.8327	2.405524014	5.902862353		-3.77479	0.144776
bta-miR-6120-3p	1.074569932	18.53633	1.05281229	4.288087668		-4.10852	0.144901
chr9_20599_mature	1.074569932	18.53633	1.05281229	4.288087668		-4.10852	0.144901
chr1_3121_mature	1.074569932	18.53633	1.05281229	4.288087668		-4.10852	0.144901
chr8_17485_mature	1.074569932	18.53633	1.05281229	4.288087668		-4.10852	0.144901
ssc-miR-450c-3p	1.074569932	18.53633	1.05281229	4.288087668		-4.10852	0.144901
chr8_17641_mature	1.074569932	18.53633	1.05281229	4.288087668		-4.10852	0.144901
chr18_35948_mature@@rno-miR-96-	2254.447717	87.04016	11.13919813	6.460089935		4.694949	0.144963
chr16_34295_star	0	8.865202	0	3.302348583		-20	0.145118
rno-miR-200b-3p	0	8.865202	0	3.302348583		-20	0.145118
hsa-miR-563	0	8.865202	0	3.302348583		-20	0.145118
chr2_5550_star	0	8.865202	0	3.302348583		-20	0.145118
hsa-miR-145-5p	0	8.865202	0	3.302348583		-20	0.145118
chr8_18841_star	0	8.865202	0	3.302348583		-20	0.145118
chr18_36088_mature	0	8.865202	0	3.302348583		-20	0.145118
chr15_33538_star	0	8.865202	0	3.302348583		-20	0.145118
chr14_29599_star	0	8.865202	0	3.302348583		-20	0.145118
chr11_25079_star	0	8.865202	0	3.302348583		-20	0.145118
chr6_14961_star	0	8.865202	0	3.302348583		-20	0.145118
chr1_3715_star	0	8.865202	0	3.302348583		-20	0.145118
chrx_22414_star	0	8.865202	0	3.302348583		-20	0.145118
hsa-miR-607	0	8.865202	0	3.302348583		-20	0.145118
hsa-miR-601	0	8.865202	0	3.302348583		-20	0.145118
chr13_27796_star	0	8.865202	0	3.302348583		-20	0.145118
mmu-miR-450a-2-3p	0	8.865202	0	3.302348583		-20	0.145118
chr1_1859_mature	0	8.865202	0	3.302348583		-20	0.145118
chr16_33720_star	0	8.865202	0	3.302348583		-20	0.145118
hsa-miR-5690	0	8.865202	0	3.302348583		-20	0.145118
chr1_2968_mature	0	8.865202	0	3.302348583		-20	0.145118
chr7_16635_mature	46.20650707	4.029637	5.560913833	2.330454351		3.519374	0.145441
chr15_33045_mature	46.20650707	4.029637	5.560913833	2.330454351		3.519374	0.145441
chr4_10445_mature	179.4531786	12.89484	7.495480746	3.796477232		3.798742	0.145468
chr5_12375_mature	5.372849659	74.95125	2.671938627	6.247001856		-3.80219	0.145539
chr2_6194_mature	27.93881823	2.417782	4.854934103	1.773060525		3.530515	0.147057
mmu-miR-3089-3p	27.93881823	2.417782	4.854934103	1.773060525		3.530515	0.147057
chr7_16567_mature	2.149139864	29.81932	1.654957834	4.94576292		-3.79442	0.147076
NW_003540337_39426_mature	12.89483918	0.805927	3.796477232	0.852739935		4	0.147491
chr4_9018_star	12.89483918	0.805927	3.796477232	0.852739935		4	0.147491
chr6_13694_mature	12.89483918	0.805927	3.796477232	0.852739935		4	0.147491
chr2_5926_mature	12.89483918	0.805927	3.796477232	0.852739935		4	0.147491
chr4_10371_star	12.89483918	0.805927	3.796477232	0.852739935		4	0.147491
chr3_7184_star	12.89483918	0.805927	3.796477232	0.852739935		4	0.147491
chr8_17976_mature	12.89483918	0.805927	3.796477232	0.852739935		4	0.147491
chr1_3032_star	12.89483918	0.805927	3.796477232	0.852739935		4	0.147491
chr1_1173_star	12.89483918	0.805927	3.796477232	0.852739935		4	0.147491
chrx_23005_mature	3.223709795	42.71415	2.078510713	5.4500286		-3.72792	0.148581
chr13_27184_mature	415.8585636	24.98375	8.703414163	4.699537803		4.057031	0.148646
chr9_20216_mature	45.13193714	4.029637	5.527693969	2.330454351		3.485427	0.149894
chr6_12549_star	1.074569932	17.7304	1.05281229	4.227310103		-4.04439	0.151467
chr13_27786_star	1.074569932	17.7304	1.05281229	4.227310103		-4.04439	0.151467
hsa-miR-4772-5p	1.074569932	17.7304	1.05281229	4.227310103		-4.04439	0.151467
chr9_19153_mature	85.96559455	7.253347	6.442372848	3.044979305		3.567041	0.152082
mmu-miR-3096b-3p	498.6004484	29.01339	8.964630961	4.907534286		4.103093	0.152323
chr1_3918_mature	19.34225877	1.611855	4.346407978	1.38507475		3.584963	0.153235
chr5_11673_mature	19.34225877	1.611855	4.346407978	1.38507475		3.584963	0.153235
chr4_10618_mature	19.34225877	1.611855	4.346407978	1.38507475		3.584963	0.153235
chrx_22228_mature	19.34225877	1.611855	4.346407978	1.38507475		3.584963	0.153235
chr14_29848_mature	19.34225877	1.611855	4.346407978	1.38507475		3.584963	0.153235
chr6_13980_mature	133.2466715	10.47706	7.068742509	3.520680821		3.668794	0.153463
chr5_11433_mature	96.71129386	8.059274	6.610453419	3.179395517		3.584963	0.153604
chr3_6639_mature	26.8642483	2.417782	4.800343329	1.773060525		3.473931	0.154231
chrx_22851_star	26.8642483	2.417782	4.800343329	1.773060525		3.473931	0.154231
chr7_15776_mature	5.372849659	70.92162	2.671938627	6.168353522		-3.72247	0.154575
hsa-miR-4652-5p	6.447419591	0	2.896740642	0		20	0.154621
chrx_22902_mature	6.447419591	0	2.896740642	0		20	0.154621
hsa-miR-6719-3p	6.447419591	0	2.896740642	0		20	0.154621
NW_003540726_39647_mature	6.447419591	0	2.896740642	0		20	0.154621
mmu-miR-3086-5p	6.447419591	0	2.896740642	0		20	0.154621
bta-miR-2399-5p	6.447419591	0	2.896740642	0		20	0.154621
hsa-miR-4782-5p	6.447419591	0	2.896740642	0		20	0.154621
chr12_26042_star	6.447419591	0	2.896740642	0		20	0.154621
chr15_33029_mature	6.447419591	0	2.896740642	0		20	0.154621
hsa-miR-3908	6.447419591	0	2.896740642	0		20	0.154621
chr5_10795_mature@@rno-miR-355	6.447419591	0	2.896740642	0		20	0.154621
chr13_27795_mature	6.447419591	0	2.896740642	0		20	0.154621
hsa-miR-1470	6.447419591	0	2.896740642	0		20	0.154621
hsa-miR-1469	6.447419591	0	2.896740642	0		20	0.154621
chr5_11222_star	6.447419591	0	2.896740642	0		20	0.154621
chr14_31065_mature	6.447419591	0	2.896740642	0		20	0.154621
chr12_26437_mature	6.447419591	0	2.896740642	0		20	0.154621
chr2_5921_mature	6.447419591	0	2.896740642	0		20	0.154621
chr13_27839_star	6.447419591	0	2.896740642	0		20	0.154621
chr18_36022_mature	6.447419591	0	2.896740642	0		20	0.154621
chr9_20339_star	6.447419591	0	2.896740642	0		20	0.154621
chr9_19911_mature	6.447419591	0	2.896740642	0		20	0.154621
NW_003539279_38689_star	6.447419591	0	2.896740642	0		20	0.154621
chr13_29176_star	6.447419591	0	2.896740642	0		20	0.154621
NW_003538922_38349_mature	6.447419591	0	2.896740642	0		20	0.154621
chr13_29234_mature	6.447419591	0	2.896740642	0		20	0.154621
mmu-miR-3079-3p	6.447419591	0	2.896740642	0		20	0.154621
chr17_35756_star	6.447419591	0	2.896740642	0		20	0.154621
chr6_13334_mature	6.447419591	0	2.896740642	0		20	0.154621
NW_003539626_38995_star	6.447419591	0	2.896740642	0		20	0.154621
chr1_3708_star	6.447419591	0	2.896740642	0		20	0.154621
rno-miR-455-3p	6.447419591	0	2.896740642	0		20	0.154621
chr15_31965_star	6.447419591	0	2.896740642	0		20	0.154621
chr6_14284_star	6.447419591	0	2.896740642	0		20	0.154621
mmu-miR-3098-3p	6.447419591	0	2.896740642	0		20	0.154621
chr5_11369_star	6.447419591	0	2.896740642	0		20	0.154621
chr17_35521_star	6.447419591	0	2.896740642	0		20	0.154621
mmu-miR-3098-5p	6.447419591	0	2.896740642	0		20	0.154621
mmu-miR-693-3p	6.447419591	0	2.896740642	0		20	0.154621
chr3_7424_star	6.447419591	0	2.896740642	0		20	0.154621
hsa-miR-4635	6.447419591	0	2.896740642	0		20	0.154621
chr14_31596_mature	6.447419591	0	2.896740642	0		20	0.154621
mmu-miR-1941-5p	6.447419591	0	2.896740642	0		20	0.154621
mmu-miR-718	6.447419591	0	2.896740642	0		20	0.154621
chr13_28676_mature	6.447419591	0	2.896740642	0		20	0.154621
chr1_1448_star	6.447419591	0	2.896740642	0		20	0.154621
chr1_2918_mature	6.447419591	0	2.896740642	0		20	0.154621
chr17_35663_mature	2.149139864	28.20746	1.654957834	4.868265031		-3.71425	0.155664
chr6_13425_mature	2.149139864	28.20746	1.654957834	4.868265031		-3.71425	0.155664
chrx_21833_mature	20.4168287	385.2333	4.420672964	8.593328822		-4.2379	0.157799
chr15_33083_mature	52.65392666	4.835565	5.745611853	2.544872269		3.444785	0.157869
chr4_8771_star	3.223709795	40.29637	2.078510713	5.367943153		-3.64386	0.157888
chr8_18468_mature	1.074569932	16.92448	1.05281229	4.163859073		-3.97728	0.158506
chr14_29552_mature	1.074569932	16.92448	1.05281229	4.163859073		-3.97728	0.158506
chr5_11269_mature	1.074569932	16.92448	1.05281229	4.163859073		-3.97728	0.158506
chrx_23076_star	1.074569932	16.92448	1.05281229	4.163859073		-3.97728	0.158506
chr1_814_star	0	8.059274	0	3.179395517		-20	0.158807
chr5_12276_mature	0	8.059274	0	3.179395517		-20	0.158807
chrx_22099_mature	0	8.059274	0	3.179395517		-20	0.158807
chr14_30180_mature	0	8.059274	0	3.179395517		-20	0.158807
chr13_27896_mature	0	8.059274	0	3.179395517		-20	0.158807
chr5_11099_mature	0	8.059274	0	3.179395517		-20	0.158807
chr16_34510_mature	0	8.059274	0	3.179395517		-20	0.158807
chr10_24174_star	0	8.059274	0	3.179395517		-20	0.158807
chr13_27566_star	0	8.059274	0	3.179395517		-20	0.158807
bta-miR-2284j	0	8.059274	0	3.179395517		-20	0.158807
hsa-miR-582-5p	0	8.059274	0	3.179395517		-20	0.158807
mmu-miR-3081-5p	0	8.059274	0	3.179395517		-20	0.158807
hsa-miR-518c-5p	0	8.059274	0	3.179395517		-20	0.158807
chr1_439_mature	0	8.059274	0	3.179395517		-20	0.158807
chr9_19649_mature	0	8.059274	0	3.179395517		-20	0.158807
NW_003539115_38548_mature	0	8.059274	0	3.179395517		-20	0.158807
hsa-miR-4298	0	8.059274	0	3.179395517		-20	0.158807
chr14_30107_star	0	8.059274	0	3.179395517		-20	0.158807
chr11_24477_star	0	8.059274	0	3.179395517		-20	0.158807
chr4_9430_star	0	8.059274	0	3.179395517		-20	0.158807
chr6_14759_mature	0	8.059274	0	3.179395517		-20	0.158807
chr6_14308_mature	42.98279727	4.029637	5.458867456	2.330454351		3.415037	0.15937
NW_003537574_37016_star	5.372849659	68.50383	2.671938627	6.11902064		-3.67243	0.160421
chr3_8448_mature	7.521989523	101.5469	3.091190278	6.680139486		-3.75489	0.16149
chrx_21804_mature	11.82026925	0.805927	3.680354657	0.852739935		3.874469	0.162517
chr1_3657_mature	11.82026925	0.805927	3.680354657	0.852739935		3.874469	0.162517
chr11_25086_mature	11.82026925	0.805927	3.680354657	0.852739935		3.874469	0.162517
chr1_3951_mature	11.82026925	0.805927	3.680354657	0.852739935		3.874469	0.162517
chr2_6308_mature	11.82026925	0.805927	3.680354657	0.852739935		3.874469	0.162517
mmu-miR-6399	11.82026925	0.805927	3.680354657	0.852739935		3.874469	0.162517
chr13_28101_mature	11.82026925	0.805927	3.680354657	0.852739935		3.874469	0.162517
chr2_4567_star@@bta-miR-6535	11.82026925	0.805927	3.680354657	0.852739935		3.874469	0.162517
chr5_12116_star@@rno-miR-200c-5	11.82026925	0.805927	3.680354657	0.852739935		3.874469	0.162517
chr8_17916_mature	11.82026925	0.805927	3.680354657	0.852739935		3.874469	0.162517
chr5_12148_mature	11.82026925	0.805927	3.680354657	0.852739935		3.874469	0.162517
chr9_20033_mature	81.66731482	7.253347	6.369245121	3.044979305		3.49304	0.162735
rno-miR-1949	18.26768884	1.611855	4.268111626	1.38507475		3.5025	0.163618
chr14_30803_mature	18.26768884	1.611855	4.268111626	1.38507475		3.5025	0.163618
mmu-miR-1955-5p	18.26768884	1.611855	4.268111626	1.38507475		3.5025	0.163618
chr9_19409_mature	103.1587135	8.865202	6.702639724	3.302348583		3.540568	0.164337
mmu-miR-6412	151.5143604	12.08891	7.25280128	3.710273245		3.647698	0.164434
chr5_11998_mature	33.31166789	3.22371	5.100627352	2.078510713		3.369234	0.164814
chr2_5244_mature	33.31166789	3.22371	5.100627352	2.078510713		3.369234	0.164814
chr14_29982_mature	2.149139864	26.59561	1.654957834	4.786366653		-3.62936	0.165052
ssc-miR-151-5p	37655.07955	734.1999	15.20059519	9.521992772		5.680528	0.165484
chr5_11434_mature	80.59274489	7.253347	6.35036897	3.044979305		3.473931	0.165555
chr6_12646_mature	1.074569932	16.11855	1.05281229	4.097488514		-3.90689	0.166063
chr5_11382_mature	1.074569932	16.11855	1.05281229	4.097488514		-3.90689	0.166063
NW_003613114_37265_mature	1.074569932	16.11855	1.05281229	4.097488514		-3.90689	0.166063
chr6_12675_star	1.074569932	16.11855	1.05281229	4.097488514		-3.90689	0.166063
chr8_18422_mature	1.074569932	16.11855	1.05281229	4.097488514		-3.90689	0.166063
chr1_1984_star	1.074569932	16.11855	1.05281229	4.097488514		-3.90689	0.166063
chr3_8071_mature@@rno-miR-409b	1.074569932	16.11855	1.05281229	4.097488514		-3.90689	0.166063
chr6_13302_mature	50.5047868	4.835565	5.686634616	2.544872269		3.384664	0.166324
chr6_14787_mature	5.372849659	66.08605	2.671938627	6.067940913		-3.62059	0.166617
chr7_16615_mature	17.19311891	290.1339	4.185320985	8.185538937		-4.07682	0.166711
chr1_384_mature	3.223709795	37.87859	2.078510713	5.280903997		-3.55459	0.168129
chr17_35548_mature	174.080329	13.70077	7.45187319	3.877819487		3.667425	0.168985
chr7_15380_mature	4.298279727	50.77343	2.405524014	5.694139975		-3.56224	0.169113
chr18_35826_mature	40.83365741	4.029637	5.38659223	2.330454351		3.341037	0.169668
chr13_29352_mature	40.83365741	4.029637	5.38659223	2.330454351		3.341037	0.169668
chr14_30864_mature	24.71510843	2.417782	4.684544332	1.773060525		3.353637	0.170051
chr18_36015_mature	24.71510843	2.417782	4.684544332	1.773060525		3.353637	0.170051
chr3_8285_mature	2.149139864	25.78968	1.654957834	4.743605355		-3.58496	0.170077
chr1_2954_mature	99.93500366	8.865202	6.657282769	3.302348583		3.494765	0.171321
chr3_8450_mature	17.19311891	282.8805	4.185320985	8.149140118		-4.04029	0.171566
chr11_25207_star	3.223709795	37.07266	2.078510713	5.250683565		-3.52356	0.17177
chr2_6439_mature	598.535452	35.46081	9.227701256	5.188274618		4.077139	0.172708
chr5_12402_mature	422.3059832	27.40153	8.72555707	4.827896911		3.94596	0.173754
chr11_24777_star	1.074569932	15.31262	1.05281229	4.027916745		-3.83289	0.174189
chr11_24821_star	1.074569932	15.31262	1.05281229	4.027916745		-3.83289	0.174189
chr1_771_star	1.074569932	15.31262	1.05281229	4.027916745		-3.83289	0.174189
chr2_4396_mature	1.074569932	15.31262	1.05281229	4.027916745		-3.83289	0.174189
chr18_36468_mature	1.074569932	15.31262	1.05281229	4.027916745		-3.83289	0.174189
chrx_21533_star	1.074569932	15.31262	1.05281229	4.027916745		-3.83289	0.174189
hsa-miR-4445-3p	0	7.253347	0	3.044979305		-20	0.174636
chr18_36120_star	0	7.253347	0	3.044979305		-20	0.174636
hsa-miR-581	0	7.253347	0	3.044979305		-20	0.174636
chr11_25109_star	0	7.253347	0	3.044979305		-20	0.174636
chr6_14945_star	0	7.253347	0	3.044979305		-20	0.174636
mmu-miR-6420	0	7.253347	0	3.044979305		-20	0.174636
mmu-miR-6387	0	7.253347	0	3.044979305		-20	0.174636
bta-miR-2285g	0	7.253347	0	3.044979305		-20	0.174636
chr1_495_star	0	7.253347	0	3.044979305		-20	0.174636
hsa-miR-518f-3p	0	7.253347	0	3.044979305		-20	0.174636
chr11_25333_star	0	7.253347	0	3.044979305		-20	0.174636
chr2_5745_mature	0	7.253347	0	3.044979305		-20	0.174636
hsa-miR-6506-3p	0	7.253347	0	3.044979305		-20	0.174636
chr16_34708_star	0	7.253347	0	3.044979305		-20	0.174636
mmu-miR-7a-2-3p	0	7.253347	0	3.044979305		-20	0.174636
ssc-miR-202-3p	0	7.253347	0	3.044979305		-20	0.174636
chr1_943_mature	0	7.253347	0	3.044979305		-20	0.174636
chr16_34089_star	0	7.253347	0	3.044979305		-20	0.174636
chr1_1197_star	0	7.253347	0	3.044979305		-20	0.174636
chrx_23205_mature	4.298279727	49.16157	2.405524014	5.648510724		-3.5157	0.174736
chr13_27034_mature	17.19311891	1.611855	4.185320985	1.38507475		3.415037	0.17496
hsa-miR-4484	17.19311891	1.611855	4.185320985	1.38507475		3.415037	0.17496
chr15_31774_mature	17.19311891	1.611855	4.185320985	1.38507475		3.415037	0.17496
chr1_1181_star	17.19311891	1.611855	4.185320985	1.38507475		3.415037	0.17496
chr3_7847_star	39.75908748	4.029637	5.349049847	2.330454351		3.302563	0.17515
chr12_26328_mature	2.149139864	24.98375	1.654957834	4.699537803		-3.53916	0.175339
chr15_33317_mature	48.35564693	4.835565	5.625143252	2.544872269		3.321928	0.175396
chr8_18995_mature	3.223709795	36.26674	2.078510713	5.21981653		-3.49185	0.175533
ssc-miR-545-3p	277.2390424	20.14819	8.120181062	4.402462031		3.782409	0.177234
mmu-miR-1964-3p	31.16252802	3.22371	5.007308904	2.078510713		3.273018	0.178171
chr8_17733_mature@@mmu-miR-30	31.16252802	3.22371	5.007308904	2.078510713		3.273018	0.178171
chr2_5595_mature	31.16252802	3.22371	5.007308904	2.078510713		3.273018	0.178171
NW_003539007_38433_star	23.6405385	2.417782	4.62296188	1.773060525		3.289507	0.178773
chr18_36348_mature	10.74569932	145.0669	3.554060706	7.190485881		-3.75489	0.179093
hsa-miR-1291	10.74569932	145.0669	3.554060706	7.190485881		-3.75489	0.179093
chr12_26382_mature	205.242857	16.11855	7.688200344	4.097488514		3.670538	0.179507
rno-miR-344b-1-3p	130.0229618	11.28298	7.033675856	3.618589217		3.526546	0.179564
chr8_18472_mature	7.521989523	91.87573	3.091190278	6.537229727		-3.6105	0.179657

chrx_22359_star	0	4.835565	0	2.544872269		-20	0.103716
hsa-miR-3148	0	4.835565	0	2.544872269		-20	0.103716
chr15_32407_star	0	4.835565	0	2.544872269		-20	0.103716
chr2_5406_star	0	4.835565	0	2.544872269		-20	0.103716
NW_003613552_39654_star	0	4.835565	0	2.544872269		-20	0.103716
hsa-miR-5581-5p	0	4.835565	0	2.544872269		-20	0.103716
chr2_5656_star	0	4.835565	0	2.544872269		-20	0.103716
rno-miR-293-5p	0	4.835565	0	2.544872269		-20	0.103716
chr4_8784_star	0	4.835565	0	2.544872269		-20	0.103716
NW_003613116_37270_star	0	4.835565	0	2.544872269		-20	0.103716
chr9_20798_mature	0	4.835565	0	2.544872269		-20	0.103716
chr13_27982_mature	0	4.835565	0	2.544872269		-20	0.103716
chr6_12824_star	0	4.835565	0	2.544872269		-20	0.103716
hsa-miR-5706	0	4.835565	0	2.544872269		-20	0.103716
chr2_4896_star	0	4.835565	0	2.544872269		-20	0.103716
chr7_16084_mature	0	4.835565	0	2.544872269		-20	0.103716
chr8_17599_star	0	4.835565	0	2.544872269		-20	0.103716
chr6_13027_star	0	4.835565	0	2.544872269		-20	0.103716
chr3_8486_star	0	4.835565	0	2.544872269		-20	0.103716
NW_003537656_37100_star	0	4.835565	0	2.544872269		-20	0.103716
mmu-miR-1929-5p	0	4.835565	0	2.544872269		-20	0.103716
chr2_4605_star	0	4.029637	0	2.330454351		-20	0.108626
NW_003539542_38921_mature	0	4.029637	0	2.330454351		-20	0.108626
chr6_14967_mature	0	4.029637	0	2.330454351		-20	0.108626
chr15_32852_mature	0	4.029637	0	2.330454351		-20	0.108626
chr7_16636_mature	0	4.029637	0	2.330454351		-20	0.108626
chr2_5077_star	0	4.029637	0	2.330454351		-20	0.108626
hsa-miR-1290	0	4.029637	0	2.330454351		-20	0.108626
hsa-miR-1267	0	4.029637	0	2.330454351		-20	0.108626
hsa-miR-4782-3p	0	4.029637	0	2.330454351		-20	0.108626
chr7_16174_mature	0	4.029637	0	2.330454351		-20	0.108626
chr2_4519_star	0	4.029637	0	2.330454351		-20	0.108626
hsa-miR-548g-3p	0	4.029637	0	2.330454351		-20	0.108626
ssc-miR-218-3p	0	4.029637	0	2.330454351		-20	0.108626
chr13_29025_mature	0	4.029637	0	2.330454351		-20	0.108626
hsa-miR-548ab	0	4.029637	0	2.330454351		-20	0.108626
chr9_19988_mature	0	4.029637	0	2.330454351		-20	0.108626
chr13_26769_mature	0	4.029637	0	2.330454351		-20	0.108626
chr8_17571_star	0	4.029637	0	2.330454351		-20	0.108626
chr13_27644_star	0	4.029637	0	2.330454351		-20	0.108626
chr14_29829_mature	0	4.029637	0	2.330454351		-20	0.108626
chr14_31584_star	0	4.029637	0	2.330454351		-20	0.108626
chr2_4213_mature	0	4.029637	0	2.330454351		-20	0.108626
NW_003541072_39884_star	0	4.029637	0	2.330454351		-20	0.108626
chr8_17864_mature	0	4.029637	0	2.330454351		-20	0.108626
NW_003538764_38206_star	0	4.029637	0	2.330454351		-20	0.108626
chr4_10620_mature	0	4.029637	0	2.330454351		-20	0.108626
chr17_35478_star	0	4.029637	0	2.330454351		-20	0.108626
chr11_24391_star	0	4.029637	0	2.330454351		-20	0.108626
chr14_30547_star	0	4.029637	0	2.330454351		-20	0.108626
chr1_3251_star	0	4.029637	0	2.330454351		-20	0.108626
rno-miR-329-5p	0	4.029637	0	2.330454351		-20	0.108626
chr17_34920_star	0	4.029637	0	2.330454351		-20	0.108626
chr3_7249_mature	0	4.029637	0	2.330454351		-20	0.108626
chr1_991_star	0	4.029637	0	2.330454351		-20	0.108626
hsa-miR-4653-3p	0	4.029637	0	2.330454351		-20	0.108626
chr17_34868_star	0	4.029637	0	2.330454351		-20	0.108626
chr11_24358_star	0	4.029637	0	2.330454351		-20	0.108626
chr18_36486_mature	0	4.029637	0	2.330454351		-20	0.108626
chr7_15704_mature	0	4.029637	0	2.330454351		-20	0.108626
chr18_36061_star	0	4.029637	0	2.330454351		-20	0.108626
chr11_24782_mature	0	4.029637	0	2.330454351		-20	0.108626
chr13_27077_mature	0	4.029637	0	2.330454351		-20	0.108626
bta-miR-195	0	4.029637	0	2.330454351		-20	0.108626
chr8_18374_star	0	4.029637	0	2.330454351		-20	0.108626
mmu-miR-3057-5p	0	4.029637	0	2.330454351		-20	0.108626
NW_003538747_38195_mature	0	4.029637	0	2.330454351		-20	0.108626
rno-miR-336-3p	0	4.029637	0	2.330454351		-20	0.108626
mmu-miR-449a-5p	0	4.029637	0	2.330454351		-20	0.108626
chr12_25620_star	0	4.029637	0	2.330454351		-20	0.108626
mmu-miR-143-3p	0	4.029637	0	2.330454351		-20	0.108626
hsa-miR-4295	0	4.029637	0	2.330454351		-20	0.108626
chrx_22854_mature	0	4.029637	0	2.330454351		-20	0.108626
chr3_8070_star	0	4.029637	0	2.330454351		-20	0.108626
chr3_7353_star	0	4.029637	0	2.330454351		-20	0.108626
chr4_9894_mature	0	4.029637	0	2.330454351		-20	0.108626
chr18_36376_mature	0	4.029637	0	2.330454351		-20	0.108626
rno-miR-3557-5p	0	4.029637	0	2.330454351		-20	0.108626
chr9_19718_mature	0	4.029637	0	2.330454351		-20	0.108626
NW_003539506_38905_star	0	4.029637	0	2.330454351		-20	0.108626
chr14_31058_star	0	4.029637	0	2.330454351		-20	0.108626
bta-miR-2473	0	4.029637	0	2.330454351		-20	0.108626
chrx_21502_star	0	4.029637	0	2.330454351		-20	0.108626
chr13_28073_mature	0	4.029637	0	2.330454351		-20	0.108626
chr13_27180_star	0	4.029637	0	2.330454351		-20	0.108626
NW_003539576_38951_star	0	4.029637	0	2.330454351		-20	0.108626
hsa-miR-645	0	4.029637	0	2.330454351		-20	0.108626
chr6_14061_star	0	4.029637	0	2.330454351		-20	0.108626
chr8_18081_mature	0	4.029637	0	2.330454351		-20	0.108626
bta-miR-2308	0	4.029637	0	2.330454351		-20	0.108626
rno-miR-582-5p	0	4.029637	0	2.330454351		-20	0.108626
chr9_19896_star	0	4.029637	0	2.330454351		-20	0.108626
mmu-miR-5129-5p	0	4.029637	0	2.330454351		-20	0.108626
mmu-miR-684	0	4.029637	0	2.330454351		-20	0.108626
NW_003538396_37820_mature	0	4.029637	0	2.330454351		-20	0.108626
chr14_29971_star	0	4.029637	0	2.330454351		-20	0.108626
chr4_9341_star	0	4.029637	0	2.330454351		-20	0.108626
chr14_29836_star	0	4.029637	0	2.330454351		-20	0.108626
chr5_12125_mature	0	4.029637	0	2.330454351		-20	0.108626
chr1_3091_mature	0	4.029637	0	2.330454351		-20	0.108626
chr4_8816_star	0	4.029637	0	2.330454351		-20	0.108626
chr8_18856_star	0	4.029637	0	2.330454351		-20	0.108626
chr15_33045_mature	0	4.029637	0	2.330454351		-20	0.108626
rno-miR-2985	0	4.029637	0	2.330454351		-20	0.108626
chr10_23316_mature	0	4.029637	0	2.330454351		-20	0.108626
hsa-miR-924	0	4.029637	0	2.330454351		-20	0.108626
hsa-miR-6085	0	4.029637	0	2.330454351		-20	0.108626
chr9_20399_star	0	4.029637	0	2.330454351		-20	0.108626
hsa-miR-4451	234.256245	63.66827	7.878089211	6.014986077		1.879441	0.110268
hsa-miR-4749-5p	0	3.22371	0	2.078510713		-20	0.114369
chr8_18464_star	0	3.22371	0	2.078510713		-20	0.114369
hsa-miR-4771	0	3.22371	0	2.078510713		-20	0.114369
chr16_34282_star	0	3.22371	0	2.078510713		-20	0.114369
hsa-miR-1302	0	3.22371	0	2.078510713		-20	0.114369
chr2_4605_mature	0	3.22371	0	2.078510713		-20	0.114369
chr1_2577_mature	0	3.22371	0	2.078510713		-20	0.114369
hsa-miR-4765	0	3.22371	0	2.078510713		-20	0.114369
hsa-miR-4668-3p	0	3.22371	0	2.078510713		-20	0.114369
chr6_12988_star	0	3.22371	0	2.078510713		-20	0.114369
chr15_32959_mature	0	3.22371	0	2.078510713		-20	0.114369
chr6_15067_mature	0	3.22371	0	2.078510713		-20	0.114369
chr6_14513_star	0	3.22371	0	2.078510713		-20	0.114369
hsa-miR-520d-3p	0	3.22371	0	2.078510713		-20	0.114369
chr3_7082_star	0	3.22371	0	2.078510713		-20	0.114369
chr7_15080_star	0	3.22371	0	2.078510713		-20	0.114369
NW_003613232_37791_mature	0	3.22371	0	2.078510713		-20	0.114369
hsa-miR-3914	0	3.22371	0	2.078510713		-20	0.114369
hsa-miR-3941	0	3.22371	0	2.078510713		-20	0.114369
mmu-miR-1938	0	3.22371	0	2.078510713		-20	0.114369
chr10_23707_star	0	3.22371	0	2.078510713		-20	0.114369
hsa-miR-5004-3p	0	3.22371	0	2.078510713		-20	0.114369
chr1_3607_star	0	3.22371	0	2.078510713		-20	0.114369
chr14_31047_star	0	3.22371	0	2.078510713		-20	0.114369
NW_003540745_39662_star	0	3.22371	0	2.078510713		-20	0.114369
hsa-miR-525-3p	0	3.22371	0	2.078510713		-20	0.114369
chr14_29827_mature	0	3.22371	0	2.078510713		-20	0.114369
mmu-miR-5110	0	3.22371	0	2.078510713		-20	0.114369
chr3_8536_star	0	3.22371	0	2.078510713		-20	0.114369
chr18_36069_mature	0	3.22371	0	2.078510713		-20	0.114369
chr1_3276_mature	0	3.22371	0	2.078510713		-20	0.114369
chr16_34446_mature	0	3.22371	0	2.078510713		-20	0.114369
rno-miR-471-5p	0	3.22371	0	2.078510713		-20	0.114369
chr15_33570_star	0	3.22371	0	2.078510713		-20	0.114369
hsa-miR-3679-3p	0	3.22371	0	2.078510713		-20	0.114369
chr15_31671_mature	0	3.22371	0	2.078510713		-20	0.114369
chr8_18754_mature	0	3.22371	0	2.078510713		-20	0.114369
chr3_7937_star	0	3.22371	0	2.078510713		-20	0.114369
chr16_34525_star@@rno-miR-449a-3p	0	3.22371	0	2.078510713		-20	0.114369
mmu-miR-3101-3p	0	3.22371	0	2.078510713		-20	0.114369
ssc-miR-494	0	3.22371	0	2.078510713		-20	0.114369
chr7_16636_star	0	3.22371	0	2.078510713		-20	0.114369
chr17_35462_star	0	3.22371	0	2.078510713		-20	0.114369
mmu-miR-5625-5p	0	3.22371	0	2.078510713		-20	0.114369
mmu-miR-6402	0	3.22371	0	2.078510713		-20	0.114369
chr6_12657_star@@mmu-miR-504-3p	0	3.22371	0	2.078510713		-20	0.114369
chr6_14428_star	0	3.22371	0	2.078510713		-20	0.114369
rno-miR-667-5p	0	3.22371	0	2.078510713		-20	0.114369
chr8_18850_mature	0	3.22371	0	2.078510713		-20	0.114369
mmu-miR-148a-3p	0	3.22371	0	2.078510713		-20	0.114369
chr12_25513_mature	0	3.22371	0	2.078510713		-20	0.114369
chrx_21746_star	0	3.22371	0	2.078510713		-20	0.114369
chr15_31928_mature	0	3.22371	0	2.078510713		-20	0.114369
bta-miR-2285d	0	3.22371	0	2.078510713		-20	0.114369
chr17_34985_mature	0	3.22371	0	2.078510713		-20	0.114369
mmu-miR-3112-3p	0	3.22371	0	2.078510713		-20	0.114369
chr13_27515_star	0	3.22371	0	2.078510713		-20	0.114369
chr8_17233_star	0	3.22371	0	2.078510713		-20	0.114369
chr18_36498_mature	0	3.22371	0	2.078510713		-20	0.114369
chrx_21569_star	0	3.22371	0	2.078510713		-20	0.114369
chr3_6870_mature	0	3.22371	0	2.078510713		-20	0.114369
chr2_5564_star@@mmu-miR-670-5p	0	3.22371	0	2.078510713		-20	0.114369
bta-miR-134	0	3.22371	0	2.078510713		-20	0.114369
chr8_17514_star	0	3.22371	0	2.078510713		-20	0.114369
chr12_25994_mature	0	3.22371	0	2.078510713		-20	0.114369
bta-miR-197	0	3.22371	0	2.078510713		-20	0.114369
chr1_1479_mature	0	3.22371	0	2.078510713		-20	0.114369
chr8_18843_star	0	3.22371	0	2.078510713		-20	0.114369
hsa-miR-4795-3p	0	3.22371	0	2.078510713		-20	0.114369
chr4_9423_mature	0	3.22371	0	2.078510713		-20	0.114369
hsa-miR-3179	0	3.22371	0	2.078510713		-20	0.114369
chr1_211_star	0	3.22371	0	2.078510713		-20	0.114369
chr7_15648_star	0	3.22371	0	2.078510713		-20	0.114369
bta-miR-30d	0	3.22371	0	2.078510713		-20	0.114369
NW_003540346_39431_star	0	3.22371	0	2.078510713		-20	0.114369
chr7_16559_mature@@rno-miR-330-5p	0	3.22371	0	2.078510713		-20	0.114369
chr1_766_star	0	3.22371	0	2.078510713		-20	0.114369
chr3_8248_mature	0	3.22371	0	2.078510713		-20	0.114369
chr5_11934_star	0	3.22371	0	2.078510713		-20	0.114369
hsa-miR-3144-3p	0	3.22371	0	2.078510713		-20	0.114369
chr8_18259_star	0	3.22371	0	2.078510713		-20	0.114369
chr18_35895_star	0	3.22371	0	2.078510713		-20	0.114369
NW_003613303_38366_mature	0	3.22371	0	2.078510713		-20	0.114369
chr1_1386_star	0	3.22371	0	2.078510713		-20	0.114369
bta-miR-2404	0	3.22371	0	2.078510713		-20	0.114369
chr8_17750_star	0	3.22371	0	2.078510713		-20	0.114369
NW_003539571_38948_mature	0	3.22371	0	2.078510713		-20	0.114369
hsa-miR-519c-3p	0	3.22371	0	2.078510713		-20	0.114369
chr2_4356_star	0	3.22371	0	2.078510713		-20	0.114369
chr7_15205_star	0	3.22371	0	2.078510713		-20	0.114369
hsa-miR-103a-2-5p	0	3.22371	0	2.078510713		-20	0.114369
chr13_28842_mature	0	3.22371	0	2.078510713		-20	0.114369
chr15_33547_star	0	3.22371	0	2.078510713		-20	0.114369
ssc-miR-376b	0	3.22371	0	2.078510713		-20	0.114369
bta-miR-2361	0	3.22371	0	2.078510713		-20	0.114369
hsa-miR-4300	0	3.22371	0	2.078510713		-20	0.114369
chr2_5776_mature	0	3.22371	0	2.078510713		-20	0.114369
chr18_36111_star	0	3.22371	0	2.078510713		-20	0.114369
chr6_13671_star	0	3.22371	0	2.078510713		-20	0.114369
chr17_35671_star	0	3.22371	0	2.078510713		-20	0.114369
chr14_31530_star	0	3.22371	0	2.078510713		-20	0.114369
chr12_25577_mature	0	3.22371	0	2.078510713		-20	0.114369
chr13_27012_star	0	3.22371	0	2.078510713		-20	0.114369
chr13_29266_mature	0	3.22371	0	2.078510713		-20	0.114369
hsa-miR-3678-3p	0	3.22371	0	2.078510713		-20	0.114369
chr3_8556_star	0	3.22371	0	2.078510713		-20	0.114369
hsa-miR-3135b	0	3.22371	0	2.078510713		-20	0.114369
chr2_5595_mature	0	3.22371	0	2.078510713		-20	0.114369
chr8_17419_star	0	3.22371	0	2.078510713		-20	0.114369
chr17_35182_star	0	3.22371	0	2.078510713		-20	0.114369
rno-miR-32-3p	0	3.22371	0	2.078510713		-20	0.114369
chr8_18569_mature	0	3.22371	0	2.078510713		-20	0.114369
hsa-miR-5697	0	3.22371	0	2.078510713		-20	0.114369
chr9_19683_mature	0	3.22371	0	2.078510713		-20	0.114369
rno-miR-379-5p	0	3.22371	0	2.078510713		-20	0.114369
NW_003613025_36805_star	0	3.22371	0	2.078510713		-20	0.114369
hsa-miR-875-5p	0	3.22371	0	2.078510713		-20	0.114369
chr5_12346_star	0	3.22371	0	2.078510713		-20	0.114369
mmu-miR-330-3p	0	3.22371	0	2.078510713		-20	0.114369
chr15_32067_mature	171.931189	444.066	7.43405428	8.797875562		-1.36894	0.120846
hsa-miR-548ar-5p	0	2.417782	0	1.773060525		-20	0.121217
chr4_10614_star	0	2.417782	0	1.773060525		-20	0.121217
chr8_18081_star	0	2.417782	0	1.773060525		-20	0.121217
chrx_23008_star	0	2.417782	0	1.773060525		-20	0.121217
rno-miR-873-3p	0	2.417782	0	1.773060525		-20	0.121217
chrx_21717_star	0	2.417782	0	1.773060525		-20	0.121217
chr10_23328_star	0	2.417782	0	1.773060525		-20	0.121217
mmu-miR-344b-3p	0	2.417782	0	1.773060525		-20	0.121217
chr15_32429_mature	0	2.417782	0	1.773060525		-20	0.121217
hsa-miR-548d-3p	0	2.417782	0	1.773060525		-20	0.121217
NW_003539103_38526_star	0	2.417782	0	1.773060525		-20	0.121217
hsa-miR-1285-5p	0	2.417782	0	1.773060525		-20	0.121217
hsa-miR-4668-5p	0	2.417782	0	1.773060525		-20	0.121217
chr6_13330_star	0	2.417782	0	1.773060525		-20	0.121217
hsa-miR-4724-3p	0	2.417782	0	1.773060525		-20	0.121217
chr3_8076_star	0	2.417782	0	1.773060525		-20	0.121217
bta-miR-2411-3p	0	2.417782	0	1.773060525		-20	0.121217
bta-miR-2397-5p	0	2.417782	0	1.773060525		-20	0.121217
chr7_16402_mature	0	2.417782	0	1.773060525		-20	0.121217
chr8_17941_star	0	2.417782	0	1.773060525		-20	0.121217
mmu-miR-669m-5p	0	2.417782	0	1.773060525		-20	0.121217
chr3_7786_star	0	2.417782	0	1.773060525		-20	0.121217
chrx_21663_star	0	2.417782	0	1.773060525		-20	0.121217
hsa-miR-6510-5p	0	2.417782	0	1.773060525		-20	0.121217
chr13_29234_star	0	2.417782	0	1.773060525		-20	0.121217
hsa-miR-575	0	2.417782	0	1.773060525		-20	0.121217
hsa-miR-578	0	2.417782	0	1.773060525		-20	0.121217
chr9_21061_mature	0	2.417782	0	1.773060525		-20	0.121217
mmu-miR-5620-3p	0	2.417782	0	1.773060525		-20	0.121217
hsa-miR-548au-3p	0	2.417782	0	1.773060525		-20	0.121217
chr13_27058_star	0	2.417782	0	1.773060525		-20	0.121217
chrx_22054_star	0	2.417782	0	1.773060525		-20	0.121217
hsa-miR-30c-5p	0	2.417782	0	1.773060525		-20	0.121217
hsa-miR-3919	0	2.417782	0	1.773060525		-20	0.121217
chr1_3348_star	0	2.417782	0	1.773060525		-20	0.121217
mmu-miR-3103-3p	0	2.417782	0	1.773060525		-20	0.121217

chr2_4097_star	1.07457	8.865202	1.05281229	3.302348583		-3.04439	0.421773
chr16_34295_star	1.07457	8.865202	1.05281229	3.302348583		-3.04439	0.421773
chr12_26249_star	1.07457	8.865202	1.05281229	3.302348583		-3.04439	0.421773
chr8_18841_star	1.07457	8.865202	1.05281229	3.302348583		-3.04439	0.421773
chr6_12645_star	1.07457	8.865202	1.05281229	3.302348583		-3.04439	0.421773
chr12_25849_star@@mmu-miR-51	11.28298	105.5765	3.618589217	6.735745493		-3.22607	0.422776
chr12_26323_star	2.14914	15.31262	1.654957834	4.027916745		-2.83289	0.422996
bta-miR-2367-3p	2.14914	15.31262	1.654957834	4.027916745		-2.83289	0.422996
chr1_771_star	2.14914	15.31262	1.654957834	4.027916745		-2.83289	0.422996
chr18_36468_mature	2.14914	15.31262	1.654957834	4.027916745		-2.83289	0.422996
chr3_7737_star	19.34226	2.417782	4.346407978	1.773060525		3	0.425593
NW_003537646_37091_star	149.9025	12.89484	7.237472949	3.796477232		3.539159	0.427257
ssc-miR-196b	6.44742	50.77343	2.896740642	5.694139975		-2.97728	0.427604
chr1_2668_star	13.43212	1.611855	3.851211749	1.38507475		3.058894	0.42951
chr13_27839_mature	13.43212	1.611855	3.851211749	1.38507475		3.058894	0.42951
chr1_3348_mature	13.43212	1.611855	3.851211749	1.38507475		3.058894	0.42951
chr18_36422_mature	13.43212	1.611855	3.851211749	1.38507475		3.058894	0.42951
chr2_6321_mature	13.43212	1.611855	3.851211749	1.38507475		3.058894	0.42951
chr1_1486_star	13.43212	1.611855	3.851211749	1.38507475		3.058894	0.42951
ssc-miR-1468	13.43212	1.611855	3.851211749	1.38507475		3.058894	0.42951
bta-miR-6122-3p	13.43212	1.611855	3.851211749	1.38507475		3.058894	0.42951
chr2_6372_star	13.43212	1.611855	3.851211749	1.38507475		3.058894	0.42951
chr14_30887_mature	40.29637	4.835565	5.367943153	2.544872269		3.058894	0.430123
chr12_25501_mature	20.41683	230.4953	4.420672964	7.854838784		-3.49691	0.430136
chr5_12092_mature	13.43212	130.5602	3.851211749	7.039579809		-3.28096	0.430689
chr6_14751_mature	4.835565	35.46081	2.544872269	5.188274618		-2.87447	0.430971
chrx_21532_mature	2.686425	18.53633	1.882222339	4.288087668		-2.7866	0.430997
chr9_20599_mature	2.686425	18.53633	1.882222339	4.288087668		-2.7866	0.430997
chr9_20263_star	4.835565	0	2.544872269	0		20	0.431264
chr15_32587_star	4.835565	0	2.544872269	0		20	0.431264
chr13_27796_mature	4.835565	0	2.544872269	0		20	0.431264
hsa-miR-1272	4.835565	0	2.544872269	0		20	0.431264
chr15_33671_star	4.835565	0	2.544872269	0		20	0.431264
mmu-miR-16-1-3p	4.835565	0	2.544872269	0		20	0.431264
chr3_7770_star	4.835565	0	2.544872269	0		20	0.431264
NW_003541081_39894_star	4.835565	0	2.544872269	0		20	0.431264
chr13_28984_mature	4.835565	0	2.544872269	0		20	0.431264
chr5_11713_mature	4.835565	0	2.544872269	0		20	0.431264
bta-miR-1301	4.835565	0	2.544872269	0		20	0.431264
chr13_28825_star	4.835565	0	2.544872269	0		20	0.431264
rno-miR-337-3p	4.835565	0	2.544872269	0		20	0.431264
mmu-miR-676-3p	4.835565	0	2.544872269	0		20	0.431264
hsa-miR-5091	4.835565	0	2.544872269	0		20	0.431264
bta-miR-1193	4.835565	0	2.544872269	0		20	0.431264
chr3_8285_star	4.835565	0	2.544872269	0		20	0.431264
chr1_526_star	4.835565	0	2.544872269	0		20	0.431264
rno-miR-764-5p	4.835565	0	2.544872269	0		20	0.431264
mmu-miR-673-5p	4.835565	0	2.544872269	0		20	0.431264
chr7_16472_star	4.835565	0	2.544872269	0		20	0.431264
chr1_3638_mature	4.835565	0	2.544872269	0		20	0.431264
chr1_990_star	4.835565	0	2.544872269	0		20	0.431264
mmu-miR-6335	4.835565	0	2.544872269	0		20	0.431264
bta-miR-2285f	4.835565	0	2.544872269	0		20	0.431264
chr2_4529_mature	4.835565	0	2.544872269	0		20	0.431264
rno-miR-708-3p	4.835565	0	2.544872269	0		20	0.431264
chr18_35940_mature	4.835565	0	2.544872269	0		20	0.431264
chr8_17858_star	4.835565	0	2.544872269	0		20	0.431264
mmu-miR-3964	4.835565	0	2.544872269	0		20	0.431264
hsa-miR-545-5p	4.835565	0	2.544872269	0		20	0.431264
hsa-miR-4711-5p	4.835565	0	2.544872269	0		20	0.431264
NW_003537297_36826_mature	4.835565	0	2.544872269	0		20	0.431264
bta-miR-194	4.835565	0	2.544872269	0		20	0.431264
chr17_35007_star	4.835565	0	2.544872269	0		20	0.431264
bta-miR-2322-3p	4.835565	0	2.544872269	0		20	0.431264
chr8_17976_star	4.835565	0	2.544872269	0		20	0.431264
rno-miR-296-3p	4.835565	0	2.544872269	0		20	0.431264
bta-miR-6522	4.835565	0	2.544872269	0		20	0.431264
chr7_15459_star	4.835565	0	2.544872269	0		20	0.431264
hsa-miR-2115-5p	4.835565	0	2.544872269	0		20	0.431264
chrx_23198_star	4.835565	0	2.544872269	0		20	0.431264
hsa-miR-4424	4.835565	0	2.544872269	0		20	0.431264
chrx_22153_mature	4.835565	0	2.544872269	0		20	0.431264
hsa-miR-3692-3p	4.835565	0	2.544872269	0		20	0.431264
hsa-miR-613	4.835565	0	2.544872269	0		20	0.431264
hsa-miR-634	4.835565	0	2.544872269	0		20	0.431264
chr6_12752_star	4.835565	0	2.544872269	0		20	0.431264
chr17_35398_star	4.835565	0	2.544872269	0		20	0.431264
chr15_33101_star	4.835565	0	2.544872269	0		20	0.431264
chr14_30956_mature	4.835565	0	2.544872269	0		20	0.431264
chr9_20875_star	4.835565	0	2.544872269	0		20	0.431264
chr16_34124_mature	4.835565	0	2.544872269	0		20	0.431264
chr6_14780_star	4.835565	0	2.544872269	0		20	0.431264
chr6_13398_mature	4.835565	0	2.544872269	0		20	0.431264
chr8_18187_mature	4.835565	0	2.544872269	0		20	0.431264
chr13_28063_star	4.835565	0	2.544872269	0		20	0.431264
chr3_8646_star	4.835565	0	2.544872269	0		20	0.431264
mmu-miR-106a-3p	4.835565	0	2.544872269	0		20	0.431264
chr9_19426_mature	4.835565	0	2.544872269	0		20	0.431264
chr4_9118_star	4.835565	0	2.544872269	0		20	0.431264
chr1_2918_mature	4.835565	0	2.544872269	0		20	0.431264
chr15_32959_mature	25.25239	3.22371	4.714377052	2.078510713		2.969626	0.431657
chr1_1949_mature	25.25239	3.22371	4.714377052	2.078510713		2.969626	0.431657
chr2_4320_mature	8.596559	0.805927	3.262517265	0.852739935		3.415037	0.431757
chr2_4773_star	8.596559	0.805927	3.262517265	0.852739935		3.415037	0.431757
chr14_30472_mature	8.596559	0.805927	3.262517265	0.852739935		3.415037	0.431757
chr12_25520_star	8.596559	0.805927	3.262517265	0.852739935		3.415037	0.431757
NW_003539698_39033_star	8.596559	0.805927	3.262517265	0.852739935		3.415037	0.431757
mmu-miR-6376	8.596559	0.805927	3.262517265	0.852739935		3.415037	0.431757
chr5_12271_star	8.596559	0.805927	3.262517265	0.852739935		3.415037	0.431757
chr13_28683_mature	8.596559	0.805927	3.262517265	0.852739935		3.415037	0.431757
chr13_28684_mature	8.596559	0.805927	3.262517265	0.852739935		3.415037	0.431757
chr1_550_mature	8.596559	0.805927	3.262517265	0.852739935		3.415037	0.431757
bta-miR-2300b-3p	8.596559	0.805927	3.262517265	0.852739935		3.415037	0.431757
chr14_30483_star	8.596559	0.805927	3.262517265	0.852739935		3.415037	0.431757
chr7_15747_star	8.596559	0.805927	3.262517265	0.852739935		3.415037	0.431757
hsa-miR-4496	8.596559	0.805927	3.262517265	0.852739935		3.415037	0.431757
mmu-miR-1930-3p	8.596559	0.805927	3.262517265	0.852739935		3.415037	0.431757
chr8_17927_mature	8.596559	0.805927	3.262517265	0.852739935		3.415037	0.431757
chr13_27252_mature	8.596559	0.805927	3.262517265	0.852739935		3.415037	0.431757
hsa-miR-3678-5p	188.587	15.31262	7.566716407	4.027916745		3.622437	0.432705
chr15_31842_mature	18.80497	2.417782	4.307790888	1.773060525		2.959358	0.432875
chr3_7274_mature	24.71511	3.22371	4.684544332	2.078510713		2.938599	0.43736
chr13_27408_mature	5.37285	39.49044	2.671938627	5.339509593		-2.87774	0.437457
chr10_24060_mature	344.9369	23.3719	8.4343653	4.607146585		3.883486	0.438506
chr7_15081_mature	1.611855	11.28298	1.38507475	3.618589217		-2.80735	0.438988
chr2_4238_mature	1.611855	11.28298	1.38507475	3.618589217		-2.80735	0.438988
chr14_29882_mature	1.611855	11.28298	1.38507475	3.618589217		-2.80735	0.438988
chr16_33845_mature	1.611855	11.28298	1.38507475	3.618589217		-2.80735	0.438988
NW_003539012_38462_mature	6.44742	49.16157	2.896740642	5.648510724		-2.93074	0.43934
hsa-miR-3646	2.14914	14.50669	1.654957834	3.954819241		-2.75489	0.439541
chr10_23328_mature	2.14914	14.50669	1.654957834	3.954819241		-2.75489	0.439541
chr13_28860_mature	2.14914	14.50669	1.654957834	3.954819241		-2.75489	0.439541
chr4_9231_star	2.14914	14.50669	1.654957834	3.954819241		-2.75489	0.439541
chr1_429_mature	12.89484	1.611855	3.796477232	1.38507475		3	0.439667
bta-miR-2464-5p	12.89484	1.611855	3.796477232	1.38507475		3	0.439667
chr17_35259_star	12.89484	1.611855	3.796477232	1.38507475		3	0.439667
chr16_34527_star@@hsa-miR-449	12.89484	1.611855	3.796477232	1.38507475		3	0.439667
mmu-miR-378c	12.89484	1.611855	3.796477232	1.38507475		3	0.439667
chr7_15292_mature	3.760995	25.78968	2.251263041	4.743605355		-2.77761	0.440525
chr3_8285_mature	3.760995	25.78968	2.251263041	4.743605355		-2.77761	0.440525
chr14_29900_mature	38.68452	4.835565	5.310504361	2.544872269		3	0.44149
bta-miR-544b	4.29828	29.81932	2.405524014	4.94576292		-2.79442	0.442976
chr3_8036_mature	24.17782	3.22371	4.654081667	2.078510713		2.906891	0.443159
chr17_34997_mature	2.686425	17.7304	1.882222339	4.227310103		-2.72247	0.444901
chr9_19622_mature	0.537285	5.641492	0.620384622	2.731507408		-3.39232	0.44508
NW_003539920_39188_star	0.537285	5.641492	0.620384622	2.731507408		-3.39232	0.44508
chr12_25395_star	0.537285	5.641492	0.620384622	2.731507408		-3.39232	0.44508
chr3_7048_star	0.537285	5.641492	0.620384622	2.731507408		-3.39232	0.44508
hsa-miR-4433-5p	0.537285	5.641492	0.620384622	2.731507408		-3.39232	0.44508
chr11_25110_mature	0.537285	5.641492	0.620384622	2.731507408		-3.39232	0.44508
chr13_28383_star	0.537285	5.641492	0.620384622	2.731507408		-3.39232	0.44508
chr9_20262_star	0.537285	5.641492	0.620384622	2.731507408		-3.39232	0.44508
chr1_816_star	0.537285	5.641492	0.620384622	2.731507408		-3.39232	0.44508
chr5_11934_mature	0.537285	5.641492	0.620384622	2.731507408		-3.39232	0.44508
chr13_27764_star	0.537285	5.641492	0.620384622	2.731507408		-3.39232	0.44508
bta-miR-2285t	15.58126	153.9321	4.051482084	7.275492671		-3.30441	0.445277
chr8_18342_mature	38.14723	4.835565	5.290838418	2.544872269		2.979822	0.445363
chr15_31776_star	8.059274	0.805927	3.179395517	0.852739935		3.321928	0.447273
chr14_31627_mature	8.059274	0.805927	3.179395517	0.852739935		3.321928	0.447273
chr1_2655_mature	8.059274	0.805927	3.179395517	0.852739935		3.321928	0.447273
chr2_6113_star	8.059274	0.805927	3.179395517	0.852739935		3.321928	0.447273
chr13_27409_star@@hsa-miR-551	8.059274	0.805927	3.179395517	0.852739935		3.321928	0.447273
chr4_9373_mature	8.059274	0.805927	3.179395517	0.852739935		3.321928	0.447273
chr2_6234_star	8.059274	0.805927	3.179395517	0.852739935		3.321928	0.447273
chr15_33376_mature	8.059274	0.805927	3.179395517	0.852739935		3.321928	0.447273
bta-miR-449d	8.059274	0.805927	3.179395517	0.852739935		3.321928	0.447273
hsa-miR-5584-5p	8.059274	0.805927	3.179395517	0.852739935		3.321928	0.447273
bta-miR-301a	8.059274	0.805927	3.179395517	0.852739935		3.321928	0.447273
chr13_27018_star	8.059274	0.805927	3.179395517	0.852739935		3.321928	0.447273
chr3_8361_mature	8.059274	0.805927	3.179395517	0.852739935		3.321928	0.447273
bta-miR-2449	8.059274	0.805927	3.179395517	0.852739935		3.321928	0.447273
bta-miR-378b	8.059274	0.805927	3.179395517	0.852739935		3.321928	0.447273
chr1_2577_star	8.059274	0.805927	3.179395517	0.852739935		3.321928	0.447273
chr14_31020_mature	11.28298	99.12908	3.618589217	6.645717165		-3.13516	0.447848
chr4_9835_mature	17.7304	2.417782	4.227310103	1.773060525		2.874469	0.447916
chr8_18166_mature	54.26578	6.44742	5.788314591	2.896740642		3.073249	0.447943
chr13_26807_mature	1.07457	8.059274	1.05281229	3.179395517		-2.90689	0.448959
bta-miR-876	1.07457	8.059274	1.05281229	3.179395517		-2.90689	0.448959
chr14_30275_mature	1.07457	8.059274	1.05281229	3.179395517		-2.90689	0.448959
chr1_439_mature	1.07457	8.059274	1.05281229	3.179395517		-2.90689	0.448959
chr9_19650_mature	1.07457	8.059274	1.05281229	3.179395517		-2.90689	0.448959
chr2_5969_mature	1.07457	8.059274	1.05281229	3.179395517		-2.90689	0.448959
chr2_6581_mature	1.07457	8.059274	1.05281229	3.179395517		-2.90689	0.448959
chrx_23221_star	1.07457	8.059274	1.05281229	3.179395517		-2.90689	0.448959
chr11_24477_star	1.07457	8.059274	1.05281229	3.179395517		-2.90689	0.448959
chr1_2966_mature	1.07457	8.059274	1.05281229	3.179395517		-2.90689	0.448959
chr1_439_star	1.07457	8.059274	1.05281229	3.179395517		-2.90689	0.448959
chr1_2659_mature	1.07457	8.059274	1.05281229	3.179395517		-2.90689	0.448959
chr17_35522_star	23.64054	3.22371	4.62296188	2.078510713		2.874469	0.449054
chr17_35478_mature	12.35755	1.611855	3.739583968	1.38507475		2.938599	0.450126
chr8_18847_mature	12.35755	1.611855	3.739583968	1.38507475		2.938599	0.450126
chr14_31446_mature	12.35755	1.611855	3.739583968	1.38507475		2.938599	0.450126
chr16_33835_star	12.35755	1.611855	3.739583968	1.38507475		2.938599	0.450126
chr1_2668_mature	114.979	11.28298	6.857719579	3.618589217		3.34915	0.450864
chr12_26249_mature	5.37285	37.87859	2.671938627	5.280903997		-2.81762	0.452002
chr2_5550_mature	4.29828	29.01339	2.405524014	4.907534286		-2.75489	0.452133
hsa-miR-4787-5p	3.22371	20.95411	2.078510713	4.456419386		-2.70044	0.452614
chr3_7600_mature	1422.731	61.25049	10.47546046	5.960013198		4.537797	0.453365
chr10_23676_mature	163.3346	14.50669	7.360492716	3.954819241		3.49304	0.45353
chr17_35140_mature	23.10325	3.22371	4.591155994	2.078510713		2.841302	0.455046
NW_003613226_37753_star	23.10325	3.22371	4.591155994	2.078510713		2.841302	0.455046
chr5_11850_mature	4.835565	33.04303	2.544872269	5.08928735		-2.77259	0.45519
chr5_12327_mature	17.19312	2.417782	4.185320985	1.773060525		2.830075	0.455677
chr1_927_mature	17.19312	2.417782	4.185320985	1.773060525		2.830075	0.455677
chr10_23316_mature	29.55067	4.029637	4.933132262	2.330454351		2.874469	0.455946
chr2_5067_mature	36.53538	4.835565	5.230179094	2.544872269		2.917538	0.457244
NW_003539576_38951_mature	2.686425	16.92448	1.882222339	4.163859073		-2.65535	0.459156
chr11_25345_mature	4.29828	0	2.405524014	0		20	0.459454
chr4_9934_star	4.29828	0	2.405524014	0		20	0.459454
hsa-miR-2116-3p	4.29828	0	2.405524014	0		20	0.459454
chr1_3728_star	4.29828	0	2.405524014	0		20	0.459454
chr9_20299_star	4.29828	0	2.405524014	0		20	0.459454
chr4_9772_star	4.29828	0	2.405524014	0		20	0.459454
hsa-miR-557	4.29828	0	2.405524014	0		20	0.459454
hsa-miR-554	4.29828	0	2.405524014	0		20	0.459454
chr5_11087_mature	4.29828	0	2.405524014	0		20	0.459454
chr4_9777_mature	4.29828	0	2.405524014	0		20	0.459454
hsa-miR-4638-5p	4.29828	0	2.405524014	0		20	0.459454
hsa-miR-3684	4.29828	0	2.405524014	0		20	0.459454
chr9_20767_star	4.29828	0	2.405524014	0		20	0.459454
rno-miR-6322	4.29828	0	2.405524014	0		20	0.459454
hsa-miR-4693-5p	4.29828	0	2.405524014	0		20	0.459454
hsa-miR-4693-3p	4.29828	0	2.405524014	0		20	0.459454
chr2_4623_mature	4.29828	0	2.405524014	0		20	0.459454
chr7_16728_mature	4.29828	0	2.405524014	0		20	0.459454
chr1_268_star	4.29828	0	2.405524014	0		20	0.459454
chr14_29583_mature	4.29828	0	2.405524014	0		20	0.459454
hsa-miR-378i	4.29828	0	2.405524014	0		20	0.459454
mmu-miR-6407	4.29828	0	2.405524014	0		20	0.459454
chr17_35680_mature	4.29828	0	2.405524014	0		20	0.459454
chr3_7521_star	4.29828	0	2.405524014	0		20	0.459454
chr13_26823_mature	4.29828	0	2.405524014	0		20	0.459454
chr9_19521_star	4.29828	0	2.405524014	0		20	0.459454
mmu-miR-6348	4.29828	0	2.405524014	0		20	0.459454
chr1_2420_star	4.29828	0	2.405524014	0		20	0.459454
chr12_25513_star	4.29828	0	2.405524014	0		20	0.459454
chr15_31855_star	4.29828	0	2.405524014	0		20	0.459454
chr7_16620_star	4.29828	0	2.405524014	0		20	0.459454
chr18_36296_star	4.29828	0	2.405524014	0		20	0.459454
chr11_25111_mature	4.29828	0	2.405524014	0		20	0.459454
chr13_26823_star	4.29828	0	2.405524014	0		20	0.459454
chr1_607_star	4.29828	0	2.405524014	0		20	0.459454
chr18_36015_star	4.29828	0	2.405524014	0		20	0.459454
chr17_35489_star	4.29828	0	2.405524014	0		20	0.459454
chr6_12983_star@@rno-miR-200b-	4.29828	0	2.405524014	0		20	0.459454
ssc-miR-4336	4.29828	0	2.405524014	0		20	0.459454
chr6_12861_star	4.29828	0	2.405524014	0		20	0.459454
rno-miR-24-2-5p	4.29828	0	2.405524014	0		20	0.459454
NW_003537967_37423_mature	4.29828	0	2.405524014	0		20	0.459454
mmu-miR-302d-3p	4.29828	0	2.405524014	0		20	0.459454
NW_003539626_38995_star	4.29828	0	2.405524014	0		20	0.459454
mmu-miR-291b-3p	4.29828	0	2.405524014	0		20	0.459454
chr13_29380_star	4.29828	0	2.405524014	0		20	0.459454
chrx_22713_star	4.29828	0	2.405524014	0		20	0.459454
chr14_30543_mature	4.29828	0	2.405524014	0		20	0.459454
hsa-miR-4438	4.29828	0	2.405524014	0		20	0.459454
hsa-miR-600	4.29828	0	2.405524014	0		20	0.459454
hsa-miR-611	4.29828	0	2.405524014	0		20	0.459454
chr18_36004_star	4.29828	0	2.405524014	0		20	0.459454
chr7_16478_star	4.29828	0	2.405524014	0		20	0.459454
chr14_31327_star	4.29828	0	2.405524014	0		20	0.459454
bta-miR-2334	4.29828	0	2.405524014	0		20	0.459454
bta-miR-2358	4.29828	0	2.405524014	0		20	0.459454
mmu-miR-3472	4.29828	0	2.405524014	0		20	0.459454
chr12_25396_star	4.29828	0	2.405524014	0		20	0.459454
NW_003538061_37480_mature	4.29828	0	2.405524014	0		20	0.459454
chr14_31524_star	4.29828	0	2.405524014	0		20	0.459454
chr1_273_star	4.29828	0	2.405524014	0		20	0.459454
chr7_16298_star	4.29828	0	2.405524014	0		20	0.459454
chr16_34140_mature	4.29828	0	2.405524014	0		20	0.459454
chr2_4860_star	4.29828	0	2.405524014	0		20	0.459454
mmu-let-7a-5p	4.29828	0	2.405524014	0		20	0.459454
chr1_785_mature	52.11664	6.44742	5.73109203	2.896740642		3.01495	0.459493
chr3_6901_star	1.611855	10.47706	1.38507475	3.520680821		-2.70044	0.460307
chr4_8767_star	1.611855	10.47706	1.38507475	3.520680821		-2.70044	0.460307

chr2_4241_mature	10.74569932	0.805927	3.554060706	0.852739935		3.736966	0.17967	hsa-miR-5187-5p	0	2.417782	0	1.773060525		-20	0.121217	NW_003540351_39435_mature	1.611855	10.47706	1.38507475	3.520680821		-2.70044	0.460307
mmu-miR-3084-3p	10.74569932	0.805927	3.554060706	0.852739935		3.736966	0.17967	mmu-miR-463-5p	0	2.417782	0	1.773060525		-20	0.121217	chr5_12267_star	1.611855	10.47706	1.38507475	3.520680821		-2.70044	0.460307
mmu-miR-5133	10.74569932	0.805927	3.554060706	0.852739935		3.736966	0.17967	chrx_23098_star	0	2.417782	0	1.773060525		-20	0.121217	chr4_9939_mature	1.611855	10.47706	1.38507475	3.520680821		-2.70044	0.460307
mmu-miR-5126	10.74569932	0.805927	3.554060706	0.852739935		3.736966	0.17967	NW_003539007_38454_star	0	2.417782	0	1.773060525		-20	0.121217	chr13_28744_mature	1.611855	10.47706	1.38507475	3.520680821		-2.70044	0.460307
chr12_26098_mature	10.74569932	0.805927	3.554060706	0.852739935		3.736966	0.17967	chr11_24562_star	0	2.417782	0	1.773060525		-20	0.121217	chr5_11735_star	1.611855	10.47706	1.38507475	3.520680821		-2.70044	0.460307
chr15_32731_mature	10.74569932	0.805927	3.554060706	0.852739935		3.736966	0.17967	hsa-miR-616-5p	0	2.417782	0	1.773060525		-20	0.121217	chr1_3025_star	11.82027	1.611855	3.680354657	1.38507475		2.874469	0.46089
hsa-miR-20b-3p	10.74569932	0.805927	3.554060706	0.852739935		3.736966	0.17967	chr3_7884_star	0	2.417782	0	1.773060525		-20	0.121217	chr4_10519_star	11.82027	1.611855	3.680354657	1.38507475		2.874469	0.46089
chr4_9665_star	10.74569932	0.805927	3.554060706	0.852739935		3.736966	0.17967	chr18_36372_star	0	2.417782	0	1.773060525		-20	0.121217	chr3_6707_star@@mmu-miR-590-5	11.82027	1.611855	3.680354657	1.38507475		2.874469	0.46089
NW_003539989_39206_star	10.74569932	0.805927	3.554060706	0.852739935		3.736966	0.17967	chr14_30569_mature	0	2.417782	0	1.773060525		-20	0.121217	chr15_33460_star	11.82027	1.611855	3.680354657	1.38507475		2.874469	0.46089
chrx_22664_star	10.74569932	0.805927	3.554060706	0.852739935		3.736966	0.17967	hsa-miR-5571-3p	0	2.417782	0	1.773060525		-20	0.121217	chr9_19408_mature@@hsa-miR-3	111.218	11.28298	6.810160141	3.618589217		3.30117	0.461081
chr17_35612_mature	10.74569932	0.805927	3.554060706	0.852739935		3.736966	0.17967	hsa-miR-3127-5p	0	2.417782	0	1.773060525		-20	0.121217	NW_003613114_37265_star	35.99809	4.835565	5.209378995	2.544872269		2.896164	0.461291
mmu-miR-1934-3p	10.74569932	0.805927	3.554060706	0.852739935		3.736966	0.17967	chr4_9150_star	0	2.417782	0	1.773060525		-20	0.121217	bta-miR-2284z	4.29828	28.20746	2.405524014	4.868265031		-2.71425	0.461441
chr2_5364_star	10.74569932	0.805927	3.554060706	0.852739935		3.736966	0.17967	chr3_7706_star	0	2.417782	0	1.773060525		-20	0.121217	chr9_20315_mature	3.760995	24.17782	2.251263041	4.654081667		-2.6845	0.461488
mmu-miR-466b-5p	10.74569932	0.805927	3.554060706	0.852739935		3.736966	0.17967	hsa-miR-4735-3p	0	2.417782	0	1.773060525		-20	0.121217	NW_003613530_39570_star	7.52199	0.805927	3.091190278	0.852739935		3.222392	0.463498
chr6_13027_mature	10.74569932	0.805927	3.554060706	0.852739935		3.736966	0.17967	NW_003537531_36983_star	0	2.417782	0	1.773060525		-20	0.121217	chr1_3951_mature	7.52199	0.805927	3.091190278	0.852739935		3.222392	0.463498
rno-miR-449c-5p	10.74569932	0.805927	3.554060706	0.852739935		3.736966	0.17967	NW_003613164_37468_star	0	2.417782	0	1.773060525		-20	0.121217	chr3_7747_mature	7.52199	0.805927	3.091190278	0.852739935		3.222392	0.463498
chr1_886_mature	10.74569932	0.805927	3.554060706	0.852739935		3.736966	0.17967	chr14_29426_mature	0	2.417782	0	1.773060525		-20	0.121217	chr16_34199_star	7.52199	0.805927	3.091190278	0.852739935		3.222392	0.463498
chr15_32938_mature	4.298279727	47.54972	2.405524014	5.601391055		-3.46761	0.180651	chr15_32377_star	0	2.417782	0	1.773060525		-20	0.121217	mmu-miR-5126	7.52199	0.805927	3.091190278	0.852739935		3.222392	0.463498
chr16_34272_mature	75.21989523	7.253347	6.25209572	3.044979305		3.374396	0.180683	rno-miR-3542	0	2.417782	0	1.773060525		-20	0.121217	chr10_24203_star	7.52199	0.805927	3.091190278	0.852739935		3.222392	0.463498
chr7_16636_mature	38.68451755	4.029637	5.310504361	2.330454351		3.263034	0.180869	hsa-miR-3668	0	2.417782	0	1.773060525		-20	0.121217	chr2_6082_mature	7.52199	0.805927	3.091190278	0.852739935		3.222392	0.463498
chr7_16231_mature	38.68451755	4.029637	5.310504361	2.330454351		3.263034	0.180869	chr2_5369_mature	0	2.417782	0	1.773060525		-20	0.121217	chr5_10827_star	7.52199	0.805927	3.091190278	0.852739935		3.222392	0.463498
bta-miR-410	777.9886306	44.32601	9.605458462	5.502267253		4.133524	0.18256	rno-miR-3552	0	2.417782	0	1.773060525		-20	0.121217	chr4_10525_star	7.52199	0.805927	3.091190278	0.852739935		3.222392	0.463498
chr13_27735_star	1.074569932	14.50669	1.05281229	3.954819241		-3.75489	0.18294	rno-miR-3553	0	2.417782	0	1.773060525		-20	0.121217	NW_003539422_38814_star	7.52199	0.805927	3.091190278	0.852739935		3.222392	0.463498
chr9_19208_mature	1.074569932	14.50669	1.05281229	3.954819241		-3.75489	0.18294	mmu-miR-5113	0	2.417782	0	1.773060525		-20	0.121217	NW_003538236_37641_star	7.52199	0.805927	3.091190278	0.852739935		3.222392	0.463498
chr2_5889_star	1.074569932	14.50669	1.05281229	3.954819241		-3.75489	0.18294	chr15_32637_star	0	2.417782	0	1.773060525		-20	0.121217	chr8_17927_star	7.52199	0.805927	3.091190278	0.852739935		3.222392	0.463498
chr6_13339_star	1.074569932	14.50669	1.05281229	3.954819241		-3.75489	0.18294	chr2_5585_mature	0	2.417782	0	1.773060525		-20	0.121217	mmu-miR-6363	7.52199	0.805927	3.091190278	0.852739935		3.222392	0.463498
chr15_33030_star	1.074569932	14.50669	1.05281229	3.954819241		-3.75489	0.18294	chr6_13734_star	0	2.417782	0	1.773060525		-20	0.121217	chr14_31484_mature	7.52199	0.805927	3.091190278	0.852739935		3.222392	0.463498
bta-miR-2350	1.074569932	14.50669	1.05281229	3.954819241		-3.75489	0.18294	hsa-miR-550a-3p	0	2.417782	0	1.773060525		-20	0.121217	chr11_24857_star	7.52199	0.805927	3.091190278	0.852739935		3.222392	0.463498
chr1_3717_star	1.074569932	14.50669	1.05281229	3.954819241		-3.75489	0.18294	chrx_21725_mature	0	2.417782	0	1.773060525		-20	0.121217	chr9_20121_star	7.52199	0.805927	3.091190278	0.852739935		3.222392	0.463498
chr15_33369_star	1.074569932	14.50669	1.05281229	3.954819241		-3.75489	0.18294	chr5_11941_star	0	2.417782	0	1.773060525		-20	0.121217	chr8_17274_mature	7.52199	0.805927	3.091190278	0.852739935		3.222392	0.463498
chr7_15959_mature	3.223709795	34.65488	2.078510713	5.156027656		-3.42626	0.183448	hsa-miR-664a-3p	0	2.417782	0	1.773060525		-20	0.121217	chr6_13572_mature	7.52199	0.805927	3.091190278	0.852739935		3.222392	0.463498
chr14_29793_mature	3.223709795	34.65488	2.078510713	5.156027656		-3.42626	0.183448	hsa-miR-5096	0	2.417782	0	1.773060525		-20	0.121217	chr16_33909_mature	4.835565	32.2371	2.544872269	5.054722516		-2.73697	0.463505
chr1_3039_star	3.223709795	34.65488	2.078510713	5.156027656		-3.42626	0.183448	chr13_26646_star	0	2.417782	0	1.773060525		-20	0.121217	ssc-miR-133a-5p	6.44742	45.93786	2.896740642	5.552680304		-2.83289	0.463645
NW_003539080_38513_mature	3.223709795	34.65488	2.078510713	5.156027656		-3.42626	0.183448	chr7_15120_star	0	2.417782	0	1.773060525		-20	0.121217	chr8_18464_mature	9.671129	77.36904	3.415640968	6.292211829		-3	0.464748
chr7_16117_mature	74.1453253	7.253347	6.231611453	3.044979305		3.353637	0.183927	chr14_31415_mature	0	2.417782	0	1.773060525		-20	0.121217	chr15_32699_mature	3.22371	20.14819	2.078510713	4.402462031		-2.64386	0.464874
chr13_28115_star	30.08795809	3.22371	4.958283956	2.078510713		3.222392	0.185387	rno-miR-802-5p	0	2.417782	0	1.773060525		-20	0.121217	chr6_15067_mature	0	3.22371	0	2.078510713		-20	0.466012
chr14_31496_mature	5.372849659	0	2.671938627	0		20	0.185725	bta-miR-2450b	0	2.417782	0	1.773060525		-20	0.121217	chr3_6912_star	0	3.22371	0	2.078510713		-20	0.466012
chr2_5426_star	5.372849659	0	2.671938627	0		20	0.185725	rno-miR-802-3p	0	2.417782	0	1.773060525		-20	0.121217	chr3_7082_star	0	3.22371	0	2.078510713		-20	0.466012
NW_003539513_38910_mature	5.372849659	0	2.671938627	0		20	0.185725	hsa-miR-378f	0	2.417782	0	1.773060525		-20	0.121217	hsa-miR-525-3p	0	3.22371	0	2.078510713		-20	0.466012
rno-let-7c-2-3p	5.372849659	0	2.671938627	0		20	0.185725	chr18_36565_star	0	2.417782	0	1.773060525		-20	0.121217	chr9_19299_mature	0	3.22371	0	2.078510713		-20	0.466012
rno-miR-664-3p	5.372849659	0	2.671938627	0		20	0.185725	chr6_15010_star	0	2.417782	0	1.773060525		-20	0.121217	chr13_28624_mature	0	3.22371	0	2.078510713		-20	0.466012
hsa-miR-566	5.372849659	0	2.671938627	0		20	0.185725	hsa-miR-5009-3p	0	2.417782	0	1.773060525		-20	0.121217	chr3_6671_mature	0	3.22371	0	2.078510713		-20	0.466012
chr10_23951_mature	5.372849659	0	2.671938627	0		20	0.185725	chr7_15080_mature	0	2.417782	0	1.773060525		-20	0.121217	chr3_7716_star	0	3.22371	0	2.078510713		-20	0.466012
chr6_15024_mature	5.372849659	0	2.671938627	0		20	0.185725	chr13_28171_star	0	2.417782	0	1.773060525		-20	0.121217	chr15_32759_star	0	3.22371	0	2.078510713		-20	0.466012
chr9_20451_mature	5.372849659	0	2.671938627	0		20	0.185725	chr5_12382_star	0	2.417782	0	1.773060525		-20	0.121217	bta-miR-1777a	0	3.22371	0	2.078510713		-20	0.466012
NW_003539588_38971_star	5.372849659	0	2.671938627	0		20	0.185725	chr5_10795_star	0	2.417782	0	1.773060525		-20	0.121217	chr12_25994_mature	0	3.22371	0	2.078510713		-20	0.466012
rno-miR-434-5p	5.372849659	0	2.671938627	0		20	0.185725	chr14_30974_star	0	2.417782	0	1.773060525		-20	0.121217	chr6_14380_star	0	3.22371	0	2.078510713		-20	0.466012
chr14_30961_mature	5.372849659	0	2.671938627	0		20	0.185725	bta-miR-2439-3p	0	2.417782	0	1.773060525		-20	0.121217	NW_003613233_37801_mature	0	3.22371	0	2.078510713		-20	0.466012
chr9_19780_star	5.372849659	0	2.671938627	0		20	0.185725	chr9_21042_mature	0	2.417782	0	1.773060525		-20	0.121217	chr3_8248_mature	0	3.22371	0	2.078510713		-20	0.466012
chr3_8495_mature	5.372849659	0	2.671938627	0		20	0.185725	chr14_29856_mature	0	2.417782	0	1.773060525		-20	0.121217	chr5_11638_mature	0	3.22371	0	2.078510713		-20	0.466012
mmu-miR-5114	5.372849659	0	2.671938627	0		20	0.185725	chr1_1637_star	0	2.417782	0	1.773060525		-20	0.121217	hsa-miR-4466	0	3.22371	0	2.078510713		-20	0.466012
mmu-miR-6392-5p	5.372849659	0	2.671938627	0		20	0.185725	chr4_9640_mature	0	2.417782	0	1.773060525		-20	0.121217	chr2_4356_star	0	3.22371	0	2.078510713		-20	0.466012
chr10_23459_mature	5.372849659	0	2.671938627	0		20	0.185725	chr1_3026_star	0	2.417782	0	1.773060525		-20	0.121217	hsa-miR-103a-2-5p	0	3.22371	0	2.078510713		-20	0.466012
chr14_29777_star	5.372849659	0	2.671938627	0		20	0.185725	chr5_12007_star	0	2.417782	0	1.773060525		-20	0.121217	rno-miR-32-3p	0	3.22371	0	2.078510713		-20	0.466012
rno-miR-3584-5p	5.372849659	0	2.671938627	0		20	0.185725	chr13_28551_star	0	2.417782	0	1.773060525		-20	0.121217	hsa-miR-875-5p	0	3.22371	0	2.078510713		-20	0.466012
rno-miR-106b-3p	5.372849659	0	2.671938627	0		20	0.185725	mmu-miR-6349	0	2.417782	0	1.773060525		-20	0.121217	chr11_24517_mature	11.82027	100.7409	3.680354657	6.668756392		-3.09132	0.466025
chr2_5371_star	5.372849659	0	2.671938627	0		20	0.185725	mmu-miR-6345	0	2.417782	0	1.773060525		-20	0.121217	chr1_861_mature	5.37285	36.26674	2.671938627	5.21981653		-2.75489	0.466939
mmu-miR-3099-3p	5.372849659	0	2.671938627	0		20	0.185725	chr8_18467_star	0	2.417782	0	1.773060525		-20	0.121217	chr11_24692_mature	22.02868	3.22371	4.525360039	2.078510713		2.77259	0.467322
mmu-miR-6407	5.372849659	0	2.671938627	0		20	0.185725	mmu-miR-6338	0	2.417782	0	1.773060525		-20	0.121217	ssc-miR-129b	88.65202	1630.391	6.486264174	10.67188709		-4.20092	0.467832
chr2_5974_star	5.372849659	0	2.671938627	0		20	0.185725	mmu-miR-6383	0	2.417782	0	1.773060525		-20	0.121217	chr7_16076_mature	42.44551	5.641492	5.441135257	2.731507408		2.911463	0.468854
chr3_6817_mature	5.372849659	0	2.671938627	0		20	0.185725	bta-miR-2285i	0	2.417782	0	1.773060525		-20	0.121217	chr8_18856_mature	34.92352	4.835565	5.166856928	2.544872269		2.852443	0.469518
chr12_26332_star	5.372849659	0	2.671938627	0		20	0.185725	bta-miR-2284y	0	2.417782	0	1.773060525		-20	0.121217	rno-miR-146a-5p	107.9943	11.28298	6.76810859	3.618589217		3.258734	0.470075
chr8_18586_mature	5.372849659	0	2.671938627	0		20	0.185725	bta-miR-2285a	0	2.417782	0	1.773060525		-20	0.121217	chr14_30186_mature	27.93882	4.029637	4.854934103	2.330454351		2.793549	0.470809
chr11_24884_star	5.372849659	0	2.671938627	0		20	0.185725	chr4_9069_star	0	2.417782	0	1.773060525		-20	0.121217	chr4_10006_mature	11.28298	93.48758	3.618589217	6.562052863		-3.05063	0.470862
chr13_27900_mature	5.372849659	0	2.671938627	0		20	0.185725	chr9_20430_star	0	2.417782	0	1.773060525		-20	0.121217	ssc-miR-145-5p	539.4341	19900.77	9.077974913	14.28060887		-5.20523	0.471417
chr12_25470_mature	5.372849659	0	2.671938627	0		20	0.185725	rno-miR-341	0	2.417782	0	1.773060525		-20	0.121217	chr1_788_star	16.11855	2.417782	4.097488514	1.773060525		2.736966	0.471688
chr5_12321_mature	5.372849659	0	2.671938627	0		20	0.185725	chr6_13506_star	0	2.417782	0	1.773060525		-20	0.121217	NW_003539866_39141_mature	4.835565	31.43117	2.544872269	5.019309189		-2.70044	0.471936
rno-miR-151-5p	5.372849659	0	2.671938627	0		20	0.185725	chrx_21689_star	0	2.417782	0	1.773060525		-20	0.121217	chr1_3642_mature	4.835565	31.43117	2.544872269	5.019309189		-2.70044	0.471936
chr16_33863_star	5.372849659	0	2.671938627	0		20	0.185725	bta-miR-654	0	2.417782	0	1.773060525		-20	0.121217	chr15_32040_mature	11.28298	1.611855	3.618589217	1.38507475		2.807355	0.471961
hsa-miR-548i	5.372849659	0	2.671938627	0		20	0.185725	chr1_1739_star	0	2.417782	0	1.773060525		-20	0.121217	chr17_35115_mature	11.28298	1.611855	3.618589217	1.38507475		2.807355	0.471961
mmu-miR-3063-3p	5.372849659	0	2.671938627	0		20	0.185725	chr2_4828_star	0	2.417782	0	1.773060525		-20	0.121217	mmu-miR-6385	11.28298	1.611855	3.618589217	1.38507475		2.807355	0.471961
chr2_4065_star	5.372849659	0	2.671938627	0		20	0.185725	chr1_153_mature	0	2.417782	0	1.773060525		-20	0.121217	chr4_10551_mature	11.28298	1.611855	3.618589217	1.38507475		2.807355	0.471961
bta-miR-223	5.372849659	0	2.671938627	0		20	0.185725	chr5_11589_star	0	2.417782	0	1.773060525		-20	0.121217	chr13_27786_mature	10.7457	87.04016	3.554060706	6.460089935		-3.01792	0.473257
chr2_3992_mature	5.372849659	0	2.671938627	0		20	0.185725	rno-miR-219-1-3p	0	2.417782	0	1.773060525		-20	0.121217	NW_003539992_39208_mature	15.04398	137.0077	4.003960082	7.108604601		-3.187	0.473595
chr17_35007_star	5.372849659	0	2.671938627	0		20	0.185725	chr6_15065_star	0	2.417782	0	1.773060525		-20	0.121217	chr8_18467_mature	2.686425	16.11855	1.882222339	4.097488514		-2.58496	0.473745
chr12_26328_star	5.372849659	0	2.671938627	0		20	0.185725	NW_003613379_38756_star	0	2.417782	0	1.773060525		-20	0.121217	chr14_29634_star	2.686425	16.11855	1.882222339	4.097488514		-2.58496	0.473745
NW_003613280_38221_star	5.372849659	0	2.671938627	0		20	0.185725	bta-miR-10a	0	2.417782	0	1.773060525		-20	0.121217	rno-miR-363-3p	2.14914	12.89484	1.654957834	3.796477232		-2.58496	0.474147
chr1_246_mature	5.372849659	0	2.671938627	0		20	0.185725	mmu-miR-5117-3p	0	2.417782	0	1.773060525		-20	0.121217	NW_003613233_37800_star	2.14914	12.89484	1.654957834	3.796477232		-2.58496	0.474147
NW_003613265_38082_mature	5.372849659	0	2.671938627	0		20	0.185725	chr4_9832_star	0	2.417782	0	1.773060525		-20	0.121217	NW_003613137_37368_star	2.14914	12.89484	1.654957834	3.796477232		-2.58496	0.474147
rno-miR-146b-5p	5.372849659	0	2.671938627	0		20	0.185725	chr5_10851_star	0	2.417782	0	1.773060525		-20	0.121217	chr9_20736_mature	2.14914	12.89484	1.654957834	3.796477232		-2.58496	0.474147
chr4_10241_mature	5.372849659	0	2.671938627	0		20	0.185725	hsa-miR-1255a	0	2.417782	0	1.773060525		-20	0.121217	bta-miR-378	49.43022	6.44742	5.656216524	2.896740642		2.938599	0.474459
mmu-miR-1955-3p	5.372849659	0	2.671938627	0		20	0.185725	chr17_35289_star	0	2.417782	0	1.773060525		-20	0.121217	chr6_13570_mature	5.37285	35.46081	2.671938627	5.188274618		-2.72247	0.474549
chr6_12911_mature	5.372849659	0	2.671938627	0		20	0.185725	hsa-miR-488-5p	0	2.417782	0	1.773060525		-20	0.121217	chr8_18596_star	27.40153	4.029637	4.827896911	2.330454351		2.765535	0.475891
hsa-miR-4307	5.372849659	0	2.671938627	0		20	0.185725	mmu-miR-679-5p	0	2.417782	0	1.773060525		-20	0.121217	chr3_7417_mature	27.40153	4.029637	4.827896911	2.330454351		2.765535	0.475891
chr15_31962_mature	5.372849659	0	2.671938627	0		20	0.185725	mmu-miR-199b-5p	0	2.417782	0	1.773060525		-20	0.121217	chr8_18282_mature	41.37094	5.641492	5.405003311	2.731507408		2.874469	0.475925
chr5_12251_star	5.372849659	0	2.671938627	0		20	0.185725	NW_003537823_37300_star	0	2.417782	0	1.773060525		-20	0.121217	chr7_15165_mature	6.44742	44.32601	2.896740642	5.502267253		-2.78136	0.476199
NW_003538681_38109_star	5.372849659	0	2.671938627	0		20	0.185725	NW_003537731_37227_star	0	2.417782	0	1.773060525		-20	0.121217	chr1_1062_mature	12.35755	103.9646	3.739583968	6.713759604		-3.07263	0.477189
chr8_18187_mature	5.372849659	0	2.671938627	0		20	0.185725	rno-miR-541-3p	0	2.417782	0	1.773060525		-20	0.121217	chr18_36120_star	1.07457	7.253347	1.05281229	3.044979305		-2.75489	0.477519
chr14_30639_star	5.372849659	0	2.671938627	0		20	0.185725	hsa-miR-320e	0	2.417782	0	1.773060525		-20	0.121217	hsa-miR-1234-5p	1.07457	7.253347	1.05281229	3.044979305		-2.75489	0.477519
chr5_11866_mature	5.372849659	0	2.671938627	0		20	0.185725	chr5_12405_star	0	2.417782	0	1.773060525		-20	0.121217	hsa-miR-6506-3p	1.07457	7.253347	1.05281229	3.044979305		-2.75489	0.477519
chr15_31760_mature	5.372849659	0	2.671938627	0		20	0.185725	bta-miR-221	0	2.417782	0	1.773060525		-20	0.121217	chr3_8179_star	1.07457	7.253347	1.05281229	3.044979305		-2.75489	0.477519
chr3_7381_mature	5.372849659	0	2.671938627	0		20	0.185725	hsa-miR-548v	0	2.417782	0	1.773060525		-20	0.121217	hsa-miR-626	1.07457	7.253347	1.05281229	3.044979305		-2.75489	0.477519
chr13_27213_star	5.372849659	0	2.671938627	0		20	0.185725	hsa-miR-548y	0	2.417782	0	1.773060525		-20	0.121217	hsa-miR-4329	1.07457	7.253347	1.05281229	3.044979305		-2.75489	0.477519
chr13_27738_mature	2.149139864	23.3719	1.654957834	4.607146585		-3.44294	0.186637	chrx_23051_star	0	2.417782	0	1.773060525		-20	0.121217	chr2_5321_star	11.28298	91.87573	3.618589217	6.537229727		-3.02554	0.477618
NW_003537216_36773_star	2.149139864	23.3719	1.654957834	4.607146585		-3.44294	0.186637	NW_003539074_38507_mature	0	2.417782	0	1.773060525		-20	0.121217	chr1_2370_star	15.58126	2.417782	4.051482084	1.773060525		2.688056	0.479939
chr15_33315_mature	37.60994761	4.029637	5.270900692	2.330454351		3.222392	0.186835	chr16_34110_star	0	2.417782	0	1.773060525		-20	0.121217	NW_003613232_37791_mature	20.95411	3.22371	4.456419386	2.078510713		2.70044	0.479992
chr4_8685_mature	16.11854898	1.611855	4.097488514	1.38507475		3.321928	0.18736	chr15_32248_star	0	2.417782	0	1.773060525		-20	0.121217	chr10_23301_mature	20.95411	3.22371	4.456419386	2.078510713		2.70044	0.479992
chr16_34832_mature	16.11854898	1.611855	4.097488514	1.38507475		3.321928	0.18736	chr7_16882_star	0	2.417782	0	1.773060525		-20	0.121217	chr13_29266_mature	20.95411	3.22371	4.456419386	2.078510713		2.70044	0.479992
chr3_7655_star	16.11854898	1.611855	4.097488514	1.38507475		3.321928	0.18736	chr9_20768_star	0	2.417782	0	1.773060525		-20	0.121217	hsa-miR-1321	6.984705	0.805927	2.997239027	0.852739935		3.115477	0.480447
chr5_11433_star	16.11854898	1.611855	4.097488514	1.38507475		3.321928	0.18736	chr1_826_star	0	2.417782	0	1.773060525		-20	0.121217	NW_003539115_38545_mature	6.984705	0.805927	2.997239027	0.852739935		3.115477	0.480447
chr5_11229_star	16.11854898	1.611855	4.097488514	1.38507475		3.321928	0.18736	bta-miR-2388-5p	0	2.417782	0	1.773060525		-20	0.121217	chr10_23828_star	6.984705	0.805927	2.997239027	0.852739935		3.115477	0.480447
chr13_26857_mature	16.11854898	1.611855	4.097488514	1.38507475		3.321928	0.18736	NW_003613233_37802_star	0	2.417782	0	1.773060525		-20	0.121217	ssc-miR-190b	6.984705	0.805927	2.997239027	0.852739935		3.115477	0.480447
chr2_4630_star	16.11854898	1.611855	4.097488514	1.38507475		3.321928	0.18736	chr6_14465_star	0	2.417782	0	1.773060525		-20	0.121217	hsa-miR-567	6.984705	0.805927	2.997239027	0.852739935		3.115477	0.480447
mmu-miR-3061-3p	16.11854898	1.611855	4.097488514	1.38507475		3.321928	0.18736	chr5_11077_mature	0	2.417782	0	1.773060525		-20	0.121217	hsa-miR-429	6.984705	0.805927	2.997239027	0.852739935		3.115477	0.480447
chr1_2937_star	16.11854898	1.611855	4.097488514	1.38507475		3.321928	0.18736	hsa-miR-3192	0	2.417782	0	1.773060525		-20	0.121217	chr18_36156_mature	6.984705	0.805927	2.997239027	0.852739935		3.115477	0.480447
chr7_15755_mature	3.223709795	33.84895	2.078510713	5.123043401		-3.39232	0.187611	ssc-miR-105-2	0	2.417782	0	1.773060525		-20	0.121217	chr17_35446_star	6.984705	0.805927	2.997239027	0.852739935		3.115477	0.480447
chr3_7890_mature	3.223709795	33.84895	2.078510713	5.123043401		-3.39232	0.187611	hsa-miR-4255	0	2.417782	0	1.773060525		-20	0.121217	chrx_22074_star	6.984705	0.805927	2.997239027	0.852739935		3.115477	0.480447
NW_003539506_38905_mature	22.56596857	2.417782	4.558633073	1.773060525		3.222392	0.18809	bta-miR-6532	0	2.417782	0	1.773060525		-20	0.121217	chr1_3025_mature	6.984705	0.805927	2.997239027	0.852739935		3.115477	0.480447
chr2_5381_star	22.56596857	2.417782	4.558633073	1.773060525		3.222392	0.18809	mmu-miR-3108-5p	0	2.417782	0	1.773060525		-20	0.121217	chr8_17498_star	6.984705	0.805927	2.997239027	0.852739935		3.115477	0.480447
chr1_3766_star	22.56596857	2.417782	4.558633073	1.773060525		3.222392	0.18809	hsa-miR-744-5p	0	2.417782	0	1.773060525		-20	0.121217	chr11_24582_star	6.984705	0.805927	2.997239027	0.852739935		3.115477	0.480447
chr9_20220_star	45.13193714	4.835565	5.527693969	2.544872269		3.222392	0.190272	hsa-miR-4285	0	2.417782	0	1.773060525		-20	0.121217	chr5_11961_star	6.984705	0.805927	2.997239027	0.852739935		3.115477	0.480447
chr15_33667_mature	6.447419591	71.72754	2.896740642	6.184429932		-3.47573	0.19087	chr1_2204_star@@hsa-miR-4695-5p	0	2.417782	0	1.773060525		-20	0.121217	chr3_7580_mature	6.984705	0.805927	2.997239027	0.852739935		3.115477	0.480447
chr4_10614_mature	112.8298428	10.47706	6.830735029	3.520680821		3.428843	0.191129	hsa-miR-4662a-5p	0	2.417782	0	1.773060525		-20	0.121217	chr2_5942_mature	6.984705	0.805927	2.997239027	0.852739935		3.115477	0.480447
chr11_25110_star	1.074569932	13.70077	1.05281229	3.877819487		-3.67243	0.192377	chr1_2954_star	0	2.417782	0	1.773060525		-20	0.121217	chrx_22133_star	6.984705	0.805927	2.997239027	0.852739935		3.115477	0.480447
chr1_3231_star	1.074569932	13.70077	1.05281229	3.877819487		-3.67243	0.192377	chr10_23951_star	0	2.417782	0	1.773060525		-20	0.121217	hsa-miR-4444	6.984705	0.805927	2.997239027	0.852739935		3.115477	0.480447
chr1_3559_mature	1.074569932	13.70077	1.05281229	3.877819487		-3.67243	0.192377	chr1_2966_star	0	2.417782	0	1.773060525		-20	0.121217	chr1_666_mature	6.984705	0.805927	2.997239027	0.852739935		3.115477	0.480447
chr18_36505_mature	1.074569932	13.70077	1.05281229	3.877819487		-3.67243	0.192377	bta-miR-320b	0	2.417782	0	1.773060525		-20	0.121217	bta-miR-2316	6.984705	0.805927	2.997239027	0.852739935		3.115477	0.480447
chr5_12411_mature	29.01338816	3.22371	4.907534286	2.078510713		3.169925	0.192988	chr1_1748_mature	0	2.417782	0	1.773060525		-20	0.121217	mmu-miR-3064-5p	6.984705	0.805927	2.997239027	0.852739935		3.115477	0.480447
chr17_35504_mature	29.01338816	3.22371	4.907534286	2.078510713		3.169925	0.192988	chr15_31881_star	0	2.417782	0	1.773060525		-20	0.121217	chr12_25719_star	6.984705	0.805927	2.997239027	0.852739935		3.115477	0.480447
chr7_15783_star	36.53537768	4.029637	5.230179094	2.330454351		3.180572	0.193062	chr12_26142_mature	0	2.417782	0	1.773060525		-20	0.121217	chr7_15294_mature	4.835565	30.62524	2.544872269	4.983004663		-2.66297	0.480479
chr17_35020_star	0	6.44742	0	2.896740642		-20	0.193074	chr3_7627_mature	0	2.417782	0	1.773060525		-20	0.121217	chr6_14501_mature	1.611855	9.671129	1.38507475	3.415640968		-2.58496	0.482369
NW_003541072_39884_mature	0	6.44742	0	2.896740642		-20	0.193074	NW_003613297_38335_mature	0	2.417782	0	1.773060525		-20	0.121217	chr17_35680_star	1.611855	9.671129	1.38507475	3.415640968		-2.58496	0.482369
hsa-miR-1258	0	6.44742	0	2.896740642		-20	0.193074	chr3_8509_star	0	2.417782	0	1.773060525		-20	0.121217	chr13_28533_star	1.611855	9.671129	1.38507475	3.415640968		-2.58496	0.482369
chr15_31774_star	0	6.44742	0	2.896740642		-20	0.193074	NW_003539453_38861_star	0	2.417782	0	1.773060525		-20	0.121217	chr15_31957_mature	1.611855	9.671129	1.38507475	3.415640968		-2.58496	0.482369
hsa-miR-3935	0	6.44742	0	2.896740642		-20	0.193074	chr9_19885_mature	0	2.417782	0	1.773060525		-20	0.121217	NW_003613231_37780_star	3.760995	22.56597	2.251263041	4.558633073		-2.58496	0.483191
chr13_27163_mature	0	6.44742	0	2.896740642		-20	0.193074	chr2_4295_star	0	2.417782	0	1.773060525		-20	0.121217	chr10_23904_star	10.7457	1.611855	3.554060706	1.38507475		2.736966	0.483342
hsa-miR-216a-3p	0	6.44742	0	2.896740642		-20	0.193074	bta-miR-2458	0	2.417782	0	1.773060525		-20	0.121217	chr15_33543_mature	10.7457	1.611855	3.554060706	1.38507475		2.736966	0.483342
chr14_31039_mature	0	6.44742	0	2.896740642		-20	0.193074	chr6_13134_star	0	2.417782	0	1.773060525		-20	0.121217	rno-miR-673-5p	10.7457	1.611855	3.554060706	1.38507475		2.736966	0.483342
chr13_28872_star	0	6.44742	0	2.896740642		-20	0.193074	hsa-miR-4472	0	2.417782	0	1.773060525		-20	0.121217	chr15_32751_mature	10.7457	1.611855	3.554060706	1.38507475		2.736966	0.483342
chr2_6442_mature	0	6.44742	0	2.896740642		-20	0.193074	NW_003613025_36806_mature	0	2.417782	0	1.773060525		-20	0.121217	chr5_11694_star	10.7457	1.611855	3.554060706	1.38507475		2.736966	0.483342
chr14_29554_star	0	6.44742	0	2.896740642		-20	0.193074	rno-miR-146b-3p	0	2.417782	0	1.773060525		-20	0.121217	chr16_34150_star	10.7457	1.611855	3.554060706	1.38507475		2.736966	0.483342
chr11_24430_star	0	6.44742	0	2.896740642		-20	0.193074	chr4_9284_star	0	2.417782	0	1.773060525		-20	0.121217	NW_003613191_37582_mature	10.7457	1.611855	3.554060706	1.38507475		2.736966	0.483342
chr1_2963_star	0	6.44742	0	2.896740642		-20	0.193074	NW_003538933_38357_star	0	2.417782	0	1.773060525		-20	0.121217	chr7_15151_mature	5.910135	38.68452	2.788713818	5.310504361		-2.71049	0.485222
chr1_569_star	0	6.44742	0	2.896740642		-20	0.193074	bta-miR-2481	0	2.417782	0	1.773060525		-20	0.121217	chr7_15943_mature	0.537285	4.835565	0.620384622	2.544872269		-3.16993	0.48575
hsa-miR-526b-5p	0	6.44742	0	2.896740642		-20	0.193074	chr4_8670_star	0	2.417782	0	1.773060525		-20	0.121217	chr4_10320_star	0.537285	4.835565	0.620384622	2.544872269		-3.16993	0.48575
hsa-miR-224-3p	0	6.44742	0	2.896740642		-20	0.193074	chr9_20275_star	0	2.417782	0	1.773060525		-20	0.121217	chr4_10242_star	0.537285	4.835565	0.620384622	2.544872269		-3.16993	0.48575
chr9_19965_star	0	6.44742	0	2.896740642		-20	0.193074	bta-miR-2486-3p	0	2.417782	0	1.773060525		-20	0.121217	NW_003537624_37070_mature	0.537285	4.835565	0.620384622	2.544872269		-3.16993	0.48575
chr6_14281_star	0	6.44742	0	2.896740642		-20	0.193074	NW_003539879_39158_mature	0	2.417782	0	1.773060525		-20	0.121217	bta-miR-1584-3p	0.537285	4.835565	0.620384622	2.544872269		-3.16993	0.48575
mmu-miR-3967	0	6.44742	0	2.896740642		-20	0.193074	chr2_6200_mature	0	2.417782	0	1.773060525		-20	0.121217	chr14_31236_star	0.537285	4.835565	0.620384622	2.544872269		-3.16993	0.48575
chr4_9649_star	0	6.44742	0	2.896740642		-20	0.193074	chr10_23618_mature	0	2.417782	0	1.773060525		-20	0.121217	mmu-miR-5136	0.537285	4.835565	0.620384622	2.544872269		-3.16993	0.48575
chr16_33724_star	0	6.44742	0	2.896740642		-20	0.193074	chr1_3835_mature	0	2.417782	0	1.773060525		-20	0.121217	chr15_33084_mature	0.537285	4.835565	0.620384622	2.544872269		-3.16993	0.48575
hsa-miR-520c-5p	0	6.44742	0	2.896740642		-20	0.193074	bta-miR-2365	0	2.417782	0	1.773060525		-20	0.121217	chr4_10054_star	0.537285	4.835565	0.620384622	2.544872269		-3.16993	0.48575
chr2_6348_mature	0	6.44742	0	2.896740642		-20	0.193074	chr11_24609_star	0	2.417782	0	1.773060525		-20	0.121217	mmu-miR-3970	0.537285	4.835565	0.620384622	2.544872269		-3.16993	0.48575
chr2_5938_mature	0	6.44742	0	2.896740642		-20	0.193074	hsa-miR-4320	0	2.417782	0	1.773060525		-20	0.121217	NW_003538795_38232_mature	0.537285	4.835565	0.620384622	2.544872269		-3.16993	0.48575
chr8_18755_mature	0	6.44742	0	2.896740642		-20	0.193074	hsa-miR-4308	0	2.417782	0	1.773060525		-20	0.121217	chr15_33146_star	0.537285	4.835565	0.620384622	2.544872269		-3.16993	0.48575
bta-miR-378	0	6.44742	0	2.896740642		-20	0.193074	mmu-miR-3471	0	2.417782	0	1.773060525		-20	0.121217	chr11_25333_mature	0.537285	4.835565	0.620384622	2.544872269		-3.16993	0.48575
chr14_30126_star	0	6.44742	0	2.896740642		-20	0.193074	chr1_662_star	0	2.417782	0	1.773060525		-20	0.121217	hsa-miR-548e	0.537285	4.835565	0.620384622	2.544872269		-3.16993	0.48575
chr5_10940_star	0	6.44742	0	2.896740642		-20	0.193074	chr13_28696_mature	0	2.417782	0	1.773060525		-20	0.121217	hsa-miR-3148	0.537285	4.835565	0.620384622	2.544872269		-3.16993	0.48575
NW_003539115_38544_star	0	6.44742	0	2.896740642		-20	0.193074	chr1_3282_mature	0	2.417782	0	1.773060525		-20	0.121217	chr13_28383_mature	0.537285	4.835565	0.620384622	2.544872269		-3.16993	0.48575
chr16_34837_star	0	6.44742	0	2.896740642		-20	0.193074	chr4_10537_star	0	2.417782	0	1.773060525		-20	0.121217	chr4_8784_star	0.537285	4.835565	0.620384622	2.544872269		-3.16993	0.48575
chr3_8070_mature	0	6.44742	0	2.896740642		-20	0.193074	chr15_32938_star	0	2.417782	0	1.773060525		-20	0.121217	chrx_22412_star	0.537285	4.835565	0.620384622	2.544872269		-3.16993	0.48575
chr9_20549_star	4.298279727	44.32601	2.405524014	5.502267253		-3.36632	0.193436	chr5_11953_star	0	2.417782	0	1.773060525		-20	0.121217	bta-miR-2403	0.537285	4.835565	0.620384622	2.544872269		-3.16993	0.48575
chr6_12705_mature	52.65392666	5.641492	5.745611853	2.731507408		3.222392	0.194743	NW_003613223_37738_mature	0	2.417782	0	1.773060525		-20	0.121217	NW_003538201_37611_star	0.537285	4.835565	0.620384622	2.544872269		-3.16993	0.48575
ssc-miR-193a-5p	98.86043373	3071.39	6.641841268	11.58514541		-4.95735	0.195077	chr15_31887_star	0	2.417782	0	1.773060525		-20	0.121217	chr6_14320_mature	0.537285	4.835565	0.620384622	2.544872269		-3.16993	0.48575
chr14_31521_mature	231.0325353	18.53633	7.858183302	4.288087668		3.639668	0.195555	chr4_10705_star	0	2.417782	0	1.773060525		-20	0.121217	chr3_7189_mature	0.537285	4.835565	0.620384622	2.544872269		-3.16993	0.48575
chr2_5564_mature@@mmu-miR-670	44.0573672	4.835565	5.493691111	2.544872269		3.187627	0.195597	bta-miR-17-5p	0	2.417782	0	1.773060525		-20	0.121217	NW_003537656_37100_star	0.537285	4.835565	0.620384622	2.544872269		-3.16993	0.48575
chr9_19536_mature	2.149139864	21.76004	1.654957834	4.508431259		-3.33985	0.199073	hsa-miR-4683	0	2.417782	0	1.773060525		-20	0.121217	ssc-miR-183	41779.28	582.6855	15.3505345	9.189047531		6.163926	0.48829
chr9_20047_mature	2.149139864	21.76004	1.654957834	4.508431259		-3.33985	0.199073	NW_003613401_38938_star	0	2.417782	0	1.773060525		-20	0.121217	ssc-miR-24-1-5p	50.50479	711.6339	5.686634616	9.47701738		-3.81664	0.488297
chr13_27363_mature	2.149139864	21.76004	1.654957834	4.508431259		-3.33985	0.199073	chr1_2014_star	0	2.417782	0	1.773060525		-20	0.121217	chr13_29017_mature	15.04398	2.417782	4.003960082	1.773060525		2.63743	0.488353
chr2_5320_star	9.671129386	0.805927	3.415640968	0.852739935		3.584963	0.199294	chr12_25581_mature	0	2.417782	0	1.773060525		-20	0.121217	chr16_34759_star	15.04398	2.417782	4.003960082	1.773060525		2.63743	0.488353
hsa-miR-569	9.671129386	0.805927	3.415640968	0.852739935		3.584963	0.199294	chr2_6128_mature	0	2.417782	0	1.773060525		-20	0.121217	rno-miR-674-3p	15.04398	2.417782	4.003960082	1.773060525		2.63743	0.488353
rno-miR-27b-5p	9.671129386	0.805927	3.415640968	0.852739935		3.584963	0.199294	chr18_36296_mature	0	2.417782	0	1.773060525		-20	0.121217	chr7_15120_mature	445.9465	30.62524	8.803958409	4.983004663		3.864078	0.488606
chr1_1242_star	9.671129386	0.805927	3.415640968	0.852739935		3.584963	0.199294	hsa-miR-625-5p	0	2.417782	0	1.773060525		-20	0.121217	mmu-miR-1899	2.686425	15.31262	1.882222339	4.027916745		-2.51096	0.488643
bta-miR-759	9.671129386	0.805927	3.415640968	0.852739935		3.584963	0.199294	hsa-miR-5684	0	2.417782	0	1.773060525		-20	0.121217	chr11_24777_star	2.686425	15.31262	1.882222339	4.027916745		-2.51096	0.488643
mmu-miR-3962	9.671129386	0.805927	3.415640968	0.852739935		3.584963	0.199294	hsa-miR-194-5p	0	2.417782	0	1.773060525		-20	0.121217	chr14_30912_mature	8.596559	62.05641	3.262517265	5.978571212		-2.85175	0.48901
chr5_11699_star	9.671129386	0.805927	3.415640968	0.852739935		3.584963	0.199294	hsa-miR-4789-5p	0	2.417782	0	1.773060525		-20	0.121217	ssc-miR-345-5p	30.62524	352.9962	4.983004663	8.467590156		-3.52686	0.489059
chr3_7464_mature	9.671129386	0.805927	3.415640968	0.852739935		3.584963	0.199294	hsa-miR-3663-5p	0	2.417782	0	1.773060525		-20	0.121217	chr9_20736_star	4.835565	29.81932	2.544872269	4.94576292		-2.62449	0.489131
mmu-miR-1247-5p	9.671129386	0.805927	3.415640968	0.852739935		3.584963	0.199294	hsa-miR-4533	0	2.417782	0	1.773060525		-20	0.121217	NW_003539007_38427_mature	4.835565	29.81932	2.544872269	4.94576292		-2.62449	0.489131
chr2_5942_mature	9.671129386	0.805927	3.415640968	0.852739935		3.584963	0.199294	chr9_20478_star	0	2.417782	0	1.773060525		-20	0.121217	chr1_3589_mature	3.22371	18.53633	2.078510713	4.288087668		-2.52356	0.490097
chr10_23333_star	9.671129386	0.805927	3.415640968	0.852739935		3.584963	0.199294	chr3_8356_mature	0	2.417782	0	1.773060525		-20	0.121217	chr6_13257_mature	3.22371	18.53633	2.078510713	4.288087668		-2.52356	0.490097
chr8_18304_mature	9.671129386	0.805927	3.415640968	0.852739935		3.584963	0.199294	chr3_7573_star	0	2.417782	0	1.773060525		-20	0.121217	NW_003613099_37159_mature	3.760995	0	2.251263041	0		20	0.490158
chr12_26428_star	9.671129386	0.805927	3.415640968	0.852739935		3.584963	0.199294	chr7_15311_mature	0	2.417782	0	1.773060525		-20	0.121217	chr7_16193_star	3.760995	0	2.251263041	0		20	0.490158
chr13_26987_star	9.671129386	0.805927	3.415640968	0.852739935		3.584963	0.199294	hsa-miR-1229-5p	0	2.417782	0	1.773060525		-20	0.121217	hsa-miR-5000-5p	3.760995	0	2.251263041	0		20	0.490158
bta-miR-378b	9.671129386	0.805927	3.415640968	0.852739935		3.584963	0.199294	chr2_6101_mature	0	1.611855	0	1.38507475		-20	0.12959	chr2_5426_star	3.760995	0	2.251263041	0		20	0.490158
chr2_4615_mature	202.0191472	16.92448	7.665471987	4.163859073		3.577309	0.199405	chr9_19886_mature	0	1.611855	0	1.38507475		-20	0.12959	bta-miR-2894	3.760995	0	2.251263041	0		20	0.490158
chr6_13633_mature	35.46080775	4.029637	5.188274618	2.330454351		3.137504	0.19956	mmu-miR-28c	0	1.611855	0	1.38507475		-20	0.12959	NW_003540726_39647_mature	3.760995	0	2.251263041	0		20	0.490158
chr15_31872_mature	4.298279727	42.71415	2.405524014	5.4500286		-3.31288	0.200349	chr15_33576_star	0	1.611855	0	1.38507475		-20	0.12959	hsa-miR-1254	3.760995	0	2.251263041	0		20	0.490158
mmu-let-7j	78.44360502	8.059274	6.311859186	3.179395517		3.282934	0.200414	hsa-miR-4790-5p	0	1.611855	0	1.38507475		-20	0.12959	chr6_13122_star	3.760995	0	2.251263041	0		20	0.490158
chr7_15459_mature	78.44360502	8.059274	6.311859186	3.179395517		3.282934	0.200414	NW_003537262_36795_star	0	1.611855	0	1.38507475		-20	0.12959	chr7_17120_star	3.760995	0	2.251263041	0		20	0.490158
chr8_18596_mature	164.4091996	14.50669	7.369895665	3.954819241		3.5025	0.200758	hsa-miR-6715b-5p	0	1.611855	0	1.38507475		-20	0.12959	chr1_2968_star	3.760995	0	2.251263041	0		20	0.490158
chr17_35115_mature	15.04397905	1.611855	4.003960082	1.38507475		3.222392	0.200925	chr1_330_mature	0	1.611855	0	1.38507475		-20	0.12959	hsa-miR-1283	3.760995	0	2.251263041	0		20	0.490158
chr6_15039_mature	15.04397905	1.611855	4.003960082	1.38507475		3.222392	0.200925	NW_003539070_38505_star	0	1.611855	0	1.38507475		-20	0.12959	chr4_9349_star	3.760995	0	2.251263041	0		20	0.490158
chr2_6308_star	15.04397905	1.611855	4.003960082	1.38507475		3.222392	0.200925	chr17_34998_star	0	1.611855	0	1.38507475		-20	0.12959	mmu-miR-5620-5p	3.760995	0	2.251263041	0		20	0.490158
chr14_31446_mature	15.04397905	1.611855	4.003960082	1.38507475		3.222392	0.200925	chrx_22905_mature	0	1.611855	0	1.38507475		-20	0.12959	chr8_17367_star	3.760995	0	2.251263041	0		20	0.490158
chr14_29971_mature	15.04397905	1.611855	4.003960082	1.38507475		3.222392	0.200925	chr13_27363_star	0	1.611855	0	1.38507475		-20	0.12959	chr6_13483_star	3.760995	0	2.251263041	0		20	0.490158
chr2_5938_star@@bta-miR-6535	15.04397905	1.611855	4.003960082	1.38507475		3.222392	0.200925	hsa-miR-4761-3p	0	1.611855	0	1.38507475		-20	0.12959	rno-miR-211-5p	3.760995	0	2.251263041	0		20	0.490158
chr7_15451_star	15.04397905	1.611855	4.003960082	1.38507475		3.222392	0.200925	chr13_26947_star	0	1.611855	0	1.38507475		-20	0.12959	chr13_27317_mature	3.760995	0	2.251263041	0		20	0.490158
NW_003539866_39141_mature	3.223709795	31.43117	2.078510713	5.019309189		-3.2854	0.200994	chr1_782_star	0	1.611855	0	1.38507475		-20	0.12959	chr13_27913_star	3.760995	0	2.251263041	0		20	0.490158
chr6_13188_mature	3.223709795	31.43117	2.078510713	5.019309189		-3.2854	0.200994	NW_003613103_37207_star	0	1.611855	0	1.38507475		-20	0.12959	hsa-miR-6504-3p	3.760995	0	2.251263041	0		20	0.490158
chr11_25073_mature	27.93881823	3.22371	4.854934103	2.078510713		3.115477	0.200998	hsa-miR-4719	0	1.611855	0	1.38507475		-20	0.12959	chr14_30807_mature	3.760995	0	2.251263041	0		20	0.490158
chr1_2879_mature	42.98279727	4.835565	5.458867456	2.544872269		3.152003	0.201117	hsa-miR-4445-5p	0	1.611855	0	1.38507475		-20	0.12959	rno-miR-760-5p	3.760995	0	2.251263041	0		20	0.490158
chr13_28673_star	1.074569932	12.89484	1.05281229	3.796477232		-3.58496	0.20257	chr15_32346_mature	0	1.611855	0	1.38507475		-20	0.12959	chr10_23298_star	3.760995	0	2.251263041	0		20	0.490158
chr10_23437_star	1.074569932	12.89484	1.05281229	3.796477232		-3.58496	0.20257	chr1_1793_star	0	1.611855	0	1.38507475		-20	0.12959	chr4_8685_star	3.760995	0	2.251263041	0		20	0.490158
chr13_26770_mature	1.074569932	12.89484	1.05281229	3.796477232		-3.58496	0.20257	hsa-miR-3620-3p	0	1.611855	0	1.38507475		-20	0.12959	chr14_30584_star	3.760995	0	2.251263041	0		20	0.490158
hsa-miR-4694-3p	1.074569932	12.89484	1.05281229	3.796477232		-3.58496	0.20257	hsa-miR-4762-5p	0	1.611855	0	1.38507475		-20	0.12959	chr8_17337_mature	3.760995	0	2.251263041	0		20	0.490158
chr13_28512_star	1.074569932	12.89484	1.05281229	3.796477232		-3.58496	0.20257	chr14_29900_star	0	1.611855	0	1.38507475		-20	0.12959	NW_003613301_38356_mature	3.760995	0	2.251263041	0		20	0.490158
rno-miR-741-3p	1.074569932	12.89484	1.05281229	3.796477232		-3.58496	0.20257	NW_003541124_39922_star	0	1.611855	0	1.38507475		-20	0.12959	chr8_17677_star	3.760995	0	2.251263041	0		20	0.490158
chr3_8153_mature	1.074569932	12.89484	1.05281229	3.796477232		-3.58496	0.20257	hsa-miR-4419b	0	1.611855	0	1.38507475		-20	0.12959	mmu-miR-669a-3p	3.760995	0	2.251263041	0		20	0.490158
NW_003538201_37612_mature	1.074569932	12.89484	1.05281229	3.796477232		-3.58496	0.20257	chr5_12006_star	0	1.611855	0	1.38507475		-20	0.12959	rno-miR-466c-5p	3.760995	0	2.251263041	0		20	0.490158
chr3_7065_mature	1.074569932	12.89484	1.05281229	3.796477232		-3.58496	0.20257	chr7_16704_mature	0	1.611855	0	1.38507475		-20	0.12959	rno-miR-30d-3p	3.760995	0	2.251263041	0		20	0.490158
chrx_22076_star	1.074569932	12.89484	1.05281229	3.796477232		-3.58496	0.20257	chr2_4626_star	0	1.611855	0	1.38507475		-20	0.12959	chr14_31486_star	3.760995	0	2.251263041	0		20	0.490158
NW_003613137_37368_star	1.074569932	12.89484	1.05281229	3.796477232		-3.58496	0.20257	hsa-miR-4724-5p	0	1.611855	0	1.38507475		-20	0.12959	chr14_30961_star	3.760995	0	2.251263041	0		20	0.490158
chrx_22424_mature@@bta-miR-2478	8873.798497	278.8509	13.11549865	8.128514563		4.991986	0.202899	chr13_28501_star	0	1.611855	0	1.38507475		-20	0.12959	rno-miR-6319	3.760995	0	2.251263041	0		20	0.490158
NW_003538820_38274_star	2.149139864	20.95411	1.654957834	4.456419386		-3.2854	0.205763	chr14_30531_star	0	1.611855	0	1.38507475		-20	0.12959	rno-miR-6327	3.760995	0	2.251263041	0		20	0.490158
chr13_28542_mature	2.149139864	20.95411	1.654957834	4.456419386		-3.2854	0.205763	hsa-miR-3620-5p	0	1.611855	0	1.38507475		-20	0.12959	chr15_33031_star	3.760995	0	2.251263041	0		20	0.490158
chr4_9796_mature	2.149139864	20.95411	1.654957834	4.456419386		-3.2854	0.205763	chr7_16069_star	0	1.611855	0	1.38507475		-20	0.12959	chr14_29507_star	3.760995	0	2.251263041	0		20	0.490158
chr7_15913_star	2.149139864	20.95411	1.654957834	4.456419386		-3.2854	0.205763	chr17_35478_mature	0	1.611855	0	1.38507475		-20	0.12959	chr5_11603_mature	3.760995	0	2.251263041	0		20	0.490158
chr18_36111_mature	2.149139864	20.95411	1.654957834	4.456419386		-3.2854	0.205763	chr3_7259_star	0	1.611855	0	1.38507475		-20	0.12959	chr14_30968_mature	3.760995	0	2.251263041	0		20	0.490158
chr7_15294_mature	3.223709795	30.62524	2.078510713	4.983004663		-3.24793	0.205775	hsa-miR-6510-3p	0	1.611855	0	1.38507475		-20	0.12959	chr9_20478_mature	3.760995	0	2.251263041	0		20	0.490158
chr2_4280_mature	7.521989523	79.78682	3.091190278	6.336047992		-3.40696	0.207151	hsa-miR-1208	0	1.611855	0	1.38507475		-20	0.12959	mmu-miR-3062-5p	3.760995	0	2.251263041	0		20	0.490158
NW_003538598_38024_mature	13.96940911	178.9159	3.90394537	7.491178829		-3.67894	0.207436	chr3_6710_star	0	1.611855	0	1.38507475		-20	0.12959	chr13_27839_star	3.760995	0	2.251263041	0		20	0.490158
mmu-miR-199b-5p	20.4168287	2.417782	4.420672964	1.773060525		3.078003	0.208689	chr5_11375_mature	0	1.611855	0	1.38507475		-20	0.12959	mmu-miR-27a-5p	3.760995	0	2.251263041	0		20	0.490158
chr6_12803_star	20.4168287	2.417782	4.420672964	1.773060525		3.078003	0.208689	chr9_20079_mature	0	1.611855	0	1.38507475		-20	0.12959	chr17_35707_star	3.760995	0	2.251263041	0		20	0.490158
bta-miR-140	16.11854898	215.9886	4.097488514	7.761475149		-3.74416	0.208923	hsa-miR-3190-5p	0	1.611855	0	1.38507475		-20	0.12959	chr12_26044_star	3.760995	0	2.251263041	0		20	0.490158
chr7_15705_mature	66.62333577	7.253347	6.079449279	3.044979305		3.199309	0.208927	mmu-miR-669a-3-3p	0	1.611855	0	1.38507475		-20	0.12959	chr1_1968_star	3.760995	0	2.251263041	0		20	0.490158
chr2_5816_mature	26.8642483	3.22371	4.800343329	2.078510713		3.058894	0.209438	hsa-miR-1270	0	1.611855	0	1.38507475		-20	0.12959	NW_003540557_39543_star	3.760995	0	2.251263041	0		20	0.490158
chr12_25693_mature	84.89102461	8.865202	6.424435476	3.302348583		3.259387	0.209711	chr9_21003_star	0	1.611855	0	1.38507475		-20	0.12959	chr14_30379_star	3.760995	0	2.251263041	0		20	0.490158
chr6_13122_mature@@mmu-miR-50	4619.576137	175.6922	12.17385704	7.465094412		4.716639	0.209717	chr10_23289_star	0	1.611855	0	1.38507475		-20	0.12959	chr4_9346_star	3.760995	0	2.251263041	0		20	0.490158
chr16_34851_mature	75.21989523	8.059274	6.25209572	3.179395517		3.222392	0.210564	NW_003539007_38438_mature	0	1.611855	0	1.38507475		-20	0.12959	chr2_6234_mature	3.760995	0	2.251263041	0		20	0.490158
NW_003538437_37870_mature	3.223709795	29.81932	2.078510713	4.94576292		-3.20945	0.210728	hsa-miR-571	0	1.611855	0	1.38507475		-20	0.12959	hsa-miR-5197-3p	3.760995	0	2.251263041	0		20	0.490158
chr9_19614_mature	4.298279727	40.29637	2.405524014	5.367943153		-3.22882	0.211436	chr15_33317_star	0	1.611855	0	1.38507475		-20	0.12959	rno-miR-667-3p	3.760995	0	2.251263041	0		20	0.490158
chr14_29727_mature	4.298279727	40.29637	2.405524014	5.367943153		-3.22882	0.211436	mmu-miR-199a-3p	0	1.611855	0	1.38507475		-20	0.12959	chr9_20370_star	3.760995	0	2.251263041	0		20	0.490158
chr7_17029_mature	56.95220639	6.44742	5.856791684	2.896740642		3.142958	0.212738	chr6_13909_star	0	1.611855	0	1.38507475		-20	0.12959	chr3_6770_star	3.760995	0	2.251263041	0		20	0.490158
chr3_7091_mature	2.149139864	20.14819	1.654957834	4.402462031		-3.22882	0.212794	chr5_11429_star	0	1.611855	0	1.38507475		-20	0.12959	chr3_6817_mature	3.760995	0	2.251263041	0		20	0.490158
chr1_2110_star	2.149139864	20.14819	1.654957834	4.402462031		-3.22882	0.212794	mmu-miR-883a-5p	0	1.611855	0	1.38507475		-20	0.12959	mmu-miR-6377	3.760995	0	2.251263041	0		20	0.490158
chr1_283_star	2.149139864	20.14819	1.654957834	4.402462031		-3.22882	0.212794	chr15_33041_star	0	1.611855	0	1.38507475		-20	0.12959	hsa-miR-4529-3p	3.760995	0	2.251263041	0		20	0.490158
chr2_6077_mature	2.149139864	20.14819	1.654957834	4.402462031		-3.22882	0.212794	chr10_24271_star	0	1.611855	0	1.38507475		-20	0.12959	NW_003540392_39464_star	3.760995	0	2.251263041	0		20	0.490158
chr10_23289_mature	2.149139864	20.14819	1.654957834	4.402462031		-3.22882	0.212794	hsa-miR-4520a-5p	0	1.611855	0	1.38507475		-20	0.12959	chr14_31499_star	3.760995	0	2.251263041	0		20	0.490158
chr1_811_mature	7.521989523	77.36904	3.091190278	6.292211829		-3.36257	0.213414	hsa-miR-2682-5p	0	1.611855	0	1.38507475		-20	0.12959	chr11_24884_star	3.760995	0	2.251263041	0		20	0.490158
chr14_31126_mature	33.31166789	4.029637	5.100627352	2.330454351		3.047306	0.213423	chrx_23193_star	0	1.611855	0	1.38507475		-20	0.12959	mmu-miR-669k-5p	3.760995	0	2.251263041	0		20	0.490158
chrx_22854_mature	33.31166789	4.029637	5.100627352	2.330454351		3.047306	0.213423	mmu-miR-453	0	1.611855	0	1.38507475		-20	0.12959	chr11_24517_star	3.760995	0	2.251263041	0		20	0.490158
chr1_2112_star	1.074569932	12.08891	1.05281229	3.710273245		-3.49185	0.213594	rno-miR-378b	0	1.611855	0	1.38507475		-20	0.12959	rno-miR-675-3p	3.760995	0	2.251263041	0		20	0.490158
chr1_3121_star	1.074569932	12.08891	1.05281229	3.710273245		-3.49185	0.213594	NW_003538773_38215_mature	0	1.611855	0	1.38507475		-20	0.12959	NW_003537879_37346_star	3.760995	0	2.251263041	0		20	0.490158
chr16_34524_star	1.074569932	12.08891	1.05281229	3.710273245		-3.49185	0.213594	chr8_17317_star	0	1.611855	0	1.38507475		-20	0.12959	chr1_1915_mature	3.760995	0	2.251263041	0		20	0.490158
rno-miR-31b	1.074569932	12.08891	1.05281229	3.710273245		-3.49185	0.213594	chr1_429_mature	0	1.611855	0	1.38507475		-20	0.12959	chr8_17350_mature	3.760995	0	2.251263041	0		20	0.490158
chr1_3142_mature	1.074569932	12.08891	1.05281229	3.710273245		-3.49185	0.213594	bta-miR-199b	0	1.611855	0	1.38507475		-20	0.12959	chr2_3992_mature	3.760995	0	2.251263041	0		20	0.490158
chr6_12657_mature	1.074569932	12.08891	1.05281229	3.710273245		-3.49185	0.213594	chr13_27900_star	0	1.611855	0	1.38507475		-20	0.12959	chr2_4005_mature	3.760995	0	2.251263041	0		20	0.490158
chr11_24684_mature	1.074569932	12.08891	1.05281229	3.710273245		-3.49185	0.213594	chr1_3836_mature	0	1.611855	0	1.38507475		-20	0.12959	mmu-miR-3070a-3p	3.760995	0	2.251263041	0		20	0.490158
hsa-miR-4463	1.074569932	12.08891	1.05281229	3.710273245		-3.49185	0.213594	chr13_26769_star	0	1.611855	0	1.38507475		-20	0.12959	chr4_10332_mature	3.760995	0	2.251263041	0		20	0.490158
chr13_28138_mature	1.074569932	12.08891	1.05281229	3.710273245		-3.49185	0.213594	chr1_1833_star	0	1.611855	0	1.38507475		-20	0.12959	hsa-miR-3182	3.760995	0	2.251263041	0		20	0.490158
chr6_12618_star	1.074569932	12.08891	1.05281229	3.710273245		-3.49185	0.213594	mmu-miR-5132-5p	0	1.611855	0	1.38507475		-20	0.12959	chr15_33216_star	3.760995	0	2.251263041	0		20	0.490158
chrx_21496_mature	467.4379203	33.04303	8.871714057	5.08928735		3.822357	0.213636	chr8_17278_star	0	1.611855	0	1.38507475		-20	0.12959	hsa-miR-3125	3.760995	0	2.251263041	0		20	0.490158
chr2_6092_mature	19.34225877	270.7916	4.346407978	8.08635718		-3.80735	0.214689	chr1_2918_star	0	1.611855	0	1.38507475		-20	0.12959	hsa-miR-24-2-5p	3.760995	0	2.251263041	0		20	0.490158
NW_003538773_38215_star	0	5.641492	0	2.731507408		-20	0.214718	chr13_27839_mature	0	1.611855	0	1.38507475		-20	0.12959	chr3_6723_mature	3.760995	0	2.251263041	0		20	0.490158
chr12_26266_mature	0	5.641492	0	2.731507408		-20	0.214718	chr13_26861_star	0	1.611855	0	1.38507475		-20	0.12959	chr8_18598_mature	3.760995	0	2.251263041	0		20	0.490158
chr1_3118_star	0	5.641492	0	2.731507408		-20	0.214718	chr11_24872_star	0	1.611855	0	1.38507475		-20	0.12959	rno-miR-702-5p	3.760995	0	2.251263041	0		20	0.490158
chr7_17039_mature	0	5.641492	0	2.731507408		-20	0.214718	mmu-miR-1907	0	1.611855	0	1.38507475		-20	0.12959	chr11_24588_star	3.760995	0	2.251263041	0		20	0.490158
hsa-miR-518d-5p	0	5.641492	0	2.731507408		-20	0.214718	chr5_12309_mature@@bta-miR-1251	0	1.611855	0	1.38507475		-20	0.12959	chr1_1312_star	3.760995	0	2.251263041	0		20	0.490158
rno-miR-222-5p	0	5.641492	0	2.731507408		-20	0.214718	chr8_17646_star	0	1.611855	0	1.38507475		-20	0.12959	bta-miR-2453	3.760995	0	2.251263041	0		20	0.490158
chr7_16671_mature	0	5.641492	0	2.731507408		-20	0.214718	chr9_20115_mature	0	1.611855	0	1.38507475		-20	0.12959	chr13_27034_star	3.760995	0	2.251263041	0		20	0.490158
NW_003537206_36767_star	0	5.641492	0	2.731507408		-20	0.214718	bta-miR-760-5p	0	1.611855	0	1.38507475		-20	0.12959	chr15_32385_star	3.760995	0	2.251263041	0		20	0.490158
chr11_25303_star	0	5.641492	0	2.731507408		-20	0.214718	rno-miR-301b-3p	0	1.611855	0	1.38507475		-20	0.12959	chr8_18702_star	3.760995	0	2.251263041	0		20	0.490158
chrx_23015_mature	0	5.641492	0	2.731507408		-20	0.214718	mmu-miR-100-5p	0	1.611855	0	1.38507475		-20	0.12959	hsa-miR-4494	3.760995	0	2.251263041	0		20	0.490158
chr3_7048_star	0	5.641492	0	2.731507408		-20	0.214718	chr15_31926_star	0	1.611855	0	1.38507475		-20	0.12959	chr2_4178_mature	3.760995	0	2.251263041	0		20	0.490158
chr4_9452_mature	0	5.641492	0	2.731507408		-20	0.214718	hsa-miR-5007-3p	0	1.611855	0	1.38507475		-20	0.12959	bta-miR-2323	3.760995	0	2.251263041	0		20	0.490158
bta-miR-181a	0	5.641492	0	2.731507408		-20	0.214718	mmu-miR-1969	0	1.611855	0	1.38507475		-20	0.12959	hsa-miR-4330	3.760995	0	2.251263041	0		20	0.490158
chrx_23196_star	0	5.641492	0	2.731507408		-20	0.214718	rno-miR-28-3p	0	1.611855	0	1.38507475		-20	0.12959	bta-miR-2364	3.760995	0	2.251263041	0		20	0.490158
NW_003613290_38304_star	0	5.641492	0	2.731507408		-20	0.214718	chr9_19886_star	0	1.611855	0	1.38507475		-20	0.12959	chr1_3355_star	3.760995	0	2.251263041	0		20	0.490158
chr5_11814_star	0	5.641492	0	2.731507408		-20	0.214718	rno-miR-28-5p	0	1.611855	0	1.38507475		-20	0.12959	bta-miR-376b	3.760995	0	2.251263041	0		20	0.490158
hsa-miR-4433-5p	0	5.641492	0	2.731507408		-20	0.214718	chr5_11433_star	0	1.611855	0	1.38507475		-20	0.12959	chr6_14314_star	3.760995	0	2.251263041	0		20	0.490158
chr7_15130_star	0	5.641492	0	2.731507408		-20	0.214718	rno-miR-673-3p	0	1.611855	0	1.38507475		-20	0.12959	chr8_17361_star	3.760995	0	2.251263041	0		20	0.490158
chr2_5656_mature	0	5.641492	0	2.731507408		-20	0.214718	chr6_13557_star	0	1.611855	0	1.38507475		-20	0.12959	chr5_11639_star	3.760995	0	2.251263041	0		20	0.490158
NW_003613264_38074_star	0	5.641492	0	2.731507408		-20	0.214718	rno-miR-673-5p	0	1.611855	0	1.38507475		-20	0.12959	mmu-miR-3110-5p	3.760995	0	2.251263041	0		20	0.490158
NW_003541083_39895_mature	0	5.641492	0	2.731507408		-20	0.214718	hsa-miR-302a-5p	0	1.611855	0	1.38507475		-20	0.12959	chr4_9868_mature	3.760995	0	2.251263041	0		20	0.490158
bta-miR-631	0	5.641492	0	2.731507408		-20	0.214718	chr8_18850_star	0	1.611855	0	1.38507475		-20	0.12959	mmu-miR-704	3.760995	0	2.251263041	0		20	0.490158

chr6_14536_star	0	5.641492	0	2.731507408		-20	0.214718	bta-miR-2483-5p	0	1.611855	0	1.38507475		-20	0.12959	chr1_2659_star	3.760995	0	2.251263041	0		20	0.490158
rno-miR-532-5p	0	5.641492	0	2.731507408		-20	0.214718	chr11_24334_mature	0	1.611855	0	1.38507475		-20	0.12959	mmu-miR-3470a	3.760995	0	2.251263041	0		20	0.490158
chr8_18895_star	0	5.641492	0	2.731507408		-20	0.214718	chr8_18571_mature	0	1.611855	0	1.38507475		-20	0.12959	chr2_4612_star	3.760995	0	2.251263041	0		20	0.490158
chr14_31047_mature	0	5.641492	0	2.731507408		-20	0.214718	chr14_30864_star	0	1.611855	0	1.38507475		-20	0.12959	chr15_32033_star	3.760995	0	2.251263041	0		20	0.490158
chr2_4728_star	0	5.641492	0	2.731507408		-20	0.214718	hsa-miR-5571-5p	0	1.611855	0	1.38507475		-20	0.12959	hsa-miR-5683	3.760995	0	2.251263041	0		20	0.490158
bta-miR-323	0	5.641492	0	2.731507408		-20	0.214718	chr13_28612_mature	0	1.611855	0	1.38507475		-20	0.12959	hsa-miR-5693	3.760995	0	2.251263041	0		20	0.490158
chr18_36022_star	0	5.641492	0	2.731507408		-20	0.214718	chr6_14888_mature	0	1.611855	0	1.38507475		-20	0.12959	mmu-miR-3106-5p	3.760995	0	2.251263041	0		20	0.490158
chr1_886_star	0	5.641492	0	2.731507408		-20	0.214718	bta-miR-181b	0	1.611855	0	1.38507475		-20	0.12959	NW_003537639_37085_star	3.760995	0	2.251263041	0		20	0.490158
chrx_22145_star	0	5.641492	0	2.731507408		-20	0.214718	hsa-miR-337-5p	0	1.611855	0	1.38507475		-20	0.12959	chrx_22025_star	3.760995	0	2.251263041	0		20	0.490158
chr8_18125_mature	0	5.641492	0	2.731507408		-20	0.214718	rno-miR-483-5p	0	1.611855	0	1.38507475		-20	0.12959	hsa-miR-4703-5p	3.760995	0	2.251263041	0		20	0.490158
chr1_816_star	0	5.641492	0	2.731507408		-20	0.214718	hsa-miR-16-2-3p	0	1.611855	0	1.38507475		-20	0.12959	chr14_30968_star	3.760995	0	2.251263041	0		20	0.490158
chr16_33909_star	0	5.641492	0	2.731507408		-20	0.214718	chr2_5585_star	0	1.611855	0	1.38507475		-20	0.12959	NW_003539426_38819_star	3.760995	0	2.251263041	0		20	0.490158
NW_003538681_38110_mature	0	5.641492	0	2.731507408		-20	0.214718	chr1_3507_star	0	1.611855	0	1.38507475		-20	0.12959	hsa-miR-6082	3.760995	0	2.251263041	0		20	0.490158
hsa-miR-3691-3p	0	5.641492	0	2.731507408		-20	0.214718	chr15_32437_star	0	1.611855	0	1.38507475		-20	0.12959	chr10_24226_star	3.760995	0	2.251263041	0		20	0.490158
chr2_6403_mature	0	5.641492	0	2.731507408		-20	0.214718	rno-miR-300-5p	0	1.611855	0	1.38507475		-20	0.12959	chrx_22200_mature	3.760995	0	2.251263041	0		20	0.490158
chr11_24683_star	0	5.641492	0	2.731507408		-20	0.214718	chr6_13898_star	0	1.611855	0	1.38507475		-20	0.12959	NW_003539350_38744_mature	3.760995	0	2.251263041	0		20	0.490158
chrx_22663_mature	166.5583394	15.31262	7.388519682	4.027916745		3.443234	0.215749	chr5_11120_mature	0	1.611855	0	1.38507475		-20	0.12959	rno-let-7a-1-3p	3.760995	0	2.251263041	0		20	0.490158
hsa-miR-4758-3p	13.96940911	1.611855	3.90394537	1.38507475		3.115477	0.215767	rno-miR-3576	0	1.611855	0	1.38507475		-20	0.12959	chr5_12211_mature	4.29828	25.78968	2.405524014	4.743605355		-2.58496	0.490215
chr8_18246_star	13.96940911	1.611855	3.90394537	1.38507475		3.115477	0.215767	chr13_27078_mature	0	1.611855	0	1.38507475		-20	0.12959	chr2_5810_mature	25.78968	4.029637	4.743605355	2.330454351		2.678072	0.491527
chr2_5245_mature	13.96940911	1.611855	3.90394537	1.38507475		3.115477	0.215767	chr1_539_star	0	1.611855	0	1.38507475		-20	0.12959	chr1_2112_star	2.14914	12.08891	1.654957834	3.710273245		-2.49185	0.492134
chr12_25892_mature	13.96940911	1.611855	3.90394537	1.38507475		3.115477	0.215767	chr1_541_star	0	1.611855	0	1.38507475		-20	0.12959	chr6_13504_mature	2.14914	12.08891	1.654957834	3.710273245		-2.49185	0.492134
chr8_18054_mature	13.96940911	1.611855	3.90394537	1.38507475		3.115477	0.215767	chr12_26317_star	0	1.611855	0	1.38507475		-20	0.12959	chr13_27994_mature	2.14914	12.08891	1.654957834	3.710273245		-2.49185	0.492134
chr1_2370_mature	13.96940911	1.611855	3.90394537	1.38507475		3.115477	0.215767	chr9_20972_star@@rno-miR-488-5p	0	1.611855	0	1.38507475		-20	0.12959	hsa-miR-3612	2.14914	12.08891	1.654957834	3.710273245		-2.49185	0.492134
chr13_28371_mature	13.96940911	1.611855	3.90394537	1.38507475		3.115477	0.215767	hsa-miR-3687	0	1.611855	0	1.38507475		-20	0.12959	chr16_34524_star	2.14914	12.08891	1.654957834	3.710273245		-2.49185	0.492134
chr13_27077_star	13.96940911	1.611855	3.90394537	1.38507475		3.115477	0.215767	hsa-miR-3683	0	1.611855	0	1.38507475		-20	0.12959	chr5_11762_star	2.14914	12.08891	1.654957834	3.710273245		-2.49185	0.492134
chr3_8036_mature	25.78967836	3.22371	4.743605355	2.078510713		3	0.218331	chr2_4579_star	0	1.611855	0	1.38507475		-20	0.12959	chr16_33920_star	2.14914	12.08891	1.654957834	3.710273245		-2.49185	0.492134
chr6_14257_mature	25.78967836	3.22371	4.743605355	2.078510713		3	0.218331	hsa-miR-2355-3p	0	1.611855	0	1.38507475		-20	0.12959	chr1_2426_mature	2.14914	12.08891	1.654957834	3.710273245		-2.49185	0.492134
chr2_5113_mature	13.96940911	170.0507	3.90394537	7.418280128		-3.60562	0.218479	rno-miR-6332	0	1.611855	0	1.38507475		-20	0.12959	NW_003539920_39178_star	19.87954	3.22371	4.384018281	2.078510713		2.624491	0.493057
chr1_1416_mature	5.372849659	49.9675	2.671938627	5.671505736		-3.21723	0.219168	chrx_22943_star	0	1.611855	0	1.38507475		-20	0.12959	chr5_11265_star	9.671129	71.72754	3.415640968	6.184429932		-2.89077	0.493218
chr1_3752_mature	4.298279727	38.68452	2.405524014	5.310504361		-3.16993	0.219345	NW_003539295_38706_star	0	1.611855	0	1.38507475		-20	0.12959	NW_003538807_38238_mature	38.68452	5.641492	5.310504361	2.731507408		2.777608	0.494167
chr4_9780_star	19.34225877	2.417782	4.346407978	1.773060525		3	0.220063	chr17_35612_star	0	1.611855	0	1.38507475		-20	0.12959	chr9_20047_mature	3.760995	21.76004	2.251263041	4.508431259		-2.5325	0.494297
chr5_12327_star	19.34225877	2.417782	4.346407978	1.773060525		3	0.220063	mmu-miR-291a-3p	0	1.611855	0	1.38507475		-20	0.12959	hsa-miR-1260a	3.760995	21.76004	2.251263041	4.508431259		-2.5325	0.494297
hsa-miR-3916	2.149139864	19.34226	1.654957834	4.346407978		-3.16993	0.220187	chrx_22204_mature	0	1.611855	0	1.38507475		-20	0.12959	chr7_16529_mature	53.7285	7.253347	5.774220321	3.044979305		2.888969	0.495016
chr2_4436_mature	2.149139864	19.34226	1.654957834	4.346407978		-3.16993	0.220187	rno-miR-144-5p	0	1.611855	0	1.38507475		-20	0.12959	NW_003539007_38438_mature	10.20841	1.611855	3.48651029	1.38507475		2.662965	0.49503
chr3_7756_mature	2.149139864	19.34226	1.654957834	4.346407978		-3.16993	0.220187	chr15_32439_star	0	1.611855	0	1.38507475		-20	0.12959	chr9_20751_star	10.20841	1.611855	3.48651029	1.38507475		2.662965	0.49503
chr4_10065_mature	2.149139864	19.34226	1.654957834	4.346407978		-3.16993	0.220187	mmu-miR-183-5p	0	1.611855	0	1.38507475		-20	0.12959	chr16_33810_mature	10.20841	1.611855	3.48651029	1.38507475		2.662965	0.49503
chr10_23893_mature	2.149139864	19.34226	1.654957834	4.346407978		-3.16993	0.220187	hsa-miR-146a-3p	0	1.611855	0	1.38507475		-20	0.12959	chr15_32458_mature	10.20841	1.611855	3.48651029	1.38507475		2.662965	0.49503
chr8_17840_mature	32.23709795	4.029637	5.054722516	2.330454351		3	0.220813	chr18_35964_star	0	1.611855	0	1.38507475		-20	0.12959	chr8_18878_star	10.20841	1.611855	3.48651029	1.38507475		2.662965	0.49503
chr8_18274_star	3.223709795	28.20746	2.078510713	4.868265031		-3.12928	0.221181	chr5_11655_star	0	1.611855	0	1.38507475		-20	0.12959	chr13_27411_star	10.20841	1.611855	3.48651029	1.38507475		2.662965	0.49503
chr15_31782_star	3.223709795	28.20746	2.078510713	4.868265031		-3.12928	0.221181	hsa-miR-95	0	1.611855	0	1.38507475		-20	0.12959	bta-miR-4286	40.29637	510.958	5.367943153	8.999881656		-3.66448	0.495309
chr3_8461_mature	8.596559455	0.805927	3.262517265	0.852739935		3.415037	0.221776	chr6_13458_mature	0	1.611855	0	1.38507475		-20	0.12959	chr13_29225_mature	6.44742	41.90823	2.896740642	5.423182395		-2.70044	0.495505
mmu-miR-93-3p	8.596559455	0.805927	3.262517265	0.852739935		3.415037	0.221776	chr12_25581_star	0	1.611855	0	1.38507475		-20	0.12959	chr3_7847_star	25.25239	4.029637	4.714377052	2.330454351		2.647698	0.496868
chr14_29634_mature	8.596559455	0.805927	3.262517265	0.852739935		3.415037	0.221776	chr9_20086_star	0	1.611855	0	1.38507475		-20	0.12959	chr7_15358_star	25.25239	4.029637	4.714377052	2.330454351		2.647698	0.496868
chr4_10242_mature	8.596559455	0.805927	3.262517265	0.852739935		3.415037	0.221776	chr15_31873_star	0	1.611855	0	1.38507475		-20	0.12959	chr14_29816_mature	25.25239	4.029637	4.714377052	2.330454351		2.647698	0.496868
chr16_34104_star	8.596559455	0.805927	3.262517265	0.852739935		3.415037	0.221776	hsa-miR-3913-3p	0	1.611855	0	1.38507475		-20	0.12959	chr1_153_mature	14.50669	2.417782	3.954819241	1.773060525		2.584963	0.49693
chr13_28700_star	8.596559455	0.805927	3.262517265	0.852739935		3.415037	0.221776	hsa-miR-2276	0	1.611855	0	1.38507475		-20	0.12959	mmu-miR-5117-5p	14.50669	2.417782	3.954819241	1.773060525		2.584963	0.49693
chr4_9772_mature	8.596559455	0.805927	3.262517265	0.852739935		3.415037	0.221776	chr9_20751_star	0	1.611855	0	1.38507475		-20	0.12959	chr4_9818_mature	14.50669	2.417782	3.954819241	1.773060525		2.584963	0.49693
rno-miR-1188-5p	8.596559455	0.805927	3.262517265	0.852739935		3.415037	0.221776	rno-miR-449a-5p	0	1.611855	0	1.38507475		-20	0.12959	chr1_2672_star	14.50669	2.417782	3.954819241	1.773060525		2.584963	0.49693
mmu-miR-6390	8.596559455	0.805927	3.262517265	0.852739935		3.415037	0.221776	hsa-miR-548ap-3p	0	1.611855	0	1.38507475		-20	0.12959	chr7_15913_mature	14.50669	2.417782	3.954819241	1.773060525		2.584963	0.49693
chr3_7118_star	8.596559455	0.805927	3.262517265	0.852739935		3.415037	0.221776	bta-miR-125b	0	1.611855	0	1.38507475		-20	0.12959	chr16_34547_star	6.44742	0.805927	2.896740642	0.852739935		3	0.49813
chr6_14282_mature	8.596559455	0.805927	3.262517265	0.852739935		3.415037	0.221776	NW_003540726_39647_star	0	1.611855	0	1.38507475		-20	0.12959	chr6_14329_mature	6.44742	0.805927	2.896740642	0.852739935		3	0.49813
chr18_35826_star	8.596559455	0.805927	3.262517265	0.852739935		3.415037	0.221776	chrx_22553_star	0	1.611855	0	1.38507475		-20	0.12959	chr9_19559_mature	6.44742	0.805927	2.896740642	0.852739935		3	0.49813
hsa-miR-6126	8.596559455	0.805927	3.262517265	0.852739935		3.415037	0.221776	chr13_28683_star	0	1.611855	0	1.38507475		-20	0.12959	rno-miR-27b-5p	6.44742	0.805927	2.896740642	0.852739935		3	0.49813
chr1_3878_star	8.596559455	0.805927	3.262517265	0.852739935		3.415037	0.221776	hsa-miR-663a	0	1.611855	0	1.38507475		-20	0.12959	chr3_6843_mature	6.44742	0.805927	2.896740642	0.852739935		3	0.49813
chr7_17076_mature	8.596559455	0.805927	3.262517265	0.852739935		3.415037	0.221776	rno-miR-1298	0	1.611855	0	1.38507475		-20	0.12959	chrx_22188_star@@mmu-miR-1298	6.44742	0.805927	2.896740642	0.852739935		3	0.49813
mmu-miR-1982.1-3p	8.596559455	0.805927	3.262517265	0.852739935		3.415037	0.221776	chr8_18863_star	0	1.611855	0	1.38507475		-20	0.12959	hsa-miR-5009-5p	6.44742	0.805927	2.896740642	0.852739935		3	0.49813
mmu-miR-669n	8.596559455	0.805927	3.262517265	0.852739935		3.415037	0.221776	chr7_16963_star	0	1.611855	0	1.38507475		-20	0.12959	chr8_18866_star	6.44742	0.805927	2.896740642	0.852739935		3	0.49813
chr7_15333_star	233.1816752	20.14819	7.871484379	4.402462031		3.532733	0.222695	rno-miR-374-5p	0	1.611855	0	1.38507475		-20	0.12959	chr3_7250_star	6.44742	0.805927	2.896740642	0.852739935		3	0.49813
ssc-miR-708-5p	41.90822734	784.9733	5.423182395	9.618336558		-4.22734	0.222899	chr2_6524_star	0	1.611855	0	1.38507475		-20	0.12959	chr11_25345_star	6.44742	0.805927	2.896740642	0.852739935		3	0.49813
chr3_7847_mature	4.298279727	37.87859	2.405524014	5.280903997		-3.13955	0.223466	rno-miR-547-3p	0	1.611855	0	1.38507475		-20	0.12959	ssc-miR-383	6.44742	0.805927	2.896740642	0.852739935		3	0.49813
chr7_15081_mature	1.074569932	11.28298	1.05281229	3.618589217		-3.39232	0.225534	hsa-miR-3064-3p	0	1.611855	0	1.38507475		-20	0.12959	chr2_6308_mature	6.44742	0.805927	2.896740642	0.852739935		3	0.49813
chr3_7220_mature	1.074569932	11.28298	1.05281229	3.618589217		-3.39232	0.225534	chr12_26222_star	0	1.611855	0	1.38507475		-20	0.12959	chr6_13702_star	6.44742	0.805927	2.896740642	0.852739935		3	0.49813
bta-miR-2352	1.074569932	11.28298	1.05281229	3.618589217		-3.39232	0.225534	chr11_24560_star	0	1.611855	0	1.38507475		-20	0.12959	mmu-miR-6372	6.44742	0.805927	2.896740642	0.852739935		3	0.49813
chr16_33845_mature	1.074569932	11.28298	1.05281229	3.618589217		-3.39232	0.225534	bta-miR-449a	0	1.611855	0	1.38507475		-20	0.12959	chrx_23090_mature	6.44742	0.805927	2.896740642	0.852739935		3	0.49813
chr16_33739_mature	1.074569932	11.28298	1.05281229	3.618589217		-3.39232	0.225534	chr6_13244_star	0	1.611855	0	1.38507475		-20	0.12959	chr14_29542_mature	6.44742	0.805927	2.896740642	0.852739935		3	0.49813
chr10_23626_star	5.372849659	48.35565	2.671938627	5.625143252		-3.16993	0.22576	hsa-miR-548am-3p	0	1.611855	0	1.38507475		-20	0.12959	rno-miR-3072	6.44742	0.805927	2.896740642	0.852739935		3	0.49813
hsa-miR-325	4.298279727	0	2.405524014	0		20	0.225995	chr6_14501_star	0	1.611855	0	1.38507475		-20	0.12959	chr1_743_mature	6.44742	0.805927	2.896740642	0.852739935		3	0.49813
chr7_16628_star	4.298279727	0	2.405524014	0		20	0.225995	NW_003538795_38232_star	0	1.611855	0	1.38507475		-20	0.12959	chr1_1734_star	6.44742	0.805927	2.896740642	0.852739935		3	0.49813
chr6_12992_mature	4.298279727	0	2.405524014	0		20	0.225995	bta-miR-450a	0	1.611855	0	1.38507475		-20	0.12959	hsa-miR-4684-3p	6.44742	0.805927	2.896740642	0.852739935		3	0.49813
chr18_35896_star	4.298279727	0	2.405524014	0		20	0.225995	chr9_19650_star	0	1.611855	0	1.38507475		-20	0.12959	bta-miR-2465	6.44742	0.805927	2.896740642	0.852739935		3	0.49813
mmu-miR-466f-5p	4.298279727	0	2.405524014	0		20	0.225995	chr6_14972_star	0	1.611855	0	1.38507475		-20	0.12959	bta-miR-2303	6.44742	0.805927	2.896740642	0.852739935		3	0.49813
rno-miR-509-5p	4.298279727	0	2.405524014	0		20	0.225995	chr2_6503_star	0	1.611855	0	1.38507475		-20	0.12959	chr18_36628_star	6.44742	0.805927	2.896740642	0.852739935		3	0.49813
chr10_23849_star	4.298279727	0	2.405524014	0		20	0.225995	rno-miR-485-3p	0	1.611855	0	1.38507475		-20	0.12959	hsa-miR-4660	6.44742	0.805927	2.896740642	0.852739935		3	0.49813
chr17_34938_star	4.298279727	0	2.405524014	0		20	0.225995	hsa-miR-548ay-3p	0	1.611855	0	1.38507475		-20	0.12959	NW_003539115_38548_star	6.44742	0.805927	2.896740642	0.852739935		3	0.49813
hsa-miR-4706	4.298279727	0	2.405524014	0		20	0.225995	rno-miR-295-3p	0	1.611855	0	1.38507475		-20	0.12959	chr9_20117_mature	6.44742	0.805927	2.896740642	0.852739935		3	0.49813
NW_003540726_39645_star	4.298279727	0	2.405524014	0		20	0.225995	chr2_5851_star	0	1.611855	0	1.38507475		-20	0.12959	chr9_21061_star	6.44742	0.805927	2.896740642	0.852739935		3	0.49813
hsa-miR-3680-5p	4.298279727	0	2.405524014	0		20	0.225995	chrx_22052_star	0	1.611855	0	1.38507475		-20	0.12959	chr14_31281_mature	31.16253	4.835565	5.007308904	2.544872269		2.688056	0.499718
chr10_23618_star	4.298279727	0	2.405524014	0		20	0.225995	chr13_26807_star	0	1.611855	0	1.38507475		-20	0.12959	chr3_7564_mature	19.34226	3.22371	4.346407978	2.078510713		2.584963	0.499736
mmu-miR-669p-3p	4.298279727	0	2.405524014	0		20	0.225995	chr18_36088_star	0	1.611855	0	1.38507475		-20	0.12959	chr9_19915_mature	4.29828	24.98375	2.405524014	4.699537803		-2.53916	0.500068
chr13_26770_star	4.298279727	0	2.405524014	0		20	0.225995	chr9_20697_star	0	1.611855	0	1.38507475		-20	0.12959	chr7_15219_star	13.43212	109.6061	3.851211749	6.789287574		-3.02857	0.500908
chr2_5134_star	4.298279727	0	2.405524014	0		20	0.225995	bta-miR-764	0	1.611855	0	1.38507475		-20	0.12959	chr6_13666_mature	11.82027	91.87573	3.680354657	6.537229727		-2.95842	0.501765
mmu-miR-1893	4.298279727	0	2.405524014	0		20	0.225995	chr14_30483_mature	0	1.611855	0	1.38507475		-20	0.12959	chr17_35096_mature	151.5144	15.31262	7.25280128	4.027916745		3.306661	0.501911
chr7_15929_star	4.298279727	0	2.405524014	0		20	0.225995	rno-miR-329-3p	0	1.611855	0	1.38507475		-20	0.12959	chr6_13308_star	3.22371	17.7304	2.078510713	4.227310103		-2.45943	0.503025
chr5_11087_mature	4.298279727	0	2.405524014	0		20	0.225995	chr17_35011_star	0	1.611855	0	1.38507475		-20	0.12959	hsa-miR-4772-5p	3.22371	17.7304	2.078510713	4.227310103		-2.45943	0.503025
hsa-miR-3943	4.298279727	0	2.405524014	0		20	0.225995	mmu-miR-6336	0	1.611855	0	1.38507475		-20	0.12959	chrx_22864_mature	7.52199	49.9675	3.091190278	5.671505736		-2.7318	0.503642
chrx_23228_star	4.298279727	0	2.405524014	0		20	0.225995	bta-miR-2285s	0	1.611855	0	1.38507475		-20	0.12959	chr5_12375_mature	10.20841	74.95125	3.48651029	6.247001856		-2.87619	0.503767
NW_003613382_38825_star	4.298279727	0	2.405524014	0		20	0.225995	NW_003613232_37791_star	0	1.611855	0	1.38507475		-20	0.12959	chr10_23277_mature	2.686425	14.50669	1.882222339	3.954819241		-2.43296	0.503819
mmu-miR-1960	4.298279727	0	2.405524014	0		20	0.225995	mmu-miR-6371	0	1.611855	0	1.38507475		-20	0.12959	chrx_21688_mature	2.686425	14.50669	1.882222339	3.954819241		-2.43296	0.503819
hsa-miR-1271-5p	4.298279727	0	2.405524014	0		20	0.225995	mmu-miR-6374	0	1.611855	0	1.38507475		-20	0.12959	rno-miR-24-3p	2.686425	14.50669	1.882222339	3.954819241		-2.43296	0.503819
hsa-miR-548ae	4.298279727	0	2.405524014	0		20	0.225995	bta-miR-2285e	0	1.611855	0	1.38507475		-20	0.12959	chr4_10016_mature	2.686425	14.50669	1.882222339	3.954819241		-2.43296	0.503819
chr4_9116_star	4.298279727	0	2.405524014	0		20	0.225995	bta-miR-2284e	0	1.611855	0	1.38507475		-20	0.12959	NW_003537641_37087_mature	2.686425	14.50669	1.882222339	3.954819241		-2.43296	0.503819
chr9_20919_star	4.298279727	0	2.405524014	0		20	0.225995	bta-miR-2284i	0	1.611855	0	1.38507475		-20	0.12959	chr4_10602_mature	30.62524	4.835565	4.983004663	2.544872269		2.662965	0.504211
chr9_20720_mature	4.298279727	0	2.405524014	0		20	0.225995	chrx_22193_mature	0	1.611855	0	1.38507475		-20	0.12959	chr2_4744_star	1.611855	8.865202	1.38507475	3.302348583		-2.45943	0.505083
rno-miR-202-3p	4.298279727	0	2.405524014	0		20	0.225995	rno-miR-217-3p	0	1.611855	0	1.38507475		-20	0.12959	rno-miR-200b-3p	1.611855	8.865202	1.38507475	3.302348583		-2.45943	0.505083
rno-miR-34c-3p	4.298279727	0	2.405524014	0		20	0.225995	chr2_4133_star	0	1.611855	0	1.38507475		-20	0.12959	chrx_22056_mature	1.611855	8.865202	1.38507475	3.302348583		-2.45943	0.505083
NW_003613246_37882_mature	4.298279727	0	2.405524014	0		20	0.225995	chr1_1435_star	0	1.611855	0	1.38507475		-20	0.12959	chr9_20563_star	1.611855	8.865202	1.38507475	3.302348583		-2.45943	0.505083
rno-miR-187-5p	4.298279727	0	2.405524014	0		20	0.225995	bta-miR-628	0	1.611855	0	1.38507475		-20	0.12959	chr8_19044_mature	1.611855	8.865202	1.38507475	3.302348583		-2.45943	0.505083
hsa-miR-5579-3p	4.298279727	0	2.405524014	0		20	0.225995	NW_003539061_38502_star	0	1.611855	0	1.38507475		-20	0.12959	NW_003613454_39203_mature	1.611855	8.865202	1.38507475	3.302348583		-2.45943	0.505083
rno-miR-3547	4.298279727	0	2.405524014	0		20	0.225995	chr15_31736_star	0	1.611855	0	1.38507475		-20	0.12959	chr16_34013_mature	239.0918	20.95411	7.90744238	4.456419386		3.512259	0.505183
chr6_14981_mature	4.298279727	0	2.405524014	0		20	0.225995	chr1_2370_mature	0	1.611855	0	1.38507475		-20	0.12959	chr1_577_mature	3.760995	20.95411	2.251263041	4.456419386		-2.47805	0.505557
hsa-miR-5588-5p	4.298279727	0	2.405524014	0		20	0.225995	chr13_27063_star	0	1.611855	0	1.38507475		-20	0.12959	chr7_16272_mature	3.760995	20.95411	2.251263041	4.456419386		-2.47805	0.505557
mmu-miR-5122	4.298279727	0	2.405524014	0		20	0.225995	chr16_33845_star	0	1.611855	0	1.38507475		-20	0.12959	NW_003537639_37085_mature	3.760995	20.95411	2.251263041	4.456419386		-2.47805	0.505557
chr7_16955_mature	4.298279727	0	2.405524014	0		20	0.225995	mmu-miR-376c-5p	0	1.611855	0	1.38507475		-20	0.12959	bta-miR-574	3.760995	20.95411	2.251263041	4.456419386		-2.47805	0.505557
mmu-miR-676-3p	4.298279727	0	2.405524014	0		20	0.225995	rno-miR-370-3p	0	1.611855	0	1.38507475		-20	0.12959	chr1_1630_mature	13.96941	2.417782	3.90394537	1.773060525		2.530515	0.505667
chr8_17569_mature	4.298279727	0	2.405524014	0		20	0.225995	chr2_4030_star	0	1.611855	0	1.38507475		-20	0.12959	chr9_21068_star	13.96941	2.417782	3.90394537	1.773060525		2.530515	0.505667
chr6_13633_star	4.298279727	0	2.405524014	0		20	0.225995	mmu-miR-3112-5p	0	1.611855	0	1.38507475		-20	0.12959	chr1_468_star	13.96941	2.417782	3.90394537	1.773060525		2.530515	0.505667
chr4_10238_mature	4.298279727	0	2.405524014	0		20	0.225995	hsa-miR-4755-3p	0	1.611855	0	1.38507475		-20	0.12959	chr14_31415_mature	13.96941	2.417782	3.90394537	1.773060525		2.530515	0.505667
chr1_3439_star	4.298279727	0	2.405524014	0		20	0.225995	chr9_19375_star	0	1.611855	0	1.38507475		-20	0.12959	chr18_36365_mature	13.96941	2.417782	3.90394537	1.773060525		2.530515	0.505667
rno-miR-126b	4.298279727	0	2.405524014	0		20	0.225995	NW_003613242_37866_star	0	1.611855	0	1.38507475		-20	0.12959	chr18_35894_star	13.96941	2.417782	3.90394537	1.773060525		2.530515	0.505667
chr16_33724_mature	4.298279727	0	2.405524014	0		20	0.225995	hsa-miR-4802-3p	0	1.611855	0	1.38507475		-20	0.12959	chr5_12356_mature	13.96941	2.417782	3.90394537	1.773060525		2.530515	0.505667
chr17_35034_star	4.298279727	0	2.405524014	0		20	0.225995	hsa-miR-556-3p	0	1.611855	0	1.38507475		-20	0.12959	chr11_25010_mature	18.80497	3.22371	4.307790888	2.078510713		2.544321	0.506513
chr1_2930_star	4.298279727	0	2.405524014	0		20	0.225995	chr1_1041_mature	0	1.611855	0	1.38507475		-20	0.12959	hsa-miR-492	4.835565	28.20746	2.544872269	4.868265031		-2.54432	0.50674
chr1_3117_star	4.298279727	0	2.405524014	0		20	0.225995	NW_003613080_37038_star	0	1.611855	0	1.38507475		-20	0.12959	chr9_19709_mature	9.671129	1.611855	3.415640968	1.38507475		2.584963	0.507023
chr1_3299_star	4.298279727	0	2.405524014	0		20	0.225995	hsa-miR-4766-3p	0	1.611855	0	1.38507475		-20	0.12959	mmu-miR-3112-5p	9.671129	1.611855	3.415640968	1.38507475		2.584963	0.507023
chr1_3638_mature	4.298279727	0	2.405524014	0		20	0.225995	mmu-miR-488-5p	0	1.611855	0	1.38507475		-20	0.12959	mmu-miR-302c-3p	9.671129	1.611855	3.415640968	1.38507475		2.584963	0.507023
rno-miR-292-5p	4.298279727	0	2.405524014	0		20	0.225995	chr6_14308_star	0	1.611855	0	1.38507475		-20	0.12959	chr12_25930_mature	9.671129	1.611855	3.415640968	1.38507475		2.584963	0.507023
chr12_25947_mature	4.298279727	0	2.405524014	0		20	0.225995	mmu-miR-700-3p	0	1.611855	0	1.38507475		-20	0.12959	chr1_1181_star	9.671129	1.611855	3.415640968	1.38507475		2.584963	0.507023
hsa-miR-3927-3p	4.298279727	0	2.405524014	0		20	0.225995	chr1_3714_star	0	1.611855	0	1.38507475		-20	0.12959	hsa-miR-3935	1.07457	6.44742	1.05281229	2.896740642		-2.58496	0.507298
chr15_33030_mature	4.298279727	0	2.405524014	0		20	0.225995	hsa-miR-6127	0	1.611855	0	1.38507475		-20	0.12959	mmu-miR-3109-5p	1.07457	6.44742	1.05281229	2.896740642		-2.58496	0.507298
chr1_1984_mature	4.298279727	0	2.405524014	0		20	0.225995	hsa-miR-6128	0	1.611855	0	1.38507475		-20	0.12959	chr17_35223_star	1.07457	6.44742	1.05281229	2.896740642		-2.58496	0.507298
mmu-miR-6367	4.298279727	0	2.405524014	0		20	0.225995	hsa-miR-6132	0	1.611855	0	1.38507475		-20	0.12959	chr13_27234_mature	1.07457	6.44742	1.05281229	2.896740642		-2.58496	0.507298
mmu-miR-6389	4.298279727	0	2.405524014	0		20	0.225995	bta-miR-2370-5p	0	1.611855	0	1.38507475		-20	0.12959	chr1_2963_star	1.07457	6.44742	1.05281229	2.896740642		-2.58496	0.507298
bta-miR-599	4.298279727	0	2.405524014	0		20	0.225995	bta-miR-302b	0	1.611855	0	1.38507475		-20	0.12959	chr9_19965_star	1.07457	6.44742	1.05281229	2.896740642		-2.58496	0.507298
chrx_21532_star	4.298279727	0	2.405524014	0		20	0.225995	bta-miR-141	0	1.611855	0	1.38507475		-20	0.12959	chr2_5938_mature	1.07457	6.44742	1.05281229	2.896740642		-2.58496	0.507298
chr15_33121_star	4.298279727	0	2.405524014	0		20	0.225995	chr1_3069_mature	0	1.611855	0	1.38507475		-20	0.12959	bta-miR-193a-5p	1.07457	6.44742	1.05281229	2.896740642		-2.58496	0.507298
ssc-miR-1296-3p	4.298279727	0	2.405524014	0		20	0.225995	mmu-miR-3094-5p	0	1.611855	0	1.38507475		-20	0.12959	chr14_29946_mature	1.07457	6.44742	1.05281229	2.896740642		-2.58496	0.507298
chr8_17350_mature	4.298279727	0	2.405524014	0		20	0.225995	bta-miR-2370-3p	0	1.611855	0	1.38507475		-20	0.12959	chr4_10607_mature	1.07457	6.44742	1.05281229	2.896740642		-2.58496	0.507298
chr9_21075_mature	4.298279727	0	2.405524014	0		20	0.225995	chr16_34520_star	0	1.611855	0	1.38507475		-20	0.12959	chr9_19985_star	1.07457	6.44742	1.05281229	2.896740642		-2.58496	0.507298
ssc-miR-1277	4.298279727	0	2.405524014	0		20	0.225995	hsa-miR-769-3p	0	1.611855	0	1.38507475		-20	0.12959	chr3_7313_mature	1.07457	6.44742	1.05281229	2.896740642		-2.58496	0.507298
chr7_16538_star	4.298279727	0	2.405524014	0		20	0.225995	chr17_35547_star	0	1.611855	0	1.38507475		-20	0.12959	NW_003537206_36767_mature	36.53538	5.641492	5.230179094	2.731507408		2.695145	0.509346
chr1_2930_mature	4.298279727	0	2.405524014	0		20	0.225995	chr9_20403_mature	0	1.611855	0	1.38507475		-20	0.12959	chr6_14620_mature	13.43212	107.1884	3.851211749	6.757401354		-2.99639	0.509577
chr8_17722_mature	4.298279727	0	2.405524014	0		20	0.225995	chr1_1390_star	0	1.611855	0	1.38507475		-20	0.12959	chr8_18593_mature	1235.218	62.05641	10.27171762	5.978571212		4.315042	0.510396
mmu-miR-3057-3p	4.298279727	0	2.405524014	0		20	0.225995	chr9_19298_star	0	1.611855	0	1.38507475		-20	0.12959	chr1_251_mature	2.14914	11.28298	1.654957834	3.618589217		-2.39232	0.510506
mmu-miR-5626-3p	4.298279727	0	2.405524014	0		20	0.225995	chr5_11103_star	0	1.611855	0	1.38507475		-20	0.12959	chr7_16939_mature	2.14914	11.28298	1.654957834	3.618589217		-2.39232	0.510506
mmu-miR-3095-5p	4.298279727	0	2.405524014	0		20	0.225995	bta-miR-185	0	1.611855	0	1.38507475		-20	0.12959	chr13_27449_mature	2.14914	11.28298	1.654957834	3.618589217		-2.39232	0.510506
chr4_9977_star	4.298279727	0	2.405524014	0		20	0.225995	hsa-miR-5196-3p	0	1.611855	0	1.38507475		-20	0.12959	chr8_17599_mature	2.14914	11.28298	1.654957834	3.618589217		-2.39232	0.510506
mmu-miR-3572-3p	4.298279727	0	2.405524014	0		20	0.225995	chr9_19522_star	0	1.611855	0	1.38507475		-20	0.12959	chr4_10714_mature	2.14914	11.28298	1.654957834	3.618589217		-2.39232	0.510506
chr8_17973_star	4.298279727	0	2.405524014	0		20	0.225995	chr9_19300_star	0	1.611855	0	1.38507475		-20	0.12959	bta-miR-193b	337.9522	9638.086	8.40493821	13.23468069		-4.83386	0.512555
hsa-miR-3173-3p	4.298279727	0	2.405524014	0		20	0.225995	mmu-miR-653-3p	0	1.611855	0	1.38507475		-20	0.12959	chr7_16048_star	29.55067	4.835565	4.933132262	2.544872269		2.611435	0.513332
ssc-miR-4336	4.298279727	0	2.405524014	0		20	0.225995	chr5_12092_star	0	1.611855	0	1.38507475		-20	0.12959	chr4_9495_mature	5.37285	31.43117	2.671938627	5.019309189		-2.54844	0.513908
chr6_13558_mature	4.298279727	0	2.405524014	0		20	0.225995	chr6_13056_mature	0	1.611855	0	1.38507475		-20	0.12959	bta-miR-543	5.37285	31.43117	2.671938627	5.019309189		-2.54844	0.513908
hsa-miR-3196	4.298279727	0	2.405524014	0		20	0.225995	rno-miR-3085	0	1.611855	0	1.38507475		-20	0.12959	chr7_16274_mature	413.1721	30.62524	8.694086697	4.983004663		3.75395	0.514147
bta-miR-433	4.298279727	0	2.405524014	0		20	0.225995	mmu-miR-669h-3p	0	1.611855	0	1.38507475		-20	0.12959	chr12_25742_mature	13.43212	2.417782	3.851211749	1.773060525		2.473931	0.514562
chrx_22793_mature	4.298279727	0	2.405524014	0		20	0.225995	chr15_31984_star	0	1.611855	0	1.38507475		-20	0.12959	chr18_36164_mature	13.43212	2.417782	3.851211749	1.773060525		2.473931	0.514562
chr15_32809_mature	4.298279727	0	2.405524014	0		20	0.225995	mmu-miR-1946a	0	1.611855	0	1.38507475		-20	0.12959	chr1_1168_mature	218.675	20.14819	7.77922676	4.402462031		3.440066	0.514901
NW_003537967_37423_mature	4.298279727	0	2.405524014	0		20	0.225995	chr4_10602_star	0	1.611855	0	1.38507475		-20	0.12959	chr8_18468_mature	3.22371	16.92448	2.078510713	4.163859073		-2.39232	0.516136
NW_003538115_37551_mature	4.298279727	0	2.405524014	0		20	0.225995	chrx_22916_mature	0	1.611855	0	1.38507475		-20	0.12959	chr2_5267_mature	3.22371	16.92448	2.078510713	4.163859073		-2.39232	0.516136
chr4_8839_star	4.298279727	0	2.405524014	0		20	0.225995	hsa-miR-1207-5p	0	1.611855	0	1.38507475		-20	0.12959	chr1_2073_star	5.910135	0.805927	2.788713818	0.852739935		2.874469	0.516547
NW_003613212_37672_mature	4.298279727	0	2.405524014	0		20	0.225995	chrx_22969_star	0	1.611855	0	1.38507475		-20	0.12959	chr9_19693_mature	5.910135	0.805927	2.788713818	0.852739935		2.874469	0.516547
chr7_15459_star	4.298279727	0	2.405524014	0		20	0.225995	mmu-miR-302c-3p	0	1.611855	0	1.38507475		-20	0.12959	chr4_10011_star	5.910135	0.805927	2.788713818	0.852739935		2.874469	0.516547
bta-miR-2413	4.298279727	0	2.405524014	0		20	0.225995	chr10_23455_mature	0	1.611855	0	1.38507475		-20	0.12959	chr5_11544_star	5.910135	0.805927	2.788713818	0.852739935		2.874469	0.516547
hsa-miR-5003-5p	4.298279727	0	2.405524014	0		20	0.225995	chrx_21505_star	0	1.611855	0	1.38507475		-20	0.12959	NW_003538449_37886_star	5.910135	0.805927	2.788713818	0.852739935		2.874469	0.516547
mmu-miR-3069-5p	4.298279727	0	2.405524014	0		20	0.225995	hsa-miR-548n	0	1.611855	0	1.38507475		-20	0.12959	hsa-miR-3614-3p	5.910135	0.805927	2.788713818	0.852739935		2.874469	0.516547
chr1_1940_mature	4.298279727	0	2.405524014	0		20	0.225995	chr5_11364_star	0	1.611855	0	1.38507475		-20	0.12959	chr4_9354_star	5.910135	0.805927	2.788713818	0.852739935		2.874469	0.516547
chr6_14899_star	4.298279727	0	2.405524014	0		20	0.225995	chr13_26704_mature	0	1.611855	0	1.38507475		-20	0.12959	chr1_1421_star	5.910135	0.805927	2.788713818	0.852739935		2.874469	0.516547
mmu-miR-467e-3p	4.298279727	0	2.405524014	0		20	0.225995	bta-miR-29b	0	1.611855	0	1.38507475		-20	0.12959	chr5_11717_mature	5.910135	0.805927	2.788713818	0.852739935		2.874469	0.516547
chr5_11343_star	4.298279727	0	2.405524014	0		20	0.225995	rno-miR-146a-3p	0	1.611855	0	1.38507475		-20	0.12959	chr6_12739_mature	5.910135	0.805927	2.788713818	0.852739935		2.874469	0.516547
chr17_35398_star	4.298279727	0	2.405524014	0		20	0.225995	chr8_17483_mature	0	1.611855	0	1.38507475		-20	0.12959	chr6_12861_mature	5.910135	0.805927	2.788713818	0.852739935		2.874469	0.516547
hsa-miR-657	4.298279727	0	2.405524014	0		20	0.225995	chr18_36561_star	0	1.611855	0	1.38507475		-20	0.12959	chr5_12345_star	5.910135	0.805927	2.788713818	0.852739935		2.874469	0.516547
chr9_20452_mature	4.298279727	0	2.405524014	0		20	0.225995	bta-miR-2388-3p	0	1.611855	0	1.38507475		-20	0.12959	chr14_30349_star	5.910135	0.805927	2.788713818	0.852739935		2.874469	0.516547
chr13_28432_mature	4.298279727	0	2.405524014	0		20	0.225995	chr3_7381_star	0	1.611855	0	1.38507475		-20	0.12959	hsa-miR-4712-5p	5.910135	0.805927	2.788713818	0.852739935		2.874469	0.516547
chr2_4097_mature	4.298279727	0	2.405524014	0		20	0.225995	NW_003613290_38304_mature	0	1.611855	0	1.38507475		-20	0.12959	chr4_9726_star	5.910135	0.805927	2.788713818	0.852739935		2.874469	0.516547
chr16_34846_mature	4.298279727	0	2.405524014	0		20	0.225995	mmu-miR-1191	0	1.611855	0	1.38507475		-20	0.12959	ssc-miR-153	5.910135	0.805927	2.788713818	0.852739935		2.874469	0.516547
mmu-miR-694	4.298279727	0	2.405524014	0		20	0.225995	rno-miR-140-3p	0	1.611855	0	1.38507475		-20	0.12959	chr3_6725_star	5.910135	0.805927	2.788713818	0.852739935		2.874469	0.516547
chr15_32263_mature	4.298279727	0	2.405524014	0		20	0.225995	bta-miR-551a	0	1.611855	0	1.38507475		-20	0.12959	chr3_6723_star	5.910135	0.805927	2.788713818	0.852739935		2.874469	0.516547
chr3_7756_star	4.298279727	0	2.405524014	0		20	0.225995	chr3_8156_star	0	1.611855	0	1.38507475		-20	0.12959	chr13_29242_mature	5.910135	0.805927	2.788713818	0.852739935		2.874469	0.516547
chr14_30041_star	4.298279727	0	2.405524014	0		20	0.225995	NW_003538331_37733_mature	0	1.611855	0	1.38507475		-20	0.12959	rno-miR-216b-5p	5.910135	0.805927	2.788713818	0.852739935		2.874469	0.516547
mmu-miR-1894-3p	4.298279727	0	2.405524014	0		20	0.225995	mmu-miR-344d-2-5p	0	1.611855	0	1.38507475		-20	0.12959	chr10_23333_star	5.910135	0.805927	2.788713818	0.852739935		2.874469	0.516547
mmu-miR-290-5p	4.298279727	0	2.405524014	0		20	0.225995	rno-miR-6216	0	1.611855	0	1.38507475		-20	0.12959	chr13_27063_mature	5.910135	0.805927	2.788713818	0.852739935		2.874469	0.516547
mmu-miR-719	4.298279727	0	2.405524014	0		20	0.225995	chr13_27994_star	0	1.611855	0	1.38507475		-20	0.12959	hsa-miR-6513-5p	5.910135	0.805927	2.788713818	0.852739935		2.874469	0.516547
NW_003541202_39972_mature	4.298279727	0	2.405524014	0		20	0.225995	chr4_10198_star	0	1.611855	0	1.38507475		-20	0.12959	mmu-miR-468-5p	5.910135	0.805927	2.788713818	0.852739935		2.874469	0.516547
chr6_14709_star	4.298279727	0	2.405524014	0		20	0.225995	hsa-miR-1273g-3p	0	1.611855	0	1.38507475		-20	0.12959	NW_003540583_39564_star	5.910135	0.805927	2.788713818	0.852739935		2.874469	0.516547
chr5_11037_star	4.298279727	0	2.405524014	0		20	0.225995	bta-miR-329a	0	1.611855	0	1.38507475		-20	0.12959	chr16_34229_star	5.910135	0.805927	2.788713818	0.852739935		2.874469	0.516547
chr4_10042_star	4.298279727	0	2.405524014	0		20	0.225995	hsa-miR-3158-5p	0	1.611855	0	1.38507475		-20	0.12959	chr2_5533_star	5.910135	0.805927	2.788713818	0.852739935		2.874469	0.516547
chr11_25077_star	4.298279727	0	2.405524014	0		20	0.225995	chrx_23005_star	0	1.611855	0	1.38507475		-20	0.12959	chr14_30584_mature	5.910135	0.805927	2.788713818	0.852739935		2.874469	0.516547
chr13_28501_mature	4.298279727	0	2.405524014	0		20	0.225995	hsa-miR-4265	0	1.611855	0	1.38507475		-20	0.12959	chr5_12148_mature	5.910135	0.805927	2.788713818	0.852739935		2.874469	0.516547
chr14_31110_star	4.298279727	0	2.405524014	0		20	0.225995	hsa-miR-4261	0	1.611855	0	1.38507475		-20	0.12959	NW_003539070_38505_mature	5.910135	0.805927	2.788713818	0.852739935		2.874469	0.516547
chr2_5388_star	4.298279727	0	2.405524014	0		20	0.225995	mmu-miR-3061-3p	0	1.611855	0	1.38507475		-20	0.12959	chr5_12388_star	5.910135	0.805927	2.788713818	0.852739935		2.874469	0.516547
chr9_21017_star	4.298279727	0	2.405524014	0		20	0.225995	chr15_31782_mature	0	1.611855	0	1.38507475		-20	0.12959	NW_003613065_36971_mature	3.760995	20.14819	2.251263041	4.402462031		-2.42146	0.516958
chr4_8920_star	4.298279727	0	2.405524014	0		20	0.225995	chr11_24756_star	0	1.611855	0	1.38507475		-20	0.12959	chr9_19210_mature	3.760995	20.14819	2.251263041	4.402462031		-2.42146	0.516958
chr1_666_star	4.298279727	0	2.405524014	0		20	0.225995	bta-miR-6528	0	1.611855	0	1.38507475		-20	0.12959	chr1_283_star	3.760995	20.14819	2.251263041	4.402462031		-2.42146	0.516958
chr2_6372_mature	4.298279727	0	2.405524014	0		20	0.225995	chr2_5690_star	0	1.611855	0	1.38507475		-20	0.12959	chr7_15333_star	3.760995	20.14819	2.251263041	4.402462031		-2.42146	0.516958
hsa-miR-3940-3p	4.298279727	0	2.405524014	0		20	0.225995	chr3_8036_star	0	1.611855	0	1.38507475		-20	0.12959	chr11_25285_mature	82.74188	10.47706	6.387877484	3.520680821		2.981384	0.517935
chr4_8705_mature	4.298279727	0	2.405524014	0		20	0.225995	chr8_18294_star	0	1.611855	0	1.38507475		-20	0.12959	chr14_31056_star	23.10325	4.029637	4.591155994	2.330454351		2.519374	0.518871
chr11_25303_mature	24.71510843	3.22371	4.684544332	2.078510713		2.938599	0.227701	NW_003538989_38414_star	0	1.611855	0	1.38507475		-20	0.12959	chr11_24560_mature	2.686425	13.70077	1.882222339	3.877819487		-2.3505	0.519236
chr3_7716_star	24.71510843	3.22371	4.684544332	2.078510713		2.938599	0.227701	rno-miR-880-3p	0	1.611855	0	1.38507475		-20	0.12959	chr1_3501_mature	2.686425	13.70077	1.882222339	3.877819487		-2.3505	0.519236
chr9_20034_mature	88.11473441	9.671129	6.477592084	3.415640968		3.187627	0.227806	chr1_1911_star	0	1.611855	0	1.38507475		-20	0.12959	chr13_28822_mature	2.686425	13.70077	1.882222339	3.877819487		-2.3505	0.519236
chr6_13257_mature	2.149139864	18.53633	1.654957834	4.288087668		-3.10852	0.227963	chr3_7264_star	0	1.611855	0	1.38507475		-20	0.12959	chr10_23892_mature	2.686425	13.70077	1.882222339	3.877819487		-2.3505	0.519236
chr6_14661_mature	2.149139864	18.53633	1.654957834	4.288087668		-3.10852	0.227963	chr15_33038_star	0	1.611855	0	1.38507475		-20	0.12959	NW_003538807_38238_star	2.686425	13.70077	1.882222339	3.877819487		-2.3505	0.519236
chr1_217_mature	2.149139864	18.53633	1.654957834	4.288087668		-3.10852	0.227963	chr1_589_star	0	1.611855	0	1.38507475		-20	0.12959	chr4_10161_star	2.686425	13.70077	1.882222339	3.877819487		-2.3505	0.519236
rno-miR-93-3p	31.16252802	4.029637	5.007308904	2.330454351		2.95109	0.228526	chr7_16088_star	0	1.611855	0	1.38507475		-20	0.12959	chr7_15701_mature	2.686425	13.70077	1.882222339	3.877819487		-2.3505	0.519236
chr10_23710_mature	31.16252802	4.029637	5.007308904	2.330454351		2.95109	0.228526	chr8_18878_star	0	1.611855	0	1.38507475		-20	0.12959	chr12_25620_mature	9.133844	1.611855	3.34110968	1.38507475		2.5025	0.519313
chr15_32628_mature	12.89483918	1.611855	3.796477232	1.38507475		3	0.232004	chr16_34150_mature	0	1.611855	0	1.38507475		-20	0.12959	chr2_4630_star	9.133844	1.611855	3.34110968	1.38507475		2.5025	0.519313
chr9_20685_mature	12.89483918	1.611855	3.796477232	1.38507475		3	0.232004	chr5_11694_star	0	1.611855	0	1.38507475		-20	0.12959	chr3_8257_star	9.133844	1.611855	3.34110968	1.38507475		2.5025	0.519313
chr1_526_mature	12.89483918	1.611855	3.796477232	1.38507475		3	0.232004	chr9_19911_star	0	1.611855	0	1.38507475		-20	0.12959	chr3_7189_star	9.133844	1.611855	3.34110968	1.38507475		2.5025	0.519313
NW_003537879_37346_mature	12.89483918	1.611855	3.796477232	1.38507475		3	0.232004	chr5_11562_mature@@mmu-miR-3059	0	1.611855	0	1.38507475		-20	0.12959	chr5_12232_star	9.133844	1.611855	3.34110968	1.38507475		2.5025	0.519313
chr14_30480_mature	12.89483918	1.611855	3.796477232	1.38507475		3	0.232004	chr17_35035_star	0	1.611855	0	1.38507475		-20	0.12959	ssc-miR-376c	9.133844	1.611855	3.34110968	1.38507475		2.5025	0.519313
bta-miR-450a	12.89483918	1.611855	3.796477232	1.38507475		3	0.232004	hsa-miR-4781-5p	0	1.611855	0	1.38507475		-20	0.12959	NW_003538873_38324_mature	9.133844	1.611855	3.34110968	1.38507475		2.5025	0.519313
mmu-miR-1946a	12.89483918	1.611855	3.796477232	1.38507475		3	0.232004	chr15_33315_star	0	1.611855	0	1.38507475		-20	0.12959	chr13_28544_mature	9.133844	1.611855	3.34110968	1.38507475		2.5025	0.519313
hsa-miR-3195	12.89483918	1.611855	3.796477232	1.38507475		3	0.232004	chr15_32159_star	0	1.611855	0	1.38507475		-20	0.12959	chr4_9919_mature	17.7304	3.22371	4.227310103	2.078510713		2.459432	0.520353
chr15_31930_mature	12.89483918	1.611855	3.796477232	1.38507475		3	0.232004	chr1_217_star	0	1.611855	0	1.38507475		-20	0.12959	mmu-miR-344b-5p	3.22371	0	2.078510713	0		20	0.523571
chr16_34150_mature	12.89483918	1.611855	3.796477232	1.38507475		3	0.232004	bta-miR-3141	0	1.611855	0	1.38507475		-20	0.12959	chr2_6500_mature	3.22371	0	2.078510713	0		20	0.523571
chr2_5353_star	12.89483918	1.611855	3.796477232	1.38507475		3	0.232004	chr1_1337_mature	0	1.611855	0	1.38507475		-20	0.12959	chr4_10638_star	3.22371	0	2.078510713	0		20	0.523571
rno-miR-141-3p	12.89483918	1.611855	3.796477232	1.38507475		3	0.232004	chr2_6321_mature	0	1.611855	0	1.38507475		-20	0.12959	chr17_35778_star	3.22371	0	2.078510713	0		20	0.523571
chr1_3232_mature	3.223709795	26.59561	2.078510713	4.786366653		-3.04439	0.232416	hsa-miR-4664-3p	0	1.611855	0	1.38507475		-20	0.12959	chr13_26892_mature	3.22371	0	2.078510713	0		20	0.523571
chr8_18162_star	5.372849659	46.74379	2.671938627	5.577241251		-3.12102	0.23265	chr13_28245_star	0	1.611855	0	1.38507475		-20	0.12959	chr13_26891_mature	3.22371	0	2.078510713	0		20	0.523571
chr13_28627_mature	5.372849659	46.74379	2.671938627	5.577241251		-3.12102	0.23265	bta-miR-496	0	1.611855	0	1.38507475		-20	0.12959	rno-miR-509-3p	3.22371	0	2.078510713	0		20	0.523571
chr4_10537_mature	68.77247564	8.059274	6.124586119	3.179395517		3.093109	0.232931	NW_003537737_37236_mature	0	1.611855	0	1.38507475		-20	0.12959	chr2_4615_star	3.22371	0	2.078510713	0		20	0.523571
ssc-miR-9-2	6114.302912	227.2715	12.57820825	7.834607195		4.749699	0.232965	chr9_20608_star	0	1.611855	0	1.38507475		-20	0.12959	hsa-miR-4706	3.22371	0	2.078510713	0		20	0.523571
chr7_15706_mature	77.36903509	8.865202	6.292211829	3.302348583		3.125531	0.233151	chr1_1486_star	0	1.611855	0	1.38507475		-20	0.12959	hsa-miR-3680-5p	3.22371	0	2.078510713	0		20	0.523571
NW_003538764_38206_mature	2.149139864	17.7304	1.654957834	4.227310103		-3.04439	0.236146	chr8_17650_star	0	1.611855	0	1.38507475		-20	0.12959	hsa-miR-4737	3.22371	0	2.078510713	0		20	0.523571
chr14_30035_star	2.149139864	17.7304	1.654957834	4.227310103		-3.04439	0.236146	chr4_9749_star	0	1.611855	0	1.38507475		-20	0.12959	chr2_5951_mature	3.22371	0	2.078510713	0		20	0.523571
hsa-miR-374a-3p	2.149139864	17.7304	1.654957834	4.227310103		-3.04439	0.236146	chr1_319_star	0	1.611855	0	1.38507475		-20	0.12959	chr7_15295_star	3.22371	0	2.078510713	0		20	0.523571
chr10_24135_mature	2.149139864	17.7304	1.654957834	4.227310103		-3.04439	0.236146	mmu-miR-1186a	0	1.611855	0	1.38507475		-20	0.12959	hsa-miR-1250	3.22371	0	2.078510713	0		20	0.523571
chr2_4844_star	2.149139864	17.7304	1.654957834	4.227310103		-3.04439	0.236146	chr15_32320_star	0	1.611855	0	1.38507475		-20	0.12959	hsa-miR-1262	3.22371	0	2.078510713	0		20	0.523571
chr1_1739_mature	2.149139864	17.7304	1.654957834	4.227310103		-3.04439	0.236146	bta-miR-2423	0	1.611855	0	1.38507475		-20	0.12959	chr14_29793_star	3.22371	0	2.078510713	0		20	0.523571
chr1_3820_star	5.372849659	45.93786	2.671938627	5.552680304		-3.09592	0.236212	chr9_20945_star	0	1.611855	0	1.38507475		-20	0.12959	mmu-miR-1892	3.22371	0	2.078510713	0		20	0.523571
chr7_16821_mature	5.372849659	45.93786	2.671938627	5.552680304		-3.09592	0.236212	chr3_7339_star	0	1.611855	0	1.38507475		-20	0.12959	chr12_25406_star	3.22371	0	2.078510713	0		20	0.523571
chr8_17676_mature	6.447419591	57.22085	2.896740642	5.863463969		-3.14975	0.236328	hsa-miR-548ao-5p	0	1.611855	0	1.38507475		-20	0.12959	chr11_25228_star	3.22371	0	2.078510713	0		20	0.523571
chr6_14751_mature	4.298279727	35.46081	2.405524014	5.188274618		-3.04439	0.236534	chr6_14576_star	0	1.611855	0	1.38507475		-20	0.12959	chr15_33130_star	3.22371	0	2.078510713	0		20	0.523571
chr2_4828_mature	4.298279727	35.46081	2.405524014	5.188274618		-3.04439	0.236534	hsa-miR-4489	0	1.611855	0	1.38507475		-20	0.12959	hsa-miR-6505-3p	3.22371	0	2.078510713	0		20	0.523571
chr14_30524_mature	30.08795809	4.029637	4.958283956	2.330454351		2.900464	0.236575	chr11_24341_star	0	1.611855	0	1.38507475		-20	0.12959	chr1_1629_star	3.22371	0	2.078510713	0		20	0.523571
chr13_27508_mature	11.82026925	124.9188	3.680354657	6.976349367		-3.40166	0.236886	hsa-miR-524-5p	0	1.611855	0	1.38507475		-20	0.12959	chr7_15701_star	3.22371	0	2.078510713	0		20	0.523571
hsa-miR-598	23.6405385	3.22371	4.62296188	2.078510713		2.874469	0.237572	chr6_14856_star	0	1.611855	0	1.38507475		-20	0.12959	chr7_16674_mature@@mmu-miR-6	3.22371	0	2.078510713	0		20	0.523571
chr17_35668_mature	23.6405385	3.22371	4.62296188	2.078510713		2.874469	0.237572	NW_003613050_36907_mature	0	1.611855	0	1.38507475		-20	0.12959	mmu-miR-3103-5p	3.22371	0	2.078510713	0		20	0.523571
chr3_7643_star	23.6405385	3.22371	4.62296188	2.078510713		2.874469	0.237572	bta-miR-2398	0	1.611855	0	1.38507475		-20	0.12959	chr13_29078_mature	3.22371	0	2.078510713	0		20	0.523571
chr7_16102_mature	23.6405385	3.22371	4.62296188	2.078510713		2.874469	0.237572	hsa-miR-561-3p	0	1.611855	0	1.38507475		-20	0.12959	chr5_11609_star	3.22371	0	2.078510713	0		20	0.523571
chr6_13308_mature	23.6405385	3.22371	4.62296188	2.078510713		2.874469	0.237572	hsa-miR-4491	0	1.611855	0	1.38507475		-20	0.12959	chr9_20079_star	3.22371	0	2.078510713	0		20	0.523571
chr8_17786_mature	3.223709795	25.78968	2.078510713	4.743605355		-3	0.238348	chr14_30313_star	0	1.611855	0	1.38507475		-20	0.12959	chr15_32789_star	3.22371	0	2.078510713	0		20	0.523571
chr14_30903_star	1.074569932	10.47706	1.05281229	3.520680821		-3.2854	0.23848	chr4_10381_mature	0	1.611855	0	1.38507475		-20	0.12959	ssc-miR-194b-3p	3.22371	0	2.078510713	0		20	0.523571
chr14_31216_mature	1.074569932	10.47706	1.05281229	3.520680821		-3.2854	0.23848	chr16_34112_star	0	1.611855	0	1.38507475		-20	0.12959	chr12_26437_mature	3.22371	0	2.078510713	0		20	0.523571
chr1_3446_star	1.074569932	10.47706	1.05281229	3.520680821		-3.2854	0.23848	chr16_33722_mature	0	1.611855	0	1.38507475		-20	0.12959	chr6_14981_mature	3.22371	0	2.078510713	0		20	0.523571
chr5_12267_star	1.074569932	10.47706	1.05281229	3.520680821		-3.2854	0.23848	chr13_27245_mature	0	1.611855	0	1.38507475		-20	0.12959	chr13_28026_star	3.22371	0	2.078510713	0		20	0.523571
chr15_33139_mature	1.074569932	10.47706	1.05281229	3.520680821		-3.2854	0.23848	NW_003538662_38098_star	0	1.611855	0	1.38507475		-20	0.12959	mmu-miR-5125	3.22371	0	2.078510713	0		20	0.523571
NW_003539061_38502_mature	1.074569932	10.47706	1.05281229	3.520680821		-3.2854	0.23848	chr4_10259_star	0	1.611855	0	1.38507475		-20	0.12959	mmu-miR-5121	3.22371	0	2.078510713	0		20	0.523571
chr9_20549_mature	1.074569932	10.47706	1.05281229	3.520680821		-3.2854	0.23848	bta-miR-22-3p	0	1.611855	0	1.38507475		-20	0.12959	chr7_17101_star	3.22371	0	2.078510713	0		20	0.523571
chr7_16877_star	1.074569932	10.47706	1.05281229	3.520680821		-3.2854	0.23848	chr18_35816_star	0	1.611855	0	1.38507475		-20	0.12959	chr9_21042_star	3.22371	0	2.078510713	0		20	0.523571
chr15_33231_mature	1.074569932	10.47706	1.05281229	3.520680821		-3.2854	0.23848	chr8_17863_mature	0	1.611855	0	1.38507475		-20	0.12959	chr7_16955_mature	3.22371	0	2.078510713	0		20	0.523571
chr13_27500_mature	1.074569932	10.47706	1.05281229	3.520680821		-3.2854	0.23848	hsa-miR-4670-3p	0	1.611855	0	1.38507475		-20	0.12959	rno-miR-298-3p	3.22371	0	2.078510713	0		20	0.523571
chr18_36575_mature	59.10134625	7.253347	5.909325402	3.044979305		3.026472	0.238487	bta-miR-2324	0	1.611855	0	1.38507475		-20	0.12959	mmu-miR-701-3p	3.22371	0	2.078510713	0		20	0.523571
ssc-miR-2411	11.82026925	123.3069	3.680354657	6.957762565		-3.38292	0.239821	hsa-miR-3187-3p	0	1.611855	0	1.38507475		-20	0.12959	chr8_18153_star	3.22371	0	2.078510713	0		20	0.523571
chr1_918_star	0	4.835565	0	2.544872269		-20	0.240333	chr15_31774_mature	0	1.611855	0	1.38507475		-20	0.12959	chr6_13633_star	3.22371	0	2.078510713	0		20	0.523571
rno-miR-7a-5p	0	4.835565	0	2.544872269		-20	0.240333	bta-miR-2351	0	1.611855	0	1.38507475		-20	0.12959	chr2_4263_star	3.22371	0	2.078510713	0		20	0.523571
chr4_10320_star	0	4.835565	0	2.544872269		-20	0.240333	chr5_11905_mature	0	1.611855	0	1.38507475		-20	0.12959	chr10_23459_mature	3.22371	0	2.078510713	0		20	0.523571
chr4_10242_star	0	4.835565	0	2.544872269		-20	0.240333	bta-miR-2371	0	1.611855	0	1.38507475		-20	0.12959	chr6_15013_star	3.22371	0	2.078510713	0		20	0.523571
chrx_22639_star	0	4.835565	0	2.544872269		-20	0.240333	chr16_34371_star	0	1.611855	0	1.38507475		-20	0.12959	chr15_33456_star	3.22371	0	2.078510713	0		20	0.523571
NW_003613114_37265_star	0	4.835565	0	2.544872269		-20	0.240333	hsa-miR-548ah-3p	0	1.611855	0	1.38507475		-20	0.12959	mmu-miR-148a-5p	3.22371	0	2.078510713	0		20	0.523571
chr2_4631_star	0	4.835565	0	2.544872269		-20	0.240333	hsa-miR-4720-3p	0	1.611855	0	1.38507475		-20	0.12959	NW_003538125_37558_star	3.22371	0	2.078510713	0		20	0.523571
chr1_1197_mature	0	4.835565	0	2.544872269		-20	0.240333	mmu-miR-686	0	1.611855	0	1.38507475		-20	0.12959	chr18_36298_mature	3.22371	0	2.078510713	0		20	0.523571
hsa-miR-1289	0	4.835565	0	2.544872269		-20	0.240333	rno-miR-879-3p	0	1.611855	0	1.38507475		-20	0.12959	mmu-miR-351-3p	3.22371	0	2.078510713	0		20	0.523571
chr4_10602_mature	0	4.835565	0	2.544872269		-20	0.240333	NW_003538173_37591_star	0	1.611855	0	1.38507475		-20	0.12959	bta-miR-2439-5p	3.22371	0	2.078510713	0		20	0.523571
chr5_11515_star	0	4.835565	0	2.544872269		-20	0.240333	chr13_26761_star	0	1.611855	0	1.38507475		-20	0.12959	rno-miR-148b-5p	3.22371	0	2.078510713	0		20	0.523571
chr5_11530_star	0	4.835565	0	2.544872269		-20	0.240333	bta-miR-2293	0	1.611855	0	1.38507475		-20	0.12959	rno-miR-205	3.22371	0	2.078510713	0		20	0.523571
chr14_31236_star	0	4.835565	0	2.544872269		-20	0.240333	bta-miR-2296	0	1.611855	0	1.38507475		-20	0.12959	chr7_16402_star	3.22371	0	2.078510713	0		20	0.523571
hsa-miR-548av-3p	0	4.835565	0	2.544872269		-20	0.240333	mmu-miR-466q	0	1.611855	0	1.38507475		-20	0.12959	chr1_607_mature	3.22371	0	2.078510713	0		20	0.523571
mmu-miR-5136	0	4.835565	0	2.544872269		-20	0.240333	chr7_16063_star	0	1.611855	0	1.38507475		-20	0.12959	chr9_19974_star	3.22371	0	2.078510713	0		20	0.523571
chr7_16829_star	0	4.835565	0	2.544872269		-20	0.240333	mmu-miR-3075-3p	0	1.611855	0	1.38507475		-20	0.12959	mmu-miR-6354	3.22371	0	2.078510713	0		20	0.523571
mmu-miR-193b-5p	0	4.835565	0	2.544872269		-20	0.240333	mmu-miR-467h	0	1.611855	0	1.38507475		-20	0.12959	mmu-miR-301b-5p	3.22371	0	2.078510713	0		20	0.523571
chr14_31127_star	0	4.835565	0	2.544872269		-20	0.240333	chr1_1198_star	0	1.611855	0	1.38507475		-20	0.12959	bta-miR-146b	3.22371	0	2.078510713	0		20	0.523571
chr15_31781_mature	0	4.835565	0	2.544872269		-20	0.240333	hsa-miR-4633-3p	0	1.611855	0	1.38507475		-20	0.12959	chr6_13459_star	3.22371	0	2.078510713	0		20	0.523571
chr8_18354_star	0	4.835565	0	2.544872269		-20	0.240333	rno-miR-20b-3p	0	1.611855	0	1.38507475		-20	0.12959	chrx_21532_star	3.22371	0	2.078510713	0		20	0.523571
bta-miR-2284b	0	4.835565	0	2.544872269		-20	0.240333	chr7_16525_mature	0	1.611855	0	1.38507475		-20	0.12959	chr7_15656_star	3.22371	0	2.078510713	0		20	0.523571
chr9_20299_mature	0	4.835565	0	2.544872269		-20	0.240333	hsa-miR-519e-5p	0	1.611855	0	1.38507475		-20	0.12959	chr9_19395_star	3.22371	0	2.078510713	0		20	0.523571
chr9_20604_star	0	4.835565	0	2.544872269		-20	0.240333	chr13_28864_mature	0	1.611855	0	1.38507475		-20	0.12959	chr8_18502_mature	3.22371	0	2.078510713	0		20	0.523571
hsa-miR-103b	0	4.835565	0	2.544872269		-20	0.240333	NW_003613326_38566_mature	0	1.611855	0	1.38507475		-20	0.12959	mmu-miR-6540-5p	3.22371	0	2.078510713	0		20	0.523571

chr14_29882_star	0	4.835565	0	2.544872269		-20	0.240333
chr10_24226_mature	0	4.835565	0	2.544872269		-20	0.240333
hsa-miR-5010-3p	0	4.835565	0	2.544872269		-20	0.240333
chr4_10404_mature	0	4.835565	0	2.544872269		-20	0.240333
mmu-miR-3970	0	4.835565	0	2.544872269		-20	0.240333
NW_003538795_38232_mature	0	4.835565	0	2.544872269		-20	0.240333
chr7_17051_star	0	4.835565	0	2.544872269		-20	0.240333
chr4_8989_star	0	4.835565	0	2.544872269		-20	0.240333
NW_003539571_38947_mature	0	4.835565	0	2.544872269		-20	0.240333
chr11_24516_mature	0	4.835565	0	2.544872269		-20	0.240333
chr6_12619_star	0	4.835565	0	2.544872269		-20	0.240333
chr15_31859_mature	0	4.835565	0	2.544872269		-20	0.240333
hsa-miR-3122	0	4.835565	0	2.544872269		-20	0.240333
hsa-miR-3148	0	4.835565	0	2.544872269		-20	0.240333
chr13_27616_mature	0	4.835565	0	2.544872269		-20	0.240333
hsa-miR-5581-5p	0	4.835565	0	2.544872269		-20	0.240333
NW_003613116_37270_star	0	4.835565	0	2.544872269		-20	0.240333
chr14_30479_star	0	4.835565	0	2.544872269		-20	0.240333
chrx_22412_star	0	4.835565	0	2.544872269		-20	0.240333
bta-miR-2403	0	4.835565	0	2.544872269		-20	0.240333
chr9_20798_mature	0	4.835565	0	2.544872269		-20	0.240333
chr6_14013_star	0	4.835565	0	2.544872269		-20	0.240333
bta-miR-2312	0	4.835565	0	2.544872269		-20	0.240333
bta-miR-2338	0	4.835565	0	2.544872269		-20	0.240333
chr10_23272_mature	0	4.835565	0	2.544872269		-20	0.240333
chr8_18856_mature	0	4.835565	0	2.544872269		-20	0.240333
NW_003613335_38593_mature	4.298279727	34.65488	2.405524014	5.156027656		-3.01123	0.241139
chr1_2204_mature	92.41301414	10.47706	6.545551653	3.520680821		3.140863	0.242029
bta-miR-2899	6.447419591	55.60899	2.896740642	5.82295938		-3.10852	0.242354
chr7_15165_mature	5.372849659	44.32601	2.671938627	5.502267253		-3.04439	0.24358
chr11_25010_star	101.0095736	11.28298	6.672560745	3.618589217		3.162271	0.243703
NW_003540770_39675_mature	3.223709795	24.98375	2.078510713	4.699537803		-2.9542	0.244502
chr15_31776_mature	3.223709795	24.98375	2.078510713	4.699537803		-2.9542	0.244502
NW_003539279_38689_mature	3.223709795	24.98375	2.078510713	4.699537803		-2.9542	0.244502
ssc-miR-1839-5p	890.8184735	57.22085	9.800606274	5.863463969		3.960519	0.244738
chr6_12563_mature	2.149139864	16.92448	1.654957834	4.163859073		-2.97728	0.24476
chr3_8628_mature	2.149139864	16.92448	1.654957834	4.163859073		-2.97728	0.24476
chr15_32700_mature	2.149139864	16.92448	1.654957834	4.163859073		-2.97728	0.24476
chr3_6868_mature	29.01338816	4.029637	4.907534286	2.330454351		2.847997	0.244974
chr18_35924_mature	29.01338816	4.029637	4.907534286	2.330454351		2.847997	0.244974
chr5_11215_mature	91.33844421	10.47706	6.52885952	3.520680821		3.123989	0.245164
chr16_34823_star	17.19311891	2.417782	4.185320985	1.773060525		2.830075	0.245195
ssc-miR-652	17.19311891	2.417782	4.185320985	1.773060525		2.830075	0.245195
chr15_31881_mature	17.19311891	2.417782	4.185320985	1.773060525		2.830075	0.245195
chr2_6095_mature	17.19311891	2.417782	4.185320985	1.773060525		2.830075	0.245195
chr3_7627_star	17.19311891	2.417782	4.185320985	1.773060525		2.830075	0.245195
chr15_33576_mature	4.298279727	33.84895	2.405524014	5.123043401		-2.97728	0.245876
chr9_19693_mature	7.521989523	0.805927	3.091190278	0.852739935		3.222392	0.247531
chr15_31776_star	7.521989523	0.805927	3.091190278	0.852739935		3.222392	0.247531
chrx_22198_mature	7.521989523	0.805927	3.091190278	0.852739935		3.222392	0.247531
mmu-miR-1928	7.521989523	0.805927	3.091190278	0.852739935		3.222392	0.247531
chr6_12747_mature	7.521989523	0.805927	3.091190278	0.852739935		3.222392	0.247531
chr17_35590_star	7.521989523	0.805927	3.091190278	0.852739935		3.222392	0.247531
hsa-miR-3648	7.521989523	0.805927	3.091190278	0.852739935		3.222392	0.247531
hsa-miR-5193	7.521989523	0.805927	3.091190278	0.852739935		3.222392	0.247531
chr2_4629_star	7.521989523	0.805927	3.091190278	0.852739935		3.222392	0.247531
chr2_4549_star	7.521989523	0.805927	3.091190278	0.852739935		3.222392	0.247531
chr1_2153_mature	7.521989523	0.805927	3.091190278	0.852739935		3.222392	0.247531
chrx_21746_mature	7.521989523	0.805927	3.091190278	0.852739935		3.222392	0.247531
bta-miR-875	7.521989523	0.805927	3.091190278	0.852739935		3.222392	0.247531
chrx_22324_mature	7.521989523	0.805927	3.091190278	0.852739935		3.222392	0.247531
chr14_30291_mature	7.521989523	0.805927	3.091190278	0.852739935		3.222392	0.247531
chr16_34188_mature	7.521989523	0.805927	3.091190278	0.852739935		3.222392	0.247531
chr1_2507_mature	7.521989523	0.805927	3.091190278	0.852739935		3.222392	0.247531
chr3_7684_star	7.521989523	0.805927	3.091190278	0.852739935		3.222392	0.247531
chr10_23976_mature	7.521989523	0.805927	3.091190278	0.852739935		3.222392	0.247531
chr12_26448_mature	7.521989523	0.805927	3.091190278	0.852739935		3.222392	0.247531
chr12_25400_mature	7.521989523	0.805927	3.091190278	0.852739935		3.222392	0.247531
chr1_1734_star	7.521989523	0.805927	3.091190278	0.852739935		3.222392	0.247531
chr7_16802_mature	7.521989523	0.805927	3.091190278	0.852739935		3.222392	0.247531
chr2_4621_mature	7.521989523	0.805927	3.091190278	0.852739935		3.222392	0.247531
chr11_24341_mature	7.521989523	0.805927	3.091190278	0.852739935		3.222392	0.247531
bta-miR-2378	7.521989523	0.805927	3.091190278	0.852739935		3.222392	0.247531
chr3_8302_mature	7.521989523	0.805927	3.091190278	0.852739935		3.222392	0.247531
mmu-miR-217-5p	7.521989523	0.805927	3.091190278	0.852739935		3.222392	0.247531
mmu-miR-3096a-3p	323.4455495	27.40153	8.341832566	4.827896911		3.561194	0.247612
bta-miR-2889	272.9407627	24.17782	8.097720146	4.654081667		3.496832	0.247805
chr3_8428_mature	9.671129386	91.0698	3.415640968	6.524656134		-3.23522	0.247841
chr4_9919_mature	22.56596857	3.22371	4.558633073	2.078510713		2.807355	0.247966
chr1_2073_mature@@bta-miR-2478	8952.242102	310.2821	13.12819448	8.282078659		4.850597	0.248464
chr5_12267_mature	30.08795809	437.6186	4.958283956	8.776823197		-3.86242	0.249359
chr2_5958_star	64.47419591	8.059274	6.032854532	3.179395517		3	0.249527
chrx_22329_star	11.82026925	1.611855	3.680354657	1.38507475		2.874469	0.249749
bta-miR-1814b	11.82026925	1.611855	3.680354657	1.38507475		2.874469	0.249749
chr14_29861_mature	11.82026925	1.611855	3.680354657	1.38507475		2.874469	0.249749
chr14_31148_mature	11.82026925	1.611855	3.680354657	1.38507475		2.874469	0.249749
chr6_14888_mature	11.82026925	1.611855	3.680354657	1.38507475		2.874469	0.249749
chr15_32437_star	11.82026925	1.611855	3.680354657	1.38507475		2.874469	0.249749
hsa-miR-3661	11.82026925	1.611855	3.680354657	1.38507475		2.874469	0.249749
chr9_20751_star	11.82026925	1.611855	3.680354657	1.38507475		2.874469	0.249749
chr2_6524_star	11.82026925	1.611855	3.680354657	1.38507475		2.874469	0.249749
chr15_31736_star	11.82026925	1.611855	3.680354657	1.38507475		2.874469	0.249749
bta-miR-138	11.82026925	1.611855	3.680354657	1.38507475		2.874469	0.249749
chr6_13355_mature	11.82026925	1.611855	3.680354657	1.38507475		2.874469	0.249749
chr1_2009_star	11.82026925	1.611855	3.680354657	1.38507475		2.874469	0.249749
chr16_34527_star@@hsa-miR-449b-	11.82026925	1.611855	3.680354657	1.38507475		2.874469	0.249749
chr9_19915_star	11.82026925	1.611855	3.680354657	1.38507475		2.874469	0.249749
chrx_22357_mature	9.671129386	90.26387	3.415640968	6.511971994		-3.22239	0.249839
ssc-miR-196b	743.6023928	50.77343	9.540326442	5.694139975		3.872386	0.250176
chr5_10760_star	4.298279727	33.04303	2.405524014	5.08928735		-2.94251	0.250748
chr5_11850_mature	4.298279727	33.04303	2.405524014	5.08928735		-2.94251	0.250748
chr5_12128_mature	4.298279727	33.04303	2.405524014	5.08928735		-2.94251	0.250748
chr5_11410_mature	37.60994761	597.1922	5.270900692	9.224465384		-3.98901	0.252108
chr7_16196_mature	1.074569932	9.671129	1.05281229	3.415640968		-3.16993	0.252531
chr15_31736_mature	1.074569932	9.671129	1.05281229	3.415640968		-3.16993	0.252531
chr2_5658_star	1.074569932	9.671129	1.05281229	3.415640968		-3.16993	0.252531
chr8_17809_star	1.074569932	9.671129	1.05281229	3.415640968		-3.16993	0.252531
chr2_4016_mature	1.074569932	9.671129	1.05281229	3.415640968		-3.16993	0.252531
chr7_17037_mature	1.074569932	9.671129	1.05281229	3.415640968		-3.16993	0.252531
chr7_16043_star	1.074569932	9.671129	1.05281229	3.415640968		-3.16993	0.252531
NW_003613481_39347_mature	1.074569932	9.671129	1.05281229	3.415640968		-3.16993	0.252531
chr16_34034_mature	1.074569932	9.671129	1.05281229	3.415640968		-3.16993	0.252531
chr6_14717_mature	1.074569932	9.671129	1.05281229	3.415640968		-3.16993	0.252531
chr2_4820_mature	1.074569932	9.671129	1.05281229	3.415640968		-3.16993	0.252531
chr1_1055_star	1.074569932	9.671129	1.05281229	3.415640968		-3.16993	0.252531
chr13_28533_star	1.074569932	9.671129	1.05281229	3.415640968		-3.16993	0.252531
chr16_33794_mature	1.074569932	9.671129	1.05281229	3.415640968		-3.16993	0.252531
chr14_31610_mature	27.93881823	4.029637	4.854934103	2.330454351		2.793549	0.253734
chr13_26618_star	2.149139864	16.11855	1.654957834	4.097488514		-2.90689	0.25383
chr1_742_mature	2.149139864	16.11855	1.654957834	4.097488514		-2.90689	0.25383
chr13_27528_mature	2.149139864	16.11855	1.654957834	4.097488514		-2.90689	0.25383
chr18_36584_mature	2.149139864	16.11855	1.654957834	4.097488514		-2.90689	0.25383
chr14_30174_mature	2.149139864	16.11855	1.654957834	4.097488514		-2.90689	0.25383
ssc-miR-326	408.3365741	33.04303	8.677143767	5.08928735		3.627341	0.254141
chr3_6737_mature	40.83365741	5.641492	5.38659223	2.731507408		2.85561	0.255206
chr6_14477_star	4.298279727	32.2371	2.405524014	5.054722516		-2.90689	0.25576
chr15_32974_mature	3.223709795	23.3719	2.078510713	4.607146585		-2.85798	0.257512
chr6_13364_mature	3.223709795	23.3719	2.078510713	4.607146585		-2.85798	0.257512
chr5_11151_mature	3.223709795	23.3719	2.078510713	4.607146585		-2.85798	0.257512
chr8_17418_mature	228.8833955	21.76004	7.844758453	4.508431259		3.39486	0.258023
chr1_1435_mature	9.671129386	87.04016	3.415640968	6.460089935		-3.16993	0.258073
chr8_17569_star	21.49139864	3.22371	4.491301473	2.078510713		2.736966	0.258905
chr7_16206_star	16.11854898	2.417782	4.097488514	1.773060525		2.736966	0.259044
chr12_25947_star	16.11854898	2.417782	4.097488514	1.773060525		2.736966	0.259044
chr12_26075_mature	16.11854898	2.417782	4.097488514	1.773060525		2.736966	0.259044
chr3_7339_mature	16.11854898	2.417782	4.097488514	1.773060525		2.736966	0.259044
chr6_14862_mature	16.11854898	2.417782	4.097488514	1.773060525		2.736966	0.259044
chr1_3559_star	16.11854898	2.417782	4.097488514	1.773060525		2.736966	0.259044
NW_003613374_38740_mature	5.372849659	41.1023	2.671938627	5.395827139		-2.93546	0.259354
bta-miR-1940	4.298279727	31.43117	2.405524014	5.019309189		-2.87036	0.260917
chrx_22054_mature	4.298279727	31.43117	2.405524014	5.019309189		-2.87036	0.260917
NW_003539814_39113_mature	6.447419591	50.77343	2.896740642	5.694139975		-2.97728	0.261842
chr15_33276_mature	26.8642483	4.029637	4.800343329	2.330454351		2.736966	0.262869
NW_003613282_38228_star	26.8642483	4.029637	4.800343329	2.330454351		2.736966	0.262869
chr13_28800_mature	2.149139864	15.31262	1.654957834	4.027916745		-2.83289	0.263381
chr13_27018_mature	2.149139864	15.31262	1.654957834	4.027916745		-2.83289	0.263381
ssc-miR-122	2.149139864	15.31262	1.654957834	4.027916745		-2.83289	0.263381
chr1_2367_mature@@mmu-miR-624	2.149139864	15.31262	1.654957834	4.027916745		-2.83289	0.263381
chr16_34626_star	2.149139864	15.31262	1.654957834	4.027916745		-2.83289	0.263381
hsa-miR-3678-5p	2.149139864	15.31262	1.654957834	4.027916745		-2.83289	0.263381
chr15_31842_star	3.223709795	22.56597	2.078510713	4.558633073		-2.80735	0.264386
chr2_5134_mature	515.7935673	40.29637	9.013444302	5.367943153		3.678072	0.266116
chr9_19965_mature	4.298279727	30.62524	2.405524014	4.983004663		-2.83289	0.266222
bta-miR-3956	1.074569932	8.865202	1.05281229	3.302348583		-3.04439	0.267789
chr10_23786_star	1.074569932	8.865202	1.05281229	3.302348583		-3.04439	0.267789
chr3_7453_mature	1.074569932	8.865202	1.05281229	3.302348583		-3.04439	0.267789
chr13_29126_mature	1.074569932	8.865202	1.05281229	3.302348583		-3.04439	0.267789
chr13_26730_mature	1.074569932	8.865202	1.05281229	3.302348583		-3.04439	0.267789
chr16_34110_mature	1.074569932	8.865202	1.05281229	3.302348583		-3.04439	0.267789
chr14_30596_star	1.074569932	8.865202	1.05281229	3.302348583		-3.04439	0.267789
chr11_24683_mature	1.074569932	8.865202	1.05281229	3.302348583		-3.04439	0.267789
chr16_34657_mature	1.074569932	8.865202	1.05281229	3.302348583		-3.04439	0.267789
chr6_13263_mature	1.074569932	8.865202	1.05281229	3.302348583		-3.04439	0.267789
chr4_9182_star	1.074569932	8.865202	1.05281229	3.302348583		-3.04439	0.267789
chr1_2545_mature	1.074569932	8.865202	1.05281229	3.302348583		-3.04439	0.267789
chrx_21506_star	1.074569932	8.865202	1.05281229	3.302348583		-3.04439	0.267789
chr3_7764_mature	1.074569932	8.865202	1.05281229	3.302348583		-3.04439	0.267789
NW_003540029_39234_mature	1.074569932	8.865202	1.05281229	3.302348583		-3.04439	0.267789
chr10_23799_star	1.074569932	8.865202	1.05281229	3.302348583		-3.04439	0.267789
bta-miR-6524	1.074569932	8.865202	1.05281229	3.302348583		-3.04439	0.267789
chr6_12645_star	1.074569932	8.865202	1.05281229	3.302348583		-3.04439	0.267789
chr10_23275_mature	1.074569932	8.865202	1.05281229	3.302348583		-3.04439	0.267789
bta-miR-2368-3p	1.074569932	8.865202	1.05281229	3.302348583		-3.04439	0.267789
hsa-miR-620	1.074569932	8.865202	1.05281229	3.302348583		-3.04439	0.267789
chr1_1041_star	1.074569932	8.865202	1.05281229	3.302348583		-3.04439	0.267789
chr14_31235_mature@@bta-miR-34	32.23709795	4.835565	5.054722516	2.544872269		2.736966	0.26875
chr7_16635_star	10.74569932	1.611855	3.554060706	1.38507475		2.736966	0.269107
chr15_33543_mature	10.74569932	1.611855	3.554060706	1.38507475		2.736966	0.269107
chr6_12506_star	10.74569932	1.611855	3.554060706	1.38507475		2.736966	0.269107
chr6_14178_mature	10.74569932	1.611855	3.554060706	1.38507475		2.736966	0.269107
chr7_16801_mature	10.74569932	1.611855	3.554060706	1.38507475		2.736966	0.269107
chr13_28475_mature	10.74569932	1.611855	3.554060706	1.38507475		2.736966	0.269107
NW_003539061_38502_star	10.74569932	1.611855	3.554060706	1.38507475		2.736966	0.269107
chr6_14380_mature	10.74569932	1.611855	3.554060706	1.38507475		2.736966	0.269107
chr17_35330_star	10.74569932	1.611855	3.554060706	1.38507475		2.736966	0.269107
chr2_4071_mature	10.74569932	1.611855	3.554060706	1.38507475		2.736966	0.269107
chr10_24294_star	10.74569932	1.611855	3.554060706	1.38507475		2.736966	0.269107
chr6_14250_star	10.74569932	1.611855	3.554060706	1.38507475		2.736966	0.269107
chr4_8920_mature	10.74569932	1.611855	3.554060706	1.38507475		2.736966	0.269107
hsa-miR-6071	10.74569932	1.611855	3.554060706	1.38507475		2.736966	0.269107
chr13_29308_mature	20.4168287	3.22371	4.420672964	2.078510713		2.662965	0.270408
ssc-miR-338	20.4168287	3.22371	4.420672964	2.078510713		2.662965	0.270408
chr8_17430_mature	20.4168287	3.22371	4.420672964	2.078510713		2.662965	0.270408
chr9_20399_mature	20.4168287	3.22371	4.420672964	2.078510713		2.662965	0.270408
NW_003538989_38422_star	20.4168287	3.22371	4.420672964	2.078510713		2.662965	0.270408
NW_003539542_38921_mature	0	4.029637	0	2.330454351		-20	0.270911
NW_003539180_38634_mature	0	4.029637	0	2.330454351		-20	0.270911
chr7_17039_star	0	4.029637	0	2.330454351		-20	0.270911
chr1_1479_star	0	4.029637	0	2.330454351		-20	0.270911
chr2_5588_mature	0	4.029637	0	2.330454351		-20	0.270911
hsa-miR-1290	0	4.029637	0	2.330454351		-20	0.270911
hsa-miR-1267	0	4.029637	0	2.330454351		-20	0.270911
chr6_13425_star	0	4.029637	0	2.330454351		-20	0.270911
chr6_14787_star	0	4.029637	0	2.330454351		-20	0.270911
hsa-miR-548ab	0	4.029637	0	2.330454351		-20	0.270911
chr2_6398_star	0	4.029637	0	2.330454351		-20	0.270911
hsa-miR-3657	0	4.029637	0	2.330454351		-20	0.270911
chr2_4213_mature	0	4.029637	0	2.330454351		-20	0.270911
chr17_35199_star	0	4.029637	0	2.330454351		-20	0.270911
hsa-miR-5092	0	4.029637	0	2.330454351		-20	0.270911
NW_003541072_39884_star	0	4.029637	0	2.330454351		-20	0.270911
chr14_30276_mature	0	4.029637	0	2.330454351		-20	0.270911
chr6_12705_star	0	4.029637	0	2.330454351		-20	0.270911
chr8_17864_mature	0	4.029637	0	2.330454351		-20	0.270911
NW_003538764_38206_star	0	4.029637	0	2.330454351		-20	0.270911
chr15_33692_mature	0	4.029637	0	2.330454351		-20	0.270911
chr4_10620_mature	0	4.029637	0	2.330454351		-20	0.270911
chr4_9687_star	0	4.029637	0	2.330454351		-20	0.270911
chr17_35478_star	0	4.029637	0	2.330454351		-20	0.270911
chr14_30547_star	0	4.029637	0	2.330454351		-20	0.270911
chr3_7249_mature	0	4.029637	0	2.330454351		-20	0.270911
hsa-miR-4653-3p	0	4.029637	0	2.330454351		-20	0.270911
chr16_34780_star	0	4.029637	0	2.330454351		-20	0.270911
bta-miR-2285j	0	4.029637	0	2.330454351		-20	0.270911
chr15_33559_star	0	4.029637	0	2.330454351		-20	0.270911
chr14_30385_star	0	4.029637	0	2.330454351		-20	0.270911
chr11_24358_mature	0	4.029637	0	2.330454351		-20	0.270911
mmu-miR-504-5p	0	4.029637	0	2.330454351		-20	0.270911
chr2_5320_mature	0	4.029637	0	2.330454351		-20	0.270911
chr8_18374_star	0	4.029637	0	2.330454351		-20	0.270911
chr10_24200_star	0	4.029637	0	2.330454351		-20	0.270911
chr2_5810_mature	0	4.029637	0	2.330454351		-20	0.270911
chr7_16325_mature	0	4.029637	0	2.330454351		-20	0.270911
bta-miR-27a-5p	0	4.029637	0	2.330454351		-20	0.270911
NW_003538747_38195_mature	0	4.029637	0	2.330454351		-20	0.270911
mmu-miR-449a-5p	0	4.029637	0	2.330454351		-20	0.270911
chr7_15656_mature	0	4.029637	0	2.330454351		-20	0.270911
chr5_10855_mature	0	4.029637	0	2.330454351		-20	0.270911
chr6_14257_star	0	4.029637	0	2.330454351		-20	0.270911
chr18_36376_mature	0	4.029637	0	2.330454351		-20	0.270911
rno-miR-3557-5p	0	4.029637	0	2.330454351		-20	0.270911
chr9_19852_star	0	4.029637	0	2.330454351		-20	0.270911
chr12_26026_star	0	4.029637	0	2.330454351		-20	0.270911
NW_003539576_38951_star	0	4.029637	0	2.330454351		-20	0.270911
hsa-miR-645	0	4.029637	0	2.330454351		-20	0.270911
hsa-miR-658	0	4.029637	0	2.330454351		-20	0.270911
chr15_33276_star	0	4.029637	0	2.330454351		-20	0.270911
chr3_8153_star	0	4.029637	0	2.330454351		-20	0.270911
mmu-miR-5129-5p	0	4.029637	0	2.330454351		-20	0.270911
bta-miR-2387	0	4.029637	0	2.330454351		-20	0.270911
chr7_16781_mature	0	4.029637	0	2.330454351		-20	0.270911
chr3_8078_star	0	4.029637	0	2.330454351		-20	0.270911
bta-miR-2330-3p	0	4.029637	0	2.330454351		-20	0.270911
chr4_9341_star	0	4.029637	0	2.330454351		-20	0.270911
chr14_30563_mature	0	4.029637	0	2.330454351		-20	0.270911
chr6_14638_mature	0	4.029637	0	2.330454351		-20	0.270911
chr6_12992_star	0	4.029637	0	2.330454351		-20	0.270911
hsa-miR-924	0	4.029637	0	2.330454351		-20	0.270911
hsa-miR-6085	0	4.029637	0	2.330454351		-20	0.270911
mmu-miR-145b	0	4.029637	0	2.330454351		-20	0.270911
chr6_15014_star	3.223709795	21.76004	2.078510713	4.508431259		-2.75489	0.271521
chr7_17161_mature	4.298279727	29.81932	2.405524014	4.94576292		-2.79442	0.271681
chr13_28823_mature	4.298279727	29.81932	2.405524014	4.94576292		-2.79442	0.271681
bta-miR-544b	4.298279727	29.81932	2.405524014	4.94576292		-2.79442	0.271681
chr6_14448_mature	7.521989523	58.8327	3.091190278	5.902862353		-2.96743	0.272377

chr17_35678_star	0	1.611855	0	1.38507475		-20	0.12959
rno-miR-31a-3p	0	1.611855	0	1.38507475		-20	0.12959
chr13_29225_star	0	1.611855	0	1.38507475		-20	0.12959
chr9_20114_star	0	1.611855	0	1.38507475		-20	0.12959
chr14_29971_mature	0	1.611855	0	1.38507475		-20	0.12959
chr2_4221_star	0	1.611855	0	1.38507475		-20	0.12959
chr15_32527_star	0	1.611855	0	1.38507475		-20	0.12959
chr9_19327_star	0	1.611855	0	1.38507475		-20	0.12959
bta-miR-6122-3p	0	1.611855	0	1.38507475		-20	0.12959
hsa-miR-4689	0	1.611855	0	1.38507475		-20	0.12959
rno-miR-23b-5p	0	1.611855	0	1.38507475		-20	0.12959
hsa-miR-516b-5p	0	1.611855	0	1.38507475		-20	0.12959
mmu-miR-24-3p	0	1.611855	0	1.38507475		-20	0.12959
chr15_32017_star	0	1.611855	0	1.38507475		-20	0.12959
chr2_4749_mature	0	1.611855	0	1.38507475		-20	0.12959
chr13_27155_mature	0	1.611855	0	1.38507475		-20	0.12959
chr9_19622_star	0	1.611855	0	1.38507475		-20	0.12959
NW_003539698_39034_star	0	1.611855	0	1.38507475		-20	0.12959
hsa-miR-5580-3p	0	1.611855	0	1.38507475		-20	0.12959
hsa-miR-4666a-5p	0	1.611855	0	1.38507475		-20	0.12959
hsa-miR-5685	0	1.611855	0	1.38507475		-20	0.12959
mmu-miR-669e-5p	0	1.611855	0	1.38507475		-20	0.12959
hsa-miR-5695	0	1.611855	0	1.38507475		-20	0.12959
chr2_5391_mature	0	1.611855	0	1.38507475		-20	0.12959
mmu-miR-28a-3p	0	1.611855	0	1.38507475		-20	0.12959
mmu-miR-669j	0	1.611855	0	1.38507475		-20	0.12959
chr1_1402_star	0	1.611855	0	1.38507475		-20	0.12959
rno-miR-291b	0	1.611855	0	1.38507475		-20	0.12959
chr5_11998_star	0	1.611855	0	1.38507475		-20	0.12959
hsa-miR-4503	0	1.611855	0	1.38507475		-20	0.12959
rno-miR-141-3p	0	1.611855	0	1.38507475		-20	0.12959
mmu-miR-5619-5p	0	1.611855	0	1.38507475		-20	0.12959
chr18_36375_mature	0	1.611855	0	1.38507475		-20	0.12959
chr13_28441_star	0	1.611855	0	1.38507475		-20	0.12959
chrx_22973_star	0	1.611855	0	1.38507475		-20	0.12959
chr11_25373_star	0	1.611855	0	1.38507475		-20	0.12959
chr1_3718_mature	0	1.611855	0	1.38507475		-20	0.12959
chr5_11967_star	0	1.611855	0	1.38507475		-20	0.12959
chr1_3837_star	0	1.611855	0	1.38507475		-20	0.12959
hsa-miR-6077	0	1.611855	0	1.38507475		-20	0.12959
chr1_1628_star	0	1.611855	0	1.38507475		-20	0.12959
chr7_16134_mature	0	1.611855	0	1.38507475		-20	0.12959
hsa-miR-506-3p	0	1.611855	0	1.38507475		-20	0.12959
chrx_22070_star	0	1.611855	0	1.38507475		-20	0.12959
chr6_13505_star	0	1.611855	0	1.38507475		-20	0.12959
chr13_29079_mature	0	1.611855	0	1.38507475		-20	0.12959
chr1_1049_mature	116.053553	12.08891	6.871024911	3.710273245		3.263034	0.132536
chr6_12650_star	0	0.805927	0	0.852739935		-20	0.140178
chr18_36239_star	0	0.805927	0	0.852739935		-20	0.140178
chr1_3261_star	0	0.805927	0	0.852739935		-20	0.140178
chr4_9551_star	0	0.805927	0	0.852739935		-20	0.140178
mmu-miR-339-3p	0	0.805927	0	0.852739935		-20	0.140178
hsa-miR-518a-3p	0	0.805927	0	0.852739935		-20	0.140178
chr2_5435_star	0	0.805927	0	0.852739935		-20	0.140178
mmu-miR-26a-5p	0	0.805927	0	0.852739935		-20	0.140178
chr18_36430_star	0	0.805927	0	0.852739935		-20	0.140178
mmu-miR-698-5p	0	0.805927	0	0.852739935		-20	0.140178
hsa-miR-4801	0	0.805927	0	0.852739935		-20	0.140178
chr13_27738_star	0	0.805927	0	0.852739935		-20	0.140178
hsa-miR-548ar-3p	0	0.805927	0	0.852739935		-20	0.140178
rno-miR-19a-5p	0	0.805927	0	0.852739935		-20	0.140178
chr3_8461_mature	0	0.805927	0	0.852739935		-20	0.140178
chr1_2073_star	0	0.805927	0	0.852739935		-20	0.140178
chr13_27228_star	0	0.805927	0	0.852739935		-20	0.140178
rno-miR-124-5p	0	0.805927	0	0.852739935		-20	0.140178
mmu-miR-466c-5p	0	0.805927	0	0.852739935		-20	0.140178
mmu-miR-1943-3p	0	0.805927	0	0.852739935		-20	0.140178
hsa-miR-130a-5p	0	0.805927	0	0.852739935		-20	0.140178
hsa-miR-4790-3p	0	0.805927	0	0.852739935		-20	0.140178
hsa-miR-4785	0	0.805927	0	0.852739935		-20	0.140178
chr9_20599_star	0	0.805927	0	0.852739935		-20	0.140178
hsa-miR-4775	0	0.805927	0	0.852739935		-20	0.140178
chr10_23315_mature	0	0.805927	0	0.852739935		-20	0.140178
chr5_11544_star	0	0.805927	0	0.852739935		-20	0.140178
rno-miR-130a-5p	0	0.805927	0	0.852739935		-20	0.140178
chr8_17916_star	0	0.805927	0	0.852739935		-20	0.140178
chr1_915_mature	0	0.805927	0	0.852739935		-20	0.140178
chr6_13733_star	0	0.805927	0	0.852739935		-20	0.140178
chr2_4023_star	0	0.805927	0	0.852739935		-20	0.140178
chr3_7274_star	0	0.805927	0	0.852739935		-20	0.140178
rno-miR-551b-3p	0	0.805927	0	0.852739935		-20	0.140178
chr6_14437_star	0	0.805927	0	0.852739935		-20	0.140178
hsa-miR-4742-5p	0	0.805927	0	0.852739935		-20	0.140178
chr15_32720_star	0	0.805927	0	0.852739935		-20	0.140178
hsa-miR-4729	0	0.805927	0	0.852739935		-20	0.140178
chr12_25625_mature	0	0.805927	0	0.852739935		-20	0.140178
hsa-miR-30a-3p	0	0.805927	0	0.852739935		-20	0.140178
hsa-miR-4742-3p	0	0.805927	0	0.852739935		-20	0.140178
hsa-miR-4748	0	0.805927	0	0.852739935		-20	0.140178
chr7_15675_star	0	0.805927	0	0.852739935		-20	0.140178
chr2_4688_star	0	0.805927	0	0.852739935		-20	0.140178
mmu-miR-194-5p	0	0.805927	0	0.852739935		-20	0.140178
hsa-miR-1224-5p	0	0.805927	0	0.852739935		-20	0.140178
rno-miR-484	0	0.805927	0	0.852739935		-20	0.140178
bta-miR-2895	0	0.805927	0	0.852739935		-20	0.140178
bta-miR-2896	0	0.805927	0	0.852739935		-20	0.140178
chr4_10619_mature	0	0.805927	0	0.852739935		-20	0.140178
chr13_29079_star	0	0.805927	0	0.852739935		-20	0.140178
NW_003613237_37814_star	0	0.805927	0	0.852739935		-20	0.140178
chr1_3451_star	0	0.805927	0	0.852739935		-20	0.140178
chr3_7219_star	0	0.805927	0	0.852739935		-20	0.140178
hsa-miR-4520b-3p	0	0.805927	0	0.852739935		-20	0.140178
chr1_3657_mature	0	0.805927	0	0.852739935		-20	0.140178
rno-miR-448-3p	0	0.805927	0	0.852739935		-20	0.140178
mmu-miR-93-3p	0	0.805927	0	0.852739935		-20	0.140178
chr11_24692_star	0	0.805927	0	0.852739935		-20	0.140178
hsa-miR-548ay-5p	0	0.805927	0	0.852739935		-20	0.140178
hsa-miR-3922-3p	0	0.805927	0	0.852739935		-20	0.140178
chrx_22989_star	0	0.805927	0	0.852739935		-20	0.140178
chr4_10081_star	0	0.805927	0	0.852739935		-20	0.140178
hsa-miR-4671-3p	0	0.805927	0	0.852739935		-20	0.140178
chr6_12905_star	0	0.805927	0	0.852739935		-20	0.140178
hsa-miR-4999-3p	0	0.805927	0	0.852739935		-20	0.140178
chr2_5753_star	0	0.805927	0	0.852739935		-20	0.140178
chr16_33881_star	0	0.805927	0	0.852739935		-20	0.140178
rno-miR-423-3p	0	0.805927	0	0.852739935		-20	0.140178
chr5_11725_star	0	0.805927	0	0.852739935		-20	0.140178
chr9_19972_star	0	0.805927	0	0.852739935		-20	0.140178
hsa-miR-3622a-3p	0	0.805927	0	0.852739935		-20	0.140178
chr1_1421_star	0	0.805927	0	0.852739935		-20	0.140178
chr16_34304_star	0	0.805927	0	0.852739935		-20	0.140178
hsa-miR-1231	0	0.805927	0	0.852739935		-20	0.140178
hsa-miR-4764-5p	0	0.805927	0	0.852739935		-20	0.140178
hsa-miR-4732-5p	0	0.805927	0	0.852739935		-20	0.140178
hsa-miR-1203	0	0.805927	0	0.852739935		-20	0.140178
hsa-miR-3136-3p	0	0.805927	0	0.852739935		-20	0.140178
hsa-miR-5589-3p	0	0.805927	0	0.852739935		-20	0.140178
chr17_35548_star	0	0.805927	0	0.852739935		-20	0.140178
hsa-miR-3681-3p	0	0.805927	0	0.852739935		-20	0.140178
chr7_15769_star	0	0.805927	0	0.852739935		-20	0.140178
hsa-miR-4764-3p	0	0.805927	0	0.852739935		-20	0.140178
NW_003613530_39570_star	0	0.805927	0	0.852739935		-20	0.140178
chr15_33376_star	0	0.805927	0	0.852739935		-20	0.140178
chr5_11956_star	0	0.805927	0	0.852739935		-20	0.140178
chr13_28125_star	0	0.805927	0	0.852739935		-20	0.140178
mmu-miR-875-3p	0	0.805927	0	0.852739935		-20	0.140178
chr10_24151_star	0	0.805927	0	0.852739935		-20	0.140178
mmu-miR-465a-3p	0	0.805927	0	0.852739935		-20	0.140178
hsa-miR-1299	0	0.805927	0	0.852739935		-20	0.140178
mmu-miR-669c-5p	0	0.805927	0	0.852739935		-20	0.140178
mmu-miR-367-3p	0	0.805927	0	0.852739935		-20	0.140178
chr5_11375_star	0	0.805927	0	0.852739935		-20	0.140178
hsa-miR-1286	0	0.805927	0	0.852739935		-20	0.140178
chr6_13565_star	0	0.805927	0	0.852739935		-20	0.140178
NW_003539771_39089_star	0	0.805927	0	0.852739935		-20	0.140178
bta-miR-1584-5p	0	0.805927	0	0.852739935		-20	0.140178
mmu-miR-5618-3p	0	0.805927	0	0.852739935		-20	0.140178
mmu-miR-31-3p	0	0.805927	0	0.852739935		-20	0.140178
hsa-miR-1279	0	0.805927	0	0.852739935		-20	0.140178
NW_003539221_38657_star	0	0.805927	0	0.852739935		-20	0.140178
chr6_14454_star	0	0.805927	0	0.852739935		-20	0.140178
hsa-miR-579	0	0.805927	0	0.852739935		-20	0.140178
NW_003539080_38513_star	0	0.805927	0	0.852739935		-20	0.140178
hsa-miR-562	0	0.805927	0	0.852739935		-20	0.140178
hsa-miR-569	0	0.805927	0	0.852739935		-20	0.140178
chr6_14217_star	0	0.805927	0	0.852739935		-20	0.140178
hsa-miR-2682-3p	0	0.805927	0	0.852739935		-20	0.140178
chr15_33121_mature	0	0.805927	0	0.852739935		-20	0.140178
hsa-miR-518d-3p	0	0.805927	0	0.852739935		-20	0.140178
chr13_28506_star	0	0.805927	0	0.852739935		-20	0.140178
chr11_25205_star	0	0.805927	0	0.852739935		-20	0.140178
chr10_23275_star	0	0.805927	0	0.852739935		-20	0.140178
mmu-miR-1898	0	0.805927	0	0.852739935		-20	0.140178
bta-miR-196a	0	0.805927	0	0.852739935		-20	0.140178
NW_003538870_38317_star	0	0.805927	0	0.852739935		-20	0.140178
mmu-miR-1a-1-5p	0	0.805927	0	0.852739935		-20	0.140178
hsa-miR-507	0	0.805927	0	0.852739935		-20	0.140178
hsa-miR-1247-3p	0	0.805927	0	0.852739935		-20	0.140178
mmu-miR-878-5p	0	0.805927	0	0.852739935		-20	0.140178
bta-miR-2284t-5p	0	0.805927	0	0.852739935		-20	0.140178
hsa-miR-3910	0	0.805927	0	0.852739935		-20	0.140178
hsa-miR-3909	0	0.805927	0	0.852739935		-20	0.140178
chr14_30061_star	0	0.805927	0	0.852739935		-20	0.140178
hsa-miR-3939	0	0.805927	0	0.852739935		-20	0.140178
chr6_13705_star	0	0.805927	0	0.852739935		-20	0.140178
hsa-miR-3923	0	0.805927	0	0.852739935		-20	0.140178
hsa-miR-3924	0	0.805927	0	0.852739935		-20	0.140178
chr9_19423_mature	0	0.805927	0	0.852739935		-20	0.140178
chr7_16183_star	0	0.805927	0	0.852739935		-20	0.140178
chr18_36542_star	0	0.805927	0	0.852739935		-20	0.140178
chr12_25591_star	0	0.805927	0	0.852739935		-20	0.140178
rno-miR-1-3p	0	0.805927	0	0.852739935		-20	0.140178
mmu-miR-1950	0	0.805927	0	0.852739935		-20	0.140178
mmu-miR-1956	0	0.805927	0	0.852739935		-20	0.140178
chr15_31808_star	0	0.805927	0	0.852739935		-20	0.140178
rno-miR-678	0	0.805927	0	0.852739935		-20	0.140178
chr10_24235_mature	0	0.805927	0	0.852739935		-20	0.140178
chr16_34240_mature	0	0.805927	0	0.852739935		-20	0.140178
chr4_9650_mature	0	0.805927	0	0.852739935		-20	0.140178
chr13_29269_star	0	0.805927	0	0.852739935		-20	0.140178
chr13_28836_star	0	0.805927	0	0.852739935		-20	0.140178
hsa-miR-4716-3p	0	0.805927	0	0.852739935		-20	0.140178
chr5_11717_mature	0	0.805927	0	0.852739935		-20	0.140178
chr4_10120_mature	0	0.805927	0	0.852739935		-20	0.140178
chr8_17643_star	0	0.805927	0	0.852739935		-20	0.140178
ssc-miR-4334-5p	0	0.805927	0	0.852739935		-20	0.140178
chrx_22329_mature	0	0.805927	0	0.852739935		-20	0.140178
chr18_36069_star	0	0.805927	0	0.852739935		-20	0.140178
mmu-miR-374b-3p	0	0.805927	0	0.852739935		-20	0.140178
mmu-miR-327	0	0.805927	0	0.852739935		-20	0.140178
chr3_8243_star	0	0.805927	0	0.852739935		-20	0.140178
chr4_8825_star	0	0.805927	0	0.852739935		-20	0.140178
chr3_8236_star	0	0.805927	0	0.852739935		-20	0.140178
chr10_23976_star	0	0.805927	0	0.852739935		-20	0.140178
mmu-miR-343	0	0.805927	0	0.852739935		-20	0.140178
chr10_24282_star	0	0.805927	0	0.852739935		-20	0.140178
hsa-miR-155-3p	0	0.805927	0	0.852739935		-20	0.140178
chr2_5207_star	0	0.805927	0	0.852739935		-20	0.140178
hsa-miR-1537	0	0.805927	0	0.852739935		-20	0.140178
rno-miR-539-3p	0	0.805927	0	0.852739935		-20	0.140178
NW_003537296_36822_star	0	0.805927	0	0.852739935		-20	0.140178
hsa-miR-548ag	0	0.805927	0	0.852739935		-20	0.140178
rno-miR-539-5p	0	0.805927	0	0.852739935		-20	0.140178
hsa-miR-6499-5p	0	0.805927	0	0.852739935		-20	0.140178
rno-miR-190a-3p	0	0.805927	0	0.852739935		-20	0.140178
hsa-miR-4738-3p	0	0.805927	0	0.852739935		-20	0.140178
chr12_26183_star	0	0.805927	0	0.852739935		-20	0.140178
hsa-miR-191-3p	0	0.805927	0	0.852739935		-20	0.140178
chrx_22905_star	0	0.805927	0	0.852739935		-20	0.140178
chr17_35590_star	0	0.805927	0	0.852739935		-20	0.140178
chr17_34991_star	0	0.805927	0	0.852739935		-20	0.140178
chr6_14448_star	0	0.805927	0	0.852739935		-20	0.140178
chr10_23892_star	0	0.805927	0	0.852739935		-20	0.140178
NW_003613326_38565_mature	0	0.805927	0	0.852739935		-20	0.140178
chr8_19043_star	0	0.805927	0	0.852739935		-20	0.140178
hsa-miR-147a	0	0.805927	0	0.852739935		-20	0.140178
NW_003541084_39898_star	0	0.805927	0	0.852739935		-20	0.140178
hsa-miR-1471	0	0.805927	0	0.852739935		-20	0.140178
chr5_11713_star	0	0.805927	0	0.852739935		-20	0.140178
chr5_12345_star	0	0.805927	0	0.852739935		-20	0.140178
chr13_27641_star	0	0.805927	0	0.852739935		-20	0.140178
chr7_16155_star	0	0.805927	0	0.852739935		-20	0.140178
hsa-miR-384	0	0.805927	0	0.852739935		-20	0.140178
mmu-miR-5107-5p	0	0.805927	0	0.852739935		-20	0.140178
chr11_25193_star	0	0.805927	0	0.852739935		-20	0.140178
chr5_11847_star	0	0.805927	0	0.852739935		-20	0.140178
chr14_29799_mature	0	0.805927	0	0.852739935		-20	0.140178
hsa-miR-5579-5p	0	0.805927	0	0.852739935		-20	0.140178
chr11_25358_star	0	0.805927	0	0.852739935		-20	0.140178
rno-miR-494-5p	0	0.805927	0	0.852739935		-20	0.140178
chr9_19660_star	0	0.805927	0	0.852739935		-20	0.140178
hsa-miR-519d	0	0.805927	0	0.852739935		-20	0.140178
rno-miR-30d-5p	0	0.805927	0	0.852739935		-20	0.140178
hsa-miR-5191	0	0.805927	0	0.852739935		-20	0.140178
chr9_20876_star	0	0.805927	0	0.852739935		-20	0.140178
hsa-miR-5190	0	0.805927	0	0.852739935		-20	0.140178
hsa-miR-5193	0	0.805927	0	0.852739935		-20	0.140178
chr14_29940_star	0	0.805927	0	0.852739935		-20	0.140178
chr12_25415_star	0	0.805927	0	0.852739935		-20	0.140178
bta-miR-130b	0	0.805927	0	0.852739935		-20	0.140178
rno-miR-3549	0	0.805927	0	0.852739935		-20	0.140178
mmu-miR-6244	0	0.805927	0	0.852739935		-20	0.140178
rno-miR-3566	0	0.805927	0	0.852739935		-20	0.140178
chr2_5745_star	0	0.805927	0	0.852739935		-20	0.140178
rno-miR-3571	0	0.805927	0	0.852739935		-20	0.140178
chr5_11928_star	0	0.805927	0	0.852739935		-20	0.140178
chr13_28489_star	0	0.805927	0	0.852739935		-20	0.140178
rno-miR-3595	0	0.805927	0	0.852739935		-20	0.140178
hsa-miR-660-3p	0	0.805927	0	0.852739935		-20	0.140178
chr1_3253_star	0	0.805927	0	0.852739935		-20	0.140178
hsa-miR-4726-3p	0	0.805927	0	0.852739935		-20	0.140178
mmu-miR-5128	0	0.805927	0	0.852739935		-20	0.140178
mmu-miR-5127	0	0.805927	0	0.852739935		-20	0.140178
chr12_26093_star	0	0.805927	0	0.852739935		-20	0.140178
hsa-miR-570-3p	0	0.805927	0	0.852739935		-20	0.140178

chr3_7065_star	3.22371	0	2.078510713	0		20	0.523571
chr7_16018_star	3.22371	0	2.078510713	0		20	0.523571
hsa-miR-556-5p	3.22371	0	2.078510713	0		20	0.523571
chr12_25470_mature	3.22371	0	2.078510713	0		20	0.523571
chrx_23200_star	3.22371	0	2.078510713	0		20	0.523571
hsa-miR-6131	3.22371	0	2.078510713	0		20	0.523571
chr2_4005_star	3.22371	0	2.078510713	0		20	0.523571
mmu-miR-196a-1-3p	3.22371	0	2.078510713	0		20	0.523571
chrx_22854_star	3.22371	0	2.078510713	0		20	0.523571
rno-miR-3559-3p	3.22371	0	2.078510713	0		20	0.523571
hsa-miR-5011-3p	3.22371	0	2.078510713	0		20	0.523571
hsa-miR-5011-5p	3.22371	0	2.078510713	0		20	0.523571
hsa-miR-3605-5p	3.22371	0	2.078510713	0		20	0.523571
chr4_10011_mature	3.22371	0	2.078510713	0		20	0.523571
chr11_24777_mature	3.22371	0	2.078510713	0		20	0.523571
bta-miR-2355-5p	3.22371	0	2.078510713	0		20	0.523571
chrx_23204_star	3.22371	0	2.078510713	0		20	0.523571
hsa-miR-132-3p	3.22371	0	2.078510713	0		20	0.523571
chr1_3523_star	3.22371	0	2.078510713	0		20	0.523571
hsa-miR-3145-3p	3.22371	0	2.078510713	0		20	0.523571
chr4_9150_mature@@rno-miR-298	3.22371	0	2.078510713	0		20	0.523571
chr15_32062_star	3.22371	0	2.078510713	0		20	0.523571
hsa-miR-3161	3.22371	0	2.078510713	0		20	0.523571
NW_003613342_38628_star	3.22371	0	2.078510713	0		20	0.523571
hsa-miR-4691-5p	3.22371	0	2.078510713	0		20	0.523571
chr6_12911_star	3.22371	0	2.078510713	0		20	0.523571
bta-miR-6536	3.22371	0	2.078510713	0		20	0.523571
hsa-miR-4267	3.22371	0	2.078510713	0		20	0.523571
hsa-miR-4276	3.22371	0	2.078510713	0		20	0.523571
chr5_11634_star	3.22371	0	2.078510713	0		20	0.523571
hsa-miR-4299	3.22371	0	2.078510713	0		20	0.523571
rno-miR-880-5p	3.22371	0	2.078510713	0		20	0.523571
chr1_3877_mature	3.22371	0	2.078510713	0		20	0.523571
chr15_33041_mature	3.22371	0	2.078510713	0		20	0.523571
chrx_22554_star	3.22371	0	2.078510713	0		20	0.523571
bta-miR-2426	3.22371	0	2.078510713	0		20	0.523571
chr9_21094_star	3.22371	0	2.078510713	0		20	0.523571
chr10_23487_star	3.22371	0	2.078510713	0		20	0.523571
chr13_27061_mature	3.22371	0	2.078510713	0		20	0.523571
chr6_14899_star	3.22371	0	2.078510713	0		20	0.523571
mmu-miR-130c	3.22371	0	2.078510713	0		20	0.523571
chr3_7766_star	3.22371	0	2.078510713	0		20	0.523571
chr3_8555_star	3.22371	0	2.078510713	0		20	0.523571
chr7_16134_star	3.22371	0	2.078510713	0		20	0.523571
chr17_35521_star	3.22371	0	2.078510713	0		20	0.523571
bta-miR-2307	3.22371	0	2.078510713	0		20	0.523571
bta-miR-2341	3.22371	0	2.078510713	0		20	0.523571
chr2_4097_mature	3.22371	0	2.078510713	0		20	0.523571
bta-miR-2375	3.22371	0	2.078510713	0		20	0.523571
chr6_14888_star	3.22371	0	2.078510713	0		20	0.523571
chr3_8361_star	3.22371	0	2.078510713	0		20	0.523571
chr12_26001_star	3.22371	0	2.078510713	0		20	0.523571
mmu-miR-5621-5p	3.22371	0	2.078510713	0		20	0.523571
mmu-miR-5621-3p	3.22371	0	2.078510713	0		20	0.523571
mmu-miR-344d-3p	3.22371	0	2.078510713	0		20	0.523571
hsa-miR-4635	3.22371	0	2.078510713	0		20	0.523571
chr10_24245_star	3.22371	0	2.078510713	0		20	0.523571
hsa-miR-1268a	3.22371	0	2.078510713	0		20	0.523571
chrx_21544_star	3.22371	0	2.078510713	0		20	0.523571
rno-miR-540-3p	3.22371	0	2.078510713	0		20	0.523571
rno-miR-125a-5p	3.22371	0	2.078510713	0		20	0.523571
mmu-miR-719	3.22371	0	2.078510713	0		20	0.523571
chr9_20053_star	3.22371	0	2.078510713	0		20	0.523571
NW_003539454_38868_star	3.22371	0	2.078510713	0		20	0.523571
chr13_28073_star	3.22371	0	2.078510713	0		20	0.523571
chr14_29649_star	3.22371	0	2.078510713	0		20	0.523571
chr15_33692_star	3.22371	0	2.078510713	0		20	0.523571
chr1_3436_star	3.22371	0	2.078510713	0		20	0.523571
NW_003538598_38024_star	3.22371	0	2.078510713	0		20	0.523571
mmu-miR-1198-5p	3.22371	0	2.078510713	0		20	0.523571
hsa-miR-3155a	3.22371	0	2.078510713	0		20	0.523571
chr7_17188_star	3.22371	0	2.078510713	0		20	0.523571
hsa-miR-302b-3p	3.22371	0	2.078510713	0		20	0.523571
hsa-miR-6078	3.22371	0	2.078510713	0		20	0.523571
chr5_11699_mature	3.22371	0	2.078510713	0		20	0.523571
chr4_9284_mature	12.89484	2.417782	3.796477232	1.773060525		2.415037	0.523612
chr18_36372_star	12.89484	2.417782	3.796477232	1.773060525		2.415037	0.523612
chr11_24909_mature	12.89484	2.417782	3.796477232	1.773060525		2.415037	0.523612
chr2_4221_mature	12.89484	2.417782	3.796477232	1.773060525		2.415037	0.523612
chr3_7573_star	12.89484	2.417782	3.796477232	1.773060525		2.415037	0.523612
ssc-miR-493-3p	6.984705	42.71415	2.997239027	5.4500286		-2.61244	0.52382
chr5_11374_mature	22.56597	4.029637	4.558633073	2.330454351		2.485427	0.524529
chr1_3091_mature	22.56597	4.029637	4.558633073	2.330454351		2.485427	0.524529
chr15_32650_mature	4.835565	26.59561	2.544872269	4.786366653		-2.45943	0.524716
chr4_9513_mature	4.835565	26.59561	2.544872269	4.786366653		-2.45943	0.524716
ssc-miR-374b-3p	89.72659	11.28298	6.503453519	3.618589217		2.991387	0.525361
chr5_11410_mature	47.28108	597.1922	5.593385953	9.224465384		-3.65886	0.526621
chr5_11418_mature	55.34035	8.059274	5.816096659	3.179395517		2.77961	0.526786
chr11_25073_mature	17.19312	3.22371	4.185320985	2.078510713		2.415037	0.527413
chr16_34089_mature	8.596559	55.60899	3.262517265	5.82295938		-2.69349	0.527829
chr2_4896_mature	1.611855	8.059274	1.38507475	3.179395517		-2.32193	0.528322
chr4_9452_star	1.611855	8.059274	1.38507475	3.179395517		-2.32193	0.528322
chr16_34510_mature	1.611855	8.059274	1.38507475	3.179395517		-2.32193	0.528322
chr3_6809_star	1.611855	8.059274	1.38507475	3.179395517		-2.32193	0.528322
chr14_31236_mature@@bta-miR-3	1.611855	8.059274	1.38507475	3.179395517		-2.32193	0.528322
bta-miR-2284j	1.611855	8.059274	1.38507475	3.179395517		-2.32193	0.528322
mmu-miR-3081-5p	1.611855	8.059274	1.38507475	3.179395517		-2.32193	0.528322
chr3_7963_mature	1.611855	8.059274	1.38507475	3.179395517		-2.32193	0.528322
chr14_31514_mature@@bta-miR-1	1.611855	8.059274	1.38507475	3.179395517		-2.32193	0.528322
chr16_33794_star	1.611855	8.059274	1.38507475	3.179395517		-2.32193	0.528322
chr10_23893_mature	3.760995	19.34226	2.251263041	4.346407978		-2.36257	0.528481
chr7_17120_mature	5.910135	33.84895	2.788713818	5.123043401		-2.51785	0.528731
chr8_18873_mature	6.44742	37.87859	2.896740642	5.280903997		-2.55459	0.528827
chr1_283_mature	33.84895	5.641492	5.123043401	2.731507408		2.584963	0.529045
rno-miR-23a-3p	0.537285	4.029637	0.620384622	2.330454351		-2.90689	0.529092
chr8_17840_star	0.537285	4.029637	0.620384622	2.330454351		-2.90689	0.529092
chr9_19114_mature	0.537285	4.029637	0.620384622	2.330454351		-2.90689	0.529092
chr6_12854_mature	0.537285	4.029637	0.620384622	2.330454351		-2.90689	0.529092
ssc-miR-296-5p	0.537285	4.029637	0.620384622	2.330454351		-2.90689	0.529092
chr2_4213_mature	0.537285	4.029637	0.620384622	2.330454351		-2.90689	0.529092
hsa-miR-1181	0.537285	4.029637	0.620384622	2.330454351		-2.90689	0.529092
hsa-miR-4653-3p	0.537285	4.029637	0.620384622	2.330454351		-2.90689	0.529092
chr17_34868_star	0.537285	4.029637	0.620384622	2.330454351		-2.90689	0.529092
mmu-miR-138-1-3p	0.537285	4.029637	0.620384622	2.330454351		-2.90689	0.529092
mmu-miR-504-5p	0.537285	4.029637	0.620384622	2.330454351		-2.90689	0.529092
bta-miR-27a-5p	0.537285	4.029637	0.620384622	2.330454351		-2.90689	0.529092
chr7_16206_mature	0.537285	4.029637	0.620384622	2.330454351		-2.90689	0.529092
chr4_9894_mature	0.537285	4.029637	0.620384622	2.330454351		-2.90689	0.529092
chr6_14390_mature	0.537285	4.029637	0.620384622	2.330454351		-2.90689	0.529092
hsa-miR-645	0.537285	4.029637	0.620384622	2.330454351		-2.90689	0.529092
NW_003538396_37820_mature	0.537285	4.029637	0.620384622	2.330454351		-2.90689	0.529092
bta-miR-2330-3p	0.537285	4.029637	0.620384622	2.330454351		-2.90689	0.529092
NW_003613297_38335_star	0.537285	4.029637	0.620384622	2.330454351		-2.90689	0.529092
chr8_18856_star	0.537285	4.029637	0.620384622	2.330454351		-2.90689	0.529092
chr11_25109_mature	0.537285	4.029637	0.620384622	2.330454351		-2.90689	0.529092
chrx_21552_star	0.537285	4.029637	0.620384622	2.330454351		-2.90689	0.529092
hsa-miR-6085	0.537285	4.029637	0.620384622	2.330454351		-2.90689	0.529092
chr5_11650_mature	10.7457	74.95125	3.554060706	6.247001856		-2.80219	0.529146
chr15_33139_mature	2.14914	10.47706	1.654957834	3.520680821		-2.2854	0.529187
NW_003540275_39376_mature	2.14914	10.47706	1.654957834	3.520680821		-2.2854	0.529187
NW_003613395_38900_mature	3.22371	16.11855	2.078510713	4.097488514		-2.32193	0.529403
chr6_12646_mature	3.22371	16.11855	2.078510713	4.097488514		-2.32193	0.529403
chr10_23348_mature	3.22371	16.11855	2.078510713	4.097488514		-2.32193	0.529403
chr3_6786_mature	40.29637	6.44742	5.367943153	2.896740642		2.643856	0.529851
chr13_28506_mature	5.37285	29.81932	2.671938627	4.94576292		-2.47249	0.530189
chr15_31842_star	4.29828	22.56597	2.405524014	4.558633073		-2.39232	0.530299
chr9_19904_mature	25.78968	251.4494	4.743605355	7.979850233		-3.2854	0.531797
chr8_17498_mature	8.596559	1.611855	3.262517265	1.38507475		2.415037	0.531889
chrx_21526_mature	8.596559	1.611855	3.262517265	1.38507475		2.415037	0.531889
chr8_17778_mature	8.596559	1.611855	3.262517265	1.38507475		2.415037	0.531889
chr9_20301_mature	8.596559	1.611855	3.262517265	1.38507475		2.415037	0.531889
chr11_25258_star	8.596559	1.611855	3.262517265	1.38507475		2.415037	0.531889
chr7_16088_star	8.596559	1.611855	3.262517265	1.38507475		2.415037	0.531889
chr1_3923_mature	8.596559	1.611855	3.262517265	1.38507475		2.415037	0.531889
chr7_16538_mature	7.52199	45.93786	3.091190278	5.552680304		-2.6105	0.532091
chr14_30470_mature	7.52199	45.93786	3.091190278	5.552680304		-2.6105	0.532091
chr1_877_mature	54.26578	8.059274	5.788314591	3.179395517		2.751321	0.532112
chr2_5682_mature	106.3824	12.89484	6.746614057	3.796477232		3.044394	0.532473
chr9_19852_mature	12.35755	2.417782	3.739583968	1.773060525		2.353637	0.53281
chr6_14862_mature	12.35755	2.417782	3.739583968	1.773060525		2.353637	0.53281
chr14_30089_star	12.35755	2.417782	3.739583968	1.773060525		2.353637	0.53281
chr2_6200_mature	12.35755	2.417782	3.739583968	1.773060525		2.353637	0.53281
chr1_1974_mature	4.835565	25.78968	2.544872269	4.743605355		-2.41504	0.533823
chr17_34881_star	16.65583	3.22371	4.142073061	2.078510713		2.369234	0.534564
chr10_23974_mature	16.65583	3.22371	4.142073061	2.078510713		2.369234	0.534564
chr4_10566_mature	2.686425	12.89484	1.882222339	3.796477232		-2.26303	0.534846
chr3_7417_star	2.686425	12.89484	1.882222339	3.796477232		-2.26303	0.534846
hsa-miR-5680	2.686425	12.89484	1.882222339	3.796477232		-2.26303	0.534846
ssc-miR-126-3p	17.7304	147.4847	4.227310103	7.214170697		-3.05627	0.535135
mmu-miR-194-5p	5.37285	0.805927	2.671938627	0.852739935		2.736966	0.535683
hsa-miR-3922-5p	5.37285	0.805927	2.671938627	0.852739935		2.736966	0.535683
chr2_5753_star	5.37285	0.805927	2.671938627	0.852739935		2.736966	0.535683
chr15_32852_star	5.37285	0.805927	2.671938627	0.852739935		2.736966	0.535683
chr4_9495_star	5.37285	0.805927	2.671938627	0.852739935		2.736966	0.535683
chr1_1242_star	5.37285	0.805927	2.671938627	0.852739935		2.736966	0.535683
mmu-miR-878-5p	5.37285	0.805927	2.671938627	0.852739935		2.736966	0.535683
mmu-miR-1935	5.37285	0.805927	2.671938627	0.852739935		2.736966	0.535683
chr14_29634_mature	5.37285	0.805927	2.671938627	0.852739935		2.736966	0.535683
chr6_12747_mature	5.37285	0.805927	2.671938627	0.852739935		2.736966	0.535683
chr17_34991_star	5.37285	0.805927	2.671938627	0.852739935		2.736966	0.535683
chr16_34104_star	5.37285	0.805927	2.671938627	0.852739935		2.736966	0.535683
chr11_25144_star	5.37285	0.805927	2.671938627	0.852739935		2.736966	0.535683
mmu-miR-676-5p	5.37285	0.805927	2.671938627	0.852739935		2.736966	0.535683
chr7_15837_star	5.37285	0.805927	2.671938627	0.852739935		2.736966	0.535683
chr15_32249_star	5.37285	0.805927	2.671938627	0.852739935		2.736966	0.535683
rno-miR-3473	5.37285	0.805927	2.671938627	0.852739935		2.736966	0.535683
mmu-miR-6414	5.37285	0.805927	2.671938627	0.852739935		2.736966	0.535683
chr16_34261_star	5.37285	0.805927	2.671938627	0.852739935		2.736966	0.535683
mmu-miR-3092-5p	5.37285	0.805927	2.671938627	0.852739935		2.736966	0.535683
chr2_5822_star	5.37285	0.805927	2.671938627	0.852739935		2.736966	0.535683
chr4_9997_star	5.37285	0.805927	2.671938627	0.852739935		2.736966	0.535683
chr18_36180_star	5.37285	0.805927	2.671938627	0.852739935		2.736966	0.535683
chr14_31415_star	5.37285	0.805927	2.671938627	0.852739935		2.736966	0.535683
mmu-miR-664-5p	5.37285	0.805927	2.671938627	0.852739935		2.736966	0.535683
chr2_6442_star	5.37285	0.805927	2.671938627	0.852739935		2.736966	0.535683
chr5_10819_mature	5.37285	0.805927	2.671938627	0.852739935		2.736966	0.535683
chr9_20751_mature	5.37285	0.805927	2.671938627	0.852739935		2.736966	0.535683
bta-miR-2456	5.37285	0.805927	2.671938627	0.852739935		2.736966	0.535683
chr2_4241_star	5.37285	0.805927	2.671938627	0.852739935		2.736966	0.535683
chr11_24341_mature	5.37285	0.805927	2.671938627	0.852739935		2.736966	0.535683
hsa-miR-3156-5p	5.37285	0.805927	2.671938627	0.852739935		2.736966	0.535683
bta-miR-2315	5.37285	0.805927	2.671938627	0.852739935		2.736966	0.535683
NW_003538125_37558_mature	5.37285	0.805927	2.671938627	0.852739935		2.736966	0.535683
chr1_3851_star	5.37285	0.805927	2.671938627	0.852739935		2.736966	0.535683
chr14_30751_mature	5.37285	0.805927	2.671938627	0.852739935		2.736966	0.535683
chr3_7100_mature	5.37285	0.805927	2.671938627	0.852739935		2.736966	0.535683
chr14_31039_mature	39.2218	6.44742	5.329905831	2.896740642		2.604862	0.536827
chr3_8118_star	20.41683	178.9159	4.420672964	7.491178829		-3.13145	0.536858
chr8_18081_star	0	2.417782	0	1.773060525		-20	0.536875
chr7_16206_star	0	2.417782	0	1.773060525		-20	0.536875
NW_003539103_38526_star	0	2.417782	0	1.773060525		-20	0.536875
chrx_21663_star	0	2.417782	0	1.773060525		-20	0.536875
hsa-miR-548au-3p	0	2.417782	0	1.773060525		-20	0.536875
chrx_22054_star	0	2.417782	0	1.773060525		-20	0.536875
chr2_6582_mature	0	2.417782	0	1.773060525		-20	0.536875
chr1_1185_mature	0	2.417782	0	1.773060525		-20	0.536875
hsa-miR-3127-5p	0	2.417782	0	1.773060525		-20	0.536875
hsa-miR-4735-3p	0	2.417782	0	1.773060525		-20	0.536875
chr3_7968_mature	0	2.417782	0	1.773060525		-20	0.536875
rno-miR-3553	0	2.417782	0	1.773060525		-20	0.536875
chr5_11653_star	0	2.417782	0	1.773060525		-20	0.536875
hsa-miR-664a-3p	0	2.417782	0	1.773060525		-20	0.536875
NW_003613518_39516_star	0	2.417782	0	1.773060525		-20	0.536875
chr18_36565_star	0	2.417782	0	1.773060525		-20	0.536875
chr7_15363_star	0	2.417782	0	1.773060525		-20	0.536875
rno-miR-433-5p	0	2.417782	0	1.773060525		-20	0.536875
chr4_9640_mature	0	2.417782	0	1.773060525		-20	0.536875
mmu-miR-6345	0	2.417782	0	1.773060525		-20	0.536875
chr16_34464_star	0	2.417782	0	1.773060525		-20	0.536875
bta-miR-654	0	2.417782	0	1.773060525		-20	0.536875
hsa-miR-4676-3p	0	2.417782	0	1.773060525		-20	0.536875
mmu-miR-3969	0	2.417782	0	1.773060525		-20	0.536875
chr7_16140_mature	0	2.417782	0	1.773060525		-20	0.536875
hsa-miR-548y	0	2.417782	0	1.773060525		-20	0.536875
chr15_32248_star	0	2.417782	0	1.773060525		-20	0.536875
chr1_826_star	0	2.417782	0	1.773060525		-20	0.536875
NW_003613233_37802_star	0	2.417782	0	1.773060525		-20	0.536875
chr13_28222_star	0	2.417782	0	1.773060525		-20	0.536875
bta-miR-320b	0	2.417782	0	1.773060525		-20	0.536875
chr15_31881_star	0	2.417782	0	1.773060525		-20	0.536875
hsa-miR-4472	0	2.417782	0	1.773060525		-20	0.536875
NW_003613025_36806_mature	0	2.417782	0	1.773060525		-20	0.536875
bta-miR-2481	0	2.417782	0	1.773060525		-20	0.536875
bta-miR-2390	0	2.417782	0	1.773060525		-20	0.536875
bta-miR-2486-3p	0	2.417782	0	1.773060525		-20	0.536875
chr11_24871_star	0	2.417782	0	1.773060525		-20	0.536875
chr15_31887_star	0	2.417782	0	1.773060525		-20	0.536875
chr4_8669_star	0	2.417782	0	1.773060525		-20	0.536875
mmu-miR-5619-3p	0	2.417782	0	1.773060525		-20	0.536875
hsa-miR-4789-5p	0	2.417782	0	1.773060525		-20	0.536875
NW_003539812_39109_mature	26.86425	4.835565	4.800343329	2.544872269		2.473931	0.5369
chr14_30596_mature	32.77438	5.641492	5.077857506	2.731507408		2.53842	0.537148
chr2_5406_mature	32.77438	5.641492	5.077857506	2.731507408		2.53842	0.537148
chr14_31575_star	1.07457	5.641492	1.05281229	2.731507408		-2.39232	0.538042
chr5_10971_mature	1.07457	5.641492	1.05281229	2.731507408		-2.39232	0.538042
NW_003613290_38304_star	1.07457	5.641492	1.05281229	2.731507408		-2.39232	0.538042
chr13_27615_mature	1.07457	5.641492	1.05281229	2.731507408		-2.39232	0.538042
chr7_16945_mature	1.07457	5.641492	1.05281229	2.731507408		-2.39232	0.538042
hsa-miR-320c	1.07457	5.641492	1.05281229	2.731507408		-2.39232	0.538042
chr15_32721_star	1.07457	5.641492	1.05281229	2.731507408		-2.39232	0.538042
chrx_22145_star	1.07457	5.641492	1.05281229	2.731507408		-2.39232	0.538042
chr16_33909_star	1.07457	5.641492	1.05281229	2.731507408		-2.39232	0.538042
hsa-miR-891b	1.07457	5.641492	1.05281229	2.731507408		-2.39232	0.538042
chr11_24683_star	1.07457	5.641492	1.05281229	2.731507408		-2.39232	0.538042
chr5_12329_mature	1.07457	5.641492	1.05281229	2.731507408		-2.39232	0.538042
chr3_8646_mature	5.37285	29.01339	2.671938627	4.907534286		-2.43296	0.538424
chr16_33920_mature	45.66922	7.253347	5.544399515	3.044979305		2.654503	0.539085
chr1_246_star	45.66922	7.253347	5.544399515	3.044979305		2.654503	0.539085
chr16_34762_mature	28.4761	283.6865	4.881473904	8.153230078		-3.31647	0.539752
bta-miR-6120-3p	3.760995	18.53633	2.251263041	4.288087668		-2.30117	0.540109
ssc-miR-450c-3p	3.760995	18.53633	2.251263041	4.288087668		-2.30117	0.540109
chr18_36565_mature	4.29828	21.76004	2.405524014	4.508431259		-2.33985	0.540561
chrx_22063_star	4.29828	21.76004	2.405524014	4.508431259		-2.33985	0.540561
chr15_32322_mature	26.32696	4.835565	4.772253246	2.544872269		2.444785	0.541742
chr1_1938_mature@@rno-miR-296	16.11855	3.22371	4.097488514	2.078510713		2.321928	0.541802

chr6_13355_star	6.447419591	48.35565	2.896740642	5.625143252		-2.90689	0.272444	bta-miR-133a	0	0.805927	0	0.852739935		-20	0.140178	chr13_27847_mature	16.11855	3.22371	4.097488514	2.078510713		2.321928	0.541802
chr1_3231_mature	2.149139864	14.50669	1.654957834	3.954819241		-2.75489	0.273439	hsa-miR-3690	0	0.805927	0	0.852739935		-20	0.140178	mmu-miR-5620-3p	11.82027	2.417782	3.680354657	1.773060525		2.289507	0.542149
chr1_397_mature	2.149139864	14.50669	1.654957834	3.954819241		-2.75489	0.273439	bta-miR-133b	0	0.805927	0	0.852739935		-20	0.140178	chr4_9294_star	11.82027	2.417782	3.680354657	1.773060525		2.289507	0.542149
chr3_8144_star	2.149139864	14.50669	1.654957834	3.954819241		-2.75489	0.273439	chr6_13529_mature	0	0.805927	0	0.852739935		-20	0.140178	chr15_32980_mature	11.82027	2.417782	3.680354657	1.773060525		2.289507	0.542149
chr4_9530_mature	9.671129386	81.39867	3.415640968	6.364549187		-3.07325	0.273467	chr9_20117_star	0	0.805927	0	0.852739935		-20	0.140178	chr4_9551_mature	11.82027	2.417782	3.680354657	1.773060525		2.289507	0.542149
chr2_6585_star	15.04397905	2.417782	4.003960082	1.773060525		2.63743	0.273798	hsa-miR-3685	0	0.805927	0	0.852739935		-20	0.140178	chr3_6716_mature	11.82027	2.417782	3.680354657	1.773060525		2.289507	0.542149
chr18_36365_star	15.04397905	2.417782	4.003960082	1.773060525		2.63743	0.273798	hsa-miR-3686	0	0.805927	0	0.852739935		-20	0.140178	chr16_33919_mature	45.13194	7.253347	5.527693969	3.044979305		2.63743	0.542172
chr5_10975_star	90.26387427	11.28298	6.511971994	3.618589217		3	0.274773	hsa-miR-4638-3p	0	0.805927	0	0.852739935		-20	0.140178	chr12_25421_mature	6.984705	40.29637	2.997239027	5.367943153		-2.52838	0.542622
ssc-miR-17-3p	2170.631262	116.8595	11.08456344	6.880923997		4.215268	0.275632	chr1_777_star	0	0.805927	0	0.852739935		-20	0.140178	chr7_17161_star	3.22371	15.31262	2.078510713	4.027916745		-2.24793	0.542795
chr2_5761_mature	6.447419591	47.54972	2.896740642	5.601391055		-2.88264	0.276114	chr8_18866_star	0	0.805927	0	0.852739935		-20	0.140178	chrx_21533_star	3.22371	15.31262	2.078510713	4.027916745		-2.24793	0.542795
chr11_24477_mature	143.9923709	16.11855	7.179833181	4.097488514		3.159199	0.276136	chr1_918_mature	0	0.805927	0	0.852739935		-20	0.140178	chrx_22000_mature	4.835565	24.98375	2.544872269	4.699537803		-2.36923	0.542999
chr7_15151_star	5.372849659	37.87859	2.671938627	5.280903997		-2.81762	0.276632	mmu-miR-3073b-3p	0	0.805927	0	0.852739935		-20	0.140178	NW_003613447_39176_star	4.835565	24.98375	2.544872269	4.699537803		-2.36923	0.542999
chr2_5658_mature	5.372849659	37.87859	2.671938627	5.280903997		-2.81762	0.276632	chr14_30846_star	0	0.805927	0	0.852739935		-20	0.140178	mmu-miR-6238	7.52199	44.32601	3.091190278	5.502267253		-2.55897	0.543723
chr8_18873_mature	5.372849659	37.87859	2.671938627	5.280903997		-2.81762	0.276632	chr15_31859_star	0	0.805927	0	0.852739935		-20	0.140178	chr6_14848_star	8.059274	1.611855	3.179395517	1.38507475		2.321928	0.544733
chr17_35550_mature	31.16252802	4.835565	5.007308904	2.544872269		2.688056	0.276927	chr6_13694_star	0	0.805927	0	0.852739935		-20	0.140178	chr4_9733_star	8.059274	1.611855	3.179395517	1.38507475		2.321928	0.544733
mmu-miR-26a-5p	6.447419591	0.805927	2.896740642	0.852739935		3	0.27697	rno-miR-6334	0	0.805927	0	0.852739935		-20	0.140178	chr12_25930_star	8.059274	1.611855	3.179395517	1.38507475		2.321928	0.544733
chr6_14191_star	6.447419591	0.805927	2.896740642	0.852739935		3	0.27697	rno-miR-6324	0	0.805927	0	0.852739935		-20	0.140178	chr6_14470_star	8.059274	1.611855	3.179395517	1.38507475		2.321928	0.544733
chr3_7274_star	6.447419591	0.805927	2.896740642	0.852739935		3	0.27697	chr10_23316_star	0	0.805927	0	0.852739935		-20	0.140178	bta-miR-2285k	8.059274	1.611855	3.179395517	1.38507475		2.321928	0.544733
chr11_24429_star	6.447419591	0.805927	2.896740642	0.852739935		3	0.27697	bta-miR-935	0	0.805927	0	0.852739935		-20	0.140178	chr10_23674_mature	8.059274	1.611855	3.179395517	1.38507475		2.321928	0.544733
chr14_30349_mature	6.447419591	0.805927	2.896740642	0.852739935		3	0.27697	chr9_20301_star	0	0.805927	0	0.852739935		-20	0.140178	chr14_30518_star	8.059274	1.611855	3.179395517	1.38507475		2.321928	0.544733
chr11_25205_star	6.447419591	0.805927	2.896740642	0.852739935		3	0.27697	hsa-miR-3200-3p	0	0.805927	0	0.852739935		-20	0.140178	chr3_8036_star	8.059274	1.611855	3.179395517	1.38507475		2.321928	0.544733
chr6_13705_star	6.447419591	0.805927	2.896740642	0.852739935		3	0.27697	mmu-miR-701-5p	0	0.805927	0	0.852739935		-20	0.140178	chr1_3478_star	8.059274	1.611855	3.179395517	1.38507475		2.321928	0.544733
chr16_34199_star	6.447419591	0.805927	2.896740642	0.852739935		3	0.27697	chr15_32156_star	0	0.805927	0	0.852739935		-20	0.140178	ssc-miR-146a-3p	31.69981	5.641492	5.03121048	2.731507408		2.490326	0.545375
chr13_27409_star@@hsa-miR-551b-	6.447419591	0.805927	2.896740642	0.852739935		3	0.27697	chr16_34334_mature	0	0.805927	0	0.852739935		-20	0.140178	chr4_9471_mature	8.059274	48.35565	3.179395517	5.625143252		-2.58496	0.545556
rno-miR-6317	6.447419591	0.805927	2.896740642	0.852739935		3	0.27697	chr2_6405_star	0	0.805927	0	0.852739935		-20	0.140178	chr14_31507_mature	8.059274	48.35565	3.179395517	5.625143252		-2.58496	0.545556
rno-miR-34b-3p	6.447419591	0.805927	2.896740642	0.852739935		3	0.27697	chr1_3835_star	0	0.805927	0	0.852739935		-20	0.140178	chr14_31449_star	2.14914	9.671129	1.654957834	3.415640968		-2.16993	0.548082
chr17_35446_star	6.447419591	0.805927	2.896740642	0.852739935		3	0.27697	hsa-miR-675-5p	0	0.805927	0	0.852739935		-20	0.140178	chr7_17037_mature	2.14914	9.671129	1.654957834	3.415640968		-2.16993	0.548082
chr10_24203_star	6.447419591	0.805927	2.896740642	0.852739935		3	0.27697	mmu-miR-224-5p	0	0.805927	0	0.852739935		-20	0.140178	chr13_27981_mature	2.14914	9.671129	1.654957834	3.415640968		-2.16993	0.548082
chr3_7875_mature	6.447419591	0.805927	2.896740642	0.852739935		3	0.27697	NW_003613497_39430_star	0	0.805927	0	0.852739935		-20	0.140178	ssc-miR-196a	32.77438	338.4895	5.077857506	8.407223265		-3.36847	0.549039
chr18_36120_mature	6.447419591	0.805927	2.896740642	0.852739935		3	0.27697	mmu-miR-183-3p	0	0.805927	0	0.852739935		-20	0.140178	chr1_1312_mature	15.58126	3.22371	4.051482084	2.078510713		2.273018	0.549124
chr7_16094_star	6.447419591	0.805927	2.896740642	0.852739935		3	0.27697	mmu-miR-3070b-3p	0	0.805927	0	0.852739935		-20	0.140178	chr13_28872_mature	15.58126	3.22371	4.051482084	2.078510713		2.273018	0.549124
chr1_3766_mature	6.447419591	0.805927	2.896740642	0.852739935		3	0.27697	bta-miR-2385-5p	0	0.805927	0	0.852739935		-20	0.140178	chr8_18908_mature	11.28298	75.75718	3.618589217	6.262229808		-2.74723	0.549197
rno-miR-30b-3p	6.447419591	0.805927	2.896740642	0.852739935		3	0.27697	rno-miR-144-3p	0	0.805927	0	0.852739935		-20	0.140178	chr7_17049_mature	202.5564	4397.946	7.669284999	12.1029422		-4.44043	0.549309
chr4_9937_mature	6.447419591	0.805927	2.896740642	0.852739935		3	0.27697	chr4_9830_star	0	0.805927	0	0.852739935		-20	0.140178	chr2_5656_mature	31.16253	5.641492	5.007308904	2.731507408		2.465664	0.549534
mmu-miR-3960	6.447419591	0.805927	2.896740642	0.852739935		3	0.27697	mmu-miR-3088-5p	0	0.805927	0	0.852739935		-20	0.140178	chr6_12619_mature	31.16253	5.641492	5.007308904	2.731507408		2.465664	0.549534
chr2_5392_mature	6.447419591	0.805927	2.896740642	0.852739935		3	0.27697	chr11_25149_star	0	0.805927	0	0.852739935		-20	0.140178	ssc-miR-299	7.52199	43.52008	3.091190278	5.476384353		-2.5325	0.549587
chr5_11160_mature	6.447419591	0.805927	2.896740642	0.852739935		3	0.27697	chr2_5583_star	0	0.805927	0	0.852739935		-20	0.140178	chr11_25215_mature	2.686425	12.08891	1.882222339	3.710273245		-2.16993	0.550591
chr1_2519_mature	6.447419591	0.805927	2.896740642	0.852739935		3	0.27697	NW_003537737_37236_star	0	0.805927	0	0.852739935		-20	0.140178	mmu-miR-3096b-5p	99.935	12.89484	6.657282769	3.796477232		2.954196	0.550616
NW_003539453_38866_star	6.447419591	0.805927	2.896740642	0.852739935		3	0.27697	mmu-miR-574-5p	0	0.805927	0	0.852739935		-20	0.140178	chr5_10912_mature	90.80116	12.08891	6.520440467	3.710273245		2.909026	0.550809
hsa-miR-520e	6.447419591	0.805927	2.896740642	0.852739935		3	0.27697	hsa-miR-3913-5p	0	0.805927	0	0.852739935		-20	0.140178	hsa-miR-517-5p	4.29828	20.95411	2.405524014	4.456419386		-2.2854	0.550894
chr7_16666_star	6.447419591	0.805927	2.896740642	0.852739935		3	0.27697	chr13_28169_star	0	0.805927	0	0.852739935		-20	0.140178	chr14_29918_mature	4.29828	20.95411	2.405524014	4.456419386		-2.2854	0.550894
chr1_3129_mature	6.447419591	0.805927	2.896740642	0.852739935		3	0.27697	hsa-miR-4433-3p	0	0.805927	0	0.852739935		-20	0.140178	chr14_30864_mature	11.28298	2.417782	3.618589217	1.773060525		2.222392	0.551622
chr4_10393_star	6.447419591	0.805927	2.896740642	0.852739935		3	0.27697	chr6_13041_star	0	0.805927	0	0.852739935		-20	0.140178	bta-miR-2285a	11.28298	2.417782	3.618589217	1.773060525		2.222392	0.551622
hsa-miR-1269b	6.447419591	0.805927	2.896740642	0.852739935		3	0.27697	chrx_22074_star	0	0.805927	0	0.852739935		-20	0.140178	bta-miR-2343	11.28298	2.417782	3.618589217	1.773060525		2.222392	0.551622
mmu-miR-703	6.447419591	0.805927	2.896740642	0.852739935		3	0.27697	rno-miR-344a	0	0.805927	0	0.852739935		-20	0.140178	hsa-miR-5701	11.28298	2.417782	3.618589217	1.773060525		2.222392	0.551622
chr15_32819_star	6.447419591	0.805927	2.896740642	0.852739935		3	0.27697	NW_003538922_38349_star	0	0.805927	0	0.852739935		-20	0.140178	chr13_28624_star	1.611855	7.253347	1.38507475	3.044979305		-2.16993	0.551908
chr10_24245_mature	6.447419591	0.805927	2.896740642	0.852739935		3	0.27697	NW_003613367_38733_star	0	0.805927	0	0.852739935		-20	0.140178	hsa-miR-581	1.611855	7.253347	1.38507475	3.044979305		-2.16993	0.551908
chr13_27007_star	6.447419591	0.805927	2.896740642	0.852739935		3	0.27697	chr14_30349_star	0	0.805927	0	0.852739935		-20	0.140178	mmu-miR-7a-2-3p	1.611855	7.253347	1.38507475	3.044979305		-2.16993	0.551908
chr13_26761_mature	6.447419591	0.805927	2.896740642	0.852739935		3	0.27697	chr11_24821_mature	0	0.805927	0	0.852739935		-20	0.140178	chr11_25077_mature	1.611855	7.253347	1.38507475	3.044979305		-2.16993	0.551908
chr9_20117_mature	6.447419591	0.805927	2.896740642	0.852739935		3	0.27697	chr3_8228_star	0	0.805927	0	0.852739935		-20	0.140178	NW_003541128_39926_star	1.611855	7.253347	1.38507475	3.044979305		-2.16993	0.551908
chr13_28621_star	6.447419591	0.805927	2.896740642	0.852739935		3	0.27697	chr12_26570_star	0	0.805927	0	0.852739935		-20	0.140178	chrx_22359_mature	4.835565	24.17782	2.544872269	4.654081667		-2.32193	0.552233
chr9_20843_mature	6.447419591	0.805927	2.896740642	0.852739935		3	0.27697	chr1_2153_mature	0	0.805927	0	0.852739935		-20	0.140178	ssc-miR-22-5p	17.7304	141.0373	4.227310103	7.150126067		-2.99178	0.552551
ssc-miR-885-3p	6.447419591	0.805927	2.896740642	0.852739935		3	0.27697	bta-miR-1284	0	0.805927	0	0.852739935		-20	0.140178	chr18_36092_mature@@hsa-miR-4	19.87954	4.029637	4.384018281	2.330454351		2.302563	0.553714
hsa-miR-6073	6.447419591	0.805927	2.896740642	0.852739935		3	0.27697	chr15_32249_star	0	0.805927	0	0.852739935		-20	0.140178	chr9_20893_mature	36.53538	6.44742	5.230179094	2.896740642		2.5025	0.554677
chr2_5590_mature	10.74569932	91.87573	3.554060706	6.537229727		-3.09592	0.278247	NW_003538331_37733_star	0	0.805927	0	0.852739935		-20	0.140178	chr13_27184_star	4.835565	0.805927	2.544872269	0.852739935		2.584963	0.555505
chr2_5068_mature	152.5889303	16.92448	7.26293043	4.163859073		3.172467	0.278332	chr1_3923_star	0	0.805927	0	0.852739935		-20	0.140178	chr5_11960_star	4.835565	0.805927	2.544872269	0.852739935		2.584963	0.555505
mmu-miR-28b	3.223709795	0	2.078510713	0		20	0.278721	hsa-miR-3679-5p	0	0.805927	0	0.852739935		-20	0.140178	chr4_9226_star	4.835565	0.805927	2.544872269	0.852739935		2.584963	0.555505
chr14_31443_star	3.223709795	0	2.078510713	0		20	0.278721	rno-miR-196c-3p	0	0.805927	0	0.852739935		-20	0.140178	chr12_25625_mature	4.835565	0.805927	2.544872269	0.852739935		2.584963	0.555505
chr4_9456_mature	3.223709795	0	2.078510713	0		20	0.278721	chr5_12276_star	0	0.805927	0	0.852739935		-20	0.140178	chr13_29079_star	4.835565	0.805927	2.544872269	0.852739935		2.584963	0.555505
chr8_17792_star	3.223709795	0	2.078510713	0		20	0.278721	hsa-miR-4707-5p	0	0.805927	0	0.852739935		-20	0.140178	hsa-miR-4762-3p	4.835565	0.805927	2.544872269	0.852739935		2.584963	0.555505
mmu-miR-467d-3p	3.223709795	0	2.078510713	0		20	0.278721	chr10_24235_star	0	0.805927	0	0.852739935		-20	0.140178	chr11_24692_star	4.835565	0.805927	2.544872269	0.852739935		2.584963	0.555505
chr9_19408_star@@bta-miR-34b	3.223709795	0	2.078510713	0		20	0.278721	chr4_9919_star	0	0.805927	0	0.852739935		-20	0.140178	chr18_36004_mature	4.835565	0.805927	2.544872269	0.852739935		2.584963	0.555505
chr17_35778_star	3.223709795	0	2.078510713	0		20	0.278721	mmu-miR-101a-3p	0	0.805927	0	0.852739935		-20	0.140178	chr13_26798_star	4.835565	0.805927	2.544872269	0.852739935		2.584963	0.555505
chr14_30949_mature	3.223709795	0	2.078510713	0		20	0.278721	chrx_21746_mature	0	0.805927	0	0.852739935		-20	0.140178	chr11_24909_star	4.835565	0.805927	2.544872269	0.852739935		2.584963	0.555505
chrx_22663_star	3.223709795	0	2.078510713	0		20	0.278721	rno-miR-487b-5p	0	0.805927	0	0.852739935		-20	0.140178	chr2_5368_mature	4.835565	0.805927	2.544872269	0.852739935		2.584963	0.555505
chr3_6736_star	3.223709795	0	2.078510713	0		20	0.278721	NW_003538922_38350_star	0	0.805927	0	0.852739935		-20	0.140178	chr2_5207_star	4.835565	0.805927	2.544872269	0.852739935		2.584963	0.555505
chr6_12438_star	3.223709795	0	2.078510713	0		20	0.278721	chr13_28323_star	0	0.805927	0	0.852739935		-20	0.140178	chr17_35299_mature	4.835565	0.805927	2.544872269	0.852739935		2.584963	0.555505
ssc-miR-676-5p	3.223709795	0	2.078510713	0		20	0.278721	chrx_21977_star@@rno-miR-384-5p	0	0.805927	0	0.852739935		-20	0.140178	chr3_6871_mature	4.835565	0.805927	2.544872269	0.852739935		2.584963	0.555505
hsa-miR-1178-5p	3.223709795	0	2.078510713	0		20	0.278721	hsa-miR-570-5p	0	0.805927	0	0.852739935		-20	0.140178	mmu-miR-701-5p	4.835565	0.805927	2.544872269	0.852739935		2.584963	0.555505
chr17_34920_mature	3.223709795	0	2.078510713	0		20	0.278721	chr4_9373_mature	0	0.805927	0	0.852739935		-20	0.140178	chr7_15959_star	4.835565	0.805927	2.544872269	0.852739935		2.584963	0.555505
chr13_26706_star	3.223709795	0	2.078510713	0		20	0.278721	hsa-miR-371b-5p	0	0.805927	0	0.852739935		-20	0.140178	mmu-miR-3088-5p	4.835565	0.805927	2.544872269	0.852739935		2.584963	0.555505
chr5_12108_star	3.223709795	0	2.078510713	0		20	0.278721	chr8_17498_star	0	0.805927	0	0.852739935		-20	0.140178	chr18_36120_mature	4.835565	0.805927	2.544872269	0.852739935		2.584963	0.555505
chr4_9318_star	3.223709795	0	2.078510713	0		20	0.278721	mmu-miR-3101-5p	0	0.805927	0	0.852739935		-20	0.140178	chr5_12276_star	4.835565	0.805927	2.544872269	0.852739935		2.584963	0.555505
chr3_7678_mature	3.223709795	0	2.078510713	0		20	0.278721	NW_003613233_37800_mature	0	0.805927	0	0.852739935		-20	0.140178	chr7_15697_mature	4.835565	0.805927	2.544872269	0.852739935		2.584963	0.555505
chr9_20033_star	3.223709795	0	2.078510713	0		20	0.278721	rno-miR-105	0	0.805927	0	0.852739935		-20	0.140178	chr16_34386_star	4.835565	0.805927	2.544872269	0.852739935		2.584963	0.555505
rno-miR-181d-3p	3.223709795	0	2.078510713	0		20	0.278721	chr13_27528_star	0	0.805927	0	0.852739935		-20	0.140178	rno-miR-105	4.835565	0.805927	2.544872269	0.852739935		2.584963	0.555505
hsa-miR-1254	3.223709795	0	2.078510713	0		20	0.278721	chr8_17349_mature	0	0.805927	0	0.852739935		-20	0.140178	chr14_30104_star	4.835565	0.805927	2.544872269	0.852739935		2.584963	0.555505
rno-miR-666-5p	3.223709795	0	2.078510713	0		20	0.278721	chr15_32263_star	0	0.805927	0	0.852739935		-20	0.140178	chr7_16875_star	4.835565	0.805927	2.544872269	0.852739935		2.584963	0.555505
chr2_5776_star	3.223709795	0	2.078510713	0		20	0.278721	bta-miR-1185	0	0.805927	0	0.852739935		-20	0.140178	chr2_6200_star	4.835565	0.805927	2.544872269	0.852739935		2.584963	0.555505
chr12_25473_star	3.223709795	0	2.078510713	0		20	0.278721	chrx_22056_star	0	0.805927	0	0.852739935		-20	0.140178	chrx_22791_star	4.835565	0.805927	2.544872269	0.852739935		2.584963	0.555505
chr13_27796_mature	3.223709795	0	2.078510713	0		20	0.278721	rno-miR-1249	0	0.805927	0	0.852739935		-20	0.140178	chr15_32065_star	4.835565	0.805927	2.544872269	0.852739935		2.584963	0.555505
mmu-miR-3087-3p	3.223709795	0	2.078510713	0		20	0.278721	hsa-miR-584-3p	0	0.805927	0	0.852739935		-20	0.140178	chr1_2402_star	4.835565	0.805927	2.544872269	0.852739935		2.584963	0.555505
chr14_30528_mature	3.223709795	0	2.078510713	0		20	0.278721	chr7_16583_star	0	0.805927	0	0.852739935		-20	0.140178	mmu-miR-6382	4.835565	0.805927	2.544872269	0.852739935		2.584963	0.555505
hsa-miR-1272	3.223709795	0	2.078510713	0		20	0.278721	rno-miR-3584-3p	0	0.805927	0	0.852739935		-20	0.140178	bta-miR-2285q	4.835565	0.805927	2.544872269	0.852739935		2.584963	0.555505
hsa-miR-580	3.223709795	0	2.078510713	0		20	0.278721	chr9_21032_star	0	0.805927	0	0.852739935		-20	0.140178	chr13_28101_mature	4.835565	0.805927	2.544872269	0.852739935		2.584963	0.555505
chrx_23200_mature	3.223709795	0	2.078510713	0		20	0.278721	hsa-miR-3129-3p	0	0.805927	0	0.852739935		-20	0.140178	chr3_7233_star	4.835565	0.805927	2.544872269	0.852739935		2.584963	0.555505
hsa-miR-555	3.223709795	0	2.078510713	0		20	0.278721	bta-miR-411c-5p	0	0.805927	0	0.852739935		-20	0.140178	chr3_6909_mature	4.835565	0.805927	2.544872269	0.852739935		2.584963	0.555505
chr15_33130_star	3.223709795	0	2.078510713	0		20	0.278721	hsa-miR-6502-3p	0	0.805927	0	0.852739935		-20	0.140178	chr10_23279_star	4.835565	0.805927	2.544872269	0.852739935		2.584963	0.555505
NW_003537225_36780_mature	3.223709795	0	2.078510713	0		20	0.278721	chr13_27252_star	0	0.805927	0	0.852739935		-20	0.140178	chrx_23097_mature	4.835565	0.805927	2.544872269	0.852739935		2.584963	0.555505
chr3_7972_mature	3.223709795	0	2.078510713	0		20	0.278721	mmu-miR-295-5p	0	0.805927	0	0.852739935		-20	0.140178	chr13_26704_star	4.835565	0.805927	2.544872269	0.852739935		2.584963	0.555505
hsa-miR-6721-5p	3.223709795	0	2.078510713	0		20	0.278721	mmu-miR-574-3p	0	0.805927	0	0.852739935		-20	0.140178	chr7_16542_star	4.835565	0.805927	2.544872269	0.852739935		2.584963	0.555505
mmu-miR-208a-3p	3.223709795	0	2.078510713	0		20	0.278721	chr14_30803_star	0	0.805927	0	0.852739935		-20	0.140178	chr3_8247_star	4.835565	0.805927	2.544872269	0.852739935		2.584963	0.555505
chr14_31505_mature	3.223709795	0	2.078510713	0		20	0.278721	chr1_3232_star	0	0.805927	0	0.852739935		-20	0.140178	chr9_19640_star	4.835565	0.805927	2.544872269	0.852739935		2.584963	0.555505
hsa-miR-92b-5p	3.223709795	0	2.078510713	0		20	0.278721	chr4_9602_star	0	0.805927	0	0.852739935		-20	0.140178	chr13_29260_star	4.835565	0.805927	2.544872269	0.852739935		2.584963	0.555505
chr6_13692_star	3.223709795	0	2.078510713	0		20	0.278721	hsa-miR-4676-5p	0	0.805927	0	0.852739935		-20	0.140178	hsa-miR-3118	4.835565	0.805927	2.544872269	0.852739935		2.584963	0.555505
chr6_12833_mature	3.223709795	0	2.078510713	0		20	0.278721	NW_003613310_38408_star	0	0.805927	0	0.852739935		-20	0.140178	chr15_33675_mature@@mmu-miR-	4.835565	0.805927	2.544872269	0.852739935		2.584963	0.555505
chr3_8026_star	3.223709795	0	2.078510713	0		20	0.278721	chr1_943_star	0	0.805927	0	0.852739935		-20	0.140178	chr1_1498_mature	4.835565	0.805927	2.544872269	0.852739935		2.584963	0.555505
rno-miR-211-3p	3.223709795	0	2.078510713	0		20	0.278721	mmu-miR-194-1-3p	0	0.805927	0	0.852739935		-20	0.140178	chr14_31627_star	4.835565	0.805927	2.544872269	0.852739935		2.584963	0.555505
mmu-miR-3103-5p	3.223709795	0	2.078510713	0		20	0.278721	mmu-miR-669d-5p	0	0.805927	0	0.852739935		-20	0.140178	chr14_30639_mature	4.835565	0.805927	2.544872269	0.852739935		2.584963	0.555505
chr14_30041_mature	3.223709795	0	2.078510713	0		20	0.278721	chr5_10827_star	0	0.805927	0	0.852739935		-20	0.140178	hsa-miR-4644	4.835565	0.805927	2.544872269	0.852739935		2.584963	0.555505
mmu-miR-1961	3.223709795	0	2.078510713	0		20	0.278721	bta-miR-1225-3p	0	0.805927	0	0.852739935		-20	0.140178	chr10_23553_star	4.835565	0.805927	2.544872269	0.852739935		2.584963	0.555505
mmu-miR-1967	3.223709795	0	2.078510713	0		20	0.278721	chr10_23941_star	0	0.805927	0	0.852739935		-20	0.140178	chr2_4343_star	4.835565	0.805927	2.544872269	0.852739935		2.584963	0.555505
chr2_6568_star	3.223709795	0	2.078510713	0		20	0.278721	rno-miR-184	0	0.805927	0	0.852739935		-20	0.140178	hsa-miR-4778-5p	4.835565	0.805927	2.544872269	0.852739935		2.584963	0.555505
chr13_27317_mature	3.223709795	0	2.078510713	0		20	0.278721	hsa-miR-301a-3p	0	0.805927	0	0.852739935		-20	0.140178	mmu-miR-215-5p	4.835565	0.805927	2.544872269	0.852739935		2.584963	0.555505
chr5_11609_star	3.223709795	0	2.078510713	0		20	0.278721	mmu-miR-6409	0	0.805927	0	0.852739935		-20	0.140178	chr1_397_mature	3.22371	14.50669	2.078510713	3.954819241		-2.16993	0.556274
chr10_23298_star	3.223709795	0	2.078510713	0		20	0.278721	chr14_31358_star	0	0.805927	0	0.852739935		-20	0.140178	chr9_19828_mature	3.22371	14.50669	2.078510713	3.954819241		-2.16993	0.556274
hsa-miR-193b-5p	3.223709795	0	2.078510713	0		20	0.278721	rno-miR-500-3p	0	0.805927	0	0.852739935		-20	0.140178	bta-miR-2350	3.22371	14.50669	2.078510713	3.954819241		-2.16993	0.556274
NW_003613301_38356_mature	3.223709795	0	2.078510713	0		20	0.278721	hsa-miR-526b-3p	0	0.805927	0	0.852739935		-20	0.140178	NW_003541277_40019_mature	6.44742	34.65488	2.896740642	5.156027656		-2.42626	0.556335
chr18_36229_star@@hsa-miR-1229-	3.223709795	0	2.078510713	0		20	0.278721	mmu-miR-3066-5p	0	0.805927	0	0.852739935		-20	0.140178	chr5_12411_mature	15.04398	3.22371	4.003960082	2.078510713		2.222392	0.556525
chrx_22279_star	3.223709795	0	2.078510713	0		20	0.278721	hsa-miR-185-5p	0	0.805927	0	0.852739935		-20	0.140178	chr13_28551_mature	15.04398	3.22371	4.003960082	2.078510713		2.222392	0.556525
chr10_23548_mature	3.223709795	0	2.078510713	0		20	0.278721	chr13_29007_star	0	0.805927	0	0.852739935		-20	0.140178	chr17_35401_star	15.04398	3.22371	4.003960082	2.078510713		2.222392	0.556525
chr4_10313_star	3.223709795	0	2.078510713	0		20	0.278721	bta-miR-449d	0	0.805927	0	0.852739935		-20	0.140178	chr13_26947_star	7.52199	1.611855	3.091190278	1.38507475		2.222392	0.557822
rno-miR-30d-3p	3.223709795	0	2.078510713	0		20	0.278721	bta-miR-449b	0	0.805927	0	0.852739935		-20	0.140178	chr6_13171_star	7.52199	1.611855	3.091190278	1.38507475		2.222392	0.557822
chr12_26042_mature	3.223709795	0	2.078510713	0		20	0.278721	chrx_22559_star	0	0.805927	0	0.852739935		-20	0.140178	chr5_10946_star	7.52199	1.611855	3.091190278	1.38507475		2.222392	0.557822
hsa-miR-3934-3p	3.223709795	0	2.078510713	0		20	0.278721	hsa-miR-20b-3p	0	0.805927	0	0.852739935		-20	0.140178	chr2_4454_mature	7.52199	1.611855	3.091190278	1.38507475		2.222392	0.557822
mmu-miR-5131	3.223709795	0	2.078510713	0		20	0.278721	hsa-miR-3689d	0	0.805927	0	0.852739935		-20	0.140178	chr3_7963_star	7.52199	1.611855	3.091190278	1.38507475		2.222392	0.557822
hsa-miR-522-3p	3.223709795	0	2.078510713	0		20	0.278721	hsa-miR-1179	0	0.805927	0	0.852739935		-20	0.140178	chr18_36189_star	7.52199	1.611855	3.091190278	1.38507475		2.222392	0.557822
rno-miR-6319	3.223709795	0	2.078510713	0		20	0.278721	chr7_16996_star	0	0.805927	0	0.852739935		-20	0.140178	rno-miR-6332	7.52199	1.611855	3.091190278	1.38507475		2.222392	0.557822
rno-miR-6314	3.223709795	0	2.078510713	0		20	0.278721	hsa-miR-1197	0	0.805927	0	0.852739935		-20	0.140178	chrx_21832_mature	7.52199	1.611855	3.091190278	1.38507475		2.222392	0.557822
bta-miR-940	3.223709795	0	2.078510713	0		20	0.278721	chr1_3640_star	0	0.805927	0	0.852739935		-20	0.140178	chr12_25892_mature	7.52199	1.611855	3.091190278	1.38507475		2.222392	0.557822
chr14_29507_star	3.223709795	0	2.078510713	0		20	0.278721	rno-miR-674-5p	0	0.805927	0	0.852739935		-20	0.140178	chr3_7564_star	7.52199	1.611855	3.091190278	1.38507475		2.222392	0.557822
chr12_26097_star	3.223709795	0	2.078510713	0		20	0.278721	hsa-miR-4731-5p	0	0.805927	0	0.852739935		-20	0.140178	chr15_33320_star	7.52199	1.611855	3.091190278	1.38507475		2.222392	0.557822
chr2_4623_mature	3.223709795	0	2.078510713	0		20	0.278721	mmu-miR-3092-5p	0	0.805927	0	0.852739935		-20	0.140178	chr2_5163_mature	7.52199	1.611855	3.091190278	1.38507475		2.222392	0.557822
chr16_34200_star	3.223709795	0	2.078510713	0		20	0.278721	chr8_17637_star	0	0.805927	0	0.852739935		-20	0.140178	chr8_17483_mature	7.52199	1.611855	3.091190278	1.38507475		2.222392	0.557822
NW_003613080_37038_mature	3.223709795	0	2.078510713	0		20	0.278721	NW_003612983_36666_star	0	0.805927	0	0.852739935		-20	0.140178	chr9_20110_mature	7.52199	1.611855	3.091190278	1.38507475		2.222392	0.557822
chr2_5373_star	3.223709795	0	2.078510713	0		20	0.278721	chr3_6641_star	0	0.805927	0	0.852739935		-20	0.140178	chr17_35182_mature@@bta-miR-6	7.52199	1.611855	3.091190278	1.38507475		2.222392	0.557822
hsa-miR-5091	3.223709795	0	2.078510713	0		20	0.278721	chr16_34699_star	0	0.805927	0	0.852739935		-20	0.140178	chr1_217_star	7.52199	1.611855	3.091190278	1.38507475		2.222392	0.557822
chr4_9373_star	3.223709795	0	2.078510713	0		20	0.278721	chr14_31221_star	0	0.805927	0	0.852739935		-20	0.140178	chr10_24294_star	7.52199	1.611855	3.091190278	1.38507475		2.222392	0.557822
chr7_15714_mature	3.223709795	0	2.078510713	0		20	0.278721	bta-miR-2461-3p	0	0.805927	0	0.852739935		-20	0.140178	chr3_8228_mature	7.52199	1.611855	3.091190278	1.38507475		2.222392	0.557822
chr8_19000_mature	3.223709795	0	2.078510713	0		20	0.278721	hsa-miR-362-5p	0	0.805927	0	0.852739935		-20	0.140178	hsa-miR-3671	19.34226	4.029637	4.346407978	2.330454351		2.263034	0.559719
chrx_22660_star	3.223709795	0	2.078510713	0		20	0.278721	chr1_3766_mature	0	0.805927	0	0.852739935		-20	0.140178	chr15_32092_mature	19.34226	4.029637	4.346407978	2.330454351		2.263034	0.559719
chr15_32720_mature	3.223709795	0	2.078510713	0		20	0.278721	bta-miR-769	0	0.805927	0	0.852739935		-20	0.140178	chr14_30563_mature	19.34226	4.029637	4.346407978	2.330454351		2.263034	0.559719
mmu-miR-493-5p	3.223709795	0	2.078510713	0		20	0.278721	chr13_27306_star	0	0.805927	0	0.852739935		-20	0.140178	chr14_30223_star	2.686425	0	1.882222339	0		20	0.559868
mmu-miR-1966-3p	3.223709795	0	2.078510713	0		20	0.278721	bta-miR-759	0	0.805927	0	0.852739935		-20	0.140178	rno-miR-138-5p	2.686425	0	1.882222339	0		20	0.559868
hsa-miR-6515-3p	3.223709795	0	2.078510713	0		20	0.278721	hsa-miR-3191-3p	0	0.805927	0	0.852739935		-20	0.140178	chr15_33603_star	2.686425	0	1.882222339	0		20	0.559868
mmu-miR-3062-3p	3.223709795	0	2.078510713	0		20	0.278721	mmu-miR-876-5p	0	0.805927	0	0.852739935		-20	0.140178	hsa-miR-518a-5p	2.686425	0	1.882222339	0		20	0.559868
mmu-miR-743b-3p	3.223709795	0	2.078510713	0		20	0.278721	chrx_23205_star	0	0.805927	0	0.852739935		-20	0.140178	hsa-miR-4659b-3p	2.686425	0	1.882222339	0		20	0.559868
chr15_32108_star	3.223709795	0	2.078510713	0		20	0.278721	bta-miR-582	0	0.805927	0	0.852739935		-20	0.140178	chr14_29661_star	2.686425	0	1.882222339	0		20	0.559868
chr1_3717_mature	3.223709795	0	2.078510713	0		20	0.278721	mmu-miR-6341	0	0.805927	0	0.852739935		-20	0.140178	chr14_30332_star	2.686425	0	1.882222339	0		20	0.559868
chr18_36298_mature	3.223709795	0	2.078510713	0		20	0.278721	chr11_25111_star	0	0.805927	0	0.852739935		-20	0.140178	chr9_19408_star@@bta-miR-34b	2.686425	0	1.882222339	0		20	0.559868
bta-miR-1193	3.223709795	0	2.078510713	0		20	0.278721	mmu-miR-6337	0	0.805927	0	0.852739935		-20	0.140178	hsa-miR-4788	2.686425	0	1.882222339	0		20	0.559868
chr12_26044_star	3.223709795	0	2.078510713	0		20	0.278721	chr13_28101_star	0	0.805927	0	0.852739935		-20	0.140178	chrx_22902_mature	2.686425	0	1.882222339	0		20	0.559868
chr16_33868_star	3.223709795	0	2.078510713	0		20	0.278721	chr8_17863_star	0	0.805927	0	0.852739935		-20	0.140178	hsa-miR-1178-3p	2.686425	0	1.882222339	0		20	0.559868
NW_003613025_36805_mature	3.223709795	0	2.078510713	0		20	0.278721	mmu-miR-6363	0	0.805927	0	0.852739935		-20	0.140178	NW_003540726_39645_star	2.686425	0	1.882222339	0		20	0.559868
NW_003613191_37580_mature	3.223709795	0	2.078510713	0		20	0.278721	NW_003613379_38761_star	0	0.805927	0	0.852739935		-20	0.140178	ssc-miR-676-5p	2.686425	0	1.882222339	0		20	0.559868
chr14_31496_star	3.223709795	0	2.078510713	0		20	0.278721	chr13_26706_mature	0	0.805927	0	0.852739935		-20	0.140178	chr18_36623_star	2.686425	0	1.882222339	0		20	0.559868
chr9_21046_star	3.223709795	0	2.078510713	0		20	0.278721	chr9_20120_star	0	0.805927	0	0.852739935		-20	0.140178	chr16_33727_star	2.686425	0	1.882222339	0		20	0.559868
chr16_33919_star	3.223709795	0	2.078510713	0		20	0.278721	mmu-miR-6353	0	0.805927	0	0.852739935		-20	0.140178	rno-miR-29b-3p	2.686425	0	1.882222339	0		20	0.559868
chr6_13297_star	3.223709795	0	2.078510713	0		20	0.278721	mmu-miR-6350	0	0.805927	0	0.852739935		-20	0.140178	rno-miR-429	2.686425	0	1.882222339	0		20	0.559868
chr3_6736_mature	3.223709795	0	2.078510713	0		20	0.278721	hsa-miR-548x-5p	0	0.805927	0	0.852739935		-20	0.140178	chr13_26706_star	2.686425	0	1.882222339	0		20	0.559868
rno-miR-500-5p	3.223709795	0	2.078510713	0		20	0.278721	mmu-miR-6382	0	0.805927	0	0.852739935		-20	0.140178	hsa-miR-5187-3p	2.686425	0	1.882222339	0		20	0.559868
chr15_33370_mature	3.223709795	0	2.078510713	0		20	0.278721	mmu-miR-6388	0	0.805927	0	0.852739935		-20	0.140178	chr1_392_mature	2.686425	0	1.882222339	0		20	0.559868
chr8_17367_mature	3.223709795	0	2.078510713	0		20	0.278721	chr3_7715_mature	0	0.805927	0	0.852739935		-20	0.140178	rno-miR-20a-3p	2.686425	0	1.882222339	0		20	0.559868
NW_003613374_38740_star	3.223709795	0	2.078510713	0		20	0.278721	mmu-miR-6376	0	0.805927	0	0.852739935		-20	0.140178	hsa-miR-4671-5p	2.686425	0	1.882222339	0		20	0.559868
hsa-miR-1180	3.223709795	0	2.078510713	0		20	0.278721	chr2_4395_star	0	0.805927	0	0.852739935		-20	0.140178	bta-miR-6121-3p	2.686425	0	1.882222339	0		20	0.559868
chr7_16402_star	3.223709795	0	2.078510713	0		20	0.278721	rno-miR-185-5p	0	0.805927	0	0.852739935		-20	0.140178	chr6_14717_star	2.686425	0	1.882222339	0		20	0.559868
chr12_26158_star	3.223709795	0	2.078510713	0		20	0.278721	bta-miR-2284q	0	0.805927	0	0.852739935		-20	0.140178	chr4_9775_star	2.686425	0	1.882222339	0		20	0.559868
mmu-miR-3104-5p	3.223709795	0	2.078510713	0		20	0.278721	chr16_34458_star	0	0.805927	0	0.852739935		-20	0.140178	chr9_20893_star	2.686425	0	1.882222339	0		20	0.559868
mmu-miR-6342	3.223709795	0	2.078510713	0		20	0.278721	bta-miR-2284s	0	0.805927	0	0.852739935		-20	0.140178	chr13_28924_star	2.686425	0	1.882222339	0		20	0.559868
chr12_25810_star	3.223709795	0	2.078510713	0		20	0.278721	rno-miR-98-3p	0	0.805927	0	0.852739935		-20	0.140178	chr18_36375_star	2.686425	0	1.882222339	0		20	0.559868
mmu-miR-6384	3.223709795	0	2.078510713	0		20	0.278721	bta-miR-2284g	0	0.805927	0	0.852739935		-20	0.140178	mmu-miR-465a-5p	2.686425	0	1.882222339	0		20	0.559868
chrx_23194_star@@mmu-miR-201-5	3.223709795	0	2.078510713	0		20	0.278721	bta-miR-2284l	0	0.805927	0	0.852739935		-20	0.140178	hsa-miR-134	2.686425	0	1.882222339	0		20	0.559868
chr6_13459_star	3.223709795	0	2.078510713	0		20	0.278721	bta-miR-6119-3p	0	0.805927	0	0.852739935		-20	0.140178	hsa-miR-1265	2.686425	0	1.882222339	0		20	0.559868
hsa-miR-5006-3p	3.223709795	0	2.078510713	0		20	0.278721	bta-miR-2284p	0	0.805927	0	0.852739935		-20	0.140178	mmu-miR-293-5p	2.686425	0	1.882222339	0		20	0.559868
rno-miR-206-5p	3.223709795	0	2.078510713	0		20	0.278721	mmu-miR-6394	0	0.805927	0	0.852739935		-20	0.140178	hsa-miR-588	2.686425	0	1.882222339	0		20	0.559868
chr7_15656_star	3.223709795	0	2.078510713	0		20	0.278721	chr7_15832_star	0	0.805927	0	0.852739935		-20	0.140178	mmu-miR-199a-5p	2.686425	0	1.882222339	0		20	0.559868
chr17_35707_mature	3.223709795	0	2.078510713	0		20	0.278721	rno-miR-218b	0	0.805927	0	0.852739935		-20	0.140178	mmu-miR-883a-3p	2.686425	0	1.882222339	0		20	0.559868
chr16_33921_star	3.223709795	0	2.078510713	0		20	0.278721	chr2_4572_star	0	0.805927	0	0.852739935		-20	0.140178	hsa-miR-555	2.686425	0	1.882222339	0		20	0.559868
chr12_26608_star	3.223709795	0	2.078510713	0		20	0.278721	rno-miR-361-5p	0	0.805927	0	0.852739935		-20	0.140178	mmu-miR-1896	2.686425	0	1.882222339	0		20	0.559868
chr12_25675_star	3.223709795	0	2.078510713	0		20	0.278721	bta-miR-2917	0	0.805927	0	0.852739935		-20	0.140178	bta-miR-2299-3p	2.686425	0	1.882222339	0		20	0.559868
chr3_7065_star	3.223709795	0	2.078510713	0		20	0.278721	chr5_12271_star	0	0.805927	0	0.852739935		-20	0.140178	chrx_21945_star	2.686425	0	1.882222339	0		20	0.559868
chr6_13925_mature	3.223709795	0	2.078510713	0		20	0.278721	chr7_16939_star	0	0.805927	0	0.852739935		-20	0.140178	chr16_34380_star	2.686425	0	1.882222339	0		20	0.559868
chr9_21075_star	3.223709795	0	2.078510713	0		20	0.278721	rno-miR-294	0	0.805927	0	0.852739935		-20	0.140178	chr1_2148_star	2.686425	0	1.882222339	0		20	0.559868
mmu-miR-188-3p	3.223709795	0	2.078510713	0		20	0.278721	chr15_31929_mature	0	0.805927	0	0.852739935		-20	0.140178	mmu-miR-190b-3p	2.686425	0	1.882222339	0		20	0.559868
chr18_36542_mature	3.223709795	0	2.078510713	0		20	0.278721	bta-miR-2903	0	0.805927	0	0.852739935		-20	0.140178	chr14_29835_star	2.686425	0	1.882222339	0		20	0.559868
bta-miR-132	3.223709795	0	2.078510713	0		20	0.278721	chr8_17371_mature	0	0.805927	0	0.852739935		-20	0.140178	NW_003613379_38755_star	2.686425	0	1.882222339	0		20	0.559868
hsa-miR-4740-5p	3.223709795	0	2.078510713	0		20	0.278721	chr7_16194_star	0	0.805927	0	0.852739935		-20	0.140178	chr13_27078_star	2.686425	0	1.882222339	0		20	0.559868
chr13_26823_star	3.223709795	0	2.078510713	0		20	0.278721	chr17_35181_star	0	0.805927	0	0.852739935		-20	0.140178	chr13_27795_mature	2.686425	0	1.882222339	0		20	0.559868
chr1_1915_star	3.223709795	0	2.078510713	0		20	0.278721	chr3_7118_star	0	0.805927	0	0.852739935		-20	0.140178	rno-miR-665	2.686425	0	1.882222339	0		20	0.559868
mmu-miR-3058-3p	3.223709795	0	2.078510713	0		20	0.278721	chr14_31199_star	0	0.805927	0	0.852739935		-20	0.140178	hsa-miR-4716-5p	2.686425	0	1.882222339	0		20	0.559868
chrx_22854_star	3.223709795	0	2.078510713	0		20	0.278721	hsa-miR-4799-3p	0	0.805927	0	0.852739935		-20	0.140178	chr16_34140_star	2.686425	0	1.882222339	0		20	0.559868
chr5_12420_star	3.223709795	0	2.078510713	0		20	0.278721	chr6_14616_mature	0	0.805927	0	0.852739935		-20	0.140178	chr18_35961_mature	2.686425	0	1.882222339	0		20	0.559868
hsa-miR-4695-3p	3.223709795	0	2.078510713	0		20	0.278721	hsa-miR-4677-5p	0	0.805927	0	0.852739935		-20	0.140178	hsa-miR-654-5p	2.686425	0	1.882222339	0		20	0.559868
bta-miR-2355-5p	3.223709795	0	2.078510713	0		20	0.278721	chr14_31063_star	0	0.805927	0	0.852739935		-20	0.140178	mmu-miR-3105-5p	2.686425	0	1.882222339	0		20	0.559868
rno-miR-362-3p	3.223709795	0	2.078510713	0		20	0.278721	rno-miR-343	0	0.805927	0	0.852739935		-20	0.140178	chr10_23548_mature	2.686425	0	1.882222339	0		20	0.559868
chr5_11609_mature	3.223709795	0	2.078510713	0		20	0.278721	chr14_30903_mature	0	0.805927	0	0.852739935		-20	0.140178	mmu-miR-3109-3p	2.686425	0	1.882222339	0		20	0.559868
mmu-miR-3080-5p	3.223709795	0	2.078510713	0		20	0.278721	chr8_17822_star	0	0.805927	0	0.852739935		-20	0.140178	chr14_31484_star	2.686425	0	1.882222339	0		20	0.559868
mmu-miR-1187	3.223709795	0	2.078510713	0		20	0.278721	chr14_30212_star	0	0.805927	0	0.852739935		-20	0.140178	hsa-miR-3666	2.686425	0	1.882222339	0		20	0.559868
chr14_29447_mature	3.223709795	0	2.078510713	0		20	0.278721	mmu-miR-708-3p	0	0.805927	0	0.852739935		-20	0.140178	mmu-miR-6237	2.686425	0	1.882222339	0		20	0.559868
chr7_16666_mature@@bta-miR-230	3.223709795	0	2.078510713	0		20	0.278721	chr4_9726_star	0	0.805927	0	0.852739935		-20	0.140178	chr3_6642_star	2.686425	0	1.882222339	0		20	0.559868
chrx_23196_mature	3.223709795	0	2.078510713	0		20	0.278721	mmu-miR-708-5p	0	0.805927	0	0.852739935		-20	0.140178	chr3_8486_mature	2.686425	0	1.882222339	0		20	0.559868
chr15_33216_star	3.223709795	0	2.078510713	0		20	0.278721	chr1_495_mature	0	0.805927	0	0.852739935		-20	0.140178	chr6_12810_star	2.686425	0	1.882222339	0		20	0.559868
mmu-miR-3091-3p	3.223709795	0	2.078510713	0		20	0.278721	chr1_2125_star	0	0.805927	0	0.852739935		-20	0.140178	rno-miR-6326	2.686425	0	1.882222339	0		20	0.559868
chr10_23272_star	3.223709795	0	2.078510713	0		20	0.278721	mmu-miR-195a-5p	0	0.805927	0	0.852739935		-20	0.140178	mmu-miR-5098	2.686425	0	1.882222339	0		20	0.559868
chrx_21708_star	3.223709795	0	2.078510713	0		20	0.278721	hsa-miR-188-3p	0	0.805927	0	0.852739935		-20	0.140178	chr13_26975_star	2.686425	0	1.882222339	0		20	0.559868
hsa-miR-3151	3.223709795	0	2.078510713	0		20	0.278721	hsa-miR-5692a	0	0.805927	0	0.852739935		-20	0.140178	chr12_26097_star	2.686425	0	1.882222339	0		20	0.559868
hsa-miR-4266	3.223709795	0	2.078510713	0		20	0.278721	chr3_6931_star	0	0.805927	0	0.852739935		-20	0.140178	chr9_19422_mature	2.686425	0	1.882222339	0		20	0.559868
hsa-miR-1225-3p	3.223709795	0	2.078510713	0		20	0.278721	hsa-miR-363-5p	0	0.805927	0	0.852739935		-20	0.140178	mmu-miR-3088-3p	2.686425	0	1.882222339	0		20	0.559868
chr5_11634_star	3.223709795	0	2.078510713	0		20	0.278721	chr14_31327_mature	0	0.805927	0	0.852739935		-20	0.140178	chr13_29181_star	2.686425	0	1.882222339	0		20	0.559868
mmu-miR-297b-5p	3.223709795	0	2.078510713	0		20	0.278721	chr14_30669_star	0	0.805927	0	0.852739935		-20	0.140178	chrx_22660_star	2.686425	0	1.882222339	0		20	0.559868
chr18_36042_star	3.223709795	0	2.078510713	0		20	0.278721	hsa-miR-888-5p	0	0.805927	0	0.852739935		-20	0.140178	rno-miR-384-3p	2.686425	0	1.882222339	0		20	0.559868
chr15_33531_star	3.223709795	0	2.078510713	0		20	0.278721	chr13_28683_mature	0	0.805927	0	0.852739935		-20	0.140178	chr4_8839_mature	2.686425	0	1.882222339	0		20	0.559868
chr9_20138_mature	3.223709795	0	2.078510713	0		20	0.278721	mmu-miR-489-3p	0	0.805927	0	0.852739935		-20	0.140178	mmu-miR-743b-3p	2.686425	0	1.882222339	0		20	0.559868
chr12_25406_mature	3.223709795	0	2.078510713	0		20	0.278721	hsa-miR-5195-5p	0	0.805927	0	0.852739935		-20	0.140178	mmu-miR-182-5p	2.686425	0	1.882222339	0		20	0.559868
mmu-miR-466l-5p	3.223709795	0	2.078510713	0		20	0.278721	chr15_33053_star	0	0.805927	0	0.852739935		-20	0.140178	hsa-miR-3194-5p	2.686425	0	1.882222339	0		20	0.559868
chr4_8756_mature	3.223709795	0	2.078510713	0		20	0.278721	chr4_9100_star	0	0.805927	0	0.852739935		-20	0.140178	chr18_36082_star	2.686425	0	1.882222339	0		20	0.559868
chr3_6642_mature	3.223709795	0	2.078510713	0		20	0.278721	bta-miR-664	0	0.805927	0	0.852739935		-20	0.140178	chr14_30043_star	2.686425	0	1.882222339	0		20	0.559868
chrx_22554_star	3.223709795	0	2.078510713	0		20	0.278721	chr4_9649_mature	0	0.805927	0	0.852739935		-20	0.140178	chr1_3508_star	2.686425	0	1.882222339	0		20	0.559868
chr5_11678_star	3.223709795	0	2.078510713	0		20	0.278721	hsa-miR-5195-3p	0	0.805927	0	0.852739935		-20	0.140178	chr14_31496_star	2.686425	0	1.882222339	0		20	0.559868
hsa-miR-4468	3.223709795	0	2.078510713	0		20	0.278721	chr18_36523_star	0	0.805927	0	0.852739935		-20	0.140178	rno-miR-295-5p	2.686425	0	1.882222339	0		20	0.559868
hsa-miR-4464	3.223709795	0	2.078510713	0		20	0.278721	chr13_27082_star	0	0.805927	0	0.852739935		-20	0.140178	chr9_21046_star	2.686425	0	1.882222339	0		20	0.559868
bta-miR-2446	3.223709795	0	2.078510713	0		20	0.278721	hsa-miR-4755-5p	0	0.805927	0	0.852739935		-20	0.140178	chr10_23305_star	2.686425	0	1.882222339	0		20	0.559868
bta-miR-2440	3.223709795	0	2.078510713	0		20	0.278721	chr4_8705_star	0	0.805927	0	0.852739935		-20	0.140178	rno-miR-292-5p	2.686425	0	1.882222339	0		20	0.559868
hsa-miR-4438	3.223709795	0	2.078510713	0		20	0.278721	mmu-miR-2861	0	0.805927	0	0.852739935		-20	0.140178	hsa-miR-1182	2.686425	0	1.882222339	0		20	0.559868
hsa-miR-611	3.223709795	0	2.078510713	0		20	0.278721	chr3_7580_mature	0	0.805927	0	0.852739935		-20	0.140178	bta-miR-2461-5p	2.686425	0	1.882222339	0		20	0.559868
bta-miR-2396	3.223709795	0	2.078510713	0		20	0.278721	NW_003540508_39511_star	0	0.805927	0	0.852739935		-20	0.140178	chr16_34016_star	2.686425	0	1.882222339	0		20	0.559868
NW_003538080_37501_star	3.223709795	0	2.078510713	0		20	0.278721	hsa-miR-4650-5p	0	0.805927	0	0.852739935		-20	0.140178	NW_003538465_37902_star	2.686425	0	1.882222339	0		20	0.559868
mmu-miR-467c-3p	3.223709795	0	2.078510713	0		20	0.278721	chr15_32446_star	0	0.805927	0	0.852739935		-20	0.140178	rno-miR-342-3p	2.686425	0	1.882222339	0		20	0.559868
chr1_2345_mature	3.223709795	0	2.078510713	0		20	0.278721	chr8_18472_star	0	0.805927	0	0.852739935		-20	0.140178	chr10_23668_star	2.686425	0	1.882222339	0		20	0.559868
chr17_35019_star	3.223709795	0	2.078510713	0		20	0.278721	hsa-miR-551b-5p	0	0.805927	0	0.852739935		-20	0.140178	chr1_551_star	2.686425	0	1.882222339	0		20	0.559868
chr7_16673_star	3.223709795	0	2.078510713	0		20	0.278721	chr9_19536_star	0	0.805927	0	0.852739935		-20	0.140178	chr2_4073_mature	2.686425	0	1.882222339	0		20	0.559868
chr1_397_star	3.223709795	0	2.078510713	0		20	0.278721	chr18_36180_star	0	0.805927	0	0.852739935		-20	0.140178	mmu-miR-6373	2.686425	0	1.882222339	0		20	0.559868
bta-miR-2346	3.223709795	0	2.078510713	0		20	0.278721	hsa-miR-4704-3p	0	0.805927	0	0.852739935		-20	0.140178	hsa-miR-339-5p	2.686425	0	1.882222339	0		20	0.559868
mmu-miR-5129-3p	3.223709795	0	2.078510713	0		20	0.278721	chr1_550_mature	0	0.805927	0	0.852739935		-20	0.140178	bta-miR-2284a	2.686425	0	1.882222339	0		20	0.559868
bta-miR-2379	3.223709795	0	2.078510713	0		20	0.278721	hsa-miR-4753-3p	0	0.805927	0	0.852739935		-20	0.140178	chr15_32429_star	2.686425	0	1.882222339	0		20	0.559868
chr6_12841_star	3.223709795	0	2.078510713	0		20	0.278721	hsa-miR-548aj-3p	0	0.805927	0	0.852739935		-20	0.140178	bta-miR-148b	2.686425	0	1.882222339	0		20	0.559868
chr15_33037_mature	3.223709795	0	2.078510713	0		20	0.278721	chr10_24040_star	0	0.805927	0	0.852739935		-20	0.140178	chr3_6725_mature	2.686425	0	1.882222339	0		20	0.559868
mmu-miR-696	3.223709795	0	2.078510713	0		20	0.278721	chr5_11270_star	0	0.805927	0	0.852739935		-20	0.140178	chr11_25350_star	2.686425	0	1.882222339	0		20	0.559868
chr15_33512_mature	3.223709795	0	2.078510713	0		20	0.278721	mmu-miR-672-3p	0	0.805927	0	0.852739935		-20	0.140178	chr13_29176_star	2.686425	0	1.882222339	0		20	0.559868
chr10_23430_star	3.223709795	0	2.078510713	0		20	0.278721	rno-miR-653-5p	0	0.805927	0	0.852739935		-20	0.140178	chr7_16262_star	2.686425	0	1.882222339	0		20	0.559868
chr6_14749_star	3.223709795	0	2.078510713	0		20	0.278721	mmu-miR-3962	0	0.805927	0	0.852739935		-20	0.140178	chr13_27900_mature	2.686425	0	1.882222339	0		20	0.559868
chr4_8979_star	3.223709795	0	2.078510713	0		20	0.278721	mmu-miR-3961	0	0.805927	0	0.852739935		-20	0.140178	chr18_36377_star	2.686425	0	1.882222339	0		20	0.559868
rno-miR-291a-5p	3.223709795	0	2.078510713	0		20	0.278721	mmu-miR-3968	0	0.805927	0	0.852739935		-20	0.140178	chr8_18847_star	2.686425	0	1.882222339	0		20	0.559868
chr5_11639_star	3.223709795	0	2.078510713	0		20	0.278721	mmu-miR-664-5p	0	0.805927	0	0.852739935		-20	0.140178	mmu-miR-489-5p	2.686425	0	1.882222339	0		20	0.559868
chr1_2992_star	3.223709795	0	2.078510713	0		20	0.278721	hsa-miR-5572	0	0.805927	0	0.852739935		-20	0.140178	ssc-miR-1296-3p	2.686425	0	1.882222339	0		20	0.559868
chr10_24245_star	3.223709795	0	2.078510713	0		20	0.278721	chr16_34426_mature	0	0.805927	0	0.852739935		-20	0.140178	chr1_485_star	2.686425	0	1.882222339	0		20	0.559868
hsa-miR-5708	3.223709795	0	2.078510713	0		20	0.278721	bta-miR-425-3p	0	0.805927	0	0.852739935		-20	0.140178	chr13_26844_star	2.686425	0	1.882222339	0		20	0.559868
chrx_22357_star	3.223709795	0	2.078510713	0		20	0.278721	hsa-miR-501-5p	0	0.805927	0	0.852739935		-20	0.140178	mmu-miR-700-5p	2.686425	0	1.882222339	0		20	0.559868
chr16_34281_mature	3.223709795	0	2.078510713	0		20	0.278721	chr2_6477_star	0	0.805927	0	0.852739935		-20	0.140178	hsa-miR-6124	2.686425	0	1.882222339	0		20	0.559868
rno-miR-3573-3p	3.223709795	0	2.078510713	0		20	0.278721	rno-miR-653-3p	0	0.805927	0	0.852739935		-20	0.140178	chr18_35822_star	2.686425	0	1.882222339	0		20	0.559868
chr9_20053_star	3.223709795	0	2.078510713	0		20	0.278721	hsa-miR-6126	0	0.805927	0	0.852739935		-20	0.140178	mmu-miR-3076-5p	2.686425	0	1.882222339	0		20	0.559868
bta-miR-2419-3p	3.223709795	0	2.078510713	0		20	0.278721	NW_003613380_38805_star	0	0.805927	0	0.852739935		-20	0.140178	chr14_30276_star	2.686425	0	1.882222339	0		20	0.559868
hsa-miR-1304-5p	3.223709795	0	2.078510713	0		20	0.278721	bta-miR-150	0	0.805927	0	0.852739935		-20	0.140178	chr7_16538_star	2.686425	0	1.882222339	0		20	0.559868
chr10_23301_star	3.223709795	0	2.078510713	0		20	0.278721	chr9_19208_star	0	0.805927	0	0.852739935		-20	0.140178	hsa-miR-27a-5p	2.686425	0	1.882222339	0		20	0.559868
chr2_5368_star	3.223709795	0	2.078510713	0		20	0.278721	hsa-miR-4714-5p	0	0.805927	0	0.852739935		-20	0.140178	chr5_11381_star	2.686425	0	1.882222339	0		20	0.559868
chr2_4013_star	3.223709795	0	2.078510713	0		20	0.278721	chr8_18153_mature	0	0.805927	0	0.852739935		-20	0.140178	chr16_34606_star	2.686425	0	1.882222339	0		20	0.559868
chr3_8064_star	3.223709795	0	2.078510713	0		20	0.278721	chr2_4701_star	0	0.805927	0	0.852739935		-20	0.140178	chr13_29242_star	2.686425	0	1.882222339	0		20	0.559868
chr15_32033_star	3.223709795	0	2.078510713	0		20	0.278721	hsa-miR-5584-5p	0	0.805927	0	0.852739935		-20	0.140178	chr7_16991_mature	2.686425	0	1.882222339	0		20	0.559868
hsa-miR-4666a-3p	3.223709795	0	2.078510713	0		20	0.278721	chr15_33101_mature	0	0.805927	0	0.852739935		-20	0.140178	hsa-miR-181a-3p	2.686425	0	1.882222339	0		20	0.559868
mmu-miR-669g	3.223709795	0	2.078510713	0		20	0.278721	chr9_20807_star	0	0.805927	0	0.852739935		-20	0.140178	hsa-miR-549a	2.686425	0	1.882222339	0		20	0.559868
chr13_27615_star	3.223709795	0	2.078510713	0		20	0.278721	chrx_22382_star	0	0.805927	0	0.852739935		-20	0.140178	chr3_7521_mature	2.686425	0	1.882222339	0		20	0.559868
chr15_32787_mature	3.223709795	0	2.078510713	0		20	0.278721	chr8_17912_star	0	0.805927	0	0.852739935		-20	0.140178	hsa-miR-1306-5p	2.686425	0	1.882222339	0		20	0.559868
chr12_25952_star	3.223709795	0	2.078510713	0		20	0.278721	NW_003613359_38685_star	0	0.805927	0	0.852739935		-20	0.140178	chrx_22193_star	2.686425	0	1.882222339	0		20	0.559868
chr16_34566_star	3.223709795	0	2.078510713	0		20	0.278721	hsa-miR-3065-3p	0	0.805927	0	0.852739935		-20	0.140178	chr1_861_star	2.686425	0	1.882222339	0		20	0.559868
chr4_10161_mature	3.223709795	0	2.078510713	0		20	0.278721	chr11_24484_star	0	0.805927	0	0.852739935		-20	0.140178	chr6_12967_star	2.686425	0	1.882222339	0		20	0.559868
chr8_17503_star	3.223709795	0	2.078510713	0		20	0.278721	mmu-miR-3094-3p	0	0.805927	0	0.852739935		-20	0.140178	chr18_35926_star	2.686425	0	1.882222339	0		20	0.559868
chr1_1968_mature	3.223709795	0	2.078510713	0		20	0.278721	hsa-miR-500a-3p	0	0.805927	0	0.852739935		-20	0.140178	mmu-miR-1195	2.686425	0	1.882222339	0		20	0.559868
chr5_12206_mature	3.223709795	0	2.078510713	0		20	0.278721	chr10_23279_star	0	0.805927	0	0.852739935		-20	0.140178	hsa-miR-4680-3p	2.686425	0	1.882222339	0		20	0.559868
mmu-miR-1231-5p	3.223709795	0	2.078510713	0		20	0.278721	hsa-miR-517c-3p	0	0.805927	0	0.852739935		-20	0.140178	chrx_21839_star	2.686425	0	1.882222339	0		20	0.559868
mmu-miR-152-5p	3.223709795	0	2.078510713	0		20	0.278721	bta-miR-15b	0	0.805927	0	0.852739935		-20	0.140178	chr13_27902_star	2.686425	0	1.882222339	0		20	0.559868
mmu-miR-155-3p	3.223709795	0	2.078510713	0		20	0.278721	chr9_20720_star	0	0.805927	0	0.852739935		-20	0.140178	hsa-miR-3171	2.686425	0	1.882222339	0		20	0.559868
hsa-miR-4511	3.223709795	20.95411	2.078510713	4.456419386		-2.70044	0.278924	chr18_35974_star@@mmu-miR-592-3p	0	0.805927	0	0.852739935		-20	0.140178	hsa-miR-3170	2.686425	0	1.882222339	0		20	0.559868
chr2_5678_star	2331.816752	124.1128	11.18785727	6.967085898		4.23173	0.279718	chr2_6343_mature	0	0.805927	0	0.852739935		-20	0.140178	chr5_11659_star	2.686425	0	1.882222339	0		20	0.559868
NW_003538949_38373_mature	5.372849659	37.07266	2.671938627	5.250683565		-2.7866	0.281205	chrx_23090_mature	0	0.805927	0	0.852739935		-20	0.140178	chr2_5163_star	2.686425	0	1.882222339	0		20	0.559868
chr7_16758_mature	64.47419591	8.865202	6.032854532	3.302348583		2.862496	0.281516	chr1_1936_star	0	0.805927	0	0.852739935		-20	0.140178	hsa-miR-3165	2.686425	0	1.882222339	0		20	0.559868

chr9_20818_star	56.95220639	8.059274	5.856791684	3.179395517		2.82103	0.282207	bta-miR-191	0	0.805927	0	0.852739935		-20	0.140178	mmu-miR-297a-5p	2.686425	0	1.882222339	0		20	0.559868
chr1_991_mature	19.34225877	3.22371	4.346407978	2.078510713		2.584963	0.282492	chr13_27018_star	0	0.805927	0	0.852739935		-20	0.140178	rno-miR-336-5p	2.686425	0	1.882222339	0		20	0.559868
chr12_25445_mature	19.34225877	3.22371	4.346407978	2.078510713		2.584963	0.282492	bta-miR-184	0	0.805927	0	0.852739935		-20	0.140178	hsa-miR-3146	2.686425	0	1.882222339	0		20	0.559868
chr12_25994_mature	19.34225877	3.22371	4.346407978	2.078510713		2.584963	0.282492	chr14_29623_star	0	0.805927	0	0.852739935		-20	0.140178	chr14_30390_mature	2.686425	0	1.882222339	0		20	0.559868
bta-miR-26a	19.34225877	3.22371	4.346407978	2.078510713		2.584963	0.282492	chr14_29542_star	0	0.805927	0	0.852739935		-20	0.140178	hsa-miR-4262	2.686425	0	1.882222339	0		20	0.559868
chr6_12830_mature	4.298279727	28.20746	2.405524014	4.868265031		-2.71425	0.283076	chr6_14927_star	0	0.805927	0	0.852739935		-20	0.140178	bta-miR-6521	2.686425	0	1.882222339	0		20	0.559868
chr13_28023_star	6.447419591	45.93786	2.896740642	5.552680304		-2.83289	0.283667	chr1_916_star	0	0.805927	0	0.852739935		-20	0.140178	mmu-miR-3080-3p	2.686425	0	1.882222339	0		20	0.559868
chr17_35115_star	2.149139864	13.70077	1.654957834	3.877819487		-2.67243	0.284028	chr14_31065_star	0	0.805927	0	0.852739935		-20	0.140178	NW_003538922_38350_mature	2.686425	0	1.882222339	0		20	0.559868
chr1_3501_mature	2.149139864	13.70077	1.654957834	3.877819487		-2.67243	0.284028	chr3_7464_mature	0	0.805927	0	0.852739935		-20	0.140178	NW_003613234_37804_star	2.686425	0	1.882222339	0		20	0.559868
chr11_24331_mature@@mmu-miR-1	2.149139864	13.70077	1.654957834	3.877819487		-2.67243	0.284028	chr11_25215_star	0	0.805927	0	0.852739935		-20	0.140178	chr9_19828_star	2.686425	0	1.882222339	0		20	0.559868
chr9_19693_star	2.149139864	13.70077	1.654957834	3.877819487		-2.67243	0.284028	hsa-miR-3682-3p	0	0.805927	0	0.852739935		-20	0.140178	chr15_33083_star	2.686425	0	1.882222339	0		20	0.559868
chr1_3174_mature	2.149139864	13.70077	1.654957834	3.877819487		-2.67243	0.284028	chrx_22108_star	0	0.805927	0	0.852739935		-20	0.140178	chr14_30331_star	2.686425	0	1.882222339	0		20	0.559868
chr1_169_star	2.149139864	13.70077	1.654957834	3.877819487		-2.67243	0.284028	NW_003613335_38593_star	0	0.805927	0	0.852739935		-20	0.140178	chr8_17941_mature	2.686425	0	1.882222339	0		20	0.559868
chr8_18587_mature	2.149139864	13.70077	1.654957834	3.877819487		-2.67243	0.284028	mmu-miR-653-5p	0	0.805927	0	0.852739935		-20	0.140178	chr6_12434_star	2.686425	0	1.882222339	0		20	0.559868
chr1_2437_star	1.074569932	8.059274	1.05281229	3.179395517		-2.90689	0.284361	chr18_36371_star	0	0.805927	0	0.852739935		-20	0.140178	chr8_18586_star	2.686425	0	1.882222339	0		20	0.559868
rno-miR-145-5p	1.074569932	8.059274	1.05281229	3.179395517		-2.90689	0.284361	chr14_31484_mature	0	0.805927	0	0.852739935		-20	0.140178	chr1_2783_mature	2.686425	0	1.882222339	0		20	0.559868
chr14_30424_star	1.074569932	8.059274	1.05281229	3.179395517		-2.90689	0.284361	mmu-miR-1247-5p	0	0.805927	0	0.852739935		-20	0.140178	hsa-miR-892c-3p	2.686425	0	1.882222339	0		20	0.559868
chr10_23893_star	1.074569932	8.059274	1.05281229	3.179395517		-2.90689	0.284361	chrx_23097_mature	0	0.805927	0	0.852739935		-20	0.140178	rno-miR-17-2-3p	2.686425	0	1.882222339	0		20	0.559868
chr9_20300_mature	1.074569932	8.059274	1.05281229	3.179395517		-2.90689	0.284361	mmu-miR-20a-5p	0	0.805927	0	0.852739935		-20	0.140178	chr4_8839_star	2.686425	0	1.882222339	0		20	0.559868
chr4_9452_star	1.074569932	8.059274	1.05281229	3.179395517		-2.90689	0.284361	chr2_4454_star	0	0.805927	0	0.852739935		-20	0.140178	mmu-miR-3083-5p	2.686425	0	1.882222339	0		20	0.559868
chr13_26807_mature	1.074569932	8.059274	1.05281229	3.179395517		-2.90689	0.284361	hsa-miR-513c-3p	0	0.805927	0	0.852739935		-20	0.140178	rno-miR-489-5p	2.686425	0	1.882222339	0		20	0.559868
bta-miR-876	1.074569932	8.059274	1.05281229	3.179395517		-2.90689	0.284361	chr12_25481_star	0	0.805927	0	0.852739935		-20	0.140178	NW_003613063_36957_mature	2.686425	0	1.882222339	0		20	0.559868
chr1_877_mature	1.074569932	8.059274	1.05281229	3.179395517		-2.90689	0.284361	hsa-miR-4786-5p	0	0.805927	0	0.852739935		-20	0.140178	chr1_1628_mature	2.686425	0	1.882222339	0		20	0.559868
bta-miR-100	1.074569932	8.059274	1.05281229	3.179395517		-2.90689	0.284361	NW_003537370_36873_star	0	0.805927	0	0.852739935		-20	0.140178	bta-miR-2460	2.686425	0	1.882222339	0		20	0.559868
chr9_20710_mature	1.074569932	8.059274	1.05281229	3.179395517		-2.90689	0.284361	hsa-miR-6511a-5p	0	0.805927	0	0.852739935		-20	0.140178	hsa-miR-2115-3p	2.686425	0	1.882222339	0		20	0.559868
chr9_19650_mature	1.074569932	8.059274	1.05281229	3.179395517		-2.90689	0.284361	chr7_16542_star	0	0.805927	0	0.852739935		-20	0.140178	hsa-miR-3121-3p	2.686425	0	1.882222339	0		20	0.559868
chr2_5969_mature	1.074569932	8.059274	1.05281229	3.179395517		-2.90689	0.284361	hsa-miR-2114-5p	0	0.805927	0	0.852739935		-20	0.140178	hsa-miR-4420	2.686425	0	1.882222339	0		20	0.559868
chr14_31645_mature	1.074569932	8.059274	1.05281229	3.179395517		-2.90689	0.284361	chr7_16992_mature	0	0.805927	0	0.852739935		-20	0.140178	hsa-miR-4699-3p	2.686425	0	1.882222339	0		20	0.559868
chr2_6581_mature	1.074569932	8.059274	1.05281229	3.179395517		-2.90689	0.284361	chr3_8247_star	0	0.805927	0	0.852739935		-20	0.140178	hsa-miR-6512-3p	2.686425	0	1.882222339	0		20	0.559868
chrx_23221_star	1.074569932	8.059274	1.05281229	3.179395517		-2.90689	0.284361	mmu-miR-5626-5p	0	0.805927	0	0.852739935		-20	0.140178	mmu-miR-1843b-3p	2.686425	0	1.882222339	0		20	0.559868
chr12_26098_star	1.074569932	8.059274	1.05281229	3.179395517		-2.90689	0.284361	hsa-miR-320d	0	0.805927	0	0.852739935		-20	0.140178	NW_003538080_37501_star	2.686425	0	1.882222339	0		20	0.559868
hsa-miR-4442	1.074569932	8.059274	1.05281229	3.179395517		-2.90689	0.284361	hsa-miR-3689b-3p	0	0.805927	0	0.852739935		-20	0.140178	chr14_29623_mature	2.686425	0	1.882222339	0		20	0.559868
chr15_33216_mature	1.074569932	8.059274	1.05281229	3.179395517		-2.90689	0.284361	hsa-miR-519a-3p	0	0.805927	0	0.852739935		-20	0.140178	chr13_29206_star	2.686425	0	1.882222339	0		20	0.559868
chr17_35178_star	1.074569932	8.059274	1.05281229	3.179395517		-2.90689	0.284361	chr9_19419_star	0	0.805927	0	0.852739935		-20	0.140178	hsa-miR-648	2.686425	0	1.882222339	0		20	0.559868
chr1_2966_mature	1.074569932	8.059274	1.05281229	3.179395517		-2.90689	0.284361	chr8_17231_star	0	0.805927	0	0.852739935		-20	0.140178	chr1_392_star	2.686425	0	1.882222339	0		20	0.559868
chr17_35035_mature	1.074569932	8.059274	1.05281229	3.179395517		-2.90689	0.284361	hsa-miR-1207-3p	0	0.805927	0	0.852739935		-20	0.140178	chr6_15067_star	2.686425	0	1.882222339	0		20	0.559868
chr16_33794_star	1.074569932	8.059274	1.05281229	3.179395517		-2.90689	0.284361	hsa-miR-548k	0	0.805927	0	0.852739935		-20	0.140178	chr13_26768_mature	2.686425	0	1.882222339	0		20	0.559868
chr2_6132_mature	1.074569932	8.059274	1.05281229	3.179395517		-2.90689	0.284361	chr15_33480_star	0	0.805927	0	0.852739935		-20	0.140178	chr15_31962_star	2.686425	0	1.882222339	0		20	0.559868
chr7_17182_mature	1.074569932	8.059274	1.05281229	3.179395517		-2.90689	0.284361	hsa-miR-548p	0	0.805927	0	0.852739935		-20	0.140178	chr6_15024_star	2.686425	0	1.882222339	0		20	0.559868
chr12_25520_mature	1.074569932	8.059274	1.05281229	3.179395517		-2.90689	0.284361	chr11_24857_star	0	0.805927	0	0.852739935		-20	0.140178	mmu-miR-3098-5p	2.686425	0	1.882222339	0		20	0.559868
hsa-miR-4497	7.521989523	55.60899	3.091190278	5.82295938		-2.88613	0.28501	hsa-miR-548z	0	0.805927	0	0.852739935		-20	0.140178	chr13_28409_star	2.686425	0	1.882222339	0		20	0.559868
rno-miR-668	30.08795809	4.835565	4.958283956	2.544872269		2.63743	0.285391	bta-miR-202	0	0.805927	0	0.852739935		-20	0.140178	chr15_31871_star	2.686425	0	1.882222339	0		20	0.559868
hsa-miR-4532	30.08795809	4.835565	4.958283956	2.544872269		2.63743	0.285391	hsa-miR-4645-3p	0	0.805927	0	0.852739935		-20	0.140178	mmu-miR-5129-3p	2.686425	0	1.882222339	0		20	0.559868
chr9_19846_mature	5.372849659	36.26674	2.671938627	5.21981653		-2.75489	0.285884	chr15_33620_mature	0	0.805927	0	0.852739935		-20	0.140178	mmu-miR-692	2.686425	0	1.882222339	0		20	0.559868
chr16_33995_mature	3.223709795	20.14819	2.078510713	4.402462031		-2.64386	0.286605	chr17_34996_star	0	0.805927	0	0.852739935		-20	0.140178	NW_003538681_38109_star	2.686425	0	1.882222339	0		20	0.559868
ssc-miR-129b	81.66731482	1630.391	6.369245121	10.67188709		-4.31932	0.287199	chr15_33043_star	0	0.805927	0	0.852739935		-20	0.140178	hsa-miR-3130-5p	2.686425	0	1.882222339	0		20	0.559868
ssc-miR-9-1	6163.733129	255.479	12.58982273	8.002696903		4.592528	0.28777	chr13_28713_star	0	0.805927	0	0.852739935		-20	0.140178	chr1_1174_star	2.686425	0	1.882222339	0		20	0.559868
chr4_9788_mature	4.298279727	27.40153	2.405524014	4.827896911		-2.67243	0.289021	hsa-miR-5002-5p	0	0.805927	0	0.852739935		-20	0.140178	hsa-miR-4636	2.686425	0	1.882222339	0		20	0.559868
chr18_36269_star	13.96940911	2.417782	3.90394537	1.773060525		2.530515	0.289487	rno-miR-30a-5p	0	0.805927	0	0.852739935		-20	0.140178	hsa-miR-5708	2.686425	0	1.882222339	0		20	0.559868
NW_003613382_38825_mature	13.96940911	2.417782	3.90394537	1.773060525		2.530515	0.289487	rno-miR-451-3p	0	0.805927	0	0.852739935		-20	0.140178	chr14_30041_star	2.686425	0	1.882222339	0		20	0.559868
chr10_23828_mature	13.96940911	2.417782	3.90394537	1.773060525		2.530515	0.289487	chr14_31020_star	0	0.805927	0	0.852739935		-20	0.140178	mmu-miR-290-5p	2.686425	0	1.882222339	0		20	0.559868
chr9_19418_star	13.96940911	2.417782	3.90394537	1.773060525		2.530515	0.289487	chr17_35646_mature	0	0.805927	0	0.852739935		-20	0.140178	chr9_19516_star	2.686425	0	1.882222339	0		20	0.559868
ssc-miR-421-3p	4371.350483	199.87	12.09419334	7.650118356		4.450945	0.290042	mmu-miR-3095-3p	0	0.805927	0	0.852739935		-20	0.140178	chr2_5267_star	2.686425	0	1.882222339	0		20	0.559868
chr1_777_mature	9.671129386	1.611855	3.415640968	1.38507475		2.584963	0.290155	chr13_27728_mature	0	0.805927	0	0.852739935		-20	0.140178	chr15_31760_star	2.686425	0	1.882222339	0		20	0.559868
chr6_13322_mature	9.671129386	1.611855	3.415640968	1.38507475		2.584963	0.290155	bta-miR-27b	0	0.805927	0	0.852739935		-20	0.140178	chr12_25473_mature	2.686425	0	1.882222339	0		20	0.559868
rno-miR-3068-5p	9.671129386	1.611855	3.415640968	1.38507475		2.584963	0.290155	chr10_24278_star	0	0.805927	0	0.852739935		-20	0.140178	chr3_8450_star	2.686425	0	1.882222339	0		20	0.559868
chr6_14501_star	9.671129386	1.611855	3.415640968	1.38507475		2.584963	0.290155	rno-miR-100-5p	0	0.805927	0	0.852739935		-20	0.140178	chr15_31760_mature	2.686425	0	1.882222339	0		20	0.559868
chr4_9327_star	9.671129386	1.611855	3.415640968	1.38507475		2.584963	0.290155	hsa-miR-3145-5p	0	0.805927	0	0.852739935		-20	0.140178	chr4_10042_star	2.686425	0	1.882222339	0		20	0.559868
NW_003538989_38414_star	9.671129386	1.611855	3.415640968	1.38507475		2.584963	0.290155	mmu-miR-1197-3p	0	0.805927	0	0.852739935		-20	0.140178	chr3_7118_mature	2.686425	0	1.882222339	0		20	0.559868
mmu-miR-1186a	9.671129386	1.611855	3.415640968	1.38507475		2.584963	0.290155	chr15_32157_star	0	0.805927	0	0.852739935		-20	0.140178	chr1_2451_star	2.686425	0	1.882222339	0		20	0.559868
chr13_26759_mature	9.671129386	1.611855	3.415640968	1.38507475		2.584963	0.290155	mmu-miR-1192	0	0.805927	0	0.852739935		-20	0.140178	chr14_31110_star	2.686425	0	1.882222339	0		20	0.559868
mmu-miR-466q	9.671129386	1.611855	3.415640968	1.38507475		2.584963	0.290155	chr7_16174_star	0	0.805927	0	0.852739935		-20	0.140178	bta-miR-3601	2.686425	0	1.882222339	0		20	0.559868
chr7_16525_mature	9.671129386	1.611855	3.415640968	1.38507475		2.584963	0.290155	chr7_16529_star	0	0.805927	0	0.852739935		-20	0.140178	chr7_16933_star	2.686425	0	1.882222339	0		20	0.559868
chr1_3928_mature	9.671129386	1.611855	3.415640968	1.38507475		2.584963	0.290155	chr6_13458_star	0	0.805927	0	0.852739935		-20	0.140178	chr8_17503_star	2.686425	0	1.882222339	0		20	0.559868
chr13_27072_star	35.46080775	5.641492	5.188274618	2.731507408		2.652077	0.290192	hsa-miR-4769-5p	0	0.805927	0	0.852739935		-20	0.140178	chr1_1968_mature	2.686425	0	1.882222339	0		20	0.559868
chr5_11762_star	93.48758407	12.08891	6.562052863	3.710273245		2.95109	0.290552	chr13_28917_star	0	0.805927	0	0.852739935		-20	0.140178	hsa-miR-1229-3p	2.686425	0	1.882222339	0		20	0.559868
ssc-miR-9	6062.723555	253.8671	12.56598827	7.993601605		4.57782	0.290762	chr1_1837_star	0	0.805927	0	0.852739935		-20	0.140178	chr15_32917_star	2.686425	0	1.882222339	0		20	0.559868
NW_003613026_36810_mature	8.596559455	64.4742	3.262517265	6.032854532		-2.90689	0.291171	chr12_26014_star	0	0.805927	0	0.852739935		-20	0.140178	chr2_4255_star	2.686425	0	1.882222339	0		20	0.559868
bta-miR-2904	10561.94786	381.2037	13.36672489	8.578197871		4.79217	0.29197	hsa-miR-422a	0	0.805927	0	0.852739935		-20	0.140178	hsa-miR-1910	2.686425	0	1.882222339	0		20	0.559868
chr6_12802_mature	92.41301414	12.08891	6.545551653	3.710273245		2.934412	0.293876	mmu-miR-133a-3p	0	0.805927	0	0.852739935		-20	0.140178	chr13_28643_mature	2.686425	0	1.882222339	0		20	0.559868
chr18_35926_mature	29.01338816	4.835565	4.907534286	2.544872269		2.584963	0.294144	rno-miR-1912-3p	0	0.805927	0	0.852739935		-20	0.140178	ssc-miR-328	18.26769	144.261	4.268111626	7.182503739		-2.98132	0.560006
chr4_9934_mature	29.01338816	4.835565	4.907534286	2.544872269		2.584963	0.294144	chr2_4310_star	0	0.805927	0	0.852739935		-20	0.140178	chr14_30569_mature	10.7457	2.417782	3.554060706	1.773060525		2.152003	0.561215
chr2_5746_star	3.223709795	19.34226	2.078510713	4.346407978		-2.58496	0.294573	mmu-miR-181a-2-3p	0	0.805927	0	0.852739935		-20	0.140178	chr4_10175_mature	10.7457	2.417782	3.554060706	1.773060525		2.152003	0.561215
chr14_31058_mature	3.223709795	19.34226	2.078510713	4.346407978		-2.58496	0.294573	rno-miR-135b-5p	0	0.805927	0	0.852739935		-20	0.140178	chr13_28551_star	10.7457	2.417782	3.554060706	1.773060525		2.152003	0.561215
chr3_7304_mature@@bta-miR-216b	4.298279727	26.59561	2.405524014	4.786366653		-2.62936	0.295137	hsa-miR-149-5p	0	0.805927	0	0.852739935		-20	0.140178	chr1_2557_star	10.7457	2.417782	3.554060706	1.773060525		2.152003	0.561215
chr9_19140_star	4.298279727	26.59561	2.405524014	4.786366653		-2.62936	0.295137	chr11_24782_star	0	0.805927	0	0.852739935		-20	0.140178	NW_003613297_38335_mature	10.7457	2.417782	3.554060706	1.773060525		2.152003	0.561215
chr7_15778_mature	4.298279727	26.59561	2.405524014	4.786366653		-2.62936	0.295137	chr7_16599_star	0	0.805927	0	0.852739935		-20	0.140178	chr1_188_mature	10.7457	2.417782	3.554060706	1.773060525		2.152003	0.561215
chr10_23700_mature	18.26768884	3.22371	4.268111626	2.078510713		2.5025	0.295167	hsa-miR-3163	0	0.805927	0	0.852739935		-20	0.140178	chr7_15311_mature	10.7457	2.417782	3.554060706	1.773060525		2.152003	0.561215
NW_003539027_38478_mature	18.26768884	3.22371	4.268111626	2.078510713		2.5025	0.295167	NW_003537967_37423_star	0	0.805927	0	0.852739935		-20	0.140178	chr3_7233_mature	4.29828	20.14819	2.405524014	4.402462031		-2.22882	0.56128
chr2_4363_mature	18.26768884	3.22371	4.268111626	2.078510713		2.5025	0.295167	chr11_25156_mature	0	0.805927	0	0.852739935		-20	0.140178	chr14_30104_mature	4.29828	20.14819	2.405524014	4.402462031		-2.22882	0.56128
chr3_7417_star	2.149139864	12.89484	1.654957834	3.796477232		-2.58496	0.295171	chr4_9937_star	0	0.805927	0	0.852739935		-20	0.140178	chr7_15107_mature	29.55067	5.641492	4.933132262	2.731507408		2.389042	0.562188
mmu-miR-5106	2.149139864	12.89484	1.654957834	3.796477232		-2.58496	0.295171	bta-miR-432	0	0.805927	0	0.852739935		-20	0.140178	chr1_782_mature	648.503	45.13194	9.343192277	5.527693969		3.844893	0.56249
chr14_30876_mature	2.149139864	12.89484	1.654957834	3.796477232		-2.58496	0.295171	chr5_11562_star@@mmu-miR-3059-3p	0	0.805927	0	0.852739935		-20	0.140178	chr14_30313_mature	5.37285	26.59561	2.671938627	4.786366653		-2.30743	0.563433
chr5_10760_mature	2.149139864	12.89484	1.654957834	3.796477232		-2.58496	0.295171	hsa-miR-3180	0	0.805927	0	0.852739935		-20	0.140178	rno-miR-450a-5p	14.50669	3.22371	3.954819241	2.078510713		2.169925	0.564
bta-miR-873	2.149139864	12.89484	1.654957834	3.796477232		-2.58496	0.295171	hsa-miR-3183	0	0.805927	0	0.852739935		-20	0.140178	chr5_11998_mature	14.50669	3.22371	3.954819241	2.078510713		2.169925	0.564
chr18_36112_mature	2.149139864	12.89484	1.654957834	3.796477232		-2.58496	0.295171	hsa-miR-3188	0	0.805927	0	0.852739935		-20	0.140178	chr18_36069_mature	14.50669	3.22371	3.954819241	2.078510713		2.169925	0.564
chr13_28125_mature@@mmu-miR-1	2.149139864	12.89484	1.654957834	3.796477232		-2.58496	0.295171	chr6_14789_star	0	0.805927	0	0.852739935		-20	0.140178	chr9_20679_mature	14.50669	3.22371	3.954819241	2.078510713		2.169925	0.564
chr9_20736_mature	2.149139864	12.89484	1.654957834	3.796477232		-2.58496	0.295171	hsa-miR-3132	0	0.805927	0	0.852739935		-20	0.140178	chr1_3439_mature@@rno-miR-872	14.50669	3.22371	3.954819241	2.078510713		2.169925	0.564
chr6_12875_mature	2.149139864	12.89484	1.654957834	3.796477232		-2.58496	0.295171	mmu-miR-365-1-5p	0	0.805927	0	0.852739935		-20	0.140178	ssc-let-7c	424.9924	11989.78	8.734683909	13.54963821		-4.81822	0.564901
chr13_28254_mature	9.671129386	74.14533	3.415640968	6.231611453		-2.9386	0.295278	bta-miR-346	0	0.805927	0	0.852739935		-20	0.140178	chr7_16088_mature	18.80497	4.029637	4.307790888	2.330454351		2.222392	0.565774
chr13_28624_star	47.281077	7.253347	5.593385953	3.044979305		2.704544	0.296151	bta-miR-335	0	0.805927	0	0.852739935		-20	0.140178	mmu-miR-3096a-5p	94.56215	12.89484	6.578367468	3.796477232		2.874469	0.566254
chr12_26422_star	200.9445773	21.76004	7.657815596	4.508431259		3.207044	0.298293	chr13_29260_star	0	0.805927	0	0.852739935		-20	0.140178	chr12_26240_mature	2.686425	11.28298	1.882222339	3.618589217		-2.07039	0.5664
chr15_31871_mature	6.447419591	42.71415	2.896740642	5.4500286		-2.72792	0.299662	hsa-miR-3159	0	0.805927	0	0.852739935		-20	0.140178	NW_003613097_37151_mature	2.686425	11.28298	1.882222339	3.618589217		-2.07039	0.5664
chr1_478_mature	10.74569932	83.81645	3.554060706	6.406272275		-2.96347	0.300116	bta-miR-340	0	0.805927	0	0.852739935		-20	0.140178	NW_003538201_37614_mature	2.686425	11.28298	1.882222339	3.618589217		-2.07039	0.5664
chr16_34306_mature	5.372849659	33.84895	2.671938627	5.123043401		-2.65535	0.30058	chr15_33675_star	0	0.805927	0	0.852739935		-20	0.140178	bta-miR-2352	2.686425	11.28298	1.882222339	3.618589217		-2.07039	0.5664
chr14_30429_mature	20.4168287	205.5115	4.420672964	7.690078309		-3.33139	0.30099	bta-miR-367	0	0.805927	0	0.852739935		-20	0.140178	hsa-miR-620	2.14914	8.865202	1.654957834	3.302348583		-2.04439	0.567068
chr7_15292_mature	4.298279727	25.78968	2.405524014	4.743605355		-2.58496	0.301428	chr9_19936_star	0	0.805927	0	0.852739935		-20	0.140178	chr1_1729_star	2.14914	8.865202	1.654957834	3.302348583		-2.04439	0.567068
chr16_34772_star	1.074569932	7.253347	1.05281229	3.044979305		-2.75489	0.302347	rno-miR-376c-3p	0	0.805927	0	0.852739935		-20	0.140178	bta-miR-2337	2.14914	8.865202	1.654957834	3.302348583		-2.04439	0.567068
chrx_22707_mature	1.074569932	7.253347	1.05281229	3.044979305		-2.75489	0.302347	hsa-miR-3664-3p	0	0.805927	0	0.852739935		-20	0.140178	chr1_1859_mature	2.14914	8.865202	1.654957834	3.302348583		-2.04439	0.567068
chr4_9687_mature	1.074569932	7.253347	1.05281229	3.044979305		-2.75489	0.302347	hsa-miR-3115	0	0.805927	0	0.852739935		-20	0.140178	chr1_2968_mature	2.14914	8.865202	1.654957834	3.302348583		-2.04439	0.567068
chr2_6087_mature	1.074569932	7.253347	1.05281229	3.044979305		-2.75489	0.302347	chr12_25400_mature	0	0.805927	0	0.852739935		-20	0.140178	chr7_16758_mature	2.14914	8.865202	1.654957834	3.302348583		-2.04439	0.567068
NW_003613217_37697_star	1.074569932	7.253347	1.05281229	3.044979305		-2.75489	0.302347	hsa-miR-4260	0	0.805927	0	0.852739935		-20	0.140178	chr7_15648_mature	1.07457	4.835565	1.05281229	2.544872269		-2.16993	0.569367
chrx_22045_star	1.074569932	7.253347	1.05281229	3.044979305		-2.75489	0.302347	chr9_20710_star	0	0.805927	0	0.852739935		-20	0.140178	chr2_4631_star	1.07457	4.835565	1.05281229	2.544872269		-2.16993	0.569367
hsa-miR-3651	1.074569932	7.253347	1.05281229	3.044979305		-2.75489	0.302347	hsa-miR-3158-3p	0	0.805927	0	0.852739935		-20	0.140178	NW_003539217_38656_mature	1.07457	4.835565	1.05281229	2.544872269		-2.16993	0.569367
chr7_16529_mature	1.074569932	7.253347	1.05281229	3.044979305		-2.75489	0.302347	hsa-miR-4274	0	0.805927	0	0.852739935		-20	0.140178	hsa-miR-548av-3p	1.07457	4.835565	1.05281229	2.544872269		-2.16993	0.569367
chr6_14103_mature	1.074569932	7.253347	1.05281229	3.044979305		-2.75489	0.302347	hsa-miR-4275	0	0.805927	0	0.852739935		-20	0.140178	NW_003537216_36773_mature	1.07457	4.835565	1.05281229	2.544872269		-2.16993	0.569367
mmu-miR-6362	1.074569932	7.253347	1.05281229	3.044979305		-2.75489	0.302347	bta-miR-6523	0	0.805927	0	0.852739935		-20	0.140178	chr7_16829_star	1.07457	4.835565	1.05281229	2.544872269		-2.16993	0.569367
chr8_17722_star	1.074569932	7.253347	1.05281229	3.044979305		-2.75489	0.302347	bta-miR-6527	0	0.805927	0	0.852739935		-20	0.140178	mmu-miR-193b-5p	1.07457	4.835565	1.05281229	2.544872269		-2.16993	0.569367
chr3_7761_mature	1.074569932	7.253347	1.05281229	3.044979305		-2.75489	0.302347	chr5_11717_star	0	0.805927	0	0.852739935		-20	0.140178	NW_003613507_39496_mature	1.07457	4.835565	1.05281229	2.544872269		-2.16993	0.569367
chrx_23209_mature	1.074569932	7.253347	1.05281229	3.044979305		-2.75489	0.302347	chr2_6095_star@@hsa-miR-513a-5p	0	0.805927	0	0.852739935		-20	0.140178	chr15_32760_mature	1.07457	4.835565	1.05281229	2.544872269		-2.16993	0.569367
chr3_7692_star	1.074569932	7.253347	1.05281229	3.044979305		-2.75489	0.302347	hsa-miR-4293	0	0.805927	0	0.852739935		-20	0.140178	chr14_30973_mature	1.07457	4.835565	1.05281229	2.544872269		-2.16993	0.569367
chr3_8179_star	1.074569932	7.253347	1.05281229	3.044979305		-2.75489	0.302347	hsa-miR-4296	0	0.805927	0	0.852739935		-20	0.140178	chrx_22359_star	1.07457	4.835565	1.05281229	2.544872269		-2.16993	0.569367
chr17_35011_mature	1.074569932	7.253347	1.05281229	3.044979305		-2.75489	0.302347	chr14_30394_star	0	0.805927	0	0.852739935		-20	0.140178	chr13_27616_mature	1.07457	4.835565	1.05281229	2.544872269		-2.16993	0.569367
bta-miR-497	1.074569932	7.253347	1.05281229	3.044979305		-2.75489	0.302347	hsa-miR-2054	0	0.805927	0	0.852739935		-20	0.140178	chr15_33317_mature	1.07457	4.835565	1.05281229	2.544872269		-2.16993	0.569367
chr11_25077_mature	1.074569932	7.253347	1.05281229	3.044979305		-2.75489	0.302347	mmu-miR-344d-1-5p	0	0.805927	0	0.852739935		-20	0.140178	rno-miR-293-5p	1.07457	4.835565	1.05281229	2.544872269		-2.16993	0.569367
chr17_34922_star	1.074569932	7.253347	1.05281229	3.044979305		-2.75489	0.302347	chr1_2519_mature	0	0.805927	0	0.852739935		-20	0.140178	hsa-miR-1236-5p	1.07457	4.835565	1.05281229	2.544872269		-2.16993	0.569367
hsa-miR-626	1.074569932	7.253347	1.05281229	3.044979305		-2.75489	0.302347	chr1_916_mature	0	0.805927	0	0.852739935		-20	0.140178	chr12_26428_mature	1.07457	4.835565	1.05281229	2.544872269		-2.16993	0.569367
chr2_4016_star	1.074569932	7.253347	1.05281229	3.044979305		-2.75489	0.302347	chrx_21688_star	0	0.805927	0	0.852739935		-20	0.140178	bta-miR-2312	1.07457	4.835565	1.05281229	2.544872269		-2.16993	0.569367
NW_003540202_39340_star	1.074569932	7.253347	1.05281229	3.044979305		-2.75489	0.302347	bta-miR-2454-5p	0	0.805927	0	0.852739935		-20	0.140178	hsa-miR-5706	1.07457	4.835565	1.05281229	2.544872269		-2.16993	0.569367
chr16_33929_star	1.074569932	7.253347	1.05281229	3.044979305		-2.75489	0.302347	mmu-miR-206-3p	0	0.805927	0	0.852739935		-20	0.140178	hsa-miR-4532	1.07457	4.835565	1.05281229	2.544872269		-2.16993	0.569367
bta-miR-208a	1.074569932	7.253347	1.05281229	3.044979305		-2.75489	0.302347	chr5_11377_star	0	0.805927	0	0.852739935		-20	0.140178	chr7_16758_star	1.07457	4.835565	1.05281229	2.544872269		-2.16993	0.569367
chr2_6281_mature	3.223709795	18.53633	2.078510713	4.288087668		-2.52356	0.302837	bta-miR-2454-3p	0	0.805927	0	0.852739935		-20	0.140178	chr14_29649_mature	1.07457	4.835565	1.05281229	2.544872269		-2.16993	0.569367
chr17_35330_mature	27.93881823	4.835565	4.854934103	2.544872269		2.530515	0.303192	mmu-miR-302a-3p	0	0.805927	0	0.852739935		-20	0.140178	chr4_10131_mature	4.835565	22.56597	2.544872269	4.558633073		-2.22239	0.570832
chr12_26134_mature	22.56596857	4.029637	4.558633073	2.330454351		2.485427	0.303343	NW_003613206_37650_star	0	0.805927	0	0.852739935		-20	0.140178	NW_003537759_37261_mature	4.835565	22.56597	2.544872269	4.558633073		-2.22239	0.570832
mmu-miR-5112	6.447419591	41.90823	2.896740642	5.423182395		-2.70044	0.303855	chr14_31467_star	0	0.805927	0	0.852739935		-20	0.140178	chr16_34823_star	10.20841	2.417782	3.48651029	1.773060525		2.078003	0.570915
chr18_36465_mature	5.372849659	33.04303	2.671938627	5.08928735		-2.62059	0.305707	chr3_7764_star	0	0.805927	0	0.852739935		-20	0.140178	NW_003613022_36785_mature	10.20841	2.417782	3.48651029	1.773060525		2.078003	0.570915
mmu-miR-6240	24345.45638	719.6932	14.57142418	9.493241447		5.080127	0.306061	bta-miR-31	0	0.805927	0	0.852739935		-20	0.140178	chr7_16542_mature@@bta-miR-17	10.20841	2.417782	3.48651029	1.773060525		2.078003	0.570915
chr6_14429_mature	12.89483918	2.417782	3.796477232	1.773060525		2.415037	0.306121	rno-miR-299b-5p	0	0.805927	0	0.852739935		-20	0.140178	chr11_24357_mature	10.20841	2.417782	3.48651029	1.773060525		2.078003	0.570915
chr6_14749_mature	12.89483918	2.417782	3.796477232	1.773060525		2.415037	0.306121	hsa-miR-29c-5p	0	0.805927	0	0.852739935		-20	0.140178	chr5_11653_mature	10.20841	2.417782	3.48651029	1.773060525		2.078003	0.570915
chr10_23553_mature	12.89483918	2.417782	3.796477232	1.773060525		2.415037	0.306121	bta-miR-96	0	0.805927	0	0.852739935		-20	0.140178	chrx_22851_star	10.20841	2.417782	3.48651029	1.773060525		2.078003	0.570915
mmu-miR-1927	12.89483918	2.417782	3.796477232	1.773060525		2.415037	0.306121	chr3_6687_star	0	0.805927	0	0.852739935		-20	0.140178	chr17_35470_star	6.984705	1.611855	2.997239027	1.38507475		2.115477	0.571121
chr2_4621_star	12.89483918	2.417782	3.796477232	1.773060525		2.415037	0.306121	hsa-miR-1236-3p	0	0.805927	0	0.852739935		-20	0.140178	chr5_11433_star	6.984705	1.611855	2.997239027	1.38507475		2.115477	0.571121
chr5_11077_mature	12.89483918	2.417782	3.796477232	1.773060525		2.415037	0.306121	chr9_21053_star	0	0.805927	0	0.852739935		-20	0.140178	chr2_5113_star	6.984705	1.611855	2.997239027	1.38507475		2.115477	0.571121
hsa-miR-4448	12.89483918	2.417782	3.796477232	1.773060525		2.415037	0.306121	chr17_35675_star	0	0.805927	0	0.852739935		-20	0.140178	NW_003537879_37346_mature	6.984705	1.611855	2.997239027	1.38507475		2.115477	0.571121
chrx_22089_star	2.149139864	12.08891	1.654957834	3.710273245		-2.49185	0.306889	rno-miR-365-3p	0	0.805927	0	0.852739935		-20	0.140178	chr11_24943_mature	6.984705	1.611855	2.997239027	1.38507475		2.115477	0.571121
chr2_5906_mature	2.149139864	12.08891	1.654957834	3.710273245		-2.49185	0.306889	hsa-miR-4781-3p	0	0.805927	0	0.852739935		-20	0.140178	chr6_14069_star	6.984705	1.611855	2.997239027	1.38507475		2.115477	0.571121
chr2_5581_mature	2.149139864	12.08891	1.654957834	3.710273245		-2.49185	0.306889	mmu-miR-298-5p	0	0.805927	0	0.852739935		-20	0.140178	chr2_5392_star	6.984705	1.611855	2.997239027	1.38507475		2.115477	0.571121
chr1_2426_mature	2.149139864	12.08891	1.654957834	3.710273245		-2.49185	0.306889	rno-miR-125b-5p	0	0.805927	0	0.852739935		-20	0.140178	bta-miR-200a	6.984705	1.611855	2.997239027	1.38507475		2.115477	0.571121
chr14_30693_star	2.149139864	12.08891	1.654957834	3.710273245		-2.49185	0.306889	chr17_35612_mature	0	0.805927	0	0.852739935		-20	0.140178	chrx_23005_star	6.984705	1.611855	2.997239027	1.38507475		2.115477	0.571121
chr8_18439_mature	2.149139864	12.08891	1.654957834	3.710273245		-2.49185	0.306889	chr8_18187_star	0	0.805927	0	0.852739935		-20	0.140178	chr15_33015_star	6.984705	1.611855	2.997239027	1.38507475		2.115477	0.571121
chr14_30700_mature	73.07075536	10.47706	6.210832145	3.520680821		2.802061	0.306928	mmu-miR-1934-3p	0	0.805927	0	0.852739935		-20	0.140178	bta-miR-2482	6.984705	1.611855	2.997239027	1.38507475		2.115477	0.571121
chr6_13171_mature	88.11473441	12.08891	6.477592084	3.710273245		2.865699	0.307627	chr11_24486_star	0	0.805927	0	0.852739935		-20	0.140178	hsa-miR-6507-3p	6.984705	1.611855	2.997239027	1.38507475		2.115477	0.571121
NW_003613327_38569_mature	0	3.22371	0	2.078510713		-20	0.307735	chr13_27616_star	0	0.805927	0	0.852739935		-20	0.140178	chr14_31514_star	6.984705	1.611855	2.997239027	1.38507475		2.115477	0.571121
mmu-miR-6537-3p	0	3.22371	0	2.078510713		-20	0.307735	chr2_6194_star	0	0.805927	0	0.852739935		-20	0.140178	hsa-miR-1246	60.7132	9.671129	5.947507226	3.415640968		2.650254	0.571433
mmu-miR-21b	0	3.22371	0	2.078510713		-20	0.307735	bta-miR-448	0	0.805927	0	0.852739935		-20	0.140178	chr17_34985_mature	13.96941	3.22371	3.90394537	2.078510713		2.115477	0.571542
hsa-miR-4771	0	3.22371	0	2.078510713		-20	0.307735	chr1_431_star	0	0.805927	0	0.852739935		-20	0.140178	chr18_36371_mature	13.96941	3.22371	3.90394537	2.078510713		2.115477	0.571542
chr1_3026_mature	0	3.22371	0	2.078510713		-20	0.307735	hsa-miR-431-5p	0	0.805927	0	0.852739935		-20	0.140178	chr15_32728_mature	13.96941	3.22371	3.90394537	2.078510713		2.115477	0.571542
chr16_34282_star	0	3.22371	0	2.078510713		-20	0.307735	chrx_21533_mature	0	0.805927	0	0.852739935		-20	0.140178	chr9_19230_mature	4.29828	19.34226	2.405524014	4.346407978		-2.16993	0.571702
hsa-miR-1302	0	3.22371	0	2.078510713		-20	0.307735	chr4_10120_star	0	0.805927	0	0.852739935		-20	0.140178	chr6_14787_mature	10.7457	66.08605	3.554060706	6.067940913		-2.62059	0.572717
chr2_4605_mature	0	3.22371	0	2.078510713		-20	0.307735	hsa-miR-520e	0	0.805927	0	0.852739935		-20	0.140178	chr1_2658_mature	8.059274	44.32601	3.179395517	5.502267253		-2.45943	0.573044
chr4_10385_mature	0	3.22371	0	2.078510713		-20	0.307735	bta-miR-490	0	0.805927	0	0.852739935		-20	0.140178	NW_003539007_38451_mature	5.910135	29.01339	2.788713818	4.907534286		-2.29546	0.57414
hsa-miR-4668-3p	0	3.22371	0	2.078510713		-20	0.307735	chr14_30483_star	0	0.805927	0	0.852739935		-20	0.140178	chr4_9000_star	0.537285	3.22371	0.620384622	2.078510713		-2.58496	0.574481
chr6_12988_star	0	3.22371	0	2.078510713		-20	0.307735	chr13_27508_star	0	0.805927	0	0.852739935		-20	0.140178	mmu-miR-1938	0.537285	3.22371	0.620384622	2.078510713		-2.58496	0.574481
chr6_13570_star	0	3.22371	0	2.078510713		-20	0.307735	bta-miR-485	0	0.805927	0	0.852739935		-20	0.140178	chr14_31047_star	0.537285	3.22371	0.620384622	2.078510713		-2.58496	0.574481
NW_003613234_37804_mature	0	3.22371	0	2.078510713		-20	0.307735	chr7_15778_star	0	0.805927	0	0.852739935		-20	0.140178	hsa-miR-5100	0.537285	3.22371	0.620384622	2.078510713		-2.58496	0.574481
NW_003540745_39662_star	0	3.22371	0	2.078510713		-20	0.307735	hsa-miR-4725-3p	0	0.805927	0	0.852739935		-20	0.140178	chr15_33570_star	0.537285	3.22371	0.620384622	2.078510713		-2.58496	0.574481
hsa-miR-525-3p	0	3.22371	0	2.078510713		-20	0.307735	NW_003539221_38657_mature	0	0.805927	0	0.852739935		-20	0.140178	hsa-miR-3679-3p	0.537285	3.22371	0.620384622	2.078510713		-2.58496	0.574481
mmu-miR-5110	0	3.22371	0	2.078510713		-20	0.307735	rno-miR-455-5p	0	0.805927	0	0.852739935		-20	0.140178	rno-miR-196c-5p	0.537285	3.22371	0.620384622	2.078510713		-2.58496	0.574481
chr4_9530_star	0	3.22371	0	2.078510713		-20	0.307735	mmu-miR-26a-2-3p	0	0.805927	0	0.852739935		-20	0.140178	chr8_18754_mature	0.537285	3.22371	0.620384622	2.078510713		-2.58496	0.574481
chr2_6199_mature	0	3.22371	0	2.078510713		-20	0.307735	chr7_17208_star	0	0.805927	0	0.852739935		-20	0.140178	mmu-miR-5625-5p	0.537285	3.22371	0.620384622	2.078510713		-2.58496	0.574481
chr1_3276_mature	0	3.22371	0	2.078510713		-20	0.307735	chr15_31930_star	0	0.805927	0	0.852739935		-20	0.140178	NW_003540275_39376_star	0.537285	3.22371	0.620384622	2.078510713		-2.58496	0.574481
chr1_1949_mature	0	3.22371	0	2.078510713		-20	0.307735	chr6_14978_star	0	0.805927	0	0.852739935		-20	0.140178	chr4_9818_star	0.537285	3.22371	0.620384622	2.078510713		-2.58496	0.574481
chr15_33570_star	0	3.22371	0	2.078510713		-20	0.307735	chr15_31817_star	0	0.805927	0	0.852739935		-20	0.140178	chr3_7643_star	0.537285	3.22371	0.620384622	2.078510713		-2.58496	0.574481
hsa-miR-3679-3p	0	3.22371	0	2.078510713		-20	0.307735	chr2_6077_star	0	0.805927	0	0.852739935		-20	0.140178	chr7_16102_mature	0.537285	3.22371	0.620384622	2.078510713		-2.58496	0.574481
chr8_18754_mature	0	3.22371	0	2.078510713		-20	0.307735	mmu-miR-468-5p	0	0.805927	0	0.852739935		-20	0.140178	chr7_15648_star	0.537285	3.22371	0.620384622	2.078510713		-2.58496	0.574481
mmu-miR-3101-3p	0	3.22371	0	2.078510713		-20	0.307735	chr2_4472_star	0	0.805927	0	0.852739935		-20	0.140178	chr1_3471_mature	0.537285	3.22371	0.620384622	2.078510713		-2.58496	0.574481
chr7_16636_star	0	3.22371	0	2.078510713		-20	0.307735	chr13_27136_star	0	0.805927	0	0.852739935		-20	0.140178	hsa-miR-3144-3p	0.537285	3.22371	0.620384622	2.078510713		-2.58496	0.574481
chr5_11215_star	0	3.22371	0	2.078510713		-20	0.307735	hsa-miR-4684-3p	0	0.805927	0	0.852739935		-20	0.140178	hsa-miR-182-5p	0.537285	3.22371	0.620384622	2.078510713		-2.58496	0.574481
chr17_35462_star	0	3.22371	0	2.078510713		-20	0.307735	rno-miR-29a-3p	0	0.805927	0	0.852739935		-20	0.140178	NW_003539571_38948_mature	0.537285	3.22371	0.620384622	2.078510713		-2.58496	0.574481
mmu-miR-5625-5p	0	3.22371	0	2.078510713		-20	0.307735	bta-miR-3578	0	0.805927	0	0.852739935		-20	0.140178	chr7_15205_star	0.537285	3.22371	0.620384622	2.078510713		-2.58496	0.574481
NW_003540275_39376_star	0	3.22371	0	2.078510713		-20	0.307735	chr17_35122_star	0	0.805927	0	0.852739935		-20	0.140178	chr17_35671_star	0.537285	3.22371	0.620384622	2.078510713		-2.58496	0.574481
chr6_12657_star@@mmu-miR-504-3	0	3.22371	0	2.078510713		-20	0.307735	bta-miR-2412	0	0.805927	0	0.852739935		-20	0.140178	chr12_25577_mature	0.537285	3.22371	0.620384622	2.078510713		-2.58496	0.574481
chr8_18850_mature	0	3.22371	0	2.078510713		-20	0.307735	bta-miR-2421	0	0.805927	0	0.852739935		-20	0.140178	mmu-miR-3110-3p	0.537285	3.22371	0.620384622	2.078510713		-2.58496	0.574481
chr12_25513_mature	0	3.22371	0	2.078510713		-20	0.307735	bta-miR-2422	0	0.805927	0	0.852739935		-20	0.140178	hsa-miR-5697	0.537285	3.22371	0.620384622	2.078510713		-2.58496	0.574481
chr3_6870_mature	0	3.22371	0	2.078510713		-20	0.307735	chr14_30485_star	0	0.805927	0	0.852739935		-20	0.140178	NW_003613025_36805_star	0.537285	3.22371	0.620384622	2.078510713		-2.58496	0.574481
bta-miR-134	0	3.22371	0	2.078510713		-20	0.307735	bta-miR-2428	0	0.805927	0	0.852739935		-20	0.140178	chr14_31142_star	0.537285	3.22371	0.620384622	2.078510713		-2.58496	0.574481
bta-miR-143	0	3.22371	0	2.078510713		-20	0.307735	chr13_27739_star	0	0.805927	0	0.852739935		-20	0.140178	chr9_20434_mature@@rno-miR-34	100.4723	13.70077	6.664941981	3.877819487		2.874469	0.574607
chr4_10437_star	0	3.22371	0	2.078510713		-20	0.307735	hsa-miR-513a-5p	0	0.805927	0	0.852739935		-20	0.140178	rno-miR-466b-5p	9.133844	52.38528	3.34110968	5.738370208		-2.51987	0.574764
chr14_30390_star	0	3.22371	0	2.078510713		-20	0.307735	chr9_19756_star	0	0.805927	0	0.852739935		-20	0.140178	chr13_28864_star	3.760995	16.11855	2.251263041	4.097488514		-2.09954	0.575374
bta-miR-23a	0	3.22371	0	2.078510713		-20	0.307735	hsa-miR-4796-3p	0	0.805927	0	0.852739935		-20	0.140178	chr5_11357_star	3.760995	16.11855	2.251263041	4.097488514		-2.09954	0.575374
chr5_11343_mature	0	3.22371	0	2.078510713		-20	0.307735	bta-miR-2400	0	0.805927	0	0.852739935		-20	0.140178	chr11_24477_mature	3.760995	16.11855	2.251263041	4.097488514		-2.09954	0.575374
chr13_28054_star	0	3.22371	0	2.078510713		-20	0.307735	bta-miR-2455	0	0.805927	0	0.852739935		-20	0.140178	chr13_27163_mature	1.611855	6.44742	1.38507475	2.896740642		-2	0.5756
rno-miR-216b-3p	0	3.22371	0	2.078510713		-20	0.307735	hsa-miR-4456	0	0.805927	0	0.852739935		-20	0.140178	chr14_29554_star	1.611855	6.44742	1.38507475	2.896740642		-2	0.5756
hsa-miR-3179	0	3.22371	0	2.078510713		-20	0.307735	mmu-miR-201-3p	0	0.805927	0	0.852739935		-20	0.140178	chr8_18597_star	1.611855	6.44742	1.38507475	2.896740642		-2	0.5756
chr7_15648_star	0	3.22371	0	2.078510713		-20	0.307735	bta-miR-2465	0	0.805927	0	0.852739935		-20	0.140178	NW_003613264_38074_mature	1.611855	6.44742	1.38507475	2.896740642		-2	0.5756
bta-miR-30d	0	3.22371	0	2.078510713		-20	0.307735	hsa-miR-4460	0	0.805927	0	0.852739935		-20	0.140178	NW_003541108_39908_mature	1.611855	6.44742	1.38507475	2.896740642		-2	0.5756
bta-miR-381	0	3.22371	0	2.078510713		-20	0.307735	bta-miR-2430	0	0.805927	0	0.852739935		-20	0.140178	chr13_27138_mature	1.611855	6.44742	1.38507475	2.896740642		-2	0.5756
chr17_35575_star	0	3.22371	0	2.078510713		-20	0.307735	bta-miR-2432	0	0.805927	0	0.852739935		-20	0.140178	chr4_8847_star	1.611855	6.44742	1.38507475	2.896740642		-2	0.5756
chr7_16559_mature@@rno-miR-330-	0	3.22371	0	2.078510713		-20	0.307735	chr2_4621_mature	0	0.805927	0	0.852739935		-20	0.140178	bta-miR-3154	1.611855	6.44742	1.38507475	2.896740642		-2	0.5756
chr3_8248_mature	0	3.22371	0	2.078510713		-20	0.307735	bta-miR-2441	0	0.805927	0	0.852739935		-20	0.140178	chr16_34837_star	1.611855	6.44742	1.38507475	2.896740642		-2	0.5756
chr1_2992_mature	0	3.22371	0	2.078510713		-20	0.307735	hsa-miR-4483	0	0.805927	0	0.852739935		-20	0.140178	chr18_36430_star	4.29828	0.805927	2.405524014	0.852739935		2.415037	0.575947
chr1_1198_mature	0	3.22371	0	2.078510713		-20	0.307735	mmu-miR-5627-5p	0	0.805927	0	0.852739935		-20	0.140178	hsa-miR-4652-3p	4.29828	0.805927	2.405524014	0.852739935		2.415037	0.575947
chr13_28026_mature	0	3.22371	0	2.078510713		-20	0.307735	chr13_26883_mature	0	0.805927	0	0.852739935		-20	0.140178	chr2_5969_star	4.29828	0.805927	2.405524014	0.852739935		2.415037	0.575947
hsa-miR-3144-3p	0	3.22371	0	2.078510713		-20	0.307735	rno-miR-875	0	0.805927	0	0.852739935		-20	0.140178	hsa-miR-4770	4.29828	0.805927	2.405524014	0.852739935		2.415037	0.575947
bta-miR-489	0	3.22371	0	2.078510713		-20	0.307735	hsa-miR-4421	0	0.805927	0	0.852739935		-20	0.140178	chr14_30718_star	4.29828	0.805927	2.405524014	0.852739935		2.415037	0.575947
chr5_11638_mature	0	3.22371	0	2.078510713		-20	0.307735	chr2_4436_star	0	0.805927	0	0.852739935		-20	0.140178	chr6_12905_star	4.29828	0.805927	2.405524014	0.852739935		2.415037	0.575947
hsa-miR-182-5p	0	3.22371	0	2.078510713		-20	0.307735	bta-miR-2470	0	0.805927	0	0.852739935		-20	0.140178	chr12_26001_mature	4.29828	0.805927	2.405524014	0.852739935		2.415037	0.575947
ssc-miR-202-5p	0	3.22371	0	2.078510713		-20	0.307735	hsa-miR-4432	0	0.805927	0	0.852739935		-20	0.140178	chr14_31502_mature	4.29828	0.805927	2.405524014	0.852739935		2.415037	0.575947
chr17_35074_star	0	3.22371	0	2.078510713		-20	0.307735	mmu-miR-201-5p	0	0.805927	0	0.852739935		-20	0.140178	chr9_20113_star	4.29828	0.805927	2.405524014	0.852739935		2.415037	0.575947
chr8_18167_star	0	3.22371	0	2.078510713		-20	0.307735	chr4_10208_star	0	0.805927	0	0.852739935		-20	0.140178	chr1_3174_star	4.29828	0.805927	2.405524014	0.852739935		2.415037	0.575947
chr8_17750_star	0	3.22371	0	2.078510713		-20	0.307735	bta-miR-2480	0	0.805927	0	0.852739935		-20	0.140178	hsa-miR-3911	4.29828	0.805927	2.405524014	0.852739935		2.415037	0.575947
hsa-miR-103a-2-5p	0	3.22371	0	2.078510713		-20	0.307735	hsa-miR-604	0	0.805927	0	0.852739935		-20	0.140178	chr3_7917_mature	4.29828	0.805927	2.405524014	0.852739935		2.415037	0.575947
ssc-miR-376b	0	3.22371	0	2.078510713		-20	0.307735	hsa-miR-603	0	0.805927	0	0.852739935		-20	0.140178	chr4_8825_star	4.29828	0.805927	2.405524014	0.852739935		2.415037	0.575947
chr2_5776_mature	0	3.22371	0	2.078510713		-20	0.307735	NW_003540392_39464_mature	0	0.805927	0	0.852739935		-20	0.140178	chr2_5745_star	4.29828	0.805927	2.405524014	0.852739935		2.415037	0.575947
chr6_13671_star	0	3.22371	0	2.078510713		-20	0.307735	hsa-miR-608	0	0.805927	0	0.852739935		-20	0.140178	mmu-miR-5120	4.29828	0.805927	2.405524014	0.852739935		2.415037	0.575947
chr17_35671_star	0	3.22371	0	2.078510713		-20	0.307735	hsa-miR-623	0	0.805927	0	0.852739935		-20	0.140178	bta-miR-133a	4.29828	0.805927	2.405524014	0.852739935		2.415037	0.575947
chr12_25577_mature	0	3.22371	0	2.078510713		-20	0.307735	mmu-miR-1843b-5p	0	0.805927	0	0.852739935		-20	0.140178	chr13_28617_star	4.29828	0.805927	2.405524014	0.852739935		2.415037	0.575947
NW_003613226_37753_star	0	3.22371	0	2.078510713		-20	0.307735	chr3_7813_star	0	0.805927	0	0.852739935		-20	0.140178	chr14_30846_star	4.29828	0.805927	2.405524014	0.852739935		2.415037	0.575947
chr13_27012_star	0	3.22371	0	2.078510713		-20	0.307735	mmu-miR-466b-3p	0	0.805927	0	0.852739935		-20	0.140178	chr2_5337_star	4.29828	0.805927	2.405524014	0.852739935		2.415037	0.575947
chr13_29266_mature	0	3.22371	0	2.078510713		-20	0.307735	mmu-miR-1930-5p	0	0.805927	0	0.852739935		-20	0.140178	chr15_32130_star	4.29828	0.805927	2.405524014	0.852739935		2.415037	0.575947
hsa-miR-3678-3p	0	3.22371	0	2.078510713		-20	0.307735	bta-miR-2395	0	0.805927	0	0.852739935		-20	0.140178	mmu-miR-295-3p	4.29828	0.805927	2.405524014	0.852739935		2.415037	0.575947
hsa-miR-3135b	0	3.22371	0	2.078510713		-20	0.307735	hsa-miR-4499	0	0.805927	0	0.852739935		-20	0.140178	chr14_30803_star	4.29828	0.805927	2.405524014	0.852739935		2.415037	0.575947
rno-miR-32-3p	0	3.22371	0	2.078510713		-20	0.307735	chr13_27068_star	0	0.805927	0	0.852739935		-20	0.140178	chrx_22707_star	4.29828	0.805927	2.405524014	0.852739935		2.415037	0.575947
hsa-miR-5697	0	3.22371	0	2.078510713		-20	0.307735	NW_003613025_36806_star	0	0.805927	0	0.852739935		-20	0.140178	chr13_27306_star	4.29828	0.805927	2.405524014	0.852739935		2.415037	0.575947
hsa-miR-5698	0	3.22371	0	2.078510713		-20	0.307735	chr12_25987_star	0	0.805927	0	0.852739935		-20	0.140178	bta-miR-665	4.29828	0.805927	2.405524014	0.852739935		2.415037	0.575947
rno-miR-379-5p	0	3.22371	0	2.078510713		-20	0.307735	chr8_18125_star	0	0.805927	0	0.852739935		-20	0.140178	chr13_28165_mature	4.29828	0.805927	2.405524014	0.852739935		2.415037	0.575947
NW_003613025_36805_star	0	3.22371	0	2.078510713		-20	0.307735	hsa-miR-1825	0	0.805927	0	0.852739935		-20	0.140178	chr2_5392_mature	4.29828	0.805927	2.405524014	0.852739935		2.415037	0.575947
chr14_31142_star	0	3.22371	0	2.078510713		-20	0.307735	mmu-miR-881-3p	0	0.805927	0	0.852739935		-20	0.140178	NW_003613217_37697_mature	4.29828	0.805927	2.405524014	0.852739935		2.415037	0.575947
mmu-miR-330-3p	0	3.22371	0	2.078510713		-20	0.307735	chr5_11345_star	0	0.805927	0	0.852739935		-20	0.140178	chr1_3878_star	4.29828	0.805927	2.405524014	0.852739935		2.415037	0.575947
chr10_23974_mature	17.19311891	3.22371	4.185320985	2.078510713		2.415037	0.308439	bta-miR-2329-3p	0	0.805927	0	0.852739935		-20	0.140178	chr3_7464_mature	4.29828	0.805927	2.405524014	0.852739935		2.415037	0.575947
chr15_33627_star	56.95220639	885.7143	5.856791684	9.792325477		-3.95902	0.310313	mmu-miR-881-5p	0	0.805927	0	0.852739935		-20	0.140178	bta-miR-222	4.29828	0.805927	2.405524014	0.852739935		2.415037	0.575947
chr7_15541_star	5.372849659	0.805927	2.671938627	0.852739935		2.736966	0.310419	chr1_319_mature	0	0.805927	0	0.852739935		-20	0.140178	NW_003613440_39149_star	4.29828	0.805927	2.405524014	0.852739935		2.415037	0.575947
NW_003540346_39431_mature	5.372849659	0.805927	2.671938627	0.852739935		2.736966	0.310419	bta-miR-2368-5p	0	0.805927	0	0.852739935		-20	0.140178	mmu-miR-3078-5p	4.29828	0.805927	2.405524014	0.852739935		2.415037	0.575947
hsa-miR-3922-5p	5.372849659	0.805927	2.671938627	0.852739935		2.736966	0.310419	chr8_17571_mature	0	0.805927	0	0.852739935		-20	0.140178	chr9_19298_mature	4.29828	0.805927	2.405524014	0.852739935		2.415037	0.575947
chrx_23097_star	5.372849659	0.805927	2.671938627	0.852739935		2.736966	0.310419	chr9_20452_star	0	0.805927	0	0.852739935		-20	0.140178	hsa-miR-302e	4.29828	0.805927	2.405524014	0.852739935		2.415037	0.575947
chr12_25921_star	5.372849659	0.805927	2.671938627	0.852739935		2.736966	0.310419	chr13_29005_star	0	0.805927	0	0.852739935		-20	0.140178	chr8_18259_mature	4.29828	0.805927	2.405524014	0.852739935		2.415037	0.575947
chr4_10519_mature	5.372849659	0.805927	2.671938627	0.852739935		2.736966	0.310419	chr1_3842_star	0	0.805927	0	0.852739935		-20	0.140178	chr3_7654_star	4.29828	0.805927	2.405524014	0.852739935		2.415037	0.575947
mmu-miR-878-5p	5.372849659	0.805927	2.671938627	0.852739935		2.736966	0.310419	mmu-miR-1196-5p	0	0.805927	0	0.852739935		-20	0.140178	chr2_4381_star	4.29828	0.805927	2.405524014	0.852739935		2.415037	0.575947
mmu-miR-1950	5.372849659	0.805927	2.671938627	0.852739935		2.736966	0.310419	mmu-miR-3082-5p	0	0.805927	0	0.852739935		-20	0.140178	bta-miR-2416	4.29828	0.805927	2.405524014	0.852739935		2.415037	0.575947
mmu-miR-1945	5.372849659	0.805927	2.671938627	0.852739935		2.736966	0.310419	chr12_25511_star	0	0.805927	0	0.852739935		-20	0.140178	chr13_29126_star	4.29828	0.805927	2.405524014	0.852739935		2.415037	0.575947
chr10_24235_mature	5.372849659	0.805927	2.671938627	0.852739935		2.736966	0.310419	chr11_24756_mature	0	0.805927	0	0.852739935		-20	0.140178	hsa-miR-4461	4.29828	0.805927	2.405524014	0.852739935		2.415037	0.575947
chr18_36156_mature	5.372849659	0.805927	2.671938627	0.852739935		2.736966	0.310419	chr8_17927_mature	0	0.805927	0	0.852739935		-20	0.140178	bta-miR-2442	4.29828	0.805927	2.405524014	0.852739935		2.415037	0.575947
chr12_26408_mature	5.372849659	0.805927	2.671938627	0.852739935		2.736966	0.310419	hsa-miR-548t-5p	0	0.805927	0	0.852739935		-20	0.140178	chrx_21844_star@@hsa-miR-660-3	4.29828	0.805927	2.405524014	0.852739935		2.415037	0.575947
mmu-miR-676-5p	5.372849659	0.805927	2.671938627	0.852739935		2.736966	0.310419	NW_003540052_39255_star	0	0.805927	0	0.852739935		-20	0.140178	chr7_17092_star	4.29828	0.805927	2.405524014	0.852739935		2.415037	0.575947
chr3_8118_mature	5.372849659	0.805927	2.671938627	0.852739935		2.736966	0.310419	chr6_14216_mature	0	0.805927	0	0.852739935		-20	0.140178	NW_003613326_38567_mature	4.29828	0.805927	2.405524014	0.852739935		2.415037	0.575947
chr15_32249_star	5.372849659	0.805927	2.671938627	0.852739935		2.736966	0.310419	rno-miR-10b-5p	0	0.805927	0	0.852739935		-20	0.140178	hsa-miR-576-3p	4.29828	0.805927	2.405524014	0.852739935		2.415037	0.575947
chr11_24884_mature	5.372849659	0.805927	2.671938627	0.852739935		2.736966	0.310419	bta-miR-2309	0	0.805927	0	0.852739935		-20	0.140178	chr1_742_star	4.29828	0.805927	2.405524014	0.852739935		2.415037	0.575947
chr13_26683_star	5.372849659	0.805927	2.671938627	0.852739935		2.736966	0.310419	bta-miR-2301	0	0.805927	0	0.852739935		-20	0.140178	chr7_15776_star	4.29828	0.805927	2.405524014	0.852739935		2.415037	0.575947
chr16_34261_star	5.372849659	0.805927	2.671938627	0.852739935		2.736966	0.310419	bta-miR-2310	0	0.805927	0	0.852739935		-20	0.140178	chr14_29836_mature	4.29828	0.805927	2.405524014	0.852739935		2.415037	0.575947
chr16_34245_star	5.372849659	0.805927	2.671938627	0.852739935		2.736966	0.310419	bta-miR-2315	0	0.805927	0	0.852739935		-20	0.140178	chr14_29516_star	4.29828	0.805927	2.405524014	0.852739935		2.415037	0.575947
chr12_26408_star	5.372849659	0.805927	2.671938627	0.852739935		2.736966	0.310419	hsa-miR-4700-3p	0	0.805927	0	0.852739935		-20	0.140178	hsa-miR-3925-3p	4.29828	0.805927	2.405524014	0.852739935		2.415037	0.575947
chr17_35169_star	5.372849659	0.805927	2.671938627	0.852739935		2.736966	0.310419	rno-miR-582-3p	0	0.805927	0	0.852739935		-20	0.140178	chr14_30718_mature	4.29828	0.805927	2.405524014	0.852739935		2.415037	0.575947
chr14_31327_mature	5.372849659	0.805927	2.671938627	0.852739935		2.736966	0.310419	mmu-miR-16-5p	0	0.805927	0	0.852739935		-20	0.140178	chr13_27012_mature	4.29828	0.805927	2.405524014	0.852739935		2.415037	0.575947
chr9_19205_star	5.372849659	0.805927	2.671938627	0.852739935		2.736966	0.310419	chr14_31643_star	0	0.805927	0	0.852739935		-20	0.140178	chr18_36465_star	4.29828	0.805927	2.405524014	0.852739935		2.415037	0.575947
rno-miR-653-3p	5.372849659	0.805927	2.671938627	0.852739935		2.736966	0.310419	bta-miR-2345	0	0.805927	0	0.852739935		-20	0.140178	NW_003613301_38356_star	4.29828	0.805927	2.405524014	0.852739935		2.415037	0.575947
chr9_19733_star	5.372849659	0.805927	2.671938627	0.852739935		2.736966	0.310419	bta-miR-2344	0	0.805927	0	0.852739935		-20	0.140178	hsa-miR-921	4.29828	0.805927	2.405524014	0.852739935		2.415037	0.575947
chr1_1936_star	5.372849659	0.805927	2.671938627	0.852739935		2.736966	0.310419	bta-miR-2349	0	0.805927	0	0.852739935		-20	0.140178	chr3_7571_mature	22.56597	4.835565	4.558633073	2.544872269		2.222392	0.576734
chr11_25035_star	5.372849659	0.805927	2.671938627	0.852739935		2.736966	0.310419	chr14_30236_star	0	0.805927	0	0.852739935		-20	0.140178	chr6_12841_mature	22.56597	4.835565	4.558633073	2.544872269		2.222392	0.576734
chr3_6725_star	5.372849659	0.805927	2.671938627	0.852739935		2.736966	0.310419	bta-miR-2347	0	0.805927	0	0.852739935		-20	0.140178	chr13_27764_mature	45.66922	8.059274	5.544399515	3.179395517		2.5025	0.576789
mmu-miR-3095-3p	5.372849659	0.805927	2.671938627	0.852739935		2.736966	0.310419	chr15_32840_mature	0	0.805927	0	0.852739935		-20	0.140178	chr5_10946_mature	33.31167	6.44742	5.100627352	2.896740642		2.369234	0.576836
chr6_12854_star	5.372849659	0.805927	2.671938627	0.852739935		2.736966	0.310419	chrx_21525_star	0	0.805927	0	0.852739935		-20	0.140178	mmu-miR-5100	19.87954	155.544	4.384018281	7.290424382		-2.96797	0.576889
chr1_743_mature	5.372849659	0.805927	2.671938627	0.852739935		2.736966	0.310419	chr12_25917_star	0	0.805927	0	0.852739935		-20	0.140178	chr3_8631_mature	17.7304	4.029637	4.227310103	2.330454351		2.137504	0.578025
chr12_26438_mature	5.372849659	0.805927	2.671938627	0.852739935		2.736966	0.310419	hsa-miR-4314	0	0.805927	0	0.852739935		-20	0.140178	chr13_27782_mature	17.7304	4.029637	4.227310103	2.330454351		2.137504	0.578025
chr9_20212_mature	5.372849659	0.805927	2.671938627	0.852739935		2.736966	0.310419	chr7_15912_mature	0	0.805927	0	0.852739935		-20	0.140178	chr9_20139_mature	13.43212	3.22371	3.851211749	2.078510713		2.058894	0.579145
chr15_32700_star	5.372849659	0.805927	2.671938627	0.852739935		2.736966	0.310419	bta-miR-2354	0	0.805927	0	0.852739935		-20	0.140178	chr8_17233_star	13.43212	3.22371	3.851211749	2.078510713		2.058894	0.579145
NW_003540965_39767_mature	5.372849659	0.805927	2.671938627	0.852739935		2.736966	0.310419	hsa-miR-3187-5p	0	0.805927	0	0.852739935		-20	0.140178	chr1_1479_mature	13.43212	3.22371	3.851211749	2.078510713		2.058894	0.579145
chr2_4882_mature	5.372849659	0.805927	2.671938627	0.852739935		2.736966	0.310419	hsa-miR-4310	0	0.805927	0	0.852739935		-20	0.140178	chr9_20394_mature	45.13194	8.059274	5.527693969	3.179395517		2.485427	0.579699
chr4_10459_mature	5.372849659	0.805927	2.671938627	0.852739935		2.736966	0.310419	chr7_17076_mature	0	0.805927	0	0.852739935		-20	0.140178	chr15_32591_star	4.835565	21.76004	2.544872269	4.508431259		-2.16993	0.58017
chr2_3980_star	5.372849659	0.805927	2.671938627	0.852739935		2.736966	0.310419	rno-miR-3580-5p	0	0.805927	0	0.852739935		-20	0.140178	chr11_25089_star	32.77438	6.44742	5.077857506	2.896740642		2.345775	0.580603
chr2_6204_star	5.372849659	0.805927	2.671938627	0.852739935		2.736966	0.310419	chr14_30644_mature	0	0.805927	0	0.852739935		-20	0.140178	NW_003539506_38905_mature	9.671129	2.417782	3.415640968	1.773060525		2	0.580703
chr15_32760_star	5.372849659	0.805927	2.671938627	0.852739935		2.736966	0.310419	bta-miR-2362	0	0.805927	0	0.852739935		-20	0.140178	chr15_32525_mature	9.671129	2.417782	3.415640968	1.773060525		2	0.580703
rno-miR-463-5p	5.372849659	0.805927	2.671938627	0.852739935		2.736966	0.310419	rno-miR-107-5p	0	0.805927	0	0.852739935		-20	0.140178	chrx_21689_star	9.671129	2.417782	3.415640968	1.773060525		2	0.580703
chr14_30639_mature	5.372849659	0.805927	2.671938627	0.852739935		2.736966	0.310419	chr9_19346_star	0	0.805927	0	0.852739935		-20	0.140178	chr1_3278_star	9.671129	2.417782	3.415640968	1.773060525		2	0.580703
NW_003613073_36998_star	5.372849659	0.805927	2.671938627	0.852739935		2.736966	0.310419	bta-miR-2378	0	0.805927	0	0.852739935		-20	0.140178	chr3_7373_mature	9.671129	2.417782	3.415640968	1.773060525		2	0.580703
chr15_33231_star	5.372849659	0.805927	2.671938627	0.852739935		2.736966	0.310419	rno-miR-3580-3p	0	0.805927	0	0.852739935		-20	0.140178	chr1_499_mature@@hsa-miR-211	9.671129	2.417782	3.415640968	1.773060525		2	0.580703

chr11_24331_star	5.372849659	0.805927	2.671938627	0.852739935		2.736966	0.310419	hsa-miR-4662b	0	0.805927	0	0.852739935		-20	0.140178	hsa-miR-3169	9.671129	2.417782	3.415640968	1.773060525		2	0.580703
NW_003613233_37801_star	5.372849659	0.805927	2.671938627	0.852739935		2.736966	0.310419	bta-miR-2286	0	0.805927	0	0.852739935		-20	0.140178	chr15_32006_star	9.671129	2.417782	3.415640968	1.773060525		2	0.580703
chr16_34849_star	5.372849659	0.805927	2.671938627	0.852739935		2.736966	0.310419	bta-miR-2289	0	0.805927	0	0.852739935		-20	0.140178	NW_003540351_39435_star	9.671129	2.417782	3.415640968	1.773060525		2	0.580703
chr7_15776_star	5.372849659	0.805927	2.671938627	0.852739935		2.736966	0.310419	chr4_9258_star	0	0.805927	0	0.852739935		-20	0.140178	chr1_3229_star	9.671129	2.417782	3.415640968	1.773060525		2	0.580703
chr7_16580_mature	5.372849659	0.805927	2.671938627	0.852739935		2.736966	0.310419	mmu-miR-683	0	0.805927	0	0.852739935		-20	0.140178	bta-miR-2383	9.671129	2.417782	3.415640968	1.773060525		2	0.580703
rno-miR-3558-5p	5.372849659	0.805927	2.671938627	0.852739935		2.736966	0.310419	mmu-miR-1947-5p	0	0.805927	0	0.852739935		-20	0.140178	chr6_13171_mature	81.13003	12.08891	6.359837917	3.710273245		2.746552	0.581857
mmu-miR-344f-3p	5.372849659	0.805927	2.671938627	0.852739935		2.736966	0.310419	mmu-miR-344i	0	0.805927	0	0.852739935		-20	0.140178	chr2_5399_mature	4.29828	18.53633	2.405524014	4.288087668		-2.10852	0.582138
mmu-miR-1198-3p	5.372849659	0.805927	2.671938627	0.852739935		2.736966	0.310419	chr14_31505_star	0	0.805927	0	0.852739935		-20	0.140178	chr3_7643_mature@@hsa-miR-47	2.686425	10.47706	1.882222339	3.520680821		-1.96347	0.582187
chr11_24484_mature	5.372849659	0.805927	2.671938627	0.852739935		2.736966	0.310419	rno-miR-351-3p	0	0.805927	0	0.852739935		-20	0.140178	chr1_3135_star	2.686425	10.47706	1.882222339	3.520680821		-1.96347	0.582187
rno-miR-770-5p	5.372849659	0.805927	2.671938627	0.852739935		2.736966	0.310419	bta-miR-2298	0	0.805927	0	0.852739935		-20	0.140178	chr3_8153_mature	3.22371	12.89484	2.078510713	3.796477232		-2	0.583304
chr14_31443_mature	37.60994761	477.915	5.270900692	8.903625744		-3.66757	0.312452	bta-miR-2297	0	0.805927	0	0.852739935		-20	0.140178	chr13_28125_mature@@mmu-miR-	3.22371	12.89484	2.078510713	3.796477232		-2	0.583304
chr12_25421_mature	6.447419591	40.29637	2.896740642	5.367943153		-2.64386	0.312482	mmu-miR-466g	0	0.805927	0	0.852739935		-20	0.140178	chr3_8007_mature	17.19312	4.029637	4.185320985	2.330454351		2.093109	0.584214
chr11_25333_mature	26.8642483	4.835565	4.800343329	2.544872269		2.473931	0.312536	mmu-miR-3097-3p	0	0.805927	0	0.852739935		-20	0.140178	chr1_2656_mature	6.44742	1.611855	2.896740642	1.38507475		2	0.584586
chr3_6786_star	8.596559455	1.611855	3.262517265	1.38507475		2.415037	0.312921	chr16_34849_star	0	0.805927	0	0.852739935		-20	0.140178	NW_003541124_39922_star	6.44742	1.611855	2.896740642	1.38507475		2	0.584586
mmu-miR-6418-3p	8.596559455	1.611855	3.262517265	1.38507475		2.415037	0.312921	mmu-miR-467g	0	0.805927	0	0.852739935		-20	0.140178	chr9_19128_star	6.44742	1.611855	2.896740642	1.38507475		2	0.584586
chr6_13171_star	8.596559455	1.611855	3.262517265	1.38507475		2.415037	0.312921	chr1_742_star	0	0.805927	0	0.852739935		-20	0.140178	chr7_16598_mature	6.44742	1.611855	2.896740642	1.38507475		2	0.584586
chr1_1948_mature	8.596559455	1.611855	3.262517265	1.38507475		2.415037	0.312921	chr7_15713_star	0	0.805927	0	0.852739935		-20	0.140178	chr2_4256_star	6.44742	1.611855	2.896740642	1.38507475		2	0.584586
mmu-miR-669a-3-3p	8.596559455	1.611855	3.262517265	1.38507475		2.415037	0.312921	chr13_26614_star	0	0.805927	0	0.852739935		-20	0.140178	chr4_9707_star	6.44742	1.611855	2.896740642	1.38507475		2	0.584586
chr11_24943_mature	8.596559455	1.611855	3.262517265	1.38507475		2.415037	0.312921	rno-miR-881-5p	0	0.805927	0	0.852739935		-20	0.140178	chr8_17337_star	6.44742	1.611855	2.896740642	1.38507475		2	0.584586
chr2_4472_mature	8.596559455	1.611855	3.262517265	1.38507475		2.415037	0.312921	hsa-miR-3130-3p	0	0.805927	0	0.852739935		-20	0.140178	rno-miR-6323	6.44742	1.611855	2.896740642	1.38507475		2	0.584586
chr6_13162_mature	8.596559455	1.611855	3.262517265	1.38507475		2.415037	0.312921	chr18_36317_mature	0	0.805927	0	0.852739935		-20	0.140178	hsa-miR-2276	6.44742	1.611855	2.896740642	1.38507475		2	0.584586
chr9_20697_mature	8.596559455	1.611855	3.262517265	1.38507475		2.415037	0.312921	mmu-miR-1839-3p	0	0.805927	0	0.852739935		-20	0.140178	chr12_25445_star	6.44742	1.611855	2.896740642	1.38507475		2	0.584586
chr2_6005_star	8.596559455	1.611855	3.262517265	1.38507475		2.415037	0.312921	rno-miR-1193-3p	0	0.805927	0	0.852739935		-20	0.140178	NW_003540989_39780_star	6.44742	1.611855	2.896740642	1.38507475		2	0.584586
ssc-miR-1468	8.596559455	1.611855	3.262517265	1.38507475		2.415037	0.312921	hsa-miR-764	0	0.805927	0	0.852739935		-20	0.140178	chr1_1941_star	6.44742	1.611855	2.896740642	1.38507475		2	0.584586
chrx_22660_mature	8.596559455	1.611855	3.262517265	1.38507475		2.415037	0.312921	hsa-miR-193a-5p	0	0.805927	0	0.852739935		-20	0.140178	chr1_2346_mature	6.44742	1.611855	2.896740642	1.38507475		2	0.584586
chr6_14141_mature	8.596559455	1.611855	3.262517265	1.38507475		2.415037	0.312921	bta-miR-376e	0	0.805927	0	0.852739935		-20	0.140178	chr16_34150_mature	6.44742	1.611855	2.896740642	1.38507475		2	0.584586
chr3_8243_mature	32.23709795	5.641492	5.054722516	2.731507408		2.514573	0.313777	hsa-miR-23a-5p	0	0.805927	0	0.852739935		-20	0.140178	chr10_23842_star	6.44742	1.611855	2.896740642	1.38507475		2	0.584586
chr8_18283_mature	50.5047868	8.059274	5.686634616	3.179395517		2.647698	0.314275	chr8_17641_star	0	0.805927	0	0.852739935		-20	0.140178	chr13_28068_mature	6.44742	1.611855	2.896740642	1.38507475		2	0.584586
chr11_25109_mature	21.49139864	4.029637	4.491301473	2.330454351		2.415037	0.314476	chr2_4676_star	0	0.805927	0	0.852739935		-20	0.140178	chr5_11866_star	6.44742	1.611855	2.896740642	1.38507475		2	0.584586
hsa-miR-1973	21.49139864	4.029637	4.491301473	2.330454351		2.415037	0.314476	hsa-miR-450b-3p	0	0.805927	0	0.852739935		-20	0.140178	chr14_30528_star	6.44742	1.611855	2.896740642	1.38507475		2	0.584586
ssc-miR-411	143.9923709	17.7304	7.179833181	4.227310103		3.021695	0.31525	NW_003613161_37463_star	0	0.805927	0	0.852739935		-20	0.140178	NW_003613562_39706_mature	6.44742	1.611855	2.896740642	1.38507475		2	0.584586
chr1_2668_mature	2.149139864	11.28298	1.654957834	3.618589217		-2.39232	0.319195	mmu-miR-1957b	0	0.805927	0	0.852739935		-20	0.140178	mmu-miR-28a-3p	6.44742	1.611855	2.896740642	1.38507475		2	0.584586
chr9_19757_star	2.149139864	11.28298	1.654957834	3.618589217		-2.39232	0.319195	chr1_3365_star	0	0.805927	0	0.852739935		-20	0.140178	chr5_11672_mature	6.44742	1.611855	2.896740642	1.38507475		2	0.584586
chr13_26809_mature	2.149139864	11.28298	1.654957834	3.618589217		-2.39232	0.319195	hsa-miR-4637	0	0.805927	0	0.852739935		-20	0.140178	chr14_30180_mature	2.14914	8.059274	1.654957834	3.179395517		-1.90689	0.585988
chr8_17599_mature	2.149139864	11.28298	1.654957834	3.618589217		-2.39232	0.319195	chr15_33667_star	0	0.805927	0	0.852739935		-20	0.140178	chr10_24174_star	2.14914	8.059274	1.654957834	3.179395517		-1.90689	0.585988
chr1_1879_star	2.149139864	11.28298	1.654957834	3.618589217		-2.39232	0.319195	hsa-miR-4644	0	0.805927	0	0.852739935		-20	0.140178	hsa-miR-518c-5p	2.14914	8.059274	1.654957834	3.179395517		-1.90689	0.585988
chr11_24816_mature	2.149139864	11.28298	1.654957834	3.618589217		-2.39232	0.319195	chr12_26570_mature	0	0.805927	0	0.852739935		-20	0.140178	chr16_34229_mature	2.14914	8.059274	1.654957834	3.179395517		-1.90689	0.585988
chr1_330_star	2.149139864	11.28298	1.654957834	3.618589217		-2.39232	0.319195	hsa-miR-4648	0	0.805927	0	0.852739935		-20	0.140178	chr12_26098_star	2.14914	8.059274	1.654957834	3.179395517		-1.90689	0.585988
chr4_10714_mature	2.149139864	11.28298	1.654957834	3.618589217		-2.39232	0.319195	NW_003538201_37612_star	0	0.805927	0	0.852739935		-20	0.140178	chr15_33216_mature	2.14914	8.059274	1.654957834	3.179395517		-1.90689	0.585988
chr3_7983_star	290.1338816	29.81932	8.185538937	4.94576292		3.2824	0.319651	hsa-miR-4651	0	0.805927	0	0.852739935		-20	0.140178	chr9_19423_star	2.14914	8.059274	1.654957834	3.179395517		-1.90689	0.585988
chrx_22097_mature	3.223709795	16.92448	2.078510713	4.163859073		-2.39232	0.320276	chr3_7910_star	0	0.805927	0	0.852739935		-20	0.140178	chr14_31302_mature	12.89484	3.22371	3.796477232	2.078510713		2	0.586798
chr15_33676_star	3.223709795	16.92448	2.078510713	4.163859073		-2.39232	0.320276	mmu-miR-703	0	0.805927	0	0.852739935		-20	0.140178	chr1_3894_mature@@bta-miR-219	12.89484	3.22371	3.796477232	2.078510713		2	0.586798
chr13_27285_star	3.223709795	16.92448	2.078510713	4.163859073		-2.39232	0.320276	hsa-miR-4657	0	0.805927	0	0.852739935		-20	0.140178	NW_003539103_38526_mature	10.20841	58.8327	3.48651029	5.902862353		-2.52686	0.58709
bta-miR-2322-5p	3.223709795	16.92448	2.078510713	4.163859073		-2.39232	0.320276	hsa-miR-4663	0	0.805927	0	0.852739935		-20	0.140178	chr6_13188_star	3.760995	15.31262	2.251263041	4.027916745		-2.02554	0.587157
chr2_4343_mature	3.223709795	16.92448	2.078510713	4.163859073		-2.39232	0.320276	hsa-miR-4660	0	0.805927	0	0.852739935		-20	0.140178	chr4_9731_mature	37.60995	7.253347	5.270900692	3.044979305		2.374396	0.587227
chr3_8165_mature	3.223709795	16.92448	2.078510713	4.163859073		-2.39232	0.320276	chr8_17864_star	0	0.805927	0	0.852739935		-20	0.140178	chr2_6005_mature	161.7228	19.34226	7.346272375	4.346407978		3.063695	0.588006
chr8_18026_star	3.223709795	16.92448	2.078510713	4.163859073		-2.39232	0.320276	NW_003538808_38240_star	0	0.805927	0	0.852739935		-20	0.140178	chr13_28823_mature	314.849	29.81932	8.303091149	4.94576292		3.400341	0.588311
NW_003613132_37331_mature	5.372849659	30.62524	2.671938627	4.983004663		-2.51096	0.321801	rno-miR-3596c	0	0.805927	0	0.852739935		-20	0.140178	ssc-let-7a	540.5087	15987.18	9.080840642	13.96471835		-4.88645	0.588858
chr5_10855_star	1.074569932	6.44742	1.05281229	2.896740642		-2.58496	0.321829	rno-miR-3596a	0	0.805927	0	0.852739935		-20	0.140178	chr5_11626_mature	78.98089	12.08891	6.32158343	3.710273245		2.707819	0.588994
chr18_36092_star	1.074569932	6.44742	1.05281229	2.896740642		-2.58496	0.321829	hsa-miR-4678	0	0.805927	0	0.852739935		-20	0.140178	chr4_9509_mature	5.910135	27.40153	2.788713818	4.827896911		-2.21299	0.589509
chr1_3589_star	1.074569932	6.44742	1.05281229	2.896740642		-2.58496	0.321829	hsa-miR-4688	0	0.805927	0	0.852739935		-20	0.140178	chr16_34112_mature	4.835565	20.95411	2.544872269	4.456419386		-2.11548	0.589515
chr14_31524_mature	1.074569932	6.44742	1.05281229	2.896740642		-2.58496	0.321829	chr15_32819_star	0	0.805927	0	0.852739935		-20	0.140178	chr13_28542_mature	4.835565	20.95411	2.544872269	4.456419386		-2.11548	0.589515
chr13_27234_mature	1.074569932	6.44742	1.05281229	2.896740642		-2.58496	0.321829	chr7_15903_star	0	0.805927	0	0.852739935		-20	0.140178	chr14_30089_mature	4.835565	20.95411	2.544872269	4.456419386		-2.11548	0.589515
chr1_480_mature	1.074569932	6.44742	1.05281229	2.896740642		-2.58496	0.321829	bta-miR-6122-5p	0	0.805927	0	0.852739935		-20	0.140178	chr2_6135_mature	4.835565	20.95411	2.544872269	4.456419386		-2.11548	0.589515
chr7_15704_star	1.074569932	6.44742	1.05281229	2.896740642		-2.58496	0.321829	hsa-miR-196a-3p	0	0.805927	0	0.852739935		-20	0.140178	chr1_1781_star	9.133844	2.417782	3.34110968	1.773060525		1.917538	0.590556
NW_003613264_38074_mature	1.074569932	6.44742	1.05281229	2.896740642		-2.58496	0.321829	chr13_29078_star	0	0.805927	0	0.852739935		-20	0.140178	chr2_5585_mature	9.133844	2.417782	3.34110968	1.773060525		1.917538	0.590556
chr2_5590_star	1.074569932	6.44742	1.05281229	2.896740642		-2.58496	0.321829	hsa-miR-4760-5p	0	0.805927	0	0.852739935		-20	0.140178	chr11_25364_star	9.133844	2.417782	3.34110968	1.773060525		1.917538	0.590556
chr8_18290_mature	1.074569932	6.44742	1.05281229	2.896740642		-2.58496	0.321829	chr4_9930_star	0	0.805927	0	0.852739935		-20	0.140178	chr14_30612_mature	9.133844	2.417782	3.34110968	1.773060525		1.917538	0.590556
chr14_30485_mature	1.074569932	6.44742	1.05281229	2.896740642		-2.58496	0.321829	bta-miR-2419-5p	0	0.805927	0	0.852739935		-20	0.140178	chr8_18647_star	20.95411	4.835565	4.456419386	2.544872269		2.115477	0.592245
chr14_30768_star	1.074569932	6.44742	1.05281229	2.896740642		-2.58496	0.321829	mmu-miR-763	0	0.805927	0	0.852739935		-20	0.140178	chr6_12549_star	4.29828	17.7304	2.405524014	4.227310103		-2.04439	0.592565
chr14_29946_mature	1.074569932	6.44742	1.05281229	2.896740642		-2.58496	0.321829	mmu-miR-3473d	0	0.805927	0	0.852739935		-20	0.140178	chr6_14257_mature	12.35755	3.22371	3.739583968	2.078510713		1.938599	0.594492
chr12_26222_mature	1.074569932	6.44742	1.05281229	2.896740642		-2.58496	0.321829	rno-miR-22-3p	0	0.805927	0	0.852739935		-20	0.140178	chr4_9286_mature	12.35755	3.22371	3.739583968	2.078510713		1.938599	0.594492
NW_003613206_37650_mature	1.074569932	6.44742	1.05281229	2.896740642		-2.58496	0.321829	mmu-miR-344f-3p	0	0.805927	0	0.852739935		-20	0.140178	chr4_9743_star	12.35755	3.22371	3.739583968	2.078510713		1.938599	0.594492
chr6_12531_mature	1.074569932	6.44742	1.05281229	2.896740642		-2.58496	0.321829	chrx_22099_star	0	0.805927	0	0.852739935		-20	0.140178	chr1_3022_mature	12.35755	3.22371	3.739583968	2.078510713		1.938599	0.594492
chr1_550_star	31.16252802	5.641492	5.007308904	2.731507408		2.465664	0.322085	chr6_12838_star	0	0.805927	0	0.852739935		-20	0.140178	chr12_25513_mature	12.35755	3.22371	3.739583968	2.078510713		1.938599	0.594492
hsa-miR-891b	31.16252802	5.641492	5.007308904	2.731507408		2.465664	0.322085	chr2_5926_star	0	0.805927	0	0.852739935		-20	0.140178	rno-miR-1843-3p	12.35755	3.22371	3.739583968	2.078510713		1.938599	0.594492
chr4_9695_star	16.11854898	3.22371	4.097488514	2.078510713		2.321928	0.322303	hsa-miR-3925-3p	0	0.805927	0	0.852739935		-20	0.140178	chr17_35668_mature	12.35755	3.22371	3.739583968	2.078510713		1.938599	0.594492
chr2_6507_mature	16.11854898	3.22371	4.097488514	2.078510713		2.321928	0.322303	chr16_34111_star	0	0.805927	0	0.852739935		-20	0.140178	chr17_34938_mature	8.059274	41.1023	3.179395517	5.395827139		-2.3505	0.595369
chr13_27409_mature@@bta-miR-55	16.11854898	3.22371	4.097488514	2.078510713		2.321928	0.322303	hsa-miR-4763-3p	0	0.805927	0	0.852739935		-20	0.140178	chr7_16875_mature	3.22371	12.08891	2.078510713	3.710273245		-1.90689	0.59674
chr7_17171_mature	16.11854898	3.22371	4.097488514	2.078510713		2.321928	0.322303	chr8_18569_star	0	0.805927	0	0.852739935		-20	0.140178	chr2_5581_mature	3.22371	12.08891	2.078510713	3.710273245		-1.90689	0.59674
chr6_14159_star	11.82026925	2.417782	3.680354657	1.773060525		2.289507	0.323687	bta-miR-6123	0	0.805927	0	0.852739935		-20	0.140178	chr2_5381_mature	3.22371	12.08891	2.078510713	3.710273245		-1.90689	0.59674
rno-miR-219-1-3p	11.82026925	2.417782	3.680354657	1.773060525		2.289507	0.323687	chr6_13572_mature	0	0.805927	0	0.852739935		-20	0.140178	chr9_19555_star	3.760995	0.805927	2.251263041	0.852739935		2.222392	0.596903
hsa-miR-1255a	11.82026925	2.417782	3.680354657	1.773060525		2.289507	0.323687	chr2_4824_star	0	0.805927	0	0.852739935		-20	0.140178	NW_003541040_39847_star	3.760995	0.805927	2.251263041	0.852739935		2.222392	0.596903
chr2_5976_mature	11.82026925	2.417782	3.680354657	1.773060525		2.289507	0.323687	mmu-miR-92b-5p	0	0.805927	0	0.852739935		-20	0.140178	chr10_23371_star	3.760995	0.805927	2.251263041	0.852739935		2.222392	0.596903
ssc-miR-4338	11.82026925	2.417782	3.680354657	1.773060525		2.289507	0.323687	chr11_24779_star	0	0.805927	0	0.852739935		-20	0.140178	hsa-miR-4784	3.760995	0.805927	2.251263041	0.852739935		2.222392	0.596903
NW_003540351_39435_star	11.82026925	2.417782	3.680354657	1.773060525		2.289507	0.323687	mmu-miR-301a-3p	0	0.805927	0	0.852739935		-20	0.140178	chr6_14191_star	3.760995	0.805927	2.251263041	0.852739935		2.222392	0.596903
chr1_3229_star	11.82026925	2.417782	3.680354657	1.773060525		2.289507	0.323687	NW_003613218_37700_star	0	0.805927	0	0.852739935		-20	0.140178	chr6_14391_mature	3.760995	0.805927	2.251263041	0.852739935		2.222392	0.596903
chr3_8091_mature	6.447419591	37.87859	2.896740642	5.280903997		-2.55459	0.326048	bta-miR-345-3p	0	0.805927	0	0.852739935		-20	0.140178	chr6_14314_mature	3.760995	0.805927	2.251263041	0.852739935		2.222392	0.596903
NW_003538347_37746_star	6.447419591	37.87859	2.896740642	5.280903997		-2.55459	0.326048	rno-miR-7a-2-3p	0	0.805927	0	0.852739935		-20	0.140178	chr18_36206_mature	3.760995	0.805927	2.251263041	0.852739935		2.222392	0.596903
chr9_20736_star	5.372849659	29.81932	2.671938627	4.94576292		-2.47249	0.32741	hsa-miR-5682	0	0.805927	0	0.852739935		-20	0.140178	chr12_25921_star	3.760995	0.805927	2.251263041	0.852739935		2.222392	0.596903
chr12_25530_star	5.372849659	29.81932	2.671938627	4.94576292		-2.47249	0.32741	hsa-miR-5691	0	0.805927	0	0.852739935		-20	0.140178	NW_003539771_39089_star	3.760995	0.805927	2.251263041	0.852739935		2.222392	0.596903
chr14_31062_mature	5.372849659	29.81932	2.671938627	4.94576292		-2.47249	0.32741	mmu-miR-669n	0	0.805927	0	0.852739935		-20	0.140178	NW_003539080_38513_star	3.760995	0.805927	2.251263041	0.852739935		2.222392	0.596903
chr13_28864_star	3.223709795	16.11855	2.078510713	4.097488514		-2.32193	0.329462	hsa-miR-4723-3p	0	0.805927	0	0.852739935		-20	0.140178	chr2_6439_star	3.760995	0.805927	2.251263041	0.852739935		2.222392	0.596903
chrx_21570_star	3.223709795	16.11855	2.078510713	4.097488514		-2.32193	0.329462	hsa-miR-4513	0	0.805927	0	0.852739935		-20	0.140178	chr8_17967_star	3.760995	0.805927	2.251263041	0.852739935		2.222392	0.596903
ssc-miR-1839-3p	128.9483918	16.92448	7.02179497	4.163859073		2.929611	0.329768	chr3_6975_star	0	0.805927	0	0.852739935		-20	0.140178	chr3_7211_star	3.760995	0.805927	2.251263041	0.852739935		2.222392	0.596903
chr15_32156_mature	53.72849659	8.865202	5.774220321	3.302348583		2.599462	0.331087	hsa-miR-4518	0	0.805927	0	0.852739935		-20	0.140178	chr18_36398_star	3.760995	0.805927	2.251263041	0.852739935		2.222392	0.596903
chr4_9733_mature	2.149139864	10.47706	1.654957834	3.520680821		-2.2854	0.332097	hsa-miR-4526	0	0.805927	0	0.852739935		-20	0.140178	chr13_26891_star	3.760995	0.805927	2.251263041	0.852739935		2.222392	0.596903
chr2_5082_star	2.149139864	10.47706	1.654957834	3.520680821		-2.2854	0.332097	hsa-miR-4527	0	0.805927	0	0.852739935		-20	0.140178	chr13_28489_star	3.760995	0.805927	2.251263041	0.852739935		2.222392	0.596903
chrx_21689_mature	10.74569932	73.3394	3.554060706	6.216055097		-2.77083	0.332335	chr2_5391_star	0	0.805927	0	0.852739935		-20	0.140178	chr12_26408_mature	3.760995	0.805927	2.251263041	0.852739935		2.222392	0.596903
mmu-miR-1194	40.83365741	7.253347	5.38659223	3.044979305		2.49304	0.334283	mmu-miR-344g-5p	0	0.805927	0	0.852739935		-20	0.140178	chr13_28605_mature	3.760995	0.805927	2.251263041	0.852739935		2.222392	0.596903
chr8_17417_star	40.83365741	7.253347	5.38659223	3.044979305		2.49304	0.334283	hsa-miR-4502	0	0.805927	0	0.852739935		-20	0.140178	chr16_33764_star	3.760995	0.805927	2.251263041	0.852739935		2.222392	0.596903
chr15_31873_mature	226.7342556	25.78968	7.831207507	4.743605355		3.136137	0.33491	hsa-miR-4500	0	0.805927	0	0.852739935		-20	0.140178	chr6_13041_star	3.760995	0.805927	2.251263041	0.852739935		2.222392	0.596903
chr6_13320_mature	8.596559455	53.19121	3.262517265	5.759986999		-2.62936	0.335737	chr14_30751_mature	0	0.805927	0	0.852739935		-20	0.140178	chr4_9772_mature	3.760995	0.805927	2.251263041	0.852739935		2.222392	0.596903
hsa-miR-4749-5p	15.04397905	3.22371	4.003960082	2.078510713		2.222392	0.33674	chr13_26883_star	0	0.805927	0	0.852739935		-20	0.140178	NW_003538922_38349_star	3.760995	0.805927	2.251263041	0.852739935		2.222392	0.596903
chr3_6912_star	15.04397905	3.22371	4.003960082	2.078510713		2.222392	0.33674	chr14_29702_star	0	0.805927	0	0.852739935		-20	0.140178	chr12_26570_star	3.760995	0.805927	2.251263041	0.852739935		2.222392	0.596903
chr18_36069_mature	15.04397905	3.22371	4.003960082	2.078510713		2.222392	0.33674	hsa-miR-4703-3p	0	0.805927	0	0.852739935		-20	0.140178	chr15_32263_star	3.760995	0.805927	2.251263041	0.852739935		2.222392	0.596903
chr9_20679_mature	15.04397905	3.22371	4.003960082	2.078510713		2.222392	0.33674	hsa-miR-4540	0	0.805927	0	0.852739935		-20	0.140178	chr9_20880_star	3.760995	0.805927	2.251263041	0.852739935		2.222392	0.596903
chr8_17733_star@@mmu-miR-3084-	15.04397905	3.22371	4.003960082	2.078510713		2.222392	0.33674	hsa-miR-3155b	0	0.805927	0	0.852739935		-20	0.140178	chr14_30876_star	3.760995	0.805927	2.251263041	0.852739935		2.222392	0.596903
ssc-miR-452	78.44360502	1299.155	6.311859186	10.34446796		-4.04977	0.337235	hsa-miR-4778-5p	0	0.805927	0	0.852739935		-20	0.140178	chrx_22191_mature	3.760995	0.805927	2.251263041	0.852739935		2.222392	0.596903
chr1_1911_mature	7.521989523	1.611855	3.091190278	1.38507475		2.222392	0.337341	chr9_19409_star	0	0.805927	0	0.852739935		-20	0.140178	chr7_16801_star	3.760995	0.805927	2.251263041	0.852739935		2.222392	0.596903
chr18_35894_mature	7.521989523	1.611855	3.091190278	1.38507475		2.222392	0.337341	bta-miR-3613	0	0.805927	0	0.852739935		-20	0.140178	hsa-miR-4482-5p	3.760995	0.805927	2.251263041	0.852739935		2.222392	0.596903
rno-miR-483-5p	7.521989523	1.611855	3.091190278	1.38507475		2.222392	0.337341	mmu-miR-1264-5p	0	0.805927	0	0.852739935		-20	0.140178	ssc-miR-325	3.760995	0.805927	2.251263041	0.852739935		2.222392	0.596903
chr6_14428_mature	7.521989523	1.611855	3.091190278	1.38507475		2.222392	0.337341	chr10_23335_star	0	0.805927	0	0.852739935		-20	0.140178	chr13_26706_mature	3.760995	0.805927	2.251263041	0.852739935		2.222392	0.596903
chr3_7586_star	7.521989523	1.611855	3.091190278	1.38507475		2.222392	0.337341	rno-miR-16-3p	0	0.805927	0	0.852739935		-20	0.140178	chr13_26824_mature	3.760995	0.805927	2.251263041	0.852739935		2.222392	0.596903
chrx_21832_mature	7.521989523	1.611855	3.091190278	1.38507475		2.222392	0.337341	chr13_27824_star	0	0.805927	0	0.852739935		-20	0.140178	chr16_34458_star	3.760995	0.805927	2.251263041	0.852739935		2.222392	0.596903
chr13_27765_star	7.521989523	1.611855	3.091190278	1.38507475		2.222392	0.337341	chr5_12007_mature	0	0.805927	0	0.852739935		-20	0.140178	mmu-miR-6390	3.760995	0.805927	2.251263041	0.852739935		2.222392	0.596903
rno-miR-295-3p	7.521989523	1.611855	3.091190278	1.38507475		2.222392	0.337341	chr14_30751_star	0	0.805927	0	0.852739935		-20	0.140178	NW_003538374_37783_star	3.760995	0.805927	2.251263041	0.852739935		2.222392	0.596903
bta-miR-744	7.521989523	1.611855	3.091190278	1.38507475		2.222392	0.337341	chr12_25719_star	0	0.805927	0	0.852739935		-20	0.140178	chr14_31199_star	3.760995	0.805927	2.251263041	0.852739935		2.222392	0.596903
chr15_32751_mature	7.521989523	1.611855	3.091190278	1.38507475		2.222392	0.337341	hsa-miR-6068	0	0.805927	0	0.852739935		-20	0.140178	chr14_31327_mature	3.760995	0.805927	2.251263041	0.852739935		2.222392	0.596903
chr15_32439_mature	7.521989523	1.611855	3.091190278	1.38507475		2.222392	0.337341	chr14_31314_star	0	0.805927	0	0.852739935		-20	0.140178	chr8_17973_mature	3.760995	0.805927	2.251263041	0.852739935		2.222392	0.596903
chr7_15773_mature	7.521989523	1.611855	3.091190278	1.38507475		2.222392	0.337341	hsa-miR-6069	0	0.805927	0	0.852739935		-20	0.140178	chr4_9100_star	3.760995	0.805927	2.251263041	0.852739935		2.222392	0.596903
ssc-miR-124a	7.521989523	1.611855	3.091190278	1.38507475		2.222392	0.337341	chr9_20117_mature	0	0.805927	0	0.852739935		-20	0.140178	chr14_30949_star	3.760995	0.805927	2.251263041	0.852739935		2.222392	0.596903
chr3_7571_star	7.521989523	1.611855	3.091190278	1.38507475		2.222392	0.337341	mmu-miR-1231-3p	0	0.805927	0	0.852739935		-20	0.140178	chr18_36633_mature	3.760995	0.805927	2.251263041	0.852739935		2.222392	0.596903
chr1_1941_star	7.521989523	1.611855	3.091190278	1.38507475		2.222392	0.337341	chr7_15425_star	0	0.805927	0	0.852739935		-20	0.140178	hsa-miR-548aj-3p	3.760995	0.805927	2.251263041	0.852739935		2.222392	0.596903
chr3_6809_mature	7.521989523	1.611855	3.091190278	1.38507475		2.222392	0.337341	chr14_29827_star	0	0.805927	0	0.852739935		-20	0.140178	rno-miR-653-5p	3.760995	0.805927	2.251263041	0.852739935		2.222392	0.596903
chr9_20512_mature	7.521989523	1.611855	3.091190278	1.38507475		2.222392	0.337341	NW_003539070_38505_mature	0	0.805927	0	0.852739935		-20	0.140178	rno-miR-653-3p	3.760995	0.805927	2.251263041	0.852739935		2.222392	0.596903
NW_003539007_38445_mature	7.521989523	1.611855	3.091190278	1.38507475		2.222392	0.337341	chr3_7552_star	0	0.805927	0	0.852739935		-20	0.140178	chr2_5880_mature	3.760995	0.805927	2.251263041	0.852739935		2.222392	0.596903
chr11_24334_star	7.521989523	1.611855	3.091190278	1.38507475		2.222392	0.337341	mmu-miR-465c-5p	0	0.805927	0	0.852739935		-20	0.140178	chr3_7091_star	3.760995	0.805927	2.251263041	0.852739935		2.222392	0.596903
chr1_79_mature	7.521989523	1.611855	3.091190278	1.38507475		2.222392	0.337341	hsa-miR-922	0	0.805927	0	0.852739935		-20	0.140178	chr1_1936_star	3.760995	0.805927	2.251263041	0.852739935		2.222392	0.596903
chr10_23842_star	7.521989523	1.611855	3.091190278	1.38507475		2.222392	0.337341	chr14_29460_star	0	0.805927	0	0.852739935		-20	0.140178	chrx_22108_star	3.760995	0.805927	2.251263041	0.852739935		2.222392	0.596903
chr4_9234_mature	7.521989523	1.611855	3.091190278	1.38507475		2.222392	0.337341	hsa-miR-5583-5p	0	0.805927	0	0.852739935		-20	0.140178	chr18_36371_star	3.760995	0.805927	2.251263041	0.852739935		2.222392	0.596903
chr2_6372_star	7.521989523	1.611855	3.091190278	1.38507475		2.222392	0.337341	rno-miR-425-3p	0	0.805927	0	0.852739935		-20	0.140178	chr2_4454_star	3.760995	0.805927	2.251263041	0.852739935		2.222392	0.596903
chr1_816_mature	7.521989523	1.611855	3.091190278	1.38507475		2.222392	0.337341	hsa-miR-6081	0	0.805927	0	0.852739935		-20	0.140178	NW_003537370_36873_star	3.760995	0.805927	2.251263041	0.852739935		2.222392	0.596903
chr1_291_star	19.34225877	4.029637	4.346407978	2.330454351		2.263034	0.337953	hsa-miR-6073	0	0.805927	0	0.852739935		-20	0.140178	chr9_19419_star	3.760995	0.805927	2.251263041	0.852739935		2.222392	0.596903
chr13_27782_mature	19.34225877	4.029637	4.346407978	2.330454351		2.263034	0.337953	chr9_19892_star	0	0.805927	0	0.852739935		-20	0.140178	rno-miR-30a-5p	3.760995	0.805927	2.251263041	0.852739935		2.222392	0.596903
chr5_10730_mature	150.4397905	19.34226	7.24260051	4.346407978		2.959358	0.338956	NW_003613364_38713_star	0	0.805927	0	0.852739935		-20	0.140178	hsa-miR-3145-5p	3.760995	0.805927	2.251263041	0.852739935		2.222392	0.596903
chr15_32764_mature	3.223709795	15.31262	2.078510713	4.027916745		-2.24793	0.338961	chr1_2742_mature	0	0.805927	0	0.852739935		-20	0.140178	chr15_33570_mature	3.760995	0.805927	2.251263041	0.852739935		2.222392	0.596903
bta-miR-2284z	5.372849659	28.20746	2.671938627	4.868265031		-2.39232	0.339005	NW_003539571_38947_star	0	0.805927	0	0.852739935		-20	0.140178	chr9_20212_mature	3.760995	0.805927	2.251263041	0.852739935		2.222392	0.596903
chr13_28971_mature	29.01338816	5.641492	4.907534286	2.731507408		2.36257	0.339374	mmu-miR-217-5p	0	0.805927	0	0.852739935		-20	0.140178	hsa-miR-3115	3.760995	0.805927	2.251263041	0.852739935		2.222392	0.596903
chr4_10247_star	29.01338816	5.641492	4.907534286	2.731507408		2.36257	0.339374	mmu-miR-491-3p	0	0.805927	0	0.852739935		-20	0.140178	chr4_9602_mature	3.760995	0.805927	2.251263041	0.852739935		2.222392	0.596903
hsa-miR-4634	29.01338816	5.641492	4.907534286	2.731507408		2.36257	0.339374	chr9_20571_star	0	0.805927	0	0.852739935		-20	0.140178	hsa-miR-659-3p	3.760995	0.805927	2.251263041	0.852739935		2.222392	0.596903
chr15_32836_mature	6.447419591	35.46081	2.896740642	5.188274618		-2.45943	0.340392	chrx_23158_star	0	0.805927	0	0.852739935		-20	0.140178	chr12_25495_star	3.760995	0.805927	2.251263041	0.852739935		2.222392	0.596903
chr13_27411_mature	10.74569932	2.417782	3.554060706	1.773060525		2.152003	0.342132	chr2_5943_star	0	0.805927	0	0.852739935		-20	0.140178	chrx_21555_star	3.760995	0.805927	2.251263041	0.852739935		2.222392	0.596903
chr3_7424_mature	10.74569932	2.417782	3.554060706	1.773060525		2.152003	0.342132	chr5_10910_star	0	0.805927	0	0.852739935		-20	0.140178	hsa-miR-766-3p	3.760995	0.805927	2.251263041	0.852739935		2.222392	0.596903
mmu-miR-486-5p	10.74569932	2.417782	3.554060706	1.773060525		2.152003	0.342132	hsa-miR-1912	0	0.805927	0	0.852739935		-20	0.140178	hsa-miR-4421	3.760995	0.805927	2.251263041	0.852739935		2.222392	0.596903
chr6_12919_mature	10.74569932	2.417782	3.554060706	1.773060525		2.152003	0.342132	mmu-miR-1929-3p	0	0.805927	0	0.852739935		-20	0.140178	hsa-miR-4422	3.760995	0.805927	2.251263041	0.852739935		2.222392	0.596903
chr18_36164_mature	10.74569932	2.417782	3.554060706	1.773060525		2.152003	0.342132	NW_003613237_37814_mature	0	0.805927	0	0.852739935		-20	0.140178	chr14_31148_star	3.760995	0.805927	2.251263041	0.852739935		2.222392	0.596903
chr9_19780_mature	10.74569932	2.417782	3.554060706	1.773060525		2.152003	0.342132	chr1_886_mature	0	0.805927	0	0.852739935		-20	0.140178	chr16_34524_mature	3.760995	0.805927	2.251263041	0.852739935		2.222392	0.596903
chr1_1185_star	10.74569932	2.417782	3.554060706	1.773060525		2.152003	0.342132	chr16_34306_star	0	0.805927	0	0.852739935		-20	0.140178	hsa-miR-548a-3p	3.760995	0.805927	2.251263041	0.852739935		2.222392	0.596903
chr8_17317_mature	10.74569932	2.417782	3.554060706	1.773060525		2.152003	0.342132	chr9_21061_star	0	0.805927	0	0.852739935		-20	0.140178	chr5_11265_mature	3.760995	0.805927	2.251263041	0.852739935		2.222392	0.596903
chr15_31672_mature	1.074569932	5.641492	1.05281229	2.731507408		-2.39232	0.342853	chr12_26534_star	4128.49768	3207.591	12.01175058	11.6477243		0.364127	0.146923	chr4_10131_star	3.760995	0.805927	2.251263041	0.852739935		2.222392	0.596903
mmu-miR-21c	1.074569932	5.641492	1.05281229	2.731507408		-2.39232	0.342853	chr3_6931_mature	77.3690351	1.611855	6.292211829	1.38507475		5.584963	0.174263	chr2_6507_star	3.760995	0.805927	2.251263041	0.852739935		2.222392	0.596903
bta-miR-3957	1.074569932	5.641492	1.05281229	2.731507408		-2.39232	0.342853	chr18_36004_mature	73.0707554	0.805927	6.210832145	0.852739935		6.5025	0.185574	chr15_31749_star	3.760995	0.805927	2.251263041	0.852739935		2.222392	0.596903
NW_003539074_38507_star	1.074569932	5.641492	1.05281229	2.731507408		-2.39232	0.342853	ssc-miR-365-5p	339.564098	689.068	8.411782547	9.430594658		-1.02096	0.198188	chr6_12838_star	3.760995	0.805927	2.251263041	0.852739935		2.222392	0.596903
chr5_12211_star	1.074569932	5.641492	1.05281229	2.731507408		-2.39232	0.342853	chr17_35521_mature	2.14913986	114.4417	1.654957834	6.851020612		-5.73471	0.205648	hsa-miR-5691	3.760995	0.805927	2.251263041	0.852739935		2.222392	0.596903
chr7_16882_mature	1.074569932	5.641492	1.05281229	2.731507408		-2.39232	0.342853	NW_003540835_39730_mature	4.29827973	121.695	2.405524014	6.938933175		-4.82337	0.218837	chr6_14372_star	3.760995	0.805927	2.251263041	0.852739935		2.222392	0.596903
chr13_26861_mature	1.074569932	5.641492	1.05281229	2.731507408		-2.39232	0.342853	hsa-miR-4531	292.283021	96.71129	8.196149744	6.610453419		1.59561	0.244937	hsa-miR-922	3.760995	0.805927	2.251263041	0.852739935		2.222392	0.596903
chr10_23470_star	1.074569932	5.641492	1.05281229	2.731507408		-2.39232	0.342853	ssc-miR-130a	318.0727	656.8309	8.317741365	9.361572903		-1.04617	0.256163	chr5_12321_star	3.760995	0.805927	2.251263041	0.852739935		2.222392	0.596903
chr5_11659_mature	1.074569932	5.641492	1.05281229	2.731507408		-2.39232	0.342853	chr14_31492_mature	2.14913986	113.6358	1.654957834	6.840913474		-5.72451	0.258947	chr7_16882_mature	25.25239	5.641492	4.714377052	2.731507408		2.162271	0.597108
chr5_12345_mature	1.074569932	5.641492	1.05281229	2.731507408		-2.39232	0.342853	chr4_9830_mature	330.967539	673.7553	8.374898366	9.398220694		-1.02554	0.306898	chr13_28990_mature	5.37285	23.3719	2.671938627	4.607146585		-2.12102	0.59717
hsa-miR-3672	1.074569932	5.641492	1.05281229	2.731507408		-2.39232	0.342853	rno-miR-185-3p	143.992371	23.3719	7.179833181	4.607146585		2.623146	0.328627	chr13_28825_mature	7.52199	37.07266	3.091190278	5.250683565		-2.30117	0.597337
chr16_33720_mature	1.074569932	5.641492	1.05281229	2.731507408		-2.39232	0.342853	chr2_6113_mature	4.29827973	120.0832	2.405524014	6.919854778		-4.80413	0.335528	chrx_22122_star	7.52199	37.07266	3.091190278	5.250683565		-2.30117	0.597337
chr17_34966_mature	1.074569932	5.641492	1.05281229	2.731507408		-2.39232	0.342853	chr16_34199_mature	330.967539	120.0832	8.374898366	6.919854778		1.462656	0.339829	chr4_9198_star	2.686425	9.671129	1.882222339	3.415640968		-1.848	0.597845
chr13_27615_mature	1.074569932	5.641492	1.05281229	2.731507408		-2.39232	0.342853	chr2_4818_mature	38.6845175	205.5115	5.310504361	7.690078309		-2.40939	0.340858	mmu-miR-1951	2.686425	9.671129	1.882222339	3.415640968		-1.848	0.597845
chr9_19579_star	1.074569932	5.641492	1.05281229	2.731507408		-2.39232	0.342853	chr12_25742_mature	79.518175	2.417782	6.331242567	1.773060525		5.039528	0.342053	chr8_17809_star	2.686425	9.671129	1.882222339	3.415640968		-1.848	0.597845
chr2_5587_mature	1.074569932	5.641492	1.05281229	2.731507408		-2.39232	0.342853	chr18_36365_mature	79.518175	2.417782	6.331242567	1.773060525		5.039528	0.342053	chr8_18878_mature	5.910135	1.611855	2.788713818	1.38507475		1.874469	0.598153
chr4_9346_mature	1.074569932	5.641492	1.05281229	2.731507408		-2.39232	0.342853	chr12_26356_mature	120.351832	14.50669	6.923052083	3.954819241		3.052467	0.357243	chr15_33576_star	5.910135	1.611855	2.788713818	1.38507475		1.874469	0.598153
chr7_16945_mature	1.074569932	5.641492	1.05281229	2.731507408		-2.39232	0.342853	chrx_22133_mature	137.544951	20.95411	7.114210328	4.456419386		2.714598	0.364367	chr13_27363_star	5.910135	1.611855	2.788713818	1.38507475		1.874469	0.598153
rno-miR-3559-5p	1.074569932	5.641492	1.05281229	2.731507408		-2.39232	0.342853	chr6_12501_mature	66.6233358	0	6.079449279	0		20	0.377819	hsa-miR-1208	5.910135	1.611855	2.788713818	1.38507475		1.874469	0.598153
chr6_14191_mature	1.074569932	5.641492	1.05281229	2.731507408		-2.39232	0.342853	chrx_21838_star@@rno-miR-188-3p	66.6233358	0	6.079449279	0		20	0.377819	chr2_6441_star	5.910135	1.611855	2.788713818	1.38507475		1.874469	0.598153
chr9_19744_star	1.074569932	5.641492	1.05281229	2.731507408		-2.39232	0.342853	chr8_17233_mature	66.6233358	0	6.079449279	0		20	0.377819	chr5_12309_mature@@bta-miR-12	5.910135	1.611855	2.788713818	1.38507475		1.874469	0.598153
hsa-miR-548f	1.074569932	5.641492	1.05281229	2.731507408		-2.39232	0.342853	chr1_3926_star	70.9216155	0.805927	6.168353522	0.852739935		6.459432	0.436013	chr17_35344_mature	5.910135	1.611855	2.788713818	1.38507475		1.874469	0.598153
chr13_27163_star	1.074569932	5.641492	1.05281229	2.731507408		-2.39232	0.342853	chr8_18166_mature	94.562154	6.44742	6.578367468	2.896740642		3.874469	0.438184	chr3_6843_star@@hsa-miR-4753-	5.910135	1.611855	2.788713818	1.38507475		1.874469	0.598153
chr13_26847_star	1.074569932	5.641492	1.05281229	2.731507408		-2.39232	0.342853	chr14_29460_mature	2.14913986	111.218	1.654957834	6.810160141		-5.69349	0.461952	chr14_30369_star	5.910135	1.611855	2.788713818	1.38507475		1.874469	0.598153
chr15_32721_star	1.074569932	5.641492	1.05281229	2.731507408		-2.39232	0.342853	ssc-miR-1271	109.606133	11.28298	6.789287574	3.618589217		3.280108	0.513184	chrx_21977_mature@@rno-miR-38	5.910135	1.611855	2.788713818	1.38507475		1.874469	0.598153
chr9_20262_star	1.074569932	5.641492	1.05281229	2.731507408		-2.39232	0.342853	chr1_1729_mature	199.870007	477.915	7.650118356	8.903625744		-1.25769	0.513609	chr2_4472_mature	5.910135	1.611855	2.788713818	1.38507475		1.874469	0.598153
chr15_32320_mature	1.074569932	5.641492	1.05281229	2.731507408		-2.39232	0.342853	chr14_29526_mature	81.6673148	3.22371	6.369245121	2.078510713		4.662965	0.518743	NW_003539061_38502_star	5.910135	1.611855	2.788713818	1.38507475		1.874469	0.598153
chr3_7580_star	1.074569932	5.641492	1.05281229	2.731507408		-2.39232	0.342853	chr11_25073_mature	81.6673148	3.22371	6.369245121	2.078510713		4.662965	0.518743	NW_003540508_39511_mature	5.910135	1.611855	2.788713818	1.38507475		1.874469	0.598153
chr13_27066_mature	1.074569932	5.641492	1.05281229	2.731507408		-2.39232	0.342853	ssc-miR-194a	96.7112939	7.253347	6.610453419	3.044979305		3.736966	0.518951	chr9_19375_star	5.910135	1.611855	2.788713818	1.38507475		1.874469	0.598153
chr5_11238_star	1.074569932	5.641492	1.05281229	2.731507408		-2.39232	0.342853	chr1_3436_mature	2.14913986	110.4121	1.654957834	6.799761604		-5.68299	0.536658	chr17_35547_star	5.910135	1.611855	2.788713818	1.38507475		1.874469	0.598153
chr5_11382_star	1.074569932	5.641492	1.05281229	2.731507408		-2.39232	0.342853	chr9_20880_mature	4.29827973	116.8595	2.405524014	6.880923997		-4.76487	0.620849	NW_003538075_37489_star	5.910135	1.611855	2.788713818	1.38507475		1.874469	0.598153
chr10_24012_star	1.074569932	5.641492	1.05281229	2.731507408		-2.39232	0.342853	chr10_24002_mature	176.229469	38.68452	7.469474698	5.310504361		2.187627	0.622634	hsa-miR-6514-3p	5.910135	1.611855	2.788713818	1.38507475		1.874469	0.598153
chr7_15474_star	1.074569932	5.641492	1.05281229	2.731507408		-2.39232	0.342853	hsa-miR-4448	77.3690351	2.417782	6.292211829	1.773060525		5	0.628133	chr13_29080_star	5.910135	1.611855	2.788713818	1.38507475		1.874469	0.598153
chr13_27764_star	1.074569932	5.641492	1.05281229	2.731507408		-2.39232	0.342853	chr14_30700_mature	105.307853	10.47706	6.732104367	3.520680821		3.329308	0.720312	mmu-miR-1191	5.910135	1.611855	2.788713818	1.38507475		1.874469	0.598153
chr7_16992_star	1.074569932	5.641492	1.05281229	2.731507408		-2.39232	0.342853	rno-miR-1188-5p	68.7724756	0.805927	6.124586119	0.852739935		6.415037	0.723518	chr4_10395_star	5.910135	1.611855	2.788713818	1.38507475		1.874469	0.598153
chr3_7067_star	1.074569932	5.641492	1.05281229	2.731507408		-2.39232	0.342853	chr1_3878_mature	68.7724756	0.805927	6.124586119	0.852739935		6.415037	0.723518	chr7_16673_mature@@mmu-miR-6	5.910135	1.611855	2.788713818	1.38507475		1.874469	0.598153
chr5_11980_star	1.074569932	5.641492	1.05281229	2.731507408		-2.39232	0.342853	ssc-miR-132	229.957965	66.08605	7.851486493	6.067940913		1.798952	0.724696	chr15_32159_star	5.910135	1.611855	2.788713818	1.38507475		1.874469	0.598153
chr10_23335_mature	1.074569932	5.641492	1.05281229	2.731507408		-2.39232	0.342853	NW_003540127_39291_mature	83.8164547	285.2983	6.406272275	8.161375379		-1.76717	0.757066	chr7_15958_mature	5.910135	1.611855	2.788713818	1.38507475		1.874469	0.598153
chr9_19234_mature	4.298279727	20.95411	2.405524014	4.456419386		-2.2854	0.343042	chr8_17892_star	85.9655945	4.835565	6.442372848	2.544872269		4.152003	0.763957	chr13_27245_mature	5.910135	1.611855	2.788713818	1.38507475		1.874469	0.598153
chr5_10849_mature	45.13193714	8.059274	5.527693969	3.179395517		2.485427	0.344068	chr17_35550_mature	85.9655945	4.835565	6.442372848	2.544872269		4.152003	0.763957	chr5_11905_mature	5.910135	1.611855	2.788713818	1.38507475		1.874469	0.598153
ssc-miR-196b-5p	776.9140607	63.66827	9.603466973	6.014986077		3.609109	0.34425	chr1_1181_mature	85.9655945	4.835565	6.442372848	2.544872269		4.152003	0.763957	chrx_21678_star	5.910135	1.611855	2.788713818	1.38507475		1.874469	0.598153
chr5_12375_star	5.372849659	27.40153	2.671938627	4.827896911		-2.3505	0.344993	chr8_18843_mature	2.14913986	106.3824	1.654957834	6.746614057		-5.62936	0.837768	rno-miR-194-5p	5.910135	1.611855	2.788713818	1.38507475		1.874469	0.598153
chr13_27155_star	5.372849659	27.40153	2.671938627	4.827896911		-2.3505	0.344993	chr2_6585_star	75.2198952	2.417782	6.25209572	1.773060525		4.959358	0.845453	chr15_33369_star	3.760995	14.50669	2.251263041	3.954819241		-1.94753	0.598891
NW_003540557_39543_mature	2.149139864	9.671129	1.654957834	3.415640968		-2.16993	0.345588	chr14_30612_mature	75.2198952	2.417782	6.25209572	1.773060525		4.959358	0.845453	chr15_31672_mature	1.611855	5.641492	1.38507475	2.731507408		-1.80735	0.599071
chr1_2964_star	2.149139864	9.671129	1.654957834	3.415640968		-2.16993	0.345588	chr10_24282_mature	75.2198952	2.417782	6.25209572	1.773060525		4.959358	0.845453	chr1_3118_star	1.611855	5.641492	1.38507475	2.731507408		-1.80735	0.599071
mmu-miR-1951	2.149139864	9.671129	1.654957834	3.415640968		-2.16993	0.345588	chr1_3365_mature	10.7456993	132.1721	3.554060706	7.057148072		-3.62059	0.866772	NW_003539074_38507_star	1.611855	5.641492	1.38507475	2.731507408		-1.80735	0.599071
chr18_36164_star	2.149139864	9.671129	1.654957834	3.415640968		-2.16993	0.345588	chr1_2672_mature	70.9216155	1.611855	6.168353522	1.38507475		5.459432	0.884579	bta-miR-2888	1.611855	5.641492	1.38507475	2.731507408		-1.80735	0.599071
chr18_35842_mature	2.149139864	9.671129	1.654957834	3.415640968		-2.16993	0.345588	chr14_30575_star	152.58893	29.81932	7.26293043	4.94576292		2.355331	0.888833	chr5_12211_star	1.611855	5.641492	1.38507475	2.731507408		-1.80735	0.599071
chr7_15714_star	2.149139864	9.671129	1.654957834	3.415640968		-2.16993	0.345588	NW_003538989_38416_mature	66.6233358	0.805927	6.079449279	0.852739935		6.369234	0.898334	chr7_15929_mature	1.611855	5.641492	1.38507475	2.731507408		-1.80735	0.599071
chr15_33605_star	2.149139864	9.671129	1.654957834	3.415640968		-2.16993	0.345588	chr15_32759_star	77.3690351	3.22371	6.292211829	2.078510713		4.584963	0.915881	hsa-miR-23b-5p	1.611855	5.641492	1.38507475	2.731507408		-1.80735	0.599071
chr8_17716_mature	2.149139864	9.671129	1.654957834	3.415640968		-2.16993	0.345588	chr14_30079_star	77.3690351	3.22371	6.292211829	2.078510713		4.584963	0.915881	hsa-miR-4715-5p	1.611855	5.641492	1.38507475	2.731507408		-1.80735	0.599071
chr2_6406_mature@@bta-miR-1814	2.149139864	9.671129	1.654957834	3.415640968		-2.16993	0.345588	chr7_17190_mature@@hsa-miR-4783-	135.395811	23.3719	7.091655531	4.607146585		2.534336	0.949826	hsa-miR-3665	1.611855	5.641492	1.38507475	2.731507408		-1.80735	0.599071
chr14_30443_star	56.95220639	9.671129	5.856791684	3.415640968		2.557995	0.346743	chr1_3087_star	79.518175	4.029637	6.331242567	2.330454351		4.302563	0.950254	chr7_15130_star	1.611855	5.641492	1.38507475	2.731507408		-1.80735	0.599071
chr8_18750_mature	63.39962598	10.47706	6.008980404	3.520680821		2.597241	0.346859	chr5_11598_star	88.1147344	6.44742	6.477592084	2.896740642		3.77259	0.954113	chr3_8521_mature	1.611855	5.641492	1.38507475	2.731507408		-1.80735	0.599071
chr12_25849_mature	50.5047868	8.865202	5.686634616	3.302348583		2.510195	0.347764	chr15_32358_mature	148.290651	384.4274	7.221980009	8.5903153		-1.37428	0.954833	chr7_17173_mature	1.611855	5.641492	1.38507475	2.731507408		-1.80735	0.599071
chr13_27738_star	4.298279727	0.805927	2.405524014	0.852739935		2.415037	0.347946	chr11_25154_mature	2.14913986	102.3528	1.654957834	6.691433469		-5.57365	0.958227	NW_003541083_39895_mature	1.611855	5.641492	1.38507475	2.731507408		-1.80735	0.599071
chr5_11960_star	4.298279727	0.805927	2.405524014	0.852739935		2.415037	0.347946	chr2_4586_mature	68.7724756	1.611855	6.124586119	1.38507475		5.415037	0.961875	chr14_29752_star	1.611855	5.641492	1.38507475	2.731507408		-1.80735	0.599071
chr13_29007_mature	4.298279727	0.805927	2.405524014	0.852739935		2.415037	0.347946	hsa-miR-4484	68.7724756	1.611855	6.124586119	1.38507475		5.415037	0.961875	chr2_4728_star	1.611855	5.641492	1.38507475	2.731507408		-1.80735	0.599071
chr14_30718_star	4.298279727	0.805927	2.405524014	0.852739935		2.415037	0.347946	chr1_3607_mature	257.896784	85.42831	8.016233232	6.433432039		1.594008	0.96542	chr13_27072_star	1.611855	5.641492	1.38507475	2.731507408		-1.80735	0.599071
NW_003539115_38545_mature	4.298279727	0.805927	2.405524014	0.852739935		2.415037	0.347946	chr14_30768_mature@@bta-miR-2478	120.351832	17.7304	6.923052083	4.227310103		2.762961	0.966355	chrx_22713_mature	1.611855	5.641492	1.38507475	2.731507408		-1.80735	0.599071
chr9_19972_star	4.298279727	0.805927	2.405524014	0.852739935		2.415037	0.347946	chr8_17750_mature	64.4741959	0.805927	6.032854532	0.852739935		6.321928	0.967488	chr5_11382_star	1.611855	5.641492	1.38507475	2.731507408		-1.80735	0.599071
chr6_14382_mature	4.298279727	0.805927	2.405524014	0.852739935		2.415037	0.347946	chr14_30385_mature	64.4741959	0.805927	6.032854532	0.852739935		6.321928	0.967488	mmu-miR-2136	1.611855	5.641492	1.38507475	2.731507408		-1.80735	0.599071
chr1_3263_star	4.298279727	0.805927	2.405524014	0.852739935		2.415037	0.347946	chr15_33317_mature	81.6673148	4.835565	6.369245121	2.544872269		4.078003	0.968133	chr10_23335_mature	1.611855	5.641492	1.38507475	2.731507408		-1.80735	0.599071
chr12_26158_mature	4.298279727	0.805927	2.405524014	0.852739935		2.415037	0.347946	chr8_18647_star	81.6673148	4.835565	6.369245121	2.544872269		4.078003	0.968133	hsa-miR-4652-5p	2.14914	0	1.654957834	0		20	0.599178
chr12_26183_star	4.298279727	0.805927	2.405524014	0.852739935		2.415037	0.347946	ssc-miR-2366	2.14913986	101.5469	1.654957834	6.680139486		-5.56224	0.968647	mmu-miR-328-3p	2.14914	0	1.654957834	0		20	0.599178
chr16_34407_mature	4.298279727	0.805927	2.405524014	0.852739935		2.415037	0.347946	chr12_25511_mature	4.29827973	107.9943	2.405524014	6.76810859		-4.65105	0.976205	mmu-miR-698-3p	2.14914	0	1.654957834	0		20	0.599178
chr13_29250_star	4.298279727	0.805927	2.405524014	0.852739935		2.415037	0.347946	ssc-miR-215	128.948392	21.76004	7.02179497	4.508431259		2.567041	0.982231	chr2_5886_mature	2.14914	0	1.654957834	0		20	0.599178
rno-miR-30d-5p	4.298279727	0.805927	2.405524014	0.852739935		2.415037	0.347946	NW_003538347_37746_mature	98.8604337	10.47706	6.641841268	3.520680821		3.23816	0.982673	chr8_17792_star	2.14914	0	1.654957834	0		20	0.599178
chr12_26093_star	4.298279727	0.805927	2.405524014	0.852739935		2.415037	0.347946	chr6_14179_mature	70.9216155	2.417782	6.168353522	1.773060525		4.874469	0.982695	hsa-miR-3152-5p	2.14914	0	1.654957834	0		20	0.599178
chr13_28692_mature	4.298279727	0.805927	2.405524014	0.852739935		2.415037	0.347946	chr18_36164_mature	70.9216155	2.417782	6.168353522	1.773060525		4.874469	0.982695	rno-miR-509-5p	2.14914	0	1.654957834	0		20	0.599178
chr4_8702_mature	4.298279727	0.805927	2.405524014	0.852739935		2.415037	0.347946	chr13_27560_mature	272.940763	95.90537	8.097720146	6.598504656		1.508904	0.986336	NW_003613107_37244_star	2.14914	0	1.654957834	0		20	0.599178
chr7_15959_star	4.298279727	0.805927	2.405524014	0.852739935		2.415037	0.347946	chr5_12291_mature	2.14913986	99.12908	1.654957834	6.645717165		-5.52748	0.986912	chr8_18247_star	2.14914	0	1.654957834	0		20	0.599178
chr11_25225_star	4.298279727	0.805927	2.405524014	0.852739935		2.415037	0.347946	ssc-miR-671-5p	597.460882	999.35	9.225113139	9.966289192		-0.74215	0.987652	hsa-miR-6719-3p	2.14914	0	1.654957834	0		20	0.599178
chr7_15837_star	4.298279727	0.805927	2.405524014	0.852739935		2.415037	0.347946	chr5_12167_mature	66.6233358	1.611855	6.079449279	1.38507475		5.369234	0.988064	chr7_15777_star	2.14914	0	1.654957834	0		20	0.599178
chr3_7048_mature	4.298279727	0.805927	2.405524014	0.852739935		2.415037	0.347946	hsa-miR-566	58.0267763	0	5.883297648	0		20	0.988662	chr5_12034_star	2.14914	0	1.654957834	0		20	0.599178
NW_003541332_40046_mature	4.298279727	0.805927	2.405524014	0.852739935		2.415037	0.347946	ssc-miR-429	58.0267763	0	5.883297648	0		20	0.988662	rno-miR-92a-1-5p	2.14914	0	1.654957834	0		20	0.599178
chr7_15697_mature	4.298279727	0.805927	2.405524014	0.852739935		2.415037	0.347946	chr1_3877_mature	58.0267763	0	5.883297648	0		20	0.988662	chrx_22247_star	2.14914	0	1.654957834	0		20	0.599178
chr13_28323_mature	4.298279727	0.805927	2.405524014	0.852739935		2.415037	0.347946	chr17_35330_mature	79.518175	4.835565	6.331242567	2.544872269		4.039528	0.989302	hsa-miR-4520b-5p	2.14914	0	1.654957834	0		20	0.599178
NW_003539422_38814_star	4.298279727	0.805927	2.405524014	0.852739935		2.415037	0.347946	NW_003613497_39427_mature	2.14913986	98.32315	1.654957834	6.634058094		-5.5157	0.990243	chr10_24135_star	2.14914	0	1.654957834	0		20	0.599178
chr4_9939_star	4.298279727	0.805927	2.405524014	0.852739935		2.415037	0.347946	chr10_24172_mature	2.14913986	98.32315	1.654957834	6.634058094		-5.5157	0.990243	chr17_35489_mature	2.14914	0	1.654957834	0		20	0.599178
chr7_15367_star	4.298279727	0.805927	2.405524014	0.852739935		2.415037	0.347946	chr3_7552_mature	6.44741959	112.0239	2.896740642	6.820484263		-4.11894	0.990486	bta-miR-2411-5p	2.14914	0	1.654957834	0		20	0.599178
chr16_34626_mature	4.298279727	0.805927	2.405524014	0.852739935		2.415037	0.347946	chr7_17178_star	73.0707554	3.22371	6.210832145	2.078510713		4.5025	0.990853	mmu-miR-501-3p	2.14914	0	1.654957834	0		20	0.599178
rno-miR-30b-5p	4.298279727	0.805927	2.405524014	0.852739935		2.415037	0.347946	chr6_14094_mature	2.14913986	97.51722	1.654957834	6.622304032		-5.50383	0.992731	chr1_1213_star	2.14914	0	1.654957834	0		20	0.599178
bta-miR-6119-3p	4.298279727	0.805927	2.405524014	0.852739935		2.415037	0.347946	chr1_1062_mature	4.29827973	103.9646	2.405524014	6.713759604		-4.59619	0.994352	hsa-miR-4797-5p	2.14914	0	1.654957834	0		20	0.599178
chr2_5822_star	4.298279727	0.805927	2.405524014	0.852739935		2.415037	0.347946	chr1_3824_star	4.29827973	103.9646	2.405524014	6.713759604		-4.59619	0.994352	hsa-miR-1253	2.14914	0	1.654957834	0		20	0.599178
chr6_13990_star	4.298279727	0.805927	2.405524014	0.852739935		2.415037	0.347946	chr7_17029_mature	83.8164547	6.44742	6.406272275	2.896740642		3.70044	0.994516	mmu-miR-146a-5p	2.14914	0	1.654957834	0		20	0.599178
chr2_4081_star	4.298279727	0.805927	2.405524014	0.852739935		2.415037	0.347946	chr13_27323_star	83.8164547	6.44742	6.406272275	2.896740642		3.70044	0.994516	chr9_19866_star	2.14914	0	1.654957834	0		20	0.599178
mmu-miR-3961	4.298279727	0.805927	2.405524014	0.852739935		2.415037	0.347946	chr14_31648_mature	2.14913986	96.71129	1.654957834	6.610453419		-5.49185	0.994588	chr5_10730_star	2.14914	0	1.654957834	0		20	0.599178
chr1_3489_mature	4.298279727	0.805927	2.405524014	0.852739935		2.415037	0.347946	chr10_23279_mature	32.237098	171.6625	5.054722516	7.431811362		-2.41278	0.995916	chr15_32751_star	2.14914	0	1.654957834	0		20	0.599178
NW_003540745_39662_mature	4.298279727	0.805927	2.405524014	0.852739935		2.415037	0.347946	chr6_14465_mature	64.4741959	1.611855	6.032854532	1.38507475		5.321928	0.996319	hsa-miR-1294	2.14914	0	1.654957834	0		20	0.599178
chr3_7091_star	4.298279727	0.805927	2.405524014	0.852739935		2.415037	0.347946	chr5_12026_mature	64.4741959	1.611855	6.032854532	1.38507475		5.321928	0.996319	chr16_34021_mature	2.14914	0	1.654957834	0		20	0.599178
chr1_608_mature	4.298279727	0.805927	2.405524014	0.852739935		2.415037	0.347946	chr7_17171_mature	70.9216155	3.22371	6.168353522	2.078510713		4.459432	0.997063	hsa-miR-1266	2.14914	0	1.654957834	0		20	0.599178
hsa-miR-1207-3p	4.298279727	0.805927	2.405524014	0.852739935		2.415037	0.347946	chr16_34200_mature	300.879581	116.0536	8.237829366	6.871024911		1.374396	0.997283	hsa-miR-558	2.14914	0	1.654957834	0		20	0.599178
hsa-miR-4645-3p	4.298279727	0.805927	2.405524014	0.852739935		2.415037	0.347946	chr13_28171_mature	6.44741959	107.9943	2.896740642	6.76810859		-4.06609	0.997699	hsa-miR-552	2.14914	0	1.654957834	0		20	0.599178
chr17_34996_star	4.298279727	0.805927	2.405524014	0.852739935		2.415037	0.347946	chr6_14848_mature	2.14913986	94.29351	1.654957834	6.57430608		-5.45533	0.997768	chr3_7972_star	2.14914	0	1.654957834	0		20	0.599178
NW_003613217_37698_mature	4.298279727	0.805927	2.405524014	0.852739935		2.415037	0.347946	chr12_26240_mature	96.7112939	11.28298	6.610453419	3.618589217		3.099536	0.997883	chr6_14458_mature	2.14914	0	1.654957834	0		20	0.599178
chr14_30332_mature	4.298279727	0.805927	2.405524014	0.852739935		2.415037	0.347946	chr11_24517_mature	4.29827973	100.7409	2.405524014	6.668756392		-4.55075	0.998225	chr8_18054_star	2.14914	0	1.654957834	0		20	0.599178
chr5_11562_star@@mmu-miR-3059-	4.298279727	0.805927	2.405524014	0.852739935		2.415037	0.347946	chr4_9294_star	66.6233358	2.417782	6.079449279	1.773060525		4.784271	0.998281	hsa-miR-511	2.14914	0	1.654957834	0		20	0.599178
chr4_9602_mature	4.298279727	0.805927	2.405524014	0.852739935		2.415037	0.347946	chr2_3979_mature	287.984742	109.6061	8.174849511	6.789287574		1.393664	0.998564	hsa-miR-3918	2.14914	0	1.654957834	0		20	0.599178
chr12_25495_star	4.298279727	0.805927	2.405524014	0.852739935		2.415037	0.347946	chr10_23333_mature	5011.79416	4094.917	12.29139928	11.99997089		0.291493	0.998598	hsa-miR-3907	2.14914	0	1.654957834	0		20	0.599178
mmu-miR-298-5p	4.298279727	0.805927	2.405524014	0.852739935		2.415037	0.347946	chr14_29991_mature	556.627225	930.8462	9.123157188	9.863948055		-0.74183	0.998713	hsa-miR-3938	2.14914	0	1.654957834	0		20	0.599178
chr2_4436_star	4.298279727	0.805927	2.405524014	0.852739935		2.415037	0.347946	chr6_13355_mature	62.325056	1.611855	5.984704544	1.38507475		5.273018	0.998867	chr6_12833_mature	2.14914	0	1.654957834	0		20	0.599178
chr3_7813_star	4.298279727	0.805927	2.405524014	0.852739935		2.415037	0.347946	chr6_13901_mature	53.7284966	0	5.774220321	0		20	0.999031	hsa-miR-3943	2.14914	0	1.654957834	0		20	0.599178
mmu-miR-1930-5p	4.298279727	0.805927	2.405524014	0.852739935		2.415037	0.347946	chr15_33346_mature	2.14913986	91.87573	1.654957834	6.537229727		-5.41785	0.99908	chr5_10795_mature@@rno-miR-35	2.14914	0	1.654957834	0		20	0.599178
chrx_21766_mature	4.298279727	0.805927	2.405524014	0.852739935		2.415037	0.347946	chr3_7813_mature	58.0267763	0.805927	5.883297648	0.852739935		6.169925	0.999105	chr2_5788_star	2.14914	0	1.654957834	0		20	0.599178
chr8_17571_mature	4.298279727	0.805927	2.405524014	0.852739935		2.415037	0.347946	chr15_32413_mature	92.4130141	10.47706	6.545551653	3.520680821		3.140863	0.999161	chr2_6568_star	2.14914	0	1.654957834	0		20	0.599178
mmu-miR-3064-5p	4.298279727	0.805927	2.405524014	0.852739935		2.415037	0.347946	chr9_19521_mature	148.290651	365.8911	7.221980009	8.519207948		-1.30299	0.999211	chrx_23237_star	2.14914	0	1.654957834	0		20	0.599178
chr7_15903_mature	4.298279727	0.805927	2.405524014	0.852739935		2.415037	0.347946	chr1_3824_mature	6.44741959	104.7706	2.896740642	6.72479443		-4.02237	0.999261	rno-miR-330-5p	2.14914	0	1.654957834	0		20	0.599178
rno-miR-26a-5p	4.298279727	0.805927	2.405524014	0.852739935		2.415037	0.347946	bta-miR-2881	70.9216155	4.029637	6.168353522	2.330454351		4.137504	0.999418	chr9_20360_star	2.14914	0	1.654957834	0		20	0.599178
chr2_4626_mature	4.298279727	0.805927	2.405524014	0.852739935		2.415037	0.347946	chr6_14411_mature	64.4741959	2.417782	6.032854532	1.773060525		4.736966	0.999458	hsa-miR-3973	2.14914	0	1.654957834	0		20	0.599178
rno-miR-26b-5p	4.298279727	0.805927	2.405524014	0.852739935		2.415037	0.347946	bta-miR-2429	2.14913986	90.26387	1.654957834	6.511971994		-5.39232	0.99949	hsa-miR-512-3p	2.14914	0	1.654957834	0		20	0.599178
chr18_36465_star	4.298279727	0.805927	2.405524014	0.852739935		2.415037	0.347946	ssc-miR-455-3p	238.554525	497.2572	7.904210254	8.960746947		-1.05967	0.999493	chr18_36229_star@@hsa-miR-122	2.14914	0	1.654957834	0		20	0.599178
chrx_23238_mature	4.298279727	0.805927	2.405524014	0.852739935		2.415037	0.347946	ssc-miR-219	266.493343	99.12908	8.063359178	6.645717165		1.426719	0.999549	chr17_34904_star	2.14914	0	1.654957834	0		20	0.599178
chr15_32446_mature	4.298279727	0.805927	2.405524014	0.852739935		2.415037	0.347946	chr12_26356_star	4.29827973	96.71129	2.405524014	6.610453419		-4.49185	0.999582	mmu-miR-871-5p	2.14914	0	1.654957834	0		20	0.599178
chr14_30751_star	4.298279727	0.805927	2.405524014	0.852739935		2.415037	0.347946	chr14_31584_mature	73.0707554	4.835565	6.210832145	2.544872269		3.917538	0.999605	rno-miR-3547	2.14914	0	1.654957834	0		20	0.599178
chr7_16598_star	4.298279727	0.805927	2.405524014	0.852739935		2.415037	0.347946	chr17_35003_mature	111.755273	18.53633	6.817051091	4.288087668		2.591915	0.999651	hsa-miR-4715-3p	2.14914	0	1.654957834	0		20	0.599178
chr5_12321_star	4.298279727	0.805927	2.405524014	0.852739935		2.415037	0.347946	chr16_34314_mature	60.1759162	1.611855	5.934891897	1.38507475		5.222392	0.999651	chr1_877_star	2.14914	0	1.654957834	0		20	0.599178
chrx_23158_star	4.298279727	0.805927	2.405524014	0.852739935		2.415037	0.347946	chr6_13900_mature	51.5793567	0	5.716424587	0		20	0.999717	hsa-miR-4423-5p	2.14914	0	1.654957834	0		20	0.599178
chr2_5884_star	4.298279727	0.805927	2.405524014	0.852739935		2.415037	0.347946	mmu-miR-466i-5p	51.5793567	0	5.716424587	0		20	0.999717	hsa-miR-514a-5p	2.14914	0	1.654957834	0		20	0.599178
mmu-miR-5624-3p	2.149139864	0	1.654957834	0		20	0.348231	hsa-miR-1268b	51.5793567	0	5.716424587	0		20	0.999717	chr6_12875_star	2.14914	0	1.654957834	0		20	0.599178
chr16_34272_star	2.149139864	0	1.654957834	0		20	0.348231	ssc-miR-18b	94.562154	12.08891	6.578367468	3.710273245		2.967579	0.999721	mmu-miR-712-5p	2.14914	0	1.654957834	0		20	0.599178
hsa-miR-376a-5p	2.149139864	0	1.654957834	0		20	0.348231	chr14_30038_star	55.8776365	0.805927	5.82978961	0.852739935		6.115477	0.999731	rno-miR-6331	2.14914	0	1.654957834	0		20	0.599178
hsa-miR-6716-5p	2.149139864	0	1.654957834	0		20	0.348231	ssc-miR-224	4.29827973	95.09944	2.405524014	6.586456103		-4.46761	0.999765	rno-miR-192-5p	2.14914	0	1.654957834	0		20	0.599178
hsa-miR-329	2.149139864	0	1.654957834	0		20	0.348231	chrx_21502_mature	2.14913986	87.84609	1.654957834	6.473236413		-5.35315	0.999789	rno-miR-6321	2.14914	0	1.654957834	0		20	0.599178
chr9_19755_star	2.149139864	0	1.654957834	0		20	0.348231	chrx_23221_mature	2.14913986	87.84609	1.654957834	6.473236413		-5.35315	0.999789	chr5_12208_star	2.14914	0	1.654957834	0		20	0.599178

chr15_32479_mature	2.149139864	0	1.654957834	0		20	0.348231	chr1_3261_mature	42.9827973	182.9455	5.458867456	7.523134815		-2.08958	0.999803	chr3_6716_star	2.14914	0	1.654957834	0		20	0.599178
hsa-miR-4659b-3p	2.149139864	0	1.654957834	0		20	0.348231	chr3_7781_mature	79.518175	7.253347	6.331242567	3.044979305		3.454566	0.999819	chr16_34200_star	2.14914	0	1.654957834	0		20	0.599178
chr2_5886_mature	2.149139864	0	1.654957834	0		20	0.348231	chr4_8977_mature	6.44741959	100.7409	2.896740642	6.668756392		-3.96578	0.99982	chr2_5373_star	2.14914	0	1.654957834	0		20	0.599178
chr1_3525_mature	2.149139864	0	1.654957834	0		20	0.348231	chr4_9685_mature	4.29827973	94.29351	2.405524014	6.57430608		-4.45533	0.999824	mmu-miR-19b-3p	2.14914	0	1.654957834	0		20	0.599178
chr14_29661_star	2.149139864	0	1.654957834	0		20	0.348231	chr14_30841_mature	219.212266	73.3394	7.782751021	6.216055097		1.579668	0.999826	chr13_29267_star	2.14914	0	1.654957834	0		20	0.599178
chr6_14212_star	2.149139864	0	1.654957834	0		20	0.348231	chr14_31575_mature	62.325056	2.417782	5.984704544	1.773060525		4.688056	0.999829	rno-miR-344g	2.14914	0	1.654957834	0		20	0.599178
chr6_13603_star	2.149139864	0	1.654957834	0		20	0.348231	chr18_36189_mature	2.14913986	87.04016	1.654957834	6.460089935		-5.33985	0.999843	chr15_32775_mature	2.14914	0	1.654957834	0		20	0.599178
chr9_20112_star	2.149139864	0	1.654957834	0		20	0.348231	chr9_19579_mature	8.59655945	105.5765	3.262517265	6.735745493		-3.61839	0.99988	bta-miR-1298	2.14914	0	1.654957834	0		20	0.599178
chr2_4615_star	2.149139864	0	1.654957834	0		20	0.348231	chr10_23437_mature	2.14913986	86.23424	1.654957834	6.446822559		-5.32643	0.999883	rno-miR-449a-3p	2.14914	0	1.654957834	0		20	0.599178
hsa-miR-4773	2.149139864	0	1.654957834	0		20	0.348231	chr1_2437_mature	2.14913986	86.23424	1.654957834	6.446822559		-5.32643	0.999883	chr14_30174_star	2.14914	0	1.654957834	0		20	0.599178
chr15_33139_star	2.149139864	0	1.654957834	0		20	0.348231	chr15_31855_mature	88.1147344	10.47706	6.477592084	3.520680821		3.07215	0.999887	mmu-miR-1966-3p	2.14914	0	1.654957834	0		20	0.599178
[illegible]	[illegible]	[illegible]	[illegible]	[illegible]		[illegible]	[illegible]	[illegible]	[illegible]	[illegible]	[illegible]	[illegible]		[illegible]	[illegible]	[illegible]	[illegible]	[illegible]	[illegible]	[illegible]		[illegible]	[illegible]

chr6_15024_star	2.149139864	0	1.654957834	0		20	0.348231	chr3_7353_mature	42.9827973	3.22371	5.458867456	2.078510713
chr9_20618_star@@bta-miR-135b	2.149139864	0	1.654957834	0		20	0.348231	chr17_34903_mature	6.44741959	66.89198	2.896740642	6.085169218
bta-miR-2307	2.149139864	0	1.654957834	0		20	0.348231	chr7_16002_mature	4.29827973	61.25049	2.405524014	5.960013198
hsa-miR-4316	2.149139864	0	1.654957834	0		20	0.348231	ssc-miR-208b	30.0879581	0	4.958283956	0
hsa-miR-4311	2.149139864	0	1.654957834	0		20	0.348231	chr3_8640_mature	30.0879581	0	4.958283956	0
bta-miR-2369	2.149139864	0	1.654957834	0		20	0.348231	chr17_35756_mature	30.0879581	0	4.958283956	0
bta-miR-2360	2.149139864	0	1.654957834	0		20	0.348231	hsa-miR-5003-5p	30.0879581	0	4.958283956	0
bta-miR-2372	2.149139864	0	1.654957834	0		20	0.348231	chr1_3554_mature	30.0879581	0	4.958283956	0
hsa-miR-4304	2.149139864	0	1.654957834	0		20	0.348231	chr16_34013_mature	85.9655945	20.95411	6.442372848	4.456419386
mmu-miR-294-5p	2.149139864	0	1.654957834	0		20	0.348231	chr14_29816_mature	45.1319371	4.029637	5.527693969	2.330454351
hsa-miR-4301	2.149139864	0	1.654957834	0		20	0.348231	chr14_31020_mature	21.4913986	99.12908	4.491301473	6.645717165
bta-miR-2287	2.149139864	0	1.654957834	0		20	0.348231	chr4_9234_star	4.29827973	60.44456	2.405524014	5.941213351
mmu-miR-682	2.149139864	0	1.654957834	0		20	0.348231	chr5_11575_mature	4.29827973	60.44456	2.405524014	5.941213351
chr7_16991_star	2.149139864	0	1.654957834	0		20	0.348231	chr3_7643_mature@@hsa-miR-4787-5[illegible]	62.325056	10.47706	5.984704544	3.520680821
chr9_20875_star	2.149139864	0	1.654957834	0		20	0.348231	chr14_29900_mature	47.281077	4.835565	5.593385953	2.544872269
mmu-miR-692	2.149139864	0	1.654957834	0		20	0.348231	chr7_16598_mature	36.5353777	1.611855	5.230179094	1.38507475
bta-miR-2295	2.149139864	0	1.654957834	0		20	0.348231	chr10_24287_mature	692.023036	986.4552	9.436759498	9.947571482
chr6_14780_star	2.149139864	0	1.654957834	0		20	0.348231	chr14_31118_mature	15.043979	85.42831	4.003960082	6.433432039
chr13_27580_star	2.149139864	0	1.654957834	0		20	0.348231	chr8_18702_mature	2.14913986	53.99714	1.654957834	5.781284667
bta-miR-2415-5p	2.149139864	0	1.654957834	0		20	0.348231	chr7_15837_mature	58.0267763	8.865202	5.883297648	3.302348583
hsa-miR-5582-3p	2.149139864	0	1.654957834	0		20	0.348231	chr5_11961_mature	58.0267763	8.865202	5.883297648	3.302348583
chr1_1174_star	2.149139864	0	1.654957834	0		20	0.348231	chr6_13158_star	51.5793567	6.44742	5.716424587	2.896740642
chr6_14314_star	2.149139864	0	1.654957834	0		20	0.348231	ssc-miR-204	68.7724756	13.70077	6.124586119	3.877819487
chr15_31967_mature	2.149139864	0	1.654957834	0		20	0.348231	chr12_25917_mature	38.6845175	2.417782	5.310504361	1.773060525
chr1_3888_mature	2.149139864	0	1.654957834	0		20	0.348231	chr17_35097_mature	126.799252	265.9561	6.997735582	8.060458479
chr6_12830_star	2.149139864	0	1.654957834	0		20	0.348231	chr9_19974_mature	6.44741959	64.4742	2.896740642	6.032854532
chr4_8848_star	2.149139864	0	1.654957834	0		20	0.348231	chr6_14448_mature	4.29827973	58.8327	2.405524014	5.902862353
mmu-miR-706	2.149139864	0	1.654957834	0		20	0.348231	chr12_25449_star	111.755273	35.46081	6.817051091	5.188274618
chr17_35344_star	2.149139864	0	1.654957834	0		20	0.348231	chr2_6585_mature	96.7112939	27.40153	6.610453419	4.827896911
mmu-miR-1894-5p	2.149139864	0	1.654957834	0		20	0.348231	chr6_14391_mature	32.237098	0.805927	5.054722516	0.852739935

[illegible]

NW_003613223_37738_mature	0	2.417782	0	1.773060525	-20	0.35246	chr12_26438_mature	23.6405385	0.805927	4.62296188	0.852739935	4.874469	1	chr11_24676_mature	7.52199	2.417782	3.091190278	1.773060525	1.63743	0.620187
chr5_12356_mature	0	2.417782	0	1.773060525	-20	0.35246	chr1_2420_mature	23.6405385	0.805927	4.62296188	0.852739935	4.874469	1	chr7_15986_star	7.52199	2.417782	3.091190278	1.773060525	1.63743	0.620187
hsa-miR-4642	0	2.417782	0	1.773060525	-20	0.35246	chr11_24484_mature	23.6405385	0.805927	4.62296188	0.852739935	4.874469	1	chrx_21664_star	0.537285	2.417782	0.620384622	1.773060525	-2.16993	0.620816
bta-miR-17-5p	0	2.417782	0	1.773060525	-20	0.35246	chr3_6956_mature	47.281077	9.671129	5.593385953	3.415640968	2.289507	1	hsa-miR-2467-5p	0.537285	2.417782	0.620384622	1.773060525	-2.16993	0.620816
hsa-miR-4683	0	2.417782	0	1.773060525	-20	0.35246	chr9_20034_mature	47.281077	9.671129	5.593385953	3.415640968	2.289507	1	hsa-miR-4668-5p	0.537285	2.417782	0.620384622	1.773060525	-2.16993	0.620816
chr14_30186_star	0	2.417782	0	1.773060525	-20	0.35246	chr8_17231_mature	2.14913986	38.68452	1.654957834	5.310504361	-4.16993	1	chr14_29702_mature	0.537285	2.417782	0.620384622	1.773060525	-2.16993	0.620816
rno-miR-540-5p	0	2.417782	0	1.773060525	-20	0.35246	NW_003539012_38462_mature	6.44741959	49.16157	2.896740642	5.648510724	-2.93074	1	chr8_17941_star	0.537285	2.417782	0.620384622	1.773060525	-2.16993	0.620816
NW_003613401_38938_star	0	2.417782	0	1.773060525	-20	0.35246	chr1_3253_mature	45.1319371	8.865202	5.527693969	3.302348583	2.347923	1	chr5_10975_mature	0.537285	2.417782	0.620384622	1.773060525	-2.16993	0.620816
chr18_36103_star	0	2.417782	0	1.773060525	-20	0.35246	chr2_6477_mature	45.1319371	8.865202	5.527693969	3.302348583	2.347923	1	hsa-miR-1297	0.537285	2.417782	0.620384622	1.773060525	-2.16993	0.620816
chr4_9813_mature	0	2.417782	0	1.773060525	-20	0.35246	chrx_22329_star	25.7896784	1.611855	4.743605355	1.38507475	4	1	chr7_16512_star	0.537285	2.417782	0.620384622	1.773060525	-2.16993	0.620816
hsa-miR-302d-5p	0	2.417782	0	1.773060525	-20	0.35246	chrx_23183_mature	25.7896784	1.611855	4.743605355	1.38507475	4	1	hsa-miR-30c-5p	0.537285	2.417782	0.620384622	1.773060525	-2.16993	0.620816
chr1_2014_star	0	2.417782	0	1.773060525	-20	0.35246	hsa-miR-3661	25.7896784	1.611855	4.743605355	1.38507475	4	1	chr1_3022_star	0.537285	2.417782	0.620384622	1.773060525	-2.16993	0.620816
chr13_27562_star	0	2.417782	0	1.773060525	-20	0.35246	chr13_28748_mature	25.7896784	1.611855	4.743605355	1.38507475	4	1	mmu-miR-463-5p	0.537285	2.417782	0.620384622	1.773060525	-2.16993	0.620816
chr12_25581_mature	0	2.417782	0	1.773060525	-20	0.35246	chr2_5245_star	25.7896784	1.611855	4.743605355	1.38507475	4	1	chr14_30472_star	0.537285	2.417782	0.620384622	1.773060525	-2.16993	0.620816
hsa-miR-4509	0	2.417782	0	1.773060525	-20	0.35246	hsa-miR-6514-3p	25.7896784	1.611855	4.743605355	1.38507475	4	1	chr7_16102_star	0.537285	2.417782	0.620384622	1.773060525	-2.16993	0.620816
chr6_13364_star	0	2.417782	0	1.773060525	-20	0.35246	chr4_10198_mature	25.7896784	1.611855	4.743605355	1.38507475	4	1	rno-miR-3572	0.537285	2.417782	0.620384622	1.773060525	-2.16993	0.620816
hsa-miR-1229-5p	0	2.417782	0	1.773060525	-20	0.35246	chr6_12506_mature	25.7896784	1.611855	4.743605355	1.38507475	4	1	chr6_13734_star	0.537285	2.417782	0.620384622	1.773060525	-2.16993	0.620816
chr3_7250_mature	0	2.417782	0	1.773060525	-20	0.35246	ssc-miR-299	4.29827973	43.52008	2.405524014	5.476384353	-3.33985	1	chrx_21725_mature	0.537285	2.417782	0.620384622	1.773060525	-2.16993	0.620816
chr13_26885_mature	0	2.417782	0	1.773060525	-20	0.35246	chr12_25587_star	4.29827973	43.52008	2.405524014	5.476384353	-3.33985	1	chr13_28998_mature	0.537285	2.417782	0.620384622	1.773060525	-2.16993	0.620816
chr3_7770_mature	22.56596857	4.835565	4.558633073	2.544872269	2.222392	0.352847	chr10_23459_mature	19.3422588	0	4.346407978	0	20	1	chr15_32658_mature	0.537285	2.417782	0.620384622	1.773060525	-2.16993	0.620816
chr8_17892_star	22.56596857	4.835565	4.558633073	2.544872269	2.222392	0.352847	chr7_16193_mature	19.3422588	0	4.346407978	0	20	1	chr13_26646_star	0.537285	2.417782	0.620384622	1.773060525	-2.16993	0.620816
chr18_36117_star	22.56596857	4.835565	4.558633073	2.544872269	2.222392	0.352847	chr2_4630_mature	19.3422588	0	4.346407978	0	20	1	mmu-miR-98-3p	0.537285	2.417782	0.620384622	1.773060525	-2.16993	0.620816
chr10_24027_mature	22.56596857	4.835565	4.558633073	2.544872269	2.222392	0.352847	chr1_246_mature	19.3422588	0	4.346407978	0	20	1	chr13_28676_star	0.537285	2.417782	0.620384622	1.773060525	-2.16993	0.620816
chr4_9749_mature	37.60994761	7.253347	5.270900692	3.044979305	2.374396	0.35523	NW_003613265_38082_mature	19.3422588	0	4.346407978	0	20	1	chr4_9959_mature	0.537285	2.417782	0.620384622	1.773060525	-2.16993	0.620816
chr10_23328_mature	95.63672393	14.50669	6.594499642	3.954819241	2.720846	0.356386	chr6_14899_star	19.3422588	0	4.346407978	0	20	1	chr7_16262_mature	0.537285	2.417782	0.620384622	1.773060525	-2.16993	0.620816
chr6_14620_mature	15.04397905	107.1884	4.003960082	6.757401354	-2.83289	0.356541	chr2_4310_mature	19.3422588	0	4.346407978	0	20	1	chr5_11087_star	0.537285	2.417782	0.620384622	1.773060525	-2.16993	0.620816
chr5_12211_mature	5.372849659	25.78968	2.671938627	4.743605355	-2.26303	0.357353	chr7_15151_star	2.14913986	37.87859	1.654957834	5.280903997	-4.13955	1	chr5_12007_star	0.537285	2.417782	0.620384622	1.773060525	-2.16993	0.620816
chr18_36042_mature	3.223709795	13.70077	2.078510713	3.877819487	-2.08746	0.358883	chr6_14946_mature	2.14913986	37.87859	1.654957834	5.280903997	-4.13955	1	mmu-miR-6338	0.537285	2.417782	0.620384622	1.773060525	-2.16993	0.620816
chr11_24560_mature	3.223709795	13.70077	2.078510713	3.877819487	-2.08746	0.358883	chr5_11533_star	2.14913986	37.87859	1.654957834	5.280903997	-4.13955	1	chr4_9731_star	0.537285	2.417782	0.620384622	1.773060525	-2.16993	0.620816
chr1_3342_mature	3.223709795	13.70077	2.078510713	3.877819487	-2.08746	0.358883	chr1_2937_mature	40.8336574	7.253347	5.38659223	3.044979305	2.49304	1	NW_003613382_38825_mature	0.537285	2.417782	0.620384622	1.773060525	-2.16993	0.620816
chr8_17856_mature	3.223709795	13.70077	2.078510713	3.877819487	-2.08746	0.358883	chr3_6687_mature	94.562154	35.46081	6.578367468	5.188274618	1.415037	1	chr5_11589_star	0.537285	2.417782	0.620384622	1.773060525	-2.16993	0.620816
chrx_22916_star	3.223709795	13.70077	2.078510713	3.877819487	-2.08746	0.358883	chr17_34991_mature	6.44741959	48.35565	2.896740642	5.625143252	-2.90689	1	bta-miR-10a	0.537285	2.417782	0.620384622	1.773060525	-2.16993	0.620816
chrx_23209_star	3.223709795	13.70077	2.078510713	3.877819487	-2.08746	0.358883	chr1_2982_mature	27.9388182	2.417782	4.854934103	1.773060525	3.530515	1	hsa-miR-488-5p	0.537285	2.417782	0.620384622	1.773060525	-2.16993	0.620816
chr15_33146_mature	3.223709795	13.70077	2.078510713	3.877819487	-2.08746	0.358883	chr14_30832_mature	27.9388182	2.417782	4.854934103	1.773060525	3.530515	1	hsa-miR-320a	0.537285	2.417782	0.620384622	1.773060525	-2.16993	0.620816
chr8_19044_star	3.223709795	13.70077	2.078510713	3.877819487	-2.08746	0.358883	chr10_23828_mature	27.9388182	2.417782	4.854934103	1.773060525	3.530515	1	chr5_12405_star	0.537285	2.417782	0.620384622	1.773060525	-2.16993	0.620816
chr2_4744_star	2.149139864	8.865202	1.654957834	3.302348583	-2.04439	0.359643	chr14_30089_star	27.9388182	2.417782	4.854934103	1.773060525	3.530515	1	chr9_20768_star	0.537285	2.417782	0.620384622	1.773060525	-2.16993	0.620816
chr12_26249_star	2.149139864	8.865202	1.654957834	3.302348583	-2.04439	0.359643	chrx_22620_mature	38.6845175	6.44742	5.310504361	2.896740642	2.584963	1	chr6_14465_star	0.537285	2.417782	0.620384622	1.773060525	-2.16993	0.620816
chr2_5082_mature	2.149139864	8.865202	1.654957834	3.302348583	-2.04439	0.359643	chr3_6786_mature	38.6845175	6.44742	5.310504361	2.896740642	2.584963	1	mmu-miR-486-5p	0.537285	2.417782	0.620384622	1.773060525	-2.16993	0.620816
chr9_20563_star	2.149139864	8.865202	1.654957834	3.302348583	-2.04439	0.359643	chr5_11482_mature	68.7724756	20.95411	6.124586119	4.456419386	1.714598	1	mmu-miR-3108-5p	0.537285	2.417782	0.620384622	1.773060525	-2.16993	0.620816
bta-miR-761	2.149139864	8.865202	1.654957834	3.302348583	-2.04439	0.359643	chr6_14284_mature	107.456993	43.52008	6.760979269	5.476384353	1.304006	1	hsa-miR-744-5p	0.537285	2.417782	0.620384622	1.773060525	-2.16993	0.620816
chr1_1729_star	2.149139864	8.865202	1.654957834	3.302348583	-2.04439	0.359643	hsa-miR-4278	107.456993	43.52008	6.760979269	5.476384353	1.304006	1	hsa-miR-4662a-5p	0.537285	2.417782	0.620384622	1.773060525	-2.16993	0.620816
mmu-miR-466f	2.149139864	8.865202	1.654957834	3.302348583	-2.04439	0.359643	chr5_12034_mature	30.0879581	3.22371	4.958283956	2.078510713	3.222392	1	chr10_23656_star	0.537285	2.417782	0.620384622	1.773060525	-2.16993	0.620816
chr5_11139_mature	6.447419591	32.2371	2.896740642	5.054722516	-2.32193	0.360772	chr4_9286_mature	30.0879581	3.22371	4.958283956	2.078510713	3.222392	1	chr2_5942_star	0.537285	2.417782	0.620384622	1.773060525	-2.16993	0.620816
chr7_16802_star	9.671129386	2.417782	3.415640968	1.773060525	2	0.361347	chr3_6671_mature	30.0879581	3.22371	4.958283956	2.078510713	3.222392	1	chr12_26184_mature	0.537285	2.417782	0.620384622	1.773060525	-2.16993	0.620816
chr14_29552_star	9.671129386	2.417782	3.415640968	1.773060525	2	0.361347	chr8_18593_star	30.0879581	3.22371	4.958283956	2.078510713	3.222392	1	rno-miR-146b-3p	0.537285	2.417782	0.620384622	1.773060525	-2.16993	0.620816
mmu-miR-3108-5p	9.671129386	2.417782	3.415640968	1.773060525	2	0.361347	chr4_9294_mature	30.0879581	3.22371	4.958283956	2.078510713	3.222392	1	chr4_8670_star	0.537285	2.417782	0.620384622	1.773060525	-2.16993	0.620816
chr1_662_star	9.671129386	2.417782	3.415640968	1.773060525	2	0.361347	chr13_28155_mature	30.0879581	3.22371	4.958283956	2.078510713	3.222392	1	hsa-miR-4498	0.537285	2.417782	0.620384622	1.773060525	-2.16993	0.620816
chr14_31221_mature	9.671129386	2.417782	3.415640968	1.773060525	2	0.361347	hsa-miR-5694	30.0879581	3.22371	4.958283956	2.078510713	3.222392	1	mmu-miR-5623-3p	0.537285	2.417782	0.620384622	1.773060525	-2.16993	0.620816
NW_003537425_36910_mature	116.0535526	16.92448	6.871024911	4.163859073	2.777608	0.362404	chr12_25609_mature	36.5353777	5.641492	5.230179094	2.731507408	2.695145	1	chr13_27132_star	0.537285	2.417782	0.620384622	1.773060525	-2.16993	0.620816
chr14_31430_star	17.19311891	4.029637	4.185320985	2.330454351	2.093109	0.362948	chr5_11356_mature	10.7456993	57.22085	3.554060706	5.863463969	-2.41278	1	chr1_3810_star	0.537285	2.417782	0.620384622	1.773060525	-2.16993	0.620816
chrx_22576_mature	6.447419591	1.611855	2.896740642	1.38507475	2	0.363189	chr7_17190_star	34.3862378	4.835565	5.145116481	2.544872269	2.830075	1	hsa-miR-345-5p	0.537285	2.417782	0.620384622	1.773060525	-2.16993	0.620816
ssc-miR-181d-3p	6.447419591	1.611855	2.896740642	1.38507475	2	0.363189	chr1_3715_mature	32.237098	4.029637	5.054722516	2.330454351	3	1	rno-let-7b-3p	0.537285	2.417782	0.620384622	1.773060525	-2.16993	0.620816
NW_003539007_38438_mature	6.447419591	1.611855	2.896740642	1.38507475	2	0.363189	rno-miR-466b-5p	8.59655945	52.38528	3.262517265	5.738370208	-2.60733	1	chrx_21800_mature	0.537285	2.417782	0.620384622	1.773060525	-2.16993	0.620816
chr5_11842_star	6.447419591	1.611855	2.896740642	1.38507475	2	0.363189	chr4_8767_star	47.281077	10.47706	5.593385953	3.520680821	2.174029	1	hsa-miR-4683	0.537285	2.417782	0.620384622	1.773060525	-2.16993	0.620816
chr8_17498_mature	6.447419591	1.611855	2.896740642	1.38507475	2	0.363189	chr1_2204_mature	47.281077	10.47706	5.593385953	3.520680821	2.174029	1	chr2_6128_mature	0.537285	2.417782	0.620384622	1.773060525	-2.16993	0.620816
chr4_9997_mature	6.447419591	1.611855	2.896740642	1.38507475	2	0.363189	chr6_13536_mature	178.378609	91.87573	7.486864046	6.537229727	0.957187	1	hsa-miR-625-5p	0.537285	2.417782	0.620384622	1.773060525	-2.16993	0.620816
chr12_26183_mature	6.447419591	1.611855	2.896740642	1.38507475	2	0.363189	chr13_27960_mature	6.44741959	46.74379	2.896740642	5.577241251	-2.85798	1	hsa-miR-5684	0.537285	2.417782	0.620384622	1.773060525	-2.16993	0.620816
chr1_2381_mature	6.447419591	1.611855	2.896740642	1.38507475	2	0.363189	chr8_18995_mature	2.14913986	36.26674	1.654957834	5.21981653	-4.07682	1	hsa-miR-4533	0.537285	2.417782	0.620384622	1.773060525	-2.16993	0.620816
chr2_5113_star	6.447419591	1.611855	2.896740642	1.38507475	2	0.363189	chr1_861_mature	2.14913986	36.26674	1.654957834	5.21981653	-4.07682	1	chr4_10240_star	0.537285	2.417782	0.620384622	1.773060525	-2.16993	0.620816
mmu-miR-5119	6.447419591	1.611855	2.896740642	1.38507475	2	0.363189	NW_003613026_36810_mature	15.043979	64.4742	4.003960082	6.032854532	-2.09954	1	hsa-miR-1229-5p	0.537285	2.417782	0.620384622	1.773060525	-2.16993	0.620816
chr8_17239_mature	6.447419591	1.611855	2.896740642	1.38507475	2	0.363189	chrx_23064_star	21.4913986	0.805927	4.491301473	0.852739935	4.736966	1	chr12_25810_mature	37.60995	8.059274	5.270900692	3.179395517	2.222392	0.621693
chrx_22204_mature	6.447419591	1.611855	2.896740642	1.38507475	2	0.363189	hsa-miR-4745-3p	21.4913986	0.805927	4.491301473	0.852739935	4.736966	1	chr13_27136_mature	1.611855	4.835565	1.38507475	2.544872269	-1.58496	0.621864
hsa-miR-378j	6.447419591	1.611855	2.896740642	1.38507475	2	0.363189	rno-miR-218a-1-3p	21.4913986	0.805927	4.491301473	0.852739935	4.736966	1	chrx_22639_star	1.611855	4.835565	1.38507475	2.544872269	-1.58496	0.621864
chr1_3928_star	6.447419591	1.611855	2.896740642	1.38507475	2	0.363189	chr7_15363_mature	21.4913986	0.805927	4.491301473	0.852739935	4.736966	1	rno-miR-3585-5p	1.611855	4.835565	1.38507475	2.544872269	-1.58496	0.621864
chr6_14892_star	6.447419591	1.611855	2.896740642	1.38507475	2	0.363189	chr12_26515_mature	21.4913986	0.805927	4.491301473	0.852739935	4.736966	1	hsa-miR-1289	1.611855	4.835565	1.38507475	2.544872269	-1.58496	0.621864
chr15_32437_mature	6.447419591	1.611855	2.896740642	1.38507475	2	0.363189	mmu-miR-363-5p	21.4913986	0.805927	4.491301473	0.852739935	4.736966	1	chr5_11515_star	1.611855	4.835565	1.38507475	2.544872269	-1.58496	0.621864
chr10_24049_mature	6.447419591	1.611855	2.896740642	1.38507475	2	0.363189	chr11_24331_star	21.4913986	0.805927	4.491301473	0.852739935	4.736966	1	NW_003613157_37452_star	1.611855	4.835565	1.38507475	2.544872269	-1.58496	0.621864
chr4_9468_mature	6.447419591	1.611855	2.896740642	1.38507475	2	0.363189	chr1_1793_mature	21.4913986	0.805927	4.491301473	0.852739935	4.736966	1	hsa-miR-2117	1.611855	4.835565	1.38507475	2.544872269	-1.58496	0.621864
NW_003541040_39849_star	6.447419591	1.611855	2.896740642	1.38507475	2	0.363189	chr7_16598_star	21.4913986	0.805927	4.491301473	0.852739935	4.736966	1	chr11_25099_star	1.611855	4.835565	1.38507475	2.544872269	-1.58496	0.621864
chr1_1041_mature	6.447419591	1.611855	2.896740642	1.38507475	2	0.363189	chr6_12549_mature	4.29827973	41.1023	2.405524014	5.395827139	-3.25739	1	NW_003539866_39141_star	1.611855	4.835565	1.38507475	2.544872269	-1.58496	0.621864
chr4_10016_star	6.447419591	1.611855	2.896740642	1.38507475	2	0.363189	chr1_1289_mature	4.29827973	41.1023	2.405524014	5.395827139	-3.25739	1	bta-miR-2284b	1.611855	4.835565	1.38507475	2.544872269	-1.58496	0.621864
hsa-miR-6514-3p	6.447419591	1.611855	2.896740642	1.38507475	2	0.363189	chr15_32726_mature	40.8336574	8.059274	5.38659223	3.179395517	2.341037	1	chr11_24964_mature	1.611855	4.835565	1.38507475	2.544872269	-1.58496	0.621864
chr2_5337_mature	6.447419591	1.611855	2.896740642	1.38507475	2	0.363189	chr14_30470_mature	6.44741959	45.93786	2.896740642	5.552680304	-2.83289	1	chr9_20604_star	1.611855	4.835565	1.38507475	2.544872269	-1.58496	0.621864
chr4_9998_mature	6.447419591	1.611855	2.896740642	1.38507475	2	0.363189	NW_003539007_38433_mature	2.14913986	35.46081	1.654957834	5.188274618	-4.04439	1	chr14_29906_star	1.611855	4.835565	1.38507475	2.544872269	-1.58496	0.621864
chr8_18294_star	6.447419591	1.611855	2.896740642	1.38507475	2	0.363189	chr6_13570_mature	2.14913986	35.46081	1.654957834	5.188274618	-4.04439	1	hsa-miR-5010-3p	1.611855	4.835565	1.38507475	2.544872269	-1.58496	0.621864
chr5_12026_mature	6.447419591	1.611855	2.896740642	1.38507475	2	0.363189	chr17_35168_star	23.6405385	1.611855	4.62296188	1.38507475	3.874469	1	chr7_17051_star	1.611855	4.835565	1.38507475	2.544872269	-1.58496	0.621864
chr11_24795_star	6.447419591	1.611855	2.896740642	1.38507475	2	0.363189	chr5_12195_mature	23.6405385	1.611855	4.62296188	1.38507475	3.874469	1	chr8_18502_star	1.611855	4.835565	1.38507475	2.544872269	-1.58496	0.621864
chr5_12195_mature	6.447419591	1.611855	2.896740642	1.38507475	2	0.363189	chr1_3923_mature	23.6405385	1.611855	4.62296188	1.38507475	3.874469	1	NW_003613552_39654_star	1.611855	4.835565	1.38507475	2.544872269	-1.58496	0.621864
chr1_319_star	6.447419591	1.611855	2.896740642	1.38507475	2	0.363189	chr12_25705_mature	23.6405385	1.611855	4.62296188	1.38507475	3.874469	1	chr15_33083_mature	1.611855	4.835565	1.38507475	2.544872269	-1.58496	0.621864
chr1_3923_mature	6.447419591	1.611855	2.896740642	1.38507475	2	0.363189	chr13_26759_mature	23.6405385	1.611855	4.62296188	1.38507475	3.874469	1	chr4_9347_star	1.611855	4.835565	1.38507475	2.544872269	-1.58496	0.621864
bta-miR-2475	6.447419591	1.611855	2.896740642	1.38507475	2	0.363189	chr9_20972_mature@@hsa-miR-488-3	23.6405385	1.611855	4.62296188	1.38507475	3.874469	1	chr6_14661_star	1.611855	4.835565	1.38507475	2.544872269	-1.58496	0.621864
chr6_14411_star	6.447419591	1.611855	2.896740642	1.38507475	2	0.363189	chr7_15451_star	23.6405385	1.611855	4.62296188	1.38507475	3.874469	1	chr13_27948_star	1.611855	4.835565	1.38507475	2.544872269	-1.58496	0.621864
chr4_10198_mature	6.447419591	1.611855	2.896740642	1.38507475	2	0.363189	chr1_3928_mature	23.6405385	1.611855	4.62296188	1.38507475	3.874469	1	chr13_27982_mature	1.611855	4.835565	1.38507475	2.544872269	-1.58496	0.621864
chr15_32017_star	6.447419591	1.611855	2.896740642	1.38507475	2	0.363189	chr18_36377_mature	38.6845175	7.253347	5.310504361	3.044979305	2.415037	1	chr8_19026_star	1.611855	4.835565	1.38507475	2.544872269	-1.58496	0.621864
mmu-miR-28a-3p	6.447419591	1.611855	2.896740642	1.38507475	2	0.363189	NW_003539698_39034_mature	38.6845175	7.253347	5.310504361	3.044979305	2.415037	1	chr6_14338_mature	1.611855	4.835565	1.38507475	2.544872269	-1.58496	0.621864
chr17_35199_mature	6.447419591	1.611855	2.896740642	1.38507475	2	0.363189	chr10_24172_star	47.281077	11.28298	5.593385953	3.618589217	2.067114	1	mmu-miR-6416-3p	1.611855	4.835565	1.38507475	2.544872269	-1.58496	0.621864
chr14_31116_mature	6.447419591	1.611855	2.896740642	1.38507475	2	0.363189	chr2_5245_star	8.59655945	49.9675	3.262517265	5.671505736	-2.53916	1	NW_003541307_40037_mature	13.96941	4.029637	3.90394537	2.330454351	1.793549	0.621899
chr17_17077_mature	467.4379203	45.93786	8.871714057	5.552680304	3.347019	0.363363	chr3_7766_mature	8.59655945	49.9675	3.262517265	5.671505736	-2.53916	1	chr15_33692_mature	13.96941	4.029637	3.90394537	2.330454351	1.793549	0.621899
chr4_10079_mature	10.74569932	64.4742	3.554060706	6.032854532	-2.58496	0.363373	chr14_29727_mature	4.29827973	40.29637	2.405524014	5.367943153	-3.22882	1	hsa-miR-5580-5p	13.96941	4.029637	3.90394537	2.330454351	1.793549	0.621899
chr7_15648_mature	21.49139864	4.835565	4.491301473	2.544872269	2.152003	0.363623	chr14_30996_mature	176.229469	296.5813	7.469476498	8.217140066	-0.75097	1	chr13_28673_star	3.760995	12.89484	2.251263041	3.796477232	-1.77761	0.622021
chr4_9347_star	21.49139864	4.835565	4.491301473	2.544872269	2.152003	0.363623	mmu-miR-6239	105.307853	201.4819	6.732104367	7.661648871	-0.93604	1	chr13_28744_star	3.760995	12.89484	2.251263041	3.796477232	-1.77761	0.622021
mmu-miR-3097-5p	5.372849659	24.98375	2.671938627	4.699537803	-2.21723	0.363725	NW_003539948_39194_star	25.7896784	2.417782	4.743605355	1.773060525	3.415037	1	chr8_17637_mature	3.760995	12.89484	2.251263041	3.796477232	-1.77761	0.622021
chr1_431_mature@@hsa-miR-4783-5	131.0975317	18.53633	7.045459699	4.288087668	2.822213	0.363843	chr12_25704_mature	25.7896784	2.417782	4.743605355	1.773060525	3.415037	1	chrx_22076_star	3.760995	12.89484	2.251263041	3.796477232	-1.77761	0.622021
chr6_13893_star	47.281077	8.865202	5.593385953	3.302348583	2.415037	0.365289	chr14_29632_mature	25.7896784	2.417782	4.743605355	1.773060525	3.415037	1	chr9_20571_mature	3.760995	12.89484	2.251263041	3.796477232	-1.77761	0.622021
chr11_24450_mature	8.596559455	46.74379	3.262517265	5.577241251	-2.44294	0.365347	chr13_28971_mature	34.3862378	5.641492	5.145116481	2.731507408	2.607683	1	chr8_18290_star	3.760995	12.89484	2.251263041	3.796477232	-1.77761	0.622021
chr12_25408_star	1.074569932	4.835565	1.05281229	2.544872269	-2.16993	0.36538	chr15_33084_star	34.3862378	5.641492	5.145116481	2.731507408	2.607683	1	chr16_34104_mature	3.760995	12.89484	2.251263041	3.796477232	-1.77761	0.622021
rno-miR-3583-5p	1.074569932	4.835565	1.05281229	2.544872269	-2.16993	0.36538	hsa-miR-3665	34.3862378	5.641492	5.145116481	2.731507408	2.607683	1	chr12_26451_star	5.37285	20.95411	2.671938627	4.456419386	-1.96347	0.622384
NW_003537189_36743_mature	1.074569932	4.835565	1.05281229	2.544872269	-2.16993	0.36538	NW_003613335_38593_mature	2.14913986	34.65488	1.654957834	5.156027656	-4.01123	1	chr8_18998_mature	5.37285	20.95411	2.671938627	4.456419386	-1.96347	0.622384
chr3_8052_mature	1.074569932	4.835565	1.05281229	2.544872269	-2.16993	0.36538	NW_003538623_38054_mature	2.14913986	34.65488	1.654957834	5.156027656	-4.01123	1	chr1_3589_star	2.14914	6.44742	1.654957834	2.896740642	-1.58496	0.622757
rno-miR-3585-5p	1.074569932	4.835565	1.05281229	2.544872269	-2.16993	0.36538	chr2_5426_star	17.1931189	0	4.185320985	0	20	1	chr5_11429_mature	2.14914	6.44742	1.654957834	2.896740642	-1.58496	0.622757
bta-miR-1584-3p	1.074569932	4.835565	1.05281229	2.544872269	-2.16993	0.36538	mmu-miR-712-5p	17.1931189	0	4.185320985	0	20	1	chr1_569_star	2.14914	6.44742	1.654957834	2.896740642	-1.58496	0.622757
rno-miR-503-5p	1.074569932	4.835565	1.05281229	2.544872269	-2.16993	0.36538	chr2_5420_star	17.1931189	0	4.185320985	0	20	1	chr15_33256_star	2.14914	6.44742	1.654957834	2.896740642	-1.58496	0.622757
chr2_6403_star	1.074569932	4.835565	1.05281229	2.544872269	-2.16993	0.36538	chr2_4844_mature	17.1931189	0	4.185320985	0	20	1	chr4_9649_star	2.14914	6.44742	1.654957834	2.896740642	-1.58496	0.622757
NW_003613440_39150_mature	1.074569932	4.835565	1.05281229	2.544872269	-2.16993	0.36538	chr2_5371_star	17.1931189	0	4.185320985	0	20	1	chr17_35223_mature	2.14914	6.44742	1.654957834	2.896740642	-1.58496	0.622757
chr8_18342_mature	1.074569932	4.835565	1.05281229	2.544872269	-2.16993	0.36538	chr8_17722_mature	17.1931189	0	4.185320985	0	20	1	chr5_10971_star	2.14914	6.44742	1.654957834	2.896740642	-1.58496	0.622757
NW_003613157_37452_star	1.074569932	4.835565	1.05281229	2.544872269	-2.16993	0.36538	chr13_28675_mature	17.1931189	0	4.185320985	0	20	1	chr7_16567_star	2.14914	6.44742	1.654957834	2.896740642	-1.58496	0.622757
chr7_17037_star	1.074569932	4.835565	1.05281229	2.544872269	-2.16993	0.36538	chr2_5163_star	17.1931189	0	4.185320985	0	20	1	chr12_26222_mature	2.14914	6.44742	1.654957834	2.896740642	-1.58496	0.622757
chr9_20315_star	1.074569932	4.835565	1.05281229	2.544872269	-2.16993	0.36538	hsa-miR-2115-3p	17.1931189	0	4.185320985	0	20	1	chr1_2367_mature@@mmu-miR-6	99.39772	15.31262	6.649582678	4.027916745	2.698491	0.622873
NW_003613480_39328_mature	1.074569932	4.835565	1.05281229	2.544872269	-2.16993	0.36538	chr1_3870_star	17.1931189	0	4.185320985	0	20	1	chr4_9733_mature	3.22371	10.47706	2.078510713	3.520680821	-1.70044	0.623079
chr15_33460_mature	1.074569932	4.835565	1.05281229	2.544872269	-2.16993	0.36538	chr4_8920_star	17.1931189	0	4.185320985	0	20	1	chr3_7219_mature	3.22371	10.47706	2.078510713	3.520680821	-1.70044	0.623079
chr13_27448_star	1.074569932	4.835565	1.05281229	2.544872269	-2.16993	0.36538	chr8_18342_mature	32.237098	4.835565	5.054722516	2.544872269	2.736966	1	chr4_10559_mature	3.22371	10.47706	2.078510713	3.520680821	-1.70044	0.623079
bta-miR-2284c	1.074569932	4.835565	1.05281229	2.544872269	-2.16993	0.36538	chr14_29906_star	32.237098	4.835565	5.054722516	2.544872269	2.736966	1	chr17_35299_star	3.22371	10.47706	2.078510713	3.520680821	-1.70044	0.623079
rno-miR-7b	1.074569932	4.835565	1.05281229	2.544872269	-2.16993	0.36538	chr15_33084_mature	32.237098	4.835565	5.054722516	2.544872269	2.736966	1	chr13_26825_mature	9.133844	44.32601	3.34110968	5.502267253	-2.27886	0.62377
chr13_29352_star	1.074569932	4.835565	1.05281229	2.544872269	-2.16993	0.36538	chr3_7189_mature	32.237098	4.835565	5.054722516	2.544872269	2.736966	1	hsa-miR-5000-3p	4.835565	1.611855	2.544872269	1.38507475	1.584963	0.625219
chr4_9349_mature	1.074569932	4.835565	1.05281229	2.544872269	-2.16993	0.36538	ssc-miR-338	27.9388182	3.22371	4.854934103	2.078510713	3.115477	1	chr15_31873_star	4.835565	1.611855	2.544872269	1.38507475	1.584963	0.625219
chr14_30973_mature	1.074569932	4.835565	1.05281229	2.544872269	-2.16993	0.36538	chr14_30424_mature	92.4130141	182.9455	6.545551653	7.523134815	-0.98525	1	chr5_11357_mature	4.835565	1.611855	2.544872269	1.38507475	1.584963	0.625219
chr15_33146_star	1.074569932	4.835565	1.05281229	2.544872269	-2.16993	0.36538	chr6_14308_mature	30.0879581	4.029637	4.958283956	2.330454351	2.900464	1	chr1_2519_star	4.835565	1.611855	2.544872269	1.38507475	1.584963	0.625219
hsa-miR-548e	1.074569932	4.835565	1.05281229	2.544872269	-2.16993	0.36538	chr15_32721_mature	30.0879581	4.029637	4.958283956	2.330454351	2.900464	1	chr2_4030_star	4.835565	1.611855	2.544872269	1.38507475	1.584963	0.625219
chr18_36050_mature	1.074569932	4.835565	1.05281229	2.544872269	-2.16993	0.36538	NW_003541040_39847_mature	45.1319371	10.47706	5.527693969	3.520680821	2.106915	1	chr10_23315_star	4.835565	1.611855	2.544872269	1.38507475	1.584963	0.625219
chr6_12750_star	1.074569932	4.835565	1.05281229	2.544872269	-2.16993	0.36538	chr13_28713_mature	51.5793567	13.70077	5.716424587	3.877819487	1.912537	1	chr15_31984_star	4.835565	1.611855	2.544872269	1.38507475	1.584963	0.625219
hsa-miR-632	1.074569932	4.835565	1.05281229	2.544872269	-2.16993	0.36538	chr13_28400_star@@hsa-miR-5585-3	90.2638743	35.46081	6.511971994	5.188274618	1.347923	1	chr4_10602_star	4.835565	1.611855	2.544872269	1.38507475	1.584963	0.625219
chr9_20694_star	1.074569932	4.835565	1.05281229	2.544872269	-2.16993	0.36538	chr7_15165_mature	6.44741959	44.32601	2.896740642	5.502267253	-2.78136	1	chr4_10198_star	4.835565	1.611855	2.544872269	1.38507475	1.584963	0.625219
chr6_12914_mature	1.074569932	4.835565	1.05281229	2.544872269	-2.16993	0.36538	mmu-miR-1931	2.14913986	33.84895	1.654957834	5.123043401	-3.97728	1	bta-miR-544a	4.835565	1.611855	2.544872269	1.38507475	1.584963	0.625219
chr6_12824_star	1.074569932	4.835565	1.05281229	2.544872269	-2.16993	0.36538	chr15_33576_mature	2.14913986	33.84895	1.654957834	5.123043401	-3.97728	1	chr3_7264_star	4.835565	1.611855	2.544872269	1.38507475	1.584963	0.625219
chrx_21721_mature	1.074569932	4.835565	1.05281229	2.544872269	-2.16993	0.36538	chr16_34306_mature	2.14913986	33.84895	1.654957834	5.123043401	-3.97728	1	chr6_12771_star	4.835565	1.611855	2.544872269	1.38507475	1.584963	0.625219
hsa-miR-5706	1.074569932	4.835565	1.05281229	2.544872269	-2.16993	0.36538	ssc-miR-1285	1343.21241	1075.107	10.39254542	10.07160611	0.321207	1	chr10_23364_star	4.835565	1.611855	2.544872269	1.38507475	1.584963	0.625219
chr2_4896_star	1.074569932	4.835565	1.05281229	2.544872269	-2.16993	0.36538	chr15_32961_mature	4.29827973	38.68452	2.405524014	5.310504361	-3.16993	1	chr4_9749_star	4.835565	1.611855	2.544872269	1.38507475	1.584963	0.625219
chr12_26324_star	1.074569932	4.835565	1.05281229	2.544872269	-2.16993	0.36538	mmu-miR-1943-5p	4.29827973	38.68452	2.405524014	5.310504361	-3.16993	1	chr1_319_star	4.835565	1.611855	2.544872269	1.38507475	1.584963	0.625219
chr15_32695_mature	1.074569932	4.835565	1.05281229	2.544872269	-2.16993	0.36538	NW_003613519_39525_star	6.44741959	43.52008	2.896740642	5.476384353	-2.75489	1	chr12_25705_mature	4.835565	1.611855	2.544872269	1.38507475	1.584963	0.625219
chr8_17599_star	1.074569932	4.835565	1.05281229	2.544872269	-2.16993	0.36538	chr8_17858_mature	38.6845175	8.059274	5.310504361	3.179395517	2.263034	1	chr4_10259_star	4.835565	1.611855	2.544872269	1.38507475	1.584963	0.625219
chr6_13027_star	1.074569932	4.835565	1.05281229	2.544872269	-2.16993	0.36538	chr7_15085_mature	2.14913986	33.04303	1.654957834	5.08928735	-3.94251	1	mmu-miR-1955-5p	4.835565	1.611855	2.544872269	1.38507475	1.584963	0.625219
chr3_7211_mature	1.074569932	4.835565	1.05281229	2.544872269	-2.16993	0.36538	ssc-miR-326	2.14913986	33.04303	1.654957834	5.08928735	-3.94251	1	chr6_14625_star	4.835565	1.611855	2.544872269	1.38507475	1.584963	0.625219
chr3_8486_star	1.074569932	4.835565	1.05281229	2.544872269	-2.16993	0.36538	chr7_15474_mature	2.14913986	33.04303	1.654957834	5.08928735	-3.94251	1	chr4_10042_mature	4.835565	1.611855	2.544872269	1.38507475	1.584963	0.625219
mmu-miR-1929-5p	1.074569932	4.835565	1.05281229	2.544872269	-2.16993	0.36538	chr5_12128_mature	2.14913986	33.04303	1.654957834	5.08928735	-3.94251	1	chr11_24439_star	4.835565	1.611855	2.544872269	1.38507475	1.584963	0.625219
chr1_3642_mature	6.447419591	31.43117	2.896740642	5.019309189	-2.2854	0.366094	chrx_21804_mature	19.3422588	0.805927	4.346407978	0.852739935	4.584963	1	NW_003537656_37099_mature	10.20841	3.22371	3.48651029	2.078510713	1.662965	0.625379
chr6_14972_mature	4.298279727	18.53633	2.405524014	4.288087668	-2.10852	0.366408	chr4_9226_star	19.3422588	0.805927	4.346407978	0.852739935	4.584963	1	chr5_11215_star	10.20841	3.22371	3.48651029	2.078510713	1.662965	0.625379
chr15_33084_star	25.78967836	5.641492	4.743605355	2.731507408	2.192645	0.366936	chr5_10752_star	19.3422588	0.805927	4.346407978	0.852739935	4.584963	1	chr11_25256_star	10.20841	3.22371	3.48651029	2.078510713	1.662965	0.625379
chr16_34309_mature	12.89483918	3.22371	3.796477232	2.078510713	2	0.367161	chr14_31502_mature	19.3422588	0.805927	4.346407978	0.852739935	4.584963	1	chr1_2086_star	10.20841	3.22371	3.48651029	2.078510713	1.662965	0.625379
chr5_12034_mature	12.89483918	3.22371	3.796477232	2.078510713	2	0.367161	chr1_1242_star	19.3422588	0.805927	4.346407978	0.852739935	4.584963	1	chr17_35572_mature	98.32315	15.31262	6.634058094	4.027916745	2.68281	0.625656
chr6_14384_mature	12.89483918	3.22371	3.796477232	2.078510713	2	0.367161	chr6_12747_mature	19.3422588	0.805927	4.346407978	0.852739935	4.584963	1	chr6_13582_mature	42.44551	8.865202	5.441135257	3.302348583	2.259387	0.626457
chrx_22864_star	12.89483918	3.22371	3.796477232	2.078510713	2	0.367161	chr11_25274_mature	19.3422588	0.805927	4.346407978	0.852739935	4.584963	1	chr13_27786_star	4.835565	17.7304	2.544872269	4.227310103	-1.87447	0.626591
chrx_21804_star	12.89483918	3.22371	3.796477232	2.078510713	2	0.367161	chr2_6308_mature	19.3422588	0.805927	4.346407978	0.852739935	4.584963	1	rno-miR-3583-3p	6.984705	29.81932	2.997239027	4.94576292	-2.09398	0.626611
chr3_7274_mature	12.89483918	3.22371	3.796477232	2.078510713	2	0.367161	chr10_23348_star	19.3422588	0.805927	4.346407978	0.852739935	4.584963	1	chr1_3232_mature	6.44742	26.59561	2.896740642	4.786366653	-2.04439	0.626635
chr2_4675_mature	12.89483918	3.22371	3.796477232	2.078510713	2	0.367161	chr4_8825_mature	19.3422588	0.805927	4.346407978	0.852739935	4.584963	1	chr4_9685_star	13.43212	4.029637	3.851211749	2.330454351	1.736966	0.628203
chr13_28744_star	3.223709795	12.89484	2.078510713	3.796477232	-2	0.369285	chr16_34525_mature@@bta-miR-449a	19.3422588	0.805927	4.346407978	0.852739935	4.584963	1	rno-miR-145-5p	2.686425	8.059274	1.882222339	3.179395517	-1.58496	0.62821
chr6_12750_mature	3.223709795	12.89484	2.078510713	3.796477232	-2	0.369285	chr4_10320_mature	19.3422588	0.805927	4.346407978	0.852739935	4.584963	1	chr5_12276_mature	2.686425	8.059274	1.882222339	3.179395517	-1.58496	0.62821
chr15_32293_star	3.223709795	12.89484	2.078510713	3.796477232	-2	0.369285	chr2_5364_star	19.3422588	0.805927	4.346407978	0.852739935	4.584963	1	chr7_15459_mature	2.686425	8.059274	1.882222339	3.179395517	-1.58496	0.62821
chr16_34104_mature	3.223709795	12.89484	2.078510713	3.796477232	-2	0.369285	chr1_3446_mature	19.3422588	0.805927	4.346407978	0.852739935	4.584963	1	chr13_29266_star	2.686425	8.059274	1.882222339	3.179395517	-1.58496	0.62821
chr16_34304_mature	5.372849659	24.17782	2.671938627	4.654081667	-2.16993	0.370223	chr3_7184_star	19.3422588	0.805927	4.346407978	0.852739935	4.584963	1	chr2_4127_mature	2.686425	8.059274	1.882222339	3.179395517	-1.58496	0.62821
chr14_30282_mature	37.60994761	390.8748	5.270900692	8.614249038	-3.37752	0.370705	chr6_13162_star	19.3422588	0.805927	4.346407978	0.852739935	4.584963	1	chr4_10537_mature	2.686425	8.059274	1.882222339	3.179395517	-1.58496	0.62821
bta-miR-483	15.04397905	100.7409	4.003960082	6.668756392	-2.74339	0.372452	chr3_7170_mature	70.9216155	24.98375	6.168353522	4.699537803	1.505235	1	chr16_34138_mature	2.686425	8.059274	1.882222339	3.179395517	-1.58496	0.62821
chr2_6208_mature	193.4225877	24.98375	7.603052029	4.699537803	2.952694	0.373075	chrx_22026_mature	8.59655945	47.54972	3.262517265	5.601391055	-2.46761	1	chr14_30107_star	2.686425	8.059274	1.882222339	3.179395517	-1.58496	0.62821
chr11_24436_mature	8.596559455	45.13194	3.262517265	5.527693969	-2.39232	0.373257	chr4_9749_mature	36.5353777	7.253347	5.230179094	3.044979305	2.332575	1	chr13_28169_mature	2.686425	8.059274	1.882222339	3.179395517	-1.58496	0.62821
chr13_26798_mature	202.0191472	25.78968	7.665471987	4.743605355	2.969626	0.373437	chr1_384_mature	4.29827973	37.87859	2.405524014	5.280903997	-3.13955	1	chr9_20394_star	21.4914	5.641492	4.491301473	2.731507408	1.929611	0.628702
chr1_398_mature	2.149139864	8.059274	1.654957834	3.179395517	-1.90689	0.374205	chr5_11550_star	4.29827973	37.87859	2.405524014	5.280903997	-3.13955	1	chr4_10605_mature	6.984705	2.417782	2.997239027	1.773060525	1.530515	0.629935
chr17_35259_mature	2.149139864	8.059274	1.654957834	3.179395517	-1.90689	0.374205	chr2_5658_mature	4.29827973	37.87859	2.405524014	5.280903997	-3.13955	1	chr2_5995_mature	6.984705	2.417782	2.997239027	1.773060525	1.530515	0.629935
chr13_29266_star	2.149139864	8.059274	1.654957834	3.179395517	-1.90689	0.374205	chr8_17278_mature	4.29827973	37.87859	2.405524014	5.280903997	-3.13955	1	chr11_24794_star	6.984705	2.417782	2.997239027	1.773060525	1.530515	0.629935
chr4_8848_mature	2.149139864	8.059274	1.654957834	3.179395517	-1.90689	0.374205	chr12_26249_mature	4.29827973	37.87859	2.405524014	5.280903997	-3.13955	1	chr7_15958_star	6.984705	2.417782	2.997239027	1.773060525	1.530515	0.629935
hsa-miR-3976	2.149139864	8.059274	1.654957834	3.179395517	-1.90689	0.374205	ssc-miR-493-3p	6.44741959	42.71415	2.896740642	5.4500286	-2.72792	1	hsa-miR-3670	6.984705	2.417782	2.997239027	1.773060525	1.530515	0.629935
chr3_6809_star	2.149139864	8.059274	1.654957834	3.179395517	-1.90689	0.374205	chr9_20853_star	118.202693	54.80307	6.897273013	5.802272499	1.108934	1	chr7_15120_star	6.984705	2.417782	2.997239027	1.773060525	1.530515	0.629935
chr2_6125_star	2.149139864	8.059274	1.654957834	3.179395517	-1.90689	0.374205	chr5_10946_mature	34.3862378	6.44742	5.145116481	2.896740642	2.415037	1	chr12_25917_mature	6.984705	2.417782	2.997239027	1.773060525	1.530515	0.629935
hsa-miR-6125	2.149139864	8.059274	1.654957834	3.179395517	-1.90689	0.374205	chr16_34089_mature	12.8948392	55.60899	3.796477232	5.82295938	-2.10852	1	chr9_20300_star	6.984705	2.417782	2.997239027	1.773060525	1.530515	0.629935
chr16_34229_mature	2.149139864	8.059274	1.654957834	3.179395517	-1.90689	0.374205	chr13_29206_mature	2.14913986	32.2371	1.654957834	5.054722516	-3.90689	1	chr2_4773_mature	6.984705	2.417782	2.997239027	1.773060525	1.530515	0.629935
chr3_8628_star	2.149139864	8.059274	1.654957834	3.179395517	-1.90689	0.374205	chr16_33909_mature	2.14913986	32.2371	1.654957834	5.054722516	-3.90689	1	chr12_26142_mature	6.984705	2.417782	2.997239027	1.773060525	1.530515	0.629935
ssc-miR-137	2.149139864	8.059274	1.654957834	3.179395517	-1.90689	0.374205	NW_003539018_38471_mature	21.4913986	1.611855	4.491301473	1.38507475	3.736966	1	chr4_9284_star	6.984705	2.417782	2.997239027	1.773060525	1.530515	0.629935
chr9_21035_mature	2.149139864	8.059274	1.654957834	3.179395517	-1.90689	0.374205	chr1_3348_mature	21.4913986	1.611855	4.491301473	1.38507475	3.736966	1	mmu-miR-1957a	6.984705	2.417782	2.997239027	1.773060525	1.530515	0.629935
mmu-miR-294-3p	2.149139864	8.059274	1.654957834	3.179395517	-1.90689	0.374205	chr4_9468_mature	21.4913986	1.611855	4.491301473	1.38507475	3.736966	1	hsa-miR-4528	6.984705	2.417782	2.997239027	1.773060525	1.530515	0.629935
chr1_1175_star	2.149139864	8.059274	1.654957834	3.179395517	-1.90689	0.374205	NW_003541040_39849_star	21.4913986	1.611855	4.491301473	1.38507475	3.736966	1	chr18_36561_mature	5.37285	20.14819	2.671938627	4.402462031	-1.90689	0.630696
chr9_19423_star	2.149139864	8.059274	1.654957834	3.179395517	-1.90689	0.374205	chr2_3985_mature	21.4913986	1.611855	4.491301473	1.38507475	3.736966	1	chr13_28917_mature	5.37285	20.14819	2.671938627	4.402462031	-1.90689	0.630696
chr1_439_star	2.149139864	8.059274	1.654957834	3.179395517	-1.90689	0.374205	chr9_20697_mature	21.4913986	1.611855	4.491301473	1.38507475	3.736966	1	chr11_24333_mature	1.07457	3.22371	1.05281229	2.078510713	-1.58496	0.631174
chr8_17483_star	2.149139864	8.059274	1.654957834	3.179395517	-1.90689	0.374205	chr11_24795_star	21.4913986	1.611855	4.491301473	1.38507475	3.736966	1	chr15_32759_mature	1.07457	3.22371	1.05281229	2.078510713	-1.58496	0.631174
chr1_2659_mature	2.149139864	8.059274	1.654957834	3.179395517	-1.90689	0.374205	chr14_31514_star	21.4913986	1.611855	4.491301473	1.38507475	3.736966	1	hsa-miR-4765	1.07457	3.22371	1.05281229	2.078510713	-1.58496	0.631174
chr14_29694_mature	20.4168287	4.835565	4.420672964	2.544872269	2.078003	0.374648	chr4_8920_mature	21.4913986	1.611855	4.491301473	1.38507475	3.736966	1	chr16_34246_star	1.07457	3.22371	1.05281229	2.078510713	-1.58496	0.631174
bta-miR-2881	16.11854898	4.029637	4.097488514	2.330454351	2	0.375939	chr9_21053_mature	206.317427	328.8184	7.695697583	8.36552807	-0.67243	1	hsa-miR-4668-3p	1.07457	3.22371	1.05281229	2.078510713	-1.58496	0.631174
chr1_2264_star	16.11854898	4.029637	4.097488514	2.330454351	2	0.375939	chr7_16620_mature	42.9827973	10.47706	5.458867456	3.520680821	2.036526	1	chr7_17178_star	1.07457	3.22371	1.05281229	2.078510713	-1.58496	0.631174
chr5_11735_mature	16.11854898	4.029637	4.097488514	2.330454351	2	0.375939	bta-miR-2888	32.237098	5.641492	5.054722516	2.731507408	2.514573	1	chr7_15080_star	1.07457	3.22371	1.05281229	2.078510713	-1.58496	0.631174
chr2_6533_mature	58.02677632	731.7821	5.883297648	9.517240499	-3.65662	0.376221	NW_003539989_39206_mature	32.237098	5.641492	5.054722516	2.731507408	2.514573	1	chr14_30569_star	1.07457	3.22371	1.05281229	2.078510713	-1.58496	0.631174
chr9_19521_mature	36.53537768	365.8911	5.230179094	8.519207948	-3.32405	0.377473	chr17_35572_mature	30.0879581	4.835565	4.958283956	2.544872269	2.63743	1	hsa-miR-3915	1.07457	3.22371	1.05281229	2.078510713	-1.58496	0.631174
NW_003540824_39708_star	3.223709795	12.08891	2.078510713	3.710273245	-1.90689	0.379952	chr2_6582_star	30.0879581	4.835565	4.958283956	2.544872269	2.63743	1	hsa-miR-3941	1.07457	3.22371	1.05281229	2.078510713	-1.58496	0.631174
chr5_10912_mature	3.223709795	12.08891	2.078510713	3.710273245	-1.90689	0.379952	chr2_5381_star	23.6405385	2.417782	4.62296188	1.773060525	3.289507	1	hsa-miR-193b-3p	1.07457	3.22371	1.05281229	2.078510713	-1.58496	0.631174
chr2_5381_mature	3.223709795	12.08891	2.078510713	3.710273245	-1.90689	0.379952	chr16_33929_mature	23.6405385	2.417782	4.62296188	1.773060525	3.289507	1	chr9_21046_mature	1.07457	3.22371	1.05281229	2.078510713	-1.58496	0.631174
chr2_5731_star	29.01338816	6.44742	4.907534286	2.896740642	2.169925	0.380244	chr7_15380_mature	10.7456993	50.77343	3.554060706	5.694139975	-2.24031	1	chr6_14384_mature	1.07457	3.22371	1.05281229	2.078510713	-1.58496	0.631174
chr7_16971_mature	8.596559455	2.417782	3.262517265	1.773060525	1.830075	0.381134	NW_003539814_39113_mature	10.7456993	50.77343	3.554060706	5.694139975	-2.24031	1	rno-miR-471-5p	1.07457	3.22371	1.05281229	2.078510713	-1.58496	0.631174
chr4_9835_star	8.596559455	2.417782	3.262517265	1.773060525	1.830075	0.381134	chr9_19114_mature	27.9388182	4.029637	4.854934103	2.330454351	2.793549	1	chr14_29715_star	1.07457	3.22371	1.05281229	2.078510713	-1.58496	0.631174
mmu-miR-5113	8.596559455	2.417782	3.262517265	1.773060525	1.830075	0.381134	chr14_29851_mature	25.7896784	3.22371	4.743605355	2.078510713	3	1	mmu-miR-1964-5p	1.07457	3.22371	1.05281229	2.078510713	-1.58496	0.631174
chr1_1981_star	8.596559455	2.417782	3.262517265	1.773060525	1.830075	0.381134	chr12_26197_mature	25.7896784	3.22371	4.743605355	2.078510713	3	1	chr6_12657_star@@mmu-miR-504	1.07457	3.22371	1.05281229	2.078510713	-1.58496	0.631174
chr4_9294_star	8.596559455	2.417782	3.262517265	1.773060525	1.830075	0.381134	chr14_30822_star	25.7896784	3.22371	4.743605355	2.078510713	3	1	chr7_15773_star	1.07457	3.22371	1.05281229	2.078510713	-1.58496	0.631174
chr15_32658_mature	8.596559455	2.417782	3.262517265	1.773060525	1.830075	0.381134	chr8_17733_star@@mmu-miR-3084-5p	25.7896784	3.22371	4.743605355	2.078510713	3	1	chrx_21804_star	1.07457	3.22371	1.05281229	2.078510713	-1.58496	0.631174
chr17_35449_star	8.596559455	2.417782	3.262517265	1.773060525	1.830075	0.381134	chr17_34936_mature	101.009574	44.32601	6.672560745	5.502267253	1.188267	1	chr13_27515_star	1.07457	3.22371	1.05281229	2.078510713	-1.58496	0.631174
rno-miR-802-5p	8.596559455	2.417782	3.262517265	1.773060525	1.830075	0.381134	ssc-miR-139-5p	23.6405385	74.14533	4.62296188	6.231611453	-1.64909	1	chr3_6673_mature	1.07457	3.22371	1.05281229	2.078510713	-1.58496	0.631174
chr13_27902_mature	8.596559455	2.417782	3.262517265	1.773060525	1.830075	0.381134	chr8_18472_mature	34.3862378	91.87573	5.145116481	6.537229727	-1.41785	1	chr4_10437_star	1.07457	3.22371	1.05281229	2.078510713	-1.58496	0.631174
chr14_30832_mature	8.596559455	2.417782	3.262517265	1.773060525	1.830075	0.381134	chr5_11037_mature	58.0267763	18.53633	5.883297648	4.288087668	1.646363	1	bta-miR-197	1.07457	3.22371	1.05281229	2.078510713	-1.58496	0.631174
chr12_26195_mature	8.596559455	2.417782	3.262517265	1.773060525	1.830075	0.381134	chr2_5399_mature	58.0267763	18.53633	5.883297648	4.288087668	1.646363	1	chr13_28054_star	1.07457	3.22371	1.05281229	2.078510713	-1.58496	0.631174
chr11_25358_mature	4.298279727	16.92448	2.405524014	4.163859073	-1.97728	0.382894	NW_003539866_39141_mature	2.14913986	31.43117	1.654957834	5.019309189	-3.87036	1	hsa-miR-4795-3p	1.07457	3.22371	1.05281229	2.078510713	-1.58496	0.631174
chr5_11634_mature	4.298279727	16.92448	2.405524014	4.163859073	-1.97728	0.382894	chr1_2947_mature	2.14913986	31.43117	1.654957834	5.019309189	-3.87036	1	chr10_23462_star	1.07457	3.22371	1.05281229	2.078510713	-1.58496	0.631174
chr15_32819_mature	11.82026925	3.22371	3.680354657	2.078510713	1.874469	0.382985	mmu-miR-1934-5p	2.14913986	31.43117	1.654957834	5.019309189	-3.87036	1	chr14_31138_mature	1.07457	3.22371	1.05281229	2.078510713	-1.58496	0.631174
hsa-miR-3676-3p	11.82026925	3.22371	3.680354657	2.078510713	1.874469	0.382985	chr1_3820_star	8.59655945	45.93786	3.262517265	5.552680304	-2.41785	1	chr17_35074_star	1.07457	3.22371	1.05281229	2.078510713	-1.58496	0.631174
chr1_3810_star	11.82026925	3.22371	3.680354657	2.078510713	1.874469	0.382985	chr15_33671_mature	15.043979	0	4.003960082	0	20	1	chr8_17750_star	1.07457	3.22371	1.05281229	2.078510713	-1.58496	0.631174
chr8_17419_star	11.82026925	3.22371	3.680354657	2.078510713	1.874469	0.382985	chr11_24729_mature	15.043979	0	4.003960082	0	20	1	chr12_26282_mature	1.07457	3.22371	1.05281229	2.078510713	-1.58496	0.631174
chr1_3052_mature	336.3403887	37.87859	8.398061248	5.280903997	3.150467	0.384251	chr5_11087_star	15.043979	0	4.003960082	0	20	1	chr6_13671_star	1.07457	3.22371	1.05281229	2.078510713	-1.58496	0.631174
ssc-miR-106a	3110.879953	185.3633	11.60357069	7.541974074	4.068895	0.384739	rno-miR-330-5p	15.043979	0	4.003960082	0	20	1	chr14_31530_star	1.07457	3.22371	1.05281229	2.078510713	-1.58496	0.631174
chr15_33084_mature	19.34225877	4.835565	4.346407978	2.544872269	2	0.385896	chr7_16788_star	15.043979	0	4.003960082	0	20	1	chr13_27012_star	1.07457	3.22371	1.05281229	2.078510713	-1.58496	0.631174
bta-miR-26c	23.6405385	5.641492	4.62296188	2.731507408	2.067114	0.386303	NW_003539588_38971_star	15.043979	0	4.003960082	0	20	1	chr4_8951_star	1.07457	3.22371	1.05281229	2.078510713	-1.58496	0.631174
NW_003539989_39206_mature	23.6405385	5.641492	4.62296188	2.731507408	2.067114	0.386303	chr14_30968_mature	15.043979	0	4.003960082	0	20	1	hsa-miR-3135b	1.07457	3.22371	1.05281229	2.078510713	-1.58496	0.631174
chr3_8352_mature	10.74569932	58.02678	3.554060706	5.883297648	-2.43296	0.388325	chr5_12116_mature@@bta-miR-200c	15.043979	0	4.003960082	0	20	1	chr2_5595_mature	1.07457	3.22371	1.05281229	2.078510713	-1.58496	0.631174
chr8_18597_star	27.93881823	6.44742	4.854934103	2.896740642	2.115477	0.388895	chr2_6234_star	15.043979	0	4.003960082	0	20	1	chr14_30822_mature	1.07457	3.22371	1.05281229	2.078510713	-1.58496	0.631174
chr8_18166_mature	27.93881823	6.44742	4.854934103	2.896740642	2.115477	0.388895	chr2_4073_mature	15.043979	0	4.003960082	0	20	1	chr3_7353_mature	1.07457	3.22371	1.05281229	2.078510713	-1.58496	0.631174
chr8_18908_star	3.223709795	0.805927	2.078510713	0.852739935	2	0.388973	chr6_15065_mature	15.043979	0	4.003960082	0	20	1	rno-miR-379-5p	1.07457	3.22371	1.05281229	2.078510713	-1.58496	0.631174
mmu-miR-1943-3p	3.223709795	0.805927	2.078510713	0.852739935	2	0.388973	chr4_9185_star	15.043979	0	4.003960082	0	20	1	chr13_29421_star	1.07457	3.22371	1.05281229	2.078510713	-1.58496	0.631174
chr10_23371_star	3.223709795	0.805927	2.078510713	0.852739935	2	0.388973	chr7_16018_star	15.043979	0	4.003960082	0	20	1	chr8_19039_star	9.671129	3.22371	3.415640968	2.078510713	1.584963	0.633028
chr5_11544_star	3.223709795	0.805927	2.078510713	0.852739935	2	0.388973	chr12_25470_mature	15.043979	0	4.003960082	0	20	1	rno-miR-216b-3p	9.671129	3.22371	3.415640968	2.078510713	1.584963	0.633028
chr2_4023_star	3.223709795	0.805927	2.078510713	0.852739935	2	0.388973	chr9_21075_mature	15.043979	0	4.003960082	0	20	1	ssc-miR-545-5p	9.671129	3.22371	3.415640968	2.078510713	1.584963	0.633028
bta-miR-2882	3.223709795	0.805927	2.078510713	0.852739935	2	0.388973	chr14_30455_mature	15.043979	0	4.003960082	0	20	1	chr12_25786_star	3.760995	12.08891	2.251263041	3.710273245	-1.6845	0.6333
hsa-miR-4767	3.223709795	0.805927	2.078510713	0.852739935	2	0.388973	ssc-miR-4336	15.043979	0	4.003960082	0	20	1	ssc-miR-490	3.760995	12.08891	2.251263041	3.710273245	-1.6845	0.6333
hsa-miR-3667-5p	3.223709795	0.805927	2.078510713	0.852739935	2	0.388973	chr7_17208_mature	15.043979	0	4.003960082	0	20	1	chr11_24519_star	3.760995	12.08891	2.251263041	3.710273245	-1.6845	0.6333
chr9_20113_star	3.223709795	0.805927	2.078510713	0.852739935	2	0.388973	hsa-miR-4632-3p	15.043979	0	4.003960082	0	20	1	chr14_30693_star	3.760995	12.08891	2.251263041	3.710273245	-1.6845	0.6333
chr10_23828_star	3.223709795	0.805927	2.078510713	0.852739935	2	0.388973	chr7_16580_star	15.043979	0	4.003960082	0	20	1	hsa-miR-4463	3.760995	12.08891	2.251263041	3.710273245	-1.6845	0.6333
chr6_14454_star	3.223709795	0.805927	2.078510713	0.852739935	2	0.388973	hsa-miR-634	15.043979	0	4.003960082	0	20	1	chr14_29447_star	4.29828	14.50669	2.405524014	3.954819241	-1.75489	0.633585
chr1_3174_star	3.223709795	0.805927	2.078510713	0.852739935	2	0.388973	hsa-miR-638	15.043979	0	4.003960082	0	20	1	chr4_8848_mature	35.46081	8.059274	5.188274618	3.179395517	2.137504	0.634053
chr13_28506_star	3.223709795	0.805927	2.078510713	0.852739935	2	0.388973	chr7_16478_star	15.043979	0	4.003960082	0	20	1	NW_003538820_38274_mature	164.4092	21.76004	7.369895665	4.508431259	2.917538	0.634391
chr5_12167_star	3.223709795	0.805927	2.078510713	0.852739935	2	0.388973	chr13_26768_mature	15.043979	0	4.003960082	0	20	1	chr14_30720_mature	16.65583	4.835565	4.142073061	2.544872269	1.784271	0.634477
NW_003539027_38478_star	3.223709795	0.805927	2.078510713	0.852739935	2	0.388973	chr12_25396_star	15.043979	0	4.003960082	0	20	1	chr7_16449_star	12.89484	4.029637	3.796477232	2.330454351	1.678072	0.634488
chr13_29016_star	3.223709795	0.805927	2.078510713	0.852739935	2	0.388973	chr16_34140_mature	15.043979	0	4.003960082	0	20	1	chr6_13633_mature	12.89484	4.029637	3.796477232	2.330454351	1.678072	0.634488
hsa-miR-3909	3.223709795	0.805927	2.078510713	0.852739935	2	0.388973	chr4_9787_mature	6.44741959	41.1023	2.896740642	5.395827139	-2.67243	1	chr6_13893_star	40.83366	8.865202	5.38659223	3.302348583	2.203533	0.634778
bta-miR-193a	3.223709795	0.805927	2.078510713	0.852739935	2	0.388973	chr3_8247_mature	4.29827973	36.26674	2.405524014	5.21981653	-3.07682	1	chr17_34922_star	30.08796	7.253347	4.958283956	3.044979305	2.052467	0.635176
rno-miR-328b-3p	3.223709795	0.805927	2.078510713	0.852739935	2	0.388973	chr10_23470_mature	55.8776365	17.7304	5.82978961	4.227310103	1.656046	1	chr16_33929_star	30.08796	7.253347	4.958283956	3.044979305	2.052467	0.635176
chr7_15658_mature@@bta-miR-230	3.223709795	0.805927	2.078510713	0.852739935	2	0.388973	NW_003613057_36932_star	2.14913986	30.62524	1.654957834	4.983004663	-3.83289	1	chr4_10705_mature	5.910135	22.56597	2.788713818	4.558633073	-1.93289	0.635441
chr10_24282_star	3.223709795	0.805927	2.078510713	0.852739935	2	0.388973	chr5_12127_mature	2.14913986	30.62524	1.654957834	4.983004663	-3.83289	1	bta-miR-2322-5p	4.835565	16.92448	2.544872269	4.163859073	-1.80735	0.63567
hsa-miR-1538	3.223709795	0.805927	2.078510713	0.852739935	2	0.388973	chr16_33806_star	2.14913986	30.62524	1.654957834	4.983004663	-3.83289	1	chr1_2051_star	3.22371	9.671129	2.078510713	3.415640968	-1.58496	0.63578
hsa-miR-520a-5p	3.223709795	0.805927	2.078510713	0.852739935	2	0.388973	chr18_35961_star	8.59655945	45.13194	3.262517265	5.527693969	-2.39232	1	chr18_36164_star	3.22371	9.671129	2.078510713	3.415640968	-1.58496	0.63578
chr6_12861_mature	3.223709795	0.805927	2.078510713	0.852739935	2	0.388973	chr14_29652_mature	36.5353777	8.059274	5.230179094	3.179395517	2.180572	1	chr1_2009_mature	3.22371	9.671129	2.078510713	3.415640968	-1.58496	0.63578
bta-miR-2320-5p	3.223709795	0.805927	2.078510713	0.852739935	2	0.388973	chr4_9182_mature	148.290651	77.36904	7.221980009	6.292211829	0.938599	1	chr7_16196_mature	3.22371	9.671129	2.078510713	3.415640968	-1.58496	0.63578
chr9_19660_star	3.223709795	0.805927	2.078510713	0.852739935	2	0.388973	hsa-miR-3653	49.4302169	14.50669	5.656216524	3.954819241	1.768674	1	chr15_33538_mature	3.22371	9.671129	2.078510713	3.415640968	-1.58496	0.63578
hsa-miR-5009-5p	3.223709795	0.805927	2.078510713	0.852739935	2	0.388973	chr13_29259_star	6.44741959	40.29637	2.896740642	5.367943153	-2.64386	1	chr9_20034_mature	3.22371	9.671129	2.078510713	3.415640968	-1.58496	0.63578
chr15_32517_star	3.223709795	0.805927	2.078510713	0.852739935	2	0.388973	chr12_25421_mature	6.44741959	40.29637	2.896740642	5.367943153	-2.64386	1	chr9_19234_star	3.22371	9.671129	2.078510713	3.415640968	-1.58496	0.63578
chr2_5745_star	3.223709795	0.805927	2.078510713	0.852739935	2	0.388973	chr15_32033_mature	90.2638743	38.68452	6.511971994	5.310504361	1.222392	1	chr5_12208_mature	20.41683	5.641492	4.420672964	2.731507408	1.85561	0.637812
chr13_28605_mature	3.223709795	0.805927	2.078510713	0.852739935	2	0.388973	chr3_6710_mature	2.14913986	29.81932	1.654957834	4.94576292	-3.79442	1	chr6_14862_star	20.41683	5.641492	4.420672964	2.731507408	1.85561	0.637812
chr14_30846_star	3.223709795	0.805927	2.078510713	0.852739935	2	0.388973	chr15_32921_mature	2.14913986	29.81932	1.654957834	4.94576292	-3.79442	1	chr6_13601_mature	12.35755	65.28012	3.739583968	6.050504383	-2.40125	0.638323
chr1_3471_star	3.223709795	0.805927	2.078510713	0.852739935	2	0.388973	chr14_30214_mature	2.14913986	29.81932	1.654957834	4.94576292	-3.79442	1	chr1_1793_star	4.29828	1.611855	2.405524014	1.38507475	1.415037	0.638433
chr11_25149_star	3.223709795	0.805927	2.078510713	0.852739935	2	0.388973	chr9_19559_mature	17.1931189	0.805927	4.185320985	0.852739935	4.415037	1	chr14_31255_mature	4.29828	1.611855	2.405524014	1.38507475	1.415037	0.638433
rno-miR-344i	3.223709795	0.805927	2.078510713	0.852739935	2	0.388973	chr2_4081_mature	17.1931189	0.805927	4.185320985	0.852739935	4.415037	1	chr3_7094_star	4.29828	1.611855	2.405524014	1.38507475	1.415037	0.638433
chr3_8228_star	3.223709795	0.805927	2.078510713	0.852739935	2	0.388973	chr2_5337_star	17.1931189	0.805927	4.185320985	0.852739935	4.415037	1	NW_003538780_38224_star	4.29828	1.611855	2.405524014	1.38507475	1.415037	0.638433
chr12_25591_mature	3.223709795	0.805927	2.078510713	0.852739935	2	0.388973	chr15_32610_mature	17.1931189	0.805927	4.185320985	0.852739935	4.415037	1	hsa-miR-4520a-5p	4.29828	1.611855	2.405524014	1.38507475	1.415037	0.638433
chr15_32130_star	3.223709795	0.805927	2.078510713	0.852739935	2	0.388973	chr11_24884_mature	17.1931189	0.805927	4.185320985	0.852739935	4.415037	1	chr17_35168_mature@@rno-miR-8	4.29828	1.611855	2.405524014	1.38507475	1.415037	0.638433
chr8_17498_star	3.223709795	0.805927	2.078510713	0.852739935	2	0.388973	chr7_17101_mature	17.1931189	0.805927	4.185320985	0.852739935	4.415037	1	bta-miR-1814a	4.29828	1.611855	2.405524014	1.38507475	1.415037	0.638433
chr7_17143_star	3.223709795	0.805927	2.078510713	0.852739935	2	0.388973	chr7_16237_mature	17.1931189	0.805927	4.185320985	0.852739935	4.415037	1	chr14_29861_mature	4.29828	1.611855	2.405524014	1.38507475	1.415037	0.638433
chr1_3525_star	3.223709795	0.805927	2.078510713	0.852739935	2	0.388973	mmu-miR-1930-3p	17.1931189	0.805927	4.185320985	0.852739935	4.415037	1	rno-miR-339-5p	4.29828	1.611855	2.405524014	1.38507475	1.415037	0.638433
chr15_32263_star	3.223709795	0.805927	2.078510713	0.852739935	2	0.388973	bta-miR-378b	17.1931189	0.805927	4.185320985	0.852739935	4.415037	1	chr9_19988_star	4.29828	1.611855	2.405524014	1.38507475	1.415037	0.638433
chr13_28924_mature	3.223709795	0.805927	2.078510713	0.852739935	2	0.388973	hsa-miR-2277-3p	17.1931189	0.805927	4.185320985	0.852739935	4.415037	1	bta-miR-181b	4.29828	1.611855	2.405524014	1.38507475	1.415037	0.638433
chr14_30104_star	3.223709795	0.805927	2.078510713	0.852739935	2	0.388973	chr6_13980_mature	40.8336574	10.47706	5.38659223	3.520680821	1.962525	1	rno-miR-483-5p	4.29828	1.611855	2.405524014	1.38507475	1.415037	0.638433
chr2_6082_mature	3.223709795	0.805927	2.078510713	0.852739935	2	0.388973	chr1_3039_star	4.29827973	34.65488	2.405524014	5.156027656	-3.01123	1	rno-miR-672-5p	4.29828	1.611855	2.405524014	1.38507475	1.415037	0.638433
chr14_30803_star	3.223709795	0.805927	2.078510713	0.852739935	2	0.388973	NW_003613157_37452_mature	4.29827973	34.65488	2.405524014	5.156027656	-3.01123	1	chr18_35964_star	4.29828	1.611855	2.405524014	1.38507475	1.415037	0.638433
chr1_3232_star	3.223709795	0.805927	2.078510713	0.852739935	2	0.388973	chr18_35894_mature	19.3422588	1.611855	4.346407978	1.38507475	3.584963	1	hsa-miR-378b	4.29828	1.611855	2.405524014	1.38507475	1.415037	0.638433
hsa-miR-4676-5p	3.223709795	0.805927	2.078510713	0.852739935	2	0.388973	chr12_25873_mature	19.3422588	1.611855	4.346407978	1.38507475	3.584963	1	chrx_22340_star	4.29828	1.611855	2.405524014	1.38507475	1.415037	0.638433
chr15_33376_mature	3.223709795	0.805927	2.078510713	0.852739935	2	0.388973	chr5_11357_mature	19.3422588	1.611855	4.346407978	1.38507475	3.584963	1	chr6_15039_mature	4.29828	1.611855	2.405524014	1.38507475	1.415037	0.638433
rno-miR-500-3p	3.223709795	0.805927	2.078510713	0.852739935	2	0.388973	chr12_25706_mature	19.3422588	1.611855	4.346407978	1.38507475	3.584963	1	chr8_17856_star	4.29828	1.611855	2.405524014	1.38507475	1.415037	0.638433
chr14_31026_star	3.223709795	0.805927	2.078510713	0.852739935	2	0.388973	chr9_19419_mature	19.3422588	1.611855	4.346407978	1.38507475	3.584963	1	chr1_2658_star	4.29828	1.611855	2.405524014	1.38507475	1.415037	0.638433
chr13_29007_star	3.223709795	0.805927	2.078510713	0.852739935	2	0.388973	chr13_29417_mature	19.3422588	1.611855	4.346407978	1.38507475	3.584963	1	chr3_6615_star	4.29828	1.611855	2.405524014	1.38507475	1.415037	0.638433
chr3_6639_star	3.223709795	0.805927	2.078510713	0.852739935	2	0.388973	chr1_3580_star	19.3422588	1.611855	4.346407978	1.38507475	3.584963	1	chr6_13056_mature	4.29828	1.611855	2.405524014	1.38507475	1.415037	0.638433
hsa-miR-4712-5p	3.223709795	0.805927	2.078510713	0.852739935	2	0.388973	chr3_6792_star	19.3422588	1.611855	4.346407978	1.38507475	3.584963	1	mmu-miR-669h-3p	4.29828	1.611855	2.405524014	1.38507475	1.415037	0.638433
chr4_10313_mature	3.223709795	0.805927	2.078510713	0.852739935	2	0.388973	chr14_30126_mature	19.3422588	1.611855	4.346407978	1.38507475	3.584963	1	chr2_5378_star	4.29828	1.611855	2.405524014	1.38507475	1.415037	0.638433
chr4_10238_star	3.223709795	0.805927	2.078510713	0.852739935	2	0.388973	NW_003613440_39150_mature	27.9388182	4.835565	4.854934103	2.544872269	2.530515	1	chr13_26893_mature	4.29828	1.611855	2.405524014	1.38507475	1.415037	0.638433
chr14_31221_star	3.223709795	0.805927	2.078510713	0.852739935	2	0.388973	mmu-miR-6416-3p	27.9388182	4.835565	4.854934103	2.544872269	2.530515	1	rno-miR-320-5p	4.29828	1.611855	2.405524014	1.38507475	1.415037	0.638433
chrx_22191_mature	3.223709795	0.805927	2.078510713	0.852739935	2	0.388973	hsa-miR-2681-3p	2.14913986	29.01339	1.654957834	4.907534286	-3.75489	1	chr6_14340_star	4.29828	1.611855	2.405524014	1.38507475	1.415037	0.638433
NW_003538623_38054_star	3.223709795	0.805927	2.078510713	0.852739935	2	0.388973	rno-miR-181a-2-3p	2.14913986	29.01339	1.654957834	4.907534286	-3.75489	1	chr5_12026_mature	4.29828	1.611855	2.405524014	1.38507475	1.415037	0.638433

chrx_23205_star	3.223709795	0.805927	2.078510713	0.852739935	2	0.388973	bta-miR-377	10.7456993	47.54972	3.554060706	5.601391055	-2.14568	1	chr11_24795_star	4.29828	1.611855	2.405524014	1.38507475	1.415037	0.638433
chr15_32249_mature	3.223709795	0.805927	2.078510713	0.852739935	2	0.388973	chr6_13908_mature	49.4302169	15.31262	5.656216524	4.027916745	1.690672	1	chr14_30803_mature	4.29828	1.611855	2.405524014	1.38507475	1.415037	0.638433
NW_003613379_38761_mature	3.223709795	0.805927	2.078510713	0.852739935	2	0.388973	chr6_14473_mature	25.7896784	4.029637	4.743605355	2.330454351	2.678072	1	chr7_17170_star	4.29828	1.611855	2.405524014	1.38507475	1.415037	0.638433
chr13_29176_mature	3.223709795	0.805927	2.078510713	0.852739935	2	0.388973	chr14_31610_mature	25.7896784	4.029637	4.743605355	2.330454351	2.678072	1	chr4_10088_star	4.29828	1.611855	2.405524014	1.38507475	1.415037	0.638433
chr3_6901_mature	3.223709795	0.805927	2.078510713	0.852739935	2	0.388973	chr3_8631_mature	25.7896784	4.029637	4.743605355	2.330454351	2.678072	1	chr2_6005_star	4.29828	1.611855	2.405524014	1.38507475	1.415037	0.638433
chr4_9960_mature	3.223709795	0.805927	2.078510713	0.852739935	2	0.388973	chr14_29702_mature	21.4913986	2.417782	4.491301473	1.773060525	3.152003	1	chr5_10732_star	4.29828	1.611855	2.405524014	1.38507475	1.415037	0.638433
bta-miR-665	3.223709795	0.805927	2.078510713	0.852739935	2	0.388973	NW_003613530_39570_mature	21.4913986	2.417782	4.491301473	1.773060525	3.152003	1	bta-miR-2475	4.29828	1.611855	2.405524014	1.38507475	1.415037	0.638433
bta-miR-653	3.223709795	0.805927	2.078510713	0.852739935	2	0.388973	chr13_28998_mature	21.4913986	2.417782	4.491301473	1.773060525	3.152003	1	chr13_26759_mature	4.29828	1.611855	2.405524014	1.38507475	1.415037	0.638433
chr13_27082_star	3.223709795	0.805927	2.078510713	0.852739935	2	0.388973	chr5_11087_star	21.4913986	2.417782	4.491301473	1.773060525	3.152003	1	bta-miR-2335	4.29828	1.611855	2.405524014	1.38507475	1.415037	0.638433
hsa-miR-4661-5p	3.223709795	0.805927	2.078510713	0.852739935	2	0.388973	chr1_3087_mature	21.4913986	2.417782	4.491301473	1.773060525	3.152003	1	chr1_2685_star	4.29828	1.611855	2.405524014	1.38507475	1.415037	0.638433
chr18_36633_mature	3.223709795	0.805927	2.078510713	0.852739935	2	0.388973	chr4_9551_mature	21.4913986	2.417782	4.491301473	1.773060525	3.152003	1	chr13_27259_star	4.29828	1.611855	2.405524014	1.38507475	1.415037	0.638433
mmu-miR-2861	3.223709795	0.805927	2.078510713	0.852739935	2	0.388973	chr13_27867_star	23.6405385	3.22371	4.62296188	2.078510713	2.874469	1	rno-miR-181b-5p	4.29828	1.611855	2.405524014	1.38507475	1.415037	0.638433
chr11_24484_star	3.223709795	0.805927	2.078510713	0.852739935	2	0.388973	rno-miR-1843-3p	23.6405385	3.22371	4.62296188	2.078510713	2.874469	1	chr13_28441_star	4.29828	1.611855	2.405524014	1.38507475	1.415037	0.638433
chr13_27018_star	3.223709795	0.805927	2.078510713	0.852739935	2	0.388973	chr3_6673_mature	23.6405385	3.22371	4.62296188	2.078510713	2.874469	1	chr17_35199_mature	4.29828	1.611855	2.405524014	1.38507475	1.415037	0.638433
chr2_4454_star	3.223709795	0.805927	2.078510713	0.852739935	2	0.388973	chr1_1111_mature	23.6405385	3.22371	4.62296188	2.078510713	2.874469	1	chr6_12862_mature	4.29828	1.611855	2.405524014	1.38507475	1.415037	0.638433
NW_003537370_36873_star	3.223709795	0.805927	2.078510713	0.852739935	2	0.388973	NW_003537189_36743_star	23.6405385	3.22371	4.62296188	2.078510713	2.874469	1	chr7_16945_star	4.29828	1.611855	2.405524014	1.38507475	1.415037	0.638433
chr4_8825_mature	3.223709795	0.805927	2.078510713	0.852739935	2	0.388973	chr6_13692_mature	58.0267763	20.14819	5.883297648	4.402462031	1.526069	1	chr1_608_star	6.44742	2.417782	2.896740642	1.773060525	1.415037	0.639506
chr15_33480_star	3.223709795	0.805927	2.078510713	0.852739935	2	0.388973	chr14_30212_mature	36.5353777	8.865202	5.230179094	3.302348583	2.043069	1	chr7_15700_mature	6.44742	2.417782	2.896740642	1.773060525	1.415037	0.639506
chr11_24857_star	3.223709795	0.805927	2.078510713	0.852739935	2	0.388973	NW_003539698_39033_mature	42.9827973	12.08891	5.458867456	3.710273245	1.830075	1	chr17_35528_star	6.44742	2.417782	2.896740642	1.773060525	1.415037	0.639506
chr5_10851_mature	3.223709795	0.805927	2.078510713	0.852739935	2	0.388973	chr4_9797_mature	15.043979	54.80307	4.003960082	5.802272499	-1.86507	1	rno-miR-383-5p	6.44742	2.417782	2.896740642	1.773060525	1.415037	0.639506
chr3_6723_star	3.223709795	0.805927	2.078510713	0.852739935	2	0.388973	chr9_19757_mature	2.14913986	28.20746	1.654957834	4.868265031	-3.71425	1	chr18_36376_star	6.44742	2.417782	2.896740642	1.773060525	1.415037	0.639506
chr1_398_star	3.223709795	0.805927	2.078510713	0.852739935	2	0.388973	chr11_24876_mature	2.14913986	28.20746	1.654957834	4.868265031	-3.71425	1	chr5_11262_mature	6.44742	2.417782	2.896740642	1.773060525	1.415037	0.639506
chr4_9973_star	3.223709795	0.805927	2.078510713	0.852739935	2	0.388973	chr6_12830_mature	2.14913986	28.20746	1.654957834	4.868265031	-3.71425	1	chr18_36226_star	6.44742	2.417782	2.896740642	1.773060525	1.415037	0.639506
chr14_29835_mature	3.223709795	0.805927	2.078510713	0.852739935	2	0.388973	ssc-miR-140-5p	414.783994	571.4026	8.69969041	9.160886317	-0.46215	1	chr12_25628_star	6.44742	2.417782	2.896740642	1.773060525	1.415037	0.639506
chr5_10819_mature	3.223709795	0.805927	2.078510713	0.852739935	2	0.388973	chr14_30997_mature	6.44741959	37.87859	2.896740642	5.280903997	-2.55459	1	bta-miR-2285m	6.44742	2.417782	2.896740642	1.773060525	1.415037	0.639506
mmu-miR-1192	3.223709795	0.805927	2.078510713	0.852739935	2	0.388973	chr8_18873_mature	6.44741959	37.87859	2.896740642	5.280903997	-2.55459	1	chr10_24002_star	6.44742	2.417782	2.896740642	1.773060525	1.415037	0.639506
chr13_28917_star	3.223709795	0.805927	2.078510713	0.852739935	2	0.388973	chr5_11850_mature	4.29827973	33.04303	2.405524014	5.08928735	-2.94251	1	chr3_6774_star	6.44742	2.417782	2.896740642	1.773060525	1.415037	0.639506
chr2_4310_star	3.223709795	0.805927	2.078510713	0.852739935	2	0.388973	chr6_12992_mature	12.8948392	0	3.796477232	0	20	1	chr6_12860_star	6.44742	2.417782	2.896740642	1.773060525	1.415037	0.639506
hsa-miR-3178	3.223709795	0.805927	2.078510713	0.852739935	2	0.388973	chrx_23064_mature	12.8948392	0	3.796477232	0	20	1	mmu-miR-679-5p	6.44742	2.417782	2.896740642	1.773060525	1.415037	0.639506
hsa-miR-3132	3.223709795	0.805927	2.078510713	0.852739935	2	0.388973	chr14_31505_mature	12.8948392	0	3.796477232	0	20	1	hsa-miR-4255	6.44742	2.417782	2.896740642	1.773060525	1.415037	0.639506
mmu-miR-3078-5p	3.223709795	0.805927	2.078510713	0.852739935	2	0.388973	chr3_7910_mature	12.8948392	0	3.796477232	0	20	1	NW_003613297_38331_mature	6.44742	2.417782	2.896740642	1.773060525	1.415037	0.639506
bta-miR-6530	3.223709795	0.805927	2.078510713	0.852739935	2	0.388973	chr3_6641_mature	12.8948392	0	3.796477232	0	20	1	bta-miR-2468	6.44742	2.417782	2.896740642	1.773060525	1.415037	0.639506
chr9_20710_star	3.223709795	0.805927	2.078510713	0.852739935	2	0.388973	chr8_17892_mature	245.001944	153.1262	7.942525909	7.26796846	0.678072	1	chr9_20275_star	6.44742	2.417782	2.896740642	1.773060525	1.415037	0.639506
chr1_1289_star	3.223709795	0.805927	2.078510713	0.852739935	2	0.388973	chr9_20720_mature	12.8948392	0	3.796477232	0	20	1	chr18_36365_star	6.44742	2.417782	2.896740642	1.773060525	1.415037	0.639506
chr14_30394_star	3.223709795	0.805927	2.078510713	0.852739935	2	0.388973	chr12_26437_mature	12.8948392	0	3.796477232	0	20	1	chrx_22000_star	6.44742	2.417782	2.896740642	1.773060525	1.415037	0.639506
chr9_20121_star	3.223709795	0.805927	2.078510713	0.852739935	2	0.388973	mmu-miR-3073b-5p	12.8948392	0	3.796477232	0	20	1	mmu-miR-21a-5p	6.984705	28.20746	2.997239027	4.868265031	-2.01381	0.639523
chrx_21688_star	3.223709795	0.805927	2.078510713	0.852739935	2	0.388973	chr2_4623_mature	12.8948392	0	3.796477232	0	20	1	chr11_24906_mature	6.984705	28.20746	2.997239027	4.868265031	-2.01381	0.639523
chr14_31467_star	3.223709795	0.805927	2.078510713	0.852739935	2	0.388973	chr12_25609_star	12.8948392	0	3.796477232	0	20	1	chr11_25269_star	2.686425	0.805927	1.882222339	0.852739935	1.736966	0.639545
bta-miR-31	3.223709795	0.805927	2.078510713	0.852739935	2	0.388973	hsa-miR-1914-3p	12.8948392	0	3.796477232	0	20	1	mmu-miR-698-5p	2.686425	0.805927	1.882222339	0.852739935	1.736966	0.639545
NW_003539217_38656_star	3.223709795	0.805927	2.078510713	0.852739935	2	0.388973	chr12_25947_mature	12.8948392	0	3.796477232	0	20	1	chr2_4241_mature	2.686425	0.805927	1.882222339	0.852739935	1.736966	0.639545
chrx_22698_mature	3.223709795	0.805927	2.078510713	0.852739935	2	0.388973	mmu-miR-6342	12.8948392	0	3.796477232	0	20	1	chr13_27228_star	2.686425	0.805927	1.882222339	0.852739935	1.736966	0.639545
chr14_30483_star	3.223709795	0.805927	2.078510713	0.852739935	2	0.388973	mmu-miR-149-3p	12.8948392	0	3.796477232	0	20	1	chr7_16671_star	2.686425	0.805927	1.882222339	0.852739935	1.736966	0.639545
chr2_4381_star	3.223709795	0.805927	2.078510713	0.852739935	2	0.388973	chr4_9149_star	12.8948392	0	3.796477232	0	20	1	chr3_7237_mature	2.686425	0.805927	1.882222339	0.852739935	1.736966	0.639545
chr9_20751_mature	3.223709795	0.805927	2.078510713	0.852739935	2	0.388973	chr14_30479_mature	12.8948392	0	3.796477232	0	20	1	mmu-miR-6418-5p	2.686425	0.805927	1.882222339	0.852739935	1.736966	0.639545
bta-miR-2465	3.223709795	0.805927	2.078510713	0.852739935	2	0.388973	NW_003538374_37783_mature	12.8948392	0	3.796477232	0	20	1	NW_003613237_37814_star	2.686425	0.805927	1.882222339	0.852739935	1.736966	0.639545
hsa-miR-4478	3.223709795	0.805927	2.078510713	0.852739935	2	0.388973	chr2_4586_star	12.8948392	0	3.796477232	0	20	1	hsa-miR-4520b-3p	2.686425	0.805927	1.882222339	0.852739935	1.736966	0.639545
rno-miR-876	3.223709795	0.805927	2.078510713	0.852739935	2	0.388973	rno-miR-6215	12.8948392	0	3.796477232	0	20	1	chrx_22989_star	2.686425	0.805927	1.882222339	0.852739935	1.736966	0.639545
chr3_7737_mature	3.223709795	0.805927	2.078510713	0.852739935	2	0.388973	NW_003613251_37928_star	12.8948392	0	3.796477232	0	20	1	hsa-miR-4671-3p	2.686425	0.805927	1.882222339	0.852739935	1.736966	0.639545
chr7_16933_mature	3.223709795	0.805927	2.078510713	0.852739935	2	0.388973	chr10_23462_mature	12.8948392	0	3.796477232	0	20	1	chr16_34304_star	2.686425	0.805927	1.882222339	0.852739935	1.736966	0.639545
chr11_24756_mature	3.223709795	0.805927	2.078510713	0.852739935	2	0.388973	NW_003537967_37423_mature	12.8948392	0	3.796477232	0	20	1	chr13_28125_star	2.686425	0.805927	1.882222339	0.852739935	1.736966	0.639545
bta-miR-2309	3.223709795	0.805927	2.078510713	0.852739935	2	0.388973	hsa-miR-1228-5p	12.8948392	0	3.796477232	0	20	1	chr13_28221_mature	2.686425	0.805927	1.882222339	0.852739935	1.736966	0.639545
rno-miR-582-3p	3.223709795	0.805927	2.078510713	0.852739935	2	0.388973	chr5_12332_star	12.8948392	0	3.796477232	0	20	1	chr3_7781_star	2.686425	0.805927	1.882222339	0.852739935	1.736966	0.639545
chr7_15912_mature	3.223709795	0.805927	2.078510713	0.852739935	2	0.388973	mmu-miR-692	12.8948392	0	3.796477232	0	20	1	chr14_30387_star	2.686425	0.805927	1.882222339	0.852739935	1.736966	0.639545
chr2_4523_mature	3.223709795	0.805927	2.078510713	0.852739935	2	0.388973	mmu-miR-3107-3p	12.8948392	0	3.796477232	0	20	1	bta-miR-2399-3p	2.686425	0.805927	1.882222339	0.852739935	1.736966	0.639545
hsa-miR-450b-3p	3.223709795	0.805927	2.078510713	0.852739935	2	0.388973	chr15_32263_mature	12.8948392	0	3.796477232	0	20	1	chr5_11356_star	2.686425	0.805927	1.882222339	0.852739935	1.736966	0.639545
chr14_29836_mature	3.223709795	0.805927	2.078510713	0.852739935	2	0.388973	chr17_34956_star	12.8948392	0	3.796477232	0	20	1	NW_003538870_38317_star	2.686425	0.805927	1.882222339	0.852739935	1.736966	0.639545
NW_003538201_37612_star	3.223709795	0.805927	2.078510713	0.852739935	2	0.388973	chr4_10381_star	12.8948392	0	3.796477232	0	20	1	chr5_12411_star	2.686425	0.805927	1.882222339	0.852739935	1.736966	0.639545
hsa-miR-4651	3.223709795	0.805927	2.078510713	0.852739935	2	0.388973	chr3_7118_mature	12.8948392	0	3.796477232	0	20	1	rno-miR-632	2.686425	0.805927	1.882222339	0.852739935	1.736966	0.639545
hsa-miR-4688	3.223709795	0.805927	2.078510713	0.852739935	2	0.388973	chr13_28676_mature	12.8948392	0	3.796477232	0	20	1	hsa-miR-3909	2.686425	0.805927	1.882222339	0.852739935	1.736966	0.639545
mmu-miR-763	3.223709795	0.805927	2.078510713	0.852739935	2	0.388973	NW_003539350_38744_mature	12.8948392	0	3.796477232	0	20	1	chr7_16183_star	2.686425	0.805927	1.882222339	0.852739935	1.736966	0.639545
chr6_12838_star	3.223709795	0.805927	2.078510713	0.852739935	2	0.388973	chr5_11699_mature	12.8948392	0	3.796477232	0	20	1	mmu-miR-1904	2.686425	0.805927	1.882222339	0.852739935	1.736966	0.639545
chr6_14296_star	3.223709795	0.805927	2.078510713	0.852739935	2	0.388973	chr4_10721_mature	8.59655945	41.90823	3.262517265	5.423182395	-2.2854	1	chr10_23649_mature	2.686425	0.805927	1.882222339	0.852739935	1.736966	0.639545
chr13_27012_mature	3.223709795	0.805927	2.078510713	0.852739935	2	0.388973	chr5_12375_star	2.14913986	27.40153	1.654957834	4.827896911	-3.67243	1	mmu-miR-1942	2.686425	0.805927	1.882222339	0.852739935	1.736966	0.639545
chr11_24779_star	3.223709795	0.805927	2.078510713	0.852739935	2	0.388973	chr6_14945_mature	2.14913986	27.40153	1.654957834	4.827896911	-3.67243	1	chr10_24235_mature	2.686425	0.805927	1.882222339	0.852739935	1.736966	0.639545
hsa-miR-4502	3.223709795	0.805927	2.078510713	0.852739935	2	0.388973	chr3_8310_mature	6.44741959	37.07266	2.896740642	5.250683565	-2.52356	1	mmu-miR-1928	2.686425	0.805927	1.882222339	0.852739935	1.736966	0.639545
chr10_23553_star	3.223709795	0.805927	2.078510713	0.852739935	2	0.388973	NW_003538949_38373_mature	6.44741959	37.07266	2.896740642	5.250683565	-2.52356	1	mmu-miR-1962	2.686425	0.805927	1.882222339	0.852739935	1.736966	0.639545
mmu-miR-1231-3p	3.223709795	0.805927	2.078510713	0.852739935	2	0.388973	NW_003541040_39849_mature	38.6845175	10.47706	5.310504361	3.520680821	1.884523	1	hsa-miR-4716-3p	2.686425	0.805927	1.882222339	0.852739935	1.736966	0.639545
hsa-miR-921	3.223709795	0.805927	2.078510713	0.852739935	2	0.388973	chr12_25849_star@@mmu-miR-5112	47.281077	105.5765	5.593385953	6.735745493	-1.15895	1	chr12_26158_mature	2.686425	0.805927	1.882222339	0.852739935	1.736966	0.639545
NW_003538342_37742_star	3.223709795	0.805927	2.078510713	0.852739935	2	0.388973	chr6_14477_star	4.29827973	32.2371	2.405524014	5.054722516	-2.90689	1	mmu-miR-327	2.686425	0.805927	1.882222339	0.852739935	1.736966	0.639545
chr15_31849_mature	2.149139864	7.253347	1.654957834	3.044979305	-1.75489	0.389175	chr17_35575_mature	30.0879581	6.44742	4.958283956	2.896740642	2.222392	1	hsa-miR-6499-5p	2.686425	0.805927	1.882222339	0.852739935	1.736966	0.639545
chr7_16274_star	2.149139864	7.253347	1.654957834	3.044979305	-1.75489	0.389175	chr3_8648_mature	8.59655945	41.1023	3.262517265	5.395827139	-2.25739	1	chr13_26857_star	2.686425	0.805927	1.882222339	0.852739935	1.736966	0.639545
chr1_3844_mature	2.149139864	7.253347	1.654957834	3.044979305	-1.75489	0.389175	chr6_13930_mature	27.9388182	5.641492	4.854934103	2.731507408	2.308122	1	hsa-miR-147a	2.686425	0.805927	1.882222339	0.852739935	1.736966	0.639545
chr1_1317_star	2.149139864	7.253347	1.654957834	3.044979305	-1.75489	0.389175	chr14_30937_mature	27.9388182	5.641492	4.854934103	2.731507408	2.308122	1	bta-miR-181d	2.686425	0.805927	1.882222339	0.852739935	1.736966	0.639545
chr2_5556_star	2.149139864	7.253347	1.654957834	3.044979305	-1.75489	0.389175	chr13_28297_star@@bta-miR-26c	51.5793567	17.7304	5.716424587	4.227310103	1.540568	1	chr3_6872_star	2.686425	0.805927	1.882222339	0.852739935	1.736966	0.639545
hsa-miR-5004-5p	2.149139864	7.253347	1.654957834	3.044979305	-1.75489	0.389175	chr10_23904_mature	2.14913986	26.59561	1.654957834	4.786366653	-3.62936	1	chr1_3090_star	2.686425	0.805927	1.882222339	0.852739935	1.736966	0.639545
chr16_34837_mature	2.149139864	7.253347	1.654957834	3.044979305	-1.75489	0.389175	chr9_19140_star	2.14913986	26.59561	1.654957834	4.786366653	-3.62936	1	chr2_4081_mature	2.686425	0.805927	1.882222339	0.852739935	1.736966	0.639545
chr12_26266_star	2.149139864	7.253347	1.654957834	3.044979305	-1.75489	0.389175	chr1_291_mature	2.14913986	26.59561	1.654957834	4.786366653	-3.62936	1	hsa-miR-5191	2.686425	0.805927	1.882222339	0.852739935	1.736966	0.639545
chr15_33558_mature	2.149139864	7.253347	1.654957834	3.044979305	-1.75489	0.389175	chr2_4631_mature	139.694091	75.75718	7.136417929	6.262229808	0.882816	1	chr6_13376_star	2.686425	0.805927	1.882222339	0.852739935	1.736966	0.639545
chr14_30762_star	2.149139864	7.253347	1.654957834	3.044979305	-1.75489	0.389175	chr4_9018_star	15.043979	0.805927	4.003960082	0.852739935	4.222392	1	hsa-miR-3934-5p	2.686425	0.805927	1.882222339	0.852739935	1.736966	0.639545
chr16_34016_mature	2.149139864	7.253347	1.654957834	3.044979305	-1.75489	0.389175	NW_003539115_38545_mature	15.043979	0.805927	4.003960082	0.852739935	4.222392	1	chr9_20117_star	2.686425	0.805927	1.882222339	0.852739935	1.736966	0.639545
chr4_10283_star	2.149139864	7.253347	1.654957834	3.044979305	-1.75489	0.389175	mmu-miR-1928	15.043979	0.805927	4.003960082	0.852739935	4.222392	1	chr7_15098_star	2.686425	0.805927	1.882222339	0.852739935	1.736966	0.639545
chr5_12382_mature	2.149139864	7.253347	1.654957834	3.044979305	-1.75489	0.389175	chr12_26408_mature	15.043979	0.805927	4.003960082	0.852739935	4.222392	1	mmu-miR-6392-3p	2.686425	0.805927	1.882222339	0.852739935	1.736966	0.639545
chr15_33029_star	2.149139864	7.253347	1.654957834	3.044979305	-1.75489	0.389175	chr14_30669_mature	15.043979	0.805927	4.003960082	0.852739935	4.222392	1	chr2_4567_mature	2.686425	0.805927	1.882222339	0.852739935	1.736966	0.639545
chr1_1120_mature	2.149139864	7.253347	1.654957834	3.044979305	-1.75489	0.389175	NW_003540770_39675_star	15.043979	0.805927	4.003960082	0.852739935	4.222392	1	chr13_27087_star	2.686425	0.805927	1.882222339	0.852739935	1.736966	0.639545
chr1_3743_star	2.149139864	7.253347	1.654957834	3.044979305	-1.75489	0.389175	chr4_10313_mature	15.043979	0.805927	4.003960082	0.852739935	4.222392	1	chrx_21746_mature	2.686425	0.805927	1.882222339	0.852739935	1.736966	0.639545
chr9_19819_mature	2.149139864	7.253347	1.654957834	3.044979305	-1.75489	0.389175	NW_003537574_37016_mature	15.043979	0.805927	4.003960082	0.852739935	4.222392	1	rno-miR-466b-1-3p	2.686425	0.805927	1.882222339	0.852739935	1.736966	0.639545
bta-miR-2393	2.149139864	7.253347	1.654957834	3.044979305	-1.75489	0.389175	chr5_12405_mature	15.043979	0.805927	4.003960082	0.852739935	4.222392	1	chr8_17349_mature	2.686425	0.805927	1.882222339	0.852739935	1.736966	0.639545
NW_003541128_39926_star	2.149139864	7.253347	1.654957834	3.044979305	-1.75489	0.389175	chr13_29176_mature	15.043979	0.805927	4.003960082	0.852739935	4.222392	1	chrx_22324_mature	2.686425	0.805927	1.882222339	0.852739935	1.736966	0.639545
hsa-miR-4329	2.149139864	7.253347	1.654957834	3.044979305	-1.75489	0.389175	chr13_28165_mature	15.043979	0.805927	4.003960082	0.852739935	4.222392	1	chr14_31026_star	2.686425	0.805927	1.882222339	0.852739935	1.736966	0.639545
chr4_9069_mature	2.149139864	7.253347	1.654957834	3.044979305	-1.75489	0.389175	chr18_35826_star	15.043979	0.805927	4.003960082	0.852739935	4.222392	1	chr4_9939_star	2.686425	0.805927	1.882222339	0.852739935	1.736966	0.639545
chr15_31926_mature	15.04397905	4.029637	4.003960082	2.330454351	1.900464	0.389192	mmu-miR-3960	15.043979	0.805927	4.003960082	0.852739935	4.222392	1	chrx_22424_star	2.686425	0.805927	1.882222339	0.852739935	1.736966	0.639545
chr7_15986_mature	15.04397905	4.029637	4.003960082	2.330454351	1.900464	0.389192	chr5_11160_mature	15.043979	0.805927	4.003960082	0.852739935	4.222392	1	chr3_6639_star	2.686425	0.805927	1.882222339	0.852739935	1.736966	0.639545
chr7_16088_mature	15.04397905	4.029637	4.003960082	2.330454351	1.900464	0.389192	NW_003612983_36666_mature	68.7724756	135.3958	6.124586119	7.091655531	-0.97728	1	chrx_23205_star	2.686425	0.805927	1.882222339	0.852739935	1.736966	0.639545
chr2_5077_star	1.074569932	4.029637	1.05281229	2.330454351	-1.90689	0.389209	chr5_10851_mature	15.043979	0.805927	4.003960082	0.852739935	4.222392	1	NW_003613379_38761_mature	2.686425	0.805927	1.882222339	0.852739935	1.736966	0.639545
chr2_4423_star	1.074569932	4.029637	1.05281229	2.330454351	-1.90689	0.389209	chr14_30124_mature	15.043979	0.805927	4.003960082	0.852739935	4.222392	1	chr15_31929_mature	2.686425	0.805927	1.882222339	0.852739935	1.736966	0.639545
chr5_11269_star	1.074569932	4.029637	1.05281229	2.330454351	-1.90689	0.389209	chr14_30639_mature	15.043979	0.805927	4.003960082	0.852739935	4.222392	1	hsa-miR-888-5p	2.686425	0.805927	1.882222339	0.852739935	1.736966	0.639545
chr8_17840_star	1.074569932	4.029637	1.05281229	2.330454351	-1.90689	0.389209	chr7_15747_star	15.043979	0.805927	4.003960082	0.852739935	4.222392	1	chr7_17101_mature	2.686425	0.805927	1.882222339	0.852739935	1.736966	0.639545
chr12_26332_mature	1.074569932	4.029637	1.05281229	2.330454351	-1.90689	0.389209	chr14_31281_mature	25.7896784	4.835565	4.743605355	2.544872269	2.415037	1	hsa-miR-4650-5p	2.686425	0.805927	1.882222339	0.852739935	1.736966	0.639545
chr8_18596_star	1.074569932	4.029637	1.05281229	2.330454351	-1.90689	0.389209	chr7_16048_star	25.7896784	4.835565	4.743605355	2.544872269	2.415037	1	chr15_32446_star	2.686425	0.805927	1.882222339	0.852739935	1.736966	0.639545
chr10_24049_star	1.074569932	4.029637	1.05281229	2.330454351	-1.90689	0.389209	chr2_6574_star	25.7896784	4.835565	4.743605355	2.544872269	2.415037	1	chr2_4523_star	2.686425	0.805927	1.882222339	0.852739935	1.736966	0.639545
hsa-miR-4782-3p	1.074569932	4.029637	1.05281229	2.330454351	-1.90689	0.389209	chr4_9934_mature	25.7896784	4.835565	4.743605355	2.544872269	2.415037	1	mmu-miR-3962	2.686425	0.805927	1.882222339	0.852739935	1.736966	0.639545
chr7_16174_mature	1.074569932	4.029637	1.05281229	2.330454351	-1.90689	0.389209	chr4_8771_star	8.59655945	40.29637	3.262517265	5.367943153	-2.22882	1	chr13_29021_star	2.686425	0.805927	1.882222339	0.852739935	1.736966	0.639545
chr2_4519_star	1.074569932	4.029637	1.05281229	2.330454351	-1.90689	0.389209	chr3_7847_star	23.6405385	4.029637	4.62296188	2.330454351	2.552541	1	chr1_916_star	2.686425	0.805927	1.882222339	0.852739935	1.736966	0.639545
hsa-miR-548g-3p	1.074569932	4.029637	1.05281229	2.330454351	-1.90689	0.389209	chr6_12854_mature	23.6405385	4.029637	4.62296188	2.330454351	2.552541	1	chr1_608_mature	2.686425	0.805927	1.882222339	0.852739935	1.736966	0.639545
chr9_19988_mature	1.074569932	4.029637	1.05281229	2.330454351	-1.90689	0.389209	NW_003538949_38371_mature	23.6405385	4.029637	4.62296188	2.330454351	2.552541	1	chr1_1062_star	2.686425	0.805927	1.882222339	0.852739935	1.736966	0.639545
chr6_12901_mature	1.074569932	4.029637	1.05281229	2.330454351	-1.90689	0.389209	chr14_29861_mature	17.1931189	1.611855	4.185320985	1.38507475	3.415037	1	chr3_7684_star	2.686425	0.805927	1.882222339	0.852739935	1.736966	0.639545
chr13_26769_mature	1.074569932	4.029637	1.05281229	2.330454351	-1.90689	0.389209	chr8_17239_mature	17.1931189	1.611855	4.185320985	1.38507475	3.415037	1	NW_003613217_37698_mature	2.686425	0.805927	1.882222339	0.852739935	1.736966	0.639545
chr9_19972_mature	1.074569932	4.029637	1.05281229	2.330454351	-1.90689	0.389209	chr9_20370_mature	17.1931189	1.611855	4.185320985	1.38507475	3.415037	1	chr4_9973_star	2.686425	0.805927	1.882222339	0.852739935	1.736966	0.639545
chr9_19300_mature	1.074569932	4.029637	1.05281229	2.330454351	-1.90689	0.389209	chrx_22145_mature	17.1931189	1.611855	4.185320985	1.38507475	3.415037	1	chr15_32289_star	2.686425	0.805927	1.882222339	0.852739935	1.736966	0.639545
chr14_29829_mature	1.074569932	4.029637	1.05281229	2.330454351	-1.90689	0.389209	chr13_26857_mature	17.1931189	1.611855	4.185320985	1.38507475	3.415037	1	chr7_16174_star	2.686425	0.805927	1.882222339	0.852739935	1.736966	0.639545
chr14_31584_star	1.074569932	4.029637	1.05281229	2.330454351	-1.90689	0.389209	chr11_24906_star	17.1931189	1.611855	4.185320985	1.38507475	3.415037	1	chr2_4310_star	2.686425	0.805927	1.882222339	0.852739935	1.736966	0.639545
chr5_11410_star	1.074569932	4.029637	1.05281229	2.330454351	-1.90689	0.389209	hsa-miR-4709-5p	17.1931189	1.611855	4.185320985	1.38507475	3.415037	1	chr15_32700_star	2.686425	0.805927	1.882222339	0.852739935	1.736966	0.639545
chr11_24910_star	1.074569932	4.029637	1.05281229	2.330454351	-1.90689	0.389209	chr14_31116_mature	17.1931189	1.611855	4.185320985	1.38507475	3.415037	1	chr2_6095_star@@hsa-miR-513a-	2.686425	0.805927	1.882222339	0.852739935	1.736966	0.639545
chr8_18251_star	1.074569932	4.029637	1.05281229	2.330454351	-1.90689	0.389209	chr6_12855_mature	21.4913986	3.22371	4.491301473	2.078510713	2.736966	1	mmu-miR-3060-3p	2.686425	0.805927	1.882222339	0.852739935	1.736966	0.639545
chr7_15734_star	1.074569932	4.029637	1.05281229	2.330454351	-1.90689	0.389209	chr3_6912_star	21.4913986	3.22371	4.491301473	2.078510713	2.736966	1	chr3_7764_star	2.686425	0.805927	1.882222339	0.852739935	1.736966	0.639545
chr11_24391_star	1.074569932	4.029637	1.05281229	2.330454351	-1.90689	0.389209	chr6_14257_mature	21.4913986	3.22371	4.491301473	2.078510713	2.736966	1	chr3_6687_star	2.686425	0.805927	1.882222339	0.852739935	1.736966	0.639545
rno-miR-329-5p	1.074569932	4.029637	1.05281229	2.330454351	-1.90689	0.389209	chr16_34188_star	21.4913986	3.22371	4.491301473	2.078510713	2.736966	1	chr9_21053_star	2.686425	0.805927	1.882222339	0.852739935	1.736966	0.639545
chr3_8007_mature	1.074569932	4.029637	1.05281229	2.330454351	-1.90689	0.389209	chr13_28624_mature	21.4913986	3.22371	4.491301473	2.078510713	2.736966	1	chr17_35675_star	2.686425	0.805927	1.882222339	0.852739935	1.736966	0.639545
chr17_34920_star	1.074569932	4.029637	1.05281229	2.330454351	-1.90689	0.389209	hsa-miR-3676-3p	21.4913986	3.22371	4.491301473	2.078510713	2.736966	1	chr6_12737_mature	2.686425	0.805927	1.882222339	0.852739935	1.736966	0.639545
chr17_34868_star	1.074569932	4.029637	1.05281229	2.330454351	-1.90689	0.389209	chr3_7643_star	21.4913986	3.22371	4.491301473	2.078510713	2.736966	1	chr2_6194_star	2.686425	0.805927	1.882222339	0.852739935	1.736966	0.639545
chr2_5587_star	1.074569932	4.029637	1.05281229	2.330454351	-1.90689	0.389209	chr7_16583_mature	21.4913986	3.22371	4.491301473	2.078510713	2.736966	1	chr7_15778_star	2.686425	0.805927	1.882222339	0.852739935	1.736966	0.639545
NW_003540745_39661_star	1.074569932	4.029637	1.05281229	2.330454351	-1.90689	0.389209	chr1_3894_mature@@bta-miR-219-3p	21.4913986	3.22371	4.491301473	2.078510713	2.736966	1	NW_003539221_38657_mature	2.686425	0.805927	1.882222339	0.852739935	1.736966	0.639545
chr18_36061_star	1.074569932	4.029637	1.05281229	2.330454351	-1.90689	0.389209	chr3_7274_mature	21.4913986	3.22371	4.491301473	2.078510713	2.736966	1	chr7_17208_star	2.686425	0.805927	1.882222339	0.852739935	1.736966	0.639545
chr6_13257_star	1.074569932	4.029637	1.05281229	2.330454351	-1.90689	0.389209	chr16_34823_star	19.3422588	2.417782	4.346407978	1.773060525	3	1	chr15_31817_star	2.686425	0.805927	1.882222339	0.852739935	1.736966	0.639545
chr11_24782_mature	1.074569932	4.029637	1.05281229	2.330454351	-1.90689	0.389209	chr12_26075_mature	19.3422588	2.417782	4.346407978	1.773060525	3	1	chr4_8784_mature	2.686425	0.805927	1.882222339	0.852739935	1.736966	0.639545
mmu-miR-138-1-3p	1.074569932	4.029637	1.05281229	2.330454351	-1.90689	0.389209	chr6_14751_mature	6.44741959	35.46081	2.896740642	5.188274618	-2.45943	1	chr13_27739_star	2.686425	0.805927	1.882222339	0.852739935	1.736966	0.639545
chr8_17522_star	1.074569932	4.029637	1.05281229	2.330454351	-1.90689	0.389209	chr9_19128_mature	19.3422588	2.417782	4.346407978	1.773060525	3	1	bta-miR-2457	2.686425	0.805927	1.882222339	0.852739935	1.736966	0.639545
chr4_9960_star	1.074569932	4.029637	1.05281229	2.330454351	-1.90689	0.389209	chr17_35504_star	19.3422588	2.417782	4.346407978	1.773060525	3	1	chr4_10208_star	2.686425	0.805927	1.882222339	0.852739935	1.736966	0.639545
rno-miR-336-3p	1.074569932	4.029637	1.05281229	2.330454351	-1.90689	0.389209	NW_003538520_37951_mature	19.3422588	2.417782	4.346407978	1.773060525	3	1	hsa-miR-604	2.686425	0.805927	1.882222339	0.852739935	1.736966	0.639545
chr2_4183_mature	1.074569932	4.029637	1.05281229	2.330454351	-1.90689	0.389209	chr14_31221_mature	19.3422588	2.417782	4.346407978	1.773060525	3	1	hsa-miR-633	2.686425	0.805927	1.882222339	0.852739935	1.736966	0.639545
mmu-miR-143-3p	1.074569932	4.029637	1.05281229	2.330454351	-1.90689	0.389209	chr8_17786_mature	2.14913986	25.78968	1.654957834	4.743605355	-3.58496	1	bta-miR-2395	2.686425	0.805927	1.882222339	0.852739935	1.736966	0.639545
hsa-miR-4295	1.074569932	4.029637	1.05281229	2.330454351	-1.90689	0.389209	chrx_21689_mature	27.9388182	73.3394	4.854934103	6.216055097	-1.39232	1	mmu-miR-466b-5p	2.686425	0.805927	1.882222339	0.852739935	1.736966	0.639545
chr3_8070_star	1.074569932	4.029637	1.05281229	2.330454351	-1.90689	0.389209	chr14_30679_star	4.29827973	30.62524	2.405524014	4.983004663	-2.83289	1	chr16_34408_star	2.686425	0.805927	1.882222339	0.852739935	1.736966	0.639545
chr3_7353_star	1.074569932	4.029637	1.05281229	2.330454351	-1.90689	0.389209	chr9_19965_mature	4.29827973	30.62524	2.405524014	4.983004663	-2.83289	1	chr5_11345_star	2.686425	0.805927	1.882222339	0.852739935	1.736966	0.639545
chr6_13229_star	1.074569932	4.029637	1.05281229	2.330454351	-1.90689	0.389209	NW_003539467_38882_mature	8.59655945	39.49044	3.262517265	5.339509593	-2.19967	1	mmu-miR-881-5p	2.686425	0.805927	1.882222339	0.852739935	1.736966	0.639545
NW_003539506_38905_star	1.074569932	4.029637	1.05281229	2.330454351	-1.90689	0.389209	chr18_36647_mature	30.0879581	7.253347	4.958283956	3.044979305	2.052467	1	chr6_14216_mature	2.686425	0.805927	1.882222339	0.852739935	1.736966	0.639545
chr8_17657_star	1.074569932	4.029637	1.05281229	2.330454351	-1.90689	0.389209	hsa-miR-4750-5p	30.0879581	7.253347	4.958283956	3.044979305	2.052467	1	hsa-miR-550a-3-5p	2.686425	0.805927	1.882222339	0.852739935	1.736966	0.639545
chrx_21502_star	1.074569932	4.029637	1.05281229	2.330454351	-1.90689	0.389209	hsa-miR-659-5p	30.0879581	7.253347	4.958283956	3.044979305	2.052467	1	rno-miR-3580-5p	2.686425	0.805927	1.882222339	0.852739935	1.736966	0.639545
chr13_28822_star	1.074569932	4.029637	1.05281229	2.330454351	-1.90689	0.389209	chr6_13311_star	2.14913986	24.98375	1.654957834	4.699537803	-3.53916	1	chr14_30644_mature	2.686425	0.805927	1.882222339	0.852739935	1.736966	0.639545
bta-miR-2308	1.074569932	4.029637	1.05281229	2.330454351	-1.90689	0.389209	chr9_19632_star	2.14913986	24.98375	1.654957834	4.699537803	-3.53916	1	mmu-miR-1947-5p	2.686425	0.805927	1.882222339	0.852739935	1.736966	0.639545
NW_003538396_37820_mature	1.074569932	4.029637	1.05281229	2.330454351	-1.90689	0.389209	chr9_19738_mature	2.14913986	24.98375	1.654957834	4.699537803	-3.53916	1	chr1_1609_star	2.686425	0.805927	1.882222339	0.852739935	1.736966	0.639545
chr4_10263_mature	1.074569932	4.029637	1.05281229	2.330454351	-1.90689	0.389209	NW_003613218_37700_mature	58.0267763	22.56597	5.883297648	4.558633073	1.36257	1	chr9_20086_mature	2.686425	0.805927	1.882222339	0.852739935	1.736966	0.639545
chr2_4882_star	1.074569932	4.029637	1.05281229	2.330454351	-1.90689	0.389209	rno-miR-3583-3p	4.29827973	29.81932	2.405524014	4.94576292	-2.79442	1	rno-miR-3593-5p	2.686425	0.805927	1.882222339	0.852739935	1.736966	0.639545
chr6_14217_mature	1.074569932	4.029637	1.05281229	2.330454351	-1.90689	0.389209	chr1_3752_mature	8.59655945	38.68452	3.262517265	5.310504361	-2.16993	1	chr13_29245_star	2.686425	0.805927	1.882222339	0.852739935	1.736966	0.639545
chr8_18856_star	1.074569932	4.029637	1.05281229	2.330454351	-1.90689	0.389209	hsa-miR-4800-3p	88.1147344	41.90823	6.477592084	5.423182395	1.07215	1	chr18_36317_mature	2.686425	0.805927	1.882222339	0.852739935	1.736966	0.639545
chr15_33061_mature	1.074569932	4.029637	1.05281229	2.330454351	-1.90689	0.389209	chr14_30949_mature	10.7456993	0	3.554060706	0	20	1	chr2_4676_star	2.686425	0.805927	1.882222339	0.852739935	1.736966	0.639545
rno-miR-2985	1.074569932	4.029637	1.05281229	2.330454351	-1.90689	0.389209	chr14_31496_mature	10.7456993	0	3.554060706	0	20	1	mmu-miR-703	2.686425	0.805927	1.882222339	0.852739935	1.736966	0.639545
chr18_36430_mature	1.074569932	4.029637	1.05281229	2.330454351	-1.90689	0.389209	NW_003613192_37588_mature	10.7456993	0	3.554060706	0	20	1	chr8_17864_star	2.686425	0.805927	1.882222339	0.852739935	1.736966	0.639545
chr10_23316_mature	1.074569932	4.029637	1.05281229	2.330454351	-1.90689	0.389209	chr3_7678_mature	10.7456993	0	3.554060706	0	20	1	chr12_26044_mature	2.686425	0.805927	1.882222339	0.852739935	1.736966	0.639545
chr9_20399_star	1.074569932	4.029637	1.05281229	2.330454351	-1.90689	0.389209	chr5_12300_star	10.7456993	0	3.554060706	0	20	1	hsa-miR-4696	2.686425	0.805927	1.882222339	0.852739935	1.736966	0.639545
chr15_32695_star	5.372849659	1.611855	2.671938627	1.38507475	1.736966	0.389952	chr13_27317_mature	10.7456993	0	3.554060706	0	20	1	chr10_24245_mature	2.686425	0.805927	1.882222339	0.852739935	1.736966	0.639545
chr3_8648_star	5.372849659	1.611855	2.671938627	1.38507475	1.736966	0.389952	rno-miR-25-5p	10.7456993	0	3.554060706	0	20	1	bta-miR-2419-5p	2.686425	0.805927	1.882222339	0.852739935	1.736966	0.639545
chr11_25386_star	5.372849659	1.611855	2.671938627	1.38507475	1.736966	0.389952	chr2_6568_mature	10.7456993	0	3.554060706	0	20	1	mmu-miR-3473d	2.686425	0.805927	1.882222339	0.852739935	1.736966	0.639545
hsa-miR-596	5.372849659	1.611855	2.671938627	1.38507475	1.736966	0.389952	chr2_5921_mature	10.7456993	0	3.554060706	0	20	1	bta-miR-502a	2.686425	0.805927	1.882222339	0.852739935	1.736966	0.639545
chr5_10910_mature	5.372849659	1.611855	2.671938627	1.38507475	1.736966	0.389952	chr9_19422_mature	10.7456993	0	3.554060706	0	20	1	bta-miR-6123	2.686425	0.805927	1.882222339	0.852739935	1.736966	0.639545
chr1_2668_star	5.372849659	1.611855	2.671938627	1.38507475	1.736966	0.389952	NW_003537262_36795_mature	10.7456993	0	3.554060706	0	20	1	chr11_24779_star	2.686425	0.805927	1.882222339	0.852739935	1.736966	0.639545
mmu-miR-1906	5.372849659	1.611855	2.671938627	1.38507475	1.736966	0.389952	chr14_29583_mature	10.7456993	0	3.554060706	0	20	1	hsa-miR-4527	2.686425	0.805927	1.882222339	0.852739935	1.736966	0.639545
chr1_2672_mature	5.372849659	1.611855	2.671938627	1.38507475	1.736966	0.389952	chr14_30174_star	10.7456993	0	3.554060706	0	20	1	chr14_30394_mature	2.686425	0.805927	1.882222339	0.852739935	1.736966	0.639545
chr17_35168_mature@@rno-miR-88	5.372849659	1.611855	2.671938627	1.38507475	1.736966	0.389952	chr15_33456_star	10.7456993	0	3.554060706	0	20	1	hsa-miR-4502	2.686425	0.805927	1.882222339	0.852739935	1.736966	0.639545
chr17_35500_star	5.372849659	1.611855	2.671938627	1.38507475	1.736966	0.389952	chr7_16155_mature	10.7456993	0	3.554060706	0	20	1	rno-miR-449c-5p	2.686425	0.805927	1.882222339	0.852739935	1.736966	0.639545
hsa-miR-151b	5.372849659	1.611855	2.671938627	1.38507475	1.736966	0.389952	chr11_25383_mature	10.7456993	0	3.554060706	0	20	1	chr13_27824_star	2.686425	0.805927	1.882222339	0.852739935	1.736966	0.639545
chr8_18571_mature	5.372849659	1.611855	2.671938627	1.38507475	1.736966	0.389952	chr4_9751_mature	10.7456993	0	3.554060706	0	20	1	chr6_12525_mature	2.686425	0.805927	1.882222339	0.852739935	1.736966	0.639545
chr15_32289_mature	5.372849659	1.611855	2.671938627	1.38507475	1.736966	0.389952	chr14_29850_mature	10.7456993	0	3.554060706	0	20	1	chr2_5884_star	2.686425	0.805927	1.882222339	0.852739935	1.736966	0.639545
chr2_6132_star	5.372849659	1.611855	2.671938627	1.38507475	1.736966	0.389952	chr12_26158_star	10.7456993	0	3.554060706	0	20	1	chr16_34306_star	2.686425	0.805927	1.882222339	0.852739935	1.736966	0.639545
chr8_17337_star	5.372849659	1.611855	2.671938627	1.38507475	1.736966	0.389952	chr11_24459_star	10.7456993	0	3.554060706	0	20	1	rno-miR-770-5p	2.686425	0.805927	1.882222339	0.852739935	1.736966	0.639545
NW_003540029_39236_mature	5.372849659	1.611855	2.671938627	1.38507475	1.736966	0.389952	chr9_19911_mature	10.7456993	0	3.554060706	0	20	1	bta-miR-3957	2.14914	5.641492	1.654957834	2.731507408	-1.39232	0.639985
chrx_22553_star	5.372849659	1.611855	2.671938627	1.38507475	1.736966	0.389952	chr18_35974_mature@@bta-miR-592	10.7456993	0	3.554060706	0	20	1	rno-miR-33-3p	2.14914	5.641492	1.654957834	2.731507408	-1.39232	0.639985
chr12_25706_mature	5.372849659	1.611855	2.671938627	1.38507475	1.736966	0.389952	chr3_7065_star	10.7456993	0	3.554060706	0	20	1	chr5_11789_mature	2.14914	5.641492	1.654957834	2.731507408	-1.39232	0.639985
chr13_29005_mature	5.372849659	1.611855	2.671938627	1.38507475	1.736966	0.389952	chr9_21075_star	10.7456993	0	3.554060706	0	20	1	chr7_17039_mature	2.14914	5.641492	1.654957834	2.731507408	-1.39232	0.639985
chr6_13576_star	5.372849659	1.611855	2.671938627	1.38507475	1.736966	0.389952	mmu-miR-700-5p	10.7456993	0	3.554060706	0	20	1	chr11_25303_star	2.14914	5.641492	1.654957834	2.731507408	-1.39232	0.639985
NW_003541083_39896_mature	5.372849659	1.611855	2.671938627	1.38507475	1.736966	0.389952	chr2_4477_mature	10.7456993	0	3.554060706	0	20	1	chr4_9665_mature	2.14914	5.641492	1.654957834	2.731507408	-1.39232	0.639985
chr1_1780_mature	5.372849659	1.611855	2.671938627	1.38507475	1.736966	0.389952	NW_003537879_37346_star	10.7456993	0	3.554060706	0	20	1	chr15_32092_star	2.14914	5.641492	1.654957834	2.731507408	-1.39232	0.639985
bta-miR-3141	5.372849659	1.611855	2.671938627	1.38507475	1.736966	0.389952	chr2_5373_mature	10.7456993	0	3.554060706	0	20	1	chr5_11814_star	2.14914	5.641492	1.654957834	2.731507408	-1.39232	0.639985
chr9_20945_star	5.372849659	1.611855	2.671938627	1.38507475	1.736966	0.389952	chr2_5742_star	10.7456993	0	3.554060706	0	20	1	chr9_19579_star	2.14914	5.641492	1.654957834	2.731507408	-1.39232	0.639985
mmu-miR-3069-3p	5.372849659	1.611855	2.671938627	1.38507475	1.736966	0.389952	chr6_14284_star	10.7456993	0	3.554060706	0	20	1	chr3_8428_star	2.14914	5.641492	1.654957834	2.731507408	-1.39232	0.639985
chr12_25705_mature	5.372849659	1.611855	2.671938627	1.38507475	1.736966	0.389952	bta-miR-2440	10.7456993	0	3.554060706	0	20	1	chr9_19744_star	2.14914	5.641492	1.654957834	2.731507408	-1.39232	0.639985
chr10_23395_mature	5.372849659	1.611855	2.671938627	1.38507475	1.736966	0.389952	chr2_5353_mature	10.7456993	0	3.554060706	0	20	1	chr9_20222_mature	2.14914	5.641492	1.654957834	2.731507408	-1.39232	0.639985
chr5_11550_mature	5.372849659	1.611855	2.671938627	1.38507475	1.736966	0.389952	chr15_33619_star	10.7456993	0	3.554060706	0	20	1	chr5_12232_mature	2.14914	5.641492	1.654957834	2.731507408	-1.39232	0.639985
chr13_27824_mature	7.521989523	34.65488	3.091190278	5.156027656	-2.20387	0.390026	chr9_21068_mature	10.7456993	0	3.554060706	0	20	1	chr15_32320_mature	2.14914	5.641492	1.654957834	2.731507408	-1.39232	0.639985
NW_003613157_37452_mature	7.521989523	34.65488	3.091190278	5.156027656	-2.20387	0.390026	mmu-miR-466k	10.7456993	0	3.554060706	0	20	1	chr3_8243_mature	2.14914	5.641492	1.654957834	2.731507408	-1.39232	0.639985
chr9_20900_mature	5.372849659	21.76004	2.671938627	4.508431259	-2.01792	0.390455	chr1_1820_mature	10.7456993	0	3.554060706	0	20	1	chr7_15474_star	2.14914	5.641492	1.654957834	2.731507408	-1.39232	0.639985
chr1_251_mature	3.223709795	11.28298	2.078510713	3.618589217	-1.80735	0.390851	chr8_18444_mature	10.7456993	0	3.554060706	0	20	1	chr2_3979_star	2.14914	5.641492	1.654957834	2.731507408	-1.39232	0.639985
chr6_13483_mature	3.223709795	11.28298	2.078510713	3.618589217	-1.80735	0.390851	chr12_25473_mature	10.7456993	0	3.554060706	0	20	1	chr3_7678_star	2.14914	5.641492	1.654957834	2.731507408	-1.39232	0.639985
chr14_30277_star	3.223709795	11.28298	2.078510713	3.618589217	-1.80735	0.390851	chr12_26049_mature	10.7456993	0	3.554060706	0	20	1	mmu-miR-2139	2.14914	5.641492	1.654957834	2.731507408	-1.39232	0.639985
chr7_16939_mature	3.223709795	11.28298	2.078510713	3.618589217	-1.80735	0.390851	chr18_36298_star	10.7456993	0	3.554060706	0	20	1	chr6_13311_star	6.44742	24.98375	2.896740642	4.699537803	-1.9542	0.640575
chr18_36225_star	3.223709795	11.28298	2.078510713	3.618589217	-1.80735	0.390851	hsa-miR-940	10.7456993	0	3.554060706	0	20	1	chr14_30730_mature	9.133844	3.22371	3.34110968	2.078510713	1.5025	0.640593
ssc-miR-532-3p	1912.734479	132.1721	10.90217496	7.057148072	3.855147	0.390902	chr10_24226_star	10.7456993	0	3.554060706	0	20	1	chr5_11343_mature	9.133844	3.22371	3.34110968	2.078510713	1.5025	0.640593
NW_003613395_38900_mature	4.298279727	16.11855	2.405524014	4.097488514	-1.90689	0.391384	chr6_14575_mature	66.6233358	28.20746	6.079449279	4.868265031	1.239951	1	ssc-miR-4339	9.133844	3.22371	3.34110968	2.078510713	1.5025	0.640593
chr17_35778_mature	4.298279727	16.11855	2.405524014	4.097488514	-1.90689	0.391384	chr13_26618_mature	6.44741959	33.84895	2.896740642	5.123043401	-2.39232	1	chr2_5776_mature	9.133844	3.22371	3.34110968	2.078510713	1.5025	0.640593
chr6_13361_star	4.298279727	16.11855	2.405524014	4.097488514	-1.90689	0.391384	NW_003539007_38439_mature	6.44741959	33.84895	2.896740642	5.123043401	-2.39232	1	chr17_35096_star	12.35755	4.029637	3.739583968	2.330454351	1.616671	0.640739
chr7_15380_star	4.298279727	16.11855	2.405524014	4.097488514	-1.90689	0.391384	chr6_14220_mature	6.44741959	33.84895	2.896740642	5.123043401	-2.39232	1	chr2_5456_star	1.611855	0	1.38507475	0	20	0.641553
chr8_17371_star	4.298279727	16.11855	2.405524014	4.097488514	-1.90689	0.391384	chr3_7259_mature	6.44741959	33.84895	2.896740642	5.123043401	-2.39232	1	NW_003540337_39426_star	1.611855	0	1.38507475	0	20	0.641553
chr8_18896_mature	24.71510843	194.2285	4.684544332	7.609019979	-2.97429	0.392342	chr3_8248_star	6.44741959	33.84895	2.896740642	5.123043401	-2.39232	1	hsa-miR-300	1.611855	0	1.38507475	0	20	0.641553
NW_003613364_38713_mature	32.23709795	7.253347	5.054722516	3.044979305	2.152003	0.392815	NW_003539279_38690_mature	2.14913986	24.17782	1.654957834	4.654081667	-3.49185	1	hsa-miR-325	1.611855	0	1.38507475	0	20	0.641553
chr3_8076_mature	6.447419591	27.40153	2.896740642	4.827896911	-2.08746	0.394055	bta-miR-199c	2.14913986	24.17782	1.654957834	4.654081667	-3.49185	1	mmu-miR-28b	1.611855	0	1.38507475	0	20	0.641553
chr7_17120_mature	7.521989523	33.84895	3.091190278	5.123043401	-2.16993	0.394944	chr10_23377_mature	2.14913986	24.17782	1.654957834	4.654081667	-3.49185	1	chr7_16628_star	1.611855	0	1.38507475	0	20	0.641553
chr9_19522_mature	40.83365741	406.9934	5.38659223	8.672401869	-3.31717	0.395129	chr16_33881_mature	2.14913986	24.17782	1.654957834	4.654081667	-3.49185	1	chr14_31443_star	1.611855	0	1.38507475	0	20	0.641553
chr8_18895_mature	22.56596857	167.6329	4.558633073	7.397742301	-2.89308	0.395638	chrx_22644_star	2.14913986	24.17782	1.654957834	4.654081667	-3.49185	1	rno-miR-214-5p	1.611855	0	1.38507475	0	20	0.641553
chr1_182_star	22.56596857	5.641492	4.558633073	2.731507408	2	0.396241	hsa-miR-4273	2.14913986	24.17782	1.654957834	4.654081667	-3.49185	1	chrx_22663_star	1.611855	0	1.38507475	0	20	0.641553
chr7_15107_mature	22.56596857	5.641492	4.558633073	2.731507408	2	0.396241	chrx_22414_mature	8.59655945	37.87859	3.262517265	5.280903997	-2.13955	1	chr9_20112_star	1.611855	0	1.38507475	0	20	0.641553
chr6_13158_mature	155.8126401	22.56597	7.292898044	4.558633073	2.787592	0.396959	chr13_27562_mature	4.29827973	29.01339	2.405524014	4.907534286	-2.75489	1	hsa-miR-548d-5p	1.611855	0	1.38507475	0	20	0.641553
chr8_18647_star	18.26768884	4.835565	4.268111626	2.544872269	1.917538	0.397332	chr2_5550_mature	4.29827973	29.01339	2.405524014	4.907534286	-2.75489	1	chr2_5312_star	1.611855	0	1.38507475	0	20	0.641553
chr14_30089_mature	5.372849659	20.95411	2.671938627	4.456419386	-1.96347	0.397431	chr15_33643_mature	4.29827973	29.01339	2.405524014	4.907534286	-2.75489	1	hsa-miR-125b-5p	1.611855	0	1.38507475	0	20	0.641553
chr12_25530_mature	19.34225877	132.1721	4.346407978	7.057148072	-2.77259	0.397957	bta-miR-6517	38.6845175	12.08891	5.310504361	3.710273245	1.678072	1	hsa-miR-1324	1.611855	0	1.38507475	0	20	0.641553
chr1_3365_mature	19.34225877	132.1721	4.346407978	7.057148072	-2.77259	0.397957	chr17_35424_mature	137.544951	77.36904	7.114210328	6.292211829	0.830075	1	NW_003537225_36780_star	1.611855	0	1.38507475	0	20	0.641553
chr11_24517_mature	16.11854898	100.7409	4.097488514	6.668756392	-2.64386	0.398338	rno-miR-24-3p	42.9827973	14.50669	5.458867456	3.954819241	1.567041	1	hsa-miR-6716-3p	1.611855	0	1.38507475	0	20	0.641553
chr7_15773_star	10.74569932	3.22371	3.554060706	2.078510713	1.736966	0.39904	chr13_29245_mature	42.9827973	14.50669	5.458867456	3.954819241	1.567041	1	rno-miR-551b-5p	1.611855	0	1.38507475	0	20	0.641553
chr14_29526_mature	10.74569932	3.22371	3.554060706	2.078510713	1.736966	0.39904	ssc-miR-491	143.992371	82.2046	7.179833181	6.378591382	0.808701	1	chr5_12108_star	1.611855	0	1.38507475	0	20	0.641553
hsa-miR-3123	10.74569932	3.22371	3.554060706	2.078510713	1.736966	0.39904	ssc-miR-218	872.550785	693.9035	9.770747768	9.440668907	0.330504	1	chr6_13322_star	1.611855	0	1.38507475	0	20	0.641553
chr15_32658_star	10.74569932	3.22371	3.554060706	2.078510713	1.736966	0.39904	chr7_15943_mature	23.6405385	4.835565	4.62296188	2.544872269	2.289507	1	hsa-miR-3667-3p	1.611855	0	1.38507475	0	20	0.641553
chrx_22026_mature	9.671129386	47.54972	3.415640968	5.601391055	-2.29768	0.399102	chr4_10674_star@@bta-miR-760-5p	23.6405385	4.835565	4.62296188	2.544872269	2.289507	1	chr3_8430_mature	1.611855	0	1.38507475	0	20	0.641553
chr6_13306_mature	6.447419591	26.59561	2.896740642	4.786366653	-2.04439	0.399908	chr14_30887_mature	23.6405385	4.835565	4.62296188	2.544872269	2.289507	1	NW_003539007_38450_star	1.611855	0	1.38507475	0	20	0.641553
chr12_26323_star	4.298279727	15.31262	2.405524014	4.027916745	-1.83289	0.400017	ssc-miR-551a	6.44741959	33.04303	2.896740642	5.08928735	-2.35755	1	chr3_7678_mature	1.611855	0	1.38507475	0	20	0.641553
chr14_31060_mature	4.298279727	15.31262	2.405524014	4.027916745	-1.83289	0.400017	hsa-miR-3922-5p	12.8948392	0.805927	3.796477232	0.852739935	4	1	hsa-miR-4800-5p	1.611855	0	1.38507475	0	20	0.641553
NW_003613251_37928_mature	7.521989523	2.417782	3.091190278	1.773060525	1.63743	0.401159	chr9_20255_star	12.8948392	0.805927	3.796477232	0.852739935	4	1	chr6_12737_star	1.611855	0	1.38507475	0	20	0.641553
chr12_26402_mature	7.521989523	2.417782	3.091190278	1.773060525	1.63743	0.401159	chr14_30349_mature	12.8948392	0.805927	3.796477232	0.852739935	4	1	chrx_23064_mature	1.611855	0	1.38507475	0	20	0.641553
hsa-miR-3662	7.521989523	2.417782	3.091190278	1.773060525	1.63743	0.401159	chr5_12114_mature@@bta-miR-141	12.8948392	0.805927	3.796477232	0.852739935	4	1	chr7_16955_star	1.611855	0	1.38507475	0	20	0.641553
chr12_25917_mature	7.521989523	2.417782	3.091190278	1.773060525	1.63743	0.401159	chr1_1977_mature	12.8948392	0.805927	3.796477232	0.852739935	4	1	chr15_32836_star	1.611855	0	1.38507475	0	20	0.641553
chr7_15797_mature	7.521989523	2.417782	3.091190278	1.773060525	1.63743	0.401159	chr4_8813_mature	12.8948392	0.805927	3.796477232	0.852739935	4	1	chr15_33671_mature	1.611855	0	1.38507475	0	20	0.641553
chr2_6281_star	7.521989523	2.417782	3.091190278	1.773060525	1.63743	0.401159	chr15_32974_mature	2.14913986	23.3719	1.654957834	4.607146585	-3.44294	1	bta-miR-1603	1.611855	0	1.38507475	0	20	0.641553
chr1_153_mature	7.521989523	2.417782	3.091190278	1.773060525	1.63743	0.401159	chr7_15658_mature@@bta-miR-2307	12.8948392	0.805927	3.796477232	0.852739935	4	1	chr3_8302_star	1.611855	0	1.38507475	0	20	0.641553
chr9_20300_star	7.521989523	2.417782	3.091190278	1.773060525	1.63743	0.401159	hsa-miR-548ak	2.14913986	23.3719	1.654957834	4.607146585	-3.44294	1	hsa-miR-1200	1.611855	0	1.38507475	0	20	0.641553
chr7_15986_star	7.521989523	2.417782	3.091190278	1.773060525	1.63743	0.401159	chr16_34407_mature	12.8948392	0.805927	3.796477232	0.852739935	4	1	rno-miR-3585-3p	1.611855	0	1.38507475	0	20	0.641553
chr1_3069_star	7.521989523	2.417782	3.091190278	1.773060525	1.63743	0.401159	chr13_27409_star@@hsa-miR-551b-5p	12.8948392	0.805927	3.796477232	0.852739935	4	1	hsa-miR-153	1.611855	0	1.38507475	0	20	0.641553
chr6_13702_mature	102.0841435	16.92448	6.687678623	4.163859073	2.592576	0.401673	chr8_17809_mature	2.14913986	23.3719	1.654957834	4.607146585	-3.44294	1	chr2_5776_star	1.611855	0	1.38507475	0	20	0.641553
chr15_32764_star	3.223709795	10.47706	2.078510713	3.520680821	-1.70044	0.401928	chr7_16094_star	12.8948392	0.805927	3.796477232	0.852739935	4	1	rno-let-7c-2-3p	1.611855	0	1.38507475	0	20	0.641553
chr13_27087_mature	3.223709795	10.47706	2.078510713	3.520680821	-1.70044	0.401928	chr13_28924_mature	12.8948392	0.805927	3.796477232	0.852739935	4	1	rno-miR-592	1.611855	0	1.38507475	0	20	0.641553
chr8_18299_mature	3.223709795	10.47706	2.078510713	3.520680821	-1.70044	0.401928	chr14_30291_mature	12.8948392	0.805927	3.796477232	0.852739935	4	1	hsa-miR-1288	1.611855	0	1.38507475	0	20	0.641553
chr8_17430_star	3.223709795	10.47706	2.078510713	3.520680821	-1.70044	0.401928	chr15_33376_mature	12.8948392	0.805927	3.796477232	0.852739935	4	1	hsa-miR-1275	1.611855	0	1.38507475	0	20	0.641553
chr5_11735_star	3.223709795	10.47706	2.078510713	3.520680821	-1.70044	0.401928	chr13_26683_star	12.8948392	0.805927	3.796477232	0.852739935	4	1	hsa-miR-1278	1.611855	0	1.38507475	0	20	0.641553
chr2_5482_mature	3.223709795	10.47706	2.078510713	3.520680821	-1.70044	0.401928	chr7_16801_star	12.8948392	0.805927	3.796477232	0.852739935	4	1	hsa-miR-573	1.611855	0	1.38507475	0	20	0.641553
chr12_25620_star	13.96940911	4.029637	3.90394537	2.330454351	1.793549	0.402627	chr17_34922_mature	12.8948392	0.805927	3.796477232	0.852739935	4	1	bta-miR-365-3p	1.611855	0	1.38507475	0	20	0.641553
ssc-miR-142-3p	13.96940911	4.029637	3.90394537	2.330454351	1.793549	0.402627	chr3_6909_mature	12.8948392	0.805927	3.796477232	0.852739935	4	1	hsa-miR-580	1.611855	0	1.38507475	0	20	0.641553
NW_003613401_38938_mature	13.96940911	4.029637	3.90394537	2.330454351	1.793549	0.402627	chr2_5880_mature	12.8948392	0.805927	3.796477232	0.852739935	4	1	hsa-miR-597	1.611855	0	1.38507475	0	20	0.641553
chr1_1977_star	13.96940911	4.029637	3.90394537	2.330454351	1.793549	0.402627	chr15_32731_star	12.8948392	0.805927	3.796477232	0.852739935	4	1	chr15_33061_star	1.611855	0	1.38507475	0	20	0.641553
ssc-miR-15a	6632.245619	329.6243	12.69549923	8.369049069	4.330603	0.402751	bta-miR-186	12.8948392	0.805927	3.796477232	0.852739935	4	1	rno-miR-871-3p	1.611855	0	1.38507475	0	20	0.641553
chrx_23198_mature	9.671129386	46.74379	3.415640968	5.577241251	-2.27302	0.402913	ssc-miR-144	12.8948392	0.805927	3.796477232	0.852739935	4	1	bta-miR-2399-5p	1.611855	0	1.38507475	0	20	0.641553
chr6_12838_mature	90169.31212	2204.212	16.46036489	11.10670136	5.354302	0.403102	chr6_12854_star	12.8948392	0.805927	3.796477232	0.852739935	4	1	chr2_5134_star	1.611855	0	1.38507475	0	20	0.641553
rno-miR-185-3p	160.1109198	23.3719	7.331910471	4.607146585	2.776225	0.404285	chr4_9602_mature	12.8948392	0.805927	3.796477232	0.852739935	4	1	chr3_6623_mature	1.611855	0	1.38507475	0	20	0.641553
chr6_14946_star	2.149139864	6.44742	1.654957834	2.896740642	-1.58496	0.404381	chr17_35047_mature	12.8948392	0.805927	3.796477232	0.852739935	4	1	hsa-miR-3189-5p	1.611855	0	1.38507475	0	20	0.641553
chr8_18304_star	2.149139864	6.44742	1.654957834	2.896740642	-1.58496	0.404381	chr2_4381_star	12.8948392	0.805927	3.796477232	0.852739935	4	1	hsa-miR-3912	1.611855	0	1.38507475	0	20	0.641553
chr13_28542_star	2.149139864	6.44742	1.654957834	2.896740642	-1.58496	0.404381	hsa-miR-4524b-3p	12.8948392	0.805927	3.796477232	0.852739935	4	1	mmu-miR-878-3p	1.611855	0	1.38507475	0	20	0.641553
chr2_4320_star	2.149139864	6.44742	1.654957834	2.896740642	-1.58496	0.404381	chr1_666_mature	12.8948392	0.805927	3.796477232	0.852739935	4	1	chr2_5147_mature	1.611855	0	1.38507475	0	20	0.641553
chr1_2060_mature	2.149139864	6.44742	1.654957834	2.896740642	-1.58496	0.404381	chr15_32407_mature	12.8948392	0.805927	3.796477232	0.852739935	4	1	bta-miR-2284t-3p	1.611855	0	1.38507475	0	20	0.641553
chr7_16063_mature	2.149139864	6.44742	1.654957834	2.896740642	-1.58496	0.404381	chr1_1936_mature	12.8948392	0.805927	3.796477232	0.852739935	4	1	hsa-miR-3937	1.611855	0	1.38507475	0	20	0.641553
hsa-miR-378d	2.149139864	6.44742	1.654957834	2.896740642	-1.58496	0.404381	chr13_28825_mature	8.59655945	37.07266	3.262517265	5.250683565	-2.10852	1	hsa-miR-548b-3p	1.611855	0	1.38507475	0	20	0.641553
chr8_17349_star	2.149139864	6.44742	1.654957834	2.896740642	-1.58496	0.404381	chr6_13134_mature	12.8948392	0.805927	3.796477232	0.852739935	4	1	chr3_6641_mature	1.611855	0	1.38507475	0	20	0.641553
chr2_6577_mature	2.149139864	6.44742	1.654957834	2.896740642	-1.58496	0.404381	chr7_16580_mature	12.8948392	0.805927	3.796477232	0.852739935	4	1	chr3_7968_star	1.611855	0	1.38507475	0	20	0.641553
chr15_33256_star	2.149139864	6.44742	1.654957834	2.896740642	-1.58496	0.404381	chr13_26761_mature	12.8948392	0.805927	3.796477232	0.852739935	4	1	mmu-miR-452-3p	1.611855	0	1.38507475	0	20	0.641553
mmu-miR-6356	2.149139864	6.44742	1.654957834	2.896740642	-1.58496	0.404381	chr6_14742_mature	12.8948392	0.805927	3.796477232	0.852739935	4	1	ssc-miR-208b	1.611855	0	1.38507475	0	20	0.641553
chrx_23192_star@@rno-miR-3585-5	2.149139864	6.44742	1.654957834	2.896740642	-1.58496	0.404381	rno-miR-23a-3p	21.4913986	4.029637	4.491301473	2.330454351	2.415037	1	chr1_1949_star	1.611855	0	1.38507475	0	20	0.641553
chr8_18198_star	2.149139864	6.44742	1.654957834	2.896740642	-1.58496	0.404381	rno-miR-598-3p	36.5353777	11.28298	5.230179094	3.618589217	1.695145	1	mmu-miR-1954	1.611855	0	1.38507475	0	20	0.641553
chr1_3435_star	2.149139864	6.44742	1.654957834	2.896740642	-1.58496	0.404381	chr14_30524_star	36.5353777	11.28298	5.230179094	3.618589217	1.695145	1	mmu-miR-1958	1.611855	0	1.38507475	0	20	0.641553
chr9_19346_mature	2.149139864	6.44742	1.654957834	2.896740642	-1.58496	0.404381	bta-miR-2479	21.4913986	4.029637	4.491301473	2.330454351	2.415037	1	hsa-miR-498	1.611855	0	1.38507475	0	20	0.641553
chrx_22620_mature	2.149139864	6.44742	1.654957834	2.896740642	-1.58496	0.404381	chr17_35663_mature	4.29827973	28.20746	2.405524014	4.868265031	-2.71425	1	rno-miR-301b-5p	1.611855	0	1.38507475	0	20	0.641553
bta-miR-193a-5p	2.149139864	6.44742	1.654957834	2.896740642	-1.58496	0.404381	mmu-miR-21a-5p	4.29827973	28.20746	2.405524014	4.868265031	-2.71425	1	hsa-miR-6504-5p	1.611855	0	1.38507475	0	20	0.641553
chr13_28070_star	2.149139864	6.44742	1.654957834	2.896740642	-1.58496	0.404381	chr9_20062_star	4.29827973	28.20746	2.405524014	4.868265031	-2.71425	1	chrx_22720_star	1.611855	0	1.38507475	0	20	0.641553
bta-miR-3154	2.149139864	6.44742	1.654957834	2.896740642	-1.58496	0.404381	ssc-miR-323	58.0267763	114.4417	5.883297648	6.851020612	-0.97982	1	hsa-miR-1271-5p	1.611855	0	1.38507475	0	20	0.641553
chr13_29380_mature	2.149139864	6.44742	1.654957834	2.896740642	-1.58496	0.404381	chr1_3842_mature	550.179805	711.6339	9.106379219	9.47701738	-0.37123	1	NW_003613480_39328_star	1.611855	0	1.38507475	0	20	0.641553
chr7_16069_mature	2.149139864	6.44742	1.654957834	2.896740642	-1.58496	0.404381	chr4_8848_mature	30.0879581	8.059274	4.958283956	3.179395517	1.900464	1	chr4_9091_star	1.611855	0	1.38507475	0	20	0.641553
chr16_34823_mature	2.149139864	6.44742	1.654957834	2.896740642	-1.58496	0.404381	chr2_5321_mature	19.3422588	3.22371	4.346407978	2.078510713	2.584963	1	hsa-miR-4746-3p	1.611855	0	1.38507475	0	20	0.641553
chr3_7586_mature	2.149139864	6.44742	1.654957834	2.896740642	-1.58496	0.404381	chr4_8682_mature	19.3422588	3.22371	4.346407978	2.078510713	2.584963	1	mmu-miR-216b-5p	1.611855	0	1.38507475	0	20	0.641553
chr7_15961_star	2.149139864	6.44742	1.654957834	2.896740642	-1.58496	0.404381	rno-miR-1843-5p	19.3422588	3.22371	4.346407978	2.078510713	2.584963	1	rno-miR-202-5p	1.611855	0	1.38507475	0	20	0.641553
chr2_4127_star	2.149139864	6.44742	1.654957834	2.896740642	-1.58496	0.404381	bta-miR-302c	19.3422588	3.22371	4.346407978	2.078510713	2.584963	1	bta-miR-1843	1.611855	0	1.38507475	0	20	0.641553
chr3_8352_star	2.149139864	6.44742	1.654957834	2.896740642	-1.58496	0.404381	chr14_31126_star	19.3422588	3.22371	4.346407978	2.078510713	2.584963	1	hsa-miR-877-3p	1.611855	0	1.38507475	0	20	0.641553

hsa-miR-1273e	2.149139864	6.44742	1.654957834	2.896740642		-1.58496	0.404381		ssc-miR-4339	19.3422588	3.22371	4.346407978	2.078510713		2.584963	1		rno-miR-187-5p	1.611855	0	1.38507475	0		20	0.641553
chr1_1213_mature	5.372849659	20.14819	2.671938627	4.402462031		-1.90689	0.404512		chr15_31863_star@@bta-miR-2881	19.3422588	3.22371	4.346407978	2.078510713		2.584963	1		chr13_28254_star	1.611855	0	1.38507475	0		20	0.641553
chr9_19210_mature	5.372849659	20.14819	2.671938627	4.402462031		-1.90689	0.404512		chr14_30822_mature	19.3422588	3.22371	4.346407978	2.078510713		2.584963	1		mmu-miR-880-3p	1.611855	0	1.38507475	0		20	0.641553
chr8_18702_mature	10.74569932	53.99714	3.554060706	5.781284667		-2.32912	0.404981		chr11_24870_star	19.3422588	3.22371	4.346407978	2.078510713		2.584963	1		chr5_11222_star	1.611855	0	1.38507475	0		20	0.641553
chr16_33863_mature	6.447419591	25.78968	2.896740642	4.743605355		-2	0.405843		chr8_17767_mature	68.7724756	30.62524	6.124586119	4.983004663		1.16711	1		NW_003613041_36883_star	1.611855	0	1.38507475	0		20	0.641553
chr9_20685_star	21.49139864	5.641492	4.491301473	2.731507408		1.929611	0.406318		chr6_13171_star	15.043979	1.611855	4.003960082	1.38507475		3.222392	1		hsa-miR-3650	1.611855	0	1.38507475	0		20	0.641553
chr5_10946_mature	25.78967836	6.44742	4.743605355	2.896740642		2	0.406593		chr11_24796_mature	15.043979	1.611855	4.003960082	1.38507475		3.222392	1		hsa-miR-3649	1.611855	0	1.38507475	0		20	0.641553
chr11_25089_star	25.78967836	6.44742	4.743605355	2.896740642		2	0.406593		chr5_11842_star	15.043979	1.611855	4.003960082	1.38507475		3.222392	1		hsa-miR-3674	1.611855	0	1.38507475	0		20	0.641553
chr8_19039_mature	16.11854898	97.51722	4.097488514	6.622304032		-2.59694	0.406634		chr6_13949_star	15.043979	1.611855	4.003960082	1.38507475		3.222392	1		hsa-miR-518b	1.611855	0	1.38507475	0		20	0.641553
chr15_33605_mature	9.671129386	45.93786	3.415640968	5.552680304		-2.24793	0.406764		chr4_9327_star	15.043979	1.611855	4.003960082	1.38507475		3.222392	1		chr16_34034_star	1.611855	0	1.38507475	0		20	0.641553
chr14_30470_star	0	1.611855	0	1.38507475		-20	0.407187		chr1_5_mature	15.043979	1.611855	4.003960082	1.38507475		3.222392	1		hsa-miR-3618	1.611855	0	1.38507475	0		20	0.641553
chr16_34446_star	0	1.611855	0	1.38507475		-20	0.407187		chr11_24795_mature	15.043979	1.611855	4.003960082	1.38507475		3.222392	1		rno-miR-3569	1.611855	0	1.38507475	0		20	0.641553
chr8_18878_mature	0	1.611855	0	1.38507475		-20	0.407187		chr14_29652_star	15.043979	1.611855	4.003960082	1.38507475		3.222392	1		rno-miR-3562	1.611855	0	1.38507475	0		20	0.641553
chr15_33576_star	0	1.611855	0	1.38507475		-20	0.407187		chr1_1941_star	15.043979	1.611855	4.003960082	1.38507475		3.222392	1		rno-miR-3587	1.611855	0	1.38507475	0		20	0.641553
NW_003537262_36795_star	0	1.611855	0	1.38507475		-20	0.407187		chr4_9998_mature	15.043979	1.611855	4.003960082	1.38507475		3.222392	1		hsa-miR-3688-3p	1.611855	0	1.38507475	0		20	0.641553
hsa-miR-6715b-5p	0	1.611855	0	1.38507475		-20	0.407187		chr6_14212_mature	15.043979	1.611855	4.003960082	1.38507475		3.222392	1		chr2_3991_star	1.611855	0	1.38507475	0		20	0.641553
chr1_480_star	0	1.611855	0	1.38507475		-20	0.407187		chr10_23842_star	15.043979	1.611855	4.003960082	1.38507475		3.222392	1		rno-miR-3591	1.611855	0	1.38507475	0		20	0.641553
chr15_33264_star	0	1.611855	0	1.38507475		-20	0.407187		chr4_9513_mature	62.325056	26.59561	5.984704544	4.786366653		1.228624	1		chr16_34281_star	1.611855	0	1.38507475	0		20	0.641553
NW_003539070_38505_star	0	1.611855	0	1.38507475		-20	0.407187		chr2_5958_mature	15.043979	1.611855	4.003960082	1.38507475		3.222392	1		chr1_3433_star	1.611855	0	1.38507475	0		20	0.641553
hsa-miR-1303	0	1.611855	0	1.38507475		-20	0.407187		chrx_22324_star	17.1931189	2.417782	4.185320985	1.773060525		2.830075	1		hsa-miR-3688-5p	1.611855	0	1.38507475	0		20	0.641553
hsa-miR-4761-3p	0	1.611855	0	1.38507475		-20	0.407187		chr7_16802_star	17.1931189	2.417782	4.185320985	1.773060525		2.830075	1		mmu-miR-5131	1.611855	0	1.38507475	0		20	0.641553
chr13_26947_star	0	1.611855	0	1.38507475		-20	0.407187		hsa-miR-298	17.1931189	2.417782	4.185320985	1.773060525		2.830075	1		chr3_6622_star	1.611855	0	1.38507475	0		20	0.641553
chr2_5894_star	0	1.611855	0	1.38507475		-20	0.407187		chr5_11657_mature	17.1931189	2.417782	4.185320985	1.773060525		2.830075	1		chr4_9471_star	1.611855	0	1.38507475	0		20	0.641553
chr8_18998_star	0	1.611855	0	1.38507475		-20	0.407187		hsa-miR-210	17.1931189	2.417782	4.185320985	1.773060525		2.830075	1		hsa-miR-522-3p	1.611855	0	1.38507475	0		20	0.641553
hsa-miR-4719	0	1.611855	0	1.38507475		-20	0.407187		hsa-miR-2861	17.1931189	2.417782	4.185320985	1.773060525		2.830075	1		chr2_5921_mature	1.611855	0	1.38507475	0		20	0.641553
chr8_18144_star	0	1.611855	0	1.38507475		-20	0.407187		chr7_16043_mature	17.1931189	2.417782	4.185320985	1.773060525		2.830075	1		rno-miR-19b-3p	1.611855	0	1.38507475	0		20	0.641553
hsa-miR-4759	0	1.611855	0	1.38507475		-20	0.407187		chr5_11336_mature	19.3422588	55.60899	4.346407978	5.82295938		-1.52356	1		mmu-miR-465b-5p	1.611855	0	1.38507475	0		20	0.641553
chr9_20798_star	0	1.611855	0	1.38507475		-20	0.407187		chr1_6_mature	17.1931189	2.417782	4.185320985	1.773060525		2.830075	1		rno-miR-6330	1.611855	0	1.38507475	0		20	0.641553
chr14_31610_star	0	1.611855	0	1.38507475		-20	0.407187		chr9_21017_mature	17.1931189	2.417782	4.185320985	1.773060525		2.830075	1		rno-miR-6325	1.611855	0	1.38507475	0		20	0.641553
hsa-miR-3620-3p	0	1.611855	0	1.38507475		-20	0.407187		chr5_12271_mature	17.1931189	2.417782	4.185320985	1.773060525		2.830075	1		chr15_32711_mature	1.611855	0	1.38507475	0		20	0.641553
chr6_12863_star	0	1.611855	0	1.38507475		-20	0.407187		chr1_1112_mature	17.1931189	2.417782	4.185320985	1.773060525		2.830075	1		chr5_12309_star@@mmu-miR-125	1.611855	0	1.38507475	0		20	0.641553
chr14_29900_star	0	1.611855	0	1.38507475		-20	0.407187		NW_003539453_38857_mature	17.1931189	2.417782	4.185320985	1.773060525		2.830075	1		chr1_2375_star	1.611855	0	1.38507475	0		20	0.641553
hsa-miR-4419b	0	1.611855	0	1.38507475		-20	0.407187		chr7_15425_mature	17.1931189	2.417782	4.185320985	1.773060525		2.830075	1		chr1_1204_star	1.611855	0	1.38507475	0		20	0.641553
chr5_12006_star	0	1.611855	0	1.38507475		-20	0.407187		chr2_4133_mature	17.1931189	2.417782	4.185320985	1.773060525		2.830075	1		chr15_31685_star	1.611855	0	1.38507475	0		20	0.641553
hsa-miR-4724-5p	0	1.611855	0	1.38507475		-20	0.407187		NW_003540351_39435_star	17.1931189	2.417782	4.185320985	1.773060525		2.830075	1		bta-miR-2425-3p	1.611855	0	1.38507475	0		20	0.641553
chr13_28501_star	0	1.611855	0	1.38507475		-20	0.407187		chrx_21800_mature	17.1931189	2.417782	4.185320985	1.773060525		2.830075	1		hsa-miR-183-5p	1.611855	0	1.38507475	0		20	0.641553
hsa-miR-3620-5p	0	1.611855	0	1.38507475		-20	0.407187		bta-miR-409b	384.696036	275.6272	8.591320507	8.111799144		0.481001	1		hsa-miR-146a-5p	1.611855	0	1.38507475	0		20	0.641553
chr7_16069_star	0	1.611855	0	1.38507475		-20	0.407187		chr9_19327_mature	34.3862378	10.47706	5.145116481	3.520680821		1.714598	1		chrx_22698_star	1.611855	0	1.38507475	0		20	0.641553
chr3_7259_star	0	1.611855	0	1.38507475		-20	0.407187		chr1_3684_mature	21.4913986	58.8327	4.491301473	5.902862353		-1.45286	1		chr3_8071_star	1.611855	0	1.38507475	0		20	0.641553
hsa-miR-6510-3p	0	1.611855	0	1.38507475		-20	0.407187		chr15_31842_star	2.14913986	22.56597	1.654957834	4.558633073		-3.39232	1		chr14_31138_star	1.611855	0	1.38507475	0		20	0.641553
hsa-miR-1208	0	1.611855	0	1.38507475		-20	0.407187		chr3_8509_mature	2.14913986	22.56597	1.654957834	4.558633073		-3.39232	1		hsa-miR-523-3p	1.611855	0	1.38507475	0		20	0.641553
hsa-miR-4999-5p	0	1.611855	0	1.38507475		-20	0.407187		chr18_35896_mature	2.14913986	22.56597	1.654957834	4.558633073		-3.39232	1		chr8_18342_star	1.611855	0	1.38507475	0		20	0.641553
hsa-miR-1205	0	1.611855	0	1.38507475		-20	0.407187		NW_003613231_37780_star	2.14913986	22.56597	1.654957834	4.558633073		-3.39232	1		chr9_20123_mature	1.611855	0	1.38507475	0		20	0.641553
chr5_11958_star	0	1.611855	0	1.38507475		-20	0.407187		chr16_34458_mature	2.14913986	22.56597	1.654957834	4.558633073		-3.39232	1		chr9_20451_star	1.611855	0	1.38507475	0		20	0.641553
chr15_33696_mature	0	1.611855	0	1.38507475		-20	0.407187		bta-miR-543	6.44741959	31.43117	2.896740642	5.019309189		-2.2854	1		chr3_8405_star	1.611855	0	1.38507475	0		20	0.641553
chr17_35470_star	0	1.611855	0	1.38507475		-20	0.407187		chr14_30841_star	25.7896784	6.44742	4.743605355	2.896740642		2	1		mmu-miR-3062-3p	1.611855	0	1.38507475	0		20	0.641553
chr10_23289_star	0	1.611855	0	1.38507475		-20	0.407187		chr3_7094_mature	25.7896784	6.44742	4.743605355	2.896740642		2	1		chr9_19846_star	1.611855	0	1.38507475	0		20	0.641553
mmu-miR-883a-5p	0	1.611855	0	1.38507475		-20	0.407187		chr15_32589_mature	4.29827973	26.59561	2.405524014	4.786366653		-2.62936	1		chr4_9351_star	1.611855	0	1.38507475	0		20	0.641553
chr5_10946_star	0	1.611855	0	1.38507475		-20	0.407187		chr9_20047_mature	2.14913986	21.76004	1.654957834	4.508431259		-3.33985	1		chr1_191_mature	1.611855	0	1.38507475	0		20	0.641553
hsa-miR-2682-5p	0	1.611855	0	1.38507475		-20	0.407187		chr18_36565_mature	2.14913986	21.76004	1.654957834	4.508431259		-3.33985	1		chr13_27448_mature	1.611855	0	1.38507475	0		20	0.641553
chrx_23193_star	0	1.611855	0	1.38507475		-20	0.407187		chr15_33603_mature	2.14913986	21.76004	1.654957834	4.508431259		-3.33985	1		rno-miR-465-5p	1.611855	0	1.38507475	0		20	0.641553
mmu-miR-453	0	1.611855	0	1.38507475		-20	0.407187		chr14_30313_mature	4.29827973	26.59561	2.405524014	4.786366653		-2.62936	1		hsa-miR-5591-3p	1.611855	0	1.38507475	0		20	0.641553
NW_003538773_38215_mature	0	1.611855	0	1.38507475		-20	0.407187		chr1_3135_mature	2.14913986	21.76004	1.654957834	4.508431259		-3.33985	1		hsa-miR-3129-5p	1.611855	0	1.38507475	0		20	0.641553
chr8_17317_star	0	1.611855	0	1.38507475		-20	0.407187		chr4_8847_mature	36.5353777	12.08891	5.230179094	3.710273245		1.59561	1		hsa-miR-1	1.611855	0	1.38507475	0		20	0.641553
chr1_429_mature	0	1.611855	0	1.38507475		-20	0.407187		chr8_18162_star	15.043979	46.74379	4.003960082	5.577241251		-1.63559	1		chr2_4834_star	1.611855	0	1.38507475	0		20	0.641553
chr15_31781_star	0	1.611855	0	1.38507475		-20	0.407187		chrx_22392_mature	15.043979	46.74379	4.003960082	5.577241251		-1.63559	1		chr15_32372_mature	1.611855	0	1.38507475	0		20	0.641553
bta-miR-199b	0	1.611855	0	1.38507475		-20	0.407187		chr7_15929_mature	23.6405385	5.641492	4.62296188	2.731507408		2.067114	1		hsa-miR-4757-3p	1.611855	0	1.38507475	0		20	0.641553
chr1_3836_mature	0	1.611855	0	1.38507475		-20	0.407187		chr3_8521_star	23.6405385	5.641492	4.62296188	2.731507408		2.067114	1		mmu-miR-532-5p	1.611855	0	1.38507475	0		20	0.641553
chr13_26769_star	0	1.611855	0	1.38507475		-20	0.407187		chr1_3715_star	30.0879581	8.865202	4.958283956	3.302348583		1.762961	1		chr7_17045_star	1.611855	0	1.38507475	0		20	0.641553
chr1_1833_star	0	1.611855	0	1.38507475		-20	0.407187		chr2_4073_star	47.281077	18.53633	5.593385953	4.288087668		1.350907	1		chr13_27449_star	1.611855	0	1.38507475	0		20	0.641553
chr1_2918_star	0	1.611855	0	1.38507475		-20	0.407187		chr5_12257_mature	66.6233358	30.62524	6.079449279	4.983004663		1.121306	1		mmu-miR-6404	1.611855	0	1.38507475	0		20	0.641553
chr13_27839_mature	0	1.611855	0	1.38507475		-20	0.407187		chr2_5456_star	8.59655945	0	3.262517265	0		20	1		chr4_10605_star	1.611855	0	1.38507475	0		20	0.641553
NW_003613223_37738_star	0	1.611855	0	1.38507475		-20	0.407187		chr4_9456_mature	8.59655945	0	3.262517265	0		20	1		chr6_13601_star	1.611855	0	1.38507475	0		20	0.641553
chr13_26861_star	0	1.611855	0	1.38507475		-20	0.407187		NW_003613290_38305_mature	8.59655945	0	3.262517265	0		20	1		rno-miR-207	1.611855	0	1.38507475	0		20	0.641553
chr11_24872_star	0	1.611855	0	1.38507475		-20	0.407187		hsa-miR-3136-5p	8.59655945	0	3.262517265	0		20	1		chr14_31500_star	1.611855	0	1.38507475	0		20	0.641553
chr18_36189_star	0	1.611855	0	1.38507475		-20	0.407187		chr15_32751_star	8.59655945	0	3.262517265	0		20	1		chr12_25947_mature	1.611855	0	1.38507475	0		20	0.641553
mmu-miR-1907	0	1.611855	0	1.38507475		-20	0.407187		chr5_11814_mature	8.59655945	0	3.262517265	0		20	1		hsa-miR-3689f	1.611855	0	1.38507475	0		20	0.641553
hsa-miR-4667-5p	0	1.611855	0	1.38507475		-20	0.407187		chr1_2128_star	8.59655945	0	3.262517265	0		20	1		chr2_4916_star	1.611855	0	1.38507475	0		20	0.641553
hsa-miR-5590-5p	0	1.611855	0	1.38507475		-20	0.407187		chr14_31358_mature	8.59655945	0	3.262517265	0		20	1		chr8_17492_star	1.611855	0	1.38507475	0		20	0.641553
chr9_20115_mature	0	1.611855	0	1.38507475		-20	0.407187		chr7_15929_star	8.59655945	0	3.262517265	0		20	1		chr9_20339_star	1.611855	0	1.38507475	0		20	0.641553
mmu-miR-1953	0	1.611855	0	1.38507475		-20	0.407187		chr12_26042_star	8.59655945	0	3.262517265	0		20	1		chr3_7724_star	1.611855	0	1.38507475	0		20	0.641553
mmu-miR-1940	0	1.611855	0	1.38507475		-20	0.407187		chr6_13898_mature	8.59655945	0	3.262517265	0		20	1		mmu-miR-3092-3p	1.611855	0	1.38507475	0		20	0.641553
rno-miR-301b-3p	0	1.611855	0	1.38507475		-20	0.407187		chr6_14470_mature	8.59655945	0	3.262517265	0		20	1		hsa-miR-4731-3p	1.611855	0	1.38507475	0		20	0.641553
mmu-miR-100-5p	0	1.611855	0	1.38507475		-20	0.407187		hsa-miR-548b-3p	8.59655945	0	3.262517265	0		20	1		chr2_5974_star	1.611855	0	1.38507475	0		20	0.641553
chrx_22435_star	0	1.611855	0	1.38507475		-20	0.407187		chr6_12833_mature	8.59655945	0	3.262517265	0		20	1		bta-miR-767	1.611855	0	1.38507475	0		20	0.641553
rno-miR-28-3p	0	1.611855	0	1.38507475		-20	0.407187		chrx_23228_star	8.59655945	0	3.262517265	0		20	1		bta-miR-763	1.611855	0	1.38507475	0		20	0.641553
mmu-miR-195b	0	1.611855	0	1.38507475		-20	0.407187		chr13_27913_star	8.59655945	0	3.262517265	0		20	1		hsa-miR-515-3p	1.611855	0	1.38507475	0		20	0.641553
chr14_30275_star	0	1.611855	0	1.38507475		-20	0.407187		mmu-miR-216b-5p	8.59655945	0	3.262517265	0		20	1		chr5_12341_star	1.611855	0	1.38507475	0		20	0.641553
rno-miR-673-3p	0	1.611855	0	1.38507475		-20	0.407187		chr9_20919_star	8.59655945	0	3.262517265	0		20	1		bta-miR-2285u	1.611855	0	1.38507475	0		20	0.641553
rno-miR-673-5p	0	1.611855	0	1.38507475		-20	0.407187		chr6_14981_mature	8.59655945	0	3.262517265	0		20	1		mmu-miR-6367	1.611855	0	1.38507475	0		20	0.641553
chr8_18850_star	0	1.611855	0	1.38507475		-20	0.407187		mmu-miR-5131	8.59655945	0	3.262517265	0		20	1		mmu-miR-6368	1.611855	0	1.38507475	0		20	0.641553
hsa-miR-520a-3p	0	1.611855	0	1.38507475		-20	0.407187		chr3_6736_mature	8.59655945	0	3.262517265	0		20	1		mmu-miR-1188-5p	1.611855	0	1.38507475	0		20	0.641553
chr8_18077_star	0	1.611855	0	1.38507475		-20	0.407187		chr1_986_mature	8.59655945	0	3.262517265	0		20	1		mmu-miR-6359	1.611855	0	1.38507475	0		20	0.641553
hsa-miR-5571-5p	0	1.611855	0	1.38507475		-20	0.407187		rno-miR-466b-2-3p	8.59655945	0	3.262517265	0		20	1		mmu-miR-6379	1.611855	0	1.38507475	0		20	0.641553
chr17_35344_mature	0	1.611855	0	1.38507475		-20	0.407187		chr7_15151_mature	10.7456993	38.68452	3.554060706	5.310504361		-1.848	1		bta-miR-2285b	1.611855	0	1.38507475	0		20	0.641553
bta-miR-181b	0	1.611855	0	1.38507475		-20	0.407187		NW_003613103_37208_star	8.59655945	0	3.262517265	0		20	1		bta-miR-2285l	1.611855	0	1.38507475	0		20	0.641553
chr16_34846_star	0	1.611855	0	1.38507475		-20	0.407187		chr17_35456_mature	8.59655945	0	3.262517265	0		20	1		hsa-miR-4713-3p	1.611855	0	1.38507475	0		20	0.641553
hsa-miR-16-2-3p	0	1.611855	0	1.38507475		-20	0.407187		mmu-miR-879-3p	8.59655945	0	3.262517265	0		20	1		chr8_17918_mature	1.611855	0	1.38507475	0		20	0.641553
chr1_811_star	0	1.611855	0	1.38507475		-20	0.407187		chr7_15656_star	8.59655945	0	3.262517265	0		20	1		bta-miR-2284k	1.611855	0	1.38507475	0		20	0.641553
chrx_23183_mature	0	1.611855	0	1.38507475		-20	0.407187		chr1_3090_mature	8.59655945	0	3.262517265	0		20	1		hsa-miR-3150b-3p	1.611855	0	1.38507475	0		20	0.641553
chr1_3507_star	0	1.611855	0	1.38507475		-20	0.407187		chr8_18586_mature	8.59655945	0	3.262517265	0		20	1		rno-miR-290	1.611855	0	1.38507475	0		20	0.641553
rno-miR-300-5p	0	1.611855	0	1.38507475		-20	0.407187		chr7_17173_star	8.59655945	0	3.262517265	0		20	1		chr4_9894_star	1.611855	0	1.38507475	0		20	0.641553
rno-miR-672-5p	0	1.611855	0	1.38507475		-20	0.407187		bta-miR-132	8.59655945	0	3.262517265	0		20	1		hsa-miR-4677-3p	1.611855	0	1.38507475	0		20	0.641553
chr6_13898_star	0	1.611855	0	1.38507475		-20	0.407187		chr4_8812_mature	8.59655945	0	3.262517265	0		20	1		hsa-miR-432-3p	1.611855	0	1.38507475	0		20	0.641553
chr5_11120_mature	0	1.611855	0	1.38507475		-20	0.407187		chr12_25704_star	8.59655945	0	3.262517265	0		20	1		hsa-miR-1238-3p	1.611855	0	1.38507475	0		20	0.641553
hsa-miR-3610	0	1.611855	0	1.38507475		-20	0.407187		chr14_29457_mature	8.59655945	0	3.262517265	0		20	1		rno-miR-130b-5p	1.611855	0	1.38507475	0		20	0.641553
rno-miR-3564	0	1.611855	0	1.38507475		-20	0.407187		chr5_11609_mature	8.59655945	0	3.262517265	0		20	1		chrx_21832_star	1.611855	0	1.38507475	0		20	0.641553
chr1_541_star	0	1.611855	0	1.38507475		-20	0.407187		chr6_13950_mature	8.59655945	0	3.262517265	0		20	1		chr16_33921_star	1.611855	0	1.38507475	0		20	0.641553
chr12_26317_star	0	1.611855	0	1.38507475		-20	0.407187		chr3_7067_mature	8.59655945	0	3.262517265	0		20	1		hsa-miR-3529-3p	1.611855	0	1.38507475	0		20	0.641553
hsa-miR-3683	0	1.611855	0	1.38507475		-20	0.407187		chr8_17677_mature	8.59655945	0	3.262517265	0		20	1		mmu-miR-674-3p	1.611855	0	1.38507475	0		20	0.641553
chr2_4579_star	0	1.611855	0	1.38507475		-20	0.407187		chr9_20053_mature	8.59655945	0	3.262517265	0		20	1		rno-miR-217-5p	1.611855	0	1.38507475	0		20	0.641553
chr9_19709_mature	0	1.611855	0	1.38507475		-20	0.407187		hsa-miR-3196	8.59655945	0	3.262517265	0		20	1		chr1_2367_star	1.611855	0	1.38507475	0		20	0.641553
hsa-miR-2355-3p	0	1.611855	0	1.38507475		-20	0.407187		chr10_23272_star	8.59655945	0	3.262517265	0		20	1		chr12_26608_star	1.611855	0	1.38507475	0		20	0.641553
rno-miR-6332	0	1.611855	0	1.38507475		-20	0.407187		chrx_22793_mature	8.59655945	0	3.262517265	0		20	1		chr12_25675_star	1.611855	0	1.38507475	0		20	0.641553
chrx_22943_star	0	1.611855	0	1.38507475		-20	0.407187		chr2_5533_mature	8.59655945	0	3.262517265	0		20	1		chr4_9832_mature	1.611855	0	1.38507475	0		20	0.641553
hsa-miR-550a-5p	0	1.611855	0	1.38507475		-20	0.407187		bta-miR-2373-3p	8.59655945	0	3.262517265	0		20	1		bta-miR-671	1.611855	0	1.38507475	0		20	0.641553
rno-miR-6323	0	1.611855	0	1.38507475		-20	0.407187		chr16_34759_mature	8.59655945	0	3.262517265	0		20	1		bta-miR-670	1.611855	0	1.38507475	0		20	0.641553
chr8_18505_star	0	1.611855	0	1.38507475		-20	0.407187		chr1_3708_star	8.59655945	0	3.262517265	0		20	1		chr12_25921_mature	1.611855	0	1.38507475	0		20	0.641553
NW_003539295_38706_star	0	1.611855	0	1.38507475		-20	0.407187		NW_003613063_36957_mature	8.59655945	0	3.262517265	0		20	1		bta-miR-652	1.611855	0	1.38507475	0		20	0.641553
mmu-miR-291a-3p	0	1.611855	0	1.38507475		-20	0.407187		chr8_18702_star	8.59655945	0	3.262517265	0		20	1		chr11_24333_star	1.611855	0	1.38507475	0		20	0.641553
mmu-miR-183-5p	0	1.611855	0	1.38507475		-20	0.407187		chr1_902_mature	8.59655945	0	3.262517265	0		20	1		chr14_31578_star	1.611855	0	1.38507475	0		20	0.641553
chr15_31872_star	0	1.611855	0	1.38507475		-20	0.407187		chr1_392_star	8.59655945	0	3.262517265	0		20	1		chr10_23450_star	1.611855	0	1.38507475	0		20	0.641553
chr18_35964_star	0	1.611855	0	1.38507475		-20	0.407187		hsa-miR-651	8.59655945	0	3.262517265	0		20	1		mmu-miR-15a-5p	1.611855	0	1.38507475	0		20	0.641553
hsa-miR-95	0	1.611855	0	1.38507475		-20	0.407187		chrx_21753_star	8.59655945	0	3.262517265	0		20	1		hsa-miR-4661-3p	1.611855	0	1.38507475	0		20	0.641553
hsa-miR-378b	0	1.611855	0	1.38507475		-20	0.407187		chr13_28155_star	8.59655945	0	3.262517265	0		20	1		chr2_4630_mature	1.611855	0	1.38507475	0		20	0.641553
chr12_25581_star	0	1.611855	0	1.38507475		-20	0.407187		hsa-miR-4330	8.59655945	0	3.262517265	0		20	1		chr12_25873_star	1.611855	0	1.38507475	0		20	0.641553
chr15_31873_star	0	1.611855	0	1.38507475		-20	0.407187		chr14_30956_mature	8.59655945	0	3.262517265	0		20	1		chr7_15081_star	1.611855	0	1.38507475	0		20	0.641553
chr8_18403_star	0	1.611855	0	1.38507475		-20	0.407187		chr16_34846_mature	8.59655945	0	3.262517265	0		20	1		chr10_23974_star	1.611855	0	1.38507475	0		20	0.641553
hsa-miR-3913-3p	0	1.611855	0	1.38507475		-20	0.407187		ssc-miR-196b-3p	8.59655945	0	3.262517265	0		20	1		chr13_29234_mature	1.611855	0	1.38507475	0		20	0.641553
rno-miR-449a-5p	0	1.611855	0	1.38507475		-20	0.407187		chr1_3355_star	8.59655945	0	3.262517265	0		20	1		bta-miR-144	1.611855	0	1.38507475	0		20	0.641553
hsa-miR-548ap-3p	0	1.611855	0	1.38507475		-20	0.407187		chr14_30937_star	8.59655945	0	3.262517265	0		20	1		chr5_12206_star	1.611855	0	1.38507475	0		20	0.641553
bta-miR-125b	0	1.611855	0	1.38507475		-20	0.407187		chr6_12830_star	8.59655945	0	3.262517265	0		20	1		hsa-miR-5584-3p	1.611855	0	1.38507475	0		20	0.641553
chr13_28683_star	0	1.611855	0	1.38507475		-20	0.407187		chr7_16512_mature	8.59655945	0	3.262517265	0		20	1		mmu-miR-5134-5p	1.611855	0	1.38507475	0		20	0.641553
chr14_30429_star	0	1.611855	0	1.38507475		-20	0.407187		hsa-miR-1268a	8.59655945	0	3.262517265	0		20	1		chrx_22034_star	1.611855	0	1.38507475	0		20	0.641553
chr8_18863_star	0	1.611855	0	1.38507475		-20	0.407187		chr9_19516_star	8.59655945	0	3.262517265	0		20	1		chr16_34832_star	1.611855	0	1.38507475	0		20	0.641553
chr8_17274_star	0	1.611855	0	1.38507475		-20	0.407187		chr6_12904_mature	8.59655945	0	3.262517265	0		20	1		chr5_12420_star	1.611855	0	1.38507475	0		20	0.641553
chr7_16963_star	0	1.611855	0	1.38507475		-20	0.407187		chr5_11866_mature	8.59655945	0	3.262517265	0		20	1		chr13_28748_star	1.611855	0	1.38507475	0		20	0.641553
NW_003613200_37636_star	0	1.611855	0	1.38507475		-20	0.407187		chr1_2829_star	8.59655945	0	3.262517265	0		20	1		mmu-miR-3113-5p	1.611855	0	1.38507475	0		20	0.641553
ssc-miR-489	0	1.611855	0	1.38507475		-20	0.407187		bta-miR-2328-3p	8.59655945	0	3.262517265	0		20	1		hsa-miR-1307-5p	1.611855	0	1.38507475	0		20	0.641553
chr6_14620_star	0	1.611855	0	1.38507475		-20	0.407187		chr16_34408_mature	8.59655945	0	3.262517265	0		20	1		chr6_13504_star	1.611855	0	1.38507475	0		20	0.641553
NW_003613507_39496_star	0	1.611855	0	1.38507475		-20	0.407187		NW_003541202_39972_star	8.59655945	0	3.262517265	0		20	1		rno-miR-3075	1.611855	0	1.38507475	0		20	0.641553
hsa-miR-6717-5p	0	1.611855	0	1.38507475		-20	0.407187		chr5_12206_mature	8.59655945	0	3.262517265	0		20	1		chr7_16234_star	1.611855	0	1.38507475	0		20	0.641553
hsa-miR-3064-3p	0	1.611855	0	1.38507475		-20	0.407187		chr1_2401_star	8.59655945	0	3.262517265	0		20	1		chr7_16275_star	1.611855	0	1.38507475	0		20	0.641553
chr5_11962_star	0	1.611855	0	1.38507475		-20	0.407187		hsa-miR-6089	8.59655945	0	3.262517265	0		20	1		chr6_12450_star	1.611855	0	1.38507475	0		20	0.641553
chr6_12646_star	0	1.611855	0	1.38507475		-20	0.407187		chr12_25647_mature	42.9827973	16.11855	5.458867456	4.097488514		1.415037	1		mmu-miR-3113-3p	1.611855	0	1.38507475	0		20	0.641553
bta-miR-449a	0	1.611855	0	1.38507475		-20	0.407187		chr16_34112_mature	2.14913986	20.95411	1.654957834	4.456419386		-3.2854	1		chr13_27779_star	1.611855	0	1.38507475	0		20	0.641553
chr6_14972_star	0	1.611855	0	1.38507475		-20	0.407187		chr4_10283_mature	2.14913986	20.95411	1.654957834	4.456419386		-3.2854	1		mmu-miR-20a-3p	1.611855	0	1.38507475	0		20	0.641553
rno-miR-485-3p	0	1.611855	0	1.38507475		-20	0.407187		chr5_12211_mature	4.29827973	25.78968	2.405524014	4.743605355		-2.58496	1		chrx_21814_star	1.611855	0	1.38507475	0		20	0.641553
hsa-miR-548ay-3p	0	1.611855	0	1.38507475		-20	0.407187		NW_003537639_37085_mature	2.14913986	20.95411	1.654957834	4.456419386		-3.2854	1		hsa-miR-544a	1.611855	0	1.38507475	0		20	0.641553
chr6_13246_mature	0	1.611855	0	1.38507475		-20	0.407187		bta-miR-574	2.14913986	20.95411	1.654957834	4.456419386		-3.2854	1		NW_003613562_39706_star	1.611855	0	1.38507475	0		20	0.641553
NW_003539007_38442_star	0	1.611855	0	1.38507475		-20	0.407187		chr7_15913_star	2.14913986	20.95411	1.654957834	4.456419386		-3.2854	1		chr14_29928_star	1.611855	0	1.38507475	0		20	0.641553
chr17_35011_star	0	1.611855	0	1.38507475		-20	0.407187		chr2_4243_mature	21.4913986	4.835565	4.491301473	2.544872269		2.152003	1		mmu-miR-3057-3p	1.611855	0	1.38507475	0		20	0.641553
bta-miR-2285s	0	1.611855	0	1.38507475		-20	0.407187		chr6_13244_mature	2.14913986	20.95411	1.654957834	4.456419386		-3.2854	1		NW_003540029_39236_star	1.611855	0	1.38507475	0		20	0.641553
mmu-miR-6366	0	1.611855	0	1.38507475		-20	0.407187		chr11_24871_mature	2.14913986	20.95411	1.654957834	4.456419386		-3.2854	1		mmu-miR-3063-3p	1.611855	0	1.38507475	0		20	0.641553
chr12_26311_star	0	1.611855	0	1.38507475		-20	0.407187		hsa-miR-649	4.29827973	25.78968	2.405524014	4.743605355		-2.58496	1		chr16_34526_star	1.611855	0	1.38507475	0		20	0.641553
bta-miR-2285e	0	1.611855	0	1.38507475		-20	0.407187		chr8_18998_mature	2.14913986	20.95411	1.654957834	4.456419386		-3.2854	1		chr9_19559_star	1.611855	0	1.38507475	0		20	0.641553
bta-miR-2285o	0	1.611855	0	1.38507475		-20	0.407187		chr2_6135_mature	2.14913986	20.95411	1.654957834	4.456419386		-3.2854	1		hsa-miR-548s	1.611855	0	1.38507475	0		20	0.641553
NW_003541332_40046_star	0	1.611855	0	1.38507475		-20	0.407187		NW_003538437_37870_mature	6.44741959	29.81932	2.896740642	4.94576292		-2.20945	1		chr8_18439_star	1.611855	0	1.38507475	0		20	0.641553
bta-miR-2284e	0	1.611855	0	1.38507475		-20	0.407187		NW_003539007_38427_mature	6.44741959	29.81932	2.896740642	4.94576292		-2.20945	1		chr14_30479_mature	1.611855	0	1.38507475	0		20	0.641553
chrx_22193_mature	0	1.611855	0	1.38507475		-20	0.407187		bta-miR-544b	6.44741959	29.81932	2.896740642	4.94576292		-2.20945	1		chr4_9977_star	1.611855	0	1.38507475	0		20	0.641553
chr9_20132_star	0	1.611855	0	1.38507475		-20	0.407187		chr13_26646_mature	27.9388182	8.059274	4.854934103	3.179395517		1.793549	1		chr3_8637_star	1.611855	0	1.38507475	0		20	0.641553
chr6_12636_star	0	1.611855	0	1.38507475		-20	0.407187		chr15_33480_mature	83.8164547	42.71415	6.406272275	5.4500286		0.972519	1		chr8_17239_star	1.611855	0	1.38507475	0		20	0.641553
chr14_29600_star	0	1.611855	0	1.38507475		-20	0.407187		chr6_12833_star	19.3422588	4.029637	4.346407978	2.330454351		2.263034	1		chr5_11275_star	1.611855	0	1.38507475	0		20	0.641553
rno-miR-217-3p	0	1.611855	0	1.38507475		-20	0.407187		NW_003541307_40037_mature	19.3422588	4.029637	4.346407978	2.330454351		2.263034	1		NW_003537295_36819_mature	1.611855	0	1.38507475	0		20	0.641553
chr2_4133_star	0	1.611855	0	1.38507475		-20	0.407187		chr15_32527_mature	19.3422588	4.029637	4.346407978	2.330454351		2.263034	1		hsa-miR-4769-3p	1.611855	0	1.38507475	0		20	0.641553
chr12_25620_mature	0	1.611855	0	1.38507475		-20	0.407187		chr12_26097_mature	19.3422588	4.029637	4.346407978	2.330454351		2.263034	1		hsa-miR-550b-3p	1.611855	0	1.38507475	0		20	0.641553
bta-miR-628	0	1.611855	0	1.38507475		-20	0.407187		chr2_5378_mature	19.3422588	4.029637	4.346407978	2.330454351		2.263034	1		hsa-miR-3166	1.611855	0	1.38507475	0		20	0.641553
mmu-miR-1968-5p	0	1.611855	0	1.38507475		-20	0.407187		chr7_15986_mature	19.3422588	4.029637	4.346407978	2.330454351		2.263034	1		hsa-miR-3198	1.611855	0	1.38507475	0		20	0.641553
chr13_27063_star	0	1.611855	0	1.38507475		-20	0.407187		chr17_35416_star	19.3422588	4.029637	4.346407978	2.330454351		2.263034	1		bta-miR-412	1.611855	0	1.38507475	0		20	0.641553
chr7_15755_star	0	1.611855	0	1.38507475		-20	0.407187		chr17_35416_mature	19.3422588	4.029637	4.346407978	2.330454351		2.263034	1		bta-miR-429	1.611855	0	1.38507475	0		20	0.641553
chr16_33845_star	0	1.611855	0	1.38507475		-20	0.407187		chr15_32699_star	19.3422588	4.029637	4.346407978	2.330454351		2.263034	1		chr13_26885_star	1.611855	0	1.38507475	0		20	0.641553
mmu-miR-376c-5p	0	1.611855	0	1.38507475		-20	0.407187		chr4_10566_mature	36.5353777	12.89484	5.230179094	3.796477232		1.5025	1		hsa-miR-3137	1.611855	0	1.38507475	0		20	0.641553
rno-miR-370-3p	0	1.611855	0	1.38507475		-20	0.407187		chr5_11615_mature	51.5793567	21.76004	5.716424587	4.508431259		1.245112	1		bta-miR-342	1.611855	0	1.38507475	0		20	0.641553
chr2_4030_star	0	1.611855	0	1.38507475		-20	0.407187		chr11_24436_mature	15.043979	45.13194	4.003960082	5.527693969		-1.58496	1		hsa-miR-4258	1.611855	0	1.38507475	0		20	0.641553
hsa-miR-4755-3p	0	1.611855	0	1.38507475		-20	0.407187		chr2_5969_star	10.7456993	0.805927	3.554060706	0.852739935		3.736966	1		chr7_15706_star	1.611855	0	1.38507475	0		20	0.641553
chr14_30974_mature	0	1.611855	0	1.38507475		-20	0.407187		hsa-miR-1343	10.7456993	0.805927	3.554060706	0.852739935		3.736966	1		hsa-miR-4279	1.611855	0	1.38507475	0		20	0.641553
chr9_19375_star	0	1.611855	0	1.38507475		-20	0.407187		chrx_22000_mature	4.29827973	24.98375	2.405524014	4.699537803		-2.53916	1		chr17_35756_star	1.611855	0	1.38507475	0		20	0.641553
NW_003613242_37866_star	0	1.611855	0	1.38507475		-20	0.407187		chr16_34839_star	17.1931189	3.22371	4.185320985	2.078510713		2.415037	1		chr15_32372_star	1.611855	0	1.38507475	0		20	0.641553
rno-miR-675-5p	0	1.611855	0	1.38507475		-20	0.407187		chr15_32567_star@@rno-miR-375-5p	17.1931189	3.22371	4.185320985	2.078510713		2.415037	1		chr15_32809_mature	1.611855	0	1.38507475	0		20	0.641553
hsa-miR-556-3p	0	1.611855	0	1.38507475		-20	0.407187		chr3_7747_mature	10.7456993	0.805927	3.554060706	0.852739935		3.736966	1		NW_003613199_37634_star	1.611855	0	1.38507475	0		20	0.641553
NW_003613080_37038_star	0	1.611855	0	1.38507475		-20	0.407187		chr1_2655_mature	10.7456993	0.805927	3.554060706	0.852739935		3.736966	1		mmu-miR-297b-3p	1.611855	0	1.38507475	0		20	0.641553
hsa-miR-4766-3p	0	1.611855	0	1.38507475		-20	0.407187		chr10_23649_mature	10.7456993	0.805927	3.554060706	0.852739935		3.736966	1		hsa-miR-4709-3p	1.611855	0	1.38507475	0		20	0.641553
mmu-miR-488-5p	0	1.611855	0	1.38507475		-20	0.407187		chr11_25383_star	10.7456993	0.805927	3.554060706	0.852739935		3.736966	1		chr17_35177_star	1.611855	0	1.38507475	0		20	0.641553
chr6_14308_star	0	1.611855	0	1.38507475		-20	0.407187		chr13_28115_star	17.1931189	3.22371	4.185320985	2.078510713		2.415037	1		chr16_33754_star	1.611855	0	1.38507475	0		20	0.641553
hsa-miR-205-5p	0	1.611855	0	1.38507475		-20	0.407187		chr16_34104_star	10.7456993	0.805927	3.554060706	0.852739935		3.736966	1		mmu-miR-467b-5p	1.611855	0	1.38507475	0		20	0.641553
bta-miR-10b	0	1.611855	0	1.38507475		-20	0.407187		chr6_14981_star	17.1931189	3.22371	4.185320985	2.078510713		2.415037	1		hsa-miR-520f	1.611855	0	1.38507475	0		20	0.641553
hsa-miR-6127	0	1.611855	0	1.38507475		-20	0.407187		chr5_12416_mature	17.1931189	3.22371	4.185320985	2.078510713		2.415037	1		hsa-miR-520h	1.611855	0	1.38507475	0		20	0.641553
hsa-miR-6128	0	1.611855	0	1.38507475		-20	0.407187		chr1_2110_star	2.14913986	20.14819	1.654957834	4.402462031		-3.22882	1		hsa-miR-520g	1.611855	0	1.38507475	0		20	0.641553
bta-miR-2370-5p	0	1.611855	0	1.38507475		-20	0.407187		chr9_19915_mature	4.29827973	24.98375	2.405524014	4.699537803		-2.53916	1		hsa-miR-4664-5p	1.611855	0	1.38507475	0		20	0.641553
chr1_3069_mature	0	1.611855	0	1.38507475		-20	0.407187		chr11_25225_star	10.7456993	0.805927	3.554060706	0.852739935		3.736966	1		mmu-miR-468-3p	1.611855	0	1.38507475	0		20	0.641553
chrx_21725_star	0	1.611855	0	1.38507475		-20	0.407187		chr11_24692_mature	17.1931189	3.22371	4.185320985	2.078510713		2.415037	1		mmu-miR-466l-3p	1.611855	0	1.38507475	0		20	0.641553
hsa-miR-769-3p	0	1.611855	0	1.38507475		-20	0.407187		chr7_15837_star	10.7456993	0.805927	3.554060706	0.852739935		3.736966	1		chr1_3198_star	1.611855	0	1.38507475	0		20	0.641553
chr9_19298_star	0	1.611855	0	1.38507475		-20	0.407187		chr3_7048_mature	10.7456993	0.805927	3.554060706	0.852739935		3.736966	1		chr4_9797_star	1.611855	0	1.38507475	0		20	0.641553
chr5_11414_star	0	1.611855	0	1.38507475		-20	0.407187		chr8_18247_mature	17.1931189	3.22371	4.185320985	2.078510713		2.415037	1		mmu-miR-499-5p	1.611855	0	1.38507475	0		20	0.641553
bta-miR-16a	0	1.611855	0	1.38507475		-20	0.407187		chrx_22324_mature	10.7456993	0.805927	3.554060706	0.852739935		3.736966	1		mmu-miR-466l-5p	1.611855	0	1.38507475	0		20	0.641553
bta-miR-185	0	1.611855	0	1.38507475		-20	0.407187		chr1_3638_star	10.7456993	0.805927	3.554060706	0.852739935		3.736966	1		mmu-miR-3090-5p	1.611855	0	1.38507475	0		20	0.641553
chr9_19522_star	0	1.611855	0	1.38507475		-20	0.407187		chr3_6639_star	10.7456993	0.805927	3.554060706	0.852739935		3.736966	1		rno-miR-702-3p	1.611855	0	1.38507475	0		20	0.641553
chr2_5163_mature	0	1.611855	0	1.38507475		-20	0.407187		chr7_15773_star	17.1931189	3.22371	4.185320985	2.078510713		2.415037	1		chr1_1736_mature	1.611855	0	1.38507475	0		20	0.641553
chr4_10602_star	0	1.611855	0	1.38507475		-20	0.407187		chr4_9960_mature	10.7456993	0.805927	3.554060706	0.852739935		3.736966	1		chr7_15107_star	1.611855	0	1.38507475	0		20	0.641553
hsa-miR-1207-5p	0	1.611855	0	1.38507475		-20	0.407187		chr4_9937_mature	10.7456993	0.805927	3.554060706	0.852739935		3.736966	1		bta-miR-2413	1.611855	0	1.38507475	0		20	0.641553
chr10_23455_mature	0	1.611855	0	1.38507475		-20	0.407187		chr9_19210_mature	2.14913986	20.14819	1.654957834	4.402462031		-3.22882	1		chr5_11678_star	1.611855	0	1.38507475	0		20	0.641553
hsa-miR-548n	0	1.611855	0	1.38507475		-20	0.407187		chr1_283_star	2.14913986	20.14819	1.654957834	4.402462031		-3.22882	1		chr13_28482_star	1.611855	0	1.38507475	0		20	0.641553
chr1_1437_star	0	1.611855	0	1.38507475		-20	0.407187		chr18_36007_mature	10.7456993	0.805927	3.554060706	0.852739935		3.736966	1		chr1_2929_star	1.611855	0	1.38507475	0		20	0.641553
chr8_17483_mature	0	1.611855	0	1.38507475		-20	0.407187		chr1_1444_star	4.29827973	24.98375	2.405524014	4.699537803		-2.53916	1		chr9_19904_star	1.611855	0	1.38507475	0		20	0.641553
bta-miR-2388-3p	0	1.611855	0	1.38507475		-20	0.407187		chr5_12388_mature	2.14913986	20.14819	1.654957834	4.402462031		-3.22882	1		hsa-miR-3619-5p	1.611855	0	1.38507475	0		20	0.641553
chr13_27530_star	0	1.611855	0	1.38507475		-20	0.407187		ssc-miR-153	10.7456993	0.805927	3.554060706	0.852739935		3.736966	1		bta-miR-2459	1.611855	0	1.38507475	0		20	0.641553
chr1_2346_mature	0	1.611855	0	1.38507475		-20	0.407187		chr14_31026_mature	10.7456993	0.805927	3.554060706	0.852739935		3.736966	1		hsa-miR-4459	1.611855	0	1.38507475	0		20	0.641553
chr3_7381_star	0	1.611855	0	1.38507475		-20	0.407187		chrx_22133_star	10.7456993	0.805927	3.554060706	0.852739935		3.736966	1		hsa-miR-4457	1.611855	0	1.38507475	0		20	0.641553
rno-miR-140-3p	0	1.611855	0	1.38507475		-20	0.407187		chr5_12116_star@@rno-miR-200c-5p	10.7456993	0.805927	3.554060706	0.852739935		3.736966	1		hsa-miR-4458	1.611855	0	1.38507475	0		20	0.641553
bta-miR-551b	0	1.611855	0	1.38507475		-20	0.407187		bta-miR-6530	10.7456993	0.805927	3.554060706	0.852739935		3.736966	1		hsa-miR-4452	1.611855	0	1.38507475	0		20	0.641553
bta-miR-551a	0	1.611855	0	1.38507475		-20	0.407187		mmu-miR-3073a-3p	10.7456993	0.805927	3.554060706	0.852739935		3.736966	1		hsa-miR-4465	1.611855	0	1.38507475	0		20	0.641553
chr3_8257_star	0	1.611855	0	1.38507475		-20	0.407187		chr1_1498_mature	10.7456993	0.805927	3.554060706	0.852739935		3.736966	1		bta-miR-2438	1.611855	0	1.38507475	0		20	0.641553
chr1_1290_star	0	1.611855	0	1.38507475		-20	0.407187		chrx_22122_mature	4.29827973	24.98375	2.405524014	4.699537803		-2.53916	1		hsa-miR-548ao-3p	1.611855	0	1.38507475	0		20	0.641553
chr13_27138_star	0	1.611855	0	1.38507475		-20	0.407187		ssc-miR-202-5p	17.1931189	3.22371	4.185320985	2.078510713		2.415037	1		bta-miR-2443	1.611855	0	1.38507475	0		20	0.641553
mmu-miR-344d-2-5p	0	1.611855	0	1.38507475		-20	0.407187		chr11_24439_mature	10.7456993	0.805927	3.554060706	0.852739935		3.736966	1		hsa-miR-4481	1.611855	0	1.38507475	0		20	0.641553
rno-miR-6216	0	1.611855	0	1.38507475		-20	0.407187		hsa-miR-4426	17.1931189	3.22371	4.185320985	2.078510713		2.415037	1		rno-miR-878	1.611855	0	1.38507475	0		20	0.641553
chr13_27994_star	0	1.611855	0	1.38507475		-20	0.407187		hsa-miR-4422	10.7456993	0.805927	3.554060706	0.852739935		3.736966	1		hsa-miR-4427	1.611855	0	1.38507475	0		20	0.641553
hsa-miR-3168	0	1.611855	0	1.38507475		-20	0.407187		chr14_31148_star	10.7456993	0.805927	3.554060706	0.852739935		3.736966	1		chr2_5353_mature	1.611855	0	1.38507475	0		20	0.641553
chr14_30518_star	0	1.611855	0	1.38507475		-20	0.407187		chrx_21844_star@@hsa-miR-660-3p	10.7456993	0.805927	3.554060706	0.852739935		3.736966	1		hsa-miR-4447	1.611855	0	1.38507475	0		20	0.641553
hsa-miR-3199	0	1.611855	0	1.38507475		-20	0.407187		NW_003539279_38689_mature	4.29827973	24.98375	2.405524014	4.699537803		-2.53916	1		hsa-miR-605	1.611855	0	1.38507475	0		20	0.641553
hsa-miR-1273g-3p	0	1.611855	0	1.38507475		-20	0.407187		chr5_11928_mature	10.7456993	0.805927	3.554060706	0.852739935		3.736966	1		rno-miR-872-3p	1.611855	0	1.38507475	0		20	0.641553
rno-miR-320-5p	0	1.611855	0	1.38507475		-20	0.407187		chr7_15367_mature	10.7456993	0.805927	3.554060706	0.852739935		3.736966	1		hsa-miR-614	1.611855	0	1.38507475	0		20	0.641553
chr11_24357_star	0	1.611855	0	1.38507475		-20	0.407187		chr8_17976_mature	10.7456993	0.805927	3.554060706	0.852739935		3.736966	1		hsa-miR-621	1.611855	0	1.38507475	0		20	0.641553
bta-miR-544a	0	1.611855	0	1.38507475		-20	0.407187		NW_003613447_39176_star	4.29827973	24.98375	2.405524014	4.699537803		-2.53916	1		hsa-miR-449a	1.611855	0	1.38507475	0		20	0.641553
mmu-miR-3078-3p	0	1.611855	0	1.38507475		-20	0.407187		chrx_22392_star	10.7456993	0.805927	3.554060706	0.852739935		3.736966	1		hsa-miR-4493	1.611855	0	1.38507475	0		20	0.641553
chr10_23674_star	0	1.611855	0	1.38507475		-20	0.407187		chr13_27007_star	10.7456993	0.805927	3.554060706	0.852739935		3.736966	1		mmu-miR-467c-5p	1.611855	0	1.38507475	0		20	0.641553
hsa-miR-3158-5p	0	1.611855	0	1.38507475		-20	0.407187		chr1_3851_star	10.7456993	0.805927	3.554060706	0.852739935		3.736966	1		NW_003613326_38566_star	1.611855	0	1.38507475	0		20	0.641553
hsa-miR-4265	0	1.611855	0	1.38507475		-20	0.407187		chr14_30394_mature	10.7456993	0.805927	3.554060706	0.852739935		3.736966	1		chr5_11343_star	1.611855	0	1.38507475	0		20	0.641553
chr15_31782_mature	0	1.611855	0	1.38507475		-20	0.407187		chr1_1173_star	10.7456993	0.805927	3.554060706	0.852739935		3.736966	1		chr14_30496_star	1.611855	0	1.38507475	0		20	0.641553
chr11_24756_star	0	1.611855	0	1.38507475		-20	0.407187		chr1_915_star	12.8948392	1.611855	3.796477232	1.38507475		3	1		NW_003613022_36785_star	1.611855	0	1.38507475	0		20	0.641553
bta-miR-6528	0	1.611855	0	1.38507475		-20	0.407187		chr6_12988_mature@@mmu-miR-6538	12.8948392	1.611855	3.796477232	1.38507475		3	1		hsa-miR-646	1.611855	0	1.38507475	0		20	0.641553
chr2_5690_star	0	1.611855	0	1.38507475		-20	0.407187		chr17_35500_star	12.8948392	1.611855	3.796477232	1.38507475		3	1		hsa-miR-647	1.611855	0	1.38507475	0		20	0.641553
chr3_8036_star	0	1.611855	0	1.38507475		-20	0.407187		hsa-miR-151b	12.8948392	1.611855	3.796477232	1.38507475		3	1		chrx_22335_star	1.611855	0	1.38507475	0		20	0.641553
rno-miR-880-3p	0	1.611855	0	1.38507475		-20	0.407187		chr9_20120_mature	15.043979	2.417782	4.003960082	1.773060525		2.63743	1		hsa-miR-662	1.611855	0	1.38507475	0		20	0.641553
chr1_1911_star	0	1.611855	0	1.38507475		-20	0.407187		chr14_30467_star@@hsa-miR-6715a-3	12.8948392	1.611855	3.796477232	1.38507475		3	1		mmu-miR-539-5p	1.611855	0	1.38507475	0		20	0.641553
chr3_7264_star	0	1.611855	0	1.38507475		-20	0.407187		hsa-miR-509-5p	12.8948392	1.611855	3.796477232	1.38507475		3	1		chr17_35019_star	1.611855	0	1.38507475	0		20	0.641553
chr15_33038_star	0	1.611855	0	1.38507475		-20	0.407187		chr6_13322_mature	12.8948392	1.611855	3.796477232	1.38507475		3	1		rno-miR-101b-5p	1.611855	0	1.38507475	0		20	0.641553
chr1_589_star	0	1.611855	0	1.38507475		-20	0.407187		chr3_7586_star	12.8948392	1.611855	3.796477232	1.38507475		3	1		chr6_14759_star	1.611855	0	1.38507475	0		20	0.641553
chr7_16088_star	0	1.611855	0	1.38507475		-20	0.407187		chr5_11729_mature	122.500972	70.92162	6.948378589	6.168353522		0.788496	1		chr11_25373_mature	1.611855	0	1.38507475	0		20	0.641553
chr17_35296_star	0	1.611855	0	1.38507475		-20	0.407187		chr4_9959_mature	15.043979	2.417782	4.003960082	1.773060525		2.63743	1		bta-miR-2314	1.611855	0	1.38507475	0		20	0.641553
chr7_16673_mature@@mmu-miR-63	0	1.611855	0	1.38507475		-20	0.407187		chr7_16597_star	15.043979	2.417782	4.003960082	1.773060525		2.63743	1		mmu-miR-1955-3p	1.611855	0	1.38507475	0		20	0.641553
chr5_11694_star	0	1.611855	0	1.38507475		-20	0.407187		ssc-miR-328	83.8164547	144.261	6.406272275	7.182503739		-0.78338	1		rno-miR-493-5p	1.611855	0	1.38507475	0		20	0.641553
chr17_35035_star	0	1.611855	0	1.38507475		-20	0.407187		chr4_10551_mature	12.8948392	1.611855	3.796477232	1.38507475		3	1		chrx_22202_mature	1.611855	0	1.38507475	0		20	0.641553
chr8_18422_star	0	1.611855	0	1.38507475		-20	0.407187		chr6_13576_star	12.8948392	1.611855	3.796477232	1.38507475		3	1		bta-miR-24-3p	1.611855	0	1.38507475	0		20	0.641553
hsa-miR-4781-5p	0	1.611855	0	1.38507475		-20	0.407187		chrx_21496_mature	68.7724756	33.04303	6.124586119	5.08928735		1.057485	1		bta-miR-2357	1.611855	0	1.38507475	0		20	0.641553
chr15_33315_star	0	1.611855	0	1.38507475		-20	0.407187		chr1_917_star	15.043979	2.417782	4.003960082	1.773060525		2.63743	1		hsa-miR-4318	1.611855	0	1.38507475	0		20	0.641553
chr15_32159_star	0	1.611855	0	1.38507475		-20	0.407187		chr18_36406_star	12.8948392	1.611855	3.796477232	1.38507475		3	1		bta-miR-2353	1.611855	0	1.38507475	0		20	0.641553
chr4_10175_star	0	1.611855	0	1.38507475		-20	0.407187		chr4_10618_mature	12.8948392	1.611855	3.796477232	1.38507475		3	1		chr4_10618_star	1.611855	0	1.38507475	0		20	0.641553
hsa-miR-4664-3p	0	1.611855	0	1.38507475		-20	0.407187		chr1_1780_star	12.8948392	1.611855	3.796477232	1.38507475		3	1		hsa-miR-5585-3p	1.611855	0	1.38507475	0		20	0.641553
chr5_12232_star	0	1.611855	0	1.38507475		-20	0.407187		chr2_5337_mature	12.8948392	1.611855	3.796477232	1.38507475		3	1		hsa-miR-4328	1.611855	0	1.38507475	0		20	0.641553
bta-miR-496	0	1.611855	0	1.38507475		-20	0.407187		chr6_12824_mature@@hsa-miR-877-3	12.8948392	1.611855	3.796477232	1.38507475		3	1		hsa-miR-4327	1.611855	0	1.38507475	0		20	0.641553
chr7_17170_star	0	1.611855	0	1.38507475		-20	0.407187		ssc-miR-4333	25.7896784	7.253347	4.743605355	3.044979305		1.830075	1		bta-miR-2369	1.611855	0	1.38507475	0		20	0.641553
chr9_20608_star	0	1.611855	0	1.38507475		-20	0.407187		chr6_12803_star	15.043979	2.417782	4.003960082	1.773060525		2.63743	1		hsa-miR-4324	1.611855	0	1.38507475	0		20	0.641553
chr1_1486_star	0	1.611855	0	1.38507475		-20	0.407187		chr12_26596_mature	15.043979	2.417782	4.003960082	1.773060525		2.63743	1		hsa-miR-5585-5p	1.611855	0	1.38507475	0		20	0.641553
chr8_17650_star	0	1.611855	0	1.38507475		-20	0.407187		chr10_23395_mature	12.8948392	1.611855	3.796477232	1.38507475		3	1		hsa-miR-4309	1.611855	0	1.38507475	0		20	0.641553
mmu-miR-1897-3p	0	1.611855	0	1.38507475		-20	0.407187		chr1_3229_star	15.043979	2.417782	4.003960082	1.773060525		2.63743	1		chrx_22749_star	1.611855	0	1.38507475	0		20	0.641553
chr1_1411_mature	0	1.611855	0	1.38507475		-20	0.407187		chr9_19744_mature	12.8948392	1.611855	3.796477232	1.38507475		3	1		chr6_12841_star	1.611855	0	1.38507475	0		20	0.641553
chr4_9749_star	0	1.611855	0	1.38507475		-20	0.407187		chr12_26324_mature	15.043979	2.417782	4.003960082	1.773060525		2.63743	1		chr12_25405_star	1.611855	0	1.38507475	0		20	0.641553
chr9_19985_mature	0	1.611855	0	1.38507475		-20	0.407187		NW_003538009_37453_star	15.043979	2.417782	4.003960082	1.773060525		2.63743	1		mmu-miR-681	1.611855	0	1.38507475	0		20	0.641553
hsa-miR-548ao-5p	0	1.611855	0	1.38507475		-20	0.407187		chr13_26988_mature	58.0267763	108.8002	5.883297648	6.778736946		-0.90689	1		mmu-miR-682	1.611855	0	1.38507475	0		20	0.641553
chr6_14576_star	0	1.611855	0	1.38507475		-20	0.407187		chr5_10760_star	8.59655945	33.04303	3.262517265	5.08928735		-1.94251	1		mmu-miR-687	1.611855	0	1.38507475	0		20	0.641553
chr9_19299_star	0	1.611855	0	1.38507475		-20	0.407187		chr17_35129_mature	42.9827973	16.92448	5.458867456	4.163859073		1.344648	1		bta-miR-2292	1.611855	0	1.38507475	0		20	0.641553
chr6_14856_star	0	1.611855	0	1.38507475		-20	0.407187		NW_003613148_37413_mature	30.0879581	9.671129	4.958283956	3.415640968		1.63743	1		chr10_23953_star	1.611855	0	1.38507475	0		20	0.641553
chr7_16903_star	0	1.611855	0	1.38507475		-20	0.407187		chr11_24430_mature	23.6405385	6.44742	4.62296188	2.896740642		1.874469	1		chr11_25257_star	1.611855	0	1.38507475	0		20	0.641553
hsa-miR-6509-5p	0	1.611855	0	1.38507475		-20	0.407187		chrx_22359_mature	4.29827973	24.17782	2.405524014	4.654081667		-2.49185	1		chr10_23277_star	1.611855	0	1.38507475	0		20	0.641553
NW_003613050_36907_mature	0	1.611855	0	1.38507475		-20	0.407187		chr9_20315_mature	4.29827973	24.17782	2.405524014	4.654081667		-2.49185	1		mmu-miR-670-3p	1.611855	0	1.38507475	0		20	0.641553
hsa-miR-4491	0	1.611855	0	1.38507475		-20	0.407187		chr2_5746_star	2.14913986	19.34226	1.654957834	4.346407978		-3.16993	1		chr13_27580_star	1.611855	0	1.38507475	0		20	0.641553
chr7_15958_mature	0	1.611855	0	1.38507475		-20	0.407187		chr2_4436_mature	2.14913986	19.34226	1.654957834	4.346407978		-3.16993	1		hsa-miR-4446-3p	1.611855	0	1.38507475	0		20	0.641553
chr16_34112_star	0	1.611855	0	1.38507475		-20	0.407187		chr9_19230_mature	2.14913986	19.34226	1.654957834	4.346407978		-3.16993	1		chr11_24466_star	1.611855	0	1.38507475	0		20	0.641553
mmu-miR-599	0	1.611855	0	1.38507475		-20	0.407187		chr9_19866_mature	2.14913986	19.34226	1.654957834	4.346407978		-3.16993	1		mmu-miR-3107-5p	1.611855	0	1.38507475	0		20	0.641553
ssc-miR-376c	0	1.611855	0	1.38507475		-20	0.407187		chr14_30846_mature	2.14913986	19.34226	1.654957834	4.346407978		-3.16993	1		chr13_26892_star	1.611855	0	1.38507475	0		20	0.641553
NW_003538662_38098_star	0	1.611855	0	1.38507475		-20	0.407187		chr14_31058_mature	2.14913986	19.34226	1.654957834	4.346407978		-3.16993	1		chr17_34956_star	1.611855	0	1.38507475	0		20	0.641553
chr14_29583_star	0	1.611855	0	1.38507475		-20	0.407187		NW_003539007_38442_mature	10.7456993	36.26674	3.554060706	5.21981653		-1.75489	1		hsa-miR-1269a	1.611855	0	1.38507475	0		20	0.641553
bta-miR-22-3p	0	1.611855	0	1.38507475		-20	0.407187		chr1_3263_mature	27.9388182	8.865202	4.854934103	3.302348583		1.656046	1		chr7_16061_star	1.611855	0	1.38507475	0		20	0.641553
chr8_17863_mature	0	1.611855	0	1.38507475		-20	0.407187		mmu-miR-1895	21.4913986	5.641492	4.491301473	2.731507408		1.929611	1		rno-miR-210-5p	1.611855	0	1.38507475	0		20	0.641553

bta-miR-2464-3p	0	1.611855	0	1.38507475	-20	0.407187	chr1_3728_mature	21.4913986	5.641492	4.491301473	2.731507408	1.929611	1	chr1_1748_star	1.611855	0	1.38507475	0	20	0.641553
chr14_31060_star	0	1.611855	0	1.38507475	-20	0.407187	chrx_22713_mature	21.4913986	5.641492	4.491301473	2.731507408	1.929611	1	chr17_35646_star	1.611855	0	1.38507475	0	20	0.641553
chr7_15096_star	0	1.611855	0	1.38507475	-20	0.407187	chr15_31782_star	6.44741959	28.20746	2.896740642	4.868265031	-2.12928	1	chr14_30543_star	1.611855	0	1.38507475	0	20	0.641553
bta-miR-2324	0	1.611855	0	1.38507475	-20	0.407187	chr4_10247_star	21.4913986	5.641492	4.491301473	2.731507408	1.929611	1	chr17_35344_star	1.611855	0	1.38507475	0	20	0.641553
chr13_28068_mature	0	1.611855	0	1.38507475	-20	0.407187	chrx_21532_mature	2.14913986	18.53633	1.654957834	4.288087668	-3.10852	1	chr13_27316_star	1.611855	0	1.38507475	0	20	0.641553
bta-miR-2332	0	1.611855	0	1.38507475	-20	0.407187	bta-miR-658	2.14913986	18.53633	1.654957834	4.288087668	-3.10852	1	hsa-miR-4672	1.611855	0	1.38507475	0	20	0.641553
hsa-miR-3187-3p	0	1.611855	0	1.38507475	-20	0.407187	chr5_11151_mature	4.29827973	23.3719	2.405524014	4.607146585	-2.44294	1	hsa-miR-4675	1.611855	0	1.38507475	0	20	0.641553
bta-miR-2348	0	1.611855	0	1.38507475	-20	0.407187	chr13_27739_mature	4.29827973	23.3719	2.405524014	4.607146585	-2.44294	1	hsa-miR-5739	1.611855	0	1.38507475	0	20	0.641553
bta-miR-2351	0	1.611855	0	1.38507475	-20	0.407187	chr15_33603_star	6.44741959	0	2.896740642	0	20	1	mmu-miR-1894-5p	1.611855	0	1.38507475	0	20	0.641553
chr5_11905_mature	0	1.611855	0	1.38507475	-20	0.407187	hsa-miR-6501-3p	6.44741959	0	2.896740642	0	20	1	chr18_36516_star	1.611855	0	1.38507475	0	20	0.641553
chr17_35263_star	0	1.611855	0	1.38507475	-20	0.407187	chr13_28104_mature	6.44741959	0	2.896740642	0	20	1	chr6_14179_star	1.611855	0	1.38507475	0	20	0.641553
hsa-miR-4321	0	1.611855	0	1.38507475	-20	0.407187	chr1_3525_mature	6.44741959	0	2.896740642	0	20	1	chr2_4310_mature	1.611855	0	1.38507475	0	20	0.641553
bta-miR-2371	0	1.611855	0	1.38507475	-20	0.407187	chrx_22663_star	6.44741959	0	2.896740642	0	20	1	mmu-miR-718	1.611855	0	1.38507475	0	20	0.641553
hsa-miR-548ah-3p	0	1.611855	0	1.38507475	-20	0.407187	hsa-miR-4780	6.44741959	0	2.896740642	0	20	1	mmu-miR-6541	1.611855	0	1.38507475	0	20	0.641553
chr7_16645_star	0	1.611855	0	1.38507475	-20	0.407187	chr13_26891_mature	6.44741959	0	2.896740642	0	20	1	mmu-miR-711	1.611855	0	1.38507475	0	20	0.641553
chr1_2685_star	0	1.611855	0	1.38507475	-20	0.407187	chr18_35896_star	6.44741959	0	2.896740642	0	20	1	mmu-miR-302b-5p	1.611855	0	1.38507475	0	20	0.641553
mmu-miR-686	0	1.611855	0	1.38507475	-20	0.407187	chr3_6736_star	6.44741959	0	2.896740642	0	20	1	rno-miR-223-5p	1.611855	0	1.38507475	0	20	0.641553
mmu-miR-691	0	1.611855	0	1.38507475	-20	0.407187	rno-miR-29b-3p	6.44741959	0	2.896740642	0	20	1	mmu-miR-767	1.611855	0	1.38507475	0	20	0.641553
NW_003538173_37591_star	0	1.611855	0	1.38507475	-20	0.407187	hsa-miR-2467-3p	6.44741959	0	2.896740642	0	20	1	bta-miR-216b	1.611855	0	1.38507475	0	20	0.641553
chr9_19649_star	0	1.611855	0	1.38507475	-20	0.407187	NW_003539513_38910_mature	6.44741959	0	2.896740642	0	20	1	bta-miR-216a	1.611855	0	1.38507475	0	20	0.641553
hsa-miR-576-5p	0	1.611855	0	1.38507475	-20	0.407187	chr15_31957_star	6.44741959	0	2.896740642	0	20	1	bta-miR-502b	1.611855	0	1.38507475	0	20	0.641553
mmu-miR-3075-3p	0	1.611855	0	1.38507475	-20	0.407187	chr17_35489_mature	6.44741959	0	2.896740642	0	20	1	chr3_8640_star	1.611855	0	1.38507475	0	20	0.641553
mmu-miR-467h	0	1.611855	0	1.38507475	-20	0.407187	chr5_10730_star	6.44741959	0	2.896740642	0	20	1	chr1_2829_star	1.611855	0	1.38507475	0	20	0.641553
mmu-miR-1947-3p	0	1.611855	0	1.38507475	-20	0.407187	chr13_28138_star	38.6845175	15.31262	5.310504361	4.027916745	1.337035	1	hsa-miR-887	1.611855	0	1.38507475	0	20	0.641553
chr1_1198_star	0	1.611855	0	1.38507475	-20	0.407187	rno-let-7c-2-3p	6.44741959	0	2.896740642	0	20	1	chr1_623_star	1.611855	0	1.38507475	0	20	0.641553
chr3_8596_star	0	1.611855	0	1.38507475	-20	0.407187	chr7_16110_star	6.44741959	0	2.896740642	0	20	1	hsa-miR-891a	1.611855	0	1.38507475	0	20	0.641553
rno-miR-20b-3p	0	1.611855	0	1.38507475	-20	0.407187	chr4_8702_star	6.44741959	0	2.896740642	0	20	1	chr3_8064_star	1.611855	0	1.38507475	0	20	0.641553
chr3_6792_star	0	1.611855	0	1.38507475	-20	0.407187	chr3_7972_mature	6.44741959	0	2.896740642	0	20	1	chr4_10714_star	1.611855	0	1.38507475	0	20	0.641553
chr6_14625_star	0	1.611855	0	1.38507475	-20	0.407187	chr8_18418_mature	6.44741959	0	2.896740642	0	20	1	chr13_28501_mature	1.611855	0	1.38507475	0	20	0.641553
hsa-miR-519e-5p	0	1.611855	0	1.38507475	-20	0.407187	hsa-miR-192-3p	6.44741959	0	2.896740642	0	20	1	hsa-miR-4523	1.611855	0	1.38507475	0	20	0.641553
chr5_11866_star	0	1.611855	0	1.38507475	-20	0.407187	chr2_5147_mature	6.44741959	0	2.896740642	0	20	1	hsa-miR-4522	1.611855	0	1.38507475	0	20	0.641553
chr7_16904_mature	0	1.611855	0	1.38507475	-20	0.407187	chr13_28482_mature	6.44741959	0	2.896740642	0	20	1	bta-miR-2313-3p	1.611855	0	1.38507475	0	20	0.641553
NW_003613326_38566_mature	0	1.611855	0	1.38507475	-20	0.407187	hsa-miR-1587	6.44741959	0	2.896740642	0	20	1	mmu-miR-669e-3p	1.611855	0	1.38507475	0	20	0.641553
rno-miR-31a-3p	0	1.611855	0	1.38507475	-20	0.407187	chr3_7968_star	6.44741959	0	2.896740642	0	20	1	bta-miR-2331-5p	1.611855	0	1.38507475	0	20	0.641553
chr1_3765_star	0	1.611855	0	1.38507475	-20	0.407187	mmu-miR-1967	6.44741959	0	2.896740642	0	20	1	mmu-miR-215-3p	1.611855	0	1.38507475	0	20	0.641553
chr6_13925_star	0	1.611855	0	1.38507475	-20	0.407187	chr11_25079_mature	6.44741959	0	2.896740642	0	20	1	hsa-miR-625-3p	1.611855	0	1.38507475	0	20	0.641553
hsa-miR-4673	0	1.611855	0	1.38507475	-20	0.407187	chr1_468_mature	6.44741959	0	2.896740642	0	20	1	chr16_34566_star	1.611855	0	1.38507475	0	20	0.641553
hsa-miR-92a-3p	0	1.611855	0	1.38507475	-20	0.407187	chr4_9777_mature	6.44741959	0	2.896740642	0	20	1	hsa-miR-941	1.611855	0	1.38507475	0	20	0.641553
hsa-miR-4686	0	1.611855	0	1.38507475	-20	0.407187	chr2_5850_star	6.44741959	0	2.896740642	0	20	1	hsa-miR-942	1.611855	0	1.38507475	0	20	0.641553
bta-miR-6122-3p	0	1.611855	0	1.38507475	-20	0.407187	rno-miR-759	6.44741959	0	2.896740642	0	20	1	hsa-miR-936	1.611855	0	1.38507475	0	20	0.641553
chr8_17767_star	0	1.611855	0	1.38507475	-20	0.407187	chr6_12823_mature@@hsa-miR-877-3	6.44741959	0	2.896740642	0	20	1	hsa-miR-138-1-3p	1.611855	0	1.38507475	0	20	0.641553
chr2_4356_mature	0	1.611855	0	1.38507475	-20	0.407187	chr5_11222_star	6.44741959	0	2.896740642	0	20	1	hsa-miR-6083	1.611855	0	1.38507475	0	20	0.641553
hsa-miR-4689	0	1.611855	0	1.38507475	-20	0.407187	chrx_23039_mature	6.44741959	0	2.896740642	0	20	1	rno-miR-24-1-5p	1.611855	0	1.38507475	0	20	0.641553
rno-miR-23b-5p	0	1.611855	0	1.38507475	-20	0.407187	rno-miR-3587	6.44741959	0	2.896740642	0	20	1	hsa-miR-6079	1.611855	0	1.38507475	0	20	0.641553
mmu-miR-24-3p	0	1.611855	0	1.38507475	-20	0.407187	chr3_8486_mature	6.44741959	0	2.896740642	0	20	1	mmu-miR-1231-5p	1.611855	0	1.38507475	0	20	0.641553
hsa-miR-6511b-3p	0	1.611855	0	1.38507475	-20	0.407187	rno-miR-6328	6.44741959	0	2.896740642	0	20	1	chr1_1049_star	1.611855	0	1.38507475	0	20	0.641553
chr14_30528_star	0	1.611855	0	1.38507475	-20	0.407187	chr8_18153_star	6.44741959	0	2.896740642	0	20	1	NW_003539318_38728_star	1.611855	0	1.38507475	0	20	0.641553
chr1_3918_star	0	1.611855	0	1.38507475	-20	0.407187	chr6_14384_star	6.44741959	0	2.896740642	0	20	1	chr6_13734_mature	1.611855	0	1.38507475	0	20	0.641553
chr14_30700_star	0	1.611855	0	1.38507475	-20	0.407187	chr8_18481_mature	6.44741959	0	2.896740642	0	20	1	chr4_8705_mature	1.611855	0	1.38507475	0	20	0.641553
chr12_26438_star	0	1.611855	0	1.38507475	-20	0.407187	chr2_5067_star	6.44741959	0	2.896740642	0	20	1	chr12_26154_star	1.611855	0	1.38507475	0	20	0.641553
hsa-miR-5580-3p	0	1.611855	0	1.38507475	-20	0.407187	chr18_36156_star	6.44741959	0	2.896740642	0	20	1	chr5_10912_star	19.87954	5.641492	4.384018281	2.731507408	1.817136	0.642367
NW_003613562_39706_mature	0	1.611855	0	1.38507475	-20	0.407187	chr1_268_star	6.44741959	0	2.896740642	0	20	1	chr15_31849_mature	2.686425	7.253347	1.882222339	3.044979305	-1.43296	0.64254
mmu-miR-669j	0	1.611855	0	1.38507475	-20	0.407187	chr14_29582_star	6.44741959	0	2.896740642	0	20	1	chr16_34272_mature	2.686425	7.253347	1.882222339	3.044979305	-1.43296	0.64254
chr1_1402_star	0	1.611855	0	1.38507475	-20	0.407187	chr14_30043_star	6.44741959	0	2.896740642	0	20	1	chr16_34837_mature	2.686425	7.253347	1.882222339	3.044979305	-1.43296	0.64254
chr5_11998_star	0	1.611855	0	1.38507475	-20	0.407187	chr4_8765_star	6.44741959	0	2.896740642	0	20	1	hsa-miR-3651	2.686425	7.253347	1.882222339	3.044979305	-1.43296	0.64254
mmu-miR-5619-5p	0	1.611855	0	1.38507475	-20	0.407187	chr14_30478_star	6.44741959	0	2.896740642	0	20	1	mmu-miR-6420	2.686425	7.253347	1.882222339	3.044979305	-1.43296	0.64254
chr18_36375_mature	0	1.611855	0	1.38507475	-20	0.407187	NW_003613191_37580_mature	6.44741959	0	2.896740642	0	20	1	mmu-miR-6362	2.686425	7.253347	1.882222339	3.044979305	-1.43296	0.64254
chr13_28441_star	0	1.611855	0	1.38507475	-20	0.407187	mmu-miR-673-5p	6.44741959	0	2.896740642	0	20	1	chr11_25333_star	2.686425	7.253347	1.882222339	3.044979305	-1.43296	0.64254
hsa-miR-2277-5p	0	1.611855	0	1.38507475	-20	0.407187	chr16_33726_mature	6.44741959	0	2.896740642	0	20	1	chr16_34708_star	2.686425	7.253347	1.882222339	3.044979305	-1.43296	0.64254
chr5_11967_star	0	1.611855	0	1.38507475	-20	0.407187	chr16_33919_star	6.44741959	0	2.896740642	0	20	1	mmu-miR-1194	2.686425	7.253347	1.882222339	3.044979305	-1.43296	0.64254
chr1_1628_star	0	1.611855	0	1.38507475	-20	0.407187	chr11_25225_mature	6.44741959	0	2.896740642	0	20	1	chr18_36575_mature	2.686425	7.253347	1.882222339	3.044979305	-1.43296	0.64254
hsa-miR-506-3p	0	1.611855	0	1.38507475	-20	0.407187	chr8_17367_mature	6.44741959	0	2.896740642	0	20	1	chrx_22620_star	2.686425	7.253347	1.882222339	3.044979305	-1.43296	0.64254
chr1_662_mature	0	1.611855	0	1.38507475	-20	0.407187	chr2_4579_mature	6.44741959	0	2.896740642	0	20	1	chr2_4016_star	2.686425	7.253347	1.882222339	3.044979305	-1.43296	0.64254
chr1_539_mature	0	1.611855	0	1.38507475	-20	0.407187	chr7_16402_star	6.44741959	0	2.896740642	0	20	1	hsa-miR-4303	2.686425	7.253347	1.882222339	3.044979305	-1.43296	0.64254
chr13_29079_mature	0	1.611855	0	1.38507475	-20	0.407187	NW_003539279_38689_star	6.44741959	0	2.896740642	0	20	1	chr15_33370_star	2.686425	7.253347	1.882222339	3.044979305	-1.43296	0.64254
ssc-miR-18a	7038.433053	347.3547	12.78124353	8.444413344	4.340773	0.408515	chr12_25810_star	6.44741959	0	2.896740642	0	20	1	chr13_28716_mature	22.02868	149.9025	4.525360039	7.237472949	-2.76657	0.642893
chr7_16587_star	30.08795809	7.253347	4.958283956	3.044979305	2.052467	0.408683	chr8_17918_mature	6.44741959	0	2.896740642	0	20	1	chr6_15014_star	5.910135	21.76004	2.788713818	4.508431259	-1.88042	0.642958
chr7_16674_star	4.298279727	14.50669	2.405524014	3.954819241	-1.75489	0.408772	mmu-miR-6396	6.44741959	0	2.896740642	0	20	1	chr11_24430_star	24.17782	6.44742	4.654081667	2.896740642	1.906891	0.643
chr4_10016_mature	4.298279727	14.50669	2.405524014	3.954819241	-1.75489	0.408772	hsa-miR-5006-3p	6.44741959	0	2.896740642	0	20	1	chr2_4605_star	1.611855	4.029637	1.38507475	2.330454351	-1.32193	0.643326
chr7_17045_mature	4.298279727	14.50669	2.405524014	3.954819241	-1.75489	0.408772	chrx_21532_star	6.44741959	0	2.896740642	0	20	1	chr2_4423_star	1.611855	4.029637	1.38507475	2.330454351	-1.32193	0.643326
chr13_27285_mature	17.19311891	106.3824	4.185320985	6.746614057	-2.62936	0.408935	chr16_33726_star	6.44741959	0	2.896740642	0	20	1	chr5_11269_star	1.611855	4.029637	1.38507475	2.330454351	-1.32193	0.643326
chr4_9495_mature	7.521989523	31.43117	3.091190278	5.019309189	-2.06301	0.410089	chrx_22097_star	6.44741959	0	2.896740642	0	20	1	chr1_2982_star	1.611855	4.029637	1.38507475	2.330454351	-1.32193	0.643326
chr13_28716_mature	2177.078682	149.9025	11.08884036	7.237472949	3.860297	0.410114	chrx_21832_star	6.44741959	0	2.896740642	0	20	1	chr10_24049_star	1.611855	4.029637	1.38507475	2.330454351	-1.32193	0.643326
NW_003540278_39378_star	67.6979057	826.0756	6.102194213	9.691875458	-3.60909	0.410473	hsa-miR-4529-5p	6.44741959	0	2.896740642	0	20	1	chr3_6868_mature	1.611855	4.029637	1.38507475	2.330454351	-1.32193	0.643326
chr4_9182_mature	13.96940911	77.36904	3.90394537	6.292211829	-2.46949	0.411361	chr12_26608_star	6.44741959	0	2.896740642	0	20	1	chr6_14787_star	1.611855	4.029637	1.38507475	2.330454351	-1.32193	0.643326
chr2_4818_mature	26.8642483	205.5115	4.800343329	7.690078309	-2.93546	0.411411	chr11_24884_star	6.44741959	0	2.896740642	0	20	1	chr17_34920_star	1.611855	4.029637	1.38507475	2.330454351	-1.32193	0.643326
ssc-miR-184	73.07075536	13.70077	6.210832145	3.877819487	2.415037	0.411622	chr13_27900_mature	6.44741959	0	2.896740642	0	20	1	chr3_7249_mature	1.611855	4.029637	1.38507475	2.330454351	-1.32193	0.643326
chr8_17643_mature	5.372849659	19.34226	2.671938627	4.346407978	-1.848	0.411689	mmu-miR-669k-5p	6.44741959	0	2.896740642	0	20	1	chr1_991_star	1.611855	4.029637	1.38507475	2.330454351	-1.32193	0.643326
chr14_30443_mature	5.372849659	19.34226	2.671938627	4.346407978	-1.848	0.411689	mmu-miR-654-5p	6.44741959	0	2.896740642	0	20	1	chr15_33559_star	1.611855	4.029637	1.38507475	2.330454351	-1.32193	0.643326
chr6_14141_star	5.372849659	19.34226	2.671938627	4.346407978	-1.848	0.411689	chr4_9832_mature	6.44741959	0	2.896740642	0	20	1	chr4_9751_star	1.611855	4.029637	1.38507475	2.330454351	-1.32193	0.643326
chr16_33741_mature	34.38623782	8.059274	5.145116481	3.179395517	2.093109	0.411941	NW_003613031_36829_mature	19.3422588	4.835565	4.346407978	2.544872269	2	1	chr14_30385_star	1.611855	4.029637	1.38507475	2.330454351	-1.32193	0.643326
chr6_14501_mature	3.223709795	9.671129	2.078510713	3.415640968	-1.58496	0.413108	chr7_17178_mature	6.44741959	0	2.896740642	0	20	1	chr6_13257_star	1.611855	4.029637	1.38507475	2.330454351	-1.32193	0.643326
chr2_4363_star	3.223709795	9.671129	2.078510713	3.415640968	-1.58496	0.413108	NW_003539007_38444_star	8.59655945	31.43117	3.262517265	5.019309189	-1.87036	1	NW_003539576_38951_star	1.611855	4.029637	1.38507475	2.330454351	-1.32193	0.643326
chr13_28700_mature	3.223709795	9.671129	2.078510713	3.415640968	-1.58496	0.413108	chr18_36542_mature	6.44741959	0	2.896740642	0	20	1	chr15_33276_star	1.611855	4.029637	1.38507475	2.330454351	-1.32193	0.643326
chr16_34013_star	3.223709795	9.671129	2.078510713	3.415640968	-1.58496	0.413108	mmu-miR-3966	6.44741959	0	2.896740642	0	20	1	chr2_4882_star	1.611855	4.029637	1.38507475	2.330454351	-1.32193	0.643326
chr8_18464_star	1.074569932	3.22371	1.05281229	2.078510713	-1.58496	0.413808	bta-miR-152	6.44741959	0	2.896740642	0	20	1	chr1_1498_star	1.611855	4.029637	1.38507475	2.330454351	-1.32193	0.643326
hsa-miR-4765	1.074569932	3.22371	1.05281229	2.078510713	-1.58496	0.413808	hsa-miR-6133	6.44741959	0	2.896740642	0	20	1	chr10_23883_star	1.611855	4.029637	1.38507475	2.330454351	-1.32193	0.643326
chr13_28551_mature	1.074569932	3.22371	1.05281229	2.078510713	-1.58496	0.413808	chr3_8236_mature	6.44741959	0	2.896740642	0	20	1	chr15_33045_mature	1.611855	4.029637	1.38507475	2.330454351	-1.32193	0.643326
chr15_32959_mature	1.074569932	3.22371	1.05281229	2.078510713	-1.58496	0.413808	ssc-miR-1277	6.44741959	0	2.896740642	0	20	1	chr9_20399_star	1.611855	4.029637	1.38507475	2.330454351	-1.32193	0.643326
chr6_14513_star	1.074569932	3.22371	1.05281229	2.078510713	-1.58496	0.413808	chr11_24429_mature	19.3422588	4.835565	4.346407978	2.544872269	2	1	chr5_10752_mature	1.611855	4.029637	1.38507475	2.330454351	-1.32193	0.643326
chr17_35522_star	1.074569932	3.22371	1.05281229	2.078510713	-1.58496	0.413808	mmu-miR-3113-3p	6.44741959	0	2.896740642	0	20	1	chr17_35548_mature	4.29828	13.70077	2.405524014	3.877819487	-1.67243	0.643479
chr9_20139_mature	1.074569932	3.22371	1.05281229	2.078510713	-1.58496	0.413808	chr16_34547_mature	6.44741959	0	2.896740642	0	20	1	chrx_23209_star	4.29828	13.70077	2.405524014	3.877819487	-1.67243	0.643479
hsa-miR-520d-3p	1.074569932	3.22371	1.05281229	2.078510713	-1.58496	0.413808	mmu-miR-3063-3p	6.44741959	0	2.896740642	0	20	1	chr18_36225_star	3.760995	11.28298	2.251263041	3.618589217	-1.58496	0.64429
chr7_15080_star	1.074569932	3.22371	1.05281229	2.078510713	-1.58496	0.413808	hsa-miR-3201	6.44741959	0	2.896740642	0	20	1	chr11_25010_star	3.760995	11.28298	2.251263041	3.618589217	-1.58496	0.64429
NW_003613109_37259_star	1.074569932	3.22371	1.05281229	2.078510713	-1.58496	0.413808	chr2_4065_star	6.44741959	0	2.896740642	0	20	1	chr2_4930_star	4.835565	16.11855	2.544872269	4.097488514	-1.73697	0.644618
chr13_27234_star	1.074569932	3.22371	1.05281229	2.078510713	-1.58496	0.413808	chr2_5564_mature@@mmu-miR-670-3	19.3422588	4.835565	4.346407978	2.544872269	2	1	mmu-miR-6413	4.835565	16.11855	2.544872269	4.097488514	-1.73697	0.644618
NW_003537656_37099_mature	1.074569932	3.22371	1.05281229	2.078510713	-1.58496	0.413808	mmu-miR-3095-5p	6.44741959	0	2.896740642	0	20	1	chr1_742_mature	4.835565	16.11855	2.544872269	4.097488514	-1.73697	0.644618
hsa-miR-3915	1.074569932	3.22371	1.05281229	2.078510713	-1.58496	0.413808	bta-miR-27a-3p	6.44741959	0	2.896740642	0	20	1	chr6_14061_mature	15.58126	4.835565	4.051482084	2.544872269	1.688056	0.64505
hsa-miR-3914	1.074569932	3.22371	1.05281229	2.078510713	-1.58496	0.413808	chr7_16666_mature@@bta-miR-2305	6.44741959	0	2.896740642	0	20	1	bta-miR-2408	49.9675	10.47706	5.671505736	3.520680821	2.253757	0.646523
chr6_12810_mature	1.074569932	3.22371	1.05281229	2.078510713	-1.58496	0.413808	NW_003540275_39376_mature	30.0879581	10.47706	4.958283956	3.520680821	1.521953	1	NW_003538949_38373_mature	8.596559	37.07266	3.262517265	5.250683565	-2.10852	0.646823
chr14_31047_star	1.074569932	3.22371	1.05281229	2.078510713	-1.58496	0.413808	hsa-miR-3197	6.44741959	0	2.896740642	0	20	1	chr15_32699_star	11.82027	4.029637	3.680354657	2.330454351	1.552541	0.64694
chr7_17077_star	1.074569932	3.22371	1.05281229	2.078510713	-1.58496	0.413808	chr6_12911_star	6.44741959	0	2.896740642	0	20	1	chr1_291_star	11.82027	4.029637	3.680354657	2.330454351	1.552541	0.64694
chr14_29827_mature	1.074569932	3.22371	1.05281229	2.078510713	-1.58496	0.413808	hsa-miR-4258	6.44741959	0	2.896740642	0	20	1	chr10_23377_mature	6.44742	24.17782	2.896740642	4.654081667	-1.90689	0.647477
chr3_8536_star	1.074569932	3.22371	1.05281229	2.078510713	-1.58496	0.413808	chr15_32809_mature	6.44741959	0	2.896740642	0	20	1	ssc-miR-9-2	6252.385	227.2715	12.61042166	7.834607195	4.781918	0.647497
chr4_9286_mature	1.074569932	3.22371	1.05281229	2.078510713	-1.58496	0.413808	chr16_34309_star	6.44741959	0	2.896740642	0	20	1	chr13_27530_mature	3.22371	8.865202	2.078510713	3.302348583	-1.45943	0.647998
chr9_19299_mature	1.074569932	3.22371	1.05281229	2.078510713	-1.58496	0.413808	chr9_20735_star	6.44741959	0	2.896740642	0	20	1	chr9_20807_mature	3.22371	8.865202	2.078510713	3.302348583	-1.45943	0.647998
rno-miR-471-5p	1.074569932	3.22371	1.05281229	2.078510713	-1.58496	0.413808	chr9_20138_mature	6.44741959	0	2.896740642	0	20	1	chr4_9182_star	3.22371	8.865202	2.078510713	3.302348583	-1.45943	0.647998
hsa-miR-2278	1.074569932	3.22371	1.05281229	2.078510713	-1.58496	0.413808	mmu-miR-101c	6.44741959	0	2.896740642	0	20	1	chr13_27796_star	3.22371	8.865202	2.078510713	3.302348583	-1.45943	0.647998
chr3_7937_star	1.074569932	3.22371	1.05281229	2.078510713	-1.58496	0.413808	NW_003613212_37672_mature	6.44741959	0	2.896740642	0	20	1	chr6_14789_mature	8.596559	3.22371	3.262517265	2.078510713	1.415037	0.648035
mmu-miR-1964-5p	1.074569932	3.22371	1.05281229	2.078510713	-1.58496	0.413808	chrx_23198_star	6.44741959	0	2.896740642	0	20	1	chr4_9294_mature	8.596559	3.22371	3.262517265	2.078510713	1.415037	0.648035
chr1_1422_mature	1.074569932	3.22371	1.05281229	2.078510713	-1.58496	0.413808	chr15_32157_mature	6.44741959	0	2.896740642	0	20	1	chr7_16583_mature	8.596559	3.22371	3.262517265	2.078510713	1.415037	0.648035
NW_003538401_37828_mature	1.074569932	3.22371	1.05281229	2.078510713	-1.58496	0.413808	chrx_22153_mature	6.44741959	0	2.896740642	0	20	1	chr13_28026_mature	8.596559	3.22371	3.262517265	2.078510713	1.415037	0.648035
chr1_1312_mature	1.074569932	3.22371	1.05281229	2.078510713	-1.58496	0.413808	chr18_36004_star	6.44741959	0	2.896740642	0	20	1	chr13_28842_mature	8.596559	3.22371	3.262517265	2.078510713	1.415037	0.648035
chr11_25256_star	1.074569932	3.22371	1.05281229	2.078510713	-1.58496	0.413808	NW_003539350_38744_star	6.44741959	0	2.896740642	0	20	1	chrx_22488_star	8.596559	3.22371	3.262517265	2.078510713	1.415037	0.648035
chrx_21746_star	1.074569932	3.22371	1.05281229	2.078510713	-1.58496	0.413808	hsa-miR-662	6.44741959	0	2.896740642	0	20	1	chr13_28070_mature	18.26769	112.0239	4.268111626	6.820484263	-2.61644	0.648409
NW_003538236_37641_mature	1.074569932	3.22371	1.05281229	2.078510713	-1.58496	0.413808	chr14_31327_star	6.44741959	0	2.896740642	0	20	1	chr10_23289_mature	138.0822	20.14819	7.119794359	4.402462031	2.776806	0.648468
chr3_7373_star	1.074569932	3.22371	1.05281229	2.078510713	-1.58496	0.413808	bta-miR-2377	6.44741959	0	2.896740642	0	20	1	chr3_6872_mature	5.910135	2.417782	2.788713818	1.773060525	1.289507	0.648807
chr12_25421_star	1.074569932	3.22371	1.05281229	2.078510713	-1.58496	0.413808	chr3_7424_star	6.44741959	0	2.896740642	0	20	1	chr9_20857_star	5.910135	2.417782	2.788713818	1.773060525	1.289507	0.648807
rno-miR-19b-2-5p	1.074569932	3.22371	1.05281229	2.078510713	-1.58496	0.413808	rno-miR-879-5p	6.44741959	0	2.896740642	0	20	1	chr13_27058_star	5.910135	2.417782	2.788713818	1.773060525	1.289507	0.648807
bta-miR-2285d	1.074569932	3.22371	1.05281229	2.078510713	-1.58496	0.413808	mmu-miR-697	6.44741959	0	2.896740642	0	20	1	chr13_27411_mature	5.910135	2.417782	2.788713818	1.773060525	1.289507	0.648807
chr6_14575_star	1.074569932	3.22371	1.05281229	2.078510713	-1.58496	0.413808	mmu-miR-694	6.44741959	0	2.896740642	0	20	1	hsa-miR-3662	5.910135	2.417782	2.788713818	1.773060525	1.289507	0.648807
chr8_17233_star	1.074569932	3.22371	1.05281229	2.078510713	-1.58496	0.413808	chr13_27058_mature	6.44741959	0	2.896740642	0	20	1	chr7_16979_mature	5.910135	2.417782	2.788713818	1.773060525	1.289507	0.648807
chr11_24488_mature	1.074569932	3.22371	1.05281229	2.078510713	-1.58496	0.413808	chr14_31235_mature@@bta-miR-346	19.3422588	4.835565	4.346407978	2.544872269	2	1	chr1_1256_star	5.910135	2.417782	2.788713818	1.773060525	1.289507	0.648807
chr18_36498_mature	1.074569932	3.22371	1.05281229	2.078510713	-1.58496	0.413808	chr4_8979_star	6.44741959	0	2.896740642	0	20	1	chr5_11905_star	5.910135	2.417782	2.788713818	1.773060525	1.289507	0.648807
chr11_25010_mature	1.074569932	3.22371	1.05281229	2.078510713	-1.58496	0.413808	chr6_14314_star	6.44741959	0	2.896740642	0	20	1	chr13_28207_mature	5.910135	2.417782	2.788713818	1.773060525	1.289507	0.648807
NW_003613063_36957_star	1.074569932	3.22371	1.05281229	2.078510713	-1.58496	0.413808	chr1_3888_mature	6.44741959	0	2.896740642	0	20	1	chr2_5754_star	5.910135	2.417782	2.788713818	1.773060525	1.289507	0.648807
NW_003537125_36714_star	1.074569932	3.22371	1.05281229	2.078510713	-1.58496	0.413808	chr15_33182_star	6.44741959	0	2.896740642	0	20	1	chr13_27902_mature	5.910135	2.417782	2.788713818	1.773060525	1.289507	0.648807
bta-miR-1777a	1.074569932	3.22371	1.05281229	2.078510713	-1.58496	0.413808	chr13_28063_star	6.44741959	0	2.896740642	0	20	1	chr2_4295_star	5.910135	2.417782	2.788713818	1.773060525	1.289507	0.648807
chr1_88_star	1.074569932	3.22371	1.05281229	2.078510713	-1.58496	0.413808	mmu-miR-302b-5p	6.44741959	0	2.896740642	0	20	1	mmu-miR-3089-3p	5.910135	2.417782	2.788713818	1.773060525	1.289507	0.648807
chr14_31127_mature	1.074569932	3.22371	1.05281229	2.078510713	-1.58496	0.413808	hsa-miR-802	6.44741959	0	2.896740642	0	20	1	chr14_29632_mature	5.910135	2.417782	2.788713818	1.773060525	1.289507	0.648807
chr4_9970_mature	1.074569932	3.22371	1.05281229	2.078510713	-1.58496	0.413808	chr8_17482_mature	6.44741959	0	2.896740642	0	20	1	chr1_79_star	5.910135	2.417782	2.788713818	1.773060525	1.289507	0.648807
chr17_35278_mature	1.074569932	3.22371	1.05281229	2.078510713	-1.58496	0.413808	mmu-miR-762	6.44741959	0	2.896740642	0	20	1	NW_003537262_36795_star	3.760995	1.611855	2.251263041	1.38507475	1.222392	0.65114
chr13_27982_star	1.074569932	3.22371	1.05281229	2.078510713	-1.58496	0.413808	chr2_5267_star	6.44741959	0	2.896740642	0	20	1	NW_003539070_38505_star	3.760995	1.611855	2.251263041	1.38507475	1.222392	0.65114
chr4_9423_mature	1.074569932	3.22371	1.05281229	2.078510713	-1.58496	0.413808	chr15_33692_star	6.44741959	0	2.896740642	0	20	1	chr17_34998_star	3.760995	1.611855	2.251263041	1.38507475	1.222392	0.65114
chr1_211_star	1.074569932	3.22371	1.05281229	2.078510713	-1.58496	0.413808	chr3_7381_mature	6.44741959	0	2.896740642	0	20	1	chr6_13246_star	3.760995	1.611855	2.251263041	1.38507475	1.222392	0.65114
bta-miR-329b	1.074569932	3.22371	1.05281229	2.078510713	-1.58496	0.413808	chr4_9100_mature	6.44741959	0	2.896740642	0	20	1	chr15_32346_mature	3.760995	1.611855	2.251263041	1.38507475	1.222392	0.65114
NW_003540346_39431_star	1.074569932	3.22371	1.05281229	2.078510713	-1.58496	0.413808	chr15_33512_star	6.44741959	0	2.896740642	0	20	1	ssc-miR-181d-3p	3.760995	1.611855	2.251263041	1.38507475	1.222392	0.65114
chr14_31138_mature	1.074569932	3.22371	1.05281229	2.078510713	-1.58496	0.413808	hsa-miR-4703-5p	6.44741959	0	2.896740642	0	20	1	chr11_25386_star	3.760995	1.611855	2.251263041	1.38507475	1.222392	0.65114
chr1_766_star	1.074569932	3.22371	1.05281229	2.078510713	-1.58496	0.413808	hsa-miR-4522	6.44741959	0	2.896740642	0	20	1	rno-miR-378b	3.760995	1.611855	2.251263041	1.38507475	1.222392	0.65114
chr6_12809_mature	1.074569932	3.22371	1.05281229	2.078510713	-1.58496	0.413808	chr18_36331_mature	6.44741959	0	2.896740642	0	20	1	chr14_29632_star	3.760995	1.611855	2.251263041	1.38507475	1.222392	0.65114
chr1_3471_mature	1.074569932	3.22371	1.05281229	2.078510713	-1.58496	0.413808	chr13_28708_star	6.44741959	0	2.896740642	0	20	1	chr8_17646_star	3.760995	1.611855	2.251263041	1.38507475	1.222392	0.65114
chr12_25994_star	1.074569932	3.22371	1.05281229	2.078510713	-1.58496	0.413808	chr12_25952_star	6.44741959	0	2.896740642	0	20	1	mmu-miR-1953	3.760995	1.611855	2.251263041	1.38507475	1.222392	0.65114
chr8_18259_star	1.074569932	3.22371	1.05281229	2.078510713	-1.58496	0.413808	chr8_18374_mature	6.44741959	0	2.896740642	0	20	1	chr15_31926_star	3.760995	1.611855	2.251263041	1.38507475	1.222392	0.65114
chr1_1390_mature	1.074569932	3.22371	1.05281229	2.078510713	-1.58496	0.413808	mmu-miR-465c-3p	6.44741959	0	2.896740642	0	20	1	mmu-miR-1969	3.760995	1.611855	2.251263041	1.38507475	1.222392	0.65114
chr7_16061_mature	1.074569932	3.22371	1.05281229	2.078510713	-1.58496	0.413808	chr18_35940_star	6.44741959	0	2.896740642	0	20	1	rno-miR-673-3p	3.760995	1.611855	2.251263041	1.38507475	1.222392	0.65114
chr1_1386_star	1.074569932	3.22371	1.05281229	2.078510713	-1.58496	0.413808	bta-miR-1388-5p	6.44741959	0	2.896740642	0	20	1	hsa-miR-151b	3.760995	1.611855	2.251263041	1.38507475	1.222392	0.65114
bta-miR-2404	1.074569932	3.22371	1.05281229	2.078510713	-1.58496	0.413808	chr4_10131_mature	4.29827973	22.56597	2.405524014	4.558633073	-2.39232	1	chr8_18571_mature	3.760995	1.611855	2.251263041	1.38507475	1.222392	0.65114
hsa-miR-4466	1.074569932	3.22371	1.05281229	2.078510713	-1.58496	0.413808	chr12_25812_star	17.1931189	4.029637	4.185320985	2.330454351	2.093109	1	chr14_31148_mature	3.760995	1.611855	2.251263041	1.38507475	1.222392	0.65114
chr3_6877_mature	1.074569932	3.22371	1.05281229	2.078510713	-1.58496	0.413808	chr13_28621_mature	17.1931189	45.93786	4.185320985	5.552680304	-1.41785	1	chr2_5585_star	3.760995	1.611855	2.251263041	1.38507475	1.222392	0.65114
hsa-miR-519c-3p	1.074569932	3.22371	1.05281229	2.078510713	-1.58496	0.413808	chr7_15712_mature	17.1931189	4.029637	4.185320985	2.330454351	2.093109	1	rno-miR-466c-3p	3.760995	1.611855	2.251263041	1.38507475	1.222392	0.65114
chr7_15205_star	1.074569932	3.22371	1.05281229	2.078510713	-1.58496	0.413808	chrx_21498_mature	85.9655945	46.74379	6.442372848	5.577241251	0.878985	1	chr2_5608_star	3.760995	1.611855	2.251263041	1.38507475	1.222392	0.65114
chr1_2086_star	1.074569932	3.22371	1.05281229	2.078510713	-1.58496	0.413808	chr1_2593_mature	17.1931189	4.029637	4.185320985	2.330454351	2.093109	1	chr8_17239_mature	3.760995	1.611855	2.251263041	1.38507475	1.222392	0.65114
bta-miR-2361	1.074569932	3.22371	1.05281229	2.078510713	-1.58496	0.413808	chrx_22025_mature	17.1931189	45.93786	4.185320985	5.552680304	-1.41785	1	chr13_27034_mature	3.760995	1.611855	2.251263041	1.38507475	1.222392	0.65114
chr10_23941_mature	1.074569932	3.22371	1.05281229	2.078510713	-1.58496	0.413808	chr1_1977_star	17.1931189	4.029637	4.185320985	2.330454351	2.093109	1	hsa-miR-146a-3p	3.760995	1.611855	2.251263041	1.38507475	1.222392	0.65114
hsa-miR-4300	1.074569932	3.22371	1.05281229	2.078510713	-1.58496	0.413808	chr10_23450_mature	15.043979	3.22371	4.003960082	2.078510713	2.222392	1	hsa-miR-378j	3.760995	1.611855	2.251263041	1.38507475	1.222392	0.65114
chr18_36111_star	1.074569932	3.22371	1.05281229	2.078510713	-1.58496	0.413808	NW_003538820_38274_star	47.281077	20.95411	5.593385953	4.456419386	1.174029	1	chr1_3928_star	3.760995	1.611855	2.251263041	1.38507475	1.222392	0.65114
chr3_8556_star	1.074569932	3.22371	1.05281229	2.078510713	-1.58496	0.413808	chr16_34246_star	15.043979	3.22371	4.003960082	2.078510713	2.222392	1	chr6_14892_star	3.760995	1.611855	2.251263041	1.38507475	1.222392	0.65114
chrx_22488_star	1.074569932	3.22371	1.05281229	2.078510713	-1.58496	0.413808	chrx_21753_mature	15.043979	3.22371	4.003960082	2.078510713	2.222392	1	chr8_17274_star	3.760995	1.611855	2.251263041	1.38507475	1.222392	0.65114
chr13_28509_star	1.074569932	3.22371	1.05281229	2.078510713	-1.58496	0.413808	chr9_20033_mature	23.6405385	7.253347	4.62296188	3.044979305	1.704544	1	hsa-miR-6717-5p	3.760995	1.611855	2.251263041	1.38507475	1.222392	0.65114
chr9_20257_mature	1.074569932	3.22371	1.05281229	2.078510713	-1.58496	0.413808	chr8_19039_star	15.043979	3.22371	4.003960082	2.078510713	2.222392	1	chr6_12646_star	3.760995	1.611855	2.251263041	1.38507475	1.222392	0.65114
hsa-miR-875-5p	1.074569932	3.22371	1.05281229	2.078510713	-1.58496	0.413808	chr15_32058_mature	2.14913986	17.7304	1.654957834	4.227310103	-3.04439	1	chr9_19650_star	3.760995	1.611855	2.251263041	1.38507475	1.222392	0.65114
chr13_29421_star	1.074569932	3.22371	1.05281229	2.078510713	-1.58496	0.413808	chr9_19233_mature	2.14913986	17.7304	1.654957834	4.227310103	-3.04439	1	chr15_32437_mature	3.760995	1.611855	2.251263041	1.38507475	1.222392	0.65114
chr5_12346_star	1.074569932	3.22371	1.05281229	2.078510713	-1.58496	0.413808	chrx_21804_star	15.043979	3.22371	4.003960082	2.078510713	2.222392	1	bta-miR-2285s	3.760995	1.611855	2.251263041	1.38507475	1.222392	0.65114
chr12_26197_mature	9.671129386	3.22371	3.415640968	2.078510713	1.584963	0.415104	chr6_13900_star	15.043979	3.22371	4.003960082	2.078510713	2.222392	1	bta-miR-2285c	3.760995	1.611855	2.251263041	1.38507475	1.222392	0.65114
chr9_21046_mature	9.671129386	3.22371	3.415640968	2.078510713	1.584963	0.415104	chr6_12549_star	2.14913986	17.7304	1.654957834	4.227310103	-3.04439	1	chr3_8052_star	3.760995	1.611855	2.251263041	1.38507475	1.222392	0.65114
chr11_24692_mature	9.671129386	3.22371	3.415640968	2.078510713	1.584963	0.415104	chr2_4019_mature	15.043979	3.22371	4.003960082	2.078510713	2.222392	1	chr1_5_mature	3.760995	1.611855	2.251263041	1.38507475	1.222392	0.65114
chr1_3113_star	9.671129386	3.22371	3.415640968	2.078510713	1.584963	0.415104	chr11_25144_mature	23.6405385	7.253347	4.62296188	3.044979305	1.704544	1	chr16_33845_star	3.760995	1.611855	2.251263041	1.38507475	1.222392	0.65114
chr4_8765_mature@@hsa-miR-4723	9.671129386	3.22371	3.415640968	2.078510713	1.584963	0.415104	chr3_7237_star	2.14913986	17.7304	1.654957834	4.227310103	-3.04439	1	chr6_14334_mature	3.760995	1.611855	2.251263041	1.38507475	1.222392	0.65114
chr15_32065_mature	60.17591618	12.08891	5.934891897	3.710273245	2.315502	0.415934	ssc-miR-135	77.3690351	41.1023	6.292211829	5.395827139	0.912537	1	chr15_32439_mature	3.760995	1.611855	2.251263041	1.38507475	1.222392	0.65114
chr1_3870_mature	12.89483918	4.029637	3.796477232	2.330454351	1.678072	0.416138	chr17_35278_mature	15.043979	3.22371	4.003960082	2.078510713	2.222392	1	hsa-miR-4802-3p	3.760995	1.611855	2.251263041	1.38507475	1.222392	0.65114
chr17_35500_mature	12.89483918	4.029637	3.796477232	2.330454351	1.678072	0.416138	chr1_1938_mature@@rno-miR-2964	15.043979	3.22371	4.003960082	2.078510713	2.222392	1	chr14_29652_star	3.760995	1.611855	2.251263041	1.38507475	1.222392	0.65114
chr6_13246_star	4.298279727	1.611855	2.405524014	1.38507475	1.415037	0.416579	bta-miR-26a	15.043979	3.22371	4.003960082	2.078510713	2.222392	1	chr15_32200_mature	3.760995	1.611855	2.251263041	1.38507475	1.222392	0.65114
chr1_902_star	4.298279727	1.611855	2.405524014	1.38507475	1.415037	0.416579	chr2_4844_star	2.14913986	17.7304	1.654957834	4.227310103	-3.04439	1	bta-miR-2388-3p	3.760995	1.611855	2.251263041	1.38507475	1.222392	0.65114
chr18_36497_star	4.298279727	1.611855	2.405524014	1.38507475	1.415037	0.416579	chr17_34881_star	15.043979	3.22371	4.003960082	2.078510713	2.222392	1	chr7_15749_star	3.760995	1.611855	2.251263041	1.38507475	1.222392	0.65114
NW_003541124_39922_star	4.298279727	1.611855	2.405524014	1.38507475	1.415037	0.416579	NW_003540202_39340_mature	6.44741959	26.59561	2.896740642	4.786366653	-2.04439	1	NW_003538949_38371_star	3.760995	1.611855	2.251263041	1.38507475	1.222392	0.65114
chr10_23904_star	4.298279727	1.611855	2.405524014	1.38507475	1.415037	0.416579	chr14_31138_mature	15.043979	3.22371	4.003960082	2.078510713	2.222392	1	chr1_589_star	3.760995	1.611855	2.251263041	1.38507475	1.222392	0.65114
chr9_20079_mature	4.298279727	1.611855	2.405524014	1.38507475	1.415037	0.416579	chr8_17733_mature@@mmu-miR-3084	15.043979	3.22371	4.003960082	2.078510713	2.222392	1	chr5_12195_mature	3.760995	1.611855	2.251263041	1.38507475	1.222392	0.65114
chr6_13334_star	4.298279727	1.611855	2.405524014	1.38507475	1.415037	0.416579	chrx_22196_mature	6.44741959	26.59561	2.896740642	4.786366653	-2.04439	1	chr15_32320_star	3.760995	1.611855	2.251263041	1.38507475	1.222392	0.65114
chr14_29876_mature	4.298279727	1.611855	2.405524014	1.38507475	1.415037	0.416579	chr2_4563_mature	15.043979	3.22371	4.003960082	2.078510713	2.222392	1	chr7_16597_mature	3.760995	1.611855	2.251263041	1.38507475	1.222392	0.65114
chr18_36331_star	4.298279727	1.611855	2.405524014	1.38507475	1.415037	0.416579	chr4_9919_mature	15.043979	3.22371	4.003960082	2.078510713	2.222392	1	chr7_16645_star	3.760995	1.611855	2.251263041	1.38507475	1.222392	0.65114
chr3_8552_star	4.298279727	1.611855	2.405524014	1.38507475	1.415037	0.416579	chr5_11678_mature	15.043979	3.22371	4.003960082	2.078510713	2.222392	1	NW_003538173_37591_star	3.760995	1.611855	2.251263041	1.38507475	1.222392	0.65114
chr5_12123_mature	4.298279727	1.611855	2.405524014	1.38507475	1.415037	0.416579	chr17_34997_mature	2.14913986	17.7304	1.654957834	4.227310103	-3.04439	1	chr3_8596_star	3.760995	1.611855	2.251263041	1.38507475	1.222392	0.65114
chr6_12563_star	4.298279727	1.611855	2.405524014	1.38507475	1.415037	0.416579	chr5_11941_mature	6.44741959	26.59561	2.896740642	4.786366653	-2.04439	1	chr14_29856_star	3.760995	1.611855	2.251263041	1.38507475	1.222392	0.65114
bta-miR-2483-5p	4.298279727	1.611855	2.405524014	1.38507475	1.415037	0.416579	chr6_14951_mature	10.7456993	1.611855	3.554060706	1.38507475	2.736966	1	chr17_35398_mature	3.760995	1.611855	2.251263041	1.38507475	1.222392	0.65114
chr3_6843_star@@hsa-miR-4753-3p	4.298279727	1.611855	2.405524014	1.38507475	1.415037	0.416579	chr5_11960_star	8.59655945	0.805927	3.262517265	0.852739935	3.415037	1	hsa-miR-4689	3.760995	1.611855	2.251263041	1.38507475	1.222392	0.65114
hsa-miR-3609	4.298279727	1.611855	2.405524014	1.38507475	1.415037	0.416579	chr6_14848_star	10.7456993	1.611855	3.554060706	1.38507475	2.736966	1	chr2_5742_mature	3.760995	1.611855	2.251263041	1.38507475	1.222392	0.65114
chr2_5608_star	4.298279727	1.611855	2.405524014	1.38507475	1.415037	0.416579	NW_003613223_37737_mature	10.7456993	1.611855	3.554060706	1.38507475	2.736966	1	rno-miR-291b	3.760995	1.611855	2.251263041	1.38507475	1.222392	0.65114
rno-miR-6315	4.298279727	1.611855	2.405524014	1.38507475	1.415037	0.416579	chr3_7237_mature	8.59655945	0.805927	3.262517265	0.852739935	3.415037	1	chr1_3928_mature	3.760995	1.611855	2.251263041	1.38507475	1.222392	0.65114
NW_003539609_38987_star	4.298279727	1.611855	2.405524014	1.38507475	1.415037	0.416579	chr5_12416_star	8.59655945	0.805927	3.262517265	0.852739935	3.415037	1	chr15_32525_star	3.760995	1.611855	2.251263041	1.38507475	1.222392	0.65114
chr17_35289_mature	4.298279727	1.611855	2.405524014	1.38507475	1.415037	0.416579	chr14_31627_mature	8.59655945	0.805927	3.262517265	0.852739935	3.415037	1	mmu-miR-1895	18.80497	5.641492	4.307790888	2.731507408	1.736966	0.651458
chr4_9333_mature	4.298279727	1.611855	2.405524014	1.38507475	1.415037	0.416579	chr9_19536_mature	4.29827973	21.76004	2.405524014	4.508431259	-2.33985	1	NW_003613325_38551_mature	18.80497	5.641492	4.307790888	2.731507408	1.736966	0.651458
bta-let-7a-5p	4.298279727	1.611855	2.405524014	1.38507475	1.415037	0.416579	chr6_15014_star	4.29827973	21.76004	2.405524014	4.508431259	-2.33985	1	hsa-miR-3691-3p	18.80497	5.641492	4.307790888	2.731507408	1.736966	0.651458
chr5_11418_star	4.298279727	1.611855	2.405524014	1.38507475	1.415037	0.416579	chr13_28152_mature	10.7456993	1.611855	3.554060706	1.38507475	2.736966	1	chrx_22196_mature	6.984705	26.59561	2.997239027	4.786366653	-1.92892	0.6523
chr13_26807_star	4.298279727	1.611855	2.405524014	1.38507475	1.415037	0.416579	hsa-miR-4763-5p	8.59655945	0.805927	3.262517265	0.852739935	3.415037	1	chrx_22664_mature	11.28298	4.029637	3.618589217	2.330454351	1.485427	0.653073
chr18_36088_star	4.298279727	1.611855	2.405524014	1.38507475	1.415037	0.416579	chr11_25086_mature	8.59655945	0.805927	3.262517265	0.852739935	3.415037	1	chrx_22034_mature	11.28298	4.029637	3.618589217	2.330454351	1.485427	0.653073
chr9_19419_mature	4.298279727	1.611855	2.405524014	1.38507475	1.415037	0.416579	chr2_4320_mature	8.59655945	0.805927	3.262517265	0.852739935	3.415037	1	chr15_33061_mature	11.28298	4.029637	3.618589217	2.330454351	1.485427	0.653073
bta-miR-6119-5p	4.298279727	1.611855	2.405524014	1.38507475	1.415037	0.416579	bta-let-7d	8.59655945	0.805927	3.262517265	0.852739935	3.415037	1	chr7_15245_mature	9.133844	39.49044	3.34110968	5.339509593	-2.11221	0.653214
chr6_14228_mature	4.298279727	1.611855	2.405524014	1.38507475	1.415037	0.416579	chr2_6574_mature	27.9388182	9.671129	4.854934103	3.415640968	1.530515	1	chr10_24200_mature	6.44742	23.3719	2.896740642	4.607146585	-1.85798	0.654319
chr1_3348_mature	4.298279727	1.611855	2.405524014	1.38507475	1.415037	0.416579	chr4_9495_star	8.59655945	0.805927	3.262517265	0.852739935	3.415037	1	chrx_21983_star	3.760995	10.47706	2.251263041	3.520680821	-1.47805	0.654899
chr14_30379_mature	4.298279727	1.611855	2.405524014	1.38507475	1.415037	0.416579	chr9_20685_mature	10.7456993	1.611855	3.554060706	1.38507475	2.736966	1	chr15_32006_mature	3.760995	10.47706	2.251263041	3.520680821	-1.47805	0.654899
NW_003540508_39511_mature	4.298279727	1.611855	2.405524014	1.38507475	1.415037	0.416579	chr3_7684_mature	10.7456993	1.611855	3.554060706	1.38507475	2.736966	1	chr14_30666_star	3.760995	10.47706	2.251263041	3.520680821	-1.47805	0.654899
chr8_17778_star	4.298279727	1.611855	2.405524014	1.38507475	1.415037	0.416579	chr12_26282_star	8.59655945	0.805927	3.262517265	0.852739935	3.415037	1	chr8_18299_mature	3.760995	10.47706	2.251263041	3.520680821	-1.47805	0.654899
chr1_3714_star	4.298279727	1.611855	2.405524014	1.38507475	1.415037	0.416579	NW_003539992_39208_star	8.59655945	0.805927	3.262517265	0.852739935	3.415037	1	chr9_19738_star	3.760995	10.47706	2.251263041	3.520680821	-1.47805	0.654899
NW_003538075_37489_star	4.298279727	1.611855	2.405524014	1.38507475	1.415037	0.416579	NW_003613251_37928_mature	12.8948392	2.417782	3.796477232	1.773060525	2.415037	1	chr8_17430_star	3.760995	10.47706	2.251263041	3.520680821	-1.47805	0.654899
mmu-miR-653-3p	4.298279727	1.611855	2.405524014	1.38507475	1.415037	0.416579	hsa-miR-624-5p	8.59655945	0.805927	3.262517265	0.852739935	3.415037	1	chr10_24085_star	8.059274	3.22371	3.179395517	2.078510713	1.321928	0.655307
chr1_3433_mature	4.298279727	1.611855	2.405524014	1.38507475	1.415037	0.416579	chr2_5482_star	8.59655945	0.805927	3.262517265	0.852739935	3.415037	1	NW_003613303_38366_mature	8.059274	3.22371	3.179395517	2.078510713	1.321928	0.655307
chr13_29080_star	4.298279727	1.611855	2.405524014	1.38507475	1.415037	0.416579	chr9_20900_mature	4.29827973	21.76004	2.405524014	4.508431259	-2.33985	1	chr1_2879_mature	14.50669	4.835565	3.954819241	2.544872269	1.584963	0.655525
NW_003613290_38304_mature	4.298279727	1.611855	2.405524014	1.38507475	1.415037	0.416579	chr7_15098_star	8.59655945	0.805927	3.262517265	0.852739935	3.415037	1	chr8_17892_star	14.50669	4.835565	3.954819241	2.544872269	1.584963	0.655525
chr12_26404_star	4.298279727	1.611855	2.405524014	1.38507475	1.415037	0.416579	chr17_35115_mature	10.7456993	1.611855	3.554060706	1.38507475	2.736966	1	chr14_31467_mature	328.2811	35.46081	8.363175959	5.188274618	3.210634	0.655742
chrx_23005_star	4.298279727	1.611855	2.405524014	1.38507475	1.415037	0.416579	chr9_19709_mature	10.7456993	1.611855	3.554060706	1.38507475	2.736966	1	chr1_918_star	2.14914	4.835565	1.654957834	2.544872269	-1.16993	0.655829
chr6_14212_mature	4.298279727	1.611855	2.405524014	1.38507475	1.415037	0.416579	rno-miR-34b-3p	8.59655945	0.805927	3.262517265	0.852739935	3.415037	1	rno-miR-3583-5p	2.14914	4.835565	1.654957834	2.544872269	-1.16993	0.655829
chr1_217_star	4.298279727	1.611855	2.405524014	1.38507475	1.415037	0.416579	chr2_4549_star	8.59655945	0.805927	3.262517265	0.852739935	3.415037	1	chr2_4529_star	2.14914	4.835565	1.654957834	2.544872269	-1.16993	0.655829
chr4_10432_mature	4.298279727	1.611855	2.405524014	1.38507475	1.415037	0.416579	chr2_5392_star	10.7456993	1.611855	3.554060706	1.38507475	2.736966	1	hsa-miR-1293	2.14914	4.835565	1.654957834	2.544872269	-1.16993	0.655829
chr8_18481_star	4.298279727	1.611855	2.405524014	1.38507475	1.415037	0.416579	chr12_25952_mature	8.59655945	0.805927	3.262517265	0.852739935	3.415037	1	chr2_6403_star	2.14914	4.835565	1.654957834	2.544872269	-1.16993	0.655829
chr15_33460_star	4.298279727	1.611855	2.405524014	1.38507475	1.415037	0.416579	chr17_35446_star	8.59655945	0.805927	3.262517265	0.852739935	3.415037	1	NW_003613440_39150_mature	2.14914	4.835565	1.654957834	2.544872269	-1.16993	0.655829
chr15_31849_star	4.298279727	1.611855	2.405524014	1.38507475	1.415037	0.416579	chr6_14428_mature	10.7456993	1.611855	3.554060706	1.38507475	2.736966	1	rno-miR-668	2.14914	4.835565	1.654957834	2.544872269	-1.16993	0.655829
hsa-miR-193a-3p	4.298279727	1.611855	2.405524014	1.38507475	1.415037	0.416579	hsa-miR-4646-5p	8.59655945	0.805927	3.262517265	0.852739935	3.415037	1	chr11_25269_mature	2.14914	4.835565	1.654957834	2.544872269	-1.16993	0.655829
chr9_19744_mature	4.298279727	1.611855	2.405524014	1.38507475	1.415037	0.416579	chr12_25591_mature	8.59655945	0.805927	3.262517265	0.852739935	3.415037	1	hsa-miR-509-3p	2.14914	4.835565	1.654957834	2.544872269	-1.16993	0.655829
NW_003539018_38471_star	4.298279727	1.611855	2.405524014	1.38507475	1.415037	0.416579	chr6_14178_mature	10.7456993	1.611855	3.554060706	1.38507475	2.736966	1	NW_003613480_39328_mature	2.14914	4.835565	1.654957834	2.544872269	-1.16993	0.655829
chr2_5742_mature	4.298279727	1.611855	2.405524014	1.38507475	1.415037	0.416579	chr11_24794_mature	10.7456993	1.611855	3.554060706	1.38507475	2.736966	1	chr8_18024_star	2.14914	4.835565	1.654957834	2.544872269	-1.16993	0.655829
chr6_12506_mature	4.298279727	1.611855	2.405524014	1.38507475	1.415037	0.416579	chr6_15039_mature	10.7456993	1.611855	3.554060706	1.38507475	2.736966	1	bta-miR-2284c	2.14914	4.835565	1.654957834	2.544872269	-1.16993	0.655829
chr6_12752_mature	4.298279727	13.70077	2.405524014	3.877819487	-1.67243	0.417619	chr3_7564_star	10.7456993	1.611855	3.554060706	1.38507475	2.736966	1	chr13_29352_star	2.14914	4.835565	1.654957834	2.544872269	-1.16993	0.655829
NW_003537687_37130_mature	138.6195212	21.76004	7.125356859	4.508431259	2.671377	0.417824	chr6_13603_mature	10.7456993	1.611855	3.554060706	1.38507475	2.736966	1	chr4_10620_star	2.14914	4.835565	1.654957834	2.544872269	-1.16993	0.655829
chrx_22359_mature	6.447419591	24.17782	2.896740642	4.654081667	-1.90689	0.417942	rno-miR-182	8.59655945	0.805927	3.262517265	0.852739935	3.415037	1	chr11_24516_mature	2.14914	4.835565	1.654957834	2.544872269	-1.16993	0.655829
chr9_20405_mature	6.447419591	24.17782	2.896740642	4.654081667	-1.90689	0.417942	chr2_4065_mature	10.7456993	1.611855	3.554060706	1.38507475	2.736966	1	chr11_24488_star	2.14914	4.835565	1.654957834	2.544872269	-1.16993	0.655829
chr5_11922_mature	6.447419591	24.17782	2.896740642	4.654081667	-1.90689	0.417942	chr4_9341_mature	10.7456993	1.611855	3.554060706	1.38507475	2.736966	1	chr2_5406_star	2.14914	4.835565	1.654957834	2.544872269	-1.16993	0.655829
chr6_14284_mature	9.671129386	43.52008	3.415640968	5.476384353	-2.16993	0.418559	hsa-miR-4482-5p	8.59655945	0.805927	3.262517265	0.852739935	3.415037	1	hsa-miR-5581-5p	2.14914	4.835565	1.654957834	2.544872269	-1.16993	0.655829
chr17_35422_star	2.149139864	5.641492	1.654957834	2.731507408	-1.39232	0.419539	chr6_13246_mature	10.7456993	1.611855	3.554060706	1.38507475	2.736966	1	hsa-miR-632	2.14914	4.835565	1.654957834	2.544872269	-1.16993	0.655829
chr9_19622_mature	2.149139864	5.641492	1.654957834	2.731507408	-1.39232	0.419539	chr4_9731_star	12.8948392	2.417782	3.796477232	1.773060525	2.415037	1	chr14_31235_mature@@bta-miR-3	2.14914	4.835565	1.654957834	2.544872269	-1.16993	0.655829
rno-miR-33-3p	2.149139864	5.641492	1.654957834	2.731507408	-1.39232	0.419539	chr4_8669_mature	8.59655945	0.805927	3.262517265	0.852739935	3.415037	1	chr6_12824_star	2.14914	4.835565	1.654957834	2.544872269	-1.16993	0.655829
chrx_22394_star	2.149139864	5.641492	1.654957834	2.731507408	-1.39232	0.419539	chr12_25620_mature	10.7456993	1.611855	3.554060706	1.38507475	2.736966	1	chr7_17190_star	2.14914	4.835565	1.654957834	2.544872269	-1.16993	0.655829
chr4_10041_mature	2.149139864	5.641492	1.654957834	2.731507408	-1.39232	0.419539	bta-miR-665	8.59655945	0.805927	3.262517265	0.852739935	3.415037	1	chr15_32695_mature	2.14914	4.835565	1.654957834	2.544872269	-1.16993	0.655829
chr13_28836_mature	2.149139864	5.641492	1.654957834	2.731507408	-1.39232	0.419539	chr15_32200_star	8.59655945	0.805927	3.262517265	0.852739935	3.415037	1	chr13_29037_star	2.14914	4.835565	1.654957834	2.544872269	-1.16993	0.655829
mmu-miR-5107-3p	2.149139864	5.641492	1.654957834	2.731507408	-1.39232	0.419539	chr11_25156_star	10.7456993	1.611855	3.554060706	1.38507475	2.736966	1	chr3_8486_star	2.14914	4.835565	1.654957834	2.544872269	-1.16993	0.655829
chr11_24398_star	2.149139864	5.641492	1.654957834	2.731507408	-1.39232	0.419539	chr17_35181_mature@@bta-miR-6121-	8.59655945	0.805927	3.262517265	0.852739935	3.415037	1	chr5_10855_star	2.686425	6.44742	1.882222339	2.896740642	-1.26303	0.655949
chr3_8074_mature	2.149139864	5.641492	1.654957834	2.731507408	-1.39232	0.419539	chr7_15773_mature	10.7456993	1.611855	3.554060706	1.38507475	2.736966	1	chr9_19632_mature	2.686425	6.44742	1.882222339	2.896740642	-1.26303	0.655949
chr15_32092_star	2.149139864	5.641492	1.654957834	2.731507408	-1.39232	0.419539	chr1_2507_mature	8.59655945	0.805927	3.262517265	0.852739935	3.415037	1	hsa-miR-526b-5p	2.686425	6.44742	1.882222339	2.896740642	-1.26303	0.655949
NW_003613031_36829_star	2.149139864	5.641492	1.654957834	2.731507408	-1.39232	0.419539	chrx_23183_star	8.59655945	0.805927	3.262517265	0.852739935	3.415037	1	mmu-miR-6356	2.686425	6.44742	1.882222339	2.896740642	-1.26303	0.655949
chrx_22877_mature	2.149139864	5.641492	1.654957834	2.731507408	-1.39232	0.419539	chr1_3489_mature	8.59655945	0.805927	3.262517265	0.852739935	3.415037	1	mmu-miR-3967	2.686425	6.44742	1.882222339	2.896740642	-1.26303	0.655949
chr13_28800_star	2.149139864	5.641492	1.654957834	2.731507408	-1.39232	0.419539	chr3_7091_star	8.59655945	0.805927	3.262517265	0.852739935	3.415037	1	chr13_28070_star	2.686425	6.44742	1.882222339	2.896740642	-1.26303	0.655949
chr7_16645_mature	2.149139864	5.641492	1.654957834	2.731507408	-1.39232	0.419539	chr1_3878_star	8.59655945	0.805927	3.262517265	0.852739935	3.415037	1	chr3_7761_mature	26.86425	7.253347	4.800343329	3.044979305	1.888969	0.656227
mmu-miR-6540-3p	2.149139864	5.641492	1.654957834	2.731507408	-1.39232	0.419539	chr5_12420_mature	8.59655945	0.805927	3.262517265	0.852739935	3.415037	1	NW_003537641_37089_mature	7.52199	29.01339	3.091190278	4.907534286	-1.94753	0.657298
NW_003613325_38551_mature	2.149139864	5.641492	1.654957834	2.731507408	-1.39232	0.419539	chr15_31671_star	8.59655945	0.805927	3.262517265	0.852739935	3.415037	1	NW_003613106_37241_mature	5.37285	2.417782	2.671938627	1.773060525	1.152003	0.657708
chr5_12232_mature	2.149139864	5.641492	1.654957834	2.731507408	-1.39232	0.419539	chr7_16903_mature	8.59655945	0.805927	3.262517265	0.852739935	3.415037	1	chr17_35445_star	5.37285	2.417782	2.671938627	1.773060525	1.152003	0.657708
chr1_283_mature	2.149139864	5.641492	1.654957834	2.731507408	-1.39232	0.419539	chr10_23976_mature	8.59655945	0.805927	3.262517265	0.852739935	3.415037	1	chr12_25947_star	5.37285	2.417782	2.671938627	1.773060525	1.152003	0.657708
NW_003541079_39891_star	2.149139864	5.641492	1.654957834	2.731507408	-1.39232	0.419539	chr13_29242_mature	8.59655945	0.805927	3.262517265	0.852739935	3.415037	1	chr1_2360_star	5.37285	2.417782	2.671938627	1.773060525	1.152003	0.657708
chr2_3979_star	2.149139864	5.641492	1.654957834	2.731507408	-1.39232	0.419539	chr12_26404_star	10.7456993	1.611855	3.554060706	1.38507475	2.736966	1	mmu-miR-1927	5.37285	2.417782	2.671938627	1.773060525	1.152003	0.657708
chr6_12619_mature	2.149139864	5.641492	1.654957834	2.731507408	-1.39232	0.419539	chr6_14892_mature	12.8948392	2.417782	3.796477232	1.773060525	2.415037	1	chr12_26323_mature	5.37285	2.417782	2.671938627	1.773060525	1.152003	0.657708
chrx_22394_mature	2.149139864	5.641492	1.654957834	2.731507408	-1.39232	0.419539	bta-miR-2300b-3p	8.59655945	0.805927	3.262517265	0.852739935	3.415037	1	chr15_31881_mature	5.37285	2.417782	2.671938627	1.773060525	1.152003	0.657708
chr12_26204_mature	2.149139864	5.641492	1.654957834	2.731507408	-1.39232	0.419539	chr14_30332_mature	8.59655945	0.805927	3.262517265	0.852739935	3.415037	1	chr1_1981_star	5.37285	2.417782	2.671938627	1.773060525	1.152003	0.657708
chr2_5406_mature	2.149139864	5.641492	1.654957834	2.731507408	-1.39232	0.419539	chr15_32587_mature	8.59655945	0.805927	3.262517265	0.852739935	3.415037	1	hsa-miR-5096	5.37285	2.417782	2.671938627	1.773060525	1.152003	0.657708
mmu-miR-2139	2.149139864	5.641492	1.654957834	2.731507408	-1.39232	0.419539	hsa-miR-3169	12.8948392	2.417782	3.796477232	1.773060525	2.415037	1	NW_003537574_37009_star	5.37285	2.417782	2.671938627	1.773060525	1.152003	0.657708
chr6_13361_mature	16.11854898	4.835565	4.097488514	2.544872269	1.736966	0.420567	chr5_11650_star	8.59655945	0.805927	3.262517265	0.852739935	3.415037	1	chr1_1120_star	5.37285	2.417782	2.671938627	1.773060525	1.152003	0.657708
chr13_27136_mature	16.11854898	4.835565	4.097488514	2.544872269	1.736966	0.420567	chr6_12860_mature	8.59655945	0.805927	3.262517265	0.852739935	3.415037	1	chr2_4589_star	5.37285	2.417782	2.671938627	1.773060525	1.152003	0.657708
chr2_4893_star	16.11854898	4.835565	4.097488514	2.544872269	1.736966	0.420567	bta-miR-30f	8.59655945	0.805927	3.262517265	0.852739935	3.415037	1	chr4_8856_mature	5.37285	2.417782	2.671938627	1.773060525	1.152003	0.657708
chr12_25593_mature	16.11854898	4.835565	4.097488514	2.544872269	1.736966	0.420567	chrx_23149_mature	8.59655945	0.805927	3.262517265	0.852739935	3.415037	1	NW_003537823_37300_star	5.37285	2.417782	2.671938627	1.773060525	1.152003	0.657708
chr2_4243_mature	16.11854898	4.835565	4.097488514	2.544872269	1.736966	0.420567	chr11_25258_star	10.7456993	1.611855	3.554060706	1.38507475	2.736966	1	chr12_25429_mature	5.37285	2.417782	2.671938627	1.773060525	1.152003	0.657708
chr7_16660_mature	16.11854898	4.835565	4.097488514	2.544872269	1.736966	0.420567	chr15_32044_mature	8.59655945	0.805927	3.262517265	0.852739935	3.415037	1	hsa-miR-3192	5.37285	2.417782	2.671938627	1.773060525	1.152003	0.657708
chrx_21498_mature	409.411144	46.74379	8.680926095	5.577241251	3.130704	0.420822	chr6_13274_star	10.7456993	1.611855	3.554060706	1.38507475	2.736966	1	chr17_35504_star	5.37285	2.417782	2.671938627	1.773060525	1.152003	0.657708
chr5_12327_mature	6.447419591	2.417782	2.896740642	1.773060525	1.415037	0.420869	chr13_27063_mature	8.59655945	0.805927	3.262517265	0.852739935	3.415037	1	ssc-miR-105-1	5.37285	2.417782	2.671938627	1.773060525	1.152003	0.657708
chr3_6817_star	6.447419591	2.417782	2.896740642	1.773060525	1.415037	0.420869	chr10_23656_star	12.8948392	2.417782	3.796477232	1.773060525	2.415037	1	chr6_12919_mature	5.37285	2.417782	2.671938627	1.773060525	1.152003	0.657708
chr9_21068_star	6.447419591	2.417782	2.896740642	1.773060525	1.415037	0.420869	mmu-miR-99a-3p	8.59655945	0.805927	3.262517265	0.852739935	3.415037	1	chr6_13134_star	5.37285	2.417782	2.671938627	1.773060525	1.152003	0.657708
chr7_15363_star	6.447419591	2.417782	2.896740642	1.773060525	1.415037	0.420869	chr11_24334_star	10.7456993	1.611855	3.554060706	1.38507475	2.736966	1	NW_003538520_37951_mature	5.37285	2.417782	2.671938627	1.773060525	1.152003	0.657708
chr1_1637_star	6.447419591	2.417782	2.896740642	1.773060525	1.415037	0.420869	chr12_26428_star	8.59655945	0.805927	3.262517265	0.852739935	3.415037	1	chr10_23710_star	5.37285	2.417782	2.671938627	1.773060525	1.152003	0.657708
chr5_11087_star	6.447419591	2.417782	2.896740642	1.773060525	1.415037	0.420869	chr9_19780_mature	12.8948392	2.417782	3.796477232	1.773060525	2.415037	1	chr11_24486_mature	226.197	28.20746	7.827799788	4.868265031	3.003431	0.658495

rno-miR-341	6.447419591	2.417782	2.896740642	1.773060525		1.415037	0.420869
chr9_21017_mature	6.447419591	2.417782	2.896740642	1.773060525		1.415037	0.420869
chr6_14513_mature	6.447419591	2.417782	2.896740642	1.773060525		1.415037	0.420869
chrx_23051_star	6.447419591	2.417782	2.896740642	1.773060525		1.415037	0.420869
chr6_14892_mature	6.447419591	2.417782	2.896740642	1.773060525		1.415037	0.420869
chr14_30612_mature	6.447419591	2.417782	2.896740642	1.773060525		1.415037	0.420869
chr2_6406_star	6.447419591	2.417782	2.896740642	1.773060525		1.415037	0.420869
chr12_26184_mature	6.447419591	2.417782	2.896740642	1.773060525		1.415037	0.420869
chr2_4133_mature	6.447419591	2.417782	2.896740642	1.773060525		1.415037	0.420869
chr9_20275_star	6.447419591	2.417782	2.896740642	1.773060525		1.415037	0.420869
bta-miR-2365	6.447419591	2.417782	2.896740642	1.773060525		1.415037	0.420869
mmu-miR-3471	6.447419591	2.417782	2.896740642	1.773060525		1.415037	0.420869
chr4_9551_mature	6.447419591	2.417782	2.896740642	1.773060525		1.415037	0.420869
chr5_10940_mature	6.447419591	2.417782	2.896740642	1.773060525		1.415037	0.420869
chr12_26324_mature	6.447419591	2.417782	2.896740642	1.773060525		1.415037	0.420869
chr10_23710_star	6.447419591	2.417782	2.896740642	1.773060525		1.415037	0.420869
chr3_7704_mature	6.447419591	2.417782	2.896740642	1.773060525		1.415037	0.420869
chr13_28138_star	82.74188475	15.31262	6.387877484	4.027916745		2.433897	0.421514
chr7_17188_mature	37.60994761	8.865202	5.270900692	3.302348583		2.084889	0.422652
chr8_17577_star	6.447419591	23.3719	2.896740642	4.607146585		-1.85798	0.424095
chr10_24060_mature	6.447419591	23.3719	2.896740642	4.607146585		-1.85798	0.424095
NW_003538922_38352_mature	3.223709795	8.865202	2.078510713	3.302348583		-1.45943	0.424286
chr16_33936_star	3.223709795	8.865202	2.078510713	3.302348583		-1.45943	0.424286
hsa-miR-320b	3.223709795	8.865202	2.078510713	3.302348583		-1.45943	0.424286
bta-miR-3432	3.223709795	8.865202	2.078510713	3.302348583		-1.45943	0.424286
rno-miR-34a-5p	3.223709795	8.865202	2.078510713	3.302348583		-1.45943	0.424286
NW_003541025_39829_mature	3.223709795	8.865202	2.078510713	3.302348583		-1.45943	0.424286
ssc-miR-376a-5p	23.6405385	6.44742	4.62296188	2.896740642		1.874469	0.424712
chr3_6786_mature	23.6405385	6.44742	4.62296188	2.896740642		1.874469	0.424712
bta-miR-677	107.4569932	1580.424	6.760979269	10.62700826		-3.87848	0.424842
chr16_34240_star	27.93881823	7.253347	4.854934103	3.044979305		1.945552	0.424927
NW_003613233_37800_star	4.298279727	12.89484	2.405524014	3.796477232		-1.58496	0.426519
chr2_5682_mature	4.298279727	12.89484	2.405524014	3.796477232		-1.58496	0.426519
chr8_18282_mature	19.34225877	5.641492	4.346407978	2.731507408		1.777608	0.426761
chr1_3220_star	148.2906506	23.3719	7.221980009	4.607146585		2.665581	0.428976
chr2_4834_mature	47.281077	452.9312	5.593385953	8.826329926		-3.25996	0.429389
chr7_16048_mature	11.82026925	4.029637	3.680354657	2.330454351		1.552541	0.429574
chr17_35416_star	11.82026925	4.029637	3.680354657	2.330454351		1.552541	0.429574
NW_003537759_37261_mature	6.447419591	22.56597	2.896740642	4.558633073		-1.80735	0.43031
chr2_5682_star	51.57935673	11.28298	5.716424587	3.618589217		2.192645	0.430336
chr3_8648_mature	9.671129386	41.1023	3.415640968	5.395827139		-2.08746	0.430705
chrx_23158_mature	8.596559455	3.22371	3.262517265	2.078510713		1.415037	0.430857
chr17_34868_mature	8.596559455	3.22371	3.262517265	2.078510713		1.415037	0.430857
chrx_21569_star	8.596559455	3.22371	3.262517265	2.078510713		1.415037	0.430857
ssc-miR-1224	8.596559455	3.22371	3.262517265	2.078510713		1.415037	0.430857
chr4_9294_mature	8.596559455	3.22371	3.262517265	2.078510713		1.415037	0.430857
ssc-miR-421-5p	8.596559455	3.22371	3.262517265	2.078510713		1.415037	0.430857
chr13_27847_mature	8.596559455	3.22371	3.262517265	2.078510713		1.415037	0.430857
chr1_814_mature	8.596559455	3.22371	3.262517265	2.078510713		1.415037	0.430857
chr3_7353_mature	8.596559455	3.22371	3.262517265	2.078510713		1.415037	0.430857
chr5_11842_mature	8.596559455	3.22371	3.262517265	2.078510713		1.415037	0.430857
chr11_24486_mature	7.521989523	28.20746	3.091190278	4.868265031		-1.90689	0.431121
chr4_9551_star	2.149139864	0.805927	1.654957834	0.852739935		1.415037	0.431307
NW_003541040_39847_star	2.149139864	0.805927	1.654957834	0.852739935		1.415037	0.431307
chrx_23237_mature	2.149139864	0.805927	1.654957834	0.852739935		1.415037	0.431307
chr16_34547_star	2.149139864	0.805927	1.654957834	0.852739935		1.415037	0.431307
chr13_27228_star	2.149139864	0.805927	1.654957834	0.852739935		1.415037	0.431307
chr15_31929_star	2.149139864	0.805927	1.654957834	0.852739935		1.415037	0.431307
chr13_27184_star	2.149139864	0.805927	1.654957834	0.852739935		1.415037	0.431307
chr15_33038_mature	2.149139864	0.805927	1.654957834	0.852739935		1.415037	0.431307
hsa-miR-4790-3p	2.149139864	0.805927	1.654957834	0.852739935		1.415037	0.431307
chr4_10011_star	2.149139864	0.805927	1.654957834	0.852739935		1.415037	0.431307
chr9_20599_star	2.149139864	0.805927	1.654957834	0.852739935		1.415037	0.431307
hsa-miR-4770	2.149139864	0.805927	1.654957834	0.852739935		1.415037	0.431307
chr3_7237_mature	2.149139864	0.805927	1.654957834	0.852739935		1.415037	0.431307
chr12_26437_star	2.149139864	0.805927	1.654957834	0.852739935		1.415037	0.431307
NW_003613290_38305_star	2.149139864	0.805927	1.654957834	0.852739935		1.415037	0.431307
chr6_14391_mature	2.149139864	0.805927	1.654957834	0.852739935		1.415037	0.431307
chr5_12416_star	2.149139864	0.805927	1.654957834	0.852739935		1.415037	0.431307
chr15_32720_star	2.149139864	0.805927	1.654957834	0.852739935		1.415037	0.431307
hsa-miR-3614-3p	2.149139864	0.805927	1.654957834	0.852739935		1.415037	0.431307
hsa-miR-4739	2.149139864	0.805927	1.654957834	0.852739935		1.415037	0.431307
bta-miR-2895	2.149139864	0.805927	1.654957834	0.852739935		1.415037	0.431307
hsa-miR-1285-3p	2.149139864	0.805927	1.654957834	0.852739935		1.415037	0.431307
chr6_14314_mature	2.149139864	0.805927	1.654957834	0.852739935		1.415037	0.431307
chr3_7219_star	2.149139864	0.805927	1.654957834	0.852739935		1.415037	0.431307
rno-miR-20a-5p	2.149139864	0.805927	1.654957834	0.852739935		1.415037	0.431307
rno-miR-448-3p	2.149139864	0.805927	1.654957834	0.852739935		1.415037	0.431307
chr6_14329_mature	2.149139864	0.805927	1.654957834	0.852739935		1.415037	0.431307
chr6_12905_star	2.149139864	0.805927	1.654957834	0.852739935		1.415037	0.431307
chr15_32840_star	2.149139864	0.805927	1.654957834	0.852739935		1.415037	0.431307
chr14_31502_mature	2.149139864	0.805927	1.654957834	0.852739935		1.415037	0.431307
chr17_35071_star	2.149139864	0.805927	1.654957834	0.852739935		1.415037	0.431307
chr7_15769_star	2.149139864	0.805927	1.654957834	0.852739935		1.415037	0.431307
hsa-miR-4764-3p	2.149139864	0.805927	1.654957834	0.852739935		1.415037	0.431307
chr15_33376_star	2.149139864	0.805927	1.654957834	0.852739935		1.415037	0.431307
chr5_11956_star	2.149139864	0.805927	1.654957834	0.852739935		1.415037	0.431307
chr10_24151_star	2.149139864	0.805927	1.654957834	0.852739935		1.415037	0.431307
NW_003539771_39089_star	2.149139864	0.805927	1.654957834	0.852739935		1.415037	0.431307
hsa-miR-567	2.149139864	0.805927	1.654957834	0.852739935		1.415037	0.431307
hsa-miR-518d-3p	2.149139864	0.805927	1.654957834	0.852739935		1.415037	0.431307
chr4_9495_star	2.149139864	0.805927	1.654957834	0.852739935		1.415037	0.431307
chr14_30387_star	2.149139864	0.805927	1.654957834	0.852739935		1.415037	0.431307
chr3_6870_star	2.149139864	0.805927	1.654957834	0.852739935		1.415037	0.431307
chr8_17967_star	2.149139864	0.805927	1.654957834	0.852739935		1.415037	0.431307
hsa-miR-504	2.149139864	0.805927	1.654957834	0.852739935		1.415037	0.431307
chr5_12411_star	2.149139864	0.805927	1.654957834	0.852739935		1.415037	0.431307
chr2_5850_mature@@rno-miR-328b-	2.149139864	0.805927	1.654957834	0.852739935		1.415037	0.431307
chrx_22228_star	2.149139864	0.805927	1.654957834	0.852739935		1.415037	0.431307
chr10_23649_mature	2.149139864	0.805927	1.654957834	0.852739935		1.415037	0.431307
chr12_25591_star	2.149139864	0.805927	1.654957834	0.852739935		1.415037	0.431307
mmu-miR-1956	2.149139864	0.805927	1.654957834	0.852739935		1.415037	0.431307
chr16_34502_star	2.149139864	0.805927	1.654957834	0.852739935		1.415037	0.431307
chr4_10120_mature	2.149139864	0.805927	1.654957834	0.852739935		1.415037	0.431307
chr11_25383_star	2.149139864	0.805927	1.654957834	0.852739935		1.415037	0.431307
chr2_6113_star	2.149139864	0.805927	1.654957834	0.852739935		1.415037	0.431307
chr4_8825_star	2.149139864	0.805927	1.654957834	0.852739935		1.415037	0.431307
chr3_8236_star	2.149139864	0.805927	1.654957834	0.852739935		1.415037	0.431307
chr3_7211_star	2.149139864	0.805927	1.654957834	0.852739935		1.415037	0.431307
chrx_22905_star	2.149139864	0.805927	1.654957834	0.852739935		1.415037	0.431307
chrx_22845_mature	2.149139864	0.805927	1.654957834	0.852739935		1.415037	0.431307
NW_003539007_38438_star	2.149139864	0.805927	1.654957834	0.852739935		1.415037	0.431307
chr18_36112_star	2.149139864	0.805927	1.654957834	0.852739935		1.415037	0.431307
chrx_22188_star@@mmu-miR-1298-	2.149139864	0.805927	1.654957834	0.852739935		1.415037	0.431307
chr5_12345_star	2.149139864	0.805927	1.654957834	0.852739935		1.415037	0.431307
chr1_3090_star	2.149139864	0.805927	1.654957834	0.852739935		1.415037	0.431307
chr11_25358_star	2.149139864	0.805927	1.654957834	0.852739935		1.415037	0.431307
hsa-miR-5190	2.149139864	0.805927	1.654957834	0.852739935		1.415037	0.431307
chr3_8637_mature	2.149139864	0.805927	1.654957834	0.852739935		1.415037	0.431307
rno-miR-3549	2.149139864	0.805927	1.654957834	0.852739935		1.415037	0.431307
NW_003537216_36774_mature	2.149139864	0.805927	1.654957834	0.852739935		1.415037	0.431307
chr6_13529_mature	2.149139864	0.805927	1.654957834	0.852739935		1.415037	0.431307
chr16_33806_mature@@bta-miR-302	2.149139864	0.805927	1.654957834	0.852739935		1.415037	0.431307
chr6_14751_star	2.149139864	0.805927	1.654957834	0.852739935		1.415037	0.431307
chr2_5851_mature	2.149139864	0.805927	1.654957834	0.852739935		1.415037	0.431307
rno-miR-6316	2.149139864	0.805927	1.654957834	0.852739935		1.415037	0.431307
chr6_13694_star	2.149139864	0.805927	1.654957834	0.852739935		1.415037	0.431307
chr15_32156_star	2.149139864	0.805927	1.654957834	0.852739935		1.415037	0.431307
hsa-miR-3913-5p	2.149139864	0.805927	1.654957834	0.852739935		1.415037	0.431307
chr13_28169_star	2.149139864	0.805927	1.654957834	0.852739935		1.415037	0.431307
bta-miR-1284	2.149139864	0.805927	1.654957834	0.852739935		1.415037	0.431307
hsa-miR-6720-3p	2.149139864	0.805927	1.654957834	0.852739935		1.415037	0.431307
chr5_12276_star	2.149139864	0.805927	1.654957834	0.852739935		1.415037	0.431307
chr13_27087_star	2.149139864	0.805927	1.654957834	0.852739935		1.415037	0.431307
NW_003613233_37800_mature	2.149139864	0.805927	1.654957834	0.852739935		1.415037	0.431307
chr8_17349_mature	2.149139864	0.805927	1.654957834	0.852739935		1.415037	0.431307
chr7_16583_star	2.149139864	0.805927	1.654957834	0.852739935		1.415037	0.431307
chr17_34966_star	2.149139864	0.805927	1.654957834	0.852739935		1.415037	0.431307
chr7_16875_star	2.149139864	0.805927	1.654957834	0.852739935		1.415037	0.431307
NW_003540770_39675_star	2.149139864	0.805927	1.654957834	0.852739935		1.415037	0.431307
bta-miR-1225-3p	2.149139864	0.805927	1.654957834	0.852739935		1.415037	0.431307
NW_003539698_39033_star	2.149139864	0.805927	1.654957834	0.852739935		1.415037	0.431307
chr14_31358_star	2.149139864	0.805927	1.654957834	0.852739935		1.415037	0.431307
chrx_22791_star	2.149139864	0.805927	1.654957834	0.852739935		1.415037	0.431307
chr1_3638_star	2.149139864	0.805927	1.654957834	0.852739935		1.415037	0.431307
chr11_25345_star	2.149139864	0.805927	1.654957834	0.852739935		1.415037	0.431307
chr7_16728_star	2.149139864	0.805927	1.654957834	0.852739935		1.415037	0.431307
hsa-miR-1184	2.149139864	0.805927	1.654957834	0.852739935		1.415037	0.431307
chr12_25405_mature	2.149139864	0.805927	1.654957834	0.852739935		1.415037	0.431307
chr3_6641_star	2.149139864	0.805927	1.654957834	0.852739935		1.415037	0.431307
chr9_20880_star	2.149139864	0.805927	1.654957834	0.852739935		1.415037	0.431307
chr13_27306_star	2.149139864	0.805927	1.654957834	0.852739935		1.415037	0.431307
chr14_30645_mature	2.149139864	0.805927	1.654957834	0.852739935		1.415037	0.431307
chr12_26572_mature	2.149139864	0.805927	1.654957834	0.852739935		1.415037	0.431307
chr9_20120_star	2.149139864	0.805927	1.654957834	0.852739935		1.415037	0.431307
chr13_26824_mature	2.149139864	0.805927	1.654957834	0.852739935		1.415037	0.431307
hsa-miR-6500-5p	2.149139864	0.805927	1.654957834	0.852739935		1.415037	0.431307
chr3_7715_mature	2.149139864	0.805927	1.654957834	0.852739935		1.415037	0.431307
mmu-miR-6372	2.149139864	0.805927	1.654957834	0.852739935		1.415037	0.431307
chr7_15832_star	2.149139864	0.805927	1.654957834	0.852739935		1.415037	0.431307
chr5_11961_star	2.149139864	0.805927	1.654957834	0.852739935		1.415037	0.431307
chr5_12405_mature	2.149139864	0.805927	1.654957834	0.852739935		1.415037	0.431307
chr1_2125_star	2.149139864	0.805927	1.654957834	0.852739935		1.415037	0.431307
chr4_10432_star	2.149139864	0.805927	1.654957834	0.852739935		1.415037	0.431307
chr9_19609_star@@bta-miR-135b	2.149139864	0.805927	1.654957834	0.852739935		1.415037	0.431307
chr14_30669_star	2.149139864	0.805927	1.654957834	0.852739935		1.415037	0.431307
chr13_28165_mature	2.149139864	0.805927	1.654957834	0.852739935		1.415037	0.431307
chr5_10819_star	2.149139864	0.805927	1.654957834	0.852739935		1.415037	0.431307
chr9_19536_star	2.149139864	0.805927	1.654957834	0.852739935		1.415037	0.431307
chr17_35181_mature@@bta-miR-61	2.149139864	0.805927	1.654957834	0.852739935		1.415037	0.431307
hsa-miR-514b-5p	2.149139864	0.805927	1.654957834	0.852739935		1.415037	0.431307
chr14_31415_star	2.149139864	0.805927	1.654957834	0.852739935		1.415037	0.431307
mmu-miR-3063-5p	2.149139864	0.805927	1.654957834	0.852739935		1.415037	0.431307
chr3_7233_star	2.149139864	0.805927	1.654957834	0.852739935		1.415037	0.431307
NW_003613217_37697_mature	2.149139864	0.805927	1.654957834	0.852739935		1.415037	0.431307
chrx_23183_star	2.149139864	0.805927	1.654957834	0.852739935		1.415037	0.431307
chr2_5880_mature	2.149139864	0.805927	1.654957834	0.852739935		1.415037	0.431307
chr15_33101_mature	2.149139864	0.805927	1.654957834	0.852739935		1.415037	0.431307
chr10_23279_star	2.149139864	0.805927	1.654957834	0.852739935		1.415037	0.431307
chr5_10731_star	2.149139864	0.805927	1.654957834	0.852739935		1.415037	0.431307
bta-miR-186	2.149139864	0.805927	1.654957834	0.852739935		1.415037	0.431307
chr6_14927_star	2.149139864	0.805927	1.654957834	0.852739935		1.415037	0.431307
chr5_12420_mature	2.149139864	0.805927	1.654957834	0.852739935		1.415037	0.431307
hsa-miR-3177-5p	2.149139864	0.805927	1.654957834	0.852739935		1.415037	0.431307
chrx_22108_star	2.149139864	0.805927	1.654957834	0.852739935		1.415037	0.431307
mmu-miR-344c-5p	2.149139864	0.805927	1.654957834	0.852739935		1.415037	0.431307
chr18_36371_star	2.149139864	0.805927	1.654957834	0.852739935		1.415037	0.431307
chr14_31484_mature	2.149139864	0.805927	1.654957834	0.852739935		1.415037	0.431307
chr13_26704_star	2.149139864	0.805927	1.654957834	0.852739935		1.415037	0.431307
chr12_25481_star	2.149139864	0.805927	1.654957834	0.852739935		1.415037	0.431307
hsa-miR-4786-5p	2.149139864	0.805927	1.654957834	0.852739935		1.415037	0.431307
chr2_4006_star	2.149139864	0.805927	1.654957834	0.852739935		1.415037	0.431307
hsa-miR-6511a-5p	2.149139864	0.805927	1.654957834	0.852739935		1.415037	0.431307
chr7_16542_star	2.149139864	0.805927	1.654957834	0.852739935		1.415037	0.431307
chr9_19419_star	2.149139864	0.805927	1.654957834	0.852739935		1.415037	0.431307
chr15_32008_star	2.149139864	0.805927	1.654957834	0.852739935		1.415037	0.431307
hsa-miR-548p	2.149139864	0.805927	1.654957834	0.852739935		1.415037	0.431307
chr13_27728_mature	2.149139864	0.805927	1.654957834	0.852739935		1.415037	0.431307
chr15_32289_star	2.149139864	0.805927	1.654957834	0.852739935		1.415037	0.431307
chr7_16529_star	2.149139864	0.805927	1.654957834	0.852739935		1.415037	0.431307
chr13_29242_mature	2.149139864	0.805927	1.654957834	0.852739935		1.415037	0.431307
chr12_26014_star	2.149139864	0.805927	1.654957834	0.852739935		1.415037	0.431307
bta-miR-2300b-3p	2.149139864	0.805927	1.654957834	0.852739935		1.415037	0.431307
chr7_16599_star	2.149139864	0.805927	1.654957834	0.852739935		1.415037	0.431307
chr5_11650_star	2.149139864	0.805927	1.654957834	0.852739935		1.415037	0.431307
hsa-miR-3188	2.149139864	0.805927	1.654957834	0.852739935		1.415037	0.431307
hsa-miR-3128	2.149139864	0.805927	1.654957834	0.852739935		1.415037	0.431307
chr6_12860_mature	2.149139864	0.805927	1.654957834	0.852739935		1.415037	0.431307
bta-miR-30f	2.149139864	0.805927	1.654957834	0.852739935		1.415037	0.431307
bta-miR-367	2.149139864	0.805927	1.654957834	0.852739935		1.415037	0.431307
NW_003537656_37100_mature	2.149139864	0.805927	1.654957834	0.852739935		1.415037	0.431307
bta-miR-2454-5p	2.149139864	0.805927	1.654957834	0.852739935		1.415037	0.431307
chr3_6687_star	2.149139864	0.805927	1.654957834	0.852739935		1.415037	0.431307
chr17_35675_star	2.149139864	0.805927	1.654957834	0.852739935		1.415037	0.431307
NW_003539820_39117_mature	2.149139864	0.805927	1.654957834	0.852739935		1.415037	0.431307
chr3_7704_star	2.149139864	0.805927	1.654957834	0.852739935		1.415037	0.431307
chr2_6194_star	2.149139864	0.805927	1.654957834	0.852739935		1.415037	0.431307
chr11_24519_mature	2.149139864	0.805927	1.654957834	0.852739935		1.415037	0.431307
mmu-miR-449c-5p	2.149139864	0.805927	1.654957834	0.852739935		1.415037	0.431307
chr14_31022_star	2.149139864	0.805927	1.654957834	0.852739935		1.415037	0.431307
chr8_17274_mature	2.149139864	0.805927	1.654957834	0.852739935		1.415037	0.431307
bta-miR-2422	2.149139864	0.805927	1.654957834	0.852739935		1.415037	0.431307
chr13_29126_star	2.149139864	0.805927	1.654957834	0.852739935		1.415037	0.431307
chr11_24439_mature	2.149139864	0.805927	1.654957834	0.852739935		1.415037	0.431307
bta-miR-2455	2.149139864	0.805927	1.654957834	0.852739935		1.415037	0.431307
rno-miR-875	2.149139864	0.805927	1.654957834	0.852739935		1.415037	0.431307
chr7_15747_star	2.149139864	0.805927	1.654957834	0.852739935		1.415037	0.431307
hsa-miR-4444	2.149139864	0.805927	1.654957834	0.852739935		1.415037	0.431307
chr3_7313_star	2.149139864	0.805927	1.654957834	0.852739935		1.415037	0.431307
mmu-miR-1930-3p	2.149139864	0.805927	1.654957834	0.852739935		1.415037	0.431307
chr7_17092_star	2.149139864	0.805927	1.654957834	0.852739935		1.415037	0.431307
chr15_32407_mature	2.149139864	0.805927	1.654957834	0.852739935		1.415037	0.431307
hsa-miR-643	2.149139864	0.805927	1.654957834	0.852739935		1.415037	0.431307
NW_003541007_39797_star	2.149139864	0.805927	1.654957834	0.852739935		1.415037	0.431307
chr1_1936_mature	2.149139864	0.805927	1.654957834	0.852739935		1.415037	0.431307
NW_003540745_39661_mature	2.149139864	0.805927	1.654957834	0.852739935		1.415037	0.431307
NW_003540583_39564_star	2.149139864	0.805927	1.654957834	0.852739935		1.415037	0.431307
hsa-miR-548t-5p	2.149139864	0.805927	1.654957834	0.852739935		1.415037	0.431307
NW_003540052_39255_star	2.149139864	0.805927	1.654957834	0.852739935		1.415037	0.431307
chr6_14216_mature	2.149139864	0.805927	1.654957834	0.852739935		1.415037	0.431307
chr14_30730_star	2.149139864	0.805927	1.654957834	0.852739935		1.415037	0.431307
rno-miR-107-5p	2.149139864	0.805927	1.654957834	0.852739935		1.415037	0.431307
mmu-miR-3097-3p	2.149139864	0.805927	1.654957834	0.852739935		1.415037	0.431307
chr9_20086_mature	2.149139864	0.805927	1.654957834	0.852739935		1.415037	0.431307
rno-miR-3593-5p	2.149139864	0.805927	1.654957834	0.852739935		1.415037	0.431307
chr17_35528_mature	2.149139864	0.805927	1.654957834	0.852739935		1.415037	0.431307
bta-miR-339a	2.149139864	0.805927	1.654957834	0.852739935		1.415037	0.431307
mmu-miR-1843a-3p	2.149139864	0.805927	1.654957834	0.852739935		1.415037	0.431307
chr6_13134_mature	2.149139864	0.805927	1.654957834	0.852739935		1.415037	0.431307
chr8_17690_star	2.149139864	0.805927	1.654957834	0.852739935		1.415037	0.431307
chr16_34229_star	2.149139864	0.805927	1.654957834	0.852739935		1.415037	0.431307
mmu-miR-1839-3p	2.149139864	0.805927	1.654957834	0.852739935		1.415037	0.431307
rno-miR-1193-3p	2.149139864	0.805927	1.654957834	0.852739935		1.415037	0.431307
chr2_6507_star	2.149139864	0.805927	1.654957834	0.852739935		1.415037	0.431307
NW_003613161_37463_star	2.149139864	0.805927	1.654957834	0.852739935		1.415037	0.431307
chr17_35576_star	2.149139864	0.805927	1.654957834	0.852739935		1.415037	0.431307
chr8_17864_star	2.149139864	0.805927	1.654957834	0.852739935		1.415037	0.431307
hsa-miR-4678	2.149139864	0.805927	1.654957834	0.852739935		1.415037	0.431307
chr13_29078_star	2.149139864	0.805927	1.654957834	0.852739935		1.415037	0.431307
chr2_5533_star	2.149139864	0.805927	1.654957834	0.852739935		1.415037	0.431307
hsa-miR-4760-5p	2.149139864	0.805927	1.654957834	0.852739935		1.415037	0.431307
chr2_4298_star	2.149139864	0.805927	1.654957834	0.852739935		1.415037	0.431307
bta-miR-2419-5p	2.149139864	0.805927	1.654957834	0.852739935		1.415037	0.431307
mmu-miR-3473a	2.149139864	0.805927	1.654957834	0.852739935		1.415037	0.431307
chr1_3851_star	2.149139864	0.805927	1.654957834	0.852739935		1.415037	0.431307

chr12_26184_mature	12.8948392	2.417782	3.796477232	1.773060525		2.415037	1
chr7_16057_mature	10.7456993	1.611855	3.554060706	1.38507475		2.736966	1
bta-miR-2444	10.7456993	1.611855	3.554060706	1.38507475		2.736966	1
hsa-miR-606	10.7456993	1.611855	3.554060706	1.38507475		2.736966	1
hsa-miR-639	10.7456993	1.611855	3.554060706	1.38507475		2.736966	1
chr10_23795_mature	8.59655945	0.805927	3.262517265	0.852739935		3.415037	1
chr4_9430_mature	10.7456993	1.611855	3.554060706	1.38507475		2.736966	1
chr7_16933_mature	8.59655945	0.805927	3.262517265	0.852739935		3.415037	1
NW_003540745_39661_mature	8.59655945	0.805927	3.262517265	0.852739935		3.415037	1
chr7_17120_mature	10.7456993	33.84895	3.554060706	5.123043401		-1.65535	1
chrx_21800_star	12.8948392	2.417782	3.796477232	1.773060525		2.415037	1
chr12_26051_mature	10.7456993	1.611855	3.554060706	1.38507475		2.736966	1
chrx_23015_star	8.59655945	0.805927	3.262517265	0.852739935		3.415037	1
chr16_34229_star	8.59655945	0.805927	3.262517265	0.852739935		3.415037	1
NW_003539018_38471_star	10.7456993	1.611855	3.554060706	1.38507475		2.736966	1
chr6_13925_star	10.7456993	1.611855	3.554060706	1.38507475		2.736966	1
chr8_17916_mature	8.59655945	0.805927	3.262517265	0.852739935		3.415037	1
chrx_21678_star	10.7456993	1.611855	3.554060706	1.38507475		2.736966	1
chr4_9234_mature	10.7456993	1.611855	3.554060706	1.38507475		2.736966	1
chr6_12905_mature	12.8948392	2.417782	3.796477232	1.773060525		2.415037	1
chrx_22000_star	12.8948392	2.417782	3.796477232	1.773060525		2.415037	1
chr3_7956_mature	8.59655945	0.805927	3.262517265	0.852739935		3.415037	1
chr14_30700_star	10.7456993	1.611855	3.554060706	1.38507475		2.736966	1
chr1_3032_star	8.59655945	0.805927	3.262517265	0.852739935		3.415037	1
chr1_3875_mature@@hsa-miR-4667-3	8.59655945	0.805927	3.262517265	0.852739935		3.415037	1
chr15_32446_mature	8.59655945	0.805927	3.262517265	0.852739935		3.415037	1
chr5_11550_mature	10.7456993	1.611855	3.554060706	1.38507475		2.736966	1
hsa-miR-6084	8.59655945	0.805927	3.262517265	0.852739935		3.415037	1
chr6_12862_mature	10.7456993	1.611855	3.554060706	1.38507475		2.736966	1
chr3_7100_mature	8.59655945	0.805927	3.262517265	0.852739935		3.415037	1
chr7_17161_mature	8.59655945	29.81932	3.262517265	4.94576292		-1.79442	1
chrx_22969_mature	21.4913986	6.44742	4.491301473	2.896740642		1.736966	1
NW_003539597_38976_mature	2.14913986	16.92448	1.654957834	4.163859073		-2.97728	1
chr8_18357_mature	6.44741959	25.78968	2.896740642	4.743605355		-2	1
chr7_15658_star	2.14913986	16.92448	1.654957834	4.163859073		-2.97728	1
chr15_33676_star	2.14913986	16.92448	1.654957834	4.163859073		-2.97728	1
chr8_18468_mature	2.14913986	16.92448	1.654957834	4.163859073		-2.97728	1
ssc-miR-136	15.043979	41.1023	4.003960082	5.395827139		-1.45003	1
chr13_28164_mature	21.4913986	6.44742	4.491301473	2.896740642		1.736966	1
chr2_5267_mature	2.14913986	16.92448	1.654957834	4.163859073		-2.97728	1
chr2_4343_mature	2.14913986	16.92448	1.654957834	4.163859073		-2.97728	1
chr11_25207_star	12.8948392	37.07266	3.796477232	5.250683565		-1.52356	1
chr15_32700_mature	2.14913986	16.92448	1.654957834	4.163859073		-2.97728	1
NW_003537425_36910_mature	2.14913986	16.92448	1.654957834	4.163859073		-2.97728	1
chr15_32017_mature	4.29827973	20.95411	2.405524014	4.456419386		-2.2854	1
chr13_28864_star	2.14913986	16.11855	1.654957834	4.097488514		-2.90689	1
ssc-miR-181a	15869.2488	15095.83	13.95403712	13.88195774		0.072084	1
chrx_23008_mature	4.29827973	20.95411	2.405524014	4.456419386		-2.2854	1
chr1_550_star	19.3422588	5.641492	4.346407978	2.731507408		1.777608	1
chr4_9796_mature	4.29827973	20.95411	2.405524014	4.456419386		-2.2854	1
chr6_12646_mature	2.14913986	16.11855	1.654957834	4.097488514		-2.90689	1
chr3_8076_mature	55.8776365	27.40153	5.82978961	4.827896911		1.028014	1
chr3_7261_star	636.1454	509.3461	9.31547883	8.995332292		0.32071	1
chr14_31118_star	19.3422588	5.641492	4.346407978	2.731507408		1.777608	1
bta-miR-656	58.0267763	29.01339	5.883297648	4.907534286		1	1
chr9_19234_mature	4.29827973	20.95411	2.405524014	4.456419386		-2.2854	1
chr18_36111_mature	4.29827973	20.95411	2.405524014	4.456419386		-2.2854	1
chr18_36584_mature	2.14913986	16.11855	1.654957834	4.097488514		-2.90689	1
chr3_7067_star	19.3422588	5.641492	4.346407978	2.731507408		1.777608	1
chr11_24705_star	32.237098	12.89484	5.054722516	3.796477232		1.321928	1
NW_003613099_37159_mature	4.29827973	0	2.405524014	0		20	1
hsa-miR-325	4.29827973	0	2.405524014	0		20	1
chr16_34282_mature	4.29827973	0	2.405524014	0		20	1
chr2_5886_mature	4.29827973	0	2.405524014	0		20	1
chr8_17792_star	4.29827973	0	2.405524014	0		20	1
chr9_20394_mature	23.6405385	8.059274	4.62296188	3.179395517		1.552541	1
chr1_2829_mature	4.29827973	0	2.405524014	0		20	1
chr17_35778_star	4.29827973	0	2.405524014	0		20	1
hsa-miR-1915-3p	4.29827973	0	2.405524014	0		20	1
chr6_14178_star	4.29827973	0	2.405524014	0		20	1
chr6_13603_star	4.29827973	0	2.405524014	0		20	1
NW_003537425_36910_star	4.29827973	0	2.405524014	0		20	1
chr2_4615_star	4.29827973	0	2.405524014	0		20	1
chr2_5312_star	4.29827973	0	2.405524014	0		20	1
chr14_29694_mature	17.1931189	4.835565	4.185320985	2.544872269		1.830075	1
chrx_22902_mature	4.29827973	0	2.405524014	0		20	1
hsa-miR-1305	4.29827973	0	2.405524014	0		20	1
chr10_23849_star	4.29827973	0	2.405524014	0		20	1
hsa-miR-532-3p	4.29827973	0	2.405524014	0		20	1
chr17_34938_star	4.29827973	0	2.405524014	0		20	1
hsa-miR-5089-3p	4.29827973	0	2.405524014	0		20	1
hsa-miR-2116-3p	4.29827973	0	2.405524014	0		20	1
chr1_3728_star	4.29827973	0	2.405524014	0		20	1
chr15_31761_star	4.29827973	0	2.405524014	0		20	1
chr15_32728_star	4.29827973	0	2.405524014	0		20	1
mmu-miR-1298-5p	4.29827973	0	2.405524014	0		20	1
rno-miR-551b-5p	4.29827973	0	2.405524014	0		20	1
chr16_34762_star	4.29827973	0	2.405524014	0		20	1
chr5_11672_star	4.29827973	0	2.405524014	0		20	1
NW_003538989_38416_star	4.29827973	0	2.405524014	0		20	1
chr14_29727_star	4.29827973	0	2.405524014	0		20	1
chr6_13600_mature	4.29827973	0	2.405524014	0		20	1
hsa-miR-4671-5p	4.29827973	0	2.405524014	0		20	1
hsa-miR-3944-5p	4.29827973	0	2.405524014	0		20	1
chr9_20033_star	4.29827973	0	2.405524014	0		20	1
chr10_23618_star	4.29827973	0	2.405524014	0		20	1
mmu-let-7k	4.29827973	0	2.405524014	0		20	1
hsa-miR-1256	4.29827973	0	2.405524014	0		20	1
bta-miR-1603	4.29827973	0	2.405524014	0		20	1
chr3_7091_mature	4.29827973	20.14819	2.405524014	4.402462031		-2.22882	1
chr9_19866_star	4.29827973	0	2.405524014	0		20	1
hsa-miR-3606-5p	4.29827973	0	2.405524014	0		20	1
chr6_13349_star	4.29827973	0	2.405524014	0		20	1
chr11_25154_star	4.29827973	0	2.405524014	0		20	1
chr7_17120_star	4.29827973	0	2.405524014	0		20	1
hsa-miR-1287	4.29827973	0	2.405524014	0		20	1
chr15_33664_star	4.29827973	0	2.405524014	0		20	1
hsa-miR-1275	4.29827973	0	2.405524014	0		20	1
chr17_35177_mature@@bta-miR-6121-	4.29827973	0	2.405524014	0		20	1
hsa-miR-1263	4.29827973	0	2.405524014	0		20	1
hsa-miR-573	4.29827973	0	2.405524014	0		20	1
chr10_23951_mature	4.29827973	0	2.405524014	0		20	1
hsa-miR-587	4.29827973	0	2.405524014	0		20	1
mmu-miR-883a-3p	4.29827973	0	2.405524014	0		20	1
hsa-miR-557	4.29827973	0	2.405524014	0		20	1
hsa-miR-552	4.29827973	0	2.405524014	0		20	1
chr2_5134_star	4.29827973	0	2.405524014	0		20	1
chr3_6623_mature	4.29827973	0	2.405524014	0		20	1
mmu-miR-5620-5p	4.29827973	0	2.405524014	0		20	1
NW_003538101_37534_star	4.29827973	0	2.405524014	0		20	1
chr6_15024_mature	4.29827973	0	2.405524014	0		20	1
mmu-miR-190b-3p	4.29827973	0	2.405524014	0		20	1
chr7_16674_mature@@mmu-miR-6399	4.29827973	0	2.405524014	0		20	1
rno-miR-221-3p	4.29827973	0	2.405524014	0		20	1
bta-miR-1814c	4.29827973	0	2.405524014	0		20	1
mmu-miR-1954	4.29827973	0	2.405524014	0		20	1
chr15_33671_star	4.29827973	0	2.405524014	0		20	1
mmu-miR-1965	4.29827973	0	2.405524014	0		20	1
chr3_7770_star	4.29827973	0	2.405524014	0		20	1
chr3_7770_mature	17.1931189	4.835565	4.185320985	2.544872269		1.830075	1
chr13_29078_mature	4.29827973	0	2.405524014	0		20	1
NW_003541081_39894_star	4.29827973	0	2.405524014	0		20	1
chr14_30807_mature	4.29827973	0	2.405524014	0		20	1
chr7_16237_star	4.29827973	0	2.405524014	0		20	1
hsa-miR-548an	4.29827973	0	2.405524014	0		20	1
chrx_23238_star	4.29827973	0	2.405524014	0		20	1
chr5_11163_star	4.29827973	0	2.405524014	0		20	1
rno-miR-434-5p	4.29827973	0	2.405524014	0		20	1
chr8_17337_mature	4.29827973	0	2.405524014	0		20	1
chr18_35938_star	4.29827973	0	2.405524014	0		20	1
hsa-miR-654-5p	4.29827973	0	2.405524014	0		20	1
chr17_34904_star	4.29827973	0	2.405524014	0		20	1
chr1_1629_mature	4.29827973	0	2.405524014	0		20	1
mmu-miR-3105-5p	4.29827973	0	2.405524014	0		20	1
chr10_23364_mature	4.29827973	0	2.405524014	0		20	1
rno-miR-300-3p	4.29827973	0	2.405524014	0		20	1
chr10_23548_mature	4.29827973	0	2.405524014	0		20	1
chr8_17677_star	4.29827973	0	2.405524014	0		20	1
mmu-miR-3109-3p	4.29827973	0	2.405524014	0		20	1
mmu-let-7g-5p	4.29827973	0	2.405524014	0		20	1
chr3_8495_mature	4.29827973	0	2.405524014	0		20	1
chr4_10313_star	4.29827973	0	2.405524014	0		20	1
rno-miR-3562	4.29827973	0	2.405524014	0		20	1
hsa-miR-514a-5p	4.29827973	0	2.405524014	0		20	1
chr14_31486_star	4.29827973	0	2.405524014	0		20	1
rno-miR-743a-3p	4.29827973	0	2.405524014	0		20	1
mmu-miR-5130	4.29827973	0	2.405524014	0		20	1
NW_003538115_37551_star	4.29827973	0	2.405524014	0		20	1
rno-miR-471-3p	4.29827973	0	2.405524014	0		20	1
mmu-miR-5098	4.29827973	0	2.405524014	0		20	1
mmu-miR-493-3p	4.29827973	0	2.405524014	0		20	1
chr15_33031_star	4.29827973	0	2.405524014	0		20	1
chr6_13911_mature	4.29827973	20.14819	2.405524014	4.402462031		-2.22882	1
chr3_6716_star	4.29827973	0	2.405524014	0		20	1
hsa-miR-202-5p	4.29827973	0	2.405524014	0		20	1
NW_003613080_37038_mature	4.29827973	0	2.405524014	0		20	1
chr2_5373_star	4.29827973	0	2.405524014	0		20	1
chr5_11603_mature	4.29827973	0	2.405524014	0		20	1
chr2_6082_star	4.29827973	0	2.405524014	0		20	1
rno-miR-344g	4.29827973	0	2.405524014	0		20	1
chr4_9373_star	4.29827973	0	2.405524014	0		20	1
chr4_9149_mature@@rno-miR-298-5p	4.29827973	0	2.405524014	0		20	1
chr4_10238_mature	4.29827973	0	2.405524014	0		20	1
chrx_22660_star	4.29827973	0	2.405524014	0		20	1
NW_003538520_37951_star	4.29827973	0	2.405524014	0		20	1
NW_003538870_38317_mature	4.29827973	0	2.405524014	0		20	1
chr14_30124_star	4.29827973	0	2.405524014	0		20	1
mmu-miR-3062-5p	4.29827973	0	2.405524014	0		20	1
chr6_15013_star	4.29827973	0	2.405524014	0		20	1
bta-miR-1256	4.29827973	0	2.405524014	0		20	1
chr7_16117_star	4.29827973	0	2.405524014	0		20	1
mmu-miR-27a-5p	4.29827973	0	2.405524014	0		20	1
chr6_13582_star	4.29827973	0	2.405524014	0		20	1
chrx_22277_star	4.29827973	0	2.405524014	0		20	1
NW_003540029_39234_star	4.29827973	0	2.405524014	0		20	1
rno-miR-344b-5p	4.29827973	0	2.405524014	0		20	1
mmu-miR-496a-5p	4.29827973	0	2.405524014	0		20	1
chr1_191_mature	4.29827973	0	2.405524014	0		20	1
chr15_32726_star	4.29827973	0	2.405524014	0		20	1
chr5_11282_star	4.29827973	0	2.405524014	0		20	1
bta-miR-2367-5p	4.29827973	0	2.405524014	0		20	1
chr3_8285_star	4.29827973	0	2.405524014	0		20	1
chr1_3439_star	4.29827973	0	2.405524014	0		20	1
chrx_21983_mature	4.29827973	0	2.405524014	0		20	1
chr16_34699_mature	4.29827973	0	2.405524014	0		20	1
rno-miR-150-3p	4.29827973	0	2.405524014	0		20	1
chr16_33868_star	4.29827973	0	2.405524014	0		20	1
chr2_4834_star	4.29827973	0	2.405524014	0		20	1
chr1_2930_star	4.29827973	0	2.405524014	0		20	1
rno-miR-764-5p	4.29827973	0	2.405524014	0		20	1
mmu-miR-351-3p	4.29827973	0	2.405524014	0		20	1
chr17_35047_star	4.29827973	0	2.405524014	0		20	1
mmu-miR-351-5p	4.29827973	0	2.405524014	0		20	1
chr9_21046_star	4.29827973	0	2.405524014	0		20	1
chr6_13297_star	4.29827973	0	2.405524014	0		20	1
chr4_10605_star	4.29827973	0	2.405524014	0		20	1
chr7_16472_star	4.29827973	0	2.405524014	0		20	1
rno-miR-205	4.29827973	0	2.405524014	0		20	1
hsa-miR-5197-3p	4.29827973	0	2.405524014	0		20	1
chr6_14371_star	4.29827973	0	2.405524014	0		20	1
hsa-miR-20b-5p	4.29827973	0	2.405524014	0		20	1
hsa-miR-1180	4.29827973	0	2.405524014	0		20	1
chr9_20370_star	4.29827973	0	2.405524014	0		20	1
bta-miR-2325c	4.29827973	0	2.405524014	0		20	1
mmu-miR-6340	4.29827973	0	2.405524014	0		20	1
chr1_1984_mature	4.29827973	0	2.405524014	0		20	1
NW_003538780_38224_mature	4.29827973	0	2.405524014	0		20	1
mmu-miR-6365	4.29827973	0	2.405524014	0		20	1
chr5_11601_star	4.29827973	0	2.405524014	0		20	1
chrx_23194_star@@mmu-miR-201-5p	4.29827973	0	2.405524014	0		20	1
bta-miR-146b	4.29827973	0	2.405524014	0		20	1
chr12_26332_star	4.29827973	0	2.405524014	0		20	1
chrx_21839_mature	4.29827973	0	2.405524014	0		20	1
rno-miR-290	4.29827973	0	2.405524014	0		20	1
NW_003539007_38456_mature	4.29827973	20.14819	2.405524014	4.402462031		-2.22882	1
hsa-miR-4799-5p	4.29827973	0	2.405524014	0		20	1
chr4_9894_star	4.29827973	0	2.405524014	0		20	1
rno-miR-206-5p	4.29827973	0	2.405524014	0		20	1
chr17_35707_mature	4.29827973	0	2.405524014	0		20	1
hsa-miR-4436b-5p	4.29827973	0	2.405524014	0		20	1
rno-miR-217-5p	4.29827973	0	2.405524014	0		20	1
chr14_31499_star	4.29827973	0	2.405524014	0		20	1
chr1_1168_mature	4.29827973	20.14819	2.405524014	4.402462031		-2.22882	1
chr17_35140_star	4.29827973	0	2.405524014	0		20	1
bta-miR-2382-3p	4.29827973	0	2.405524014	0		20	1
chr1_2420_star	4.29827973	0	2.405524014	0		20	1
chr3_7453_star	4.29827973	0	2.405524014	0		20	1
chr9_19733_mature	4.29827973	0	2.405524014	0		20	1
chr7_16196_star	4.29827973	0	2.405524014	0		20	1
ssc-miR-1296-3p	4.29827973	0	2.405524014	0		20	1
chr12_25873_star	4.29827973	0	2.405524014	0		20	1
chr1_485_star	4.29827973	0	2.405524014	0		20	1
hsa-miR-33b-3p	4.29827973	0	2.405524014	0		20	1
chr6_14288_star	4.29827973	0	2.405524014	0		20	1
chr16_33810_star	4.29827973	0	2.405524014	0		20	1
chr10_23974_star	4.29827973	0	2.405524014	0		20	1
chrx_23200_star	4.29827973	0	2.405524014	0		20	1
hsa-miR-545-5p	4.29827973	0	2.405524014	0		20	1
hsa-miR-6130	4.29827973	0	2.405524014	0		20	1
chr12_25593_mature	17.1931189	4.835565	4.185320985	2.544872269		1.830075	1
chr1_1915_mature	4.29827973	0	2.405524014	0		20	1
chr8_17350_mature	4.29827973	0	2.405524014	0		20	1
chr18_36548_mature	4.29827973	0	2.405524014	0		20	1
chr5_11515_mature	4.29827973	0	2.405524014	0		20	1
chr6_12987_star	4.29827973	0	2.405524014	0		20	1
chr13_26823_star	4.29827973	0	2.405524014	0		20	1
rno-miR-151-5p	4.29827973	0	2.405524014	0		20	1
ssc-miR-137	23.6405385	8.059274	4.62296188	3.179395517		1.552541	1
mmu-miR-3113-5p	4.29827973	0	2.405524014	0		20	1
hsa-miR-1307-5p	4.29827973	0	2.405524014	0		20	1
chr16_34606_star	4.29827973	0	2.405524014	0		20	1
chr17_35808_star	4.29827973	0	2.405524014	0		20	1
rno-miR-3559-3p	4.29827973	0	2.405524014	0		20	1
mmu-miR-20a-3p	4.29827973	0	2.405524014	0		20	1
chrx_21814_star	4.29827973	0	2.405524014	0		20	1

chr8_18081_mature	10.7457	4.029637	3.554060706	2.330454351		1.415037	0.659114
hsa-miR-548ar-5p	1.07457	2.417782	1.05281229	1.773060525		-1.16993	0.659416
chrx_23008_star	1.07457	2.417782	1.05281229	1.773060525		-1.16993	0.659416
NW_003541277_40020_star	1.07457	2.417782	1.05281229	1.773060525		-1.16993	0.659416
chr14_30973_star	1.07457	2.417782	1.05281229	1.773060525		-1.16993	0.659416
mmu-miR-344b-3p	1.07457	2.417782	1.05281229	1.773060525		-1.16993	0.659416
chr2_5921_star	1.07457	2.417782	1.05281229	1.773060525		-1.16993	0.659416
hsa-miR-1285-5p	1.07457	2.417782	1.05281229	1.773060525		-1.16993	0.659416
chr4_8756_star	1.07457	2.417782	1.05281229	1.773060525		-1.16993	0.659416
chr8_18853_star	1.07457	2.417782	1.05281229	1.773060525		-1.16993	0.659416
chr3_8076_star	1.07457	2.417782	1.05281229	1.773060525		-1.16993	0.659416
bta-miR-2411-3p	1.07457	2.417782	1.05281229	1.773060525		-1.16993	0.659416
chr17_35010_star	1.07457	2.417782	1.05281229	1.773060525		-1.16993	0.659416
hsa-miR-6505-5p	1.07457	2.417782	1.05281229	1.773060525		-1.16993	0.659416
chr1_3348_star	1.07457	2.417782	1.05281229	1.773060525		-1.16993	0.659416
rno-miR-330-3p	1.07457	2.417782	1.05281229	1.773060525		-1.16993	0.659416
hsa-miR-5571-3p	1.07457	2.417782	1.05281229	1.773060525		-1.16993	0.659416
hsa-miR-3655	1.07457	2.417782	1.05281229	1.773060525		-1.16993	0.659416
rno-miR-3552	1.07457	2.417782	1.05281229	1.773060525		-1.16993	0.659416
chr3_8500_mature	1.07457	2.417782	1.05281229	1.773060525		-1.16993	0.659416
chr12_25573_star	1.07457	2.417782	1.05281229	1.773060525		-1.16993	0.659416
bta-miR-2450b	1.07457	2.417782	1.05281229	1.773060525		-1.16993	0.659416
chr3_7627_star	1.07457	2.417782	1.05281229	1.773060525		-1.16993	0.659416
hsa-miR-5009-3p	1.07457	2.417782	1.05281229	1.773060525		-1.16993	0.659416
chr7_15080_mature	1.07457	2.417782	1.05281229	1.773060525		-1.16993	0.659416
chr4_10006_star	1.07457	2.417782	1.05281229	1.773060525		-1.16993	0.659416
mmu-miR-6349	1.07457	2.417782	1.05281229	1.773060525		-1.16993	0.659416
NW_003539542_38921_star	1.07457	2.417782	1.05281229	1.773060525		-1.16993	0.659416
mmu-miR-6361	1.07457	2.417782	1.05281229	1.773060525		-1.16993	0.659416
bta-miR-2285i	1.07457	2.417782	1.05281229	1.773060525		-1.16993	0.659416
bta-miR-2284y	1.07457	2.417782	1.05281229	1.773060525		-1.16993	0.659416
mmu-miR-6397	1.07457	2.417782	1.05281229	1.773060525		-1.16993	0.659416
NW_003539948_39194_star	1.07457	2.417782	1.05281229	1.773060525		-1.16993	0.659416
bta-miR-2900	1.07457	2.417782	1.05281229	1.773060525		-1.16993	0.659416
chr8_18587_star	1.07457	2.417782	1.05281229	1.773060525		-1.16993	0.659416
chr2_4828_star	1.07457	2.417782	1.05281229	1.773060525		-1.16993	0.659416
chr12_26594_mature	1.07457	2.417782	1.05281229	1.773060525		-1.16993	0.659416
hsa-miR-589-3p	1.07457	2.417782	1.05281229	1.773060525		-1.16993	0.659416
chr5_11789_star	1.07457	2.417782	1.05281229	1.773060525		-1.16993	0.659416
hsa-miR-6514-5p	1.07457	2.417782	1.05281229	1.773060525		-1.16993	0.659416
chr4_9832_star	1.07457	2.417782	1.05281229	1.773060525		-1.16993	0.659416
chr3_7424_mature	1.07457	2.417782	1.05281229	1.773060525		-1.16993	0.659416
chr5_10851_star	1.07457	2.417782	1.05281229	1.773060525		-1.16993	0.659416
hsa-miR-320e	1.07457	2.417782	1.05281229	1.773060525		-1.16993	0.659416
chr6_14179_mature	1.07457	2.417782	1.05281229	1.773060525		-1.16993	0.659416
hsa-miR-548v	1.07457	2.417782	1.05281229	1.773060525		-1.16993	0.659416
chr5_11474_star	1.07457	2.417782	1.05281229	1.773060525		-1.16993	0.659416
chr5_11077_mature	1.07457	2.417782	1.05281229	1.773060525		-1.16993	0.659416
bta-miR-6532	1.07457	2.417782	1.05281229	1.773060525		-1.16993	0.659416
chr1_1112_mature	1.07457	2.417782	1.05281229	1.773060525		-1.16993	0.659416
chr6_12803_star	1.07457	2.417782	1.05281229	1.773060525		-1.16993	0.659416
chr4_10559_star	1.07457	2.417782	1.05281229	1.773060525		-1.16993	0.659416
chr1_3877_star	1.07457	2.417782	1.05281229	1.773060525		-1.16993	0.659416
NW_003539513_38909_star	1.07457	2.417782	1.05281229	1.773060525		-1.16993	0.659416
chr1_3087_mature	1.07457	2.417782	1.05281229	1.773060525		-1.16993	0.659416
chr3_8509_star	1.07457	2.417782	1.05281229	1.773060525		-1.16993	0.659416
hsa-miR-4708-5p	1.07457	2.417782	1.05281229	1.773060525		-1.16993	0.659416
NW_003538933_38357_star	1.07457	2.417782	1.05281229	1.773060525		-1.16993	0.659416
chr6_14320_star	1.07457	2.417782	1.05281229	1.773060525		-1.16993	0.659416
chr12_26596_mature	1.07457	2.417782	1.05281229	1.773060525		-1.16993	0.659416
chrx_21800_star	1.07457	2.417782	1.05281229	1.773060525		-1.16993	0.659416
hsa-miR-4312	1.07457	2.417782	1.05281229	1.773060525		-1.16993	0.659416
bta-miR-2365	1.07457	2.417782	1.05281229	1.773060525		-1.16993	0.659416
hsa-miR-4308	1.07457	2.417782	1.05281229	1.773060525		-1.16993	0.659416
chr4_10537_star	1.07457	2.417782	1.05281229	1.773060525		-1.16993	0.659416
chr15_32938_star	1.07457	2.417782	1.05281229	1.773060525		-1.16993	0.659416
NW_003613223_37738_mature	1.07457	2.417782	1.05281229	1.773060525		-1.16993	0.659416
NW_003613401_38938_star	1.07457	2.417782	1.05281229	1.773060525		-1.16993	0.659416
chr18_36103_star	1.07457	2.417782	1.05281229	1.773060525		-1.16993	0.659416
chr12_25581_mature	1.07457	2.417782	1.05281229	1.773060525		-1.16993	0.659416
chr14_29526_star	1.07457	2.417782	1.05281229	1.773060525		-1.16993	0.659416
hsa-miR-4509	1.07457	2.417782	1.05281229	1.773060525		-1.16993	0.659416
hsa-miR-548h-5p	1.07457	2.417782	1.05281229	1.773060525		-1.16993	0.659416
hsa-miR-4778-3p	1.07457	2.417782	1.05281229	1.773060525		-1.16993	0.659416
chr6_13364_star	1.07457	2.417782	1.05281229	1.773060525		-1.16993	0.659416
chr3_7704_mature	1.07457	2.417782	1.05281229	1.773060525		-1.16993	0.659416
rno-miR-222-3p	3.22371	8.059274	2.078510713	3.179395517		-1.32193	0.659559
chr16_34851_mature	3.22371	8.059274	2.078510713	3.179395517		-1.32193	0.659559
NW_003613481_39347_mature	41.37094	9.671129	5.405003311	3.415640968		2.096862	0.659612
rno-miR-344b-1-3p	52.65393	11.28298	5.745611853	3.618589217		2.222392	0.660004
chr4_9551_star	2.14914	0.805927	1.654957834	0.852739935		1.415037	0.66047
chr8_18908_star	2.14914	0.805927	1.654957834	0.852739935		1.415037	0.66047
chr13_27738_star	2.14914	0.805927	1.654957834	0.852739935		1.415037	0.66047
chr6_13733_star	2.14914	0.805927	1.654957834	0.852739935		1.415037	0.66047
rno-miR-551b-3p	2.14914	0.805927	1.654957834	0.852739935		1.415037	0.66047
hsa-miR-4729	2.14914	0.805927	1.654957834	0.852739935		1.415037	0.66047
chr16_34636_star	2.14914	0.805927	1.654957834	0.852739935		1.415037	0.66047
chr17_35073_star	2.14914	0.805927	1.654957834	0.852739935		1.415037	0.66047
rno-miR-20a-5p	2.14914	0.805927	1.654957834	0.852739935		1.415037	0.66047
chr14_31063_mature	2.14914	0.805927	1.654957834	0.852739935		1.415037	0.66047
hsa-miR-4764-5p	2.14914	0.805927	1.654957834	0.852739935		1.415037	0.66047
chr6_13565_star	2.14914	0.805927	1.654957834	0.852739935		1.415037	0.66047
hsa-miR-4727-5p	2.14914	0.805927	1.654957834	0.852739935		1.415037	0.66047
chr14_31022_mature	2.14914	0.805927	1.654957834	0.852739935		1.415037	0.66047
chr13_28617_mature@@mmu-miR	2.14914	0.805927	1.654957834	0.852739935		1.415037	0.66047
chr5_12114_mature@@bta-miR-14	2.14914	0.805927	1.654957834	0.852739935		1.415037	0.66047
mmu-miR-1898	2.14914	0.805927	1.654957834	0.852739935		1.415037	0.66047
chr5_12125_star	2.14914	0.805927	1.654957834	0.852739935		1.415037	0.66047
chr2_6441_mature	2.14914	0.805927	1.654957834	0.852739935		1.415037	0.66047
hsa-miR-3923	2.14914	0.805927	1.654957834	0.852739935		1.415037	0.66047
hsa-miR-3929	2.14914	0.805927	1.654957834	0.852739935		1.415037	0.66047
hsa-miR-3928	2.14914	0.805927	1.654957834	0.852739935		1.415037	0.66047
chrx_22120_star	2.14914	0.805927	1.654957834	0.852739935		1.415037	0.66047
NW_003538808_38240_mature	2.14914	0.805927	1.654957834	0.852739935		1.415037	0.66047
chr8_17482_star	2.14914	0.805927	1.654957834	0.852739935		1.415037	0.66047
chr1_3263_star	2.14914	0.805927	1.654957834	0.852739935		1.415037	0.66047
chr10_24282_star	2.14914	0.805927	1.654957834	0.852739935		1.415037	0.66047
chr16_33995_star	2.14914	0.805927	1.654957834	0.852739935		1.415037	0.66047
chr12_26183_star	2.14914	0.805927	1.654957834	0.852739935		1.415037	0.66047
hsa-miR-4746-5p	2.14914	0.805927	1.654957834	0.852739935		1.415037	0.66047
NW_003539007_38438_star	2.14914	0.805927	1.654957834	0.852739935		1.415037	0.66047
rno-miR-30d-5p	2.14914	0.805927	1.654957834	0.852739935		1.415037	0.66047
hsa-miR-5193	2.14914	0.805927	1.654957834	0.852739935		1.415037	0.66047
chr9_19683_star	2.14914	0.805927	1.654957834	0.852739935		1.415037	0.66047
mmu-miR-6244	2.14914	0.805927	1.654957834	0.852739935		1.415037	0.66047
chr1_188_star	2.14914	0.805927	1.654957834	0.852739935		1.415037	0.66047
rno-miR-3566	2.14914	0.805927	1.654957834	0.852739935		1.415037	0.66047
hsa-miR-660-3p	2.14914	0.805927	1.654957834	0.852739935		1.415037	0.66047
hsa-miR-4745-3p	2.14914	0.805927	1.654957834	0.852739935		1.415037	0.66047
chr6_14250_mature	2.14914	0.805927	1.654957834	0.852739935		1.415037	0.66047
chr16_34334_mature	2.14914	0.805927	1.654957834	0.852739935		1.415037	0.66047
NW_003538867_38314_mature	2.14914	0.805927	1.654957834	0.852739935		1.415037	0.66047
bta-miR-2385-5p	2.14914	0.805927	1.654957834	0.852739935		1.415037	0.66047
NW_003613367_38733_star	2.14914	0.805927	1.654957834	0.852739935		1.415037	0.66047
bta-miR-1281	2.14914	0.805927	1.654957834	0.852739935		1.415037	0.66047
chr13_28323_star	2.14914	0.805927	1.654957834	0.852739935		1.415037	0.66047
chrx_21977_star@@rno-miR-384-5	2.14914	0.805927	1.654957834	0.852739935		1.415037	0.66047
chr7_16583_star	2.14914	0.805927	1.654957834	0.852739935		1.415037	0.66047
bta-miR-877	2.14914	0.805927	1.654957834	0.852739935		1.415037	0.66047
chr9_21032_star	2.14914	0.805927	1.654957834	0.852739935		1.415037	0.66047
hsa-miR-5008-3p	2.14914	0.805927	1.654957834	0.852739935		1.415037	0.66047
mmu-miR-574-3p	2.14914	0.805927	1.654957834	0.852739935		1.415037	0.66047
bta-miR-1225-3p	2.14914	0.805927	1.654957834	0.852739935		1.415037	0.66047
rno-miR-184	2.14914	0.805927	1.654957834	0.852739935		1.415037	0.66047
chr14_31358_star	2.14914	0.805927	1.654957834	0.852739935		1.415037	0.66047
chr5_11374_star	2.14914	0.805927	1.654957834	0.852739935		1.415037	0.66047
chr15_32731_mature	2.14914	0.805927	1.654957834	0.852739935		1.415037	0.66047
chr1_2562_star	2.14914	0.805927	1.654957834	0.852739935		1.415037	0.66047
chr4_10238_star	2.14914	0.805927	1.654957834	0.852739935		1.415037	0.66047
hsa-miR-362-5p	2.14914	0.805927	1.654957834	0.852739935		1.415037	0.66047
chr8_17863_star	2.14914	0.805927	1.654957834	0.852739935		1.415037	0.66047
NW_003613231_37780_mature	2.14914	0.805927	1.654957834	0.852739935		1.415037	0.66047
chr16_34626_mature	2.14914	0.805927	1.654957834	0.852739935		1.415037	0.66047
mmu-miR-6381	2.14914	0.805927	1.654957834	0.852739935		1.415037	0.66047
chr2_4395_star	2.14914	0.805927	1.654957834	0.852739935		1.415037	0.66047
rno-miR-30b-3p	2.14914	0.805927	1.654957834	0.852739935		1.415037	0.66047
chr8_18283_star	2.14914	0.805927	1.654957834	0.852739935		1.415037	0.66047
chr11_25228_mature	2.14914	0.805927	1.654957834	0.852739935		1.415037	0.66047
chr1_191_star	2.14914	0.805927	1.654957834	0.852739935		1.415037	0.66047
hsa-miR-548x-3p	2.14914	0.805927	1.654957834	0.852739935		1.415037	0.66047
chr7_17055_star	2.14914	0.805927	1.654957834	0.852739935		1.415037	0.66047
chr5_10819_star	2.14914	0.805927	1.654957834	0.852739935		1.415037	0.66047
chr18_35826_star	2.14914	0.805927	1.654957834	0.852739935		1.415037	0.66047
chr6_13990_star	2.14914	0.805927	1.654957834	0.852739935		1.415037	0.66047
rno-miR-344a-5p	2.14914	0.805927	1.654957834	0.852739935		1.415037	0.66047
chr2_5926_mature	2.14914	0.805927	1.654957834	0.852739935		1.415037	0.66047
chr10_24040_star	2.14914	0.805927	1.654957834	0.852739935		1.415037	0.66047
mmu-miR-3965	2.14914	0.805927	1.654957834	0.852739935		1.415037	0.66047
chr9_19208_star	2.14914	0.805927	1.654957834	0.852739935		1.415037	0.66047
chr9_20807_star	2.14914	0.805927	1.654957834	0.852739935		1.415037	0.66047
hsa-miR-3065-3p	2.14914	0.805927	1.654957834	0.852739935		1.415037	0.66047
bta-miR-16b	2.14914	0.805927	1.654957834	0.852739935		1.415037	0.66047
bta-miR-186	2.14914	0.805927	1.654957834	0.852739935		1.415037	0.66047
chr6_14927_star	2.14914	0.805927	1.654957834	0.852739935		1.415037	0.66047
NW_003613335_38593_star	2.14914	0.805927	1.654957834	0.852739935		1.415037	0.66047
mmu-miR-1247-5p	2.14914	0.805927	1.654957834	0.852739935		1.415037	0.66047
chr15_33480_star	2.14914	0.805927	1.654957834	0.852739935		1.415037	0.66047
chr17_35663_star	2.14914	0.805927	1.654957834	0.852739935		1.415037	0.66047
chr17_35646_mature	2.14914	0.805927	1.654957834	0.852739935		1.415037	0.66047
mmu-miR-3095-3p	2.14914	0.805927	1.654957834	0.852739935		1.415037	0.66047
bta-miR-2355-3p	2.14914	0.805927	1.654957834	0.852739935		1.415037	0.66047
chr4_10721_star	2.14914	0.805927	1.654957834	0.852739935		1.415037	0.66047
chr12_25827_star	2.14914	0.805927	1.654957834	0.852739935		1.415037	0.66047
chr12_26448_mature	2.14914	0.805927	1.654957834	0.852739935		1.415037	0.66047
chr5_12116_star@@rno-miR-200c-	2.14914	0.805927	1.654957834	0.852739935		1.415037	0.66047
NW_003537967_37423_star	2.14914	0.805927	1.654957834	0.852739935		1.415037	0.66047
bta-miR-30f	2.14914	0.805927	1.654957834	0.852739935		1.415037	0.66047
NW_003540965_39767_mature	2.14914	0.805927	1.654957834	0.852739935		1.415037	0.66047
bta-miR-30b-5p	2.14914	0.805927	1.654957834	0.852739935		1.415037	0.66047
bta-miR-2454-3p	2.14914	0.805927	1.654957834	0.852739935		1.415037	0.66047
mmu-miR-298-5p	2.14914	0.805927	1.654957834	0.852739935		1.415037	0.66047
chr6_14978_star	2.14914	0.805927	1.654957834	0.852739935		1.415037	0.66047
chr7_16084_star	2.14914	0.805927	1.654957834	0.852739935		1.415037	0.66047
chr14_31022_star	2.14914	0.805927	1.654957834	0.852739935		1.415037	0.66047
rno-miR-29a-3p	2.14914	0.805927	1.654957834	0.852739935		1.415037	0.66047
chr17_35122_star	2.14914	0.805927	1.654957834	0.852739935		1.415037	0.66047
bta-miR-2401	2.14914	0.805927	1.654957834	0.852739935		1.415037	0.66047
hsa-miR-4455	2.14914	0.805927	1.654957834	0.852739935		1.415037	0.66047
NW_003538201_37614_star	2.14914	0.805927	1.654957834	0.852739935		1.415037	0.66047
chr6_14951_star	2.14914	0.805927	1.654957834	0.852739935		1.415037	0.66047
bta-miR-2432	2.14914	0.805927	1.654957834	0.852739935		1.415037	0.66047
hsa-miR-4478	2.14914	0.805927	1.654957834	0.852739935		1.415037	0.66047
chr2_4621_mature	2.14914	0.805927	1.654957834	0.852739935		1.415037	0.66047
chr13_28165_star	2.14914	0.805927	1.654957834	0.852739935		1.415037	0.66047
NW_003613025_36806_star	2.14914	0.805927	1.654957834	0.852739935		1.415037	0.66047
chr3_6909_star	2.14914	0.805927	1.654957834	0.852739935		1.415037	0.66047
chr15_32407_mature	2.14914	0.805927	1.654957834	0.852739935		1.415037	0.66047
chr2_6533_star	2.14914	0.805927	1.654957834	0.852739935		1.415037	0.66047
bta-miR-2301	2.14914	0.805927	1.654957834	0.852739935		1.415037	0.66047
rno-miR-582-3p	2.14914	0.805927	1.654957834	0.852739935		1.415037	0.66047
chr12_25917_star	2.14914	0.805927	1.654957834	0.852739935		1.415037	0.66047
bta-miR-2354	2.14914	0.805927	1.654957834	0.852739935		1.415037	0.66047
hsa-miR-3187-5p	2.14914	0.805927	1.654957834	0.852739935		1.415037	0.66047
hsa-miR-4310	2.14914	0.805927	1.654957834	0.852739935		1.415037	0.66047
chr14_31507_star	2.14914	0.805927	1.654957834	0.852739935		1.415037	0.66047
chr6_13320_star	2.14914	0.805927	1.654957834	0.852739935		1.415037	0.66047
chrx_22392_star	2.14914	0.805927	1.654957834	0.852739935		1.415037	0.66047
chr12_26547_mature	2.14914	0.805927	1.654957834	0.852739935		1.415037	0.66047
chr12_26570_mature	2.14914	0.805927	1.654957834	0.852739935		1.415037	0.66047
hsa-miR-4678	2.14914	0.805927	1.654957834	0.852739935		1.415037	0.66047
chr15_32819_star	2.14914	0.805927	1.654957834	0.852739935		1.415037	0.66047
hsa-miR-4698	2.14914	0.805927	1.654957834	0.852739935		1.415037	0.66047
NW_003541025_39829_star	2.14914	0.805927	1.654957834	0.852739935		1.415037	0.66047
mmu-miR-717	2.14914	0.805927	1.654957834	0.852739935		1.415037	0.66047
mmu-miR-1933-3p	2.14914	0.805927	1.654957834	0.852739935		1.415037	0.66047
chr6_14296_star	2.14914	0.805927	1.654957834	0.852739935		1.415037	0.66047
hsa-miR-1911-5p	2.14914	0.805927	1.654957834	0.852739935		1.415037	0.66047
hsa-miR-5001-3p	2.14914	0.805927	1.654957834	0.852739935		1.415037	0.66047
chr2_4824_star	2.14914	0.805927	1.654957834	0.852739935		1.415037	0.66047
hsa-miR-5001-5p	2.14914	0.805927	1.654957834	0.852739935		1.415037	0.66047
rno-miR-7a-2-3p	2.14914	0.805927	1.654957834	0.852739935		1.415037	0.66047
chr7_16194_mature	2.14914	0.805927	1.654957834	0.852739935		1.415037	0.66047
chr3_6975_star	2.14914	0.805927	1.654957834	0.852739935		1.415037	0.66047
hsa-miR-3942-3p	2.14914	0.805927	1.654957834	0.852739935		1.415037	0.66047
hsa-miR-4703-3p	2.14914	0.805927	1.654957834	0.852739935		1.415037	0.66047
chr5_12007_mature	2.14914	0.805927	1.654957834	0.852739935		1.415037	0.66047
chr14_30751_star	2.14914	0.805927	1.654957834	0.852739935		1.415037	0.66047
chr9_19145_star	2.14914	0.805927	1.654957834	0.852739935		1.415037	0.66047
hsa-miR-6068	2.14914	0.805927	1.654957834	0.852739935		1.415037	0.66047
chr9_19892_star	2.14914	0.805927	1.654957834	0.852739935		1.415037	0.66047
chr9_20571_star	2.14914	0.805927	1.654957834	0.852739935		1.415037	0.66047
mmu-miR-1929-3p	2.14914	0.805927	1.654957834	0.852739935		1.415037	0.66047
bta-miR-1248	58.56406	12.08891	5.89637022	3.710273245		2.276331	0.660568
chr17_35330_mature	13.96941	4.835565	3.90394537	2.544872269		1.530515	0.660701
chr4_10263_star	13.96941	4.835565	3.90394537	2.544872269		1.530515	0.660701
chr9_20397_mature	13.96941	4.835565	3.90394537	2.544872269		1.530515	0.660701
chr2_5244_star	7.52199	3.22371	3.091190278	2.078510713		1.222392	0.662349
chr15_31928_mature	7.52199	3.22371	3.091190278	2.078510713		1.222392	0.662349
chr4_9657_star	7.52199	3.22371	3.091190278	2.078510713		1.222392	0.662349
chr1_1386_star	7.52199	3.22371	3.091190278	2.078510713		1.222392	0.662349
chr8_18167_star	7.52199	3.22371	3.091190278	2.078510713		1.222392	0.662349
hsa-miR-4749-5p	1.611855	3.22371	1.38507475	2.078510713		-1	0.662466
hsa-miR-4771	1.611855	3.22371	1.38507475	2.078510713		-1	0.662466
chr1_2577_mature	1.611855	3.22371	1.38507475	2.078510713		-1	0.662466
chr2_5321_mature	1.611855	3.22371	1.38507475	2.078510713		-1	0.662466
chr8_18128_star	1.611855	3.22371	1.38507475	2.078510713		-1	0.662466
chr5_11598_mature	1.611855	3.22371	1.38507475	2.078510713		-1	0.662466
hsa-miR-5004-3p	1.611855	3.22371	1.38507475	2.078510713		-1	0.662466
chr3_8536_star	1.611855	3.22371	1.38507475	2.078510713		-1	0.662466
chr17_34868_mature	1.611855	3.22371	1.38507475	2.078510713		-1	0.662466
chr8_18247_mature	1.611855	3.22371	1.38507475	2.078510713		-1	0.662466
chr1_1422_mature	1.611855	3.22371	1.38507475	2.078510713		-1	0.662466
chr11_25303_mature	1.611855	3.22371	1.38507475	2.078510713		-1	0.662466
chr3_7373_star	1.611855	3.22371	1.38507475	2.078510713		-1	0.662466
bta-miR-2285d	1.611855	3.22371	1.38507475	2.078510713		-1	0.662466
ssc-miR-1224	1.611855	3.22371	1.38507475	2.078510713		-1	0.662466
NW_003613063_36957_star	1.611855	3.22371	1.38507475	2.078510713		-1	0.662466
chr15_31697_mature	1.611855	3.22371	1.38507475	2.078510713		-1	0.662466
chr8_17514_star	1.611855	3.22371	1.38507475	2.078510713		-1	0.662466
chr15_32589_star	1.611855	3.22371	1.38507475	2.078510713		-1	0.662466
bta-miR-23a	1.611855	3.22371	1.38507475	2.078510713		-1	0.662466
hsa-miR-3179	1.611855	3.22371	1.38507475	2.078510713		-1	0.662466
chr2_5244_mature	1.611855	3.22371	1.38507475	2.078510713		-1	0.662466
chr14_29940_mature	1.611855	3.22371	1.38507475	2.078510713		-1	0.662466
chr3_6750_mature	1.611855	3.22371	1.38507475	2.078510713		-1	0.662466

chr3_7956_mature	2.149139864	0.805927	1.654957834	0.852739935		1.415037	0.431307		chr4_9813_star	4.29827973	0	2.405524014	0		20	1		hsa-miR-519c-3p	1.611855	3.22371	1.38507475	2.078510713		-1	0.662466
chr2_5926_star	2.149139864	0.805927	1.654957834	0.852739935		1.415037	0.431307		chr16_33863_star	4.29827973	0	2.405524014	0		20	1		chr12_26006_star	1.611855	3.22371	1.38507475	2.078510713		-1	0.662466
chr14_30718_mature	2.149139864	0.805927	1.654957834	0.852739935		1.415037	0.431307		chr3_7747_star	4.29827973	0	2.405524014	0		20	1		chr17_35182_star	1.611855	3.22371	1.38507475	2.078510713		-1	0.662466
NW_003537531_36983_mature	2.149139864	0.805927	1.654957834	0.852739935		1.415037	0.431307		chr12_25742_star	4.29827973	0	2.405524014	0		20	1		chr1_2060_mature	21.4914	6.44742	4.491301473	2.896740642		1.736966	0.662845
hsa-miR-5001-5p	2.149139864	0.805927	1.654957834	0.852739935		1.415037	0.431307		chr2_3992_mature	4.29827973	0	2.405524014	0		20	1		chr3_7094_mature	21.4914	6.44742	4.491301473	2.896740642		1.736966	0.662845
hsa-miR-211-3p	2.149139864	0.805927	1.654957834	0.852739935		1.415037	0.431307		chr17_35007_star	4.29827973	0	2.405524014	0		20	1		chr2_6101_mature	3.22371	1.611855	2.078510713	1.38507475		1	0.662987
hsa-miR-5682	2.149139864	0.805927	1.654957834	0.852739935		1.415037	0.431307		chr1_384_star	4.29827973	0	2.405524014	0		20	1		NW_003613223_37737_mature	3.22371	1.611855	2.078510713	1.38507475		1	0.662987
chr14_30584_mature	2.149139864	0.805927	1.654957834	0.852739935		1.415037	0.431307		chr3_7521_mature	4.29827973	0	2.405524014	0		20	1		chr16_34314_mature	3.22371	1.611855	2.078510713	1.38507475		1	0.662987
hsa-miR-4518	2.149139864	0.805927	1.654957834	0.852739935		1.415037	0.431307		hsa-miR-4695-3p	4.29827973	0	2.405524014	0		20	1		chr14_29900_star	3.22371	1.611855	2.078510713	1.38507475		1	0.662987
hsa-miR-4526	2.149139864	0.805927	1.654957834	0.852739935		1.415037	0.431307		chrx_23204_star	4.29827973	0	2.405524014	0		20	1		chr3_6710_star	3.22371	1.611855	2.078510713	1.38507475		1	0.662987
chr1_3875_mature@@hsa-miR-4667	2.149139864	0.805927	1.654957834	0.852739935		1.415037	0.431307		chr15_31697_star	4.29827973	0	2.405524014	0		20	1		chr5_11958_star	3.22371	1.611855	2.078510713	1.38507475		1	0.662987
chr14_30394_mature	2.149139864	0.805927	1.654957834	0.852739935		1.415037	0.431307		chr4_9977_star	4.29827973	0	2.405524014	0		20	1		chr9_20079_mature	3.22371	1.611855	2.078510713	1.38507475		1	0.662987
hsa-miR-4538	2.149139864	0.805927	1.654957834	0.852739935		1.415037	0.431307		chr6_12983_star@@rno-miR-200b-5p	4.29827973	0	2.405524014	0		20	1		chr5_11575_star	3.22371	1.611855	2.078510713	1.38507475		1	0.662987
chr14_29702_star	2.149139864	0.805927	1.654957834	0.852739935		1.415037	0.431307		mmu-miR-3572-3p	4.29827973	0	2.405524014	0		20	1		chr1_3837_mature	3.22371	1.611855	2.078510713	1.38507475		1	0.662987
chr4_8682_star	2.149139864	0.805927	1.654957834	0.852739935		1.415037	0.431307		chr8_17973_star	4.29827973	0	2.405524014	0		20	1		chr15_31781_star	3.22371	1.611855	2.078510713	1.38507475		1	0.662987
chr12_25719_star	2.149139864	0.805927	1.654957834	0.852739935		1.415037	0.431307		chr6_12967_star	4.29827973	0	2.405524014	0		20	1		chrx_22435_star	3.22371	1.611855	2.078510713	1.38507475		1	0.662987
hsa-miR-1972	2.149139864	0.805927	1.654957834	0.852739935		1.415037	0.431307		hsa-miR-3145-3p	4.29827973	0	2.405524014	0		20	1		chr5_11673_mature	3.22371	1.611855	2.078510713	1.38507475		1	0.662987
chr3_7552_star	2.149139864	0.805927	1.654957834	0.852739935		1.415037	0.431307		chr14_30644_star	4.29827973	0	2.405524014	0		20	1		chr15_32437_star	3.22371	1.611855	2.078510713	1.38507475		1	0.662987
hsa-miR-6084	2.149139864	0.805927	1.654957834	0.852739935		1.415037	0.431307		chr1_2440_star	4.29827973	0	2.405524014	0		20	1		chr8_18863_star	3.22371	1.611855	2.078510713	1.38507475		1	0.662987
chr9_19892_star	2.149139864	0.805927	1.654957834	0.852739935		1.415037	0.431307		chr4_9150_mature@@rno-miR-298-5p	4.29827973	0	2.405524014	0		20	1		chr8_18873_star	3.22371	1.611855	2.078510713	1.38507475		1	0.662987
NW_003613377_38747_star	2.149139864	0.805927	1.654957834	0.852739935		1.415037	0.431307		chrx_23196_mature	4.29827973	0	2.405524014	0		20	1		chr17_35168_star	3.22371	1.611855	2.078510713	1.38507475		1	0.662987
chr14_29900_mature	15.04397905	4.835565	4.003960082	2.544872269		1.63743	0.432225		chr3_7937_mature	163.33463	236.1367	7.360492716	7.889575404		-0.53179	1		chr4_9509_star	3.22371	1.611855	2.078510713	1.38507475		1	0.662987
rno-miR-345-3p	15.04397905	4.835565	4.003960082	2.544872269		1.63743	0.432225		chr8_17976_star	4.29827973	0	2.405524014	0		20	1		chr9_19419_mature	3.22371	1.611855	2.078510713	1.38507475		1	0.662987
chr1_2451_mature	15.04397905	4.835565	4.003960082	2.544872269		1.63743	0.432225		chr5_11364_mature	4.29827973	0	2.405524014	0		20	1		NW_003613232_37791_star	3.22371	1.611855	2.078510713	1.38507475		1	0.662987
chr16_34708_mature	15.04397905	4.835565	4.003960082	2.544872269		1.63743	0.432225		chr10_23498_star	4.29827973	0	2.405524014	0		20	1		mmu-miR-6374	3.22371	1.611855	2.078510713	1.38507475		1	0.662987
chr13_28383_mature	15.04397905	4.835565	4.003960082	2.544872269		1.63743	0.432225		mmu-miR-297a-5p	4.29827973	0	2.405524014	0		20	1		chr8_18054_mature	3.22371	1.611855	2.078510713	1.38507475		1	0.662987
NW_003539698_39034_mature	26.8642483	7.253347	4.800343329	3.044979305		1.888969	0.433158		hsa-miR-3142	4.29827973	0	2.405524014	0		20	1		chr6_12636_star	3.22371	1.611855	2.078510713	1.38507475		1	0.662987
chr5_12332_mature	10.74569932	47.54972	3.554060706	5.601391055		-2.14568	0.433327		chr3_6871_star	4.29827973	0	2.405524014	0		20	1		chr15_31736_star	3.22371	1.611855	2.078510713	1.38507475		1	0.662987
chr2_5267_mature	5.372849659	16.92448	2.671938627	4.163859073		-1.65535	0.433665		mmu-miR-3091-3p	4.29827973	0	2.405524014	0		20	1		chr4_9468_mature	3.22371	1.611855	2.078510713	1.38507475		1	0.662987
chr7_15943_mature	2.149139864	4.835565	1.654957834	2.544872269		-1.16993	0.434181		rno-miR-195-5p	4.29827973	0	2.405524014	0		20	1		chr14_30974_mature	3.22371	1.611855	2.078510713	1.38507475		1	0.662987
NW_003537624_37070_mature	2.149139864	4.835565	1.654957834	2.544872269		-1.16993	0.434181		hsa-miR-3116	4.29827973	0	2.405524014	0		20	1		chr7_17049_star	3.22371	1.611855	2.078510713	1.38507475		1	0.662987
chr2_4529_star	2.149139864	4.835565	1.654957834	2.544872269		-1.16993	0.434181		chr6_12861_star	4.29827973	0	2.405524014	0		20	1		rno-miR-1949	3.22371	1.611855	2.078510713	1.38507475		1	0.662987
hsa-miR-1293	2.149139864	4.835565	1.654957834	2.544872269		-1.16993	0.434181		NW_003613310_38408_mature	4.29827973	0	2.405524014	0		20	1		chr6_14308_star	3.22371	1.611855	2.078510713	1.38507475		1	0.662987
chr7_16140_star	2.149139864	4.835565	1.654957834	2.544872269		-1.16993	0.434181		chr8_17650_mature	4.29827973	0	2.405524014	0		20	1		chr14_30996_star	3.22371	1.611855	2.078510713	1.38507475		1	0.662987
chr14_31521_star	2.149139864	4.835565	1.654957834	2.544872269		-1.16993	0.434181		hsa-miR-4267	4.29827973	0	2.405524014	0		20	1		hsa-miR-6134	3.22371	1.611855	2.078510713	1.38507475		1	0.662987
NW_003613246_37882_star	2.149139864	4.835565	1.654957834	2.544872269		-1.16993	0.434181		chr3_7761_star	4.29827973	0	2.405524014	0		20	1		hsa-miR-5196-3p	3.22371	1.611855	2.078510713	1.38507475		1	0.662987
chr5_11064_mature	2.149139864	4.835565	1.654957834	2.544872269		-1.16993	0.434181		chr5_11634_star	4.29827973	0	2.405524014	0		20	1		mmu-miR-653-3p	3.22371	1.611855	2.078510713	1.38507475		1	0.662987
chr5_12346_mature	2.149139864	4.835565	1.654957834	2.544872269		-1.16993	0.434181		bta-miR-6520	4.29827973	0	2.405524014	0		20	1		chrx_21505_star	3.22371	1.611855	2.078510713	1.38507475		1	0.662987
chr4_10263_star	2.149139864	4.835565	1.654957834	2.544872269		-1.16993	0.434181		bta-miR-6521	4.29827973	0	2.405524014	0		20	1		chr13_27530_star	3.22371	1.611855	2.078510713	1.38507475		1	0.662987
hsa-miR-509-3p	2.149139864	4.835565	1.654957834	2.544872269		-1.16993	0.434181		chr2_5399_star	4.29827973	0	2.405524014	0		20	1		NW_003613522_39530_mature	3.22371	1.611855	2.078510713	1.38507475		1	0.662987
chr4_10650_star	2.149139864	4.835565	1.654957834	2.544872269		-1.16993	0.434181		hsa-miR-4291	4.29827973	0	2.405524014	0		20	1		mmu-miR-3093-3p	3.22371	1.611855	2.078510713	1.38507475		1	0.662987
chr15_31984_mature	2.149139864	4.835565	1.654957834	2.544872269		-1.16993	0.434181		hsa-miR-770-5p	4.29827973	0	2.405524014	0		20	1		chr17_35035_star	3.22371	1.611855	2.078510713	1.38507475		1	0.662987
chr1_2545_star	2.149139864	4.835565	1.654957834	2.544872269		-1.16993	0.434181		chr14_30563_star	4.29827973	0	2.405524014	0		20	1		chr8_18481_star	3.22371	1.611855	2.078510713	1.38507475		1	0.662987
chr1_1318_star	2.149139864	4.835565	1.654957834	2.544872269		-1.16993	0.434181		NW_003613234_37804_star	4.29827973	0	2.405524014	0		20	1		chrx_22973_mature	3.22371	1.611855	2.078510713	1.38507475		1	0.662987
chr17_35550_star	2.149139864	4.835565	1.654957834	2.544872269		-1.16993	0.434181		chr13_27210_star	4.29827973	0	2.405524014	0		20	1		chr7_16057_mature	3.22371	1.611855	2.078510713	1.38507475		1	0.662987
NW_003539866_39141_star	2.149139864	4.835565	1.654957834	2.544872269		-1.16993	0.434181		chr15_33558_star	4.29827973	0	2.405524014	0		20	1		hsa-miR-3156-3p	3.22371	1.611855	2.078510713	1.38507475		1	0.662987
mmu-miR-466p-5p	2.149139864	4.835565	1.654957834	2.544872269		-1.16993	0.434181		hsa-miR-3677-5p	4.29827973	0	2.405524014	0		20	1		bta-miR-2464-3p	3.22371	1.611855	2.078510713	1.38507475		1	0.662987
chr5_12148_star	2.149139864	4.835565	1.654957834	2.544872269		-1.16993	0.434181		bta-miR-541	4.29827973	0	2.405524014	0		20	1		chr6_14250_star	3.22371	1.611855	2.078510713	1.38507475		1	0.662987
chr4_10054_star	2.149139864	4.835565	1.654957834	2.544872269		-1.16993	0.434181		chr15_33531_star	4.29827973	0	2.405524014	0		20	1		mmu-miR-686	3.22371	1.611855	2.078510713	1.38507475		1	0.662987
chr17_35802_star	2.149139864	4.835565	1.654957834	2.544872269		-1.16993	0.434181		chr13_28352_star	4.29827973	0	2.405524014	0		20	1		rno-miR-879-3p	3.22371	1.611855	2.078510713	1.38507475		1	0.662987
chr14_29876_star	2.149139864	4.835565	1.654957834	2.544872269		-1.16993	0.434181		NW_003537759_37261_star	4.29827973	0	2.405524014	0		20	1		rno-miR-20b-3p	3.22371	1.611855	2.078510713	1.38507475		1	0.662987
hsa-miR-4795-5p	2.149139864	4.835565	1.654957834	2.544872269		-1.16993	0.434181		chr3_7078_star	4.29827973	0	2.405524014	0		20	1		chr6_12809_star	3.22371	1.611855	2.078510713	1.38507475		1	0.662987
chr1_2751_star	2.149139864	4.835565	1.654957834	2.544872269		-1.16993	0.434181		mmu-miR-291b-5p	4.29827973	0	2.405524014	0		20	1		chr5_11967_star	3.22371	1.611855	2.078510713	1.38507475		1	0.662987
chrx_22359_star	2.149139864	4.835565	1.654957834	2.544872269		-1.16993	0.434181		chr4_8839_star	4.29827973	0	2.405524014	0		20	1		chr1_662_mature	3.22371	1.611855	2.078510713	1.38507475		1	0.662987
chr11_24488_star	2.149139864	4.835565	1.654957834	2.544872269		-1.16993	0.434181		mmu-miR-466l-5p	4.29827973	0	2.405524014	0		20	1		chr17_35446_mature	7.52199	28.20746	3.091190278	4.868265031		-1.90689	0.663158
chr2_5406_star	2.149139864	4.835565	1.654957834	2.544872269		-1.16993	0.434181		chr15_33041_mature	4.29827973	0	2.405524014	0		20	1		chr8_18274_star	7.52199	28.20746	3.091190278	4.868265031		-1.90689	0.663158
chr2_5656_star	2.149139864	4.835565	1.654957834	2.544872269		-1.16993	0.434181		chr7_15107_star	4.29827973	0	2.405524014	0		20	1		chr9_19632_star	6.984705	24.98375	2.997239027	4.699537803		-1.83872	0.664879
chr13_28840_mature	2.149139864	4.835565	1.654957834	2.544872269		-1.16993	0.434181		NW_003538437_37870_star	4.29827973	0	2.405524014	0		20	1		chr12_26204_mature	17.19312	5.641492	4.185320985	2.731507408		1.607683	0.664976
hsa-miR-1236-5p	2.149139864	4.835565	1.654957834	2.544872269		-1.16993	0.434181		mmu-miR-1186b	4.29827973	0	2.405524014	0		20	1		chr2_5746_star	5.910135	19.34226	2.788713818	4.346407978		-1.71049	0.664994
chr4_8784_star	2.149139864	4.835565	1.654957834	2.544872269		-1.16993	0.434181		chr8_17814_star	4.29827973	0	2.405524014	0		20	1		chr8_18510_mature	5.910135	19.34226	2.788713818	4.346407978		-1.71049	0.664994
chr6_14661_star	2.149139864	4.835565	1.654957834	2.544872269		-1.16993	0.434181		hsa-miR-4468	4.29827973	0	2.405524014	0		20	1		NW_003539007_38456_star	3.760995	9.671129	2.251263041	3.415640968		-1.36257	0.665017
chr12_26428_mature	2.149139864	4.835565	1.654957834	2.544872269		-1.16993	0.434181		chr1_577_star	4.29827973	0	2.405524014	0		20	1		chr15_33015_mature	3.760995	9.671129	2.251263041	3.415640968		-1.36257	0.665017
chr9_20397_mature	2.149139864	4.835565	1.654957834	2.544872269		-1.16993	0.434181		chr2_4623_star	4.29827973	0	2.405524014	0		20	1		chr2_5658_star	3.760995	9.671129	2.251263041	3.415640968		-1.36257	0.665017
bta-miR-3431	2.149139864	4.835565	1.654957834	2.544872269		-1.16993	0.434181		hsa-miR-4488	4.29827973	0	2.405524014	0		20	1		chr7_16043_star	3.760995	9.671129	2.251263041	3.415640968		-1.36257	0.665017
chr8_19026_star	2.149139864	4.835565	1.654957834	2.544872269		-1.16993	0.434181		chr13_27061_mature	4.29827973	0	2.405524014	0		20	1		chr2_6406_mature@@bta-miR-181	3.760995	9.671129	2.251263041	3.415640968		-1.36257	0.665017
NW_003538201_37611_star	2.149139864	4.835565	1.654957834	2.544872269		-1.16993	0.434181		hsa-miR-602	4.29827973	0	2.405524014	0		20	1		chr1_1055_star	3.760995	9.671129	2.251263041	3.415640968		-1.36257	0.665017
chr15_33009_star	2.149139864	4.835565	1.654957834	2.544872269		-1.16993	0.434181		chr1_3342_star	4.29827973	0	2.405524014	0		20	1		chr12_26332_mature	10.20841	4.029637	3.48651029	2.330454351		1.341037	0.665039
chr3_7189_mature	2.149139864	4.835565	1.654957834	2.544872269		-1.16993	0.434181		chr10_24287_star	4.29827973	0	2.405524014	0		20	1		chr13_26769_mature	10.20841	4.029637	3.48651029	2.330454351		1.341037	0.665039
chr13_29037_star	2.149139864	4.835565	1.654957834	2.544872269		-1.16993	0.434181		rno-miR-872-3p	4.29827973	0	2.405524014	0		20	1		chr3_7884_mature	10.20841	4.029637	3.48651029	2.330454351		1.341037	0.665039
NW_003613440_39149_mature	2.149139864	4.835565	1.654957834	2.544872269		-1.16993	0.434181		chr9_19770_mature	38.6845175	16.92448	5.310504361	4.163859073		1.192645	1		NW_003540745_39661_star	10.20841	4.029637	3.48651029	2.330454351		1.341037	0.665039
NW_003539007_38439_mature	8.596559455	33.84895	3.262517265	5.123043401		-1.97728	0.434344		chr5_11139_mature	10.7456993	32.2371	3.554060706	5.054722516		-1.58496	1		chr11_24358_mature	10.20841	4.029637	3.48651029	2.330454351		1.341037	0.665039
chr12_25810_mature	3.223709795	8.059274	2.078510713	3.179395517		-1.32193	0.435308		hsa-miR-4494	4.29827973	0	2.405524014	0		20	1		chr5_11735_mature	10.20841	4.029637	3.48651029	2.330454351		1.341037	0.665039
chr2_5207_mature	3.223709795	8.059274	2.078510713	3.179395517		-1.32193	0.435308		NW_003538080_37501_star	4.29827973	0	2.405524014	0		20	1		ssc-miR-182	76719.99	1181.49	16.22733381	10.20761183		6.020924	0.665495
chr10_23348_mature	84.89102461	16.11855	6.424435476	4.097488514		2.39689	0.435365		chr6_12752_star	4.29827973	0	2.405524014	0		20	1		chr3_7211_mature	13.43212	4.835565	3.851211749	2.544872269		1.473931	0.665822
hsa-miR-3612	4.298279727	12.08891	2.405524014	3.710273245		-1.49185	0.435419		chr14_30496_star	4.29827973	0	2.405524014	0		20	1		chr3_6817_star	4.835565	2.417782	2.544872269	1.773060525		1	0.666037
chr13_29260_mature	4.298279727	12.08891	2.405524014	3.710273245		-1.49185	0.435419		NW_003613022_36785_star	4.29827973	0	2.405524014	0		20	1		chr17_35178_mature@@bta-miR-6	4.835565	2.417782	2.544872269	1.773060525		1	0.666037
chr7_16875_mature	4.298279727	12.08891	2.405524014	3.710273245		-1.49185	0.435419		chr2_5690_mature	4.29827973	0	2.405524014	0		20	1		hsa-miR-298	4.835565	2.417782	2.544872269	1.773060525		1	0.666037
chr6_12979_mature	4.298279727	12.08891	2.405524014	3.710273245		-1.49185	0.435419		chr6_15024_star	4.29827973	0	2.405524014	0		20	1		NW_003539115_38544_mature	4.835565	2.417782	2.544872269	1.773060525		1	0.666037
ssc-miR-216	4.298279727	12.08891	2.405524014	3.710273245		-1.49185	0.435419		chr9_20618_star@@bta-miR-135b	4.29827973	0	2.405524014	0		20	1		chr1_2982_mature	4.835565	2.417782	2.544872269	1.773060525		1	0.666037
hsa-miR-4286	4.298279727	12.08891	2.405524014	3.710273245		-1.49185	0.435419		mmu-miR-3098-5p	4.29827973	0	2.405524014	0		20	1		hsa-miR-3654	4.835565	2.417782	2.544872269	1.773060525		1	0.666037
chr5_11414_mature	4.298279727	12.08891	2.405524014	3.710273245		-1.49185	0.435419		hsa-miR-4670-5p	4.29827973	0	2.405524014	0		20	1		chr15_32637_star	4.835565	2.417782	2.544872269	1.773060525		1	0.666037
chr4_8847_mature	4.298279727	12.08891	2.405524014	3.710273245		-1.49185	0.435419		bta-miR-2356	4.29827973	0	2.405524014	0		20	1		chr2_5381_star	4.835565	2.417782	2.544872269	1.773060525		1	0.666037
NW_003539626_38995_mature	55.87763645	571.4026	5.82978961	9.160886317		-3.35416	0.435523		mmu-miR-5129-3p	4.29827973	0	2.405524014	0		20	1		chr5_11567_star	4.835565	2.417782	2.544872269	1.773060525		1	0.666037
rno-miR-17-5p	7.521989523	27.40153	3.091190278	4.827896911		-1.86507	0.436511		hsa-miR-4328	4.29827973	0	2.405524014	0		20	1		chr2_6095_mature	4.835565	2.417782	2.544872269	1.773060525		1	0.666037
chr9_20121_mature	6.447419591	21.76004	2.896740642	4.508431259		-1.75489	0.436576		bta-miR-2364	4.29827973	0	2.405524014	0		20	1		NW_003537297_36826_star	4.835565	2.417782	2.544872269	1.773060525		1	0.666037
bta-miR-487a	18.26768884	5.641492	4.268111626	2.731507408		1.695145	0.437042		bta-miR-2360	4.29827973	0	2.405524014	0		20	1		rno-miR-802-3p	4.835565	2.417782	2.544872269	1.773060525		1	0.666037
NW_003538009_37453_mature	18.26768884	5.641492	4.268111626	2.731507408		1.695145	0.437042		bta-miR-2372	4.29827973	0	2.405524014	0		20	1		chr1_888_star	4.835565	2.417782	2.544872269	1.773060525		1	0.666037
chr17_35617_mature	18.26768884	5.641492	4.268111626	2.731507408		1.695145	0.437042		bta-miR-2375	4.29827973	0	2.405524014	0		20	1		ssc-miR-451	4.835565	2.417782	2.544872269	1.773060525		1	0.666037
chr6_12434_mature	18.26768884	5.641492	4.268111626	2.731507408		1.695145	0.437042		chr6_14282_star	4.29827973	0	2.405524014	0		20	1		mmu-miR-6383	4.835565	2.417782	2.544872269	1.773060525		1	0.666037
ssc-miR-708-3p	92.41301414	1212.115	6.545551653	10.24450047		-3.71329	0.437606		chr9_20875_star	4.29827973	0	2.405524014	0		20	1		chr1_1739_star	4.835565	2.417782	2.544872269	1.773060525		1	0.666037
rno-miR-873-3p	1.074569932	2.417782	1.05281229	1.773060525		-1.16993	0.437931		mmu-miR-696	4.29827973	0	2.405524014	0		20	1		NW_003613379_38756_star	4.835565	2.417782	2.544872269	1.773060525		1	0.666037
chrx_21717_star	1.074569932	2.417782	1.05281229	1.773060525		-1.16993	0.437931		chr16_34124_mature	4.29827973	0	2.405524014	0		20	1		chr9_19614_star	4.835565	2.417782	2.544872269	1.773060525		1	0.666037
chr8_17418_star	1.074569932	2.417782	1.05281229	1.773060525		-1.16993	0.437931		chr15_33512_mature	4.29827973	0	2.405524014	0		20	1		chr6_14390_star	4.835565	2.417782	2.544872269	1.773060525		1	0.666037
NW_003539103_38526_star	1.074569932	2.417782	1.05281229	1.773060525		-1.16993	0.437931		chr10_23430_star	4.29827973	0	2.405524014	0		20	1		chr2_5993_star	4.835565	2.417782	2.544872269	1.773060525		1	0.666037
hsa-miR-4724-3p	1.074569932	2.417782	1.05281229	1.773060525		-1.16993	0.437931		chr6_14749_star	4.29827973	0	2.405524014	0		20	1		chr1_3039_mature	4.835565	2.417782	2.544872269	1.773060525		1	0.666037
chr4_9513_star	1.074569932	2.417782	1.05281229	1.773060525		-1.16993	0.437931		hsa-miR-711	4.29827973	0	2.405524014	0		20	1		NW_003537656_37099_star	4.835565	2.417782	2.544872269	1.773060525		1	0.666037
chr1_2982_mature	1.074569932	2.417782	1.05281229	1.773060525		-1.16993	0.437931		chr10_23395_star	4.29827973	0	2.405524014	0		20	1		chr5_11862_star	4.835565	2.417782	2.544872269	1.773060525		1	0.666037
chr3_7786_star	1.074569932	2.417782	1.05281229	1.773060525		-1.16993	0.437931		chr6_13451_mature	240.703665	170.8566	7.917095537	7.425061609		0.494472	1		NW_003541277_40019_star	4.835565	2.417782	2.544872269	1.773060525		1	0.666037
hsa-miR-1206	1.074569932	2.417782	1.05281229	1.773060525		-1.16993	0.437931		chr14_29928_mature	4.29827973	20.14819	2.405524014	4.402462031		-2.22882	1		chr14_30180_star	4.835565	2.417782	2.544872269	1.773060525		1	0.666037
hsa-miR-6510-5p	1.074569932	2.417782	1.05281229	1.773060525		-1.16993	0.437931		NW_003539513_38909_mature	4.29827973	0	2.405524014	0		20	1		chr13_28696_mature	4.835565	2.417782	2.544872269	1.773060525		1	0.666037
hsa-miR-1297	1.074569932	2.417782	1.05281229	1.773060525		-1.16993	0.437931		chr15_31967_mature	4.29827973	0	2.405524014	0		20	1		chrx_22576_mature	0.537285	1.611855	0.620384622	1.38507475		-1.58496	0.666201
chr9_21061_mature	1.074569932	2.417782	1.05281229	1.773060525		-1.16993	0.437931		chr9_20930_star	4.29827973	0	2.405524014	0		20	1		NW_003537105_36696_star	0.537285	1.611855	0.620384622	1.38507475		-1.58496	0.666201
chr1_3022_star	1.074569932	2.417782	1.05281229	1.773060525		-1.16993	0.437931		chr13_27434_mature	4.29827973	0	2.405524014	0		20	1		hsa-miR-4790-5p	0.537285	1.611855	0.620384622	1.38507475		-1.58496	0.666201
chrx_23098_star	1.074569932	2.417782	1.05281229	1.773060525		-1.16993	0.437931		chr4_8848_star	4.29827973	0	2.405524014	0		20	1		hsa-miR-6715b-5p	0.537285	1.611855	0.620384622	1.38507475		-1.58496	0.666201
chr4_9284_mature	1.074569932	2.417782	1.05281229	1.773060525		-1.16993	0.437931		hsa-miR-4643	4.29827973	0	2.405524014	0		20	1		chr9_20255_mature	0.537285	1.611855	0.620384622	1.38507475		-1.58496	0.666201
chr11_25375_star	1.074569932	2.417782	1.05281229	1.773060525		-1.16993	0.437931		bta-miR-545-3p	4.29827973	0	2.405524014	0		20	1		chr2_5906_star	0.537285	1.611855	0.620384622	1.38507475		-1.58496	0.666201
hsa-miR-548o-3p	1.074569932	2.417782	1.05281229	1.773060525		-1.16993	0.437931		chr14_31596_mature	4.29827973	0	2.405524014	0		20	1		chrx_22905_mature	0.537285	1.611855	0.620384622	1.38507475		-1.58496	0.666201
chr9_20120_mature	1.074569932	2.417782	1.05281229	1.773060525		-1.16993	0.437931		chr4_9868_mature	4.29827973	0	2.405524014	0		20	1		hsa-miR-4761-3p	0.537285	1.611855	0.620384622	1.38507475		-1.58496	0.666201
chr3_7706_star	1.074569932	2.417782	1.05281229	1.773060525		-1.16993	0.437931		mmu-miR-704	4.29827973	0	2.405524014	0		20	1		NW_003613103_37207_star	0.537285	1.611855	0.620384622	1.38507475		-1.58496	0.666201
chr1_1781_star	1.074569932	2.417782	1.05281229	1.773060525		-1.16993	0.437931		mmu-miR-709	4.29827973	0	2.405524014	0		20	1		hsa-miR-4759	0.537285	1.611855	0.620384622	1.38507475		-1.58496	0.666201
NW_003613164_37468_star	1.074569932	2.417782	1.05281229	1.773060525		-1.16993	0.437931		chr17_35344_star	4.29827973	0	2.405524014	0		20	1		hsa-miR-3620-3p	0.537285	1.611855	0.620384622	1.38507475		-1.58496	0.666201
hsa-miR-3655	1.074569932	2.417782	1.05281229	1.773060525		-1.16993	0.437931		chr18_36516_star	4.29827973	0	2.405524014	0		20	1		chr2_4626_star	0.537285	1.611855	0.620384622	1.38507475		-1.58496	0.666201
rno-miR-3552	1.074569932	2.417782	1.05281229	1.773060525		-1.16993	0.437931		NW_003537297_36825_mature	4.29827973	0	2.405524014	0		20	1		chr1_3642_star	0.537285	1.611855	0.620384622	1.38507475		-1.58496	0.666201
rno-miR-3553	1.074569932	2.417782	1.05281229	1.773060525		-1.16993	0.437931		chr7_16725_mature	4.29827973	0	2.405524014	0		20	1		chr7_16069_star	0.537285	1.611855	0.620384622	1.38507475		-1.58496	0.666201
rno-miR-3572	1.074569932	2.417782	1.05281229	1.773060525		-1.16993	0.437931		mmu-miR-6541	4.29827973	0	2.405524014	0		20	1		chrx_22329_star	0.537285	1.611855	0.620384622	1.38507475		-1.58496	0.666201
chr12_25429_star	1.074569932	2.417782	1.05281229	1.773060525		-1.16993	0.437931		mmu-miR-710	4.29827973	0	2.405524014	0		20	1		chrx_22335_mature	0.537285	1.611855	0.620384622	1.38507475		-1.58496	0.666201
chrx_21725_mature	1.074569932	2.417782	1.05281229	1.773060525		-1.16993	0.437931		mmu-miR-3473b	4.29827973	0	2.405524014	0		20	1		chr9_21003_star	0.537285	1.611855	0.620384622	1.38507475		-1.58496	0.666201
chr14_30452_mature	1.074569932	2.417782	1.05281229	1.773060525		-1.16993	0.437931		hsa-miR-3135a	4.29827973	0	2.405524014	0		20	1		chr10_23289_star	0.537285	1.611855	0.620384622	1.38507475		-1.58496	0.666201
chr3_8500_mature	1.074569932	2.417782	1.05281229	1.773060525		-1.16993	0.437931		chr2_6398_mature	4.29827973	0	2.405524014	0		20	1		hsa-miR-361-5p	0.537285	1.611855	0.620384622	1.38507475		-1.58496	0.666201
chr1_3915_mature	1.074569932	2.417782	1.05281229	1.773060525		-1.16993	0.437931		chr1_3945_mature	4.29827973	0	2.405524014	0		20	1		chr6_13909_star	0.537285	1.611855	0.620384622	1.38507475		-1.58496	0.666201
chr18_36565_star	1.074569932	2.417782	1.05281229	1.773060525		-1.16993	0.437931		hsa-miR-1304-5p	4.29827973	0	2.405524014	0		20	1		hsa-miR-553	0.537285	1.611855	0.620384622	1.38507475		-1.58496	0.666201
chr6_15010_star	1.074569932	2.417782	1.05281229	1.773060525		-1.16993	0.437931		chr13_28073_star	4.29827973	0	2.405524014	0		20	1		chr15_32628_mature	0.537285	1.611855	0.620384622	1.38507475		-1.58496	0.666201
chr7_15080_mature	1.074569932	2.417782	1.05281229	1.773060525		-1.16993	0.437931		hsa-miR-3663-3p	4.29827973	0	2.405524014	0		20	1		chr11_24872_star	0.537285	1.611855	0.620384622	1.38507475		-1.58496	0.666201
chr1_3536_star	1.074569932	2.417782	1.05281229	1.773060525		-1.16993	0.437931		mmu-miR-106a-3p	4.29827973	0	2.405524014	0		20	1		chr14_31648_star	0.537285	1.611855	0.620384622	1.38507475		-1.58496	0.666201
chr5_12382_star	1.074569932	2.417782	1.05281229	1.773060525		-1.16993	0.437931		chr7_16877_mature	4.29827973	0	2.405524014	0		20	1		chr3_7684_mature	0.537285	1.611855	0.620384622	1.38507475		-1.58496	0.666201
chr5_10795_star	1.074569932	2.417782	1.05281229	1.773060525		-1.16993	0.437931		NW_003538922_38352_star	4.29827973	0	2.405524014	0		20	1		bta-miR-760-5p	0.537285	1.611855	0.620384622	1.38507475		-1.58496	0.666201
chr1_3752_star	1.074569932	2.417782	1.05281229	1.773060525		-1.16993	0.437931		chr14_29649_star	4.29827973	0	2.405524014	0		20	1		mmu-miR-100-5p	0.537285	1.611855	0.620384622	1.38507475		-1.58496	0.666201
chr14_29856_mature	1.074569932	2.417782	1.05281229	1.773060525		-1.16993	0.437931		chr2_5368_star	4.29827973	0	2.405524014	0		20	1		chr4_9327_mature	0.537285	1.611855	0.620384622	1.38507475		-1.58496	0.666201
mmu-miR-6338	1.074569932	2.417782	1.05281229	1.773060525		-1.16993	0.437931		chr3_8450_star	4.29827973	0	2.405524014	0		20	1		mmu-miR-195b	0.537285	1.611855	0.620384622	1.38507475		-1.58496	0.666201
bta-miR-2285m	1.074569932	2.417782	1.05281229	1.773060525		-1.16993	0.437931		chr15_32358_star	4.29827973	0	2.405524014	0		20	1		chr9_19886_star	0.537285	1.611855	0.620384622	1.38507475		-1.58496	0.666201
bta-miR-2284y	1.074569932	2.417782	1.05281229	1.773060525		-1.16993	0.437931		hsa-miR-5683	4.29827973	0	2.405524014	0		20	1		chr12_26183_mature	0.537285	1.611855	0.620384622	1.38507475		-1.58496	0.666201
chr13_28207_mature	1.074569932	2.417782	1.05281229	1.773060525		-1.16993	0.437931		chr1_2086_mature	4.29827973	0	2.405524014	0		20	1		bta-miR-2483-5p	0.537285	1.611855	0.620384622	1.38507475		-1.58496	0.666201
chr9_20430_star	1.074569932	2.417782	1.05281229	1.773060525		-1.16993	0.437931		hsa-miR-4514	4.29827973	0	2.405524014	0		20	1		chr1_743_star	0.537285	1.611855	0.620384622	1.38507475		-1.58496	0.666201
chr8_18587_star	1.074569932	2.417782	1.05281229	1.773060525		-1.16993	0.437931		hsa-miR-4517	4.29827973	0	2.405524014	0		20	1		hsa-miR-5571-5p	0.537285	1.611855	0.620384622	1.38507475		-1.58496	0.666201
chr1_2557_star	1.074569932	2.417782	1.05281229	1.773060525		-1.16993	0.437931		rno-miR-141-5p	4.29827973	0	2.405524014	0		20	1		NW_003537295_36819_star	0.537285	1.611855	0.620384622	1.38507475		-1.58496	0.666201
chr10_24002_star	1.074569932	2.417782	1.05281229	1.773060525		-1.16993	0.437931		chr13_27615_star	4.29827973	0	2.405524014	0		20	1		chrx_23183_mature	0.537285	1.611855	0.620384622	1.38507475		-1.58496	0.666201
chr3_6774_star	1.074569932	2.417782	1.05281229	1.773060525		-1.16993	0.437931		bta-miR-2331-5p	4.29827973	0	2.405524014	0		20	1		NW_003613545_39630_star	0.537285	1.611855	0.620384622	1.38507475		-1.58496	0.666201
chrx_21689_star	1.074569932	2.417782	1.05281229	1.773060525		-1.16993	0.437931		chr14_31486_mature	4.29827973	0	2.405524014	0		20	1		mmu-miR-5119	0.537285	1.611855	0.620384622	1.38507475		-1.58496	0.666201
chr11_24816_star	1.074569932	2.417782	1.05281229	1.773060525		-1.16993	0.437931		hsa-miR-4535	4.29827973	0	2.405524014	0		20	1		hsa-miR-3610	0.537285	1.611855	0.620384622	1.38507475		-1.58496	0.666201
chr3_7654_mature	1.074569932	2.417782	1.05281229	1.773060525		-1.16993	0.437931		hsa-miR-3155a	4.29827973	0	2.405524014	0		20	1		chr8_18753_mature	0.537285	1.611855	0.620384622	1.38507475		-1.58496	0.666201
chr1_3094_star	1.074569932	2.417782	1.05281229	1.773060525		-1.16993	0.437931		chr7_16933_star	4.29827973	0	2.405524014	0		20	1		chr12_26317_star	0.537285	1.611855	0.620384622	1.38507475		-1.58496	0.666201
chr2_5680_star	1.074569932	2.417782	1.05281229	1.773060525		-1.16993	0.437931		chr7_16758_star	17.1931189	4.835565	4.185320985	2.544872269		1.830075	1		chr9_20086_star	0.537285	1.611855	0.620384622	1.38507475		-1.58496	0.666201
chr5_11789_star	1.074569932	2.417782	1.05281229	1.773060525		-1.16993	0.437931		chr16_34566_star	4.29827973	0	2.405524014	0		20	1		chr3_7586_star	0.537285	1.611855	0.620384622	1.38507475		-1.58496	0.666201
chr6_15065_star	1.074569932	2.417782	1.05281229	1.773060525		-1.16993	0.437931		chr7_17188_star	4.29827973	0	2.405524014	0		20	1		chr4_10041_star	0.537285	1.611855	0.620384622	1.38507475		-1.58496	0.666201
NW_003613379_38756_star	1.074569932	2.417782	1.05281229	1.773060525		-1.16993	0.437931		hsa-miR-942	4.29827973	0	2.405524014	0		20	1		NW_003540726_39647_star	0.537285	1.611855	0.620384622	1.38507475		-1.58496	0.666201
chr9_19614_star	1.074569932	2.417782	1.05281229	1.773060525		-1.16993	0.437931		hsa-miR-1909-3p	4.29827973	0	2.405524014	0		20	1		chr14_30429_star	0.537285	1.611855	0.620384622	1.38507475		-1.58496	0.666201
chr1_3153_star	1.074569932	2.417782	1.05281229	1.773060525		-1.16993	0.437931		hsa-miR-875-3p	4.29827973	0	2.405524014	0		20	1		rno-miR-1298	0.537285	1.611855	0.620384622	1.38507475		-1.58496	0.666201
chr4_9832_star	1.074569932	2.417782	1.05281229	1.773060525		-1.16993	0.437931		hsa-miR-936	4.29827973	0	2.405524014	0		20	1		rno-miR-374-5p	0.537285	1.611855	0.620384622	1.38507475		-1.58496	0.666201
chr2_5993_star	1.074569932	2.417782	1.05281229	1.773060525		-1.16993	0.437931		chr1_666_star	4.29827973	0	2.405524014	0		20	1		chr12_26222_star	0.537285	1.611855	0.620384622	1.38507475		-1.58496	0.666201
chr6_12860_star	1.074569932	2.417782	1.05281229	1.773060525		-1.16993	0.437931		NW_003613212_37672_star	4.29827973	0	2.405524014	0		20	1		chr1_3091_star	0.537285	1.611855	0.620384622	1.38507475		-1.58496	0.666201
hsa-miR-320e	1.074569932	2.417782	1.05281229	1.773060525		-1.16993	0.437931		hsa-miR-6079	4.29827973	0	2.405524014	0		20	1		chr6_13244_star	0.537285	1.611855	0.620384622	1.38507475		-1.58496	0.666201
hsa-miR-548v	1.074569932	2.417782	1.05281229	1.773060525		-1.16993	0.437931		chr2_6372_mature	4.29827973	0	2.405524014	0		20	1		chr6_14972_star	0.537285	1.611855	0.620384622	1.38507475		-1.58496	0.666201
chr5_11474_star	1.074569932	2.417782	1.05281229	1.773060525		-1.16993	0.437931		chr1_2742_star	4.29827973	0	2.405524014	0		20	1		rno-miR-485-3p	0.537285	1.611855	0.620384622	1.38507475		-1.58496	0.666201
chr1_826_star	1.074569932	2.417782	1.05281229	1.773060525		-1.16993	0.437931		hsa-miR-3940-3p	4.29827973	0	2.405524014	0		20	1		chr2_5851_star	0.537285	1.611855	0.620384622	1.38507475		-1.58496	0.666201
bta-miR-2388-5p	1.074569932	2.417782	1.05281229	1.773060525		-1.16993	0.437931		hsa-miR-1908	4.29827973	0	2.405524014	0		20	1		bta-miR-744	0.537285	1.611855	0.620384622	1.38507475		-1.58496	0.666201
NW_003613303_38366_star	1.074569932	2.417782	1.05281229	1.773060525		-1.16993	0.437931		chr13_29017_star	4.29827973	0	2.405524014	0		20	1		bta-miR-6119-5p	0.537285	1.611855	0.620384622	1.38507475		-1.58496	0.666201
hsa-miR-3169	1.074569932	2.417782	1.05281229	1.773060525		-1.16993	0.437931		chr4_9963_star	2.14913986	0	1.654957834	0		20	1		chr12_26311_star	0.537285	1.611855	0.620384622	1.38507475		-1.58496	0.666201
hsa-miR-3664-5p	1.074569932	2.417782	1.05281229	1.773060525		-1.16993	0.437931		mmu-miR-344b-5p	2.14913986	0	1.654957834	0		20	1		NW_003537603_37043_mature	0.537285	1.611855	0.620384622	1.38507475		-1.58496	0.666201
hsa-miR-4255	1.074569932	2.417782	1.05281229	1.773060525		-1.16993	0.437931		NW_003538747_38195_star	2.14913986	0	1.654957834	0		20	1		chr6_14228_mature	0.537285	1.611855	0.620384622	1.38507475		-1.58496	0.666201
chr13_27783_star	1.074569932	2.417782	1.05281229	1.773060525		-1.16993	0.437931		rno-miR-218a-5p	2.14913986	0	1.654957834	0		20	1		chr1_1435_star	0.537285	1.611855	0.620384622	1.38507475		-1.58496	0.666201
rno-miR-93-5p	1.074569932	2.417782	1.05281229	1.773060525		-1.16993	0.437931		chr6_13980_star	2.14913986	0	1.654957834	0		20	1		chrx_23149_star	0.537285	1.611855	0.620384622	1.38507475		-1.58496	0.666201
chr8_17350_star	1.074569932	2.417782	1.05281229	1.773060525		-1.16993	0.437931		chr2_6500_mature	2.14913986	0	1.654957834	0		20	1		bta-miR-628	0.537285	1.611855	0.620384622	1.38507475		-1.58496	0.666201
NW_003539453_38857_mature	1.074569932	2.417782	1.05281229	1.773060525		-1.16993	0.437931		hsa-miR-328	2.14913986	0	1.654957834	0		20	1		rno-miR-370-3p	0.537285	1.611855	0.620384622	1.38507475		-1.58496	0.666201
chr15_32775_star	1.074569932	2.417782	1.05281229	1.773060525		-1.16993	0.437931		mmu-miR-365-2-5p	2.14913986	0	1.654957834	0		20	1		NW_003539609_38987_mature	0.537285	1.611855	0.620384622	1.38507475		-1.58496	0.666201
bta-miR-500	1.074569932	2.417782	1.05281229	1.773060525		-1.16993	0.437931		hsa-miR-3150a-5p	2.14913986	0	1.654957834	0		20	1		hsa-miR-6127	0.537285	1.611855	0.620384622	1.38507475		-1.58496	0.666201
chr1_2966_star	1.074569932	2.417782	1.05281229	1.773060525		-1.16993	0.437931		chr14_31443_star	2.14913986	0	1.654957834	0		20	1		bta-miR-2370-3p	0.537285	1.611855	0.620384622	1.38507475		-1.58496	0.666201
chr1_1748_mature	1.074569932	2.417782	1.05281229	1.773060525		-1.16993	0.437931		chr15_32479_mature	2.14913986	0	1.654957834	0		20	1		chrx_21725_star	0.537285	1.611855	0.620384622	1.38507475		-1.58496	0.666201
chr6_12823_star	1.074569932	2.417782	1.05281229	1.773060525		-1.16993	0.437931		hsa-miR-4659b-3p	2.14913986	0	1.654957834	0		20	1		chr2_5369_star	0.537285	1.611855	0.620384622	1.38507475		-1.58496	0.666201
chr1_244_star	1.074569932	2.417782	1.05281229	1.773060525		-1.16993	0.437931		chrx_23192_mature	2.14913986	0	1.654957834	0		20	1		mmu-miR-1247-3p	0.537285	1.611855	0.620384622	1.38507475		-1.58496	0.666201
chr12_26142_star	1.074569932	2.417782	1.05281229	1.773060525		-1.16993	0.437931		hsa-miR-150-5p	2.14913986	0	1.654957834	0		20	1		hsa-miR-1207-5p	0.537285	1.611855	0.620384622	1.38507475		-1.58496	0.666201
chr1_3087_mature	1.074569932	2.417782	1.05281229	1.773060525		-1.16993	0.437931		chr14_29661_star	2.14913986	0	1.654957834	0		20	1		hsa-miR-548n	0.537285	1.611855	0.620384622	1.38507475		-1.58496	0.666201
chr3_7627_mature	1.074569932	2.417782	1.05281229	1.773060525		-1.16993	0.437931		hsa-miR-150-3p	2.14913986	0	1.654957834	0		20	1		chr2_5337_mature	0.537285	1.611855	0.620384622	1.38507475		-1.58496	0.666201
chr3_8509_star	1.074569932	2.417782	1.05281229	1.773060525		-1.16993	0.437931		rno-miR-380-3p	2.14913986	0	1.654957834	0		20	1		chr14_29851_star	0.537285	1.611855	0.620384622	1.38507475		-1.58496	0.666201
bta-miR-2405	1.074569932	2.417782	1.05281229	1.773060525		-1.16993	0.437931		chr14_30332_star	2.14913986	0	1.654957834	0		20	1		chr9_20512_mature	0.537285	1.611855	0.620384622	1.38507475		-1.58496	0.666201
bta-miR-2468	1.074569932	2.417782	1.05281229	1.773060525		-1.16993	0.437931		mmu-miR-666-5p	2.14913986	0	1.654957834	0		20	1		mmu-miR-3078-3p	0.537285	1.611855	0.620384622	1.38507475		-1.58496	0.666201
chr6_13134_star	1.074569932	2.417782	1.05281229	1.773060525		-1.16993	0.437931		chr6_14212_star	2.14913986	0	1.654957834	0		20	1		chr5_10732_mature	0.537285	1.611855	0.620384622	1.38507475		-1.58496	0.666201
bta-miR-2433	1.074569932	2.417782	1.05281229	1.773060525		-1.16993	0.437931		chr9_20112_star	2.14913986	0	1.654957834	0		20	1		chr11_24756_star	0.537285	1.611855	0.620384622	1.38507475		-1.58496	0.666201
hsa-miR-4472	1.074569932	2.417782	1.05281229	1.773060525		-1.16993	0.437931		chr2_4178_star	2.14913986	0	1.654957834	0		20	1		chr2_5690_star	0.537285	1.611855	0.620384622	1.38507475		-1.58496	0.666201
chr4_8670_star	1.074569932	2.417782	1.05281229	1.773060525		-1.16993	0.437931		chr3_7631_star	2.14913986	0	1.654957834	0		20	1		chr14_29848_mature	0.537285	1.611855	0.620384622	1.38507475		-1.58496	0.666201
chr14_30455_star	1.074569932	2.417782	1.05281229	1.773060525		-1.16993	0.437931		rno-miR-509-5p	2.14913986	0	1.654957834	0		20	1		hsa-miR-4709-5p	0.537285	1.611855	0.620384622	1.38507475		-1.58496	0.666201
hsa-miR-4498	1.074569932	2.417782	1.05281229	1.773060525		-1.16993	0.437931		chr15_33139_star	2.14913986	0	1.654957834	0		20	1		hsa-miR-4665-5p	0.537285	1.611855	0.620384622	1.38507475		-1.58496	0.666201
chr6_14320_star	1.074569932	2.417782	1.05281229	1.773060525		-1.16993	0.437931		chr7_16503_star	2.14913986	0	1.654957834	0		20	1		chr17_35296_star	0.537285	1.611855	0.620384622	1.38507475		-1.58496	0.666201
chr5_11862_star	1.074569932	2.417782	1.05281229	1.773060525		-1.16993	0.437931		NW_003613107_37244_star	2.14913986	0	1.654957834	0		20	1		chr1_2783_star	0.537285	1.611855	0.620384622	1.38507475		-1.58496	0.666201
chr14_30089_star	1.074569932	2.417782	1.05281229	1.773060525		-1.16993	0.437931		hsa-miR-4761-5p	2.14913986	0	1.654957834	0		20	1		chr8_18422_star	0.537285	1.611855	0.620384622	1.38507475		-1.58496	0.666201
chrx_22198_star	1.074569932	2.417782	1.05281229	1.773060525		-1.16993	0.437931		rno-miR-873-5p	2.14913986	0	1.654957834	0		20	1		bta-miR-496	0.537285	1.611855	0.620384622	1.38507475		-1.58496	0.666201
chr1_3810_star	1.074569932	2.417782	1.05281229	1.773060525		-1.16993	0.437931		hsa-miR-1323	2.14913986	0	1.654957834	0		20	1		bta-miR-488	0.537285	1.611855	0.620384622	1.38507475		-1.58496	0.666201
chr2_6200_mature	1.074569932	2.417782	1.05281229	1.773060525		-1.16993	0.437931		chr6_12438_star	2.14913986	0	1.654957834	0		20	1		chr4_10432_mature	0.537285	1.611855	0.620384622	1.38507475		-1.58496	0.666201
chr1_3835_mature	1.074569932	2.417782	1.05281229	1.773060525		-1.16993	0.437931		hsa-miR-1224-3p	2.14913986	0	1.654957834	0		20	1		chr9_20034_star	0.537285	1.611855	0.620384622	1.38507475		-1.58496	0.666201
chr17_35073_mature	1.074569932	2.417782	1.05281229	1.773060525		-1.16993	0.437931		mmu-miR-344h-3p	2.14913986	0	1.654957834	0		20	1		bta-miR-2423	0.537285	1.611855	0.620384622	1.38507475		-1.58496	0.666201
chr11_24609_star	1.074569932	2.417782	1.05281229	1.773060525		-1.16993	0.437931		chr7_15912_star	2.14913986	0	1.654957834	0		20	1		chr6_14576_star	0.537285	1.611855	0.620384622	1.38507475		-1.58496	0.666201
hsa-miR-4320	1.074569932	2.417782	1.05281229	1.773060525		-1.16993	0.437931		hsa-miR-4706	2.14913986	0	1.654957834	0		20	1		hsa-miR-4425	0.537285	1.611855	0.620384622	1.38507475		-1.58496	0.666201
hsa-miR-4308	1.074569932	2.417782	1.05281229	1.773060525		-1.16993	0.437931		NW_003540726_39645_star	2.14913986	0	1.654957834	0		20	1		hsa-miR-4665-3p	0.537285	1.611855	0.620384622	1.38507475		-1.58496	0.666201
chr13_28696_mature	1.074569932	2.417782	1.05281229	1.773060525		-1.16993	0.437931		mmu-miR-3084-5p	2.14913986	0	1.654957834	0		20	1		chr16_34526_mature@@bta-miR-4	0.537285	1.611855	0.620384622	1.38507475		-1.58496	0.666201
hsa-miR-345-5p	1.074569932	2.417782	1.05281229	1.773060525		-1.16993	0.437931		chr9_20299_star	2.14913986	0	1.654957834	0		20	1		NW_003538662_38098_star	0.537285	1.611855	0.620384622	1.38507475		-1.58496	0.666201
chr1_3282_mature	1.074569932	2.417782	1.05281229	1.773060525		-1.16993	0.437931		chr7_15777_star	2.14913986	0	1.654957834	0		20	1		chr18_35816_star	0.537285	1.611855	0.620384622	1.38507475		-1.58496	0.666201
chr15_32938_star	1.074569932	2.417782	1.05281229	1.773060525		-1.16993	0.437931		chr18_36623_star	2.14913986	0	1.654957834	0		20	1		hsa-miR-4670-3p	0.537285	1.611855	0.620384622	1.38507475		-1.58496	0.666201
chr5_11953_star	1.074569932	2.417782	1.05281229	1.773060525		-1.16993	0.437931		chr16_33727_star	2.14913986	0	1.654957834	0		20	1		chr14_31060_star	0.537285	1.611855	0.620384622	1.38507475		-1.58496	0.666201
chr15_31887_star	1.074569932	2.417782	1.05281229	1.773060525		-1.16993	0.437931		rno-miR-23a-5p	2.14913986	0	1.654957834	0		20	1		chr2_5353_star	0.537285	1.611855	0.620384622	1.38507475		-1.58496	0.666201
chrx_22153_star	1.074569932	2.417782	1.05281229	1.773060525		-1.16993	0.437931		rno-miR-429	2.14913986	0	1.654957834	0		20	1		hsa-miR-3187-3p	0.537285	1.611855	0.620384622	1.38507475		-1.58496	0.666201
hsa-miR-5684	1.074569932	2.417782	1.05281229	1.773060525		-1.16993	0.437931		rno-miR-33-5p	2.14913986	0	1.654957834	0		20	1		bta-miR-2371	0.537285	1.611855	0.620384622	1.38507475		-1.58496	0.666201
chr14_29526_star	1.074569932	2.417782	1.05281229	1.773060525		-1.16993	0.437931		hsa-miR-4741	2.14913986	0	1.654957834	0		20	1		bta-miR-2389	0.537285	1.611855	0.620384622	1.38507475		-1.58496	0.666201
hsa-miR-3663-5p	1.074569932	2.417782	1.05281229	1.773060525		-1.16993	0.437931		bta-miR-2886	2.14913986	0	1.654957834	0		20	1		chr18_36628_mature	0.537285	1.611855	0.620384622	1.38507475		-1.58496	0.666201
hsa-miR-4533	1.074569932	2.417782	1.05281229	1.773060525		-1.16993	0.437931		chr16_34380_mature	2.14913986	0	1.654957834	0		20	1		chr16_34371_star	0.537285	1.611855	0.620384622	1.38507475		-1.58496	0.666201
chr14_29632_mature	1.074569932	2.417782	1.05281229	1.773060525		-1.16993	0.437931		hsa-miR-4737	2.14913986	0	1.654957834	0		20	1		mmu-miR-1947-3p	0.537285	1.611855	0.620384622	1.38507475		-1.58496	0.666201
chr9_20478_star	1.074569932	2.417782	1.05281229	1.773060525		-1.16993	0.437931		hsa-miR-4736	2.14913986	0	1.654957834	0		20	1		hsa-miR-519e-3p	0.537285	1.611855	0.620384622	1.38507475		-1.58496	0.666201
chr15_32322_star	1.074569932	2.417782	1.05281229	1.773060525		-1.16993	0.437931		chr9_20263_star	2.14913986	0	1.654957834	0		20	1		NW_003613326_38566_mature	0.537285	1.611855	0.620384622	1.38507475		-1.58496	0.666201
chr4_10240_star	1.074569932	2.417782	1.05281229	1.773060525		-1.16993	0.437931		bta-miR-149-3p	2.14913986	0	1.654957834	0		20	1		rno-miR-31a-3p	0.537285	1.611855	0.620384622	1.38507475		-1.58496	0.666201
chr1_6_star	1.074569932	2.417782	1.05281229	1.773060525		-1.16993	0.437931		chr13_26706_star	2.14913986	0	1.654957834	0		20	1		mmu-miR-5616-3p	0.537285	1.611855	0.620384622	1.38507475		-1.58496	0.666201
chr7_15311_mature	1.074569932	2.417782	1.05281229	1.773060525		-1.16993	0.437931		rno-miR-326-5p	2.14913986	0	1.654957834	0		20	1		chr2_4221_star	0.537285	1.611855	0.620384622	1.38507475		-1.58496	0.666201
chrx_21936_mature	1.074569932	2.417782	1.05281229	1.773060525		-1.16993	0.437931		hsa-miR-4752	2.14913986	0	1.654957834	0		20	1		rno-miR-23b-3p	0.537285	1.611855	0.620384622	1.38507475		-1.58496	0.666201
chr7_15913_mature	1.074569932	2.417782	1.05281229	1.773060525		-1.16993	0.437931		bta-miR-2892	2.14913986	0	1.654957834	0		20	1		hsa-miR-92a-3p	0.537285	1.611855	0.620384622	1.38507475		-1.58496	0.666201
NW_003613106_37241_mature	5.372849659	2.417782	2.671938627	1.773060525		1.152003	0.439341		bta-miR-2894	2.14913986	0	1.654957834	0		20	1		chr15_32017_star	0.537285	1.611855	0.620384622	1.38507475		-1.58496	0.666201
chr6_13330_star	5.372849659	2.417782	2.671938627	1.773060525		1.152003	0.439341		rno-miR-542-3p	2.14913986	0	1.654957834	0		20	1		mmu-miR-669e-5p	0.537285	1.611855	0.620384622	1.38507475		-1.58496	0.666201
chr3_7956_star	5.372849659	2.417782	2.671938627	1.773060525		1.152003	0.439341		chr4_9318_star	2.14913986	0	1.654957834	0		20	1		chr1_3129_star	0.537285	1.611855	0.620384622	1.38507475		-1.58496	0.666201
chr14_29702_mature	5.372849659	2.417782	2.671938627	1.773060525		1.152003	0.439341		chr10_24135_star	2.14913986	0	1.654957834	0		20	1		rno-miR-141-3p	0.537285	1.611855	0.620384622	1.38507475		-1.58496	0.666201
chr14_30720_star	5.372849659	2.417782	2.671938627	1.773060525		1.152003	0.439341		chr6_14256_star	2.14913986	0	1.654957834	0		20	1		chr9_19915_star	0.537285	1.611855	0.620384622	1.38507475		-1.58496	0.666201
chr9_20132_mature	5.372849659	2.417782	2.671938627	1.773060525		1.152003	0.439341		chr14_30645_star	2.14913986	0	1.654957834	0		20	1		chr2_5958_mature	0.537285	1.611855	0.620384622	1.38507475		-1.58496	0.666201
rno-miR-383-5p	5.372849659	2.417782	2.671938627	1.773060525		1.152003	0.439341		chr13_27960_star	2.14913986	0	1.654957834	0		20	1		chr1_3837_star	0.537285	1.611855	0.620384622	1.38507475		-1.58496	0.666201
chr13_29017_mature	5.372849659	2.417782	2.671938627	1.773060525		1.152003	0.439341		rno-miR-3594-5p	2.14913986	0	1.654957834	0		20	1		chr15_31749_mature	25.25239	7.253347	4.714377052	3.044979305		1.799701	0.666757
chr12_26323_mature	5.372849659	2.417782	2.671938627	1.773060525		1.152003	0.439341		hsa-let-7d-5p	2.14913986	0	1.654957834	0		20	1		chr13_27566_mature	39.75909	9.671129	5.349049847	3.415640968		2.039528	0.66729
NW_003537297_36826_star	5.372849659	2.417782	2.671938627	1.773060525		1.152003	0.439341		chr13_28423_mature	2.14913986	0	1.654957834	0		20	1		chr13_26975_mature	39.75909	9.671129	5.349049847	3.415640968		2.039528	0.66729
chr16_33929_mature	5.372849659	2.417782	2.671938627	1.773060525		1.152003	0.439341		NW_003540726_39647_mature	2.14913986	0	1.654957834	0		20	1		chr15_31855_mature	45.13194	10.47706	5.527693969	3.520680821		2.106915	0.66742
chr1_6_mature	5.372849659	2.417782	2.671938627	1.773060525		1.152003	0.439341		chr18_36468_star	2.14913986	0	1.654957834	0		20	1		chr5_12127_mature	8.059274	30.62524	3.179395517	4.983004663		-1.926	0.667836
chr11_25364_star	5.372849659	2.417782	2.671938627	1.773060525		1.152003	0.439341		rno-miR-449c-3p	2.14913986	0	1.654957834	0		20	1		hsa-miR-518d-5p	2.686425	5.641492	1.882222339	2.731507408		-1.07039	0.668056
chr13_28551_star	5.372849659	2.417782	2.671938627	1.773060525		1.152003	0.439341		chr14_29829_star	2.14913986	0	1.654957834	0		20	1		chr5_11659_mature	2.686425	5.641492	1.882222339	2.731507408		-1.07039	0.668056
chr12_25742_mature	5.372849659	2.417782	2.671938627	1.773060525		1.152003	0.439341		chr3_7984_star	2.14913986	0	1.654957834	0		20	1		mmu-miR-5107-3p	2.686425	5.641492	1.882222339	2.731507408		-1.07039	0.668056
chr2_4221_mature	5.372849659	2.417782	2.671938627	1.773060525		1.152003	0.439341		chr6_14717_star	2.14913986	0	1.654957834	0		20	1		chr10_23487_mature	2.686425	5.641492	1.882222339	2.731507408		-1.07039	0.668056
chr2_5754_star	5.372849659	2.417782	2.671938627	1.773060525		1.152003	0.439341		chr7_16955_star	2.14913986	0	1.654957834	0		20	1		chr6_13572_star	2.686425	5.641492	1.882222339	2.731507408		-1.07039	0.668056
chr1_917_star	5.372849659	2.417782	2.671938627	1.773060525		1.152003	0.439341		chr2_5147_star	2.14913986	0	1.654957834	0		20	1		chr13_28800_star	2.686425	5.641492	1.882222339	2.731507408		-1.07039	0.668056
NW_003537624_37070_star	5.372849659	2.417782	2.671938627	1.773060525		1.152003	0.439341		chr1_1213_star	2.14913986	0	1.654957834	0		20	1		mmu-miR-6540-3p	2.686425	5.641492	1.882222339	2.731507408		-1.07039	0.668056
chr1_3039_mature	5.372849659	2.417782	2.671938627	1.773060525		1.152003	0.439341		hsa-miR-4797-5p	2.14913986	0	1.654957834	0		20	1		rno-miR-3559-5p	2.686425	5.641492	1.882222339	2.731507408		-1.07039	0.668056
hsa-miR-320a	5.372849659	2.417782	2.671938627	1.773060525		1.152003	0.439341		hsa-miR-1257	2.14913986	0	1.654957834	0		20	1		chr13_26847_star	2.686425	5.641492	1.882222339	2.731507408		-1.07039	0.668056
chr1_499_mature@@hsa-miR-2113	5.372849659	2.417782	2.671938627	1.773060525		1.152003	0.439341		hsa-miR-1250	2.14913986	0	1.654957834	0		20	1		bta-miR-26c	2.686425	5.641492	1.882222339	2.731507408		-1.07039	0.668056
chr14_31128_star	5.372849659	2.417782	2.671938627	1.773060525		1.152003	0.439341		hsa-miR-1253	2.14913986	0	1.654957834	0		20	1		chr6_12705_mature	2.686425	5.641492	1.882222339	2.731507408		-1.07039	0.668056
chr12_26142_mature	5.372849659	2.417782	2.671938627	1.773060525		1.152003	0.439341		hsa-miR-1254	2.14913986	0	1.654957834	0		20	1		hsa-miR-4634	2.686425	5.641492	1.882222339	2.731507408		-1.07039	0.668056
chr1_2672_star	5.372849659	2.417782	2.671938627	1.773060525		1.152003	0.439341		chr6_13122_star	2.14913986	0	1.654957834	0		20	1		chr3_7067_star	2.686425	5.641492	1.882222339	2.731507408		-1.07039	0.668056
NW_003541277_40019_star	5.372849659	2.417782	2.671938627	1.773060525		1.152003	0.439341		mmu-miR-146a-5p	2.14913986	0	1.654957834	0		20	1		chr6_13411_star	2.686425	5.641492	1.882222339	2.731507408		-1.07039	0.668056
hsa-miR-939-5p	5.372849659	2.417782	2.671938627	1.773060525		1.152003	0.439341		hsa-miR-130b-5p	2.14913986	0	1.654957834	0		20	1		chr6_12855_mature	6.984705	3.22371	2.997239027	2.078510713		1.115477	0.669088
ssc-miR-432-3p	5.372849659	2.417782	2.671938627	1.773060525		1.152003	0.439341		hsa-miR-1204	2.14913986	0	1.654957834	0		20	1		chr14_29850_star	6.984705	3.22371	2.997239027	2.078510713		1.115477	0.669088
NW_003539295_38706_mature	5.372849659	2.417782	2.671938627	1.773060525		1.152003	0.439341		hsa-miR-1202	2.14913986	0	1.654957834	0		20	1		chrx_21753_mature	6.984705	3.22371	2.997239027	2.078510713		1.115477	0.669088
NW_003538009_37453_star	5.372849659	2.417782	2.671938627	1.773060525		1.152003	0.439341		chr3_8302_star	2.14913986	0	1.654957834	0		20	1		chr8_18593_star	6.984705	3.22371	2.997239027	2.078510713		1.115477	0.669088
chr11_25345_mature	1.074569932	0	1.05281229	0		20	0.439617		rno-miR-666-3p	2.14913986	0	1.654957834	0		20	1		chr6_14575_star	6.984705	3.22371	2.997239027	2.078510713		1.115477	0.669088
chr2_5456_star	1.074569932	0	1.05281229	0		20	0.439617		chr6_14386_star	2.14913986	0	1.654957834	0		20	1		bta-miR-143	6.984705	3.22371	2.997239027	2.078510713		1.115477	0.669088
chr4_9963_star	1.074569932	0	1.05281229	0		20	0.439617		chr15_32587_star	2.14913986	0	1.654957834	0		20	1		chr17_35278_mature	6.984705	3.22371	2.997239027	2.078510713		1.115477	0.669088
NW_003538747_38195_star	1.074569932	0	1.05281229	0		20	0.439617		rno-miR-200b-5p	2.14913986	0	1.654957834	0		20	1		chr7_16559_star	6.984705	3.22371	2.997239027	2.078510713		1.115477	0.669088
rno-miR-138-5p	1.074569932	0	1.05281229	0		20	0.439617		chr15_32058_star	2.14913986	0	1.654957834	0		20	1		chr5_12346_star	6.984705	3.22371	2.997239027	2.078510713		1.115477	0.669088
hsa-miR-300	1.074569932	0	1.05281229	0		20	0.439617		chr14_31446_star	2.14913986	0	1.654957834	0		20	1		chr14_31118_star	16.65583	5.641492	4.142073061	2.731507408		1.561879	0.669429
hsa-miR-518a-5p	1.074569932	0	1.05281229	0		20	0.439617		rno-miR-3585-3p	2.14913986	0	1.654957834	0		20	1		chr6_12833_star	2.14914	4.029637	1.654957834	2.330454351		-0.90689	0.669559
hsa-miR-328	1.074569932	0	1.05281229	0		20	0.439617		hsa-miR-1227-3p	2.14913986	0	1.654957834	0		20	1		NW_003539180_38634_mature	2.14914	4.029637	1.654957834	2.330454351		-0.90689	0.669559
mmu-miR-5624-5p	1.074569932	0	1.05281229	0		20	0.439617		chr2_5776_star	2.14913986	0	1.654957834	0		20	1		chr6_14967_mature	2.14914	4.029637	1.654957834	2.330454351		-0.90689	0.669559
mmu-miR-365-2-5p	1.074569932	0	1.05281229	0		20	0.439617		chr7_16057_star	2.14913986	0	1.654957834	0		20	1		chr2_6521_mature	2.14914	4.029637	1.654957834	2.330454351		-0.90689	0.669559
chr7_16193_star	1.074569932	0	1.05281229	0		20	0.439617		chr13_27796_mature	2.14913986	0	1.654957834	0		20	1		hsa-miR-1267	2.14914	4.029637	1.654957834	2.330454351		-0.90689	0.669559
chr4_10638_star	1.074569932	0	1.05281229	0		20	0.439617		chr14_31645_star	2.14913986	0	1.654957834	0		20	1		hsa-miR-4782-3p	2.14914	4.029637	1.654957834	2.330454351		-0.90689	0.669559
hsa-miR-5000-5p	1.074569932	0	1.05281229	0		20	0.439617		chr11_25309_star	2.14913986	0	1.654957834	0		20	1		chr13_29218_star	2.14914	4.029637	1.654957834	2.330454351		-0.90689	0.669559
chrx_23192_mature	1.074569932	0	1.05281229	0		20	0.439617		rno-miR-592	2.14913986	0	1.654957834	0		20	1		hsa-miR-548ab	2.14914	4.029637	1.654957834	2.330454351		-0.90689	0.669559
rno-miR-214-5p	1.074569932	0	1.05281229	0		20	0.439617		chr4_9349_star	2.14913986	0	1.654957834	0		20	1		chr9_19988_mature	2.14914	4.029637	1.654957834	2.330454351		-0.90689	0.669559
hsa-miR-212-5p	1.074569932	0	1.05281229	0		20	0.439617		hsa-miR-1272	2.14913986	0	1.654957834	0		20	1		chr2_6398_star	2.14914	4.029637	1.654957834	2.330454351		-0.90689	0.669559
rno-miR-380-3p	1.074569932	0	1.05281229	0		20	0.439617		chr4_9772_star	2.14913986	0	1.654957834	0		20	1		NW_003613367_38732_star	2.14914	4.029637	1.654957834	2.330454351		-0.90689	0.669559
hsa-miR-1915-5p	1.074569932	0	1.05281229	0		20	0.439617		mmu-miR-293-5p	2.14913986	0	1.654957834	0		20	1		chr1_2264_star	2.14914	4.029637	1.654957834	2.330454351		-0.90689	0.669559
chr14_29982_star	1.074569932	0	1.05281229	0		20	0.439617		hsa-miR-597	2.14913986	0	1.654957834	0		20	1		chr11_24910_star	2.14914	4.029637	1.654957834	2.330454351		-0.90689	0.669559
chr3_7967_star	1.074569932	0	1.05281229	0		20	0.439617		hsa-miR-599	2.14913986	0	1.654957834	0		20	1		NW_003541072_39884_star	2.14914	4.029637	1.654957834	2.330454351		-0.90689	0.669559
mmu-miR-666-5p	1.074569932	0	1.05281229	0		20	0.439617		hsa-miR-595	2.14913986	0	1.654957834	0		20	1		chr1_2346_star	2.14914	4.029637	1.654957834	2.330454351		-0.90689	0.669559
hsa-miR-1915-3p	1.074569932	0	1.05281229	0		20	0.439617		hsa-miR-585	2.14913986	0	1.654957834	0		20	1		NW_003538764_38206_star	2.14914	4.029637	1.654957834	2.330454351		-0.90689	0.669559
hsa-miR-3152-3p	1.074569932	0	1.05281229	0		20	0.439617		chr4_9437_star	2.14913986	0	1.654957834	0		20	1		chr2_5378_mature	2.14914	4.029637	1.654957834	2.330454351		-0.90689	0.669559
hsa-miR-4788	1.074569932	0	1.05281229	0		20	0.439617		NW_003537216_36774_star	2.14913986	0	1.654957834	0		20	1		chr18_35826_mature	2.14914	4.029637	1.654957834	2.330454351		-0.90689	0.669559

chr12_26195_star	1.074569932	0	1.05281229	0		20	0.439617	bta-miR-2399-5p	2.14913986	0	1.654957834	0		20	1		bta-miR-2285j	2.14914	4.029637	1.654957834	2.330454351		-0.90689	0.669559
chr1_2593_star	1.074569932	0	1.05281229	0		20	0.439617	mmu-miR-764-5p	2.14913986	0	1.654957834	0		20	1		chr8_18293_star	2.14914	4.029637	1.654957834	2.330454351		-0.90689	0.669559
chr2_5312_star	1.074569932	0	1.05281229	0		20	0.439617	hsa-miR-558	2.14913986	0	1.654957834	0		20	1		chr12_25812_star	2.14914	4.029637	1.654957834	2.330454351		-0.90689	0.669559
mmu-miR-292-5p	1.074569932	0	1.05281229	0		20	0.439617	chr3_7972_star	2.14913986	0	1.654957834	0		20	1		chr1_888_mature	2.14914	4.029637	1.654957834	2.330454351		-0.90689	0.669559
chr7_16503_star	1.074569932	0	1.05281229	0		20	0.439617	hsa-miR-555	2.14913986	0	1.654957834	0		20	1		chr10_24200_star	2.14914	4.029637	1.654957834	2.330454351		-0.90689	0.669559
hsa-miR-1305	1.074569932	0	1.05281229	0		20	0.439617	chr6_14458_mature	2.14913986	0	1.654957834	0		20	1		chr7_17076_star	2.14914	4.029637	1.654957834	2.330454351		-0.90689	0.669559
rno-miR-181a-5p	1.074569932	0	1.05281229	0		20	0.439617	chr14_29793_star	2.14913986	0	1.654957834	0		20	1		chr8_17657_star	2.14914	4.029637	1.654957834	2.330454351		-0.90689	0.669559
mmu-miR-496b	1.074569932	0	1.05281229	0		20	0.439617	mmu-miR-1896	2.14913986	0	1.654957834	0		20	1		bta-miR-2479	2.14914	4.029637	1.654957834	2.330454351		-0.90689	0.669559
hsa-miR-1322	1.074569932	0	1.05281229	0		20	0.439617	mmu-miR-1893	2.14913986	0	1.654957834	0		20	1		chr13_28822_star	2.14914	4.029637	1.654957834	2.330454351		-0.90689	0.669559
hsa-miR-532-3p	1.074569932	0	1.05281229	0		20	0.439617	mmu-miR-1899	2.14913986	15.31262	1.654957834	4.027916745		-2.83289	1		NW_003613401_38938_mature	2.14914	4.029637	1.654957834	2.330454351		-0.90689	0.669559
NW_003537225_36780_star	1.074569932	0	1.05281229	0		20	0.439617	NW_003537225_36780_mature	2.14913986	0	1.654957834	0		20	1		chr7_15783_star	2.14914	4.029637	1.654957834	2.330454351		-0.90689	0.669559
hsa-miR-450a-5p	1.074569932	0	1.05281229	0		20	0.439617	chr13_28643_star	2.14913986	0	1.654957834	0		20	1		chr4_9341_star	2.14914	4.029637	1.654957834	2.330454351		-0.90689	0.669559
hsa-miR-642a-3p	1.074569932	0	1.05281229	0		20	0.439617	hsa-miR-511	2.14913986	0	1.654957834	0		20	1		chr14_29836_star	2.14914	4.029637	1.654957834	2.330454351		-0.90689	0.669559
chrx_22497_star	1.074569932	0	1.05281229	0		20	0.439617	hsa-miR-548aq-3p	2.14913986	0	1.654957834	0		20	1		chr8_18750_star	2.14914	4.029637	1.654957834	2.330454351		-0.90689	0.669559
hsa-miR-4744	1.074569932	0	1.05281229	0		20	0.439617	hsa-miR-3918	2.14913986	0	1.654957834	0		20	1		rno-miR-2985	2.14914	4.029637	1.654957834	2.330454351		-0.90689	0.669559
hsa-miR-2467-3p	1.074569932	0	1.05281229	0		20	0.439617	chr17_35010_mature	2.14913986	0	1.654957834	0		20	1		chr18_36377_mature	3.22371	7.253347	2.078510713	3.044979305		-1.16993	0.670241
bta-miR-2886	1.074569932	0	1.05281229	0		20	0.439617	mmu-miR-878-3p	2.14913986	0	1.654957834	0		20	1		hsa-miR-1252	3.22371	7.253347	2.078510713	3.044979305		-1.16993	0.670241
chr5_12034_star	1.074569932	0	1.05281229	0		20	0.439617	chr6_13692_star	2.14913986	0	1.654957834	0		20	1		chr3_7692_star	3.22371	7.253347	2.078510713	3.044979305		-1.16993	0.670241
bta-miR-2885	1.074569932	0	1.05281229	0		20	0.439617	hsa-miR-192-5p	2.14913986	0	1.654957834	0		20	1		chr1_1120_mature	3.22371	7.253347	2.078510713	3.044979305		-1.16993	0.670241
mmu-miR-9-5p	1.074569932	0	1.05281229	0		20	0.439617	chr1_1629_star	2.14913986	0	1.654957834	0		20	1		chr6_14856_mature	4.835565	13.70077	2.544872269	3.877819487		-1.5025	0.6703
hsa-miR-4734	1.074569932	0	1.05281229	0		20	0.439617	hsa-miR-3907	2.14913986	0	1.654957834	0		20	1		chr9_20114_mature	5.37285	16.11855	2.671938627	4.097488514		-1.58496	0.670652
bta-miR-149-3p	1.074569932	0	1.05281229	0		20	0.439617	hsa-miR-483-5p	2.14913986	0	1.654957834	0		20	1		chr8_18198_star	20.41683	6.44742	4.420672964	2.896740642		1.662965	0.67072
rno-miR-326-5p	1.074569932	0	1.05281229	0		20	0.439617	chr7_17092_mature	2.14913986	0	1.654957834	0		20	1		chr9_19972_mature	9.671129	4.029637	3.415640968	2.330454351		1.263034	0.670814
rno-miR-92a-1-5p	1.074569932	0	1.05281229	0		20	0.439617	hsa-miR-92b-3p	2.14913986	0	1.654957834	0		20	1		NW_003540978_39774_mature	9.671129	4.029637	3.415640968	2.330454351		1.263034	0.670814
bta-miR-2892	1.074569932	0	1.05281229	0		20	0.439617	chr7_15701_star	2.14913986	0	1.654957834	0		20	1		chr14_31430_star	9.671129	4.029637	3.415640968	2.330454351		1.263034	0.670814
bta-miR-2894	1.074569932	0	1.05281229	0		20	0.439617	hsa-miR-1247-5p	2.14913986	0	1.654957834	0		20	1		chr1_3251_star	9.671129	4.029637	3.415640968	2.330454351		1.263034	0.670814
chr16_34762_star	1.074569932	0	1.05281229	0		20	0.439617	chr14_29835_star	2.14913986	0	1.654957834	0		20	1		ssc-miR-205	9.671129	4.029637	3.415640968	2.330454351		1.263034	0.670814
mmu-miR-466e-5p	1.074569932	0	1.05281229	0		20	0.439617	chr9_20088_star	2.14913986	0	1.654957834	0		20	1		mmu-miR-378d	9.671129	4.029637	3.415640968	2.330454351		1.263034	0.670814
hsa-miR-508-3p	1.074569932	0	1.05281229	0		20	0.439617	NW_003613379_38755_star	2.14913986	0	1.654957834	0		20	1		rno-miR-883-3p	4.29828	11.28298	2.405524014	3.618589217		-1.39232	0.671436
chr15_31957_star	1.074569932	0	1.05281229	0		20	0.439617	hsa-miR-1245b-3p	2.14913986	0	1.654957834	0		20	1		chr12_25520_mature	29.01339	8.059274	4.907534286	3.179395517		1.847997	0.671606
chr2_5951_mature	1.074569932	0	1.05281229	0		20	0.439617	mmu-miR-463-3p	2.14913986	0	1.654957834	0		20	1		chr5_12128_mature	8.596559	33.04303	3.262517265	5.08928735		-1.94251	0.67258
bta-miR-2397-3p	1.074569932	0	1.05281229	0		20	0.439617	chr3_7785_star	2.14913986	0	1.654957834	0		20	1		chr7_17161_mature	8.059274	29.81932	3.179395517	4.94576292		-1.88753	0.673251
chr14_31548_star	1.074569932	0	1.05281229	0		20	0.439617	mmu-miR-452-3p	2.14913986	0	1.654957834	0		20	1		chr1_1948_star	2.686425	1.611855	1.882222339	1.38507475		0.736966	0.673425
chr13_27960_star	1.074569932	0	1.05281229	0		20	0.439617	chr9_20451_mature	2.14913986	0	1.654957834	0		20	1		chr10_23455_star	2.686425	1.611855	1.882222339	1.38507475		0.736966	0.673425
chr14_29727_star	1.074569932	0	1.05281229	0		20	0.439617	mmu-miR-1952	2.14913986	0	1.654957834	0		20	1		hsa-miR-3614-5p	2.686425	1.611855	1.882222339	1.38507475		0.736966	0.673425
rno-miR-450a-3p	1.074569932	0	1.05281229	0		20	0.439617	chr12_25838_mature	2.14913986	0	1.654957834	0		20	1		chr13_27981_star	2.686425	1.611855	1.882222339	1.38507475		0.736966	0.673425
chr3_8429_star	1.074569932	0	1.05281229	0		20	0.439617	chr6_14834_mature	2.14913986	0	1.654957834	0		20	1		chr8_18144_star	2.686425	1.611855	1.882222339	1.38507475		0.736966	0.673425
chr6_14103_star	1.074569932	0	1.05281229	0		20	0.439617	hsa-miR-498	2.14913986	0	1.654957834	0		20	1		chr14_31610_star	2.686425	1.611855	1.882222339	1.38507475		0.736966	0.673425
chr6_12737_star	1.074569932	0	1.05281229	0		20	0.439617	chr10_24300_star	2.14913986	0	1.654957834	0		20	1		chr6_13349_mature	2.686425	1.611855	1.882222339	1.38507475		0.736966	0.673425
bta-miR-9-3p	1.074569932	0	1.05281229	0		20	0.439617	mmu-miR-1912-5p	2.14913986	0	1.654957834	0		20	1		chr5_12006_star	2.686425	1.611855	1.882222339	1.38507475		0.736966	0.673425
mmu-miR-501-5p	1.074569932	0	1.05281229	0		20	0.439617	hsa-miR-518e-5p	2.14913986	0	1.654957834	0		20	1		hsa-miR-3620-5p	2.686425	1.611855	1.882222339	1.38507475		0.736966	0.673425
chrx_23064_mature	1.074569932	0	1.05281229	0		20	0.439617	mmu-miR-1960	2.14913986	0	1.654957834	0		20	1		hsa-miR-1270	2.686425	1.611855	1.882222339	1.38507475		0.736966	0.673425
bta-miR-6121-3p	1.074569932	0	1.05281229	0		20	0.439617	chr5_11345_mature	2.14913986	0	1.654957834	0		20	1		chr18_36331_star	2.686425	1.611855	1.882222339	1.38507475		0.736966	0.673425
hsa-miR-483-3p	1.074569932	0	1.05281229	0		20	0.439617	mmu-miR-7a-5p	2.14913986	0	1.654957834	0		20	1		chr13_26769_star	2.686425	1.611855	1.882222339	1.38507475		0.736966	0.673425
chr14_29829_star	1.074569932	0	1.05281229	0		20	0.439617	chr11_24777_star	2.14913986	15.31262	1.654957834	4.027916745		-2.83289	1		chr1_1833_star	2.686425	1.611855	1.882222339	1.38507475		0.736966	0.673425
chr15_32836_star	1.074569932	0	1.05281229	0		20	0.439617	chrx_22720_star	2.14913986	0	1.654957834	0		20	1		NW_003613223_37738_star	2.686425	1.611855	1.882222339	1.38507475		0.736966	0.673425
chr1_1213_star	1.074569932	0	1.05281229	0		20	0.439617	chr14_29853_mature	2.14913986	0	1.654957834	0		20	1		chr5_12167_mature	2.686425	1.611855	1.882222339	1.38507475		0.736966	0.673425
hsa-miR-1257	1.074569932	0	1.05281229	0		20	0.439617	hsa-miR-3126-3p	2.14913986	0	1.654957834	0		20	1		chr8_18251_mature	2.686425	1.611855	1.882222339	1.38507475		0.736966	0.673425
hsa-miR-1250	1.074569932	0	1.05281229	0		20	0.439617	hsa-miR-5586-5p	2.14913986	0	1.654957834	0		20	1		chr5_11229_star	2.686425	1.611855	1.882222339	1.38507475		0.736966	0.673425
hsa-miR-1253	1.074569932	0	1.05281229	0		20	0.439617	hsa-miR-1271-5p	2.14913986	0	1.654957834	0		20	1		chr2_6132_star	2.686425	1.611855	1.882222339	1.38507475		0.736966	0.673425
chr15_33045_star	1.074569932	0	1.05281229	0		20	0.439617	hsa-miR-4716-5p	2.14913986	0	1.654957834	0		20	1		rno-miR-300-5p	2.686425	1.611855	1.882222339	1.38507475		0.736966	0.673425
hsa-miR-130b-5p	1.074569932	0	1.05281229	0		20	0.439617	NW_003613480_39328_star	2.14913986	0	1.654957834	0		20	1		chr5_11120_mature	2.686425	1.611855	1.882222339	1.38507475		0.736966	0.673425
chr18_36505_star	1.074569932	0	1.05281229	0		20	0.439617	chr4_8749_star	2.14913986	0	1.654957834	0		20	1		chr5_11045_star	2.686425	1.611855	1.882222339	1.38507475		0.736966	0.673425
chr15_32587_star	1.074569932	0	1.05281229	0		20	0.439617	NW_003613454_39203_star	2.14913986	0	1.654957834	0		20	1		rno-miR-3564	2.686425	1.611855	1.882222339	1.38507475		0.736966	0.673425
chr9_20893_star	1.074569932	0	1.05281229	0		20	0.439617	chr4_9091_star	2.14913986	0	1.654957834	0		20	1		chr1_539_star	2.686425	1.611855	1.882222339	1.38507475		0.736966	0.673425
hsa-miR-3186-3p	1.074569932	0	1.05281229	0		20	0.439617	chr2_5291_mature	2.14913986	0	1.654957834	0		20	1		chr1_541_star	2.686425	1.611855	1.882222339	1.38507475		0.736966	0.673425
chr15_32058_star	1.074569932	0	1.05281229	0		20	0.439617	hsa-miR-519b-3p	2.14913986	0	1.654957834	0		20	1		hsa-miR-3683	2.686425	1.611855	1.882222339	1.38507475		0.736966	0.673425
chr10_24076_star	1.074569932	0	1.05281229	0		20	0.439617	chr9_20360_star	2.14913986	0	1.654957834	0		20	1		NW_003540029_39236_mature	2.686425	1.611855	1.882222339	1.38507475		0.736966	0.673425
NW_003538115_37552_star	1.074569932	0	1.05281229	0		20	0.439617	hsa-miR-3977	2.14913986	0	1.654957834	0		20	1		NW_003539609_38987_star	2.686425	1.611855	1.882222339	1.38507475		0.736966	0.673425
hsa-miR-4732-3p	1.074569932	0	1.05281229	0		20	0.439617	chr10_23298_star	2.14913986	0	1.654957834	0		20	1		hsa-miR-550a-5p	2.686425	1.611855	1.882222339	1.38507475		0.736966	0.673425
chr7_16057_star	1.074569932	0	1.05281229	0		20	0.439617	hsa-miR-4746-3p	2.14913986	0	1.654957834	0		20	1		mmu-miR-183-5p	2.686425	1.611855	1.882222339	1.38507475		0.736966	0.673425
chr15_32751_star	1.074569932	0	1.05281229	0		20	0.439617	chr4_8685_star	2.14913986	0	1.654957834	0		20	1		chr7_16788_mature	2.686425	1.611855	1.882222339	1.38507475		0.736966	0.673425
mmu-miR-465a-5p	1.074569932	0	1.05281229	0		20	0.439617	chr15_32044_star	2.14913986	0	1.654957834	0		20	1		chr6_14428_mature	2.686425	1.611855	1.882222339	1.38507475		0.736966	0.673425
chr2_5437_star	1.074569932	0	1.05281229	0		20	0.439617	hsa-miR-4738-5p	2.14913986	0	1.654957834	0		20	1		NW_003613326_38565_star	2.686425	1.611855	1.882222339	1.38507475		0.736966	0.673425
hsa-miR-141-3p	1.074569932	0	1.05281229	0		20	0.439617	hsa-miR-548aw	2.14913986	0	1.654957834	0		20	1		chr13_27560_star	2.686425	1.611855	1.882222339	1.38507475		0.736966	0.673425
mmu-miR-146a-3p	1.074569932	0	1.05281229	0		20	0.439617	hsa-miR-512-3p	2.14913986	0	1.654957834	0		20	1		chr12_25706_mature	2.686425	1.611855	1.882222339	1.38507475		0.736966	0.673425
hsa-miR-1283	1.074569932	0	1.05281229	0		20	0.439617	mmu-miR-467a-5p	2.14913986	0	1.654957834	0		20	1		bta-miR-449a	2.686425	1.611855	1.882222339	1.38507475		0.736966	0.673425
chr4_9349_star	1.074569932	0	1.05281229	0		20	0.439617	chr14_30584_star	2.14913986	0	1.654957834	0		20	1		chr6_14501_star	2.686425	1.611855	1.882222339	1.38507475		0.736966	0.673425
hsa-miR-141-5p	1.074569932	0	1.05281229	0		20	0.439617	hsa-miR-548ad	2.14913986	0	1.654957834	0		20	1		bta-miR-450a	2.686425	1.611855	1.882222339	1.38507475		0.736966	0.673425
chr15_33664_star	1.074569932	0	1.05281229	0		20	0.439617	chr14_29853_star	2.14913986	0	1.654957834	0		20	1		chr16_33722_star	2.686425	1.611855	1.882222339	1.38507475		0.736966	0.673425
hsa-miR-1275	1.074569932	0	1.05281229	0		20	0.439617	mmu-miR-669a-5p	2.14913986	0	1.654957834	0		20	1		chrx_22052_star	2.686425	1.611855	1.882222339	1.38507475		0.736966	0.673425
chr17_35177_mature@@bta-miR-612	1.074569932	0	1.05281229	0		20	0.439617	chr9_19422_star	2.14913986	0	1.654957834	0		20	1		chr13_26807_star	2.686425	1.611855	1.882222339	1.38507475		0.736966	0.673425
hsa-miR-1278	1.074569932	0	1.05281229	0		20	0.439617	chr11_25215_mature	30.0879581	12.08891	4.958283956	3.710273245		1.315502	1		chr13_27728_star	2.686425	1.611855	1.882222339	1.38507475		0.736966	0.673425
chr4_9772_star	1.074569932	0	1.05281229	0		20	0.439617	chr1_2018_star	2.14913986	0	1.654957834	0		20	1		NW_003541332_40046_star	2.686425	1.611855	1.882222339	1.38507475		0.736966	0.673425
NW_003613148_37413_star	1.074569932	0	1.05281229	0		20	0.439617	NW_003613301_38356_mature	2.14913986	0	1.654957834	0		20	1		chrx_22193_mature	2.686425	1.611855	1.882222339	1.38507475		0.736966	0.673425
hsa-miR-1264	1.074569932	0	1.05281229	0		20	0.439617	hsa-miR-7-5p	2.14913986	0	1.654957834	0		20	1		chr9_20132_star	2.686425	1.611855	1.882222339	1.38507475		0.736966	0.673425
hsa-miR-1266	1.074569932	0	1.05281229	0		20	0.439617	hsa-miR-147b	2.14913986	0	1.654957834	0		20	1		chr6_14380_mature	2.686425	1.611855	1.882222339	1.38507475		0.736966	0.673425
hsa-miR-1262	1.074569932	0	1.05281229	0		20	0.439617	chr5_11713_mature	2.14913986	0	1.654957834	0		20	1		NW_003541040_39849_star	2.686425	1.611855	1.882222339	1.38507475		0.736966	0.673425
chr5_11814_mature	1.074569932	0	1.05281229	0		20	0.439617	chr9_19780_star	2.14913986	0	1.654957834	0		20	1		hsa-miR-4650-3p	2.686425	1.611855	1.882222339	1.38507475		0.736966	0.673425
hsa-miR-595	1.074569932	0	1.05281229	0		20	0.439617	chr8_18753_star	2.14913986	0	1.654957834	0		20	1		rno-miR-675-5p	2.686425	1.611855	1.882222339	1.38507475		0.736966	0.673425
chr15_33061_star	1.074569932	0	1.05281229	0		20	0.439617	hsa-miR-1469	2.14913986	0	1.654957834	0		20	1		chr7_15773_mature	2.686425	1.611855	1.882222339	1.38507475		0.736966	0.673425
hsa-miR-521	1.074569932	0	1.05281229	0		20	0.439617	chr13_27018_mature	2.14913986	15.31262	1.654957834	4.027916745		-2.83289	1		hsa-miR-205-5p	2.686425	1.611855	1.882222339	1.38507475		0.736966	0.673425
hsa-miR-106b-3p	1.074569932	0	1.05281229	0		20	0.439617	mmu-miR-871-5p	2.14913986	0	1.654957834	0		20	1		hsa-miR-4711-3p	2.686425	1.611855	1.882222339	1.38507475		0.736966	0.673425
rno-miR-598-5p	1.074569932	0	1.05281229	0		20	0.439617	hsa-miR-4524a-5p	2.14913986	0	1.654957834	0		20	1		chr10_24040_mature	2.686425	1.611855	1.882222339	1.38507475		0.736966	0.673425
hsa-miR-543	1.074569932	0	1.05281229	0		20	0.439617	rno-miR-483-3p	2.14913986	0	1.654957834	0		20	1		mmu-miR-5615-5p	2.686425	1.611855	1.882222339	1.38507475		0.736966	0.673425
chr14_29793_star	1.074569932	0	1.05281229	0		20	0.439617	hsa-miR-4777-3p	2.14913986	0	1.654957834	0		20	1		chr3_7571_star	2.686425	1.611855	1.882222339	1.38507475		0.736966	0.673425
mmu-miR-1892	1.074569932	0	1.05281229	0		20	0.439617	hsa-miR-5579-3p	2.14913986	0	1.654957834	0		20	1		mmu-miR-5615-3p	2.686425	1.611855	1.882222339	1.38507475		0.736966	0.673425
hsa-miR-4804-5p	1.074569932	0	1.05281229	0		20	0.439617	mmu-miR-669a-3p	2.14913986	0	1.654957834	0		20	1		rno-miR-3085	2.686425	1.611855	1.882222339	1.38507475		0.736966	0.673425
chr8_18054_star	1.074569932	0	1.05281229	0		20	0.439617	hsa-miR-3656	2.14913986	0	1.654957834	0		20	1		chr10_23455_mature	2.686425	1.611855	1.882222339	1.38507475		0.736966	0.673425
chr13_28643_star	1.074569932	0	1.05281229	0		20	0.439617	chr13_28115_mature	51.5793567	25.78968	5.716424587	4.743605355		1	1		chr13_26704_mature	2.686425	1.611855	1.882222339	1.38507475		0.736966	0.673425
chr18_36617_star	1.074569932	0	1.05281229	0		20	0.439617	chr7_15292_star	2.14913986	0	1.654957834	0		20	1		rno-miR-146a-3p	2.686425	1.611855	1.882222339	1.38507475		0.736966	0.673425
hsa-miR-510	1.074569932	0	1.05281229	0		20	0.439617	chr13_28825_star	2.14913986	0	1.654957834	0		20	1		chr1_1386_mature	2.686425	1.611855	1.882222339	1.38507475		0.736966	0.673425
mmu-miR-432	1.074569932	0	1.05281229	0		20	0.439617	hsa-miR-518b	2.14913986	0	1.654957834	0		20	1		chr7_15660_star	2.686425	1.611855	1.882222339	1.38507475		0.736966	0.673425
bta-miR-2299-3p	1.074569932	0	1.05281229	0		20	0.439617	rno-miR-3544	2.14913986	0	1.654957834	0		20	1		chr3_8156_star	2.686425	1.611855	1.882222339	1.38507475		0.736966	0.673425
chr6_13898_mature	1.074569932	0	1.05281229	0		20	0.439617	rno-miR-3543	2.14913986	0	1.654957834	0		20	1		rno-miR-6216	2.686425	1.611855	1.882222339	1.38507475		0.736966	0.673425
chr8_18418_mature	1.074569932	0	1.05281229	0		20	0.439617	hsa-miR-4715-3p	2.14913986	0	1.654957834	0		20	1		hsa-miR-3168	2.686425	1.611855	1.882222339	1.38507475		0.736966	0.673425
chr6_12901_star@@hsa-miR-1233-1	1.074569932	0	1.05281229	0		20	0.439617	hsa-miR-3666	2.14913986	0	1.654957834	0		20	1		hsa-miR-3199	2.686425	1.611855	1.882222339	1.38507475		0.736966	0.673425
bta-miR-2467-3p	1.074569932	0	1.05281229	0		20	0.439617	mmu-miR-6241	2.14913986	0	1.654957834	0		20	1		NW_003537687_37130_star	2.686425	1.611855	1.882222339	1.38507475		0.736966	0.673425
chr2_5147_mature	1.074569932	0	1.05281229	0		20	0.439617	hsa-miR-5588-5p	2.14913986	0	1.654957834	0		20	1		rno-miR-880-3p	2.686425	1.611855	1.882222339	1.38507475		0.736966	0.673425
chr1_1629_star	1.074569932	0	1.05281229	0		20	0.439617	hsa-miR-3618	2.14913986	0	1.654957834	0		20	1		chr9_19911_star	2.686425	1.611855	1.882222339	1.38507475		0.736966	0.673425
hsa-miR-3907	1.074569932	0	1.05281229	0		20	0.439617	rno-miR-3550	2.14913986	0	1.654957834	0		20	1		chr13_28245_star	2.686425	1.611855	1.882222339	1.38507475		0.736966	0.673425
mmu-miR-3067-3p	1.074569932	0	1.05281229	0		20	0.439617	mmu-miR-6237	2.14913986	0	1.654957834	0		20	1		chr9_20608_star	2.686425	1.611855	1.882222339	1.38507475		0.736966	0.673425
chr1_2060_star	1.074569932	0	1.05281229	0		20	0.439617	mmu-miR-5101	2.14913986	0	1.654957834	0		20	1		chr1_1411_mature	2.686425	1.611855	1.882222339	1.38507475		0.736966	0.673425
hsa-miR-3938	1.074569932	0	1.05281229	0		20	0.439617	chr13_28026_star	2.14913986	0	1.654957834	0		20	1		mmu-miR-1186a	2.686425	1.611855	1.882222339	1.38507475		0.736966	0.673425
hsa-miR-3937	1.074569932	0	1.05281229	0		20	0.439617	hsa-miR-4726-5p	2.14913986	0	1.654957834	0		20	1		chr4_9347_mature	2.686425	1.611855	1.882222339	1.38507475		0.736966	0.673425
mmu-miR-190b-3p	1.074569932	0	1.05281229	0		20	0.439617	chr6_14742_star	2.14913986	0	1.654957834	0		20	1		chr14_29999_star	2.686425	1.611855	1.882222339	1.38507475		0.736966	0.673425
chr3_6641_mature	1.074569932	0	1.05281229	0		20	0.439617	chr14_31596_star	2.14913986	0	1.654957834	0		20	1		NW_003539812_39109_star	2.686425	1.611855	1.882222339	1.38507475		0.736966	0.673425
NW_003613379_38755_star	1.074569932	0	1.05281229	0		20	0.439617	chr10_23895_star	2.14913986	0	1.654957834	0		20	1		chr14_29861_star	2.686425	1.611855	1.882222339	1.38507475		0.736966	0.673425
bta-miR-760-3p	1.074569932	0	1.05281229	0		20	0.439617	chr9_19811_star	2.14913986	0	1.654957834	0		20	1		chr4_9430_mature	2.686425	1.611855	1.882222339	1.38507475		0.736966	0.673425
rno-miR-211-5p	1.074569932	0	1.05281229	0		20	0.439617	chr12_26042_mature	2.14913986	0	1.654957834	0		20	1		chr14_29589_star	2.686425	1.611855	1.882222339	1.38507475		0.736966	0.673425
chr1_1949_star	1.074569932	0	1.05281229	0		20	0.439617	chr16_34281_star	2.14913986	0	1.654957834	0		20	1		chr14_29583_star	2.686425	1.611855	1.882222339	1.38507475		0.736966	0.673425
hsa-miR-1245b-5p	1.074569932	0	1.05281229	0		20	0.439617	chr1_3433_star	2.14913986	0	1.654957834	0		20	1		bta-miR-2293	2.686425	1.611855	1.882222339	1.38507475		0.736966	0.673425
chr2_5788_star	1.074569932	0	1.05281229	0		20	0.439617	chr2_6343_star	2.14913986	0	1.654957834	0		20	1		bta-miR-2296	2.686425	1.611855	1.882222339	1.38507475		0.736966	0.673425
hsa-miR-5590-3p	1.074569932	0	1.05281229	0		20	0.439617	chr13_27833_mature	2.14913986	0	1.654957834	0		20	1		hsa-miR-576-5p	2.686425	1.611855	1.882222339	1.38507475		0.736966	0.673425
hsa-miR-498	1.074569932	0	1.05281229	0		20	0.439617	mmu-miR-5121	2.14913986	0	1.654957834	0		20	1		chr12_26051_mature	2.686425	1.611855	1.882222339	1.38507475		0.736966	0.673425
mmu-miR-497-5p	1.074569932	0	1.05281229	0		20	0.439617	chr4_9471_star	2.14913986	0	1.654957834	0		20	1		chr7_16797_star	2.686425	1.611855	1.882222339	1.38507475		0.736966	0.673425
mmu-miR-122-5p	1.074569932	0	1.05281229	0		20	0.439617	chr3_6642_star	2.14913986	0	1.654957834	0		20	1		chr13_28990_star	2.686425	1.611855	1.882222339	1.38507475		0.736966	0.673425
rno-miR-665	1.074569932	0	1.05281229	0		20	0.439617	chr7_17101_star	2.14913986	0	1.654957834	0		20	1		chr3_6792_star	2.686425	1.611855	1.882222339	1.38507475		0.736966	0.673425
rno-miR-3590-5p	1.074569932	0	1.05281229	0		20	0.439617	bta-miR-135b	2.14913986	0	1.654957834	0		20	1		chr13_28371_mature	2.686425	1.611855	1.882222339	1.38507475		0.736966	0.673425
rno-miR-671	1.074569932	0	1.05281229	0		20	0.439617	chr9_20767_star	2.14913986	0	1.654957834	0		20	1		chr8_17767_star	2.686425	1.611855	1.882222339	1.38507475		0.736966	0.673425
hsa-miR-518e-5p	1.074569932	0	1.05281229	0		20	0.439617	hsa-miR-4745-5p	2.14913986	0	1.654957834	0		20	1		chr7_15943_star	2.686425	1.611855	1.882222339	1.38507475		0.736966	0.673425
mmu-miR-1963	1.074569932	0	1.05281229	0		20	0.439617	rno-miR-19b-3p	2.14913986	0	1.654957834	0		20	1		chr9_19622_star	2.686425	1.611855	1.882222339	1.38507475		0.736966	0.673425
chr1_468_mature	1.074569932	0	1.05281229	0		20	0.439617	rno-miR-6319	2.14913986	0	1.654957834	0		20	1		NW_003539698_39034_star	2.686425	1.611855	1.882222339	1.38507475		0.736966	0.673425
mmu-miR-1a-3p	1.074569932	0	1.05281229	0		20	0.439617	rno-miR-6318	2.14913986	0	1.654957834	0		20	1		hsa-miR-5695	2.686425	1.611855	1.882222339	1.38507475		0.736966	0.673425
chr3_7770_star	1.074569932	0	1.05281229	0		20	0.439617	mmu-miR-465b-5p	2.14913986	0	1.654957834	0		20	1		chr2_5391_mature	2.686425	1.611855	1.882222339	1.38507475		0.736966	0.673425
chr18_35998_star	1.074569932	0	1.05281229	0		20	0.439617	rno-miR-6314	2.14913986	0	1.654957834	0		20	1		chr1_1402_star	2.686425	1.611855	1.882222339	1.38507475		0.736966	0.673425
chr4_9777_mature	1.074569932	0	1.05281229	0		20	0.439617	rno-miR-3068-3p	2.14913986	0	1.654957834	0		20	1		hsa-miR-4503	2.686425	1.611855	1.882222339	1.38507475		0.736966	0.673425
hsa-miR-421	1.074569932	0	1.05281229	0		20	0.439617	hsa-miR-33a-5p	2.14913986	0	1.654957834	0		20	1		hsa-miR-6070	2.686425	1.611855	1.882222339	1.38507475		0.736966	0.673425
hsa-miR-3126-3p	1.074569932	0	1.05281229	0		20	0.439617	chr9_21042_star	2.14913986	0	1.654957834	0		20	1		chr1_1628_star	2.686425	1.611855	1.882222339	1.38507475		0.736966	0.673425
mmu-miR-374b-5p	1.074569932	0	1.05281229	0		20	0.439617	rno-miR-6330	2.14913986	0	1.654957834	0		20	1		chrx_22324_star	4.29828	2.417782	2.405524014	1.773060525		0.830075	0.673554
chr15_33620_star	1.074569932	0	1.05281229	0		20	0.439617	rno-miR-6325	2.14913986	0	1.654957834	0		20	1		hsa-miR-1206	4.29828	2.417782	2.405524014	1.773060525		0.830075	0.673554
hsa-miR-6504-3p	1.074569932	0	1.05281229	0		20	0.439617	chr15_32711_mature	2.14913986	0	1.654957834	0		20	1		chr14_30720_star	4.29828	2.417782	2.405524014	1.773060525		0.830075	0.673554
mmu-miR-880-5p	1.074569932	0	1.05281229	0		20	0.439617	rno-miR-192-5p	2.14913986	0	1.654957834	0		20	1		chr9_21061_mature	4.29828	2.417782	2.405524014	1.773060525		0.830075	0.673554
chr9_20079_star	1.074569932	0	1.05281229	0		20	0.439617	rno-miR-6321	2.14913986	0	1.654957834	0		20	1		hsa-miR-3919	4.29828	2.417782	2.405524014	1.773060525		0.830075	0.673554
hsa-miR-3977	1.074569932	0	1.05281229	0		20	0.439617	rno-miR-6322	2.14913986	0	1.654957834	0		20	1		NW_003613164_37468_star	4.29828	2.417782	2.405524014	1.773060525		0.830075	0.673554
chr3_6720_star	1.074569932	0	1.05281229	0		20	0.439617	hsa-miR-516a-3p	2.14913986	0	1.654957834	0		20	1		rno-miR-3542	4.29828	2.417782	2.405524014	1.773060525		0.830075	0.673554
rno-miR-25-5p	1.074569932	0	1.05281229	0		20	0.439617	chr5_12309_star@@mmu-miR-1251-3p	2.14913986	0	1.654957834	0		20	1		hsa-miR-3668	4.29828	2.417782	2.405524014	1.773060525		0.830075	0.673554
chr15_32044_star	1.074569932	0	1.05281229	0		20	0.439617	chr4_8813_star	2.14913986	0	1.654957834	0		20	1		chr2_5369_mature	4.29828	2.417782	2.405524014	1.773060525		0.830075	0.673554
hsa-miR-4738-5p	1.074569932	0	1.05281229	0		20	0.439617	mmu-miR-701-3p	2.14913986	0	1.654957834	0		20	1		chr5_11941_star	4.29828	2.417782	2.405524014	1.773060525		0.830075	0.673554
hsa-miR-548ax	1.074569932	0	1.05281229	0		20	0.439617	chr1_1204_star	2.14913986	0	1.654957834	0		20	1		rno-miR-802-5p	4.29828	2.417782	2.405524014	1.773060525		0.830075	0.673554
rno-miR-34c-5p	1.074569932	0	1.05281229	0		20	0.439617	mmu-miR-676-3p	2.14913986	0	1.654957834	0		20	1		chr1_3536_star	4.29828	2.417782	2.405524014	1.773060525		0.830075	0.673554
hsa-miR-302a-3p	1.074569932	0	1.05281229	0		20	0.439617	chr16_34138_star	2.14913986	0	1.654957834	0		20	1		chr1_6_mature	4.29828	2.417782	2.405524014	1.773060525		0.830075	0.673554
hsa-miR-548ai	1.074569932	0	1.05281229	0		20	0.439617	hsa-miR-4693-3p	2.14913986	0	1.654957834	0		20	1		chr1_3752_star	4.29828	2.417782	2.405524014	1.773060525		0.830075	0.673554
hsa-miR-548al	1.074569932	0	1.05281229	0		20	0.439617	rno-miR-188-5p	2.14913986	0	1.654957834	0		20	1		chr3_7732_star	4.29828	2.417782	2.405524014	1.773060525		0.830075	0.673554
hsa-miR-548ad	1.074569932	0	1.05281229	0		20	0.439617	hsa-miR-34b-5p	2.14913986	0	1.654957834	0		20	1		chr2_6194_mature	4.29828	2.417782	2.405524014	1.773060525		0.830075	0.673554
chr14_29853_star	1.074569932	0	1.05281229	0		20	0.439617	hsa-miR-183-5p	2.14913986	0	1.654957834	0		20	1		chr9_19396_star	4.29828	2.417782	2.405524014	1.773060525		0.830075	0.673554
NW_003537589_37025_star	1.074569932	0	1.05281229	0		20	0.439617	chr6_13396_star	2.14913986	0	1.654957834	0		20	1		ssc-miR-105-2	4.29828	2.417782	2.405524014	1.773060525		0.830075	0.673554
hsa-miR-2909	1.074569932	0	1.05281229	0		20	0.439617	chr7_16724_star	2.14913986	0	1.654957834	0		20	1		chr13_27783_star	4.29828	2.417782	2.405524014	1.773060525		0.830075	0.673554
chr2_6568_mature	1.074569932	0	1.05281229	0		20	0.439617	hsa-miR-3713	2.14913986	0	1.654957834	0		20	1		chr1_3069_star	4.29828	2.417782	2.405524014	1.773060525		0.830075	0.673554
chr2_6335_star	1.074569932	0	1.05281229	0		20	0.439617	hsa-miR-3714	2.14913986	0	1.654957834	0		20	1		chr1_2966_star	4.29828	2.417782	2.405524014	1.773060525		0.830075	0.673554
chr15_32789_star	1.074569932	0	1.05281229	0		20	0.439617	hsa-miR-34b-3p	2.14913986	0	1.654957834	0		20	1		chr12_26608_mature	4.29828	2.417782	2.405524014	1.773060525		0.830075	0.673554
bta-miR-1835	1.074569932	0	1.05281229	0		20	0.439617	chr14_29507_star	2.14913986	0	1.654957834	0		20	1		chr5_12327_star	4.29828	2.417782	2.405524014	1.773060525		0.830075	0.673554
chr13_28254_star	1.074569932	0	1.05281229	0		20	0.439617	mmu-miR-3070b-5p	2.14913986	0	1.654957834	0		20	1		mmu-miR-3471	4.29828	2.417782	2.405524014	1.773060525		0.830075	0.673554
chr8_18753_star	1.074569932	0	1.05281229	0		20	0.439617	chr12_26097_star	2.14913986	0	1.654957834	0		20	1		chr13_27562_star	4.29828	2.417782	2.405524014	1.773060525		0.830075	0.673554
hsa-miR-1468	1.074569932	0	1.05281229	0		20	0.439617	chr4_9970_star	2.14913986	0	1.654957834	0		20	1		chr10_24282_mature	4.29828	2.417782	2.405524014	1.773060525		0.830075	0.673554
mmu-miR-871-5p	1.074569932	0	1.05281229	0		20	0.439617	mmu-miR-3088-3p	2.14913986	0	1.654957834	0		20	1		hsa-miR-3663-5p	4.29828	2.417782	2.405524014	1.773060525		0.830075	0.673554
hsa-miR-34a-5p	1.074569932	0	1.05281229	0		20	0.439617	mmu-miR-465b-3p	2.14913986	0	1.654957834	0		20	1		chr3_8356_mature	4.29828	2.417782	2.405524014	1.773060525		0.830075	0.673554
hsa-miR-4777-3p	1.074569932	0	1.05281229	0		20	0.439617	hsa-miR-4649-5p	2.14913986	0	1.654957834	0		20	1		chr13_28245_mature	10.20841	42.71415	3.48651029	5.4500286		-2.06496	0.674012
hsa-miR-4685-5p	1.074569932	0	1.05281229	0		20	0.439617	hsa-miR-4701-3p	2.14913986	0	1.654957834	0		20	1		chrx_23008_mature	6.44742	20.95411	2.896740642	4.456419386		-1.70044	0.674358
hsa-miR-3650	1.074569932	0	1.05281229	0		20	0.439617	hsa-miR-5091	2.14913986	0	1.654957834	0		20	1		bta-miR-3956	3.760995	8.865202	2.251263041	3.302348583		-1.23704	0.674501
hsa-miR-3656	1.074569932	0	1.05281229	0		20	0.439617	hsa-miR-5087	2.14913986	0	1.654957834	0		20	1		chr15_33538_star	3.760995	8.865202	2.251263041	3.302348583		-1.23704	0.674501
bta-miR-1307	1.074569932	0	1.05281229	0		20	0.439617	chr14_31138_star	2.14913986	0	1.654957834	0		20	1		chr1_2954_mature	3.760995	8.865202	2.251263041	3.302348583		-1.23704	0.674501
chr4_9679_mature	1.074569932	0	1.05281229	0		20	0.439617	hsa-miR-4783-5p	2.14913986	0	1.654957834	0		20	1		bta-miR-761	3.760995	8.865202	2.251263041	3.302348583		-1.23704	0.674501
hsa-miR-3674	1.074569932	0	1.05281229	0		20	0.439617	chr13_29267_star	2.14913986	0	1.654957834	0		20	1		chr5_11725_mature	3.760995	8.865202	2.251263041	3.302348583		-1.23704	0.674501
rno-miR-3544	1.074569932	0	1.05281229	0		20	0.439617	hsa-miR-1234-3p	2.14913986	0	1.654957834	0		20	1		chr6_14961_star	3.760995	8.865202	2.251263041	3.302348583		-1.23704	0.674501
hsa-miR-3666	1.074569932	0	1.05281229	0		20	0.439617	chr9_20478_mature	2.14913986	0	1.654957834	0		20	1		hsa-miR-601	3.760995	8.865202	2.251263041	3.302348583		-1.23704	0.674501
mmu-miR-6241	1.074569932	0	1.05281229	0		20	0.439617	chr7_16728_mature	2.14913986	0	1.654957834	0		20	1		chr2_5590_star	19.87954	6.44742	4.384018281	2.896740642		1.624491	0.674631
chr2_4918_star	1.074569932	0	1.05281229	0		20	0.439617	chr7_17161_star	2.14913986	15.31262	1.654957834	4.027916745		-2.83289	1		chr2_5822_mature	6.44742	3.22371	2.896740642	2.078510713		1	0.675428
rno-miR-3569	1.074569932	0	1.05281229	0		20	0.439617	hsa-miR-4743-3p	2.14913986	0	1.654957834	0		20	1		NW_003613327_38569_mature	6.44742	3.22371	2.896740642	2.078510713		1	0.675428
mmu-miR-5101	1.074569932	0	1.05281229	0		20	0.439617	chr7_15714_mature	2.14913986	0	1.654957834	0		20	1		chr2_4605_mature	6.44742	3.22371	2.896740642	2.078510713		1	0.675428
rno-miR-3587	1.074569932	0	1.05281229	0		20	0.439617	hsa-miR-548ap-5p	2.14913986	0	1.654957834	0		20	1		chr1_2051_mature	6.44742	3.22371	2.896740642	2.078510713		1	0.675428
chr3_7715_star	1.074569932	0	1.05281229	0		20	0.439617	bta-miR-2450a	2.14913986	0	1.654957834	0		20	1		chrx_21932_mature	6.44742	3.22371	2.896740642	2.078510713		1	0.675428
chr10_23895_star	1.074569932	0	1.05281229	0		20	0.439617	bta-miR-1287	2.14913986	0	1.654957834	0		20	1		chr6_13900_star	6.44742	3.22371	2.896740642	2.078510713		1	0.675428
chr2_3991_star	1.074569932	0	1.05281229	0		20	0.439617	chr12_25706_star	2.14913986	0	1.654957834	0		20	1		chr3_6737_star	6.44742	3.22371	2.896740642	2.078510713		1	0.675428
chr9_19811_star	1.074569932	0	1.05281229	0		20	0.439617	mmu-miR-448-3p	2.14913986	0	1.654957834	0		20	1		chr1_927_star	6.44742	3.22371	2.896740642	2.078510713		1	0.675428
mmu-miR-7b-5p	1.074569932	0	1.05281229	0		20	0.439617	chr15_33543_star	2.14913986	0	1.654957834	0		20	1		chr15_32921_star	6.44742	3.22371	2.896740642	2.078510713		1	0.675428
chr6_13903_mature	1.074569932	0	1.05281229	0		20	0.439617	chr18_36191_star	2.14913986	0	1.654957834	0		20	1		chr13_28627_mature	473.3481	46.74379	8.889802221	5.577241251		3.340055	0.675798
mmu-miR-135a-1-3p	1.074569932	0	1.05281229	0		20	0.439617	chr9_20123_mature	2.14913986	0	1.654957834	0		20	1		rno-miR-7a-5p	12.35755	4.835565	3.739583968	2.544872269		1.353637	0.675838
rno-miR-743a-3p	1.074569932	0	1.05281229	0		20	0.439617	chr3_8405_star	2.14913986	0	1.654957834	0		20	1		NW_003537189_36743_mature	12.35755	4.835565	3.739583968	2.544872269		1.353637	0.675838
mmu-miR-5135	1.074569932	0	1.05281229	0		20	0.439617	chr13_27839_star	2.14913986	0	1.654957834	0		20	1		NW_003541090_39901_mature	12.35755	4.835565	3.739583968	2.544872269		1.353637	0.675838
rno-let-7d-5p	1.074569932	0	1.05281229	0		20	0.439617	mmu-miR-5046	2.14913986	0	1.654957834	0		20	1		chr3_8156_mature	9.133844	4.029637	3.34110968	2.330454351		1.180572	0.676403
chr5_11695_star	1.074569932	0	1.05281229	0		20	0.439617	mmu-miR-148a-5p	2.14913986	0	1.654957834	0		20	1		chrx_21868_star	9.133844	4.029637	3.34110968	2.330454351		1.180572	0.676403
chr7_17101_star	1.074569932	0	1.05281229	0		20	0.439617	hsa-miR-378g	2.14913986	0	1.654957834	0		20	1		chr14_30524_mature	9.133844	4.029637	3.34110968	2.330454351		1.180572	0.676403
hsa-miR-4745-5p	1.074569932	0	1.05281229	0		20	0.439617	hsa-miR-378i	2.14913986	0	1.654957834	0		20	1		chr4_10263_mature	9.133844	4.029637	3.34110968	2.330454351		1.180572	0.676403
rno-miR-6318	1.074569932	0	1.05281229	0		20	0.439617	chr15_32108_star	2.14913986	0	1.654957834	0		20	1		chrx_22045_star	23.64054	7.253347	4.62296188	3.044979305		1.704544	0.67723
mmu-miR-465b-5p	1.074569932	0	1.05281229	0		20	0.439617	chr15_32591_mature	2.14913986	0	1.654957834	0		20	1		chr7_16587_star	23.64054	7.253347	4.62296188	3.044979305		1.704544	0.67723
bta-miR-424-5p	1.074569932	0	1.05281229	0		20	0.439617	NW_003613116_37269_star	2.14913986	0	1.654957834	0		20	1		chr4_10614_star	1.611855	2.417782	1.38507475	1.773060525		-0.58496	0.677642
chr6_12810_star	1.074569932	0	1.05281229	0		20	0.439617	NW_003538125_37558_star	2.14913986	0	1.654957834	0		20	1		chr6_14429_mature	1.611855	2.417782	1.38507475	1.773060525		-0.58496	0.677642
NW_003541307_40037_star	1.074569932	0	1.05281229	0		20	0.439617	chr1_3523_mature	2.14913986	0	1.654957834	0		20	1		chr8_17418_star	1.611855	2.417782	1.38507475	1.773060525		-0.58496	0.677642
rno-miR-6331	1.074569932	0	1.05281229	0		20	0.439617	chr4_9351_star	2.14913986	0	1.654957834	0		20	1		hsa-miR-548d-3p	1.611855	2.417782	1.38507475	1.773060525		-0.58496	0.677642
bta-miR-92b	1.074569932	0	1.05281229	0		20	0.439617	chr6_13425_mature	8.59655945	28.20746	3.262517265	4.868265031		-1.71425	1		chr7_16802_star	1.611855	2.417782	1.38507475	1.773060525		-0.58496	0.677642
chr8_17485_star	1.074569932	0	1.05281229	0		20	0.439617	chr8_17361_mature	2.14913986	0	1.654957834	0		20	1		chr6_13330_star	1.611855	2.417782	1.38507475	1.773060525		-0.58496	0.677642
chr4_8813_star	1.074569932	0	1.05281229	0		20	0.439617	chr5_12402_star	2.14913986	0	1.654957834	0		20	1		hsa-miR-210	1.611855	2.417782	1.38507475	1.773060525		-0.58496	0.677642
mmu-miR-701-3p	1.074569932	0	1.05281229	0		20	0.439617	chr17_35707_star	2.14913986	0	1.654957834	0		20	1		chr16_34839_mature	1.611855	2.417782	1.38507475	1.773060525		-0.58496	0.677642
bta-miR-2425-3p	1.074569932	0	1.05281229	0		20	0.439617	chr7_15100_star	2.14913986	0	1.654957834	0		20	1		bta-miR-2397-5p	1.611855	2.417782	1.38507475	1.773060525		-0.58496	0.677642
bta-miR-2385-3p	1.074569932	0	1.05281229	0		20	0.439617	bta-miR-1193	2.14913986	0	1.654957834	0		20	1		hsa-miR-6510-5p	1.611855	2.417782	1.38507475	1.773060525		-0.58496	0.677642
hsa-miR-181a-2-3p	1.074569932	0	1.05281229	0		20	0.439617	chr15_33696_star	2.14913986	0	1.654957834	0		20	1		mmu-miR-3103-3p	1.611855	2.417782	1.38507475	1.773060525		-0.58496	0.677642
chr6_13396_star	1.074569932	0	1.05281229	0		20	0.439617	hsa-miR-24-3p	2.14913986	0	1.654957834	0		20	1		hsa-miR-2861	1.611855	2.417782	1.38507475	1.773060525		-0.58496	0.677642
chr3_6716_star	1.074569932	0	1.05281229	0		20	0.439617	chr13_27448_mature	2.14913986	0	1.654957834	0		20	1		NW_003539007_38454_star	1.611855	2.417782	1.38507475	1.773060525		-0.58496	0.677642
chr9_19422_mature	1.074569932	0	1.05281229	0		20	0.439617	rno-miR-743a-5p	2.14913986	0	1.654957834	0		20	1		hsa-miR-616-5p	1.611855	2.417782	1.38507475	1.773060525		-0.58496	0.677642
hsa-miR-202-5p	1.074569932	0	1.05281229	0		20	0.439617	hsa-miR-5591-3p	2.14913986	0	1.654957834	0		20	1		hsa-miR-616-3p	1.611855	2.417782	1.38507475	1.773060525		-0.58496	0.677642
chr4_9970_star	1.074569932	0	1.05281229	0		20	0.439617	mmu-miR-344d-3-5p	2.14913986	0	1.654957834	0		20	1		chr18_36015_mature	1.611855	2.417782	1.38507475	1.773060525		-0.58496	0.677642
mmu-miR-3088-3p	1.074569932	0	1.05281229	0		20	0.439617	ssc-miR-484	2.14913986	0	1.654957834	0		20	1		NW_003613251_37928_mature	1.611855	2.417782	1.38507475	1.773060525		-0.58496	0.677642
hsa-miR-126-3p	1.074569932	0	1.05281229	0		20	0.439617	hsa-miR-5008-5p	2.14913986	0	1.654957834	0		20	1		hsa-miR-548o-3p	1.611855	2.417782	1.38507475	1.773060525		-0.58496	0.677642
NW_003537262_36795_mature	1.074569932	0	1.05281229	0		20	0.439617	chr12_26044_star	2.14913986	0	1.654957834	0		20	1		chr4_9150_star	1.611855	2.417782	1.38507475	1.773060525		-0.58496	0.677642
mmu-miR-465b-3p	1.074569932	0	1.05281229	0		20	0.439617	chr1_1968_star	2.14913986	0	1.654957834	0		20	1		chr9_20120_mature	1.611855	2.417782	1.38507475	1.773060525		-0.58496	0.677642
hsa-miR-126-5p	1.074569932	0	1.05281229	0		20	0.439617	rno-miR-374-3p	2.14913986	0	1.654957834	0		20	1		chr12_25429_star	1.611855	2.417782	1.38507475	1.773060525		-0.58496	0.677642
chr4_10566_star	1.074569932	0	1.05281229	0		20	0.439617	rno-miR-126b	2.14913986	0	1.654957834	0		20	1		hsa-miR-550a-3p	1.611855	2.417782	1.38507475	1.773060525		-0.58496	0.677642
hsa-miR-4701-5p	1.074569932	0	1.05281229	0		20	0.439617	chr5_12150_star	2.14913986	0	1.654957834	0		20	1		hsa-miR-3140-5p	1.611855	2.417782	1.38507475	1.773060525		-0.58496	0.677642
chr5_11603_mature	1.074569932	0	1.05281229	0		20	0.439617	bta-miR-411c-3p	2.14913986	0	1.654957834	0		20	1		chr5_10795_star	1.611855	2.417782	1.38507475	1.773060525		-0.58496	0.677642
hsa-miR-378e	1.074569932	0	1.05281229	0		20	0.439617	hsa-miR-3129-5p	2.14913986	0	1.654957834	0		20	1		chr14_30974_star	1.611855	2.417782	1.38507475	1.773060525		-0.58496	0.677642
chr4_9423_star	1.074569932	0	1.05281229	0		20	0.439617	chr4_9787_star	2.14913986	0	1.654957834	0		20	1		chr8_18467_star	1.611855	2.417782	1.38507475	1.773060525		-0.58496	0.677642
hsa-miR-5093	1.074569932	0	1.05281229	0		20	0.439617	hsa-miR-6502-5p	2.14913986	0	1.654957834	0		20	1		chr3_6639_mature	1.611855	2.417782	1.38507475	1.773060525		-0.58496	0.677642
NW_003613050_36907_star	1.074569932	0	1.05281229	0		20	0.439617	chr15_31967_star	2.14913986	0	1.654957834	0		20	1		mmu-miR-6391	1.611855	2.417782	1.38507475	1.773060525		-0.58496	0.677642
hsa-miR-4694-5p	1.074569932	0	1.05281229	0		20	0.439617	mmu-miR-3099-3p	2.14913986	0	1.654957834	0		20	1		chr9_20430_star	1.611855	2.417782	1.38507475	1.773060525		-0.58496	0.677642
chr10_23834_star	1.074569932	0	1.05281229	0		20	0.439617	mmu-miR-532-3p	2.14913986	0	1.654957834	0		20	1		hsa-miR-5692c	1.611855	2.417782	1.38507475	1.773060525		-0.58496	0.677642
hsa-miR-4649-3p	1.074569932	0	1.05281229	0		20	0.439617	mmu-miR-669d-3p	2.14913986	0	1.654957834	0		20	1		chr13_26947_mature	1.611855	2.417782	1.38507475	1.773060525		-0.58496	0.677642
chr1_268_star	1.074569932	0	1.05281229	0		20	0.439617	chr15_32372_mature	2.14913986	0	1.654957834	0		20	1		chr2_4621_star	1.611855	2.417782	1.38507475	1.773060525		-0.58496	0.677642
bta-miR-1287	1.074569932	0	1.05281229	0		20	0.439617	hsa-miR-4757-3p	2.14913986	0	1.654957834	0		20	1		NW_003613481_39347_star	1.611855	2.417782	1.38507475	1.773060525		-0.58496	0.677642
hsa-miR-3140-3p	1.074569932	0	1.05281229	0		20	0.439617	bta-miR-2284aa-1	2.14913986	0	1.654957834	0		20	1		chr1_3153_star	1.611855	2.417782	1.38507475	1.773060525		-0.58496	0.677642
chr12_25706_star	1.074569932	0	1.05281229	0		20	0.439617	bta-miR-2284aa-2	2.14913986	0	1.654957834	0		20	1		chr7_16079_star	1.611855	2.417782	1.38507475	1.773060525		-0.58496	0.677642
chr18_36191_star	1.074569932	0	1.05281229	0		20	0.439617	chr1_526_star	2.14913986	0	1.654957834	0		20	1		hsa-miR-1255a	1.611855	2.417782	1.38507475	1.773060525		-0.58496	0.677642
bta-miR-125a	1.074569932	0	1.05281229	0		20	0.439617	chr4_9640_star	2.14913986	0	1.654957834	0		20	1		NW_003537731_37227_star	1.611855	2.417782	1.38507475	1.773060525		-0.58496	0.677642
chr6_15013_star	1.074569932	0	1.05281229	0		20	0.439617	chr7_17045_star	2.14913986	0	1.654957834	0		20	1		rno-miR-541-3p	1.611855	2.417782	1.38507475	1.773060525		-0.58496	0.677642
chr14_30174_star	1.074569932	0	1.05281229	0		20	0.439617	chr2_4071_star	2.14913986	0	1.654957834	0		20	1		chr8_17595_mature	1.611855	2.417782	1.38507475	1.773060525		-0.58496	0.677642
bta-miR-1256	1.074569932	0	1.05281229	0		20	0.439617	chr14_31496_star	2.14913986	0	1.654957834	0		20	1		NW_003539422_38814_mature	1.611855	2.417782	1.38507475	1.773060525		-0.58496	0.677642
bta-miR-1249	1.074569932	0	1.05281229	0		20	0.439617	chr14_30379_star	2.14913986	0	1.654957834	0		20	1		chr15_33346_star	1.611855	2.417782	1.38507475	1.773060525		-0.58496	0.677642
chr3_8405_star	1.074569932	0	1.05281229	0		20	0.439617	mmu-miR-423-5p	2.14913986	0	1.654957834	0		20	1		chr16_34110_star	1.611855	2.417782	1.38507475	1.773060525		-0.58496	0.677642
chr7_16117_star	1.074569932	0	1.05281229	0		20	0.439617	chr1_3117_star	2.14913986	0	1.654957834	0		20	1		chr3_7339_mature	1.611855	2.417782	1.38507475	1.773060525		-0.58496	0.677642
hsa-miR-30e-3p	1.074569932	0	1.05281229	0		20	0.439617	hsa-miR-548az-3p	2.14913986	0	1.654957834	0		20	1		chr7_16882_star	1.611855	2.417782	1.38507475	1.773060525		-0.58496	0.677642
mmu-miR-5046	1.074569932	0	1.05281229	0		20	0.439617	chr6_13983_mature	2.14913986	0	1.654957834	0		20	1		bta-miR-2388-5p	1.611855	2.417782	1.38507475	1.773060525		-0.58496	0.677642
chr3_7797_star	1.074569932	0	1.05281229	0		20	0.439617	hsa-miR-4798-5p	2.14913986	0	1.654957834	0		20	1		hsa-miR-3133	1.611855	2.417782	1.38507475	1.773060525		-0.58496	0.677642
hsa-miR-371b-3p	1.074569932	0	1.05281229	0		20	0.439617	chr13_27449_star	2.14913986	0	1.654957834	0		20	1		hsa-miR-3664-5p	1.611855	2.417782	1.38507475	1.773060525		-0.58496	0.677642
rno-miR-381-3p	1.074569932	0	1.05281229	0		20	0.439617	chr15_32040_star	2.14913986	0	1.654957834	0		20	1		hsa-miR-31-5p	1.611855	2.417782	1.38507475	1.773060525		-0.58496	0.677642
chr9_19846_star	1.074569932	0	1.05281229	0		20	0.439617	mmu-miR-6401	2.14913986	0	1.654957834	0		20	1		bta-miR-6531	1.611855	2.417782	1.38507475	1.773060525		-0.58496	0.677642
hsa-miR-663b	1.074569932	0	1.05281229	0		20	0.439617	mmu-miR-6403	2.14913986	0	1.654957834	0		20	1		NW_003539453_38857_mature	1.611855	2.417782	1.38507475	1.773060525		-0.58496	0.677642
hsa-miR-135a-5p	1.074569932	0	1.05281229	0		20	0.439617	bta-miR-2439-5p	2.14913986	0	1.654957834	0		20	1		chr10_23951_star	1.611855	2.417782	1.38507475	1.773060525		-0.58496	0.677642
NW_003613116_37269_star	1.074569932	0	1.05281229	0		20	0.439617	chr1_3299_star	2.14913986	0	1.654957834	0		20	1		chr2_5788_mature	1.611855	2.417782	1.38507475	1.773060525		-0.58496	0.677642
chr12_25609_star	1.074569932	0	1.05281229	0		20	0.439617	chr1_2655_star	2.14913986	0	1.654957834	0		20	1		chr3_7732_mature	1.611855	2.417782	1.38507475	1.773060525		-0.58496	0.677642
NW_003540029_39234_star	1.074569932	0	1.05281229	0		20	0.439617	hsa-miR-548am-5p	2.14913986	0	1.654957834	0		20	1		NW_003539453_38861_star	1.611855	2.417782	1.38507475	1.773060525		-0.58496	0.677642
chr18_36082_star	1.074569932	0	1.05281229	0		20	0.439617	hsa-miR-21-3p	2.14913986	0	1.654957834	0		20	1		chr9_19885_mature	1.611855	2.417782	1.38507475	1.773060525		-0.58496	0.677642
chr11_25383_mature	1.074569932	0	1.05281229	0		20	0.439617	mmu-miR-130b-5p	2.14913986	0	1.654957834	0		20	1		bta-miR-2405	1.611855	2.417782	1.38507475	1.773060525		-0.58496	0.677642
chr15_32726_star	1.074569932	0	1.05281229	0		20	0.439617	chr4_9346_star	2.14913986	0	1.654957834	0		20	1		bta-miR-2433	1.611855	2.417782	1.38507475	1.773060525		-0.58496	0.677642
chr5_12402_star	1.074569932	0	1.05281229	0		20	0.439617	chr15_33120_star	2.14913986	0	1.654957834	0		20	1		chr9_19205_mature	1.611855	2.417782	1.38507475	1.773060525		-0.58496	0.677642
bta-miR-2367-5p	1.074569932	0	1.05281229	0		20	0.439617	rno-miR-30c-2-3p	2.14913986	0	1.654957834	0		20	1		chr1_2055_star	1.611855	2.417782	1.38507475	1.773060525		-0.58496	0.677642
chr6_14458_star	1.074569932	0	1.05281229	0		20	0.439617	chr13_29223_star	2.14913986	0	1.654957834	0		20	1		chrx_22198_star	1.611855	2.417782	1.38507475	1.773060525		-0.58496	0.677642
hsa-miR-5008-5p	1.074569932	0	1.05281229	0		20	0.439617	rno-miR-148b-5p	2.14913986	0	1.654957834	0		20	1		chr1_3835_mature	1.611855	2.417782	1.38507475	1.773060525		-0.58496	0.677642
chr14_30478_star	1.074569932	0	1.05281229	0		20	0.439617	rno-miR-19b-1-5p	2.14913986	0	1.654957834	0		20	1		chr1_662_star	1.611855	2.417782	1.38507475	1.773060525		-0.58496	0.677642
hsa-miR-642b-3p	1.074569932	0	1.05281229	0		20	0.439617	mmu-miR-6413	36.5353777	16.11855	5.230179094	4.097488514		1.180572	1		hsa-miR-939-5p	1.611855	2.417782	1.38507475	1.773060525		-0.58496	0.677642
chr4_9751_mature	1.074569932	0	1.05281229	0		20	0.439617	mmu-miR-6415	2.14913986	0	1.654957834	0		20	1		ssc-miR-432-3p	1.611855	2.417782	1.38507475	1.773060525		-0.58496	0.677642
chr1_3508_star	1.074569932	0	1.05281229	0		20	0.439617	rno-miR-207	2.14913986	0	1.654957834	0		20	1		chrx_22153_star	1.611855	2.417782	1.38507475	1.773060525		-0.58496	0.677642
chrx_21983_mature	1.074569932	0	1.05281229	0		20	0.439617	NW_003538396_37820_star	2.14913986	0	1.654957834	0		20	1		chr4_9813_mature	1.611855	2.417782	1.38507475	1.773060525		-0.58496	0.677642
NW_003613223_37737_star	1.074569932	0	1.05281229	0		20	0.439617	hsa-miR-486-3p	2.14913986	0	1.654957834	0		20	1		NW_003613522_39530_star	1.611855	2.417782	1.38507475	1.773060525		-0.58496	0.677642
chr14_29848_star	1.074569932	0	1.05281229	0		20	0.439617	hsa-miR-382-5p	2.14913986	0	1.654957834	0		20	1		chr9_20478_star	1.611855	2.417782	1.38507475	1.773060525		-0.58496	0.677642
hsa-miR-3129-5p	1.074569932	0	1.05281229	0		20	0.439617	mmu-miR-30b-5p	2.14913986	0	1.654957834	0		20	1		chr3_8596_mature	27.93882	8.059274	4.854934103	3.179395517		1.793549	0.677864
hsa-miR-3613-3p	1.074569932	0	1.05281229	0		20	0.439617	rno-miR-667-3p	2.14913986	0	1.654957834	0		20	1		chr1_3278_mature	27.93882	8.059274	4.854934103	3.179395517		1.793549	0.677864
chr7_15622_star	1.074569932	0	1.05281229	0		20	0.439617	rno-miR-485-5p	2.14913986	0	1.654957834	0		20	1		chr9_19640_mature	5.37285	15.31262	2.671938627	4.027916745		-1.51096	0.678134
hsa-miR-6502-5p	1.074569932	0	1.05281229	0		20	0.439617	hsa-miR-3180-5p	2.14913986	0	1.654957834	0		20	1		chrx_22191_star	5.37285	15.31262	2.671938627	4.027916745		-1.51096	0.678134
chr3_8500_star	1.074569932	0	1.05281229	0		20	0.439617	hsa-miR-1181	15.043979	4.029637	4.003960082	2.330454351		1.900464	1		chr13_26861_mature	15.58126	5.641492	4.051482084	2.731507408		1.465664	0.678213
rno-miR-150-3p	1.074569932	0	1.05281229	0		20	0.439617	chr1_251_star	15.043979	4.029637	4.003960082	2.330454351		1.900464	1		chr15_32248_mature	4.835565	12.89484	2.544872269	3.796477232		-1.41504	0.678316
hsa-miR-548az-5p	1.074569932	0	1.05281229	0		20	0.439617	chr18_36022_mature	2.14913986	0	1.654957834	0		20	1		chr6_12750_mature	4.835565	12.89484	2.544872269	3.796477232		-1.41504	0.678316
bta-miR-30a-5p	1.074569932	0	1.05281229	0		20	0.439617	hsa-miR-1193	2.14913986	0	1.654957834	0		20	1		chr12_25408_star	2.686425	4.835565	1.882222339	2.544872269		-0.848	0.678316
mmu-miR-3099-5p	1.074569932	0	1.05281229	0		20	0.439617	chr13_26823_mature	2.14913986	0	1.654957834	0		20	1		chr7_16140_star	2.686425	4.835565	1.882222339	2.544872269		-0.848	0.678316
rno-miR-543-3p	1.074569932	0	1.05281229	0		20	0.439617	chr1_990_star	2.14913986	0	1.654957834	0		20	1		chr18_35926_mature	2.686425	4.835565	1.882222339	2.544872269		-0.848	0.678316
chr1_526_star	1.074569932	0	1.05281229	0		20	0.439617	chr16_34016_star	2.14913986	0	1.654957834	0		20	1		chr1_2451_mature	2.686425	4.835565	1.882222339	2.544872269		-0.848	0.678316

bta-miR-2284aa-4	1.074569932	0	1.05281229	0		20	0.439617	hsa-miR-5197-5p	2.14913986	0	1.654957834	0		20	1		chr14_31127_star	2.686425	4.835565	1.882222339	2.544872269		-0.848	0.678316
chr14_30379_star	1.074569932	0	1.05281229	0		20	0.439617	chr3_6623_star	2.14913986	0	1.654957834	0		20	1		chr1_1318_star	2.686425	4.835565	1.882222339	2.544872269		-0.848	0.678316
mmu-miR-673-5p	1.074569932	0	1.05281229	0		20	0.439617	chr13_29315_star	2.14913986	0	1.654957834	0		20	1		hsa-miR-3160-5p	2.686425	4.835565	1.882222339	2.544872269		-0.848	0.678316
rno-miR-1839-3p	1.074569932	0	1.05281229	0		20	0.439617	NW_003613552_39654_mature	2.14913986	0	1.654957834	0		20	1		chr9_20299_mature	2.686425	4.835565	1.882222339	2.544872269		-0.848	0.678316
rno-miR-150-5p	1.074569932	0	1.05281229	0		20	0.439617	chr4_10265_mature	25.7896784	9.671129	4.743605355	3.415640968		1.415037	1		mmu-miR-466p-5p	2.686425	4.835565	1.882222339	2.544872269		-0.848	0.678316
chr6_13983_mature	1.074569932	0	1.05281229	0		20	0.439617	hsa-miR-4757-5p	2.14913986	0	1.654957834	0		20	1		chr12_26184_star	2.686425	4.835565	1.882222339	2.544872269		-0.848	0.678316
chr15_32040_star	1.074569932	0	1.05281229	0		20	0.439617	hsa-miR-4728-5p	2.14913986	0	1.654957834	0		20	1		chr9_19153_star	2.686425	4.835565	1.882222339	2.544872269		-0.848	0.678316
mmu-miR-130b-3p	1.074569932	0	1.05281229	0		20	0.439617	hsa-miR-1237-5p	2.14913986	0	1.654957834	0		20	1		chr14_29876_star	2.686425	4.835565	1.882222339	2.544872269		-0.848	0.678316
chr16_33726_mature	1.074569932	0	1.05281229	0		20	0.439617	chr10_23668_star	2.14913986	0	1.654957834	0		20	1		mmu-miR-3079-5p	2.686425	4.835565	1.882222339	2.544872269		-0.848	0.678316
mmu-miR-133b-3p	1.074569932	0	1.05281229	0		20	0.439617	mmu-miR-6347	2.14913986	0	1.654957834	0		20	1		hsa-miR-4795-5p	2.686425	4.835565	1.882222339	2.544872269		-0.848	0.678316
hsa-miR-21-3p	1.074569932	0	1.05281229	0		20	0.439617	mmu-miR-6343	2.14913986	0	1.654957834	0		20	1		chr2_6524_mature	2.686425	4.835565	1.882222339	2.544872269		-0.848	0.678316
rno-miR-547-5p	1.074569932	0	1.05281229	0		20	0.439617	chr9_19640_mature	2.14913986	15.31262	1.654957834	4.027916745		-2.83289	1		chr2_4549_mature	2.686425	4.835565	1.882222339	2.544872269		-0.848	0.678316
chr4_10605_star	1.074569932	0	1.05281229	0		20	0.439617	hsa-miR-4713-5p	2.14913986	0	1.654957834	0		20	1		chr6_12914_mature	2.686425	4.835565	1.882222339	2.544872269		-0.848	0.678316
hsa-miR-6722-5p	1.074569932	0	1.05281229	0		20	0.439617	mmu-miR-6339	2.14913986	0	1.654957834	0		20	1		chr2_4896_star	2.686425	4.835565	1.882222339	2.544872269		-0.848	0.678316
hsa-miR-4712-3p	1.074569932	0	1.05281229	0		20	0.439617	bta-miR-2285v	2.14913986	0	1.654957834	0		20	1		chr13_26683_mature	2.686425	4.835565	1.882222339	2.544872269		-0.848	0.678316
chr5_11025_star	1.074569932	0	1.05281229	0		20	0.439617	bta-miR-562	2.14913986	0	1.654957834	0		20	1		NW_003613440_39149_mature	2.686425	4.835565	1.882222339	2.544872269		-0.848	0.678316
chr7_16472_star	1.074569932	0	1.05281229	0		20	0.439617	chr9_19527_star	2.14913986	0	1.654957834	0		20	1		ssc-miR-664-5p	13.43212	63.66827	3.851211749	6.014986077		-2.24489	0.678541
chr14_29850_mature	1.074569932	0	1.05281229	0		20	0.439617	mmu-miR-6359	2.14913986	0	1.654957834	0		20	1		chr4_10147_star	65.54877	13.70077	6.056340006	3.877819487		2.258312	0.678916
chr6_13536_star	1.074569932	0	1.05281229	0		20	0.439617	chr1_2152_star	2.14913986	0	1.654957834	0		20	1		chr10_24135_mature	5.910135	17.7304	2.788713818	4.227310103		-1.58496	0.679063
rno-miR-207	1.074569932	0	1.05281229	0		20	0.439617	mmu-miR-6386	2.14913986	0	1.654957834	0		20	1		chr3_7237_star	5.910135	17.7304	2.788713818	4.227310103		-1.58496	0.679063
hsa-miR-486-3p	1.074569932	0	1.05281229	0		20	0.439617	bta-miR-2285f	2.14913986	0	1.654957834	0		20	1		chr1_1739_mature	5.910135	17.7304	2.788713818	4.227310103		-1.58496	0.679063
chr1_986_mature	1.074569932	0	1.05281229	0		20	0.439617	bta-miR-2285h	2.14913986	0	1.654957834	0		20	1		chr7_15077_mature	47.81836	11.28298	5.609351983	3.618589217		2.083416	0.679264
rno-miR-292-3p	1.074569932	0	1.05281229	0		20	0.439617	hsa-miR-3927-5p	2.14913986	0	1.654957834	0		20	1		chr17_35020_star	3.22371	6.44742	2.078510713	2.896740642		-1	0.679743
hsa-miR-3180-5p	1.074569932	0	1.05281229	0		20	0.439617	bta-miR-2285l	2.14913986	0	1.654957834	0		20	1		chr2_4320_star	3.22371	6.44742	2.078510713	2.896740642		-1	0.679743
chr8_17492_star	1.074569932	0	1.05281229	0		20	0.439617	bta-miR-2284v	2.14913986	0	1.654957834	0		20	1		chr14_31524_mature	3.22371	6.44742	2.078510713	2.896740642		-1	0.679743
bta-miR-2461-5p	1.074569932	0	1.05281229	0		20	0.439617	bta-miR-2284d	2.14913986	0	1.654957834	0		20	1		chrx_21932_star	3.22371	6.44742	2.078510713	2.896740642		-1	0.679743
chr3_7170_star	1.074569932	0	1.05281229	0		20	0.439617	bta-miR-2284f	2.14913986	0	1.654957834	0		20	1		chr18_36229_mature	3.22371	6.44742	2.078510713	2.896740642		-1	0.679743
chr16_33853_mature	1.074569932	0	1.05281229	0		20	0.439617	bta-miR-146a	2.14913986	0	1.654957834	0		20	1		chr16_33724_star	3.22371	6.44742	2.078510713	2.896740642		-1	0.679743
ssc-miR-381	1.074569932	0	1.05281229	0		20	0.439617	chr14_30956_star	2.14913986	0	1.654957834	0		20	1		chr7_16628_mature	3.22371	6.44742	2.078510713	2.896740642		-1	0.679743
hsa-miR-452-5p	1.074569932	0	1.05281229	0		20	0.439617	chr6_13018_star	2.14913986	0	1.654957834	0		20	1		NW_003539115_38544_star	3.22371	6.44742	2.078510713	2.896740642		-1	0.679743
rno-miR-99a-3p	1.074569932	0	1.05281229	0		20	0.439617	rno-miR-297	2.14913986	0	1.654957834	0		20	1		chr15_31797_star	3.22371	6.44742	2.078510713	2.896740642		-1	0.679743
rno-let-7a-5p	1.074569932	0	1.05281229	0		20	0.439617	chrx_22793_star	2.14913986	0	1.654957834	0		20	1		chr3_7586_mature	3.22371	6.44742	2.078510713	2.896740642		-1	0.679743
hsa-miR-339-3p	1.074569932	0	1.05281229	0		20	0.439617	chr7_16076_star	2.14913986	0	1.654957834	0		20	1		chr3_8352_star	3.22371	6.44742	2.078510713	2.896740642		-1	0.679743
bta-miR-2325a	1.074569932	0	1.05281229	0		20	0.439617	hsa-miR-4677-3p	2.14913986	0	1.654957834	0		20	1		chr14_30467_mature@@hsa-miR-6	3.760995	2.417782	2.251263041	1.773060525		0.63743	0.679914
NW_003613552_39654_mature	1.074569932	0	1.05281229	0		20	0.439617	chr1_3844_star	2.14913986	0	1.654957834	0		20	1		chr3_7956_star	3.760995	2.417782	2.251263041	1.773060525		0.63743	0.679914
mmu-miR-5617-5p	1.074569932	0	1.05281229	0		20	0.439617	chr3_6725_mature	2.14913986	0	1.654957834	0		20	1		chr4_9513_star	3.760995	2.417782	2.251263041	1.773060525		0.63743	0.679914
hsa-miR-515-5p	1.074569932	0	1.05281229	0		20	0.439617	hsa-miR-432-3p	2.14913986	0	1.654957834	0		20	1		chr3_7786_star	3.760995	2.417782	2.251263041	1.773060525		0.63743	0.679914
NW_003613161_37463_mature	1.074569932	0	1.05281229	0		20	0.439617	chr3_7082_mature	2.14913986	0	1.654957834	0		20	1		hsa-miR-575	3.760995	2.417782	2.251263041	1.773060525		0.63743	0.679914
hsa-miR-515-3p	1.074569932	0	1.05281229	0		20	0.439617	chr9_19770_star	2.14913986	0	1.654957834	0		20	1		chr13_28164_star	3.760995	2.417782	2.251263041	1.773060525		0.63743	0.679914
chr11_24459_star	1.074569932	0	1.05281229	0		20	0.439617	rno-miR-350	2.14913986	0	1.654957834	0		20	1		chr17_35449_star	3.760995	2.417782	2.251263041	1.773060525		0.63743	0.679914
hsa-miR-1237-5p	1.074569932	0	1.05281229	0		20	0.439617	hsa-miR-6722-3p	2.14913986	0	1.654957834	0		20	1		chr5_12382_star	3.760995	2.417782	2.251263041	1.773060525		0.63743	0.679914
chr10_23668_star	1.074569932	0	1.05281229	0		20	0.439617	chr15_33121_star	2.14913986	0	1.654957834	0		20	1		chr7_16597_star	3.760995	2.417782	2.251263041	1.773060525		0.63743	0.679914
rno-miR-190b-3p	1.074569932	0	1.05281229	0		20	0.439617	chr4_8670_mature	2.14913986	0	1.654957834	0		20	1		chr5_11601_mature	3.760995	2.417782	2.251263041	1.773060525		0.63743	0.679914
mmu-miR-6347	1.074569932	0	1.05281229	0		20	0.439617	chr18_35940_mature	2.14913986	0	1.654957834	0		20	1		chr6_13506_star	3.760995	2.417782	2.251263041	1.773060525		0.63743	0.679914
chr1_551_star	1.074569932	0	1.05281229	0		20	0.439617	hsa-miR-548c-5p	2.14913986	0	1.654957834	0		20	1		chr6_15065_star	3.760995	2.417782	2.251263041	1.773060525		0.63743	0.679914
chr17_35456_mature	1.074569932	0	1.05281229	0		20	0.439617	chr8_18299_star	2.14913986	0	1.654957834	0		20	1		chr5_11119_mature	3.760995	2.417782	2.251263041	1.773060525		0.63743	0.679914
mmu-miR-6368	1.074569932	0	1.05281229	0		20	0.439617	chr1_788_mature	2.14913986	0	1.654957834	0		20	1		chr5_12271_mature	3.760995	2.417782	2.251263041	1.773060525		0.63743	0.679914
hsa-miR-4653-5p	1.074569932	0	1.05281229	0		20	0.439617	chr1_2367_star	2.14913986	0	1.654957834	0		20	1		chr9_19233_star	3.760995	2.417782	2.251263041	1.773060525		0.63743	0.679914
mmu-miR-6359	1.074569932	0	1.05281229	0		20	0.439617	chr7_16262_star	2.14913986	0	1.654957834	0		20	1		chr1_1748_mature	3.760995	2.417782	2.251263041	1.773060525		0.63743	0.679914
mmu-miR-6354	1.074569932	0	1.05281229	0		20	0.439617	NW_003540828_39718_star	2.14913986	0	1.654957834	0		20	1		chr14_31302_star	3.760995	2.417782	2.251263041	1.773060525		0.63743	0.679914
mmu-miR-3104-3p	1.074569932	0	1.05281229	0		20	0.439617	chr11_25285_star	2.14913986	0	1.654957834	0		20	1		chr14_30455_star	3.760995	2.417782	2.251263041	1.773060525		0.63743	0.679914
bta-miR-2285f	1.074569932	0	1.05281229	0		20	0.439617	hsa-miR-518f-5p	2.14913986	0	1.654957834	0		20	1		chr5_11953_star	3.760995	2.417782	2.251263041	1.773060525		0.63743	0.679914
bta-miR-2285l	1.074569932	0	1.05281229	0		20	0.439617	chrx_21884_star	2.14913986	0	1.654957834	0		20	1		chr1_3766_star	3.760995	2.417782	2.251263041	1.773060525		0.63743	0.679914
bta-miR-2284r	1.074569932	0	1.05281229	0		20	0.439617	chr9_20754_star	2.14913986	0	1.654957834	0		20	1		NW_003538009_37453_star	3.760995	2.417782	2.251263041	1.773060525		0.63743	0.679914
bta-miR-2284v	1.074569932	0	1.05281229	0		20	0.439617	chr5_11099_star	2.14913986	0	1.654957834	0		20	1		chr15_32322_star	3.760995	2.417782	2.251263041	1.773060525		0.63743	0.679914
bta-miR-2284x	1.074569932	0	1.05281229	0		20	0.439617	hsa-miR-221-5p	2.14913986	0	1.654957834	0		20	1		chr18_36007_star	3.760995	2.417782	2.251263041	1.773060525		0.63743	0.679914
bta-miR-2284a	1.074569932	0	1.05281229	0		20	0.439617	chr8_18293_star	15.043979	4.029637	4.003960082	2.330454351		1.900464	1		chr14_29799_star	3.760995	2.417782	2.251263041	1.773060525		0.63743	0.679914
bta-miR-2284k	1.074569932	0	1.05281229	0		20	0.439617	bta-miR-670	2.14913986	0	1.654957834	0		20	1		mmu-miR-2137	3.760995	2.417782	2.251263041	1.773060525		0.63743	0.679914
mmu-miR-6393	1.074569932	0	1.05281229	0		20	0.439617	hsa-miR-4436a	2.14913986	0	1.654957834	0		20	1		chr8_18464_star	2.14914	3.22371	1.654957834	2.078510713		-0.58496	0.680031
hsa-miR-218-2-3p	1.074569932	0	1.05281229	0		20	0.439617	mmu-miR-489-5p	2.14913986	0	1.654957834	0		20	1		mmu-miR-6537-3p	2.14914	3.22371	1.654957834	2.078510713		-0.58496	0.680031
chr14_30956_star	1.074569932	0	1.05281229	0		20	0.439617	chr6_13925_mature	2.14913986	0	1.654957834	0		20	1		hsa-miR-1302	2.14914	3.22371	1.654957834	2.078510713		-0.58496	0.680031
chr6_13018_star	1.074569932	0	1.05281229	0		20	0.439617	chr11_24333_star	2.14913986	0	1.654957834	0		20	1		chr15_32567_star@@rno-miR-375-	2.14914	3.22371	1.654957834	2.078510713		-0.58496	0.680031
hsa-miR-6500-3p	1.074569932	0	1.05281229	0		20	0.439617	chr18_36427_star@@mmu-miR-592-3p	2.14913986	0	1.654957834	0		20	1		chr10_24278_mature	2.14914	3.22371	1.654957834	2.078510713		-0.58496	0.680031
rno-miR-290	1.074569932	0	1.05281229	0		20	0.439617	mmu-miR-181b-5p	2.14913986	0	1.654957834	0		20	1		hsa-miR-520d-3p	2.14914	3.22371	1.654957834	2.078510713		-0.58496	0.680031
bta-miR-1468	1.074569932	0	1.05281229	0		20	0.439617	chr1_5_star	2.14913986	0	1.654957834	0		20	1		chr8_17595_star	2.14914	3.22371	1.654957834	2.078510713		-0.58496	0.680031
rno-miR-347	1.074569932	0	1.05281229	0		20	0.439617	chr14_31578_star	2.14913986	0	1.654957834	0		20	1		chr1_3607_star	2.14914	3.22371	1.654957834	2.078510713		-0.58496	0.680031
chr3_7082_mature	1.074569932	0	1.05281229	0		20	0.439617	chr10_23450_star	2.14913986	0	1.654957834	0		20	1		chr12_25445_mature	2.14914	3.22371	1.654957834	2.078510713		-0.58496	0.680031
chr9_19770_star	1.074569932	0	1.05281229	0		20	0.439617	chr1_2879_star	2.14913986	0	1.654957834	0		20	1		chr14_29827_mature	2.14914	3.22371	1.654957834	2.078510713		-0.58496	0.680031
chr4_9185_star	1.074569932	0	1.05281229	0		20	0.439617	chr12_26594_star	2.14913986	0	1.654957834	0		20	1		mmu-miR-5103	2.14914	3.22371	1.654957834	2.078510713		-0.58496	0.680031
chr6_13901_star	1.074569932	0	1.05281229	0		20	0.439617	hsa-miR-4787-3p	2.14913986	0	1.654957834	0		20	1		chr6_14473_star	2.14914	3.22371	1.654957834	2.078510713		-0.58496	0.680031
chrx_22097_star	1.074569932	0	1.05281229	0		20	0.439617	mmu-miR-675-3p	2.14913986	0	1.654957834	0		20	1		mmu-miR-3101-3p	2.14914	3.22371	1.654957834	2.078510713		-0.58496	0.680031
chrx_21832_star	1.074569932	0	1.05281229	0		20	0.439617	chr8_17858_star	2.14913986	0	1.654957834	0		20	1		mmu-miR-6402	2.14914	3.22371	1.654957834	2.078510713		-0.58496	0.680031
chr14_31128_mature	1.074569932	0	1.05281229	0		20	0.439617	chr7_16620_star	2.14913986	0	1.654957834	0		20	1		chr1_1411_star	2.14914	3.22371	1.654957834	2.078510713		-0.58496	0.680031
bta-miR-2319a	1.074569932	0	1.05281229	0		20	0.439617	NW_003613217_37698_star	2.14913986	0	1.654957834	0		20	1		ssc-miR-338	2.14914	3.22371	1.654957834	2.078510713		-0.58496	0.680031
hsa-miR-4436b-5p	1.074569932	0	1.05281229	0		20	0.439617	chr5_12127_star	2.14913986	0	1.654957834	0		20	1		chr6_12855_star	2.14914	3.22371	1.654957834	2.078510713		-0.58496	0.680031
hsa-miR-26a-1-3p	1.074569932	0	1.05281229	0		20	0.439617	chr8_18271_star	2.14913986	0	1.654957834	0		20	1		hsa-miR-3676-3p	2.14914	3.22371	1.654957834	2.078510713		-0.58496	0.680031
hsa-miR-338-5p	1.074569932	0	1.05281229	0		20	0.439617	chr14_30067_mature	186.975168	128.1425	7.554398282	7.012819653		0.545098	1		chr3_6750_star	2.14914	3.22371	1.654957834	2.078510713		-0.58496	0.680031
chr3_8026_mature	1.074569932	0	1.05281229	0		20	0.439617	hsa-miR-551a	2.14913986	0	1.654957834	0		20	1		chr14_31126_star	2.14914	3.22371	1.654957834	2.078510713		-0.58496	0.680031
bta-miR-2957	1.074569932	0	1.05281229	0		20	0.439617	chr18_36296_star	2.14913986	0	1.654957834	0		20	1		chr4_9423_mature	2.14914	3.22371	1.654957834	2.078510713		-0.58496	0.680031
chr8_18299_star	1.074569932	0	1.05281229	0		20	0.439617	mmu-miR-541-5p	2.14913986	0	1.654957834	0		20	1		hsa-miR-3174	2.14914	3.22371	1.654957834	2.078510713		-0.58496	0.680031
hsa-miR-4529-3p	1.074569932	0	1.05281229	0		20	0.439617	mmu-miR-3963	2.14913986	0	1.654957834	0		20	1		chr8_17569_star	2.14914	3.22371	1.654957834	2.078510713		-0.58496	0.680031
NW_003540392_39464_star	1.074569932	0	1.05281229	0		20	0.439617	mmu-miR-188-5p	2.14913986	0	1.654957834	0		20	1		chr17_35675_mature	2.14914	3.22371	1.654957834	2.078510713		-0.58496	0.680031
chr14_31499_star	1.074569932	0	1.05281229	0		20	0.439617	NW_003613497_39428_star	2.14913986	0	1.654957834	0		20	1		chr12_25994_star	2.14914	3.22371	1.654957834	2.078510713		-0.58496	0.680031
mmu-miR-669k-5p	1.074569932	0	1.05281229	0		20	0.439617	bta-miR-127	2.14913986	0	1.654957834	0		20	1		chr13_27409_mature@@bta-miR-5	2.14914	3.22371	1.654957834	2.078510713		-0.58496	0.680031
chr5_11099_star	1.074569932	0	1.05281229	0		20	0.439617	hsa-miR-138-5p	2.14913986	0	1.654957834	0		20	1		chr8_18259_star	2.14914	3.22371	1.654957834	2.078510713		-0.58496	0.680031
chr18_36377_star	1.074569932	0	1.05281229	0		20	0.439617	hsa-miR-6124	2.14913986	0	1.654957834	0		20	1		ssc-miR-376b	2.14914	3.22371	1.654957834	2.078510713		-0.58496	0.680031
chr4_9832_mature	1.074569932	0	1.05281229	0		20	0.439617	hsa-miR-6131	2.14913986	0	1.654957834	0		20	1		chr15_32658_star	2.14914	3.22371	1.654957834	2.078510713		-0.58496	0.680031
bta-miR-669	1.074569932	0	1.05281229	0		20	0.439617	NW_003540978_39774_star	2.14913986	0	1.654957834	0		20	1		chr14_30214_star	2.14914	3.22371	1.654957834	2.078510713		-0.58496	0.680031
bta-miR-2382-3p	1.074569932	0	1.05281229	0		20	0.439617	chr2_4005_star	2.14913986	0	1.654957834	0		20	1		chr15_31863_star@@bta-miR-2881	2.14914	3.22371	1.654957834	2.078510713		-0.58496	0.680031
hsa-miR-1255b-5p	1.074569932	0	1.05281229	0		20	0.439617	chr18_35822_star	2.14913986	0	1.654957834	0		20	1		hsa-miR-3678-3p	2.14914	3.22371	1.654957834	2.078510713		-0.58496	0.680031
chrx_22747_star	1.074569932	0	1.05281229	0		20	0.439617	chr17_35169_mature	2.14913986	0	1.654957834	0		20	1		chr8_18569_mature	2.14914	3.22371	1.654957834	2.078510713		-0.58496	0.680031
hsa-miR-219-5p	1.074569932	0	1.05281229	0		20	0.439617	chr5_12206_star	2.14913986	0	1.654957834	0		20	1		chr5_11842_mature	2.14914	3.22371	1.654957834	2.078510713		-0.58496	0.680031
chr7_16018_star	1.074569932	0	1.05281229	0		20	0.439617	bta-miR-1777b	2.14913986	0	1.654957834	0		20	1		chr6_12650_star	1.611855	0.805927	1.38507475	0.852739935		1	0.680133
chr14_31578_star	1.074569932	0	1.05281229	0		20	0.439617	chr6_12449_mature	2.14913986	0	1.654957834	0		20	1		chr18_36239_star	1.611855	0.805927	1.38507475	0.852739935		1	0.680133
hsa-miR-518c-3p	1.074569932	0	1.05281229	0		20	0.439617	chr13_27312_star	2.14913986	0	1.654957834	0		20	1		chr1_3261_star	1.611855	0.805927	1.38507475	0.852739935		1	0.680133
chr7_16196_star	1.074569932	0	1.05281229	0		20	0.439617	hsa-miR-205-3p	2.14913986	0	1.654957834	0		20	1		chr5_10979_mature	1.611855	0.805927	1.38507475	0.852739935		1	0.680133
chr12_26594_star	1.074569932	0	1.05281229	0		20	0.439617	bta-miR-101	2.14913986	0	1.654957834	0		20	1		chrx_23237_mature	1.611855	0.805927	1.38507475	0.852739935		1	0.680133
hsa-miR-1255b-2-3p	1.074569932	0	1.05281229	0		20	0.439617	mmu-miR-1981-5p	2.14913986	0	1.654957834	0		20	1		chr7_15541_star	1.611855	0.805927	1.38507475	0.852739935		1	0.680133
mmu-miR-15a-3p	1.074569932	0	1.05281229	0		20	0.439617	chr7_16725_star	2.14913986	0	1.654957834	0		20	1		hsa-miR-130a-5p	1.611855	0.805927	1.38507475	0.852739935		1	0.680133
chr8_17858_star	1.074569932	0	1.05281229	0		20	0.439617	hsa-miR-4740-5p	2.14913986	0	1.654957834	0		20	1		hsa-miR-23c	1.611855	0.805927	1.38507475	0.852739935		1	0.680133
chr7_16620_star	1.074569932	0	1.05281229	0		20	0.439617	mmu-miR-5134-3p	2.14913986	0	1.654957834	0		20	1		chr11_24429_star	1.611855	0.805927	1.38507475	0.852739935		1	0.680133
mmu-miR-488-3p	1.074569932	0	1.05281229	0		20	0.439617	chr7_16538_star	2.14913986	0	1.654957834	0		20	1		hsa-miR-4742-3p	1.611855	0.805927	1.38507475	0.852739935		1	0.680133
chr9_20122_star	1.074569932	0	1.05281229	0		20	0.439617	chr2_4006_mature	2.14913986	0	1.654957834	0		20	1		mmu-miR-147-5p	1.611855	0.805927	1.38507475	0.852739935		1	0.680133
chr8_18271_star	1.074569932	0	1.05281229	0		20	0.439617	hsa-miR-423-3p	2.14913986	0	1.654957834	0		20	1		chr17_35034_mature	1.611855	0.805927	1.38507475	0.852739935		1	0.680133
rno-miR-328a-5p	1.074569932	0	1.05281229	0		20	0.439617	NW_003613065_36971_star	2.14913986	0	1.654957834	0		20	1		hsa-miR-3944-3p	1.611855	0.805927	1.38507475	0.852739935		1	0.680133
hsa-miR-4753-5p	1.074569932	0	1.05281229	0		20	0.439617	chr2_4526_star	109.606133	67.69791	6.789287574	6.102194213		0.695145	1		chr15_32840_star	1.611855	0.805927	1.38507475	0.852739935		1	0.680133
chr18_36296_star	1.074569932	0	1.05281229	0		20	0.439617	chr2_5367_star	2.14913986	0	1.654957834	0		20	1		chr5_11638_star	1.611855	0.805927	1.38507475	0.852739935		1	0.680133
hsa-miR-548aj-5p	1.074569932	0	1.05281229	0		20	0.439617	mmu-miR-3058-5p	2.14913986	0	1.654957834	0		20	1		chr2_5320_star	1.611855	0.805927	1.38507475	0.852739935		1	0.680133
hsa-miR-1245a	1.074569932	0	1.05281229	0		20	0.439617	chr14_31600_mature	2.14913986	0	1.654957834	0		20	1		hsa-miR-3681-5p	1.611855	0.805927	1.38507475	0.852739935		1	0.680133
mmu-miR-3966	1.074569932	0	1.05281229	0		20	0.439617	chrx_22034_star	2.14913986	0	1.654957834	0		20	1		hsa-miR-4732-5p	1.611855	0.805927	1.38507475	0.852739935		1	0.680133
mmu-miR-188-5p	1.074569932	0	1.05281229	0		20	0.439617	chr3_8495_star	2.14913986	0	1.654957834	0		20	1		hsa-miR-3136-3p	1.611855	0.805927	1.38507475	0.852739935		1	0.680133
chr16_33810_star	1.074569932	0	1.05281229	0		20	0.439617	chr12_26455_star	2.14913986	0	1.654957834	0		20	1		hsa-miR-5589-3p	1.611855	0.805927	1.38507475	0.852739935		1	0.680133
chr14_29816_star	1.074569932	0	1.05281229	0		20	0.439617	chr13_28748_star	2.14913986	0	1.654957834	0		20	1		hsa-miR-3681-3p	1.611855	0.805927	1.38507475	0.852739935		1	0.680133
chr2_4477_mature	1.074569932	0	1.05281229	0		20	0.439617	chr8_17983_star@@mmu-miR-302b-5p	2.14913986	0	1.654957834	0		20	1		chr7_15769_star	1.611855	0.805927	1.38507475	0.852739935		1	0.680133
bta-miR-136	1.074569932	0	1.05281229	0		20	0.439617	chr14_30518_mature	2.14913986	0	1.654957834	0		20	1		chr15_33376_star	1.611855	0.805927	1.38507475	0.852739935		1	0.680133
NW_003613497_39428_star	1.074569932	0	1.05281229	0		20	0.439617	hsa-miR-27a-5p	2.14913986	0	1.654957834	0		20	1		mmu-miR-3086-3p	1.611855	0.805927	1.38507475	0.852739935		1	0.680133
hsa-miR-6124	1.074569932	0	1.05281229	0		20	0.439617	mmu-miR-3079-3p	2.14913986	0	1.654957834	0		20	1		hsa-miR-1299	1.611855	0.805927	1.38507475	0.852739935		1	0.680133
bta-miR-152	1.074569932	0	1.05281229	0		20	0.439617	chr6_12987_mature@@mmu-miR-6538	2.14913986	0	1.654957834	0		20	1		hsa-miR-1279	1.611855	0.805927	1.38507475	0.852739935		1	0.680133
hsa-miR-6131	1.074569932	0	1.05281229	0		20	0.439617	mmu-miR-669h-5p	2.14913986	0	1.654957834	0		20	1		NW_003539221_38657_star	1.611855	0.805927	1.38507475	0.852739935		1	0.680133
bta-miR-144	1.074569932	0	1.05281229	0		20	0.439617	chr2_5867_mature	15.043979	4.029637	4.003960082	2.330454351		1.900464	1		hsa-miR-562	1.611855	0.805927	1.38507475	0.852739935		1	0.680133
chr2_4005_star	1.074569932	0	1.05281229	0		20	0.439617	chr7_16234_star	2.14913986	0	1.654957834	0		20	1		bta-let-7d	1.611855	0.805927	1.38507475	0.852739935		1	0.680133
mmu-miR-149-5p	1.074569932	0	1.05281229	0		20	0.439617	chr13_27500_star	2.14913986	0	1.654957834	0		20	1		chr5_12167_star	1.611855	0.805927	1.38507475	0.852739935		1	0.680133
mmu-miR-3076-5p	1.074569932	0	1.05281229	0		20	0.439617	rno-miR-201-5p	2.14913986	0	1.654957834	0		20	1		bta-miR-196a	1.611855	0.805927	1.38507475	0.852739935		1	0.680133
hsa-miR-520c-3p	1.074569932	0	1.05281229	0		20	0.439617	chr13_27779_star	2.14913986	0	1.654957834	0		20	1		hsa-miR-507	1.611855	0.805927	1.38507475	0.852739935		1	0.680133
bta-miR-1777b	1.074569932	0	1.05281229	0		20	0.439617	rno-miR-743b-5p	2.14913986	0	1.654957834	0		20	1		hsa-miR-504	1.611855	0.805927	1.38507475	0.852739935		1	0.680133
chr14_30276_star	1.074569932	0	1.05281229	0		20	0.439617	chr6_14460_mature	2.14913986	0	1.654957834	0		20	1		chr10_23377_star	1.611855	0.805927	1.38507475	0.852739935		1	0.680133
bta-miR-101	1.074569932	0	1.05281229	0		20	0.439617	chr8_18755_star	2.14913986	0	1.654957834	0		20	1		bta-miR-2284t-5p	1.611855	0.805927	1.38507475	0.852739935		1	0.680133
chr6_12987_star	1.074569932	0	1.05281229	0		20	0.439617	NW_003538989_38414_mature	2.14913986	0	1.654957834	0		20	1		hsa-miR-3910	1.611855	0.805927	1.38507475	0.852739935		1	0.680133
mmu-miR-149-3p	1.074569932	0	1.05281229	0		20	0.439617	mmu-miR-3057-3p	2.14913986	0	1.654957834	0		20	1		chr13_28971_star	1.611855	0.805927	1.38507475	0.852739935		1	0.680133
chr9_19709_star	1.074569932	0	1.05281229	0		20	0.439617	NW_003540029_39236_star	2.14913986	0	1.654957834	0		20	1		chr2_5850_mature@@rno-miR-328	1.611855	0.805927	1.38507475	0.852739935		1	0.680133
chr10_23459_star	1.074569932	0	1.05281229	0		20	0.439617	mmu-miR-5626-3p	2.14913986	0	1.654957834	0		20	1		chr7_15451_mature	1.611855	0.805927	1.38507475	0.852739935		1	0.680133
bta-miR-182	1.074569932	0	1.05281229	0		20	0.439617	chr4_10011_mature	2.14913986	0	1.654957834	0		20	1		chr15_31808_star	1.611855	0.805927	1.38507475	0.852739935		1	0.680133
chr16_34832_star	1.074569932	0	1.05281229	0		20	0.439617	chr7_15077_star	2.14913986	0	1.654957834	0		20	1		chr13_28836_star	1.611855	0.805927	1.38507475	0.852739935		1	0.680133
chr12_26455_star	1.074569932	0	1.05281229	0		20	0.439617	bta-miR-224	2.14913986	0	1.654957834	0		20	1		chr18_36069_star	1.611855	0.805927	1.38507475	0.852739935		1	0.680133
mmu-miR-743a-3p	1.074569932	0	1.05281229	0		20	0.439617	bta-miR-223	2.14913986	0	1.654957834	0		20	1		chr3_8243_star	1.611855	0.805927	1.38507475	0.852739935		1	0.680133
chr14_30518_mature	1.074569932	0	1.05281229	0		20	0.439617	mmu-miR-3074-5p	2.14913986	0	1.654957834	0		20	1		hsa-miR-1537	1.611855	0.805927	1.38507475	0.852739935		1	0.680133
chr4_10404_star	1.074569932	0	1.05281229	0		20	0.439617	bta-miR-218	2.14913986	0	1.654957834	0		20	1		hsa-miR-518e-3p	1.611855	0.805927	1.38507475	0.852739935		1	0.680133
chr5_11381_star	1.074569932	0	1.05281229	0		20	0.439617	bta-miR-210	2.14913986	0	1.654957834	0		20	1		hsa-miR-4738-3p	1.611855	0.805927	1.38507475	0.852739935		1	0.680133
chr6_13504_star	1.074569932	0	1.05281229	0		20	0.439617	hsa-miR-548m	2.14913986	0	1.654957834	0		20	1		chr10_23892_star	1.611855	0.805927	1.38507475	0.852739935		1	0.680133
NW_003613325_38551_star	1.074569932	0	1.05281229	0		20	0.439617	chr3_8091_star	2.14913986	0	1.654957834	0		20	1		chr8_19043_star	1.611855	0.805927	1.38507475	0.852739935		1	0.680133
mmu-miR-669h-5p	1.074569932	0	1.05281229	0		20	0.439617	hsa-miR-143-5p	2.14913986	0	1.654957834	0		20	1		NW_003541084_39898_star	1.611855	0.805927	1.38507475	0.852739935		1	0.680133
chr16_33739_star	1.074569932	0	1.05281229	0		20	0.439617	chrx_22193_star	2.14913986	0	1.654957834	0		20	1		chr18_36112_star	1.611855	0.805927	1.38507475	0.852739935		1	0.680133
chr7_16234_star	1.074569932	0	1.05281229	0		20	0.439617	hsa-miR-3177-3p	2.14913986	0	1.654957834	0		20	1		chr2_6135_star	1.611855	0.805927	1.38507475	0.852739935		1	0.680133
mmu-miR-3113-3p	1.074569932	0	1.05281229	0		20	0.439617	bta-miR-29e	2.14913986	0	1.654957834	0		20	1		chr5_11847_star	1.611855	0.805927	1.38507475	0.852739935		1	0.680133
rno-miR-3559-3p	1.074569932	0	1.05281229	0		20	0.439617	mmu-miR-3572-5p	2.14913986	0	1.654957834	0		20	1		hsa-miR-525-5p	1.611855	0.805927	1.38507475	0.852739935		1	0.680133
chrx_21814_star	1.074569932	0	1.05281229	0		20	0.439617	chr12_26328_star	2.14913986	0	1.654957834	0		20	1		chr1_3915_star	1.611855	0.805927	1.38507475	0.852739935		1	0.680133
chr6_14460_mature	1.074569932	0	1.05281229	0		20	0.439617	chr11_24910_mature	2.14913986	0	1.654957834	0		20	1		chr9_19660_star	1.611855	0.805927	1.38507475	0.852739935		1	0.680133
hsa-miR-5011-3p	1.074569932	0	1.05281229	0		20	0.439617	chr1_861_star	2.14913986	0	1.654957834	0		20	1		hsa-miR-5190	1.611855	0.805927	1.38507475	0.852739935		1	0.680133
hsa-miR-544a	1.074569932	0	1.05281229	0		20	0.439617	hsa-miR-4695-5p	2.14913986	0	1.654957834	0		20	1		chr3_8637_mature	1.611855	0.805927	1.38507475	0.852739935		1	0.680133
chr1_607_star	1.074569932	0	1.05281229	0		20	0.439617	chr14_30887_star	2.14913986	0	1.654957834	0		20	1		bta-miR-130b	1.611855	0.805927	1.38507475	0.852739935		1	0.680133
NW_003613562_39706_star	1.074569932	0	1.05281229	0		20	0.439617	NW_003539513_38910_star	2.14913986	0	1.654957834	0		20	1		rno-miR-3549	1.611855	0.805927	1.38507475	0.852739935		1	0.680133
chr8_18755_star	1.074569932	0	1.05281229	0		20	0.439617	chr1_3523_star	2.14913986	0	1.654957834	0		20	1		chr15_32517_star	1.611855	0.805927	1.38507475	0.852739935		1	0.680133
hsa-miR-3605-5p	1.074569932	0	1.05281229	0		20	0.439617	NW_003537295_36819_mature	2.14913986	0	1.654957834	0		20	1		chr5_11928_star	1.611855	0.805927	1.38507475	0.852739935		1	0.680133
chr6_13396_mature	1.074569932	0	1.05281229	0		20	0.439617	mmu-miR-1190	2.14913986	0	1.654957834	0		20	1		chr17_35617_star	1.611855	0.805927	1.38507475	0.852739935		1	0.680133
chr13_28675_mature	1.074569932	0	1.05281229	0		20	0.439617	hsa-miR-4769-3p	2.14913986	0	1.654957834	0		20	1		chr1_3253_star	1.611855	0.805927	1.38507475	0.852739935		1	0.680133
chr9_19559_star	1.074569932	0	1.05281229	0		20	0.439617	chr9_20138_star	2.14913986	0	1.654957834	0		20	1		chr17_35097_star	1.611855	0.805927	1.38507475	0.852739935		1	0.680133
chr7_15077_star	1.074569932	0	1.05281229	0		20	0.439617	chr13_28533_mature	2.14913986	0	1.654957834	0		20	1		chr11_24729_star	1.611855	0.805927	1.38507475	0.852739935		1	0.680133
bta-miR-20b	1.074569932	0	1.05281229	0		20	0.439617	chr14_29447_mature	2.14913986	0	1.654957834	0		20	1		hsa-miR-3685	1.611855	0.805927	1.38507475	0.852739935		1	0.680133
bta-miR-212	1.074569932	0	1.05281229	0		20	0.439617	chr14_30036_star	2.14913986	0	1.654957834	0		20	1		chr13_28692_mature	1.611855	0.805927	1.38507475	0.852739935		1	0.680133
chr4_9149_star	1.074569932	0	1.05281229	0		20	0.439617	chrx_21839_star	2.14913986	0	1.654957834	0		20	1		chr6_14751_star	1.611855	0.805927	1.38507475	0.852739935		1	0.680133
chr1_384_star	1.074569932	0	1.05281229	0		20	0.439617	hsa-miR-550b-3p	2.14913986	0	1.654957834	0		20	1		chr2_5851_mature	1.611855	0.805927	1.38507475	0.852739935		1	0.680133
hsa-miR-6503-3p	1.074569932	0	1.05281229	0		20	0.439617	ssc-miR-4335	2.14913986	0	1.654957834	0		20	1		rno-miR-34b-3p	1.611855	0.805927	1.38507475	0.852739935		1	0.680133
rno-miR-18a-5p	1.074569932	0	1.05281229	0		20	0.439617	chr13_26759_star	2.14913986	0	1.654957834	0		20	1		hsa-miR-3200-3p	1.611855	0.805927	1.38507475	0.852739935		1	0.680133
NW_003613327_38569_star	1.074569932	0	1.05281229	0		20	0.439617	chr15_32062_star	2.14913986	0	1.654957834	0		20	1		chr17_34985_star	1.611855	0.805927	1.38507475	0.852739935		1	0.680133
chr8_18439_star	1.074569932	0	1.05281229	0		20	0.439617	hsa-miR-425-3p	2.14913986	0	1.654957834	0		20	1		rno-miR-144-3p	1.611855	0.805927	1.38507475	0.852739935		1	0.680133
chr17_35489_star	1.074569932	0	1.05281229	0		20	0.439617	hsa-miR-3161	2.14913986	0	1.654957834	0		20	1		chr11_25149_star	1.611855	0.805927	1.38507475	0.852739935		1	0.680133
chr8_18345_star	1.074569932	0	1.05281229	0		20	0.439617	hsa-miR-3166	2.14913986	0	1.654957834	0		20	1		mmu-miR-574-5p	1.611855	0.805927	1.38507475	0.852739935		1	0.680133
mmu-miR-326-5p	1.074569932	0	1.05281229	0		20	0.439617	bta-miR-421	2.14913986	0	1.654957834	0		20	1		rno-miR-344i	1.611855	0.805927	1.38507475	0.852739935		1	0.680133
rno-miR-129-5p	1.074569932	0	1.05281229	0		20	0.439617	hsa-miR-3193	2.14913986	0	1.654957834	0		20	1		chr12_25591_mature	1.611855	0.805927	1.38507475	0.852739935		1	0.680133
rno-miR-216a-5p	1.074569932	0	1.05281229	0		20	0.439617	bta-miR-433	2.14913986	0	1.654957834	0		20	1		chr9_19527_mature	1.611855	0.805927	1.38507475	0.852739935		1	0.680133
hsa-miR-132-5p	1.074569932	0	1.05281229	0		20	0.439617	hsa-miR-3181	2.14913986	0	1.654957834	0		20	1		chr7_16971_star	1.611855	0.805927	1.38507475	0.852739935		1	0.680133
chr11_24910_mature	1.074569932	0	1.05281229	0		20	0.439617	chr7_16206_mature	15.043979	4.029637	4.003960082	2.330454351		1.900464	1		rno-miR-196c-3p	1.611855	0.805927	1.38507475	0.852739935		1	0.680133
chr1_861_star	1.074569932	0	1.05281229	0		20	0.439617	hsa-miR-3182	2.14913986	0	1.654957834	0		20	1		NW_003541332_40046_mature	1.611855	0.805927	1.38507475	0.852739935		1	0.680133
chr6_13950_mature	1.074569932	0	1.05281229	0		20	0.439617	bta-miR-429	2.14913986	0	1.654957834	0		20	1		chr10_24235_star	1.611855	0.805927	1.38507475	0.852739935		1	0.680133
bta-miR-296	1.074569932	0	1.05281229	0		20	0.439617	chr15_33216_star	2.14913986	0	1.654957834	0		20	1		chr6_12449_star	1.611855	0.805927	1.38507475	0.852739935		1	0.680133
bta-miR-292	1.074569932	0	1.05281229	0		20	0.439617	bta-miR-328	2.14913986	0	1.654957834	0		20	1		mmu-miR-1966-5p	1.611855	0.805927	1.38507475	0.852739935		1	0.680133
chr14_30887_star	1.074569932	0	1.05281229	0		20	0.439617	NW_003613342_38628_star	2.14913986	0	1.654957834	0		20	1		chrx_22056_star	1.611855	0.805927	1.38507475	0.852739935		1	0.680133
bta-miR-2322-3p	1.074569932	0	1.05281229	0		20	0.439617	rno-miR-336-5p	2.14913986	0	1.654957834	0		20	1		chr2_5886_star	1.611855	0.805927	1.38507475	0.852739935		1	0.680133
chr2_5742_star	1.074569932	0	1.05281229	0		20	0.439617	chr4_10259_mature	2.14913986	0	1.654957834	0		20	1		chr6_14382_star	1.611855	0.805927	1.38507475	0.852739935		1	0.680133
NW_003539513_38910_star	1.074569932	0	1.05281229	0		20	0.439617	hsa-miR-3134	2.14913986	0	1.654957834	0		20	1		rno-miR-465-3p	1.611855	0.805927	1.38507475	0.852739935		1	0.680133
chr1_3523_star	1.074569932	0	1.05281229	0		20	0.439617	hsa-miR-3137	2.14913986	0	1.654957834	0		20	1		chr4_9602_star	1.611855	0.805927	1.38507475	0.852739935		1	0.680133
NW_003537295_36819_mature	1.074569932	0	1.05281229	0		20	0.439617	hsa-miR-3139	2.14913986	0	1.654957834	0		20	1		NW_003613310_38408_star	1.611855	0.805927	1.38507475	0.852739935		1	0.680133
chr8_17677_mature	1.074569932	0	1.05281229	0		20	0.439617	ssc-miR-139-3p	8.59655945	28.20746	3.262517265	4.868265031		-1.71425	1		chr18_36422_star	1.611855	0.805927	1.38507475	0.852739935		1	0.680133
hsa-miR-4687-3p	1.074569932	0	1.05281229	0		20	0.439617	chr9_20405_mature	6.44741959	24.17782	2.896740642	4.654081667		-1.90689	1		mmu-miR-194-1-3p	1.611855	0.805927	1.38507475	0.852739935		1	0.680133
chr9_20138_star	1.074569932	0	1.05281229	0		20	0.439617	chr14_30390_mature	2.14913986	0	1.654957834	0		20	1		chr7_15311_star	1.611855	0.805927	1.38507475	0.852739935		1	0.680133
chr13_28533_mature	1.074569932	0	1.05281229	0		20	0.439617	mmu-miR-511-3p	2.14913986	0	1.654957834	0		20	1		chr8_18418_star	1.611855	0.805927	1.38507475	0.852739935		1	0.680133
chr12_26457_star	1.074569932	0	1.05281229	0		20	0.439617	hsa-miR-4691-5p	2.14913986	0	1.654957834	0		20	1		chr3_7967_mature	1.611855	0.805927	1.38507475	0.852739935		1	0.680133
hsa-miR-299-5p	1.074569932	0	1.05281229	0		20	0.439617	mmu-miR-133a-5p	2.14913986	0	1.654957834	0		20	1		hsa-miR-3689d	1.611855	0.805927	1.38507475	0.852739935		1	0.680133
chr4_10332_mature	1.074569932	0	1.05281229	0		20	0.439617	rno-miR-296-5p	2.14913986	0	1.654957834	0		20	1		hsa-miR-676-5p	1.611855	0.805927	1.38507475	0.852739935		1	0.680133
ssc-miR-4335	1.074569932	0	1.05281229	0		20	0.439617	chr1_1940_star	2.14913986	0	1.654957834	0		20	1		chr4_9665_star	1.611855	0.805927	1.38507475	0.852739935		1	0.680133
chr13_26759_star	1.074569932	0	1.05281229	0		20	0.439617	hsa-miR-503-5p	2.14913986	0	1.654957834	0		20	1		chr1_3640_star	1.611855	0.805927	1.38507475	0.852739935		1	0.680133
chr8_17976_star	1.074569932	0	1.05281229	0		20	0.439617	hsa-miR-4259	2.14913986	0	1.654957834	0		20	1		hsa-miR-676-3p	1.611855	0.805927	1.38507475	0.852739935		1	0.680133
chr11_24688_star	1.074569932	0	1.05281229	0		20	0.439617	hsa-miR-4251	2.14913986	0	1.654957834	0		20	1		hsa-miR-4776-3p	1.611855	0.805927	1.38507475	0.852739935		1	0.680133
hsa-miR-3170	1.074569932	0	1.05281229	0		20	0.439617	bta-miR-6536	2.14913986	0	1.654957834	0		20	1		mmu-miR-876-5p	1.611855	0.805927	1.38507475	0.852739935		1	0.680133
hsa-miR-3176	1.074569932	0	1.05281229	0		20	0.439617	bta-miR-6533	2.14913986	0	1.654957834	0		20	1		NW_003613379_38761_star	1.611855	0.805927	1.38507475	0.852739935		1	0.680133
chr15_32062_star	1.074569932	0	1.05281229	0		20	0.439617	hsa-miR-1225-5p	2.14913986	0	1.654957834	0		20	1		chr9_20120_star	1.611855	0.805927	1.38507475	0.852739935		1	0.680133
chr1_2401_mature	1.074569932	0	1.05281229	0		20	0.439617	hsa-miR-4253	2.14913986	0	1.654957834	0		20	1		hsa-miR-548x-5p	1.611855	0.805927	1.38507475	0.852739935		1	0.680133
hsa-miR-149-3p	1.074569932	0	1.05281229	0		20	0.439617	hsa-miR-4256	2.14913986	0	1.654957834	0		20	1		chr12_26408_star	1.611855	0.805927	1.38507475	0.852739935		1	0.680133
chr5_11659_star	1.074569932	0	1.05281229	0		20	0.439617	hsa-miR-4264	2.14913986	0	1.654957834	0		20	1		bta-miR-6119-3p	1.611855	0.805927	1.38507475	0.852739935		1	0.680133
chr10_23498_star	1.074569932	0	1.05281229	0		20	0.439617	hsa-miR-4268	2.14913986	0	1.654957834	0		20	1		mmu-miR-6399	1.611855	0.805927	1.38507475	0.852739935		1	0.680133
hsa-miR-3164	1.074569932	0	1.05281229	0		20	0.439617	hsa-miR-4266	2.14913986	0	1.654957834	0		20	1		hsa-miR-4659a-5p	1.611855	0.805927	1.38507475	0.852739935		1	0.680133
bta-miR-421	1.074569932	0	1.05281229	0		20	0.439617	hsa-miR-4279	2.14913986	0	1.654957834	0		20	1		chr5_12123_star	1.611855	0.805927	1.38507475	0.852739935		1	0.680133
mmu-miR-363-3p	1.074569932	0	1.05281229	0		20	0.439617	hsa-miR-1225-3p	2.14913986	0	1.654957834	0		20	1		chr17_35181_star	1.611855	0.805927	1.38507475	0.852739935		1	0.680133
hsa-miR-3198	1.074569932	0	1.05281229	0		20	0.439617	bta-miR-6518	2.14913986	0	1.654957834	0		20	1		chr14_31063_star	1.611855	0.805927	1.38507475	0.852739935		1	0.680133
chr7_15294_star	1.074569932	0	1.05281229	0		20	0.439617	hsa-miR-4271	2.14913986	0	1.654957834	0		20	1		chr3_6901_mature	1.611855	0.805927	1.38507475	0.852739935		1	0.680133
hsa-miR-3182	1.074569932	0	1.05281229	0		20	0.439617	bta-miR-6519	2.14913986	0	1.654957834	0		20	1		chr1_2125_star	1.611855	0.805927	1.38507475	0.852739935		1	0.680133
bta-miR-193a-3p	1.074569932	0	1.05281229	0		20	0.439617	bta-miR-6525	2.14913986	0	1.654957834	0		20	1		chr17_35169_star	1.611855	0.805927	1.38507475	0.852739935		1	0.680133
bta-miR-328	1.074569932	0	1.05281229	0		20	0.439617	hsa-miR-4280	2.14913986	0	1.654957834	0		20	1		chr4_10432_star	1.611855	0.805927	1.38507475	0.852739935		1	0.680133
rno-miR-336-5p	1.074569932	0	1.05281229	0		20	0.439617	hsa-miR-4290	2.14913986	0	1.654957834	0		20	1		hsa-miR-888-3p	1.611855	0.805927	1.38507475	0.852739935		1	0.680133
hsa-miR-3134	1.074569932	0	1.05281229	0		20	0.439617	hsa-miR-4297	2.14913986	0	1.654957834	0		20	1		chr14_30669_star	1.611855	0.805927	1.38507475	0.852739935		1	0.680133
hsa-miR-490-3p	1.074569932	0	1.05281229	0		20	0.439617	mmu-miR-544-5p	2.14913986	0	1.654957834	0		20	1		hsa-miR-5195-5p	1.611855	0.805927	1.38507475	0.852739935		1	0.680133
chr3_6871_star	1.074569932	0	1.05281229	0		20	0.439617	bta-miR-30b-3p	2.14913986	0	1.654957834	0		20	1		chr15_33053_star	1.611855	0.805927	1.38507475	0.852739935		1	0.680133
hsa-miR-3149	1.074569932	0	1.05281229	0		20	0.439617	rno-miR-880-5p	2.14913986	0	1.654957834	0		20	1		bta-miR-660	1.611855	0.805927	1.38507475	0.852739935		1	0.680133
bta-miR-34a	1.074569932	0	1.05281229	0		20	0.439617	chr15_32372_star	2.14913986	0	1.654957834	0		20	1		bta-miR-653	1.611855	0.805927	1.38507475	0.852739935		1	0.680133
hsa-miR-1273g-5p	1.074569932	0	1.05281229	0		20	0.439617	NW_003613199_37634_star	2.14913986	0	1.654957834	0		20	1		hsa-miR-4755-5p	1.611855	0.805927	1.38507475	0.852739935		1	0.680133
chr6_12861_star	1.074569932	0	1.05281229	0		20	0.439617	mmu-miR-297a-3p	2.14913986	0	1.654957834	0		20	1		chr18_36007_mature	1.611855	0.805927	1.38507475	0.852739935		1	0.680133
hsa-miR-503-5p	1.074569932	0	1.05281229	0		20	0.439617	chr4_8677_star	2.14913986	0	1.654957834	0		20	1		mmu-miR-2861	1.611855	0.805927	1.38507475	0.852739935		1	0.680133
hsa-miR-3119	1.074569932	0	1.05281229	0		20	0.439617	chr6_14338_star	2.14913986	0	1.654957834	0		20	1		NW_003540508_39511_star	1.611855	0.805927	1.38507475	0.852739935		1	0.680133
hsa-miR-4691-3p	1.074569932	0	1.05281229	0		20	0.439617	chr6_13903_star	2.14913986	0	1.654957834	0		20	1		chr6_13263_star	1.611855	0.805927	1.38507475	0.852739935		1	0.680133
hsa-miR-4259	1.074569932	0	1.05281229	0		20	0.439617	bta-miR-24	2.14913986	0	1.654957834	0		20	1		hsa-miR-5010-5p	1.611855	0.805927	1.38507475	0.852739935		1	0.680133
bta-miR-6536	1.074569932	0	1.05281229	0		20	0.439617	hsa-miR-367-5p	2.14913986	0	1.654957834	0		20	1		chr5_11270_star	1.611855	0.805927	1.38507475	0.852739935		1	0.680133
hsa-miR-4253	1.074569932	0	1.05281229	0		20	0.439617	mmu-miR-470-3p	2.14913986	0	1.654957834	0		20	1		mmu-miR-3063-5p	1.611855	0.805927	1.38507475	0.852739935		1	0.680133
hsa-miR-4257	1.074569932	0	1.05281229	0		20	0.439617	hsa-miR-424-5p	2.14913986	0	1.654957834	0		20	1		chrx_23183_star	1.611855	0.805927	1.38507475	0.852739935		1	0.680133
mmu-miR-3073a-5p	1.074569932	0	1.05281229	0		20	0.439617	hsa-miR-1185-5p	2.14913986	0	1.654957834	0		20	1		chr8_17912_star	1.611855	0.805927	1.38507475	0.852739935		1	0.680133
chr7_15706_star	1.074569932	0	1.05281229	0		20	0.439617	mmu-miR-3100-5p	2.14913986	0	1.654957834	0		20	1		NW_003613359_38685_star	1.611855	0.805927	1.38507475	0.852739935		1	0.680133
hsa-miR-4279	1.074569932	0	1.05281229	0		20	0.439617	rno-miR-410-5p	2.14913986	0	1.654957834	0		20	1		chr11_24484_star	1.611855	0.805927	1.38507475	0.852739935		1	0.680133
mmu-miR-1b-5p	1.074569932	0	1.05281229	0		20	0.439617	rno-miR-3561-5p	2.14913986	0	1.654957834	0		20	1		hsa-miR-517c-3p	1.611855	0.805927	1.38507475	0.852739935		1	0.680133
chr6_14391_star	1.074569932	0	1.05281229	0		20	0.439617	chr15_33083_star	2.14913986	0	1.654957834	0		20	1		chr1_3926_star	1.611855	0.805927	1.38507475	0.852739935		1	0.680133
chr4_9650_star	1.074569932	0	1.05281229	0		20	0.439617	chr1_1168_star	2.14913986	0	1.654957834	0		20	1		chr15_32731_star	1.611855	0.805927	1.38507475	0.852739935		1	0.680133
hsa-miR-24-2-5p	1.074569932	0	1.05281229	0		20	0.439617	rno-miR-293-3p	2.14913986	0	1.654957834	0		20	1		ssc-miR-144	1.611855	0.805927	1.38507475	0.852739935		1	0.680133
hsa-miR-4289	1.074569932	0	1.05281229	0		20	0.439617	chr1_3765_mature	2.14913986	0	1.654957834	0		20	1		chr7_16903_mature	1.611855	0.805927	1.38507475	0.852739935		1	0.680133
hsa-miR-4287	1.074569932	0	1.05281229	0		20	0.439617	mmu-miR-302a-5p	2.14913986	15.31262	1.654957834	4.027916745		-2.83289	1		chr12_25481_star	1.611855	0.805927	1.38507475	0.852739935		1	0.680133
bta-miR-6525	1.074569932	0	1.05281229	0		20	0.439617	NW_003539626_38995_star	2.14913986	0	1.654957834	0		20	1		chrx_22664_star	1.611855	0.805927	1.38507475	0.852739935		1	0.680133
mmu-miR-1197-5p	1.074569932	0	1.05281229	0		20	0.439617	hsa-miR-5587-3p	2.14913986	0	1.654957834	0		20	1		mmu-miR-5626-5p	1.611855	0.805927	1.38507475	0.852739935		1	0.680133
mmu-miR-3108-3p	1.074569932	0	1.05281229	0		20	0.439617	chr8_17941_mature	2.14913986	0	1.654957834	0		20	1		chr13_27408_star	1.611855	0.805927	1.38507475	0.852739935		1	0.680133
hsa-miR-4299	1.074569932	0	1.05281229	0		20	0.439617	chr15_32458_star	21.4913986	7.253347	4.491301473	3.044979305		1.567041	1		chr17_34996_star	1.611855	0.805927	1.38507475	0.852739935		1	0.680133
rno-miR-880-5p	1.074569932	0	1.05281229	0		20	0.439617	hsa-miR-302f	2.14913986	0	1.654957834	0		20	1		chr13_28713_star	1.611855	0.805927	1.38507475	0.852739935		1	0.680133
hsa-miR-203a	1.074569932	0	1.05281229	0		20	0.439617	chr1_1416_star	2.14913986	0	1.654957834	0		20	1		chr14_31020_star	1.611855	0.805927	1.38507475	0.852739935		1	0.680133
NW_003613199_37634_star	1.074569932	0	1.05281229	0		20	0.439617	chr1_2783_mature	2.14913986	0	1.654957834	0		20	1		chr6_12854_star	1.611855	0.805927	1.38507475	0.852739935		1	0.680133
chr4_8677_star	1.074569932	0	1.05281229	0		20	0.439617	chr6_14032_star	2.14913986	0	1.654957834	0		20	1		chr5_10851_mature	1.611855	0.805927	1.38507475	0.852739935		1	0.680133
mmu-miR-883b-5p	1.074569932	0	1.05281229	0		20	0.439617	hsa-miR-892c-3p	2.14913986	0	1.654957834	0		20	1		chr14_29835_mature	1.611855	0.805927	1.38507475	0.852739935		1	0.680133
chr6_13903_star	1.074569932	0	1.05281229	0		20	0.439617	hsa-miR-6513-3p	2.14913986	0	1.654957834	0		20	1		chr10_24278_star	1.611855	0.805927	1.38507475	0.852739935		1	0.680133
chr15_32650_star	1.074569932	0	1.05281229	0		20	0.439617	hsa-miR-5587-5p	2.14913986	0	1.654957834	0		20	1		mmu-miR-1197-3p	1.611855	0.805927	1.38507475	0.852739935		1	0.680133
bta-miR-24	1.074569932	0	1.05281229	0		20	0.439617	hsa-miR-520g	2.14913986	0	1.654957834	0		20	1		chr13_28917_star	1.611855	0.805927	1.38507475	0.852739935		1	0.680133
chr6_14325_star	1.074569932	0	1.05281229	0		20	0.439617	mmu-miR-466l-3p	2.14913986	0	1.654957834	0		20	1		hsa-miR-422a	1.611855	0.805927	1.38507475	0.852739935		1	0.680133
mmu-miR-470-3p	1.074569932	0	1.05281229	0		20	0.439617	rno-miR-17-2-3p	2.14913986	0	1.654957834	0		20	1		mmu-miR-133a-3p	1.611855	0.805927	1.38507475	0.852739935		1	0.680133
hsa-miR-770-5p	1.074569932	0	1.05281229	0		20	0.439617	chrx_22713_star	2.14913986	0	1.654957834	0		20	1		chr5_11720_star	1.611855	0.805927	1.38507475	0.852739935		1	0.680133
hsa-miR-424-5p	1.074569932	0	1.05281229	0		20	0.439617	chr1_1486_mature	2.14913986	0	1.654957834	0		20	1		chr12_26438_mature	1.611855	0.805927	1.38507475	0.852739935		1	0.680133
chr4_10638_mature	1.074569932	0	1.05281229	0		20	0.439617	mmu-miR-499-5p	2.14913986	0	1.654957834	0		20	1		chr5_11650_star	1.611855	0.805927	1.38507475	0.852739935		1	0.680133
chr13_28544_star	1.074569932	0	1.05281229	0		20	0.439617	chr1_3501_star	2.14913986	0	1.654957834	0		20	1		chr4_9937_star	1.611855	0.805927	1.38507475	0.852739935		1	0.680133
hsa-miR-4662a-3p	1.074569932	0	1.05281229	0		20	0.439617	chr16_34314_star	2.14913986	0	1.654957834	0		20	1		hsa-miR-3132	1.611855	0.805927	1.38507475	0.852739935		1	0.680133
mmu-miR-297b-3p	1.074569932	0	1.05281229	0		20	0.439617	chr13_29417_star	2.14913986	0	1.654957834	0		20	1		mmu-miR-365-1-5p	1.611855	0.805927	1.38507475	0.852739935		1	0.680133
hsa-miR-181c-3p	1.074569932	0	1.05281229	0		20	0.439617	hsa-miR-4725-5p	2.14913986	0	1.654957834	0		20	1		hsa-miR-3138	1.611855	0.805927	1.38507475	0.852739935		1	0.680133
chr15_33083_star	1.074569932	0	1.05281229	0		20	0.439617	mmu-miR-3090-3p	2.14913986	0	1.654957834	0		20	1		bta-miR-346	1.611855	0.805927	1.38507475	0.852739935		1	0.680133
mmu-miR-3093-5p	1.074569932	0	1.05281229	0		20	0.439617	hsa-miR-296-5p	2.14913986	0	1.654957834	0		20	1		hsa-miR-3159	1.611855	0.805927	1.38507475	0.852739935		1	0.680133
hsa-miR-892c-5p	1.074569932	0	1.05281229	0		20	0.439617	mmu-miR-3090-5p	2.14913986	0	1.654957834	0		20	1		chr9_20710_star	1.611855	0.805927	1.38507475	0.852739935		1	0.680133
mmu-miR-302d-5p	1.074569932	0	1.05281229	0		20	0.439617	chr12_25892_star	2.14913986	0	1.654957834	0		20	1		bta-miR-6527	1.611855	0.805927	1.38507475	0.852739935		1	0.680133
chr8_17941_mature	1.074569932	0	1.05281229	0		20	0.439617	hsa-miR-4796-5p	2.14913986	0	1.654957834	0		20	1		hsa-miR-4296	1.611855	0.805927	1.38507475	0.852739935		1	0.680133
NW_003539151_38597_star	1.074569932	0	1.05281229	0		20	0.439617	chr14_30543_mature	2.14913986	0	1.654957834	0		20	1		chr1_2656_star	1.611855	0.805927	1.38507475	0.852739935		1	0.680133
bta-miR-2373-3p	1.074569932	0	1.05281229	0		20	0.439617	mmu-miR-3083-5p	2.14913986	0	1.654957834	0		20	1		chr18_36498_star	1.611855	0.805927	1.38507475	0.852739935		1	0.680133
mmu-miR-3077-3p	1.074569932	0	1.05281229	0		20	0.439617	hsa-miR-4639-5p	2.14913986	0	1.654957834	0		20	1		NW_003539453_38866_star	1.611855	0.805927	1.38507475	0.852739935		1	0.680133
mmu-miR-24-2-5p	1.074569932	0	1.05281229	0		20	0.439617	chr12_25400_star	2.14913986	0	1.654957834	0		20	1		chr11_24486_star	1.611855	0.805927	1.38507475	0.852739935		1	0.680133
chr8_18586_star	1.074569932	0	1.05281229	0		20	0.439617	bta-miR-2410	2.14913986	0	1.654957834	0		20	1		chrx_22698_mature	1.611855	0.805927	1.38507475	0.852739935		1	0.680133
chr6_14032_star	1.074569932	0	1.05281229	0		20	0.439617	bta-miR-2413	2.14913986	0	1.654957834	0		20	1		hsa-miR-520e	1.611855	0.805927	1.38507475	0.852739935		1	0.680133
chr3_7078_star	1.074569932	0	1.05281229	0		20	0.439617	chr3_8429_mature	2.14913986	0	1.654957834	0		20	1		chr12_26428_star	1.611855	0.805927	1.38507475	0.852739935		1	0.680133

chr16_33754_star	1.074569932	0	1.05281229	0		20	0.439617
hsa-miR-5587-5p	1.074569932	0	1.05281229	0		20	0.439617
hsa-miR-520h	1.074569932	0	1.05281229	0		20	0.439617
mmu-miR-499-3p	1.074569932	0	1.05281229	0		20	0.439617
rno-miR-208b-3p	1.074569932	0	1.05281229	0		20	0.439617
chr1_3501_star	1.074569932	0	1.05281229	0		20	0.439617
chr16_34314_star	1.074569932	0	1.05281229	0		20	0.439617
hsa-miR-4725-5p	1.074569932	0	1.05281229	0		20	0.439617
chr7_17208_mature	1.074569932	0	1.05281229	0		20	0.439617
chr15_33041_mature	1.074569932	0	1.05281229	0		20	0.439617
hsa-miR-539-5p	1.074569932	0	1.05281229	0		20	0.439617
chr12_25892_star	1.074569932	0	1.05281229	0		20	0.439617
NW_003538437_37870_star	1.074569932	0	1.05281229	0		20	0.439617
chr2_5761_star	1.074569932	0	1.05281229	0		20	0.439617
chr14_30543_mature	1.074569932	0	1.05281229	0		20	0.439617
bta-miR-2409	1.074569932	0	1.05281229	0		20	0.439617
rno-miR-489-5p	1.074569932	0	1.05281229	0		20	0.439617
bta-miR-2414	1.074569932	0	1.05281229	0		20	0.439617
bta-miR-2418	1.074569932	0	1.05281229	0		20	0.439617
hsa-miR-3619-3p	1.074569932	0	1.05281229	0		20	0.439617
chrx_23198_star	1.074569932	0	1.05281229	0		20	0.439617
rno-miR-489-3p	1.074569932	0	1.05281229	0		20	0.439617
chr11_24588_star	1.074569932	0	1.05281229	0		20	0.439617
chrx_21496_star	1.074569932	0	1.05281229	0		20	0.439617
bta-miR-2407	1.074569932	0	1.05281229	0		20	0.439617
bta-miR-2459	1.074569932	0	1.05281229	0		20	0.439617
hsa-miR-4459	1.074569932	0	1.05281229	0		20	0.439617
hsa-miR-4452	1.074569932	0	1.05281229	0		20	0.439617
bta-miR-2463	1.074569932	0	1.05281229	0		20	0.439617
hsa-miR-4465	1.074569932	0	1.05281229	0		20	0.439617
chr9_21094_star	1.074569932	0	1.05281229	0		20	0.439617
chr10_23487_star	1.074569932	0	1.05281229	0		20	0.439617
hsa-miR-4473	1.074569932	0	1.05281229	0		20	0.439617
hsa-miR-4476	1.074569932	0	1.05281229	0		20	0.439617
bta-miR-2443	1.074569932	0	1.05281229	0		20	0.439617
chr9_19426_star	1.074569932	0	1.05281229	0		20	0.439617
hsa-miR-593-3p	1.074569932	0	1.05281229	0		20	0.439617
hsa-miR-4639-3p	1.074569932	0	1.05281229	0		20	0.439617
mmu-miR-3083-3p	1.074569932	0	1.05281229	0		20	0.439617
mmu-miR-130a-5p	1.074569932	0	1.05281229	0		20	0.439617
rno-miR-878	1.074569932	0	1.05281229	0		20	0.439617
hsa-miR-4437	1.074569932	0	1.05281229	0		20	0.439617
hsa-miR-4439	1.074569932	0	1.05281229	0		20	0.439617
hsa-miR-4431	1.074569932	0	1.05281229	0		20	0.439617
mmu-miR-5622-3p	1.074569932	0	1.05281229	0		20	0.439617
mmu-miR-133c	1.074569932	0	1.05281229	0		20	0.439617
hsa-miR-602	1.074569932	0	1.05281229	0		20	0.439617
hsa-miR-600	1.074569932	0	1.05281229	0		20	0.439617
chr7_15713_mature	1.074569932	0	1.05281229	0		20	0.439617
hsa-miR-3692-3p	1.074569932	0	1.05281229	0		20	0.439617
chr7_16580_star	1.074569932	0	1.05281229	0		20	0.439617
hsa-miR-627	1.074569932	0	1.05281229	0		20	0.439617
hsa-miR-638	1.074569932	0	1.05281229	0		20	0.439617
hsa-miR-937-3p	1.074569932	0	1.05281229	0		20	0.439617
hsa-miR-631	1.074569932	0	1.05281229	0		20	0.439617
hsa-miR-4493	1.074569932	0	1.05281229	0		20	0.439617
mmu-miR-546	1.074569932	0	1.05281229	0		20	0.439617
chr14_29623_mature	1.074569932	0	1.05281229	0		20	0.439617
chr13_26824_star	1.074569932	0	1.05281229	0		20	0.439617
mmu-miR-329-5p	1.074569932	0	1.05281229	0		20	0.439617
rno-miR-872-5p	1.074569932	0	1.05281229	0		20	0.439617
mmu-miR-466o-3p	1.074569932	0	1.05281229	0		20	0.439617
hsa-miR-199a-3p	1.074569932	0	1.05281229	0		20	0.439617
chr3_7766_star	1.074569932	0	1.05281229	0		20	0.439617
hsa-miR-6509-3p	1.074569932	0	1.05281229	0		20	0.439617
chr18_36004_star	1.074569932	0	1.05281229	0		20	0.439617
NW_003537297_36825_star	1.074569932	0	1.05281229	0		20	0.439617
hsa-miR-561-5p	1.074569932	0	1.05281229	0		20	0.439617
hsa-miR-640	1.074569932	0	1.05281229	0		20	0.439617
hsa-miR-648	1.074569932	0	1.05281229	0		20	0.439617
mmu-miR-539-3p	1.074569932	0	1.05281229	0		20	0.439617
chr1_392_star	1.074569932	0	1.05281229	0		20	0.439617
chr6_15067_star	1.074569932	0	1.05281229	0		20	0.439617
chrx_22335_star	1.074569932	0	1.05281229	0		20	0.439617
hsa-miR-650	1.074569932	0	1.05281229	0		20	0.439617
hsa-miR-937-5p	1.074569932	0	1.05281229	0		20	0.439617
hsa-miR-665	1.074569932	0	1.05281229	0		20	0.439617
chr15_33619_star	1.074569932	0	1.05281229	0		20	0.439617
hsa-miR-4640-3p	1.074569932	0	1.05281229	0		20	0.439617
bta-miR-2486-5p	1.074569932	0	1.05281229	0		20	0.439617
chr18_36328_star	1.074569932	0	1.05281229	0		20	0.439617
rno-miR-183-3p	1.074569932	0	1.05281229	0		20	0.439617
chr3_7600_star	1.074569932	0	1.05281229	0		20	0.439617
chr15_33101_star	1.074569932	0	1.05281229	0		20	0.439617
hsa-miR-548t-3p	1.074569932	0	1.05281229	0		20	0.439617
chr7_16662_star	1.074569932	0	1.05281229	0		20	0.439617
hsa-miR-590-3p	1.074569932	0	1.05281229	0		20	0.439617
chr6_14759_star	1.074569932	0	1.05281229	0		20	0.439617
chr9_20723_star	1.074569932	0	1.05281229	0		20	0.439617
chr13_28155_star	1.074569932	0	1.05281229	0		20	0.439617
chr15_31962_star	1.074569932	0	1.05281229	0		20	0.439617
bta-miR-2304	1.074569932	0	1.05281229	0		20	0.439617
bta-miR-2306	1.074569932	0	1.05281229	0		20	0.439617
chr2_6128_star	1.074569932	0	1.05281229	0		20	0.439617
bta-miR-2311	1.074569932	0	1.05281229	0		20	0.439617
bta-miR-2314	1.074569932	0	1.05281229	0		20	0.439617
rno-miR-186-5p	1.074569932	0	1.05281229	0		20	0.439617
bta-miR-2336	1.074569932	0	1.05281229	0		20	0.439617
bta-miR-2341	1.074569932	0	1.05281229	0		20	0.439617
bta-miR-2342	1.074569932	0	1.05281229	0		20	0.439617
hsa-miR-4315	1.074569932	0	1.05281229	0		20	0.439617
bta-miR-2358	1.074569932	0	1.05281229	0		20	0.439617
hsa-miR-5585-3p	1.074569932	0	1.05281229	0		20	0.439617
hsa-miR-3617-5p	1.074569932	0	1.05281229	0		20	0.439617
bta-miR-2364	1.074569932	0	1.05281229	0		20	0.439617
hsa-miR-590-5p	1.074569932	0	1.05281229	0		20	0.439617
bta-miR-2375	1.074569932	0	1.05281229	0		20	0.439617
bta-miR-2386	1.074569932	0	1.05281229	0		20	0.439617
bta-miR-2415-3p	1.074569932	0	1.05281229	0		20	0.439617
mmu-miR-3472	1.074569932	0	1.05281229	0		20	0.439617
mmu-miR-687	1.074569932	0	1.05281229	0		20	0.439617
mmu-miR-688	1.074569932	0	1.05281229	0		20	0.439617
mmu-miR-697	1.074569932	0	1.05281229	0		20	0.439617
hsa-miR-548ah-5p	1.074569932	0	1.05281229	0		20	0.439617
mmu-miR-542-3p	1.074569932	0	1.05281229	0		20	0.439617
mmu-miR-20b-3p	1.074569932	0	1.05281229	0		20	0.439617
hsa-miR-345-3p	1.074569932	0	1.05281229	0		20	0.439617
bta-miR-2294	1.074569932	0	1.05281229	0		20	0.439617
mmu-miR-466j	1.074569932	0	1.05281229	0		20	0.439617
mmu-miR-466k	1.074569932	0	1.05281229	0		20	0.439617
chr2_5756_star	1.074569932	0	1.05281229	0		20	0.439617
rno-miR-20b-5p	1.074569932	0	1.05281229	0		20	0.439617
NW_003541081_39894_mature	1.074569932	0	1.05281229	0		20	0.439617
NW_003539696_39032_star	1.074569932	0	1.05281229	0		20	0.439617
chr10_23277_star	1.074569932	0	1.05281229	0		20	0.439617
chr12_26001_star	1.074569932	0	1.05281229	0		20	0.439617
mmu-miR-670-3p	1.074569932	0	1.05281229	0		20	0.439617
chr1_408_star	1.074569932	0	1.05281229	0		20	0.439617
mmu-miR-5621-5p	1.074569932	0	1.05281229	0		20	0.439617
hsa-miR-6508-5p	1.074569932	0	1.05281229	0		20	0.439617
chr2_3992_star	1.074569932	0	1.05281229	0		20	0.439617
chr17_34956_star	1.074569932	0	1.05281229	0		20	0.439617
hsa-miR-4446-5p	1.074569932	0	1.05281229	0		20	0.439617
hsa-miR-765	1.074569932	0	1.05281229	0		20	0.439617
mmu-miR-5621-3p	1.074569932	0	1.05281229	0		20	0.439617
chr9_19638_mature	1.074569932	0	1.05281229	0		20	0.439617
hsa-miR-1269a	1.074569932	0	1.05281229	0		20	0.439617
chr1_3355_star	1.074569932	0	1.05281229	0		20	0.439617
hsa-miR-6511b-5p	1.074569932	0	1.05281229	0		20	0.439617
chr8_17361_star	1.074569932	0	1.05281229	0		20	0.439617
mmu-miR-192-5p	1.074569932	0	1.05281229	0		20	0.439617
mmu-miR-503-5p	1.074569932	0	1.05281229	0		20	0.439617
chr13_27898_star	1.074569932	0	1.05281229	0		20	0.439617
bta-miR-545-5p	1.074569932	0	1.05281229	0		20	0.439617
chr1_1748_star	1.074569932	0	1.05281229	0		20	0.439617
rno-miR-291a-3p	1.074569932	0	1.05281229	0		20	0.439617
chr17_35646_star	1.074569932	0	1.05281229	0		20	0.439617
hsa-miR-125a-3p	1.074569932	0	1.05281229	0		20	0.439617
bta-miR-545-3p	1.074569932	0	1.05281229	0		20	0.439617
hsa-miR-5704	1.074569932	0	1.05281229	0		20	0.439617
hsa-miR-5703	1.074569932	0	1.05281229	0		20	0.439617
chr14_30543_star	1.074569932	0	1.05281229	0		20	0.439617
NW_003538061_37480_mature	1.074569932	0	1.05281229	0		20	0.439617
hsa-miR-4669	1.074569932	0	1.05281229	0		20	0.439617
hsa-miR-4675	1.074569932	0	1.05281229	0		20	0.439617
chr1_2659_star	1.074569932	0	1.05281229	0		20	0.439617
chr9_19752_star	1.074569932	0	1.05281229	0		20	0.439617
mmu-miR-181d-5p	1.074569932	0	1.05281229	0		20	0.439617
hsa-miR-5739	1.074569932	0	1.05281229	0		20	0.439617
chr15_33182_star	1.074569932	0	1.05281229	0		20	0.439617
hsa-miR-4692	1.074569932	0	1.05281229	0		20	0.439617
chrx_21544_mature	1.074569932	0	1.05281229	0		20	0.439617
NW_003538867_38314_star	1.074569932	0	1.05281229	0		20	0.439617
rno-miR-3558-3p	1.074569932	0	1.05281229	0		20	0.439617
mmu-miR-290-3p	1.074569932	0	1.05281229	0		20	0.439617
chr8_18444_mature	1.074569932	0	1.05281229	0		20	0.439617
mmu-miR-6541	1.074569932	0	1.05281229	0		20	0.439617
mmu-miR-713	1.074569932	0	1.05281229	0		20	0.439617
mmu-miR-711	1.074569932	0	1.05281229	0		20	0.439617
chr17_35278_star	1.074569932	0	1.05281229	0		20	0.439617
hsa-miR-4793-5p	1.074569932	0	1.05281229	0		20	0.439617
chr13_28998_star	1.074569932	0	1.05281229	0		20	0.439617
mmu-miR-106a-5p	1.074569932	0	1.05281229	0		20	0.439617
bta-miR-345-5p	1.074569932	0	1.05281229	0		20	0.439617
chr14_31524_star	1.074569932	0	1.05281229	0		20	0.439617
chr9_19516_star	1.074569932	0	1.05281229	0		20	0.439617
rno-miR-22-5p	1.074569932	0	1.05281229	0		20	0.439617
rno-miR-223-3p	1.074569932	0	1.05281229	0		20	0.439617
mmu-miR-762	1.074569932	0	1.05281229	0		20	0.439617
mmu-miR-767	1.074569932	0	1.05281229	0		20	0.439617
hsa-miR-3135a	1.074569932	0	1.05281229	0		20	0.439617
mmu-miR-1933-5p	1.074569932	0	1.05281229	0		20	0.439617
chr1_2829_star	1.074569932	0	1.05281229	0		20	0.439617
mmu-miR-125b-5p	1.074569932	0	1.05281229	0		20	0.439617
chr7_16298_star	1.074569932	0	1.05281229	0		20	0.439617
chr16_34140_mature	1.074569932	0	1.05281229	0		20	0.439617
ssc-miR-490-5p	1.074569932	0	1.05281229	0		20	0.439617
chr3_8450_star	1.074569932	0	1.05281229	0		20	0.439617
hsa-miR-891a	1.074569932	0	1.05281229	0		20	0.439617
chr15_32358_star	1.074569932	0	1.05281229	0		20	0.439617
hsa-miR-892b	1.074569932	0	1.05281229	0		20	0.439617
chr2_4612_star	1.074569932	0	1.05281229	0		20	0.439617
hsa-miR-1233-1-5p	1.074569932	0	1.05281229	0		20	0.439617
rno-miR-883-5p	1.074569932	0	1.05281229	0		20	0.439617
hsa-miR-3942-5p	1.074569932	0	1.05281229	0		20	0.439617
chr14_30427_star	1.074569932	0	1.05281229	0		20	0.439617
mmu-miR-669i	1.074569932	0	1.05281229	0		20	0.439617
NW_003537639_37085_star	1.074569932	0	1.05281229	0		20	0.439617
rno-miR-152-5p	1.074569932	0	1.05281229	0		20	0.439617
chr9_19718_star	1.074569932	0	1.05281229	0		20	0.439617
NW_003538598_38024_star	1.074569932	0	1.05281229	0		20	0.439617
hsa-miR-4523	1.074569932	0	1.05281229	0		20	0.439617
chr18_36298_star	1.074569932	0	1.05281229	0		20	0.439617
hsa-miR-4521	1.074569932	0	1.05281229	0		20	0.439617
bta-miR-2313-3p	1.074569932	0	1.05281229	0		20	0.439617
rno-miR-141-5p	1.074569932	0	1.05281229	0		20	0.439617
chr16_33778_star	1.074569932	0	1.05281229	0		20	0.439617
bta-miR-362-3p	1.074569932	0	1.05281229	0		20	0.439617
chr18_36331_mature	1.074569932	0	1.05281229	0		20	0.439617
chr1_2451_star	1.074569932	0	1.05281229	0		20	0.439617
rno-miR-2964	1.074569932	0	1.05281229	0		20	0.439617
chr16_34408_mature	1.074569932	0	1.05281229	0		20	0.439617
hsa-miR-451b	1.074569932	0	1.05281229	0		20	0.439617
hsa-miR-1273f	1.074569932	0	1.05281229	0		20	0.439617
bta-miR-2328-5p	1.074569932	0	1.05281229	0		20	0.439617
mmu-miR-802-5p	1.074569932	0	1.05281229	0		20	0.439617
chr12_26547_star	1.074569932	0	1.05281229	0		20	0.439617
bta-miR-3602	1.074569932	0	1.05281229	0		20	0.439617
chr9_20355_star	1.074569932	0	1.05281229	0		20	0.439617
chr7_16933_star	1.074569932	0	1.05281229	0		20	0.439617
mmu-miR-802-3p	1.074569932	0	1.05281229	0		20	0.439617
chr7_15541_mature@@bta-miR-230	1.074569932	0	1.05281229	0		20	0.439617
hsa-miR-625-3p	1.074569932	0	1.05281229	0		20	0.439617
mmu-miR-465c-3p	1.074569932	0	1.05281229	0		20	0.439617
hsa-miR-1909-3p	1.074569932	0	1.05281229	0		20	0.439617
mmu-miR-882	1.074569932	0	1.05281229	0		20	0.439617
mmu-miR-1193-3p	1.074569932	0	1.05281229	0		20	0.439617
mmu-miR-155-5p	1.074569932	0	1.05281229	0		20	0.439617
hsa-miR-1909-5p	1.074569932	0	1.05281229	0		20	0.439617
hsa-miR-936	1.074569932	0	1.05281229	0		20	0.439617
bta-miR-2471-5p	1.074569932	0	1.05281229	0		20	0.439617
mmu-miR-466h-3p	1.074569932	0	1.05281229	0		20	0.439617
hsa-miR-302b-3p	1.074569932	0	1.05281229	0		20	0.439617
chr1_2401_star	1.074569932	0	1.05281229	0		20	0.439617
chr2_4918_mature	1.074569932	0	1.05281229	0		20	0.439617
hsa-miR-708-3p	1.074569932	0	1.05281229	0		20	0.439617
mmu-miR-421-5p	1.074569932	0	1.05281229	0		20	0.439617
hsa-miR-664b-5p	1.074569932	0	1.05281229	0		20	0.439617
chr10_24226_star	1.074569932	0	1.05281229	0		20	0.439617
hsa-miR-6078	1.074569932	0	1.05281229	0		20	0.439617
chr5_11637_star	1.074569932	0	1.05281229	0		20	0.439617
mmu-miR-491-5p	1.074569932	0	1.05281229	0		20	0.439617
chr2_5291_star	1.074569932	0	1.05281229	0		20	0.439617
hsa-miR-506-5p	1.074569932	0	1.05281229	0		20	0.439617
chr1_3371_star	1.074569932	0	1.05281229	0		20	0.439617
chr11_25086_star	1.074569932	0	1.05281229	0		20	0.439617
chr5_11407_star	1.074569932	0	1.05281229	0		20	0.439617
chr2_4255_star	1.074569932	0	1.05281229	0		20	0.439617
mmu-miR-2183	1.074569932	0	1.05281229	0		20	0.439617
NW_003539350_38744_mature	1.074569932	0	1.05281229	0		20	0.439617
chr6_13734_mature	1.074569932	0	1.05281229	0		20	0.439617
chrx_21606_star	1.074569932	0	1.05281229	0		20	0.439617
chr12_26154_star	1.074569932	0	1.05281229	0		20	0.439617
rno-miR-664-1-5p	1.074569932	0	1.05281229	0		20	0.439617
chr13_29017_star	1.074569932	0	1.05281229	0		20	0.439617
NW_003537105_36696_star	3.223709795	1.611855	2.078510713	1.38507475		1	0.440959
mmu-miR-28c	3.223709795	1.611855	2.078510713	1.38507475		1	0.440959
chr6_13990_mature	3.223709795	1.611855	2.078510713	1.38507475		1	0.440959
chr10_23455_star	3.223709795	1.611855	2.078510713	1.38507475		1	0.440959
chr6_14951_mature	3.223709795	1.611855	2.078510713	1.38507475		1	0.440959
chr2_5906_star	3.223709795	1.611855	2.078510713	1.38507475		1	0.440959
chr1_915_star	3.223709795	1.611855	2.078510713	1.38507475		1	0.440959
chr13_26740_star	3.223709795	1.611855	2.078510713	1.38507475		1	0.440959
hsa-miR-4419a	3.223709795	1.611855	2.078510713	1.38507475		1	0.440959
chr4_9733_star	3.223709795	1.611855	2.078510713	1.38507475		1	0.440959

bta-miR-2426	2.14913986	0	1.654957834	0		20	1	
chr6_12597_star	2.14913986	0	1.654957834	0		20	1	
NW_003613359_38685_mature	2.14913986	0	1.654957834	0		20	1	
hsa-miR-5003-3p	2.14913986	0	1.654957834	0		20	1	
chr13_28482_star	2.14913986	0	1.654957834	0		20	1	
chrx_21496_star	2.14913986	0	1.654957834	0		20	1	
chr1_1312_star	2.14913986	0	1.654957834	0		20	1	
hsa-miR-4459	2.14913986	0	1.654957834	0		20	1	
chr1_1628_mature	2.14913986	0	1.654957834	0		20	1	
hsa-miR-4452	2.14913986	0	1.654957834	0		20	1	
bta-miR-2469	2.14913986	0	1.654957834	0		20	1	
hsa-miR-4467	2.14913986	0	1.654957834	0		20	1	
mmu-miR-5627-3p	2.14913986	0	1.654957834	0		20	1	
chr13_27034_star	2.14913986	0	1.654957834	0		20	1	
chr9_21094_star	2.14913986	0	1.654957834	0		20	1	
hsa-miR-4470	2.14913986	0	1.654957834	0		20	1	
hsa-miR-4475	2.14913986	0	1.654957834	0		20	1	
mmu-miR-3069-5p	2.14913986	0	1.654957834	0		20	1	
chr9_19426_star	2.14913986	0	1.654957834	0		20	1	
hsa-miR-4487	2.14913986	0	1.654957834	0		20	1	
chr1_1940_mature	2.14913986	0	1.654957834	0		20	1	
chr15_32385_star	2.14913986	0	1.654957834	0		20	1	
mmu-miR-3083-3p	2.14913986	0	1.654957834	0		20	1	
rno-miR-878	2.14913986	0	1.654957834	0		20	1	
hsa-miR-4427	2.14913986	0	1.654957834	0		20	1	
hsa-miR-4438	2.14913986	0	1.654957834	0		20	1	
hsa-miR-4435	2.14913986	0	1.654957834	0		20	1	
hsa-miR-4431	2.14913986	0	1.654957834	0		20	1	
chr6_12627_star	2.14913986	0	1.654957834	0		20	1	
hsa-miR-4447	2.14913986	0	1.654957834	0		20	1	
rno-miR-146b-5p	2.14913986	0	1.654957834	0		20	1	
hsa-miR-1295b-5p	2.14913986	0	1.654957834	0		20	1	
chr7_15713_mature	2.14913986	0	1.654957834	0		20	1	
hsa-miR-613	2.14913986	0	1.654957834	0		20	1	
hsa-miR-619	2.14913986	0	1.654957834	0		20	1	
bta-miR-2394	2.14913986	0	1.654957834	0		20	1	
mmu-miR-3072-5p	2.14913986	0	1.654957834	0		20	1	
hsa-miR-4490	2.14913986	0	1.654957834	0		20	1	
hsa-miR-4492	2.14913986	0	1.654957834	0		20	1	
mmu-miR-546	2.14913986	0	1.654957834	0		20	1	
chr14_29623_mature	2.14913986	0	1.654957834	0		20	1	
hsa-miR-4632-5p	2.14913986	0	1.654957834	0		20	1	
hsa-miR-26b-3p	2.14913986	0	1.654957834	0		20	1	
chr3_8555_star	2.14913986	0	1.654957834	0		20	1	
NW_003541083_39895_star	2.14913986	0	1.654957834	0		20	1	
chr7_16134_star	2.14913986	0	1.654957834	0		20	1	
bta-miR-2329-5p	2.14913986	0	1.654957834	0		20	1	
chr16_34626_star	2.14913986	15.31262	1.654957834	4.027916745		-2.83289	1	
chr13_29206_star	2.14913986	0	1.654957834	0		20	1	
chr7_16969_star	2.14913986	0	1.654957834	0		20	1	
hsa-miR-640	2.14913986	0	1.654957834	0		20	1	
mmu-miR-1948-3p	2.14913986	0	1.654957834	0		20	1	
mmu-miR-5623-5p	2.14913986	0	1.654957834	0		20	1	
hsa-miR-646	2.14913986	0	1.654957834	0		20	1	
hsa-miR-647	2.14913986	0	1.654957834	0		20	1	
chr6_15067_star	2.14913986	0	1.654957834	0		20	1	
chr13_27643_star	2.14913986	0	1.654957834	0		20	1	
hsa-miR-937-5p	2.14913986	0	1.654957834	0		20	1	
chr4_10241_mature	2.14913986	0	1.654957834	0		20	1	
hsa-miR-665	2.14913986	0	1.654957834	0		20	1	
chr4_9118_mature	2.14913986	0	1.654957834	0		20	1	
chr7_16662_star	2.14913986	0	1.654957834	0		20	1	
chr7_16673_star	2.14913986	0	1.654957834	0		20	1	
chr6_14759_star	2.14913986	0	1.654957834	0		20	1	
hsa-miR-3124-3p	2.14913986	0	1.654957834	0		20	1	
chr9_20723_star	2.14913986	0	1.654957834	0		20	1	
hsa-miR-6508-3p	2.14913986	0	1.654957834	0		20	1	
hsa-miR-548a-5p	2.14913986	0	1.654957834	0		20	1	
chr15_31962_star	2.14913986	0	1.654957834	0		20	1	
chr13_28432_mature	2.14913986	0	1.654957834	0		20	1	
chr5_11369_star	2.14913986	0	1.654957834	0		20	1	
chr17_35521_star	2.14913986	0	1.654957834	0		20	1	
hsa-miR-3124-5p	2.14913986	0	1.654957834	0		20	1	
bta-miR-2304	2.14913986	0	1.654957834	0		20	1	
bta-miR-2306	2.14913986	0	1.654957834	0		20	1	
hsa-miR-1295a	2.14913986	0	1.654957834	0		20	1	
rno-miR-186-5p	2.14913986	0	1.654957834	0		20	1	
bta-miR-2321	2.14913986	0	1.654957834	0		20	1	
bta-miR-2327	2.14913986	0	1.654957834	0		20	1	
chr2_5951_star	2.14913986	0	1.654957834	0		20	1	
rno-miR-493-5p	2.14913986	0	1.654957834	0		20	1	
bta-miR-2339	2.14913986	0	1.654957834	0		20	1	
bta-miR-22-5p	2.14913986	0	1.654957834	0		20	1	
chr13_28409_star	2.14913986	0	1.654957834	0		20	1	
chr15_31871_star	2.14913986	0	1.654957834	0		20	1	
hsa-miR-4325	2.14913986	0	1.654957834	0		20	1	
hsa-miR-4327	2.14913986	0	1.654957834	0		20	1	
chr2_4097_mature	2.14913986	0	1.654957834	0		20	1	
bta-miR-2379	2.14913986	0	1.654957834	0		20	1	
hsa-miR-4301	2.14913986	0	1.654957834	0		20	1	
hsa-miR-4302	2.14913986	0	1.654957834	0		20	1	
mmu-miR-3474	2.14913986	0	1.654957834	0		20	1	
mmu-miR-678	2.14913986	0	1.654957834	0		20	1	
chr12_25405_star	2.14913986	0	1.654957834	0		20	1	
chr12_26555_mature	2.14913986	0	1.654957834	0		20	1	
mmu-miR-687	2.14913986	0	1.654957834	0		20	1	
chr11_24937_star	2.14913986	0	1.654957834	0		20	1	
mmu-miR-20b-3p	2.14913986	0	1.654957834	0		20	1	
bta-miR-2292	2.14913986	0	1.654957834	0		20	1	
bta-miR-2294	2.14913986	0	1.654957834	0		20	1	
chr2_5756_star	2.14913986	0	1.654957834	0		20	1	
chr4_9932_star	2.14913986	0	1.654957834	0		20	1	
NW_003541081_39894_mature	2.14913986	0	1.654957834	0		20	1	
chr16_34426_star	2.14913986	0	1.654957834	0		20	1	
chr3_8361_star	2.14913986	0	1.654957834	0		20	1	
NW_003539696_39032_star	2.14913986	0	1.654957834	0		20	1	
chrx_22063_mature	2.14913986	0	1.654957834	0		20	1	
chr11_24876_star	2.14913986	0	1.654957834	0		20	1	
chr2_3992_star	2.14913986	0	1.654957834	0		20	1	
bta-miR-2415-5p	2.14913986	0	1.654957834	0		20	1	
chr1_1174_star	2.14913986	0	1.654957834	0		20	1	
mmu-miR-5616-5p	2.14913986	0	1.654957834	0		20	1	
chr6_14334_star	2.14913986	0	1.654957834	0		20	1	
mmu-miR-192-5p	2.14913986	0	1.654957834	0		20	1	
rno-miR-291a-5p	2.14913986	0	1.654957834	0		20	1	
hsa-miR-939-3p	2.14913986	0	1.654957834	0		20	1	
chr5_11639_star	2.14913986	0	1.654957834	0		20	1	
hsa-miR-125a-5p	2.14913986	0	1.654957834	0		20	1	
bta-miR-545-5p	2.14913986	0	1.654957834	0		20	1	
hsa-miR-125a-3p	2.14913986	0	1.654957834	0		20	1	
mmu-miR-706	2.14913986	0	1.654957834	0		20	1	
hsa-miR-5708	2.14913986	0	1.654957834	0		20	1	
hsa-miR-5704	2.14913986	0	1.654957834	0		20	1	
hsa-miR-4658	2.14913986	0	1.654957834	0		20	1	
hsa-miR-5703	2.14913986	0	1.654957834	0		20	1	
chr14_30543_star	2.14913986	0	1.654957834	0		20	1	
NW_003538061_37480_mature	2.14913986	0	1.654957834	0		20	1	
hsa-miR-4672	2.14913986	0	1.654957834	0		20	1	
hsa-miR-4674	2.14913986	0	1.654957834	0		20	1	
chr8_18187_mature	2.14913986	0	1.654957834	0		20	1	
hsa-miR-4681	2.14913986	0	1.654957834	0		20	1	
chr17_35007_mature	12.8948392	35.46081	3.796477232	5.188274618		-1.45943	1	
chrx_21544_mature	2.14913986	0	1.654957834	0		20	1	
mmu-miR-1894-5p	2.14913986	0	1.654957834	0		20	1	
rno-miR-3558-3p	2.14913986	0	1.654957834	0		20	1	
mmu-miR-1894-3p	2.14913986	0	1.654957834	0		20	1	
mmu-miR-711	2.14913986	0	1.654957834	0		20	1	
rno-miR-3573-3p	2.14913986	0	1.654957834	0		20	1	
NW_003538681_38110_star	2.14913986	0	1.654957834	0		20	1	
rno-miR-223-5p	2.14913986	0	1.654957834	0		20	1	
chr13_26825_star	2.14913986	0	1.654957834	0		20	1	
chr4_8977_star	2.14913986	0	1.654957834	0		20	1	
chr14_30639_star	2.14913986	0	1.654957834	0		20	1	
chrx_21682_star	2.14913986	0	1.654957834	0		20	1	
mmu-miR-3473e	2.14913986	0	1.654957834	0		20	1	
chr3_8631_star	2.14913986	0	1.654957834	0		20	1	
chr3_8646_star	2.14913986	0	1.654957834	0		20	1	
chr10_23883_mature	2.14913986	0	1.654957834	0		20	1	
bta-miR-216b	2.14913986	0	1.654957834	0		20	1	
chr3_8640_star	2.14913986	0	1.654957834	0		20	1	
hsa-miR-373-5p	2.14913986	0	1.654957834	0		20	1	
chr5_11037_star	2.14913986	0	1.654957834	0		20	1	
hsa-miR-4655-3p	2.14913986	0	1.654957834	0		20	1	
chr15_31760_star	2.14913986	0	1.654957834	0		20	1	
chr10_23301_star	2.14913986	0	1.654957834	0		20	1	
hsa-miR-890	2.14913986	0	1.654957834	0		20	1	
rno-miR-3551-3p	2.14913986	0	1.654957834	0		20	1	
mmu-miR-186-3p	2.14913986	0	1.654957834	0		20	1	
chr1_623_star	2.14913986	0	1.654957834	0		20	1	
chr1_3436_star	2.14913986	0	1.654957834	0		20	1	
chr9_19210_star	2.14913986	0	1.654957834	0		20	1	
chr3_8064_star	2.14913986	0	1.654957834	0		20	1	
mmu-let-7a-5p	2.14913986	0	1.654957834	0		20	1	
mmu-miR-742-3p	2.14913986	0	1.654957834	0		20	1	
chr15_31760_mature	2.14913986	0	1.654957834	0		20	1	
chr9_20843_star	2.14913986	0	1.654957834	0		20	1	
hsa-miR-1233-1-5p	2.14913986	0	1.654957834	0		20	1	
chr4_10714_star	2.14913986	0	1.654957834	0		20	1	
rno-miR-883-5p	2.14913986	0	1.654957834	0		20	1	
chr5_11262_star	2.14913986	0	1.654957834	0		20	1	
hsa-miR-5689	2.14913986	0	1.654957834	0		20	1	
chr11_25077_star	2.14913986	0	1.654957834	0		20	1	
chr11_25073_star	2.14913986	0	1.654957834	0		20	1	
mmu-miR-1264-3p	2.14913986	0	1.654957834	0		20	1	
hsa-miR-5687	2.14913986	0	1.654957834	0		20	1	
mmu-miR-669i	2.14913986	0	1.654957834	0		20	1	
hsa-miR-5693	2.14913986	0	1.654957834	0		20	1	
NW_003613109_37254_mature	2.14913986	0	1.654957834	0		20	1	
chr7_17077_mature	19.3422588	45.93786	4.346407978	5.552680304		-1.24793	1	
mmu-miR-669l-3p	2.14913986	0	1.654957834	0		20	1	
chr9_19718_star	2.14913986	0	1.654957834	0		20	1	
hsa-miR-4756-5p	2.14913986	0	1.654957834	0		20	1	
bta-miR-2313-3p	2.14913986	0	1.654957834	0		20	1	
bta-miR-362-5p	2.14913986	0	1.654957834	0		20	1	
chr7_15085_star	2.14913986	0	1.654957834	0		20	1	
chr16_33778_star	2.14913986	0	1.654957834	0		20	1	
chr16_34606_mature	2.14913986	0	1.654957834	0		20	1	
hsa-miR-4504	2.14913986	0	1.654957834	0		20	1	
chr1_2451_star	2.14913986	0	1.654957834	0		20	1	
rno-miR-2964	2.14913986	0	1.654957834	0		20	1	
hsa-miR-451b	2.14913986	0	1.654957834	0		20	1	
hsa-miR-4539	2.14913986	0	1.654957834	0		20	1	
hsa-miR-4537	2.14913986	0	1.654957834	0		20	1	
chr1_1835_star	2.14913986	0	1.654957834	0		20	1	
chr9_20355_star	2.14913986	0	1.654957834	0		20	1	
chr7_16660_star	2.14913986	0	1.654957834	0		20	1	
chr7_15541_mature@@bta-miR-2305	2.14913986	0	1.654957834	0		20	1	
chr13_28421_star	2.14913986	0	1.654957834	0		20	1	
NW_003539426_38819_star	2.14913986	0	1.654957834	0		20	1	
mmu-miR-741-3p	2.14913986	0	1.654957834	0		20	1	
chr4_10161_mature	2.14913986	0	1.654957834	0		20	1	
NW_003539820_39117_star	2.14913986	0	1.654957834	0		20	1	
hsa-miR-574-3p	2.14913986	0	1.654957834	0		20	1	
hsa-miR-1976	2.14913986	0	1.654957834	0		20	1	
mmu-miR-1193-3p	2.14913986	0	1.654957834	0		20	1	
mmu-miR-155-5p	2.14913986	0	1.654957834	0		20	1	
chr5_11528_star	2.14913986	0	1.654957834	0		20	1	
bta-miR-2471-5p	2.14913986	0	1.654957834	0		20	1	
chr1_1242_mature	25.7896784	9.671129	4.743605355	3.415640968		1.415037	1	
chr1_3718_star	2.14913986	0	1.654957834	0		20	1	
hsa-miR-1229-3p	2.14913986	0	1.654957834	0		20	1	
hsa-miR-6083	2.14913986	0	1.654957834	0		20	1	
hsa-miR-6082	2.14913986	0	1.654957834	0		20	1	
hsa-miR-6086	2.14913986	0	1.654957834	0		20	1	
chr2_4918_mature	2.14913986	0	1.654957834	0		20	1	
hsa-miR-708-3p	2.14913986	0	1.654957834	0		20	1	
NW_003613395_38900_star	2.14913986	0	1.654957834	0		20	1	
hsa-miR-6072	2.14913986	0	1.654957834	0		20	1	
hsa-miR-6075	2.14913986	0	1.654957834	0		20	1	
hsa-miR-4699-5p	2.14913986	0	1.654957834	0		20	1	
chr4_9118_star	2.14913986	0	1.654957834	0		20	1	
chrx_22200_mature	2.14913986	0	1.654957834	0		20	1	
chr2_5291_star	2.14913986	0	1.654957834	0		20	1	
hsa-miR-506-5p	2.14913986	0	1.654957834	0		20	1	
chr10_23649_star	2.14913986	0	1.654957834	0		20	1	
chr5_11407_star	2.14913986	0	1.654957834	0		20	1	
chr2_4255_star	2.14913986	0	1.654957834	0		20	1	
mmu-miR-2183	2.14913986	0	1.654957834	0		20	1	
bta-miR-1388-3p	2.14913986	0	1.654957834	0		20	1	
chr4_8705_mature	2.14913986	0	1.654957834	0		20	1	
chr1_2918_mature	2.14913986	0	1.654957834	0		20	1	
chr8_18597_mature	2.14913986	0	1.654957834	0		20	1	
chr9_20430_mature	2.14913986	14.50669	1.654957834	3.954819241		-2.75489	1	
chr8_18878_mature	8.59655945	1.611855	3.262517265	1.38507475		2.415037	1	
NW_003613377_38747_mature	6.44741959	0.805927	2.896740642	0.852739935		3	1	
chr9_20893_mature	19.3422588	6.44742	4.346407978	2.896740642		1.584963	1	
chr13_27738_mature	6.44741959	23.3719	2.896740642	4.607146585		-1.85798	1	
NW_003613326_38567_star	6.44741959	0.805927	2.896740642	0.852739935		3	1	
chr3_6786_star	8.59655945	1.611855	3.262517265	1.38507475		2.415037	1	
chr12_26437_star	6.44741959	0.805927	2.896740642	0.852739935		3	1	
chr6_14191_star	6.44741959	0.805927	2.896740642	0.852739935		3	1	
chr11_24429_star	6.44741959	0.805927	2.896740642	0.852739935		3	1	
NW_003540346_39431_mature	6.44741959	0.805927	2.896740642	0.852739935		3	1	
chr2_5398_star	6.44741959	0.805927	2.896740642	0.852739935		3	1	
chr5_11377_mature	6.44741959	0.805927	2.896740642	0.852739935		3	1	
hsa-miR-3667-5p	6.44741959	0.805927	2.896740642	0.852739935		3	1	
chr16_34832_mature	8.59655945	1.611855	3.262517265	1.38507475		2.415037	1	
chr3_7956_star	10.7456993	2.417782	3.554060706	1.773060525		2.152003	1	
chr6_13349_mature	8.59655945	1.611855	3.262517265	1.38507475		2.415037	1	
chr4_9513_star	10.7456993	2.417782	3.554060706	1.773060525		2.152003	1	
chr10_23904_star	8.59655945	1.611855	3.262517265	1.38507475		2.415037	1	
chr1_1911_mature	8.59655945	1.611855	3.262517265	1.38507475		2.415037	1	
chr2_6101_star	8.59655945	1.611855	3.262517265	1.38507475		2.415037	1	
chr2_5320_star	6.44741959	0.805927	2.896740642	0.852739935		3	1	
chr1_1948_mature	8.59655945	1.611855	3.262517265	1.38507475		2.415037	1	
chr2_5368_mature	6.44741959	0.805927	2.896740642	0.852739935		3	1	
chr15_33696_mature	8.59655945	1.611855	3.262517265	1.38507475		2.415037	1	
chr5_11575_star	8.59655945	1.611855	3.262517265	1.38507475		2.415037	1	
chr11_25386_star	8.59655945	1.611855	3.262517265	1.38507475		2.415037	1	
chr5_11998_mature	12.8948392	3.22371	3.796477232	2.078510713		2	1	
chr12_25921_star	6.44741959	0.805927	2.896740642	0.852739935		3	1	
chr14_31022_mature	6.44741959	0.805927	2.896740642	0.852739935		3	1	
hsa-miR-567	6.44741959	0.805927	2.896740642	0.852739935		3	1	
chr7_15700_mature	10.7456993	2.417782	3.554060706	1.773060525		2.152003	1	
chrx_22367_star	8.59655945	1.611855	3.262517265	1.38507475		2.415037	1	

rno-miR-463-5p	1.611855	0.805927	1.38507475	0.852739935		1	0.680133
bta-miR-2412	1.611855	0.805927	1.38507475	0.852739935		1	0.680133
bta-miR-2400	1.611855	0.805927	1.38507475	0.852739935		1	0.680133
hsa-miR-4460	1.611855	0.805927	1.38507475	0.852739935		1	0.680133
hsa-miR-4524b-3p	1.611855	0.805927	1.38507475	0.852739935		1	0.680133
bta-miR-2441	1.611855	0.805927	1.38507475	0.852739935		1	0.680133
chr13_26883_mature	1.611855	0.805927	1.38507475	0.852739935		1	0.680133
rno-miR-875	1.611855	0.805927	1.38507475	0.852739935		1	0.680133
hsa-miR-4432	1.611855	0.805927	1.38507475	0.852739935		1	0.680133
NW_003613073_36998_star	1.611855	0.805927	1.38507475	0.852739935		1	0.680133
hsa-miR-603	1.611855	0.805927	1.38507475	0.852739935		1	0.680133
hsa-miR-6512-5p	1.611855	0.805927	1.38507475	0.852739935		1	0.680133
mmu-miR-1843b-5p	1.611855	0.805927	1.38507475	0.852739935		1	0.680133
mmu-miR-1930-5p	1.611855	0.805927	1.38507475	0.852739935		1	0.680133
hsa-miR-4499	1.611855	0.805927	1.38507475	0.852739935		1	0.680133
chr12_25987_star	1.611855	0.805927	1.38507475	0.852739935		1	0.680133
chr2_4571_star	1.611855	0.805927	1.38507475	0.852739935		1	0.680133
chr15_33434_star	1.611855	0.805927	1.38507475	0.852739935		1	0.680133
chr9_20452_star	1.611855	0.805927	1.38507475	0.852739935		1	0.680133
mmu-miR-1196-5p	1.611855	0.805927	1.38507475	0.852739935		1	0.680133
mmu-miR-3082-5p	1.611855	0.805927	1.38507475	0.852739935		1	0.680133
mmu-miR-1196-3p	1.611855	0.805927	1.38507475	0.852739935		1	0.680133
NW_003540052_39255_star	1.611855	0.805927	1.38507475	0.852739935		1	0.680133
rno-miR-10b-5p	1.611855	0.805927	1.38507475	0.852739935		1	0.680133
chr14_30730_star	1.611855	0.805927	1.38507475	0.852739935		1	0.680133
chr15_32840_mature	1.611855	0.805927	1.38507475	0.852739935		1	0.680133
hsa-miR-4314	1.611855	0.805927	1.38507475	0.852739935		1	0.680133
chr6_13027_mature	1.611855	0.805927	1.38507475	0.852739935		1	0.680133
bta-miR-2362	1.611855	0.805927	1.38507475	0.852739935		1	0.680133
hsa-miR-4323	1.611855	0.805927	1.38507475	0.852739935		1	0.680133
chr12_26572_star	1.611855	0.805927	1.38507475	0.852739935		1	0.680133
bta-miR-2289	1.611855	0.805927	1.38507475	0.852739935		1	0.680133
mmu-miR-683	1.611855	0.805927	1.38507475	0.852739935		1	0.680133
mmu-miR-344i	1.611855	0.805927	1.38507475	0.852739935		1	0.680133
mmu-miR-466g	1.611855	0.805927	1.38507475	0.852739935		1	0.680133
chr17_35808_mature	1.611855	0.805927	1.38507475	0.852739935		1	0.680133
chr16_34849_star	1.611855	0.805927	1.38507475	0.852739935		1	0.680133
mmu-miR-1843a-3p	1.611855	0.805927	1.38507475	0.852739935		1	0.680133
chr14_30038_star	1.611855	0.805927	1.38507475	0.852739935		1	0.680133
chr8_17690_star	1.611855	0.805927	1.38507475	0.852739935		1	0.680133
chrx_23015_star	1.611855	0.805927	1.38507475	0.852739935		1	0.680133
rno-miR-1193-3p	1.611855	0.805927	1.38507475	0.852739935		1	0.680133
hsa-miR-1269b	1.611855	0.805927	1.38507475	0.852739935		1	0.680133
NW_003613161_37463_star	1.611855	0.805927	1.38507475	0.852739935		1	0.680133
chr6_14460_star	1.611855	0.805927	1.38507475	0.852739935		1	0.680133
chr8_17916_mature	1.611855	0.805927	1.38507475	0.852739935		1	0.680133
hsa-miR-4656	1.611855	0.805927	1.38507475	0.852739935		1	0.680133
chr2_4298_star	1.611855	0.805927	1.38507475	0.852739935		1	0.680133
mmu-miR-3473a	1.611855	0.805927	1.38507475	0.852739935		1	0.680133
rno-miR-3551-5p	1.611855	0.805927	1.38507475	0.852739935		1	0.680133
rno-miR-26b-5p	1.611855	0.805927	1.38507475	0.852739935		1	0.680133
chr1_3657_star	1.611855	0.805927	1.38507475	0.852739935		1	0.680133
chr1_3032_star	1.611855	0.805927	1.38507475	0.852739935		1	0.680133
hsa-miR-4723-3p	1.611855	0.805927	1.38507475	0.852739935		1	0.680133
chr3_8302_mature	1.611855	0.805927	1.38507475	0.852739935		1	0.680133
hsa-miR-509-3-5p	1.611855	0.805927	1.38507475	0.852739935		1	0.680133
chrx_23238_mature	1.611855	0.805927	1.38507475	0.852739935		1	0.680133
hsa-miR-1273c	1.611855	0.805927	1.38507475	0.852739935		1	0.680133
chr11_24484_mature	1.611855	0.805927	1.38507475	0.852739935		1	0.680133
chr14_31314_star	1.611855	0.805927	1.38507475	0.852739935		1	0.680133
mmu-miR-465c-5p	1.611855	0.805927	1.38507475	0.852739935		1	0.680133
mmu-miR-217-5p	1.611855	0.805927	1.38507475	0.852739935		1	0.680133
NW_003613377_38747_star	1.611855	0.805927	1.38507475	0.852739935		1	0.680133
hsa-miR-1912	1.611855	0.805927	1.38507475	0.852739935		1	0.680133
chr17_34991_mature	11.28298	48.35565	3.618589217	5.625143252		-2.09954	0.680697
chr17_35802_star	11.82027	4.835565	3.680354657	2.544872269		1.289507	0.680698
chr9_19819_mature	23.10325	7.253347	4.591155994	3.044979305		1.671377	0.680699
hsa-miR-659-5p	23.10325	7.253347	4.591155994	3.044979305		1.671377	0.680699
chr1_1213_mature	6.44742	20.14819	2.896740642	4.402462031		-1.64386	0.680826
chr10_23450_mature	5.910135	3.22371	2.788713818	2.078510713		0.874469	0.681246
chr15_32346_star	5.910135	3.22371	2.788713818	2.078510713		0.874469	0.681246
chr5_12114_star@@hsa-miR-141-	5.910135	3.22371	2.788713818	2.078510713		0.874469	0.681246
chr1_3276_mature	5.910135	3.22371	2.788713818	2.078510713		0.874469	0.681246
NW_003539007_38439_star	5.910135	3.22371	2.788713818	2.078510713		0.874469	0.681246
NW_003538236_37641_mature	5.910135	3.22371	2.788713818	2.078510713		0.874469	0.681246
chr6_14228_star	5.910135	3.22371	2.788713818	2.078510713		0.874469	0.681246
chr14_30822_star	5.910135	3.22371	2.788713818	2.078510713		0.874469	0.681246
chr8_18843_star	5.910135	3.22371	2.788713818	2.078510713		0.874469	0.681246
chr8_17786_star	5.910135	3.22371	2.788713818	2.078510713		0.874469	0.681246
chr8_17733_mature@@mmu-miR-	5.910135	3.22371	2.788713818	2.078510713		0.874469	0.681246
chr7_15705_star	5.910135	3.22371	2.788713818	2.078510713		0.874469	0.681246
chr2_4019_star	5.910135	3.22371	2.788713818	2.078510713		0.874469	0.681246
chr10_23941_mature	5.910135	3.22371	2.788713818	2.078510713		0.874469	0.681246
chr3_7100_star	5.910135	3.22371	2.788713818	2.078510713		0.874469	0.681246
chr9_19327_mature	41.90823	10.47706	5.423182395	3.520680821		2	0.68146
chr15_31761_mature	2.14914	1.611855	1.654957834	1.38507475		0.415037	0.681555
mmu-miR-28c	2.14914	1.611855	1.654957834	1.38507475		0.415037	0.681555
chr4_8685_mature	2.14914	1.611855	1.654957834	1.38507475		0.415037	0.681555
chr6_13990_mature	2.14914	1.611855	1.654957834	1.38507475		0.415037	0.681555
chr1_330_mature	2.14914	1.611855	1.654957834	1.38507475		0.415037	0.681555
chr15_33264_star	2.14914	1.611855	1.654957834	1.38507475		0.415037	0.681555
hsa-miR-1303	2.14914	1.611855	1.654957834	1.38507475		0.415037	0.681555
chr1_782_star	2.14914	1.611855	1.654957834	1.38507475		0.415037	0.681555
hsa-miR-4719	2.14914	1.611855	1.654957834	1.38507475		0.415037	0.681555
chr13_26740_star	2.14914	1.611855	1.654957834	1.38507475		0.415037	0.681555
chr16_34832_mature	2.14914	1.611855	1.654957834	1.38507475		0.415037	0.681555
hsa-miR-4762-5p	2.14914	1.611855	1.654957834	1.38507475		0.415037	0.681555
hsa-miR-4419a	2.14914	1.611855	1.654957834	1.38507475		0.415037	0.681555
chr2_6101_star	2.14914	1.611855	1.654957834	1.38507475		0.415037	0.681555
mmu-miR-3085-5p	2.14914	1.611855	1.654957834	1.38507475		0.415037	0.681555
chr1_1820_star	2.14914	1.611855	1.654957834	1.38507475		0.415037	0.681555
NW_003539007_38445_star	2.14914	1.611855	1.654957834	1.38507475		0.415037	0.681555
hsa-miR-571	2.14914	1.611855	1.654957834	1.38507475		0.415037	0.681555
chr15_33317_star	2.14914	1.611855	1.654957834	1.38507475		0.415037	0.681555
chr5_11429_star	2.14914	1.611855	1.654957834	1.38507475		0.415037	0.681555
chrx_22367_star	2.14914	1.611855	1.654957834	1.38507475		0.415037	0.681555
hsa-miR-2682-5p	2.14914	1.611855	1.654957834	1.38507475		0.415037	0.681555
chr14_29876_mature	2.14914	1.611855	1.654957834	1.38507475		0.415037	0.681555
chr1_3918_mature	2.14914	1.611855	1.654957834	1.38507475		0.415037	0.681555
mmu-miR-5132-5p	2.14914	1.611855	1.654957834	1.38507475		0.415037	0.681555
chr1_2918_star	2.14914	1.611855	1.654957834	1.38507475		0.415037	0.681555
chr13_26861_star	2.14914	1.611855	1.654957834	1.38507475		0.415037	0.681555
mmu-miR-1906	2.14914	1.611855	1.654957834	1.38507475		0.415037	0.681555
chr5_10731_mature	2.14914	1.611855	1.654957834	1.38507475		0.415037	0.681555
mmu-miR-1940	2.14914	1.611855	1.654957834	1.38507475		0.415037	0.681555
chr14_30275_star	2.14914	1.611855	1.654957834	1.38507475		0.415037	0.681555
chr11_24334_mature	2.14914	1.611855	1.654957834	1.38507475		0.415037	0.681555
chr13_28612_mature	2.14914	1.611855	1.654957834	1.38507475		0.415037	0.681555
chr5_11704_mature@@rno-miR-35	2.14914	1.611855	1.654957834	1.38507475		0.415037	0.681555
chr14_30467_star@@hsa-miR-671	2.14914	1.611855	1.654957834	1.38507475		0.415037	0.681555
hsa-miR-3661	2.14914	1.611855	1.654957834	1.38507475		0.415037	0.681555
chr7_15700_star	2.14914	1.611855	1.654957834	1.38507475		0.415037	0.681555
chr15_31883_star	2.14914	1.611855	1.654957834	1.38507475		0.415037	0.681555
rno-miR-6315	2.14914	1.611855	1.654957834	1.38507475		0.415037	0.681555
chrx_22943_star	2.14914	1.611855	1.654957834	1.38507475		0.415037	0.681555
NW_003539295_38706_star	2.14914	1.611855	1.654957834	1.38507475		0.415037	0.681555
chr15_31872_star	2.14914	1.611855	1.654957834	1.38507475		0.415037	0.681555
chrx_21498_star	2.14914	1.611855	1.654957834	1.38507475		0.415037	0.681555
chr15_33043_mature	2.14914	1.611855	1.654957834	1.38507475		0.415037	0.681555
chr13_27765_star	2.14914	1.611855	1.654957834	1.38507475		0.415037	0.681555
ssc-miR-489	2.14914	1.611855	1.654957834	1.38507475		0.415037	0.681555
chr16_34527_mature@@bta-miR-4	2.14914	1.611855	1.654957834	1.38507475		0.415037	0.681555
NW_003613507_39496_star	2.14914	1.611855	1.654957834	1.38507475		0.415037	0.681555
chr11_24560_star	2.14914	1.611855	1.654957834	1.38507475		0.415037	0.681555
chr4_9327_star	2.14914	1.611855	1.654957834	1.38507475		0.415037	0.681555
bta-miR-764	2.14914	1.611855	1.654957834	1.38507475		0.415037	0.681555
mmu-miR-6364	2.14914	1.611855	1.654957834	1.38507475		0.415037	0.681555
bta-miR-2284i	2.14914	1.611855	1.654957834	1.38507475		0.415037	0.681555
mmu-miR-1968-5p	2.14914	1.611855	1.654957834	1.38507475		0.415037	0.681555
chr14_31548_mature	2.14914	1.611855	1.654957834	1.38507475		0.415037	0.681555
chr6_13576_star	2.14914	1.611855	1.654957834	1.38507475		0.415037	0.681555
chr9_19885_star	2.14914	1.611855	1.654957834	1.38507475		0.415037	0.681555
ssc-miR-1249	2.14914	1.611855	1.654957834	1.38507475		0.415037	0.681555
chr6_13265_star	2.14914	1.611855	1.654957834	1.38507475		0.415037	0.681555
chr18_36406_star	2.14914	1.611855	1.654957834	1.38507475		0.415037	0.681555
chr5_11414_star	2.14914	1.611855	1.654957834	1.38507475		0.415037	0.681555
chr5_12092_star	2.14914	1.611855	1.654957834	1.38507475		0.415037	0.681555
chr13_26857_mature	2.14914	1.611855	1.654957834	1.38507475		0.415037	0.681555
chr2_4563_star	2.14914	1.611855	1.654957834	1.38507475		0.415037	0.681555
chr17_35330_star	2.14914	1.611855	1.654957834	1.38507475		0.415037	0.681555
chr3_7381_star	2.14914	1.611855	1.654957834	1.38507475		0.415037	0.681555
chr5_11695_mature	2.14914	1.611855	1.654957834	1.38507475		0.415037	0.681555
chr4_8929_star	2.14914	1.611855	1.654957834	1.38507475		0.415037	0.681555
chr12_26404_star	2.14914	1.611855	1.654957834	1.38507475		0.415037	0.681555
bta-miR-551b	2.14914	1.611855	1.654957834	1.38507475		0.415037	0.681555
chr1_1290_star	2.14914	1.611855	1.654957834	1.38507475		0.415037	0.681555
ssc-miR-4337	2.14914	1.611855	1.654957834	1.38507475		0.415037	0.681555
chr10_23674_star	2.14914	1.611855	1.654957834	1.38507475		0.415037	0.681555
hsa-miR-4269	2.14914	1.611855	1.654957834	1.38507475		0.415037	0.681555
chr3_8430_star	2.14914	1.611855	1.654957834	1.38507475		0.415037	0.681555
chr6_13274_star	2.14914	1.611855	1.654957834	1.38507475		0.415037	0.681555
chr15_33643_star	2.14914	1.611855	1.654957834	1.38507475		0.415037	0.681555
chr5_11562_mature@@mmu-miR-	2.14914	1.611855	1.654957834	1.38507475		0.415037	0.681555
chr3_7339_star	2.14914	1.611855	1.654957834	1.38507475		0.415037	0.681555
bta-miR-2444	2.14914	1.611855	1.654957834	1.38507475		0.415037	0.681555
chr9_19299_star	2.14914	1.611855	1.654957834	1.38507475		0.415037	0.681555
chr15_31849_star	2.14914	1.611855	1.654957834	1.38507475		0.415037	0.681555
hsa-miR-4491	2.14914	1.611855	1.654957834	1.38507475		0.415037	0.681555
chr14_30313_star	2.14914	1.611855	1.654957834	1.38507475		0.415037	0.681555
chr16_34112_star	2.14914	1.611855	1.654957834	1.38507475		0.415037	0.681555
chr6_14411_star	2.14914	1.611855	1.654957834	1.38507475		0.415037	0.681555
chr7_15096_star	2.14914	1.611855	1.654957834	1.38507475		0.415037	0.681555
chr13_26761_star	2.14914	1.611855	1.654957834	1.38507475		0.415037	0.681555
chr9_19649_star	2.14914	1.611855	1.654957834	1.38507475		0.415037	0.681555
chr13_29225_star	2.14914	1.611855	1.654957834	1.38507475		0.415037	0.681555
chr15_32809_star	2.14914	1.611855	1.654957834	1.38507475		0.415037	0.681555
chr14_29971_mature	2.14914	1.611855	1.654957834	1.38507475		0.415037	0.681555
hsa-miR-516b-5p	2.14914	1.611855	1.654957834	1.38507475		0.415037	0.681555
chr14_30126_mature	2.14914	1.611855	1.654957834	1.38507475		0.415037	0.681555
chr2_4749_mature	2.14914	1.611855	1.654957834	1.38507475		0.415037	0.681555
chr13_27155_mature	2.14914	1.611855	1.654957834	1.38507475		0.415037	0.681555
hsa-miR-4793-3p	2.14914	1.611855	1.654957834	1.38507475		0.415037	0.681555
chr14_30700_star	2.14914	1.611855	1.654957834	1.38507475		0.415037	0.681555
hsa-miR-5685	2.14914	1.611855	1.654957834	1.38507475		0.415037	0.681555
chr16_34398_star	2.14914	1.611855	1.654957834	1.38507475		0.415037	0.681555
chr18_36375_mature	2.14914	1.611855	1.654957834	1.38507475		0.415037	0.681555
chr6_13505_star	2.14914	1.611855	1.654957834	1.38507475		0.415037	0.681555
chr1_1859_star	8.596559	4.029637	3.262517265	2.330454351		1.093109	0.681759
chr11_24588_mature	8.596559	4.029637	3.262517265	2.330454351		1.093109	0.681759
chr18_35924_mature	8.596559	4.029637	3.262517265	2.330454351		1.093109	0.681759
chr13_27210_mature	15.04398	5.641492	4.003960082	2.731507408		1.415037	0.682526
chr18_36088_mature	31.69981	8.865202	5.03121048	3.302348583		1.838249	0.682641
chr15_32695_star	1.07457	1.611855	1.05281229	1.38507475		-0.58496	0.683014
chr16_34446_star	1.07457	1.611855	1.05281229	1.38507475		-0.58496	0.683014
chr9_19886_mature	1.07457	1.611855	1.05281229	1.38507475		-0.58496	0.683014
chr8_17244_star	1.07457	1.611855	1.05281229	1.38507475		-0.58496	0.683014
chr8_18998_star	1.07457	1.611855	1.05281229	1.38507475		-0.58496	0.683014
hsa-miR-4445-5p	1.07457	1.611855	1.05281229	1.38507475		-0.58496	0.683014
hsa-miR-4724-5p	1.07457	1.611855	1.05281229	1.38507475		-0.58496	0.683014
chr13_28501_star	1.07457	1.611855	1.05281229	1.38507475		-0.58496	0.683014
chr14_30531_star	1.07457	1.611855	1.05281229	1.38507475		-0.58496	0.683014
hsa-miR-3190-5p	1.07457	1.611855	1.05281229	1.38507475		-0.58496	0.683014
chr6_12988_mature@@mmu-miR-6	1.07457	1.611855	1.05281229	1.38507475		-0.58496	0.683014
mmu-miR-669a-3-3p	1.07457	1.611855	1.05281229	1.38507475		-0.58496	0.683014
chr3_7655_star	1.07457	1.611855	1.05281229	1.38507475		-0.58496	0.683014
chr8_17317_star	1.07457	1.611855	1.05281229	1.38507475		-0.58496	0.683014
chr5_12123_mature	1.07457	1.611855	1.05281229	1.38507475		-0.58496	0.683014
chr13_27900_star	1.07457	1.611855	1.05281229	1.38507475		-0.58496	0.683014
chr12_25395_mature	1.07457	1.611855	1.05281229	1.38507475		-0.58496	0.683014
mmu-miR-1907	1.07457	1.611855	1.05281229	1.38507475		-0.58496	0.683014
hsa-miR-4667-5p	1.07457	1.611855	1.05281229	1.38507475		-0.58496	0.683014
bta-miR-1814b	1.07457	1.611855	1.05281229	1.38507475		-0.58496	0.683014
rno-miR-301b-3p	1.07457	1.611855	1.05281229	1.38507475		-0.58496	0.683014
hsa-miR-5007-3p	1.07457	1.611855	1.05281229	1.38507475		-0.58496	0.683014
chr3_6880_mature	1.07457	1.611855	1.05281229	1.38507475		-0.58496	0.683014
rno-miR-28-5p	1.07457	1.611855	1.05281229	1.38507475		-0.58496	0.683014
chr6_13557_star	1.07457	1.611855	1.05281229	1.38507475		-0.58496	0.683014
hsa-miR-520a-3p	1.07457	1.611855	1.05281229	1.38507475		-0.58496	0.683014
chr8_18077_star	1.07457	1.611855	1.05281229	1.38507475		-0.58496	0.683014
chr16_34846_star	1.07457	1.611855	1.05281229	1.38507475		-0.58496	0.683014
hsa-miR-16-2-3p	1.07457	1.611855	1.05281229	1.38507475		-0.58496	0.683014
chr6_13949_star	1.07457	1.611855	1.05281229	1.38507475		-0.58496	0.683014
hsa-miR-3609	1.07457	1.611855	1.05281229	1.38507475		-0.58496	0.683014
chr2_4579_star	1.07457	1.611855	1.05281229	1.38507475		-0.58496	0.683014
chrx_22204_mature	1.07457	1.611855	1.05281229	1.38507475		-0.58496	0.683014
chr15_32439_star	1.07457	1.611855	1.05281229	1.38507475		-0.58496	0.683014
chr5_11655_star	1.07457	1.611855	1.05281229	1.38507475		-0.58496	0.683014
chr12_25873_mature	1.07457	1.611855	1.05281229	1.38507475		-0.58496	0.683014
chr6_13458_mature	1.07457	1.611855	1.05281229	1.38507475		-0.58496	0.683014
chr6_13578_star	1.07457	1.611855	1.05281229	1.38507475		-0.58496	0.683014
chr4_9333_mature	1.07457	1.611855	1.05281229	1.38507475		-0.58496	0.683014
chr6_14178_mature	1.07457	1.611855	1.05281229	1.38507475		-0.58496	0.683014
chr2_5245_mature	1.07457	1.611855	1.05281229	1.38507475		-0.58496	0.683014
chr7_16801_mature	1.07457	1.611855	1.05281229	1.38507475		-0.58496	0.683014
chr6_14620_star	1.07457	1.611855	1.05281229	1.38507475		-0.58496	0.683014
bta-let-7a-5p	1.07457	1.611855	1.05281229	1.38507475		-0.58496	0.683014
chr5_11418_star	1.07457	1.611855	1.05281229	1.38507475		-0.58496	0.683014
chr18_36088_star	1.07457	1.611855	1.05281229	1.38507475		-0.58496	0.683014
chr17_35011_star	1.07457	1.611855	1.05281229	1.38507475		-0.58496	0.683014
mmu-miR-6336	1.07457	1.611855	1.05281229	1.38507475		-0.58496	0.683014
chr10_24049_mature	1.07457	1.611855	1.05281229	1.38507475		-0.58496	0.683014
rno-miR-217-3p	1.07457	1.611855	1.05281229	1.38507475		-0.58496	0.683014
chr2_4133_star	1.07457	1.611855	1.05281229	1.38507475		-0.58496	0.683014
chr1_2370_mature	1.07457	1.611855	1.05281229	1.38507475		-0.58496	0.683014
NW_003613242_37866_star	1.07457	1.611855	1.05281229	1.38507475		-0.58496	0.683014
NW_003613080_37038_star	1.07457	1.611855	1.05281229	1.38507475		-0.58496	0.683014
chr14_31255_star	1.07457	1.611855	1.05281229	1.38507475		-0.58496	0.683014
NW_003541083_39896_mature	1.07457	1.611855	1.05281229	1.38507475		-0.58496	0.683014
mmu-miR-488-5p	1.07457	1.611855	1.05281229	1.38507475		-0.58496	0.683014
rno-let-7c-5p	1.07457	1.611855	1.05281229	1.38507475		-0.58496	0.683014
hsa-miR-6132	1.07457	1.611855	1.05281229	1.38507475		-0.58496	0.683014
bta-miR-2370-5p	1.07457	1.611855	1.05281229	1.38507475		-0.58496	0.683014
mmu-miR-3094-5p	1.07457	1.611855	1.05281229	1.38507475		-0.58496	0.683014
chr6_13302_star	1.07457	1.611855	1.05281229	1.38507475		-0.58496	0.683014
chr9_19298_star	1.07457	1.611855	1.05281229	1.38507475		-0.58496	0.683014
mmu-miR-1946a	1.07457	1.611855	1.05281229	1.38507475		-0.58496	0.683014
chr1_3433_mature	1.07457	1.611855	1.05281229	1.38507475		-0.58496	0.683014
chrx_22969_star	1.07457	1.611855	1.05281229	1.38507475		-0.58496	0.683014
hsa-miR-6503-5p	1.07457	1.611855	1.05281229	1.38507475		-0.58496	0.683014
chr6_12824_mature@@hsa-miR-87	1.07457	1.611855	1.05281229	1.38507475		-0.58496	0.683014
chr18_36575_star	1.07457	1.611855	1.05281229	1.38507475		-0.58496	0.683014
rno-miR-140-3p	1.07457	1.611855	1.05281229	1.38507475		-0.58496	0.683014
chr13_27994_star	1.07457	1.611855	1.05281229	1.38507475		-0.58496	0.683014
hsa-miR-3195	1.07457	1.611855	1.05281229	1.38507475		-0.58496	0.683014
hsa-miR-4261	1.07457	1.611855	1.05281229	1.38507475		-0.58496	0.683014

chr5_11575_star	3.223709795	1.611855	2.078510713	1.38507475		1	0.440959	chrx_22791_mature	8.59655945	1.611855	3.262517265	1.38507475		2.415037	1	mmu-miR-3061-3p	1.07457	1.611855	1.05281229	1.38507475		-0.58496	0.683014
chr2_6441_star	3.223709795	1.611855	2.078510713	1.38507475		1	0.440959	chr8_17522_mature	6.44741959	0.805927	2.896740642	0.852739935		3	1	bta-miR-6528	1.07457	1.611855	1.05281229	1.38507475		-0.58496	0.683014
chrx_22089_mature	3.223709795	1.611855	2.078510713	1.38507475		1	0.440959	chr3_8552_star	8.59655945	1.611855	3.262517265	1.38507475		2.415037	1	chr8_18294_star	1.07457	1.611855	1.05281229	1.38507475		-0.58496	0.683014
hsa-miR-1270	3.223709795	1.611855	2.078510713	1.38507475		1	0.440959	chr5_10910_mature	8.59655945	1.611855	3.262517265	1.38507475		2.415037	1	chr15_33038_star	1.07457	1.611855	1.05281229	1.38507475		-0.58496	0.683014
chr7_16598_mature	3.223709795	1.611855	2.078510713	1.38507475		1	0.440959	chr5_12411_star	6.44741959	0.805927	2.896740642	0.852739935		3	1	chr15_33315_star	1.07457	1.611855	1.05281229	1.38507475		-0.58496	0.683014
chr5_11429_star	3.223709795	1.611855	2.078510713	1.38507475		1	0.440959	hsa-miR-3916	4.29827973	19.34226	2.405524014	4.346407978		-2.16993	1	hsa-miR-4664-3p	1.07457	1.611855	1.05281229	1.38507475		-0.58496	0.683014
chr5_12167_mature	3.223709795	1.611855	2.078510713	1.38507475		1	0.440959	hsa-miR-3911	6.44741959	0.805927	2.896740642	0.852739935		3	1	chr5_11151_star	1.07457	1.611855	1.05281229	1.38507475		-0.58496	0.683014
chr3_7963_star	3.223709795	1.611855	2.078510713	1.38507475		1	0.440959	chr7_15451_mature	6.44741959	0.805927	2.896740642	0.852739935		3	1	chr8_17650_star	1.07457	1.611855	1.05281229	1.38507475		-0.58496	0.683014
chr12_25395_mature	3.223709795	1.611855	2.078510713	1.38507475		1	0.440959	rno-miR-328b-3p	6.44741959	0.805927	2.896740642	0.852739935		3	1	chr1_3580_star	1.07457	1.611855	1.05281229	1.38507475		-0.58496	0.683014
chr12_25930_star	3.223709795	1.611855	2.078510713	1.38507475		1	0.440959	chr7_16635_star	8.59655945	1.611855	3.262517265	1.38507475		2.415037	1	hsa-miR-4684-5p	1.07457	1.611855	1.05281229	1.38507475		-0.58496	0.683014
rno-miR-339-5p	3.223709795	1.611855	2.078510713	1.38507475		1	0.440959	mmu-miR-1905	6.44741959	0.805927	2.896740642	0.852739935		3	1	chr15_32787_star	1.07457	1.611855	1.05281229	1.38507475		-0.58496	0.683014
chr14_29474_star	3.223709795	1.611855	2.078510713	1.38507475		1	0.440959	hsa-miR-4667-5p	8.59655945	1.611855	3.262517265	1.38507475		2.415037	1	chr9_20945_star	1.07457	1.611855	1.05281229	1.38507475		-0.58496	0.683014
chr2_5585_star	3.223709795	1.611855	2.078510713	1.38507475		1	0.440959	chr15_33543_mature	8.59655945	1.611855	3.262517265	1.38507475		2.415037	1	hsa-miR-4489	1.07457	1.611855	1.05281229	1.38507475		-0.58496	0.683014
chr7_15700_star	3.223709795	1.611855	2.078510713	1.38507475		1	0.440959	chr13_27163_mature	19.3422588	6.44742	4.346407978	2.896740642		1.584963	1	chr11_24341_star	1.07457	1.611855	1.05281229	1.38507475		-0.58496	0.683014
chr13_27434_star	3.223709795	1.611855	2.078510713	1.38507475		1	0.440959	chr14_29634_mature	6.44741959	0.805927	2.896740642	0.852739935		3	1	hsa-miR-524-5p	1.07457	1.611855	1.05281229	1.38507475		-0.58496	0.683014
chr6_13458_mature	3.223709795	1.611855	2.078510713	1.38507475		1	0.440959	chr13_28612_star	12.8948392	3.22371	3.796477232	2.078510713		2	1	chr6_14856_star	1.07457	1.611855	1.05281229	1.38507475		-0.58496	0.683014
chr1_3025_star	3.223709795	1.611855	2.078510713	1.38507475		1	0.440959	chr4_10242_mature	6.44741959	0.805927	2.896740642	0.852739935		3	1	ssc-miR-155-3p	1.07457	1.611855	1.05281229	1.38507475		-0.58496	0.683014
chr4_10041_star	3.223709795	1.611855	2.078510713	1.38507475		1	0.440959	chr15_32289_mature	8.59655945	1.611855	3.262517265	1.38507475		2.415037	1	hsa-miR-606	1.07457	1.611855	1.05281229	1.38507475		-0.58496	0.683014
chr4_10519_star	3.223709795	1.611855	2.078510713	1.38507475		1	0.440959	chr15_32525_mature	10.7456993	2.417782	3.554060706	1.773060525		2.152003	1	hsa-miR-612	1.07457	1.611855	1.05281229	1.38507475		-0.58496	0.683014
chr6_13578_star	3.223709795	1.611855	2.078510713	1.38507475		1	0.440959	chr13_26857_star	6.44741959	0.805927	2.896740642	0.852739935		3	1	hsa-miR-6509-5p	1.07457	1.611855	1.05281229	1.38507475		-0.58496	0.683014
hsa-miR-663a	3.223709795	1.611855	2.078510713	1.38507475		1	0.440959	chr2_6132_star	8.59655945	1.611855	3.262517265	1.38507475		2.415037	1	NW_003613050_36907_mature	1.07457	1.611855	1.05281229	1.38507475		-0.58496	0.683014
chr11_24794_mature	3.223709795	1.611855	2.078510713	1.38507475		1	0.440959	NW_003539007_38438_star	6.44741959	0.805927	2.896740642	0.852739935		3	1	bta-miR-2398	1.07457	1.611855	1.05281229	1.38507475		-0.58496	0.683014
chr13_27560_star	3.223709795	1.611855	2.078510713	1.38507475		1	0.440959	chr11_25144_star	6.44741959	0.805927	2.896740642	0.852739935		3	1	chr11_24450_star	1.07457	1.611855	1.05281229	1.38507475		-0.58496	0.683014
rno-miR-547-3p	3.223709795	1.611855	2.078510713	1.38507475		1	0.440959	chr18_36112_star	6.44741959	0.805927	2.896740642	0.852739935		3	1	hsa-miR-193a-3p	1.07457	1.611855	1.05281229	1.38507475		-0.58496	0.683014
chr3_7564_star	3.223709795	1.611855	2.078510713	1.38507475		1	0.440959	chr2_6135_star	6.44741959	0.805927	2.896740642	0.852739935		3	1	bta-miR-2332	1.07457	1.611855	1.05281229	1.38507475		-0.58496	0.683014
chr11_24560_star	3.223709795	1.611855	2.078510713	1.38507475		1	0.440959	chr1_2381_mature	8.59655945	1.611855	3.262517265	1.38507475		2.415037	1	bta-miR-2348	1.07457	1.611855	1.05281229	1.38507475		-0.58496	0.683014
chr6_13244_star	3.223709795	1.611855	2.078510713	1.38507475		1	0.440959	chr7_15958_star	10.7456993	2.417782	3.554060706	1.773060525		2.152003	1	hsa-miR-4321	1.07457	1.611855	1.05281229	1.38507475		-0.58496	0.683014
mmu-miR-6385	3.223709795	1.611855	2.078510713	1.38507475		1	0.440959	chr1_3090_star	6.44741959	0.805927	2.896740642	0.852739935		3	1	chr2_4749_star	1.07457	1.611855	1.05281229	1.38507475		-0.58496	0.683014
bta-miR-2285k	3.223709795	1.611855	2.078510713	1.38507475		1	0.440959	hsa-miR-5009-5p	6.44741959	0.805927	2.896740642	0.852739935		3	1	chr7_16063_star	1.07457	1.611855	1.05281229	1.38507475		-0.58496	0.683014
chr3_8052_star	3.223709795	1.611855	2.078510713	1.38507475		1	0.440959	chr7_15700_star	8.59655945	1.611855	3.262517265	1.38507475		2.415037	1	mmu-miR-467h	1.07457	1.611855	1.05281229	1.38507475		-0.58496	0.683014
chr4_10551_mature	3.223709795	1.611855	2.078510713	1.38507475		1	0.440959	NW_003537216_36774_mature	6.44741959	0.805927	2.896740642	0.852739935		3	1	hsa-miR-762	1.07457	1.611855	1.05281229	1.38507475		-0.58496	0.683014
chr1_1435_star	3.223709795	1.611855	2.078510713	1.38507475		1	0.440959	chr14_30480_mature	8.59655945	1.611855	3.262517265	1.38507475		2.415037	1	chr7_16904_mature	1.07457	1.611855	1.05281229	1.38507475		-0.58496	0.683014
chr7_17049_star	3.223709795	1.611855	2.078510713	1.38507475		1	0.440959	chr11_24729_star	6.44741959	0.805927	2.896740642	0.852739935		3	1	bta-miR-2330-5p	1.07457	1.611855	1.05281229	1.38507475		-0.58496	0.683014
hsa-miR-4650-3p	3.223709795	1.611855	2.078510713	1.38507475		1	0.440959	chr2_4629_star	6.44741959	0.805927	2.896740642	0.852739935		3	1	chr9_19327_star	1.07457	1.611855	1.05281229	1.38507475		-0.58496	0.683014
chr13_28432_star	3.223709795	1.611855	2.078510713	1.38507475		1	0.440959	chr13_28692_mature	6.44741959	0.805927	2.896740642	0.852739935		3	1	hsa-miR-4673	1.07457	1.611855	1.05281229	1.38507475		-0.58496	0.683014
chr10_24040_mature	3.223709795	1.611855	2.078510713	1.38507475		1	0.440959	chr6_14751_star	6.44741959	0.805927	2.896740642	0.852739935		3	1	hsa-miR-4686	1.07457	1.611855	1.05281229	1.38507475		-0.58496	0.683014
chrx_22145_mature	3.223709795	1.611855	2.078510713	1.38507475		1	0.440959	NW_003540029_39236_mature	8.59655945	1.611855	3.262517265	1.38507475		2.415037	1	rno-miR-23b-5p	1.07457	1.611855	1.05281229	1.38507475		-0.58496	0.683014
chr18_36561_star	3.223709795	1.611855	2.078510713	1.38507475		1	0.440959	chr2_5851_mature	6.44741959	0.805927	2.896740642	0.852739935		3	1	hsa-miR-6511b-3p	1.07457	1.611855	1.05281229	1.38507475		-0.58496	0.683014
chr18_36422_mature	3.223709795	1.611855	2.078510713	1.38507475		1	0.440959	chr14_30452_mature	10.7456993	2.417782	3.554060706	1.773060525		2.152003	1	chr12_26438_star	1.07457	1.611855	1.05281229	1.38507475		-0.58496	0.683014
chr11_25258_star	3.223709795	1.611855	2.078510713	1.38507475		1	0.440959	NW_003539609_38987_star	8.59655945	1.611855	3.262517265	1.38507475		2.415037	1	hsa-miR-4666a-5p	1.07457	1.611855	1.05281229	1.38507475		-0.58496	0.683014
chr6_13274_star	3.223709795	1.611855	2.078510713	1.38507475		1	0.440959	chr7_15959_star	6.44741959	0.805927	2.896740642	0.852739935		3	1	chr6_14141_mature	1.07457	1.611855	1.05281229	1.38507475		-0.58496	0.683014
chr6_12771_star	3.223709795	1.611855	2.078510713	1.38507475		1	0.440959	chr13_27434_star	8.59655945	1.611855	3.262517265	1.38507475		2.415037	1	chrx_22973_star	1.07457	1.611855	1.05281229	1.38507475		-0.58496	0.683014
NW_003537737_37236_mature	3.223709795	1.611855	2.078510713	1.38507475		1	0.440959	rno-miR-298-5p	6.44741959	0.805927	2.896740642	0.852739935		3	1	chr11_25373_star	1.07457	1.611855	1.05281229	1.38507475		-0.58496	0.683014
chr9_20034_star	3.223709795	1.611855	2.078510713	1.38507475		1	0.440959	chr7_16788_mature	8.59655945	1.611855	3.262517265	1.38507475		2.415037	1	chr7_15451_star	1.07457	1.611855	1.05281229	1.38507475		-0.58496	0.683014
chr15_32787_star	3.223709795	1.611855	2.078510713	1.38507475		1	0.440959	chr12_25573_star	10.7456993	2.417782	3.554060706	1.773060525		2.152003	1	hsa-miR-6077	1.07457	1.611855	1.05281229	1.38507475		-0.58496	0.683014
hsa-miR-4425	3.223709795	1.611855	2.078510713	1.38507475		1	0.440959	NW_003537297_36826_star	10.7456993	2.417782	3.554060706	1.773060525		2.152003	1	chrx_22070_star	1.07457	1.611855	1.05281229	1.38507475		-0.58496	0.683014
bta-miR-2482	3.223709795	1.611855	2.078510713	1.38507475		1	0.440959	chr12_25520_star	6.44741959	0.805927	2.896740642	0.852739935		3	1	ssc-miR-29a	400.2773	8630.677	8.648455734	13.07542517		-4.4304	0.683152
chr14_29861_star	3.223709795	1.611855	2.078510713	1.38507475		1	0.440959	hsa-miR-3140-5p	10.7456993	2.417782	3.554060706	1.773060525		2.152003	1	chr14_30424_star	3.760995	8.059274	2.251263041	3.179395517		-1.09954	0.683171
chr7_16597_mature	3.223709795	1.611855	2.078510713	1.38507475		1	0.440959	rno-miR-344b-1-3p	27.9388182	11.28298	4.854934103	3.618589217		1.308122	1	hsa-miR-6125	3.760995	8.059274	2.251263041	3.179395517		-1.09954	0.683171
chr12_26515_star	3.223709795	1.611855	2.078510713	1.38507475		1	0.440959	NW_003541332_40046_mature	6.44741959	0.805927	2.896740642	0.852739935		3	1	chr3_8628_star	3.760995	8.059274	2.251263041	3.179395517		-1.09954	0.683171
chr18_36628_mature	3.223709795	1.611855	2.078510713	1.38507475		1	0.440959	chr6_12501_star	6.44741959	0.805927	2.896740642	0.852739935		3	1	chr5_11482_star	3.760995	8.059274	2.251263041	3.179395517		-1.09954	0.683171
chr2_4749_star	3.223709795	1.611855	2.078510713	1.38507475		1	0.440959	chr2_6577_mature	19.3422588	6.44742	4.346407978	2.896740642		1.584963	1	chr3_8509_mature	6.984705	22.56597	2.997239027	4.558633073		-1.69188	0.683206
rno-miR-879-3p	3.223709795	1.611855	2.078510713	1.38507475		1	0.440959	chr1_3928_star	8.59655945	1.611855	3.262517265	1.38507475		2.415037	1	chr12_25822_mature	15.58126	78.17496	4.051482084	6.306972375		-2.32689	0.683677
chr14_31514_star	3.223709795	1.611855	2.078510713	1.38507475		1	0.440959	chr5_11058_mature	10.7456993	2.417782	3.554060706	1.773060525		2.152003	1	chr11_24876_mature	8.059274	28.20746	3.179395517	4.868265031		-1.80735	0.683932
mmu-miR-5616-3p	3.223709795	1.611855	2.078510713	1.38507475		1	0.440959	chr7_16801_mature	8.59655945	1.611855	3.262517265	1.38507475		2.415037	1	chr7_16971_mature	3.22371	2.417782	2.078510713	1.773060525		0.415037	0.684606
chr2_5974_mature	3.223709795	1.611855	2.078510713	1.38507475		1	0.440959	chr2_6234_star	6.44741959	0.805927	2.896740642	0.852739935		3	1	chr5_11673_star	3.22371	2.417782	2.078510713	1.773060525		0.415037	0.684606
chr13_27155_mature	3.223709795	1.611855	2.078510713	1.38507475		1	0.440959	chr17_34966_star	6.44741959	0.805927	2.896740642	0.852739935		3	1	chr15_33676_mature@@mmu-miR-	3.22371	2.417782	2.078510713	1.773060525		0.415037	0.684606
chr11_24439_star	3.223709795	1.611855	2.078510713	1.38507475		1	0.440959	chr12_25892_mature	8.59655945	1.611855	3.262517265	1.38507475		2.415037	1	chr9_20512_star	3.22371	2.417782	2.078510713	1.773060525		0.415037	0.684606
chr6_14281_mature	3.223709795	1.611855	2.078510713	1.38507475		1	0.440959	bta-miR-409a	6.44741959	23.3719	2.896740642	4.607146585		-1.85798	1	chr5_11657_mature	3.22371	2.417782	2.078510713	1.773060525		0.415037	0.684606
rno-miR-291b	3.223709795	1.611855	2.078510713	1.38507475		1	0.440959	chr18_36422_star	6.44741959	0.805927	2.896740642	0.852739935		3	1	NW_003538236_37642_mature	3.22371	2.417782	2.078510713	1.773060525		0.415037	0.684606
chr16_34398_star	3.223709795	1.611855	2.078510713	1.38507475		1	0.440959	chr15_33030_star	2.14913986	14.50669	1.654957834	3.954819241		-2.75489	1	hsa-miR-578	3.22371	2.417782	2.078510713	1.773060525		0.415037	0.684606
chrx_22973_star	3.223709795	1.611855	2.078510713	1.38507475		1	0.440959	chr14_31026_star	6.44741959	0.805927	2.896740642	0.852739935		3	1	ssc-miR-95	3.22371	2.417782	2.078510713	1.773060525		0.415037	0.684606
chr6_12862_mature	3.223709795	1.611855	2.078510713	1.38507475		1	0.440959	chr4_9939_star	6.44741959	0.805927	2.896740642	0.852739935		3	1	hsa-miR-5187-5p	3.22371	2.417782	2.078510713	1.773060525		0.415037	0.684606
chrx_22070_star	3.223709795	1.611855	2.078510713	1.38507475		1	0.440959	hsa-miR-6724-5p	6.44741959	0.805927	2.896740642	0.852739935		3	1	NW_003537531_36983_star	3.22371	2.417782	2.078510713	1.773060525		0.415037	0.684606
chr6_13505_star	3.223709795	1.611855	2.078510713	1.38507475		1	0.440959	hsa-miR-486-5p	6.44741959	0.805927	2.896740642	0.852739935		3	1	chr13_28860_star	3.22371	2.417782	2.078510713	1.773060525		0.415037	0.684606
chr2_4930_star	5.372849659	16.11855	2.671938627	4.097488514		-1.58496	0.441075	hsa-miR-4712-5p	6.44741959	0.805927	2.896740642	0.852739935		3	1	chr7_15165_star	3.22371	2.417782	2.078510713	1.773060525		0.415037	0.684606
bta-miR-1247-5p	25.78967836	7.253347	4.743605355	3.044979305		1.830075	0.441438	chr13_27765_mature	8.59655945	1.611855	3.262517265	1.38507475		2.415037	1	chr13_28171_star	3.22371	2.417782	2.078510713	1.773060525		0.415037	0.684606
chr5_10849_star	30.08795809	8.059274	4.958283956	3.179395517		1.900464	0.441629	hsa-miR-1183	6.44741959	0.805927	2.896740642	0.852739935		3	1	chr5_10827_mature	3.22371	2.417782	2.078510713	1.773060525		0.415037	0.684606
ssc-miR-769-3p	34.38623782	267.5679	5.145116481	8.06914314		-2.96	0.442482	hsa-miR-1184	6.44741959	0.805927	2.896740642	0.852739935		3	1	chr1_1637_star	3.22371	2.417782	2.078510713	1.773060525		0.415037	0.684606
chr13_28744_mature	44.0573672	10.47706	5.493691111	3.520680821		2.07215	0.442596	chr9_20818_mature	8.59655945	1.611855	3.262517265	1.38507475		2.415037	1	chr1_3026_star	3.22371	2.417782	2.078510713	1.773060525		0.415037	0.684606
chr3_7884_mature	10.74569932	4.029637	3.554060706	2.330454351		1.415037	0.442734	chr7_15367_star	6.44741959	0.805927	2.896740642	0.852739935		3	1	chr7_15797_mature	3.22371	2.417782	2.078510713	1.773060525		0.415037	0.684606
chrx_21868_star	10.74569932	4.029637	3.554060706	2.330454351		1.415037	0.442734	chr8_17856_star	8.59655945	1.611855	3.262517265	1.38507475		2.415037	1	chr6_14296_mature	3.22371	2.417782	2.078510713	1.773060525		0.415037	0.684606
chr12_26451_star	6.447419591	20.95411	2.896740642	4.456419386		-1.70044	0.442884	chrx_22191_mature	6.44741959	0.805927	2.896740642	0.852739935		3	1	chr2_4519_mature	3.22371	2.417782	2.078510713	1.773060525		0.415037	0.684606
chr1_429_star	6.447419591	20.95411	2.896740642	4.456419386		-1.70044	0.442884	chr6_13702_star	6.44741959	0.805927	2.896740642	0.852739935		3	1	chr14_30481_star	3.22371	2.417782	2.078510713	1.773060525		0.415037	0.684606
chr13_27782_star	34.38623782	8.865202	5.145116481	3.302348583		1.955606	0.443094	mmu-miR-6364	8.59655945	1.611855	3.262517265	1.38507475		2.415037	1	chr4_9069_star	3.22371	2.417782	2.078510713	1.773060525		0.415037	0.684606
chr6_13983_star	13.96940911	67.69791	3.90394537	6.102194213		-2.27684	0.443226	NW_003613231_37780_mature	6.44741959	0.805927	2.896740642	0.852739935		3	1	chr14_29552_star	3.22371	2.417782	2.078510713	1.773060525		0.415037	0.684606
chr7_15245_star	13.96940911	4.835565	3.90394537	2.544872269		1.530515	0.443778	chr13_27728_star	8.59655945	1.611855	3.262517265	1.38507475		2.415037	1	chr13_28297_mature@@rno-miR-2	3.22371	2.417782	2.078510713	1.773060525		0.415037	0.684606
NW_003539812_39109_mature	13.96940911	4.835565	3.90394537	2.544872269		1.530515	0.443778	chr13_26824_mature	6.44741959	0.805927	2.896740642	0.852739935		3	1	chr11_24816_star	3.22371	2.417782	2.078510713	1.773060525		0.415037	0.684606
chr11_25099_star	13.96940911	4.835565	3.90394537	2.544872269		1.530515	0.443778	hsa-miR-6500-5p	6.44741959	0.805927	2.896740642	0.852739935		3	1	chr14_29426_star	3.22371	2.417782	2.078510713	1.773060525		0.415037	0.684606
chr11_24964_mature	13.96940911	4.835565	3.90394537	2.544872269		1.530515	0.443778	chr15_32249_mature	6.44741959	0.805927	2.896740642	0.852739935		3	1	chr2_5680_star	3.22371	2.417782	2.078510713	1.773060525		0.415037	0.684606
mmu-miR-6416-3p	13.96940911	4.835565	3.90394537	2.544872269		1.530515	0.443778	bta-miR-2285q	6.44741959	0.805927	2.896740642	0.852739935		3	1	chr13_29302_star	3.22371	2.417782	2.078510713	1.773060525		0.415037	0.684606
chr4_9980_star	4.298279727	11.28298	2.405524014	3.618589217		-1.39232	0.444252	chr10_24049_mature	8.59655945	1.611855	3.262517265	1.38507475		2.415037	1	chr17_35289_star	3.22371	2.417782	2.078510713	1.773060525		0.415037	0.684606
chr17_35470_mature	38.68451755	9.671129	5.310504361	3.415640968		2	0.445455	chr12_26408_star	6.44741959	0.805927	2.896740642	0.852739935		3	1	chr2_5976_mature	3.22371	2.417782	2.078510713	1.773060525		0.415037	0.684606
chr5_12114_star@@hsa-miR-141-5p	7.521989523	3.22371	3.091190278	2.078510713		1.222392	0.445813	chr5_11961_star	6.44741959	0.805927	2.896740642	0.852739935		3	1	NW_003613303_38366_star	3.22371	2.417782	2.078510713	1.773060525		0.415037	0.684606
chr5_12251_mature	7.521989523	3.22371	3.091190278	2.078510713		1.222392	0.445813	chr6_12855_star	12.8948392	3.22371	3.796477232	2.078510713		2	1	chr14_31142_mature	3.22371	2.417782	2.078510713	1.773060525		0.415037	0.684606
chr7_17051_mature	7.521989523	3.22371	3.091190278	2.078510713		1.222392	0.445813	chr4_8831_mature	8.59655945	1.611855	3.262517265	1.38507475		2.415037	1	chr15_32775_star	3.22371	2.417782	2.078510713	1.773060525		0.415037	0.684606
chr17_35401_star	7.521989523	3.22371	3.091190278	2.078510713		1.222392	0.445813	chr2_5822_star	6.44741959	0.805927	2.896740642	0.852739935		3	1	chr14_31128_star	3.22371	2.417782	2.078510713	1.773060525		0.415037	0.684606
chr8_18843_star	7.521989523	3.22371	3.091190278	2.078510713		1.222392	0.445813	chr6_13364_mature	6.44741959	23.3719	2.896740642	4.607146585		-1.85798	1	chr1_244_star	3.22371	2.417782	2.078510713	1.773060525		0.415037	0.684606
ssc-miR-4339	7.521989523	3.22371	3.091190278	2.078510713		1.222392	0.445813	chr9_19609_star@@bta-miR-135b	6.44741959	0.805927	2.896740642	0.852739935		3	1	chr2_6406_star	3.22371	2.417782	2.078510713	1.773060525		0.415037	0.684606
chr17_35140_mature	7.521989523	3.22371	3.091190278	2.078510713		1.222392	0.445813	chr13_28297_mature@@rno-miR-26a-3	10.7456993	2.417782	3.554060706	1.773060525		2.152003	1	bta-miR-2406	3.22371	2.417782	2.078510713	1.773060525		0.415037	0.684606
chr5_11678_mature	7.521989523	3.22371	3.091190278	2.078510713		1.222392	0.445813	chr6_14380_mature	8.59655945	1.611855	3.262517265	1.38507475		2.415037	1	hsa-miR-4429	3.22371	2.417782	2.078510713	1.773060525		0.415037	0.684606
chr14_31530_star	7.521989523	3.22371	3.091190278	2.078510713		1.222392	0.445813	chr9_19205_star	6.44741959	0.805927	2.896740642	0.852739935		3	1	hsa-miR-4448	3.22371	2.417782	2.078510713	1.773060525		0.415037	0.684606
chr15_31863_star@@bta-miR-2881	7.521989523	3.22371	3.091190278	2.078510713		1.222392	0.445813	chrx_21708_mature	30.0879581	61.25049	4.958283956	5.960013198		-1.02554	1	chr1_3282_mature	3.22371	2.417782	2.078510713	1.773060525		0.415037	0.684606
chr12_25687_star	7.521989523	3.22371	3.091190278	2.078510713		1.222392	0.445813	chrx_22644_mature	10.7456993	2.417782	3.554060706	1.773060525		2.152003	1	hsa-miR-541-3p	3.22371	2.417782	2.078510713	1.773060525		0.415037	0.684606
NW_003539920_39188_mature	7.521989523	3.22371	3.091190278	2.078510713		1.222392	0.445813	chr2_4523_star	6.44741959	0.805927	2.896740642	0.852739935		3	1	chr6_12598_star	3.22371	2.417782	2.078510713	1.773060525		0.415037	0.684606
chr15_32593_star	7.521989523	3.22371	3.091190278	2.078510713		1.222392	0.445813	ssc-miR-124a	8.59655945	1.611855	3.262517265	1.38507475		2.415037	1	hsa-miR-302d-5p	3.22371	2.417782	2.078510713	1.773060525		0.415037	0.684606
chr8_17822_mature	3.223709795	7.253347	2.078510713	3.044979305		-1.16993	0.445955	NW_003539814_39113_star	19.3422588	6.44742	4.346407978	2.896740642		1.584963	1	NW_003539295_38706_mature	3.22371	2.417782	2.078510713	1.773060525		0.415037	0.684606
chr17_34956_mature	3.223709795	7.253347	2.078510713	3.044979305		-1.16993	0.445955	NW_003613440_39150_star	27.9388182	11.28298	4.854934103	3.618589217		1.308122	1	chr18_36296_mature	3.22371	2.417782	2.078510713	1.773060525		0.415037	0.684606
ssc-miR-4333	3.223709795	7.253347	2.078510713	3.044979305		-1.16993	0.445955	hsa-miR-4786-3p	6.44741959	0.805927	2.896740642	0.852739935		3	1	chr2_4298_mature	3.22371	2.417782	2.078510713	1.773060525		0.415037	0.684606
chr1_3720_star	3.223709795	7.253347	2.078510713	3.044979305		-1.16993	0.445955	chr7_15294_mature	10.7456993	30.62524	3.554060706	4.983004663		-1.51096	1	chr1_6_star	3.22371	2.417782	2.078510713	1.773060525		0.415037	0.684606
chr2_6087_star	3.223709795	7.253347	2.078510713	3.044979305		-1.16993	0.445955	chr4_10016_mature	2.14913986	14.50669	1.654957834	3.954819241		-2.75489	1	chr13_26885_mature	3.22371	2.417782	2.078510713	1.773060525		0.415037	0.684606
chr17_34997_star	3.223709795	7.253347	2.078510713	3.044979305		-1.16993	0.445955	chr15_32567_mature@@bta-miR-375	6.44741959	0.805927	2.896740642	0.852739935		3	1	chr12_25675_mature	76.29447	15.31262	6.272293205	4.027916745		2.316857	0.684758
chr17_35787_mature	3.223709795	7.253347	2.078510713	3.044979305		-1.16993	0.445955	chr1_1780_mature	8.59655945	1.611855	3.262517265	1.38507475		2.415037	1	chr13_28800_mature	76.29447	15.31262	6.272293205	4.027916745		2.316857	0.684758
chr15_33370_star	3.223709795	7.253347	2.078510713	3.044979305		-1.16993	0.445955	chr13_28840_star	4.29827973	19.34226	2.405524014	4.346407978		-2.16993	1	ssc-miR-149	40.29637	320.7591	5.367943153	8.329837252		-2.99277	0.68512
chr9_20889_star	3.223709795	7.253347	2.078510713	3.044979305		-1.16993	0.445955	chr13_27664_star	6.44741959	0.805927	2.896740642	0.852739935		3	1	rno-miR-873-3p	2.14914	2.417782	1.654957834	1.773060525		-0.16993	0.685285
chr7_15675_mature	17.19311891	5.641492	4.185320985	2.731507408		1.607683	0.447286	chr5_11725_mature	23.6405385	8.865202	4.62296188	3.302348583		1.415037	1	chrx_21717_star	2.14914	2.417782	1.654957834	1.773060525		-0.16993	0.685285
chr2_4605_star	2.149139864	4.029637	1.654957834	2.330454351		-0.90689	0.447517	chr17_35668_mature	12.8948392	3.22371	3.796477232	2.078510713		2	1	chr1_2507_star	2.14914	2.417782	1.654957834	1.773060525		-0.16993	0.685285
chr15_32527_mature	2.149139864	4.029637	1.654957834	2.330454351		-0.90689	0.447517	bta-miR-222	6.44741959	0.805927	2.896740642	0.852739935		3	1	chr1_3888_star	2.14914	2.417782	1.654957834	1.773060525		-0.16993	0.685285
chr1_2982_star	2.149139864	4.029637	1.654957834	2.330454351		-0.90689	0.447517	hsa-miR-548w	4.29827973	18.53633	2.405524014	4.288087668		-2.10852	1	chr5_11120_star	2.14914	2.417782	1.654957834	1.773060525		-0.16993	0.685285
chr8_18282_star	2.149139864	4.029637	1.654957834	2.330454351		-0.90689	0.447517	chr5_11343_mature	12.8948392	3.22371	3.796477232	2.078510713		2	1	chr11_25362_star	2.14914	2.417782	1.654957834	1.773060525		-0.16993	0.685285
chr14_31578_mature	2.149139864	4.029637	1.654957834	2.330454351		-0.90689	0.447517	chr6_14513_mature	10.7456993	2.417782	3.554060706	1.773060525		2.152003	1	hsa-miR-4724-3p	2.14914	2.417782	1.654957834	1.773060525		-0.16993	0.685285
hsa-miR-1181	2.149139864	4.029637	1.654957834	2.330454351		-0.90689	0.447517	NW_003539422_38814_mature	10.7456993	2.417782	3.554060706	1.773060525		2.152003	1	chr7_16402_mature	2.14914	2.417782	1.654957834	1.773060525		-0.16993	0.685285
chr1_3251_star	2.149139864	4.029637	1.654957834	2.330454351		-0.90689	0.447517	chr13_26893_mature	8.59655945	1.611855	3.262517265	1.38507475		2.415037	1	mmu-miR-669m-5p	2.14914	2.417782	1.654957834	1.773060525		-0.16993	0.685285
chr6_14536_mature	2.149139864	4.029637	1.654957834	2.330454351		-0.90689	0.447517	chr4_9973_star	6.44741959	0.805927	2.896740642	0.852739935		3	1	chr9_20132_mature	2.14914	2.417782	1.654957834	1.773060525		-0.16993	0.685285
NW_003539007_38427_star	2.149139864	4.029637	1.654957834	2.330454351		-0.90689	0.447517	chr4_10721_star	6.44741959	0.805927	2.896740642	0.852739935		3	1	chr3_6956_star	2.14914	2.417782	1.654957834	1.773060525		-0.16993	0.685285
chr11_24358_star	2.149139864	4.029637	1.654957834	2.330454351		-0.90689	0.447517	chr14_30485_mature	19.3422588	6.44742	4.346407978	2.896740642		1.584963	1	mmu-miR-1902	2.14914	2.417782	1.654957834	1.773060525		-0.16993	0.685285
chr4_9751_star	2.149139864	4.029637	1.654957834	2.330454351		-0.90689	0.447517	mmu-miR-486-5p	10.7456993	2.417782	3.554060706	1.773060525		2.152003	1	chrx_23098_star	2.14914	2.417782	1.654957834	1.773060525		-0.16993	0.685285
chr13_27077_mature	2.149139864	4.029637	1.654957834	2.330454351		-0.90689	0.447517	chr9_19640_star	6.44741959	0.805927	2.896740642	0.852739935		3	1	chr11_24562_star	2.14914	2.417782	1.654957834	1.773060525		-0.16993	0.685285
chr11_25386_mature	2.149139864	4.029637	1.654957834	2.330454351		-0.90689	0.447517	chr6_14972_mature	4.29827973	18.53633	2.405524014	4.288087668		-2.10852	1	NW_003539007_38433_star	2.14914	2.417782	1.654957834	1.773060525		-0.16993	0.685285
chrx_21526_star	2.149139864	4.029637	1.654957834	2.330454351		-0.90689	0.447517	hsa-miR-3168	8.59655945	1.611855	3.262517265	1.38507475		2.415037	1	chr3_7706_star	2.14914	2.417782	1.654957834	1.773060525		-0.16993	0.685285
chr7_17076_star	2.149139864	4.029637	1.654957834	2.330454351		-0.90689	0.447517	chr18_36422_mature	8.59655945	1.611855	3.262517265	1.38507475		2.415037	1	chr14_30282_star	2.14914	2.417782	1.654957834	1.773060525		-0.16993	0.685285
NW_003540989_39780_mature	2.149139864	4.029637	1.654957834	2.330454351		-0.90689	0.447517	rno-miR-320-5p	8.59655945	1.611855	3.262517265	1.38507475		2.415037	1	NW_003538949_38373_star	2.14914	2.417782	1.654957834	1.773060525		-0.16993	0.685285
NW_003613545_39630_mature	2.149139864	4.029637	1.654957834	2.330454351		-0.90689	0.447517	chr10_23462_star	12.8948392	3.22371	3.796477232	2.078510713		2	1	chr15_32377_star	2.14914	2.417782	1.654957834	1.773060525		-0.16993	0.685285
chr9_19718_mature	2.149139864	4.029637	1.654957834	2.330454351		-0.90689	0.447517	chr15_32700_star	6.44741959	0.805927	2.896740642	0.852739935		3	1	rno-miR-3541	2.14914	2.417782	1.654957834	1.773060525		-0.16993	0.685285
chr3_8631_mature	2.149139864	4.029637	1.654957834	2.330454351		-0.90689	0.447517	NW_003540965_39767_mature	6.44741959	0.805927	2.896740642	0.852739935		3	1	mmu-miR-5113	2.14914	2.417782	1.654957834	1.773060525		-0.16993	0.685285
bta-miR-2473	2.149139864	4.029637	1.654957834	2.330454351		-0.90689	0.447517	chr2_4882_mature	6.44741959	0.805927	2.896740642	0.852739935		3	1	chr15_31807_star	2.14914	2.417782	1.654957834	1.773060525		-0.16993	0.685285
chr13_28073_mature	2.149139864	4.029637	1.654957834	2.330454351		-0.90689	0.447517	hsa-miR-3118	6.44741959	0.805927	2.896740642	0.852739935		3	1	bta-miR-2439-3p	2.14914	2.417782	1.654957834	1.773060525		-0.16993	0.685285
NW_003538949_38371_mature	2.149139864	4.029637	1.654957834	2.330454351		-0.90689	0.447517	chr4_10459_mature	6.44741959	0.805927	2.896740642	0.852739935		3	1	chrx_21721_star	2.14914	2.417782	1.654957834	1.773060525		-0.16993	0.685285
rno-miR-582-5p	2.149139864	4.029637	1.654957834	2.330454351		-0.90689	0.447517	chr2_4263_mature	6.44741959	0.805927	2.896740642	0.852739935		3	1	chr1_989_star	2.14914	2.417782	1.654957834	1.773060525		-0.16993	0.685285
chr15_32092_mature	2.149139864	4.029637	1.654957834	2.330454351		-0.90689	0.447517	chr9_20112_mature	8.59655945	1.611855	3.262517265	1.38507475		2.415037	1	chr17_35456_star	2.14914	2.417782	1.654957834	1.773060525		-0.16993	0.685285
chr1_1421_mature	2.149139864	4.029637	1.654957834	2.330454351		-0.90689	0.447517	chr18_36498_star	6.44741959	0.805927	2.896740642	0.852739935		3	1	chr18_36269_star	2.14914	2.417782	1.654957834	1.773060525		-0.16993	0.685285
NW_003613297_38335_star	2.149139864	4.029637	1.654957834	2.330454351		-0.90689	0.447517	chr9_20121_star	6.44741959	0.805927	2.896740642	0.852739935		3	1	chr1_3094_star	2.14914	2.417782	1.654957834	1.773060525		-0.16993	0.685285
chr17_35096_star	2.149139864	4.029637	1.654957834	2.330454351		-0.90689	0.447517	rno-miR-488-3p	6.44741959	0.805927	2.896740642	0.852739935		3	1	NW_003541090_39901_star	2.14914	2.417782	1.654957834	1.773060525		-0.16993	0.685285
NW_003613226_37753_mature	2.149139864	4.029637	1.654957834	2.330454351		-0.90689	0.447517	chr14_30612_star	8.59655945	1.611855	3.262517265	1.38507475		2.415037	1	rno-miR-219-1-3p	2.14914	2.417782	1.654957834	1.773060525		-0.16993	0.685285
chr1_1175_mature	2.149139864	4.029637	1.654957834	2.330454351		-0.90689	0.447517	chr9_19298_mature	6.44741959	0.805927	2.896740642	0.852739935		3	1	chrx_23051_star	2.14914	2.417782	1.654957834	1.773060525		-0.16993	0.685285
hsa-miR-5580-5p	2.149139864	4.029637	1.654957834	2.330454351		-0.90689	0.447517	chr4_9780_mature	6.44741959	0.805927	2.896740642	0.852739935		3	1	chr13_27489_star	2.14914	2.417782	1.654957834	1.773060525		-0.16993	0.685285
chr10_23883_star	2.149139864	4.029637	1.654957834	2.330454351		-0.90689	0.447517	chr14_31022_star	6.44741959	0.805927	2.896740642	0.852739935		3	1	chr14_31235_star@@mmu-miR-34	2.14914	2.417782	1.654957834	1.773060525		-0.16993	0.685285
chr4_9685_star	2.149139864	4.029637	1.654957834	2.330454351		-0.90689	0.447517	chr3_7732_mature	10.7456993	2.417782	3.554060706	1.773060525		2.152003	1	rno-miR-93-5p	2.14914	2.417782	1.654957834	1.773060525		-0.16993	0.685285
chr3_7417_mature	2.149139864	4.029637	1.654957834	2.330454351		-0.90689	0.447517	chr16_34743_star	10.7456993	2.417782	3.554060706	1.773060525		2.152003	1	chr8_17350_star	2.14914	2.417782	1.654957834	1.773060525		-0.16993	0.685285
chr3_7739_star	15.04397905	74.14533	4.003960082	6.231611453		-2.30117	0.448428	chr14_29982_mature	8.59655945	26.59561	3.262517265	4.786366653		-1.62936	1	chr12_26049_star	2.14914	2.417782	1.654957834	1.773060525		-0.16993	0.685285
chr7_15096_mature	5.372849659	15.31262	2.671938627	4.027916745		-1.51096	0.448485	bta-miR-2401	6.44741959	0.805927	2.896740642	0.852739935		3	1	chr1_2954_star	2.14914	2.417782	1.654957834	1.773060525		-0.16993	0.685285
chr9_19640_mature	5.372849659	15.31262	2.671938627	4.027916745		-1.51096	0.448485	chr1_3121_mature	4.29827973	18.53633	2.405524014	4.288087668		-2.10852	1	bta-miR-500	2.14914	2.417782	1.654957834	1.773060525		-0.16993	0.685285
chr7_17143_mature	67.6979057	731.7821	6.102194213	9.517240499		-3.43423	0.448918	NW_003538201_37614_star	6.44741959	0.805927	2.896740642	0.852739935		3	1	chr6_12823_star	2.14914	2.417782	1.654957834	1.773060525		-0.16993	0.685285
chr6_14444_star	33.31166789	8.865202	5.100627352	3.302348583		1.909802	0.450005	chr6_14951_star	6.44741959	0.805927	2.896740642	0.852739935		3	1	bta-miR-2458	2.14914	2.417782	1.654957834	1.773060525		-0.16993	0.685285
chr7_16996_mature	7.521989523	24.98375	3.091190278	4.699537803		-1.7318	0.452927	hsa-miR-4466	12.8948392	3.22371	3.796477232	2.078510713		2	1	chr2_4133_mature	2.14914	2.417782	1.654957834	1.773060525		-0.16993	0.685285
NW_003540351_39435_mature	4.298279727	10.47706	2.405524014	3.520680821		-1.2854	0.452929	NW_003539812_39109_star	8.59655945	1.611855	3.262517265	1.38507475		2.415037	1	chr1_1185_star	2.14914	2.417782	1.654957834	1.773060525		-0.16993	0.685285
chr11_25285_mature	4.298279727	10.47706	2.405524014	3.520680821		-1.2854	0.452929	chr2_6203_star	8.59655945	1.611855	3.262517265	1.38507475		2.415037	1	hsa-miR-641	2.14914	2.417782	1.654957834	1.773060525		-0.16993	0.685285
chr17_35802_mature	4.298279727	10.47706	2.405524014	3.520680821		-1.2854	0.452929	hsa-miR-4524b-5p	6.44741959	0.805927	2.896740642	0.852739935		3	1	chr2_6503_mature	2.14914	2.417782	1.654957834	1.773060525		-0.16993	0.685285
chr14_30666_star	4.298279727	10.47706	2.405524014	3.520680821		-1.2854	0.452929	chr9_19205_mature	10.7456993	2.417782	3.554060706	1.773060525		2.152003	1	chr10_23618_mature	2.14914	2.417782	1.654957834	1.773060525		-0.16993	0.685285
chr10_23778_mature	4.298279727	10.47706	2.405524014	3.520680821		-1.2854	0.452929	chr8_18510_mature	4.29827973	19.34226	2.405524014	4.346407978		-2.16993	1	hsa-miR-4642	2.14914	2.417782	1.654957834	1.773060525		-0.16993	0.685285
chr13_27546_mature	4.298279727	10.47706	2.405524014	3.520680821		-1.2854	0.452929	chr13_28165_star	6.44741959	0.805927	2.896740642	0.852739935		3	1	bta-miR-17-5p	2.14914	2.417782	1.654957834	1.773060525		-0.16993	0.685285
bta-miR-2408	4.298279727	10.47706	2.405524014	3.520680821		-1.2854	0.452929	hsa-miR-635	6.44741959	0.805927	2.896740642	0.852739935		3	1	rno-miR-540-5p	2.14914	2.417782	1.654957834	1.773060525		-0.16993	0.685285
chr4_9657_mature	4.298279727	10.47706	2.405524014	3.520680821		-1.2854	0.452929	chr18_36577_mature	12.8948392	3.22371	3.796477232	2.078510713		2	1	chr6_12905_mature	2.14914	2.417782	1.654957834	1.773060525		-0.16993	0.685285
bta-miR-380-5p	4.298279727	10.47706	2.405524014	3.520680821		-1.2854	0.452929	chr6_14320_star	10.7456993	2.417782	3.554060706	1.773060525		2.152003	1	chr1_2014_star	2.14914	2.417782	1.654957834	1.773060525		-0.16993	0.685285
chr5_11356_mature	12.89483918	57.22085	3.796477232	5.863463969		-2.14975	0.453558	NW_003613326_38567_mature	6.44741959	0.805927	2.896740642	0.852739935		3	1	chr2_6447_star	2.14914	2.417782	1.654957834	1.773060525		-0.16993	0.685285
chrx_21606_mature	4.298279727	2.417782	2.405524014	1.773060525		0.830075	0.454997	chr1_927_mature	10.7456993	2.417782	3.554060706	1.773060525		2.152003	1	hsa-miR-194-5p	2.14914	2.417782	1.654957834	1.773060525		-0.16993	0.685285
hsa-miR-298	4.298279727	2.417782	2.405524014	1.773060525		0.830075	0.454997	hsa-miR-4640-5p	6.44741959	0.805927	2.896740642	0.852739935		3	1	chr16_33817_star	2.14914	2.417782	1.654957834	1.773060525		-0.16993	0.685285
chr11_25362_star	4.298279727	2.417782	2.405524014	1.773060525		0.830075	0.454997	hsa-miR-548a-3p	6.44741959	0.805927	2.896740642	0.852739935		3	1	chr1_3231_mature	5.37285	14.50669	2.671938627	3.954819241		-1.43296	0.685365
chr5_11657_mature	4.298279727	2.417782	2.405524014	1.773060525		0.830075	0.454997	NW_003540583_39564_star	6.44741959	0.805927	2.896740642	0.852739935		3	1	chr2_4893_star	11.28298	4.835565	3.618589217	2.544872269		1.222392	0.685432
chr4_8756_star	4.298279727	2.417782	2.405524014	1.773060525		0.830075	0.454997	chr6_14576_mature	58.0267763	30.62524	5.883297648	4.983004663		0.921997	1	chr4_9349_mature	11.28298	4.835565	3.618589217	2.544872269		1.222392	0.685432
chr17_35528_star	4.298279727	2.417782	2.405524014	1.773060525		0.830075	0.454997	chr12_26572_star	6.44741959	0.805927	2.896740642	0.852739935		3	1	chr13_27782_star	31.16253	8.865202	5.007308904	3.302348583		1.813587	0.685458
chr11_24794_star	4.298279727	2.417782	2.405524014	1.773060525		0.830075	0.454997	chr5_11265_mature	6.44741959	0.805927	2.896740642	0.852739935		3	1	chr14_30470_star	1.611855	1.611855	1.38507475	1.38507475		0	0.685789
chr11_24562_star	4.298279727	2.417782	2.405524014	1.773060525		0.830075	0.454997	chr9_19892_mature	6.44741959	0.805927	2.896740642	0.852739935		3	1	chr1_480_star	1.611855	1.611855	1.38507475	1.38507475		0	0.685789
chr15_32479_star	4.298279727	2.417782	2.405524014	1.773060525		0.830075	0.454997	chr9_20086_mature	6.44741959	0.805927	2.896740642	0.852739935		3	1	mmu-miR-6418-3p	1.611855	1.611855	1.38507475	1.38507475		0	0.685789
NW_003613530_39570_mature	4.298279727	2.417782	2.405524014	1.773060525		0.830075	0.454997	chr17_35528_mature	6.44741959	0.805927	2.896740642	0.852739935		3	1	chr2_5894_star	1.611855	1.611855	1.38507475	1.38507475		0	0.685789
chr5_11262_mature	4.298279727	2.417782	2.405524014	1.773060525		0.830075	0.454997	chr10_23974_mature	12.8948392	3.22371	3.796477232	2.078510713		2	1	rno-miR-181b-1-3p	1.611855	1.611855	1.38507475	1.38507475		0	0.685789
chr13_28998_mature	4.298279727	2.417782	2.405524014	1.773060525		0.830075	0.454997	chr4_10131_star	6.44741959	0.805927	2.896740642	0.852739935		3	1	chr18_36497_star	1.611855	1.611855	1.38507475	1.38507475		0	0.685789
chr4_9959_mature	4.298279727	2.417782	2.405524014	1.773060525		0.830075	0.454997	chr2_4523_mature	6.44741959	0.805927	2.896740642	0.852739935		3	1	chr4_10081_mature	1.611855	1.611855	1.38507475	1.38507475		0	0.685789
chr8_18754_star	4.298279727	2.417782	2.405524014	1.773060525		0.830075	0.454997	chr18_36365_star	10.7456993	2.417782	3.554060706	1.773060525		2.152003	1	chr6_12863_star	1.611855	1.611855	1.38507475	1.38507475		0	0.685789
NW_003539542_38921_star	4.298279727	2.417782	2.405524014	1.773060525		0.830075	0.454997	chr1_2370_star	10.7456993	2.417782	3.554060706	1.773060525		2.152003	1	hsa-miR-4419b	1.611855	1.611855	1.38507475	1.38507475		0	0.685789
chr1_3278_star	4.298279727	2.417782	2.405524014	1.773060525		0.830075	0.454997	bta-miR-378c	6.44741959	0.805927	2.896740642	0.852739935		3	1	chr13_28152_mature	1.611855	1.611855	1.38507475	1.38507475		0	0.685789
chr9_20110_star	4.298279727	2.417782	2.405524014	1.773060525		0.830075	0.454997	chr18_36628_star	6.44741959	0.805927	2.896740642	0.852739935		3	1	chr1_1911_mature	1.611855	1.611855	1.38507475	1.38507475		0	0.685789
chr4_8856_mature	4.298279727	2.417782	2.405524014	1.773060525		0.830075	0.454997	chr2_5974_mature	8.59655945	1.611855	3.262517265	1.38507475		2.415037	1	hsa-miR-4999-5p	1.611855	1.611855	1.38507475	1.38507475		0	0.685789
chr12_25429_mature	4.298279727	2.417782	2.405524014	1.773060525		0.830075	0.454997	chr7_15903_mature	6.44741959	0.805927	2.896740642	0.852739935		3	1	hsa-miR-1205	1.611855	1.611855	1.38507475	1.38507475		0	0.685789
chr11_24676_mature	4.298279727	2.417782	2.405524014	1.773060525		0.830075	0.454997	chr2_5533_star	6.44741959	0.805927	2.896740642	0.852739935		3	1	hsa-miR-4758-3p	1.611855	1.611855	1.38507475	1.38507475		0	0.685789
chr14_31235_star@@mmu-miR-346-	4.298279727	2.417782	2.405524014	1.773060525		0.830075	0.454997	rno-miR-3558-5p	6.44741959	0.805927	2.896740642	0.852739935		3	1	mmu-miR-199a-3p	1.611855	1.611855	1.38507475	1.38507475		0	0.685789
chr17_35504_star	4.298279727	2.417782	2.405524014	1.773060525		0.830075	0.454997	chr2_6372_star	8.59655945	1.611855	3.262517265	1.38507475		2.415037	1	chr15_33041_star	1.611855	1.611855	1.38507475	1.38507475		0	0.685789
chr9_19233_star	4.298279727	2.417782	2.405524014	1.773060525		0.830075	0.454997	chr18_36191_mature	10.7456993	2.417782	3.554060706	1.773060525		2.152003	1	chrx_22791_mature	1.611855	1.611855	1.38507475	1.38507475		0	0.685789
chr2_4295_star	4.298279727	2.417782	2.405524014	1.773060525		0.830075	0.454997	chr5_11103_mature	6.44741959	0.805927	2.896740642	0.852739935		3	1	chrx_23193_star	1.611855	1.611855	1.38507475	1.38507475		0	0.685789
chr4_9835_mature	4.298279727	2.417782	2.405524014	1.773060525		0.830075	0.454997	chr4_10393_mature	4.29827973	19.34226	2.405524014	4.346407978		-2.16993	1	chr6_14465_mature	1.611855	1.611855	1.38507475	1.38507475		0	0.685789
mmu-miR-5623-3p	4.298279727	2.417782	2.405524014	1.773060525		0.830075	0.454997	hsa-miR-302d-5p	10.7456993	2.417782	3.554060706	1.773060525		2.152003	1	chr15_33434_mature	1.611855	1.611855	1.38507475	1.38507475		0	0.685789
chr1_2055_star	4.298279727	2.417782	2.405524014	1.773060525		0.830075	0.454997	bta-miR-502a	6.44741959	0.805927	2.896740642	0.852739935		3	1	chr7_16635_star	1.611855	1.611855	1.38507475	1.38507475		0	0.685789
chr2_6503_mature	4.298279727	2.417782	2.405524014	1.773060525		0.830075	0.454997	chr14_30718_mature	6.44741959	0.805927	2.896740642	0.852739935		3	1	chr9_20115_mature	1.611855	1.611855	1.38507475	1.38507475		0	0.685789
chrx_21800_star	4.298279727	2.417782	2.405524014	1.773060525		0.830075	0.454997	chr12_26195_mature	10.7456993	2.417782	3.554060706	1.773060525		2.152003	1	NW_003613056_36922_star	1.611855	1.611855	1.38507475	1.38507475		0	0.685789
bta-miR-2383	4.298279727	2.417782	2.405524014	1.773060525		0.830075	0.454997	hsa-miR-211-3p	6.44741959	0.805927	2.896740642	0.852739935		3	1	chr6_12563_star	1.611855	1.611855	1.38507475	1.38507475		0	0.685789
chr6_12598_star	4.298279727	2.417782	2.405524014	1.773060525		0.830075	0.454997	chr7_16194_mature	6.44741959	0.805927	2.896740642	0.852739935		3	1	chr8_18850_star	1.611855	1.611855	1.38507475	1.38507475		0	0.685789
chr9_20563_mature	4.298279727	2.417782	2.405524014	1.773060525		0.830075	0.454997	ssc-miR-133b	152.58893	101.5469	7.26293043	6.680139486		0.587505	1	chr18_36050_star	1.611855	1.611855	1.38507475	1.38507475		0	0.685789
chrx_22000_star	4.298279727	2.417782	2.405524014	1.773060525		0.830075	0.454997	chr9_19145_star	6.44741959	0.805927	2.896740642	0.852739935		3	1	chr14_30864_star	1.611855	1.611855	1.38507475	1.38507475		0	0.685789
chr18_36296_mature	4.298279727	2.417782	2.405524014	1.773060525		0.830075	0.454997	chr6_12525_star@@bta-miR-2890	38.6845175	17.7304	5.310504361	4.227310103		1.125531	1	chr6_14888_mature	1.611855	1.611855	1.38507475	1.38507475		0	0.685789
NW_003613522_39530_star	4.298279727	2.417782	2.405524014	1.773060525		0.830075	0.454997	chr13_28621_star	6.44741959	0.805927	2.896740642	0.852739935		3	1	chr1_3507_star	1.611855	1.611855	1.38507475	1.38507475		0	0.685789
chr10_24282_mature	4.298279727	2.417782	2.405524014	1.773060525		0.830075	0.454997	chr9_20843_mature	6.44741959	0.805927	2.896740642	0.852739935		3	1	chr14_30480_mature	1.611855	1.611855	1.38507475	1.38507475		0	0.685789
hsa-miR-548h-5p	4.298279727	2.417782	2.405524014	1.773060525		0.830075	0.454997	chr1_2577_star	6.44741959	0.805927	2.896740642	0.852739935		3	1	hsa-miR-2355-3p	1.611855	1.611855	1.38507475	1.38507475		0	0.685789
chr3_7573_star	4.298279727	2.417782	2.405524014	1.773060525		0.830075	0.454997	chr4_8765_mature@@hsa-miR-4723-3	12.8948392	3.22371	3.796477232	2.078510713		2	1	chr8_18505_star	1.611855	1.611855	1.38507475	1.38507475		0	0.685789
chr3_6716_mature	4.298279727	2.417782	2.405524014	1.773060525		0.830075	0.454997	chr2_5884_star	6.44741959	0.805927	2.896740642	0.852739935		3	1	chr17_35612_star	1.611855	1.611855	1.38507475	1.38507475		0	0.685789
chr9_19114_mature	9.671129386	4.029637	3.415640968	2.330454351		1.263034	0.455331	chrx_21936_mature	10.7456993	2.417782	3.554060706	1.773060525		2.152003	1	chr13_27434_star	1.611855	1.611855	1.38507475	1.38507475		0	0.685789
hsa-miR-3917	9.671129386	4.029637	3.415640968	2.330454351		1.263034	0.455331	chr17_35115_star	2.14913986	13.70077	1.654957834	3.877819487		-2.67243	1	chr12_25581_star	1.611855	1.611855	1.38507475	1.38507475		0	0.685789
chr12_26097_mature	9.671129386	4.029637	3.415640968	2.330454351		1.263034	0.455331	ssc-miR-676-3p	92.4130141	141.8432	6.545551653	7.158288862		-0.61813	1	hsa-miR-3913-3p	1.611855	1.611855	1.38507475	1.38507475		0	0.685789
chr2_6092_star	9.671129386	4.029637	3.415640968	2.330454351		1.263034	0.455331	chr5_11544_mature	2.14913986	12.89484	1.654957834	3.796477232		-2.58496	1	chrx_22553_star	1.611855	1.611855	1.38507475	1.38507475		0	0.685789
chr15_32721_mature	9.671129386	4.029637	3.415640968	2.330454351		1.263034	0.455331	chr9_20275_mature	15.043979	4.835565	4.003960082	2.544872269		1.63743	1	chr7_16963_star	1.611855	1.611855	1.38507475	1.38507475		0	0.685789
chr2_6125_mature	9.671129386	4.029637	3.415640968	2.330454351		1.263034	0.455331	chr15_32248_mature	2.14913986	12.89484	1.654957834	3.796477232		-2.58496	1	NW_003613200_37636_star	1.611855	1.611855	1.38507475	1.38507475		0	0.685789
chr11_24856_star	9.671129386	4.029637	3.415640968	2.330454351		1.263034	0.455331	chr10_23437_star	2.14913986	12.89484	1.654957834	3.796477232		-2.58496	1	NW_003539115_38545_star	1.611855	1.611855	1.38507475	1.38507475		0	0.685789
chr8_18081_mature	9.671129386	4.029637	3.415640968	2.330454351		1.263034	0.455331	NW_003539007_38444_mature	10.7456993	29.81932	3.554060706	4.94576292		-1.47249	1	rno-miR-547-3p	1.611855	1.611855	1.38507475	1.38507475		0	0.685789
chr13_26645_mature	9.671129386	4.029637	3.415640968	2.330454351		1.263034	0.455331	chr13_28744_star	2.14913986	12.89484	1.654957834	3.796477232		-2.58496	1	chr6_13603_mature	1.611855	1.611855	1.38507475	1.38507475		0	0.685789
chr6_12967_mature	171.9311891	27.40153	7.43405428	4.827896911		2.649503	0.455337	chr1_3501_mature	2.14913986	13.70077	1.654957834	3.877819487		-2.67243	1	chr3_7786_mature	1.611855	1.611855	1.38507475	1.38507475		0	0.685789
NW_003613193_37595_mature	5.372849659	14.50669	2.671938627	3.954819241		-1.43296	0.455859	chr11_24331_mature@@mmu-miR-119	2.14913986	13.70077	1.654957834	3.877819487		-2.67243	1	chr18_35816_mature	1.611855	1.611855	1.38507475	1.38507475		0	0.685789
chr14_31039_star	3.223709795	6.44742	2.078510713	2.896740642		-1	0.455906	mmu-miR-5106	2.14913986	12.89484	1.654957834	3.796477232		-2.58496	1	chr2_4065_mature	1.611855	1.611855	1.38507475	1.38507475		0	0.685789
chrx_22969_mature	3.223709795	6.44742	2.078510713	2.896740642		-1	0.455906	chr6_12752_mature	2.14913986	13.70077	1.654957834	3.877819487		-2.67243	1	chr2_6503_star	1.611855	1.611855	1.38507475	1.38507475		0	0.685789
chr6_14709_mature	3.223709795	6.44742	2.078510713	2.896740642		-1	0.455906	chr11_25110_star	2.14913986	13.70077	1.654957834	3.877819487		-2.67243	1	mmu-miR-6366	1.611855	1.611855	1.38507475	1.38507475		0	0.685789
bta-miR-2285p	3.223709795	6.44742	2.078510713	2.896740642		-1	0.455906	chr5_10760_mature	2.14913986	12.89484	1.654957834	3.796477232		-2.58496	1	bta-miR-2285e	1.611855	1.611855	1.38507475	1.38507475		0	0.685789
NW_003541108_39908_mature	3.223709795	6.44742	2.078510713	2.896740642		-1	0.455906	chr13_26770_mature	2.14913986	12.89484	1.654957834	3.796477232		-2.58496	1	bta-miR-2285o	1.611855	1.611855	1.38507475	1.38507475		0	0.685789
chr2_5746_mature	3.223709795	6.44742	2.078510713	2.896740642		-1	0.455906	chr3_8074_mature	17.1931189	5.641492	4.185320985	2.731507408		1.607683	1	chr11_24465_star	1.611855	1.611855	1.38507475	1.38507475		0	0.685789
chr9_19985_star	3.223709795	6.44742	2.078510713	2.896740642		-1	0.455906	chr12_26134_star	15.043979	4.835565	4.003960082	2.544872269		1.63743	1	chr13_27063_star	1.611855	1.611855	1.38507475	1.38507475		0	0.685789
chr13_27323_star	3.223709795	6.44742	2.078510713	2.896740642		-1	0.455906	chr8_17856_mature	2.14913986	13.70077	1.654957834	3.877819487		-2.67243	1	mmu-miR-376c-5p	1.611855	1.611855	1.38507475	1.38507475		0	0.685789
chr9_19723_mature	3.223709795	6.44742	2.078510713	2.896740642		-1	0.455906	chr13_28512_star	2.14913986	12.89484	1.654957834	3.796477232		-2.58496	1	hsa-miR-4755-3p	1.611855	1.611855	1.38507475	1.38507475		0	0.685789
chr3_7094_mature	3.223709795	6.44742	2.078510713	2.896740642		-1	0.455906	chr15_32322_mature	15.043979	4.835565	4.003960082	2.544872269		1.63743	1	chr7_15100_mature	1.611855	1.611855	1.38507475	1.38507475		0	0.685789
NW_003539180_38634_star	3.223709795	6.44742	2.078510713	2.896740642		-1	0.455906	chr1_3174_mature	2.14913986	13.70077	1.654957834	3.877819487		-2.67243	1	mmu-miR-700-3p	1.611855	1.611855	1.38507475	1.38507475		0	0.685789
chr13_28690_mature	3.223709795	6.44742	2.078510713	2.896740642		-1	0.455906	chr11_24450_mature	21.4913986	46.74379	4.491301473	5.577241251		-1.12102	1	chr1_3714_star	1.611855	1.611855	1.38507475	1.38507475		0	0.685789
chr14_31236_mature@@bta-miR-34	27.93881823	8.059274	4.854934103	3.179395517		1.793549	0.456762	chr13_28696_star	6.44741959	22.56597	2.896740642	4.558633073		-1.80735	1	hsa-miR-6128	1.611855	1.611855	1.38507475	1.38507475		0	0.685789
chr3_7963_mature	27.93881823	8.059274	4.854934103	3.179395517		1.793549	0.456762	chrx_22916_star	2.14913986	13.70077	1.654957834	3.877819487		-2.67243	1	hsa-miR-769-3p	1.611855	1.611855	1.38507475	1.38507475		0	0.685789
ssc-miR-146a-3p	16.11854898	5.641492	4.097488514	2.731507408		1.514573	0.457418	chrx_22063_star	6.44741959	21.76004	2.896740642	4.508431259		-1.75489	1	chr1_1390_star	1.611855	1.611855	1.38507475	1.38507475		0	0.685789
chr15_33627_mature	208.4665668	31.43117	7.710576182	5.019309189		2.729548	0.457537	NW_003541277_40019_mature	62.325056	34.65488	5.984704544	5.156027656		0.846754	1	chr4_10016_star	1.611855	1.611855	1.38507475	1.38507475		0	0.685789
chr4_9000_star	2.149139864	3.22371	1.654957834	2.078510713		-0.58496	0.458169	ssc-miR-184	2.14913986	13.70077	1.654957834	3.877819487		-2.67243	1	bta-miR-16a	1.611855	1.611855	1.38507475	1.38507475		0	0.685789
chr15_32346_star	2.149139864	3.22371	1.654957834	2.078510713		-0.58496	0.458169	NW_003538009_37453_mature	17.1931189	5.641492	4.185320985	2.731507408		1.607683	1	chr9_19522_star	1.611855	1.611855	1.38507475	1.38507475		0	0.685789
chr13_27867_star	2.149139864	3.22371	1.654957834	2.078510713		-0.58496	0.458169	chr6_13265_mature	15.043979	4.835565	4.003960082	2.544872269		1.63743	1	chr3_6809_mature	1.611855	1.611855	1.38507475	1.38507475		0	0.685789
chr1_2577_mature	2.149139864	3.22371	1.654957834	2.078510713		-0.58496	0.458169	chr8_18510_star	4.29827973	17.7304	2.405524014	4.227310103		-2.04439	1	chr13_29417_mature	1.611855	1.611855	1.38507475	1.38507475		0	0.685789
chr16_34246_star	2.149139864	3.22371	1.654957834	2.078510713		-0.58496	0.458169	NW_003538807_38238_star	2.14913986	13.70077	1.654957834	3.877819487		-2.67243	1	chr1_1437_star	1.611855	1.611855	1.38507475	1.38507475		0	0.685789
chr6_12855_mature	2.149139864	3.22371	1.654957834	2.078510713		-0.58496	0.458169	NW_003613137_37368_star	2.14913986	12.89484	1.654957834	3.796477232		-2.58496	1	NW_003613290_38304_mature	1.611855	1.611855	1.38507475	1.38507475		0	0.685789
rno-miR-450a-5p	2.149139864	3.22371	1.654957834	2.078510713		-0.58496	0.458169	chr8_18290_star	2.14913986	12.89484	1.654957834	3.796477232		-2.58496	1	chr16_34293_star	1.611855	1.611855	1.38507475	1.38507475		0	0.685789
chrx_21932_mature	2.149139864	3.22371	1.654957834	2.078510713		-0.58496	0.458169	chr7_16660_mature	15.043979	4.835565	4.003960082	2.544872269		1.63743	1	chr14_31530_mature	1.611855	1.611855	1.38507475	1.38507475		0	0.685789
chr17_35074_mature	2.149139864	3.22371	1.654957834	2.078510713		-0.58496	0.458169	chrx_21678_mature	2.14913986	12.89484	1.654957834	3.796477232		-2.58496	1	chr11_24357_star	1.611855	1.611855	1.38507475	1.38507475		0	0.685789
chrx_21753_mature	2.149139864	3.22371	1.654957834	2.078510713		-0.58496	0.458169	chr8_17694_mature	2.14913986	13.70077	1.654957834	3.877819487		-2.67243	1	chr9_20112_mature	1.611855	1.611855	1.38507475	1.38507475		0	0.685789
chr5_12291_star	2.149139864	3.22371	1.654957834	2.078510713		-0.58496	0.458169	chr1_1781_mature	4.29827973	17.7304	2.405524014	4.227310103		-2.04439	1	chr1_2009_star	1.611855	1.611855	1.38507475	1.38507475		0	0.685789
chr13_28221_star	2.149139864	3.22371	1.654957834	2.078510713		-0.58496	0.458169	chr15_32695_mature	15.043979	4.835565	4.003960082	2.544872269		1.63743	1	chr2_4071_mature	1.611855	1.611855	1.38507475	1.38507475		0	0.685789
NW_003613232_37791_mature	2.149139864	3.22371	1.654957834	2.078510713		-0.58496	0.458169	chr4_10705_mature	6.44741959	22.56597	2.896740642	4.558633073		-1.80735	1	chr16_34464_mature	1.611855	1.611855	1.38507475	1.38507475		0	0.685789
chr2_5321_mature	2.149139864	3.22371	1.654957834	2.078510713		-0.58496	0.458169	chr7_15287_star	15.043979	4.835565	4.003960082	2.544872269		1.63743	1	chr14_30612_star	1.611855	1.611855	1.38507475	1.38507475		0	0.685789
chr8_18128_star	2.149139864	3.22371	1.654957834	2.078510713		-0.58496	0.458169	chr8_17840_mature	12.8948392	4.029637	3.796477232	2.330454351		1.678072	1	hsa-miR-4781-5p	1.611855	1.611855	1.38507475	1.38507475		0	0.685789
hsa-miR-3941	2.149139864	3.22371	1.654957834	2.078510713		-0.58496	0.458169	chr11_25269_star	2.14913986	0.805927	1.654957834	0.852739935		1.415037	1	chr1_1337_mature	1.611855	1.611855	1.38507475	1.38507475		0	0.685789
mmu-miR-1938	2.149139864	3.22371	1.654957834	2.078510713		-0.58496	0.458169	chr4_9456_star	2.14913986	0.805927	1.654957834	0.852739935		1.415037	1	chr4_10175_star	1.611855	1.611855	1.38507475	1.38507475		0	0.685789
chr10_23707_star	2.149139864	3.22371	1.654957834	2.078510713		-0.58496	0.458169	chr12_26154_mature	2.14913986	0.805927	1.654957834	0.852739935		1.415037	1	NW_003537737_37236_mature	1.611855	1.611855	1.38507475	1.38507475		0	0.685789
chr7_16503_mature	2.149139864	3.22371	1.654957834	2.078510713		-0.58496	0.458169	chr6_13306_star	2.14913986	0.805927	1.654957834	0.852739935		1.415037	1	chr5_11637_mature	1.611855	1.611855	1.38507475	1.38507475		0	0.685789
hsa-miR-193b-3p	2.149139864	3.22371	1.654957834	2.078510713		-0.58496	0.458169	chr5_10979_mature	4.29827973	0.805927	2.405524014	0.852739935		2.415037	1	mmu-miR-3069-3p	1.611855	1.611855	1.38507475	1.38507475		0	0.685789
hsa-miR-5004-3p	2.149139864	3.22371	1.654957834	2.078510713		-0.58496	0.458169	chr1_1579_star	2.14913986	0.805927	1.654957834	0.852739935		1.415037	1	chr7_16903_star	1.611855	1.611855	1.38507475	1.38507475		0	0.685789
chr2_5807_star	2.149139864	3.22371	1.654957834	2.078510713		-0.58496	0.458169	chr9_19555_star	2.14913986	0.805927	1.654957834	0.852739935		1.415037	1	hsa-miR-4495	1.611855	1.611855	1.38507475	1.38507475		0	0.685789
chr14_31302_mature	2.149139864	3.22371	1.654957834	2.078510713		-0.58496	0.458169	chr6_14429_mature	8.59655945	2.417782	3.262517265	1.773060525		1.830075	1	hsa-miR-561-3p	1.611855	1.611855	1.38507475	1.38507475		0	0.685789
chr6_14789_mature	2.149139864	3.22371	1.654957834	2.078510713		-0.58496	0.458169	hsa-miR-4652-3p	4.29827973	0.805927	2.405524014	0.852739935		2.415037	1	chr16_33722_mature	1.611855	1.611855	1.38507475	1.38507475		0	0.685789
rno-miR-196c-5p	2.149139864	3.22371	1.654957834	2.078510713		-0.58496	0.458169	NW_003540337_39426_mature	2.14913986	0.805927	1.654957834	0.852739935		1.415037	1	chr15_31774_mature	1.611855	1.611855	1.38507475	1.38507475		0	0.685789
chr14_29715_star	2.149139864	3.22371	1.654957834	2.078510713		-0.58496	0.458169	chr8_18908_star	4.29827973	0.805927	2.405524014	0.852739935		2.415037	1	chr17_35263_star	1.611855	1.611855	1.38507475	1.38507475		0	0.685789
chr15_31671_mature	2.149139864	3.22371	1.654957834	2.078510713		-0.58496	0.458169	chr13_26975_mature	23.6405385	9.671129	4.62296188	3.415640968		1.289507	1	mmu-miR-691	1.611855	1.611855	1.38507475	1.38507475		0	0.685789
ssc-miR-494	2.149139864	3.22371	1.654957834	2.078510713		-0.58496	0.458169	chr16_34547_star	2.14913986	0.805927	1.654957834	0.852739935		1.415037	1	mmu-miR-466q	1.611855	1.611855	1.38507475	1.38507475		0	0.685789
chr8_18247_mature	2.149139864	3.22371	1.654957834	2.078510713		-0.58496	0.458169	hsa-miR-3680-3p	4.29827973	0.805927	2.405524014	0.852739935		2.415037	1	mmu-miR-3075-3p	1.611855	1.611855	1.38507475	1.38507475		0	0.685789
chr14_30730_mature	2.149139864	3.22371	1.654957834	2.078510713		-0.58496	0.458169	chr17_35440_mature	2.14913986	0.805927	1.654957834	0.852739935		1.415037	1	chr7_16525_mature	1.611855	1.611855	1.38507475	1.38507475		0	0.685789
chr12_25827_mature	2.149139864	3.22371	1.654957834	2.078510713		-0.58496	0.458169	chr5_11673_star	8.59655945	2.417782	3.262517265	1.773060525		1.830075	1	hsa-miR-519e-5p	1.611855	1.611855	1.38507475	1.38507475		0	0.685789
chr4_8682_mature	2.149139864	3.22371	1.654957834	2.078510713		-0.58496	0.458169	chr7_15541_star	2.14913986	0.805927	1.654957834	0.852739935		1.415037	1	chr13_28864_mature	1.611855	1.611855	1.38507475	1.38507475		0	0.685789
mmu-miR-148a-3p	2.149139864	3.22371	1.654957834	2.078510713		-0.58496	0.458169	chr15_31929_star	2.14913986	0.805927	1.654957834	0.852739935		1.415037	1	chr17_35678_star	1.611855	1.611855	1.38507475	1.38507475		0	0.685789
chr10_24085_star	2.149139864	3.22371	1.654957834	2.078510713		-0.58496	0.458169	chr9_19693_mature	4.29827973	0.805927	2.405524014	0.852739935		2.415037	1	chr1_3765_star	1.611855	1.611855	1.38507475	1.38507475		0	0.685789
chr6_14228_star	2.149139864	3.22371	1.654957834	2.078510713		-0.58496	0.458169	chr7_16671_star	2.14913986	0.805927	1.654957834	0.852739935		1.415037	1	chr9_20114_star	1.611855	1.611855	1.38507475	1.38507475		0	0.685789
chr13_27515_star	2.149139864	3.22371	1.654957834	2.078510713		-0.58496	0.458169	chr13_27184_star	2.14913986	0.805927	1.654957834	0.852739935		1.415037	1	chr15_32527_star	1.611855	1.611855	1.38507475	1.38507475		0	0.685789
chr12_26402_star	2.149139864	3.22371	1.654957834	2.078510713		-0.58496	0.458169	chr15_33038_mature	2.14913986	0.805927	1.654957834	0.852739935		1.415037	1	chrx_22660_mature	1.611855	1.611855	1.38507475	1.38507475		0	0.685789
chr2_5564_star@@mmu-miR-670-5p	2.149139864	3.22371	1.654957834	2.078510713		-0.58496	0.458169	chr10_23371_star	2.14913986	0.805927	1.654957834	0.852739935		1.415037	1	chr15_32637_mature	1.611855	1.611855	1.38507475	1.38507475		0	0.685789
chr1_376_star	2.149139864	3.22371	1.654957834	2.078510713		-0.58496	0.458169	chr1_1111_star	6.44741959	1.611855	2.896740642	1.38507475		2	1	chr6_12506_mature	1.611855	1.611855	1.38507475	1.38507475		0	0.685789
bta-miR-197	2.149139864	3.22371	1.654957834	2.078510713		-0.58496	0.458169	chr4_10011_star	2.14913986	0.805927	1.654957834	0.852739935		1.415037	1	chr9_19145_mature@@hsa-miR-47	1.611855	1.611855	1.38507475	1.38507475		0	0.685789
chr1_1479_mature	2.149139864	3.22371	1.654957834	2.078510713		-0.58496	0.458169	chr4_9639_star	4.29827973	0.805927	2.405524014	0.852739935		2.415037	1	chr6_14281_mature	1.611855	1.611855	1.38507475	1.38507475		0	0.685789
chr8_17786_star	2.149139864	3.22371	1.654957834	2.078510713		-0.58496	0.458169	chr6_14824_star	2.14913986	0.805927	1.654957834	0.852739935		1.415037	1	chr13_26893_star	1.611855	1.611855	1.38507475	1.38507475		0	0.685789
chr1_3894_mature@@bta-miR-219-3	2.149139864	3.22371	1.654957834	2.078510713		-0.58496	0.458169	hsa-miR-4784	2.14913986	0.805927	1.654957834	0.852739935		1.415037	1	chr7_15101_star	1.611855	1.611855	1.38507475	1.38507475		0	0.685789
chr5_11934_star	2.149139864	3.22371	1.654957834	2.078510713		-0.58496	0.458169	chr6_14749_mature	8.59655945	2.417782	3.262517265	1.773060525		1.830075	1	hsa-miR-211-5p	1.611855	1.611855	1.38507475	1.38507475		0	0.685789

chr7_15705_star	2.149139864	3.22371	1.654957834	2.078510713		-0.58496	0.458169		hsa-miR-4770	2.14913986	0.805927	1.654957834	0.852739935		1.415037	1		hsa-miR-2277-5p	1.611855	1.611855	1.38507475	1.38507475		0	0.685789
chr18_35895_star	2.149139864	3.22371	1.654957834	2.078510713		-0.58496	0.458169		chr15_33276_mature	12.8948392	4.029637	3.796477232	2.330454351		1.678072	1		bta-miR-2331-3p	1.611855	1.611855	1.38507475	1.38507475		0	0.685789
chr2_4019_star	2.149139864	3.22371	1.654957834	2.078510713		-0.58496	0.458169		mmu-miR-6418-5p	2.14913986	0.805927	1.654957834	0.852739935		1.415037	1		hsa-miR-506-3p	1.611855	1.611855	1.38507475	1.38507475		0	0.685789
NW_003613507_39500_star	2.149139864	3.22371	1.654957834	2.078510713		-0.58496	0.458169		hsa-miR-4791	6.44741959	1.611855	2.896740642	1.38507475		2	1		NW_003539542_38921_mature	2.686425	4.029637	1.882222339	2.330454351		-0.58496	0.685909
hsa-miR-4418	2.149139864	3.22371	1.654957834	2.078510713		-0.58496	0.458169		NW_003613290_38305_star	2.14913986	0.805927	1.654957834	0.852739935		1.415037	1		chr2_5588_mature	2.686425	4.029637	1.882222339	2.330454351		-0.58496	0.685909
chr10_24174_mature	2.149139864	3.22371	1.654957834	2.078510713		-0.58496	0.458169		chr6_13246_star	6.44741959	1.611855	2.896740642	1.38507475		2	1		chr13_29025_mature	2.686425	4.029637	1.882222339	2.330454351		-0.58496	0.685909
NW_003539571_38948_mature	2.149139864	3.22371	1.654957834	2.078510713		-0.58496	0.458169		chrx_21651_star	2.14913986	0.805927	1.654957834	0.852739935		1.415037	1		chr6_12901_mature	2.686425	4.029637	1.882222339	2.330454351		-0.58496	0.685909
chr2_4356_star	2.149139864	3.22371	1.654957834	2.078510713		-0.58496	0.458169		NW_003538449_37886_star	2.14913986	0.805927	1.654957834	0.852739935		1.415037	1		chr8_17571_star	2.686425	4.029637	1.882222339	2.330454351		-0.58496	0.685909
chr3_8536_mature	2.149139864	3.22371	1.654957834	2.078510713		-0.58496	0.458169		hsa-miR-1321	2.14913986	0.805927	1.654957834	0.852739935		1.415037	1		chr14_29829_mature	2.686425	4.029637	1.882222339	2.330454351		-0.58496	0.685909
chr12_26006_star	2.149139864	3.22371	1.654957834	2.078510713		-0.58496	0.458169		NW_003538371_37782_star	2.14913986	0.805927	1.654957834	0.852739935		1.415037	1		chr17_35199_star	2.686425	4.029637	1.882222339	2.330454351		-0.58496	0.685909
chr14_30822_mature	2.149139864	3.22371	1.654957834	2.078510713		-0.58496	0.458169		hsa-miR-3150a-3p	2.14913986	0.805927	1.654957834	0.852739935		1.415037	1		chr7_15734_star	2.686425	4.029637	1.882222339	2.330454351		-0.58496	0.685909
mmu-miR-3110-3p	2.149139864	3.22371	1.654957834	2.078510713		-0.58496	0.458169		chr2_4930_star	4.29827973	16.11855	2.405524014	4.097488514		-1.90689	1		chr7_16231_mature	2.686425	4.029637	1.882222339	2.330454351		-0.58496	0.685909
chr17_35182_star	2.149139864	3.22371	1.654957834	2.078510713		-0.58496	0.458169		hsa-miR-23c	4.29827973	0.805927	2.405524014	0.852739935		2.415037	1		chr14_29474_mature	2.686425	4.029637	1.882222339	2.330454351		-0.58496	0.685909
chr3_7100_star	2.149139864	3.22371	1.654957834	2.078510713		-0.58496	0.458169		chrx_21606_mature	8.59655945	2.417782	3.262517265	1.773060525		1.830075	1		chr4_8769_mature	2.686425	4.029637	1.882222339	2.330454351		-0.58496	0.685909
chr14_30832_star	2.149139864	3.22371	1.654957834	2.078510713		-0.58496	0.458169		chr9_20062_mature	2.14913986	11.28298	1.654957834	3.618589217		-2.39232	1		chr2_5320_mature	2.686425	4.029637	1.882222339	2.330454351		-0.58496	0.685909
chr16_33921_mature	8.596559455	29.81932	3.262517265	4.94576292		-1.79442	0.458307		chr16_34636_star	4.29827973	0.805927	2.405524014	0.852739935		2.415037	1		mmu-miR-3057-5p	2.686425	4.029637	1.882222339	2.330454351		-0.58496	0.685909
chr8_17244_star	2.149139864	1.611855	1.654957834	1.38507475		0.415037	0.458605		bta-miR-2882	2.14913986	0.805927	1.654957834	0.852739935		1.415037	1		chr8_17522_star	2.686425	4.029637	1.882222339	2.330454351		-0.58496	0.685909
chr16_34314_mature	2.149139864	1.611855	1.654957834	1.38507475		0.415037	0.458605		hsa-miR-3614-3p	2.14913986	0.805927	1.654957834	0.852739935		1.415037	1		chrx_21526_star	2.686425	4.029637	1.882222339	2.330454351		-0.58496	0.685909
chrx_21735_star	2.149139864	1.611855	1.654957834	1.38507475		0.415037	0.458605		hsa-miR-4739	2.14913986	0.805927	1.654957834	0.852739935		1.415037	1		chr4_9960_star	2.686425	4.029637	1.882222339	2.330454351		-0.58496	0.685909
chr5_12026_star	2.149139864	1.611855	1.654957834	1.38507475		0.415037	0.458605		chr2_5921_star	8.59655945	2.417782	3.262517265	1.773060525		1.830075	1		chr2_4183_mature	2.686425	4.029637	1.882222339	2.330454351		-0.58496	0.685909
hsa-miR-4445-5p	2.149139864	1.611855	1.654957834	1.38507475		0.415037	0.458605		hsa-miR-4767	2.14913986	0.805927	1.654957834	0.852739935		1.415037	1		hsa-miR-4295	2.686425	4.029637	1.882222339	2.330454351		-0.58496	0.685909
chr6_13349_mature	2.149139864	1.611855	1.654957834	1.38507475		0.415037	0.458605		NW_003613022_36785_mature	8.59655945	2.417782	3.262517265	1.773060525		1.830075	1		rno-miR-93-3p	2.686425	4.029637	1.882222339	2.330454351		-0.58496	0.685909
chr9_19128_star	2.149139864	1.611855	1.654957834	1.38507475		0.415037	0.458605		chr8_17814_mature	4.29827973	0.805927	2.405524014	0.852739935		2.415037	1		chr3_8070_star	2.686425	4.029637	1.882222339	2.330454351		-0.58496	0.685909
chr2_4626_star	2.149139864	1.611855	1.654957834	1.38507475		0.415037	0.458605		chr17_35073_star	2.14913986	0.805927	1.654957834	0.852739935		1.415037	1		chr3_7353_star	2.686425	4.029637	1.882222339	2.330454351		-0.58496	0.685909
chr14_30531_star	2.149139864	1.611855	1.654957834	1.38507475		0.415037	0.458605		NW_003539920_39178_mature	143.992371	97.51722	7.179833181	6.622304032		0.562263	1		chr18_36376_mature	2.686425	4.029637	1.882222339	2.330454351		-0.58496	0.685909
chr2_6101_star	2.149139864	1.611855	1.654957834	1.38507475		0.415037	0.458605		hsa-miR-1285-3p	4.29827973	0.805927	2.405524014	0.852739935		2.415037	1		chrx_21531_star	2.686425	4.029637	1.882222339	2.330454351		-0.58496	0.685909
chr11_24796_mature	2.149139864	1.611855	1.654957834	1.38507475		0.415037	0.458605		chr6_14314_mature	4.29827973	0.805927	2.405524014	0.852739935		2.415037	1		chr11_24856_star	2.686425	4.029637	1.882222339	2.330454351		-0.58496	0.685909
chr17_35478_mature	2.149139864	1.611855	1.654957834	1.38507475		0.415037	0.458605		chr13_29007_mature	2.14913986	0.805927	1.654957834	0.852739935		1.415037	1		mmu-miR-145b	2.686425	4.029637	1.882222339	2.330454351		-0.58496	0.685909
hsa-miR-361-5p	2.149139864	1.611855	1.654957834	1.38507475		0.415037	0.458605		hsa-miR-4762-3p	2.14913986	0.805927	1.654957834	0.852739935		1.415037	1		bta-miR-2299-5p	4.835565	12.08891	2.544872269	3.710273245		-1.32193	0.685959
chr15_33317_star	2.149139864	1.611855	1.654957834	1.38507475		0.415037	0.458605		rno-miR-20a-5p	2.14913986	0.805927	1.654957834	0.852739935		1.415037	1		chr1_3121_star	4.835565	12.08891	2.544872269	3.710273245		-1.32193	0.685959
chrx_22367_star	2.149139864	1.611855	1.654957834	1.38507475		0.415037	0.458605		mmu-miR-147-5p	2.14913986	0.805927	1.654957834	0.852739935		1.415037	1		chr5_11615_star	4.835565	12.08891	2.544872269	3.710273245		-1.32193	0.685959
chrx_22791_mature	2.149139864	1.611855	1.654957834	1.38507475		0.415037	0.458605		chr15_31842_mature	8.59655945	2.417782	3.262517265	1.773060525		1.830075	1		hsa-miR-618	4.835565	12.08891	2.544872269	3.710273245		-1.32193	0.685959
chr1_3837_mature	2.149139864	1.611855	1.654957834	1.38507475		0.415037	0.458605		chr14_30718_star	4.29827973	0.805927	2.405524014	0.852739935		2.415037	1		chr14_31039_star	18.26769	6.44742	4.268111626	2.896740642		1.5025	0.686205
bta-miR-2464-5p	2.149139864	1.611855	1.654957834	1.38507475		0.415037	0.458605		chr7_17029_star	2.14913986	0.805927	1.654957834	0.852739935		1.415037	1		chr8_18841_mature	18.26769	6.44742	4.268111626	2.896740642		1.5025	0.686205
chr2_4454_mature	2.149139864	1.611855	1.654957834	1.38507475		0.415037	0.458605		ssc-miR-181d-3p	6.44741959	1.611855	2.896740642	1.38507475		2	1		ssc-miR-106a	4023.727	185.3633	11.97467525	7.541974074		4.440105	0.686296
mmu-miR-5132-5p	2.149139864	1.611855	1.654957834	1.38507475		0.415037	0.458605		chr6_12863_star	6.44741959	1.611855	2.896740642	1.38507475		2	1		chr5_12291_star	5.37285	3.22371	2.671938627	2.078510713		0.736966	0.686376
NW_003539018_38471_mature	2.149139864	1.611855	1.654957834	1.38507475		0.415037	0.458605		chr6_14329_mature	4.29827973	0.805927	2.405524014	0.852739935		2.415037	1		chr12_25827_mature	5.37285	3.22371	2.671938627	2.078510713		0.736966	0.686376
chr14_31648_star	2.149139864	1.611855	1.654957834	1.38507475		0.415037	0.458605		NW_003539115_38544_mature	8.59655945	2.417782	3.262517265	1.773060525		1.830075	1		chr1_3113_star	5.37285	3.22371	2.671938627	2.078510713		0.736966	0.686376
chr3_7684_mature	2.149139864	1.611855	1.654957834	1.38507475		0.415037	0.458605		chr17_35034_mature	2.14913986	0.805927	1.654957834	0.852739935		1.415037	1		chr7_16979_star	5.37285	3.22371	2.671938627	2.078510713		0.736966	0.686376
bta-miR-1814a	2.149139864	1.611855	1.654957834	1.38507475		0.415037	0.458605		chr4_9354_star	4.29827973	0.805927	2.405524014	0.852739935		2.415037	1		chr13_27982_star	5.37285	3.22371	2.671938627	2.078510713		0.736966	0.686376
chr8_17646_star	2.149139864	1.611855	1.654957834	1.38507475		0.415037	0.458605		hsa-miR-3944-3p	4.29827973	0.805927	2.405524014	0.852739935		2.415037	1		NW_003539012_38462_star	5.37285	3.22371	2.671938627	2.078510713		0.736966	0.686376
chr15_31926_star	2.149139864	1.611855	1.654957834	1.38507475		0.415037	0.458605		chr12_26001_mature	4.29827973	0.805927	2.405524014	0.852739935		2.415037	1		chr9_20399_mature	5.37285	3.22371	2.671938627	2.078510713		0.736966	0.686376
hsa-miR-5007-3p	2.149139864	1.611855	1.654957834	1.38507475		0.415037	0.458605		mmu-miR-92a-2-5p	2.14913986	0.805927	1.654957834	0.852739935		1.415037	1		bta-miR-2404	5.37285	3.22371	2.671938627	2.078510713		0.736966	0.686376
mmu-miR-1969	2.149139864	1.611855	1.654957834	1.38507475		0.415037	0.458605		chr15_32840_star	4.29827973	0.805927	2.405524014	0.852739935		2.415037	1		ssc-miR-421-5p	5.37285	3.22371	2.671938627	2.078510713		0.736966	0.686376
NW_003613056_36922_star	2.149139864	1.611855	1.654957834	1.38507475		0.415037	0.458605		chr5_11638_star	2.14913986	0.805927	1.654957834	0.852739935		1.415037	1		chr15_32593_star	5.37285	3.22371	2.671938627	2.078510713		0.736966	0.686376
chr11_24334_mature	2.149139864	1.611855	1.654957834	1.38507475		0.415037	0.458605		chr16_34391_star	4.29827973	0.805927	2.405524014	0.852739935		2.415037	1		chr4_8765_mature@@hsa-miR-472	5.37285	3.22371	2.671938627	2.078510713		0.736966	0.686376
chr5_11704_mature@@rno-miR-355	2.149139864	1.611855	1.654957834	1.38507475		0.415037	0.458605		chr14_31063_mature	4.29827973	0.805927	2.405524014	0.852739935		2.415037	1		NW_003539989_39206_mature	14.50669	5.641492	3.954819241	2.731507408		1.36257	0.686774
hsa-miR-337-5p	2.149139864	1.611855	1.654957834	1.38507475		0.415037	0.458605		chr17_35071_star	2.14913986	0.805927	1.654957834	0.852739935		1.415037	1		chr15_32527_mature	8.059274	4.029637	3.179395517	2.330454351		1	0.686824
chr15_32040_mature	2.149139864	1.611855	1.654957834	1.38507475		0.415037	0.458605		chr9_20113_star	4.29827973	0.805927	2.405524014	0.852739935		2.415037	1		chr1_986_star	8.059274	4.029637	3.179395517	2.330454351		1	0.686824
chr4_9707_star	2.149139864	1.611855	1.654957834	1.38507475		0.415037	0.458605		chr10_23828_star	4.29827973	0.805927	2.405524014	0.852739935		2.415037	1		hsa-miR-4802-5p	8.059274	4.029637	3.179395517	2.330454351		1	0.686824
hsa-miR-509-5p	2.149139864	1.611855	1.654957834	1.38507475		0.415037	0.458605		chrx_23097_star	2.14913986	0.805927	1.654957834	0.852739935		1.415037	1		chr15_32974_star	8.059274	4.029637	3.179395517	2.330454351		1	0.686824
chr1_539_star	2.149139864	1.611855	1.654957834	1.38507475		0.415037	0.458605		chr1_2051_star	23.6405385	9.671129	4.62296188	3.415640968		1.289507	1		NW_003613282_38228_star	8.059274	4.029637	3.179395517	2.330454351		1	0.686824
chr6_14069_star	2.149139864	1.611855	1.654957834	1.38507475		0.415037	0.458605		mmu-miR-1199-5p	2.14913986	0.805927	1.654957834	0.852739935		1.415037	1		chr12_26404_mature	2.686425	2.417782	1.882222339	1.773060525		0.152003	0.686833
chr2_5392_star	2.149139864	1.611855	1.654957834	1.38507475		0.415037	0.458605		chr14_31255_mature	6.44741959	1.611855	2.896740642	1.38507475		2	1		chr10_23328_star	2.686425	2.417782	1.882222339	1.773060525		0.152003	0.686833
chr7_16788_mature	2.149139864	1.611855	1.654957834	1.38507475		0.415037	0.458605		chr5_12356_star	2.14913986	0.805927	1.654957834	0.852739935		1.415037	1		chr13_29234_star	2.686425	2.417782	1.882222339	1.773060525		0.152003	0.686833
chr14_30369_star	2.149139864	1.611855	1.654957834	1.38507475		0.415037	0.458605		chr15_32852_star	4.29827973	0.805927	2.405524014	0.852739935		2.415037	1		chr2_6585_star	2.686425	2.417782	1.882222339	1.773060525		0.152003	0.686833
chr5_11655_star	2.149139864	1.611855	1.654957834	1.38507475		0.415037	0.458605		mmu-miR-1199-3p	4.29827973	0.805927	2.405524014	0.852739935		2.415037	1		rno-miR-679	2.686425	2.417782	1.882222339	1.773060525		0.152003	0.686833
chr8_18354_mature	2.149139864	1.611855	1.654957834	1.38507475		0.415037	0.458605		chr5_11410_mature	485.705609	597.1922	8.926905591	9.224465384		-0.29811	1		mmu-miR-1970	2.686425	2.417782	1.882222339	1.773060525		0.152003	0.686833
chrx_21498_star	2.149139864	1.611855	1.654957834	1.38507475		0.415037	0.458605		hsa-miR-3681-5p	2.14913986	0.805927	1.654957834	0.852739935		1.415037	1		chr11_25375_star	2.686425	2.417782	1.882222339	1.773060525		0.152003	0.686833
chr9_20086_star	2.149139864	1.611855	1.654957834	1.38507475		0.415037	0.458605		chr13_27663_star	2.14913986	0.805927	1.654957834	0.852739935		1.415037	1		chr3_7884_star	2.686425	2.417782	1.882222339	1.773060525		0.152003	0.686833
hsa-miR-2276	2.149139864	1.611855	1.654957834	1.38507475		0.415037	0.458605		NW_003613367_38733_mature	19.3422588	7.253347	4.346407978	3.044979305		1.415037	1		chr15_32479_star	2.686425	2.417782	1.882222339	1.773060525		0.152003	0.686833
chr5_11357_mature	2.149139864	1.611855	1.654957834	1.38507475		0.415037	0.458605		chr13_26798_star	4.29827973	0.805927	2.405524014	0.852739935		2.415037	1		chr9_20405_star	2.686425	2.417782	1.882222339	1.773060525		0.152003	0.686833
NW_003539115_38545_star	2.149139864	1.611855	1.654957834	1.38507475		0.415037	0.458605		chr11_24909_star	2.14913986	0.805927	1.654957834	0.852739935		1.415037	1		hsa-miR-378f	2.686425	2.417782	1.882222339	1.773060525		0.152003	0.686833
chr3_7786_mature	2.149139864	1.611855	1.654957834	1.38507475		0.415037	0.458605		chr2_4824_mature	2.14913986	11.28298	1.654957834	3.618589217		-2.39232	1		chr9_21042_mature	2.686425	2.417782	1.882222339	1.773060525		0.152003	0.686833
chr17_35259_star	2.149139864	1.611855	1.654957834	1.38507475		0.415037	0.458605		chr1_3951_mature	2.14913986	0.805927	1.654957834	0.852739935		1.415037	1		chr8_18754_star	2.686425	2.417782	1.882222339	1.773060525		0.152003	0.686833
chr1_3091_star	2.149139864	1.611855	1.654957834	1.38507475		0.415037	0.458605		rno-miR-17-1-3p	2.14913986	0.805927	1.654957834	0.852739935		1.415037	1		chr18_36647_star	2.686425	2.417782	1.882222339	1.773060525		0.152003	0.686833
chr6_14470_star	2.149139864	1.611855	1.654957834	1.38507475		0.415037	0.458605		mmu-miR-3086-3p	2.14913986	0.805927	1.654957834	0.852739935		1.415037	1		NW_003538342_37742_mature	2.686425	2.417782	1.882222339	1.773060525		0.152003	0.686833
chr2_4065_mature	2.149139864	1.611855	1.654957834	1.38507475		0.415037	0.458605		chr5_11603_star	2.14913986	0.805927	1.654957834	0.852739935		1.415037	1		chr3_7654_mature	2.686425	2.417782	1.882222339	1.773060525		0.152003	0.686833
chr4_9341_mature	2.149139864	1.611855	1.654957834	1.38507475		0.415037	0.458605		NW_003539007_38445_star	6.44741959	1.611855	2.896740642	1.38507475		2	1		chr14_30036_mature	2.686425	2.417782	1.882222339	1.773060525		0.152003	0.686833
chr8_18873_star	2.149139864	1.611855	1.654957834	1.38507475		0.415037	0.458605		chrx_22198_mature	2.14913986	0.805927	1.654957834	0.852739935		1.415037	1		NW_003537624_37070_star	2.686425	2.417782	1.882222339	1.773060525		0.152003	0.686833
chr9_20697_star	2.149139864	1.611855	1.654957834	1.38507475		0.415037	0.458605		mmu-miR-669c-3p	2.14913986	0.805927	1.654957834	0.852739935		1.415037	1		bta-miR-221	2.686425	2.417782	1.882222339	1.773060525		0.152003	0.686833
bta-miR-764	2.149139864	1.611855	1.654957834	1.38507475		0.415037	0.458605		hsa-miR-4727-5p	2.14913986	0.805927	1.654957834	0.852739935		1.415037	1		chr2_5371_mature	2.686425	2.417782	1.882222339	1.773060525		0.152003	0.686833
chr14_30483_mature	2.149139864	1.611855	1.654957834	1.38507475		0.415037	0.458605		chr5_12128_star	2.14913986	12.08891	1.654957834	3.710273245		-2.49185	1		chr3_7875_star	2.686425	2.417782	1.882222339	1.773060525		0.152003	0.686833
mmu-miR-6336	2.149139864	1.611855	1.654957834	1.38507475		0.415037	0.458605		chr3_7094_star	6.44741959	1.611855	2.896740642	1.38507475		2	1		ssc-miR-4338	2.686425	2.417782	1.882222339	1.773060525		0.152003	0.686833
chr7_15747_mature	2.149139864	1.611855	1.654957834	1.38507475		0.415037	0.458605		chr4_10519_mature	4.29827973	0.805927	2.405524014	0.852739935		2.415037	1		chr6_13908_star	2.686425	2.417782	1.882222339	1.773060525		0.152003	0.686833
chr15_33320_star	2.149139864	1.611855	1.654957834	1.38507475		0.415037	0.458605		hsa-miR-572	4.29827973	0.805927	2.405524014	0.852739935		2.415037	1		chr14_30832_mature	2.686425	2.417782	1.882222339	1.773060525		0.152003	0.686833
bta-miR-2285c	2.149139864	1.611855	1.654957834	1.38507475		0.415037	0.458605		rno-miR-871-5p	2.14913986	0.805927	1.654957834	0.852739935		1.415037	1		chr7_16707_star	2.686425	2.417782	1.882222339	1.773060525		0.152003	0.686833
bta-miR-2284i	2.149139864	1.611855	1.654957834	1.38507475		0.415037	0.458605		hsa-miR-596	6.44741959	1.611855	2.896740642	1.38507475		2	1		hsa-miR-4285	2.686425	2.417782	1.882222339	1.773060525		0.152003	0.686833
chr11_24465_star	2.149139864	1.611855	1.654957834	1.38507475		0.415037	0.458605		chr2_6439_star	4.29827973	0.805927	2.405524014	0.852739935		2.415037	1		chr1_2204_star@@hsa-miR-4695-	2.686425	2.417782	1.882222339	1.773060525		0.152003	0.686833
chr1_5_mature	2.149139864	1.611855	1.654957834	1.38507475		0.415037	0.458605		chr1_3174_star	2.14913986	0.805927	1.654957834	0.852739935		1.415037	1		bta-miR-2451	2.686425	2.417782	1.882222339	1.773060525		0.152003	0.686833
chr4_8831_mature	2.149139864	1.611855	1.654957834	1.38507475		0.415037	0.458605		hsa-miR-586	2.14913986	0.805927	1.654957834	0.852739935		1.415037	1		chr11_24609_star	2.686425	2.417782	1.882222339	1.773060525		0.152003	0.686833
chr11_25156_star	2.149139864	1.611855	1.654957834	1.38507475		0.415037	0.458605		hsa-miR-583	2.14913986	0.805927	1.654957834	0.852739935		1.415037	1		hsa-miR-4320	2.686425	2.417782	1.882222339	1.773060525		0.152003	0.686833
chr10_23315_star	2.149139864	1.611855	1.654957834	1.38507475		0.415037	0.458605		chr3_7781_star	4.29827973	0.805927	2.405524014	0.852739935		2.415037	1		bta-miR-2384	2.686425	2.417782	1.882222339	1.773060525		0.152003	0.686833
chr9_20370_mature	2.149139864	1.611855	1.654957834	1.38507475		0.415037	0.458605		chr16_33995_mature	6.44741959	20.14819	2.896740642	4.402462031		-1.64386	1		chr6_12914_star	2.686425	2.417782	1.882222339	1.773060525		0.152003	0.686833
chr16_34520_star	2.149139864	1.611855	1.654957834	1.38507475		0.415037	0.458605		chr9_20132_mature	8.59655945	2.417782	3.262517265	1.773060525		1.830075	1		chr14_30186_star	2.686425	2.417782	1.882222339	1.773060525		0.152003	0.686833
chr7_16615_star	2.149139864	1.611855	1.654957834	1.38507475		0.415037	0.458605		hsa-miR-559	2.14913986	0.805927	1.654957834	0.852739935		1.415037	1		chr5_10940_mature	2.686425	2.417782	1.882222339	1.773060525		0.152003	0.686833
hsa-miR-4711-3p	2.149139864	1.611855	1.654957834	1.38507475		0.415037	0.458605		chr5_12167_star	4.29827973	0.805927	2.405524014	0.852739935		2.415037	1		chr5_11704_star	2.686425	2.417782	1.882222339	1.773060525		0.152003	0.686833
chr18_36406_star	2.149139864	1.611855	1.654957834	1.38507475		0.415037	0.458605		chr14_30387_star	4.29827973	0.805927	2.405524014	0.852739935		2.415037	1		chr3_7250_mature	2.686425	2.417782	1.882222339	1.773060525		0.152003	0.686833
rno-miR-3085	2.149139864	1.611855	1.654957834	1.38507475		0.415037	0.458605		chr3_6870_star	2.14913986	0.805927	1.654957834	0.852739935		1.415037	1		chrx_21936_mature	2.686425	2.417782	1.882222339	1.773060525		0.152003	0.686833
mmu-miR-669h-3p	2.149139864	1.611855	1.654957834	1.38507475		0.415037	0.458605		bta-miR-2399-3p	2.14913986	0.805927	1.654957834	0.852739935		1.415037	1		mmu-miR-5624-3p	1.07457	0	1.05281229	0		20	0.686912
chr2_5378_star	2.149139864	1.611855	1.654957834	1.38507475		0.415037	0.458605		NW_003539027_38478_star	2.14913986	0.805927	1.654957834	0.852739935		1.415037	1		NW_003538747_38195_star	1.07457	0	1.05281229	0		20	0.686912
chrx_21505_star	2.149139864	1.611855	1.654957834	1.38507475		0.415037	0.458605		rno-miR-3065-3p	2.14913986	0.805927	1.654957834	0.852739935		1.415037	1		hsa-miR-6501-5p	1.07457	0	1.05281229	0		20	0.686912
chr1_1780_star	2.149139864	1.611855	1.654957834	1.38507475		0.415037	0.458605		chr5_11356_star	2.14913986	0.805927	1.654957834	0.852739935		1.415037	1		mmu-miR-9-3p	1.07457	0	1.05281229	0		20	0.686912
chr2_4563_star	2.149139864	1.611855	1.654957834	1.38507475		0.415037	0.458605		chr8_17967_star	2.14913986	0.805927	1.654957834	0.852739935		1.415037	1		hsa-miR-328	1.07457	0	1.05281229	0		20	0.686912
chr14_29851_star	2.149139864	1.611855	1.654957834	1.38507475		0.415037	0.458605		chr5_12125_star	2.14913986	0.805927	1.654957834	0.852739935		1.415037	1		hsa-miR-3150a-5p	1.07457	0	1.05281229	0		20	0.686912
chr5_11695_mature	2.149139864	1.611855	1.654957834	1.38507475		0.415037	0.458605		hsa-miR-504	4.29827973	0.805927	2.405524014	0.852739935		2.415037	1		hsa-miR-4803	1.07457	0	1.05281229	0		20	0.686912
chr4_8929_star	2.149139864	1.611855	1.654957834	1.38507475		0.415037	0.458605		chr5_12123_mature	6.44741959	1.611855	2.896740642	1.38507475		2	1		chr4_9456_mature	1.07457	0	1.05281229	0		20	0.686912
chr7_15749_star	2.149139864	1.611855	1.654957834	1.38507475		0.415037	0.458605		chr11_24937_mature	15.043979	5.641492	4.003960082	2.731507408		1.415037	1		hsa-miR-150-5p	1.07457	0	1.05281229	0		20	0.686912
bta-miR-329a	2.149139864	1.611855	1.654957834	1.38507475		0.415037	0.458605		chr7_15077_mature	2.14913986	11.28298	1.654957834	3.618589217		-2.39232	1		chr6_12531_star	1.07457	0	1.05281229	0		20	0.686912
chr3_8430_star	2.149139864	1.611855	1.654957834	1.38507475		0.415037	0.458605		chr2_4773_star	4.29827973	0.805927	2.405524014	0.852739935		2.415037	1		rno-miR-92a-2-5p	1.07457	0	1.05281229	0		20	0.686912
chr6_14340_star	2.149139864	1.611855	1.654957834	1.38507475		0.415037	0.458605		chr6_12641_star	15.043979	5.641492	4.003960082	2.731507408		1.415037	1		chr3_7967_star	1.07457	0	1.05281229	0		20	0.686912
chr8_18878_star	2.149139864	1.611855	1.654957834	1.38507475		0.415037	0.458605		chr2_6441_mature	2.14913986	0.805927	1.654957834	0.852739935		1.415037	1		chr3_7249_star	1.07457	0	1.05281229	0		20	0.686912
chr9_19911_star	2.149139864	1.611855	1.654957834	1.38507475		0.415037	0.458605		chrx_21570_star	4.29827973	16.11855	2.405524014	4.097488514		-1.90689	1		NW_003537425_36910_star	1.07457	0	1.05281229	0		20	0.686912
mmu-miR-3093-3p	2.149139864	1.611855	1.654957834	1.38507475		0.415037	0.458605		chr10_23377_star	2.14913986	0.805927	1.654957834	0.852739935		1.415037	1		chr11_25311_star	1.07457	0	1.05281229	0		20	0.686912
chr5_11151_star	2.149139864	1.611855	1.654957834	1.38507475		0.415037	0.458605		mmu-miR-5132-3p	2.14913986	0.805927	1.654957834	0.852739935		1.415037	1		hsa-miR-4780	1.07457	0	1.05281229	0		20	0.686912
chr4_10088_star	2.149139864	1.611855	1.654957834	1.38507475		0.415037	0.458605		bta-miR-2467-5p	4.29827973	0.805927	2.405524014	0.852739935		2.415037	1		chr1_2593_star	1.07457	0	1.05281229	0		20	0.686912
chr1_3580_star	2.149139864	1.611855	1.654957834	1.38507475		0.415037	0.458605		chr13_29016_star	2.14913986	0.805927	1.654957834	0.852739935		1.415037	1		chr8_18343_star	1.07457	0	1.05281229	0		20	0.686912
hsa-miR-4684-5p	2.149139864	1.611855	1.654957834	1.38507475		0.415037	0.458605		rno-miR-632	2.14913986	0.805927	1.654957834	0.852739935		1.415037	1		hsa-miR-532-5p	1.07457	0	1.05281229	0		20	0.686912
chr16_34150_star	2.149139864	1.611855	1.654957834	1.38507475		0.415037	0.458605		chr7_16961_star	4.29827973	0.805927	2.405524014	0.852739935		2.415037	1		rno-miR-181a-5p	1.07457	0	1.05281229	0		20	0.686912
chr4_9347_mature	2.149139864	1.611855	1.654957834	1.38507475		0.415037	0.458605		bta-miR-193a	4.29827973	0.805927	2.405524014	0.852739935		2.415037	1		rno-miR-130a-3p	1.07457	0	1.05281229	0		20	0.686912
chr3_7339_star	2.149139864	1.611855	1.654957834	1.38507475		0.415037	0.458605		chr13_28971_star	4.29827973	0.805927	2.405524014	0.852739935		2.415037	1		hsa-miR-1323	1.07457	0	1.05281229	0		20	0.686912
bta-miR-2444	2.149139864	1.611855	1.654957834	1.38507475		0.415037	0.458605		chr9_19757_star	2.14913986	11.28298	1.654957834	3.618589217		-2.39232	1		chr6_12438_star	1.07457	0	1.05281229	0		20	0.686912
chr5_10732_star	2.149139864	1.611855	1.654957834	1.38507475		0.415037	0.458605		hsa-miR-3929	2.14913986	0.805927	1.654957834	0.852739935		1.415037	1		chr17_34938_star	1.07457	0	1.05281229	0		20	0.686912
hsa-miR-639	2.149139864	1.611855	1.654957834	1.38507475		0.415037	0.458605		hsa-miR-3928	2.14913986	0.805927	1.654957834	0.852739935		1.415037	1		mmu-miR-1306-3p	1.07457	0	1.05281229	0		20	0.686912
hsa-miR-4495	2.149139864	1.611855	1.654957834	1.38507475		0.415037	0.458605		chr13_29017_mature	8.59655945	2.417782	3.262517265	1.773060525		1.830075	1		mmu-miR-3084-5p	1.07457	0	1.05281229	0		20	0.686912
hsa-miR-561-3p	2.149139864	1.611855	1.654957834	1.38507475		0.415037	0.458605		chr12_25593_star	2.14913986	0.805927	1.654957834	0.852739935		1.415037	1		hsa-miR-1178-5p	1.07457	0	1.05281229	0		20	0.686912
bta-miR-2389	2.149139864	1.611855	1.654957834	1.38507475		0.415037	0.458605		chr11_25358_mature	4.29827973	16.92448	2.405524014	4.163859073		-1.97728	1		chrx_22497_star	1.07457	0	1.05281229	0		20	0.686912
chr16_34371_star	2.149139864	1.611855	1.654957834	1.38507475		0.415037	0.458605		chrx_22228_star	4.29827973	0.805927	2.405524014	0.852739935		2.415037	1		hsa-miR-4710	1.07457	0	1.05281229	0		20	0.686912
chr13_27259_star	2.149139864	1.611855	1.654957834	1.38507475		0.415037	0.458605		chr2_4256_star	6.44741959	1.611855	2.896740642	1.38507475		2	1		hsa-miR-4751	1.07457	0	1.05281229	0		20	0.686912
rno-miR-181b-5p	2.149139864	1.611855	1.654957834	1.38507475		0.415037	0.458605		chr18_36427_mature@@bta-miR-592	4.29827973	0.805927	2.405524014	0.852739935		2.415037	1		bta-miR-2893	1.07457	0	1.05281229	0		20	0.686912
mmu-miR-378c	2.149139864	1.611855	1.654957834	1.38507475		0.415037	0.458605		mmu-miR-1904	2.14913986	0.805927	1.654957834	0.852739935		1.415037	1		chr16_34762_star	1.07457	0	1.05281229	0		20	0.686912
chrx_21678_star	2.149139864	1.611855	1.654957834	1.38507475		0.415037	0.458605		mmu-miR-1906	6.44741959	1.611855	2.896740642	1.38507475		2	1		hsa-miR-508-3p	1.07457	0	1.05281229	0		20	0.686912
chr9_19145_mature@@hsa-miR-476	2.149139864	1.611855	1.654957834	1.38507475		0.415037	0.458605		chr5_12251_mature	10.7456993	3.22371	3.554060706	2.078510713		1.736966	1		chr5_11672_star	1.07457	0	1.05281229	0		20	0.686912
NW_003539698_39034_star	2.149139864	1.611855	1.654957834	1.38507475		0.415037	0.458605		chrx_22120_star	2.14913986	0.805927	1.654957834	0.852739935		1.415037	1		chr4_9318_star	1.07457	0	1.05281229	0		20	0.686912
NW_003538873_38324_mature	2.149139864	1.611855	1.654957834	1.38507475		0.415037	0.458605		chr2_5281_star	2.14913986	0.805927	1.654957834	0.852739935		1.415037	1		hsa-miR-508-5p	1.07457	0	1.05281229	0		20	0.686912
hsa-miR-4666a-5p	2.149139864	1.611855	1.654957834	1.38507475		0.415037	0.458605		chr5_10731_mature	6.44741959	1.611855	2.896740642	1.38507475		2	1		rno-miR-3594-3p	1.07457	0	1.05281229	0		20	0.686912
chr2_5391_mature	2.149139864	1.611855	1.654957834	1.38507475		0.415037	0.458605		chr1_3894_star@@bta-miR-219-5p	4.29827973	0.805927	2.405524014	0.852739935		2.415037	1		hsa-miR-6715a-3p	1.07457	0	1.05281229	0		20	0.686912
NW_003613191_37582_mature	2.149139864	1.611855	1.654957834	1.38507475		0.415037	0.458605		chr14_31056_star	12.8948392	4.029637	3.796477232	2.330454351		1.678072	1		chr6_14256_star	1.07457	0	1.05281229	0		20	0.686912
chr1_3718_mature	2.149139864	1.611855	1.654957834	1.38507475		0.415037	0.458605		chr8_17482_star	2.14913986	0.805927	1.654957834	0.852739935		1.415037	1		rno-miR-3594-5p	1.07457	0	1.05281229	0		20	0.686912
chr6_12809_star	2.149139864	1.611855	1.654957834	1.38507475		0.415037	0.458605		mmu-miR-1942	2.14913986	0.805927	1.654957834	0.852739935		1.415037	1		chr18_36468_star	1.07457	0	1.05281229	0		20	0.686912
chr13_27411_star	2.149139864	1.611855	1.654957834	1.38507475		0.415037	0.458605		mmu-miR-1945	2.14913986	0.805927	1.654957834	0.852739935		1.415037	1		chr6_13900_mature	1.07457	0	1.05281229	0		20	0.686912
hsa-miR-6070	2.149139864	1.611855	1.654957834	1.38507475		0.415037	0.458605		hsa-miR-6718-5p	2.14913986	0.805927	1.654957834	0.852739935		1.415037	1		mmu-let-7k	1.07457	0	1.05281229	0		20	0.686912
chr13_28544_mature	2.149139864	1.611855	1.654957834	1.38507475		0.415037	0.458605		mmu-miR-1935	4.29827973	0.805927	2.405524014	0.852739935		2.415037	1		chr3_7984_star	1.07457	0	1.05281229	0		20	0.686912
chr15_31761_mature	1.074569932	1.611855	1.05281229	1.38507475		-0.58496	0.458644		mmu-miR-1936	4.29827973	0.805927	2.405524014	0.852739935		2.415037	1		chr2_5147_star	1.07457	0	1.05281229	0		20	0.686912
chr1_1948_star	1.074569932	1.611855	1.05281229	1.38507475		-0.58496	0.458644		NW_003612993_36688_star	2.14913986	0.805927	1.654957834	0.852739935		1.415037	1		chr6_14386_mature	1.07457	0	1.05281229	0		20	0.686912
chr9_19886_mature	1.074569932	1.611855	1.05281229	1.38507475		-0.58496	0.458644		chr14_31216_star	4.29827973	0.805927	2.405524014	0.852739935		2.415037	1		mmu-miR-669p-5p	1.07457	0	1.05281229	0		20	0.686912
hsa-miR-4790-5p	1.074569932	1.611855	1.05281229	1.38507475		-0.58496	0.458644		mmu-miR-1962	2.14913986	0.805927	1.654957834	0.852739935		1.415037	1		hsa-miR-376b-5p	1.07457	0	1.05281229	0		20	0.686912
chr6_14325_mature	1.074569932	1.611855	1.05281229	1.38507475		-0.58496	0.458644		mmu-miR-1971	4.29827973	0.805927	2.405524014	0.852739935		2.415037	1		hsa-miR-873-5p	1.07457	0	1.05281229	0		20	0.686912
NW_003613223_37737_mature	1.074569932	1.611855	1.05281229	1.38507475		-0.58496	0.458644		chr3_6880_mature	6.44741959	1.611855	2.896740642	1.38507475		2	1		chrx_22200_star	1.07457	0	1.05281229	0		20	0.686912
hsa-miR-3614-5p	1.074569932	1.611855	1.05281229	1.38507475		-0.58496	0.458644		chr6_12979_star	2.14913986	0.805927	1.654957834	0.852739935		1.415037	1		chr6_13349_star	1.07457	0	1.05281229	0		20	0.686912
chr17_34998_star	1.074569932	1.611855	1.05281229	1.38507475		-0.58496	0.458644		chr12_26158_mature	4.29827973	0.805927	2.405524014	0.852739935		2.415037	1		hsa-miR-1202	1.07457	0	1.05281229	0		20	0.686912
chr13_27981_star	1.074569932	1.611855	1.05281229	1.38507475		-0.58496	0.458644		chr16_34502_star	2.14913986	0.805927	1.654957834	0.852739935		1.415037	1		chr5_12329_star	1.07457	0	1.05281229	0		20	0.686912
chr13_27363_star	1.074569932	1.611855	1.05281229	1.38507475		-0.58496	0.458644		hsa-miR-429	4.29827973	0.805927	2.405524014	0.852739935		2.415037	1		rno-miR-200b-5p	1.07457	0	1.05281229	0		20	0.686912
chr1_2656_mature	1.074569932	1.611855	1.05281229	1.38507475		-0.58496	0.458644		chrx_22210_star	4.29827973	0.805927	2.405524014	0.852739935		2.415037	1		chr10_24076_star	1.07457	0	1.05281229	0		20	0.686912
rno-miR-181b-1-3p	1.074569932	1.611855	1.05281229	1.38507475		-0.58496	0.458644		chr2_6113_star	4.29827973	0.805927	2.405524014	0.852739935		2.415037	1		hsa-miR-4727-3p	1.07457	0	1.05281229	0		20	0.686912
chr1_782_star	1.074569932	1.611855	1.05281229	1.38507475		-0.58496	0.458644		chr6_12563_star	6.44741959	1.611855	2.896740642	1.38507475		2	1		hsa-miR-4732-3p	1.07457	0	1.05281229	0		20	0.686912
NW_003613103_37207_star	1.074569932	1.611855	1.05281229	1.38507475		-0.58496	0.458644		chr4_9468_star	2.14913986	0.805927	1.654957834	0.852739935		1.415037	1		chr7_16057_star	1.07457	0	1.05281229	0		20	0.686912
chr1_1736_star	1.074569932	1.611855	1.05281229	1.38507475		-0.58496	0.458644		chr1_1185_mature	8.59655945	2.417782	3.262517265	1.773060525		1.830075	1		chr2_5437_star	1.07457	0	1.05281229	0		20	0.686912
hsa-miR-4762-5p	1.074569932	1.611855	1.05281229	1.38507475		-0.58496	0.458644		chr6_13557_mature	2.14913986	0.805927	1.654957834	0.852739935		1.415037	1		chr11_25309_star	1.07457	0	1.05281229	0		20	0.686912
chr7_16704_mature	1.074569932	1.611855	1.05281229	1.38507475		-0.58496	0.458644		hsa-miR-1538	4.29827973	0.805927	2.405524014	0.852739935		2.415037	1		hsa-miR-1287	1.07457	0	1.05281229	0		20	0.686912
chr1_3642_star	1.074569932	1.611855	1.05281229	1.38507475		-0.58496	0.458644		hsa-miR-520a-5p	2.14913986	0.805927	1.654957834	0.852739935		1.415037	1		hsa-miR-141-5p	1.07457	0	1.05281229	0		20	0.686912
chr13_28152_mature	1.074569932	1.611855	1.05281229	1.38507475		-0.58496	0.458644		hsa-miR-518e-3p	4.29827973	0.805927	2.405524014	0.852739935		2.415037	1		NW_003613148_37413_star	1.07457	0	1.05281229	0		20	0.686912
mmu-miR-3085-5p	1.074569932	1.611855	1.05281229	1.38507475		-0.58496	0.458644		chr3_7211_star	4.29827973	0.805927	2.405524014	0.852739935		2.415037	1		chr3_7917_star	1.07457	0	1.05281229	0		20	0.686912
chr3_6710_star	1.074569932	1.611855	1.05281229	1.38507475		-0.58496	0.458644		chr9_21046_mature	10.7456993	3.22371	3.554060706	2.078510713		1.736966	1		hsa-miR-566	1.07457	0	1.05281229	0		20	0.686912
chr5_11375_mature	1.074569932	1.611855	1.05281229	1.38507475		-0.58496	0.458644		chr16_33995_star	4.29827973	0.805927	2.405524014	0.852739935		2.415037	1		hsa-miR-564	1.07457	0	1.05281229	0		20	0.686912
hsa-miR-3190-5p	1.074569932	1.611855	1.05281229	1.38507475		-0.58496	0.458644		chr14_29793_mature	15.043979	34.65488	4.003960082	5.156027656		-1.20387	1		chr10_23951_mature	1.07457	0	1.05281229	0		20	0.686912
chr1_1820_star	1.074569932	1.611855	1.05281229	1.38507475		-0.58496	0.458644		chr2_4766_mature	4.29827973	0.805927	2.405524014	0.852739935		2.415037	1		chr5_11814_mature	1.07457	0	1.05281229	0		20	0.686912
NW_003613106_37241_star	1.074569932	1.611855	1.05281229	1.38507475		-0.58496	0.458644		chr5_11229_star	6.44741959	1.611855	2.896740642	1.38507475		2	1		chr4_9437_star	1.07457	0	1.05281229	0		20	0.686912
NW_003539007_38445_star	1.074569932	1.611855	1.05281229	1.38507475		-0.58496	0.458644		chr6_12861_mature	4.29827973	0.805927	2.405524014	0.852739935		2.415037	1		hsa-miR-521	1.07457	0	1.05281229	0		20	0.686912
chr9_21003_star	1.074569932	1.611855	1.05281229	1.38507475		-0.58496	0.458644		chr17_35299_mature	2.14913986	0.805927	1.654957834	0.852739935		1.415037	1		chr13_26770_star	1.07457	0	1.05281229	0		20	0.686912
chr3_7094_star	1.074569932	1.611855	1.05281229	1.38507475		-0.58496	0.458644		hsa-miR-4746-5p	2.14913986	0.805927	1.654957834	0.852739935		1.415037	1		hsa-miR-543	1.07457	0	1.05281229	0		20	0.686912
hsa-miR-571	1.074569932	1.611855	1.05281229	1.38507475		-0.58496	0.458644		chrx_22845_mature	4.29827973	0.805927	2.405524014	0.852739935		2.415037	1		hsa-miR-510	1.07457	0	1.05281229	0		20	0.686912
NW_003538780_38224_star	1.074569932	1.611855	1.05281229	1.38507475		-0.58496	0.458644		hsa-miR-3127-3p	4.29827973	0.805927	2.405524014	0.852739935		2.415037	1		hsa-miR-187-5p	1.07457	0	1.05281229	0		20	0.686912
mmu-miR-199a-3p	1.074569932	1.611855	1.05281229	1.38507475		-0.58496	0.458644		bta-miR-181d	2.14913986	0.805927	1.654957834	0.852739935		1.415037	1		chr4_8702_star	1.07457	0	1.05281229	0		20	0.686912
chr6_13909_star	1.074569932	1.611855	1.05281229	1.38507475		-0.58496	0.458644		chr4_9679_star	2.14913986	0.805927	1.654957834	0.852739935		1.415037	1		mmu-miR-432	1.07457	0	1.05281229	0		20	0.686912
chr15_33041_star	1.074569932	1.611855	1.05281229	1.38507475		-0.58496	0.458644		chr3_6843_mature	4.29827973	0.805927	2.405524014	0.852739935		2.415037	1		chr3_7972_mature	1.07457	0	1.05281229	0		20	0.686912
chr10_24271_star	1.074569932	1.611855	1.05281229	1.38507475		-0.58496	0.458644		chrx_22188_star@@mmu-miR-1298-3p	2.14913986	0.805927	1.654957834	0.852739935		1.415037	1		hsa-miR-4782-5p	1.07457	0	1.05281229	0		20	0.686912
hsa-miR-4520a-5p	1.074569932	1.611855	1.05281229	1.38507475		-0.58496	0.458644		chr6_12901_mature	12.8948392	4.029637	3.796477232	2.330454351		1.678072	1		hsa-miR-6721-5p	1.07457	0	1.05281229	0		20	0.686912
hsa-miR-553	1.074569932	1.611855	1.05281229	1.38507475		-0.58496	0.458644		chr13_27994_mature	2.14913986	12.08891	1.654957834	3.710273245		-2.49185	1		chr4_8783_star	1.07457	0	1.05281229	0		20	0.686912
rno-miR-378b	1.074569932	1.611855	1.05281229	1.38507475		-0.58496	0.458644		chr13_29250_star	4.29827973	0.805927	2.405524014	0.852739935		2.415037	1		bta-miR-2467-3p	1.07457	0	1.05281229	0		20	0.686912
chr13_27900_star	1.074569932	1.611855	1.05281229	1.38507475		-0.58496	0.458644		chr15_32040_mature	6.44741959	1.611855	2.896740642	1.38507475		2	1		chr15_33029_mature	1.07457	0	1.05281229	0		20	0.686912
chr8_17278_star	1.074569932	1.611855	1.05281229	1.38507475		-0.58496	0.458644		chr4_10065_star	2.14913986	11.28298	1.654957834	3.618589217		-2.39232	1		mmu-miR-3067-3p	1.07457	0	1.05281229	0		20	0.686912
chr2_4256_star	1.074569932	1.611855	1.05281229	1.38507475		-0.58496	0.458644		chr3_6872_star	2.14913986	0.805927	1.654957834	0.852739935		1.415037	1		chr13_28482_mature	1.07457	0	1.05281229	0		20	0.686912
chr15_33434_mature	1.074569932	1.611855	1.05281229	1.38507475		-0.58496	0.458644		chr13_28164_star	8.59655945	2.417782	3.262517265	1.773060525		1.830075	1		chrx_21766_star	1.07457	0	1.05281229	0		20	0.686912
chr5_12309_mature@@bta-miR-125	1.074569932	1.611855	1.05281229	1.38507475		-0.58496	0.458644		hsa-miR-525-5p	2.14913986	0.805927	1.654957834	0.852739935		1.415037	1		chr9_20088_star	1.07457	0	1.05281229	0		20	0.686912
bta-miR-760-5p	1.074569932	1.611855	1.05281229	1.38507475		-0.58496	0.458644		chr1_3915_star	2.14913986	0.805927	1.654957834	0.852739935		1.415037	1		chr3_8026_star	1.07457	0	1.05281229	0		20	0.686912
chr5_10731_mature	1.074569932	1.611855	1.05281229	1.38507475		-0.58496	0.458644		hsa-miR-3648	2.14913986	0.805927	1.654957834	0.852739935		1.415037	1		chr9_20451_mature	1.07457	0	1.05281229	0		20	0.686912
chr9_19886_star	1.074569932	1.611855	1.05281229	1.38507475		-0.58496	0.458644		chr13_28700_star	2.14913986	0.805927	1.654957834	0.852739935		1.415037	1		mmu-miR-100-3p	1.07457	0	1.05281229	0		20	0.686912
rno-miR-28-5p	1.074569932	1.611855	1.05281229	1.38507475		-0.58496	0.458644		chr2_4699_mature	2.14913986	0.805927	1.654957834	0.852739935		1.415037	1		NW_003613382_38825_star	1.07457	0	1.05281229	0		20	0.686912
chr6_13557_star	1.074569932	1.611855	1.05281229	1.38507475		-0.58496	0.458644		mmu-miR-335-3p	4.29827973	0.805927	2.405524014	0.852739935		2.415037	1		mmu-miR-1932	1.07457	0	1.05281229	0		20	0.686912
chr18_36050_star	1.074569932	1.611855	1.05281229	1.38507475		-0.58496	0.458644		chr9_19683_star	2.14913986	0.805927	1.654957834	0.852739935		1.415037	1		bta-miR-6120-5p	1.07457	0	1.05281229	0		20	0.686912
chr9_19988_star	1.074569932	1.611855	1.05281229	1.38507475		-0.58496	0.458644		chr6_13376_star	2.14913986	0.805927	1.654957834	0.852739935		1.415037	1		chr10_24300_star	1.07457	0	1.05281229	0		20	0.686912
chr1_743_star	1.074569932	1.611855	1.05281229	1.38507475		-0.58496	0.458644		chr15_32517_star	4.29827973	0.805927	2.405524014	0.852739935		2.415037	1		bta-miR-365-5p	1.07457	0	1.05281229	0		20	0.686912
chr14_30864_star	1.074569932	1.611855	1.05281229	1.38507475		-0.58496	0.458644		chr5_11045_star	6.44741959	1.611855	2.896740642	1.38507475		2	1		hsa-miR-5007-5p	1.07457	0	1.05281229	0		20	0.686912
NW_003537295_36819_star	1.074569932	1.611855	1.05281229	1.38507475		-0.58496	0.458644		chr1_188_star	2.14913986	0.805927	1.654957834	0.852739935		1.415037	1		mmu-miR-1960	1.07457	0	1.05281229	0		20	0.686912
NW_003613545_39630_star	1.074569932	1.611855	1.05281229	1.38507475		-0.58496	0.458644		rno-miR-3568	2.14913986	0.805927	1.654957834	0.852739935		1.415037	1		mmu-miR-1963	1.07457	0	1.05281229	0		20	0.686912
chr14_30467_star@@hsa-miR-6715a	1.074569932	1.611855	1.05281229	1.38507475		-0.58496	0.458644		chr18_36398_star	4.29827973	0.805927	2.405524014	0.852739935		2.415037	1		chr11_24964_star	1.07457	0	1.05281229	0		20	0.686912
chr5_11045_star	1.074569932	1.611855	1.05281229	1.38507475		-0.58496	0.458644		rno-miR-3577	2.14913986	0.805927	1.654957834	0.852739935		1.415037	1		mmu-miR-1a-3p	1.07457	0	1.05281229	0		20	0.686912
rno-miR-3576	1.074569932	1.611855	1.05281229	1.38507475		-0.58496	0.458644		chr13_26891_star	2.14913986	0.805927	1.654957834	0.852739935		1.415037	1		hsa-miR-421	1.07457	0	1.05281229	0		20	0.686912
chr15_31883_star	1.074569932	1.611855	1.05281229	1.38507475		-0.58496	0.458644		chr11_24943_mature	6.44741959	1.611855	2.896740642	1.38507475		2	1		rno-miR-339-3p	1.07457	0	1.05281229	0		20	0.686912
chr13_27078_mature	1.074569932	1.611855	1.05281229	1.38507475		-0.58496	0.458644		mmu-miR-291a-5p	2.14913986	0.805927	1.654957834	0.852739935		1.415037	1		chrx_21717_mature	1.07457	0	1.05281229	0		20	0.686912
chr8_18753_mature	1.074569932	1.611855	1.05281229	1.38507475		-0.58496	0.458644		chr12_25693_star	2.14913986	0.805927	1.654957834	0.852739935		1.415037	1		hsa-miR-548g-5p	1.07457	0	1.05281229	0		20	0.686912
chr13_27213_mature	1.074569932	1.611855	1.05281229	1.38507475		-0.58496	0.458644		chr2_5608_star	6.44741959	1.611855	2.896740642	1.38507475		2	1		rno-miR-30c-5p	1.07457	0	1.05281229	0		20	0.686912
chr17_35612_star	1.074569932	1.611855	1.05281229	1.38507475		-0.58496	0.458644		chr17_35617_star	2.14913986	0.805927	1.654957834	0.852739935		1.415037	1		mmu-miR-467a-3p	1.07457	0	1.05281229	0		20	0.686912
rno-miR-144-5p	1.074569932	1.611855	1.05281229	1.38507475		-0.58496	0.458644		chr14_31578_mature	12.8948392	4.029637	3.796477232	2.330454351		1.678072	1		hsa-miR-3978	1.07457	0	1.05281229	0		20	0.686912
chr15_32439_star	1.074569932	1.611855	1.05281229	1.38507475		-0.58496	0.458644		chr3_6871_mature	4.29827973	0.805927	2.405524014	0.852739935		2.415037	1		chr3_6720_star	1.07457	0	1.05281229	0		20	0.686912
hsa-miR-146a-3p	1.074569932	1.611855	1.05281229	1.38507475		-0.58496	0.458644		chr13_27034_mature	6.44741959	1.611855	2.896740642	1.38507475		2	1		hsa-miR-512-5p	1.07457	0	1.05281229	0		20	0.686912
chr12_25873_mature	1.074569932	1.611855	1.05281229	1.38507475		-0.58496	0.458644		mmu-miR-5133	2.14913986	0.805927	1.654957834	0.852739935		1.415037	1		hsa-miR-4738-5p	1.07457	0	1.05281229	0		20	0.686912
NW_003613326_38565_star	1.074569932	1.611855	1.05281229	1.38507475		-0.58496	0.458644		hsa-miR-3622b-5p	2.14913986	0.805927	1.654957834	0.852739935		1.415037	1		hsa-miR-548ai	1.07457	0	1.05281229	0		20	0.686912
NW_003540726_39647_star	1.074569932	1.611855	1.05281229	1.38507475		-0.58496	0.458644		mmu-miR-5126	4.29827973	0.805927	2.405524014	0.852739935		2.415037	1		chrx_23238_star	1.07457	0	1.05281229	0		20	0.686912
chr15_33043_mature	1.074569932	1.611855	1.05281229	1.38507475		-0.58496	0.458644		chr17_35097_star	2.14913986	0.805927	1.654957834	0.852739935		1.415037	1		hsa-miR-548aa	1.07457	0	1.05281229	0		20	0.686912
chrx_22340_star	1.074569932	1.611855	1.05281229	1.38507475		-0.58496	0.458644		mmu-miR-5120	2.14913986	0.805927	1.654957834	0.852739935		1.415037	1		hsa-miR-548ad	1.07457	0	1.05281229	0		20	0.686912
rno-miR-1298	1.074569932	1.611855	1.05281229	1.38507475		-0.58496	0.458644		chr8_17918_star	2.14913986	0.805927	1.654957834	0.852739935		1.415037	1		chr6_12739_star	1.07457	0	1.05281229	0		20	0.686912
NW_003538173_37591_mature	1.074569932	1.611855	1.05281229	1.38507475		-0.58496	0.458644		chr13_28617_star	2.14913986	0.805927	1.654957834	0.852739935		1.415037	1		chr4_9116_star	1.07457	0	1.05281229	0		20	0.686912
rno-miR-374-5p	1.074569932	1.611855	1.05281229	1.38507475		-0.58496	0.458644		chr13_28605_mature	2.14913986	0.805927	1.654957834	0.852739935		1.415037	1		NW_003537589_37025_star	1.07457	0	1.05281229	0		20	0.686912
chr8_18847_mature	1.074569932	1.611855	1.05281229	1.38507475		-0.58496	0.458644		chr16_33806_mature@@bta-miR-302b	4.29827973	0.805927	2.405524014	0.852739935		2.415037	1		rno-miR-103-3p	1.07457	0	1.05281229	0		20	0.686912
chr6_13603_mature	1.074569932	1.611855	1.05281229	1.38507475		-0.58496	0.458644		hsa-miR-1233-3p	2.14913986	0.805927	1.654957834	0.852739935		1.415037	1		rno-miR-202-3p	1.07457	0	1.05281229	0		20	0.686912
chrx_21526_mature	1.074569932	1.611855	1.05281229	1.38507475		-0.58496	0.458644		rno-miR-6316	4.29827973	0.805927	2.405524014	0.852739935		2.415037	1		rno-miR-34c-3p	1.07457	0	1.05281229	0		20	0.686912
chr12_25445_star	1.074569932	1.611855	1.05281229	1.38507475		-0.58496	0.458644		NW_003539453_38857_star	8.59655945	2.417782	3.262517265	1.773060525		1.830075	1		chr1_2018_star	1.07457	0	1.05281229	0		20	0.686912
chr12_26222_star	1.074569932	1.611855	1.05281229	1.38507475		-0.58496	0.458644		chr3_8500_mature	8.59655945	2.417782	3.262517265	1.773060525		1.830075	1		rno-miR-301a-5p	1.07457	0	1.05281229	0		20	0.686912
chr18_35816_mature	1.074569932	1.611855	1.05281229	1.38507475		-0.58496	0.458644		rno-miR-6333	4.29827973	0.805927	2.405524014	0.852739935		2.415037	1		bta-miR-1839	1.07457	0	1.05281229	0		20	0.686912
hsa-miR-548am-3p	1.074569932	1.611855	1.05281229	1.38507475		-0.58496	0.458644		chr1_3471_star	2.14913986	0.805927	1.654957834	0.852739935		1.415037	1		chr18_35938_mature	1.07457	0	1.05281229	0		20	0.686912
NW_003538795_38232_star	1.074569932	1.611855	1.05281229	1.38507475		-0.58496	0.458644		hsa-miR-3200-5p	4.29827973	0.805927	2.405524014	0.852739935		2.415037	1		chr14_30961_mature	1.07457	0	1.05281229	0		20	0.686912
chr9_19650_star	1.074569932	1.611855	1.05281229	1.38507475		-0.58496	0.458644		rno-miR-6329	4.29827973	0.805927	2.405524014	0.852739935		2.415037	1		hsa-miR-147b	1.07457	0	1.05281229	0		20	0.686912
chr13_27765_mature	1.074569932	1.611855	1.05281229	1.38507475		-0.58496	0.458644		mmu-miR-676-5p	2.14913986	0.805927	1.654957834	0.852739935		1.415037	1		hsa-miR-337-3p	1.07457	0	1.05281229	0		20	0.686912
chr2_6503_star	1.074569932	1.611855	1.05281229	1.38507475		-0.58496	0.458644		chr14_30876_mature	27.9388182	12.89484	4.854934103	3.796477232		1.115477	1		bta-miR-1835	1.07457	0	1.05281229	0		20	0.686912
chr9_20818_mature	1.074569932	1.611855	1.05281229	1.38507475		-0.58496	0.458644		rno-miR-6323	6.44741959	1.611855	2.896740642	1.38507475		2	1		hsa-miR-4735-5p	1.07457	0	1.05281229	0		20	0.686912
chr16_33722_star	1.074569932	1.611855	1.05281229	1.38507475		-0.58496	0.458644		chr4_8702_mature	4.29827973	0.805927	2.405524014	0.852739935		2.415037	1		chr8_17694_star	1.07457	0	1.05281229	0		20	0.686912
chr2_5851_star	1.074569932	1.611855	1.05281229	1.38507475		-0.58496	0.458644		chr4_9743_star	10.7456993	3.22371	3.554060706	2.078510713		1.736966	1		chr6_12823_mature@@hsa-miR-87	1.07457	0	1.05281229	0		20	0.686912
chrx_22052_star	1.074569932	1.611855	1.05281229	1.38507475		-0.58496	0.458644		chr3_7250_star	2.14913986	0.805927	1.654957834	0.852739935		1.415037	1		hsa-miR-1469	1.07457	0	1.05281229	0		20	0.686912
chr8_17856_star	1.074569932	1.611855	1.05281229	1.38507475		-0.58496	0.458644		chr16_33764_star	4.29827973	0.805927	2.405524014	0.852739935		2.415037	1		hsa-miR-1468	1.07457	0	1.05281229	0		20	0.686912
chr17_35168_star	1.074569932	1.611855	1.05281229	1.38507475		-0.58496	0.458644		chr3_8118_mature	4.29827973	0.805927	2.405524014	0.852739935		2.415037	1		hsa-miR-4524a-5p	1.07457	0	1.05281229	0		20	0.686912
chr4_9509_star	1.074569932	1.611855	1.05281229	1.38507475		-0.58496	0.458644		NW_003538867_38314_mature	4.29827973	0.805927	2.405524014	0.852739935		2.415037	1		chr10_23364_mature	1.07457	0	1.05281229	0		20	0.686912
rno-miR-329-3p	1.074569932	1.611855	1.05281229	1.38507475		-0.58496	0.458644		mmu-miR-6392-3p	2.14913986	0.805927	1.654957834	0.852739935		1.415037	1		chr14_31065_mature	1.07457	0	1.05281229	0		20	0.686912
chr1_2519_star	1.074569932	1.611855	1.05281229	1.38507475		-0.58496	0.458644		chr10_24203_star	4.29827973	0.805927	2.405524014	0.852739935		2.415037	1		hsa-miR-4777-3p	1.07457	0	1.05281229	0		20	0.686912
mmu-miR-6364	1.074569932	1.611855	1.05281229	1.38507475		-0.58496	0.458644		chr14_29946_star	2.14913986	0.805927	1.654957834	0.852739935		1.415037	1		hsa-miR-5579-3p	1.07457	0	1.05281229	0		20	0.686912
NW_003613232_37791_star	1.074569932	1.611855	1.05281229	1.38507475		-0.58496	0.458644		chr17_34985_star	2.14913986	0.805927	1.654957834	0.852739935		1.415037	1		chr1_3489_star	1.07457	0	1.05281229	0		20	0.686912
chr13_27728_star	1.074569932	1.611855	1.05281229	1.38507475		-0.58496	0.458644		chr2_4567_mature	4.29827973	0.805927	2.405524014	0.852739935		2.415037	1		chr13_29037_mature	1.07457	0	1.05281229	0		20	0.686912
mmu-miR-6371	1.074569932	1.611855	1.05281229	1.38507475		-0.58496	0.458644		chr3_7875_mature	2.14913986	0.805927	1.654957834	0.852739935		1.415037	1		hsa-miR-3658	1.07457	0	1.05281229	0		20	0.686912
mmu-miR-6374	1.074569932	1.611855	1.05281229	1.38507475		-0.58496	0.458644		chr15_31872_star	6.44741959	1.611855	2.896740642	1.38507475		2	1		hsa-miR-642b-5p	1.07457	0	1.05281229	0		20	0.686912
chr15_32789_mature	1.074569932	1.611855	1.05281229	1.38507475		-0.58496	0.458644		chr14_30472_mature	2.14913986	0.805927	1.654957834	0.852739935		1.415037	1		chr3_8590_star	1.07457	0	1.05281229	0		20	0.686912
chrx_23149_star	1.074569932	1.611855	1.05281229	1.38507475		-0.58496	0.458644		chr18_36120_mature	2.14913986	0.805927	1.654957834	0.852739935		1.415037	1		bta-miR-503-3p	1.07457	0	1.05281229	0		20	0.686912
chr3_7078_mature	1.074569932	1.611855	1.05281229	1.38507475		-0.58496	0.458644		chr15_32959_star	4.29827973	0.805927	2.405524014	0.852739935		2.415037	1		hsa-miR-5186	1.07457	0	1.05281229	0		20	0.686912
chr14_31548_mature	1.074569932	1.611855	1.05281229	1.38507475		-0.58496	0.458644		chr17_34966_mature	15.043979	5.641492	4.003960082	2.731507408		1.415037	1		hsa-miR-5189	1.07457	0	1.05281229	0		20	0.686912
mmu-miR-3112-5p	1.074569932	1.611855	1.05281229	1.38507475		-0.58496	0.458644		chr9_20772_star	4.29827973	0.805927	2.405524014	0.852739935		2.415037	1		rno-miR-3546	1.07457	0	1.05281229	0		20	0.686912
chr6_14334_mature	1.074569932	1.611855	1.05281229	1.38507475		-0.58496	0.458644		NW_003538329_37725_mature	2.14913986	0.805927	1.654957834	0.852739935		1.415037	1		chr9_21088_star	1.07457	0	1.05281229	0		20	0.686912
NW_003540989_39780_star	1.074569932	1.611855	1.05281229	1.38507475		-0.58496	0.458644		rno-miR-344i	4.29827973	0.805927	2.405524014	0.852739935		2.415037	1		chr2_4918_star	1.07457	0	1.05281229	0		20	0.686912
chr1_2658_star	1.074569932	1.611855	1.05281229	1.38507475		-0.58496	0.458644		chr4_10519_star	6.44741959	1.611855	2.896740642	1.38507475		2	1		mmu-miR-5114	1.07457	0	1.05281229	0		20	0.686912
hsa-miR-4802-3p	1.074569932	1.611855	1.05281229	1.38507475		-0.58496	0.458644		chr8_17991_mature@@bta-miR-367	6.44741959	1.611855	2.896740642	1.38507475		2	1		mmu-miR-5118	1.07457	0	1.05281229	0		20	0.686912
chr3_6615_star	1.074569932	1.611855	1.05281229	1.38507475		-0.58496	0.458644		chr18_36618_star	2.14913986	0.805927	1.654957834	0.852739935		1.415037	1		chr3_7715_star	1.07457	0	1.05281229	0		20	0.686912
chr14_31255_star	1.074569932	1.611855	1.05281229	1.38507475		-0.58496	0.458644		chr1_3025_mature	2.14913986	0.805927	1.654957834	0.852739935		1.415037	1		hsa-miR-3621	1.07457	0	1.05281229	0		20	0.686912
chr14_30996_star	1.074569932	1.611855	1.05281229	1.38507475		-0.58496	0.458644		bta-miR-1282	2.14913986	0.805927	1.654957834	0.852739935		1.415037	1		hsa-miR-3934-3p	1.07457	0	1.05281229	0		20	0.686912
chr9_19885_star	1.074569932	1.611855	1.05281229	1.38507475		-0.58496	0.458644		bta-miR-1281	2.14913986	0.805927	1.654957834	0.852739935		1.415037	1		chr2_6343_star	1.07457	0	1.05281229	0		20	0.686912
chr11_24795_mature	1.074569932	1.611855	1.05281229	1.38507475		-0.58496	0.458644		hsa-miR-6720-3p	2.14913986	0.805927	1.654957834	0.852739935		1.415037	1		chr13_27833_mature	1.07457	0	1.05281229	0		20	0.686912
hsa-miR-6132	1.074569932	1.611855	1.05281229	1.38507475		-0.58496	0.458644		rno-miR-3473	2.14913986	0.805927	1.654957834	0.852739935		1.415037	1		chr14_31110_mature	1.07457	0	1.05281229	0		20	0.686912
bta-miR-302b	1.074569932	1.611855	1.05281229	1.38507475		-0.58496	0.458644		hsa-miR-4707-3p	2.14913986	0.805927	1.654957834	0.852739935		1.415037	1		hsa-miR-514a-3p	1.07457	0	1.05281229	0		20	0.686912
bta-miR-141	1.074569932	1.611855	1.05281229	1.38507475		-0.58496	0.458644		chr9_19527_mature	2.14913986	0.805927	1.654957834	0.852739935		1.415037	1		chr4_9777_star	1.07457	0	1.05281229	0		20	0.686912
mmu-miR-3094-5p	1.074569932	1.611855	1.05281229	1.38507475		-0.58496	0.458644		chr7_16971_star	4.29827973	0.805927	2.405524014	0.852739935		2.415037	1		chr10_23700_star	1.07457	0	1.05281229	0		20	0.686912
bta-miR-2370-3p	1.074569932	1.611855	1.05281229	1.38507475		-0.58496	0.458644		chr7_15697_mature	2.14913986	0.805927	1.654957834	0.852739935		1.415037	1		rno-miR-192-3p	1.07457	0	1.05281229	0		20	0.686912
chr6_13302_star	1.074569932	1.611855	1.05281229	1.38507475		-0.58496	0.458644		chr13_27087_star	4.29827973	0.805927	2.405524014	0.852739935		2.415037	1		chr6_13949_mature	1.07457	0	1.05281229	0		20	0.686912
chr17_35547_star	1.074569932	1.611855	1.05281229	1.38507475		-0.58496	0.458644		chr6_12449_star	4.29827973	0.805927	2.405524014	0.852739935		2.415037	1		bta-miR-940	1.07457	0	1.05281229	0		20	0.686912
chr9_20403_mature	1.074569932	1.611855	1.05281229	1.38507475		-0.58496	0.458644		chr13_27530_mature	21.4913986	8.865202	4.491301473	3.302348583		1.277534	1		hsa-miR-516a-3p	1.07457	0	1.05281229	0		20	0.686912
chr6_13265_star	1.074569932	1.611855	1.05281229	1.38507475		-0.58496	0.458644		mmu-miR-1966-5p	2.14913986	0.805927	1.654957834	0.852739935		1.415037	1		chr8_17485_star	1.07457	0	1.05281229	0		20	0.686912
chr1_1390_star	1.074569932	1.611855	1.05281229	1.38507475		-0.58496	0.458644		hsa-miR-378j	6.44741959	1.611855	2.896740642	1.38507475		2	1		chr4_8813_star	1.07457	0	1.05281229	0		20	0.686912
chr5_11103_star	1.074569932	1.611855	1.05281229	1.38507475		-0.58496	0.458644		chr4_9333_mature	6.44741959	1.611855	2.896740642	1.38507475		2	1		chr16_34138_star	1.07457	0	1.05281229	0		20	0.686912
hsa-miR-5196-3p	1.074569932	1.611855	1.05281229	1.38507475		-0.58496	0.458644		hsa-miR-5047	2.14913986	0.805927	1.654957834	0.852739935		1.415037	1		mmu-miR-6392-5p	1.07457	0	1.05281229	0		20	0.686912
chr9_19300_star	1.074569932	1.611855	1.05281229	1.38507475		-0.58496	0.458644		chr15_32130_star	2.14913986	0.805927	1.654957834	0.852739935		1.415037	1		hsa-miR-135b-5p	1.07457	0	1.05281229	0		20	0.686912
chr5_12092_star	1.074569932	1.611855	1.05281229	1.38507475		-0.58496	0.458644		chr16_34386_star	2.14913986	0.805927	1.654957834	0.852739935		1.415037	1		chr6_13396_star	1.07457	0	1.05281229	0		20	0.686912
chr6_13056_mature	1.074569932	1.611855	1.05281229	1.38507475		-0.58496	0.458644		chrx_21832_mature	6.44741959	1.611855	2.896740642	1.38507475		2	1		rno-miR-135a-5p	1.07457	0	1.05281229	0		20	0.686912
mmu-miR-1247-3p	1.074569932	1.611855	1.05281229	1.38507475		-0.58496	0.458644		chr2_5880_star	17.1931189	6.44742	4.185320985	2.896740642		1.415037	1		hsa-miR-3714	1.07457	0	1.05281229	0		20	0.686912
chr14_29652_star	1.074569932	1.611855	1.05281229	1.38507475		-0.58496	0.458644		chr7_15363_star	8.59655945	2.417782	3.262517265	1.773060525		1.830075	1		chr6_14384_star	1.07457	0	1.05281229	0		20	0.686912
chr15_31984_star	1.074569932	1.611855	1.05281229	1.38507475		-0.58496	0.458644		chr1_1283_star	4.29827973	0.805927	2.405524014	0.852739935		2.415037	1		hsa-miR-34b-3p	1.07457	0	1.05281229	0		20	0.686912
chrx_22916_mature	1.074569932	1.611855	1.05281229	1.38507475		-0.58496	0.458644		chr7_17143_star	2.14913986	0.805927	1.654957834	0.852739935		1.415037	1		chr6_13131_mature	1.07457	0	1.05281229	0		20	0.686912
chr2_6450_star	1.074569932	1.611855	1.05281229	1.38507475		-0.58496	0.458644		chr1_3525_star	2.14913986	0.805927	1.654957834	0.852739935		1.415037	1		hsa-miR-675-3p	1.07457	0	1.05281229	0		20	0.686912
chrx_22969_star	1.074569932	1.611855	1.05281229	1.38507475		-0.58496	0.458644		NW_003538173_37591_mature	6.44741959	1.611855	2.896740642	1.38507475		2	1		bta-miR-2425-5p	1.07457	0	1.05281229	0		20	0.686912

chr9_20301_mature	1.074569932	1.611855	1.05281229	1.38507475		-0.58496	0.458644		chr13_28409_mature	25.7896784	11.28298	4.743605355	3.618589217		1.192645	1		chr2_5067_star	1.07457	0	1.05281229	0		20	0.686912
mmu-miR-302c-3p	1.074569932	1.611855	1.05281229	1.38507475		-0.58496	0.458644		chr7_16875_mature	2.14913986	12.08891	1.654957834	3.710273245		-2.49185	1		mmu-miR-465b-3p	1.07457	0	1.05281229	0		20	0.686912
hsa-miR-6503-5p	1.074569932	1.611855	1.05281229	1.38507475		-0.58496	0.458644		hsa-miR-1292-3p	2.14913986	0.805927	1.654957834	0.852739935		1.415037	1		hsa-miR-126-5p	1.07457	0	1.05281229	0		20	0.686912
chr14_30481_mature	1.074569932	1.611855	1.05281229	1.38507475		-0.58496	0.458644		chr2_5886_star	4.29827973	0.805927	2.405524014	0.852739935		2.415037	1		chr4_10566_star	1.07457	0	1.05281229	0		20	0.686912
chr13_26893_mature	1.074569932	1.611855	1.05281229	1.38507475		-0.58496	0.458644		mmu-miR-295-3p	4.29827973	0.805927	2.405524014	0.852739935		2.415037	1		hsa-miR-378c	1.07457	0	1.05281229	0		20	0.686912
bta-miR-29b	1.074569932	1.611855	1.05281229	1.38507475		-0.58496	0.458644		chr14_30104_star	2.14913986	0.805927	1.654957834	0.852739935		1.415037	1		mmu-miR-466d-5p	1.07457	0	1.05281229	0		20	0.686912
rno-miR-146a-3p	1.074569932	1.611855	1.05281229	1.38507475		-0.58496	0.458644		chr6_14382_star	2.14913986	0.805927	1.654957834	0.852739935		1.415037	1		hsa-miR-5095	1.07457	0	1.05281229	0		20	0.686912
chr10_23674_mature	1.074569932	1.611855	1.05281229	1.38507475		-0.58496	0.458644		rno-miR-465-3p	4.29827973	0.805927	2.405524014	0.852739935		2.415037	1		hsa-miR-4701-3p	1.07457	0	1.05281229	0		20	0.686912
chr6_12824_mature@@hsa-miR-877	1.074569932	1.611855	1.05281229	1.38507475		-0.58496	0.458644		bta-miR-877	2.14913986	0.805927	1.654957834	0.852739935		1.415037	1		hsa-miR-5093	1.07457	0	1.05281229	0		20	0.686912
chr18_36575_star	1.074569932	1.611855	1.05281229	1.38507475		-0.58496	0.458644		bta-miR-875	4.29827973	0.805927	2.405524014	0.852739935		2.415037	1		hsa-miR-5087	1.07457	0	1.05281229	0		20	0.686912
chr7_15660_star	1.074569932	1.611855	1.05281229	1.38507475		-0.58496	0.458644		hsa-miR-5008-3p	2.14913986	0.805927	1.654957834	0.852739935		1.415037	1		mmu-miR-466d-3p	1.07457	0	1.05281229	0		20	0.686912
chr16_34293_star	1.074569932	1.611855	1.05281229	1.38507475		-0.58496	0.458644		chr2_6082_mature	4.29827973	0.805927	2.405524014	0.852739935		2.415037	1		chr10_23834_star	1.07457	0	1.05281229	0		20	0.686912
chr3_8156_star	1.074569932	1.611855	1.05281229	1.38507475		-0.58496	0.458644		chr3_7220_mature	2.14913986	11.28298	1.654957834	3.618589217		-2.39232	1		hsa-miR-4649-3p	1.07457	0	1.05281229	0		20	0.686912
NW_003538331_37733_mature	1.074569932	1.611855	1.05281229	1.38507475		-0.58496	0.458644		chr1_429_star	6.44741959	20.95411	2.896740642	4.456419386		-1.70044	1		hsa-miR-1234-3p	1.07457	0	1.05281229	0		20	0.686912
ssc-miR-4337	1.074569932	1.611855	1.05281229	1.38507475		-0.58496	0.458644		NW_003613097_37151_star	2.14913986	0.805927	1.654957834	0.852739935		1.415037	1		NW_003538520_37951_star	1.07457	0	1.05281229	0		20	0.686912
chr4_10198_star	1.074569932	1.611855	1.05281229	1.38507475		-0.58496	0.458644		chr7_16875_star	2.14913986	0.805927	1.654957834	0.852739935		1.415037	1		NW_003538870_38317_mature	1.07457	0	1.05281229	0		20	0.686912
chr17_35182_mature@@bta-miR-61	1.074569932	1.611855	1.05281229	1.38507475		-0.58496	0.458644		chrx_23090_star	2.14913986	0.805927	1.654957834	0.852739935		1.415037	1		chrx_21838_star@@rno-miR-188-3	1.07457	0	1.05281229	0		20	0.686912
chr5_10732_mature	1.074569932	1.611855	1.05281229	1.38507475		-0.58496	0.458644		chr11_24582_star	2.14913986	0.805927	1.654957834	0.852739935		1.415037	1		chr12_25706_star	1.07457	0	1.05281229	0		20	0.686912
hsa-miR-4269	1.074569932	1.611855	1.05281229	1.38507475		-0.58496	0.458644		rno-miR-30e-3p	2.14913986	0.805927	1.654957834	0.852739935		1.415037	1		mmu-miR-493-5p	1.07457	0	1.05281229	0		20	0.686912
hsa-miR-4261	1.074569932	1.611855	1.05281229	1.38507475		-0.58496	0.458644		NW_003539698_39033_star	4.29827973	0.805927	2.405524014	0.852739935		2.415037	1		mmu-miR-466i-5p	1.07457	0	1.05281229	0		20	0.686912
chr9_20112_mature	1.074569932	1.611855	1.05281229	1.38507475		-0.58496	0.458644		chr4_10525_star	2.14913986	0.805927	1.654957834	0.852739935		1.415037	1		hsa-miR-6515-3p	1.07457	0	1.05281229	0		20	0.686912
chr4_10395_star	1.074569932	1.611855	1.05281229	1.38507475		-0.58496	0.458644		NW_003539422_38814_star	2.14913986	0.805927	1.654957834	0.852739935		1.415037	1		chr7_16117_star	1.07457	0	1.05281229	0		20	0.686912
NW_003538949_38371_star	1.074569932	1.611855	1.05281229	1.38507475		-0.58496	0.458644		mmu-miR-6405	4.29827973	0.805927	2.405524014	0.852739935		2.415037	1		chr10_23548_star	1.07457	0	1.05281229	0		20	0.686912
hsa-miR-4665-5p	1.074569932	1.611855	1.05281229	1.38507475		-0.58496	0.458644		chr7_15311_star	2.14913986	0.805927	1.654957834	0.852739935		1.415037	1		chr15_31928_star	1.07457	0	1.05281229	0		20	0.686912
hsa-miR-4772-3p	1.074569932	1.611855	1.05281229	1.38507475		-0.58496	0.458644		chr2_4916_mature	2.14913986	0.805927	1.654957834	0.852739935		1.415037	1		hsa-miR-378h	1.07457	0	1.05281229	0		20	0.686912
chr1_2783_star	1.074569932	1.611855	1.05281229	1.38507475		-0.58496	0.458644		chr12_26240_star	15.043979	5.641492	4.003960082	2.731507408		1.415037	1		chr3_7797_star	1.07457	0	1.05281229	0		20	0.686912
chr14_30612_star	1.074569932	1.611855	1.05281229	1.38507475		-0.58496	0.458644		chr2_6200_star	2.14913986	0.805927	1.654957834	0.852739935		1.415037	1		chr3_7464_star	1.07457	0	1.05281229	0		20	0.686912
chr1_766_mature	1.074569932	1.611855	1.05281229	1.38507475		-0.58496	0.458644		chr3_8590_mature	4.29827973	0.805927	2.405524014	0.852739935		2.415037	1		rno-miR-344b-5p	1.07457	0	1.05281229	0		20	0.686912
chr15_33643_star	1.074569932	1.611855	1.05281229	1.38507475		-0.58496	0.458644		chr5_11374_star	4.29827973	0.805927	2.405524014	0.852739935		2.415037	1		chr11_25383_mature	1.07457	0	1.05281229	0		20	0.686912
chr3_7189_star	1.074569932	1.611855	1.05281229	1.38507475		-0.58496	0.458644		chrx_22791_star	2.14913986	0.805927	1.654957834	0.852739935		1.415037	1		chr7_15100_star	1.07457	0	1.05281229	0		20	0.686912
chr1_551_mature	1.074569932	1.611855	1.05281229	1.38507475		-0.58496	0.458644		chr8_18418_star	2.14913986	0.805927	1.654957834	0.852739935		1.415037	1		rno-miR-743a-5p	1.07457	0	1.05281229	0		20	0.686912
chr2_6321_mature	1.074569932	1.611855	1.05281229	1.38507475		-0.58496	0.458644		chr3_7786_mature	6.44741959	1.611855	2.896740642	1.38507475		2	1		NW_003613109_37259_mature	1.07457	0	1.05281229	0		20	0.686912
chr10_23364_star	1.074569932	1.611855	1.05281229	1.38507475		-0.58496	0.458644		hsa-miR-4690-5p	2.14913986	0.805927	1.654957834	0.852739935		1.415037	1		mmu-miR-505-3p	1.07457	0	1.05281229	0		20	0.686912
chr13_28245_star	1.074569932	1.611855	1.05281229	1.38507475		-0.58496	0.458644		chr11_25345_star	2.14913986	0.805927	1.654957834	0.852739935		1.415037	1		chr4_9751_mature	1.07457	0	1.05281229	0		20	0.686912
bta-miR-488	1.074569932	1.611855	1.05281229	1.38507475		-0.58496	0.458644		chr6_14781_mature	2.14913986	0.805927	1.654957834	0.852739935		1.415037	1		rno-miR-126b	1.07457	0	1.05281229	0		20	0.686912
chr5_11637_mature	1.074569932	1.611855	1.05281229	1.38507475		-0.58496	0.458644		chr15_32065_star	4.29827973	0.805927	2.405524014	0.852739935		2.415037	1		bta-miR-411c-3p	1.07457	0	1.05281229	0		20	0.686912
chr15_32320_star	1.074569932	1.611855	1.05281229	1.38507475		-0.58496	0.458644		chr6_14470_star	6.44741959	1.611855	2.896740642	1.38507475		2	1		chr14_29848_star	1.07457	0	1.05281229	0		20	0.686912
bta-miR-2423	1.074569932	1.611855	1.05281229	1.38507475		-0.58496	0.458644		mmu-miR-6419	2.14913986	0.805927	1.654957834	0.852739935		1.415037	1		hsa-miR-6502-5p	1.07457	0	1.05281229	0		20	0.686912
bta-miR-30e-5p	1.074569932	1.611855	1.05281229	1.38507475		-0.58496	0.458644		mmu-miR-6414	4.29827973	0.805927	2.405524014	0.852739935		2.415037	1		hsa-miR-19b-3p	1.07457	0	1.05281229	0		20	0.686912
chr15_33015_star	1.074569932	1.611855	1.05281229	1.38507475		-0.58496	0.458644		chrx_22707_star	4.29827973	0.805927	2.405524014	0.852739935		2.415037	1		chr3_8500_star	1.07457	0	1.05281229	0		20	0.686912
chr7_16057_mature	1.074569932	1.611855	1.05281229	1.38507475		-0.58496	0.458644		NW_003538236_37641_star	2.14913986	0.805927	1.654957834	0.852739935		1.415037	1		hsa-miR-548az-5p	1.07457	0	1.05281229	0		20	0.686912
chr14_29999_star	1.074569932	1.611855	1.05281229	1.38507475		-0.58496	0.458644		chr3_7967_mature	4.29827973	0.805927	2.405524014	0.852739935		2.415037	1		bta-miR-30a-5p	1.07457	0	1.05281229	0		20	0.686912
NW_003539812_39109_star	1.074569932	1.611855	1.05281229	1.38507475		-0.58496	0.458644		chr12_25705_star	4.29827973	0.805927	2.405524014	0.852739935		2.415037	1		mmu-miR-532-3p	1.07457	0	1.05281229	0		20	0.686912
hsa-miR-4489	1.074569932	1.611855	1.05281229	1.38507475		-0.58496	0.458644		chr10_24027_star	2.14913986	0.805927	1.654957834	0.852739935		1.415037	1		mmu-miR-669d-3p	1.07457	0	1.05281229	0		20	0.686912
chr2_6203_star	1.074569932	1.611855	1.05281229	1.38507475		-0.58496	0.458644		chr13_29218_mature	2.14913986	11.28298	1.654957834	3.618589217		-2.39232	1		NW_003613191_37580_mature	1.07457	0	1.05281229	0		20	0.686912
chr11_24341_star	1.074569932	1.611855	1.05281229	1.38507475		-0.58496	0.458644		chr1_2562_star	2.14913986	0.805927	1.654957834	0.852739935		1.415037	1		rno-miR-543-5p	1.07457	0	1.05281229	0		20	0.686912
hsa-miR-524-5p	1.074569932	1.611855	1.05281229	1.38507475		-0.58496	0.458644		chr7_16728_star	2.14913986	0.805927	1.654957834	0.852739935		1.415037	1		mmu-miR-3066-3p	1.07457	0	1.05281229	0		20	0.686912
hsa-miR-606	1.074569932	1.611855	1.05281229	1.38507475		-0.58496	0.458644		hsa-miR-676-5p	2.14913986	0.805927	1.654957834	0.852739935		1.415037	1		hsa-miR-3157-5p	1.07457	0	1.05281229	0		20	0.686912
hsa-miR-612	1.074569932	1.611855	1.05281229	1.38507475		-0.58496	0.458644		chr12_25405_mature	4.29827973	0.805927	2.405524014	0.852739935		2.415037	1		rno-miR-764-3p	1.07457	0	1.05281229	0		20	0.686912
chr7_16272_star	1.074569932	1.611855	1.05281229	1.38507475		-0.58496	0.458644		chr4_9665_star	4.29827973	0.805927	2.405524014	0.852739935		2.415037	1		mmu-miR-6408	1.07457	0	1.05281229	0		20	0.686912
bta-miR-2398	1.074569932	1.611855	1.05281229	1.38507475		-0.58496	0.458644		chr16_34261_star	4.29827973	0.805927	2.405524014	0.852739935		2.415037	1		chr1_3299_star	1.07457	0	1.05281229	0		20	0.686912
chr13_27833_star	1.074569932	1.611855	1.05281229	1.38507475		-0.58496	0.458644		chr4_10238_star	2.14913986	0.805927	1.654957834	0.852739935		1.415037	1		chr1_2655_star	1.07457	0	1.05281229	0		20	0.686912
chr4_9430_mature	1.074569932	1.611855	1.05281229	1.38507475		-0.58496	0.458644		chr16_34245_star	4.29827973	0.805927	2.405524014	0.852739935		2.415037	1		chr16_33919_star	1.07457	0	1.05281229	0		20	0.686912
chr14_30313_star	1.074569932	1.611855	1.05281229	1.38507475		-0.58496	0.458644		chr17_35096_mature	32.237098	15.31262	5.054722516	4.027916745		1.074001	1		rno-miR-547-5p	1.07457	0	1.05281229	0		20	0.686912
hsa-miR-3156-3p	1.074569932	1.611855	1.05281229	1.38507475		-0.58496	0.458644		ssc-miR-383	2.14913986	0.805927	1.654957834	0.852739935		1.415037	1		chr11_25225_mature	1.07457	0	1.05281229	0		20	0.686912
chr4_10381_mature	1.074569932	1.611855	1.05281229	1.38507475		-0.58496	0.458644		bta-miR-744	6.44741959	1.611855	2.896740642	1.38507475		2	1		chr14_29850_mature	1.07457	0	1.05281229	0		20	0.686912
chr13_28063_mature	1.074569932	1.611855	1.05281229	1.38507475		-0.58496	0.458644		mmu-miR-301b-3p	4.29827973	0.805927	2.405524014	0.852739935		2.415037	1		rno-miR-148b-3p	1.07457	0	1.05281229	0		20	0.686912
chr11_24450_star	1.074569932	1.611855	1.05281229	1.38507475		-0.58496	0.458644		bta-miR-2448-3p	4.29827973	0.805927	2.405524014	0.852739935		2.415037	1		mmu-miR-6415	1.07457	0	1.05281229	0		20	0.686912
chr16_33722_mature	1.074569932	1.611855	1.05281229	1.38507475		-0.58496	0.458644		hsa-miR-676-3p	2.14913986	0.805927	1.654957834	0.852739935		1.415037	1		mmu-miR-6417	1.07457	0	1.05281229	0		20	0.686912
hsa-miR-6507-3p	1.074569932	1.611855	1.05281229	1.38507475		-0.58496	0.458644		chr9_20880_star	2.14913986	0.805927	1.654957834	0.852739935		1.415037	1		rno-miR-215	1.07457	0	1.05281229	0		20	0.686912
chr4_10259_star	1.074569932	1.611855	1.05281229	1.38507475		-0.58496	0.458644		chr3_7890_star	4.29827973	0.805927	2.405524014	0.852739935		2.415037	1		mmu-miR-30b-5p	1.07457	0	1.05281229	0		20	0.686912
chr18_35816_star	1.074569932	1.611855	1.05281229	1.38507475		-0.58496	0.458644		chr14_30876_star	2.14913986	0.805927	1.654957834	0.852739935		1.415037	1		hsa-miR-200b-5p	1.07457	0	1.05281229	0		20	0.686912
hsa-miR-4670-3p	1.074569932	1.611855	1.05281229	1.38507475		-0.58496	0.458644		chr4_9509_star	6.44741959	1.611855	2.896740642	1.38507475		2	1		hsa-miR-5197-5p	1.07457	0	1.05281229	0		20	0.686912
bta-miR-2335	1.074569932	1.611855	1.05281229	1.38507475		-0.58496	0.458644		chr2_4612_mature	15.043979	5.641492	4.003960082	2.731507408		1.415037	1		chr9_20036_star	1.07457	0	1.05281229	0		20	0.686912
chr1_785_star	1.074569932	1.611855	1.05281229	1.38507475		-0.58496	0.458644		chr14_30645_mature	4.29827973	0.805927	2.405524014	0.852739935		2.415037	1		chr16_33853_mature	1.07457	0	1.05281229	0		20	0.686912
hsa-miR-4720-3p	1.074569932	1.611855	1.05281229	1.38507475		-0.58496	0.458644		hsa-miR-4776-3p	4.29827973	0.805927	2.405524014	0.852739935		2.415037	1		chr13_29315_star	1.07457	0	1.05281229	0		20	0.686912
chr13_26761_star	1.074569932	1.611855	1.05281229	1.38507475		-0.58496	0.458644		chr1_2519_star	6.44741959	1.611855	2.896740642	1.38507475		2	1		mmu-miR-340-5p	1.07457	0	1.05281229	0		20	0.686912
bta-miR-2293	1.074569932	1.611855	1.05281229	1.38507475		-0.58496	0.458644		chr1_2402_star	2.14913986	0.805927	1.654957834	0.852739935		1.415037	1		bta-miR-2325b	1.07457	0	1.05281229	0		20	0.686912
bta-miR-2296	1.074569932	1.611855	1.05281229	1.38507475		-0.58496	0.458644		NW_003538623_38054_star	2.14913986	0.805927	1.654957834	0.852739935		1.415037	1		hsa-miR-452-3p	1.07457	0	1.05281229	0		20	0.686912
hsa-miR-718	1.074569932	1.611855	1.05281229	1.38507475		-0.58496	0.458644		chr8_17927_star	2.14913986	0.805927	1.654957834	0.852739935		1.415037	1		hsa-miR-4728-3p	1.07457	0	1.05281229	0		20	0.686912
chr12_26051_mature	1.074569932	1.611855	1.05281229	1.38507475		-0.58496	0.458644		ssc-miR-325	4.29827973	0.805927	2.405524014	0.852739935		2.415037	1		mmu-miR-509-3p	1.07457	0	1.05281229	0		20	0.686912
chr7_16797_star	1.074569932	1.611855	1.05281229	1.38507475		-0.58496	0.458644		chr12_26572_mature	4.29827973	0.805927	2.405524014	0.852739935		2.415037	1		NW_003613497_39427_star	1.07457	0	1.05281229	0		20	0.686912
chr13_28990_star	1.074569932	1.611855	1.05281229	1.38507475		-0.58496	0.458644		hsa-miR-5006-5p	2.14913986	0.805927	1.654957834	0.852739935		1.415037	1		bta-miR-758	1.07457	0	1.05281229	0		20	0.686912
hsa-miR-519e-3p	1.074569932	1.611855	1.05281229	1.38507475		-0.58496	0.458644		mmu-miR-6352	2.14913986	0.805927	1.654957834	0.852739935		1.415037	1		NW_003613161_37463_mature	1.07457	0	1.05281229	0		20	0.686912
hsa-miR-4633-3p	1.074569932	1.611855	1.05281229	1.38507475		-0.58496	0.458644		mmu-miR-6381	2.14913986	0.805927	1.654957834	0.852739935		1.415037	1		mmu-miR-509-5p	1.07457	0	1.05281229	0		20	0.686912
chr17_35678_star	1.074569932	1.611855	1.05281229	1.38507475		-0.58496	0.458644		rno-miR-30b-5p	2.14913986	0.805927	1.654957834	0.852739935		1.415037	1		chr11_24459_star	1.07457	0	1.05281229	0		20	0.686912
chr13_29225_star	1.074569932	1.611855	1.05281229	1.38507475		-0.58496	0.458644		mmu-miR-6372	2.14913986	0.805927	1.654957834	0.852739935		1.415037	1		rno-miR-190b-3p	1.07457	0	1.05281229	0		20	0.686912
bta-miR-2330-5p	1.074569932	1.611855	1.05281229	1.38507475		-0.58496	0.458644		NW_003613057_36932_mature	2.14913986	0.805927	1.654957834	0.852739935		1.415037	1		NW_003613103_37208_star	1.07457	0	1.05281229	0		20	0.686912
chr15_32809_star	1.074569932	1.611855	1.05281229	1.38507475		-0.58496	0.458644		mmu-miR-6378	4.29827973	0.805927	2.405524014	0.852739935		2.415037	1		mmu-miR-6344	1.07457	0	1.05281229	0		20	0.686912
chr9_20114_star	1.074569932	1.611855	1.05281229	1.38507475		-0.58496	0.458644		bta-miR-2285k	6.44741959	1.611855	2.896740642	1.38507475		2	1		mmu-miR-6347	1.07457	0	1.05281229	0		20	0.686912
chr16_33835_star	1.074569932	1.611855	1.05281229	1.38507475		-0.58496	0.458644		bta-miR-2285o	6.44741959	1.611855	2.896740642	1.38507475		2	1		hsa-miR-3150b-5p	1.07457	0	1.05281229	0		20	0.686912
chr15_31887_mature	1.074569932	1.611855	1.05281229	1.38507475		-0.58496	0.458644		bta-miR-2284z	10.7456993	28.20746	3.554060706	4.868265031		-1.39232	1		mmu-miR-6369	1.07457	0	1.05281229	0		20	0.686912
chr2_4221_star	1.074569932	1.611855	1.05281229	1.38507475		-0.58496	0.458644		rno-miR-30b-3p	2.14913986	0.805927	1.654957834	0.852739935		1.415037	1		chr5_11601_star	1.07457	0	1.05281229	0		20	0.686912
chr15_32527_star	1.074569932	1.611855	1.05281229	1.38507475		-0.58496	0.458644		mmu-miR-6390	2.14913986	0.805927	1.654957834	0.852739935		1.415037	1		mmu-miR-6355	1.07457	0	1.05281229	0		20	0.686912
chr17_35398_mature	1.074569932	1.611855	1.05281229	1.38507475		-0.58496	0.458644		chr8_18283_star	2.14913986	0.805927	1.654957834	0.852739935		1.415037	1		bta-miR-568	1.07457	0	1.05281229	0		20	0.686912
chr9_19327_star	1.074569932	1.611855	1.05281229	1.38507475		-0.58496	0.458644		chr1_3137_star	4.29827973	0.805927	2.405524014	0.852739935		2.415037	1		chr18_35924_star	1.07457	0	1.05281229	0		20	0.686912
rno-miR-23b-3p	1.074569932	1.611855	1.05281229	1.38507475		-0.58496	0.458644		mmu-miR-6395	4.29827973	0.805927	2.405524014	0.852739935		2.415037	1		hsa-miR-664a-5p	1.07457	0	1.05281229	0		20	0.686912
hsa-miR-516b-5p	1.074569932	1.611855	1.05281229	1.38507475		-0.58496	0.458644		mmu-miR-6399	2.14913986	0.805927	1.654957834	0.852739935		1.415037	1		mmu-miR-6384	1.07457	0	1.05281229	0		20	0.686912
chr14_30126_mature	1.074569932	1.611855	1.05281229	1.38507475		-0.58496	0.458644		hsa-miR-4659a-5p	2.14913986	0.805927	1.654957834	0.852739935		1.415037	1		bta-miR-2285h	1.07457	0	1.05281229	0		20	0.686912
hsa-miR-4793-3p	1.074569932	1.611855	1.05281229	1.38507475		-0.58496	0.458644		chr5_12123_star	4.29827973	0.805927	2.405524014	0.852739935		2.415037	1		bta-miR-2284x	1.07457	0	1.05281229	0		20	0.686912
chr7_15943_star	1.074569932	1.611855	1.05281229	1.38507475		-0.58496	0.458644		chr14_30481_star	8.59655945	2.417782	3.262517265	1.773060525		1.830075	1		chr5_12257_star	1.07457	0	1.05281229	0		20	0.686912
chr9_19622_star	1.074569932	1.611855	1.05281229	1.38507475		-0.58496	0.458644		NW_003613379_38761_mature	2.14913986	0.805927	1.654957834	0.852739935		1.415037	1		bta-miR-2284m	1.07457	0	1.05281229	0		20	0.686912
chr4_10042_mature	1.074569932	1.611855	1.05281229	1.38507475		-0.58496	0.458644		chr13_28101_mature	4.29827973	0.805927	2.405524014	0.852739935		2.415037	1		chr14_30956_star	1.07457	0	1.05281229	0		20	0.686912
chr13_26893_star	1.074569932	1.611855	1.05281229	1.38507475		-0.58496	0.458644		chrx_22202_star	2.14913986	0.805927	1.654957834	0.852739935		1.415037	1		chr2_4396_star	1.07457	0	1.05281229	0		20	0.686912
mmu-miR-28a-5p	1.074569932	1.611855	1.05281229	1.38507475		-0.58496	0.458644		chr15_33536_star	4.29827973	0.805927	2.405524014	0.852739935		2.415037	1		hsa-miR-6500-3p	1.07457	0	1.05281229	0		20	0.686912
hsa-miR-5685	1.074569932	1.611855	1.05281229	1.38507475		-0.58496	0.458644		chr8_18054_mature	6.44741959	1.611855	2.896740642	1.38507475		2	1		NW_003539146_38590_star	1.07457	0	1.05281229	0		20	0.686912
hsa-miR-5695	1.074569932	1.611855	1.05281229	1.38507475		-0.58496	0.458644		NW_003538374_37783_star	2.14913986	0.805927	1.654957834	0.852739935		1.415037	1		mmu-miR-879-3p	1.07457	0	1.05281229	0		20	0.686912
chr7_15101_star	1.074569932	1.611855	1.05281229	1.38507475		-0.58496	0.458644		chr18_36225_star	2.14913986	11.28298	1.654957834	3.618589217		-2.39232	1		bta-miR-2901	1.07457	0	1.05281229	0		20	0.686912
hsa-miR-211-5p	1.074569932	1.611855	1.05281229	1.38507475		-0.58496	0.458644		rno-miR-352	2.14913986	0.805927	1.654957834	0.852739935		1.415037	1		mmu-miR-490-5p	1.07457	0	1.05281229	0		20	0.686912
chr1_3129_star	1.074569932	1.611855	1.05281229	1.38507475		-0.58496	0.458644		rno-miR-130b-3p	4.29827973	0.805927	2.405524014	0.852739935		2.415037	1		rno-miR-206-5p	1.07457	0	1.05281229	0		20	0.686912
hsa-miR-4503	1.074569932	1.611855	1.05281229	1.38507475		-0.58496	0.458644		rno-miR-31b	2.14913986	12.08891	1.654957834	3.710273245		-2.49185	1		rno-miR-327	1.07457	0	1.05281229	0		20	0.686912
chr11_25373_star	1.074569932	1.611855	1.05281229	1.38507475		-0.58496	0.458644		chr3_6901_mature	2.14913986	0.805927	1.654957834	0.852739935		1.415037	1		chr6_13901_star	1.07457	0	1.05281229	0		20	0.686912
chr2_5958_mature	1.074569932	1.611855	1.05281229	1.38507475		-0.58496	0.458644		chr4_8831_star	4.29827973	0.805927	2.405524014	0.852739935		2.415037	1		chr9_19808_star	1.07457	0	1.05281229	0		20	0.686912
rno-miR-194-5p	1.074569932	1.611855	1.05281229	1.38507475		-0.58496	0.458644		chr11_25228_mature	4.29827973	0.805927	2.405524014	0.852739935		2.415037	1		chr4_8670_mature	1.07457	0	1.05281229	0		20	0.686912
chr1_3478_star	1.074569932	1.611855	1.05281229	1.38507475		-0.58496	0.458644		chr18_36584_star	2.14913986	0.805927	1.654957834	0.852739935		1.415037	1		bta-miR-2319b	1.07457	0	1.05281229	0		20	0.686912
chr3_8228_mature	1.074569932	1.611855	1.05281229	1.38507475		-0.58496	0.458644		chr4_9997_star	4.29827973	0.805927	2.405524014	0.852739935		2.415037	1		bta-miR-2319a	1.07457	0	1.05281229	0		20	0.686912
chr1_3837_star	1.074569932	1.611855	1.05281229	1.38507475		-0.58496	0.458644		chr17_35456_star	8.59655945	2.417782	3.262517265	1.773060525		1.830075	1		hsa-miR-4436b-5p	1.07457	0	1.05281229	0		20	0.686912
hsa-miR-6077	1.074569932	1.611855	1.05281229	1.38507475		-0.58496	0.458644		chr17_35169_star	4.29827973	0.805927	2.405524014	0.852739935		2.415037	1		hsa-miR-1238-5p	1.07457	0	1.05281229	0		20	0.686912
chr7_16134_mature	1.074569932	1.611855	1.05281229	1.38507475		-0.58496	0.458644		chr7_15697_star	2.14913986	0.805927	1.654957834	0.852739935		1.415037	1		hsa-miR-338-5p	1.07457	0	1.05281229	0		20	0.686912
chr17_35760_star	1.074569932	1.611855	1.05281229	1.38507475		-0.58496	0.458644		chr4_10432_star	2.14913986	0.805927	1.654957834	0.852739935		1.415037	1		hsa-miR-3529-5p	1.07457	0	1.05281229	0		20	0.686912
chr7_16945_star	1.074569932	1.611855	1.05281229	1.38507475		-0.58496	0.458644		chr1_191_star	4.29827973	0.805927	2.405524014	0.852739935		2.415037	1		mmu-miR-669k-3p	1.07457	0	1.05281229	0		20	0.686912
chr15_32525_star	1.074569932	1.611855	1.05281229	1.38507475		-0.58496	0.458644		bta-miR-615	4.29827973	0.805927	2.405524014	0.852739935		2.415037	1		chr2_6577_star	1.07457	0	1.05281229	0		20	0.686912
chr10_24278_mature	6.447419591	3.22371	2.896740642	2.078510713		1	0.459239		chr8_17973_mature	4.29827973	0.805927	2.405524014	0.852739935		2.415037	1		chr11_25285_star	1.07457	0	1.05281229	0		20	0.686912
chr16_34525_star@@rno-miR-449a-	6.447419591	3.22371	2.896740642	2.078510713		1	0.459239		hsa-miR-888-3p	2.14913986	0.805927	1.654957834	0.852739935		1.415037	1		mmu-miR-654-3p	1.07457	0	1.05281229	0		20	0.686912
chr6_12855_star	6.447419591	3.22371	2.896740642	2.078510713		1	0.459239		chr9_20355_mature	4.29827973	0.805927	2.405524014	0.852739935		2.415037	1		chr5_11099_star	1.07457	0	1.05281229	0		20	0.686912
chr17_34985_mature	6.447419591	3.22371	2.896740642	2.078510713		1	0.459239		hsa-miR-548x-3p	2.14913986	0.805927	1.654957834	0.852739935		1.415037	1		chr1_3945_star	1.07457	0	1.05281229	0		20	0.686912
chr3_7564_mature	6.447419591	3.22371	2.896740642	2.078510713		1	0.459239		chr5_11357_star	4.29827973	16.11855	2.405524014	4.097488514		-1.90689	1		bta-miR-669	1.07457	0	1.05281229	0		20	0.686912
chr3_6750_star	6.447419591	3.22371	2.896740642	2.078510713		1	0.459239		bta-miR-660	4.29827973	0.805927	2.405524014	0.852739935		2.415037	1		hsa-miR-4436a	1.07457	0	1.05281229	0		20	0.686912
chr14_31126_star	6.447419591	3.22371	2.896740642	2.078510713		1	0.459239		chr9_20123_star	2.14913986	0.805927	1.654957834	0.852739935		1.415037	1		hsa-miR-1255b-5p	1.07457	0	1.05281229	0		20	0.686912
hsa-miR-4795-3p	6.447419591	3.22371	2.896740642	2.078510713		1	0.459239		chr7_17055_star	4.29827973	0.805927	2.405524014	0.852739935		2.415037	1		chr7_16231_star	1.07457	0	1.05281229	0		20	0.686912
NW_003539012_38462_star	6.447419591	3.22371	2.896740642	2.078510713		1	0.459239		bta-miR-653	4.29827973	0.805927	2.405524014	0.852739935		2.415037	1		chr11_24436_star	1.07457	0	1.05281229	0		20	0.686912
chr4_9657_star	6.447419591	3.22371	2.896740642	2.078510713		1	0.459239		chr16_33810_mature	6.44741959	1.611855	2.896740642	1.38507475		2	1		chr6_13925_mature	1.07457	0	1.05281229	0		20	0.686912
chr1_927_star	6.447419591	3.22371	2.896740642	2.078510713		1	0.459239		chr14_30949_star	4.29827973	0.805927	2.405524014	0.852739935		2.415037	1		chr7_17178_mature	1.07457	0	1.05281229	0		20	0.686912
hsa-miR-4426	6.447419591	3.22371	2.896740642	2.078510713		1	0.459239		hsa-miR-4661-5p	4.29827973	0.805927	2.405524014	0.852739935		2.415037	1		chr18_36427_star@@mmu-miR-59	1.07457	0	1.05281229	0		20	0.686912
chr2_4563_mature	6.447419591	3.22371	2.896740642	2.078510713		1	0.459239		chr18_36486_star	2.14913986	0.805927	1.654957834	0.852739935		1.415037	1		chrx_22747_star	1.07457	0	1.05281229	0		20	0.686912
chr18_35994_star	6.447419591	3.22371	2.896740642	2.078510713		1	0.459239		chr8_17778_mature	6.44741959	1.611855	2.896740642	1.38507475		2	1		chr3_7453_star	1.07457	0	1.05281229	0		20	0.686912
NW_003539920_39178_star	6.447419591	3.22371	2.896740642	2.078510713		1	0.459239		NW_003539597_38976_star	2.14913986	0.805927	1.654957834	0.852739935		1.415037	1		hsa-miR-6506-5p	1.07457	0	1.05281229	0		20	0.686912
chr13_26975_mature	4.298279727	9.671129	2.405524014	3.415640968		-1.16993	0.461329		chr6_14334_mature	6.44741959	1.611855	2.896740642	1.38507475		2	1		chrx_23039_star	1.07457	0	1.05281229	0		20	0.686912
chr18_36633_star	4.298279727	9.671129	2.405524014	3.415640968		-1.16993	0.461329		chr13_29005_mature	6.44741959	1.611855	2.896740642	1.38507475		2	1		rno-miR-328a-3p	1.07457	0	1.05281229	0		20	0.686912
NW_003539007_38456_star	4.298279727	9.671129	2.405524014	3.415640968		-1.16993	0.461329		chr6_13263_star	2.14913986	0.805927	1.654957834	0.852739935		1.415037	1		chr9_20122_star	1.07457	0	1.05281229	0		20	0.686912
chr5_12006_mature	4.298279727	9.671129	2.405524014	3.415640968		-1.16993	0.461329		chr13_28684_mature	2.14913986	0.805927	1.654957834	0.852739935		1.415037	1		hsa-miR-1245a	1.07457	0	1.05281229	0		20	0.686912
chr4_10265_mature	4.298279727	9.671129	2.405524014	3.415640968		-1.16993	0.461329		chr6_13990_star	4.29827973	0.805927	2.405524014	0.852739935		2.415037	1		chr4_9959_star	1.07457	0	1.05281229	0		20	0.686912
chr15_31957_mature	4.298279727	9.671129	2.405524014	3.415640968		-1.16993	0.461329		chr3_7716_star	10.7456993	3.22371	3.554060706	2.078510713		1.736966	1		chr14_29816_star	1.07457	0	1.05281229	0		20	0.686912
chr14_30912_star	4.298279727	9.671129	2.405524014	3.415640968		-1.16993	0.461329		hsa-miR-5010-5p	2.14913986	0.805927	1.654957834	0.852739935		1.415037	1		NW_003613497_39428_star	1.07457	0	1.05281229	0		20	0.686912
chrx_22543_mature	19.34225877	6.44742	4.346407978	2.896740642		1.584963	0.461375		NW_003540989_39780_star	6.44741959	1.611855	2.896740642	1.38507475		2	1		bta-miR-152	1.07457	0	1.05281229	0		20	0.686912
chr8_18291_mature	19.34225877	6.44742	4.346407978	2.896740642		1.584963	0.461375		rno-miR-344a-5p	2.14913986	0.805927	1.654957834	0.852739935		1.415037	1		bta-miR-153	1.07457	0	1.05281229	0		20	0.686912
chr1_1168_mature	107.4569932	20.14819	6.760979269	4.402462031		2.415037	0.461534		chr2_5926_mature	4.29827973	0.805927	2.405524014	0.852739935		2.415037	1		NW_003540978_39774_star	1.07457	0	1.05281229	0		20	0.686912
chr12_25647_star	6.447419591	18.53633	2.896740642	4.288087668		-1.52356	0.461927		chr8_17778_star	6.44741959	1.611855	2.896740642	1.38507475		2	1		hsa-miR-545-3p	1.07457	0	1.05281229	0		20	0.686912
chr8_18647_mature	5.372849659	13.70077	2.671938627	3.877819487		-1.3505	0.46315		chr15_32439_mature	6.44741959	1.611855	2.896740642	1.38507475		2	1		hsa-miR-500a-5p	1.07457	0	1.05281229	0		20	0.686912
chr6_14824_mature	5.372849659	13.70077	2.671938627	3.877819487		-1.3505	0.46315		chr15_31965_mature	4.29827973	0.805927	2.405524014	0.852739935		2.415037	1		hsa-miR-520c-3p	1.07457	0	1.05281229	0		20	0.686912
chr2_4280_star	5.372849659	13.70077	2.671938627	3.877819487		-1.3505	0.46315		NW_003539989_39206_star	4.29827973	0.805927	2.405524014	0.852739935		2.415037	1		chr1_2381_star	1.07457	0	1.05281229	0		20	0.686912
chr3_8646_mature	8.596559455	29.01339	3.262517265	4.907534286		-1.75489	0.463194		chr1_2658_star	6.44741959	1.611855	2.896740642	1.38507475		2	1		bta-miR-1777b	1.07457	0	1.05281229	0		20	0.686912
NW_003537641_37089_mature	8.596559455	29.01339	3.262517265	4.907534286		-1.75489	0.463194		NW_003613114_37265_mature	4.29827973	16.11855	2.405524014	4.097488514		-1.90689	1		chr13_27312_star	1.07457	0	1.05281229	0		20	0.686912
chrx_22324_star	2.149139864	2.417782	1.654957834	1.773060525		-0.16993	0.463586		chr18_36103_mature	19.3422588	41.90823	4.346407978	5.423182395		-1.11548	1		ssc-miR-1277	1.07457	0	1.05281229	0		20	0.686912
chr4_10614_star	2.149139864	2.417782	1.654957834	1.773060525		-0.16993	0.463586		hsa-miR-514b-5p	2.14913986	0.805927	1.654957834	0.852739935		1.415037	1		bta-miR-19b	1.07457	0	1.05281229	0		20	0.686912
chr8_18081_star	2.149139864	2.417782	1.654957834	1.773060525		-0.16993	0.463586		chr2_4081_star	2.14913986	0.805927	1.654957834	0.852739935		1.415037	1		hsa-miR-3065-5p	1.07457	0	1.05281229	0		20	0.686912
chr12_26404_mature	2.149139864	2.417782	1.654957834	1.773060525		-0.16993	0.463586		chr14_31415_star	4.29827973	0.805927	2.405524014	0.852739935		2.415037	1		hsa-miR-4740-5p	1.07457	0	1.05281229	0		20	0.686912
NW_003541277_40020_star	2.149139864	2.417782	1.654957834	1.773060525		-0.16993	0.463586		chr11_25274_star	4.29827973	0.805927	2.405524014	0.852739935		2.415037	1		bta-miR-423-5p	1.07457	0	1.05281229	0		20	0.686912
chr4_10605_mature	2.149139864	2.417782	1.654957834	1.773060525		-0.16993	0.463586		chr2_5754_star	8.59655945	2.417782	3.262517265	1.773060525		1.830075	1		NW_003613065_36971_star	1.07457	0	1.05281229	0		20	0.686912
chr14_30467_mature@@hsa-miR-67	2.149139864	2.417782	1.654957834	1.773060525		-0.16993	0.463586		chr2_5392_mature	2.14913986	0.805927	1.654957834	0.852739935		1.415037	1		chr15_33037_star	1.07457	0	1.05281229	0		20	0.686912
chr9_20857_star	2.149139864	2.417782	1.654957834	1.773060525		-0.16993	0.463586		mmu-miR-3965	2.14913986	0.805927	1.654957834	0.852739935		1.415037	1		rno-miR-3102	1.07457	0	1.05281229	0		20	0.686912
chr17_35445_star	2.149139864	2.417782	1.654957834	1.773060525		-0.16993	0.463586		mmu-miR-3063-5p	2.14913986	0.805927	1.654957834	0.852739935		1.415037	1		rno-miR-151-5p	1.07457	0	1.05281229	0		20	0.686912
chr2_5921_star	2.149139864	2.417782	1.654957834	1.773060525		-0.16993	0.463586		chr14_30996_star	6.44741959	1.611855	2.896740642	1.38507475		2	1		mmu-miR-3058-5p	1.07457	0	1.05281229	0		20	0.686912
NW_003613022_36785_mature	2.149139864	2.417782	1.654957834	1.773060525		-0.16993	0.463586		hsa-miR-205-5p	6.44741959	1.611855	2.896740642	1.38507475		2	1		chr10_23459_star	1.07457	0	1.05281229	0		20	0.686912
chr9_20512_star	2.149139864	2.417782	1.654957834	1.773060525		-0.16993	0.463586		hsa-miR-4714-3p	4.29827973	0.805927	2.405524014	0.852739935		2.415037	1		mmu-miR-3058-3p	1.07457	0	1.05281229	0		20	0.686912
hsa-miR-1285-5p	2.149139864	2.417782	1.654957834	1.773060525		-0.16993	0.463586		chr3_7233_star	4.29827973	0.805927	2.405524014	0.852739935		2.415037	1		chr12_26455_star	1.07457	0	1.05281229	0		20	0.686912
chr15_31842_mature	2.149139864	2.417782	1.654957834	1.773060525		-0.16993	0.463586		NW_003613217_37697_mature	2.14913986	0.805927	1.654957834	0.852739935		1.415037	1		chr15_32628_star	1.07457	0	1.05281229	0		20	0.686912
bta-miR-2397-5p	2.149139864	2.417782	1.654957834	1.773060525		-0.16993	0.463586		chr11_24676_star	4.29827973	0.805927	2.405524014	0.852739935		2.415037	1		chr4_10404_star	1.07457	0	1.05281229	0		20	0.686912
chrx_21663_star	2.149139864	2.417782	1.654957834	1.773060525		-0.16993	0.463586		chr16_34452_star	2.14913986	0.805927	1.654957834	0.852739935		1.415037	1		chr13_28692_star	1.07457	0	1.05281229	0		20	0.686912
chr5_10975_mature	2.149139864	2.417782	1.654957834	1.773060525		-0.16993	0.463586		chr4_10371_star	4.29827973	0.805927	2.405524014	0.852739935		2.415037	1		mmu-miR-669h-5p	1.07457	0	1.05281229	0		20	0.686912
mmu-miR-5620-3p	2.149139864	2.417782	1.654957834	1.773060525		-0.16993	0.463586		chr9_19733_star	2.14913986	0.805927	1.654957834	0.852739935		1.415037	1		rno-miR-201-5p	1.07457	0	1.05281229	0		20	0.686912
chr7_15700_mature	2.149139864	2.417782	1.654957834	1.773060525		-0.16993	0.463586		NW_003540745_39662_mature	2.14913986	0.805927	1.654957834	0.852739935		1.415037	1		mmu-miR-429-5p	1.07457	0	1.05281229	0		20	0.686912
hsa-miR-548au-3p	2.149139864	2.417782	1.654957834	1.773060525		-0.16993	0.463586		chr5_11699_star	4.29827973	0.805927	2.405524014	0.852739935		2.415037	1		rno-miR-3099	1.07457	0	1.05281229	0		20	0.686912
chr13_27058_star	2.149139864	2.417782	1.654957834	1.773060525		-0.16993	0.463586		chr2_4621_star	8.59655945	2.417782	3.262517265	1.773060525		1.830075	1		hsa-miR-222-5p	1.07457	0	1.05281229	0		20	0.686912
chr3_6956_star	2.149139864	2.417782	1.654957834	1.773060525		-0.16993	0.463586		chr5_10731_star	2.14913986	0.805927	1.654957834	0.852739935		1.415037	1		hsa-miR-3162-5p	1.07457	0	1.05281229	0		20	0.686912
chr1_3348_star	2.149139864	2.417782	1.654957834	1.773060525		-0.16993	0.463586		bta-miR-192	2.14913986	0.805927	1.654957834	0.852739935		1.415037	1		mmu-miR-679-3p	1.07457	0	1.05281229	0		20	0.686912
mmu-miR-1902	2.149139864	2.417782	1.654957834	1.773060525		-0.16993	0.463586		chr13_28207_star	4.29827973	0.805927	2.405524014	0.852739935		2.415037	1		rno-miR-743b-5p	1.07457	0	1.05281229	0		20	0.686912
NW_003539007_38454_star	2.149139864	2.417782	1.654957834	1.773060525		-0.16993	0.463586		chr13_29021_star	2.14913986	0.805927	1.654957834	0.852739935		1.415037	1		chr6_14460_mature	1.07457	0	1.05281229	0		20	0.686912
hsa-miR-3127-5p	2.149139864	2.417782	1.654957834	1.773060525		-0.16993	0.463586		chr2_4567_star@@bta-miR-6535	2.14913986	0.805927	1.654957834	0.852739935		1.415037	1		hsa-miR-544b	1.07457	0	1.05281229	0		20	0.686912
chr14_30331_mature	2.149139864	2.417782	1.654957834	1.773060525		-0.16993	0.463586		chr4_9818_mature	8.59655945	2.417782	3.262517265	1.773060525		1.830075	1		chr16_33863_star	1.07457	0	1.05281229	0		20	0.686912
hsa-miR-4735-3p	2.149139864	2.417782	1.654957834	1.773060525		-0.16993	0.463586		chr2_6442_star	2.14913986	0.805927	1.654957834	0.852739935		1.415037	1		chr12_25704_star	1.07457	0	1.05281229	0		20	0.686912
chr14_30472_star	2.149139864	2.417782	1.654957834	1.773060525		-0.16993	0.463586		chr8_17676_star	2.14913986	0.805927	1.654957834	0.852739935		1.415037	1		hsa-miR-3201	1.07457	0	1.05281229	0		20	0.686912
chr18_36376_star	2.149139864	2.417782	1.654957834	1.773060525		-0.16993	0.463586		chr11_25035_star	2.14913986	0.805927	1.654957834	0.852739935		1.415037	1		rno-miR-362-5p	1.07457	0	1.05281229	0		20	0.686912
chr3_7968_mature	2.149139864	2.417782	1.654957834	1.773060525		-0.16993	0.463586		hsa-miR-3177-5p	4.29827973	0.805927	2.405524014	0.852739935		2.415037	1		chr2_4065_star	1.07457	0	1.05281229	0		20	0.686912
hsa-miR-3668	2.149139864	2.417782	1.654957834	1.773060525		-0.16993	0.463586		chr14_29542_mature	2.14913986	0.805927	1.654957834	0.852739935		1.415037	1		chr12_25742_star	1.07457	0	1.05281229	0		20	0.686912
chr2_5369_mature	2.149139864	2.417782	1.654957834	1.773060525		-0.16993	0.463586		chr4_8856_mature	8.59655945	2.417782	3.262517265	1.773060525		1.830075	1		bta-miR-215	1.07457	0	1.05281229	0		20	0.686912
chr7_16102_star	2.149139864	2.417782	1.654957834	1.773060525		-0.16993	0.463586		chr2_6281_mature	36.5353777	18.53633	5.230179094	4.288087668		0.978938	1		bta-miR-218	1.07457	0	1.05281229	0		20	0.686912
chr7_16298_mature	2.149139864	2.417782	1.654957834	1.773060525		-0.16993	0.463586		mmu-miR-344c-5p	4.29827973	0.805927	2.405524014	0.852739935		2.415037	1		rno-miR-18a-5p	1.07457	0	1.05281229	0		20	0.686912
chr13_28860_star	2.149139864	2.417782	1.654957834	1.773060525		-0.16993	0.463586		chr1_608_mature	4.29827973	0.805927	2.405524014	0.852739935		2.415037	1		chr3_8091_star	1.07457	0	1.05281229	0		20	0.686912
chr5_11567_star	2.149139864	2.417782	1.654957834	1.773060525		-0.16993	0.463586		rno-miR-3072	2.14913986	0.805927	1.654957834	0.852739935		1.415037	1		NW_003613327_38569_star	1.07457	0	1.05281229	0		20	0.686912
chr7_16979_mature	2.149139864	2.417782	1.654957834	1.773060525		-0.16993	0.463586		chr1_3039_mature	8.59655945	2.417782	3.262517265	1.773060525		1.830075	1		hsa-miR-4695-3p	1.07457	0	1.05281229	0		20	0.686912
chr13_26646_star	2.149139864	2.417782	1.654957834	1.773060525		-0.16993	0.463586		chr13_26704_star	2.14913986	0.805927	1.654957834	0.852739935		1.415037	1		chr8_18345_star	1.07457	0	1.05281229	0		20	0.686912
NW_003537574_37009_star	2.149139864	2.417782	1.654957834	1.773060525		-0.16993	0.463586		chr2_5942_mature	4.29827973	0.805927	2.405524014	0.852739935		2.415037	1		mmu-miR-326-5p	1.07457	0	1.05281229	0		20	0.686912
chr7_16597_star	2.149139864	2.417782	1.654957834	1.773060525		-0.16993	0.463586		NW_003537603_37043_star	2.14913986	0.805927	1.654957834	0.852739935		1.415037	1		bta-miR-27a-3p	1.07457	0	1.05281229	0		20	0.686912
chrx_21721_star	2.149139864	2.417782	1.654957834	1.773060525		-0.16993	0.463586		chr1_3113_mature	2.14913986	0.805927	1.654957834	0.852739935		1.415037	1		rno-miR-30a-3p	1.07457	0	1.05281229	0		20	0.686912
chr5_12007_star	2.149139864	2.417782	1.654957834	1.773060525		-0.16993	0.463586		rno-miR-499-5p	4.29827973	0.805927	2.405524014	0.852739935		2.415037	1		hsa-miR-132-5p	1.07457	0	1.05281229	0		20	0.686912
chr8_18467_star	2.149139864	2.417782	1.654957834	1.773060525		-0.16993	0.463586		chr12_25396_mature	2.14913986	0.805927	1.654957834	0.852739935		1.415037	1		hsa-miR-519a-5p	1.07457	0	1.05281229	0		20	0.686912
bta-miR-2285a	2.149139864	2.417782	1.654957834	1.773060525		-0.16993	0.463586		chr2_4006_star	4.29827973	0.805927	2.405524014	0.852739935		2.415037	1		mmu-miR-466n-3p	1.07457	0	1.05281229	0		20	0.686912
chr14_30481_star	2.149139864	2.417782	1.654957834	1.773060525		-0.16993	0.463586		chr1_1062_star	2.14913986	0.805927	1.654957834	0.852739935		1.415037	1		mmu-miR-1190	1.07457	0	1.05281229	0		20	0.686912
chr6_13506_star	2.149139864	2.417782	1.654957834	1.773060525		-0.16993	0.463586		chr14_29634_star	4.29827973	16.11855	2.405524014	4.097488514		-1.90689	1		chr9_20138_star	1.07457	0	1.05281229	0		20	0.686912
chr16_34464_star	2.149139864	2.417782	1.654957834	1.773060525		-0.16993	0.463586		chr3_7684_star	2.14913986	0.805927	1.654957834	0.852739935		1.415037	1		hsa-miR-4687-5p	1.07457	0	1.05281229	0		20	0.686912
chr1_1739_star	2.149139864	2.417782	1.654957834	1.773060525		-0.16993	0.463586		chr2_6321_star	2.14913986	0.805927	1.654957834	0.852739935		1.415037	1		mmu-miR-486-3p	1.07457	0	1.05281229	0		20	0.686912
NW_003541090_39901_star	2.149139864	2.417782	1.654957834	1.773060525		-0.16993	0.463586		chr2_6450_star	6.44741959	1.611855	2.896740642	1.38507475		2	1		chr14_30036_star	1.07457	0	1.05281229	0		20	0.686912
chr14_29426_star	2.149139864	2.417782	1.654957834	1.773060525		-0.16993	0.463586		chr14_29991_star	4.29827973	0.805927	2.405524014	0.852739935		2.415037	1		mmu-miR-341-3p	1.07457	0	1.05281229	0		20	0.686912
bta-miR-10a	2.149139864	2.417782	1.654957834	1.773060525		-0.16993	0.463586		chr3_6725_star	4.29827973	0.805927	2.405524014	0.852739935		2.415037	1		bta-miR-106a	1.07457	0	1.05281229	0		20	0.686912
hsa-miR-6514-5p	2.149139864	2.417782	1.654957834	1.773060525		-0.16993	0.463586		chr11_25010_star	25.7896784	11.28298	4.743605355	3.618589217		1.192645	1		chr5_12427_star	1.07457	0	1.05281229	0		20	0.686912
chr15_32980_mature	2.149139864	2.417782	1.654957834	1.773060525		-0.16993	0.463586		rno-miR-146a-5p	2.14913986	11.28298	1.654957834	3.618589217		-2.39232	1		ssc-miR-4335	1.07457	0	1.05281229	0		20	0.686912
chr7_16140_mature	2.149139864	2.417782	1.654957834	1.773060525		-0.16993	0.463586		chr13_27902_mature	8.59655945	2.417782	3.262517265	1.773060525		1.830075	1		mmu-miR-3070a-5p	1.07457	0	1.05281229	0		20	0.686912
chr5_12405_star	2.149139864	2.417782	1.654957834	1.773060525		-0.16993	0.463586		chr15_32008_star	2.14913986	0.805927	1.654957834	0.852739935		1.415037	1		chr13_26759_star	1.07457	0	1.05281229	0		20	0.686912
bta-miR-221	2.149139864	2.417782	1.654957834	1.773060525		-0.16993	0.463586		hsa-miR-6503-5p	6.44741959	1.611855	2.896740642	1.38507475		2	1		chr11_24688_star	1.07457	0	1.05281229	0		20	0.686912
chr5_12271_mature	2.149139864	2.417782	1.654957834	1.773060525		-0.16993	0.463586		chr6_12675_star	4.29827973	16.11855	2.405524014	4.097488514		-1.90689	1		chr5_11364_mature	1.07457	0	1.05281229	0		20	0.686912
chr15_33346_star	2.149139864	2.417782	1.654957834	1.773060525		-0.16993	0.463586		chr2_4630_star	6.44741959	1.611855	2.896740642	1.38507475		2	1		hsa-miR-3176	1.07457	0	1.05281229	0		20	0.686912
chr3_7732_star	2.149139864	2.417782	1.654957834	1.773060525		-0.16993	0.463586		chr13_27408_star	2.14913986	0.805927	1.654957834	0.852739935		1.415037	1		hsa-miR-3175	1.07457	0	1.05281229	0		20	0.686912
chr2_5371_mature	2.149139864	2.417782	1.654957834	1.773060525		-0.16993	0.463586		hsa-miR-548u	4.29827973	0.805927	2.405524014	0.852739935		2.415037	1		hsa-miR-3164	1.07457	0	1.05281229	0		20	0.686912
ssc-miR-105-1	2.149139864	2.417782	1.654957834	1.773060525		-0.16993	0.463586		NW_003538236_37642_star	2.14913986	0.805927	1.654957834	0.852739935		1.415037	1		hsa-miR-3193	1.07457	0	1.05281229	0		20	0.686912
chr6_13908_star	2.149139864	2.417782	1.654957834	1.773060525		-0.16993	0.463586		chr17_35663_star	4.29827973	0.805927	2.405524014	0.852739935		2.415037	1		chr7_15294_star	1.07457	0	1.05281229	0		20	0.686912
chr1_2954_star	2.149139864	2.417782	1.654957834	1.773060525		-0.16993	0.463586		NW_003613217_37698_mature	4.29827973	0.805927	2.405524014	0.852739935		2.415037	1		hsa-miR-490-3p	1.07457	0	1.05281229	0		20	0.686912
chr10_23951_star	2.149139864	2.417782	1.654957834	1.773060525		-0.16993	0.463586		chr13_27528_mature	4.29827973	16.11855	2.405524014	4.097488514		-1.90689	1		hsa-miR-3139	1.07457	0	1.05281229	0		20	0.686912
chr15_32006_star	2.149139864	2.417782	1.654957834	1.773060525		-0.16993	0.463586		bta-miR-2355-3p	2.14913986	0.805927	1.654957834	0.852739935		1.415037	1		chr3_6871_star	1.07457	0	1.05281229	0		20	0.686912
NW_003539513_38909_star	2.149139864	2.417782	1.654957834	1.773060525		-0.16993	0.463586		chr10_23674_mature	6.44741959	1.611855	2.896740642	1.38507475		2	1		chr10_23462_mature	1.07457	0	1.05281229	0		20	0.686912
chr18_35894_star	2.149139864	2.417782	1.654957834	1.773060525		-0.16993	0.463586		chr3_6723_star	4.29827973	0.805927	2.405524014	0.852739935		2.415037	1		bta-miR-33b	1.07457	0	1.05281229	0		20	0.686912
chr2_5788_mature	2.149139864	2.417782	1.654957834	1.773060525		-0.16993	0.463586		chrx_22228_mature	6.44741959	1.611855	2.896740642	1.38507475		2	1		chr9_19723_star	1.07457	0	1.05281229	0		20	0.686912
chr3_7732_mature	2.149139864	2.417782	1.654957834	1.773060525		-0.16993	0.463586		chr14_29851_star	6.44741959	1.611855	2.896740642	1.38507475		2	1		chr7_15488_star	1.07457	0	1.05281229	0		20	0.686912
bta-miR-2406	2.149139864	2.417782	1.654957834	1.773060525		-0.16993	0.463586		chr1_398_star	2.14913986	0.805927	1.654957834	0.852739935		1.415037	1		hsa-miR-2681-5p	1.07457	0	1.05281229	0		20	0.686912
bta-miR-2481	2.149139864	2.417782	1.654957834	1.773060525		-0.16993	0.463586		chr14_29835_mature	2.14913986	0.805927	1.654957834	0.852739935		1.415037	1		mmu-miR-511-3p	1.07457	0	1.05281229	0		20	0.686912
chr1_927_mature	2.149139864	2.417782	1.654957834	1.773060525		-0.16993	0.463586		chr15_32610_star	4.29827973	0.805927	2.405524014	0.852739935		2.415037	1		mmu-miR-434-3p	1.07457	0	1.05281229	0		20	0.686912
NW_003539879_39158_mature	2.149139864	2.417782	1.654957834	1.773060525		-0.16993	0.463586		chr15_32699_mature	6.44741959	20.14819	2.896740642	4.402462031		-1.64386	1		chr1_1940_star	1.07457	0	1.05281229	0		20	0.686912
bta-miR-2343	2.149139864	2.417782	1.654957834	1.773060525		-0.16993	0.463586		chr5_10819_mature	2.14913986	0.805927	1.654957834	0.852739935		1.415037	1		bta-miR-383	1.07457	0	1.05281229	0		20	0.686912
chr6_12914_star	2.149139864	2.417782	1.654957834	1.773060525		-0.16993	0.463586		hsa-miR-5002-3p	2.14913986	0.805927	1.654957834	0.852739935		1.415037	1		hsa-miR-4691-3p	1.07457	0	1.05281229	0		20	0.686912
chr4_9695_mature	2.149139864	2.417782	1.654957834	1.773060525		-0.16993	0.463586		chr1_3142_mature	2.14913986	12.08891	1.654957834	3.710273245		-2.49185	1		NW_003613310_38408_mature	1.07457	0	1.05281229	0		20	0.686912
chr1_2370_star	2.149139864	2.417782	1.654957834	1.773060525		-0.16993	0.463586		chr15_32289_star	4.29827973	0.805927	2.405524014	0.852739935		2.415037	1		hsa-miR-4251	1.07457	0	1.05281229	0		20	0.686912
mmu-miR-1957a	2.149139864	2.417782	1.654957834	1.773060525		-0.16993	0.463586		chr12_26382_star	4.29827973	0.805927	2.405524014	0.852739935		2.415037	1		chr18_36348_star	1.07457	0	1.05281229	0		20	0.686912
chr6_12905_mature	2.149139864	2.417782	1.654957834	1.773060525		-0.16993	0.463586		chr12_25827_star	2.14913986	0.805927	1.654957834	0.852739935		1.415037	1		hsa-miR-4270	1.07457	0	1.05281229	0		20	0.686912
chr4_8669_star	2.149139864	2.417782	1.654957834	1.773060525		-0.16993	0.463586		chr3_7875_star	8.59655945	2.417782	3.262517265	1.773060525		1.830075	1		chr3_8007_star	1.07457	0	1.05281229	0		20	0.686912
chr2_6128_mature	2.149139864	2.417782	1.654957834	1.773060525		-0.16993	0.463586		chrx_22070_mature	2.14913986	0.805927	1.654957834	0.852739935		1.415037	1		bta-miR-6525	1.07457	0	1.05281229	0		20	0.686912
chr5_11704_star	2.149139864	2.417782	1.654957834	1.773060525		-0.16993	0.463586		chr1_743_mature	4.29827973	0.805927	2.405524014	0.852739935		2.415037	1		bta-miR-6526	1.07457	0	1.05281229	0		20	0.686912
hsa-miR-194-5p	2.149139864	2.417782	1.654957834	1.773060525		-0.16993	0.463586		chr2_6077_mature	6.44741959	20.14819	2.896740642	4.402462031		-1.64386	1		hsa-miR-4280	1.07457	0	1.05281229	0		20	0.686912
mmu-miR-5619-3p	2.149139864	2.417782	1.654957834	1.773060525		-0.16993	0.463586		chr16_33920_star	2.14913986	12.08891	1.654957834	3.710273245		-2.49185	1		hsa-miR-4292	1.07457	0	1.05281229	0		20	0.686912
hsa-miR-4789-5p	2.149139864	2.417782	1.654957834	1.773060525		-0.16993	0.463586		chr8_18271_mature	23.6405385	10.47706	4.62296188	3.520680821		1.174029	1		mmu-miR-297a-3p	1.07457	0	1.05281229	0		20	0.686912
chr1_79_star	2.149139864	2.417782	1.654957834	1.773060525		-0.16993	0.463586		bta-miR-21-5p	4.29827973	0.805927	2.405524014	0.852739935		2.415037	1		chr4_8677_star	1.07457	0	1.05281229	0		20	0.686912
chr14_29799_star	2.149139864	2.417782	1.654957834	1.773060525		-0.16993	0.463586		chr12_25822_star	2.14913986	0.805927	1.654957834	0.852739935		1.415037	1		NW_003613280_38221_star	1.07457	0	1.05281229	0		20	0.686912
mmu-miR-2137	2.149139864	2.417782	1.654957834	1.773060525		-0.16993	0.463586		chr14_31373_star	2.14913986	0.805927	1.654957834	0.852739935		1.415037	1		chr6_13911_star	1.07457	0	1.05281229	0		20	0.686912
chr9_19395_mature	7.521989523	23.3719	3.091190278	4.607146585		-1.63559	0.464012		chr5_11720_star	2.14913986	0.805927	1.654957834	0.852739935		1.415037	1		mmu-miR-297b-5p	1.07457	0	1.05281229	0		20	0.686912
bta-miR-377	11.82026925	47.54972	3.680354657	5.601391055		-2.00817	0.464303		rno-miR-216b-5p	2.14913986	0.805927	1.654957834	0.852739935		1.415037	1		chr13_28544_star	1.07457	0	1.05281229	0		20	0.686912
chr12_25470_star	26.8642483	8.059274	4.800343329	3.179395517		1.736966	0.464349		chr3_8361_mature	2.14913986	0.805927	1.654957834	0.852739935		1.415037	1		hsa-miR-1226-3p	1.07457	0	1.05281229	0		20	0.686912
NW_003537206_36767_mature	3.223709795	5.641492	2.078510713	2.731507408		-0.80735	0.464687		hsa-miR-3178	2.14913986	0.805927	1.654957834	0.852739935		1.415037	1		rno-miR-3561-3p	1.07457	0	1.05281229	0		20	0.686912
chr4_10265_star	3.223709795	5.641492	2.078510713	2.731507408		-0.80735	0.464687		chr15_33570_mature	2.14913986	0.805927	1.654957834	0.852739935		1.415037	1		hsa-miR-3677-5p	1.07457	0	1.05281229	0		20	0.686912
chr12_25395_star	3.223709795	5.641492	2.078510713	2.731507408		-0.80735	0.464687		NW_003613440_39149_star	4.29827973	0.805927	2.405524014	0.852739935		2.415037	1		mmu-miR-3093-5p	1.07457	0	1.05281229	0		20	0.686912
chr13_27948_mature	3.223709795	5.641492	2.078510713	2.731507408		-0.80735	0.464687		chr10_23333_star	2.14913986	0.805927	1.654957834	0.852739935		1.415037	1		hsa-miR-1226-5p	1.07457	0	1.05281229	0		20	0.686912
hsa-miR-23b-5p	3.223709795	5.641492	2.078510713	2.731507408		-0.80735	0.464687		hsa-miR-3123	10.7456993	3.22371	3.554060706	2.078510713		1.736966	1		hsa-miR-892c-5p	1.07457	0	1.05281229	0		20	0.686912
chr5_10971_mature	3.223709795	5.641492	2.078510713	2.731507408		-0.80735	0.464687		hsa-miR-3128	2.14913986	0.805927	1.654957834	0.852739935		1.415037	1		chr1_3765_mature	1.07457	0	1.05281229	0		20	0.686912
NW_003537461_36946_mature	3.223709795	5.641492	2.078510713	2.731507408		-0.80735	0.464687		hsa-miR-3133	8.59655945	2.417782	3.262517265	1.773060525		1.830075	1		chr16_34309_star	1.07457	0	1.05281229	0		20	0.686912
chr6_13572_star	3.223709795	5.641492	2.078510713	2.731507408		-0.80735	0.464687		mmu-miR-3078-5p	2.14913986	0.805927	1.654957834	0.852739935		1.415037	1		chr14_30931_star	1.07457	0	1.05281229	0		20	0.686912
chr3_8428_star	3.223709795	5.641492	2.078510713	2.731507408		-0.80735	0.464687		hsa-miR-3138	2.14913986	0.805927	1.654957834	0.852739935		1.415037	1		chr6_13666_star	1.07457	0	1.05281229	0		20	0.686912
chr8_18291_star	3.223709795	5.641492	2.078510713	2.731507408		-0.80735	0.464687		hsa-miR-3141	2.14913986	0.805927	1.654957834	0.852739935		1.415037	1		mmu-miR-300-3p	1.07457	0	1.05281229	0		20	0.686912
chr13_28383_star	3.223709795	5.641492	2.078510713	2.731507408		-0.80735	0.464687		chr8_18571_star	2.14913986	0.805927	1.654957834	0.852739935		1.415037	1		chr6_14032_star	1.07457	0	1.05281229	0		20	0.686912
chr2_5816_star	3.223709795	5.641492	2.078510713	2.731507408		-0.80735	0.464687		chr11_25035_mature	2.14913986	0.805927	1.654957834	0.852739935		1.415037	1		chr3_7078_star	1.07457	0	1.05281229	0		20	0.686912
mmu-miR-2136	3.223709795	5.641492	2.078510713	2.731507408		-0.80735	0.464687		chr7_15749_star	6.44741959	1.611855	2.896740642	1.38507475		2	1		bta-miR-142-3p	1.07457	0	1.05281229	0		20	0.686912
chrx_22045_mature	3.223709795	2.417782	2.078510713	1.773060525		0.415037	0.464973		chr15_31790_mature	12.8948392	4.029637	3.796477232	2.330454351		1.678072	1		mmu-miR-291b-5p	1.07457	0	1.05281229	0		20	0.686912
chr1_3888_star	3.223709795	2.417782	2.078510713	1.773060525		0.415037	0.464973		bta-miR-1179	2.14913986	0.805927	1.654957834	0.852739935		1.415037	1		mmu-miR-500-5p	1.07457	0	1.05281229	0		20	0.686912
chr15_33676_mature@@mmu-miR-6	3.223709795	2.417782	2.078510713	1.773060525		0.415037	0.464973		hsa-miR-6723-5p	8.59655945	2.417782	3.262517265	1.773060525		1.830075	1		bta-miR-2373-5p	1.07457	0	1.05281229	0		20	0.686912
chr1_608_star	3.223709795	2.417782	2.078510713	1.773060525		0.415037	0.464973		chr1_2426_mature	2.14913986	12.08891	1.654957834	3.710273245		-2.49185	1		chr13_27097_star	1.07457	0	1.05281229	0		20	0.686912
NW_003538236_37642_mature	3.223709795	2.417782	2.078510713	1.773060525		0.415037	0.464973		chr14_30997_star	2.14913986	0.805927	1.654957834	0.852739935		1.415037	1		bta-miR-484	1.07457	0	1.05281229	0		20	0.686912
chr8_17941_star	3.223709795	2.417782	2.078510713	1.773060525		0.415037	0.464973		bta-miR-6534	4.29827973	0.805927	2.405524014	0.852739935		2.415037	1		chr13_29417_star	1.07457	0	1.05281229	0		20	0.686912
chr7_16542_mature@@bta-miR-172	3.223709795	2.417782	2.078510713	1.773060525		0.415037	0.464973		NW_003539279_38690_star	2.14913986	0.805927	1.654957834	0.852739935		1.415037	1		mmu-miR-3090-3p	1.07457	0	1.05281229	0		20	0.686912
hsa-miR-578	3.223709795	2.417782	2.078510713	1.773060525		0.415037	0.464973		hsa-miR-5581-3p	2.14913986	0.805927	1.654957834	0.852739935		1.415037	1		hsa-miR-296-5p	1.07457	0	1.05281229	0		20	0.686912
mmu-miR-3103-3p	3.223709795	2.417782	2.078510713	1.773060525		0.415037	0.464973		hsa-miR-4277	2.14913986	0.805927	1.654957834	0.852739935		1.415037	1		chr7_17208_mature	1.07457	0	1.05281229	0		20	0.686912
chr16_34759_star	3.223709795	2.417782	2.078510713	1.773060525		0.415037	0.464973		bta-miR-30b-5p	2.14913986	0.805927	1.654957834	0.852739935		1.415037	1		chr12_25892_star	1.07457	0	1.05281229	0		20	0.686912
mmu-miR-1970	3.223709795	2.417782	2.078510713	1.773060525		0.415037	0.464973		chr1_1289_star	2.14913986	0.805927	1.654957834	0.852739935		1.415037	1		NW_003613212_37672_mature	1.07457	0	1.05281229	0		20	0.686912
chr2_6582_mature	3.223709795	2.417782	2.078510713	1.773060525		0.415037	0.464973		chr1_2656_star	4.29827973	0.805927	2.405524014	0.852739935		2.415037	1		rno-miR-29a-5p	1.07457	0	1.05281229	0		20	0.686912
rno-miR-330-3p	3.223709795	2.417782	2.078510713	1.773060525		0.415037	0.464973		hsa-miR-4768-3p	2.14913986	0.805927	1.654957834	0.852739935		1.415037	1		chr12_25400_star	1.07457	0	1.05281229	0		20	0.686912
chr7_15958_star	3.223709795	2.417782	2.078510713	1.773060525		0.415037	0.464973		hsa-miR-4722-5p	2.14913986	0.805927	1.654957834	0.852739935		1.415037	1		bta-miR-2410	1.07457	0	1.05281229	0		20	0.686912
chr13_28164_star	3.223709795	2.417782	2.078510713	1.773060525		0.415037	0.464973		chr4_10559_star	8.59655945	2.417782	3.262517265	1.773060525		1.830075	1		hsa-miR-3619-3p	1.07457	0	1.05281229	0		20	0.686912
NW_003537531_36983_star	3.223709795	2.417782	2.078510713	1.773060525		0.415037	0.464973		hsa-miR-659-3p	2.14913986	0.805927	1.654957834	0.852739935		1.415037	1		chr8_18166_star	1.07457	0	1.05281229	0		20	0.686912
chr5_11941_star	3.223709795	2.417782	2.078510713	1.773060525		0.415037	0.464973		NW_003537656_37100_mature	2.14913986	0.805927	1.654957834	0.852739935		1.415037	1		NW_003613359_38685_mature	1.07457	0	1.05281229	0		20	0.686912

chr13_28676_star	3.223709795	2.417782	2.078510713	1.773060525		0.415037	0.464973		mmu-miR-3060-3p	4.29827973	0.805927	2.405524014	0.852739935		2.415037	1		hsa-miR-5003-3p	1.07457	0	1.05281229	0		20	0.686912
chr5_11058_mature	3.223709795	2.417782	2.078510713	1.773060525		0.415037	0.464973		chr14_30535_star	2.14913986	0.805927	1.654957834	0.852739935		1.415037	1		rno-miR-489-3p	1.07457	0	1.05281229	0		20	0.686912
chr4_10175_mature	3.223709795	2.417782	2.078510713	1.773060525		0.415037	0.464973		chr1_282_mature	4.29827973	0.805927	2.405524014	0.852739935		2.415037	1		chrx_21496_star	1.07457	0	1.05281229	0		20	0.686912
chr6_12675_mature	3.223709795	2.417782	2.078510713	1.773060525		0.415037	0.464973		chr5_11241_star	2.14913986	0.805927	1.654957834	0.852739935		1.415037	1		hsa-miR-629-3p	1.07457	0	1.05281229	0		20	0.686912
chr9_21042_mature	3.223709795	2.417782	2.078510713	1.773060525		0.415037	0.464973		chr2_3980_star	4.29827973	0.805927	2.405524014	0.852739935		2.415037	1		hsa-miR-4708-3p	1.07457	0	1.05281229	0		20	0.686912
chr4_9731_star	3.223709795	2.417782	2.078510713	1.773060525		0.415037	0.464973		chr12_25495_star	4.29827973	0.805927	2.405524014	0.852739935		2.415037	1		chr6_14284_star	1.07457	0	1.05281229	0		20	0.686912
chr6_14296_mature	3.223709795	2.417782	2.078510713	1.773060525		0.415037	0.464973		NW_003539453_38866_star	2.14913986	0.805927	1.654957834	0.852739935		1.415037	1		bta-miR-2469	1.07457	0	1.05281229	0		20	0.686912
mmu-miR-6361	3.223709795	2.417782	2.078510713	1.773060525		0.415037	0.464973		bta-miR-2431-5p	2.14913986	0.805927	1.654957834	0.852739935		1.415037	1		bta-miR-2463	1.07457	0	1.05281229	0		20	0.686912
NW_003539948_39194_star	3.223709795	2.417782	2.078510713	1.773060525		0.415037	0.464973		chr8_18304_mature	2.14913986	0.805927	1.654957834	0.852739935		1.415037	1		hsa-miR-4464	1.07457	0	1.05281229	0		20	0.686912
chr2_4589_star	3.223709795	2.417782	2.078510713	1.773060525		0.415037	0.464973		chr2_6121_mature	2.14913986	0.805927	1.654957834	0.852739935		1.415037	1		hsa-miR-4467	1.07457	0	1.05281229	0		20	0.686912
chr14_30036_mature	3.223709795	2.417782	2.078510713	1.773060525		0.415037	0.464973		NW_003539217_38656_star	2.14913986	0.805927	1.654957834	0.852739935		1.415037	1		rno-miR-154-3p	1.07457	0	1.05281229	0		20	0.686912
chr7_16079_star	3.223709795	2.417782	2.078510713	1.773060525		0.415037	0.464973		NW_003539820_39117_mature	2.14913986	0.805927	1.654957834	0.852739935		1.415037	1		hsa-miR-4471	1.07457	0	1.05281229	0		20	0.686912
chr16_34110_star	3.223709795	2.417782	2.078510713	1.773060525		0.415037	0.464973		NW_003540830_39720_mature	4.29827973	0.805927	2.405524014	0.852739935		2.415037	1		hsa-miR-4476	1.07457	0	1.05281229	0		20	0.686912
chr3_7875_star	3.223709795	2.417782	2.078510713	1.773060525		0.415037	0.464973		chr15_33675_mature@@mmu-miR-639	4.29827973	0.805927	2.405524014	0.852739935		2.415037	1		chr15_32980_star	1.07457	0	1.05281229	0		20	0.686912
chr9_20768_star	3.223709795	2.417782	2.078510713	1.773060525		0.415037	0.464973		chr3_7704_star	4.29827973	0.805927	2.405524014	0.852739935		2.415037	1		hsa-miR-454-5p	1.07457	0	1.05281229	0		20	0.686912
chr6_14465_star	3.223709795	2.417782	2.078510713	1.773060525		0.415037	0.464973		hsa-miR-302e	2.14913986	0.805927	1.654957834	0.852739935		1.415037	1		bta-miR-2474	1.07457	0	1.05281229	0		20	0.686912
chr4_9998_star	3.223709795	2.417782	2.078510713	1.773060525		0.415037	0.464973		chr2_6204_star	2.14913986	0.805927	1.654957834	0.852739935		1.415037	1		bta-miR-2472	1.07457	0	1.05281229	0		20	0.686912
ssc-miR-105-2	3.223709795	2.417782	2.078510713	1.773060525		0.415037	0.464973		chrx_22698_mature	2.14913986	0.805927	1.654957834	0.852739935		1.415037	1		hsa-miR-4430	1.07457	0	1.05281229	0		20	0.686912
chr7_16707_star	3.223709795	2.417782	2.078510713	1.773060525		0.415037	0.464973		chr11_24519_mature	4.29827973	0.805927	2.405524014	0.852739935		2.415037	1		hsa-miR-4431	1.07457	0	1.05281229	0		20	0.686912
chr4_10559_star	3.223709795	2.417782	2.078510713	1.773060525		0.415037	0.464973		chr1_1112_star	2.14913986	0.805927	1.654957834	0.852739935		1.415037	1		chr6_12627_star	1.07457	0	1.05281229	0		20	0.686912
chr12_25704_mature	3.223709795	2.417782	2.078510713	1.773060525		0.415037	0.464973		mmu-miR-449c-5p	2.14913986	0.805927	1.654957834	0.852739935		1.415037	1		bta-miR-2485	1.07457	0	1.05281229	0		20	0.686912
chr7_15425_mature	3.223709795	2.417782	2.078510713	1.773060525		0.415037	0.464973		chr5_11151_star	6.44741959	1.611855	2.896740642	1.38507475		2	1		rno-miR-146b-5p	1.07457	0	1.05281229	0		20	0.686912
NW_003613025_36806_mature	3.223709795	2.417782	2.078510713	1.773060525		0.415037	0.464973		chr2_5682_mature	27.9388182	12.89484	4.854934103	3.796477232		1.115477	1		mmu-miR-133c	1.07457	0	1.05281229	0		20	0.686912
chr4_9284_star	3.223709795	2.417782	2.078510713	1.773060525		0.415037	0.464973		chr14_30186_mature	12.8948392	4.029637	3.796477232	2.330454351		1.678072	1		hsa-miR-609	1.07457	0	1.05281229	0		20	0.686912
bta-miR-2390	3.223709795	2.417782	2.078510713	1.773060525		0.415037	0.464973		hsa-miR-6513-5p	4.29827973	0.805927	2.405524014	0.852739935		2.415037	1		hsa-miR-617	1.07457	0	1.05281229	0		20	0.686912
NW_003538520_37951_mature	3.223709795	2.417782	2.078510713	1.773060525		0.415037	0.464973		chr8_18259_mature	2.14913986	0.805927	1.654957834	0.852739935		1.415037	1		hsa-miR-454-3p	1.07457	0	1.05281229	0		20	0.686912
hsa-miR-641	3.223709795	2.417782	2.078510713	1.773060525		0.415037	0.464973		chr3_7654_star	2.14913986	0.805927	1.654957834	0.852739935		1.415037	1		hsa-miR-638	1.07457	0	1.05281229	0		20	0.686912
chr12_26596_mature	3.223709795	2.417782	2.078510713	1.773060525		0.415037	0.464973		chr7_16084_star	2.14913986	0.805927	1.654957834	0.852739935		1.415037	1		bta-miR-2394	1.07457	0	1.05281229	0		20	0.686912
chrx_21800_mature	3.223709795	2.417782	2.078510713	1.773060525		0.415037	0.464973		chr5_11637_mature	6.44741959	1.611855	2.896740642	1.38507475		2	1		hsa-miR-181d	1.07457	0	1.05281229	0		20	0.686912
chr2_4676_mature	3.223709795	2.417782	2.078510713	1.773060525		0.415037	0.464973		hsa-miR-517-5p	6.44741959	20.95411	2.896740642	4.456419386		-1.70044	1		rno-miR-872-5p	1.07457	0	1.05281229	0		20	0.686912
chr4_10705_star	3.223709795	2.417782	2.078510713	1.773060525		0.415037	0.464973		chrx_21555_star	2.14913986	0.805927	1.654957834	0.852739935		1.415037	1		NW_003537297_36825_star	1.07457	0	1.05281229	0		20	0.686912
hsa-miR-5701	3.223709795	2.417782	2.078510713	1.773060525		0.415037	0.464973		chr14_31627_star	4.29827973	0.805927	2.405524014	0.852739935		2.415037	1		hsa-miR-561-5p	1.07457	0	1.05281229	0		20	0.686912
chr14_30374_mature	3.223709795	2.417782	2.078510713	1.773060525		0.415037	0.464973		chr2_6125_mature	12.8948392	4.029637	3.796477232	2.330454351		1.678072	1		NW_003539350_38744_star	1.07457	0	1.05281229	0		20	0.686912
chr18_36191_mature	3.223709795	2.417782	2.078510713	1.773060525		0.415037	0.464973		chr4_8784_mature	2.14913986	0.805927	1.654957834	0.852739935		1.415037	1		mmu-miR-1948-3p	1.07457	0	1.05281229	0		20	0.686912
chr2_6447_star	3.223709795	2.417782	2.078510713	1.773060525		0.415037	0.464973		chr2_4423_mature	2.14913986	0.805927	1.654957834	0.852739935		1.415037	1		chr6_14429_star	1.07457	0	1.05281229	0		20	0.686912
hsa-miR-625-5p	3.223709795	2.417782	2.078510713	1.773060525		0.415037	0.464973		rno-miR-511-3p	2.14913986	0.805927	1.654957834	0.852739935		1.415037	1		hsa-miR-651	1.07457	0	1.05281229	0		20	0.686912
hsa-miR-4528	3.223709795	2.417782	2.078510713	1.773060525		0.415037	0.464973		chr15_32760_star	2.14913986	0.805927	1.654957834	0.852739935		1.415037	1		hsa-miR-661	1.07457	0	1.05281229	0		20	0.686912
chr16_33817_star	3.223709795	2.417782	2.078510713	1.773060525		0.415037	0.464973		chr9_20751_mature	2.14913986	0.805927	1.654957834	0.852739935		1.415037	1		hsa-miR-670	1.07457	0	1.05281229	0		20	0.686912
hsa-miR-4778-3p	3.223709795	2.417782	2.078510713	1.773060525		0.415037	0.464973		rno-miR-463-5p	2.14913986	0.805927	1.654957834	0.852739935		1.415037	1		rno-miR-501-3p	1.07457	0	1.05281229	0		20	0.686912
chr18_36007_star	3.223709795	2.417782	2.078510713	1.773060525		0.415037	0.464973		bta-miR-2416	2.14913986	0.805927	1.654957834	0.852739935		1.415037	1		rno-miR-128-2-5p	1.07457	0	1.05281229	0		20	0.686912
chr14_29906_mature	11.82026925	4.835565	3.680354657	2.544872269		1.289507	0.465983		NW_003541083_39896_star	2.14913986	0.805927	1.654957834	0.852739935		1.415037	1		mmu-miR-669b-5p	1.07457	0	1.05281229	0		20	0.686912
[illegible]	[illegible]	[illegible]	[illegible]	[illegible]		[illegible]	[illegible]		[illegible]	[illegible]	[illegible]	[illegible]	[illegible]		[illegible]	[illegible]		[illegible]	[illegible]	[illegible]	[illegible]	[illegible]		[illegible]	[illegible]

chr4_8831_star	1.074569932	0.805927	1.05281229	0.852739935		0.415037	0.468172
chr18_36584_star	1.074569932	0.805927	1.05281229	0.852739935		0.415037	0.468172
chr7_15697_star	1.074569932	0.805927	1.05281229	0.852739935		0.415037	0.468172
chr1_191_star	1.074569932	0.805927	1.05281229	0.852739935		0.415037	0.468172
bta-miR-615	1.074569932	0.805927	1.05281229	0.852739935		0.415037	0.468172
chr8_17973_mature	1.074569932	0.805927	1.05281229	0.852739935		0.415037	0.468172
hsa-miR-888-3p	1.074569932	0.805927	1.05281229	0.852739935		0.415037	0.468172
chr9_20355_mature	1.074569932	0.805927	1.05281229	0.852739935		0.415037	0.468172
chr13_28683_mature	1.074569932	0.805927	1.05281229	0.852739935		0.415037	0.468172
bta-miR-660	1.074569932	0.805927	1.05281229	0.852739935		0.415037	0.468172
bta-miR-664	1.074569932	0.805927	1.05281229	0.852739935		0.415037	0.468172
chr18_36523_star	1.074569932	0.805927	1.05281229	0.852739935		0.415037	0.468172
hsa-miR-4755-5p	1.074569932	0.805927	1.05281229	0.852739935		0.415037	0.468172
chr18_36486_star	1.074569932	0.805927	1.05281229	0.852739935		0.415037	0.468172
NW_003539597_38976_star	1.074569932	0.805927	1.05281229	0.852739935		0.415037	0.468172
chr18_36007_mature	1.074569932	0.805927	1.05281229	0.852739935		0.415037	0.468172
chr13_28684_mature	1.074569932	0.805927	1.05281229	0.852739935		0.415037	0.468172
hsa-miR-5010-5p	1.074569932	0.805927	1.05281229	0.852739935		0.415037	0.468172
chr10_24040_star	1.074569932	0.805927	1.05281229	0.852739935		0.415037	0.468172
chr2_4523_star	1.074569932	0.805927	1.05281229	0.852739935		0.415037	0.468172
rno-miR-653-5p	1.074569932	0.805927	1.05281229	0.852739935		0.415037	0.468172
mmu-miR-3968	1.074569932	0.805927	1.05281229	0.852739935		0.415037	0.468172
mmu-miR-664-5p	1.074569932	0.805927	1.05281229	0.852739935		0.415037	0.468172
mmu-miR-3965	1.074569932	0.805927	1.05281229	0.852739935		0.415037	0.468172
chr16_34426_mature	1.074569932	0.805927	1.05281229	0.852739935		0.415037	0.468172
rno-miR-218a-1-3p	1.074569932	0.805927	1.05281229	0.852739935		0.415037	0.468172
bta-miR-150	1.074569932	0.805927	1.05281229	0.852739935		0.415037	0.468172
hsa-miR-4714-5p	1.074569932	0.805927	1.05281229	0.852739935		0.415037	0.468172
chr9_20807_star	1.074569932	0.805927	1.05281229	0.852739935		0.415037	0.468172
hsa-miR-4786-3p	1.074569932	0.805927	1.05281229	0.852739935		0.415037	0.468172
chr8_17912_star	1.074569932	0.805927	1.05281229	0.852739935		0.415037	0.468172
NW_003613359_38685_star	1.074569932	0.805927	1.05281229	0.852739935		0.415037	0.468172
mmu-miR-3094-3p	1.074569932	0.805927	1.05281229	0.852739935		0.415037	0.468172
chr2_6343_mature	1.074569932	0.805927	1.05281229	0.852739935		0.415037	0.468172
chrx_23090_mature	1.074569932	0.805927	1.05281229	0.852739935		0.415037	0.468172
bta-miR-192	1.074569932	0.805927	1.05281229	0.852739935		0.415037	0.468172
chr13_28207_star	1.074569932	0.805927	1.05281229	0.852739935		0.415037	0.468172
chr15_32731_star	1.074569932	0.805927	1.05281229	0.852739935		0.415037	0.468172
chr14_29623_star	1.074569932	0.805927	1.05281229	0.852739935		0.415037	0.468172
chr13_29021_star	1.074569932	0.805927	1.05281229	0.852739935		0.415037	0.468172
chr14_29542_star	1.074569932	0.805927	1.05281229	0.852739935		0.415037	0.468172
chr2_6442_star	1.074569932	0.805927	1.05281229	0.852739935		0.415037	0.468172
hsa-miR-3682-3p	1.074569932	0.805927	1.05281229	0.852739935		0.415037	0.468172
chr7_16903_mature	1.074569932	0.805927	1.05281229	0.852739935		0.415037	0.468172
mmu-miR-653-5p	1.074569932	0.805927	1.05281229	0.852739935		0.415037	0.468172
rno-miR-3072	1.074569932	0.805927	1.05281229	0.852739935		0.415037	0.468172
chr12_25396_mature	1.074569932	0.805927	1.05281229	0.852739935		0.415037	0.468172
chr2_6321_star	1.074569932	0.805927	1.05281229	0.852739935		0.415037	0.468172
chr3_8247_star	1.074569932	0.805927	1.05281229	0.852739935		0.415037	0.468172
mmu-miR-5626-5p	1.074569932	0.805927	1.05281229	0.852739935		0.415037	0.468172
bta-miR-222	1.074569932	0.805927	1.05281229	0.852739935		0.415037	0.468172
hsa-miR-548u	1.074569932	0.805927	1.05281229	0.852739935		0.415037	0.468172
chr15_33620_mature	1.074569932	0.805927	1.05281229	0.852739935		0.415037	0.468172
NW_003538236_37642_star	1.074569932	0.805927	1.05281229	0.852739935		0.415037	0.468172
chr15_33043_star	1.074569932	0.805927	1.05281229	0.852739935		0.415037	0.468172
chr13_28713_star	1.074569932	0.805927	1.05281229	0.852739935		0.415037	0.468172
bta-miR-2355-3p	1.074569932	0.805927	1.05281229	0.852739935		0.415037	0.468172
chr10_24278_star	1.074569932	0.805927	1.05281229	0.852739935		0.415037	0.468172
chrx_22133_star	1.074569932	0.805927	1.05281229	0.852739935		0.415037	0.468172
hsa-miR-3145-5p	1.074569932	0.805927	1.05281229	0.852739935		0.415037	0.468172
chr7_16174_star	1.074569932	0.805927	1.05281229	0.852739935		0.415037	0.468172
hsa-miR-422a	1.074569932	0.805927	1.05281229	0.852739935		0.415037	0.468172
chr14_31373_star	1.074569932	0.805927	1.05281229	0.852739935		0.415037	0.468172
chr5_11720_star	1.074569932	0.805927	1.05281229	0.852739935		0.415037	0.468172
rno-miR-1912-3p	1.074569932	0.805927	1.05281229	0.852739935		0.415037	0.468172
chr9_19640_star	1.074569932	0.805927	1.05281229	0.852739935		0.415037	0.468172
chr3_8361_mature	1.074569932	0.805927	1.05281229	0.852739935		0.415037	0.468172
chr11_24782_star	1.074569932	0.805927	1.05281229	0.852739935		0.415037	0.468172
hsa-miR-3163	1.074569932	0.805927	1.05281229	0.852739935		0.415037	0.468172
NW_003613440_39149_star	1.074569932	0.805927	1.05281229	0.852739935		0.415037	0.468172
hsa-miR-3183	1.074569932	0.805927	1.05281229	0.852739935		0.415037	0.468172
chr6_14789_star	1.074569932	0.805927	1.05281229	0.852739935		0.415037	0.468172
chr11_25035_mature	1.074569932	0.805927	1.05281229	0.852739935		0.415037	0.468172
chr14_30997_star	1.074569932	0.805927	1.05281229	0.852739935		0.415037	0.468172
bta-miR-6534	1.074569932	0.805927	1.05281229	0.852739935		0.415037	0.468172
hsa-miR-4275	1.074569932	0.805927	1.05281229	0.852739935		0.415037	0.468172
bta-miR-30b-5p	1.074569932	0.805927	1.05281229	0.852739935		0.415037	0.468172
chr2_6095_star@@hsa-miR-513a-5p	1.074569932	0.805927	1.05281229	0.852739935		0.415037	0.468172
hsa-miR-4722-5p	1.074569932	0.805927	1.05281229	0.852739935		0.415037	0.468172
chr7_16237_mature	1.074569932	0.805927	1.05281229	0.852739935		0.415037	0.468172
chr18_36498_star	1.074569932	0.805927	1.05281229	0.852739935		0.415037	0.468172
hsa-miR-659-3p	1.074569932	0.805927	1.05281229	0.852739935		0.415037	0.468172
bta-miR-2454-3p	1.074569932	0.805927	1.05281229	0.852739935		0.415037	0.468172
chr17_35047_mature	1.074569932	0.805927	1.05281229	0.852739935		0.415037	0.468172
chr1_282_mature	1.074569932	0.805927	1.05281229	0.852739935		0.415037	0.468172
rno-miR-488-3p	1.074569932	0.805927	1.05281229	0.852739935		0.415037	0.468172
chr9_19298_mature	1.074569932	0.805927	1.05281229	0.852739935		0.415037	0.468172
rno-miR-125b-5p	1.074569932	0.805927	1.05281229	0.852739935		0.415037	0.468172
chr15_33675_mature@@mmu-miR-6	1.074569932	0.805927	1.05281229	0.852739935		0.415037	0.468172
chr11_24486_star	1.074569932	0.805927	1.05281229	0.852739935		0.415037	0.468172
bta-miR-448	1.074569932	0.805927	1.05281229	0.852739935		0.415037	0.468172
chr1_431_star	1.074569932	0.805927	1.05281229	0.852739935		0.415037	0.468172
chr1_1498_mature	1.074569932	0.805927	1.05281229	0.852739935		0.415037	0.468172
chrx_21533_mature	1.074569932	0.805927	1.05281229	0.852739935		0.415037	0.468172
chr4_10120_star	1.074569932	0.805927	1.05281229	0.852739935		0.415037	0.468172
chr1_1112_star	1.074569932	0.805927	1.05281229	0.852739935		0.415037	0.468172
bta-miR-490	1.074569932	0.805927	1.05281229	0.852739935		0.415037	0.468172
chr8_18259_mature	1.074569932	0.805927	1.05281229	0.852739935		0.415037	0.468172
hsa-miR-4725-3p	1.074569932	0.805927	1.05281229	0.852739935		0.415037	0.468172
mmu-miR-26a-2-3p	1.074569932	0.805927	1.05281229	0.852739935		0.415037	0.468172
chr3_7654_star	1.074569932	0.805927	1.05281229	0.852739935		0.415037	0.468172
chr15_31930_star	1.074569932	0.805927	1.05281229	0.852739935		0.415037	0.468172
chr4_9780_mature	1.074569932	0.805927	1.05281229	0.852739935		0.415037	0.468172
chr4_8784_mature	1.074569932	0.805927	1.05281229	0.852739935		0.415037	0.468172
chr2_4472_star	1.074569932	0.805927	1.05281229	0.852739935		0.415037	0.468172
bta-miR-2412	1.074569932	0.805927	1.05281229	0.852739935		0.415037	0.468172
bta-miR-2416	1.074569932	0.805927	1.05281229	0.852739935		0.415037	0.468172
chr14_30124_mature	1.074569932	0.805927	1.05281229	0.852739935		0.415037	0.468172
bta-miR-2400	1.074569932	0.805927	1.05281229	0.852739935		0.415037	0.468172
bta-miR-2401	1.074569932	0.805927	1.05281229	0.852739935		0.415037	0.468172
bta-miR-2456	1.074569932	0.805927	1.05281229	0.852739935		0.415037	0.468172
hsa-miR-4456	1.074569932	0.805927	1.05281229	0.852739935		0.415037	0.468172
NW_003538201_37614_star	1.074569932	0.805927	1.05281229	0.852739935		0.415037	0.468172
mmu-miR-201-3p	1.074569932	0.805927	1.05281229	0.852739935		0.415037	0.468172
chr6_14951_star	1.074569932	0.805927	1.05281229	0.852739935		0.415037	0.468172
hsa-miR-766-3p	1.074569932	0.805927	1.05281229	0.852739935		0.415037	0.468172
hsa-miR-4461	1.074569932	0.805927	1.05281229	0.852739935		0.415037	0.468172
mmu-miR-5627-5p	1.074569932	0.805927	1.05281229	0.852739935		0.415037	0.468172
hsa-miR-4422	1.074569932	0.805927	1.05281229	0.852739935		0.415037	0.468172
bta-miR-2470	1.074569932	0.805927	1.05281229	0.852739935		0.415037	0.468172
chr4_10208_star	1.074569932	0.805927	1.05281229	0.852739935		0.415037	0.468172
hsa-miR-604	1.074569932	0.805927	1.05281229	0.852739935		0.415037	0.468172
chr14_31148_star	1.074569932	0.805927	1.05281229	0.852739935		0.415037	0.468172
NW_003540392_39464_mature	1.074569932	0.805927	1.05281229	0.852739935		0.415037	0.468172
hsa-miR-6512-5p	1.074569932	0.805927	1.05281229	0.852739935		0.415037	0.468172
hsa-miR-623	1.074569932	0.805927	1.05281229	0.852739935		0.415037	0.468172
chr16_34524_mature	1.074569932	0.805927	1.05281229	0.852739935		0.415037	0.468172
chr13_28165_star	1.074569932	0.805927	1.05281229	0.852739935		0.415037	0.468172
hsa-miR-633	1.074569932	0.805927	1.05281229	0.852739935		0.415037	0.468172
hsa-miR-636	1.074569932	0.805927	1.05281229	0.852739935		0.415037	0.468172
hsa-miR-635	1.074569932	0.805927	1.05281229	0.852739935		0.415037	0.468172
bta-miR-2395	1.074569932	0.805927	1.05281229	0.852739935		0.415037	0.468172
hsa-miR-4499	1.074569932	0.805927	1.05281229	0.852739935		0.415037	0.468172
NW_003613025_36806_star	1.074569932	0.805927	1.05281229	0.852739935		0.415037	0.468172
chr12_25987_star	1.074569932	0.805927	1.05281229	0.852739935		0.415037	0.468172
chr8_18125_star	1.074569932	0.805927	1.05281229	0.852739935		0.415037	0.468172
chr2_4241_star	1.074569932	0.805927	1.05281229	0.852739935		0.415037	0.468172
NW_003613326_38567_mature	1.074569932	0.805927	1.05281229	0.852739935		0.415037	0.468172
chr2_6533_star	1.074569932	0.805927	1.05281229	0.852739935		0.415037	0.468172
bta-miR-2368-5p	1.074569932	0.805927	1.05281229	0.852739935		0.415037	0.468172
chr3_7716_mature	1.074569932	0.805927	1.05281229	0.852739935		0.415037	0.468172
chr12_25526_star	1.074569932	0.805927	1.05281229	0.852739935		0.415037	0.468172
mmu-miR-1196-3p	1.074569932	0.805927	1.05281229	0.852739935		0.415037	0.468172
hsa-miR-548a-3p	1.074569932	0.805927	1.05281229	0.852739935		0.415037	0.468172
chr14_31281_star	1.074569932	0.805927	1.05281229	0.852739935		0.415037	0.468172
chr9_20130_star	1.074569932	0.805927	1.05281229	0.852739935		0.415037	0.468172
bta-miR-2310	1.074569932	0.805927	1.05281229	0.852739935		0.415037	0.468172
chr4_10385_star	1.074569932	0.805927	1.05281229	0.852739935		0.415037	0.468172
bta-miR-2333	1.074569932	0.805927	1.05281229	0.852739935		0.415037	0.468172
chr14_31643_star	1.074569932	0.805927	1.05281229	0.852739935		0.415037	0.468172
chrx_21525_star	1.074569932	0.805927	1.05281229	0.852739935		0.415037	0.468172
chr12_25917_star	1.074569932	0.805927	1.05281229	0.852739935		0.415037	0.468172
hsa-miR-550a-3-5p	1.074569932	0.805927	1.05281229	0.852739935		0.415037	0.468172
bta-miR-2354	1.074569932	0.805927	1.05281229	0.852739935		0.415037	0.468172
hsa-miR-4310	1.074569932	0.805927	1.05281229	0.852739935		0.415037	0.468172
hsa-miR-4326	1.074569932	0.805927	1.05281229	0.852739935		0.415037	0.468172
hsa-miR-4662b	1.074569932	0.805927	1.05281229	0.852739935		0.415037	0.468172
chr4_9258_star	1.074569932	0.805927	1.05281229	0.852739935		0.415037	0.468172
mmu-miR-344i	1.074569932	0.805927	1.05281229	0.852739935		0.415037	0.468172
chr5_11265_mature	1.074569932	0.805927	1.05281229	0.852739935		0.415037	0.468172
chr1_1609_star	1.074569932	0.805927	1.05281229	0.852739935		0.415037	0.468172
chr2_4675_star	1.074569932	0.805927	1.05281229	0.852739935		0.415037	0.468172
hsa-miR-576-3p	1.074569932	0.805927	1.05281229	0.852739935		0.415037	0.468172
chr1_742_star	1.074569932	0.805927	1.05281229	0.852739935		0.415037	0.468172
bta-miR-339b	1.074569932	0.805927	1.05281229	0.852739935		0.415037	0.468172
chrx_23015_star	1.074569932	0.805927	1.05281229	0.852739935		0.415037	0.468172
hsa-miR-764	1.074569932	0.805927	1.05281229	0.852739935		0.415037	0.468172
bta-miR-376d	1.074569932	0.805927	1.05281229	0.852739935		0.415037	0.468172
chr7_16704_star	1.074569932	0.805927	1.05281229	0.852739935		0.415037	0.468172
mmu-miR-1957b	1.074569932	0.805927	1.05281229	0.852739935		0.415037	0.468172
hsa-miR-4637	1.074569932	0.805927	1.05281229	0.852739935		0.415037	0.468172
chr6_14460_star	1.074569932	0.805927	1.05281229	0.852739935		0.415037	0.468172
chr15_33667_star	1.074569932	0.805927	1.05281229	0.852739935		0.415037	0.468172
chr3_6671_star	1.074569932	0.805927	1.05281229	0.852739935		0.415037	0.468172
rno-miR-3596a	1.074569932	0.805927	1.05281229	0.852739935		0.415037	0.468172
hsa-miR-4698	1.074569932	0.805927	1.05281229	0.852739935		0.415037	0.468172
hsa-miR-4696	1.074569932	0.805927	1.05281229	0.852739935		0.415037	0.468172
chr14_29516_star	1.074569932	0.805927	1.05281229	0.852739935		0.415037	0.468172
chr6_13162_star	1.074569932	0.805927	1.05281229	0.852739935		0.415037	0.468172
chr6_14069_mature	1.074569932	0.805927	1.05281229	0.852739935		0.415037	0.468172
rno-miR-22-3p	1.074569932	0.805927	1.05281229	0.852739935		0.415037	0.468172
hsa-miR-3925-3p	1.074569932	0.805927	1.05281229	0.852739935		0.415037	0.468172
chr16_34111_star	1.074569932	0.805927	1.05281229	0.852739935		0.415037	0.468172
rno-miR-3573-5p	1.074569932	0.805927	1.05281229	0.852739935		0.415037	0.468172
chr8_18569_star	1.074569932	0.805927	1.05281229	0.852739935		0.415037	0.468172
chr6_14742_mature	1.074569932	0.805927	1.05281229	0.852739935		0.415037	0.468172
bta-miR-3660	1.074569932	0.805927	1.05281229	0.852739935		0.415037	0.468172
hsa-miR-4527	1.074569932	0.805927	1.05281229	0.852739935		0.415037	0.468172
hsa-miR-3942-3p	1.074569932	0.805927	1.05281229	0.852739935		0.415037	0.468172
chr2_5391_star	1.074569932	0.805927	1.05281229	0.852739935		0.415037	0.468172
hsa-miR-4505	1.074569932	0.805927	1.05281229	0.852739935		0.415037	0.468172
hsa-miR-1273c	1.074569932	0.805927	1.05281229	0.852739935		0.415037	0.468172
chr2_4343_star	1.074569932	0.805927	1.05281229	0.852739935		0.415037	0.468172
hsa-miR-3155b	1.074569932	0.805927	1.05281229	0.852739935		0.415037	0.468172
chr9_19409_star	1.074569932	0.805927	1.05281229	0.852739935		0.415037	0.468172
rno-miR-16-3p	1.074569932	0.805927	1.05281229	0.852739935		0.415037	0.468172
chr6_12525_mature	1.074569932	0.805927	1.05281229	0.852739935		0.415037	0.468172
chr9_20397_star	1.074569932	0.805927	1.05281229	0.852739935		0.415037	0.468172
hsa-miR-943	1.074569932	0.805927	1.05281229	0.852739935		0.415037	0.468172
chr14_31314_star	1.074569932	0.805927	1.05281229	0.852739935		0.415037	0.468172
NW_003539070_38505_mature	1.074569932	0.805927	1.05281229	0.852739935		0.415037	0.468172
ssc-miR-885-5p	1.074569932	0.805927	1.05281229	0.852739935		0.415037	0.468172
chr18_36497_mature	1.074569932	0.805927	1.05281229	0.852739935		0.415037	0.468172
hsa-miR-6081	1.074569932	0.805927	1.05281229	0.852739935		0.415037	0.468172
chr5_12388_star	1.074569932	0.805927	1.05281229	0.852739935		0.415037	0.468172
NW_003613364_38713_star	1.074569932	0.805927	1.05281229	0.852739935		0.415037	0.468172
chr1_2577_star	1.074569932	0.805927	1.05281229	0.852739935		0.415037	0.468172
NW_003539571_38947_star	1.074569932	0.805927	1.05281229	0.852739935		0.415037	0.468172
chr2_5943_star	1.074569932	0.805927	1.05281229	0.852739935		0.415037	0.468172
chr5_10910_star	1.074569932	0.805927	1.05281229	0.852739935		0.415037	0.468172
mmu-miR-1929-3p	1.074569932	0.805927	1.05281229	0.852739935		0.415037	0.468172
chr13_28104_star	6.447419591	17.7304	2.896740642	4.227310103		-1.45943	0.468253
chr3_7655_mature	9.671129386	33.84895	3.415640968	5.123043401		-1.80735	0.468993
chrx_22056_mature	4.298279727	8.865202	2.405524014	3.302348583		-1.04439	0.469294
chr5_11725_mature	4.298279727	8.865202	2.405524014	3.302348583		-1.04439	0.469294
bta-miR-2337	4.298279727	8.865202	2.405524014	3.302348583		-1.04439	0.469294
chr7_17055_mature	7.521989523	22.56597	3.091190278	4.558633073		-1.58496	0.469571
chr13_28696_star	7.521989523	22.56597	3.091190278	4.558633073		-1.58496	0.469571
chr10_23450_mature	5.372849659	3.22371	2.671938627	2.078510713		0.736966	0.469979
chr18_36082_mature	5.372849659	3.22371	2.671938627	2.078510713		0.736966	0.469979
chr8_18339_mature	5.372849659	3.22371	2.671938627	2.078510713		0.736966	0.469979
chr14_29850_star	5.372849659	3.22371	2.671938627	2.078510713		0.736966	0.469979
chr14_30569_star	5.372849659	3.22371	2.671938627	2.078510713		0.736966	0.469979
chr1_3607_star	5.372849659	3.22371	2.671938627	2.078510713		0.736966	0.469979
chr6_14981_star	5.372849659	3.22371	2.671938627	2.078510713		0.736966	0.469979
chr1_3022_mature	5.372849659	3.22371	2.671938627	2.078510713		0.736966	0.469979
rno-miR-667-5p	5.372849659	3.22371	2.671938627	2.078510713		0.736966	0.469979
chr3_6673_mature	5.372849659	3.22371	2.671938627	2.078510713		0.736966	0.469979
chr7_16234_mature	5.372849659	3.22371	2.671938627	2.078510713		0.736966	0.469979
chr7_16583_mature	5.372849659	3.22371	2.671938627	2.078510713		0.736966	0.469979
chr9_20679_star	5.372849659	3.22371	2.671938627	2.078510713		0.736966	0.469979
NW_003537189_36743_star	5.372849659	3.22371	2.671938627	2.078510713		0.736966	0.469979
chr15_32921_star	5.372849659	3.22371	2.671938627	2.078510713		0.736966	0.469979
chr8_18569_mature	5.372849659	3.22371	2.671938627	2.078510713		0.736966	0.469979
chr11_24705_star	5.372849659	12.89484	2.671938627	3.796477232		-1.26303	0.470303
chrx_22367_mature	5.372849659	12.89484	2.671938627	3.796477232		-1.26303	0.470303
chr13_27138_mature	18.26768884	6.44742	4.268111626	2.896740642		1.5025	0.470391
NW_003540726_39645_mature	30.08795809	8.865202	4.958283956	3.302348583		1.762961	0.470891
NW_003613379_38756_mature	45.13193714	367.5029	5.527693969	8.525532228		-3.02554	0.471013
chr14_30720_mature	3.223709795	4.835565	2.078510713	2.544872269		-0.58496	0.471574
chr7_16599_mature	3.223709795	4.835565	2.078510713	2.544872269		-0.58496	0.471574
NW_003540835_39730_star	3.223709795	4.835565	2.078510713	2.544872269		-0.58496	0.471574
hsa-miR-2117	3.223709795	4.835565	2.078510713	2.544872269		-0.58496	0.471574
chr3_6622_mature	3.223709795	4.835565	2.078510713	2.544872269		-0.58496	0.471574
chr8_18024_star	3.223709795	4.835565	2.078510713	2.544872269		-0.58496	0.471574
NW_003537641_37087_star	3.223709795	4.835565	2.078510713	2.544872269		-0.58496	0.471574
chr11_24796_star	3.223709795	4.835565	2.078510713	2.544872269		-0.58496	0.471574
chr5_11313_mature@@hsa-miR-141	3.223709795	4.835565	2.078510713	2.544872269		-0.58496	0.471574
chr6_13265_mature	3.223709795	4.835565	2.078510713	2.544872269		-0.58496	0.471574
NW_003613552_39654_star	3.223709795	4.835565	2.078510713	2.544872269		-0.58496	0.471574
rno-miR-293-5p	3.223709795	4.835565	2.078510713	2.544872269		-0.58496	0.471574
chr2_6574_star	3.223709795	4.835565	2.078510713	2.544872269		-0.58496	0.471574
chrx_22797_star	3.223709795	4.835565	2.078510713	2.544872269		-0.58496	0.471574
chr14_30207_star	3.223709795	4.835565	2.078510713	2.544872269		-0.58496	0.471574
chr13_26683_mature	3.223709795	4.835565	2.078510713	2.544872269		-0.58496	0.471574
chr6_14338_mature	3.223709795	4.835565	2.078510713	2.544872269		-0.58496	0.471574
chr7_16758_star	3.223709795	4.835565	2.078510713	2.544872269		-0.58496	0.471574
chr13_27580_mature	8.596559455	27.40153	3.262517265	4.827896911		-1.67243	0.473026
ssc-miR-139-5p	16.11854898	74.14533	4.097488514	6.231611453		-2.20163	0.473279
chr7_15777_mature	9.671129386	33.04303	3.415640968	5.08928735		-1.77259	0.473383
chr1_2051_mature	3.223709795	3.22371	2.078510713	2.078510713		0	0.474337
chr6_15067_mature	3.223709795	3.22371	2.078510713	2.078510713		0	0.474337
chr3_7082_star	3.223709795	3.22371	2.078510713	2.078510713		0	0.474337
chr6_14473_star	3.223709795	3.22371	2.078510713	2.078510713		0	0.474337
chr16_34446_mature	3.223709795	3.22371	2.078510713	2.078510713		0	0.474337

chr14_29447_star	4.29827973	14.50669	2.405524014	3.954819241		-1.75489	1
hsa-miR-3614-5p	2.14913986	1.611855	1.654957834	1.38507475		0.415037	1
chr3_8596_mature	2.14913986	8.059274	1.654957834	3.179395517		-1.90689	1
chr9_20857_star	2.14913986	2.417782	1.654957834	1.773060525		-0.16993	1
mmu-miR-6418-3p	4.29827973	1.611855	2.405524014	1.38507475		1.415037	1
chr3_6817_star	2.14913986	2.417782	1.654957834	1.773060525		-0.16993	1
chr12_25408_star	12.8948392	4.835565	3.796477232	2.544872269		1.415037	1
hsa-miR-1303	2.14913986	1.611855	1.654957834	1.38507475		0.415037	1
chr13_27981_star	4.29827973	1.611855	2.405524014	1.38507475		1.415037	1
chr2_5906_star	4.29827973	1.611855	2.405524014	1.38507475		1.415037	1
chr1_3888_star	6.44741959	2.417782	2.896740642	1.773060525		1.415037	1
bta-miR-3956	2.14913986	8.865202	1.654957834	3.302348583		-2.04439	1
chr1_902_star	4.29827973	1.611855	2.405524014	1.38507475		1.415037	1
chr5_11120_star	2.14913986	2.417782	1.654957834	1.773060525		-0.16993	1
chr15_33676_mature@@mmu-miR-639	2.14913986	2.417782	1.654957834	1.773060525		-0.16993	1
chr1_2656_mature	2.14913986	1.611855	1.654957834	1.38507475		0.415037	1
chr2_5894_star	2.14913986	1.611855	1.654957834	1.38507475		0.415037	1
chr17_35445_star	2.14913986	2.417782	1.654957834	1.773060525		-0.16993	1
rno-miR-181b-1-3p	2.14913986	1.611855	1.654957834	1.38507475		0.415037	1
chr8_18998_star	2.14913986	1.611855	1.654957834	1.38507475		0.415037	1
chr18_36497_star	2.14913986	1.611855	1.654957834	1.38507475		0.415037	1
chrx_21735_star	2.14913986	1.611855	1.654957834	1.38507475		0.415037	1
chr5_12026_star	4.29827973	1.611855	2.405524014	1.38507475		1.415037	1
chr17_35178_mature@@bta-miR-6121-	2.14913986	2.417782	1.654957834	1.773060525		-0.16993	1
chr3_8648_star	2.14913986	1.611855	1.654957834	1.38507475		0.415037	1
chr11_25362_star	2.14913986	2.417782	1.654957834	1.773060525		-0.16993	1
chr8_18144_star	2.14913986	1.611855	1.654957834	1.38507475		0.415037	1
chr9_20512_star	6.44741959	2.417782	2.896740642	1.773060525		1.415037	1
hsa-miR-4759	2.14913986	1.611855	1.654957834	1.38507475		0.415037	1
chr4_10081_mature	2.14913986	1.611855	1.654957834	1.38507475		0.415037	1
chr13_26740_star	2.14913986	1.611855	1.654957834	1.38507475		0.415037	1
chr9_20798_star	2.14913986	1.611855	1.654957834	1.38507475		0.415037	1
chr4_8989_mature	2.14913986	8.059274	1.654957834	3.179395517		-1.90689	1
chr1_398_mature	2.14913986	8.059274	1.654957834	3.179395517		-1.90689	1
chr14_31610_star	4.29827973	1.611855	2.405524014	1.38507475		1.415037	1
chr3_8626_star	4.29827973	14.50669	2.405524014	3.954819241		-1.75489	1
chr1_1736_star	2.14913986	1.611855	1.654957834	1.38507475		0.415037	1
chr4_8756_star	2.14913986	2.417782	1.654957834	1.773060525		-0.16993	1
chr3_8156_mature	10.7456993	4.029637	3.554060706	2.330454351		1.415037	1
chr9_19128_star	2.14913986	1.611855	1.654957834	1.38507475		0.415037	1
hsa-miR-4419a	2.14913986	1.611855	1.654957834	1.38507475		0.415037	1
chr3_7219_mature	2.14913986	10.47706	1.654957834	3.520680821		-2.2854	1
chr1_3642_star	4.29827973	1.611855	2.405524014	1.38507475		1.415037	1
chr6_13570_star	8.59655945	3.22371	3.262517265	2.078510713		1.415037	1
chr1_608_star	2.14913986	2.417782	1.654957834	1.773060525		-0.16993	1
chr1_2360_star	2.14913986	2.417782	1.654957834	1.773060525		-0.16993	1
chr17_35010_star	2.14913986	2.417782	1.654957834	1.773060525		-0.16993	1
chr8_18505_mature	19.3422588	8.865202	4.346407978	3.302348583		1.125531	1
mmu-miR-3085-5p	2.14913986	1.611855	1.654957834	1.38507475		0.415037	1
NW_003538236_37642_mature	2.14913986	2.417782	1.654957834	1.773060525		-0.16993	1
chrx_21932_mature	8.59655945	3.22371	3.262517265	2.078510713		1.415037	1
hsa-miR-4999-5p	2.14913986	1.611855	1.654957834	1.38507475		0.415037	1
hsa-miR-1206	2.14913986	2.417782	1.654957834	1.773060525		-0.16993	1
chr6_13893_mature	51.5793567	29.81932	5.716424587	4.94576292		0.790547	1
hsa-miR-1205	2.14913986	1.611855	1.654957834	1.38507475		0.415037	1
chr4_9733_star	4.29827973	1.611855	2.405524014	1.38507475		1.415037	1
chrx_22335_mature	4.29827973	1.611855	2.405524014	1.38507475		1.415037	1
chr5_11958_star	4.29827973	1.611855	2.405524014	1.38507475		1.415037	1
chr14_30424_star	2.14913986	8.059274	1.654957834	3.179395517		-1.90689	1
chr2_6441_star	4.29827973	1.611855	2.405524014	1.38507475		1.415037	1
chrx_21983_star	2.14913986	10.47706	1.654957834	3.520680821		-2.2854	1
hsa-miR-1297	2.14913986	2.417782	1.654957834	1.773060525		-0.16993	1
chr1_1820_star	2.14913986	1.611855	1.654957834	1.38507475		0.415037	1
NW_003613106_37241_star	2.14913986	1.611855	1.654957834	1.38507475		0.415037	1
chrx_22089_mature	2.14913986	1.611855	1.654957834	1.38507475		0.415037	1
chr17_35470_star	2.14913986	1.611855	1.654957834	1.38507475		0.415037	1
hsa-miR-4758-3p	4.29827973	1.611855	2.405524014	1.38507475		1.415037	1
hsa-miR-361-5p	2.14913986	1.611855	1.654957834	1.38507475		0.415037	1
NW_003538780_38224_star	4.29827973	1.611855	2.405524014	1.38507475		1.415037	1
chr13_27735_mature	10.7456993	4.029637	3.554060706	2.330454351		1.415037	1
NW_003538662_38098_mature	6.44741959	2.417782	2.896740642	1.773060525		1.415037	1
chr6_13334_star	4.29827973	1.611855	2.405524014	1.38507475		1.415037	1
chr13_27735_star	4.29827973	14.50669	2.405524014	3.954819241		-1.75489	1
chr5_10946_star	4.29827973	1.611855	2.405524014	1.38507475		1.415037	1
hsa-miR-553	4.29827973	1.611855	2.405524014	1.38507475		1.415037	1
chr15_32628_mature	2.14913986	1.611855	1.654957834	1.38507475		0.415037	1
chr17_35259_mature	19.3422588	8.059274	4.346407978	3.179395517		1.263034	1
chr13_28221_star	8.59655945	3.22371	3.262517265	2.078510713		1.415037	1
chr3_6956_star	2.14913986	2.417782	1.654957834	1.773060525		-0.16993	1
chr17_35528_star	2.14913986	2.417782	1.654957834	1.773060525		-0.16993	1
chr3_7655_star	4.29827973	1.611855	2.405524014	1.38507475		1.415037	1
NW_003539007_38456_star	2.14913986	9.671129	1.654957834	3.415640968		-2.16993	1
chr14_29876_mature	4.29827973	1.611855	2.405524014	1.38507475		1.415037	1
chr1_3837_mature	4.29827973	1.611855	2.405524014	1.38507475		1.415037	1
chr18_36331_star	2.14913986	1.611855	1.654957834	1.38507475		0.415037	1
bta-miR-2464-5p	2.14913986	1.611855	1.654957834	1.38507475		0.415037	1
chr7_16512_star	2.14913986	2.417782	1.654957834	1.773060525		-0.16993	1
chr2_4454_mature	2.14913986	1.611855	1.654957834	1.38507475		0.415037	1
chr2_5995_mature	6.44741959	2.417782	2.896740642	1.773060525		1.415037	1
ssc-miR-499-3p	17.1931189	8.059274	4.185320985	3.179395517		1.093109	1
chr1_2668_star	2.14913986	1.611855	1.654957834	1.38507475		0.415037	1
rno-miR-383-5p	2.14913986	2.417782	1.654957834	1.773060525		-0.16993	1
chr13_29126_mature	2.14913986	8.865202	1.654957834	3.302348583		-2.04439	1
NW_003613223_37738_star	4.29827973	1.611855	2.405524014	1.38507475		1.415037	1
chr4_9997_mature	4.29827973	1.611855	2.405524014	1.38507475		1.415037	1
chr1_3022_star	2.14913986	2.417782	1.654957834	1.773060525		-0.16993	1
chr3_7963_star	2.14913986	1.611855	1.654957834	1.38507475		0.415037	1
chr14_31648_star	4.29827973	1.611855	2.405524014	1.38507475		1.415037	1
chr11_24794_star	6.44741959	2.417782	2.896740642	1.773060525		1.415037	1
chr18_36189_star	2.14913986	1.611855	1.654957834	1.38507475		0.415037	1
chr12_25395_mature	2.14913986	1.611855	1.654957834	1.38507475		0.415037	1
mmu-miR-1902	2.14913986	2.417782	1.654957834	1.773060525		-0.16993	1
hsa-miR-5590-5p	4.29827973	1.611855	2.405524014	1.38507475		1.415037	1
chr4_10041_mature	12.8948392	5.641492	3.796477232	2.731507408		1.192645	1
bta-miR-1814b	4.29827973	1.611855	2.405524014	1.38507475		1.415037	1
bta-miR-1814a	4.29827973	1.611855	2.405524014	1.38507475		1.415037	1
mmu-miR-1951	2.14913986	9.671129	1.654957834	3.415640968		-2.16993	1
mmu-miR-1953	4.29827973	1.611855	2.405524014	1.38507475		1.415037	1
chr16_34759_star	6.44741959	2.417782	2.896740642	1.773060525		1.415037	1
mmu-miR-1940	2.14913986	1.611855	1.654957834	1.38507475		0.415037	1
chr4_9284_mature	2.14913986	2.417782	1.654957834	1.773060525		-0.16993	1
chr9_21068_star	6.44741959	2.417782	2.896740642	1.773060525		1.415037	1
rno-miR-679	2.14913986	2.417782	1.654957834	1.773060525		-0.16993	1
chrx_22435_star	2.14913986	1.611855	1.654957834	1.38507475		0.415037	1
mmu-miR-195b	2.14913986	1.611855	1.654957834	1.38507475		0.415037	1
NW_003613056_36922_star	2.14913986	1.611855	1.654957834	1.38507475		0.415037	1
chr11_25375_star	2.14913986	2.417782	1.654957834	1.773060525		-0.16993	1
hsa-miR-616-3p	2.14913986	2.417782	1.654957834	1.773060525		-0.16993	1
chr12_26183_mature	2.14913986	1.611855	1.654957834	1.38507475		0.415037	1
chr12_25930_star	2.14913986	1.611855	1.654957834	1.38507475		0.415037	1
chr14_30275_star	2.14913986	1.611855	1.654957834	1.38507475		0.415037	1
chr2_6582_mature	2.14913986	2.417782	1.654957834	1.773060525		-0.16993	1
rno-miR-330-3p	6.44741959	2.417782	2.896740642	1.773060525		1.415037	1
chr7_16272_mature	8.59655945	20.95411	3.262517265	4.456419386		-1.2854	1
chr18_36015_mature	2.14913986	2.417782	1.654957834	1.773060525		-0.16993	1
chr18_36050_star	2.14913986	1.611855	1.654957834	1.38507475		0.415037	1
NW_003539007_38433_star	2.14913986	2.417782	1.654957834	1.773060525		-0.16993	1
hsa-miR-766-5p	4.29827973	13.70077	2.405524014	3.877819487		-1.67243	1
rno-miR-339-5p	2.14913986	1.611855	1.654957834	1.38507475		0.415037	1
hsa-miR-3976	19.3422588	8.059274	4.346407978	3.179395517		1.263034	1
chr8_18251_mature	4.29827973	1.611855	2.405524014	1.38507475		1.415037	1
chr9_19988_star	2.14913986	1.611855	1.654957834	1.38507475		0.415037	1
chr1_743_star	2.14913986	1.611855	1.654957834	1.38507475		0.415037	1
chr11_24909_mature	2.14913986	2.417782	1.654957834	1.773060525		-0.16993	1
hsa-miR-520a-3p	2.14913986	1.611855	1.654957834	1.38507475		0.415037	1
chr12_26402_mature	6.44741959	2.417782	2.896740642	1.773060525		1.415037	1
chr8_18077_star	2.14913986	1.611855	1.654957834	1.38507475		0.415037	1
chr1_3446_star	2.14913986	10.47706	1.654957834	3.520680821		-2.2854	1
chr14_30331_mature	6.44741959	2.417782	2.896740642	1.773060525		1.415037	1
NW_003537295_36819_star	2.14913986	1.611855	1.654957834	1.38507475		0.415037	1
chr17_35344_mature	4.29827973	1.611855	2.405524014	1.38507475		1.415037	1
chr4_9452_mature	12.8948392	5.641492	3.796477232	2.731507408		1.192645	1
chr5_11704_mature@@rno-miR-3550	4.29827973	1.611855	2.405524014	1.38507475		1.415037	1
chr14_29474_star	2.14913986	1.611855	1.654957834	1.38507475		0.415037	1
chr16_34846_star	2.14913986	1.611855	1.654957834	1.38507475		0.415037	1
chr18_36376_star	2.14913986	2.417782	1.654957834	1.773060525		-0.16993	1
chr13_27896_mature	2.14913986	8.059274	1.654957834	3.179395517		-1.90689	1
chr1_811_star	2.14913986	1.611855	1.654957834	1.38507475		0.415037	1
chr4_9707_star	2.14913986	1.611855	1.654957834	1.38507475		0.415037	1
chr1_1781_star	2.14913986	2.417782	1.654957834	1.773060525		-0.16993	1
chr3_6843_star@@hsa-miR-4753-3p	2.14913986	1.611855	1.654957834	1.38507475		0.415037	1
NW_003613545_39630_star	2.14913986	1.611855	1.654957834	1.38507475		0.415037	1
NW_003538949_38373_star	2.14913986	2.417782	1.654957834	1.773060525		-0.16993	1
chr16_34110_mature	2.14913986	8.865202	1.654957834	3.302348583		-2.04439	1
hsa-miR-3655	6.44741959	2.417782	2.896740642	1.773060525		1.415037	1
chr3_7968_mature	6.44741959	2.417782	2.896740642	1.773060525		1.415037	1
rno-miR-672-5p	2.14913986	1.611855	1.654957834	1.38507475		0.415037	1
chr2_5113_star	4.29827973	1.611855	2.405524014	1.38507475		1.415037	1
hsa-miR-3670	2.14913986	2.417782	1.654957834	1.773060525		-0.16993	1
NW_003538201_37611_mature	4.29827973	13.70077	2.405524014	3.877819487		-1.67243	1
rno-miR-3541	2.14913986	2.417782	1.654957834	1.773060525		-0.16993	1
hsa-miR-3662	6.44741959	2.417782	2.896740642	1.773060525		1.415037	1
mmu-miR-5119	2.14913986	1.611855	1.654957834	1.38507475		0.415037	1
hsa-miR-3610	4.29827973	1.611855	2.405524014	1.38507475		1.415037	1
rno-miR-3564	2.14913986	1.611855	1.654957834	1.38507475		0.415037	1
chr7_16102_star	6.44741959	2.417782	2.896740642	1.773060525		1.415037	1
rno-miR-466c-3p	4.29827973	1.611855	2.405524014	1.38507475		1.415037	1
hsa-miR-3609	2.14913986	1.611855	1.654957834	1.38507475		0.415037	1
rno-miR-3572	2.14913986	2.417782	1.654957834	1.773060525		-0.16993	1
chr15_31881_mature	6.44741959	2.417782	2.896740642	1.773060525		1.415037	1
chr6_14473_star	8.59655945	3.22371	3.262517265	2.078510713		1.415037	1
chr15_31883_star	4.29827973	1.611855	2.405524014	1.38507475		1.415037	1
chr12_25429_star	6.44741959	2.417782	2.896740642	1.773060525		1.415037	1
chr8_18753_mature	2.14913986	1.611855	1.654957834	1.38507475		0.415037	1
chr13_26807_mature	2.14913986	8.059274	1.654957834	3.179395517		-1.90689	1
chr9_19300_mature	10.7456993	4.029637	3.554060706	2.330454351		1.415037	1
chr15_31807_star	2.14913986	2.417782	1.654957834	1.773060525		-0.16993	1
chr13_28860_star	6.44741959	2.417782	2.896740642	1.773060525		1.415037	1
chr8_17337_star	2.14913986	1.611855	1.654957834	1.38507475		0.415037	1
chr14_31060_mature	4.29827973	15.31262	2.405524014	4.027916745		-1.83289	1
chr4_8856_star	8.59655945	3.22371	3.262517265	2.078510713		1.415037	1
rno-miR-6315	2.14913986	1.611855	1.654957834	1.38507475		0.415037	1
rno-miR-3068-5p	4.29827973	1.611855	2.405524014	1.38507475		1.415037	1
chr7_15832_mature	2.14913986	8.059274	1.654957834	3.179395517		-1.90689	1
chr5_11653_star	2.14913986	2.417782	1.654957834	1.773060525		-0.16993	1
hsa-miR-550a-5p	2.14913986	1.611855	1.654957834	1.38507475		0.415037	1
chr13_29260_mature	25.7896784	12.08891	4.743605355	3.710273245		1.093109	1
chr1_397_mature	4.29827973	14.50669	2.405524014	3.954819241		-1.75489	1
chr13_27213_mature	2.14913986	1.611855	1.654957834	1.38507475		0.415037	1
chr6_14069_star	2.14913986	1.611855	1.654957834	1.38507475		0.415037	1
chr5_11262_mature	2.14913986	2.417782	1.654957834	1.773060525		-0.16993	1
chr8_18505_star	2.14913986	1.611855	1.654957834	1.38507475		0.415037	1
chr5_11567_star	2.14913986	2.417782	1.654957834	1.773060525		-0.16993	1
chr2_6405_mature@@bta-miR-1814c	2.14913986	8.865202	1.654957834	3.302348583		-2.04439	1
chr1_408_mature	4.29827973	1.611855	2.405524014	1.38507475		1.415037	1
chr1_3915_mature	6.44741959	2.417782	2.896740642	1.773060525		1.415037	1
chr3_7884_mature	10.7456993	4.029637	3.554060706	2.330454351		1.415037	1
chr9_20405_star	2.14913986	2.417782	1.654957834	1.773060525		-0.16993	1
chr8_18841_star	2.14913986	8.865202	1.654957834	3.302348583		-2.04439	1
chr6_13601_mature	38.6845175	65.28012	5.310504361	6.050504383		-0.75489	1
chr14_30369_star	2.14913986	1.611855	1.654957834	1.38507475		0.415037	1
chr8_18354_mature	2.14913986	1.611855	1.654957834	1.38507475		0.415037	1
hsa-miR-378b	2.14913986	1.611855	1.654957834	1.38507475		0.415037	1
chrx_21977_mature@@rno-miR-384-3p	2.14913986	1.611855	1.654957834	1.38507475		0.415037	1
chrx_21498_star	2.14913986	1.611855	1.654957834	1.38507475		0.415037	1
chr12_25573_mature	6.44741959	19.34226	2.896740642	4.346407978		-1.58496	1
NW_003613326_38565_star	4.29827973	1.611855	2.405524014	1.38507475		1.415037	1
NW_003613518_39516_star	2.14913986	2.417782	1.654957834	1.773060525		-0.16993	1
chr1_3025_star	4.29827973	1.611855	2.405524014	1.38507475		1.415037	1
chr4_10041_star	2.14913986	1.611855	1.654957834	1.38507475		0.415037	1
chr8_18403_star	4.29827973	1.611855	2.405524014	1.38507475		1.415037	1
NW_003537574_37009_star	6.44741959	2.417782	2.896740642	1.773060525		1.415037	1
NW_003540387_39459_mature	30.0879581	14.50669	4.958283956	3.954819241		1.052467	1
hsa-miR-1234-5p	17.1931189	7.253347	4.185320985	3.044979305		1.245112	1
chr6_13339_star	4.29827973	14.50669	2.405524014	3.954819241		-1.75489	1
NW_003613480_39328_mature	12.8948392	4.835565	3.796477232	2.544872269		1.415037	1
chr13_28676_star	2.14913986	2.417782	1.654957834	1.773060525		-0.16993	1
chr17_35289_mature	4.29827973	1.611855	2.405524014	1.38507475		1.415037	1
chr1_1866_mature	27.9388182	14.50669	4.854934103	3.954819241		0.945552	1
chr15_33043_mature	2.14913986	1.611855	1.654957834	1.38507475		0.415037	1
chr6_13578_star	2.14913986	1.611855	1.654957834	1.38507475		0.415037	1
chr14_30429_star	2.14913986	1.611855	1.654957834	1.38507475		0.415037	1
chrx_22340_star	4.29827973	1.611855	2.405524014	1.38507475		1.415037	1
chr6_12506_star	4.29827973	1.611855	2.405524014	1.38507475		1.415037	1
chr6_14892_star	2.14913986	1.611855	1.654957834	1.38507475		0.415037	1
chr10_23301_mature	8.59655945	3.22371	3.262517265	2.078510713		1.415037	1
chr2_4363_star	2.14913986	9.671129	1.654957834	3.415640968		-2.16993	1
chr13_27765_star	4.29827973	1.611855	2.405524014	1.38507475		1.415037	1
chr8_17274_star	2.14913986	1.611855	1.654957834	1.38507475		0.415037	1
NW_003613200_37636_star	4.29827973	1.611855	2.405524014	1.38507475		1.415037	1
ssc-miR-489	4.29827973	1.611855	2.405524014	1.38507475		1.415037	1
chr10_24174_star	2.14913986	8.059274	1.654957834	3.179395517		-1.90689	1
chr6_14620_star	4.29827973	1.611855	2.405524014	1.38507475		1.415037	1
bta-miR-876	2.14913986	8.059274	1.654957834	3.179395517		-1.90689	1
chr13_27560_star	2.14913986	1.611855	1.654957834	1.38507475		0.415037	1
NW_003613507_39496_star	2.14913986	1.611855	1.654957834	1.38507475		0.415037	1
NW_003539115_38545_star	2.14913986	1.611855	1.654957834	1.38507475		0.415037	1
hsa-miR-6717-5p	4.29827973	1.611855	2.405524014	1.38507475		1.415037	1
chr1_3536_star	2.14913986	2.417782	1.654957834	1.773060525		-0.16993	1
bta-let-7a-5p	4.29827973	1.611855	2.405524014	1.38507475		1.415037	1
chr1_785_mature	15.043979	6.44742	4.003960082	2.896740642		1.222392	1
chr8_17809_star	2.14913986	9.671129	1.654957834	3.415640968		-2.16993	1
chr7_16262_mature	6.44741959	2.417782	2.896740642	1.773060525		1.415037	1
chr4_10006_star	2.14913986	2.417782	1.654957834	1.773060525		-0.16993	1
chr4_10175_mature	2.14913986	2.417782	1.654957834	1.773060525		-0.16993	1
chr8_18847_mature	4.29827973	1.611855	2.405524014	1.38507475		1.415037	1
chr2_6125_star	2.14913986	8.059274	1.654957834	3.179395517		-1.90689	1
ssc-miR-411	34.3862378	17.7304	5.145116481	4.227310103		0.955606	1
chr5_11962_star	4.29827973	1.611855	2.405524014	1.38507475		1.415037	1
chrx_21526_mature	2.14913986	1.611855	1.654957834	1.38507475		0.415037	1
chr12_25445_star	2.14913986	1.611855	1.654957834	1.38507475		0.415037	1
chr1_2153_star	4.29827973	13.70077	2.405524014	3.877819487		-1.67243	1
chr1_3752_star	2.14913986	2.417782	1.654957834	1.773060525		-0.16993	1
chr6_12646_star	2.14913986	1.611855	1.654957834	1.38507475		0.415037	1
chr5_11418_star	2.14913986	1.611855	1.654957834	1.38507475		0.415037	1
chr17_35259_star	2.14913986	1.611855	1.654957834	1.38507475		0.415037	1
chr18_35816_mature	2.14913986	1.611855	1.654957834	1.38507475		0.415037	1
rno-miR-433-5p	2.14913986	2.417782	1.654957834	1.773060525		-0.16993	1
chr1_3091_star	2.14913986	1.611855	1.654957834	1.38507475		0.415037	1
chr5_11601_mature	2.14913986	2.417782	1.654957834	1.773060525		-0.16993	1
chr14_31449_star	2.14913986	9.671129	1.654957834	3.415640968		-2.16993	1
chr8_18873_star	2.14913986	1.611855	1.654957834	1.38507475		0.415037	1
chr8_18754_star	2.14913986	2.417782	1.654957834	1.773060525		-0.16993	1
chr6_13162_mature	2.14913986	1.611855	1.654957834	1.38507475		0.415037	1
chr16_34657_mature	2.14913986	8.865202	1.654957834	3.302348583		-2.04439	1

chr6_12619_star	3.22371	4.835565	2.078510713	2.544872269		-0.58496	0.693361
chr8_17514_mature	3.22371	4.835565	2.078510713	2.544872269		-0.58496	0.693361
chr2_4557_mature	3.22371	4.835565	2.078510713	2.544872269		-0.58496	0.693361
chr17_35550_mature	3.22371	4.835565	2.078510713	2.544872269		-0.58496	0.693361
chr7_16084_mature	3.22371	4.835565	2.078510713	2.544872269		-0.58496	0.693361
chr8_17599_star	3.22371	4.835565	2.078510713	2.544872269		-0.58496	0.693361
chr14_31584_mature	3.22371	4.835565	2.078510713	2.544872269		-0.58496	0.693361
chr8_18339_mature	4.29828	3.22371	2.405524014	2.078510713		0.415037	0.693592
chr15_33130_mature	4.29828	3.22371	2.405524014	2.078510713		0.415037	0.693592
NW_003613109_37259_star	4.29828	3.22371	2.405524014	2.078510713		0.415037	0.693592
chr13_28221_star	4.29828	3.22371	2.405524014	2.078510713		0.415037	0.693592
mmu-miR-1903	4.29828	3.22371	2.405524014	2.078510713		0.415037	0.693592
chrx_23158_mature	4.29828	3.22371	2.405524014	2.078510713		0.415037	0.693592
chr6_12810_mature	4.29828	3.22371	2.405524014	2.078510713		0.415037	0.693592
chr6_14981_star	4.29828	3.22371	2.405524014	2.078510713		0.415037	0.693592
ssc-miR-494	4.29828	3.22371	2.405524014	2.078510713		0.415037	0.693592
chr7_16636_star	4.29828	3.22371	2.405524014	2.078510713		0.415037	0.693592
rno-miR-667-5p	4.29828	3.22371	2.405524014	2.078510713		0.415037	0.693592
chr18_36498_mature	4.29828	3.22371	2.405524014	2.078510713		0.415037	0.693592
chr2_5564_star@@mmu-miR-670-5	4.29828	3.22371	2.405524014	2.078510713		0.415037	0.693592
chr1_88_star	4.29828	3.22371	2.405524014	2.078510713		0.415037	0.693592
chr8_17430_mature	4.29828	3.22371	2.405524014	2.078510713		0.415037	0.693592
chr17_35575_star	4.29828	3.22371	2.405524014	2.078510713		0.415037	0.693592
chr1_766_star	4.29828	3.22371	2.405524014	2.078510713		0.415037	0.693592
chr1_2992_mature	4.29828	3.22371	2.405524014	2.078510713		0.415037	0.693592
chr3_6877_mature	4.29828	3.22371	2.405524014	2.078510713		0.415037	0.693592
chr14_30387_mature	4.29828	3.22371	2.405524014	2.078510713		0.415037	0.693592
chr8_17419_star	4.29828	3.22371	2.405524014	2.078510713		0.415037	0.693592
chr13_28421_mature	3.22371	3.22371	2.078510713	2.078510713		0	0.693907
hsa-miR-3914	3.22371	3.22371	2.078510713	2.078510713		0	0.693907
chr13_28612_star	3.22371	3.22371	2.078510713	2.078510713		0	0.693907
chr13_28115_star	3.22371	3.22371	2.078510713	2.078510713		0	0.693907
chr16_34188_star	3.22371	3.22371	2.078510713	2.078510713		0	0.693907
NW_003540745_39662_star	3.22371	3.22371	2.078510713	2.078510713		0	0.693907
chr2_5807_star	3.22371	3.22371	2.078510713	2.078510713		0	0.693907
chr13_29308_mature	3.22371	3.22371	2.078510713	2.078510713		0	0.693907
chr7_17077_star	3.22371	3.22371	2.078510713	2.078510713		0	0.693907
mmu-miR-5110	3.22371	3.22371	2.078510713	2.078510713		0	0.693907
chr16_34446_mature	3.22371	3.22371	2.078510713	2.078510713		0	0.693907
chr13_28222_mature	3.22371	3.22371	2.078510713	2.078510713		0	0.693907
chr3_7937_star	3.22371	3.22371	2.078510713	2.078510713		0	0.693907
mmu-miR-148a-3p	3.22371	3.22371	2.078510713	2.078510713		0	0.693907
chr12_26596_star	3.22371	3.22371	2.078510713	2.078510713		0	0.693907
mmu-miR-3112-3p	3.22371	3.22371	2.078510713	2.078510713		0	0.693907
chr11_24488_mature	3.22371	3.22371	2.078510713	2.078510713		0	0.693907
chr14_30480_star	3.22371	3.22371	2.078510713	2.078510713		0	0.693907
chr3_6870_mature	3.22371	3.22371	2.078510713	2.078510713		0	0.693907
chr14_31127_mature	3.22371	3.22371	2.078510713	2.078510713		0	0.693907
chr14_30390_star	3.22371	3.22371	2.078510713	2.078510713		0	0.693907
chr4_9970_mature	3.22371	3.22371	2.078510713	2.078510713		0	0.693907
chr10_23743_star	3.22371	3.22371	2.078510713	2.078510713		0	0.693907
chr8_17733_star@@mmu-miR-308	3.22371	3.22371	2.078510713	2.078510713		0	0.693907
chr9_20679_star	3.22371	3.22371	2.078510713	2.078510713		0	0.693907
chr2_4563_mature	3.22371	3.22371	2.078510713	2.078510713		0	0.693907
chr6_14256_mature	3.22371	3.22371	2.078510713	2.078510713		0	0.693907
chr18_35994_star	3.22371	3.22371	2.078510713	2.078510713		0	0.693907
chr18_36111_star	3.22371	3.22371	2.078510713	2.078510713		0	0.693907
NW_003537189_36743_star	3.22371	3.22371	2.078510713	2.078510713		0	0.693907
chr3_8536_mature	3.22371	3.22371	2.078510713	2.078510713		0	0.693907
chr11_24870_star	3.22371	3.22371	2.078510713	2.078510713		0	0.693907
chr13_28509_star	3.22371	3.22371	2.078510713	2.078510713		0	0.693907
chr9_20257_mature	3.22371	3.22371	2.078510713	2.078510713		0	0.693907
chr6_12627_mature	3.22371	3.22371	2.078510713	2.078510713		0	0.693907
chr14_30832_star	3.22371	3.22371	2.078510713	2.078510713		0	0.693907
chr15_31859_mature	10.20841	4.835565	3.48651029	2.544872269		1.078003	0.694414
chr6_12967_mature	186.9752	27.40153	7.554398282	4.827896911		2.770518	0.694907
chr16_34839_star	3.760995	3.22371	2.251263041	2.078510713		0.222392	0.694919
chr18_36082_mature	3.760995	3.22371	2.251263041	2.078510713		0.222392	0.694919
NW_003613234_37804_mature	3.760995	3.22371	2.251263041	2.078510713		0.222392	0.694919
chr13_27234_star	3.760995	3.22371	2.251263041	2.078510713		0.222392	0.694919
chr7_16503_mature	3.760995	3.22371	2.251263041	2.078510713		0.222392	0.694919
chr2_5816_mature	3.760995	3.22371	2.251263041	2.078510713		0.222392	0.694919
chrx_21552_mature	3.760995	3.22371	2.251263041	2.078510713		0.222392	0.694919
chr6_14428_star	3.760995	3.22371	2.251263041	2.078510713		0.222392	0.694919
chr8_18850_mature	3.760995	3.22371	2.251263041	2.078510713		0.222392	0.694919
chrx_21746_star	3.760995	3.22371	2.251263041	2.078510713		0.222392	0.694919
chr12_25421_star	3.760995	3.22371	2.251263041	2.078510713		0.222392	0.694919
chr2_6507_mature	3.760995	3.22371	2.251263041	2.078510713		0.222392	0.694919
chr10_23700_mature	3.760995	3.22371	2.251263041	2.078510713		0.222392	0.694919
hsa-miR-3123	3.760995	3.22371	2.251263041	2.078510713		0.222392	0.694919
chr6_13308_mature	3.760995	3.22371	2.251263041	2.078510713		0.222392	0.694919
chr6_12809_mature	3.760995	3.22371	2.251263041	2.078510713		0.222392	0.694919
chr5_11934_star	3.760995	3.22371	2.251263041	2.078510713		0.222392	0.694919
chr1_1198_mature	3.760995	3.22371	2.251263041	2.078510713		0.222392	0.694919
NW_003613507_39500_star	3.760995	3.22371	2.251263041	2.078510713		0.222392	0.694919
chr15_33547_star	3.760995	3.22371	2.251263041	2.078510713		0.222392	0.694919
chr9_19683_mature	3.760995	3.22371	2.251263041	2.078510713		0.222392	0.694919
bta-miR-2300a-5p	3.760995	3.22371	2.251263041	2.078510713		0.222392	0.694919
chr13_29126_mature	4.29828	8.865202	2.405524014	3.302348583		-1.04439	0.694959
chr2_6405_mature@@bta-miR-181	4.29828	8.865202	2.405524014	3.302348583		-1.04439	0.694959
NW_003537647_37094_star	4.29828	8.865202	2.405524014	3.302348583		-1.04439	0.694959
chr6_13263_mature	4.29828	8.865202	2.405524014	3.302348583		-1.04439	0.694959
chr7_15706_mature	4.29828	8.865202	2.405524014	3.302348583		-1.04439	0.694959
chr4_10265_star	13.43212	5.641492	3.851211749	2.731507408		1.251539	0.695023
chr4_8979_mature	13.43212	5.641492	3.851211749	2.731507408		1.251539	0.695023
chr7_16645_mature	13.43212	5.641492	3.851211749	2.731507408		1.251539	0.695023
chr15_32961_mature	10.20841	38.68452	3.48651029	5.310504361		-1.922	0.69514
chr15_32852_mature	6.984705	4.029637	2.997239027	2.330454351		0.793549	0.695779
chr15_31926_mature	6.984705	4.029637	2.997239027	2.330454351		0.793549	0.695779
chr7_15712_mature	6.984705	4.029637	2.997239027	2.330454351		0.793549	0.695779
chr8_18374_star	6.984705	4.029637	2.997239027	2.330454351		0.793549	0.695779
chr14_31126_mature	6.984705	4.029637	2.997239027	2.330454351		0.793549	0.695779
chrx_22854_mature	6.984705	4.029637	2.997239027	2.330454351		0.793549	0.695779
ssc-miR-142-3p	6.984705	4.029637	2.997239027	2.330454351		0.793549	0.695779
chr13_27180_star	6.984705	4.029637	2.997239027	2.330454351		0.793549	0.695779
chr3_8153_star	6.984705	4.029637	2.997239027	2.330454351		0.793549	0.695779
chr6_14217_mature	6.984705	4.029637	2.997239027	2.330454351		0.793549	0.695779
chr13_26645_mature	6.984705	4.029637	2.997239027	2.330454351		0.793549	0.695779
chr17_35005_star	3.22371	4.029637	2.078510713	2.330454351		-0.32193	0.695947
chr15_33276_mature	3.22371	4.029637	2.078510713	2.330454351		-0.32193	0.695947
chr2_5077_star	3.22371	4.029637	2.078510713	2.330454351		-0.32193	0.695947
chr4_9773_star	3.22371	4.029637	2.078510713	2.330454351		-0.32193	0.695947
chrx_21833_star	3.22371	4.029637	2.078510713	2.330454351		-0.32193	0.695947
chr8_17244_mature	3.22371	4.029637	2.078510713	2.330454351		-0.32193	0.695947
chr14_31610_mature	3.22371	4.029637	2.078510713	2.330454351		-0.32193	0.695947
ssc-miR-218-3p	3.22371	4.029637	2.078510713	2.330454351		-0.32193	0.695947
hsa-miR-3657	3.22371	4.029637	2.078510713	2.330454351		-0.32193	0.695947
chr9_19300_mature	3.22371	4.029637	2.078510713	2.330454351		-0.32193	0.695947
chr14_31584_star	3.22371	4.029637	2.078510713	2.330454351		-0.32193	0.695947
chr3_7785_mature	3.22371	4.029637	2.078510713	2.330454351		-0.32193	0.695947
chr1_3580_mature	3.22371	4.029637	2.078510713	2.330454351		-0.32193	0.695947
chr2_4255_mature	3.22371	4.029637	2.078510713	2.330454351		-0.32193	0.695947
chr7_15656_mature	3.22371	4.029637	2.078510713	2.330454351		-0.32193	0.695947
NW_003540989_39780_mature	3.22371	4.029637	2.078510713	2.330454351		-0.32193	0.695947
bta-miR-2447	3.22371	4.029637	2.078510713	2.330454351		-0.32193	0.695947
chr1_1421_mature	3.22371	4.029637	2.078510713	2.330454351		-0.32193	0.695947
chr14_29971_star	3.22371	4.029637	2.078510713	2.330454351		-0.32193	0.695947
NW_003613226_37753_mature	3.22371	4.029637	2.078510713	2.330454351		-0.32193	0.695947
chr1_1977_star	3.22371	4.029637	2.078510713	2.330454351		-0.32193	0.695947
hsa-miR-3976	24.71511	8.059274	4.684544332	3.179395517		1.616671	0.69644
chr1_88_mature	24.71511	8.059274	4.684544332	3.179395517		1.616671	0.69644
ssc-miR-9	6368.976	253.8671	12.63707222	7.993601605		4.648916	0.696617
NW_003540726_39645_mature	29.01339	8.865202	4.907534286	3.302348583		1.710493	0.696665
chr4_9456_star	1.07457	0.805927	1.05281229	0.852739935		0.415037	0.696907
chr12_26154_mature	1.07457	0.805927	1.05281229	0.852739935		0.415037	0.696907
mmu-miR-339-3p	1.07457	0.805927	1.05281229	0.852739935		0.415037	0.696907
hsa-miR-4801	1.07457	0.805927	1.05281229	0.852739935		0.415037	0.696907
rno-miR-19a-5p	1.07457	0.805927	1.05281229	0.852739935		0.415037	0.696907
chr15_31929_star	1.07457	0.805927	1.05281229	0.852739935		0.415037	0.696907
chrx_21804_mature	1.07457	0.805927	1.05281229	0.852739935		0.415037	0.696907
chr12_26437_star	1.07457	0.805927	1.05281229	0.852739935		0.415037	0.696907
NW_003613290_38305_star	1.07457	0.805927	1.05281229	0.852739935		0.415037	0.696907
rno-miR-130a-5p	1.07457	0.805927	1.05281229	0.852739935		0.415037	0.696907
chr8_17916_star	1.07457	0.805927	1.05281229	0.852739935		0.415037	0.696907
chr6_14437_star	1.07457	0.805927	1.05281229	0.852739935		0.415037	0.696907
hsa-miR-4742-5p	1.07457	0.805927	1.05281229	0.852739935		0.415037	0.696907
chr15_32720_star	1.07457	0.805927	1.05281229	0.852739935		0.415037	0.696907
chr7_15675_star	1.07457	0.805927	1.05281229	0.852739935		0.415037	0.696907
chr2_5398_star	1.07457	0.805927	1.05281229	0.852739935		0.415037	0.696907
hsa-miR-3667-5p	1.07457	0.805927	1.05281229	0.852739935		0.415037	0.696907
chr7_17029_star	1.07457	0.805927	1.05281229	0.852739935		0.415037	0.696907
chr4_9018_star	1.07457	0.805927	1.05281229	0.852739935		0.415037	0.696907
hsa-miR-3922-3p	1.07457	0.805927	1.05281229	0.852739935		0.415037	0.696907
chr5_10752_star	1.07457	0.805927	1.05281229	0.852739935		0.415037	0.696907
chr17_35071_star	1.07457	0.805927	1.05281229	0.852739935		0.415037	0.696907
chrx_23097_star	1.07457	0.805927	1.05281229	0.852739935		0.415037	0.696907
hsa-miR-1231	1.07457	0.805927	1.05281229	0.852739935		0.415037	0.696907
hsa-miR-1203	1.07457	0.805927	1.05281229	0.852739935		0.415037	0.696907
hsa-miR-4763-5p	1.07457	0.805927	1.05281229	0.852739935		0.415037	0.696907
hsa-miR-4764-3p	1.07457	0.805927	1.05281229	0.852739935		0.415037	0.696907
mmu-miR-367-3p	1.07457	0.805927	1.05281229	0.852739935		0.415037	0.696907
mmu-miR-5618-3p	1.07457	0.805927	1.05281229	0.852739935		0.415037	0.696907
chr6_14454_star	1.07457	0.805927	1.05281229	0.852739935		0.415037	0.696907
hsa-miR-569	1.07457	0.805927	1.05281229	0.852739935		0.415037	0.696907
chr15_33121_mature	1.07457	0.805927	1.05281229	0.852739935		0.415037	0.696907
hsa-miR-518d-3p	1.07457	0.805927	1.05281229	0.852739935		0.415037	0.696907
chr13_28506_star	1.07457	0.805927	1.05281229	0.852739935		0.415037	0.696907
hsa-miR-559	1.07457	0.805927	1.05281229	0.852739935		0.415037	0.696907
chr3_6870_star	1.07457	0.805927	1.05281229	0.852739935		0.415037	0.696907
NW_003539027_38478_star	1.07457	0.805927	1.05281229	0.852739935		0.415037	0.696907
rno-miR-3065-3p	1.07457	0.805927	1.05281229	0.852739935		0.415037	0.696907
chr8_17522_mature	1.07457	0.805927	1.05281229	0.852739935		0.415037	0.696907
mmu-miR-1a-1-5p	1.07457	0.805927	1.05281229	0.852739935		0.415037	0.696907
chr9_20130_mature	1.07457	0.805927	1.05281229	0.852739935		0.415037	0.696907
chr13_29016_star	1.07457	0.805927	1.05281229	0.852739935		0.415037	0.696907
chr14_30061_star	1.07457	0.805927	1.05281229	0.852739935		0.415037	0.696907
chr6_13705_star	1.07457	0.805927	1.05281229	0.852739935		0.415037	0.696907
chrx_22228_star	1.07457	0.805927	1.05281229	0.852739935		0.415037	0.696907
chr2_5281_star	1.07457	0.805927	1.05281229	0.852739935		0.415037	0.696907
chr12_25591_star	1.07457	0.805927	1.05281229	0.852739935		0.415037	0.696907
mmu-miR-1945	1.07457	0.805927	1.05281229	0.852739935		0.415037	0.696907
mmu-miR-1936	1.07457	0.805927	1.05281229	0.852739935		0.415037	0.696907
chr16_34240_mature	1.07457	0.805927	1.05281229	0.852739935		0.415037	0.696907
chr13_29269_star	1.07457	0.805927	1.05281229	0.852739935		0.415037	0.696907
chr6_12979_star	1.07457	0.805927	1.05281229	0.852739935		0.415037	0.696907
chr8_17643_star	1.07457	0.805927	1.05281229	0.852739935		0.415037	0.696907
chrx_22329_mature	1.07457	0.805927	1.05281229	0.852739935		0.415037	0.696907
chr3_8236_star	1.07457	0.805927	1.05281229	0.852739935		0.415037	0.696907
NW_003613326_38565_mature	1.07457	0.805927	1.05281229	0.852739935		0.415037	0.696907
chr4_9679_star	1.07457	0.805927	1.05281229	0.852739935		0.415037	0.696907
chr13_29250_star	1.07457	0.805927	1.05281229	0.852739935		0.415037	0.696907
chr7_16155_star	1.07457	0.805927	1.05281229	0.852739935		0.415037	0.696907
chr13_28700_star	1.07457	0.805927	1.05281229	0.852739935		0.415037	0.696907
chr14_29940_star	1.07457	0.805927	1.05281229	0.852739935		0.415037	0.696907
rno-miR-3571	1.07457	0.805927	1.05281229	0.852739935		0.415037	0.696907
hsa-miR-4726-3p	1.07457	0.805927	1.05281229	0.852739935		0.415037	0.696907
chr12_26093_star	1.07457	0.805927	1.05281229	0.852739935		0.415037	0.696907
chr8_17918_star	1.07457	0.805927	1.05281229	0.852739935		0.415037	0.696907
hsa-miR-570-3p	1.07457	0.805927	1.05281229	0.852739935		0.415037	0.696907
hsa-miR-3690	1.07457	0.805927	1.05281229	0.852739935		0.415037	0.696907
chr2_4629_star	1.07457	0.805927	1.05281229	0.852739935		0.415037	0.696907
chr1_777_star	1.07457	0.805927	1.05281229	0.852739935		0.415037	0.696907
mmu-miR-3073b-3p	1.07457	0.805927	1.05281229	0.852739935		0.415037	0.696907
rno-miR-6316	1.07457	0.805927	1.05281229	0.852739935		0.415037	0.696907
chr6_13694_star	1.07457	0.805927	1.05281229	0.852739935		0.415037	0.696907
chr15_32156_star	1.07457	0.805927	1.05281229	0.852739935		0.415037	0.696907
chr12_25952_mature	1.07457	0.805927	1.05281229	0.852739935		0.415037	0.696907
chr11_25225_star	1.07457	0.805927	1.05281229	0.852739935		0.415037	0.696907
hsa-miR-675-5p	1.07457	0.805927	1.05281229	0.852739935		0.415037	0.696907
mmu-miR-183-3p	1.07457	0.805927	1.05281229	0.852739935		0.415037	0.696907
hsa-miR-4433-3p	1.07457	0.805927	1.05281229	0.852739935		0.415037	0.696907
chr1_2153_mature	1.07457	0.805927	1.05281229	0.852739935		0.415037	0.696907
hsa-miR-6720-3p	1.07457	0.805927	1.05281229	0.852739935		0.415037	0.696907
chr1_3923_star	1.07457	0.805927	1.05281229	0.852739935		0.415037	0.696907
chr3_7048_mature	1.07457	0.805927	1.05281229	0.852739935		0.415037	0.696907
rno-miR-487b-5p	1.07457	0.805927	1.05281229	0.852739935		0.415037	0.696907
chr1_1283_star	1.07457	0.805927	1.05281229	0.852739935		0.415037	0.696907
mmu-miR-3101-5p	1.07457	0.805927	1.05281229	0.852739935		0.415037	0.696907
chr1_3525_star	1.07457	0.805927	1.05281229	0.852739935		0.415037	0.696907
bta-miR-1185	1.07457	0.805927	1.05281229	0.852739935		0.415037	0.696907
chr2_5976_star	1.07457	0.805927	1.05281229	0.852739935		0.415037	0.696907
rno-miR-1249	1.07457	0.805927	1.05281229	0.852739935		0.415037	0.696907
rno-miR-3584-3p	1.07457	0.805927	1.05281229	0.852739935		0.415037	0.696907
bta-miR-411c-5p	1.07457	0.805927	1.05281229	0.852739935		0.415037	0.696907
mmu-miR-295-5p	1.07457	0.805927	1.05281229	0.852739935		0.415037	0.696907
NW_003613097_37151_star	1.07457	0.805927	1.05281229	0.852739935		0.415037	0.696907
chrx_23090_star	1.07457	0.805927	1.05281229	0.852739935		0.415037	0.696907
chr11_24884_mature	1.07457	0.805927	1.05281229	0.852739935		0.415037	0.696907
chr3_8590_mature	1.07457	0.805927	1.05281229	0.852739935		0.415037	0.696907
chr7_16996_star	1.07457	0.805927	1.05281229	0.852739935		0.415037	0.696907
chr3_6641_star	1.07457	0.805927	1.05281229	0.852739935		0.415037	0.696907
chr16_34699_star	1.07457	0.805927	1.05281229	0.852739935		0.415037	0.696907
bta-miR-2461-3p	1.07457	0.805927	1.05281229	0.852739935		0.415037	0.696907
chr1_3766_mature	1.07457	0.805927	1.05281229	0.852739935		0.415037	0.696907
bta-miR-769	1.07457	0.805927	1.05281229	0.852739935		0.415037	0.696907
mmu-miR-6337	1.07457	0.805927	1.05281229	0.852739935		0.415037	0.696907
mmu-miR-6350	1.07457	0.805927	1.05281229	0.852739935		0.415037	0.696907
hsa-miR-6500-5p	1.07457	0.805927	1.05281229	0.852739935		0.415037	0.696907
bta-miR-2284q	1.07457	0.805927	1.05281229	0.852739935		0.415037	0.696907
mmu-miR-6394	1.07457	0.805927	1.05281229	0.852739935		0.415037	0.696907
chr7_15832_star	1.07457	0.805927	1.05281229	0.852739935		0.415037	0.696907
chr2_4572_star	1.07457	0.805927	1.05281229	0.852739935		0.415037	0.696907
chr15_33536_star	1.07457	0.805927	1.05281229	0.852739935		0.415037	0.696907
chr8_17371_mature	1.07457	0.805927	1.05281229	0.852739935		0.415037	0.696907
chr13_27180_mature	1.07457	0.805927	1.05281229	0.852739935		0.415037	0.696907
chr7_16194_star	1.07457	0.805927	1.05281229	0.852739935		0.415037	0.696907
hsa-miR-4799-3p	1.07457	0.805927	1.05281229	0.852739935		0.415037	0.696907
rno-miR-352	1.07457	0.805927	1.05281229	0.852739935		0.415037	0.696907
chr14_30212_star	1.07457	0.805927	1.05281229	0.852739935		0.415037	0.696907
mmu-miR-708-5p	1.07457	0.805927	1.05281229	0.852739935		0.415037	0.696907
mmu-miR-195a-5p	1.07457	0.805927	1.05281229	0.852739935		0.415037	0.696907
chr9_19609_star@@bta-miR-135b	1.07457	0.805927	1.05281229	0.852739935		0.415037	0.696907
chr18_36486_star	1.07457	0.805927	1.05281229	0.852739935		0.415037	0.696907
chr9_19205_star	1.07457	0.805927	1.05281229	0.852739935		0.415037	0.696907
chr9_19536_star	1.07457	0.805927	1.05281229	0.852739935		0.415037	0.696907
hsa-miR-4704-3p	1.07457	0.805927	1.05281229	0.852739935		0.415037	0.696907
bta-miR-150	1.07457	0.805927	1.05281229	0.852739935		0.415037	0.696907
hsa-miR-4714-5p	1.07457	0.805927	1.05281229	0.852739935		0.415037	0.696907
chr8_18153_mature	1.07457	0.805927	1.05281229	0.852739935		0.415037	0.696907
chr16_34452_star	1.07457	0.805927	1.05281229	0.852739935		0.415037	0.696907
bta-miR-15b	1.07457	0.805927	1.05281229	0.852739935		0.415037	0.696907
chr18_35974_star@@mmu-miR-59	1.07457	0.805927	1.05281229	0.852739935		0.415037	0.696907
chr5_10731_star	1.07457	0.805927	1.05281229	0.852739935		0.415037	0.696907
chr8_17676_star	1.07457	0.805927	1.05281229	0.852739935		0.415037	0.696907
chr11_25035_star	1.07457	0.805927	1.05281229	0.852739935		0.415037	0.696907

chr4_9743_star	3.223709795	3.22371	2.078510713	2.078510713		0	0.474337		chr16_33722_star	4.29827973	1.611855	2.405524014	1.38507475		1.415037	1		hsa-miR-3177-5p	1.07457	0.805927	1.05281229	0.852739935		0.415037	0.696907
chr2_5244_star	3.223709795	3.22371	2.078510713	2.078510713		0	0.474337		chr14_29599_star	2.14913986	8.865202	1.654957834	3.302348583		-2.04439	1		mmu-miR-20a-5p	1.07457	0.805927	1.05281229	0.852739935		0.415037	0.696907
chr10_23301_mature	3.223709795	3.22371	2.078510713	2.078510713		0	0.474337		chr14_30443_mature	6.44741959	19.34226	2.896740642	4.346407978		-1.58496	1		hsa-miR-4786-5p	1.07457	0.805927	1.05281229	0.852739935		0.415037	0.696907
chr1_1411_star	3.223709795	3.22371	2.078510713	2.078510713		0	0.474337		chr6_14536_mature	10.7456993	4.029637	3.554060706	2.330454351		1.415037	1		rno-miR-499-5p	1.07457	0.805927	1.05281229	0.852739935		0.415037	0.696907
chr8_18593_star	3.223709795	3.22371	2.078510713	2.078510713		0	0.474337		rno-miR-674-3p	2.14913986	2.417782	1.654957834	1.773060525		-0.16993	1		chr12_25396_mature	1.07457	0.805927	1.05281229	0.852739935		0.415037	0.696907
chr2_4019_mature	3.223709795	3.22371	2.078510713	2.078510713		0	0.474337		chrx_21721_star	2.14913986	2.417782	1.654957834	1.773060525		-0.16993	1		chr2_4006_star	1.07457	0.805927	1.05281229	0.852739935		0.415037	0.696907
chr15_32759_star	3.223709795	3.22371	2.078510713	2.078510713		0	0.474337		chr7_15797_mature	2.14913986	2.417782	1.654957834	1.773060525		-0.16993	1		hsa-miR-2114-5p	1.07457	0.805927	1.05281229	0.852739935		0.415037	0.696907
chr14_30079_star	3.223709795	3.22371	2.078510713	2.078510713		0	0.474337		chr2_4381_mature	19.3422588	39.49044	4.346407978	5.339509593		-1.02975	1		chr14_29991_star	1.07457	0.805927	1.05281229	0.852739935		0.415037	0.696907
chr15_31697_mature	3.223709795	3.22371	2.078510713	2.078510713		0	0.474337		NW_003538236_37641_mature	8.59655945	3.22371	3.262517265	2.078510713		1.415037	1		chr8_17231_star	1.07457	0.805927	1.05281229	0.852739935		0.415037	0.696907
chr8_17514_star	3.223709795	3.22371	2.078510713	2.078510713		0	0.474337		chr15_32437_mature	4.29827973	1.611855	2.405524014	1.38507475		1.415037	1		chr1_3878_mature	1.07457	0.805927	1.05281229	0.852739935		0.415037	0.696907
chr10_23743_star	3.223709795	3.22371	2.078510713	2.078510713		0	0.474337		NW_003539007_38442_star	2.14913986	1.611855	1.654957834	1.38507475		0.415037	1		hsa-miR-548k	1.07457	0.805927	1.05281229	0.852739935		0.415037	0.696907
chr4_9818_star	3.223709795	3.22371	2.078510713	2.078510713		0	0.474337		chr13_27566_star	2.14913986	8.059274	1.654957834	3.179395517		-1.90689	1		bta-miR-202	1.07457	0.805927	1.05281229	0.852739935		0.415037	0.696907
chr6_14380_star	3.223709795	3.22371	2.078510713	2.078510713		0	0.474337		bta-miR-6119-5p	2.14913986	1.611855	1.654957834	1.38507475		0.415037	1		hsa-miR-4645-3p	1.07457	0.805927	1.05281229	0.852739935		0.415037	0.696907
hsa-miR-3174	3.223709795	3.22371	2.078510713	2.078510713		0	0.474337		NW_003539542_38921_star	2.14913986	2.417782	1.654957834	1.773060525		-0.16993	1		NW_003538236_37642_star	1.07457	0.805927	1.05281229	0.852739935		0.415037	0.696907
chr10_23462_star	3.223709795	3.22371	2.078510713	2.078510713		0	0.474337		chr7_15747_mature	2.14913986	1.611855	1.654957834	1.38507475		0.415037	1		hsa-miR-5002-5p	1.07457	0.805927	1.05281229	0.852739935		0.415037	0.696907
chr17_35675_mature	3.223709795	3.22371	2.078510713	2.078510713		0	0.474337		chr15_33320_star	2.14913986	1.611855	1.654957834	1.38507475		0.415037	1		rno-miR-451-3p	1.07457	0.805927	1.05281229	0.852739935		0.415037	0.696907
NW_003613303_38366_mature	3.223709795	3.22371	2.078510713	2.078510713		0	0.474337		mmu-miR-6366	2.14913986	1.611855	1.654957834	1.38507475		0.415037	1		mmu-miR-1192	1.07457	0.805927	1.05281229	0.852739935		0.415037	0.696907
chr3_6750_mature	3.223709795	3.22371	2.078510713	2.078510713		0	0.474337		chr6_14296_mature	6.44741959	2.417782	2.896740642	1.773060525		1.415037	1		chr7_16529_star	1.07457	0.805927	1.05281229	0.852739935		0.415037	0.696907
chr18_36577_mature	3.223709795	3.22371	2.078510713	2.078510713		0	0.474337		chr12_26311_star	4.29827973	1.611855	2.405524014	1.38507475		1.415037	1		chr12_26014_star	1.07457	0.805927	1.05281229	0.852739935		0.415037	0.696907
chr14_30035_mature	3.223709795	3.22371	2.078510713	2.078510713		0	0.474337		mmu-miR-6361	2.14913986	2.417782	1.654957834	1.773060525		-0.16993	1		chr11_24782_star	1.07457	0.805927	1.05281229	0.852739935		0.415037	0.696907
chr15_33547_star	3.223709795	3.22371	2.078510713	2.078510713		0	0.474337		chr18_36647_star	2.14913986	2.417782	1.654957834	1.773060525		-0.16993	1		chr7_16599_star	1.07457	0.805927	1.05281229	0.852739935		0.415037	0.696907
chr14_30214_star	3.223709795	3.22371	2.078510713	2.078510713		0	0.474337		chr18_36226_star	2.14913986	2.417782	1.654957834	1.773060525		-0.16993	1		hsa-miR-3163	1.07457	0.805927	1.05281229	0.852739935		0.415037	0.696907
chr4_8951_star	3.223709795	3.22371	2.078510713	2.078510713		0	0.474337		mmu-miR-6385	2.14913986	1.611855	1.654957834	1.38507475		0.415037	1		hsa-miR-3180	1.07457	0.805927	1.05281229	0.852739935		0.415037	0.696907
chr7_16559_star	3.223709795	3.22371	2.078510713	2.078510713		0	0.474337		chr12_25628_star	2.14913986	2.417782	1.654957834	1.773060525		-0.16993	1		chr8_18571_star	1.07457	0.805927	1.05281229	0.852739935		0.415037	0.696907
NW_003537823_37300_mature	3.223709795	3.22371	2.078510713	2.078510713		0	0.474337		bta-miR-2285c	2.14913986	1.611855	1.654957834	1.38507475		0.415037	1		chr15_33675_star	1.07457	0.805927	1.05281229	0.852739935		0.415037	0.696907
bta-miR-2300a-5p	3.223709795	3.22371	2.078510713	2.078510713		0	0.474337		chr1_989_star	2.14913986	2.417782	1.654957834	1.773060525		-0.16993	1		hsa-miR-3664-3p	1.07457	0.805927	1.05281229	0.852739935		0.415037	0.696907
chr4_9456_star	0	0.805927	0	0.852739935		-20	0.474469		NW_003541332_40046_star	2.14913986	1.611855	1.654957834	1.38507475		0.415037	1		bta-miR-6530	1.07457	0.805927	1.05281229	0.852739935		0.415037	0.696907
chr12_26154_mature	0	0.805927	0	0.852739935		-20	0.474469		NW_003537603_37043_mature	2.14913986	1.611855	1.654957834	1.38507475		0.415037	1		hsa-miR-4260	1.07457	0.805927	1.05281229	0.852739935		0.415037	0.696907
chr1_3261_star	0	0.805927	0	0.852739935		-20	0.474469		mmu-miR-6397	2.14913986	2.417782	1.654957834	1.773060525		-0.16993	1		hsa-miR-4277	1.07457	0.805927	1.05281229	0.852739935		0.415037	0.696907
hsa-miR-518a-3p	0	0.805927	0	0.852739935		-20	0.474469		chr3_8052_star	4.29827973	1.611855	2.405524014	1.38507475		1.415037	1		hsa-miR-4274	1.07457	0.805927	1.05281229	0.852739935		0.415037	0.696907
chr5_10979_mature	0	0.805927	0	0.852739935		-20	0.474469		chr6_14967_star	8.59655945	3.22371	3.262517265	2.078510713		1.415037	1		hsa-miR-4275	1.07457	0.805927	1.05281229	0.852739935		0.415037	0.696907
hsa-miR-4652-3p	0	0.805927	0	0.852739935		-20	0.474469		chr6_12802_star	12.8948392	5.641492	3.796477232	2.731507408		1.192645	1		bta-miR-6523	1.07457	0.805927	1.05281229	0.852739935		0.415037	0.696907
mmu-miR-698-5p	0	0.805927	0	0.852739935		-20	0.474469		chr6_14228_mature	4.29827973	1.611855	2.405524014	1.38507475		1.415037	1		hsa-miR-4293	1.07457	0.805927	1.05281229	0.852739935		0.415037	0.696907
hsa-miR-548ar-3p	0	0.805927	0	0.852739935		-20	0.474469		chr11_25079_star	2.14913986	8.865202	1.654957834	3.302348583		-2.04439	1		chr7_16237_mature	1.07457	0.805927	1.05281229	0.852739935		0.415037	0.696907
hsa-miR-3680-3p	0	0.805927	0	0.852739935		-20	0.474469		bta-miR-2900	2.14913986	2.417782	1.654957834	1.773060525		-0.16993	1		mmu-miR-344d-1-5p	1.07457	0.805927	1.05281229	0.852739935		0.415037	0.696907
chr17_35440_mature	0	0.805927	0	0.852739935		-20	0.474469		chr11_24465_star	2.14913986	1.611855	1.654957834	1.38507475		0.415037	1		chrx_21688_star	1.07457	0.805927	1.05281229	0.852739935		0.415037	0.696907
chr1_2073_star	0	0.805927	0	0.852739935		-20	0.474469		chr9_20132_star	2.14913986	1.611855	1.654957834	1.38507475		0.415037	1		bta-miR-2454-5p	1.07457	0.805927	1.05281229	0.852739935		0.415037	0.696907
chrx_23064_star	0	0.805927	0	0.852739935		-20	0.474469		chr8_18587_star	2.14913986	2.417782	1.654957834	1.773060525		-0.16993	1		chr14_31467_star	1.07457	0.805927	1.05281229	0.852739935		0.415037	0.696907
rno-miR-124-5p	0	0.805927	0	0.852739935		-20	0.474469		chr15_32789_mature	2.14913986	1.611855	1.654957834	1.38507475		0.415037	1		chr2_3980_star	1.07457	0.805927	1.05281229	0.852739935		0.415037	0.696907
chr7_16671_star	0	0.805927	0	0.852739935		-20	0.474469		chr12_25470_star	17.1931189	8.059274	4.185320985	3.179395517		1.093109	1		bta-miR-31	1.07457	0.805927	1.05281229	0.852739935		0.415037	0.696907
hsa-miR-130a-5p	0	0.805927	0	0.852739935		-20	0.474469		chr6_12636_star	2.14913986	1.611855	1.654957834	1.38507475		0.415037	1		bta-miR-96	1.07457	0.805927	1.05281229	0.852739935		0.415037	0.696907
hsa-miR-4785	0	0.805927	0	0.852739935		-20	0.474469		chr14_29600_star	2.14913986	1.611855	1.654957834	1.38507475		0.415037	1		hsa-miR-1236-3p	1.07457	0.805927	1.05281229	0.852739935		0.415037	0.696907
hsa-miR-1343	0	0.805927	0	0.852739935		-20	0.474469		chr10_23676_mature	30.0879581	14.50669	4.958283956	3.954819241		1.052467	1		bta-miR-2431-5p	1.07457	0.805927	1.05281229	0.852739935		0.415037	0.696907
chr10_23315_mature	0	0.805927	0	0.852739935		-20	0.474469		NW_003538342_37742_mature	2.14913986	2.417782	1.654957834	1.773060525		-0.16993	1		chr7_16802_mature	1.07457	0.805927	1.05281229	0.852739935		0.415037	0.696907
rno-miR-130a-5p	0	0.805927	0	0.852739935		-20	0.474469		chr15_33139_mature	2.14913986	10.47706	1.654957834	3.520680821		-2.2854	1		chr8_18187_star	1.07457	0.805927	1.05281229	0.852739935		0.415037	0.696907
chr1_915_mature	0	0.805927	0	0.852739935		-20	0.474469		chrx_23149_star	2.14913986	1.611855	1.654957834	1.38507475		0.415037	1		bta-miR-448	1.07457	0.805927	1.05281229	0.852739935		0.415037	0.696907
chr6_13733_star	0	0.805927	0	0.852739935		-20	0.474469		hsa-miR-5692c	2.14913986	2.417782	1.654957834	1.773060525		-0.16993	1		chr1_431_star	1.07457	0.805927	1.05281229	0.852739935		0.415037	0.696907
chrx_21651_star	0	0.805927	0	0.852739935		-20	0.474469		chr2_4221_mature	2.14913986	2.417782	1.654957834	1.773060525		-0.16993	1		chr4_10120_star	1.07457	0.805927	1.05281229	0.852739935		0.415037	0.696907
NW_003538449_37886_star	0	0.805927	0	0.852739935		-20	0.474469		chr3_7078_mature	2.14913986	1.611855	1.654957834	1.38507475		0.415037	1		chr1_1112_star	1.07457	0.805927	1.05281229	0.852739935		0.415037	0.696907
hsa-miR-23c	0	0.805927	0	0.852739935		-20	0.474469		mmu-miR-1968-5p	4.29827973	1.611855	2.405524014	1.38507475		1.415037	1		chr13_27508_star	1.07457	0.805927	1.05281229	0.852739935		0.415037	0.696907
rno-miR-551b-3p	0	0.805927	0	0.852739935		-20	0.474469		chr1_771_star	4.29827973	15.31262	2.405524014	4.027916745		-1.83289	1		chr2_6077_star	1.07457	0.805927	1.05281229	0.852739935		0.415037	0.696907
hsa-miR-4742-5p	0	0.805927	0	0.852739935		-20	0.474469		chr1_2557_star	2.14913986	2.417782	1.654957834	1.773060525		-0.16993	1		chr4_9780_mature	1.07457	0.805927	1.05281229	0.852739935		0.415037	0.696907
hsa-miR-4729	0	0.805927	0	0.852739935		-20	0.474469		chr6_14228_star	8.59655945	3.22371	3.262517265	2.078510713		1.415037	1		NW_003541083_39896_star	1.07457	0.805927	1.05281229	0.852739935		0.415037	0.696907
chr16_34636_star	0	0.805927	0	0.852739935		-20	0.474469		chr14_31548_mature	4.29827973	1.611855	2.405524014	1.38507475		1.415037	1		bta-miR-2422	1.07457	0.805927	1.05281229	0.852739935		0.415037	0.696907
hsa-miR-4742-3p	0	0.805927	0	0.852739935		-20	0.474469		chr3_8646_mature	51.5793567	29.01339	5.716424587	4.907534286		0.830075	1		chr14_30485_star	1.07457	0.805927	1.05281229	0.852739935		0.415037	0.696907
hsa-miR-4748	0	0.805927	0	0.852739935		-20	0.474469		chr10_24002_star	6.44741959	2.417782	2.896740642	1.773060525		1.415037	1		bta-miR-2428	1.07457	0.805927	1.05281229	0.852739935		0.415037	0.696907
chr7_15675_star	0	0.805927	0	0.852739935		-20	0.474469		chr13_27489_mature	4.29827973	14.50669	2.405524014	3.954819241		-1.75489	1		hsa-miR-4796-3p	1.07457	0.805927	1.05281229	0.852739935		0.415037	0.696907
chr2_4688_star	0	0.805927	0	0.852739935		-20	0.474469		chr14_30379_mature	2.14913986	1.611855	1.654957834	1.38507475		0.415037	1		bta-miR-2462	1.07457	0.805927	1.05281229	0.852739935		0.415037	0.696907
hsa-miR-1224-5p	0	0.805927	0	0.852739935		-20	0.474469		chr10_24226_mature	12.8948392	4.835565	3.796477232	2.544872269		1.415037	1		hsa-miR-4483	1.07457	0.805927	1.05281229	0.852739935		0.415037	0.696907
rno-miR-484	0	0.805927	0	0.852739935		-20	0.474469		chr3_6774_star	2.14913986	2.417782	1.654957834	1.773060525		-0.16993	1		hsa-miR-4524b-5p	1.07457	0.805927	1.05281229	0.852739935		0.415037	0.696907
bta-miR-2896	0	0.805927	0	0.852739935		-20	0.474469		chr7_15755_star	2.14913986	1.611855	1.654957834	1.38507475		0.415037	1		bta-miR-2470	1.07457	0.805927	1.05281229	0.852739935		0.415037	0.696907
chr4_10619_mature	0	0.805927	0	0.852739935		-20	0.474469		chr1_3278_star	2.14913986	2.417782	1.654957834	1.773060525		-0.16993	1		bta-miR-2480	1.07457	0.805927	1.05281229	0.852739935		0.415037	0.696907
chr13_29079_star	0	0.805927	0	0.852739935		-20	0.474943		rno-miR-370-5p	6.44741959	17.7304	2.896740642	4.227310103		-1.45943	1		hsa-miR-635	1.07457	0.805927	1.05281229	0.852739935		0.415037	0.696907
chr17_35073_star	0	0.805927	0	0.852739935		-20	0.474469		hsa-miR-4676-3p	2.14913986	2.417782	1.654957834	1.773060525		-0.16993	1		mmu-miR-466b-3p	1.07457	0.805927	1.05281229	0.852739935		0.415037	0.696907
chr5_11377_mature	0	0.805927	0	0.852739935		-20	0.474469		NW_003540052_39255_mature	2.14913986	10.47706	1.654957834	3.520680821		-2.2854	1		chr8_18125_star	1.07457	0.805927	1.05281229	0.852739935		0.415037	0.696907
chr1_3451_star	0	0.805927	0	0.852739935		-20	0.474469		NW_003613382_38825_mature	2.14913986	2.417782	1.654957834	1.773060525		-0.16993	1		bta-miR-2329-3p	1.07457	0.805927	1.05281229	0.852739935		0.415037	0.696907
hsa-miR-4520b-3p	0	0.805927	0	0.852739935		-20	0.474469		chr13_26947_mature	2.14913986	2.417782	1.654957834	1.773060525		-0.16993	1		hsa-miR-653	1.07457	0.805927	1.05281229	0.852739935		0.415037	0.696907
chr11_24692_star	0	0.805927	0	0.852739935		-20	0.474469		chr14_30974_mature	2.14913986	1.611855	1.654957834	1.38507475		0.415037	1		bta-miR-2368-5p	1.07457	0.805927	1.05281229	0.852739935		0.415037	0.696907
chr7_17029_star	0	0.805927	0	0.852739935		-20	0.474469		chr7_17049_star	2.14913986	1.611855	1.654957834	1.38507475		0.415037	1		hsa-miR-371a-3p	1.07457	0.805927	1.05281229	0.852739935		0.415037	0.696907
hsa-miR-548ay-5p	0	0.805927	0	0.852739935		-20	0.474469		hsa-miR-4650-3p	2.14913986	1.611855	1.654957834	1.38507475		0.415037	1		chr14_31281_star	1.07457	0.805927	1.05281229	0.852739935		0.415037	0.696907
hsa-miR-3922-3p	0	0.805927	0	0.852739935		-20	0.474469		rno-miR-675-5p	2.14913986	1.611855	1.654957834	1.38507475		0.415037	1		chr9_20130_star	1.07457	0.805927	1.05281229	0.852739935		0.415037	0.696907
chrx_22989_star	0	0.805927	0	0.852739935		-20	0.474469		chr13_29421_mature	12.8948392	5.641492	3.796477232	2.731507408		1.192645	1		hsa-miR-4700-3p	1.07457	0.805927	1.05281229	0.852739935		0.415037	0.696907
chr4_10081_star	0	0.805927	0	0.852739935		-20	0.474469		NW_003541090_39901_star	2.14913986	2.417782	1.654957834	1.773060525		-0.16993	1		bta-miR-2333	1.07457	0.805927	1.05281229	0.852739935		0.415037	0.696907
hsa-miR-4671-3p	0	0.805927	0	0.852739935		-20	0.474469		chr13_28432_star	4.29827973	1.611855	2.405524014	1.38507475		1.415037	1		chr14_31643_star	1.07457	0.805927	1.05281229	0.852739935		0.415037	0.696907
hsa-miR-3944-3p	0	0.805927	0	0.852739935		-20	0.474469		chr3_6615_star	2.14913986	1.611855	1.654957834	1.38507475		0.415037	1		chrx_21525_star	1.07457	0.805927	1.05281229	0.852739935		0.415037	0.696907
hsa-miR-4999-3p	0	0.805927	0	0.852739935		-20	0.474469		chr14_31255_star	2.14913986	1.611855	1.654957834	1.38507475		0.415037	1		chr3_7184_star	1.07457	0.805927	1.05281229	0.852739935		0.415037	0.696907
rno-miR-423-3p	0	0.805927	0	0.852739935		-20	0.474469		NW_003541083_39896_mature	4.29827973	1.611855	2.405524014	1.38507475		1.415037	1		hsa-miR-4326	1.07457	0.805927	1.05281229	0.852739935		0.415037	0.696907
chr5_11725_star	0	0.805927	0	0.852739935		-20	0.474469		chr7_15100_mature	2.14913986	1.611855	1.654957834	1.38507475		0.415037	1		chr7_17076_mature	1.07457	0.805927	1.05281229	0.852739935		0.415037	0.696907
mmu-miR-92a-2-5p	0	0.805927	0	0.852739935		-20	0.474469		mmu-miR-3969	2.14913986	2.417782	1.654957834	1.773060525		-0.16993	1		rno-miR-3580-3p	1.07457	0.805927	1.05281229	0.852739935		0.415037	0.696907
chr5_11638_star	0	0.805927	0	0.852739935		-20	0.474469		hsa-miR-589-3p	2.14913986	2.417782	1.654957834	1.773060525		-0.16993	1		hsa-miR-4662b	1.07457	0.805927	1.05281229	0.852739935		0.415037	0.696907
chr16_34391_star	0	0.805927	0	0.852739935		-20	0.474469		chr9_19885_star	4.29827973	1.611855	2.405524014	1.38507475		1.415037	1		bta-miR-2297	1.07457	0.805927	1.05281229	0.852739935		0.415037	0.696907
hsa-miR-3622a-3p	0	0.805927	0	0.852739935		-20	0.474469		chr1_439_mature	2.14913986	8.059274	1.654957834	3.179395517		-1.90689	1		chr2_4675_star	1.07457	0.805927	1.05281229	0.852739935		0.415037	0.696907
mmu-miR-1199-3p	0	0.805927	0	0.852739935		-20	0.474469		chr9_20300_star	6.44741959	2.417782	2.896740642	1.773060525		1.415037	1		chr8_17976_mature	1.07457	0.805927	1.05281229	0.852739935		0.415037	0.696907
hsa-miR-3681-5p	0	0.805927	0	0.852739935		-20	0.474469		chr10_23315_star	4.29827973	1.611855	2.405524014	1.38507475		1.415037	1		chr7_15713_star	1.07457	0.805927	1.05281229	0.852739935		0.415037	0.696907
hsa-miR-4764-5p	0	0.805927	0	0.852739935		-20	0.474469		bta-miR-10b	2.14913986	1.611855	1.654957834	1.38507475		0.415037	1		chr13_26614_star	1.07457	0.805927	1.05281229	0.852739935		0.415037	0.696907
chr13_27663_star	0	0.805927	0	0.852739935		-20	0.474469		chr17_35802_star	12.8948392	4.835565	3.796477232	2.544872269		1.415037	1		rno-miR-881-5p	1.07457	0.805927	1.05281229	0.852739935		0.415037	0.696907
hsa-miR-1203	0	0.805927	0	0.852739935		-20	0.474469		chr5_11119_mature	2.14913986	2.417782	1.654957834	1.773060525		-0.16993	1		chr4_10393_star	1.07457	0.805927	1.05281229	0.852739935		0.415037	0.696907
hsa-miR-5589-3p	0	0.805927	0	0.852739935		-20	0.474469		rno-let-7c-5p	4.29827973	1.611855	2.405524014	1.38507475		1.415037	1		hsa-miR-23a-5p	1.07457	0.805927	1.05281229	0.852739935		0.415037	0.696907
chr17_35548_star	0	0.805927	0	0.852739935		-20	0.474469		hsa-miR-6134	2.14913986	1.611855	1.654957834	1.38507475		0.415037	1		hsa-miR-450b-3p	1.07457	0.805927	1.05281229	0.852739935		0.415037	0.696907
hsa-miR-3681-3p	0	0.805927	0	0.852739935		-20	0.474469		chrx_21725_star	2.14913986	1.611855	1.654957834	1.38507475		0.415037	1		chr15_33667_star	1.07457	0.805927	1.05281229	0.852739935		0.415037	0.696907
chr13_26798_star	0	0.805927	0	0.852739935		-20	0.474469		chr6_13302_star	2.14913986	1.611855	1.654957834	1.38507475		0.415037	1		hsa-miR-4648	1.07457	0.805927	1.05281229	0.852739935		0.415037	0.696907
chr2_5368_mature	0	0.805927	0	0.852739935		-20	0.474469		chr6_13265_star	4.29827973	1.611855	2.405524014	1.38507475		1.415037	1		chr3_7910_star	1.07457	0.805927	1.05281229	0.852739935		0.415037	0.696907
rno-miR-17-1-3p	0	0.805927	0	0.852739935		-20	0.474469		chr9_19614_star	2.14913986	2.417782	1.654957834	1.773060525		-0.16993	1		hsa-miR-4657	1.07457	0.805927	1.05281229	0.852739935		0.415037	0.696907
chr5_11603_star	0	0.805927	0	0.852739935		-20	0.474469		hsa-miR-6514-5p	2.14913986	2.417782	1.654957834	1.773060525		-0.16993	1		NW_003538808_38240_star	1.07457	0.805927	1.05281229	0.852739935		0.415037	0.696907
mmu-miR-367-3p	0	0.805927	0	0.852739935		-20	0.474469		chr7_16615_star	2.14913986	1.611855	1.654957834	1.38507475		0.415037	1		chr7_15903_mature	1.07457	0.805927	1.05281229	0.852739935		0.415037	0.696907
chr6_13565_star	0	0.805927	0	0.852739935		-20	0.474469		hsa-miR-4711-3p	4.29827973	1.611855	2.405524014	1.38507475		1.415037	1		hsa-miR-4688	1.07457	0.805927	1.05281229	0.852739935		0.415037	0.696907
bta-miR-1584-5p	0	0.805927	0	0.852739935		-20	0.474469		NW_003538075_37489_star	4.29827973	1.611855	2.405524014	1.38507475		1.415037	1		chr7_15903_star	1.07457	0.805927	1.05281229	0.852739935		0.415037	0.696907
mmu-miR-31-3p	0	0.805927	0	0.852739935		-20	0.474469		chr11_24357_mature	6.44741959	2.417782	2.896740642	1.773060525		1.415037	1		rno-miR-26a-5p	1.07457	0.805927	1.05281229	0.852739935		0.415037	0.696907
NW_003539221_38657_star	0	0.805927	0	0.852739935		-20	0.474469		chr12_26422_star	8.59655945	21.76004	3.262517265	4.508431259		-1.33985	1		mmu-miR-344f-3p	1.07457	0.805927	1.05281229	0.852739935		0.415037	0.696907
chr6_14217_star	0	0.805927	0	0.852739935		-20	0.474469		chr5_11414_star	4.29827973	1.611855	2.405524014	1.38507475		1.415037	1		chr3_7956_mature	1.07457	0.805927	1.05281229	0.852739935		0.415037	0.696907
hsa-miR-2682-3p	0	0.805927	0	0.852739935		-20	0.474469		chr15_32764_mature	4.29827973	15.31262	2.405524014	4.027916745		-1.83289	1		chr13_26761_mature	1.07457	0.805927	1.05281229	0.852739935		0.415037	0.696907
hsa-miR-586	0	0.805927	0	0.852739935		-20	0.474469		chr10_24040_mature	2.14913986	1.611855	1.654957834	1.38507475		0.415037	1		chr8_18569_star	1.07457	0.805927	1.05281229	0.852739935		0.415037	0.696907
hsa-miR-583	0	0.805927	0	0.852739935		-20	0.474469		NW_003613481_39347_star	6.44741959	2.417782	2.896740642	1.773060525		1.415037	1		chr3_6880_star@@hsa-miR-197-5	1.07457	0.805927	1.05281229	0.852739935		0.415037	0.696907
chr3_7781_star	0	0.805927	0	0.852739935		-20	0.474469		chr2_5082_star	2.14913986	10.47706	1.654957834	3.520680821		-2.2854	1		mmu-miR-92b-5p	1.07457	0.805927	1.05281229	0.852739935		0.415037	0.696907
hsa-miR-559	0	0.805927	0	0.852739935		-20	0.474469		chr14_30036_mature	6.44741959	2.417782	2.896740642	1.773060525		1.415037	1		hsa-miR-5682	1.07457	0.805927	1.05281229	0.852739935		0.415037	0.696907
chr10_23275_star	0	0.805927	0	0.852739935		-20	0.474469		rno-miR-151-3p	2.14913986	1.611855	1.654957834	1.38507475		0.415037	1		hsa-miR-4505	1.07457	0.805927	1.05281229	0.852739935		0.415037	0.696907
bta-miR-196a	0	0.805927	0	0.852739935		-20	0.474469		chr9_20110_star	2.14913986	2.417782	1.654957834	1.773060525		-0.16993	1		chr4_8682_star	1.07457	0.805927	1.05281229	0.852739935		0.415037	0.696907
rno-miR-3065-3p	0	0.805927	0	0.852739935		-20	0.474469		chr1_3153_star	2.14913986	2.417782	1.654957834	1.773060525		-0.16993	1		hsa-miR-4540	1.07457	0.805927	1.05281229	0.852739935		0.415037	0.696907
chr5_11356_star	0	0.805927	0	0.852739935		-20	0.474469		chr4_10016_star	4.29827973	1.611855	2.405524014	1.38507475		1.415037	1		chr9_19409_star	1.07457	0.805927	1.05281229	0.852739935		0.415037	0.696907
chr8_17522_mature	0	0.805927	0	0.852739935		-20	0.474469		bta-miR-16a	2.14913986	1.611855	1.654957834	1.38507475		0.415037	1		mmu-miR-1264-5p	1.07457	0.805927	1.05281229	0.852739935		0.415037	0.696907
NW_003538870_38317_star	0	0.805927	0	0.852739935		-20	0.474469		NW_003613481_39347_mature	2.14913986	9.671129	1.654957834	3.415640968		-2.16993	1		hsa-miR-943	1.07457	0.805927	1.05281229	0.852739935		0.415037	0.696907
mmu-miR-1a-1-5p	0	0.805927	0	0.852739935		-20	0.474469		chr15_32980_mature	2.14913986	2.417782	1.654957834	1.773060525		-0.16993	1		chr2_6199_star	1.07457	0.805927	1.05281229	0.852739935		0.415037	0.696907
hsa-miR-507	0	0.805927	0	0.852739935		-20	0.474469		chr6_13257_mature	6.44741959	18.53633	2.896740642	4.288087668		-1.52356	1		chr3_7552_star	1.07457	0.805927	1.05281229	0.852739935		0.415037	0.696907
chr2_6441_mature	0	0.805927	0	0.852739935		-20	0.474469		ssc-miR-122	4.29827973	15.31262	2.405524014	4.027916745		-1.83289	1		chr14_29460_star	1.07457	0.805927	1.05281229	0.852739935		0.415037	0.696907
hsa-miR-1247-3p	0	0.805927	0	0.852739935		-20	0.474469		chr15_32961_star	4.29827973	15.31262	2.405524014	4.027916745		-1.83289	1		chr9_20843_mature	1.07457	0.805927	1.05281229	0.852739935		0.415037	0.696907
mmu-miR-5132-3p	0	0.805927	0	0.852739935		-20	0.474469		chr2_5077_mature	2.14913986	2.417782	1.654957834	1.773060525		-0.16993	1		ssc-miR-885-3p	1.07457	0.805927	1.05281229	0.852739935		0.415037	0.696907
bta-miR-2284t-5p	0	0.805927	0	0.852739935		-20	0.474469		chr1_2545_mature	2.14913986	8.865202	1.654957834	3.302348583		-2.04439	1		NW_003538342_37742_star	1.07457	0.805927	1.05281229	0.852739935		0.415037	0.696907
hsa-miR-3910	0	0.805927	0	0.852739935		-20	0.474469		chr1_273_mature	6.44741959	19.34226	2.896740642	4.346407978		-1.58496	1		NW_003541072_39884_mature	3.760995	6.44742	2.251263041	2.896740642		-0.77761	0.697036
chr14_30061_star	0	0.805927	0	0.852739935		-20	0.474469		chr3_7571_star	2.14913986	1.611855	1.654957834	1.38507475		0.415037	1		chr13_27068_mature	3.760995	6.44742	2.251263041	2.896740642		-0.77761	0.697036
hsa-miR-3939	0	0.805927	0	0.852739935		-20	0.474469		chr7_16140_mature	2.14913986	2.417782	1.654957834	1.773060525		-0.16993	1		chr8_18304_star	3.760995	6.44742	2.251263041	2.896740642		-0.77761	0.697036
chr13_28971_star	0	0.805927	0	0.852739935		-20	0.474469		chr2_5993_star	2.14913986	2.417782	1.654957834	1.773060525		-0.16993	1		chr13_28872_star	3.760995	6.44742	2.251263041	2.896740642		-0.77761	0.697036
hsa-miR-3923	0	0.805927	0	0.852739935		-20	0.474469		chr2_5369_star	4.29827973	1.611855	2.405524014	1.38507475		1.415037	1		chrx_23192_star@@rno-miR-3585-	3.760995	6.44742	2.251263041	2.896740642		-0.77761	0.697036
hsa-miR-3929	0	0.805927	0	0.852739935		-20	0.474469		chr2_5163_mature	4.29827973	1.611855	2.405524014	1.38507475		1.415037	1		chr13_28164_mature	3.760995	6.44742	2.251263041	2.896740642		-0.77761	0.697036
chr9_19423_mature	0	0.805927	0	0.852739935		-20	0.474469		chr3_7373_mature	6.44741959	2.417782	2.896740642	1.773060525		1.415037	1		NW_003541277_40020_mature	8.596559	29.01339	3.262517265	4.907534286		-1.75489	0.697431
chr7_16183_star	0	0.805927	0	0.852739935		-20	0.474469		chr1_3433_mature	2.14913986	1.611855	1.654957834	1.38507475		0.415037	1		chr3_7781_mature	20.41683	7.253347	4.420672964	3.044979305		1.49304	0.697751
chr18_36542_star	0	0.805927	0	0.852739935		-20	0.474469		NW_003537656_37099_star	2.14913986	2.417782	1.654957834	1.773060525		-0.16993	1		chr6_13361_mature	9.671129	4.835565	3.415640968	2.544872269		1	0.698595
mmu-miR-1905	0	0.805927	0	0.852739935		-20	0.474469		rno-miR-541-5p	2.14913986	1.611855	1.654957834	1.38507475		0.415037	1		chr14_29694_mature	9.671129	4.835565	3.415640968	2.544872269		1	0.698595
chrx_22120_star	0	0.805927	0	0.852739935		-20	0.474469		chr7_15292_mature	10.7456993	25.78968	3.554060706	4.743605355		-1.26303	1		chr10_24226_mature	9.671129	4.835565	3.415640968	2.544872269		1	0.698595
chr2_5281_star	0	0.805927	0	0.852739935		-20	0.474469		chr15_32200_mature	2.14913986	1.611855	1.654957834	1.38507475		0.415037	1		chr6_13265_mature	9.671129	4.835565	3.415640968	2.544872269		1	0.698595
rno-miR-1-3p	0	0.805927	0	0.852739935		-20	0.474469		chr9_20301_mature	2.14913986	1.611855	1.654957834	1.38507475		0.415037	1		chr1_244_mature	5.37285	12.89484	2.671938627	3.796477232		-1.26303	0.69873
chr1_3894_star@@bta-miR-219-5p	0	0.805927	0	0.852739935		-20	0.474469		bta-miR-23a	8.59655945	3.22371	3.262517265	2.078510713		1.415037	1		NW_003539394_39194_mature	6.44742	17.7304	2.896740642	4.227310103		-1.45943	0.69934
rno-miR-678	0	0.805927	0	0.852739935		-20	0.474469		chrx_21506_star	2.14913986	8.865202	1.654957834	3.302348583		-2.04439	1		chr8_17840_mature	6.44742	4.029637	2.896740642	2.330454351		0.678072	0.699464
mmu-miR-1936	0	0.805927	0	0.852739935		-20	0.474469		chr8_17595_mature	2.14913986	2.417782	1.654957834	1.773060525		-0.16993	1		chr7_16636_mature	6.44742	4.029637	2.896740642	2.330454351		0.678072	0.699464
NW_003612993_36688_star	0	0.805927	0	0.852739935		-20	0.474469		chr2_5378_star	4.29827973	1.611855	2.405524014	1.38507475		1.415037	1		chr7_16048_mature	6.44742	4.029637	2.896740642	2.330454351		0.678072	0.699464
chr14_31216_star	0	0.805927	0	0.852739935		-20	0.474469		chr13_29080_star	2.14913986	1.611855	1.654957834	1.38507475		0.415037	1		chr4_9796_star	6.44742	4.029637	2.896740642	2.330454351		0.678072	0.699464
chr4_9650_mature	0	0.805927	0	0.852739935		-20	0.474469		chr1_1609_mature	10.7456993	4.029637	3.554060706	2.330454351		1.415037	1		rno-miR-329-5p	6.44742	4.029637	2.896740642	2.330454351		0.678072	0.699464
chr13_29269_star	0	0.805927	0	0.852739935		-20	0.474469		chr1_1437_star	2.14913986	1.611855	1.654957834	1.38507475		0.415037	1		chr11_24358_star	6.44742	4.029637	2.896740642	2.330454351		0.678072	0.699464
mmu-miR-1971	0	0.805927	0	0.852739935		-20	0.474469		chr14_30481_mature	2.14913986	1.611855	1.654957834	1.38507475		0.415037	1		chr7_15704_mature	6.44742	4.029637	2.896740642	2.330454351		0.678072	0.699464
chr6_12979_star	0	0.805927	0	0.852739935		-20	0.474469		chr2_4563_star	2.14913986	1.611855	1.654957834	1.38507475		0.415037	1		rno-miR-336-3p	6.44742	4.029637	2.896740642	2.330454351		0.678072	0.699464
hsa-miR-4716-3p	0	0.805927	0	0.852739935		-20	0.474469		chr2_6521_star	66.6233358	41.1023	6.079449279	5.395827139		0.696808	1		chr6_14257_star	6.44742	4.029637	2.896740642	2.330454351		0.678072	0.699464
chr5_11717_mature	0	0.805927	0	0.852739935		-20	0.474469		chr15_33346_star	6.44741959	2.417782	2.896740642	1.773060525		1.415037	1		chr15_32721_mature	6.44742	4.029637	2.896740642	2.330454351		0.678072	0.699464
chr8_17643_star	0	0.805927	0	0.852739935		-20	0.474469		chr2_5976_mature	6.44741959	2.417782	2.896740642	1.773060525		1.415037	1		chr7_16781_mature	6.44742	4.029637	2.896740642	2.330454351		0.678072	0.699464
hsa-miR-429	0	0.805927	0	0.852739935		-20	0.474469		chr13_27489_star	2.14913986	2.417782	1.654957834	1.773060525		-0.16993	1		chr9_20222_star	6.44742	4.029637	2.896740642	2.330454351		0.678072	0.699464
chrx_22210_star	0	0.805927	0	0.852739935		-20	0.474469		chr3_7732_star	6.44741959	2.417782	2.896740642	1.773060525		1.415037	1		chr13_27642_star	6.44742	4.029637	2.896740642	2.330454351		0.678072	0.699464
ssc-miR-4334-5p	0	0.805927	0	0.852739935		-20	0.474469		chr18_36575_star	2.14913986	1.611855	1.654957834	1.38507475		0.415037	1		chr3_7184_mature	15.58126	73.3394	4.051482084	6.216055097		-2.23478	0.699784
chr18_36069_star	0	0.805927	0	0.852739935		-20	0.474469		chr1_1386_mature	2.14913986	1.611855	1.654957834	1.38507475		0.415037	1		chr2_5807_mature	4.835565	10.47706	2.544872269	3.520680821		-1.11548	0.699795
mmu-miR-374b-3p	0	0.805927	0	0.852739935		-20	0.474443		chr13_27786_star	6.44741959	17.7304	2.896740642	4.227310103		-1.45943	1		NW_003540052_39255_mature	4.835565	10.47706	2.544872269	3.520680821		-1.11548	0.699795
chr4_9468_star	0	0.805927	0	0.852739935		-20	0.474469		chr5_11474_star	2.14913986	2.417782	1.654957834	1.773060525		-0.16993	1		chr1_2204_mature	4.835565	10.47706	2.544872269	3.520680821		-1.11548	0.699795
mmu-miR-327	0	0.805927	0	0.852739935		-20	0.474469		chr2_5371_mature	6.44741959	2.417782	2.896740642	1.773060525		1.415037	1		chr6_13306_star	0	0.805927	0	0.852739935		-20	0.699942
chr3_8243_star	0	0.805927	0	0.852739935		-20	0.474469		chr13_27530_star	4.29827973	1.611855	2.405524014	1.38507475		1.415037	1		hsa-miR-518a-3p	0	0.805927	0	0.852739935		-20	0.699942
mmu-miR-343	0	0.805927	0	0.852739935		-20	0.474469		chr1_2346_mature	4.29827973	1.611855	2.405524014	1.38507475		1.415037	1		hsa-miR-548ar-3p	0	0.805927	0	0.852739935		-20	0.699942
hsa-miR-155-3p	0	0.805927	0	0.852739935		-20	0.474469		chr9_20512_mature	2.14913986	1.611855	1.654957834	1.38507475		0.415037	1		rno-miR-124-5p	0	0.805927	0	0.852739935		-20	0.699942
hsa-miR-1537	0	0.805927	0	0.852739935		-20	0.474469		chr5_11695_mature	4.29827973	1.611855	2.405524014	1.38507475		1.415037	1		mmu-miR-466c-5p	0	0.805927	0	0.852739935		-20	0.699942
rno-miR-539-3p	0	0.805927	0	0.852739935		-20	0.474469		chr16_34293_star	4.29827973	1.611855	2.405524014	1.38507475		1.415037	1		chr15_33038_mature	0	0.805927	0	0.852739935		-20	0.699942
NW_003537296_36822_star	0	0.805927	0	0.852739935		-20	0.474469		chr6_14380_star	8.59655945	3.22371	3.262517265	2.078510713		1.415037	1		hsa-miR-4790-3p	0	0.805927	0	0.852739935		-20	0.699942
rno-miR-539-5p	0	0.805927	0	0.852739935		-20	0.474469		chr4_8929_star	2.14913986	1.611855	1.654957834	1.38507475		0.415037	1		hsa-miR-1343	0	0.805927	0	0.852739935		-20	0.699942
hsa-miR-6499-5p	0	0.805927	0	0.852739935		-20	0.474469		chr9_19738_star	2.14913986	10.47706	1.654957834	3.520680821		-2.2854	1		chr5_12416_star	0	0.805927	0	0.852739935		-20	0.699942
rno-miR-190a-3p	0	0.805927	0	0.852739935		-20	0.474469		chr2_6194_mature	2.14913986	2.417782	1.654957834	1.773060525		-0.16993	1		chr2_4688_star	0	0.805927	0	0.852739935		-20	0.699942
hsa-miR-4738-3p	0	0.805927	0	0.852739935		-20	0.474469		chr15_32458_mature	4.29827973	1.611855	2.405524014	1.38507475		1.415037	1		hsa-miR-4739	0	0.805927	0	0.852739935		-20	0.699942
hsa-miR-624-5p	0	0.805927	0	0.852739935		-20	0.474469		chr8_17716_mature	2.14913986	9.671129	1.654957834	3.415640968		-2.16993	1		hsa-miR-1224-5p	0	0.805927	0	0.852739935		-20	0.699942
hsa-miR-191-3p	0	0.805927	0	0.852739935		-20	0.474469		chr4_9960_star	10.7456993	4.029637	3.554060706	2.330454351		1.415037	1		rno-miR-484	0	0.805927	0	0.852739935		-20	0.699942
chr17_34991_star	0	0.805927	0	0.852739935		-20	0.474469		bta-miR-551b	2.14913986	1.611855	1.654957834	1.38507475		0.415037	1		hsa-miR-4767	0	0.805927	0	0.852739935		-20	0.699942
hsa-miR-4746-5p	0	0.805927	0	0.852739935		-20	0.474469		chr13_26614_mature	2.14913986	9.671129	1.654957834	3.415640968		-2.16993	1		chr4_10619_mature	0	0.805927	0	0.852739935		-20	0.699942
NW_003613326_38565_mature	0	0.805927	0	0.852739935		-20	0.474469		chr3_8257_star	2.14913986	1.611855	1.654957834	1.38507475		0.415037	1		mmu-miR-93-3p	0	0.805927	0	0.852739935		-20	0.699942
hsa-miR-147a	0	0.805927	0	0.852739935		-20	0.474469		chr9_19555_mature	2.14913986	2.417782	1.654957834	1.773060525		-0.16993	1		hsa-miR-548ay-5p	0	0.805927	0	0.852739935		-20	0.699942
hsa-miR-1471	0	0.805927	0	0.852739935		-20	0.474469		chr6_14661_mature	6.44741959	18.53633	2.896740642	4.288087668		-1.52356	1		hsa-miR-4999-3p	0	0.805927	0	0.852739935		-20	0.699942
chr11_25144_star	0	0.805927	0	0.852739935		-20	0.474469		chr1_1290_star	2.14913986	1.611855	1.654957834	1.38507475		0.415037	1		rno-miR-423-3p	0	0.805927	0	0.852739935		-20	0.699942
bta-miR-181d	0	0.805927	0	0.852739935		-20	0.474469		chr13_27138_star	2.14913986	1.611855	1.654957834	1.38507475		0.415037	1		chr9_19972_star	0	0.805927	0	0.852739935		-20	0.699942
chr4_9679_star	0	0.805927	0	0.852739935		-20	0.474469		ssc-miR-4338	2.14913986	2.417782	1.654957834	1.773060525		-0.16993	1		hsa-miR-3622a-3p	0	0.805927	0	0.852739935		-20	0.699942
chr3_6843_mature	0	0.805927	0	0.852739935		-20	0.474469		ssc-miR-4337	4.29827973	1.611855	2.405524014	1.38507475		1.415037	1		mmu-miR-1199-3p	0	0.805927	0	0.852739935		-20	0.699942
chr5_11713_star	0	0.805927	0	0.852739935		-20	0.474469		NW_003613303_38366_star	2.14913986	2.417782	1.654957834	1.773060525		-0.16993	1		mmu-miR-875-3p	0	0.805927	0	0.852739935		-20	0.699942
chr2_6135_star	0	0.805927	0	0.852739935		-20	0.474469		chr2_4557_mature	12.8948392	4.835565	3.796477232	2.544872269		1.415037	1		mmu-miR-465a-3p	0	0.805927	0	0.852739935		-20	0.699942
chr13_27641_star	0	0.805927	0	0.852739935		-20	0.474469		chr14_31235_star@@mmu-miR-346-3	6.44741959	2.417782	2.896740642	1.773060525		1.415037	1		chr5_11375_star	0	0.805927	0	0.852739935		-20	0.699942
chr3_6872_star	0	0.805927	0	0.852739935		-20	0.474469		chr14_30518_star	2.14913986	1.611855	1.654957834	1.38507475		0.415037	1		bta-miR-1584-5p	0	0.805927	0	0.852739935		-20	0.699942
hsa-miR-384	0	0.805927	0	0.852739935		-20	0.474469		hsa-miR-3199	2.14913986	1.611855	1.654957834	1.38507475		0.415037	1		mmu-miR-31-3p	0	0.805927	0	0.852739935		-20	0.699942
mmu-miR-5107-5p	0	0.805927	0	0.852739935		-20	0.474469		chr14_31530_mature	4.29827973	1.611855	2.405524014	1.38507475		1.415037	1		hsa-miR-579	0	0.805927	0	0.852739935		-20	0.699942
chr11_25193_star	0	0.805927	0	0.852739935		-20	0.474469		NW_003613522_39530_mature	4.29827973	1.611855	2.405524014	1.38507475		1.415037	1		rno-miR-871-5p	0	0.805927	0	0.852739935		-20	0.699942
hsa-miR-5579-5p	0	0.805927	0	0.852739935		-20	0.474469		hsa-miR-3664-5p	2.14913986	2.417782	1.654957834	1.773060525		-0.16993	1		hsa-miR-2682-3p	0	0.805927	0	0.852739935		-20	0.699942
hsa-miR-525-5p	0	0.805927	0	0.852739935		-20	0.474469		chr11_24357_star	2.14913986	1.611855	1.654957834	1.38507475		0.415037	1		hsa-miR-586	0	0.805927	0	0.852739935		-20	0.699942
rno-miR-494-5p	0	0.805927	0	0.852739935		-20	0.474469		bta-miR-544a	2.14913986	1.611855	1.654957834	1.38507475		0.415037	1		hsa-miR-583	0	0.805927	0	0.852739935		-20	0.699942
hsa-miR-519d	0	0.805927	0	0.852739935		-20	0.474469		mmu-miR-3078-3p	2.14913986	1.611855	1.654957834	1.38507475		0.415037	1		chr12_25593_star	0	0.805927	0	0.852739935		-20	0.699942
hsa-miR-5191	0	0.805927	0	0.852739935		-20	0.474439		chr14_31502_star	2.14913986	8.865202	1.654957834	3.302348583		-2.04439	1		mmu-miR-1950	0	0.805927	0	0.852739935		-20	0.699942
chr9_20876_star	0	0.805927	0	0.852739935		-20	0.474469		chr9_20110_mature	2.14913986	1.611855	1.654957834	1.38507475		0.415037	1		mmu-miR-1956	0	0.805927	0	0.852739935		-20	0.699942
chr14_29940_star	0	0.805927	0	0.852739935		-20	0.474469		ssc-miR-105-1	6.44741959	2.417782	2.896740642	1.773060525		1.415037	1		NW_003612993_36688_star	0	0.805927	0	0.852739935		-20	0.699942
chr12_25415_star	0	0.805927	0	0.852739935		-20	0.474469		hsa-miR-31-5p	2.14913986	2.417782	1.654957834	1.773060525		-0.16993	1		mmu-miR-1971	0	0.805927	0	0.852739935		-20	0.699942
chr9_19683_star	0	0.805927	0	0.852739935		-20	0.474469		chr17_35182_mature@@bta-miR-6121	2.14913986	1.611855	1.654957834	1.38507475		0.415037	1		ssc-miR-4334-5p	0	0.805927	0	0.852739935		-20	0.699942
bta-miR-130b	0	0.805927	0	0.852739935		-20	0.474469		chr10_23674_star	2.14913986	1.611855	1.654957834	1.38507475		0.415037	1		mmu-miR-343	0	0.805927	0	0.852739935		-20	0.699942
mmu-miR-6244	0	0.805927	0	0.852739935		-20	0.474469		chr5_10732_mature	2.14913986	1.611855	1.654957834	1.38507475		0.415037	1		rno-miR-539-3p	0	0.805927	0	0.852739935		-20	0.699942
chr1_188_star	0	0.805927	0	0.852739935		-20	0.474469		bta-miR-6531	2.14913986	2.417782	1.654957834	1.773060525		-0.16993	1		hsa-miR-548ag	0	0.805927	0	0.852739935		-20	0.699942
rno-miR-3568	0	0.805927	0	0.852739935		-20	0.474469		chr6_13908_star	2.14913986	2.417782	1.654957834	1.773060525		-0.16993	1		rno-miR-539-5p	0	0.805927	0	0.852739935		-20	0.699942
rno-miR-3577	0	0.805927	0	0.852739935		-20	0.474469		hsa-miR-4269	4.29827973	1.611855	2.405524014	1.38507475		1.415037	1		hsa-miR-624-5p	0	0.805927	0	0.852739935		-20	0.699942
chr13_26891_star	0	0.805927	0	0.852739935		-20	0.474469		chr7_16707_star	2.14913986	2.417782	1.654957834	1.773060525		-0.16993	1		chr4_10242_mature	0	0.805927	0	0.852739935		-20	0.699942
mmu-miR-291a-5p	0	0.805927	0	0.852739935		-20	0.474469		rno-miR-93-5p	6.44741959	2.417782	2.896740642	1.773060525		1.415037	1		chr17_35590_star	0	0.805927	0	0.852739935		-20	0.699942
rno-miR-3571	0	0.805927	0	0.852739935		-20	0.474469		chr7_15986_star	2.14913986	2.417782	1.654957834	1.773060525		-0.16993	1		hsa-miR-1471	0	0.805927	0	0.852739935		-20	0.699942
chr13_28489_star	0	0.805927	0	0.852739935		-20	0.474469		chr4_10395_star	4.29827973	1.611855	2.405524014	1.38507475		1.415037	1		chr5_11713_star	0	0.805927	0	0.852739935		-20	0.699942
chr12_25693_star	0	0.805927	0	0.852739935		-20	0.474469		NW_003539007_38445_mature	2.14913986	1.611855	1.654957834	1.38507475		0.415037	1		bta-miR-2320-5p	0	0.805927	0	0.852739935		-20	0.699942
rno-miR-3595	0	0.805927	0	0.852739935		-20	0.474469		chr1_3069_star	2.14913986	2.417782	1.654957834	1.773060525		-0.16993	1		hsa-miR-384	0	0.805927	0	0.852739935		-20	0.699942
hsa-miR-660-3p	0	0.805927	0	0.852739935		-20	0.474469		chr1_2009_star	2.14913986	1.611855	1.654957834	1.38507475		0.415037	1		mmu-miR-5107-5p	0	0.805927	0	0.852739935		-20	0.699942
chr3_6871_mature	0	0.805927	0	0.852739935		-20	0.474469		chr4_10161_star	4.29827973	13.70077	2.405524014	3.877819487		-1.67243	1		chr11_25193_star	0	0.805927	0	0.852739935		-20	0.699942
chr1_3253_star	0	0.805927	0	0.852739935		-20	0.474469		chr3_8430_star	2.14913986	1.611855	1.654957834	1.38507475		0.415037	1		hsa-miR-5579-5p	0	0.805927	0	0.852739935		-20	0.699942
hsa-miR-3622b-5p	0	0.805927	0	0.852739935		-20	0.474469		chr16_34527_star@@hsa-miR-449b-3	2.14913986	1.611855	1.654957834	1.38507475		0.415037	1		rno-miR-494-5p	0	0.805927	0	0.852739935		-20	0.699942
hsa-miR-4726-3p	0	0.805927	0	0.852739935		-20	0.474469		chr6_14340_star	2.14913986	1.611855	1.654957834	1.38507475		0.415037	1		hsa-miR-519d	0	0.805927	0	0.852739935		-20	0.699942
mmu-miR-5127	0	0.805927	0	0.852739935		-20	0.474469		chr5_11369_mature	17.1931189	7.253347	4.185320985	3.044979305		1.245112	1		chr9_20876_star	0	0.805927	0	0.852739935		-20	0.699942
chr8_17918_star	0	0.805927	0	0.852739935		-20	0.474469		NW_003538949_38371_star	4.29827973	1.611855	2.405524014	1.38507475		1.415037	1		rno-miR-3577	0	0.805927	0	0.852739935		-20	0.699942
bta-miR-133a	0	0.805927	0	0.852739935		-20	0.474469		chr17_34938_mature	21.4913986	41.1023	4.491301473	5.395827139		-0.93546	1		mmu-let-7g-3p	0	0.805927	0	0.852739935		-20	0.699942
hsa-miR-3690	0	0.805927	0	0.852739935		-20	0.474469		hsa-miR-4665-5p	4.29827973	1.611855	2.405524014	1.38507475		1.415037	1		chr12_25693_star	0	0.805927	0	0.852739935		-20	0.699942
bta-miR-133b	0	0.805927	0	0.852739935		-20	0.474469		hsa-miR-4772-3p	2.14913986	1.611855	1.654957834	1.38507475		0.415037	1		rno-miR-3595	0	0.805927	0	0.852739935		-20	0.699942
hsa-miR-4745-3p	0	0.805927	0	0.852739935		-20	0.474469		chr17_35296_star	4.29827973	1.611855	2.405524014	1.38507475		1.415037	1		hsa-miR-3622b-5p	0	0.805927	0	0.852739935		-20	0.699942
chr9_20117_star	0	0.805927	0	0.852739935		-20	0.474469		chr15_32775_star	2.14913986	2.417782	1.654957834	1.773060525		-0.16993	1		mmu-miR-5128	0	0.805927	0	0.852739935		-20	0.699942
hsa-miR-3686	0	0.805927	0	0.852739935		-20	0.474469		chr15_31930_mature	2.14913986	1.611855	1.654957834	1.38507475		0.415037	1		bta-miR-133b	0	0.805927	0	0.852739935		-20	0.699942
chr13_28617_star	0	0.805927	0	0.852739935		-20	0.474469		chr14_29940_mature	8.59655945	3.22371	3.262517265	2.078510713		1.415037	1		bta-miR-935	0	0.805927	0	0.852739935		-20	0.699942
hsa-miR-4638-3p	0	0.805927	0	0.852739935		-20	0.474469		chr1_2783_star	4.29827973	1.611855	2.405524014	1.38507475		1.415037	1		chr2_4549_star	0	0.805927	0	0.852739935		-20	0.699942
chr8_18866_star	0	0.805927	0	0.852739935		-20	0.474469		chr1_3471_mature	8.59655945	3.22371	3.262517265	2.078510713		1.415037	1		NW_003537737_37236_star	0	0.805927	0	0.852739935		-20	0.699942
chr1_918_mature	0	0.805927	0	0.852739935		-20	0.474469		chr7_16673_mature@@mmu-miR-6399	2.14913986	1.611855	1.654957834	1.38507475		0.415037	1		chr13_28169_star	0	0.805927	0	0.852739935		-20	0.699942
mmu-miR-3073b-3p	0	0.805927	0	0.852739935		-20	0.474469		chr15_33146_mature	4.29827973	13.70077	2.405524014	3.877819487		-1.67243	1		chr3_8228_star	0	0.805927	0	0.852739935		-20	0.699942
rno-miR-6334	0	0.805927	0	0.852739935		-20	0.474469		chr1_766_mature	4.29827973	1.611855	2.405524014	1.38507475		1.415037	1		hsa-miR-4707-3p	0	0.805927	0	0.852739935		-20	0.699942
rno-miR-6333	0	0.805927	0	0.852739935		-20	0.474469		bta-miR-500	2.14913986	2.417782	1.654957834	1.773060525		-0.16993	1		mmu-miR-101a-3p	0	0.805927	0	0.852739935		-20	0.699942
rno-miR-6324	0	0.805927	0	0.852739935		-20	0.474469		chr15_33643_star	2.14913986	1.611855	1.654957834	1.38507475		0.415037	1		chr6_12501_star	0	0.805927	0	0.852739935		-20	0.699942
chr10_23316_star	0	0.805927	0	0.852739935		-20	0.474469		chr6_12771_star	4.29827973	1.611855	2.405524014	1.38507475		1.415037	1		chr7_17143_star	0	0.805927	0	0.852739935		-20	0.699942
bta-miR-935	0	0.805927	0	0.852739935		-20	0.474469		chrx_22414_star	2.14913986	8.865202	1.654957834	3.302348583		-2.04439	1		hsa-miR-1292-3p	0	0.805927	0	0.852739935		-20	0.699942
hsa-miR-3200-3p	0	0.805927	0	0.852739935		-20	0.474469		chr14_31128_star	2.14913986	2.417782	1.654957834	1.773060525		-0.16993	1		hsa-miR-6502-3p	0	0.805927	0	0.852739935		-20	0.699942
mmu-miR-701-5p	0	0.805927	0	0.852739935		-20	0.474469		mmu-miR-3093-3p	4.29827973	1.611855	2.405524014	1.38507475		1.415037	1		chr13_27252_star	0	0.805927	0	0.852739935		-20	0.699942
chr2_6405_star	0	0.805927	0	0.852739935		-20	0.474469		chr8_18422_star	2.14913986	1.611855	1.654957834	1.38507475		0.415037	1		chr1_943_star	0	0.805927	0	0.852739935		-20	0.699942
chr1_3835_star	0	0.805927	0	0.852739935		-20	0.474469		chr3_7189_star	4.29827973	1.611855	2.405524014	1.38507475		1.415037	1		mmu-miR-669d-5p	0	0.805927	0	0.852739935		-20	0.699942
hsa-miR-675-5p	0	0.805927	0	0.852739935		-20	0.474469		chr17_35675_mature	8.59655945	3.22371	3.262517265	2.078510713		1.415037	1		chr10_23941_star	0	0.805927	0	0.852739935		-20	0.699942
NW_003613497_39430_star	0	0.805927	0	0.852739935		-20	0.474469		chr1_551_mature	4.29827973	1.611855	2.405524014	1.38507475		1.415037	1		rno-miR-30e-3p	0	0.805927	0	0.852739935		-20	0.699942
mmu-miR-183-3p	0	0.805927	0	0.852739935		-20	0.474469		chr9_20549_mature	2.14913986	10.47706	1.654957834	3.520680821		-2.2854	1		hsa-miR-301a-3p	0	0.805927	0	0.852739935		-20	0.699942
mmu-miR-3070b-3p	0	0.805927	0	0.852739935		-20	0.474469		NW_003539513_38909_star	2.14913986	2.417782	1.654957834	1.773060525		-0.16993	1		rno-miR-500-3p	0	0.805927	0	0.852739935		-20	0.699942
mmu-miR-6392-3p	0	0.805927	0	0.852739935		-20	0.474469		chr18_35894_star	6.44741959	2.417782	2.896740642	1.773060525		1.415037	1		hsa-miR-526b-3p	0	0.805927	0	0.852739935		-20	0.699942
bta-miR-2385-5p	0	0.805927	0	0.852739935		-20	0.474469		chr18_36180_mature	4.29827973	14.50669	2.405524014	3.954819241		-1.75489	1		hsa-miR-4690-5p	0	0.805927	0	0.852739935		-20	0.699942
chr4_9830_star	0	0.805927	0	0.852739935		-20	0.474469		chr4_10175_star	2.14913986	1.611855	1.654957834	1.38507475		0.415037	1		hsa-miR-185-5p	0	0.805927	0	0.852739935		-20	0.699942
chr14_30472_mature	0	0.805927	0	0.852739935		-20	0.474469		chr6_12823_star	2.14913986	2.417782	1.654957834	1.773060525		-0.16993	1		chr13_29007_star	0	0.805927	0	0.852739935		-20	0.699942
chr2_5583_star	0	0.805927	0	0.852739935		-20	0.474469		chr2_4773_mature	2.14913986	2.417782	1.654957834	1.773060525		-0.16993	1		bta-miR-449b	0	0.805927	0	0.852739935		-20	0.699942
chr12_25520_star	0	0.805927	0	0.852739935		-20	0.474469		chr10_23364_star	2.14913986	1.611855	1.654957834	1.38507475		0.415037	1		hsa-miR-20b-3p	0	0.805927	0	0.852739935		-20	0.699942
NW_003537737_37236_star	0	0.805927	0	0.852739935		-20	0.474469		chr14_31302_star	2.14913986	2.417782	1.654957834	1.773060525		-0.16993	1		chr12_25705_star	0	0.805927	0	0.852739935		-20	0.699942
mmu-miR-574-5p	0	0.805927	0	0.852739935		-20	0.474469		chr5_12232_star	4.29827973	1.611855	2.405524014	1.38507475		1.415037	1		hsa-miR-1183	0	0.805927	0	0.852739935		-20	0.699942
hsa-miR-4646-5p	0	0.805927	0	0.852739935		-20	0.474469		chr7_17170_star	2.14913986	1.611855	1.654957834	1.38507475		0.415037	1		chr12_25405_mature	0	0.805927	0	0.852739935		-20	0.699942
hsa-miR-4433-3p	0	0.805927	0	0.852739935		-20	0.474469		bta-miR-488	2.14913986	1.611855	1.654957834	1.38507475		0.415037	1		hsa-miR-1197	0	0.805927	0	0.852739935		-20	0.699942
chr9_20772_star	0	0.805927	0	0.852739935		-20	0.474469		chr4_10088_star	4.29827973	1.611855	2.405524014	1.38507475		1.415037	1		chr7_15367_star	0	0.805927	0	0.852739935		-20	0.699942
chrx_22074_star	0	0.805927	0	0.852739935		-20	0.474469		chr1_244_star	2.14913986	2.417782	1.654957834	1.773060525		-0.16993	1		chr14_31221_star	0	0.805927	0	0.852739935		-20	0.699942
NW_003538329_37725_mature	0	0.805927	0	0.852739935		-20	0.474469		chr12_26142_star	2.14913986	2.417782	1.654957834	1.773060525		-0.16993	1		mmu-miR-301b-3p	0	0.805927	0	0.852739935		-20	0.699942
rno-miR-344a	0	0.805927	0	0.852739935		-20	0.474469		chr1_79_mature	4.29827973	1.611855	2.405524014	1.38507475		1.415037	1		hsa-miR-3191-3p	0	0.805927	0	0.852739935		-20	0.699942
NW_003538922_38349_star	0	0.805927	0	0.852739935		-20	0.474469		mmu-miR-1897-3p	2.14913986	1.611855	1.654957834	1.38507475		0.415037	1		bta-miR-582	0	0.805927	0	0.852739935		-20	0.699942
NW_003613367_38733_star	0	0.805927	0	0.852739935		-20	0.474469		chr2_5942_star	2.14913986	2.417782	1.654957834	1.773060525		-0.16993	1		mmu-miR-6341	0	0.805927	0	0.852739935		-20	0.699942
chr12_26570_star	0	0.805927	0	0.852739935		-20	0.474469		chr9_20034_star	2.14913986	1.611855	1.654957834	1.38507475		0.415037	1		chr11_25111_star	0	0.805927	0	0.852739935		-20	0.699942
chr1_3025_mature	0	0.805927	0	0.852739935		-20	0.474469		chr2_6406_star	2.14913986	2.417782	1.654957834	1.773060525		-0.16993	1		hsa-miR-5006-5p	0	0.805927	0	0.852739935		-20	0.699942
NW_003538331_37733_star	0	0.805927	0	0.852739935		-20	0.474469		chr1_1411_mature	2.14913986	1.611855	1.654957834	1.38507475		0.415037	1		mmu-miR-6353	0	0.805927	0	0.852739935		-20	0.699942
hsa-miR-4707-3p	0	0.805927	0	0.852739935		-20	0.474469		chr9_19985_mature	2.14913986	1.611855	1.654957834	1.38507475		0.415037	1		NW_003613057_36932_mature	0	0.805927	0	0.852739935		-20	0.699942
chr1_3923_star	0	0.805927	0	0.852739935		-20	0.474469		hsa-miR-4684-5p	2.14913986	1.611855	1.654957834	1.38507475		0.415037	1		rno-miR-185-5p	0	0.805927	0	0.852739935		-20	0.699942
hsa-miR-3679-5p	0	0.805927	0	0.852739935		-20	0.474469		chr1_927_star	8.59655945	3.22371	3.262517265	2.078510713		1.415037	1		rno-miR-98-3p	0	0.805927	0	0.852739935		-20	0.699942
hsa-miR-4707-5p	0	0.805927	0	0.852739935		-20	0.474469		chr7_16061_mature	8.59655945	3.22371	3.262517265	2.078510713		1.415037	1		bta-miR-2284l	0	0.805927	0	0.852739935		-20	0.699942
chr4_9919_star	0	0.805927	0	0.852739935		-20	0.474469		chr17_34922_star	17.1931189	7.253347	4.185320985	3.044979305		1.245112	1		bta-miR-2284p	0	0.805927	0	0.852739935		-20	0.699942
mmu-miR-101a-3p	0	0.805927	0	0.852739935		-20	0.474469		chr16_34150_star	4.29827973	1.611855	2.405524014	1.38507475		1.415037	1		rno-miR-218b	0	0.805927	0	0.852739935		-20	0.699942
chr6_12449_star	0	0.805927	0	0.852739935		-20	0.474469		chr8_18481_star	2.14913986	1.611855	1.654957834	1.38507475		0.415037	1		rno-miR-361-5p	0	0.805927	0	0.852739935		-20	0.699942
mmu-miR-1966-5p	0	0.805927	0	0.852739935		-20	0.474469		chr15_32787_star	2.14913986	1.611855	1.654957834	1.38507475		0.415037	1		bta-miR-2903	0	0.805927	0	0.852739935		-20	0.699942
NW_003538922_38350_star	0	0.805927	0	0.852739935		-20	0.474469		bta-miR-2405	2.14913986	2.417782	1.654957834	1.773060525		-0.16993	1		rno-miR-130b-3p	0	0.805927	0	0.852739935		-20	0.699942
hsa-miR-5047	0	0.805927	0	0.852739935		-20	0.474469		bta-miR-2406	2.14913986	2.417782	1.654957834	1.773060525		-0.16993	1		mmu-miR-708-3p	0	0.805927	0	0.852739935		-20	0.699942
chr13_28323_star	0	0.805927	0	0.852739935		-20	0.474469		bta-miR-30e-5p	2.14913986	1.611855	1.654957834	1.38507475		0.415037	1		hsa-miR-188-3p	0	0.805927	0	0.852739935		-20	0.699942
hsa-miR-570-5p	0	0.805927	0	0.852739935		-20	0.474469		chrx_22973_mature	2.14913986	1.611855	1.654957834	1.38507475		0.415037	1		chr3_6931_star	0	0.805927	0	0.852739935		-20	0.699942
hsa-miR-371b-5p	0	0.805927	0	0.852739935		-20	0.474469		bta-miR-2451	2.14913986	2.417782	1.654957834	1.773060525		-0.16993	1		mmu-miR-489-3p	0	0.805927	0	0.852739935		-20	0.699942
rno-miR-105	0	0.805927	0	0.852739935		-20	0.474469		chr15_33015_star	2.14913986	1.611855	1.654957834	1.38507475		0.415037	1		chr18_36523_star	0	0.805927	0	0.852739935		-20	0.699942
bta-miR-1185	0	0.805927	0	0.852739935		-20	0.474469		bta-miR-2468	2.14913986	2.417782	1.654957834	1.773060525		-0.16993	1		chr2_4081_star	0	0.805927	0	0.852739935		-20	0.699942
rno-miR-1249	0	0.805927	0	0.852739935		-20	0.474469		mmu-miR-3069-3p	4.29827973	1.611855	2.405524014	1.38507475		1.415037	1		hsa-miR-5572	0	0.805927	0	0.852739935		-20	0.699942
chr6_14382_star	0	0.805927	0	0.852739935		-20	0.474469		ssc-miR-421-5p	8.59655945	3.22371	3.262517265	2.078510713		1.415037	1		hsa-miR-501-5p	0	0.805927	0	0.852739935		-20	0.699942
chr2_6234_star	0	0.805927	0	0.852739935		-20	0.474469		chr14_29999_star	2.14913986	1.611855	1.654957834	1.38507475		0.415037	1		chr2_6477_star	0	0.805927	0	0.852739935		-20	0.699942

rno-miR-3584-3p	0	0.805927	0	0.852739935		-20	0.474469	hsa-miR-4708-5p	2.14913986	2.417782	1.654957834	1.773060525		-0.16993	1		hsa-miR-6126	0	0.805927	0	0.852739935		-20	0.699942
chr9_21032_star	0	0.805927	0	0.852739935		-20	0.474469	chr13_27847_mature	8.59655945	3.22371	3.262517265	2.078510713		1.415037	1		NW_003613380_38805_star	0	0.805927	0	0.852739935		-20	0.699942
hsa-miR-5008-3p	0	0.805927	0	0.852739935		-20	0.474469	chr9_19299_star	4.29827973	1.611855	2.405524014	1.38507475		1.415037	1		chr2_4701_star	0	0.805927	0	0.852739935		-20	0.699942
hsa-miR-6502-3p	0	0.805927	0	0.852739935		-20	0.474469	chr5_10732_star	4.29827973	1.611855	2.405524014	1.38507475		1.415037	1		chr4_10371_star	0	0.805927	0	0.852739935		-20	0.699942
chr13_27252_star	0	0.805927	0	0.852739935		-20	0.474469	hsa-miR-4425	2.14913986	1.611855	1.654957834	1.38507475		0.415037	1		hsa-miR-500a-3p	0	0.805927	0	0.852739935		-20	0.699942
mmu-miR-295-5p	0	0.805927	0	0.852739935		-20	0.474469	chr6_13459_mature	17.1931189	7.253347	4.185320985	3.044979305		1.245112	1		bta-miR-191	0	0.805927	0	0.852739935		-20	0.699942
mmu-miR-574-3p	0	0.805927	0	0.852739935		-20	0.474469	bta-miR-2475	2.14913986	1.611855	1.654957834	1.38507475		0.415037	1		chr14_29542_star	0	0.805927	0	0.852739935		-20	0.699942
NW_003613097_37151_star	0	0.805927	0	0.852739935		-20	0.474469	bta-miR-2482	4.29827973	1.611855	2.405524014	1.38507475		1.415037	1		chr11_25215_star	0	0.805927	0	0.852739935		-20	0.699942
chr11_24582_star	0	0.805927	0	0.852739935		-20	0.474469	hsa-miR-601	2.14913986	8.865202	1.654957834	3.302348583		-2.04439	1		mmu-miR-344c-5p	0	0.805927	0	0.852739935		-20	0.699942
NW_003613310_38408_star	0	0.805927	0	0.852739935		-20	0.474469	bta-miR-2368-3p	2.14913986	8.865202	1.654957834	3.302348583		-2.04439	1		chr14_31026_mature	0	0.805927	0	0.852739935		-20	0.699942
chr1_943_star	0	0.805927	0	0.852739935		-20	0.474469	chr15_31849_star	2.14913986	1.611855	1.654957834	1.38507475		0.415037	1		hsa-miR-6511a-5p	0	0.805927	0	0.852739935		-20	0.699942
mmu-miR-194-1-3p	0	0.805927	0	0.852739935		-20	0.474469	hsa-miR-612	4.29827973	1.611855	2.405524014	1.38507475		1.415037	1		hsa-miR-3689b-3p	0	0.805927	0	0.852739935		-20	0.699942
rno-miR-184	0	0.805927	0	0.852739935		-20	0.474469	hsa-miR-6509-5p	2.14913986	1.611855	1.654957834	1.38507475		0.415037	1		hsa-miR-519a-3p	0	0.805927	0	0.852739935		-20	0.699942
hsa-miR-301a-3p	0	0.805927	0	0.852739935		-20	0.474469	chr14_29861_star	2.14913986	1.611855	1.654957834	1.38507475		0.415037	1		hsa-miR-548z	0	0.805927	0	0.852739935		-20	0.699942
mmu-miR-6405	0	0.805927	0	0.852739935		-20	0.474469	chr1_1185_star	2.14913986	2.417782	1.654957834	1.773060525		-0.16993	1		chr13_27728_mature	0	0.805927	0	0.852739935		-20	0.699942
chr7_15311_star	0	0.805927	0	0.852739935		-20	0.474469	chr14_30455_star	2.14913986	2.417782	1.654957834	1.773060525		-0.16993	1		bta-miR-27b	0	0.805927	0	0.852739935		-20	0.699942
chr2_4916_mature	0	0.805927	0	0.852739935		-20	0.474469	hsa-miR-620	2.14913986	8.865202	1.654957834	3.302348583		-2.04439	1		rno-miR-100-5p	0	0.805927	0	0.852739935		-20	0.699942
hsa-miR-526b-3p	0	0.805927	0	0.852739935		-20	0.474469	bta-miR-2390	2.14913986	2.417782	1.654957834	1.773060525		-0.16993	1		hsa-miR-5002-3p	0	0.805927	0	0.852739935		-20	0.699942
chr5_11374_star	0	0.805927	0	0.852739935		-20	0.474469	chr13_27833_star	4.29827973	1.611855	2.405524014	1.38507475		1.415037	1		chr12_26382_star	0	0.805927	0	0.852739935		-20	0.699942
hsa-miR-185-5p	0	0.805927	0	0.852739935		-20	0.474469	hsa-miR-4665-3p	4.29827973	1.611855	2.405524014	1.38507475		1.415037	1		chr15_32157_star	0	0.805927	0	0.852739935		-20	0.699942
bta-miR-449b	0	0.805927	0	0.852739935		-20	0.474469	chr12_26515_star	2.14913986	1.611855	1.654957834	1.38507475		0.415037	1		chr1_1837_star	0	0.805927	0	0.852739935		-20	0.699942
chrx_22559_star	0	0.805927	0	0.852739935		-20	0.474469	hsa-miR-4495	2.14913986	1.611855	1.654957834	1.38507475		0.415037	1		mmu-miR-181a-2-3p	0	0.805927	0	0.852739935		-20	0.699942
chrx_22707_star	0	0.805927	0	0.852739935		-20	0.474469	hsa-miR-4498	2.14913986	2.417782	1.654957834	1.773060525		-0.16993	1		hsa-miR-149-5p	0	0.805927	0	0.852739935		-20	0.699942
chr3_7967_mature	0	0.805927	0	0.852739935		-20	0.474469	chr7_15958_mature	2.14913986	1.611855	1.654957834	1.38507475		0.415037	1		bta-miR-432	0	0.805927	0	0.852739935		-20	0.699942
hsa-miR-486-5p	0	0.805927	0	0.852739935		-20	0.474469	chr1_3559_star	2.14913986	2.417782	1.654957834	1.773060525		-0.16993	1		bta-miR-335	0	0.805927	0	0.852739935		-20	0.699942
hsa-miR-3689d	0	0.805927	0	0.852739935		-20	0.474469	chrx_22620_star	17.1931189	7.253347	4.185320985	3.044979305		1.245112	1		bta-miR-340	0	0.805927	0	0.852739935		-20	0.699942
chr10_24027_star	0	0.805927	0	0.852739935		-20	0.474469	hsa-miR-3156-3p	2.14913986	1.611855	1.654957834	1.38507475		0.415037	1		chr9_19936_star	0	0.805927	0	0.852739935		-20	0.699942
hsa-miR-1179	0	0.805927	0	0.852739935		-20	0.474469	chr13_28063_mature	4.29827973	1.611855	2.405524014	1.38507475		1.415037	1		chr12_25400_mature	0	0.805927	0	0.852739935		-20	0.699942
chr1_2562_star	0	0.805927	0	0.852739935		-20	0.474469	ssc-miR-376a-5p	15.043979	6.44742	4.003960082	2.896740642		1.222392	1		chr4_10459_mature	0	0.805927	0	0.852739935		-20	0.699942
hsa-miR-1183	0	0.805927	0	0.852739935		-20	0.474469	mmu-miR-599	2.14913986	1.611855	1.654957834	1.38507475		0.415037	1		chr1_1289_star	0	0.805927	0	0.852739935		-20	0.699942
hsa-miR-676-5p	0	0.805927	0	0.852739935		-20	0.474469	chr5_12327_star	2.14913986	2.417782	1.654957834	1.773060525		-0.16993	1		chr5_11717_star	0	0.805927	0	0.852739935		-20	0.699942
chr7_16996_star	0	0.805927	0	0.852739935		-20	0.474469	mmu-miR-5623-3p	2.14913986	2.417782	1.654957834	1.773060525		-0.16993	1		hsa-miR-2054	0	0.805927	0	0.852739935		-20	0.699942
hsa-miR-1197	0	0.805927	0	0.852739935		-20	0.474469	chr11_24450_star	4.29827973	1.611855	2.405524014	1.38507475		1.415037	1		mmu-miR-206-3p	0	0.805927	0	0.852739935		-20	0.699942
hsa-miR-4731-5p	0	0.805927	0	0.852739935		-20	0.474469	chr6_14411_star	4.29827973	1.611855	2.405524014	1.38507475		1.415037	1		chr14_30535_star	0	0.805927	0	0.852739935		-20	0.699942
bta-miR-2448-3p	0	0.805927	0	0.852739935		-20	0.474469	chr14_29589_star	2.14913986	1.611855	1.654957834	1.38507475		0.415037	1		NW_003613206_37650_star	0	0.805927	0	0.852739935		-20	0.699942
hsa-miR-362-5p	0	0.805927	0	0.852739935		-20	0.474469	chrx_22851_star	6.44741959	2.417782	2.896740642	1.773060525		1.415037	1		rno-miR-488-3p	0	0.805927	0	0.852739935		-20	0.699942
hsa-miR-676-3p	0	0.805927	0	0.852739935		-20	0.474469	hsa-miR-193a-3p	2.14913986	1.611855	1.654957834	1.38507475		0.415037	1		rno-miR-299b-5p	0	0.805927	0	0.852739935		-20	0.699942
chr14_30876_star	0	0.805927	0	0.852739935		-20	0.474469	chr13_27132_star	2.14913986	2.417782	1.654957834	1.773060525		-0.16993	1		hsa-miR-29c-5p	0	0.805927	0	0.852739935		-20	0.699942
hsa-miR-3191-3p	0	0.805927	0	0.852739935		-20	0.474469	ssc-miR-376c	2.14913986	1.611855	1.654957834	1.38507475		0.415037	1		rno-miR-365-3p	0	0.805927	0	0.852739935		-20	0.699942
mmu-miR-876-5p	0	0.805927	0	0.852739935		-20	0.474469	chr7_16797_mature	2.14913986	9.671129	1.654957834	3.415640968		-2.16993	1		rno-miR-125b-5p	0	0.805927	0	0.852739935		-20	0.699942
chr1_2402_star	0	0.805927	0	0.852739935		-20	0.474469	chrx_22198_star	2.14913986	2.417782	1.654957834	1.773060525		-0.16993	1		NW_003613233_37802_mature	0	0.805927	0	0.852739935		-20	0.699942
chr7_16801_star	0	0.805927	0	0.852739935		-20	0.474469	chr14_29583_star	4.29827973	1.611855	2.405524014	1.38507475		1.415037	1		rno-miR-511-3p	0	0.805927	0	0.852739935		-20	0.699942
hsa-miR-4482-5p	0	0.805927	0	0.852739935		-20	0.474469	ssc-miR-126-3p	103.158713	147.4847	6.702639724	7.214170697		-0.5157	1		bta-miR-3578	0	0.805927	0	0.852739935		-20	0.699942
chr11_25111_star	0	0.805927	0	0.852739935		-20	0.474469	chr1_2672_star	2.14913986	2.417782	1.654957834	1.773060525		-0.16993	1		chr9_19756_star	0	0.805927	0	0.852739935		-20	0.699942
hsa-miR-5006-5p	0	0.805927	0	0.852739935		-20	0.474469	chr11_24871_star	2.14913986	2.417782	1.654957834	1.773060525		-0.16993	1		bta-miR-2455	0	0.805927	0	0.852739935		-20	0.699942
NW_003613379_38761_star	0	0.805927	0	0.852739935		-20	0.474469	chr16_34452_mature	15.043979	6.44742	4.003960082	2.896740642		1.222392	1		mmu-miR-201-3p	0	0.805927	0	0.852739935		-20	0.699942
NW_003613231_37780_mature	0	0.805927	0	0.852739935		-20	0.474469	chr1_2055_mature	17.1931189	8.059274	4.185320985	3.179395517		1.093109	1		mmu-miR-5627-5p	0	0.805927	0	0.852739935		-20	0.699942
hsa-miR-548x-5p	0	0.805927	0	0.852739935		-20	0.474469	chr1_3810_star	2.14913986	2.417782	1.654957834	1.773060525		-0.16993	1		chr2_5364_star	0	0.805927	0	0.852739935		-20	0.699942
mmu-miR-6381	0	0.805927	0	0.852739935		-20	0.474469	bta-miR-3431	12.8948392	4.835565	3.796477232	2.544872269		1.415037	1		hsa-miR-623	0	0.805927	0	0.852739935		-20	0.699942
mmu-miR-6382	0	0.805927	0	0.852739935		-20	0.474469	bta-miR-2464-3p	4.29827973	1.611855	2.405524014	1.38507475		1.415037	1		chr3_7313_star	0	0.805927	0	0.852739935		-20	0.699942
mmu-miR-6378	0	0.805927	0	0.852739935		-20	0.474469	chr14_31060_star	4.29827973	1.611855	2.405524014	1.38507475		1.415037	1		chr13_27068_star	0	0.805927	0	0.852739935		-20	0.699942
mmu-miR-6376	0	0.805927	0	0.852739935		-20	0.474469	NW_003541277_40019_star	2.14913986	2.417782	1.654957834	1.773060525		-0.16993	1		rno-miR-338-5p	0	0.805927	0	0.852739935		-20	0.699942
chr2_4395_star	0	0.805927	0	0.852739935		-20	0.474469	chr7_15096_star	4.29827973	1.611855	2.405524014	1.38507475		1.415037	1		mmu-miR-881-3p	0	0.805927	0	0.852739935		-20	0.699942
rno-miR-185-5p	0	0.805927	0	0.852739935		-20	0.474469	chr13_28068_mature	2.14913986	1.611855	1.654957834	1.38507475		0.415037	1		chr7_15367_mature	0	0.805927	0	0.852739935		-20	0.699942
chr16_34458_star	0	0.805927	0	0.852739935		-20	0.474469	bta-miR-2332	2.14913986	1.611855	1.654957834	1.38507475		0.415037	1		bta-miR-2309	0	0.805927	0	0.852739935		-20	0.699942
bta-miR-2284s	0	0.805927	0	0.852739935		-20	0.474469	bta-miR-2335	4.29827973	1.611855	2.405524014	1.38507475		1.415037	1		bta-miR-2347	0	0.805927	0	0.852739935		-20	0.699942
rno-miR-98-3p	0	0.805927	0	0.852739935		-20	0.474469	bta-miR-2337	2.14913986	8.865202	1.654957834	3.302348583		-2.04439	1		rno-miR-107-5p	0	0.805927	0	0.852739935		-20	0.699942
bta-miR-2284g	0	0.805927	0	0.852739935		-20	0.474469	bta-miR-2348	2.14913986	1.611855	1.654957834	1.38507475		0.415037	1		bta-miR-2378	0	0.805927	0	0.852739935		-20	0.699942
bta-miR-2284l	0	0.805927	0	0.852739935		-20	0.474469	chr6_13330_mature	2.14913986	2.417782	1.654957834	1.773060525		-0.16993	1		chr13_26987_star	0	0.805927	0	0.852739935		-20	0.699942
bta-miR-2284p	0	0.805927	0	0.852739935		-20	0.474469	chr11_24588_mature	10.7456993	4.029637	3.554060706	2.330454351		1.415037	1		chr14_31505_star	0	0.805927	0	0.852739935		-20	0.699942
mmu-miR-6394	0	0.805927	0	0.852739935		-20	0.474469	chr17_35073_mature	6.44741959	2.417782	2.896740642	1.773060525		1.415037	1		bta-miR-2298	0	0.805927	0	0.852739935		-20	0.699942
chr1_3137_star	0	0.805927	0	0.852739935		-20	0.474469	chr7_16069_mature	15.043979	6.44742	4.003960082	2.896740642		1.222392	1		mmu-miR-467g	0	0.805927	0	0.852739935		-20	0.699942
rno-miR-218b	0	0.805927	0	0.852739935		-20	0.474469	chr17_35263_star	2.14913986	1.611855	1.654957834	1.38507475		0.415037	1		rno-miR-3593-3p	0	0.805927	0	0.852739935		-20	0.699942
chr5_12123_star	0	0.805927	0	0.852739935		-20	0.474469	chr14_30180_star	2.14913986	2.417782	1.654957834	1.773060525		-0.16993	1		hsa-miR-193a-5p	0	0.805927	0	0.852739935		-20	0.699942
rno-miR-361-5p	0	0.805927	0	0.852739935		-20	0.474469	bta-miR-2384	2.14913986	2.417782	1.654957834	1.773060525		-0.16993	1		bta-miR-2436-3p	0	0.805927	0	0.852739935		-20	0.699942
[illegible]	[illegible]	[illegible]	[illegible]	[illegible]		[illegible]	[illegible]	[illegible]	[illegible]	[illegible]	[illegible]	[illegible]		[illegible]	[illegible]		[illegible]	[illegible]	[illegible]	[illegible]	[illegible]		[illegible]	[illegible]

NW_003541083_39896_star	0	0.805927	0	0.852739935	-20	0.474469	NW_003537189_36743_mature	10.7456993	4.835565	3.554060706	2.544872269	1.152003	1	chr6_14448_star	0.537285	0.805927	0.620384622	0.852739935	-0.58496	0.70711
bta-miR-2421	0	0.805927	0	0.852739935	-20	0.474469	chr8_18339_mature	2.14913986	3.22371	1.654957834	2.078510713	-0.58496	1	hsa-miR-3127-3p	0.537285	0.805927	0.620384622	0.852739935	-0.58496	0.70711
chr14_30485_star	0	0.805927	0	0.852739935	-20	0.474469	chr13_29308_star	2.14913986	4.835565	1.654957834	2.544872269	-1.16993	1	hsa-miR-3648	0.537285	0.805927	0.620384622	0.852739935	-0.58496	0.70711
bta-miR-2428	0	0.805927	0	0.852739935	-20	0.474469	chr6_12771_mature	4.29827973	8.059274	2.405524014	3.179395517	-0.90689	1	chr12_25415_star	0.537285	0.805927	0.620384622	0.852739935	-0.58496	0.70711
chr13_27739_star	0	0.805927	0	0.852739935	-20	0.474469	chr14_31039_star	4.29827973	6.44742	2.405524014	2.896740642	-0.58496	1	mmu-miR-335-3p	0.537285	0.805927	0.620384622	0.852739935	-0.58496	0.70711
hsa-miR-513a-5p	0	0.805927	0	0.852739935	-20	0.474469	NW_003539007_38450_mature	4.29827973	8.059274	2.405524014	3.179395517	-0.90689	1	NW_003537216_36774_mature	0.537285	0.805927	0.620384622	0.852739935	-0.58496	0.70711
chr9_19756_star	0	0.805927	0	0.852739935	-20	0.474469	chr2_5889_mature	8.59655945	8.059274	3.262517265	3.179395517	0.093109	1	rno-miR-3568	0.537285	0.805927	0.620384622	0.852739935	-0.58496	0.70711
hsa-miR-4796-3p	0	0.805927	0	0.852739935	-20	0.474469	chr9_20900_star	4.29827973	4.029637	2.405524014	2.330454351	0.093109	1	mmu-miR-291a-5p	0.537285	0.805927	0.620384622	0.852739935	-0.58496	0.70711
bta-miR-2457	0	0.805927	0	0.852739935	-20	0.474469	chr3_8448_star	17.1931189	8.865202	4.185320985	3.302348583	0.955606	1	mmu-miR-5133	0.537285	0.805927	0.620384622	0.852739935	-0.58496	0.70711
hsa-miR-4455	0	0.805927	0	0.852739935	-20	0.474469	chr2_4529_star	2.14913986	4.835565	1.654957834	2.544872269	-1.16993	1	mmu-miR-5127	0.537285	0.805927	0.620384622	0.852739935	-0.58496	0.70711
bta-miR-2462	0	0.805927	0	0.852739935	-20	0.474469	chr5_11789_mature	6.44741959	5.641492	2.896740642	2.731507408	0.192645	1	hsa-miR-4638-3p	0.537285	0.805927	0.620384622	0.852739935	-0.58496	0.70711
hsa-miR-4460	0	0.805927	0	0.852739935	-20	0.474469	chr9_19516_mature	2.14913986	4.835565	1.654957834	2.544872269	-1.16993	1	hsa-miR-1233-3p	0.537285	0.805927	0.620384622	0.852739935	-0.58496	0.70711
bta-miR-2430	0	0.805927	0	0.852739935	-20	0.474469	NW_003540557_39543_mature	15.043979	9.671129	4.003960082	3.415640968	0.63743	1	rno-miR-6334	0.537285	0.805927	0.620384622	0.852739935	-0.58496	0.70711
bta-miR-2432	0	0.805927	0	0.852739935	-20	0.474469	chr1_1479_star	8.59655945	4.029637	3.262517265	2.330454351	1.093109	1	rno-miR-6333	0.537285	0.805927	0.620384622	0.852739935	-0.58496	0.70711
bta-miR-2449	0	0.805927	0	0.852739935	-20	0.474469	chr15_33130_mature	2.14913986	3.22371	1.654957834	2.078510713	-0.58496	1	hsa-miR-3200-5p	0.537285	0.805927	0.620384622	0.852739935	-0.58496	0.70711
hsa-miR-4524b-3p	0	0.805927	0	0.852739935	-20	0.474469	chr3_8052_mature	4.29827973	4.835565	2.405524014	2.544872269	-0.16993	1	rno-miR-6324	0.537285	0.805927	0.620384622	0.852739935	-0.58496	0.70711
bta-miR-2442	0	0.805927	0	0.852739935	-20	0.474469	chr1_3875_star	4.29827973	4.029637	2.405524014	2.330454351	0.093109	1	chr10_23316_star	0.537285	0.805927	0.620384622	0.852739935	-0.58496	0.70711
bta-miR-2441	0	0.805927	0	0.852739935	-20	0.474469	hsa-miR-1258	4.29827973	6.44742	2.405524014	2.896740642	-0.58496	1	chr9_20301_star	0.537285	0.805927	0.620384622	0.852739935	-0.58496	0.70711
hsa-miR-4483	0	0.805927	0	0.852739935	-20	0.474469	chr5_12114_star@@hsa-miR-141-5p	6.44741959	3.22371	2.896740642	2.078510713	1	1	chr4_8702_mature	0.537285	0.805927	0.620384622	0.852739935	-0.58496	0.70711
chr13_26883_mature	0	0.805927	0	0.852739935	-20	0.474469	chr17_35548_mature	6.44741959	13.70077	2.896740642	3.877819487	-1.08746	1	chr2_6405_star	0.537285	0.805927	0.620384622	0.852739935	-0.58496	0.70711
hsa-miR-4421	0	0.805927	0	0.852739935	-20	0.474469	hsa-miR-1246	4.29827973	9.671129	2.405524014	3.415640968	-1.16993	1	chr1_3835_star	0.537285	0.805927	0.620384622	0.852739935	-0.58496	0.70711
hsa-miR-4432	0	0.805927	0	0.852739935	-20	0.474469	chr5_12276_mature	4.29827973	8.059274	2.405524014	3.179395517	-0.90689	1	mmu-miR-224-5p	0.537285	0.805927	0.620384622	0.852739935	-0.58496	0.70711
mmu-miR-201-5p	0	0.805927	0	0.852739935	-20	0.474469	NW_003540351_39435_mature	4.29827973	10.47706	2.405524014	3.520680821	-1.2854	1	NW_003613497_39430_star	0.537285	0.805927	0.620384622	0.852739935	-0.58496	0.70711
bta-miR-2480	0	0.805927	0	0.852739935	-20	0.474469	chrx_22099_mature	8.59655945	8.059274	3.262517265	3.179395517	0.093109	1	mmu-miR-3070b-3p	0.537285	0.805927	0.620384622	0.852739935	-0.58496	0.70711
hsa-miR-603	0	0.805927	0	0.852739935	-20	0.474469	chr15_31926_mature	4.29827973	4.029637	2.405524014	2.330454351	0.093109	1	chr14_29946_star	0.537285	0.805927	0.620384622	0.852739935	-0.58496	0.70711
hsa-miR-608	0	0.805927	0	0.852739935	-20	0.474469	chr7_16542_mature@@bta-miR-1721	4.29827973	2.417782	2.405524014	1.773060525	0.830075	1	chr4_9830_star	0.537285	0.805927	0.620384622	0.852739935	-0.58496	0.70711
chr10_23795_mature	0	0.805927	0	0.852739935	-20	0.474469	chr2_4423_star	4.29827973	4.029637	2.405524014	2.330454351	0.093109	1	chr2_5583_star	0.537285	0.805927	0.620384622	0.852739935	-0.58496	0.70711
hsa-miR-4496	0	0.805927	0	0.852739935	-20	0.474469	chr4_9773_star	8.59655945	4.029637	3.262517265	2.330454351	1.093109	1	hsa-miR-3913-5p	0.537285	0.805927	0.620384622	0.852739935	-0.58496	0.70711
chr13_27068_star	0	0.805927	0	0.852739935	-20	0.474469	chr1_2014_mature	8.59655945	11.28298	3.262517265	3.618589217	-0.39232	1	hsa-miR-4646-5p	0.537285	0.805927	0.620384622	0.852739935	-0.58496	0.70711
rno-miR-338-5p	0	0.805927	0	0.852739935	-20	0.474469	chr6_14501_mature	6.44741959	9.671129	2.896740642	3.415640968	-0.58496	1	NW_003538329_37725_mature	0.537285	0.805927	0.620384622	0.852739935	-0.58496	0.70711
chr16_34408_star	0	0.805927	0	0.852739935	-20	0.474469	hsa-miR-23b-5p	6.44741959	5.641492	2.896740642	2.731507408	0.192645	1	rno-miR-344a	0.537285	0.805927	0.620384622	0.852739935	-0.58496	0.70711
chr3_6909_star	0	0.805927	0	0.852739935	-20	0.474469	chr5_11269_star	4.29827973	4.029637	2.405524014	2.330454351	0.093109	1	chr11_24821_mature	0.537285	0.805927	0.620384622	0.852739935	-0.58496	0.70711
hsa-miR-1825	0	0.805927	0	0.852739935	-20	0.474469	chr2_6087_mature	2.14913986	7.253347	1.654957834	3.044979305	-1.75489	1	chr18_36618_star	0.537285	0.805927	0.620384622	0.852739935	-0.58496	0.70711
chr5_11345_star	0	0.805927	0	0.852739935	-20	0.474469	chr2_4631_star	2.14913986	4.835565	1.654957834	2.544872269	-1.16993	1	hsa-miR-3679-5p	0.537285	0.805927	0.620384622	0.852739935	-0.58496	0.70711
bta-miR-2329-3p	0	0.805927	0	0.852739935	-20	0.474469	chr14_29850_star	4.29827973	3.22371	2.405524014	2.078510713	0.415037	1	hsa-miR-4707-5p	0.537285	0.805927	0.620384622	0.852739935	-0.58496	0.70711
mmu-miR-881-5p	0	0.805927	0	0.852739935	-20	0.474469	chr6_12563_mature	8.59655945	16.92448	3.262517265	4.163859073	-0.97728	1	chr4_9919_star	0.537285	0.805927	0.620384622	0.852739935	-0.58496	0.70711
chr2_3985_star	0	0.805927	0	0.852739935	-20	0.474469	chr2_5588_mature	6.44741959	4.029637	2.896740642	2.330454351	0.678072	1	NW_003538922_38350_star	0.537285	0.805927	0.620384622	0.852739935	-0.58496	0.70711
hsa-miR-4640-5p	0	0.805927	0	0.852739935	-20	0.474469	chr5_10975_mature	4.29827973	2.417782	2.405524014	1.773060525	0.830075	1	hsa-miR-5047	0.537285	0.805927	0.620384622	0.852739935	-0.58496	0.70711
chr15_33434_star	0	0.805927	0	0.852739935	-20	0.474469	chr17_35522_star	4.29827973	3.22371	2.405524014	2.078510713	0.415037	1	hsa-miR-570-5p	0.537285	0.805927	0.620384622	0.852739935	-0.58496	0.70711
hsa-miR-653	0	0.805927	0	0.852739935	-20	0.474469	bta-miR-1584-3p	2.14913986	4.835565	1.654957834	2.544872269	-1.16993	1	hsa-miR-371b-5p	0.537285	0.805927	0.620384622	0.852739935	-0.58496	0.70711
chr9_20452_star	0	0.805927	0	0.852739935	-20	0.474469	chrx_22394_star	4.29827973	5.641492	2.405524014	2.731507408	-0.39232	1	chr7_16094_star	0.537285	0.805927	0.620384622	0.852739935	-0.58496	0.70711
chr13_29005_star	0	0.805927	0	0.852739935	-20	0.474469	chr17_35074_mature	4.29827973	3.22371	2.405524014	2.078510713	0.415037	1	hsa-miR-584-3p	0.537285	0.805927	0.620384622	0.852739935	-0.58496	0.70711
hsa-miR-3156-5p	0	0.805927	0	0.852739935	-20	0.474469	chr11_25285_mature	8.59655945	10.47706	3.262517265	3.520680821	-0.2854	1	bta-miR-875	0.537285	0.805927	0.620384622	0.852739935	-0.58496	0.70711
chr1_3842_star	0	0.805927	0	0.852739935	-20	0.474469	chrx_21833_star	6.44741959	4.029637	2.896740642	2.330454351	0.678072	1	hsa-miR-3129-3p	0.537285	0.805927	0.620384622	0.852739935	-0.58496	0.70711
chr5_11928_mature	0	0.805927	0	0.852739935	-20	0.474469	hsa-miR-873-3p	8.59655945	4.029637	3.262517265	2.330454351	1.093109	1	hsa-miR-4676-5p	0.537285	0.805927	0.620384622	0.852739935	-0.58496	0.70711
mmu-miR-3082-5p	0	0.805927	0	0.852739935	-20	0.474469	chr15_32819_mature	4.29827973	3.22371	2.405524014	2.078510713	0.415037	1	mmu-miR-6405	0.537285	0.805927	0.620384622	0.852739935	-0.58496	0.70711
chr12_25511_star	0	0.805927	0	0.852739935	-20	0.474469	chr9_20139_mature	4.29827973	3.22371	2.405524014	2.078510713	0.415037	1	chr2_4916_mature	0.537285	0.805927	0.620384622	0.852739935	-0.58496	0.70711
chr8_17927_mature	0	0.805927	0	0.852739935	-20	0.474469	chr7_16140_star	6.44741959	4.835565	2.896740642	2.544872269	0.415037	1	chr6_14781_mature	0.537285	0.805927	0.620384622	0.852739935	-0.58496	0.70711
hsa-miR-371a-3p	0	0.805927	0	0.852739935	-20	0.474469	chr10_23470_star	6.44741959	5.641492	2.896740642	2.731507408	0.192645	1	hsa-miR-6724-5p	0.537285	0.805927	0.620384622	0.852739935	-0.58496	0.70711
hsa-miR-3675-3p	0	0.805927	0	0.852739935	-20	0.474469	chr8_17840_star	8.59655945	4.029637	3.262517265	2.330454351	1.093109	1	chrx_22559_star	0.537285	0.805927	0.620384622	0.852739935	-0.58496	0.70711
rno-miR-10b-5p	0	0.805927	0	0.852739935	-20	0.474469	chr13_28673_star	4.29827973	12.89484	2.405524014	3.796477232	-1.58496	1	hsa-miR-486-5p	0.537285	0.805927	0.620384622	0.852739935	-0.58496	0.70711
bta-miR-2301	0	0.805927	0	0.852739935	-20	0.474469	chr14_31521_star	4.29827973	4.835565	2.405524014	2.544872269	-0.16993	1	chr10_24027_star	0.537285	0.805927	0.620384622	0.852739935	-0.58496	0.70711
bta-miR-2315	0	0.805927	0	0.852739935	-20	0.474469	NW_003613395_38900_mature	8.59655945	16.11855	3.262517265	4.097488514	-0.90689	1	hsa-miR-4731-5p	0.537285	0.805927	0.620384622	0.852739935	-0.58496	0.70711
hsa-miR-4700-3p	0	0.805927	0	0.852739935	-20	0.474469	chr7_15459_mature	4.29827973	8.059274	2.405524014	3.179395517	-0.90689	1	chr3_7890_star	0.537285	0.805927	0.620384622	0.852739935	-0.58496	0.70711
mmu-miR-16-5p	0	0.805927	0	0.852739935	-20	0.474469	rno-miR-345-3p	6.44741959	4.835565	2.896740642	2.544872269	0.415037	1	bta-miR-759	0.537285	0.805927	0.620384622	0.852739935	-0.58496	0.70711
bta-miR-2345	0	0.805927	0	0.852739935	-20	0.474469	chr1_2982_star	2.14913986	4.029637	1.654957834	2.330454351	-0.90689	1	chr10_23348_star	0.537285	0.805927	0.620384622	0.852739935	-0.58496	0.70711
bta-miR-2344	0	0.805927	0	0.852739935	-20	0.474469	hsa-miR-6505-5p	4.29827973	2.417782	2.405524014	1.773060525	0.830075	1	mmu-miR-6352	0.537285	0.805927	0.620384622	0.852739935	-0.58496	0.70711
bta-miR-2349	0	0.805927	0	0.852739935	-20	0.474469	chr11_24609_mature	6.44741959	5.641492	2.896740642	2.731507408	0.192645	1	mmu-miR-6388	0.537285	0.805927	0.620384622	0.852739935	-0.58496	0.70711
chr14_30236_star	0	0.805927	0	0.852739935	-20	0.474469	chr16_34334_star	4.29827973	7.253347	2.405524014	3.044979305	-0.75489	1	chr3_7715_mature	0.537285	0.805927	0.620384622	0.852739935	-0.58496	0.70711
bta-miR-2347	0	0.805927	0	0.852739935	-20	0.474469	chr12_26332_mature	2.14913986	4.029637	1.654957834	2.330454351	-0.90689	1	mmu-miR-6378	0.537285	0.805927	0.620384622	0.852739935	-0.58496	0.70711
hsa-miR-4314	0	0.805927	0	0.852739935	-20	0.474469	chr13_28800_mature	10.7456993	15.31262	3.554060706	4.027916745	-0.51096	1	bta-miR-2284s	0.537285	0.805927	0.620384622	0.852739935	-0.58496	0.70711
hsa-miR-3187-5p	0	0.805927	0	0.852739935	-20	0.474469	chr5_12291_star	2.14913986	3.22371	1.654957834	2.078510713	-0.58496	1	bta-miR-2284g	0.537285	0.805927	0.620384622	0.852739935	-0.58496	0.70711
rno-miR-3580-5p	0	0.805927	0	0.852739935	-20	0.474469	chr16_33936_mature	25.7896784	44.32601	4.743605355	5.502267253	-0.78136	1	chr1_3137_star	0.537285	0.805927	0.620384622	0.852739935	-0.58496	0.70711
chr14_30644_mature	0	0.805927	0	0.852739935	-20	0.474469	chr14_30569_star	4.29827973	3.22371	2.405524014	2.078510713	0.415037	1	mmu-miR-6395	0.537285	0.805927	0.620384622	0.852739935	-0.58496	0.70711
bta-miR-2362	0	0.805927	0	0.852739935	-20	0.474469	chr8_18596_star	6.44741959	4.029637	2.896740642	2.330454351	0.678072	1	bta-miR-2917	0.537285	0.805927	0.620384622	0.852739935	-0.58496	0.70711
hsa-miR-4323	0	0.805927	0	0.852739935	-20	0.474469	NW_003613234_37804_mature	4.29827973	3.22371	2.405524014	2.078510713	0.415037	1	rno-miR-294	0.537285	0.805927	0.620384622	0.852739935	-0.58496	0.70711
chr14_31507_star	0	0.805927	0	0.852739935	-20	0.474469	chr10_24049_star	4.29827973	4.029637	2.405524014	2.330454351	0.093109	1	chrx_22202_star	0.537285	0.805927	0.620384622	0.852739935	-0.58496	0.70711
chr9_19346_star	0	0.805927	0	0.852739935	-20	0.474469	chr4_10602_mature	2.14913986	4.835565	1.654957834	2.544872269	-1.16993	1	chr4_8669_mature	0.537285	0.805927	0.620384622	0.852739935	-0.58496	0.70711
chr12_26572_star	0	0.805927	0	0.852739935	-20	0.474469	NW_003613364_38713_mature	8.59655945	7.253347	3.262517265	3.044979305	0.245112	1	rno-miR-343	0.537285	0.805927	0.620384622	0.852739935	-0.58496	0.70711
rno-miR-3580-3p	0	0.805927	0	0.852739935	-20	0.474469	chr9_20300_mature	6.44741959	8.059274	2.896740642	3.179395517	-0.32193	1	chr13_29176_mature	0.537285	0.805927	0.620384622	0.852739935	-0.58496	0.70711
bta-miR-2286	0	0.805927	0	0.852739935	-20	0.474469	NW_003613109_37259_star	2.14913986	3.22371	1.654957834	2.078510713	-0.58496	1	chr18_36584_star	0.537285	0.805927	0.620384622	0.852739935	-0.58496	0.70711
bta-miR-2289	0	0.805927	0	0.852739935	-20	0.474469	chr8_18841_mature	2.14913986	6.44742	1.654957834	2.896740642	-1.58496	1	hsa-miR-5692a	0.537285	0.805927	0.620384622	0.852739935	-0.58496	0.70711
mmu-miR-683	0	0.805927	0	0.852739935	-20	0.474469	chr6_13983_star	40.8336574	67.69791	5.38659223	6.102194213	-0.72935	1	chr7_15697_star	0.537285	0.805927	0.620384622	0.852739935	-0.58496	0.70711
chr9_19892_mature	0	0.805927	0	0.852739935	-20	0.474469	chr13_27234_star	2.14913986	3.22371	1.654957834	2.078510713	-0.58496	1	hsa-miR-363-5p	0.537285	0.805927	0.620384622	0.852739935	-0.58496	0.70711
chr14_31505_star	0	0.805927	0	0.852739935	-20	0.474469	chr7_15096_mature	6.44741959	15.31262	2.896740642	4.027916745	-1.24793	1	chr4_9960_mature	0.537285	0.805927	0.620384622	0.852739935	-0.58496	0.70711
bta-miR-2298	0	0.805927	0	0.852739935	-20	0.474469	chr6_13702_mature	30.0879581	16.92448	4.958283956	4.163859073	0.830075	1	bta-miR-615	0.537285	0.805927	0.620384622	0.852739935	-0.58496	0.70711
bta-miR-2297	0	0.805927	0	0.852739935	-20	0.474469	chr3_7453_mature	4.29827973	8.865202	2.405524014	3.302348583	-1.04439	1	chr9_20123_star	0.537285	0.805927	0.620384622	0.852739935	-0.58496	0.70711
mmu-miR-467g	0	0.805927	0	0.852739935	-20	0.474469	chr13_27411_mature	4.29827973	2.417782	2.405524014	1.773060525	0.830075	1	chr4_9649_mature	0.537285	0.805927	0.620384622	0.852739935	-0.58496	0.70711
chr7_15713_star	0	0.805927	0	0.852739935	-20	0.474469	chr7_16671_mature	2.14913986	5.641492	1.654957834	2.731507408	-1.39232	1	hsa-miR-5195-3p	0.537285	0.805927	0.620384622	0.852739935	-0.58496	0.70711
chr13_26614_star	0	0.805927	0	0.852739935	-20	0.474469	chr15_31774_star	6.44741959	6.44742	2.896740642	2.896740642	0	1	chr13_27082_star	0.537285	0.805927	0.620384622	0.852739935	-0.58496	0.70711
mmu-miR-670-5p	0	0.805927	0	0.852739935	-20	0.474469	chr17_35778_mature	8.59655945	16.11855	3.262517265	4.097488514	-0.90689	1	hsa-miR-4661-5p	0.537285	0.805927	0.620384622	0.852739935	-0.58496	0.70711
chr13_29245_star	0	0.805927	0	0.852739935	-20	0.474469	chr14_30443_star	4.29827973	9.671129	2.405524014	3.415640968	-1.16993	1	hsa-miR-551b-5p	0.537285	0.805927	0.620384622	0.852739935	-0.58496	0.70711
rno-miR-881-5p	0	0.805927	0	0.852739935	-20	0.474469	chr5_11689_star	12.8948392	21.76004	3.796477232	4.508431259	-0.75489	1	hsa-miR-514b-5p	0.537285	0.805927	0.620384622	0.852739935	-0.58496	0.70711
chr4_10131_star	0	0.805927	0	0.852739935	-20	0.474469	chr18_36092_star	2.14913986	6.44742	1.654957834	2.896740642	-1.58496	1	hsa-miR-4753-3p	0.537285	0.805927	0.620384622	0.852739935	-0.58496	0.70711
hsa-miR-3130-3p	0	0.805927	0	0.852739935	-20	0.474469	chr1_3589_star	2.14913986	6.44742	1.654957834	2.896740642	-1.58496	1	mmu-miR-672-3p	0.537285	0.805927	0.620384622	0.852739935	-0.58496	0.70711
chr18_36317_mature	0	0.805927	0	0.852739935	-20	0.474469	chr9_20139_star	2.14913986	4.029637	1.654957834	2.330454351	-0.90689	1	mmu-miR-3960	0.537285	0.805927	0.620384622	0.852739935	-0.58496	0.70711
rno-miR-3593-3p	0	0.805927	0	0.852739935	-20	0.474469	chr3_6868_mature	2.14913986	4.029637	1.654957834	2.330454351	-0.90689	1	mmu-miR-3961	0.537285	0.805927	0.620384622	0.852739935	-0.58496	0.70711
hsa-miR-193a-5p	0	0.805927	0	0.852739935	-20	0.474469	chr5_11659_mature	6.44741959	5.641492	2.896740642	2.731507408	0.192645	1	mmu-miR-3968	0.537285	0.805927	0.620384622	0.852739935	-0.58496	0.70711
bta-miR-2436-3p	0	0.805927	0	0.852739935	-20	0.474469	chr6_14625_mature@@rno-miR-298-5	4.29827973	4.029637	2.405524014	2.330454351	0.093109	1	chr16_34426_mature	0.537285	0.805927	0.620384622	0.852739935	-0.58496	0.70711
bta-miR-376e	0	0.805927	0	0.852739935	-20	0.474469	chr8_18128_star	4.29827973	3.22371	2.405524014	2.078510713	0.415037	1	hsa-miR-4714-3p	0.537285	0.805927	0.620384622	0.852739935	-0.58496	0.70711
chr6_13320_star	0	0.805927	0	0.852739935	-20	0.474469	NW_003537656_37099_mature	4.29827973	3.22371	2.405524014	2.078510713	0.415037	1	rno-miR-218a-1-3p	0.537285	0.805927	0.620384622	0.852739935	-0.58496	0.70711
hsa-miR-23a-5p	0	0.805927	0	0.852739935	-20	0.474469	chr3_7417_star	4.29827973	12.89484	2.405524014	3.796477232	-1.58496	1	chr9_19733_star	0.537285	0.805927	0.620384622	0.852739935	-0.58496	0.70711
chr8_17641_star	0	0.805927	0	0.852739935	-20	0.474469	hsa-miR-3917	6.44741959	4.029637	2.896740642	2.330454351	0.678072	1	hsa-miR-4786-3p	0.537285	0.805927	0.620384622	0.852739935	-0.58496	0.70711
chr2_4676_star	0	0.805927	0	0.852739935	-20	0.474469	hsa-miR-3915	2.14913986	3.22371	1.654957834	2.078510713	-0.58496	1	NW_003540745_39662_mature	0.537285	0.805927	0.620384622	0.852739935	-0.58496	0.70711
chr1_3365_star	0	0.805927	0	0.852739935	-20	0.474469	chr4_10559_mature	15.043979	10.47706	4.003960082	3.520680821	0.521953	1	chr5_11699_star	0.537285	0.805927	0.620384622	0.852739935	-0.58496	0.70711
bta-miR-378c	0	0.805927	0	0.852739935	-20	0.474469	bta-miR-1940	19.3422588	31.43117	4.346407978	5.019309189	-0.70044	1	mmu-miR-3094-3p	0.537285	0.805927	0.620384622	0.852739935	-0.58496	0.70711
chrx_22392_star	0	0.805927	0	0.852739935	-20	0.474469	chr17_35440_star	10.7456993	5.641492	3.554060706	2.731507408	0.929611	1	chr9_20720_star	0.537285	0.805927	0.620384622	0.852739935	-0.58496	0.70711
chr12_26547_mature	0	0.805927	0	0.852739935	-20	0.474469	chr13_28684_star	6.44741959	4.029637	2.896740642	2.330454351	0.678072	1	bta-miR-192	0.537285	0.805927	0.620384622	0.852739935	-0.58496	0.70711
hsa-miR-4644	0	0.805927	0	0.852739935	-20	0.474469	chr13_29218_star	6.44741959	4.029637	2.896740642	2.330454351	0.678072	1	bta-miR-184	0.537285	0.805927	0.620384622	0.852739935	-0.58496	0.70711
chr12_26570_mature	0	0.805927	0	0.852739935	-20	0.474469	mmu-miR-1903	4.29827973	3.22371	2.405524014	2.078510713	0.415037	1	chr14_29623_star	0.537285	0.805927	0.620384622	0.852739935	-0.58496	0.70711
hsa-miR-4648	0	0.805927	0	0.852739935	-20	0.474469	chr13_28542_star	6.44741959	6.44742	2.896740642	2.896740642	0	1	chr2_4567_star@@bta-miR-6535	0.537285	0.805927	0.620384622	0.852739935	-0.58496	0.70711
chr3_7910_star	0	0.805927	0	0.852739935	-20	0.474469	chr13_29266_star	6.44741959	8.059274	2.896740642	3.179395517	-0.32193	1	chr14_31065_star	0.537285	0.805927	0.620384622	0.852739935	-0.58496	0.70711
hsa-miR-4657	0	0.805927	0	0.852739935	-20	0.474469	chr11_25303_star	2.14913986	5.641492	1.654957834	2.731507408	-1.39232	1	chr7_15363_mature	0.537285	0.805927	0.620384622	0.852739935	-0.58496	0.70711
hsa-miR-4663	0	0.805927	0	0.852739935	-20	0.474469	chr14_31116_star	2.14913986	4.029637	1.654957834	2.330454351	-0.90689	1	hsa-miR-3682-3p	0.537285	0.805927	0.620384622	0.852739935	-0.58496	0.70711
hsa-miR-4660	0	0.805927	0	0.852739935	-20	0.474469	chr13_26809_mature	15.043979	11.28298	4.003960082	3.618589217	0.415037	1	chr5_12420_mature	0.537285	0.805927	0.620384622	0.852739935	-0.58496	0.70711
NW_003538808_38240_star	0	0.805927	0	0.852739935	-20	0.474469	chr1_1213_mature	8.59655945	20.14819	3.262517265	4.402462031	-1.22882	1	chr15_31671_star	0.537285	0.805927	0.620384622	0.852739935	-0.58496	0.70711
rno-miR-3596c	0	0.805927	0	0.852739935	-20	0.474469	chr6_13131_star	4.29827973	3.22371	2.405524014	2.078510713	0.415037	1	chr14_30385_mature	0.537285	0.805927	0.620384622	0.852739935	-0.58496	0.70711
mmu-miR-181d-3p	0	0.805927	0	0.852739935	-20	0.474469	hsa-miR-492	15.043979	28.20746	4.003960082	4.868265031	-0.90689	1	chr12_26515_mature	0.537285	0.805927	0.620384622	0.852739935	-0.58496	0.70711
hsa-miR-4682	0	0.805927	0	0.852739935	-20	0.474469	chr5_11530_star	2.14913986	4.835565	1.654957834	2.544872269	-1.16993	1	mmu-miR-653-5p	0.537285	0.805927	0.620384622	0.852739935	-0.58496	0.70711
NW_003541025_39829_star	0	0.805927	0	0.852739935	-20	0.474469	chr5_11429_mature	2.14913986	6.44742	1.654957834	2.896740642	-1.58496	1	NW_003537603_37043_star	0.537285	0.805927	0.620384622	0.852739935	-0.58496	0.70711
chr7_15903_star	0	0.805927	0	0.852739935	-20	0.474469	chr1_1637_mature	19.3422588	11.28298	4.346407978	3.618589217	0.777608	1	hsa-miR-513c-3p	0.537285	0.805927	0.620384622	0.852739935	-0.58496	0.70711
bta-miR-6122-5p	0	0.805927	0	0.852739935	-20	0.474469	chr13_28836_mature	4.29827973	5.641492	2.405524014	2.731507408	-0.39232	1	chr2_6321_star	0.537285	0.805927	0.620384622	0.852739935	-0.58496	0.70711
hsa-miR-196a-3p	0	0.805927	0	0.852739935	-20	0.474469	chr5_12345_mature	2.14913986	5.641492	1.654957834	2.731507408	-1.39232	1	hsa-miR-320d	0.537285	0.805927	0.620384622	0.852739935	-0.58496	0.70711
mmu-miR-717	0	0.805927	0	0.852739935	-20	0.474469	chr8_17595_star	2.14913986	3.22371	1.654957834	2.078510713	-0.58496	1	chr15_32008_star	0.537285	0.805927	0.620384622	0.852739935	-0.58496	0.70711
chr4_9930_star	0	0.805927	0	0.852739935	-20	0.474469	mmu-miR-1927	4.29827973	2.417782	2.405524014	1.773060525	0.830075	1	hsa-miR-1207-3p	0.537285	0.805927	0.620384622	0.852739935	-0.58496	0.70711
chr5_11103_mature	0	0.805927	0	0.852739935	-20	0.474469	chr11_25269_mature	2.14913986	4.835565	1.654957834	2.544872269	-1.16993	1	hsa-miR-548p	0.537285	0.805927	0.620384622	0.852739935	-0.58496	0.70711
hsa-miR-3925-5p	0	0.805927	0	0.852739935	-20	0.474469	chr7_16503_mature	6.44741959	3.22371	2.896740642	2.078510713	1	1	chr10_23976_mature	0.537285	0.805927	0.620384622	0.852739935	-0.58496	0.70711
chrx_22099_star	0	0.805927	0	0.852739935	-20	0.474469	mmu-miR-1970	4.29827973	2.417782	2.405524014	1.773060525	0.830075	1	chr15_33043_star	0.537285	0.805927	0.620384622	0.852739935	-0.58496	0.70711
hsa-miR-4763-3p	0	0.805927	0	0.852739935	-20	0.474469	chr6_13425_star	6.44741959	4.029637	2.896740642	2.330454351	0.678072	1	chr1_398_star	0.537285	0.805927	0.620384622	0.852739935	-0.58496	0.70711
bta-miR-6123	0	0.805927	0	0.852739935	-20	0.474469	chr17_35547_mature	15.043979	11.28298	4.003960082	3.618589217	0.415037	1	chrx_22070_mature	0.537285	0.805927	0.620384622	0.852739935	-0.58496	0.70711
rno-miR-3551-5p	0	0.805927	0	0.852739935	-20	0.474469	chr6_12810_mature	4.29827973	3.22371	2.405524014	2.078510713	0.415037	1	chr6_13458_star	0.537285	0.805927	0.620384622	0.852739935	-0.58496	0.70711
chr6_13572_mature	0	0.805927	0	0.852739935	-20	0.474469	chr18_36164_star	19.3422588	9.671129	4.346407978	3.415640968	1	1	hsa-miR-4769-5p	0.537285	0.805927	0.620384622	0.852739935	-0.58496	0.70711
chr1_3657_star	0	0.805927	0	0.852739935	-20	0.474469	chr13_26844_mature	8.59655945	5.641492	3.262517265	2.731507408	0.607683	1	bta-miR-21-5p	0.537285	0.805927	0.620384622	0.852739935	-0.58496	0.70711
chr3_6880_star@@hsa-miR-197-5p	0	0.805927	0	0.852739935	-20	0.474469	chr8_18282_star	4.29827973	4.029637	2.405524014	2.330454351	0.093109	1	chr16_34525_mature@@bta-miR-4	0.537285	0.805927	0.620384622	0.852739935	-0.58496	0.70711
hsa-miR-1911-5p	0	0.805927	0	0.852739935	-20	0.474469	chr6_12811_mature	6.44741959	5.641492	2.896740642	2.731507408	0.192645	1	rno-miR-135b-5p	0.537285	0.805927	0.620384622	0.852739935	-0.58496	0.70711
hsa-miR-5001-3p	0	0.805927	0	0.852739935	-20	0.474469	chr1_1402_mature	8.59655945	11.28298	3.262517265	3.618589217	-0.39232	1	hsa-miR-3178	0.537285	0.805927	0.620384622	0.852739935	-0.58496	0.70711
chr2_4824_star	0	0.805927	0	0.852739935	-20	0.474469	chr8_18144_mature	2.14913986	5.641492	1.654957834	2.731507408	-1.39232	1	hsa-miR-3188	0.537285	0.805927	0.620384622	0.852739935	-0.58496	0.70711
mmu-miR-92b-5p	0	0.805927	0	0.852739935	-20	0.474469	chr6_14787_star	2.14913986	4.029637	1.654957834	2.330454351	-0.90689	1	chr6_14789_star	0.537285	0.805927	0.620384622	0.852739935	-0.58496	0.70711
mmu-miR-301a-3p	0	0.805927	0	0.852739935	-20	0.474469	hsa-miR-193b-3p	4.29827973	3.22371	2.405524014	2.078510713	0.415037	1	hsa-miR-3141	0.537285	0.805927	0.620384622	0.852739935	-0.58496	0.70711
NW_003613218_37700_star	0	0.805927	0	0.852739935	-20	0.474469	chr5_11064_mature	4.29827973	4.835565	2.405524014	2.544872269	-0.16993	1	bta-miR-367	0.537285	0.805927	0.620384622	0.852739935	-0.58496	0.70711
NW_003613301_38356_star	0	0.805927	0	0.852739935	-20	0.474469	NW_003538922_38352_mature	10.7456993	8.865202	3.554060706	3.302348583	0.277534	1	bta-miR-1179	0.537285	0.805927	0.620384622	0.852739935	-0.58496	0.70711
bta-miR-345-3p	0	0.805927	0	0.852739935	-20	0.474469	chr6_12750_mature	8.59655945	12.89484	3.262517265	3.796477232	-0.58496	1	rno-miR-376c-3p	0.537285	0.805927	0.620384622	0.852739935	-0.58496	0.70711
rno-miR-7a-2-3p	0	0.805927	0	0.852739935	-20	0.474469	chr3_7571_mature	6.44741959	4.835565	2.896740642	2.544872269	0.415037	1	chr14_30997_star	0.537285	0.805927	0.620384622	0.852739935	-0.58496	0.70711
hsa-miR-5691	0	0.805927	0	0.852739935	-20	0.474469	chr4_10263_star	6.44741959	4.835565	2.896740642	2.544872269	0.415037	1	NW_003539279_38690_star	0.537285	0.805927	0.620384622	0.852739935	-0.58496	0.70711
hsa-miR-4756-3p	0	0.805927	0	0.852739935	-20	0.474469	chr15_32479_star	4.29827973	2.417782	2.405524014	1.773060525	0.830075	1	hsa-miR-5581-3p	0.537285	0.805927	0.620384622	0.852739935	-0.58496	0.70711
hsa-miR-4723-3p	0	0.805927	0	0.852739935	-20	0.474469	chr14_31236_star	2.14913986	4.835565	1.654957834	2.544872269	-1.16993	1	hsa-miR-3158-3p	0.537285	0.805927	0.620384622	0.852739935	-0.58496	0.70711
hsa-miR-4513	0	0.805927	0	0.852739935	-20	0.474469	NW_003539097_38524_mature	4.29827973	8.865202	2.405524014	3.302348583	-1.04439	1	chrx_23149_mature	0.537285	0.805927	0.620384622	0.852739935	-0.58496	0.70711
chr3_6975_star	0	0.805927	0	0.852739935	-20	0.474469	hsa-miR-548o-3p	4.29827973	2.417782	2.405524014	1.773060525	0.830075	1	mmu-miR-3073a-3p	0.537285	0.805927	0.620384622	0.852739935	-0.58496	0.70711
chr13_27252_mature	0	0.805927	0	0.852739935	-20	0.474469	chr14_31449_mature	6.44741959	7.253347	2.896740642	3.044979305	-0.16993	1	hsa-miR-4722-5p	0.537285	0.805927	0.620384622	0.852739935	-0.58496	0.70711
mmu-miR-344g-5p	0	0.805927	0	0.852739935	-20	0.474469	chr5_11655_mature	8.59655945	8.865202	3.262517265	3.302348583	-0.04439	1	chr5_11377_star	0.537285	0.805927	0.620384622	0.852739935	-0.58496	0.70711
hsa-miR-331-5p	0	0.805927	0	0.852739935	-20	0.474469	chr7_16599_mature	6.44741959	4.835565	2.896740642	2.544872269	0.415037	1	chr8_18304_mature	0.537285	0.805927	0.620384622	0.852739935	-0.58496	0.70711
hsa-miR-4500	0	0.805927	0	0.852739935	-20	0.474469	chr3_7048_star	6.44741959	5.641492	2.896740642	2.731507408	0.192645	1	hsa-miR-4781-3p	0.537285	0.805927	0.620384622	0.852739935	-0.58496	0.70711
mmu-miR-804	0	0.805927	0	0.852739935	-20	0.474469	chr12_26451_star	12.8948392	20.95411	3.796477232	4.456419386	-0.70044	1	NW_003539217_38656_star	0.537285	0.805927	0.620384622	0.852739935	-0.58496	0.70711
chr14_30751_mature	0	0.805927	0	0.852739935	-20	0.474469	chr2_6398_star	2.14913986	4.029637	1.654957834	2.330454351	-0.90689	1	chr3_7704_star	0.537285	0.805927	0.620384622	0.852739935	-0.58496	0.70711
hsa-miR-509-3-5p	0	0.805927	0	0.852739935	-20	0.474469	chr4_9665_mature	8.59655945	5.641492	3.262517265	2.731507408	0.607683	1	chr2_6204_star	0.537285	0.805927	0.620384622	0.852739935	-0.58496	0.70711
mmu-miR-665-5p	0	0.805927	0	0.852739935	-20	0.474469	chr18_35842_mature	4.29827973	9.671129	2.405524014	3.415640968	-1.16993	1	hsa-miR-431-5p	0.537285	0.805927	0.620384622	0.852739935	-0.58496	0.70711
chr13_26883_star	0	0.805927	0	0.852739935	-20	0.474469	chr2_6450_mature	2.14913986	4.029637	1.654957834	2.330454351	-0.90689	1	mmu-miR-449c-5p	0.537285	0.805927	0.620384622	0.852739935	-0.58496	0.70711
hsa-miR-1273a	0	0.805927	0	0.852739935	-20	0.474469	chr12_25445_mature	2.14913986	3.22371	1.654957834	2.078510713	-0.58496	1	bta-miR-490	0.537285	0.805927	0.620384622	0.852739935	-0.58496	0.70711
hsa-miR-4703-3p	0	0.805927	0	0.852739935	-20	0.474469	chr14_30472_star	4.29827973	2.417782	2.405524014	1.773060525	0.830075	1	mmu-miR-99a-3p	0.537285	0.805927	0.620384622	0.852739935	-0.58496	0.70711
hsa-miR-4540	0	0.805927	0	0.852739935	-20	0.474469	chr14_30035_star	10.7456993	17.7304	3.554060706	4.227310103	-0.72247	1	bta-miR-485	0.537285	0.805927	0.620384622	0.852739935	-0.58496	0.70711
hsa-miR-4778-5p	0	0.805927	0	0.852739935	-20	0.474469	mmu-miR-3105-3p	2.14913986	6.44742	1.654957834	2.896740642	-1.58496	1	hsa-miR-4725-3p	0.537285	0.805927	0.620384622	0.852739935	-0.58496	0.70711
mmu-miR-215-5p	0	0.805927	0	0.852739935	-20	0.474469	chr9_20113_mature	6.44741959	12.89484	2.896740642	3.796477232	-1	1	rno-miR-455-5p	0.537285	0.805927	0.620384622	0.852739935	-0.58496	0.70711
hsa-miR-671-3p	0	0.805927	0	0.852739935	-20	0.474469	chr12_26266_star	4.29827973	7.253347	2.405524014	3.044979305	-0.75489	1	mmu-miR-26a-2-3p	0.537285	0.805927	0.620384622	0.852739935	-0.58496	0.70711
bta-miR-3613	0	0.805927	0	0.852739935	-20	0.474469	chr14_30282_star	4.29827973	2.417782	2.405524014	1.773060525	0.830075	1	chr15_31930_star	0.537285	0.805927	0.620384622	0.852739935	-0.58496	0.70711
mmu-miR-1264-5p	0	0.805927	0	0.852739935	-20	0.474469	chr10_23834_mature	4.29827973	5.641492	2.405524014	2.731507408	-0.39232	1	chr13_27136_star	0.537285	0.805927	0.620384622	0.852739935	-0.58496	0.70711
chr6_14372_star	0	0.805927	0	0.852739935	-20	0.474469	chr2_5807_star	2.14913986	3.22371	1.654957834	2.078510713	-0.58496	1	chr15_32760_star	0.537285	0.805927	0.620384622	0.852739935	-0.58496	0.70711
chr16_33727_mature	0	0.805927	0	0.852739935	-20	0.474469	chr13_29308_mature	2.14913986	3.22371	1.654957834	2.078510713	-0.58496	1	bta-miR-2421	0.537285	0.805927	0.620384622	0.852739935	-0.58496	0.70711
chr13_27824_star	0	0.805927	0	0.852739935	-20	0.474469	ssc-miR-194b-5p	4.29827973	5.641492	2.405524014	2.731507408	-0.39232	1	hsa-miR-4456	0.537285	0.805927	0.620384622	0.852739935	-0.58496	0.70711
chr9_19145_star	0	0.805927	0	0.852739935	-20	0.474469	NW_003613367_38732_star	4.29827973	4.029637	2.405524014	2.330454351	0.093109	1	chr2_4436_star	0.537285	0.805927	0.620384622	0.852739935	-0.58496	0.70711
chr2_6199_star	0	0.805927	0	0.852739935	-20	0.474469	chr8_18423_star	21.4913986	14.50669	4.491301473	3.954819241	0.567041	1	bta-miR-2478	0.537285	0.805927	0.620384622	0.852739935	-0.58496	0.70711
hsa-miR-6068	0	0.805927	0	0.852739935	-20	0.474469	chr11_24398_star	10.7456993	5.641492	3.554060706	2.731507408	0.929611	1	mmu-miR-201-5p	0.537285	0.805927	0.620384622	0.852739935	-0.58496	0.70711
hsa-miR-6069	0	0.805927	0	0.852739935	-20	0.474469	chr12_26323_mature	4.29827973	2.417782	2.405524014	1.773060525	0.830075	1	hsa-miR-608	0.537285	0.805927	0.620384622	0.852739935	-0.58496	0.70711
chr7_15425_star	0	0.805927	0	0.852739935	-20	0.474469	chr9_19632_mature	4.29827973	6.44742	2.405524014	2.896740642	-0.58496	1	hsa-miR-636	0.537285	0.805927	0.620384622	0.852739935	-0.58496	0.70711
chr14_29827_star	0	0.805927	0	0.852739935	-20	0.474469	mmu-miR-3109-5p	4.29827973	6.44742	2.405524014	2.896740642	-0.58496	1	chr3_7813_star	0.537285	0.805927	0.620384622	0.852739935	-0.58496	0.70711
mmu-miR-465c-5p	0	0.805927	0	0.852739935	-20	0.474469	chrx_22045_star	2.14913986	7.253347	1.654957834	3.044979305	-1.75489	1	chr15_33231_star	0.537285	0.805927	0.620384622	0.852739935	-0.58496	0.70711
hsa-miR-922	0	0.805927	0	0.852739935	-20	0.474469	chr7_17077_star	2.14913986	3.22371	1.654957834	2.078510713	-0.58496	1	chr1_3446_mature	0.537285	0.805927	0.620384622	0.852739935	-0.58496	0.70711
chr4_9980_mature	0	0.805927	0	0.852739935	-20	0.474469	hsa-miR-3651	4.29827973	7.253347	2.405524014	3.044979305	-0.75489	1	hsa-miR-1825	0.537285	0.805927	0.620384622	0.852739935	-0.58496	0.70711
chr14_29460_star	0	0.805927	0	0.852739935	-20	0.474469	hsa-miR-3654	4.29827973	2.417782	2.405524014	1.773060525	0.830075	1	chr3_7737_mature	0.537285	0.805927	0.620384622	0.852739935	-0.58496	0.70711
hsa-miR-5583-5p	0	0.805927	0	0.852739935	-20	0.474469	hsa-miR-3657	4.29827973	4.029637	2.405524014	2.330454351	0.093109	1	chr1_319_mature	0.537285	0.805927	0.620384622	0.852739935	-0.58496	0.70711
rno-miR-425-3p	0	0.805927	0	0.852739935	-20	0.474469	chr9_19660_mature	4.29827973	10.47706	2.405524014	3.520680821	-1.2854	1	hsa-miR-4524a-3p	0.537285	0.805927	0.620384622	0.852739935	-0.58496	0.70711
hsa-miR-6074	0	0.805927	0	0.852739935	-20	0.474469	chr15_32293_star	6.44741959	12.89484	2.896740642	3.796477232	-1	1	hsa-miR-4640-5p	0.537285	0.805927	0.620384622	0.852739935	-0.58496	0.70711
chr1_2742_mature	0	0.805927	0	0.852739935	-20	0.474469	hsa-miR-2117	2.14913986	4.835565	1.654957834	2.544872269	-1.16993	1	chr3_7716_mature	0.537285	0.805927	0.620384622	0.852739935	-0.58496	0.70711
mmu-miR-491-3p	0	0.805927	0	0.852739935	-20	0.474469	hsa-miR-4715-5p	8.59655945	5.641492	3.262517265	2.731507408	0.607683	1	NW_003541007_39797_star	0.537285	0.805927	0.620384622	0.852739935	-0.58496	0.70711
chr9_20571_star	0	0.805927	0	0.852739935	-20	0.474469	hsa-miR-3672	2.14913986	5.641492	1.654957834	2.731507408	-1.39232	1	chr13_29005_star	0.537285	0.805927	0.620384622	0.852739935	-0.58496	0.70711
hsa-miR-1912	0	0.805927	0	0.852739935	-20	0.474469	chr9_19972_mature	4.29827973	4.029637	2.405524014	2.330454351	0.093109	1	chr1_1936_mature	0.537285	0.805927	0.620384622	0.852739935	-0.58496	0.70711
rno-miR-770-3p	0	0.805927	0	0.852739935	-20	0.474469	hsa-miR-3671	6.44741959	4.029637	2.896740642	2.330454351	0.678072	1	chr12_25526_star	0.537285	0.805927	0.620384622	0.852739935	-0.58496	0.70711
chr16_34306_star	0	0.805927	0	0.852739935	-20	0.474469	chr1_2264_star	8.59655945	4.029637	3.262517265	2.330454351	1.093109	1	chr12_25511_star	0.537285	0.805927	0.620384622	0.852739935	-0.58496	0.70711
chr9_21061_star	0	0.805927	0	0.852739935	-20	0.474469	rno-miR-672-3p	6.44741959	9.671129	2.896740642	3.415640968	-0.58496	1	chr7_16666_star	0.537285	0.805927	0.620384622	0.852739935	-0.58496	0.70711
chr1_1941_mature	7.521989523	21.76004	3.091190278	4.508431259	-1.5325	0.475126	NW_003537461_36946_mature	4.29827973	5.641492	2.405524014	2.731507408	-0.39232	1	hsa-miR-548t-5p	0.537285	0.805927	0.620384622	0.852739935	-0.58496	0.70711
chr15_32852_mature	3.223709795	4.029637	2.078510713	2.330454351	-0.32193	0.475423	chr1_1974_mature	38.6845175	25.78968	5.310504361	4.743605355	0.584963	1	chr12_25987_mature	0.537285	0.805927	0.620384622	0.852739935	-0.58496	0.70711
chrx_21833_star	3.223709795	4.029637	2.078510713	2.330454351	-0.32193	0.475423	mmu-miR-5103	6.44741959	3.22371	2.896740642	2.078510713	1	1	hsa-miR-3675-3p	0.537285	0.805927	0.620384622	0.852739935	-0.58496	0.70711
chr13_27735_mature	3.223709795	4.029637	2.078510713	2.330454351	-0.32193	0.475423	chr1_2060_mature	6.44741959	6.44742	2.896740642	2.896740642	0	1	bta-miR-2310	0.537285	0.805927	0.620384622	0.852739935	-0.58496	0.70711
chr14_31116_star	3.223709795	4.029637	2.078510713	2.330454351	-0.32193	0.475423	chr7_16063_mature	6.44741959	6.44742	2.896740642	2.896740642	0	1	mmu-miR-16-5p	0.537285	0.805927	0.620384622	0.852739935	-0.58496	0.70711
chr14_31056_star	3.223709795	4.029637	2.078510713	2.330454351	-0.32193	0.475423	chr4_9530_star	2.14913986	3.22371	1.654957834	2.078510713	-0.58496	1	bta-miR-2345	0.537285	0.805927	0.620384622	0.852739935	-0.58496	0.70711
chr8_17571_star	3.223709795	4.029637	2.078510713	2.330454351	-0.32193	0.475423	chr14_31302_mature	4.29827973	3.22371	2.405524014	2.078510713	0.415037	1	bta-miR-2344	0.537285	0.805927	0.620384622	0.852739935	-0.58496	0.70711
chr6_12863_mature	3.223709795	4.029637	2.078510713	2.330454351	-0.32193	0.475423	chr6_12863_mature	8.59655945	4.029637	3.262517265	2.330454351	1.093109	1	chr14_30236_star	0.537285	0.805927	0.620384622	0.852739935	-0.58496	0.70711
NW_003540978_39774_mature	3.223709795	4.029637	2.078510713	2.330454351	-0.32193	0.475423	chr1_3121_star	4.29827973	12.08891	2.405524014	3.710273245	-1.49185	1	chr7_15912_mature	0.537285	0.805927	0.620384622	0.852739935	-0.58496	0.70711
chr4_10551_star	3.223709795	4.029637	2.078510713	2.330454351	-0.32193	0.475423	chr9_19693_star	8.59655945	13.70077	3.262517265	3.877819487	-0.67243	1	NW_003613233_37801_star	0.537285	0.805927	0.620384622	0.852739935	-0.58496	0.70711
bta-miR-195	3.223709795	4.029637	2.078510713	2.330454351	-0.32193	0.475423	chr2_6199_mature	2.14913986	3.22371	1.654957834	2.078510713	-0.58496	1	chr9_19346_star	0.537285	0.805927	0.620384622	0.852739935	-0.58496	0.70711
chr2_5867_mature	3.223709795	4.029637	2.078510713	2.330454351	-0.32193	0.475423	chr7_16298_mature	4.29827973	2.417782	2.405524014	1.773060525	0.830075	1	bta-miR-2286	0.537285	0.805927	0.620384622	0.852739935	-0.58496	0.70711
chr4_9894_mature	3.223709795	4.029637	2.078510713	2.330454351	-0.32193	0.475423	chr7_17051_mature	4.29827973	3.22371	2.405524014	2.078510713	0.415037	1	chr4_9258_star	0.537285	0.805927	0.620384622	0.852739935	-0.58496	0.70711
chr14_31058_star	3.223709795	4.029637	2.078510713	2.330454351	-0.32193	0.475423	chr7_17171_star	8.59655945	18.53633	3.262517265	4.288087668	-1.10852	1	rno-miR-351-3p	0.537285	0.805927	0.620384622	0.852739935	-0.58496	0.70711
hsa-miR-622	3.223709795	4.029637	2.078510713	2.330454351	-0.32193	0.475423	mmu-miR-5136	2.14913986	4.835565	1.654957834	2.544872269	-1.16993	1	bta-miR-339a	0.537285	0.805927	0.620384622	0.852739935	-0.58496	0.70711
chr9_19896_star	3.223709795	4.029637	2.078510713	2.330454351	-0.32193	0.475423	chr7_15380_star	6.44741959	16.11855	2.896740642	4.097488514	-1.32193	1	chr6_12919_star	0.537285	0.805927	0.620384622	0.852739935	-0.58496	0.70711
mmu-miR-684	3.223709795	4.029637	2.078510713	2.330454351	-0.32193	0.475423	chr14_31039_mature	12.8948392	6.44742	3.796477232	2.896740642	1	1	chr6_13134_mature	0.537285	0.805927	0.620384622	0.852739935	-0.58496	0.70711
chrx_22034_mature	3.223709795	4.029637	2.078510713	2.330454351	-0.32193	0.475423	chr16_33720_mature	2.14913986	5.641492	1.654957834	2.731507408	-1.39232	1	mmu-miR-1839-3p	0.537285	0.805927	0.620384622	0.852739935	-0.58496	0.70711
chr14_29971_star	3.223709795	4.029637	2.078510713	2.330454351	-0.32193	0.475423	NW_003540770_39680_star	4.29827973	4.029637	2.405524014	2.330454351	0.093109	1	chr2_4523_mature	0.537285	0.805927	0.620384622	0.852739935	-0.58496	0.70711
chr9_20222_star	3.223709795	4.029637	2.078510713	2.330454351	-0.32193	0.475423	NW_003540978_39774_mature	2.14913986	4.029637	1.654957834	2.330454351	-0.90689	1	hsa-miR-764	0.537285	0.805927	0.620384622	0.852739935	-0.58496	0.70711
chr14_29836_star	3.223709795	4.029637	2.078510713	2.330454351	-0.32193	0.475423	chr17_35668_star	10.7456993	23.3719	3.554060706	4.607146585	-1.12102	1	bta-miR-376d	0.537285	0.805927	0.620384622	0.852739935	-0.58496	0.70711
chr4_8816_star	3.223709795	4.029637	2.078510713	2.330454351	-0.32193	0.475423	chr1_2009_mature	8.59655945	9.671129	3.262517265	3.415640968	-0.16993	1	chr7_16704_star	0.537285	0.805927	0.620384622	0.852739935	-0.58496	0.70711
chrx_21552_star	3.223709795	4.029637	2.078510713	2.330454351	-0.32193	0.475423	NW_003537216_36773_mature	10.7456993	4.835565	3.554060706	2.544872269	1.152003	1	mmu-miR-1957b	0.537285	0.805927	0.620384622	0.852739935	-0.58496	0.70711
chr11_24333_mature	4.298279727	3.22371	2.405524014	2.078510713	0.415037	0.476129	chr1_1949_mature	4.29827973	3.22371	2.405524014	2.078510713	0.415037	1	chr1_3365_star	0.537285	0.805927	0.620384622	0.852739935	-0.58496	0.70711
chr13_28421_mature	4.298279727	3.22371	2.405524014	2.078510713	0.415037	0.476129	chr13_28872_star	2.14913986	6.44742	1.654957834	2.896740642	-1.58496	1	NW_003538201_37612_star	0.537285	0.805927	0.620384622	0.852739935	-0.58496	0.70711
chr15_33130_mature	4.298279727	3.22371	2.405524014	2.078510713	0.415037	0.476129	chr9_20114_mature	6.44741959	16.11855	2.896740642	4.097488514	-1.32193	1	bta-miR-6122-5p	0.537285	0.805927	0.620384622	0.852739935	-0.58496	0.70711
mmu-miR-1903	4.298279727	3.22371	2.405524014	2.078510713	0.415037	0.476129	chr9_19299_mature	2.14913986	3.22371	1.654957834	2.078510713	-0.58496	1	chr13_29078_star	0.537285	0.805927	0.620384622	0.852739935	-0.58496	0.70711
chr6_13131_star	4.298279727	3.22371	2.405524014	2.078510713	0.415037	0.476129	chr2_5082_mature	17.1931189	8.865202	4.185320985	3.302348583	0.955606	1	rno-miR-22-3p	0.537285	0.805927	0.620384622	0.852739935	-0.58496	0.70711
chr13_28612_star	4.298279727	3.22371	2.405524014	2.078510713	0.415037	0.476129	chrx_22576_star	10.7456993	18.53633	3.554060706	4.288087668	-0.7866	1	chrx_22099_star	0.537285	0.805927	0.620384622	0.852739935	-0.58496	0.70711
chr16_34188_star	4.298279727	3.22371	2.405524014	2.078510713	0.415037	0.476129	chr2_4728_mature	4.29827973	2.417782	2.405524014	1.773060525	0.830075	1	hsa-miR-4763-3p	0.537285	0.805927	0.620384622	0.852739935	-0.58496	0.70711
chr5_12416_mature	4.298279727	3.22371	2.405524014	2.078510713	0.415037	0.476129	NW_003537296_36822_mature	12.8948392	9.671129	3.796477232	3.415640968	0.415037	1	rno-miR-3573-5p	0.537285	0.805927	0.620384622	0.852739935	-0.58496	0.70711
chr4_8856_star	4.298279727	3.22371	2.405524014	2.078510713	0.415037	0.476129	chr6_14789_mature	4.29827973	3.22371	2.405524014	2.078510713	0.415037	1	mmu-miR-301a-3p	0.537285	0.805927	0.620384622	0.852739935	-0.58496	0.70711
chr8_19039_star	4.298279727	3.22371	2.405524014	2.078510713	0.415037	0.476129	chrx_21932_star	4.29827973	6.44742	2.405524014	2.896740642	-0.58496	1	NW_003613218_37700_star	0.537285	0.805927	0.620384622	0.852739935	-0.58496	0.70711
NW_003539007_38439_star	4.298279727	3.22371	2.405524014	2.078510713	0.415037	0.476129	chr9_19828_mature	8.59655945	14.50669	3.262517265	3.954819241	-0.75489	1	hsa-miR-211-3p	0.537285	0.805927	0.620384622	0.852739935	-0.58496	0.70711
chr2_3991_mature	4.298279727	3.22371	2.405524014	2.078510713	0.415037	0.476129	chr3_8356_star	2.14913986	4.029637	1.654957834	2.330454351	-0.90689	1	mmu-miR-1198-3p	0.537285	0.805927	0.620384622	0.852739935	-0.58496	0.70711
chr13_28624_mature	4.298279727	3.22371	2.405524014	2.078510713	0.415037	0.476129	chr9_20219_mature	15.043979	9.671129	4.003960082	3.415640968	0.63743	1	bta-miR-3660	0.537285	0.805927	0.620384622	0.852739935	-0.58496	0.70711
mmu-miR-6402	4.298279727	3.22371	2.405524014	2.078510713	0.415037	0.476129	chrx_23076_mature	6.44741959	16.11855	2.896740642	4.097488514	-1.32193	1	hsa-miR-4513	0.537285	0.805927	0.620384622	0.852739935	-0.58496	0.70711
chr6_14428_star	4.298279727	3.22371	2.405524014	2.078510713	0.415037	0.476129	chr3_6975_mature	146.141511	188.587	7.201060499	7.566716407	-0.36786	1	hsa-miR-4526	0.537285	0.805927	0.620384622	0.852739935	-0.58496	0.70711
chr3_6671_mature	4.298279727	3.22371	2.405524014	2.078510713	0.415037	0.476129	chr11_24430_star	6.44741959	6.44742	2.896740642	2.896740642	0	1	chr1_3875_mature@@hsa-miR-466	0.537285	0.805927	0.620384622	0.852739935	-0.58496	0.70711
chr15_31928_mature	4.298279727	3.22371	2.405524014	2.078510713	0.415037	0.476129	chr7_16979_mature	4.29827973	2.417782	2.405524014	1.773060525	0.830075	1	hsa-miR-2277-3p	0.537285	0.805927	0.620384622	0.852739935	-0.58496	0.70711
chr6_14967_star	4.298279727	3.22371	2.405524014	2.078510713	0.415037	0.476129	chr17_35680_star	4.29827973	9.671129	2.405524014	3.415640968	-1.16993	1	chr13_26883_star	0.537285	0.805927	0.620384622	0.852739935	-0.58496	0.70711
chr7_16979_star	4.298279727	3.22371	2.405524014	2.078510713	0.415037	0.476129	chr1_3342_mature	6.44741959	13.70077	2.896740642	3.877819487	-1.08746	1	hsa-miR-1273a	0.537285	0.805927	0.620384622	0.852739935	-0.58496	0.70711
chr6_13900_star	4.298279727	3.22371	2.405524014	2.078510713	0.415037	0.476129	chr3_7220_star	8.59655945	17.7304	3.262517265	4.227310103	-1.04439	1	chr14_29702_star	0.537285	0.805927	0.620384622	0.852739935	-0.58496	0.70711
mmu-miR-3112-3p	4.298279727	3.22371	2.405524014	2.078510713	0.415037	0.476129	chr6_12645_mature	8.59655945	17.7304	3.262517265	4.227310103	-1.04439	1	chr10_23335_star	0.537285	0.805927	0.620384622	0.852739935	-0.58496	0.70711
chr14_30480_star	4.298279727	3.22371	2.405524014	2.078510713	0.415037	0.476129	chr15_32092_star	8.59655945	5.641492	3.262517265	2.731507408	0.607683	1	hsa-miR-1972	0.537285	0.805927	0.620384622	0.852739935	-0.58496	0.70711
chr1_1938_mature@@rno-miR-2964	4.298279727	3.22371	2.405524014	2.078510713	0.415037	0.476129	chr1_1859_star	8.59655945	4.029637	3.262517265	2.330454351	1.093109	1	ssc-miR-885-5p	0.537285	0.805927	0.620384622	0.852739935	-0.58496	0.70711
chr17_34881_star	4.298279727	3.22371	2.405524014	2.078510713	0.415037	0.476129	chr17_35199_star	4.29827973	4.029637	2.405524014	2.330454351	0.093109	1	rno-miR-425-3p	0.537285	0.805927	0.620384622	0.852739935	-0.58496	0.70711
chr3_6737_star	4.298279727	3.22371	2.405524014	2.078510713	0.415037	0.476129	hsa-miR-378d	12.8948392	6.44742	3.796477232	2.896740642	1	1	hsa-miR-6081	0.537285	0.805927	0.620384622	0.852739935	-0.58496	0.70711
chr12_26282_mature	4.298279727	3.22371	2.405524014	2.078510713	0.415037	0.476129	chr2_5244_star	2.14913986	3.22371	1.654957834	2.078510713	-0.58496	1	hsa-miR-6073	0.537285	0.805927	0.620384622	0.852739935	-0.58496	0.70711
hsa-miR-5694	4.298279727	3.22371	2.405524014	2.078510713	0.415037	0.476129	chr2_6500_star	2.14913986	4.029637	1.654957834	2.330454351	-0.90689	1	NW_003539571_38947_star	0.537285	0.805927	0.620384622	0.852739935	-0.58496	0.70711
chr9_19516_mature	10.74569932	4.835565	3.554060706	2.544872269	1.152003	0.476211	NW_003538807_38238_mature	10.7456993	5.641492	3.554060706	2.731507408	0.929611	1	chr5_10910_star	0.537285	0.805927	0.620384622	0.852739935	-0.58496	0.70711
chr4_10674_star@@bta-miR-760-5p	10.74569932	4.835565	3.554060706	2.544872269	1.152003	0.476211	chr5_11410_star	6.44741959	4.029637	2.896740642	2.330454351	0.678072	1	NW_003613237_37814_mature	0.537285	0.805927	0.620384622	0.852739935	-0.58496	0.70711
chr14_30887_mature	10.74569932	4.835565	3.554060706	2.544872269	1.152003	0.476211	chr12_26204_star	19.3422588	29.81932	4.346407978	4.94576292	-0.62449	1	chr9_19752_mature	11.82027	45.93786	3.680354657	5.552680304	-1.95842	0.70747
chr6_14320_mature	10.74569932	4.835565	3.554060706	2.544872269	1.152003	0.476211	chr11_24910_star	4.29827973	4.029637	2.405524014	2.330454351	0.093109	1	chr9_19536_mature	7.52199	21.76004	3.091190278	4.508431259	-1.5325	0.707569
chr3_7797_mature	10.74569932	4.835565	3.554060706	2.544872269	1.152003	0.476211	hsa-miR-5092	4.29827973	4.029637	2.405524014	2.330454351	0.093109	1	chr5_11544_mature	51.04207	12.89484	5.701606491	3.796477232	1.984893	0.707616
chr7_15287_star	10.74569932	4.835565	3.554060706	2.544872269	1.152003	0.476211	chr8_17349_star	6.44741959	6.44742	2.896740642	2.896740642	0	1	NW_003538430_37856_star	18.80497	7.253347	4.307790888	3.044979305	1.374396	0.707621
chr15_32385_mature	4.298279727	8.059274	2.405524014	3.179395517	-0.90689	0.476605	chr18_36229_mature	8.59655945	6.44742	3.262517265	2.896740642	0.415037	1	chr7_15837_mature	26.86425	8.865202	4.800343329	3.302348583	1.599462	0.707719
chr2_4896_mature	4.298279727	8.059274	2.405524014	3.179395517	-0.90689	0.476605	chr18_36088_mature	10.7456993	8.865202	3.554060706	3.302348583	0.277534	1	NW_003539097_38524_mature	26.86425	8.865202	4.800343329	3.302348583	1.599462	0.707719
chr12_25510_mature	4.298279727	8.059274	2.405524014	3.179395517	-0.90689	0.476605	chr7_15622_mature	6.44741959	4.029637	2.896740642	2.330454351	0.678072	1	NW_003540965_39767_star	15.04398	6.44742	4.003960082	2.896740642	1.222392	0.708111
chr2_5889_mature	4.298279727	8.059274	2.405524014	3.179395517	-0.90689	0.476605	chr5_11814_star	4.29827973	5.641492	2.405524014	2.731507408	-0.39232	1	chr4_9286_star	15.04398	6.44742	4.003960082	2.896740642	1.222392	0.708111
chr7_15145_star	4.298279727	8.059274	2.405524014	3.179395517	-0.90689	0.476605	chr17_34868_mature	2.14913986	3.22371	1.654957834	2.078510713	-0.58496	1	ssc-miR-491	17.19312	82.2046	4.185320985	6.378591382	-2.25739	0.709023
chr17_35071_mature	4.298279727	8.059274	2.405524014	3.179395517	-0.90689	0.476605	chr9_20315_star	4.29827973	4.835565	2.405524014	2.544872269	-0.16993	1	chr17_35572_star	8.059274	4.835565	3.179395517	2.544872269	0.736966	0.709328
chr6_12802_star	13.96940911	5.641492	3.90394537	2.731507408	1.308122	0.476935	chr4_9796_star	4.29827973	4.029637	2.405524014	2.330454351	0.093109	1	chr5_11064_mature	8.059274	4.835565	3.179395517	2.544872269	0.736966	0.709328
chr7_16076_mature	13.96940911	5.641492	3.90394537	2.731507408	1.308122	0.476935	chr1_1256_star	4.29827973	2.417782	2.405524014	1.773060525	0.830075	1	chr5_12346_mature	8.059274	4.835565	3.179395517	2.544872269	0.736966	0.709328
NW_003541307_40037_mature	7.521989523	4.029637	3.091190278	2.330454351	0.900464	0.477062	chr17_35576_mature	12.8948392	6.44742	3.796477232	2.896740642	1	1	NW_003537641_37087_star	8.059274	4.835565	3.179395517	2.544872269	0.736966	0.709328
chr13_29218_star	7.521989523	4.029637	3.091190278	2.330454351	0.900464	0.477062	chr15_33538_star	6.44741959	8.865202	2.896740642	3.302348583	-0.45943	1	chr6_14638_star	8.059274	4.835565	3.179395517	2.544872269	0.736966	0.709328
chr3_8356_star	7.521989523	4.029637	3.091190278	2.330454351	0.900464	0.477062	hsa-miR-2278	2.14913986	3.22371	1.654957834	2.078510713	-0.58496	1	chr2_5564_mature@@mmu-miR-67	8.059274	4.835565	3.179395517	2.544872269	0.736966	0.709328
chr1_888_mature	7.521989523	4.029637	3.091190278	2.330454351	0.900464	0.477062	chr14_30276_mature	6.44741959	4.029637	2.896740642	2.330454351	0.678072	1	chr8_18077_mature	8.059274	4.835565	3.179395517	2.544872269	0.736966	0.709328
chr2_5581_star	7.521989523	4.029637	3.091190278	2.330454351	0.900464	0.477062	chr6_12705_star	8.59655945	4.029637	3.262517265	2.330454351	1.093109	1	chr5_11313_mature@@hsa-miR-14	8.059274	4.835565	3.179395517	2.544872269	0.736966	0.709328
chr2_4255_mature	7.521989523	4.029637	3.091190278	2.330454351	0.900464	0.477062	chr5_11374_mature	8.59655945	4.029637	3.262517265	2.330454351	1.093109	1	chr15_33009_star	8.059274	4.835565	3.179395517	2.544872269	0.736966	0.709328
chr7_16206_mature	7.521989523	4.029637	3.091190278	2.330454351	0.900464	0.477062	rno-miR-196c-5p	2.14913986	3.22371	1.654957834	2.078510713	-0.58496	1	chr7_15098_mature	8.059274	24.17782	3.179395517	4.654081667	-1.58496	0.709443
chr4_9977_mature	7.521989523	4.029637	3.091190278	2.330454351	0.900464	0.477062	mmu-miR-98-3p	4.29827973	2.417782	2.405524014	1.773060525	0.830075	1	mmu-miR-3105-3p	4.29828	6.44742	2.405524014	2.896740642	-0.58496	0.709699
chr11_24588_mature	7.521989523	4.029637	3.091190278	2.330454351	0.900464	0.477062	chr7_16196_mature	4.29827973	9.671129	2.405524014	3.415640968	-1.16993	1	hsa-miR-3160-3p	4.29828	6.44742	2.405524014	2.896740642	-0.58496	0.709699
chr15_32974_star	7.521989523	4.029637	3.091190278	2.330454351	0.900464	0.477062	chr1_1843_mature	40.8336574	24.17782	5.38659223	4.654081667	0.756074	1	chrx_22620_mature	4.29828	6.44742	2.405524014	2.896740642	-0.58496	0.709699
chr3_8074_star	7.521989523	4.029637	3.091190278	2.330454351	0.900464	0.477062	chr1_2584_star	2.14913986	7.253347	1.654957834	3.044979305	-1.75489	1	chr14_30768_star	4.29828	6.44742	2.405524014	2.896740642	-0.58496	0.709699
chr8_18750_star	7.521989523	4.029637	3.091190278	2.330454351	0.900464	0.477062	chr1_2451_mature	2.14913986	4.835565	1.654957834	2.544872269	-1.16993	1	hsa-miR-4288	4.29828	6.44742	2.405524014	2.896740642	-0.58496	0.709699
chr11_24870_mature	5.372849659	12.08891	2.671938627	3.710273245	-1.16993	0.477247	NW_003539007_38439_star	2.14913986	3.22371	1.654957834	2.078510713	-0.58496	1	chr4_8989_star	4.29828	4.835565	2.405524014	2.544872269	-0.16993	0.70984
ssc-miR-490	5.372849659	12.08891	2.671938627	3.710273245	-1.16993	0.477247	chr15_31736_mature	6.44741959	9.671129	2.896740642	3.415640968	-0.58496	1	chr15_32407_star	4.29828	4.835565	2.405524014	2.544872269	-0.16993	0.70984
chr4_10147_star	56.95220639	13.70077	5.856791684	3.877819487	2.055495	0.477418	chr14_29715_star	2.14913986	3.22371	1.654957834	2.078510713	-0.58496	1	chr18_36050_mature	4.29828	4.835565	2.405524014	2.544872269	-0.16993	0.70984
chr16_33936_mature	11.82026925	44.32601	3.680354657	5.502267253	-1.90689	0.478505	chr17_35223_star	2.14913986	6.44742	1.654957834	2.896740642	-1.58496	1	chr6_12750_star	4.29828	4.835565	2.405524014	2.544872269	-0.16993	0.70984
chr1_953_mature	807.0020188	83.81645	9.658215087	6.406272275	3.267267	0.479289	chr7_17055_mature	38.6845175	22.56597	5.310504361	4.558633073	0.777608	1	chr14_30479_star	4.29828	4.835565	2.405524014	2.544872269	-0.16993	0.70984
NW_003538873_38324_star	24.71510843	8.059274	4.684544332	3.179395517	1.616671	0.479461	chr8_18024_star	2.14913986	4.835565	1.654957834	2.544872269	-1.16993	1	chr6_14013_star	4.29828	4.835565	2.405524014	2.544872269	-0.16993	0.70984
chr13_27779_mature	6.447419591	16.11855	2.896740642	4.097488514	-1.32193	0.480727	chr1_2545_star	8.59655945	4.835565	3.262517265	2.544872269	0.830075	1	chrx_22797_star	4.29828	4.835565	2.405524014	2.544872269	-0.16993	0.70984
chr14_29554_mature	6.447419591	16.11855	2.896740642	4.097488514	-1.32193	0.480727	chrx_22877_mature	4.29827973	5.641492	2.405524014	2.731507408	-0.39232	1	bta-miR-2338	4.29828	4.835565	2.405524014	2.544872269	-0.16993	0.70984
chr9_21032_mature	6.447419591	16.11855	2.896740642	4.097488514	-1.32193	0.480727	chr7_16821_mature	30.0879581	45.93786	4.958283956	5.552680304	-0.6105	1	chr10_24027_mature	4.29828	4.835565	2.405524014	2.544872269	-0.16993	0.70984
chr6_14340_mature	20.4168287	7.253347	4.420672964	3.044979305	1.49304	0.48268	chr1_480_mature	4.29827973	6.44742	2.405524014	2.896740642	-0.58496	1	chr7_16671_mature	11.28298	5.641492	3.618589217	2.731507408	1	0.71008
chr15_31749_mature	20.4168287	7.253347	4.420672964	3.044979305	1.49304	0.48268	chr2_4818_star	6.44741959	16.92448	2.896740642	4.163859073	-1.39232	1	ssc-miR-194b-5p	11.28298	5.641492	3.618589217	2.731507408	1	0.71008

chr1_1579_mature	4.298279727	7.253347	2.405524014	3.044979305	-0.75489	0.482964
NW_003613367_38733_mature	4.298279727	7.253347	2.405524014	3.044979305	-0.75489	0.482964
chr4_10332_star	4.298279727	7.253347	2.405524014	3.044979305	-0.75489	0.482964
chr5_12300_mature	4.298279727	7.253347	2.405524014	3.044979305	-0.75489	0.482964
chr3_8256_star	4.298279727	7.253347	2.405524014	3.044979305	-0.75489	0.482964
chr10_24172_star	5.372849659	11.28298	2.671938627	3.618589217	-1.07039	0.483889
chr5_12150_mature	5.372849659	11.28298	2.671938627	3.618589217	-1.07039	0.483889
rno-miR-883-3p	5.372849659	11.28298	2.671938627	3.618589217	-1.07039	0.483889
chr12_25449_mature	32.23709795	9.671129	5.054722516	3.415640968	1.736966	0.483987
chr6_14473_mature	6.447419591	4.029637	2.896740642	2.330454351	0.678072	0.484763
chr18_36092_mature@@hsa-miR-44	6.447419591	4.029637	2.896740642	2.330454351	0.678072	0.484763
hsa-miR-3671	6.447419591	4.029637	2.896740642	2.330454351	0.678072	0.484763
chr1_251_star	6.447419591	4.029637	2.896740642	2.330454351	0.678072	0.484763
mmu-miR-203-5p	6.447419591	4.029637	2.896740642	2.330454351	0.678072	0.484763
chr11_25207_mature	6.447419591	4.029637	2.896740642	2.330454351	0.678072	0.484763
chr5_12125_mature	6.447419591	4.029637	2.896740642	2.330454351	0.678072	0.484763
chr14_31281_mature	9.671129386	4.835565	3.415640968	2.544872269	1	0.485449
chr7_16048_star	9.671129386	4.835565	3.415640968	2.544872269	1	0.485449
chr2_4557_mature	9.671129386	4.835565	3.415640968	2.544872269	1	0.485449
chr13_27982_mature	9.671129386	4.835565	3.415640968	2.544872269	1	0.485449
chr6_14780_mature	12.89483918	5.641492	3.796477232	2.731507408	1.192645	0.486042
chr5_12127_mature	9.671129386	30.62524	3.415640968	4.983004663	-1.66297	0.486643
chr14_30061_mature	420.1568433	53.99714	8.718213798	5.781284667	2.959973	0.486731
chr15_32961_star	6.447419591	15.31262	2.896740642	4.027916745	-1.24793	0.486805
chr6_12833_star	4.298279727	4.029637	2.405524014	2.330454351	0.093109	0.486868
rno-miR-23a-3p	4.298279727	4.029637	2.405524014	2.330454351	0.093109	0.486868
chr1_3875_star	4.298279727	4.029637	2.405524014	2.330454351	0.093109	0.486868
hsa-miR-873-3p	4.298279727	4.029637	2.405524014	2.330454351	0.093109	0.486868
NW_003613367_38732_star	4.298279727	4.029637	2.405524014	2.330454351	0.093109	0.486868
NW_003540770_39680_star	4.298279727	4.029637	2.405524014	2.330454351	0.093109	0.486868
chr1_1859_star	4.298279727	4.029637	2.405524014	2.330454351	0.093109	0.486868
chr2_6500_star	4.298279727	4.029637	2.405524014	2.330454351	0.093109	0.486868
chr7_15622_mature	4.298279727	4.029637	2.405524014	2.330454351	0.093109	0.486868
chr4_9796_star	4.298279727	4.029637	2.405524014	2.330454351	0.093109	0.486868
chr1_2346_star	4.298279727	4.029637	2.405524014	2.330454351	0.093109	0.486868
rno-miR-193-3p	4.298279727	4.029637	2.405524014	2.330454351	0.093109	0.486868
chr1_991_star	4.298279727	4.029637	2.405524014	2.330454351	0.093109	0.486868
chr14_29474_mature	4.298279727	4.029637	2.405524014	2.330454351	0.093109	0.486868
chr12_25812_star	4.298279727	4.029637	2.405524014	2.330454351	0.093109	0.486868
hsa-miR-4802-5p	4.298279727	4.029637	2.405524014	2.330454351	0.093109	0.486868
chr18_36486_mature	4.298279727	4.029637	2.405524014	2.330454351	0.093109	0.486868
chr3_7785_mature	4.298279727	4.029637	2.405524014	2.330454351	0.093109	0.486868
chr7_15712_mature	4.298279727	4.029637	2.405524014	2.330454351	0.093109	0.486868
chr7_15704_mature	4.298279727	4.029637	2.405524014	2.330454351	0.093109	0.486868
chr14_30277_mature	4.298279727	4.029637	2.405524014	2.330454351	0.093109	0.486868
chrx_21531_star	4.298279727	4.029637	2.405524014	2.330454351	0.093109	0.486868
bta-miR-2447	4.298279727	4.029637	2.405524014	2.330454351	0.093109	0.486868
bta-miR-2479	4.298279727	4.029637	2.405524014	2.330454351	0.093109	0.486868
chr13_27180_star	4.298279727	4.029637	2.405524014	2.330454351	0.093109	0.486868
chr6_14061_star	4.298279727	4.029637	2.405524014	2.330454351	0.093109	0.486868
mmu-miR-378d	4.298279727	4.029637	2.405524014	2.330454351	0.093109	0.486868
chr5_11904_star	4.298279727	4.029637	2.405524014	2.330454351	0.093109	0.486868
chr13_27642_star	4.298279727	4.029637	2.405524014	2.330454351	0.093109	0.486868
chr1_1498_star	4.298279727	4.029637	2.405524014	2.330454351	0.093109	0.486868
chr13_29048_mature	4.298279727	4.029637	2.405524014	2.330454351	0.093109	0.486868
chr1_153_star	4.298279727	4.029637	2.405524014	2.330454351	0.093109	0.486868
chr9_21035_star	16.11854898	6.44742	4.097488514	2.896740642	1.321928	0.487796
chr17_35223_mature	16.11854898	6.44742	4.097488514	2.896740642	1.321928	0.487796
chr8_18841_mature	4.298279727	6.44742	2.405524014	2.896740642	-0.58496	0.487948
mmu-miR-3105-3p	4.298279727	6.44742	2.405524014	2.896740642	-0.58496	0.487948
chr9_19632_mature	4.298279727	6.44742	2.405524014	2.896740642	-0.58496	0.487948
chr4_8847_star	4.298279727	6.44742	2.405524014	2.896740642	-0.58496	0.487948
chr7_16628_mature	4.298279727	6.44742	2.405524014	2.896740642	-0.58496	0.487948
hsa-miR-4288	4.298279727	6.44742	2.405524014	2.896740642	-0.58496	0.487948
chr4_9018_mature	4.298279727	6.44742	2.405524014	2.896740642	-0.58496	0.487948
NW_003539576_38951_mature	75.21989523	16.92448	6.25209572	4.163859073	2.152003	0.488175
NW_003539467_38882_star	5.372849659	4.029637	2.671938627	2.330454351	0.415037	0.488747
chr7_16449_star	5.372849659	4.029637	2.671938627	2.330454351	0.415037	0.488747
chr6_14675_star	5.372849659	4.029637	2.671938627	2.330454351	0.415037	0.488747
chr3_8156_mature	5.372849659	4.029637	2.671938627	2.330454351	0.415037	0.488747
chr9_20900_star	5.372849659	4.029637	2.671938627	2.330454351	0.415037	0.488747
chr4_9773_star	5.372849659	4.029637	2.671938627	2.330454351	0.415037	0.488747
chr2_6450_mature	5.372849659	4.029637	2.671938627	2.330454351	0.415037	0.488747
chr13_27644_star	5.372849659	4.029637	2.671938627	2.330454351	0.415037	0.488747
chr3_7692_mature	5.372849659	4.029637	2.671938627	2.330454351	0.415037	0.488747
chr17_35424_star	5.372849659	4.029637	2.671938627	2.330454351	0.415037	0.488747
chr13_29025_star	5.372849659	4.029637	2.671938627	2.330454351	0.415037	0.488747
chr1_2593_mature	5.372849659	4.029637	2.671938627	2.330454351	0.415037	0.488747
chr17_35416_mature	5.372849659	4.029637	2.671938627	2.330454351	0.415037	0.488747
chr15_32699_star	5.372849659	4.029637	2.671938627	2.330454351	0.415037	0.488747
chr1_569_mature	5.372849659	4.029637	2.671938627	2.330454351	0.415037	0.488747
chr1_3091_mature	5.372849659	4.029637	2.671938627	2.330454351	0.415037	0.488747
chr15_32336_star	12.89483918	48.35565	3.796477232	5.625143252	-1.90689	0.488945
chr9_19844_mature	11.82026925	41.90823	3.680354657	5.423182395	-1.82597	0.489344
chr9_20122_mature	5.372849659	10.47706	2.671938627	3.520680821	-0.96347	0.490115
chr4_10559_mature	5.372849659	10.47706	2.671938627	3.520680821	-0.96347	0.490115
chr9_19660_mature	5.372849659	10.47706	2.671938627	3.520680821	-0.96347	0.490115
NW_003540052_39255_mature	5.372849659	10.47706	2.671938627	3.520680821	-0.96347	0.490115
chr4_9939_mature	5.372849659	10.47706	2.671938627	3.520680821	-0.96347	0.490115
chr9_19738_star	5.372849659	10.47706	2.671938627	3.520680821	-0.96347	0.490115
chr15_32008_mature	5.372849659	10.47706	2.671938627	3.520680821	-0.96347	0.490115
chr4_8767_mature	39.75908748	11.28298	5.349049847	3.618589217	1.817136	0.490787
chr8_17419_mature	4.298279727	5.641492	2.405524014	2.731507408	-0.39232	0.490947
chr11_24609_mature	4.298279727	5.641492	2.405524014	2.731507408	-0.39232	0.490947
chr6_12811_mature	4.298279727	5.641492	2.405524014	2.731507408	-0.39232	0.490947
chr4_9665_mature	4.298279727	5.641492	2.405524014	2.731507408	-0.39232	0.490947
ssc-miR-194b-5p	4.298279727	5.641492	2.405524014	2.731507408	-0.39232	0.490947
chr10_23487_mature	4.298279727	5.641492	2.405524014	2.731507408	-0.39232	0.490947
chr14_31118_star	4.298279727	5.641492	2.405524014	2.731507408	-0.39232	0.490947
chr4_8979_mature	4.298279727	5.641492	2.405524014	2.731507408	-0.39232	0.490947
hsa-miR-4423-3p	4.298279727	5.641492	2.405524014	2.731507408	-0.39232	0.490947
chr14_29752_star	4.298279727	5.641492	2.405524014	2.731507408	-0.39232	0.490947
NW_003613518_39516_mature	4.298279727	5.641492	2.405524014	2.731507408	-0.39232	0.490947
chr11_25375_mature	4.298279727	5.641492	2.405524014	2.731507408	-0.39232	0.490947
chr2_5810_star	4.298279727	5.641492	2.405524014	2.731507408	-0.39232	0.490947
chr9_19844_star	4.298279727	5.641492	2.405524014	2.731507408	-0.39232	0.490947
chr14_30038_mature	972.4857883	97.51722	9.927016107	6.622304032	3.317948	0.490963
chr14_31492_star	4.298279727	4.835565	2.405524014	2.544872269	-0.16993	0.491054
chr13_29308_star	4.298279727	4.835565	2.405524014	2.544872269	-0.16993	0.491054
chr11_25269_mature	4.298279727	4.835565	2.405524014	2.544872269	-0.16993	0.491054
NW_003537216_36773_mature	4.298279727	4.835565	2.405524014	2.544872269	-0.16993	0.491054
chr7_16525_star	4.298279727	4.835565	2.405524014	2.544872269	-0.16993	0.491054
hsa-miR-3160-5p	4.298279727	4.835565	2.405524014	2.544872269	-0.16993	0.491054
NW_003613031_36829_mature	4.298279727	4.835565	2.405524014	2.544872269	-0.16993	0.491054
chr17_35296_mature	4.298279727	4.835565	2.405524014	2.544872269	-0.16993	0.491054
chr2_6524_mature	4.298279727	4.835565	2.405524014	2.544872269	-0.16993	0.491054
chr13_27948_star	4.298279727	4.835565	2.405524014	2.544872269	-0.16993	0.491054
chr16_33714_star	4.298279727	4.835565	2.405524014	2.544872269	-0.16993	0.491054
rno-miR-3583-3p	9.671129386	29.81932	3.415640968	4.94576292	-1.62449	0.49108
NW_003539007_38433_mature	10.74569932	35.46081	3.554060706	5.188274618	-1.72247	0.491345
NW_003613507_39500_mature	26.8642483	8.865202	4.800343329	3.302348583	1.599462	0.491714
chr4_10445_star	6.447419591	14.50669	2.896740642	3.954819241	-1.16993	0.492719
chr14_30427_mature	29.01338816	171.6625	4.907534286	7.431811362	-2.56478	0.493137
chr13_26740_mature	8.596559455	4.835565	3.262517265	2.544872269	0.830075	0.493241
chr17_35572_star	8.596559455	4.835565	3.262517265	2.544872269	0.830075	0.493241
chr3_7571_mature	8.596559455	4.835565	3.262517265	2.544872269	0.830075	0.493241
NW_003541090_39901_mature	8.596559455	4.835565	3.262517265	2.544872269	0.830075	0.493241
chr15_32322_mature	8.596559455	4.835565	3.262517265	2.544872269	0.830075	0.493241
chr2_6582_star	8.596559455	4.835565	3.262517265	2.544872269	0.830075	0.493241
chr14_29649_mature	8.596559455	4.835565	3.262517265	2.544872269	0.830075	0.493241
NW_003540278_39378_mature	193.4225877	32.2371	7.603052029	5.054722516	2.584963	0.493974
chr3_8521_mature	11.82026925	5.641492	3.680354657	2.731507408	1.067114	0.494459
chr2_5364_mature	11.82026925	5.641492	3.680354657	2.731507408	1.067114	0.494459
chr18_35998_mature	11.82026925	5.641492	3.680354657	2.731507408	1.067114	0.494459
chr9_20219_mature	5.372849659	9.671129	2.671938627	3.415640968	-0.848	0.495771
chr17_35680_star	5.372849659	9.671129	2.671938627	3.415640968	-0.848	0.495771
chr15_33538_mature	5.372849659	9.671129	2.671938627	3.415640968	-0.848	0.495771
chr15_31672_star	5.372849659	9.671129	2.671938627	3.415640968	-0.848	0.495771
chr15_32413_star	5.372849659	9.671129	2.671938627	3.415640968	-0.848	0.495771
chr9_19234_star	5.372849659	9.671129	2.671938627	3.415640968	-0.848	0.495771
chr11_24430_mature	15.04397905	6.44742	4.003960082	2.896740642	1.222392	0.495991
chr17_35575_mature	15.04397905	6.44742	4.003960082	2.896740642	1.222392	0.495991
chr8_17989_star@@mmu-miR-302d-	38.68451755	11.28298	5.310504361	3.618589217	1.777608	0.496393
chr5_11037_mature	7.521989523	18.53633	3.091190278	4.288087668	-1.30117	0.496996
chr17_35003_mature	7.521989523	18.53633	3.091190278	4.288087668	-1.30117	0.496996
chr17_35668_star	8.596559455	23.3719	3.262517265	4.607146585	-1.44294	0.497646
chr13_27739_mature	8.596559455	23.3719	3.262517265	4.607146585	-1.44294	0.497646
chr1_2937_mature	18.26768884	7.253347	4.268111626	3.044979305	1.332575	0.498376
chr4_10161_star	6.447419591	13.70077	2.896740642	3.877819487	-1.08746	0.498412
chr6_14856_mature	6.447419591	13.70077	2.896740642	3.877819487	-1.08746	0.498412
chr9_19752_mature	12.89483918	45.93786	3.796477232	5.552680304	-1.83289	0.498934
chrx_22025_mature	12.89483918	45.93786	3.796477232	5.552680304	-1.83289	0.498934
chr6_14638_star	7.521989523	4.835565	3.091190278	2.544872269	0.63743	0.498939
mmu-miR-3079-5p	7.521989523	4.835565	3.091190278	2.544872269	0.63743	0.498939
chr8_18077_mature	7.521989523	4.835565	3.091190278	2.544872269	0.63743	0.498939
chr3_6720_mature	7.521989523	4.835565	3.091190278	2.544872269	0.63743	0.498939
chr12_25501_star	5.372849659	4.835565	2.671938627	2.544872269	0.152003	0.499741
chr2_4557_star	5.372849659	4.835565	2.671938627	2.544872269	0.152003	0.499741
chr4_10620_star	5.372849659	4.835565	2.671938627	2.544872269	0.152003	0.499741
NW_003613507_39496_mature	5.372849659	4.835565	2.671938627	2.544872269	0.152003	0.499741
chr9_19153_star	5.372849659	4.835565	2.671938627	2.544872269	0.152003	0.499741
chr15_31883_mature	5.372849659	4.835565	2.671938627	2.544872269	0.152003	0.499741
chr8_18502_star	5.372849659	4.835565	2.671938627	2.544872269	0.152003	0.499741
chr8_17514_mature	5.372849659	4.835565	2.671938627	2.544872269	0.152003	0.499741
chr15_32407_star	5.372849659	4.835565	2.671938627	2.544872269	0.152003	0.499741
NW_003537656_37100_star	5.372849659	4.835565	2.671938627	2.544872269	0.152003	0.499741
chr4_10525_mature	9.671129386	28.20746	3.415640968	4.868265031	-1.54432	0.49995
chr8_18167_mature	88.11473441	19.34226	6.477592084	4.346407978	2.187627	0.499972
chr2_5051_star	5.372849659	8.865202	2.671938627	3.302348583	-0.72247	0.50066
chr5_11655_mature	5.372849659	8.865202	2.671938627	3.302348583	-0.72247	0.50066
chr15_31790_star	5.372849659	8.865202	2.671938627	3.302348583	-0.72247	0.50066
chr14_31502_star	5.372849659	8.865202	2.671938627	3.302348583	-0.72247	0.50066
chr1_88_mature	21.49139864	8.059274	4.491301473	3.179395517	1.415037	0.501538
chr7_16707_mature	21.49139864	8.059274	4.491301473	3.179395517	1.415037	0.501538
chr9_20275_mature	6.447419591	4.835565	2.896740642	2.544872269	0.415037	0.501591
chr12_26134_star	6.447419591	4.835565	2.896740642	2.544872269	0.415037	0.501591
chr6_14061_mature	6.447419591	4.835565	2.896740642	2.544872269	0.415037	0.501591
chr14_29906_star	6.447419591	4.835565	2.896740642	2.544872269	0.415037	0.501591
chr2_6342_star	10.74569932	5.641492	3.554060706	2.731507408	0.929611	0.501917
chr13_29315_mature	10.74569932	5.641492	3.554060706	2.731507408	0.929611	0.501917
chr5_12208_mature	10.74569932	5.641492	3.554060706	2.731507408	0.929611	0.501917
chr11_24937_mature	10.74569932	5.641492	3.554060706	2.731507408	0.929611	0.501917
chr14_30596_mature	10.74569932	5.641492	3.554060706	2.731507408	0.929611	0.501917
chr12_26240_star	10.74569932	5.641492	3.554060706	2.731507408	0.929611	0.501917
chr6_14862_star	10.74569932	5.641492	3.554060706	2.731507408	0.929611	0.501917
chr11_25110_mature	10.74569932	5.641492	3.554060706	2.731507408	0.929611	0.501917
chr9_21094_mature	10.74569932	5.641492	3.554060706	2.731507408	0.929611	0.501917
chr12_25609_mature	10.74569932	5.641492	3.554060706	2.731507408	0.929611	0.501917
rno-miR-146a-5p	37.60994761	11.28298	5.270900692	3.618589217	1.736966	0.501993
chr8_18128_mature	616.8031409	72.53347	9.271003395	6.200329169	3.088091	0.502482
ssc-miR-215	104.2332834	21.76004	6.717447265	4.508431259	2.260063	0.502978
chr9_20113_mature	6.447419591	12.89484	2.896740642	3.796477232	-1	0.503815
chr8_17858_mature	5.372849659	8.059274	2.671938627	3.179395517	-0.58496	0.504514
chr14_31514_mature@@bta-miR-18	5.372849659	8.059274	2.671938627	3.179395517	-0.58496	0.504514
chr14_31575_star	5.372849659	5.641492	2.671938627	2.731507408	-0.07039	0.50547
chr5_11789_mature	5.372849659	5.641492	2.671938627	2.731507408	-0.07039	0.50547
NW_003538807_38238_mature	5.372849659	5.641492	2.671938627	2.731507408	-0.07039	0.50547
chrx_21936_star	5.372849659	5.641492	2.671938627	2.731507408	-0.07039	0.50547
chr7_16002_star	5.372849659	5.641492	2.671938627	2.731507408	-0.07039	0.50547
chr13_27847_star	5.372849659	5.641492	2.671938627	2.731507408	-0.07039	0.50547
hsa-miR-320c	5.372849659	5.641492	2.671938627	2.731507408	-0.07039	0.50547
chr9_20222_mature	5.372849659	5.641492	2.671938627	2.731507408	-0.07039	0.50547
NW_003613109_37260_star	5.372849659	5.641492	2.671938627	2.731507408	-0.07039	0.50547
chr3_7678_star	5.372849659	5.641492	2.671938627	2.731507408	-0.07039	0.50547
chr6_13411_star	5.372849659	5.641492	2.671938627	2.731507408	-0.07039	0.50547
chr7_17182_star	17.19311891	7.253347	4.185320985	3.044979305	1.245112	0.505817
hsa-miR-1252	5.372849659	7.253347	2.671938627	3.044979305	-0.43296	0.506971
chr1_2584_star	5.372849659	7.253347	2.671938627	3.044979305	-0.43296	0.506971
hsa-miR-659-5p	5.372849659	7.253347	2.671938627	3.044979305	-0.43296	0.506971
chr15_32458_star	5.372849659	7.253347	2.671938627	3.044979305	-0.43296	0.506971
chrx_22620_star	5.372849659	7.253347	2.671938627	3.044979305	-0.43296	0.506971
chr8_17373_star	5.372849659	7.253347	2.671938627	3.044979305	-0.43296	0.506971
chr5_11729_star	5.372849659	7.253347	2.671938627	3.044979305	-0.43296	0.506971
chr12_26451_mature	8.596559455	21.76004	3.262517265	4.508431259	-1.33985	0.50731
chrx_22063_star	8.596559455	21.76004	3.262517265	4.508431259	-1.33985	0.50731
chr16_34743_mature	7.521989523	16.92448	3.091190278	4.163859073	-1.16993	0.507416
chr5_11862_mature	7.521989523	16.92448	3.091190278	4.163859073	-1.16993	0.507416
chrx_21932_star	5.372849659	6.44742	2.671938627	2.896740642	-0.26303	0.507529
chr18_36229_mature	5.372849659	6.44742	2.671938627	2.896740642	-0.26303	0.507529
chr17_35223_star	5.372849659	6.44742	2.671938627	2.896740642	-0.26303	0.507529
hsa-miR-3160-3p	5.372849659	6.44742	2.671938627	2.896740642	-0.26303	0.507529
NW_003540965_39767_star	5.372849659	6.44742	2.671938627	2.896740642	-0.26303	0.507529
chr13_27546_star	5.372849659	6.44742	2.671938627	2.896740642	-0.26303	0.507529
chr4_10607_mature	5.372849659	6.44742	2.671938627	2.896740642	-0.26303	0.507529
chr16_34452_mature	5.372849659	6.44742	2.671938627	2.896740642	-0.26303	0.507529
chr14_30841_star	5.372849659	6.44742	2.671938627	2.896740642	-0.26303	0.507529
chr16_34295_mature	5.372849659	6.44742	2.671938627	2.896740642	-0.26303	0.507529
NW_003539920_39188_star	9.671129386	5.641492	3.415640968	2.731507408	0.777608	0.508053
bta-miR-2888	9.671129386	5.641492	3.415640968	2.731507408	0.777608	0.508053
chr10_23834_mature	9.671129386	5.641492	3.415640968	2.731507408	0.777608	0.508053
chr2_6283_star	9.671129386	5.641492	3.415640968	2.731507408	0.777608	0.508053
chr13_29421_mature	9.671129386	5.641492	3.415640968	2.731507408	0.777608	0.508053
chr7_15734_mature	9.671129386	5.641492	3.415640968	2.731507408	0.777608	0.508053
chr5_11934_mature	9.671129386	5.641492	3.415640968	2.731507408	0.777608	0.508053
chr4_8989_mature	20.4168287	8.059274	4.420672964	3.179395517	1.341037	0.508586
chr13_28169_mature	20.4168287	8.059274	4.420672964	3.179395517	1.341037	0.508586
chr7_16587_mature	6.447419591	12.08891	2.896740642	3.710273245	-0.90689	0.508841
hsa-miR-3676-5p	809.1511587	88.65202	9.662047302	6.486264174	3.190184	0.509226
mmu-miR-5105	13176.3765	641.5182	13.68577555	9.327593621	4.36032	0.509349
bta-miR-1434-5p	59.10134625	15.31262	5.909325402	4.027916745	1.94847	0.509387
chr9_19936_mature	1656.986835	146.6788	10.69521684	7.206318883	3.49783	0.510598
chr6_12951_star	68.77247564	16.92448	6.124586119	4.163859073	2.02272	0.510633
chr17_35576_mature	12.89483918	6.44742	3.796477232	2.896740642	1	0.510687
chr6_13928_star	12.89483918	6.44742	3.796477232	2.896740642	1	0.510687
chr2_5420_mature	190.1988779	33.04303	7.578930247	5.08928735	2.525091	0.510811
chr6_13451_star	66.62333577	584.2974	6.079449279	9.193026061	-3.1326	0.51141
chr1_1837_mature	21.49139864	99.12908	4.491301473	6.645717165	-2.20555	0.511658
chr5_11961_mature	23.6405385	8.865202	4.62296188	3.302348583	1.415037	0.511925
hsa-miR-4715-5p	8.596559455	5.641492	3.262517265	2.731507408	0.607683	0.512368
hsa-miR-3665	8.596559455	5.641492	3.262517265	2.731507408	0.607683	0.512368
hsa-miR-4666b	8.596559455	5.641492	3.262517265	2.731507408	0.607683	0.512368
chr14_30937_mature	8.596559455	5.641492	3.262517265	2.731507408	0.607683	0.512368
chrx_21673_star	8.596559455	5.641492	3.262517265	2.731507408	0.607683	0.512368
chr3_8521_star	6.447419591	5.641492	2.896740642	2.731507408	0.192645	0.512378
chr17_35440_star	6.447419591	5.641492	2.896740642	2.731507408	0.192645	0.512378
chr6_13930_mature	6.447419591	5.641492	2.896740642	2.731507408	0.192645	0.512378
chr15_32159_mature	6.447419591	5.641492	2.896740642	2.731507408	0.192645	0.512378
chr9_19230_star	6.447419591	5.641492	2.896740642	2.731507408	0.192645	0.512378
chrx_21675_star	6.447419591	5.641492	2.896740642	2.731507408	0.192645	0.512378
chr1_412_star	487.854749	62.86234	8.933262058	5.996893534	2.956184	0.5124
chr4_9789_star	16.11854898	7.253347	4.097488514	3.044979305	1.152003	0.512868
chr9_20062_mature	6.447419591	11.28298	2.896740642	3.618589217	-0.80735	0.51338
chr4_9726_mature	6.447419591	11.28298	2.896740642	3.618589217	-0.80735	0.51338
bta-miR-1291	29.01338816	158.7677	4.907534286	7.319832027	-2.45213	0.513442
chr7_16620_mature	31.16252802	10.47706	5.007308904	3.520680821	1.572579	0.513984

chr13_27449_mature	4.29827973	11.28298	2.405524014	3.618589217	-1.39232	1
chr1_3022_mature	4.29827973	3.22371	2.405524014	2.078510713	0.415037	1
chr1_2346_star	2.14913986	4.029637	1.654957834	2.330454351	-0.90689	1
chr8_19039_mature	70.9216155	97.51722	6.168353522	6.622304032	-0.45943	1
chrx_22089_star	6.44741959	12.08891	2.896740642	3.710273245	-0.90689	1
chr15_33558_mature	4.29827973	7.253347	2.405524014	3.044979305	-0.75489	1
chr7_15704_star	4.29827973	6.44742	2.405524014	2.896740642	-0.58496	1
chr8_18251_star	2.14913986	4.029637	1.654957834	2.330454351	-0.90689	1
chr15_33692_mature	2.14913986	4.029637	1.654957834	2.330454351	-0.90689	1
chr3_6809_star	10.7456993	8.059274	3.554060706	3.179395517	0.415037	1
chr7_15734_star	6.44741959	4.029637	2.896740642	2.330454351	0.678072	1
NW_003541090_39901_mature	8.59655945	4.835565	3.262517265	2.544872269	0.830075	1
bta-miR-1197	30.0879581	21.76004	4.958283956	4.508431259	0.467505	1
mmu-miR-193b-5p	2.14913986	4.835565	1.654957834	2.544872269	-1.16993	1
ssc-miR-490	6.44741959	12.08891	2.896740642	3.710273245	-0.90689	1
chr15_33460_mature	4.29827973	4.835565	2.405524014	2.544872269	-0.16993	1
chrx_21552_mature	4.29827973	3.22371	2.405524014	2.078510713	0.415037	1
mmu-miR-1964-5p	6.44741959	3.22371	2.896740642	2.078510713	1	1
chr13_27615_mature	2.14913986	5.641492	1.654957834	2.731507408	-1.39232	1
chr5_11215_star	2.14913986	3.22371	1.654957834	2.078510713	-0.58496	1
chr7_16525_star	2.14913986	4.835565	1.654957834	2.544872269	-1.16993	1
chr2_5906_mature	4.29827973	12.08891	2.405524014	3.710273245	-1.49185	1
chr5_11639_mature	8.59655945	8.059274	3.262517265	3.179395517	0.093109	1
bta-miR-873	6.44741959	12.89484	2.896740642	3.796477232	-1	1
chr1_2954_mature	8.59655945	8.865202	3.262517265	3.302348583	-0.04439	1
rno-miR-193-3p	8.59655945	4.029637	3.262517265	2.330454351	1.093109	1
chr1_376_mature	6.44741959	8.059274	2.896740642	3.179395517	-0.32193	1
chr4_9687_star	2.14913986	4.029637	1.654957834	2.330454351	-0.90689	1
mmu-miR-1964-3p	6.44741959	3.22371	2.896740642	2.078510713	1	1
chr13_27448_star	4.29827973	4.835565	2.405524014	2.544872269	-0.16993	1
chr1_569_star	4.29827973	6.44742	2.405524014	2.896740642	-0.58496	1
chr1_888_star	4.29827973	2.417782	2.405524014	1.773060525	0.830075	1
chr17_35671_mature	19.3422588	35.46081	4.346407978	5.188274618	-0.87447	1
chr1_1422_mature	2.14913986	3.22371	1.654957834	2.078510713	-0.58496	1
chr11_25303_mature	4.29827973	3.22371	2.405524014	2.078510713	0.415037	1
chr4_10551_star	8.59655945	4.029637	3.262517265	2.330454351	1.093109	1
chr2_3991_mature	4.29827973	3.22371	2.405524014	2.078510713	0.415037	1
chr12_25510_star	8.59655945	14.50669	3.262517265	3.954819241	-0.75489	1
chr15_33256_star	8.59655945	6.44742	3.262517265	2.896740642	0.415037	1
chr7_16635_mature	2.14913986	4.029637	1.654957834	2.330454351	-0.90689	1
chr14_31236_mature@@bta-miR-346	8.59655945	8.059274	3.262517265	3.179395517	0.093109	1
chr5_11275_mature	4.29827973	4.835565	2.405524014	2.544872269	-0.16993	1
chr5_12006_mature	12.8948392	9.671129	3.796477232	3.415640968	0.415037	1
chr1_1120_star	4.29827973	2.417782	2.405524014	1.773060525	0.830075	1
chr6_12675_mature	4.29827973	2.417782	2.405524014	1.773060525	0.830075	1
chr7_15130_star	2.14913986	5.641492	1.654957834	2.731507408	-1.39232	1
NW_003540275_39376_star	4.29827973	3.22371	2.405524014	2.078510713	0.415037	1
chr7_16002_star	2.14913986	5.641492	1.654957834	2.731507408	-1.39232	1
chr15_33538_mature	6.44741959	9.671129	2.896740642	3.415640968	-0.58496	1
chr5_11905_star	4.29827973	2.417782	2.405524014	1.773060525	0.830075	1
chr2_4557_star	10.7456993	4.835565	3.554060706	2.544872269	1.152003	1
NW_003538401_37828_mature	2.14913986	3.22371	1.654957834	2.078510713	-0.58496	1
chr14_31127_star	2.14913986	4.835565	1.654957834	2.544872269	-1.16993	1
chr4_9780_star	4.29827973	2.417782	2.405524014	1.773060525	0.830075	1
chr1_1312_mature	2.14913986	3.22371	1.654957834	2.078510713	-0.58496	1
chr1_88_mature	6.44741959	8.059274	2.896740642	3.179395517	-0.32193	1
chr5_11407_mature	6.44741959	8.865202	2.896740642	3.302348583	-0.45943	1
chr8_18423_mature	47.281077	33.84895	5.593385953	5.123043401	0.482152	1
NW_003539812_39109_mature	4.29827973	4.835565	2.405524014	2.544872269	-0.16993	1
chr14_30730_mature	2.14913986	3.22371	1.654957834	2.078510713	-0.58496	1
chr6_14103_mature	2.14913986	7.253347	1.654957834	3.044979305	-1.75489	1
chr15_32836_mature	21.4956793	35.46081	4.491301473	5.188274618	-0.72247	1
chr14_30762_star	6.44741959	7.253347	2.896740642	3.044979305	-0.16993	1
chr13_28823_mature	15.043979	29.81932	4.003960082	4.94576292	-0.98706	1
NW_003613264_38074_mature	2.14913986	6.44742	1.654957834	2.896740642	-1.58496	1
chr1_1411_star	4.29827973	3.22371	2.405524014	2.078510713	0.415037	1
chr12_25827_mature	4.29827973	3.22371	2.405524014	2.078510713	0.415037	1
chr15_32764_star	4.29827973	10.47706	2.405524014	3.520680821	-1.2854	1
chr11_25256_star	2.14913986	3.22371	1.654957834	2.078510713	-0.58496	1
chr4_8771_mature	8.59655945	12.08891	3.262517265	3.710273245	-0.49185	1
chr2_6204_mature	113.904413	148.2907	6.844290394	7.221980009	-0.3806	1
chr4_9346_mature	2.14913986	5.641492	1.654957834	2.731507408	-1.39232	1
chr3_8007_mature	8.59655945	4.029637	3.262517265	2.330454351	1.093109	1
chr8_18354_star	4.29827973	4.835565	2.405524014	2.544872269	-0.16993	1
chr16_34743_mature	23.6405385	16.92448	4.62296188	4.163859073	0.482152	1
NW_003539866_39141_star	4.29827973	4.835565	2.405524014	2.544872269	-0.16993	1
ssc-miR-146a-3p	2.14913986	5.641492	1.654957834	2.731507408	-1.39232	1
chr3_7373_star	2.14913986	3.22371	1.654957834	2.078510713	-0.58496	1
bta-miR-584	12.8948392	6.44742	3.796477232	2.896740642	1	1
chr1_3113_star	6.44741959	3.22371	2.896740642	2.078510713	1	1
chr12_25421_star	4.29827973	3.22371	2.405524014	2.078510713	0.415037	1
chr6_13572_star	2.14913986	5.641492	1.654957834	2.731507408	-1.39232	1
chr10_24085_star	6.44741959	3.22371	2.896740642	2.078510713	1	1
NW_003539007_38427_star	2.14913986	4.029637	1.654957834	2.330454351	-0.90689	1
chr18_36465_mature	17.1931189	33.04303	4.185320985	5.08928735	-0.94251	1
mmu-miR-6362	4.29827973	7.253347	2.405524014	3.044979305	-0.75489	1
chr12_25647_star	8.59655945	18.53633	3.262517265	4.288087668	-1.10852	1
mmu-miR-6356	2.14913986	6.44742	1.654957834	2.896740642	-1.58496	1
chr18_36112_mature	8.59655945	12.89484	3.262517265	3.796477232	-0.58496	1
chr16_34780_star	6.44741959	4.029637	2.896740642	2.330454351	0.678072	1
rno-miR-19b-2-5p	2.14913986	3.22371	1.654957834	2.078510713	-0.58496	1
NW_003541083_39895_mature	2.14913986	5.641492	1.654957834	2.731507408	-1.39232	1
bta-miR-2285j	2.14913986	4.029637	1.654957834	2.330454351	-0.90689	1
bta-miR-2285m	4.29827973	2.417782	2.405524014	1.773060525	0.830075	1
mmu-miR-6391	4.29827973	2.417782	2.405524014	1.773060525	0.830075	1
chrx_22864_star	2.14913986	3.22371	1.654957834	2.078510713	-0.58496	1
chr4_8979_mature	8.59655945	5.641492	3.262517265	2.731507408	0.607683	1
chr13_28207_mature	4.29827973	2.417782	2.405524014	1.773060525	0.830075	1
hsa-miR-3160-5p	6.44741959	4.835565	2.896740642	2.544872269	0.415037	1
chr6_13188_star	8.59655945	15.31262	3.262517265	4.027916745	-0.83289	1
chr4_10332_star	8.59655945	7.253347	3.262517265	3.044979305	0.245112	1
chr7_15712_star	2.14913986	3.22371	1.654957834	2.078510713	-0.58496	1
chr6_14061_mature	8.59655945	4.835565	3.262517265	2.544872269	0.830075	1
chr8_18596_mature	8.59655945	14.50669	3.262517265	3.954819241	-0.75489	1
chr2_5587_star	2.14913986	4.029637	1.654957834	2.330454351	-0.90689	1
chr1_244_mature	4.29827973	12.89484	2.405524014	3.796477232	-1.58496	1
chr13_28800_star	4.29827973	5.641492	2.405524014	2.731507408	-0.39232	1
NW_003540965_39767_star	10.7456993	6.44742	3.554060706	2.896740642	0.736966	1
NW_003540770_39675_mature	15.043979	24.98375	4.003960082	4.699537803	-0.7318	1
chr7_16979_star	4.29827973	3.22371	2.405524014	2.078510713	0.415037	1
chr1_1879_star	6.44741959	11.28298	2.896740642	3.618589217	-0.80735	1
chr4_9731_mature	15.043979	7.253347	4.003960082	3.044979305	1.052467	1
chr2_6507_mature	2.14913986	3.22371	1.654957834	2.078510713	-0.58496	1
chr5_12300_mature	6.44741959	7.253347	2.896740642	3.044979305	-0.16993	1
chr7_16645_mature	4.29827973	5.641492	2.405524014	2.731507408	-0.39232	1
chr6_14575_star	2.14913986	3.22371	1.654957834	2.078510713	-0.58496	1
mmu-miR-3081-5p	6.44741959	8.059274	2.896740642	3.179395517	-0.32193	1
chr14_29474_mature	8.59655945	4.029637	3.262517265	2.330454351	1.093109	1
NW_003539007_38451_mature	19.3422588	29.01339	4.346407978	4.907534286	-0.58496	1
chr9_19965_star	2.14913986	6.44742	1.654957834	2.896740642	-1.58496	1
rno-miR-7b	2.14913986	4.835565	1.654957834	2.544872269	-1.16993	1
chr14_30275_mature	10.7456993	8.059274	3.554060706	3.179395517	0.415037	1
chr9_20807_mature	17.1931189	8.865202	4.185320985	3.302348583	0.955606	1
chr6_14281_star	4.29827973	6.44742	2.405524014	2.896740642	-0.58496	1
bta-miR-631	4.29827973	5.641492	2.405524014	2.731507408	-0.39232	1
chr5_12148_star	10.7456993	4.835565	3.554060706	2.544872269	1.152003	1
NW_003540745_39661_star	2.14913986	4.029637	1.654957834	2.330454351	-0.90689	1
chr2_4589_star	4.29827973	2.417782	2.405524014	1.773060525	0.830075	1
chr17_35401_star	6.44741959	3.22371	2.896740642	2.078510713	1	1
chr14_29552_star	4.29827973	2.417782	2.405524014	1.773060525	0.830075	1
chr16_34464_star	4.29827973	2.417782	2.405524014	1.773060525	0.830075	1
NW_003541108_39908_mature	8.59655945	6.44742	3.262517265	2.896740642	0.415037	1
chr6_13263_mature	17.1931189	8.865202	4.185320985	3.302348583	0.955606	1
chr15_33559_star	2.14913986	4.029637	1.654957834	2.330454351	-0.90689	1
chr12_25570_mature	19.3422588	12.89484	4.346407978	3.796477232	0.584963	1
hsa-miR-4423-3p	2.14913986	5.641492	1.654957834	2.731507408	-1.39232	1
chr2_5581_mature	4.29827973	12.08891	2.405524014	3.710273245	-1.49185	1
chr4_9751_star	4.29827973	4.029637	2.405524014	2.330454351	0.093109	1
chr4_10054_star	4.29827973	4.835565	2.405524014	2.544872269	-0.16993	1
chr2_6092_star	6.44741959	4.029637	2.896740642	2.330454351	0.678072	1
chrx_22191_star	6.44741959	15.31262	2.896740642	4.027916745	-1.24793	1
chr18_36269_star	4.29827973	2.417782	2.405524014	1.773060525	0.830075	1
chr11_24816_star	4.29827973	2.417782	2.405524014	1.773060525	0.830075	1
chr8_17822_mature	4.29827973	7.253347	2.405524014	3.044979305	-0.75489	1
chr4_10283_star	6.44741959	7.253347	2.896740642	3.044979305	-0.16993	1
hsa-miR-589-5p	15.043979	8.059274	4.003960082	3.179395517	0.900464	1
chr4_9182_star	6.44741959	8.865202	2.896740642	3.302348583	-0.45943	1
hsa-miR-518c-5p	4.29827973	8.059274	2.405524014	3.179395517	-0.90689	1
chr11_24488_mature	4.29827973	3.22371	2.405524014	2.078510713	0.415037	1
chr11_25010_mature	2.14913986	3.22371	1.654957834	2.078510713	-0.58496	1
chr1_3094_star	4.29827973	2.417782	2.405524014	1.773060525	0.830075	1
chr1_888_mature	2.14913986	4.029637	1.654957834	2.330454351	-0.90689	1
chr16_34240_star	4.29827973	7.253347	2.405524014	3.044979305	-0.75489	1
hsa-miR-4802-5p	6.44741959	4.029637	2.896740642	2.330454351	0.678072	1
chr18_36372_mature	15.043979	8.059274	4.003960082	3.179395517	0.900464	1
chr12_26402_star	4.29827973	3.22371	2.405524014	2.078510713	0.415037	1
chr14_29752_star	10.7456993	5.641492	3.554060706	2.731507408	0.929611	1
chr2_5745_mature	12.8948392	7.253347	3.796477232	3.044979305	0.830075	1
chr3_8628_mature	10.7456993	16.92448	3.554060706	4.163859073	-0.65535	1
hsa-miR-6506-3p	4.29827973	7.253347	2.405524014	3.044979305	-0.75489	1
chr4_9939_mature	4.29827973	10.47706	2.405524014	3.520680821	-1.2854	1
chr14_30480_star	2.14913986	3.22371	1.654957834	2.078510713	-0.58496	1
chr14_30666_star	4.29827973	10.47706	2.405524014	3.520680821	-1.2854	1
chr4_10404_mature	6.44741959	4.835565	2.896740642	2.544872269	0.415037	1
chr14_29426_star	4.29827973	2.417782	2.405524014	1.773060525	0.830075	1
NW_003613325_38551_mature	2.14913986	5.641492	1.654957834	2.731507408	-1.39232	1
NW_003539007_38454_mature	10.7456993	20.14819	3.554060706	4.402462031	-0.90689	1
chr10_23778_mature	6.44741959	10.47706	2.896740642	3.520680821	-0.70044	1
chr13_27847_star	4.29827973	5.641492	2.405524014	2.731507408	-0.39232	1
chr3_7785_mature	2.14913986	4.029637	1.654957834	2.330454351	-0.90689	1
chr14_30385_star	2.14913986	4.029637	1.654957834	2.330454351	-0.90689	1
ssc-miR-1224	4.29827973	3.22371	2.405524014	2.078510713	0.415037	1
chr3_7564_mature	6.44741959	3.22371	2.896740642	2.078510713	1	1
chr14_30236_mature	4.29827973	4.029637	2.405524014	2.330454351	0.093109	1
NW_003613507_39496_mature	10.7456993	4.835565	3.554060706	2.544872269	1.152003	1
chr11_24816_mature	6.44741959	11.28298	2.896740642	3.618589217	-0.80735	1
NW_003613063_36957_star	2.14913986	3.22371	1.654957834	2.078510713	-0.58496	1
chr8_18198_star	10.7456993	6.44742	3.554060706	2.896740642	0.736966	1
chr17_34956_mature	2.14913986	7.253347	1.654957834	3.044979305	-1.75489	1
chr4_9649_star	2.14913986	6.44742	1.654957834	2.896740642	-1.58496	1
chr2_5680_star	4.29827973	2.417782	2.405524014	1.773060525	0.830075	1
chr5_11789_star	4.29827973	2.417782	2.405524014	1.773060525	0.830075	1
NW_003537125_36714_star	2.14913986	3.22371	1.654957834	2.078510713	-0.58496	1
chr15_32159_mature	4.29827973	5.641492	2.405524014	2.731507408	-0.39232	1
hsa-miR-6125	6.44741959	8.059274	2.896740642	3.179395517	-0.32193	1
chr15_31697_mature	2.14913986	3.22371	1.654957834	2.078510713	-0.58496	1
NW_003613519_39525_mature	6.44741959	9.671129	2.896740642	3.415640968	-0.58496	1
bta-miR-143	2.14913986	3.22371	1.654957834	2.078510713	-0.58496	1
chr6_13257_star	2.14913986	4.029637	1.654957834	2.330454351	-0.90689	1
chr10_23700_mature	4.29827973	3.22371	2.405524014	2.078510713	0.415037	1
bta-miR-1777a	2.14913986	3.22371	1.654957834	2.078510713	-0.58496	1
chr15_32589_star	2.14913986	3.22371	1.654957834	2.078510713	-0.58496	1
chr17_35424_star	8.59655945	4.029637	3.262517265	2.330454351	1.093109	1
NW_003613518_39516_mature	8.59655945	5.641492	3.262517265	2.731507408	0.607683	1
chr1_88_star	4.29827973	3.22371	2.405524014	2.078510713	0.415037	1
chr8_17722_star	6.44741959	7.253347	2.896740642	3.044979305	-0.16993	1
chr14_31127_mature	2.14913986	3.22371	1.654957834	2.078510713	-0.58496	1
chr1_376_star	4.29827973	3.22371	2.405524014	2.078510713	0.415037	1
chr11_24358_mature	4.29827973	4.029637	2.405524014	2.330454351	0.093109	1
chr16_33724_star	2.14913986	6.44742	1.654957834	2.896740642	-1.58496	1
chr4_10437_star	2.14913986	3.22371	1.654957834	2.078510713	-0.58496	1
mmu-miR-138-1-3p	2.14913986	4.029637	1.654957834	2.330454351	-0.90689	1
chr14_29876_star	2.14913986	4.835565	1.654957834	2.544872269	-1.16993	1
chr3_7761_mature	2.14913986	7.253347	1.654957834	3.044979305	-1.75489	1
chr17_35223_mature	2.14913986	6.44742	1.654957834	2.896740642	-1.58496	1
chr14_30390_star	2.14913986	3.22371	1.654957834	2.078510713	-0.58496	1
chr16_34229_mature	4.29827973	8.059274	2.405524014	3.179395517	-0.90689	1
chr13_29302_star	4.29827973	2.417782	2.405524014	1.773060525	0.830075	1
bta-miR-1260b	113.904413	83.01053	6.844290394	6.392498216	0.456457	1
NW_003537624_37070_star	4.29827973	2.417782	2.405524014	1.773060525	0.830075	1
chr8_18299_mature	4.29827973	10.47706	2.405524014	3.520680821	-1.2854	1
chr4_9970_mature	2.14913986	3.22371	1.654957834	2.078510713	-0.58496	1
chr1_3198_mature	4.29827973	11.28298	2.405524014	3.618589217	-1.39232	1
mmu-miR-504-5p	2.14913986	4.029637	1.654957834	2.330454351	-0.90689	1
chr7_16079_star	4.29827973	2.417782	2.405524014	1.773060525	0.830075	1
chr7_16234_mature	2.14913986	3.22371	1.654957834	2.078510713	-0.58496	1
chr6_14390_star	4.29827973	2.417782	2.405524014	1.773060525	0.830075	1
chr3_7424_mature	4.29827973	2.417782	2.405524014	1.773060525	0.830075	1
chr10_23743_star	6.44741959	3.22371	2.896740642	2.078510713	1	1
chr2_5320_mature	6.44741959	4.029637	2.896740642	2.330454351	0.678072	1
chr3_8628_star	4.29827973	8.059274	2.405524014	3.179395517	-0.90689	1
chr12_25719_mature	15.043979	9.671129	4.003960082	3.415640968	0.63743	1
chr13_27138_mature	12.8948392	6.44742	3.796477232	2.896740642	1	1
chr9_19852_mature	4.29827973	2.417782	2.405524014	1.773060525	0.830075	1
chr8_17786_star	6.44741959	3.22371	2.896740642	2.078510713	1	1
chr11_24796_star	4.29827973	4.835565	2.405524014	2.544872269	-0.16993	1
chr10_24200_star	6.44741959	4.029637	2.896740642	2.330454351	0.678072	1
chr16_33863_mature	12.8948392	25.78968	3.796477232	4.743605355	-1	1
chr3_7065_mature	4.29827973	12.89484	2.405524014	3.796477232	-1.58496	1
chr2_5958_star	6.44741959	8.059274	2.896740642	3.179395517	-0.32193	1
chr8_17430_mature	2.14913986	3.22371	1.654957834	2.078510713	-0.58496	1
chr11_25386_mature	4.29827973	4.029637	2.405524014	2.330454351	0.093109	1
chr2_5581_star	2.14913986	4.029637	1.654957834	2.330454351	-0.90689	1
chr6_12860_star	4.29827973	2.417782	2.405524014	1.773060525	0.830075	1
chr6_14191_mature	8.59655945	5.641492	3.262517265	2.731507408	0.607683	1
chr8_18895_star	2.14913986	5.641492	1.654957834	2.731507408	-1.39232	1
chr2_5810_mature	2.14913986	4.029637	1.654957834	2.330454351	-0.90689	1
chr4_9726_mature	6.44741959	11.28298	2.896740642	3.618589217	-0.80735	1
chr7_16325_mature	2.14913986	4.029637	1.654957834	2.330454351	-0.90689	1
chr8_18502_star	2.14913986	4.835565	1.654957834	2.544872269	-1.16993	1
chrx_22076_star	4.29827973	12.89484	2.405524014	3.796477232	-1.58496	1
hsa-miR-320c	4.29827973	5.641492	2.405524014	2.731507408	-0.39232	1
chr13_27982_star	4.29827973	3.22371	2.405524014	2.078510713	0.415037	1
hsa-miR-548e	4.29827973	4.835565	2.405524014	2.544872269	-0.16993	1
hsa-miR-548f	4.29827973	5.641492	2.405524014	2.731507408	-0.39232	1
chr7_15734_mature	8.59655945	5.641492	3.262517265	2.731507408	0.607683	1
chr4_10065_mature	12.8948392	19.34226	3.796477232	4.346407978	-0.58496	1
chr4_9818_star	2.14913986	3.22371	1.654957834	2.078510713	-0.58496	1
chr9_19650_mature	12.8948392	8.059274	3.796477232	3.179395517	0.678072	1
chr13_28822_mature	8.59655945	13.70077	3.262517265	3.877819487	-0.67243	1
hsa-miR-320a	4.29827973	2.417782	2.405524014	1.773060525	0.830075	1
hsa-miR-320b	10.7456993	8.865202	3.554060706	3.302348583	0.277534	1
chr6_12979_mature	4.29827973	12.08891	2.405524014	3.710273245	-1.49185	1
chr8_18291_star	8.59655945	5.641492	3.262517265	2.731507408	0.607683	1
chr3_7692_star	10.7456993	7.253347	3.554060706	3.044979305	0.567041	1
chr13_27163_star	4.29827973	5.641492	2.405524014	2.731507408	-0.39232	1
chr1_1120_mature	6.44741959	7.253347	2.896740642	3.044979305	-0.16993	1
chr16_34261_mature	32.237098	20.95411	5.054722516	4.456419386	0.621488	1
chr1_499_mature@@hsa-miR-2113	4.29827973	2.417782	2.405524014	1.773060525	0.830075	1
ssc-miR-212	23.6405385	12.08891	4.62296188	3.710273245	0.967579	1
chr9_19649_mature	4.29827973	8.059274	2.405524014	3.179395517	-0.90689	1
chr13_26847_star	2.14913986	5.641492	1.654957834	2.731507408	-1.39232	1
chr2_6348_mature	2.14913986	6.44742	1.654957834	2.896740642	-1.58496	1
bta-miR-27a-5p	2.14913986	4.029637	1.654957834	2.330454351	-0.90689	1
hsa-miR-4795-5p	2.14913986	4.835565	1.654957834	2.544872269	-1.16993	1

chr3_7220_mature	5.37285	11.28298	2.671938627	3.618589217	-1.07039	0.710681
chr1_1879_star	5.37285	11.28298	2.671938627	3.618589217	-1.07039	0.710681
ssc-miR-1271	5.37285	11.28298	2.671938627	3.618589217	-1.07039	0.710681
chr6_12675_star	6.44742	16.11855	2.896740642	4.097488514	-1.32193	0.7107
chr13_27528_mature	6.44742	16.11855	2.896740642	4.097488514	-1.32193	0.7107
chr1_182_mature	6.44742	16.11855	2.896740642	4.097488514	-1.32193	0.7107
chr1_1579_mature	18.26769	7.253347	4.268111626	3.044979305	1.332575	0.710823
chr16_34016_mature	18.26769	7.253347	4.268111626	3.044979305	1.332575	0.710823
chr3_8448_star	4.835565	8.865202	2.544872269	3.302348583	-0.87447	0.710916
chr5_11407_mature	4.835565	8.865202	2.544872269	3.302348583	-0.87447	0.710916
chr15_31790_star	4.835565	8.865202	2.544872269	3.302348583	-0.87447	0.710916
chr14_31502_star	4.835565	8.865202	2.544872269	3.302348583	-0.87447	0.710916
chr2_4256_mature	4.835565	8.865202	2.544872269	3.302348583	-0.87447	0.710916
chrx_22394_star	4.29828	5.641492	2.405524014	2.731507408	-0.39232	0.711156
chr10_23470_star	4.29828	5.641492	2.405524014	2.731507408	-0.39232	0.711156
chr4_10041_mature	4.29828	5.641492	2.405524014	2.731507408	-0.39232	0.711156
chr4_9452_mature	4.29828	5.641492	2.405524014	2.731507408	-0.39232	0.711156
chr11_24398_star	4.29828	5.641492	2.405524014	2.731507408	-0.39232	0.711156
chr6_14536_star	4.29828	5.641492	2.405524014	2.731507408	-0.39232	0.711156
hsa-miR-548f	4.29828	5.641492	2.405524014	2.731507408	-0.39232	0.711156
chr8_18291_star	4.29828	5.641492	2.405524014	2.731507408	-0.39232	0.711156
hsa-miR-4666b	4.29828	5.641492	2.405524014	2.731507408	-0.39232	0.711156
ssc-miR-126-5p	4.29828	5.641492	2.405524014	2.731507408	-0.39232	0.711156
chr7_16992_star	4.29828	5.641492	2.405524014	2.731507408	-0.39232	0.711156
chr5_11980_star	4.29828	5.641492	2.405524014	2.731507408	-0.39232	0.711156
chr1_1866_mature	60.7132	14.50669	5.947507226	3.954819241	2.065291	0.711962
chr18_36117_star	7.52199	4.835565	3.091190278	2.544872269	0.63743	0.712077
chr14_30616_mature	45.13194	348.9666	5.527693969	8.451073371	-2.95087	0.712433
chr12_26051_star	7.52199	20.95411	3.091190278	4.456419386	-1.47805	0.712656
ssc-miR-486	173.0058	27.40153	7.442991245	4.827896911	2.658492	0.712869
NW_003537296_36822_mature	30.08796	9.671129	4.958283956	3.415640968	1.63743	0.713147
chr6_14444_star	25.78968	8.865202	4.743605355	3.302348583	1.540568	0.71316
chr2_6283_star	10.7457	5.641492	3.554060706	2.731507408	0.929611	0.713414
chr5_11530_star	4.835565	4.835565	2.544872269	2.544872269	0	0.713748
chr7_15245_star	4.835565	4.835565	2.544872269	2.544872269	0	0.713748
chr5_11275_mature	4.835565	4.835565	2.544872269	2.544872269	0	0.713748
chr2_4557_star	4.835565	4.835565	2.544872269	2.544872269	0	0.713748
NW_003613031_36829_mature	4.835565	4.835565	2.544872269	2.544872269	0	0.713748
chr6_14899_mature	4.835565	4.835565	2.544872269	2.544872269	0	0.713748
chr15_31883_mature	4.835565	4.835565	2.544872269	2.544872269	0	0.713748
chr3_6720_mature	4.835565	4.835565	2.544872269	2.544872269	0	0.713748
chr2_5656_star	4.835565	4.835565	2.544872269	2.544872269	0	0.713748
chr10_23272_mature	4.835565	4.835565	2.544872269	2.544872269	0	0.713748
ssc-miR-194a	17.7304	7.253347	4.227310103	3.044979305	1.289507	0.713972
chr5_12148_star	6.984705	4.835565	2.997239027	2.544872269	0.530515	0.714253
mmu-miR-1929-5p	6.984705	4.835565	2.997239027	2.544872269	0.530515	0.714253
chr8_18283_mature	21.4914	8.059274	4.491301473	3.179395517	1.415037	0.714387
chr5_11160_star	4.835565	8.059274	2.544872269	3.179395517	-0.73697	0.715034
chr15_32726_mature	4.835565	8.059274	2.544872269	3.179395517	-0.73697	0.715034
NW_003539007_38450_mature	4.835565	8.059274	2.544872269	3.179395517	-0.73697	0.715034
chr1_376_mature	4.835565	8.059274	2.544872269	3.179395517	-0.73697	0.715034
chr12_25470_star	4.835565	8.059274	2.544872269	3.179395517	-0.73697	0.715034
chr9_20710_mature	4.835565	8.059274	2.544872269	3.179395517	-0.73697	0.715034
NW_003538873_38324_star	4.835565	8.059274	2.544872269	3.179395517	-0.73697	0.715034
NW_003539115_38548_mature	4.835565	8.059274	2.544872269	3.179395517	-0.73697	0.715034
chr13_29302_mature	6.44742	4.835565	2.896740642	2.544872269	0.415037	0.715718
chr12_25593_mature	6.44742	4.835565	2.896740642	2.544872269	0.415037	0.715718
chr16_33714_star	6.44742	4.835565	2.896740642	2.544872269	0.415037	0.715718
chr4_9934_mature	6.44742	4.835565	2.896740642	2.544872269	0.415037	0.715718
chr7_15287_star	6.44742	4.835565	2.896740642	2.544872269	0.415037	0.715718
chr15_32764_star	5.37285	10.47706	2.671938627	3.520680821	-0.96347	0.71573
chr1_1197_mature	5.37285	4.835565	2.671938627	2.544872269	0.152003	0.715748
NW_003613246_37882_star	5.37285	4.835565	2.671938627	2.544872269	0.152003	0.715748
chr3_7770_mature	5.37285	4.835565	2.671938627	2.544872269	0.152003	0.715748
hsa-miR-3122	5.37285	4.835565	2.671938627	2.544872269	0.152003	0.715748
chr16_34708_mature	5.37285	4.835565	2.671938627	2.544872269	0.152003	0.715748
chr14_30207_star	5.37285	4.835565	2.671938627	2.544872269	0.152003	0.715748
bta-miR-2340	5.910135	12.89484	2.788713818	3.796477232	-1.12553	0.715784
chr14_31521_star	5.910135	4.835565	2.788713818	2.544872269	0.289507	0.716296
chr12_26134_star	5.910135	4.835565	2.788713818	2.544872269	0.289507	0.716296
chr2_6582_star	5.910135	4.835565	2.788713818	2.544872269	0.289507	0.716296
chr3_7797_mature	5.910135	4.835565	2.788713818	2.544872269	0.289507	0.716296
bta-miR-380-5p	33.84895	10.47706	5.123043401	3.520680821	1.691878	0.716374
chr7_15675_mature	10.20841	5.641492	3.48651029	2.731507408	0.85561	0.716514
chr13_27066_mature	10.20841	5.641492	3.48651029	2.731507408	0.85561	0.716514
chr17_35617_mature	10.20841	5.641492	3.48651029	2.731507408	0.85561	0.716514
chr8_18510_star	6.984705	17.7304	2.997239027	4.227310103	-1.34395	0.716585
chr12_26266_star	4.835565	7.253347	2.544872269	3.044979305	-0.58496	0.717848
chr8_17822_mature	4.835565	7.253347	2.544872269	3.044979305	-0.58496	0.717848
chr15_33029_star	4.835565	7.253347	2.544872269	3.044979305	-0.58496	0.717848
chr2_6342_star	4.835565	5.641492	2.544872269	2.731507408	-0.22239	0.71788
chr8_18024_mature	4.835565	5.641492	2.544872269	2.731507408	-0.22239	0.71788
hsa-miR-3607-5p	4.835565	5.641492	2.544872269	2.731507408	-0.22239	0.71788
chr17_34966_mature	4.835565	5.641492	2.544872269	2.731507408	-0.22239	0.71788
chrx_22877_mature	4.835565	5.641492	2.544872269	2.731507408	-0.22239	0.71788
chr6_13930_mature	4.835565	5.641492	2.544872269	2.731507408	-0.22239	0.71788
chr3_6620_mature	4.835565	5.641492	2.544872269	2.731507408	-0.22239	0.71788
chr8_18895_star	4.835565	5.641492	2.544872269	2.731507408	-0.22239	0.71788
bta-miR-2887	17.7304	83.01053	4.227310103	6.392498216	-2.22707	0.718097
chr8_19035_star	12.35755	46.74379	3.739583968	5.577241251	-1.91938	0.718141
chr8_18505_mature	24.71511	8.865202	4.684544332	3.302348583	1.479168	0.718522
chr4_9091_mature	69.84705	16.11855	6.146635787	4.097488514	2.115477	0.718694
chr7_15704_star	4.835565	6.44742	2.544872269	2.896740642	-0.41504	0.718978
chr6_14281_star	4.835565	6.44742	2.544872269	2.896740642	-0.41504	0.718978
chr9_19346_mature	4.835565	6.44742	2.544872269	2.896740642	-0.41504	0.718978
chrx_21735_mature	4.835565	6.44742	2.544872269	2.896740642	-0.41504	0.718978
chr9_19723_mature	4.835565	6.44742	2.544872269	2.896740642	-0.41504	0.718978
chr4_9182_mature	899.9523	77.36904	9.815306944	6.292211829	3.54002	0.719259
chr13_28971_mature	9.671129	5.641492	3.415640968	2.731507408	0.777608	0.719338
mmu-miR-21c	9.671129	5.641492	3.415640968	2.731507408	0.777608	0.719338
chr12_25609_mature	9.671129	5.641492	3.415640968	2.731507408	0.777608	0.719338
chr1_3094_mature	5.910135	12.08891	2.788713818	3.710273245	-1.03242	0.720638
mmu-miR-6412	5.910135	12.08891	2.788713818	3.710273245	-1.03242	0.720638
chr13_27184_mature	8.596559	24.98375	3.262517265	4.699537803	-1.53916	0.7207
chr10_24060_star	24.17782	8.865202	4.654081667	3.302348583	1.447459	0.721169
chr8_18026_star	6.984705	16.92448	2.997239027	4.163859073	-1.27684	0.721507
rno-miR-532-5p	9.133844	5.641492	3.34110968	2.731507408	0.695145	0.721837
NW_003613109_37260_star	9.133844	5.641492	3.34110968	2.731507408	0.695145	0.721837
chr3_8247_mature	10.7457	36.26674	3.554060706	5.21981653	-1.75489	0.722327
chr3_7756_mature	7.52199	19.34226	3.091190278	4.346407978	-1.36257	0.722369
chr6_12641_star	5.37285	5.641492	2.671938627	2.731507408	-0.07039	0.722379
chr8_18144_mature	5.37285	5.641492	2.671938627	2.731507408	-0.07039	0.722379
NW_003537461_36946_mature	5.37285	5.641492	2.671938627	2.731507408	-0.07039	0.722379
chr2_5884_mature	5.37285	5.641492	2.671938627	2.731507408	-0.07039	0.722379
chr1_3728_mature	5.37285	5.641492	2.671938627	2.731507408	-0.07039	0.722379
chr8_18125_mature	5.37285	5.641492	2.671938627	2.731507408	-0.07039	0.722379
hsa-miR-4497	13.96941	55.60899	3.90394537	5.82295938	-1.99305	0.723003
chr8_17722_star	16.11855	7.253347	4.097488514	3.044979305	1.152003	0.723026
chr17_35011_mature	16.11855	7.253347	4.097488514	3.044979305	1.152003	0.723026
chr12_26356_mature	56.95221	14.50669	5.856791684	3.954819241	1.973033	0.72312
chr15_32008_mature	32.2371	10.47706	5.054722516	3.520680821	1.621488	0.723208
chr2_5051_star	5.37285	8.865202	2.671938627	3.302348583	-0.72247	0.723463
chr2_5550_star	5.37285	8.865202	2.671938627	3.302348583	-0.72247	0.723463
chr5_11689_star	8.059274	21.76004	3.179395517	4.508431259	-1.43296	0.723572
chr8_17646_mature	8.059274	21.76004	3.179395517	4.508431259	-1.43296	0.723572
chr14_29928_mature	98.86043	20.14819	6.641841268	4.402462031	2.294743	0.723786
chr8_18026_mature	23.64054	8.865202	4.62296188	3.302348583	1.415037	0.72379
hsa-miR-563	23.64054	8.865202	4.62296188	3.302348583	1.415037	0.72379
chr3_8521_star	8.596559	5.641492	3.262517265	2.731507408	0.607683	0.723949
chr13_28836_mature	8.596559	5.641492	3.262517265	2.731507408	0.607683	0.723949
chr2_5364_mature	8.596559	5.641492	3.262517265	2.731507408	0.607683	0.723949
NW_003538009_37453_mature	8.596559	5.641492	3.262517265	2.731507408	0.607683	0.723949
chr4_10721_mature	11.82027	41.90823	3.680354657	5.423182395	-1.82597	0.724833
chr5_10975_star	5.910135	11.28298	2.788713818	3.618589217	-0.93289	0.724947
chr17_35547_mature	5.910135	11.28298	2.788713818	3.618589217	-0.93289	0.724947
chr9_20405_mature	8.596559	24.17782	3.262517265	4.654081667	-1.49185	0.725084
chr17_35422_star	5.910135	5.641492	2.788713818	2.731507408	0.067114	0.725179
chr5_12345_mature	5.910135	5.641492	2.788713818	2.731507408	0.067114	0.725179
NW_003613031_36829_star	5.910135	5.641492	2.788713818	2.731507408	0.067114	0.725179
chr7_16002_star	5.910135	5.641492	2.788713818	2.731507408	0.067114	0.725179
chr3_7580_star	5.910135	5.641492	2.788713818	2.731507408	0.067114	0.725179
chr15_31672_star	27.40153	9.671129	4.827896911	3.415640968	1.5025	0.725443
ssc-miR-184	6.44742	13.70077	2.896740642	3.877819487	-1.08746	0.725595
chr13_29315_mature	8.059274	5.641492	3.179395517	2.731507408	0.514573	0.725596
chr13_27847_star	8.059274	5.641492	3.179395517	2.731507408	0.514573	0.725596
chrx_21673_star	8.059274	5.641492	3.179395517	2.731507408	0.514573	0.725596
chr15_31774_star	5.37285	6.44742	2.671938627	2.896740642	-0.26303	0.725722
chr6_13158_star	5.37285	6.44742	2.671938627	2.896740642	-0.26303	0.725722
chr7_16063_mature	5.37285	6.44742	2.671938627	2.896740642	-0.26303	0.725722
hsa-miR-1273e	5.37285	6.44742	2.671938627	2.896740642	-0.26303	0.725722
chr4_8989_mature	5.37285	8.059274	2.671938627	3.179395517	-0.58496	0.725765
chr1_398_mature	5.37285	8.059274	2.671938627	3.179395517	-0.58496	0.725765
chr9_20300_mature	5.37285	8.059274	2.671938627	3.179395517	-0.58496	0.725765
ssc-miR-499-3p	5.37285	8.059274	2.671938627	3.179395517	-0.58496	0.725765
chr5_10849_star	5.37285	8.059274	2.671938627	3.179395517	-0.58496	0.725765
chrx_21570_star	6.984705	16.11855	2.997239027	4.097488514	-1.20645	0.726175
chr8_18755_mature	11.82027	6.44742	3.680354657	2.896740642	0.874469	0.726602
chr14_31449_mature	5.37285	7.253347	2.671938627	3.044979305	-0.43296	0.726655
chr17_35787_mature	5.37285	7.253347	2.671938627	3.044979305	-0.43296	0.726655
chr14_30616_star	5.37285	7.253347	2.671938627	3.044979305	-0.43296	0.726655
chr13_27815_mature	5.37285	7.253347	2.671938627	3.044979305	-0.43296	0.726655
chr15_33084_star	6.44742	5.641492	2.896740642	2.731507408	0.192645	0.726657
chrx_23196_star	6.44742	5.641492	2.896740642	2.731507408	0.192645	0.726657
chr6_12803_mature	6.44742	5.641492	2.896740642	2.731507408	0.192645	0.726657
chr4_10247_star	6.44742	5.641492	2.896740642	2.731507408	0.192645	0.726657
chr2_6403_mature	6.44742	5.641492	2.896740642	2.731507408	0.192645	0.726657
chr14_30937_mature	6.44742	5.641492	2.896740642	2.731507408	0.192645	0.726657
chr12_26266_mature	7.52199	5.641492	3.091190278	2.731507408	0.415037	0.726684
chr17_35440_star	7.52199	5.641492	3.091190278	2.731507408	0.415037	0.726684
chr13_28475_star	7.52199	5.641492	3.091190278	2.731507408	0.415037	0.726684
chr1_550_star	7.52199	5.641492	3.091190278	2.731507408	0.415037	0.726684
chr2_4612_mature	7.52199	5.641492	3.091190278	2.731507408	0.415037	0.726684
NW_003613518_39516_mature	7.52199	5.641492	3.091190278	2.731507408	0.415037	0.726684
chr2_5816_star	7.52199	5.641492	3.091190278	2.731507408	0.415037	0.726684
chrx_22394_mature	7.52199	5.641492	3.091190278	2.731507408	0.415037	0.726684
chr6_13274_mature	9.133844	26.59561	3.34110968	4.786366653	-1.54189	0.726859
chr3_6615_mature	7.52199	18.53633	3.091190278	4.288087668	-1.30117	0.726955
chr12_25647_star	7.52199	18.53633	3.091190278	4.288087668	-1.30117	0.726955
chr3_7631_mature	7.52199	18.53633	3.091190278	4.288087668	-1.30117	0.726955
chr15_32159_mature	6.984705	5.641492	2.997239027	2.731507408	0.308122	0.72709
chr9_20685_star	6.984705	5.641492	2.997239027	2.731507408	0.308122	0.72709
chr9_19844_star	6.984705	5.641492	2.997239027	2.731507408	0.308122	0.72709
chr15_33053_mature	35.46081	11.28298	5.188274618	3.618589217	1.652077	0.728354
chr5_11369_mature	15.04398	7.253347	4.003960082	3.044979305	1.052467	0.728645
chr12_25849_mature	22.56597	8.865202	4.558633073	3.302348583	1.347923	0.728942
chr7_17029_mature	11.28298	6.44742	3.618589217	2.896740642	0.807355	0.729102
chr6_13364_mature	8.596559	23.3719	3.262517265	4.607146585	-1.44294	0.729356
rno-miR-741-3p	6.44742	12.89484	2.896740642	3.796477232	-1	0.729797
bta-miR-873	6.44742	12.89484	2.896740642	3.796477232	-1	0.729797
ssc-miR-214	448.0957	9044.924	8.810878975	13.14305212	-4.33523	0.729997
chr7_16464_star	20.41683	98.32315	4.420672964	6.634058094	-2.26777	0.730123
chr13_28542_star	5.910135	6.44742	2.788713818	2.896740642	-0.12553	0.730529
chr8_18290_mature	5.910135	6.44742	2.788713818	2.896740642	-0.12553	0.730529
chrx_22747_mature	5.910135	6.44742	2.788713818	2.896740642	-0.12553	0.730529
ssc-miR-376a-5p	5.910135	6.44742	2.788713818	2.896740642	-0.12553	0.730529
chr8_18291_mature	5.910135	6.44742	2.788713818	2.896740642	-0.12553	0.730529
chr7_15961_star	5.910135	6.44742	2.788713818	2.896740642	-0.12553	0.730529
chr13_28690_mature	5.910135	6.44742	2.788713818	2.896740642	-0.12553	0.730529
chr2_4013_mature	6.984705	15.31262	2.997239027	4.027916745	-1.13245	0.730553
chr14_31430_mature	52.11664	406.9934	5.73109203	8.672401869	-2.96519	0.731106
chr7_16069_mature	10.7457	6.44742	3.554060706	2.896740642	0.736966	0.731363
chr11_25207_star	11.28298	37.07266	3.618589217	5.250683565	-1.71621	0.732265
chr6_13692_mature	8.059274	20.14819	3.179395517	4.402462031	-1.32193	0.7323
chr5_11482_mature	100.4723	20.95411	6.664941981	4.456419386	2.261492	0.733286
NW_003613367_38733_mature	5.910135	7.253347	2.788713818	3.044979305	-0.29546	0.733288
chr15_33558_mature	5.910135	7.253347	2.788713818	3.044979305	-0.29546	0.733288
chr3_8256_star	5.910135	7.253347	2.788713818	3.044979305	-0.29546	0.733288
chr1_1197_star	5.910135	7.253347	2.788713818	3.044979305	-0.29546	0.733288
chr2_5746_mature	10.20841	6.44742	3.48651029	2.896740642	0.662965	0.733347
chr8_19054_star	10.20841	6.44742	3.48651029	2.896740642	0.662965	0.733347
chr2_5082_mature	5.910135	8.865202	2.788713818	3.302348583	-0.58496	0.733382
chr15_32062_mature	5.910135	8.865202	2.788713818	3.302348583	-0.58496	0.733382
chr2_6477_mature	5.910135	8.865202	2.788713818	3.302348583	-0.58496	0.733382
chr7_16587_mature	6.44742	12.08891	2.896740642	3.710273245	-0.90689	0.733507
chr11_24870_mature	6.44742	12.08891	2.896740642	3.710273245	-0.90689	0.733507
chr8_18439_mature	6.44742	12.08891	2.896740642	3.710273245	-0.90689	0.733507
chr6_12618_star	6.44742	12.08891	2.896740642	3.710273245	-0.90689	0.733507
chr2_5889_mature	17.7304	8.059274	4.227310103	3.179395517	1.137504	0.733639
chr6_14946_star	6.44742	6.44742	2.896740642	2.896740642	0	0.733824
chr8_17349_star	6.44742	6.44742	2.896740642	2.896740642	0	0.733824
chr7_16110_mature	6.44742	6.44742	2.896740642	2.896740642	0	0.733824
NW_003613206_37650_mature	6.44742	6.44742	2.896740642	2.896740642	0	0.733824
chrx_22707_mature	13.96941	7.253347	3.90394537	3.044979305	0.945552	0.733825
chr9_20889_star	13.96941	7.253347	3.90394537	3.044979305	0.945552	0.733825
chr16_33806_star	10.20841	30.62524	3.48651029	4.983004663	-1.58496	0.734742
mmu-miR-339-5p	0.537285	0	0.620384622	0	20	0.734972
hsa-miR-203b-3p	0.537285	0	0.620384622	0	20	0.734972
chr4_9963_star	0.537285	0	0.620384622	0	20	0.734972
rno-miR-218a-5p	0.537285	0	0.620384622	0	20	0.734972
chr3_7072_star	0.537285	0	0.620384622	0	20	0.734972
mmu-miR-135a-5p	0.537285	0	0.620384622	0	20	0.734972
mmu-miR-18b-3p	0.537285	0	0.620384622	0	20	0.734972
hsa-miR-16-5p	0.537285	0	0.620384622	0	20	0.734972
mmu-miR-744-5p	0.537285	0	0.620384622	0	20	0.734972
hsa-miR-4659b-5p	0.537285	0	0.620384622	0	20	0.734972
hsa-miR-6501-3p	0.537285	0	0.620384622	0	20	0.734972
hsa-miR-6716-5p	0.537285	0	0.620384622	0	20	0.734972
hsa-miR-329	0.537285	0	0.620384622	0	20	0.734972
mmu-miR-328-5p	0.537285	0	0.620384622	0	20	0.734972
mmu-miR-6537-5p	0.537285	0	0.620384622	0	20	0.734972
mmu-miR-494-5p	0.537285	0	0.620384622	0	20	0.734972
chr15_32479_mature	0.537285	0	0.620384622	0	20	0.734972
chr16_34282_mature	0.537285	0	0.620384622	0	20	0.734972
NW_003613026_36810_star	0.537285	0	0.620384622	0	20	0.734972
hsa-miR-150-3p	0.537285	0	0.620384622	0	20	0.734972
hsa-miR-130a-3p	0.537285	0	0.620384622	0	20	0.734972
chr1_2829_mature	0.537285	0	0.620384622	0	20	0.734972
mmu-miR-207	0.537285	0	0.620384622	0	20	0.734972
mmu-miR-666-5p	0.537285	0	0.620384622	0	20	0.734972
chr6_14212_star	0.537285	0	0.620384622	0	20	0.734972
mmu-miR-467d-5p	0.537285	0	0.620384622	0	20	0.734972
chr6_13603_star	0.537285	0	0.620384622	0	20	0.734972
mmu-miR-1298-3p	0.537285	0	0.620384622	0	20	0.734972
hsa-miR-195-3p	0.537285	0	0.620384622	0	20	0.734972
chr12_26195_star	0.537285	0	0.620384622	0	20	0.734972
hsa-miR-4779	0.537285	0	0.620384622	0	20	0.734972
hsa-miR-133a	0.537285	0	0.620384622	0	20	0.734972
chr13_28675_star	0.537285	0	0.620384622	0	20	0.734972
hsa-miR-4773	0.537285	0	0.620384622	0	20	0.734972
bta-miR-1721	0.537285	0	0.620384622	0	20	0.734972
hsa-miR-4794	0.537285	0	0.620384622	0	20	0.734972
chr7_16503_star	0.537285	0	0.620384622	0	20	0.734972
chr16_33853_star	0.537285	0	0.620384622	0	20	0.734972
hsa-miR-1305	0.537285	0	0.620384622	0	20	0.734972
rno-miR-3586-3p	0.537285	0	0.620384622	0	20	0.734972

chr9_20394_star	7.521989523	5.641492	3.091190278	2.731507408		0.415037
mmu-miR-1895	7.521989523	5.641492	3.091190278	2.731507408		0.415037
chr6_12641_star	7.521989523	5.641492	3.091190278	2.731507408		0.415037
chr8_18144_mature	7.521989523	5.641492	3.091190278	2.731507408		0.415037
chr2_5884_mature	7.521989523	5.641492	3.091190278	2.731507408		0.415037
chr6_13930_star	7.521989523	5.641492	3.091190278	2.731507408		0.415037
chr8_17690_mature	102.0841435	1112.18	6.687678623	10.12047102		-3.44556
bta-miR-1247-3p	11.82026925	36.26674	3.680354657	5.21981653		-1.61738
rno-miR-222-3p	19.34225877	8.059274	4.346407978	3.179395517		1.263034
chr1_1866_mature	52.65392666	14.50669	5.745611853	3.954819241		1.859822
chr5_12354_mature	29.01338816	157.1559	4.907534286	7.305203133		-2.43741
chr8_17792_mature	82.74188475	19.34226	6.387877484	4.346407978		2.096862
chr1_785_mature	11.82026925	6.44742	3.680354657	2.896740642		0.874469
chr13_29407_star	6.447419591	10.47706	2.896740642	3.520680821		-0.70044
chr2_4013_mature	56.95220639	15.31262	5.856791684	4.027916745		1.89503
chr14_31199_mature	264.3442032	41.90823	8.051721221	5.423182395		2.657112
mmu-miR-3109-5p	6.447419591	6.44742	2.896740642	2.896740642		0
chr1_623_mature	6.447419591	6.44742	2.896740642	2.896740642		0
chr17_35760_mature	6.447419591	6.44742	2.896740642	2.896740642		0
chr3_7313_mature	6.447419591	6.44742	2.896740642	2.896740642		0
chr4_9731_mature	15.04397905	7.253347	4.003960082	3.044979305		1.052467
chr2_5750_mature	36.53537768	223.2419	5.230179094	7.808912085		-2.61124
chr11_25362_mature	87.04016448	20.14819	6.460089935	4.402462031		2.111031
chr4_9198_star	6.447419591	9.671129	2.896740642	3.415640968		-0.58496
NW_003537296_36822_mature	6.447419591	9.671129	2.896740642	3.415640968		-0.58496
NW_003613519_39525_mature	6.447419591	9.671129	2.896740642	3.415640968		-0.58496
chr12_25719_mature	6.447419591	9.671129	2.896740642	3.415640968		-0.58496
chr12_25481_mature	6.447419591	9.671129	2.896740642	3.415640968		-0.58496
chr14_30679_mature	6.447419591	9.671129	2.896740642	3.415640968		-0.58496
chr8_19043_mature	6.447419591	9.671129	2.896740642	3.415640968		-0.58496

[illegible]

chr13_27560_mature	719.9618543	95.90537	9.493779119	6.598504656	2.908237	0.593505	chr3_8074_star	2.14913986	4.029637	1.654957834	2.330454351	-0.90689	1	bta-miR-2367-5p	0.537285	0	0.620384622	0	20	0.734972
chr17_34991_mature	18.26768884	48.35565	4.268111626	5.625143252	-1.40439	0.593512	chrx_22034_mature	2.14913986	4.029637	1.654957834	2.330454351	-0.90689	1	chr6_14458_star	0.537285	0	0.620384622	0	20	0.734972
chr12_25786_mature	12.89483918	20.95411	3.796477232	4.456419386	-0.70044	0.593598	mmu-miR-466f	4.29827973	8.865202	2.405524014	3.302348583	-1.04439	1	mmu-miR-344d-3-5p	0.537285	0	0.620384622	0	20	0.734972
chr8_18872_star	243.9273745	46.74379	7.936210217	5.577241251	2.383605	0.593952	chr8_19026_star	2.14913986	4.835565	1.654957834	2.544872269	-1.16993	1	ssc-miR-484	0.537285	0	0.620384622	0	20	0.734972
chr9_20853_star	310.5507103	54.80307	8.283323196	5.802272499	2.5025	0.59461	chr15_32377_mature	17.1931189	11.28298	4.185320985	3.618589217	0.607683	1	rno-miR-3584-5p	0.537285	0	0.620384622	0	20	0.734972
chr7_15488_mature	12.89483918	13.70077	3.796477232	3.877819487	-0.08746	0.594635	chr1_1175_star	4.29827973	8.059274	2.405524014	3.179395517	-0.90689	1	hsa-miR-5008-5p	0.537285	0	0.620384622	0	20	0.734972
bta-miR-504	12.89483918	13.70077	3.796477232	3.877819487	-0.08746	0.594635	NW_003538201_37611_star	2.14913986	4.835565	1.654957834	2.544872269	-1.16993	1	rno-miR-1224	0.537285	0	0.620384622	0	20	0.734972
chr4_8771_mature	16.11854898	12.08891	4.097488514	3.710273245	0.415037	0.595241	chr5_11934_mature	10.7456993	5.641492	3.554060706	2.731507408	0.929611	1	hsa-miR-98-5p	0.537285	0	0.620384622	0	20	0.734972
bta-miR-380-3p	198.7954374	41.1023	7.642379827	5.395827139	2.273994	0.595344	chr10_23272_mature	2.14913986	4.835565	1.654957834	2.544872269	-1.16993	1	bta-miR-874	0.537285	0	0.620384622	0	20	0.734972
NW_003539698_39033_mature	17.19311891	12.08891	4.185320985	3.710273245	0.508147	0.595403	chr1_1859_mature	4.29827973	8.865202	2.405524014	3.302348583	-1.04439	1	rno-miR-106b-5p	0.537285	0	0.620384622	0	20	0.734972
NW_003539007_38456_mature	61.25048611	20.14819	5.960013198	4.402462031	1.604071	0.595449	chr6_12914_mature	6.44741959	4.835565	2.896740642	2.544872269	0.415037	1	rno-miR-374-3p	0.537285	0	0.620384622	0	20	0.734972
chrx_22851_mature	27.93881823	13.70077	4.854934103	3.877819487	1.028014	0.595503	chr17_35299_star	12.8948392	10.47706	3.796477232	3.520680821	0.29956	1	chr1_3439_star	0.537285	0	0.620384622	0	20	0.734972
chr9_19230_mature	12.89483918	19.34226	3.796477232	4.346407978	-0.58496	0.596554	chr7_16781_mature	6.44741959	4.029637	2.896740642	2.330454351	0.678072	1	chr6_13950_star	0.537285	0	0.620384622	0	20	0.734972
chr9_20876_mature	12.89483918	14.50669	3.796477232	3.954819241	-0.16993	0.596596	chr4_10263_mature	6.44741959	4.029637	2.896740642	2.330454351	0.678072	1	hsa-miR-642b-3p	0.537285	0	0.620384622	0	20	0.734972
chr4_10650_mature	35.46080775	154.7381	5.188274618	7.282977844	-2.12553	0.597469	chr3_8078_star	2.14913986	4.029637	1.654957834	2.330454351	-0.90689	1	mmu-miR-877-3p	0.537285	0	0.620384622	0	20	0.734972
chr13_28297_star@@bta-miR-26c	12.89483918	17.7304	3.796477232	4.227310103	-0.45943	0.598315	chr1_1055_star	4.29827973	9.671129	2.405524014	3.415640968	-1.16993	1	chr6_12437_star	0.537285	0	0.620384622	0	20	0.734972
chr11_24906_mature	107.4569932	28.20746	6.760979269	4.868265031	1.929611	0.598315	chr9_20257_star	6.44741959	16.11855	2.896740642	4.097488514	-1.32193	1	rno-miR-7a-1-3p	0.537285	0	0.620384622	0	20	0.734972
ssc-miR-183	80.59274489	582.6855	6.35036897	9.189047531	-2.854	0.598829	chr13_27500_mature	4.29827973	10.47706	2.405524014	3.520680821	-1.2854	1	chr18_35842_star	0.537285	0	0.620384622	0	20	0.734972
chr5_12257_mature	122.5009722	30.62524	6.948378589	4.983004663	2	0.599209	chr6_14675_mature	6.44741959	6.44742	2.896740642	2.896740642	0	1	chr1_1155_star	0.537285	0	0.620384622	0	20	0.734972
ssc-miR-363	39919.1984	1648.122	15.28483127	10.6874821	4.598188	0.599322	chr2_4363_mature	6.44741959	3.22371	2.896740642	2.078510713	1	1	mmu-miR-3099-3p	0.537285	0	0.620384622	0	20	0.734972
ssc-miR-190a	347.086088	59.63863	8.443300345	5.922165283	2.540974	0.599855	chr1_3810_mature	6.44741959	3.22371	2.896740642	2.078510713	1	1	NW_003613025_36805_mature	0.537285	0	0.620384622	0	20	0.734972
ssc-miR-361-5p	1693.522213	174.8863	10.72666283	7.458498946	3.275538	0.600861	bta-miR-2330-3p	2.14913986	4.029637	1.654957834	2.330454351	-0.90689	1	mmu-miR-322-5p	0.537285	0	0.620384622	0	20	0.734972
chr12_25408_mature	16.11854898	12.89484	4.097488514	3.796477232	0.321928	0.602111	chrx_21721_mature	4.29827973	4.835565	2.405524014	2.544872269	-0.16993	1	bta-let-7a-3p	0.537285	0	0.620384622	0	20	0.734972
chr14_29661_mature	23.6405385	74.14533	4.62296188	6.231611453	-1.64909	0.60289	chr15_33009_star	8.59655945	4.835565	3.262517265	2.544872269	0.830075	1	bta-miR-2284aa-3	0.537285	0	0.620384622	0	20	0.734972
ssc-miR-450b-3p	18.26768884	12.89484	4.268111626	3.796477232	0.5025	0.603403	chr7_15474_star	2.14913986	5.641492	1.654957834	2.731507408	-1.39232	1	bta-miR-2284aa-2	0.537285	0	0.620384622	0	20	0.734972
ssc-miR-664-3p	13.96940911	14.50669	3.90394537	3.954819241	-0.05445	0.603414	chr3_8144_star	8.59655945	14.50669	3.262517265	3.954819241	-0.75489	1	mmu-miR-3099-5p	0.537285	0	0.620384622	0	20	0.734972
chr7_16536_mature	16.11854898	33.84895	4.097488514	5.123043401	-1.07039	0.604026	chr16_34089_star	2.14913986	7.253347	1.654957834	3.044979305	-1.75489	1	rno-miR-543-3p	0.537285	0	0.620384622	0	20	0.734972
chr13_27259_mature	16.11854898	33.84895	4.097488514	5.123043401	-1.07039	0.604026	NW_003613297_38335_star	4.29827973	4.029637	2.405524014	2.330454351	0.093109	1	chr4_9640_star	0.537285	0	0.620384622	0	20	0.734972
chr8_18423_star	29.01338816	14.50669	4.907534286	3.954819241	1	0.604454	NW_003541025_39829_mature	10.7456993	8.865202	3.554060706	3.302348583	0.277534	1	mmu-miR-322-3p	0.537285	0	0.620384622	0	20	0.734972
chr1_1981_mature	15.04397905	13.70077	4.003960082	3.877819487	0.13493	0.604786	chr9_19723_mature	6.44741959	6.44742	2.896740642	2.896740642	0	1	hsa-miR-4798-3p	0.537285	0	0.620384622	0	20	0.734972
chr17_35096_mature	13.96940911	15.31262	3.90394537	4.027916745	-0.13245	0.605578	chr6_12875_mature	4.29827973	12.89484	2.405524014	3.796477232	-1.58496	1	rno-miR-1839-3p	0.537285	0	0.620384622	0	20	0.734972
chr13_27084_star	40.83365741	16.92448	5.38659223	4.163859073	1.270648	0.605962	chr15_32008_mature	6.44741959	10.47706	2.896740642	3.520680821	-0.70044	1	rno-miR-150-5p	0.537285	0	0.620384622	0	20	0.734972
chr5_11482_mature	13.96940911	20.95411	3.90394537	4.456419386	-0.58496	0.606519	chr3_8536_mature	2.14913986	3.22371	1.654957834	2.078510713	-0.58496	1	mmu-miR-673-3p	0.537285	0	0.620384622	0	20	0.734972
chr4_9509_mature	15.04397905	27.40153	4.003960082	4.827896911	-0.86507	0.606633	chr9_20222_star	6.44741959	4.029637	2.896740642	2.330454351	0.678072	1	mmu-miR-351-5p	0.537285	0	0.620384622	0	20	0.734972
chr2_6585_mature	15.04397905	27.40153	4.003960082	4.827896911	-0.86507	0.606633	NW_003613109_37260_star	2.14913986	5.641492	1.654957834	2.731507408	-1.39232	1	hsa-miR-548am-5p	0.537285	0	0.620384622	0	20	0.734972
chr12_26356_mature	27.93881823	14.50669	4.854934103	3.954819241	0.945552	0.607266	chr2_3979_star	6.44741959	5.641492	2.896740642	2.731507408	0.192645	1	hsa-miR-21-3p	0.537285	0	0.620384622	0	20	0.734972
chr15_33547_mature	243.9273745	3136.67	7.936210217	11.61547774	-3.68471	0.608511	chr14_30214_star	2.14913986	3.22371	1.654957834	2.078510713	-0.58496	1	rno-miR-1188-3p	0.537285	0	0.620384622	0	20	0.734972
chr13_29245_mature	15.04397905	14.50669	4.003960082	3.954819241	0.052467	0.608614	chr6_13930_star	4.29827973	5.641492	2.405524014	2.731507408	-0.39232	1	rno-miR-500-5p	0.537285	0	0.620384622	0	20	0.734972
chr2_5398_mature	39.75908748	16.92448	5.349049847	4.163859073	1.232173	0.609121	chr12_26006_star	4.29827973	3.22371	2.405524014	2.078510713	0.415037	1	mmu-miR-5625-3p	0.537285	0	0.620384622	0	20	0.734972
chr10_23904_mature	92.41301414	26.59561	6.545551653	4.786366653	1.796908	0.609418	NW_003537641_37087_mature	6.44741959	14.50669	2.896740642	3.954819241	-1.16993	1	hsa-miR-4712-3p	0.537285	0	0.620384622	0	20	0.734972
chr8_17694_mature	21.49139864	13.70077	4.491301473	3.877819487	0.649503	0.609631	NW_003613226_37753_star	2.14913986	3.22371	1.654957834	2.078510713	-0.58496	1	chr13_29223_star	0.537285	0	0.620384622	0	20	0.734972
chr6_14454_mature	8848.008819	568.1789	13.11130015	9.152738248	3.960936	0.609738	chr3_8165_star	4.29827973	4.029637	2.405524014	2.330454351	0.093109	1	chr9_20047_star	0.537285	0	0.620384622	0	20	0.734972
chr5_11847_mature	18.26768884	13.70077	4.268111626	3.877819487	0.415037	0.610753	chr3_7586_mature	6.44741959	6.44742	2.896740642	2.896740642	0	1	chr15_33370_mature	0.537285	0	0.620384622	0	20	0.734972
chr18_35822_mature@@mmu-miR-5	2703.617948	246.6138	11.4012091	7.951947907	3.454566	0.610833	chr17_35787_mature	15.043979	7.253347	4.003960082	3.044979305	1.052467	1	rno-miR-19b-1-5p	0.537285	0	0.620384622	0	20	0.734972
rno-miR-203b-3p	21.49139864	58.8327	4.491301473	5.902862353	-1.45286	0.610847	chr16_33714_star	4.29827973	4.835565	2.405524014	2.544872269	-0.16993	1	rno-miR-147	0.537285	0	0.620384622	0	20	0.734972
chr14_30768_mature@@bta-miR-247	42.98279727	17.7304	5.458867456	4.227310103	1.277534	0.61162	mmu-miR-378d	4.29827973	4.029637	2.405524014	2.330454351	0.093109	1	mmu-miR-30b-3p	0.537285	0	0.620384622	0	20	0.734972
chr12_25510_star	25.78967836	14.50669	4.743605355	3.954819241	0.830075	0.612155	chr15_33370_star	4.29827973	7.253347	2.405524014	3.044979305	-0.75489	1	NW_003538396_37820_star	0.537285	0	0.620384622	0	20	0.734972
chrx_22412_mature	220.286836	45.93786	7.78977382	5.552680304	2.261628	0.614613	chr7_16969_mature	10.7456993	9.671129	3.554060706	3.415640968	0.152003	1	chr18_36398_mature@@hsa-miR-1	0.537285	0	0.620384622	0	20	0.734972
chr10_23470_mature	41.90822734	17.7304	5.423182395	4.227310103	1.241008	0.61464	chr3_7797_mature	4.29827973	4.835565	2.405524014	2.544872269	-0.16993	1	bta-miR-450b	0.537285	0	0.620384622	0	20	0.734972
chr5_11533_mature	38.68451755	165.2151	5.310504361	7.376907876	-2.09452	0.615061	chr5_12427_mature	15.043979	12.08891	4.003960082	3.710273245	0.315502	1	NW_003613374_38740_star	0.537285	0	0.620384622	0	20	0.734972
chr16_34780_mature	17.19311891	14.50669	4.185320985	3.954819241	0.245112	0.615203	chr3_7631_mature	8.59655945	18.53633	3.262517265	4.288087668	-1.10852	1	hsa-miR-3180-5p	0.537285	0	0.620384622	0	20	0.734972
chr10_23676_mature	17.19311891	14.50669	4.185320985	3.954819241	0.245112	0.615203	hsa-miR-1260b	12.8948392	10.47706	3.796477232	3.520680821	0.29956	1	hsa-miR-1180	0.537285	0	0.620384622	0	20	0.734972
chr17_35129_mature	15.04397905	16.92448	4.003960082	4.163859073	-0.16993	0.615638	chr2_4882_star	2.14913986	4.029637	1.654957834	2.330454351	-0.90689	1	rno-let-7a-2-3p	0.537285	0	0.620384622	0	20	0.734972
ssc-miR-193a-3p	6944.945469	484.3624	12.76195537	8.92291853	3.841805	0.616948	chr14_31102_mature	8.59655945	13.70077	3.262517265	3.877819487	-0.67243	1	hsa-miR-1193	0.537285	0	0.620384622	0	20	0.734972
chr5_11336_mature	21.49139864	55.60899	4.491301473	5.82295938	-1.37156	0.618628	chr5_11904_star	2.14913986	4.029637	1.654957834	2.330454351	-0.90689	1	chr3_7170_star	0.537285	0	0.620384622	0	20	0.734972
chr12_26026_mature	61.25048611	21.76004	5.960013198	4.508431259	1.49304	0.61884	chr4_8951_star	6.44741959	3.22371	2.896740642	2.078510713	1	1	chr9_19638_star	0.537285	0	0.620384622	0	20	0.734972
chr16_34245_mature	65.54876584	22.56597	6.056340006	4.558633073	1.53842	0.618895	chr14_30207_star	6.44741959	4.835565	2.896740642	2.544872269	0.415037	1	chr3_6623_star	0.537285	0	0.620384622	0	20	0.734972
chr1_270_star	52.65392666	20.14819	5.745611853	4.402462031	1.385891	0.619164	chr9_19396_mature	21.4913986	15.31262	4.491301473	4.027916745	0.489038	1	rno-miR-377-3p	0.537285	0	0.620384622	0	20	0.734972
ssc-miR-345-3p	84.89102461	586.7152	6.424435476	9.198973359	-2.78898	0.619361	ssc-miR-432-3p	4.29827973	2.417782	2.405524014	1.773060525	0.830075	1	hsa-miR-4776-5p	0.537285	0	0.620384622	0	20	0.734972
chr14_30535_mature	17.19311891	15.31262	4.185320985	4.027916745	0.16711	0.619762	chr3_7678_star	10.7456993	5.641492	3.554060706	2.731507408	0.929611	1	rno-miR-99a-3p	0.537285	0	0.620384622	0	20	0.734972
chr10_23430_mature	17.19311891	15.31262	4.185320985	4.027916745	0.16711	0.619762	chr13_27642_star	4.29827973	4.029637	2.405524014	2.330454351	0.093109	1	rno-let-7a-5p	0.537285	0	0.620384622	0	20	0.734972
chr2_4571_mature	35.46080775	16.92448	5.188274618	4.163859073	1.067114	0.620772	chr2_6403_mature	8.59655945	5.641492	3.262517265	2.731507408	0.607683	1	hsa-miR-515-5p	0.537285	0	0.620384622	0	20	0.734972
chr14_30942_mature@@bta-miR-301	141.843231	35.46081	7.158288862	5.188274618	2	0.620832	chr12_26324_star	6.44741959	4.835565	2.896740642	2.544872269	0.415037	1	chr1_1669_star	0.537285	0	0.620384622	0	20	0.734972
chr6_14927_mature	16.11854898	16.92448	4.097488514	4.163859073	-0.07039	0.621753	chr6_14217_mature	4.29827973	4.029637	2.405524014	2.330454351	0.093109	1	mmu-miR-3074-1-3p	0.537285	0	0.620384622	0	20	0.734972
chr1_3451_mature	18.26768884	15.31262	4.268111626	4.027916745	0.254573	0.622257	chr15_32921_star	2.14913986	3.22371	1.654957834	2.078510713	-0.58496	1	mmu-miR-33-3p	0.537285	0	0.620384622	0	20	0.734972
chr2_6509_mature	17.19311891	16.11855	4.185320985	4.097488514	0.093109	0.623531	chr9_20563_mature	4.29827973	2.417782	2.405524014	1.773060525	0.830075	1	chr1_1974_star	0.537285	0	0.620384622	0	20	0.734972
chr6_12525_star@@bta-miR-2890	16.11854898	17.7304	4.097488514	4.227310103	-0.1375	0.623601	chr1_3439_mature@@rno-miR-872-5p	4.29827973	3.22371	2.405524014	2.078510713	0.415037	1	hsa-miR-4713-5p	0.537285	0	0.620384622	0	20	0.734972
chr8_18510_mature	16.11854898	19.34226	4.097488514	4.346407978	-0.26303	0.625874	chr7_16559_star	2.14913986	3.22371	1.654957834	2.078510713	-0.58496	1	chr15_33030_mature	0.537285	0	0.620384622	0	20	0.734972
chr4_9141_mature	62.32505605	22.56597	5.984704544	4.558633073	1.465664	0.626777	chr9_20889_star	2.14913986	7.253347	1.654957834	3.044979305	-1.75489	1	chr9_19527_star	0.537285	0	0.620384622	0	20	0.734972
NW_003537647_37094_mature	2522.01563	242.5842	11.30093343	7.928276522	3.37802	0.628178	chr3_7313_mature	2.14913986	6.44742	1.654957834	2.896740642	-1.58496	1	mmu-miR-6365	0.537285	0	0.620384622	0	20	0.734972
chr13_29407_mature	102.0841435	29.81932	6.687678623	4.94576292	1.77544	0.628821	chr17_35096_star	4.29827973	4.029637	2.405524014	2.330454351	0.093109	1	mmu-miR-6360	0.537285	0	0.620384622	0	20	0.734972
mmu-miR-6238	20.4168287	44.32601	4.420672964	5.502267253	-1.11839	0.632065	NW_003539180_38634_star	4.29827973	6.44742	2.405524014	2.896740642	-0.58496	1	mmu-miR-547-3p	0.537285	0	0.620384622	0	20	0.734972
chr5_11941_mature	17.19311891	26.59561	4.185320985	4.786366653	-0.62936	0.632065	chr3_8071_mature@@rno-miR-409b	23.6405385	16.11855	4.62296188	4.097488514	0.552541	1	hsa-miR-4653-5p	0.537285	0	0.620384622	0	20	0.734972
chr6_14878_star	42.98279727	19.34226	5.458867456	4.346407978	1.152003	0.63303	chr6_14856_mature	6.44741959	13.70077	2.896740642	3.877819487	-1.08746	1	mmu-miR-6351	0.537285	0	0.620384622	0	20	0.734972
NW_003613519_39525_star	20.4168287	43.52008	4.420672964	5.476384353	-1.09192	0.633854	chr1_1498_star	4.29827973	4.029637	2.405524014	2.330454351	0.093109	1	mmu-miR-6357	0.537285	0	0.620384622	0	20	0.734972
chr9_19770_mature	29.01338816	16.92448	4.907534286	4.163859073	0.777608	0.633906	chr3_8626_mature	8.59655945	14.50669	3.262517265	3.954819241	-0.75489	1	mmu-miR-6380	0.537285	0	0.620384622	0	20	0.734972
chr14_30067_star	54.80306652	21.76004	5.802272499	4.508431259	1.332575	0.634638	chr1_1981_mature	19.3422588	13.70077	4.346407978	3.877819487	0.4975	1	hsa-miR-3927-5p	0.537285	0	0.620384622	0	20	0.734972
chr6_12598_mature	108.5315631	31.43117	6.775202853	5.019309189	1.787847	0.634686	chrx_21673_star	6.44741959	5.641492	2.896740642	2.731507408	0.192645	1	bta-miR-2284r	0.537285	0	0.620384622	0	20	0.734972
chr4_10149_star	46.20650707	20.14819	5.560913833	4.402462031	1.197446	0.635248	NW_003613226_37753_mature	4.29827973	4.029637	2.405524014	2.330454351	0.093109	1	bta-miR-2284u	0.537285	0	0.620384622	0	20	0.734972
chrx_22266_star	41.90822734	19.34226	5.423182395	4.346407978	1.115477	0.635508	chr4_8669_star	4.29827973	2.417782	2.405524014	1.773060525	0.830075	1	bta-miR-2284v	0.537285	0	0.620384622	0	20	0.734972
ssc-miR-185	589.9388926	10970.28	9.206865143	13.42144481	-4.21689	0.63602	chr12_25687_star	4.29827973	3.22371	2.405524014	2.078510713	0.415037	1	bta-miR-2284w	0.537285	0	0.620384622	0	20	0.734972
chr2_5750_star	25.78967836	16.92448	4.743605355	4.163859073	0.607683	0.637398	mmu-miR-3110-3p	2.14913986	3.22371	1.654957834	2.078510713	-0.58496	1	bta-miR-2284d	0.537285	0	0.620384622	0	20	0.734972
ssc-miR-499-5p	136.4703813	36.26674	7.102977006	5.21981653	1.911869	0.637685	chr14_30563_mature	6.44741959	4.029637	2.896740642	2.330454351	0.678072	1	bta-miR-2284f	0.537285	0	0.620384622	0	20	0.734972
chrx_22576_star	36.53537768	18.53633	5.230179094	4.288087668	0.978938	0.637795	chr8_18750_star	8.59655945	4.029637	3.262517265	2.330454351	1.093109	1	mmu-miR-6398	0.537285	0	0.620384622	0	20	0.734972
chr3_7261_mature	260.0459235	53.99714	8.028159819	5.781284667	2.267812	0.637975	chr15_32413_star	4.29827973	9.671129	2.405524014	3.415640968	-1.16993	1	mmu-miR-184-3p	0.537285	0	0.620384622	0	20	0.734972
NW_003539007_38451_mature	92.41301414	29.01339	6.545551653	4.907534286	1.671377	0.638473	chr17_35071_mature	15.043979	8.059274	4.003960082	3.179395517	0.900464	1	mmu-miR-879-5p	0.537285	0	0.620384622	0	20	0.734972
ssc-miR-30b-3p	140.7686611	37.07266	7.14739484	5.250683565	1.924899	0.638943	chr3_7890_mature	21.4913986	33.84895	4.491301473	5.123043401	-0.65535	1	chr6_13018_star	0.537285	0	0.620384622	0	20	0.734972
bta-miR-431	96.71129386	29.81932	6.610453419	4.94576292	1.697437	0.639085	chr1_1175_mature	2.14913986	4.029637	1.654957834	2.330454351	-0.90689	1	rno-miR-361-3p	0.537285	0	0.620384622	0	20	0.734972
chr7_15749_mature	19.34225877	19.34226	4.346407978	4.346407978	0	0.642569	chr13_28690_mature	4.29827973	6.44742	2.405524014	2.896740642	-0.58496	1	hsa-miR-5006-3p	0.537285	0	0.620384622	0	20	0.734972
chr1_2128_mature	1260.47053	154.7381	10.30089079	7.282977844	3.026062	0.642621	chr2_6208_mature	36.5353777	24.98375	5.230179094	4.699537803	0.548304	1	hsa-miR-4799-5p	0.537285	0	0.620384622	0	20	0.734972
chr15_32058_mature	22.56596857	17.7304	4.558633073	4.227310103	0.347923	0.642894	chrx_22488_star	2.14913986	3.22371	1.654957834	2.078510713	-0.58496	1	bta-miR-148a	0.537285	0	0.620384622	0	20	0.734972
chr2_4073_star	20.4168287	18.53633	4.420672964	4.288087668	0.139403	0.64318	chr7_15701_mature	8.59655945	13.70077	3.262517265	3.877819487	-0.67243	1	rno-miR-346	0.537285	0	0.620384622	0	20	0.734972
chr18_36226_mature	32.23709795	103.1587	5.054722516	6.702639724	-1.67807	0.64332	chr7_16449_mature	47.281077	31.43117	5.593385953	5.019309189	0.589067	1	hsa-miR-6722-3p	0.537285	0	0.620384622	0	20	0.734972
chr1_3491_mature	26.8642483	17.7304	4.800343329	4.227310103	0.599462	0.643544	chr6_14638_mature	2.14913986	4.029637	1.654957834	2.330454351	-0.90689	1	mmu-miR-450b-3p	0.537285	0	0.620384622	0	20	0.734972
chr2_5399_mature	33.31166789	18.53633	5.100627352	4.288087668	0.845672	0.643616	chr17_35035_mature	15.043979	8.059274	4.003960082	3.179395517	0.900464	1	hsa-miR-432-5p	0.537285	0	0.620384622	0	20	0.734972
chr5_12399_mature	16513.99071	940.5173	14.01148854	9.878843844	4.13409	0.643644	NW_003539920_39188_mature	6.44741959	3.22371	2.896740642	2.078510713	1	1	chrx_22097_star	0.537285	0	0.620384622	0	20	0.734972
chrx_22435_mature	25.78967836	17.7304	4.743605355	4.227310103	0.540568	0.643979	chr16_33794_star	4.29827973	8.059274	2.405524014	3.179395517	-0.90689	1	chr15_33121_star	0.537285	0	0.620384622	0	20	0.734972
chr13_26988_mature	33.31166789	108.8002	5.100627352	6.778736946	-1.70758	0.644554	chr4_9069_mature	2.14913986	7.253347	1.654957834	3.044979305	-1.75489	1	hsa-miR-221-3p	0.537285	0	0.620384622	0	20	0.734972
chr4_9797_mature	23.6405385	54.80307	4.62296188	5.802272499	-1.21299	0.644941	chr18_35998_mature	2.14913986	5.641492	1.654957834	2.731507408	-1.39232	1	bta-miR-2957	0.537285	0	0.620384622	0	20	0.734972
bta-miR-2890	42.98279727	170.8566	5.458867456	7.425061609	-1.99095	0.645292	hsa-miR-5698	2.14913986	3.22371	1.654957834	2.078510713	-0.58496	1	hsa-miR-5692b	0.537285	0	0.620384622	0	20	0.734972
chr1_3684_mature	24.71510843	58.8327	4.684544332	5.902862353	-1.25123	0.647994	chr6_14338_mature	2.14913986	4.835565	1.654957834	2.544872269	-1.16993	1	hsa-miR-548c-3p	0.537285	0	0.620384622	0	20	0.734972
chr5_11953_mature	142.9178009	1200.832	7.169101237	10.2310194	-3.07078	0.648299	chr14_29526_star	4.29827973	2.417782	2.405524014	1.773060525	0.830075	1	mmu-miR-1968-3p	0.537285	0	0.620384622	0	20	0.734972
chr2_4631_mature	27.93881823	75.75718	4.854934103	6.262229808	-1.43911	0.648385	hsa-miR-4511	12.8948392	20.95411	3.796477232	4.456419386	-0.70044	1	rno-miR-103-2-5p	0.537285	0	0.620384622	0	20	0.734972
chr7_17171_star	29.01338816	18.53633	4.907534286	4.288087668	0.646363	0.64875	chr16_34104_mature	6.44741959	12.89484	2.896740642	3.796477232	-1	1	rno-miR-652-5p	0.537285	0	0.620384622	0	20	0.734972
chr17_35446_mature	19.34225877	28.20746	4.346407978	4.868265031	-0.54432	0.648974	hsa-miR-5580-5p	6.44741959	4.029637	2.896740642	2.330454351	0.678072	1	chr12_26197_star	0.537285	0	0.620384622	0	20	0.734972
ssc-miR-424-3p	23.6405385	52.38528	4.62296188	5.738370208	-1.1479	0.649715	chr1_153_star	2.14913986	4.029637	1.654957834	2.330454351	-0.90689	1	chr17_35140_star	0.537285	0	0.620384622	0	20	0.734972
chr1_1843_mature	19.34225877	24.17782	4.346407978	4.654081667	-0.32193	0.649848	chr6_13506_mature	146.141511	186.9752	7.201060499	7.554398282	-0.35548	1	bta-miR-655	0.537285	0	0.620384622	0	20	0.734972
NW_003613065_36971_mature	38.68451755	20.14819	5.310504361	4.402462031	0.941106	0.650719	chr13_28509_star	6.44741959	3.22371	2.896740642	2.078510713	1	1	bta-miR-2382-3p	0.537285	0	0.620384622	0	20	0.734972
chr16_34261_mature	20.4168287	20.95411	4.420672964	4.456419386	-0.03747	0.650914	chr7_16758_mature	4.29827973	8.865202	2.405524014	3.302348583	-1.04439	1	mmu-let-7f-2-3p	0.537285	0	0.620384622	0	20	0.734972
chr16_33741_star	33.31166789	19.34226	5.100627352	4.346407978	0.784271	0.651256	chr10_23883_star	2.14913986	4.029637	1.654957834	2.330454351	-0.90689	1	chr15_33361_star	0.537285	0	0.620384622	0	20	0.734972
chr4_10393_mature	25.78967836	19.34226	4.743605355	4.346407978	0.415037	0.655004	chr6_13411_star	4.29827973	5.641492	2.405524014	2.731507408	-0.39232	1	mmu-miR-376c-3p	0.537285	0	0.620384622	0	20	0.734972
chr7_15660_mature	35.46080775	20.14819	5.188274618	4.402462031	0.815575	0.65567	chr3_8352_star	2.14913986	6.44742	1.654957834	2.896740642	-1.58496	1	bta-miR-2382-5p	0.537285	0	0.620384622	0	20	0.734972
chr14_31622_star	39.75908748	20.95411	5.349049847	4.456419386	0.924051	0.656704	chr1_439_star	10.7456993	8.059274	3.554060706	3.179395517	0.415037	1	hsa-miR-4477b	0.537285	0	0.620384622	0	20	0.734972
chr14_29918_mature	39.75908748	20.95411	5.349049847	4.456419386	0.924051	0.656704	hsa-miR-4532	4.29827973	4.835565	2.405524014	2.544872269	-0.16993	1	hsa-miR-4477a	0.537285	0	0.620384622	0	20	0.734972
chr14_29752_mature	20.4168287	26.59561	4.420672964	4.786366653	-0.38143	0.6571	chr15_33061_mature	2.14913986	4.029637	1.654957834	2.330454351	-0.90689	1	chr15_32711_star	0.537285	0	0.620384622	0	20	0.734972
ssc-miR-376a-3p	52.65392666	23.3719	5.745611853	4.607146585	1.171766	0.657175	chr6_12992_star	2.14913986	4.029637	1.654957834	2.330454351	-0.90689	1	chr9_21075_star	0.537285	0	0.620384622	0	20	0.734972
chr2_6203_mature	39.75908748	139.4254	5.349049847	7.133660602	-1.81014	0.657459	ssc-miR-450b-3p	23.6405385	12.89484	4.62296188	3.796477232	0.874469	1	hsa-miR-4766-5p	0.537285	0	0.620384622	0	20	0.734972
chrx_22074_mature	95.63672393	31.43117	6.594499642	5.019309189	1.605369	0.657661	chr13_27764_mature	10.7456993	8.059274	3.554060706	3.179395517	0.415037	1	hsa-miR-3689a-3p	0.537285	0	0.620384622	0	20	0.734972
chr2_5894_mature	22.56596857	20.95411	4.558633073	4.456419386	0.106915	0.657771	chr4_9685_star	4.29827973	4.029637	2.405524014	2.330454351	0.093109	1	hsa-miR-4704-5p	0.537285	0	0.620384622	0	20	0.734972
chr1_2562_mature	38.68451755	132.1721	5.310504361	7.057148072	-1.77259	0.658101	chr15_31957_mature	6.44741959	9.671129	2.896740642	3.415640968	-0.58496	1	mmu-miR-107-5p	0.537285	0	0.620384622	0	20	0.734972
chr16_34013_mature	38.68451755	20.95411	5.310504361	4.456419386	0.884523	0.658363	chr3_7211_mature	6.44741959	4.835565	2.896740642	2.544872269	0.415037	1	hsa-miR-493-5p	0.537285	0	0.620384622	0	20	0.734972
NW_003539007_38454_mature	24.71510843	20.14819	4.684544332	4.402462031	0.294743	0.658482	bta-miR-2300a-5p	4.29827973	3.22371	2.405524014	2.078510713	0.415037	1	chr1_2879_star	0.537285	0	0.620384622	0	20	0.734972
NW_003613218_37700_mature	21.49139864	22.56597	4.491301473	4.558633073	-0.07039	0.658645	chr16_33868_mature	6.44741959	8.865202	2.896740642	3.302348583	-0.45943	1	chr12_26594_star	0.537285	0	0.620384622	0	20	0.734972
NW_003538820_38274_mature	22.56596857	21.76004	4.558633073	4.508431259	0.052467	0.660315	chr15_32322_star	4.29827973	2.417782	2.405524014	1.773060525	0.830075	1	hsa-miR-1255b-2-3p	0.537285	0	0.620384622	0	20	0.734972
chr6_12597_mature	89.18930434	30.62524	6.494884448	4.983004663	1.542149	0.661103	chr18_36430_mature	2.14913986	4.029637	1.654957834	2.330454351	-0.90689	1	mmu-miR-675-3p	0.537285	0	0.620384622	0	20	0.734972
NW_003537731_37227_mature	50.5047868	23.3719	5.686634616	4.607146585	1.111645	0.6612	chr9_20257_mature	4.29827973	3.22371	2.405524014	2.078510713	0.415037	1	mmu-miR-15a-3p	0.537285	0	0.620384622	0	20	0.734972
NW_003538080_37501_mature	21.49139864	32.2371	4.491301473	5.054722516	-0.58496	0.662058	chr18_36007_star	4.29827973	2.417782	2.405524014	1.773060525	0.830075	1	chr18_36542_mature	0.537285	0	0.620384622	0	20	0.734972
ssc-miR-30e-5p	519.0172771	8322.007	9.022415746	13.02288911	-4.00308	0.662195	chr14_31142_star	2.14913986	3.22371	1.654957834	2.078510713	-0.58496	1	bta-miR-425-5p	0.537285	0	0.620384622	0	20	0.734972
mmu-miR-1934-5p	21.49139864	31.43117	4.491301473	5.019309189	-0.54844	0.662656	chr2_5482_mature	8.59655945	10.47706	3.262517265	3.520680821	-0.2854	1	mmu-miR-675-5p	0.537285	0	0.620384622	0	20	0.734972
chrx_22133_mature	31.16252802	20.95411	5.007308904	4.456419386	0.572579	0.665617	chr4_10240_star	4.29827973	2.417782	2.405524014	1.773060525	0.830075	1	NW_003613217_37698_star	0.537285	0	0.620384622	0	20	0.734972
ssc-miR-126-3p	1089.613911	147.4847	10.09092475	7.214170697	2.88518	0.66704	chr11_25109_mature	2.14913986	4.029637	1.654957834	2.330454351	-0.90689	1	chr5_12127_star	0.537285	0	0.620384622	0	20	0.734972
chr3_7170_mature	22.56596857	24.98375	4.558633073	4.699537803	-0.14684	0.667106	chr15_32593_star	4.29827973	3.22371	2.405524014	2.078510713	0.415037	1	chr8_18271_star	0.537285	0	0.620384622	0	20	0.734972
chr7_15358_mature	208.4665668	50.77343	7.710576182	5.694139975	2.03767	0.667461	chr1_2659_mature	8.59655945	8.059274	3.262517265	3.179395517	0.093109	1	chr13_27867_mature	0.537285	0	0.620384622	0	20	0.734972
ssc-miR-331-5p	224.5851158	53.19121	7.817528071	5.759986999	2.078003	0.667893	chr10_24027_mature	2.14913986	4.835565	1.654957834	2.544872269	-1.16993	1	hsa-miR-3689b-5p	0.537285	0	0.620384622	0	20	0.734972
chr16_33754_mature	1530.187583	186.1692	10.58043532	7.548199552	3.039022	0.668488	chr3_7100_star	2.14913986	3.22371	1.654957834	2.078510713	-0.58496	1	chr6_14288_star	0.537285	0	0.620384622	0	20	0.734972
chr4_10131_mature	40.83365741	22.56597	5.38659223	4.558633073	0.85561	0.66928	chr5_11729_star	8.59655945	7.253347	3.262517265	3.044979305	0.245112	1	mmu-miR-188-5p	0.537285	0	0.620384622	0	20	0.734972
bta-miR-1197	34.38623782	21.76004	5.145116481	4.508431259	0.66015	0.669567	chr13_29037_star	8.59655945	4.835565	3.262517265	2.544872269	0.830075	1	chr2_4477_mature	0.537285	0	0.620384622	0	20	0.734972
chr13_28371_star	22.56596857	29.01339	4.558633073	4.907534286	-0.36257	0.670067	chrx_21552_star	6.44741959	4.029637	2.896740642	2.330454351	0.678072	1	bta-miR-127	0.537285	0	0.620384622	0	20	0.734972
chr8_17646_mature	33.31166789	21.76004	5.100627352	4.508431259	0.614346	0.670183	chr9_20216_mature	4.29827973	4.029637	2.405524014	2.330454351	0.093109	1	hsa-miR-138-5p	0.537285	0	0.620384622	0	20	0.734972
chr6_13536_mature	33.31166789	91.87573	5.100627352	6.537229727	-1.46366	0.670311	chr6_12618_star	6.44741959	12.08891	2.896740642	3.710273245	-0.90689	1	bta-miR-128	0.537285	0	0.620384622	0	20	0.734972
chr5_11689_star	30.08795809	21.76004	4.958283956	4.508431259	0.467505	0.670697	chr16_33794_mature	8.59655945	9.671129	3.262517265	3.415640968	-0.16993	1	bta-miR-155	0.537285	0	0.620384622	0	20	0.734972
rno-miR-664-2-5p	23.6405385	25.78968	4.62296188	4.743605355	-0.12553	0.672599	chr9_20263_mature	6.44741959	10.47706	2.896740642	3.520680821	-0.70044	1	hsa-miR-6133	0.537285	0	0.620384622	0	20	0.734972
ssc-miR-503	68.77247564	332.0421	6.124586119	8.379560789	-2.27146	0.67386	chr13_29421_star	4.29827973	3.22371	2.405524014	2.078510713	0.415037	1	hsa-miR-4733-3p	0.537285	0	0.620384622	0	20	0.734972
ssc-miR-493-5p	31.16252802	22.56597	5.007308904	4.558633073	0.465664	0.675431	chr3_7704_mature	4.29827973	2.417782	2.405524014	1.773060525	0.830075	1	bta-miR-302d	0.537285	0	0.620384622	0	20	0.734972
chr2_6283_mature	50.5047868	24.98375	5.686634616	4.699537803	1.01543	0.675705	mmu-miR-145b	2.14913986	4.029637	1.654957834	2.330454351	-0.90689	1	chr3_8236_mature	0.537285	0	0.620384622	0	20	0.734972
NW_003540770_39680_mature	139.6940911	41.1023	7.136417929	5.395827139	1.76498	0.675877	chr9_20220_mature	8.59655945	8.059274	3.262517265	3.179395517	0.093109	1	mmu-miR-702-5p	0.537285	0	0.620384622	0	20	0.734972
hsa-miR-4508	130.0229618	39.49044	7.033675856	5.339509593	1.719191	0.676027	chr3_7250_mature	4.29827973	2.417782	2.405524014	1.773060525	0.830075	1	chr6_12449_mature	0.537285	0	0.620384622	0	20	0.734972
hsa-miR-1261	25.78967836	47.54972	4.743605355	5.601391055	-0.88264	0.676598	chr14_30912_star	8.59655945	9.671129	3.262517265	3.415640968	-0.16993	1	bta-miR-107	0.537285	0	0.620384622	0	20	0.734972
chr9_20945_mature	29.01338816	64.4742	4.907534286	6.032854532	-1.152	0.676987	mmu-miR-2136	2.14913986	5.641492	1.654957834	2.731507408	-1.39232	1	bta-miR-101	0.537285	0	0.620384622	0	20	0.734972
bta-miR-1434-3p	49.43021686	24.98375	5.656216524	4.699537803	0.984403	0.67728	chr3_6716_mature	4.29827973	2.417782	2.405524014	1.773060525	0.830075	1	bta-miR-423-3p	0.537285	0	0.620384622	0	20	0.734972
chr15_32336_mature	81.66731482	31.43117	6.369245121	5.019309189	1.377563	0.680911	mmu-miR-2139	4.29827973	5.641492	2.405524014	2.731507408	-0.39232	1	mmu-miR-141-5p	0.537285	0	0.620384622	0	20	0.734972
chr9_20193_mature	24.71510843	29.81932	4.684544332	4.94576292	-0.27085	0.68116	chr8_18856_mature	2.14913986	4.835565	1.654957834	2.544872269	-1.16993	1	chr5_11077_star	0.537285	0	0.620384622	0	20	0.734972
chr1_2148_mature	27.93881823	54.80307	4.854934103	5.802272499	-0.97199	0.682832	chr13_28104_star	10.7456993	17.7304	3.554060706	4.227310103	-0.72247	1	mmu-miR-1981-3p	0.537285	0	0.620384622	0	20	0.734972
chr14_31467_mature	102.0841435	35.46081	6.687678623	5.188274618	1.525461	0.683116	chr8_17792_mature	30.0879581	19.34226	4.958283956	4.346407978	0.63743	1	bta-miR-2284h-5p	0.537285	0	0.620384622	0	20	0.734972
chr14_30942_star@@rno-miR-301b-	31.16252802	24.17782	5.007308904	4.654081667	0.366128	0.68346	chr16_34295_mature	4.29827973	6.44742	2.405524014	2.896740642	-0.58496	1	chr1_2930_mature	0.537285	0	0.620384622	0	20	0.734972
hsa-miR-4705	67.6979057	29.81932	6.102194213	4.94576292	1.182864	0.689155	chr14_30832_star	6.44741959	3.22371	2.896740642	2.078510713	1	1	chr2_5367_star	0.537285	0	0.620384622	0	20	0.734972
chr18_36225_mature	40.83365741	120.0832	5.38659223	6.919854778	-1.5562	0.689248	chr8_19054_star	4.29827973	6.44742	2.405524014	2.896740642	-0.58496	1	bta-miR-183	0.537285	0	0.620384622	0	20	0.734972
chr1_1835_mature	36.53537768	95.09944	5.230179094	6.586456103	-1.38014	0.690398	chr3_7417_mature	4.29827973	4.029637	2.405524014	2.330454351	0.093109	1	bta-miR-187	0.537285	0	0.620384622	0	20	0.734972
chr16_34246_mature	62.32505605	29.01339	5.984704544	4.907534286	1.103093	0.690641	chr10_23335_mature	4.29827973	5.641492	2.405524014	2.731507408	-0.39232	1	rno-miR-191a-5p	0.537285	0	0.620384622	0	20	0.734972
bta-miR-2484	26.8642483	38.68452	4.800343329	5.310504361	-0.52607	0.692013	chr7_16464_star	85.9655945	98.32315	6.442372848	6.634058094	-0.19377	1	chr4_8812_mature	0.537285	0	0.620384622	0	20	0.734972
chr12_25781_mature	34.38623782	25.78968	5.145116481	4.743605355	0.415037	0.692416	chr14_30535_mature	19.3422588	15.31262	4.346407978	4.027916745	0.337035	1	mmu-miR-344c-3p	0.537285	0	0.620384622	0	20	0.734972
NW_003612983_36666_mature	44.0573672	135.3958	5.493691111	7.091655531	-1.61973	0.693208	chr1_3231_mature	15.043979	14.50669	4.003960082	3.954819241	0.052467	1	mmu-miR-141-3p	0.537285	0	0.620384622	0	20	0.734972
NW_003539007_38444_mature	27.93881823	29.81932	4.854934103	4.94576292	-0.09398	0.693316	chrx_23005_mature	30.0879581	42.71415	4.958283956	5.4500286	-0.50553	1	mmu-miR-3079-3p	0.537285	0	0.620384622	0	20	0.734972
bta-miR-2424	54.80306652	28.20746	5.802272499	4.868265031	0.95818	0.694549	chr8_18162_mature	68.7724756	74.14533	6.124586119	6.231611453	-0.10852	1	NW_003613325_38551_star	0.537285	0	0.620384622	0	20	0.734972
chr14_30932_mature	37.60994761	97.51722	5.270900692	6.622304032	-1.37454	0.69477	chr13_27824_mature	36.5353777	34.65488	5.230179094	5.156027656	0.076236	1	chr16_33739_star	0.537285	0	0.620384622	0	20	0.734972
bta-miR-454	29.01338816	29.01339	4.907534286	4.907534286	0	0.694991	chr6_13505_mature	161.18549	177.304	7.341500942	7.478195571	-0.1375	1	hsa-miR-513c-5p	0.537285	0	0.620384622	0	20	0.734972
chr12_26057_mature	27.93881823	31.43117	4.854934103	5.019309189	-0.16993	0.695262	chrx_22797_mature	21.4913986	24.98375	4.491301473	4.699537803	-0.21723	1	hsa-miR-142-5p	0.537285	0	0.620384622	0	20	0.734972
chr6_12636_mature	38.68451755	100.7409	5.310504361	6.668756392	-1.38082	0.698014	chr7_15721_mature	32.237098	28.20746	5.054722516	4.868265031	0.192645	1	chr13_27500_star	0.537285	0	0.620384622	0	20	0.734972
ssc-miR-551a	78.44360502	33.04303	6.311859186	5.08928735	1.24731	0.69823	rno-miR-17-5p	34.3862378	27.40153	5.145116481	4.827896911	0.327575	1	hsa-miR-3162-3p	0.537285	0	0.620384622	0	20	0.734972
chr15_33480_mature	131.0975317	42.71415	7.045459699	5.4500286	1.617854	0.69865	chr11_24459_mature	17.1931189	24.98375	4.185320985	4.699537803	-0.53916	1	mmu-miR-345-5p	0.537285	0	0.620384622	0	20	0.734972
ssc-miR-491	35.46080775	82.2046	5.188274618	6.378591382	-1.21299	0.699478	chr9_20875_mature	10.7456993	14.50669	3.554060706	3.954819241	-0.43296	1	hsa-miR-3117-3p	0.537285	0	0.620384622	0	20	0.734972
mmu-miR-21a-5p	33.31166789	28.20746	5.100627352	4.868265031	0.239951	0.700718	chr14_30616_mature	330.967539	348.9666	8.374898366	8.451073371	-0.0764	1	chr17_35808_star	0.537285	0	0.620384622	0	20	0.734972
chr2_5731_mature	152.5889303	46.74379	7.26293043	5.577241251	1.706804	0.700806	NW_003538075_37489_mature	10.7456993	13.70077	3.554060706	3.877819487	-0.3505	1	chr4_9813_star	0.537285	0	0.620384622	0	20	0.734972
bta-miR-656	53.72849659	29.01339	5.774220321	4.907534286	0.888969	0.701095	chr7_16996_mature	17.1931189	24.98375	4.185320985	4.699537803	-0.53916	1	chr3_7747_star	0.537285	0	0.620384622	0	20	0.734972
hsa-miR-4451	32.23709795	63.66827	5.054722516	6.014986077	-0.98185	0.701139	chr14_30932_mature	94.562154	97.51722	6.578367468	6.622304032	-0.04439	1	mmu-miR-466a-5p	0.537285	0	0.620384622	0	20	0.734972
chr15_32961_mature	29.01338816	38.68452	4.907534286	5.310504361	-0.41504	0.702642	chrx_22435_mature	17.1931189	17.7304	4.185320985	4.227310103	-0.04439	1	chr10_24300_mature	0.537285	0	0.620384622	0	20	0.734972
NW_003541277_40020_mature	51.57935673	29.01339	5.716424587	4.907534286	0.830075	0.703132	chr9_20876_mature	12.8948392	14.50669	3.796477232	3.954819241	-0.16993	1	mmu-miR-302c-5p	0.537285	0	0.620384622	0	20	0.734972
mmu-miR-677-3p	61.25048611	30.62524	5.960013198	4.983004663	1	0.703358	NW_003539007_38451_star	15.043979	20.95411	4.003960082	4.456419386	-0.47805	1	bta-miR-199a-3p	0.537285	0	0.620384622	0	20	0.734972
chr6_14575_mature	36.53537768	28.20746	5.230179094	4.868265031	0.373217	0.703461	chr12_26026_mature	27.9388182	21.76004	4.854934103	4.508431259	0.36059	1	hsa-miR-517b-3p	0.537285	0	0.620384622	0	20	0.734972
chr6_13893_mature	32.23709795	29.81932	5.054722516	4.94576292	0.112475	0.703582	chrx_21688_mature	10.7456993	14.50669	3.554060706	3.954819241	-0.43296	1	mmu-miR-5626-3p	0.537285	0	0.620384622	0	20	0.734972
chr13_26691_star	50.5047868	29.01339	5.686634616	4.907534286	0.799701	0.704044	chr1_2128_mature	131.097532	154.7381	7.045459699	7.282977844	-0.23919	1	hsa-miR-501-3p	0.537285	0	0.620384622	0	20	0.734972
bta-let-7b	356.7572174	78.17496	8.482837062	6.306972375	2.190164	0.70506	chr1_3232_mature	21.4913986	26.59561	4.491301473	4.786366653	-0.30743	1	mmu-miR-668-3p	0.537285	0	0.620384622	0	20	0.734972
chr17_35422_mature	35.46080775	77.36904	5.188274618	6.292211829	-1.12553	0.705345	chr3_8091_mature	36.5353777	37.87859	5.230179094	5.280903997	-0.05209	1	hsa-miR-488-3p	0.537285	0	0.620384622	0	20	0.734972
chr8_19035_mature	32.23709795	58.8327	5.054722516	5.902862353	-0.8679	0.706448	hsa-miR-3612	10.7456993	12.08891	3.554060706	3.710273245	-0.16993	1	bta-miR-224	0.537285	0	0.620384622	0	20	0.734972
chr15_32921_mature	34.38623782	29.81932	5.145116481	4.94576292	0.205584	0.70684	mmu-miR-5100	182.676888	155.544	7.521026297	7.290424382	0.231971	1	chr14_29457_mature	0.537285	0	0.620384622	0	20	0.734972
chr13_27913_mature	31.16252802	35.46081	5.007308904	5.188274618	-0.18641	0.709585	mmu-miR-6238	40.8336574	44.32601	5.38659223	5.502267253	-0.11839	1	bta-miR-214	0.537285	0	0.620384622	0	20	0.734972
ssc-miR-129a	40.83365741	101.5469	5.38659223	6.680139486	-1.31431	0.710563	chr2_5889_star	10.7456993	14.50669	3.554060706	3.954819241	-0.43296	1	hsa-miR-3616-5p	0.537285	0	0.620384622	0	20	0.734972
hsa-miR-4800-3p	116.0535526	41.90823	6.871024911	5.423182395	1.469485	0.711153	ssc-miR-361-3p	281.537322	327.2065	8.142297645	8.358460195	-0.21688	1	hsa-miR-548l	0.537285	0	0.620384622	0	20	0.734972
chr13_28842_star	63.39962598	32.2371	6.008980404	5.054722516	0.975752	0.711165	chr2_6203_mature	109.606133	139.4254	6.789287574	7.133660602	-0.34717	1	hsa-miR-548m	0.537285	0	0.620384622	0	20	0.734972
chr14_30575_star	45.13193714	29.81932	5.527693969	4.94576292	0.597902	0.711242	chr4_9963_mature	42.9827973	48.35565	5.458867456	5.625143252	-0.16993	1	hsa-miR-6503-3p	0.537285	0	0.620384622	0	20	0.734972
chr12_25587_star	31.16252802	43.52008	5.007308904	5.476384353	-0.48187	0.711506	chr8_18896_star	12.8948392	18.53633	3.796477232	4.288087668	-0.52356	1	chr12_26448_star	0.537285	0	0.620384622	0	20	0.734972
chr8_18458_mature@@mmu-miR-62	220.286836	1860.886	7.78977382	10.8625494	-3.07853	0.711842	chr13_26987_mature	90.2638743	116.0536	6.511971994	6.871024911	-0.36257	1	hsa-miR-181a-5p	0.537285	0	0.620384622	0	20	0.734972
chr14_29600_mature	47.281077	136.2017	5.593385953	7.100154956	-1.52641	0.711845	ssc-miR-1839-3p	21.4913986	16.92448	4.491301473	4.163859073	0.344648	1	chr6_12904_star	0.537285	0	0.620384622	0	20	0.734972
ssc-miR-30c-1-3p	316.9981299	74.14533	8.312874471	6.231611453	2.096047	0.712105	chr14_30277_star	10.7456993	11.28298	3.554060706	3.618589217	-0.07039	1	hsa-miR-4645-5p	0.537285	0	0.620384622	0	20	0.734972
NW_003541277_40019_mature	33.31166789	34.65488	5.100627352	5.156027656	-0.05703	0.714646	ssc-miR-30a-3p	500.749588	465.0201	8.970823717	8.864248489	0.106796	1	mmu-miR-216a-3p	0.537285	0	0.620384622	0	20	0.734972
chr14_30931_mature	38.68451755	86.23424	5.310504361	6.446822559	-1.1565	0.71471	chr3_7739_star	68.7724756	74.14533	6.124586119	6.231611453	-0.10852	1	hsa-miR-3177-3p	0.537285	0	0.620384622	0	20	0.734972
chr6_14576_mature	45.13193714	30.62524	5.527693969	4.983004663	0.559427	0.714881	chr4_8854_star	10.7456993	12.08891	3.554060706	3.710273245	-0.16993	1	rno-miR-362-3p	0.537285	0	0.620384622	0	20	0.734972
bta-miR-543	37.60994761	31.43117	5.270900692	5.019309189	0.258918	0.715017	chr14_30478_mature	12.8948392	11.28298	3.796477232	3.618589217	0.192645	1	bta-miR-29e	0.537285	0	0.620384622	0	20	0.734972
ssc-miR-339-3p	73.07075536	34.65488	6.210832145	5.156027656	1.076236	0.71547	chrx_23051_mature	23.6405385	32.2371	4.62296188	5.054722516	-0.44746	1	mmu-miR-3572-5p	0.537285	0	0.620384622	0	20	0.734972
chr4_10247_mature	405.1128643	87.04016	8.665736918	6.460089935	2.218571	0.715486	chrx_21833_mature	328.818399	385.2333	8.365528072	8.593328822	-0.22844	1	mmu-miR-3572-3p	0.537285	0	0.620384622	0	20	0.734972
chr4_9437_mature	41.90822734	31.43117	5.423182395	5.019309189	0.415037	0.717756	ssc-miR-92b-5p	96.7112939	121.695	6.610453419	6.938933175	-0.33151	1	chr6_13950_mature	0.537285	0	0.620384622	0	20	0.734972
chr1_826_mature	42.98279727	31.43117	5.458867456	5.019309189	0.451563	0.718055	chr4_10079_mature	64.4741959	64.4742	6.032854532	6.032854532	0	1	bta-miR-299	0.537285	0	0.620384622	0	20	0.734972
rno-miR-3074	52.65392666	160.3796	5.745611853	7.334314072	-1.60688	0.718446	chr13_27285_star	12.8948392	16.92448	3.796477232	4.163859073	-0.39232	1	mmu-miR-466n-5p	0.537285	0	0.620384622	0	20	0.734972
chr13_27306_mature	33.31166789	48.35565	5.100627352	5.625143252	-0.53766	0.719446	hsa-miR-4787-5p	23.6405385	20.95411	4.62296188	4.456419386	0.174029	1	chr1_3554_star	0.537285	0	0.620384622	0	20	0.734972
chr8_17278_mature	34.38623782	37.87859	5.145116481	5.280903997	-0.13955	0.720993	chr11_24688_mature	23.6405385	29.81932	4.62296188	4.94576292	-0.33498	1	chr2_5742_star	0.537285	0	0.620384622	0	20	0.734972
chr12_25570_star	55.87763645	33.04303	5.82978961	5.08928735	0.757925	0.721803	chr1_1941_mature	19.3422588	21.76004	4.346407978	4.508431259	-0.16993	1	chr14_30644_star	0.537285	0	0.620384622	0	20	0.734972
ssc-miR-135	102.0841435	41.1023	6.687678623	5.395827139	1.312468	0.721964	NW_003539588_38971_mature	17.1931189	19.34226	4.185320985	4.346407978	-0.16993	1	hsa-miR-208b	0.537285	0	0.620384622	0	20	0.734972
ssc-miR-107	4232.730961	420.6941	12.04771388	8.720053123	3.330745	0.723601	chr13_27779_mature	12.8948392	16.11855	3.796477232	4.097488514	-0.32193	1	rno-miR-1912-5p	0.537285	0	0.620384622	0	20	0.734972
chr13_29250_mature	74.1453253	36.26674	6.231611453	5.21981653	1.031709	0.723642	chr11_24486_mature	21.4913986	28.20746	4.491301473	4.868265031	-0.39232	1	hsa-miR-4687-3p	0.537285	0	0.620384622	0	20	0.734972
ssc-miR-335	4190.822734	418.2763	12.03336199	8.711757631	3.324705	0.724109	chr4_10525_mature	25.7896784	28.20746	4.743605355	4.868265031	-0.12928	1	mmu-miR-1187	0.537285	0	0.620384622	0	20	0.734972
chr3_6687_mature	66.62333577	35.46081	6.079449279	5.188274618	0.909802	0.726116	chr6_13928_mature	502.898728	544.001	8.976990005	9.090115141	-0.11334	1	bta-miR-21-3p	0.537285	0	0.620384622	0	20	0.734972
NW_003539007_38442_mature	70.9216155	36.26674	6.168353522	5.21981653	0.967579	0.726624	NW_003541007_39797_mature	12.8948392	12.08891	3.796477232	3.710273245	0.093109	1	mmu-miR-450a-5p	0.537285	0	0.620384622	0	20	0.734972
chr15_32033_mature	36.53537768	38.68452	5.230179094	5.310504361	-0.08246	0.727047	chr6_13308_star	12.8948392	17.7304	3.796477232	4.227310103	-0.45943	1	chr12_26457_star	0.537285	0	0.620384622	0	20	0.734972
ssc-miR-493-3p	35.46080775	42.71415	5.188274618	5.4500286	-0.26849	0.727221	chr5_12332_mature	42.9827973	47.54972	5.458867456	5.601391055	-0.14568	1	chr7_16666_mature@@bta-miR-23	0.537285	0	0.620384622	0	20	0.734972
chr13_28400_star@@hsa-miR-5585-	39.75908748	35.46081	5.349049847	5.188274618	0.165059	0.727424	chr13_28371_star	27.9388182	29.01339	4.854934103	4.907534286	-0.05445	1	chr6_13056_star	0.537285	0	0.620384622	0	20	0.734972
chr4_8929_mature	35.46080775	43.52008	5.188274618	5.476384353	-0.29546	0.727523	chr6_13018_mature	122.500972	118.4713	6.948378589	6.900520701	0.048255	1	chr9_20053_mature	0.537285	0	0.620384622	0	20	0.734972
ssc-miR-20b	3915.732832	402.9637	11.93543501	8.658081936	3.28056	0.728435	bta-miR-431	23.6405385	29.81932	4.62296188	4.94576292	-0.33498	1	mmu-miR-17-3p	0.537285	0	0.620384622	0	20	0.734972
chr12_25449_star	40.83365741	35.46081	5.38659223	5.188274618	0.203533	0.728652	chr18_36623_mature	15.043979	13.70077	4.003960082	3.877819487	0.13493	1	mmu-miR-500-3p	0.537285	0	0.620384622	0	20	0.734972
hsa-miR-4278	36.53537768	43.52008	5.230179094	5.476384353	-0.25239	0.730728	chr2_5398_mature	19.3422588	16.92448	4.346407978	4.163859073	0.192645	1	rno-miR-6215	0.537285	0	0.620384622	0	20	0.734972
chr14_29516_mature	88.11473441	40.29637	6.477592084	5.367943153	1.128733	0.731805	chr7_15488_mature	10.7456993	13.70077	3.554060706	3.877819487	-0.3505	1	mmu-miR-17-5p	0.537285	0	0.620384622	0	20	0.734972
chr17_35671_mature	55.87763645	35.46081	5.82978961	5.188274618	0.656046	0.732532	chr5_11720_mature	124.650112	121.695	6.973268148	6.938933175	0.034614	1	hsa-miR-3167	0.537285	0	0.620384622	0	20	0.734972
chr3_7739_mature	53.72849659	35.46081	5.774220321	5.188274618	0.599462	0.733113	chr14_30693_star	10.7456993	12.08891	3.554060706	3.710273245	-0.16993	1	bta-miR-421	0.537285	0	0.620384622	0	20	0.734972
chr12_26534_mature	676.979057	126.5306	9.405096899	6.994699749	2.419625	0.734025	chr8_17577_star	27.9388182	23.3719	4.854934103	4.607146585	0.257496	1	chr7_15273_star	0.537285	0	0.620384622	0	20	0.734972
chr17_34936_mature	37.60994761	44.32601	5.270900692	5.502267253	-0.23704	0.734098	bta-miR-504	12.8948392	13.70077	3.796477232	3.877819487	-0.08746	1	hsa-miR-3196	0.537285	0	0.620384622	0	20	0.734972
chr7_15101_mature	1633.346296	225.6597	10.67449799	7.824384002	2.85561	0.7347	chr12_25786_mature	15.043979	20.95411	4.003960082	4.456419386	-0.47805	1	mmu-miR-3091-5p	0.537285	0	0.620384622	0	20	0.734972
chr2_5993_mature	37.60994761	50.77343	5.270900692	5.694139975	-0.43296	0.735362	chr6_14824_mature	12.8948392	13.70077	3.796477232	3.877819487	-0.08746	1	bta-miR-324	0.537285	0	0.620384622	0	20	0.734972
ssc-miR-136	39.75908748	41.1023	5.349049847	5.395827139	-0.04793	0.736463	NW_003537759_37261_mature	25.7896784	22.56597	4.743605355	4.558633073	0.192645	1	hsa-miR-3131	0.537285	0	0.620384622	0	20	0.734972
chr10_24002_mature	42.98279727	38.68452	5.458867456	5.310504361	0.152003	0.73776	NW_003538347_37746_star	27.9388182	37.87859	4.854934103	5.280903997	-0.43911	1	mmu-miR-434-5p	0.537285	0	0.620384622	0	20	0.734972
chr15_33456_mature	568.4474939	114.4417	9.153419014	6.851020612	2.312414	0.737785	chr13_27285_mature	122.500972	106.3824	6.948378589	6.746614057	0.203533	1	bta-miR-30c	0.537285	0	0.620384622	0	20	0.734972
NW_003539318_38728_mature	38.68451755	53.19121	5.310504361	5.759986999	-0.45943	0.738749	chr7_16538_mature	51.5793567	45.93786	5.716424587	5.552680304	0.16711	1	mmu-miR-3091-3p	0.537285	0	0.620384622	0	20	0.734972
ssc-miR-299	39.75908748	43.52008	5.349049847	5.476384353	-0.1304	0.738815	bta-miR-2484	32.237098	38.68452	5.054722516	5.310504361	-0.26303	1	chr10_23272_star	0.537285	0	0.620384622	0	20	0.734972
chr12_25687_mature	39.75908748	43.52008	5.349049847	5.476384353	-0.1304	0.738815	chr6_12636_mature	92.4130141	100.7409	6.545551653	6.668756392	-0.12448	1	chrx_21708_star	0.537285	0	0.620384622	0	20	0.734972
chr17_35020_mature	69.84704557	38.68452	6.146635787	5.310504361	0.852443	0.739005	chr5_11589_mature	141.843231	138.6195	7.158288862	7.125356859	0.033167	1	hsa-miR-3149	0.537285	0	0.620384622	0	20	0.734972
chr18_36103_mature	41.90822734	41.90823	5.423182395	5.423182395	0	0.741275	chr7_17143_mature	676.979057	731.7821	9.405096899	9.517240499	-0.1123	1	bta-miR-338	0.537285	0	0.620384622	0	20	0.734972
chr7_15245_mature	45.13193714	39.49044	5.527693969	5.339509593	0.192645	0.741613	chr10_24060_mature	17.1931189	23.3719	4.185320985	4.607146585	-0.44294	1	rno-miR-320-3p	0.537285	0	0.620384622	0	20	0.734972
chr2_5426_mature	2388.768958	296.5813	11.22265543	8.217140066	3.009768	0.74192	NW_003539454_38868_mature	588.864323	671.3376	9.204239342	9.393041948	-0.1891	1	mmu-miR-133a-5p	0.537285	0	0.620384622	0	20	0.734972
mmu-miR-1943-5p	53.72849659	38.68452	5.774220321	5.310504361	0.473931	0.744028	chr17_35003_star	60.1759162	52.38528	5.934891897	5.738370208	0.200025	1	hsa-miR-330-3p	0.537285	0	0.620384622	0	20	0.734972
ssc-miR-132	217.0631262	66.08605	7.768602025	6.067940913	1.715697	0.745422	chr8_17646_mature	15.043979	21.76004	4.003960082	4.508431259	-0.5325	1	hsa-miR-1273g-5p	0.537285	0	0.620384622	0	20	0.734972
chr3_6703_mature	53.72849659	39.49044	5.774220321	5.339509593	0.444184	0.746367	chr14_31056_mature	829.567987	901.0269	9.697954457	9.817026628	-0.11921	1	hsa-miR-490-5p	0.537285	0	0.620384622	0	20	0.734972
NW_003613380_38805_mature	127.8738219	50.77343	7.009815429	5.694139975	1.332575	0.747267	chr13_28627_mature	38.6845175	46.74379	5.310504361	5.577241251	-0.27302	1	hsa-miR-3119	0.537285	0	0.620384622	0	20	0.734972
chr2_6521_star	49.43021686	41.1023	5.656216524	5.395827139	0.266174	0.748469	NW_003539992_39208_mature	139.694091	137.0077	7.136417929	7.108604601	0.028014	1	hsa-miR-4259	0.537285	0	0.620384622	0	20	0.734972
chr3_7983_mature	1435.625429	214.3767	10.48846824	7.750718383	2.743459	0.748482	chr14_30931_mature	83.8164547	86.23424	6.406272275	6.446822559	-0.04103	1	mmu-miR-671-3p	0.537285	0	0.620384622	0	20	0.734972
hsa-miR-4531	48.35564693	96.71129	5.625143252	6.610453419	-1	0.751576	chr4_9788_mature	21.4913986	27.40153	4.491301473	4.827896911	-0.3505	1	mmu-miR-3061-5p	0.537285	0	0.620384622	0	20	0.734972
chr4_9973_mature	42.98279727	57.22085	5.458867456	5.863463969	-0.41278	0.751682	chr2_5750_mature	191.273448	223.2419	7.587015736	7.808912085	-0.22297	1	hsa-miR-4254	0.537285	0	0.620384622	0	20	0.734972
NW_003539426_38819_mature	281.5373221	78.17496	8.142297645	6.306972375	1.848548	0.751735	chr14_29600_mature	120.351832	136.2017	6.923052083	7.100154956	-0.17849	1	hsa-miR-1225-5p	0.537285	0	0.620384622	0	20	0.734972
chr13_27132_mature	126.799252	51.57936	6.997735582	5.716424587	1.297681	0.751738	ssc-miR-146b	236.405385	214.3767	7.891208849	7.750718383	0.141115	1	hsa-miR-4257	0.537285	0	0.620384622	0	20	0.734972
chr2_4589_mature	90.26387427	348.9666	6.511971994	8.451073371	-1.95087	0.752267	bta-miR-380-3p	51.5793567	41.1023	5.716424587	5.395827139	0.327575	1	mmu-miR-3073a-5p	0.537285	0	0.620384622	0	20	0.734972
bta-miR-3596	402.9637244	96.71129	8.658081936	6.610453419	2.058894	0.752429	chr14_29752_mature	36.5353777	26.59561	5.230179094	4.786366653	0.458106	1	hsa-miR-4263	0.537285	0	0.620384622	0	20	0.734972
bta-miR-1260b	46.20650707	83.01053	5.560913833	6.392498216	-0.8452	0.752528	hsa-miR-4508	47.281077	39.49044	5.593385953	5.339509593	0.259759	1	rno-miR-3556b	0.537285	0	0.620384622	0	20	0.734972
chr17_35019_mature	54.80306652	41.90823	5.802272499	5.423182395	0.387023	0.752983	NW_003613193_37595_mature	17.1931189	14.50669	4.185320985	3.954819241	0.245112	1	chr3_7761_star	0.537285	0	0.620384622	0	20	0.734972
chr8_18853_mature	62.32505605	41.90823	5.984704544	5.423182395	0.572579	0.753722	chr6_14620_mature	90.2638743	107.1884	6.511971994	6.757401354	-0.24793	1	bta-miR-6518	0.537285	0	0.620384622	0	20	0.734972
chr13_26825_mature	50.5047868	44.32601	5.686634616	5.502267253	0.188267	0.755437	chr5_11922_mature	17.1931189	24.17782	4.185320985	4.654081667	-0.49185	1	hsa-miR-4271	0.537285	0	0.620384622	0	20	0.734972
chr17_35424_mature	46.20650707	77.36904	5.560913833	6.292211829	-0.74366	0.755733	chr17_35263_mature	12.8948392	12.89484	3.796477232	3.796477232	0	1	chr6_14391_star	0.537285	0	0.620384622	0	20	0.734972
chr6_13297_mature	217.0631262	1499.831	7.768602025	10.5515458	-2.78861	0.755878	chr6_13355_star	60.1759162	48.35565	5.934891897	5.625143252	0.315502	1	hsa-miR-4287	0.537285	0	0.620384622	0	20	0.734972
chr4_10674_mature@@bta-miR-760-	60.17591618	42.71415	5.934891897	5.4500286	0.494472	0.756075	chr9_19395_mature	19.3422588	23.3719	4.346407978	4.607146585	-0.27302	1	hsa-miR-4284	0.537285	0	0.620384622	0	20	0.734972
chr1_782_mature	51.57935673	45.13194	5.716424587	5.527693969	0.192645	0.757714								hsa-miR-4282	0.537285	0	0.620384622	0	20	0.734972
hsa-miR-4454	558.7763645	120.0832	9.128706763	6.919854778	2.218237	0.757963								mmu-miR-3108-3p	0.537285	0	0.620384622	0	20	0.734972
chr8_17888_mature	59.10134625	145.0669	5.909325402	7.190485881	-1.29546	0.758742								hsa-miR-4294	0.537285	0	0.620384622	0	20	0.734972
chr4_9000_mature	108.5315631	49.9675	6.775202853	5.671505736	1.119053	0.758931								hsa-miR-4297	0.537285	0	0.620384622	0	20	0.734972
chr15_33009_mature	68.77247564	195.8404	6.124586119	7.620882324	-1.50978	0.761027								bta-miR-30b-3p	0.537285	0	0.620384622	0	20	0.734972
chr1_2584_mature	50.5047868	93.48758	5.686634616	6.562052863	-0.88835	0.761602								mmu-miR-883b-3p	0.537285	0	0.620384622	0	20	0.734972
chr12_26224_mature	84.89102461	46.74379	6.424435476	5.577241251	0.860837	0.761746								hsa-miR-2053	0.537285	0	0.620384622	0	20	0.734972
ssc-miR-92a	536735.8607	14602.6	19.03385544	13.83403638	5.199915	0.761972								chr1_1843_star	0.537285	0	0.620384622	0	20	0.734972
chr2_6291_mature	355.6826474	92.68166	8.47849722	6.549694683	1.940235	0.762072								hsa-miR-124-5p	0.537285	0	0.620384622	0	20	0.734972
ssc-miR-199b-5p	919.8318616	13757.99	9.846793943	13.74808669	-3.90276	0.763696								mmu-miR-206-5p	0.537285	0	0.620384622	0	20	0.734972

chr2_6342_mature	109.606133	51.57936	6.789287574	5.716424587		1.087463	0.76436
chr7_16538_mature	69.84704557	45.93786	6.146635787	5.552680304		0.604515	0.764453
chr7_15331_star	309.4761404	86.23424	8.278338593	6.446822559		1.843496	0.764465
ssc-miR-98	14227.3059	1075.107	13.79647628	10.07160611		3.72611	0.764538
chr4_9471_mature	53.72849659	48.35565	5.774220321	5.625143252		0.152003	0.764574
chr7_16464_star	52.65392666	98.32315	5.745611853	6.634058094		-0.90099	0.766048
ssc-miR-10a-3p	63.39962598	157.1559	6.008980404	7.305203133		-1.30965	0.766207
chr4_9354_mature	68.77247564	186.1692	6.124586119	7.548199552		-1.43671	0.766784
ssc-miR-324	780.1377705	152.3203	9.609433211	7.260404801		2.356621	0.767675
chr7_17017_star	94.562154	49.9675	6.578367468	5.671505736		0.920273	0.767691
chr13_27084_mature	445.9465217	107.9943	8.803958409	6.76810859		2.045916	0.767816
chr1_3195_star	56.95220639	117.6654	5.856791684	6.890755622		-1.04687	0.768875
ssc-miR-152	1957.866416	42144.36	10.9358033	15.36308633		-4.42799	0.769056
ssc-miR-30c-5p	22443.4676	1500.637	14.45407225	10.5523203		3.902649	0.769431
chr2_3980_mature	59.10134625	127.3365	5.909325402	7.003788148		-1.10738	0.770131
chr4_9963_mature	73.07075536	48.35565	6.210832145	5.625143252		0.59561	0.770503
chr18_36406_mature	99.93500366	52.38528	6.657282769	5.738370208		0.931828	0.772801
chr15_33664_mature	51.57935673	66.89198	5.716424587	6.085169218		-0.37504	0.773089
chr8_17503_mature	51.57935673	68.50383	5.716424587	6.11902064		-0.40939	0.773144
NW_003539920_39178_mature	54.80306652	97.51722	5.802272499	6.622304032		-0.8314	0.773196
chr14_31374_mature	426.6042629	107.1884	8.740132426	6.757401354		1.99275	0.773437
chr9_20723_mature	64.47419591	150.7084	6.032854532	7.245157472		-1.22497	0.773616
chr9_20919_mature	3619.15153	426.3356	11.82183437	8.739225767		3.08559	0.774371
chr7_15273_mature	1157.311817	199.87	10.17780796	7.650118356		2.533644	0.774372
ssc-miR-22-5p	667.3079277	141.0373	9.384369178	7.150126067		2.242276	0.774439
chr12_26213_mature	1634.420866	250.6434	10.67544624	7.97523716		2.705071	0.774805
chr7_15219_star	438.4245322	109.6061	8.779471605	6.789287574		2	0.775234
chr16_34200_mature	59.10134625	116.0536	5.909325402	6.871024911		-0.97353	0.776539
chr14_29599_mature	66.62333577	157.1559	6.079449279	7.305203133		-1.2381	0.776627
NW_003613116_37269_mature	61.25048611	53.19121	5.960013198	5.759986999		0.203533	0.777141
chr3_7184_mature	207.3919968	73.3394	7.703156063	6.216055097		1.4997	0.778431
chr17_35003_star	70.9216155	52.38528	6.168353522	5.738370208		0.437064	0.77954
chr14_31373_mature	459.9159308	114.4417	8.848359823	6.851020612		2.006757	0.779545
chr1_2976_star	74.1453253	52.38528	6.231611453	5.738370208		0.501194	0.779857
chr5_11729_mature	54.80306652	70.92162	5.802272499	6.168353522		-0.37197	0.77999
ssc-miR-664-5p	55.87763645	63.66827	5.82978961	6.014986077		-0.1883	0.780231
chr8_18593_mature	56.95220639	62.05641	5.856791684	5.978571212		-0.12383	0.781045
chr5_11904_mature	110.680703	58.02678	6.803236118	5.883297648		0.931613	0.7845
chr4_9318_mature	68.77247564	55.60899	6.124586119	5.82295938		0.306513	0.784534
chr3_6703_star	60.17591618	104.7706	5.934891897	6.72479443		-0.79998	0.78482
chr6_14094_mature	59.10134625	97.51722	5.909325402	6.622304032		-0.72247	0.784846
chr8_17892_mature	68.77247564	153.1262	6.124586119	7.26796846		-1.15482	0.785034
chr6_13671_mature	3762.069331	450.5134	11.87769415	8.818625135		3.061884	0.785256
ssc-miR-455-3p	124.6501121	497.2572	6.973268148	8.960746947		-1.99611	0.786444
chr1_3118_mature@@mmu-miR-195	125.724682	61.25049	6.985553734	5.960013198		1.037475	0.786521
bta-miR-495	59.10134625	82.2046	5.909325402	6.378591382		-0.47603	0.788345
NW_003613116_37270_mature	74.1453253	57.22085	6.231611453	5.863463969		0.373815	0.788765
chr18_36516_mature	844.6119664	171.6625	9.723851982	7.431811362		2.298713	0.79
chrx_21708_mature	114.9789827	61.25049	6.857719579	5.960013198		0.908577	0.791278
chr18_36618_mature	96.71129386	58.8327	6.610453419	5.902862353		0.717066	0.791338
chr13_27228_mature	61.25048611	82.2046	5.960013198	6.378591382		-0.4245	0.792416
NW_003540583_39564_mature	1304.527897	228.0775	10.35041757	7.839691752		2.515932	0.79261
chr3_6912_mature	914.459012	11960.77	9.838351483	13.54614317		-3.70925	0.794028
NW_003538371_37782_mature	77.36903509	180.5277	6.292211829	7.504046287		-1.22239	0.794431
ssc-miR-542-5p	63.39962598	74.95125	6.008980404	6.247001856		-0.24148	0.795032
chr3_7984_mature	1366.852953	236.9427	10.41769743	7.894470201		2.528248	0.795201
chr6_14220_star	82.74188475	207.9293	6.387877484	7.706870893		-1.3294	0.795428
ssc-miR-671-3p	938.0995505	186.9752	9.875134291	7.554398282		2.326894	0.796016
chr14_30067_mature	68.77247564	128.1425	6.124586119	7.012819653		-0.89785	0.796417
ssc-miR-92b-5p	67.6979057	121.695	6.102194213	6.938933175		-0.84609	0.796419
chr4_10149_mature	73.07075536	149.9025	6.210832145	7.237472949		-1.03666	0.797521
ssc-miR-874	67.6979057	117.6654	6.102194213	6.890755622		-0.79751	0.797853
ssc-miR-32	529.7629764	132.978	9.051923928	7.065852624		1.994159	0.798167
chr18_36617_mature	89.18930434	62.05641	6.494884448	5.978571212		0.52329	0.798585
chr3_8127_star	92.41301414	62.86234	6.545551653	5.996893534		0.5559	0.799941
chr5_11589_mature	557.7017946	138.6195	9.125934644	7.125356859		2.008363	0.801192
ssc-miR-1306-3p	197.7208675	1001.768	7.634599566	9.969771887		-2.34101	0.801874
chr1_2125_mature	75.21989523	67.69791	6.25209572	6.102194213		0.152003	0.802165
chr2_4526_mature	67.6979057	95.90537	6.102194213	6.598504656		-0.5025	0.802899
chr2_4526_star	120.3518324	67.69791	6.923052083	6.102194213		0.830075	0.804256
chr6_13505_mature	818.822288	177.304	9.679167402	7.478195571		2.207325	0.805184
chr1_3299_mature	206.3174269	83.81645	7.695697583	6.406272275		1.29956	0.807259
chr9_20360_mature	183.7514583	79.78682	7.529441942	6.336047992		1.203533	0.807402
ssc-miR-676-3p	554.4780848	141.8432	9.117586184	7.158288862		1.966833	0.808145
chr14_30000_mature	1387.269782	249.8375	10.43907224	7.970609288		2.473186	0.808686
ssc-miR-328	564.1492142	144.261	9.142488017	7.182503739		1.967395	0.809892
chr14_29999_mature	1326.019296	244.196	10.37397363	7.937791734		2.44099	0.810468
chr3_7145_star	175.1548989	79.78682	7.460700787	6.336047992		1.134409	0.810796
chr1_3117_mature@@mmu-miR-195	133.2466715	73.3394	7.068742509	6.216055097		0.861439	0.811851
chr6_13506_mature	857.5068056	186.9752	9.745685761	7.554398282		2.197301	0.812084
chr9_21003_mature	78.44360502	78.98089	6.311859186	6.32158343		-0.00985	0.812442
chr11_25350_mature	112.8298428	333.654	6.830735029	8.386526292		-1.5642	0.812796
chr15_33536_mature	1600.034628	278.8509	10.6447888	8.128514563		2.520537	0.814167
chr14_30841_mature	103.1587135	73.3394	6.702639724	6.216055097		0.492205	0.815966
chr2_5068_star	308.4015704	106.3824	8.273336709	6.746614057		1.53555	0.816396
chr5_11650_mature	93.48758407	74.95125	6.562052863	6.247001856		0.318822	0.816516
chr2_6335_mature	857.5068056	9704.978	9.745685761	13.24465793		-3.5005	0.816666
chr12_25849_star@@mmu-miR-5112	301.9541508	105.5765	8.242955662	6.735745493		1.516041	0.817035
ssc-miR-24-2-5p	87.04016448	78.17496	6.460089935	6.306972375		0.154975	0.817125
chr4_10079_star	135.3958114	76.56311	7.091655531	6.277298702		0.822462	0.817169
NW_003539992_39208_mature	81.66731482	137.0077	6.369245121	7.108604601		-0.74643	0.818682
chr4_10147_mature	82.74188475	142.6492	6.387877484	7.166405731		-0.78578	0.819068
chr9_20605_star	79.51817495	99.12908	6.331242567	6.645717165		-0.31802	0.819388
ssc-miR-29b	358.9063572	2412.141	8.491477775	11.23669641		-2.74863	0.819597
ssc-miR-342	2481.181973	378.7859	11.27739317	8.569042538		2.711573	0.820445
chr13_26633_star	189.124308	87.84609	7.570799187	6.473236413		1.106285	0.821843
chr2_3979_mature	82.74188475	109.6061	6.387877484	6.789287574		-0.40564	0.823655
ssc-miR-30b-5p	18492.27396	1505.472	14.17471303	10.5569586		3.618634	0.823736
mmu-miR-5115	26251.74343	1928.584	14.68018057	10.91407442		3.766799	0.82423
chr15_33256_mature	102.0841435	83.01053	6.687678623	6.392498216		0.298393	0.826301
chr16_34199_mature	85.96559455	120.0832	6.442372848	6.919854778		-0.4822	0.827245
chr1_270_mature	447.0210916	137.0077	8.807422842	7.108604601		1.706086	0.82865
chr2_5756_mature@@bta-miR-2898	9130.620711	936.4877	13.15665522	9.872655945		3.285381	0.828891
ssc-miR-4334-3p	733.9312634	182.9455	9.521465514	7.523134815		2.004231	0.830468
chr1_3607_mature	120.3518324	85.42831	6.923052083	6.433432039		0.494472	0.830987
ssc-miR-186	57446.50856	3437.281	15.80995671	11.74747156		4.06288	0.8321
[truncated: 145,298 more chars]
