# Supplementary material for: Common microRNA–mRNA interactions exist among distinct porcine iPSC lines independent of their metastable pluripotent states
Source: Cell Death Dis. 2017 Aug 31;8(8):e3027–. doi: 10.1038/cddis.2017.426 (PMC5596602; doi:10.1038/cddis.2017.426)
Supplement: Supplementary Table 2 [file cddis2017426x3.pdf]

Common differential miRNAs

miRNA	miRNApreName	mirSequence	piPS-L	piPS-F	piPS-LF	PEFs	Style
chr1_1155_mature	chr1_1155	aaagatcagaggttctg	2.149139864	0	2.68642483	1000.155964	Low
chr1_1317_mature	chr1_1317	ttgagattggattgctc	2.149139864	0	24.71510843	2365.397062	Low
chr1_1318_mature	chr1_1318	ttgagattggattgctc	0	0	23.6405385	2350.890368	Low
chr1_169_mature	chr1_169	aaaggaaaatgaagtac	0	2.149139864	5.372849659	386.8451755	Low
chr1_211_mature	chr1_211	tctggatgttttgcttc	0	0	0	209.5411367	Low
chr1_2110_mature	chr1_2110	attggattgaattgtgt	0	0	0.537284966	52.38528418	Low
chr1_2264_mature	chr1_2264	actgcagtgaacgtagg	3.223709795	2.149139864	4.835564693	193.4225877	Low
chr1_2360_mature	chr1_2360	aaggctgagaaatactc	2.149139864	0	12.35755422	922.786929	Low
chr1_2557_mature	chr1_2557	aatggactgaattattcc	1.074569932	0	15.04397905	949.3825348	Low
chr1_268_mature	chr1_268	tctgtgtctgaatctgt	7.521989523	4.298279727	4.835564693	684.2324041	Low
chr1_2963_mature	chr1_2963	agaacatcctgatcttg	4.298279727	6.447419591	8.059274489	476.3031223	Low
chr1_2964_mature	chr1_2964	agaacatcctgatcttg	1.074569932	4.298279727	4.835564693	463.4082831	Low
chr1_3153_mature	chr1_3153	ccagaacctctgatctttc	0	0	2.149139864	967.1129386	Low
chr1_3371_mature	chr1_3371	taaatgatgagttctctt	3.223709795	0	8.596559455	529.4943339	Low
chr1_3435_mature	chr1_3435	gaatagaaggtgctgaa	0	0	3.760994761	138.6195212	Low
chr1_3708_mature	chr1_3708	tgagttggttcagttct	2.149139864	0	1.611854898	78.17496254	Low
chr1_3720_mature	chr1_3720	cttggtagaggtctttgag	0	0	19.87954374	5863.928118	Low
chr1_485_mature	chr1_485	aagataggaagttgcct	0	0	4.298279727	1921.331038	Low
chr1_541_mature	chr1_541	tctgcaactgatagagc	0	2.149139864	4.835564693	772.8844235	Low
chr10_23795_star	chr10_23795	tgtttcacgggctttgcttgcag	1.074569932	2.149139864	3.223709795	186.9751681	Low
chr10_24012_mature	chr10_24012	gcgatctgagatttcttc	0	0	0.537284966	91.06980172	Low
chr10_24151_mature	chr10_24151	aaggaatggatgacttg	0	0	2.68642483	346.548803	Low
chr11_24398_mature	chr11_24398	tctggattttctgcatc	1.074569932	0	4.835564693	849.4475311	Low
chr11_24582_mature	chr11_24582	aatgagtgtgaacatct	0	4.298279727	16.11854898	1636.032721	Low
chr11_25089_mature	chr11_25089	aggatgtggaattgctg	3.223709795	17.19311891	22.0286836	1072.689434	Low
chr11_25205_mature	chr11_25205	aggagaaagaacagaaaac	5.372849659	4.298279727	9.133844421	362.667352	Low
chr11_25257_mature	chr11_25257	tgtgaatctgatcctgc	9.671129386	2.149139864	3.760994761	416.6644911	Low
chr12_25534_mature	chr12_25534	cagatctttgatagttt	3.223709795	4.298279727	5.372849659	3712.907757	Low
chr12_25534_star	chr12_25534	agttatctatgggttgga	1.074569932	0	2.68642483	89.45794682	Low
chr12_25625_star	chr12_25625	tgggggggaagccagaatc	0	0	2.68642483	91.87572917	Low
chr12_25628_mature	chr12_25628	tgcgatcctgattctgg	0	8.596559455	7.521989523	371.5325539	Low
chr12_26093_mature	chr12_26093	cttgtctgtaaagcttt	2.149139864	4.298279727	3.760994761	182.1396034	Low
chr12_26317_mature	chr12_26317	aaattcaggatcaggaa	8.596559455	0	7.521989523	369.920699	Low
chr12_26457_mature	chr12_26457	tttggtgaaattcttcc	0	0	8.596559455	390.8748127	Low
chr13_27072_mature	chr13_27072	aaggaaaaccagaatga	11.82026925	4.298279727	9.133844421	728.5584138	Low
chr13_27097_mature	chr13_27097	tctggagttgaacttta	1.074569932	0	1.611854898	65.28012336	Low
chr13_27515_mature	chr13_27515	cccaagaggctgaagggc	4.298279727	2.149139864	3.760994761	560.119577	Low
chr13_27783_mature	chr13_27783	aggaagttgaaggaaga	4.298279727	2.149139864	3.223709795	139.4254487	Low
chr13_28441_mature	chr13_28441	tctgtggtagattttgtc	5.372849659	0	6.447419591	1987.417089	Low
chr13_28509_mature	chr13_28509	gttgattgtgaatgcat	0	0	1.074569932	45.13193714	Low
chr13_28708_mature	chr13_28708	tcaaactgcatttcctg	3.223709795	2.149139864	0.537284966	294.9694463	Low
chr13_29267_mature	chr13_29267	tgaattaggaccaagatc	0	2.149139864	2.149139864	430.3652577	Low
chr13_29269_mature	chr13_29269	tttgagattgaatttttcc	0	4.298279727	5.372849659	461.7964282	Low
chr13_29304_mature	chr13_29304	aatgatatggaaggtagag	2.149139864	0	2.149139864	164.4091996	Low
chr14_29715_mature	chr14_29715	aaaggatttgaagaaac	3.223709795	8.596559455	8.596559455	381.2036833	Low
chr14_30207_mature	chr14_30207	catctttatcaactgtt	4.298279727	2.149139864	5.372849659	339.295456	Low
chr14_30223_mature	chr14_30223	ttggctgtggggcaaac	8.596559455	0	2.149139864	344.1310207	Low
chr14_30497_mature	chr14_30497	tgtgaatctgatcctgt	0	0	3.223709795	550.4484476	Low
chr14_30531_mature	chr14_30531	aaatggtgggaacgaat	1.074569932	0	1.611854898	133.7839565	Low
chr14_31042_mature	chr14_31042	agtgtgattactaggac	1.074569932	0	1.611854898	135.3958114	Low
chr14_31042_star	chr14_31042	ttctatgaagacagcat	0	0	1.074569932	68.50383315	Low
chr14_31044_mature	chr14_31044	agtgtgattactaggac	1.074569932	2.149139864	2.149139864	137.8135938	Low
chr14_31044_star	chr14_31044	ttctatgaagacagcat	0	0	1.611854898	70.11568805	Low
chr14_31314_mature	chr14_31314	ttggctgtggggcaaac	7.521989523	0	3.760994761	297.3872286	Low
chr14_31463_star	chr14_31463	aagaagaactat	2.149139864	2.149139864	17.19311891	2371.844482	Low
chr14_31500_mature	chr14_31500	tttgccccacagccacc	0	0	1.074569932	77.36903509	Low
chr15_31797_mature	chr15_31797	ataatgcagagtgagaat	33.31166789	85.96559455	87.57744944	0	High
chr15_31863_mature	chr15_31863	ctgctctcctgtctccgctcag	3.223709795	27.93881823	9.133844421	685.844259	Low
chr15_31936_mature	chr15_31936	agtggttcagttctctt	7.521989523	4.298279727	4.835564693	938.9054779	Low
chr15_32293_mature	chr15_32293	actgcagtgaacgtagg	1.074569932	0	1.611854898	137.0076663	Low
chr15_32517_mature	chr15_32517	ccaatttgtctaccatc	1.074569932	2.149139864	2.149139864	125.724682	Low
chr15_32593_mature	chr15_32593	taaaaagtgaactatagat	1.074569932	0	0.537284966	359.4436422	Low
chr15_33031_mature	chr15_33031	attttgattgatcttgac	0	6.447419591	4.298279727	195.8403701	Low
chr15_33120_mature	chr15_33120	ctaggtagagatctttg	1.074569932	2.149139864	4.298279727	686.6501864	Low
chr15_33221_mature	chr15_33221	caagtgtttgagttcttc	3.223709795	0	12.89483918	502.0928006	Low
chr15_33320_mature	chr15_33320	gtagctgtcataactcc	3.223709795	4.298279727	4.835564693	277.2390424	Low
chr16_33714_mature	chr16_33714	gcaaactgcatttcctg	0	4.298279727	5.372849659	520.629132	Low
chr16_34017_mature	chr16_34017	gaagaataaaagctgaaa	0	2.149139864	13.96940911	3926.478531	Low
chr16_34386_mature	chr16_34386	tgtagatctaattgaatc	5.372849659	0	8.596559455	1477.265014	Low
chr16_34502_mature	chr16_34502	tctgtgtgaattttttc	1.074569932	2.149139864	1.074569932	159.5736349	Low
chr17_34881_mature	chr17_34881	tacagatggtagagctg	3.223709795	0	5.910134625	630.235265	Low
chr17_35005_mature	chr17_35005	tttgttgacattgcagg	0	2.149139864	2.68642483	127.3365369	Low
chr18_36061_mature	chr18_36061	tcagcttttactctatt	1.074569932	0	0	103.1587135	Low
chr18_36117_mature	chr18_36117	ggaggacaacaacaaag	1.074569932	6.447419591	13.43212415	13979.61753	Low
chr18_36269_mature	chr18_36269	aaagaataaaagctgaac	1.074569932	2.149139864	17.19311891	3564.617106	Low
chr2_4023_mature	chr2_4023	aatgcagttggtcttcc	1.074569932	0	0.537284966	57.22084887	Low
chr2_4295_mature	chr2_4295	ttacttttggtctttgat	6.447419591	6.447419591	1.611854898	576.2381259	Low
chr2_4477_star	chr2_4477	tggtgggtaggaaagaatgaa	1.074569932	0	9.133844421	2107.500279	Low
chr2_4744_mature	chr2_4744	ttaggaattgacagact	3.223709795	0	2.68642483	294.1635188	Low
chr2_4930_mature	chr2_4930	caagtgtttgagttcttc	9.671129386	0	10.74569932	559.3136495	Low
chr2_5680_mature	chr2_5680	agaatgtgaagtttgagc	3.223709795	4.298279727	2.68642483	701.1568805	Low
chr3_7072_mature	chr3_7072	attgtaggccaagcagg	1.074569932	0	1.074569932	261.9264209	Low
chr3_7573_mature	chr3_7573	acagatgaagaacttga	2.149139864	17.19311891	18.26768884	1579.6178	Low
chr3_8078_mature	chr3_8078	cctgcttggcctacaatat	0	0	0.537284966	265.9560581	Low
chr3_8256_mature	chr3_8256	agagttaaatattttgga	1.074569932	2.149139864	3.760994761	417.4704185	Low
chr4_10088_mature	chr4_10088	catggactgaattattcc	0	2.149139864	33.84895285	9929.832097	Low
chr4_10244_mature	chr4_10244	aaaggaaaatgaagttc	1.074569932	0	4.298279727	394.0985225	Low
chr4_10395_mature	chr4_10395	tctgcagccactgctcc	2.149139864	6.447419591	4.298279727	984.0374151	Low
chr4_8854_mature	chr4_8854	tcttggtgctatgaacc	1.074569932	8.596559455	7.521989523	1067.047942	Low
chr4_9198_mature	chr4_9198	acagctgccaaccttgacactg	3.223709795	6.447419591	3.760994761	257.8967836	Low
chr4_9231_mature	chr4_9231	atggaacacctcctttc	0	0	2.149139864	660.8605081	Low
chr4_9743_mature	chr4_9743	tttcttgttaaaatcttc	5.372849659	8.596559455	8.596559455	552.0603025	Low
chr4_9789_mature	chr4_9789	cttgatcactgtgaaaa	4.298279727	4.298279727	10.20841435	514.9876398	Low

chr4_9930_mature	chr4_9930	tgcttgttgagctgctc	0	0	3.223709795	108.8002056	Low
chr4_9932_mature	chr4_9932	tgcttgttgagctgctc	0	0	2.68642483	99.93500366	Low
chr5_11474_mature	chr5_11474	gaagaatcaaagctgaaa	2.149139864	4.298279727	9.133844421	676.979057	Low
chr5_11530_mature	chr5_11530	attctagtagattctttg	1.074569932	2.149139864	6.447419591	1885.064303	Low
chr5_11958_mature	chr5_11958	tttgagattgtgctgat	0	2.149139864	3.760994761	321.5650521	Low
chr5_11967_mature	chr5_11967	tttacaaacacggagtt	1.074569932	4.298279727	4.298279727	429.5593302	Low
ssc-miR-371-5p	chr6_12932	actcaaactgtgggggcactt	103988.2814	1111.10531	7189.410129	16.92447643	High
chr6_12951_mature	chr6_12951	actggaggacagactgg	1.074569932	0	5.372849659	1104.120605	Low
chr6_13041_mature	chr6_13041	tttgaatctggagctga	0	4.298279727	2.149139864	195.8403701	Low
chr6_13229_mature	chr6_13229	ctagagcctctgatctttc	9.671129386	0	3.760994761	724.5287765	Low
chr6_13339_mature	chr6_13339	caagattctggagcaaa	3.223709795	6.447419591	10.74569932	656.0249434	Low
chr6_13565_mature	chr6_13565	aaaggatttgaagaaac	2.149139864	4.298279727	7.521989523	325.5946893	Low
chr6_13566_mature	chr6_13566	aaaggatttgaagaaac	1.074569932	15.04397905	5.910134625	375.5621912	Low
chr6_13576_mature	chr6_13576	gttgttttggattttggag	1.074569932	0	3.760994761	265.9560581	Low
chr6_13578_mature	chr6_13578	gttgttttggattttggag	1.074569932	4.298279727	1.611854898	252.2552915	Low
chr6_13600_star	chr6_13600	ataataaatttcaaata	1.074569932	0	0.537284966	123.3068997	Low
chr6_14477_mature@@bta-miR-483	chr6_14477	aggacgggaagagaggaggga	8.596559455	8.596559455	3.223709795	888.9379761	Low
chr6_14978_mature	chr6_14978	aggaccatcacatgctg	4.298279727	4.298279727	5.910134625	3488.859926	Low
chr6_15013_mature	chr6_15013	caatggcagcaggacgg	0	0	0.537284966	59.63863122	Low
chr6_15014_mature	chr6_15014	caatggcagcaggacgg	1.074569932	0	0.537284966	58.83270377	Low
chr7_15145_mature	chr7_15145	aggctgaagaggacgcgc	1.074569932	2.149139864	7.521989523	2137.319594	Low
chr7_15797_star	chr7_15797	ctgaataaattaagagaaag	6.447419591	6.447419591	2.68642483	241.7782347	Low
chr7_16963_mature	chr7_16963	tggacaaactaatacag	1.074569932	0	11.28298428	930.040276	Low
chr8_17989_mature@@bta-miR-302b	chr8_17989	taagtgcttccatgttttagtg	2325.369332	90.26387427	17222.66958	1.611854898	High
chr8_18343_mature	chr8_18343	tttggtggaaaagatgg	1.074569932	2.149139864	2.149139864	238.5545249	Low
chr9_19375_mature	chr9_19375	tttgttgggttttttgt	2.149139864	4.298279727	5.910134625	406.9933617	Low
chr9_19755_mature	chr9_19755	agtgattcaagttcttg	0	0	0	128.1424644	Low
chr9_19756_mature	chr9_19756	agtgattcaagttcttg	1.074569932	0	0.537284966	157.96178	Low
chr9_20694_mature	chr9_20694	ttttttaagagttgact	0	0	1.611854898	89.45794682	Low
chr9_20753_mature	chr9_20753	agtgattcaagttcttg	2.149139864	0	1.611854898	170.0506917	Low
chr9_20754_mature	chr9_20754	agtgattcaagttcttg	0	0	0.537284966	145.8728682	Low
chr9_20772_mature	chr9_20772	tatcagttggaactctgt	2.149139864	0	3.760994761	170.0506917	Low
chrx_21555_mature	chrx_21555	aaagagaatcaactctg	0	2.149139864	48.8929319	5591.52464	Low
chrx_21569_mature	chrx_21569	agtaattgggattgggttg	0	0	1.611854898	159.5736349	Low
chrx_21570_mature	chrx_21570	agtaattgggattgggttg	3.223709795	0	1.611854898	193.4225877	Low
chrx_21663_mature	chrx_21663	caagttttaaatcctgt	0	0	1.074569932	49.96750183	Low
chrx_21682_mature	chrx_21682	agtgtggttgactgcaaa	2.149139864	12.89483918	11.28298428	510.9580026	Low
chrx_21884_mature	chrx_21884	cagcagctctagctcttc	3.223709795	2.149139864	5.910134625	253.0612189	Low
chrx_22076_mature	chrx_22076	tttgggcttgattcattct	0	2.149139864	5.910134625	1105.73246	Low
chrx_22120_mature	chrx_22120	tgtgcattggattgttt	1.074569932	0	1.074569932	365.8910618	Low
chrx_22340_mature	chrx_22340	aagaatcaaagctgaac	1.074569932	2.149139864	3.223709795	610.0870788	Low
chrx_22382_mature	chrx_22382	tctgcagatacattgggaat	0	2.149139864	3.223709795	124.1128271	Low
chrx_22488_mature	chrx_22488	acaggaatgaattcaga	1.074569932	0	6.984704557	290.939809	Low
chrx_22497_mature	chrx_22497	tttgctttggattttggag	0	8.596559455	3.760994761	178.9158936	Low
chrx_22553_mature	chrx_22553	agaggctataaaattggat	1.074569932	0	7.521989523	1729.520305	Low
chrx_22554_mature	chrx_22554	agaggctataaaattggat	1.074569932	2.149139864	4.298279727	1641.674213	Low
chrx_22639_mature	chrx_22639	actggaggtcagtctgg	1.074569932	4.298279727	8.059274489	1014.662658	Low
chrx_22720_mature	chrx_22720	aagagctagagctgctgc	0	2.149139864	6.447419591	299.805011	Low
chrx_22902_star	chrx_22902	ttagttatttcctttc	0	0	0.537284966	64.47419591	Low
chrx_22943_mature	chrx_22943	tgtgcattggattgttt	0	2.149139864	3.223709795	362.667352	Low
chrx_23228_mature	chrx_23228	aaggctgagaaatactc	2.149139864	4.298279727	13.96940911	982.4255602	Low
hsa-miR-1298	hsa-mir-1298	ttcattcggctgtccagatgta	0	0	0	54.80306652	Low
hsa-miR-184	hsa-mir-184	tggacggagaactgataagggt	0	0	0.537284966	104.7705684	Low
hsa-miR-3659	hsa-mir-3659	tgagtgttgtctacgagggca	0	0	59.10134625	13122.91665	Low
hsa-miR-4685-3p	hsa-mir-4685	tctcccttcctgccctggctag	0	2.149139864	0	134.589884	Low
hsa-miR-516a-5p	mir-516a-1/hsa-mir-51	ttctcgaggaaagaagcactttc	1.074569932	0	2.149139864	473.8853399	Low
mmu-miR-5108	mmu-mir-5108	gtagagcactggatggttt	1.074569932	0	2.149139864	104.7705684	Low
mmu-miR-5124b	mmu-mir-5124b	tggctcagtgactaagaagca	0	0	2.68642483	107.9942781	Low
NW_003537574_37009_mature	NW_003537574_3700	aaggaatagaaaactct	3.223709795	8.596559455	5.372849659	8313.141635	Low
NW_003537694_37136_mature	NW_003537694_3713	gctagagtggattctgc	2.149139864	0	3.223709795	346.548803	Low
NW_003537990_37435_mature	NW_003537990_3743	ctggtggtggacacggag	1.074569932	0	2.149139864	108.8002056	Low
NW_003538465_37902_mature	NW_003538465_3790	tttgttttggattttggat	2.149139864	0	2.149139864	205.5114995	Low
NW_003541079_39891_mature	NW_003541079_3989	taggaatagaaaacttt	2.149139864	0	4.835564693	7881.164522	Low
NW_003541201_39964_mature	NW_003541201_3996	tggtgcctgacgtcttggcag	2.149139864	12.89483918	1.611854898	365.8910618	Low
NW_003613041_36883_mature	NW_003613041_3688	aaaggaaaatgaagttc	1.074569932	0	5.910134625	384.4273931	Low
NW_003613056_36922_mature	NW_003613056_3692	aatagagtaaaagctga	0	0	0.537284966	86.23423703	Low
NW_003613137_37368_mature	NW_003613137_3736	cttgggttggatttgag	1.074569932	0	1.611854898	1938.255515	Low
NW_003613242_37864_mature	NW_003613242_3786	attgtaggccaagcagg	0	0	2.149139864	266.7619856	Low
NW_003613242_37866_mature	NW_003613242_3786	cctgcttggcctacaatat	2.149139864	0	1.074569932	257.0908562	Low
ssc-miR-206	ssc-mir-206	tggaatgtaaggaagtgtgtga	1.074569932	0	75.75718019	11796.36007	Low
ssc-miR-370	ssc-mir-370	gcctgctggggtggaacctggt	4.298279727	6.447419591	31.69981299	2733.705907	Low
ssc-miR-432-5p	ssc-mir-432	tcttggagtaggtcattgggt	1.074569932	0	2.149139864	260.314566	Low

piPS-L Vs PEFs

AccID	piPS-L	PEF	piPS-L_log2(count+1)	PEF_log2(count+1)	PEF_log2(count+1)<0.05	Log2FC	FDR
rno-miR-351-5p	27370.370733	0.805927	14.740380	0.852740	0.852740	15.051605	0.000000
rno-miR-298-5p	14916.105224	0.805927	13.864680	0.852740	0.852740	14.175861	0.000000
mmu-miR-5099	6044.455867	0.000000	12.561635	0.000000	0.000000	20.000000	0.000000
chr6_12933_star@@hsa-miR-520e	29877.342384	4.029637	14.866812	2.330454	2.330454	12.856114	0.000000
rno-miR-872-3p	4087.664021	0.000000	11.997414	0.000000	0.000000	20.000000	0.000001
ssc-miR-371-5p	103988.281442	16.924476	16.666075	4.163859	4.163859	12.585022	0.000001
chr8_18357_star	2904.562526	0.000000	11.504602	0.000000	0.000000	20.000000	0.000001
chr6_12933_mature	2181.376962	0.000000	11.091685	0.000000	0.000000	20.000000	0.000003
hsa-miR-3659	0.000000	13122.916650	0.000000	13.679911	13.679911	-20.000000	0.000003
chr4_10088_mature	0.000000	9929.832097	0.000000	13.277699	13.277699	-20.000000	0.000005
chr2_4320_mature	2915.308225	0.805927	11.509927	0.852740	0.852740	11.820711	0.000005
chr13_28872_mature	6854.681595	3.223710	12.743084	2.078511	2.078511	11.054152	0.000006
chr4_9150_mature@@rno-miR-298-5p	1419.506880	0.000000	10.472190	0.000000	0.000000	20.000000	0.000007
chr4_9149_mature@@rno-miR-298-5p	1391.568062	0.000000	10.443532	0.000000	0.000000	20.000000	0.000007
chr1_3439_mature@@rno-miR-872-5p	6064.872695	3.223710	12.566500	2.078511	2.078511	10.877540	0.000008
NW_003538989_38416_mature	2325.369332	0.805927	11.183864	0.852740	0.852740	11.494522	0.000008
chr18_36117_mature	1.074570	13979.617528	1.052812	13.771140	13.771140	-13.667278	0.000008
ssc-miR-126-5p	10080.540530	5.641492	13.299428	2.731507	2.731507	10.803209	0.000008

ssc-miR-206	1.074570	11796.360069	1.052812	13.526176	13.526176	-13.422295	0.000010
chr1_3720_mature	0.000000	5863.928118	0.000000	12.517898	12.517898	-20.000000	0.000011
chrx_21555_mature	0.000000	5591.524640	0.000000	12.449284	12.449284	-20.000000	0.000012
mmu-miR-883a-3p	1090.688481	0.000000	10.092346	0.000000	0.000000	20.000000	0.000012
chr15_32852_star	1884.795660	0.805927	10.880958	0.852740	0.852740	11.191471	0.000012
chr5_11699_mature	971.411218	0.000000	9.925423	0.000000	0.000000	20.000000	0.000015
chr1_2086_mature	959.590949	0.000000	9.907778	0.000000	0.000000	20.000000	0.000015
chr7_16402_mature	3175.354149	2.417782	11.633156	1.773061	1.773061	10.359017	0.000018
chr8_17989_mature@@bta-miR-302b	2325.369332	1.611855	11.183864	1.385075	1.385075	10.494522	0.000018
chr16_34017_mature	0.000000	3926.478531	0.000000	11.939388	11.939388	-20.000000	0.000020
bta-miR-379	0.000000	3912.777764	0.000000	11.934346	11.934346	-20.000000	0.000020
chr6_12932_star@@bta-miR-292	1486.130216	0.805927	10.538315	0.852740	0.852740	10.848623	0.000020
rno-miR-322-3p	804.852879	0.000000	9.654373	0.000000	0.000000	20.000000	0.000022
chr8_17983_mature@@bta-miR-302b	2088.963947	1.611855	11.029262	1.385075	1.385075	10.339850	0.000023
rno-miR-3586-5p	1200.294614	0.805927	10.230374	0.852740	0.852740	10.540451	0.000032
rno-miR-182	1188.474345	0.805927	10.216108	0.852740	0.852740	10.526173	0.000032
chr2_4263_mature	1143.342407	0.805927	10.160303	0.852740	0.852740	10.470320	0.000035
NW_003613310_38408_mature	638.294540	0.000000	9.320337	0.000000	0.000000	20.000000	0.000035
mmu-miR-344d-3p	620.026851	0.000000	9.278512	0.000000	0.000000	20.000000	0.000037
NW_003541079_39891_mature	2.149140	7881.164522	1.654958	12.944376	12.944376	-11.840434	0.000040
NW_003538989_38422_mature	2056.726850	2.417782	11.006836	1.773061	1.773061	9.732450	0.000044
chr1_1318_mature	0.000000	2350.890368	0.000000	11.199605	11.199605	-20.000000	0.000045
ssc-miR-153	999.350037	0.805927	9.966289	0.852740	0.852740	10.276124	0.000046
chr1_319_mature	949.919820	0.805927	9.893180	0.852740	0.852740	10.202940	0.000052
rno-miR-350	524.390127	0.000000	9.037245	0.000000	0.000000	20.000000	0.000053
chr1_485_mature	0.000000	1921.331038	0.000000	10.908641	10.908641	-20.000000	0.000061
NW_003537574_37009_mature	3.223710	8313.141635	2.078511	13.021352	13.021352	-11.332456	0.000063
chr4_10011_mature	476.034480	0.000000	8.897950	0.000000	0.000000	20.000000	0.000065
ssc-miR-455-5p	0.000000	1852.021277	0.000000	10.855664	10.855664	-20.000000	0.000065
chr18_36269_mature	1.074570	3564.617106	1.052812	11.799936	11.799936	-11.695772	0.000068
mmu-miR-467a-5p	456.692221	0.000000	8.838234	0.000000	0.000000	20.000000	0.000070
chr11_24857_mature	0.000000	1671.493529	0.000000	10.707785	10.707785	-20.000000	0.000076
chr11_24582_mature	0.000000	1636.032721	0.000000	10.676867	10.676867	-20.000000	0.000079
chr7_15777_star	431.977113	0.000000	8.758147	0.000000	0.000000	20.000000	0.000079
chr9_19733_mature	428.753403	0.000000	8.747365	0.000000	0.000000	20.000000	0.000080
rno-miR-151-3p	1108.956170	1.611855	10.116287	1.385075	1.385075	9.426265	0.000085
ssc-miR-1249	1028.363425	1.611855	10.007537	1.385075	1.385075	9.317413	0.000099
chr8_18597_mature	363.204637	0.000000	8.508605	0.000000	0.000000	20.000000	0.000113
chr8_18598_mature	352.458938	0.000000	8.465399	0.000000	0.000000	20.000000	0.000120
chr15_33570_mature	615.728571	0.805927	9.268492	0.852740	0.852740	9.577429	0.000127
chr2_4298_mature	1162.684666	2.417782	10.184484	1.773061	1.773061	8.909560	0.000144
chrx_22076_mature	0.000000	1105.732460	0.000000	10.112091	10.112091	-20.000000	0.000146
chr7_15145_mature	1.074570	2137.319594	1.052812	11.062262	11.062262	-10.957828	0.000152
chr2_4477_star	1.074570	2107.500279	1.052812	11.042001	11.042001	-10.937558	0.000155
rno-miR-674-3p	1120.776439	2.417782	10.131569	1.773061	1.773061	8.856598	0.000156
mmu-miR-669a-3p	309.476140	0.000000	8.278339	0.000000	0.000000	20.000000	0.000157
NW_003613137_37368_mature	1.074570	1938.255515	1.052812	10.921287	10.921287	-10.816784	0.000177
chr1_3153_mature	0.000000	967.112939	0.000000	9.919032	9.919032	-20.000000	0.000180
rno-miR-3068-3p	289.059312	0.000000	8.180204	0.000000	0.000000	20.000000	0.000181
chr5_11530_mature	1.074570	1885.064303	1.052812	10.881163	10.881163	-10.776639	0.000185
chrx_22553_mature	1.074570	1729.520305	1.052812	10.756990	10.756990	-10.652397	0.000211
mmu-miR-467d-5p	267.567913	0.000000	8.069143	0.000000	0.000000	20.000000	0.000212
chr8_18153_mature	467.437920	0.805927	8.871714	0.852740	0.852740	9.179909	0.000224
NW_003538125_37558_mature	467.437920	0.805927	8.871714	0.852740	0.852740	9.179909	0.000224
chr12_25534_mature	3.223710	3712.907757	2.078511	11.858722	11.858722	-10.169612	0.000224
mmu-miR-669a-5p	260.045924	0.000000	8.028160	0.000000	0.000000	20.000000	0.000225
chrx_22554_mature	1.074570	1641.674213	1.052812	10.681831	10.681831	-10.577193	0.000229
chr6_12627_mature	1176.654075	3.223710	10.201700	2.078511	2.078511	8.511753	0.000234
chr5_11364_mature	251.449364	0.000000	7.979850	0.000000	0.000000	20.000000	0.000241
chr6_12983_mature@@rno-miR-200b-3p	249.300224	0.000000	7.967516	0.000000	0.000000	20.000000	0.000246
chr1_541_mature	0.000000	772.884423	0.000000	9.595974	9.595974	-20.000000	0.000255
chr14_31463_star	2.149140	2371.844482	1.654958	11.212402	11.212402	-10.108034	0.000263
chr16_33920_mature	2508.046221	7.253347	11.292923	3.044979	3.044979	8.433701	0.000264
chr1_1317_mature	2.149140	2365.397062	1.654958	11.208476	11.208476	-10.104107	0.000264
chrx_21552_mature	1105.732460	3.223710	10.112091	2.078511	2.078511	8.422065	0.000266
mmu-miR-690	238.554525	0.000000	7.904210	0.000000	0.000000	20.000000	0.000269
chr16_33919_mature	2479.032833	7.253347	11.276144	3.044979	3.044979	8.416915	0.000270
mmu-miR-1198-5p	234.256245	0.000000	7.878089	0.000000	0.000000	20.000000	0.000279
chr18_36371_mature	1077.793642	3.223710	10.075203	2.078511	2.078511	8.385143	0.000281
rno-miR-296-3p	229.957965	0.000000	7.851486	0.000000	0.000000	20.000000	0.000290
chr4_8839_mature	225.659686	0.000000	7.824384	0.000000	0.000000	20.000000	0.000301
NW_003538989_38414_mature	224.585116	0.000000	7.817528	0.000000	0.000000	20.000000	0.000304
chr2_6234_mature	221.361406	0.000000	7.796763	0.000000	0.000000	20.000000	0.000314
chr4_9231_mature	0.000000	660.860508	0.000000	9.370383	9.370383	-20.000000	0.000325
rno-miR-339-3p	215.988556	0.000000	7.761475	0.000000	0.000000	20.000000	0.000330
chr13_28643_mature	211.690277	0.000000	7.732610	0.000000	0.000000	20.000000	0.000344
rno-miR-1843-5p	964.963799	3.223710	9.915825	2.078511	2.078511	8.225609	0.000353
chr6_15010_mature	208.466567	0.000000	7.710576	0.000000	0.000000	20.000000	0.000355
chr9_20972_mature@@hsa-miR-488-3p	548.030665	1.611855	9.100743	1.385075	1.385075	8.409391	0.000365
chr10_23883_mature	205.242857	0.000000	7.688200	0.000000	0.000000	20.000000	0.000366
chr6_14978_mature	4.298280	3488.859926	2.405524	11.768953	11.768953	-9.664781	0.000381
chr5_10827_mature	723.185564	2.417782	9.500216	1.773061	1.773061	8.224538	0.000385
rno-miR-466b-2-3p	191.273448	0.000000	7.587016	0.000000	0.000000	20.000000	0.000423
chr1_1915_mature	191.273448	0.000000	7.587016	0.000000	0.000000	20.000000	0.000423
chr6_12951_mature	1.074570	1104.120605	1.052812	10.109988	10.109988	-10.004923	0.000426
chr14_30497_mature	0.000000	550.448448	0.000000	9.107082	9.107082	-20.000000	0.000433
chr4_8854_mature	1.074570	1067.047942	1.052812	10.060761	10.060761	-9.955650	0.000449
chr16_34772_mature	0.000000	535.135826	0.000000	9.066455	9.066455	-20.000000	0.000452
chr10_24300_mature	183.751458	0.000000	7.529442	0.000000	0.000000	20.000000	0.000459
chr12_26282_star	327.743829	0.805927	8.360820	0.852740	0.852740	8.667703	0.000464
chr16_33714_mature	0.000000	520.629132	0.000000	9.026881	9.026881	-20.000000	0.000472
chrx_22639_mature	1.074570	1014.662658	1.052812	9.988206	9.988206	-9.883025	0.000486
chr1_3819_mature	1.074570	1007.409311	1.052812	9.977866	9.977866	-9.872675	0.000491
chr13_28482_mature	177.304039	0.000000	7.478196	0.000000	0.000000	20.000000	0.000494
chr3_7573_mature	2.149140	1579.617800	1.654958	10.626273	10.626273	-9.521600	0.000497
chrx_21838_star@@rno-miR-188-3p	174.080329	0.000000	7.451873	0.000000	0.000000	20.000000	0.000513

bta-miR-302c	800.554599	3.223710	9.646657	2.078511	2.078511	7.956134	0.000518
chr5_11562_mature@@mmu-miR-3059-5p	454.543081	1.611855	8.831444	1.385075	1.385075	8.139551	0.000536
chr1_2557_mature	1.074570	949.382535	1.052812	9.892365	9.892365	-9.787086	0.000539
chr7_15832_mature	1991.178084	8.059274	10.960131	3.179396	3.179396	7.948757	0.000540
chr4_10241_star	2433.900896	9.671129	11.249647	3.415641	3.415641	7.975370	0.000547
mmu-miR-467e-5p	167.632909	0.000000	7.397742	0.000000	0.000000	20.000000	0.000554
chr7_16963_mature	1.074570	930.040276	1.052812	9.862700	9.862700	-9.757390	0.000556
ssc-miR-370	4.298280	2733.705907	2.405524	11.417170	11.417170	-9.312883	0.000558
chr13_29269_mature	0.000000	461.796428	0.000000	8.854234	8.854234	-20.000000	0.000569
mmu-miR-3082-3p	163.334630	0.000000	7.360493	0.000000	0.000000	20.000000	0.000584
mmu-miR-467c-5p	162.260060	0.000000	7.351028	0.000000	0.000000	20.000000	0.000592
bta-miR-877	290.133882	0.805927	8.185539	0.852740	0.852740	8.491853	0.000596
chr13_27061_mature	161.185490	0.000000	7.341501	0.000000	0.000000	20.000000	0.000600
mmu-miR-351-5p	159.036350	0.000000	7.322256	0.000000	0.000000	20.000000	0.000616
ssc-miR-145-5p	23.640539	19900.766495	4.622962	14.280609	14.280609	-9.717345	0.000626
chr13_29267_mature	0.000000	430.365258	0.000000	8.752766	8.752766	-20.000000	0.000635
chr11_24398_mature	1.074570	849.447531	1.052812	9.732078	9.732078	-9.626622	0.000641
chr15_32372_mature	155.812640	0.000000	7.292898	0.000000	0.000000	20.000000	0.000642
chr8_17778_mature	413.709424	1.611855	8.695957	1.385075	1.385075	8.003752	0.000650
ssc-miR-296-5p	877.923634	4.029637	9.779594	2.330454	2.330454	7.767302	0.000657
chr12_25526_mature	0.000000	419.888201	0.000000	8.717293	8.717293	-20.000000	0.000659
chr3_8430_mature	153.663500	0.000000	7.272989	0.000000	0.000000	20.000000	0.000661
chr18_35940_mature	153.663500	0.000000	7.272989	0.000000	0.000000	20.000000	0.000661
chr18_35938_mature	149.365221	0.000000	7.232327	0.000000	0.000000	20.000000	0.000700
NW_003613237_37813_mature	147.216081	0.000000	7.211558	0.000000	0.000000	20.000000	0.000721
chr12_26457_mature	0.000000	390.874813	0.000000	8.614249	8.614249	-20.000000	0.000737
chr16_34293_mature	1.074570	774.496278	1.052812	9.598976	9.598976	-9.493355	0.000740
chr1_169_mature	0.000000	386.845175	0.000000	8.599337	8.599337	-20.000000	0.000749
chr3_8429_mature	143.992371	0.000000	7.179833	0.000000	0.000000	20.000000	0.000754
chr1_468_star	512.569857	2.417782	9.004417	1.773061	1.773061	7.727920	0.000780
chr12_25628_mature	0.000000	371.532554	0.000000	8.541223	8.541223	-20.000000	0.000797
chr1_3926_mature	139.694091	0.000000	7.136418	0.000000	0.000000	20.000000	0.000802
chr6_14625_mature@@rno-miR-298-5p	791.958040	4.029637	9.631101	2.330454	2.330454	7.618630	0.000812
chrx_22943_mature	0.000000	362.667352	0.000000	8.506476	8.506476	-20.000000	0.000828
chr16_34839_mature	492.153029	2.417782	8.945892	1.773061	1.773061	7.669279	0.000848
chr2_6101_mature	358.906357	1.611855	8.491478	1.385075	1.385075	7.798742	0.000869
chr14_30693_mature	0.000000	348.966585	0.000000	8.451073	8.451073	-20.000000	0.000879
chr10_24151_mature	0.000000	346.548803	0.000000	8.441072	8.441072	-20.000000	0.000888
chr15_33120_mature	1.074570	686.650186	1.052812	9.425531	9.425531	-9.319672	0.000892
chrx_22070_mature	235.330815	0.805927	7.884664	0.852740	0.852740	8.189825	0.000913
ssc-miR-150	342.787808	1.611855	8.425375	1.385075	1.385075	7.732450	0.000954
chr4_9327_mature	339.564098	1.611855	8.411783	1.385075	1.385075	7.718818	0.000973
chr1_3820_mature	2.149140	1015.468586	1.654958	9.989350	9.989350	-8.884171	0.000988
chr18_35948_star@@rno-miR-96-3p	125.724682	0.000000	6.985554	0.000000	0.000000	20.000000	0.000993
chr5_11958_mature	0.000000	321.565052	0.000000	8.333446	8.333446	-20.000000	0.000998
chr15_32458_mature	335.265819	1.611855	8.393458	1.385075	1.385075	7.700440	0.000998
ssc-miR-192	4378.872472	20.148186	12.096673	4.402462	4.402462	7.763766	0.001003
chr1_1155_mature	2.149140	1000.155964	1.654958	9.967451	9.967451	-8.862250	0.001012
chr4_10395_mature	2.149140	984.037415	1.654958	9.944035	9.944035	-8.838810	0.001038
chrx_23228_mature	2.149140	982.425560	1.654958	9.941672	9.941672	-8.836445	0.001040
chr9_20478_mature	121.426402	0.000000	6.935771	0.000000	0.000000	20.000000	0.001065
chrx_22340_mature	1.074570	610.087079	1.052812	9.255234	9.255234	-9.149112	0.001072
ssc-miR-145-3p	2.149140	955.829954	1.654958	9.902119	9.902119	-8.796851	0.001086
rno-miR-466c-5p	119.277262	0.000000	6.910220	0.000000	0.000000	20.000000	0.001104
mmu-miR-3470b	119.277262	0.000000	6.910220	0.000000	0.000000	20.000000	0.001104
chrx_22720_mature	0.000000	299.805011	0.000000	8.232685	8.232685	-20.000000	0.001112
ssc-miR-362	1.074570	588.327038	1.052812	9.202925	9.202925	-9.096715	0.001134
rno-miR-466b-1-3p	210.615707	0.805927	7.725303	0.852740	0.852740	8.029747	0.001144
chr1_2360_mature	2.149140	922.786929	1.654958	9.851416	9.851416	-8.746094	0.001147
mmu-miR-1948-3p	116.053553	0.000000	6.871025	0.000000	0.000000	20.000000	0.001167
chr4_8769_mature	659.785938	4.029637	9.368039	2.330454	2.330454	7.355204	0.001179
chr13_27180_mature	206.317427	0.805927	7.695698	0.852740	0.852740	8.000000	0.001193
chr16_34762_mature	0.000000	283.686462	0.000000	8.153230	8.153230	-20.000000	0.001212
rno-miR-674-5p	199.870007	0.805927	7.650118	0.852740	0.852740	7.954196	0.001272
rno-miR-6215	110.680703	0.000000	6.803236	0.000000	0.000000	20.000000	0.001283
ssc-miR-486	5661.908971	27.401533	12.467328	4.827897	4.827897	7.690888	0.001310
chr13_28441_mature	5.372850	1987.417089	2.671939	10.957405	10.957405	-8.530991	0.001318
NW_003613242_37864_mature	0.000000	266.761986	0.000000	8.064807	8.064807	-20.000000	0.001333
chr3_8078_mature	0.000000	265.956058	0.000000	8.060458	8.060458	-20.000000	0.001339
ssc-miR-378	19.342259	9122.292794	4.346408	13.155339	13.155339	-8.881496	0.001392
chrx_22424_star	182.676888	0.805927	7.521026	0.852740	0.852740	7.824428	0.001525
mmu-miR-1196-5p	182.676888	0.805927	7.521026	0.852740	0.852740	7.824428	0.001525
chr12_26049_mature	101.009574	0.000000	6.672561	0.000000	0.000000	20.000000	0.001542
chr8_18345_mature	0.000000	241.778235	0.000000	7.923495	7.923495	-20.000000	0.001551
chr11_25089_mature	3.223710	1072.689434	2.078511	10.068361	10.068361	-8.378295	0.001552
hsa-miR-516a-5p	1.074570	473.885340	1.052812	8.891435	8.891435	-8.784635	0.001586
bta-miR-449c	98.860434	0.000000	6.641841	0.000000	0.000000	20.000000	0.001610
chrx_22153_mature	98.860434	0.000000	6.641841	0.000000	0.000000	20.000000	0.001610
chr1_2964_mature	1.074570	463.408283	1.052812	8.859250	8.859250	-8.752381	0.001642
chr14_31530_mature	260.045924	1.611855	8.028160	1.385075	1.385075	7.333901	0.001671
chr5_11275_mature	647.965669	4.835565	9.341998	2.544872	2.544872	7.066089	0.001765
chr6_14899_mature	640.443679	4.835565	9.325179	2.544872	2.544872	7.049244	0.001808
chr1_3446_mature	166.558339	0.805927	7.388520	0.852740	0.852740	7.691162	0.001836
chr5_11967_mature	1.074570	429.559330	1.052812	8.750068	8.750068	-8.642954	0.001846
chr2_4519_mature	335.265819	2.417782	8.393458	1.773061	1.773061	7.115477	0.001850
chr5_11474_mature	2.149140	676.979057	1.654958	9.405097	9.405097	-8.299208	0.001854
chr3_8256_mature	1.074570	417.470419	1.052812	8.708982	8.708982	-8.601771	0.001929
mmu-miR-125b-2-3p	90.263874	0.000000	6.511972	0.000000	0.000000	20.000000	0.001930
rno-miR-3102	90.263874	0.000000	6.511972	0.000000	0.000000	20.000000	0.001930
chr1_211_mature	0.000000	209.541137	0.000000	7.717958	7.717958	-20.000000	0.001935
chr1_589_mature	0.000000	207.929282	0.000000	7.706871	7.706871	-20.000000	0.001958
ssc-miR-429	89.189304	0.000000	6.494884	0.000000	0.000000	20.000000	0.001977
chr16_34240_mature	156.887210	0.805927	7.302750	0.852740	0.852740	7.604862	0.002070
chr16_34386_mature	5.372850	1477.265014	2.671939	10.529689	10.529689	-8.103025	0.002089
chr18_36372_mature	1029.437995	8.059274	10.009042	3.179396	3.179396	6.996991	0.002089
chr4_10244_mature	1.074570	394.098522	1.052812	8.626069	8.626069	-8.518653	0.002109

chr6_13041_mature	0.000000	195.840370	0.000000	7.620882	7.620882	-20.000000	0.002147
chr15_33031_mature	0.000000	195.840370	0.000000	7.620882	7.620882	-20.000000	0.002147
rno-miR-24-2-5p	84.891025	0.000000	6.424435	0.000000	0.000000	20.000000	0.002181
mmu-miR-350-5p	84.891025	0.000000	6.424435	0.000000	0.000000	20.000000	0.002181
NW_003613041_36883_mature	1.074570	384.427393	1.052812	8.590315	8.590315	-8.482808	0.002191
hsa-miR-6087	1593.587209	12.088912	10.638967	3.710273	3.710273	7.042450	0.002228
rno-miR-503-3p	83.816455	0.000000	6.406272	0.000000	0.000000	20.000000	0.002237
chr1_986_star	481.407329	4.029637	8.914108	2.330454	2.330454	6.900464	0.002237
mmu-miR-339-3p	150.439790	0.805927	7.242601	0.852740	0.852740	7.544321	0.002252
chrx_22905_mature	223.510546	1.611855	7.810639	1.385075	1.385075	7.115477	0.002266
chr6_13566_mature	1.074570	375.562191	1.052812	8.556744	8.556744	-8.449149	0.002271
chr13_28748_mature	222.435976	1.611855	7.803718	1.385075	1.385075	7.108524	0.002288
chr8_17233_mature	82.741885	0.000000	6.387877	0.000000	0.000000	20.000000	0.002295
chrx_22120_mature	1.074570	365.891062	1.052812	8.519208	8.519208	-8.411511	0.002365
chr14_30387_mature	377.174046	3.223710	8.562907	2.078511	2.078511	6.870365	0.002403
chr15_32593_mature	1.074570	359.443642	1.052812	8.493630	8.493630	-8.385862	0.002430
NW_003613237_37814_mature	143.992371	0.805927	7.179833	0.852740	0.852740	7.481127	0.002457
chrx_22497_mature	0.000000	178.915894	0.000000	7.491179	7.491179	-20.000000	0.002467
ssc-miR-7	69.847046	37072.662648	6.146636	15.178107	15.178107	-9.051941	0.002474
bta-miR-33b	79.518175	0.000000	6.331243	0.000000	0.000000	20.000000	0.002483
chr9_20036_mature	0.000000	178.109966	0.000000	7.484702	7.484702	-20.000000	0.002484
rno-miR-344a-3p	78.443605	0.000000	6.311859	0.000000	0.000000	20.000000	0.002551
rno-miR-3560	77.369035	0.000000	6.292212	0.000000	0.000000	20.000000	0.002621
chr1_666_mature	137.544951	0.805927	7.114210	0.852740	0.852740	7.415037	0.002692
mmu-miR-5097	13593.309638	70.921616	13.730715	6.168354	6.168354	7.582456	0.002724
chrx_22196_star	0.000000	167.632909	0.000000	7.397742	7.397742	-20.000000	0.002726
chr2_6477_star	135.395811	0.805927	7.091656	0.852740	0.852740	7.392317	0.002778
chrx_22713_mature	601.759162	5.641492	9.235438	2.731507	2.731507	6.736966	0.002828
chrx_21682_mature	2.149140	510.958003	1.654958	8.999882	8.999882	-7.893302	0.002863
chr8_17991_mature@@bta-miR-367	198.795437	1.611855	7.642380	1.385075	1.385075	6.946419	0.002865
chr15_32917_mature	1186.325205	10.477057	10.213499	3.520681	3.520681	6.823122	0.002883
mmu-miR-466b-3p	132.172102	0.805927	7.057148	0.852740	0.852740	7.357552	0.002914
chr4_9236_mature	1.074570	319.147270	1.052812	8.322592	8.322592	-8.214319	0.002918
hsa-miR-762	196.646298	1.611855	7.626777	1.385075	1.385075	6.930737	0.002928
rno-miR-702-3p	73.070755	0.000000	6.210832	0.000000	0.000000	20.000000	0.002934
chrx_21569_mature	0.000000	159.573635	0.000000	7.327091	7.327091	-20.000000	0.002940
chr2_5680_mature	3.223710	701.156881	2.078511	9.455650	9.455650	-7.764872	0.002996
mmu-miR-1935	130.022962	0.805927	7.033676	0.852740	0.852740	7.333901	0.003011
ssc-miR-187	262.195063	2.417782	8.039989	1.773061	1.773061	6.760812	0.003033
chr15_31863_mature	3.223710	685.844259	2.078511	9.423839	9.423839	-7.733015	0.003100
mmu-miR-5121	70.921616	0.000000	6.168354	0.000000	0.000000	20.000000	0.003111
chr11_25111_mature	70.921616	0.000000	6.168354	0.000000	0.000000	20.000000	0.003111
chr6_14250_mature	127.873822	0.805927	7.009815	0.852740	0.852740	7.309855	0.003112
chr13_29417_mature	190.198878	1.611855	7.578930	1.385075	1.385075	6.882643	0.003129
chr6_12650_mature	2.149140	477.914977	1.654958	8.903626	8.903626	-7.796851	0.003173
rno-miR-871-3p	69.847046	0.000000	6.146636	0.000000	0.000000	20.000000	0.003206
rno-miR-501-3p	69.847046	0.000000	6.146636	0.000000	0.000000	20.000000	0.003206
mmu-miR-5117-3p	252.523934	2.417782	7.985978	1.773061	1.773061	6.706592	0.003270
chr6_13339_mature	3.223710	656.024943	2.078511	9.359804	9.359804	-7.668885	0.003319
chrx_22488_mature	1.074570	290.939809	1.052812	8.189527	8.189527	-8.080818	0.003364
chr9_20754_mature	0.000000	145.872868	0.000000	7.198424	7.198424	-20.000000	0.003372
mmu-miR-700-5p	67.697906	0.000000	6.102194	0.000000	0.000000	20.000000	0.003409
bta-miR-1246	5.372850	1074.301289	2.671939	10.070525	10.070525	-7.643495	0.003418
chr12_25546_mature	0.000000	142.649158	0.000000	7.166406	7.166406	-20.000000	0.003489
mmu-miR-1965	66.623336	0.000000	6.079449	0.000000	0.000000	20.000000	0.003517
chr17_34881_mature	3.223710	630.235265	2.078511	9.302034	9.302034	-7.611025	0.003531
NW_003612993_36688_mature	2.149140	444.066024	1.654958	8.797876	8.797876	-7.690871	0.003552
NW_003537574_37016_mature	119.277262	0.805927	6.910220	0.852740	0.852740	7.209453	0.003571
chr12_26075_star	65.548766	0.000000	6.056340	0.000000	0.000000	20.000000	0.003631
chr1_3435_mature	0.000000	138.619521	0.000000	7.125357	7.125357	-20.000000	0.003645
chr6_12448_star	10816.620934	66.891978	13.401096	6.085169	6.085169	7.337201	0.003718
chr1_1444_mature	1.074570	271.597550	1.052812	8.090629	8.090629	-7.981567	0.003737
chr6_13600_mature	64.474196	0.000000	6.032855	0.000000	0.000000	20.000000	0.003750
mmu-miR-712-5p	64.474196	0.000000	6.032855	0.000000	0.000000	20.000000	0.003750
hsa-miR-4685-3p	0.000000	134.589884	0.000000	7.083106	7.083106	-20.000000	0.003813
chr3_8310_star	0.000000	134.589884	0.000000	7.083106	7.083106	-20.000000	0.003813
chr16_34839_star	298.730441	3.223710	8.227522	2.078511	2.078511	6.533979	0.003835
chr6_13576_mature	1.074570	265.956058	1.052812	8.060458	8.060458	-7.951285	0.003859
mmu-miR-450b-5p	63.399626	0.000000	6.008980	0.000000	0.000000	20.000000	0.003875
hsa-miR-4524a-3p	113.904413	0.805927	6.844290	0.852740	0.852740	7.142958	0.003911
chr3_7072_mature	1.074570	261.926421	1.052812	8.038515	8.038515	-7.929258	0.003951
chr14_30236_mature	362.130067	4.029637	8.504343	2.330454	2.330454	6.489714	0.003964
NW_003613522_39530_mature	168.707479	1.611855	7.406906	1.385075	1.385075	6.709658	0.003970
ssc-miR-432-5p	1.074570	260.314566	1.052812	8.029644	8.029644	-7.920353	0.003988
chr9_19375_mature	2.149140	406.993362	1.654958	8.672402	8.672402	-7.565102	0.004060
hsa-miR-4524b-5p	111.755273	0.805927	6.817051	0.852740	0.852740	7.115477	0.004060
chr13_27312_mature	1.074570	257.090856	1.052812	8.011735	8.011735	-7.902375	0.004065
chr3_8533_star	908.011592	9.671129	9.828155	3.415641	3.415641	6.552883	0.004100
chr9_19755_mature	0.000000	128.142464	0.000000	7.012820	7.012820	-20.000000	0.004109
mmu-miR-669o-5p	61.250486	0.000000	5.960013	0.000000	0.000000	20.000000	0.004145
chr17_35005_mature	0.000000	127.336537	0.000000	7.003788	7.003788	-20.000000	0.004148
ssc-miR-195	731.782124	8.059274	9.517240	3.179396	3.179396	6.504620	0.004166
chrx_21977_mature@@rno-miR-384-3p	164.409200	1.611855	7.369896	1.385075	1.385075	6.672425	0.004178
chr6_13578_mature	1.074570	252.255291	1.052812	7.984449	7.984449	-7.874981	0.004184
chr16_34380_mature	60.175916	0.000000	5.934892	0.000000	0.000000	20.000000	0.004290
chr17_35010_mature	60.175916	0.000000	5.934892	0.000000	0.000000	20.000000	0.004290
chrx_22382_mature	0.000000	124.112827	0.000000	6.967086	6.967086	-20.000000	0.004313
hsa-miR-3202	0.000000	123.306900	0.000000	6.957763	6.957763	-20.000000	0.004356
mmu-miR-669c-5p	107.456993	0.805927	6.760979	0.852740	0.852740	7.058894	0.004386
chr15_32108_mature	3.223710	545.612883	2.078511	9.094376	9.094376	-7.403012	0.004406
NW_003540835_39730_mature	0.000000	121.695045	0.000000	6.938933	6.938933	-20.000000	0.004444
ssc-miR-182	456053.926486	1181.489640	18.798848	10.207612	10.207612	8.592454	0.004473
chr10_23799_mature	2.149140	381.203683	1.654958	8.578198	8.578198	-7.470659	0.004488
chr2_6113_mature	0.000000	120.083190	0.000000	6.919855	6.919855	-20.000000	0.004535
chr8_18343_mature	1.074570	238.554525	1.052812	7.904210	7.904210	-7.794416	0.004557
chr1_3371_mature	3.223710	529.494334	2.078511	9.051194	9.051194	-7.359750	0.004613

chr15_33531_mature	0.000000	116.859480	0.000000	6.880924	6.880924	-20.000000	0.004726
chr9_20880_mature	0.000000	116.859480	0.000000	6.880924	6.880924	-20.000000	0.004726
mmu-miR-466g	103.158713	0.805927	6.702640	0.852740	0.852740	7.000000	0.004751
ssc-miR-18b	1096.061330	12.088912	10.099428	3.710273	3.710273	6.502500	0.004755
chr5_12116_mature@@bta-miR-200c	56.952206	0.000000	5.856792	0.000000	0.000000	20.000000	0.004773
NW_003541201_39964_mature	2.149140	365.891062	1.654958	8.519208	8.519208	-7.411511	0.004779
chr3_8552_mature	2033.086311	20.148186	10.990165	4.402462	4.402462	6.656878	0.004812
chr17_35521_mature	0.000000	114.441698	0.000000	6.851021	6.851021	-20.000000	0.004878
ssc-miR-181c	7996.949433	58.832704	12.965414	5.902862	5.902862	7.086688	0.004884
chr15_31749_star	101.009574	0.805927	6.672561	0.852740	0.852740	6.969626	0.004950
chr15_32711_mature	55.877636	0.000000	5.829790	0.000000	0.000000	20.000000	0.004953
chr13_27434_mature	55.877636	0.000000	5.829790	0.000000	0.000000	20.000000	0.004953
ssc-miR-1	8.596559	1441.804206	3.262517	10.494660	10.494660	-7.389900	0.004954
chr15_33221_mature	3.223710	502.092801	2.078511	8.974681	8.974681	-7.283088	0.005004
chr13_28070_mature	0.000000	112.023915	0.000000	6.820484	6.820484	-20.000000	0.005039
chr3_6707_mature@@mmu-miR-590-3p	1320.646446	14.506694	10.368121	3.954819	3.954819	6.508382	0.005079
chr7_16724_mature	7662.758184	58.026776	12.903836	5.883298	5.883298	7.045001	0.005135
mmu-miR-669l-5p	54.803067	0.000000	5.802272	0.000000	0.000000	20.000000	0.005142
chr13_28475_star	444.871952	5.641492	8.800486	2.731507	2.731507	6.301170	0.005181
NW_003537694_37136_mature	2.149140	346.548803	1.654958	8.441072	8.441072	-7.333155	0.005192
chr4_9930_mature	0.000000	108.800206	0.000000	6.778737	6.778737	-20.000000	0.005266
chrx_22335_mature	146.141511	1.611855	7.201060	1.385075	1.385075	6.502500	0.005268
mmu-miR-5124b	0.000000	107.994278	0.000000	6.768109	6.768109	-20.000000	0.005326
NW_003538870_38317_mature	53.728497	0.000000	5.774220	0.000000	0.000000	20.000000	0.005342
chr18_35974_mature@@bta-miR-592	53.728497	0.000000	5.774220	0.000000	0.000000	20.000000	0.005342
NW_003538374_37783_mature	53.728497	0.000000	5.774220	0.000000	0.000000	20.000000	0.005342
hsa-miR-4760-3p	53.728497	0.000000	5.774220	0.000000	0.000000	20.000000	0.005342
chr3_6615_mature	1718.237321	18.536331	10.747553	4.288088	4.288088	6.534430	0.005481
chr12_26608_mature	193.422588	2.417782	7.603052	1.773061	1.773061	6.321928	0.005546
mmu-miR-677-5p	52.653927	0.000000	5.745612	0.000000	0.000000	20.000000	0.005554
hsa-miR-184	0.000000	104.770568	0.000000	6.724794	6.724794	-20.000000	0.005576
hsa-miR-210	192.348018	2.417782	7.595056	1.773061	1.773061	6.313891	0.005607
chr1_3229_mature	0.000000	103.964641	0.000000	6.713760	6.713760	-20.000000	0.005641
chr1_3824_star	0.000000	103.964641	0.000000	6.713760	6.713760	-20.000000	0.005641
NW_003613107_37244_mature	0.000000	103.964641	0.000000	6.713760	6.713760	-20.000000	0.005641
chr6_13949_star	140.768661	1.611855	7.147395	1.385075	1.385075	6.448461	0.005669
chr13_27316_mature	1.074570	206.317427	1.052812	7.695698	7.695698	-7.584963	0.005683
chr10_23656_mature	301.954151	4.029637	8.242956	2.330454	2.330454	6.227536	0.005687
chr14_30616_star	556.627225	7.253347	9.123157	3.044979	3.044979	6.261921	0.005692
chr6_13565_mature	2.149140	325.594689	1.654958	8.351358	8.351358	-7.243174	0.005709
chr13_27664_mature	1.074570	204.705572	1.052812	7.684437	7.684437	-7.573647	0.005751
hsa-miR-1287	51.579357	0.000000	5.716425	0.000000	0.000000	20.000000	0.005778
ssc-miR-497	756.497232	9.671129	9.565097	3.415641	3.415641	6.289507	0.005912
chr4_9932_mature	0.000000	99.935004	0.000000	6.657283	6.657283	-20.000000	0.005988
ssc-miR-545-5p	236.405385	3.223710	7.891209	2.078511	2.078511	6.196397	0.006096
NW_003537589_37025_mature	1.074570	196.646298	1.052812	7.626777	7.626777	-7.515700	0.006112
NW_003613497_39427_mature	0.000000	98.323149	0.000000	6.634058	6.634058	-20.000000	0.006136
chr9_20605_mature	0.000000	98.323149	0.000000	6.634058	6.634058	-20.000000	0.006136
chr10_24172_mature	0.000000	98.323149	0.000000	6.634058	6.634058	-20.000000	0.006136
chr4_9116_mature	1.074570	195.840370	1.052812	7.620882	7.620882	-7.509775	0.006150
chr9_19559_mature	90.263874	0.805927	6.511972	0.852740	0.852740	6.807355	0.006160
chr1_1386_mature	134.321241	1.611855	7.080245	1.385075	1.385075	6.380822	0.006212
chr13_29417_star	49.430217	0.000000	5.656217	0.000000	0.000000	20.000000	0.006269
mmu-miR-344b-3p	181.602318	2.417782	7.512561	1.773061	1.773061	6.230954	0.006276
chr12_26356_star	0.000000	96.711294	0.000000	6.610453	6.610453	-20.000000	0.006291
NW_003538101_37534_mature	0.000000	95.905366	0.000000	6.598505	6.598505	-20.000000	0.006371
chr5_11238_mature	462.065071	6.447420	8.855071	2.896741	2.896741	6.163230	0.006377
chr13_27515_mature	4.298280	560.119577	2.405524	9.132164	9.132164	-7.025832	0.006485
chr10_23795_star	1.074570	186.975168	1.052812	7.554398	7.554398	-7.442943	0.006597
chr16_34525_mature@@bta-miR-449a	87.040164	0.805927	6.460090	0.852740	0.852740	6.754888	0.006609
chr4_10006_mature	0.000000	93.487584	0.000000	6.562053	6.562053	-20.000000	0.006620
chr15_33053_mature	855.357666	11.282984	9.742070	3.618589	3.618589	6.244307	0.006631
chr15_33346_mature	0.000000	91.875729	0.000000	6.537230	6.537230	-20.000000	0.006795
chr12_25625_star	0.000000	91.875729	0.000000	6.537230	6.537230	-20.000000	0.006795
chr17_35756_mature	47.281077	0.000000	5.593386	0.000000	0.000000	20.000000	0.006824
chr13_27058_mature	47.281077	0.000000	5.593386	0.000000	0.000000	20.000000	0.006824
chr1_2742_star	47.281077	0.000000	5.593386	0.000000	0.000000	20.000000	0.006824
chr10_24012_mature	0.000000	91.069802	0.000000	6.524656	6.524656	-20.000000	0.006886
chr3_8118_star	1.074570	178.915894	1.052812	7.491179	7.491179	-7.379378	0.007051
chr9_20694_mature	0.000000	89.457947	0.000000	6.499175	6.499175	-20.000000	0.007073
chr8_17373_mature	0.000000	89.457947	0.000000	6.499175	6.499175	-20.000000	0.007073
rno-miR-3473	83.816455	0.805927	6.406272	0.852740	0.852740	6.700440	0.007108
NW_003613233_37802_mature	83.816455	0.805927	6.406272	0.852740	0.852740	6.700440	0.007108
mmu-miR-669p-5p	46.206507	0.000000	5.560914	0.000000	0.000000	20.000000	0.007129
chr16_34021_mature	46.206507	0.000000	5.560914	0.000000	0.000000	20.000000	0.007129
hsa-miR-3663-3p	46.206507	0.000000	5.560914	0.000000	0.000000	20.000000	0.007129
hsa-miR-3687	124.650112	1.611855	6.973268	1.385075	1.385075	6.273018	0.007183
chr4_9789_mature	4.298280	514.987640	2.405524	9.011193	9.011193	-6.904635	0.007370
mmu-miR-532-5p	45.131937	0.000000	5.527694	0.000000	0.000000	20.000000	0.007454
mmu-miR-539-5p	45.131937	0.000000	5.527694	0.000000	0.000000	20.000000	0.007454
NW_003613056_36922_mature	0.000000	86.234237	0.000000	6.446823	6.446823	-20.000000	0.007472
chr10_23437_mature	0.000000	86.234237	0.000000	6.446823	6.446823	-20.000000	0.007472
chr1_2437_mature	0.000000	86.234237	0.000000	6.446823	6.446823	-20.000000	0.007472
chr12_26455_mature	3.223710	385.233321	2.078511	8.593329	8.593329	-6.900867	0.007491
chr15_31936_mature	7.521990	938.905478	3.091190	9.876372	9.876372	-6.963722	0.007507
chr14_29715_mature	3.223710	381.203683	2.078511	8.578198	8.578198	-6.885696	0.007612
chr15_33121_mature	80.592745	0.805927	6.350369	0.852740	0.852740	6.643856	0.007664
chr10_23786_mature	0.000000	84.622382	0.000000	6.419916	6.419916	-20.000000	0.007686
ssc-miR-146b	35437.167212	214.376701	15.113016	7.750718	7.750718	7.368971	0.007753
chr5_11528_mature	0.000000	83.816455	0.000000	6.406272	6.406272	-20.000000	0.007797
chr6_13705_mature	0.000000	83.816455	0.000000	6.406272	6.406272	-20.000000	0.007797
mmu-miR-467b-5p	44.057367	0.000000	5.493691	0.000000	0.000000	20.000000	0.007802
chr3_7600_mature	6661.259007	61.250486	12.701796	5.960013	5.960013	6.764930	0.007879
chr1_1337_mature	118.202693	1.611855	6.897273	1.385075	1.385075	6.196397	0.007960
chr5_11672_mature	117.128123	1.611855	6.884209	1.385075	1.385075	6.183222	0.008101
NW_003613242_37866_mature	2.149140	257.090856	1.654958	8.011735	8.011735	-6.902375	0.008168

rno-miR-879-5p	42.982797	0.000000	5.458867	0.000000	0.000000	20.000000	0.008174
chr8_17595_mature	157.961780	2.417782	7.312536	1.773061	1.773061	6.029747	0.008234
chr16_34601_mature	9.671129	1191.966697	3.415641	10.220338	10.220338	-6.945444	0.008244
chr1_3926_star	77.369035	0.805927	6.292212	0.852740	0.852740	6.584963	0.008287
chr1_2963_mature	4.298280	476.303122	2.405524	8.898762	8.898762	-6.791977	0.008297
chr16_34502_mature	1.074570	159.573635	1.052812	7.327091	7.327091	-7.214319	0.008376
chr9_19904_mature	2.149140	251.449364	1.654958	7.979850	7.979850	-6.870365	0.008445
chr1_1185_mature	155.812640	2.417782	7.292898	1.773061	1.773061	6.009984	0.008455
chr9_19756_mature	1.074570	157.961780	1.052812	7.312536	7.312536	-7.199672	0.008505
chr18_36523_mature	0.000000	78.980890	0.000000	6.321583	6.321583	-20.000000	0.008521
mmu-miR-466c-3p	41.908227	0.000000	5.423182	0.000000	0.000000	20.000000	0.008574
chr3_7706_mature	41.908227	0.000000	5.423182	0.000000	0.000000	20.000000	0.008574
mmu-miR-1981-3p	41.908227	0.000000	5.423182	0.000000	0.000000	20.000000	0.008574
chr6_12641_mature	41.908227	0.000000	5.423182	0.000000	0.000000	20.000000	0.008574
mmu-miR-3470a	41.908227	0.000000	5.423182	0.000000	0.000000	20.000000	0.008574
chr8_18374_mature	41.908227	0.000000	5.423182	0.000000	0.000000	20.000000	0.008574
chr9_19519_mature	0.000000	78.174963	0.000000	6.306972	6.306972	-20.000000	0.008652
chr13_28627_star	153.663500	2.417782	7.272989	1.773061	1.773061	5.989946	0.008685
chr1_3052_star	0.000000	77.369035	0.000000	6.292212	6.292212	-20.000000	0.008786
chr4_9333_star	0.000000	77.369035	0.000000	6.292212	6.292212	-20.000000	0.008786
chr14_31500_mature	0.000000	77.369035	0.000000	6.292212	6.292212	-20.000000	0.008786
chr15_33015_mature	618.952281	9.671129	9.276013	3.415641	3.415641	6.000000	0.008802
chr13_28222_star	152.588930	2.417782	7.262930	1.773061	1.773061	5.979822	0.008803
bta-miR-2285t	1.074570	153.932143	1.052812	7.275493	7.275493	-7.162391	0.008841
chr4_9639_mature	40.833657	0.000000	5.386592	0.000000	0.000000	20.000000	0.009002
chr13_29078_mature	40.833657	0.000000	5.386592	0.000000	0.000000	20.000000	0.009002
mmu-miR-664-3p	40.833657	0.000000	5.386592	0.000000	0.000000	20.000000	0.009002
mmu-miR-1195	40.833657	0.000000	5.386592	0.000000	0.000000	20.000000	0.009002
chr5_11626_mature	786.585190	12.088912	9.621292	3.710273	3.710273	6.023847	0.009214
chr13_28489_mature	0.000000	74.145325	0.000000	6.231611	6.231611	-20.000000	0.009360
chr4_9743_mature	5.372850	552.060302	2.671939	9.111293	9.111293	-6.682995	0.009462
chr1_3877_mature	39.759087	0.000000	5.349050	0.000000	0.000000	20.000000	0.009463
chr15_31808_mature	0.000000	73.339398	0.000000	6.216055	6.216055	-20.000000	0.009513
chr14_31044_star	0.000000	70.115688	0.000000	6.152096	6.152096	-20.000000	0.010169
ssc-miR-504	2.149140	221.630048	1.654958	7.798505	7.798505	-6.688250	0.010210
chr1_1112_mature	140.768661	2.417782	7.147395	1.773061	1.773061	5.863498	0.010280
chr14_31499_mature	0.000000	69.309761	0.000000	6.135653	6.135653	-20.000000	0.010344
chr18_36239_mature	0.000000	69.309761	0.000000	6.135653	6.135653	-20.000000	0.010344
chr12_26422_mature	0.000000	69.309761	0.000000	6.135653	6.135653	-20.000000	0.010344
chr9_20130_mature	68.772476	0.805927	6.124586	0.852740	0.852740	6.415037	0.010363
chr6_14477_mature@@bta-miR-483	8.596559	888.937976	3.262517	9.797561	9.797561	-6.692180	0.010370
chr14_31044_mature	1.074570	137.813594	1.052812	7.117005	7.117005	-7.002815	0.010430
mmu-miR-5130	37.609948	0.000000	5.270901	0.000000	0.000000	20.000000	0.010495
hsa-miR-5095	37.609948	0.000000	5.270901	0.000000	0.000000	20.000000	0.010495
bta-miR-2317	37.609948	0.000000	5.270901	0.000000	0.000000	20.000000	0.010495
chr1_3945_mature	37.609948	0.000000	5.270901	0.000000	0.000000	20.000000	0.010495
chr15_32293_mature	1.074570	137.007666	1.052812	7.108605	7.108605	-6.994353	0.010522
chr14_31042_star	0.000000	68.503833	0.000000	6.119021	6.119021	-20.000000	0.010525
chr13_28617_mature@@mmu-miR-698-5p	67.697906	0.805927	6.102194	0.852740	0.852740	6.392317	0.010675
chrx_21977_star@@rno-miR-384-5p	67.697906	0.805927	6.102194	0.852740	0.852740	6.392317	0.010675
mmu-miR-1947-5p	67.697906	0.805927	6.102194	0.852740	0.852740	6.392317	0.010675
chr14_31042_mature	1.074570	135.395811	1.052812	7.091656	7.091656	-6.977280	0.010709
chr6_14094_star	0.000000	67.697906	0.000000	6.102194	6.102194	-20.000000	0.010710
chr15_33264_mature	1572.095810	22.565969	10.619391	4.558633	4.558633	6.122397	0.010711
chr1_3507_mature	0.000000	66.891978	0.000000	6.085169	6.085169	-20.000000	0.010901
chr14_30531_mature	1.074570	133.783957	1.052812	7.074505	7.074505	-6.960002	0.010901
chr14_29507_mature	1.074570	132.978029	1.052812	7.065853	7.065853	-6.951285	0.011000
chr13_28708_mature	3.223710	294.969446	2.078511	8.209304	8.209304	-6.515700	0.011205
chr2_4744_mature	3.223710	294.163519	2.078511	8.205371	8.205371	-6.511753	0.011251
chr16_33764_mature	0.000000	65.280123	0.000000	6.050504	6.050504	-20.000000	0.011300
chr5_12092_mature	1.074570	130.560247	1.052812	7.039580	7.039580	-6.924813	0.011304
chr13_28221_mature	65.548766	0.805927	6.056340	0.852740	0.852740	6.345775	0.011343
NW_003538465_37902_mature	2.149140	205.511499	1.654958	7.690078	7.690078	-6.579316	0.011430
chrx_22279_mature	0.000000	64.474196	0.000000	6.032855	6.032855	-20.000000	0.011509
chrx_22902_star	0.000000	64.474196	0.000000	6.032855	6.032855	-20.000000	0.011509
chrx_22973_mature	96.711294	1.611855	6.610453	1.385075	1.385075	5.906891	0.011678
NW_003538989_38416_star	35.460808	0.000000	5.188275	0.000000	0.000000	20.000000	0.011703
chr7_16877_mature	35.460808	0.000000	5.188275	0.000000	0.000000	20.000000	0.011703
chr15_31807_mature	0.000000	63.668268	0.000000	6.014986	6.014986	-20.000000	0.011724
chr10_23842_mature	4.298280	377.979974	2.405524	8.565978	8.565978	-6.458407	0.011759
chr15_32517_mature	1.074570	125.724682	1.052812	6.985554	6.985554	-6.870365	0.011955
chr2_4295_mature	6.447420	576.238126	2.896741	9.173023	9.173023	-6.481799	0.012028
chr1_268_mature	7.521990	684.232404	3.091190	9.420450	9.420450	-6.507228	0.012130
NW_003540828_39718_mature	0.000000	62.056414	0.000000	5.978571	5.978571	-20.000000	0.012174
chr14_30912_mature	0.000000	62.056414	0.000000	5.978571	5.978571	-20.000000	0.012174
chr15_33320_mature	3.223710	277.239042	2.078511	8.120181	8.120181	-6.426265	0.012294
chr6_13600_star	1.074570	123.306900	1.052812	6.957763	6.957763	-6.842350	0.012304
NW_003537425_36910_star	34.386238	0.000000	5.145116	0.000000	0.000000	20.000000	0.012384
chr7_15205_mature	34.386238	0.000000	5.145116	0.000000	0.000000	20.000000	0.012384
chr16_34526_mature@@bta-miR-449a	93.487584	1.611855	6.562053	1.385075	1.385075	5.857981	0.012449
chrx_22499_mature	0.000000	60.444559	0.000000	5.941213	5.941213	-20.000000	0.012653
chr1_3508_mature	0.000000	60.444559	0.000000	5.941213	5.941213	-20.000000	0.012653
rno-miR-101b-3p	281.537322	5.641492	8.142298	2.731507	2.731507	5.641106	0.012699
chr14_29632_star	92.413014	1.611855	6.545552	1.385075	1.385075	5.841302	0.012723
chr14_31142_mature	125.724682	2.417782	6.985554	1.773061	1.773061	5.700440	0.012749
chr6_15013_mature	0.000000	59.638631	0.000000	5.922165	5.922165	-20.000000	0.012905
chrx_22559_mature	0.000000	59.638631	0.000000	5.922165	5.922165	-20.000000	0.012905
chr3_6620_mature	278.313612	5.641492	8.125742	2.731507	2.731507	5.624491	0.012983
chr15_31797_mature	33.311668	0.000000	5.100627	0.000000	0.000000	20.000000	0.013126
chr13_29302_mature	235.330815	4.835565	7.884664	2.544872	2.544872	5.604862	0.013165
chr4_9707_mature	1.074570	116.053553	1.052812	6.871025	6.871025	-6.754888	0.013457
chr5_12329_mature	271.866193	5.641492	8.092050	2.731507	2.731507	5.590676	0.013581
NW_003537105_36696_mature	1520.516454	24.177823	10.571294	4.654082	4.654082	5.974733	0.013595
chr12_26093_mature	2.149140	182.139603	1.654958	7.516800	7.516800	-6.405141	0.013678
chr4_9198_mature	3.223710	257.896784	2.078511	8.016233	8.016233	-6.321928	0.013693
chr10_23626_mature	0.000000	57.220849	0.000000	5.863464	5.863464	-20.000000	0.013709

chr7_15363_mature	59.101346	0.805927	5.909325	0.852740	0.852740	6.196397	0.013759
chr5_11567_mature	6.447420	525.464697	2.896741	9.040193	9.040193	-6.348728	0.013815
chr14_30207_mature	4.298280	339.295456	2.405524	8.410644	8.410644	-6.302639	0.013820
ssc-miR-935	491.078459	9.671129	8.942745	3.415641	3.415641	5.666125	0.013836
mmu-miR-5620-5p	32.237098	0.000000	5.054723	0.000000	0.000000	20.000000	0.013935
mmu-miR-1981-5p	32.237098	0.000000	5.054723	0.000000	0.000000	20.000000	0.013935
chr1_1283_mature	0.000000	56.414921	0.000000	5.843354	5.843354	-20.000000	0.013996
chr17_35122_mature	2.149140	178.915894	1.654958	7.491179	7.491179	-6.379378	0.014044
chr11_24694_mature	440.573672	8.865202	8.786510	3.302349	3.302349	5.635086	0.014047
chrx_21884_mature	3.223710	253.061219	2.078511	7.989032	7.989032	-6.294621	0.014084
chr9_20889_mature	1.074570	112.023915	1.052812	6.820484	6.820484	-6.703904	0.014175
chr11_25309_mature	0.000000	55.608994	0.000000	5.822959	5.822959	-20.000000	0.014292
chr16_33868_mature	436.275392	8.865202	8.772398	3.302349	3.302349	5.620942	0.014316
hsa-miR-4750-5p	347.086088	7.253347	8.443300	3.044979	3.044979	5.580503	0.014357
chr17_35678_mature	1411.984890	23.371896	10.464530	4.607147	4.607147	5.916806	0.014451
chr8_17577_mature	21.491399	2302.534721	4.491301	11.169634	11.169634	-6.743320	0.014463
chr14_29940_mature	150.439790	3.223710	7.242601	2.078511	2.078511	5.544321	0.014587
hsa-miR-1298	0.000000	54.803067	0.000000	5.802272	5.802272	-20.000000	0.014599
chrx_22747_mature	301.954151	6.447420	8.242956	2.896741	2.896741	5.549464	0.014622
chr5_11313_star@@bta-miR-141	56.952206	0.805927	5.856792	0.852740	0.852740	6.142958	0.014734
NW_003537990_37435_mature	1.074570	108.800206	1.052812	6.778737	6.778737	-6.661778	0.014797
mmu-miR-3109-3p	31.162528	0.000000	5.007309	0.000000	0.000000	20.000000	0.014819
chr14_31110_mature	31.162528	0.000000	5.007309	0.000000	0.000000	20.000000	0.014819
rno-miR-322-5p	31.162528	0.000000	5.007309	0.000000	0.000000	20.000000	0.014819
chr7_16155_mature	31.162528	0.000000	5.007309	0.000000	0.000000	20.000000	0.014819
chr9_20370_star	31.162528	0.000000	5.007309	0.000000	0.000000	20.000000	0.014819
bta-miR-211	31.162528	0.000000	5.007309	0.000000	0.000000	20.000000	0.014819
chr16_34124_mature	31.162528	0.000000	5.007309	0.000000	0.000000	20.000000	0.014819
chr14_30043_mature	31.162528	0.000000	5.007309	0.000000	0.000000	20.000000	0.014819
mmu-miR-700-3p	84.891025	1.611855	6.424435	1.385075	1.385075	5.718818	0.014914
chrx_21664_mature	0.000000	53.997139	0.000000	5.781285	5.781285	-20.000000	0.014917
chr8_18026_mature	426.604263	8.865202	8.740132	3.302349	3.302349	5.588601	0.014948
chr13_28171_mature	1.074570	107.994278	1.052812	6.768109	6.768109	-6.651052	0.014959
hsa-miR-4750-3p	5514.692890	67.697906	12.429326	6.102194	6.102194	6.348026	0.014996
ssc-miR-194a	338.489529	7.253347	8.407223	3.044979	3.044979	5.544321	0.015065
chr9_20772_mature	2.149140	170.050692	1.654958	7.418280	7.418280	-6.306062	0.015139
chr9_20753_mature	2.149140	170.050692	1.654958	7.418280	7.418280	-6.306062	0.015139
chr9_20339_mature@@mmu-miR-3096a-5p	1434.550859	24.177823	10.487389	4.654082	4.654082	5.890771	0.015242
chr1_3819_star	0.000000	53.191212	0.000000	5.759987	5.759987	-20.000000	0.015246
chr3_7813_mature	55.877636	0.805927	5.829790	0.852740	0.852740	6.115477	0.015260
hsa-miR-4791	83.816455	1.611855	6.406272	1.385075	1.385075	5.700440	0.015272
chr6_14288_mature	180.527749	4.029637	7.504046	2.330454	2.330454	5.485427	0.015407
chr9_19579_mature	1.074570	105.576496	1.052812	6.735745	6.735745	-6.618386	0.015464
chr1_2110_mature	0.000000	52.385284	0.000000	5.738370	5.738370	-20.000000	0.015588
NW_003541128_39926_mature	0.000000	52.385284	0.000000	5.738370	5.738370	-20.000000	0.015588
chr7_16094_mature	0.000000	52.385284	0.000000	5.738370	5.738370	-20.000000	0.015588
mmu-miR-5108	1.074570	104.770568	1.052812	6.724794	6.724794	-6.607330	0.015638
chr10_23743_mature	1009.021166	18.536331	9.980170	4.288088	4.288088	5.766457	0.015759
chr13_27448_mature	30.087958	0.000000	4.958284	0.000000	0.000000	20.000000	0.015789
chr7_16991_mature	30.087958	0.000000	4.958284	0.000000	0.000000	20.000000	0.015789
hsa-miR-4264	30.087958	0.000000	4.958284	0.000000	0.000000	20.000000	0.015789
chr5_12332_star	30.087958	0.000000	4.958284	0.000000	0.000000	20.000000	0.015789
chr7_16061_star	30.087958	0.000000	4.958284	0.000000	0.000000	20.000000	0.015789
bta-miR-301a	54.803067	0.805927	5.802272	0.852740	0.852740	6.087463	0.015814
NW_003539115_38548_star	54.803067	0.805927	5.802272	0.852740	0.852740	6.087463	0.015814
chr13_29304_mature	2.149140	164.409200	1.654958	7.369896	7.369896	-6.257388	0.015911
chr18_36061_mature	1.074570	103.158713	1.052812	6.702640	6.702640	-6.584963	0.015997
chr10_23298_mature	3.223710	231.301178	2.078511	7.859853	7.859853	-6.164907	0.016090
chr12_25501_mature	3.223710	230.495250	2.078511	7.854839	7.854839	-6.159871	0.016173
chr16_34398_mature	1.074570	102.352786	1.052812	6.691433	6.691433	-6.573647	0.016181
chr11_25154_mature	1.074570	102.352786	1.052812	6.691433	6.691433	-6.573647	0.016181
chr1_1437_mature	1881.571951	30.625243	10.878489	4.983005	4.983005	5.941073	0.016203
mmu-miR-3096b-5p	636.145400	12.894839	9.315479	3.796477	3.796477	5.624491	0.016233
ssc-miR-382	2.149140	161.991417	1.654958	7.348652	7.348652	-6.236014	0.016261
chr9_20262_mature	0.000000	50.773429	0.000000	5.694140	5.694140	-20.000000	0.016309
mmu-miR-669d-5p	53.728497	0.805927	5.774220	0.852740	0.852740	6.058894	0.016398
chr3_6880_mature	80.592745	1.611855	6.350369	1.385075	1.385075	5.643856	0.016423
chr13_27663_mature	3.223710	226.465613	2.078511	7.829505	7.829505	-6.134426	0.016600
chr2_5245_star	0.000000	49.967502	0.000000	5.671506	5.671506	-20.000000	0.016690
chrx_21663_mature	0.000000	49.967502	0.000000	5.671506	5.671506	-20.000000	0.016690
chr3_6956_mature	443.797382	9.671129	8.797004	3.415641	3.415641	5.520073	0.016816
chr18_35961_mature	29.013388	0.000000	4.907534	0.000000	0.000000	20.000000	0.016854
mmu-miR-466p-3p	29.013388	0.000000	4.907534	0.000000	0.000000	20.000000	0.016854
chr8_17983_star@@mmu-miR-302b-5p	29.013388	0.000000	4.907534	0.000000	0.000000	20.000000	0.016854
mmu-miR-3473c	29.013388	0.000000	4.907534	0.000000	0.000000	20.000000	0.016854
chr6_13229_mature	9.671129	724.528777	3.415641	9.502889	9.502889	-6.227215	0.017483
chr1_2112_mature	0.000000	48.355647	0.000000	5.625143	5.625143	-20.000000	0.017497
chr14_31507_mature	0.000000	48.355647	0.000000	5.625143	5.625143	-20.000000	0.017497
chr11_25205_mature	5.372850	362.667352	2.671939	8.506476	8.506476	-6.076816	0.017753
NW_003613103_37208_mature	0.000000	47.549719	0.000000	5.601391	5.601391	-20.000000	0.017925
chr8_18481_mature	27.938818	0.000000	4.854934	0.000000	0.000000	20.000000	0.018029
chr1_2783_mature	27.938818	0.000000	4.854934	0.000000	0.000000	20.000000	0.018029
mmu-miR-215-3p	27.938818	0.000000	4.854934	0.000000	0.000000	20.000000	0.018029
chr6_14848_mature	1.074570	94.293512	1.052812	6.574306	6.574306	-6.455327	0.018234
chrx_22989_mature	2.149140	149.096578	1.654958	7.229747	7.229747	-6.116344	0.018363
bta-miR-2897	1.074570	93.487584	1.052812	6.562053	6.562053	-6.442943	0.018462
chr12_25415_mature	132.172102	3.223710	7.057148	2.078511	2.078511	5.357552	0.018592
chr13_28612_mature	75.219895	1.611855	6.252096	1.385075	1.385075	5.544321	0.018647
chr6_14444_mature	2.149140	147.484723	1.654958	7.214171	7.214171	-6.100662	0.018657
chr4_10240_mature	8.596559	599.610022	3.262517	9.230285	9.230285	-6.124121	0.018731
chrx_22664_mature	162.260060	4.029637	7.351028	2.330454	2.330454	5.331514	0.018827
chr1_2685_mature	0.000000	45.937865	0.000000	5.552680	5.552680	-20.000000	0.018834
chr14_30470_mature	0.000000	45.937865	0.000000	5.552680	5.552680	-20.000000	0.018834
chr13_28673_mature	1.074570	91.875729	1.052812	6.537230	6.537230	-6.417853	0.018934
chr18_36427_mature@@bta-miR-592	49.430217	0.805927	5.656217	0.852740	0.852740	5.938599	0.019082
chr6_14465_mature	74.145325	1.611855	6.231611	1.385075	1.385075	5.523562	0.019144

chr14_29589_mature	9.671129	679.396839	3.415641	9.410233	9.410233	-6.134426	0.019240
chr13_28509_mature	0.000000	45.131937	0.000000	5.527694	5.527694	-20.000000	0.019317
chr17_34904_mature	0.000000	45.131937	0.000000	5.527694	5.527694	-20.000000	0.019317
chr3_6868_star	0.000000	45.131937	0.000000	5.527694	5.527694	-20.000000	0.019317
chr13_27833_mature	26.864248	0.000000	4.800343	0.000000	0.000000	20.000000	0.019328
mmu-miR-3076-3p	26.864248	0.000000	4.800343	0.000000	0.000000	20.000000	0.019328
chr6_12987_mature@@mmu-miR-6538	26.864248	0.000000	4.800343	0.000000	0.000000	20.000000	0.019328
chr4_9118_mature	26.864248	0.000000	4.800343	0.000000	0.000000	20.000000	0.019328
chr16_34636_mature	452.393941	10.477057	8.824621	3.520681	3.520681	5.432274	0.019398
NW_003539609_38987_mature	73.070755	1.611855	6.210832	1.385075	1.385075	5.502500	0.019661
chr12_25534_star	1.074570	89.457947	1.052812	6.499175	6.499175	-6.379378	0.019678
hsa-miR-591	0.000000	44.326010	0.000000	5.502267	5.502267	-20.000000	0.019821
chr1_2658_mature	0.000000	44.326010	0.000000	5.502267	5.502267	-20.000000	0.019821
ssc-miR-628	705.992445	15.312622	9.465551	4.027917	4.027917	5.526860	0.019884
chr6_12448_mature	3885.644873	58.026776	11.924310	5.883298	5.883298	6.065291	0.020044
chr16_34336_mature	0.000000	43.520082	0.000000	5.476384	5.476384	-20.000000	0.020346
chr7_15130_mature	0.000000	43.520082	0.000000	5.476384	5.476384	-20.000000	0.020346
chr6_14179_mature	97.785864	2.417782	6.626233	1.773061	1.773061	5.337870	0.020372
chr2_5337_star	47.281077	0.805927	5.593386	0.852740	0.852740	5.874469	0.020671
chr8_18246_mature	70.921616	1.611855	6.168354	1.385075	1.385075	5.459432	0.020758
chr6_14717_star	25.789678	0.000000	4.743605	0.000000	0.000000	20.000000	0.020769
chr16_34371_mature	25.789678	0.000000	4.743605	0.000000	0.000000	20.000000	0.020769
mmu-miR-669f-5p	25.789678	0.000000	4.743605	0.000000	0.000000	20.000000	0.020769
chr4_10437_mature	0.000000	42.714155	0.000000	5.450029	5.450029	-20.000000	0.020894
chr14_31463_mature	0.000000	42.714155	0.000000	5.450029	5.450029	-20.000000	0.020894
chr18_35994_mature	0.000000	42.714155	0.000000	5.450029	5.450029	-20.000000	0.020894
chr1_2264_mature	3.223710	193.422588	2.078511	7.603052	7.603052	-5.906891	0.020920
chrx_21570_mature	3.223710	193.422588	2.078511	7.603052	7.603052	-5.906891	0.020920
chr10_23953_mature	4.298280	255.479001	2.405524	8.002697	8.002697	-5.893302	0.021020
chr14_29694_star	1.074570	85.428310	1.052812	6.433432	6.433432	-6.312883	0.021030
chr7_15367_mature	46.206507	0.805927	5.560914	0.852740	0.852740	5.841302	0.021540
chr5_11222_mature	2.149140	132.978029	1.654958	7.065853	7.065853	-5.951285	0.021686
chr16_33817_mature	3.223710	188.587023	2.078511	7.566716	7.566716	-5.870365	0.021705
chr16_34391_mature	1.074570	83.010527	1.052812	6.392498	6.392498	-6.271463	0.021915
chr1_1289_mature	0.000000	41.102300	0.000000	5.395827	5.395827	-20.000000	0.022066
chr13_27815_mature	276.164472	7.253347	8.114599	3.044979	3.044979	5.250737	0.022155
chr1_412_mature	2.149140	130.560247	1.654958	7.039580	7.039580	-5.924813	0.022268
chr1_191_mature	24.715108	0.000000	4.684544	0.000000	0.000000	20.000000	0.022374
chr8_18866_mature	24.715108	0.000000	4.684544	0.000000	0.000000	20.000000	0.022374
NW_003613192_37588_star	24.715108	0.000000	4.684544	0.000000	0.000000	20.000000	0.022374
bta-miR-302a	24.715108	0.000000	4.684544	0.000000	0.000000	20.000000	0.022374
chr11_24876_star	24.715108	0.000000	4.684544	0.000000	0.000000	20.000000	0.022374
chr1_788_star	92.413014	2.417782	6.545552	1.773061	1.773061	5.256340	0.022587
chr15_33182_mature	0.000000	40.296372	0.000000	5.367943	5.367943	-20.000000	0.022693
bta-miR-2305	66.623336	1.611855	6.079449	1.385075	1.385075	5.369234	0.023239
chr14_29856_star	66.623336	1.611855	6.079449	1.385075	1.385075	5.369234	0.023239
chrx_21838_mature@@bta-miR-188	3146.340760	52.385284	11.619918	5.738370	5.738370	5.908370	0.023272
chr13_27408_mature	0.000000	39.490445	0.000000	5.339510	5.339510	-20.000000	0.023349
NW_003539771_39089_mature	0.000000	39.490445	0.000000	5.339510	5.339510	-20.000000	0.023349
NW_003539467_38882_mature	0.000000	39.490445	0.000000	5.339510	5.339510	-20.000000	0.023349
chr5_12114_mature@@bta-miR-141	44.057367	0.805927	5.493691	0.852740	0.852740	5.772590	0.023444
chr13_26706_mature	44.057367	0.805927	5.493691	0.852740	0.852740	5.772590	0.023444
chr13_29025_mature	143.992371	4.029637	7.179833	2.330454	2.330454	5.159199	0.023487
chrx_22416_mature	1.074570	78.980890	1.052812	6.321583	6.321583	-6.199672	0.023532
mmu-miR-466e-3p	23.640539	0.000000	4.622962	0.000000	0.000000	20.000000	0.024167
rno-miR-6328	23.640539	0.000000	4.622962	0.000000	0.000000	20.000000	0.024167
mmu-miR-547-3p	23.640539	0.000000	4.622962	0.000000	0.000000	20.000000	0.024167
chr4_8670_mature	23.640539	0.000000	4.622962	0.000000	0.000000	20.000000	0.024167
chr16_34759_mature	23.640539	0.000000	4.622962	0.000000	0.000000	20.000000	0.024167
mmu-miR-1948-5p	23.640539	0.000000	4.622962	0.000000	0.000000	20.000000	0.024167
mmu-miR-3473b	23.640539	0.000000	4.622962	0.000000	0.000000	20.000000	0.024167
chr8_18464_mature	1.074570	77.369035	1.052812	6.292212	6.292212	-6.169925	0.024234
chr2_5281_mature	13.969409	920.369147	3.903945	9.847635	9.847635	-6.041870	0.024382
chr1_3878_mature	42.982797	0.805927	5.458867	0.852740	0.852740	5.736966	0.024490
chr5_12007_mature	42.982797	0.805927	5.458867	0.852740	0.852740	5.736966	0.024490
chr14_30997_mature	0.000000	37.878590	0.000000	5.280904	5.280904	-20.000000	0.024758
chr6_14946_mature	0.000000	37.878590	0.000000	5.280904	5.280904	-20.000000	0.024758
chr12_26249_mature	0.000000	37.878590	0.000000	5.280904	5.280904	-20.000000	0.024758
chr5_11533_star	0.000000	37.878590	0.000000	5.280904	5.280904	-20.000000	0.024758
ssc-miR-221-5p	34.386238	3056.882814	5.145116	11.578317	11.578317	-6.474086	0.024812
chr13_27072_mature	11.820269	728.558414	3.680355	9.510880	9.510880	-5.945710	0.025114
chr5_11598_mature	111.755273	3.223710	6.817051	2.078511	2.078511	5.115477	0.025302
chr9_19408_mature@@hsa-miR-34b-3p	427.678833	11.282984	8.743753	3.618589	3.618589	5.244307	0.025476
NW_003541124_39922_mature	7.521990	415.052636	3.091190	8.700622	8.700622	-5.786036	0.025488
NW_003613200_37636_mature	0.000000	37.072663	0.000000	5.250684	5.250684	-20.000000	0.025515
chr2_4930_mature	9.671129	559.313650	3.415641	9.130091	9.130091	-5.853829	0.025636
chr15_32759_mature	110.680703	3.223710	6.803236	2.078511	2.078511	5.101538	0.025749
chr1_1833_mature	2.149140	116.859480	1.654958	6.880924	6.880924	-5.764872	0.026112
chr6_14834_mature	22.565969	0.000000	4.558633	0.000000	0.000000	20.000000	0.026179
mmu-miR-669o-3p	22.565969	0.000000	4.558633	0.000000	0.000000	20.000000	0.026179
chr8_17361_mature	22.565969	0.000000	4.558633	0.000000	0.000000	20.000000	0.026179
rno-miR-3099	22.565969	0.000000	4.558633	0.000000	0.000000	20.000000	0.026179
mmu-miR-669b-5p	22.565969	0.000000	4.558633	0.000000	0.000000	20.000000	0.026179
chr8_18251_mature	62.325056	1.611855	5.984705	1.385075	1.385075	5.273018	0.026180
chr9_19519_star	0.000000	36.266735	0.000000	5.219817	5.219817	-20.000000	0.026310
chr17_35299_mature	40.833657	0.805927	5.386592	0.852740	0.852740	5.662965	0.026797
chr17_35663_star	40.833657	0.805927	5.386592	0.852740	0.852740	5.662965	0.026797
chr5_11265_star	1.074570	71.727543	1.052812	6.184430	6.184430	-6.060696	0.026981
chr2_3985_mature	61.250486	1.611855	5.960013	1.385075	1.385075	5.247928	0.027000
chr1_1111_mature	107.456993	3.223710	6.760979	2.078511	2.078511	5.058894	0.027161
chr1_376_mature	278.313612	8.059274	8.125742	3.179396	3.179396	5.109918	0.027253
chr1_1734_mature	2.149140	111.217988	1.654958	6.810160	6.810160	-5.693487	0.028019
chr4_9773_mature	39.759087	0.805927	5.349050	0.852740	0.852740	5.624491	0.028072
hsa-miR-4485	39.759087	0.805927	5.349050	0.852740	0.852740	5.624491	0.028072
NW_003613297_38335_mature	81.667315	2.417782	6.369245	1.773061	1.773061	5.078003	0.028214
bta-miR-2435	1.074570	69.309761	1.052812	6.135653	6.135653	-6.011227	0.028316

chr8_18247_star	21.491399	0.000000	4.491301	0.000000	0.000000	20.000000	0.028446
mmu-miR-182-5p	21.491399	0.000000	4.491301	0.000000	0.000000	20.000000	0.028446
mmu-miR-3061-5p	21.491399	0.000000	4.491301	0.000000	0.000000	20.000000	0.028446
chr2_4178_mature	21.491399	0.000000	4.491301	0.000000	0.000000	20.000000	0.028446
ssc-miR-424-5p	9037.133127	125.724682	13.141809	6.985554	6.985554	6.167525	0.028704
chr16_34527_mature@@bta-miR-449b	59.101346	1.611855	5.909325	1.385075	1.385075	5.196397	0.028756
chr1_3137_mature	4.298280	205.511499	2.405524	7.690078	7.690078	-5.579316	0.028813
chr16_34510_star	2.149140	107.994278	1.654958	6.768109	6.768109	-5.651052	0.029212
chr2_5398_star	38.684518	0.805927	5.310504	0.852740	0.852740	5.584963	0.029437
chr15_32567_mature@@bta-miR-375	38.684518	0.805927	5.310504	0.852740	0.852740	5.584963	0.029437
chr14_30385_mature	38.684518	0.805927	5.310504	0.852740	0.852740	5.584963	0.029437
chr17_34903_mature	1.074570	66.891978	1.052812	6.085169	6.085169	-5.960002	0.029763
chr8_18843_mature	2.149140	106.382423	1.654958	6.746614	6.746614	-5.629357	0.029840
chr7_15085_mature	0.000000	33.043025	0.000000	5.089287	5.089287	-20.000000	0.029936
chr9_19808_mature	0.000000	33.043025	0.000000	5.089287	5.089287	-20.000000	0.029936
chr7_15474_mature	0.000000	33.043025	0.000000	5.089287	5.089287	-20.000000	0.029936
chr1_3824_mature	2.149140	104.770568	1.654958	6.724794	6.724794	-5.607330	0.030491
rno-miR-466c-3p	56.952206	1.611855	5.856792	1.385075	1.385075	5.142958	0.030684
chr13_27097_mature	1.074570	65.280123	1.052812	6.050504	6.050504	-5.924813	0.030795
chr1_1062_mature	2.149140	103.964641	1.654958	6.713760	6.713760	-5.596190	0.030825
bta-miR-16b	37.609948	0.805927	5.270901	0.852740	0.852740	5.544321	0.030901
mmu-miR-3473d	37.609948	0.805927	5.270901	0.852740	0.852740	5.544321	0.030901
bta-miR-502a	37.609948	0.805927	5.270901	0.852740	0.852740	5.544321	0.030901
chr16_34111_mature	0.000000	32.237098	0.000000	5.054723	5.054723	-20.000000	0.030970
chr1_392_mature	20.416829	0.000000	4.420673	0.000000	0.000000	20.000000	0.031012
mmu-miR-466d-3p	20.416829	0.000000	4.420673	0.000000	0.000000	20.000000	0.031012
chr16_34520_mature	20.416829	0.000000	4.420673	0.000000	0.000000	20.000000	0.031012
chr6_12434_star	20.416829	0.000000	4.420673	0.000000	0.000000	20.000000	0.031012
chr6_12752_star	20.416829	0.000000	4.420673	0.000000	0.000000	20.000000	0.031012
hsa-miR-1268b	20.416829	0.000000	4.420673	0.000000	0.000000	20.000000	0.031012
chr6_13692_mature	783.361480	20.148186	9.615375	4.402462	4.402462	5.280956	0.031262
chr9_19974_mature	1.074570	64.474196	1.052812	6.032855	6.032855	-5.906891	0.031334
bta-miR-584	199.870007	6.447420	7.650118	2.896741	2.896741	4.954196	0.031604
rno-miR-541-5p	55.877636	1.611855	5.829790	1.385075	1.385075	5.115477	0.031720
NW_003613497_39428_mature	2.149140	101.546859	1.654958	6.680139	6.680139	-5.562242	0.031863
ssc-miR-2366	2.149140	101.546859	1.654958	6.680139	6.680139	-5.562242	0.031863
chr6_14390_mature	121.426402	4.029637	6.935771	2.330454	2.330454	4.913288	0.031979
chr1_2947_mature	0.000000	31.431171	0.000000	5.019309	5.019309	-20.000000	0.032064
chr14_30452_star	0.000000	31.431171	0.000000	5.019309	5.019309	-20.000000	0.032064
chr9_19140_mature	3856.631485	69.309761	11.913500	6.135653	6.135653	5.798139	0.032119
chr14_31026_mature	36.535378	0.805927	5.230179	0.852740	0.852740	5.502500	0.032473
chr4_8816_mature	5.372850	239.360452	2.671939	7.909056	7.909056	-5.477354	0.032504
rno-miR-203a-3p	11.820269	608.475224	3.680355	9.251424	9.251424	-5.685864	0.032669
NW_003613233_37801_mature	96.711294	3.223710	6.610453	2.078511	2.078511	4.906891	0.032780
chr13_28716_star	1.074570	62.056414	1.052812	5.978571	5.978571	-5.851749	0.033044
chr12_26184_star	142.917801	4.835565	7.169101	2.544872	2.544872	4.885357	0.033235
chr7_17092_mature	19.342259	0.000000	4.346408	0.000000	0.000000	20.000000	0.033932
mmu-miR-467a-3p	19.342259	0.000000	4.346408	0.000000	0.000000	20.000000	0.033932
mmu-miR-17-3p	19.342259	0.000000	4.346408	0.000000	0.000000	20.000000	0.033932
chr1_1736_mature	19.342259	0.000000	4.346408	0.000000	0.000000	20.000000	0.033932
bta-miR-502b	19.342259	0.000000	4.346408	0.000000	0.000000	20.000000	0.033932
mmu-miR-5615-5p	53.728497	1.611855	5.774220	1.385075	1.385075	5.058894	0.033949
mmu-miR-1933-3p	35.460808	0.805927	5.188275	0.852740	0.852740	5.459432	0.034165
chr18_35895_mature	0.000000	29.819316	0.000000	4.945763	4.945763	-20.000000	0.034450
chr12_26204_star	0.000000	29.819316	0.000000	4.945763	4.945763	-20.000000	0.034450
chr13_28506_mature	0.000000	29.819316	0.000000	4.945763	4.945763	-20.000000	0.034450
chr10_24294_mature	7.521990	332.848036	3.091190	8.383048	8.383048	-5.467606	0.035062
chr2_4749_mature	52.653927	1.611855	5.745612	1.385075	1.385075	5.029747	0.035150
chr4_9685_mature	2.149140	94.293512	1.654958	6.574306	6.574306	-5.455327	0.035344
ssc-miR-29a	84.891025	8630.677050	6.424435	13.075425	13.075425	-6.667718	0.035390
chr12_25999_mature	3.223710	133.783957	2.078511	7.074505	7.074505	-5.375039	0.035464
chr6_15014_mature	1.074570	58.832704	1.052812	5.902862	5.902862	-5.774787	0.035571
hsa-miR-5702	0.000000	29.013388	0.000000	4.907534	4.907534	-20.000000	0.035753
chr8_18908_mature	4101.633430	75.757180	12.002335	6.262230	6.262230	5.758672	0.035802
chr7_17173_mature	161.185490	5.641492	7.341501	2.731507	2.731507	4.836501	0.035842
chr14_31022_mature	34.386238	0.805927	5.145116	0.852740	0.852740	5.415037	0.035989
NW_003539217_38656_mature	136.470381	4.835565	7.102977	2.544872	2.544872	4.818760	0.036082
mmu-miR-5117-5p	70.921616	2.417782	6.168354	1.773061	1.773061	4.874469	0.036149
chr14_31575_mature	70.921616	2.417782	6.168354	1.773061	1.773061	4.874469	0.036149
chrx_22845_star	1.074570	58.026776	1.052812	5.883298	5.883298	-5.754888	0.036252
hsa-miR-6134	51.579357	1.611855	5.716425	1.385075	1.385075	5.000000	0.036414
NW_003613447_39176_mature	2.149140	91.875729	1.654958	6.537230	6.537230	-5.417853	0.036643
chr6_13666_mature	2.149140	91.875729	1.654958	6.537230	6.537230	-5.417853	0.036643
chr12_25812_mature	2.149140	91.875729	1.654958	6.537230	6.537230	-5.417853	0.036643
chr5_11598_star	183.751458	6.447420	7.529442	2.896741	2.896741	4.832890	0.036768
chr2_4023_mature	1.074570	57.220849	1.052812	5.863464	5.863464	-5.734710	0.036955
chr9_19757_mature	0.000000	28.207461	0.000000	4.868265	4.868265	-20.000000	0.037138
chr1_990_mature	0.000000	28.207461	0.000000	4.868265	4.868265	-20.000000	0.037138
NW_003539453_38866_mature	0.000000	28.207461	0.000000	4.868265	4.868265	-20.000000	0.037138
chr3_8556_mature	0.000000	28.207461	0.000000	4.868265	4.868265	-20.000000	0.037138
chr10_23279_mature	4.298280	171.662547	2.405524	7.431811	7.431811	-5.319672	0.037204
rno-miR-326-3p	18.267689	0.000000	4.268112	0.000000	0.000000	20.000000	0.037270
hsa-miR-377-3p	18.267689	0.000000	4.268112	0.000000	0.000000	20.000000	0.037270
chr7_16275_star	18.267689	0.000000	4.268112	0.000000	0.000000	20.000000	0.037270
chr11_24777_mature	18.267689	0.000000	4.268112	0.000000	0.000000	20.000000	0.037270
chr1_3554_mature	18.267689	0.000000	4.268112	0.000000	0.000000	20.000000	0.037270
chr8_18863_mature	289.059312	9.671129	8.180204	3.415641	3.415641	4.901537	0.037435
chr7_16961_mature	2.149140	90.263874	1.654958	6.511972	6.511972	-5.392317	0.037553
bta-miR-2429	2.149140	90.263874	1.654958	6.511972	6.511972	-5.392317	0.037553
chr1_1111_star	50.504787	1.611855	5.686635	1.385075	1.385075	4.969626	0.037745
chr3_6931_mature	50.504787	1.611855	5.686635	1.385075	1.385075	4.969626	0.037745
chr12_26317_mature	8.596559	369.920699	3.262517	8.534967	8.534967	-5.427313	0.037866
chr16_34089_mature	1.074570	55.608994	1.052812	5.822959	5.822959	-5.693487	0.038428
chr6_14945_mature	0.000000	27.401533	0.000000	4.827897	4.827897	-20.000000	0.038612
chr1_1669_mature	0.000000	27.401533	0.000000	4.827897	4.827897	-20.000000	0.038612
hsa-miR-2861	67.697906	2.417782	6.102194	1.773061	1.773061	4.807355	0.039166

chr11_25257_mature	9.671129	416.664491	3.415641	8.706201	8.706201	-5.429058	0.039236
chr17_35522_mature	3.223710	123.306900	2.078511	6.957763	6.957763	-5.257388	0.039737
NW_003541201_39964_star	1.074570	53.997139	1.052812	5.781285	5.781285	-5.651052	0.039998
chr9_20255_star	32.237098	0.805927	5.054723	0.852740	0.852740	5.321928	0.040087
chrx_22196_mature	0.000000	26.595606	0.000000	4.786367	4.786367	-20.000000	0.040184
chr7_16275_mature	0.000000	26.595606	0.000000	4.786367	4.786367	-20.000000	0.040184
chr12_25999_star	0.000000	26.595606	0.000000	4.786367	4.786367	-20.000000	0.040184
ssc-miR-148b-5p	2035.235451	46.743792	10.991689	5.577241	5.577241	5.444277	0.040220
chr9_19205_mature	66.623336	2.417782	6.079449	1.773061	1.773061	4.784271	0.040253
chr9_19683_mature	85.965595	3.223710	6.442373	2.078511	2.078511	4.736966	0.040267
chr14_31118_mature	2.149140	85.428310	1.654958	6.433432	6.433432	-5.312883	0.040514
chr12_26014_mature	48.355647	3535.603718	5.625143	11.788149	11.788149	-6.192128	0.040515
chrx_22749_mature	304.103291	10.477057	8.253154	3.520681	3.520681	4.859256	0.040533
mmu-miR-5615-3p	48.355647	1.611855	5.625143	1.385075	1.385075	4.906891	0.040629
chr9_20434_mature@@rno-miR-34b-3p	420.156843	13.700767	8.718214	3.877819	3.877819	4.938599	0.040640
chr6_13376_mature	2.149140	84.622382	1.654958	6.419916	6.419916	-5.299208	0.041045
chr8_18274_mature	2.149140	84.622382	1.654958	6.419916	6.419916	-5.299208	0.041045
rno-miR-345-5p	17.193119	0.000000	4.185321	0.000000	0.000000	20.000000	0.041110
chr13_28152_star	17.193119	0.000000	4.185321	0.000000	0.000000	20.000000	0.041110
chr2_4630_mature	17.193119	0.000000	4.185321	0.000000	0.000000	20.000000	0.041110
chr13_27867_mature	17.193119	0.000000	4.185321	0.000000	0.000000	20.000000	0.041110
chr6_14288_star	17.193119	0.000000	4.185321	0.000000	0.000000	20.000000	0.041110
chr4_10618_star	17.193119	0.000000	4.185321	0.000000	0.000000	20.000000	0.041110
ssc-miR-196b-3p	17.193119	0.000000	4.185321	0.000000	0.000000	20.000000	0.041110
chr14_31314_mature	7.521990	297.387229	3.091190	8.221042	8.221042	-5.305085	0.041143
rno-miR-203b-5p	13.969409	640.712322	3.903945	9.325783	9.325783	-5.519334	0.041291
chr11_24779_mature	2044.906580	47.549719	10.998525	5.601391	5.601391	5.426454	0.041585
chr1_1290_mature	1.074570	52.385284	1.052812	5.738370	5.738370	-5.607330	0.041676
hsa-miR-5707	1.074570	52.385284	1.052812	5.738370	5.738370	-5.607330	0.041676
rno-miR-466b-5p	1.074570	52.385284	1.052812	5.738370	5.738370	-5.607330	0.041676
ssc-miR-487b	0.000000	25.789678	0.000000	4.743605	4.743605	-20.000000	0.041862
chr6_12811_star	0.000000	25.789678	0.000000	4.743605	4.743605	-20.000000	0.041862
chr8_18357_mature	0.000000	25.789678	0.000000	4.743605	4.743605	-20.000000	0.041862
chr2_5753_mature	0.000000	25.789678	0.000000	4.743605	4.743605	-20.000000	0.041862
chr2_5754_mature	0.000000	25.789678	0.000000	4.743605	4.743605	-20.000000	0.041862
hsa-miR-649	0.000000	25.789678	0.000000	4.743605	4.743605	-20.000000	0.041862
chr14_30223_mature	8.596559	344.131021	3.262517	8.431000	8.431000	-5.323055	0.041953
chr6_13411_mature	6.447420	245.001944	2.896741	7.942526	7.942526	-5.247928	0.041983
chr16_34464_mature	47.281077	1.611855	5.593386	1.385075	1.385075	4.874469	0.042191
chr4_8813_mature	31.162528	0.805927	5.007309	0.852740	0.852740	5.273018	0.042394
chr16_34334_mature	31.162528	0.805927	5.007309	0.852740	0.852740	5.273018	0.042394
chr7_16992_mature	31.162528	0.805927	5.007309	0.852740	0.852740	5.273018	0.042394
chr13_26947_mature	64.474196	2.417782	6.032855	1.773061	1.773061	4.736966	0.042563
chr7_15797_star	6.447420	241.778235	2.896741	7.923495	7.923495	-5.228819	0.042770
mmu-miR-6236	2903.487956	62.862341	11.504068	5.996894	5.996894	5.529447	0.043081
chr7_15783_mature	465.288780	15.312622	8.865080	4.027917	4.027917	4.925333	0.043326
NW_003613103_37207_mature	1.074570	50.773429	1.052812	5.694140	5.694140	-5.562242	0.043470
chr15_32760_mature	122.500972	4.835565	6.948379	2.544872	2.544872	4.662965	0.043602
hsa-miR-1248	0.000000	24.983751	0.000000	4.699538	4.699538	-20.000000	0.043657
NW_003613367_38732_mature	0.000000	24.983751	0.000000	4.699538	4.699538	-20.000000	0.043657
chr9_19915_mature	0.000000	24.983751	0.000000	4.699538	4.699538	-20.000000	0.043657
chr3_8555_mature	0.000000	24.983751	0.000000	4.699538	4.699538	-20.000000	0.043657
mmu-miR-222-3p	0.000000	24.983751	0.000000	4.699538	4.699538	-20.000000	0.043657
chr3_6707_star@@mmu-miR-590-5p	46.206507	1.611855	5.560914	1.385075	1.385075	4.841302	0.043843
chr14_29589_star	46.206507	1.611855	5.560914	1.385075	1.385075	4.841302	0.043843
rno-miR-598-3p	318.072700	11.282984	8.317741	3.618589	3.618589	4.817136	0.043849
chrx_22864_mature	1.074570	49.967502	1.052812	5.671506	5.671506	-5.539159	0.044415
chr8_19054_mature	1.074570	49.967502	1.052812	5.671506	5.671506	-5.539159	0.044415
mmu-miR-466c-5p	30.087958	0.805927	4.958284	0.852740	0.852740	5.222392	0.044899
mmu-miR-3066-5p	30.087958	0.805927	4.958284	0.852740	0.852740	5.222392	0.044899
bta-miR-2478	30.087958	0.805927	4.958284	0.852740	0.852740	5.222392	0.044899
chr2_6291_star	30.087958	0.805927	4.958284	0.852740	0.852740	5.222392	0.044899
chr17_35401_mature	80.592745	3.223710	6.350369	2.078511	2.078511	4.643856	0.044972
chr1_1609_mature	99.935004	4.029637	6.657283	2.330454	2.330454	4.632268	0.044972
chr11_25215_mature	340.638668	12.088912	8.416327	3.710273	3.710273	4.816486	0.045030
rno-miR-760-5p	16.118549	0.000000	4.097489	0.000000	0.000000	20.000000	0.045554
rno-miR-300-3p	16.118549	0.000000	4.097489	0.000000	0.000000	20.000000	0.045554
mmu-miR-5123	16.118549	0.000000	4.097489	0.000000	0.000000	20.000000	0.045554
chrx_21884_star	16.118549	0.000000	4.097489	0.000000	0.000000	20.000000	0.045554
chr4_8812_mature	16.118549	0.000000	4.097489	0.000000	0.000000	20.000000	0.045554
mmu-miR-466n-5p	16.118549	0.000000	4.097489	0.000000	0.000000	20.000000	0.045554
chr1_3765_mature	16.118549	0.000000	4.097489	0.000000	0.000000	20.000000	0.045554
chr6_13566_star	16.118549	0.000000	4.097489	0.000000	0.000000	20.000000	0.045554
rno-miR-881-3p	16.118549	0.000000	4.097489	0.000000	0.000000	20.000000	0.045554
hsa-miR-4697-5p	16.118549	0.000000	4.097489	0.000000	0.000000	20.000000	0.045554
NW_003539279_38690_mature	0.000000	24.177823	0.000000	4.654082	4.654082	-20.000000	0.045580
chr10_23377_mature	0.000000	24.177823	0.000000	4.654082	4.654082	-20.000000	0.045580
chr6_13311_mature	0.000000	24.177823	0.000000	4.654082	4.654082	-20.000000	0.045580
hsa-miR-4665-3p	45.131937	1.611855	5.527694	1.385075	1.385075	4.807355	0.045590
chr1_3708_mature	2.149140	78.174963	1.654958	6.306972	6.306972	-5.184875	0.045726
chr1_3436_mature	3.223710	110.412060	2.078511	6.799762	6.799762	-5.098032	0.046262
chr2_5388_mature	1.074570	48.355647	1.052812	5.625143	5.625143	-5.491853	0.046406
chr14_30524_star	306.252431	11.282984	8.263281	3.618589	3.618589	4.762501	0.046909
chr9_20604_mature	2.149140	76.563108	1.654958	6.277299	6.277299	-5.154818	0.047032
bta-miR-2887	4011.369555	83.010527	11.970239	6.392498	6.392498	5.594657	0.047055
chr6_12988_mature@@mmu-miR-6538	44.057367	1.611855	5.493691	1.385075	1.385075	4.772590	0.047439
mmu-miR-6370	1.074570	47.549719	1.052812	5.601391	5.601391	-5.467606	0.047457
NW_003539097_38524_star	1.074570	47.549719	1.052812	5.601391	5.601391	-5.467606	0.047457
chr9_20212_star	29.013388	0.805927	4.907534	0.852740	0.852740	5.169925	0.047624
hsa-miR-548ak	0.000000	23.371896	0.000000	4.607147	4.607147	-20.000000	0.047644
chr11_24562_mature	0.000000	23.371896	0.000000	4.607147	4.607147	-20.000000	0.047644
chr17_35097_mature	7.521990	265.956058	3.091190	8.060458	8.060458	-5.143930	0.048106
chr7_17178_star	77.369035	3.223710	6.292212	2.078511	2.078511	4.584963	0.048186
chr11_25193_mature	1.074570	46.743792	1.052812	5.577241	5.577241	-5.442943	0.048547
ssc-miR-451	59.101346	2.417782	5.909325	1.773061	1.773061	4.611435	0.049249
hsa-miR-5000-3p	42.982797	1.611855	5.458867	1.385075	1.385075	4.736966	0.049399

chr1_330_mature	42.982797	1.611855	5.458867	1.385075	1.385075	4.736966	0.049399
chr9_20255_mature	42.982797	1.611855	5.458867	1.385075	1.385075	4.736966	0.049399
chr1_3087_star	94.562154	4.029637	6.578367	2.330454	2.330454	4.552541	0.049410
chr2_5067_mature	113.904413	4.835565	6.844290	2.544872	2.544872	4.557995	0.049410
chr13_27783_mature	4.298280	139.425449	2.405524	7.133661	7.133661	-5.019591	0.049627
ssc-miR-133a-5p	1.074570	45.937865	1.052812	5.552680	5.552680	-5.417853	0.049677
chr13_28621_mature	1.074570	45.937865	1.052812	5.552680	5.552680	-5.417853	0.049677
chr13_29181_mature	1.074570	45.937865	1.052812	5.552680	5.552680	-5.417853	0.049677
chr11_24465_mature	1.074570	45.937865	1.052812	5.552680	5.552680	-5.417853	0.049677
chr3_8509_mature	0.000000	22.565969	0.000000	4.558633	4.558633	-20.000000	0.049864
chr11_24872_mature	0.000000	22.565969	0.000000	4.558633	4.558633	-20.000000	0.049864
chr18_35896_mature	0.000000	22.565969	0.000000	4.558633	4.558633	-20.000000	0.049864
bta-miR-103	0.000000	22.565969	0.000000	4.558633	4.558633	-20.000000	0.049864
chr5_11025_mature	0.000000	22.565969	0.000000	4.558633	4.558633	-20.000000	0.049864
chr4_10705_mature	0.000000	22.565969	0.000000	4.558633	4.558633	-20.000000	0.049864

piPS-F Vs PEFs

AccID	piPS-F	PEF	piPS-F_log2(count+1)	PEF_log2(count+1)	PEF_log2(count+1)<0.05	Log2FC	FDR
chr11_25345_mature	251.4493640	0.000000	7.98	0.00	0.00	20.00	0.00
chr18_35948_star@@rno-miR-96-3p	245.0019445	0.000000	9.44	4.40	4.40	20.00	0.00
chr13_29176_star	339.5640985	0.000000	11.66	4.56	4.56	20.00	0.00
chr7_15205_mature	202.0191472	0.000000	7.40	2.08	2.08	20.00	0.00
bta-miR-211	156.8872100	0.000000	3.80	9.00	9.00	20.00	0.00
bta-miR-33b	444.8719518	0.000000	9.45	6.79	6.79	20.00	0.00
chr12_26075_star	365.3537768	0.000000	1.65	11.06	11.06	20.00	0.00
chr6_12983_mature@@rno-miR-200b-3p	120.3518324	0.000000	2.41	9.51	9.51	20.00	0.00
NW_003613280_38221_mature	113.9044128	0.000000	3.55	8.77	8.77	20.00	0.00
hsa-miR-4760-3p	98.8604337	0.000000	7.52	0.85	0.85	20.00	0.00
chr8_18502_mature	90.2638743	0.000000	4.00	8.15	8.15	20.00	0.00
chr15_31797_mature	85.9655945	0.000000	2.41	8.63	8.63	20.00	0.00
chr11_25373_mature	83.8164547	0.000000	8.32	2.73	2.73	20.00	0.00
chr6_13566_star	81.6673148	0.000000	11.56	4.56	4.56	20.00	0.00
mmu-miR-532-5p	77.3690351	0.000000	11.26	8.39	8.39	20.00	0.00
chr6_12502_mature	75.2198952	0.000000	7.57	2.08	2.08	20.00	0.00
chr1_392_mature	73.0707554	0.000000	11.44	6.75	6.75	20.00	0.01
chr6_13901_star	70.9216155	0.000000	14.02	10.55	10.55	20.00	0.05
chr5_10827_mature	92576.3487665	2.417782	10.71	13.24	13.24	15.22	0.00
chr2_4298_mature	67700.0548447	2.417782	8.98	6.10	6.10	14.77	0.00
chr6_12627_mature	67551.7641942	3.223710	13.45	7.69	7.69	14.35	0.00
chr13_28872_mature	22675.5747013	3.223710	2.41	8.86	8.86	12.78	0.00
chr1_468_star	8330.0661115	2.417782	11.58	3.30	3.30	11.75	0.00
chr1_986_star	7979.7563137	4.029637	12.54	7.94	7.94	10.95	0.00
ssc-miR-1249	2149.1398636	1.611855	8.58	19.30	19.30	10.38	0.00
ssc-miR-486	29978.3519580	27.401533	1.65	9.26	9.26	10.10	0.00
ssc-miR-142-5p	3853.4077755	4.029637	9.57	6.72	6.72	9.90	0.00
chr13_27180_mature	765.0937915	0.805927	10.77	9.74	9.74	9.89	0.00
ssc-miR-182	1030542.6525769	1181.489640	3.55	11.18	11.18	9.77	0.00
chr2_5067_mature	3672.8800270	4.835565	4.00	8.56	8.56	9.57	0.00
chr16_33920_mature	5119.2511552	7.253347	8.99	4.95	4.95	9.46	0.00
chr16_33919_mature	5069.8209383	7.253347	5.31	12.76	12.76	9.45	0.00
chr7_16724_mature	35828.3106669	58.026776	2.90	8.02	8.02	9.27	0.00
chrx_22424_star	464.2142105	0.805927	9.32	5.19	5.19	9.17	0.00
hsa-miR-762	870.4016448	1.611855	3.26	8.54	8.54	9.08	0.00
chr9_20339_mature@@mmu-miR-3096a-5p	12267.2903417	24.177823	11.55	9.36	9.36	8.99	0.00
NW_003537105_36696_mature	12058.8237749	24.177823	16.88	13.33	13.33	8.96	0.00
ssc-miR-205	1972.9103948	4.029637	7.80	0.85	0.85	8.94	0.00
mmu-miR-5097	31104.5012466	70.921616	10.09	7.39	7.39	8.78	0.00
chr13_27815_mature	3060.3751658	7.253347	1.65	10.11	10.11	8.72	0.00
bta-miR-1247-5p	2877.6982774	7.253347	8.72	4.65	4.65	8.63	0.00
chr3_6707_mature@@mmu-miR-590-3p	5645.7904218	14.506694	9.11	6.39	6.39	8.60	0.00
chr13_28716_mature	52742.0413938	149.902505	8.14	2.73	2.73	8.46	0.00
chr3_7304_star@@mmu-miR-216b-3p	3053.9277462	8.865202	9.86	3.30	3.30	8.43	0.00
chr4_10241_star	3032.4363476	9.671129	9.95	8.10	8.10	8.29	0.00
ssc-miR-545-5p	980.0077778	3.223710	8.05	3.42	3.42	8.25	0.00
chr13_28221_mature	236.4053850	0.805927	12.33	9.02	9.02	8.20	0.00
chr4_9773_mature	221.3614060	0.805927	10.99	5.34	5.34	8.10	0.00
chr2_5437_mature	55033.0244884	215.988556	8.78	5.98	5.98	7.99	0.00
chr12_25987_mature	204.1682870	0.805927	16.73	13.03	13.03	7.98	0.00
chr2_5435_mature	55538.0723564	221.630048	20.68	13.83	13.83	7.97	0.00
ssc-miR-296-5p	984.3060576	4.029637	7.30	1.77	1.77	7.93	0.00
chr5_11626_mature	2899.1896761	12.088912	9.18	4.83	4.83	7.91	0.00
chr7_16102_mature	752.1989523	3.223710	3.26	9.07	9.07	7.87	0.00
chrx_22664_mature	937.0249806	4.029637	10.43	6.67	6.67	7.86	0.00
chr2_4241_mature	182.6768884	0.805927	9.23	3.52	3.52	7.82	0.00
chr2_5068_mature	3806.1266985	16.924476	8.20	12.91	12.91	7.81	0.00
chr13_27210_mature	1237.9045615	5.641492	15.61	13.52	13.52	7.78	0.00
chr12_25415_mature	696.3213158	3.223710	8.69	2.73	2.73	7.75	0.00
chr15_32917_mature	2224.3597589	10.477057	1.65	9.60	9.60	7.73	0.00
chr1_1437_mature	6419.4807727	30.625243	12.72	8.63	8.63	7.71	0.00
chr3_7304_mature@@bta-miR-216b	5458.8152537	26.595606	10.09	7.54	7.54	7.68	0.00
NW_003541078_39887_mature	7653.0870544	39.490445	4.19	8.83	8.83	7.60	0.00
NW_003541078_39885_mature	7786.3337260	40.296372	8.96	4.83	4.83	7.59	0.00
hsa-miR-4449	307.3270005	1.611855	1.65	11.94	11.94	7.57	0.00
chr3_8533_star	1689.2239328	9.671129	16.22	9.52	9.52	7.45	0.00
chr5_12329_mature	952.0689596	5.641492	15.04	11.11	11.11	7.40	0.00
chr5_11672_mature	268.6424830	1.611855	8.41	2.33	2.33	7.38	0.00
chr12_26534_mature	20784.3316213	126.530609	10.21	4.95	4.95	7.36	0.00
chr3_6707_star@@mmu-miR-590-5p	257.8967836	1.611855	10.72	3.42	3.42	7.32	0.00
chr1_788_star	380.3977559	2.417782	15.13	5.88	5.88	7.30	0.00
ssc-miR-126-5p	885.4456238	5.641492	9.79	3.62	3.62	7.29	0.00
chr2_4519_mature	373.9503363	2.417782	8.14	4.40	4.40	7.27	0.00
chr8_18853_mature	6430.2264720	41.908227	4.19	8.41	8.41	7.26	0.00
mmu-miR-5615-5p	238.5545249	1.611855	8.80	5.69	5.69	7.21	0.00
chr6_13158_mature	3232.3063549	22.565969	2.41	8.15	8.15	7.16	0.00

chr12_26213_mature	35815.4158277	250.643437	15.94	14.27	14.27	7.16	0.00
chr15_32731_mature	113.9044128	0.805927	13.21	8.42	8.42	7.14	0.00
chr1_412_star	8779.2363430	62.862341	2.41	9.99	9.99	7.13	0.00
chr12_26608_mature	330.9675390	2.417782	7.89	4.70	4.70	7.10	0.00
ssc-miR-192	2757.3464451	20.148186	7.89	0.85	0.85	7.10	0.00
chr13_27580_mature	3743.8016425	27.401533	10.29	7.19	7.19	7.09	0.00
chr13_28627_star	324.5201194	2.417782	8.05	4.03	4.03	7.07	0.00
chr15_33264_mature	3023.8397882	22.565969	12.66	7.82	7.82	7.07	0.00
chr3_8637_mature	107.4569932	0.805927	9.49	4.03	4.03	7.06	0.00
ssc-miR-216	1575.3195201	12.088912	13.06	7.89	7.89	7.03	0.00
mmu-miR-5615-3p	206.3174269	1.611855	1.65	7.99	7.99	7.00	0.00
rno-miR-27b-5p	103.1587135	0.805927	12.51	8.00	8.00	7.00	0.00
ssc-miR-142-3p	496.4513085	4.029637	12.56	7.83	7.83	6.94	0.00
ssc-miR-885-5p	98.8604337	0.805927	11.06	5.02	5.02	6.94	0.00
chr7_16018_mature	8841.5613990	73.339398	9.21	3.62	3.62	6.91	0.00
hsa-miR-4454	14371.2982682	120.083190	2.41	8.85	8.85	6.90	0.00
ssc-miR-150	189.1243080	1.611855	15.17	13.43	13.43	6.87	0.00
ssc-miR-92a	1684551.7027630	14602.599446	8.93	6.31	6.31	6.85	0.00
chr11_24694_mature	1020.8414352	8.865202	20.16	21.09	21.09	6.85	0.00
chr2_6291_star	92.4130141	0.805927	1.65	8.09	8.09	6.84	0.00
chr6_14250_mature	88.1147344	0.805927	7.80	3.71	3.71	6.77	0.00
ssc-miR-95	257.8967836	2.417782	3.80	9.23	9.23	6.74	0.00
chr8_18026_mature	930.5775610	8.865202	9.73	7.07	7.07	6.71	0.00
ssc-miR-151-5p	76582.4499012	734.199906	13.68	12.69	12.69	6.70	0.00
rno-miR-92a-3p	754.3480921	7.253347	17.56	11.22	11.22	6.70	0.00
ssc-miR-18a	35987.3470168	347.354730	12.21	10.75	10.75	6.69	0.00
chr6_12597_mature	3172.1304387	30.625243	10.64	6.89	6.89	6.69	0.00
chr6_12598_mature	3159.2355996	31.431171	9.39	5.22	5.22	6.65	0.00
chr7_15358_mature	5052.6278194	50.773429	10.77	5.96	5.96	6.64	0.00
hsa-miR-4485	79.5181750	0.805927	10.48	7.15	7.15	6.62	0.01
chr12_26204_mature	554.4780848	5.641492	9.80	8.39	8.39	6.62	0.00
ssc-miR-148b-5p	4586.2644690	46.743792	8.87	3.71	3.71	6.62	0.00
chrx_22394_mature	550.1798051	5.641492	8.02	1.77	1.77	6.61	0.00
chr4_9327_mature	152.5889303	1.611855	1.65	13.28	13.28	6.56	0.00
chr7_15705_mature	681.2773368	7.253347	19.07	14.37	14.37	6.55	0.00
ssc-miR-628	1429.1780093	15.312622	16.22	10.07	10.07	6.54	0.00
chr6_12933_star@@hsa-miR-520e	371.8011964	4.029637	11.85	9.44	9.44	6.53	0.00
ssc-miR-181c	5265.3926659	58.832704	14.26	15.86	15.86	6.48	0.00
NW_003537879_37346_mature	143.9923709	1.611855	1.65	10.71	10.71	6.48	0.00
chr13_28222_mature	287.9847417	3.223710	9.33	5.69	5.69	6.48	0.00
bta-miR-147	4130.6468179	46.743792	16.26	13.23	13.23	6.47	0.00
chr11_24779_mature	4169.3313355	47.549719	8.67	6.00	6.00	6.45	0.00
chr12_25666_mature	14485.2026810	166.826982	9.51	7.21	7.21	6.44	0.00
chr2_5678_star	10685.5234021	124.112827	7.75	13.02	13.02	6.43	0.00
ssc-miR-652	206.3174269	2.417782	11.24	12.51	12.51	6.42	0.00
chr10_23656_mature	339.5640985	4.029637	12.36	5.90	5.90	6.40	0.00
chr15_33434_mature	135.3958114	1.611855	8.17	5.31	5.31	6.39	0.00
chr9_19153_mature	603.9083017	7.253347	13.03	8.69	8.69	6.38	0.00
ssc-miR-92b-3p	60988.2910506	744.676963	2.90	8.90	8.90	6.36	0.00
ssc-miR-16	193293.6393364	2383.933394	11.68	8.98	8.98	6.34	0.00
chr15_33053_mature	883.2964840	11.282984	9.30	5.72	5.72	6.29	0.00
ssc-miR-195	623.2505605	8.059274	11.63	7.93	7.93	6.27	0.00
chr10_23299_mature	324947.7982436	4222.253905	13.35	7.85	7.85	6.27	0.00
mmu-miR-5117-5p	184.8260283	2.417782	12.11	8.29	8.29	6.26	0.00
chrx_22660_mature	122.5009722	1.611855	9.87	2.33	2.33	6.25	0.00
chr18_35948_mature@@rno-miR-96-5p	6503.2972274	87.040164	17.70	16.29	16.29	6.22	0.00
chr12_25930_mature	118.2026925	1.611855	16.61	14.00	14.00	6.20	0.00
chr5_10912_star	412.6348538	5.641492	12.41	4.79	4.79	6.19	0.00
chr15_31781_star	116.0535526	1.611855	13.82	7.39	7.39	6.17	0.00
chr13_27323_mature	116.0535526	1.611855	18.27	14.30	14.30	6.17	0.00
chr5_11762_mature	694.1721760	9.671129	7.26	1.39	1.39	6.17	0.00
chr7_15331_mature	8555.7257972	119.277262	14.43	11.92	11.92	6.16	0.00
NW_003537687_37130_mature	1553.8281214	21.760041	14.52	13.55	13.55	6.16	0.00
chr1_246_star	515.7935673	7.253347	18.04	13.96	13.96	6.15	0.00
ssc-miR-98	76169.8150474	1075.107217	9.51	6.85	6.85	6.15	0.00
chr13_26798_mature	1803.1283456	25.789678	8.20	2.73	2.73	6.13	0.00
ssc-miR-451	167.6329094	2.417782	11.85	10.08	10.08	6.12	0.00
ssc-miR-138	6342.1117376	92.681657	8.25	2.54	2.54	6.10	0.00
chr7_15706_mature	606.0574415	8.865202	11.41	8.15	8.15	6.10	0.00
chr6_14390_mature	275.0899025	4.029637	9.96	5.02	5.02	6.09	0.00
chr4_9437_mature	2138.3941643	31.431171	1.65	10.88	10.88	6.09	0.00
bta-miR-2305	109.6061330	1.611855	1.65	11.21	11.21	6.09	0.00
chr14_30387_mature	217.0631262	3.223710	10.74	7.52	7.52	6.07	0.00
chr8_18750_mature	700.6195955	10.477057	8.20	5.02	5.02	6.06	0.00
hsa-miR-3195	107.4569932	1.611855	1.65	8.60	8.60	6.06	0.00
ssc-miR-497	638.2945395	9.671129	9.38	4.16	4.16	6.04	0.00
chr6_12932_mature@@hsa-miR-371b-3p	1111.1053095	16.924476	13.02	11.00	11.00	6.04	0.00
chr12_25947_star	156.8872100	2.417782	16.06	13.42	13.42	6.02	0.00
mmu-miR-3096a-5p	814.5240083	12.894839	13.01	7.93	7.93	5.98	0.00
NW_003539217_38656_mature	303.0287208	4.835565	5.88	8.62	8.62	5.97	0.00
chr8_18246_mature	96.7112939	1.611855	14.05	11.17	11.17	5.91	0.00
chr6_14250_star	96.7112939	1.611855	10.45	7.81	7.81	5.91	0.00
chr1_188_mature	141.8432310	2.417782	9.85	7.45	7.45	5.87	0.00
chr15_32759_mature	189.1243080	3.223710	8.35	10.12	10.12	5.87	0.00
mmu-miR-3096b-5p	750.0498124	12.894839	14.51	10.52	10.52	5.86	0.00
chr9_20255_mature	92.4130141	1.611855	9.51	11.62	11.62	5.84	0.00
chr13_29407_mature	1708.5661916	29.819316	5.39	9.62	9.62	5.84	0.00
chr3_6901_star	597.4608821	10.477057	6.29	10.24	10.24	5.83	0.00
chr8_18024_mature	318.0726998	5.641492	10.33	8.86	8.86	5.82	0.00
chr8_17989_mature@@bta-miR-302b	90.2638743	1.611855	2.41	8.67	8.67	5.81	0.00
ssc-miR-187	133.2466715	2.417782	13.02	1.77	1.77	5.78	0.00
hsa-miR-4792	11197.0186896	205.511499	2.90	9.94	9.94	5.77	0.00
ssc-miR-15b	11560.2233266	212.764847	11.55	6.09	6.09	5.76	0.00
chrx_22747_mature	350.3097978	6.447420	9.94	2.33	2.33	5.76	0.00
chr3_6639_mature	131.0975317	2.417782	9.60	6.34	6.34	5.76	0.00
chr16_34245_mature	1220.7114426	22.565969	10.48	4.03	4.03	5.76	0.00

mmu-miR-6412	651.1893787	12.088912	1.65	11.80	11.80	5.75	0.00
ssc-miR-4332	3247.3503340	60.444559	7.70	1.77	1.77	5.75	0.00
chrx_22663_mature	816.6731482	15.312622	7.18	1.39	1.39	5.74	0.00
ssc-miR-374b-3p	593.1626024	11.282984	7.97	9.88	9.88	5.72	0.00
chr2_5822_mature	167.6329094	3.223710	13.95	10.91	10.91	5.70	0.00
chr13_28475_star	292.2830215	5.641492	12.90	5.34	5.34	5.70	0.00
chr7_15245_mature	2037.3845907	39.490445	11.10	6.00	6.00	5.69	0.00
chr12_26006_mature@@mmu-miR-744-3p	739.3041131	14.506694	12.45	9.33	9.33	5.67	0.00
chr14_29632_star	81.6673148	1.611855	10.95	12.87	12.87	5.66	0.02
hsa-miR-3607-5p	281.5373221	5.641492	10.31	7.55	7.55	5.64	0.00
chr3_7654_mature	120.3518324	2.417782	12.67	9.00	9.00	5.64	0.00
chr4_8769_mature	199.8700073	4.029637	11.28	7.46	7.46	5.63	0.00
chr16_34839_mature	118.2026925	2.417782	9.45	3.52	3.52	5.61	0.00
hsa-miR-6087	588.8643226	12.088912	9.70	8.10	8.10	5.61	0.00
chr17_35504_mature	154.7380702	3.223710	9.24	3.04	3.04	5.58	0.00
ssc-miR-27a	106126.6756068	2232.419033	4.19	9.23	9.23	5.57	0.00
ssc-miR-17-3p	5527.5877293	116.859480	4.35	8.19	8.19	5.56	0.00
chr3_8533_mature	719.9618543	15.312622	10.71	7.43	7.43	5.56	0.00
chr7_17173_mature	262.1950634	5.641492	7.97	4.95	4.95	5.54	0.00
chr13_29352_mature	186.9751681	4.029637	3.26	10.73	10.73	5.54	0.00
chr12_26594_mature	111.7552729	2.417782	4.35	10.22	10.22	5.53	0.00
hsa-miR-1973	184.8260283	4.029637	2.41	9.41	9.41	5.52	0.00
chrx_21844_mature@@hsa-miR-660-5p	10434.0740380	230.495250	4.35	8.84	8.84	5.50	0.00
chr4_9471_mature	2164.1838427	48.355647	6.97	9.85	9.85	5.48	0.00
chr6_12448_star	2993.7518301	66.891978	2.90	9.36	9.36	5.48	0.00
NW_003537647_37094_star	391.1434552	8.865202	8.17	2.08	2.08	5.46	0.00
rno-let-7b-3p	105.3078533	2.417782	9.68	4.03	4.03	5.44	0.00
chr12_26596_star	139.6940911	3.223710	10.00	3.30	3.30	5.44	0.00
chr3_6620_mature	242.8528046	5.641492	4.19	10.07	10.07	5.43	0.00
chr3_6770_mature	171.9311891	4.029637	14.92	6.17	6.17	5.42	0.00
chr16_34246_mature	1218.5623027	29.013388	8.50	3.71	3.71	5.39	0.00
chr15_32760_mature	202.0191472	4.835565	11.54	7.71	7.71	5.38	0.00
bta-miR-33a	771.5412110	18.536331	3.80	8.78	8.78	5.38	0.00
chr12_26134_mature	167.6329094	4.029637	8.47	5.92	5.92	5.38	0.00
chrx_22749_mature	434.1262525	10.477057	8.36	4.40	4.40	5.37	0.00
chr6_13122_mature@@mmu-miR-5097	7257.6453195	175.692184	9.32	5.19	5.19	5.37	0.00
ssc-miR-3613	397.5908748	9.671129	11.57	3.42	3.42	5.36	0.00
chr2_6095_mature	98.8604337	2.417782	8.77	4.79	4.79	5.35	0.00
chr5_11238_mature	262.1950634	6.447420	8.25	5.58	5.58	5.35	0.00
chr2_5244_mature	131.0975317	3.223710	9.14	5.58	5.58	5.35	0.00
chr3_7983_star	1186.3252047	29.819316	4.00	9.04	9.04	5.31	0.00
chr2_4615_mature	664.0842179	16.924476	9.52	6.25	6.25	5.29	0.00
chr16_34498_mature	752.1989523	19.342259	9.71	7.80	7.80	5.28	0.00
chr8_18908_mature	2944.3216132	75.757180	11.38	9.33	9.33	5.28	0.00
chr1_2879_mature	186.9751681	4.835565	2.41	8.38	8.38	5.27	0.00
chr6_12802_mature	466.3633504	12.088912	13.07	10.38	10.38	5.27	0.00
chr14_30942_mature@@bta-miR-301b	1362.5546736	35.460808	13.96	8.74	8.74	5.26	0.00
chr3_6615_mature	702.7687354	18.536331	11.03	6.14	6.14	5.24	0.00
chr9_20919_mature	15950.9160680	426.335620	15.69	7.24	7.24	5.23	0.00
chr3_8036_mature	118.2026925	3.223710	1.65	7.90	7.90	5.20	0.00
chr15_32156_mature	324.5201194	8.865202	11.32	7.32	7.32	5.19	0.00
chr11_24694_star	206.3174269	5.641492	12.83	7.47	7.47	5.19	0.00
chr2_4893_mature	646.8910990	17.730404	9.74	5.37	5.37	5.19	0.00
chr14_30864_mature	88.1147344	2.417782	3.80	10.07	10.07	5.19	0.00
chr7_15333_mature	8544.9800979	236.942670	9.53	3.95	3.95	5.17	0.00
chr13_27072_star	202.0191472	5.641492	7.28	2.08	2.08	5.16	0.00
mmu-miR-6236	2198.5700805	62.862341	13.67	12.63	12.63	5.13	0.00
chr16_33741_mature	279.3881823	8.059274	8.65	5.67	5.67	5.12	0.00
chr14_30524_mature	139.6940911	4.029637	11.66	9.94	9.94	5.12	0.00
ssc-miR-545-3p	694.1721760	20.148186	5.05	8.28	8.28	5.11	0.00
chr7_16478_mature	8729.8061261	253.867146	9.72	14.03	14.03	5.10	0.00
chr13_29302_mature	165.4837695	4.835565	9.46	4.29	4.29	5.10	0.00
chr7_16464_mature	8252.6970764	242.584162	9.32	3.42	3.42	5.09	0.00
ssc-miR-421-3p	6769.7905705	199.870007	14.87	4.83	4.83	5.08	0.00
NW_003539948_39194_mature	595.3117422	17.730404	2.41	8.80	8.80	5.07	0.00
ssc-miR-186	114020.4663259	3437.280569	7.40	2.33	2.33	5.05	0.00
chr6_14454_mature	18794.2281076	568.178851	7.93	2.73	2.73	5.05	0.00
ssc-miR-193a-3p	16002.4954247	484.362397	6.58	9.69	9.69	5.05	0.00
chr2_4549_mature	159.0363499	4.835565	12.46	3.95	3.95	5.04	0.00
chr16_34309_mature	105.3078533	3.223710	12.50	9.25	9.25	5.03	0.00
chr7_15333_star	651.1893787	20.148186	13.10	6.00	6.00	5.01	0.00
NW_003540278_39378_mature	1038.0345541	32.237098	10.25	4.56	4.56	5.01	0.00
chr5_10971_star	204.1682870	6.447420	9.65	5.19	5.19	4.98	0.00
chr15_33627_mature	992.9026170	31.431171	20.56	15.71	15.71	4.98	0.00
chr1_2618_mature	12475.7569085	397.322232	7.75	10.63	10.63	4.97	0.00
chr7_16048_mature	122.5009722	4.029637	8.03	4.29	4.29	4.93	0.00
chr9_19140_mature	2086.8148076	69.309761	11.96	8.82	8.82	4.91	0.00
chr9_20679_mature	96.7112939	3.223710	7.40	1.77	1.77	4.91	0.00
chr8_17417_mature	361.0554971	12.088912	3.26	10.34	10.34	4.90	0.00
chr1_3870_mature	120.3518324	4.029637	8.52	4.40	4.40	4.90	0.00
chr14_31199_mature	1246.5011209	41.908227	7.94	0.00	0.00	4.89	0.00
ssc-miR-10b	1021266.9649254	34525.125982	3.26	10.06	10.06	4.89	0.00
chr2_5426_mature	8695.4198883	296.581301	6.12	9.57	9.57	4.87	0.00
ssc-miR-27b-3p	1541784.3416210	53677.991804	8.21	3.88	3.88	4.84	0.00
chr7_15101_mature	6471.0601294	225.659686	8.13	12.10	12.10	4.84	0.00
chr15_33243_star	277.2390424	9.671129	10.64	7.95	7.95	4.84	0.00
chr17_35401_mature	92.4130141	3.223710	4.85	9.42	9.42	4.84	0.00
hsa-miR-3676-5p	2538.1341790	88.652019	16.50	1.77	1.77	4.84	0.00
chr7_15107_mature	161.1854898	5.641492	2.41	8.51	8.51	4.84	0.00
chr3_7600_mature	1742.9524294	61.250486	1.65	12.45	12.45	4.83	0.00
ssc-let-7d-3p	9501.3473372	342.519166	8.42	10.41	10.41	4.79	0.00
chr18_35826_mature	111.7552729	4.029637	8.86	0.85	0.85	4.79	0.00
ssc-miR-107	11500.0474104	420.694128	9.35	3.71	3.71	4.77	0.00
ssc-miR-935	264.3442032	9.671129	8.90	4.51	4.51	4.77	0.00
chr9_19408_mature@@hsa-miR-34b-3p	307.3270005	11.282984	8.04	2.90	2.90	4.77	0.00
chr2_6521_mature	107.4569932	4.029637	2.90	8.67	8.67	4.74	0.00

ssc-miR-9-2	6039.0830168	227.271541	2.41	9.09	9.09	4.73	0.00
chr3_7983_mature	5641.4921421	214.376701	2.90	7.92	7.92	4.72	0.00
chr4_10371_mature@@bta-miR-2478	6443.1213112	245.001944	1.65	8.75	8.75	4.72	0.00
chr14_30038_mature	2563.9238573	97.517221	8.54	2.33	2.33	4.72	0.00
ssc-miR-22-3p	550998.6273817	21109.657668	11.00	7.65	7.65	4.71	0.00
chr2_5068_star	2772.3904241	106.382423	2.90	9.17	9.17	4.70	0.00
bta-miR-1434-5p	395.4417349	15.312622	7.63	11.05	11.05	4.69	0.00
mmu-miR-3096a-3p	702.7687354	27.401533	9.90	6.41	6.41	4.68	0.00
chr10_23371_mature	20365.2493479	798.674102	8.12	3.42	3.42	4.67	0.00
chr1_2148_mature	1392.6426316	54.803067	7.54	3.42	3.42	4.67	0.00
ssc-miR-301	973.5603582	38.684518	9.14	4.61	4.61	4.65	0.00
NW_003538080_37501_mature	808.0765887	32.237098	15.01	10.72	10.72	4.65	0.00
chrx_21838_mature@@bta-miR-188	1310.9753168	52.385284	12.30	5.69	5.69	4.65	0.00
chr3_7984_mature	5910.1346250	236.942670	8.10	4.35	4.35	4.64	0.00
chr8_18128_mature	1805.2774855	72.533470	11.52	8.57	8.57	4.64	0.00
chr14_29999_mature	5970.3105412	244.196017	11.06	8.32	8.32	4.61	0.00
chr4_10147_star	333.1166789	13.700767	7.73	13.73	13.73	4.60	0.00
chr1_3220_star	563.0746443	23.371896	13.40	10.36	10.36	4.59	0.00
chr3_7692_mature	96.7112939	4.029637	6.76	13.16	13.16	4.58	0.00
chr14_30000_mature	5993.9510797	249.837509	11.56	9.93	9.93	4.58	0.00
chr14_30616_star	169.7820492	7.253347	2.90	11.42	11.42	4.55	0.00
NW_003613507_39500_mature	206.3174269	8.865202	12.46	8.71	8.71	4.54	0.00
ssc-miR-15a	7599.3585579	329.624327	9.17	10.69	10.69	4.53	0.00
ssc-miR-9	5845.6604291	253.867146	9.96	14.24	14.24	4.53	0.00
ssc-miR-9-1	5845.6604291	255.479001	10.08	7.26	7.26	4.52	0.00
chr7_17188_mature	202.0191472	8.865202	12.74	13.44	13.44	4.51	0.00
chr14_30079_mature	384.6960356	16.924476	6.17	8.71	8.71	4.51	0.00
chr9_20220_star	109.6061330	4.835565	11.44	8.50	8.50	4.50	0.00
bta-miR-410	1003.6483163	44.326010	4.85	11.58	11.58	4.50	0.00
chr2_6439_mature	801.6291691	35.460808	9.93	5.31	5.31	4.50	0.00
chr7_16274_mature	687.7247564	30.625243	12.32	9.37	9.37	4.49	0.00
mmu-miR-3096b-3p	642.5928192	29.013388	3.55	8.90	8.90	4.47	0.00
chr9_20768_mature	2336.1150318	105.576496	12.96	2.33	2.33	4.47	0.00
chr12_25849_mature	195.5717276	8.865202	8.04	2.73	2.73	4.46	0.00
chr8_17418_mature	477.1090497	21.760041	13.87	14.95	14.95	4.45	0.00
chr7_15120_mature	670.5316375	30.625243	2.41	11.86	11.86	4.45	0.00
chr9_19409_mature	193.4225877	8.865202	8.55	1.77	1.77	4.45	0.00
chr14_30596_mature	122.5009722	5.641492	11.12	3.52	3.52	4.44	0.00
chr9_20434_mature@@rno-miR-34b-3p	294.4321613	13.700767	1.65	8.21	8.21	4.43	0.00
chr7_15245_star	103.1587135	4.835565	14.47	2.08	2.08	4.42	0.00
chr2_5134_mature	853.2085259	40.296372	8.77	3.52	3.52	4.40	0.00
chr6_12967_mature	578.1186233	27.401533	7.55	2.54	2.54	4.40	0.00
chrx_22851_mature	287.9847417	13.700767	3.80	8.72	8.72	4.39	0.00
chr13_27782_star	184.8260283	8.865202	12.44	8.58	8.58	4.38	0.00
ssc-miR-133a-3p	8347.2592304	412.634854	11.89	4.16	4.16	4.34	0.00
chr17_35617_mature	113.9044128	5.641492	8.37	12.28	12.28	4.34	0.00
hsa-miR-4634	113.9044128	5.641492	7.38	2.54	2.54	4.34	0.00
chr6_12841_mature	96.7112939	4.835565	1.65	7.85	7.85	4.32	0.01
chr5_10849_mature	161.1854898	8.059274	9.58	0.85	0.85	4.32	0.00
chr12_25693_mature	176.2294688	8.865202	13.06	6.91	6.91	4.31	0.00
chr2_5731_star	126.7992520	6.447420	2.41	9.42	9.42	4.30	0.00
chr9_20767_mature	2273.7899757	116.053553	6.87	8.96	8.96	4.29	0.00
ssc-miR-34a	32929.1209908	1682.776513	2.41	8.75	8.75	4.29	0.00
ssc-let-7d-5p	39888.0358693	2043.832010	10.02	5.05	5.05	4.29	0.00
chr1_2976_mature	105393.8189133	5434.368788	3.26	7.91	7.91	4.28	0.00
chr17_35678_mature	451.3193714	23.371896	14.31	9.64	9.64	4.27	0.00
chr6_12448_mature	1113.2544494	58.026776	8.41	0.00	0.00	4.26	0.00
chr17_35470_mature	184.8260283	9.671129	8.35	1.77	1.77	4.26	0.00
chr1_3118_mature@@mmu-miR-1957a	1166.9829460	61.250486	9.65	6.38	6.38	4.25	0.00
chr9_21035_star	122.5009722	6.447420	3.26	8.58	8.58	4.25	0.00
chrx_22424_mature@@bta-miR-2478	5289.0332044	278.850897	3.26	13.02	13.02	4.25	0.00
ssc-miR-30b-5p	28506.1911514	1505.472474	8.62	3.30	3.30	4.24	0.00
chr6_13893_star	167.6329094	8.865202	15.90	9.54	9.54	4.24	0.00
chrx_22543_star	212.7648465	11.282984	7.34	11.24	11.24	4.24	0.00
chr16_33754_mature	3475.1591595	186.169241	4.49	13.08	13.08	4.22	0.00
chr6_14444_star	165.4837695	8.865202	11.52	6.26	6.26	4.22	0.00
chr10_23498_mature	135.3958114	7.253347	11.31	6.49	6.49	4.22	0.00
chr3_6737_mature	105.3078533	5.641492	8.96	5.90	5.90	4.22	0.00
chr9_20360_mature	1485.0556458	79.786817	16.08	13.86	13.86	4.22	0.00
ssc-miR-499-5p	670.5316375	36.266735	16.70	11.13	11.13	4.21	0.00
chr6_13171_mature	221.3614060	12.088912	16.71	14.34	14.34	4.19	0.00
chr16_34601_star	365.3537768	20.148186	2.90	14.28	14.28	4.18	0.00
chr5_12402_mature	496.4513085	27.401533	10.44	5.80	5.80	4.18	0.00
chr14_31467_mature	638.2945395	35.460808	15.88	12.59	12.59	4.17	0.00
chr3_7739_mature	638.2945395	35.460808	15.12	12.99	12.99	4.17	0.00
chr1_2073_mature@@bta-miR-2478	5551.2282678	310.282068	13.58	4.65	4.65	4.16	0.00
rno-miR-203b-3p	1050.9293933	58.832704	8.95	5.53	5.53	4.16	0.00
chr3_7261_mature	962.8146589	53.997139	7.65	2.33	2.33	4.16	0.00
ssc-miR-217	1048.7802535	58.832704	13.73	14.36	14.36	4.16	0.00
ssc-miR-20a	46629.8876216	2634.576830	15.51	11.36	11.36	4.15	0.00
chr7_15219_mature	15245.9981927	867.983862	12.31	3.04	3.04	4.13	0.00
bta-miR-2889	421.2314133	24.177823	12.67	6.46	6.46	4.12	0.00
chr15_31873_mature	447.0210916	25.789678	10.96	7.46	7.46	4.12	0.00
NW_003537731_37227_mature	404.0382944	23.371896	8.63	4.03	4.03	4.11	0.00
chr7_15783_mature	264.3442032	15.312622	9.94	6.45	6.45	4.11	0.00
chr7_17170_mature	6804.1768083	397.322232	2.41	11.77	11.77	4.10	0.00
chr7_16183_mature	6737.5534725	394.098522	10.27	2.73	2.73	4.10	0.00
hsa-miR-4705	507.1970078	29.819316	8.31	5.86	5.86	4.09	0.00
chr4_9141_mature	382.5468957	22.565969	12.89	9.82	9.82	4.08	0.00
ssc-let-7a	270157.6265598	15987.182803	13.38	6.97	6.97	4.08	0.00
chr5_12399_mature	15622.0976689	940.517333	8.72	6.23	6.23	4.05	0.00
chr15_33536_mature	4590.5627488	278.850897	9.80	7.91	7.91	4.04	0.00
chr6_13306_mature	436.2753923	26.595606	6.76	9.19	9.19	4.04	0.00
bta-miR-2427	354.6080775	21.760041	10.26	7.18	7.18	4.03	0.00
chr7_15660_mature	326.6692593	20.148186	8.73	5.98	5.98	4.02	0.00
ssc-miR-2483	352.4589376	21.760041	7.36	11.17	11.17	4.02	0.00

bta-miR-1247-3p	586.7151828	36.266735	2.41	8.35	8.35	4.02	0.00
bta-miR-1291	2551.0290181	158.767707	7.62	3.30	3.30	4.01	0.00
chr6_13928_star	103.1587135	6.447420	8.81	4.74	4.74	4.00	0.01
ssc-miR-2320-5p	23337.5097793	1463.564247	16.07	13.44	13.44	4.00	0.00
chr1_3117_mature@@mmu-miR-1957a	1154.0881068	73.339398	1.65	8.71	8.71	3.98	0.00
chr12_26382_mature	253.5985039	16.118549	8.38	3.88	3.88	3.98	0.00
chr13_27084_mature	1689.2239328	107.994278	15.22	11.41	11.41	3.97	0.00
ssc-let-7f	315534.5656408	20199.765578	16.32	14.88	14.88	3.97	0.00
chr3_6703_mature	614.6540010	39.490445	11.07	1.39	1.39	3.96	0.00
chr6_12838_mature	33582.4595093	2204.211573	13.93	9.88	9.88	3.93	0.00
chr14_31374_mature	1631.1971565	107.188351	12.01	5.58	5.58	3.93	0.00
ssc-miR-17-5p	63737.0409362	4266.579914	9.93	7.31	7.31	3.90	0.00
bta-miR-2904	5572.7196664	381.203683	9.55	10.67	10.67	3.87	0.00
chr18_36406_mature	760.7955117	52.385284	9.50	6.68	6.68	3.86	0.00
chr2_5682_star	163.3346296	11.282984	16.69	12.41	12.41	3.86	0.00
chr6_14220_star	2978.7078510	207.929282	7.02	9.20	9.20	3.84	0.00
ssc-miR-361-5p	2490.8531020	174.886256	7.67	0.00	0.00	3.83	0.00
ssc-miR-1306-5p	4425.0789792	311.893923	7.54	1.77	1.77	3.83	0.00
chr16_33741_star	272.9407627	19.342259	10.43	8.47	8.47	3.82	0.00
ssc-miR-125a	38263.2861324	2722.422922	8.59	4.16	4.16	3.81	0.00
chr14_31373_mature	1607.5566180	114.441698	14.88	11.55	11.55	3.81	0.00
chr10_23743_mature	260.0459235	18.536331	13.28	10.25	10.25	3.81	0.00
chr4_10149_star	281.5373221	20.148186	7.67	2.54	2.54	3.80	0.00
bta-miR-93	36576.2113394	2620.070136	3.26	9.80	9.80	3.80	0.00
ssc-miR-19a	2606.9066546	187.781096	8.13	3.18	3.18	3.80	0.00
chr13_28823_star	1375.4495127	100.740931	7.70	2.73	2.73	3.77	0.00
mmu-let-7j	109.6061330	8.059274	2.41	9.03	9.03	3.77	0.01
bta-miR-25	114452.4434385	8425.165550	12.89	8.37	8.37	3.76	0.00
ssc-miR-874	1590.3634991	117.665408	13.50	7.74	7.74	3.76	0.00
ssc-miR-34c	5622.1498833	416.664491	9.35	4.40	4.40	3.75	0.00
rno-miR-203a-5p	988.6043373	74.145325	7.90	1.39	1.39	3.74	0.00
chr3_7963_mature	107.4569932	8.059274	12.03	5.60	5.60	3.74	0.02
NW_003613073_36998_mature	180.5277485	13.700767	12.03	8.33	8.33	3.72	0.00
chr5_10975_star	148.2906506	11.282984	7.57	1.39	1.39	3.72	0.00
ssc-miR-149	4186.5244544	320.759125	9.77	15.36	15.36	3.71	0.00
chr7_15287_mature	109066.6989402	8365.526919	9.61	7.50	7.50	3.70	0.00
chr1_3195_mature@@bta-miR-2887	3159.2355996	243.390090	10.62	7.55	7.55	3.70	0.00
chr13_29250_mature	468.5124903	36.266735	19.96	15.08	15.08	3.69	0.00
chr12_25449_mature	124.6501121	9.671129	8.02	1.39	1.39	3.69	0.00
bta-miR-4286	6511.8937869	510.958003	6.64	9.79	9.79	3.67	0.00
chr1_270_mature	1742.9524294	137.007666	13.58	11.07	11.07	3.67	0.00
NW_003613380_38805_mature	640.4436794	50.773429	9.34	13.54	13.54	3.66	0.00
chr2_5420_mature	404.0382944	33.043025	1.65	8.33	8.33	3.61	0.00
chr13_27132_mature	629.6979800	51.579357	12.63	6.55	6.55	3.61	0.00
chr2_4572_mature	176.2294688	14.506694	16.54	13.46	13.46	3.60	0.00
ssc-miR-532-3p	1598.9600586	132.172102	16.12	14.48	14.48	3.60	0.00
chr8_18872_star	563.0746443	46.743792	14.68	16.65	16.65	3.59	0.00
chr6_12951_star	199.8700073	16.924476	13.49	8.72	8.72	3.56	0.00
chr2_6291_mature	1091.7630507	92.681657	7.70	1.39	1.39	3.56	0.00
chr13_27084_star	197.7208675	16.924476	9.25	3.30	3.30	3.55	0.00
chr6_12438_mature	120403.4117210	10329.572112	3.26	9.11	9.11	3.54	0.00
chr1_3491_mature	206.3174269	17.730404	9.44	7.33	7.33	3.54	0.00
chr13_26691_star	335.2658187	29.013388	12.65	4.98	4.98	3.53	0.00
chr6_12437_mature	119232.1304953	10347.302516	13.09	8.22	8.22	3.53	0.00
NW_003613193_37595_star	176.2294688	15.312622	12.88	10.98	10.98	3.52	0.00
ssc-miR-365-3p	28665.2275013	2520.941060	11.35	7.56	7.56	3.51	0.00
ssc-miR-1296-5p	1987.9543739	174.886256	10.65	6.85	6.85	3.51	0.00
chr7_15331_star	980.0077778	86.234237	2.41	9.99	9.99	3.51	0.00
chr1_953_mature	952.0689596	83.816455	11.76	7.55	7.55	3.51	0.00
ssc-miR-331-5p	595.3117422	53.191212	19.97	10.21	10.21	3.48	0.00
ssc-miR-30c-5p	16638.6408244	1500.636910	11.03	13.42	13.42	3.47	0.00
chr10_23348_mature	178.3786087	16.118549	16.80	11.75	11.75	3.47	0.00
chr4_10445_mature	141.8432310	12.894839	15.14	8.44	8.44	3.46	0.00
chr5_11956_mature	176.2294688	16.118549	11.43	4.40	4.40	3.45	0.00
chr1_782_mature	492.1530288	45.131937	9.29	3.18	3.18	3.45	0.00
chr1_270_star	219.2122661	20.148186	2.41	8.32	8.32	3.44	0.00
NW_003537647_37094_mature	2634.8454728	242.584162	12.55	7.97	7.97	3.44	0.00
chr2_4013_mature	165.4837695	15.312622	7.92	10.86	10.86	3.43	0.00
hsa-miR-3615	135.3958114	12.894839	7.30	0.00	0.00	3.39	0.00
chr14_30575_mature	1401.2391911	134.589884	11.63	5.02	5.02	3.38	0.00
bta-miR-1248	124.6501121	12.088912	9.56	3.04	3.04	3.37	0.00
chr2_4571_mature	174.0803290	16.924476	12.19	10.53	10.53	3.36	0.00
chr7_15273_mature	2048.1302901	199.870007	10.62	3.71	3.71	3.36	0.00
ssc-miR-22-5p	1431.3271492	141.037304	10.04	5.90	5.90	3.34	0.00
chr3_7724_mature	30236.2487417	2997.244182	5.39	13.14	13.14	3.33	0.00
chr2_5456_mature	5162.2339525	517.405422	10.95	2.33	2.33	3.32	0.00
chr5_11650_mature	735.0058334	74.951253	12.51	7.99	7.99	3.29	0.00
ssc-miR-23b	60410.1724273	6179.851678	9.45	15.18	15.18	3.29	0.00
chr13_27228_mature	801.6291691	82.204600	3.26	10.49	10.49	3.29	0.00
chr18_36516_mature	1672.0308139	171.662547	1.65	10.68	10.68	3.28	0.00
chr3_7145_star	775.8394908	79.786817	8.58	4.56	4.56	3.28	0.00
chr9_20930_mature	2720.8110674	282.880535	11.50	3.71	3.71	3.27	0.00
NW_003538820_38274_mature	208.4665668	21.760041	7.16	10.23	10.23	3.26	0.00
chr4_8769_star	169.7820492	17.730404	15.13	7.98	7.98	3.26	0.00
ssc-miR-27b-5p	5800.5284920	608.475224	9.86	8.11	8.11	3.25	0.00
chr14_30067_star	206.3174269	21.760041	5.39	9.52	9.52	3.25	0.00
chr2_6283_mature	236.4053850	24.983751	1.65	8.71	8.71	3.24	0.00
chr3_8552_mature	189.1243080	20.148186	7.67	3.30	3.30	3.23	0.00
chr14_31622_mature	1435.6254289	153.126215	10.17	6.22	6.22	3.23	0.00
ssc-miR-4334-3p	1706.4170517	182.945531	13.66	10.66	10.66	3.22	0.00
chrx_22074_mature	292.2830215	31.431171	2.90	13.77	13.77	3.22	0.00
ssc-miR-374a-5p	2899.1896761	313.505778	9.56	2.08	2.08	3.21	0.00
chr14_30061_mature	498.6004484	53.997139	10.82	6.20	6.20	3.21	0.00
chrx_22266_star	178.3786087	19.342259	14.81	11.30	11.30	3.21	0.00
ssc-miR-664-3p	133.2466715	14.506694	10.82	8.52	8.52	3.20	0.00
bta-miR-1434-3p	225.6596857	24.983751	11.67	5.94	5.94	3.18	0.00

chr9_20723_mature	1360.4055337	150.708433	9.97	5.50	5.50	3.17	0.00
chr4_9000_mature	447.0210916	49.967502	10.74	4.95	4.95	3.16	0.00
chr2_5894_mature	186.9751681	20.954114	2.41	9.41	9.41	3.16	0.00
chr6_13671_mature	3995.2510065	450.513444	15.75	7.76	7.76	3.15	0.00
ssc-miR-196b	444.8719518	50.773429	9.40	6.70	6.70	3.13	0.00
chr14_31521_mature	161.1854898	18.536331	9.22	5.76	5.76	3.12	0.00
mmu-miR-5105	5577.0179462	641.518249	10.04	5.90	5.90	3.12	0.00
chr2_5750_star	146.1415107	16.924476	9.72	6.98	6.98	3.11	0.00
hsa-miR-1291	1250.7994006	145.066941	9.59	4.29	4.29	3.11	0.00
chr8_18872_mature	1225.0097223	144.261013	8.80	0.00	0.00	3.09	0.00
chr13_26633_star	745.7515327	87.846092	8.58	1.77	1.77	3.09	0.00
ssc-miR-103	95445.4504844	11249.941259	12.25	9.98	9.98	3.08	0.00
chr18_36618_mature	498.6004484	58.832704	2.41	10.68	10.68	3.08	0.00
ssc-miR-671-3p	1577.4686599	186.975168	10.49	7.27	7.27	3.08	0.00
chr2_4155_mature@@hsa-miR-5701	7571.4197396	901.832815	11.05	9.33	9.33	3.07	0.00
chr9_20193_mature	249.3002242	29.819316	11.87	4.83	4.83	3.06	0.00
chr1_3851_mature	199.8700073	24.177823	2.41	8.12	8.12	3.05	0.00
ssc-miR-340	10820.9192135	1312.855814	11.15	6.87	6.87	3.04	0.00
mmu-miR-5115	15869.2487532	1928.584385	1.65	8.67	8.67	3.04	0.00
NW_003537370_36873_mature	9965.5615477	1218.562303	7.55	2.33	2.33	3.03	0.00
bta-miR-193b	78512.3774988	9638.086361	7.68	2.90	2.90	3.03	0.00
chr2_6342_mature	419.0822734	51.579357	15.21	12.63	12.63	3.02	0.00
ssc-miR-339-5p	6516.1920666	811.568941	10.60	4.51	4.51	3.01	0.00
chr7_17017_star	399.7400146	49.967502	7.67	2.73	2.73	3.00	0.00
bta-miR-106b	12914.1814407	1622.331955	9.57	5.74	5.74	2.99	0.00
chr7_15749_mature	152.5889303	19.342259	2.41	8.58	8.58	2.98	0.00
ssc-miR-342	2931.4267740	378.785901	10.45	7.08	7.08	2.95	0.00
chr15_33572_star	5108.5054559	661.666436	11.15	10.19	10.19	2.95	0.00
ssc-miR-339	2776.6887038	361.861425	16.80	13.04	13.04	2.94	0.00
chr17_35020_mature	287.9847417	38.684518	10.48	13.55	13.55	2.90	0.00
chr1_2125_mature	502.8987281	67.697906	2.41	9.46	9.46	2.89	0.00
ssc-miR-744	16961.0118039	2297.699157	12.73	8.64	8.64	2.88	0.00
chr11_25350_mature	2456.4668641	333.653964	13.82	14.10	14.10	2.88	0.00
chr5_10730_mature	141.8432310	19.342259	11.21	8.53	8.53	2.87	0.01
NW_003537646_37091_mature	2828.2680606	388.457030	10.44	12.04	12.04	2.86	0.00
chr3_6703_star	760.7955117	104.770568	15.69	14.94	14.94	2.86	0.00
ssc-miR-324	1083.1664913	152.320288	7.43	2.33	2.33	2.83	0.00
ssc-miR-129a	722.1109942	101.546859	14.49	16.60	16.60	2.83	0.00
ssc-miR-24-1-5p	5035.4347005	711.633937	8.46	2.90	2.90	2.82	0.00
chr18_36617_mature	438.4245322	62.056414	13.64	12.36	12.36	2.82	0.00
chr11_24906_mature	193.4225877	28.207461	10.09	11.07	11.07	2.78	0.00
chr8_18593_mature	423.3805531	62.056414	1.65	8.23	8.23	2.77	0.00
chr6_14159_mature	1265.8433797	186.169241	15.16	11.36	11.36	2.77	0.00
chrx_22073_star	844.6119664	124.918755	10.64	7.06	7.06	2.76	0.00
chr7_17017_mature	1162.6846662	172.468474	11.32	6.62	6.62	2.75	0.00
ssc-miR-130b	2490.8531020	371.532554	12.43	6.88	6.88	2.75	0.00
chrx_21868_mature	2134.0958846	318.341342	8.37	1.77	1.77	2.74	0.00
bta-miR-2887	550.1798051	83.010527	9.54	6.47	6.47	2.73	0.00
chr13_26825_mature	292.2830215	44.326010	12.46	7.75	7.75	2.72	0.00
NW_003538371_37782_mature	1186.3252047	180.527749	12.44	8.28	8.28	2.72	0.00
chr15_33664_mature	438.4245322	66.891978	15.96	12.06	12.06	2.71	0.00
chr18_36226_mature	674.8299172	103.158713	11.19	6.74	6.74	2.71	0.00
hsa-miR-3607-3p	1087.4647710	166.826982	9.10	6.92	6.92	2.70	0.00
mmu-miR-1900	3268.8417326	502.898728	12.37	8.13	8.13	2.70	0.00
chr2_5731_mature	303.0287208	46.743792	12.79	13.66	13.66	2.70	0.00
chr3_8127_star	406.1874342	62.862341	10.19	5.96	5.96	2.69	0.00
chr18_35822_mature@@mmu-miR-5115	1592.5126390	246.613799	11.84	2.54	2.54	2.69	0.00
ssc-miR-500	8587.9628951	1333.809928	8.82	4.61	4.61	2.69	0.00
chr3_8144_mature	2372.6504095	369.920699	8.39	4.91	4.91	2.68	0.00
chr17_35019_mature	268.6424830	41.908227	4.49	8.70	8.70	2.68	0.00
ssc-miR-32	851.0593860	132.978029	8.52	0.00	0.00	2.68	0.00
chr15_33456_mature	728.5584138	114.441698	8.94	6.39	6.39	2.67	0.00
chr12_26057_mature	199.8700073	31.431171	7.74	3.62	3.62	2.67	0.00
chr12_25587_mature	696.3213158	109.606133	1.65	8.41	8.41	2.67	0.00
chr2_5756_mature@@bta-miR-2898	5895.0906460	936.487696	10.67	6.76	6.76	2.65	0.00
ssc-miR-196b-5p	399.7400146	63.668268	10.49	9.15	9.15	2.65	0.00
ssc-miR-2320-3p	1399.0900512	224.047831	15.76	7.80	7.80	2.64	0.00
bta-let-7b	487.8547490	78.174963	8.11	2.33	2.33	2.64	0.00
chr10_23895_mature	2424.2297662	389.262958	12.65	7.94	7.94	2.64	0.00
chr17_35449_mature	68198.6552931	10954.971812	11.74	10.09	10.09	2.64	0.00
chrx_22266_mature	68574.7547693	11104.068390	3.55	9.98	9.98	2.63	0.00
chr7_15295_mature	971.4112184	157.961780	12.63	13.34	13.34	2.62	0.00
ssc-let-7g	99756.6250509	16362.744994	10.41	7.25	7.25	2.61	0.00
bta-miR-6529	37852.8004184	6316.859344	12.32	3.04	3.04	2.58	0.00
bta-miR-3596	575.9694835	96.711294	18.31	12.04	12.04	2.57	0.00
ssc-miR-582	354.6080775	59.638631	13.90	9.76	9.76	2.57	0.00
bta-miR-2424	167.6329094	28.207461	11.70	14.75	14.75	2.57	0.00
bta-miR-454	171.9311891	29.013388	12.07	11.07	11.07	2.57	0.00
chr12_25570_star	195.5717276	33.043025	2.41	8.21	8.21	2.57	0.00
chr15_33256_mature	490.0038889	83.010527	10.72	6.77	6.77	2.56	0.00
ssc-miR-1343	3036.7346273	514.987640	8.66	5.09	5.09	2.56	0.00
chr3_7145_mature	1087.4647710	184.557386	12.16	5.58	5.58	2.56	0.00
ssc-miR-339-3p	204.1682870	34.654880	9.17	6.61	6.61	2.56	0.00
ssc-let-7e	22069.5172598	3867.645827	8.89	14.73	14.73	2.51	0.00
chr1_3251_mature	2434.9754655	427.947475	8.47	4.51	4.51	2.51	0.00
ssc-miR-128	12245.7989431	2152.632216	14.37	12.85	12.85	2.51	0.00
ssc-miR-30c-1-3p	421.2314133	74.145325	13.81	6.92	6.92	2.51	0.00
NW_003539151_38597_mature	232.1071053	41.102300	11.47	8.61	8.61	2.50	0.00
NW_003613116_37270_mature	315.9235600	57.220849	13.73	11.48	11.48	2.46	0.00
chr14_31374_star	597.4608821	109.606133	12.73	7.65	7.65	2.45	0.00
ssc-miR-30e-3p	3696.5205655	692.291679	9.66	7.39	7.39	2.42	0.00
NW_003541078_39887_star	924.1301414	174.080329	8.27	3.62	3.62	2.41	0.00
ssc-miR-30b-3p	193.4225877	37.072663	8.96	2.33	2.33	2.38	0.00
ssc-miR-26a	107575.1958749	20739.736969	2.41	8.59	8.59	2.37	0.00
NW_003539318_38728_mature	275.0899025	53.191212	11.91	2.33	2.33	2.37	0.00
NW_003540770_39680_mature	206.3174269	41.102300	8.27	1.39	1.39	2.33	0.00

chr9_19936_mature	726.4092739	146.678796	14.20	9.15	9.15	2.31	0.00
ssc-miR-4331	1800.9792057	365.085134	1.65	9.13	9.13	2.30	0.00
chr17_34996_mature	810.2257286	166.826982	7.54	3.30	3.30	2.28	0.00
hsa-miR-4700-5p	2142.6924441	442.454169	13.11	6.22	6.22	2.28	0.00
chr18_36328_mature	229.9579654	47.549719	8.35	3.30	3.30	2.27	0.00
ssc-miR-331-3p	4882.8457702	1010.633021	2.41	9.01	9.01	2.27	0.00
ssc-miR-769-5p	13548.1777004	2857.012806	3.80	8.52	8.52	2.25	0.00
chr1_3299_mature	393.2925950	83.816455	9.41	7.97	7.97	2.23	0.00
NW_003613116_37269_mature	249.3002242	53.191212	2.41	7.98	7.98	2.23	0.00
chr1_3220_mature	69155.0225324	14904.822239	13.83	11.93	11.93	2.21	0.00
chr3_8257_mature@@bta-miR-2898	2993.7518301	656.830871	7.60	3.30	3.30	2.19	0.00
chr18_36225_mature	545.8815254	120.083190	8.88	5.22	5.22	2.18	0.00
chr5_11904_mature	260.0459235	58.026776	11.54	13.75	13.75	2.16	0.00
ssc-miR-23a	35645.6337785	8160.821347	8.78	6.09	6.09	2.13	0.00
NW_003541078_39885_star	782.2869104	180.527749	10.70	13.75	13.75	2.12	0.00
rno-miR-3074	692.0230361	160.379562	10.41	5.19	5.19	2.11	0.00
chr3_7184_mature	315.9235600	73.339398	7.18	9.22	9.22	2.11	0.00
chr4_9318_mature	238.5545249	55.608994	7.68	0.85	0.85	2.10	0.00
ssc-miR-99b	49872.9396758	11746.392567	9.34	4.23	4.23	2.09	0.00
ssc-miR-24-2-5p	330.9675390	78.174963	9.94	10.86	10.86	2.08	0.00
ssc-miR-505	2664.9334309	642.324177	14.80	10.56	10.56	2.05	0.00
chr4_10079_star	311.6252802	76.563108	9.79	2.73	2.73	2.03	0.00
chr16_34600_star	8306.4255730	2051.891285	7.32	2.54	2.54	2.02	0.00
ssc-miR-664-5p	255.7476438	63.668268	11.07	8.79	8.79	2.01	0.00
ssc-miR-345-5p	1375.4495127	352.996223	13.93	14.83	14.83	1.96	0.00
chrx_22247_mature	369.6520565	95.905366	9.56	4.35	4.35	1.95	0.00
chr3_8127_mature	468.5124903	124.112827	11.08	12.15	12.15	1.92	0.00
ssc-miR-504	836.0154070	221.630048	10.21	7.50	7.50	1.92	0.00
chr2_4526_mature	361.0554971	95.905366	7.34	2.73	2.73	1.91	0.00
chr7_15219_star	410.4857140	109.606133	9.66	5.05	5.05	1.91	0.00
ssc-miR-296-3p	14519.5889188	3888.599941	11.08	5.63	5.63	1.90	0.00
ssc-miR-19b	7526.2878025	2019.654187	11.00	9.25	9.25	1.90	0.00
chr17_35129_star	891.8930434	240.166380	10.82	4.74	4.74	1.89	0.00
bta-miR-2898	990.7534771	274.015333	8.65	6.01	6.01	1.85	0.00
chr15_33572_mature	16857.8530904	4667.125856	10.77	7.11	7.11	1.85	0.00
chr4_10247_mature	313.7744201	87.040164	10.54	6.34	6.34	1.85	0.00
chr9_20601_star	391.1434552	111.217988	8.17	3.88	3.88	1.81	0.00
mmu-miR-1983	3702.9679851	1081.554636	9.95	6.23	6.23	1.78	0.00
chr9_20605_star	337.4149586	99.129076	15.13	13.93	13.93	1.77	0.00
chr6_14878_mature	926.2792812	274.821260	3.80	8.38	8.38	1.75	0.00
rno-miR-203a-3p	2043.8320103	608.475224	8.81	5.67	5.67	1.75	0.00
ssc-miR-423-3p	36963.0565149	11045.235687	8.62	6.41	6.41	1.74	0.00
rno-miR-203b-5p	2125.4993251	640.712322	10.25	4.91	4.91	1.73	0.00
NW_003541084_39898_mature	3240.9029144	980.813705	8.71	5.72	5.72	1.72	0.00
ssc-miR-151-3p	62737.6908996	19804.861128	10.28	5.42	5.42	1.66	0.00
ssc-miR-210	4667.9317838	1474.041304	12.53	9.87	9.87	1.66	0.00
ssc-miR-28-5p	3423.5798028	1092.031693	8.96	5.78	5.78	1.65	0.00
ssc-miR-101	71233.2407806	22860.132087	9.54	10.55	10.55	1.64	0.00
chr13_28352_mature	3010.9449490	971.142576	12.53	7.89	7.89	1.63	0.00
chr13_26691_mature	831.7171272	274.015333	12.93	5.37	5.37	1.60	0.00
chr1_3195_star	354.6080775	117.665408	11.36	7.93	7.93	1.59	0.00
chr8_18895_mature	500.7495882	167.632909	9.01	3.04	3.04	1.58	0.00
ssc-miR-148b-3p	21244.2475522	7378.265794	9.43	4.98	4.98	1.53	0.00
chr7_16829_mature	367.5029167	131.366174	9.77	1.39	1.39	1.48	0.00
chr8_18896_mature	541.5832456	194.228515	9.91	5.78	5.78	1.48	0.00
chr5_11960_mature	1287.3347783	462.602356	9.67	3.80	3.80	1.48	0.00
chr14_29777_mature	4734.5551196	1721.461031	10.36	5.74	5.74	1.46	0.00
ssc-miR-125b	81819.9037489	30049.810858	12.16	8.13	8.13	1.45	0.00
chr15_33243_mature	676.9790570	249.031582	9.45	2.08	2.08	1.44	0.00
chr1_2618_star	584.5660429	215.988556	9.46	4.83	4.83	1.44	0.00
ssc-miR-374b-5p	891.8930434	333.653964	9.94	2.08	2.08	1.42	0.00
ssc-let-7i	212218.9649757	79993.134865	7.70	4.23	4.23	1.41	0.00
chr14_29582_mature	709.2161550	275.627188	1.65	9.43	9.43	1.36	0.00
chrx_21651_mature	1437.7745688	568.984779	2.41	8.90	8.90	1.34	0.00
ssc-miR-1307	8839.4122592	3506.590330	7.47	3.30	3.30	1.33	0.00
NW_003540583_39564_mature	560.9255044	228.077468	14.89	13.75	13.75	1.30	0.00
ssc-miR-532-5p	12774.4873495	5248.199547	9.44	3.42	3.42	1.28	0.00
chr4_9280_mature	640.4436794	265.956058	16.86	13.34	13.34	1.27	0.00
chr11_24705_mature	494.3021686	214.376701	3.26	9.44	9.44	1.21	0.02
chr11_25256_mature	672.6807773	291.745736	8.66	4.61	4.61	1.21	0.00
ssc-miR-24-3p	35774.5821703	15580.995369	10.12	4.16	4.16	1.20	0.00
chr14_30762_mature	30343.7057348	13748.316350	14.34	6.99	6.99	1.14	0.00
ssc-miR-425-5p	12993.6996156	6349.902370	13.56	4.65	4.65	1.03	0.00
chr5_10765_star	1749.3998490	855.089023	11.49	3.04	3.04	1.03	0.00
ssc-miR-155-5p	4300.4288672	2150.214434	15.28	11.00	11.00	1.00	0.00
ssc-miR-423-5p	13111.9023081	6627.141412	8.20	5.50	5.50	0.98	0.00
ssc-let-7c	23511.5901083	11989.782657	1.65	8.51	8.51	0.97	0.00
chr13_28054_mature	2275.9391156	1166.982946	2.41	7.98	7.98	0.96	0.00
chr2_5678_mature	909.0861623	475.497195	9.20	5.22	5.22	0.93	0.00
chr14_30369_mature	1033.7362744	564.149214	9.22	4.23	4.23	0.87	0.00
mmu-miR-6240	1240.0537013	719.693212	7.77	2.08	2.08	0.78	0.00
chr14_30414_mature	52864.5423660	31351.383688	2.41	9.88	9.88	0.75	0.00
ssc-miR-28-3p	14427.1759047	17500.714552	8.64	3.42	3.42	-0.28	0.00
ssc-miR-191	4375.6487624	5628.597303	9.11	2.73	2.73	-0.36	0.00
chr16_34600_mature	58254.5851440	79653.839409	11.50	8.30	8.30	-0.45	0.00
ssc-miR-222	13629.8450152	20983.932986	11.63	4.98	4.98	-0.62	0.00
ssc-miR-320	6825.6682069	11150.006255	7.70	3.30	3.30	-0.71	0.00
chr10_23299_star	6318.4711991	10370.674412	11.28	8.54	8.54	-0.71	0.00
chr5_11282_mature	3028.1380679	5234.498780	7.42	3.04	3.04	-0.79	0.00
chrx_21505_mature	1147.6406872	2024.489752	11.57	9.01	9.01	-0.82	0.00
chr15_32130_mature	7074.9684311	12920.628860	11.25	8.74	8.74	-0.87	0.00
ssc-miR-140-3p	15650.0364871	29085.115702	1.65	10.27	10.27	-0.89	0.00
chr13_26847_mature	980.0077778	1859.274625	11.56	12.35	12.35	-0.92	0.00
ssc-miR-21	1168614.1431169	2238508.621167	11.58	3.04	3.04	-0.94	0.00
ssc-miR-1306-3p	515.7935673	1001.767819	9.55	3.80	3.80	-0.96	0.00
chr8_18403_mature	449.1702315	881.684629	13.11	11.78	11.78	-0.97	0.00

chrx_21506_mature	1085.3156311	2153.438143	9.27	5.34	5.34	-0.99	0.00
chr6_13297_mature	741.4532530	1499.830982	11.24	8.61	8.61	-1.02	0.00
chr17_35351_mature	2170.6312623	4530.118190	9.23	6.79	6.79	-1.06	0.00
ssc-miR-221-3p	15007.4436678	31697.126564	9.12	2.73	2.73	-1.08	0.00
ssc-miR-129b	747.9006725	1630.391229	14.04	12.19	12.19	-1.12	0.00
ssc-miR-181b	2413.4840669	5820.408036	7.78	4.40	4.40	-1.27	0.00
chr13_28400_mature	206.3174269	527.076552	9.90	2.73	2.73	-1.35	0.00
ssc-miR-363	575.9694835	1648.121633	6.21	9.04	9.04	-1.52	0.00
ssc-miR-10a-5p	19675.3754517	59396.047054	8.47	4.51	4.51	-1.59	0.00
chr2_5312_mature	221.3614060	671.337565	4.19	10.63	10.63	-1.60	0.00
ssc-miR-148a-5p	1392.6426316	4224.671687	9.39	4.98	4.98	-1.60	0.00
chr2_5556_mature	113.9044128	377.174046	9.33	4.91	4.91	-1.73	0.00
chr8_17690_mature	324.5201194	1112.179879	13.09	7.99	7.99	-1.78	0.00
chr9_19522_mature	113.9044128	406.993362	4.49	8.57	8.57	-1.84	0.00
mmu-miR-6243	249.3002242	939.711405	10.18	7.44	7.44	-1.91	0.00
mmu-miR-5109	1972.9103948	7493.513420	16.04	2.08	2.08	-1.93	0.00
ssc-miR-193a-5p	795.1817495	3071.389508	12.67	9.67	9.67	-1.95	0.00
ssc-miR-100	26230.2520358	102701.752592	7.54	2.33	2.33	-1.97	0.00
NW_003613379_38756_mature	92.4130141	367.502917	16.05	1.77	1.77	-1.99	0.00
chrx_22210_mature	341.7132383	1359.599606	8.88	6.97	6.97	-1.99	0.00
NW_003613379_38755_mature	94.5621540	383.621466	15.83	16.28	16.28	-2.02	0.00
chr1_1204_mature	143.9923709	596.386312	7.99	4.10	4.10	-2.05	0.00
chr8_18458_star	116.0535526	496.451309	9.64	11.59	11.59	-2.10	0.00
chr15_33547_mature	726.4092739	3136.669631	8.07	1.39	1.39	-2.11	0.00
ssc-miR-148a-3p	22989.3491214	99491.743563	13.97	8.92	8.92	-2.11	0.00
chr2_4589_mature	79.5181750	348.966585	9.20	3.71	3.71	-2.13	0.00
chr13_29223_mature	66.6233358	302.222793	13.61	8.64	8.64	-2.18	0.00
ssc-miR-345-3p	128.9483918	586.715183	12.65	5.42	5.42	-2.19	0.00
ssc-miR-199b-5p	2967.9621517	13757.987480	8.30	11.79	11.79	-2.21	0.00
chr14_30693_mature	70.9216155	348.966585	10.17	10.98	10.98	-2.30	0.00
ssc-miR-185	2088.9639475	10970.284434	10.12	5.88	5.88	-2.39	0.00
chr6_13451_star	107.4569932	584.297400	1.65	8.16	8.16	-2.44	0.00
chr2_6335_mature	1672.0308139	9704.978339	2.41	9.94	9.94	-2.54	0.00
ssc-miR-335	70.9216155	418.276346	10.09	6.55	6.55	-2.56	0.00
ssc-miR-758	58.0267763	392.486668	1.65	9.60	9.60	-2.76	0.00
ssc-miR-769-3p	38.6845175	267.567913	12.30	9.48	9.48	-2.79	0.00
chr5_11567_mature	73.0707554	525.464697	9.41	3.04	3.04	-2.85	0.00
bta-miR-677	214.9139864	1580.423727	1.65	7.71	7.71	-2.88	0.00
chr14_30429_mature	27.9388182	205.511499	7.82	4.70	4.70	-2.88	0.00
chr2_5281_mature	124.6501121	920.369147	1.65	7.70	7.70	-2.88	0.00
chr8_18458_mature@@mmu-miR-6243	240.7036647	1860.886479	3.26	7.86	7.86	-2.95	0.00
ssc-miR-199a-3p	3326.8685089	27535.317218	7.80	9.39	9.39	-3.05	0.00
ssc-miR-199b-3p	1659.1359747	13762.823044	7.16	1.77	1.77	-3.05	0.00
chr3_6912_mature	1431.3271492	11960.769269	7.65	4.16	4.16	-3.06	0.00
chr5_11953_mature	141.8432310	1200.831899	8.07	5.42	5.42	-3.08	0.00
chr5_12341_mature	21.4913986	186.169241	7.09	1.39	1.39	-3.11	0.00
NW_003540278_39378_star	94.5621540	826.075635	9.47	8.11	8.11	-3.13	0.00
chr15_33627_star	98.8604337	885.714266	7.63	4.16	4.16	-3.16	0.00
ssc-miR-425-3p	32.2370980	309.476140	7.40	3.30	3.30	-3.26	0.00
chr9_20853_mature@@mmu-miR-6243	197.7208675	2113.141771	1.65	7.65	7.65	-3.42	0.00
chr9_20088_mature	17.1931189	186.169241	1.65	7.64	7.64	-3.44	0.00
bta-miR-154c	68.7724756	759.183657	7.38	3.30	3.30	-3.46	0.00
chr12_26014_mature	313.7744201	3535.603718	1.65	7.63	7.63	-3.49	0.00
chr2_5113_mature	15.0439790	170.050692	7.71	4.51	4.51	-3.50	0.00
chr8_17577_mature	163.3346296	2302.534721	8.68	6.79	6.79	-3.82	0.00
chr16_33817_mature	12.8948392	188.587023	7.34	3.18	3.18	-3.87	0.00
ssc-miR-29b	161.1854898	2412.140854	7.14	2.08	2.08	-3.90	0.00
chr7_16615_mature	19.3422588	290.133882	6.92	0.00	0.00	-3.91	0.00
chr4_10236_mature	330.9675390	4977.407924	7.50	3.88	3.88	-3.91	0.00
ssc-miR-708-3p	77.3690351	1212.114883	7.70	4.51	4.51	-3.97	0.00
chr7_17049_mature	279.3881823	4397.946088	1.65	7.60	7.60	-3.98	0.00
chr11_25149_mature	10.7456993	170.050692	7.07	1.77	1.77	-3.98	0.00
chr10_23842_mature	21.4913986	377.979974	7.14	2.33	2.33	-4.14	0.00
chr2_6533_mature	40.8336574	731.782124	7.05	1.77	1.77	-4.16	0.00
ssc-miR-127	649.0402388	11950.292212	2.90	7.68	7.68	-4.20	0.00
chr3_8450_mature	15.0439790	282.880535	2.41	7.62	7.62	-4.23	0.00
ssc-miR-708-5p	40.8336574	784.973335	1.65	7.55	7.55	-4.26	0.00
NW_003541124_39922_mature	21.4913986	415.052636	6.84	0.00	0.00	-4.27	0.00
ssc-miR-30d	992.9026170	19309.215747	7.47	3.95	3.95	-4.28	0.00
ssc-miR-196a	17.1931189	338.489529	8.53	6.60	6.60	-4.30	0.00
ssc-miR-30a-5p	844.6119664	16701.234523	9.19	7.76	7.76	-4.31	0.00
chrx_22497_mature	8.5965595	178.915894	7.05	2.08	2.08	-4.38	0.00
chr17_35590_mature	6.4474196	136.201739	6.95	1.39	1.39	-4.40	0.02
ssc-miR-615	19.3422588	456.154936	8.97	7.40	7.40	-4.56	0.00
bta-miR-2285t	6.4474196	153.932143	7.36	3.62	3.62	-4.58	0.00
chr1_3137_mature	8.5965595	205.511499	3.26	7.69	7.69	-4.58	0.00
chr15_31863_mature	27.9388182	685.844259	8.37	6.31	6.31	-4.62	0.00
chr6_13566_mature	15.0439790	375.562191	7.47	4.03	4.03	-4.64	0.00
chr10_24294_mature	12.8948392	332.848036	7.49	4.10	4.10	-4.69	0.00
chrx_22196_star	6.4474196	167.632909	8.11	5.76	5.76	-4.70	0.00
ssc-miR-99a	292.2830215	7699.024919	2.90	7.62	7.62	-4.72	0.00
chr2_4834_mature	17.1931189	452.931226	9.08	7.61	7.61	-4.72	0.00
chr10_23298_mature	8.5965595	231.301178	7.57	4.40	4.40	-4.75	0.00
chr4_8816_mature	8.5965595	239.360452	7.47	4.10	4.10	-4.80	0.00
NW_003541201_39964_mature	12.8948392	365.891062	6.84	0.85	0.85	-4.83	0.00
chr15_33031_mature	6.4474196	195.840370	7.65	4.65	4.65	-4.92	0.00
chr12_25530_mature	4.2982797	132.172102	6.90	1.39	1.39	-4.94	0.01
chr12_25999_mature	4.2982797	133.783957	1.65	7.47	7.47	-4.96	0.00
chr13_27664_mature	6.4474196	204.705572	8.31	6.22	6.22	-4.99	0.00
chr12_25526_mature	12.8948392	419.888201	8.62	6.81	6.81	-5.03	0.00
chr5_12267_mature	12.8948392	437.618605	8.50	6.60	6.60	-5.08	0.00
chr7_15769_mature	15.0439790	523.852842	6.17	8.45	8.45	-5.12	0.00
chr4_10240_mature	17.1931189	599.610022	2.41	7.52	7.52	-5.12	0.00
chr7_15797_star	6.4474196	241.778235	6.87	1.39	1.39	-5.23	0.00
ssc-miR-30e-5p	214.9139864	8322.006837	6.87	1.39	1.39	-5.28	0.00
chrx_21682_mature	12.8948392	510.958003	6.92	1.77	1.77	-5.31	0.00

chr4_9198_mature	6.4474196	257.896784	7.55	4.46	4.46	-5.32	0.00
chr9_19418_mature@@bta-miR-2452	10.7456993	436.812677	7.45	4.16	4.16	-5.35	0.00
chr3_8118_star	4.2982797	178.915894	2.41	7.49	7.49	-5.38	0.00
chr12_26093_mature	4.2982797	182.139603	6.90	1.77	1.77	-5.41	0.00
chr12_25628_mature	8.5965595	371.532554	7.49	4.35	4.35	-5.43	0.00
chr14_29715_mature	8.5965595	381.203683	6.76	0.85	0.85	-5.47	0.00
chr14_31443_mature	10.7456993	477.914977	7.38	4.03	4.03	-5.47	0.00
chr6_13041_mature	4.2982797	195.840370	1.65	7.39	7.39	-5.51	0.00
ssc-miR-31	12.8948392	601.221877	9.33	8.06	8.06	-5.54	0.00
ssc-miR-152	870.4016448	42144.364084	8.29	6.28	6.28	-5.60	0.00
ssc-miR-7	700.6195955	37072.662648	6.95	2.33	2.33	-5.73	0.00
chr6_14961_mature	2.1491399	122.500972	6.58	8.59	8.59	-5.83	0.01
hsa-miR-3202	2.1491399	123.306900	5.31	8.07	8.07	-5.84	0.01
ssc-miR-199a-5p	472.8107700	27248.407046	7.09	3.04	3.04	-5.85	0.00
chrx_22382_mature	2.1491399	124.112827	6.90	2.08	2.08	-5.85	0.01
chr9_19904_mature	4.2982797	251.449364	7.86	5.40	5.40	-5.87	0.00
chr15_32517_mature	2.1491399	125.724682	6.79	1.39	1.39	-5.87	0.00
chr6_13578_mature	4.2982797	252.255291	7.42	4.23	4.23	-5.87	0.00
chr17_35005_mature	2.1491399	127.336537	6.64	0.00	0.00	-5.89	0.00
chr5_12092_mature	2.1491399	130.560247	6.92	2.33	2.33	-5.92	0.00
chr16_34601_mature	19.3422588	1191.966697	7.22	3.62	3.62	-5.95	0.00
chr16_34772_mature	8.5965595	535.135826	6.70	0.85	0.85	-5.96	0.00
chrx_21673_mature	2.1491399	133.783957	6.33	8.45	8.45	-5.96	0.00
chr11_25089_mature	17.1931189	1072.689434	6.82	1.77	1.77	-5.96	0.00
hsa-miR-4685-3p	2.1491399	134.589884	9.40	8.19	8.19	-5.97	0.00
chr3_8310_star	2.1491399	134.589884	6.76	1.39	1.39	-5.97	0.00
chr14_31430_mature	6.4474196	406.993362	1.65	7.33	7.33	-5.98	0.00
chr8_19026_mature	2.1491399	137.813594	1.65	7.31	7.31	-6.00	0.00
chr14_31044_mature	2.1491399	137.813594	7.00	2.90	2.90	-6.00	0.00
chr4_9743_mature	8.5965595	552.060302	3.26	7.49	7.49	-6.00	0.00
chr15_33320_mature	4.2982797	277.239042	7.60	4.87	4.87	-6.01	0.00
chr6_14437_mature	2.1491399	138.619521	6.55	8.53	8.53	-6.01	0.00
ssc-miR-146a-5p	210.6157066	13622.591668	6.84	8.67	8.67	-6.02	0.00
chr13_27783_mature	2.1491399	139.425449	6.95	2.73	2.73	-6.02	0.00
chr16_34762_mature	4.2982797	283.686462	6.64	0.85	0.85	-6.04	0.00
chr12_25546_mature	2.1491399	142.649158	7.65	5.02	5.02	-6.05	0.00
ssc-miR-450a	4.2982797	294.969446	3.80	7.57	7.57	-6.10	0.00
chr6_14444_mature	2.1491399	147.484723	8.03	5.88	5.88	-6.10	0.00
chrx_22989_mature	2.1491399	149.096578	7.97	5.76	5.76	-6.12	0.00
chr5_12354_mature	2.1491399	157.155853	2.90	7.40	7.40	-6.19	0.00
chr1_2963_mature	6.4474196	476.303122	9.13	7.84	7.84	-6.21	0.00
chr4_9236_mature	4.2982797	319.147270	6.73	1.77	1.77	-6.21	0.00
chr16_34502_mature	2.1491399	159.573635	6.95	2.90	2.90	-6.21	0.00
chr6_13565_mature	4.2982797	325.594689	7.68	5.16	5.16	-6.24	0.00
ssc-miR-503	4.2982797	332.042109	6.82	2.33	2.33	-6.27	0.00
chrx_22108_mature	2.1491399	166.826982	1.65	7.23	7.23	-6.28	0.00
ssc-miR-450c-5p	8.5965595	694.709461	7.34	4.29	4.29	-6.34	0.00
chr11_25099_mature	2.1491399	176.498111	6.51	0.00	0.00	-6.36	0.00
bta-miR-1246	12.8948392	1074.301289	8.40	6.65	6.65	-6.38	0.00
chr11_25205_mature	4.2982797	362.667352	8.30	6.46	6.46	-6.40	0.00
ssc-miR-378	107.4569932	9122.292794	1.65	7.21	7.21	-6.41	0.00
chr10_23795_star	2.1491399	186.975168	12.10	12.46	12.46	-6.44	0.00
chr10_23799_mature	4.2982797	381.203683	9.01	9.97	9.97	-6.47	0.00
chr2_4295_mature	6.4474196	576.238126	7.16	3.80	3.80	-6.48	0.00
chr12_26455_mature	4.2982797	385.233321	7.85	5.60	5.60	-6.49	0.00
chr1_2264_mature	2.1491399	193.422588	6.73	2.08	2.08	-6.49	0.00
chr11_25258_mature	4.2982797	394.098522	7.62	5.09	5.09	-6.52	0.00
chr3_7573_mature	17.1931189	1579.617800	6.55	0.85	0.85	-6.52	0.00
chr5_11980_mature	2.1491399	197.452225	6.08	8.24	8.24	-6.52	0.00
chr6_14013_mature	2.1491399	199.064080	6.61	1.39	1.39	-6.53	0.00
chr14_30666_mature	2.1491399	199.870007	6.61	1.39	1.39	-6.54	0.00
chr1_3819_mature	10.7456993	1007.409311	10.02	9.14	9.14	-6.55	0.00
chr9_19375_mature	4.2982797	406.993362	6.76	2.33	2.33	-6.57	0.00
chr13_27316_mature	2.1491399	206.317427	6.84	2.73	2.73	-6.58	0.00
chr1_589_mature	2.1491399	207.929282	6.84	2.73	2.73	-6.60	0.00
chr5_11967_mature	4.2982797	429.559330	1.65	7.17	7.17	-6.64	0.00
chr6_13339_mature	6.4474196	656.024943	10.28	9.49	9.49	-6.67	0.00
NW_003612993_36688_mature	4.2982797	444.066024	6.44	0.00	0.00	-6.69	0.00
chr6_14477_mature@@bta-miR-483	8.5965595	888.937976	9.83	8.90	8.90	-6.69	0.00
chr12_25501_mature	2.1491399	230.495250	3.55	7.42	7.42	-6.74	0.00
chr13_29269_mature	4.2982797	461.796428	6.79	2.54	2.54	-6.75	0.00
chr1_2964_mature	4.2982797	463.408283	6.64	1.77	1.77	-6.75	0.00
ssc-miR-221-5p	27.9388182	3056.882814	4.19	7.55	7.55	-6.77	0.00
chr8_18343_mature	2.1491399	238.554525	1.65	7.13	7.13	-6.79	0.00
chr6_12650_mature	4.2982797	477.914977	6.41	0.00	0.00	-6.80	0.00
chrx_21884_mature	2.1491399	253.061219	2.90	7.28	7.28	-6.88	0.00
chr4_9789_mature	4.2982797	514.987640	1.65	7.13	7.13	-6.90	0.00
chr16_33714_mature	4.2982797	520.629132	1.65	7.12	7.12	-6.92	0.00
chr4_8854_mature	8.5965595	1067.047942	1.65	7.12	7.12	-6.96	0.00
chr1_1444_mature	2.1491399	271.597550	6.48	0.85	0.85	-6.98	0.00
chr15_32108_mature	4.2982797	545.612883	7.09	3.80	3.80	-6.99	0.00
chr14_30497_star	2.1491399	284.492389	6.55	1.39	1.39	-7.05	0.00
chr13_28708_mature	2.1491399	294.969446	6.97	3.42	3.42	-7.10	0.00
chrx_22720_mature	2.1491399	299.805011	8.00	6.01	6.01	-7.12	0.00
chr5_11958_mature	2.1491399	321.565052	7.90	5.82	5.82	-7.23	0.00
ssc-miR-452	8.5965595	1299.155048	7.20	4.16	4.16	-7.24	0.00
chr4_10395_mature	6.4474196	984.037415	6.37	0.00	0.00	-7.25	0.00
chr5_11474_mature	4.2982797	676.979057	7.26	4.35	4.35	-7.30	0.00
chr14_30207_mature	2.1491399	339.295456	7.70	5.40	5.40	-7.30	0.00
chr14_29589_mature	4.2982797	679.396839	6.84	8.56	8.56	-7.30	0.00
chr1_268_mature	4.2982797	684.232404	8.47	6.89	6.89	-7.31	0.00
chr2_5680_mature	4.2982797	701.156881	1.65	7.08	7.08	-7.35	0.00
ssc-miR-1	8.5965595	1441.804206	1.65	7.08	7.08	-7.39	0.00
chrx_22943_mature	2.1491399	362.667352	6.51	1.39	1.39	-7.40	0.00
chr13_27072_mature	4.2982797	728.558414	1.65	7.07	7.07	-7.41	0.00
ssc-miR-542-3p	38.6845175	6947.094609	7.70	9.04	9.04	-7.49	0.00

chr1_169_mature	2.1491399	386.845175	8.81	9.79	9.79	-7.49	0.00
chr1_2018_mature	2.1491399	406.187434	4.85	7.69	7.69	-7.56	0.00
chr11_25257_mature	2.1491399	416.664491	6.61	2.08	2.08	-7.60	0.00
chr3_8256_mature	2.1491399	417.470419	6.70	2.54	2.54	-7.60	0.00
chr6_14032_mature	8.5965595	1699.700990	7.60	5.25	5.25	-7.63	0.00
chr13_29267_mature	2.1491399	430.365258	6.73	2.73	2.73	-7.65	0.00
ssc-miR-143-5p	10.7456993	2311.399923	1.65	7.04	7.04	-7.75	0.00
chr15_31936_mature	4.2982797	938.905478	4.00	7.42	7.42	-7.77	0.00
ssc-miR-214	40.8336574	9044.923759	4.49	7.55	7.55	-7.79	0.00
chrx_23228_mature	4.2982797	982.425560	6.29	0.00	0.00	-7.84	0.00
chrx_22639_mature	4.2982797	1014.662658	7.43	4.91	4.91	-7.88	0.00
chr1_3820_mature	4.2982797	1015.468586	7.07	3.95	3.95	-7.88	0.00
chr13_27515_mature	2.1491399	560.119577	6.61	2.33	2.33	-8.03	0.00
chrx_22340_mature	2.1491399	610.087079	1.65	7.00	7.00	-8.15	0.00
chr15_33120_mature	2.1491399	686.650186	6.97	3.71	3.71	-8.32	0.00
chr1_541_mature	2.1491399	772.884423	6.55	2.08	2.08	-8.49	0.00
chr16_34293_mature	2.1491399	774.496278	7.40	4.87	4.87	-8.49	0.00
chr11_24582_mature	4.2982797	1636.032721	0.00	13.68	13.68	-8.57	0.00
ssc-miR-29a	21.4913986	8630.677050	8.53	7.05	7.05	-8.65	0.00
ssc-miR-370	6.4474196	2733.705907	2.41	7.07	7.07	-8.73	0.00
chrx_22076_mature	2.1491399	1105.732460	0.00	13.53	13.53	-9.01	0.00
ssc-miR-450b-5p	2.1491399	1233.874924	1.65	6.99	6.99	-9.17	0.00
chrx_22554_mature	2.1491399	1641.674213	6.25	0.00	0.00	-9.58	0.00
chr11_24857_mature	2.1491399	1671.493529	0.00	12.94	12.94	-9.60	0.00
chr6_14978_mature	4.2982797	3488.859926	6.48	1.77	1.77	-9.66	0.00
chr12_25534_mature	4.2982797	3712.907757	0.00	12.52	12.52	-9.75	0.00
chr5_11530_mature	2.1491399	1885.064303	6.33	0.85	0.85	-9.78	0.00
NW_003537574_37009_mature	8.5965595	8313.141635	0.00	11.93	11.93	-9.92	0.00
chr7_15145_mature	2.1491399	2137.319594	2.41	7.06	7.06	-9.96	0.00
chr14_31463_star	2.1491399	2371.844482	6.79	3.18	3.18	-10.11	0.00
chr18_36269_mature	2.1491399	3564.617106	1.65	6.97	6.97	-10.70	0.00
ssc-miR-143-3p	382.5468957	647476.470928	0.00	11.21	11.21	-10.72	0.00
chr16_34017_mature	2.1491399	3926.478531	0.00	11.20	11.20	-10.84	0.00
chr18_36117_mature	6.4474196	13979.617528	0.00	11.04	11.04	-11.08	0.00
chrx_21555_mature	2.1491399	5591.524640	0.00	10.96	10.96	-11.35	0.00
ssc-miR-145-5p	6.4474196	19900.766495	0.00	10.92	10.92	-11.59	0.00
chr4_10088_mature	2.1491399	9929.832097	0.00	10.91	10.91	-12.17	0.00
hsa-miR-3659	0.0000000	13122.916650	0.00	10.86	10.86	-20.00	0.00
ssc-miR-206	0.0000000	11796.360069	0.00	10.76	10.76	-20.00	0.00
NW_003541079_39891_mature	0.0000000	7881.164522	6.70	2.90	2.90	-20.00	0.00
chr1_3720_mature	0.0000000	5863.928118	0.00	10.53	10.53	-20.00	0.00
bta-miR-379	0.0000000	3912.777764	1.65	6.96	6.96	-20.00	0.01
chr1_1317_mature	0.0000000	2365.397062	0.00	10.11	10.11	-20.00	0.01
chr1_1318_mature	0.0000000	2350.890368	6.61	2.54	2.54	-20.00	0.01
chr2_4477_star	0.0000000	2107.500279	0.00	9.97	9.97	-20.00	0.01
chr13_28441_mature	0.0000000	1987.417089	0.00	9.92	9.92	-20.00	0.01
NW_003613137_37368_mature	0.0000000	1938.255515	0.00	9.90	9.90	-20.00	0.01
chr1_485_mature	0.0000000	1921.331038	0.00	9.89	9.89	-20.00	0.01
ssc-miR-455-5p	0.0000000	1852.021277	0.00	9.86	9.86	-20.00	0.01
chrx_22553_mature	0.0000000	1729.520305	0.00	9.85	9.85	-20.00	0.01
chr16_34386_mature	0.0000000	1477.265014	0.00	9.73	9.73	-20.00	0.01
chr6_12951_mature	0.0000000	1104.120605	1.65	6.95	6.95	-20.00	0.01
chr1_1155_mature	0.0000000	1000.155964	7.16	4.35	4.35	-20.00	0.01
chr1_3153_mature	0.0000000	967.112939	0.00	9.50	9.50	-20.00	0.01
ssc-miR-145-3p	0.0000000	955.829954	0.00	9.37	9.37	-20.00	0.01
chr1_2557_mature	0.0000000	949.382535	0.00	9.33	9.33	-20.00	0.01
chr7_16963_mature	0.0000000	930.040276	0.00	9.30	9.30	-20.00	0.01
chr1_2360_mature	0.0000000	922.786929	6.21	0.00	0.00	-20.00	0.01
chr11_24398_mature	0.0000000	849.447531	0.00	9.20	9.20	-20.00	0.01
chr6_13229_mature	0.0000000	724.528777	0.00	9.19	9.19	-20.00	0.01
chr4_9231_mature	0.0000000	660.860508	0.00	9.16	9.16	-20.00	0.01
chr2_6447_mature	0.0000000	642.324177	0.00	9.13	9.13	-20.00	0.01
chr17_34881_mature	0.0000000	630.235265	0.00	9.11	9.11	-20.00	0.01
ssc-miR-362	0.0000000	588.327038	0.00	9.05	9.05	-20.00	0.01
chr12_26311_mature	0.0000000	584.297400	0.00	8.97	8.97	-20.00	0.01
NW_003539626_38995_mature	0.0000000	571.402561	0.00	8.89	8.89	-20.00	0.01
chr2_4930_mature	0.0000000	559.313650	0.00	8.63	8.63	-20.00	0.01
chr14_30497_mature	0.0000000	550.448448	0.00	8.61	8.61	-20.00	0.01
chr1_3371_mature	0.0000000	529.494334	0.00	8.61	8.61	-20.00	0.02
chr15_33221_mature	0.0000000	502.092801	0.00	8.59	8.59	-20.00	0.02
hsa-miR-516a-5p	0.0000000	473.885340	2.90	7.10	7.10	-20.00	0.02
chr4_10244_mature	0.0000000	394.098522	0.00	8.53	8.53	-20.00	0.02
chr14_30282_mature	0.0000000	390.874813	0.00	8.52	8.52	-20.00	0.02
chr12_26457_mature	0.0000000	390.874813	0.00	8.49	8.49	-20.00	0.02
NW_003613041_36883_mature	0.0000000	384.427393	0.00	8.44	8.44	-20.00	0.02
chr12_26317_mature	0.0000000	369.920699	0.00	8.44	8.44	-20.00	0.02
chrx_22120_mature	0.0000000	365.891062	0.00	8.43	8.43	-20.00	0.02
chr15_32593_mature	0.0000000	359.443642	6.76	3.18	3.18	-20.00	0.02
NW_003537694_37136_mature	0.0000000	346.548803	8.95	7.75	7.75	-20.00	0.02
chr10_24151_mature	0.0000000	346.548803	6.37	1.39	1.39	-20.00	0.02
chr14_30223_mature	0.0000000	344.131021	0.00	8.22	8.22	-20.00	0.02
chr14_31314_mature	0.0000000	297.387229	0.00	8.21	8.21	-20.00	0.02
chr2_4744_mature	0.0000000	294.163519	0.00	8.19	8.19	-20.00	0.02
chrx_22488_mature	0.0000000	290.939809	0.00	8.09	8.09	-20.00	0.02
chr2_6092_mature	0.0000000	270.791623	0.00	8.06	8.06	-20.00	0.02
NW_003613242_37864_mature	0.0000000	266.761986	0.00	8.06	8.06	-20.00	0.02
chr3_8078_mature	0.0000000	265.956058	0.00	8.06	8.06	-20.00	0.02
chr6_13576_mature	0.0000000	265.956058	0.00	8.04	8.04	-20.00	0.02
chr3_7072_mature	0.0000000	261.926421	0.00	8.03	8.03	-20.00	0.02
ssc-miR-432-5p	0.0000000	260.314566	0.00	8.01	8.01	-20.00	0.02
chr13_27312_mature	0.0000000	257.090856	0.00	8.01	8.01	-20.00	0.02
NW_003613242_37866_mature	0.0000000	257.090856	0.00	8.00	8.00	-20.00	0.02
chr10_23953_mature	0.0000000	255.479001	0.00	7.94	7.94	-20.00	0.02
chr6_13411_mature	0.0000000	245.001944	0.00	7.92	7.92	-20.00	0.02
chr8_18345_mature	0.0000000	241.778235	0.00	7.87	7.87	-20.00	0.02
rno-miR-143-3p	0.0000000	233.718960	0.00	7.83	7.83	-20.00	0.02

chr13_27663_mature	0.0000000	226.465613	0.00	7.72	7.72	-20.00	0.02
chr1_211_mature	0.0000000	209.541137	0.00	7.69	7.69	-20.00	0.02
NW_003538465_37902_mature	0.0000000	205.511499	0.00	7.63	7.63	-20.00	0.02
NW_003537589_37025_mature	0.0000000	196.646298	0.00	7.62	7.62	-20.00	0.02
chr4_9116_mature	0.0000000	195.840370	0.00	7.60	7.60	-20.00	0.02
chrx_21570_mature	0.0000000	193.422588	0.00	7.49	7.49	-20.00	0.02
chr17_35122_mature	0.0000000	178.915894	0.00	7.48	7.48	-20.00	0.02
chr9_20036_mature	0.0000000	178.109966	0.00	7.42	7.42	-20.00	0.02
chr9_20772_mature	0.0000000	170.050692	0.00	7.42	7.42	-20.00	0.03
chr9_20753_mature	0.0000000	170.050692	0.00	7.38	7.38	-20.00	0.03
chr5_11533_mature	0.0000000	165.215127	0.00	7.37	7.37	-20.00	0.03
chr13_29304_mature	0.0000000	164.409200	0.00	7.35	7.35	-20.00	0.03
ssc-miR-382	0.0000000	161.991417	0.00	7.33	7.33	-20.00	0.03
chrx_21569_mature	0.0000000	159.573635	0.00	7.31	7.31	-20.00	0.03
chr9_19756_mature	0.0000000	157.961780	0.00	7.31	7.31	-20.00	0.03
ssc-miR-10a-3p	0.0000000	157.155853	0.00	7.20	7.20	-20.00	0.03
chr9_20754_mature	0.0000000	145.872868	0.00	7.13	7.13	-20.00	0.03
chr1_3435_mature	0.0000000	138.619521	0.00	7.11	7.11	-20.00	0.03
chr15_32293_mature	0.0000000	137.007666	0.00	7.09	7.09	-20.00	0.03
chr14_31042_mature	0.0000000	135.395811	0.00	7.07	7.07	-20.00	0.03
chr14_30531_mature	0.0000000	133.783957	0.00	7.07	7.07	-20.00	0.03
chr14_29507_mature	0.0000000	132.978029	0.00	7.04	7.04	-20.00	0.03
chr1_412_mature	0.0000000	130.560247	0.00	7.01	7.01	-20.00	0.03
chr9_19755_mature	0.0000000	128.142464	0.00	6.98	6.98	-20.00	0.03
chr13_27508_mature	0.0000000	124.918755	0.00	6.96	6.96	-20.00	0.03
chr17_35522_mature	0.0000000	123.306900	0.00	6.96	6.96	-20.00	0.03
chr6_13600_star	0.0000000	123.306900	0.00	6.82	6.82	-20.00	0.03
chr13_28070_mature	0.0000000	112.023915	0.00	6.81	6.81	-20.00	0.03
chr1_1734_mature	0.0000000	111.217988	0.00	6.78	6.78	-20.00	0.03
NW_003537990_37435_mature	0.0000000	108.800206	0.00	6.78	6.78	-20.00	0.03
chr4_9930_mature	0.0000000	108.800206	0.00	6.77	6.77	-20.00	0.03
mmu-miR-5124b	0.0000000	107.994278	0.00	6.77	6.77	-20.00	0.03
chr16_34510_star	0.0000000	107.994278	0.00	6.72	6.72	-20.00	0.03
hsa-miR-184	0.0000000	104.770568	0.00	6.72	6.72	-20.00	0.03
mmu-miR-5108	0.0000000	104.770568	0.00	6.71	6.71	-20.00	0.03
NW_003613107_37244_mature	0.0000000	103.964641	0.00	6.70	6.70	-20.00	0.03
chr18_36061_mature	0.0000000	103.158713	0.00	6.68	6.68	-20.00	0.03
NW_003613497_39428_mature	0.0000000	101.546859	0.00	6.67	6.67	-20.00	0.03
bta-miR-483	0.0000000	100.740931	0.00	6.66	6.66	-20.00	0.03
chr4_9932_mature	0.0000000	99.935004	0.00	6.60	6.60	-20.00	0.03
NW_003538101_37534_mature	0.0000000	95.905366	0.00	6.56	6.56	-20.00	0.03
chr4_10006_mature	0.0000000	93.487584	0.00	6.54	6.54	-20.00	0.03
NW_003613447_39176_mature	0.0000000	91.875729	0.00	6.54	6.54	-20.00	0.03
chr6_13666_mature	0.0000000	91.875729	0.00	6.54	6.54	-20.00	0.03
chr2_5590_mature	0.0000000	91.875729	0.00	6.54	6.54	-20.00	0.03
chr12_25625_star	0.0000000	91.875729	0.00	6.52	6.52	-20.00	0.03
chr10_24012_mature	0.0000000	91.069802	0.00	6.52	6.52	-20.00	0.03
chr3_8428_mature	0.0000000	91.069802	0.00	6.52	6.52	-20.00	0.03
chrx_22188_mature@@bta-miR-1298	0.0000000	91.069802	0.00	6.51	6.51	-20.00	0.03
chr7_16961_mature	0.0000000	90.263874	0.00	6.51	6.51	-20.00	0.03
chr2_5583_mature	0.0000000	90.263874	0.00	6.50	6.50	-20.00	0.03
chr9_20694_mature	0.0000000	89.457947	0.00	6.50	6.50	-20.00	0.03
chr12_25534_star	0.0000000	89.457947	0.00	6.50	6.50	-20.00	0.03
chr8_17373_mature	0.0000000	89.457947	0.00	6.45	6.45	-20.00	0.03
NW_003613056_36922_mature	0.0000000	86.234237	0.00	6.41	6.41	-20.00	0.03
chr5_11528_mature	0.0000000	83.816455	0.00	6.41	6.41	-20.00	0.04
chr1_478_mature	0.0000000	83.816455	0.00	6.38	6.38	-20.00	0.04
chr7_16556_mature	0.0000000	82.204600	0.00	6.32	6.32	-20.00	0.04
chr7_16555_mature	0.0000000	78.980890	0.00	6.32	6.32	-20.00	0.04
chrx_22416_mature	0.0000000	78.980890	0.00	6.31	6.31	-20.00	0.04
chr12_25822_mature	0.0000000	78.174963	0.00	6.31	6.31	-20.00	0.04
chr1_3708_mature	0.0000000	78.174963	0.00	6.29	6.29	-20.00	0.04
chr4_9333_star	0.0000000	77.369035	0.00	6.29	6.29	-20.00	0.04
chr14_31500_mature	0.0000000	77.369035	0.00	6.29	6.29	-20.00	0.04
chr5_12108_mature	0.0000000	77.369035	0.00	6.28	6.28	-20.00	0.04
chr9_20604_mature	0.0000000	76.563108	0.00	6.25	6.25	-20.00	0.04
ssc-miR-542-5p	0.0000000	74.951253	0.00	6.25	6.25	-20.00	0.04
chr5_12375_mature	0.0000000	74.951253	0.00	6.23	6.23	-20.00	0.04
chr13_28489_mature	0.0000000	74.145325	0.00	6.17	6.17	-20.00	0.04
chr7_15776_mature	0.0000000	70.921616	0.00	6.15	6.15	-20.00	0.04
chr14_31044_star	0.0000000	70.115688	0.00	6.14	6.14	-20.00	0.04
chr18_36239_mature	0.0000000	69.309761	0.00	6.12	6.12	-20.00	0.04
chr14_31042_star	0.0000000	68.503833	0.00	6.09	6.09	-20.00	0.04
chr1_3507_mature	0.0000000	66.891978	0.00	6.05	6.05	-20.00	0.04
chr13_27097_mature	0.0000000	65.280123	0.00	6.03	6.03	-20.00	0.04
chrx_22902_star	0.0000000	64.474196	0.00	5.98	5.98	-20.00	0.04
chr7_16781_star	0.0000000	62.056414	0.00	5.98	5.98	-20.00	0.04
chr9_20857_mature	0.0000000	62.056414	0.00	5.98	5.98	-20.00	0.04
chr13_28716_star	0.0000000	62.056414	0.00	5.98	5.98	-20.00	0.04
NW_003540828_39718_mature	0.0000000	62.056414	0.00	5.94	5.94	-20.00	0.04
chrx_22499_mature	0.0000000	60.444559	0.00	5.94	5.94	-20.00	0.04
chr1_3508_mature	0.0000000	60.444559	0.00	5.92	5.92	-20.00	0.04
chr6_15013_mature	0.0000000	59.638631	0.00	5.92	5.92	-20.00	0.04
hsa-miR-4474-5p	0.0000000	59.638631	0.00	5.90	5.90	-20.00	0.04
NW_003539103_38526_mature	0.0000000	58.832704	0.00	5.90	5.90	-20.00	0.04
chr6_15014_mature	0.0000000	58.832704	0.00	5.90	5.90	-20.00	0.04
chr4_9258_mature	0.0000000	58.832704	0.00	5.88	5.88	-20.00	0.04
chrx_22845_star	0.0000000	58.026776	0.00	5.88	5.88	-20.00	0.04
chr3_8352_mature	0.0000000	58.026776	0.00	5.86	5.86	-20.00	0.04
chr2_4023_mature	0.0000000	57.220849	0.00	5.86	5.86	-20.00	0.04
chr10_23626_mature	0.0000000	57.220849	0.00	5.86	5.86	-20.00	0.04
chr13_26768_star	0.0000000	57.220849	0.00	5.84	5.84	-20.00	0.04
chr1_1283_mature	0.0000000	56.414921	0.00	5.80	5.80	-20.00	0.04
hsa-miR-1298	0.0000000	54.803067	0.00	5.76	5.76	-20.00	0.04
chr6_13320_mature	0.0000000	53.191212	0.00	5.76	5.76	-20.00	0.04
chr11_24466_mature	0.0000000	53.191212	0.00	5.74	5.74	-20.00	0.04

chr1_2110_mature	0.0000000	52.385284	0.00	5.74	5.74	-20.00	0.04
chr1_1290_mature	0.0000000	52.385284	0.00	5.74	5.74	-20.00	0.04
hsa-miR-5707	0.0000000	52.385284	0.00	5.67	5.67	-20.00	0.04
chrx_22864_mature	0.0000000	49.967502	0.00	5.67	5.67	-20.00	0.04
chrx_21663_mature	0.0000000	49.967502	0.00	5.65	5.65	-20.00	0.04
chrx_23205_mature	0.0000000	49.161574	0.00	5.63	5.63	-20.00	0.04
chr2_5388_mature	0.0000000	48.355647	0.00	5.60	5.60	-20.00	0.04
chr2_5761_mature	0.0000000	47.549719	0.00	5.60	5.60	-20.00	0.05
NW_003613103_37208_mature	0.0000000	47.549719	0.00	5.60	5.60	-20.00	0.05
NW_003539097_38524_star	0.0000000	47.549719	0.00	5.58	5.58	-20.00	0.05
chr11_25193_mature	0.0000000	46.743792	0.00	5.55	5.55	-20.00	0.05
ssc-miR-133a-5p	0.0000000	45.937865	0.00	5.55	5.55	-20.00	0.05
chr13_28023_star	0.0000000	45.937865	0.00	5.53	5.53	-20.00	0.05
chr13_28509_mature	0.0000000	45.131937	0.00	5.53	5.53	-20.00	0.05
chr11_25311_mature	0.0000000	45.131937	0.00	5.50	5.50	-20.00	0.05
chr9_20549_star	0.0000000	44.326010	0.00	5.48	5.48	-20.00	0.05
chr16_34336_mature	0.0000000	43.520082	0.00	5.48	5.48	-20.00	0.05
chr7_15130_mature	0.0000000	43.520082	0.00	5.45	5.45	-20.00	0.05
chr14_31463_mature	0.0000000	42.714155	0.00	5.45	5.45	-20.00	0.05
chr15_31872_mature	0.0000000	42.714155	0.00	5.45	5.45	-20.00	0.05
chr15_31871_mature	0.0000000	42.714155	0.00	5.45	5.45	-20.00	0.05
chr18_35994_mature	0.0000000	42.714155	0.00	5.45	5.45	-20.00	0.05
chr13_28245_mature	0.0000000	42.714155	0.00	5.42	5.42	-20.00	0.05
mmu-miR-695	0.0000000	41.908227	0.00	5.42	5.42	-20.00	0.05
NW_003613164_37468_mature	0.0000000	41.908227	6.17	0.00	0.00	-20.00	0.05
chr5_11434_star	0.0000000	41.102300	0.00	5.40	5.40	-20.00	0.05
chr15_33182_mature	0.0000000	40.296372	0.00	5.37	5.37	-20.00	0.05
chr14_29516_mature	0.0000000	40.296372	0.00	5.37	5.37	-20.00	0.05
chr16_33835_mature	0.0000000	37.878590	0.00	5.28	5.28	-20.00	0.05
chr16_34498_star	0.0000000	37.878590	0.00	5.28	5.28	-20.00	0.05
mmu-miR-6406	0.0000000	37.878590	0.00	5.28	5.28	-20.00	0.05
hsa-miR-526a	0.0000000	37.878590	0.00	5.28	5.28	-20.00	0.05

piPS-LF Vs PEFs

AccID	piPS-LF	PEF	piPS-LF_log2(count+1)	PEF_log2(count+1)	PEF_log2(count+1)<0.05	Log2FC	FDR
NW_003541079_39891_mature	4.83556469	7881.16452248	2.54	12.94	12.94	-10.67	0.00
NW_003537574_37009_mature	5.37284966	8313.14163507	2.67	13.02	13.02	-10.60	0.00
NW_003538989_38416_mature	17292.51662787	0.80592745	14.08	0.85	0.85	14.39	0.00
NW_003613137_37368_mature	1.61185490	1938.25551453	1.39	10.92	10.92	-10.23	0.00
chr8_17989_mature@@bta-miR-302b	17222.66958230	1.61185490	14.07	1.39	1.39	13.38	0.00
chr8_17983_mature@@bta-miR-302b	16849.25653099	1.61185490	14.04	1.39	1.39	13.35	0.00
chr18_36117_mature	13.43212415	13979.61752806	3.85	13.77	13.77	-10.02	0.00
NW_003538989_38422_mature	16620.37313551	2.41778235	14.02	1.77	1.77	12.75	0.00
bta-miR-302c	19119.28551197	3.22370980	14.22	2.08	2.08	12.53	0.00
chr12_25534_mature	5.37284966	3712.90775693	2.67	11.86	11.86	-9.43	0.00
chr6_14978_mature	5.91013463	3488.85992615	2.79	11.77	11.77	-9.21	0.00
chr1_485_mature	4.29827973	1921.33103810	2.41	10.91	10.91	-8.80	0.00
chr1_3153_mature	2.14913986	967.11293864	1.65	9.92	9.92	-8.81	0.00
chrx_22554_mature	4.29827973	1641.67421334	2.41	10.68	10.68	-8.58	0.00
chr1_1155_mature	2.68642483	1000.15596404	1.88	9.97	9.97	-8.54	0.00
chr15_32593_mature	0.53728497	359.44364219	0.62	8.49	8.49	-9.39	0.00
chr1_211_mature	0.00000000	209.54113671	0.00	7.72	7.72	-20.00	0.00
chr12_26311_mature	1.61185490	584.29740043	1.39	9.19	9.19	-8.50	0.00
chr2_4295_mature	1.61185490	576.23812594	1.39	9.17	9.17	-8.48	0.00
chr5_12267_mature	1.07456993	437.61860474	1.05	8.78	8.78	-8.67	0.00
chr13_28441_mature	6.44741959	1987.41708891	2.90	10.96	10.96	-8.27	0.00
chr13_28708_mature	0.53728497	294.96944629	0.62	8.21	8.21	-9.10	0.00
chr4_9231_mature	2.14913986	660.86050807	1.65	9.37	9.37	-8.26	0.00
chr5_11530_mature	6.44741959	1885.06430290	2.90	10.88	10.88	-8.19	0.00
chr6_14477_mature@@bta-miR-483	3.22370980	888.93797610	2.08	9.80	9.80	-8.11	0.00
chr7_15145_mature	7.52198952	2137.31959440	3.09	11.06	11.06	-8.15	0.00
chr3_8078_mature	0.53728497	265.95605813	0.62	8.06	8.06	-8.95	0.00
chrx_22120_mature	1.07456993	365.89106179	1.05	8.52	8.52	-8.41	0.00
chr2_5680_mature	2.68642483	701.15688051	1.88	9.46	9.46	-8.03	0.00
chr16_34017_mature	13.96940911	3926.47853088	3.90	11.94	11.94	-8.13	0.00
bta-miR-154c	3.22370980	759.18365683	2.08	9.57	9.57	-7.88	0.00
chr1_3720_mature	19.87954374	5863.92811796	4.38	12.52	12.52	-8.20	0.00
chr4_10395_mature	4.29827973	984.03741507	2.41	9.94	9.94	-7.84	0.00
chrx_22553_mature	7.52198952	1729.52030527	3.09	10.76	10.76	-7.85	0.00
chr2_4477_star	9.13384442	2107.50027879	3.34	11.04	11.04	-7.85	0.00
hsa-miR-4685-3p	0.00000000	134.58988396	0.00	7.08	7.08	-20.00	0.00
hsa-miR-516a-5p	2.14913986	473.88533993	1.65	8.89	8.89	-7.78	0.00
chr6_12951_mature	5.37284966	1104.12060495	2.67	10.11	10.11	-7.68	0.00
NW_003541201_39964_mature	1.61185490	365.89106179	1.39	8.52	8.52	-7.83	0.00
chr9_19755_mature	0.00000000	128.14246437	0.00	7.01	7.01	-20.00	0.00
chr4_10088_mature	33.84895285	9929.83209750	5.12	13.28	13.28	-8.20	0.00
chr15_31936_mature	4.83556469	938.90547793	2.54	9.88	9.88	-7.60	0.00
chr6_13229_mature	3.76099476	724.52877653	2.25	9.50	9.50	-7.59	0.00
chr13_29267_mature	2.14913986	430.36525770	1.65	8.75	8.75	-7.65	0.00
chrx_22340_mature	3.22370980	610.08707879	2.08	9.26	9.26	-7.56	0.00
chr3_7072_mature	1.07456993	261.92642088	1.05	8.04	8.04	-7.93	0.00
chrx_22076_mature	5.91013463	1105.73245985	2.79	10.11	10.11	-7.55	0.00
NW_003613242_37866_mature	1.07456993	257.09085619	1.05	8.01	8.01	-7.90	0.00
chr11_24398_mature	4.83556469	849.44753111	2.54	9.73	9.73	-7.46	0.00
chr14_30497_mature	3.22370980	550.44844758	2.08	9.11	9.11	-7.42	0.00
chr18_36269_mature	17.19311891	3564.61710634	4.19	11.80	11.80	-7.70	0.00
chr16_34386_mature	8.59655945	1477.26501377	3.26	10.53	10.53	-7.42	0.00
chr1_3824_star	0.00000000	103.96464090	0.00	6.71	6.71	-20.00	0.00
chr18_36061_mature	0.00000000	103.15871346	0.00	6.70	6.70	-20.00	0.00
chr15_33120_mature	4.29827973	686.65018644	2.41	9.43	9.43	-7.32	0.00
chr1_541_mature	4.83556469	772.88442346	2.54	9.60	9.60	-7.32	0.00
chr9_19756_mature	0.53728497	157.96177998	0.62	7.31	7.31	-8.20	0.00
chr14_30223_mature	2.14913986	344.13102067	1.65	8.43	8.43	-7.32	0.00

chr13_27515_mature	3.76099476	560.11957696	2.25	9.13	9.13	-7.22	0.00
chr9_20754_mature	0.53728497	145.87286825	0.62	7.20	7.20	-8.08	0.00
chr1_268_mature	4.83556469	684.23240409	2.54	9.42	9.42	-7.14	0.00
bta-miR-2429	0.00000000	90.26387427	0.00	6.51	6.51	-20.00	0.00
mmu-miR-5099	332.04210893	0.00000000	8.38	0.00	0.00	20.00	0.00
chr6_13578_mature	1.61185490	252.25529150	1.39	7.98	7.98	-7.29	0.00
chr4_8854_mature	7.52198952	1067.04794230	3.09	10.06	10.06	-7.15	0.00
chr10_24151_mature	2.68642483	346.54880301	1.88	8.44	8.44	-7.01	0.00
hsa-miR-3659	59.10134625	13122.91664991	5.91	13.68	13.68	-7.79	0.00
chr6_13600_star	0.53728497	123.30689968	0.62	6.96	6.96	-7.84	0.00
chrx_22639_mature	8.05927449	1014.66265812	3.18	9.99	9.99	-6.98	0.00
ssc-miR-708-5p	6.44741959	784.97333520	2.90	9.62	9.62	-6.93	0.00
NW_003613242_37864_mature	2.14913986	266.76198558	1.65	8.06	8.06	-6.96	0.00
ssc-miR-432-5p	2.14913986	260.31456598	1.65	8.03	8.03	-6.92	0.00
chr16_34502_mature	1.07456993	159.57363488	1.05	7.33	7.33	-7.21	0.00
chr14_31492_mature	0.53728497	113.63577029	0.62	6.84	6.84	-7.72	0.00
chrx_22943_mature	3.22370980	362.66735199	2.08	8.51	8.51	-6.81	0.00
chr14_31463_star	17.19311891	2371.84448202	4.19	11.21	11.21	-7.11	0.00
chr3_8256_mature	3.76099476	417.47041851	2.25	8.71	8.71	-6.79	0.00
chr11_25257_mature	3.76099476	416.66449106	2.25	8.71	8.71	-6.79	0.00
chr5_11980_mature	1.61185490	197.45222497	1.39	7.63	7.63	-6.94	0.00
chr12_26422_mature	0.00000000	69.30976060	0.00	6.14	6.14	-20.00	0.00
chr1_2018_mature	3.76099476	406.18743423	2.25	8.67	8.67	-6.75	0.00
chr2_4744_mature	2.68642483	294.16351884	1.88	8.21	8.21	-6.77	0.00
NW_003537694_37136_mature	3.22370980	346.54880301	2.08	8.44	8.44	-6.75	0.00
chrx_21570_mature	1.61185490	193.42258773	1.39	7.60	7.60	-6.91	0.00
chr6_12932_star@@bta-miR-292	385.77060552	0.80592745	8.60	0.85	0.85	8.90	0.00
chr8_18343_mature	2.14913986	238.55452486	1.65	7.90	7.90	-6.79	0.00
chr17_34881_mature	5.91013463	630.23526501	2.79	9.30	9.30	-6.74	0.00
hsa-miR-184	0.53728497	104.77056835	0.62	6.72	6.72	-7.61	0.00
rno-miR-300-3p	214.91398636	0.00000000	7.75	0.00	0.00	20.00	0.00
ssc-miR-371-5p	7189.41012887	16.92447643	12.81	4.16	4.16	8.73	0.00
chr5_11967_mature	4.29827973	429.55933025	2.41	8.75	8.75	-6.64	0.00
bta-miR-302a	199.33272235	0.00000000	7.65	0.00	0.00	20.00	0.00
chr11_25099_mature	1.61185490	176.49811130	1.39	7.47	7.47	-6.77	0.00
chr16_33714_mature	5.37284966	520.62913197	2.67	9.03	9.03	-6.60	0.00
chr1_2964_mature	4.83556469	463.40828310	2.54	8.86	8.86	-6.58	0.00
NW_003538989_38414_mature	188.58702304	0.00000000	7.57	0.00	0.00	20.00	0.00
chr9_20753_mature	1.61185490	170.05069171	1.39	7.42	7.42	-6.72	0.01
chr4_10244_mature	4.29827973	394.09852250	2.41	8.63	8.63	-6.52	0.01
chr10_24012_mature	0.53728497	91.06980172	0.62	6.52	6.52	-7.41	0.01
chr14_30282_mature	4.29827973	390.87481270	2.41	8.61	8.61	-6.51	0.01
NW_003538465_37902_mature	2.14913986	205.51149946	1.65	7.69	7.69	-6.58	0.01
chr7_15797_star	2.68642483	241.77823466	1.88	7.92	7.92	-6.49	0.01
NW_003538989_38416_star	169.24476426	0.00000000	7.41	0.00	0.00	20.00	0.01
chrx_21569_mature	1.61185490	159.57363488	1.39	7.33	7.33	-6.63	0.01
bta-miR-2899	0.00000000	55.60899397	0.00	5.82	5.82	-20.00	0.01
chr6_13041_mature	2.14913986	195.84037007	1.65	7.62	7.62	-6.51	0.01
NW_003613056_36922_mature	0.53728497	86.23423703	0.62	6.45	6.45	-7.33	0.01
hsa-miR-1298	0.00000000	54.80306652	0.00	5.80	5.80	-20.00	0.01
chr5_11958_mature	3.76099476	321.56505210	2.25	8.33	8.33	-6.42	0.01
chr13_29269_mature	5.37284966	461.79642820	2.67	8.85	8.85	-6.43	0.01
chr11_24582_mature	16.11854898	1636.03272120	4.10	10.68	10.68	-6.67	0.01
chr11_25258_mature	4.83556469	394.09852250	2.54	8.63	8.63	-6.35	0.01
ssc-miR-206	75.75718019	11796.36006908	6.26	13.53	13.53	-7.28	0.01
NW_003613103_37207_mature	0.00000000	50.77342928	0.00	5.69	5.69	-20.00	0.01
chr18_36523_mature	0.53728497	78.98088999	0.62	6.32	6.32	-7.20	0.01
chr14_31314_mature	3.76099476	297.38722863	2.25	8.22	8.22	-6.31	0.01
chr16_33806_mature@@bta-miR-302b	239.62909480	0.80592745	7.91	0.85	0.85	8.22	0.01
chr15_32293_mature	1.61185490	137.00766631	1.39	7.11	7.11	-6.41	0.01
ssc-miR-455-3p	6.44741959	497.25723595	2.90	8.96	8.96	-6.27	0.01
mmu-miR-6370	0.00000000	47.54971948	0.00	5.60	5.60	-20.00	0.01
rno-miR-434-5p	133.24667155	0.00000000	7.07	0.00	0.00	20.00	0.01
chr14_31042_mature	1.61185490	135.39581141	1.39	7.09	7.09	-6.39	0.01
chr13_27072_mature	9.13384442	728.55841378	3.34	9.51	9.51	-6.32	0.01
chr14_30531_mature	1.61185490	133.78395651	1.39	7.07	7.07	-6.38	0.01
chr1_1318_mature	23.64053850	2350.89036835	4.62	11.20	11.20	-6.64	0.01
chr7_16963_mature	11.28298428	930.04027599	3.62	9.86	9.86	-6.37	0.01
ssc-miR-2366	1.07456993	101.54685856	1.05	6.68	6.68	-6.56	0.01
chr13_29304_mature	2.14913986	164.40919957	1.65	7.37	7.37	-6.26	0.01
chrx_23196_mature	127.87382189	0.00000000	7.01	0.00	0.00	20.00	0.01
bta-miR-483	1.07456993	100.74093111	1.05	6.67	6.67	-6.55	0.01
chr1_169_mature	5.37284966	386.84517546	2.67	8.60	8.60	-6.17	0.01
chr6_13576_mature	3.76099476	265.95605813	2.25	8.06	8.06	-6.14	0.01
chr2_4586_mature	320.75912465	1.61185490	8.33	1.39	1.39	7.64	0.01
chr1_1317_mature	24.71510843	2365.39706243	4.68	11.21	11.21	-6.58	0.01
chr15_31863_mature	9.13384442	685.84425899	3.34	9.42	9.42	-6.23	0.01
rno-miR-541-5p	312.16256519	1.61185490	8.29	1.39	1.39	7.60	0.01
chr1_282_mature	206.31742691	0.80592745	7.70	0.85	0.85	8.00	0.01
chr4_9198_mature	3.76099476	257.89678364	2.25	8.02	8.02	-6.10	0.01
chr5_11474_mature	9.13384442	676.97905705	3.34	9.41	9.41	-6.21	0.01
chr3_7573_mature	18.26768884	1579.61779978	4.27	10.63	10.63	-6.43	0.01
chr9_19375_mature	5.91013463	406.99336168	2.79	8.67	8.67	-6.11	0.01
chrx_21555_mature	48.89293190	5591.52464024	5.64	12.45	12.45	-6.84	0.01
chr17_34903_mature	0.53728497	66.89197826	0.62	6.09	6.09	-6.96	0.01
hsa-miR-302a-5p	294.43216132	1.61185490	8.21	1.39	1.39	7.51	0.01
chr6_14032_mature	19.87954374	1699.70098966	4.38	10.73	10.73	-6.42	0.01
chr1_2360_mature	12.35755422	922.78692895	3.74	9.85	9.85	-6.22	0.01
chrx_22902_star	0.53728497	64.47419591	0.62	6.03	6.03	-6.91	0.01
chr9_20880_mature	1.61185490	116.85948009	1.39	6.88	6.88	-6.18	0.01
chr9_20403_mature	277.77632738	1.61185490	8.12	1.39	1.39	7.43	0.01
NW_003613041_36883_mature	5.91013463	384.42739311	2.79	8.59	8.59	-6.02	0.01
chr6_12933_mature	104.23328339	0.00000000	6.72	0.00	0.00	20.00	0.01
chr14_30207_mature	5.37284966	339.29545597	2.67	8.41	8.41	-5.98	0.01
chr6_13566_mature	5.91013463	375.56219117	2.79	8.56	8.56	-5.99	0.01
chr14_29460_mature	1.61185490	111.21798794	1.39	6.81	6.81	-6.11	0.01

chr8_18274_mature	1.07456993	84.62238213	1.05	6.42	6.42	-6.30	0.01
chr14_31044_mature	2.14913986	137.81359376	1.65	7.12	7.12	-6.00	0.01
chr5_11533_mature	2.68642483	165.21512702	1.88	7.38	7.38	-5.94	0.01
chr6_15013_mature	0.53728497	59.63863122	0.62	5.92	5.92	-6.79	0.01
hsa-miR-4474-5p	0.53728497	59.63863122	0.62	5.92	5.92	-6.79	0.01
chr4_9743_mature	8.59655945	552.06030247	3.26	9.11	9.11	-6.00	0.01
chr9_19709_star	95.63672393	0.00000000	6.59	0.00	0.00	20.00	0.01
chr6_15014_mature	0.53728497	58.83270377	0.62	5.90	5.90	-6.77	0.01
chrx_23228_mature	13.96940911	982.42556017	3.90	9.94	9.94	-6.14	0.01
chr4_8920_mature	243.39008956	1.61185490	7.93	1.39	1.39	7.24	0.01
ssc-miR-370	31.69981299	2733.70590656	5.03	11.42	11.42	-6.43	0.01
chr10_23795_star	3.22370980	186.97516814	2.08	7.55	7.55	-5.86	0.01
chr2_4023_mature	0.53728497	57.22084887	0.62	5.86	5.86	-6.73	0.01
chr8_18995_mature	0.00000000	36.26673520	0.00	5.22	5.22	-20.00	0.01
chr1_3371_mature	8.59655945	529.49433391	3.26	9.05	9.05	-5.94	0.01
chr15_33320_mature	4.83556469	277.23904241	2.54	8.12	8.12	-5.84	0.01
chr2_6447_mature	10.20841435	642.32417675	3.49	9.33	9.33	-5.98	0.02
chr1_3052_star	1.07456993	77.36903509	1.05	6.29	6.29	-6.17	0.02
chr14_31500_mature	1.07456993	77.36903509	1.05	6.29	6.29	-6.17	0.02
chr15_31797_mature	87.57744944	0.00000000	6.47	0.00	0.00	20.00	0.02
NW_003539007_38445_mature	228.88339548	1.61185490	7.84	1.39	1.39	7.15	0.02
chr15_32517_mature	2.14913986	125.72468202	1.65	6.99	6.99	-5.87	0.02
chr1_2963_mature	8.05927449	476.30312228	3.18	8.90	8.90	-5.89	0.02
chrx_22228_mature	224.58511575	1.61185490	7.82	1.39	1.39	7.12	0.02
chr14_30429_mature	3.76099476	205.51149946	2.25	7.69	7.69	-5.77	0.02
chr6_13339_mature	10.74569932	656.02494338	3.55	9.36	9.36	-5.93	0.02
chr7_16788_star	83.27916972	0.00000000	6.40	0.00	0.00	20.00	0.02
chr8_17991_mature@@bta-miR-367	216.52584126	1.61185490	7.77	1.39	1.39	7.07	0.02
chr12_26356_star	1.61185490	96.71129386	1.39	6.61	6.61	-5.91	0.02
chr1_2110_mature	0.53728497	52.38528418	0.62	5.74	5.74	-6.61	0.02
chr1_2557_mature	15.04397905	949.38253477	4.00	9.89	9.89	-5.98	0.02
chr16_34017_star	1.07456993	70.92161550	1.05	6.17	6.17	-6.04	0.02
chr14_31499_mature	1.07456993	69.30976060	1.05	6.14	6.14	-6.01	0.02
chrx_23205_mature	0.53728497	49.16157438	0.62	5.65	5.65	-6.52	0.02
chr14_31042_star	1.07456993	68.50383315	1.05	6.12	6.12	-5.99	0.02
chr9_20694_mature	1.61185490	89.45794682	1.39	6.50	6.50	-5.79	0.02
chr4_10238_mature	73.60804033	0.00000000	6.22	0.00	0.00	20.00	0.02
ssc-miR-615	8.59655945	456.15493606	3.26	8.84	8.84	-5.73	0.02
chr1_2112_mature	0.53728497	48.35564693	0.62	5.63	5.63	-6.49	0.02
chr12_26093_mature	3.76099476	182.13960344	2.25	7.52	7.52	-5.60	0.02
NW_003537990_37435_mature	2.14913986	108.80020560	1.65	6.78	6.78	-5.66	0.02
chr7_16615_mature	5.91013463	290.13388159	2.79	8.19	8.19	-5.62	0.02
chr8_17417_mature	1543.08242210	12.08891173	10.59	3.71	3.71	7.00	0.02
chr9_20601_mature	1.07456993	66.08605081	1.05	6.07	6.07	-5.94	0.02
chr10_23437_mature	1.61185490	86.23423703	1.39	6.45	6.45	-5.74	0.02
chrx_22497_mature	3.76099476	178.91589365	2.25	7.49	7.49	-5.57	0.02
chr7_16567_mature	0.00000000	29.81931561	0.00	4.95	4.95	-20.00	0.02
NW_003539626_38995_mature	10.74569932	571.40256125	3.55	9.16	9.16	-5.73	0.02
chr12_25628_mature	7.52198952	371.53255393	3.09	8.54	8.54	-5.63	0.02
chr9_19579_mature	2.14913986	105.57649580	1.65	6.74	6.74	-5.62	0.02
chr12_26317_mature	7.52198952	369.92069903	3.09	8.53	8.53	-5.62	0.02
chr3_8179_mature	0.53728497	45.93786459	0.62	5.55	5.55	-6.42	0.02
chr17_35005_mature	2.68642483	127.33653692	1.88	7.00	7.00	-5.57	0.02
mmu-miR-5108	2.14913986	104.77056835	1.65	6.72	6.72	-5.61	0.02
mmu-miR-193b-3p	0.00000000	29.01338816	0.00	4.91	4.91	-20.00	0.02
hsa-miR-5702	0.00000000	29.01338816	0.00	4.91	4.91	-20.00	0.02
rno-miR-181a-2-3p	0.00000000	29.01338816	0.00	4.91	4.91	-20.00	0.02
chr2_4930_mature	10.74569932	559.31364951	3.55	9.13	9.13	-5.70	0.02
chr3_6868_star	0.53728497	45.13193714	0.62	5.53	5.53	-6.39	0.02
chr3_7304_star@@mmu-miR-216b-3p	963.35194388	8.86520194	9.91	3.30	3.30	6.76	0.02
chr15_33031_mature	4.29827973	195.84037007	2.41	7.62	7.62	-5.51	0.02
chr9_20220_mature	850.52210104	8.05927449	9.73	3.18	3.18	6.72	0.02
chr16_34398_mature	2.14913986	102.35278601	1.65	6.69	6.69	-5.57	0.02
chr4_9789_mature	10.20841435	514.98763983	3.49	9.01	9.01	-5.66	0.02
chr9_20772_mature	3.76099476	170.05069171	2.25	7.42	7.42	-5.50	0.02
chrx_22720_mature	6.44741959	299.80501098	2.90	8.23	8.23	-5.54	0.02
chr12_26098_mature	110.68070298	0.80592745	6.80	0.85	0.85	7.10	0.03
chr7_16002_mature	1.07456993	61.25048611	1.05	5.96	5.96	-5.83	0.03
chr14_31496_mature	62.32505605	0.00000000	5.98	0.00	0.00	20.00	0.03
chr9_19418_mature@@bta-miR-2452	9.13384442	436.81267729	3.34	8.77	8.77	-5.58	0.03
chr4_9234_star	1.07456993	60.44455867	1.05	5.94	5.94	-5.81	0.03
rno-miR-6317	107.99427815	0.80592745	6.77	0.85	0.85	7.07	0.03
chr10_24172_mature	2.14913986	98.32314876	1.65	6.63	6.63	-5.52	0.03
chr9_19423_mature	106.91970822	0.80592745	6.75	0.85	0.85	7.05	0.03
chr1_3708_mature	1.61185490	78.17496254	1.39	6.31	6.31	-5.60	0.03
chrx_22559_mature	1.07456993	59.63863122	1.05	5.92	5.92	-5.79	0.03
chr6_14094_mature	2.14913986	97.51722131	1.65	6.62	6.62	-5.50	0.03
chr13_27783_mature	3.22370980	139.42544865	2.08	7.13	7.13	-5.43	0.03
chr5_11058_mature	217.60041119	2.41778235	7.77	1.77	1.77	6.49	0.03
ssc-miR-758	8.59655945	392.48666760	3.26	8.62	8.62	-5.51	0.03
chr12_26457_mature	8.59655945	390.87481270	3.26	8.61	8.61	-5.51	0.03
chrx_21884_mature	5.91013463	253.06121894	2.79	7.99	7.99	-5.42	0.03
chr5_11434_star	0.53728497	41.10229989	0.62	5.40	5.40	-6.26	0.03
chr14_29715_mature	8.59655945	381.20368331	3.26	8.58	8.58	-5.47	0.03
chr14_29516_mature	0.53728497	40.29637244	0.62	5.37	5.37	-6.23	0.03
chr6_13565_mature	7.52198952	325.59468934	3.09	8.35	8.35	-5.44	0.03
chr7_15769_mature	11.28298428	523.85284176	3.62	9.04	9.04	-5.54	0.03
chr3_8428_mature	2.14913986	91.06980172	1.65	6.52	6.52	-5.41	0.03
hsa-miR-1248	0.00000000	24.98375091	0.00	4.70	4.70	-20.00	0.03
chr11_24459_mature	0.00000000	24.98375091	0.00	4.70	4.70	-20.00	0.03
NW_003613367_38732_mature	0.00000000	24.98375091	0.00	4.70	4.70	-20.00	0.03
chr8_18354_mature	142.38051597	1.61185490	7.16	1.39	1.39	6.46	0.03
chr12_25693_mature	800.01731424	8.86520194	9.65	3.30	3.30	6.50	0.03
chr9_20219_mature	893.50489831	9.67112939	9.80	3.42	3.42	6.53	0.03
chrx_22488_mature	6.98470456	290.93980904	3.00	8.19	8.19	-5.38	0.03
chr1_3478_mature	94.02486903	0.80592745	6.57	0.85	0.85	6.87	0.03

chr1_2264_mature	4.83556469	193.42258773	2.54	7.60	7.60	-5.32	0.03
chrx_21682_mature	11.28298428	510.95800258	3.62	9.00	9.00	-5.50	0.03
mmu-miR-5124b	2.68642483	107.99427815	1.88	6.77	6.77	-5.33	0.03
rno-miR-1188-5p	92.41301414	0.80592745	6.55	0.85	0.85	6.84	0.03
chr2_4844_mature	52.65392666	0.00000000	5.75	0.00	0.00	20.00	0.03
chr14_31044_star	1.61185490	70.11568805	1.39	6.15	6.15	-5.44	0.03
chr14_30497_star	6.98470456	284.49238945	3.00	8.16	8.16	-5.35	0.03
chr1_3069_mature	137.00766631	1.61185490	7.11	1.39	1.39	6.41	0.03
chr18_36189_mature	2.14913986	87.04016448	1.65	6.46	6.46	-5.34	0.03
chrx_22382_mature	3.22370980	124.11282713	2.08	6.97	6.97	-5.27	0.03
rno-miR-434-3p	51.04207176	0.00000000	5.70	0.00	0.00	20.00	0.03
chr6_14094_star	1.61185490	67.69790570	1.39	6.10	6.10	-5.39	0.04
hsa-miR-548ak	0.00000000	23.37189602	0.00	4.61	4.61	-20.00	0.04
chr7_16939_star	85.96559455	0.80592745	6.44	0.85	0.85	6.74	0.04
chr1_3435_mature	3.76099476	138.61952121	2.25	7.13	7.13	-5.20	0.04
chr8_19026_mature	3.76099476	137.81359376	2.25	7.12	7.12	-5.20	0.04
chr4_9932_mature	2.68642483	99.93500366	1.88	6.66	6.66	-5.22	0.04
chrx_21663_mature	1.07456993	49.96750183	1.05	5.67	5.67	-5.54	0.04
chr5_10910_mature	124.65011209	1.61185490	6.97	1.39	1.39	6.27	0.04
bta-miR-103	0.00000000	22.56596857	0.00	4.56	4.56	-20.00	0.04
chr13_27097_mature	1.61185490	65.28012336	1.39	6.05	6.05	-5.34	0.04
chr11_25205_mature	9.13384442	362.66735199	3.34	8.51	8.51	-5.31	0.04
chr4_9530_mature	2.14913986	81.39867234	1.65	6.36	6.36	-5.24	0.04
chr8_17677_mature	46.74379203	0.00000000	5.58	0.00	0.00	20.00	0.04
chr11_25089_mature	22.02868360	1072.68943444	4.53	10.07	10.07	-5.61	0.04
bta-miR-2308	271.32890779	4.02963724	8.09	2.33	2.33	6.07	0.04
chrx_22145_mature	119.27726243	1.61185490	6.91	1.39	1.39	6.21	0.04
NW_003539597_38976_star	78.44360502	0.80592745	6.31	0.85	0.85	6.60	0.04
rno-miR-341	162.26005971	2.41778235	7.35	1.77	1.77	6.07	0.04
chr11_24729_mature	44.59465217	0.00000000	5.51	0.00	0.00	20.00	0.04
mmu-miR-1931	0.53728497	33.84895285	0.62	5.12	5.12	-5.98	0.04
chr3_7655_mature	0.53728497	33.84895285	0.62	5.12	5.12	-5.98	0.04
chr13_28643_star	44.05736720	0.00000000	5.49	0.00	0.00	20.00	0.04
chr3_7082_mature	44.05736720	0.00000000	5.49	0.00	0.00	20.00	0.04
chr1_3436_mature	3.22370980	110.41206049	2.08	6.80	6.80	-5.10	0.04
chr2_6092_mature	7.52198952	270.79162282	3.09	8.09	8.09	-5.17	0.04
chr8_18586_mature	42.98279727	0.00000000	5.46	0.00	0.00	20.00	0.04
chr12_25625_star	2.68642483	91.87572917	1.88	6.54	6.54	-5.10	0.04
chr4_9930_mature	3.22370980	108.80020560	2.08	6.78	6.78	-5.08	0.04
chr15_33605_mature	1.07456993	45.93786459	1.05	5.55	5.55	-5.42	0.05
chr11_24465_mature	1.07456993	45.93786459	1.05	5.55	5.55	-5.42	0.05
chr15_31817_mature	73.60804033	0.80592745	6.22	0.85	0.85	6.51	0.05
chr17_35576_mature	420.69412831	6.44741959	8.72	2.90	2.90	6.03	0.05
chr14_30496_mature	1.61185490	59.63863122	1.39	5.92	5.92	-5.21	0.05
chr17_35575_mature	416.39584858	6.44741959	8.71	2.90	2.90	6.01	0.05
chr4_10161_mature	41.37094238	0.00000000	5.41	0.00	0.00	20.00	0.05
chr13_28509_mature	1.07456993	45.13193714	1.05	5.53	5.53	-5.39	0.05
chr15_33221_mature	12.89483918	502.09280064	3.80	8.97	8.97	-5.28	0.05
chr4_9258_mature	1.61185490	58.83270377	1.39	5.90	5.90	-5.19	0.05
chr12_25534_star	2.68642483	89.45794682	1.88	6.50	6.50	-5.06	0.05
NW_003539007_38454_mature	0.00000000	20.14818622	0.00	4.40	4.40	-20.00	0.05
chr11_25362_mature	0.00000000	20.14818622	0.00	4.40	4.40	-20.00	0.05
chr1_3525_mature	40.29637244	0.00000000	5.37	0.00	0.00	20.00	0.05
mmu-miR-467a-5p	40.29637244	0.00000000	5.37	0.00	0.00	20.00	0.05
chr1_3523_mature	40.29637244	0.00000000	5.37	0.00	0.00	20.00	0.05
bta-miR-433	40.29637244	0.00000000	5.37	0.00	0.00	20.00	0.05
