# Supplementary material for: Common microRNA–mRNA interactions exist among distinct porcine iPSC lines independent of their metastable pluripotent states
Source: Cell Death Dis. 2017 Aug 31;8(8):e3027–. doi: 10.1038/cddis.2017.426 (PMC5596602; doi:10.1038/cddis.2017.426)
Supplement: Supplementary Table 3 [file cddis2017426x4.pdf]

miRNA	Style	target Symbol
ssc-miR-371-5p	High	UPI00025E168D
ssc-miR-371-5p	High	LOC100739080
ssc-miR-371-5p	High	F1RSZ8
ssc-miR-371-5p	High	CYP2E1
ssc-miR-432-5p	Low	USE1
ssc-miR-432-5p	Low	TESK1
ssc-miR-432-5p	Low	SASH3
ssc-miR-432-5p	Low	PTGIS
ssc-miR-432-5p	Low	LOC100511765
ssc-miR-432-5p	Low	IL12RB2
ssc-miR-432-5p	Low	F1SES8
ssc-miR-432-5p	Low	DHX34
ssc-miR-432-5p	Low	CLUH
ssc-miR-370	Low	ZNF335
ssc-miR-370	Low	ZFYVE27
ssc-miR-370	Low	ZBP1
ssc-miR-370	Low	YIPF2
ssc-miR-370	Low	YIF1B
ssc-miR-370	Low	XPO6
ssc-miR-370	Low	WDR90
ssc-miR-370	Low	WDR62
ssc-miR-370	Low	WDR6
ssc-miR-370	Low	WDR38
ssc-miR-370	Low	VPS53
ssc-miR-370	Low	VPS51
ssc-miR-370	Low	UPK3A
ssc-miR-370	Low	UPI00028F4B8D
ssc-miR-370	Low	UPI00028F48F0
ssc-miR-370	Low	UPI00025E1926
ssc-miR-370	Low	UPI00025E1901
ssc-miR-370	Low	UPI00025E13B9
ssc-miR-370	Low	UPI00025E0715
ssc-miR-370	Low	UPI00025E0343
ssc-miR-370	Low	UPI00025E0118
ssc-miR-370	Low	UPI00025DFE83
ssc-miR-370	Low	UPI00025DFC99
ssc-miR-370	Low	UPI00025DF2CD
ssc-miR-370	Low	UPI0001C9826D
ssc-miR-370	Low	UPI0001C981DF
ssc-miR-370	Low	UPI0001C972CB
ssc-miR-370	Low	UPI0001C96BBD
ssc-miR-370	Low	UPI0001C9672C
ssc-miR-370	Low	UPI0001C96357
ssc-miR-370	Low	UBL7
ssc-miR-370	Low	UBA7
ssc-miR-370	Low	TXN2
ssc-miR-370	Low	TUFT1
ssc-miR-370	Low	TUBGCP6
ssc-miR-370	Low	TTLL12
ssc-miR-370	Low	TTC19
ssc-miR-370	Low	TSC2
ssc-miR-370	Low	TRABD

ssc-miR-370	Low	TMEM43
ssc-miR-370	Low	TMEM204
ssc-miR-370	Low	TM9SF4
ssc-miR-370	Low	TM4SF20
ssc-miR-370	Low	TINAGL1
ssc-miR-370	Low	THBS3
ssc-miR-370	Low	TEPP
ssc-miR-370	Low	SYTL3
ssc-miR-370	Low	SYT13
ssc-miR-370	Low	STAT6
ssc-miR-370	Low	SPRYD3
ssc-miR-370	Low	SPNS3
ssc-miR-370	Low	SPI1
ssc-miR-370	Low	SPECC1L
ssc-miR-370	Low	SNRNP25
ssc-miR-370	Low	SNAP47
ssc-miR-370	Low	SLC7A4
ssc-miR-370	Low	SLC6A12
ssc-miR-370	Low	SLC24A6
ssc-miR-370	Low	SIN3B
ssc-miR-370	Low	SHROOM2
ssc-miR-370	Low	SH2D3C
ssc-miR-370	Low	SETD1B
ssc-miR-370	Low	SDF4
ssc-miR-370	Low	SCRN2
ssc-miR-370	Low	SASH3
ssc-miR-370	Low	SARS
ssc-miR-370	Low	RSAD1
ssc-miR-370	Low	RPS19BP1
ssc-miR-370	Low	RPL35
ssc-miR-370	Low	RNF31
ssc-miR-370	Low	RNF121
ssc-miR-370	Low	RHO
ssc-miR-370	Low	RFX1
ssc-miR-370	Low	RELT
ssc-miR-370	Low	RBFA
ssc-miR-370	Low	RASGRF1
ssc-miR-370	Low	RALY
ssc-miR-370	Low	RADIL
ssc-miR-370	Low	PTK7
ssc-miR-370	Low	PTGIS
ssc-miR-370	Low	PSMG4
ssc-miR-370	Low	PRMT1
ssc-miR-370	Low	PRKAB1
ssc-miR-370	Low	PREP
ssc-miR-370	Low	PRCC
ssc-miR-370	Low	PPP1R32
ssc-miR-370	Low	PPOX
ssc-miR-370	Low	PPM1M
ssc-miR-370	Low	PPAP2C
ssc-miR-370	Low	POLD1
ssc-miR-370	Low	PLEKHM2
ssc-miR-370	Low	PIN1
ssc-miR-370	Low	PCPA1

ssc-miR-370	Low	PARP10
ssc-miR-370	Low	OSBPL7
ssc-miR-370	Low	NPFF
ssc-miR-370	Low	NME3
ssc-miR-370	Low	NFIX
ssc-miR-370	Low	NEURL
ssc-miR-370	Low	NDUFS3
ssc-miR-370	Low	NDUFA3
ssc-miR-370	Low	NCLN
ssc-miR-370	Low	NCK2
ssc-miR-370	Low	MYO7B
ssc-miR-370	Low	MTMR1
ssc-miR-370	Low	MRPS2
ssc-miR-370	Low	MRPL38
ssc-miR-370	Low	MIOX
ssc-miR-370	Low	MIF4GD
ssc-miR-370	Low	MICAL1
ssc-miR-370	Low	MIB2
ssc-miR-370	Low	MGAT4B
ssc-miR-370	Low	MECR
ssc-miR-370	Low	MBD6
ssc-miR-370	Low	LY6H
ssc-miR-370	Low	LRP5
ssc-miR-370	Low	LPCAT4
ssc-miR-370	Low	LONRF3
ssc-miR-370	Low	LOC733662
ssc-miR-370	Low	LOC733611
ssc-miR-370	Low	LOC102166542
ssc-miR-370	Low	LOC102163641
ssc-miR-370	Low	LOC100738900
ssc-miR-370	Low	LOC100737905
ssc-miR-370	Low	LOC100623380
ssc-miR-370	Low	LOC100526035
ssc-miR-370	Low	LOC100525790
ssc-miR-370	Low	LOC100525039
ssc-miR-370	Low	LOC100521376
ssc-miR-370	Low	LOC100520244
ssc-miR-370	Low	LOC100519531
ssc-miR-370	Low	LOC100519448
ssc-miR-370	Low	LOC100518132
ssc-miR-370	Low	LOC100518128
ssc-miR-370	Low	LOC100516662
ssc-miR-370	Low	LOC100156892
ssc-miR-370	Low	LOC100156764
ssc-miR-370	Low	LASS4
ssc-miR-370	Low	L3MBTL2
ssc-miR-370	Low	KDM6B
ssc-miR-370	Low	JAM3
ssc-miR-370	Low	ITGB4
ssc-miR-370	Low	INPP5A
ssc-miR-370	Low	INCA1
ssc-miR-370	Low	IGHMBP2
ssc-miR-370	Low	IFT43
ssc-miR-370	Low	IFT140

ssc-miR-370	Low	IFITM1
ssc-miR-370	Low	IFFO1
ssc-miR-370	Low	HCRT
ssc-miR-370	Low	HBZ
ssc-miR-370	Low	GSX2
ssc-miR-370	Low	GSDMD
ssc-miR-370	Low	GRK4
ssc-miR-370	Low	GRIK5
ssc-miR-370	Low	GPT
ssc-miR-370	Low	GPR161
ssc-miR-370	Low	GNAZ
ssc-miR-370	Low	GNAQ
ssc-miR-370	Low	GLTPD2
ssc-miR-370	Low	GGT5
ssc-miR-370	Low	GALNT18
ssc-miR-370	Low	GALM
ssc-miR-370	Low	FXYD2
ssc-miR-370	Low	FKBP2
ssc-miR-370	Low	FBXW4
ssc-miR-370	Low	FAM113A
ssc-miR-370	Low	F1SI01
ssc-miR-370	Low	F1S9Z4
ssc-miR-370	Low	F1RJA6
ssc-miR-370	Low	ESRRA
ssc-miR-370	Low	EPHB2
ssc-miR-370	Low	DUSP13
ssc-miR-370	Low	DNAJC4
ssc-miR-370	Low	DENND5A
ssc-miR-370	Low	DBNDD1
ssc-miR-370	Low	DAB2IP
ssc-miR-370	Low	CYP2D25
ssc-miR-370	Low	CYB5B
ssc-miR-370	Low	CUTA
ssc-miR-370	Low	CTSW
ssc-miR-370	Low	CTSF
ssc-miR-370	Low	CRYBB3
ssc-miR-370	Low	CPSF3L
ssc-miR-370	Low	CPR2
ssc-miR-370	Low	COPRS
ssc-miR-370	Low	COL4A2
ssc-miR-370	Low	CNDP1
ssc-miR-370	Low	CLDN7
ssc-miR-370	Low	CIB4
ssc-miR-370	Low	CHP1
ssc-miR-370	Low	CHAC1
ssc-miR-370	Low	CENPT
ssc-miR-370	Low	CDK16
ssc-miR-370	Low	CDK11B
ssc-miR-370	Low	CDCA3
ssc-miR-370	Low	CCDC61
ssc-miR-370	Low	CCDC150
ssc-miR-370	Low	CCDC124
ssc-miR-370	Low	CC2D1B
ssc-miR-370	Low	CAPS

ssc-miR-370	Low	LIN28A
ssc-miR-370	Low	CAMSAP3
ssc-miR-370	Low	CALY
ssc-miR-370	Low	CACNB3
ssc-miR-370	Low	CABP1
ssc-miR-370	Low	CABLES2
ssc-miR-370	Low	C4
ssc-miR-370	Low	C2H19orf60
ssc-miR-370	Low	C12H17orf62
ssc-miR-370	Low	BRI3BP
ssc-miR-370	Low	BIN3
ssc-miR-370	Low	BCL7C
ssc-miR-370	Low	ATL3
ssc-miR-370	Low	ARPC4
ssc-miR-370	Low	ARL2
ssc-miR-370	Low	ARHGAP4
ssc-miR-370	Low	APOA2
ssc-miR-370	Low	ANKZF1
ssc-miR-370	Low	ANHX
ssc-miR-370	Low	ADPRHL2
ssc-miR-370	Low	ADAMTS7
ssc-miR-370	Low	AAR2
ssc-miR-206	Low	VAT1
ssc-miR-206	Low	UNC50
ssc-miR-206	Low	TNPO2
ssc-miR-206	Low	TERF2
ssc-miR-206	Low	SNX2
ssc-miR-206	Low	SMIM14
ssc-miR-206	Low	SMARCB1
ssc-miR-206	Low	SLC29A3
ssc-miR-206	Low	SH3GL1
ssc-miR-206	Low	RNF38
ssc-miR-206	Low	OTX2
ssc-miR-206	Low	NR4A2
ssc-miR-206	Low	NCOA1
ssc-miR-206	Low	LIN7C
ssc-miR-206	Low	KIF2A
ssc-miR-206	Low	HDAC4
ssc-miR-206	Low	FNBP1L
ssc-miR-206	Low	DGKZ
ssc-miR-206	Low	CTTNBP2NL
ssc-miR-206	Low	CDC42
ssc-miR-206	Low	CD164
ssc-miR-206	Low	ATG13
ssc-miR-206	Low	AMOT
NW_003613242_37866_mature	Low	UPI00025E11F1
NW_003613242_37866_mature	Low	SASH3
NW_003613242_37866_mature	Low	LRP5
NW_003613242_37866_mature	Low	F1SBJ5
NW_003613242_37864_mature	Low	UPI00025E1926
NW_003613242_37864_mature	Low	SPATA21
NW_003613242_37864_mature	Low	LAMP5
NW_003613242_37864_mature	Low	JPH4
NW_003613242_37864_mature	Low	ELMO3

NW_003613242_37864_mature	Low	CHID1
NW_003613137_37368_mature	Low	LOC100524424
NW_003541201_39964_mature	Low	YIPF2
NW_003541201_39964_mature	Low	UPI00025E189D
NW_003541201_39964_mature	Low	UPI00025E047F
NW_003541201_39964_mature	Low	UPI00025DFC0E
NW_003541201_39964_mature	Low	SLC22A17
NW_003541201_39964_mature	Low	SASH3
NW_003541201_39964_mature	Low	RGS14
NW_003541201_39964_mature	Low	RASL11B
NW_003541201_39964_mature	Low	MAP2K7
NW_003541201_39964_mature	Low	MAGED2
NW_003541201_39964_mature	Low	LOC100739011
NW_003541201_39964_mature	Low	LOC100525790
NW_003541201_39964_mature	Low	LOC100512091
NW_003541201_39964_mature	Low	L3MBTL2
NW_003541201_39964_mature	Low	FBXO31
NW_003541201_39964_mature	Low	DUS3L
NW_003541201_39964_mature	Low	CDPF1
NW_003541201_39964_mature	Low	CCHCR1
NW_003541201_39964_mature	Low	CABLES2
NW_003541201_39964_mature	Low	C1QTNF2
NW_003541201_39964_mature	Low	C19orf70
NW_003541201_39964_mature	Low	ASB13
NW_003541201_39964_mature	Low	AGRP
NW_003538465_37902_mature	Low	C1H9orf100
NW_003537990_37435_mature	Low	UPI00025DED9B
NW_003537990_37435_mature	Low	TOMM20L
NW_003537990_37435_mature	Low	STX1A
NW_003537990_37435_mature	Low	SLC25A36
NW_003537990_37435_mature	Low	RLN3
NW_003537990_37435_mature	Low	PACS1
NW_003537990_37435_mature	Low	HBM
NW_003537990_37435_mature	Low	FGD5
NW_003537990_37435_mature	Low	F1S8Y0
NW_003537990_37435_mature	Low	DHX58
NW_003537990_37435_mature	Low	CABLES2
NW_003537694_37136_mature	Low	INTS9
mmu-miR-5124b	Low	SSR4
mmu-miR-5108	Low	UPI00025DFB2F
mmu-miR-5108	Low	SLC25A28
mmu-miR-5108	Low	PSMC3
mmu-miR-5108	Low	PRSS50
mmu-miR-5108	Low	EEF1E1
mmu-miR-5108	Low	C13H3orf17
hsa-miR-4685-3p	Low	ZFPL1
hsa-miR-4685-3p	Low	ZFAND2B
hsa-miR-4685-3p	Low	ZBTB12
hsa-miR-4685-3p	Low	ZAP70
hsa-miR-4685-3p	Low	WNT10B
hsa-miR-4685-3p	Low	WNK1
hsa-miR-4685-3p	Low	WDR91
hsa-miR-4685-3p	Low	WDR46
hsa-miR-4685-3p	Low	WDR45

hsa-miR-4685-3p	Low	WDR24
hsa-miR-4685-3p	Low	UPI00025E11F9
hsa-miR-4685-3p	Low	UPI00025E11F1
hsa-miR-4685-3p	Low	UPI00025E116A
hsa-miR-4685-3p	Low	UPI00025E1021
hsa-miR-4685-3p	Low	UPI00025E0C15
hsa-miR-4685-3p	Low	UPI00025E08B2
hsa-miR-4685-3p	Low	UPI00025E0134
hsa-miR-4685-3p	Low	UPI00025DFE7C
hsa-miR-4685-3p	Low	UPI00025DF30D
hsa-miR-4685-3p	Low	UBR1
hsa-miR-4685-3p	Low	TSPAN18
hsa-miR-4685-3p	Low	TRPC4AP
hsa-miR-4685-3p	Low	TREM1
hsa-miR-4685-3p	Low	TRAP1
hsa-miR-4685-3p	Low	TPCN2
hsa-miR-4685-3p	Low	TMEM8A
hsa-miR-4685-3p	Low	TMEM130
hsa-miR-4685-3p	Low	TIMM44
hsa-miR-4685-3p	Low	THOP1
hsa-miR-4685-3p	Low	TGOLN2
hsa-miR-4685-3p	Low	TGM1
hsa-miR-4685-3p	Low	TGFBR2
hsa-miR-4685-3p	Low	TEC
hsa-miR-4685-3p	Low	TEAD4
hsa-miR-4685-3p	Low	TBC1D17
hsa-miR-4685-3p	Low	TAB1
hsa-miR-4685-3p	Low	STOML2
hsa-miR-4685-3p	Low	STK32C
hsa-miR-4685-3p	Low	STK11
hsa-miR-4685-3p	Low	SPAG7
hsa-miR-4685-3p	Low	SNTA1
hsa-miR-4685-3p	Low	SLC6A8
hsa-miR-4685-3p	Low	SLC39A1
hsa-miR-4685-3p	Low	SLC35E4
hsa-miR-4685-3p	Low	SLC22A17
hsa-miR-4685-3p	Low	SHARPIN
hsa-miR-4685-3p	Low	SH3GLB2
hsa-miR-4685-3p	Low	SFTPD
hsa-miR-4685-3p	Low	RQCD1
hsa-miR-4685-3p	Low	RNF25
hsa-miR-4685-3p	Low	RHBDD3
hsa-miR-4685-3p	Low	RCN1
hsa-miR-4685-3p	Low	RBM10
hsa-miR-4685-3p	Low	RASL11B
hsa-miR-4685-3p	Low	RAB6B
hsa-miR-4685-3p	Low	RAB4B
hsa-miR-4685-3p	Low	PYCR2
hsa-miR-4685-3p	Low	PTK2B
hsa-miR-4685-3p	Low	PSME2
hsa-miR-4685-3p	Low	PRRC2C
hsa-miR-4685-3p	Low	PPP2R5B
hsa-miR-4685-3p	Low	PPCS
hsa-miR-4685-3p	Low	PLA2G4B

hsa-miR-4685-3p	Low	PISD
hsa-miR-4685-3p	Low	PHF12
hsa-miR-4685-3p	Low	PFDN6
hsa-miR-4685-3p	Low	PEX16
hsa-miR-4685-3p	Low	PEX11G
hsa-miR-4685-3p	Low	NUP188
hsa-miR-4685-3p	Low	NOP9
hsa-miR-4685-3p	Low	NMRAL1
hsa-miR-4685-3p	Low	NKIRAS2
hsa-miR-4685-3p	Low	NKAIN1
hsa-miR-4685-3p	Low	NGB
hsa-miR-4685-3p	Low	NDUFA10
hsa-miR-4685-3p	Low	NAT9
hsa-miR-4685-3p	Low	MXD4
hsa-miR-4685-3p	Low	MMS19
hsa-miR-4685-3p	Low	MFAP5
hsa-miR-4685-3p	Low	MED27
hsa-miR-4685-3p	Low	MECR
hsa-miR-4685-3p	Low	MCAT
hsa-miR-4685-3p	Low	MATN4
hsa-miR-4685-3p	Low	MAPK8IP3
hsa-miR-4685-3p	Low	MAPK8IP1
hsa-miR-4685-3p	Low	MAP3K6
hsa-miR-4685-3p	Low	LPIN3
hsa-miR-4685-3p	Low	LOC102163641
hsa-miR-4685-3p	Low	LOC100737129
hsa-miR-4685-3p	Low	LOC100736962
hsa-miR-4685-3p	Low	LOC100627857
hsa-miR-4685-3p	Low	LOC100626825
hsa-miR-4685-3p	Low	LOC100620305
hsa-miR-4685-3p	Low	LOC100525039
hsa-miR-4685-3p	Low	LOC100524940
hsa-miR-4685-3p	Low	LOC100524682
hsa-miR-4685-3p	Low	LOC100522856
hsa-miR-4685-3p	Low	LOC100522360
hsa-miR-4685-3p	Low	LOC100521975
hsa-miR-4685-3p	Low	LOC100518681
hsa-miR-4685-3p	Low	LOC100513601
hsa-miR-4685-3p	Low	LOC100512723
hsa-miR-4685-3p	Low	LOC100512091
hsa-miR-4685-3p	Low	LOC100156358
hsa-miR-4685-3p	Low	LOC100152291
hsa-miR-4685-3p	Low	LOC100137083
hsa-miR-4685-3p	Low	LAMTOR1
hsa-miR-4685-3p	Low	LAMP5
hsa-miR-4685-3p	Low	L1CAM
hsa-miR-4685-3p	Low	KLHL40
hsa-miR-4685-3p	Low	KLHL3
hsa-miR-4685-3p	Low	INSIG1
hsa-miR-4685-3p	Low	IGF1R
hsa-miR-4685-3p	Low	IFT140
hsa-miR-4685-3p	Low	HP
hsa-miR-4685-3p	Low	HDHD2
hsa-miR-4685-3p	Low	GSTK1

hsa-miR-4685-3p	Low	GSK3A
hsa-miR-4685-3p	Low	GRK4
hsa-miR-4685-3p	Low	GLB1L2
hsa-miR-4685-3p	Low	GEMIN8
hsa-miR-4685-3p	Low	FDPS
hsa-miR-4685-3p	Low	FAM160B2
hsa-miR-4685-3p	Low	FADS2
hsa-miR-4685-3p	Low	FABP6
hsa-miR-4685-3p	Low	F1SL63
hsa-miR-4685-3p	Low	F1S8T8
hsa-miR-4685-3p	Low	F1S852
hsa-miR-4685-3p	Low	F1S7H6
hsa-miR-4685-3p	Low	F1RQL1
hsa-miR-4685-3p	Low	EXOC3L1
hsa-miR-4685-3p	Low	ERP29
hsa-miR-4685-3p	Low	EPB41L4A
hsa-miR-4685-3p	Low	EMC9
hsa-miR-4685-3p	Low	EIF4A1
hsa-miR-4685-3p	Low	EFS
hsa-miR-4685-3p	Low	EFEMP2
hsa-miR-4685-3p	Low	EEF2K
hsa-miR-4685-3p	Low	E4F1
hsa-miR-4685-3p	Low	DUSP26
hsa-miR-4685-3p	Low	DUS3L
hsa-miR-4685-3p	Low	DTX1
hsa-miR-4685-3p	Low	DNAJC7
hsa-miR-4685-3p	Low	DHX58
hsa-miR-4685-3p	Low	DENND5A
hsa-miR-4685-3p	Low	DDR1
hsa-miR-4685-3p	Low	DAB2IP
hsa-miR-4685-3p	Low	CSNK2A1
hsa-miR-4685-3p	Low	CREB3L3
hsa-miR-4685-3p	Low	CPR2
hsa-miR-4685-3p	Low	COMTD1
hsa-miR-4685-3p	Low	COMP
hsa-miR-4685-3p	Low	CNP
hsa-miR-4685-3p	Low	CHGA
hsa-miR-4685-3p	Low	CEP250
hsa-miR-4685-3p	Low	CELSR3
hsa-miR-4685-3p	Low	CAMTA2
hsa-miR-4685-3p	Low	C9orf24
hsa-miR-4685-3p	Low	C7H14orf2
hsa-miR-4685-3p	Low	C6H1orf210
hsa-miR-4685-3p	Low	C6H19orf33
hsa-miR-4685-3p	Low	C4
hsa-miR-4685-3p	Low	C1QTNF2
hsa-miR-4685-3p	Low	C14H10orf76
hsa-miR-4685-3p	Low	BRSK1
hsa-miR-4685-3p	Low	BIN2
hsa-miR-4685-3p	Low	AVPR2
hsa-miR-4685-3p	Low	AUP1
hsa-miR-4685-3p	Low	ATP1A1
hsa-miR-4685-3p	Low	ATG4D
hsa-miR-4685-3p	Low	ASNS

hsa-miR-4685-3p	Low	ASNA1
hsa-miR-4685-3p	Low	ARMC6
hsa-miR-4685-3p	Low	ARL2
hsa-miR-4685-3p	Low	APBB1
hsa-miR-4685-3p	Low	AP5Z1
hsa-miR-4685-3p	Low	ANXA8
hsa-miR-4685-3p	Low	ANXA13
hsa-miR-4685-3p	Low	ACTR1B
hsa-miR-4685-3p	Low	ACE
hsa-miR-4685-3p	Low	ABLIM2
hsa-miR-3659	Low	MRPL46
hsa-miR-3659	Low	LOC100516792
hsa-miR-3659	Low	GOLIM4
hsa-miR-184	Low	RBBP5
hsa-miR-1298	Low	TYRO3
chrx_22720_mature	Low	UBA7
chrx_22720_mature	Low	SLC1A5
chrx_22720_mature	Low	SASH3
chrx_22720_mature	Low	RHO
chrx_22720_mature	Low	LOC100626252
chrx_22720_mature	Low	FAH
chrx_22720_mature	Low	F1S7X0
chrx_22720_mature	Low	ALOX5AP
chrx_22639_mature	Low	SGSM2
chrx_22639_mature	Low	RNF207
chrx_22639_mature	Low	POLR1D
chrx_22639_mature	Low	GRIK5
chrx_22639_mature	Low	F1SIY2
chrx_22639_mature	Low	EYA2
chrx_22639_mature	Low	CHID1
chrx_22639_mature	Low	C9orf24
chrx_22639_mature	Low	C3H7orf43
chrx_22639_mature	Low	BANF2
chrx_22639_mature	Low	ANKRD46
chrx_22382_mature	Low	TARSL2
chrx_21884_mature	Low	UPI00025E1093
chrx_21884_mature	Low	UPI00025DF673
chrx_21884_mature	Low	TUSC5
chrx_21884_mature	Low	TUFT1
chrx_21884_mature	Low	TAP2
chrx_21884_mature	Low	NAGA
chrx_21884_mature	Low	GRTP1
chrx_21884_mature	Low	EMC9
chrx_21884_mature	Low	CARS
chrx_21884_mature	Low	ARHGEF1
chrx_21682_mature	Low	ORAI3
chrx_21682_mature	Low	MLC1
chrx_21570_mature	Low	LFNG
chrx_21569_mature	Low	LFNG
chrx_21555_mature	Low	GTF2A1
chr9_19375_mature	Low	SLC4A1
chr9_19375_mature	Low	C12H17orf104
chr7_15145_mature	Low	WNT16
chr7_15145_mature	Low	UPI00025DFC99

chr7_15145_mature	Low	MBOAT7
chr7_15145_mature	Low	ATP8B2
chr6_15014_mature	Low	LOC100515475
chr6_15014_mature	Low	F1SKK5
chr6_15014_mature	Low	F1SES8
chr6_15014_mature	Low	EYA2
chr6_15014_mature	Low	BCL7C
chr6_15013_mature	Low	LOC100515475
chr6_15013_mature	Low	F1SKK5
chr6_15013_mature	Low	F1SES8
chr6_15013_mature	Low	EYA2
chr6_15013_mature	Low	BCL7C
chr6_14477_mature@@bta-miR-483	Low	UROC1
chr6_14477_mature@@bta-miR-483	Low	UPI00025E0143
chr6_14477_mature@@bta-miR-483	Low	UPI00025DFB2F
chr6_14477_mature@@bta-miR-483	Low	UPI00025DE797
chr6_14477_mature@@bta-miR-483	Low	TMEM86A
chr6_14477_mature@@bta-miR-483	Low	SNX15
chr6_14477_mature@@bta-miR-483	Low	RNF167
chr6_14477_mature@@bta-miR-483	Low	RAB6B
chr6_14477_mature@@bta-miR-483	Low	RAB33A
chr6_14477_mature@@bta-miR-483	Low	NMRAL1
chr6_14477_mature@@bta-miR-483	Low	NKIRAS2
chr6_14477_mature@@bta-miR-483	Low	MAP4K4
chr6_14477_mature@@bta-miR-483	Low	MAP4
chr6_14477_mature@@bta-miR-483	Low	LRRC71
chr6_14477_mature@@bta-miR-483	Low	LOC100519531
chr6_14477_mature@@bta-miR-483	Low	LOC100519025
chr6_14477_mature@@bta-miR-483	Low	LOC100516662
chr6_14477_mature@@bta-miR-483	Low	LOC100514194
chr6_14477_mature@@bta-miR-483	Low	LOC100513248
chr6_14477_mature@@bta-miR-483	Low	LOC100512626
chr6_14477_mature@@bta-miR-483	Low	LGALS12
chr6_14477_mature@@bta-miR-483	Low	LAMTOR4
chr6_14477_mature@@bta-miR-483	Low	JPH4
chr6_14477_mature@@bta-miR-483	Low	JAG1
chr6_14477_mature@@bta-miR-483	Low	GNAQ
chr6_14477_mature@@bta-miR-483	Low	F1SNT4
chr6_14477_mature@@bta-miR-483	Low	ETHE1
chr6_14477_mature@@bta-miR-483	Low	EIF4E2
chr6_14477_mature@@bta-miR-483	Low	E4
chr6_14477_mature@@bta-miR-483	Low	DTX3
chr6_14477_mature@@bta-miR-483	Low	DTNBP1
chr6_14477_mature@@bta-miR-483	Low	DNAL4
chr6_14477_mature@@bta-miR-483	Low	DNAJC4
chr6_14477_mature@@bta-miR-483	Low	CENPV
chr6_14477_mature@@bta-miR-483	Low	C1QA
chr6_14477_mature@@bta-miR-483	Low	AKR1B1
chr6_13578_mature	Low	C1H9orf100
chr6_13576_mature	Low	C1H9orf100
chr6_12951_mature	Low	NDOR1
chr6_12951_mature	Low	LYPD1
chr6_12951_mature	Low	LOC100515475
chr6_12951_mature	Low	GTF2F1

chr6_12951_mature	Low	F1SIY2
chr6_12951_mature	Low	C14H10orf137
chr4_9932_mature	Low	ZBP1
chr4_9932_mature	Low	UPI00025DEBAD
chr4_9932_mature	Low	TMEM204
chr4_9932_mature	Low	SLC45A4
chr4_9932_mature	Low	SASH3
chr4_9932_mature	Low	NRP1
chr4_9932_mature	Low	LOC100626252
chr4_9932_mature	Low	F1RL74
chr4_9932_mature	Low	CST11
chr4_9932_mature	Low	CENPQ
chr4_9932_mature	Low	ACADSB
chr4_9930_mature	Low	ZBP1
chr4_9930_mature	Low	UPI00025DEBAD
chr4_9930_mature	Low	TMEM204
chr4_9930_mature	Low	SLC45A4
chr4_9930_mature	Low	SASH3
chr4_9930_mature	Low	NRP1
chr4_9930_mature	Low	LOC100626252
chr4_9930_mature	Low	F1RL74
chr4_9930_mature	Low	CST11
chr4_9930_mature	Low	CENPQ
chr4_9930_mature	Low	ACADSB
chr4_9231_mature	Low	EMID1
chr4_9198_mature	Low	SLC16A13
chr4_9198_mature	Low	SESN3
chr4_9198_mature	Low	GGT1
chr4_9198_mature	Low	FLAD1
chr4_10395_mature	Low	ZBP1
chr4_10395_mature	Low	UPI00025E080D
chr4_10395_mature	Low	UPI00025DFB8B
chr4_10395_mature	Low	TMEM101
chr4_10395_mature	Low	ROMO1
chr4_10395_mature	Low	RGS4
chr4_10395_mature	Low	NR2F6
chr4_10395_mature	Low	NAGA
chr4_10395_mature	Low	MMAB
chr4_10395_mature	Low	LOC100156775
chr4_10395_mature	Low	IGHMBP2
chr4_10395_mature	Low	HINFP
chr4_10395_mature	Low	GSTP1
chr4_10395_mature	Low	GLT25D1
chr4_10395_mature	Low	EMID1
chr4_10395_mature	Low	CRTAM
chr4_10395_mature	Low	CD274
chr4_10395_mature	Low	C14H10orf137
chr4_10395_mature	Low	ANKRD39
chr3_8078_mature	Low	UPI00025E11F1
chr3_8078_mature	Low	SASH3
chr3_8078_mature	Low	LRP5
chr3_8078_mature	Low	F1SBJ5
chr3_7072_mature	Low	UPI00025E1926
chr3_7072_mature	Low	SPATA21

chr3_7072_mature	Low	LAMP5
chr3_7072_mature	Low	JPH4
chr3_7072_mature	Low	ELMO3
chr3_7072_mature	Low	CHID1
chr2_4477_star	Low	UPI00028F476E
chr2_4295_mature	Low	PAPP-A
chr2_4023_mature	Low	UPI0001C9826D
chr2_4023_mature	Low	RGS14
chr2_4023_mature	Low	LGALS12
chr2_4023_mature	Low	FGF10
chr17_34881_mature	Low	RBBP5
chr17_34881_mature	Low	ACCSL
chr15_33320_mature	Low	PTGS1
chr15_33320_mature	Low	NAGA
chr15_32293_mature	Low	F2
chr15_31936_mature	Low	RIBC1
chr15_31936_mature	Low	PIP5K1A
chr15_31863_mature	Low	WDR46
chr15_31863_mature	Low	WDR24
chr15_31863_mature	Low	WASH1
chr15_31863_mature	Low	USP11
chr15_31863_mature	Low	UPI00025E11F1
chr15_31863_mature	Low	UPI00025E1093
chr15_31863_mature	Low	UPI00025DFEBE
chr15_31863_mature	Low	UPI0001C96BBD
chr15_31863_mature	Low	UPI0001C96357
chr15_31863_mature	Low	UPI0001C95F6C
chr15_31863_mature	Low	UPI0001C958DC
chr15_31863_mature	Low	UFBP
chr15_31863_mature	Low	UBTD1
chr15_31863_mature	Low	TULP3
chr15_31863_mature	Low	TTC13
chr15_31863_mature	Low	TSPAN9
chr15_31863_mature	Low	TSPAN18
chr15_31863_mature	Low	TRIM15
chr15_31863_mature	Low	TMEM55B
chr15_31863_mature	Low	TIPRL
chr15_31863_mature	Low	TEX261
chr15_31863_mature	Low	TCTE1
chr15_31863_mature	Low	STRA8
chr15_31863_mature	Low	STOML2
chr15_31863_mature	Low	SFTPC
chr15_31863_mature	Low	SDF2L1
chr15_31863_mature	Low	SDC4
chr15_31863_mature	Low	RPS19BP1
chr15_31863_mature	Low	RNF25
chr15_31863_mature	Low	RGL2
chr15_31863_mature	Low	RETN
chr15_31863_mature	Low	RASSF1
chr15_31863_mature	Low	RAD54L
chr15_31863_mature	Low	PPP2R5B
chr15_31863_mature	Low	PLA2G4B
chr15_31863_mature	Low	PHF1
chr15_31863_mature	Low	PFDN6

chr15_31863_mature	Low	PEX16
chr15_31863_mature	Low	NT5C2
chr15_31863_mature	Low	NOP9
chr15_31863_mature	Low	NFKBIL1
chr15_31863_mature	Low	NDUFA10
chr15_31863_mature	Low	NAGK
chr15_31863_mature	Low	NAGA
chr15_31863_mature	Low	MXD4
chr15_31863_mature	Low	MMS19
chr15_31863_mature	Low	MDP1
chr15_31863_mature	Low	MARCKSL1
chr15_31863_mature	Low	MAPT
chr15_31863_mature	Low	MAPK8IP3
chr15_31863_mature	Low	MAPK8IP1
chr15_31863_mature	Low	LOC396700
chr15_31863_mature	Low	LOC100737129
chr15_31863_mature	Low	LOC100627857
chr15_31863_mature	Low	LOC100622764
chr15_31863_mature	Low	LOC100525790
chr15_31863_mature	Low	LOC100524254
chr15_31863_mature	Low	LOC100522856
chr15_31863_mature	Low	LOC100522360
chr15_31863_mature	Low	LOC100153684
chr15_31863_mature	Low	LIN28A
chr15_31863_mature	Low	LENG1
chr15_31863_mature	Low	ITM2C
chr15_31863_mature	Low	ITIH3
chr15_31863_mature	Low	IGF1R
chr15_31863_mature	Low	HOXD4
chr15_31863_mature	Low	HID1
chr15_31863_mature	Low	HEXA
chr15_31863_mature	Low	GUCA2A
chr15_31863_mature	Low	GSTP1
chr15_31863_mature	Low	GEMIN8
chr15_31863_mature	Low	FAM198B
chr15_31863_mature	Low	FAM129A
chr15_31863_mature	Low	FADS2
chr15_31863_mature	Low	F1S852
chr15_31863_mature	Low	EXOSC5
chr15_31863_mature	Low	DHX58
chr15_31863_mature	Low	DENND5A
chr15_31863_mature	Low	DDX56
chr15_31863_mature	Low	DAXX
chr15_31863_mature	Low	DAB2IP
chr15_31863_mature	Low	CLEC3B
chr15_31863_mature	Low	CCRL1
chr15_31863_mature	Low	C7H14orf2
chr15_31863_mature	Low	C1QTNF2
chr15_31863_mature	Low	ANKRD39
chr15_31863_mature	Low	ANHX
chr15_31863_mature	Low	AMDHD2
chr15_31863_mature	Low	ACTR1B
chr14_31500_mature	Low	UPI00025E11F1
chr14_31500_mature	Low	UPI00025E116A

chr14_31500_mature	Low	UPI00025E0B25
chr14_31500_mature	Low	UPI00025DF32D
chr14_31500_mature	Low	TNIP1
chr14_31500_mature	Low	TMEM204
chr14_31500_mature	Low	SETD3
chr14_31500_mature	Low	S100A9
chr14_31500_mature	Low	RNF25
chr14_31500_mature	Low	RASL11B
chr14_31500_mature	Low	PSMG4
chr14_31500_mature	Low	POLD1
chr14_31500_mature	Low	PLD4
chr14_31500_mature	Low	PGLYRP4
chr14_31500_mature	Low	MYOM1
chr14_31500_mature	Low	MXD4
chr14_31500_mature	Low	MAP3K6
chr14_31500_mature	Low	IGHMBP2
chr14_31500_mature	Low	FOLR1
chr14_31500_mature	Low	EMC9
chr14_31500_mature	Low	ALDH2
chr14_31500_mature	Low	ACOT9
chr14_31314_mature	Low	WDR38
chr14_31314_mature	Low	UPI00025DFB2F
chr14_31314_mature	Low	UPI00025DF1FA
chr14_31314_mature	Low	UBA52
chr14_31314_mature	Low	UACA
chr14_31314_mature	Low	TTC38
chr14_31314_mature	Low	TPM2
chr14_31314_mature	Low	TENC1
chr14_31314_mature	Low	SYT6
chr14_31314_mature	Low	STX1A
chr14_31314_mature	Low	SPPL2B
chr14_31314_mature	Low	SPNS3
chr14_31314_mature	Low	SPATC1
chr14_31314_mature	Low	SFRP5
chr14_31314_mature	Low	NT5M
chr14_31314_mature	Low	NDOR1
chr14_31314_mature	Low	LYZL4
chr14_31314_mature	Low	LOC100737048
chr14_31314_mature	Low	LOC100626135
chr14_31314_mature	Low	LOC100623380
chr14_31314_mature	Low	HPS6
chr14_31314_mature	Low	GTF2F1
chr14_31314_mature	Low	GSX2
chr14_31314_mature	Low	EPHB4
chr14_31314_mature	Low	CPR2
chr14_31314_mature	Low	CCL27
chr14_31314_mature	Low	BAG6
chr14_31314_mature	Low	APBA1
chr14_30223_mature	Low	WDR38
chr14_30223_mature	Low	UPI00025DFB2F
chr14_30223_mature	Low	UPI00025DF1FA
chr14_30223_mature	Low	UBA52
chr14_30223_mature	Low	UACA
chr14_30223_mature	Low	TTC38

chr14_30223_mature	Low	TPM2
chr14_30223_mature	Low	TENC1
chr14_30223_mature	Low	SYT6
chr14_30223_mature	Low	STX1A
chr14_30223_mature	Low	SPPL2B
chr14_30223_mature	Low	SPNS3
chr14_30223_mature	Low	SPATC1
chr14_30223_mature	Low	SFRP5
chr14_30223_mature	Low	NT5M
chr14_30223_mature	Low	NDOR1
chr14_30223_mature	Low	LYZL4
chr14_30223_mature	Low	LOC100737048
chr14_30223_mature	Low	LOC100626135
chr14_30223_mature	Low	LOC100623380
chr14_30223_mature	Low	HPS6
chr14_30223_mature	Low	GTF2F1
chr14_30223_mature	Low	GSX2
chr14_30223_mature	Low	EPHB4
chr14_30223_mature	Low	CPR2
chr14_30223_mature	Low	CCL27
chr14_30223_mature	Low	BAG6
chr14_30223_mature	Low	APBA1
chr13_28441_mature	Low	ELMO3
chr13_27515_mature	Low	WNT16
chr13_27515_mature	Low	UPI00025E1021
chr13_27515_mature	Low	UPI00025E077F
chr13_27515_mature	Low	TTC38
chr13_27515_mature	Low	TMEM63B
chr13_27515_mature	Low	TBC1D13
chr13_27515_mature	Low	PTK2B
chr13_27515_mature	Low	NDUFS5
chr13_27515_mature	Low	MED16
chr13_27515_mature	Low	IFFO1
chr13_27515_mature	Low	ESPN
chr13_27515_mature	Low	DTNBP1
chr13_27515_mature	Low	DHX58
chr13_27515_mature	Low	C6H19orf54
chr13_27515_mature	Low	C6H19orf33
chr13_27515_mature	Low	BANF2
chr12_26093_mature	Low	NRP1
chr12_25628_mature	Low	FSCN3
chr12_25625_star	Low	WDR62
chr12_25625_star	Low	UPI00025E0420
chr12_25625_star	Low	TMEM86A
chr12_25625_star	Low	TAGLN2
chr12_25625_star	Low	S100A12
chr12_25625_star	Low	POLD2
chr12_25625_star	Low	PGLYRP4
chr12_25625_star	Low	PGLYRP1
chr12_25625_star	Low	PDGFRB
chr12_25625_star	Low	PCPA1
chr12_25625_star	Low	OBFC1
chr12_25625_star	Low	NRSN2
chr12_25625_star	Low	NDUFA3

chr12_25625_star	Low	MRPL10
chr12_25625_star	Low	METAP1
chr12_25625_star	Low	MAK
chr12_25625_star	Low	LOC100737129
chr12_25625_star	Low	LOC100513248
chr12_25625_star	Low	LOC100512723
chr12_25625_star	Low	LOC100512091
chr12_25625_star	Low	LOC100511695
chr12_25625_star	Low	KRT8
chr12_25625_star	Low	KPTN
chr12_25625_star	Low	IRF7
chr12_25625_star	Low	IFFO1
chr12_25625_star	Low	GTF2F1
chr12_25625_star	Low	GFI1
chr12_25625_star	Low	FUT2
chr12_25625_star	Low	DUS3L
chr12_25625_star	Low	DNAJC4
chr12_25625_star	Low	CSAD
chr12_25625_star	Low	BRI3BP
chr12_25625_star	Low	AP1G1
chr12_25625_star	Low	AOC1
chr12_25625_star	Low	ANAPC5
chr12_25625_star	Low	ACSL1
chr12_25625_star	Low	ACAP1
chr12_25625_star	Low	ABCA3
chr11_25257_mature	Low	SLC39A1
chr10_24151_mature	Low	LOC396877
chr10_23795_star	Low	UROC1
chr10_23795_star	Low	UBL7
chr10_23795_star	Low	TINAGL1
chr10_23795_star	Low	SMOC1
chr10_23795_star	Low	LOC100525790
chr10_23795_star	Low	HINFP
chr10_23795_star	Low	HAND2
chr10_23795_star	Low	AKAP8L
chr1_3720_mature	Low	MYF6
chr1_3708_mature	Low	PRPF19
chr1_2264_mature	Low	F2
chr1_211_mature	Low	SCG3
chr1_211_mature	Low	LOC100525790
chr1_211_mature	Low	ABCA3
chr1_1318_mature	Low	GOLIM4
chr1_1317_mature	Low	GOLIM4
