# Supplementary material for: Common microRNA–mRNA interactions exist among distinct porcine iPSC lines independent of their metastable pluripotent states
Source: Cell Death Dis. 2017 Aug 31;8(8):e3027–. doi: 10.1038/cddis.2017.426 (PMC5596602; doi:10.1038/cddis.2017.426)
Supplement: Supplementary Table 4 [file cddis2017426x5.pdf]

| GO | Category | GO Term | p-Value | FDR | (-log2P) |
|---|---|---|---|---|---|
| | GOTERM_BP_DIRECT | in utero embryonic development | 1.20E-02 | 1.00E+00 | 6.380821784 |
| | GOTERM_BP_DIRECT | positive regulation of peptidyl-tyrosine phosphorylation | 2.40E-02 | 1.00E+00 | 5.380821784 |
| | GOTERM_BP_DIRECT | actin cytoskeleton reorganization | 3.30E-02 | 1.00E+00 | 4.921390165 |
| | GOTERM_BP_DIRECT | axonal fasciculation | 3.90E-02 | 1.00E+00 | 4.680382066 |
| | GOTERM_BP_DIRECT | autophagy | 4.30E-02 | 1.00E+00 | 4.53951953 |
| | GOTERM_BP_DIRECT | positive regulation of Notch signaling pathway | 4.30E-02 | 1.00E+00 | 4.53951953 |
| | GOTERM_BP_DIRECT | glutathione metabolic process | 4.30E-02 | 1.00E+00 | 4.53951953 |
| | GOTERM_BP_DIRECT | retrograde transport, endosome to Golgi | 4.70E-02 | 1.00E+00 | 4.411195433 |
| | | | | | |
| | | | | | |
| | | | | | |
| **KEGG** | Category | pathway Term | p-Value | FDR | (-log2P) |
| | KEGG_PATHWAY | Metabolic pathways | 1.10E-02 | 9.20E-01 | 6.506352666 |
| | KEGG_PATHWAY | Histidine metabolism | 2.60E-02 | 9.50E-01 | 5.265344567 |
| | KEGG_PATHWAY | MAPK signaling pathway | 3.20E-02 | 9.20E-01 | 4.965784285 |
| | KEGG_PATHWAY | Arachidonic acid metabolism | 3.60E-02 | 8.80E-01 | 4.795859283 |
| | KEGG_PATHWAY | Taurine and hypotaurine metabolism | 4.10E-02 | 8.50E-01 | 4.60823228 |
| | KEGG_PATHWAY | Glycerophospholipid metabolism | 4.20E-02 | 8.10E-01 | 4.573466862 |
| | KEGG_PATHWAY | Fatty acid metabolism | 4.40E-02 | 7.70E-01 | 4.506352666 |
