# Supplementary material for: Common microRNA–mRNA interactions exist among distinct porcine iPSC lines independent of their metastable pluripotent states
Source: Cell Death Dis. 2017 Aug 31;8(8):e3027–. doi: 10.1038/cddis.2017.426 (PMC5596602; doi:10.1038/cddis.2017.426)
Supplement: Supplementary Table 5 [file cddis2017426x6.pdf]

piPS-L Vs PEFs

AccID	piPS-L	PEF	piPS-L_log2(count+1	PEF_log2(count+1	PEF(FDR<0.05)	Log2FC	FDR
FOS	53500.8579	135.50965	15.70730137	7.092859162	7.092859162	8.625023	0
CCK	1006.61037	0	9.97672216	0	0	20	0
MFNG	8947.64774	14.873011	13.12745397	3.98850389	3.98850389	9.232668	0
LOC100622539	2537.92848	4.1313919	11.31000404	2.359350201	2.359350201	9.262808	0
ASPN	0	15025.046	0	13.87517779	13.87517779	-20	0
POSTN	5.52323934	55326.773	2.705588563	15.75571625	15.75571625	-13.2902	0
SLC52A3	7405.28315	1.6525567	12.85453399	1.407383613	1.407383613	12.12964	0
LOC100518318	2441.27179	0	11.25400805	0	0	20	0
DARC	1654.21018	0	10.69279871	0	0	20	0
KRT5	1668.01828	0	10.70478404	0	0	20	0
LOC102159782	1353.19364	1.6525567	10.40321833	1.407383613	1.407383613	9.677453	0
GPX6	1299.34206	0	10.34467546	0	0	20	0
KIT	5454.19885	12.394176	12.41341607	3.74353388	3.74353388	8.781561	0
OTX2	1931.75296	0.8262784	10.91644153	0.868906686	0.868906686	11.191	0
LUM	0	13427.85	0	13.71304812	13.71304812	-20	0
MGP	1.38080984	26806.949	1.251452392	14.71037324	14.71037324	-14.2448	0
RAB3C	2071.21475	4.9576702	11.01695781	2.574748269	2.574748269	8.706599	0
HENMT1	48853.052	4.9576702	15.5761906	2.574748269	2.574748269	13.2665	0
EMSP1	1854.42761	0.8262784	10.857536	0.868906686	0.868906686	11.13206	0
XDH	901.668823	0	9.818052969	0	0	20	0
ANGPTL2	2.76161967	18276.451	1.911353989	14.15777729	14.15777729	-12.6922	0
LOC102164762	2756.09643	0.8262784	11.42893401	0.868906686	0.868906686	11.70371	0
PRKCZ	2528.26281	0	11.30450124	0	0	20	0
DIRAS2	1036.98819	0	10.01957431	0	0	20	0
LOC100738213	161.554751	387901.34	7.34478191	18.56533395	18.56533395	-11.2295	0
TNC	2.76161967	18393.783	1.911353989	14.16700902	14.16700902	-12.7014	0
POU2F3	1030.08414	0	10.00994635	0	0	20	0
IBSP	4230.80134	0	12.04705619	0	0	20	0
ITGA8	1.38080984	16505.737	1.251452392	14.01076732	14.01076732	-13.5452	0
RAC2	1122.5984	0	10.13391075	0	0	20	0
ERBB4	1303.48449	1.6525567	10.34926407	1.407383613	1.407383613	9.623458	0
ANO9	2490.98094	4.1313919	11.28307732	2.359350201	2.359350201	9.23587	0
TRIML2	2685.67513	0	11.39160617	0	0	20	0
TAGLN	38.6626754	120386.28	5.309710091	16.87732344	16.87732344	-11.6044	0
VRTN	2573.82953	0	11.33026121	0	0	20	0
BPIFB6	2134.73201	0	11.06051491	0	0	20	0
TCL1A	17978.1441	0	14.13403672	0	0	20	0
LOC100621401	0	8792.4282	0	13.10221	13.10221	-20	0
LOC102160657	2.76161967	22024.45	1.911353989	14.42688387	14.42688387	-12.9613	0
PKP1	1083.93572	0	10.08339385	0	0	20	0
FOXD4	2089.16528	1.6525567	11.02940131	1.407383613	1.407383613	10.30401	0
CALB2	817.439423	0	9.676731829	0	0	20	0
SOX21	2307.33324	3.3051135	11.17263579	2.106051269	2.106051269	9.447311	0
LOC396757	3612.19853	6.610227	11.81906081	2.927939483	2.927939483	9.093962	0
PNCK	1168.16512	0	10.19126298	0	0	20	0
LOC102164968	33008.2591	1.6525567	15.01058314	1.407383613	1.407383613	14.28584	0
LOC100152743	2735.38429	0.8262784	11.41805513	0.868906686	0.868906686	11.69283	0
LOC102160289	2177.53711	0.8262784	11.08914397	0.868906686	0.868906686	11.36378	0
THBS1	62.1364426	211976.76	5.98040107	17.69355337	17.69355337	-11.7362	0
FMOD	0	59302.825	0	15.85583754	15.85583754	-20	0
CCR1	2275.57461	0	11.15264903	0	0	20	0
LOC102158452	2606.96897	3.3051135	11.34871099	2.106051269	2.106051269	9.623458	0
CDH1	4965.39217	3.3051135	12.27798247	2.106051269	2.106051269	10.55299	0
CRYBA4	987.279033	0	9.948774622	0	0	20	0
TMEM171	1239.96723	0	10.27724931	0	0	20	0
NOXA1	2326.66457	3.3051135	11.18466746	2.106051269	2.106051269	9.459348	0
SNCA	1273.10667	0	10.31527035	0	0	20	0
FAM43B	5131.08935	6.610227	12.32533057	2.927939483	2.927939483	9.60035	0
LMO1	875.433436	0	9.775500714	0	0	20	0
LOC100737072	2039.45613	0	10.99467598	0	0	20	0
SOX2	4460.01577	14.046732	12.12315653	3.911378308	3.911378308	8.31067	0
KRT18	4650.56753	4.1313919	12.18350126	2.359350201	2.359350201	10.13656	0
KRT36	1601.73941	0	10.64632416	0	0	20	0
KRT6A	112193.561	1.6525567	16.77564321	1.407383613	1.407383613	16.05093	0
UTF1	1198.54294	0	10.22826908	0	0	20	0
ACTA2	16.569718	109145.59	4.135019133	16.73590752	16.73590752	-12.6854	0
LOC100523609	1297.96125	0	10.34314267	0	0	20	0
ST14	1615.54751	0	10.65870019	0	0	20	0
LOC100519925	4896.35168	0.8262784	12.25778608	0.868906686	0.868906686	12.53279	0
LOC100511106	2604.20735	3.3051135	11.34718249	2.106051269	2.106051269	9.621929	0
DOCK8	6331.0131	16.525567	12.62844852	4.13138925	4.13138925	8.581593	0
LCP2	2467.50718	4.9576702	11.26942312	2.574748269	2.574748269	8.959176	0
LOC100521510	4598.09675	0	12.16713483	0	0	20	0
COL15A1	0	16447.071	0	14.00563078	14.00563078	-20	0
COL8A2	0	16593.322	0	14.01840209	14.01840209	-20	0
CP	47742.8809	213.17982	15.54302822	7.742678745	7.742678745	7.807071	1.1E-16
FAM198B	1.38080984	11644.741	1.251452392	13.50751483	13.50751483	-13.0419	1.1E-16
PDZD4	1763.29416	4.1313919	10.78487541	2.359350201	2.359350201	8.73743	1.1E-16
MXRA5	2.76161967	14656.526	1.911353989	13.83935397	13.83935397	-12.3737	1.1E-16
LOC100512967	1861.33166	4.9576702	10.86289431	2.574748269	2.574748269	8.552457	1.1E-16
LOC733603	1.38080984	10361.531	1.251452392	13.33908877	13.33908877	-12.8734	1.1E-16
LOC100620394	0	8025.6418	0	12.97058081	12.97058081	-20	1.1E-16
ELN	0	7319.1738	0	12.83766219	12.83766219	-20	2.2E-16
LOC100738461	0	7119.2145	0	12.79770498	12.79770498	-20	2.2E-16
LOC100520836	735.971642	0	9.525465297	0	0	20	2.2E-16
tRNA-Ala	1106.02868	1.6525567	10.11247688	1.407383613	1.407383613	9.386473	2.2E-16
LINGO1	3011.54625	10.741619	11.55676768	3.553559423	3.553559423	8.131149	2.2E-16
BATF3	2856.89555	9.0890621	11.48073747	3.334720158	3.334720158	8.296101	2.2E-16
LAMA4	1.38080984	9344.3821	1.251452392	13.19003794	13.19003794	-12.7244	2.2E-16
IRG6	0	6658.1511	0	12.70112256	12.70112256	-20	3.3E-16
MMP1	2.76161967	11619.953	1.911353989	13.50444073	13.50444073	-12.0388	3.3E-16
LOC102166564	8.28485901	21479.106	3.214880004	14.39071351	14.39071351	-11.3402	3.3E-16
LTBP2	27.6161967	56402.588	4.838760036	15.78349932	15.78349932	-10.996	3.3E-16
LOC100153689	726.305974	0	9.506418615	0	0	20	3.3E-16
LOC100523909	709.736256	0	9.473170485	0	0	20	3.3E-16
DRD2	2948.029	11.567897	11.52602429	3.65167138	3.65167138	7.99348	4.4E-16
COL8A1	9.66566885	22202.926	3.414902536	14.43852718	14.43852718	-11.1656	4.4E-16
VCAM1	0	6099.5869	0	12.57473234	12.57473234	-20	5.6E-16
KCNK5	3093.01403	12.394176	11.59526402	3.74353388	3.74353388	7.963207	5.6E-16
NRARP	1306.24611	3.3051135	10.35231506	2.106051269	2.106051269	8.626511	6.7E-16
NPTX1	6434.57384	31.398578	12.65185308	5.017858594	5.017858594	7.679002	7.8E-16
LOC102160189	11.0464787	22683.82	3.590539588	14.4694396	14.4694396	-11.0039	7.8E-16
EGR2	2919.03199	12.394176	11.51176846	3.74353388	3.74353388	7.879684	8.9E-16
LOC102157779	654.503862	0	9.35646047	0	0	20	8.9E-16
PTF-BETA	1.38080984	7819.0722	1.251452392	12.93296622	12.93296622	-12.4673	8.9E-16
LRAT	973.470934	1.6525567	9.928475344	1.407383613	1.407383613	9.202294	8.9E-16
RAX	958.282026	1.6525567	9.905811215	1.407383613	1.407383613	9.179607	1E-15
CITED4	5965.09849	29.746021	12.54257208	4.942327829	4.942327829	7.647705	1E-15
LOC100627277	0	5187.3756	0	12.34106721	12.34106721	-20	1.6E-15
LOC100522691	1904.13676	7.4365053	10.89567885	3.076645515	3.076645515	8.000297	1.7E-15
PVRL4	1477.46652	4.9576702	10.52988586	2.574748269	2.574748269	8.219247	1.8E-15
LOC100156689	15.1889082	25125.473	4.016933786	14.61692054	14.61692054	-10.6919	1.8E-15
LOC100514847	593.748229	0	9.216135261	0	0	20	2.1E-15
GRM3	585.46337	0	9.195897192	0	0	20	2.4E-15
PRELP	0	4799.8511	0	12.22907446	12.22907446	-20	2.6E-15
WDR72	575.797702	0	9.171921605	0	0	20	2.9E-15
DUOXA1	729.067593	0.8262784	9.511886232	0.868906686	0.868906686	9.785209	2.9E-15
HOXB13	861.625338	1.6525567	9.752590282	1.407383613	1.407383613	9.026217	3.1E-15
IFIT2	0	4632.1166	0	12.17776726	12.17776726	-20	3.2E-15
CLDN5	8011.45867	47.097867	12.96802929	5.587901017	5.587901017	7.410259	3.6E-15
C1QL4	1742.58201	7.4365053	10.76783851	3.076645515	3.076645515	7.872386	4.1E-15
NUP210	13085.9348	80.149002	13.67583961	6.342501448	6.342501448	7.351117	4.3E-15
FBN1	15.1889082	21241.964	4.016933786	14.37469748	14.37469748	-10.4497	4.8E-15
HSPA2	17157.943	108.24247	14.06667307	6.771389985	6.771389985	7.308466	5.4E-15
CDA	532.992597	0	9.06067593	0	0	20	6E-15
COLL11A1	22.0929574	27569.604	4.529381037	14.75084326	14.75084326	-10.2853	6.4E-15
LOC102163958	1027.32252	3.3051135	10.0060771	2.106051269	2.106051269	8.279974	8.2E-15
FOLR1	1500.94029	6.610227	10.55261174	2.927939483	2.927939483	7.826951	8.3E-15
LOC102164256	0	3954.5683	0	11.94966926	11.94966926	-20	8.3E-15
VWC2	3395.41139	19.830681	11.7297955	4.380638094	4.380638094	7.419708	8.3E-15
LOXL4	8.28485901	12123.156	3.214880004	13.56559673	13.56559673	-10.515	9.6E-15
TPD52L1	505.3764	0	8.984066358	0	0	20	1E-14
VIT	1230.30156	4.9576702	10.26596843	2.574748269	2.574748269	7.955134	1.1E-14
LOC100738383	759.44541	1.6525567	9.570700874	1.407383613	1.407383613	8.844103	1.1E-14
ARL4D	11884.6303	80.149002	13.53693079	6.342501448	6.342501448	7.212197	1.1E-14
SYBU	2580.73358	14.873011	11.33412442	3.98850389	3.98850389	7.438941	1.2E-14
CORO1A	3904.93022	24.788351	11.93145046	4.688647628	4.688647628	7.299491	1.4E-14
LOC100739233	0	3576.1328	0	11.80458796	11.80458796	-20	1.6E-14
EIF2S3	0	3510.0305	0	11.77767882	11.77767882	-20	1.7E-14
NPW	3008.78463	19.004403	11.55544454	4.322245637	4.322245637	7.306703	2E-14
LOC100628118	0	3442.2757	0	11.74956598	11.74956598	-20	2E-14
ACTL8	469.475344	0	8.877975309	0	0	20	2.1E-14
VSNL1	693.166538	1.6525567	9.439138011	1.407383613	1.407383613	8.712358	2.7E-14
MX2	0	3252.2317	0	11.66765785	11.66765785	-20	2.8E-14
PROK1	455.667246	0	8.834999507	0	0	20	2.8E-14
COL4A1	52.4707738	47585.371	5.740678649	15.53826083	15.53826083	-9.82479	2.9E-14
LOC102163104	4.14242951	6692.8548	2.362450112	12.70862154	12.70862154	-10.6579	3E-14
TTC22	452.905626	0	8.82624856	0	0	20	3E-14
LOC100519889	1518.89082	8.2627837	10.56975198	3.211445828	3.211445828	7.522175	3.2E-14
LEFTY2	996.944701	4.1313919	9.962816064	2.359350201	2.359350201	7.914742	3.3E-14
LOC102164134	0	3124.9848	0	11.61009505	11.61009505	-20	3.5E-14
COL4A2	49.7091541	43357.305	5.664174303	15.40402074	15.40402074	-9.76855	3.7E-14
DUOXA2	1284.15315	6.610227	10.32772458	2.927939483	2.927939483	7.601902	4E-14
TRIM29	769.111079	2.4788351	9.588922741	1.798604302	1.798604302	8.277386	4.2E-14
LOC102165673	9.66566885	10474.731	3.414902536	13.35476329	13.35476329	-10.0818	4.5E-14
LOC100738003	0	2990.3014	0	11.54655758	11.54655758	-20	4.6E-14
COL3A1	564.751223	487725.68	9.144023987	18.89571337	18.89571337	-9.75424	4.9E-14
LOC100738704	1353.19364	7.4365053	10.40321833	3.076645515	3.076645515	7.507528	5E-14
PEX5L	644.838193	1.6525567	9.335028951	1.407383613	1.407383613	8.608094	5.6E-14
KIAA1244	1521.65244	9.0890621	10.57237095	3.334720158	3.334720158	7.387292	6.1E-14
KLK15	419.76619	0	8.716874974	0	0	20	6.4E-14
LOX	12.4272885	11478.659	3.747096094	13.48679218	13.48679218	-9.85123	7.8E-14
LOC100157228	4.14242951	5598.8622	2.362450112	12.45117562	12.45117562	-10.4004	8.9E-14
PAPP-A	0	2684.5784	0	11.39101714	11.39101714	-20	8.9E-14
DUSP9	1543.7454	9.9153405	10.59315336	3.448285225	3.448285225	7.282557	9.7E-14
LOC100511328	401.815662	0	8.653975969	0	0	20	9.8E-14
LOC102161103	2.76161967	4513.9587	1.911353989	12.14049709	12.14049709	-10.6747	1.1E-13
DPPA4	393.530803	0	8.623994134	0	0	20	1.2E-13
LOC100737363	596.509849	1.6525567	9.222818684	1.407383613	1.407383613	8.495702	1.2E-13
LOC102160384	966.566885	4.9576702	9.918217584	2.574748269	2.574748269	7.607063	1.2E-13
FRMPD4	589.6058	1.6525567	9.206051714	1.407383613	1.407383613	8.478907	1.3E-13
NID2	33.1394361	23899.276	5.093367322	14.54473964	14.54473964	-9.4942	1.4E-13
COL1A2	534.373406	385650.56	9.064401668	18.55693841	18.55693841	-9.49523	1.4E-13
LOC102166965	0	2473.8774	0	11.27314137	11.27314137	-20	1.5E-13
LOC100738989	244.403341	170068.75	7.93901108	17.37576699	17.37576699	-9.44264	1.5E-13
XCR1	383.865134	0	8.58820917	0	0	20	1.5E-13
LOC100737120	4.14242951	5022.9462	2.362450112	12.29460531	12.29460531	-10.2438	1.7E-13
LOC100736872	2.76161967	4152.8751	1.911353989	12.02024212	12.02024212	-10.5544	1.8E-13
FOXA3	570.274462	1.6525567	9.158040228	1.407383613	1.407383613	8.430813	1.9E-13
INHBE	0	2371.4189	0	11.21214307	11.21214307	-20	1.9E-13
LOC100153335	0	2349.9357	0	11.19901936	11.19901936	-20	2E-13
E4	2292.14433	18.178124	11.16311144	4.261389712	4.261389712	6.978351	2E-13
LOC100626687	0	2323.4948	0	11.18270147	11.18270147	-20	2.1E-13
NID1	0	2274.7444	0	11.15212279	11.15212279	-20	2.4E-13
SLC6A17	0	2253.2611	0	11.13843892	11.13843892	-20	2.6E-13
STMN2	5.52323934	5444.3482	2.705588563	12.41080859	12.41080859	-9.94503	2.7E-13
LRRN4CL	5.52323934	5439.3905	2.705588563	12.4094945	12.4094945	-9.94371	2.7E-13
CCL2	74.5637311	45810.525	6.239622035	15.48342298	15.48342298	-9.26299	2.9E-13
LOC100516162	356.248938	0	8.480785911	0	0	20	3.2E-13
CAMSAP3	2024.26722	16.525567	10.98389656	4.13138925	4.13138925	6.936556	3.2E-13
DSE	2.76161967	3738.0834	1.911353989	11.86846892	11.86846892	-10.4026	3.4E-13
LOC100511351	0	2149.9763	0	11.07077593	11.07077593	-20	3.4E-13
LOC100153940	2.76161967	3705.8585	1.911353989	11.85598133	11.85598133	-10.3901	3.5E-13
ADAM19	26.2353869	16526.394	4.767410456	14.01257162	14.01257162	-9.29904	3.6E-13
KCNAB1	2078.1188	17.351846	11.02175648	4.19785327	4.19785327	6.904045	3.7E-13
LOC102163946	350.725698	0	8.458306937	0	0	20	3.7E-13
LOC100620960	350.725698	0	8.458306937	0	0	20	3.7E-13
LOC100621417	0	2088.8317	0	11.02917106	11.02917106	-20	4.1E-13
LOC100524999	1012.13361	6.610227	9.984608731	2.927939483	2.927939483	7.258484	4.2E-13
MOBP	342.440839	0	8.423917793	0	0	20	4.6E-13
SLC8A1	0	2005.3776	0	10.97037744	10.97037744	-20	5.2E-13
ALPL	1143.31054	8.2627837	10.16026291	3.211445828	3.211445828	7.112374	5.3E-13
LOC100155138	5082.76101	49.576702	12.31168049	5.660401068	5.660401068	6.679806	5.4E-13
NFE2L3	2936.98252	27.267186	11.5206101	4.821056379	4.821056379	6.751025	5.4E-13
LOC100738735	664.169531	3.3051135	9.377578275	2.106051269	2.106051269	7.650708	6.1E-13
DNAJC12	4542.86436	44.619032	12.14970405	5.511563931	5.511563931	6.669799	6.2E-13
SEMA3C	0	1944.233	0	10.92572726	10.92572726	-20	6.3E-13
DAAM2	23.4737672	13680.691	4.613164285	13.73995893	13.73995893	-9.18688	6.3E-13
C12H17orf67	0	1926.0549	0	10.91218195	10.91218195	-20	6.7E-13
SULF2	1.38080984	2634.1754	1.251452392	11.3636833	11.3636833	-10.8976	6.9E-13
LOC100152082	16.569718	9950.0442	4.135019133	13.2806322	13.2806322	-9.23001	7.4E-13
MMP23B	5.52323934	4607.3282	2.705588563	12.17002775	12.17002775	-9.7042	7.5E-13
LOC100621838	5.52323934	4604.8494	2.705588563	12.16925151	12.16925151	-9.70342	7.5E-13
LOC100525680	2.76161967	3270.4098	1.911353989	11.67569678	11.67569678	-10.2097	7.6E-13
SDC2	15.1889082	9156.8169	4.016933786	13.16078801	13.16078801	-9.23568	8E-13
DDX60	0	1853.3424	0	10.85669193	10.85669193	-20	8.4E-13
DUOX2	867.148577	5.7839486	9.761798159	2.762125239	2.762125239	7.228081	8.5E-13
AWN	0	1846.7322	0	10.85153993	10.85153993	-20	8.6E-13
PTGFR	0	1842.6008	0	10.84831056	10.84831056	-20	8.7E-13
PLN	320.347882	0	8.327992153	0	0	20	8.9E-13
LOC100512873	318.967072	0	8.321779634	0	0	20	9.2E-13
LOC100738123	423.90862	219619.01	8.7310088	17.74464996	17.74464996	-9.01703	1E-12
LOC100157992	31.7586262	16447.897	5.033802952	14.00570325	14.00570325	-9.01654	1.1E-12
LOC102165390	313.443833	0	8.29665853	0	0	20	1.1E-12
MFAP2	23.4737672	12445.405	4.613164285	13.60344146	13.60344146	-9.05035	1.1E-12
OCLN	469.475344	1.6525567	8.877975309	1.407383613	1.407383613	8.150206	1.3E-12
F7	755.30298	4.9576702	9.562820493	2.574748269	2.574748269	7.251249	1.4E-12
GABRA5	463.952105	1.6525567	8.8609383	1.407383613	1.407383613	8.133132	1.4E-12
NPY	0	1688.0867	0	10.72202768	10.72202768	-20	1.5E-12
SPOCK3	0	1679.8239	0	10.71495289	10.71495289	-20	1.5E-12
MYBPC2	2078.1188	20.656959	11.02175648	4.436758793	4.436758793	6.652506	1.5E-12
LOC100626400	1159.88026	9.9153405	10.18100346	3.448285225	3.448285225	6.870098	1.6E-12
GREM1	0	1662.4721	0	10.69998194	10.69998194	-20	1.6E-12
COL1A1	265.115489	124736.64	8.055908671	16.92853729	16.92853729	-8.87805	1.6E-12
CRHR1	1017.65685	8.2627837	9.992452423	3.211445828	3.211445828	6.944408	1.7E-12
CCL11	0	1648.4254	0	10.68774777	10.68774777	-20	1.7E-12
FAP	1.38080984	2238.3881	1.251452392	11.12888887	11.12888887	-10.6627	1.9E-12
NTRK2	450.144007	1.6525567	8.817444209	1.407383613	1.407383613	8.089543	1.9E-12
tRNA-Cys	3075.0635	33.051135	11.58686957	5.089630977	5.089630977	6.539773	1.9E-12
KCNE4	0	1620.3319	0	10.66296373	10.66296373	-20	1.9E-12
SRPX2	0	1613.7217	0	10.65706978	10.65706978	-20	2E-12
LOC102167439	0	1613.7217	0	10.65706978	10.65706978	-20	2E-12
AVPR2	294.112495	0	8.205121195	0	0	20	2E-12
ARG1	515.042069	2.4788351	9.011344871	1.798604302	1.798604302	7.698884	2.1E-12
DUSP2	2823.75611	30.5723	11.4639106	4.980587446	4.980587446	6.529247	2.2E-12
CHI3L1	5.52323934	3854.5886	2.705588563	11.91273541	11.91273541	-9.44685	2.2E-12
NKX2-5	291.350875	0	8.191557101	0	0	20	2.2E-12
LOC102167666	441.859147	1.6525567	8.790704108	1.407383613	1.407383613	8.062743	2.3E-12
LOC100624590	2.76161967	2715.977	1.911353989	11.40778664	11.40778664	-9.94174	2.3E-12
POU5F1	705.593826	4.9576702	9.464737333	2.574748269	2.574748269	7.153032	2.7E-12
TDGF1	433.574288	1.6525567	8.76345901	1.407383613	1.407383613	8.035436	2.7E-12
AIF1	967.947695	8.2627837	9.920274979	3.211445828	3.211445828	6.872157	2.7E-12
LOC100737939	2.76161967	2620.1287	1.911353989	11.35597249	11.35597249	-9.88991	2.9E-12
PPP1R1A	2003.55507	21.483238	10.96906634	4.490777898	4.490777898	6.543207	3.1E-12
CREB3L1	24.854577	10908.527	4.692347799	13.41330094	13.41330094	-8.77773	3.4E-12
GBX2	622.745236	4.1313919	9.284813081	2.359350201	2.359350201	7.23587	3.4E-12
CAMKV	2613.87302	29.746021	11.35252517	4.942327829	4.942327829	6.457349	3.7E-12
MYLK	0	1434.4193	0	10.48725646	10.48725646	-20	4E-12
POF1B	273.400348	0	8.100138498	0	0	20	4.1E-12
CHGA	2006.31669	22.309516	10.97105253	4.542847146	4.542847146	6.490746	4.1E-12
NT5E	12.4272885	5935.9838	3.747096094	12.53551447	12.53551447	-8.89983	4.3E-12
CSF1	4.14242951	2943.2036	2.362450112	11.52366171	11.52366171	-9.47269	4.4E-12
CARP	8.28485901	4422.2418	3.214880004	12.11088841	12.11088841	-9.06009	4.4E-12
COL5A3	0	1383.19	0	10.43482627	10.43482627	-20	5E-12
GADL1	266.496298	0	8.063375117	0	0	20	5.2E-12
CXCL10	2.76161967	2351.5882	1.911353989	11.20003312	11.20003312	-9.73391	5.6E-12
LOC100513671	0	1343.5286	0	10.39288476	10.39288476	-20	5.9E-12
LOC102158737	262.353869	0	8.040858843	0	0	20	6.1E-12
CCN2	125.653695	46688.859	6.984745358	15.51082162	15.51082162	-8.53748	6.2E-12
LOC100739307	1.38080984	1824.4226	1.251452392	10.83401482	10.83401482	-10.3677	6.4E-12
LOC102164667	258.211439	0	8.017985577	0	0	20	7.1E-12
LOC100521530	0	1300.5622	0	10.34602849	10.34602849	-20	7.2E-12
LOC100517050	1578.26564	18.178124	10.62503815	4.261389712	4.261389712	6.439993	8E-12
LOC102164776	1.38080984	1751.7101	1.251452392	10.77537172	10.77537172	-10.309	8.2E-12
TENM4	2.76161967	2188.8114	1.911353989	11.09659091	11.09659091	-9.63042	8.6E-12
IFIT3	24.854577	9279.1061	4.692347799	13.17992559	13.17992559	-8.54433	9E-12

piPS-F Vs PEFs

AccID	piPS-F	PEF	piPS-F_log2(count+1	PEF_log2(count+1	PEF_log2(count+1)_FDR<0.0	Log2FC	FDR
POSTN	1.057359087	55326.773	1.04079362	15.75571625	15.75571625	-15.6752	0
OTX2	6196.124247	0.8262784	12.59738318	0.868906686	0.868906686	12.87245	0
MGP	0	26806.949	0	14.71037324	14.71037324	-20	0
AEBP1	0	41982.378	0	15.35753062	15.35753062	-20	0
LOC100738123	0	219619.01	0	17.74464996	17.74464996	-20	0
LOC100738213	0	387901.34	0	18.56533395	18.56533395	-20	0
COL1A1	0	124736.64	0	16.92853729	16.92853729	-20	0
CCL2	0	45810.525	0	15.48342298	15.48342298	-20	0
ACTL8	7079.019085	0	12.78953753	0	0	20	0
LIN28A	5810.188181	0	12.50461746	0	0	20	0
COL3A1	7.401513606	487725.68	3.070649265	18.89571337	18.89571337	-16.0079	0
GABRA5	13109.13796	1.6525567	13.67839525	1.407383614	1.407383614	12.95359	0
COL4A2	3.17207726	43357.305	2.060765874	15.40402074	15.40402074	-13.7386	1.1E-16
COL4A1	5.286795433	47585.371	2.65232482	15.53826083	15.53826083	-13.1358	3.3E-16
ERAS	3455.449495	0	11.75507513	0	0	20	4.4E-16
LOC100513556	0	13236.153	0	13.69230527	13.69230527	-20	7.8E-16
MFAP2	0	12445.405	0	13.60344146	13.60344146	-20	1E-15
CSPG4	0	12812.272	0	13.64535136	13.64535136	-20	1E-15
LOC100156358	7.401513606	43927.437	3.070649265	15.42286755	15.42286755	-12.535	1.3E-15
MMP1	0	11619.953	0	13.50444073	13.50444073	-20	1.6E-15
HSD17B2	2799.886861	0	11.45166799	0	0	20	1.6E-15
COL1A2	87.76080419	385650.56	6.471850834	18.55693841	18.55693841	-12.1014	1.8E-15
PXDN	2.114718173	19766.231	1.639101631	14.27082319	14.27082319	-13.1903	1.9E-15
SOX21	5971.964121	3.3051135	12.54423134	2.106051269	2.106051269	10.81929	2.2E-15
FMOD	12.68830904	59302.825	3.774872332	15.85583754	15.85583754	-12.1904	2.2E-15
DES	10.57359087	49798.145	3.532764645	15.60383335	15.60383335	-12.2014	2.6E-15
LOC733603	0	10361.531	0	13.33908877	13.33908877	-20	2.8E-15
LOC100623720	4.229436346	24788.351	2.386655454	14.59743289	14.59743289	-12.5169	3.3E-15
PRRX2	1.057359087	13710.437	1.04079362	13.74309216	13.74309216	-13.6625	3.4E-15
COL6A3	3.17207726	20982.513	2.060765874	14.35696861	14.35696861	-12.6915	3.6E-15
LOC100738989	44.40908164	170068.75	5.504908955	17.37576699	17.37576699	-11.903	3.7E-15
LUM	1.057359087	13427.85	1.04079362	13.71304812	13.71304812	-13.6325	3.8E-15
VRTN	2408.663999	0	11.23461628	0	0	20	4E-15
ST8SIA6	2379.057945	0	11.21678098	0	0	20	4.4E-15
ST14	2351.566609	0	11.20001986	0	0	20	4.8E-15
DCN	0	8943.6371	0	13.12680723	13.12680723	-20	5.8E-15
LOC100621401	0	8792.4282	0	13.10221	13.10221	-20	6.2E-15
PTGES	0	7529.0485	0	12.87844345	12.87844345	-20	1.4E-14
BACE2	0	7459.6411	0	12.8650839	12.8650839	-20	1.5E-14
ELN	0	7319.1738	0	12.83766219	12.83766219	-20	1.6E-14
LOC102160657	6.344154519	22024.45	2.876596413	14.42688387	14.42688387	-11.7614	2.4E-14
LOC100622870	3240.8056	2.4788351	11.66258186	1.798604302	1.798604302	10.35247	4.7E-14
ENTPD3	0	5839.3093	0	12.51182905	12.51182905	-20	5.1E-14
COL8A2	5.286795433	16593.322	2.65232482	14.01840209	14.01840209	-11.6159	5.4E-14
GAS7	0	5731.8931	0	12.48504765	12.48504765	-20	5.6E-14
LOC100157992	5.286795433	16447.897	2.65232482	14.00570325	14.00570325	-11.6032	5.7E-14
SALL4	2628.594689	1.6525567	11.36062473	1.407383614	1.407383614	10.63538	5.9E-14
TNC	6.344154519	18393.783	2.876596413	14.16700902	14.16700902	-11.5015	5.9E-14
LOC100152082	2.114718173	9950.0442	1.639101631	13.2806322	13.2806322	-12.2	6E-14
AMCF-II	2.114718173	9804.6192	1.639101631	13.25939301	13.25939301	-12.1788	6.5E-14
CDH1	3527.349913	3.3051135	11.78477793	2.106051269	2.106051269	10.05967	7E-14
LOC102159782	2541.891244	1.6525567	11.31225405	1.407383614	1.407383614	10.58699	7.3E-14
TMEM171	1541.629548	0	10.59117593	0	0	20	7.4E-14
LOC100511106	3464.965727	3.3051135	11.75904167	2.106051269	2.106051269	10.03393	7.8E-14
LOC100628205	3404.696259	3.3051135	11.73373406	2.106051269	2.106051269	10.00861	8.8E-14
LOC100627277	0	5187.3756	0	12.34106721	12.34106721	-20	9.3E-14
LOC100623527	1486.646876	0	10.5388164	0	0	20	9.4E-14
CRMP1	0	5056.8236	0	12.30430102	12.30430102	-20	1.1E-13
SVEP1	0	4956.844	0	12.27549715	12.27549715	-20	1.2E-13
AHNAK	25.37661808	49721.301	4.721187694	15.60160544	15.60160544	-10.9361	1.3E-13
PRELP	0	4799.8511	0	12.22907446	12.22907446	-20	1.4E-13
LOC100622539	3575.988431	4.1313919	11.80452974	2.359350201	2.359350201	9.757498	1.5E-13
LEFTY2	3472.36724	4.1313919	11.76211924	2.359350201	2.359350201	9.715076	1.8E-13
LOC100736763	0	4545.3573	0	12.15049536	12.15049536	-20	1.8E-13
FAM198B	5.286795433	11644.741	2.65232482	13.50751483	13.50751483	-11.105	3.3E-13
TMEM163	2003.695469	1.6525567	10.96916738	1.407383614	1.407383614	10.24375	3.4E-13
GPR124	21.14718173	34432.672	4.46905122	15.07153243	15.07153243	-10.6691	3.5E-13
CASP4	0	3914.9069	0	11.93513076	11.93513076	-20	3.9E-13
LOC100736983	1190.586331	0	10.21866776	0	0	20	4E-13
CHI3L1	0	3854.5886	0	11.91273541	11.91273541	-20	4.2E-13
LOC100513452	1169.43915	0	10.19283422	0	0	20	4.4E-13
SIX1	5.286795433	10886.218	2.65232482	13.41034767	13.41034767	-11.0078	4.6E-13
IRG6	2.114718173	6658.1511	1.639101631	12.70112256	12.70112256	-11.6204	4.7E-13
LTBP2	39.1222862	56402.588	5.326331909	15.78349932	15.78349932	-10.4936	5.4E-13
C1QTNF5	0	3612.489	0	11.8191768	11.8191768	-20	5.9E-13
ADM	1.057359087	4836.2073	1.04079362	12.23995865	12.23995865	-12.1592	7E-13
LOC100628118	0	3442.2757	0	11.74956598	11.74956598	-20	7.5E-13
SLC6A1	1067.932677	0	10.06195528	0	0	20	8E-13
CMPK2	0	3366.2581	0	11.71735859	11.71735859	-20	8.4E-13
MCF2	1057.359087	0	10.04761348	0	0	20	8.5E-13
IGFBP6	1.057359087	4610.6333	1.04079362	12.17106209	12.17106209	-12.0903	8.9E-13
LOC100525680	0	3270.4098	0	11.67569678	11.67569678	-20	9.8E-13
MX2	0	3252.2317	0	11.66765785	11.66765785	-20	1E-12
EHBP1L1	3.17207726	6902.7295	2.060765874	12.75316023	12.75316023	-11.0875	1E-12
OBSL1	3.17207726	6779.614	2.060765874	12.72720021	12.72720021	-11.0616	1.1E-12
CRLF1	1.057359087	4342.9191	1.04079362	12.08478153	12.08478153	-12.004	1.2E-12
LOC102166006	973.8277187	0	9.929003464	0	0	20	1.5E-12
MCSF	3.17207726	6173.1257	2.060765874	12.59201914	12.59201914	-10.9264	1.8E-12
LOC100737434	13.74566813	17070.085	3.882219287	14.05926713	14.05926713	-10.2783	1.8E-12
LOC100737120	2.114718173	5022.9462	1.639101631	12.29460531	12.29460531	-11.2139	2E-12
NPYR5	919.9024053	0	9.846904461	0	0	20	2.1E-12
LOC100156689	22.20454082	25125.473	4.536335244	14.61692054	14.61692054	-10.1441	2.2E-12
HOXB6	0	2758.9435	0	11.43042301	11.43042301	-20	2.3E-12
KIF1A	1.057359087	3651.3241	1.04079362	11.83459909	11.83459909	-11.7537	2.9E-12
LOC100621838	2.114718173	4604.8494	1.639101631	12.16925151	12.16925151	-11.0885	3E-12
CDC42EP5	2.114718173	4564.3617	1.639101631	12.15651346	12.15651346	-11.0757	3.2E-12
TIMP-3	5.286795433	7434.0265	2.65232482	12.86012217	12.86012217	-10.4575	3.2E-12
INSM1	842.715192	0	9.720612269	0	0	20	3.7E-12
SERPINH1	57.09739067	55440.8	5.860401464	15.75868647	15.75868647	-9.92331	3.9E-12
PDLIM4	0	2430.0847	0	11.24738444	11.24738444	-20	4.4E-12
ADAM33	0	2406.9489	0	11.23358906	11.23358906	-20	4.6E-12
LOC100152170	0	2376.3766	0	11.21515474	11.21515474	-20	5E-12
LOC102166259	0	2373.0715	0	11.21314766	11.21314766	-20	5E-12
MAB21L2	1.057359087	3284.4565	1.04079362	11.68187814	11.68187814	-11.601	5E-12
FABP7	11260.87427	33.877413	13.45915933	5.124221138	5.124221138	8.376779	5.1E-12
CXCL10	0	2351.5882	0	11.20003312	11.20003312	-20	5.2E-12
LOC100153335	0	2349.9357	0	11.19901936	11.19901936	-20	5.2E-12
SLC2A1	3.17207726	5001.463	2.060765874	12.28842287	12.28842287	-10.6227	5.3E-12
THY1	48.63851798	44528.141	5.633388138	15.44246218	15.44246218	-9.8384	5.3E-12
PRRX1	0	2323.4948	0	11.18270147	11.18270147	-20	5.6E-12
C1QTNF6	0	2309.448	0	11.17395694	11.17395694	-20	5.7E-12
LOC102161109	7.401513606	8165.2829	3.070649265	12.99546383	12.99546383	-10.1075	6.5E-12
LOC100622285	7.401513606	7905.8315	3.070649265	12.94888396	12.94888396	-10.0609	7.7E-12
KLHDC8A	1697.061334	3.3051135	10.72967285	2.106051269	2.106051269	9.004123	8E-12
POU5F1	2148.553664	4.9576702	11.06982141	2.57474827	2.57474827	8.759488	8.3E-12
ASPN	16.91774539	15025.046	4.163317207	13.8751778	13.8751778	-9.79462	8.7E-12
LOC100622125	738.0366424	0	9.529502087	0	0	20	8.7E-12
EOMES	738.0366424	0	9.529502087	0	0	20	8.7E-12
LPPR5	2583.128248	6.610227	11.33546196	2.927939483	2.927939483	8.610204	8.8E-12
ACTA2	131.1125267	109145.59	7.045623457	16.73590752	16.73590752	-9.70123	9.2E-12
AKR1CL1	6.344154519	6820.928	2.876596413	12.7359638	12.7359638	-10.0703	9.3E-12
C3	3.17207726	4449.509	2.060765874	12.11975464	12.11975464	-10.454	9.6E-12
PLA2G4A	2.114718173	3654.6292	1.639101631	11.83590404	11.83590404	-10.755	9.9E-12
LOC100519925	963.2541279	0.8262784	9.913269607	0.868906686	0.868906686	10.18707	9.9E-12
SERTAD4	1.057359087	2809.3465	1.04079362	11.45653228	11.45653228	-11.3756	1.1E-11
SLA-3	6.344154519	6546.6035	2.876596413	12.67675125	12.67675125	-10.0111	1.2E-11
PARP12	3.17207726	4273.5117	2.060765874	12.06154392	12.06154392	-10.3958	1.2E-11
LOC100513601	5.286795433	5755.8551	2.65232482	12.49106519	12.49106519	-10.0884	1.2E-11
PDGFRA	2.114718173	3512.5094	1.639101631	11.77869703	11.77869703	-10.6978	1.2E-11
MAP7D2	1586.03863	3.3051135	10.63212153	2.106051269	2.106051269	8.906512	1.2E-11
KCNJ3	1366.10794	2.4788351	10.41691144	1.798604302	1.798604302	9.106193	1.3E-11
HOXB3	1.057359087	2740.7654	1.04079362	11.42088939	11.42088939	-11.3399	1.3E-11
SOX11	0	1957.4535	0	10.93549913	10.93549913	-20	1.3E-11
C12H17orf67	0	1926.0549	0	10.91218195	10.91218195	-20	1.4E-11
COL5A1	144.8581949	110737.83	7.188422634	16.75680162	16.75680162	-9.57829	1.4E-11
LOC102165113	1962.458465	4.9576702	10.93918136	2.57474827	2.57474827	8.628784	1.5E-11
LOC102161974	1726.667388	4.1313919	10.75460978	2.359350201	2.359350201	8.707147	1.6E-11
NFE2L3	7627.788451	27.267186	12.89723824	4.821056378	4.821056378	8.127955	1.7E-11
AWN	0	1846.7322	0	10.85153993	10.85153993	-20	1.8E-11
PLAC9	1.057359087	2550.7213	1.04079362	11.31725507	11.31725507	-11.2362	1.8E-11
LOC100153981	2307.157527	6.610227	11.17252597	2.927939483	2.927939483	8.447201	1.8E-11
ZNF275	0	1793.0241	0	10.80898353	10.80898353	-20	2.1E-11
LOC102164776	0	1751.7101	0	10.77537172	10.77537172	-20	2.3E-11
INPP5D	1042.556059	1.6525567	10.02729239	1.407383614	1.407383614	9.301209	2.4E-11
DAAM2	19.03246356	13680.691	4.324267947	13.73995893	13.73995893	-9.48946	2.4E-11
TSHZ3	0	1718.659	0	10.74790681	10.74790681	-20	2.6E-11
tRNA-Ala	1025.638314	1.6525567	10.00371229	1.407383614	1.407383614	9.277607	2.6E-11
LOC100514841	1.057359087	2366.4613	1.04079362	11.2091251	11.2091251	-11.1281	2.7E-11
LOC100620394	10.57359087	8025.6418	3.532764645	12.97058081	12.97058081	-9.56801	2.8E-11
LOC100627044	0	1683.9553	0	10.71849462	10.71849462	-20	2.9E-11
NFIX	1.057359087	2329.2787	1.04079362	11.18628681	11.18628681	-11.1052	2.9E-11
LOC100739233	3.17207726	3576.1328	2.060765874	11.80458796	11.80458796	-10.1388	2.9E-11
GM2A	3.17207726	3566.2175	2.060765874	11.80058345	11.80058345	-10.1348	3E-11
NECD	0	1658.3407	0	10.69639441	10.69639441	-20	3.1E-11
LOC102157759	1749.929288	4.9576702	10.77390511	2.57474827	2.57474827	8.463419	3.1E-11
CCL11	0	1648.4254	0	10.68774777	10.68774777	-20	3.2E-11
TPD52L1	598.465243	0	9.227532298	0	0	20	3.4E-11
SLC6A17	1.057359087	2253.2611	1.04079362	11.13843892	11.13843892	-11.0573	3.4E-11
KCNE4	0	1620.3319	0	10.66296373	10.66296373	-20	3.5E-11
TAC3	593.1784476	0	9.214752466	0	0	20	3.6E-11
CRABP2	24.31925899	15784.396	4.662163277	13.94630281	13.94630281	-9.34218	3.6E-11
PHF2	1.057359087	2227.6465	1.04079362	11.12195208	11.12195208	-11.0408	3.6E-11
LOC100525528	0	1597.1961	0	10.64222872	10.64222872	-20	3.7E-11
KIFC3	1.057359087	2210.2946	1.04079362	11.11067555	11.11067555	-11.0296	3.8E-11
LOC102159390	3.17207726	3391.8727	2.060765874	11.72829159	11.72829159	-10.0624	3.8E-11
LOC100518132	22.20454082	14235.124	4.536335244	13.79726876	13.79726876	-9.32438	3.9E-11
PLEKHF1	1.057359087	2174.7647	1.04079362	11.08730681	11.08730681	-11.0062	4.1E-11
ACTG2	35.95020894	22116.993	5.207510617	14.43293287	14.43293287	-9.26494	4.2E-11
AIF1	2388.574177	8.2627837	11.22253784	3.211445828	3.211445828	8.175306	4.2E-11
LOC102166564	34.89284986	21479.106	5.165624571	14.39071351	14.39071351	-9.26579	4.2E-11
PPP1R26	0	1550.9245	0	10.59984266	10.59984266	-20	4.3E-11
LOC100525396	0	1536.8778	0	10.58672513	10.58672513	-20	4.5E-11
CXCL2	1.057359087	2125.188	1.04079362	11.05405343	11.05405343	-10.9729	4.6E-11
MVP	14.80302721	9626.143	3.982129041	13.23289201	13.23289201	-9.34492	4.6E-11
PAPP-A	2.114718173	2684.5784	1.639101631	11.39101714	11.39101714	-10.31	4.7E-11
LOC733579	1.057359087	2098.7471	1.04079362	11.03599984	11.03599984	-10.9548	4.9E-11
LOC100515546	0	1513.742	0	10.56485635	10.56485635	-20	4.9E-11
SQRDL	0	1509.6106	0	10.56091609	10.56091609	-20	5E-11
TINAGL1	0	1483.996	0	10.53624329	10.53624329	-20	5.4E-11
TAGLN	203.0129446	120386.28	7.672516884	16.87732344	16.87732344	-9.21188	5.6E-11
TNS1	1.057359087	1972.3265	1.04079362	10.94641394	10.94641394	-10.8652	6.7E-11
LOC100624156	0	1416.2411	0	10.46886952	10.46886952	-20	6.9E-11
LOC100525721	0	1404.6732	0	10.45704554	10.45704554	-20	7.2E-11
LOC100627123	10.57359087	6652.3672	3.532764645	12.69986894	12.69986894	-9.29726	7.4E-11
LOC102164419	1.057359087	1927.7074	1.04079362	10.91341861	10.91341861	-10.8322	7.6E-11
CSF1	3.17207726	2943.2036	2.060765874	11.52366171	11.52366171	-9.85774	7.9E-11
ACKR3	6.344154519	4399.1061	2.876596413	12.10332258	12.10332258	-9.43757	8.7E-11
WFDC1	11.63094995	6949.8274	3.658891241	12.762969	12.762969	-9.22286	8.7E-11
LOC100513671	0	1343.5286	0	10.39288476	10.39288476	-20	9E-11
ODF4	511.7617979	0	9.002144971	0	0	20	9.2E-11
LOC102165697	678.8245336	0.8262784	9.409018617	0.868906686	0.868906686	9.682195	9.4E-11
SLIT3	9.516231779	5827.7414	3.394545939	12.50896867	12.50896867	-9.25833	9.5E-11
KCNK13	507.5323616	0	8.990195776	0	0	20	9.7E-11
BOK	0	1291.4731	0	10.33591853	10.33591853	-20	1.1E-10
LOC102161219	0	1290.6468	0	10.33499592	10.33499592	-20	1.1E-10
CLDN10	812.0517785	1.6525567	9.667203422	1.407383614	1.407383614	8.940728	1.2E-10
UROC1	965.368846	2.4788351	9.916430135	1.798604302	1.798604302	8.605274	1.2E-10
FAP	2.114718173	2238.3881	1.639101631	11.12888887	11.12888887	-10.0478	1.2E-10
EPAS1	7.401513606	4575.1033	3.070649265	12.15990392	12.15990392	-9.27177	1.2E-10
LRAT	802.5355467	1.6525567	9.650218037	1.407383614	1.407383614	8.923722	1.3E-10
IER5L	9.516231779	5495.5775	3.394545939	12.42431786	12.42431786	-9.17366	1.3E-10
LOC100049650	14.80302721	7884.3482	3.982129041	12.94495875	12.94495875	-9.05696	1.3E-10
LOC100738990	1.057359087	1729.4006	1.04079362	10.75689038	10.75689038	-10.6756	1.3E-10
KIAA1244	2139.037432	9.0890621	11.06342032	3.334720158	3.334720158	7.878615	1.4E-10
DPT	0	1233.6336	0	10.26986725	10.26986725	-20	1.4E-10
C1QL4	1831.345938	7.4365053	10.83947619	3.076645515	3.076645515	7.944064	1.4E-10
LOC100738662	8616.419196	43.792754	13.07304015	5.485193456	5.485193456	7.620252	1.4E-10
RBP4	0	1226.1971	0	10.26115127	10.26115127	-20	1.4E-10
LOC100625416	0	1223.7183	0	10.2582342	10.2582342	-20	1.4E-10
LOC102164256	6.344154519	3954.5683	2.876596413	11.94966926	11.94966926	-9.28388	1.5E-10
HSPA12B	0	1210.4978	0	10.24257609	10.24257609	-20	1.5E-10
RFX4	2391.746254	10.741619	11.22445169	3.553559423	3.553559423	7.798709	1.6E-10
C1QTNF1	2.114718173	2121.0566	1.639101631	11.05124741	11.05124741	-9.9701	1.6E-10
LOC100153689	470.5247935	0	8.881189822	0	0	20	1.6E-10
HOXC6	0	1198.9299	0	10.22873443	10.22873443	-20	1.6E-10
ARAP3	7.401513606	4325.5673	3.070649265	12.07900712	12.07900712	-9.19085	1.7E-10
TRIM44	1.057359087	1645.1202	1.04079362	10.684854	10.684854	-10.6035	1.7E-10
GLI3	2.114718173	2078.9164	1.639101631	11.02230982	11.02230982	-9.94115	1.7E-10
ENPP2	4402.843236	22.309516	12.1045474	4.542847146	4.542847146	7.624632	1.8E-10
VSNL1	761.2985423	1.6525567	9.574212307	1.407383614	1.407383614	8.847619	1.8E-10
OCLN	760.2411832	1.6525567	9.572209803	1.407383614	1.407383614	8.845614	1.8E-10
LOC100628220	0	1165.8788	0	10.18843898	10.18843898	-20	1.8E-10
LOC100739007	3.17207726	2482.9665	2.060765874	11.27843001	11.27843001	-9.61242	1.9E-10
GALNTL2	4.229436346	2873.7962	2.386655454	11.48924396	11.48924396	-9.40828	2E-10
LOC100623776	0	1146.0481	0	10.16371018	10.16371018	-20	2E-10
LOC100625207	0	1140.2642	0	10.15641704	10.15641704	-20	2.1E-10
EPHB1	1442.237794	5.7839486	10.49509331	2.76212524	2.76212524	7.962039	2.1E-10
HOXA5	3.17207726	2425.127	2.060765874	11.24443937	11.24443937	-9.57842	2.1E-10
ACTC1	11.63094995	5781.4698	3.658891241	12.4974701	12.4974701	-8.95732	2.2E-10
LOC102165673	23.2618999	10474.731	4.600620625	13.35476329	13.35476329	-8.81473	2.3E-10
LOC100627141	1.057359087	1538.5303	1.04079362	10.58827457	10.58827457	-10.5069	2.4E-10
LOC100514326	0	1101.4291	0	10.10647012	10.10647012	-20	2.5E-10
GNG11	0	1091.5137	0	10.09343569	10.09343569	-20	2.6E-10
LOC102164134	5.286795433	3124.9848	2.65232482	11.61009505	11.61009505	-9.20724	2.7E-10
ZNF503	2.114718173	1911.1819	1.639101631	10.90100403	10.90100403	-9.81978	2.7E-10
IL33	2.114718173	1905.3979	1.639101631	10.89663357	10.89663357	-9.81541	2.7E-10
LOC102160289	572.0312658	0.8262784	9.162470047	0.868906686	0.868906686	9.43525	2.8E-10
LOXL4	28.54869534	12123.156	4.885022527	13.56559673	13.56559673	-8.73012	2.9E-10
MMP23B	9.516231779	4607.3282	3.394545939	12.17002775	12.17002775	-8.91932	3.1E-10
SAMD9	8.458872693	4197.4941	3.241668254	12.03565625	12.03565625	-8.95485	3.2E-10
LOC100739307	2.114718173	1824.4226	1.639101631	10.83401482	10.83401482	-9.75276	3.4E-10
PLAU	8.458872693	4118.9977	3.241668254	12.00842781	12.00842781	-8.92761	3.5E-10
BCL3	2.114718173	1805.4182	1.639101631	10.81891625	10.81891625	-9.73765	3.6E-10
WISP1	0	1014.6698	0	9.988215792	9.988215792	-20	3.7E-10
ZNF12	0	1012.191	0	9.984690459	9.984690459	-20	3.8E-10
CCL20	0	1005.5808	0	9.975247238	9.975247238	-20	3.9E-10
ELOVL2	2573.612017	14.046732	11.33013933	3.911378308	3.911378308	7.517416	4.1E-10
COL5A3	1.057359087	1383.19	1.04079362	10.43482627	10.43482627	-10.3533	4.1E-10
LOC100514082	404.9685302	0	8.665224087	0	0	20	4.1E-10
IFFO1	2.114718173	1750.8839	1.639101631	10.77469143	10.77469143	-9.6934	4.2E-10
LOC102161033	0	987.40265	0	9.948955075	9.948955075	-20	4.3E-10
SLC26A7	0	986.57638	0	9.947748515	9.947748515	-20	4.3E-10
EVC2	0	985.7501	0	9.946540946	9.946540946	-20	4.3E-10
FBXO32	0	970.05081	0	9.923402974	9.923402974	-20	4.7E-10
S1PR3	0	961.78802	0	9.911074387	9.911074387	-20	4.9E-10
LOC100517050	3121.324024	18.178124	11.60840455	4.261389711	4.261389711	7.423811	4.9E-10
P4HA3	25.37661808	9791.3987	4.721187694	13.25744658	13.25744658	-8.59187	4.9E-10
SEMA3B	8.458872693	3839.7156	3.241668254	11.90715942	11.90715942	-8.82632	5E-10
ADAMTSL3	0	946.91501	0	9.888613909	9.888613909	-20	5.3E-10
NLRP12	388.0507848	0	8.60381468	0	0	20	5.4E-10
LOC100739662	758.1264651	2.4788351	9.568196438	1.798604302	1.798604302	8.256632	5.6E-10
LOC102166331	636.5301701	1.6525567	9.316349807	1.407383614	1.407383614	8.589385	5.6E-10
LMO3	379.5919121	0	8.572101093	0	0	20	6.2E-10
OAS1	0	912.21132	0	9.834804937	9.834804937	-20	6.4E-10
PEG3	0	882.4653	0	9.787029662	9.787029662	-20	7.6E-10
ROBO2	3033.563219	19.004403	11.56727316	4.322245637	4.322245637	7.318536	7.6E-10
GIMAP8	944.2216643	4.1313919	9.884508886	2.359350201	2.359350201	7.836354	8E-10
NSUN7	2430.86854	14.873011	11.24784953	3.98850389	3.98850389	7.352631	8E-10
ZSWIM5	2541.891244	15.699289	11.31225405	4.061714779	4.061714779	7.339059	8.1E-10
FAM111A	1.057359087	1205.5401	1.04079362	10.2366602	10.2366602	-10.155	8.2E-10
LOC100513263	0	868.41857	0	9.763907098	9.763907098	-20	8.2E-10
TNNT2	0	868.41857	0	9.763907098	9.763907098	-20	8.2E-10
TRIM25	0	860.15578	0	9.750130438	9.750130438	-20	8.6E-10
LOC102166965	5.286795433	2473.8774	2.65232482	11.27314137	11.27314137	-8.87016	8.7E-10
TWIST2	0	857.67695	0	9.745971655	9.745971655	-20	8.8E-10
LOC100519889	1486.646876	8.2627837	10.5388164	3.211445828	3.211445828	7.491218	8.9E-10

piPS-LF Vs PEFs

AccID	piPS-LF	PEF	piPS-LF_log2(count+1)	PEF_log2(count+1	PEF_log2(count+1)_FDR<0.0	Log2FC	FDR
SLC6A1	1663.66015	0	10.70101196	0	0	20	0
LOC100736983	3771.62615	0	11.88135343	0	0	20	0
NLRP12	925.084567	0	9.855000132	0	0	20	0
LOC100514082	933.373855	0	9.867856097	0	0	20	0
C1QL2	2182.56959	0	11.0924728	0	0	20	0
TRIM71	859.59919	0	9.749197672	0	0	20	0
LOC100623527	2132.83386	0	11.05923214	0	0	20	0
VRTN	852.138831	0	9.736636719	0	0	20	0
BPIFB6	5035.7426	0	12.29827529	0	0	20	0
NXNL2	2489.27326	0	11.28208834	0	0	20	0
SNCA	2962.59162	0	11.53313094	0	0	20	0
LOC102165604	877.006695	0	9.778088131	0	0	20	0
UTF1	2461.91861	0	11.26615324	0	0	20	0
ACTL8	5309.28912	0	12.37457469	0	0	20	0
LIN28A	10943.5183	0	13.41792084	0	0	20	0
ST14	1661.17336	0	10.69885515	0	0	20	0
HSD17B2	3343.06995	0	11.70738931	0	0	20	0
GABRR2	1272.40574	0	10.31447646	0	0	20	0
INPP5D	9428.23644	1.6525567	13.20292523	1.407383613	1.407383613	12.47807	0
GABRA5	3870.26868	1.6525567	11.91859072	1.407383613	1.407383613	11.19352	0
OTX2	1613.92442	0.8262784	10.65725093	0.868906686	0.868906686	10.93166	0
KRT6A	2853.17301	1.6525567	11.47885707	1.407383613	1.407383613	10.75365	0
SALL4	2776.91156	1.6525567	11.43978495	1.407383613	1.407383613	10.71457	0
SOX21	5280.27661	3.3051135	12.36667099	2.106051269	2.106051269	10.6417	0
HENMT1	6600.76022	4.9576702	12.68863503	2.574748269	2.574748269	10.37875	0
CDH1	3384.51639	3.3051135	11.72516019	2.106051269	2.106051269	10.00003	0
LOC100511106	3238.62491	3.3051135	11.66161107	2.106051269	2.106051269	9.936466	0
AIF1	7267.219	8.2627837	12.82738617	3.211445828	3.211445828	9.78056	0
ESRRB	1307.22076	1.6525567	10.35339029	1.407383613	1.407383613	9.627587	0
LOC100622539	3264.32171	4.1313919	11.67300942	2.359350201	2.359350201	9.62594	0
LOC100524089	1293.12897	1.6525567	10.33776568	1.407383613	1.407383613	9.611951	0
NTRK2	1223.49894	1.6525567	10.25797581	1.407383613	1.407383613	9.532097	0
TDH	2137.80744	3.3051135	11.06259088	2.106051269	2.106051269	9.337216	0
NUTM1	1051.08175	1.6525567	10.03903109	1.407383613	1.407383613	9.312959	0
MAP7D2	2070.6642	3.3051135	11.01657446	2.106051269	2.106051269	9.291178	0
LOC100519889	4690.07928	8.2627837	12.19570417	3.211445828	3.211445828	9.148769	0
ANO9	2135.32065	4.1313919	11.06091249	2.359350201	2.359350201	9.013609	0
LOC100521657	2499.2204	4.9576702	11.28783956	2.574748269	2.574748269	8.9776	0
NPTX1	13338.2937	31.398578	13.70339466	5.017858594	5.017858594	8.730659	0
NRARP	1315.51004	3.3051135	10.36250281	2.106051269	2.106051269	8.636707	0
FAM43B	2572.16614	6.610227	11.32932889	2.927939483	2.927939483	8.604068	0
SAMD5	2104.65028	5.7839486	11.04005013	2.762125239	2.762125239	8.50731	0
SOX2	5045.68975	14.046732	12.30112168	3.911378308	3.911378308	8.488673	0
C1QL4	2426.27467	7.4365053	11.24512166	3.076645515	3.076645515	8.349902	0
ALPL	2573.824	8.2627837	11.3302581	3.211445828	3.211445828	8.28307	0
NUP210	21174.9868	80.149002	14.37014158	6.342501448	6.342501448	8.045461	0
ELMOD1	6216.13725	28.093465	12.60203471	4.862623207	4.862623207	7.78964	0
PODXL	6663.75881	30.5723	12.70233695	4.980587446	4.980587446	7.767967	0
COL6A2	363.899754	73816.405	8.511356367	16.17167339	16.17167339	-7.66426	0
PDGFRB	58.8539465	13038.673	5.90337447	13.67062004	13.67062004	-7.79144	0
MCSF	25.6967935	6173.1257	4.73859457	12.59201914	12.59201914	-7.90827	0
SERPINF1	103.616103	26974.684	6.708961125	14.71937191	14.71937191	-8.02421	0
LOC102159390	12.4339324	3391.8727	3.747809764	11.72829159	11.72829159	-8.09166	0
LOC100624590	9.94714588	2715.977	3.452482877	11.40778664	11.40778664	-8.09297	0
CRABP2	56.36716	15784.396	5.842153193	13.94630281	13.94630281	-8.12943	0
LOC100627123	23.2100071	6652.3672	4.597531595	12.69986894	12.69986894	-8.16298	0
IGFBP4	8.28928824	2432.5635	3.215568059	11.24885472	11.24885472	-8.19701	0
ITGB5	23.2100071	7134.9137	4.597531595	12.80088246	12.80088246	-8.26401	0
PDLIM4	7.46035941	2430.0847	3.080718953	11.24738444	11.24738444	-8.34755	0
LOC100515628	95.3268147	31142.432	6.589865555	14.92664031	14.92664031	-8.35178	0
DHX58	25.6967935	8611.4732	4.73859457	13.07221187	13.07221187	-8.38853	0
LOC100515260	43.1042988	14486.312	5.462847376	13.82250236	13.82250236	-8.39264	0
BAHCC1	9.94714588	3346.4274	3.452482877	11.70883705	11.70883705	-8.39412	0
NPY	4.97357294	1688.0867	2.578594101	10.72202768	10.72202768	-8.40689	0
GBP1	10.7760747	3798.4017	3.557786824	11.89155653	11.89155653	-8.46142	0
LOC100157228	15.7496477	5598.8622	4.066058842	12.45117562	12.45117562	-8.47367	0
IFIT3	25.6967935	9279.1061	4.73859457	13.17992559	13.17992559	-8.49625	0
LRRN4CL	14.9207188	5439.3905	3.99283357	12.4094945	12.4094945	-8.50998	0
RPS6KA2	17.4075053	6651.5409	4.202222212	12.69968976	12.69968976	-8.57784	0
SLC6A17	5.80250177	2253.2611	2.766065426	11.13843892	11.13843892	-8.60112	0
LOC102167439	4.14464412	1613.7217	2.363071282	10.65706978	10.65706978	-8.60493	0
CSF1	7.46035941	2943.2036	3.080718953	11.52366171	11.52366171	-8.62393	0
GREM1	4.14464412	1662.4721	2.363071282	10.69998194	10.69998194	-8.64787	0
LOC100623720	61.340733	24788.351	5.962103212	14.59743289	14.59743289	-8.6586	0
PCDH7	11.6050035	4780.0204	3.655924617	12.22310284	12.22310284	-8.68613	0
HOXA5	5.80250177	2425.127	2.766065426	11.24443937	11.24443937	-8.70717	0
LOC100525721	3.31571529	1404.6732	2.109599694	10.45704554	10.45704554	-8.7267	0
TBX2	7.46035941	3531.5138	3.080718953	11.78647947	11.78647947	-8.88683	0
THBS1	442.647992	211976.76	8.793271628	17.69355337	17.69355337	-8.90353	0
VEGFC	3.31571529	1636.0312	2.109599694	10.67686608	10.67686608	-8.94666	0
COL1A1	247.849718	124736.64	7.959130944	16.92853729	16.92853729	-8.9752	0
GPR124	65.4853771	34432.672	6.054965161	15.07153243	15.07153243	-9.03839	0
BGN	370.531184	198659.63	8.537339497	17.59994647	17.59994647	-9.06649	0
ISG15	44.7621565	24180.21	5.516083133	14.56159883	14.56159883	-9.07733	0
NES	42.27537	23472.09	5.435474249	14.51872013	14.51872013	-9.11691	0
LOC100624156	2.48678647	1416.2411	1.801898017	10.46886952	10.46886952	-9.15357	0
ACTC1	9.94714588	5781.4698	3.452482877	12.4974701	12.4974701	-9.18294	0
DES	85.3796688	49798.145	6.43261988	15.60383335	15.60383335	-9.18798	0
THY1	76.2614518	44528.141	6.271676881	15.44246218	15.44246218	-9.18955	0
SEMA3C	3.31571529	1944.233	2.109599694	10.92572726	10.92572726	-9.19567	0
ITGA8	26.5257224	16505.737	4.78270852	14.01076732	14.01076732	-9.28136	0
SAMD9	6.63143059	4197.4941	2.93195353	12.03565625	12.03565625	-9.30599	0
IGLON5	1.65785765	1059.2889	1.410263837	10.05024166	10.05024166	-9.31956	0
CXCL2	3.31571529	2125.188	2.109599694	11.05405343	11.05405343	-9.32405	0
CNN1	9.94714588	6544.1247	3.452482877	12.67620496	12.67620496	-9.3617	0
HOXB3	4.14464412	2740.7654	2.363071282	11.42088939	11.42088939	-9.36911	0
CARP	6.63143059	4422.2418	2.93195353	12.11088841	12.11088841	-9.38124	0
LOXL1	19.8942918	13617.894	4.385036953	13.73332191	13.73332191	-9.41893	0
EPAS1	6.63143059	4575.1033	2.93195353	12.15990392	12.15990392	-9.43027	0
LOC100622618	1.65785765	1144.3955	1.410263837	10.16163018	10.16163018	-9.43105	0
LOC102165913	11.6050035	8517.2775	3.655924617	13.05634601	13.05634601	-9.5195	0
LOC100157992	22.3810782	16447.897	4.547269558	14.00570325	14.00570325	-9.52141	0
CTHRC1	8.28928824	6120.2439	3.215568059	12.57960914	12.57960914	-9.52813	0
DKK2	1.65785765	1229.5022	1.410263837	10.26503154	10.26503154	-9.53454	0
ARNT2	2.48678647	1853.3424	1.801898017	10.85669193	10.85669193	-9.54163	0
LOC100525680	4.14464412	3270.4098	2.363071282	11.67569678	11.67569678	-9.62401	0
COL4A2	54.7093024	43357.305	5.799846344	15.40402074	15.40402074	-9.63027	0
MX1	65.4853771	52119.161	6.054965161	15.66955392	15.66955392	-9.63643	0
LOC100152082	12.4339324	9950.0442	3.747809764	13.2806322	13.2806322	-9.64428	0
LTBP2	69.6300212	56402.588	6.142209624	15.78349932	15.78349932	-9.66184	0
LOC100736872	4.97357294	4152.8751	2.578594101	12.02024212	12.02024212	-9.70561	0
SVEP1	5.80250177	4956.844	2.766065426	12.27549715	12.27549715	-9.73853	0
PRRX2	15.7496477	13710.437	4.066058842	13.74309216	13.74309216	-9.76574	0
FAP	2.48678647	2238.3881	1.801898017	11.12888887	11.12888887	-9.81396	0
LOC100621838	4.97357294	4604.8494	2.578594101	12.16925151	12.16925151	-9.85466	0
LOC100525396	1.65785765	1536.8778	1.410263837	10.58672513	10.58672513	-9.85647	0
IFIT2	4.97357294	4632.1166	2.578594101	12.17776726	12.17776726	-9.86317	0
LOC100738123	233.757928	219619.01	7.875030072	17.74464996	17.74464996	-9.87577	0
LOC100525528	1.65785765	1597.1961	1.410263837	10.64222872	10.64222872	-9.91201	0
SRPX2	1.65785765	1613.7217	1.410263837	10.65706978	10.65706978	-9.92686	0
LOC102161109	8.28928824	8165.2829	3.215568059	12.99546383	12.99546383	-9.94404	0
LOC100627920	0.82892882	970.87709	0.870998931	9.924630058	9.924630058	-10.1938	0
ST3GAL1	1.65785765	1969.8476	1.410263837	10.94460053	10.94460053	-10.2145	0
HR	0.82892882	1042.7633	0.870998931	10.02757887	10.02757887	-10.2969	0
FAM198B	9.11821706	11644.741	3.338883189	13.50751483	13.50751483	-10.3186	0
ACKR3	3.31571529	4399.1061	2.109599694	12.10332258	12.10332258	-10.3737	0
AEBP1	30.6703665	41982.378	4.985061656	15.35753062	15.35753062	-10.4187	0
COL8A2	11.6050035	16593.322	3.655924617	14.01840209	14.01840209	-10.4816	0
LOXL4	8.28928824	12123.156	3.215568059	13.56559673	13.56559673	-10.5142	0
LOC100518095	4.14464412	6110.3286	2.363071282	12.57727033	12.57727033	-10.5258	0
KIF12	1.65785765	2577.9885	1.410263837	11.33258964	11.33258964	-10.6027	0
LOC100049650	4.97357294	7884.3482	2.578594101	12.94495875	12.94495875	-10.6305	0
HOXB6	1.65785765	2758.9435	1.410263837	11.43042301	11.43042301	-10.7006	0
ELN	4.14464412	7319.1738	2.363071282	12.83766219	12.83766219	-10.7862	0
ACTA2	59.6828753	109145.59	5.92321754	16.73590752	16.73590752	-10.8366	0
LOC100738989	90.3532418	170068.75	6.51338402	17.37576699	17.37576699	-10.8783	0
CCL11	0.82892882	1648.4254	0.870998931	10.68774777	10.68774777	-10.9576	0
TSHZ3	0.82892882	1718.659	0.870998931	10.74790681	10.74790681	-11.0177	0
LOC100628118	1.65785765	3442.2757	1.410263837	11.74956598	11.74956598	-11.0198	0
COL1A2	173.246124	385650.56	7.444982755	18.55693841	18.55693841	-11.1203	0
MMP19	1.65785765	3729.8206	1.410263837	11.86527726	11.86527726	-11.1356	0
ASPN	6.63143059	15025.046	2.93195353	13.87517779	13.87517779	-11.1458	0
IL33	0.82892882	1905.3979	0.870998931	10.89663357	10.89663357	-11.1666	0
C12H17orf67	0.82892882	1926.0549	0.870998931	10.91218195	10.91218195	-11.1821	0
LOC100736763	1.65785765	4545.3573	1.410263837	12.15049536	12.15049536	-11.4209	0
LOC100738213	130.970754	387901.34	7.044074441	18.56533395	18.56533395	-11.5322	0
CSPG4	3.31571529	12812.272	2.109599694	13.64535136	13.64535136	-11.9159	0
IRG6	1.65785765	6658.1511	1.410263837	12.70112256	12.70112256	-11.9716	0
MGP	4.97357294	26806.949	2.578594101	14.71037324	14.71037324	-12.396	0
PRELP	0.82892882	4799.8511	0.870998931	12.22907446	12.22907446	-12.4995	0
LOC100737120	0.82892882	5022.9462	0.870998931	12.29460531	12.29460531	-12.565	0
LOC100627277	0.82892882	5187.3756	0.870998931	12.34106721	12.34106721	-12.6115	0
LOC102165673	1.65785765	10474.731	1.410263837	13.35476329	13.35476329	-12.6253	0
COL3A1	56.36716	487725.68	5.842153193	18.89571337	18.89571337	-13.0789	0
LOC100621401	0.82892882	8792.4282	0.870998931	13.10221	13.10221	-13.3727	0
LOC102166564	1.65785765	21479.106	1.410263837	14.39071351	14.39071351	-13.6613	0
POSTN	3.31571529	55326.773	2.109599694	15.75571625	15.75571625	-14.0264	0
LOC102160657	0.82892882	22024.45	0.870998931	14.42688387	14.42688387	-14.6975	0
COL5A1	1.65785765	110737.83	1.410263837	16.75680162	16.75680162	-16.0275	0
IL8	0	789.09585	0	9.625883864	9.625883864	-20	0
CXCL10	0	2351.5882	0	11.20003312	11.20003312	-20	0
GAS7	0	5731.8931	0	12.48504765	12.48504765	-20	0
LOC100628220	0	1165.8788	0	10.18843898	10.18843898	-20	0
ISM1	0	838.67255	0	9.71368301	9.71368301	-20	0
HOXB2	0	796.53235	0	9.63939923	9.63939923	-20	0
DPT	0	1233.6336	0	10.26986725	10.26986725	-20	0
SERTAD4	0	2809.3465	0	11.45653228	11.45653228	-20	0
LOC100622513	0	1071.683	0	10.06700814	10.06700814	-20	0
DDX60	0	1853.3424	0	10.85669193	10.85669193	-20	0
VCAM1	0	6099.5869	0	12.57473234	12.57473234	-20	0
MMP1	0	11619.953	0	13.50444073	13.50444073	-20	0
ZBP1	0	911.38504	0	9.833498988	9.833498988	-20	0
BACE2	0	7459.6411	0	12.8650839	12.8650839	-20	0
LOC102161103	0	4513.9587	0	12.14049709	12.14049709	-20	0
LUM	0	13427.85	0	13.71304812	13.71304812	-20	0
TMEM98	0	5284.0502	0	12.36770146	12.36770146	-20	0
MX2	0	3252.2317	0	11.66765785	11.66765785	-20	0
LOC102164776	0	1751.7101	0	10.77537172	10.77537172	-20	0
LOC100626687	0	2323.4948	0	11.18270147	11.18270147	-20	0
KCNE4	0	1620.3319	0	10.66296373	10.66296373	-20	0
CHI3L1	0	3854.5886	0	11.91273541	11.91273541	-20	0
TNC	0	18393.783	0	14.16700902	14.16700902	-20	0
LOC102157763	0	854.37184	0	9.740407896	9.740407896	-20	0
LOC100516442	0	709.77312	0	9.473245315	9.473245315	-20	0
LOC100738461	0	7119.2145	0	12.79770498	12.79770498	-20	0
LOC100738990	0	1729.4006	0	10.75689038	10.75689038	-20	0
PEG3	0	882.4653	0	9.787029662	9.787029662	-20	0
LOC102166259	0	2373.0715	0	11.21314766	11.21314766	-20	0
NECD	0	1658.3407	0	10.69639441	10.69639441	-20	0
LOC102158099	0	1097.2977	0	10.10105341	10.10105341	-20	0
LOC100623769	0	782.48562	0	9.613762982	9.613762982	-20	0
FOXS1	0	1942.5805	0	10.92450111	10.92450111	-20	0
AMCF-II	0	9804.6192	0	13.25939301	13.25939301	-20	0
LOC102160189	0	22683.82	0	14.4694396	14.4694396	-20	0
MXRA5	0	14656.526	0	13.83935397	13.83935397	-20	0
LOC100627044	0	1683.9553	0	10.71849462	10.71849462	-20	0
FMOD	0	59302.825	0	15.85583754	15.85583754	-20	0
CCL2	0	45810.525	0	15.48342298	15.48342298	-20	0
EIF2S3	0	3510.0305	0	11.77767882	11.77767882	-20	0
TRIM4	0	818.84187	0	9.679201855	9.679201855	-20	0
LOC100737137	0	729.6038	0	9.512945452	9.512945452	-20	0
TSPAN18	0	1655.0356	0	10.69351795	10.69351795	-20	0
LOC100153335	0	2349.9357	0	11.19901936	11.19901936	-20	0
LOC100513671	0	1343.5286	0	10.39288476	10.39288476	-20	0
LOC733603	0	10361.531	0	13.33908877	13.33908877	-20	0
LOC733579	0	2098.7471	0	11.03599984	11.03599984	-20	0
LOC100625300	0	1055.9838	0	10.04573749	10.04573749	-20	0
LOC100625207	0	1140.2642	0	10.15641704	10.15641704	-20	0
LOC100620730	0	789.09585	0	9.625883864	9.625883864	-20	0
OAS1	0	912.21132	0	9.834804937	9.834804937	-20	0
CCL20	0	1005.5808	0	9.975247238	9.975247238	-20	0
AWN	0	1846.7322	0	10.85153993	10.85153993	-20	0
COL6A3	0	20982.513	0	14.35696861	14.35696861	-20	0
SLC8A1	0	2005.3776	0	10.97037744	10.97037744	-20	0
LOC100737434	0	17070.085	0	14.05926713	14.05926713	-20	0
POF1B	675.576991	0	9.402110305	0	0	20	1.1E-16
LOC102164762	816.494891	0.8262784	9.675065905	0.868906686	0.868906686	9.9486	1.1E-16
LOC100152743	814.837034	0.8262784	9.672137187	0.868906686	0.868906686	9.945668	1.1E-16
LOC102164968	1026.21388	1.6525567	10.00452089	1.407383613	1.407383613	9.278416	1.1E-16
PCNXL2	1153.03999	2.4788351	10.17247751	1.798604302	1.798604302	8.861565	1.1E-16
LOC100620918	1448.13866	4.1313919	10.50097992	2.359350201	2.359350201	8.453356	1.1E-16
GREB1L	3600.03788	17.351846	11.81419706	4.19785327	4.19785327	7.696779	1.1E-16
HSPA2	19714.4142	108.24247	14.2670364	6.771389985	6.771389985	7.50884	1.1E-16
SDC2	48.9068006	9156.8169	5.641164514	13.16078801	13.16078801	-7.54867	1.1E-16
COL5A2	450.93728	86391.535	8.819978759	16.39861904	16.39861904	-7.58182	1.1E-16
IFIT1	35.6439394	6919.2551	5.195502704	12.7566095	12.7566095	-7.60082	1.1E-16
C1R	33.9860818	6800.271	5.128709195	12.73158866	12.73158866	-7.6445	1.1E-16
ARAP3	19.0653629	4325.5673	4.326635347	12.07900712	12.07900712	-7.82579	1.1E-16
MAB21L2	13.2628612	3284.4565	3.834191515	11.68187814	11.68187814	-7.95212	1.1E-16
LOC100525856	4.14464412	1469.9492	2.363071282	10.52253173	10.52253173	-8.4703	1.1E-16
EVC2	1.65785765	985.7501	1.410263837	9.946540946	9.946540946	-9.21576	1.1E-16
LOC102164335	0	656.89131	0	9.361705436	9.361705436	-20	1.1E-16
COL27A1	0	651.93364	0	9.350792552	9.350792552	-20	1.1E-16
ZNF814	0	636.23435	0	9.315680218	9.315680218	-20	1.1E-16
GDF10	0	688.28988	0	9.428967032	9.428967032	-20	1.1E-16
ZNF134	0	647.80224	0	9.341634998	9.341634998	-20	1.1E-16
LOC100739844	0	689.94244	0	9.43242172	9.43242172	-20	1.1E-16
LOC100624681	630.814835	0	9.303358001	0	0	20	2.2E-16
SLC26A5	612.578401	0	9.261103887	0	0	20	2.2E-16
LOC100155551	610.091614	0	9.255244873	0	0	20	2.2E-16
LOC100523750	911.821706	1.6525567	9.834189288	1.407383613	1.407383613	9.107908	2.2E-16
GPR150	911.821706	1.6525567	9.834189288	1.407383613	1.407383613	9.107908	2.2E-16
EMILIN1	159.983263	27857.975	7.330766893	14.76585457	14.76585457	-7.44403	2.2E-16
LOC102165344	40.6175124	7593.4982	5.379118827	12.89073893	12.89073893	-7.54652	2.2E-16
SERPINE1	52.2225159	10062.418	5.733964805	13.29683278	13.29683278	-7.59009	2.2E-16
CDC42EP5	23.2100071	4564.3617	4.597531595	12.15651346	12.15651346	-7.61952	2.2E-16
PTGS2	17.4075053	3762.0454	4.202222212	11.87768499	11.87768499	-7.75566	2.2E-16
C1QTNF6	9.94714588	2309.448	3.452482877	11.17395694	11.17395694	-7.85905	2.2E-16
VGF	12.4339324	2927.5043	3.747809764	11.51594828	11.51594828	-7.87924	2.2E-16
TENM4	8.28928824	2188.8114	3.215568059	11.09659091	11.09659091	-8.04468	2.2E-16
ERG	3.31571529	1231.9811	2.109599694	10.26793491	10.26793491	-8.53744	2.2E-16
BST2	0.82892882	829.58349	0.870998931	9.697981376	9.697981376	-9.96692	2.2E-16
COLQ	0	618.05622	0	9.273926629	9.273926629	-20	2.2E-16
ECEL1	0	623.01389	0	9.285434338	9.285434338	-20	2.2E-16
LOC100737503	0	617.22994	0	9.271999722	9.271999722	-20	2.2E-16
BMP6	1001.34602	2.4788351	9.969164911	1.798604302	1.798604302	8.658063	3.3E-16
FAM65B	1832.76163	7.4365053	10.8405904	3.076645515	3.076645515	7.945179	3.3E-16
CAMKV	5327.52555	29.746021	12.37952067	4.942327829	4.942327829	7.484625	3.3E-16
HOXB7	1.65785765	892.38064	1.410263837	9.803131183	9.803131183	-9.0722	3.3E-16
RUNX1	0.82892882	738.69286	0.870998931	9.530782548	9.530782548	-9.79951	3.3E-16
SP100	0	598.22554	0	9.226955308	9.226955308	-20	3.3E-16
LOC100513699	736.088795	0.8262784	9.525694618	0.868906686	0.868906686	9.799036	4.4E-16
LOC102160252	1254.16931	4.1313919	10.29366627	2.359350201	2.359350201	8.245889	4.4E-16
TNNT2	1.65785765	868.41857	1.410263837	9.763907098	9.763907098	-9.03293	4.4E-16
ZNF418	0	573.43719	0	9.166005344	9.166005344	-20	4.4E-16
COL4A1	289.296159	47585.371	8.181381675	15.53826083	15.53826083	-7.36183	5.6E-16
LOC100513556	79.5771671	13236.153	6.332299179	13.69230527	13.69230527	-7.37791	5.6E-16
LOC100152170	11.6050035	2376.3766	3.655924617	11.21515474	11.21515474	-7.67787	5.6E-16
LOC100737566	696.300212	0.8262784	9.44563611	0.868906686	0.868906686	9.718866	6.7E-16
LOC100738626	1094.18605	3.3051135	10.09696026	2.106051269	2.106051269	8.370943	6.7E-16
LOC100153981	1575.79369	6.610227	10.6227782	2.927939483	2.927939483	7.897163	6.7E-16
ZSWIM5	2973.36769	15.699289	11.53836729	4.061714779	4.061714779	7.565255	6.7E-16
VSTM4	14.09179	2702.7566	3.915692025	11.40074954	11.40074954	-7.58343	7.8E-16
LOC100514326	3.31571529	1101.4291	2.109599694	10.10647012	10.10647012	-8.37584	7.8E-16
KCNK12	0	548.64884	0	9.10236639	9.10236639	-20	7.8E-16
FIBIN	4.14464412	1193.9722	2.363071282	10.2227614	10.2227614	-8.17031	8.9E-16

KCNH3	382.484325	1.6525567	8.583023796	1.407383613	1.407383613	7.854557	9.2E-12
EPCAM	381.103515	1.6525567	8.577819718	1.407383613	1.407383613	7.849339	9.5E-12
LOC100623720	71.8021115	24788.351	6.185908388	14.59743289	14.59743289	-8.43142	9.7E-12
EGR3	499.853161	3.3051135	8.968243888	2.106051269	2.106051269	7.240661	9.8E-12
DPT	0	1233.6336	0	10.26986725	10.26986725	-20	9.9E-12
ERG	0	1231.9811	0	10.26793491	10.26793491	-20	1E-11
LOC102166492	314.824643	0.8262784	8.302979933	0.868906686	0.868906686	8.573705	1.1E-11
TGM1	2172.01387	27.267186	11.08548167	4.821056379	4.821056379	6.315724	1.1E-11
GBP1	8.28485901	3798.4017	3.214880004	11.89155653	11.89155653	-8.8407	1.1E-11
ADAMTS2	0	1203.8876	0	10.23468284	10.23468284	-20	1.2E-11
FN1	2339.09186	881757.18	11.19234945	19.75002353	19.75002353	-8.55829	1.2E-11
LOC100037970	1978.7005	24.788351	10.95106647	4.688647628	4.688647628	6.318747	1.2E-11
ITGB5	19.3313377	7134.9137	4.345633235	12.80088246	12.80088246	-8.52781	1.2E-11
FIBIN	0	1193.9722	0	10.2227614	10.2227614	-20	1.2E-11
KLHDC8A	488.806682	3.3051135	8.936068645	2.106051269	2.106051269	7.20842	1.2E-11
VEGFC	1.38080984	1636.0312	1.251452392	10.67686608	10.67686608	-10.2105	1.2E-11
LOC100623930	370.057036	1.6525567	8.535497154	1.407383613	1.407383613	7.806904	1.3E-11
ACTC1	15.1889082	5781.4698	4.016933786	12.4974701	12.4974701	-8.57227	1.3E-11
ITGA11	11.0464787	4512.3062	3.590539588	12.13996894	12.13996894	-8.67413	1.3E-11
LOC100628220	0	1165.8788	0	10.18843898	10.18843898	-20	1.4E-11
LOC100737327	1996.65102	25.61463	10.96408886	4.734147579	4.734147579	6.28447	1.4E-11
PROC	592.36742	4.9576702	9.212781904	2.574748269	2.574748269	6.900686	1.5E-11
SALL4	361.772177	1.6525567	8.502920002	1.407383613	1.407383613	7.774238	1.6E-11
LUZP2	1136.4065	13.220454	10.15153223	3.829895615	3.829895615	6.425563	1.6E-11
LOC100621284	0	1129.5225	0	10.14277403	10.14277403	-20	1.7E-11
CLDN10	356.248938	1.6525567	8.480785911	1.407383613	1.407383613	7.752042	1.8E-11
SERPINF1	86.9910197	26974.684	6.459284385	14.71937191	14.71937191	-8.27652	1.8E-11
SAMD5	632.410905	5.7839486	9.306997895	2.762125239	2.762125239	6.772664	1.9E-11
KDR	737.352452	7.4365053	9.52816584	3.076645515	3.076645515	6.631586	2E-11
LOC100523870	2.76161967	1878.957	1.911353989	10.87648396	10.87648396	-9.4102	2.2E-11
KRT8	30781.0129	442.05893	14.90979995	8.791354786	8.791354786	6.121658	2.2E-11
LOC100626686	229.214433	0	7.846834472	0	0	20	2.2E-11
LOC102159390	8.28485901	3391.8727	3.214880004	11.72829159	11.72829159	-8.67739	2.2E-11
LOC100622513	0	1071.683	0	10.06700814	10.06700814	-20	2.3E-11
LOC100525856	1.38080984	1469.9492	1.251452392	10.52253173	10.52253173	-10.056	2.4E-11
LOC100624640	345.202459	1.6525567	8.435472161	1.407383613	1.407383613	7.706599	2.5E-11
VGF	6.90404918	2927.5043	2.982591923	11.51594828	11.51594828	-8.72801	2.6E-11
TRPC6	0	1048.5473	0	10.03555141	10.03555141	-20	2.7E-11
LOC102166331	342.440839	1.6525567	8.423917793	1.407383613	1.407383613	7.695011	2.7E-11
PLAC9	5.52323934	2550.7213	2.705588563	11.31725507	11.31725507	-8.85117	2.7E-11
EDIL3	0	1045.2421	0	10.03100107	10.03100107	-20	2.7E-11
PDGFRB	44.1859147	13038.673	5.497801224	13.67062004	13.67062004	-8.20499	2.8E-11
BPIFB1	283.066016	0.8262784	8.150082438	0.868906686	0.868906686	8.420295	2.9E-11
DAB2	40.0434852	11665.398	5.359081339	13.51007158	13.51007158	-8.18645	3.1E-11
MATK	854.721288	9.9153405	9.740997172	3.448285225	3.448285225	6.429648	3.2E-11
ITGB8	0	1013.8436	0	9.987041638	9.987041638	-20	3.2E-11
GLIS1	2.76161967	1754.189	1.911353989	10.77741066	10.77741066	-9.31107	3.3E-11
LOC100739460	1056.31952	13.220454	10.04619571	3.829895615	3.829895615	6.320131	3.3E-11
FLRT2	17.9505279	5674.0536	4.24416613	12.4704183	12.4704183	-8.30421	3.3E-11
SLC16A6	332.77517	1.6525567	8.382732826	1.407383613	1.407383613	7.653704	3.5E-11
CLDN7	1197.16213	15.699289	10.22660742	4.061714779	4.061714779	6.252776	3.7E-11
LOC102165477	216.787144	0	7.766774985	0	0	20	3.8E-11
ELMOD1	1952.46511	28.093465	10.93181977	4.862623207	4.862623207	6.118918	3.9E-11
SEMA5B	215.406334	0	7.757598918	0	0	20	4E-11
CERS3	215.406334	0	7.757598918	0	0	20	4E-11
GRIK2	481.902633	4.1313919	8.915588519	2.359350201	2.359350201	6.86597	4.1E-11
ATP2A3	1888.94786	27.267186	10.88413071	4.821056379	4.821056379	6.114273	4.1E-11
ROBO2	1367.00174	19.004403	10.41785435	4.322245637	4.322245637	6.168538	4.7E-11
LOC780430	209.883095	0	7.720299639	0	0	20	5.1E-11
MSRB3	41.4242951	11075.435	5.406818783	13.43520604	13.43520604	-8.06267	5.2E-11
LOC102157888	208.502285	0	7.71082217	0	0	20	5.4E-11
GPR37	1933.13377	28.919743	10.91747186	4.903025878	4.903025878	6.062743	5.5E-11
LOC100736763	15.1889082	4545.3573	4.016933786	12.15049536	12.15049536	-8.22523	5.6E-11
COL6A3	81.4677803	20982.513	6.365758672	14.35696861	14.35696861	-8.00874	5.7E-11
CCR8	207.121475	0	7.70128203	0	0	20	5.8E-11
TMPRSS5	205.740666	0	7.691678382	0	0	20	6.2E-11
LOC102159987	1.38080984	1254.2906	1.251452392	10.29380563	10.29380563	-9.82714	6.2E-11
ENPEP	4674.04129	75.191332	12.19076339	6.251554969	6.251554969	5.95796	6.2E-11
CDH8	694.547347	8.2627837	9.442004915	3.211445828	3.211445828	6.393301	6.9E-11
UPTI	202.979046	0	7.672277146	0	0	20	7E-11
APBB1IP	0	892.38064	0	9.803131183	9.803131183	-20	7E-11
LOC100739007	6.90404918	2482.9665	2.982591923	11.27843001	11.27843001	-8.49041	7.1E-11
LOC100516994	8.28485901	2778.7742	3.214880004	11.44075196	11.44075196	-8.38976	7.4E-11
PHACTR1	1.38080984	1215.4555	1.251452392	10.24846781	10.24846781	-9.78177	7.5E-11
PEG3	0	882.4653	0	9.787029662	9.787029662	-20	7.5E-11
CDH2	0	880.81274	0	9.784328517	9.784328517	-20	7.6E-11
LOC102164249	306.539784	1.6525567	8.26462924	1.407383613	1.407383613	7.535231	7.7E-11
LOC102166491	200.217426	0	7.652611443	0	0	20	8E-11
INPP5D	305.158974	1.6525567	8.25813716	1.407383613	1.407383613	7.528717	8E-11
LOC100515546	2.76161967	1513.742	1.911353989	10.56485635	10.56485635	-9.09839	8.1E-11
ENTPD3	22.0929574	5839.3093	4.529381037	12.51182905	12.51182905	-8.04607	8.1E-11
ATP12A	198.836616	0	7.642677144	0	0	20	8.5E-11
LOC100739808	198.836616	0	7.642677144	0	0	20	8.5E-11
LOC100513415	198.836616	0	7.642677144	0	0	20	8.5E-11
LOC100737477	84.2294	20208.29	6.413279271	14.30273103	14.30273103	-7.90641	8.7E-11
LOC100158194	4.14242951	1785.5876	2.362450112	10.80299091	10.80299091	-8.75171	9.1E-11
BEST2	396.292423	3.3051135	8.634057469	2.106051269	2.106051269	6.905722	9.2E-11
FOXH1	3232.47583	53.708094	11.65887011	5.773682393	5.773682393	5.911356	9.6E-11
KCND3	196.074997	0	7.62260094	0	0	20	9.7E-11
EVPL	759.44541	9.9153405	9.570700874	3.448285225	3.448285225	6.25914	1E-10
ISM1	0	838.67255	0	9.71368301	9.71368301	-20	1E-10
LOC100739059	0	832.06232	0	9.702280616	9.702280616	-20	1.1E-10
CTHRC1	24.854577	6120.2439	4.692347799	12.57960914	12.57960914	-7.94393	1.1E-10
LOC100517681	929.28502	13.220454	9.861528985	3.829895615	3.829895615	6.135278	1.2E-10
DNMT3B	12390.0067	214.0061	13.59700578	7.74823377	7.74823377	5.855381	1.2E-10
GUCY1B3	0	804.79513	0	9.654269283	9.654269283	-20	1.3E-10
CNN1	27.6161967	6544.1247	4.838760036	12.67620496	12.67620496	-7.88854	1.3E-10
LOC100626688	8.28485901	2512.7125	3.214880004	11.29560396	11.29560396	-8.24455	1.4E-10
C1QTNF5	13.8080984	3612.489	3.888314478	11.8191768	11.8191768	-8.03133	1.4E-10
PLXDC2	2.76161967	1360.8805	1.911353989	10.41138438	10.41138438	-8.49481	1.5E-10
ETNK2	375.580275	3.3051135	8.556813626	2.106051269	2.106051269	6.828278	1.5E-10
DPP10	1604.50103	26.440908	10.64880787	4.77825631	4.77825631	5.923209	1.5E-10
C18H7orf58	0	775.87539	0	9.601539402	9.601539402	-20	1.6E-10
CLEC14A	0	775.04911	0	9.600004147	9.600004147	-20	1.6E-10
HSD17B2	185.028518	0	7.539379992	0	0	20	1.7E-10
GAS7	24.854577	5731.8931	4.692347799	12.48504765	12.48504765	-7.84936	1.7E-10
MMP19	15.1889082	3729.8206	4.016933786	11.86527726	11.86527726	-7.93994	1.9E-10
MCSF	27.6161967	6173.1257	4.838760036	12.59201914	12.59201914	-7.80434	1.9E-10
LOC100514524	182.266898	0	7.517802419	0	0	20	1.9E-10
FXYD3	182.266898	0	7.517802419	0	0	20	1.9E-10
LIN28A	182.266898	0	7.517802419	0	0	20	1.9E-10
EGFR	2.76161967	1304.6935	1.911353989	10.35060062	10.35060062	-8.88398	2E-10
LOC102161608	998.325511	15.699289	9.964810875	4.061714779	4.061714779	5.990739	2.1E-10
SVEP1	22.0929574	4956.844	4.529381037	12.27549715	12.27549715	-7.80969	2.2E-10
LOC100737434	82.8485901	17070.085	6.389714621	14.05926713	14.05926713	-7.68678	2.2E-10
LOC102161270	179.505279	0	7.495897217	0	0	20	2.2E-10
LOC100738350	657.265482	9.0890621	9.362525737	3.334720158	3.334720158	6.176201	2.2E-10
GBX1	2441.27179	43.792754	11.25400805	5.485193456	5.485193456	5.800797	2.3E-10
LOC102159296	0	732.90892	0	9.519457213	9.519457213	-20	2.3E-10
LOC100736874	1369.76336	23.135794	10.42076382	4.593102408	4.593102408	5.887656	2.3E-10
LOC100152528	1311.76934	22.309516	10.35839774	4.542847146	4.542847146	5.877711	2.6E-10
LOC100524089	269.257918	1.6525567	8.078193076	1.407383613	1.407383613	7.348145	2.6E-10
SLC15A2	3812.41596	71.886218	11.89686819	6.187574144	6.187574144	5.728847	2.9E-10
GRAP2	306.539784	2.4788351	8.26462924	1.798604302	1.798604302	6.950268	3.2E-10
LOC102163185	306.539784	2.4788351	8.26462924	1.798604302	1.798604302	6.950268	3.2E-10
CSPG4	66.2788721	12812.272	6.072081615	13.64535136	13.64535136	-7.59476	3.3E-10
LOC100518132	74.5637311	14235.124	6.239622035	13.79726876	13.79726876	-7.57677	3.5E-10
GLT1D1	260.973059	1.6525567	8.033274644	1.407383613	1.407383613	7.303057	3.5E-10
GPR124	179.505279	34432.672	7.495897217	15.07153243	15.07153243	-7.58361	3.6E-10
LOC100736865	169.83961	0	7.416498698	0	0	20	3.7E-10
LOC100155551	169.83961	0	7.416498698	0	0	20	3.7E-10
LOC100519956	169.83961	0	7.416498698	0	0	20	3.7E-10
NDST3	341.060029	3.3051135	8.418105721	2.106051269	2.106051269	6.689182	3.9E-10
PARM1	3244.90311	62.797156	11.66440422	5.995420212	5.995420212	5.691332	3.9E-10
ARSJ	0	666.80665	0	9.383286641	9.383286641	-20	4E-10
INPP4B	0	665.15409	0	9.379712118	9.379712118	-20	4.1E-10
NOL4	255.44982	1.6525567	8.002532746	1.407383613	1.407383613	7.272196	4.3E-10
LOC102164335	0	656.89131	0	9.361705436	9.361705436	-20	4.4E-10
LOC100523750	254.06901	1.6525567	7.994743816	1.407383613	1.407383613	7.264377	4.6E-10
ACVRL1	2.76161967	1132.8276	1.911353989	10.14698564	10.14698564	-8.6802	4.6E-10
LOC100622870	294.112495	2.4788351	8.205121195	1.798604302	1.798604302	6.890562	4.7E-10
LOC100515628	171.22042	31142.432	7.428112398	14.92664031	14.92664031	-7.50688	4.9E-10
LOC102163398	289.970066	2.4788351	8.184726929	1.798604302	1.798604302	6.870098	5.3E-10
LOC100739671	0	632.10295	0	9.306296318	9.306296318	-20	5.6E-10
NXPH3	248.54577	1.6525567	7.963160641	1.407383613	1.407383613	7.232668	5.6E-10
CHST15	2.76161967	1094.8188	1.911353989	10.0977936	10.0977936	-8.63096	5.7E-10
ANGPT2	0	629.62412	0	9.300636539	9.300636539	-20	5.7E-10
ARHGAP4	441.859147	5.7839486	8.790704108	2.762125239	2.762125239	6.255388	5.8E-10
MYBPH	0	625.49273	0	9.291153952	9.291153952	-20	5.9E-10
ADM	26.2353869	4836.2073	4.767410456	12.23995865	12.23995865	-7.52622	6.2E-10
LOC100738990	6.90404918	1729.4006	2.982591923	10.75689038	10.75689038	-7.96861	6.3E-10
DPP4	1.38080984	851.893	1.251452392	9.736220951	9.736220951	-9.26901	6.4E-10
COLQ	0	618.05622	0	9.273926629	9.273926629	-20	6.4E-10
CCDC85C	2075.35718	41.313919	11.01983893	5.403060391	5.403060391	5.650588	6.4E-10
CNTN5	473.617774	6.610227	8.890622318	2.927939483	2.927939483	6.16288	6.6E-10
LOC100738803	0	610.61972	0	9.256491105	9.256491105	-20	6.9E-10
LOC102165808	158.793131	0	7.320061583	0	0	20	6.9E-10
CLSTN2	1633.49804	32.224857	10.67463193	5.054191063	5.054191063	5.663647	7.1E-10
PNP	35485.432	722.99358	15.1149799	9.499833085	9.499833085	5.6171	7.5E-10
LOC733579	9.66566885	2098.7471	3.414902536	11.03599984	11.03599984	-7.76244	7.6E-10
LOXL1	81.4677803	13617.894	6.365758672	13.73332191	13.73332191	-7.38506	7.8E-10
EGR1	33577.1578	688.28988	15.03523525	9.428967032	9.428967032	5.60832	7.8E-10
CLMP	24.854577	4428.0258	4.692347799	12.11277368	12.11277368	-7.47701	8E-10
CYP7B1	0	594.09415	0	9.216974124	9.216974124	-20	8.1E-10
LOC100622713	0	587.48392	0	9.20085919	9.20085919	-20	8.6E-10
THBS4	0	585.83137	0	9.196802174	9.196802174	-20	8.8E-10
SCN4A	531.611787	8.2627837	9.056940546	3.211445828	3.211445828	6.007601	9E-10
CCL20	2.76161967	1005.5808	1.911353989	9.975247238	9.975247238	-8.5083	9.5E-10
GH1	153.269892	0	7.269312714	0	0	20	9.6E-10
SORBS2	5.52323934	1409.6309	2.705588563	10.46212483	10.46212483	-7.99559	9.7E-10
FAM65B	490.187492	7.4365053	8.940130012	3.076645515	3.076645515	6.042565	9.9E-10
PLA2R1	2.76161967	996.49172	1.911353989	9.96216105	9.96216105	-8.4952	1E-09
IFNGR1	5.52323934	1398.8893	2.705588563	10.45109701	10.45109701	-7.98455	1E-09
GBP7	0	568.47952	0	9.153500149	9.153500149	-20	1.1E-09
SLC26A7	2.76161967	986.57638	1.911353989	9.947748515	9.947748515	-8.48077	1.1E-09
LOC100525452	0	563.52185	0	9.140885611	9.140885611	-20	1.1E-09
LOC102162332	4.14242951	1172.489	2.362450112	10.19658861	10.19658861	-8.14488	1.2E-09
LOC100513259	302.397354	3.3051135	8.245064693	2.106051269	2.106051269	6.515602	1.2E-09
CKMT1B	372.818656	4.9576702	8.546194759	2.574748269	2.574748269	6.232668	1.2E-09
VDR	4.14242951	1154.3109	2.362450112	10.17406541	10.17406541	-8.12234	1.3E-09
LOC100525045	2577.97196	56.186929	11.33258037	5.837613535	5.837613535	5.519858	1.3E-09
CDH10	147.746652	0	7.21671339	0	0	20	1.3E-09
CCDC80	66.2788721	10149.177	6.072081615	13.3092173	13.3092173	-7.2586	1.3E-09
LOC102161596	2.76161967	947.74129	1.911353989	9.889870929	9.889870929	-8.42283	1.4E-09
MASP1	23.4737672	3815.7535	4.613164285	11.89813031	11.89813031	-7.34477	1.5E-09
PPP1R16B	747.018121	14.046732	9.54692941	3.911378308	3.911378308	5.732837	1.5E-09
TJP3	146.365843	0	7.203258356	0	0	20	1.5E-09
RGS4	3075.0635	68.581105	11.58686957	6.120623682	6.120623682	5.486661	1.5E-09
AGT	0	533.77583	0	9.062790447	9.062790447	-20	1.5E-09
LOC100514326	4.14242951	1101.4291	2.362450112	10.10647012	10.10647012	-8.05468	1.7E-09
C1H15orf48	2154.06334	47.924146	11.07351456	5.61247475	5.61247475	5.490164	1.7E-09
LOC102161294	0	524.68677	0	9.038059606	9.038059606	-20	1.7E-09
ZBP1	2.76161967	911.38504	1.911353989	9.833498988	9.833498988	-8.3664	1.7E-09
LOC102158099	4.14242951	1097.2977	2.362450112	10.10105341	10.10105341	-8.04926	1.7E-09
LOC100152144	0	521.38165	0	9.028960416	9.028960416	-20	1.8E-09
EHF	219.548764	1.6525567	7.784953864	1.407383613	1.407383613	7.053698	1.8E-09
STOX1	560.608793	9.9153405	9.133421714	3.448285225	3.448285225	5.821188	1.8E-09
KAZALD1	2134.73201	47.924146	11.06051491	5.61247475	5.61247475	5.477158	1.8E-09
ATP8A2	1785.38712	39.661362	10.80282904	5.34558663	5.34558663	5.492359	1.9E-09
FAM151A	142.223413	0	7.162123543	0	0	20	1.9E-09
IL1A	0	507.33492	0	8.98963553	8.98963553	-20	2.1E-09
GRTP1	618.602806	11.567897	9.275199868	3.65167138	3.65167138	5.740815	2.1E-09
NR5A2	1119.83678	23.962073	10.13036048	4.641665831	4.641665831	5.546392	2.1E-09
KCNA6	0	503.20353	0	8.977862404	8.977862404	-20	2.2E-09
AIM1	20.7121475	3200.1761	4.440430524	11.64438634	11.64438634	-7.27153	2.2E-09
COL5A1	751.160551	110737.83	9.554896832	16.75680162	16.75680162	-7.20381	2.4E-09
EFCAB4B	280.304397	3.3051135	8.135988291	2.106051269	2.106051269	6.406151	2.4E-09
IL33	11.0464787	1905.3979	3.590539588	10.89663357	10.89663357	-7.43036	2.4E-09
ADRB3	138.080984	0	7.119781365	0	0	20	2.5E-09
LOC102159289	138.080984	0	7.119781365	0	0	20	2.5E-09
LOC102159469	0	486.67796	0	8.929784965	8.929784965	-20	2.7E-09
LOC102159598	0	485.85168	0	8.927338518	8.927338518	-20	2.7E-09
CD14	2.76161967	841.15138	1.911353989	9.717935781	9.717935781	-8.25071	2.8E-09
LOC102163523	0	482.54657	0	8.917511028	8.917511028	-20	2.8E-09
USP18	102.179928	13783.15	6.689018532	13.75072264	13.75072264	-7.07565	2.8E-09
ISLR2	0	480.89401	0	8.912572065	8.912572065	-20	2.9E-09
PLCH2	208.502285	1.6525567	7.71082217	1.407383613	1.407383613	6.97922	2.9E-09
CACNG6	274.781157	3.3051135	8.107380078	2.106051269	2.106051269	6.377439	2.9E-09
CHL1	2391.56264	57.013208	11.22434098	5.858309486	5.858309486	5.390514	3E-09
PHOSPHO1	466.713725	8.2627837	8.869481953	3.211445828	3.211445828	5.819766	3.1E-09
LOC100519663	0	473.45751	0	8.890135073	8.890135073	-20	3.1E-09
SNX22	205.740666	1.6525567	7.691678382	1.407383613	1.407383613	6.959983	3.3E-09
LOC102157807	133.938554	0	7.076158798	0	0	20	3.3E-09
TRIM44	9.66566885	1645.1202	3.414902536	10.684854	10.684854	-7.41111	3.3E-09
LOC102165705	0	467.67356	0	8.872439593	8.872439593	-20	3.4E-09
LOC100625207	5.52323934	1140.2642	2.705588563	10.15641704	10.15641704	-7.68964	3.5E-09
LOC100737066	204.359856	1.6525567	7.682010377	1.407383613	1.407383613	6.950268	3.5E-09
CRABP1	0	463.54217	0	8.859665746	8.859665746	-20	3.6E-09
KRT2	132.557744	0	7.061319821	0	0	20	3.6E-09
KIF26B	2.76161967	801.49002	1.911353989	9.648339641	9.648339641	-8.18103	3.7E-09
TFAP2C	1213.73185	28.093465	10.24642216	4.862623207	4.862623207	5.433071	3.7E-09
PDZD3	519.184498	9.9153405	9.022879597	3.448285225	3.448285225	5.710441	3.8E-09
COL16A1	33.1394361	4378.4491	5.093367322	12.09653368	12.09653368	-7.04573	4E-09
GDNF	0	453.62683	0	8.828539005	8.828539005	-20	4E-09
KIAA1598	0	451.97427	0	8.823285292	8.823285292	-20	4.1E-09
LOC100623179	200.217426	1.6525567	7.652611443	1.407383613	1.407383613	6.920724	4.2E-09
LOC100621648	129.796125	0	7.031175985	0	0	20	4.4E-09
LOC102159043	2.76161967	772.57028	1.911353989	9.595388554	9.595388554	-8.12801	4.6E-09
BNC2	6.90404918	1240.2438	2.982591923	10.27757084	10.27757084	-7.48897	4.7E-09
LOC102166634	197.455807	1.6525567	7.632673964	1.407383613	1.407383613	6.900686	4.8E-09
PRSS55	353.487318	5.7839486	8.469590204	2.762125239	2.762125239	5.93346	4.8E-09
LOC100158001	196.074997	1.6525567	7.62260094	1.407383613	1.407383613	6.890562	5.1E-09
KCNK4	227.833623	2.4788351	7.838155235	1.798604302	1.798604302	6.522175	5.1E-09
TRIM50	258.211439	3.3051135	8.017985577	2.106051269	2.106051269	6.287709	5.2E-09
ST8SIA1	2.76161967	752.7396	1.911353989	9.557922374	9.557922374	-8.09049	5.4E-09
LOC100739699	38.6626754	4813.0715	5.309710091	12.23304186	12.23304186	-6.95987	5.4E-09
POU3F3	194.694187	1.6525567	7.61245709	1.407383613	1.407383613	6.880366	5.4E-09
SERPINB7	977.613364	23.135794	9.934595174	4.593102408	4.593102408	5.401065	5.7E-09
LOC100516568	193.313377	1.6525567	7.602241413	1.407383613	1.407383613	6.870098	5.8E-09
GALNTL2	22.0929574	2873.7962	4.529381037	11.48924396	11.48924396	-7.02323	5.8E-09
WSCD2	0	426.35964	0	8.739306855	8.739306855	-20	5.8E-09
LOC100525793	125.653695	0	6.984745358	0	0	20	5.9E-09
LOC100625479	23.4737672	3020.0474	4.613164285	11.56083313	11.56083313	-7.00738	5.9E-09
NIPAL4	16.569718	2246.6509	4.135019133	11.13420226	11.13420226	-7.08308	6E-09
LOC100626567	0	423.05453	0	8.728105973	8.728105973	-20	6.1E-09
LOC100512657	2.76161967	730.43008	1.911353989	9.51457615	9.51457615	-8.04709	6.4E-09
CRYBB1	124.272885	0	6.968930373	0	0	20	6.5E-09
LOC100524147	124.272885	0	6.968930373	0	0	20	6.5E-09
COL6A2	596.509849	73816.405	9.222818684	16.17167339	16.17167339	-6.95125	6.5E-09
THSD7B	2259.00489	58.665764	11.14211018	5.89883146	5.89883146	5.267025	6.7E-09
MAP1LC3A	33.1394361	4021.4968	5.093367322	11.97387557	11.97387557	-6.92304	6.7E-09
NRK	0	415.61802	0	8.702581435	8.702581435	-20	6.8E-09
MYL9	260.973059	30984.613	8.033274644	14.91931087	14.91931087	-6.89151	6.9E-09
TRIM63	0	414.79174	0	8.699717297	8.699717297	-20	6.9E-09
LOC100525778	189.170948	1.6525567	7.571153052	1.407383613	1.407383613	6.838847	7.1E-09
LOC100515181	0	409.00779	0	8.679507525	8.679507525	-20	7.5E-09
LOC100737080	1067.366	26.440908	10.06119026	4.77825631	4.77825631	5.335139	7.5E-09
P4HA3	85.6102098	9791.3987	6.436465198	13.25744658	13.25744658	-6.83759	7.6E-09
CLDN4	1063.22357	26.440908	10.05558555	4.77825631	4.77825631	5.329529	7.8E-09
LOC100514340	0	405.70268	0	8.667830689	8.667830689	-20	7.8E-09
EIF1AY	0	405.70268	0	8.667830689	8.667830689	-20	7.8E-09
COL11A2	245.784151	3.3051135	7.947105933	2.106051269	2.106051269	6.216548	8.3E-09
CGN	1168.16512	29.746021	10.19126298	4.942327829	4.942327829	5.295404	8.6E-09
MYL1	0	399.09245	0	8.644189607	8.644189607	-20	8.6E-09
APOA2	120.130456	0	6.920417836	0	0	20	8.8E-09
NXF2B	120.130456	0	6.920417836	0	0	20	8.8E-09
GDF10	2.76161967	688.28988	1.911353989	9.428967032	9.428967032	-7.96136	9.2E-09

LOC102157763	0	854.37184	0	9.740407896	9.740407896	-20	8.9E-10
LOC100621315	358.4447303	0	8.489626143	0	0	20	8.9E-10
NUP210	11747.25945	80.149002	13.52015941	6.342501448	6.342501448	7.195424	9E-10
VPAC1	474.7542299	0.8262784	8.894072673	0.868906686	0.868906686	9.166337	9.3E-10
LOC100519601	2249.002777	14.046732	11.13571107	3.911378308	3.911378308	7.322907	9.6E-10
MYO5C	695.742279	2.4788351	9.4444813	1.798604302	1.798604302	8.132747	9.7E-10
LOC100525856	2.114718173	1469.9492	1.639101631	10.52253173	10.52253173	-9.44109	1E-09
LOC100517681	2113.660814	13.220454	11.04621056	3.829895615	3.829895615	7.320828	1E-09
C1R	20.08982264	6800.271	4.398475058	12.73158866	12.73158866	-8.40298	1.1E-09
COL6A2	222.0454082	73816.405	7.801193638	16.17167339	16.17167339	-8.37694	1.1E-09
TRIM4	0	818.84187	0	9.679201855	9.679201855	-20	1.1E-09
PTGS1	3.17207726	1743.4474	2.060765874	10.76855435	10.76855435	-9.1023	1.1E-09
TSPO	7.401513606	2946.5087	3.070649265	11.52528034	11.52528034	-8.63697	1.2E-09
LOC102165099	2.114718173	1430.2879	1.639101631	10.48309814	10.48309814	-9.40162	1.2E-09
LOC102159856	672.4803791	2.4788351	9.395492105	1.798604302	1.798604302	8.083686	1.2E-09
PTGIS	5.286795433	2316.0583	2.65232482	11.17807861	11.17807861	-8.77506	1.2E-09
MATN4	0	803.14258	0	9.651307509	9.651307509	-20	1.2E-09
GREB1L	2590.529762	17.351846	11.33958825	4.19785327	4.19785327	7.222014	1.3E-09
ZSCAN10	339.4122668	0	8.411139215	0	0	20	1.3E-09
HOXB2	0	796.53235	0	9.63939923	9.63939923	-20	1.3E-09
COL16A1	12.68830904	4378.4491	3.774872332	12.09653368	12.09653368	-8.43078	1.3E-09
LOC102158099	1.057359087	1097.2977	1.04079362	10.10105341	10.10105341	-10.0193	1.3E-09
IL8	0	789.09585	0	9.625883864	9.625883864	-20	1.3E-09
LOC100620730	0	789.09585	0	9.625883864	9.625883864	-20	1.3E-09
LRRC32	3.17207726	1681.4765	2.060765874	10.71637063	10.71637063	-9.05008	1.4E-09
CDC42EP1	12.68830904	4337.1352	3.774872332	12.08285929	12.08285929	-8.4171	1.4E-09
SYNPO	4.229436346	1963.2374	2.386655454	10.9397536	10.9397536	-8.85855	1.4E-09
LOC100623769	0	782.48562	0	9.613762982	9.613762982	-20	1.4E-09
DAB1	550.8840841	1.6525567	9.10822147	1.407383614	1.407383614	8.380905	1.4E-09
CLEC14A	0	775.04911	0	9.600004147	9.600004147	-20	1.5E-09
FXYD5	5.286795433	2213.5998	2.65232482	11.11283027	11.11283027	-8.70979	1.5E-09
IQCJ	328.8386759	0	8.365616765	0	0	20	1.5E-09
PRKCZ	328.8386759	0	8.365616765	0	0	20	1.5E-09
GADD45B	7.401513606	2772.1639	3.070649265	11.43731719	11.43731719	-8.54898	1.6E-09
LOC100513362	0	757.69727	0	9.56738053	9.56738053	-20	1.6E-09
TRPC6	1.057359087	1048.5473	1.04079362	10.03555141	10.03555141	-9.95371	1.7E-09
CLU	435.6319437	136815.17	8.77027387	17.06187925	17.06187925	-8.2949	1.7E-09
LOC100520893	322.4945214	0	8.337597469	0	0	20	1.7E-09
NID2	81.41664967	23899.276	6.364863912	14.54473964	14.54473964	-8.19743	1.7E-09
LGALS1	268.569208	82013.912	8.074511901	16.32359863	16.32359863	-8.25443	1.8E-09
LOC100515902	3.17207726	1595.5435	2.060765874	10.64073618	10.64073618	-8.9744	1.8E-09
EMSP1	429.2877891	0.8262784	8.749158089	0.868906686	0.868906686	9.021102	1.8E-09
LOC100511351	5.286795433	2149.9763	2.65232482	11.07077593	11.07077593	-8.66771	1.8E-09
EMILIN1	95.16231779	27857.975	6.587399765	14.76585457	14.76585457	-8.19348	1.8E-09
DNMT3B	28611.07952	214.0061	14.80433674	7.74823377	7.74823377	7.062778	1.9E-09
ONECUT1	319.3224441	0	8.323381077	0	0	20	1.9E-09
PODNL1	1.057359087	1022.9326	1.04079362	9.999905072	9.999905072	-9.91803	1.9E-09
HOXB4	0	732.08264	0	9.517832026	9.517832026	-20	1.9E-09
LOC102160493	0	732.08264	0	9.517832026	9.517832026	-20	1.9E-09
LOC100737137	0	729.6038	0	9.512945452	9.512945452	-20	2E-09
LOC100620681	0	727.12497	0	9.508042269	9.508042269	-20	2E-09
ACSL5	0	723.81985	0	9.501478663	9.501478663	-20	2.1E-09
SLC35F1	2290.239782	16.525567	11.16191273	4.131389251	4.131389251	7.114655	2.1E-09
ALDH3B1	3.17207726	1522.0048	2.060765874	10.57270474	10.57270474	-8.90633	2.2E-09
LOC100516442	0	709.77312	0	9.473245315	9.473245315	-20	2.3E-09
FNDC1	796.1913922	4.1313919	9.638782322	2.359350201	2.359350201	7.590344	2.4E-09
DRD2	1654.76697	11.567897	10.69328393	3.651671381	3.651671381	7.160358	2.4E-09
LOC100623748	0	697.37895	0	9.447866256	9.447866256	-20	2.5E-09
HOXC4	0	694.90011	0	9.442736427	9.442736427	-20	2.5E-09
GDF10	0	688.28988	0	9.428967032	9.428967032	-20	2.7E-09
GBP1	12.68830904	3798.4017	3.774872332	11.89155653	11.89155653	-8.22575	2.7E-09
VASN	33.83549077	9142.7702	5.122485983	13.15857343	13.15857343	-8.07795	2.8E-09
SLA-1	29.60605442	8040.5148	4.935745167	12.97325158	12.97325158	-8.08525	2.8E-09
IRF7	15.8603863	4557.7515	4.075565686	12.15442305	12.15442305	-8.16675	2.8E-09
VWA5A	7.401513606	2446.6103	3.070649265	11.25715814	11.25715814	-8.36875	3E-09
NPY	4.229436346	1688.0067	2.386655454	10.72202768	10.72202768	-8.64071	3E-09
MGAT5B	2348.394531	18.178124	11.19807329	4.261389711	4.261389711	7.013328	3.1E-09
CNN1	24.31925899	6544.1247	4.662163277	12.67620496	12.67620496	-8.07196	3.1E-09
LOC100739808	293.9458261	0	8.204306183	0	0	20	3.1E-09
S100A16	11.63094995	3415.0085	3.658891241	11.73809585	11.73809585	-8.19778	3.2E-09
LOC102164335	0	656.89131	0	9.361705436	9.361705436	-20	3.4E-09
LOC100621630	3628.856385	29.746021	11.82569675	4.942327829	4.942327829	6.930674	3.4E-09
ARSI	0	653.58619	0	9.35443936	9.35443936	-20	3.5E-09
SLAIN1	11985.16525	100.80596	13.54908255	6.669678232	6.669678232	6.893525	3.5E-09
ZNF134	0	647.80224	0	9.341634998	9.341634998	-20	3.6E-09
MASP1	13.74566813	3815.7535	3.882219287	11.89813031	11.89813031	-8.11685	3.7E-09
LOC100153751	1.057359087	893.20692	1.04079362	9.8044649	9.8044649	-9.72239	3.7E-09
LOC102164249	472.6395117	1.6525567	8.887645627	1.407383614	1.407383614	8.159897	3.7E-09
DPYSL5	6025.889434	51.229259	12.55719788	5.706786332	5.706786332	6.878062	3.8E-09
ACVRL1	2.114718173	1132.8276	1.639101631	10.14698564	10.14698564	-9.06525	3.8E-09
ZNF814	0	636.23435	0	9.315680218	9.315680218	-20	4E-09
CYGB	2.114718173	1117.9546	1.639101631	10.12793583	10.12793583	-9.04618	4E-09
LOC100626670	5299.483742	45.44531	12.37190832	5.537461031	5.537461031	6.865577	4E-09
CDH13	0	629.62412	0	9.300636539	9.300636539	-20	4.2E-09
LYSMD2	728.5204106	4.1313919	9.510804532	2.359350201	2.359350201	7.462198	4.2E-09
SYT6	901.9273008	5.7839486	9.818466024	2.76212524	2.76212524	7.284813	4.2E-09
NFIB	10.57359087	2991.954	3.532764645	11.54735438	11.54735438	-8.14448	4.3E-09
MYBPH	0	625.49273	0	9.291153952	9.291153952	-20	4.3E-09
ITGB5	28.54869534	7134.9137	4.885022527	12.80088246	12.80088246	-7.96533	4.3E-09
ECEL1	0	623.01389	0	9.285434338	9.285434338	-20	4.4E-09
VGF	10.57359087	2927.5043	3.532764645	11.51594828	11.51594828	-8.11306	4.8E-09
LOC100738803	0	610.61972	0	9.256491105	9.256491105	-20	4.9E-09
LTBP3	25.37661808	6236.7491	4.721187694	12.60680982	12.60680982	-7.94115	4.9E-09
TLR3	2.114718173	1075.8144	1.639101631	10.07255395	10.07255395	-8.99075	4.9E-09
NRCAM	3505.145372	30.5723	11.7756701	4.980587447	4.980587447	6.841105	5E-09
CHRNG	1.057359087	841.97766	1.04079362	9.71935059	9.71935059	-9.63717	5E-09
LOC100737260	535.0236978	2.4788351	9.066152974	1.798604302	1.798604302	7.753797	5.2E-09
CAMKV	3389.893232	29.746021	11.72744964	4.942327829	4.942327829	6.832399	5.2E-09
IGLON5	2.114718173	1059.2889	1.639101631	10.05024166	10.05024166	-8.96841	5.3E-09
BGN	768.7000559	198659.63	9.588152542	17.59994647	17.59994647	-8.01366	5.4E-09
SP100	0	598.22554	0	9.226955308	9.226955308	-20	5.4E-09
LOC100627133	0	588.3102	0	9.202883429	9.202883429	-20	5.9E-09
CHL1	6229.959738	57.013208	12.60523868	5.858309485	5.858309485	6.771783	6E-09
LOC102161796	2.114718173	1031.1954	1.639101631	10.0115004	10.0115004	-8.92964	6.1E-09
UTF1	264.3397716	0	8.051697126	0	0	20	6.1E-09
GUCY1B3	1.057359087	804.79513	1.04079362	9.654269283	9.654269283	-9.57201	6.3E-09
SDC4	8.458872693	2324.3211	3.241668254	11.18321421	11.18321421	-8.10213	6.4E-09
ZNF606	0	575.91603	0	9.172217528	9.172217528	-20	6.5E-09
NR0B1	255.880899	0	8.004955809	0	0	20	7.5E-09
SRPX2	5.286795433	1613.7217	2.65232482	10.65706978	10.65706978	-8.25378	7.6E-09
FIBIN	3.17207726	1193.9722	2.060765874	10.2227614	10.2227614	-8.55613	7.6E-09
HOXB8	6.344154519	1809.5496	2.876596413	10.82221201	10.82221201	-8.15599	7.9E-09
PPP1R3D	2.114718173	976.66104	1.639101631	9.933190545	9.933190545	-8.85125	8E-09
ID3	21.14718173	4783.3255	4.46905122	12.22409983	12.22409983	-7.8214	8E-09
LOC100525868	498.0161298	2.4788351	8.962942639	1.798604302	1.798604302	7.650386	8.1E-09
KCNK12	0	548.64884	0	9.10236639	9.10236639	-20	8.3E-09
LOC102165782	251.6514626	0	7.981004722	0	0	20	8.4E-09
LOC100620576	0	547.82256	0	9.100195977	9.100195977	-20	8.4E-09
COL15A1	77.18721332	16447.071	6.288860784	14.00563078	14.00563078	-7.73525	8.8E-09
CLEC11A	2.114718173	949.39385	1.639101631	9.892381689	9.892381689	-8.8104	9.2E-09
GLIS1	6.344154519	1754.189	2.876596413	10.77741066	10.77741066	-8.11116	9.2E-09
ZNF473	0	536.25466	0	9.06946229	9.06946229	-20	9.4E-09
CARP	20.08982264	4422.2418	4.398475058	12.11088841	12.11088841	-7.78217	9.4E-09
ZNF667	0	532.94955	0	9.060559625	9.060559625	-20	9.7E-09
RUNX1	1.057359087	738.69286	1.04079362	9.530782548	9.530782548	-9.44837	9.7E-09
SYBU	1629.390352	14.873011	10.6710017	3.98850389	3.98850389	6.775492	1E-08
LOC100515404	1.057359087	729.6038	1.04079362	9.512945452	9.512945452	-9.4305	1E-08
NETO2	1088.0225	9.0890621	10.08881805	3.334720158	3.334720158	6.903361	1E-08
LUZP4	242.1352308	0	7.925615148	0	0	20	1.1E-08
LOC102165344	37.00756803	7593.4982	5.24821481	12.89073893	12.89073893	-7.6808	1.1E-08
NID1	9.516231779	2274.7444	3.394545939	11.15212279	11.15212279	-7.9011	1.1E-08
NBL1	39.1222862	7981.8491	5.326331909	12.96268802	12.96268802	-7.67259	1.1E-08
LOC100736977	0	516.42398	0	9.015203114	9.015203114	-20	1.1E-08
ZBP1	2.114718173	911.38504	1.639101631	9.833498988	9.833498988	-8.75145	1.1E-08
HOXA9	0	513.94515	0	9.008274952	9.008274952	-20	1.2E-08
LGALS9	0	513.11887	0	9.005958152	9.005958152	-20	1.2E-08
IL1A	0	507.33492	0	8.98963553	8.98963553	-20	1.2E-08
OSR1	0	507.33492	0	8.98963553	8.98963553	-20	1.2E-08
HOXB7	2.114718173	892.38064	1.639101631	9.803131183	9.803131183	-8.72105	1.3E-08
KCNA6	0	503.20353	0	8.977862404	8.977862404	-20	1.3E-08
CDH10	234.7337172	0	7.881014313	0	0	20	1.3E-08
ECM1	3.17207726	1061.7677	2.060765874	10.05361058	10.05361058	-8.38682	1.4E-08
TARSL2	231.56164	0	7.86146934	0	0	20	1.4E-08
LOC102166536	231.56164	0	7.86146934	0	0	20	1.4E-08
LOC100623939	0	494.11447	0	8.951618292	8.951618292	-20	1.4E-08
FOXL1	0	493.28819	0	8.94920862	8.94920862	-20	1.4E-08
DKK2	4.229436346	1229.5022	2.386655454	10.26503154	10.26503154	-8.18339	1.5E-08
MAMLD1	1.057359087	679.20082	1.04079362	9.409816938	9.409816938	-9.32723	1.5E-08
NDRG1	15.8603863	3261.3207	4.075565686	11.67168291	11.67168291	-7.68388	1.5E-08
DPP4	2.114718173	851.893	1.639101631	9.736220951	9.736220951	-8.65406	1.6E-08
LOC100737092	0	482.54657	0	8.917511028	8.917511028	-20	1.6E-08
ISLR2	0	480.89401	0	8.912572065	8.912572065	-20	1.6E-08
LOC100513632	7.401513606	1742.6211	3.070649265	10.76787084	10.76787084	-7.87922	1.7E-08
LOC100512163	0	478.41518	0	8.905131772	8.905131772	-20	1.7E-08
LOC100738561	0	478.41518	0	8.905131772	8.905131772	-20	1.7E-08
GRIK1	224.1601264	0	7.814807553	0	0	20	1.7E-08
LOC100521659	2.114718173	837.01999	1.639101631	9.710840849	9.710840849	-8.62865	1.7E-08
SLC7A3	8938.913718	96.674569	13.12604519	6.609911087	6.609911087	6.530819	1.7E-08
LOC494560	40.17964529	7495.1711	5.363859498	12.87193817	12.87193817	-7.54335	1.8E-08
LOC102165604	223.1027673	0	7.808016653	0	0	20	1.8E-08
SCMH1	0	461.88961	0	8.854524369	8.854524369	-20	2E-08
LOC100515735	0	461.88961	0	8.854524369	8.854524369	-20	2E-08
VDR	4.229436346	1154.3109	2.386655454	10.17406541	10.17406541	-8.09235	2E-08
FOLR2	2.114718173	806.44769	1.639101631	9.65722499	9.65722499	-8.57497	2.1E-08
CHGA	2074.538528	22.309516	11.01927	4.542847146	4.542847146	6.538987	2.2E-08
MDFI	14.80302721	2858.0969	3.982129041	11.48134379	11.48134379	-7.59302	2.2E-08
SPEG	8.458872693	1794.6766	3.241668254	10.81031185	10.81031185	-7.72904	2.3E-08
UGT1A10	0	445.36404	0	8.802077003	8.802077003	-20	2.4E-08
GLCCI1	13417.88681	152.03522	13.71197737	7.257719911	7.257719911	6.463608	2.5E-08
PDGFRB	75.07249515	13038.673	6.249303021	13.67062004	13.67062004	-7.4403	2.5E-08
CALB2	211.4718173	0	7.731127682	0	0	20	2.5E-08
ZNF333	0	440.40637	0	8.785963646	8.785963646	-20	2.5E-08
LOC100622872	611.153552	4.9576702	9.257749772	2.57474827	2.57474827	6.945729	2.6E-08
LOC100512967	611.153552	4.9576702	9.257749772	2.57474827	2.57474827	6.945729	2.6E-08
POU2F3	209.3570991	0	7.716696697	0	0	20	2.6E-08
LOC100521657	609.0388339	4.9576702	9.252757274	2.57474827	2.57474827	6.940728	2.7E-08
LOC100737813	279.1427989	0.8262784	8.130018598	0.868906686	0.868906686	8.40016	2.7E-08
AHNAK2	0	434.62242	0	8.766934408	8.766934408	-20	2.7E-08
LOC100513741	0	434.62242	0	8.766934408	8.766934408	-20	2.7E-08
CRISPLD2	3.17207726	915.51644	2.060765874	9.840016943	9.840016943	-8.17301	2.9E-08
IFI35	2.114718173	755.21843	1.639101631	9.562659203	9.562659203	-8.48028	2.9E-08
WSCD2	0	426.35964	0	8.739306855	8.739306855	-20	3E-08
CLDN11	0	426.35964	0	8.739306855	8.739306855	-20	3E-08
CEBPD	188.2099174	32373.587	7.563843899	14.98257415	14.98257415	-7.42633	3E-08
LOC100626567	0	423.05453	0	8.728105973	8.728105973	-20	3.1E-08
CD86	0	423.05453	0	8.728105973	8.728105973	-20	3.1E-08
SOWAHB	204.0703037	0	7.67997478	0	0	20	3.1E-08
ZNF181	0	422.22825	0	8.725292112	8.725292112	-20	3.1E-08
HOXB9	0	420.57569	0	8.719647871	8.719647871	-20	3.2E-08
LOC100623028	27.49133625	4631.2903	4.832451381	12.17750994	12.17750994	-7.39629	3.2E-08
COL9A1	2745.961548	32.224856	11.423621	5.054191063	5.054191063	6.412994	3.2E-08
BHLHE41	2.114718173	738.69286	1.639101631	9.530782548	9.530782548	-8.44837	3.3E-08
RBAK	0	416.4443	0	8.705439898	8.705439898	-20	3.3E-08
NRK	0	415.61802	0	8.702581435	8.702581435	-20	3.4E-08
TRIM63	0	414.79174	0	8.699717297	8.699717297	-20	3.4E-08
LOC100623035	0	414.79174	0	8.699717297	8.699717297	-20	3.4E-08
LOC100737413	0	414.79174	0	8.699717297	8.699717297	-20	3.4E-08
LOC100519278	5.286795433	1194.7985	2.65232482	10.22375862	10.22375862	-7.82016	3.4E-08
EHF	332.0107532	1.6525567	8.379424954	1.407383614	1.407383614	7.650386	3.5E-08
RAB3C	582.6048567	4.9576702	9.188848079	2.57474827	2.57474827	6.876712	3.5E-08
FUT9	199.8408674	0	7.64990905	0	0	20	3.5E-08
GBP7	1.057359087	568.47952	1.04079362	9.153500149	9.153500149	-9.0705	3.6E-08
LOC100515181	0	409.00779	0	8.679507525	8.679507525	-20	3.6E-08
LOC102161103	27.49133625	4513.9587	4.832451381	12.14049709	12.14049709	-7.35927	3.6E-08
TBX3	2.114718173	721.34102	1.639101631	9.496536287	9.496536287	-8.41407	3.7E-08
HMGCLL1	197.7261492	0	7.63463791	0	0	20	3.8E-08
LOC100514340	0	405.70268	0	8.667830689	8.667830689	-20	3.8E-08
EIF1AY	0	405.70268	0	8.667830689	8.667830689	-20	3.8E-08
LRRN4CL	33.83549077	5439.3905	5.122485983	12.4094945	12.4094945	-7.32876	3.8E-08
LOC100521069	0	404.05012	0	8.661956638	8.661956638	-20	3.9E-08
SERPINE1	63.44154519	10062.418	6.009919183	13.29683278	13.29683278	-7.30933	3.9E-08
LOC100518586	0	400.74501	0	8.650136295	8.650136295	-20	4E-08
LOC100518738	0	399.91873	0	8.647166015	8.647166015	-20	4.1E-08
MYL1	0	399.09245	0	8.644189607	8.644189607	-20	4.1E-08
MXRA5	93.04759962	14656.526	6.555319217	13.83935397	13.83935397	-7.29936	4.1E-08
ZFP28	0	398.26618	0	8.641207045	8.641207045	-20	4.2E-08
COL8A1	139.5713994	22202.926	7.135159284	14.43852718	14.43852718	-7.3136	4.2E-08
PTGS2	23.2618999	3762.0454	4.600620625	11.87768499	11.87768499	-7.3374	4.2E-08
ZNF268	0	397.4399	0	8.638218305	8.638218305	-20	4.2E-08
SOX2	1237.110131	14.046732	10.27392393	3.911378308	3.911378308	6.460596	4.3E-08
PARP14	16.91774539	2802.7362	4.163317207	11.45313492	11.45313492	-7.37215	4.3E-08
ZBTB4	9.516231779	1736.8371	3.394545939	10.76307717	10.76307717	-7.51186	4.4E-08
THBS1	1211.733513	211976.76	10.24404685	17.69355337	17.69355337	-7.45069	4.4E-08
PDZD4	499.0734889	4.1313919	8.965996313	2.359350201	2.359350201	6.916481	4.6E-08
UNC45B	1.057359087	542.03861	1.04079362	9.084910972	9.084910972	-9.00179	4.6E-08
CYYR1	1470.786489	17.351846	10.52335268	4.19785327	4.19785327	6.405355	4.6E-08
LOC102165956	191.3819947	0	7.587829971	0	0	20	4.6E-08
CCNA1	498.0161298	4.1313919	8.962942639	2.359350201	2.359350201	6.913421	4.6E-08
RIPK3	2.114718173	686.63733	1.639101631	9.42550405	9.42550405	-8.34294	4.7E-08
NNAT	54.9826725	8396.6408	5.806908455	13.03576837	13.03576837	-7.25469	4.7E-08
LOC102159197	435.6319437	3.3051135	8.77027387	2.106051269	2.106051269	7.042266	4.8E-08
GPR68	0	386.69828	0	8.598790517	8.598790517	-20	4.8E-08
LMNA	549.826725	90316.357	9.105454748	16.46271565	16.46271565	-7.35987	4.9E-08
LOC100514506	0	385.872	0	8.595712505	8.595712505	-20	4.9E-08
EDNRA	0	385.04572	0	8.592627913	8.592627913	-20	4.9E-08
IGF2	0	382.56689	0	8.583334366	8.583334366	-20	5.1E-08
LOC102166950	0	382.56689	0	8.583334366	8.583334366	-20	5.1E-08
RNF128	899.8125827	9.9153405	9.815083169	3.448285225	3.448285225	6.503818	5.3E-08
SCARA3	179.7510447	27463.841	7.497860176	14.74529829	14.74529829	-7.25539	5.3E-08
LOC102161294	1.057359087	524.68677	1.04079362	9.038059606	9.038059606	-8.95485	5.4E-08
PARP10	12.68830904	2094.6157	3.774872332	11.03315844	11.03315844	-7.36704	5.4E-08
MYOG	0	376.78294	0	8.561413734	8.561413734	-20	5.5E-08
HOXB5	0	375.13038	0	8.55508903	8.55508903	-20	5.6E-08
PDE1A	1.057359087	518.90282	1.04079362	9.022098163	9.022098163	-8.93886	5.7E-08
LOC100515339	0	373.47782	0	8.548736477	8.548736477	-20	5.7E-08
FAM65B	712.6600243	7.4365053	9.479093152	3.076645515	3.076645515	6.582445	5.8E-08
PAX1	0	370.99899	0	8.53915489	8.53915489	-20	5.9E-08
LOC102164895	0	370.17271	0	8.535946834	8.535946834	-20	6E-08
SLA-5	1.057359087	513.11887	1.04079362	9.005958152	9.005958152	-8.92268	6.1E-08
SNED1	3.17207726	786.61701	2.060765874	9.621350459	9.621350459	-7.95409	6.2E-08
LOC100523560	0	366.8676	0	8.523042794	8.523042794	-20	6.3E-08
CHRM4	587.8916521	5.7839486	9.201858412	2.76212524	2.76212524	6.667352	6.3E-08
LOC100739847	1983.605646	25.61463	10.95463665	4.734147579	4.734147579	6.275013	6.5E-08
LOC100738461	49.69587707	7119.2145	5.663796517	12.79770498	12.79770498	-7.16245	6.5E-08
LOC102167050	180.8084038	0	7.506275077	0	0	20	6.6E-08
WNT5B	6.344154519	1177.4467	2.876596413	10.20267077	10.20267077	-7.53602	6.9E-08
LOC100620451	0	358.60481	0	8.490268521	8.490268521	-20	7E-08
LRRC4	238.9631536	0.8262784	7.906669086	0.868906686	0.868906686	8.175945	7.1E-08
LOC100517243	0	354.47342	0	8.473597884	8.473597884	-20	7.4E-08
ISG12%28A%29	153.3170676	21800.529	7.269753823	14.41214167	14.41214167	-7.1517	7.5E-08
FRMD5	740.1513606	8.2627837	9.533624395	3.211445828	3.211445828	6.485049	7.5E-08
LOC102165913	61.32682702	8517.2775	5.961781364	13.05634601	13.05634601	-7.11773	7.6E-08
UNC5B	31.7207726	4459.4244	5.032134908	12.12296526	12.12296526	-7.13529	7.7E-08
LOC100622791	32.77813168	4600.718	5.078017628	12.16795685	12.16795685	-7.13298	7.7E-08
LOC100623474	175.5216084	0	7.463700987	0	0	20	7.9E-08
LOC102167067	175.5216084	0	7.463700987	0	0	20	7.9E-08
COLQ	2.114718173	618.05622	1.639101631	9.273926629	9.273926629	-8.19113	8E-08
LOC100626144	1461.270258	19.004403	10.51399426	4.322245637	4.322245637	6.264746	8E-08
RHOJ	0	348.68947	0	8.449930555	8.449930555	-20	8.1E-08
LOC100526171	2.114718173	613.92483	1.639101631	9.264266253	9.264266253	-8.18145	8.2E-08
LOC100620529	1.057359087	480.89401	1.04079362	8.912572065	8.912572065	-8.82911	8.4E-08
TSPAN18	10.57359087	1655.0356	3.532764645	10.69351795	10.69351795	-7.29025	8.4E-08
KRT18	451.49233	4.1313919	8.821749528	2.359350201	2.359350201	6.77193	8.6E-08
LOC100622576	0	342.07925	0	8.422398044	8.422398044	-20	8.9E-08
ACPT	2.114718173	604.00949	1.639101631	9.240813961	9.240813961	-8.15796	8.9E-08
BTBD11	4416.588905	61.970878	12.10904345	5.976612875	5.976612875	6.155198	9E-08
ALPL	717.9468198	8.2627837	9.489741249	3.211445828	3.211445828	6.441105	9E-08

LOC102166331	795.771671	1.6525567	9.638022543	1.407383613	1.407383613	8.911511	1E-15
GABRB2	793.284884	1.6525567	9.633512738	1.407383613	1.407383613	8.906995	1E-15
MATN4	1.65785765	803.14258	1.410263837	9.651307509	9.651307509	-8.92019	1E-15
EPHA3	0	531.29699	0	9.056087606	9.056087606	-20	1E-15
ZNF667	0	532.94955	0	9.060559625	9.060559625	-20	1E-15
ZNF473	0	536.25466	0	9.06946229	9.06946229	-20	1E-15
LOC100622870	912.650635	2.4788351	9.835498797	1.798604302	1.798604302	8.524257	1.1E-15
LOC102161294	0	524.68677	0	9.038059606	9.038059606	-20	1.2E-15
COL14A1	150.036117	22555.747	7.238749771	14.4612714	14.4612714	-7.23204	1.3E-15
PDE1A	0	518.90282	0	9.022098163	9.022098163	-20	1.3E-15
FOLR1	1461.40152	6.610227	10.51412376	2.927939483	2.927939483	7.788437	1.4E-15
WISP1	3.31571529	1014.6698	2.109599694	9.988215791	9.988215791	-8.25747	1.4E-15
CLEC14A	1.65785765	775.04911	1.410263837	9.600004147	9.600004147	-8.86882	1.4E-15
LOC100511846	1328.7729	5.7839486	10.37696417	2.762125239	2.762125239	7.843824	1.7E-15
CHGA	3634.02396	22.309516	11.82774916	4.542847146	4.542847146	7.347765	1.7E-15
KCNA6	0	503.20353	0	8.977862404	8.977862404	-20	1.8E-15
PLN	497.357294	0	8.961036634	0	0	20	1.9E-15
MFAP2	85.3796688	12445.405	6.43261988	13.60344146	13.60344146	-7.1875	1.9E-15
C1QTNF1	11.6050035	2121.0566	3.655924617	11.05124741	11.05124741	-7.51389	1.9E-15
KCNH3	741.062368	1.6525567	9.535396636	1.407383613	1.407383613	8.808751	2E-15
GIMAP8	1080.92319	4.1313919	10.07938236	2.359350201	2.359350201	8.03142	2E-15
DRD2	2058.23027	11.567897	11.00788945	3.65167138	3.65167138	7.475134	2.3E-15
SQRDL	7.46035941	1509.6106	3.080718953	10.56091609	10.56091609	-7.66072	2.6E-15
LOC100515404	1.65785765	729.6038	1.410263837	9.512945452	9.512945452	-8.78165	2.6E-15
LOC100738803	0.82892882	610.61972	0.870998931	9.256491105	9.256491105	-9.52481	2.7E-15
ACSL5	1.65785765	723.81985	1.410263837	9.501478663	9.501478663	-8.77017	2.9E-15
ST8SIA6	474.976216	0	8.894745675	0	0	20	3E-15
LOC100512163	0	478.41518	0	8.905131772	8.905131772	-20	3.1E-15
LOC100738561	0	478.41518	0	8.905131772	8.905131772	-20	3.1E-15
JPH1	1770.59197	9.9153405	10.79083065	3.448285225	3.448285225	7.480354	3.3E-15
LOC100739422	16.5785765	2648.2222	4.135746339	11.37135313	11.37135313	-7.31956	3.3E-15
LOC100624693	16.5785765	2649.0485	4.135746339	11.37180303	11.37180303	-7.32001	3.3E-15
LOC100519663	0	473.45751	0	8.890135073	8.890135073	-20	3.3E-15
LOC102160246	466.686928	0	8.869399294	0	0	20	3.4E-15
ID3	33.1571529	4783.3255	5.094115824	12.22409983	12.22409983	-7.17255	3.6E-15
THBS4	0.82892882	585.83137	0.870998931	9.196802174	9.196802174	-9.46502	3.9E-15
PTGFR	10.7760747	1842.6008	3.557786824	10.84831056	10.84831056	-7.41777	4.6E-15
ZNF606	0.82892882	575.91603	0.870998931	9.172217528	9.172217528	-9.44039	4.8E-15
TPD52L1	445.134778	0	8.801335807	0	0	20	5.6E-15
SLC26A7	4.14464412	986.57638	2.363071282	9.947748515	9.947748515	-7.89504	5.8E-15
DAB2	89.524313	11665.398	6.500233417	13.51007158	13.51007158	-7.02574	5.9E-15
TINAGL1	8.28928824	1483.996	3.215568059	10.53624329	10.53624329	-7.48402	6.2E-15
KIAA1244	1562.53083	9.0890621	10.61059195	3.334720158	3.334720158	7.425538	6.3E-15
LOC100739007	16.5785765	2482.9665	4.135746339	11.27843001	11.27843001	-7.2266	6.3E-15
CACNB4	434.358704	0	8.766060755	0	0	20	7E-15
COL8A1	174.075053	22202.926	7.451829714	14.43852718	14.43852718	-6.9949	7E-15
MYO5C	754.32523	2.4788351	9.560954167	1.798604302	1.798604302	8.249381	7.2E-15
LOC100513741	0	434.62242	0	8.766934408	8.766934408	-20	7.7E-15
PNCK	430.21406	0	8.752260407	0	0	20	7.8E-15
FRMPD4	647.393411	1.6525567	9.34072562	1.407383613	1.407383613	8.613799	7.8E-15
VDR	5.80250177	1154.3109	2.766065426	10.17406541	10.17406541	-7.63614	7.8E-15
LOC100620576	0.82892882	547.82256	0.870998931	9.100195978	9.100195978	-9.36824	7.8E-15
KCNJ3	746.86487	2.4788351	9.546633806	1.798604302	1.798604302	8.235041	8E-15
LRAT	639.104123	1.6525567	9.322162791	1.407383613	1.407383613	8.595207	8.7E-15
WSCD2	0	426.35964	0	8.739306855	8.739306855	-20	9.3E-15
DPPA4	421.095842	0	8.721426809	0	0	20	9.6E-15
DDAH1	137.602185	16977.542	7.114806188	14.05142493	14.05142493	-6.94698	9.7E-15
LOC102167710	0	422.22825	0	8.725292112	8.725292112	-20	1E-14
TRPC3	0	421.40197	0	8.722472752	8.722472752	-20	1E-14
NFE2L3	3566.0518	27.267186	11.80051645	4.821056379	4.821056379	7.031018	1.1E-14
TNS1	13.2628612	1972.3265	3.834191515	10.94641394	10.94641394	-7.21636	1.1E-14
LOC100625479	22.3810782	3020.0474	4.547269558	11.56083313	11.56083313	-7.07615	1.1E-14
LOC102157946	721.168077	2.4788351	9.496190837	1.798604302	1.798604302	8.184529	1.1E-14
ITGB8	4.97357294	1013.8436	2.578594101	9.987041638	9.987041638	-7.67134	1.2E-14
LOC100737785	29.0125088	3757.914	4.907492018	11.87610021	11.87610021	-7.01711	1.2E-14
NRK	0	415.61802	0	8.702581435	8.702581435	-20	1.2E-14
MYBPH	1.65785765	625.49273	1.410263837	9.291153952	9.291153952	-8.55953	1.2E-14
BEND4	411.148697	0	8.687021122	0	0	20	1.2E-14
TRIM63	0	414.79174	0	8.699717297	8.699717297	-20	1.2E-14
LOC100623035	0	414.79174	0	8.699717297	8.699717297	-20	1.2E-14
CMPK2	25.6967935	3366.2581	4.73859457	11.71735859	11.71735859	-7.03341	1.2E-14
TPM2	467.515857	57079.31	8.871954065	15.80070555	15.80070555	-6.93181	1.3E-14
MCF2	407.832981	0	8.675367775	0	0	20	1.3E-14
LOC100515181	0	409.00779	0	8.679507525	8.679507525	-20	1.4E-14
THEM5	402.030479	0	8.654745137	0	0	20	1.5E-14
EIF1AY	0	405.70268	0	8.667830689	8.667830689	-20	1.5E-14
LOC100521069	0	404.05012	0	8.661956638	8.661956638	-20	1.6E-14
SLC24A4	601.802326	1.6525567	9.235541173	1.407383613	1.407383613	8.508446	1.6E-14
LOC100521383	399.543693	0	8.645815816	0	0	20	1.6E-14
OSR1	0.82892882	507.33492	0.870998931	8.98963553	8.98963553	-9.25747	1.7E-14
LOC100518586	0	400.74501	0	8.650136295	8.650136295	-20	1.7E-14
LOC100518738	0	399.91873	0	8.647166015	8.647166015	-20	1.7E-14
LOC100519221	395.399049	0	8.630809691	0	0	20	1.8E-14
MYL1	0	399.09245	0	8.644189607	8.644189607	-20	1.8E-14
TFAP2E	586.052678	1.6525567	9.197346157	1.407383613	1.407383613	8.470187	2.1E-14
LOC100627133	1.65785765	588.3102	1.410263837	9.202883429	9.202883429	-8.47111	2.2E-14
SLC52A3	578.592319	1.6525567	9.178894665	1.407383613	1.407383613	8.451704	2.3E-14
VIT	929.229211	4.9576702	9.861442434	2.574748269	2.574748269	7.550228	2.5E-14
EDNRA	0	385.04572	0	8.592627913	8.592627913	-20	2.5E-14
PODNL1	5.80250177	1022.9326	2.766065426	9.999905072	9.999905072	-7.46182	2.6E-14
LOC102161998	380.47833	0	8.575457296	0	0	20	2.6E-14
FAM184B	571.960888	1.6525567	9.16229285	1.407383613	1.407383613	8.435073	2.6E-14
IGF2	0	382.56689	0	8.583334365	8.583334365	-20	2.7E-14
LOC102166950	0	382.56689	0	8.583334365	8.583334365	-20	2.7E-14
LOC100521510	377.162615	0	8.562862935	0	0	20	2.8E-14
ID4	7.46035941	1186.5357	3.080718953	10.21375522	10.21375522	-7.31329	2.8E-14
TMEM163	566.158387	1.6525567	9.147607873	1.407383613	1.407383613	8.420362	2.9E-14
LOC102159856	654.024842	2.4788351	9.355405812	1.798604302	1.798604302	8.043539	2.9E-14
PDGFRA	29.8414377	3512.5094	4.946798112	11.77869703	11.77869703	-6.87904	3E-14
LOC100524999	1080.09426	6.610227	10.0782766	2.927939483	2.927939483	7.352242	3.1E-14
GBP7	1.65785765	568.47952	1.410263837	9.153500149	9.153500149	-8.42164	3.1E-14
FGF10	0.82892882	475.93634	0.870998931	8.897652909	8.897652909	-9.1653	3.1E-14
LOC100523100	736.917724	3.3051135	9.527316158	2.106051269	2.106051269	7.80066	3.1E-14
CDH8	1245.88002	8.2627837	10.28410694	3.211445828	3.211445828	7.236322	3.1E-14
LOC102162332	7.46035941	1172.489	3.080718953	10.19658861	10.19658861	-7.29611	3.2E-14
RAX	559.526956	1.6525567	9.130639944	1.407383613	1.407383613	8.403364	3.3E-14
HOXB5	0	375.13038	0	8.55508903	8.55508903	-20	3.3E-14
ODF4	368.044398	0	8.527650579	0	0	20	3.6E-14
PAX1	0	370.99899	0	8.53915489	8.53915489	-20	3.6E-14
LOC102159296	3.31571529	732.90892	2.109599694	9.519457213	9.519457213	-7.78817	3.6E-14
PTGS1	13.2628612	1743.4474	3.834191515	10.76855435	10.76855435	-7.03841	3.7E-14
KIAA1199	0	370.17271	0	8.535946834	8.535946834	-20	3.7E-14
LOC100623028	41.4464412	4631.2903	5.407571694	12.17750994	12.17750994	-6.80402	3.8E-14
ITGA3	52.2225159	5752.55	5.733964805	12.49023668	12.49023668	-6.78339	3.8E-14
LOC100512657	3.31571529	730.43008	2.109599694	9.51457615	9.51457615	-7.78328	3.8E-14
TMEM171	365.557611	0	8.517896152	0	0	20	3.8E-14
LOC100511574	635.788408	2.4788351	9.314670263	1.798604302	1.798604302	8.002741	3.9E-14
ATP12A	363.899754	0	8.511356367	0	0	20	4E-14
LOC100523560	0	366.8676	0	8.523042794	8.523042794	-20	4.1E-14
LOC102161270	359.755109	0	8.494876018	0	0	20	4.5E-14
TRPC6	6.63143059	1048.5473	2.93195353	10.03555141	10.03555141	-7.30486	4.5E-14
LOC102157888	358.926181	0	8.491557236	0	0	20	4.6E-14
LOC102159782	540.461593	1.6525567	9.080715198	1.407383613	1.407383613	8.353348	4.6E-14
HOXC6	8.28928824	1198.9299	3.215568059	10.22873443	10.22873443	-7.17628	5.1E-14
SYBU	1826.95913	14.873011	10.8360181	3.98850389	3.98850389	6.940604	5.2E-14
CR2	353.952608	0	8.471482603	0	0	20	5.3E-14
NDST3	697.129141	3.3051135	9.447350122	2.106051269	2.106051269	7.720582	5.4E-14
LOC100622674	352.29475	0	8.464728501	0	0	20	5.5E-14
LOC100517243	0	354.47342	0	8.473597884	8.473597884	-20	5.7E-14
AGT	1.65785765	533.77583	1.410263837	9.062790447	9.062790447	-8.33077	5.7E-14
HOXB8	14.9207188	1809.5496	3.99283357	10.82221201	10.82221201	-6.92217	6.4E-14
VSNL1	520.567301	1.6525567	9.026709615	1.407383613	1.407383613	8.299241	6.6E-14
LOC102166344	345.663319	0	8.437391382	0	0	20	6.6E-14
LOC100738735	678.063778	3.3051135	9.407403269	2.106051269	2.106051269	7.680577	7.1E-14
HTRA3	6.63143059	999.79683	2.93195353	9.966933409	9.966933409	-7.23617	7.3E-14
PLEKHF1	19.0653629	2174.7647	4.326635347	11.08730681	11.08730681	-6.83376	7.4E-14
IER3	66.3143059	6745.7366	6.072841239	12.71997413	12.71997413	-6.66851	7.5E-14
EHBP1L1	67.9721635	6902.7295	6.107942317	12.75316023	12.75316023	-6.66608	7.5E-14
SELM	61.340733	6239.228	5.962103212	12.60738302	12.60738302	-6.66838	7.7E-14
LOC100622576	0	342.07925	0	8.422398044	8.422398044	-20	8E-14
RIN2	0	341.25297	0	8.41891924	8.41891924	-20	8.2E-14
ZNF211	0	340.42669	0	8.415432027	8.415432027	-20	8.4E-14
DIRAS2	337.374031	0	8.402475042	0	0	20	8.4E-14
LOC100739594	0	338.77413	0	8.408432212	8.408432212	-20	8.8E-14
GPR56	9.94714588	1284.8629	3.452482877	10.32852108	10.32852108	-7.01312	9.1E-14
UNC5B	43.9332277	4459.4244	5.489710792	12.12296526	12.12296526	-6.6654	9.4E-14
LOC100627012	420.266914	0.8262784	8.718590801	0.868906686	0.868906686	8.990462	9.5E-14
LOC100736853	0	335.46902	0	8.394329862	8.394329862	-20	9.7E-14
BARHL2	332.400458	0	8.381112277	0	0	20	9.7E-14
CREB3L1	111.905391	10908.527	6.818970566	13.41330094	13.41330094	-6.60703	1E-13
ZNF613	0	332.99018	0	8.383661891	8.383661891	-20	1E-13
LOC100628205	649.880198	3.3051135	9.346248212	2.106051269	2.106051269	7.61933	1.1E-13
HOXB4	4.14464412	732.08264	2.363071282	9.517832026	9.517832026	-7.46461	1.1E-13
LOC102160493	4.14464412	732.08264	2.363071282	9.517832026	9.517832026	-7.46461	1.1E-13
TNNI1	0	331.33763	0	8.376505833	8.376505833	-20	1.1E-13
ARSI	3.31571529	653.58619	2.109599694	9.35443936	9.35443936	-7.62291	1.1E-13
LOC102157467	0	330.51135	0	8.372914449	8.372914449	-20	1.1E-13
LOC102165808	326.597957	0	8.355782547	0	0	20	1.2E-13
ZNF227	0	328.85879	0	8.365704748	8.365704748	-20	1.2E-13
LOC100737756	0	328.85879	0	8.365704748	8.365704748	-20	1.2E-13
LOC100737413	0.82892882	414.79174	0.870998931	8.699717297	8.699717297	-8.96692	1.2E-13
FOXL1	1.65785765	493.28819	1.410263837	8.94920862	8.94920862	-8.21697	1.2E-13
RBM46	2115.42636	19.830681	11.04741457	4.380638094	4.380638094	6.73707	1.3E-13
ZNF503	17.4075053	1911.1819	4.202222212	10.90100403	10.90100403	-6.77861	1.3E-13
UNC-6	0	326.37996	0	8.354822188	8.354822188	-20	1.3E-13
COL5A3	11.6050035	1383.19	3.655924617	10.43482627	10.43482627	-6.89711	1.3E-13
LOC100626722	0	324.7274	0	8.347521274	8.347521274	-20	1.3E-13
ZNF584	0	324.7274	0	8.347521274	8.347521274	-20	1.3E-13
LOC100513452	319.966526	0	8.326279034	0	0	20	1.4E-13
ANGPTL2	193.969345	18276.451	7.607103495	14.15777729	14.15777729	-6.55801	1.4E-13
PLAC9	24.8678647	2550.7213	4.693089065	11.31725507	11.31725507	-6.68048	1.4E-13
SLC16A12	993.056731	7.4365053	9.957184378	3.076645515	3.076645515	7.061108	1.5E-13
MMP23B	48.0778718	4607.3282	5.617000784	12.17002775	12.17002775	-6.58241	1.6E-13
DPP4	5.80250177	851.893	2.766065426	9.736220951	9.736220951	-7.19785	1.6E-13
ZNF577	0	318.94345	0	8.321673128	8.321673128	-20	1.6E-13
LOC100739751	0	318.94345	0	8.321673128	8.321673128	-20	1.6E-13
LOC100622121	315.821882	0	8.307528171	0	0	20	1.6E-13
GPX6	315.821882	0	8.307528171	0	0	20	1.6E-13
NMUR1	314.164024	0	8.299959051	0	0	20	1.7E-13
CD14	5.80250177	841.15138	2.766065426	9.717935781	9.717935781	-7.17955	1.8E-13
CACNG7	3.31571529	623.84017	2.109599694	9.287343396	9.287343396	-7.55571	1.8E-13
GBX1	4117.28947	43.792754	12.00782952	5.485193456	5.485193456	6.554859	1.9E-13
APOA1	11.6050035	1337.7447	3.655924617	10.38666513	10.38666513	-6.84891	1.9E-13
HOMER3	131.799683	12073.58	7.053107893	13.55968534	13.55968534	-6.51736	1.9E-13
PDZD4	685.524137	4.1313919	9.423166634	2.359350201	2.359350201	7.374436	1.9E-13
PSTPIP2	309.190451	0	8.277010465	0	0	20	2E-13
LOC100153689	309.190451	0	8.277010465	0	0	20	2E-13
EXO5	0	311.50695	0	8.287744447	8.287744447	-20	2E-13
PYGM	4145.47305	44.619032	12.017669	5.511563931	5.511563931	6.537734	2.1E-13
CRYBB1	307.532594	0	8.269279095	0	0	20	2.1E-13
LOC102158081	2713.91297	28.093465	11.40669023	4.862623207	4.862623207	6.593996	2.1E-13
ZNF329	0	309.85439	0	8.28009514	8.28009514	-20	2.1E-13
ELAVL2	305.874736	0	8.261506068	0	0	20	2.2E-13
MAMLD1	4.14464412	679.20082	2.363071282	9.409816938	9.409816938	-7.35645	2.3E-13
PLAU	44.7621565	4118.9977	5.516083133	12.00842781	12.00842781	-6.52387	2.4E-13
TDRD1	300.901163	0	8.237932504	0	0	20	2.6E-13
OSBPL3	2.48678647	523.86049	1.801898017	9.035790183	9.035790183	-7.71876	2.9E-13
TAGLN	1317.99683	120386.28	10.36522538	16.87732344	16.87732344	-6.51318	3E-13
LOC494560	85.3796688	7495.1711	6.43261988	12.87193817	12.87193817	-6.45593	3E-13
LOC102163456	0	298.28649	0	8.225383356	8.225383356	-20	3E-13
PTX3	21.5521494	2081.3952	4.495193036	11.02402819	11.02402819	-6.59358	3.2E-13
LOC100515380	294.269732	0	8.205889666	0	0	20	3.2E-13
LOC100622713	3.31571529	587.48392	2.109599694	9.20085919	9.20085919	-7.46909	3.2E-13
KCNK2	0	296.63394	0	8.217395218	8.217395218	-20	3.2E-13
TLR4	0	296.63394	0	8.217395218	8.217395218	-20	3.2E-13
LOC102162293	293.440804	0	8.201833804	0	0	20	3.3E-13
NPAS1	10984.1358	128.07315	13.42326509	7.012045083	7.012045083	6.42231	3.3E-13
LOC100736658	0	293.32882	0	8.201285014	8.201285014	-20	3.6E-13
ADAMTS4	5.80250177	781.65934	2.766065426	9.612240686	9.612240686	-7.07372	3.6E-13
LOC102165782	287.638302	0	8.173118945	0	0	20	4E-13
CPVL	1288.15539	12.394176	10.33221046	3.74353388	3.74353388	6.699501	4.1E-13
CD248	87.8664553	7462.9463	6.473567038	12.86572288	12.86572288	-6.40829	4.1E-13
BEND6	12.4339324	1298.9096	3.747809764	10.34419558	10.34419558	-6.70687	4.1E-13
LINGO1	1153.03999	10.741619	10.17247751	3.553559423	3.553559423	6.746087	4.2E-13
PDZRN4	360.584038	0.8262784	8.498187183	0.868906686	0.868906686	8.769492	4.3E-13
DAB1	427.727273	1.6525567	8.743916385	1.407383613	1.407383613	8.015848	4.5E-13
LOC102165358	0	285.89232	0	8.164365521	8.164365521	-20	4.6E-13
ZNF568	0	285.06604	0	8.160204421	8.160204421	-20	4.7E-13
HOXC4	4.97357294	694.90011	2.578594101	9.442736426	9.442736426	-7.12638	4.8E-13
LOC100620451	0.82892882	358.60481	0.870998931	8.490268521	8.490268521	-8.75693	4.9E-13
SPEG	19.0653629	1794.6766	4.326635347	10.81031185	10.81031185	-6.55663	4.9E-13
VWA5A	27.3546512	2446.6103	4.825513503	11.25715814	11.25715814	-6.48285	5E-13
FN1	9553.40469	881757.18	13.22195027	19.75002353	19.75002353	-6.52822	5E-13
LOC100623939	2.48678647	494.11447	1.801898017	8.951618292	8.951618292	-7.63442	5.1E-13
LOC100158194	19.0653629	1785.5876	4.326635347	10.80299091	10.80299091	-6.5493	5.2E-13
LOC102157546	0	281.76092	0	8.143438955	8.143438955	-20	5.3E-13
LOC100515669	278.520085	0	8.12680814	0	0	20	5.4E-13
ELOVL2	1374.36399	14.046732	10.42559776	3.911378308	3.911378308	6.612386	5.5E-13
LOC100626132	54.7093024	4564.3617	5.799846344	12.15651346	12.15651346	-6.38248	5.7E-13
LOC100736668	0	279.28209	0	8.130735747	8.130735747	-20	5.7E-13
LOC100523909	276.862227	0	8.118225916	0	0	20	5.8E-13
HOXB9	1.65785765	420.57569	1.410263837	8.719647871	8.719647871	-7.9869	5.9E-13
RHOJ	0.82892882	348.68947	0.870998931	8.449930555	8.449930555	-8.71648	6.4E-13
CXCL14	3.31571529	545.34373	2.109599694	9.09366508	9.09366508	-7.3617	6.6E-13
SR-PSOX	19.8942918	1803.7657	4.385036953	10.81759583	10.81759583	-6.50251	6.7E-13
HGF	2.48678647	478.41518	1.801898017	8.905131772	8.905131772	-7.58784	6.9E-13
PTPRB	4745.61752	58.665764	12.21268409	5.89883146	5.89883146	6.337933	7E-13
NOX4	21.5521494	1915.3133	4.495193036	10.90411771	10.90411771	-6.4736	7.2E-13
DMRTB1	1152.21107	11.567897	10.17144087	3.65167138	3.65167138	6.638135	7.3E-13
LOC102166405	270.230797	0	8.083377186	0	0	20	7.3E-13
DAAM2	172.417195	13680.691	7.438103147	13.73995893	13.73995893	-6.31009	7.8E-13
ID2	22.3810782	1948.3644	4.547269558	10.92878809	10.92878809	-6.44384	8.4E-13
ZNF226	0	268.54047	0	8.074358096	8.074358096	-20	8.4E-13
LOC100737260	464.200141	2.4788351	8.861707724	1.798604302	1.798604302	7.548941	8.5E-13
PTGES	96.1557435	7529.0485	6.602227381	12.87844345	12.87844345	-6.29095	9E-13
LOC102159043	6.63143059	772.57028	2.93195353	9.595388554	9.595388554	-6.8642	9.1E-13
LOC102167727	0	265.23536	0	8.05655837	8.05655837	-20	9.4E-13
OLFM1	0	264.40908	0	8.052073912	8.052073912	-20	9.7E-13
TMEM132E	0	264.40908	0	8.052073912	8.052073912	-20	9.7E-13
LOC100738589	26.5257224	2205.337	4.78270852	11.10743744	11.10743744	-6.37746	1.1E-12
EPSTI1	25.6967935	2124.3617	4.73859457	11.05349266	11.05349266	-6.3693	1.2E-12
GGT5	4.14464412	573.43719	2.363071282	9.166005344	9.166005344	-7.11224	1.2E-12
CAMSAP3	1440.6783	16.525567	10.49353355	4.13138925	4.13138925	6.445905	1.3E-12
TPM1	1618.89799	128639.97	10.66168725	16.97299072	16.97299072	-6.31218	1.3E-12
POU2F3	255.310078	0	8.001746394	0	0	20	1.3E-12
FRMD3	384.622974	1.6525567	8.591047195	1.407383613	1.407383613	7.862601	1.3E-12
TRIML2	254.481149	0	7.997073033	0	0	20	1.3E-12
CEBPD	424.411558	32373.587	8.732715419	14.98257415	14.98257415	-6.25321	1.3E-12
DNMT3B	16000.8131	214.0061	13.96594776	7.74823377	7.74823377	6.224349	1.3E-12
LOC100737912	0	256.1463	0	8.006445558	8.006445558	-20	1.3E-12
ZFP112	0	255.32002	0	8.001802338	8.001802338	-20	1.4E-12
LOC100621315	252.823291	0	7.987680649	0	0	20	1.4E-12
BATF3	906.019204	9.0890621	9.824989287	3.334720158	3.334720158	6.639266	1.4E-12
MYL4	0	254.49374	0	7.997144125	7.997144125	-20	1.4E-12
LOC100521619	0	254.49374	0	7.997144125	7.997144125	-20	1.4E-12
NR0B1	249.507576	0	7.968710424	0	0	20	1.6E-12
CCDC129	374.675828	1.6525567	8.553344483	1.407383613	1.407383613	7.824799	1.7E-12
LOC100621169	605.118041	4.9576702	9.243454975	2.574748269	2.574748269	6.93141	1.7E-12
LOC100736640	0.82892882	313.98578	0.870998931	8.299142895	8.299142895	-8.56524	1.8E-12
SLIT2	33.9860818	2606.9083	5.128709195	11.34867741	11.34867741	-6.26125	1.8E-12
LOC100736898	6.63143059	720.51474	2.93195353	9.494885058	9.494885058	-6.76356	1.8E-12
NTN4	22.3810782	1795.5029	4.547269558	10.81097555	10.81097555	-6.32596	1.9E-12
POU3F2	1102.47534	12.394176	10.10783867	3.74353388	3.74353388	6.47494	1.9E-12
IRX2	0	246.23095	0	7.949715578	7.949715578	-20	1.9E-12
LOC100519459	3153.24525	42.966475	11.62307912	5.458331975	5.458331975	6.197482	2.2E-12
LOC100155368	1249.19574	14.873011	10.28793827	3.98850389	3.98850389	6.392159	2.2E-12
DIRAS3	6.63143059	705.64173	2.93195353	9.464835137	9.464835137	-6.73347	2.2E-12

SMOC1	4.14242951	825.45209	2.362450112	9.690787384	9.690787384	-7.63856	9.4E-09	ZNF211	0	340.42669	0	8.415432027	8.415432027	-20	9.1E-08	SDK2	0	242.09956	0	7.925403489	7.925403489	-20	2.3E-12
LOC100158081	1097.743827	28.093465	10.10163833	4.862623207	4.862623207	5.288163	9.6E-09	[illegible]	[illegible]	[illegible]	[illegible]	[illegible]	[illegible]	[illegible]	[illegible]	[illegible]	[illegible]	[illegible]	[illegible]	[illegible]	[illegible]	[illegible]	[illegible]
CDHR1	118.749646	0	6.90387758	[illegible]	[illegible]	-20	9.7E-09	[illegible]	[illegible]	[illegible]	[illegible]	[illegible]	[illegible]	[illegible]	[illegible]	[illegible]	[illegible]	[illegible]	[illegible]	[illegible]	[illegible]	[illegible]	[illegible]
[illegible]	[illegible]	[illegible]	[illegible]	[illegible]	[illegible]	[illegible]	[illegible]	[illegible]	[illegible]	[illegible]	[illegible]	[illegible]	[illegible]	[illegible]	[illegible]	[illegible]	[illegible]	[illegible]	[illegible]	[illegible]	[illegible]	[illegible]	[illegible]

LGR5	0	208.22215	0	7.708891783	7.708891783	-20	3.9E-07
LOC102160246	77.3253508	0	6.291407421	0	0	20	4E-07
CRYM	788.442416	30.5723	9.624690226	4.980587446	4.980587446	4.688708	4.1E-07
LOC100621578	0	206.56959	0	7.697451307	7.697451307	-20	4.1E-07
PTHLH	0	205.74331	0	7.691696868	7.691696868	-20	4.2E-07
LOC100621672	5.52323934	507.33492	2.705588563	8.98963553	8.98963553	-6.52128	4.2E-07
TIMP-3	127.034505	7434.0265	7.000388854	12.86012217	12.86012217	-5.87085	4.2E-07
CHRM1	118.749646	1.6525567	6.90387758	1.407383613	1.407383613	6.16708	4.5E-07
ANPEP	70.4213016	4080.1626	6.158282521	11.99476448	11.99476448	-5.85647	4.5E-07
TSTD1	12518.422	508.98748	13.61188033	8.994318011	8.994318011	4.620279	4.6E-07
LOC100626014	2.76161967	353.64714	1.911353989	8.470240516	8.470240516	-7.00065	4.7E-07
PAPSS2	42.8051049	2503.6235	5.453027102	11.29037802	11.29037802	-5.87009	4.7E-07
LOC102166772	75.944541	0	6.265747069	0	0	20	4.7E-07
LOC102160606	75.944541	0	6.265747069	0	0	20	4.7E-07
LOC100514098	4.14242951	424.70708	2.362450112	8.733717284	8.733717284	-6.67985	4.8E-07
IL1RL1	0	200.78564	0	7.65667973	7.65667973	-20	4.8E-07
LOC100517161	0	199.95937	0	7.650760008	7.650760008	-20	5E-07
LOC100622715	0	199.13309	0	7.644815895	7.644815895	-20	5.1E-07
LOC102158748	0	199.13309	0	7.644815895	7.644815895	-20	5.1E-07
C1QTNF3	0	199.13309	0	7.644815895	7.644815895	-20	5.1E-07
FBLN5	103.560738	5856.6611	6.708197415	12.51610901	12.51610901	-5.82153	5.1E-07
LOC102168112	487.425872	18.178124	8.931995812	4.261389712	4.261389712	4.744908	5.2E-07
CHRDL1	28.9970066	1716.1802	4.906746634	10.74582571	10.74582571	-5.88715	5.2E-07
SAMD9	74.5637311	4197.4941	6.239622035	12.03565625	12.03565625	-5.81491	5.3E-07
ITGA9	0	197.48053	0	7.632853688	7.632853688	-20	5.3E-07
CHRNG	12.4272885	841.97766	3.747096094	9.71935059	9.71935059	-6.0822	5.3E-07
LOC100525180	265.115489	8.2627837	8.055908671	3.211445828	3.211445828	5.003849	5.4E-07
LOC100621315	74.5637311	0	6.239622035	0	0	20	5.5E-07
LOC100521229	74.5637311	0	6.239622035	0	0	20	5.5E-07
LOC100049650	140.842603	7884.3482	7.148147109	12.94495875	12.94495875	-5.80684	5.6E-07
LOC100525848	1400.14117	58.665764	10.45238661	5.89883146	5.89883146	4.57691	5.6E-07
LOC100626977	2.76161967	342.90552	1.911353989	8.42586848	8.42586848	-6.95615	5.6E-07
PRICKLE2	5.52323934	481.72029	2.705588563	8.91504366	8.91504366	-6.44654	5.7E-07
LOC100739844	9.66566885	689.94244	3.414902536	9.43242172	9.43242172	-6.15746	5.8E-07
LOC102158049	353.487318	12.394176	8.469590204	3.74353388	3.74353388	4.833924	5.8E-07
PENK	0	194.17542	0	7.608627545	7.608627545	-20	5.9E-07
PPFIA4	17.9505279	1108.0393	4.24416613	10.11509477	10.11509477	-5.94784	5.9E-07
DFNA5	9.66566885	686.63733	3.414902536	9.42550405	9.42550405	-6.15053	6E-07
ANO1	2.76161967	338.77413	1.911353989	8.408432212	8.408432212	-6.93867	6E-07
NR2E1	189.170948	4.9576702	7.571153052	2.574748269	2.574748269	5.253884	6E-07
TRIM72	0	193.34914	0	7.602506906	7.602506906	-20	6E-07
ANG1	2145.77849	91.716899	11.06795762	6.534760414	6.534760414	4.54817	6E-07
LOC100515260	258.211439	14486.312	8.017985577	13.82250236	13.82250236	-5.80999	6E-07
SLC28A3	73.1829213	0	6.213015177	0	0	20	6.4E-07
TBX5	2.76161967	332.99018	1.911353989	8.383661891	8.383661891	-6.91382	6.6E-07
LOC102165620	11.0464787	741.1717	3.590539588	9.535609179	9.535609179	-6.06815	6.7E-07
WDR17	0	189.21775	0	7.571508044	7.571508044	-20	6.8E-07
HSPB7	0	189.21775	0	7.571508044	7.571508044	-20	6.8E-07
ADCY2	0	189.21775	0	7.571508044	7.571508044	-20	6.8E-07
LOC100513619	272.019538	14958.117	8.092860386	13.86873743	13.86873743	-5.78107	6.9E-07
LOC102163961	9.66566885	669.28548	3.414902536	9.388631874	9.388631874	-6.11361	6.9E-07
LOC100127164	2.76161967	330.51135	1.911353989	8.372914449	8.372914449	-6.90304	6.9E-07
CPXM2	8.28485901	598.22595	3.214880004	9.226955308	9.226955308	-6.17407	7.2E-07
LOC100516621	2114.01986	92.543178	11.04645549	6.547560532	6.547560532	4.513718	7.4E-07
ZBTB8B	71.8021115	0	6.185908388	0	0	20	7.5E-07
LOC102165956	71.8021115	0	6.185908388	0	0	20	7.5E-07
SLC13A5	682.120059	28.093465	9.415995345	4.862623207	4.862623207	4.601719	7.5E-07
IGFBP7	608.937138	34102.987	9.252516751	15.0576528	15.0576528	-5.80746	7.5E-07
LOC100737747	6.90404918	523.86049	2.982591923	9.035790183	9.035790183	-6.2456	7.7E-07
BATF2	4.14242951	390.82967	2.362450112	8.614082834	8.614082834	-6.55992	7.8E-07
LOC102165113	183.647708	4.9576702	7.528631546	2.574748269	2.574748269	5.211135	7.8E-07
MSLN	767.730269	32.224857	9.586333665	5.054191063	5.054191063	4.574354	7.9E-07
SYNJ2	23.4737672	1326.1768	4.613164285	10.37414484	10.37414484	-5.82008	7.9E-07
LOC100738589	41.4242951	2205.337	5.406818783	11.10743744	11.10743744	-5.73438	8.3E-07
LOC100517484	374.199466	14.046732	8.551513962	3.911378308	3.911378308	4.735501	8.4E-07
LOC100739751	2.76161967	318.94345	1.911353989	8.321673128	8.321673128	-6.85164	8.5E-07
LOC100622637	109.083977	5660.0068	6.782460686	12.46684295	12.46684295	-5.69729	8.5E-07
LOC100737392	146.365843	3.3051135	7.203258356	2.106051269	2.106051269	5.468735	8.5E-07
LOC102159094	70.4213016	0	6.158282521	0	0	20	8.8E-07
GIMAP4	70.4213016	0	6.158282521	0	0	20	8.8E-07
DIRAS3	16274.2247	705.64173	13.99038984	9.464835137	9.464835137	4.527509	8.9E-07
tRNA-Tyr	509.518829	20.656959	8.995820363	4.436758793	4.436758793	4.624436	9.2E-07
LOC100623058	2.76161967	314.81206	1.911353989	8.302922453	8.302922453	-6.83283	9.2E-07
TSPAN1	109.083977	1.6525567	6.782460686	1.407383613	1.407383613	6.044596	9.3E-07
IRX2	1.38080984	246.23095	1.251452392	7.949715578	7.949715578	-7.47835	9.8E-07
CASQ1	0	177.64985	0	7.480990892	7.480990892	-20	9.8E-07
LOC102160748	13.8080984	820.49442	3.888314478	9.682106971	9.682106971	-5.89291	1E-06
DMRTB1	314.824643	11.567897	8.302979933	3.65167138	3.65167138	4.76635	1E-06
LOC100626670	1025.94171	45.44531	10.00413858	5.537461031	5.537461031	4.496674	1E-06
CELA1	69.0404918	0	6.130117309	0	0	20	1E-06
LOC100519780	69.0404918	0	6.130117309	0	0	20	1E-06
LOC100514323	1441.56547	65.275991	10.49442108	6.05041444	6.05041444	4.46494	1.1E-06
LOC100523405	0	174.34474	0	7.454050313	7.454050313	-20	1.1E-06
PROM1	1.38080984	241.27328	1.251452392	7.920491517	7.920491517	-7.44901	1.1E-06
PODXL	702.832206	30.5723	9.459087721	4.980587446	4.980587446	4.522883	1.1E-06
TMEM246	2.76161967	303.24416	1.911353989	8.249085772	8.249085772	-6.77882	1.1E-06
C1H14orf37	45.5667246	2280.5283	5.541227504	11.15578484	11.15578484	-5.64524	1.2E-06
LOC100623862	2.76161967	300.76533	1.911353989	8.23728324	8.23728324	-6.76698	1.2E-06
FHL2	160.173941	7891.7847	7.332474693	12.94631869	12.94631869	-5.62264	1.2E-06
LOC102161109	165.69718	8165.2829	7.381085891	12.99546383	12.99546383	-5.62288	1.2E-06
LOC100737693	0	171.03962	0	7.426597064	7.426597064	-20	1.2E-06
ZBTB32	67.659682	0	6.101391269	0	0	20	1.2E-06
SLC46A2	4.14242951	361.08365	2.362450112	8.500179216	8.500179216	-6.44571	1.2E-06
CRISPLD2	16.569718	915.51644	4.135019133	9.840016943	9.840016943	-5.78796	1.2E-06
RCVRN	0	170.21334	0	7.419651342	7.419651342	-20	1.3E-06
TCF21	0	170.21334	0	7.419651342	7.419651342	-20	1.3E-06
LOC100511846	190.551757	5.7839486	7.581590451	2.762125239	2.762125239	5.041984	1.3E-06
HOXB9	5.52323934	420.57569	2.705588563	8.719647871	8.719647871	-6.25071	1.3E-06
SLCO4C1	207.121475	6.610227	7.70128203	2.927939483	2.927939483	4.969634	1.3E-06
GPR56	24.854577	1284.8629	4.692347799	10.32852108	10.32852108	-5.69196	1.3E-06
KIAA0319	273.400348	9.9153405	8.100138498	3.448285225	3.448285225	4.785209	1.3E-06
ARMC2	0	169.38707	0	7.412672017	7.412672017	-20	1.3E-06
tRNA-Thr	760.82622	33.877413	9.573318131	5.124221138	5.124221138	4.489171	1.3E-06
PRR19	1271.72586	58.665764	10.31370599	5.89883146	5.89883146	4.438125	1.3E-06
SLITRK2	11.0464787	656.06503	3.590539588	9.359892345	9.359892345	-5.89218	1.4E-06
BEX1	3534.87318	166.08195	11.78785082	7.384412099	7.384412099	4.411691	1.4E-06
IL20RA	138.080984	3.3051135	7.119781365	2.106051269	2.106051269	5.384671	1.4E-06
EPHA3	8.28485901	531.29699	3.214880004	9.056087606	9.056087606	-6.0029	1.4E-06
SLITRK5	970.709315	44.619032	9.924380988	5.511563931	5.511563931	4.443308	1.4E-06
LOC100515962	5.52323934	411.48663	2.705588563	8.688203544	8.688203544	-6.21919	1.4E-06
KIAA1210	0	166.08195	0	7.384412099	7.384412099	-20	1.4E-06
PRRX1	48.3283443	2323.4948	5.624344958	11.18270147	11.18270147	-5.58728	1.4E-06
LOC100516442	12.4272885	709.77312	3.747096094	9.473245315	9.473245315	-5.83577	1.5E-06
RASL10A	66.2788721	0	6.072081615	0	0	20	1.5E-06
LOC100737239	1.38080984	229.70539	1.251452392	7.849907883	7.849907883	-7.37813	1.5E-06
NR4A1	1717.72744	80.97528	10.74712506	6.357117027	6.357117027	4.406876	1.5E-06
DDR2	276.161967	13338.612	8.11458549	13.70342906	13.70342906	-5.59395	1.5E-06
TNFAIP6	5042.71752	237.96817	12.30027176	7.900674664	7.900674664	4.405361	1.5E-06
LOC100153981	201.598236	6.610227	7.662477803	2.927939483	2.927939483	4.930639	1.6E-06
LOC102165358	2.76161967	285.89232	1.911353989	8.164365521	8.164365521	-6.69381	1.6E-06
LOC102161654	0	162.77684	0	7.35558754	7.35558754	-20	1.6E-06
LOC102159728	102.179928	1.6525567	6.689018532	1.407383613	1.407383613	5.950268	1.6E-06
LOC100524545	13.8080984	752.7396	3.888314478	9.557922374	9.557922374	-5.76856	1.7E-06
PPP1R17	64.8980623	0	6.042164139	0	0	20	1.7E-06
LOC102161046	34.5202459	1641.8151	5.150569664	10.68195442	10.68195442	-5.57171	1.7E-06
TPM2	1140.54892	57079.31	10.15677698	15.80070555	15.80070555	-5.64517	1.8E-06
PCSK6	0	160.298	0	7.333584776	7.333584776	-20	1.8E-06
LOC102163369	0	160.298	0	7.333584776	7.333584776	-20	1.8E-06
LMCD1	5.52323934	394.96106	2.705588563	8.629214754	8.629214754	-6.16005	1.8E-06
LOC100627808	4.14242951	334.64274	2.362450112	8.390782629	8.390782629	-6.336	1.9E-06
ZNF503	41.4242951	1911.1819	5.406818783	10.90100403	10.90100403	-5.52784	1.9E-06
TLR3	22.0929574	1075.8144	4.529381037	10.07255395	10.07255395	-5.6057	2E-06
PSAT1	610.317947	28975.93	9.255779113	14.82261713	14.82261713	-5.56915	2E-06
LOC102159412	0	156.16661	0	7.296150961	7.296150961	-20	2E-06
LOC102159293	63.5172524	0	6.011613095	0	0	20	2.1E-06
MNX1	63.5172524	0	6.011613095	0	0	20	2.1E-06
LOC100621416	26.2353869	1238.5913	4.767410456	10.27564879	10.27564879	-5.56104	2.1E-06
MEGF10	1.38080984	215.65866	1.251452392	7.75928006	7.75928006	-7.28709	2.1E-06
ITGBL1	0	155.34033	0	7.288546214	7.288546214	-20	2.1E-06
LOC100625270	0	155.34033	0	7.288546214	7.288546214	-20	2.1E-06
COL9A2	33860.2188	1578.1917	15.04734628	10.62497059	10.62497059	4.423247	2.2E-06
LOC102163032	81.4677803	0.8262784	6.365758672	0.868906686	0.868906686	6.623458	2.2E-06
PLA2G4A	82.8485901	3654.6292	6.389714621	11.83590404	11.83590404	-5.4631	2.2E-06
SERTAD4	63.5172524	2809.3465	6.011613095	11.45653228	11.45653228	-5.46694	2.2E-06
LOC102163538	4.14242951	325.55368	2.362450112	8.35117635	8.35117635	-6.29627	2.2E-06
LOC100737186	4.14242951	324.7274	2.362450112	8.347521274	8.347521274	-6.29261	2.3E-06
MAPK4	0	152.8615	0	7.265488457	7.265488457	-20	2.3E-06
LOC100622976	2.76161967	266.88791	1.911353989	8.065485684	8.065485684	-6.59458	2.4E-06
NEURL1B	238.880102	9.0890621	7.906169679	3.334720158	3.334720158	4.716011	2.4E-06
SSC5D	20.7121475	981.61871	4.440430524	9.940487893	9.940487893	-5.56661	2.4E-06
NLRP3	0	151.20894	0	7.249909307	7.249909307	-20	2.5E-06
LOC100621009	0	151.20894	0	7.249909307	7.249909307	-20	2.5E-06
LOC100620561	0	151.20894	0	7.249909307	7.249909307	-20	2.5E-06
LOC102164132	62.1364426	0	5.98040107	0	0	20	2.5E-06
LOC100157629	62.1364426	0	5.98040107	0	0	20	2.5E-06
PHYH	86.9910197	3767.0031	6.459284385	11.87958444	11.87958444	-5.43641	2.5E-06
SLPI	144.985033	4.1313919	7.189676653	2.359350201	2.359350201	5.133132	2.5E-06
SYNPR	0	150.38266	0	7.242056188	7.242056188	-20	2.5E-06
DDX58	125.653695	5427.8226	6.984745358	12.40642363	12.40642363	-5.43285	2.5E-06
TMEM229A	6.90404918	426.35964	2.982591923	8.739306855	8.739306855	-5.94848	2.6E-06
UNC13A	11.0464787	587.48392	3.590539588	9.20085919	9.20085919	-5.73289	2.6E-06
CD44	563.370413	25303.949	9.140498547	14.62713194	14.62713194	-5.48913	2.7E-06
BOK	28.9970066	1291.4731	4.906746634	10.33591853	10.33591853	-5.47697	2.8E-06
GRIA4	0	147.90383	0	7.218237038	7.218237038	-20	2.8E-06
TRIM9	2.76161967	259.45141	1.911353989	8.02487043	8.02487043	-6.55381	2.8E-06
TDRKH	509.518829	23.962073	8.995820363	4.641665831	4.641665831	4.410311	2.8E-06
LOC102163288	6.90404918	418.09686	2.982591923	8.711139889	8.711139889	-5.92025	2.9E-06
SLA-DMA	95.2758787	1.6525567	6.58910248	1.407383613	1.407383613	5.849339	2.9E-06
LOC102166360	864.386957	42.966475	9.757201567	5.458331975	5.458331975	4.330394	2.9E-06
LOC100627578	1973.17726	100.80596	10.94703582	6.669678233	6.669678233	4.290868	2.9E-06
MCF2	60.7556328	0	5.948498827	0	0	20	2.9E-06
LOC102159104	60.7556328	0	5.948498827	0	0	20	2.9E-06
CLDN3	60.7556328	0	5.948498827	0	0	20	2.9E-06
LOC102158037	60.7556328	0	5.948498827	0	0	20	2.9E-06
SOWAHB	60.7556328	0	5.948498827	0	0	20	2.9E-06
CDH18	1.38080984	203.26448	1.251452392	7.674294538	7.674294538	-7.2017	2.9E-06
OMD	0	146.25127	0	7.202136284	7.202136284	-20	3E-06
TPMT	2692.57918	137.98849	11.39530876	7.118821585	7.118821585	4.286369	3E-06
BMP6	110.464787	2.4788351	6.800444207	1.798604302	1.798604302	5.47778	3.1E-06
SEMA3E	231.976052	9.0890621	7.864037858	3.334720158	3.334720158	4.673701	3.1E-06
LOC100624220	1.38080984	199.95937	1.251452392	7.650760008	7.650760008	-7.17805	3.2E-06
PPAP2B	96.6566885	3982.6618	6.609646952	11.95987944	11.95987944	-5.36472	3.3E-06
LOC100739745	8.28485901	458.5845	3.214880004	8.84418632	8.84418632	-5.79057	3.4E-06
RPRML	109.083977	2.4788351	6.782460686	1.798604302	1.798604302	5.459633	3.4E-06
LOC100510930	2080.88042	108.24247	11.02367149	6.771389985	6.771389985	4.264856	3.4E-06
LOC100620918	139.461793	4.1313919	7.13403395	2.359350201	2.359350201	5.077098	3.4E-06
LOC102162556	124.272885	3.3051135	6.968930373	2.106051269	2.106051269	5.232668	3.5E-06
B4GALNT2	363.152987	16.525567	8.508400868	4.13138925	4.13138925	4.457806	3.5E-06
LOC100158117	214.025525	8.2627837	7.748364114	3.211445828	3.211445828	4.695011	3.5E-06
OPRK1	59.3748229	0	5.915875148	0	0	20	3.5E-06
MPP3	183.647708	6.610227	7.528631546	2.927939483	2.927939483	4.796097	3.6E-06
PODNL1	23.4737672	1022.9326	4.613164285	9.999905072	9.999905072	-5.44552	3.6E-06
LOC102161846	92.514259	1.6525567	6.547114458	1.407383613	1.407383613	5.806904	3.7E-06
LOC102163456	4.14242951	298.28649	2.362450112	8.225383356	8.225383356	-6.17008	3.7E-06
LOC100152091	93.8950688	3790.1389	6.568261215	11.8884156	11.8884156	-5.33506	3.7E-06
LOC100516232	0	140.46732	0	7.144325041	7.144325041	-20	3.7E-06
LOC100524008	165.69718	6728.3848	7.381085891	12.7162589	12.7162589	-5.34364	3.8E-06
LOC102158176	11.0464787	550.3014	3.590539588	9.106697443	9.106697443	-5.63856	3.8E-06
TRANK1	13.8080984	651.10736	3.888314478	9.348965686	9.348965686	-5.55931	3.8E-06
KCNK12	11.0464787	548.64884	3.590539588	9.10236639	9.10236639	-5.63422	3.9E-06
PTGS2	93.8950688	3762.0454	6.568261215	11.87768499	11.87768499	-5.32432	3.9E-06
LOC100511782	760.82622	38.835083	9.573318131	5.315967693	5.315967693	4.292134	3.9E-06
KCNQ5	12.4272885	595.74671	3.747096094	9.220974888	9.220974888	-5.58312	4E-06
ENPP6	2.76161967	242.92584	1.911353989	7.930298794	7.930298794	-6.45886	4.1E-06
FOXS1	48.3283443	1942.5805	5.624344958	10.92450111	10.92450111	-5.32896	4.1E-06
CCL5	1.38080984	191.69658	1.251452392	7.590187173	7.590187173	-7.11717	4.1E-06
ARHGAP24	0	137.98849	0	7.118821585	7.118821585	-20	4.1E-06
GABRE	0	137.98849	0	7.118821585	7.118821585	-20	4.1E-06
PCNXL2	106.322357	2.4788351	6.745806839	1.798604302	1.798604302	5.422639	4.2E-06
KLF7	5.52323934	341.25297	2.705588563	8.41891924	8.41891924	-5.94918	4.2E-06
LOC102158418	0	137.16221	0	7.110219252	7.110219252	-20	4.2E-06
TRIM34	2.76161967	240.44701	1.911353989	7.915562765	7.915562765	-6.44406	4.3E-06
PLAU	104.941548	4118.9977	6.727124677	12.00842781	12.00842781	-5.29464	4.4E-06
LOC102159188	0	136.33593	0	7.101565318	7.101565318	-20	4.4E-06
LOC100627356	52.4707738	2076.4375	5.740678649	11.02058939	11.02058939	-5.30645	4.4E-06
LOC100520702	1.38080984	189.21775	1.251452392	7.571508044	7.571508044	-7.09839	4.4E-06
PLLP	164.316371	5.7839486	7.369085785	2.762125239	2.762125239	4.828278	4.5E-06
LOC100620286	4.14242951	288.37115	2.362450112	8.176777292	8.176777292	-6.12131	4.5E-06
SNAI2	1.38080984	188.39147	1.251452392	7.565227535	7.565227535	-7.09208	4.5E-06
PTER	1.38080984	188.39147	1.251452392	7.565227535	7.565227535	-7.09208	4.5E-06
SYT7	74.5637311	0.8262784	6.239622035	0.868906686	0.868906686	6.495702	4.5E-06
AHNAK2	8.28485901	434.62242	3.214880004	8.766934408	8.766934408	-5.71314	4.6E-06
ZC3H12D	4.14242951	287.54487	2.362450112	8.172651889	8.172651889	-6.11717	4.6E-06
RFTN1	4.14242951	287.54487	2.362450112	8.172651889	8.172651889	-6.11717	4.6E-06
SIGIRR	3740.61385	200.78564	11.86944496	7.65667973	7.65667973	4.219547	4.6E-06
RNF43	0	134.68337	0	7.084100147	7.084100147	-20	4.7E-06
GPNMB	0	134.68337	0	7.084100147	7.084100147	-20	4.7E-06
LOC100513943	89.7526393	1.6525567	6.503867696	1.407383613	1.407383613	5.763183	4.8E-06
LOC102164569	205.740666	8.2627837	7.691678382	3.211445828	3.211445828	4.638055	4.9E-06
LOC100736608	0	133.8571	0	7.075287628	7.075287628	-20	4.9E-06
KCNQ4	262.353869	11.567897	8.040858843	3.65167138	3.65167138	4.503316	5E-06
ECEL1	13.8080984	623.01389	3.888314478	9.285434338	9.285434338	-5.49568	5E-06
LOC100620852	2.76161967	233.83678	1.911353989	7.875514565	7.875514565	-6.40384	5.1E-06
CLDN11	8.28485901	426.35964	3.214880004	8.739306855	8.739306855	-5.68545	5.1E-06
SDS	548.181505	28.093465	9.101139229	4.862623207	4.862623207	4.286347	5.1E-06
TFAP2D	56.6132033	0	5.848327568	0	0	20	5.1E-06
LOC100513452	56.6132033	0	5.848327568	0	0	20	5.1E-06
LOC102163860	0	132.20454	0	7.057499439	7.057499439	-20	5.2E-06
LOC100624585	0	132.20454	0	7.057499439	7.057499439	-20	5.2E-06
BCL6B	103.560738	2.4788351	6.708197415	1.798604302	1.798604302	5.384671	5.3E-06
CABLES1	16.569718	712.25196	4.135019133	9.478267989	9.478267989	-5.42577	5.3E-06
TMEM140	8.28485901	423.05453	3.214880004	8.728105973	8.728105973	-5.67422	5.4E-06
LOC100525572	19.3313377	808.92653	4.345633235	9.661647227	9.661647227	-5.387	5.4E-06
MYOM1	13.8080984	614.75111	3.888314478	9.26620351	9.26620351	-5.47642	5.4E-06
FOLR2	19.3313377	806.44769	4.345633235	9.65722499	9.65722499	-5.38257	5.4E-06
ADAM3A	173.982039	6.610227	7.451063037	2.927939483	2.927939483	4.718095	5.6E-06
RAB27B	1222.01671	66.928548	10.25622839	6.085946114	6.085946114	4.190498	5.7E-06
CCRN4L	4103.76683	225.574	12.00308456	7.823838478	7.823838478	4.185276	5.8E-06
C8H4orf19	370.057036	18.178124	8.535497154	4.261389712	4.261389712	4.347472	5.8E-06
tRNA-Pro	158.793131	5.7839486	7.320061583	2.762125239	2.762125239	4.77895	6E-06
LOC100736999	86.9910197	1.6525567	6.459284385	1.407383613	1.407383613	5.718095	6.1E-06
WNT11	255.44982	11.567897	8.002532746	3.65167138	3.65167138	4.464841	6.2E-06
PALM2	0	128.07315	0	7.012045083	7.012045083	-20	6.2E-06
DPEP1	0	128.07315	0	7.012045083	7.012045083	-20	6.2E-06
CGNL1	2.76161967	225.574	1.911353989	7.823838478	7.823838478	-6.35194	6.2E-06
LOC100522769	2.76161967	225.574	1.911353989	7.823838478	7.823838478	-6.35194	6.2E-06
LOC100518491	55.2323934	0	5.813329549	0	0	20	6.3E-06
GCS	1107.40949	61.1446	10.11427525	5.957557119	5.957557119	4.17882	6.3E-06
LOC100510899	2.76161967	224.74772	1.911353989	7.818567588	7.818567588	-6.34665	6.4E-06
FAR2	19.3313377	783.3119	4.345633235	9.615283673	9.615283673	-5.34057	6.4E-06
NTNG2	0	127.24687	0	7.002779797	7.002779797	-20	6.5E-06
LOC102160132	0	127.24687	0	7.002779797	7.002779797	-20	6.5E-06
ADAMTS9	2.76161967	223.92144	1.911353989	7.81327737	7.81327737	-6.34133	6.5E-06
AQP3	2107.11581	118.15781	11.04173841	6.89672967	6.89672967	4.156483	6.6E-06
ANGPT4	2149.92091	120.63664	11.07073876	6.926434087	6.926434087	4.155544	6.6E-06
LOC100154987	13.8080984	589.96276	3.888314478	9.206923404	9.206923404	-5.41704	6.8E-06
CBWD2	8179.91747	451.14799	12.99804693	8.820651243	8.820651243	4.180414	6.8E-06
ARHGEF6	67.659682	2463.9621	6.101391269	11.26734975	11.26734975	-5.18654	6.9E-06
LOC100522935	156.031511	5.7839486	7.294910283	2.762125239	2.762125239	4.753639	6.9E-06
UNC45B	12.4272885	542.03861	3.747096094	9.084910972	9.084910972	-5.44681	6.9E-06
TMPRSS11F	0	125.59431	0	6.98406878	6.98406878	-20	7E-06
PIEZO2	0	125.59431	0	6.98406878	6.98406878	-20	7E-06
LOC102159707	17.9505279	726.29869	4.24416613	9.506404165	9.506404165	-5.33846	7E-06
LOC100525814	20.7121475	817.18931	4.440430524	9.676290877	9.676290877	-5.30212	7.1E-06
LOC102164037	16.569718	677.54826	4.135019133	9.406307627	9.406307627	-5.3537	7.1E-06
LOC102159872	70.4213016	0.8262784	6.158282521	0.868906686	0.868906686	6.41324	7.2E-06
LHFPL2	8.28485901	401.57129	3.214880004	8.653100473	8.653100473	-5.59904	7.2E-06

HSPB7	0	189.21775	0	7.571508044	7.571508044	-20	1.7E-06
ADCY2	0	189.21775	0	7.571508044	7.571508044	-20	1.7E-06
LOC100520570	77.18721332	5802.1267	6.288860784	12.50261472	12.50261472	-6.23208	1.7E-06
LOC100625132	2108.374019	47.097867	11.04259921	5.587901017	5.587901017	5.484325	1.7E-06
ESM1	2.114718173	334.64274	1.639101631	8.390782629	8.390782629	-7.30601	1.7E-06
SNX22	176.5789675	1.6525567	7.472316908	1.407383614	1.407383614	6.73947	1.7E-06
DIRAS2	105.7359087	0	6.737901807	0	0	20	1.7E-06
LOC100626788	0	187.56519	0	7.558919565	7.558919565	-20	1.7E-06
HOXA10	0	187.56519	0	7.558919565	7.558919565	-20	1.7E-06
CSGALNACT1	15.8603863	1265.8585	4.075565686	10.30703964	10.30703964	-6.31854	1.7E-06
LOC100524748	0	185.91263	0	7.546220276	7.546220276	-20	1.8E-06
NTRK1	2.114718173	328.03251	1.639101631	8.362086342	8.362086342	-7.27723	1.9E-06
AK5	609.0388339	12.394176	9.252757274	3.74353388	3.74353388	5.6188	1.9E-06
PTPRU	21.14718173	1613.7217	4.46905122	10.65706978	10.65706978	-6.25378	1.9E-06
LOC100626135	10.57359087	889.90181	3.532764645	9.799122619	9.799122619	-6.39511	1.9E-06
HERC6	58.15474976	4262.7701	5.886422108	12.05791394	12.05791394	-6.19575	1.9E-06
LOC100737711	0	184.26008	0	7.533408207	7.533408207	-20	1.9E-06
EPSTI1	28.54869534	2124.3617	4.885022527	11.05349266	11.05349266	-6.21746	1.9E-06
LOC100738115	1.057359087	256.1463	1.04079362	8.006445558	8.006445558	-7.92036	1.9E-06
LOC102159817	173.4068902	1.6525567	7.446313227	1.407383614	1.407383614	6.713318	1.9E-06
LOC102157989	2.114718173	325.55368	1.639101631	8.351176349	8.351176349	-7.26629	1.9E-06
SLC26A5	103.6211905	0	6.709031281	0	0	20	2E-06
TSPAN33	5761.549663	131.37826	12.49249156	7.048522415	7.048522415	5.454658	2E-06
HTR1B	14.80302721	1159.2686	3.982129041	10.18024305	10.18024305	-6.29118	2E-06
TMEM8C	0	181.78124	0	7.513974209	7.513974209	-20	2E-06
NR6A1	1484.532158	33.877413	10.53676412	5.124221138	5.124221138	5.453541	2E-06
LY96	9.516231779	801.49002	3.394545939	9.648339641	9.648339641	-6.39615	2.1E-06
ADAMTS2	15.8603863	1203.8876	4.075565686	10.23468284	10.23468284	-6.24613	2.2E-06
LOC100519519	16.91774539	1271.6424	4.163317207	10.31361139	10.31361139	-6.23201	2.3E-06
UBE2L6	71.90041789	5069.2178	6.187855179	12.30783201	12.30783201	-6.13962	2.3E-06
LOC100624847	4.229436346	447.84288	2.386655454	8.810066691	8.810066691	-6.72638	2.3E-06
ST3GAL1	27.49133625	1969.8476	4.832451381	10.94460053	10.94460053	-6.16296	2.3E-06
NANOG	100.4491132	0	6.664612444	0	0	20	2.4E-06
LOC100622075	37.00756803	2604.4294	5.24821481	11.34730546	11.34730546	-6.137	2.4E-06
FGFBP3	167.0627357	1.6525567	7.392856063	1.407383614	1.407383614	6.659546	2.4E-06
LOC100621092	3.17207726	376.78294	2.060765874	8.561413734	8.561413734	-6.89216	2.4E-06
ZNF418	6.344154519	573.43719	2.876596413	9.166005344	9.166005344	-6.49806	2.5E-06
GREM1	23.2618999	1662.4721	4.600620625	10.69998194	10.69998194	-6.15922	2.5E-06
LOC100738686	99.39175414	0	6.649496966	0	0	20	2.5E-06
BRINP2	904.042019	20.656959	9.821840965	4.436758793	4.436758793	5.45169	2.5E-06
ADAP1	0	173.51846	0	7.447235822	7.447235822	-20	2.5E-06
TMEM151A	0	173.51846	0	7.447235822	7.447235822	-20	2.5E-06
A2M	1.057359087	241.27328	1.04079362	7.920491517	7.920491517	-7.83406	2.6E-06
TENC1	108.9079859	7562.9259	6.780152406	12.88491952	12.88491952	-6.11776	2.6E-06
COL2A1	1097.538732	25.61463	10.10137002	4.734147579	4.734147579	5.42116	2.6E-06
WNT2B	9.516231779	765.96005	3.394545939	9.583007622	9.583007622	-6.33073	2.6E-06
LOC100736962	1.057359087	239.62073	1.04079362	7.910617116	7.910617116	-7.82414	2.6E-06
FAM180A	1.057359087	239.62073	1.04079362	7.910617116	7.910617116	-7.82414	2.6E-06
PCOLCE	132.1698858	9169.2111	7.057124067	13.16273923	13.16273923	-6.11633	2.6E-06
KIAA1199	3.17207726	370.17271	2.060765874	8.535946834	8.535946834	-6.86663	2.6E-06
EPB41L1	107.8506268	7434.0265	6.766205905	12.86012217	12.86012217	-6.10704	2.7E-06
LOC100512533	98.33439505	0	6.63422144	0	0	20	2.7E-06
LOC396583	5.286795433	499.07214	2.65232482	8.965992412	8.965992412	-6.56071	2.7E-06
LOC100624942	17.97510447	1294.7782	4.246035924	10.33960309	10.33960309	-6.17056	2.7E-06
TNFSF10	0	171.03962	0	7.426597064	7.426597064	-20	2.7E-06
LOC100737693	0	171.03962	0	7.426597064	7.426597064	-20	2.7E-06
CRELD1	17.97510447	1290.6468	4.246035924	10.33499592	10.33499592	-6.16595	2.7E-06
SYNE3	8.458872693	690.76872	3.241668254	9.434145967	9.434145967	-6.35159	2.8E-06
RCVRN	0	170.21334	0	7.419651341	7.419651341	-20	2.8E-06
LOC100737919	0	170.21334	0	7.419651341	7.419651341	-20	2.8E-06
TCF21	0	170.21334	0	7.419651341	7.419651341	-20	2.8E-06
ABI3BP	3.17207726	366.04132	2.060765874	8.519798669	8.519798669	-6.85043	2.8E-06
LOC100736904	225.2174854	3.3051135	7.821566636	2.106051269	2.106051269	6.090475	2.8E-06
LOC100739541	2.114718173	299.93905	1.639101631	8.233327508	8.233327508	-7.14806	2.9E-06
DKK1	0	168.56079	0	7.405658765	7.405658765	-20	2.9E-06
ZNF582	0	168.56079	0	7.405658765	7.405658765	-20	2.9E-06
CCDC69	0	168.56079	0	7.405658765	7.405658765	-20	2.9E-06
LOC100736876	975.9424369	23.135794	9.932129748	4.593102407	4.593102407	5.398598	3E-06
ALPK1	7.401513606	614.75111	3.070649265	9.26620351	9.26620351	-6.37604	3E-06
LOC102158081	1166.267072	28.093465	10.18891897	4.862623207	4.862623207	5.37552	3E-06
LOC102164919	96.21967688	0	6.603176434	0	0	20	3E-06
LOC100521071	96.21967688	0	6.603176434	0	0	20	3E-06
CYLC1	96.21967688	0	6.603176434	0	0	20	3E-06
FN1	10314.53789	881757.18	13.33253143	19.75002353	19.75002353	-6.41763	3.1E-06
LYL1	22.20454082	1522.831	4.536335244	10.57348723	10.57348723	-6.09976	3.1E-06
MFSD6	4967.472989	122.2892	12.27858681	6.945902605	6.945902605	5.344143	3.1E-06
SLC16A12	374.3051166	7.4365053	8.551920149	3.076645515	3.076645515	5.653446	3.1E-06
CES1	0	165.25567	0	7.377259771	7.377259771	-20	3.2E-06
LOC100738970	127.9404495	0.8262784	7.010561107	0.868906686	0.868906686	7.274629	3.2E-06
ATP12A	95.16231779	0	6.587399765	0	0	20	3.2E-06
CACNA2D3	95.16231779	0	6.587399765	0	0	20	3.2E-06
LOC100523701	12.68830904	923.77922	3.774872332	9.85296517	9.85296517	-6.18598	3.2E-06
KIF12	39.1222862	2577.9885	5.326331909	11.33258964	11.33258964	-6.04211	3.2E-06
LOC100620398	1.057359087	229.70539	1.04079362	7.849907883	7.849907883	-7.76318	3.2E-06
LOC100153543	463.1232799	9.9153405	8.858364253	3.448285225	3.448285225	5.54559	3.3E-06
LOC100521749	1.057359087	228.87911	1.04079362	7.844731552	7.844731552	-7.75798	3.3E-06
COL18A1	62.38418611	4068.5947	5.986051038	11.99066941	11.99066941	-6.02721	3.3E-06
SSC5D	13.74566813	981.61871	3.882219287	9.940487893	9.940487893	-6.15811	3.3E-06
PARP9	37.00756803	2427.6059	5.24821481	11.24591266	11.24591266	-6.03557	3.4E-06
LOC100624297	26.43397716	1748.405	4.777891883	10.77264864	10.77264864	-6.0475	3.5E-06
FBLN2	30.66341351	2011.1616	4.98474489	10.97453043	10.97453043	-6.03537	3.5E-06
ITGA8	245.3073081	16505.737	7.944315624	14.01076732	14.01076732	-6.07223	3.5E-06
PHLDA2	14.80302721	1036.9794	3.982129041	10.01956204	10.01956204	-6.13035	3.5E-06
PVR	8.458872693	657.71758	3.241668254	9.363516252	9.363516252	-6.28086	3.5E-06
SELM	96.21967688	6239.228	6.603176434	12.60738302	12.60738302	-6.01889	3.5E-06
TEC	3.17207726	348.68947	2.060765874	8.449930555	8.449930555	-6.78037	3.5E-06
TIMP2	948.4511007	68241.504	9.890949889	16.05838297	16.05838297	-6.16893	3.6E-06
LOC102162518	82.47400875	5319.5802	6.383255151	12.37736785	12.37736785	-6.01123	3.6E-06
TFEB	4.229436346	408.18152	2.386655454	8.676597164	8.676597164	-6.5926	3.6E-06
LOC100736654	186.0951992	2.4788351	7.54762873	1.798604302	1.798604302	6.230235	3.6E-06
PWWP2B	2.114718173	285.89232	1.639101631	8.164365521	8.164365521	-7.07886	3.6E-06
GNAT1	1.057359087	223.92144	1.04079362	7.81327737	7.81327737	-7.72638	3.7E-06
LCN2	8.458872693	651.10736	3.241668254	9.348965686	9.348965686	-6.26629	3.7E-06
FBXO43	155.4317857	1.6525567	7.289389877	1.407383614	1.407383614	6.555438	3.7E-06
PARP4	4.229436346	405.70268	2.386655454	8.667830689	8.667830689	-6.58381	3.7E-06
CALML4	4.229436346	405.70268	2.386655454	8.667830689	8.667830689	-6.58381	3.7E-06
LOC100525848	2296.583936	58.665764	11.16590185	5.89883146	5.89883146	5.290827	3.8E-06
IL4R	58.15474976	3674.4599	5.886422108	11.84370907	11.84370907	-5.98149	3.9E-06
LOC606745	5.286795433	461.88961	2.65232482	8.854524369	8.854524369	-6.44901	3.9E-06
ABCC8	212.5291764	3.3051135	7.738289401	2.106051269	2.106051269	6.006817	4E-06
WIF1	2.114718173	280.10837	1.639101631	8.13498259	8.13498259	-7.04938	4E-06
LOC100737517	21.14718173	1380.7112	4.46905122	10.43224034	10.43224034	-6.0288	4E-06
NOR-1	864.9197328	21.483238	9.758089489	4.490777899	4.490777899	5.331283	4.1E-06
SNAP25	3026.161706	78.496445	11.56375003	6.312818447	6.312818447	5.268718	4.1E-06
LOC100513699	122.653654	0.8262784	6.950161063	0.868906686	0.868906686	7.213747	4.1E-06
LOC100522153	28.54869534	1814.5073	4.885022527	10.82615702	10.82615702	-5.99001	4.1E-06
LOC100511616	1.057359087	218.13749	1.04079362	7.775692512	7.775692512	-7.68863	4.2E-06
LOC100739750	12.68830904	875.85507	3.774872332	9.776194605	9.776194605	-6.10912	4.2E-06
ARHGAP23	34.89284986	2189.6377	5.165624571	11.09713518	11.09713518	-5.97162	4.2E-06
RHPN2	2538.719167	66.10227	11.31045326	6.068289661	6.068289661	5.263257	4.2E-06
ZNF350	0	156.16661	0	7.296150961	7.296150961	-20	4.2E-06
GGT5	7.401513606	573.43719	3.070649265	9.166005344	9.166005344	-6.27567	4.2E-06
CCN2	685.1686881	46688.859	9.422419483	15.51082162	15.51082162	-6.09048	4.3E-06
TCIRG1	61.32682702	3802.5331	5.961781364	11.89312443	11.89312443	-5.9543	4.3E-06
MGAT3	0	155.34033	0	7.288546214	7.288546214	-20	4.3E-06
ITGBL1	0	155.34033	0	7.288546214	7.288546214	-20	4.3E-06
PPL	21.14718173	1360.0542	4.46905122	10.4105088	10.4105088	-6.00705	4.3E-06
DEXI	12.68830904	870.07113	3.774872332	9.766646714	9.766646714	-6.09956	4.4E-06
LGALS3	172.3495311	10919.269	7.437540124	13.41472073	13.41472073	-5.9854	4.4E-06
MYL9	467.3527163	30984.613	8.871451622	14.91931087	14.91931087	-6.0509	4.4E-06
LOC102167053	0	154.51406	0	7.280901168	7.280901168	-20	4.5E-06
BPIFB6	89.87552236	0	6.505819847	0	0	20	4.5E-06
LOC100736923	0	153.68778	0	7.273215395	7.273215395	-20	4.6E-06
LOC102163933	15.8603863	1040.2845	4.075565686	10.02414854	10.02414854	-6.03541	4.6E-06
LOC100519934	116921.7104	2704.4091	16.83519565	11.40163106	11.40163106	5.434086	4.6E-06
HSPG2	271.7412852	17333.668	8.091389289	14.08137256	14.08137256	-5.9952	4.7E-06
TNNC1	5.286795433	445.36404	2.65232482	8.802077003	8.802077003	-6.39645	4.7E-06
BAHCC1	54.9826725	3346.4274	5.806908455	11.70883705	11.70883705	-5.9275	4.7E-06
HS3ST5	378.534553	8.2627837	8.568087425	3.211445828	3.211445828	5.517653	4.7E-06
LOC100738767	0	152.03522	0	7.257719911	7.257719911	-20	4.8E-06
RASGRF2	870.2065282	22.309516	9.766870954	4.542847146	4.542847146	5.285627	4.9E-06
LOC100522330	22978.52767	576.7423	14.48806152	9.174282325	9.174282325	5.316216	4.9E-06
MYOM1	8.458872693	614.75111	3.241668254	9.26620351	9.26620351	-6.18339	4.9E-06
KCNK6	0	151.20894	0	7.249909307	7.249909307	-20	4.9E-06
NLRP3	0	151.20894	0	7.249909307	7.249909307	-20	4.9E-06
LOC100621009	0	151.20894	0	7.249909307	7.249909307	-20	4.9E-06
LOC100620561	0	151.20894	0	7.249909307	7.249909307	-20	4.9E-06
LOC102161872	148.0302721	1.6525567	7.219461601	1.407383614	1.407383614	6.485049	5E-06
NTF3	0	150.38266	0	7.242056187	7.242056187	-20	5.1E-06
GYG2	8.458872693	609.79344	3.241668254	9.254540753	9.254540753	-6.17171	5.1E-06
STX11	6.344154519	494.11447	2.876596413	8.951618292	8.951618292	-6.28327	5.1E-06
SH2D3C	4.229436346	379.26177	2.386655454	8.570849103	8.570849103	-6.48658	5.2E-06
ANXA8	15.8603863	1014.6698	4.075565686	9.988215792	9.988215792	-5.99944	5.2E-06
LOC102157888	87.76080419	0	6.471850834	0	0	20	5.2E-06
NXNL2	87.76080419	0	6.471850834	0	0	20	5.2E-06
LMO1	87.76080419	0	6.471850834	0	0	20	5.2E-06
CCNJL	1210.676154	32.224856	10.24278844	5.054191063	5.054191063	5.231495	5.3E-06
LOC102160711	7.401513606	546.99628	3.070649265	9.098022295	9.098022295	-6.20757	5.3E-06
LOC102159968	0	148.73011	0	7.22622053	7.22622053	-20	5.4E-06
PRDM16	0	148.73011	0	7.22622053	7.22622053	-20	5.4E-06
ARID5A	7.401513606	546.17	3.070649265	9.095845332	9.095845332	-6.20539	5.4E-06
LOC100157228	94.1049587	5598.8622	6.571448659	12.45117562	12.45117562	-5.89472	5.4E-06
LOC100518095	102.5638314	6110.3286	6.694376434	12.57727033	12.57727033	-5.89666	5.4E-06
KIT	510.7044388	12.394176	8.999166938	3.74353388	3.74353388	5.364754	5.5E-06
LOC100621301	0	147.90383	0	7.218237038	7.218237038	-20	5.5E-06
LOC100523068	0	147.07755	0	7.210209122	7.210209122	-20	5.7E-06
DPYSL4	0	147.07755	0	7.210209122	7.210209122	-20	5.7E-06
CEP170B	50.75323616	2975.4284	5.693577173	11.53936648	11.53936648	-5.87345	5.7E-06
SLC25A53	790.9045968	20.656959	9.629182825	4.436758793	4.436758793	5.258804	5.7E-06
ICOSLG	31.7207726	1877.3045	5.032134908	10.87521522	10.87521522	-5.88709	5.7E-06
FAM217B	0	146.25127	0	7.202136284	7.202136284	-20	5.8E-06
WIPF3	12094.07323	319.76973	13.56213188	8.325394195	8.325394195	5.241123	5.8E-06
TRIM9	2.114718173	259.45141	1.639101631	8.02487043	8.02487043	-6.93886	5.9E-06
PODN	1.057359087	203.26448	1.04079362	7.674294538	7.674294538	-7.58675	5.9E-06
IGFBP3	0	145.42499	0	7.194018019	7.194018019	-20	6E-06
LOC100522595	0	145.42499	0	7.194018019	7.194018019	-20	6E-06
LOC100737264	45.46644072	2640.7857	5.538117235	11.36729771	11.36729771	-5.86002	6E-06
SLC40A1	45.46644072	2637.4806	5.538117235	11.36549164	11.36549164	-5.85821	6E-06
ELOVL3	85.64608601	0	6.437062676	0	0	20	6E-06
TRPC3	5.286795433	421.40197	2.65232482	8.722472752	8.722472752	-6.31666	6.1E-06
KCNH3	142.7434767	1.6525567	7.167352675	1.407383614	1.407383614	6.432581	6.2E-06
NOL4	142.7434767	1.6525567	7.167352675	1.407383614	1.407383614	6.432581	6.2E-06
LOC102163597	3.17207726	310.68067	2.060765874	8.283924863	8.283924863	-6.61386	6.2E-06
LOC100517809	892.4110691	23.962073	9.803180319	4.641665831	4.641665831	5.218884	6.3E-06
RSPO3	0	143.77244	0	7.177643143	7.177643143	-20	6.3E-06
AFAP1	27.49133625	1600.5012	4.832451381	10.64520917	10.64520917	-5.8634	6.5E-06
C1QTNF3	1.057359087	199.13309	1.04079362	7.644815895	7.644815895	-7.55712	6.5E-06
PCDHB10	358.4447303	8.2627837	8.489626143	3.211445828	3.211445828	5.438979	6.6E-06
PDK2	15.8603863	967.57197	4.075565686	9.919715447	9.919715447	-5.93087	6.6E-06
TMEM45A	17.97510447	1079.1196	4.246035924	10.07697529	10.07697529	-5.90771	6.6E-06
PPFIBP2	0	142.11988	0	7.161080272	7.161080272	-20	6.7E-06
NYNRIN	62.38418611	3536.4714	5.986051038	11.78850278	11.78850278	-5.82499	6.7E-06
MREG	1022.466237	28.093465	9.999247795	4.862623207	4.862623207	5.185675	6.7E-06
JAM3	99.39175414	5652.5703	6.649496966	12.46494653	12.46494653	-5.82964	6.8E-06
LOC100626048	0	141.2936	0	7.15272698	7.15272698	-20	6.9E-06
DNAJC22	83.53136784	0	6.40141489	0	0	20	7E-06
LOC100739615	193.4967128	3.3051135	7.603601962	2.106051269	2.106051269	5.871465	7E-06
DDX58	96.21967688	5427.8226	6.603176434	12.40642363	12.40642363	-5.8179	7.1E-06
LOC100516232	0	140.46732	0	7.144325041	7.144325041	-20	7.1E-06
ALDH1L1	843.7725511	23.135794	9.722419148	4.593102407	4.593102407	5.188656	7.2E-06
LOC102166594	7.401513606	513.94515	3.070649265	9.008274952	9.008274952	-6.11765	7.3E-06
LRRC4B	1.057359087	194.17542	1.04079362	7.608627544	7.608627544	-7.52075	7.4E-06
LOC100521600	5.286795433	404.8764	2.65232482	8.664896653	8.664896653	-6.25894	7.5E-06
LOC102165837	82.47400875	0	6.383255151	0	0	20	7.5E-06
GABRE	0	137.98849	0	7.118821584	7.118821584	-20	7.7E-06
FAM184B	137.4566813	1.6525567	7.113290863	1.407383614	1.407383614	6.378133	7.7E-06
LOC100737490	163.8906584	2.4788351	7.365365858	1.798604302	1.798604302	6.046928	7.8E-06
LOC102163451	268.569208	5.7839486	8.074511901	2.76212524	2.76212524	5.537095	7.9E-06
PLD5	1411.574381	40.48764	10.46411112	5.374609695	5.374609695	5.12368	7.9E-06
ENPP6	2.114718173	242.92584	1.639101631	7.930298794	7.930298794	-6.84391	8.1E-06
LTBR	99.39175414	5448.4796	6.649496966	12.41190275	12.41190275	-5.77658	8.2E-06
SDK2	2.114718173	242.09956	1.639101631	7.925403489	7.925403489	-6.83899	8.2E-06
LOC100737780	108.9079859	0.8262784	6.780152406	0.868906686	0.868906686	7.042266	8.4E-06
LOC100737019	10.57359087	656.06503	3.532764645	9.359892345	9.359892345	-5.9553	8.4E-06
TNFAIP3	37.00756803	2016.1192	5.24821481	10.97808065	10.97808065	-5.76762	8.4E-06
LOC100623510	10.57359087	655.23875	3.532764645	9.358076973	9.358076973	-5.95348	8.4E-06
NPHS2	135.3419631	1.6525567	7.09108585	1.407383614	1.407383614	6.355766	8.5E-06
PARP3	141.6861176	7787.6737	7.156701167	12.92716195	12.92716195	-5.78042	8.5E-06
FOXC2	12.68830904	753.56588	3.774872332	9.559503046	9.559503046	-5.89216	8.8E-06
LOC100627460	8.458872693	544.51745	3.241668254	9.091481528	9.091481528	-6.00837	8.9E-06
SCARA5	0	133.8571	0	7.075287628	7.075287628	-20	8.9E-06
LOC100620286	3.17207726	288.37115	2.060765874	8.176777292	8.176777292	-6.50636	9E-06
HYAL1	1.057359087	185.91263	1.04079362	7.546220276	7.546220276	-7.45802	9.1E-06
SOCS3	1.057359087	185.91263	1.04079362	7.546220276	7.546220276	-7.45802	9.1E-06
LOC100521595	339.4122668	8.2627837	8.411139215	3.211445828	3.211445828	5.360267	9.1E-06
ZNF792	0	133.03082	0	7.066420949	7.066420949	-20	9.2E-06
LOC102162351	0	133.03082	0	7.066420949	7.066420949	-20	9.2E-06
LOC100739500	7.401513606	489.98307	3.070649265	8.939529481	8.939529481	-6.04877	9.2E-06
ADAMTS19	468.4100754	12.394176	8.874704998	3.74353388	3.74353388	5.240038	9.2E-06
MYLK	26.43397716	1434.4193	4.777891883	10.48725646	10.48725646	-5.76193	9.2E-06
KCP	1.057359087	185.08636	1.04079362	7.539828464	7.539828464	-7.45159	9.3E-06
FZD5	835.3136784	23.962073	9.707900349	4.641665831	4.641665831	5.123493	9.4E-06
ACOT12	79.30193149	0	6.327362784	0	0	20	9.4E-06
LOC100736853	4.229436346	335.46902	2.386655454	8.394329862	8.394329862	-6.30957	9.5E-06
SERPINF1	474.7542299	26974.684	8.894072673	14.71937191	14.71937191	-5.82828	9.5E-06
AIPL1	0	131.37826	0	7.048522415	7.048522415	-20	9.7E-06
NT5E	112.0800632	5935.9838	6.821200784	12.53551447	12.53551447	-5.72689	9.8E-06
LOC100626812	1040.441341	30.5723	10.02436587	4.980587447	4.980587447	5.088827	9.8E-06
ELTD1	0	130.55198	0	7.039489183	7.039489183	-20	1E-05
NXF2B	78.24457241	0	6.308240221	0	0	20	1E-05
APOE	57.09739067	2928.3305	5.860401464	11.51635528	11.51635528	-5.68051	1.1E-05
LOC102163538	4.229436346	325.55368	2.386655454	8.351176349	8.351176349	-6.26629	1.1E-05
TMEM140	6.344154519	423.05453	2.876596413	8.728105973	8.728105973	-6.05927	1.1E-05
IRX4	0	128.07315	0	7.012045083	7.012045083	-20	1.1E-05
LOC100516179	2.114718173	227.22655	1.639101631	7.834322837	7.834322837	-6.74752	1.1E-05
PTPLAD2	51.81059524	2645.7433	5.722755497	11.37000259	11.37000259	-5.67428	1.1E-05
LOC102157535	428.2304301	11.567897	8.745608549	3.651671381	3.651671381	5.210189	1.1E-05
LOC100621484	3.17207726	275.1507	2.060765874	8.109311963	8.109311963	-6.43865	1.1E-05
NDST3	178.6936856	3.3051135	7.489395904	2.106051269	2.106051269	5.756645	1.1E-05
NTNG2	0	127.24687	0	7.002779797	7.002779797	-20	1.1E-05
LOC100625548	127.9404495	1.6525567	7.010561107	1.407383614	1.407383614	6.274629	1.2E-05
ANKRD6	324.6092396	8.2627837	8.346997828	3.211445828	3.211445828	5.295932	1.2E-05
HPRT1	26233.07894	751.91332	14.67915452	9.556339969	9.556339969	5.124677	1.2E-05
ANKS1B	76.12985423	0	6.269217479	0	0	20	1.2E-05
TMPRSS11F	0	125.59431	0	6.98406878	6.98406878	-20	1.2E-05
FAM162B	1057.359087	32.224856	10.04761348	5.054191063	5.054191063	5.036148	1.2E-05
BEX1	5428.48155	166.08195	12.40659873	7.3844121	7.3844121	5.030581	1.2E-05
SRPX	19.03246356	999.79683	4.324267947	9.966933409	9.966933409	-5.7151	1.2E-05
LOC100523405	1.057359087	174.34474	1.04079362	7.454050314	7.454050314	-7.36533	1.2E-05
HOMER3	232.618999	12073.58	7.868013796	13.55968534	13.55968534	-5.69774	1.2E-05
C2H19orf66	15.8603863	848.58789	4.075565686	9.730619388	9.730619388	-5.74156	1.3E-05
TRNAG-UCC	101.5064723	0.8262784	6.679571195	0.868906686	0.868906686	6.940728	1.3E-05
LOC100521298	101.5064723	0.8262784	6.679571195	0.868906686	0.868906686	6.940728	1.3E-05
LOC102161895	0	123.94176	0	6.965111899	6.965111899	-20	1.3E-05
OTUD7A	1.057359087	172.69218	1.04079362	7.440388989	7.440388989	-7.35159	1.3E-05
GABRB3	125.8257313	1.6525567	6.986703669	1.407383614	1.407383614	6.250583	1.3E-05
AFF3	614.3256293	18.178124	9.265206274	4.261389711	4.261389711	5.078728	1.3E-05
MX1	943.1643052	52119.161	9.882894132	15.66955392	15.66955392	-5.78816	1.3E-05
LOC100517194	1.057359087	171.8659	1.04079362	7.433509508	7.433509508	-7.34467	1.3E-05
CDH15	1.057359087	171.8659	1.04079362	7.433509508	7.433509508	-7.34467	1.3E-05
OSTF1	71.90041789	3543.0817	6.187855179	11.79119613	11.79119613	-5.62286	1.3E-05
LOC100524949	0	122.2892	0	6.945902605	6.945902605	-20	1.4E-05
CXCL14	9.516231779	545.34373	3.394545939	9.09366508	9.09366508	-5.84063	1.4E-05
LOC100627693	17.97510447	927.91061	4.246035924	9.859395963	9.859395963	-5.68991	1.4E-05
SEMA3E	340.4696259	9.0890621	8.415613445	3.334720158	3.334720158	5.227251	1.4E-05
PLAUR	50.75323616	2475.53	5.693577173	11.27410438	11.27410438	-5.60809	1.4E-05
LOC100738076	3.17207726	262.75652	2.060765874	8.043062959	8.043062959	-6.37215	1.4E-05

ZNF541	145.891473	0	7.19860684	0	0	20	2.6E-10
ERAS	145.891473	0	7.19860684	0	0	20	2.6E-10
LOC102167451	2524.9172	57.013208	11.30259163	5.858309486	5.858309486	5.468796	2.6E-10
LOC102162054	10.7760747	599.8781	3.557786824	9.230928526	9.230928526	-5.79877	2.6E-10
FGFR4	1988.60025	44.619032	10.95826288	5.511563931	5.511563931	5.47795	2.7E-10
PHLDA2	21.5521494	1036.9794	4.495193036	10.01956204	10.01956204	-5.58841	2.8E-10
IGFBP3	0	145.42499	0	7.194018019	7.194018019	-20	2.8E-10
FAM179A	144.233615	0	7.182231603	0	0	20	2.8E-10
NID1	51.3935871	2274.7444	5.711318333	11.15212279	11.15212279	-5.46797	2.9E-10
ALOX12B	755.983087	14.873011	9.564117257	3.98850389	3.98850389	5.667585	2.9E-10
ZNF180	2.48678647	255.32002	1.801898017	8.001802338	8.001802338	-6.68188	2.9E-10
NOV	0	144.59872	0	7.185853814	7.185853814	-20	3E-10
LOC102157759	353.952608	4.9576702	8.471482603	2.574748269	2.574748269	6.15775	3E-10
GPRIN3	1186.19715	25.61463	10.21334381	4.734147579	4.734147579	5.533232	3.1E-10
EFEMP2	256.967935	11063.041	8.011047944	13.4335908	13.4335908	-5.42802	3.2E-10
TRIM29	249.507576	2.4788351	7.968710424	1.798604302	1.798604302	6.653278	3.4E-10
INHBE	54.7093024	2371.4189	5.799846344	11.21214307	11.21214307	-5.43782	3.4E-10
LOC100626974	1.65785765	216.48493	1.410263837	7.764771649	7.764771649	-7.0288	3.4E-10
SLA-3	154.180761	6546.6035	7.277805898	12.67675125	12.67675125	-5.40805	3.4E-10
PPFIBP2	0	142.11988	0	7.161080272	7.161080272	-20	3.5E-10
LOC100736977	9.11821706	516.42398	3.338883189	9.015203113	9.015203113	-5.82366	3.5E-10
SPOCK3	38.1307259	1679.8239	5.290229968	10.71495289	10.71495289	-5.46121	3.6E-10
LOC100519925	178.219697	0.8262784	7.485585395	0.868906686	0.868906686	7.752813	3.6E-10
LOC100520570	137.602185	5802.1267	7.114806188	12.50261472	12.50261472	-5.39801	3.7E-10
GRB14	1190.34179	26.440908	10.21837166	4.77825631	4.77825631	5.49246	3.9E-10
LOC100516232	0	140.46732	0	7.144325041	7.144325041	-20	3.9E-10
SPRY4	37.3017971	1631.0735	5.259340178	10.67249032	10.67249032	-5.45043	3.9E-10
LOC102157497	139.260042	0	7.131960259	0	0	20	3.9E-10
LOC102166521	139.260042	0	7.131960259	0	0	20	3.9E-10
LOC100513263	18.2364341	868.41857	4.265769485	9.763907098	9.763907098	-5.5735	4E-10
EVPL	536.316949	9.9153405	9.069629536	3.448285225	3.448285225	5.75728	4.1E-10
MEIS3	43.9332277	1887.2198	5.489710792	10.882811	10.882811	-5.42481	4.1E-10
GABRQ	138.431114	0	7.123408719	0	0	20	4.2E-10
LOC100511351	50.5646582	2149.9763	5.688310694	11.07077593	11.07077593	-5.41005	4.2E-10
C12H17orf72	469.173714	8.2627837	8.877050075	3.211445828	3.211445828	5.82735	4.3E-10
PCOLCE	220.495067	9169.2111	7.791130759	13.16273923	13.16273923	-5.37798	4.3E-10
PHACTR1	27.3546512	1215.4555	4.825513503	10.24846781	10.24846781	-5.47357	4.5E-10
SYNPO	46.4200141	1963.2374	5.567424187	10.9397536	10.9397536	-5.40234	4.7E-10
HOXA3	6.63143059	404.8764	2.93195353	8.664896653	8.664896653	-5.93202	4.7E-10
PKHD1L1	623.354475	12.394176	9.286221537	3.74353388	3.74353388	5.652319	4.7E-10
LOC100620419	70.45895	2921.7203	6.159042811	11.51309607	11.51309607	-5.37389	4.7E-10
SNED1	16.5785765	786.61701	4.135746339	9.621350459	9.621350459	-5.56827	4.8E-10
PLEKHG4	239.56043	2.4788351	7.910255542	1.798604302	1.798604302	6.594584	4.9E-10
CCN2	1097.50176	46688.859	10.10132147	15.51082162	15.51082162	-5.41078	5E-10
LOC100522153	43.1042988	1814.5073	5.462847376	10.82615702	10.82615702	-5.3956	5.1E-10
LOC102158049	617.551974	12.394176	9.272751012	3.74353388	3.74353388	5.638826	5.1E-10
LOC100517194	0.82892882	171.8659	0.870998931	7.433509508	7.433509508	-7.69582	5.4E-10
LOC100521789	770.074877	16.525567	9.590727153	4.13138925	4.13138925	5.542227	5.5E-10
MYLPF	14.9207188	713.07823	3.99283357	9.479938335	9.479938335	-5.57867	5.5E-10
LIN28B	893.585272	19.830681	9.805075196	4.380638094	4.380638094	5.493799	5.6E-10
GPNMB	0	134.68337	0	7.084100147	7.084100147	-20	5.7E-10
NOXA1	267.74401	3.3051135	8.070088789	2.106051269	2.106051269	6.340011	5.7E-10
AMY2A	765.101304	16.525567	9.581391367	4.13138925	4.13138925	5.532879	5.8E-10
LOC102167854	133.457541	0	7.071006856	0	0	20	5.8E-10
LOC100739427	4.97357294	332.16391	2.578594101	8.380088299	8.380088299	-6.06147	5.9E-10
SLC15A3	23.2100071	1022.1063	4.597531595	9.998740397	9.998740397	-5.46065	5.9E-10
ERBB4	202.258633	1.6525567	7.667172819	1.407383613	1.407383613	6.935358	6E-10
SLC5A7	545.435166	10.741619	9.093906521	3.553559423	3.553559423	5.666124	6E-10
ABCC8	266.086152	3.3051135	8.061161368	2.106051269	2.106051269	6.33105	6.1E-10
LOC100624511	3.31571529	267.71419	2.109599694	8.06992871	8.06992871	-6.33523	6.1E-10
TFAP2D	132.628612	0	7.062085132	0	0	20	6.2E-10
TMEM174	132.628612	0	7.062085132	0	0	20	6.2E-10
LOC100515546	36.4728682	1513.742	5.227774503	10.56485635	10.56485635	-5.37515	6.6E-10
HOXA9	9.94714588	513.94515	3.452482877	9.008274952	9.008274952	-5.69119	6.6E-10
LOC100627460	10.7760747	544.51745	3.557786824	9.091481528	9.091481528	-5.65907	6.6E-10
ARL4C	87.0375265	3445.5808	6.460046706	11.75095013	11.75095013	-5.30697	7E-10
MPP7	688.839852	14.873011	9.430117666	3.98850389	3.98850389	5.5334	7E-10
GPR68	6.63143059	386.69828	2.93195353	8.598790517	8.598790517	-5.86574	7.2E-10
LOC100624329	9.94714588	508.98748	3.452482877	8.994318011	8.994318011	-5.6772	7.2E-10
SLITRK3	322.453312	4.9576702	8.337413677	2.574748269	2.574748269	6.023284	7.3E-10
LOC100622715	1.65785765	199.13309	1.410263837	7.644815895	7.644815895	-6.90827	7.4E-10
C1QTNF3	1.65785765	199.13309	1.410263837	7.644815895	7.644815895	-6.90827	7.4E-10
PPP1R14C	4759.70931	122.2892	12.21696082	6.945902605	6.945902605	5.282505	7.5E-10
ELTD1	0	130.55198	0	7.039489182	7.039489182	-20	7.6E-10
ELK3	153.351832	5977.2977	7.2700788	12.54551903	12.54551903	-5.28458	7.8E-10
LOC100525496	196.456131	1.6525567	7.625388355	1.407383613	1.407383613	6.893364	7.9E-10
FAM111A	29.0125088	1205.5401	4.907492018	10.2366602	10.2366602	-5.37686	8E-10
LOC102163734	319.137597	4.9576702	8.322548307	2.574748269	2.574748269	6.008372	8E-10
GFI1	128.483968	0	7.016629669	0	0	20	8.3E-10
LOC100739473	162.470049	0.8262784	7.352882522	0.868906686	0.868906686	7.61933	8.6E-10
LOC100737813	162.470049	0.8262784	7.352882522	0.868906686	0.868906686	7.61933	8.6E-10
LOC100511383	13.2628612	618.8825	3.834191515	9.275850966	9.275850966	-5.5442	8.7E-10
SORL1	193.969345	1.6525567	7.607103495	1.407383613	1.407383613	6.874985	8.9E-10
PCDH17	83.7218112	3234.0535	6.404661526	11.65957388	11.65957388	-5.2716	8.9E-10
IRX4	0	128.07315	0	7.012045083	7.012045083	-20	9E-10
PTPN20B	126.82611	0	6.998038744	0	0	20	9.3E-10
LOC100157667	126.82611	0	6.998038744	0	0	20	9.3E-10
LRRC4B	1.65785765	194.17542	1.410263837	7.608627545	7.608627545	-6.8719	9.4E-10
LOC100739480	0.82892882	161.95056	0.870998931	7.348290508	7.348290508	-7.61009	9.4E-10
HHAT	8.28928824	434.62242	3.215568059	8.766934408	8.766934408	-5.71237	9.6E-10
LOC100521600	7.46035941	404.8764	3.080718953	8.664896653	8.664896653	-5.76209	9.6E-10
NTNG2	0	127.24687	0	7.002779797	7.002779797	-20	9.6E-10
UROC1	222.981854	2.4788351	7.807238043	1.798604302	1.798604302	6.49112	9.7E-10
LOC102164256	103.616103	3954.5683	6.708961125	11.94966926	11.94966926	-5.2542	9.7E-10
ENTPD3	153.351832	5839.3093	7.2700788	12.51182905	12.51182905	-5.25088	9.8E-10
FAM151A	125.997181	0	6.988652665	0	0	20	9.9E-10
LOC102164341	125.997181	0	6.988652665	0	0	20	9.9E-10
SLA-DRB1	125.997181	0	6.988652665	0	0	20	9.9E-10
LOC100737780	159.983263	0.8262784	7.330766893	0.868906686	0.868906686	7.597077	9.9E-10
NPTX2	0	126.42059	0	6.993454623	6.993454623	-20	1E-09
LOC100515458	159.154334	0.8262784	7.323319031	0.868906686	0.868906686	7.589583	1E-09
CAMK2B	281.006871	4.1313919	8.139586505	2.359350201	2.359350201	6.087834	1E-09
LOC102159297	125.168252	0	6.979205122	0	0	20	1.1E-09
LOC100626448	748.522728	17.351846	9.549828415	4.19785327	4.19785327	5.430885	1.1E-09
PLCXD2	746.86487	17.351846	9.546633806	4.19785327	4.19785327	5.427686	1.1E-09
TMPRSS11F	0	125.59431	0	6.98406878	6.98406878	-20	1.1E-09
PIEZO2	0	125.59431	0	6.98406878	6.98406878	-20	1.1E-09
ESR2	158.325405	0.8262784	7.315832521	0.868906686	0.868906686	7.582049	1.1E-09
ZAP70	279.349014	4.1313919	8.131080184	2.359350201	2.359350201	6.079297	1.1E-09
CLIC2	1.65785765	190.8703	1.410263837	7.58398763	7.58398763	-6.84713	1.1E-09
ABAT	366.38654	6.610227	8.521154961	2.927939483	2.927939483	5.792523	1.1E-09
LOC100511763	124.339324	0	6.969695301	0	0	20	1.1E-09
SHE	124.339324	0	6.969695301	0	0	20	1.1E-09
IFI44	237.073644	8936.2006	7.895264103	13.12560729	13.12560729	-5.23625	1.1E-09
LOC102165792	1182.0525	29.746021	10.20829838	4.942327829	4.942327829	5.312454	1.1E-09
LOC102160134	6249.2944	167.73451	12.60970843	7.398611253	7.398611253	5.219442	1.2E-09
LOC102164058	0	124.76803	0	6.974621475	6.974621475	-20	1.2E-09
CRYAB	32.3282241	1274.9475	5.058672541	10.31735329	10.31735329	-5.3015	1.2E-09
LOC100625443	2.48678647	219.79005	1.801898017	7.786531327	7.786531327	-6.4657	1.2E-09
LOC100739808	123.510395	0	6.96012238	0	0	20	1.2E-09
JAM3	151.693975	5652.5703	7.254499324	12.46494653	12.46494653	-5.21967	1.2E-09
HERC6	114.392178	4262.7701	6.850401618	12.05791394	12.05791394	-5.21973	1.2E-09
RNF213	342.347604	12863.502	8.423526086	13.65110795	13.65110795	-5.23168	1.2E-09
LOC102165620	17.4075053	741.1717	4.202222212	9.535609179	9.535609179	-5.41203	1.2E-09
PDE10A	907.677062	22.309516	9.82762385	4.542847146	4.542847146	5.346448	1.3E-09
LOC102163451	332.400458	5.7839486	8.381112277	2.762125239	2.762125239	5.844724	1.3E-09
LOC100623776	29.0125088	1146.0481	4.907492018	10.16371018	10.16371018	-5.30385	1.3E-09
CYP7B1	13.2628612	594.09415	3.834191515	9.216974124	9.216974124	-5.48523	1.3E-09
GPR63	818.152749	19.830681	9.677988689	4.380638094	4.380638094	5.366564	1.3E-09
LOC100737127	16.5785765	708.94684	4.135746339	9.471567197	9.471567197	-5.41829	1.3E-09
LOC102159412	0.82892882	156.16661	0.870998931	7.296150961	7.296150961	-7.55762	1.3E-09
VWCE	1654.54193	43.792754	10.69308784	5.485193456	5.485193456	5.239596	1.3E-09
LOC100154530	41.4464412	1579.018	5.407571694	10.62572525	10.62572525	-5.25164	1.4E-09
FBLN1	30.6703665	1198.9299	4.985061656	10.22873443	10.22873443	-5.28876	1.4E-09
GLIS1	46.4200141	1754.189	5.567424187	10.77741066	10.77741066	-5.23991	1.4E-09
LOC100736715	272.717583	4.1313919	8.096544303	2.359350201	2.359350201	6.044636	1.4E-09
LOC100517699	272.717583	4.1313919	8.096544303	2.359350201	2.359350201	6.044636	1.4E-09
RAPGEF4	1446.4808	38.008805	10.49932849	5.285727902	5.285727902	5.25007	1.4E-09
PLA2G4A	99.4714588	3654.6292	6.65064192	11.83590404	11.83590404	-5.1993	1.4E-09
PPAT	6843.63637	187.56519	12.74075818	7.558919565	7.558919565	5.189299	1.4E-09
TNNC1	9.11821706	445.36404	3.338883189	8.802077003	8.802077003	-5.61009	1.4E-09
MYOM1	14.09179	614.75111	3.915692025	9.26620351	9.26620351	-5.44708	1.5E-09
LOC100515328	8.28928824	415.61802	3.215568059	8.702581435	8.702581435	-5.64787	1.5E-09
LOC100623866	35.6439394	1360.8805	5.195502704	10.41138438	10.41138438	-5.25474	1.5E-09
LOC102160883	120.194679	0	6.921182554	0	0	20	1.5E-09
MYHC	108.589676	3943.0004	6.775968083	11.94544398	11.94544398	-5.18234	1.6E-09
LOC102164566	0	120.63664	0	6.926434087	6.926434087	-20	1.6E-09
KCNMA1	2.48678647	213.17982	1.801898017	7.742678745	7.742678745	-6.42164	1.6E-09
LOC100622791	127.655039	4600.718	7.007364151	12.16795685	12.16795685	-5.17154	1.7E-09
LOC100513356	24.0389359	945.26246	4.646101346	9.886096578	9.886096578	-5.29727	1.7E-09
CCDC42	0	119.81036	0	6.916600414	6.916600414	-20	1.7E-09
CA14	4646.14606	129.7257	12.18212927	7.030399034	7.030399034	5.162498	1.7E-09
TSPAN8	209.718992	2.4788351	7.719176542	1.798604302	1.798604302	6.402651	1.7E-09
CD79B	118.536822	0	6.90131128	0	0	20	1.7E-09
LOC102160758	237.902572	3.3051135	7.900278578	2.106051269	2.106051269	6.169527	1.7E-09
LOC102166489	0	118.98409	0	6.906699252	6.906699252	-20	1.8E-09
FAM83A	117.707893	0	6.891272054	0	0	20	1.9E-09
LOC102160402	117.707893	0	6.891272054	0	0	20	1.9E-09
HNF4A	117.707893	0	6.891272054	0	0	20	1.9E-09
ACVR2B	1430.73115	38.835083	10.48354489	5.315967693	5.315967693	5.203248	1.9E-09
NFIX	64.6564482	2329.2787	6.036864803	11.18628681	11.18628681	-5.17095	1.9E-09
HOXA1	0	118.15781	0	6.89672967	6.89672967	-20	1.9E-09
CHRNA1	0	118.15781	0	6.89672967	6.89672967	-20	1.9E-09
ALDH3B1	41.4464412	1522.0048	5.407571694	10.57270474	10.57270474	-5.19858	1.9E-09
CD36	2.48678647	208.22215	1.801898017	7.708891783	7.708891783	-6.3877	2E-09
CACNA1I	344.834391	6.610227	8.433937531	2.927939483	2.927939483	5.70506	2E-09
PFAS	13290.2158	373.47782	13.69818546	8.548736477	8.548736477	5.153198	2E-09
RBP5	0	117.33153	0	6.886690713	6.886690713	-20	2E-09
LOC102161872	177.390768	1.6525567	7.478897148	1.407383613	1.407383613	6.746087	2E-09
REEP3	132.628612	4670.9516	7.062085132	12.18980962	12.18980962	-5.13825	2.1E-09
LOC102159968	0.82892882	148.73011	0.870998931	7.226220529	7.226220529	-7.48723	2.1E-09
CRYM	1135.63249	30.5723	10.15055014	4.980587446	4.980587446	5.215127	2.1E-09
LOC100621648	116.050035	0	6.870981559	0	0	20	2.1E-09
LOC100627331	147.549331	0.8262784	7.214798294	0.868906686	0.868906686	7.480354	2.1E-09
LOC102167737	2.48678647	206.56959	1.801898017	7.697451307	7.697451307	-6.3762	2.1E-09
AMY2	1380.16649	38.008805	10.43167152	5.285727902	5.285727902	5.182365	2.2E-09
LOC100736904	232.100071	3.3051135	7.864805631	2.106051269	2.106051269	6.133903	2.2E-09
LOC102160150	12.4339324	533.77583	3.747809764	9.062790447	9.062790447	-5.42388	2.2E-09
TMEM200A	6.63143059	342.07925	2.93195353	8.422398044	8.422398044	-5.68887	2.3E-09
LOC100621905	0	115.67897	0	6.86640077	6.86640077	-20	2.3E-09
SLC16A6	174.903982	1.6525567	7.45864433	1.407383613	1.407383613	6.72572	2.3E-09
DPPA2	174.075053	1.6525567	7.451829714	1.407383613	1.407383613	6.718866	2.4E-09
LOC100153940	106.931818	3705.8585	6.753976422	11.85598133	11.85598133	-5.11504	2.4E-09
SEMA3A	0	114.85269	0	6.856147779	6.856147779	-20	2.4E-09
MYL9	870.375265	30984.613	9.767150351	14.91931087	14.91931087	-5.15377	2.6E-09
LOC100625694	0	114.02642	0	6.845821397	6.845821397	-20	2.6E-09
SV2C	172.417195	1.6525567	7.438103147	1.407383613	1.407383613	6.70506	2.7E-09
LOC100518938	7590.50124	222.26888	12.8901695	7.80263838	7.80263838	5.093817	2.7E-09
IL1R1	60.5118041	2099.5733	5.942791385	11.03656744	11.03656744	-5.11674	2.7E-09
ZNF467	22.3810782	843.63022	4.547269558	9.722176052	9.722176052	-5.23626	2.7E-09
NETO2	411.977625	9.0890621	8.68991981	3.334720158	3.334720158	5.502291	2.8E-09
STX11	11.6050035	494.11447	3.655924617	8.951618292	8.951618292	-5.41203	2.8E-09
LOC100737762	16.5785765	653.58619	4.135746339	9.35443936	9.35443936	-5.30099	2.8E-09
ZFP57	358.097252	7.4365053	8.488230802	3.076645515	3.076645515	5.589583	2.8E-09
KCND2	251.994362	4.1313919	7.982961427	2.359350201	2.359350201	5.93062	2.9E-09
NT5DC3	645.735554	16.525567	9.337032113	4.13138925	4.13138925	5.288172	2.9E-09
LOC102161753	111.905391	0	6.818970566	0	0	20	2.9E-09
CDHR1	111.905391	0	6.818970566	0	0	20	2.9E-09
EPS8L3	111.905391	0	6.818970566	0	0	20	2.9E-09
IFITM1	68.8010924	2355.7196	6.125177709	11.20256443	11.20256443	-5.09759	3E-09
F3	21.5521494	808.10025	4.495193036	9.660174654	9.660174654	-5.22863	3E-09
LOC100738565	510.620155	12.394176	8.99892929	3.74353388	3.74353388	5.364516	3.1E-09
LOC102157775	111.076462	0	6.808339514	0	0	20	3.1E-09
LOC100737374	218.008281	7370.4031	7.774841608	12.84772353	12.84772353	-5.07929	3.1E-09
LOC100620538	196.456131	2.4788351	7.625388355	1.798604302	1.798604302	6.308401	3.1E-09
POU4F1	222.981854	3.3051135	7.807238043	2.106051269	2.106051269	6.076083	3.2E-09
PSP-I	0	111.54758	0	6.814391229	6.814391229	-20	3.2E-09
LOC100521376	6945.59461	207.39587	12.76209019	7.703182885	7.703182885	5.065639	3.2E-09
RBP4	34.8150106	1226.1971	5.162492464	10.26115127	10.26115127	-5.13834	3.2E-09
LOC102167733	1.65785765	169.38707	1.410263837	7.412672017	7.412672017	-6.67486	3.3E-09
CX3CL1	4.97357294	275.97698	2.578594101	8.113622247	8.113622247	-5.79412	3.4E-09
LOC100624137	0	110.7213	0	6.803760479	6.803760479	-20	3.4E-09
AKAP5	1130.65892	32.224857	10.14422348	5.054191063	5.054191063	5.132846	3.4E-09
RBP1	3382.0296	101.63224	11.72410008	6.681340183	6.681340183	5.056459	3.4E-09
CLMP	132.628612	4428.0258	7.062085132	12.11277368	12.11277368	-5.0612	3.4E-09
LOC100738546	48.9068006	1669.9086	5.641164514	10.70641709	10.70641709	-5.09359	3.5E-09
TRPC4	4.14464412	248.70979	2.363071282	7.964108573	7.964108573	-5.90707	3.5E-09
LOC100623862	5.80250177	300.76533	2.766065426	8.23728324	8.23728324	-5.69582	3.5E-09
SPRN	55.5382312	1881.4359	5.821154844	10.87838499	10.87838499	-5.08221	3.5E-09
AK5	503.159796	12.394176	8.977737265	3.74353388	3.74353388	5.343282	3.6E-09
SLC6A3	109.418605	0	6.786839466	0	0	20	3.6E-09
ALOXE3	109.418605	0	6.786839466	0	0	20	3.6E-09
CMKLR1	0	109.89502	0	6.793050813	6.793050813	-20	3.6E-09
CRLF1	130.970754	4342.9191	7.044074441	12.08478153	12.08478153	-5.05135	3.7E-09
IRF7	137.602185	4557.7515	7.114806188	12.15442305	12.15442305	-5.04975	3.7E-09
LOC100626670	1536.00511	45.44531	10.58590625	5.537461031	5.537461031	5.078908	3.8E-09
LOC100514841	71.2878788	2366.4613	6.175681852	11.2091251	11.2091251	-5.05293	3.9E-09
LOC102161875	107.760747	0	6.765014156	0	0	20	4.1E-09
LOC102166597	107.760747	0	6.765014156	0	0	20	4.1E-09
ZSCAN10	107.760747	0	6.765014156	0	0	20	4.1E-09
IQCA1P1	107.760747	0	6.765014156	0	0	20	4.1E-09
LOC100620517	0	108.24247	0	6.771389985	6.771389985	-20	4.2E-09
ST8SIA1	20.7232206	752.7396	4.441166102	9.557922374	9.557922374	-5.18283	4.3E-09
MAPRE3	21.5521494	775.87539	4.495193036	9.601539402	9.601539402	-5.16992	4.4E-09
IFIH1	77.0903806	2520.9753	6.287072939	11.30033844	11.30033844	-5.03129	4.4E-09
ARID5A	14.09179	546.17	3.915692025	9.095845332	9.095845332	-5.27642	4.5E-09
CCDC85A	1252.51145	37.182527	10.29175946	5.254840671	5.254840671	5.074055	4.5E-09
GLS2	566.158387	14.873011	9.147607873	3.98850389	3.98850389	5.250437	4.5E-09
LOC100737630	565.329458	14.873011	9.145497764	3.98850389	3.98850389	5.248323	4.6E-09
CACNA2D2	3328.97816	103.2848	11.701297	6.704385035	6.704385035	5.01038	4.6E-09
HMHA1	5879.59215	182.60752	12.52174572	7.52048134	7.52048134	5.008898	4.7E-09
LOC100523440	106.102889	0	6.742853591	0	0	20	4.7E-09
PTPRE	1.65785765	162.77684	1.410263837	7.35558754	7.35558754	-6.61743	4.8E-09
CXCL11	0	106.58991	0	6.749398975	6.749398975	-20	4.8E-09
LOC102159413	0	106.58991	0	6.749398975	6.749398975	-20	4.8E-09
LOC102164998	0	106.58991	0	6.749398975	6.749398975	-20	4.8E-09
PARP14	87.0375265	2802.7362	6.460046706	11.45313492	11.45313492	-5.00905	5E-09
SLA-5	13.2628612	513.11887	3.834191515	9.005958152	9.005958152	-5.27383	5.1E-09
SGCD	4.97357294	263.5828	2.578594101	8.047575471	8.047575471	-5.72783	5.1E-09
ANK1	806.547745	23.135794	9.65740375	4.593102408	4.593102408	5.123561	5.2E-09
M1AP	335.716174	7.4365053	8.39538921	3.076645515	3.076645515	5.496473	5.2E-09
LOC100518541	185.680057	2.4788351	7.544423999	1.798604302	1.798604302	6.227013	5.3E-09
ZNF311	185.680057	2.4788351	7.544423999	1.798604302	1.798604302	6.227013	5.3E-09
LOC102164089	5.80250177	287.54487	2.766065426	8.172651889	8.172651889	-5.63097	5.3E-09
WT1	1379.33756	42.140197	10.43080541	5.430960858	5.430960858	5.032635	5.4E-09
LOC100736608	0.82892882	133.8571	0.870998931	7.075287628	7.075287628	-7.33523	5.4E-09
CKMT2	104.445032	0	6.720347312	0	0	20	5.5E-09
C2CD4D	104.445032	0	6.720347312	0	0	20	5.5E-09
LOC102165956	104.445032	0	6.720347312	0	0	20	5.5E-09
POPDC3	0	104.93735	0	6.727067559	6.727067559	-20	5.5E-09
FOXH1	1727.48767	53.708094	10.7552946	5.773682393	5.773682393	5.007392	5.6E-09
MIR214	8.28928824	360.25737	3.215568059	8.49688321	8.49688321	-5.44164	5.6E-09
FOLR2	23.2100071	806.44769	4.597531595	9.65722499	9.65722499	-5.11876	5.6E-09
WNT5B	35.6439394	1177.4467	5.195502704	10.20267077	10.20267077	-5.04586	5.8E-09
JAM2	0.82892882	132.20454	0.870998931	7.057499439	7.057499439	-7.31731	6.1E-09
ITGA1	15.7496477	575.91603	4.066058842	9.172217528	9.172217528	-5.19247	6.2E-09
LRP2	102.787174	0	6.697484358	0	0	20	6.3E-09
FAT4	11.6050035	452.80055	3.655924617	8.82591454	8.82591454	-5.28606	6.3E-09
FOXA3	156.667548	1.6525567	7.300741934	1.407383613	1.407383613	6.566863	6.4E-09
LOC102165113	254.481149	4.9576702	7.997073033	2.574748269	2.574748269	5.681753	6.7E-09
ECM1	32.3282241	1061.7677	5.058672541	10.05361058	10.05361058	-5.03753	6.7E-09
LOC102163342	101.958245	0	6.685915561	0	0	20	6.8E-09
FAM71E1	9.11821706	376.78294	3.338883189	8.561413734	8.561413734	-5.36884	6.8E-09
LOC100515902	50.5646582	1595.5435	5.688310694	10.64073618	10.64073618	-4.97977	7.2E-09
TNFAIP3	64.6564482	2016.1192	6.036864803	10.97808065	10.97808065	-4.96264	7.3E-09
SOX7	101.129317	0	6.674253245	0	0	20	7.3E-09
RNASEL	16.5785765	589.96276	4.135746339	9.206923404	9.206923404	-5.15323	7.3E-09

LOC100524114	291.350875	14.046732	8.191557101	3.911378308	3.911378308	4.374451	7.4E-06
LOC100737399	2.76161967	218.96377	1.911353989	7.781122098	7.781122098	-6.30903	7.4E-06
LOC102163792	0	123.94176	0	6.965111899	6.965111899	-20	7.5E-06
FAM83F	386.626754	19.830681	8.598524339	4.380638094	4.380638094	4.285135	7.5E-06
KNDC1	2.76161967	218.13749	1.911353989	7.775692512	7.775692512	-6.30358	7.6E-06
KIF1A	102.179928	3651.3241	6.689018532	11.83459909	11.83459909	-5.15924	7.6E-06
LOC100628233	53.8515836	0	5.777461367	0	0	20	7.6E-06
LOC102166884	53.8515836	0	5.777461367	0	0	20	7.6E-06
LOC100621290	53.8515836	0	5.777461367	0	0	20	7.6E-06
LOC100623445	619.983616	33.877413	9.278411395	5.124221138	5.124221138	4.193834	7.7E-06
TMEM45A	28.9970066	1079.1196	4.906746634	10.07697529	10.07697529	-5.21781	7.9E-06
TPM1	3149.62724	128639.97	11.62142336	16.97299072	16.97299072	-5.35201	8E-06
MLXIPL	84.2294	1.6525567	6.413279271	1.407383613	1.407383613	5.671552	8E-06
LOC100737919	1.38080984	170.21334	1.251452392	7.419651342	7.419651342	-6.94569	8E-06
TMEM26	0	122.2892	0	6.945902605	6.945902605	-20	8.1E-06
LOC100515339	6609.93668	373.47782	12.69063898	8.548736477	8.548736477	4.145542	8.1E-06
RNASE1	247.164961	11.567897	7.95515562	3.65167138	3.65167138	4.417276	8.2E-06
XIRP2	0	121.46292	0	6.936201185	6.936201185	-20	8.4E-06
LOC100522432	1891.70948	109.06875	10.88623726	6.782261052	6.782261052	4.116381	8.4E-06
LOC100515458	69.0404918	0.8262784	6.130117309	0.868906686	0.868906686	6.384671	8.4E-06
GARNL3	151.889082	5.7839486	7.256341575	2.762125239	2.762125239	4.71482	8.7E-06
ADM2	0	120.63664	0	6.926434087	6.926434087	-20	8.7E-06
LOC102164566	0	120.63664	0	6.926434087	6.926434087	-20	8.7E-06
PLEKHF1	62.1364426	2174.7647	5.98040107	11.08730681	11.08730681	-5.12928	8.7E-06
HS3ST5	191.932567	8.2627837	7.591952882	3.211445828	3.211445828	4.537828	8.7E-06
FN3K	1540.98378	89.238064	10.59057187	6.495664213	6.495664213	4.110049	8.8E-06
LOC102162560	9.66566885	431.31731	3.414902536	8.755946793	8.755946793	-5.47974	8.9E-06
GUCY1A2	258.211439	12.394176	8.017985577	3.74353388	3.74353388	4.380819	8.9E-06
STEAP2	1.38080984	166.90823	1.251452392	7.391529145	7.391529145	-6.9174	9E-06
CCDC42	0	119.81036	0	6.916600414	6.916600414	-20	9E-06
LOC100514839	851.959669	31767.098	9.736333717	14.95529111	14.95529111	-5.2206	9.1E-06
LOC100157489	110.464787	3.3051135	6.800444207	2.106051269	2.106051269	5.062743	9.2E-06
LOC100738690	96.6566885	2.4788351	6.609646952	1.798604302	1.798604302	5.285135	9.3E-06
LOC100622674	52.4707738	0	5.740678649	0	0	20	9.3E-06
LOC102159414	52.4707738	0	5.740678649	0	0	20	9.3E-06
LOC100626938	52.4707738	0	5.740678649	0	0	20	9.3E-06
LOC100512117	52.4707738	0	5.740678649	0	0	20	9.3E-06
UBASH3A	52.4707738	0	5.740678649	0	0	20	9.3E-06
CSF2RB	52.4707738	0	5.740678649	0	0	20	9.3E-06
KRT13	52.4707738	0	5.740678649	0	0	20	9.3E-06
LIPC	52.4707738	0	5.740678649	0	0	20	9.3E-06
LOC102166489	0	118.98409	0	6.906699252	6.906699252	-20	9.4E-06
GDAP1	0	118.98409	0	6.906699252	6.906699252	-20	9.4E-06
LOC100737142	5.52323934	296.63394	2.705588563	8.217395218	8.217395218	-5.74703	9.4E-06
TNFRSF1B	77.3253508	2657.3112	6.291407421	11.37629431	11.37629431	-5.10288	9.5E-06
CYBRD1	115.988026	3986.7931	6.870217066	11.96137486	11.96137486	-5.10318	9.5E-06
FAM212A	28.9970066	1042.7633	4.906746634	10.02757887	10.02757887	-5.16836	9.6E-06
LOC100625277	806.392944	46.271589	9.657127169	5.56290145	5.56290145	4.123284	9.7E-06
CHRNA1	0	118.15781	0	6.89672967	6.89672967	-20	9.8E-06
ADCY4	1148.83378	66.928548	10.16720961	6.085946114	6.085946114	4.101405	9.8E-06
LOC100521067	30.3778164	1082.4247	4.971673052	10.08138313	10.08138313	-5.1551	9.8E-06
FZD3	1118.45597	65.275991	10.12858207	6.05041444	6.05041444	4.098812	1E-05
LOC102160653	40.0434852	1390.6265	5.359081339	10.44255634	10.44255634	-5.11802	1E-05
FILIP1L	40.0434852	1389.8002	5.359081339	10.44169949	10.44169949	-5.11717	1E-05
FAM65C	0	117.33153	0	6.886690713	6.886690713	-20	1E-05
PTH1R	2.76161967	206.56959	1.911353989	7.697451307	7.697451307	-6.22497	1E-05
LOC100525496	81.4677803	1.6525567	6.365758672	1.407383613	1.407383613	5.623458	1E-05
LOC100621915	582.701751	33.051135	9.189087585	5.089630977	5.089630977	4.139986	1.1E-05
CDH13	16.569718	629.62412	4.135019133	9.300636539	9.300636539	-5.24787	1.1E-05
LOC102166397	9.66566885	415.61802	3.414902536	8.702581435	8.702581435	-5.42624	1.1E-05
LOC100621905	0	115.67897	0	6.86640077	6.86640077	-20	1.1E-05
KBTBD11	0	115.67897	0	6.86640077	6.86640077	-20	1.1E-05
LOC102165781	474.998584	26.440908	8.89481347	4.77825631	4.77825631	4.16708	1.1E-05
ARHGEF26	1763.29416	104.93735	10.78487541	6.727067559	6.727067559	4.070673	1.1E-05
MYLPF	19.3313377	713.07823	4.345633235	9.479938335	9.479938335	-5.20505	1.1E-05
IL17RD	8.28485901	371.82527	3.214880004	8.542355828	8.542355828	-5.488	1.1E-05
THEMIS2	394.911613	21.483238	8.629034575	4.490777898	4.490777898	4.200246	1.1E-05
FBXO32	27.6161967	970.05081	4.838760036	9.923402974	9.923402974	-5.13447	1.1E-05
LOC102164089	5.52323934	287.54487	2.705588563	8.172651889	8.172651889	-5.70213	1.1E-05
LOC102167554	107.703167	3.3051135	6.764250165	2.106051269	2.106051269	5.026217	1.1E-05
GABRA1	133.938554	4.9576702	7.076158798	2.574748269	2.574748269	4.755765	1.1E-05
SEMA3A	0	114.85269	0	6.856147779	6.856147779	-20	1.1E-05
LOC100739707	0	114.85269	0	6.856147779	6.856147779	-20	1.1E-05
LOC100622474	51.0899639	0	5.702933533	0	0	20	1.2E-05
SLC6A1	51.0899639	0	5.702933533	0	0	20	1.2E-05
CLDN8	51.0899639	0	5.702933533	0	0	20	1.2E-05
LOC100628123	51.0899639	0	5.702933533	0	0	20	1.2E-05
ASB18	51.0899639	0	5.702933533	0	0	20	1.2E-05
LOC102160070	51.0899639	0	5.702933533	0	0	20	1.2E-05
LOC100037976	2522.73957	151.20894	11.30134733	7.249909307	7.249909307	4.060376	1.2E-05
ARPP21	0	114.02642	0	6.845821397	6.845821397	-20	1.2E-05
CALD1	1986.98535	73879.202	10.95709141	16.17290019	16.17290019	-5.21652	1.2E-05
LOC102158695	1.38080984	158.64545	1.251452392	7.318727602	7.318727602	-6.84415	1.2E-05
LOC102157425	80.0869705	1.6525567	6.341398207	1.407383613	1.407383613	5.598796	1.2E-05
LOC100155404	80.0869705	1.6525567	6.341398207	1.407383613	1.407383613	5.598796	1.2E-05
TSPAN7	9.66566885	409.00779	3.414902536	8.679507525	8.679507525	-5.40311	1.2E-05
NMB	274.781157	14.046732	8.107380078	3.911378308	3.911378308	4.289977	1.2E-05
PDE8B	13.8080984	532.94955	3.888314478	9.060559625	9.060559625	-5.27041	1.2E-05
FBN2	403.196472	13763.319	8.658912919	13.7486456	13.7486456	-5.0932	1.2E-05
LOC100737019	17.9505279	656.06503	4.24416613	9.359892345	9.359892345	-5.19174	1.3E-05
CTSC	182.266898	6028.527	7.517802419	12.55782912	12.55782912	-5.04768	1.3E-05
LOC100626363	4.14242951	240.44701	2.362450112	7.915562765	7.915562765	-5.8591	1.3E-05
16S	13826.0489	564649.72	13.75520565	19.1069992	19.1069992	-5.3519	1.3E-05
FBP1	157.412321	6.610227	7.307540742	2.927939483	2.927939483	4.573705	1.3E-05
LOC100523785	157.412321	6.610227	7.307540742	2.927939483	2.927939483	4.573705	1.3E-05
IL7	2.76161967	197.48053	1.911353989	7.632853688	7.632853688	-6.16005	1.3E-05
PSP-I	0	111.54758	0	6.814391229	6.814391229	-20	1.3E-05
LOC102164681	4.14242951	237.96817	2.362450112	7.900674664	7.900674664	-5.84415	1.3E-05
LOC100521565	131.176934	4.9576702	7.04632663	2.574748269	2.574748269	4.725708	1.4E-05
GJA1	980.374983	34378.138	9.938660685	15.06924575	15.06924575	-5.13201	1.4E-05
PTGIS	71.8021115	2316.0583	6.185908388	11.17807861	11.17807861	-5.0115	1.4E-05
MPP7	283.066016	14.873011	8.150082438	3.98850389	3.98850389	4.25037	1.4E-05
GVIN1	2.76161967	195.82797	1.911353989	7.620791468	7.620791468	-6.14793	1.4E-05
SLC5A5	104.941548	3.3051135	6.727124677	2.106051269	2.106051269	4.988742	1.4E-05
GALNT12	2485.4577	152.03522	11.27987617	7.257719911	7.257719911	4.031034	1.4E-05
TBX18	11.0464787	437.92754	3.590539588	8.777838973	8.777838973	-5.30903	1.4E-05
SGIP1	49.7091541	0	5.664174303	0	0	20	1.4E-05
ACVR1C	49.7091541	0	5.664174303	0	0	20	1.4E-05
S100A7	49.7091541	0	5.664174303	0	0	20	1.4E-05
CD70	6616.84073	398.26618	12.69214486	8.641207046	8.641207046	4.054338	1.4E-05
MSMP	122.892075	3930.6062	6.9529401	11.94090311	11.94090311	-4.99929	1.5E-05
CMKLR1	0	109.89502	0	6.793050813	6.793050813	-20	1.5E-05
SHC4	0	109.89502	0	6.793050813	6.793050813	-20	1.5E-05
CCDC88B	255.44982	13.220454	8.002532746	3.829895615	3.829895615	4.272196	1.5E-05
FLNC	636.553334	21473.322	9.316402225	14.39032498	14.39032498	-5.07612	1.5E-05
LOC100511379	662.787861	39.661362	9.374580306	5.34558663	5.34558663	4.062743	1.5E-05
LOC100737860	1.38080984	152.03522	1.251452392	7.257719911	7.257719911	-6.78275	1.5E-05
RUSC2	128.415315	4077.6838	7.015864542	11.99388794	11.99388794	-4.98886	1.5E-05
AARD	103.560738	3.3051135	6.708197415	2.106051269	2.106051269	4.969634	1.6E-05
LAMA2	0	108.24247	0	6.771389985	6.771389985	-20	1.6E-05
KLK1	115.988026	4.1313919	6.870217066	2.359350201	2.359350201	4.811204	1.6E-05
LOC100153192	30.3778164	992.36032	4.971673052	9.956173315	9.956173315	-5.02977	1.6E-05
LOC100154071	190.551757	9.0890621	7.581590451	3.334720158	3.334720158	4.389908	1.6E-05
LOC100737780	63.5172524	0.8262784	6.011613095	0.868906686	0.868906686	6.264377	1.6E-05
LAMB1	865.767767	29156.059	9.759501694	14.83155757	14.83155757	-5.07367	1.7E-05
GLIPR1	0	107.41619	0	6.76043638	6.76043638	-20	1.7E-05
ADAMTS4	23.4737672	781.65934	4.613164285	9.612240686	9.612240686	-5.05742	1.7E-05
LOC574051	251.30739	13.220454	7.979038653	3.829895615	3.829895615	4.248609	1.7E-05
LOC102166598	1.38080984	148.73011	1.251452392	7.226220529	7.226220529	-6.75104	1.7E-05
CXCL11	0	106.58991	0	6.749398975	6.749398975	-20	1.7E-05
LOC102159413	0	106.58991	0	6.749398975	6.749398975	-20	1.7E-05
LOC100738449	0	106.58991	0	6.749398975	6.749398975	-20	1.7E-05
LOC102165979	0	106.58991	0	6.749398975	6.749398975	-20	1.7E-05
C13H3orf56	48.3283443	0	5.624344958	0	0	20	1.8E-05
IL23RA	48.3283443	0	5.624344958	0	0	20	1.8E-05
LOC100738968	0	105.76363	0	6.738276475	6.738276475	-20	1.8E-05
LOC100525666	26.2353869	852.71928	4.767410456	9.73761795	9.73761795	-5.02248	1.8E-05
DENND3	2449.55665	154.51406	11.25889378	7.280901168	7.280901168	3.986711	1.8E-05
HS3ST1	187.790138	9.0890621	7.560639591	3.334720158	3.334720158	4.368846	1.8E-05
ZNF211	8.28485901	340.42669	3.214880004	8.415432027	8.415432027	-5.36072	1.9E-05
FOXL1	13.8080984	493.28819	3.888314478	8.94920862	8.94920862	-5.15884	1.9E-05
CAMK2B	113.226407	4.1313919	6.835752397	2.359350201	2.359350201	4.776439	1.9E-05
DLC1	52.4707738	1607.1114	5.740678649	10.65115167	10.65115167	-4.93681	1.9E-05
WNT5A	11.0464787	413.96546	3.590539588	8.696847462	8.696847462	-5.22785	2E-05
CPNE5	186.409328	9.0890621	7.550048951	3.334720158	3.334720158	4.358199	2E-05
LOC102165933	0	104.11107	0	6.715770874	6.715770874	-20	2E-05
LOC100518848	480.521823	28.919743	8.911457373	4.903025878	4.903025878	4.054475	2E-05
IER3	219.548764	6745.7366	7.784953864	12.71997413	12.71997413	-4.94136	2E-05
YPEL2	35.9010557	1114.6495	5.205590187	10.12366817	10.12366817	-4.95642	2E-05
ID3	157.412321	4783.3255	7.307540742	12.22409983	12.22409983	-4.92539	2E-05
VSTM4	89.7526393	2702.7566	6.503867696	11.40074954	11.40074954	-4.91233	2E-05
CMPK2	111.845597	3366.2581	6.818206315	11.71735859	11.71735859	-4.91157	2.1E-05
DCHS1	325.871121	10073.986	8.352578112	13.2984902	13.2984902	-4.95019	2.1E-05
LOC100626144	330.013551	19.004403	8.370746468	4.322245637	4.322245637	4.11812	2.1E-05
TMEM8C	2.76161967	181.78124	1.911353989	7.513974209	7.513974209	-6.04054	2.1E-05
SLC36A4	26.2353869	829.58349	4.767410456	9.697981376	9.697981376	-4.9828	2.1E-05
AMPD3	5.52323934	256.97257	2.705588563	8.011073883	8.011073883	-5.53996	2.1E-05
PDZK1IP1	439.097528	26.440908	8.781679457	4.77825631	4.77825631	4.053698	2.1E-05
LOC100628148	0	102.45852	0	6.692908622	6.692908622	-20	2.1E-05
LOC100623513	0	102.45852	0	6.692908622	6.692908622	-20	2.1E-05
VASN	298.254925	9142.7702	8.225231178	13.15857343	13.15857343	-4.93801	2.2E-05
LOC100738012	3552.82371	227.22655	11.7951564	7.834322836	7.834322836	3.966763	2.2E-05
FRMD4A	2.76161967	180.95496	1.911353989	7.507437596	7.507437596	-6.03397	2.2E-05
JAM3	187.790138	5652.5703	7.560639591	12.46494653	12.46494653	-4.91171	2.2E-05
RNASE6	46.9475344	0	5.583384725	0	0	20	2.2E-05
LOC102168166	46.9475344	0	5.583384725	0	0	20	2.2E-05
LOC100524561	46.9475344	0	5.583384725	0	0	20	2.2E-05
LOC102162598	46.9475344	0	5.583384725	0	0	20	2.2E-05
RHPN2	1032.84576	66.10227	10.01380525	6.068289661	6.068289661	3.965781	2.2E-05
NTRK1	8.28485901	328.03251	3.214880004	8.362086342	8.362086342	-5.30722	2.3E-05
PRAP1	60.7556328	0.8262784	5.948498827	0.868906686	0.868906686	6.200246	2.3E-05
LOC100627133	17.9505279	588.3102	4.24416613	9.202883429	9.202883429	-5.03448	2.3E-05
LOC102166035	0	100.80596	0	6.669678233	6.669678233	-20	2.3E-05
LOC102161934	24.854577	776.70167	4.692347799	9.603073025	9.603073025	-4.96578	2.4E-05
LOC100515533	110.464787	4.1313919	6.800444207	2.359350201	2.359350201	4.740815	2.4E-05
PDE5	9.66566885	361.08365	3.414902536	8.500179216	8.500179216	-5.22332	2.4E-05
SYNPO2L	4.14242951	214.0061	2.362450112	7.74823377	7.74823377	-5.69103	2.5E-05
PCDH19	0	99.979683	0	6.657921243	6.657921243	-20	2.5E-05
LOC100739139	73.1829213	1.6525567	6.213015177	1.407383613	1.407383613	5.468735	2.5E-05
CD248	251.30739	7462.9463	7.979038653	12.86572288	12.86572288	-4.89222	2.5E-05
PEA15	658.646292	20362.804	9.365548835	14.31371948	14.31371948	-4.95029	2.5E-05
RASSF10	121.511266	4.9576702	6.936770608	2.574748269	2.574748269	4.615284	2.5E-05
AFGF	0	99.153405	0	6.646067655	6.646067655	-20	2.6E-05
RPS6KA2	226.452813	6651.5409	7.829423468	12.69968976	12.69968976	-4.87641	2.6E-05
LOC100739291	2.76161967	175.17101	1.911353989	7.460832769	7.460832769	-5.98711	2.6E-05
TINAGL1	51.0899639	1483.996	5.702933533	10.53624329	10.53624329	-4.8603	2.6E-05
SDK1	0	98.327126	0	6.634115866	6.634115866	-20	2.7E-05
GGT5	17.9505279	573.43719	4.24416613	9.166005344	9.166005344	-4.99754	2.7E-05
FSHR	45.5667246	0	5.541227504	0	0	20	2.7E-05
LOC102162836	45.5667246	0	5.541227504	0	0	20	2.7E-05
WAP-3	45.5667246	0	5.541227504	0	0	20	2.7E-05
LOC102158429	45.5667246	0	5.541227504	0	0	20	2.7E-05
LOC100517891	45.5667246	0	5.541227504	0	0	20	2.7E-05
LOC100625208	45.5667246	0	5.541227504	0	0	20	2.7E-05
OBSCN	9.66566885	353.64714	3.414902536	8.470240516	8.470240516	-5.1933	2.7E-05
LOC102161923	59.3748229	0.8262784	5.915875148	0.868906686	0.868906686	6.16708	2.8E-05
CELF5	59.3748229	0.8262784	5.915875148	0.868906686	0.868906686	6.16708	2.8E-05
KIAA1211	17.9505279	570.13208	4.24416613	9.157680603	9.157680603	-4.9892	2.8E-05
LOC102157883	84.2294	2.4788351	6.413279271	1.798604302	1.798604302	5.08659	2.8E-05
AR	0	97.500848	0	6.622064238	6.622064238	-20	2.8E-05
FGF19	0	97.500848	0	6.622064238	6.622064238	-20	2.8E-05
GULO	6.90404918	280.10837	2.982591923	8.13498259	8.13498259	-5.3424	2.8E-05
ROR2	131.176934	3742.2147	7.04632663	11.8700621	11.8700621	-4.83431	2.8E-05
CHAC1	175.362849	5014.6834	7.462402878	12.29223058	12.29223058	-4.83774	2.9E-05
LOC102165476	0	96.674569	0	6.609911086	6.609911086	-20	3E-05
LOC100623420	122.892075	3470.3692	6.9529401	11.76128908	11.76128908	-4.81963	3E-05
LOC100621773	430.812669	12664.369	8.75426176	13.62860144	13.62860144	-4.87757	3E-05
EBF2	8.28485901	312.33322	3.214880004	8.291553945	8.291553945	-5.23647	3E-05
ID4	41.4242951	1186.5357	5.406818783	10.21375522	10.21375522	-4.84013	3E-05
LOC100622925	95.2758787	3.3051135	6.58910248	2.106051269	2.106051269	4.849339	3E-05
SEMA3D	225.072003	6419.3567	7.820638529	12.64843773	12.64843773	-4.83397	3.1E-05
LOC100518411	1.38080984	133.8571	1.251452392	7.075287628	7.075287628	-6.59904	3.1E-05
LOC100622678	0	95.848291	0	6.597654688	6.597654688	-20	3.1E-05
ZNF804A	0	95.848291	0	6.597654688	6.597654688	-20	3.1E-05
LOC102158140	0	95.848291	0	6.597654688	6.597654688	-20	3.1E-05
LOC102159096	338.29841	20.656959	8.406410858	4.436758793	4.436758793	4.033597	3.1E-05
LOC102167733	2.76161967	169.38707	1.911353989	7.412672017	7.412672017	-5.93867	3.1E-05
PDE3B	820.201042	54.534373	9.681591649	5.795309087	5.795309087	3.91074	3.2E-05
MICAL2	49.7091541	1398.063	5.664174303	10.45024522	10.45024522	-4.81377	3.2E-05
CCDC69	2.76161967	168.56079	1.911353989	7.405658764	7.405658764	-5.93161	3.2E-05
ANGPT1	12.4272885	413.96546	3.747096094	8.696847462	8.696847462	-5.05793	3.2E-05
LOC102161276	0	95.022013	0	6.585293272	6.585293272	-20	3.2E-05
LOC102162312	0	95.022013	0	6.585293272	6.585293272	-20	3.2E-05
SLC2A13	0	95.022013	0	6.585293272	6.585293272	-20	3.2E-05
ENTPD8	70.4213016	1.6525567	6.158282521	1.407383613	1.407383613	5.41324	3.3E-05
LOC100512920	70.4213016	1.6525567	6.158282521	1.407383613	1.407383613	5.41324	3.3E-05
SERPINE1	352.106508	10062.418	8.463959601	13.29683278	13.29683278	-4.83682	3.4E-05
SWAP70	115.988026	3205.9601	6.870217066	11.64699068	11.64699068	-4.78871	3.4E-05
SPEG	64.8980623	1794.6766	6.042164139	10.81031185	10.81031185	-4.7894	3.4E-05
MAOB	415.623761	26.440908	8.70260131	4.77825631	4.77825631	3.974435	3.4E-05
LOC100517858	46.9475344	1310.4775	5.583384725	10.35697734	10.35697734	-4.8029	3.4E-05
LOC100513982	0	94.195734	0	6.572825024	6.572825024	-20	3.4E-05
KLHL30	0	94.195734	0	6.572825024	6.572825024	-20	3.4E-05
LOC100511475	93.8950688	3.3051135	6.568261215	2.106051269	2.106051269	4.828278	3.4E-05
ATP6V0D2	44.1859147	0	5.497801224	0	0	20	3.4E-05
LOC100624460	44.1859147	0	5.497801224	0	0	20	3.4E-05
LOC102164033	44.1859147	0	5.497801224	0	0	20	3.4E-05
TNS4	44.1859147	0	5.497801224	0	0	20	3.4E-05
LOC100515669	44.1859147	0	5.497801224	0	0	20	3.4E-05
GRB7	44.1859147	0	5.497801224	0	0	20	3.4E-05
KCTD12	2.76161967	166.08195	1.911353989	7.384412099	7.384412099	-5.91024	3.5E-05
ACSL5	24.854577	723.81985	4.692347799	9.501478663	9.501478663	-4.86405	3.5E-05
SLIT2	95.2758787	2606.9083	6.58910248	11.34867741	11.34867741	-4.77408	3.5E-05
LOC102163771	0	93.369456	0	6.560248082	6.560248082	-20	3.6E-05
LOC102157709	150.508272	7.4365053	7.243252754	3.076645515	3.076645515	4.339074	3.6E-05
LOC102165155	207.121475	11.567897	7.70128203	3.65167138	3.65167138	4.162279	3.6E-05
SOX11	71.8021115	1957.4535	6.185908388	10.93549913	10.93549913	-4.76881	3.6E-05
THY1	1473.3241	44528.141	10.52583799	15.44246218	15.44246218	-4.91757	3.6E-05
LOC102161162	81.4677803	2.4788351	6.365758672	1.798604302	1.798604302	5.038495	3.6E-05
RANBP3L	2.76161967	164.4294	1.911353989	7.370071807	7.370071807	-5.89581	3.7E-05
LOC102157822	2.76161967	164.4294	1.911353989	7.370071807	7.370071807	-5.89581	3.7E-05
SCG2	0	92.543178	0	6.547560532	6.547560532	-20	3.7E-05
LOC100624621	4.14242951	198.30681	2.362450112	7.63884719	7.63884719	-5.58111	3.7E-05
TRIM24	8280.71659	552.78023	13.01571412	9.113169742	9.113169742	3.904978	3.8E-05
LOC100525624	2.76161967	163.60312	1.911353989	7.362847851	7.362847851	-5.88854	3.8E-05
ADAM22	238.880102	14.046732	7.906169679	3.911378308	3.911378308	4.08798	3.9E-05
LOC102164270	13.8080984	434.62242	3.888314478	8.766934408	8.766934408	-4.97618	3.9E-05
LOC100518277	8.28485901	298.28649	3.214880004	8.225383356	8.225383356	-5.17008	3.9E-05
PTPRE	2.76161967	162.77684	1.911353989	7.35558754	7.35558754	-5.88124	3.9E-05
B3GNT9	2.76161967	162.77684	1.911353989	7.35558754	7.35558754	-5.88124	3.9E-05
IRX4	1.38080984	128.07315	1.251452392	7.012045083	7.012045083	-6.53531	3.9E-05
SLC7A2	1.38080984	128.07315	1.251452392	7.012045083	7.012045083	-6.53531	3.9E-05
PIK3CD	1056.31952	72.712497	10.04619571	6.20383732	6.20383732	3.860699	4E-05
LOC100519977	5.52323934	229.70539	2.705588563	7.849907883	7.849907883	-5.37813	0.00004
TIMP2	2250.72003	68241.504	11.13681175	16.05838297	16.05838297	-4.92219	4.1E-05
LOC102161722	0	90.890621	0	6.521845711	6.521845711	-20	4.1E-05
LOC100626465	0	90.890621	0	6.521845711	6.521845711	-20	4.1E-05
LOC102162178	0	90.890621	0	6.521845711	6.521845711	-20	4.1E-05
LOC102166299	0	90.890621	0	6.521845711	6.521845711	-20	4.1E-05
CLU	4364.73989	136815.17	12.09201046	17.06187925	17.06187925	-4.97019	4.1E-05
LOC100738391	2.76161967	161.12428	1.911353989	7.340956379	7.340956379	-5.86652	4.1E-05
MAN1C1	164.316371	4399.1061	7.369085785	12.10332258	12.10332258	-4.74266	4.2E-05
LOC102162237	1.38080984	126.42059	1.251452392	6.993454623	6.993454623	-6.51657	4.2E-05
CDCA7	11873.5838	795.70607	13.53558933	9.63790376	9.63790376	3.899376	4.3E-05
ARHGAP22	11.0464787	360.25737	3.590539588	8.49688321	8.49688321	-5.02737	4.3E-05
PTGIR	0	90.064343	0	6.508814353	6.508814353	-20	4.3E-05
LOC100514834	0	90.064343	0	6.508814353	6.508814353	-20	4.3E-05

LOC100520093	385.9360666	10.741619	8.5959514	3.553559423	3.553559423	5.167079	1.5E-05
LOC100623829	3.17207726	261.10397	2.060765874	8.033995371	8.033995371	-6.36305	1.5E-05
LOC102164566	0	120.63664	0	6.926434087	6.926434087	-20	1.5E-05
EFEMP2	220.9880491	11063.041	7.7943382	13.4335908	13.4335908	-5.64564	1.5E-05
JUP	247.4220263	12395.828	7.956649285	13.59768342	13.59768342	-5.64674	1.5E-05
CCDC42	0	119.81036	0	6.916600414	6.916600414	-20	1.5E-05
LOC100625208	72.95777697	0	6.208629956	0	0	20	1.5E-05
LAMA1	429.2877891	12.394176	8.749158089	3.74353388	3.74353388	5.114211	1.6E-05
LOC100524517	1997.351315	64.449713	10.96459452	6.032314961	6.032314961	4.95377	1.6E-05
LOC102166489	0	118.98409	0	6.906699252	6.906699252	-20	1.6E-05
GDAP1	0	118.98409	0	6.906699252	6.906699252	-20	1.6E-05
COL5A2	1571.235603	86391.535	10.61860171	16.39861904	16.39861904	-5.78092	1.6E-05
HTRA3	20.08982264	999.79683	4.398475058	9.966933409	9.966933409	-5.6371	1.6E-05
AXL	189.2672765	9277.4536	7.571883648	13.17966866	13.17966866	-5.61523	1.6E-05
SH2D6	34.89284986	1673.2137	5.165624571	10.70926798	10.70926798	-5.58355	1.6E-05
LOC102160907	121.596295	1.6525567	6.937771569	1.407383614	1.407383614	6.201256	1.6E-05
KRT6A	121.596295	1.6525567	6.937771569	1.407383614	1.407383614	6.201256	1.6E-05
LOC100153163	40.17964529	1916.1395	5.363859498	10.90473964	10.90473964	-5.57559	1.6E-05
CMAH	1.057359087	165.25567	1.04079362	7.377259771	7.377259771	-7.28809	1.6E-05
MYLPF	13.74566813	713.07823	3.882219287	9.479938335	9.479938335	-5.69701	1.6E-05
CHRNA1	0	118.15781	0	6.896729669	6.896729669	-20	1.6E-05
ASB9	5.286795433	344.55808	2.65232482	8.432784407	8.432784407	-6.02621	1.6E-05
RAPGEF5	1801.739884	58.665764	10.81597553	5.89883146	5.89883146	4.940728	1.6E-05
LOC100737535	4.229436346	299.11277	2.386655454	8.2293609	8.2293609	-6.14408	1.7E-05
LOC100623234	40.17964529	1897.9614	5.363859498	10.89099488	10.89099488	-5.56184	1.7E-05
RBP5	0	117.33153	0	6.886690713	6.886690713	-20	1.7E-05
LOC100627840	0	117.33153	0	6.886690713	6.886690713	-20	1.7E-05
MFAP3L	281.257517	7.4365053	8.140868193	3.076645515	3.076645515	5.241123	1.7E-05
LOC100511337	2.114718173	206.56959	1.639101631	7.697451307	7.697451307	-6.61002	1.8E-05
LOC102167737	2.114718173	206.56959	1.639101631	7.697451307	7.697451307	-6.61002	1.8E-05
PCDH17	69.7853665	3234.0535	6.145386028	11.65957388	11.65957388	-5.53427	1.8E-05
CCDC38	701.0290744	22.309516	9.45538697	4.542847146	4.542847146	4.973743	1.8E-05
LOC100622219	70.8430588	0	6.166776871	0	0	20	1.8E-05
LOC102163689	70.8430588	0	6.166776871	0	0	20	1.8E-05
LOC100157853	70.8430588	0	6.166776871	0	0	20	1.8E-05
RELB	20.08982264	970.05081	4.398475058	9.923402974	9.923402974	-5.59352	1.8E-05
SEMA3A	0	114.85269	0	6.856147778	6.856147778	-20	1.9E-05
RXFP4	0	114.85269	0	6.856147778	6.856147778	-20	1.9E-05
LOC100623937	187.1525583	4.1313919	7.555759096	2.359350201	2.359350201	5.501443	1.9E-05
LOC102164968	118.4242177	1.6525567	6.899951616	1.407383614	1.407383614	6.163121	1.9E-05
COL14A1	455.7217663	22555.747	8.835171737	14.4612714	14.4612714	-5.6292	1.9E-05
LOC102161702	370.0756803	10.741619	8.535569642	3.553559423	3.553559423	5.106537	1.9E-05
SPINT2	2.114718173	203.26448	1.639101631	7.674294538	7.674294538	-6.58675	1.9E-05
ENO2	58.15474976	2659.7901	5.886422108	11.37763898	11.37763898	-5.51527	1.9E-05
LOC100625694	0	114.02642	0	6.845821397	6.845821397	-20	1.9E-05
LOC102167708	322.4945214	9.0890621	8.337597469	3.334720158	3.334720158	5.148999	1.9E-05
LOC100512657	14.80302721	730.43008	3.982129041	9.51457615	9.51457615	-5.62478	1.9E-05
FOXH1	1605.071093	53.708094	10.64932004	5.773682393	5.773682393	4.901354	1.9E-05
LOC102158695	1.057359087	158.64545	1.04079362	7.318727602	7.318727602	-7.2292	2E-05
CLDN1	10.57359087	551.12767	3.532764645	9.108858104	9.108858104	-5.70385	2E-05
CCDC85C	1238.16749	41.313919	10.27515549	5.403060391	5.403060391	4.905435	2E-05
FSTL3	0	113.20014	0	6.83542057	6.83542057	-20	2E-05
LOC102163288	7.401513606	418.09686	3.070649265	8.711139889	8.711139889	-5.81987	2E-05
CCDC85A	1116.571195	37.182527	10.12615103	5.254840671	5.254840671	4.908307	2E-05
LOC100739238	15.8603863	770.91772	4.075565686	9.592303268	9.592303268	-5.60308	2E-05
LOC100511825	433.5172255	13.220454	8.76326956	3.829895615	3.829895615	5.035246	2E-05
IL16	7.401513606	416.4443	3.070649265	8.705439898	8.705439898	-5.81416	2E-05
CAMSAP3	524.4501069	16.525567	9.037409972	4.131389251	4.131389251	4.988034	2E-05
LOC102165358	4.229436346	285.89232	2.386655454	8.164365521	8.164365521	-6.07886	2.1E-05
JUNB	67.67098154	3046.4884	6.10162868	11.57340499	11.57340499	-5.49247	2.1E-05
KCNJ14	476.868948	14.873011	8.900471214	3.98850389	3.98850389	5.002824	2.1E-05
SIPA1L2	823.6827284	27.267186	9.687695383	4.821056378	4.821056378	4.916851	2.1E-05
LOC100738401	89.87552236	4055.3742	6.505819847	11.98597505	11.98597505	-5.49576	2.1E-05
VSTM4	60.26946793	2702.7566	5.937096418	11.40074954	11.40074954	-5.48686	2.1E-05
LOC100512258	10.57359087	1392.2791	4.98474489	10.44426853	10.44426853	-5.50479	2.1E-05
CKMT1B	205.1276628	4.9576702	7.687394321	2.57474827	2.57474827	5.370716	2.1E-05
PSP-I	0	111.54758	0	6.814391228	6.814391228	-20	2.1E-05
PTK2B	28.54869534	1297.257	4.885022527	10.34236034	10.34236034	-5.5059	2.2E-05
TRIM34	3.17207726	240.44701	2.060765874	7.915562765	7.915562765	-6.24415	2.2E-05
LOC100624137	0	110.7213	0	6.803760479	6.803760479	-20	2.2E-05
PDE3A	0	110.7213	0	6.803760479	6.803760479	-20	2.2E-05
LOC100525798	746.4955151	24.788351	9.545921113	4.688647628	4.688647628	4.912399	2.2E-05
MT-2B	49.69587707	2206.1633	5.663796517	11.10797763	11.10797763	-5.47227	2.2E-05
LOC100738863	26.43397716	1196.4511	4.777891883	10.22575101	10.22575101	-5.50022	2.2E-05
WNT8B	203.0129446	4.9576702	7.672516884	2.57474827	2.57474827	5.355766	2.3E-05
EN1	0	109.89502	0	6.793050814	6.793050814	-20	2.3E-05
CMKLR1	0	109.89502	0	6.793050814	6.793050814	-20	2.3E-05
OSR2	7.401513606	404.8764	3.070649265	8.664896653	8.664896653	-5.77352	2.3E-05
LOC780439	32.77813168	1455.9025	5.078017628	10.50868861	10.50868861	-5.47304	2.3E-05
MMP19	84.58872693	3729.8206	6.419348884	11.86527726	11.86527726	-5.4625	2.3E-05
ADAM22	445.1481754	14.046732	8.80137913	3.911378308	3.911378308	4.985979	2.3E-05
GUCY2C	1083.793064	37.182527	10.08320414	5.254840671	5.254840671	4.865321	2.4E-05
TNN	3.17207726	235.48934	2.060765874	7.885631319	7.885631319	-6.21409	2.4E-05
LOC100628185	15.8603863	741.1717	4.075565686	9.535609179	9.535609179	-5.54631	2.4E-05
LOC100620844	2983.867342	103.2848	11.5434511	6.704385034	6.704385034	4.852484	2.4E-05
ANKRD35	17.97510447	825.45209	4.246035924	9.690787384	9.690787384	-5.52111	2.5E-05
LOC100152707	0	108.24247	0	6.771389985	6.771389985	-20	2.5E-05
LOC100620517	0	108.24247	0	6.771389985	6.771389985	-20	2.5E-05
ARHGEF19	14.80302721	695.72639	3.982129041	9.444448397	9.444448397	-5.55456	2.5E-05
TNFRSF1B	61.32682702	2657.3112	5.961781364	11.37629431	11.37629431	-5.43731	2.5E-05
SCG3	286.5443125	8.2627837	8.167640492	3.211445828	3.211445828	5.115987	2.5E-05
NRG3	462.0659208	14.873011	8.855073776	3.98850389	3.98850389	4.95733	2.5E-05
STXBP5L	264.3397716	7.4365053	8.051697126	3.076645515	3.076645515	5.151625	2.5E-05
LOC102158596	66.61362245	0	6.079242038	0	0	20	2.6E-05
PDLIM1	196.6687901	8727.1522	7.626941292	13.09146054	13.09146054	-5.47167	2.6E-05
FRAS1	2664.544898	93.369456	11.38021477	6.560248082	6.560248082	4.834795	2.6E-05
MYO7A	133.2272449	2.4788351	7.068533723	1.798604302	1.798604302	5.748083	2.6E-05
CXCL11	0	106.58991	0	6.749398975	6.749398975	-20	2.6E-05
LOC102165979	0	106.58991	0	6.749398975	6.749398975	-20	2.6E-05
C-JUN	62.38418611	2668.8791	5.986051038	11.38255872	11.38255872	-5.41891	2.7E-05
LOC100738921	326.7239578	9.9153405	8.356337332	3.448285225	3.448285225	5.042266	2.7E-05
LOC100737466	21.14718173	936.99967	4.46905122	9.87344361	9.87344361	-5.46951	2.7E-05
CTBS	111.0227041	1.6525567	6.807647348	1.407383614	1.407383614	6.070011	2.7E-05
GLDC	3314.820736	116.50525	11.6951503	6.876581411	6.876581411	4.830464	2.7E-05
LOC100738968	0	105.76363	0	6.738276475	6.738276475	-20	2.7E-05
CEBPB	27.49133625	1190.6671	4.832451381	10.21876559	10.21876559	-5.43665	2.7E-05
PTER	2.114718173	188.39147	1.639101631	7.565227535	7.565227535	-6.47712	2.8E-05
ANGPTL1	5.286795433	309.02811	2.65232482	8.276255224	8.276255224	-5.8692	2.8E-05
ATP6V1G3	65.55626337	0	6.056502534	0	0	20	2.8E-05
GAD2	65.55626337	0	6.056502534	0	0	20	2.8E-05
GGN	4.229436346	267.71419	2.386655454	8.069928711	8.069928711	-5.98408	2.8E-05
PLEKHH1	650.2758382	22.309516	9.347124895	4.542847146	4.542847146	4.865321	2.8E-05
LEPREL2	233.6763581	10254.115	7.874528698	13.32405599	13.32405599	-5.45555	2.8E-05
POPDC3	0	104.93735	0	6.727067559	6.727067559	-20	2.8E-05
FOXC1	13.74566813	633.75551	3.882219287	9.310057206	9.310057206	-5.52688	2.8E-05
PPARG	17.97510447	797.35863	4.246035924	9.640893151	9.640893151	-5.47116	2.9E-05
SPOCD1	87.76080419	0.8262784	6.471850834	0.868906686	0.868906686	6.730805	2.9E-05
LOC102167736	0	104.11107	0	6.715770874	6.715770874	-20	2.9E-05
FLNC	475.811589	21473.322	8.897275491	14.39032498	14.39032498	-5.49601	3E-05
SP140	154.3744266	6587.9175	7.279605257	12.68582574	12.68582574	-5.41532	3E-05
LOC100627814	108.9079859	1.6525567	6.780152406	1.407383614	1.407383614	6.042266	3E-05
LOC100152976	130.0551676	2.4788351	7.034030432	1.798604302	1.798604302	5.713318	3E-05
PAX6	2363.197558	85.106672	11.20713488	6.428053129	6.428053129	4.795324	3E-05
LOC102161685	24430.2817	813.8842	14.57644193	9.670451241	9.670451241	4.907703	3E-05
CHD5	1.057359087	144.59872	1.04079362	7.185853813	7.185853813	-7.09545	3E-05
LRRC70	555.1135205	19.004403	9.119235603	4.322245637	4.322245637	4.868377	3.1E-05
LOC100523418	1485.589517	53.708094	10.53779062	5.773682393	5.773682393	4.789752	3.1E-05
MSC	9.516231779	461.06333	3.394545939	8.851946794	8.851946794	-5.59843	3.1E-05
RIC3	3.17207726	223.09516	2.060765874	7.807967682	7.807967682	-6.13609	3.1E-05
ZIC1	37.00756803	1539.3566	5.24821481	10.58904867	10.58904867	-5.37836	3.1E-05
LOC100622513	25.37661808	1071.683	4.721187694	10.06700814	10.06700814	-5.40023	3.2E-05
LOC100628148	0	102.45852	0	6.692908622	6.692908622	-20	3.2E-05
LOC100623513	0	102.45852	0	6.692908622	6.692908622	-20	3.2E-05
LOC102162044	274.9133625	8.2627837	8.108071518	3.211445828	3.211445828	5.056205	3.2E-05
LOC102160748	19.03246356	820.49442	4.324267947	9.682106971	9.682106971	-5.42996	3.2E-05
LOC100737503	13.74566813	617.22994	3.882219287	9.271999722	9.271999722	-5.48876	3.2E-05
CAPG	556.1708795	24799.093	9.121976047	14.5980579	14.5980579	-5.47862	3.3E-05
RBMS3	1.057359087	142.11988	1.04079362	7.161080272	7.161080272	-7.0705	3.3E-05
LOC102166397	8.458872693	415.61802	3.241668254	8.702581435	8.702581435	-5.61865	3.3E-05
ZNF256	0	101.63224	0	6.681340184	6.681340184	-20	3.3E-05
LOC100624765	440.9187391	14.873011	8.787637298	3.98850389	3.98850389	4.889744	3.3E-05
LOC100512853	63.44154519	0	6.009919183	0	0	20	3.4E-05
PROX1	630.1860156	22.309516	9.301921431	4.542847146	4.542847146	4.820047	3.4E-05
LHFPL3	312.9782896	9.9153405	8.294520996	3.448285225	3.448285225	4.980256	3.4E-05
LOC100621722	61.32682702	2484.6191	5.961781364	11.2793895	11.2793895	-5.34036	3.5E-05
TGFB3	10.57359087	487.50424	3.532764645	8.932227272	8.932227272	-5.52688	3.5E-05
DTX3L	25.37661808	1046.8947	4.721187694	10.03327803	10.03327803	-5.36647	3.6E-05
FABP3	2295.526577	85.106672	11.16523776	6.428053129	6.428053129	4.753409	3.6E-05
PLXND1	189.2672765	7831.4664	7.571883648	12.93525096	12.93525096	-5.37079	3.6E-05
LOC100624035	0	99.153405	0	6.646067655	6.646067655	-20	3.7E-05
ANKRD63	0	99.153405	0	6.646067655	6.646067655	-20	3.7E-05
SEMA5B	62.38418611	0	5.986051038	0	0	20	3.7E-05
LOC100156194	62.38418611	0	5.986051038	0	0	20	3.7E-05
SLC24A5	5030.914534	184.26008	12.2968917	7.533408207	7.533408207	4.771005	3.7E-05
LOC100154753	620.6697838	22.309516	9.280004648	4.542847146	4.542847146	4.798095	3.7E-05
SPATA20	6.344154519	328.03251	2.876596413	8.362086342	8.362086342	-5.69227	3.8E-05
C16H5orf49	4.229436346	251.18863	2.386655454	7.978359394	7.978359394	-5.89216	3.8E-05
LOC102157883	124.7683722	2.4788351	6.974625354	1.798604302	1.798604302	5.653446	3.9E-05
KCNMA1	3.17207726	213.17982	2.060765874	7.742678745	7.742678745	-6.0705	3.9E-05
LOC100621279	0	98.327126	0	6.634115866	6.634115866	-20	3.9E-05
SDK1	0	98.327126	0	6.634115866	6.634115866	-20	3.9E-05
ZC3H12D	5.286795433	287.54487	2.65232482	8.172651888	8.172651888	-5.76525	3.9E-05
LOC102163961	15.8603863	669.28548	4.075565686	9.388631874	9.388631874	-5.39912	4E-05
VAMP8	75.07249515	2961.3817	6.249303021	11.53254182	11.53254182	-5.30184	4E-05
LOC102164445	3.17207726	211.52726	2.060765874	7.731504113	7.731504113	-6.05927	4E-05
STARD8	0	97.500848	0	6.622064238	6.622064238	-20	4E-05
LOC102159188	1.057359087	136.33593	1.04079362	7.101565318	7.101565318	-7.01056	4E-05
APOA1	33.83549077	1337.7447	5.122485983	10.38666513	10.38666513	-5.30512	4.1E-05
LOC102162014	61.32682702	0	5.961781364	0	0	20	4.1E-05
LOC100154928	61.32682702	0	5.961781364	0	0	20	4.1E-05
LOC100621385	61.32682702	0	5.961781364	0	0	20	4.1E-05
MAGIX	1.057359087	135.50965	1.04079362	7.092859161	7.092859161	-7.00179	4.1E-05
TSPAN13	6370.588497	235.48934	12.63743738	7.885631319	7.885631319	4.757693	4.1E-05
ZNF286A	0	96.674569	0	6.609911087	6.609911087	-20	4.2E-05
PDE4A	34.89284986	1369.9695	5.165624571	10.4209808	10.4209808	-5.29507	4.2E-05
SMTN	81.41664967	3178.6929	6.364863912	11.63467172	11.63467172	-5.28697	4.2E-05
C6H19orf33	15.8603863	661.0227	4.075565686	9.37073687	9.37073687	-5.3812	4.2E-05
LOC102161934	19.03246356	776.70167	4.324267947	9.603073025	9.603073025	-5.35083	4.2E-05
LOC100738735	142.7434767	3.3051135	7.167352675	2.106051269	2.106051269	5.432581	4.2E-05
BEX5	142.7434767	3.3051135	7.167352675	2.106051269	2.106051269	5.432581	4.2E-05
LOC100627489	122.653654	2.4788351	6.950161063	1.798604302	1.798604302	5.628784	4.3E-05
GPNMB	1.057359087	134.68337	1.04079362	7.084100147	7.084100147	-6.99296	4.3E-05
CD36	3.17207726	208.22215	2.060765874	7.708891783	7.708891783	-6.03655	4.3E-05
tRNA-Tyr	563.5723931	20.656959	9.141014774	4.436758793	4.436758793	4.769901	4.4E-05
ICAM-1	169.1774539	6696.9862	7.410896102	12.70951169	12.70951169	-5.3069	4.4E-05
BATF2	8.458872693	390.82967	3.241668254	8.614082834	8.614082834	-5.52993	4.4E-05
HHIPL1	16.91774539	690.76872	4.163317207	9.434145967	9.434145967	-5.35159	4.5E-05
LOC100627331	81.41664967	0.8262784	6.364863912	0.868906686	0.868906686	6.622552	4.5E-05
LOC102159258	60.26946793	0	5.937096418	0	0	20	4.5E-05
LOC102165390	60.26946793	0	5.937096418	0	0	20	4.5E-05
LRIT3	60.26946793	0	5.937096418	0	0	20	4.5E-05
AREG	0	95.022013	0	6.585293272	6.585293272	-20	4.5E-05
LOC102157992	0	95.022013	0	6.585293272	6.585293272	-20	4.5E-05
LOC102162312	0	95.022013	0	6.585293272	6.585293272	-20	4.5E-05
LOC102159707	17.97510447	726.29869	4.246035924	9.506404165	9.506404165	-5.33649	4.6E-05
LOC100522644	2.114718173	169.38707	1.639101631	7.412672017	7.412672017	-6.32371	4.6E-05
TMEM74	457.8364845	16.525567	8.841836302	4.131389251	4.131389251	4.792061	4.6E-05
AHDC1	67.67098154	2587.0776	6.10162868	11.33766515	11.33766515	-5.25664	4.6E-05
LOC100624675	541.3678523	22451.636	9.083127858	14.45459721	14.45459721	-5.37407	4.6E-05
LOC100739178	20.08982264	799.83746	4.398475058	9.645365656	9.645365656	-5.31517	4.6E-05
TPMT	3601.365049	137.98849	11.81472867	7.118821584	7.118821584	4.705924	4.6E-05
GRB14	694.9717153	26.440908	9.453212421	4.778256309	4.778256309	4.726453	4.6E-05
LOC100514871	515.9912342	19.004403	9.013996009	4.322245637	4.322245637	4.762941	4.7E-05
LOC100513982	0	94.195734	0	6.572825024	6.572825024	-20	4.7E-05
LOC100517213	0	94.195734	0	6.572825024	6.572825024	-20	4.7E-05
KLHL30	0	94.195734	0	6.572825024	6.572825024	-20	4.7E-05
LOC100620869	0	94.195734	0	6.572825024	6.572825024	-20	4.7E-05
LOC100516787	454.6644072	16.525567	8.831827873	4.131389251	4.131389251	4.78203	4.8E-05
LOC102161205	31.7207726	1217.108	5.032134908	10.25042639	10.25042639	-5.26189	4.8E-05
LOC100155098	860.6902965	33.051135	9.751025627	5.089630977	5.089630977	4.702722	4.8E-05
ERBB4	100.4491132	1.6525567	6.664612444	1.407383614	1.407383614	5.925621	4.8E-05
RAB33A	473.6968708	17.351846	8.890862729	4.19785327	4.19785327	4.770803	4.8E-05
KRBA2	354.215294	12.394176	8.472549889	3.74353388	3.74353388	4.836892	4.8E-05
LOC100514231	2914.081643	113.20014	11.50932057	6.83542057	6.83542057	4.686094	4.9E-05
ZNF77	0	93.369456	0	6.560248082	6.560248082	-20	4.9E-05
LOC100627927	0	93.369456	0	6.560248082	6.560248082	-20	4.9E-05
LOC102161634	876.5506828	33.877413	9.77733864	5.124221138	5.124221138	4.693442	4.9E-05
LOC100523918	9.516231779	418.09686	3.394545939	8.711139889	8.711139889	-5.4573	5E-05
ELAVL2	59.21210885	0	5.911981742	0	0	20	5E-05
SLFN14	59.21210885	0	5.911981742	0	0	20	5E-05
NELL1	59.21210885	0	5.911981742	0	0	20	5E-05
SIGLEC10	52.86795433	1983.8944	5.751355374	10.95484652	10.95484652	-5.2298	5E-05
LOC100037974	0	92.543178	0	6.547560532	6.547560532	-20	5.1E-05
PADI1	0	92.543178	0	6.547560532	6.547560532	-20	5.1E-05
JOSD2	51.81059524	1932.6651	5.722755497	10.91712224	10.91712224	-5.2212	5.2E-05
LOC780424	137.4566813	3.3051135	7.113290863	2.106051269	2.106051269	5.378133	5.2E-05
LOC102167439	43.35172255	1613.7217	5.470918232	10.65706978	10.65706978	-5.21816	5.3E-05
LOC100511614	407.0832483	14.873011	8.672179679	3.98850389	3.98850389	4.774555	5.3E-05
LOC100622715	3.17207726	199.13309	2.060765874	7.644815895	7.644815895	-5.97216	5.3E-05
OSMR	67.67098154	2507.7549	6.10162868	11.29275579	11.29275579	-5.21172	5.4E-05
LOC100516615	0	91.716899	0	6.534760414	6.534760414	-20	5.4E-05
LOC102164954	0	91.716899	0	6.534760414	6.534760414	-20	5.4E-05
LOC100521431	0	91.716899	0	6.534760414	6.534760414	-20	5.4E-05
DPEP1	1.057359087	128.07315	1.04079362	7.012045083	7.012045083	-6.92036	5.4E-05
EMID1	90.93288145	3378.6523	6.522509054	11.7226591	11.7226591	-5.2155	5.4E-05
DLX2	4.229436346	233.0105	2.386655454	7.87042946	7.87042946	-5.78379	5.5E-05
LOC100625116	58.15474976	0	5.886422108	0	0	20	5.5E-05
GPRC5C	68.72834063	2527.5855	6.123673244	11.30411487	11.30411487	-5.20071	5.6E-05
LOC102159568	938.9348689	37.182527	9.876416981	5.254840671	5.254840671	4.658328	5.6E-05
LOC102165939	16.91774539	660.19642	4.163317207	9.368935101	9.368935101	-5.28629	5.6E-05
LOC100626465	0	90.890621	0	6.521845711	6.521845711	-20	5.6E-05
LOC102162178	0	90.890621	0	6.521845711	6.521845711	-20	5.6E-05
ZNF432	0	90.890621	0	6.521845711	6.521845711	-20	5.6E-05
LMX1A	78.24457241	0.8262784	6.308240221	0.868906686	0.868906686	6.565219	5.7E-05
LOC100738381	1300.551676	52.055537	10.34601688	5.729431428	5.729431428	4.642928	5.7E-05
LOC100624327	5.286795433	266.06164	2.65232482	8.061028933	8.061028933	-5.65322	5.7E-05
LOC100622991	6.344154519	300.76533	2.876596413	8.23728324	8.23728324	-5.56707	5.7E-05
MAFB	7.401513606	335.46902	3.070649265	8.394329862	8.394329862	-5.50222	5.7E-05
LOC100522842	596.3505248	23.135794	9.222433942	4.593102407	4.593102407	4.687962	5.8E-05
LOC100153177	97.27703596	1.6525567	6.618782442	1.407383614	1.407383614	5.879328	5.8E-05
RIMBP2	362.6741667	13.220454	8.506502639	3.829895615	3.829895615	4.77783	5.8E-05
PTGIR	0	90.064343	0	6.508814353	6.508814353	-20	5.8E-05
SALL1	0	90.064343	0	6.508814353	6.508814353	-20	5.8E-05
LOC102160134	4230.493705	167.73451	12.04695131	7.398611253	7.398611253	4.656575	5.9E-05
LOC102167673	1.057359087	125.59431	1.04079362	6.98406878	6.98406878	-6.89216	5.9E-05
CD99L2	59.21210885	2145.8449	5.911981742	11.06800227	11.06800227	-5.17951	5.9E-05
LOC100620963	9.516231779	402.39757	3.394545939	8.656058573	8.656058573	-5.40209	6E-05
RAB3IL1	85.64608601	3108.4592	6.437062676	11.60244799	11.60244799	-5.18167	6E-05
LPAR3	171.292172	4.9576702	7.428713345	2.57474827	2.57474827	5.110653	6.1E-05
LOC100623149	340.4696259	12.394176	8.415613445	3.74353388	3.74353388	4.779792	6.1E-05
PRSS8	1.057359087	124.76803	1.04079362	6.974621476	6.974621476	-6.88264	6.1E-05
XIRP1	1.057359087	124.76803	1.04079362	6.974621476	6.974621476	-6.88264	6.1E-05
LOC100624356	57.09739067	0	5.860401464	0	0	20	6.1E-05
LOC100152658	57.09739067	0	5.860401464	0	0	20	6.1E-05
LOC100737042	57.09739067	0	5.860401464	0	0	20	6.1E-05
KCND1	12.68830904	504.85609	3.774872332	8.98258319	8.98258319	-5.3143	6.2E-05
C14H10orf54	5.286795433	261.10397	2.65232482	8.033995371	8.033995371	-5.62609	6.2E-05
SLC8A3	2.114718173	158.64545	1.639101631	7.318727602	7.318727602	-6.2292	6.2E-05
LOC100622258	499.0734889	19368.791	8.965996313	14.24152079	14.24152079	-5.27834	6.3E-05
FST	6456.234583	255.32002	12.65670072	8.001802338	8.001802338	4.660314	6.3E-05
LOC100153517	0	88.411786	0	6.482393107	6.482393107	-20	6.4E-05
ADAMTSL2	1.057359087	123.11548	1.04079362	6.955539223	6.955539223	-6.8634	6.5E-05
WFDC3	1.057359087	123.11548	1.04079362	6.955539223	6.955539223	-6.8634	6.5E-05
DGKB	169.1774539	4.9576702	7.410896102	2.57474827	2.57474827	5.092731	6.5E-05
REPS2	677.7671745	27.267186	9.406772987	4.821056378	4.821056378	4.635552	6.7E-05
LOC100737468	1.057359087	122.2892	1.04079362	6.945902605	6.945902605	-6.85369	6.7E-05
CD2	56.04003159	0	5.833902876	0	0	20	6.8E-05

ZBED2	4.97357294	253.66746	2.578594101	7.992470822	7.992470822	-5.67251	7.3E-09
ZNF256	0	101.63224	0	6.681340183	6.681340183	-20	7.4E-09
PSTPIP1	179.048626	2.4788351	7.492242779	1.798604302	1.798604302	6.174545	7.4E-09
KCNQ4	441.819063	11.567897	8.79057352	3.65167138	3.65167138	5.255257	7.4E-09
TMEM158	30.6703665	997.31799	4.985061656	9.963355621	9.963355621	-5.02314	7.7E-09
ADAMTS5	6.63143059	299.93905	2.93195353	8.233327508	8.233327508	-5.49921	7.8E-09
TEX15	1887.47093	61.1446	10.88300286	5.957557119	5.957557119	4.948086	7.8E-09
NIPAL4	72.9457365	2246.6509	6.208395063	11.13420226	11.13420226	-4.94481	7.9E-09
LGALS9	14.09179	513.11887	3.915692025	9.005958152	9.005958152	-5.18637	8E-09
APBB1	125.168252	3802.5331	6.979205122	11.89312443	11.89312443	-4.92502	8.4E-09
IL12RB2	176.561839	2.4788351	7.472177749	1.798604302	1.798604302	6.154367	8.4E-09
RAB33A	600.144468	17.351846	9.231567934	4.19785327	4.19785327	5.112149	8.5E-09
KCNK5	456.739782	12.394176	8.838383871	3.74353388	3.74353388	5.203638	8.9E-09
TDRKH	784.995596	23.962073	9.618377419	4.641665831	4.641665831	5.03386	8.9E-09
LOC100624487	14.9207188	529.64444	3.99283357	9.051601682	9.051601682	-5.14964	9.1E-09
ANO4	0.82892882	126.42059	0.870998931	6.993454623	6.993454623	-7.25277	9.1E-09
FAM19A4	98.64253	0	6.638689748	0	0	20	9.1E-09
CITED4	944.978859	29.746021	9.885664132	4.942327829	4.942327829	4.989513	9.4E-09
C6H16orf74	65.4853771	1981.4155	6.054965161	10.95304368	10.95304368	-4.91921	9.6E-09
LPPR5	290.954017	6.610227	8.189597352	2.927939483	2.927939483	5.459848	9.6E-09
LOC102157983	5.80250177	269.36675	2.766065426	8.078773924	8.078773924	-5.53675	9.7E-09
LOC102165469	197.28506	3.3051135	7.63143217	2.106051269	2.106051269	5.899438	9.8E-09
LOC100621279	0	98.327126	0	6.634115866	6.634115866	-20	9.9E-09
SDK1	0	98.327126	0	6.634115866	6.634115866	-20	9.9E-09
FLT4	266.086152	5.7839486	8.061161368	2.762125239	2.762125239	5.523695	1E-08
LOC102167117	123.510395	0.8262784	6.96012238	0.868906686	0.868906686	7.223789	1.1E-08
RSPO2	379.649401	9.9153405	8.572318998	3.448285225	3.448285225	5.258862	1.1E-08
PTGDS	0	97.500848	0	6.622064238	6.622064238	-20	1.1E-08
LOC100627081	608.433757	18.178124	9.251325603	4.261389712	4.261389712	5.064825	1.1E-08
LOC100153213	263.599366	5.7839486	8.047665794	2.762125239	2.762125239	5.510148	1.1E-08
MPZL2	3.31571529	195.82797	2.109599694	7.620791468	7.620791468	-5.88412	1.1E-08
LOC100519458	96.1557435	0	6.602227381	0	0	20	1.1E-08
LOC102165476	0	96.674569	0	6.609911086	6.609911086	-20	1.2E-08
ZNF286A	0	96.674569	0	6.609911086	6.609911086	-20	1.2E-08
ASTN1	671.432347	20.656959	9.393245316	4.436758793	4.436758793	5.022542	1.2E-08
TBX3	22.3810782	721.34102	4.547269558	9.496536287	9.496536287	-5.01033	1.2E-08
NFKBIA	152.522904	4461.0769	7.262310092	12.12349967	12.12349967	-4.87029	1.2E-08
LOC100523068	1.65785765	147.07755	1.410263837	7.210209122	7.210209122	-6.47111	1.2E-08
DPYSL4	1.65785765	147.07755	1.410263837	7.210209122	7.210209122	-6.47111	1.2E-08
AMDHD1	372.189042	9.9153405	8.543762814	3.448285225	3.448285225	5.230229	1.3E-08
NID2	795.771671	23899.276	9.638022543	14.54473964	14.54473964	-4.90847	1.3E-08
LOC100511782	1166.30286	38.835083	10.1889632	5.315967693	5.315967693	4.908438	1.3E-08
LOC102163689	94.4978859	0	6.57739689	0	0	20	1.3E-08
LOC100522595	1.65785765	145.42499	1.410263837	7.194018019	7.194018019	-6.45481	1.3E-08
AREG	0	95.022013	0	6.585293272	6.585293272	-20	1.3E-08
LOC102157992	0	95.022013	0	6.585293272	6.585293272	-20	1.3E-08
LOC102162312	0	95.022013	0	6.585293272	6.585293272	-20	1.3E-08
LOC100515735	13.2628612	461.88961	3.834191515	8.854524369	8.854524369	-5.12208	1.4E-08
STOX1	368.873327	9.9153405	8.530887454	3.448285225	3.448285225	5.217319	1.4E-08
LOC100623579	590.197322	18.178124	9.207495925	4.261389712	4.261389712	5.020922	1.4E-08
LOC102165266	143.404687	1.6525567	7.173973754	1.407383613	1.407383613	6.439249	1.4E-08
TSPAN33	3750.90293	131.37826	11.87340679	7.048522415	7.048522415	4.835439	1.4E-08
LOC100524773	93.6689571	0	6.564819524	0	0	20	1.5E-08
LOC100513982	0	94.195734	0	6.572825024	6.572825024	-20	1.5E-08
WIF1	6.63143059	280.10837	2.93195353	8.13498259	8.13498259	-5.40052	1.5E-08
RSPO3	1.65785765	143.77244	1.410263837	7.177643143	7.177643143	-6.43832	1.5E-08
PRSS33	1324.62826	45.44531	10.37246055	5.537461031	5.537461031	4.865312	1.5E-08
GATA3	55.5382312	1609.5903	5.821154844	10.65337381	10.65337381	-4.85707	1.5E-08
TWIST2	28.18358	857.67695	4.867084967	9.745971655	9.745971655	-4.92751	1.6E-08
PRR16	5.80250177	256.1463	2.766065426	8.006445558	8.006445558	-5.46415	1.6E-08
FNDC7	92.8400282	0	6.552131542	0	0	20	1.6E-08
CACNA1E	232.100071	4.9576702	7.864805631	2.574748269	2.574748269	5.548941	1.6E-08
ZNF77	0	93.369456	0	6.560248082	6.560248082	-20	1.6E-08
DDX58	190.653629	5427.8226	7.582357509	12.40642363	12.40642363	-4.83135	1.6E-08
LOC100520275	0.82892882	118.98409	0.870998931	6.906699252	6.906699252	-7.1653	1.6E-08
LOC102163193	208.890064	4.1313919	7.713490059	2.359350201	2.359350201	5.659972	1.6E-08
LOC102160653	48.0778718	1390.6265	5.617000784	10.44255634	10.44255634	-4.85422	1.7E-08
CYP2C49	140.9179	1.6525567	7.148912757	1.407383613	1.407383613	6.414011	1.7E-08
FILIP1L	48.0778718	1389.8002	5.617000784	10.44169949	10.44169949	-4.85336	1.7E-08
SLC41A2	27.3546512	827.93093	4.825513503	9.695108082	9.695108082	-4.91965	1.7E-08
HSPA12A	4141.3284	147.90383	12.01622622	7.218237038	7.218237038	4.807362	1.7E-08
LOC100626048	1.65785765	141.2936	1.410263837	7.152726981	7.152726981	-6.41323	1.7E-08
LOC100152091	135.115398	3790.1389	7.088666472	11.8884156	11.8884156	-4.80999	1.8E-08
SYT5	4.97357294	230.53167	2.578594101	7.855065709	7.855065709	-5.53454	1.8E-08
LOC100513632	61.340733	1742.6211	5.962103212	10.76787084	10.76787084	-4.82827	1.8E-08
LOC100627840	0.82892882	117.33153	0.870998931	6.886690713	6.886690713	-7.14513	1.8E-08
SOCS3	3.31571529	185.91263	2.109599694	7.546220275	7.546220275	-5.80916	1.8E-08
ARSJ	21.5521494	666.80665	4.495193036	9.383286641	9.383286641	-4.95136	1.8E-08
LOC100516615	0	91.716899	0	6.534760414	6.534760414	-20	1.8E-08
LOC102157807	91.1821706	0	6.526415834	0	0	20	1.8E-08
LOC100522330	16423.5668	576.7423	14.0035677	9.174282325	9.174282325	4.831697	1.8E-08
PDE8B	16.5785765	532.94955	4.135746339	9.060559625	9.060559625	-5.00661	1.9E-08
LOC100737711	3.31571529	184.26008	2.109599694	7.533408207	7.533408207	-5.79628	2E-08
LOC100626465	0	90.890621	0	6.521845711	6.521845711	-20	2E-08
LOC102162178	0	90.890621	0	6.521845711	6.521845711	-20	2E-08
ZNF432	0	90.890621	0	6.521845711	6.521845711	-20	2E-08
VENTX	90.3532418	0	6.51338402	0	0	20	2E-08
TJP3	90.3532418	0	6.51338402	0	0	20	2E-08
CALB2	90.3532418	0	6.51338402	0	0	20	2E-08
SLC1A1	1508.65246	53.708094	10.55999883	5.773682393	5.773682393	4.811975	2E-08
METRNL	116.050035	3214.2229	6.870981559	11.65070303	11.65070303	-4.79165	2E-08
TLR3	37.3017971	1075.8144	5.259340178	10.07255395	10.07255395	-4.85004	2E-08
LOC100514169	482.436575	14.873011	8.917182817	3.98850389	3.98850389	5.019571	2E-08
LOC102162114	14.9207188	483.37285	3.99283357	8.919974184	8.919974184	-5.01775	2.1E-08
PTGIR	0	90.064343	0	6.508814353	6.508814353	-20	2.2E-08
LOC102167305	0	90.064343	0	6.508814353	6.508814353	-20	2.2E-08
LOC100623608	19.8942918	611.446	4.385036953	9.258438824	9.258438824	-4.9418	2.2E-08
LOC100739707	0.82892882	114.85269	0.870998931	6.856147779	6.856147779	-7.11432	2.2E-08
FAM212A	36.4728682	1042.7633	5.227774503	10.02757887	10.02757887	-4.83744	2.2E-08
SPINT2	4.14464412	203.26448	2.363071282	7.674294538	7.674294538	-5.61597	2.2E-08
LOC102166581	113.563249	0.8262784	6.8400005	0.868906686	0.868906686	7.102652	2.3E-08
LMX1A	113.563249	0.8262784	6.8400005	0.868906686	0.868906686	7.102652	2.3E-08
BRINP2	624.183404	20.656959	9.288135672	4.436758793	4.436758793	4.91727	2.3E-08
CASP4	143.404687	3914.9069	7.173973754	11.93513076	11.93513076	-4.77081	2.3E-08
LOC100525045	1550.92583	56.186929	10.59984389	5.837613535	5.837613535	4.786751	2.3E-08
LOC100738946	29.8414377	865.11346	4.946798112	9.758412211	9.758412211	-4.8575	2.3E-08
GNAT1	4.97357294	223.92144	2.578594101	7.81327737	7.81327737	-5.49257	2.3E-08
C17H20orf174	135.944327	1.6525567	7.097445694	1.407383613	1.407383613	6.362172	2.3E-08
LOC102161556	88.6953841	0	6.486961839	0	0	20	2.3E-08
IGDCC3	1284.83968	46.271589	10.32849506	5.56290145	5.56290145	4.795318	2.4E-08
LOC102159119	4.97357294	223.09516	2.578594101	7.807967682	7.807967682	-5.48723	2.4E-08
IL10RA	5.80250177	243.75212	2.766065426	7.935177544	7.935177544	-5.3926	2.4E-08
FBXO41	1383.48221	50.402981	10.4351308	5.683780114	5.683780114	4.778651	2.5E-08
LOC100621773	461.884426	12664.369	8.851388091	13.62860144	13.62860144	-4.78023	2.6E-08
EHF	134.286469	1.6525567	7.079873746	1.407383613	1.407383613	6.34447	2.6E-08
CRISPLD2	32.3282241	915.51644	5.058672541	9.840016943	9.840016943	-4.82372	2.7E-08
LOC100738350	322.453312	9.0890621	8.337413677	3.334720158	3.334720158	5.148815	2.7E-08
LOC100519542	13.2628612	428.83847	3.834191515	8.747650814	8.747650814	-5.01497	2.7E-08
LOC100517809	696.300212	23.962073	9.44563611	4.641665831	4.641665831	4.860885	2.7E-08
TMEM45B	133.457541	1.6525567	7.071006856	1.407383613	1.407383613	6.335537	2.8E-08
KCNG3	87.0375265	0	6.460046706	0	0	20	2.8E-08
SPRED3	100.300388	2686.231	6.662495885	11.39190462	11.39190462	-4.74318	2.8E-08
SIX1	401.201551	10886.218	8.651774833	13.41034767	13.41034767	-4.76203	2.8E-08
STK17B	100.300388	2682.9259	6.662495885	11.39012911	11.39012911	-4.74141	2.8E-08
LOC100625270	2.48678647	155.34033	1.801898017	7.288546214	7.288546214	-5.96501	2.9E-08
OSR2	12.4339324	404.8764	3.747809764	8.664896653	8.664896653	-5.02513	2.9E-08
LOC100522115	0	86.759229	0	6.455478947	6.455478947	-20	3E-08
TPH1	86.2085977	0	6.446398468	0	0	20	3E-08
TCL1A	86.2085977	0	6.446398468	0	0	20	3E-08
ADRA1B	4.97357294	217.31121	2.578594101	7.770242414	7.770242414	-5.44934	3E-08
SUSD2	3.31571529	174.34474	2.109599694	7.454050313	7.454050313	-5.71648	3.2E-08
F5	0	85.932951	0	6.441831208	6.441831208	-20	3.3E-08
GARNL3	234.586857	5.7839486	7.880115246	2.762125239	2.762125239	5.341924	3.3E-08
LOC102166947	85.3796688	0	6.43261988	0	0	20	3.3E-08
LOC102160384	213.863637	4.9576702	7.747277532	2.574748269	2.574748269	5.430885	3.3E-08
OMG	193.140416	4.1313919	7.600956677	2.359350201	2.359350201	5.546878	3.3E-08
LOC100737938	1.65785765	131.37826	1.410263837	7.048522415	7.048522415	-6.30826	3.3E-08
APBB1IP	32.3282241	892.38064	5.058672541	9.803131183	9.803131183	-4.78679	3.4E-08
LOC102158959	0.82892882	109.06875	0.870998931	6.782261052	6.782261052	-7.03977	3.4E-08
KLHL41	9.94714588	336.2953	3.452482877	8.397868394	8.397868394	-5.0793	3.5E-08
AFAP1	60.5118041	1600.5012	5.942791385	10.64520917	10.64520917	-4.72516	3.5E-08
CCDC141	8.28928824	294.98138	3.215568059	8.209362603	8.209362603	-5.15323	3.5E-08
LOC100737661	272.717583	7.4365053	8.096544303	3.076645515	3.076645515	5.196639	3.6E-08
ATP8A2	1065.17354	39.661362	10.05822657	5.34558663	5.34558663	4.74721	3.6E-08
ACSS3	35.6439394	968.39825	5.195502704	9.92094567	9.92094567	-4.76387	3.6E-08
LOC100152707	0.82892882	108.24247	0.870998931	6.771389985	6.771389985	-7.0288	3.7E-08
LOC100739215	330.742601	9.9153405	8.373920477	3.448285225	3.448285225	5.059903	3.8E-08
SEMA3B	148.378259	3839.7156	7.222826382	11.90715942	11.90715942	-4.69365	3.8E-08
HERC5	116.050035	3002.6956	6.870981559	11.5525229	11.5525229	-4.69344	3.8E-08
LOC100623748	24.8678647	697.37895	4.693089065	9.447866256	9.447866256	-4.80959	3.8E-08
SLC7A3	2492.58897	96.674569	11.28400796	6.609911086	6.609911086	4.688365	3.8E-08
TMSB15A	22.3810782	635.40807	4.547269558	9.313808315	9.313808315	-4.82733	3.8E-08
HS3ST5	290.125088	8.2627837	8.185495362	3.211445828	3.211445828	5.133903	3.9E-08
LOC102161274	83.7218112	0	6.404661526	0	0	20	3.9E-08
GLIPR1	0.82892882	107.41619	0.870998931	6.76043638	6.76043638	-7.01775	3.9E-08
PDGFRL	27.3546512	755.21843	4.825513503	9.562659203	9.562659203	-4.78704	4E-08
LOC100737466	34.8150106	936.99967	5.162492464	9.87344361	9.87344361	-4.75027	4E-08
LOC102161846	127.655039	1.6525567	7.007364151	1.407383613	1.407383613	6.271407	4.1E-08
LOC102163653	106.102889	0.8262784	6.742853591	0.868906686	0.868906686	7.00462	4.2E-08
LOC100512381	406.175124	13.220454	8.669505612	3.829895615	3.829895615	4.941258	4.2E-08
LOC100737582	0	83.454116	0	6.400095824	6.400095824	-20	4.2E-08
ITM2A	227.955427	5.7839486	7.838922949	2.762125239	2.762125239	5.300553	4.2E-08
LOC102163946	82.8928824	0	6.39047651	0	0	20	4.3E-08
LOC100623553	326.597957	9.9153405	8.355782547	3.448285225	3.448285225	5.04171	4.3E-08
LOC100626442	543.777308	19.004403	9.089522802	4.322245637	4.322245637	4.83861	4.3E-08
KIRREL	116.050035	2962.208	6.870981559	11.53294417	11.53294417	-4.67385	4.3E-08
ODF3B	126.82611	1.6525567	6.998038744	1.407383613	1.407383613	6.262008	4.4E-08
LOC100620681	26.5257224	727.12497	4.78270852	9.508042269	9.508042269	-4.77674	4.4E-08
LOC102165647	8.28928824	288.37115	3.215568059	8.176777292	8.176777292	-5.12053	4.4E-08
PDLIM2	126.82611	3231.5747	6.998038744	11.658468	11.658468	-4.67131	4.4E-08
DKK1	3.31571529	168.56079	2.109599694	7.405658764	7.405658764	-5.66781	4.4E-08
CLDN11	14.09179	426.35964	3.915692025	8.739306855	8.739306855	-4.91914	4.4E-08
LOC100621301	2.48678647	147.90383	1.801898017	7.218237038	7.218237038	-5.89423	4.5E-08
LOC100157017	2884.67231	114.02642	11.49469176	6.845821397	6.845821397	4.660967	4.5E-08
EMX2	0	82.627837	0	6.385911347	6.385911347	-20	4.6E-08
UPK1B	1460.57259	57.013208	10.51330577	5.858309486	5.858309486	4.679094	4.6E-08
LOC780430	82.0639535	0	6.376150635	0	0	20	4.6E-08
TWIST1	247.020789	6279.7156	7.954317244	12.61671323	12.61671323	-4.66799	4.7E-08
LOC100511379	1034.50317	39.661362	10.01611626	5.34558663	5.34558663	4.70506	4.7E-08
LOC102162237	1.65785765	126.42059	1.410263837	6.993454623	6.993454623	-6.25277	4.7E-08
LOC100737186	9.94714588	324.7274	3.452482877	8.347521274	8.347521274	-5.0288	4.8E-08
OMD	2.48678647	146.25127	1.801898017	7.202136284	7.202136284	-5.87802	4.9E-08
LOC102159425	340.689747	10.741619	8.416543145	3.553559423	3.553559423	4.987175	4.9E-08
LOC100514469	0	81.801559	0	6.371586023	6.371586023	-20	5E-08
NKX2-2	0	81.801559	0	6.371586023	6.371586023	-20	5E-08
CGN	788.311311	29.746021	9.624450614	4.942327829	4.942327829	4.727997	5.1E-08
LOC100152487	92.8400282	2331.7576	6.552131542	11.18782067	11.18782067	-4.65053	5.1E-08
LOC100737020	3759.19222	102321.36	11.8765907	16.64276186	16.64276186	-4.76654	5.1E-08
P4HA3	385.451903	9791.3987	8.59414506	13.25744658	13.25744658	-4.66689	5.1E-08
NPHS2	124.339324	1.6525567	6.969695301	1.407383613	1.407383613	6.233439	5.2E-08
LOC102164431	80.4060959	0	6.347064926	0	0	20	5.5E-08
PPP1R17	80.4060959	0	6.347064926	0	0	20	5.5E-08
LOC100739471	24.0389359	649.4548	4.646101346	9.345304998	9.345304998	-4.75578	5.6E-08
NR2F1	72.9457365	1822.7701	6.208395063	10.83270815	10.83270815	-4.64317	5.6E-08
NNAT	335.716174	8396.6408	8.39538921	13.03576837	13.03576837	-4.6445	5.8E-08
LXN	42.27537	1078.2933	5.435474249	10.07587122	10.07587122	-4.67279	5.8E-08
LOC100512967	200.600775	4.9576702	7.655357377	2.574748269	2.574748269	5.338521	5.9E-08
LOC102163368	0	80.149002	0	6.342501448	6.342501448	-20	6E-08
ARGFX	79.5771671	0	6.332299179	0	0	20	6.1E-08
LOC100627431	79.5771671	0	6.332299179	0	0	20	6.1E-08
LOC100524424	121.852537	1.6525567	6.940783842	1.407383613	1.407383613	6.204293	6.2E-08
TGFB3	17.4075053	487.50424	4.202222212	8.932227272	8.932227272	-4.80763	6.2E-08
DPYD	37.3017971	951.87268	5.259340178	9.896139655	9.896139655	-4.67345	6.2E-08
SERPINB8	30.6703665	794.87979	4.985061656	9.636406738	9.636406738	-4.69582	6.3E-08
CELF5	101.129317	0.8262784	6.674253245	0.868906686	0.868906686	6.935358	6.4E-08
SLC15A1	198.942918	4.9576702	7.643444369	2.574748269	2.574748269	5.326548	6.4E-08
LOC100514323	1603.97727	65.275991	10.64833715	6.05041444	6.05041444	4.618957	6.4E-08
LOC100622354	140.9179	2.4788351	7.148912757	1.798604302	1.798604302	5.829049	6.5E-08
CWH43	78.7482382	0	6.317380743	0	0	20	6.6E-08
CACNA2D3	78.7482382	0	6.317380743	0	0	20	6.6E-08
C1QTNF5	148.378259	3612.489	7.222826382	11.8191768	11.8191768	-4.60564	6.7E-08
COLL11A1	1085.06783	27569.604	10.08489849	14.75084326	14.75084326	-4.66722	6.7E-08
LOC100511616	5.80250177	218.13749	2.766065426	7.775692512	7.775692512	-5.23242	6.7E-08
SMOX	92.8400282	2263.1765	6.552131542	11.14477068	11.14477068	-4.60746	6.7E-08
LOC100512568	120.194679	1.6525567	6.921182554	1.407383613	1.407383613	6.184529	7E-08
GAS6	81.2350247	1974.8053	6.361681078	10.94822508	10.94822508	-4.60346	7E-08
LOC100737024	533.001234	19.830681	9.060699264	4.380638094	4.380638094	4.748333	7.2E-08
LOC100738422	77.9193094	0	6.302306427	0	0	20	7.3E-08
DENND1C	531.343376	19.830681	9.056213313	4.380638094	4.380638094	4.743838	7.4E-08
LOC102166594	19.0653629	513.94515	4.326635347	9.008274952	9.008274952	-4.75259	7.7E-08
AOAH	509.791227	19.004403	8.996589935	4.322245637	4.322245637	4.745501	7.8E-08
LOC100522650	0	77.670167	0	6.29774474	6.29774474	-20	7.8E-08
ZKSCAN2	0	77.670167	0	6.29774474	6.29774474	-20	7.8E-08
LOC102160712	0	77.670167	0	6.29774474	6.29774474	-20	7.8E-08
GDAP1	1.65785765	118.98409	1.410263837	6.906699252	6.906699252	-6.1653	8.1E-08
FZD3	1563.35976	65.275991	10.61135662	6.05041444	6.05041444	4.581954	8.2E-08
SIM1	230.442213	6.610227	7.854508213	2.927939483	2.927939483	5.123561	8.2E-08
SH2D3C	13.2628612	379.26177	3.834191515	8.570849103	8.570849103	-4.83773	8.4E-08
MASP1	160.812192	3815.7535	7.338176502	11.89813031	11.89813031	-4.56852	8.5E-08
CD82	27.3546512	694.90011	4.825513503	9.442736426	9.442736426	-4.66695	8.5E-08
LOC100737474	168.272551	3981.0092	7.403204238	11.95928084	11.95928084	-4.56426	8.8E-08
PGA	76.2614518	0	6.271676881	0	0	20	8.8E-08
KIAA1958	1475.49331	61.970878	10.5279591	5.976612875	5.976612875	4.573463	8.8E-08
LOC102165788	337.374031	11.567897	8.402475042	3.65167138	3.65167138	4.86615	9E-08
SERPINB7	593.513038	23.135794	9.215564638	4.593102408	4.593102408	4.681081	9E-08
C3	188.995772	4449.509	7.569823503	12.11975464	12.11975464	-4.55722	9.3E-08
LOC102164235	0	76.01761	0	6.267116453	6.267116453	-20	9.4E-08
ZNF709	0	76.01761	0	6.267116453	6.267116453	-20	9.4E-08
LOC102166686	116.050035	1.6525567	6.870981559	1.407383613	1.407383613	6.133903	9.6E-08
ETV1	244.534003	5750.8975	7.939779021	12.48982224	12.48982224	-4.55568	9.6E-08
LTBP1	139.260042	3253.0579	7.131960259	11.66802423	11.66802423	-4.54594	9.8E-08
DUSP27	0.82892882	96.674569	0.870998931	6.609911086	6.609911086	-6.86574	9.9E-08
MAFB	11.6050035	335.46902	3.655924617	8.394329862	8.394329862	-4.85336	1E-07
LOC102160003	1734.1191	74.365053	10.76081898	6.2358238	6.2358238	4.543434	1E-07
LOC100737535	9.94714588	299.11277	3.452482877	8.229360901	8.229360901	-4.91026	1E-07
LOC100625609	296.756519	9.9153405	8.217989283	3.448285225	3.448285225	4.903474	1E-07
OTX1	1041.96353	43.792754	10.026473	5.485193456	5.485193456	4.572469	1E-07
MAGEL2	57.1960888	1347.66	5.862850292	10.397311	10.397311	-4.5584	1E-07
C1H15orf43	74.6035941	0	6.240382915	0	0	20	1.1E-07
LOC102158432	151.693975	3.3051135	7.254499324	2.106051269	2.106051269	5.52032	1.1E-07
EFCC1	114.392178	1.6525567	6.850401618	1.407383613	1.407383613	6.113145	1.1E-07
OTOG	114.392178	1.6525567	6.850401618	1.407383613	1.407383613	6.113145	1.1E-07
WNT2B	31.4992953	765.96005	5.022336531	9.583007622	9.583007622	-4.60388	1.1E-07
RXFP4	1.65785765	114.85269	1.410263837	6.856147779	6.856147779	-6.11432	1.1E-07
LOC100523457	2064.03277	90.064343	11.01194896	6.508814353	6.508814353	4.518366	1.2E-07
DCC	113.563249	1.6525567	6.8400005	1.407383613	1.407383613	6.102652	1.2E-07
NSUN7	398.714764	14.873011	8.642827052	3.98850389	3.98850389	4.744588	1.2E-07
LOC100738544	154.180761	3536.4714	7.277805898	11.78850278	11.78850278	-4.51962	1.2E-07
NLRC5	6.63143059	223.09516	2.93195353	7.807967682	7.807967682	-5.0722	1.2E-07
LOC100037976	3.31571529	151.20894	2.109599694	7.249909307	7.249909307	-5.51108	1.2E-07
ESRRG	327.426885	11.567897	8.359428422	3.65167138	3.65167138	4.822974	1.2E-07
PPARG	33.1571529	797.35863	5.094115824	9.640893151	9.640893151	-4.58784	1.2E-07
NR2E1	185.680057	4.9576702	7.544423999	2.574748269	2.574748269	5.227013	1.2E-07
ACTG2	930.887069	22116.993	9.864011322	14.43293287	14.43293287	-4.57041	1.2E-07
LOC100738758	256.139007	8.2627837	8.006404665	3.211445828	3.211445828	4.954155	1.2E-07
LOC100737139	860.428119	36.356248	9.750586607	5.223277669	5.223277669	4.564779	1.2E-07
LOC102166137	7.46035941	239.62073	3.080718953	7.910617116	7.910617116	-5.00536	1.2E-07
HOXA4	3.31571529	150.38266	2.109599694	7.242056188	7.242056188	-5.50317	1.2E-07
PARP12	187.337914	4273.5117	7.557179646	12.06154392	12.06154392	-4.51171	1.2E-07
LOC100624658	0	73.538775	0	6.219919206	6.219919206	-20	1.3E-07
LOC100517751	0	73.538775	0	6.219919206	6.219919206	-20	1.3E-07
PDZD3	289.296159	9.9153405	8.181381675	3.448285225	3.448285225	4.866741	1.3E-07
IGFBPL1	72.9457365	0	6.208395063	0	0	20	1.3E-07
LOC100624606	72.9457365	0	6.208395063	0	0	20	1.3E-07
LOC100621379	16.5785765	431.31731	4.135746339	8.755946793	8.755946793	-4.70136	1.3E-07
ERBB3	409.490839	15.699289	8.681206215	4.061714779	4.061714779	4.70506	1.4E-07
PPFIA4	48.0778718	1108.0393	5.617000784	10.11509477	10.11509477	-4.52649	1.4E-07

LOC102167305	0	90.064343	0	6.508814353	6.508814353	-20	4.3E-05	
LOC102159210	42.8051049	0	5.453027102	0	0	20	4.4E-05	
LOC102157580	42.8051049	0	5.453027102	0	0	20	4.4E-05	
LOC100155246	42.8051049	0	5.453027102	0	0	20	4.4E-05	
SOX7	42.8051049	0	5.453027102	0	0	20	4.4E-05	
TSPAN33	1875.13976	131.37826	10.87355159	7.048522415	7.048522415	3.8352	4.4E-05	
LOC100738887	1.38080984	124.76803	1.251452392	6.974621475	6.974621475	-6.49759	4.5E-05	
LOC102157630	0	89.238064	0	6.495664213	6.495664213	-20	4.6E-05	
PCPA1	0	89.238064	0	6.495664213	6.495664213	-20	4.6E-05	
IGF2BP2	230.595243	6145.8585	7.855461808	12.58563357	12.58563357	-4.73618	4.6E-05	
LOC102162895	13.8080984	422.22825	3.888314478	8.725292112	8.725292112	-4.93444	4.6E-05	
CILP2	8.28485901	289.19743	3.214880004	8.180890933	8.180890933	-5.12543	4.6E-05	
LOC102165157	793.965656	55.360651	9.634748723	5.816616368	5.816616368	3.842144	4.7E-05	
IRK1	2.76161967	156.99289	1.911353989	7.303715831	7.303715831	-5.82904	4.8E-05	
PRSS16	122.892075	5.7839486	6.9529401	2.762125239	2.762125239	4.409193	4.9E-05	
SLC15A3	38.6626754	1022.1063	5.309710091	9.998740397	9.998740397	-4.72446	4.9E-05	
LOC100739389	243.022531	14.873011	7.930870551	3.98850389	3.98850389	4.030321	4.9E-05	
RHCG	2.76161967	156.16661	1.911353989	7.296150961	7.296150961	-5.82143	4.9E-05	
MYO16	209.883095	12.394176	7.720299639	3.74353388	3.74353388	4.081852	5E-05	
ITGB2	834.009141	58.665764	9.705648181	5.89883146	5.89883146	3.829472	5E-05	
OCA2	0	87.585507	0	6.468998788	6.468998788	-20	5E-05	
PLSCR4	22.0929574	610.61972	4.529381037	9.256491105	9.256491105	-4.78862	5.1E-05	
42432	34.5202459	910.55877	5.150569664	9.832191855	9.832191855	-4.72124	5.2E-05	
ATOH8	8.28485901	283.41348	3.214880004	8.151846042	8.151846042	-5.09629	5.2E-05	
ACACB	75.944541	1940.9279	6.265747069	10.92327392	10.92327392	-4.67566	5.2E-05	
LOC100736679	1.38080984	121.46292	1.251452392	6.936201185	6.936201185	-6.45886	5.2E-05	
LRP1	1110.17111	30988.744	10.11786528	14.91950322	14.91950322	-4.80289	5.3E-05	
LOC100523833	5.52323934	218.13749	2.705588563	7.775692512	7.775692512	-5.30358	5.3E-05	
ARNT2	73.1829213	1853.3424	6.213015177	10.85669193	10.85669193	-4.66248	5.5E-05	
LOC102157775	41.4242951	0	5.406818783	0	0	20	5.5E-05	
SP6	41.4242951	0	5.406818783	0	0	20	5.5E-05	
ALOXE3	41.4242951	0	5.406818783	0	0	20	5.5E-05	
TOX	31.7586262	832.8886	5.033802952	9.703710853	9.703710853	-4.7129	5.5E-05	
PEAK1	44.1859147	1132.8276	5.497801224	10.14698564	10.14698564	-4.6802	5.6E-05	
F5	0	85.932951	0	6.441831208	6.441831208	-20	5.6E-05	
LOC100623096	49.7091541	1265.8585	5.664174303	10.30703964	10.30703964	-4.67046	5.6E-05	
HEPH	2.76161967	152.03522	1.911353989	7.257719911	7.257719911	-5.78275	5.7E-05	
ATP8B1	16.569718	470.15239	4.135019133	8.880049963	8.880049963	-4.82651	5.7E-05	
GPX3	33.1394361	849.41417	5.093367322	9.732021819	9.732021819	-4.67985	6.2E-05	
LOC100156874	0	84.280394	0	6.414142197	6.414142197	-20	6.2E-05	
LOC100736623	0	84.280394	0	6.414142197	6.414142197	-20	6.2E-05	
LOC100512907	0	84.280394	0	6.414142197	6.414142197	-20	6.2E-05	
LOC102163142	0	84.280394	0	6.414142197	6.414142197	-20	6.2E-05	
LOC100525036	5.52323934	212.35354	2.705588563	7.737102248	7.737102248	-5.26481	6.2E-05	
FOXF2	11.0464787	337.12158	3.590539588	8.401398268	8.401398268	-4.93161	6.2E-05	
LOC100519756	129.796125	6.610227	7.031175985	2.927939483	2.927939483	4.295404	6.2E-05	
LY6H	64.8980623	1.6525567	6.042164139	1.407383613	1.407383613	5.295404	6.2E-05	
ATCAY	64.8980623	1.6525567	6.042164139	1.407383613	1.407383613	5.295404	6.2E-05	
KLHL41	11.0464787	336.2953	3.590539588	8.397868394	8.397868394	-4.92807	6.3E-05	
RBP5	1.38080984	117.33153	1.251452392	6.886690713	6.886690713	-6.40893	6.3E-05	
LOC102159968	2.76161967	148.73011	1.911353989	7.226220529	7.226220529	-5.75104	6.4E-05	
LOC100620787	62.1364426	1531.0938	5.98040107	10.58128893	10.58128893	-4.62298	6.5E-05	
LOC102164464	0	83.454116	0	6.400095824	6.400095824	-20	6.5E-05	
SLAIN1	1371.14417	100.80596	10.42221635	6.669678233	6.669678233	3.765727	6.6E-05	
ZNF613	11.0464787	332.99018	3.590539588	8.383661891	8.383661891	-4.91382	6.6E-05	
ACTN2	12.4272885	363.56248	3.747096094	8.510022294	8.510022294	-4.87062	6.7E-05	
MADCAM1	96.6566885	4.1313919	6.609646952	2.359350201	2.359350201	4.54817	6.8E-05	
LOC100523068	2.76161967	147.07755	1.911353989	7.210209122	7.210209122	-5.73492	6.8E-05	
RGS7BP	0	82.627837	0	6.385911347	6.385911347	-20	6.9E-05	
EMILIN2	357.629748	9008.9131	8.486351351	13.13729747	13.13729747	-4.65481	6.9E-05	
PPIF	3083.34836	226.40027	11.59075	7.829090182	7.829090182	3.76755	6.9E-05	
LOC102167710	15.1889082	422.22825	4.016933786	8.725292112	8.725292112	-4.79693	0.00007	
VPAC1	52.4707738	0.8262784	5.740678649	0.868906686	0.868906686	5.988742	7E-05	
SPP1	57.9940131	1412.1097	5.882496648	10.46465779	10.46465779	-4.6058	7E-05	
LOC100627899	2.76161967	146.25127	1.911353989	7.202136284	7.202136284	-5.72679	7.1E-05	
LOC102161206	40.0434852	0	5.359081339	0	0	20	7.1E-05	
LOC100737191	40.0434852	0	5.359081339	0	0	20	7.1E-05	
LOC100737075	40.0434852	0	5.359081339	0	0	20	7.1E-05	
LOC102162614	8.28485901	267.71419	3.214880004	8.06992871	8.06992871	-5.01407	7.1E-05	
LOC100737584	20.7121475	544.51745	4.440430524	9.091481528	9.091481528	-4.71643	7.2E-05	
MDGA2	0	81.801559	0	6.371586023	6.371586023	-20	7.3E-05	
NKX2-2	0	81.801559	0	6.371586023	6.371586023	-20	7.3E-05	
LOC100739353	0	81.801559	0	6.371586023	6.371586023	-20	7.3E-05	
ANKRD35	33.1394361	825.45209	5.093367322	9.690787384	9.690787384	-4.63856	7.3E-05	
ADAMTS12	2.76161967	145.42499	1.911353989	7.194018019	7.194018019	-5.71862	7.3E-05	
GLIS3	6.90404918	236.31561	2.982591923	7.890663216	7.890663216	-5.09713	7.3E-05	
LOC100517249	63.5172524	1.6525567	6.011613095	1.407383613	1.407383613	5.264377	7.3E-05	
AKAP6	8.28485901	266.06164	3.214880004	8.061028933	8.061028933	-5.00514	7.4E-05	
LGI2	168.4588	9.9153405	7.404790748	3.448285225	3.448285225	4.08659	7.4E-05	
HOXC4	27.6161967	694.90011	4.838760036	9.442736426	9.442736426	-4.65322	7.5E-05	
LOC102163193	95.2758787	4.1313919	6.58910248	2.359350201	2.359350201	4.527411	7.6E-05	
MFSD6	1634.87885	122.2892	10.67585019	6.945902605	6.945902605	3.740815	7.6E-05	
LOC100515328	15.1889082	415.61802	4.016933786	8.702581435	8.702581435	-4.77417	7.7E-05	
LOC100525978	0	80.97528	0	6.357117027	6.357117027	-20	7.7E-05	
LOC100625107	5.52323934	204.09076	2.705588563	7.68011867	7.68011867	-5.20755	7.7E-05	
PODN	5.52323934	203.26448	2.705588563	7.674294538	7.674294538	-5.2017	7.9E-05	
NKAIN3	84.2294	3.3051135	6.413279271	2.106051269	2.106051269	4.671552	8E-05	
LOC102163118	84.2294	3.3051135	6.413279271	2.106051269	2.106051269	4.671552	8E-05	
CXCL2	89.7526393	2125.188	6.503867696	11.05405343	11.05405343	-4.56549	8E-05	
LOC100622435	9.66566885	291.67627	3.414902536	8.193161944	8.193161944	-4.91535	8.1E-05	
FNDC5	0	80.149002	0	6.342501448	6.342501448	-20	8.1E-05	
LOC102163368	0	80.149002	0	6.342501448	6.342501448	-20	8.1E-05	
LOC102157645	4.14242951	171.8659	2.362450112	7.433509508	7.433509508	-5.37466	8.3E-05	
QPCT	8.28485901	260.27769	3.214880004	8.029440115	8.029440115	-4.97343	8.4E-05	
LOC102160320	114.607216	5.7839486	6.853087646	2.762125239	2.762125239	4.308499	8.5E-05	
ANO5	0	79.322724	0	6.327736286	6.327736286	-20	8.6E-05	
C3	187.790138	4449.509	7.560639591	12.11975464	12.11975464	-4.56645	8.6E-05	
LOC102158609	51.0899639	0.8262784	5.702933533	0.868906686	0.868906686	5.950268	8.6E-05	
LOC102162369	74.5637311	1743.4474	6.239622035	10.76855435	10.76855435	-4.54732	8.6E-05	
CEBPE	62.1364426	1.6525567	5.98040107	1.407383613	1.407383613	5.232668	8.7E-05	
LOC100154755	62.1364426	1.6525567	5.98040107	1.407383613	1.407383613	5.232668	8.7E-05	
EXOC3L1	62.1364426	1.6525567	5.98040107	1.407383613	1.407383613	5.232668	8.7E-05	
LOC100625994	62.1364426	1.6525567	5.98040107	1.407383613	1.407383613	5.232668	8.7E-05	
SORD	8440.89053	620.53506	13.04335041	9.279691957	9.279691957	3.765811	8.7E-05	
LOC100153281	59.3748229	1390.6265	5.915875148	10.44255634	10.44255634	-4.54974	8.7E-05	
LOC102158647	124.272885	6.610227	6.968930373	2.927939483	2.927939483	4.232668	8.8E-05	
LOC100623939	19.3313377	494.11447	4.345633235	8.951618292	8.951618292	-4.67583	8.9E-05	
LOC100522644	4.14242951	169.38707	2.362450112	7.412672017	7.412672017	-5.3537	9E-05	
LOC100621868	4.14242951	169.38707	2.362450112	7.412672017	7.412672017	-5.3537	9E-05	
LOC102159468	0	78.496445	0	6.312818447	6.312818447	-20	9E-05	
NTS	0	78.496445	0	6.312818447	6.312818447	-20	9E-05	
LOC102164702	0	78.496445	0	6.312818447	6.312818447	-20	9E-05	
LOC100622236	38.6626754	0	5.309710091	0	0	20	9.1E-05	
LOC102157622	38.6626754	0	5.309710091	0	0	20	9.1E-05	
LOC100511891	38.6626754	0	5.309710091	0	0	20	9.1E-05	
PTAFR	38.6626754	0	5.309710091	0	0	20	9.1E-05	
LOC102166434	38.6626754	0	5.309710091	0	0	20	9.1E-05	
SLC6A20	38.6626754	0	5.309710091	0	0	20	9.1E-05	
AVP	38.6626754	0	5.309710091	0	0	20	9.1E-05	
LOC100737912	8.28485901	256.1463	3.214880004	8.006445558	8.006445558	-4.95035	9.1E-05	
ESRP1	731.829213	55.360651	9.517333205	5.816616368	5.816616368	3.724574	9.2E-05	
LOC102160929	113.226407	5.7839486	6.835752397	2.762125239	2.762125239	4.291012	9.3E-05	
IFI44L	370.057036	8855.2253	8.535497154	13.11247621	13.11247621	-4.58071	9.4E-05	
HOXB8	78.7061606	1809.5496	6.316619332	10.82221201	10.82221201	-4.52301	9.5E-05	
HLF	153.269892	9.0890621	7.269312714	3.334720158	3.334720158	4.075799	9.5E-05	
NETO2	153.269892	9.0890621	7.269312714	3.334720158	3.334720158	4.075799	9.5E-05	
LOC100622492	0	77.670167	0	6.29774474	6.29774474	-20	9.6E-05	
C1QTNF7	0	77.670167	0	6.29774474	6.29774474	-20	9.6E-05	
LOC102166831	2.76161967	137.98849	1.911353989	7.118821585	7.118821585	-5.64289	9.7E-05	
HSPG2	706.974636	17333.668	9.467553865	14.08137256	14.08137256	-4.61577	9.7E-05	
LOC102167451	747.018121	57.013208	9.54692941	5.858309486	5.858309486	3.711775	9.8E-05	
GPM6B	46.9475344	1083.2509	5.583384725	10.08248299	10.08248299	-4.52817	9.9E-05	
LOC102165939	27.6161967	660.19642	4.838760036	9.368935101	9.368935101	-4.57931	0.0001	
FSCN2	13.8080984	366.8676	3.888314478	8.523042794	8.523042794	-4.73167	0.0001	
SLC1A6	1027.32252	79.322724	10.0060771	6.327736286	6.327736286	3.695011	0.0001	
LOC102161033	42.8051049	987.40265	5.453027102	9.948955075	9.948955075	-4.52778	0.0001	
PAQR5	111.845597	5.7839486	6.818206315	2.762125239	2.762125239	4.27331	0.0001	
C14H10orf82	60.7556328	1.6525567	5.948498827	1.407383613	1.407383613	5.200246	0.0001	
LOC100738560	60.7556328	1.6525567	5.948498827	1.407383613	1.407383613	5.200246	0.0001	
ACAP1	372.818656	27.267186	8.546194759	4.821056379	4.821056379	3.773236	0.0001	
LOC100516238	121.511266	6.610227	6.936770608	2.927939483	2.927939483	4.200246	0.0001	
LOC100624711	1.38080984	106.58991	1.251452392	6.749398975	6.749398975	-6.27041	0.00011	
LOC102163074	6.90404918	220.61633	2.982591923	7.791920351	7.791920351	-4.99795	0.00011	
DOCK10	2.76161967	135.50965	1.911353989	7.092859162	7.092859162	-5.61674	0.00011	
SSTR1	0	76.01761	0	6.267116453	6.267116453	-20	0.00011	
CCDC110	0	76.01761	0	6.267116453	6.267116453	-20	0.00011	
LEPREL2	436.335908	10254.115	8.772597998	13.32405599	13.32405599	-4.55462	0.00011	
LOC100516650	26.2353869	621.36134	4.767410456	9.281608625	9.281608625	-4.56585	0.00011	
LOC100515976	6.90404918	219.79005	2.982591923	7.786531327	7.786531327	-4.99254	0.00011	
GAS6	88.3718295	1974.8053	6.481748252	10.94822508	10.94822508	-4.48198	0.00011	
LOC100621566	5.52323934	190.8703	2.705588563	7.58398763	7.58398763	-5.11093	0.00011	
TNNI1	12.4272885	331.33763	3.747096094	8.376505833	8.376505833	-4.73672	0.00011	
SRPX	44.1859147	999.79683	5.497801224	9.966933409	9.966933409	-4.49998	0.00011	
KCNIP4	179.505279	11.567897	7.495897217	3.65167138	3.65167138	3.955828	0.00011	
FAM185A	1400.14117	109.89502	10.45238661	6.793050813	6.793050813	3.671374	0.00011	
MYOT	0	75.191332	0	6.251554969	6.251554969	-20	0.00011	
LOC100156578	0	75.191332	0	6.251554969	6.251554969	-20	0.00011	
LOC102164159	0	75.191332	0	6.251554969	6.251554969	-20	0.00011	
LOC100736635	0	75.191332	0	6.251554969	6.251554969	-20	0.00011	
LOC100623148	5.52323934	190.04403	2.705588563	7.577761331	7.577761331	-5.10468	0.00011	
FRMPD1	120.130456	6.610227	6.920417836	2.927939483	2.927939483	4.183758	0.00011	
BICC1	28.9970066	672.59059	4.906746634	9.395728184	9.395728184	-4.53575	0.00012	
VCAN	374.199466	8627.9988	8.551513692	13.07497745	13.07497745	-4.52715	0.00012	
ARHGAP8	792.584846	61.970878	9.632240666	5.976612875	5.976612875	3.676903	0.00012	
SNED1	34.5202459	786.61701	5.150569664	9.621350459	9.621350459	-4.51015	0.00012	
LOC102162351	2.76161967	133.03082	1.911353989	7.066420948	7.066420948	-5.5901	0.00012	
LOC100525734	37.2818656	0	5.258589232	0	0	20	0.00012	
LOC102164369	37.2818656	0	5.258589232	0	0	20	0.00012	
LOC100521071	37.2818656	0	5.258589232	0	0	20	0.00012	
MET	5.52323934	188.39147	2.705588563	7.565227535	7.565227535	-5.09208	0.00012	
LOC102166540	0	74.365053	0	6.2358238	6.2358238	-20	0.00012	
LOC102167766	0	74.365053	0	6.2358238	6.2358238	-20	0.00012	
PBX1	194.694187	4349.5293	7.61245709	12.08697524	12.08697524	-4.48158	0.00012	
LOC100156557	89.7526393	4.1313919	6.503867696	2.359350201	2.359350201	4.441255	0.00012	
LOC100623335	1.38080984	104.11107	1.251452392	6.715770874	6.715770874	-6.23647	0.00012	
CCDC147	59.3748229	1.6525567	5.915875148	1.407383613	1.407383613	5.16708	0.00012	
LOC100155350	110.464787	2431.7372	6.800444207	11.24836479	11.24836479	-4.46033	0.00012	
LOC102164964	109.083977	5.7839486	6.782460686	2.762125239	2.762125239	4.237241	0.00012	
SDK2	8.28485901	242.09956	3.214880004	7.925403489	7.925403489	-4.86898	0.00012	
PLXNB3	664.169531	52.055537	9.377578275	5.729431428	5.729431428	3.673428	0.00013	
SEMA7A	1.38080984	103.2848	1.251452392	6.704385035	6.704385035	-6.22497	0.00013	
LOC100626467	0	73.538775	0	6.219919206	6.219919206	-20	0.00013	
GPR123	0	73.538775	0	6.219919206	6.219919206	-20	0.00013	
FUT4	11.0464787	295.80766	3.590539588	8.213384501	8.213384501	-4.743	0.00013	
KLF6	287.208446	6422.6618	8.170968803	12.64918022	12.64918022	-4.483	0.00013	
ELTD1	2.76161967	130.55198	1.911353989	7.039489182	7.039489182	-5.56297	0.00013	
DRP2	1054.93872	84.280394	10.04431039	6.414142197	6.414142197	3.645818	0.00013	
BMP5	78.7061606	3.3051135	6.316619332	2.106051269	2.106051269	4.573705	0.00013	
PTCHD1	0	72.712497	0	6.20383732	6.20383732	-20	0.00014	
[illegible]	[illegible]	[illegible]	[illegible]	[illegible]	[illegible]	[illegible]	[illegible]	

MORC1	34.5202459	0	5.150569664	0	0	20	0.0002
LOC102163381	34.5202459	0	5.150569664	0	0	20	0.0002
TNFSF10	5.52323934	171.03962	2.705588563	7.426597064	7.426597064	-4.95267	0.0002
LOC100522595	4.14242951	145.42499	2.362450112	7.194018019	7.194018019	-5.13365	0.00021
KCNN1	397.673233	32.224857	8.639062934	5.054191063	5.054191063	3.625338	0.00021
MAGI2	2.76161967	119.81036	1.911353989	6.916600414	6.916600414	-5.43909	0.00021
TRIM55	0	66.928548	0	6.085946114	6.085946114	-20	0.00021
LRFN5	0	66.928548	0	6.085946114	6.085946114	-20	0.00021
NOR-1	276.161967	21.483238	8.11458549	4.490777898	4.490777898	3.684231	0.00021
TNK1	359.010557	28.919743	8.491895404	4.903025878	4.903025878	3.6339	0.00021
CADM3	621.364426	52.055537	9.281615789	5.729431428	5.729431428	3.577316	0.00021
SLC16A10	508.13802	42.140197	8.991912992	5.430960858	5.430960858	3.591951	0.00021
LOC102163300	19.3313377	421.40197	4.345633235	8.722472752	8.722472752	-4.44618	0.00021
GULP1	52.4707738	1048.5473	5.740678649	10.03555141	10.03555141	-4.32073	0.00022
LOC102162054	28.9970066	599.8781	4.906746634	9.230928526	9.230928526	-4.37069	0.00022
DKK1	5.52323934	168.56079	2.705588563	7.405658764	7.405658764	-4.93161	0.00022
LOC100622865	6.90404918	193.34914	2.982591923	7.602506906	7.602506906	-4.80762	0.00022
LOC100738804	23.4737672	495.76702	4.613164285	8.956425596	8.956425596	-4.40054	0.00022
TENM1	0	66.10227	0	6.068289661	6.068289661	-20	0.00022
PRRT1	2.76161967	118.15781	1.911353989	6.89672967	6.89672967	-5.41906	0.00022
TPST2	3221.42935	271.84558	11.65393301	8.091940885	8.091940885	3.566842	0.00022
LOC100523100	73.1829213	3.3051135	6.213015177	2.106051269	2.106051269	4.468735	0.00023
MAP2K6	327.251931	26.440908	8.358659688	4.77825631	4.77825631	3.629558	0.00023
LRRC37A	317.586262	25.61463	8.315540247	4.734147579	4.734147579	3.632109	0.00024
OSAP	307.920593	24.788351	8.271092238	4.688647628	4.688647628	3.634824	0.00024
LOC100737121	262.353869	20.656959	8.040858843	4.436758793	4.436758793	3.666814	0.00024
FRZB	4.14242951	141.2936	2.362450112	7.152726981	7.152726981	-5.09208	0.00024
CASZ1	144.985033	9.9153405	7.189676653	3.448285225	3.448285225	3.870098	0.00024
CD200	1169.54593	100.80596	10.19296583	6.669678233	6.669678233	3.536296	0.00024
LOC100037967	1746.72444	150.38266	10.77126202	7.242056188	7.242056188	3.537942	0.00024
C7H15orf27	316.205452	25.61463	8.309273759	4.734147579	4.734147579	3.625822	0.00024
CMAH	5.52323934	165.25567	2.705588563	7.37725977	7.37725977	-4.90304	0.00024
NNAT	414.242951	8396.6408	8.697811867	13.03576837	13.03576837	-4.34126	0.00025
LOC100511764	44.1859147	0.8262784	5.497801224	0.868906686	0.868906686	5.740815	0.00025
MAST1	44.1859147	0.8262784	5.497801224	0.868906686	0.868906686	5.740815	0.00025
LOC102167117	44.1859147	0.8262784	5.497801224	0.868906686	0.868906686	5.740815	0.00025
ITGB3	118.749646	2304.4904	6.90387758	11.17085793	11.17085793	-4.27845	0.00025

[illegible]

LOC100624264	2.76161967	95.848291	1.911353989	6.597654688	6.597654688	-5.11717	0.00067
LOC100513233	2.76161967	95.848291	1.911353989	6.597654688	6.597654688	-5.11717	0.00067
TRABD2A	8.28485901	176.82357	3.214880004	7.474302765	7.474302765	-4.41569	0.00068
LOC100625416	75.944541	1223.7183	6.265747069	10.2582342	10.2582342	-4.01018	0.00068
LOC100737535	16.569718	299.11277	4.135019133	8.229360901	8.229360901	-4.17407	0.00069
LOC102168145	157.412321	13.220454	7.307540742	3.829895615	3.829895615	3.573705	0.00069
AXL	544.039075	9277.4536	9.090215854	13.17966866	13.17966866	-4.09195	0.00069
C7H15orf59	0	52.881816	0	5.751726565	5.751726565	-20	0.00069
ADAMTSL1	0	52.881816	0	5.751726565	5.751726565	-20	0.00069
SLC38A4	0	52.881816	0	5.751726565	5.751726565	-20	0.00069
PID1	0	52.881816	0	5.751726565	5.751726565	-20	0.00069
LOC100627583	0	52.881816	0	5.751726565	5.751726565	-20	0.00069
LOC100515572	0	52.881816	0	5.751726565	5.751726565	-20	0.00069
ABCA13	526.088547	51.229259	9.041901535	5.706786332	5.706786332	3.360266	0.0007
PPFIA2	379.722705	36.356248	8.572596799	5.223277669	5.223277669	3.384671	0.0007
ABLIM1	1349.05121	133.03082	10.39879842	7.066420948	7.066420948	3.342113	0.0007
LOC100521998	661.407911	65.275991	9.371576093	6.05041444	6.05041444	3.340916	0.00073
LYSMD2	70.4213016	4.1313919	6.158282521	2.359350201	2.359350201	4.091312	0.00073
MPPED2	1679.06476	166.08195	10.71430113	7.384412099	7.384412099	3.337691	0.00073
CYP4V2	24.854577	418.09686	4.692347799	8.711139889	8.711139889	-4.07225	0.00074
SLITRK4	156.031511	13.220454	7.294910283	3.829895615	3.829895615	3.560994	0.00074
LOC100524058	376.961085	36.356248	8.562093892	5.223277669	5.223277669	3.37414	0.00074
LOC100737021	5.52323934	133.8571	2.705588563	7.075287628	7.075287628	-4.59904	0.00075
LOC100514491	613.079567	10365.662	9.262281789	13.33966383	13.33966383	-4.07959	0.00075
LOC100736817	0	52.055537	0	5.729431428	5.729431428	-20	0.00075
LRRC31	0	52.055537	0	5.729431428	5.729431428	-20	0.00075
KCNF1	109.083977	8.2627837	6.782460686	3.211445828	3.211445828	3.722667	0.00075
LOC100737137	45.5667246	729.6038	5.541227504	9.512945452	9.512945452	-4.00106	0.00075
LOC102158261	62.1364426	3.3051135	5.98040107	2.106051269	2.106051269	4.232668	0.00076
TMEM177	1687.34962	167.73451	10.72139797	7.398611253	7.398611253	3.330507	0.00076
UNC5B	274.781157	4459.4244	8.107380078	12.12296526	12.12296526	-4.0205	0.00077
LOC100623035	24.854577	414.79174	4.692347799	8.699717297	8.699717297	-4.0608	0.00077
NOTCH2	531.611787	8880.0137	9.056940546	13.11650864	13.11650864	-4.06212	0.00077
LOC100738863	75.944541	1196.4511	6.265747069	10.22575101	10.22575101	-3.97767	0.00077
LOC102162528	22.0929574	373.47782	4.529381037	8.548736477	8.548736477	-4.07936	0.00077
LOC100628061	33.1394361	537.90722	5.093367322	9.073893105	9.073893105	-4.02074	0.00077
LOC100739847	272.019538	25.61463	8.092860386	4.734147579	4.734147579	3.40867	0.00078
CRABP2	921.00016	15784.396	9.848623191	13.94630281	13.94630281	-4.09915	0.00078
SYNM	182.266898	2902.7159	7.517802419	11.5036846	11.5036846	-3.99328	0.00078
LOC100622846	1.38080984	72.712497	1.251452392	6.20383732	6.20383732	-5.71862	0.00079
LOC100739371	1.38080984	72.712497	1.251452392	6.20383732	6.20383732	-5.71862	0.00079
VCL	2587.63763	46834.284	11.33797731	15.5153082	15.5153082	-4.17786	0.00079
LOC102157528	37.2818656	0.8262784	5.258589232	0.868906686	0.868906686	5.495702	0.0008
LOC102161349	37.2818656	0.8262784	5.258589232	0.868906686	0.868906686	5.495702	0.0008
LOC100156741	37.2818656	0.8262784	5.258589232	0.868906686	0.868906686	5.495702	0.0008
LOC100737408	37.2818656	0.8262784	5.258589232	0.868906686	0.868906686	5.495702	0.0008
LOC100154508	169.83961	14.873011	7.416498698	3.98850389	3.98850389	3.513404	0.0008
CASP4	244.403341	3914.9069	7.93901108	11.93513076	11.93513076	-4.00164	0.0008
SLC44A5	2513.0739	249.53607	11.29581134	7.968874505	7.968874505	3.332133	0.00081
LOC100738373	4.14242951	112.37386	2.362450112	6.824944216	6.824944216	-4.76169	0.00081
LOC100516350	0	51.229259	0	5.706786332	5.706786332	-20	0.00081
LOC100626805	0	51.229259	0	5.706786332	5.706786332	-20	0.00081
LOC102166113	0	51.229259	0	5.706786332	5.706786332	-20	0.00081
UCMA	0	51.229259	0	5.706786332	5.706786332	-20	0.00081
LOC100737841	0	51.229259	0	5.706786332	5.706786332	-20	0.00081
SULT2B1	92.514259	6.610227	6.547114458	2.927939483	2.927939483	3.806904	0.00082
LOC100736741	967.947695	97.500848	9.920274979	6.622064238	6.622064238	3.311442	0.00082
LOC102158761	107.703167	8.2627837	6.764250165	3.211445828	3.211445828	3.704289	0.00082
KRT14	107.703167	8.2627837	6.764250165	3.211445828	3.211445828	3.704289	0.00082
LOC102165647	16.569718	288.37115	4.135019133	8.176777292	8.176777292	-4.12131	0.00083
LOC100626318	69.0404918	4.1313919	6.130117309	2.359350201	2.359350201	4.062743	0.00084
SPINT1	153.269892	13.220454	7.269312714	3.829895615	3.829895615	3.535231	0.00084
MID1	12.4272885	228.05283	3.747096094	7.839536581	7.839536581	-4.19778	0.00085
UNC-6	19.3313377	326.37996	4.345633235	8.354822188	8.354822188	-4.07754	0.00086
LOC102157895	0	50.402981	0	5.683780114	5.683780114	-20	0.00088
HOXD1	5.52323934	129.7257	2.705588563	7.030399034	7.030399034	-4.55381	0.00088
ZNFX1	473.617774	7672.821	8.890622318	12.90572939	12.90572939	-4.01796	0.00089
LOC102159499	60.7556328	3.3051135	5.948498827	2.106051269	2.106051269	4.200246	0.00089
LOC100520832	60.7556328	3.3051135	5.948498827	2.106051269	2.106051269	4.200246	0.00089
LOC100155159	1.38080984	71.05994	1.251452392	6.171125545	6.171125545	-5.68545	0.00089
LOC100622412	27.6161967	0	4.838760036	0	0	20	0.0009
TDRD1	27.6161967	0	4.838760036	0	0	20	0.0009
LOC102159671	27.6161967	0	4.838760036	0	0	20	0.0009
LOC102161389	27.6161967	0	4.838760036	0	0	20	0.0009
PRDM14	27.6161967	0	4.838760036	0	0	20	0.0009
MIP	27.6161967	0	4.838760036	0	0	20	0.0009
FAM179A	27.6161967	0	4.838760036	0	0	20	0.0009
P2RY14	27.6161967	0	4.838760036	0	0	20	0.0009
MARVELD3	27.6161967	0	4.838760036	0	0	20	0.0009
IGLON5	69.0404918	1059.2889	6.130117309	10.05024166	10.05024166	-3.93951	0.0009
RNF213	776.015128	12863.502	9.601798876	13.65110795	13.65110795	-4.05105	0.0009
LOC100620141	251.30739	23.962073	7.979038653	4.641665831	4.641665831	3.390628	0.0009
LOC102163243	197.455807	18.178124	7.632673964	4.261389712	4.261389712	3.441255	0.00091
KBTBD8	785.680797	80.149002	9.619634556	6.342501448	6.342501448	3.293187	0.00091
TRPC3	26.2353869	421.40197	4.767410456	8.722472752	8.722472752	-4.00561	0.00091
C1QTNF1	138.080984	2121.0566	7.119781365	11.05124741	11.05124741	-3.9412	0.00092
LOC102158142	8.28485901	166.90823	3.214880004	7.391529145	7.391529145	-4.33243	0.00092
SRSF12	847.817239	86.759229	9.729310147	6.455478947	6.455478947	3.288664	0.00093
PIP4K2C	791.204036	80.97528	9.629728241	6.357117027	6.357117027	3.288496	0.00094
LOC102157511	249.92658	23.962073	7.971121491	4.641665831	4.641665831	3.38268	0.00094
LOC102159425	128.415315	10.741619	7.015864542	3.553559423	3.553559423	3.579534	0.00094
DENND2D	128.415315	10.741619	7.015864542	3.553559423	3.553559423	3.579534	0.00094
LOC102166308	1.38080984	70.233662	1.251452392	6.154487245	6.154487245	-5.66858	0.00095
LOC100627126	1.38080984	70.233662	1.251452392	6.154487245	6.154487245	-5.66858	0.00095
42430	52.4707738	2.4788351	5.740678649	1.798604302	1.798604302	4.40378	0.00095
KCP	9.66566885	185.08636	3.414902536	7.539828464	7.539828464	-4.25919	0.00095
LOC100520518	0	49.576702	0	5.660401068	5.660401068	-20	0.00096
OMG	67.659682	4.1313919	6.101391269	2.359350201	2.359350201	4.033597	0.00097
IRAK3	8.28485901	165.25567	3.214880004	7.37725977	7.37725977	-4.31808	0.00097
FOXC2	49.7091541	753.56588	5.664174303	9.559503046	9.559503046	-3.92215	0.00099
LOC100620268	104.941548	8.2627837	6.727164247	3.211445828	3.211445828	3.666814	0.001
FNDC3A	15.1889082	259.45141	4.016933786	8.02487043	8.02487043	-4.09437	0.001
HYDIN	44.1859147	1.6525567	5.497801224	1.407383613	1.407383613	4.740815	0.001
LOC100624271	44.1859147	671.76432	5.497801224	9.393957376	9.393957376	-3.9263	0.001
CACNA1I	89.7526393	6.610227	6.503867696	2.927939483	2.927939483	3.763183	0.00101
LOC100520306	941.712308	97.500848	9.880673754	6.622064238	6.622064238	3.2718	0.00101
LOC100520093	127.034505	10.741619	7.000388854	3.553559423	3.553559423	3.563937	0.00102
TRIO	69.0404918	1034.5005	6.130117309	10.01611256	10.01611256	-3.90535	0.00102
ZIC2	44.1859147	669.28548	5.497801224	9.388631874	9.388631874	-3.92096	0.00102
LOC100157899	35.9010557	0.8262784	5.205590187	0.868906686	0.868906686	5.441255	0.00103
TSPAN18	110.464787	1655.0356	6.800444207	10.69351795	10.69351795	-3.9052	0.00103
LOC100515900	74.5637311	4.9576702	6.239622035	2.574748269	2.574748269	3.91074	0.00103
F3	53.8515836	808.10025	5.777461367	9.660174654	9.660174654	-3.90747	0.00104
LOC102161728	0	48.750424	0	5.636636914	5.636636914	-20	0.00104
GUCA1B	0	48.750424	0	5.636636914	5.636636914	-20	0.00104
FAM133A	0	48.750424	0	5.636636914	5.636636914	-20	0.00104
PNOC	0	48.750424	0	5.636636914	5.636636914	-20	0.00104
LOC102158919	0	48.750424	0	5.636636914	5.636636914	-20	0.00104
LOC100737190	0	48.750424	0	5.636636914	5.636636914	-20	0.00104
ZNF583	11.0464787	200.78564	3.590539588	7.65667973	7.65667973	-4.184	0.00104
ITPKA	59.3748229	3.3051135	5.915875148	2.106051269	2.106051269	4.16708	0.00105
ACSL6	59.3748229	3.3051135	5.915875148	2.106051269	2.106051269	4.16708	0.00105
LOC100737760	646.219003	66.928548	9.338110157	6.085946114	6.085946114	3.27133	0.00105
ABCA9	19.3313377	313.98578	4.345633235	8.299142895	8.299142895	-4.02169	0.00106
LRFN1	951.377977	99.153405	9.895390449	6.646067655	6.646067655	3.262284	0.00107
LOC100155449	63.5172524	943.6099	6.011613095	9.883574846	9.883574846	-3.89297	0.00107
LOC100620317	81.4677803	5.7839486	6.365758672	2.762125239	2.762125239	3.816103	0.00109
OAS2	563.370413	8866.7932	9.140498547	13.11435941	13.11435941	-3.97626	0.00109
DEPDC1B	2105.735	218.13749	11.04079314	7.775692512	7.775692512	3.271014	0.00109
ZBTB4	117.368836	1736.8371	6.88714549	10.76307717	10.76307717	-3.88734	0.0011
PARP12	281.685207	4273.5117	8.143052576	12.06154392	12.06154392	-3.92327	0.00112
GNB3	24.854577	386.69828	4.692347799	8.598790517	8.598790517	-3.95962	0.00112
LOC100739452	132.557744	11.567897	7.061319821	3.65167138	3.65167138	3.518422	0.00113
LOC102164820	0	47.924146	0	5.61247475	5.61247475	-20	0.00113
IFNE	0	47.924146	0	5.61247475	5.61247475	-20	0.00113
LOC102160618	0	47.924146	0	5.61247475	5.61247475	-20	0.00113
LOC100627982	0	47.924146	0	5.61247475	5.61247475	-20	0.00113
TTNLOC1006202	0	47.924146	0	5.61247475	5.61247475	-20	0.00113
CACNA1A	0	47.924146	0	5.61247475	5.61247475	-20	0.00113
LOC100739107	1909.66	199.13309	10.89985536	7.644815895	7.644815895	3.261511	0.00113
TMEM117	26.2353869	404.8764	4.767410456	8.664896653	8.664896653	-3.9479	0.00113
ACTA1	1.38080984	67.754826	1.251452392	6.103389087	6.103389087	-5.61674	0.00114
UBA7	288.589256	4366.8812	8.177864267	12.0927179	12.0927179	-3.91951	0.00114
LOC102166134	51.0899639	2.4788351	5.702933533	1.798604302	1.798604302	4.365306	0.00114
KLF12	9.66566885	178.47613	3.414902536	7.487648157	7.487648157	-4.20672	0.00115
LOC100739776	22.0929574	345.38436	4.529381037	8.436229978	8.436229978	-3.96654	0.00117
PTPN14	33.1394361	497.41958	5.093367322	8.961216934	8.961216934	-3.90784	0.00118
LOC100737799	197.455807	19.004403	7.632673964	4.322245637	4.322245637	3.377124	0.0012
RNF39	1.38080984	66.928548	1.251452392	6.085946114	6.085946114	-5.59904	0.00121
TTLL7	1.38080984	66.928548	1.251452392	6.085946114	6.085946114	-5.59904	0.00121
LOC102161930	0	47.097867	0	5.587901017	5.587901017	-20	0.00123
RGSL1	0	47.097867	0	5.587901017	5.587901017	-20	0.00123
LOC100621405	0	47.097867	0	5.587901017	5.587901017	-20	0.00123
LOC102158175	0	47.097867	0	5.587901017	5.587901017	-20	0.00123
CCL19	0	47.097867	0	5.587901017	5.587901017	-20	0.00123
ICA	57.9940131	3.3051135	5.882496648	2.106051269	2.106051269	4.133132	0.00123
LOC100622779	26.2353869	0	4.767410456	0	0	20	0.00124
NMUR1	26.2353869	0	4.767410456	0	0	20	0.00124
FAM83A	26.2353869	0	4.767410456	0	0	20	0.00124
LOC102157666	26.2353869	0	4.767410456	0	0	20	0.00124
MSLNL	26.2353869	0	4.767410456	0	0	20	0.00124
KCNG3	26.2353869	0	4.767410456	0	0	20	0.00124
LOC100156285	26.2353869	0	4.767410456	0	0	20	0.00124
LOC100621683	26.2353869	0	4.767410456	0	0	20	0.00124
ABCG8	26.2353869	0	4.767410456	0	0	20	0.00124
NXNL2	26.2353869	0	4.767410456	0	0	20	0.00124
LOC100623337	26.2353869	0	4.767410456	0	0	20	0.00124
TMIGD2	26.2353869	0	4.767410456	0	0	20	0.00124
SDCBP2	404.577282	42.140197	8.663833033	5.430960858	5.430960858	3.263146	0.00124
GNG8	2.76161967	85.106672	1.911353989	6.428053129	6.428053129	-4.94569	0.00124
LOC100624937	2.76161967	85.106672	1.911353989	6.428053129	6.428053129	-4.94569	0.00124
LOC102166014	4.14242951	103.2848	2.362450112	6.704385035	6.704385035	-4.64001	0.00125
NPR3	4.14242951	103.2848	2.362450112	6.704385035	6.704385035	-4.64001	0.00125
SOX15	240.260911	23.962073	7.914450382	4.641665831	4.641665831	3.325777	0.00126
FOXJ1	115.988026	9.9153405	6.870217066	3.448285225	3.448285225	3.54817	0.00126
LOC102158789	115.988026	9.9153405	6.870217066	3.448285225	3.448285225	3.54817	0.00126
GEM	128.415315	1855.8212	7.015864542	10.8586192	10.8586192	-3.85317	0.00127
LOC100523704	11.0464787	193.34914	3.590539588	7.602506906	7.602506906	-4.12955	0.00127
LOC100621888	247.164961	24.788351	7.95515562	4.688647628	4.688647628	3.31774	0.00127
LOC102167192	15.1889082	247.88351	4.016933786	7.959326845	7.959326845	-4.02857	0.00127
STEAP3	705.593826	75.191332	9.464737333	6.251554969	6.251554969	3.2302	0.00128
LOC102164189	19.3313377	302.41788	4.345633235	8.245162313	8.245162313	-3.96753	0.00129
ZFP57	93.8950688	7.4365053	6.568261215	3.076645515	3.076645515	3.658353	0.0013
LOC100623836	9.66566885	174.34474	3.414902536	7.454050313	7.454050313	-4.17293	0.0013
LOC100521038	129.796125	11.567897	7.031175985	3.65167138	3.65167138	3.488049	0.00131
HOXB5	24.854577	375.13038	4.692347799	8.55508903	8.55508903	-3.91581	0.00131
LOC100626218	35.9010557	524.68677	5.205590187	9.038059606	9.038059606	-3.86936	0.00132
SLC38A1	48.3283443	694.90011	5.624344958	9.442736426	9.442736426	-3.84586	0.00133
LOC100624348	20.7121475	318.94345	4.440430524	8.321673128	8.321673128	-3.94475	0.00133
DMPK	34.5202459	504.85609	5.150569664	8.98258319	8.98258319	-3.87036	0.00133
LOC102167359	5.52323934	119.81036	2.705588563	6.916600414	6.916600414	-4.43909	0.00134
IGFBP2	108753.964	10062.418	16.73072172	13.29683278	13.29683278	3.434019	0.00134
TBX4	0	46.271589	0	5.56290145	5.56290145	-20	0.00134
LOC102159229	0	46.271589	0	5.56290145	5.56290145	-20	0.00134
LOC102167982	0	46.271589	0	5.56290145	5.56290145	-20	0.00134
WFDC1	463.952105	6949.8274	8.8609383	12.762969	12.762969	-3.90493	0.00135
SHC3	4.14242951	101.63224	2.362450112	6.681340183	6.681340183	-4.61674	0.00136
SYNPO2	24.854577	372.65155	4.692347799	8.54554968	8.54554968	-3.90624	0.00136
LOC100737382	53.8515836	767.61261	5.777461367	9.58611283	9.58611283	-3.83332	0.00137
LOC100153492	49.7091541	2.4788351	5.664174303	1.798604302	1.798604302	4.325777	0.00137
CDH11	2971.50277	49085.067	11.53746244	15.58302595	15.58302595	-4.04602	0.00138
LOC100520275	5.52323934	118.95909	2.705588563	6.906699252	6.906699252	-4.42911	0.00138
KCNG1	842.294	90.890621	9.719891879	6.521845711	6.521845711	3.212121	0.00139
DBNDD2	2844.46826	302.41788	11.47445037	8.245162313	8.245162313	3.233544	0.00142
CTTNBP2	53.8515836	761.82866	5.777461367	9.575215235	9.575215235	-3.82241	0.00142
PRUNE2	167.07799	2372.2452	7.392987005	11.21264545	11.21264545	-3.82766	0.00144
ARHGAP28	157.412321	2227.6465	7.307540742	11.12195208	11.12195208	-3.8229	0.00146
LOC100739736	1.38080984	64.449713	1.251452392	6.032314961	6.032314961	-5.54459	0.00146
REEP3	321.728692	4670.9516	8.334178034	12.18980962	12.18980962	-3.8598	0.00146
LOC102161524	0	45.44531	0	5.537461031	5.537461031	-20	0.00146
KLHL38	0	45.44531	0	5.537461031	5.537461031	-20	0.00147
CCL3L1	0	45.44531	0	5.537461031	5.537461031	-20	0.00147
SERPINB8	56.6132033	794.87979	5.848327568	9.636406738	9.636406738	-3.81153	0.00147
SYT1	12.4272885	204.91704	3.747096094	7.685919384	7.685919384	-4.04346	0.0015
LOC100624650	327.251931	4731.27	8.358659688	12.20831666	12.20831666	-3.85375	0.0015
EXO5	20.7121475	311.50695	4.440430524	8.287744447	8.287744447	-3.91072	0.00151
LOC102163252	63.5172524	4.1313919	6.011613095	2.359350201	2.359350201	3.942449	0.00151
LOC100626459	9.66566885	169.38707	3.414902536	7.412672017	7.412672017	-4.13131	0.00151
SLC16A11	120.130456	10.741619	6.920417836	3.553559423	3.553559423	3.483319	0.00152
LOC102166137	15.1889082	239.62073	4.016933786	7.910617116	7.910617116	-3.97966	0.00152
LOC100524679	41.4242951	1.6525567	5.406818783	1.407383613	1.407383613	4.647705	0.00155
LOC100625548	41.4242951	1.6525567	5.406818783	1.407383613	1.407383613	4.647705	0.00155
LEPR	190.551757	19.004403	7.581590451	4.322245637	4.322245637	3.325777	0.00155
LOC102166743	1.38080984	63.623435	1.251452392	6.013985524	6.013985524	-5.52597	0.00156
KLHL29	49.7091541	690.76872	5.664174303	9.434145968	9.434145968	-3.79662	0.00158
TSHZ3	124.272885	1718.659	6.968930373	10.74790681	10.74790681	-3.7897	0.0016
LOC102162177	2.76161967	80.97528	1.911353989	6.357117027	6.357117027	-4.8739	0.0016
LOC100522267	2.76161967	80.97528	1.911353989	6.357117027	6.357117027	-4.8739	0.0016
LOC102160052	0	44.619032	0	5.511563931	5.511563931	-20	0.0016
LOC102162300	0	44.619032	0	5.511563931	5.511563931	-20	0.0016
LOC100513736	0	44.619032	0	5.511563931	5.511563931	-20	0.0016
CSRP3	0	44.619032	0	5.511563931	5.511563931	-20	0.0016
C6H1orf233	3079.20593	332.16391	11.58881109	8.380088299	8.380088299	3.212591	0.00162
LOC100738475	13.8080984	218.96377	3.888314478	7.781122098	7.781122098	-3.98711	0.00163
ARX	111.845597	9.9153405	6.818206315	3.448285225	3.448285225	3.495702	0.00164
LOC102159586	26.2353869	377.60922	4.767410456	8.564565719	8.564565719	-3.84731	0.00164
JAM2	6.90404918	132.20454	2.982591923	7.057499439	7.057499439	-4.25919	0.00165
C1QTNF4	118.749646	10.741619	6.90387758	3.553559423	3.553559423	3.46664	0.00165
GRB14	252.6882	26.440908	7.986912605	4.77825631	4.77825631	3.256515	0.00165
DENND6A	5284.35924	562.69557	12.36778582	9.138772422	9.138772422	3.231302	0.00166
ENO3	127.034505	1743.4474	7.000388854	10.76855435	10.76855435	-3.77865	0.00167
LOC102160994	4.14242951	97.500848	2.362450112	6.622064238	6.622064238	-4.55687	0.00167
SNPH	4.14242951	97.500848	2.362450112	6.622064238	6.622064238	-4.55687	0.00167
IGFBP3	1317.29258	145.42499	10.36445488	7.194018019	7.194018019	3.179229	0.00168
ZNRF2	2742.28833	298.28649	11.42169055	8.225383356	8.225383356	3.20061	0.00168
NAPSA	621.364426	68.581105	9.281615789	6.120623682	6.120623682	3.179557	0.00168
GNS	273.400338	3838.8893	8.100138498	11.90684901	11.90684901	-3.8116	0.00169
LOC100525748	12.4272885	199.95937	3.747096094	7.650760008	7.650760008	-4.00812	0.0017
LOC100625867	52.4707738	718.03591	5.740678649	9.489920003	9.489920003	-3.77447	0.0017
LOC102160758	55.2323934	3.3051135	5.813329549	2.106051269	2.106051269	4.062743	0.00172
LOC102159424	24.854577	0	4.692347799	0	0	20	0.00173
LOC102159402	24.854577	0	4.692347799	0	0	20	0.00173
LOC100516308	24.854577	0	4.692347799	0	0	20	0.00173
LOC102166765	24.854577	0	4.692347799	0	0	20	0.00173
LOC100156977	24.854577	0	4.692347799	0	0	20	0.00173
TCL1B	24.854577	0	4.692347799	0	0	20	0.00173
ARGFX	24.854577	0	4.692347799	0	0	20	0.00173
LOC100739411	24.854577	0	4.692347799	0	0	20	0.00173
WNT8A	24.854577	0	4.692347799	0	0	20	0.00173
LOC100739117	24.854577	0	4.692347799	0	0	20	0.00173
LOC100513450	24.854577	0	4.692347799	0	0	20	0.00173
LOC102165082	24.854577	0	4.692347799	0	0	20	0.00173
DEFB123	24.854577	0	4.692347799	0	0	20	0.00173
LOC100737333	24.854577	0	4.692347799	0	0	20	0.00173
FBXL7	27.6161967	390.82967	4.838760036	8.614082834	8.614082834	-3.82295	0.00174
LOC100626660	11.0464787	181.78124	3.590539588	7.513974209	7.513974209	-4.04054	0.00175
ZNF660	0	43.792754	0	5.485193456	5.485193456	-20	0.00175
CADPS	0	43.792754	0	5.485193456	5.485193456	-20	0.00175
LMOD3	0	43.792754	0	5.485193456	5.485193456	-20	0.00175
NR2F1	133.938554	1822.7701	7.076158798	10.83270815	10.83270815	-3.76649	0.00176
GPR19	559.227984	61.970878	9.129870238	5.976612875	5.976612875	3.173774	0.00176
NPFF	15.1889082	233.0105	4.016933786	7.870429459	7.870429459	-3.9393	0.00176
RSPH9	69.0404918	4.9576702	6.130117309	2.574748269	2.574748269	3.799709	0.00177
RELB	71.8021115	970.05081	6.185908388	9.923402974	9.923402974	-3.75596	0.00177
KCNN3	2.76161967	79.322724	1.911353989	6.327736286	6.327736286	-4.84415	0.00178
LOC100737389	40.0434852	549.47512	5.359081339	9.104533542	9.104533542	-3.77841	0.00178
PRR15L	82.8485901	6.610227	6.389714621	2.927939483	2.927939483	3.647705	0.00178

LOC100153559	355.2726531	20.656959	8.476837939	4.436758793	4.436758793	4.104227	0.00062
FRMD4B	751.7823106	45.44531	9.556088917	5.537461031	5.537461031	4.048112	0.00062
LOC102164089	11.63094995	287.54487	3.658891241	8.172651888	8.172651888	-4.62775	0.00062
SULT1C4	16.91774539	395.78734	4.163317207	8.632222185	8.632222185	-4.54812	0.00063
LOC102159415	37.00756803	0	5.24821481	0	0	20	0.00063
LOC102161632	37.00756803	0	5.24821481	0	0	20	0.00063
MYOT	1.057359087	75.191332	1.04079362	6.251554969	6.251554969	-6.15203	0.00063
TMEM176B	4.229436346	137.98849	2.386655454	7.118821584	7.118821584	-5.02794	0.00063
C13H21orf63	4.229436346	137.98849	2.386655454	7.118821584	7.118821584	-5.02794	0.00063
SDC3	224.1601264	5130.3624	7.814807553	12.32512621	12.32512621	-4.51646	0.00064
IFI30	191.3819947	4342.9191	7.587829971	12.08478153	12.08478153	-4.50414	0.00065
RNF24	16.91774539	391.65595	4.163317207	8.617121942	8.617121942	-4.53298	0.00066
GFRA4	0	52.881816	0	5.751726565	5.751726565	-20	0.00066
PID1	0	52.881816	0	5.751726565	5.751726565	-20	0.00066
LOC102165762	0	52.881816	0	5.751726565	5.751726565	-20	0.00066
LOC100515572	0	52.881816	0	5.751726565	5.751726565	-20	0.00066
LOC102157481	6.344154519	178.47613	2.876596413	7.487648157	7.487648157	-4.81416	0.00066
LZTS1	6.344154519	178.47613	2.876596413	7.487648157	7.487648157	-4.81416	0.00066
HGF	21.14718173	478.41518	4.46905122	8.905131772	8.905131772	-4.49973	0.00066
LOC102166540	1.057359087	74.365053	1.04079362	6.2358238	6.2358238	-6.13609	0.00066
LOC100525112	2153.840459	130.55198	11.07336534	7.039489183	7.039489183	4.044215	0.00066
HSPA2	1776.363265	108.24247	10.79552286	6.771389985	6.771389985	4.036588	0.00066
LOC102163869	2.114718173	95.022013	1.639101631	6.585293272	6.585293272	-5.48972	0.00067
LOC102159147	3.17207726	115.67897	2.060765874	6.866400771	6.866400771	-5.18855	0.00067
PION	14.80302721	346.21064	3.982129041	8.439667339	8.439667339	-4.54769	0.00068
LOC100623503	49.69587707	0.8262784	5.663796517	0.868906686	0.868906686	5.910354	0.00068
C15H2orf66	49.69587707	0.8262784	5.663796517	0.868906686	0.868906686	5.910354	0.00068
LOC100737186	13.74566813	324.7274	3.882219287	8.347521274	8.347521274	-4.56218	0.00068
LOC100515210	20.08982264	452.80055	4.398475058	8.82591454	8.82591454	-4.49434	0.00068
PER2	5.286795433	156.16661	2.65232482	7.296150961	7.296150961	-4.88455	0.00069
MGLL	44.40908164	962.6143	5.504908955	9.912311998	9.912311998	-4.43803	0.00069
LOC102159369	16.91774539	387.52456	4.163317207	8.601861976	8.601861976	-4.51768	0.00069
LOC100155350	111.0227041	2431.7372	6.807647348	11.24836479	11.24836479	-4.45306	0.00069
ZHX3	22.20454082	494.94074	4.536335244	8.954023947	8.954023947	-4.47833	0.00069
WNT10B	12.68830904	301.59161	3.774872332	8.241228155	8.241228155	-4.57102	0.0007
LOC102162336	2.114718173	94.195734	1.639101631	6.572825024	6.572825024	-5.47712	0.0007
LOC102164225	135.3419631	6.610227	7.09108585	2.927939483	2.927939483	4.355766	0.0007
DLX1	0	52.055537	0	5.729431428	5.729431428	-20	0.0007
LOC100627144	0	52.055537	0	5.729431428	5.729431428	-20	0.0007
LOC102165534	0	52.055537	0	5.729431428	5.729431428	-20	0.0007
LRRC31	0	52.055537	0	5.729431428	5.729431428	-20	0.0007
LOC100620473	0	52.055537	0	5.729431428	5.729431428	-20	0.0007
LOC102164797	334.1254714	19.830681	8.388557533	4.380638094	4.380638094	4.074584	0.0007
LOC102160069	2192.962746	134.68337	11.09932331	7.084100147	7.084100147	4.025238	0.00072
DFNB59	459.9512027	28.093465	8.848470222	4.862623207	4.862623207	4.033174	0.00072
LGI4	2.114718173	93.369456	1.639101631	6.560248082	6.560248082	-5.46441	0.00072
JDP2	49.69587707	1063.4203	5.663796517	10.05585217	10.05585217	-4.41944	0.00073
LOC100622674	35.95020894	0	5.207510617	0	0	20	0.00073
LOC100738821	35.95020894	0	5.207510617	0	0	20	0.00073
LOC102162482	13.74566813	318.94345	3.882219287	8.321673128	8.321673128	-4.53625	0.00074
MRC2	1392.541917	34358.307	10.44454068	15.06841333	15.06841333	-4.62487	0.00074
TMEM40	9.516231779	235.48934	3.394545939	7.885631319	7.885631319	-4.62913	0.00074
SCML2	2428.753822	149.55639	11.24659443	7.234160086	7.234160086	4.021455	0.00074
LOC100628093	12.68830904	297.46021	3.774872332	8.221394816	8.221394816	-4.55112	0.00074
ACN9	883.9521964	55.360651	9.789455715	5.816616368	5.816616368	3.997036	0.00075
LOC100520228	6.344154519	173.51846	2.876596413	7.447235822	7.447235822	-4.77352	0.00075
ZNRF2	4957.956757	298.28649	12.27582093	8.225383356	8.225383356	4.054975	0.00075
ADA	23.2618999	507.33492	4.600620625	8.98963553	8.98963553	-4.4469	0.00075
LOC102166660	2.114718173	92.543178	1.639101631	6.547560532	6.547560532	-5.45159	0.00075
CPNE4	280.2001579	16.525567	8.135453595	4.131389251	4.131389251	4.083686	0.00076
FGB	0	51.229259	0	5.706786332	5.706786332	-20	0.00076
LOC102166113	0	51.229259	0	5.706786332	5.706786332	-20	0.00076
AZGP1	0	51.229259	0	5.706786332	5.706786332	-20	0.00076
LOC100739734	48.63851798	0.8262784	5.633388138	0.868906686	0.868906686	5.879328	0.00076
LOC100517801	90.93288145	1935.9702	6.522509054	10.91958606	10.91958606	-4.41211	0.00077
PLCB4	1168.381791	73.538775	10.19153032	6.219919206	6.219919206	3.989863	0.00077
APOBEC3F	11.63094995	274.32442	3.658891241	8.104988763	8.104988763	-4.55984	0.00077
CGN	478.9836662	29.746021	8.906841502	4.942327829	4.942327829	4.009208	0.00077
LOC100737860	5.286795433	152.03522	2.65232482	7.257719911	7.257719911	-4.84587	0.00078
KIAA1958	978.0571551	61.970878	9.935249273	5.976612875	5.976612875	3.980256	0.00079
RPS6KA6	1305.838472	82.627837	10.35186512	6.385911347	6.385911347	3.982205	0.0008
TRIM62	29.60605442	627.97156	4.935745167	9.29685098	9.29685098	-4.40674	0.0008
LOC102162660	60.26946793	1.6525567	5.937096418	1.407383614	1.407383614	5.188656	0.00081
LOC100737862	60.26946793	1.6525567	5.937096418	1.407383614	1.407383614	5.188656	0.00081
SLC9A3R2	633.3580929	14609.428	9.309153656	13.83471081	13.83471081	-4.52773	0.00081
LOC102157895	0	50.402981	0	5.683780113	5.683780113	-20	0.00081
MYH14	0	50.402981	0	5.683780113	5.683780113	-20	0.00081
KRT81	0	50.402981	0	5.683780113	5.683780113	-20	0.00081
LOC100512321	1340.731322	85.106672	10.38988009	6.428053129	6.428053129	3.977604	0.00081
ARHGAP6	11.63094995	271.01931	3.658891241	8.087565237	8.087565237	-4.54235	0.00082
LOC100627393	1420.033253	90.064343	10.4727246	6.508814353	6.508814353	3.978825	0.00082
HOXA4	5.286795433	150.38266	2.65232482	7.242056187	7.242056187	-4.8301	0.00082
LOC102161722	2.114718173	90.890621	1.639101631	6.521845711	6.521845711	-5.42559	0.00082
SYNPO2	16.91774539	372.65155	4.163317207	8.54554968	8.54554968	-4.46122	0.00082
SIM1	131.1125267	6.610227	7.045623457	2.927939483	2.927939483	4.309962	0.00083
RSPH4A	71.90041789	2.4788351	6.187855179	1.798604302	1.798604302	4.858266	0.00083
LOC102159423	17.97510447	392.48223	4.246035924	8.620154661	8.620154661	-4.44856	0.00083
TOR4A	14.80302721	330.51135	3.982129041	8.372914449	8.372914449	-4.48074	0.00084
MAB21L1	11.63094995	269.36675	3.658891241	8.078773924	8.078773924	-4.53353	0.00084
LOC100738242	360.5594485	22.309516	8.498089068	4.542847146	4.542847146	4.014506	0.00084
LOC100738305	360.5594485	22.309516	8.498089068	4.542847146	4.542847146	4.014506	0.00084
GPR160	190.3246356	10.741619	7.579878842	3.553559423	3.553559423	4.147179	0.00084
MXRA8	376.4198348	8351.1955	8.560026434	13.02793977	13.02793977	-4.47157	0.00085
PROSER2	1384.083044	88.411786	10.43575676	6.482393107	6.482393107	3.968548	0.00085
TEP1	254.8235399	5533.5863	7.999005211	12.43425976	12.43425976	-4.44064	0.00085
MYRF	17.97510447	390.82967	4.246035924	8.614082834	8.614082834	-4.44247	0.00085
KCNV1	34.89284986	0	5.165624571	0	0	20	0.00085
FAM19A4	34.89284986	0	5.165624571	0	0	20	0.00085
GPR55	34.89284986	0	5.165624571	0	0	20	0.00085
LOC102164859	34.89284986	0	5.165624571	0	0	20	0.00085
LOC102163452	43.35172255	897.33831	5.470918232	9.811115052	9.811115052	-4.37149	0.00085
PRMT8	1.057359087	70.233662	1.04079362	6.154487245	6.154487245	-6.05363	0.00085
LOC102164150	6.344154519	168.56079	2.876596413	7.405658765	7.405658765	-4.7317	0.00085
LOC102163734	106.7932677	4.9576702	6.752123267	2.57474827	2.57474827	4.429015	0.00086
PDSS1	3289.444118	205.74331	11.68406661	7.691696868	7.691696868	3.998926	0.00086
SEMA3G	371.1330394	23.135794	8.539674674	4.593102407	4.593102407	4.003738	0.00086
NEURL2	12.68830904	287.54487	3.774872332	8.172651888	8.172651888	-4.50222	0.00087
LOC102157611	0	49.576702	0	5.660401069	5.660401069	-20	0.00087
LOC100515686	0	49.576702	0	5.660401069	5.660401069	-20	0.00087
PDGFD	12.68830904	286.71859	3.774872332	8.168514654	8.168514654	-4.49806	0.00088
FAM98C	76.12985423	1567.4501	6.269217479	10.61512389	10.61512389	-4.36381	0.00088
PCPA1	2.114718173	89.238064	1.639101631	6.495664213	6.495664213	-5.39912	0.00089
LOC100628155	2135.865355	136.33593	11.06128029	7.101565318	7.101565318	3.969583	0.00089
LOC102166317	48.63851798	994.83916	5.633388138	9.959768938	9.959768938	-4.35429	0.0009
IFI44L	402.853812	8855.2253	8.657689347	13.11247621	13.11247621	-4.4582	0.0009
LOC100524058	564.6295722	36.356248	9.143714198	5.223277669	5.223277669	3.95703	0.00091
LOC396904	5837.679517	150749.53	12.51142641	17.20180356	17.20180356	-4.69061	0.00091
LOC100737139	562.5150341	36.356248	9.13831029	5.223277669	5.223277669	3.951616	0.00092
IFT27	24.31925899	505.68236	4.662163277	8.984937802	8.984937802	-4.37806	0.00093
NPAS2	72.95777697	1483.1697	6.208629956	10.53544032	10.53544032	-4.34548	0.00093
LOC100621949	0	48.750424	0	5.636636914	5.636636914	-20	0.00094
ZNF614	0	48.750424	0	5.636636914	5.636636914	-20	0.00094
TMEM150C	1786.936856	115.67897	10.80408007	6.866400771	6.866400771	3.94929	0.00094
LOC100624418	1.057359087	68.581105	1.04079362	6.120623682	6.120623682	-6.01927	0.00095
SRSF12	1330.157731	86.759229	10.37846581	6.455478947	6.455478947	3.938436	0.00095
SOCS1	15.8603863	341.25297	4.075565686	8.41891924	8.41891924	-4.42734	0.00095
LOC100622291	46.52379981	0.8262784	5.570578288	0.868906686	0.868906686	5.815197	0.00095
ZAP70	93.04759962	4.1313919	6.555319217	2.359350201	2.359350201	4.493269	0.00096
SCARF2	189.2672765	3943.0004	7.571883648	11.94544398	11.94544398	-4.3808	0.00096
POLR3G	4931.52278	309.85439	12.26811	8.28009514	8.28009514	3.992371	0.00097
IGSF1	411.3126847	26.440908	8.687595036	4.778256309	4.778256309	3.959392	0.00097
LOC100524679	58.15474976	1.6525567	5.886422108	1.407383614	1.407383614	5.137125	0.00097
SIDT1	58.15474976	1.6525567	5.886422108	1.407383614	1.407383614	5.137125	0.00097
LOC102164866	33.83549077	0	5.122485983	0	0	20	0.00099
CPPED1	11.63094995	299.45141	3.658891241	8.02487043	8.02487043	-4.47942	0.00099
ELF4	78.24457241	1569.9289	6.308240221	10.61740218	10.61740218	-4.32656	0.001
B3GALT4	24.31925899	497.41958	4.662163277	8.961216934	8.961216934	-4.35429	0.001
LSR	409.1979665	26.440908	8.68017653	4.778256309	4.778256309	3.951955	0.001
LOC102167381	5.286795433	143.77244	2.65232482	7.177643143	7.177643143	-4.76525	0.001
MFGE8	818.395933	18321.07	9.678416922	14.16129491	14.16129491	-4.48456	0.001
ITFG3	126.8830904	2577.1622	6.998681703	11.33212734	11.33212734	-4.34421	0.001
LOC100623747	103.6211905	4.9576702	6.709031281	2.57474827	2.57474827	4.385513	0.00101
PPM1J	2.114718173	86.759229	1.639101631	6.455478947	6.455478947	-5.35848	0.00101
LOC102164820	0	47.924146	0	5.61247475	5.61247475	-20	0.00101
IFNE	0	47.924146	0	5.61247475	5.61247475	-20	0.00101
LOC102160618	0	47.924146	0	5.61247475	5.61247475	-20	0.00101
KIAA1755	0	47.924146	0	5.61247475	5.61247475	-20	0.00101
TTNLOC100620261	0	47.924146	0	5.61247475	5.61247475	-20	0.00101
CACNA1A	0	47.924146	0	5.61247475	5.61247475	-20	0.00101
TRIM36	372.1903985	23.962073	8.543768058	4.641665831	4.641665831	3.957216	0.00101
LOC102162418	24.31925899	495.76702	4.662163277	8.956425596	8.956425596	-4.34949	0.00101
LOC100155572	601.6373253	39.661362	9.235146209	5.34558663	5.34558663	3.923088	0.00102
MFSD12	172.3495311	3533.1663	7.437540124	11.78715422	11.78715422	-4.35755	0.00102
NELL2	661.9067882	43.792754	9.372662216	5.485193456	5.485193456	3.917864	0.00103
LOC100737847	4.229436346	123.94176	2.386655454	6.965111899	6.965111899	-4.87305	0.00103
MAP1LC3A	195.611431	4021.4968	7.619203392	11.97387557	11.97387557	-4.36167	0.00103
ATP8	164794.7004	8846.1362	17.33031908	13.11099483	13.11099483	4.219479	0.00103
LOC102163871	80.35929058	3.3051135	6.346235194	2.106051269	2.106051269	4.603693	0.00103
LOC102159288	3.17207726	104.93735	2.060765874	6.727067559	6.727067559	-5.04796	0.00104
LOC102161477	171.292172	9.9153405	7.428713345	3.448285225	3.448285225	4.110653	0.00105
TRPM4	6.344154519	161.12428	2.876596413	7.340956379	7.340956379	-4.6666	0.00105
MYEF2	8570.952756	532.94955	13.06540818	9.060559625	9.060559625	4.007385	0.00105
CSDC2	13.74566813	294.98138	3.882219287	8.209362603	8.209362603	-4.42357	0.00105
AKAP17B	1293.150163	85.932951	10.33778931	6.441831208	6.441831208	3.911535	0.00105
TRIM55	1.057359087	66.928548	1.04079362	6.085946114	6.085946114	-5.98408	0.00106
RNF39	1.057359087	66.928548	1.04079362	6.085946114	6.085946114	-5.98408	0.00106
PSMB8	111.0227041	2214.426	6.807647348	11.11336845	11.11336845	-4.31801	0.00107
LOC102157456	45.46644072	0.8262784	5.538117235	0.868906686	0.868906686	5.78203	0.00107
LOC102164296	45.46644072	0.8262784	5.538117235	0.868906686	0.868906686	5.78203	0.00107
LOC100739677	45.46644072	0.8262784	5.538117235	0.868906686	0.868906686	5.78203	0.00107
CADM2	181.8657629	10.741619	7.514641181	3.553559423	3.553559423	4.081591	0.00108
LOC100625123	38.06492712	751.08704	5.287802017	9.554755826	9.554755826	-4.30245	0.00108
TMEM61	0	47.097867	0	5.587901017	5.587901017	-20	0.00109
LOC100624393	0	47.097867	0	5.587901017	5.587901017	-20	0.00109
MEOX2	0	47.097867	0	5.587901017	5.587901017	-20	0.00109
LOC102160923	0	47.097867	0	5.587901017	5.587901017	-20	0.00109
LOC100621405	0	47.097867	0	5.587901017	5.587901017	-20	0.00109
LOC100524378	0	47.097867	0	5.587901017	5.587901017	-20	0.00109
B2R	0	47.097867	0	5.587901017	5.587901017	-20	0.00109
LOC100738669	219.93069	4485.039	7.787450031	12.13122645	12.13122645	-4.35	0.0011
FCGRT	953.7378961	21103.976	9.898960915	14.36529556	14.36529556	-4.46778	0.0011
ARNT2	94.1049587	1853.3424	6.571448659	10.85669193	10.85669193	-4.29971	0.00111
NPR3	3.17207726	103.2848	2.060765874	6.704385034	6.704385034	-5.02506	0.00112
GVIN1	8.458872693	195.82797	3.241668254	7.620791467	7.620791467	-4.53298	0.00113
FSCN2	17.97510447	366.8676	4.246035924	8.523042794	8.523042794	-4.35119	0.00113
GPR37	434.5745846	28.919743	8.766775966	4.903025878	4.903025878	3.909477	0.00114
LOC100627770	67.67098154	2.4788351	6.10162868	1.798604302	1.798604302	4.770803	0.00114
ADAMTSL5	2.114718173	84.280394	1.639101631	6.414142197	6.414142197	-5.31666	0.00114
SHPK	7.401513606	175.99729	3.070649265	7.467583487	7.467583487	-4.57159	0.00116
ADM2	4.229436346	120.63664	2.386655454	6.926434087	6.926434087	-4.83406	0.00116
ECT2L	32.77813168	0	5.078017628	0	0	20	0.00116
LOC102168070	32.77813168	0	5.078017628	0	0	20	0.00116
LOC102160718	32.77813168	0	5.078017628	0	0	20	0.00116
LOC100521810	32.77813168	0	5.078017628	0	0	20	0.00116
C1H15orf43	32.77813168	0	5.078017628	0	0	20	0.00116
RBP1	1505.679339	101.63224	10.55715669	6.681340184	6.681340184	3.888985	0.00117
PIGL	32.77813168	637.8869	5.078017628	9.319416755	9.319416755	-4.2825	0.00117
LOC102167710	21.14718173	422.22825	4.46905122	8.725292112	8.725292112	-4.31949	0.00117
LOC100523351	0	46.271589	0	5.56290145	5.56290145	-20	0.00118
LOC102158339	1.057359087	65.275991	1.04079362	6.05041444	6.05041444	-5.94802	0.00118
LOC100627924	1.057359087	65.275991	1.04079362	6.05041444	6.05041444	-5.94802	0.00118
LOC100738148	56.04003159	1.6525567	5.833902876	1.407383614	1.407383614	5.083686	0.00118
MAL2	431.4025073	28.919743	8.756231078	4.903025878	4.903025878	3.898908	0.00119
COLL11A1	1245.559004	27569.604	10.28374703	14.75084326	14.75084326	-4.4682	0.00119
S100A14	5.286795433	137.98849	2.65232482	7.118821584	7.118821584	-4.70601	0.0012
KDELR3	286.5443125	5807.9107	8.167640492	12.50405193	12.50405193	-4.34119	0.0012
IQCA1	44.40908164	0.8262784	5.504908955	0.868906686	0.868906686	5.748083	0.00121
FGF10	24.31925899	475.93634	4.662163277	8.897652909	8.897652909	-4.2906	0.00122
LOC100520275	4.229436346	118.98409	2.386655454	6.906699252	6.906699252	-4.81416	0.00124
NKX3-2	14.80302721	303.24416	3.982129041	8.249085772	8.249085772	-4.35652	0.00124
LOC100520244	21.14718173	416.4443	4.46905122	8.705439898	8.705439898	-4.29959	0.00125
LOC100627628	244.249949	15.699289	7.938109026	4.061714779	4.061714779	3.959587	0.00125
CACNA1S	2.114718173	82.627837	1.639101631	6.385911347	6.385911347	-5.28809	0.00125
CHCHD6	41.23700438	784.13817	5.400435611	9.616802763	9.616802763	-4.2491	0.00126
KLF4	158.603863	3090.2811	7.31835176	11.59398914	11.59398914	-4.28424	0.00127
MEG3	5.286795433	136.33593	2.65232482	7.101565318	7.101565318	-4.68863	0.00127
SEC24D	217.8159718	4304.9103	7.773574237	12.07210256	12.07210256	-4.3048	0.00127
ME3	43.35172255	822.97326	5.470918232	9.686453706	9.686453706	-4.24668	0.00127
LOC100627863	219.93069	4347.0505	7.787450031	12.08615299	12.08615299	-4.30492	0.00127
LOC100524524	24.31925899	471.80495	4.662163277	8.88510133	8.88510133	-4.27802	0.00127
PRSS33	0	45.44531	0	5.537461031	5.537461031	-20	0.00127
LOC100739222	0	45.44531	0	5.537461031	5.537461031	-20	0.00127
EREG	0	45.44531	0	5.537461031	5.537461031	-20	0.00127
CCL3L1	0	45.44531	0	5.537461031	5.537461031	-20	0.00127
LOC100515260	688.3407654	14486.312	9.429073524	13.82250236	13.82250236	-4.39542	0.00127
LOC100627728	243.1925899	15.699289	7.931875612	4.061714779	4.061714779	3.953328	0.00128
LOC102158067	77.18721332	3.3051135	6.288860784	2.106051269	2.106051269	4.54559	0.00128
LOC100626168	20.08982264	394.96106	4.398475058	8.629214754	8.629214754	-4.29717	0.00128
LOC100625903	48.63851798	920.47411	5.633388138	9.847799816	9.847799816	-4.24221	0.00128
PQLC3	43.35172255	820.49442	5.470918232	9.682106971	9.682106971	-4.24233	0.00129
LOC102168044	33.83549077	642.84457	5.122485983	9.330568647	9.330568647	-4.24786	0.0013
LOC102162560	22.20454082	431.31731	4.536335244	8.755946793	8.755946793	-4.27982	0.0013
LOC102162575	54.9826725	1.6525567	5.806908455	1.407383614	1.407383614	5.056205	0.0013
LOC100739353	2.114718173	81.801559	1.639101631	6.371586023	6.371586023	-5.27359	0.00131
LOC102166028	1438.008358	99.153405	10.49085926	6.646067655	6.646067655	3.858266	0.00131
DSP	3907.999184	261.10397	11.93258357	8.033995371	8.033995371	3.903734	0.00131
LOC102165736	19.03246356	373.47782	4.324267947	8.548736477	8.548736477	-4.29449	0.00132
SIRT2	204.0703037	3982.6618	7.67997478	11.95987944	11.95987944	-4.28659	0.00133
AFGF	3.17207726	99.153405	2.060765874	6.646067655	6.646067655	-4.96616	0.00134
LRRN4	108.9079859	5.7839486	6.780152406	2.76212524	2.76212524	4.234911	0.00135
ITM2C	1080.620986	23078.781	10.07897933	14.49434193	14.49434193	-4.41663	0.00135
TOP1MT	15.8603863	315.63834	4.075565686	8.306692135	8.306692135	-4.31477	0.00136
PTHLH	9.516231779	205.74331	3.394545939	7.691696868	7.691696868	-4.43431	0.00136
LOC102158647	119.4815768	6.610227	6.912668746	2.927939483	2.927939483	4.175945	0.00137
SOX13	466.2953572	32.224856	8.868190893	5.054191063	5.054191063	3.854998	0.00137
OPALIN	31.7207726	0	5.032134908	0	0	20	0.00137
TJP3	31.7207726	0	5.032134908	0	0	20	0.00137
TMEM215	31.7207726	0	5.032134908	0	0	20	0.00137
LOC100736881	0	44.619032	0	5.511563931	5.511563931	-20	0.00138
LOC102163702	0	44.619032	0	5.511563931	5.511563931	-20	0.00138
LOC102160052	0	44.619032	0	5.511563931	5.511563931	-20	0.00138
LOC102162300	0	44.619032	0	5.511563931	5.511563931	-20	0.00138
EDN1	7.401513606	169.38707	3.070649265	7.412672017	7.412672017	-4.51636	0.00138
SULF2	138.5140403	2634.1754	7.124266508	11.3636833	11.3636833	-4.24925	0.00138
HOGA1	3.17207726	98.327126	2.060765874	6.634115866	6.634115866	-4.95409	0.00139
SYT1	9.516231779	204.91704	3.394545939	7.685919384	7.685919384	-4.42851	0.00139
LOC100624471	295.0031852	19.830681	8.20946889	4.380638094	4.380638094	3.894924	0.00139
LOC102161978	140.6287585	8.2627837	7.145970432	3.211445828	3.211445828	4.08912	0.0014
LOC100738135	140.6287585	8.2627837	7.145970432	3.211445828	3.211445828	4.08912	0.0014
FAM101A	1.057359087	62.797156	1.04079362	5.995420212	5.995420212	-5.89216	0.0014
CD207	1.057359087	62.797156	1.04079362	5.995420212	5.995420212	-5.89216	0.0014
LOC102158551	1.057359087	62.797156	1.04079362	5.995420212	5.995420212	-5.89216	0.0014
BCO2	1.057359087	62.797156	1.04079362	5.995420212	5.995420212	-5.89216	0.0014
TMEFF1	3609.823922	244.5784	11.81811235	7.940039851	7.940039851	3.88356	0.0014
IRF1	142.7434767	2710.1931	7.167352675	11.40471213	11.40471213	-4.2469	0.0014
LOC100621905	4.229436346	115.67897	2.386655454	6.866400771	6.866400771	-4.77352	0.0014
FRMPD3	20.08982264	386.69828	4.398475058	8.598790517	8.598790517	-4.26667	0.00141
B3GALNT1	2498.539522	171.8659	11.28744662	7.433509508	7.433509508	3.86173	0.00141
LOC102166068	107.8506268	5.7839486	6.766205905	2.76212524	2.76212524	4.220836	0.00142
ARPC1B	322.4945214	6348.2967	8.337597469	12.63238109	12.63238109	-4.29902	0.00142
HEPH	2197.192182	152.03522	11.10210181	7.257719911	7.257719911	3.853184	0.00143
LOC100521170	270.6839262	18.178124	8.085785403	4.261389711	4.261389711	3.896334	0.00145
RHBDL2	14.80302721	292.50254	3.982129041	8.197229196	8.197229196	-4.30449	0.00146
CD44	1196.930486	25303.949	10.22632848	14.62713194	14.62713194	-4.40195	0.00146
IGFBP5	23.2618999	438.75382	4.600620625	8.780552285	8.780552285	-4.23737	0.00147
LOC100514709	204.0703037	13.220454	7.67997478	3.829895615	3.829895615	3.948223	0.00147
TBX18	23.2618999	437.92754	4.600620625	8.777838973	8.777838973	-4.23465	0.00148

LOC102166984	1.65785765	71.05994	1.410263837	6.171125545	6.171125545	-5.42164	6.6E-06
SHROOM4	0.82892882	58.665764	0.870998931	5.89883146	5.89883146	-6.14513	6.6E-06
HSD3B7	116.050035	1697.1758	6.870981559	10.72977008	10.72977008	-3.87032	6.6E-06
LDHC	116.878964	4.9576702	6.881162477	2.574748269	2.574748269	4.559209	6.7E-06
ICOSLG	128.483968	1877.3045	7.016629669	10.87521522	10.87521522	-3.869	6.7E-06
NEURL2	17.4075053	287.54487	4.202222212	8.172651889	8.172651889	-4.04601	6.8E-06
LOC100620869	3.31571529	94.195734	2.109599694	6.572825024	6.572825024	-4.82827	6.8E-06
GYG2	40.6175124	609.79344	5.379118827	9.254540753	9.254540753	-3.90815	6.9E-06
MVP	641.59091	9626.143	9.327756761	13.23289201	13.23289201	-3.90723	7E-06
TARSL2	44.7621565	0	5.516083133	0	0	20	7.2E-06
LOC100738279	44.7621565	0	5.516083133	0	0	20	7.2E-06
PDE6A	44.7621565	0	5.516083133	0	0	20	7.2E-06
LOC102167207	44.7621565	0	5.516083133	0	0	20	7.2E-06
SH3YL1	4.14464412	104.93735	2.363071282	6.727067559	6.727067559	-4.66214	7.3E-06
LOC100512258	96.1557435	1392.2791	6.602227381	10.44426852	10.44426852	-3.85593	7.3E-06
LOC100738662	647.393411	43.792754	9.34072562	5.485193456	5.485193456	3.885879	7.3E-06
NHSL1	3911.71512	269.36675	11.93395436	8.078773924	8.078773924	3.860158	7.4E-06
ABCA4	406.175124	26.440908	8.669505612	4.77825631	4.77825631	3.941258	7.5E-06
LOC102163702	0	44.619032	0	5.511563931	5.511563931	-20	7.6E-06
LOC102162300	0	44.619032	0	5.511563931	5.511563931	-20	7.6E-06
FBLN7	828.928824	57.013208	9.696843803	5.858309486	5.858309486	3.86188	7.6E-06
LOC100525848	852.138831	58.665764	9.736636719	5.89883146	5.89883146	3.860498	7.6E-06
LOC102162112	632.472692	42.966475	9.307138619	5.458331975	5.458331975	3.87972	7.7E-06
LOC100153163	133.457541	1916.1395	7.071006856	10.90473964	10.90473964	-3.84375	7.8E-06
NKX2-3	57.1960888	0.8262784	5.862850292	0.868906686	0.868906686	6.113145	7.8E-06
LOC102160550	57.1960888	0.8262784	5.862850292	0.868906686	0.868906686	6.113145	7.8E-06
LOC100516245	456.739782	6696.1599	8.838383871	12.7093337	12.7093337	-3.87389	7.9E-06
SCG2	3.31571529	92.543178	2.109599694	6.547560532	6.547560532	-4.80273	7.9E-06
SLC46A2	23.2100071	361.08365	4.597531595	8.500179216	8.500179216	-3.95951	7.9E-06
RYR3	1.65785765	69.407383	1.410263837	6.137654819	6.137654819	-5.3877	7.9E-06
PN-1	1401.71864	21228.744	10.45400995	14.37379935	14.37379935	-3.92075	8E-06
LOC100739399	594.341967	40.48764	9.217574785	5.374609695	5.374609695	3.87574	8.1E-06
LOC102159402	43.9332277	0	5.489710792	0	0	20	8.3E-06
HAPLN2	102.787174	4.1313919	6.697484358	2.359350201	2.359350201	4.636889	8.3E-06
LOC102160786	14.09179	236.31561	3.915692025	7.890663216	7.890663216	-4.06779	8.3E-06
LOC100154002	2989.11734	209.87471	11.54598638	7.72024225	7.72024225	3.832119	8.5E-06
LOC102163119	124.339324	5.7839486	6.969695301	2.762125239	2.762125239	4.426084	8.6E-06
CD19	1.65785765	68.581105	1.410263837	6.120623682	6.120623682	-5.37042	8.8E-06
LOC100622512	0	43.792754	0	5.485193456	5.485193456	-20	8.8E-06
ZNF660	0	43.792754	0	5.485193456	5.485193456	-20	8.8E-06
STX3	75.432523	1073.3356	6.256114748	10.06922902	10.06922902	-3.83077	8.8E-06
LOC100738887	5.80250177	124.76803	2.766065426	6.974621475	6.974621475	-4.42643	8.8E-06
LOC102161046	116.050035	1641.8151	6.870981559	10.68195442	10.68195442	-3.82247	8.9E-06
SORD	8995.53559	620.53506	13.13515384	9.279691957	9.279691957	3.857625	9.1E-06
RASGEF1B	3.31571529	90.890621	2.109599694	6.521845711	6.521845711	-4.77674	9.2E-06
LOC102158317	90.3532418	3.3051135	6.51338402	2.106051269	2.106051269	4.772805	9.3E-06
TNXB	708.734144	49.576702	9.471134904	5.660401068	5.660401068	3.83751	9.3E-06
ROBO2	297.585448	19.004403	8.222000044	4.322245637	4.322245637	3.968898	9.3E-06
CEP152	6117.49472	428.0122	12.57896105	8.744874853	8.744874853	3.837217	9.4E-06
LOC100623103	188.166843	10.741619	7.563515426	3.553559423	3.553559423	4.130729	9.4E-06
LOC100620159	101.129317	4.1313919	6.674253245	2.359350201	2.359350201	4.61343	9.5E-06
LOC100737799	296.756519	19.004403	8.217989283	4.322245637	4.322245637	3.964874	9.5E-06
IGSF11	660.656272	46.271589	9.369938128	5.56290145	5.56290145	3.835701	9.7E-06
TNMD	1.65785765	67.754826	1.410263837	6.103389087	6.103389087	-5.35293	9.7E-06
LOC102166578	1.65785765	67.754826	1.410263837	6.103389087	6.103389087	-5.35293	9.7E-06
LOC102158142	9.11821706	166.90823	3.338883189	7.391529145	7.391529145	-4.19416	9.7E-06
LOC100738373	4.97357294	112.37386	2.578594101	6.824944216	6.824944216	-4.49788	9.7E-06
LRMP	1008.80638	71.886218	9.979862979	6.187574144	6.187574144	3.81079	9.7E-06
LOC100738167	155.00969	8.2627837	7.28549183	3.211445828	3.211445828	4.229587	9.7E-06
LOC100525798	372.189042	24.788351	8.543762814	4.688647628	4.688647628	3.908301	9.8E-06
PTER	10.7760747	188.39147	3.557786824	7.565227535	7.565227535	-4.12783	9.8E-06
LOC102166369	11.6050035	199.13309	3.655924617	7.644815895	7.644815895	-4.10091	9.8E-06
LOC102166065	404.517266	27.267186	8.663619532	4.821056379	4.821056379	3.890964	9.9E-06
LOC102157594	55.5382312	0.8262784	5.821154844	0.868906686	0.868906686	6.07071	9.9E-06
LOC100514834	3.31571529	90.064343	2.109599694	6.508814353	6.508814353	-4.76356	9.9E-06
PTGDR	89.524313	3.3051135	6.500233417	2.106051269	2.106051269	4.759508	1E-05
MSC	31.4992953	461.06333	5.022336531	8.851946794	8.851946794	-3.87157	1E-05
LOC102159666	0	42.966475	0	5.458331975	5.458331975	-20	1E-05
NTS	2.48678647	78.496445	1.801898017	6.312818447	6.312818447	-4.98027	1E-05
DAPP1	314.164024	20.656959	8.299959051	4.436758793	4.436758793	3.926818	1.1E-05
ARHGAP27	586.881607	41.313919	9.199381831	5.403060391	5.403060391	3.82837	1.1E-05
JAG2	3432.59426	247.05723	11.74550385	7.954529215	7.954529215	3.796382	1.1E-05
FOS	1869.2345	135.50965	10.86900346	7.092859162	7.092859162	3.78598	1.1E-05
HPX	4.97357294	110.7213	2.578594101	6.803760479	6.803760479	-4.47651	1.1E-05
LOC102165705	32.3282241	467.67356	5.058672541	8.872439593	8.872439593	-3.85464	1.1E-05
STMND1	42.27537	0	5.435474249	0	0	20	1.1E-05
LOC102164307	42.27537	0	5.435474249	0	0	20	1.1E-05
SLFN14	42.27537	0	5.435474249	0	0	20	1.1E-05
LOC102160718	42.27537	0	5.435474249	0	0	20	1.1E-05
LOC102162598	42.27537	0	5.435474249	0	0	20	1.1E-05
LOC102165105	42.27537	0	5.435474249	0	0	20	1.1E-05
EXOC3L1	66.3143059	1.6525567	6.072841239	1.407383613	1.407383613	5.326548	1.1E-05
LOC100739138	628.328048	44.619032	9.297668433	5.511563931	5.511563931	3.815787	1.1E-05
LOC396583	34.8150106	499.07214	5.162492464	8.965992412	8.965992412	-3.84147	1.1E-05
NFKBIZ	132.628612	1825.2489	7.062085132	10.83466771	10.83466771	-3.78263	1.1E-05
PHLDA1	306.703665	4271.8592	8.265397816	12.06098606	12.06098606	-3.79995	1.1E-05
LPAR3	109.418605	4.9576702	6.786839466	2.574748269	2.574748269	4.464052	1.2E-05
TENM1	1.65785765	66.10227	1.410263837	6.068289661	6.068289661	-5.31731	1.2E-05
SCN4B	0	42.140197	0	5.430960858	5.430960858	-20	1.2E-05
SST	0	42.140197	0	5.430960858	5.430960858	-20	1.2E-05
CLCA1	65.4853771	1.6525567	6.054965161	1.407383613	1.407383613	5.308401	1.2E-05
LOC100517850	65.4853771	1.6525567	6.054965161	1.407383613	1.407383613	5.308401	1.2E-05
PACSIN1	150.865046	8.2627837	7.246646039	3.211445828	3.211445828	4.190487	1.2E-05
PHYH	273.546512	3767.0031	8.100906771	11.87958444	11.87958444	-3.78356	1.2E-05
TAL1	7171.89218	514.77143	12.80833923	9.010588038	9.010588038	3.80035	1.2E-05
PHYHD1	3093.56237	226.40027	11.59551968	7.829090182	7.829090182	3.772322	1.2E-05
EGR2	203.087562	12.394176	7.673044449	3.74353388	3.74353388	4.034368	1.2E-05
LOC100524517	881.980268	64.449713	9.786237389	6.032314961	6.032314961	3.774501	1.2E-05
TSPO	215.521494	2946.5087	7.758366438	11.52528034	11.52528034	-3.7731	1.3E-05
LOC100622032	53.8803735	0.8262784	5.778218396	0.868906686	0.868906686	6.026988	1.3E-05
TUB	80.4060959	1095.6451	6.347064926	10.09888102	10.09888102	-3.76833	1.3E-05
LOC100623510	47.248943	655.23875	5.592425431	9.358076973	9.358076973	-3.79367	1.3E-05
SLC13A5	402.859408	28.093465	8.657709339	4.862623207	4.862623207	3.84197	1.3E-05
PAOX	3362.96424	247.05723	11.71594665	7.954529215	7.954529215	3.766816	1.3E-05
PEAR1	186.508985	2534.1958	7.55081592	11.30788144	11.30788144	-3.76421	1.3E-05
LOC102157540	41.4464412	0	5.407571694	0	0	20	1.3E-05
LOC102161357	41.4464412	0	5.407571694	0	0	20	1.3E-05
LOC102157836	41.4464412	0	5.407571694	0	0	20	1.3E-05
LOC102158737	41.4464412	0	5.407571694	0	0	20	1.3E-05
LOC102163174	41.4464412	0	5.407571694	0	0	20	1.3E-05
LOC102158339	1.65785765	65.275991	1.410263837	6.05041444	6.05041444	-5.29916	1.3E-05
LOC100621769	843.020614	61.970878	9.721134424	5.976612875	5.976612875	3.765906	1.3E-05
LOXL3	92.0110994	1244.3752	6.539330984	10.28236477	10.28236477	-3.75747	1.3E-05
LOC100520252	54.7093024	749.43448	5.799846344	9.551582312	9.551582312	-3.77594	1.3E-05
LOC100626660	10.7760747	181.78124	3.557786824	7.513974209	7.513974209	-4.0763	1.3E-05
NUDT7	35.6439394	499.89841	5.195502704	8.968374236	8.968374236	-3.80991	1.3E-05
HS3ST2	625.012333	45.44531	9.29004727	5.537461031	5.537461031	3.781681	1.3E-05
LOC100623570	3.31571529	86.759229	2.109599694	6.455478947	6.455478947	-4.70963	1.3E-05
SGSM1	0.82892882	53.708094	0.870998931	5.773682393	5.773682393	-6.01775	1.3E-05
ENTPD8	64.6564482	1.6525567	6.036864803	1.407383613	1.407383613	5.290023	1.4E-05
LOC100525229	86.2085977	3.3051135	6.446398468	2.106051269	2.106051269	4.70506	1.4E-05
LOC102167069	86.2085977	3.3051135	6.446398468	2.106051269	2.106051269	4.70506	1.4E-05
LOC102167347	0	41.313919	0	5.403060391	5.403060391	-20	1.4E-05
LOC102167351	0	41.313919	0	5.403060391	5.403060391	-20	1.4E-05
ZDHHC8	150.865046	2027.6871	7.246646039	10.98633067	10.98633067	-3.7485	1.4E-05
IFT27	36.4728682	505.68236	5.227774503	8.984937802	8.984937802	-3.79334	1.5E-05
DUSP10	9.94714588	169.38707	3.452482877	7.412672017	7.412672017	-4.0899	1.5E-05
CD27	168.272551	9.9153405	7.403204238	3.448285225	3.448285225	4.084994	1.5E-05
FLT3	106.102889	4.9576702	6.742853591	2.574748269	2.574748269	4.419658	1.5E-05
ARSB	36.4728682	504.02981	5.227774503	8.980224728	8.980224728	-3.78861	1.5E-05
LOC100525748	12.4339324	199.95937	3.747809764	7.650760008	7.650760008	-4.00735	1.5E-05
SMARCD3	225.46864	3019.2212	7.823167478	11.56043849	11.56043849	-3.74318	1.5E-05
TRHDE	4.97357294	106.58991	2.578594101	6.749398975	6.749398975	-4.42164	1.5E-05
C7H6orf132	40.6175124	0	5.379118827	0	0	20	1.5E-05
LOC100158041	40.6175124	0	5.379118827	0	0	20	1.5E-05
HSD17B3	40.6175124	0	5.379118827	0	0	20	1.5E-05
RRAGD	2083.09813	156.99289	11.0252075	7.303715831	7.303715831	3.72996	1.5E-05
CYP4V2	29.8414377	418.09686	4.946798112	8.711139889	8.711139889	-3.80845	1.5E-05
IGFBP5	31.4992953	438.75382	5.022336531	8.780552285	8.780552285	-3.80002	1.5E-05
LOC100738154	3.31571529	85.106672	2.109599694	6.428053129	6.428053129	-4.68188	1.6E-05
LOC100518295	0	40.48764	0	5.374609695	5.374609695	-20	1.6E-05
LOC102159522	0	40.48764	0	5.374609695	5.374609695	-20	1.6E-05
MYH2	0	40.48764	0	5.374609695	5.374609695	-20	1.6E-05
PPP2R2B	0	40.48764	0	5.374609695	5.374609695	-20	1.6E-05
SRPX	75.432523	999.79683	6.256114748	9.966933409	9.966933409	-3.72838	1.6E-05
SULF1	314.164024	4198.3204	8.299959051	12.03594015	12.03594015	-3.74022	1.6E-05
EPB41L5	2286.1857	173.51846	11.15935779	7.447235822	7.447235822	3.719782	1.6E-05
MEG3	7.46035941	136.33593	3.080718953	7.101565318	7.101565318	-4.19178	1.6E-05
SATB1	3382.0296	255.32002	11.72410008	8.001802338	8.001802338	3.727511	1.6E-05
LOC100622375	896.072058	67.754826	9.809080066	6.103389087	6.103389087	3.725219	1.6E-05
FNDC1	94.4978859	4.1313919	6.57739689	2.359350201	2.359350201	4.515582	1.7E-05
IFNAR2	165.785765	2186.3326	7.381852349	11.09495688	11.09495688	-3.72112	1.7E-05
AMOTL2	368.044398	4927.9242	8.527650579	12.26705708	12.26705708	-3.74303	1.7E-05
STEAP1	104.445032	1372.4484	6.720347312	10.42358697	10.42358697	-3.71594	1.7E-05
CAP2	745.207012	56.186929	9.543432108	5.837613535	5.837613535	3.729335	1.7E-05
PLBD2	397.885835	5323.7115	8.639832083	12.37848766	12.37848766	-3.74201	1.7E-05
CHRNA9	104.445032	4.9576702	6.720347312	2.574748269	2.574748269	4.396938	1.7E-05
IRF1	205.574348	2710.1931	7.690517306	11.40471213	11.40471213	-3.72066	1.7E-05
LOC100737939	198.942918	2620.1287	7.643444369	11.35597249	11.35597249	-3.71921	1.7E-05
H2AFY2	33.1571529	454.4531	5.094115824	8.831158705	8.831158705	-3.77674	1.7E-05
LOC102167180	93.6689571	4.1313919	6.564819524	2.359350201	2.359350201	4.502871	1.8E-05
CYP2C91	19.0653629	276.80325	4.326635347	8.117919691	8.117919691	-3.85984	1.8E-05
LOC102160408	103.616103	4.9576702	6.708961125	2.574748269	2.574748269	4.385442	1.8E-05
LOC100738797	4.97357294	104.11107	2.578594101	6.715770874	6.715770874	-4.3877	1.8E-05
LGALS7	244.534003	16.525567	7.939779021	4.13138925	4.13138925	3.887263	1.8E-05
FAM49A	62.9985906	827.10465	5.999968229	9.693669287	9.693669287	-3.71468	1.8E-05
LOC100621509	51.3935871	0.8262784	5.711318333	0.868906686	0.868906686	5.958817	1.8E-05
LOC100738527	1632.98978	22616.892	10.67418325	14.46517683	14.46517683	-3.79181	1.8E-05
EGLN3	14866.8385	1092.34	13.8599073	10.0945264	10.0945264	3.766604	1.9E-05
LOC102157898	50.5646582	667.63292	5.688310694	9.385070587	9.385070587	-3.72285	1.9E-05
LOC102159458	0	39.661362	0	5.34558663	5.34558663	-20	1.9E-05
SERPINB2	0	39.661362	0	5.34558663	5.34558663	-20	1.9E-05
ZSCAN4	0	39.661362	0	5.34558663	5.34558663	-20	1.9E-05
CCDC64	324.11117	23.135794	8.344789314	4.593102408	4.593102408	3.80829	1.9E-05
LOC102163771	4.14464412	93.369456	2.363071282	6.560248082	6.560248082	-4.49363	1.9E-05
LOC100521253	102.787174	4.9576702	6.697484358	2.574748269	2.574748269	4.373854	1.9E-05
MAOB	363.899754	26.440908	8.511356367	4.77825631	4.77825631	3.782697	1.9E-05
PDE1C	18.2364341	264.40908	4.265769485	8.052073912	8.052073912	-3.85788	1.9E-05
GJA5	0.82892882	51.229259	0.870998931	5.706786332	5.706786332	-5.94958	1.9E-05
LOC100738836	23.2100071	324.7274	4.597531595	8.347521274	8.347521274	-3.80641	2E-05
LOC100628155	1754.01339	136.33593	10.77726632	7.101565318	7.101565318	3.685422	2E-05
LOC396858	464.200141	34.703692	8.861707724	5.158001345	5.158001345	3.741586	2E-05
SCN1B	13.2628612	203.26448	3.834191515	7.674294538	7.674294538	-3.93789	2E-05
LOC100624234	271.888654	19.004403	8.092168603	4.322245637	4.322245637	3.83861	2E-05
LOC100511223	2616.09937	203.26448	11.35375299	7.674294538	7.674294538	3.685987	2E-05
LOC102165895	211.37685	14.046732	7.730482704	3.911378308	3.911378308	3.911511	2E-05
B2M	991.398873	13340.264	9.954776287	13.70360777	13.70360777	-3.75018	2E-05
LOC100037974	4.14464412	92.543178	2.363071282	6.547560532	6.547560532	-4.48081	2E-05
S1PR3	74.6035941	961.78802	6.240382915	9.911074387	9.911074387	-3.6884	2.1E-05
LOC100516519	61.340733	1.6525567	5.962103212	1.407383613	1.407383613	5.214074	2.1E-05
RGAG1	61.340733	1.6525567	5.962103212	1.407383613	1.407383613	5.214074	2.1E-05
LOC100738971	121.852537	6.610227	6.940783842	2.927939483	2.927939483	4.204293	2.1E-05
C10H1orf95	50.5646582	0.8262784	5.688310694	0.868906686	0.868906686	5.935358	2.1E-05
SLC14A1	38.9596547	0	5.320472212	0	0	20	2.1E-05
LOC102164571	38.9596547	0	5.320472212	0	0	20	2.1E-05
LOC102158818	38.9596547	0	5.320472212	0	0	20	2.1E-05
PRSS48	38.9596547	0	5.320472212	0	0	20	2.1E-05
LOC100737937	38.9596547	0	5.320472212	0	0	20	2.1E-05
LOC100513619	1109.9357	14958.117	10.1175596	13.86873743	13.86873743	-3.75238	2.1E-05
LOC100737860	9.11821706	152.03522	3.338883189	7.257719911	7.257719911	-4.05951	2.1E-05
LOC102163860	7.46035941	132.20454	3.080718953	7.057499439	7.057499439	-4.14738	2.1E-05
RGS9BP	359.755109	26.440908	8.494876018	4.77825631	4.77825631	3.766172	2.1E-05
RAD51C	879.493482	68.581105	9.782168514	6.120623682	6.120623682	3.68079	2.1E-05
TMEM82	71.2878788	2.4788351	6.175681852	1.798604302	1.798604302	4.845923	2.2E-05
LOC100152059	71.2878788	2.4788351	6.175681852	1.798604302	1.798604302	4.845923	2.2E-05
LOC102165794	71.2878788	2.4788351	6.175681852	1.798604302	1.798604302	4.845923	2.2E-05
LOC102165247	229.613284	15.699289	7.849331809	4.061714779	4.061714779	3.870435	2.2E-05
LOC102163494	1105.79105	86.759229	10.11216717	6.455478947	6.455478947	3.671918	2.2E-05
LOC100517362	479.12086	36.356248	8.907253808	5.223277669	5.223277669	3.720114	2.2E-05
XAF1	222.981854	2859.7494	7.807238043	11.48217743	11.48217743	-3.68089	2.2E-05
MYCBPAP	722.825934	56.186929	9.499498989	5.837613535	5.837613535	3.685342	2.2E-05
HECW2	38.9596547	512.29259	5.320472212	9.003637625	9.003637625	-3.71692	2.2E-05
LOC102167526	1.65785765	61.1446	1.410263837	5.957557119	5.957557119	-5.20483	2.2E-05
LOC102164190	101.129317	4.9576702	6.674253245	2.574748269	2.574748269	4.350395	2.2E-05
RAB11FIP4	447.621565	33.877413	8.80935516	5.124221138	5.124221138	3.723884	2.2E-05
MCCC2	2858.97551	224.74772	11.48178708	7.818567588	7.818567588	3.66912	2.3E-05
REN	60.5118041	1.6525567	5.942791385	1.407383613	1.407383613	5.194445	2.3E-05
LOC102163428	60.5118041	1.6525567	5.942791385	1.407383613	1.407383613	5.194445	2.3E-05
LOC102162786	218.008281	14.873011	7.774841608	3.98850389	3.98850389	3.873614	2.3E-05
TBC1D4	901.87456	71.05994	9.818381752	6.171125545	6.171125545	3.665818	2.3E-05
TDRD7	133.457541	1689.7393	7.071006856	10.72343848	10.72343848	-3.66235	2.3E-05
LOC102158232	3107.65416	244.5784	11.60207441	7.940039851	7.940039851	3.667457	2.3E-05
LOC102167582	100.300388	4.9576702	6.662495885	2.574748269	2.574748269	4.338521	2.4E-05
LOC100520903	655.6827	51.229259	9.359052637	5.706786332	5.706786332	3.677958	2.4E-05
LOC100516621	1166.30286	92.543178	10.1889632	6.547560532	6.547560532	3.655672	2.4E-05
LOC100623661	70.45895	2.4788351	6.159042811	1.798604302	1.798604302	4.829049	2.4E-05
CXCR5	216.350423	2744.8968	7.763879093	11.42306166	11.42306166	-3.66531	2.4E-05
LOC100627550	80.4060959	3.3051135	6.347064926	2.106051269	2.106051269	4.604533	2.4E-05
NOS3	334.887245	24.788351	8.3918332	4.688647628	4.688647628	3.755941	2.4E-05
LOC100738511	117.707893	1483.1697	6.891272054	10.53544032	10.53544032	-3.6554	2.4E-05
PLCG2	927.571354	73.538775	9.858868964	6.219919206	6.219919206	3.656881	2.4E-05
LOC102167200	57.1960888	728.77752	5.862850292	9.511312908	9.511312908	-3.67149	2.4E-05
PAPSS2	198.113989	2503.6235	7.637450771	11.29037802	11.29037802	-3.65961	2.4E-05
RRH	38.1307259	0	5.290229968	0	0	20	2.5E-05
SKOR2	38.1307259	0	5.290229968	0	0	20	2.5E-05
LOC102165954	38.1307259	0	5.290229968	0	0	20	2.5E-05
LOC102157511	324.11117	23.962073	8.344789314	4.641665831	4.641665831	3.757664	2.5E-05
COL9A1	422.7537	32.224857	8.727082155	5.054191063	5.054191063	3.713572	2.5E-05
FGF12	1058.54211	84.280394	10.04922521	6.414142197	6.414142197	3.650738	2.5E-05
ICAM-1	518.909444	6696.9862	9.02211655	12.70951169	12.70951169	-3.68996	2.5E-05
P2RX7	177.390768	11.567897	7.478897148	3.65167138	3.65167138	3.938732	2.5E-05
LOC102165830	109.418605	5.7839486	6.786839466	2.762125239	2.762125239	4.24166	2.5E-05
LOC100523418	681.379493	53.708094	9.414430481	5.773682393	5.773682393	3.665247	2.5E-05
PRMT8	2.48678647	70.233662	1.801898017	6.154487245	6.154487245	-4.81981	2.5E-05
LOC100621791	2.48678647	70.233662	1.801898017	6.154487245	6.154487245	-4.81981	2.5E-05
LOC100511392	89.524313	4.1313919	6.500233417	2.359350201	2.359350201	4.43758	2.6E-05
C3H16orf54	7.46035941	128.89943	3.080718953	7.021251246	7.021251246	-4.11086	2.6E-05
LOC100625114	0	38.008805	0	5.285727902	5.285727902	-20	2.6E-05
SCMH1	35.6439394	461.88961	5.195502704	8.854524369	8.854524369	-3.69582	2.6E-05
LDLRAD4	1510.30832	121.46292	10.56158229	6.936201185	6.936201185	3.636255	2.6E-05
PRAP1	48.9068006	0.8262784	5.641164514	0.868906686	0.868906686	5.887263	2.7E-05
FSCN1	1315.51004	17303.095	10.36250281	14.0788259	14.0788259	-3.71734	2.7E-05
LOC102158670	1.65785765	59.492043	1.410263837	5.918673475	5.918673475	-5.1653	2.7E-05
HOXC10	1.65785765	59.492043	1.410263837	5.918673475	5.918673475	-5.1653	2.7E-05
SYT11	26.5257224	351.16831	4.78270852	8.460121275	8.460121275	-3.7267	2.7E-05
LOC100624297	140.9179	1748.405	7.148912757	10.77264864	10.77264864	-3.63311	2.8E-05
LOC100512652	116.878964	1448.466	6.881162477	10.50130576	10.50130576	-3.63144	2.8E-05
TSHZ2	3.31571529	79.322724	2.109599694	6.327736286	6.327736286	-4.58034	2.8E-05
KCNN3	3.31571529	79.322724	2.109599694	6.327736286	6.327736286	-4.58034	2.8E-05
LAMA2	5.80250177	108.24247	2.766065426	6.771389985	6.771389985	-4.22145	2.8E-05
PDE3B	680.550564	54.534373	9.412676884	5.795309087	5.795309087	3.641465	2.9E-05
LOC102161901	68.8010924	2.4788351	6.125177709	1.798604302	1.798604302	4.794697	2.9E-05
TMEFF2	7.46035941	127.24687	3.080718953	7.002779797	7.002779797	-4.09224	2.9E-05
LOC100622802	37.3017971	0	5.259340178	0	0	20	2.9E-05
LOC102159633	37.3017971	0	5.259340178	0	0	20	2.9E-05
PDIA2	37.3017971	0	5.259340178	0	0	20	2.9E-05
LOC102158259	37.3017971	0	5.259340178	0	0	20	2.9E-05
CAPG	1880.8395	24799.093	10.87792787	14.5980579	14.5980579	-3.72084	3E-05
LOC100153517	4.14464412	88.411786	2.363071282	6.482393107	6.482393107	-4.41492	3E-05
KIF17	1105.79105	90.064343	10.11216717	6.508814353	6.508814353	3.617979	3E-05
TPMT	1691.84373	137.98849	10.72523308	7.118821585	7.118821585	3.615976	3E-05
OSMR	203.087562	2507.7549	7.673044449	11.29275579	11.29275579	-3.62622	3E-05
LOC102160475	716.194504	57.839486	9.486220622	5.878712739	5.878712739	3.630225	0.00003
VAMP8	239.56043	2961.3817	7.910255542	11.53254182	11.53254182	-3.62781	3E-05
C3H2orf40	1.65785765	58.665764	1.410263837	5.89883146	5.89883146	-5.14513	3E-05
TOX2	2.48678647	68.581105	1.801898017	6.120623682	6.120623682	-4.78546	3.1E-05
LOC102161426	0	37.182527	0	5.254840671	5.254840671	-20	3.1E-05

DNM1	324.490311	4540.3997	8.34647079	12.14892129	12.14892129	-3.80657	0.00179
KIAA1033	11.0464787	180.95496	3.590539588	7.507437596	7.507437596	-4.03397	0.00179
TMEM55A	9.66566885	163.60312	3.414902536	7.362847851	7.362847851	-4.08119	0.00181
NAV3	27.6161967	386.69828	4.838760036	8.598790517	8.598790517	-3.80762	0.00185
LOC102164565	459.809675	6495.3743	8.848027198	12.66541904	12.66541904	-3.8203	0.00186
DLG4	104.941548	1406.3258	6.727124677	10.45874063	10.45874063	-3.74427	0.00186
LOC100516675	2.76161967	78.496445	1.911353989	6.312818447	6.312818447	-4.82904	0.00187
SNTB1	6.90404918	128.89943	2.982591923	7.021251246	7.021251246	-4.22266	0.00188
DAPK2	8.28485901	145.42499	3.214880004	7.194018019	7.194018019	-4.13365	0.00189
LOC102159666	0	42.966475	0	5.458331975	5.458331975	-20	0.00192
DMKN	40.0434852	1.6525567	5.359081339	1.407383613	1.407383613	4.598796	0.00194
GYPC	6.90404918	128.07315	2.982591923	7.012045083	7.012045083	-4.21338	0.00194
COL7A1	566.132033	8015.7265	9.147540835	12.96879754	12.96879754	-3.82362	0.00194
LOC100514810	14084.2603	1478.212	13.78189861	10.53061313	10.53061313	3.252159	0.00196
BZW2	23070.5707	2376.3766	14.49382861	11.21515474	11.21515474	3.279218	0.00196
LOXL3	93.8950688	1244.3752	6.568261215	10.28236477	10.28236477	-3.72823	0.00197
AMCF-II	687.643298	9804.6192	9.427613082	13.25939301	13.25939301	-3.83373	0.00197
LOC100739584	2.76161967	77.670167	1.911353989	6.29774474	6.29774474	-4.81377	0.00197
KIF5C	6391.76873	691.595	12.64222519	9.435868156	9.435868156	3.208216	0.00199
LOC100739030	6.90404918	127.24687	2.982591923	7.002779797	7.002779797	-4.20404	0.002
LOC100625312	46.9475344	2.4788351	5.583384725	1.798604302	1.798604302	4.243315	0.00202
CAPNS2	46.9475344	2.4788351	5.583384725	1.798604302	1.798604302	4.243315	0.00202
DIAPH2	16.569718	243.75212	4.135019133	7.935177544	7.935177544	-3.87879	0.00202
LOC100518476	41.4242951	551.95395	5.406818783	9.111015534	9.111015534	-3.736	0.00206
UBE2L6	370.057036	5069.2178	8.535497154	12.30783201	12.30783201	-3.77594	0.00207
CTSK	450.144007	6223.5287	8.817444209	12.60374889	12.60374889	-3.78927	0.00207
SPON1	2.76161967	76.843889	1.911353989	6.282511874	6.282511874	-4.79834	0.00208
SCN4B	0	42.140197	0	5.430960858	5.430960858	-20	0.00211
LOC102158540	0	42.140197	0	5.430960858	5.430960858	-20	0.00211
SPMI	0	42.140197	0	5.430960858	5.430960858	-20	0.00211
LOC102167277	0	42.140197	0	5.430960858	5.430960858	-20	0.00211
DCTPP1	3170.33938	353.64714	11.63087656	8.470240516	8.470240516	3.164255	0.00211
LOC100738192	56.6132033	741.99798	5.848327568	9.537214474	9.537214474	-3.7122	0.00212
LOC100622576	24.854577	342.07925	4.692347799	8.422398044	8.422398044	-3.78275	0.00214
LOC100739399	361.772177	40.48764	8.502920002	5.374609695	5.374609695	3.159528	0.00214
LOC100154916	107.703167	9.9153405	6.764250165	3.448285225	3.448285225	3.441255	0.00214
ROR1	27.6161967	375.95666	4.838760036	8.558254848	8.558254848	-3.76698	0.00214
HAUS1	6934.427	756.04471	12.75976899	9.564234696	9.564234696	3.197233	0.00216
SIPA1L1	392.149993	5339.4108	8.61893602	12.38273502	12.38273502	-3.7672	0.00217
LOC100037974	4.14242951	92.543178	2.362450112	6.547560532	6.547560532	-4.48158	0.00218
KLB	277.542777	30.5723	8.121755095	4.980587446	4.980587446	3.182413	0.00219
VIM	30631.8854	533350.29	14.90279364	19.02472656	19.02472656	-4.12198	0.00219
C6H19orf68	13.8080984	206.56959	3.888314478	7.697451307	7.697451307	-3.90304	0.00221
EPB41L4A	948.616357	109.06875	9.891200975	6.782261052	6.782261052	3.120587	0.00223
LOC100515642	133.938554	13.220454	7.076158798	3.829895615	3.829895615	3.340728	0.00223
LOC100152707	5.52323934	108.24247	2.705588563	6.771389985	6.771389985	-4.29261	0.00224
WDR63	386.626754	43.792754	8.598524339	5.485193456	5.485193456	3.142177	0.00226
WISP1	78.7061606	1014.6698	6.316619332	9.988215791	9.988215791	-3.68839	0.00227
LOC102158578	120.130456	11.567897	6.920417836	3.65167138	3.65167138	3.376403	0.00228
LOC102165965	248.54577	27.267186	7.963160641	4.821056379	4.821056379	3.188274	0.00228
MAB21L3	4.14242951	91.716899	2.362450112	6.534760414	6.534760414	-4.46864	0.00228
MLKL	16.569718	237.96817	4.135019133	7.900674664	7.900674664	-3.84415	0.00229
ZNF350	9.66566885	156.16661	3.414902536	7.296150961	7.296150961	-4.01407	0.0023
ZBED3	0	41.313919	0	5.403060391	5.403060391	-20	0.00232
HSD17B13	0	41.313919	0	5.403060391	5.403060391	-20	0.00232
LOC102167347	0	41.313919	0	5.403060391	5.403060391	-20	0.00232
LOC100620105	2.76161967	75.191332	1.911353989	6.251554969	6.251554969	-4.76698	0.00232
LOC100515588	1.38080984	58.665764	1.251452392	5.89883146	5.89883146	-5.40893	0.00232
LOC102167736	900.288013	104.11107	9.815844393	6.715770874	6.715770874	3.112263	0.00232
LOC102164190	66.2788721	4.9576702	6.072081615	2.574748269	2.574748269	3.740815	0.00234
SYT2	106.322357	9.9153405	6.745806839	3.448285225	3.448285225	3.422639	0.00234
LOC100156401	3283.56579	370.99899	11.68148695	8.53915489	8.53915489	3.145776	0.00235
LOC102157983	19.3313377	269.36675	4.345633235	8.078773924	8.078773924	-3.80056	0.00236
LOC100737024	186.409328	19.830681	7.550048951	4.380638094	4.380638094	3.232668	0.00237
DENND1C	186.409328	19.830681	7.550048951	4.380638094	4.380638094	3.232668	0.00237
ESPN	59.3748229	4.1313919	5.915875148	2.359350201	2.359350201	3.845151	0.00239
LOC100517234	22.0929574	301.59161	4.529381037	8.241228156	8.241228156	-3.77094	0.00239
ITGA10	154.650702	1992.1572	7.28216827	10.96083975	10.96083975	-3.68725	0.0024
BOC	202.979046	2637.4806	7.672277146	11.36549164	11.36549164	-3.69976	0.00241
STX3	84.2294	1073.3356	6.413279271	10.06922902	10.06922902	-3.67163	0.00241
DTL	3233.85664	366.8676	11.65948606	8.523042794	8.523042794	3.139924	0.00242
LOC100737431	22.0929574	300.76533	4.529381037	8.23728324	8.23728324	-3.76698	0.00243
OPRL1	52.4707738	3.3051135	5.740678649	2.106051269	2.106051269	3.988742	0.00243
LAMC3	52.4707738	3.3051135	5.740678649	2.106051269	2.106051269	3.988742	0.00243
HRC	9.66566885	154.51406	3.414902536	7.280901168	7.280901168	-3.99872	0.00243
LOC102165764	38.6626754	1.6525567	5.309710091	1.407383613	1.407383613	4.54817	0.00243
LOC100519278	93.8950688	1194.7985	6.568261215	10.22375862	10.22375862	-3.66957	0.00244
ARSI	51.0899639	653.58619	5.702933533	9.35443936	9.35443936	-3.67727	0.00244
ACN9	479.141013	55.360651	8.907314364	5.816616368	5.816616368	3.113517	0.00245
PTI	23.4737672	0	4.613164285	0	0	20	0.00245
LOC102164656	23.4737672	0	4.613164285	0	0	20	0.00245
LOC100736733	23.4737672	0	4.613164285	0	0	20	0.00245
RGS9	23.4737672	0	4.613164285	0	0	20	0.00245
OPRM1	23.4737672	0	4.613164285	0	0	20	0.00245
DNAJC22	23.4737672	0	4.613164285	0	0	20	0.00245
WNT9A	6.90404918	122.2892	2.982591923	6.945902605	6.945902605	-4.14671	0.00245
LOC100737468	6.90404918	122.2892	2.982591923	6.945902605	6.945902605	-4.14671	0.00245
LOC102160150	41.4242951	533.77583	5.406818783	9.062790447	9.062790447	-3.68768	0.00246
TAL1	4576.0038	514.77143	12.16018777	9.010588038	9.010588038	3.152084	0.00247
LOC100739471	51.0899639	649.4548	5.702933533	9.345304998	9.345304998	-3.66812	0.00253
LOC100518295	0	40.48764	0	5.374609695	5.374609695	-20	0.00255
LOC102166786	0	40.48764	0	5.374609695	5.374609695	-20	0.00255
MYH2	0	40.48764	0	5.374609695	5.374609695	-20	0.00255
PPP2R2B	0	40.48764	0	5.374609695	5.374609695	-20	0.00255
PLD5	0	40.48764	0	5.374609695	5.374609695	-20	0.00255
CLDN19	0	40.48764	0	5.374609695	5.374609695	-20	0.00255
LOC100517085	0	40.48764	0	5.374609695	5.374609695	-20	0.00255
SHAS2	2.76161967	73.538775	1.911353989	6.219919206	6.219919206	-4.73492	0.00259
LOC100623769	62.1364426	782.48562	5.98040107	9.613762982	9.613762982	-3.65455	0.00259
LRRK2	92.514259	1163.3999	6.547114458	10.18537096	10.18537096	-3.65253	0.00259
ZNF567	16.569718	232.18422	4.135019133	7.865326367	7.865326367	-3.80865	0.0026
LOC100739118	86.9910197	1092.34	6.459284385	10.0945264	10.0945264	-3.65041	0.00261
LOC102165757	5.52323934	104.93735	2.705588563	6.727067559	6.727067559	-4.24787	0.00263
CHAD	15.1889082	215.65866	4.016933786	7.75928006	7.75928006	-3.82766	0.00263
RGAG4	38.6626754	492.46191	5.309710091	8.946794917	8.946794917	-3.671	0.00266
MYPN	1.38080984	57.013208	1.251452392	5.858309486	5.858309486	-5.36771	0.00267
MIR214	27.6161967	360.25737	4.838760036	8.49688321	8.49688321	-3.70544	0.00267
ZNF385C	35.9010557	456.93194	5.205590187	8.838989383	8.838989383	-3.66988	0.00273
MAPK10	5.52323934	104.11107	2.705588563	6.715770874	6.715770874	-4.23647	0.00273
PAOX	2118.16229	247.05723	11.04927836	7.954529215	7.954529215	3.099896	0.00273
MSI1	822.962662	97.500848	9.686435153	6.622064238	6.622064238	3.07734	0.00277
LOC102167902	9.66566885	150.38266	3.414902536	7.242056188	7.242056188	-3.95962	0.00279
LOC100736715	57.9940131	4.1313919	5.882496648	2.359350201	2.359350201	3.811204	0.00279
LOC100517699	57.9940131	4.1313919	5.882496648	2.359350201	2.359350201	3.811204	0.00279
DCX	0	39.661362	0	5.34558663	5.34558663	-20	0.00281
LOC102159458	0	39.661362	0	5.34558663	5.34558663	-20	0.00281
LOC102159747	0	39.661362	0	5.34558663	5.34558663	-20	0.00281
LOC100154648	0	39.661362	0	5.34558663	5.34558663	-20	0.00281
ZSCAN4	0	39.661362	0	5.34558663	5.34558663	-20	0.00281
LOC100510952	0	39.661362	0	5.34558663	5.34558663	-20	0.00281
LOC100625959	0	39.661362	0	5.34558663	5.34558663	-20	0.00281
CLVS2	0	39.661362	0	5.34558663	5.34558663	-20	0.00281
LOC102165051	0	39.661362	0	5.34558663	5.34558663	-20	0.00281
TCP11	103.560738	9.9153405	6.708197415	3.448285225	3.448285225	3.384671	0.00281
[illegible]							

LOC100523504	75.07249515	3.3051135	6.249303021	2.106051269	2.106051269	4.505513	0.00148
MYLK2	1.057359087	61.970878	1.04079362	5.976612875	5.976612875	-5.87305	0.00148
HTR2A	1.057359087	61.970878	1.04079362	5.976612875	5.976612875	-5.87305	0.00148
EPS8L1	1.057359087	61.970878	1.04079362	5.976612875	5.976612875	-5.87305	0.00148
STAT1	612.2109111	12381.781	9.260239557	13.59604779	13.59604779	-4.33805	0.00148
LOC100520085	22.20454082	418.92313	4.536335244	8.713981461	8.713981461	-4.23776	0.00149
LOC100622512	0	43.792754	0	5.485193456	5.485193456	-20	0.00149
ZNF660	0	43.792754	0	5.485193456	5.485193456	-20	0.00149
CADPS	0	43.792754	0	5.485193456	5.485193456	-20	0.00149
EXOC6	872.3212464	61.970878	9.770368629	5.976612875	5.976612875	3.815197	0.0015
ARPP21	4.229436346	114.02642	2.386655454	6.845821397	6.845821397	-4.75276	0.0015
TSHZ2	2.114718173	79.322724	1.639101631	6.327736286	6.327736286	-5.2292	0.0015
NYX	2.114718173	79.322724	1.639101631	6.327736286	6.327736286	-5.2292	0.0015
LOC100627666	6.344154519	147.90383	2.876596413	7.218237038	7.218237038	-4.54309	0.00154
LOC100522323	6607.436932	443.71149	12.69009336	8.796725856	8.796725856	3.896397	0.00154
LOC102165468	5675.903577	384.21944	12.47088852	8.589536711	8.589536711	3.884848	0.00155
STX3	59.21210885	1073.3356	5.911981742	10.06922902	10.06922902	-4.18007	0.00156
PAICS	60846.786	3678.5913	15.89291715	11.84532982	11.84532982	4.047956	0.00157
MTHFD2L	1.057359087	61.1446	1.04079362	5.957557118	5.957557118	-5.85369	0.00157
TNFRSF21	4573.078049	313.1595	12.15926527	8.29535341	8.29535341	3.868196	0.00157
CHSY3	6.344154519	147.07755	2.876596413	7.210209122	7.210209122	-4.53501	0.00158
PSMB9	20.08982264	376.78294	4.398475058	8.561413734	8.561413734	-4.2292	0.00159
LOC100739537	25.37661808	467.67356	4.721187694	8.872439593	8.872439593	-4.20393	0.00159
CAMTA2	178.6936856	3334.0332	7.489395904	11.70348542	11.70348542	-4.22171	0.00159
TRIM50	74.01513606	3.3051135	6.229109817	2.106051269	2.106051269	4.485049	0.00159
LOC100737157	74.01513606	3.3051135	6.229109817	2.106051269	2.106051269	4.485049	0.00159
LOC100737319	25.37661808	466.84728	4.721187694	8.869893856	8.869893856	-4.20138	0.0016
PPM1E	1455.983462	104.11107	10.50876879	6.715770874	6.715770874	3.805799	0.00161
NES	1137.718377	23472.09	10.15319527	14.51872013	14.51872013	-4.36673	0.00161
LOC102159666	0	42.966475	0	5.458331975	5.458331975	-20	0.00162
HAS1	0	42.966475	0	5.458331975	5.458331975	-20	0.00162
C1RL	0	42.966475	0	5.458331975	5.458331975	-20	0.00162
LOC100626234	30.66341351	0	4.98474489	0	0	20	0.00162
PDIA2	30.66341351	0	4.98474489	0	0	20	0.00162
LOC102166344	30.66341351	0	4.98474489	0	0	20	0.00162
LOC100623257	30.66341351	0	4.98474489	0	0	20	0.00162
LOC102165082	30.66341351	0	4.98474489	0	0	20	0.00162
LOC100623608	33.83549077	611.446	5.122485983	9.258438824	9.258438824	-4.17562	0.00163
LIN28B	285.4869534	19.830681	8.16232563	4.380638094	4.380638094	3.847619	0.00166
LOC102157733	628.0712974	45.44531	9.297079728	5.537461031	5.537461031	3.788725	0.00167
IFIT3	477.9263071	9279.1061	8.903659874	13.17992559	13.17992559	-4.27913	0.00168
RMND5B	3.17207726	94.195734	2.060765874	6.572825024	6.572825024	-4.89216	0.00168
EGFR	72.95777697	1304.6935	6.208629956	10.35060062	10.35060062	-4.16051	0.00168
GPR56	71.90041789	1284.8629	6.187855179	10.32852108	10.32852108	-4.15947	0.00169
WNK3	1466.557053	105.76363	10.51920088	6.738276475	6.738276475	3.793518	0.00169
LMTK3	6.344154519	144.59872	2.876596413	7.185853813	7.185853813	-4.51048	0.0017
NRROS	197.7261492	13.220454	7.63463791	3.829895615	3.829895615	3.90266	0.00175
LOC102163771	3.17207726	93.369456	2.060765874	6.560248082	6.560248082	-4.87945	0.00175
LOC102163119	103.6211905	5.7839486	6.709031281	2.76212524	2.76212524	4.163121	0.00175
SCN4B	0	42.140197	0	5.430960858	5.430960858	-20	0.00176
SST	0	42.140197	0	5.430960858	5.430960858	-20	0.00176
LOC102165507	0	42.140197	0	5.430960858	5.430960858	-20	0.00176
SPMI	0	42.140197	0	5.430960858	5.430960858	-20	0.00176
TMEM151B	10.57359087	211.52726	3.532764645	7.731504113	7.731504113	-4.32231	0.00176
PDE2A	4.229436346	109.89502	2.386655454	6.793050814	6.793050814	-4.69952	0.00176
LOC100512333	41.23700438	0.8262784	5.400435611	0.868906686	0.868906686	5.641168	0.00177
RNF17	1.057359087	59.492043	1.04079362	5.918673475	5.918673475	-5.81416	0.00177
LOC100522117	51.81059524	1.6525567	5.722755497	1.407383614	1.407383614	4.970475	0.00177
FAM129B	861.7476556	17118.009	9.752794838	14.06331157	14.06331157	-4.31211	0.00178
LOC100625740	115.2521404	2060.7383	6.861113469	11.00964548	11.00964548	-4.1603	0.00178
LOC102161422	388.0507848	7343.9622	8.60381468	12.84253934	12.84253934	-4.24224	0.00179
LOC100737519	6.344154519	142.94616	2.876596413	7.169385476	7.169385476	-4.4939	0.00179
ZNF704	123.7110131	7.4365053	6.962445064	3.076645515	3.076645515	4.056205	0.0018
ANO9	82.47400875	4.1313919	6.383255151	2.359350201	2.359350201	4.31924	0.00181
CTSA	794.076674	15585.263	9.634950185	13.92798741	13.92798741	-4.29476	0.00184
PAPLN	6.344154519	142.11988	2.876596413	7.161080272	7.161080272	-4.48554	0.00184
NUDT22	90.93288145	1602.1538	6.522509054	10.64669709	10.64669709	-4.13907	0.00184
LOC100510895	11.63094995	226.40027	3.658891241	7.829090181	7.829090181	-4.28283	0.00184
KCNK10	71.90041789	3.3051135	6.187855179	2.106051269	2.106051269	4.443228	0.00185
BTBD17	8.458872693	175.17101	3.241668254	7.460832769	7.460832769	-4.37215	0.00186
LOC102167052	25.37661808	451.14799	4.721187694	8.820651243	8.820651243	-4.15203	0.00187
LOC102161016	1.057359087	58.665764	1.04079362	5.89883146	5.89883146	-5.79398	0.00188
LAMA2	4.229436346	108.24247	2.386655454	6.771389985	6.771389985	-4.67766	0.00188
LOC100622295	27.49133625	484.19913	4.832451381	8.922433141	8.922433141	-4.13855	0.00191
LOC102167347	0	41.313919	0	5.403060391	5.403060391	-20	0.00191
LOC102167351	0	41.313919	0	5.403060391	5.403060391	-20	0.00191
LOC100620566	0	41.313919	0	5.403060391	5.403060391	-20	0.00191
TCP11L2	113.1374223	1988.852	6.834628076	10.95844545	10.95844545	-4.13579	0.00192
EMILIN2	477.9263071	9008.9131	8.903659874	13.13729747	13.13729747	-4.23649	0.00192
RBCK1	95.16231779	1661.6458	6.587399765	10.69926515	10.69926515	-4.12608	0.00193
LOC102159120	29.60605442	0	4.935745167	0	0	20	0.00193
LOC102161287	29.60605442	0	4.935745167	0	0	20	0.00193
PNLIPRP2	29.60605442	0	4.935745167	0	0	20	0.00193
LOC100738781	29.60605442	0	4.935745167	0	0	20	0.00193
LOC100627398	29.60605442	0	4.935745167	0	0	20	0.00193
LOC100623228	29.60605442	0	4.935745167	0	0	20	0.00193
LOC102167676	29.60605442	0	4.935745167	0	0	20	0.00193
DYNC1I1	1101.768168	81.801559	10.10691381	6.371586023	6.371586023	3.751549	0.00193
FSD1L	2313.501681	168.56079	11.1764859	7.405658765	7.405658765	3.778737	0.00193
LOC100520265	13.74566813	257.79885	3.882219287	8.015687407	8.015687407	-4.2292	0.00193
LOC100152091	210.4144582	3790.1389	7.723930233	11.8884156	11.8884156	-4.17094	0.00193
LOC102161873	81.41664967	4.1313919	6.364863912	2.359350201	2.359350201	4.300624	0.00194
LOC102164231	65.55626337	1134.4802	6.056502534	10.14908684	10.14908684	-4.11315	0.00194
E2F5	5354.466414	377.60922	12.38679651	8.564565718	8.564565718	3.825777	0.00194
PDE10A	308.7488533	22.309516	8.274955131	4.542847146	4.542847146	3.790703	0.00195
LOC100521376	2862.271047	207.39587	11.48344853	7.703182885	7.703182885	3.786701	0.00195
RUNX2	3.17207726	90.890621	2.060765874	6.521845711	6.521845711	-4.84063	0.00196
LOC100738501	1176.840663	87.585507	10.20192867	6.468998788	6.468998788	3.748083	0.00197
LOC102166805	9.516231779	189.21775	3.394545939	7.571508044	7.571508044	-4.31351	0.00199
LONRF3	68.72834063	1183.2306	6.123673244	10.20973436	10.20973436	-4.10568	0.00199
LOC100621853	488.499898	36.356248	8.935164749	5.223277669	5.223277669	3.748083	0.002
LOC100154020	90.93288145	4.9576702	6.522509054	2.57474827	2.57474827	4.197068	0.002
LOC100624372	40.17964529	0.8262784	5.363859498	0.868906686	0.868906686	5.603693	0.00201
LOC100621908	40.17964529	0.8262784	5.363859498	0.868906686	0.868906686	5.603693	0.00201
LOC102166444	40.17964529	0.8262784	5.363859498	0.868906686	0.868906686	5.603693	0.00201
PPP1R14A	4.229436346	106.58991	2.386655454	6.749398975	6.749398975	-4.65546	0.00202
KBTBD8	1070.047396	80.149002	10.06480661	6.342501448	6.342501448	3.738846	0.00202
JPH1	151.2023494	9.9153405	7.249846818	3.448285225	3.448285225	3.930674	0.00204
LOC102167305	3.17207726	90.064343	2.060765874	6.508814353	6.508814353	-4.82746	0.00204
TRIM24	7903.759172	552.78023	12.94850579	9.113169742	9.113169742	3.837761	0.00205
LOC102163367	421.8862755	31.398578	8.724125929	5.017858594	5.017858594	3.748083	0.00205
ZNF784	8.458872693	171.03962	3.241668254	7.426597064	7.426597064	-4.33772	0.00207
MIEN1	115.2521404	1992.1572	6.861113469	10.96083975	10.96083975	-4.11147	0.00208
LOC102162058	7.401513606	154.51406	3.070649265	7.280901168	7.280901168	-4.38377	0.00208
LOC100518295	0	40.48764	0	5.374609695	5.374609695	-20	0.00208
TLR1	0	40.48764	0	5.374609695	5.374609695	-20	0.00208
CRB2	60.26946793	2.4788351	5.937096418	1.798604302	1.798604302	4.603693	0.00208
LOC102161865	2.114718173	73.538775	1.639101631	6.219919206	6.219919206	-5.11997	0.00208
DSCC1	1683.315666	125.59431	10.71794683	6.98406878	6.98406878	3.744463	0.00209
BTBD19	100.4491132	1726.0955	6.664612444	10.75413216	10.75413216	-4.10298	0.00209
[illegible]							

LOC100738788	0	37.182527	0	5.254840671	5.254840671	-20	3.1E-05
SLC25A41	286.809373	21.483238	8.168969766	4.490777898	4.490777898	3.738809	3.1E-05
LOC100523827	77.9193094	3.3051135	6.302306427	2.106051269	2.106051269	4.559209	3.1E-05
LOC102162369	142.575758	1743.4474	7.165668365	10.76855435	10.76855435	-3.61214	3.1E-05
LOC100623149	181.535412	12.394176	7.512032568	3.74353388	3.74353388	3.872517	3.2E-05
LOC100623245	58.0250177	715.55707	5.883254663	9.484937802	9.484937802	-3.62432	3.2E-05
TSPAN1	58.0250177	1.6525567	5.883254663	1.407383613	1.407383613	5.133903	3.2E-05
LOC100739139	58.0250177	1.6525567	5.883254663	1.407383613	1.407383613	5.133903	3.2E-05
ETV4	1075.12068	13773.234	10.07162417	13.74968449	13.74968449	-3.6793	3.2E-05
TTNLOC100620261	0.82892882	47.924146	0.870998931	5.61247475	5.61247475	-5.85336	3.2E-05
CACNA1A	0.82892882	47.924146	0.870998931	5.61247475	5.61247475	-5.85336	3.2E-05
LOC102159646	370.531184	28.919743	8.537339497	4.903025878	4.903025878	3.679468	3.3E-05
LOC100737177	169.930409	2069.001	7.417265267	11.01541578	11.01541578	-3.60592	3.3E-05
NFATC4	374.675828	4628.8114	8.553344483	12.17673772	12.17673772	-3.62693	3.3E-05
VWF	96.1557435	4.9576702	6.602227381	2.574748269	2.574748269	4.277639	3.3E-05
LOC102164625	9.11821706	143.77244	3.338883189	7.177643143	7.177643143	-3.97889	3.3E-05
EGFL7	100.300388	1213.8029	6.662495885	10.24650658	10.24650658	-3.59714	3.4E-05
TMEM249	608.433757	49.576702	9.251325603	5.660401068	5.660401068	3.617366	3.4E-05
LOC100525247	36.4728682	0	5.227774503	0	0	20	3.4E-05
LOC100525793	36.4728682	0	5.227774503	0	0	20	3.4E-05
LOC102163735	36.4728682	0	5.227774503	0	0	20	3.4E-05
TNP2	36.4728682	0	5.227774503	0	0	20	3.4E-05
RASL10A	36.4728682	0	5.227774503	0	0	20	3.4E-05
ABCC4	36.4728682	0	5.227774503	0	0	20	3.4E-05
HMGCLL1	36.4728682	0	5.227774503	0	0	20	3.4E-05
LOC102160749	36.4728682	0	5.227774503	0	0	20	3.4E-05
LOC100523671	37.3017971	466.021	5.259340178	8.867343618	8.867343618	-3.64308	3.5E-05
LOC100622473	396.227978	31.398578	8.633823429	5.017858594	5.017858594	3.65756	3.5E-05
HIST1H2BD	83.7218112	1012.191	6.404661526	9.984690459	9.984690459	-3.59573	3.5E-05
C18H7orf31	95.3268147	4.9576702	6.589865555	2.574748269	2.574748269	4.265148	3.6E-05
PLEKHG1	3.31571529	76.843889	2.109599694	6.282511874	6.282511874	-4.53454	3.6E-05
RIMBP2	188.166843	13.220454	7.563515426	3.829895615	3.829895615	3.831169	3.6E-05
SLA-DQA1	0	36.356248	0	5.223277669	5.223277669	-20	3.6E-05
LOC102166264	0	36.356248	0	5.223277669	5.223277669	-20	3.6E-05
PPP1R9A	375.504757	29.746021	8.556524283	4.942327829	4.942327829	3.658063	3.6E-05
DTX3L	87.0375265	1046.8947	6.460046706	10.03327803	10.03327803	-3.58833	3.6E-05
LOC100627004	1824.47234	23463.001	10.83405409	14.5181614	14.5181614	-3.68484	3.6E-05
CAR	5526.46847	451.97427	12.43240317	8.823285292	8.823285292	3.612045	3.7E-05
LOC100522421	15.7496477	216.48493	4.066058842	7.764771649	7.764771649	-3.78088	3.7E-05
LOC102160506	122.681466	7.4365053	6.950485513	3.076645515	3.076645515	4.044149	3.7E-05
LOX	915.96635	11478.659	9.840724982	13.48679218	13.48679218	-3.64752	3.7E-05
KCNK6	9.94714588	151.20894	3.452482877	7.249909307	7.249909307	-3.92612	3.7E-05
DLX4	9.94714588	151.20894	3.452482877	7.249909307	7.249909307	-3.92612	3.7E-05
MEOX2	0.82892882	47.097867	0.870998931	5.587901017	5.587901017	-5.82827	3.7E-05
SLC35F1	223.810782	16.525567	7.812567422	4.13138925	4.13138925	3.759508	3.8E-05
FAM114A1	464.200141	5671.5747	8.861707724	12.469788	12.469788	-3.61093	3.8E-05
PAX6	1013.77995	85.106672	9.986951206	6.428053129	6.428053129	3.574328	3.9E-05
ASS1	848.823115	71.05994	9.731018776	6.171125545	6.171125545	3.578356	3.9E-05
THBS3	330.742601	4000.0136	8.373920477	11.96614982	11.96614982	-3.59622	3.9E-05
CXXC5	474.147287	5788.9063	8.892230982	12.49932428	12.49932428	-3.60988	3.9E-05
LOC100513288	306.703665	23.962073	8.265397816	4.641665831	4.641665831	3.678021	3.9E-05
LOC102159510	3.31571529	76.01761	2.109599694	6.267116453	6.267116453	-4.51894	3.9E-05
KLF6	525.540874	6422.6618	9.040401719	12.64918022	12.64918022	-3.6113	3.9E-05
NRSN1	75.432523	3.3051135	6.256114748	2.106051269	2.106051269	4.512415	4E-05
LOC102163525	75.432523	3.3051135	6.256114748	2.106051269	2.106051269	4.512415	4E-05
UNC5CL	56.36716	1.6525567	5.842153193	1.407383613	1.407383613	5.092083	0.00004
RPL3L	56.36716	1.6525567	5.842153193	1.407383613	1.407383613	5.092083	0.00004
GVIN1	14.09179	195.82797	3.915692025	7.620791468	7.620791468	-3.79666	4E-05
PRKAB2	8384.61505	684.15849	13.03370089	9.420293943	9.420293943	3.615342	4E-05
PRICKLE1	187.337914	2230.1253	7.557179646	11.12355584	11.12355584	-3.57341	4E-05
LOC102161699	35.6439394	0	5.195502704	0	0	20	4.1E-05
TMPRSS5	35.6439394	0	5.195502704	0	0	20	4.1E-05
LOC100156689	1974.50846	25125.473	10.94800831	14.61692054	14.61692054	-3.66959	4.1E-05
CPEB2	99.4714588	1177.4467	6.65064192	10.20267077	10.20267077	-3.56524	4.1E-05
CCDC88C	3038.85307	255.32002	11.56978588	8.001802338	8.001802338	3.573148	4.1E-05
LOC102166021	304.216878	23.962073	8.253690934	4.641665831	4.641665831	3.666276	4.2E-05
GBP5	1.65785765	56.186929	1.410263837	5.837613535	5.837613535	-5.08284	4.2E-05
IFI30	362.241896	4342.9191	8.504786801	12.08478153	12.08478153	-3.58364	4.3E-05
STEAP2	11.6050035	166.90823	3.655924617	7.391529145	7.391529145	-3.84624	4.3E-05
ADCYAP1	0	35.52997	0	5.191008663	5.191008663	-20	4.3E-05
LOC100627616	0	35.52997	0	5.191008663	5.191008663	-20	4.3E-05
FLI1	0	35.52997	0	5.191008663	5.191008663	-20	4.3E-05
CCDC60	0	35.52997	0	5.191008663	5.191008663	-20	4.3E-05
ADAMTS6	159.154334	1876.4782	7.323319031	10.87458043	10.87458043	-3.55953	4.3E-05
TLE2	51.3935871	613.92483	5.711318333	9.264266253	9.264266253	-3.5784	4.3E-05
LOC100512907	4.14464412	84.280394	2.363071282	6.414142197	6.414142197	-4.34588	4.3E-05
COL4A6	770.074877	65.275991	9.590727153	6.05041444	6.05041444	3.560374	4.4E-05
MADCAM1	83.7218112	4.1313919	6.404661526	2.359350201	2.359350201	4.340904	4.4E-05
LOC100736921	101.958245	5.7839486	6.685915561	2.762125239	2.762125239	4.13978	4.4E-05
HHEX	402.859408	33.051135	8.657709339	5.089630977	5.089630977	3.607505	4.5E-05
GUCY1A2	174.075053	12.394176	7.451829714	3.74353388	3.74353388	3.811975	4.5E-05
LOC100626903	16.5785765	220.61633	4.135746339	7.791920351	7.791920351	-3.73415	4.5E-05
CDKL2	300.901163	23.962073	8.237932504	4.641665831	4.641665831	3.650465	4.6E-05
LOC100524687	200.600775	14.873011	7.655357377	3.98850389	3.98850389	3.753559	4.6E-05
SAMD14	48.0778718	571.78463	5.617000784	9.161848978	9.161848978	-3.57203	4.6E-05
C1H14orf37	194.798274	2280.5283	7.613224233	11.15578484	11.15578484	-3.54932	4.7E-05
FXYD6	368.044398	4360.271	8.527650579	12.09053291	12.09053291	-3.56647	4.7E-05
LOC100513259	73.7746653	3.3051135	6.224477643	2.106051269	2.106051269	4.480354	4.8E-05
RAB27A	2232.30532	191.69658	11.12496478	7.590187173	7.590187173	3.541638	4.8E-05
EFHD1	1.65785765	55.360651	1.410263837	5.816616368	5.816616368	-5.06147	4.8E-05
TMED8	818.152749	70.233662	9.677988689	6.154487245	6.154487245	3.542136	4.8E-05
CKM	34.8150106	0	5.162492464	0	0	20	4.8E-05
LOC100153139	34.8150106	0	5.162492464	0	0	20	4.8E-05
SIT1	34.8150106	0	5.162492464	0	0	20	4.8E-05
SLAIN1	1167.13178	100.80596	10.18998733	6.669678233	6.669678233	3.533315	4.8E-05
LOC102159737	226.297569	17.351846	7.828438443	4.19785327	4.19785327	3.70506	4.9E-05
CCL3L1	0.82892882	45.44531	0.870998931	5.537461031	5.537461031	-5.77674	4.9E-05
GLCCI1	1758.15804	152.03522	10.78066938	7.257719911	7.257719911	3.531587	4.9E-05
EFNB2	27.3546512	336.2953	4.825513503	8.397868394	8.397868394	-3.61987	4.9E-05
BCL2L14	109.418605	6.610227	6.786839466	2.927939483	2.927939483	4.049014	5E-05
TMEM8C	13.2628612	181.78124	3.834191515	7.513974209	7.513974209	-3.77674	5E-05
WNT16	145.062544	9.9153405	7.190442454	3.448285225	3.448285225	3.870869	5E-05
TSHR	54.7093024	1.6525567	5.799846344	1.407383613	1.407383613	5.049014	5E-05
LOC100738354	4.97357294	91.716899	2.578594101	6.534760414	6.534760414	-4.20483	5E-05
DUOX1	261.112579	20.656959	8.034042783	4.436758793	4.436758793	3.659972	5.1E-05
C14H10orf116	0	34.703692	0	5.158001345	5.158001345	-20	5.1E-05
RIMBP3	82.0639535	4.1313919	6.376150635	2.359350201	2.359350201	4.312049	5.2E-05
LOC100737660	124.339324	1431.1141	6.969695301	10.48393077	10.48393077	-3.52478	5.2E-05
TCP11L2	172.417195	1988.852	7.438103147	10.95844545	10.95844545	-3.52796	5.2E-05
LOC102159756	242.047217	19.004403	7.925092802	4.322245637	4.322245637	3.670883	5.2E-05
RRBP1	1722.5141	21178.341	10.75113738	14.37037008	14.37037008	-3.62	5.3E-05
MYEF2	6280.7937	532.94955	12.61696085	9.060559625	9.060559625	3.558876	5.3E-05
AOX1	44.7621565	0.8262784	5.516083133	0.868906686	0.868906686	5.759508	5.3E-05
CNTNAP1	58.0250177	672.59059	5.883254663	9.395728184	9.395728184	-3.53498	5.3E-05
TMEM176A	1.65785765	54.534373	1.410263837	5.795309087	5.795309087	-5.03977	5.3E-05
GPX3	73.7746653	849.41417	6.224477643	9.732021819	9.732021819	-3.52527	5.4E-05
ITPR2	1595.68799	139.64104	10.6408667	7.135873883	7.135873883	3.514384	5.4E-05
CHD7	10572.1582	887.42297	13.36811876	9.795102886	9.795102886	3.574504	5.5E-05
STON2	959.899578	84.280394	9.908241855	6.414142197	6.414142197	3.509615	5.6E-05
LOC100512841	213.863637	2451.5679	7.747277532	11.26007738	11.26007738	-3.51894	5.6E-05
[illegible]							

LOC102165291	34.5202459	1.6525567	5.150569664	1.407383613	1.407383613	4.384671	0.00497
GRIP1	9.66566885	133.8571	3.414902536	7.075287628	7.075287628	-3.79168	0.00501
LOC102163494	673.8352	86.759229	9.398391417	6.455478947	6.455478947	2.957307	0.00505
TNNI3	539.896646	69.407383	9.079209141	6.137654819	6.137654819	2.959522	0.00507
GIP	20.7121475	0	4.440430524	0	0	20	0.0051
LOC102161699	20.7121475	0	4.440430524	0	0	20	0.0051
MDH1B	20.7121475	0	4.440430524	0	0	20	0.0051
USH2A	20.7121475	0	4.440430524	0	0	20	0.0051
LOC102161179	20.7121475	0	4.440430524	0	0	20	0.0051
LOC102161013	20.7121475	0	4.440430524	0	0	20	0.0051
LOC100522379	20.7121475	0	4.440430524	0	0	20	0.0051
LOC102166200	20.7121475	0	4.440430524	0	0	20	0.0051
LOC102158025	20.7121475	0	4.440430524	0	0	20	0.0051
LOC102162201	20.7121475	0	4.440430524	0	0	20	0.0051
CDH9	20.7121475	0	4.440430524	0	0	20	0.0051
LOC102167461	20.7121475	0	4.440430524	0	0	20	0.0051
LOC100523792	20.7121475	0	4.440430524	0	0	20	0.0051
LOC100737801	20.7121475	0	4.440430524	0	0	20	0.0051
LOC100514709	118.749646	13.220454	6.90387758	3.829895615	3.829895615	3.16708	0.00511
LOC100516615	5.52323934	91.716899	2.705588563	6.534760414	6.534760414	-4.0536	0.00512
NHSL2	17.9505279	217.31121	4.24416613	7.770242414	7.770242414	-3.59766	0.00513
TRIL	11.0464787	147.07755	3.590539588	7.210209122	7.210209122	-3.73492	0.00514
LOC100627762	20.7121475	245.40468	4.440430524	7.944885826	7.944885826	-3.56661	0.00516
LOC100522650	4.14242951	77.670167	2.362450112	6.29774474	6.29774474	-4.22881	0.00519
LOC100516594	4.14242951	77.670167	2.362450112	6.29774474	6.29774474	-4.22881	0.00519
LOC100520600	10897.3512	1307.9987	13.41182227	10.35424791	10.35424791	3.058545	0.0052
PRICKLE1	198.836616	2230.1253	7.642677144	11.12355584	11.12355584	-3.48747	0.00521
GP9	0	34.703692	0	5.158001345	5.158001345	-20	0.00521
PTGER1	0	34.703692	0	5.158001345	5.158001345	-20	0.00521
LOC100738540	0	34.703692	0	5.158001345	5.158001345	-20	0.00521
LOC102167614	0	34.703692	0	5.158001345	5.158001345	-20	0.00521
LOC102157611	1.38080984	49.576702	1.251452392	5.660401068	5.660401068	-5.16608	0.00521
LOC102162488	1.38080984	49.576702	1.251452392	5.660401068	5.660401068	-5.16608	0.00521
ELFN2	106.322357	11.567897	6.745806839	3.65167138	3.65167138	3.200246	0.00525
ITGA5	2089.16528	26341.754	11.02940131	14.68511859	14.68511859	-3.65635	0.00533
LOC102160731	70.4213016	6.610227	6.158282521	2.927939483	2.927939483	3.41324	0.00534
ZNF432	5.52323934	90.890621	2.705588563	6.521845711	6.521845711	-4.04054	0.00535
SPOCD1	27.6161967	0.8262784	4.838760036	0.868906686	0.868906686	5.062743	0.00536
GGT1	38.6626754	428.83847	5.309710091	8.747650814	8.747650814	-3.47142	0.00543
LOC100516424	19.3313377	228.87911	4.345633235	7.844731552	7.844731552	-3.56557	0.00543
PLEKHG1	4.14242951	76.843889	2.362450112	6.282511874	6.282511874	-4.21338	0.00546
LOC100738797	6.90404918	104.11107	2.982591923	6.715770874	6.715770874	-3.91454	0.00548
TBX3	66.2788721	721.34102	6.072081615	9.496536287	9.496536287	-3.44406	0.00551
LOC100523082	80.0869705	869.24485	6.341398207	9.765277556	9.765277556	-3.44012	0.00557
FEZ1	346.583269	44.619032	8.441214828	5.511563931	5.511563931	2.957471	0.00558
SPIC	40.0434852	2.4788351	5.359081339	1.798604302	1.798604302	4.013833	0.00561
LOC102162264	22.0929574	255.32002	4.529381037	8.001802338	8.001802338	-3.53065	0.00562
MYCL	457.048056	59.492043	8.839355155	5.918673475	5.918673475	2.941577	0.00564
LOC102164425	1.38080984	48.750424	1.251452392	5.636636914	5.636636914	-5.14183	0.00564
LOC102157816	1.38080984	48.750424	1.251452392	5.636636914	5.636636914	-5.14183	0.00564
ACBD3	57.9940131	627.97156	5.882496648	9.29685098	9.29685098	-3.43672	0.00571
LOC100739594	30.3778164	338.77413	4.971673052	8.408432212	8.408432212	-3.47923	0.00573
FAM184A	8.28485901	116.50525	3.214880004	6.876581411	6.876581411	-3.81377	0.00578
LOC100154674	241.641721	30.5723	7.922683827	4.980587446	4.980587446	2.982573	0.00579
LOC100037951	0	33.877413	0	5.124221138	5.124221138	-20	0.00581
LOC102157549	0	33.877413	0	5.124221138	5.124221138	-20	0.00581
LOC102161387	0	33.877413	0	5.124221138	5.124221138	-20	0.00581
LOC102161064	0	33.877413	0	5.124221138	5.124221138	-20	0.00581
COLGALT2	0	33.877413	0	5.124221138	5.124221138	-20	0.00581
LOC102166942	0	33.877413	0	5.124221138	5.124221138	-20	0.00581
LOC100625760	0	33.877413	0	5.124221138	5.124221138	-20	0.00581
COX17	22411.9244	2652.3536	14.45204328	11.37360122	11.37360122	3.078922	0.00582
CA14	9.66566885	129.7257	3.414902536	7.030399034	7.030399034	-3.74645	0.00585
PIF1	411.481331	53.708094	8.688185014	5.773682393	5.773682393	2.937616	0.00586
RYR2	27.6161967	309.02811	4.838760036	8.276255224	8.276255224	-3.48415	0.00586
MAP1A	557.847174	6397.8734	9.126309997	12.64360222	12.64360222	-3.51965	0.00594
EFNB2	30.3778164	336.2953	4.971673052	8.397868394	8.397868394	-3.46864	0.00594
LOC102163830	2.76161967	61.970878	1.911353989	5.976612875	5.976612875	-4.488	0.00595
HTR2A	2.76161967	61.970878	1.911353989	5.976612875	5.976612875	-4.488	0.00595
TNFRSF10B	46.9475344	506.50864	5.583384725	8.987288579	8.987288579	-3.43147	0.00598
LOC100622807	17.9505279	210.70098	4.24416613	7.72588417	7.72588417	-3.5531	0.00599
LOC102157722	45.5667246	3.3051135	5.541227504	2.106051269	2.106051269	3.785209	0.00605
LOC102160764	4.14242951	75.191332	2.362450112	6.251554969	6.251554969	-4.18202	0.00607
LOC100739138	342.440839	44.619032	8.423917793	5.511563931	5.511563931	2.940124	0.00607
SAMD4A	85.6102098	913.86388	6.436465198	9.837413293	9.837413293	-3.41612	0.00607
S100A14	1046.65386	137.98849	10.03294641	7.118821585	7.118821585	2.923165	0.00609
ATF5	466.713725	5278.2662	8.869481953	12.36612171	12.36612171	-3.49945	0.00609
EPS8	365.914607	4086.7728	8.519300528	11.99709931	11.99709931	-3.48138	0.0061
LOC102162629	1.38080984	47.924146	1.251452392	5.61247475	5.61247475	-5.11717	0.00611
LOC102159034	5.52323934	88.411786	2.705588563	6.482393107	6.482393107	-4.00065	0.00612
LOC100158075	1633.49804	214.0061	10.67463193	7.74823377	7.74823377	2.932241	0.00619
RXFP4	8.28485901	114.85269	3.214880004	6.856147779	6.856147779	-3.79316	0.0062
LOC100737060	26.2353869	291.67627	4.767410456	8.193161944	8.193161944	-3.47478	0.00621
LOC102165247	132.557744	15.699289	7.061319821	4.061714779	4.061714779	3.07785	0.0063
CYP2C91	24.854577	276.80325	4.692347799	8.117919691	8.117919691	-3.47728	0.00633
SGCB	75.944541	803.14258	6.265747069	9.651307509	9.651307509	-3.40264	0.00636
ITGB7	51.0899639	4.1313919	5.702933533	2.359350201	2.359350201	3.62834	0.00636
OTOG	33.1394361	1.6525567	5.093367322	1.407383613	1.407383613	4.325777	0.00638
LOC100516234	33.1394361	1.6525567	5.093367322	1.407383613	1.407383613	4.325777	0.00638
LOC100521567	33.1394361	1.6525567	5.093367322	1.407383613	1.407383613	4.325777	0.00638
LRRN1	35.9010557	386.69828	5.205590187	8.598790517	8.598790517	-3.42911	0.00643
CD40	46.9475344	499.07214	5.583384725	8.965992412	8.965992412	-3.41013	0.00645
LOC100622016	0	33.051135	0	5.089630977	5.089630977	-20	0.00649
LOC102162837	0	33.051135	0	5.089630977	5.089630977	-20	0.00649
MYO18B	0	33.051135	0	5.089630977	5.089630977	-20	0.00649
LOC102167726	0	33.051135	0	5.089630977	5.089630977	-20	0.00649
ACE	0	33.051135	0	5.089630977	5.089630977	-20	0.00649
PARD3B	15.1889082	180.12869	4.016933786	7.500871231	7.500871231	-3.56794	0.00654
RPIA	5128.32773	653.58619	12.32455404	9.35443936	9.35443936	2.972039	0.0066
B2R	1.38080984	47.097867	1.251452392	5.587901017	5.587901017	-5.09208	0.00663
C1H15orf52	110.464787	1162.5737	6.800444207	10.18434684	10.18434684	-3.39566	0.00664
LAMC2	9.66566885	126.42059	3.414902536	6.993454623	6.993454623	-3.70922	0.00664
MX1	4136.90627	52119.161	12.01468525	15.66955392	15.66955392	-3.65519	0.00664
LOC100524899	1951.0843	256.97257	10.93079964	8.011073883	8.011073883	2.92459	0.00666
LOC102167038	13.8080984	166.08195	3.888314478	7.384412099	7.384412099	-3.58831	0.00667
EPHB3	138.080984	1459.2076	7.119781365	10.51195778	10.51195778	-3.4016	0.00667
STK32C	421.147	56.186929	8.721601651	5.837613535	5.837613535	2.906017	0.00676
ITM2A	62.1364426	5.7839486	5.98040107	2.762125239	2.762125239	3.425313	0.00678
LOC102159565	6.90404918	99.153405	2.982591923	6.646067655	6.646067655	-3.84415	0.00695
GIPR	38.6626754	2.4788351	5.309710091	1.798604302	1.798604302	3.963207	0.00697
SLC19A3	38.6626754	2.4788351	5.309710091	1.798604302	1.798604302	3.963207	0.00697
RIPPLY3	38.6626754	2.4788351	5.309710091	1.798604302	1.798604302	3.963207	0.00697
CD55	86.9910197	901.4697	6.459284385	9.817734691	9.817734691	-3.37334	0.00707
LOC100519345	305.158974	40.48764	8.25813716	5.374609695	5.374609695	2.914008	0.00709
SH2D3C	35.9010557	379.26177	5.205590187	8.570849103	8.570849103	-3.4011	0.0071
LOC100737455	84.2294	871.72368	6.413279271	9.769381136	9.769381136	-3.37148	0.00711
LOC100621670	4.14242951	72.712497	2.362450112	6.20383732	6.20383732	-4.13365	0.00712
FAM166B	1.38080984	46.271589	1.251452392	5.56290145	5.56290145	-5.06654	0.00719
CHST1	2.76161967	59.492043	1.911353989	5.918673475	5.918673475	-4.42911	0.00721
LOC100622805	26.2353869	0.8262784	4.767410456	0.868906686	0.868906686	4.988742	0.00725
MFRP	0	32.224857	0	5.054191063	5.054191063	-20	0.00727
ADAMDEC1	0	32.224857	0	5.054191063	5.054191063	-20	0.00727
CXCL9	0	32.224857	0	5.054191063	5.054191063	-20	0.00727
LOC102163603	0	32.224857	0	5.054191063	5.054191063	-20	0.00727
LOC102166048	0	32.224857	0	5.054191063	5.054191063	-20	0.00727
CTNNA2	0	32.224857	0	5.054191063	5.054191063	-20	0.00727
LOC102158668	0	32.224857	0	5.054191063	5.054191063	-20	0.00727
LOC100739358	0	32.224857	0	5.054191063	5.054191063	-20	0.00727
LOC102161149	150.508272	1566.6238	7.243252754	10.61436366	10.61436366	-3.37974	0.0073
LOC100621092	35.9010557	376.78294	5.205590187	8.561413734	8.561413734	-3.39164	0.00733
LOC100738154	5.52323934	85.106672	2.705588563	6.428053129	6.428053129	-3.94569	0.00736
LOC100512321	5.52323934	85.106672	2.705588563	6.428053129	6.428053129	-3.94569	0.00736
LOC100526189	12.4272885	149.55639	3.747096094	7.234160086	7.234160086	-3.58911	0.00741
FGFR3	361.772177	48.750424	8.502920002	5.636636914	5.636636914	2.891595	0.00746
DSCC1	923.76178	125.59431	9.852937963	6.98406878	6.98406878	2.87875	0.00749
LOC100517087	186.409328	23.962073	7.550048951	4.641665831	4.641665831	2.959649	0.00749
SLC22A16	19.3313377	0	4.345633235	0	0	20	0.0075
TCTEX1D1	19.3313377	0	4.345633235	0	0	20	0.0075
SMIM18	19.3313377	0	4.345633235	0	0	20	0.0075
LOC102159232	19.3313377	0	4.345633235	0	0	20	0.0075
LOC102157403	19.3313377	0	4.345633235	0	0	20	0.0075
LOC102159763	19.3313377	0	4.345633235	0	0	20	0.0075
FAM71A	19.3313377	0	4.345633235	0	0	20	0.0075
LCK	19.3313377	0	4.345633235	0	0	20	0.0075
LTF	19.3313377	0	4.345633235	0	0	20	0.0075
SPN	19.3313377	0	4.345633235	0	0	20	0.0075
TRIM15	19.3313377	0	4.345633235	0	0	20	0.0075
LOC100623474	19.3313377	0	4.345633235	0	0	20	0.0075
LOC100519721	19.3313377	0	4.345633235	0	0	20	0.0075
CCNO	19.3313377	0	4.345633235	0	0	20	0.0075
LOC102158406	19.3313377	0	4.345633235	0	0	20	0.0075
LOC102167637	19.3313377	0	4.345633235	0	0	20	0.0075
BIN2	19.3313377	0	4.345633235	0	0	20	0.0075
LOC100513607	19.3313377	0	4.345633235	0	0	20	0.0075
LOC100153882	19.3313377	0	4.345633235	0	0	20	0.0075
LOC100515722	19.3313377	0	4.345633235	0	0	20	0.0075
CXHXorf48	19.3313377	0	4.345633235	0	0	20	0.0075
LOC100515519	19.3313377	0	4.345633235	0	0	20	0.0075
LOC100525236	6.90404918	97.500848	2.982591923	6.622064238	6.622064238	-3.8199	0.00754
LOC100621366	30.3778164	320.59601	4.971673052	8.329105689	8.329105689	-3.39967	0.00756
CAPRIN2	2341.85348	312.33322	11.19405102	8.291553945	8.291553945	2.906493	0.00758
LOC100736917	26.2353869	280.10837	4.767410456	8.13498259	8.13498259	-3.4164	0.0076
LOC100739014	26.2353869	280.10837	4.767410456	8.13498259	8.13498259	-3.4164	0.0076
NRAP	197.455807	25.61463	7.632673964	4.734147579	4.734147579	2.94649	0.00761
LOC100620844	755.30298	103.2848	9.562820493	6.704385035	6.704385035	2.870428	0.0077
C3H2orf40	2.76161967	58.665764	1.911353989	5.89883146	5.89883146	-4.40893	0.0077
LOC102159377	5.52323934	84.280394	2.705588563	6.414142197	6.414142197	-3.93161	0.00771
GLS2	122.892075	14.873011	6.9529401	3.98850389	3.98850389	3.046623	0.00772
LOC100737630	122.892075	14.873011	6.9529401	3.98850389	3.98850389	3.046623	0.00772
RAB20	128.415315	15.699289	7.015864542	4.061714779	4.061714779	3.032046	0.00778
LAMB3	60.7556328	5.7839486	5.948498827	2.762125239	2.762125239	3.392892	0.00782
AIF1L	24.854577	265.23536	4.692347799	8.05655837	8.05655837	-3.41569	0.00783
LOC100154173	122.892075	1251.8117	6.9529401	10.29095391	10.29095391	-3.34855	0.00793
LOC102166571	4.14242951	71.05994	2.362450112	6.171125545	6.171125545	-4.10049	0.00794
LOC100736836	16.569718	185.91263	4.135019133	7.546220275	7.546220275	-3.488	0.00798
CHSY3	12.4272885	147.07755	3.747096094	7.210209122	7.210209122	-3.56499	0.00804
LOC100621248	249.92658	2599.4718	7.971121491	11.34455765	11.34455765	-3.37864	0.00806
C3H16orf54	937.569879	128.89943	9.874320351	7.021251246	7.021251246	2.86268	0.00811
EDN3	0	31.398578	0	5.017858594	5.017858594	-20	0.00816
LOC102164680	0	31.398578	0	5.017858594	5.017858594	-20	0.00816
LOC100626991	0	31.398578	0	5.017858594	5.017858594	-20	0.00816
LOC100738453	0	31.398578	0	5.017858594	5.017858594	-20	0.00816
SLFNL1	0	31.398578	0	5.017858594	5.017858594	-20	0.00816
ABCC9	0	31.398578	0	5.017858594	5.017858594	-20	0.00816
LMX1B	0	31.398578	0	5.017858594	5.017858594	-20	0.00816
LOC100625402	0	31.398578	0	5.017858594	5.017858594	-20	0.00816
SLC9B2	0	31.398578	0	5.017858594	5.017858594	-20	0.00816
HMHA1	1333.8623	182.60752	10.38247521	7.52048134	7.52048134	2.868792	0.00822
PTPRC	31.7586262	1.6525567	5.033802952	1.407383613	1.407383613	4.264377	0.00823
TM4SF5	31.7586262	1.6525567	5.033802952	1.407383613	1.407383613	4.264377	0.00823
ATP2B3	31.7586262	1.6525567	5.033802952	1.407383613	1.407383613	4.264377	0.00823
RNF182	31.7586262	1.6525567	5.033802952	1.407383613	1.407383613	4.264377	0.00823
KIAA1958	450.144007	61.970878	8.817444209	5.976612875	5.976612875	2.860724	0.00825
DSTN	2444.03341	28625.588	11.25563846	14.8050681	14.8050681	-3.54997	0.00827
LOC100519396	41.4242951	420.57569	5.406818783	8.719647871	8.719647871	-3.34382	0.00834
TRPC4	23.4737672	248.70979	4.613164285	7.964108573	7.964108573	-3.40534	0.00836
LOC102168045	4.14242951	70.233662	2.362450112	6.154487245	6.154487245	-4.08361	0.0084
LOC100624776	52.4707738	527.1656	5.740678649	9.044846533	9.044846533	-3.32867	0.00843
LOC100736922	15.1889082	171.03962	4.016933786	7.426597064	7.426597064	-3.49324	0.00844
PFAS	2774.04696	373.47782	11.43829647	8.548736477	8.548736477	2.892898	0.00844
C14H10orf54	24.854577	261.10397	4.692347799	8.033995371	8.033995371	-3.39304	0.00846
LOC100627892	4330.21965	574.26347	12.08055762	9.168079046	9.168079046	2.914656	0.00847
TMEM198	33.1394361	339.60041	5.093367322	8.411936365	8.411936365	-3.35722	0.00848
LOC100626623	5.52323934	82.627837	2.705588563	6.385911347	6.385911347	-3.90304	0.00848
LOC100519366	5.52323934	82.627837	2.705588563	6.385911347	6.385911347	-3.90304	0.00848
LOC102160944	1.38080984	44.619032	1.251452392	5.511563931	5.511563931	-5.01407	0.0085
TNNC1	44.1859147	445.36404	5.497801224	8.802077003	8.802077003	-3.33333	0.00853
CCDC38	171.22042	22.309516	7.428112398	4.542847146	4.542847146	2.940124	0.00863
NRIP3	313.443833	42.966475	8.29665853	5.458331975	5.458331975	2.866924	0.00867
LOC100519519	127.034505	1271.6424	7.000388854	10.31361139	10.31361139	-3.3234	0.0087
LOC100737124	1193.0197	165.25567	10.22161092	7.37725977	7.37725977	2.851846	0.00879
CCDC153	446.001577	61.970878	8.804136111	5.976612875	5.976612875	2.847387	0.00879
LOC102159735	2.76161967	57.013208	1.911353989	5.858309486	5.858309486	-4.36771	0.0088
LOC100516384	2.76161967	57.013208	1.911353989	5.858309486	5.858309486	-4.36771	0.0088
LOC102165421	2.76161967	57.013208	1.911353989	5.858309486	5.858309486	-4.36771	0.0088
RAPGEF5	422.52781	58.665764	8.726312893	5.89883146	5.89883146	2.848456	0.00881
LOC100520570	546.800695	5802.1267	9.097507286	12.50261472	12.50261472	-3.40749	0.00885
LOC100155273	871.291006	121.46292	9.768665705	6.936201185	6.936201185	2.842639	0.00888
TRHDE	8.28485901	106.58991	3.214880004	6.749398975	6.749398975	-3.68545	0.00891
CCNA1	48.3283443	4.1313919	5.624344958	2.359350201	2.359350201	3.54817	0.00899
LOC100623000	15.1889082	168.56079	4.016933786	7.405658764	7.405658764	-3.47218	0.00907
ELMO3	1909.66	262.75652	10.89985536	8.043062959	8.043062959	2.861517	0.00909
PDSS1	1484.37057	205.74331	10.53660719	7.691696868	7.691696868	2.850934	0.00914
LOC100736982	0	30.5723	0	4.980587446	4.980587446	-20	0.00917
LOC102164187	0	30.5723	0	4.980587446	4.980587446	-20	0.00917
RNF180	0	30.5723	0	4.980587446	4.980587446	-20	0.00917
INHBB	0	30.5723	0	4.980587446	4.980587446	-20	0.00917
LOC102158359	0	30.5723	0	4.980587446	4.980587446	-20	0.00917
KLHL34	0	30.5723	0	4.980587446	4.980587446	-20	0.00917
MIR145	0	30.5723	0	4.980587446	4.980587446	-20	0.00917
LOC100737745	0	30.5723	0	4.980587446	4.980587446	-20	0.00917
IER2	6879.1946	906.42737	12.74823366	9.825638371	9.825638371	2.923976	0.00918
ARHGAP9	11.0464787	130.55198	3.590539588	7.039489182	7.039489182	-3.56297	0.00923
LOC100622512	1.38080984	43.792754	1.251452392	5.485193456	5.485193456	-4.98711	0.00926
LOC100738599	1.38080984	43.792754	1.251452392	5.485193456	5.485193456	-4.98711	0.00926
SPARC	2270.05137	25894.738	11.14914462	14.66042705	14.66042705	-3.51186	0.00928
SYNPO	196.074997	1963.2374	7.62260094	10.9397536	10.9397536	-3.32376	0.00932
PPA2	2191.34521	301.59161	11.09825927	8.241228156	8.241228156	2.861149	0.00938
TOX2	4.14242951	68.581105	2.362450112	6.120623682	6.120623682	-4.04926	0.00939
LOC100513133	4118.95574	48519.892	12.00841312	15.56631846	15.56631846	-3.55823	0.00946
TMEM38A	193.313377	1928.5337	7.602241413	10.91403654	10.91403654	-3.31849	0.00947
CHDH	527.469357	74.365053	9.045676011	6.2358238	6.2358238	2.826391	0.00953
CPEB2	120.130456	1177.4467	6.920417836	10.20267077	10.20267077	-3.29299	0.00962
SLC29A4	291.350875	40.48764	8.191557101	5.374609695	5.374609695	2.847204	0.00969
ZNF70	5.52323934	80.149002	2.705588563	6.342501448	6.342501448	-3.8591	0.0098
MYB	313.443833	43.792754	8.29665853	5.485193456	5.485193456	2.839443	0.0098
ZNF827	34.5202459	342.07925	5.150569664	8.422398044	8.422398044	-3.30882	0.00988
LOC100156509	8.28485901	104.11107	3.214880004	6.715770874	6.715770874	-3.6515	0.00997
LOC100738923	71.8021115	693.24755	6.185908388	9.439306379	9.439306379	-3.27127	0.01008
LOC102157484	2.76161967	55.360651	1.911353989	5.816616368	5.816616368	-4.32527	0.01009
LOC100737045	2.76161967	55.360651	1.911353989	5.816616368	5.816616368	-4.32527	0.01009
TIMM8A	4645.04429	629.62412	12.18178719	9.300636539	9.300636539	2.88313	0.01013
RASGRF2	167.07799	22.309516	7.392987005	4.542847146	4.542847146	2.904791	0.01014
B3GNT3	334.15598	47.097867	8.388688866	5.587901017	5.587901017	2.826788	0.01019
UGP2	784.299987	8256.9998	9.617100062	13.01157666	13.01157666	-3.39614	0.0102
LRRC70	144.985033	19.004403	7.189676653	4.322245637	4.322245637	2.931498	0.01021
HTRA3	103.560738	999.79683	6.708197415	9.966933409	9.966933409	-3.27116	0.01023
LOC102158232	1743.96282	244.5784	10.76898058	7.940039851	7.940039851	2.834	0.01023
LOC102159568	266.496298	37.182527	8.063375117	5.254840671	5.254840671	2.841419	0.01025
NYX	5.52323934	79.322724	2.705588563	6.327736286	6.327736286	-3.84415	0.01029
PIM2	2351.51915	327.20624	11.19999075	8.358458837	8.358458837	2.845321	0.01031
LOC100626135	92.514259	889.90181	6.547114458	9.799122619	9.799122619	-3.2659	0.01033
KCNN4	0	29.746021	0	4.942327829	4.942327829	-20	0.01033
LOC100621628	0	29.746021	0	4.942327829	4.942327829	-20	0.01033
LOC100621630	0	29.746021	0	4.942327829	4.942327829	-20	0.01033
LOC100624808	0	29.746021	0	4.942327829	4.942327829	-20	0.01033
UNCX	0	29.746021	0	4.942327829	4.942327829	-20	0.01033
CSMD2	0	29.746021	0	4.942327829	4.942327829	-20	0.01033
BLNK	57.9940131	5.7839486	5.882496648	2.762125239	2.762125239	3.325777	0.01045
ANKLE1	698.689777	99.979683	9.450571603	6.657921243	6.657921243	2.804945	0.0105
LOC100518964	20.7121475	212.35354	4.440430524	7.737102248	7.737102248	-3.35792	0.01058
LOC102164131	198.836616	27.267186	7.642677144	4.821056379	4.821056379	2.866346	0.01059
LOC100519123	46.9475344	451.97427	5.583384725	8.823285292	8.823285292	-3.26712	0.0106
MDGA1	593.748229	85.106672	9.216135261	6.428053129	6.428053129	2.802507	0.01061
XKR5	35.9010557	349.51575	5.205590187	8.453335466	8.453335466	-3.28326	0.01066
LOC102161872	30.3778164	1.6525567	4.971673052	1.407383613	1.407383613	4.200246	0.01068
ARHGAP30	30.3778164	1.6525567	4.971673052	1.407383613	1.407383613	4.200246	0.01068

LOC102162373	56.04003159	2.4788351	5.833902876	1.798604302	1.798604302	4.498724	0.00301
LOC102162699	96.21967688	1522.831	6.603176434	10.57348723	10.57348723	-3.98428	0.00302
LOC102161046	103.6211905	1641.8151	6.709031281	10.68195442	10.68195442	-3.9859	0.00303
IL12A	46.52379981	1.6525567	5.570578288	1.407383614	1.407383614	4.815197	0.00305
FAM227B	6.344154519	126.42059	2.876596413	6.993454622	6.993454622	-4.31666	0.00308
LOC100521274	7.401513606	141.2936	3.070649265	7.15272698	7.15272698	-4.25473	0.00308
TENM4	137.4566813	2188.8114	7.113290863	11.09659091	11.09659091	-3.9931	0.00311
LOC100736817	1.057359087	52.055537	1.04079362	5.729431428	5.729431428	-5.62151	0.00313
AMZ1	1.057359087	52.055537	1.04079362	5.729431428	5.729431428	-5.62151	0.00313
SPNS2	1.057359087	52.055537	1.04079362	5.729431428	5.729431428	-5.62151	0.00313
LOC100623746	1.057359087	52.055537	1.04079362	5.729431428	5.729431428	-5.62151	0.00313
CBX7	1.057359087	52.055537	1.04079362	5.729431428	5.729431428	-5.62151	0.00313
GSTO2	111.0227041	7.4365053	6.807647348	3.076645515	3.076645515	3.900086	0.00316
GPD1L	7106.510421	538.7335	12.79512859	9.07610342	9.07610342	3.721498	0.00317
MYB	537.138416	43.792754	9.07183349	5.485193456	5.485193456	3.61653	0.00318
LOC100517224	101.5064723	6.610227	6.679571195	2.927939483	2.927939483	3.940728	0.0032
MPZL3	4.229436346	95.848291	2.386655454	6.597654688	6.597654688	-4.50222	0.00321
ZFP92	19.03246356	306.54928	4.324267947	8.264673769	8.264673769	-4.00958	0.00321
LOC102165900	64.49890428	3.3051135	6.033398867	2.106051269	2.106051269	4.286503	0.00323
DDIT3	150.1449903	2378.0292	7.239789351	11.21615724	11.21615724	-3.98534	0.00324
SP8	91.99024053	5.7839486	6.539007406	2.76212524	2.76212524	3.991354	0.00325
MSI1	1195.873127	97.500848	10.22505451	6.622064238	6.622064238	3.616506	0.00325
ITGA10	126.8830904	1992.1572	6.998681703	10.96083975	10.96083975	-3.97276	0.00326
SLA-DQA1	0	36.356248	0	5.223277669	5.223277669	-20	0.00327
LOC102166264	0	36.356248	0	5.223277669	5.223277669	-20	0.00327
PUS10	7221.762561	550.3014	12.81833503	9.106697443	9.106697443	3.714057	0.00327
LOC102160955	376.4198348	30.5723	8.560026434	4.980587447	4.980587447	3.622046	0.00329
LOC100739788	2739.617393	218.13749	11.42028522	7.775692512	7.775692512	3.650665	0.0033
LOC100737556	2126.349123	171.03962	11.0548411	7.426597064	7.426597064	3.635976	0.00332
LOC100155975	4.229436346	95.022013	2.386655454	6.585293272	6.585293272	-4.48972	0.00333
GC	26.43397716	0	4.777891883	0	0	20	0.00333
GPRC6A	26.43397716	0	4.777891883	0	0	20	0.00333
LOC100523434	26.43397716	0	4.777891883	0	0	20	0.00333
LOC100739530	26.43397716	0	4.777891883	0	0	20	0.00333
MMRN1	26.43397716	0	4.777891883	0	0	20	0.00333
BDKRB1	1.057359087	51.229259	1.04079362	5.706786332	5.706786332	-5.59843	0.00335
FGF9	1.057359087	51.229259	1.04079362	5.706786332	5.706786332	-5.59843	0.00335
DEPTOR	1147.234609	94.195734	10.16520173	6.572825024	6.572825024	3.606355	0.00337
MAML2	33.83549077	519.7291	5.122485983	9.02438921	9.02438921	-3.94115	0.00339
LHFPL1	45.46644072	1.6525567	5.538117235	1.407383614	1.407383614	4.78203	0.00341
LOC100153771	12.68830904	211.52726	3.774872332	7.731504113	7.731504113	-4.05927	0.00344
LOC102160986	90.93288145	5.7839486	6.522509054	2.76212524	2.76212524	3.974675	0.00345
CA5B	15.8603863	256.1463	4.075565686	8.006445558	8.006445558	-4.01347	0.00345
LOC100624568	286.5443125	23.135794	8.167640492	4.593102407	4.593102407	3.63056	0.00346
NAGLU	105.7359087	1625.2896	6.737901807	10.66736843	10.66736843	-3.94216	0.00349
LOC102168075	35.95020894	0.8262784	5.207510617	0.868906686	0.868906686	5.443228	0.00349
LOC100737566	35.95020894	0.8262784	5.207510617	0.868906686	0.868906686	5.443228	0.00349
LOC102159538	5.286795433	108.24247	2.65232482	6.771389985	6.771389985	-4.35573	0.00351
LOC102162175	438.8040209	36.356248	8.780716984	5.223277669	5.223277669	3.593302	0.00353
LTBP4	241.0778717	3829.8003	7.919327399	11.90343009	11.90343009	-3.9897	0.00354
RGS4	822.6253694	68.581105	9.685844458	6.120623682	6.120623682	3.584353	0.00356
DLX4	8.458872693	151.20894	3.241668254	7.249909307	7.249909307	-4.15993	0.00356
LOC102165490	8.458872693	151.20894	3.241668254	7.249909307	7.249909307	-4.15993	0.00356
UPK1B	681.9966108	57.013208	9.415734609	5.858309485	5.858309485	3.580396	0.00359
IRF5	0	35.52997	0	5.191008663	5.191008663	-20	0.00359
LOC102166114	0	35.52997	0	5.191008663	5.191008663	-20	0.00359
ADCYAP1	0	35.52997	0	5.191008663	5.191008663	-20	0.00359
PON1	0	35.52997	0	5.191008663	5.191008663	-20	0.00359
LOC100627616	0	35.52997	0	5.191008663	5.191008663	-20	0.00359
LOC100736946	12557.19651	946.91501	13.61634167	9.888613909	9.888613909	3.729136	0.0036
IL-6	7.401513606	146.33593	3.070649265	7.101565318	7.101565318	-4.2032	0.0036
ADCY9	17.97510447	283.41348	4.246035924	8.151846042	8.151846042	-3.97884	0.00361
ATOH8	17.97510447	283.41348	4.246035924	8.151846042	8.151846042	-3.97884	0.00361
LOC100522418	107.8506268	7.4365053	6.766205905	3.076645515	3.076645515	3.858266	0.00367
MND1	2357.910763	192.52286	11.20390513	7.59636019	7.59636019	3.614407	0.00368
CCDC159	9.516231779	164.4294	3.394545939	7.370071807	7.370071807	-4.11093	0.00369
PAFAH2	117.3668586	1786.4138	6.887121389	10.80365798	10.80365798	-3.92797	0.0037
EPN3	3.17207726	78.496445	2.060765874	6.312818447	6.312818447	-4.62913	0.00371
SCG2	4.229436346	92.543178	2.386655454	6.547560532	6.547560532	-4.45159	0.00373
DTL	[illegible]	366.8676	12.17022894	8.523042794	8.523042794	3.6508	0.00373
LOC102159413	5.286795433	106.58991	2.65232482	6.749398975	6.749398975	-4.33353	0.00375
PLSCR3	407.0832483	6569.7393	8.672719679	12.68184	12.68184	-4.01244	0.00375
MZT2B	34.89284986	522.20793	5.165624571	9.0312406	9.0312406	-3.90362	0.00379
LOC102160306	7.401513606	134.68337	3.070649265	7.084100147	7.084100147	-4.18561	0.0038
LOC100738836	21.14718173	324.7274	4.46905122	8.347521274	8.347521274	-3.94069	0.0038
LOC102159151	44.40908164	1.6525567	5.504908955	1.407383614	1.407383614	4.748083	0.00383
FSTL4	44.40908164	1.6525567	5.504908955	1.407383614	1.407383614	4.748083	0.00383
LOC102167316	44.40908164	1.6525567	5.504908955	1.407383614	1.407383614	4.748083	0.00383
ETV4	822.6253694	13773.234	9.685844458	13.74968449	13.74968449	-4.06549	0.00385
ZNF8	31.7207726	474.28379	5.032134908	8.892645374	8.892645374	-3.90225	0.00386
LOC102158885	3.17207726	77.670167	2.060765874	6.29774474	6.29774474	-4.61386	0.00388
C1QTNF7	3.17207726	77.670167	2.060765874	6.29774474	6.29774474	-4.61386	0.00388
LOC102163435	88.81816327	5.7839486	6.488935315	2.76212524	2.76212524	3.940728	0.00388
BHLHE40	282.3148761	4428.8521	8.146262546	12.11304281	12.11304281	-3.97156	0.00389
PPA2	3724.018703	301.59161	11.86303195	8.241228155	8.241228155	3.626192	0.0039
GLRX	132.1698858	1995.4623	7.057124067	10.96323009	10.96323009	-3.91626	0.00392
PTGER1	0	34.703692	0	5.158001345	5.158001345	-20	0.00396
LOC102167614	0	34.703692	0	5.158001345	5.158001345	-20	0.00396
LOC102165475	0	34.703692	0	5.158001345	5.158001345	-20	0.00396
C14H10orf116	0	34.703692	0	5.158001345	5.158001345	-20	0.00396
LOC100513464	62.38418611	920.47411	5.986051038	9.847799816	9.847799816	-3.88312	0.00396
KLHL41	22.20454082	336.2953	4.536335244	8.397868394	8.397868394	-3.9208	0.00397
SNX18	49.69587707	732.08264	5.663796517	9.517832026	9.517832026	-3.88081	0.00397
LIPT2	447.2628936	38.008805	8.80820127	5.285727902	5.285727902	3.556717	0.00401
SORD	7892.128222	620.53506	12.94638147	9.279691957	9.279691957	3.66683	0.00401
CNKSR2	570.9739067	48.750424	9.159805523	5.636636914	5.636636914	3.549938	0.00403
ZNHIT2	62.38418611	917.16899	5.986051038	9.842615902	9.842615902	-3.87794	0.00403
GPR83	34.89284986	0.8262784	5.165624571	0.868906686	0.868906686	5.40016	0.00403
PTPRO	25.37661808	0	4.721187694	0	0	20	0.00404
CDA	25.37661808	0	4.721187694	0	0	20	0.00404
IGFBPL1	25.37661808	0	4.721187694	0	0	20	0.00404
LOC102157691	25.37661808	0	4.721187694	0	0	20	0.00404
LOC100624756	25.37661808	0	4.721187694	0	0	20	0.00404
FOXB2	25.37661808	0	4.721187694	0	0	20	0.00404
SBSPON	25.37661808	0	4.721187694	0	0	20	0.00404
CKMT2	25.37661808	0	4.721187694	0	0	20	0.00404
LOC100622742	33.83549077	499.07214	5.122485983	8.965992412	8.965992412	-3.88264	0.00406
LOC100627808	22.20454082	334.64274	4.536335244	8.390782629	8.390782629	-3.9137	0.00406
GRIN3B	3.17207726	76.843889	2.060765874	6.282511874	6.282511874	-4.59843	0.00406
LOC100523276	20.08982264	304.89672	4.398475058	8.256900823	8.256900823	-3.92378	0.00408
ARHGDIB	1.057359087	48.750424	1.04079362	5.636636914	5.636636914	-5.52688	0.00413
LOC102160403	1.057359087	48.750424	1.04079362	5.636636914	5.636636914	-5.52688	0.00413
LOC100737190	1.057359087	48.750424	1.04079362	5.636636914	5.636636914	-5.52688	0.00413
HUNK	114.1947813	8.2627837	6.84793155	3.211445828	3.211445828	3.788725	0.00413
MROH1	19.03246356	289.19743	4.324267947	8.180890933	8.180890933	-3.92552	0.00414
LOC100623471	1427.434767	121.46292	10.48021944	6.936201186	6.936201186	3.554837	0.00421
SORBS3	762.3559014	12447.884	9.576212035	13.60372876	13.60372876	-4.02929	0.00422
NYAP1	44.40908164	645.32341	5.504908955	9.336112433	9.336112433	-3.8611	0.00423
OSAP	293.9458261	24.788351	8.204306183	4.688647628	4.688647628	3.567816	0.00424
ITGB3	154.3744266	2304.4904	7.279605257	11.17085793	11.17085793	-3.89994	0.00425
LOC100623304	19.03246356	287.54487	4.324267947	8.172651888	8.172651888	-3.91725	0.00425
RFTN1	19.03246356	287.54487	4.324267947	8.172651888	8.172651888	-3.91725	0.00425
SEMA3D	409.1979665	6419.3567	8.68017653	12.64843773	12.64843773	-3.97156	0.00427
BLM	3691.240571	304.07044	11.85028084	8.252998589	8.252998589	3.601628	0.00428
MPST	9.516231779	158.64545	3.394545939	7.318727602	7.318727602	-4.05927	0.00431
MEOX1	9.516231779	158.64545	3.394545939	7.318727602	7.318727602	-4.05927	0.00431
PTPRC	43.35172255	1.6525567	5.470918232	1.407383614	1.407383614	4.713318	0.00431
LOC100521953	43.35172255	1.6525567	5.470918232	1.407383614	1.407383614	4.713318	0.00431
LOC100037951	0	33.877413	0	5.124221138	5.124221138	-20	0.00437
LOC102157549	0	33.877413	0	5.124221138	5.124221138	-20	0.00437
LOC102158116	0	33.877413	0	5.124221138	5.124221138	-20	0.00437
LOC100625760	0	33.877413	0	5.124221138	5.124221138	-20	0.00437
LOC100739000	148.0302721	11.567897	7.219461601	3.651671381	3.651671381	3.677694	0.00439
TBC1D10C	12.68830904	199.95937	3.774872332	7.650760007	7.650760007	-3.97814	0.00439
OTUD1	9.516231779	157.81917	3.394545939	7.311241242	7.311241242	-4.05174	0.00441
TMEM102	34.89284986	504.02981	5.165624571	8.980224728	8.980224728	-3.85251	0.00443
PYCARD	1.057359087	47.924146	1.04079362	5.61247475	5.61247475	-5.50222	0.00443
MOV10	614.3256293	9789.7461	9.265206274	13.2572031	13.2572031	-3.9942	0.00444
LOC100515348	51.81059524	2.4788351	5.722755497	1.798604302	1.798604302	4.385513	0.00445
LOC102158083	3.17207726	75.191332	2.060765874	6.251554969	6.251554969	-4.56707	0.00445
LOC100624749	254.8235399	3857.0674	7.999005211	11.91366265	11.91366265	-3.91993	0.00445
ITGA3	372.1903985	5752.55	8.543768058	12.49023668	12.49023668	-3.95009	0.00446
N4BP2	7045.183594	567.65324	12.78262635	9.151405371	9.151405371	3.633555	0.00447
RRAGD	1838.747452	156.99289	10.84529202	7.303715831	7.303715831	3.549952	0.00449
LOC100737472	391.222862	33.877413	8.61552982	5.124221138	5.124221138	3.529595	0.0045
SLC35F6	103.6211905	1502.1741	6.709031281	10.55379638	10.55379638	-3.85766	0.00452
LOC102164731	51.81059524	741.1717	5.722755497	9.535609179	9.535609179	-3.83849	0.00452
NRSN2	217.8159718	18.178124	7.773574237	4.261389711	4.261389711	3.582835	0.00456
TAF4B	1883.156533	161.12428	10.87970311	7.340956379	7.340956379	3.546907	0.00456
C3H16orf58	10.57359087	170.21334	3.532764645	7.419651341	7.419651341	-4.00881	0.00458
PKD1	549.826725	8640.3929	9.105454748	13.07704817	13.07704817	-3.97405	0.00459
GCS	2.114718173	61.1446	1.639101631	5.957557118	5.957557118	-4.85369	0.00459
GBP6	2.114718173	61.1446	1.639101631	5.957557118	5.957557118	-4.85369	0.00459
MMP9	2.114718173	61.1446	1.639101631	5.957557118	5.957557118	-4.85369	0.00459
LOC102160223	2.114718173	61.1446	1.639101631	5.957557118	5.957557118	-4.85369	0.00459
LOC102167526	2.114718173	61.1446	1.639101631	5.957557118	5.957557118	-4.85369	0.00459
DUS4L	4109.95477	341.25297	12.00525778	8.41891924	8.41891924	3.590209	0.0046
LOC102159412	9.516231779	156.16661	3.394545939	7.296150961	7.296150961	-4.03655	0.00461
LOC102159410	77.18721332	4.9576702	6.288860784	2.57474827	2.57474827	3.960628	0.00463
tRNA-Thr	389.1081439	33.877413	8.607730306	5.124221138	5.124221138	3.521776	0.00463
IL7	12.68830904	197.48053	3.774872332	7.632853689	7.632853689	-3.96014	0.00464
LOC102163843	33.83549077	0.8262784	5.122485983	0.868906686	0.868906686	5.355766	0.00467
LOC100515458	33.83549077	0.8262784	5.122485983	0.868906686	0.868906686	5.355766	0.00467
SLC5A7	137.4566813	10.741619	7.113290863	3.553559423	3.553559423	3.677694	0.00468
RXRG	297.1179033	25.61463	8.219739208	4.734147579	4.734147579	3.535996	0.0047
LOC100520252	52.86795433	749.43448	5.751355374	9.551582312	9.551582312	-3.82534	0.00471
LDB3	9.516231779	155.34033	3.394545939	7.288546214	7.288546214	-4.0289	0.00472
CALD1	4163.880083	73879.202	12.02405924	16.17290019	16.17290019	-4.14917	0.00476
LOC102158175	1.057359087	47.097867	1.04079362	5.587901017	5.587901017	-5.47712	0.00477
CCL19	1.057359087	47.097867	1.04079362	5.587901017	5.587901017	-5.47712	0.00477
NPEPL1	175.5216084	2566.4206	7.463700987	11.32610396	11.32610396	-3.87004	0.00478
CXCR4	1392.541917	121.46292	10.44454068	6.936201186	6.936201186	3.519133	0.0048
CAST	427.173071	6543.2984	8.742050253	12.67602282	12.67602282	-3.93713	0.00481
LOC100622016	0	33.051135	0	5.089630977	5.089630977	-20	0.00483
LOC102166930	0	33.051135	0	5.089630977	5.089630977	-20	0.00483
LOC100626667	0	33.051135	0	5.089630977	5.089630977	-20	0.00483
AMPH	0	33.051135	0	5.089630977	5.089630977	-20	0.00483
LOC102162837	0	33.051135	0	5.089630977	5.089630977	-20	0.00483
MYO18B	0	33.051135	0	5.089630977	5.089630977	-20	0.00483
LOC102167726	0	33.051135	0	5.089630977	5.089630977	-20	0.00483
RUSC2	273.8560034	4077.6838	8.102532181	11.99388794	11.99388794	-3.89626	0.00488
LOC100737894	19.03246356	278.45581	4.324267947	8.126476366	8.126476366	-3.87091	0.00489
LOC100517487	2767.10873	236.31561	11.4346849	7.890663216	7.890663216	3.549592	0.00489
RNF213	810.9944194	12863.502	9.665326002	13.65110795	13.65110795	-3.98745	0.0049
GHDC	60.26946793	846.93533	5.937096418	9.72781043	9.72781043	-3.81275	0.00491
LOC102163494	4.229436346	86.759229	2.386655454	6.455478947	6.455478947	-4.35848	0.00491
LOC102157411	24.31925899	0	4.662163277	0	0	20	0.00492
LOC780430	24.31925899	0	4.662163277	0	0	20	0.00492
LRRTM3	24.31925899	0	4.662163277	0	0	20	0.00492
HS1BP3	175.5216084	2549.8951	7.463700987	11.31678783	11.31678783	-3.86072	0.00492
ZNF618	46.52379981	652.75991	5.570578288	9.352617108	9.352617108	-3.81051	0.00493
AS-A	124.7683722	1783.935	6.974625354	10.80165583	10.80165583	-3.83774	0.00495
LOC100738335	6.344154519	113.20014	2.876596413	6.83542057	6.83542057	-4.1573	0.00496
LRP10	512.819157	7880.2168	9.00511687	12.94420268	12.94420268	-3.94171	0.00498
CDKL2	275.9707216	23.962073	8.113589668	4.641665831	4.641665831	3.525691	0.00498
RHBDF1	332.0107532	4967.5856	8.379424954	12.2786195	12.2786195	-3.90324	0.00501
C1QTNF2	87.76080419	1236.1124	6.471850834	10.27276092	10.27276092	-3.81609	0.00502
LOC100512370	48.63748768	679.20082	5.633388138	9.409816938	9.409816938	-3.80367	0.00503
ZNF362	11.63094995	180.12869	3.658891241	7.500871231	7.500871231	-3.95299	0.00504
DNASE1L1	64.49890428	900.64343	6.033398867	9.81641319	9.81641319	-3.80361	0.00507
TSPAN2	656.6199928	58.665764	9.36111035	5.89883146	5.89883146	3.484468	0.00509
PPIP5K2	14118.91588	1125.3911	13.78544387	10.13749218	10.13749218	3.649131	0.00511
TRIM26	271.7412852	4000.0136	8.091389289	11.96614982	11.96614982	-3.8797	0.00513
TBX4	1.057359087	46.271589	1.04079362	5.56290145	5.56290145	-5.45159	0.00513
FAM166B	1.057359087	46.271589	1.04079362	5.56290145	5.56290145	-5.45159	0.00513
LOC100627548	2.114718173	59.492043	1.639101631	5.918673475	5.918673475	-4.81416	0.00515
SH2D4A	9.516231779	152.03522	3.394545939	7.257719911	7.257719911	-3.99787	0.00518
PUS7	2201.421618	191.69658	11.10487496	7.590187173	7.590187173	3.521539	0.00518
CECR2	4694.674344	396.61362	12.19711664	8.63522336	8.63522336	3.565219	0.00524
LOC100624822	2255.346932	196.65425	11.1397732	7.626835184	7.626835184	3.519616	0.00525
LOC102163625	125.8257313	9.9153405	6.986703669	3.448285225	3.448285225	3.665621	0.00526
LRRC1	1735.126261	152.8615	10.76165616	7.265488457	7.265488457	3.504744	0.00527
FAM43A	97.27703596	1359.2279	6.618782442	10.4096327	10.4096327	-3.80454	0.00527
LOC102167780	75.07249515	4.9576702	6.249303021	2.57474827	2.57474827	3.92055	0.00531
MFRP	0	32.224856	0	5.054191063	5.054191063	-20	0.00535
LOC100514309	0	32.224856	0	5.054191063	5.054191063	-20	0.00535
LOC100736857	0	32.224856	0	5.054191063	5.054191063	-20	0.00535
LOC102158668	0	32.224856	0	5.054191063	5.054191063	-20	0.00535
PPAPDC1A	66.61362245	4.1313919	6.079242038	2.359350201	2.359350201	4.011117	0.00535
BAIAP2L1	1183.184818	105.76363	10.20967855	6.738276475	6.738276475	3.48376	0.00535
ZFPM2	19.03246356	272.67186	4.324267947	8.096303303	8.096303303	-3.84063	0.00536
LOC102167705	5678.018295	477.5889	12.47142584	8.902643123	8.902643123	3.571546	0.00537
MAOB	298.1752624	26.440908	8.224847079	4.778256309	4.778256309	3.495317	0.00538
LOC102157527	3.17207726	71.886218	2.060765874	6.187574144	6.187574144	-4.50222	0.00539
RASGRP3	3.17207726	71.886218	2.060765874	6.187574144	6.187574144	-4.50222	0.00539
LOC102167620	3.17207726	71.886218	2.060765874	6.187574144	6.187574144	-4.50222	0.00539
UCP2	2796.714784	242.92584	11.45003318	7.930298794	7.930298794	3.525145	0.00539
MICAL2	100.4491132	1398.063	6.664612444	10.45024522	10.45024522	-3.79889	0.00539
RPS6KA4	124.7683722	1747.5788	6.974625354	10.77196706	10.77196706	-3.80803	0.00542
HEY2	9.516231779	150.38266	3.394545939	7.242056187	7.242056187	-3.9821	0.00543
PPFIA2	403.9111711	36.356248	8.661461636	5.223277669	5.223277669	3.473763	0.00544
LOC102165667	17.97510447	257.79885	4.246035924	8.015687407	8.015687407	-3.84217	0.00546
B3GAT3	168.1200948	2381.3343	7.40190428	11.21816014	11.21816014	-3.82421	0.00546
STC1	2.114718173	58.665764	1.639101631	5.89883146	5.89883146	-4.79398	0.00546
PIEZO1	43.35172255	594.09415	5.470918232	9.216974124	9.216974124	-3.77653	0.00548
CTNNA3	41.23704228	1.6525567	5.400435611	1.407383614	1.407383614	4.641168	0.0055
TTC39B	19.03246356	271.01931	4.324267947	8.087565237	8.087565237	-3.83186	0.0055
LOC100157320	21.14718173	298.28649	4.46905122	8.225383356	8.225383356	-3.81816	0.00551
NPR2	391.222862	5780.6435	8.61552982	12.49726394	12.49726394	-3.88517	0.00553
MIOX	1.057359087	45.44531	1.04079362	5.537461031	5.537461031	-5.42559	0.00553
CBLN3	1.057359087	45.44531	1.04079362	5.537461031	5.537461031	-5.42559	0.00553
LOC102159841	1.057359087	45.44531	1.04079362	5.537461031	5.537461031	-5.42559	0.00553
LOC100525621	1.057359087	45.44531	1.04079362	5.537461031	5.537461031	-5.42559	0.00553
PHC2	1.057359087	45.44531	1.04079362	5.537461031	5.537461031	-5.42559	0.00553
AMPD3	17.97510447	256.97257	4.246035924	8.011073883	8.011073883	-3.83754	0.00554
PLEKHH3	85.64608601	1179.0992	6.436762676	10.20469247	10.20469247	-3.78315	0.00554
C1H9orf116	4.229436346	84.280394	2.386655454	6.414142197	6.414142197	-4.31666	0.00556
LOC100515505	4.229436346	84.280394	2.386655454	6.414142197	6.414142197	-4.31666	0.00556
HS3ST3A1	8.458872693	136.33593	3.241668254	7.101565318	7.101565318	-4.01056	0.00557
BBC3	42.29436346	577.56858	5.436107306	9.176344172	9.176344172	-3.77145	0.00558
CNOT6	7016.634898	588.3102	12.77676918	9.202883429	9.202883429	3.57613	0.00559
SYN1	6.344154519	109.89502	2.876596413	6.793050814	6.793050814	-4.11455	0.00563
tRNA-Pro	82.47400875	5.7839486	6.383255151	2.76212524	2.76212524	3.833813	0.00563
CENPL	1086.965141	98.327126	10.08741662	6.634115866	6.634115866	3.466572	0.00564
LOC100155159	3.17207726	71.05994	2.060765874	6.171125545	6.171125545	-4.48554	0.00565
DMPK	37.00756803	504.85609	5.24821481	8.98258319	8.98258319	-3.76998	0.00566
NEFL	1234.995413	111.54758	10.27145767	6.814391228	6.814391228	3.468775	0.0057
CD200	1112.341759	100.80596	10.1206808	6.669678232	6.669678232	3.463947	0.00572
HMOX1	653.4479155	9883.1156	9.354134569	13.27089617	13.27089617	-3.91882	0.00572
LOC100622843	1803.854602	161.12428	10.8176669	7.340956379	7.340956379	3.484837	0.00572
ATAD5	3315.878095	289.19743	11.69561027	8.180890933	8.180890933	3.519264	0.00573
ARHGEF6	175.5216084	2463.9621	7.463700987	11.26734975	11.26734975	-3.81126	0.00573
TRAPPC5	228.3895627	3247.274	7.84165594	11.66545762	11.66545762	-3.82966	0.00574
WNT9A	7.401513606	122.2892	3.070649265	6.945902605	6.945902605	-4.04633	0.00575
MICALL2	152.2597085	2120.2303	7.259834656	11.05068555	11.05068555	-3.79961	0.00577
NDUFAF4	2671.946412	235.48934	11.3842152	7.885631319	7.885631319	3.504157	0.00578
LOC102160753	2.114718173	57.839486	1.639101631	5.878712739	5.878712739	-4.77352	0.00579
MRAP2	2.114718173	57.839486	1.639101631	5.878712739	5.878712739	-4.77352	0.00579
KLF6	436.6893028	6422.6618	8.773763314	12.64918022	12.64918022	-3.87849	0.00581
LOC100627559	77.18721332	1048.5473	6.288860784	10.03555141	10.03555141	-3.76389	0.00581
VIM	27757.79074	533350.29	14.76061571	19.02472656	19.02472656	-4.26412	0.00585
PCDH7	330.9533941	4780.0204	8.374836893	12.22310284	12.22310284	-3.85232	0.00586
IL6	23.2618999	320.59601	4.600620625	8.329105689	8.329105689	-3.78472	0.00591
CAMK4	89.87552236	6.610227	6.505819847	2.927939483	2.927939483	3.765157	0.00591
LOC100736998	0	31.398578	0	5.017858594	5.017858594	-20	0.00594
LOC102164680	0	31.398578	0	5.017858594	5.017858594	-20	0.00594
LOC100626991	0	31.398578	0	5.017858594	5.017858594	-20	0.00594

CUEDC1	234.586857	2463.1358	7.880115246	11.26686607	11.26686607	-3.3923	0.00012
CYP7A1	30.6703665	0	4.985061656	0	0	20	0.00012
LOC100620374	57.1960888	2.4788351	5.862850292	1.798604302	1.798604302	4.528182	0.00012
LOC100625123	72.1168077	751.08704	6.192131177	9.554755826	9.554755826	-3.38057	0.00012
LOC102160096	413.635483	38.835083	8.695699772	5.315967693	5.315967693	3.412927	0.00012
ARHGDIB	1.65785765	48.750424	1.410263837	5.636636914	5.636636914	-4.87802	0.00012
EMP2	1.65785765	48.750424	1.410263837	5.636636914	5.636636914	-4.87802	0.00012
LOC100511091	49.7357294	523.03421	5.664930183	9.033517185	9.033517185	-3.39455	0.00013
STRBP	5349.0777	504.02981	12.38534413	8.980224728	8.980224728	3.407709	0.00013
LOC100738968	7.46035941	105.76363	3.080718953	6.738276475	6.738276475	-3.82545	0.00013
LOC100736982	0	30.5723	0	4.980587446	4.980587446	-20	0.00013
DCTPP1	3711.11434	353.64714	11.85802543	8.470240516	8.470240516	3.39147	0.00013
LOC102161750	39.7885835	0.8262784	5.350093502	0.868906686	0.868906686	5.589583	0.00013
LOC102166494	39.7885835	0.8262784	5.350093502	0.868906686	0.868906686	5.589583	0.00013
PGM2	4630.39641	438.75382	12.17723153	8.780552285	8.780552285	3.399652	0.00013
SADC	2.48678647	57.013208	1.801898017	5.858309486	5.858309486	-4.51894	0.00013
LOC100523505	176.561839	14.873011	7.472177749	3.98850389	3.98850389	3.569405	0.00013
SH2D5	1087.55462	105.76363	10.08819808	6.738276475	6.738276475	3.362172	0.00013
HSP70.2	1001.34602	10857.298	9.969164911	13.40651034	13.40651034	-3.43865	0.00013
USH1G	435.187632	41.313919	8.768805054	5.403060391	5.403060391	3.396938	0.00013
ARAP2	8.28928824	113.20014	3.215568059	6.83542057	6.83542057	-3.77148	0.00013
LOC102167148	15.7496477	185.08636	4.066058842	7.539828464	7.539828464	-3.55481	0.00013
SNTB1	9.94714588	128.89943	3.452482877	7.021251246	7.021251246	-3.69582	0.00013
PANK3	2537.35113	245.40468	11.30967594	7.944885826	7.944885826	3.370089	0.00013
NRIP3	450.108351	42.966475	8.817330184	5.458331975	5.458331975	3.388989	0.00013
ITIH2	48.0778718	1.6525567	5.617000784	1.407383613	1.407383613	4.862601	0.00013
LOC102158304	215.521494	19.004403	7.758366438	4.322245637	4.322245637	3.503426	0.00013
LOC102167532	63.8275194	659.37014	6.018534465	9.367131078	9.367131078	-3.36884	0.00013
TIAM1	819.810607	80.149002	9.680905563	6.342501448	6.342501448	3.354534	0.00014
LOC102158822	9.94714588	128.07315	3.452482877	7.012045083	7.012045083	-3.68654	0.00014
DNAJC12	463.371212	44.619032	8.859134728	5.511563931	5.511563931	3.376437	0.00014
LOC102167199	2.48678647	56.186929	1.801898017	5.837613535	5.837613535	-4.49788	0.00014
LOC100156509	7.46035941	104.11107	3.080718953	6.715770874	6.715770874	-3.80273	0.00014
LOC102167158	857.941332	84.280394	9.746415785	6.414142197	6.414142197	3.34761	0.00014
LOC100523701	9847.67442	923.77922	13.26571384	9.85296517	9.85296517	3.414163	0.00014
GADD45G	1862.60307	182.60752	10.86387889	7.52048134	7.52048134	3.350502	0.00014
LOC102157728	594.341967	6259.0587	9.217574785	12.61196046	12.61196046	-3.39658	0.00014
LOC102167359	9.11821706	119.81036	3.338883189	6.916600414	6.916600414	-3.71586	0.00014
LOC102161787	29.8414377	0	4.946798112	0	0	20	0.00015
DAO1	29.8414377	0	4.946798112	0	0	20	0.00015
NPHS1	29.8414377	0	4.946798112	0	0	20	0.00015
LOC100516162	29.8414377	0	4.946798112	0	0	20	0.00015
SCTR	29.8414377	0	4.946798112	0	0	20	0.00015
LOC100515312	29.8414377	0	4.946798112	0	0	20	0.00015
LOC102165288	29.8414377	0	4.946798112	0	0	20	0.00015
LOC100738827	33.1571529	350.34203	5.094115824	8.45673236	8.45673236	-3.40137	0.00015
LIMD1	2060.71706	202.4382	11.00963064	7.6684468	7.6684468	3.347593	0.00015
POU5F1	79.5771671	4.9576702	6.332299179	2.574748269	2.574748269	4.00462	0.00015
LOC102161720	38.9596547	0.8262784	5.320472212	0.868906686	0.868906686	5.559209	0.00015
CYGB	110.247534	1117.9546	6.79762954	10.12793583	10.12793583	-3.34204	0.00015
LOC100737264	257.796864	2640.7857	8.015676326	11.36729771	11.36729771	-3.35666	0.00015
SLC40A1	257.796864	2637.4806	8.015676326	11.36549164	11.36549164	-3.35485	0.00015
LOC100514340	38.9596547	405.70268	5.320472212	8.667830689	8.667830689	-3.38037	0.00015
FAM78A	102.787174	7.4365053	6.697484358	3.076645515	3.076645515	3.788892	0.00015
MDK	2364.93393	25947.62	11.20819407	14.66337018	14.66337018	-3.45573	0.00015
TAPBP	634.959479	6649.8883	9.312791035	12.69933133	12.69933133	-3.38859	0.00015
CCDC155	47.248943	1.6525567	5.592425431	1.407383613	1.407383613	4.83751	0.00015
LOC100624808	0	29.746021	0	4.942327829	4.942327829	-20	0.00015
LOC100152800	0	29.746021	0	4.942327829	4.942327829	-20	0.00015
UNCX	0	29.746021	0	4.942327829	4.942327829	-20	0.00015
CSMD2	0	29.746021	0	4.942327829	4.942327829	-20	0.00015
LOC102160529	3.31571529	63.623435	2.109599694	6.013985524	6.013985524	-4.26217	0.00015
SDCBP2	434.358704	42.140197	8.766060755	5.430960858	5.430960858	3.365618	0.00015
PLEKHH3	116.878964	1179.0992	6.881162477	10.20469247	10.20469247	-3.3346	0.00015
SYT1	18.2364341	204.91704	4.265769485	7.685919384	7.685919384	-3.49014	0.00016
LOC100738039	46.4200141	476.76262	5.567424187	8.900150173	8.900150173	-3.36045	0.00016
LOC100621472	1296.44468	128.89943	10.34145731	7.021251246	7.021251246	3.330243	0.00016
LOC100628224	35.6439394	371.82527	5.195502704	8.542355828	8.542355828	-3.3829	0.00016
HRH1	914.308492	90.890621	9.838114256	6.521845711	6.521845711	3.330478	0.00016
KANK3	156.667548	13.220454	7.300741934	3.829895615	3.829895615	3.566863	0.00016
SRCIN1	94.4978859	6.610227	6.57739689	2.927939483	2.927939483	3.83751	0.00016
TCIRG1	370.531184	3802.5331	8.537339497	11.89312443	11.89312443	-3.35929	0.00016
LOC100153854	561.184814	55.360651	9.134900672	5.816616368	5.816616368	3.341543	0.00016
LOC100624979	878.664553	87.585507	9.780809668	6.468998788	6.468998788	3.326548	0.00016
TMEM61	1.65785765	47.097867	1.410263837	5.587901017	5.587901017	-4.82827	0.00016
B2R	1.65785765	47.097867	1.410263837	5.587901017	5.587901017	-4.82827	0.00016
FLNA	717.852361	7493.5186	9.489551688	12.87162009	12.87162009	-3.38388	0.00016
FRZB	11.6050035	141.2936	3.655924617	7.152726981	7.152726981	-3.60588	0.00016
ENPP6	22.3810782	242.92584	4.547269558	7.930298794	7.930298794	-3.44016	0.00016
ZIC1	153.351832	1539.3566	7.2700788	10.58904867	10.58904867	-3.32741	0.00016
APC2	2238.10782	222.26888	11.12870829	7.80263838	7.80263838	3.331902	0.00016
APOBEC3F	25.6967935	274.32442	4.73859457	8.104988763	8.104988763	-3.41622	0.00016
LOC100627126	704.5895	70.233662	9.462685282	6.154487245	6.154487245	3.326548	0.00016
LEF1	3284.216	323.90112	11.68177251	8.343856914	8.343856914	3.341924	0.00017
LOC102160168	327.426885	31.398578	8.359428422	5.017858594	5.017858594	3.382402	0.00017
LOC102167659	225.46864	20.656959	7.823167478	4.436758793	4.436758793	3.448227	0.00017
LOC102162329	54.7093024	2.4788351	5.799846344	1.798604302	1.798604302	4.464052	0.00017
LOC100738892	3.31571529	62.797156	2.109599694	5.995420212	5.995420212	-4.24331	0.00017
LOC100153054	3.31571529	62.797156	2.109599694	5.995420212	5.995420212	-4.24331	0.00017
DENND3	1538.4919	154.51406	10.58823856	7.280901168	7.280901168	3.315707	0.00017
PRSS8	9.94714588	124.76603	3.452482877	6.974621475	6.974621475	-3.64882	0.00017
PINK1	239.56043	2406.1226	7.910255542	11.23309392	11.23309392	-3.32825	0.00017
LOC102161702	131.799683	10.741619	7.053107893	3.553559423	3.553559423	3.617064	0.00017
GCK	756.812016	76.01761	9.565696205	6.267116453	6.267116453	3.315529	0.00017
LOC102160289	38.1307259	0.8262784	5.290229968	0.868906686	0.868906686	5.528182	0.00017
LOC102623141	38.1307259	0.8262784	5.290229968	0.868906686	0.868906686	5.528182	0.00017
TGFBI	73.7746653	735.38775	6.224477643	9.524321819	9.524321819	-3.31731	0.00018
ALDH1L1	247.020789	23.135794	7.954317244	4.593102408	4.593102408	3.416434	0.00018
LOC102161807	562.013742	56.186929	9.137026327	5.837613535	5.837613535	3.322299	0.00018
LOC100515054	85.3796688	5.7839486	6.43261988	2.762125239	2.762125239	3.883766	0.00018
LOC102168065	29.0125088	0	4.907492018	0	0	20	0.00018
CAPN9	29.0125088	0	4.907492018	0	0	20	0.00018
LOC102163405	29.0125088	0	4.907492018	0	0	20	0.00018
LOC100523921	29.0125088	0	4.907492018	0	0	20	0.00018
NANOG	29.0125088	0	4.907492018	0	0	20	0.00018
MFSD6	1206.92037	122.2892	10.23830963	6.945902605	6.945902605	3.302962	0.00018
LOC102163538	31.4992953	325.55368	5.022336531	8.35117635	8.35117635	-3.3695	0.00018
TMEM108	199.771847	18.178124	7.649413169	4.261389712	4.261389712	3.458078	0.00018
CECR2	3993.77907	396.61362	11.9639	8.63522336	8.63522336	3.331948	0.00018
FBN1	1992.74489	21241.964	10.96126511	14.37469748	14.37469748	-3.41409	0.00018
HSP27	653.195913	6690.376	9.353578936	12.70808719	12.70808719	-3.3565	0.00018
LOC100152887	107.760747	8.2627837	6.765014156	3.211445828	3.211445828	3.70506	0.00019
LTBP4	380.47833	3829.8003	8.575457296	11.90343009	11.90343009	-3.33138	0.00019
LOC102164195	0	28.919743	0	4.903025878	4.903025878	-20	0.00019
IDO1	0	28.919743	0	4.903025878	4.903025878	-20	0.00019
CCL8	0	28.919743	0	4.903025878	4.903025878	-20	0.00019
LOC102163138	0	28.919743	0	4.903025878	4.903025878	-20	0.00019
LOC100739460	153.351832	13.220454	7.2700788	3.829895615	3.829895615	3.536002	0.00019
LOC102163981	1056.05532	107.41619	10.04583517	6.76043638	6.76043638	3.297402	0.00019
APBA1	14.9207188	169.38707	3.99283357	7.412672017	7.412672017	-3.50493	0.00019
TMEM180	621.696618	62.797156	9.282385633	5.995420212	5.995420212	3.30744	0.00019
LOC100737457	152.522904	1505.4792	7.262310092	10.55696503	10.55696503	-3.30312	0.00019
RTN4RL1	1080.09426	109.89502	10.0782766	6.793050813	6.793050813	3.296959	0.00019
ARHGAP8	3.31571529	61.970878	2.109599694	5.976612875	5.976612875	-4.2242	0.00019
LOC100627089	3.31571529	61.970878	2.109599694	5.976612875	5.976612875	-4.2242	0.00019
LOC102164246	1288.98432	131.37826	10.33313782	7.048522415	7.048522415	3.294436	0.00019
PADI1	6.63143059	92.543178	2.93195353	6.547560532	6.547560532	-3.80273	0.00019
UCP2	2402.23573	242.92584	11.23076245	7.930298794	7.930298794	3.30579	0.00019
42432	92.8400282	910.55877	6.552131542	9.832191855	9.832191855	-3.29393	0.0002
C7H15orf27	266.915081	25.61463	8.065631984	4.734147579	4.734147579	3.381341	0.0002
MFAP3L	99.4714588	7.4365053	6.65064192	3.076645515	3.076645515	3.741586	0.0002
CDCA7	8111.89747	795.70607	12.98600154	9.63790376	9.63790376	3.349732	0.0002
TRAF1	4.97357294	76.843889	2.578594101	6.282511874	6.282511874	-3.94958	0.0002
RGS7BP	805.718817	82.627837	9.655922097	6.385911347	6.385911347	3.285577	0.0002
ATP10A	113.563249	1108.0393	6.8400005	10.11509477	10.11509477	-3.28644	0.0002
LOC100736623	5.80250177	84.280394	2.766065426	6.414142197	6.414142197	-3.86045	0.0002
LOC100737407	5.80250177	84.280394	2.766065426	6.414142197	6.414142197	-3.86045	0.0002
LOC100624812	1383.48221	14376.417	10.4351308	13.81151693	13.81151693	-3.37733	0.0002
LOC100737961	6.63143059	91.716899	2.93195353	6.534760414	6.534760414	-3.7898	0.00021
LOC102159457	83.7218112	5.7839486	6.404661526	2.762125239	2.762125239	3.855477	0.00021
MAP3K9	256.967935	24.788351	8.011047944	4.688647628	4.688647628	3.373854	0.00021
NFE2	1.65785765	45.44531	1.410263837	5.537461031	5.537461031	-4.77674	0.00021
PCDHB8	121.023608	9.9153405	6.931016486	3.448285225	3.448285225	3.609482	0.00021
KAL1	707.076287	72.712497	9.467760991	6.20383732	6.20383732	3.281591	0.00021
BCL6B	53.0514447	2.4788351	5.756261274	1.798604302	1.798604302	4.419658	0.00021
LOC100739491	261.112579	2560.6367	8.034042783	11.32285015	11.32285015	-3.29376	0.00021
PRR14L	6407.61981	638.71318	12.64579797	9.3212814	9.3212814	3.326548	0.00021
SH3BP5	405.346195	4019.8443	8.666565574	11.97328275	11.97328275	-3.30991	0.00021
LOC100624418	4.14464412	68.581105	2.363071282	6.120623682	6.120623682	-4.04849	0.00021
WASF3	12.4339324	143.77244	3.747809764	7.177643143	7.177643143	-3.53143	0.00022
HIC2	1762.30268	181.78124	10.78406443	7.513974209	7.513974209	3.277186	0.00022
KLK15	28.18358	0	4.867084967	0	0	20	0.00022
EGF	28.18358	0	4.867084967	0	0	20	0.00022
NMU	28.18358	0	4.867084967	0	0	20	0.00022
LOC100514667	28.18358	0	4.867084967	0	0	20	0.00022
LOC102162884	28.18358	0	4.867084967	0	0	20	0.00022
PGLYRP3	28.18358	0	4.867084967	0	0	20	0.00022
LOC100519278	123.510395	1194.7985	6.96012238	10.22375862	10.22375862	-3.27406	0.00022
FAM155B	3462.4357	352.82086	11.75798817	8.466875316	8.466875316	3.294779	0.00022
TBX5	33.1571529	332.09018	5.094115824	8.383661891	8.383661891	-3.32809	0.00022
USH1C	105.273961	8.2627837	6.731644339	3.211445828	3.211445828	3.671377	0.00022
DUOX2	82.8928824	5.7839486	6.39047651	2.762125239	2.762125239	3.841122	0.00022
LOC102163336	82.8928824	5.7839486	6.39047651	2.762125239	2.762125239	3.841122	0.00022
LOC100737804	1280.69503	133.03082	10.32383731	7.066420948	7.066420948	3.267095	0.00022
PDGFD	28.18358	286.7186	4.867084967	8.168514654	8.168514654	-3.34671	0.00022
ETV5	267.74401	2606.9083	8.070088789	11.34867741	11.34867741	-3.28341	0.00023
LOC100626511	0	28.093465	0	4.862623207	4.862623207	-20	0.00023
LOC100516062	0	28.093465	0	4.862623207	4.862623207	-20	0.00023
LOC100738280	0	28.093465	0	4.862623207	4.862623207	-20	0.00023
ISG20	0	28.093465	0	4.862623207	4.862623207	-20	0.00023
BMP3	0	28.093465	0	4.862623207	4.862623207	-20	0.00023
MOG	44.7621565	1.6525567	5.516083133	1.407383613	1.407383613	4.759508	0.00023
LOC102160907	44.7621565	1.6525567	5.516083133	1.407383613	1.407383613	4.759508	0.00023
ZNF423	77.9193094	751.08704	6.302306427	9.554755826	9.554755826	-3.26893	0.00023
LOC102159216	521.39623	53.708094	9.029000675	5.773682393	5.773682393	3.279169	0.00023
CD2AP	1101.64641	114.85269	10.10675451	6.856147779	6.856147779	3.261805	0.00023
STOM	159.154334	1552.7464	7.323319031	10.58284422	10.58284422	-3.26762	0.00023
SERINC2	78.7482382	757.69727	6.317380743	9.56738053	9.56738053	-3.2663	0.00023
CCNB3	5935.13038	599.05182	12.53530706	9.228943285	9.228943285	3.308527	0.00023
AK1	245.362932	2376.3766	7.944641392	11.21515474	11.21515474	-3.27577	0.00023
LOC100626258	627.49912	65.275991	9.295766913	6.05041444	6.05041444	3.264989	0.00024
LOC100620105	4.97357294	75.191332	2.578594101	6.251554969	6.251554969	-3.91821	0.00024
LOC100156578	4.97357294	75.191332	2.578594101	6.251554969	6.251554969	-3.91821	0.00024
CYP2C32	96.9846724	7.4365053	6.614484182	3.076645515	3.076645515	3.70506	0.00024
SEMA3G	237.902572	23.135794	7.900278578	4.593102408	4.593102408	3.362172	0.00024
HCLS1	1.65785765	44.619032	1.410263837	5.511563931	5.511563931	-4.75027	0.00024
LOC102160052	1.65785765	44.619032	1.410263837	5.511563931	5.511563931	-4.75027	0.00024
CSRP3	1.65785765	44.619032	1.410263837	5.511563931	5.511563931	-4.75027	0.00024
EFEMP1	8980.61488	898.16459	13.13275915	9.812441412	9.812441412	3.321762	0.00024
LOC100154987	61.340733	589.96276	5.962103212	9.206923404	9.206923404	-3.26571	0.00024
DNAH1	140.9179	12.394176	7.148912757	3.74353388	3.74353388	3.507121	0.00024
LOC102165438	834.731325	87.585507	9.706895403	6.468998788	6.468998788	3.252548	0.00024
LOC100738469	974.820297	102.45852	9.930471681	6.692908622	6.692908622	3.250096	0.00024
MST4	3090.24665	319.76973	11.59397306	8.325394195	8.325394195	3.272617	0.00025
SEMA3D	649.880198	6419.3567	9.346248212	12.64843773	12.64843773	-3.30418	0.00025
LDB2	155.00969	14.046732	7.28549183	3.911378308	3.911378308	3.464052	0.00025
LOC100623335	8.28928824	104.11107	3.215568059	6.715770874	6.715770874	-3.65073	0.00025
LOC100627144	2.48678647	52.055537	1.801898017	5.729431428	5.729431428	-4.3877	0.00025
CADM3	2.48678647	52.055537	1.801898017	5.729431428	5.729431428	-4.3877	0.00025
LOC100737760	637.446265	66.928548	9.318421391	6.085946114	6.085946114	3.25161	0.00025
HOOK1	583.565892	61.1446	9.191221843	5.957557119	5.957557119	3.254599	0.00025
DTL	3544.49965	366.8676	11.79177324	8.523042794	8.523042794	3.272251	0.00025
ZNF287	24.8678647	252.0149	4.693089065	7.983078557	7.983078557	-3.34115	0.00025
ELFN2	132.628612	11.567897	7.062085132	3.65167138	3.65167138	3.519193	0.00026
ACOX2	50.5646582	485.85168	5.688310694	8.927338518	8.927338518	-3.26431	0.00026
NR1I2	81.2350247	5.7839486	6.361681078	2.762125239	2.762125239	3.811975	0.00026
LOC100739348	43.9332277	1.6525567	5.489710792	1.407383613	1.407383613	4.732541	0.00026
RAB3D	523.054088	5090.701	9.03357191	12.313932	12.313932	-3.28283	0.00027
LOC102166835	35.6439394	347.86319	5.195502704	8.446517589	8.446517589	-3.28679	0.00027
TMEM140	43.9332277	423.05453	5.489710792	8.728105973	8.728105973	-3.26746	0.00027
LOC100624257	27.3546512	0	4.825513503	0	0	20	0.00027
LOC102164438	27.3546512	0	4.825513503	0	0	20	0.00027
ONECUT1	27.3546512	0	4.825513503	0	0	20	0.00027
LOC102158768	27.3546512	0	4.825513503	0	0	20	0.00027
LOC102165746	27.3546512	0	4.825513503	0	0	20	0.00027
LHX1	27.3546512	0	4.825513503	0	0	20	0.00027
LOC102165228	51.3935871	2.4788351	5.711318333	1.798604302	1.798604302	4.373854	0.00027
PKDCC	547.921952	5333.6269	9.100457226	12.38117166	12.38117166	-3.28307	0.00027
FLYWCH2	28.18358	280.10837	4.867084967	8.13498259	8.13498259	-3.31306	0.00027
GTF2IRD2	109.418605	1031.1954	6.786839466	10.0115004	10.0115004	-3.23639	0.00027
PLXDC2	144.233615	1360.8805	7.182231603	10.41138438	10.41138438	-3.23806	0.00027
ADAM19	1635.47657	16526.394	10.67637723	14.01257162	14.01257162	-3.33699	0.00027
TNNT1	39.7885835	384.21944	5.350093502	8.589536711	8.589536711	-3.2715	0.00027
YARS2	1444.82294	153.68778	10.49767517	7.273215394	7.273215394	3.232818	0.00027
MSRB3	1114.08034	11075.435	10.12293194	13.43520604	13.43520604	-3.31344	0.00027
LOC100515642	145.891473	13.220454	7.19860684	3.829895615	3.829895615	3.464052	0.00028
SLC2A1	517.251586	5001.463	9.017508816	12.28842287	12.28842287	-3.27341	0.00028
LOC102161240	0	27.267186	0	4.821056379	4.821056379	-20	0.00028
TM4SF18	0	27.267186	0	4.821056379	4.821056379	-20	0.00028
LOC102158724	0	27.267186	0	4.821056379	4.821056379	-20	0.00028
FAM26F	0	27.267186	0	4.821056379	4.821056379	-20	0.00028
LOC100737032	0	27.267186	0	4.821056379	4.821056379	-20	0.00028
LOC100524359	314.164024	32.224857	8.299959051	5.054191063	5.054191063	3.285272	0.00028
LOC102167098	35.6439394	0.8262784	5.195502704	0.868906686	0.868906686	5.430885	0.00028
LOC100737835	80.4060959	5.7839486	6.347064926	2.762125239	2.762125239	3.797178	0.00028
LOC100519934	27641.4606	2704.4091	14.75459842	11.40163106	11.40163106	3.353449	0.00028
LOC100626247	258.625793	2447.4365	8.020289907	11.25764509	11.25764509	-3.24233	0.00028
MAN1C1	458.39764	4399.1061	8.843599634	12.10332258	12.10332258	-3.26254	0.00029
SLC29A4	386.280832	40.48764	8.597236288	5.374609695	5.374609695	3.254097	0.00029
PON1	0.82892882	35.52997	0.870998931	5.191008663	5.191008663	-5.42164	0.00029
MAB21L1	27.3546512	269.36675	4.825513503	8.078773924	8.078773924	-3.29971	0.0003
LOC102166268	238.731501	23.962073	7.905275684	4.641665831	4.641665831	3.316564	0.0003
ARHGEF19	74.6035941	695.72639	6.240382915	9.444448397	9.444448397	-3.2212	0.0003
CA8	43.1042988	1.6525567	5.462847376	1.407383613	1.407383613	4.70506	0.0003
LOC100626715	43.1042988	1.6525567	5.462847376	1.407383613	1.407383613	4.70506	0.0003
LOC102165140	481.607647	51.229259	8.914706964	5.706786332	5.706786332	3.232818	0.0003
OBSL1	700.444856	6779.614	9.454185881	12.72720021	12.72720021	-3.27486	0.0003
LOC100625132	444.30585	47.097867	8.798652753	5.587901017	5.587901017	3.23782	0.0003
LOC100627889	165.785765	15.699289	7.381852349	4.061714779	4.061714779	3.400549	0.0003
LOC102167380	50.5646582	2.4788351	5.688310694	1.798604302	1.798604302	4.350395	0.0003
PTCHD1	4.97357294	72.712497	2.578594101	6.20383732	6.20383732	-3.86985	0.0003
LOC100627808	34.8150106	334.64274	5.162492464	8.390782629	8.390782629	-3.26484	0.0003
AIFM2	297.585448	2802.7362	8.222000044	11.45313492	11.45313492	-3.23546	0.0003
LOC100621484	28.18358	275.1507	4.867084967	8.109311963	8.109311963	-3.2873	0.00031
LOC100736584	3235.3092	343.7318	11.66013373	8.429330588	8.429330588	3.234548	0.00031
LOC100158003	741.891297	80.149002	9.537007315	6.342501448	6.342501448	3.210451	0.00031
CNN2	1996.88954	19993.458	10.9642611	14.28731254	14.28731254	-3.3237	0.00031
GUCY2C	353.952608	37.182527	8.471482603	5.254840671	5.254840671	3.250859	0.00031
FRMD4B	426.898344	45.44531	8.741124286	5.537461031	5.537461031	3.231689	0.00031
LOC100154430	915.96635	8889.929	9.840724982	13.11811846	13.11811846	-3.27881	0.00032
LOC102157895	2.48678647	50.402981	1.801898017	5.683780114	5.683780114	-4.34115	0.00032
LRFN1	912.650635	99.153405	9.835498797	6.646067655	6.646067655	3.202328	0.00032
LOC100737965	42.27537	398.26618	5.435474249	8.641207046	8.641207046	-3.23584	0.00032
BCHE	1108.27784	120.63664	10.11540504	6.926434087	6.926434087	3.19958	0.00032
CLDN7	164.127907	15.699289	7.36744015	4.061714779	4.061714779	3.386049	0.00033
LOC102159547	26.5257224	0	4.78270852	0	0	20	0.00033
LOC102161712	26.5257224	0	4.78270852	0	0	20	0.00033
LOC102161639	26.5257224	0	4.78270852	0	0	20	0.00033
OLIG2	26.5257224	0	4.78270852	0	0	20	0.00033
LOC102162285	26.5257224	0	4.78270852	0	0	20	0.00033
LOC102158347	26.5257224	0	4.78270852	0	0	20	0.00033
LOC102165047	26.5257224	0	4.78270852	0	0	20	0.00033
TRIM72	19.0653629	193.34914	4.326635347	7.602506906	7.602506906	-3.34218	0.00033
CACNG1	34.8150106	0.8262784	5.162492464	0.868906686	0.868906686	5.396938	0.00033
LOC100738931	57.1960888	3.3051135	5.862850292	2.106051269	2.106051269	4.113145	0.00033
SLC1A6	727.799507	79.322724	9.509378173	6.327736286	6.327736286	3.197735	0.00033
LOC100739211	4.14464412	64.449713	2.363071282	6.032314961	6.032314961	-3.95885	0.00034

ARHGEF16	30.3778164	1.6525567	4.971673052	1.407383613	1.407383613	4.200246	0.01068
LOC100626715	30.3778164	1.6525567	4.971673052	1.407383613	1.407383613	4.200246	0.01068
RPL3L	30.3778164	1.6525567	4.971673052	1.407383613	1.407383613	4.200246	0.01068
SPRP	30.3778164	1.6525567	4.971673052	1.407383613	1.407383613	4.200246	0.01068
RAB6B	4651.94834	635.40807	12.18392945	9.313808315	9.313808315	2.87208	0.01072
LOC100622083	46.9475344	4.1313919	5.583384725	2.359350201	2.359350201	3.50635	0.01074
GNAT1	22.0929574	223.92144	4.529381037	7.81327737	7.81327737	-3.34133	0.01076
PDLIM1	835.389951	8727.1522	9.708031918	13.09146054	13.09146054	-3.38499	0.01081
LOC102159729	2.76161967	54.534373	1.911353989	5.795309087	5.795309087	-4.30358	0.01081
ZWINT	1216.49347	173.51846	10.24969831	7.447235822	7.447235822	2.809568	0.01083
LOC102164486	41.4242951	3.3051135	5.406818783	2.106051269	2.106051269	3.647705	0.01085
LOC100517279	17.9505279	186.73891	4.24416613	7.552583893	7.552583893	-3.37892	0.01086
LOC102167215	35.9010557	2.4788351	5.205590187	1.798604302	1.798604302	3.856292	0.01088
LOC100515829	52.4707738	500.72469	5.740678649	8.970752135	8.970752135	-3.25443	0.0109
LOC102157672	44.1859147	423.8808	5.497801224	8.730914357	8.730914357	-3.262	0.01091
EML5	230.595243	32.224857	7.855461808	5.054191063	5.054191063	2.839117	0.011
TLR7	84.2294	9.9153405	6.413279271	3.448285225	3.448285225	3.08659	0.011
LOC733663	15.1889082	161.95056	4.016933786	7.348290508	7.348290508	-3.41446	0.01101
RAPSN	6.90404918	90.064343	2.982591923	6.508814353	6.508814353	-3.70544	0.01101
SST	1.38080984	42.140197	1.251452392	5.430960858	5.430960858	-4.93161	0.01102
LIMS2	102.179928	971.70337	6.689018532	9.925856099	9.925856099	-3.2494	0.01102
SCUBE3	8.28485901	101.63224	3.214880004	6.681340183	6.681340183	-3.61674	0.01118
SLC6A3	17.9505279	0	4.24416613	0	0	20	0.01119
PTCHD4	17.9505279	0	4.24416613	0	0	20	0.01119
LOC102161687	17.9505279	0	4.24416613	0	0	20	0.01119
PLA2G4F	17.9505279	0	4.24416613	0	0	20	0.01119
LOC100154181	17.9505279	0	4.24416613	0	0	20	0.01119
LOC100624606	17.9505279	0	4.24416613	0	0	20	0.01119
LOC102164094	17.9505279	0	4.24416613	0	0	20	0.01119
GFI1B	17.9505279	0	4.24416613	0	0	20	0.01119
LOC102165799	17.9505279	0	4.24416613	0	0	20	0.01119
LOC102167231	17.9505279	0	4.24416613	0	0	20	0.01119
GABRR2	17.9505279	0	4.24416613	0	0	20	0.01119
PSORS1C2	110.464787	14.046732	6.800444207	3.911378308	3.911378308	2.97528	0.01135
PN-1	1952.46511	21228.744	10.93181977	14.37379935	14.37379935	-3.44265	0.01137
PTGER4	2.76161967	53.708094	1.911353989	5.773682393	5.773682393	-4.28155	0.0116
TMEM132C	2.76161967	53.708094	1.911353989	5.773682393	5.773682393	-4.28155	0.0116
LOC102157560	0	28.919743	0	4.903025878	4.903025878	-20	0.01167
42440	0	28.919743	0	4.903025878	4.903025878	-20	0.01167
LOC102164195	0	28.919743	0	4.903025878	4.903025878	-20	0.01167
IDO1	0	28.919743	0	4.903025878	4.903025878	-20	0.01167
BHLHE22	0	28.919743	0	4.903025878	4.903025878	-20	0.01167
LOC102158984	0	28.919743	0	4.903025878	4.903025878	-20	0.01167
CCL8	0	28.919743	0	4.903025878	4.903025878	-20	0.01167
LOC100627386	0	28.919743	0	4.903025878	4.903025878	-20	0.01167
LOC102163138	0	28.919743	0	4.903025878	4.903025878	-20	0.01167
LDLRAD4	836.770761	121.46292	9.710411723	6.936201185	6.936201185	2.784316	0.0117
PKC	4.14242951	65.275991	2.362450112	6.05041444	6.05041444	-3.978	0.01182
LOC100516787	125.653695	16.525567	6.984745358	4.13138925	4.13138925	2.926681	0.01186
LIF	24.854577	243.75212	4.692347799	7.935177544	7.935177544	-3.29383	0.01186
TTC28	198.836616	1893.83	7.642677144	10.88785272	10.88785272	-3.25165	0.01203
LOC102158553	1.38080984	41.313919	1.251452392	5.403060391	5.403060391	-4.90304	0.01204
APRIL	13.8080984	147.07755	3.888314478	7.210209122	7.210209122	-3.41299	0.01205
C10H1orf115	303.778164	43.792754	8.251615733	5.485193456	5.485193456	2.794254	0.01206
TMEM173	151.889082	20.656959	7.256341575	4.436758793	4.436758793	2.878318	0.0121
LOC102157898	71.8021115	667.63292	6.185908388	9.385070587	9.385070587	-3.21696	0.01216
LOC102166840	30.3778164	290.84999	4.971673052	8.189083193	8.189083193	-3.25919	0.01222
LOC100511616	22.0929574	218.13749	4.529381037	7.775692512	7.775692512	-3.30358	0.01222
PARP8	270.638728	2599.4718	8.08554537	11.34455765	11.34455765	-3.26378	0.01231
KCNN2	2.76161967	52.881816	1.911353989	5.751726565	5.751726565	-4.25919	0.01245
LOC100738205	2.76161967	52.881816	1.911353989	5.751726565	5.751726565	-4.25919	0.01245
PPGRP-S	51.0899639	4.9576702	5.702933533	2.574748269	2.574748269	3.365306	0.01246
ZNF711	1383.57146	200.78564	10.43522379	7.65667973	7.65667973	2.784669	0.01247
ADRA1D	544.039075	80.149002	9.090215854	6.342501448	6.342501448	2.762954	0.01277
NUAK1	361.772177	3493.505	8.502920002	11.77087238	11.77087238	-3.27152	0.01284
RBP1	689.024108	101.63224	9.430502957	6.681340183	6.681340183	2.761196	0.01289
LOC396904	12685.5	150749.53	13.63100648	17.20180356	17.20180356	-3.5709	0.01295
HOMER2	2085.02285	300.76533	11.02653925	8.23728324	8.23728324	2.793353	0.01297
FHOD3	135.319364	1252.638	7.090846698	10.29190511	10.29190511	-3.21053	0.01301
SETBP1	9.66566885	109.89502	3.414902536	6.793050813	6.793050813	-3.50711	0.01304
LOC100621279	8.28485901	98.327126	3.214880004	6.634115866	6.634115866	-3.56904	0.01307
LOC100737092	52.4707738	482.54657	5.740678649	8.917511028	8.917511028	-3.20108	0.01308
STK36	66.2788721	606.48832	6.072081615	9.246712872	9.246712872	-3.19386	0.01318
LOC100626511	0	28.093465	0	4.862623207	4.862623207	-20	0.01321
ISG20	0	28.093465	0	4.862623207	4.862623207	-20	0.01321
BMP3	0	28.093465	0	4.862623207	4.862623207	-20	0.01321
ARHGAP42	182.266898	1698.0021	7.517802419	10.73047188	10.73047188	-3.21971	0.01322
KLHL40	4.14242951	63.623435	2.362450112	6.013985524	6.013985524	-3.94101	0.01331
SYPL2	2.76161967	52.055537	1.911353989	5.729431428	5.729431428	-4.23647	0.01337
WASF3	13.8080984	143.77244	3.888314478	7.177643143	7.177643143	-3.3802	0.01343
LOC102162901	15.1889082	155.34033	4.016933786	7.288546214	7.288546214	-3.35434	0.01345
SNAP91	9.66566885	109.06875	3.414902536	6.782261052	6.782261052	-3.49622	0.01351
SLC22A17	305.158974	2892.8006	8.25813716	11.49874979	11.49874979	-3.24483	0.01352
CYP26A1	23.4737672	0.8262784	4.613164285	0.868906686	0.868906686	4.828278	0.01359
SAMD12	23.4737672	0.8262784	4.613164285	0.868906686	0.868906686	4.828278	0.01359
VAV1	23.4737672	0.8262784	4.613164285	0.868906686	0.868906686	4.828278	0.01359
SLC7A9	23.4737672	0.8262784	4.613164285	0.868906686	0.868906686	4.828278	0.01359
PTGDS	8.28485901	97.500848	3.214880004	6.622064238	6.622064238	-3.55687	0.0136
DAPK1	1271.72586	187.56519	10.31370599	7.558919565	7.558919565	2.761324	0.01376
SLC4A11	169.83961	23.962073	7.416498698	4.641665831	4.641665831	2.825348	0.01378
LOC100738338	12.4272885	131.37826	3.747096094	7.048522415	7.048522415	-3.40214	0.01384
LOC100737078	22.0929574	212.35354	4.529381037	7.737102248	7.737102248	-3.26481	0.01391
CDH24	37.2818656	342.90552	5.258589232	8.42586848	8.42586848	-3.20127	0.01393
HCK	28.9970066	1.6525567	4.906746634	1.407383613	1.407383613	4.133132	0.01394
CACNA1F	28.9970066	1.6525567	4.906746634	1.407383613	1.407383613	4.133132	0.01394
GCNT3	75.944541	9.0890621	6.265747069	3.334720158	3.334720158	3.062743	0.01394
NT5C	7245.10921	1008.8859	12.82299083	9.979976575	9.979976575	2.844244	0.01398
LOC100739719	96.6566885	875.0288	6.609646952	9.774834483	9.774834483	-3.17839	0.01402
APOA1	146.365843	1337.7447	7.203258356	10.38666513	10.38666513	-3.19215	0.01402
LOC100737413	45.5667246	414.79174	5.541227504	8.699717297	8.699717297	-3.18633	0.01403
ICA1	4.14242951	62.797156	2.362450112	5.995420212	5.995420212	-3.92215	0.01413
KLF9	77.3253508	697.37895	6.291407421	9.447866256	9.447866256	-3.17293	0.01414
LOC100627081	132.557744	18.178124	7.061319821	4.261389712	4.261389712	2.866346	0.01417
LOC100621406	1713.58501	252.0149	10.74364372	7.983078557	7.983078557	2.765437	0.01425
BDKRB1	2.76161967	51.229259	1.911353989	5.706786332	5.706786332	-4.21338	0.01437
AZGP1	2.76161967	51.229259	1.911353989	5.706786332	5.706786332	-4.21338	0.01437
FAM154B	127.034505	17.351846	7.000388854	4.19785327	4.19785327	2.872059	0.0144
LOC102164007	1.38080984	39.661362	1.251452392	5.34558663	5.34558663	-4.84415	0.01443
ELOVL2	106.322357	14.046732	6.745806839	3.911378308	3.911378308	2.920139	0.01444
LOC100524954	106.322357	14.046732	6.745806839	3.911378308	3.911378308	2.920139	0.01444
CDHR4	60.7556328	546.17	5.948498827	9.095845332	9.095845332	-3.16826	0.01444
USP44	1433.28061	212.35354	10.48611159	7.737102248	7.737102248	2.754781	0.01449
ICOSLG	204.359856	1877.3045	7.682010377	10.87521522	10.87521522	-3.19948	0.01449
LOC102159265	24.854577	233.83678	4.692347799	7.875514565	7.875514565	-3.23392	0.0145
LOC100736682	180.886089	1653.383	7.506891393	10.69207757	10.69207757	-3.19227	0.01452
LOC100624658	5.52323934	73.538775	2.705588563	6.219919206	6.219919206	-3.73492	0.01463
LOC100736744	121.511266	16.525567	6.936770608	4.13138925	4.13138925	2.878318	0.01466
ADM5	510.899639	76.843889	8.999717179	6.282511874	6.282511874	2.733037	0.01466
VWF	49.7091541	4.9576702	5.664174303	2.574748269	2.574748269	3.325777	0.01468
CCR7	49.7091541	4.9576702	5.664174303	2.574748269	2.574748269	3.325777	0.01468
LPAR3	49.7091541	4.9576702	5.664174303	2.574748269	2.574748269	3.325777	0.01468
LOC100518639	12.4272885	129.7257	3.747096094	7.030399034	7.030399034	-3.38388	0.0147
LOC102167807	20.7121475	198.30681	4.440430524	7.63884719	7.63884719	-3.25919	0.01472
LOC100155572	267.877108	39.661362	8.070803121	5.34558663	5.34558663	2.755765	0.01479
LOC100739077	143.604223	1297.257	7.175965874	10.34236034	10.34236034	-3.17529	0.01481
HHIPL1	77.3253508	690.76872	6.291407421	9.434145968	9.434145968	-3.15919	0.01482
RAVER2	537.135026	80.97528	9.071824402	6.357117027	6.357117027	2.729731	0.01486
WSCD1	0	27.267186	0	4.821056379	4.821056379	-20	0.015
LOC102161240	0	27.267186	0	4.821056379	4.821056379	-20	0.015
CNTN4	0	27.267186	0	4.821056379	4.821056379	-20	0.015
TM4SF18	0	27.267186	0	4.821056379	4.821056379	-20	0.015
LOC100522814	0	27.267186	0	4.821056379	4.821056379	-20	0.015
LOC102160144	0	27.267186	0	4.821056379	4.821056379	-20	0.015
LOC102158661	0	27.267186	0	4.821056379	4.821056379	-20	0.015
LOC102158724	0	27.267186	0	4.821056379	4.821056379	-20	0.015
FAM26F	0	27.267186	0	4.821056379	4.821056379	-20	0.015
LOC100737032	0	27.267186	0	4.821056379	4.821056379	-20	0.015
LOC100737008	0	27.267186	0	4.821056379	4.821056379	-20	0.015
LOC100737091	19.3313377	185.91263	4.345633235	7.546220275	7.546220275	-3.26561	0.01505
LOC100622791	484.664252	4600.718	8.923815489	12.16795685	12.16795685	-3.2468	0.01511
LOC100513486	272.019538	40.48764	8.092860386	5.374609695	5.374609695	2.748157	0.0152
MPC2	9417.12308	1312.9563	13.20122386	10.35970162	10.35970162	2.842467	0.01523
CDON	4110.67088	592.44159	12.00550907	9.212962234	9.212962234	2.794629	0.01525
ASXL3	573.036082	86.759229	9.164997612	6.455478947	6.455478947	2.723537	0.01528
LOC102163869	8.28485901	95.022013	3.214880004	6.585293272	6.585293272	-3.51971	0.01533
PHF11	662.788721	6380.5216	9.374580306	12.63968474	12.63968474	-3.26705	0.01534
KAL1	5.52323934	72.712497	2.705588563	6.20383732	6.20383732	-3.71862	0.01541
LOC102160564	2863.7996	418.92313	11.48421851	8.713981461	8.713981461	2.773173	0.01543
LOC100622375	447.382387	67.754826	8.808585798	6.103389087	6.103389087	2.723113	0.01545
CMYA5	2.76161967	50.402981	1.911353989	5.683780114	5.683780114	-4.18992	0.01546
BCAR3	298.254925	2749.0281	8.225231178	11.42523067	11.42523067	-3.2043	0.01548
LOC102158822	12.4272885	128.07315	3.747096094	7.012045083	7.012045083	-3.36538	0.01561
LOC100513211	100.799118	889.90181	6.669581252	9.799122619	9.799122619	-3.14216	0.01591
SLC41A2	93.8950688	827.93093	6.568261215	9.695108082	9.695108082	-3.14039	0.01592
LOC100624689	143.604223	1278.2526	7.175965874	10.3210855	10.3210855	-3.154	0.01593
LOC102162364	4.14242951	61.1446	2.362450112	5.957557119	5.957557119	-3.88368	0.01595
PLXNB1	99.4183082	875.85507	6.649878514	9.776194605	9.776194605	-3.13911	0.01606
LOC102166921	742.875692	113.20014	9.538917744	6.83542057	6.83542057	2.714245	0.01609
LOC100736995	22.0929574	205.74331	4.529381037	7.691696868	7.691696868	-3.21919	0.01618
CAMK1D	31.7586262	285.89232	5.033802952	8.164365521	8.164365521	-3.17025	0.01621
LOC102159363	573.036082	87.585507	9.164997612	6.468998788	6.468998788	2.709862	0.01626
LOC102167481	243.022531	36.356248	7.930870551	5.223277669	5.223277669	2.740815	0.01628
LOC102168047	38.6626754	3.3051135	5.309710091	2.106051269	2.106051269	3.54817	0.01628
PSRC1	7704.91888	1095.6451	12.91175128	10.09888102	10.09888102	2.813999	0.01655
LOC100620668	2.76161967	49.576702	1.911353989	5.660401068	5.660401068	-4.16608	0.01664
LOC102166028	646.219003	99.153405	9.338110157	6.646067655	6.646067655	2.704289	0.01672
LOC100513915	16.569718	159.47173	4.135019133	7.326175314	7.326175314	-3.26668	0.01673
BTG2	7499.17822	1069.2042	12.87270916	10.0636704	10.0636704	2.810195	0.01673
LOC102158304	133.938554	19.004403	7.076158798	4.322245637	4.322245637	2.817166	0.01691
LOC100737501	118.749646	16.525567	6.90387758	4.13138925	4.13138925	2.845151	0.01692
TFF1	16.569718	0	4.135019133	0	0	20	0.01694
KLHL6	16.569718	0	4.135019133	0	0	20	0.01694
EPHA6	16.569718	0	4.135019133	0	0	20	0.01694
TARSL2	16.569718	0	4.135019133	0	0	20	0.01694
LOC102161998	16.569718	0	4.135019133	0	0	20	0.01694
PGA	16.569718	0	4.135019133	0	0	20	0.01694
LOC102159894	16.569718	0	4.135019133	0	0	20	0.01694
RXFP2	16.569718	0	4.135019133	0	0	20	0.01694
LOC100514940	16.569718	0	4.135019133	0	0	20	0.01694
ADAM18	16.569718	0	4.135019133	0	0	20	0.01694
LOC100621138	16.569718	0	4.135019133	0	0	20	0.01694
THEM5	16.569718	0	4.135019133	0	0	20	0.01694
LOC102160414	16.569718	0	4.135019133	0	0	20	0.01694
LOC102158704	16.569718	0	4.135019133	0	0	20	0.01694
ZSCAN10	16.569718	0	4.135019133	0	0	20	0.01694
CACNB4	16.569718	0	4.135019133	0	0	20	0.01694
LOC102163198	16.569718	0	4.135019133	0	0	20	0.01694
PAPPA2	16.569718	0	4.135019133	0	0	20	0.01694
LOC102163242	16.569718	0	4.135019133	0	0	20	0.01694
LOC100157667	16.569718	0	4.135019133	0	0	20	0.01694
KRTDAP	16.569718	0	4.135019133	0	0	20	0.01694
FCGR2B	16.569718	0	4.135019133	0	0	20	0.01694
LCN8	16.569718	0	4.135019133	0	0	20	0.01694
LOC102162060	4.14242951	60.318321	2.362450112	5.938246292	5.938246292	-3.86405	0.01696
MLC2V	0	26.440908	0	4.77825631	4.77825631	-20	0.01706
ZBTB16	0	26.440908	0	4.77825631	4.77825631	-20	0.01706
LOC102160855	0	26.440908	0	4.77825631	4.77825631	-20	0.01706
LOC102160410	0	26.440908	0	4.77825631	4.77825631	-20	0.01706
LOC102158399	0	26.440908	0	4.77825631	4.77825631	-20	0.01706
LOC102162226	0	26.440908	0	4.77825631	4.77825631	-20	0.01706
LOC102163370	0	26.440908	0	4.77825631	4.77825631	-20	0.01706
LOC100738691	69.0404918	599.05182	6.130117309	9.228943285	9.228943285	-3.11717	0.01708
RNF207	637.934144	98.327126	9.319523428	6.634115866	6.634115866	2.697746	0.01722
MEG3	13.8080984	136.33593	3.888314478	7.101565318	7.101565318	-3.30358	0.01726
LOC100623747	48.3283443	4.9576702	5.624344958	2.574748269	2.574748269	3.285135	0.01734
LOC100517709	48.3283443	4.9576702	5.624344958	2.574748269	2.574748269	3.285135	0.01734
HFE2	1.38080984	38.008805	1.251452392	5.285727902	5.285727902	-4.78275	0.01738
LOC100624749	421.147	3857.0674	8.721601651	11.91366265	11.91366265	-3.19511	0.01741
LOC100625694	11.0464787	114.02642	3.590539588	6.845821397	6.845821397	-3.36771	0.01753
CHADL	255.44982	38.835083	8.002532746	5.315967693	5.315967693	2.717607	0.01764
TRIB3	397.673233	3618.273	8.639062934	11.82148421	11.82148421	-3.18565	0.01773
LOC102162975	13.8080984	135.50965	3.888314478	7.092859162	7.092859162	-3.29481	0.01776
ALOX12B	107.703167	14.873011	6.764250165	3.98850389	3.98850389	2.856292	0.01788
ARHGDIB	2.76161967	48.750424	1.911353989	5.636636914	5.636636914	-4.14183	0.01793
CNKSR2	2.76161967	48.750424	1.911353989	5.636636914	5.636636914	-4.14183	0.01793
LOC102163693	6.90404918	80.97528	2.982591923	6.357117027	6.357117027	-3.55197	0.01805
TRAM2	309.301403	2768.8588	8.277526411	11.43559673	11.43559673	-3.16221	0.01805
PDZD2	13.8080984	134.68337	3.888314478	7.084100147	7.084100147	-3.28599	0.01827
MAFB	38.6626754	335.46902	5.309710091	8.394329862	8.394329862	-3.11717	0.01828
BAALC	27.6161967	1.6525567	4.838760036	1.407383613	1.407383613	4.062743	0.01829
LOC102164563	27.6161967	1.6525567	4.838760036	1.407383613	1.407383613	4.062743	0.01829
LOC100152383	27.6161967	1.6525567	4.838760036	1.407383613	1.407383613	4.062743	0.01829
LOC102165191	27.6161967	1.6525567	4.838760036	1.407383613	1.407383613	4.062743	0.01829
ZNF185	33.1394361	290.02371	5.093367322	8.184992878	8.184992878	-3.12955	0.01829
LOC102165339	67.659682	8.2627837	6.101391269	3.211445828	3.211445828	3.033597	0.01833
LOC100737119	67.659682	8.2627837	6.101391269	3.211445828	3.211445828	3.033597	0.01833
LOC100622991	34.5202459	300.76533	5.150569664	8.23728324	8.23728324	-3.12312	0.01846
NRN1	167.07799	24.788351	7.392987005	4.688647628	4.688647628	2.752787	0.01854
LOC100518152	120.130456	1031.1954	6.920417836	10.0115004	10.0115004	-3.10164	0.01858
SPARCL1	618.602806	96.674569	9.275199868	6.609911086	6.609911086	2.677805	0.01883
CHD5	15.1889082	144.59872	4.016933786	7.185853814	7.185853814	-3.25096	0.01885
FAM50B	22.0929574	0.8262784	4.529381037	0.868906686	0.868906686	4.740815	0.01886
LOC102161067	22.0929574	0.8262784	4.529381037	0.868906686	0.868906686	4.740815	0.01886
C15H2orf66	22.0929574	0.8262784	4.529381037	0.868906686	0.868906686	4.740815	0.01886
SCARA3	2732.62267	27463.841	11.4165984	14.74529829	14.74529829	-3.32918	0.01888
LOC102158952	86.9910197	11.567897	6.459284385	3.65167138	3.65167138	2.91074	0.01889
NTM	6.90404918	80.149002	2.982591923	6.342501448	6.342501448	-3.53717	0.01891
LOC102161148	1.38080984	37.182527	1.251452392	5.254840671	5.254840671	-4.75104	0.01911
MYH1	1.38080984	37.182527	1.251452392	5.254840671	5.254840671	-4.75104	0.01911
LOC100624445	1688.73043	259.45141	10.72257739	8.02487043	8.02487043	2.702403	0.0192
GCK	484.664252	76.01761	8.923815489	6.267116453	6.267116453	2.67258	0.01927
CLDN9	537.135026	84.280394	9.071824402	6.414142197	6.414142197	2.672016	0.01928
KIAA1755	2.76161967	47.924146	1.911353989	5.61247475	5.61247475	-4.11717	0.01934
LOC102165555	62.1364426	7.4365053	5.98040107	3.076645515	3.076645515	3.062743	0.01938
TBC1D30	16.569718	154.51406	4.135019133	7.280901168	7.280901168	-3.22112	0.0194
RAB3A	1289.67639	199.95937	10.3339116	7.650760008	7.650760008	2.68923	0.0194
HIF3A	0	25.61463	0	4.734147579	4.734147579	-20	0.01946
LOC102157470	0	25.61463	0	4.734147579	4.734147579	-20	0.01946
LOC100624399	0	25.61463	0	4.734147579	4.734147579	-20	0.01946
GPRIN3	0	25.61463	0	4.734147579	4.734147579	-20	0.01946
TRPA1	0	25.61463	0	4.734147579	4.734147579	-20	0.01946
LOC100625061	0	25.61463	0	4.734147579	4.734147579	-20	0.01946
LOC102162967	0	25.61463	0	4.734147579	4.734147579	-20	0.01946
LOC100737228	0	25.61463	0	4.734147579	4.734147579	-20	0.01946
LOC102163531	24.854577	219.79005	4.692347799	7.786531327	7.786531327	-3.14454	0.01947
LOC100620996	175.362849	1507.958	7.462402878	10.55933696	10.55933696	-3.10418	0.01951
LOC100738612	7285.15269	1062.594	12.83094151	10.05473181	10.05473181	2.777369	0.01951
IL6ST	388.007564	3457.1487	8.603654397	11.75578419	11.75578419	-3.15543	0.01954
LOC100514841	270.638728	2366.4613	8.08554537	11.2091251	11.2091251	-3.12829	0.01965
LOC100623234	219.548764	1897.9614	7.784953864	10.89099488	10.89099488	-3.11184	0.01986
LOC100520605	15.1889082	142.94616	4.016933786	7.169385476	7.169385476	-3.23438	0.01989
LOC100736655	37.2818656	3.3051135	5.258589232	2.106051269	2.106051269	3.495702	0.02004
LOC102165654	37.2818656	3.3051135	5.258589232	2.106051269	2.106051269	3.495702	0.02004
SYT17	100.799118	14.046732	6.669581252	3.911378308	3.911378308	2.843177	0.02006
SLC39A8	5.52323934	68.581105	2.705588563	6.120623682	6.120623682	-3.63422	0.0201
LOC100627741	144.985033	21.483238	7.189676653	4.490777898	4.490777898	2.754621	0.02017
SMAD3	196.074997	1681.4765	7.62260094	10.71637063	10.71637063	-3.10025	0.02019
TP53INP1	95.2758787	799.83746	6.58910248	9.645365656	9.645365656	-3.06952	0.02024
LOC102166259	273.400348	2373.0715	8.100138498	11.21314766	11.21314766	-3.11767	0.02042
PCDH11X	474.998584	75.191332	8.89481347	6.251554969	6.251554969	2.659285	0.02045
MAML2	62.1364426	519.7291	5.98040107	9.02438921	9.02438921	-3.06425	0.02045
FABP5	10437.5416	1510.4369	13.34963254	10.561705	10.561705	2.788744	0.02052
LOC100157017	720.782734	114.02642	9.495420823	6.845821397	6.845821397	2.660196	0.02056
TET1	12.4272885	120.63664	3.747096094	6.926434087	6.926434087	-3.27908	0.02062
SGPP2	56.6132033	6.610227	5.848327568	2.927939483	2.927939483	3.098367	0.02067
KIAA1324L	672.45439	106.58991	9.395492632	6.749398975	6.749398975	2.657366	0.02073
ULBP1	75.944541	9.9153405	6.265747069	3.448285225	3.448285225	2.937212	0.02076

LMX1B	0	31.398578	0	5.017858594	5.017858594	-20	0.00594
LOC100625402	0	31.398578	0	5.017858594	5.017858594	-20	0.00594
LOC102167414	0	31.398578	0	5.017858594	5.017858594	-20	0.00594
WFIKKN2	1.057359087	44.619032	1.04079362	5.511563931	5.511563931	-5.39912	0.00596
LOC102158225	1.057359087	44.619032	1.04079362	5.511563931	5.511563931	-5.39912	0.00596
MIR214	26.43397716	360.25737	4.777891883	8.49688321	8.49688321	-3.76856	0.006
GLIS2	300.2899806	4285.9059	8.235008887	12.06572104	12.06572104	-3.83517	0.00603
CST3	1002.376414	15387.782	9.970647215	13.90959144	13.90959144	-3.94029	0.00603
KLHL6	23.2618999	0	4.600620625	0	0	20	0.00604
PTCHD4	23.2618999	0	4.600620625	0	0	20	0.00604
LOC102161637	23.2618999	0	4.600620625	0	0	20	0.00604
LOC100511763	23.2618999	0	4.600620625	0	0	20	0.00604
LOC102164021	23.2618999	0	4.600620625	0	0	20	0.00604
LOC100158105	23.2618999	0	4.600620625	0	0	20	0.00604
HAO1	23.2618999	0	4.600620625	0	0	20	0.00604
LRRC18	23.2618999	0	4.600620625	0	0	20	0.00604
SGSH	168.1200948	2326.7999	7.40190428	11.18475133	11.18475133	-3.79078	0.00605
LOC100515974	4411.302109	384.21944	12.10731586	8.589536711	8.589536711	3.521202	0.00611
OLFM1	19.03246356	264.40908	4.324267947	8.052073912	8.052073912	-3.79624	0.00612
LOC100625482	72.95777697	4.9576702	6.208629956	2.57474827	2.57474827	3.879328	0.00612
RPS6KA2	456.7791254	6651.5409	8.838507868	12.69968976	12.69968976	-3.86412	0.00614
UMODL1	138.5140403	11.567897	7.124266508	3.651671381	3.651671381	3.581834	0.00615
CDRT1	2.114718173	57.013208	1.639101631	5.858309485	5.858309485	-4.75276	0.00615
GDNF	33.83549077	453.62683	5.122485983	8.828539005	8.828539005	-3.7449	0.00616
LOC100525679	324.6092396	29.746021	8.346997828	4.942327829	4.942327829	3.447935	0.00618
WDR66	255.880899	23.135794	8.004955809	4.593102407	4.593102407	3.467274	0.0062
YARS2	1690.717179	153.68778	10.72427268	7.273215395	7.273215395	3.459561	0.00622
RYR3	3.17207726	69.407383	2.060765874	6.137654819	6.137654819	-4.45159	0.00624
LOC100519123	33.83549077	451.97427	5.122485983	8.823285292	8.823285292	-3.73963	0.00626
RHBDL1	77.18721332	1030.3691	6.288860784	10.01034505	10.01034505	-3.73866	0.00627
MEIS2	19.03246356	262.75652	4.324267947	8.043062959	8.043062959	-3.78719	0.00629
TAF5	2025.90001	183.4338	10.9850592	7.526959252	7.526959252	3.465232	0.0063
RPF1	14114.68645	1168.3576	13.78501167	10.19150049	10.19150049	3.594643	0.00632
LOC102166581	31.7207726	0.8262784	5.032134908	0.868906686	0.868906686	5.262656	0.00634
LOC100739369	611.153552	57.013208	9.257749772	5.858309485	5.858309485	3.422167	0.00637
SR-PSOX	133.2272449	1803.7657	7.068533723	10.81759583	10.81759583	-3.75905	0.00637
PPP1R13B	17.97510447	248.70979	4.246035924	7.964108573	7.964108573	-3.79039	0.00638
CCDC73	530.7942615	49.576702	9.054724399	5.660401069	5.660401069	3.420419	0.00641
TAGAP	1.057359087	43.792754	1.04079362	5.485193456	5.485193456	-5.37215	0.00643
LOC102160827	1.057359087	43.792754	1.04079362	5.485193456	5.485193456	-5.37215	0.00643
TMEM81	56.04003159	3.3051135	5.833902876	2.106051269	2.106051269	4.083686	0.00647
SYCP3	161.7759402	14.046732	7.346743662	3.911378308	3.911378308	3.525691	0.00647
LOC100156943	120.5389359	9.9153405	6.925274755	3.448285225	3.448285225	3.603693	0.00652
SOAT1	12542.39348	1050.1998	13.61464009	10.03782121	10.03782121	3.578077	0.00652
TUBA8	3.17207726	68.581105	2.060765874	6.120623682	6.120623682	-4.43431	0.00656
ARSJ	50.75323616	666.80665	5.693577173	9.383286641	9.383286641	-3.7157	0.00657
ITPKB	4.229436346	80.97528	2.386655454	6.357117027	6.357117027	-4.25894	0.00657
GABRA1	71.90041789	4.9576702	6.187855179	2.57474827	2.57474827	3.858266	0.00657
MESP2	27.49133625	366.04132	4.832451381	8.519798669	8.519798669	-3.73496	0.00658
LOC102161478	5.286795433	93.369456	2.65232482	6.560248082	6.560248082	-4.14249	0.00659
RTKN2	606.9241157	57.013208	9.24774744	5.858309485	5.858309485	3.412148	0.0066
LOC100624161	0	30.5723	0	4.980587447	4.980587447	-20	0.0066
XPNPEP2	0	30.5723	0	4.980587447	4.980587447	-20	0.0066
INHBB	0	30.5723	0	4.980587447	4.980587447	-20	0.0066
LOC102158359	0	30.5723	0	4.980587447	4.980587447	-20	0.0066
MIR145	0	30.5723	0	4.980587447	4.980587447	-20	0.0066
LOC100620819	337.2975486	31.398578	8.402148913	5.017858594	5.017858594	3.425251	0.00662
NPM2	169.1774539	14.873011	7.410896102	3.98850389	3.98850389	3.507769	0.00664
LOC100736869	817.3385739	76.843889	9.676554047	6.282511874	6.282511874	3.410931	0.00672
GPR126	161.7759402	2179.7223	7.346743662	11.09059038	11.09059038	-3.75208	0.00676
C5H12orf35	12281.22579	1036.9794	13.58428441	10.01956204	10.01956204	3.565995	0.0068
ART5	63.44154519	4.1313919	6.009919183	2.359350201	2.359350201	3.940728	0.0068
ABCD1	128.9978086	1718.659	7.022343493	10.74790681	10.74790681	-3.73586	0.0068
PPAP2B	287.6016715	3982.6618	8.172935846	11.95987944	11.95987944	-3.79159	0.00682
C7H15orf60	2302.928091	210.70098	11.16987997	7.72588417	7.72588417	3.4502	0.00686
FNDC5	4.229436346	80.149002	2.386655454	6.342501448	6.342501448	-4.24415	0.00686
STRIP2	411.3126847	38.835083	8.687595036	5.315967693	5.315967693	3.404803	0.00689
HRC	10.57359087	154.51406	3.532764645	7.280901168	7.280901168	-3.8692	0.00694
EFHD1	2.114718173	55.360651	1.639101631	5.816616368	5.816616368	-4.71032	0.00695
LOC102162112	1.057359087	42.966475	1.04079362	5.458331975	5.458331975	-5.34467	0.00695
NUP54	10013.19055	860.15578	13.28975819	9.750130438	9.750130438	3.54116	0.00706
G2E3	3876.278411	348.68947	11.92082862	8.449930555	8.449930555	3.474657	0.00707
LOC102162556	54.9826725	3.3051135	5.806908455	2.106051269	2.106051269	4.056205	0.0071
CCDC114	22.20454082	294.1551	4.536335244	8.205329462	8.205329462	-3.72765	0.00711
LOC100622845	118.4242177	9.9153405	6.899951616	3.448285225	3.448285225	3.578158	0.00712
SKAP2	27.49133625	358.60481	4.832451381	8.490268521	8.490268521	-3.70535	0.00719
TIMP1	687.2834063	9904.5988	9.426858918	13.27402848	13.27402848	-3.84912	0.0072
LOC100037967	1606.128453	150.38266	10.65026953	7.242056187	7.242056187	3.416877	0.00722
LOC100628067	56.04003159	720.51474	5.833902876	9.494885058	9.494885058	-3.6845	0.00723
ADCY4	3.17207726	66.928548	2.060765874	6.085946114	6.085946114	-4.39912	0.00726
HSP70.2	750.7249515	10857.298	9.554061081	13.40651034	13.40651034	-3.85424	0.00726
EPHA2	340.4696259	4693.2612	8.415613445	12.19668239	12.19668239	-3.78499	0.00726
UBASH3B	2914.081643	266.88791	11.50932057	8.065485684	8.065485684	3.448735	0.00727
DAPP1	223.1027673	20.656959	7.808016653	4.436758793	4.436758793	3.433009	0.00729
KCNN4	0	29.746021	0	4.942327829	4.942327829	-20	0.00736
LOC100152800	0	29.746021	0	4.942327829	4.942327829	-20	0.00736
LOC102160261	0	29.746021	0	4.942327829	4.942327829	-20	0.00736
LOC102165740	0	29.746021	0	4.942327829	4.942327829	-20	0.00736
UNCX	0	29.746021	0	4.942327829	4.942327829	-20	0.00736
CSMD2	0	29.746021	0	4.942327829	4.942327829	-20	0.00736
LOC102159364	30.66341351	0.8262784	4.98474489	0.868906686	0.868906686	5.213747	0.00743
GDF3	22.20454082	0	4.536335244	0	0	20	0.00745
LOC100626482	22.20454082	0	4.536335244	0	0	20	0.00745
LOC100519009	22.20454082	0	4.536335244	0	0	20	0.00745
LOC100620123	4.229436346	78.496445	2.386655454	6.312818447	6.312818447	-4.21409	0.00748
LOC102161798	514.9338752	49.576702	9.011042364	5.660401069	5.660401069	3.376653	0.00749
LOC100626354	1.057359087	42.140197	1.04079362	5.430960858	5.430960858	-5.31666	0.00752
LOC100520855	1.057359087	42.140197	1.04079362	5.430960858	5.430960858	-5.31666	0.00752
LOC102166568	1.057359087	42.140197	1.04079362	5.430960858	5.430960858	-5.31666	0.00752
RAVER2	841.6578329	80.97528	9.718803123	6.357117027	6.357117027	3.37768	0.0076
PROCR	21.14718173	276.80325	4.46905122	8.117919691	8.117919691	-3.71032	0.0076
KLF2	10.57359087	151.20894	3.532764645	7.249909307	7.249909307	-3.83801	0.00761
RNF144B	6118.937034	546.17	12.57930109	9.095845332	9.095845332	3.485859	0.00763
PEX11G	9.516231779	138.81477	3.394545939	7.127372928	7.127372928	-3.86663	0.00763
ACER3	4772.918917	431.31731	12.22095835	8.755946793	8.755946793	3.46805	0.00765
LOC100523056	259.0529762	3474.5006	8.022661739	11.76300506	11.76300506	-3.74549	0.00766
AFAP1L1	5.286795433	90.064343	2.65232482	6.508814353	6.508814353	-4.09049	0.00767
LOC100625094	93.04759962	7.4365053	6.555319217	3.076645515	3.076645515	3.645272	0.00768
LOC100623777	289.7163897	3903.339	8.183468598	11.93086262	11.93086262	-3.752	0.00772
LOC100154430	630.1860156	8889.929	9.301921431	13.11811846	13.11811846	-3.81832	0.00772
CNNM1	4980.161298	450.32171	12.28226641	8.818012376	8.818012376	3.467165	0.00776
IFIT2	341.526985	4632.1166	8.420073841	12.17776726	12.17776726	-3.7616	0.00781
CCNE2	1277.289777	122.2892	10.3199992	6.945902605	6.945902605	3.384717	0.00782
LOC100739584	4.229436346	77.670167	2.386655454	6.29774474	6.29774474	-4.19882	0.00782
TFAM	4473.686295	407.35524	12.12757083	8.673680921	8.673680921	3.457105	0.00784
MITF	2.114718173	53.708094	1.639101631	5.773682393	5.773682393	-4.6666	0.00787
PTK7	2569.38258	39515.937	11.32776739	15.2701835	15.2701835	-3.94294	0.00788
LINGO1	123.7110131	10.741619	6.962445064	3.553559423	3.553559423	3.525691	0.00794
PPIF	2409.721358	226.40027	11.23524919	7.829090181	7.829090181	3.411919	0.008
LOC100737177	159.6612221	2069.001	7.327877945	11.01541578	11.01541578	-3.69585	0.008
DBH	38.06492712	1.6525567	5.287802017	1.407383614	1.407383614	4.525691	0.00804
ASB4	38.06492712	1.6525567	5.287802017	1.407383614	1.407383614	4.525691	0.00804
CCDC146	38.06492712	1.6525567	5.287802017	1.407383614	1.407383614	4.525691	0.00804
LOC100153710	38.06492712	1.6525567	5.287802017	1.407383614	1.407383614	4.525691	0.00804
LOC102161288	432.4598664	5902.9327	8.759754612	12.52746056	12.52746056	-3.77079	0.00807
LOC100519871	131.1125267	11.567897	7.045623457	3.651671381	3.651671381	3.502607	0.00808
TRIM3	171.292172	2218.5574	7.428713345	11.11605632	11.11605632	-3.69509	0.00814
LOC102167735	115.2521404	9.9153405	6.861113469	3.448285225	3.448285225	3.538987	0.00814
HSD17B13	1.057359087	41.313919	1.04079362	5.403060391	5.403060391	-5.28809	0.00815
TRPV4	1.057359087	41.313919	1.04079362	5.403060391	5.403060391	-5.28809	0.00815
LOC102159125	0	28.919743	0	4.903025878	4.903025878	-20	0.00823
ZNF536	0	28.919743	0	4.903025878	4.903025878	-20	0.00823
CCL8	0	28.919743	0	4.903025878	4.903025878	-20	0.00823
LOC102163138	0	28.919743	0	4.903025878	4.903025878	-20	0.00823
MMP15	44.40908164	553.60651	5.504908955	9.115320738	9.115320738	-3.63993	0.00826
LOC102159034	5.286795433	88.411786	2.65232482	6.482393107	6.482393107	-4.06377	0.00828
FMO5	5.286795433	88.411786	2.65232482	6.482393107	6.482393107	-4.06377	0.00828
ATG9B	487.4425389	47.924146	8.932045041	5.61247475	5.61247475	3.346408	0.00834
LOC100621534	45.46644072	2.4788351	5.538117235	1.798604302	1.798604302	4.197068	0.00836
NRGN	45.46644072	2.4788351	5.538117235	1.798604302	1.798604302	4.197068	0.00836
LOC102165005	45.46644072	2.4788351	5.538117235	1.798604302	1.798604302	4.197068	0.00836
LOC100623841	3516.776322	328.03251	11.78044804	8.362086342	8.362086342	3.422343	0.00841
PNPLA2	302.4046988	3995.0559	8.245099618	11.96436106	11.96436106	-3.72366	0.0085
CDKN2B	64.49890428	799.83746	6.033398867	9.645365656	9.645365656	-3.63236	0.00851
LRRN3	435.6319437	42.966475	8.77027387	5.458331975	5.458331975	3.341826	0.00853
LOC102164131	280.2001579	27.267186	8.135453595	4.821056378	4.821056378	3.36122	0.00855
LOC100739477	2313.501681	220.61633	11.1764859	7.79192035	7.79192035	3.390467	0.00859
PLOD1	1172.611227	16785.019	10.19673886	14.03497248	14.03497248	-3.83938	0.00863
LOC100626165	1148.291968	112.37386	10.16652963	6.824944216	6.824944216	3.353111	0.00863
LAMB2	763.4132605	10629.245	9.578208995	13.37588722	13.37588722	-3.79943	0.00864
WASF1	956.9099733	94.195734	9.903746264	6.572825024	6.572825024	3.34465	0.00869
DDX18	28491.59795	2406.9489	14.79829955	11.23358906	11.23358906	3.565259	0.00871
tRNA-Lys	12005.25507	1060.9414	13.5514986	10.05248848	10.05248848	3.500249	0.00871
CSF3R	29.60605442	0.8262784	4.935745167	0.868906686	0.868906686	5.163121	0.00873
LOC100738517	29.60605442	0.8262784	4.935745167	0.868906686	0.868906686	5.163121	0.00873
LOC102166078	29.60605442	0.8262784	4.935745167	0.868906686	0.868906686	5.163121	0.00873
PTPRM	12.68830904	170.21334	3.774872332	7.419651341	7.419651341	-3.74577	0.00876
TMEM26	8.458872693	122.2892	3.241668254	6.945902605	6.945902605	-3.85369	0.00882
LOC102159522	1.057359087	40.48764	1.04079362	5.374609695	5.374609695	-5.25894	0.00884
LOC102166786	1.057359087	40.48764	1.04079362	5.374609695	5.374609695	-5.25894	0.00884
MYH2	1.057359087	40.48764	1.04079362	5.374609695	5.374609695	-5.25894	0.00884
GSDMD	238.9631536	3087.8023	7.906669086	11.59283181	11.59283181	-3.69172	0.00885
FLRT2	425.0583528	5674.0536	8.734907225	12.4704183	12.4704183	-3.73865	0.00886
IFFO2	23.2618999	291.67627	4.600620625	8.193161944	8.193161944	-3.64833	0.00887
DUOX2	75.07249515	5.7839486	6.249303021	2.76212524	2.76212524	3.698158	0.00892
LOC102164273	19.03246356	241.27328	4.324267947	7.920491517	7.920491517	-3.66413	0.00905
NMB	151.2023494	14.046732	7.249846818	3.911378308	3.911378308	3.428174	0.00906
PKDCC	402.853812	5333.6269	8.657689347	12.38117166	12.38117166	-3.72679	0.00906
NHSL1	2818.919325	269.36675	11.46143817	8.078773924	8.078773924	3.387499	0.00909
TRIM47	187.1525583	2372.2452	7.555759096	11.21264545	11.21264545	-3.66397	0.00911
APBB1IP	72.95777697	892.38064	6.208629956	9.803131183	9.803131183	-3.61253	0.00912
MPP7	158.603863	14.873011	7.31835176	3.98850389	3.98850389	3.414659	0.00916
ETNPPL	37.00756803	1.6525567	5.24821481	1.407383614	1.407383614	4.485049	0.00917
LOC100624854	185.0378402	2339.1941	7.539452286	11.19241246	11.19241246	-3.66012	0.00919
FHL2	584.7195749	7891.7847	9.1940663	12.94631869	12.94631869	-3.75453	0.0092
LOC100516062	0	28.093465	0	4.862623207	4.862623207	-20	0.00922
LOC102162702	0	28.093465	0	4.862623207	4.862623207	-20	0.00922
ZNF215	0	28.093465	0	4.862623207	4.862623207	-20	0.00922
SPP1	114.1947813	1412.1097	6.84793155	10.46465779	10.46465779	-3.62828	0.00922
LOC100620998	44.40908164	539.55978	5.504908955	9.078310354	9.078310354	-3.60286	0.00923
LOC102161687	21.14718173	0	4.46905122	0	0	20	0.00926
LOC102157412	21.14718173	0	4.46905122	0	0	20	0.00926
LOC102163618	21.14718173	0	4.46905122	0	0	20	0.00926
MYO3A	21.14718173	0	4.46905122	0	0	20	0.00926
SHE	21.14718173	0	4.46905122	0	0	20	0.00926
GP91-PHOX	21.14718173	0	4.46905122	0	0	20	0.00926
LOC102167163	21.14718173	0	4.46905122	0	0	20	0.00926
LOC100620631	21.14718173	0	4.46905122	0	0	20	0.00926
ITGB4	102.5638314	1260.9008	6.694376434	10.30138278	10.30138278	-3.61986	0.0093
CHPF2	510.7044388	6815.144	8.999166938	12.7347401	12.7347401	-3.73818	0.00932
RHOB	138.5140403	1721.9641	7.124266508	10.75067695	10.75067695	-3.63595	0.00933
GPAT	8533.945188	777.52795	13.05916617	9.60460502	9.60460502	3.456246	0.00936
STAT2	255.880899	3272.0624	8.004955809	11.67642537	11.67642537	-3.67666	0.00941
LOC102163118	51.81059524	3.3051135	5.722755497	2.106051269	2.106051269	3.970475	0.00943
LOC100738582	3.17207726	62.797156	2.060765874	5.995420212	5.995420212	-4.3072	0.00943
THBD	3.17207726	62.797156	2.060765874	5.995420212	5.995420212	-4.3072	0.00943
LOC100153054	3.17207726	62.797156	2.060765874	5.995420212	5.995420212	-4.3072	0.00943
LOC102157994	59.21210885	4.1313919	5.911981742	2.359350201	2.359350201	3.841192	0.00948
LOC102160252	59.21210885	4.1313919	5.911981742	2.359350201	2.359350201	3.841192	0.00948
LOC102163264	59.21210885	4.1313919	5.911981742	2.359350201	2.359350201	3.841192	0.00948
ERAP1	322.4945214	4171.0532	8.337597469	12.02654185	12.02654185	-3.69307	0.00948
NPAS1	1289.978086	128.07315	10.3342488	7.012045083	7.012045083	3.332307	0.00949
GJA5	2.114718173	51.229259	1.639101631	5.706786332	5.706786332	-4.59843	0.00953
LOC102165035	2.114718173	51.229259	1.639101631	5.706786332	5.706786332	-4.59843	0.00953
SLC18B1	2329.362068	226.40027	11.18633841	7.829090181	7.829090181	3.362987	0.00953
LOC102159592	1.057359087	39.661362	1.04079362	5.34558663	5.34558663	-5.2292	0.0096
FBXO41	498.0161298	50.402981	8.962942639	5.683780113	5.683780113	3.304612	0.00964
DARS2	3160.44631	304.07044	11.626369	8.252998589	8.252998589	3.377651	0.0097
LOC100625965	5.286795433	85.106672	2.65232482	6.428053129	6.428053129	-4.00881	0.0097
HLF	103.6211905	9.0890621	6.709031281	3.334720158	3.334720158	3.511044	0.00973
LOC100620831	19.03246356	237.14189	4.324267947	7.895677624	7.895677624	-3.63922	0.00973
LOC100627685	70.8430588	852.71928	6.166776871	9.73761795	9.73761795	-3.58937	0.00975
NUS1	24657.6139	2146.6712	14.58980409	11.06855743	11.06855743	3.52186	0.00987
TRIB3	284.4295943	3618.273	8.156991116	11.82148421	11.82148421	-3.66916	0.00988
GPX3	70.8430588	849.41417	6.166776871	9.732021819	9.732021819	-3.58377	0.00991
PPP1R1B	24.31925899	295.80766	4.662163277	8.213384501	8.213384501	-3.60449	0.00997
LOC100516621	914.6156099	92.543178	9.838598249	6.547560532	6.547560532	3.304967	0.00997
LOC100624264	6.344154519	95.848291	2.876596413	6.597654688	6.597654688	-3.91725	0.00999
B3GNTL1	90.93288145	1093.9926	6.522509054	10.09670536	10.09670536	-3.58866	0.01004
ZNF793	8.458872693	118.15781	3.241668254	6.896729669	6.896729669	-3.80411	0.01017
LIMCH1	2326.18999	228.87911	11.18437328	7.844731552	7.844731552	3.345311	0.01018
CEACAM1	2.114718173	50.402981	1.639101631	5.683780113	5.683780113	-4.57497	0.01018
LOC100513188	2.114718173	50.402981	1.639101631	5.683780113	5.683780113	-4.57497	0.01018
FBLIM1	541.3678523	7099.3838	9.083127858	12.79368129	12.79368129	-3.71301	0.01023
LOC102167091	340.4696259	34.703692	8.415613445	5.158001345	5.158001345	3.294365	0.01028
LOC102167744	6.344154519	95.022013	2.876596413	6.585293272	6.585293272	-3.90476	0.01035
LOC102161240	0	27.267186	0	4.821056378	4.821056378	-20	0.01035
TM4SF18	0	27.267186	0	4.821056378	4.821056378	-20	0.01035
LOC100522814	0	27.267186	0	4.821056378	4.821056378	-20	0.01035
LOC404701	0	27.267186	0	4.821056378	4.821056378	-20	0.01035
LOC102158724	0	27.267186	0	4.821056378	4.821056378	-20	0.01035
FAM26F	0	27.267186	0	4.821056378	4.821056378	-20	0.01035
LOC100737008	0	27.267186	0	4.821056378	4.821056378	-20	0.01035
LOC100620373	0	27.267186	0	4.821056378	4.821056378	-20	0.01035
GPRASP1	882.8948373	90.064343	9.787730923	6.508814353	6.508814353	3.293214	0.01036
LOC102161569	50.75323616	3.3051135	5.693577173	2.106051269	2.106051269	3.940728	0.01039
KCTD7	61.32682702	725.47241	5.961781364	9.504764199	9.504764199	-3.56433	0.01039
LOC100738277	159.6612221	1947.5381	7.327877945	10.92817644	10.92817644	-3.60857	0.01041
CCND3	84.58872693	1006.4071	6.419348884	9.976431027	9.976431027	-3.5726	0.01044
LOC100517092	154.3744266	14.873011	7.279605257	3.98850389	3.98850389	3.375665	0.01046
LOC100521440	159.6612221	1945.0593	7.327877945	10.92633995	10.92633995	-3.60673	0.01047
ACE2	35.95020894	1.6525567	5.207510617	1.407383614	1.407383614	4.443228	0.01049
LOC100623751	161.7759402	15.699289	7.346743662	4.061714779	4.061714779	3.365226	0.0105
LOC102161128	5.286795433	83.434116	2.65232482	6.400095824	6.400095824	-3.98052	0.01051
LOC100518417	3.17207726	61.1446	2.060765874	5.957557118	5.957557118	-4.26873	0.01051
LOC100625759	12.68830904	162.77684	3.774872332	7.35558754	7.35558754	-3.68132	0.01056
RPF2	6088.27362	576.7423	12.57205443	9.174282325	9.174282325	3.400034	0.01056
tRNA-Arg	4123.700484	398.26618	12.01007363	8.641207045	8.641207045	3.372135	0.01058
REC8	8906.135586	827.93093	13.12074584	9.695108082	9.695108082	3.427217	0.01058
KCTD17	124.7683722	1498.869	6.974625354	10.55062075	10.55062075	-3.58655	0.01061
KCTD11	100.4491132	1196.4511	6.664612444	10.22575101	10.22575101	-3.57422	0.01063
LOC100521530	108.9079859	1300.5622	6.780152406	10.3460285	10.3460285	-3.57795	0.01064
ITGA9	1976.204133	197.48053	10.94924611	7.632853689	7.632853689	3.32295	0.01066
ANO1	28.54869534	338.77413	4.885022527	8.408432212	8.408432212	-3.56883	0.01068
PALM2	9.516231779	128.07315	3.394545939	7.012045083	7.012045083	-3.75043	0.01069
PDGFA	96.21967688	1142.743	6.603176434	10.15954718	10.15954718	-3.57003	0.0107
XYLT1	19.03246356	231.35794	4.324267947	7.86020516	7.86020516	-3.60359	0.0108
AMMECR1	2325.132631	231.35794	11.18371764	7.86020516	7.86020516	3.329114	0.0108
LOC100738786	2.114718173	49.576702	1.639101631	5.660401069	5.660401069	-4.55112	0.01088
ABAT	79.30193149	6.610227	6.327362784	2.927939483	2.927939483	3.584584	0.01088
LOC102163221	10.57359087	138.81477	3.532764645	7.127372928	7.127372928	-3.71462	0.01089
LOC100518021	31.7207726	372.65155	5.032134908	8.54554968	8.54554968	-3.55433	0.01095
COL19A1	71.90041789	5.7839486	6.187855179	2.76212524	2.76212524	3.635874	0.01097
PDE8A	71.90041789	841.97766	6.187855179	9.71935059	9.71935059	-3.54971	0.01098
MEGF6	35.95020894	420.57569	5.207510617	8.719647871	8.719647871	-3.54829	0.01098
E4F1	124.7683722	1483.996	6.974625354	10.53624329	10.53624329	-3.57216	0.01108
LOC100518848	281.257517	28.919743	8.140868193	4.903025878	4.903025878	3.281765	0.01109
EPB41L4A	1059.473805	109.06875	10.05049327	6.782261052	6.782261052	3.280038	0.01112
PLCD3	145.9155539	1742.6211	7.198843332	10.76787084	10.76787084	-3.57805	0.0112
SEPP1	7073.732289	672.59059	12.78845984	9.395728184	9.395728184	3.394671	0.01123
FGF11	279.1427989	3438.1443	8.130018598	11.74783394	11.74783394	-3.62255	0.01132
LOC100623959	1.057359087	38.008805	1.04079362	5.285727902	5.285727902	-5.1678	0.01136
FOXP2	1.057359087	38.008805	1.04079362	5.285727902	5.285727902	-5.1678	0.01136
LOC102167165	1.057359087	38.008805	1.04079362	5.285727902	5.285727902	-5.1678	0.01136
LAD1	1.057359087	38.008805	1.04079362	5.285727902	5.285727902	-5.1678	0.01136
LDLRAP1	284.4295943	3502.594	8.156991116	11.7746199	11.7746199	-3.62228	0.01138
SLCO2B1	5.286795433	81.801559	2.65232482	6.371586023	6.371586023	-3.95166	0.01141

LOC102167243	99.4714588	8.2627837	6.65064192	3.211445828	3.211445828	3.589583	0.00034
ZNF500	14.09179	149.55639	3.915692025	7.234160086	7.234160086	-3.40776	0.00034
PDZK1IP1	0	26.440908	0	4.77825631	4.77825631	-20	0.00034
LOC102160410	0	26.440908	0	4.77825631	4.77825631	-20	0.00034
BCL2L10	42.27537	1.6525567	5.435474249	1.407383613	1.407383613	4.677046	0.00034
C6H19orf68	20.7232206	206.56959	4.441166102	7.697451307	7.697451307	-3.31731	0.00034
LOC100739439	9.94714588	114.02642	3.452482877	6.845821397	6.845821397	-3.51894	0.00034
DEXI	95.3268147	870.07113	6.589865555	9.766646714	9.766646714	-3.19018	0.00035
LOC102159735	3.31571529	57.013208	2.109599694	5.858309486	5.858309486	-4.1039	0.00035
PLCH1	894.414201	98.327126	9.806411388	6.634115866	6.634115866	3.185282	0.00035
TMEM54	67.9721635	622.18761	6.107942317	9.283522749	9.283522749	-3.19433	0.00035
LOC100621139	1271.57682	12318.984	10.31353703	13.58871279	13.58871279	-3.27619	0.00035
LOC100525418	204.745419	20.656959	7.684716501	4.436758793	4.436758793	3.309131	0.00036
CMTM8	304.216878	32.224857	8.253690934	5.054191063	5.054191063	3.238854	0.00036
LOC100624745	397.885835	42.966475	8.639832083	5.458331975	5.458331975	3.211071	0.00036
LOC100156854	1213.5518	133.8571	10.2462083	7.075287628	7.075287628	3.18047	0.00036
LOC100157320	31.4992953	298.28649	5.022336531	8.225383356	8.225383356	-3.24331	0.00036
CKAP4	1473.83545	14307.01	10.52633828	13.80453541	13.80453541	-3.27907	0.00036
LOC102164934	19.0653629	190.8703	4.326635347	7.58398763	7.58398763	-3.32357	0.00036
INSR	23.2100071	226.40027	4.597531595	7.829090182	7.829090182	-3.28606	0.00037
FLVCR1	993.88566	109.89502	9.958386918	6.793050813	6.793050813	3.176954	0.00037
DUOXA2	84.55074	6.610227	6.418708429	2.927939483	2.927939483	3.677046	0.00037
LOC100513717	56.36716	3.3051135	5.842153193	2.106051269	2.106051269	4.092083	0.00037
LOC100738475	22.3810782	218.96377	4.547269558	7.781122098	7.781122098	-3.29034	0.00037
SALL1	814.008105	90.064343	9.670670596	6.508814353	6.508814353	3.176015	0.00037
ONECUT2	70.45895	4.9576702	6.159042811	2.574748269	2.574748269	3.829049	0.00037
LOC100623430	12.4339324	133.8571	3.747809764	7.075287628	7.075287628	-3.42834	0.00037
ZKSCAN7	5.80250177	77.670167	2.766065426	6.29774474	6.29774474	-3.74261	0.00038
LOC100152206	281.006871	29.746021	8.139586505	4.942327829	4.942327829	3.239837	0.00038
CLEC11A	105.273961	949.39385	6.731644339	9.892381689	9.892381689	-3.17286	0.00038
SLC23A2	825.613108	91.716899	9.691068432	6.534760414	6.534760414	3.170206	0.00038
ANXA5	4985.17795	50369.103	12.28371865	15.62028007	15.62028007	-3.33682	0.00038
FAM46A	295.92759	2706.0617	8.213967342	11.40251204	11.40251204	-3.19288	0.00039
LOC100516098	344.005462	37.182527	8.430475391	5.254840671	5.254840671	3.209735	0.00039
ARHGAP18	4821.05004	520.55537	12.23543091	9.026676623	9.026676623	3.211224	0.00039
MDM4	8415.28542	892.38064	13.03896792	9.803131183	9.803131183	3.237281	0.00039
P4HA1	926.742425	8770.1186	9.857580506	13.09854514	13.09854514	-3.24236	0.00039
IQCA1	33.9860818	0.8262784	5.128709195	0.868906686	0.868906686	5.362172	0.00039
LOC100737062	2184.22745	241.27328	11.09356774	7.920491517	7.920491517	3.178383	0.00039
CREG2	41.4464412	1.6525567	5.407571694	1.407383613	1.407383613	4.648477	0.0004
LOC396679	1057.71318	118.15781	10.04809608	6.89672967	6.89672967	3.162162	0.0004
LOC102158578	125.168252	11.567897	6.979205122	3.65167138	3.65167138	3.43567	0.0004
BTG4	257.796864	27.267186	8.015676326	4.821056379	4.821056379	3.240997	0.0004
SEMA6A	85.3796688	765.96005	6.43261988	9.583007622	9.583007622	-3.1653	0.0004
SPATS2L	428.556202	3938.869	8.746703091	11.94393195	11.94393195	-3.20023	0.0004
SLC22A4	69.6300212	4.9576702	6.142209624	2.574748269	2.574748269	3.811975	0.00041
CCRN4L	2031.70455	225.574	10.98918482	7.823838478	7.823838478	3.171018	0.00041
SH3BGRL2	951.61029	106.58991	9.895742322	6.749398975	6.749398975	3.1583	0.00041
LOC100628123	25.6967935	0	4.73859457	0	0	20	0.00041
LOC100522352	25.6967935	0	4.73859457	0	0	20	0.00041
LOC102164667	25.6967935	0	4.73859457	0	0	20	0.00041
ELOVL3	25.6967935	0	4.73859457	0	0	20	0.00041
ZBTB32	25.6967935	0	4.73859457	0	0	20	0.00041
FOXB2	25.6967935	0	4.73859457	0	0	20	0.00041
LOC102160165	25.6967935	0	4.73859457	0	0	20	0.00041
IKZF3	25.6967935	0	4.73859457	0	0	20	0.00041
CRMP1	547.093024	5056.8236	9.098276961	12.30430102	12.30430102	-3.20837	0.00041
C14H10orf2	5397.9845	584.17881	12.39847236	9.192733716	9.192733716	3.207939	0.00041
PDLIM1	927.571354	8727.1522	9.858868964	13.09146054	13.09146054	-3.23398	0.00041
LOC102167245	0.82892882	33.877413	0.870998931	5.124221138	5.124221138	-5.35293	0.00041
CLDN23	55.5382312	3.3051135	5.821154844	2.106051269	2.106051269	4.07071	0.00041
LOC100739178	89.524313	799.83746	6.500233417	9.645365656	9.645365656	-3.15936	0.00041
LOC100515261	312.506167	33.877413	8.29235001	5.124221138	5.124221138	3.205489	0.00042
PPP1R3G	186.508985	19.004403	7.55081592	4.322245637	4.322245637	3.29484	0.00042
LOC100521355	6085.99542	658.54386	12.57151457	9.365324797	9.365324797	3.208142	0.00042
MSH5	227.955427	23.962073	7.838922949	4.641665831	4.641665831	3.249927	0.00042
MCM10	4434.76921	485.0254	12.11496859	8.924887914	8.924887914	3.192727	0.00042
LOC102157470	0	25.61463	0	4.734147579	4.734147579	-20	0.00043
LOC102160901	0	25.61463	0	4.734147579	4.734147579	-20	0.00043
TRPA1	0	25.61463	0	4.734147579	4.734147579	-20	0.00043
LOC100623327	0	25.61463	0	4.734147579	4.734147579	-20	0.00043
LOC100517057	0	25.61463	0	4.734147579	4.734147579	-20	0.00043
LOC100621406	2262.14676	252.0149	11.14411443	7.983078557	7.983078557	3.166112	0.00043
CBFA2T3	503.159796	56.186929	8.977737265	5.837613535	5.837613535	3.16271	0.00043
LOC100739750	98.64253	875.85507	6.638689748	9.776194605	9.776194605	-3.15041	0.00043
LOC102159434	34.8150106	319.76973	5.162492464	8.325394195	8.325394195	-3.19925	0.00043
S1PR2	189.824701	1693.8707	7.576104118	10.72695947	10.72695947	-3.15758	0.00043
FRMD7	3.31571529	55.360651	2.109599694	5.816616368	5.816616368	-4.06147	0.00043
LOC100157806	3.31571529	55.360651	2.109599694	5.816616368	5.816616368	-4.06147	0.00043
LOC102161634	310.848309	33.877413	8.284700625	5.124221138	5.124221138	3.197815	0.00044
ROBO1	1019.58245	9523.6845	9.995177027	13.21745559	13.21745559	-3.22354	0.00045
AGRN	205.574348	1829.3803	7.690517306	10.83792773	10.83792773	-3.15362	0.00045
RIN3	114.392178	1010.5384	6.850401618	9.982335443	9.982335443	-3.14306	0.00045
SLC24A5	1631.33193	184.26008	10.67271874	7.533408207	7.533408207	3.146235	0.00045
PION	38.1307259	346.21064	5.290229968	8.439667339	8.439667339	-3.18262	0.00045
LOC100524931	599.31554	67.754826	9.229577203	6.103389087	6.103389087	3.14492	0.00045
LOC102163597	33.9860818	310.68067	5.128709195	8.283924863	8.283924863	-3.19242	0.00046
LOC102166841	40.6175124	1.6525567	5.379118827	1.407383613	1.407383613	4.61933	0.00046
LOC102166076	364.728682	40.48764	8.514629965	5.374609695	5.374609695	3.17127	0.00046
LOC100155273	1066.8314	121.46292	10.06046816	6.936201185	6.936201185	3.134744	0.00046
LOC102157456	33.1571529	0.8262784	5.094115824	0.868906686	0.868906686	5.326548	0.00047
DGAT2	33.1571529	0.8262784	5.094115824	0.868906686	0.868906686	5.326548	0.00047
LOC100622678	8.28928824	95.848291	3.215568059	6.597654688	6.597654688	-3.53143	0.00047
LOC100624264	8.28928824	95.848291	3.215568059	6.597654688	6.597654688	-3.53143	0.00047
RASSF4	87.0375265	765.13377	6.460046706	9.581452508	9.581452508	-3.136	0.00047
HK2	5851.40857	642.84457	12.51481477	9.330568647	9.330568647	3.186242	0.00047
IRK1	15.7496477	156.99289	4.066058842	7.303715831	7.303715831	-3.31731	0.00048
REPS2	251.994362	27.267186	7.982961427	4.821056379	4.821056379	3.208154	0.00048
LOC100625071	101.958245	9.0890621	6.685915561	3.334720158	3.334720158	3.487703	0.00048
MATN2	101.958245	9.0890621	6.685915561	3.334720158	3.334720158	3.487703	0.00048
LOC100738418	67.9721635	4.9576702	6.107942317	2.574748269	2.574748269	3.77721	0.00049
IGSF3	1402.54757	160.298	10.45486225	7.333584776	7.333584776	3.129221	0.00049
OAZ3	16.5785765	162.77684	4.135746339	7.35558754	7.35558754	-3.2955	0.0005
BCL6	186.508985	1634.3786	7.55081592	10.67540897	10.67540897	-3.13143	0.0005
ADRB3	24.8678647	0	4.693089065	0	0	20	0.00051
LOC102159682	24.8678647	0	4.693089065	0	0	20	0.00051
LOC102164429	24.8678647	0	4.693089065	0	0	20	0.00051
LOC102157713	24.8678647	0	4.693089065	0	0	20	0.00051
LOC102166554	24.8678647	0	4.693089065	0	0	20	0.00051
LOC102158667	24.8678647	0	4.693089065	0	0	20	0.00051
LOC102167081	24.8678647	0	4.693089065	0	0	20	0.00051
ANKRD34B	24.8678647	0	4.693089065	0	0	20	0.00051
LOC100521071	24.8678647	0	4.693089065	0	0	20	0.00051
CXHXorf48	24.8678647	0	4.693089065	0	0	20	0.00051
GZMA	24.8678647	0	4.693089065	0	0	20	0.00051
LOC100620529	54.7093024	480.89401	5.799846344	8.912572065	8.912572065	-3.13586	0.00051
TNFRSF21	2763.6487	313.1595	11.43288045	8.29535341	8.29535341	3.141605	0.00051
VIM	49905.6598	533350.29	15.60694473	19.02472656	19.02472656	-3.41781	0.00051
PRDM16	14.9207188	148.73011	3.99283357	7.226220529	7.226220529	-3.31731	0.00051
LOC102160923	2.48678647	47.097867	1.801898017	5.587901017	5.587901017	-4.24331	0.00052
LOC102158175	2.48678647	47.097867	1.801898017	5.587901017	5.587901017	-4.24331	0.00052
LOC102168145	134.286469	13.220454	7.079873746	3.829895615	3.829895615	3.34447	0.00052
DPEP1	12.4339324	128.07315	3.747809764	7.012045083	7.012045083	-3.36461	0.00052
GAP43	2962.59162	336.2953	11.53313094	8.397868394	8.397868394	3.139059	0.00052
CDC6	1208.57823	139.64104	10.24028836	7.135873883	7.135873883	3.113516	0.00053
JOSD2	221.323996	1932.6651	7.79651986	10.91712224	10.91712224	-3.12636	0.00053
LOC100622303	0	24.788351	0	4.688647628	4.688647628	-20	0.00053
PLA2G7	0	24.788351	0	4.688647628	4.688647628	-20	0.00053
LOC100511329	0	24.788351	0	4.688647628	4.688647628	-20	0.00053
LOC100737673	0	24.788351	0	4.688647628	4.688647628	-20	0.00053
PLOD1	1797.11769	16785.019	10.81227174	14.03497248	14.03497248	-3.22342	0.00053
LOC100736946	109.418605	946.91501	6.786839466	9.888613909	9.888613909	-3.11338	0.00053
SLC12A8	39.7885835	1.6525567	5.350093502	1.407383613	1.407383613	4.589583	0.00053
LOC102158629	214.692565	23.135794	7.752832639	4.593102408	4.593102408	3.214074	0.00053
LOC102159922	268.572939	29.746021	8.074531868	4.942327829	4.942327829	3.174545	0.00054
HRC	15.7496477	154.51406	4.066058842	7.280901168	7.280901168	-3.29435	0.00054
MDN1	28066.701	2960.5554	14.77662328	11.53213936	11.53213936	3.24492	0.00054
LOC102159647	1147.33149	133.03082	10.16520535	7.066420948	7.066420948	3.108332	0.00054
ARHGEF10L	126.82611	1095.6451	6.998038744	10.09888102	10.09888102	-3.11086	0.00054
KIFAP3	442.647992	3914.0806	8.793271628	11.93482631	11.93482631	-3.14444	0.00055
LOC102164483	4.14464412	60.318321	2.363071282	5.938246292	5.938246292	-3.86328	0.00055
NAGK	468.344785	4147.0911	8.87450432	12.01823188	12.01823188	-3.14646	0.00055
LOC102165406	1293.95789	150.38266	10.33868947	7.242056188	7.242056188	3.105081	0.00055
LOC100737877	1.65785765	39.661362	1.410263837	5.34558663	5.34558663	-4.58034	0.00056
LOC100523520	1448.13866	13321.26	10.50097992	13.70155121	13.70155121	-3.20146	0.00056
LOC100736842	300.901163	33.877413	8.237932504	5.124221138	5.124221138	3.150894	0.00056
CNNM1	3963.10871	450.32171	11.95278081	8.818012376	8.818012376	3.137605	0.00057
LOC100515962	47.248943	411.48663	5.592425431	8.688203544	8.688203544	-3.12249	0.00057
LOC100736655	53.0514447	3.3051135	5.756261274	2.106051269	2.106051269	4.00462	0.00057
PKLR	53.0514447	3.3051135	5.756261274	2.106051269	2.106051269	4.00462	0.00057
LOC102158761	92.8400282	8.2627837	6.552131542	3.211445828	3.211445828	3.490047	0.00058
LOC102167634	59.6828753	4.1313919	5.92321754	2.359350201	2.359350201	3.852617	0.00058
LOC100514309	0.82892882	32.224857	0.870998931	5.054191063	5.054191063	-5.28078	0.00058
LOC100522935	72.9457365	5.7839486	6.208395063	2.762125239	2.762125239	3.656697	0.00058
ADAMTS12	14.9207188	145.42499	3.99283357	7.194018019	7.194018019	-3.28489	0.00061
BOK	151.693975	1291.4731	7.254499324	10.33591853	10.33591853	-3.08978	0.00061
GRID1	4.97357294	66.10227	2.578594101	6.068289661	6.068289661	-3.73235	0.00061
FBXO43	38.9596547	1.6525567	5.320472212	1.407383613	1.407383613	4.559209	0.00061
SNX31	38.9596547	1.6525567	5.320472212	1.407383613	1.407383613	4.559209	0.00061
LOC102160627	38.9596547	1.6525567	5.320472212	1.407383613	1.407383613	4.559209	0.00061
CDH7	38.9596547	1.6525567	5.320472212	1.407383613	1.407383613	4.559209	0.00061
LOC100620361	38.9596547	1.6525567	5.320472212	1.407383613	1.407383613	4.559209	0.00061
LOC102164420	150.865046	15.699289	7.246646039	4.061714779	4.061714779	3.264487	0.00062
LOC102167649	150.865046	15.699289	7.246646039	4.061714779	4.061714779	3.264487	0.00062
KCTD11	140.9179	1196.4511	7.148912757	10.22575101	10.22575101	-3.08583	0.00062
LOC102159415	24.0389359	0	4.646101346	0	0	20	0.00063
C17H20orf26	24.0389359	0	4.646101346	0	0	20	0.00063
AGXT2	24.0389359	0	4.646101346	0	0	20	0.00063
LOC102158664	24.0389359	0	4.646101346	0	0	20	0.00063
TMEM132D	24.0389359	0	4.646101346	0	0	20	0.00063
LOC100523842	58.8539465	501.55097	5.90337447	8.97312612	8.97312612	-3.09119	0.00064
LOC100738338	13.2628612	131.37826	3.834191515	7.048522415	7.048522415	-3.30826	0.00064
LOC100514384	58.8539465	4.1313919	5.90337447	2.359350201	2.359350201	3.832439	0.00064
STEAP4	1.65785765	38.835083	1.410263837	5.315967693	5.315967693	-4.54997	0.00064
LMNA	9298.09461	90316.357	13.18287454	16.46271565	16.46271565	-3.27998	0.00065
LOC100511614	143.404687	14.873011	7.173973754	3.98850389	3.98850389	3.269324	0.00065
TSTD3	2001.03418	235.48934	10.96725089	7.885631319	7.885631319	3.087012	0.00065
LOC102162310	97.8136012	9.0890621	6.62663773	3.334720158	3.334720158	3.427832	0.00065
ANO6	977.307083	8688.3171	9.934143577	13.08502708	13.08502708	-3.15219	0.00066
NKAPL	0	23.962073	0	4.641665831	4.641665831	-20	0.00066
LOC100738421	0	23.962073	0	4.641665831	4.641665831	-20	0.00066
SCML1	1981.96882	233.83678	10.95344628	7.875514565	7.875514565	3.08336	0.00066
LOC100516650	73.7746653	621.36134	6.224477643	9.281608625	9.281608625	-3.07424	0.00067
LOC100626629	4380.0599	506.50864	12.09706423	8.987288579	8.987288579	3.112292	0.00067
LOC102160331	4.97357294	65.275991	2.578594101	6.05041444	6.05041444	-3.7142	0.00067
LOC100627924	4.97357294	65.275991	2.578594101	6.05041444	6.05041444	-3.7142	0.00067
SQSTM1	4569.05568	42939.208	12.15799603	15.39004156	15.39004156	-3.23233	0.00068
LGALS3	1222.67002	10919.269	10.25699885	13.41472073	13.41472073	-3.15877	0.00068
SCP2	32.3282241	281.76092	5.058672541	8.143438955	8.143438955	-3.12361	0.00068
LOC100620963	47.248943	402.39757	5.592425431	8.656058572	8.656058572	-3.09027	0.00068
ICK	3968.08228	461.06333	11.95458975	8.851946794	8.851946794	3.105405	0.00068
LOC100738921	103.616103	9.9153405	6.708961125	3.448285225	3.448285225	3.385442	0.00069
KCNIP3	0.82892882	31.398578	0.870998931	5.017858594	5.017858594	-5.24331	0.00069
HCN1	0.82892882	31.398578	0.870998931	5.017858594	5.017858594	-5.24331	0.00069
HTR1B	138.431114	1159.2686	7.123408719	10.18024306	10.18024306	-3.06597	0.00069
IL-6	14.09179	136.33593	3.915692025	7.101565318	7.101565318	-3.27424	0.0007
FCHSD1	175.732911	1475.7332	7.465426908	10.52819346	10.52819346	-3.06998	0.0007
LOC102161722	8.28928824	90.890621	3.215568059	6.521845711	6.521845711	-3.45481	0.0007
RASSF10	64.6564482	4.9576702	6.036864803	2.574748269	2.574748269	3.70506	0.0007
CD70	3399.43711	398.26618	11.73150449	8.641207046	8.641207046	3.093491	0.0007
LOC102160423	148.378259	15.699289	7.222826382	4.061714779	4.061714779	3.240509	0.0007
BRSK1	12.4339324	123.11548	3.747809764	6.955539223	6.955539223	-3.30766	0.0007
LOC102161596	113.563249	947.74129	6.8400005	9.889870929	9.889870929	-3.061	0.00071
LOC102158163	1373.53506	164.4294	10.42472799	7.370071807	7.370071807	3.062354	0.00071
MFNG	141.746829	14.873011	7.157314885	3.98850389	3.98850389	3.252548	0.00071
TLX1	58.0250177	4.1313919	5.883254663	2.359350201	2.359350201	3.811975	0.00071
LOC102158355	58.0250177	4.1313919	5.883254663	2.359350201	2.359350201	3.811975	0.00071
LDB3	16.5785765	155.34033	4.135746339	7.288546214	7.288546214	-3.22804	0.00071
LOC102162964	38.1307259	1.6525567	5.290229968	1.407383613	1.407383613	4.528182	0.00071
LOC100627814	38.1307259	1.6525567	5.290229968	1.407383613	1.407383613	4.528182	0.00071
LOC100515857	38.1307259	1.6525567	5.290229968	1.407383613	1.407383613	4.528182	0.00071
LOC102163041	38.1307259	1.6525567	5.290229968	1.407383613	1.407383613	4.528182	0.00071
SYNPO2L	24.0389359	214.0061	4.646101346	7.74823377	7.74823377	-3.15421	0.00072
MGA	1099.15962	132.20454	10.10349714	7.057499439	7.057499439	3.055557	0.00072
COL11A2	51.3935871	3.3051135	5.711318333	2.106051269	2.106051269	3.958817	0.00072
ITGA5	2878.8698	26341.754	11.49178788	14.68511859	14.68511859	-3.19378	0.00072
LOC102157793	44.7621565	2.4788351	5.516083133	1.798604302	1.798604302	4.174545	0.00072
LOC102166014	9.94714588	103.2848	3.452482877	6.704385035	6.704385035	-3.3762	0.00073
LOC100155159	5.80250177	71.05994	2.766065426	6.171125545	6.171125545	-3.61429	0.00073
CCDC92	34.8150106	299.11277	5.162492464	8.229360901	8.229360901	-3.10291	0.00073
ADAM11	1242.56431	149.55639	10.2802654	7.234160086	7.234160086	3.054559	0.00073
TMEM150C	959.070649	115.67897	9.906996763	6.86640077	6.86640077	3.05151	0.00073
NPR2	668.116632	5780.6435	9.386113895	12.49726394	12.49726394	-3.11306	0.00073
TNFRSF1A	678.063778	5869.0553	9.407403269	12.51915837	12.51915837	-3.11364	0.00073
SCAI	496.528365	59.492043	8.958634969	5.918673475	5.918673475	3.061108	0.00073
LAMA1	121.852537	12.394176	6.940783842	3.74353388	3.74353388	3.297402	0.00074
LOC100738036	536.316949	64.449713	9.069629536	6.032314961	6.032314961	3.05684	0.00074
LOC100515685	16.5785765	154.51406	4.135746339	7.280901168	7.280901168	-3.22035	0.00074
OLFML2A	33.9860818	291.67627	5.128709195	8.193161944	8.193161944	-3.10135	0.00074
LOC102160753	4.14464412	57.839486	2.363071282	5.878712739	5.878712739	-3.80273	0.00075
LOC102168189	70.45895	5.7839486	6.159042811	2.762125239	2.762125239	3.606656	0.00075
ATP10D	1880.8395	225.574	10.87792787	7.823838478	7.823838478	3.059704	0.00075
COL16A1	512.278013	4378.4491	9.003596652	12.09653368	12.09653368	-3.09542	0.00075
EDA	1009.63531	122.2892	9.981046772	6.945902605	6.945902605	3.045465	0.00076
LOC100625680	63.8275194	4.9576702	6.018534465	2.574748269	2.574748269	3.686444	0.00077
LOC102161259	121.023608	12.394176	6.931016486	3.74353388	3.74353388	3.287554	0.00077
PLXND1	901.87456	7831.4664	9.818381752	12.93525096	12.93525096	-3.11828	0.00077
AS3MT	140.088971	14.873011	7.140461408	3.98850389	3.98850389	3.235575	0.00078
PLXDC1	275.204369	32.224857	8.109592332	5.054191063	5.054191063	3.094258	0.00078
MB21D1	23.2100071	204.91704	4.597531595	7.685919384	7.685919384	-3.14222	0.00079
BRI3BP	672.261276	81.801559	9.395022677	6.371586023	6.371586023	3.038822	0.00079
SEMA3E	95.3268147	9.0890621	6.589865555	3.334720158	3.334720158	3.390679	0.00079
LOC102166276	112.73432	927.08433	6.829523851	9.858112096	9.858112096	-3.03977	0.00079
LRPPRC	42518.2462	4621.3749	15.3758284	12.17441857	12.17441857	3.201688	0.00079
LOC102161605	76.2614518	6.610227	6.271676881	2.927939483	2.927939483	3.528182	0.00079
MAST1	30.6703665	0.8262784	4.985061656	0.868906686	0.868906686	5.214074	0.00079
LOC102159645	23.2100071	0	4.597531595	0	0	20	0.0008
LOC102157403	23.2100071	0	4.597531595	0	0	20	0.0008
LOC102161139	23.2100071	0	4.597531595	0	0	20	0.0008
LOC100626203	23.2100071	0	4.597531595	0	0	20	0.0008
DRD1	23.2100071	0	4.597531595	0	0	20	0.0008
SERPINE3	23.2100071	0	4.597531595	0	0	20	0.0008
LOC100621043	23.2100071	0	4.597531595	0	0	20	0.0008
LOC102165901	23.2100071	0	4.597531595	0	0	20	0.0008
LOC100521617	23.2100071	0	4.597531595	0	0	20	0.0008
LOC102165269	23.2100071	0	4.597531595	0	0	20	0.0008
HPRT1	6447.40839	751.91332	12.6547274	9.556339969	9.556339969	3.100081	0.0008
LOC102164161	385.451903	46.271589	8.59414506	5.56290145	5.56290145	3.058352	0.0008
FAM169A	1117.39605	136.33593	10.12721546	7.101565318	7.101565318	3.034903	0.00081
MFGE8	2056.57241	18321.07	11.00672749	14.16129491	14.16129491	-3.15519	0.00081
PAPP-A	321.624384	2684.5784	8.33371167	11.39101714	11.39101714	-3.06125	0.00081
LOC102161711	139.260042	14.873011	7.131960259	3.98850389	3.98850389	3.227013	0.00081
LOC100520391	33.1571529	281.76092	5.094115824	8.143438955	8.143438955	-3.08708	0.00081
PLCB4	602.631255	73.538775	9.237523697	6.219919206	6.219919206	3.034698	0.00082
NAGA	80.4060959	659.37014	6.347064926	9.367131078	9.367131078	-3.03571	0.00082
JAKMIP2	2221.52925	268.54047	11.11798669	8.074358096	8.074358096	3.048342	0.00083
LOC100737306	43.9332277	2.4788351	5.489710792	1.798604302	1.798604302	4.147578	0.00083
LOC100626333	1025.38496	125.59431	10.00335621	6.98406878	6.98406878	3.029323	0.00083
LOC100154674	260.283651	30.5723	8.029473043	4.980587446	4.980587446	3.089788	0.00083
LOC100626249	0	23.135794	0	4.593102408	4.593102408	-20	0.00083
LOC100624568	0	23.135794	0	4.593102408	4.593102408	-20	0.00083
MAP3K7CL	0	23.135794	0	4.593102408	4.593102408	-20	0.00083
LOC100522946	0	23.135794	0	4.593102408	4.593102408	-20	0.00083
LOC100623534	0	23.135794	0	4.593102408	4.593102408	-20	0.00083
LOC100627897	0.82892882	30.5723	0.870998931	4.980587446	4.980587446	-5.20483	0.00083
LOC100739315	37.3017971	1.6525567	5.259340178	1.407383613	1.407383613	4.496473	0.00083
PTCRA	164.127907	18.178124	7.36744015	4.261389712	4.261389712	3.174545	0.00083

CALN1	11.0464787	109.89502	3.590539588	6.793050813	6.793050813	-3.31447	0.02079
LOC100512171	114.607216	960.13547	6.853087646	9.908595977	9.908595977	-3.06654	0.0208
BCAN	85.6102098	11.567897	6.436465198	3.65167138	3.65167138	2.887656	0.02081
TEC	41.4242951	348.68947	5.406818783	8.449930555	8.449930555	-3.07339	0.02081
ARHGEF10	106.322357	889.07553	6.745806839	9.797783952	9.797783952	-3.06386	0.02082
MYOF	1586.5505	15124.199	10.63258677	13.88466653	13.88466653	-3.25289	0.02085
LOC100624393	2.76161967	47.097867	1.911353989	5.587901017	5.587901017	-4.09208	0.02087
LOC100524378	2.76161967	47.097867	1.911353989	5.587901017	5.587901017	-4.09208	0.02087
NTNG1	57.9940131	483.37285	5.882496648	8.919974184	8.919974184	-3.05916	0.02088
LOC100525578	24.854577	216.48493	4.692347799	7.764771649	7.764771649	-3.12268	0.02091
MAP4K5	37.2818656	314.81206	5.258589232	8.302922453	8.302922453	-3.07794	0.02099
LOC102162398	9.66566885	99.153405	3.414902536	6.646067655	6.646067655	-3.35872	0.02102
PTPN21	440.478338	3892.5974	8.786198839	11.926888	11.926888	-3.14359	0.02103
LOC102159206	1.38080984	36.356248	1.251452392	5.223277669	5.223277669	-4.71862	0.02105
CTSA	1637.64047	15585.263	10.67828363	13.92798741	13.92798741	-3.25049	0.02123
LOC100521307	955.520406	151.20894	9.901651937	7.249909307	7.249909307	2.659743	0.02123
NAP1L3	115.988026	967.57197	6.870217066	9.919715447	9.919715447	-3.06039	0.02126
LOC100625305	20.7121475	183.4338	4.440430524	7.526959252	7.526959252	-3.14671	0.02127
LOC100739516	8.28485901	88.411786	3.214880004	6.482393107	6.482393107	-3.41569	0.02135
FAM169A	858.863718	136.33593	9.747964211	7.101565318	7.101565318	2.655263	0.02139
OTUD1	996.944701	157.81917	9.962816064	7.311241242	7.311241242	2.659241	0.0214
TLN1	5331.30678	54472.402	12.38054407	15.73326434	15.73326434	-3.35296	0.02141
ZBED6	31.7586262	269.36675	5.033802952	8.078773924	8.078773924	-3.08435	0.02151
LOC102158959	11.0464787	109.06875	3.590539588	6.782261052	6.782261052	-3.30358	0.02152
LOC100738662	277.542777	43.792754	8.121755095	5.485193456	5.485193456	2.663946	0.02156
HDDC2	2745.04995	422.22825	11.42314215	8.725292112	8.725292112	2.700737	0.02165
EPM2A	22.0929574	193.34914	4.529381037	7.602506906	7.602506906	-3.12955	0.02169
LOC100525245	545.419885	4842.8175	9.093866176	12.24192881	12.24192881	-3.15041	0.02176
SOX9	51.0899639	423.05453	5.702933533	8.728105973	8.728105973	-3.04973	0.02178
ZC3HAV1	182.266898	1534.3989	7.517802419	10.58439784	10.58439784	-3.07355	0.02179
CRSP-2	4.14242951	57.013208	2.362450112	5.858309486	5.858309486	-3.78275	0.02181
LOC100739537	56.6132033	467.67356	5.848327568	8.872439593	8.872439593	-3.04629	0.02182
LOC100621698	9.66566885	98.327126	3.414902536	6.634115866	6.634115866	-3.34665	0.02184
LOC100737211	42.8051049	356.12598	5.453027102	8.480289273	8.480289273	-3.05653	0.02186
LOC100519468	31.7586262	2.4788351	5.033802952	1.798604302	1.798604302	3.679414	0.0219
HOMER3	1295.19963	12073.58	10.34007221	13.55968534	13.55968534	-3.22061	0.02193
LOC100523560	44.1859147	366.8676	5.497801224	8.523042794	8.523042794	-3.0536	0.02195
JHDM1D	2129.20877	331.33763	11.05677911	8.376505833	8.376505833	2.683943	0.02205
EVA1B	151.889082	1266.6847	7.256341575	10.3079803	10.3079803	-3.05997	0.0221
LOC100511492	1815.76493	284.23976	10.82715605	8.156031285	8.156031285	2.675397	0.02215
HRK	0	24.788351	0	4.688647628	4.688647628	-20	0.02225
TCEAL7	0	24.788351	0	4.688647628	4.688647628	-20	0.02225
LOC102163993	0	24.788351	0	4.688647628	4.688647628	-20	0.02225
TNFSF15	0	24.788351	0	4.688647628	4.688647628	-20	0.02225
LOC102160363	0	24.788351	0	4.688647628	4.688647628	-20	0.02225
LOC102162241	0	24.788351	0	4.688647628	4.688647628	-20	0.02225
SOGA3	0	24.788351	0	4.688647628	4.688647628	-20	0.02225
LOC102167440	0	24.788351	0	4.688647628	4.688647628	-20	0.02225
LOC100737220	167.07799	25.61463	7.392987005	4.734147579	4.734147579	2.705482	0.02228
LOC100513317	80.0869705	657.71758	6.341398207	9.363516252	9.363516252	-3.03783	0.02229
HAUS4	2174.77549	338.77413	11.08731398	8.408432212	8.408432212	2.682471	0.02231
HAPLN2	41.4242951	4.1313919	5.406818783	2.359350201	2.359350201	3.325777	0.02236
LOC102160991	38.6626754	321.42229	5.309710091	8.33280766	8.33280766	-3.05546	0.02239
LOC100520821	5.52323934	66.928548	2.705588563	6.085946114	6.085946114	-3.59904	0.02242
LOC100038019	201.598236	1693.8707	7.662477803	10.72695947	10.72695947	-3.07077	0.02242
NEDD4	34.5202459	288.37115	5.150569664	8.176777292	8.176777292	-3.06241	0.02253
LOC102162209	2.76161967	46.271589	1.911353989	5.56290145	5.56290145	-4.06654	0.02254
LOC100737550	2.76161967	46.271589	1.911353989	5.56290145	5.56290145	-4.06654	0.02254
LOC102164546	132.557744	19.830681	7.061319821	4.380638094	4.380638094	2.740815	0.02266
GCOM1	324.490311	52.055537	8.34647079	5.729431428	5.729431428	2.640052	0.02307
HCN1	200.217426	31.398578	7.652611443	5.017858594	5.017858594	2.672796	0.02319
LOC102166276	113.226407	927.08433	6.835752397	9.858112096	9.858112096	-3.03349	0.02319
PCNA	20629.299	2951.4663	14.33247711	11.5277049	11.5277049	2.805191	0.02329
PDXK	4411.68743	674.24315	12.10744184	9.399263292	9.399263292	2.70999	0.02347
IL16	51.0899639	416.4443	5.702933533	8.705439898	8.705439898	-3.02701	0.02347
APC	156.031511	1285.6891	7.294910283	10.32944784	10.32944784	-3.04263	0.02352
COL17A1	263.734679	42.140197	8.048403381	5.430960858	5.430960858	2.645818	0.02355
ZNF582	19.3313377	168.56079	4.345633235	7.405658764	7.405658764	-3.12426	0.02383
LOC100627071	55.2323934	6.610227	5.813329549	2.927939483	2.927939483	3.062743	0.02385
LOC102165346	55.2323934	6.610227	5.813329549	2.927939483	2.927939483	3.062743	0.02385
SCUBE2	1307.62691	209.04843	10.35383813	7.714578179	7.714578179	2.645042	0.02388
LOC102166805	22.0929574	189.21775	4.529381037	7.571508044	7.571508044	-3.09839	0.02398
GADD45B	327.251931	2772.1639	8.358659688	11.43731719	11.43731719	-3.08254	0.024
LIAS	2651.15489	414.79174	11.37294932	8.699717297	8.699717297	2.676162	0.02411
LOC102158450	131.176934	19.830681	7.04632663	4.380638094	4.380638094	2.725708	0.02414
LSDP5	26.2353869	1.6525567	4.767410456	1.407383613	1.407383613	3.988742	0.02415
LOC102158611	26.2353869	1.6525567	4.767410456	1.407383613	1.407383613	3.988742	0.02415
LOC102162275	26.2353869	1.6525567	4.767410456	1.407383613	1.407383613	3.988742	0.02415
ALOX15B	26.2353869	1.6525567	4.767410456	1.407383613	1.407383613	3.988742	0.02415
ADAMTS17	1383.57146	221.4426	10.43522379	7.797289318	7.797289318	2.643392	0.02432
LOC102166958	45.5667246	4.9576702	5.541227504	2.574748269	2.574748269	3.200246	0.02433
LOC100621169	45.5667246	4.9576702	5.541227504	2.574748269	2.574748269	3.200246	0.02433
SH3BGR	102.179928	826.27837	6.689018532	9.692229055	9.692229055	-3.01552	0.02437
SLC35D2	38.6626754	315.63834	5.309710091	8.306692135	8.306692135	-3.02926	0.02439
LOC100620506	40.0434852	326.37996	5.359081339	8.354822188	8.354822188	-3.02691	0.02439
LOC100156854	825.724282	133.8571	9.691262451	7.075287628	7.075287628	2.624967	0.02442
LOC100521848	35.9010557	3.3051135	5.205590187	2.106051269	2.106051269	3.441255	0.02474
NFIX	278.923587	2329.2787	8.128889246	11.18628681	11.18628681	-3.06194	0.02477
LOC102164768	218.167954	1801.2869	7.775893057	10.81561293	10.81561293	-3.04552	0.0248
PEAR1	302.397354	2534.1958	8.245064693	11.30788144	11.30788144	-3.06701	0.02481
LOC100623829	31.7586262	261.10397	5.033802952	8.033995371	8.033995371	-3.0394	0.02488
FGF11	404.577282	3438.1443	8.663833033	11.74783394	11.74783394	-3.08714	0.02492
LOC100514227	5.52323934	65.275991	2.705588563	6.05041444	6.05041444	-3.56297	0.02504
LOC102164214	167.07799	1361.7068	7.392987005	10.41225942	10.41225942	-3.02682	0.02508
LOC100739101	651.742243	106.58991	9.350369597	6.749398975	6.749398975	2.612231	0.02531
LOC100625781	30.3778164	249.53607	4.971673052	7.968874505	7.968874505	-3.03816	0.02535
IFFO2	35.9010557	291.67627	5.205590187	8.193161944	8.193161944	-3.02227	0.02539
EVI2A	0	23.962073	0	4.641665831	4.641665831	-20	0.0255
LOC102164456	0	23.962073	0	4.641665831	4.641665831	-20	0.0255
KCNRG	0	23.962073	0	4.641665831	4.641665831	-20	0.0255
LOC100738421	0	23.962073	0	4.641665831	4.641665831	-20	0.0255
RAI2	0	23.962073	0	4.641665831	4.641665831	-20	0.0255
C1QL3	0	23.962073	0	4.641665831	4.641665831	-20	0.0255
LOC102165701	0	23.962073	0	4.641665831	4.641665831	-20	0.0255
LOC100524214	19.3313377	166.08195	4.345633235	7.384412099	7.384412099	-3.10288	0.02551
LOC102166908	1.38080984	34.703692	1.251452392	5.158001345	5.158001345	-4.6515	0.02562
MYBPHL	1.38080984	34.703692	1.251452392	5.158001345	5.158001345	-4.6515	0.02562
RAB3IL1	370.057036	3108.4592	8.535497154	11.60244799	11.60244799	-3.07038	0.02577
LOC102161631	191.932567	30.5723	7.591952882	4.980587446	4.980587446	2.650303	0.0258
LOC100517767	28.9970066	237.96817	4.906746634	7.900674664	7.900674664	-3.03679	0.02588
LOC102164058	13.8080984	124.76803	3.888314478	6.974621475	6.974621475	-3.17566	0.02597
WASF1	573.036082	94.195734	9.164997612	6.572825024	6.572825024	2.604892	0.02598
LOC100620251	15.1889082	0	4.016933786	0	0	20	0.02603
GNAL	15.1889082	0	4.016933786	0	0	20	0.02603
LOC102164431	15.1889082	0	4.016933786	0	0	20	0.02603
NLRP12	15.1889082	0	4.016933786	0	0	20	0.02603
LOC100621822	15.1889082	0	4.016933786	0	0	20	0.02603
LOC102166344	15.1889082	0	4.016933786	0	0	20	0.02603
HES2	15.1889082	0	4.016933786	0	0	20	0.02603
LOC102162972	15.1889082	0	4.016933786	0	0	20	0.02603
CD79B	15.1889082	0	4.016933786	0	0	20	0.02603
LOC102162510	15.1889082	0	4.016933786	0	0	20	0.02603
LOC100739323	15.1889082	0	4.016933786	0	0	20	0.02603
LOC102165746	15.1889082	0	4.016933786	0	0	20	0.02603
LOC102167219	15.1889082	0	4.016933786	0	0	20	0.02603
LOC100625116	15.1889082	0	4.016933786	0	0	20	0.02603
OTOL1	15.1889082	0	4.016933786	0	0	20	0.02603
CACNA2D3	15.1889082	0	4.016933786	0	0	20	0.02603
SLCO2A1	49.7091541	396.61362	5.664174303	8.63522336	8.63522336	-2.99615	0.02603
LRRN4	49.7091541	5.7839486	5.664174303	2.762125239	2.762125239	3.103385	0.02605
TLL1	20.7121475	175.17101	4.440430524	7.460832769	7.460832769	-3.08022	0.02632
LOC100621387	2.76161967	44.619032	1.911353989	5.511563931	5.511563931	-4.01407	0.02636
LOC100739607	2.76161967	44.619032	1.911353989	5.511563931	5.511563931	-4.01407	0.02636
ZMYND12	20.7121475	0.8262784	4.440430524	0.868906686	0.868906686	4.647705	0.02639
AOX1	20.7121475	0.8262784	4.440430524	0.868906686	0.868906686	4.647705	0.02639
LOC100621509	20.7121475	0.8262784	4.440430524	0.868906686	0.868906686	4.647705	0.02639
LOC100152671	20.7121475	0.8262784	4.440430524	0.868906686	0.868906686	4.647705	0.02639
LOC102158743	20.7121475	0.8262784	4.440430524	0.868906686	0.868906686	4.647705	0.02639
LOC100625374	20.7121475	0.8262784	4.440430524	0.868906686	0.868906686	4.647705	0.02639
LOC100518827	1130.88326	184.26008	10.14450945	7.533408207	7.533408207	2.617635	0.02639
LOC102162827	35.9010557	289.19743	5.205590187	8.180890933	8.180890933	-3.00996	0.02642
GSTO1	18606.4125	2722.5872	14.18358983	11.41129236	11.41129236	2.77275	0.02646
LOC102164245	5.52323934	64.449713	2.705588563	6.032314961	6.032314961	-3.54459	0.02649
APH1B	5.52323934	64.449713	2.705588563	6.032314961	6.032314961	-3.54459	0.02649
LOC102157563	63.5172524	8.2627837	6.011613095	3.211445828	3.211445828	2.942449	0.02655
LOC102158094	63.5172524	8.2627837	6.011613095	3.211445828	3.211445828	2.942449	0.02655
ARHGEF40	1014.89523	8967.5992	9.988535907	13.13066695	13.13066695	-3.14339	0.02659
LOC100521211	12.4272885	114.02642	3.747096094	6.845821397	6.845821397	-3.19778	0.02666
HOXA3	51.0899639	404.8764	5.702933533	8.664896653	8.664896653	-2.98637	0.02679
KIF12	312.063023	2577.9885	8.290309306	11.33258964	11.33258964	-3.04634	0.02679
MFSD2A	1107.40949	180.95496	10.11427525	7.507437596	7.507437596	2.613486	0.0268
LRRC8D	176.743659	1422.8514	7.473654282	10.47558283	10.47558283	-3.00905	0.02688
LOC100157320	37.2818656	298.28649	5.258589232	8.225383356	8.225383356	-3.00015	0.02702
DEPDC4	40.0434852	4.1313919	5.359081339	2.359350201	2.359350201	3.276868	0.02702
ZFP36	2468.88799	394.13478	11.2702299	8.626201041	8.626201041	2.6471	0.02715
LOC102159432	31.7586262	256.1463	5.033802952	8.006445558	8.006445558	-3.01175	0.0272
PEX3	2638.7276	420.57569	11.36617334	8.719647871	8.719647871	2.649405	0.0273
PYCARD	291.350875	47.924146	8.191557101	5.61247475	5.61247475	2.603933	0.02736
LRIG1	265.115489	2159.8917	8.055908671	11.07741103	11.07741103	-3.02627	0.02758
PPP1R12A	272.019538	2218.5574	8.092860386	11.11605632	11.11605632	-3.02784	0.0276
LOC100521647	185.028518	29.746021	7.539379992	4.942327829	4.942327829	2.636979	0.02762
CDKN2A	6.90404918	73.538775	2.982591923	6.219919206	6.219919206	-3.41299	0.02781
LOC100737490	30.3778164	2.4788351	4.971673052	1.798604302	1.798604302	3.615284	0.02788
RFC3	4203.18514	660.19642	12.03761049	9.368935101	9.368935101	2.670516	0.02794
LOC100624195	22.0929574	182.60752	4.529381037	7.52048134	7.52048134	-3.04709	0.02823
LOC102160967	1.38080984	33.877413	1.251452392	5.124221138	5.124221138	-4.61674	0.02833
LOC102160047	1.38080984	33.877413	1.251452392	5.124221138	5.124221138	-4.61674	0.02833
F2	2.76161967	43.792754	1.911353989	5.485193456	5.485193456	-3.98711	0.02855
CACNG7	80.0869705	623.84017	6.341398207	9.287343396	9.287343396	-2.96154	0.02864
ESCO2	1358.71688	223.09516	10.40909057	7.807967682	7.807967682	2.606514	0.02869
SCIN	160.173941	25.61463	7.332474693	4.734147579	4.734147579	2.6446	0.02878
ASS1	425.289429	71.05994	8.735689471	6.171125545	6.171125545	2.581337	0.02879
LOC100514541	84.2294	655.23875	6.413279271	9.358076973	9.358076973	-2.95962	0.02888
LOC100625039	24.854577	201.61192	4.692347799	7.662575262	7.662575262	-3.02	0.02906
LOC102166021	150.508272	23.962073	7.243252754	4.641665831	4.641665831	2.651018	0.02909
LOC100524801	82.8485901	642.84457	6.389714621	9.330568647	9.330568647	-2.95592	0.02921
ZFPM2	34.5202459	272.67186	5.150569664	8.096303303	8.096303303	-2.98165	0.02923
LOC100520746	3826.22406	607.3146	11.90208265	9.248673828	9.248673828	2.655405	0.02924
LOC102164797	127.034505	19.830681	7.000388854	4.380638094	4.380638094	2.679414	0.02924
PRDM8	0	23.135794	0	4.593102408	4.593102408	-20	0.02932
SPTLC3	0	23.135794	0	4.593102408	4.593102408	-20	0.02932
MUSK	0	23.135794	0	4.593102408	4.593102408	-20	0.02932
RGS22	0	23.135794	0	4.593102408	4.593102408	-20	0.02932
LOC100522946	0	23.135794	0	4.593102408	4.593102408	-20	0.02932
CD40LG	0	23.135794	0	4.593102408	4.593102408	-20	0.02932
LOC102167616	0	23.135794	0	4.593102408	4.593102408	-20	0.02932
LOC102165354	0	23.135794	0	4.593102408	4.593102408	-20	0.02932
LOC102165408	0	23.135794	0	4.593102408	4.593102408	-20	0.02932
LOC100516485	27.6161967	221.4426	4.838760036	7.797289318	7.797289318	-3.00335	0.02933
LOC100623625	2600.06492	419.74941	11.34488669	8.716817448	8.716817448	2.630947	0.02964
LOC102164420	103.560738	15.699289	6.708197415	4.061714779	4.061714779	2.721706	0.02971
SH2D4B	131.176934	20.656959	7.04632663	4.436758793	4.436758793	2.666814	0.02992
CCNE2	729.067593	122.2892	9.511886232	6.945902605	6.945902605	2.575756	0.03001
LOC102164708	22.0929574	180.12869	4.529381037	7.500871231	7.500871231	-3.02737	0.03004
LOC100154002	1266.20262	209.87471	10.30743151	7.72024225	7.72024225	2.592908	0.03011
LOC100626399	4.14242951	52.881816	2.362450112	5.751726565	5.751726565	-3.67422	0.03025
GFRA4	4.14242951	52.881816	2.362450112	5.751726565	5.751726565	-3.67422	0.03025
LOC102165762	4.14242951	52.881816	2.362450112	5.751726565	5.751726565	-3.67422	0.03025
LOC100523914	26.2353869	209.87471	4.767410456	7.72024225	7.72024225	-2.99994	0.03026
LOC100622080	272.019538	45.44531	8.092860386	5.537461031	5.537461031	2.581507	0.03041
LOC100521295	12.4272885	110.7213	3.747096094	6.803760479	6.803760479	-3.15535	0.03041
SLC17A7	382.484325	64.449713	8.583023796	6.032314961	6.032314961	2.569155	0.0305
EID1	457.048056	3748.825	8.839355155	11.87260754	11.87260754	-3.03602	0.03053
HPN	34.5202459	3.3051135	5.150569664	2.106051269	2.106051269	3.384671	0.03063
LOC100512875	34.5202459	3.3051135	5.150569664	2.106051269	2.106051269	3.384671	0.03063
S100A9	34.5202459	3.3051135	5.150569664	2.106051269	2.106051269	3.384671	0.03063
LOC102166369	24.854577	199.13309	4.692347799	7.644815895	7.644815895	-3.00215	0.03075
LOC100512382	80.0869705	11.567897	6.341398207	3.65167138	3.65167138	2.791441	0.03078
ADGB	2.76161967	42.966475	1.911353989	5.458331975	5.458331975	-3.95962	0.03094
PAK7	2.76161967	42.966475	1.911353989	5.458331975	5.458331975	-3.95962	0.03094
LOC102162112	2.76161967	42.966475	1.911353989	5.458331975	5.458331975	-3.95962	0.03094
ADAMTS6	237.499292	1876.4782	7.897841171	10.87458043	10.87458043	-2.98203	0.03117
FAM110B	88.3718295	676.72199	6.481748252	9.404549765	9.404549765	-2.9369	0.03117
TMEM180	371.437846	62.797156	8.540855872	5.995420212	5.995420212	2.56435	0.03119
BAI3	66.2788721	9.0890621	6.072081615	3.334720158	3.334720158	2.866346	0.03119
LOC100522323	2732.62267	443.71149	11.4165984	8.796725856	8.796725856	2.622592	0.03119
SLC9B1	139.461793	22.309516	7.13403395	4.542847146	4.542847146	2.644139	0.03123
LOC100626667	1.38080984	33.051135	1.251452392	5.089630977	5.089630977	-4.58111	0.03136
LOC100739482	1.38080984	33.051135	1.251452392	5.089630977	5.089630977	-4.58111	0.03136
LOC102163519	5.52323934	61.970878	2.705588563	5.976612875	5.976612875	-3.488	0.03141
LOC100737880	5.52323934	61.970878	2.705588563	5.976612875	5.976612875	-3.488	0.03141
RAB11FIP4	204.359856	33.877413	7.682010377	5.124221138	5.124221138	2.592716	0.03153
LOC102163221	16.569718	138.81477	4.135019133	7.127372928	7.127372928	-3.06654	0.03166
JUNB	20373.8491	3046.4884	14.31450176	11.57340499	11.57340499	2.741499	0.03167
KLHL25	1362.85931	227.22655	10.41347911	7.834322836	7.834322836	2.584433	0.03172
LOC100737398	62.1364426	473.45751	5.98040107	8.890135073	8.890135073	-2.92972	0.03173
SFRP1	994.183082	8455.3066	9.958818149	13.04581197	13.04581197	-3.08827	0.03184
LOC100738126	52.4707738	6.610227	5.740678649	2.927939483	2.927939483	2.988742	0.03187
ZNF135	27.6161967	217.31121	4.838760036	7.770242414	7.770242414	-2.97618	0.03196
LOC100520944	49.7091541	379.26177	5.664174303	8.570849103	8.570849103	-2.93161	0.03205
MYO3B	24.854577	1.6525567	4.692347799	1.407383613	1.407383613	3.91074	0.03205
LOC100524449	24.854577	1.6525567	4.692347799	1.407383613	1.407383613	3.91074	0.03205
LOC100517257	24.854577	1.6525567	4.692347799	1.407383613	1.407383613	3.91074	0.03205
MIR935	24.854577	1.6525567	4.692347799	1.407383613	1.407383613	3.91074	0.03205
RIMKLA	24.854577	1.6525567	4.692347799	1.407383613	1.407383613	3.91074	0.03205
LOC102159769	272.019538	2149.9763	8.092860386	11.07077593	11.07077593	-2.98254	0.03207
NIP7	5650.27385	894.85948	12.46436039	9.80712864	9.80712864	2.658588	0.03209
VLDLR	999.706321	8486.7055	9.966802931	13.05115883	13.05115883	-3.08563	0.03217
AMDHD1	70.4213016	9.9153405	6.158282521	3.448285225	3.448285225	2.828278	0.0322
HIC1	55.2323934	419.74941	5.813329549	8.716817448	8.716817448	-2.92594	0.03232
LOC100738919	4.14242951	52.055537	2.362450112	5.729431428	5.729431428	-3.6515	0.03235
FAM131B	1003.84875	169.38707	9.972762649	7.412672017	7.412672017	2.567146	0.03248
VSTM5	28.9970066	226.40027	4.906746634	7.829090182	7.829090182	-2.9649	0.03253
MMD	1199.92375	201.61192	10.22992883	7.662575262	7.662575262	2.57329	0.03256
LOC100157418	1463.65843	244.5784	10.51634854	7.940039851	7.940039851	2.58121	0.03264
NFASC	38.6626754	4.1313919	5.309710091	2.359350201	2.359350201	3.226242	0.03273
CX3CL1	35.9010557	275.97698	5.205590187	8.113622247	8.113622247	-2.94245	0.03277
DBI	38394.7983	5612.0827	15.22866082	12.4545776	12.4545776	2.774303	0.03286
LOC100739477	1314.53096	220.61633	10.36142949	7.791920351	7.791920351	2.574937	0.03287
LOC100622387	74.5637311	10.741619	6.239622035	3.553559423	3.553559423	2.795263	0.03312
ZNF793	13.8080984	118.15781	3.888314478	6.89672967	6.89672967	-3.09713	0.03315
LOC100623627	5.52323934	61.1446	2.705588563	5.957557119	5.957557119	-3.46864	0.03328
LOC100621170	5.52323934	61.1446	2.705588563	5.957557119	5.957557119	-3.46864	0.03328
LOC100625901	16.569718	137.16221	4.135019133	7.110219252	7.110219252	-3.04926	0.0334
GREB1L	110.464787	17.351846	6.800444207	4.19785327	4.19785327	2.670426	0.03355
LOC100626354	2.76161967	42.140197	1.911353989	5.430960858	5.430960858	-3.93161	0.03356
LOC102166568	2.76161967	42.140197	1.911353989	5.430960858	5.430960858	-3.93161	0.03356
ADAMTS14	193.313377	1488.9536	7.602241413	10.54105171	10.54105171	-2.94529	0.03371
SCN2B	0	22.309516	0	4.542847146	4.542847146	-20	0.0338
LOC102159543	0	22.309516	0	4.542847146	4.542847146	-20	0.0338
LOC102157538	0	22.309516	0	4.542847146	4.542847146	-20	0.0338
LOC102159960	0	22.309516	0	4.542847146	4.542847146	-20	0.0338
LOC102164750	0	22.309516	0	4.542847146	4.542847146	-20	0.0338
LOC100516820	0	22.309516	0	4.542847146	4.542847146	-20	0.0338
LOC100624148	0	22.309516	0	4.542847146	4.542847146	-20	0.0338
LOC100736739	0	22.309516	0	4.542847146	4.542847146	-20	0.0338
GPR75	0	22.309516	0	4.542847146	4.542847146	-20	0.0338
RHBDL3	0	22.309516	0	4.542847146	4.542847146	-20	0.0338
SGCG	0	22.309516	0	4.542847146	4.542847146	-20	0.0338
LOC100739700	24.854577	195.0017	4.692347799	7.614722326	7.614722326	-2.9719	0.0338
LOC100155802	24.854577	195.0017	4.692347799	7.614722326	7.614722326	-2.9719	0.0338
LOC100737465	457.048056	78.496445	8.839355155	6.312818447	6.312818447	2.541647	0.03399
LOC100515974	2321.14133	384.21944	11.18124007	8.589536711	8.589536711	2.594832	0.03403
HOGA1	11.0464787	98.327126	3.590539588	6.634115866	6.634115866	-3.154	0.03425
LRRC29	11.0464787	98.327126	3.590539588	6.634115866	6.634115866	-3.154	0.03425
EFCAB4A	27.6161967	214.0061	4.838760036	7.74823377	7.74823377	-2.95407	0.03425
NKX3-2	40.0434852	303.24416	5.359081339	8.249085772	8.249085772	-2.92084	0.03426

WDR43	17412.58944	1585.6282	14.08792599	10.63174838	10.63174838	3.457004	0.01145
LOC100620491	49.69587707	3.3051135	5.663796517	2.106051269	2.106051269	3.910354	0.01146
SLC2A11	45.46644072	524.68677	5.538117235	9.038059606	9.038059606	-3.52858	0.01148
BCAS3	87.76080419	1022.1063	6.471850834	9.998740397	9.998740397	-3.54182	0.01149
TNFRSF1A	464.180639	5869.0553	8.861647242	12.51915837	12.51915837	-3.66037	0.01151
ZIC3	647.103761	67.754826	9.340080996	6.103389087	6.103389087	3.255601	0.01151
MIOS	4936.809575	480.06773	12.26965549	8.910096228	8.910096228	3.362269	0.01151
LOC102159210	20.08982264	0	4.398475058	0	0	20	0.01159
LOC102159797	20.08982264	0	4.398475058	0	0	20	0.01159
SUN3	20.08982264	0	4.398475058	0	0	20	0.01159
LOC100626755	20.08982264	0	4.398475058	0	0	20	0.01159
LOC102164033	20.08982264	0	4.398475058	0	0	20	0.01159
LOC100621043	20.08982264	0	4.398475058	0	0	20	0.01159
LOC102167745	20.08982264	0	4.398475058	0	0	20	0.01159
LOC102165751	20.08982264	0	4.398475058	0	0	20	0.01159
DEFB123	20.08982264	0	4.398475058	0	0	20	0.01159
HOXD13	20.08982264	0	4.398475058	0	0	20	0.01159
LOC100520029	1040.441341	13820.332	10.02436587	13.75460904	13.75460904	-3.73152	0.01161
MAP3K15	15771.56814	1447.6397	13.94512996	10.50048311	10.50048311	3.445552	0.01162
CCDC170	2.114718173	48.750424	1.639101631	5.636636914	5.636636914	-4.52688	0.01163
DPP10	0	26.440908	0	4.778256309	4.778256309	-20	0.01165
MLC2V	0	26.440908	0	4.778256309	4.778256309	-20	0.01165
CLDN4	0	26.440908	0	4.778256309	4.778256309	-20	0.01165
ANO2	0	26.440908	0	4.778256309	4.778256309	-20	0.01165
PDZK1IP1	0	26.440908	0	4.778256309	4.778256309	-20	0.01165
LOC102162791	0	26.440908	0	4.778256309	4.778256309	-20	0.01165
LOC102160410	0	26.440908	0	4.778256309	4.778256309	-20	0.01165
LOC102162226	0	26.440908	0	4.778256309	4.778256309	-20	0.01165
LOC102163370	0	26.440908	0	4.778256309	4.778256309	-20	0.01165
ZNF575	31.7207726	366.04132	5.032134908	8.519798669	8.519798669	-3.52851	0.01181
PIK3R3	3591.848817	356.95226	11.81091251	8.483623364	8.483623364	3.330924	0.01192
APLP1	22.20454082	260.27769	4.536335244	8.029440115	8.029440115	-3.55112	0.01194
ZNF396	1289.978086	133.8571	10.3342488	7.075287628	7.075287628	3.268581	0.01196
LOC100738354	6.344154519	91.716899	2.876596413	6.534760414	6.534760414	-3.85369	0.01197
LOC100515261	322.4945214	33.877413	8.337597469	5.124221138	5.124221138	3.250879	0.01198
HCRTR1	34.89284986	1.6525567	5.165624571	1.407383614	1.407383614	4.40016	0.01202
FLNA	590.0063703	7493.5186	9.207029871	12.87162009	12.87162009	-3.66684	0.01204
DYDC1	7.401513606	102.45852	3.070649265	6.692908622	6.692908622	-3.79108	0.01205
LOC100515204	735.9219243	77.670167	9.525367966	6.29774474	6.29774474	3.24412	0.0121
SFMBT1	775.0442105	81.801559	9.599995033	6.371586023	6.371586023	3.244078	0.01216
TIMM8A	6497.471587	629.62412	12.66588473	9.300636539	9.300636539	3.367316	0.01216
LOC102164238	18776.58866	1722.7904	14.19672373	10.75136865	10.75136865	3.446115	0.0122
LOC100513950	27.49133625	0.8262784	4.832451381	0.868906686	0.868906686	5.056205	0.01221
LOC100522050	27.49133625	0.8262784	4.832451381	0.868906686	0.868906686	5.056205	0.01221
ABCG4	29.60605442	339.60041	4.935745167	8.411936365	8.411936365	-3.51987	0.01224
CERKL	765.5279787	80.97528	9.582194642	6.357117027	6.357117027	3.240902	0.01228
C9H11orf88	56.04003159	4.1313919	5.833902876	2.359350201	2.359350201	3.761758	0.01228
LOC100738016	2367.426995	241.27328	11.20971349	7.920491517	7.920491517	3.29458	0.01232
LENG9	34.89284986	397.4399	5.165624571	8.638218305	8.638218305	-3.50973	0.01233
LOC100155621	1994.179237	204.91704	10.96230264	7.685919384	7.685919384	3.282683	0.01239
MYH1	1.057359087	37.182527	1.04079362	5.254840671	5.254840671	-5.13609	0.01239
LOC100738788	1.057359087	37.182527	1.04079362	5.254840671	5.254840671	-5.13609	0.01239
ZNF70	5.286795433	80.149002	2.65232482	6.342501448	6.342501448	-3.92222	0.01239
LOC102159969	2.114718173	47.924146	1.639101631	5.61247475	5.61247475	-4.50222	0.01245
LOC100623625	37.00756803	419.74941	5.24821481	8.716817448	8.716817448	-3.50364	0.01248
EGFLAM	14.80302721	178.47613	3.982129041	7.487648157	7.487648157	-3.59177	0.01251
TPM1	8558.264447	128639.97	13.06327111	16.97299072	16.97299072	-3.90988	0.01251
AMD1	32603.66743	2919.2415	14.99279088	11.51187196	11.51187196	3.481369	0.01253
DLGAP4	367.9609621	4495.7806	8.52732437	12.13467679	12.13467679	-3.61095	0.01256
EMP3	779.2736468	9964.9172	9.607836365	13.28278687	13.28278687	-3.67666	0.01263
LOC102161059	1365.050581	142.94616	10.41579519	7.169385476	7.169385476	3.255411	0.01268
ZNF711	1943.426001	200.78564	10.92512862	7.65667973	7.65667973	3.274874	0.01268
CDH8	90.93288145	8.2627837	6.522509054	3.211445828	3.211445828	3.460102	0.01269
TMEM79	11.63094995	144.59872	3.658891241	7.185853813	7.185853813	-3.63601	0.01273
PMM1	100.4491132	1146.8744	6.664612444	10.16474905	10.16474905	-3.51317	0.01274
LOC100625270	12.68830904	155.34033	3.774872332	7.288546214	7.288546214	-3.61386	0.01282
LOC100737431	26.43397716	300.76533	4.777891883	8.23728324	8.23728324	-3.50817	0.01294
LOC100624077	62.38418611	4.9576702	5.986051038	2.57474827	2.57474827	3.653446	0.01297
KIAA1875	31.7207726	357.77863	5.032134908	8.486949769	8.486949769	-3.49557	0.013
LOC102164625	11.63094995	143.77244	3.658891241	7.177643143	7.177643143	-3.62775	0.01304
LOC100737543	4294.99261	430.49103	12.0687758	8.753186764	8.753186764	3.318601	0.01306
LOC100738027	152.2597085	1757.4941	7.259834656	10.78012478	10.78012478	-3.52891	0.01307
HS6ST1	1333.329080	17511.318	10.38189959	14.0960824	14.0960824	-3.71518	0.0131
LOC100515685	12.68830904	154.51406	3.774872332	7.280901168	7.280901168	-3.60617	0.0131
NT5DC3	161.7759402	16.525567	7.346743662	4.131389251	4.131389251	3.291225	0.01311
LOC102161907	0	25.61463	0	4.734147579	4.734147579	-20	0.01316
HIF3A	0	25.61463	0	4.734147579	4.734147579	-20	0.01316
LOC102157470	0	25.61463	0	4.734147579	4.734147579	-20	0.01316
AMICA1	0	25.61463	0	4.734147579	4.734147579	-20	0.01316
LOC100624399	0	25.61463	0	4.734147579	4.734147579	-20	0.01316
AIRE	0	25.61463	0	4.734147579	4.734147579	-20	0.01316
LOC102160901	0	25.61463	0	4.734147579	4.734147579	-20	0.01316
LOC100623023	0	25.61463	0	4.734147579	4.734147579	-20	0.01316
LOC100623327	0	25.61463	0	4.734147579	4.734147579	-20	0.01316
LOC100737228	0	25.61463	0	4.734147579	4.734147579	-20	0.01316
ZPBP	0	25.61463	0	4.734147579	4.734147579	-20	0.01316
LOC100737327	0	25.61463	0	4.734147579	4.734147579	-20	0.01316
LOC102165794	41.23700438	2.4788351	5.400435611	1.798604302	1.798604302	4.056205	0.01317
NAGK	344.6990622	4147.0911	8.43337288	12.01823188	12.01823188	-3.58869	0.01321
LOC100626982	26.43397716	299.11277	4.777891883	8.2293609	8.2293609	-3.50022	0.01324
LOC100628219	71.90041789	805.62141	6.187855179	9.655747894	9.655747894	-3.48603	0.01325
EXTL1	9.516231779	121.46292	3.394545939	6.936201186	6.936201186	-3.67398	0.01329
TMEM101	125.8257313	1432.7667	6.986703669	10.48559457	10.48559457	-3.5093	0.01337
NASP	41747.70882	3732.2994	15.34944396	11.8662355	11.8662355	3.48356	0.01337
RGS14	54.9826725	4.1313919	5.806908455	2.359350201	2.359350201	3.734277	0.01342
ITGA1	51.81059524	575.91603	5.722755497	9.172217528	9.172217528	-3.47454	0.01345
C3H16orf71	5.286795433	78.496445	2.65232482	6.312818447	6.312818447	-3.89216	0.01349
PRAF2	326.7239578	3897.5551	8.356337332	11.9287238	11.9287238	-3.57643	0.01351
LOC102167722	1.057359087	36.356248	1.04079362	5.223277669	5.223277669	-5.10367	0.01353
RORA	21.14718173	241.27328	4.46905122	7.920491517	7.920491517	-3.51213	0.01356
CHN1	1177.898022	125.59431	10.20322321	6.98406878	6.98406878	3.229372	0.01356
KIAA0020	13403.08378	1276.6001	13.71038499	10.3192206	10.3192206	3.392186	0.01358
LOC100737938	10.57359087	131.37826	3.532764645	7.048522415	7.048522415	-3.63519	0.01366
LOC102163664	8.458872693	109.89502	3.241668254	6.793050814	6.793050814	-3.69952	0.0137
TBC1D4	655.5626337	71.05994	9.358788835	6.171125545	6.171125545	3.205625	0.01371
LOC102159565	7.401513606	99.153405	3.070649265	6.646067655	6.646067655	-3.74377	0.01377
MLXIPL	33.83549077	1.6525567	5.122485983	1.407383614	1.407383614	4.355766	0.01382
C8H4orf48	22.20454082	251.18863	4.536335244	7.978359394	7.978359394	-3.49984	0.01384
LDB2	138.5140403	14.046732	7.124266508	3.911378308	3.911378308	3.301726	0.01386
LOC100524931	622.784502	67.754826	9.284903899	6.103389087	6.103389087	3.200337	0.01392
LOC100514966	3.17207726	57.013208	2.060765874	5.858309485	5.858309485	-4.1678	0.01393
LOC100155040	5118.675338	514.77143	12.32183661	9.010588038	9.010588038	3.313767	0.01393
LOC102158317	47.5811589	3.3051135	5.602325001	2.106051269	2.106051269	3.847769	0.01402
LOC102164141	5.286795433	77.670167	2.65232482	6.29774474	6.29774474	-3.8769	0.01407
ARHGAP27	378.534553	41.313919	8.568087425	5.403060391	5.403060391	3.195725	0.01414
LOC100624332	3920.687493	400.74501	11.93725886	8.650136295	8.650136295	3.29035	0.01416
LOC100736959	173.4026892	18.178124	7.446313227	4.261389711	4.261389711	3.253886	0.01418
LOC102159229	2.114718173	46.271589	1.639101631	5.56290145	5.56290145	-4.45159	0.01431
LOC102162209	2.114718173	46.271589	1.639101631	5.56290145	5.56290145	-4.45159	0.01431
ELANE	12.68830904	151.20894	3.774872332	7.249909307	7.249909307	-3.57497	0.01432
BICC1	61.32682702	672.59059	5.961781364	9.395728184	9.395728184	-3.45514	0.01433
YPEL2	100.4491132	1114.6495	6.664612444	10.12366817	10.12366817	-3.47205	0.01437
LOC100624284	10.57359087	129.7257	3.532764645	7.030399033	7.030399033	-3.61693	0.01438
LOC100516279	4270.673351	436.27498	12.06058561	8.772396993	8.772396993	3.291154	0.01444
WDHD1	6720.574354	671.76432	12.71458347	9.393957376	9.393957376	3.322557	0.01451
LOC100625846	26.43397716	0.8262784	4.777891883	0.868906686	0.868906686	4.999622	0.01453
PAQR5	67.67098154	5.7839486	6.10162868	2.76212524	2.76212524	3.548411	0.01459
SERTAD1	187.1525583	2126.0143	7.555759096	11.05461398	11.05461398	-3.50586	0.01461
LOC102161863	19.03246356	0	4.324267947	0	0	20	0.01461
ACSBG1	19.03246356	0	4.324267947	0	0	20	0.01461
LOC100525247	19.03246356	0	4.324267947	0	0	20	0.01461
LOC102161224	19.03246356	0	4.324267947	0	0	20	0.01461
ASB11	19.03246356	0	4.324267947	0	0	20	0.01461
LOC102166082	19.03246356	0	4.324267947	0	0	20	0.01461
LOC102166417	19.03246356	0	4.324267947	0	0	20	0.01461
CALY	19.03246356	0	4.324267947	0	0	20	0.01461
LOC100625927	19.03246356	0	4.324267947	0	0	20	0.01461
LOC100737086	19.03246356	0	4.324267947	0	0	20	0.01461
LOC100737534	19.03246356	0	4.324267947	0	0	20	0.01461
TRPM6	12.68830904	150.38266	3.774872332	7.242056187	7.242056187	-3.56707	0.01464
GJA1	2571.497299	34378.138	11.32895385	15.06924575	15.06924575	-3.74081	0.01468
LOC102160528	7.401513606	97.500848	3.070649265	6.622064238	6.622064238	-3.71952	0.01474
CDKN1A	1703.405488	22121.951	10.73505289	14.43325621	14.43325621	-3.69898	0.01477
TSPAN4	230.5042809	2638.3068	7.854895061	11.36594337	11.36594337	-3.51675	0.01482
PAOX	22.20454082	247.05723	4.536335244	7.954529215	7.954529215	-3.47592	0.01483
LOC102161333	1250.855799	135.50965	10.28985267	7.092859161	7.092859161	3.206448	0.01487
LOC100620234	0	24.788351	0	4.688647628	4.688647628	-20	0.01489
ASTN2	0	24.788351	0	4.688647628	4.688647628	-20	0.01489
LOC100511329	0	24.788351	0	4.688647628	4.688647628	-20	0.01489
HSPB3	0	24.788351	0	4.688647628	4.688647628	-20	0.01489
LOC100737673	0	24.788351	0	4.688647628	4.688647628	-20	0.01489
EFCAB4A	19.03246356	214.0061	4.324267947	7.74823377	7.74823377	-3.49112	0.01493
LOC100738036	583.6622158	64.449713	9.191459549	6.032314961	6.032314961	3.178888	0.01493
IGDCC3	418.7141983	46.271589	8.713263458	5.56290145	5.56290145	3.177767	0.01494
LOC100623570	6.344154519	86.759229	2.876596413	6.455478947	6.455478947	-3.77352	0.01499
TMEM141	77.18721332	841.15138	6.288860784	9.717935781	9.717935781	-3.44593	0.015
LOC100523138	26.43397716	290.02371	4.777891883	8.184992878	8.184992878	-3.45571	0.01505
DYRK4	115.2521404	11.567897	6.861113469	3.651671381	3.651671381	3.316595	0.01506
CHCHD4	9053.108499	898.16459	13.14435688	9.812441412	9.812441412	3.333361	0.01515
LOC100739435	17.97510447	202.4382	4.246035924	7.6684468	7.6684468	-3.49341	0.01516
LOC100153192	90.93288145	992.36032	6.522509054	9.956173315	9.956173315	-3.44799	0.01521
LOC102158833	142.7434347	14.873011	7.167352675	3.98850389	3.98850389	3.262656	0.01525
AGPAT3	41.23700438	444.53776	5.400435611	8.79940391	8.79940391	-3.43029	0.01534
OSBPL3	48.63851798	523.86049	5.633388138	9.035790183	9.035790183	-3.42901	0.01534
LOC100522668	156.4891448	16.525567	7.299108582	4.131389251	4.131389251	3.243291	0.01537
LOC100738613	723.2336152	80.149002	9.500311331	6.342501448	6.342501448	3.173705	0.01545
CHD7	8902.963509	887.42297	13.12023196	9.795102886	9.795102886	3.326592	0.01546
LOC102160467	37.00756803	398.26618	5.24821481	8.641207045	8.641207045	-3.42784	0.01555
PTPN6	13.74566813	158.64545	3.882219287	7.318727602	7.318727602	-3.52876	0.01557
PLXNC1	2136.922714	228.87911	11.06199399	7.844731552	7.844731552	3.222877	0.01564
LOC102164447	3.17207726	55.360651	2.060765874	5.816616368	5.816616368	-4.12536	0.01565
RAPGEF4	341.526985	38.008805	8.420073841	5.285727902	5.285727902	3.167594	0.01566
RNASE1	114.1947813	11.567897	6.84793155	3.651671381	3.651671381	3.303298	0.01573
CYP2D25	494.8440525	55.360651	8.953742641	5.816616368	5.816616368	3.160041	0.01583
LOC100516519	32.77813168	1.6525567	5.078017628	1.407383614	1.407383614	4.309962	0.01592
CRHBP	32.77813168	1.6525567	5.078017628	1.407383614	1.407383614	4.309962	0.01592
LOC102160710	32.77813168	1.6525567	5.078017628	1.407383614	1.407383614	4.309962	0.01592
VWA3B	32.77813168	1.6525567	5.078017628	1.407383614	1.407383614	4.309962	0.01592
LOC102158296	32.77813168	1.6525567	5.078017628	1.407383614	1.407383614	4.309962	0.01592
LOC100739139	32.77813168	1.6525567	5.078017628	1.407383614	1.407383614	4.309962	0.01592
PWWP2A	6813.621954	693.24755	12.73441791	9.439306379	9.439306379	3.296979	0.01602
LOC102166621	310.8635715	34.703692	8.284771232	5.158001345	5.158001345	3.163121	0.01606
OMA1	1497.220467	163.60312	10.54903422	7.362847851	7.362847851	3.194015	0.01608
TMEM169	507.5323616	57.013208	8.990195776	5.858309485	5.858309485	3.154132	0.01616
SLC38A3	1.057359087	34.703692	1.04079362	5.158001345	5.158001345	-5.03655	0.01619
LOC100624593	168.1200948	1853.3424	7.40190428	10.85669193	10.85669193	-3.46257	0.01622
ZFP36	37.00756803	394.13478	5.24821481	8.626201041	8.626201041	-3.4128	0.01623
LOC100521355	6434.030042	658.54386	12.65173117	9.365324797	9.365324797	3.288371	0.01628
LOC100621139	1005.548491	12318.984	9.975200962	13.58871279	13.58871279	-3.61483	0.01635
LIX1	140.6287585	14.873011	7.145970432	3.98850389	3.98850389	3.241123	0.01637
LOC100620861	6781.901181	693.24755	12.72768676	9.439306379	9.439306379	3.290247	0.0164
RNF207	880.7801191	98.327126	9.78427514	6.634115866	6.634115866	3.163121	0.01641
LOC102160944	2.114718173	44.619032	1.639101631	5.511563931	5.511563931	-4.39912	0.0165
LOC100739623	2.114718173	44.619032	1.639101631	5.511563931	5.511563931	-4.39912	0.0165
DMTN	42.29436346	447.84288	5.436107306	8.810066691	8.810066691	-3.40446	0.0165
HENMT1	59.21210885	4.9576702	5.911981742	2.57474827	2.57474827	3.578158	0.01652
LOC100737474	348.9284986	3981.0092	8.450916354	11.95928084	11.95928084	-3.51213	0.01663
LOC100156520	160.7185812	17.351846	7.337341641	4.19785327	4.19785327	3.211376	0.01667
LOC100521743	4.229436346	64.449713	2.386655454	6.032314961	6.032314961	-3.92964	0.01667
SUMF2	96.21967688	1027.064	6.603176434	10.00571439	10.00571439	-3.41605	0.01682
VSIG8	0	23.962073	0	4.641665831	4.641665831	-20	0.01691
LOC102157475	0	23.962073	0	4.641665831	4.641665831	-20	0.01691
NKAPL	0	23.962073	0	4.641665831	4.641665831	-20	0.01691
KCNC3	0	23.962073	0	4.641665831	4.641665831	-20	0.01691
LOC100739318	0	23.962073	0	4.641665831	4.641665831	-20	0.01691
LHX9	0	23.962073	0	4.641665831	4.641665831	-20	0.01691
SDPR	0	23.962073	0	4.641665831	4.641665831	-20	0.01691
ALDH1A2	0	23.962073	0	4.641665831	4.641665831	-20	0.01691
JAKMIP1	0	23.962073	0	4.641665831	4.641665831	-20	0.01691
CTSD	546.6546478	6375.5639	9.097122603	12.63856351	12.63856351	-3.54385	0.01704
LOC100737316	560.4003159	63.623435	9.132886065	6.013985524	6.013985524	3.138828	0.01709
EPB41L4B	91.99024053	9.0890621	6.539007406	3.334720158	3.334720158	3.339277	0.01712
LOC100511785	4397.556441	463.54217	12.10281441	8.859665746	8.859665746	3.24593	0.01718
LOC100512875	45.46644072	3.3051135	5.538117235	2.106051269	2.106051269	3.78203	0.01724
GNL3	22115.72265	2143.3661	14.43285	11.06633552	11.06633552	3.367122	0.01725
LOC100624503	64.49890428	676.72199	6.033398867	9.404549765	9.404549765	-3.39122	0.0173
LOC100739107	1810.198756	199.13309	10.82272916	7.644815895	7.644815895	3.184343	0.01732
MST1R	50.75323616	530.47071	5.693577173	9.053846388	9.053846388	-3.3857	0.01739
SNX10	5779.524767	602.35693	12.49698475	9.236867912	9.236867912	3.26226	0.01741
ATP6AP1	58.15474976	608.14088	5.886422108	9.250632122	9.250632122	-3.38644	0.01743
CACNA2D2	914.6156099	103.2848	9.838598249	6.704385034	6.704385034	3.146538	0.01748
BMPER	48.63851798	507.33492	5.633388138	8.98963553	8.98963553	-3.38277	0.01753
LOC102160736	4.229436346	63.623435	2.386655454	6.013985524	6.013985524	-3.91102	0.01754
LOC100518118	27.49133625	290.02371	4.832451381	8.184992878	8.184992878	-3.39912	0.01755
SGSM1	3.17207726	53.708094	2.060765874	5.773682393	5.773682393	-4.08164	0.01763
PLEKHG6	3.17207726	53.708094	2.060765874	5.773682393	5.773682393	-4.08164	0.01763
LOC100511112	234.7337172	2581.2936	7.881014313	11.33443734	11.33443734	-3.459	0.01765
ZNF580	82.47400875	865.11346	6.383255151	9.758412211	9.758412211	-3.39088	0.01773
WDR63	2.114718173	43.792754	1.639101631	5.485193456	5.485193456	-4.37215	0.01774
MBNL2	81.41664967	853.54556	6.364863912	9.739013597	9.739013597	-3.39007	0.01774
MBOAT1	133.2272449	1422.0251	7.068533723	10.47474537	10.47474537	-3.41599	0.01775
LOC102166942	1.057359087	33.877413	1.04079362	5.124221138	5.124221138	-5.00179	0.01776
ATP10D	2051.276628	225.574	11.00300949	7.823838478	7.823838478	3.184849	0.01777
ABCB9	21.14718173	225.574	4.46905122	7.823838478	7.823838478	-3.41506	0.01788
LOC102165085	481.0983844	55.360651	8.913183785	5.816616368	5.816616368	3.119399	0.01815
THAP8	33.83549077	351.16831	5.122485983	8.460121275	8.460121275	-3.37555	0.0182
LOC100739646	9.516231779	112.37386	3.394545939	6.824944216	6.824944216	-3.56177	0.01821
LOC100526069	1923.336178	213.17982	10.91014514	7.742678745	7.742678745	3.173468	0.01825
UBALD2	318.2650851	3526.5561	8.318610978	11.78445331	11.78445331	-3.46996	0.0184
TRAF2	364.7888849	4075.2049	8.514867427	11.99301087	11.99301087	-3.48174	0.0184
GLT8D2	218.8733309	2373.8978	7.780528816	11.21364969	11.21364969	-3.43909	0.0184
BCAS1	31.7207726	1.6525567	5.032134908	1.407383614	1.407383614	4.262656	0.0184
LOC102157584	31.7207726	1.6525567	5.032134908	1.407383614	1.407383614	4.262656	0.0184
GPR4	31.7207726	1.6525567	5.032134908	1.407383614	1.407383614	4.262656	0.0184
SP4	1664.283202	185.91263	10.70155183	7.546220276	7.546220276	3.162204	0.01841
EEF1E1	9643.11487	989.05521	13.23543312	9.95136517	9.95136517	3.285376	0.01843
TGFBR3	30.66341351	318.11717	4.98474489	8.317942438	8.317942438	-3.37497	0.01846
LOC100623211	13.74566813	152.03522	3.882219287	7.257719911	7.257719911	-3.46736	0.01851
C8H4orf21	421.8862755	48.750424	8.724125929	5.636636914	5.636636914	3.113367	0.01851
EGF	17.97510447	0	4.246035924	0	0	20	0.01857
LOC100511328	17.97510447	0	4.246035924	0	0	20	0.01857
RRH	17.97510447	0	4.246035924	0	0	20	0.01857
PAQR9	17.97510447	0	4.246035924	0	0	20	0.01857
LOC100626259	17.97510447	0	4.246035924	0	0	20	0.01857
LOC102164431	17.97510447	0	4.246035924	0	0	20	0.01857
LOC102166773	17.97510447	0	4.246035924	0	0	20	0.01857
KCND3	17.97510447	0	4.246035924	0	0	20	0.01857
CPB2	17.97510447	0	4.246035924	0	0	20	0.01857
DNAJB7	17.97510447	0	4.246035924	0	0	20	0.01857
FOXB1	17.97510447	0	4.246035924	0	0	20	0.01857
LOC102162630	17.97510447	0	4.246035924	0	0	20	0.01857
LHX1	17.97510447	0	4.246035924	0	0	20	0.01857
LOC100737801	17.97510447	0	4.246035924	0	0	20	0.01857
PLBD2	470.5247935	5323.7115	8.881189822	12.37848766	12.37848766	-3.50009	0.01863
C7H15orf59	3.17207726	52.881816	2.060765874	5.751726565	5.751726565	-4.05927	0.01873
TNFRSF12A	464.180639	5236.9523	8.861647242	12.35478721	12.35478721	-3.49597	0.01879
LOC100514839	2520.744062	31767.098	11.30020615	14.95529111	14.95529111	-3.65561	0.01888
LOC100738371	1034.097187	118.15781	10.01555052	6.896729669	6.896729669	3.129585	0.01889
ID2	182.923122	1948.3644	7.52295905	10.92878809	10.92878809	-3.41295	0.01908
LOC100511757	2.114718173	42.966475	1.639101631	5.458331975	5.458331975	-4.34467	0.01909
SHD	2.114718173	42.966475	1.639101631	5.458331975	5.458331975	-4.34467	0.01909
PRSS36	2.114718173	42.966475	1.639101631	5.458331975	5.458331975	-4.34467	0.01909
LOC100155517	685.1686881	79.322724	9.422419483	6.327736286	6.327736286	3.110653	0.0191
LYPD6	306.6341351	35.52997	8.265071784	5.191008663	5.191008663	3.10941	0.01914
LOC100620901	44.40908164	3.3051135	5.504908955	2.106051269	2.106051269	3.748083	0.01916
LOC100624862	16.91774539	180.95496	4.163317207	7.507437596	7.507437596	-3.41902	0.01917
TLN1	4192.428778	54472.402	12.03391464	15.73326434	15.73326434	-3.69967	0.01921

LOC102167974	23.2100071	203.26448	4.597531595	7.674294538	7.674294538	-3.13054	0.00084
LOC100513611	144.233615	1179.9255	7.182231603	10.20570226	10.20570226	-3.03222	0.00084
TUBA4A	3545.32858	423.8808	11.7921105	8.730914357	8.730914357	3.064189	0.00084
LOC100127144	6.63143059	76.01761	2.93195353	6.267116453	6.267116453	-3.51894	0.00084
TMEM145	455.081924	55.360651	8.833149183	5.816616368	5.816616368	3.039193	0.00084
LOC100520194	337.374031	40.48764	8.402475042	5.374609695	5.374609695	3.058795	0.00084
CTSD	746.86487	6375.5639	9.546633806	12.63856351	12.63856351	-3.09363	0.00084
LOC100739618	94.4978859	9.0890621	6.57739689	3.334720158	3.334720158	3.378079	0.00084
MECOM	284.322587	2351.5882	8.156450147	11.20003312	11.20003312	-3.04803	0.00085
AIM1	383.794045	3200.1761	8.587942663	11.64438634	11.64438634	-3.05975	0.00085
LOC100524058	304.216878	36.356248	8.253690934	5.223277669	5.223277669	3.064825	0.00086
LOC100513808	141.746829	1155.1372	7.157314885	10.17509685	10.17509685	-3.02668	0.00086
LOC102157556	401.201551	48.750424	8.651774833	5.636636914	5.636636914	3.040841	0.00086
SCN9A	201.429704	23.135794	7.661277193	4.593102408	4.593102408	3.122078	0.00086
DDN	355.610465	42.966475	8.478205232	5.458331975	5.458331975	3.049014	0.00086
LOC102164731	91.1821706	741.1717	6.526415834	9.535609179	9.535609179	-3.02298	0.00087
CADPS	2.48678647	43.792754	1.801898017	5.485193456	5.485193456	-4.13834	0.00087
MT1A	1.65785765	37.182527	1.410263837	5.254840671	5.254840671	-4.48723	0.00087
LOC102167801	1.65785765	37.182527	1.410263837	5.254840671	5.254840671	-4.48723	0.00087
ZBTB4	212.205779	1736.8371	7.736102732	10.76307717	10.76307717	-3.03293	0.00087
THPO	439.332277	53.708094	8.782448788	5.773682393	5.773682393	3.032101	0.00088
MDGA2	7.46035941	81.801559	3.080718953	6.371586023	6.371586023	-3.45481	0.00088
LOC102163580	137.602185	14.873011	7.114806188	3.98850389	3.98850389	3.209735	0.00089
LOC100620687	247.020789	2022.7295	7.954317244	10.98280072	10.98280072	-3.0336	0.00089
ZNF396	1080.09426	133.8571	10.0782766	7.075287628	7.075287628	3.012392	0.00091
OCA2	8.28928824	87.585507	3.215568059	6.468998788	6.468998788	-3.40137	0.00092
OSBPL10	831.41561	103.2848	9.70116021	6.704385035	6.704385035	3.008942	0.00092
KHDRBS3	31.4992953	264.40908	5.022336531	8.052073912	8.052073912	-3.06938	0.00092
EPB41L4A	877.835624	109.06875	9.779449541	6.782261052	6.782261052	3.008713	0.00092
LOC102158723	250.336505	29.746021	7.973476417	4.942327829	4.942327829	3.0731	0.00092
TP53I3	1422.44186	175.99729	10.47516785	7.467583487	7.467583487	3.014745	0.00092
WDR65	99.4714588	9.9153405	6.65064192	3.448285225	3.448285225	3.326548	0.00093
COCH	62.1696618	4.9576702	5.981159942	2.574748269	2.574748269	3.648477	0.00093
ZNF684	11.6050035	112.37386	3.655924617	6.824944216	6.824944216	-3.27549	0.00093
CARD14	149.207188	16.525567	7.230810045	4.13138925	4.13138925	3.174545	0.00093
LOC100522999	64.6564482	523.03421	6.036864803	9.033517185	9.033517185	-3.01604	0.00093
ATP6V1G2	34.8150106	289.19743	5.162492464	8.180890933	8.180890933	-3.05427	0.00094
LOC102158445	748.522728	93.369456	9.549828415	6.560248082	6.560248082	3.003024	0.00095
LOC100625542	415.293341	51.229259	8.70145667	5.706786332	5.706786332	3.019091	0.00095
LOC100525036	24.8678647	212.35354	4.693089065	7.737102248	7.737102248	-3.09411	0.00095
LOC100154114	29.8414377	0.8262784	4.946798112	0.868906686	0.868906686	5.174545	0.00095
LOC100518635	250.336505	2032.6448	7.973476417	10.989852	10.989852	-3.02142	0.00096
MYO3B	36.4728682	1.6525567	5.227774503	1.407383613	1.407383613	4.464052	0.00097
CTBS	36.4728682	1.6525567	5.227774503	1.407383613	1.407383613	4.464052	0.00097
LOC100515463	67.9721635	5.7839486	6.107942317	2.762125239	2.762125239	3.554817	0.00097
LOC100736869	615.065187	76.843889	9.266939203	6.282511874	6.282511874	3.000737	0.00097
TMSB10	17135.6166	162224.06	14.06479468	17.30763717	17.30763717	-3.24292	0.00098
MYO9A	247.849718	29.746021	7.959130944	4.942327829	4.942327829	3.058697	0.00099
LOC100737472	279.349014	33.877413	8.131080184	5.124221138	5.124221138	3.043673	0.00099
HHIP	3050.45807	374.3041	11.57528305	8.55191625	8.55191625	3.026743	0.001
LOC100625253	116.878964	12.394176	6.881162477	3.74353388	3.74353388	3.237281	0.001
LOC100524712	272.717583	33.051135	8.096544303	5.089630977	5.089630977	3.044636	0.001
DDX21	55173.5025	6108.676	15.7517141	12.57688016	12.57688016	3.175044	0.00101
HAL	22.3810782	0	4.547269558	0	0	20	0.00101
LOC102159232	22.3810782	0	4.547269558	0	0	20	0.00101
EDAR	22.3810782	0	4.547269558	0	0	20	0.00101
LOC100738215	22.3810782	0	4.547269558	0	0	20	0.00101
FAM163B	22.3810782	0	4.547269558	0	0	20	0.00101
LOC102160606	22.3810782	0	4.547269558	0	0	20	0.00101
FAM228A	22.3810782	0	4.547269558	0	0	20	0.00101
LOC102165254	22.3810782	0	4.547269558	0	0	20	0.00101
PRKAG2	266.086152	32.224857	8.061161368	5.054191063	5.054191063	3.045648	0.00101
RNF224	1.65785765	36.356248	1.410263837	5.223277669	5.223277669	-4.45481	0.00102
LOC100627304	431.042988	53.708094	8.755031058	5.773682393	5.773682393	3.00462	0.00102
BTBD11	494.870508	61.970878	8.953819612	5.976612875	5.976612875	2.997389	0.00102
BIRC6	322.453312	39.661362	8.337413677	5.34558663	5.34558663	3.023284	0.00102
LOC102166272	48.9068006	3.3051135	5.641164514	2.106051269	2.106051269	3.887263	0.00102
LOC102162854	48.9068006	3.3051135	5.641164514	2.106051269	2.106051269	3.887263	0.00102
SCARF2	480.778718	3943.0004	8.912226855	11.94544398	11.94544398	-3.03585	0.00103
KLB	252.823291	30.5723	7.987680649	4.980587446	4.980587446	3.047832	0.00103
RORA	29.0125088	241.27328	4.907492018	7.920491517	7.920491517	-3.05592	0.00104
SHMT2	739.404511	93.369456	9.532169874	6.560248082	6.560248082	2.985341	0.00104
SCN2B	0	22.309516	0	4.542847146	4.542847146	-20	0.00105
CDH12	0	22.309516	0	4.542847146	4.542847146	-20	0.00105
CYP11A1	0	22.309516	0	4.542847146	4.542847146	-20	0.00105
OXCT2	97.8136012	9.9153405	6.62663773	3.448285225	3.448285225	3.302301	0.00105
LOC102163982	8.28928824	85.932951	3.215568059	6.441831208	6.441831208	-3.37389	0.00105
QTRTD1	1544.2944	194.17542	10.593666	7.608627545	7.608627545	2.991515	0.00106
GUCA1B	3.31571529	48.750424	2.109599694	5.636636914	5.636636914	-3.87802	0.00108
GBP6	4.97357294	61.1446	2.578594101	5.957557119	5.957557119	-3.61987	0.00108
LOC102159805	358.097252	44.619032	8.488230802	5.511563931	5.511563931	3.00462	0.00108
LOC102168054	54.7093024	4.1313919	5.799846344	2.359350201	2.359350201	3.727086	0.00108
SCUBE1	402.030479	50.402981	8.654745137	5.683780114	5.683780114	2.995724	0.00108
LOC102157733	363.899754	45.44531	8.511356367	5.537461031	5.537461031	3.001338	0.00109
LOC102166279	91.1821706	9.0890621	6.526415834	3.334720158	3.334720158	3.326548	0.00109
LOC100623058	38.9596547	314.81206	5.320472212	8.302922453	8.302922453	-3.01444	0.0011
LOC100511112	321.624384	2581.2936	8.33371167	11.33443734	11.33443734	-3.00465	0.0011
LOC100515730	810.69239	103.2848	9.664789277	6.704385035	6.704385035	2.972527	0.00112
LOC100510895	27.3546512	226.40027	4.825513503	7.829090182	7.829090182	-3.04902	0.00112
DLGAP4	552.066597	4495.7806	9.1113094	12.13467679	12.13467679	-3.02566	0.00112
NLRP14	60.5118041	4.9576702	5.942791385	2.574748269	2.574748269	3.609482	0.00113
SLC17A7	507.30444	64.449713	8.989549022	6.032314961	6.032314961	2.976606	0.00113
XBP1	537.145878	4365.2286	9.071853494	12.09217196	12.09217196	-3.02267	0.00113
LOC100157542	35.6439394	1.6525567	5.195502704	1.407383613	1.407383613	4.430885	0.00113
AKR1CL1	828.099895	6820.928	9.695402126	12.7359638	12.7359638	-3.04209	0.00115
THEMIS2	181.535412	21.483238	7.512032568	4.490777898	4.490777898	3.078968	0.00115
TTBK2	1177.90786	150.38266	10.20323525	7.242056188	7.242056188	2.969517	0.00116
LOC102161200	323.282241	40.48764	8.341106209	5.374609695	5.374609695	2.997241	0.00116
KCNJ11	114.392178	12.394176	6.850401618	3.74353388	3.74353388	3.206254	0.00117
LOC100738143	7.46035941	78.496445	3.080718953	6.312818447	6.312818447	-3.39531	0.00118
BNC1	416.122269	52.881816	8.704326527	5.751726565	5.751726565	2.976164	0.00119
LOC100516635	1.65785765	35.52997	1.410263837	5.191008663	5.191008663	-4.42164	0.00119
EDN1	19.8942918	169.38707	4.385036953	7.412672017	7.412672017	-3.0899	0.00119
LOC100737584	69.6300212	544.51745	6.142209624	9.091481528	9.091481528	-2.9672	0.00119
LOC102161631	247.849718	30.5723	7.959130944	4.980587446	4.980587446	3.019169	0.0012
TMEM117	51.3935871	404.8764	5.711318333	8.664896653	8.664896653	-2.97782	0.0012
KIAA1239	53.8803735	4.1313919	5.778218396	2.359350201	2.359350201	3.70506	0.0012
AK7	3.31571529	47.924146	2.109599694	5.61247475	5.61247475	-3.85336	0.00121
LOC100737517	176.561839	1380.7112	7.472177749	10.43224034	10.43224034	-2.96717	0.00121
TDRD12	9959.57982	1205.5401	13.28201401	10.2366602	10.2366602	3.046405	0.00123
LOC102158014	41.4464412	2.4788351	5.407571694	1.798604302	1.798604302	4.063514	0.00124
TOP1MT	39.7885835	315.63834	5.350093502	8.306692135	8.306692135	-2.98785	0.00125
AKAP17B	664.800917	85.932951	9.378947046	6.441831208	6.441831208	2.951639	0.00125
ARHGAP24	15.7496477	137.98849	4.066058842	7.118821585	7.118821585	-3.13116	0.00125
C13H21orf63	15.7496477	137.98849	4.066058842	7.118821585	7.118821585	-3.13116	0.00125
RELB	125.168252	970.05081	6.979205122	9.923402974	9.923402974	-2.95419	0.00126
KBTBD8	620.03876	80.149002	9.278539502	6.342501448	6.342501448	2.951602	0.00126
GTSF1L	21.5521494	0	4.495193036	0	0	20	0.00128
LOC102161484	21.5521494	0	4.495193036	0	0	20	0.00128
LOC100518709	21.5521494	0	4.495193036	0	0	20	0.00128
ATOH7	21.5521494	0	4.495193036	0	0	20	0.00128
LOC102162525	21.5521494	0	4.495193036	0	0	20	0.00128
LOC102163222	21.5521494	0	4.495193036	0	0	20	0.00128
MARVELD3	21.5521494	0	4.495193036	0	0	20	0.00128
LOC100739530	21.5521494	0	4.495193036	0	0	20	0.00128
C1QTNF7	7.46035941	77.670167	3.080718953	6.29774474	6.29774474	-3.38004	0.00128
AXL	1129.00106	9277.4536	10.14210841	13.17966866	13.17966866	-3.03868	0.00128
INSRR	160.812192	19.004403	7.338176502	4.322245637	4.322245637	3.080971	0.00129
LOC102158067	47.248943	3.3051135	5.592425431	2.106051269	2.106051269	3.83751	0.0013
LONRF2	251.165434	31.398578	7.978226717	5.017858594	5.017858594	2.999867	0.0013
ITM2C	2721.37333	23078.781	11.41064921	14.49434193	14.49434193	-3.08416	0.00131
PDSS1	1594.03013	205.74331	10.63936796	7.691696868	7.691696868	2.953761	0.00131
KCP	22.3810782	185.08636	4.547269558	7.539828464	7.539828464	-3.04785	0.00132
LOC100520605	16.5785765	142.94616	4.135746339	7.169385476	7.169385476	-3.10808	0.00132
KLHL1	0	21.483238	0	4.490777898	4.490777898	-20	0.00133
HEPHL1	0	21.483238	0	4.490777898	4.490777898	-20	0.00133
LOC100738798	0	21.483238	0	4.490777898	4.490777898	-20	0.00133
SKAP1	0	21.483238	0	4.490777898	4.490777898	-20	0.00133
LOC102167375	0	21.483238	0	4.490777898	4.490777898	-20	0.00133
LOC100737944	0	21.483238	0	4.490777898	4.490777898	-20	0.00133
LOC102165903	640.761981	83.454116	9.325894513	6.400095824	6.400095824	2.940733	0.00133
ETNPPL	34.8150106	1.6525567	5.162492464	1.407383613	1.407383613	4.396938	0.00133
LOC100626746	88.6953841	9.0890621	6.486961839	3.334720158	3.334720158	3.286656	0.00134
LOC102167744	9.94714588	95.022013	3.452482877	6.585293272	6.585293272	-3.25591	0.00134
LSR	213.863637	26.440908	7.747277532	4.77825631	4.77825631	3.015848	0.00135
LOC102159808	100.300388	10.741619	6.662495885	3.553559423	3.553559423	3.223044	0.00136
PDZD2	1031.18746	134.68337	10.01148929	7.084100147	7.084100147	2.936663	0.00137
SLC38A3	1.65785765	34.703692	1.410263837	5.158001345	5.158001345	-4.3877	0.00139
LOC102163557	1.65785765	34.703692	1.410263837	5.158001345	5.158001345	-4.3877	0.00139
FA2H	28.18358	0.8262784	4.867084967	0.868906686	0.868906686	5.092083	0.00139
LOC102158893	28.18358	0.8262784	4.867084967	0.868906686	0.868906686	5.092083	0.00139
LOC100519366	8.28928824	82.627837	3.215568059	6.385911347	6.385911347	-3.31731	0.0014
LOC100739077	169.10148	1297.257	7.410251883	10.34236034	10.34236034	-2.9395	0.0014
LOC100516844	4.14464412	52.881816	2.363071282	5.751726565	5.751726565	-3.67345	0.00141
ADA	66.3143059	507.33492	6.072841239	8.98963553	8.98963553	-2.93555	0.00142
LOC102159172	40.6175124	2.4788351	5.379118827	1.798604302	1.798604302	4.034368	0.00142
LOC100157933	40.6175124	2.4788351	5.379118827	1.798604302	1.798604302	4.034368	0.00142
LOC100521521	4.97357294	58.665764	2.578594101	5.89883146	5.89883146	-3.56016	0.00144
LOC102166414	277.691156	35.52997	8.12252341	5.191008663	5.191008663	2.966373	0.00145
CORIN	0.82892882	28.093465	0.870998931	4.862623207	4.862623207	-5.08284	0.00145
PCDH1	271.059725	2089.658	8.087779591	11.02974136	11.02974136	-2.94658	0.00146
LOC102164895	48.0778718	370.17271	5.617000784	8.535946834	8.535946834	-2.94475	0.00146
SEZ6	169.930409	20.656959	7.417265267	4.436758793	4.436758793	3.040244	0.00146
LOC100156757	933.373855	123.11548	9.867856097	6.955539223	6.955539223	2.922443	0.00146
LOC102168181	122.681466	14.046732	6.950485513	3.911378308	3.911378308	3.126611	0.00147
DUSP2	241.218288	30.5723	7.920163984	4.980587446	4.980587446	2.980042	0.00147
LOC102164486	46.4200141	3.3051135	5.567424187	2.106051269	2.106051269	3.811975	0.00147
STPG1	46.4200141	3.3051135	5.567424187	2.106051269	2.106051269	3.811975	0.00147
FIGN	1051.91068	138.81477	10.04016734	7.127372928	7.127372928	2.921779	0.00148
DDR1	407.004052	3165.4724	8.672439671	11.62866081	11.62866081	-2.95931	0.00149
CD97	789.969169	6278.8893	9.627477651	12.61652342	12.61652342	-2.99064	0.0015
GSDMD	397.885835	3087.8023	8.639832083	11.59283181	11.59283181	-2.95615	0.00151
DYNC1I1	617.551974	81.801559	9.272751012	6.371586023	6.371586023	2.91636	0.00151
AK3L1	7413.11047	930.38945	12.8560579	9.863240725	9.863240725	2.994172	0.00152
LOC100625463	218.837209	1669.0823	7.780291786	10.70570349	10.70570349	-2.93113	0.00152
PLLP	63.8275194	5.7839486	6.018534465	2.762125239	2.762125239	3.464052	0.00152
WIPI1	361.412967	2790.3421	8.501490766	11.44674321	11.44674321	-2.94872	0.00153
RUNDC3B	98.64253	10.741619	6.638689748	3.553559423	3.553559423	3.198998	0.00153
LOC100156657	121.852537	919.64783	6.940783842	9.846505582	9.846505582	-2.91595	0.00154
MYLIP	169.930409	1287.3417	7.417265267	10.33129957	10.33129957	-2.92138	0.00154
LOC100152184	625.012333	4908.9198	9.29004727	12.26148375	12.26148375	-2.97345	0.00154
LOC100523351	3.31571529	46.271589	2.109599694	5.56290145	5.56290145	-3.80273	0.00154
LOC100627892	4485.33387	574.26347	12.13132127	9.168079046	9.168079046	2.965431	0.00155
BARX1	664.800917	88.411786	9.378947046	6.482393107	6.482393107	2.910612	0.00155
LOC100516198	33.9860818	1.6525567	5.128709195	1.407383613	1.407383613	4.362172	0.00156
LOC102164563	33.9860818	1.6525567	5.128709195	1.407383613	1.407383613	4.362172	0.00156
PITX2	3676.29933	474.28379	11.8444309	8.892645374	8.892645374	2.954432	0.00156
UBASH3B	2033.3624	266.88791	10.99036099	8.065485684	8.065485684	2.929561	0.00156
LOC102165046	162.470049	19.830681	7.352882522	4.380638094	4.380638094	3.034368	0.00157
CHST12	70.45895	531.29699	6.159042811	9.056087606	9.056087606	-2.91466	0.00157
LOC102165618	16.5785765	139.64104	4.135746339	7.135873883	7.135873883	-3.07433	0.00157
CTNS	169.930409	1279.0789	7.417265267	10.32201704	10.32201704	-2.91209	0.00162
ARHGEF28	11.6050035	104.11107	3.655924617	6.715770874	6.715770874	-3.1653	0.00162
LOC102165933	11.6050035	104.11107	3.655924617	6.715770874	6.715770874	-3.1653	0.00162
EN1	12.4339324	109.89502	3.747809764	6.793050813	6.793050813	-3.14377	0.00162
LOC100622125	20.7232206	0	4.441166102	0	0	20	0.00163
LOC102168196	20.7232206	0	4.441166102	0	0	20	0.00163
SASH3	20.7232206	0	4.441166102	0	0	20	0.00163
EOMES	20.7232206	0	4.441166102	0	0	20	0.00163
LOC102162983	20.7232206	0	4.441166102	0	0	20	0.00163
LOC100623257	20.7232206	0	4.441166102	0	0	20	0.00163
GLB1L2	20.7232206	0	4.441166102	0	0	20	0.00163
LOC100737451	20.7232206	0	4.441166102	0	0	20	0.00163
LOC100151957	243.705074	31.398578	7.934900207	5.017858594	5.017858594	2.956365	0.00163
LOC100621946	39.7885835	2.4788351	5.350093502	1.798604302	1.798604302	4.00462	0.00164
RUNDC3A	15.7496477	133.03082	4.066058842	7.066420948	7.066420948	-3.07837	0.00164
LOC102167875	61.340733	461.06333	5.962103212	8.851946794	8.851946794	-2.91005	0.00164
LOC102160391	80.4060959	8.2627837	6.347064926	3.211445828	3.211445828	3.282605	0.00165
LOC100622933	213.863637	27.267186	7.747277532	4.821056379	4.821056379	2.971453	0.00165
LOC102160929	62.9985906	5.7839486	5.999968229	2.762125239	2.762125239	3.445193	0.00167
RASSF2	21.5521494	173.51846	4.495193036	7.447235822	7.447235822	-3.00919	0.00167
NEBL	867.888478	116.50525	9.763027209	6.876581411	6.876581411	2.897115	0.00167
HIPK4	101.129317	754.39215	6.674253245	9.561081988	9.561081988	-2.89911	0.00167
LOC100622673	57.1960888	4.9576702	5.862850292	2.574748269	2.574748269	3.528182	0.00167
ARSD	135.944327	1014.6698	7.097445694	9.988215791	9.988215791	-2.89992	0.00168
LOC102159603	0	20.656959	0	4.436758793	4.436758793	-20	0.00169
LOC102163989	0	20.656959	0	4.436758793	4.436758793	-20	0.00169
PTPRCAP	0	20.656959	0	4.436758793	4.436758793	-20	0.00169
LOC100520139	0	20.656959	0	4.436758793	4.436758793	-20	0.00169
ZNF132	0	20.656959	0	4.436758793	4.436758793	-20	0.00169
E2F7	1916.48344	254.49374	10.9049984	7.997144125	7.997144125	2.912759	0.0017
LOC102167381	17.4075053	143.77244	4.202222212	7.177643143	7.177643143	-3.04601	0.00171
EXO1	2115.42636	280.93465	11.04741457	8.139216969	8.139216969	2.912642	0.00173
C1QTNF2	165.785765	1236.1124	7.381852349	10.27276092	10.27276092	-2.89842	0.00173
F2RL2	7.46035941	74.365053	3.080718953	6.2358238	6.2358238	-3.31731	0.00174
CBLN3	3.31571529	45.44531	2.109599694	5.537461031	5.537461031	-3.77674	0.00175
SFRP1	1072.6339	8455.3066	10.06828641	13.04581197	13.04581197	-2.9787	0.00175
SLC9A3R2	1817.01198	14609.428	10.82814599	13.83471081	13.83471081	-3.00726	0.00176
LOC100737008	0.82892882	27.267186	0.870998931	4.821056379	4.821056379	-5.03977	0.00176
LOC102157990	830.586681	112.37386	9.699722842	6.824944216	6.824944216	2.885824	0.00177
LDOC1L	76.2614518	565.17441	6.271676881	9.145102724	9.145102724	-2.88967	0.00177
SLA-7	313.335095	2363.1561	8.296159547	11.20710961	11.20710961	-2.91494	0.00178
GRAMD1B	2568.0215	340.42669	11.32700325	8.415432027	8.415432027	2.915241	0.00178
LOC102157928	73.7746653	7.4365053	6.224477643	3.076645515	3.076645515	3.310429	0.00179
CD44	3089.41773	25303.949	11.59358614	14.62713194	14.62713194	-3.03396	0.00179
LOC100739537	62.9985906	467.67356	5.999968229	8.872439593	8.872439593	-2.89211	0.0018
UGP2	1052.73961	8256.9998	10.04130269	13.01157666	13.01157666	-2.97147	0.00181
LOC100625326	30.6703665	235.48934	4.985061656	7.885631319	7.885631319	-2.94074	0.00182
SEPP1	91.1821706	672.59059	6.526415834	9.395728184	9.395728184	-2.8829	0.00182
FHL2	1008.80638	7891.7847	9.979862979	12.94631869	12.94631869	-2.9677	0.00182
CUX1	369.702255	2791.1683	8.534117082	11.44717021	11.44717021	-2.91643	0.00183
CHRM4	62.1696618	5.7839486	5.981159942	2.762125239	2.762125239	3.426084	0.00183
TM4SF5	33.1571529	1.6525567	5.094115824	1.407383613	1.407383613	4.326548	0.00184
LOC102167610	33.1571529	1.6525567	5.094115824	1.407383613	1.407383613	4.326548	0.00184
ST6GALNAC2	583.565892	4467.6872	9.191221843	12.12563533	12.12563533	-2.93656	0.00184
LOC102163288	56.36716	418.09686	5.842153193	8.711139889	8.711139889	-2.89091	0.00185
EFNB3	9.11821706	85.106672	3.338883189	6.428053129	6.428053129	-3.22245	0.00185
CMAH	20.7232206	165.25567	4.441166102	7.37725977	7.37725977	-2.99538	0.00185
SGK1	157.496477	1162.5737	7.308306958	10.18434684	10.18434684	-2.88393	0.00186
LOC100739105	90.3532418	9.9153405	6.51338402	3.448285225	3.448285225	3.187842	0.00186
NOR-1	169.930409	21.483238	7.417265267	4.490777898	4.490777898	2.983661	0.00188
LOC100515829	67.9721635	500.72469	6.107942317	8.970752135	8.970752135	-2.881	0.00188
LOC102163881	84.55074	9.0890621	6.418708429	3.334720158	3.334720158	3.217614	0.00188
ADI1	900.216702	6982.0522	9.815730241	12.76964205	12.76964205	-2.95531	0.00188
LOC102166544	102.787174	754.39215	6.697484358	9.561081988	9.561081988	-2.87565	0.00189
LOC100739615	44.7621565	3.3051135	5.516083133	2.106051269	2.106051269	3.759508	0.00189
NTRK1	43.9332277	328.03251	5.489710792	8.362086342	8.362086342	-2.90045	0.00189
CD99L2	287.638302	2145.8449	8.173118945	11.06800227	11.06800227	-2.89922	0.00189
LOC100739698	279.349014	2080.5689	8.131080184	11.02345562	11.02345562	-2.89684	0.00191
KATNAL2	146.720402	18.178124	7.206725282	4.261389712	4.261389712	3.012794	0.00191
UNG	6071.0747	789.92212	12.56797382	9.627391839	9.627391839	2.94217	0.00192
COL2A1	198.113989	25.61463	7.637450771	4.734147579	4.734147579	2.951291	0.00192
LOC100622016	1.65785765	33.051135	1.410263837	5.089630977	5.089630977	-4.31731	0.00192
LOC102167726	1.65785765	33.051135	1.410263837	5.089630977	5.089630977	-4.31731	0.00192
MEOX1	19.8942918	158.64545	4.385036953	7.318727602	7.318727602	-2.99538	0.00193
DMPK	68.8010924	504.85609	6.125177709	8.98258319	8.98258319	-2.87537	0.00193
DOCK10	16.5785765	135.50965	4.135746339	7.092859162	7.092859162	-3.031	0.00195
APCDD1	123.510395	14.873011	6.96012238	3.98850389	3.98850389	3.053864	0.00196
GCNT2	3127.54845	417.27058	11.61127773	8.708292708	8.708292708	2.905977	0.00196
IGFBP6	606.775893	4610.6333	9.247395656	12.17106209	12.17106209	-2.92573	0.00197
BTBD17	22.3810782	175.17101	4.547269558	7.460832769	7.460832769	-2.96841	0.00198
ZNF793	14.09179	118.15781	3.915692025	6.89672967	6.89672967	-3.06779	0.00199
EML5	242.876145	32.224857	7.930004838	5.054191063	5.054191063	2.913975	0.002
ZNF624	36.4728682	272.67186	5.227774503	8.096303303	8.096303303	-2.90227	0.00202
LOC102163074	29.0125088	220.61633	4.907492018	7.791920351	7.791920351	-2.92679	0.00202
LIPG	671.432347	5103.9215	9.393245316	12.31767306	12.31767306	-2.92629	0.00202
LOC100517788	1463.05937	199.95937	10.51575835	7.650760008	7.650760008	2.87121	0.00202

LOC100516665	127.034505	960.13547	7.000388854	9.908595977	9.908595977	-2.91802	0.03436	TAPBPL	233.6763581	2517.6702	7.874528698	11.29844651	11.29844651	-3.42951	0.01921	PAIP2B	1229.30145	168.56079	10.26479613	7.405658764	7.405658764	2.866498	0.00202
LDB3	19.3313377	155.34033	4.345633235	7.288546214	7.288546214	-3.00642	0.03453	MFI2	35.95020894	367.69388	5.207510617	8.526279641	8.526279641	-3.35443	0.01922	GPR19	451.766209	61.970878	8.82262248	5.976612875	5.976612875	2.865914	0.00203
TTLL6	8.28485901	79.322724	3.214880004	6.327736286	6.327736286	-3.25919	0.03455	LOC100622310	0	23.135794	0	4.593102407	4.593102407	-20	0.01926	GAREML	1889.95772	256.97257	10.88490139	8.011073883	8.011073883	2.878668	0.00203
EXOC6	360.391367	61.970878	8.497418233	5.976612875	5.976612875	2.539902	0.03461	SPTLC3	0	23.135794	0	4.593102407	4.593102407	-20	0.01926	DSTN	3532.89465	28625.588	11.78704331	14.8050681	14.8050681	-3.01838	0.00204
NXPH4	256.830629	43.792754	8.010279851	5.485193456	5.485193456	2.552053	0.03466	LOC100626249	0	23.135794	0	4.593102407	4.593102407	-20	0.01926	LOC100738135	77.9193094	8.2627837	6.302306427	3.211445828	3.211445828	3.237281	0.00205
GPR143	207.121475	1589.7596	7.70128203	10.6355001	10.6355001	-2.94026	0.03474	MUSK	0	23.135794	0	4.593102407	4.593102407	-20	0.01926	LOC102161349	26.5257224	0.8262784	4.78270852	0.868906686	0.868906686	5.00462	0.00206
LOC102162973	1.38080984	32.224857	1.251452392	5.054191063	5.054191063	-4.54459	0.03477	LOC102164504	0	23.135794	0	4.593102407	4.593102407	-20	0.01926	SOX3	26.5257224	0.8262784	4.78270852	0.868906686	0.868906686	5.00462	0.00206
LOC100737173	1.38080984	32.224857	1.251452392	5.054191063	5.054191063	-4.54459	0.03477	VTN	0	23.135794	0	4.593102407	4.593102407	-20	0.01926	CHRND	19.8942918	156.99289	4.385036953	7.303715831	7.303715831	-2.98027	0.00208
TOMM20	25725.8681	3859.5463	14.65098822	11.91458929	11.91458929	2.736717	0.03482	MAP3K7CL	0	23.135794	0	4.593102407	4.593102407	-20	0.01926	SPHK1	73.7746653	535.42838	6.224477643	9.067241769	9.067241769	-2.8595	0.00208
MDFI	363.152987	2858.0969	8.508400868	11.48134379	11.48134379	-2.97641	0.03506	LOC102167616	0	23.135794	0	4.593102407	4.593102407	-20	0.01926	LOC100621940	94.4978859	10.741619	6.57739689	3.553559423	3.553559423	3.137071	0.00208
GOLGA3	216.787144	1663.2984	7.766774985	10.70069838	10.70069838	-2.9397	0.03512	LOC100737823	0	23.135794	0	4.593102407	4.593102407	-20	0.01926	FAM65C	14.09179	117.33153	3.915692025	6.886690713	6.886690713	-3.05766	0.00209
LOC100738084	5.52323934	60.318321	2.705588563	5.938246292	5.938246292	-3.44901	0.03527	LOC102165147	0	23.135794	0	4.593102407	4.593102407	-20	0.01926	ZIC5	890.269557	123.11548	9.799718018	6.955539223	6.955539223	2.85423	0.00209
NOD1	85.6102098	637.8869	6.436465198	9.319416755	9.319416755	-2.89745	0.03533	URGCP	141.6861176	1486.4748	7.156701167	10.5386495	10.5386495	-3.39112	0.0193	GNAL	19.8942918	0	4.385036953	0	0	20	0.0021
APC2	28.9970066	222.26888	4.906746634	7.80263838	7.80263838	-2.93833	0.03535	LOC100517788	1778.477984	199.95937	10.79723837	7.650760007	7.650760007	3.152864	0.01931	LOC102168035	19.8942918	0	4.385036953	0	0	20	0.0021
B4GALNT4	1383.57146	234.66306	10.43522379	7.88058181	7.88058181	2.559735	0.03554	LOC100515242	50.75323616	4.1313919	5.693577173	2.359350201	2.359350201	3.6188	0.01934	LOC100628139	19.8942918	0	4.385036953	0	0	20	0.0021
LOC100157933	28.9970066	2.4788351	4.906746634	1.798604302	1.798604302	3.54817	0.03563	LRRC27	31.7207726	324.7274	5.032134908	8.347521274	8.347521274	-3.35573	0.0194	LOC100739063	19.8942918	0	4.385036953	0	0	20	0.0021
LIPI	28.9970066	2.4788351	4.906746634	1.798604302	1.798604302	3.54817	0.03563	ASB6	295.0031852	3210.9178	8.20946889	11.64921923	11.64921923	-3.44418	0.01947	PAQR9	19.8942918	0	4.385036953	0	0	20	0.0021
PAX6	491.568302	85.106672	8.944179978	6.428053129	6.428053129	2.530048	0.03571	STMN2	485.3278207	5444.3482	8.925785314	12.41080859	12.41080859	-3.48773	0.01948	LOC102157880	19.8942918	0	4.385036953	0	0	20	0.0021
THSD7A	6.90404918	69.407383	2.982591923	6.137654819	6.137654819	-3.32957	0.03575	LOC100739776	33.83549077	345.38436	5.122485983	8.436229978	8.436229978	-3.35159	0.01948	PVALB1	19.8942918	0	4.385036953	0	0	20	0.0021
LOC100623494	15.1889082	125.59431	4.016933786	6.98406878	6.98406878	-3.04768	0.03576	LOC100152601	209.3570991	23.962073	7.716696697	4.641665831	4.641665831	3.127141	0.0195	LOC100623946	19.8942918	0	4.385036953	0	0	20	0.0021
ABCA4	158.793131	26.440908	7.320061583	4.77825631	4.77825631	2.586305	0.03595	DYDC2	1.057359087	33.051135	1.04079362	5.089630977	5.089630977	-4.96616	0.01951	LOC102158173	19.8942918	0	4.385036953	0	0	20	0.0021
SERPINB9	69.0404918	9.9153405	6.130117309	3.448285225	3.448285225	2.799709	0.036	HMGA2	232.618999	2496.187	7.868013796	11.28608812	11.28608812	-3.42369	0.01952	CRYBA4	19.8942918	0	4.385036953	0	0	20	0.0021
LOC100738857	78.7061606	582.52625	6.316619332	9.188653752	9.188653752	-2.88777	0.0363	OLFML2B	51.81059524	526.33932	5.722755497	9.04258777	9.04258777	-3.34467	0.01956	SLC9A4	19.8942918	0	4.385036953	0	0	20	0.0021
OTUD7A	22.0929574	172.69218	4.529381037	7.44038899	7.44038899	-2.96654	0.03632	IFI44	773.9868514	8936.2006	9.598028023	13.12560729	13.12560729	-3.52928	0.01958	ARMC4	49.7357294	4.1313919	5.664930183	2.359350201	2.359350201	3.589583	0.0021
FABP3	490.187492	85.106672	8.940130012	6.428053129	6.428053129	2.52599	0.03632	GUF1	4683.043394	504.85609	12.19353873	8.98258319	8.98258319	3.213502	0.01966	WIPF3	2356.64465	319.76973	11.20313057	8.325394195	8.325394195	2.881629	0.0021
KLHL42	122.892075	915.51644	6.9529401	9.840016943	9.840016943	-2.89719	0.03658	LOC102163337	181.8657629	20.656959	7.514641181	4.436758793	4.436758793	3.138174	0.01972	LOC102163933	143.404687	1040.2845	7.173973754	10.02414854	10.02414854	-2.85881	0.0021
OAZ3	20.7121475	162.77684	4.440430524	7.35558754	7.35558754	-2.97435	0.03666	GAS1	347.8711395	3811.6221	8.446550445	11.89656784	11.89656784	-3.45378	0.01973	KPNA5	315.821882	42.966475	8.307528171	5.458331975	5.458331975	2.877828	0.00211
BST2	111.845597	829.58349	6.818206315	9.697981376	9.697981376	-2.89088	0.03691	LOC100522267	6.344154519	80.97528	2.876596413	6.357117027	6.357117027	-3.67398	0.01973	NHS	1553.41262	213.17982	10.6021538	7.742678745	7.742678745	2.865298	0.00211
STEAP1	182.266898	1372.4484	7.517802419	10.42358697	10.42358697	-2.91263	0.03706	SLA-DOB2	11.63094995	129.7257	3.658891241	7.030399033	7.030399033	-3.47942	0.01978	LOC102167027	88.6953841	9.9153405	6.486961839	3.448285225	3.448285225	3.161125	0.00212
KRT81	4.14242951	50.402981	2.362450112	5.683780114	5.683780114	-3.60496	0.03708	DHFR	7680.656405	808.92653	12.90720172	9.661647227	9.661647227	3.247149	0.0199	FNDC9	88.6953841	9.9153405	6.486961839	3.448285225	3.448285225	3.161125	0.00212
ZNF226	35.9010557	268.54047	5.205590187	8.074358096	8.074358096	-2.90304	0.03709	LOC100516420	3.17207726	52.055537	2.060765874	5.729431428	5.729431428	-4.03655	0.01991	LOC100153751	123.510395	893.20692	6.96012238	9.8044649	9.8044649	-2.85436	0.00212
RECK	197.455807	1491.4325	7.632673964	10.54344993	10.54344993	-2.9171	0.03709	LOC102160060	5.286795433	71.05994	2.65232482	6.171125545	6.171125545	-3.74857	0.02004	LOC100738813	9.11821706	83.454116	3.338883189	6.400095824	6.400095824	-3.19416	0.00213
LOC100518000	19.3313377	0.8262784	4.345633235	0.868906686	0.868906686	4.54817	0.03721	SASS6	1726.667388	195.82797	10.75460978	7.620791467	7.620791467	3.140331	0.02005	LOC100627669	1087.55462	150.38266	10.08819808	7.242056188	7.242056188	2.854378	0.00213
LOC102164525	19.3313377	0.8262784	4.345633235	0.868906686	0.868906686	4.54817	0.03721	ENG	252.7088217	2706.8879	7.987029874	11.40295233	11.40295233	-3.42109	0.02006	EPHA2	623.354475	4693.2612	9.286221537	12.19668239	12.19668239	-2.91247	0.00213
LOC100514688	19.3313377	0.8262784	4.345633235	0.868906686	0.868906686	4.54817	0.03721	MBTD1	9130.295713	955.1778	13.15660387	9.901135097	9.901135097	3.25682	0.02016	LOC102167627	43.9332277	3.3051135	5.489710792	2.106051269	2.106051269	3.732541	0.00214
LOC102158079	19.3313377	0.8262784	4.345633235	0.868906686	0.868906686	4.54817	0.03721	LOC100627892	5335.433951	574.26347	12.38166027	9.168079046	9.168079046	3.215821	0.02019	LOC102163179	43.9332277	3.3051135	5.489710792	2.106051269	2.106051269	3.732541	0.00214
LOC102160118	19.3313377	0.8262784	4.345633235	0.868906686	0.868906686	4.54817	0.03721	LOC102165360	13.74566813	148.73011	3.882219287	7.22622053	7.22622053	-3.43565	0.02021	MTMR9	1488.75617	204.91704	10.5408605	7.685919384	7.685919384	2.860996	0.00214
LOC100627180	19.3313377	0.8262784	4.345633235	0.868906686	0.868906686	4.54817	0.03721	LOC100620269	729.5777697	8327.2334	9.512894045	13.02379479	13.02379479	-3.5127	0.02023	LOC100514339	436.84549	60.318321	8.774278041	5.938246292	5.938246292	2.856455	0.00215
LOC100625977	19.3313377	0.8262784	4.345633235	0.868906686	0.868906686	4.54817	0.03721	TMEM200A	33.83549077	342.07925	5.122485983	8.422398044	8.422398044	-3.33772	0.02026	LOC100152536	1139.77713	157.81917	10.15580125	7.311241242	7.311241242	2.852407	0.00216
DGAT2	19.3313377	0.8262784	4.345633235	0.868906686	0.868906686	4.54817	0.03721	CAMK2N2	441.9760982	52.055537	8.791085047	5.729431428	5.729431428	3.085845	0.02028	LOC102162575	32.3282241	1.6525567	5.058672541	1.407383613	1.407383613	4.290023	0.00217
WNK3	12.4272885	105.76363	3.747096094	6.738276475	6.738276475	-3.08926	0.03723	tRNA-His	3285.214682	362.73621	11.68221102	8.506748724	8.506748724	3.178995	0.02032	LOC102167675	32.3282241	1.6525567	5.058672541	1.407383613	1.407383613	4.290023	0.00217
RASSF4	103.560738	765.13377	6.708197415	9.581452508	9.581452508	-2.88523	0.03728	LOC100737169	476.868948	5288.1816	8.900471214	12.36882879	12.36882879	-3.47111	0.02036	LOC100515353	32.3282241	1.6525567	5.058672541	1.407383613	1.407383613	4.290023	0.00217
LOC102157981	153.269892	25.61463	7.269312714	4.734147579	4.734147579	2.581034	0.03731	GAMT	70.8430588	715.55707	6.166776871	9.484937802	9.484937802	-3.33637	0.0204	LOC102159118	0	19.830681	0	4.380638094	4.380638094	-20	0.00217
SLCO3A1	95.2758787	702.33662	6.58910248	9.458071516	9.458071516	-2.88198	0.03739	CUX1	261.1676944	2791.1683	8.03434611	11.44717021	11.44717021	-3.41782	0.02041	LOC100736919	0	19.830681	0	4.380638094	4.380638094	-20	0.00217
PGM2	2624.9195	438.75382	11.35860697	8.780552285	8.780552285	2.58079	0.03739	FAM46B	77.18721332	780.83306	6.288860784	9.610716783	9.610716783	-3.33858	0.02045	LOC102164546	0	19.830681	0	4.380638094	4.380638094	-20	0.00217
RNF17	5.52323934	59.492043	2.705588563	5.918673475	5.918673475	-3.42911	0.0374	LOC100737799	167.0627357	19.004403	7.392856063	4.322245637	4.322245637	3.135984	0.02049	LOC102158147	0	19.830681	0	4.380638094	4.380638094	-20	0.00217
ASF1B	3891.12212	640.36574	11.92634126	9.325003477	9.325003477	2.603218	0.03741	YBX2	4.229436346	61.1446	2.386655454	5.957557118	5.957557118	-3.85369	0.0205	LOC102165393	0	19.830681	0	4.380638094	4.380638094	-20	0.00217
LDHD	1441.56547	246.23095	10.49442108	7.949715578	7.949715578	2.549552	0.03749	LOC102167822	4.229436346	61.1446	2.386655454	5.957557118	5.957557118	-3.85369	0.0205	MIR221	0	19.830681	0	4.380638094	4.380638094	-20	0.00217
LCA5	299.635734	52.055537	8.231872692	5.729431428	5.729431428	2.525086	0.03753	LOC102164444	107.8506268	11.567897	6.766205905	3.651671381	3.651671381	3.220836	0.02053	LOC100620821	0	19.830681	0	4.380638094	4.380638094	-20	0.00217
TLE6	77.3253508	11.567897	6.291407421	3.65167138	3.65167138	2.740815	0.03755	LOC102165489	6.344154519	80.149002	2.876596413	6.342501448	6.342501448	-3.65918	0.02054	ADAMTS7	616.723045	4623.8538	9.270816343	12.17519203	12.17519203	-2.9064	0.0022
LOC100626258	374.199466	65.275991	8.551513962	6.05041444	6.05041444	2.519183	0.03758	LOC100738639	2.114718173	42.140197	1.639101631	5.430960858	5.430960858	-4.31666	0.02057	LOC100739082	290.954017	39.661362	8.189597352	5.34558663	5.34558663	2.874985	0.00221
IFITM1	306.539784	2355.7196	8.26462924	11.20256443	11.20256443	-2.94202	0.03765	LOC100737149	1118.685914	129.7257	10.12887838	7.030399033	7.030399033	3.108269	0.02062	LOC102162476	149.207188	19.004403	7.230810045	4.322245637	4.322245637	2.972911	0.00223
LOC100622645	157.412321	26.440908	7.307540742	4.77825631	4.77825631	2.573705	0.03783	MLKL	23.2618999	237.96817	4.600620625	7.900674664	7.900674664	-3.35473	0.02063	LOC100628093	40.6175124	297.46021	5.379118827	8.221394816	8.221394816	-2.87252	0.00224
FAM54A	1379.42903	236.31561	10.430901	7.890663216	7.890663216	2.545284	0.03787	LOC102164270	43.35172255	434.62242	5.470918232	8.766934408	8.766934408	-3.3256	0.02065	LOC100513927	149.207188	1073.3356	7.230810045	10.06922902	10.06922902	-2.84671	0.00224
LOC100628205	33.1394361	3.3051135	5.093367322	2.106051269	2.106051269	3.325777	0.03803	TAP1	530.7942615	5905.4115	9.054724399	12.52806616	12.52806616	-3.47581	0.02066	LOC100737118	3.31571529	43.792754	2.109599694	5.485193456	5.485193456	-3.7233	0.00224
LOC100739496	33.1394361	3.3051135	5.093367322	2.106051269	2.106051269	3.325777	0.03803	AGRN	175.5216084	1829.3803	7.463700987	10.83792773	10.83792773	-3.38163	0.02071	LMOD3	3.31571529	43.792754	2.109599694	5.485193456	5.485193456	-3.7233	0.00224
NFKBIA	563.370413	4461.0769	9.140498547	12.12349967	12.12349967	-2.98524	0.03819	ARHGAP24	12.68830904	137.98849	3.774872332	7.118821584	7.118821584	-3.44298	0.02073	C2H19orf67	216.350423	28.919743	7.763879093	4.903025878	4.903025878	2.903243	0.00225
HHAT	59.3748229	434.62242	5.915875148	8.766934408	8.766934408	-2.87184	0.03822	LOC100737115	12.68830904	137.98849	3.774872332	7.118821584	7.118821584	-3.44298	0.02073	ADAMDEC1	1.65785765	32.224857	1.410263837	5.054191063	5.054191063	-4.28078	0.00227
DDIT3	310.682213	2378.0292	8.283932016	11.21615724	11.21615724	-2.93625	0.03848	LOC102157421	24.31925899	0.8262784	4.662163277	0.868906686	0.868906686	4.879328	0.02083	LIN7A	1.65785765	32.224857	1.410263837	5.054191063	5.054191063	-4.28078	0.00227
LOC100523668	1.38080984	31.398578	1.251452392	5.017858594	5.017858594	-4.50711	0.0386	LOC100621509	24.31925899	0.8262784	4.662163277	0.868906686	0.868906686	4.879328	0.02083	LOC102158582	1.65785765	32.224857	1.410263837	5.054191063	5.054191063	-4.28078	0.00227
CMTM5	59.3748229	8.2627837	5.915875148	3.211445828	3.211445828	2.845151	0.03878	LOC100624812	1225.479181	14376.417	10.26030703	13.81151693	13.81151693	-3.55229	0.02083	LOC100623995	84.55074	605.66205	6.418708429	9.244749247	9.244749247	-2.84062	0.00227
LOC102163855	19.3313377	151.20894	4.345633235	7.249909307	7.249909307	-2.96753	0.03891	LOC102167134	273.8560034	32.224856	8.102532181	5.054191063	5.054191063	3.087172	0.02086	ATP2C2	71.2878788	7.4365053	6.175681852	3.076645515	3.076645515	3.26096	0.00227
URGCP	198.836616	1486.4748	7.642677144	10.5386495	10.5386495	-2.90224	0.03897	DDX21	64471.41294	6108.676	15.97639436	12.57688016	12.57688016	3.399728	0.02092	PUS7	1377.67971	191.69658	10.42907161	7.590187173	7.590187173	2.845344	0.0023
LOC100522672	15.1889082	123.11548	4.016933786	6.955539223	6.955539223	-3.01892	0.03903	LOC102163704	34.89284986	349.51575	5.165624571	8.453335466	8.453335466	-3.32435	0.02098	ZFAND4	98.64253	11.567897	6.638689748	3.65167138	3.65167138	3.092083	0.00231
TCEAL5	0	21.483238	0	4.490777898	4.490777898	-20	0.03907	LAPTM4B	47883.56359	4616.4173	15.54727303	12.1728704	12.1728704	3.374685	0.02105	LOC100739621	148.378259	19.004403	7.222826382	4.322245637	4.322245637	2.964874	0.00232
LOC102161784	0	21.483238	0	4.490777898	4.490777898	-20	0.03907	LOC100625581	48.63851798	485.0254	5.633388138	8.924887914	8.924887914	-3.31789	0.02108	PLK2	1333.74648	10222.716	10.38235003	13.31963205	13.31963205	-2.93822	0.00232
LOC102163527	0	21.483238	0	4.490777898	4.490777898	-20	0.03907	PTH1R	20.08982264	206.56959	4.398475058	7.697451307	7.697451307	-3.36209	0.02108	LOC100737851	43.1042988	313.1595	5.462847376	8.29535341	8.29535341	-2.86099	0.00232
SLC18A1	0	21.483238	0	4.490777898	4.490777898	-20	0.03907	WDR93	212.5291764	24.788351	7.738289401	4.688647628	4.688647628	3.099927	0.02111	USP49	237.902572	32.224857	7.900278578	5.054191063	5.054191063	2.884125	0.00233
HEPHL1	0	21.483238	0	4.490777898	4.490777898	-20	0.03907	LOC100516238	68.72834063	6.610227	6.123673244	2.927939483	2.927939483	3.378133	0.02116	ELAC2	6565.11628	873.37624	12.68082458	9.772110386	9.772110386	2.910145	0.00234
LOC102160773	0	21.483238	0	4.490777898	4.490777898	-20	0.03907	LOC100737841	3.17207726	51.229259	2.060765874	5.706786332	5.706786332	-4.01347	0.02118	MROH6	142.575758	18.178124	7.165668365	4.261389712	4.261389712	2.971453	0.00235
LOC102160716	0	21.483238	0	4.490777898	4.490777898	-20	0.03907	LOC100522521	1104.940245	128.89943	10.11105772	7.021251245	7.021251245	3.099651	0.0212	LOC100517681	109.418605	13.220454	6.786839466	3.829895615	3.829895615	3.049014	0.00235
LOC102158216	0	21.483238	0	4.490777898	4.490777898	-20	0.03907	ZER1	203.0129446	2118.5777	7.672516884	11.04956117	11.04956117	-3.38345	0.02127	LOC100626701	2035.84919	15834.799	10.99212345	13.95090201	13.95090201	-2.9594	0.00236
LOC102167375	0	21.483238	0	4.490777898	4.490777898	-20	0.03907	SORL1	30.66341351	1.6525567	4.98474489	1.407383614	1.407383614	4.213747	0.02133	LOC100622645	198.113989	26.440908	7.637450771	4.77825631	4.77825631	2.905487	0.00236
MBOAT1	8889.65372	1422.0251	13.11807379	10.47474537	10.47474537	2.64418	0.03914	LOC100512568	30.66341351	1.6525567	4.98474489	1.407383614	1.407383614	4.213747	0.02133	TGFBR3	43.9332277	318.11717	5.489710792	8.317942438	8.317942438	-2.85617	0.00237
MYCBPAP	320.347882	56.186929	8.327992153	5.837613535	5.837613535	2.511333	0.03933	LOC102163191	30.66341351	1.6525567	4.98474489	1.407383614	1.407383614	4.213747	0.02133	LRP3	718.68129	5360.0678	9.491214342	12.38830466	12.38830466	-2.89883	0.00239
PTPRB	334.15598	58.665764	8.388688866	5.89883146	5.89883146	2.509931	0.03938	LOC102168050	354.215294	42.140197	8.472549889	5.430960858	5.430960858	3.071358	0.02138	SERTAD1	294.269732	2126.0143	8.205889666	11.05461398	11.05461398	-2.85294	0.00243
LOC100737262	63.5172524	461.06333	6.011613095	8.851946794	8.851946794	-2.85974	0.03961	LOC102167215	37.00756803	2.4788351	5.24821481	1.798604302	1.798604302	3.900086	0.02138	PNMA2	43.1042988	3.3051135	5.462847376	2.106051269	2.106051269	3.70506	0.00243
LOC100737774	323.109502	2462.3095	8.340337505	11.26638222	11.26638222	-2.92992	0.03965	KCNN3	6.344154519	79.322724	2.876596413	6.327736286	6.327736286	-3.64423	0.02139	LOC102158261	43.1042988	3.3051135	5.462847376	2.106051269	2.106051269	3.70506	0.00243
LOC102161016	5.52323934	58.665764	2.705588563	5.89883146	5.89883146	-3.40893	0.03968	LAMTOR4	255.880899	2699.4514	8.004955809	11.39898489	11.39898489	-3.39912	0.02144	IRF6	11.6050035	98.327126	3.655924617	6.634115866	6.634115866	-3.08284	0.00244
LOC102163005	37.2818656	4.1313919	5.258589232	2.359350201	2.359350201	3.173774	0.03973	CXCL9	1.057359087	32.224856	1.04079362	5.054191063	5.054191063	-4.92964	0.02146	LRRC29	11.6050035	98.327126	3.655924617	6.634115866	6.634115866	-3.08284	0.00244
LOC102162807	19.3313377	150.38266	4.345633235	7.242056188	7.242056188	-2.95962	0.03986	KCNN1	1.057359087	32.224856	1.04079362	5.054191063	5.054191063	-4.92964	0.02146	LOC100623982	70.45895	7.4365053	6.159042811	3.076645515	3.076645515	3.244086	0.00247
LOC100626701	1876.52057	15834.799	10.874613	13.95090201	13.95090201	-3.07697	0.03992	ICAM5	1.057359087	32.224856	1.04079362	5.054191063	5.054191063	-4.92964	0.02146	LOC100622980	543.777308	76.843889	9.089522802	6.282511874	6.282511874	2.823014	0.00247
WWC1	13.8080984	113.20014	3.888314478	6.83542057	6.83542057	-3.03529	0.04002	LOC102166231	1.057359087	32.224856	1.04079362	5.054191063	5.054191063	-4.92964	0.02146	MRAS	440.161205	3210.9178	8.785162119	11.64921924	11.64921924	-2.86688	0.00248
LOC102161587	109.083977	794.05352	6.782460686	9.634908162	9.634908162	-2.8638	0.04013	ZNF555	1.057359087	32.224856	1.04079362	5.054191063	5.054191063	-4.92964	0.02146	LOC100517940	27.3546512	203.26448	4.825513503	7.674294538	7.674294538	-2.8935	0.00248
HSD11B2	67.659682	9.9153405	6.101391269	3.448285225	3.448285225	2.770562	0.04027	LOC102158582	1.057359087	32.224856	1.04079362	5.054191063	5.054191063	-4.92964	0.02146	MARCKS	2181.74066	16901.512	11.09192502	14.04495108	14.04495108	-2.9536	0.00248
TDRD7	226.452813	1689.7393	7.829423468	10.72343848	10.72343848	-2.89952	0.04037	LOC100515384	106.7932677	11.567897	6.752123267	3.651671381	3.651671381	3.206622	0.02148	LRFN5	474.147287	66.928548	8.892230982	6.085946114	6.085946114	2.824642	0.00248
LOC102159605	13.8080984	0	3.888314478	0	0	20	0.04057	SPRY1	158.603863	1629.4209	7.319670341	10.67102878	10.67102878	-3.36086	0.02153	TOM1L1	348.150106	48.750424	8.4477036	5.636636914	5.636636914	2.836223	0.0025
LOC102168014	13.8080984	0	3.888314478	0	0	20	0.04057	ZFP112	25.37661808	255.32002	4.721187694	8.001802338	8.001802338	-3.33073	0.02162	LOC102166801	382.136188	53.708094	8.581713486	5.773682393	5.773682393	2.830875	0.00251
LOC102161357	13.8080984	0	3.888314478	0	0	20	0.04057	SCUBE1	421.8862755	50.402981	8.724125929	5.683780113	5.683780113	3.065273	0.0217	ZSCAN9	92.0110994	10.741619	6.539330984	3.553559423	3.553559423	3.098596	0.00252
LOC102159869	13.8080984	0	3.888314478	0	0	20	0.04057	RCN3	1903.246356	22778.016	10.89500442	14.4754178	14.4754178	-3.58111	0.02172	EMSP1	25.6967935	0.8262784	4.73859457	0.868906686	0.868906686	4.958817	0.00252
SERPINB11	13.8080984	0	3.888314478	0	0	20	0.04057	LOC100739855	431.4025073	4680.867	8.756231078	12.19286823	12.19286823	-3.43967	0.02173	LOC102162937	25.6967935	0.8262784	4.73859457	0.868906686	0.868906686	4.958817	0.00252
LOC102163657	13.8080984	0	3.888314478	0	0	20	0.04057	PGAM2	20.08982264	204.91704	4.398475058	7.685919384	7.685919384	-3.3505	0.02177	LOC100625977	25.6967935	0.8262784	4.73859457	0.868906686	0.868906686	4.958817	0.00252
LOC102163658	13.8080984	0	3.888314478	0	0	20	0.04057	SCN2B	0	22.309516	0	4.542847146	4.542847146	-20	0.022	GPD1L	3939.06977	538.7335	11.94400546	9.07610342	9.07610342	2.870211	0.00253
LOC102166688	13.8080984	0	3.888314478	0	0	20	0.04057	LOC102157538	0	22.309516	0	4.542847146	4.542847146	-20	0.022	EMP3	1316.33897	9964.9172	10.36341091	13.28278687	13.28278687	-2.92033	0.00254
LOC102166698	13.8080984	0	3.888314478	0	0	20	0.04057	LOC102164750	0	22.309516	0	4.542847146	4.542847146	-20	0.022	LOC102164768	251.994362	1801.2869	7.982961427	10.81561293	10.81561293	-2.83756	0.00255
ELOVL3	13.8080984	0	3.888314478	0	0	20	0.04057	MYLK4	0	22.309516	0	4.542847146	4.542847146	-20	0.022	EFHC2	113.563249	14.046732	6.8400005	3.911378308	3.911378308	3.01519	0.00256
DLL3	13.8080984	0	3.888314478	0	0	20	0.04057	LOC100516820	0	22.309516	0	4.542847146	4.542847146	-20	0.022	ATF5	714.536646	5278.2662	9.482881846	12.36612171	12.36612171	-2.88498	0.00256
LOC100520863	13.8080984	0	3.888314478	0	0	20	0.04057	LOC100623732	0	22.309516	0	4.542847146	4.542847146	-20	0.022	UCP3	31.4992953	1.6525567	5.022336531	1.407383613	1.407383613	4.252548	0.00257
LOC102166129	13.8080984	0	3.888314478	0	0	20	0.04057	LOC100623790	0	22.309516	0	4.542847146	4.542847146	-20	0.022	LOC100155404	31.4992953	1.6525567	5.022336531	1.407383613	1.407383613	4.252548	0.00257
ANKRD65	13.8080984	0	3.888314478	0	0	20	0.04057	LOC100739181	0	22.309516	0	4.542847146	4.542847146	-20	0.022	NDRG4	152.522904	1077.467	7.262310092	10.07476631	10.07476631	-2.82055	0.00258
CREB3L3	13.8080984	0	3.888314478	0	0	20	0.04057	CYP11A1	0	22.309516	0	4.542847146	4.542847146	-20	0.022	ANKDD1A	75.432523	8.2627837	6.256114748	3.211445828	3.211445828	3.190487	0.00258
IL12B	13.8080984	0	3.888314478	0	0	20	0.04057	SGCG	0	22.309516	0	4.542847146	4.542847146	-20	0.022	LOC100621092	53.0514447	376.78294	5.756261274	8.561413734	8.561413734	-2.82827	0.00258
LOC100623615	13.8080984	0	3.888314478	0	0	20	0.04057	IFT140	141.6861176	1438.5506	7.156701167	10.49140283	10.49140283	-3.34385	0.02212	C7H14orf50	386.280832	54.534373	8.597236288	5.795309087	5.795309087	2.824412	0.00258
LOC102160807	13.8080984	0	3.888314478	0	0	20	0.04057	SYNPO2L	21.14718173	214.0061	4.46905122	7.74823377	7.74823377	-3.33911	0.02212	LOC102163527	162.470049	21.483238	7.352882522	4.490777898	4.490777898	2.91889	0.0026
LOC102160718	13.8080984	0	3.888314478	0	0	20	0.04057	PALM	109.965345	1103.9079	6.793965376	10.10971041	10.10971041	-3.3275	0.02215	LOC100737316	448.450494	63.623435	8.812018403	6.013985524	6.013985524	2.817319	0.0026
LOC102160138	13.8080984	0	3.888314478	0	0	20	0.04057	CELF4	256.938258	30.5723	8.010881963	4.980587447	4.980587447	3.071125	0.02216	LOC102162929	173.246124	23.135794	7.444982755	4.593102408	4.593102408	2.904625	0.00262
TMEM130	13.8080984	0	3.888314478	0	0	20	0.04057	LOC100512652	142.7434767	1448.466	7.167352675	10.50130576	10.50130576	-3.34303	0.0222	LOC102161907	0.82892882	25.61463	0.870998931	4.734147579	4.734147579	-4.94958	0.00262
LOC102163175	13.8080984	0	3.888314478	0	0	20	0.04057	LOC100738449	9.516231779	106.58991	3.394545939	6.749398975	6.749398975	-3.48554	0.02246	LOC100623023	0.82892882	25.61463	0.870998931	4.734147579	4.734147579	-4.94958	0.00262
HSH2D	13.8080984	0	3.888314478	0	0	20	0.04057	PHYHIPL	112.0800632	12.394176	6.821200784	3.74353388	3.74353388	3.176795	0.02252	FAM107A	9.11821706	80.97528	3.338883189	6.357117027	6.357117027	-3.15066	0.00263
CWH43	13.8080984	0	3.888314478	0	0	20	0.04057	HAUS3	5351.294337	588.3102	12.38594174	9.202883429	9.202883429	3.185239	0.02258	LOC100621145	986.4253	140.46732	9.9475278	7.144325041	7.144325041	2.811975	0.00264
LOC100737290	13.8080984	0	3.888314478	0	0	20	0.04057	LOC102165474	7.401513606	87.585507	3.070649265	6.468998788	6.468998788	-3.5648	0.02259	ART5	48.0778718	4.1313919	5.617000784	2.359350201	2.359350201	3.540673	0.00264
LGR6	13.8080984	0	3.888314478	0	0	20	0.04057	PLAG1	17.97510447	183.4338	4.246035924	7.526959252	7.526959252	-3.35119	0.02259	TSTD1	3692.87791	508.98748	11.85092047	8.994318011	8.994318011	2.859044	0.00264
ZSWIM5	98.0374983	15.699289	6.629902968	4.061714779	4.061714779	2.642634	0.04068	LOC102162199	67.67098154	6.610227	6.10162868	2.927939483	2.927939483	3.355766	0.0227	UNC13A	83.7218112	587.48392	6.404661526	9.20085919	9.20085919	-2.81087	0.00265
NFAT5	189.170948	1397.2367	7.571153052	10.44939292	10.44939292	-2.88481	0.04082	FAM136A	21417.86566	2189.6377	14.38659446	11.09713518	11.09713518	3.290051	0.02278	PTGER4	4.97357294	53.708094	2.578594101	5.773682393	5.773682393	-3.43278	0.00265
SPAG9	44.1859147	319.76973	5.497801224	8.325394195	8.325394195	-2.85537	0.04136	LOC733634	4.229436346	59.492043	2.386655454	5.918673475	5.918673475	-3.81416	0.02279	NBEA	2048.28312	287.54487	11.0009036	8.172651889	8.172651889	2.832556	0.00265
GUCY1A3	135.319364	984.09754	7.090846698	9.944122772	9.944122772	-2.86243	0.04142	LOC100624577	61.32682702	5.7839486	5.961781364	2.76212524	2.76212524	3.406392	0.02285	LOC102166590	167.443622	22.309516	7.396121996	4.542847146	4.542847146	2.907944	0.00266
SLIT1	150.508272	25.61463	7.243252754	4.734147579	4.734147579	2.554803	0.04143	LOC102157639	14.80302721	153.68778	3.982129041	7.273215395	7.273215395	-3.37604	0.02294	ANKRD35	117.707893	825.45209	6.891272054	9.690787384	9.690787384	-2.80997	0.00266
LOC102159348	34.5202459	252.0149	5.150569664	7.983078557	7.983078557	-2.86799	0.04175	CHODL	195.611431	23.135794	7.619203392	4.593102407	4.593102407	3.079792	0.02296	IFI35	107.760747	755.21843	6.765014156	9.562659203	9.562659203	-2.80906	0.00267
SEMA6D	31.7586262	233.0105	5.033802952	7.870429459	7.870429459	-2.87517	0.04177	GPRC5A	17.97510447	182.60752	4.246035924	7.520481339	7.520481339	-3.34467	0.02301	LOC100515984	69.6300212	7.4365053	6.142209624	3.076645515	3.076645515	3.227013	0.00268
LOC100624376	24.854577	185.91263	4.692347799	7.546220275	7.546220275	-2.90304	0.04181	PDLIM3	5.286795433	68.581105	2.65232482	6.120623682	6.120623682	-3.69735	0.02301	LOC100738689	6.63143059	64.449713	2.93195353	6.032314961	6.032314961	-3.28078	0.00269
LOC102168189	45.5667246	5.7839486	5.541227504	2.762125239	2.762125239	2.977854	0.042	LOC102164151	1434.83628	168.56079	10.48767554	7.405658765	7.405658765	3.089545	0.02306	ZCCHC12	6.63143059	64.449713	2.93195353	6.032314961	6.032314961	-3.28078	0.00269
LOC100152103	80.0869705	12.394176	6.341398207	3.74353388	3.74353388	2.691905	0.04208	LOC102166840	29.60605442	290.84894	4.935745167	8.189083193	8.189083193	-3.29631	0.02314	DOCK4	977.307083	139.64104	9.934143577	7.135873883	7.135873883	2.807089	0.0027
KCNJ8	5.52323934	57.839486	2.705588563	5.878712739	5.878712739	-3.38847	0.04211	USP20	159.6612221	1610.4165	7.327877945	10.65411376	10.65411376	-3.33435	0.02327	LOC100620095	19.0653629	0	4.326635347	0	0	20	0.00271
LOC100737241	5.52323934	57.839486	2.705588563	5.878712739	5.878712739	-3.38847	0.04211	LOC100736715	48.63851798	4.1313919	5.633388138	2.359350201	2.359350201	3.557399	0.02337	TDRD9	19.0653629	0	4.326635347	0	0	20	0.00271
DACT3	665.550341	5208.8589	9.380570028	12.34702857	12.34702857	-2.96835	0.04225	LOC100517699	48.63851798	4.1313919	5.633388138	2.359350201	2.359350201	3.557399	0.02337	LOC102161829	19.0653629	0	4.326635347	0	0	20	0.00271
LOC100621470	111.845597	804.79513	6.818206315	9.654269283	9.654269283	-2.84711	0.04242	HSD17B1	12.68830904	133.8571	3.774872332	7.075287628	7.075287628	-3.39912	0.02338	LOC102161675	19.0653629	0	4.326635347	0	0	20	0.00271
PCDH1	280.304397	2089.658	8.135988291	11.02974136	11.02974136	-2.8982	0.04249	LOC100620994	83.53136784	819.66814	6.40141489	9.680655144	9.680655144	-3.29465	0.02338	LOC102159758	19.0653629	0	4.326635347	0	0	20	0.00271
EFNA5	15.1889082	120.63664	4.016933786	6.926434087	6.926434087	-2.98958	0.04264	ISG15	2047.047192	24180.21	11.00003324	14.56159883	14.56159883	-3.56221	0.02346	LOC102159995	19.0653629	0	4.326635347	0	0	20	0.00271
SCN1A	23.4737672	1.6525567	4.613164285	1.407383613	1.407383613	3.828278	0.04274	FASTKD5	2355.796045	271.84558	11.2026112	8.091940885	8.091940885	3.115355	0.0235	USH2A	19.0653629	0	4.326635347	0	0	20	0.00271
SPAG6	23.4737672	1.6525567	4.613164285	1.407383613	1.407383613	3.828278	0.04274	LOC102162280	130.0551676	14.873011	7.034030432	3.98850389	3.98850389	3.128355	0.02355	LOC102166688	19.0653629	0	4.326635347	0	0	20	0.00271
EML6	23.4737672	1.6525567	4.613164285	1.407383613	1.407383613	3.828278	0.04274	LOC100736899	1.057359087	31.398578	1.04079362	5.017858594	5.017858594	-4.89216	0.02364	LOC102164534	19.0653629	0	4.326635347	0	0	20	0.00271
GABRB3	23.4737672	1.6525567	4.613164285	1.407383613	1.407383613	3.828278	0.04274	EDN3	1.057359087	31.398578	1.04079362	5.017858594	5.017858594	-4.89216	0.02364	NT5C1A	19.0653629	0	4.326635347	0	0	20	0.00271
LOC100739034	17.9505279	138.81477	4.24416613	7.127372928	7.127372928	-2.95106	0.04276	LOC102163868	1.057359087	31.398578	1.04079362	5.017858594	5.017858594	-4.89216	0.02364	ZBTB8B	19.0653629	0	4.326635347	0	0	20	0.00271
LOC102157752	49.7091541	6.610227	5.664174303	2.927939483	2.927939483	2.91074	0.04281	NPTX1	1.057359087	31.398578	1.04079362	5.017858594	5.017858594	-4.89216	0.02364	CPB2	19.0653629	0	4.326635347	0	0	20	0.00271
LOC102162573	70.4213016	502.37725	6.158282521	8.975496206	8.975496206	-2.83469	0.04282	ABCC9	1.057359087	31.398578	1.04079362	5.017858594	5.017858594	-4.89216	0.02364	MOV10L1	19.0653629	0	4.326635347	0	0	20	0.00271
CEP170	57.9940131	413.96546	5.882496648	8.696847462	8.696847462	-2.83553	0.04286	LOC100737344	39.1222862	379.26177	5.326331909	8.570849103	8.570849103	-3.27713	0.02375	LOC100739117	19.0653629	0	4.326635347	0	0	20	0.00271
LOC102160497	1.38080984	30.5723	1.251452392	4.980587446	4.980587446	-4.46864	0.04292	OPRL1	42.29436346	3.3051135	5.436107306	2.106051269	2.106051269	3.677694	0.02376	LOC102163079	19.0653629	0	4.326635347	0	0	20	0.00271
SERPINB2	2.76161967	39.661362	1.911353989	5.34558663	5.34558663	-3.84415	0.04306	LOC100519977	23.2618999	229.70539	4.600620625	7.849907883	7.849907883	-3.30374	0.02378	LOC102165193	19.0653629	0	4.326635347	0	0	20	0.00271
LOC102165012	2.76161967	39.661362	1.911353989	5.34558663	5.34558663	-3.84415	0.04306	SCML1	2006.867546	233.83678	10.97144839	7.875514565	7.875514565	3.101372	0.02379	LOC102159708	8.28928824	75.191332	3.215568059	6.251554969	6.251554969	-3.18125	0.00271
ZFP37	9.66566885	84.280394	3.414902536	6.414142197	6.414142197	-3.12426	0.04309	PRDX6	52679.74441	5168.3712	15.68498811	12.33577309	12.33577309	3.349467	0.02379	ZMYND15	8.28928824	75.191332	3.215568059	6.251554969	6.251554969	-3.18125	0.00271
SETD7	683.508869	5331.1481	9.418908562	12.38050112	12.38050112	-2.96343	0.04325	LOC100622296	16.91774539	0	4.163317207	0	0	20	0.0238	LOC100511572	19.8942918	151.20894	4.385036953	7.249909307	7.249909307	-2.92612	0.00272
LOC102166427	74.5637311	530.47071	6.239622035	9.053846388	9.053846388	-2.83073	0.04339	ATP6V0D2	16.91774539	0	4.163317207	0	0	20	0.0238	CD40	71.2878788	499.07214	6.175681852	8.965992412	8.965992412	-2.80752	0.00273
ZMYND15	8.28485901	75.191332	3.214880004	6.251554969	6.251554969	-3.18202	0.04345	DLGAP1	16.91774539	0	4.163317207	0	0	20	0.0238	LOC396904	18071.4773	150749.53	14.14150666	17.20180356	17.20180356	-3.06037	0.00275
DNAH9	8.28485901	75.191332	3.214880004	6.251554969	6.251554969	-3.18202	0.04345	LOC102159146	16.91774539	0	4.163317207	0	0	20	0.0238	LOC100515339	53.0514447	373.47782	5.756261274	8.548736477	8.548736477	-2.81556	0.00275
GSTO2	53.8515836	7.4365053	5.777461367	3.076645515	3.076645515	2.856292	0.04348	EYS	16.91774539	0	4.163317207	0	0	20	0.0238	SYNC	315.821882	2248.3034	8.307528171	11.13526259	11.13526259	-2.83165	0.00276
VWA5B2	53.8515836	7.4365053	5.777461367	3.076645515	3.076645515	2.856292	0.04348	LOC102162153	16.91774539	0	4.163317207	0	0	20	0.0238	LOC100516409	869.546336	124.76803	9.765777279	6.974621475	6.974621475	2.801015	0.00276
LOC102167547	15.1889082	119.81036	4.016933786	6.916600414	6.916600414	-2.97966	0.04393	LOC100516399	16.91774539	0	4.163317207	0	0	20	0.0238	BLM	2156.8728	304.07044	11.07539411	8.252998589	8.252998589	2.826464	0.00277
LOC100519128	3122.01104	531.29699	11.60872195	9.056087606	9.056087606	2.554885	0.04393	LOC100738107	16.91774539	0	4.163317207	0	0	20	0.0238	FADS3	760.95666	5576.5527	9.57356513	12.44541653	12.44541653	-2.87349	0.00277
HPX	13.8080984	110.7213	3.888314478	6.803760479	6.803760479	-3.00335	0.04404	PPEF2	16.91774539	0	4.163317207	0	0	20	0.0238	SPATA20	46.4200141	328.03251	5.567424187	8.362086342	8.362086342	-2.82102	0.00278
RINL	13.8080984	110.7213	3.888314478	6.803760479	6.803760479	-3.00335	0.04404	LOC100522181	16.91774539	0	4.163317207	0	0	20	0.0238	LOC100624220	27.3546512	199.95937	4.825513503	7.650760008	7.650760008	-2.86985	0.00279
PDE3A	13.8080984	110.7213	3.888314478	6.803760479	6.803760479	-3.00335	0.04404	LOC102157915	16.91774539	0	4.163317207	0	0	20	0.0238	C5AR2	0	19.004403	0	4.322245637	4.322245637	-20	0.00281
LOC100152184	635.172524	4908.9198	9.313274255	12.26148375	12.26148375	-2.95019	0.04425	LOC100514051	16.91774539	0	4.163317207	0	0	20	0.0238	WDR16	0	19.004403	0	4.322245637	4.322245637	-20	0.00281
BRI3BP	455.667246	81.801559	8.834999507	6.371586023	6.371586023	2.47778	0.04433	RFX6	16.91774539	0	4.163317207	0	0	20	0.0238	LOC102163875	0	19.004403	0	4.322245637	4.322245637	-20	0.00281
SMOX	305.158974	2263.1765	8.25813716	11.14477068	11.14477068	-2.89072	0.04439	LOC102162801	16.91774539	0	4.163317207	0	0	20	0.0238	DLX3	0	19.004403	0	4.322245637	4.322245637	-20	0.00281
LOC102158716	259.592249	46.271589	8.025650364	5.56290145	5.56290145	2.488049	0.04449	LOC102158914	16.91774539	0	4.163317207	0	0	20	0.0238	LOC102160975	0	19.004403	0	4.322245637	4.322245637	-20	0.00281
LOC100624745	241.641721	42.966475	7.922683827	5.458331975	5.458331975	2.491586	0.04452	LOC102158923	16.91774539	0	4.163317207	0	0	20	0.0238	LOC102160101	0	19.004403	0	4.322245637	4.322245637	-20	0.00281
LOC100514211	459.809675	82.627837	8.848027198	6.385911347	6.385911347	2.476337	0.0446	LOC102158700	16.91774539	0	4.163317207	0	0	20	0.0238	LOC102158370	0	19.004403	0	4.322245637	4.322245637	-20	0.00281
LOC100738006	2521.35876	433.79615	11.30055777	8.76419534	8.76419534	2.539112	0.04463	LOC100627657	16.91774539	0	4.163317207	0	0	20	0.0238	RSPO1	0	19.004403	0	4.322245637	4.322245637	-20	0.00281
ZNF606	81.4677803	575.91603	6.365758672	9.172217528	9.172217528	-2.82156	0.0448	LOC102167553	16.91774539	0	4.163317207	0	0	20	0.0238	LOC100627895	0	19.004403	0	4.322245637	4.322245637	-20	0.00281
LOC100737020	11093.4262	102321.36	13.43754744	16.64276186	16.64276186	-3.20533	0.04482	LOC100155636	16.91774539	0	4.163317207	0	0	20	0.0238	ZNF8	67.9721635	474.28379	6.107942317	8.892645374	8.892645374	-2.80273	0.00281
LOC100736687	104.941548	17.351846	6.727124677	4.19785327	4.19785327	2.596425	0.0449	ZNF703	253.7661808	2608.5608	7.993029968	11.34959131	11.34959131	-3.36168	0.02384	MSN	2543.98256	19515.043	11.31344005	14.25237292	14.25237292	-2.93943	0.00281
LOC102166544	106.322357	754.39215	6.745806839	9.561081988	9.561081988	-2.82687	0.04497	B3GNT8	2.114718173	40.48764	1.639101631	5.374609695	5.374609695	-4.25894	0.02395	IQGAP2	539.632664	77.670167	9.07850487	6.29774474	6.29774474	2.796545	0.00282
TMEM169	317.586262	57.013208	8.315540247	5.858309486	5.858309486	2.47778	0.04502	PPP2R2B	2.114718173	40.48764	1.639101631	5.374609695	5.374609695	-4.25894	0.02395	C9H11orf82	1429.07329	204.09076	10.48187337	7.68011867	7.68011867	2.807797	0.00283
RABEPK	2234.15031	386.69828	11.12615614	8.598790517	8.598790517	2.530446	0.04503	THSD7B	4.229436346	58.665764	2.386655454	5.89883146	5.89883146	-3.79398	0.02405	PDE8A	121.023608	841.97766	6.931016486	9.71935059	9.71935059	-2.79849	0.00283
ALDH3A1	249.92658	44.619032	7.971121491	5.511563931	5.511563931	2.485773	0.04521	LOC102160349	5.286795433	67.754826	2.65232482	6.103389087	6.103389087	-3.67986	0.02412	ERICH2	111.905391	14.046732	6.818970566	3.911378308	3.911378308	2.993973	0.00284
C14H10orf71	0	20.656959	0	4.436758793	4.436758793	-20	0.04529	TMOD2	232.618999	28.093465	7.868013796	4.862623207	4.862623207	3.049662	0.02412	FUCA1	316.650811	2244.9983	8.31129789	11.13314114	11.13314114	-2.82575	0.00285
LOC102159603	0	20.656959	0	4.436758793	4.436758793	-20	0.04529	ATG2A	265.3971307	2724.2398	8.057434734	11.41216746	11.41216746	-3.35963	0.02424	ADAMTS3	62.1696618	433.79615	5.981159942	8.76419534	8.76419534	-2.80273	0.00285
LOC102163989	0	20.656959	0	4.436758793	4.436758793	-20	0.04529	LOC100738129	35.95020894	347.03692	5.207510617	8.44309653	8.44309653	-3.27102	0.0243	PRAF2	540.461593	3897.5551	9.080715198	11.9287238	11.9287238	-2.85031	0.00285
LOC100513913	0	20.656959	0	4.436758793	4.436758793	-20	0.04529	LOC100524893	7879.439913	860.98206	12.94406045	9.751514039	9.751514039	3.194038	0.02432	XRCC2	767.588091	110.7213	9.586066812	6.803760479	6.803760479	2.7934	0.00285
PTPRCAP	0	20.656959	0	4.436758793	4.436758793	-20	0.04529	ZNF467	86.7034451	843.63022	6.45456161	9.722176052	9.722176052	-3.28245	0.02432	LOC100626128	646.564482	93.369456	9.338880049	6.560248082	6.560248082	2.791772	0.00287
NKX6-1	0	20.656959	0	4.436758793	4.436758793	-20	0.04529	C1H9orf41	16252.66652	1707.9174	13.98847758	10.73886695	10.73886695	3.250366	0.02433	CHD5	19.0653629	144.59872	4.326635347	7.185853814	7.185853814	-2.92303	0.00288
COMP	0	20.656959	0	4.436758793	4.436758793	-20	0.04529	LOC102160850	66.61362245	6.610227	6.079242038	2.927939483	2.927939483	3.333046	0.02435	LOC102158821	79.5771671	9.0890621	6.332299179	3.334720158	3.334720158	3.130151	0.00288
LOC100736567	0	20.656959	0	4.436758793	4.436758793	-20	0.04529	LOC102160131	66.61362245	6.610227	6.079242038	2.927939483	2.927939483	3.333046	0.02435	RAB39B	20.7232206	155.34033	4.441166102	7.288546214	7.288546214	-2.90611	0.00289
LOC102166377	0	20.656959	0	4.436758793	4.436758793	-20	0.04529	POLE2	1996.293955	233.83678	10.96383096	7.875514565	7.875514565	3.09375	0.0244	LOC100518132	1885.81307	14235.124	10.88173579	13.79726876	13.79726876	-2.9162	0.0029
LOC102158398	0	20.656959	0	4.436758793	4.436758793	-20	0.04529	LOC102166369	20.08982264	199.13309	4.398475058	7.644815895	7.644815895	-3.3092	0.02441	TXNIP	372.189042	2644.9171	8.543762814	11.36955213	11.36955213	-2.82912	0.0029
LOC100739192	0	20.656959	0	4.436758793	4.436758793	-20	0.04529	LOC100739472	355.2726531	3702.5534	8.476837939	11.85469442	11.85469442	-3.38152	0.02446	LOC100621534	36.4728682	2.4788351	5.227774503	1.798604302	1.798604302	3.879089	0.00292
CCDC176	0	20.656959	0	4.436758793	4.436758793	-20	0.04529	LOC102165153	14.80302721	151.20894	3.982129041	7.249909307	7.249909307	-3.35258	0.02447	GNG8	9.94714588	85.106672	3.452482877	6.428053129	6.428053129	-3.09692	0.00292
SEZ6	0	20.656959	0	4.436758793	4.436758793	-20	0.04529	LOC100621998	23.2618999	228.05283	4.600620625	7.839536581	7.839536581	-3.29333	0.02447	LOC100625965	9.94714588	85.106672	3.452482877	6.428053129	6.428053129	-3.09692	0.00292
ZMYND10	289.970066	52.055537	8.184726929	5.729431428	5.729431428	2.47778	0.0455	OXTR	42.29436346	406.52896	5.436107306	8.67075877	8.67075877	-3.26482	0.02451	LOC102159363	605.118041	87.585507	9.243454975	6.468998788	6.468998788	2.788453	0.00292
IRAK2	8.28485901	74.365053	3.214880004	6.2358238	6.2358238	-3.16608	0.04552	PNPLA4	1285.748649	153.68778	10.32951455	7.273215395	7.273215395	3.064534	0.02462	STRIP2	274.375441	38.835083	8.105256088	5.315967693	5.315967693	2.820719	0.00293
RGS7	8.28485901	74.365053	3.214880004	6.2358238	6.2358238	-3.16608	0.04552	LOC100623437	112.0800632	1095.6451	6.821200784	10.09888102	10.09888102	-3.28918	0.02478	TMEM229A	61.340733	426.35964	5.962103212	8.739306855	8.739306855	-2.79715	0.00293
TNFAIP3	274.781157	2016.1192	8.107380078	10.97808065	10.97808065	-2.87523	0.04552	CHST9	29.60605442	1.6525567	4.935745167	1.407383614	1.407383614	4.163121	0.02479	TMEM182	95.3268147	11.567897	6.589865555	3.65167138	3.65167138	3.042755	0.00294
LOC100523822	27.6161967	2.4788351	4.838760036	1.798604302	1.798604302	3.47778	0.04568	TMIGD1	29.60605442	1.6525567	4.935745167	1.407383614	1.407383614	4.163121	0.02479	COL19A1	58.0250177	5.7839486	5.883254663	2.762125239	2.762125239	3.326548	0.00294
LOC100525801	4.14242951	47.924146	2.362450112	5.61247475	5.61247475	-3.5322	0.04568	SELRC1	2128.463841	249.53607	11.05627452	7.968874505	7.968874505	3.092492	0.02487	ZNF774	34.8150106	247.88351	5.162492464	7.959326845	7.959326845	-2.83188	0.00294
LOC100622259	30.3778164	218.96377	4.971673052	7.781122098	7.781122098	-2.8496	0.04577	CTSZ	256.938258	2615.9973	8.010881963	11.35369673	11.35369673	-3.34787	0.02488	FGF11	480.778718	3438.1443	8.912226855	11.74783394	11.74783394	-2.83819	0.00295
ARNTL2	70.4213016	494.94074	6.158282521	8.954023947	8.954023947	-2.81317	0.04579	NUCB1	1272.002981	14340.887	10.31402008	13.80794728	13.80794728	-3.49496	0.02492	IL7R	47.248943	4.1313919	5.592425431	2.359350201	2.359350201	3.515582	0.00297
LOC102160806	40.0434852	284.23976	5.359081339	8.156031285	8.156031285	-2.82747	0.0458	TERF1	4855.392926	546.99628	12.24566944	9.098022295	9.098022295	3.149985	0.02497	TMEM136	47.248943	4.1313919	5.592425431	2.359350201	2.359350201	3.515582	0.00297
LOC396903	3268.37688	27752.212	11.67479998	14.76036711	14.76036711	-3.08596	0.04584	LOC100526059	975.9424369	118.15781	9.932129748	6.896729669	6.896729669	3.046081	0.02497	LOC102162075	84.55074	9.9153405	6.418708429	3.448285225	3.448285225	3.092083	0.00297
LOC780415	741.494882	5739.3296	9.536237271	12.48691785	12.48691785	-2.95237	0.04586	LOC102161198	23.2618999	0.8262784	4.600620625	0.868906686	0.868906686	4.815197	0.02511	LOC100737349	474.147287	3382.7837	8.892230982	11.72442162	11.72442162	-2.8348	0.00299
CDCP1	63.5172524	446.19032	6.011613095	8.804745152	8.804745152	-2.81244	0.04591	LOC102164762	23.2618999	0.8262784	4.600620625	0.868906686	0.868906686	4.815197	0.02511	SELENBP1	7339.3358	1003.1019	12.84163035	9.971690033	9.971690033	2.871181	0.003
LOC102167078	74.5637311	11.567897	6.239622035	3.65167138	3.65167138	2.688347	0.04591	LOC100152743	23.2618999	0.8262784	4.600620625	0.868906686	0.868906686	4.815197	0.02511	LOC100513945	227.955427	1590.5859	7.838922949	10.63624928	10.63624928	-2.80273	0.003
RALGAPA2	1288.29558	228.05283	10.33236733	7.839536581	7.839536581	2.498024	0.04595	SESN1	4877.597466	550.3014	12.25225074	9.106697443	9.106697443	3.147877	0.02519	NDUFAF4	1641.27907	235.48934	10.68148359	7.885631319	7.885631319	2.801087	0.003
LOC102167727	37.2818656	265.23536	5.258589232	8.05655837	8.05655837	-2.83073	0.04599	KLHL1	0	21.483238	0	4.490777899	4.490777899	-20	0.02521	PCDHB10	73.7746653	8.2627837	6.224477643	3.211445828	3.211445828	3.158426	0.00301
PXDN	2377.75454	19766.231	11.21599069	14.27082319	14.27082319	-3.05537	0.04606	TCEAL5	0	21.483238	0	4.490777899	4.490777899	-20	0.02521	PDGFA	164.956836	1142.743	7.374664246	10.15954718	10.15954718	-2.79234	0.00301
JAZF1	1350.43202	238.79445	10.40027323	7.905654454	7.905654454	2.49958	0.04607	LOC102161784	0	21.483238	0	4.490777899	4.490777899	-20	0.02521	LOC100738512	36.4728682	257.79885	5.227774503	8.015687408	8.015687408	-2.82135	0.00304
DYNC2H1	23.4737672	172.69218	4.613164285	7.44038899	7.44038899	-2.87908	0.04609	LOC100626194	0	21.483238	0	4.490777899	4.490777899	-20	0.02521	LOC102164705	961.557435	139.64104	9.910728818	7.135873883	7.135873883	2.78365	0.00305
LOC100739570	23.4737672	172.69218	4.613164285	7.44038899	7.44038899	-2.87908	0.04609	LOC102163805	0	21.483238	0	4.490777899	4.490777899	-20	0.02521	GPR62	2.48678647	36.356248	1.801898017	5.223277669	5.223277669	-3.86985	0.00305
LOC100738110	34.5202459	246.23095	5.150569664	7.949715578	7.949715578	-2.8345	0.04626	LOC100156469	0	21.483238	0	4.490777899	4.490777899	-20	0.02521	SIGLEC15	30.6703665	1.6525567	4.985061656	1.407383613	1.407383613	4.214074	0.00305
FBXO15	151.889082	26.440908	7.256341575	4.77825631	4.77825631	2.522175	0.0464	LOC102164355	0	21.483238	0	4.490777899	4.490777899	-20	0.02521	VWA3B	30.6703665	1.6525567	4.985061656	1.407383613	1.407383613	4.214074	0.00305
NPM3	17037.8126	2715.977	14.05653718	11.40778664	11.40778664	2.649197	0.04642	HEPHL1	0	21.483238	0	4.490777899	4.490777899	-20	0.02521	LOC102159131	266.915081	38.008805	8.065631984	5.285727902	5.285727902	2.811975	0.00309
LOC100526005	650.361433	117.33153	9.347314489	6.886690713	6.886690713	2.470651	0.04658	LOC102166296	0	21.483238	0	4.490777899	4.490777899	-20	0.02521	HOXA11	5.80250177	57.839486	2.766065426	5.878712739	5.878712739	-3.31731	0.00309
OPHN1	15.1889082	118.15781	4.016933786	6.89672967	6.89672967	-2.95962	0.04663	LOC100623643	0	21.483238	0	4.490777899	4.490777899	-20	0.02521	LOC100738709	24.8678647	0.8262784	4.693089065	0.868906686	0.868906686	4.911511	0.00309
LOC102164300	701.451397	126.42059	9.456254597	6.993454623	6.993454623	2.472112	0.04666	LOC102167375	0	21.483238	0	4.490777899	4.490777899	-20	0.02521	LOC102163869	11.6050035	95.022013	3.655924617	6.585293272	6.585293272	-3.03351	0.0031
LOC102157933	82.8485901	13.220454	6.389714621	3.829895615	3.829895615	2.647705	0.04667	LOC100737944	0	21.483238	0	4.490777899	4.490777899	-20	0.02521	LOC102158104	78.7482382	9.0890621	6.317380743	3.334720158	3.334720158	3.115044	0.0031
PRSS22	140.842603	998.97055	7.148147109	9.965741799	9.965741799	-2.82636	0.0467	LOC100737685	0	21.483238	0	4.490777899	4.490777899	-20	0.02521	LOC102161161	4.14464412	47.097867	2.363071282	5.587901017	5.587901017	-3.50634	0.00311
HPRT1	4440.68443	751.91332	12.11689118	9.556339969	9.556339969	2.562144	0.04674	SYNGR1	62.38418611	596.57298	5.986051038	9.222971117	9.222971117	-3.25745	0.02524	AS-A	256.139007	1783.935	8.006404665	10.80165583	10.80165583	-2.80006	0.00311
ADAT2	1422.23413	252.0149	10.4749573	7.983078557	7.983078557	2.496578	0.04717	EPHA4	37.00756803	353.64714	5.24821481	8.470240516	8.470240516	-3.25642	0.02525	AHCY	37346.5592	4814.7241	15.18872634	12.23353702	12.23353702	2.95545	0.00311
ABHD6	102.179928	716.38335	6.689018532	9.486600448	9.486600448	-2.80962	0.04727	LOC102160451	5.286795433	66.928548	2.65232482	6.085946114	6.085946114	-3.66216	0.02528	PCDHB4	493.21265	71.886218	8.948988129	6.187574144	6.187574144	2.778423	0.00312
SLC13A4	19.3313377	144.59872	4.345633235	7.185853814	7.185853814	-2.90304	0.04728	LOC102166411	13.74566813	140.46732	3.882219287	7.144325041	7.144325041	-3.35319	0.02536	GLIS2	599.31554	4285.9059	9.229577203	12.06572104	12.06572104	-2.83821	0.00313
LOC100738956	31.7586262	3.3051135	5.033802952	2.106051269	2.106051269	3.264377	0.04733	LOC102160864	4.229436346	57.839486	2.386655454	5.878712739	5.878712739	-3.77352	0.02539	EGFR	188.995772	1304.6935	7.569823503	10.35060062	10.35060062	-2.78729	0.00315
LOC102167627	31.7586262	3.3051135	5.033802952	2.106051269	2.106051269	3.264377	0.04733	LOC102166014	9.516231779	103.2848	3.394545939	6.704385034	6.704385034	-3.44009	0.02541	LOC100621171	110.247534	14.046732	6.79762954	3.911378308	3.911378308	2.97244	0.00315
LOC100512447	811.916183	146.25127	9.666962799	7.202136284	7.202136284	2.472882	0.04735	KCNJ8	468.4100754	57.839486	8.874704998	5.878712739	5.878712739	3.017645	0.02542	PLB1	41.4464412	3.3051135	5.407571694	2.106051269	2.106051269	3.648477	0.00316
LOC102160623	5.52323934	56.186929	2.705588563	5.837613535	5.837613535	-3.34665	0.04748	HAP1	204.0703037	2039.255	7.67997478	10.99453378	10.99453378	-3.3209	0.02548	LOC102158452	41.4464412	3.3051135	5.407571694	2.106051269	2.106051269	3.648477	0.00316
FAM114A1	738.733262	5671.5747	9.530861338	12.469788	12.469788	-2.94062	0.04759	METTL13	7899.529736	870.8974	12.94773367	9.768014573	9.768014573	3.181192	0.02549	VWA5B2	67.9721635	7.4365053	6.107942317	3.076645515	3.076645515	3.192247	0.00316
DCP2	1574.12321	278.45581	10.62124897	8.126476366	8.126476366	2.499028	0.04768	PRRT2	31.7207726	303.24416	5.032134908	8.249085772	8.249085772	-3.25698	0.02558	LOC100738652	184.851128	25.61463	7.538003632	4.734147579	4.734147579	2.851324	0.00316
LOC100623810	95.2758787	15.699289	6.58910248	4.061714779	4.061714779	2.601412	0.04769	LOC102160629	2693.093593	313.98578	11.39558426	8.299142895	8.299142895	3.100493	0.02558	LOC102164231	164.956836	1134.4802	7.374664246	10.14908684	10.14908684	-2.78187	0.00318
USP51	1.38080984	29.746021	1.251452392	4.942327829	4.942327829	-4.42911	0.04779	LOC102161728	3.17207726	48.750424	2.060765874	5.636636914	5.636636914	-3.94191	0.02559	XPNPEP2	1.65785765	30.5723	1.410263837	4.980587446	4.980587446	-4.20483	0.00319
LOC100152800	1.38080984	29.746021	1.251452392	4.942327829	4.942327829	-4.42911	0.04779	GUCA1B	3.17207726	48.750424	2.060765874	5.636636914	5.636636914	-3.94191	0.02559	INHBB	1.65785765	30.5723	1.410263837	4.980587446	4.980587446	-4.20483	0.00319
LOC102160261	1.38080984	29.746021	1.251452392	4.942327829	4.942327829	-4.42911	0.04779	LOC100739553	3.17207726	48.750424	2.060765874	5.636636914	5.636636914	-3.94191	0.02559	LOC102167836	1.65785765	30.5723	1.410263837	4.980587446	4.980587446	-4.20483	0.00319
ELK3	777.395938	5977.2977	9.60436037	12.54551903	12.54551903	-2.94277	0.04794	LRP3	508.5897206	5360.0678	8.993192366	12.38830466	12.38830466	-3.39768	0.02564	MIR145	1.65785765	30.5723	1.410263837	4.980587446	4.980587446	-4.20483	0.00319
SMG1	209.883095	1500.5215	7.720299639	10.55220944	10.55220944	-2.83781	0.04838	NPL	108.9079859	12.394176	6.780152406	3.74353388	3.74353388	3.135376	0.02566	ROBO3	1.65785765	30.5723	1.410263837	4.980587446	4.980587446	-4.20483	0.00319
NFIC	822.962662	6331.7712	9.686435153	12.62862123	12.62862123	-2.94371	0.04856	NFASC	47.5811589	4.1313919	5.602325001	2.359350201	2.359350201	3.525691	0.02574	NPL	99.4714588	12.394176	6.65064192	3.74353388	3.74353388	3.00462	0.00319
IRX6	6.90404918	64.449713	2.982591923	6.032314961	6.032314961	-3.22266	0.04886	FAM114A1	537.138416	5671.5747	9.07183349	12.469788	12.469788	-3.40038	0.02582	LOC100524077	8992.21988	1230.3285	13.13462203	10.26599998	10.26599998	2.869634	0.00321
LOC102158240	4.14242951	47.097867	2.362450112	5.587901017	5.587901017	-3.50711	0.04903	LOC100153934	137.4566813	1341.8761	7.113290863	10.39111046	10.39111046	-3.2872	0.02584	LHX2	232.928999	33.051135	7.869926909	5.089630977	5.089630977	2.817119	0.00321
LOC102165529	4.14242951	47.097867	2.362450112	5.587901017	5.587901017	-3.50711	0.04903	LOC100522777	2.114718173	39.661362	1.639101631	5.34558663	5.34558663	-4.2292	0.02588	ZNF334	0.82892882	24.788351	0.870998931	4.688647628	4.688647628	-4.90227	0.00322
ZFYVE26	440.478338	3260.4945	8.786198839	11.67131746	11.67131746	-2.88795	0.04904	SDR42E2	2.114718173	39.661362	1.639101631	5.34558663	5.34558663	-4.2292	0.02588	LOC100517269	1045.27925	152.8615	10.03105224	7.265488457	7.265488457	2.773591	0.00324
MAP4K1	263.734679	47.924146	8.048403381	5.61247475	5.61247475	2.460263	0.0494	LOC102165051	2.114718173	39.661362	1.639101631	5.34558663	5.34558663	-4.2292	0.02588	RBMS3	19.0653629	142.11988	4.326635347	7.161080272	7.161080272	-2.89808	0.00325
LOC100515885	20.7121475	152.03522	4.440430524	7.257719911	7.257719911	-2.87586	0.04941	NOX1	1045.728137	127.24687	10.03167107	7.002779797	7.002779797	3.038806	0.0259	EPN3	9.11821706	78.496445	3.338883189	6.312818447	6.312818447	-3.1058	0.00326
FLRT3	2818.23288	490.80935	11.46108694	8.941955361	8.941955361	2.521556	0.04962	CEP78	3433.244954	396.61362	11.74577723	8.63522336	8.63522336	3.113767	0.02591	LOC100515214	109.418605	14.046732	6.786839466	3.911378308	3.911378308	2.961552	0.00332
ARHGEF3	27.6161967	196.65425	4.838760036	7.626835185	7.626835185	-2.83207	0.04979	SCG5	905.0993781	110.7213	9.823525479	6.803760479	6.803760479	3.031143	0.02596	LOC102167753	7.46035941	67.754826	3.080718953	6.103389087	6.103389087	-3.18301	0.00332
STXBP5L	52.4707738	7.4365053	5.740678649	3.076645515	3.076645515	2.818817	0.04992	ADAMTS7	444.0908164	4623.8538	8.797955923	12.17519203	12.17519203	-3.38017	0.02602	LOC100515690	973.162439	142.94616	9.928018548	7.169385476	7.169385476	2.767209	0.00332
EGFLAM	24.854577	178.47613	4.692347799	7.487648157	7.487648157	-2.84415	0.04996	LOC102160006	1.057359087	30.5723	1.04079362	4.980587447	4.980587447	-4.85369	0.0261	PADI3	46.4200141	4.1313919	5.567424187	2.359350201	2.359350201	3.490047	0.00334
LPHN2	610.317947	4582.5399	9.255779113	12.1622465		-2.90851	0.05006	NOS3	203.0129446	24.788351	7.672516884	4.688647628	4.688647628	3.033838	0.0261	SPO11	1206.92037	176.82357	10.23830963	7.474302765	7.474302765	2.770948	0.00335
SLC8A2	128.415315	22.309516	7.015864542	4.542847146		2.525086	0.05008	KCNAB2	31.7207726	301.59161	5.032134908	8.241228155	8.241228155	-3.2491	0.02614	PIGA	1293.12897	189.21775	10.33776568	7.571508044	7.571508044	2.772747	0.00335
FNDC3B	422.52781	3105.1541	8.726312893	11.6009137		-2.87755	0.05015	ELMO3	27.49133625	262.75652	4.832451381	8.043062959	8.043062959	-3.25668	0.02614	LOC102164681	33.9860818	237.96817	5.128709195	7.900674664	7.900674664	-2.80775	0.00336
LOC100623842	938.950688	170.21334	9.876441262	7.419651342		2.463705	0.05029	CGREF1	41.23700438	390.00339	5.400435611	8.611037311	8.611037311	-3.24148	0.02617	PNAS-5	7223.28577	1003.1019	12.81863925	9.971690033	9.971690033	2.848187	0.00338
LOC100625071	60.7556328	9.0890621	5.948498827	3.334720158		2.740815	0.0503	LOC100737528	14104.11286	1514.5683	13.78393059	10.56564311	10.56564311	3.219137	0.02618	ACPL2	1906.53629	276.80325	10.89749479	8.117919691	8.117919691	2.784021	0.00339
SLA-5	74.5637311	513.11887	6.239622035	9.005958152		-2.78275	0.05036	LOC102159908	372.1903959	46.271589	8.543768058	5.56290145	5.56290145	3.007842	0.02619	LOC102161782	35.6439394	2.4788351	5.195502704	1.798604302	1.798604302	3.845923	0.00339
LOC100153854	5.52323934	55.360651	2.705588563	5.816616368		-3.32527	0.05045	ATG4A	43.35172255	409.00779	5.470918232	8.679507525	8.679507525	-3.23797	0.0264	LOC100739662	35.6439394	2.4788351	5.195502704	1.798604302	1.798604302	3.845923	0.00339
LOC100624822	1088.07815	196.65425	10.08889177	7.626835185		2.468049	0.05063	LOC100514431	2042.817755	242.92584	10.99705084	7.930298794	7.930298794	3.071973	0.02647	CITED1	514.7648	76.01761	9.010569504	6.267116453	6.267116453	2.759508	0.00342
LOC102159808	69.0404918	10.741619	6.130117309	3.553559423		2.684231	0.05063	LOX	1046.785496	11478.659	10.03312768	13.48679218	13.48679218	-3.45492	0.0265	GPR146	21.5521494	156.99289	4.495193036	7.303715831	7.303715831	-2.8648	0.00343
LOC100623321	73.1829213	11.567897	6.213015177	3.65167138		2.66138	0.0508	ANXA4	220.9880491	2195.4216	7.7943382	11.10093931	11.10093931	-3.31246	0.02658	FAM214B	207.232206	1416.2411	7.702049408	10.46886952	10.46886952	-2.77275	0.00344
LGI3	198.836616	1403.0207	7.642677144	10.45534846		-2.81888	0.0508	CARHSP1	218.8733309	2171.4596	7.780528816	11.08511361	11.08511361	-3.3105	0.02667	GPC1	867.05955	6204.5243	9.761650206	12.59933739	12.59933739	-2.83912	0.00345
LOC100624115	2.76161967	38.008805	1.911353989	5.285727902		-3.78275	0.05105	RNASET2	450.4349709	4665.994	8.818374373	12.18827789	12.18827789	-3.37279	0.02668	LOC100514190	1855.14271	270.19303	10.85809192	8.083176277	8.083176277	2.779468	0.00345
LOC100625530	60.7556328	416.4443	5.948498827	8.705439898		-2.77703	0.05125	ZCWPW2	107.8506268	12.394176	6.766205905	3.74353388	3.74353388	3.1213	0.02682	DCP2	1911.50987	278.45581	10.90125148	8.126476366	8.126476366	2.779192	0.00348
ALDH3B1	215.406334	1522.0048	7.757598918	10.57270474		-2.82084	0.0513	LOC100624791	1147.234609	12617.271	10.16520173	13.62322659	13.62322659	-3.45917	0.02687	TMEM169	387.109761	57.013208	8.600320906	5.858309486	5.858309486	2.763375	0.00348
LOC102161634	187.790138	33.877413	7.560639591	5.124221138		2.470726	0.05149	FGD6	1205.389359	147.07755	10.23647989	7.210209122	7.210209122	3.03485	0.02693	DNA2	1165.47393	171.8659	10.18793834	7.433509508	7.433509508	2.761561	0.0035
LOC100627810	1183.35403	214.0061	10.20988468	7.74823377		2.467158	0.05161	LOC102166675	576.2607022	71.886218	9.173079205	6.187574144	6.187574144	3.002935	0.02702	LOC100737985	573.618746	85.106672	9.166461249	6.428053129	6.428053129	2.752748	0.00351
SOCS3	1023.18009	185.91263	10.0002537	7.546220275		2.460363	0.05174	CDHR4	58.15474976	546.17	5.886422108	9.095845332	9.095845332	-3.23138	0.02703	MYC	16086.1928	2177.2435	13.97362497	11.08894953	11.08894953	2.885248	0.00352
CDK1	6607.17506	1117.1284	12.69003619	10.1268701		2.564239	0.05176	TNFSF12	44.40908164	416.4443	5.504908955	8.705439898	8.705439898	-3.2292	0.02704	LOC102161593	18.2364341	0	4.265769485	0	0	20	0.00352
SPRY4	230.595243	1631.0735	7.855461808	10.67249032		-2.82239	0.05178	SLC48A1	8.458872693	92.543178	3.241668254	6.547560532	6.547560532	-3.45159	0.02712	RGR	18.2364341	0	4.265769485	0	0	20	0.00352
LOC102164265	26.2353869	185.91263	4.767410456	7.546220275		-2.82504	0.0518	LOC102158177	8.458872693	92.543178	3.241668254	6.547560532	6.547560532	-3.45159	0.02712	LOC100512242	18.2364341	0	4.265769485	0	0	20	0.00352
SIM2	11.0464787	89.238064	3.590539588	6.495664213		-3.01407	0.0519	LOC100628231	6094.617775	689.94244	12.57355673	9.43242172	9.43242172	3.142988	0.02722	CCR2	18.2364341	0	4.265769485	0	0	20	0.00352
CALML4	59.3748229	405.70268	5.915875148	8.667830689		-2.7725	0.052	LOC100626204	895.5831463	110.7213	9.80829357	6.803760479	6.803760479	3.015895	0.0273	OSBP2	18.2364341	0	4.265769485	0	0	20	0.00352
LOC102161843	1025.94171	7883.5219	10.00413858	12.94480757		-2.94189	0.05201	LOC102162629	3.17207726	47.924146	2.060765874	5.61247475	5.61247475	-3.91725	0.0273	LOC102163106	18.2364341	0	4.265769485	0	0	20	0.00352
LOC100737896	8.28485901	71.886218	3.214880004	6.187574144		-3.11717	0.05243	LOC100626096	964.311487	118.98409	9.914850736	6.906699252	6.906699252	3.018731	0.02736	LOC102167670	18.2364341	0	4.265769485	0	0	20	0.00352
LOC100736898	4165.90327	720.51474	12.0247599	9.494885058		2.531529	0.05264	LOC100518209	10168.62234	1121.2598	13.31197848	10.13219092	10.13219092	3.180932	0.02739	LOC102165684	18.2364341	0	4.265769485	0	0	20	0.00352
LOC102159118	0	19.830681	0	4.380638094		-20	0.05265	PNAS-5	9038.305472	1003.1019	13.14199621	9.971690034	9.971690034	3.171584	0.0274	LOC102166114	2.48678647	35.52997	1.801898017	5.191008663	5.191008663	-3.83668	0.00354
LOC100736919	0	19.830681	0	4.380638094		-20	0.05265	LOC102160130	52.86795433	4.9576702	5.751355374	2.57474827	2.57474827	3.414659	0.02743	SFN	251.165434	36.356248	7.978226717	5.223277669	5.223277669	2.788363	0.00354
IFN-DELTA-1	0	19.830681	0	4.380638094		-20	0.05265	RABAC1	969.5982824	10491.256	9.922730497	13.35703736	13.35703736	-3.43566	0.02743	LOC100517371	129.312897	17.351846	7.025836058	4.19785327	4.19785327	2.897705	0.00354
FAM169B	0	19.830681	0	4.380638094		-20	0.05265	IRAK2	6.344154519	74.365053	2.876596413	6.2358238	6.2358238	-3.55112	0.02747	SLA-1	1116.56713	8040.5148	10.12614577	12.97325158	12.97325158	-2.84822	0.00354
LOC100738697	0	19.830681	0	4.380638094		-20	0.05265	CYP7B1	63.44154519	594.09415	6.009919183	9.216974124	9.216974124	-3.22719	0.0275	LOC100523056	498.186223	3474.5006	8.963434308	11.76300506	11.76300506	-2.80205	0.00359
LOC100154599	0	19.830681	0	4.380638094		-20	0.05265	LOC100627454	859.6329374	106.58991	9.749254245	6.749398975	6.749398975	3.01165	0.02752	LOC100624749	551.237668	3857.0674	9.109145486	11.91366265	11.91366265	-2.80676	0.0036
LOC102158147	0	19.830681	0	4.380638094		-20	0.05265	LOC100524744	34.89284986	2.4788351	5.165624571	1.798604302	1.798604302	3.815197	0.02757	DEF8	314.164024	2155.7603	8.299959051	11.07465011	11.07465011	-2.77861	0.00363
BCL2L11	0	19.830681	0	4.380638094		-20	0.05265	LOC100627470	17.97510447	174.34474	4.246035924	7.454050314	7.454050314	-3.27787	0.02763	LOC100516568	29.8414377	1.6525567	4.946798112	1.407383613	1.407383613	4.174545	0.00364
LOC102165817	0	19.830681	0	4.380638094		-20	0.05265	DMRTA1	88.81816327	9.9153405	6.488935315	3.448285225	3.448285225	3.163121	0.02769	EDIL3	155.00969	1045.2421	7.28549183	10.03100107	10.03100107	-2.75341	0.00365
LIN28B	0	19.830681	0	4.380638094		-20	0.05265	SMAD6	154.3744266	1488.9536	7.279605257	10.54105171	10.54105171	-3.26979	0.02777	LOC100156073	0	18.178124	0	4.261389712	4.261389712	-20	0.00365
LOC100620445	0	19.830681	0	4.380638094		-20	0.05265	TUFT1	186.0951992	1812.0285	7.54762873	10.82418586	10.82418586	-3.28349	0.02777	LOC100520170	0	18.178124	0	4.261389712	4.261389712	-20	0.00365
SYTL1	118.749646	20.656959	6.90387758	4.436758793		2.523223	0.05277	TNNT1	41.23700438	384.21944	5.400435611	8.589536711	8.589536711	-3.21992	0.02778	HTR3C	0	18.178124	0	4.261389712	4.261389712	-20	0.00365
DNAJA4	70.4213016	479.24146	6.158282521	8.907616136		-2.76667	0.05284	TRIM21	168.1200948	1627.7684	7.40190428	10.66956575	10.66956575	-3.27533	0.02782	LOC100512775	0	18.178124	0	4.261389712	4.261389712	-20	0.00365
LOC102166078	17.9505279	0.8262784	4.24416613	0.868906686		4.441255	0.05285	LOC100622637	545.5972887	5660.0068	9.094333493	12.46684295	12.46684295	-3.3749	0.02791	LOC100517129	0	18.178124	0	4.261389712	4.261389712	-20	0.00365
LOC100621908	17.9505279	0.8262784	4.24416613	0.868906686		4.441255	0.05285	LOC100737748	897.6978645	111.54758	9.811692364	6.814391228	6.814391228	3.008571	0.02799	LOC102162278	0	18.178124	0	4.261389712	4.261389712	-20	0.00365
LOC100158121	17.9505279	0.8262784	4.24416613	0.868906686		4.441255	0.05285	LOC100519903	2.114718173	38.835083	1.639101631	5.315967693	5.315967693	-4.19882	0.02799	LOC102165168	0	18.178124	0	4.261389712	4.261389712	-20	0.00365
ZNF500	20.7121475	149.55639	4.440430524	7.234160086		-2.85214	0.05302	LOC102158861	1068.990037	132.20454	10.06338165	7.057499439	7.057499439	3.015405	0.02816	CNIH2	33.1571529	229.70539	5.094115824	7.849907883	7.849907883	-2.79239	0.00367
LOC102159125	1.38080984	28.919743	1.251452392	4.903025878		-4.38847	0.05328	CCDC112	1221.249745	150.38266	10.25532339	7.242056187	7.242056187	3.021648	0.02825	ANPEP	583.565892	4080.1626	9.191221843	11.99476448	11.99476448	-2.80566	0.00368
LOC102163532	1.38080984	28.919743	1.251452392	4.903025878		-4.38847	0.05328	PRMT3	964.311487	119.81036	9.914850736	6.916600414	6.916600414	3.008747	0.0283	LOC100627110	168.272551	1134.4802	7.403204238	10.14908684	10.14908684	-2.75316	0.00369
LOC102164642	1.38080984	28.919743	1.251452392	4.903025878		-4.38847	0.05328	LOC102158578	100.4491132	11.567897	6.664612444	3.651671381	3.651671381	3.118266	0.02835	RBCK1	244.534003	1661.6458	7.939779021	10.69926515	10.69926515	-2.76451	0.0037

PTK2B	186.409328	1297.257	7.550048951	10.34236034		-2.79892	0.0534
MATN4	117.368836	803.14258	6.88714549	9.651307509		-2.77461	0.05351
ERAP1	566.132033	4171.0532	9.147540835	12.02654185		-2.8812	0.05352
LOC102167822	330.013551	61.1446	8.370746468	5.957557119		2.432228	0.05368
LOC100516260	11.0464787	88.411786	3.590539588	6.482393107		-3.00065	0.05395
LOC100517307	11.0464787	88.411786	3.590539588	6.482393107		-3.00065	0.05395
LOC100518245	66.2788721	448.66916	6.072081615	8.812720118		-2.75903	0.05407
ZNRD1	1075.65086	196.65425	10.07233477	7.626835185		2.451477	0.05419
KCNE1L	8.28485901	71.05994	3.214880004	6.171125545		-3.10049	0.05498
LOC102166984	8.28485901	71.05994	3.214880004	6.171125545		-3.10049	0.05498
LOC100739867	383.865134	2748.2019	8.58820917	11.42479713		-2.83982	0.05516
EPHA7	7584.78843	1287.3417	12.88908342	10.33129957		2.558714	0.05519
LOC100621455	6082.46733	1042.7633	12.57067812	10.02757887		2.544245	0.05527
LOC100516216	13.8080984	104.93735	3.888314478	6.727067559		-2.92594	0.05529
LOC100511579	85.6102098	577.56858	6.436465198	9.176344172		-2.75414	0.05531
MFSD12	487.425872	3533.1663	8.931995812	11.78715422		-2.85771	0.05541
BCL9L	690.404918	5106.4003	9.433387055	12.31837343		-2.88679	0.05549
TCF19	3184.14748	561.86929	11.63714446	9.136656134		2.502601	0.05564
ITGA3	773.253508	5752.55	9.596662204	12.49023668		-2.89519	0.05575
LOC102166395	92.514259	15.699289	6.547114458	4.061714779		2.558977	0.05595
LOC100622572	80.0869705	13.220454	6.341398207	3.829895615		2.598796	0.05603
LOC100522360	80.0869705	13.220454	6.341398207	3.829895615		2.598796	0.05603
LOC100738501	11.0464787	87.585507	3.590539588	6.468998788		-2.98711	0.05609
LOC102160975	109.083977	19.004403	6.782460686	4.322245637		2.521034	0.0561
LOC102158027	75.944541	12.394176	6.265747069	3.74353388		2.615284	0.05611
RBM38	1701.15772	308.20183	10.733149	8.27240506		2.46457	0.05626
AURKB	5883.63071	1013.0173	12.52273617	9.985866528		2.538048	0.05628
LOC100739123	16.569718	121.46292	4.135019133	6.936201185		-2.8739	0.05653
LOC100622965	4.14242951	45.44531	2.362450112	5.537461031		-3.45558	0.05657
NOS3	138.080984	24.788351	7.119781365	4.688647628		2.47778	0.05676
ZIC3	361.772177	67.754826	8.502920002	6.103389087		2.416686	0.05677
RPA2	5943.00553	1025.4115	12.53721974	10.00339347		2.53499	0.05721
FSTL4	22.0929574	1.6525567	4.529381037	1.407383613		3.740815	0.05724
SSFA2	6.90404918	61.970878	2.982591923	5.976612875		-3.16608	0.05737
GYG2	91.1334492	609.79344	6.525653118	9.254540753		-2.74227	0.0576
HOXA9	2884.51175	513.94515	11.49461149	9.008274952		2.488641	0.05763
ATOX1	2288.0019	411.48663	11.16050294	8.688203544		2.475171	0.05763
DHRS3	27.6161967	190.04403	4.838760036	7.577761331		-2.78275	0.05766
LOC100738678	41.4242951	278.45581	5.406818783	8.126476366		-2.7489	0.05775
LOC100512812	9.66566885	78.496445	3.414902536	6.312818447		-3.02169	0.05799
PERP	183.647708	33.877413	7.528631546	5.124221138		2.438545	0.05829
PRSS56	368.676226	69.407383	8.530118456	6.137654819		2.409193	0.05842
MYO7A	26.2353869	2.4788351	4.767410456	1.798604302		3.40378	0.05871
LOC100510883	26.2353869	2.4788351	4.767410456	1.798604302		3.40378	0.05871
SSX2IP	2118.16229	383.39316	11.04927836	8.586438871		2.465917	0.05886
LOC780424	30.3778164	3.3051135	4.971673052	2.106051269		3.200246	0.05902
LOC100513717	30.3778164	3.3051135	4.971673052	2.106051269		3.200246	0.05902
DYNLT3	223.691193	1533.5727	7.81179977	10.58362124		-2.77732	0.0592
LOC100525830	328.632741	61.970878	8.364715736	5.976612875		2.406814	0.05925
LOC100516062	1.38080984	28.093465	1.251452392	4.862623207		-4.34665	0.05948
LOC100737397	1.38080984	28.093465	1.251452392	4.862623207		-4.34665	0.05948
GINS2	3822.08163	676.72199	11.90052029	9.404549765		2.497723	0.05965
LOC100625384	70.4213016	466.021	6.158282521	8.867343618		-2.72631	0.05972
PKDCC	731.829213	5333.6269	9.517333205	12.38117166		-2.86554	0.0603
PTPN6	846.436429	158.64545	9.726961338	7.318727602		2.415596	0.06046
LOC102158885	9.66566885	77.670167	3.414902536	6.29774474		-3.00642	0.06055
LOC100518417	6.90404918	61.1446	2.982591923	5.957557119		-3.14671	0.06056
LOC102160069	715.259495	134.68337	9.484338548	7.084100147		2.408895	0.06071
FBL	33160.1482	5340.2371	15.01720634	12.38295822		2.634475	0.06074
LOC100523135	2.76161967	36.356248	1.911353989	5.223277669		-3.71862	0.06074
HHLA2	2.76161967	36.356248	1.911353989	5.223277669		-3.71862	0.06074
ALK	2.76161967	36.356248	1.911353989	5.223277669		-3.71862	0.06074
SYN1	581.320941	109.89502	9.18567069	6.793050813		2.403209	0.06076
LOC100519113	4.14242951	44.619032	2.362450112	5.511563931		-3.42911	0.06082
LOC100512960	795.346465	149.55639	9.637252429	7.234160086		2.410894	0.06108
C5AR2	0	19.004403	0	4.322245637		-20	0.06136
MYRIP	0	19.004403	0	4.322245637		-20	0.06136
LOC102159557	0	19.004403	0	4.322245637		-20	0.06136
LOC102163875	0	19.004403	0	4.322245637		-20	0.06136
INHA	0	19.004403	0	4.322245637		-20	0.06136
LOC102166882	0	19.004403	0	4.322245637		-20	0.06136
LOC100621844	0	19.004403	0	4.322245637		-20	0.06136
LOC100154914	0	19.004403	0	4.322245637		-20	0.06136
LOC100519891	0	19.004403	0	4.322245637		-20	0.06136
LOC100627140	0	19.004403	0	4.322245637		-20	0.06136
RSPO1	0	19.004403	0	4.322245637		-20	0.06136
LOC102165502	0	19.004403	0	4.322245637		-20	0.06136
LOC100627895	0	19.004403	0	4.322245637		-20	0.06136
LOC102166880	513.661259	3650.4978	9.007479378	11.83427266		-2.8292	0.06144
FEN1	6286.82718	1094.8188	12.61834585	10.0977936		2.52164	0.06164
PROSER2	465.332915	88.411786	8.865216451	6.482393107		2.395953	0.06164
ZMIZ1	802.250515	5850.8772	9.64970619	12.51468377		-2.86653	0.06166
C12H17orf97	74.5637311	12.394176	6.239622035	3.74353388		2.588812	0.06179
ACSM5	30.3778164	204.09076	4.971673052	7.68011867		-2.74812	0.06196
LOC100524239	11731.3604	1988.852	13.51820567	10.95844545		2.560362	0.06219
LOC100737965	60.7556328	398.26618	5.948498827	8.641207046		-2.71264	0.06224
GTF2IRD2	154.650702	1031.1954	7.28216827	10.0115004		-2.73723	0.06246
LOC100522455	20.7121475	143.77244	4.440430524	7.177643143		-2.79524	0.0626
LOC100625132	248.54577	47.097867	7.963160641	5.587901017		2.399778	0.06269
TRIM14	62.1364426	406.52896	5.98040107	8.67075877		-2.70985	0.06274
TMTC3	64.6980623	423.8808	6.042164139	8.730914357		-2.70741	0.06316
HYAL1	27.6161967	185.91263	4.838760036	7.546220275		-2.75104	0.06327
POLE2	1252.39452	233.83678	10.29162488	7.875514565		2.421115	0.06331
SLC16A8	62.1364426	9.9153405	5.98040107	3.448285225		2.647705	0.06336
CEL	8.28485901	68.581105	3.214880004	6.120623682		-3.04926	0.06351
LOC100521619	38.6626754	254.49374	5.309710091	7.997144125		-2.71862	0.0639
LOC100738306	6.90404918	60.318321	2.982591923	5.938246292		-3.12708	0.06394
LOC100518732	41.4242951	271.84558	5.406818783	8.091940885		-2.71424	0.06399
BNP	12.4272885	0	3.747096094	0		20	0.06404
LOC102159158	12.4272885	0	3.747096094	0		20	0.06404
EGF	12.4272885	0	3.747096094	0		20	0.06404
LOC102159508	12.4272885	0	3.747096094	0		20	0.06404
LOC102159513	12.4272885	0	3.747096094	0		20	0.06404
RGR	12.4272885	0	3.747096094	0		20	0.06404
LOC102164610	12.4272885	0	3.747096094	0		20	0.06404
DNAH8	12.4272885	0	3.747096094	0		20	0.06404
DRD1	12.4272885	0	3.747096094	0		20	0.06404
TRPM3	12.4272885	0	3.747096094	0		20	0.06404
LOC100512853	12.4272885	0	3.747096094	0		20	0.06404
LOC102162884	12.4272885	0	3.747096094	0		20	0.06404
LOC102160396	12.4272885	0	3.747096094	0		20	0.06404
LOC100519692	12.4272885	0	3.747096094	0		20	0.06404
LOC102158541	12.4272885	0	3.747096094	0		20	0.06404
LOC102163174	12.4272885	0	3.747096094	0		20	0.06404
LOC102167848	12.4272885	0	3.747096094	0		20	0.06404
ITLN2	12.4272885	0	3.747096094	0		20	0.06404
LOC100627811	222.310384	42.140197	7.802906526	5.430960858		2.399306	0.06406
SALL1	470.856154	90.064343	8.882203309	6.508814353		2.386258	0.06407
MATN2	57.9940131	9.0890621	5.882496648	3.334720158		2.673701	0.06408
LOC100514269	238.880102	45.44531	7.906169679	5.537461031		2.394083	0.06444
SLA-3	900.288013	6546.6035	9.815844393	12.67675125		-2.86229	0.06453
LOC100739391	5.52323934	52.055537	2.705588563	5.729431428		-3.23647	0.06458
ERCC6L	1554.79188	289.19743	10.60343336	8.180890933		2.426595	0.06474
HSPB2	48.3283443	314.81206	5.624344958	8.302922453		-2.70355	0.0648
CHMP4C	53.8515836	8.2627837	5.777461367	3.211445828		2.704289	0.06491
LOC102157728	864.386957	6259.0587	9.757201567	12.61196046		-2.8562	0.06503
LOC100620858	773.253508	147.07755	9.596662204	7.210209122		2.394364	0.06506
PRIM1	2891.4158	525.51304	11.49805924	9.040325464		2.459978	0.06522
RGS17	40.0434852	261.93024	5.359081339	8.038536289		-2.70954	0.06523
LOC100738143	408.719711	78.496445	8.678493492	6.312818447		2.380413	0.06528
TAGAP	4.14242951	43.792754	2.362450112	5.485193456		-3.40214	0.06542
DOCK7	230.595243	1547.6194	7.855461808	10.5967669		-2.74662	0.06547
MFAP3L	49.7091541	7.4365053	5.664174303	3.076645515		2.740815	0.0659
LOC100624528	13464.2767	2289.6174	13.71695626	11.16152077		2.555958	0.06603
HHEX	175.362849	33.051135	7.462402878	5.089630977		2.407571	0.06603
LOC100737457	225.072003	1505.4792	7.820638529	10.55696503		-2.74176	0.06611
MEOX1	23.4737672	158.64545	4.613164285	7.318727602		-2.75668	0.06612
LOC100155040	2822.3753	514.77143	11.4632052	9.010588038		2.454906	0.06623
LOC102157979	2.76161967	35.52997	1.911353989	5.191008663		-3.68545	0.06633
LOC100622884	1.38080984	27.267186	1.251452392	4.821056379		-4.30358	0.06648
GORAB	46.9475344	304.07044	5.583384725	8.252998589		-2.69528	0.06659
HOXD8	4161.76085	747.78193	12.02332496	9.548401802		2.476505	0.06689
ATP6V1C2	3286.32741	596.57298	11.68269944	9.222971117		2.461706	0.06692
FAM129C	15.1889082	108.24247	4.016933786	6.771389985		-2.83318	0.06729
FCGR1A	77.3253508	13.220454	6.291407421	3.829895615		2.54817	0.06734
FMNL3	243.022531	1623.637	7.930870551	10.66590169		-2.74007	0.06753
LOC102165201	6.90404918	59.492043	2.982591923	5.918673475		-3.10718	0.06754
C4H8orf47	121.511266	22.309516	6.936770608	4.542847146		2.445359	0.06755
ACER3	2337.71105	431.31731	11.19149791	8.755946793		2.438275	0.06778
LOC100519683	429.431859	2949.8138	8.749641054	11.52689717		-2.78012	0.0683
LOC100737582	11.0464787	83.454116	3.590539588	6.400095824		-2.9174	0.06831
DUSP6	436.335908	2999.3905	8.772597998	11.55093456		-2.78116	0.06836
FGF2	55.2323934	354.47342	5.813329549	8.473597884		-2.68209	0.06837
LOC100514151	669.69277	128.89943	9.38950824	7.021251246		2.377254	0.06839
TRNP1	499.853161	96.674569	8.968423888	6.609911086		2.370296	0.06842
ZNF382	38.6626754	250.36235	5.309710091	7.973624744		-2.695	0.06846
FBXO41	260.973059	50.402981	8.033274644	5.683780114		2.37232	0.06892
FOXO3	77.3253508	494.94074	6.291407421	8.954023947		-2.67824	0.06911
ZHX3	77.3253508	494.94074	6.291407421	8.954023947		-2.67824	0.06911
MTBP	1201.30456	228.05283	10.23158668	7.839536581		2.397162	0.06931
ANGPTL4	3943.59289	714.73079	11.9456607	9.483273237		2.464039	0.06953
MRPS27	10147.5715	1763.278	13.30898905	10.78486223		2.524803	0.06972
MOAP1	40.0434852	257.79885	5.359081339	8.015687408		-2.68661	0.06974
LOC102159768	35.9010557	232.18422	5.205590187	7.865326367		-2.69317	0.06979
SLC15A1	37.2818656	4.9576702	5.258589232	2.574748269		2.91074	0.06996
LOC102167582	37.2818656	4.9576702	5.258589232	2.574748269		2.91074	0.06996
LOC102163800	8.28485901	66.928548	3.214880004	6.085946114		-3.01407	0.06999
AAK1	301.016544	2016.1192	8.238483771	10.97808065		-2.74367	0.07006
SACS	56.6132033	361.08365	5.848327568	8.500179216		-2.67312	0.07011
PRSS35	260.973059	1734.3583	8.033274644	10.76101785		-2.73243	0.07019
LOC100517801	289.970066	1935.9702	8.184726929	10.91958606		-2.73908	0.07043
CARD14	92.514259	16.525567	6.547114458	4.13138925		2.484976	0.0705
PER2	23.4737672	156.16661	4.613164285	7.296150961		-2.73396	0.07056
LOC102159119	34.5202459	223.09516	5.150569664	7.807967682		-2.69214	0.07058
CDC20	16732.6536	2847.3553	14.03046485	11.47591339		2.554972	0.07069
IFIT5	1010.7528	7249.7664	9.982641126	12.82391779		-2.8425	0.07091
NCOA2	85.6102098	545.34373	6.436465198	9.09366508		-2.67131	0.07093
WDR38	1017.65685	195.0017	9.992452423	7.614722326		2.383693	0.07097
LOC102160600	60.7556328	9.9153405	5.948498827	3.448285225		2.615284	0.07102
LOC100514644	205.740666	39.661362	7.691678382	5.34558663		2.375021	0.07107
RPS6KA6	11.0464787	82.627837	3.590539588	6.385911347		-2.90304	0.07109
CREB5	11.0464787	82.627837	3.590539588	6.385911347		-2.90304	0.07109
LYPD6	185.028518	35.52997	7.539379992	5.191008663		2.380639	0.07148
BLCAP	675.21601	4713.9181	9.401340363	12.203017		-2.80351	0.07156
FOXC1	99.4183082	633.75551	6.649878514	9.310057206		-2.67234	0.07158
LOC100739394	1677.68395	316.46462	10.71311492	8.310451992		2.406355	0.0716
SGSH	346.583269	2326.7999	8.441214828	11.18475133		-2.74707	0.07167
NRSN2	0	18.178124	0	4.261389712		-20	0.07171
CLCNKA	0	18.178124	0	4.261389712		-20	0.07171
METTL11B	0	18.178124	0	4.261389712		-20	0.07171
LOC100152036	0	18.178124	0	4.261389712		-20	0.07171
DISP2	0	18.178124	0	4.261389712		-20	0.07171
LOC100512345	0	18.178124	0	4.261389712		-20	0.07171
LOC102166285	0	18.178124	0	4.261389712		-20	0.07171
HTR3C	0	18.178124	0	4.261389712		-20	0.07171
LOC100512775	0	18.178124	0	4.261389712		-20	0.07171
TMPRSS7	0	18.178124	0	4.261389712		-20	0.07171
LOC102158616	0	18.178124	0	4.261389712		-20	0.07171
LOC100517129	0	18.178124	0	4.261389712		-20	0.07171
LOC102162278	0	18.178124	0	4.261389712		-20	0.07171
LOC102158558	0	18.178124	0	4.261389712		-20	0.07171
LOC100524250	0	18.178124	0	4.261389712		-20	0.07171
LOC102165168	0	18.178124	0	4.261389712		-20	0.07171
FBXO36	33.1394361	4.1313919	5.093367322	2.359350201		3.003849	0.07174
LOC102164492	724.925164	140.46732	9.503677017	7.144325041		2.367598	0.07178
ZHX2	93.8950688	597.39926	6.568261215	9.224964588		-2.66957	0.07179
FHDC1	672.45439	130.55198	9.395436432	7.039489182		2.364812	0.07188
LSMD1	2754.71562	509.8176	11.4282113	8.996653565		2.433861	0.07193
PLXDC1	168.4588	32.224857	7.404790748	5.054191063		2.38615	0.07205
ARHGEF19	109.083977	695.72639	6.782460686	9.444448397		-2.67308	0.07212
LOC100736766	164.316371	31.398578	7.369085785	5.017858594		2.387705	0.07224
HERC2	30.3778164	196.65425	4.971673052	7.626835185		-2.69457	0.07234
NPR2	820.201042	5780.6435	9.681591649	12.49726394		-2.81718	0.07235
LOC100624428	56.6132033	9.0890621	5.848327568	3.334720158		2.638935	0.07235
MB21D1	31.7586262	204.91704	5.033802952	7.685919384		-2.68982	0.07248
LOC100511598	2.76161967	34.703692	1.911353989	5.158001345		-3.6515	0.07249
LOC102168140	2.76161967	34.703692	1.911353989	5.158001345		-3.6515	0.07249
LOC102164120	2.76161967	34.703692	1.911353989	5.158001345		-3.6515	0.07249
GZMK	2.76161967	34.703692	1.911353989	5.158001345		-3.6515	0.07249
LOC100525237	19.3313377	130.55198	4.345633235	7.039489182		-2.75561	0.07256
PLXND1	1092.22058	7831.4664	10.09436881	12.93525096		-2.84202	0.07258
C2H5orf45	379.722705	74.365053	8.572596799	6.2358238		2.35225	0.07268
LOC100627393	12.4272885	90.064343	3.747096094	6.508814353		-2.85744	0.07296
CCDC88C	1338.00473	255.32002	10.38694534	8.001802338		2.389705	0.07301
LASS6	5.52323934	50.402981	2.705588563	5.683780114		-3.18992	0.07323
SLC29A1	12206.359	2120.2303	13.57546349	11.05068555		2.52534	0.07338
LOC102159133	8.28485901	66.10227	3.214880004	6.068289661		-2.99615	0.0735
GLTPD2	8.28485901	66.10227	3.214880004	6.068289661		-2.99615	0.0735
SMIM13	62.1364426	391.65595	5.98040107	8.617121942		-2.65608	0.07351
MYOZ1	28.9970066	3.3051135	4.906746634	2.106051269		3.133132	0.07369
LOC102162072	52.4707738	8.2627837	5.740678649	3.211445828		2.666814	0.07391
LOC102165471	11.0464787	81.801559	3.590539588	6.371586023		-2.88605	0.07399
NEFM	62.1364426	390.82967	5.98040107	8.614082834		-2.65303	0.07416
ICAM-1	947.235547	6696.9862	9.889101667	12.70951169		-2.82172	0.07425
LOC102166432	1.38080984	26.440908	1.251452392	4.77825631		-4.25919	0.07441
CCNB3	3240.76068	599.05182	11.66256188	9.228943285		2.43558	0.07445
HEY1	131.176934	24.788351	7.04632663	4.688647628		2.40378	0.07464
INADL	33.1394361	211.52726	5.093367322	7.731504113		-2.67422	0.075
TMEM261	314.824643	61.970878	8.302979933	5.976612875		2.344886	0.07506
TMEM158	156.031511	997.31799	7.294910283	9.963355621		-2.67622	0.07515
LOC102163992	6.90404918	57.839486	2.982591923	5.878712739		-3.06654	0.0754
LOC102160753	6.90404918	57.839486	2.982591923	5.878712739		-3.06654	0.0754
LOC102164756	16.569718	0.8262784	4.135019133	0.868906686		4.325777	0.07543
LOC100738970	16.569718	0.8262784	4.135019133	0.868906686		4.325777	0.07543
LMX1A	16.569718	0.8262784	4.135019133	0.868906686		4.325777	0.07543
C7H14orf159	16.569718	0.8262784	4.135019133	0.868906686		4.325777	0.07543
CDHR5	16.569718	0.8262784	4.135019133	0.868906686		4.325777	0.07543
LOC102162203	16.569718	0.8262784	4.135019133	0.868906686		4.325777	0.07543
TRNAG-UCC	16.569718	0.8262784	4.135019133	0.868906686		4.325777	0.07543
LOC100623190	16.569718	0.8262784	4.135019133	0.868906686		4.325777	0.07543
LOC100739108	16.569718	0.8262784	4.135019133	0.868906686		4.325777	0.07543
LOC100627705	2551.73658	477.5889	11.31782895	8.902643123		2.417638	0.0755
LOC102167189	24.854577	2.4788351	4.692347799	1.798604302		3.325777	0.07561
P4HA1	1226.15913	8770.1186	10.26110663	13.09854514		-2.83845	0.07583
GABRP	532.992597	104.93735	9.06067593	6.727067559		2.344587	0.07596
LOC102167019	53.8515836	337.12158	5.777461367	8.401398268		-2.64621	0.07599
LOC100521652	91.1334492	16.525567	6.525653118	4.13138925		2.463281	0.07616
LOC100739750	138.080984	875.85507	7.119781365	9.776194605		-2.66518	0.07616
LOC100736576	186.409328	1196.4511	7.550048951	10.22575101		-2.68222	0.07619
APBA1	26.2353869	169.38707	4.767410456	7.412672017		-2.69074	0.07651
BCAS1	20.7121475	1.6525567	4.440430524	1.407383613		3.647705	0.07686
PAX2	20.7121475	1.6525567	4.440430524	1.407383613		3.647705	0.07686
MSTN	20.7121475	1.6525567	4.440430524	1.407383613		3.647705	0.07686
KCNC2	20.7121475	1.6525567	4.440430524	1.407383613		3.647705	0.07686
HCRTR1	20.7121475	1.6525567	4.440430524	1.407383613		3.647705	0.07686
NUTM1	20.7121475	1.6525567	4.440430524	1.407383613		3.647705	0.07686
TMEM45B	20.7121475	1.6525567	4.440430524	1.407383613		3.647705	0.07686
CRYBB3	20.7121475	1.6525567	4.440430524	1.407383613		3.647705	0.07686
LOC100523649	118.749646	747.78193	6.90387758	9.548401802		-2.65469	0.07692
CAMK2N2	263.734679	52.055537	8.048403381	5.729431428		2.340964	0.0772
LOC102160331	8.28485901	65.275991	3.214880004	6.05041444		-2.978	0.0772
HMCN1	33.1394361	209.87471	5.093367322	7.72024225		-2.66291	0.07745
KMT2E	912.715301	6367.3011	9.835600905	12.63669284		-2.80245	0.07796
LOC100037969	5.52323934	49.576702	2.705588563	5.660401068		-3.16608	0.07802
LOC100523163	5.52323934	49.576702	2.705588563	5.660401068		-3.16608	0.07802
LOC102165992	526.088547	104.11107	9.041901535	6.715770874		2.337182	0.07807
SELRC1	1291.0572	249.53607	10.33545422	7.968874505		2.371233	0.07811

LOC100736638	160.7185812	1545.1406	7.337341641	10.59445576	10.59445576	-3.26513	0.02836
ADAM19	1496.163107	16526.394	10.54801569	14.01257162	14.01257162	-3.46543	0.02846
PHLDB1	244.249949	2399.5124	7.938109026	11.22912667	11.22912667	-3.29631	0.02848
CHPF	993.9175414	10653.207	9.95843315	13.3791356	13.3791356	-3.42202	0.02873
LOC100626660	19.03246356	181.78124	4.324267947	7.513974209	7.513974209	-3.25567	0.02876
LOC100523964	120.5389359	1139.4379	6.925274755	10.15537214	10.15537214	-3.24075	0.02876
LOC100624808	1.057359087	29.746021	1.04079362	4.942327829	4.942327829	-4.81416	0.02885
LOC100737709	1.057359087	29.746021	1.04079362	4.942327829	4.942327829	-4.81416	0.02885
PLEKHA7	871.2638873	109.06875	9.768620852	6.782261052	6.782261052	2.997872	0.02888
LOC102166061	28.54869534	1.6525567	4.885022527	1.407383614	1.407383614	4.110653	0.0289
LOC102158314	28.54869534	1.6525567	4.885022527	1.407383614	1.407383614	4.110653	0.0289
PANK1	2690.978875	321.42229	11.39445137	8.33280766	8.33280766	3.065589	0.02891
C14H10orf71	0	20.656959	0	4.436758793	4.436758793	-20	0.02898
LOC102159603	0	20.656959	0	4.436758793	4.436758793	-20	0.02898
LOC102163989	0	20.656959	0	4.436758793	4.436758793	-20	0.02898
PTPRCAP	0	20.656959	0	4.436758793	4.436758793	-20	0.02898
LOC100520139	0	20.656959	0	4.436758793	4.436758793	-20	0.02898
LOC100624548	0	20.656959	0	4.436758793	4.436758793	-20	0.02898
NKX6-1	0	20.656959	0	4.436758793	4.436758793	-20	0.02898
COMP	0	20.656959	0	4.436758793	4.436758793	-20	0.02898
LOC102166377	0	20.656959	0	4.436758793	4.436758793	-20	0.02898
LOC102160315	0	20.656959	0	4.436758793	4.436758793	-20	0.02898
ZNF132	0	20.656959	0	4.436758793	4.436758793	-20	0.02898
TMEM173	0	20.656959	0	4.436758793	4.436758793	-20	0.02898
LOC100739192	0	20.656959	0	4.436758793	4.436758793	-20	0.02898
SGCA	0	20.656959	0	4.436758793	4.436758793	-20	0.02898
LOC100525890	8.458872693	90.890621	3.241668254	6.521845711	6.521845711	-3.42559	0.02907
PM20D2	3855.13123	452.80055	11.91293843	8.82591454	8.82591454	3.089832	0.02908
SLC9B2	248.4793853	31.398578	7.962776799	5.017858594	5.017858594	2.984355	0.02915
MAPRE3	83.53136784	775.87539	6.40141489	9.601539402	9.601539402	-3.21544	0.02921
MAP3K14	133.2272449	1260.0745	7.068533723	10.30043781	10.30043781	-3.24155	0.02922
PTPN18	76.12985423	704.81545	6.269217479	9.463147202	9.463147202	-3.21071	0.02928
RFC3	5728.771531	660.19642	12.4842619	9.368935101	9.368935101	3.117259	0.02933
LOC102158232	2011.096983	244.5784	10.97448413	7.940039851	7.940039851	3.039614	0.02952
NRARP	40.17964529	3.3051135	5.363859498	2.106051269	2.106051269	3.603693	0.02966
PNP	6291.286565	722.99358	12.61936866	9.499833085	9.499833085	3.1213	0.02966
LOC102162960	2502.768958	301.59161	11.28988572	8.241228155	8.241228155	3.052857	0.02969
ABCC3	472.6395117	4783.3255	8.887645627	12.22409983	12.22409983	-3.3392	0.02975
16S	41120.69488	564649.72	15.32761211	19.1069992	19.1069992	-3.77942	0.02976
OLFML2A	31.7207726	291.67627	5.032134908	8.193161944	8.193161944	-3.20087	0.02984
WDR17	20.08982264	189.21775	4.398475058	7.571508044	7.571508044	-3.23551	0.02985
NCKIPSD	86.7034451	801.49002	6.45456161	9.648339641	9.648339641	-3.20852	0.02992
LOC100153854	4.229436346	55.360651	2.386655454	5.816616368	5.816616368	-3.71032	0.02998
LOC100625063	166.0053766	20.656959	7.38375074	4.436758793	4.436758793	3.00653	0.03
GSTZ1	87.76080419	809.7528	6.471850834	9.663118299	9.663118299	-3.20583	0.03019
LOC102167612	63.44154519	6.610227	6.009919183	2.927939483	2.927939483	3.262656	0.0302
TRIM13	2579.956171	311.50695	11.33368993	8.287744447	8.287744447	3.05001	0.03021
TMEM170B	3107.578355	371.82527	11.60203923	8.542355828	8.542355828	3.063094	0.03023
LOC102159321	2.114718173	38.008805	1.639101631	5.285727902	5.285727902	-4.1678	0.03031
NHLRC1	2.114718173	38.008805	1.639101631	5.285727902	5.285727902	-4.1678	0.03031
LOC100625114	2.114718173	38.008805	1.639101631	5.285727902	5.285727902	-4.1678	0.03031
LOC102159404	22.20454082	0.8262784	4.536335244	0.868906686	0.868906686	4.748083	0.03041
LOC100518808	22.20454082	0.8262784	4.536335244	0.868906686	0.868906686	4.748083	0.03041
ACHE	7.401513606	80.97528	3.070649265	6.357117027	6.357117027	-3.45159	0.03058
LOC100626258	508.5897206	65.275991	8.993192366	6.05041444	6.05041444	2.961878	0.03062
ART3	104.6785496	12.394176	6.723538761	3.74353388	3.74353388	3.078232	0.03064
KLHL40	5.286795433	63.623435	2.65232482	6.013985524	6.013985524	-3.58909	0.03066
LOC100737628	93.04759962	856.85067	6.555319217	9.744582725	9.744582725	-3.203	0.03068
FGG	15.8603863	0	4.075565686	0	0	20	0.03075
STMND1	15.8603863	0	4.075565686	0	0	20	0.03075
C6H1orf177	15.8603863	0	4.075565686	0	0	20	0.03075
LOC102168169	15.8603863	0	4.075565686	0	0	20	0.03075
LOC100525793	15.8603863	0	4.075565686	0	0	20	0.03075
LOC100520278	15.8603863	0	4.075565686	0	0	20	0.03075
DLL3	15.8603863	0	4.075565686	0	0	20	0.03075
LOC100516636	15.8603863	0	4.075565686	0	0	20	0.03075
LOC100516605	15.8603863	0	4.075565686	0	0	20	0.03075
LOC102157755	15.8603863	0	4.075565686	0	0	20	0.03075
LOC100621571	15.8603863	0	4.075565686	0	0	20	0.03075
LOC100623971	15.8603863	0	4.075565686	0	0	20	0.03075
LOC102158492	15.8603863	0	4.075565686	0	0	20	0.03075
LOC100627431	15.8603863	0	4.075565686	0	0	20	0.03075
LOC100625468	15.8603863	0	4.075565686	0	0	20	0.03075
PPP1R17	15.8603863	0	4.075565686	0	0	20	0.03075
SYNDIG1L	15.8603863	0	4.075565686	0	0	20	0.03075
LOC100737710	3437.47439	410.66035	11.74755289	8.685310691	8.685310691	3.065331	0.03077
SLA-6	396.5096575	3934.7376	8.634846101	11.94241832	11.94241832	-3.3184	0.0308
PHF16	5723.484736	665.98037	12.48293013	9.381500486	9.381500486	3.103342	0.03081
LOC102165713	251.6514626	32.224856	7.981004722	5.054191063	5.054191063	2.965181	0.03085
LOC100621575	67.67098154	616.40367	6.10162868	9.270070238	9.270070238	-3.18727	0.03089
LOC100511684	103.6211905	956.00408	6.709031281	9.902381259	9.902381259	-3.2057	0.03094
ANGPTL2	1675.914152	18276.451	10.71159312	14.15777729	14.15777729	-3.44697	0.03101
PMCH	189.2672765	23.962073	7.571883648	4.641665831	4.641665831	2.9816	0.03104
LOC404703	116.3094995	14.046732	6.874176035	3.911378308	3.911378308	3.049662	0.03107
LOC100510918	3.17207726	46.271589	2.060765874	5.56290145	5.56290145	-3.86663	0.03113
SH3RF2	45.46644072	4.1313919	5.538117235	2.359350201	2.359350201	3.460102	0.03133
HCN4	45.46644072	4.1313919	5.538117235	2.359350201	2.359350201	3.460102	0.03133
LOC100620089	1565.948807	195.0017	10.61374233	7.614722326	7.614722326	3.005478	0.0314
STAP1	170.2348129	21.483238	7.419832229	4.490777899	4.490777899	2.986243	0.03156
CEP152	3570.701635	428.0122	11.80239585	8.744874853	8.744874853	3.060484	0.0316
TGM5	4.229436346	54.534373	2.386655454	5.795309087	5.795309087	-3.68863	0.03172
LOC102160662	4.229436346	54.534373	2.386655454	5.795309087	5.795309087	-3.68863	0.03172
HEATR4	163.8906584	20.656959	7.365365858	4.436758793	4.436758793	2.988034	0.03178
C3H9orf89	65.55626337	592.44159	6.056502534	9.212962234	9.212962234	-3.17587	0.03179
HSPB2	34.89284986	314.81206	5.165624571	8.302922453	8.302922453	-3.17349	0.03186
LOC102160307	121.596295	14.873011	6.937771569	3.98850389	3.98850389	3.031331	0.03189
LOC102164620	6707.886045	779.1805	12.71185752	9.607664138	9.607664138	3.105829	0.03189
LOC100525472	6502.758382	756.87099	12.66705795	9.565808471	9.565808471	3.102933	0.03194
C7H14orf79	25.37661808	231.35794	4.721187694	7.86020516	7.86020516	-3.18855	0.03196
TRABD2A	19.03246356	176.82357	4.324267947	7.474302764	7.474302764	-3.15078	0.03203
SLC1A1	413.4274028	53.708094	8.69497559	5.773682393	5.773682393	2.944423	0.03209
PXDC1	498.0161298	4962.6279	8.962942639	12.27717925	12.27717925	-3.31684	0.03214
JAK1	219.93069	2083.8741	7.787450031	11.02574452	11.02574452	-3.24415	0.0322
LOC100522672	12.68830904	123.11548	3.774872332	6.955539223	6.955539223	-3.27844	0.03238
PLXDC2	146.972913	1360.8805	7.209189299	10.41138438	10.41138438	-3.21092	0.03244
LOC100621280	50.75323616	454.4531	5.693577173	8.831158705	8.831158705	-3.16256	0.03251
CAMK2N1	84.58872693	765.13377	6.419438884	9.581452508	9.581452508	-3.17717	0.03253
NENF	146.972913	1359.2279	7.209189299	10.4096327	10.4096327	-3.20917	0.03259
HOMER1	463.1232799	60.318321	8.858364253	5.938246292	5.938246292	2.940728	0.03261
LOC102167488	1082.735705	137.98849	10.08179725	7.118821584	7.118821584	2.972061	0.03267
LOC100627784	72.95777987	656.06503	6.208629956	9.359892345	9.359892345	-3.16871	0.03274
LOC102165476	9.516231779	96.674569	3.394545939	6.609911087	6.609911087	-3.34467	0.03278
TLDC1	57.09739067	510.64003	5.860401464	8.998985343	8.998985343	-3.16081	0.03282
IL21R	2.114718173	37.182527	1.639101631	5.254840671	5.254840671	-4.13609	0.03286
STK36	67.67098154	606.48832	6.10162868	9.246712872	9.246712872	-3.16387	0.03294
NOSIP	175.5216084	1632.7261	7.463700987	10.67395038	10.67395038	-3.21756	0.03301
LOC100737465	603.7520384	78.496445	9.240199917	6.312818447	6.312818447	2.943257	0.03301
PIK3IP1	133.2272449	1222.0657	7.068533723	10.2562862	10.2562862	-3.19736	0.03306
ND2	470238.2492	43491.988	18.84303544	15.40849522	15.40849522	3.43457	0.03307
PGR	39.1222862	3.3051135	5.326331909	2.106051269	2.106051269	3.565219	0.03321
LOC100514211	635.472811	82.627837	9.313955076	6.385911347	6.385911347	2.943131	0.03321
PHLDB2	265.3971307	2522.6279	8.057434734	11.30128347	11.30128347	-3.2487	0.03322
LOC100155756	10.57359087	104.93735	3.532764645	6.727067559	6.727067559	-3.31099	0.03324
KLHL38	3.17207726	45.44531	2.060765874	5.537461031	5.537461031	-3.84063	0.03328
LOC100739358	247.4220263	32.224856	7.956649285	5.054191063	5.054191063	2.940728	0.03331
LOC100513647	166.0053766	1536.0515	7.38375074	10.58594978	10.58594978	-3.20992	0.03333
LOC102165406	1178.955382	150.38266	10.20451659	7.242056187	7.242056187	2.970799	0.03334
LOC100622368	0	19.830681	0	4.380638094	4.380638094	-20	0.03344
LOC102159118	0	19.830681	0	4.380638094	4.380638094	-20	0.03344
LOC102161858	0	19.830681	0	4.380638094	4.380638094	-20	0.03344
SNX20	0	19.830681	0	4.380638094	4.380638094	-20	0.03344
FAM83F	0	19.830681	0	4.380638094	4.380638094	-20	0.03344
LOC102164546	0	19.830681	0	4.380638094	4.380638094	-20	0.03344
LOC100738697	0	19.830681	0	4.380638094	4.380638094	-20	0.03344
LOC102158147	0	19.830681	0	4.380638094	4.380638094	-20	0.03344
LOC100623646	0	19.830681	0	4.380638094	4.380638094	-20	0.03344
LOC102158584	0	19.830681	0	4.380638094	4.380638094	-20	0.03344
LOC100517727	0	19.830681	0	4.380638094	4.380638094	-20	0.03344
LOC102165393	0	19.830681	0	4.380638094	4.380638094	-20	0.03344
MIR221	0	19.830681	0	4.380638094	4.380638094	-20	0.03344
LOC100620821	0	19.830681	0	4.380638094	4.380638094	-20	0.03344
LOC100620445	0	19.830681	0	4.380638094	4.380638094	-20	0.03344
LOC100623686	32.77813168	292.50254	5.078017628	8.197229196	8.197229196	-3.15764	0.03345
PGAP1	6506.987819	764.30749	12.66799584	9.579895716	9.579895716	3.089765	0.03347
LOC102166858	8.458872693	87.585507	3.241668254	6.468998788	6.468998788	-3.37215	0.03348
ZBTB44	4131.101951	497.41958	12.01266013	8.961216934	8.961216934	3.053991	0.03358
DENND5B	259.0529762	33.877413	8.022661739	5.124221138	5.124221138	2.934852	0.03372
LOC102160785	27.49133625	1.6525567	4.832451381	1.407383614	1.407383614	4.056205	0.03379
CORO1B	746.4955151	7541.4427	9.545921113	12.88081612	12.88081612	-3.33664	0.0339
LOC102159647	1034.097187	133.03082	10.01555052	7.066420949	7.066420949	2.958539	0.03391
LOC102164364	56.04003159	5.7839486	5.833902876	2.76212524	2.76212524	3.276331	0.03391
AHCY	45523.53811	4814.7241	15.47435676	12.23353702	12.23353702	3.241088	0.03395
C4H1orf85	773.9868514	7824.8562	9.598028023	12.93403288	12.93403288	-3.33768	0.03413
ARHGAP18	4317.19715	520.55537	12.0762134	9.026676623	9.026676623	3.051971	0.03421
LOC100627437	4078.233997	493.28819	11.99408255	8.94920862	8.94920862	3.047442	0.03423
RAB11FIP5	166.0053766	1525.3099	7.38375074	10.57583218	10.57583218	-3.1998	0.03428
LOC100626687	247.4220263	2323.4948	7.956649285	11.18270147	11.18270147	-3.23125	0.03431
ACOX2	54.9826725	485.85168	5.806908455	8.927338518	8.927338518	-3.14347	0.03435
LOC100627468	93.04759962	832.8886	6.555319217	9.703710853	9.703710853	-3.16208	0.03435
42432	101.5064723	910.55877	6.679571195	9.832191856	9.832191856	-3.16518	0.0345
LOC100521366	85.64608601	763.48122	6.437062676	9.578337242	9.578337242	-3.15613	0.03453
KLHL23	2103.087223	263.5828	11.0389788	8.047575471	8.047575471	2.996181	0.03463
RANBP3L	17.97510447	164.4294	4.246035924	7.370071807	7.370071807	-3.1934	0.03468
LOC100627251	3237.633523	397.4399	11.66116951	8.638218305	8.638218305	3.026131	0.03473
CDK8	507.5323616	66.928548	8.990195776	6.085946114	6.085946114	2.922806	0.03474
VRK1	6574.6588	777.52795	12.68291973	9.60460502	9.60460502	3.07995	0.03474
LOC102163775	622.784502	81.801559	9.284903899	6.371586023	6.371586023	2.928533	0.03475
MSN	1828.173861	19515.043	10.83697649	14.25237292	14.25237292	-3.41611	0.03475
PHYHD1	25.37661808	226.40027	4.721187694	7.829090181	7.829090181	-3.1573	0.03476
IL17RD	42.29436346	371.82527	5.436107306	8.542355828	8.542355828	-3.13609	0.03492
SLC5A4	34.89284986	307.37555	5.165624571	8.268544593	8.268544593	-3.139	0.03498
PAQR4	34.89284986	307.37555	5.165624571	8.268544593	8.268544593	-3.139	0.03498
NDUFAF6	1543.744266	196.65425	10.5931523	7.626835184	7.626835184	2.9727	0.03499
FBXO5	2276.494113	285.06604	11.15323161	8.160204421	8.160204421	2.997446	0.03513
COBLL1	3270.411655	402.39757	11.6756976	8.656058573	8.656058573	3.022779	0.03522
LOC102162332	130.0551676	1172.489	7.034030432	10.19658861	10.19658861	-3.17238	0.03527
CNTNAP1	76.12985423	672.59059	6.269217479	9.395728184	9.395728184	-3.14319	0.03527
CMTM8	244.249949	32.224856	7.938109026	5.054191063	5.054191063	2.922112	0.0353
LOC100738280	1.057359087	28.093465	1.04079362	4.862623207	4.862623207	-4.7317	0.03545
SDS	1.057359087	28.093465	1.04079362	4.862623207	4.862623207	-4.7317	0.03545
CCDC166	1.057359087	28.093465	1.04079362	4.862623207	4.862623207	-4.7317	0.03545
MRPL47	4514.9233	546.99628	12.14080527	9.098022295	9.098022295	3.045099	0.03545
SLC6A15	775.0442105	101.63224	9.599995033	6.681340184	6.681340184	2.93092	0.03546
HCLS1	3.17207726	44.619032	2.060765874	5.511563931	5.511563931	-3.81416	0.0356
LOC100621492	3.17207726	44.619032	2.060765874	5.511563931	5.511563931	-3.81416	0.0356
LOC100739607	3.17207726	44.619032	2.060765874	5.511563931	5.511563931	-3.81416	0.0356
ARHGEF15	6.344154519	69.407383	2.876596413	6.137654819	6.137654819	-3.45159	0.03566
GCK	574.145984	76.01761	9.167784378	6.267116453	6.267116453	2.917012	0.03577
LOC100627397	6518.618769	775.87539	12.67057189	9.601539402	9.601539402	3.070669	0.0358
LOC733662	8.458872693	85.932951	3.241668254	6.441831208	6.441831208	-3.34467	0.03597
STAC	225.2174854	2075.6113	7.821566636	11.02001546	11.02001546	-3.20415	0.0362
SKIDA1	674.5950972	89.238064	9.400015048	6.495664213	6.495664213	2.918291	0.03627
PGM5	10.57359087	102.45852	3.532764645	6.692908622	6.692908622	-3.2765	0.03639
LOC100522989	194.5540719	25.61463	7.611423767	4.734147579	4.734147579	2.925131	0.0364
LOC100739654	19.03246356	171.03962	4.324267947	7.426597064	7.426597064	-3.1678	0.0364
TMTC1	1793.281011	228.87911	10.80919014	7.844731552	7.844731552	2.969944	0.0365
LOC102166396	200.8982264	1835.1643	7.657484427	10.84247941	10.84247941	-3.19137	0.03656
LOC100153723	3062.111915	380.91433	11.58078236	8.57710524	8.57710524	3.006989	0.03657
TOMM20	35464.88112	3859.5463	15.11414417	11.91458929	11.91458929	3.199888	0.03662
SORCS1	146.972913	19.004403	7.209189299	4.322245637	4.322245637	2.951145	0.03664
LOC100520559	111.0227041	983.27126	6.807647348	9.942912163	9.942912163	-3.14673	0.03681
LOC102166971	21.14718173	0.8262784	4.46905122	0.868906686	0.868906686	4.677694	0.037
LOC102167388	21.14718173	0.8262784	4.46905122	0.868906686	0.868906686	4.677694	0.037
LOC100739082	296.0605442	39.661362	8.214613188	5.34558663	5.34558663	2.900086	0.03707
LOC100737457	167.0627357	1505.4792	7.392856063	10.55696503	10.55696503	-3.17176	0.0371
CCNB3	4922.006548	599.05182	12.26532394	9.228943285	9.228943285	3.038494	0.03712
LOC100625751	30.66341351	266.88791	4.98474489	8.065485684	8.065485684	-3.12164	0.03712
EVI5L	195.611431	1777.3248	7.619203392	10.79630311	10.79630311	-3.18364	0.03713
BRINP3	38.06492712	3.3051135	5.287802017	2.106051269	2.106051269	3.525691	0.03724
LOC102160217	38.06492712	3.3051135	5.287802017	2.106051269	2.106051269	3.525691	0.03724
C7H15orf29	3737.764371	461.88961	11.86834584	8.854524369	8.854524369	3.016556	0.03724
LOC100523505	121.596295	14.873011	6.887121389	3.98850389	3.98850389	2.980256	0.03726
TREX1	66.61362245	577.56858	6.079242038	9.176344172	9.176344172	-3.1161	0.03748
LOC100737760	499.0734889	66.928548	8.965996313	6.085946114	6.085946114	2.898559	0.03749
RASA4	10.57359087	101.63224	3.532764645	6.681340184	6.681340184	-3.26482	0.03752
CENPQ	2954.261288	370.99899	11.52906998	8.53915489	8.53915489	2.99331	0.03798
TAS1R3	3.17207726	43.792754	2.060765874	5.485193456	5.485193456	-3.78719	0.03811
LOC100738599	3.17207726	43.792754	2.060765874	5.485193456	5.485193456	-3.78719	0.03811
HMHA1	1402.058149	182.60752	10.45435909	7.520481339	7.520481339	2.940728	0.03818
LOC100620307	82.47400875	9.9153405	6.383255151	3.448285225	3.448285225	3.056205	0.03829
PCDHB4	533.9663387	71.886218	9.063304307	6.187574144	6.187574144	2.892962	0.03838
LOC100623307	4830.016307	592.44159	12.23811101	9.212962234	9.212962234	3.027283	0.03839
LOC102165223	685.1686881	91.716899	9.422419483	6.534760414	6.534760414	2.9012	0.03842
LOC102162469	34.89284986	299.93905	5.165624571	8.233327508	8.233327508	-3.10367	0.03845
CCDC58	5853.539903	711.42568	12.51534008	9.476595706	9.476595706	3.040524	0.03862
LOC100627210	9.516231779	92.543178	3.394545939	6.547560532	6.547560532	-3.28166	0.03865
C5AR2	0	19.004403	0	4.322245637	4.322245637	-20	0.03872
WDR16	0	19.004403	0	4.322245637	4.322245637	-20	0.03872
A3GALT2	0	19.004403	0	4.322245637	4.322245637	-20	0.03872
LOC102159557	0	19.004403	0	4.322245637	4.322245637	-20	0.03872
LOC102163875	0	19.004403	0	4.322245637	4.322245637	-20	0.03872
SLC34A3	0	19.004403	0	4.322245637	4.322245637	-20	0.03872
INHA	0	19.004403	0	4.322245637	4.322245637	-20	0.03872
LOC100626840	0	19.004403	0	4.322245637	4.322245637	-20	0.03872
LOC100621844	0	19.004403	0	4.322245637	4.322245637	-20	0.03872
WNT4	0	19.004403	0	4.322245637	4.322245637	-20	0.03872
LOC100154914	0	19.004403	0	4.322245637	4.322245637	-20	0.03872
LOC102160975	0	19.004403	0	4.322245637	4.322245637	-20	0.03872
LOC102160101	0	19.004403	0	4.322245637	4.322245637	-20	0.03872
LOC102165502	0	19.004403	0	4.322245637	4.322245637	-20	0.03872
HIVEP3	0	19.004403	0	4.322245637	4.322245637	-20	0.03872
LOC102165893	0	19.004403	0	4.322245637	4.322245637	-20	0.03872
KIF19	0	19.004403	0	4.322245637	4.322245637	-20	0.03872
LOC100621187	2.114718173	35.52997	1.639101631	5.191008663	5.191008663	-4.0705	0.03874
LOC102158449	2.114718173	35.52997	1.639101631	5.191008663	5.191008663	-4.0705	0.03874
POLK	80.35929058	694.07383	6.346235194	9.441022422	9.441022422	-3.11055	0.03879
LOC100739565	274.9133625	37.182527	8.108071518	5.254840671	5.254840671	2.88628	0.03886
LOC100524940	390.1655029	52.881816	8.611635334	5.751726565	5.751726565	2.883243	0.03888
TNRC18	431.4025073	4063.637	8.756231078	11.98891081	11.98891081	-3.23567	0.0389
LOC100518088	21104.88707	2394.5547	14.36535787	11.22614405	11.22614405	3.139748	0.03907
EME2	29.60605442	254.49374	4.935745167	7.997144125	7.997144125	-3.10367	0.03911
ZFPL1	242.1352308	2197.9005	7.925615148	11.10256659	11.10256659	-3.18224	0.03911
COCH	48.63851798	4.9576702	5.633388138	2.57474827	2.57474827	3.294365	0.03917
LOC396709	389.1081439	3634.7986	8.607730306	11.82805655	11.82805655	-3.22363	0.03917
LOC100736819	1.057359087	27.267186	1.04079362	4.821056378	4.821056378	-4.68863	0.0394
LOC100737032	1.057359087	27.267186	1.04079362	4.821056378	4.821056378	-4.68863	0.0394
LYPD3	1.057359087	27.267186	1.04079362	4.821056378	4.821056378	-4.68863	0.0394
LOC100737558	1.057359087	27.267186	1.04079362	4.821056378	4.821056378	-4.68863	0.0394
NKRF	3783.230812	472.63123	11.88578437	8.887620397	8.887620397	3.000832	0.03943
SUV39H2	1850.378402	239.62073	10.85438408	7.910617116	7.910617116	2.948996	0.03945
TMSB10	13628.30127	162224.06	13.73442398	17.30763717	17.30763717	-3.57331	0.03955
AAMDC	56.04003159	477.5889	5.833902876	8.902643123	8.902643123	-3.09124	0.0396
SUMO1	26.43397716	1.6525567	4.777891883	1.407383614	1.407383614	3.999622	0.03962
LOC102163428	26.43397716	1.6525567	4.777891883	1.407383614	1.407383614	3.999622	0.03962
CDC42SE2	14903.47633	1729.4006	13.86345807	10.75689038	10.75689038	3.107305	0.03967
MAN2B1	530.7942615	5042.7769	9.054724399	12.30028875	12.30028875	-3.24799	0.03968
LOC100512447	1101.768168	146.25127	10.10691381	7.202136284	7.202136284	2.9133	0.03984
ETV2	11.63094995	108.24247	3.658891241	6.771389985	6.771389985	-3.21823	0.03989
LOC100516109	131.1125267	1146.0481	7.045623457	10.16371018	10.16371018	-3.12779	0.03992
UCMA	4.229436346	51.229259	2.386655454	5.706786332	5.706786332	-3.59843	0.03995
CAPN12	4.229436346	51.229259	2.386655454	5.706786332	5.706786332	-3.59843	0.03995
HIPK4	87.76080419	754.39215	6.471850834	9.561081988	9.561081988	-3.10367	0.03997
LOC102161383	14.80302721	0	3.982129041	0	0	20	0.04009
LOC102164700	14.80302721	0	3.982129041	0	0	20	0.04009
LOC100522152	14.80302721	0	3.982129041	0	0	20	0.04009
LOC100512349	14.80302721	0	3.982129041	0	0	20	0.04009
CC2D2B	14.80302721	0	3.982129041	0	0	20	0.04009

WDR66	164.956836	23.135794	7.374664246	4.593102408	4.593102408	2.83389	0.00372
LOC100623488	93.6689571	627.14528	6.564819524	9.294954469	9.294954469	-2.74316	0.00372
PHF21B	19.8942918	144.59872	4.385036953	7.185853814	7.185853814	-2.86163	0.00373
PERP	232.928999	33.877413	7.869926909	5.124221138	5.124221138	2.781495	0.00379
SORBS3	1714.22481	12447.884	10.74418196	13.60372876	13.60372876	-2.86027	0.00379
LOC102162451	899.387774	134.68337	9.814402656	7.084100147	7.084100147	2.739371	0.00381
LOC102160118	24.0389359	0.8262784	4.646101346	0.868906686	0.868906686	4.862601	0.00381
LOC102165574	24.0389359	0.8262784	4.646101346	0.868906686	0.868906686	4.862601	0.00381
TTC39B	39.7885835	271.01931	5.350093502	8.087565237	8.087565237	-2.76797	0.00382
KCTD17	222.152925	1498.869	7.801888905	10.55062075	10.55062075	-2.75425	0.00383
AIF1L	38.9596547	265.23536	5.320472212	8.05655837	8.05655837	-2.76722	0.00387
CSRNP3	50.5646582	4.9576702	5.688310694	2.574748269	2.574748269	3.350395	0.00387
PIK3CD	483.265504	72.712497	8.919654429	6.20383732	6.20383732	2.732541	0.00393
PLEKHA7	723.653863	109.06875	9.501150225	6.782261052	6.782261052	2.730064	0.00394
LOC102166423	34.8150106	2.4788351	5.162492464	1.798604302	1.798604302	3.811975	0.00394
LOC102167189	34.8150106	2.4788351	5.162492464	1.798604302	1.798604302	3.811975	0.00394
PROX1	158.325405	22.309516	7.315832521	4.542847146	4.542847146	2.827162	0.00396
LOC102164153	86.2085977	10.741619	6.446398468	3.553559423	3.553559423	3.00462	0.00396
LOC100155322	0.82892882	23.962073	0.870998931	4.641665831	4.641665831	-4.85336	0.00396
RTN4R	493.21265	74.365053	8.948988129	6.2358238	6.2358238	2.729513	0.00398
IKZF2	231.271142	33.877413	7.859666109	5.124221138	5.124221138	2.77119	0.00398
PTPLAD2	387.938689	2645.7433	8.603398943	11.37000259	11.37000259	-2.76977	0.00399
ACPT	91.1821706	604.00949	6.526415834	9.240813961	9.240813961	-2.72775	0.00403
LOC100517200	4.97357294	50.402981	2.578594101	5.683780114	5.683780114	-3.34115	0.00405
LOC102165199	9.11821706	76.01761	3.338883189	6.267116453	6.267116453	-3.05951	0.00406
LOC100526125	2291.15927	338.77413	11.16249158	8.408432212	8.408432212	2.757682	0.00406
SEMA4D	3488.13249	508.98748	11.76865267	8.994318011	8.994318011	2.776753	0.00407
E2F5	2561.39007	377.60922	11.32327439	8.564565719	8.564565719	2.761961	0.00407
GEMIN5	11113.4487	1554.2296	13.44014877	10.60291188	10.60291188	2.838035	0.00407
LOC102165146	319.966526	47.924146	8.326279034	5.61247475	5.61247475	2.739096	0.0041
MTTP	70.45895	8.2627837	6.159042811	3.211445828	3.211445828	3.092083	0.00412
LOC102164274	933.373855	141.2936	9.867856097	7.152726981	7.152726981	2.723759	0.00414
ZNF366	5.80250177	55.360651	2.766065426	5.816616368	5.816616368	-3.25411	0.00414
C18H7orf41	232.100071	1550.0982	7.864805631	10.59907433	10.59907433	-2.73954	0.00417
HELLS	3697.02255	540.38606	11.85253831	9.080513917	9.080513917	2.774301	0.00418
DMXL2	3397.77925	498.24586	11.73080095	8.963606649	8.963606649	2.769662	0.00419
REG4	250.336505	37.182527	7.973476417	5.254840671	5.254840671	2.751172	0.00422
LOC102160087	41.4464412	277.62953	5.407571694	8.122204372	8.122204372	-2.74384	0.00424
LOC100625662	125.997181	17.351846	6.988652665	4.19785327	4.19785327	2.86023	0.00425
SMYD2	104.445032	686.63733	6.720347312	9.42550405	9.42550405	-2.7168	0.00426
NEIL2	552.895525	84.280394	9.113470073	6.414142197	6.414142197	2.713738	0.00428
MAT1A	13.2628612	100.80596	3.834191515	6.669678233	6.669678233	-2.92612	0.00429
DCX	3.31571529	39.661362	2.109599694	5.34558663	5.34558663	-3.58034	0.00431
LOC102165012	3.31571529	39.661362	2.109599694	5.34558663	5.34558663	-3.58034	0.00431
C9H11orf52	3.31571529	39.661362	2.109599694	5.34558663	5.34558663	-3.58034	0.00431
FGFBP3	29.0125088	1.6525567	4.907492018	1.407383613	1.407383613	4.133903	0.00434
SPAG6	29.0125088	1.6525567	4.907492018	1.407383613	1.407383613	4.133903	0.00434
LOC100739429	105.273961	14.046732	6.731644339	3.911378308	3.911378308	2.905842	0.00434
ANGPT1	62.9985906	413.96546	5.999968293	8.696847462	8.696847462	-2.71612	0.00439
LOC100521443	411.148697	2772.1639	8.687021122	11.43731719	11.43731719	-2.75328	0.0044
LOC102164478	1101.64641	167.73451	10.10675451	7.398611253	7.398611253	2.71541	0.00441
ISOC1	3403.58175	502.37725	11.73326186	8.975496206	8.975496206	2.760211	0.00441
MOB3C	95.3268147	623.01389	6.589865555	9.285434338	9.285434338	-2.70831	0.00444
LOC102158225	4.14464412	44.619032	2.363071282	5.511563931	5.511563931	-3.42834	0.00444
BEST4	69.6300212	8.2627837	6.142209624	3.211445828	3.211445828	3.07501	0.00446
LOC100738208	69.6300212	8.2627837	6.142209624	3.211445828	3.211445828	3.07501	0.00446
LOC102164565	936.689571	6495.3743	9.872966576	12.66541904	12.66541904	-2.79377	0.00447
KIAA1644	25.6967935	176.82357	4.73859457	7.474302765	7.474302765	-2.78265	0.00447
FAM117B	814.008105	124.76803	9.670670596	6.974621475	6.974621475	2.705795	0.00449
USP44	1398.40293	212.35354	10.4505957	7.737102248	7.737102248	2.71924	0.00449
LOC102160100	79.5771671	9.9153405	6.332299179	3.448285225	3.448285225	3.00462	0.0045
PLAT	618.380902	4215.6723	9.27468309	12.04188918	12.04188918	-2.7692	0.00451
TRNP1	629.156977	96.674569	9.29956745	6.609911086	6.609911086	2.702212	0.00452
MYCL	388.767618	59.492043	8.606470427	5.918673475	5.918673475	2.708139	0.00455
LOC100516938	64.6564482	422.22825	6.036864803	8.725292112	8.725292112	-2.70716	0.00457
LOC100622731	2169.30673	326.37996	11.08368324	8.354822188	8.354822188	2.73261	0.00457
NPFF	34.8150106	233.0105	5.162492464	7.870429459	7.870429459	-2.74261	0.00458
LOC102165992	676.40592	104.11107	9.403876785	6.715770874	6.715770874	2.699766	0.00458
LOC100157370	122.681466	799.01119	6.950485513	9.643876361	9.643876361	-2.7033	0.00459
PON2	119.365751	776.70167	6.911281129	9.603073025	9.603073025	-2.70197	0.00462
LOC102159132	17.4075053	0	4.202222212	0	0	20	0.00462
LOC102157412	17.4075053	0	4.202222212	0	0	20	0.00462
LOC102157590	17.4075053	0	4.202222212	0	0	20	0.00462
LOC100738052	17.4075053	0	4.202222212	0	0	20	0.00462
LOC100524824	17.4075053	0	4.202222212	0	0	20	0.00462
MYCT1	17.4075053	0	4.202222212	0	0	20	0.00462
OLIG3	17.4075053	0	4.202222212	0	0	20	0.00462
IGSF9B	17.4075053	0	4.202222212	0	0	20	0.00462
LOC102167179	17.4075053	0	4.202222212	0	0	20	0.00462
LOC102165997	17.4075053	0	4.202222212	0	0	20	0.00462
LOC733664	17.4075053	0	4.202222212	0	0	20	0.00462
IFITM3	1319.65469	9234.4871	10.36703758	13.17297234	13.17297234	-2.80687	0.00462
LOC100739187	122.681466	798.18491	6.950485513	9.642385527	9.642385527	-2.70181	0.00463
LOC100737646	621.696618	95.848291	9.282385633	6.597654688	6.597654688	2.697386	0.00463
LOC100739295	702.102714	108.24247	9.457591652	6.771389985	6.771389985	2.697415	0.00464
LOC102162418	76.2614518	495.76702	6.271676881	8.956425596	8.956425596	-2.70064	0.00464
ACN9	361.412967	55.360651	8.501490766	5.816616368	5.816616368	2.706715	0.00465
LOC100737806	3861.97359	571.78463	11.91549826	9.161848978	9.161848978	2.755797	0.00467
LOC102161831	23.2100071	0.8262784	4.597531595	0.868906686	0.868906686	4.811975	0.00472
LOC102161463	23.2100071	0.8262784	4.597531595	0.868906686	0.868906686	4.811975	0.00472
LOC102164756	23.2100071	0.8262784	4.597531595	0.868906686	0.868906686	4.811975	0.00472
C12H17orf100	23.2100071	0.8262784	4.597531595	0.868906686	0.868906686	4.811975	0.00472
BARX2	23.2100071	0.8262784	4.597531595	0.868906686	0.868906686	4.811975	0.00472
LOC396634	23.2100071	0.8262784	4.597531595	0.868906686	0.868906686	4.811975	0.00472
LOC100622302	534.659091	82.627837	9.06517131	6.385911347	6.385911347	2.693919	0.00472
LRP10	1136.46142	7880.2168	10.15160189	12.94420268	12.94420268	-2.79369	0.00473
ITPR1	2989.94627	447.84288	11.54638628	8.810066691	8.810066691	2.739055	0.00478
EPHB6	0	17.351846	0	4.19785327	4.19785327	-20	0.00478
CLEC1A	0	17.351846	0	4.19785327	4.19785327	-20	0.00478
LOC102159826	0	17.351846	0	4.19785327	4.19785327	-20	0.00478
LOC100626866	0	17.351846	0	4.19785327	4.19785327	-20	0.00478
POU3F4	0	17.351846	0	4.19785327	4.19785327	-20	0.00478
LOC102164918	0	17.351846	0	4.19785327	4.19785327	-20	0.00478
LAMB4	0	17.351846	0	4.19785327	4.19785327	-20	0.00478
KRT83	0	17.351846	0	4.19785327	4.19785327	-20	0.00478
LOC100515100	0	17.351846	0	4.19785327	4.19785327	-20	0.00478
FABP3	549.57981	85.106672	9.104807896	6.428053129	6.428053129	2.690985	0.00479
LOC100515533	43.9332277	4.1313919	5.489710792	2.359350201	2.359350201	3.410613	0.00479
LOC102159614	2.48678647	33.877413	1.801898017	5.124221138	5.124221138	-3.76797	0.0048
LOC102163714	13.2628612	99.153405	3.834191515	6.646067655	6.646067655	-2.90227	0.00481
LOC100620580	73.7746653	9.0890621	6.224477643	3.334720158	3.334720158	3.020922	0.00483
LOC102157596	53.8803735	5.7839486	5.778218396	2.762125239	2.762125239	3.219633	0.00483
TRNAM-CAU	53.8803735	5.7839486	5.778218396	2.762125239	2.762125239	3.219633	0.00483
LOC102157941	63.8275194	7.4365053	6.018534465	3.076645515	3.076645515	3.101482	0.00484
SFTPC	58.8539465	6.610227	5.90337447	2.927939483	2.927939483	3.154367	0.00484
LOC574051	98.64253	13.220454	6.638689748	3.829895615	3.829895615	2.899438	0.00484
DARS2	1999.37632	304.07044	10.96605572	8.252998589	8.252998589	2.717073	0.00487
LOC102157822	24.0389359	164.4294	4.646101346	7.370071807	7.370071807	-2.77402	0.00489
ZC3H12D	43.9332277	287.54487	5.489710792	8.172651889	8.172651889	-2.7104	0.0049
LOC102161895	17.4075053	123.94176	4.202222212	6.965111899	6.965111899	-2.83188	0.0049
PRDM8	0.82892882	23.135794	0.870998931	4.593102408	4.593102408	-4.80273	0.0049
SPTLC3	0.82892882	23.135794	0.870998931	4.593102408	4.593102408	-4.80273	0.0049
MUSK	0.82892882	23.135794	0.870998931	4.593102408	4.593102408	-4.80273	0.0049
LOC100737823	0.82892882	23.135794	0.870998931	4.593102408	4.593102408	-4.80273	0.0049
MBTD1	6526.98556	955.1778	12.67242215	9.901135097	9.901135097	2.772576	0.00495
CAMK2N2	337.374031	52.055537	8.402475042	5.729431428	5.729431428	2.696225	0.00496
LOC100520746	4074.18517	607.3146	11.9926499	9.248673828	9.248673828	2.745996	0.00499
ENOPH1	11354.667	1630.2472	13.47112483	10.67175973	10.67175973	2.800123	0.00504
ABLIM1	854.625617	133.03082	9.740835867	7.066420948	7.066420948	2.683532	0.00505
LOC102160740	4.97357294	48.750424	2.578594101	5.636636914	5.636636914	-3.29306	0.00505
PLEKHG2	197.28506	1278.2526	7.63143217	10.3210855	10.3210855	-2.69582	0.00505
LOC100739199	197.28506	1277.4264	7.63143217	10.32015335	10.32015335	-2.69489	0.00508
LOC102161478	12.4339324	93.369456	3.747809764	6.560248082	6.560248082	-2.90867	0.00508
PPA2	1971.19274	301.59161	10.94558484	8.241228156	8.241228156	2.708401	0.00509
ADCK1	639.104123	99.979683	9.322162791	6.657921243	6.657921243	2.676344	0.00515
LOC100038019	260.283651	1693.8707	8.029473043	10.72695947	10.72695947	-2.70217	0.00515
LOC102167980	167.443622	24.788351	7.396121996	4.688647628	4.688647628	2.755941	0.00516
AKR1C4	1007.14852	6869.6784	9.977492478	12.74623684	12.74623684	-2.76997	0.00516
CFI	19.8942918	137.98849	4.385036953	7.118821585	7.118821585	-2.79412	0.00517
RAB6B	4249.08915	635.40807	12.05327739	9.313808315	9.313808315	2.741398	0.00518
LOC606745	72.1168077	461.88961	6.192131177	8.854524369	8.854524369	-2.67914	0.00519
LOC102163537	28.18358	1.6525567	4.867084967	1.407383613	1.407383613	4.092083	0.0052
LOC102166020	28.18358	1.6525567	4.867084967	1.407383613	1.407383613	4.092083	0.0052
GPR37L1	28.18358	1.6525567	4.867084967	1.407383613	1.407383613	4.092083	0.0052
LOC102162606	28.18358	1.6525567	4.867084967	1.407383613	1.407383613	4.092083	0.0052
LOC102164986	278.520085	42.966475	8.12680814	5.458331975	5.458331975	2.696498	0.00522
MYO1G	558.698027	87.585507	9.128504852	6.468998788	6.468998788	2.673305	0.00523
LOC100738326	556.211241	3700.9008	9.122080551	11.85405053	11.85405053	-2.73417	0.00525
LOC102162530	37.3017971	243.75212	5.259340178	7.935177544	7.935177544	-2.7081	0.00526
LOC100739541	46.4200141	299.93905	5.567424187	8.233327508	8.233327508	-2.69185	0.00528
B3GALT4	77.9193094	497.41958	6.302306427	8.961216934	8.961216934	-2.67441	0.00528
TBC1D20	1142.26392	7801.7204	10.15894277	12.92976149	12.92976149	-2.7719	0.00531
CCDC17	121.852537	17.351846	6.940783842	4.19785327	4.19785327	2.811975	0.00535
LOC100737091	1191.17072	185.91263	10.21937513	7.546220275	7.546220275	2.679683	0.00536
LOC100524949	17.4075053	122.2892	4.202222212	6.945902605	6.945902605	-2.81252	0.00538
GPR15	106.931818	14.873011	6.753976422	3.98850389	3.98850389	2.845923	0.00539
LOC100738281	48.0778718	4.9576702	5.617000784	2.574748269	2.574748269	3.277639	0.00539
LOC102165796	20.7232206	142.11988	4.441166102	7.161080272	7.161080272	-2.77779	0.0054
LOC102162958	43.1042988	4.1313919	5.462847376	2.359350201	2.359350201	3.383132	0.00542
CAPN2	2569.67935	18084.755	11.32793396	14.14256618	14.14256618	-2.81511	0.00544
MAP6	404.517266	63.623435	8.663619532	6.013985524	6.013985524	2.668571	0.00548
CCSAP	818.152749	128.89943	9.677988689	7.021251246	7.021251246	2.666124	0.00549
SNCG	87.0375265	11.567897	6.460046706	3.65167138	3.65167138	2.911511	0.00549
LOC100623642	155.838619	23.135794	7.293137032	4.593102408	4.593102408	2.751854	0.00553
LOC100623296	1554.24154	242.09956	10.60292295	7.925403489	7.925403489	2.682538	0.00554
LOC100738678	1793.80197	278.45581	10.80960896	8.126476366	8.126476366	2.6875	0.00556
ANKH	668.116632	4438.7674	9.386113895	12.11626838	12.11626838	-2.73199	0.00558
DYDC2	2.48678647	33.051135	1.801898017	5.089630977	5.089630977	-3.73235	0.00561
LOC102166930	2.48678647	33.051135	1.801898017	5.089630977	5.089630977	-3.73235	0.00561
SMAD7	365.557611	2373.8978	8.517896152	11.21364969	11.21364969	-2.69909	0.00564
LOC100624376	28.18358	185.91263	4.867084967	7.546220275	7.546220275	-2.7217	0.00566
SERPING1	881.15134	5902.1064	9.784882372	12.52725863	12.52725863	-2.74377	0.00568
SHC3	14.09179	101.63224	3.915692025	6.681340183	6.681340183	-2.85043	0.00569
LOC100738987	67.1432347	8.2627837	6.090498528	3.211445828	3.211445828	3.022542	0.00569
UPF0639	179.877555	27.267186	7.498869583	4.821056379	4.821056379	2.721777	0.00571
LGR5	1325.45719	208.22215	10.3733624	7.708891783	7.708891783	2.670295	0.00573
LOC100624395	1316.33897	8948.5948	10.36341091	13.12760664	13.12760664	-2.76513	0.00574
WNK3	665.629845	105.76363	9.380742099	6.738276475	6.738276475	2.653877	0.00576
PAICS	26015.1022	3678.5913	14.66711721	11.84532982	11.84532982	2.822124	0.0058
GRN	2742.92548	19193.62	11.42202558	14.2284144	14.2284144	-2.80684	0.0058
42616	26.5257224	175.17101	4.78270852	7.460832769	7.460832769	-2.7233	0.00583
SLC12A5	19.0653629	130.55198	4.326633547	7.039489182	7.039489182	-2.7756	0.00587
LOC100519237	984.767442	156.16661	9.945103523	7.296150961	7.296150961	2.656697	0.00587
PDE4A	215.521494	1369.9695	7.758366438	10.4209808	10.4209808	-2.66824	0.00589
LOC733637	3325.66244	23411.771	11.69985977	14.5150081	14.5150081	-2.81552	0.00589
GABARAP	1307.22076	8849.4414	10.35339029	13.11153369	13.11153369	-2.75908	0.0059
HSPB8	52.2225159	330.51135	5.733964805	8.372914449	8.372914449	-2.66196	0.00593
CADPS2	2510.82541	389.17711	11.29452047	8.607985345	8.607985345	2.689663	0.00593
LOC102158625	159.154334	23.962073	7.323319031	4.641665831	4.641665831	2.731602	0.00594
C18H7orf58	123.510395	775.87539	6.96012238	9.601539402	9.601539402	-2.65119	0.00596
GRASP	268.572939	1714.5276	8.074531868	10.74443664	10.74443664	-2.67443	0.00596
LOC100626459	25.6967935	169.38707	4.73859457	7.412672017	7.412672017	-2.72066	0.00603
PLXNC1	1449.79651	228.87911	10.50262947	7.844731552	7.844731552	2.663193	0.00604
LOC102157538	0.82892882	22.309516	0.870998931	4.542847146	4.542847146	-4.75027	0.00609
LOC100516820	0.82892882	22.309516	0.870998931	4.542847146	4.542847146	-4.75027	0.00609
LOC100739181	0.82892882	22.309516	0.870998931	4.542847146	4.542847146	-4.75027	0.00609
LOC102161519	16.5785765	0	4.135746339	0	0	20	0.00609
LOC102159917	16.5785765	0	4.135746339	0	0	20	0.00609
LOC100514940	16.5785765	0	4.135746339	0	0	20	0.00609
LOC102166773	16.5785765	0	4.135746339	0	0	20	0.00609
LOC100520863	16.5785765	0	4.135746339	0	0	20	0.00609
LOC102166149	16.5785765	0	4.135746339	0	0	20	0.00609
NPYR5	16.5785765	0	4.135746339	0	0	20	0.00609
MORN5	16.5785765	0	4.135746339	0	0	20	0.00609
LOC102158871	16.5785765	0	4.135746339	0	0	20	0.00609
LOC102160350	16.5785765	0	4.135746339	0	0	20	0.00609
CLDN18	16.5785765	0	4.135746339	0	0	20	0.00609
LOC102158428	16.5785765	0	4.135746339	0	0	20	0.00609
LOC102165972	16.5785765	0	4.135746339	0	0	20	0.00609
SPTSSB	16.5785765	0	4.135746339	0	0	20	0.00609
LOC102157933	95.3268147	13.220454	6.589865555	3.829895615	3.829895615	2.85011	0.0061
RNF43	19.8942918	134.68337	4.385036953	7.084100147	7.084100147	-2.75915	0.00611
TPK1	233.757928	1481.5171	7.875030072	10.53383305	10.53383305	-2.66399	0.00611
SHOX2	79.5771671	497.41958	6.332299179	8.961216934	8.961216934	-2.64404	0.00612
LOC102168083	42.27537	4.1313919	5.435474249	2.359350201	2.359350201	3.355118	0.00614
LRCH1	114.392178	714.73079	6.850401618	9.483273237	9.483273237	-2.64341	0.00616
CCBL1	1354.4697	214.83238	10.40457715	7.753767487	7.753767487	2.656445	0.00617
LOC100516420	5.80250177	52.055537	2.766065426	5.729431428	5.729431428	-3.1653	0.00621
BEST2	37.3017971	3.3051135	5.259340178	2.106051269	2.106051269	3.496473	0.00624
BEX5	37.3017971	3.3051135	5.259340178	2.106051269	2.106051269	3.496473	0.00624
LOC102161009	586.052678	94.195734	9.197346157	6.572825024	6.572825024	2.637297	0.00624
EPCAM	27.3546512	1.6525567	4.825513503	1.407383613	1.407383613	4.049014	0.00624
CCDC146	27.3546512	1.6525567	4.825513503	1.407383613	1.407383613	4.049014	0.00624
LOC100628113	187.337914	28.919743	7.557179646	4.903025878	4.903025878	2.695516	0.00625
LOC100622904	109.418605	15.699289	6.786839466	4.061714779	4.061714779	2.801087	0.00626
F11R	87.0375265	542.03861	6.460046706	9.084910972	9.084910972	-2.63869	0.00627
GFPT2	4273.12809	655.23875	12.06141443	9.358076973	9.358076973	2.7052	0.00628
LOC102163998	0	16.525567	0	4.13138925	4.13138925	-20	0.0063
IL15	0	16.525567	0	4.13138925	4.13138925	-20	0.0063
LOC100738049	0	16.525567	0	4.13138925	4.13138925	-20	0.0063
KCNA3	0	16.525567	0	4.13138925	4.13138925	-20	0.0063
LOC100520680	0	16.525567	0	4.13138925	4.13138925	-20	0.0063
HPGD	0	16.525567	0	4.13138925	4.13138925	-20	0.0063
FAM211B	0	16.525567	0	4.13138925	4.13138925	-20	0.0063
LOC102165745	0	16.525567	0	4.13138925	4.13138925	-20	0.0063
SELE	0	16.525567	0	4.13138925	4.13138925	-20	0.0063
LOC102164099	133.457541	19.830681	7.071006856	4.380638094	4.380638094	2.750575	0.00631
EFR3B	3241.94063	502.37725	11.6630869	8.975496206	8.975496206	2.690015	0.00632
SLC6A15	630.814835	101.63224	9.303358001	6.681340183	6.681340183	2.633858	0.00635
RTKN2	355.610465	57.013208	8.478205232	5.858309486	5.858309486	2.64093	0.00638
LOC100158075	1342.86469	214.0061	10.39217217	7.74823377	7.74823377	2.64959	0.00639
LOC100626354	4.14464412	42.140197	2.363071282	5.430960858	5.430960858	-3.34588	0.00641
CELSR2	380.47833	61.1446	8.575457296	5.957557119	5.957557119	2.637517	0.00641
LOC102162199	56.36716	6.610227	5.842153193	2.927939483	2.927939483	3.092083	0.00642
SLCO4C1	56.36716	6.610227	5.842153193	2.927939483	2.927939483	3.092083	0.00642
LOC100518740	334.887245	53.708094	8.3918332	5.773682393	5.773682393	2.640464	0.00647
RAPSN	12.4339324	90.064343	3.747809764	6.508814353	6.508814353	-2.85667	0.00648
LOC100522814	1.65785765	27.267186	1.410263837	4.821056379	4.821056379	-4.03977	0.00649
MYH1	3.31571529	37.182527	2.109599694	5.254840671	5.254840671	-3.48723	0.00651
EYA2	3.31571529	37.182527	2.109599694	5.254840671	5.254840671	-3.48723	0.00651
LOC102158961	2.48678647	32.224857	1.801898017	5.054191063	5.054191063	-3.69582	0.00656
DEPTOR	581.908034	94.195734	9.187124476	6.572825024	6.572825024	2.627058	0.00656
LOC102164786	51.3935871	5.7839486	5.711318333	2.762125239	2.762125239	3.151462	0.00658
MYH6	51.3935871	5.7839486	5.711318333	2.762125239	2.762125239	3.151462	0.00658
LOC102164531	132.628612	19.830681	7.062085132	4.380638094	4.380638094	2.741586	0.00658
TMED3	193.140416	1205.5401	7.600956677	10.2366602	10.2366602	-2.64196	0.00659
ZDHHC23	418.609056	67.754826	8.712902004	6.103389087	6.103389087	2.627208	0.00666
TLN1	7592.98802	54472.402	12.89064201	15.73326434	15.73326434	-2.84279	0.00666
MEGF10	1345.35148	215.65866	10.39483938	7.75928006	7.75928006	2.641162	0.00667
LOC102161970	1909.85201	303.24416	10.90000033	8.249085772	8.249085772	2.654909	0.00667
ZNFX1	1158.01357	7672.821	10.17868174	12.90572939	12.90572939	-2.7281	0.00667
ZNF215	180.706484	28.093465	7.505466087	4.862623207	4.862623207	2.685342	0.00668
ANKRD63	14.09179	99.153405	3.915692025	6.646067655	6.646067655	-2.81481	0.00671
LOC102164779	46.4200141	4.9576702	5.567424187	2.574748269	2.574748269	3.227013	0.00676
CTSB	7797.73344	55891.948	12.92902413	15.77037865	15.77037865	-2.84151	0.00676
SGCB	130.141825	803.14258	7.03498407	9.651307509	9.651307509	-2.62557	0.00679
LOC102164151	1042.79246	168.56079	10.02761917	7.405658764	7.405658764	2.629111	0.0068
KCND1	82.0639535	504.85609	6.376150635	8.98258319	8.98258319	-2.62105	0.00684
LOC102161185	5.80250177	51.229259	2.766065426	5.706786332	5.706786332	-3.14222	0.00688
FOXL2	41.4464412	4.1313919	5.407571694	2.359350201	2.359350201	3.326548	0.00696
ECE2	1828.61699	292.50254	10.83732595	8.197229196	8.197229196	2.644232	0.00698
LOC102159472	116.050035	712.25196	6.870981559	9.478267989	9.478267989	-2.61764	0.007
LOC100621853	227.955427	36.356248	7.838922949	5.223277669	5.223277669	2.648477	0.00701
LOC100627990	996.372446	161.95056	9.961988537	7.348290508	7.348290508	2.621132	0.00704
LOC100622769	27.3546512	175.17101	4.825513503	7.460832769	7.460832769	-2.67891	0.00707
EFCAB4A	33.9860818	214.0061	5.128709195	7.74823377	7.74823377	-2.65464	0.00708
LOC100623841	2054.08563	328.03251	11.00498279	8.362086342	8.362086342	2.646586	0.00708

DEXI	138.080984	870.07113	7.119781365	9.766646714		-2.65562	0.07834		LOC102158037	14.80302721	0	3.982129041	0	0	20	0.04009		MND1	1187.855	192.52286	10.21535706	7.59636019	7.59636019	2.625257	0.00708
LOC100624791	1738.43958	12617.271	10.76440685	13.62322659		-2.85954	0.07851		LOC102158071	14.80302721	0	3.982129041	0	0	20	0.04009		EGR3	36.4728682	3.3051135	5.227774503	2.106051269	2.106051269	3.464052	0.00718
FMO5	12.4272885	88.411786	3.747096094	6.482393107		-2.83073	0.07852		ABCA6	14.80302721	0	3.982129041	0	0	20	0.04009		BPIFB2	36.4728682	3.3051135	5.227774503	2.106051269	2.106051269	3.464052	0.00718
LOC102158159	2.76161967	33.877413	1.911353989	5.124221138		-3.61674	0.07927		LOC100623122	14.80302721	0	3.982129041	0	0	20	0.04009		LOC100517234	48.9068006	301.59161	5.641164514	8.241228156	8.241228156	-2.62449	0.00721
LOC100737472	2.76161967	33.877413	1.911353989	5.124221138		-3.61674	0.07927		WNT8A	14.80302721	0	3.982129041	0	0	20	0.04009		ARHGEF26	639.104123	104.93735	9.322162791	6.727067559	6.727067559	2.606523	0.00728
LOC100738725	157.412321	30.5723	7.307540742	4.980587446		2.364251	0.07935		LOC102163470	14.80302721	0	3.982129041	0	0	20	0.04009		LOC100622851	92.8400282	13.220454	6.552131542	3.829895615	3.829895615	2.811975	0.00728
LOC100739541	48.3283443	299.93905	5.624344958	8.233327508		-2.63373	0.07936		LOC100513690	14.80302721	0	3.982129041	0	0	20	0.04009		LOC100522888	21.5521494	0.8262784	4.495193036	0.868906686	0.868906686	4.70506	0.00733
LOC100736802	1737.05877	332.99018	10.76326115	8.383661891		2.383095	0.07937		LOC100513764	14.80302721	0	3.982129041	0	0	20	0.04009		DDX31	6628.9438	1020.4538	12.69478093	9.996408222	9.996408222	2.699568	0.00734
LOC100519601	78.7061606	14.046732	6.316619332	3.911378308		2.486242	0.07951		LOC100517804	14.80302721	0	3.982129041	0	0	20	0.04009		LGALS1	11382.8506	82013.912	13.47470101	16.32359863	16.32359863	-2.84901	0.00735
LOC100739369	6.90404918	57.013208	2.982591923	5.858309486		-3.04578	0.07971		SLA-DOA	14.80302721	0	3.982129041	0	0	20	0.04009		LOC100738337	31.4992953	2.4788351	5.022336531	1.798604302	1.798604302	3.667585	0.00739
LOC100520655	20.7121475	135.50965	4.440430524	7.092859162		-2.70985	0.07974		NUMBL	433.5172255	4052.0691	8.76326956	11.98479907	11.98479907	-3.2245	0.04018		GRAP2	31.4992953	2.4788351	5.022336531	1.798604302	1.798604302	3.667585	0.00739
LOC100627044	260.973059	1683.9553	8.033274644	10.71849462		-2.68988	0.07976		NUP160	8959.003541	1072.5093	13.12928359	10.06811901	10.06811901	3.062348	0.04024		LRP5	421.095842	2646.5696	8.721426809	11.37045291	11.37045291	-2.6519	0.0074
AHCY	28447.4442	4814.7241	14.79606214	12.23353702		2.562774	0.0799		CD109	283.3722352	2577.1622	8.151636803	11.33212734	11.33212734	-3.18501	0.04029		LOC100156943	73.7746653	9.9153405	6.224477643	3.448285225	3.448285225	2.895391	0.00746
PKD1L3	13.8080984	95.848291	3.888314478	6.597654688		-2.79524	0.07992		C4	7.401513606	75.191332	3.070649265	6.251554969	6.251554969	-3.34467	0.04038		NT5C2	1201.11787	7847.1657	10.23136264	12.93813979	12.93813979	-2.70779	0.00748
LOC102159655	121.511266	23.135794	6.936770608	4.593102408		2.392892	0.08014		FAM54A	1814.428193	236.31561	10.82609415	7.890663216	7.890663216	2.940728	0.04038		LOC102167053	24.0389359	154.51406	4.646101346	7.280901168	7.280901168	-2.68429	0.00749
CCDC64	121.511266	23.135794	6.936770608	4.593102408		2.392892	0.08014		JMJD8	180.8084038	1601.3275	7.506275077	10.64595332	10.64595332	-3.14673	0.04041		LOC100738148	26.5257224	1.6525567	4.78270852	1.407383613	1.407383613	4.00462	0.00752
LOC102167158	422.52781	84.280394	8.726312893	6.414142197		2.325777	0.08048		C10H9orf72	3092.775328	392.48223	11.59515272	8.620154661	8.620154661	2.978203	0.04046		LOC102162724	26.5257224	1.6525567	4.78270852	1.407383613	1.407383613	4.00462	0.00752
TMC4	318.967072	63.623435	8.321779634	6.013985524		2.325777	0.08049		CTSH	739.0940015	7132.4349	9.531564713	12.80038122	12.80038122	-3.27056	0.04073		CXHXorf57	26.5257224	1.6525567	4.78270852	1.407383613	1.407383613	4.00462	0.00752
TXNIP	401.815662	2644.9171	8.653975969	11.36955213		-2.71862	0.08095		LOC102159784	3.17207726	42.966475	2.060765874	5.458331975	5.458331975	-3.75971	0.04083		CACNA1F	26.5257224	1.6525567	4.78270852	1.407383613	1.407383613	4.00462	0.00752
LOC100738689	8.28485901	64.449713	3.214880004	6.032314961		-2.95962	0.08111		NCCRP1	3.17207726	42.966475	2.060765874	5.458331975	5.458331975	-3.75971	0.04083		CDC42EP4	257.796864	1591.4121	8.015676326	10.63699806	10.63699806	-2.626	0.00753
ABLIM2	55.2323934	9.0890621	5.813329549	3.334720158		2.603311	0.0817		SUSD2	20.08982264	174.34474	4.398475058	7.454050314	7.454050314	-3.11741	0.04089		LOC100737964	125.168252	19.004403	6.979205122	4.322245637	4.322245637	2.719463	0.00758
LOC102164934	30.3778164	190.8703	4.971673052	7.58398763		-2.6515	0.08173		CLIP2	268.569208	2425.127	8.074511901	11.24443937	11.24443937	-3.17469	0.04092		LOC100526222	45.5910853	4.9576702	5.541982032	2.574748269	2.574748269	3.201018	0.00758
PLAT	625.506856	4215.6723	9.291186486	12.04188918		-2.75267	0.08185		LOC102164112	31.7207726	2.4788351	5.032134908	1.798604302	1.798604302	3.677694	0.04097		ATP2B4	2688.21618	18131.853	11.39297002	14.14631828	14.14631828	-2.75381	0.00758
LOC102162904	48.3283443	297.46021	5.624344958	8.221394816		-2.62176	0.08212		LOC102165555	64.49890428	7.4365053	6.033398867	3.076645515	3.076645515	3.116578	0.04118		LOC102161784	0.82892882	21.483238	0.870998931	4.490777898	4.490777898	-4.69582	0.0076
LOC100622731	1690.11124	326.37996	10.72375585	8.354822188		2.372494	0.08234		NARS2	3388.835872	429.66475	11.72699971	8.750421444	8.750421444	2.979506	0.04123		LOC100626194	0.82892882	21.483238	0.870998931	4.490777898	4.490777898	-4.69582	0.0076
LOC102157527	9.66566885	71.886218	3.414902536	6.187574144		-2.89477	0.08235		LOC102160340	35.95020894	303.24416	5.207510617	8.249085772	8.249085772	-3.07641	0.04126		LOC102160773	0.82892882	21.483238	0.870998931	4.490777898	4.490777898	-4.69582	0.0076
CLIP4	102.179928	630.4504	6.689018532	9.3025256		-2.62527	0.08238		LOC102167200	85.64608601	728.77752	6.437062676	9.511312908	9.511312908	-3.08902	0.04145		LOC100737685	0.82892882	21.483238	0.870998931	4.490777898	4.490777898	-4.69582	0.0076
CCNYL1	401.815662	2634.1754	8.653975969	11.3636833		-2.71275	0.08238		SVCT1	142.7434767	19.004403	7.167352675	4.322245637	4.322245637	2.909019	0.04162		GFRA1	19.0653629	125.59431	4.326635347	6.98406878	6.98406878	-2.71975	0.00763
LOC100622810	70.4213016	12.394176	6.158282521	3.74353388		2.50635	0.0826		LEPR	142.7434767	19.004403	7.167352675	4.322245637	4.322245637	2.909019	0.04162		SMPDL3B	18.2364341	120.63664	4.265769485	6.926434087	6.926434087	-2.72577	0.00772
LOC100512726	13.8080984	95.022013	3.888314478	6.585293272		-2.78275	0.08269		C2H2orf81	8.458872693	82.627837	3.241668254	6.385911347	6.385911347	-3.28809	0.04163		C1H9orf9	67.1432347	406.52896	6.090498528	8.67075877	8.67075877	-2.59804	0.00776
USP53	330.013551	2137.5821	8.370746468	11.06243891		-2.69538	0.08275		LOC102165339	69.78569971	8.2627837	6.145386028	3.211445828	3.211445828	3.078232	0.04174		C2H11orf24	565.329458	3566.2175	9.145497764	11.80058345	11.80058345	-2.65723	0.00778

LOC102166581	15.1889082	0.8262784	4.016933786	0.868906686		4.200246	0.1079
IFITM5	15.1889082	0.8262784	4.016933786	0.868906686		4.200246	0.1079
GAB2	27.6161967	162.77684	4.838760036	7.35558754		-2.55931	0.1079
LOC100515210	2260.3857	452.80055	11.14299136	8.82591454		2.319621	0.10861
STOX2	1089.45896	224.74772	10.09071976	7.818567588		2.277233	0.1089
LOC100517135	1792.29117	362.73621	10.80839403	8.506748724		2.304812	0.10891
SLC22A7	99.4183082	19.830681	6.649878514	4.380638094		2.325777	0.10893
EPHB6	88.3718295	17.351846	6.481748252	4.19785327		2.348497	0.10899
LOC102163580	77.3253508	14.873011	6.291407421	3.98850389		2.378245	0.10941
FBXO5	1393.23712	285.06604	10.44526023	8.160204421		2.289073	0.10943
LOC100737660	240.260911	1431.1141	7.914450382	10.48393077		-2.57446	0.10944
LOC100739312	153.269892	894.0332	7.269312714	9.805797385		-2.54425	0.10952
TBC1D4	335.53679	71.05994	8.394620418	6.171125545		2.239363	0.10954
LOC102162469	52.4707738	299.93905	5.740678649	8.233327508		-2.51508	0.10976
ARHGEF2	2424.70207	16653.641	11.24418665	14.0236366		-2.77996	0.10977
DPYSL4	24.854577	147.07755	4.692347799	7.210209122		-2.56499	0.1098
LOC100628221	132.557744	27.267186	7.061319821	4.821056379		2.281383	0.10997
LRRFIP1	1101.88625	7180.3591	10.10706828	12.81004118		-2.70408	0.11006
CCDC37	37.2818656	214.83238	5.258589232	7.753767487		-2.52667	0.11015
ZFHX3	498.472351	3087.8023	8.964261007	11.59283181		-2.63099	0.11033
LOC102159321	4.14242951	38.008805	2.362450112	5.285727902		-3.19778	0.11041
LOC100736719	180.886089	1060.1152	7.506891393	10.05136551		-2.55107	0.11057
CXCL14	95.2758787	545.34373	6.58910248	9.09366508		-2.51698	0.11102
PARP14	455.667246	2802.7362	8.834999507	11.45313492		-2.62078	0.11109
IKBIP	617.221997	3866.9828	9.271981177	11.91736566		-2.64735	0.11115
ZNF169	412.862141	87.585507	8.69300647	6.468998788		2.236896	0.11115
TEX14	154.650702	32.224857	7.28216827	5.054191063		2.262768	0.11119
ITGAV	560.608793	3490.1998	9.133421714	11.76950723		-2.63824	0.11131
LOC100622776	603.413898	127.24687	9.239393025	7.002779797		2.245518	0.11147
LOC102163336	37.2818656	5.7839486	5.258589232	2.762125239		2.688347	0.11169
LOC102165830	37.2818656	5.7839486	5.258589232	2.762125239		2.688347	0.11169
LOC100736972	6.90404918	52.055537	2.982591923	5.729431428		-2.91454	0.1117
CXCR5	447.382387	2744.8968	8.808585798	11.42306166		-2.61717	0.11174
ABCC3	755.30298	4783.3255	9.562820493	12.22409983		-2.66289	0.11204
FBLIM1	1094.9822	7099.3838	10.09800865	12.79368129		-2.69679	0.11222
STRBP	2511.69309	504.02981	11.29501875	8.980224728		2.317079	0.11251
LOC102165438	13.8080984	87.585507	3.888314478	6.468998788		-2.66518	0.11276
PDE1A	91.1334492	518.90282	6.525653118	9.022098163		-2.50941	0.1129
LOC100737723	187.790138	39.661362	7.560639591	5.34558663		2.243315	0.11335
LOC100737340	187.790138	39.661362	7.560639591	5.34558663		2.243315	0.11335
C6H16orf74	330.013551	1981.4155	8.370746468	10.95304368		-2.58593	0.11356
DUOX1	102.179928	20.656959	6.689018532	4.436758793		2.306412	0.1137
LOC100737595	1187.49646	246.23095	10.21492189	7.949715578		2.269839	0.11386
ACTR3B	1320.0542	272.67186	10.36747395	8.096303303		2.275359	0.11395
SYCP2	40.0434852	228.05283	5.359081339	7.839536581		-2.50973	0.11403
JAKMIP3	2.76161967	30.5723	1.911353989	4.980587446		-3.46864	0.11408
CDC42EP2	441.859147	94.195734	8.790704108	6.572825024		2.229853	0.11442
CALCB	24.854577	145.42499	4.692347799	7.194018019		-2.54869	0.11459
NOP56	14459.8406	2682.0996	13.8198638	11.38968489		2.430617	0.11461
LOC102166696	33.1394361	190.04403	5.093367322	7.577761331		-2.51971	0.11461
LOC100623509	23.4737672	137.98849	4.613164285	7.118821585		-2.55543	0.11481
LOC100736881	5.52323934	44.619032	2.705588563	5.511563931		-3.01407	0.11483
HCLS1	5.52323934	44.619032	2.705588563	5.511563931		-3.01407	0.11483
SCN5A	26.2353869	3.3051135	4.767410456	2.106051269		2.988742	0.1149
LOC100739476	8.28485901	58.665764	3.214880004	5.89883146		-2.82397	0.11509
STC1	8.28485901	58.665764	3.214880004	5.89883146		-2.82397	0.11509
LOC102163205	295.493305	1756.6678	8.211855717	10.77944673		-2.57164	0.11531
SLC37A2	103.560738	588.3102	6.708197415	9.202883429		-2.5061	0.11532
LOC100157683	77.3253508	436.27498	6.291407421	8.772396993		-2.49622	0.11571
PDGFC	501.23397	3069.6242	8.972215804	11.58431622		-2.61451	0.11578
CAST	1021.79928	6543.2984	9.998307332	12.67602282		-2.67891	0.11595
PITX3	0	15.699289	0	4.061714779		-20	0.11602
CFP	0	15.699289	0	4.061714779		-20	0.11602
LOC100522375	0	15.699289	0	4.061714779		-20	0.11602
LOC102162002	0	15.699289	0	4.061714779		-20	0.11602
LOC102157735	0	15.699289	0	4.061714779		-20	0.11602
DOK7	0	15.699289	0	4.061714779		-20	0.11602
LOC100738778	0	15.699289	0	4.061714779		-20	0.11602
GPHB5	0	15.699289	0	4.061714779		-20	0.11602
LOC102158137	0	15.699289	0	4.061714779		-20	0.11602
TRNAA-AGC	0	15.699289	0	4.061714779		-20	0.11602
FOXQ1	0	15.699289	0	4.061714779		-20	0.11602
LOC102165637	0	15.699289	0	4.061714779		-20	0.11602
LOC102165671	0	15.699289	0	4.061714779		-20	0.11602
TNFAIP8L2	0	15.699289	0	4.061714779		-20	0.11602
VAT1L	0	15.699289	0	4.061714779		-20	0.11602
HELLS	2685.67513	540.38606	11.39160617	9.080513917		2.313222	0.11608
LOC100737338	30.3778164	174.34474	4.971673052	7.454050313		-2.52085	0.11647
LOC100522115	13.8080984	86.759229	3.888314478	6.455478947		-2.6515	0.11675
LOC100154715	2419.17883	489.98307	11.24089794	8.939529481		2.303714	0.11735
ZNF696	1097.74382	229.70539	10.10163933	7.849907883		2.256685	0.11773
PARK2	115.988026	23.962073	6.870217066	4.641665831		2.275151	0.11783
LOC100522128	44.1859147	248.70979	5.497801224	7.964108573		-2.4928	0.11792
LOC100737522	81.4677803	457.75822	6.365758672	8.841590191		-2.49028	0.11792
MAT1A	16.569718	100.80596	4.135019133	6.669678233		-2.60496	0.11796
WNT1	1.38080984	23.135794	1.251452392	4.593102408		-4.06654	0.11798
PDCD2L	1060.46195	222.26888	10.05183695	7.80263838		2.254315	0.11799
PDGFRA	571.655272	3512.5094	9.161523114	11.77869703		-2.61928	0.11804
LOC102166296	104.941548	21.483238	6.727124677	4.490777898		2.288303	0.11835
LOC100518592	12.4272885	79.322724	3.747096094	6.327736286		-2.67422	0.11843
SERINC4	158.793131	908.90621	7.320061583	9.829574033		-2.51698	0.11876
CLHC1	349.344888	75.191332	8.452632037	6.251554969		2.216014	0.11879
THPO	249.92658	53.708094	7.971121491	5.773682393		2.218293	0.11881
LOC100627304	249.92658	53.708094	7.971121491	5.773682393		2.218293	0.11881
LOC102160832	4.14242951	37.182527	2.362450112	5.254840671		-3.16608	0.11916
HDAC9	24.854577	143.77244	4.692347799	7.177643143		-2.5322	0.11959
C6H1orf172	33.1394361	4.9576702	5.093367322	2.574748269		2.740815	0.11968
LOC102158933	33.1394361	4.9576702	5.093367322	2.574748269		2.740815	0.11968
LOC102162520	33.1394361	4.9576702	5.093367322	2.574748269		2.740815	0.11968
LOC102164931	46.9475344	262.75652	5.583384725	8.043062959		-2.48461	0.11987
HS3ST3A1	23.4737672	136.33593	4.613164285	7.101565318		-2.53804	0.12009
GNRHR	111.845597	23.135794	6.818206315	4.593102408		2.27331	0.12025
PCBP4	740.114072	4599.0654	9.533551808	12.16743866		-2.63552	0.12047
TMEM74	82.8485901	16.525567	6.389714621	4.13138925		2.325777	0.12079
HIP1	539.896646	3286.1091	9.079209141	11.68260362		-2.60563	0.12092
HOXA11	8.28485901	57.839486	3.214880004	5.878712739		-2.80351	0.12105
EPHA4	63.5172524	353.64714	6.011613095	8.470240516		-2.47709	0.12107
LOC102166065	129.796125	27.267186	7.031175985	4.821056379		2.25101	0.12153
BRIX1	5604.70712	1099.7765	12.45268066	10.10430588		2.349429	0.12194
SNRPE	15057.7313	2817.6092	13.8783126	11.46076777		2.417961	0.12195
LOC100522421	38.6626754	216.48493	5.309710091	7.764771649		-2.48525	0.12219
CAMK4	40.0434852	6.610227	5.359081339	2.927939483		2.598796	0.12228
TLR2	34.5202459	194.17542	5.150569664	7.608627545		-2.49185	0.12231
LOC102167001	5.52323934	43.792754	2.705588563	5.485193456		-2.98711	0.12256
LOC102165059	5.52323934	43.792754	2.705588563	5.485193456		-2.98711	0.12256
ARHGEF9	15.1889082	92.543178	4.016933786	6.547560532		-2.60711	0.12316
LOC102167584	15.1889082	92.543178	4.016933786	6.547560532		-2.60711	0.12316
LOC100626367	9.66566885	64.449713	3.414902536	6.032314961		-2.73723	0.12351
PKD2	1292.43801	8270.2202	10.33699519	13.01388447		-2.67783	0.12391
TEX40	46.9475344	8.2627837	5.583384725	3.211445828		2.50635	0.12405
LOC100739251	169.83961	963.44058	7.416498698	9.913548547		-2.50402	0.12443
LOC100736901	157.412321	889.90181	7.307540742	9.799122619		-2.4991	0.1245
PPFIBP2	24.854577	142.11988	4.692347799	7.161080272		-2.51552	0.12482
B3GALT4	89.7526393	497.41958	6.503867696	8.961216934		-2.47044	0.12523
LOC102163854	22.0929574	2.4788351	4.529381037	1.798604302		3.155852	0.12538
LOC100623214	22.0929574	2.4788351	4.529381037	1.798604302		3.155852	0.12538
LOC100620265	53.8515836	9.9153405	5.777461367	3.448285225		2.441255	0.12549
LOC102161477	53.8515836	9.9153405	5.777461367	3.448285225		2.441255	0.12549
C2H11orf91	53.8515836	9.9153405	5.777461367	3.448285225		2.441255	0.12549
LOC102167126	53.8515836	9.9153405	5.777461367	3.448285225		2.441255	0.12549
FERMT2	1384.95227	8870.9246	10.43666185	13.11503139		-2.67925	0.12575
SPHK1	96.6566885	535.42838	6.609646952	9.067241769		-2.46975	0.12629
SMAD7	401.815662	2373.8978	8.653975969	11.21364969		-2.56265	0.12683
RPL36AL	23886.6294	4405.7163	14.54397606	12.1054883		2.438755	0.12768
PM20D2	2191.34521	452.80055	11.09825927	8.82591454		2.274869	0.12779
LOC100515432	412.862141	90.064343	8.69300647	6.508814353		2.196632	0.12783
CPT1C	1010.7528	215.65866	9.982641126	7.75928006		2.228609	0.12842
TROP	4.14242951	36.356248	2.362450112	5.223277669		-3.13365	0.12862
RNF224	4.14242951	36.356248	2.362450112	5.223277669		-3.13365	0.12862
LOC102167980	117.368836	24.788351	6.88714549	4.688647628		2.243315	0.12884
FANCB	531.611787	115.67897	9.056940546	6.86640077		2.200246	0.129
AATK	74.5637311	14.873011	6.239622035	3.98850389		2.325777	0.12918
PDLIM2	539.896646	3231.5747	9.079209141	11.658468		-2.58148	0.12932
LOC100520965	2917.65118	596.57298	11.51108608	9.222971117		2.290037	0.12937
NHS	38.6626754	213.17982	5.309710091	7.742678745		-2.46306	0.12941
LOC100522904	961.043646	205.74331	9.909958537	7.691696868		2.223756	0.12948
LOC100512700	6876.43298	1350.9651	12.74765446	10.40084224		2.34767	0.12965
LOC100623937	28.9970066	4.1313919	4.906746634	2.359350201		2.811204	0.12987
LOC100738314	263.734679	1512.0894	8.048403381	10.56328153		-2.51938	0.12993
LOC102158538	426.670239	93.369456	8.740355006	6.560248082		2.192099	0.1301
CITED1	346.583269	76.01761	8.441214828	6.267116453		2.188796	0.13025
LOC100158114	457.048056	99.979683	8.839355155	6.657921243		2.192639	0.13054
MEOX2	215.406334	47.097867	7.757598918	5.587901017		2.193327	0.13057
DDN	5.52323934	42.966475	2.705588563	5.458331975		-2.95962	0.13083
LOC100620172	17.9505279	104.93735	4.24416613	6.727067559		-2.54743	0.13094
AREG	433.574288	95.022013	8.76345901	6.585293272		2.189946	0.13121
SLA-DOB	35.9010557	5.7839486	5.205590187	2.762125239		2.6339	0.13133
RUNX2	15.1889082	90.890621	4.016933786	6.521845711		-2.58111	0.1314
LOC100623591	96.6566885	529.64444	6.609646952	9.051601682		-2.45408	0.13164
LOC100738308	394.911613	2308.6218	8.629034575	11.1734409		-2.54743	0.1317
ATXN1L	53.8515836	293.32882	5.777461367	8.201285014		-2.44546	0.1318
RFC4	5557.75959	1106.3867	12.44054727	10.11294344		2.328648	0.1318
LOC100622780	973.470934	209.04843	9.928475344	7.714578179		2.219301	0.13187
GPR1	2082.26123	433.79615	11.02462804	8.76419534		2.263062	0.13193
LOC102161809	42.8051049	7.4365053	5.453027102	3.076645515		2.525086	0.132
LOC102162148	42.8051049	7.4365053	5.453027102	3.076645515		2.525086	0.132
NASP	19931.99	3732.2994	14.28287051	11.8662355		2.416949	0.13237
BAI2	857.482908	185.08636	9.745645601	7.539828464		2.211909	0.13241
LOC100739181	1.38080984	22.309516	1.251452392	4.542847146		-4.01407	0.13265
LOC102166093	28.9970066	161.12428	4.906746634	7.340956379		-2.4742	0.13278
C4H8orf34	229.214433	50.402981	7.846834472	5.683780114		2.185117	0.13326
BTBD17	31.7586262	175.17101	5.033802952	7.460832769		-2.46354	0.13365
PPAT	867.148577	187.56519	9.761798159	7.558919565		2.208887	0.13409
CCDC92	55.2323934	299.11277	5.813329549	8.229390901		-2.4371	0.13457
LOC100738384	484.664252	106.58991	8.923815489	6.749398975		2.184915	0.13473
SH3KBP1	247.164961	1398.063	7.95551562	10.45024522		-2.49988	0.13512
LOC102160467	1894.4711	398.26618	10.88834074	8.641207046		2.24999	0.13544
LOC102163187	84.2294	17.351846	6.413279271	4.19785327		2.279235	0.13577
ARRDC3	474.998584	2784.5581	8.89481347	11.4437507		-2.55145	0.1361
LOC100513756	578.559321	3433.1866	9.178812527	11.74575273		-2.56901	0.13614
TMEFF1	1137.78731	244.5784	10.1532826	7.940039851		2.217862	0.13651
LOC102159010	0	14.873011	0	3.98850389		-20	0.13667
LOC102159493	0	14.873011	0	3.98850389		-20	0.13667
LOC100518346	0	14.873011	0	3.98850389		-20	0.13667
LOC102159701	0	14.873011	0	3.98850389		-20	0.13667
LOC102168172	0	14.873011	0	3.98850389		-20	0.13667
LOC102163886	0	14.873011	0	3.98850389		-20	0.13667
LOC100156142	0	14.873011	0	3.98850389		-20	0.13667
LOC102164457	0	14.873011	0	3.98850389		-20	0.13667
LOC102157810	0	14.873011	0	3.98850389		-20	0.13667
LOC102157791	0	14.873011	0	3.98850389		-20	0.13667
LOC102166319	0	14.873011	0	3.98850389		-20	0.13667
SUPT20H	0	14.873011	0	3.98850389		-20	0.13667
LOC102158833	0	14.873011	0	3.98850389		-20	0.13667
LTC4S	0	14.873011	0	3.98850389		-20	0.13667
LOC102165347	0	14.873011	0	3.98850389		-20	0.13667
IZUMO2	0	14.873011	0	3.98850389		-20	0.13667
LOC102167383	0	14.873011	0	3.98850389		-20	0.13667
GPR113	0	14.873011	0	3.98850389		-20	0.13667
BMP7	0	14.873011	0	3.98850389		-20	0.13667
LOC100620514	0	14.873011	0	3.98850389		-20	0.13667
LOC100739711	135.319364	741.99798	7.090846698	9.537214474		-2.45505	0.13679
LOC100736725	2.76161967	28.919743	1.911353989	4.903025878		-3.38847	0.13721
GNL3	11049.2403	2143.3661	13.43179012	11.06633551		2.365997	0.13729
RHBDF2	332.77517	1905.3979	8.382732826	10.89663357		-2.51747	0.13737
ZIC1	272.019538	1539.3566	8.092860386	10.58904867		-2.50055	0.13759
RCAN1	370.057036	2130.9719	8.535497154	11.05797272		-2.52569	0.13762
LOC102157741	17.9505279	1.6525567	4.24416613	1.407383613		3.441255	0.13859
CTDSPL	104.941548	568.47952	6.727124677	9.153500149		-2.43752	0.13864
LOC100739713	1462.27762	312.33322	10.51498779	8.291553945		2.227059	0.13902
LOC100627432	349.344888	77.670167	8.452632037	6.29774474		2.16922	0.13905
MSC	85.6102098	461.06333	6.436465198	8.851946794		-2.42911	0.13907
HIPK4	138.080984	754.39215	7.119781365	9.561081988		-2.4498	0.1391
DPYD	172.60123	951.87268	7.439633355	9.896139655		-2.46333	0.13914
TRIB1	111.845597	23.962073	6.818206315	4.641665831		2.222684	0.13922
LOC100624525	96.6566885	521.38165	6.609646952	9.028960416		-2.4314	0.13968
LOC102163617	64.8980623	347.86319	6.042164139	8.446517589		-2.42227	0.13972
SNCG	59.3748229	11.567897	5.915875148	3.65167138		2.359725	0.14014
LOC100739553	6.90404918	48.750424	2.982591923	5.636636914		-2.8199	0.1403
TMEM79	26.2353869	144.59872	4.767410456	7.185853814		-2.46247	0.14036
OSBPL10	465.332915	103.2848	8.865216451	6.704385035		2.171635	0.1404
DAPK3	1231.68237	7616.634	10.26758539	12.89512726		-2.62852	0.14043
SH3BP5	676.59682	4019.8443	9.404283293	11.97328775		-2.57077	0.14089
GNB4	86.9910197	466.84728	6.459284385	8.869893856		-2.42401	0.14108
DHFR	3946.35451	808.92653	11.94667038	9.661647227		2.28644	0.14141
LMO2	56.6132033	302.41788	5.848327568	8.245162313		-2.41733	0.14149
ZNF33B	9.66566885	61.970878	3.414902536	5.976612875		-2.68065	0.14158
LOC100160923	211.263905	47.097867	7.729715254	5.587901017		2.165313	0.14291
MVB12B	12.4272885	75.191332	3.747096094	6.251554969		-2.59705	0.1431
CXCL13	24.854577	3.3051135	4.692347799	2.106051269		2.91074	0.14314
LOC100625155	24.854577	3.3051135	4.692347799	2.106051269		2.91074	0.14314
LOC102164625	26.2353869	143.77244	4.767410456	7.177643143		-2.4542	0.14326
LOC102161674	70.4213016	375.13038	6.158282521	8.55508903		-2.41331	0.14327
SLC22A15	225.072003	1247.6803	7.820638529	10.28618848		-2.47079	0.14329
DCBLD2	294.112495	1653.383	8.205121195	10.69207757		-2.49098	0.14352
PLEKHA7	19.3313377	109.06875	4.345633235	6.782261052		-2.49622	0.14369
C2H19orf71	332.77517	1883.0884	8.382732826	10.87965095		-2.50048	0.14376
LOC102161367	13.8080984	81.801559	3.888314478	6.371586023		-2.56661	0.14393
LRRN2	218.167954	48.750424	7.775893057	5.636636914		2.161953	0.14398
TOR1AIP2	241.641721	1342.7024	7.922683827	10.39199788		-2.4742	0.14402
SLC25A51	71.8021115	381.74061	6.185908388	8.580223165		-2.41049	0.14441
LOC102164618	462.571295	103.2848	8.856647425	6.704385035		2.163048	0.14443
CYCS	20757.7143	3944.6529	14.34142947	11.94604835		2.395677	0.14543
LOC100511091	98.0374983	523.03421	6.629902968	9.033517185		-2.4155	0.14574
CAR	2130.58958	451.97427	11.05771397	8.823285292		2.23694	0.14589
LOC102159205	875.433436	192.52286	9.775500714	7.59636019		2.184968	0.14604
UNG	3820.70082	789.92212	11.89999912	9.627391839		2.274055	0.14682
LOC100738268	23.4737672	128.89943	4.613164285	7.021251246		-2.45712	0.14707
MRPS17	3116.4878	651.10736	11.6061682	9.348985686		2.258954	0.1475
A2M	45.5667246	241.27328	5.541227504	7.920491517		-2.40462	0.14752
LOC102164887	19.3313377	108.24247	4.345633235	6.771389985		-2.48525	0.14757
LOC100621345	55.2323934	291.67627	5.813329549	8.193161944		-2.40078	0.1476
NLGN1	8.28485901	54.534373	3.214880004	5.795309087		-2.71862	0.14824
GPR162	374.199466	84.280394	8.551513162	6.414142197		2.150539	0.14827
LOC102164953	11.0464787	67.754826	3.590539588	6.103389087		-2.61674	0.14832
GPR126	385.245944	2179.7223	8.593375974	11.09059038		-2.50029	0.14866
MAMDC2	584.082561	130.55198	9.192496406	7.039489182		2.161548	0.14887
HAPLN3	190.551757	1036.9794	7.581590451	10.01956204		-2.44413	0.1489
LOC100514785	4132.76384	27326.606	12.01324025	14.73807528		-2.72513	0.14899
DYX1C1	13.8080984	80.97528	3.888314478	6.357117027		-2.55197	0.14905
TP53BP1	5.52323934	41.313919	2.705588563	5.403060391		-2.90304	0.14912
SPRN	335.53679	1881.4359	8.394620418	10.87838499		-2.48729	0.14915
LOC100628117	223.691193	50.402981	7.81179977	5.683780114		2.149928	0.14916
C1H6orf163	1.38080984	21.483238	1.251452392	4.490777898		-3.95962	0.14919
LOC100626194	1.38080984	21.483238	1.251452392	4.490777898		-3.95962	0.14919
MMRN2	1.38080984	21.483238	1.251452392	4.490777898		-3.95962	0.14919
LOC100157925	48.3283443	9.0890621	5.624344958	3.334720158		2.410666	0.14925
LRRC56	886.479915	195.82797	9.793570658	7.620791468		2.178501	0.14966
LOC102163557	4.14242951	34.703692	2.362450112	5.158001345		-3.06654	0.1499
LOC100627072	4.14242951	34.703692	2.362450112	5.158001345		-3.06654	0.1499
LOC100739234	16.569718	94.195734	4.135019133	6.572825024		-2.50711	0.15018
SEMA4A	2.76161967	28.093465	1.911353989	4.862623207		-3.34665	0.15052
LOC100522818	2.76161967	28.093465	1.911353989	4.862623207		-3.34665	0.15052
RNF150	2.76161967	28.093465	1.911353989	4.862623207		-3.34665	0.15052
LOC100738465	277.542777	62.797156	8.121755095	5.995420212		2.143939	0.15054
LOC102166675	317.586262	71.886218	8.315540247	6.187574144		2.143361	0.15086
LOC100525798	113.226407	24.788351	6.835752397	4.688647628		2.191476	0.15134

MFSD4	2.114718173	32.224856	1.639101631	5.054191063		-3.92964	0.05455
LOC102158881	127.9404495	1030.3691	7.010561107	10.01034505		-3.00962	0.05456
LOC100627015	644.9890428	93.369456	9.335365884	6.560248082		2.788252	0.05457
LOC100037955	1.057359087	24.788351	1.04079362	4.688647628		-4.55112	0.05469
LOC102162241	1.057359087	24.788351	1.04079362	4.688647628		-4.55112	0.05469
FAM160B2	434.5745846	3757.0878	8.766775966	11.87578304		-3.11194	0.05471
LOC100738954	14.80302721	122.2892	3.982129041	6.945902605		-3.04633	0.05474
AGPAT5	9808.062887	1243.5489	13.2598996	10.28140726		2.979505	0.05483
HID1	124.7683722	1002.2757	6.974625354	9.970502347		-3.00596	0.05484
LOC102168008	198.7835083	28.919743	7.642293686	4.903025878		2.781071	0.05485
LOC102167020	22.20454082	176.82357	4.536335244	7.474302764		-2.99338	0.05486
NAP1L5	1043.613418	148.73011	10.02875343	7.22622053		2.810819	0.055
LOC102163714	11.63094995	99.153405	3.658891241	6.646067655		-3.09169	0.05504
LOC102165672	54.9826725	6.610227	5.806908455	2.927939483		3.056205	0.05504
TUBA4A	3136.127051	423.8808	11.61522824	8.730914357		2.887253	0.05506
LPIN3	138.5140403	1117.1284	7.124266508	10.1268701		-3.01169	0.05506
LOC100739029	2685.69208	366.04132	11.39161527	8.519798669		2.875215	0.0551
RAB3D	579.4327794	5090.701	9.180985186	12.313932		-3.13515	0.05537
BARX2	19.03246356	0.8262784	4.324267947	0.868906686		4.525691	0.05551
LOC102160692	19.03246356	0.8262784	4.324267947	0.868906686		4.525691	0.05551
LOC100627012	19.03246356	0.8262784	4.324267947	0.868906686		4.525691	0.05551
LOC100739014	35.95020894	280.10837	5.207510617	8.13498259		-2.96191	0.05561
ITM2B	2559.866349	24916.424	11.32241625	14.60486733		-3.28296	0.05567
LOC100623579	127.9404495	18.178124	7.010561107	4.261389711		2.815197	0.05571
NUDT11	133.2272449	19.004403	7.068533723	4.322245637		2.809484	0.05579
ANKLE1	688.3407654	99.979683	9.429073524	6.657921243		2.783416	0.0559
GPN3	3331.738482	450.32171	11.7024924	8.818012376		2.887247	0.05591
LOC102164532	14.80302721	121.46292	3.982129041	6.936201186		-3.03655	0.05609
LOC102166311	256.938258	2133.4508	8.010881963	11.05964916		-3.0537	0.0562
LOC102159434	41.23700438	319.76973	5.400435611	8.325394195		-2.95502	0.05632
CPNE9	80.35929058	10.741619	6.346235194	3.553559423		2.903253	0.05637
LOC100155494	15.8603863	128.89943	4.075565686	7.021251245		-3.02275	0.05647
ARID3B	2328.304709	321.42229	11.18568366	8.33280766		2.856738	0.05653
ATIC	19508.27515	2394.5547	14.25187256	11.22614405		3.026257	0.05669
LOC102159726	44.40908164	4.9576702	5.504908955	2.57474827		3.163121	0.05674
NR2E1	44.40908164	4.9576702	5.504908955	2.57474827		3.163121	0.05674
LOC102161256	5.286795433	53.708094	2.65232482	5.773682393		-3.34467	0.05681
LOC102164824	16.91774539	136.33593	4.163317207	7.101565318		-3.01056	0.05685
LOC100512871	1983.605646	276.80325	10.95463665	8.117919691		2.841192	0.05724
ARHGAP11A	7675.369609	995.66544	12.90620846	9.96096549		2.946503	0.05727
TFPI2	187.1525583	1517.8734	7.555759096	10.56878588		-3.01976	0.05728
LOC102159254	21.14718173	166.90823	4.46905122	7.391529145		-2.98052	0.05734
LRPPRC	39087.39335	4621.3749	15.25445267	12.17441857		3.080309	0.05736
GRIP1	925.1892008	133.8571	9.855163125	7.075287628		2.789055	0.05763
LOC100153260	6602.150137	865.11346	12.68893873	9.758412211		2.931975	0.05771
PITX2	3493.514422	474.28379	11.77087629	8.892645374		2.880857	0.05779
SELO	317.207726	2649.0485	8.313825054	11.37180303		-3.06197	0.05781
PACSIN3	200.8982264	1631.8998	7.657484427	10.67322054		-3.02202	0.05781
CTHRC1	695.742279	6120.2439	9.4444813	12.57960914		-3.13696	0.05784
LOXL3	155.4317857	1244.3752	7.289389877	10.28236477		-3.00107	0.0579
SPIRE1	9126.066276	1174.9678	13.15593549	10.1996329		2.957372	0.0579
CENPA	4605.856181	616.40367	12.16956684	9.270070238		2.901522	0.058
TMEM187	27.49133625	213.17982	4.832451381	7.742678745		-2.95502	0.05811
LOC102168145	95.16231779	13.220454	6.587399765	3.829895615		2.847619	0.05836
NDOR1	152.2597085	1214.6292	7.259834656	10.24748753		-2.99591	0.05845
LOC102162528	48.63851798	373.47782	5.633388138	8.548736477		-2.94085	0.05845
ZBTB7A	203.0129446	1645.1202	7.672516884	10.684854		-3.01855	0.05848
LOC102160764	8.458872693	75.191332	3.241668254	6.251554969		-3.15203	0.05853
TOPOII	41196.82473	4881.6526	15.33028054	12.25344942		3.077092	0.05887
SLC25A13	11405.73247	1455.0762	13.47759796	10.50787015		2.970592	0.0589
PRKCI	9709.728492	1250.1592	13.24536381	10.28904963		2.957319	0.05891
LOC100514190	1919.106742	270.19303	10.9069708	8.083176277		2.828373	0.0592
LOC102167660	2086.169478	292.50254	11.02733204	8.197229196		2.834335	0.05922
LOC100514491	1145.119891	10365.662	10.16254225	13.33966383		-3.17824	0.05923
LOC100621115	9187.393103	1188.1883	13.16559686	10.21576146		2.950892	0.05928
SRPK1	23718.67903	2906.021	14.53379687	11.50532579		3.028907	0.05936
FAM174B	1102.825527	159.47173	10.10829644	7.326175314		2.789832	0.05941
SLFNL1	2.114718173	31.398578	1.639101631	5.017858594		-3.89216	0.05959
CMTM7	1602.956375	228.05283	10.64741919	7.839536581		2.813295	0.05964
PARPBP	988.6307459	143.77244	9.950746513	7.177643143		2.781645	0.05971
LOC102159356	120.5389359	17.351846	6.925274755	4.19785327		2.796338	0.0598
IKBKE	105.7359087	823.79954	6.737901807	9.687899712		-2.96183	0.05984
LOC100626128	631.2433747	93.369456	9.304336204	6.560248082		2.757174	0.0599
PABPC1L2A	5.286795433	52.881816	2.65232482	5.751726565		-3.32231	0.05997
LOC100621250	5.286795433	52.881816	2.65232482	5.751726565		-3.32231	0.05997
EDA	834.2563193	122.2892	9.706075182	6.945902605		2.770194	0.06004
LOC102159970	42.29436346	322.24856	5.436107306	8.336500155		-2.92964	0.06008
LOC100627058	9847.185173	1272.4687	13.26564217	10.31454778		2.952081	0.06018
CHST11	34.89284986	266.06164	5.165624571	8.061028933		-2.93076	0.06031
RPA3	2288.125063	320.59601	11.16058057	8.329105689		2.835338	0.06034
CFB	305.576776	2516.8439	8.260104603	11.29797314		-3.04201	0.0604
LOC102161367	9.516231779	81.801559	3.394545939	6.371586023		-3.10367	0.06052
TFDP2	3367.688691	462.71589	11.7179714	8.857097348		2.86356	0.06059
LIN7B	33.83549077	257.79885	5.122485983	8.015687407		-2.92964	0.0606
ERCC6L	2050.219269	289.19743	11.002266	8.180890933		2.825652	0.06065
LOC100524737	144.8581949	1141.0904	7.188422634	10.15746117		-2.9777	0.06071
LOC100522935	48.63851798	5.7839486	5.633388138	2.76212524		3.071973	0.06073
LOC100620317	48.63851798	5.7839486	5.633388138	2.76212524		3.071973	0.06073
LOC102167015	68.72834063	9.0890621	6.123673244	3.334720158		2.918702	0.0608
LOC102157645	22.20454082	171.8659	4.536335244	7.433509508		-2.95236	0.06083
PREI2	384.8787075	57.839486	8.592003629	5.878712739		2.734277	0.0612
HHIP	2685.69208	374.3041	11.39161527	8.55191625		2.843011	0.0612
PARK2	1.057359087	23.962073	1.04079362	4.641665831		-4.50222	0.06123
LOC102161400	1.057359087	23.962073	1.04079362	4.641665831		-4.50222	0.06123
LOC100738421	1.057359087	23.962073	1.04079362	4.641665831		-4.50222	0.06123
LOC102166021	1.057359087	23.962073	1.04079362	4.641665831		-4.50222	0.06123
NR5A2	1.057359087	23.962073	1.04079362	4.641665831		-4.50222	0.06123
LOC102165701	1.057359087	23.962073	1.04079362	4.641665831		-4.50222	0.06123
AP1AR	3726.133421	510.64003	11.86385075	8.998985343		2.867301	0.06142
CA4	0	16.525567	0	4.131389251		-20	0.06143
LOC100622546	0	16.525567	0	4.131389251		-20	0.06143
LOC102168017	0	16.525567	0	4.131389251		-20	0.06143
LOC102163998	0	16.525567	0	4.131389251		-20	0.06143
B4GALNT2	0	16.525567	0	4.131389251		-20	0.06143
IL15	0	16.525567	0	4.131389251		-20	0.06143
KCNA3	0	16.525567	0	4.131389251		-20	0.06143
LOC100520680	0	16.525567	0	4.131389251		-20	0.06143
LGALS7	0	16.525567	0	4.131389251		-20	0.06143
SERPINF2	0	16.525567	0	4.131389251		-20	0.06143
LOC102162885	0	16.525567	0	4.131389251		-20	0.06143
CD5L	0	16.525567	0	4.131389251		-20	0.06143
LOC100627544	0	16.525567	0	4.131389251		-20	0.06143
FAM211B	0	16.525567	0	4.131389251		-20	0.06143
LOC102165745	0	16.525567	0	4.131389251		-20	0.06143
SELE	0	16.525567	0	4.131389251		-20	0.06143
MIS18BP1	1949.770156	276.80325	10.92982809	8.117919691		2.816371	0.06175
DAPK1	1295.264881	187.56519	10.34014484	7.558919565		2.787783	0.06178
MIC-1	14.80302721	118.15781	3.982129041	6.896729669		-2.99675	0.06192
TMA16	9151.442894	1194.7985	13.15994115	10.22375862		2.937232	0.06198
LOC100626363	31.7207726	240.44701	5.032134908	7.915562765		-2.92222	0.06207
ZNF280C	4041.226429	552.78023	11.98093442	9.113169742		2.870015	0.06217
RPS6KB2	260.1103353	2103.7047	8.028515753	11.03940214		-3.01574	0.06231
LOC102165247	108.9079859	15.699289	6.780152406	4.061714779		2.794339	0.06256
HCST	3.17207726	38.008805	2.060765874	5.285727902		-3.58283	0.06271
ZFP57	58.15474976	7.4365053	5.886422108	3.076645515		2.9672	0.06286
LOC100515833	1307.95319	190.04403	10.35419779	7.577761331		2.782905	0.06286
ATP8A1	272.7986443	41.313919	8.096971493	5.403060391		2.723137	0.06294
LOC100623323	1716.093798	246.23095	10.74575313	7.949715578		2.801044	0.06297
LOC100622718	75.07249515	569.3058	6.249303021	9.15559189		-2.92285	0.06314
MPHOSPH10	7696.516791	1018.8012	12.91017739	9.994072271		2.917333	0.06315
YIF1A	317.207726	2584.5987	8.313825054	11.33628269		-3.02644	0.0635
CHAD	28.54869534	215.65866	4.885022527	7.75928006		-2.91725	0.06357
SNRPA1	10314.53789	1345.1812	13.33253143	10.39465689		2.938807	0.06371
NLGN2	169.1774539	1325.3505	7.410896102	10.37324636		-2.96976	0.06392
VPS37D	45.46644072	340.42669	5.538117235	8.415432027		-2.90447	0.06404
HSPD1	122136.6054	13845.947	16.89814794	13.75728027		3.14096	0.06439
ACTN2	48.63851798	363.56248	5.633388138	8.510022294		-2.90203	0.06452
LOC100621768	38.06492712	4.1313919	5.287802017	2.359350201		3.203763	0.06453
DCT	23.2618999	1.6525567	4.600620625	1.407383614		3.815197	0.06495
EFCC1	23.2618999	1.6525567	4.600620625	1.407383614		3.815197	0.06495
LOC102166686	23.2618999	1.6525567	4.600620625	1.407383614		3.815197	0.06495
LOC100623930	23.2618999	1.6525567	4.600620625	1.407383614		3.815197	0.06495
PROKR2	23.2618999	1.6525567	4.600620625	1.407383614		3.815197	0.06495
LOC100155404	23.2618999	1.6525567	4.600620625	1.407383614		3.815197	0.06495
LOC102165083	23.2618999	1.6525567	4.600620625	1.407383614		3.815197	0.06495
MLF1IP	5864.113494	793.22724	12.51794331	9.633408027		2.886107	0.06505
BARD1	761.2985423	114.02642	9.574212307	6.845821397		2.739094	0.06511
LOC100152387	2.114718173	30.5723	1.639101631	4.980587447		-3.85369	0.06516
C2H19orf57	6.344154519	58.665764	2.876596413	5.89883146		-3.20902	0.06524
LOC100737349	411.3126847	3382.7837	8.687595036	11.72442162		-3.0399	0.06535
LOC102165321	938.9348689	139.64104	9.876416981	7.135873883		2.749302	0.06542
LOC100624490	133.2272449	19.830681	7.068533723	4.380638094		2.748083	0.06556
NTF4	8.458872693	72.712497	3.241668254	6.20383732		-3.10367	0.06582
UGT1A3	86.7034451	652.75991	6.45456161	9.352617108		-2.91239	0.06601
FNDC3A	34.89284986	259.45141	5.165624571	8.02487043		-2.89446	0.06607
AGTRAP	286.5443159	2295.4013	8.167640492	11.16515907		-3.00192	0.06609
CCDC9	461.0085617	3809.1433	8.851775777	11.89562954		-3.0466	0.06609
LOC102166831	17.97510447	137.98849	4.246035924	7.118821584		-2.94048	0.06629
LOC100737062	1660.053766	241.27328	10.69788306	7.920491517		2.78249	0.06629
LOC100513945	203.0129446	1590.5859	7.672516884	10.63624928		-2.96991	0.0664
LOC102161248	39.1222862	290.02371	5.326331909	8.184992878		-2.89011	0.06646
LOC102160271	15.8603863	123.11548	4.075565686	6.955539223		-2.95651	0.06652
FURIN	1011.892646	8809.78	9.984265559	13.10505403		-3.12205	0.06659
LYRM1	74.01513606	552.78023	6.229109817	9.113169742		-2.90081	0.06668
PPARGC1B	1472.901208	215.65866	10.52542411	7.75928006		2.771839	0.06676
GPC2	79.30193149	593.26787	6.327362784	9.214969574		-2.90326	0.0668
LOC102161145	939.992228	140.46732	9.878038997	7.144325041		2.742414	0.06684
ARMCX2	265.3971307	2109.4887	8.057434734	11.04336138		-2.99067	0.06684
LOC102161185	5.286795433	51.229259	2.65232482	5.706786332		-3.2765	0.06689
tRNA-Val	91.99024053	13.220454	6.539007406	3.829895615		2.798709	0.06692
ADAMTS13	7.401513606	65.275991	3.070649265	6.05041444		-3.14066	0.06694
CASP10	7.401513606	65.275991	3.070649265	6.05041444		-3.14066	0.06694
LOC102165578	220.9880491	33.877413	7.7943382	5.124221138		2.705573	0.06696
HSP27	783.5030832	6690.376	9.615635307	12.70808719		-3.09408	0.06696
FAT3	371.1330394	57.013208	8.539674674	5.858309485		2.702568	0.06707
LOC100516409	830.026683	124.76803	9.698751337	6.974621476		2.73391	0.06711
HSD17B14	138.5140403	1059.2889	7.124266508	10.05024166		-2.93499	0.06723
TIPIN	2425.581745	347.03692	11.24470975	8.44309653		2.80517	0.06741
CTDSP2	603.7520384	5054.3448	9.240199917	12.30359378		-3.0655	0.06742
MAPT	68.72834063	510.64003	6.123673244	8.998985343		-2.89333	0.06743
NOXA1	32.77813168	3.3051135	5.078017628	2.106051269		3.309962	0.06744
LOC100518548	120.5389359	914.6916	6.925274755	9.838715705		-2.92378	0.06747
LOC102161849	3.17207726	37.182527	2.060765874	5.254840671		-3.55112	0.06753
LOC100515885	20.08982264	152.0322	4.398475058	7.257719911		-2.91987	0.06754
TRMT11	1738.298338	253.66746	10.7642897	7.992470822		2.776665	0.06823
CCDC88C	1749.929286	255.32002	10.77390511	8.001802338		2.776918	0.06827
TMEM54	83.53136784	622.18761	6.40141489	9.283522749		-2.89696	0.06831
LOC100737589	860.6902965	129.7257	9.751025627	7.030399033		2.73003	0.06834
LOC102161463	17.97510447	0.8262784	4.246035924	0.868906686		4.443228	0.06843
LOC100513836	17.97510447	0.8262784	4.246035924	0.868906686		4.443228	0.06843
LOC102162103	17.97510447	0.8262784	4.246035924	0.868906686		4.443228	0.06843
MAST1	17.97510447	0.8262784	4.246035924	0.868906686		4.443228	0.06843
LOC102160225	17.97510447	0.8262784	4.246035924	0.868906686		4.443228	0.06843
SAMD12	17.97510447	0.8262784	4.246035924	0.868906686		4.443228	0.06843
LOC102159328	1.057359087	23.135794	1.04079362	4.593102407		-4.45159	0.06867
LOC100156062	1.057359087	23.135794	1.04079362	4.593102407		-4.45159	0.06867
LOC100522946	1.057359087	23.135794	1.04079362	4.593102407		-4.45159	0.06867
FIGNL1	1735.126261	253.66746	10.76165616	7.992470822		2.77403	0.06877
GRASP	219.93069	1714.5276	7.787450031	10.74443664		-2.96269	0.06886
WASF3	19.03246356	143.77244	4.324267947	7.177643143		-2.91725	0.06898
LOC100623894	1822.887805	266.06164	10.83280069	8.061028933		2.776393	0.06903
SGOL1	2447.786285	351.99459	11.25785115	8.463502248		2.797852	0.06915
LOC100737118	4.229436346	43.792754	2.386655454	5.485193456		-3.37215	0.06926
LOC100737277	3935.49052	551.95395	11.94269429	9.111015534		2.833924	0.0694
EDEM1	40.17964529	294.1551	5.363859498	8.205329462		-2.87204	0.06948
CCDC148	136.3993222	20.656959	7.102231077	4.436758793		2.723137	0.06949
NPFF	31.7207726	233.0105	5.032134908	7.87042946		-2.8769	0.0695
tRNA-Gln	367.9609621	57.013208	8.52732437	5.858309485		2.690185	0.06951
SEMA6C	43.35172255	317.29089	5.470918232	8.314202077		-2.87164	0.06951
VEGFA	153.3170676	1167.5313	7.269753823	10.19048071		-2.92887	0.06963
LOC102163666	76.12985423	561.86929	6.269217479	9.136656134		-2.8837	0.06985
SPAG11B	12.68830904	0	3.774872332	0		20	0.06986
LOC100622164	12.68830904	0	3.774872332	0		20	0.06986
TCTEX1D1	12.68830904	0	3.774872332	0		20	0.06986
LOC102159289	12.68830904	0	3.774872332	0		20	0.06986
LOC102159318	12.68830904	0	3.774872332	0		20	0.06986
LOC102159341	12.68830904	0	3.774872332	0		20	0.06986
LOC100511315	12.68830904	0	3.774872332	0		20	0.06986
GPX6	12.68830904	0	3.774872332	0		20	0.06986
LOC102159890	12.68830904	0	3.774872332	0		20	0.06986
LOC100626148	12.68830904	0	3.774872332	0		20	0.06986
LOC102166821	12.68830904	0	3.774872332	0		20	0.06986
LOC100626910	12.68830904	0	3.774872332	0		20	0.06986
TAT	12.68830904	0	3.774872332	0		20	0.06986
LOC102158871	12.68830904	0	3.774872332	0		20	0.06986
NPPC	12.68830904	0	3.774872332	0		20	0.06986
LRP2	12.68830904	0	3.774872332	0		20	0.06986
LOC102162596	12.68830904	0	3.774872332	0		20	0.06986
LOC102158260	12.68830904	0	3.774872332	0		20	0.06986
LOC100270682	12.68830904	0	3.774872332	0		20	0.06986
LOC102167250	12.68830904	0	3.774872332	0		20	0.06986
LOC102167183	12.68830904	0	3.774872332	0		20	0.06986
LOC100739652	12.68830904	0	3.774872332	0		20	0.06986
IQCA1P1	12.68830904	0	3.774872332	0		20	0.06986
HSD17B3	12.68830904	0	3.774872332	0		20	0.06986
LOC102165169	12.68830904	0	3.774872332	0		20	0.06986
RNF32	7.401513606	64.449713	3.070649265	6.032314961		-3.12228	0.06993
LOC100155802	26.43397716	195.0017	4.777891883	7.614722326		-2.88302	0.06993
ACSL3	17252.92922	2227.6465	14.07463724	11.12195208		2.953249	0.07
CSWD2	3172.07726	451.14799	11.63166693	8.820651243		2.813755	0.07008
LOC102164615	11.63094995	92.543178	3.658891241	6.547560532		-2.99216	0.07022
MRPS36	4423.990418	618.05622	12.11145862	9.273926629		2.839538	0.07023
LOC102157769	747.5528742	114.02642	9.547960415	6.845821397		2.712808	0.07034
FAM69B	233.6763851	1816.9861	7.874528698	10.82812548		-2.95896	0.07047
THUMPD3	3369.803409	478.41518	11.71887677	8.905131772		2.816329	0.07058
POFUT2	799.3634694	6740.779	9.64451151	12.71891361		-3.07599	0.07062
ARSE	5.286795433	50.402981	2.65232482	5.683780113		-3.25304	0.07068
MGMT	642.8743246	5335.2794	9.330635311	12.3816185		-3.05296	0.07083
LOC100154278	10.57359087	85.106672	3.532764645	6.428053129		-3.00881	0.0712
LOC102166571	8.458872693	71.05994	3.241668254	6.171125545		-3.0705	0.07125
FBXO40	2.114718173	29.746021	1.639101631	4.942327829		-3.81416	0.07134
PPID	10641.26185	1418.72	13.37751719	10.47139067		2.907007	0.07136
MAP1A	763.4132605	6397.8734	9.578208995	12.64360222		-3.06706	0.07142
ZACN	96.21967688	711.42568	6.603176434	9.476595706		-2.88631	0.07156
LOC100626088	338.3549077	52.881816	8.406651067	5.751726565		2.677694	0.0718
LOC100625773	222.0454082	1712.0488	7.801193638	10.74235052		-2.9468	0.07189
ETFB	502.2455661	4081.8152	8.975118746	11.99534854		-3.02275	0.07191
PABPC1L2B	6.344154519	57.013208	2.876596413	5.858309485		-3.1678	0.07193
SULT4A1	0	15.699289	0	4.061714779		-20	0.07217
IFN-OMEGA-2	0	15.699289	0	4.061714779		-20	0.07217
SLC35F3	0	15.699289	0	4.061714779		-20	0.07217
RAB20	0	15.699289	0	4.061714779		-20	0.07217
LOC100522375	0	15.699289	0	4.061714779		-20	0.07217
LOC102157735	0	15.699289	0	4.061714779		-20	0.07217
DOK7	0	15.699289	0	4.061714779		-20	0.07217
LOC102166142	0	15.699289	0	4.061714779		-20	0.07217
HAND1	0	15.699289	0	4.061714779		-20	0.07217
MIR24-2	0	15.699289	0	4.061714779		-20	0.07217
LOC100621514	0	15.699289	0	4.061714779		-20	0.07217
PSD2	0	15.699289	0	4.061714779		-20	0.07217
FOXQ1	0	15.699289	0	4.061714779		-20	0.07217
LOC102162641	0	15.699289	0	4.061714779		-20	0.07217
LOC102163218	0	15.699289	0	4.061714779		-20	0.07217
MIIP	140.6287585	1055.1575	7.145970432	10.04460925		-2.90749	0.07232
RAD17	933.6480734	142.11988	9.868279434	7.161080272		2.71577	0.07244
LOC100737477	2242.675823	20208.29	11.13163747	14.30273103		-3.17167	0.07259
LOC100513919	222.0454082	1707.0911	7.801193638	10.73816922		-2.94261	0.07266

CEP85	2769.4512	470.97867	11.43590524	8.882577857	8.882577857	2.555866	0.01211
FAM129C	17.4075053	108.24247	4.202222212	6.771389985	6.771389985	-2.63649	0.01212
LOXHD1	29.0125088	2.4788351	4.907492018	1.798604302	1.798604302	3.548941	0.01212
LOC100511579	101.958245	577.56858	6.685915561	9.176344172	9.176344172	-2.50201	0.01213
PIK3IP1	213.034708	1222.0657	7.741700952	10.2562862	10.2562862	-2.52016	0.01215
LOC102161615	702.102714	123.94176	9.457591652	6.965111899	6.965111899	2.50202	0.01217
LOC100152254	702.102714	123.94176	9.457591652	6.965111899	6.965111899	2.50202	0.01217
YPEL5	413.635483	2420.9956	8.695699772	11.24198055	11.24198055	-2.54917	0.01223
LOC102165314	138.431114	23.135794	7.123408719	4.593102408	4.593102408	2.58097	0.01228
LOC102165099	248.678647	1430.2879	7.963928635	10.48309814	10.48309814	-2.52395	0.0123
LOC102158208	164.956836	28.093465	7.374664246	4.862623207	4.862623207	2.553782	0.01233
LOC100154532	331.571529	1920.2709	8.377520858	10.90784527	10.90784527	-2.53392	0.01248
POLA1	851.309902	150.38266	9.735234283	7.242056188	7.242056188	2.501046	0.01249
TSPAN2	331.571529	58.665764	8.377520858	5.89883146	5.89883146	2.498729	0.01251
DSCC1	708.734144	125.59431	9.471134904	6.98406878	6.98406878	2.496473	0.01251
LOC100739472	625.012333	3702.5534	9.29004727	11.85469442	11.85469442	-2.56656	0.01253
LOC100511825	85.3796688	13.220454	6.43261988	3.829895615	3.829895615	2.691121	0.01258
LOC102166534	4.97357294	42.140197	2.578594101	5.430960858	5.430960858	-3.08284	0.01265
ZNF554	613.40733	109.06875	9.263051616	6.782261052	6.782261052	2.491608	0.01267
IFT140	251.165434	1438.5506	7.978226717	10.49140283	10.49140283	-2.51791	0.01269
DRP2	473.318358	84.280394	8.889711898	6.414142197	6.414142197	2.489542	0.01271
RP2	339.860818	60.318321	8.41303896	5.938246292	5.938246292	2.494276	0.01272
PCSK6	27.3546512	160.298	4.825513503	7.333584776	7.333584776	-2.5509	0.0128
PLEK2	97.8136012	549.47512	6.62663773	9.104533542	9.104533542	-2.48995	0.01282
LOC100738956	33.1571529	3.3051135	5.094115824	2.106051269	2.106051269	3.326548	0.01284
LOC102158276	33.1571529	3.3051135	5.094115824	2.106051269	2.106051269	3.326548	0.01284
NEK10	320.795455	57.013208	8.330000138	5.858309486	5.858309486	2.492286	0.01294
LOC102159370	54.7093024	7.4365053	5.799846344	3.076645515	3.076645515	2.879089	0.01306
SLC43A2	220.495067	1252.638	7.791130759	10.29190511	10.29190511	-2.50615	0.01308
ZSCAN23	115.221107	19.004403	6.860728285	4.322245637	4.322245637	2.599999	0.01309
RIMS1	2587.91579	446.19032	11.33813232	8.804745152	8.804745152	2.53606	0.01315
CCDC114	52.2225159	294.1551	5.733964805	8.205329462	8.205329462	-2.49383	0.01316
LOC100621058	27.3546512	159.47173	4.825513503	7.326175314	7.326175314	-2.54344	0.01324
LOC102166380	433.529775	77.670167	8.763311226	6.29774474	6.29774474	2.480699	0.01327
LOC100737652	6047.8647	1011.3647	12.56244868	9.983513431	9.983513431	2.580122	0.0133
PKC	364.728682	65.275991	8.514629965	6.05041444	6.05041444	2.482199	0.01333
TMPRSS6	37.3017971	4.1313919	5.259340178	2.359350201	2.359350201	3.174545	0.01334
ESPN	37.3017971	4.1313919	5.259340178	2.359350201	2.359350201	3.174545	0.01334
SLC38A6	204.745419	1157.616	7.684716501	10.17818678	10.17818678	-2.49925	0.01334
LOC100517249	24.0389359	1.6525567	4.646101346	1.407383613	1.407383613	3.862601	0.01338
LOC100512171	170.759338	960.13547	7.424244723	9.908595977	9.908595977	-2.49127	0.01344
LOC100524511	176.561839	993.1866	7.472177749	9.957372852	9.957372852	-2.49189	0.01347
PABPC5	62.9985906	9.0890621	5.999968229	3.334720158	3.334720158	2.793116	0.01348
LOC100620700	298.414377	1702.9597	8.225999684	10.73467552	10.73467552	-2.51266	0.0135
LOC100625970	923.42671	5498.0563	9.852415134	12.42496834	12.42496834	-2.57385	0.0135
LOC100627705	2763.6487	477.5889	11.43288045	8.902643123	8.902643123	2.532733	0.0136
ULBP1	67.1432347	9.9153405	6.090498528	3.448285225	3.448285225	2.759508	0.01365
DMRTA1	67.1432347	9.9153405	6.090498528	3.448285225	3.448285225	2.759508	0.01365
LOC100620819	179.877555	31.398578	7.498869583	5.017858594	5.017858594	2.518244	0.01371
GCKR	629.156977	113.20014	9.29956745	6.83542057	6.83542057	2.474544	0.01376
RAI2	1.65785765	23.962073	1.410263837	4.641665831	4.641665831	-3.85336	0.01387
LOC100739318	1.65785765	23.962073	1.410263837	4.641665831	4.641665831	-3.85336	0.01387
ZIC4	113.563249	631.27668	6.8400005	9.304412191	9.304412191	-2.47478	0.01388
CA11	42.27537	237.96817	5.435474249	7.900674664	7.900674664	-2.49288	0.0139
ARHGAP29	775.04845	4562.7092	9.600002915	12.15599114	12.15599114	-2.55753	0.0139
LOC102158109	75.432523	11.567897	6.256114748	3.65167138	3.65167138	2.70506	0.01397
SH2D6	295.098661	1673.2137	8.209934157	10.70926798	10.70926798	-2.50335	0.01408
LOC102158716	258.625793	46.271589	8.020289907	5.56290145	5.56290145	2.482668	0.01409
LOC102166675	397.885835	71.886218	8.639832083	6.187574144	6.187574144	2.468567	0.0141
LOC100515833	1061.02889	190.04403	10.0526073	7.577761331	7.577761331	2.481058	0.01422
LOC100514456	38.1307259	214.83238	5.290229968	7.753767487	7.753767487	-2.49419	0.01427
LOC100739376	341.518675	1941.7542	8.420038841	10.92388765	10.92388765	-2.50732	0.01428
LRRC8D	252.823291	1422.8514	7.987680649	10.47558283	10.47558283	-2.49258	0.01434
LOC102161347	53.8803735	7.4365053	5.778218396	3.076645515	3.076645515	2.857063	0.01435
LOC100524408	410.319768	74.365053	8.684116598	6.2358238	6.2358238	2.464052	0.01437
LOC100152144	94.4978859	521.38165	6.57739689	9.028960416	9.028960416	-2.46399	0.01447
TRIM36	139.260042	23.962073	7.131960259	4.641665831	4.641665831	2.538957	0.0145
LOC102158867	58.0250177	8.2627837	5.883254663	3.211445828	3.211445828	2.811975	0.0145
LOC102164640	707.076287	128.07315	9.467760991	7.012045083	7.012045083	2.464898	0.01455
SLC5A9	14.09179	0	3.915692025	0	0	20	0.01464
LOC102159070	14.09179	0	3.915692025	0	0	20	0.01464
LOC102159289	14.09179	0	3.915692025	0	0	20	0.01464
LOC102159341	14.09179	0	3.915692025	0	0	20	0.01464
LOC100525238	14.09179	0	3.915692025	0	0	20	0.01464
LOC102164452	14.09179	0	3.915692025	0	0	20	0.01464
LOC102164248	14.09179	0	3.915692025	0	0	20	0.01464
LOC102166194	14.09179	0	3.915692025	0	0	20	0.01464
LOC102160990	14.09179	0	3.915692025	0	0	20	0.01464
LOC100621290	14.09179	0	3.915692025	0	0	20	0.01464
FOXB1	14.09179	0	3.915692025	0	0	20	0.01464
LOC102160621	14.09179	0	3.915692025	0	0	20	0.01464
LOC102162810	14.09179	0	3.915692025	0	0	20	0.01464
LOC102160074	14.09179	0	3.915692025	0	0	20	0.01464
LOC100519633	14.09179	0	3.915692025	0	0	20	0.01464
C15H2orf82	14.09179	0	3.915692025	0	0	20	0.01464
LOC102162647	14.09179	0	3.915692025	0	0	20	0.01464
LOC100521169	14.09179	0	3.915692025	0	0	20	0.01464
GSDMA	14.09179	0	3.915692025	0	0	20	0.01464
LOC102159404	19.0653629	0.8262784	4.326635347	0.868906686	0.868906686	4.528182	0.01467
LOC102164525	19.0653629	0.8262784	4.326635347	0.868906686	0.868906686	4.528182	0.01467
LOC102166555	19.0653629	0.8262784	4.326635347	0.868906686	0.868906686	4.528182	0.01467
LOC102160240	19.0653629	0.8262784	4.326635347	0.868906686	0.868906686	4.528182	0.01467
PNP	4205.15592	722.99358	12.03828662	9.499833085	9.499833085	2.540105	0.01468
ENG	472.48943	2706.8879	8.887188408	11.40295233	11.40295233	-2.51828	0.01469
LOC100525818	196.456131	1093.9926	7.625388355	10.09670536	10.09670536	-2.47732	0.01469
LOC100622725	3522.11857	609.79344	11.78263731	9.254540753	9.254540753	2.530051	0.0147
LOC102164603	217.179352	38.835083	7.769370763	5.315967693	5.315967693	2.483454	0.01477
LOC100623650	169.10148	29.746021	7.410251883	4.942327829	4.942327829	2.507121	0.0148
TFR2	70.45895	10.741619	6.159042811	3.553559423	3.553559423	2.713572	0.01487
SCUBE2	37.3017971	209.04843	5.259340178	7.714578179	7.714578179	-2.48652	0.01489
ASB15	32.3282241	3.3051135	5.058672541	2.106051269	2.106051269	3.290023	0.01491
LOC102162757	32.3282241	3.3051135	5.058672541	2.106051269	2.106051269	3.290023	0.01491
LOC100620843	32.3282241	3.3051135	5.058672541	2.106051269	2.106051269	3.290023	0.01491
RRAS	1193.65751	7079.5531	10.22238136	12.78964634	12.78964634	-2.56827	0.01497
LOC102164286	74.6035941	11.567897	6.240382915	3.65167138	3.65167138	2.689118	0.01497
LOC102160237	74.6035941	11.567897	6.240382915	3.65167138	3.65167138	2.689118	0.01497
DLGAP2	2725.51797	477.5889	11.41284395	8.902643123	8.902643123	2.512689	0.01498
LOC102159409	194.798274	34.703692	7.613224233	5.158001345	5.158001345	2.48882	0.01501
RBM38	1730.80338	308.20183	10.75805943	8.27240506	8.27240506	2.489495	0.01504
FAM111B	164.956836	911.38504	7.374664246	9.833498988	9.833498988	-2.46597	0.01507
LOC100622585	0	14.046732	0	3.911378308	3.911378308	-20	0.01508
FREM1	0	14.046732	0	3.911378308	3.911378308	-20	0.01508
LOC100739463	0	14.046732	0	3.911378308	3.911378308	-20	0.01508
LOC102165274	0	14.046732	0	3.911378308	3.911378308	-20	0.01508
LOC100738992	203.916491	1132.0014	7.678892279	10.14593389	10.14593389	-2.47283	0.01511
LOC100621844	0.82892882	19.004403	0.870998931	4.322245637	4.322245637	-4.51894	0.01516
LOC102161819	82.8928824	13.220454	6.39047651	3.829895615	3.829895615	2.648477	0.01517
LOC100739587	1915.65451	340.42669	10.90437459	8.415432027	8.415432027	2.492421	0.01517
LOC100518021	67.9721635	372.65155	6.107942317	8.54554968	8.54554968	-2.45481	0.01522
LOC100739813	330.742601	60.318321	8.373920477	5.938246292	5.938246292	2.455041	0.01525
MRVI1	9.11821706	61.970878	3.338883189	5.976612875	5.976612875	-2.76477	0.01529
GAS2L3	1327.11505	238.79445	10.37516441	7.905654454	7.905654454	2.474452	0.0153
PKD1L3	15.7496477	95.848291	4.066058842	6.597654688	6.597654688	-2.60543	0.01531
ITGA4	2960.93376	518.90282	11.53232366	9.022098163	9.022098163	2.512516	0.01532
MSRA	53.0514447	291.67627	5.756261274	8.193161944	8.193161944	-2.4589	0.01541
LOC102164843	40.6175124	4.9576702	5.379118827	2.574748269	2.574748269	3.034368	0.01546
NBL1	1345.35148	7981.8491	10.39483938	12.96268802	12.96268802	-2.56874	0.01548
CTNNA2	3.31571529	32.224857	2.109599694	5.054191063	5.054191063	-3.28078	0.0155
LOC102162973	3.31571529	32.224857	2.109599694	5.054191063	5.054191063	-3.28078	0.0155
SDC4	411.148697	2324.3211	8.687021122	11.18321421	11.18321421	-2.49908	0.01554
LOC102160850	48.9068006	6.610227	5.641164514	2.927939483	2.927939483	2.887263	0.0157
LOC102162222	107.760747	18.178124	6.765014156	4.261389712	4.261389712	2.567557	0.0157
STAT1	2054.08563	12381.781	11.00498279	13.59604779	13.59604779	-2.59165	0.01575
GAL3ST1	53.0514447	7.4365053	5.756261274	3.076645515	3.076645515	2.834695	0.01578
TET1	657.340557	120.63664	9.362690268	6.926434087	6.926434087	2.445973	0.01581
OMA1	896.072058	163.60312	9.809080066	7.362847851	7.362847851	2.453415	0.01582
GNAS	8859.59127	56922.317	13.11318726	15.79673211	15.79673211	-2.68368	0.01584
FRMD4A	32.3282241	180.95496	5.058672541	7.507437596	7.507437596	-2.48476	0.01588
EPB41L4B	61.340733	9.0890621	5.962103212	3.334720158	3.334720158	2.754642	0.0159
C8H4orf29	493.21265	90.890621	8.948988129	6.521845711	6.521845711	2.440006	0.01601
LOC102162607	73.7746653	11.567897	6.224477643	3.65167138	3.65167138	2.672999	0.01606
LOC102161732	664.800917	122.2892	9.378947046	6.945902605	6.945902605	2.442625	0.01607
LOC100621128	4.97357294	40.48764	2.578594101	5.374609695	5.374609695	-3.02513	0.01609
CES3	240.389359	43.792754	7.915218269	5.485193456	5.485193456	2.456609	0.0161
LOC102163227	77.9193094	12.394176	6.302306427	3.74353388	3.74353388	2.652319	0.01611
RSPH6A	82.0639535	13.220454	6.376150635	3.829895615	3.829895615	2.633977	0.01616
SULT1C4	72.9457365	395.78734	6.208395063	8.632222186	8.632222186	-2.43983	0.01623
CDKN2B	146.720402	799.83746	7.206725282	9.645365656	9.645365656	-2.44664	0.01624
LOC100518456	5.80250177	44.619032	2.766065426	5.511563931	5.511563931	-2.94291	0.01625
NPM2	90.3532418	14.873011	6.51338402	3.98850389	3.98850389	2.60288	0.01627
LOC102164377	23.2100071	1.6525567	4.597531595	1.407383613	1.407383613	3.811975	0.01632
UOX	23.2100071	1.6525567	4.597531595	1.407383613	1.407383613	3.811975	0.01632
LOC102165355	23.2100071	1.6525567	4.597531595	1.407383613	1.407383613	3.811975	0.01632
XKRX	23.2100071	1.6525567	4.597531595	1.407383613	1.407383613	3.811975	0.01632
PIWIL4	213.863637	38.835083	7.747277532	5.315967693	5.315967693	2.461259	0.01633
CCDC170	6.63143059	48.750424	2.93195353	5.636636914	5.636636914	-2.87802	0.01637
LOC100624174	145.062544	25.61463	7.190442454	4.734147579	4.734147579	2.501635	0.01643
LOC100736859	639.933052	118.15781	9.324029859	6.89672967	6.89672967	2.437206	0.01644
NUDT18	137.602185	747.78193	7.114806188	9.548401802	9.548401802	-2.44211	0.01645
ADK	4826.02361	838.67255	12.23691817	9.71368301	9.71368301	2.524655	0.01651
LOC102160067	97.8136012	528.81816	6.62663773	9.049353478	9.049353478	-2.43466	0.01659
LOC102157897	9.11821706	61.1446	3.338883189	5.957557119	5.957557119	-2.7454	0.0166
LOC102158271	183.19327	33.051135	7.525076539	5.089630977	5.089630977	2.470595	0.01662
LOC100157413	1366.90363	248.70979	10.41775088	7.964108573	7.964108573	2.458376	0.01664
GRPEL2	1087.55462	199.13309	10.08819808	7.644815895	7.644815895	2.449283	0.01665
MRPS27	10453.6214	1763.278	13.3518532	10.78486223	10.78486223	2.567671	0.01671
TMEM37	123.510395	21.483238	6.96012238	4.490777898	4.490777898	2.523349	0.01673
BTN2A2	1132.31677	207.39587	10.14633545	7.703182885	7.703182885	2.448819	0.01681
KLF17	299.243305	55.360651	8.229988267	5.816616368	5.816616368	2.434386	0.01696
AARS2	3706.9697	653.58619	11.85641374	9.35443936	9.35443936	2.503791	0.01701
LOC102166211	27.3546512	2.4788351	4.825513503	1.798604302	1.798604302	3.464052	0.01705
LIPI	27.3546512	2.4788351	4.825513503	1.798604302	1.798604302	3.464052	0.01705
SELRC1	1366.0747	249.53607	10.41687636	7.968874505	7.968874505	2.452716	0.0171
TM7SF2	169.10148	917.16899	7.410251883	9.842615902	9.842615902	-2.4393	0.01714
LOC100624822	1068.48925	196.65425	10.06270627	7.626835185	7.626835185	2.441839	0.0172
LYRM1	102.787174	552.78023	6.697484358	9.113169742	9.113169742	-2.42705	0.01722
42614	85.3796688	14.046732	6.43261988	3.911378308	3.911378308	2.603658	0.01722
UMODL1	72.9457365	11.567897	6.208395063	3.65167138	3.65167138	2.656697	0.01722
LOC100511417	89.524313	14.873011	6.500233417	3.98850389	3.98850389	2.589583	0.01723
ANO10	218.008281	1189.0146	7.774841608	10.21676353	10.21676353	-2.44731	0.01724
TAF1B	2799.29264	499.89841	11.45136188	8.968374236	8.968374236	2.485355	0.01728
LOC102164310	60.5118041	9.0890621	5.942791385	3.334720158	3.334720158	2.735013	0.01729
LRRN2	263.599366	48.750424	8.047665794	5.636636914	5.636636914	2.43486	0.01732
RINL	19.0653629	110.7213	4.326635347	6.803760479	6.803760479	-2.53791	0.01733
LOC102162301	31.4992953	3.3051135	5.022336531	2.106051269	2.106051269	3.252548	0.01735
LOC100737267	31.4992953	3.3051135	5.022336531	2.106051269	2.106051269	3.252548	0.01735
LOC100038017	31.4992953	3.3051135	5.022336531	2.106051269	2.106051269	3.252548	0.01735
CD72	52.2225159	7.4365053	5.733964805	3.076645515	3.076645515	2.811975	0.01736
DAPK1	1016.26674	187.56519	9.990482303	7.558919565	7.558919565	2.437815	0.01739
FGD6	792.455955	147.07755	9.632006331	7.210209122	7.210209122	2.429754	0.01741
LRRN4	43.9332277	5.7839486	5.489710792	2.762125239	2.762125239	2.925186	0.01745
SLC2A11	97.8136012	524.68677	6.62663773	9.038059606	9.038059606	-2.42335	0.01748
LOC102167252	39.7885835	4.9576702	5.350093502	2.574748269	2.574748269	3.00462	0.01748
LOC100525876	11122.567	1883.0884	13.44133187	10.87965095	10.87965095	2.562317	0.01751
LOC102159265	43.1042988	233.83678	5.462847376	7.875514565	7.875514565	-2.4396	0.0176
TNNC2	181.535412	33.051135	7.512032568	5.089630977	5.089630977	2.457479	0.01762
LOC100738431	215.521494	39.661362	7.758366438	5.34558663	5.34558663	2.442026	0.01768
LOC102158399	147.549331	26.440908	7.214798294	4.77825631	4.77825631	2.480354	0.01774
LOC100737465	419.437985	78.496445	8.715749206	6.312818447	6.312818447	2.417758	0.01775
FOXA1	193.969345	35.52997	7.607103495	5.191008663	5.191008663	2.44872	0.01782
ERAP1	736.088795	4171.0532	9.525694618	12.02654185	12.02654185	-2.50246	0.01787
GRIA4	26.5257224	147.90383	4.78270852	7.218237038	7.218237038	-2.4792	0.01794
TMEM9B	1008.80638	5788.08	9.979862979	12.49911838	12.49911838	-2.52044	0.01795
PCP4L1	528.027661	2949.8138	9.047199346	11.52689717	11.52689717	-2.48194	0.01799
LOC100521743	9.94714588	64.449713	3.452482877	6.032314961	6.032314961	-2.69582	0.01802
LOC100738657	3.31571529	31.398578	2.109599694	5.017858594	5.017858594	-3.24331	0.01802
LOC100738884	8.28928824	56.186929	3.215568059	5.837613535	5.837613535	-2.76091	0.01807
HES1	172.417195	927.91061	7.438103147	9.859395963	9.859395963	-2.42808	0.01811
LOC100513627	1535.17618	281.76092	10.58512797	8.143438955	8.143438955	2.445861	0.01812
LOC102164820	6.63143059	47.924146	2.93195353	5.61247475	5.61247475	-2.85336	0.01814
LOC100738599	5.80250177	43.792754	2.766065426	5.485193456	5.485193456	-2.91595	0.01817
CLVS2	4.97357294	39.661362	2.578594101	5.34558663	5.34558663	-2.99538	0.01818
LOC100737927	105.273961	18.178124	6.731644339	4.261389712	4.261389712	2.533873	0.0182
LYPD4	1027.04281	190.8703	10.00568463	7.58398763	7.58398763	2.427832	0.01827
LOC100517015	130.141825	23.135794	7.03498407	4.593102408	4.593102408	2.491886	0.01827
TCEAL4	60.5118041	323.07484	5.942791385	8.340183224	8.340183224	-2.41658	0.01832
LOC102167025	138.431114	24.788351	7.123408719	4.688647628	4.688647628	2.481434	0.01832
LRRC41	668.116632	3756.2615	9.386113895	11.87546581	11.87546581	-2.49113	0.01836
FAM54A	1276.55039	236.31561	10.31916448	7.890663216	7.890663216	2.433464	0.01851
FSBP	67.9721635	10.741619	6.107942317	3.553559423	3.553559423	2.661733	0.01857
LOC100627376	44.7621565	240.44701	5.516083133	7.915562765	7.915562765	-2.42537	0.01858
NCKIPSD	150.036117	801.49002	7.238749771	9.648339641	9.648339641	-2.41737	0.01865
CSF3R	18.2364341	0.8262784	4.265769485	0.868906686	0.868906686	4.464052	0.01865
LOC102157528	18.2364341	0.8262784	4.265769485	0.868906686	0.868906686	4.464052	0.01865
TCP11	63.8275194	9.9153405	6.018534465	3.448285225	3.448285225	2.686444	0.01867
LOC102161733	649.880198	122.2892	9.346248212	6.945902605	6.945902605	2.409877	0.0187
LOC102166969	59.6828753	9.0890621	5.92321754	3.334720158	3.334720158	2.715114	0.0188
ANKRD34A	872.862051	163.60312	9.771261742	7.362847851	7.362847851	2.415553	0.01887
FLNC	3566.88073	21473.322	11.80085167	14.39032498	14.39032498	-2.58981	0.01888
ATRNL1	1090.0414	203.26448	10.09149013	7.674294538	7.674294538	2.422953	0.0189
ZNF358	910.992777	5164.2398	9.832878588	12.33461962	12.33461962	-2.50304	0.01894
KCNA5	55.5382312	8.2627837	5.821154844	3.211445828	3.211445828	2.748781	0.01895
MGPD	6237.6894	1093.9926	12.60702727	10.09670536	10.09670536	2.511409	0.01901
SNAP91	19.0653629	109.06875	4.326635347	6.782261052	6.782261052	-2.51621	0.01905
LOC100627210	15.7496477	92.543178	4.066058842	6.547560532	6.547560532	-2.55481	0.01907
NUAK1	626.670191	3493.505	9.293862884	11.77087238	11.77087238	-2.4789	0.01912
CHST6	2163.50423	395.78734	11.0798209	8.632222186	8.632222186	2.450573	0.01919
METTL11B	0.82892882	18.178124	0.870998931	4.261389712	4.261389712	-4.45481	0.01926
LOC100512345	0.82892882	18.178124	0.870998931	4.261389712	4.261389712	-4.45481	0.01926
TMPRSS7	0.82892882	18.178124	0.870998931	4.261389712	4.261389712	-4.45481	0.01926
LOC102157527	11.6050035	71.886218	3.655924617	6.187574144	6.187574144	-2.63097	0.0193
LOC100624445	1396.74507	259.45141	10.44888554	8.02487043	8.02487043	2.428532	0.01931
PSAP	8456.73186	52725.649	13.04605511	15.68624469	15.68624469	-2.64033	0.01937
AHNAK	7997.50529	49721.301	12.96551471	15.60160544	15.60160544	-2.63624	0.01941
LOC100519435	151.693975	805.62141	7.254499324	9.655747894	9.655747894	-2.40894	0.01942
AVEN	67.9721635	358.60481	6.107942317	8.490268521	8.490268521	-2.39938	0.01959
LOC100627730	1676.92301	310.68067	10.71246081	8.283924863	8.283924863	2.432312	0.01974
SNX18	138.431114	732.08264	7.123408719	9.517832026	9.517832026	-2.40284	0.01974
LOC102159180	38.1307259	204.09076	5.290229968	7.68011867	7.68011867	-2.42018	0.0199
LOC100737841	7.46035941	51.229259	3.080718953	5.706786332	5.706786332	-2.77965	0.01992
LOC102159430	13.2628612	0	3.834191515	0	0	20	0.01993
LOC102168198	13.2628612	0	3.834191515	0	0	20	0.01993
LOC102161625	13.2628612	0	3.834191515	0	0	20	0.01993
LOC100524527	13.2628612	0	3.834191515	0	0	20	0.01993
LOC102164330	13.2628612	0	3.834191515	0	0	20	0.01993
LOC102164340	13.2628612	0	3.834191515	0	0	20	0.01993
LOC100738693	13.2628612	0	3.834191515	0	0	20	0.01993
LOC100736688	13.2628612	0	3.834191515	0	0	20	0.01993
ANKRD65	13.2628612	0	3.834191515	0	0	20	0.01993
LOC102166491	13.2628612	0	3.834191515	0	0	20	0.01993
LOC100627297	13.2628612	0	3.834191515	0	0	20	0.01993
TEX11	13.2628612	0	3.834191515	0	0	20	0.01993
LOC102158197	13.2628612	0	3.834191515	0	0	20	0.01993
LOC102162625	13.2628612	0	3.834191515	0	0	20	0.01993
LOC102162603	13.2628612	0	3.834191515	0	0	20	0.01993
LOC102163108	13.2628612	0	3.834191515	0	0	20	0.01993
PRSS42	13.2628612	0	3.834191515	0	0	20	0.01993
BIN2	13.2628612	0	3.834191515	0	0	20	0.01993
LOC102167228	13.2628612	0	3.834191515	0	0	20	0.01993
LOC100153663	13.2628612	0	3.834191515	0	0	20	0.01993
LOC100739539	13.2628612	0	3.834191515	0	0	20	0.01993
GPR151	13.2628612	0	3.834191515	0	0	20	0.01993
URB2	4864.15434	867.59229	12.24826986	9.762535337	9.762535337	2.4871	0.01996
HEXA	1633.81871	9426.1837	10.67491495	13.20261112	13.20261112	-2.52843	0.01996
KIF6	22.3810782	1.6525567	4.547269558	1.407383613	1.407383613	3.759508	0.01996
LOC100738196	22.3810782	1.6525567	4.547269558	1.407383613	1.407383613	3.759508	0.01996
LOC102158385	22.3810782	1.6525567	4.547269558	1.407383613	1.407383613	3.759508	0.01996

TMEM2	165.69718	891.55436	7.381085891	9.801796232		-2.42777	0.15161
CHAF1A	5995.47631	1221.2394	12.54989927	10.25531122		2.295528	0.15221
TECTA	59.3748229	310.68067	5.915875148	8.283924863		-2.38751	0.15247
C7H15orf60	40.0434852	210.70098	5.359081339	7.72588417		-2.39556	0.1527
SLC24A5	828.485901	184.26008	9.69607365	7.533408207		2.168734	0.15297
LOC100625660	22.0929574	120.63664	4.529381037	6.926434087		-2.44901	0.15306
GPR3	64.8980623	13.220454	6.042164139	3.829895615		2.295404	0.15328
LOC102167356	41.4242951	217.31121	5.406818783	7.770242414		-2.39121	0.15379
KLHL3	23.4737672	127.24687	4.613164285	7.002779797		-2.43851	0.15385
LOC102159595	13.8080984	0.8262784	3.888314478	0.868906686		4.062743	0.1539
LOC100525750	13.8080984	0.8262784	3.888314478	0.868906686		4.062743	0.1539
LOC100621936	13.8080984	0.8262784	3.888314478	0.868906686		4.062743	0.1539
FA2H	13.8080984	0.8262784	3.888314478	0.868906686		4.062743	0.1539
MYH6	34.5202459	5.7839486	5.150569664	2.762125239		2.577316	0.15416
FSIP1	13.8080984	80.149002	3.888314478	6.342501448		-2.53717	0.15435
LOC100622420	12.4272885	73.538775	3.747096094	6.219919206		-2.56499	0.15436
PLCB4	12.4272885	73.538775	3.747096094	6.219919206		-2.56499	0.15436
MED13	104.941548	551.95395	6.727124677	9.111015534		-2.39496	0.15466
QKI	269.257918	1476.5595	8.078193076	10.52900046		-2.45518	0.15476
TRO	9.66566885	60.318321	3.414902536	5.938246292		-2.64165	0.15509
LOC100153113	9.66566885	60.318321	3.414902536	5.938246292		-2.64165	0.15509
HOXC11	9.66566885	60.318321	3.414902536	5.938246292		-2.64165	0.15509
LOC100739690	75.944541	395.78734	6.265747069	8.632222186		-2.38171	0.15571
NCAPH	3782.03814	791.57468	11.88532961	9.630403069		2.256367	0.15618
AVEN	69.0404918	358.60481	6.130117309	8.490268521		-2.37688	0.15694
LOC102162673	6.90404918	47.097867	2.982591923	5.587901017		-2.77015	0.15728
GPR64	842.294	188.39147	9.719891879	7.565227535		2.16059	0.1577
MT1A	164.316371	37.182527	7.369085785	5.254840671		2.14378	0.15778
POLD2	3811.03515	799.01119	11.89634571	9.643876361		2.253895	0.15787
TRPS1	24.854577	133.03082	4.692347799	7.066420948		-2.42018	0.15801
EPHA2	805.012134	4693.2612	9.654657748	12.19668239		-2.54351	0.15837
MXRA7	227.833623	1230.3285	7.838155235	10.26599998		-2.43299	0.15849
PIR	251.30739	1363.3593	7.979038653	10.41400792		-2.43964	0.15886
BTBD11	270.638728	61.970878	8.08554537	5.976612875		2.126706	0.15895
KIAA0226L	42.8051049	222.26888	5.453027102	7.80263838		-2.37645	0.15895
RAD51	2641.48922	564.34813	11.36768187	9.142995708		2.226694	0.15915
LOC100736913	5.52323934	40.48764	2.705588563	5.374609695		-2.8739	0.1592
UBE3D	386.626754	88.411786	8.598524339	6.482393107		2.128631	0.15944
PADI1	16.569718	92.543178	4.135019133	6.547560532		-2.48158	0.15953
LOC100152564	15.1889082	85.932951	4.016933786	6.441831208		-2.50019	0.15959
GLIPR2	868.529387	5077.4806	9.764090976	12.31018121		-2.54747	0.15975
LOC102160007	13.8080984	79.322724	3.888314478	6.327736286		-2.52222	0.15983
LOC100525224	150.508272	794.87979	7.243252754	9.636406738		-2.40089	0.15989
LOC102157958	619.983616	3544.7342	9.278411395	11.79186868		-2.51538	0.1599
LOC100622075	463.952105	2604.4294	8.8609383	11.34730546		-2.48892	0.15991
PAIP2B	748.398931	168.56079	9.549590109	7.405658764		2.150539	0.16001
MANBA	57.9940131	299.11277	5.882496648	8.229360901		-2.36671	0.16054
ANTXR1	407.338902	2265.6553	8.673623206	11.14634929		-2.47563	0.16064
MKKS	3258.71121	690.76872	11.67052844	9.434145968		2.238027	0.16078
LOC102162694	51.0899639	263.5828	5.702933533	8.047575471		-2.36714	0.16081
C1QA	20.7121475	2.4788351	4.440430524	1.798604302		3.062743	0.16083
SNRPA1	6562.98915	1345.1812	12.68035714	10.39465689		2.286553	0.16102
TAC1	0	14.046732	0	3.911378308		-20	0.16113
LOC100622585	0	14.046732	0	3.911378308		-20	0.16113
LOC100518260	0	14.046732	0	3.911378308		-20	0.16113
LOC102168181	0	14.046732	0	3.911378308		-20	0.16113
LOC102157660	0	14.046732	0	3.911378308		-20	0.16113
LOC100738033	0	14.046732	0	3.911378308		-20	0.16113
LOC102157701	0	14.046732	0	3.911378308		-20	0.16113
LOC102160904	0	14.046732	0	3.911378308		-20	0.16113
LOC100621268	0	14.046732	0	3.911378308		-20	0.16113
FREM1	0	14.046732	0	3.911378308		-20	0.16113
LOC100512793	0	14.046732	0	3.911378308		-20	0.16113
CHRM3	0	14.046732	0	3.911378308		-20	0.16113
MIR199A-2	0	14.046732	0	3.911378308		-20	0.16113
LOC102158412	0	14.046732	0	3.911378308		-20	0.16113
LOC102158451	0	14.046732	0	3.911378308		-20	0.16113
LOC100623155	0	14.046732	0	3.911378308		-20	0.16113
LOC100521043	0	14.046732	0	3.911378308		-20	0.16113
MIR143	0	14.046732	0	3.911378308		-20	0.16113
MIR421	0	14.046732	0	3.911378308		-20	0.16113
LOC102165274	0	14.046732	0	3.911378308		-20	0.16113
LOC100512344	35.9010557	186.73891	5.205590187	7.552583893		-2.37892	0.16127
LOC102164723	118.749646	619.70878	6.90387758	9.27777274		-2.38367	0.16147
ABHD17C	5364.44621	1111.3444	12.38948244	10.11938784		2.271123	0.1617
MTHFD1	18183.8847	3546.3868	14.15045216	11.79254092		2.358239	0.16173
RPP25	180.886089	41.313919	7.506891393	5.403060391		2.130382	0.16175
LOC102161795	9.66566885	0	3.414902536	0		20	0.16188
LOC102159645	9.66566885	0	3.414902536	0		20	0.16188
KCNJ10	9.66566885	0	3.414902536	0		20	0.16188
LOC102159945	9.66566885	0	3.414902536	0		20	0.16188
LOC100626259	9.66566885	0	3.414902536	0		20	0.16188
MYL7	9.66566885	0	3.414902536	0		20	0.16188
LOC102166085	9.66566885	0	3.414902536	0		20	0.16188
LOC102158923	9.66566885	0	3.414902536	0		20	0.16188
LOC102158285	9.66566885	0	3.414902536	0		20	0.16188
LOC100519221	9.66566885	0	3.414902536	0		20	0.16188
FNDC7	9.66566885	0	3.414902536	0		20	0.16188
LOC100739779	9.66566885	0	3.414902536	0		20	0.16188
LOC100521899	9.66566885	0	3.414902536	0		20	0.16188
LCN5	9.66566885	0	3.414902536	0		20	0.16188
LOC102166221	9.66566885	59.492043	3.414902536	5.918673475		-2.62176	0.16232
LOC100737775	9.66566885	59.492043	3.414902536	5.918673475		-2.62176	0.16232
GPRASP1	392.149993	90.064343	8.61893602	6.508814353		2.122378	0.1628
ADAR	1466.42005	8834.5684	10.51906618	13.10910722		-2.59086	0.16284
IRF5	156.031511	35.52997	7.294910283	5.191008663		2.134729	0.16348
LOC100627730	1405.66441	310.68067	10.45806247	8.283924863		2.177748	0.16392
GLB1L	332.77517	1818.6387	8.382732826	10.8294363		-2.45024	0.16397
AQP1	53.8515836	10.741619	5.777461367	3.553559423		2.325777	0.1641
ABCA8	37.2818656	6.610227	5.258589232	2.927939483		2.495702	0.16419
KCNK1	2988.07249	638.71318	11.54548217	9.3212814		2.225975	0.16432
PRKCE	22.0929574	118.15781	4.529381037	6.89627967		-2.41906	0.16435
LOC102161584	23.4737672	124.76803	4.613164285	6.974621475		-2.41013	0.1646
LOC102164599	190.551757	43.792754	7.581590451	5.485193456		2.121419	0.16503
LOC100738858	2.76161967	27.267186	1.911353989	4.821056379		-3.30358	0.16512
IKZF4	26.2353869	137.98849	4.767410456	7.118821585		-2.39496	0.1653
RAD51AP1	2444.03341	527.99188	11.25563846	9.047101766		2.210676	0.16532
SNAP25	13.8080984	78.496445	3.888314478	6.312818447		-2.50711	0.16551
LOC100736851	13.8080984	78.496445	3.888314478	6.312818447		-2.50711	0.16551
LOC100518698	996.944701	5825.2625	9.962816064	12.50835499		-2.54674	0.16574
LOC100620342	6.90404918	46.271589	2.982591923	5.56290145		-2.74461	0.16651
ZDBF2	651.742243	148.73011	9.350369597	7.226220529		2.131605	0.16686
BAHCC1	593.748229	3346.4274	9.216135261	11.70883705		-2.4947	0.16702
LOC100154532	352.106508	1920.2709	8.463959601	10.90784527		-2.44723	0.1673
LOC100620364	38.6626754	198.30681	5.309710091	7.63884719		-2.35872	0.16765
LOC102161984	1.38080984	20.656959	1.251452392	4.436758793		-3.90304	0.1678
LOC102161567	1.38080984	20.656959	1.251452392	4.436758793		-3.90304	0.1678
SPHKAP	1.38080984	20.656959	1.251452392	4.436758793		-3.90304	0.1678
LOC102160315	1.38080984	20.656959	1.251452392	4.436758793		-3.90304	0.1678
LOC102165987	46.9475344	9.0890621	5.583384725	3.334720158		2.368846	0.16789
PHACTR2	117.368836	605.66205	6.88714549	9.244749247		-2.36746	0.16789
LOC102158339	11.0464787	65.275991	3.590539588	6.05041444		-2.56297	0.16791
AD014	9128.53382	1855.8212	13.15632548	10.8586192		2.298325	0.16806
LOC100737528	7370.7629	1514.5683	12.84779396	10.56564311		2.282907	0.16812
ATF6	167.07799	874.20252	7.392987005	9.773473077		-2.38745	0.16821
GPRIN1	207.121475	47.924146	7.70128203	5.61247475		2.111653	0.1685
NDUFAF4	1046.65386	235.48934	10.03294641	7.885631319		2.152051	0.1687
CST3	2486.83851	15387.782	11.28067713	13.90959144		-2.6294	0.16877
METTL23	4744.4626	997.31799	12.21233302	9.963355621		2.250119	0.169
LOC100522842	103.560738	23.135794	6.708197415	4.593102408		2.162279	0.1691
LOC102160374	103.560738	23.135794	6.708197415	4.593102408		2.162279	0.1691
GM2A	632.410905	3566.2175	9.306997895	11.80058345		-2.49546	0.16924
tRNA-Lys	5060.66805	1060.9414	12.30539718	10.05248848		2.253983	0.16942
RASGEF1B	16.569718	90.890621	4.135019133	6.521845711		-2.45558	0.16945
EFCAB5	16.569718	90.890621	4.135019133	6.521845711		-2.45558	0.16945
LOC100623278	16.569718	90.890621	4.135019133	6.521845711		-2.45558	0.16945
TBX2	626.887665	3531.5138	9.294362661	11.78647947		-2.49401	0.16952
LOC102160080	9.66566885	58.665764	3.414902536	5.89883146		-2.60158	0.16987
LOC102162069	30.3778164	4.9576702	4.971673052	2.574748269		2.615284	0.17001
LOC102167417	30.3778164	4.9576702	4.971673052	2.574748269		2.615284	0.17001
LOC100627036	976.232554	220.61633	9.932558114	7.791920351		2.145685	0.17013
MSH6	5436.24832	1137.7853	12.408661	10.15328008		2.256383	0.17104
ROCK2	818.820233	4677.5619	9.679163785	12.19184941		-2.51414	0.17178
STS	22.0929574	116.50525	4.529381037	6.876581411		-2.39874	0.17233
LOC100152428	67.659682	342.07925	6.101391269	8.422398044		-2.33796	0.17255
LOC102159978	8.28485901	52.055537	3.214880004	5.729431428		-2.6515	0.17257
PRKX	342.440839	1848.3847	8.423917793	10.85282966		-2.43234	0.17271
NEIL2	361.772177	84.280394	8.502920002	6.414142197		2.101812	0.17283
CXCL12	109.083977	556.91162	6.782460686	9.123892796		-2.35201	0.17288
UPK1B	244.403341	57.013208	7.93901108	5.858309486		2.099896	0.17289
LOC102157934	12.4272885	71.05994	3.747096094	6.171125545		-2.51552	0.17292
LOC100623982	40.0434852	7.4365053	5.359081339	3.076645515		2.428871	0.17314
NEURL	33.1394361	169.38707	5.093367322	7.412672017		-2.3537	0.17339
NOVA2	66.2788721	14.046732	6.072081615	3.911378308		2.238314	0.1734
LRCH2	2468.88799	538.7335	11.2702299	9.07610342		2.196218	0.17423
AMIGO2	104.941548	533.77583	6.727124677	9.062790447		-2.34665	0.17441
PDE10A	99.4183082	22.309516	6.649878514	4.542847146		2.155852	0.17457
LOC100514971	4.14242951	33.051135	2.362450112	5.089630977		-2.99615	0.17471
C6H19orf40	741.494882	170.21334	9.536237271	7.419651342		2.123093	0.1749
LOC102161361	389.388374	90.890621	8.608766278	6.521845711		2.099006	0.17515
CHEK1	1458.13519	326.37996	10.51089784	8.354822188		2.1595	0.17572
UNC93B1	545.419885	3015.0898	9.093866176	11.55846366		-2.46676	0.17587
MAP4K3	63.5172524	318.94345	6.011613095	8.321673128		-2.32808	0.17642
LOC102157989	64.8980623	325.55368	6.042164139	8.35117635		-2.32665	0.1771
LOC100626222	23.4737672	3.3051135	4.613164285	2.106051269		2.828278	0.17766
ASB15	23.4737672	3.3051135	4.613164285	2.106051269		2.828278	0.17766
LOC100623841	1462.27762	328.03251	10.51498779	8.362086342		2.156307	0.17771
NPC1	610.317947	3388.5676	9.255779113	11.72688553		-2.47304	0.17775
LOC100516109	219.548764	1146.0481	7.784953864	10.16371018		-2.38405	0.17807
PIK3IP1	233.356862	1222.0657	7.872563229	10.2562862		-2.38871	0.17808
LCTL	675.21601	156.16661	9.401340363	7.296150961		2.112263	0.17857
C1QTNF2	236.118482	1236.1124	7.889464306	10.27276092		-2.38823	0.1787
LOC100514456	42.8051049	214.83238	5.453027102	7.753767487		-2.32736	0.17877
LOC102162786	69.0404918	14.873011	6.130117309	3.98850389		2.214746	0.17896
UBE2C	10115.8129	2071.4799	13.30446724	11.01714238		2.287879	0.1792
MCM2	16297.6985	3261.3207	13.99246914	11.67168291		2.32114	0.18004
NPAS3	2.76161967	26.440908	1.911353989	4.77825631		-3.25919	0.1811
TOX3	2.76161967	26.440908	1.911353989	4.77825631		-3.25919	0.1811
LOC102166111	2.76161967	26.440908	1.911353989	4.77825631		-3.25919	0.1811
KLHL35	2.76161967	26.440908	1.911353989	4.77825631		-3.25919	0.1811
PRKCG	214.025525	50.402981	7.748364114	5.683780114		2.086202	0.18113
MTTP	42.8051049	8.2627837	5.453027102	3.211445828		2.373083	0.18121
LOC102161469	5.52323934	38.835083	2.705588563	5.315967693		-2.81377	0.18141
LOC100738904	5.52323934	38.835083	2.705588563	5.315967693		-2.81377	0.18141
LOC100510989	5.52323934	38.835083	2.705588563	5.315967693		-2.81377	0.18141
LOC100624238	8.28485901	51.229259	3.214880004	5.706786332		-2.62842	0.18151
ADCY5	8.28485901	51.229259	3.214880004	5.706786332		-2.62842	0.18151
MBD5	15.1889082	82.627837	4.016933786	6.385911347		-2.44361	0.18161
HOXB2	156.031511	796.53235	7.294910283	9.63939923		-2.3519	0.18164
FSBP	52.4707738	10.741619	5.740678649	3.553559423		2.288303	0.18194
HAT1	5989.95307	1263.3796	12.54856982	10.30421398		2.245256	0.18227
LOC100155089	11.0464787	63.623435	3.590539588	6.013985524		-2.52597	0.18235
LOC102163816	66.2788721	329.68507	6.072081615	8.369314102		-2.31447	0.18241
LOC100515902	302.397354	1595.5435	8.245064693	10.64073618		-2.39953	0.18262
JAKMIP1	104.941548	23.962073	6.727124677	4.641665831		2.130761	0.18287
LOC102164351	26.2353869	133.8571	4.767410456	7.075287628		-2.35111	0.18303
LOC100049690	49.7091541	247.05723	5.664174303	7.954529215		-2.31326	0.18314
TMEM176A	230.595243	54.534373	7.855461808	5.795309087		2.080125	0.18367
AP1AR	2307.33324	510.64003	11.17263579	8.998985343		2.175848	0.18386
LOC100517259	70.4213016	349.51575	6.158282521	8.453335466		-2.31127	0.18418
DNAJC15	4012.63338	865.11346	11.97069313	9.758412211		2.213588	0.18423
LOC102161992	71.8021115	15.699289	6.185908388	4.061714779		2.193327	0.18423
LOC100512496	513.661259	120.63664	9.007479378	6.926434087		2.090149	0.18476
ATP6V0A4	16.569718	1.6525567	4.135019133	1.407383613		3.325777	0.18489
SERPINB10	16.569718	1.6525567	4.135019133	1.407383613		3.325777	0.18489
LOC100525661	16.569718	1.6525567	4.135019133	1.407383613		3.325777	0.18489
LOC100524609	16.569718	1.6525567	4.135019133	1.407383613		3.325777	0.18489
FAM184B	16.569718	1.6525567	4.135019133	1.407383613		3.325777	0.18489
METRN	16.569718	1.6525567	4.135019133	1.407383613		3.325777	0.18489
LOC102160785	16.569718	1.6525567	4.135019133	1.407383613		3.325777	0.18489
CCDC155	16.569718	1.6525567	4.135019133	1.407383613		3.325777	0.18489
CSMD1	16.569718	1.6525567	4.135019133	1.407383613		3.325777	0.18489
LOC100521603	240.260911	1246.8541	7.914450382	10.28523351		-2.37562	0.18497
RGS9BP	114.607216	26.440908	6.853087646	4.77825631		2.115854	0.18508
FKBP4	24306.3955	4795.7197	14.5691077	12.22783241		2.341517	0.18572
LOC100511711	6.90404918	44.619032	2.982591923	5.511563931		-2.69214	0.1866
LOC100523888	6.90404918	44.619032	2.982591923	5.511563931		-2.69214	0.1866
LOC100523736	6.90404918	44.619032	2.982591923	5.511563931		-2.69214	0.1866
LOC100739623	6.90404918	44.619032	2.982591923	5.511563931		-2.69214	0.1866
LOC102163158	330.013551	1738.4897	8.370746468	10.76444842		-2.39724	0.18706
GTDC1	62.1364426	306.54928	5.98040107	8.264673769		-2.30261	0.18731
EIF4E3	57.9940131	285.89232	5.882496648	8.164365521		-2.3015	0.1877
LOC100737652	4709.94235	1011.3647	12.20179996	9.983513431		2.219406	0.18779
UPK3A	164.316371	38.835083	7.369085785	5.315967693		2.081044	0.18867
LOC102161019	1.38080984	19.830681	1.251452392	4.380638094		-3.84415	0.18871
LOC100511883	1.38080984	19.830681	1.251452392	4.380638094		-3.84415	0.18871
LOC100624490	1.38080984	19.830681	1.251452392	4.380638094		-3.84415	0.18871
LOC100517727	1.38080984	19.830681	1.251452392	4.380638094		-3.84415	0.18871
TMEM249	208.502285	49.576702	7.71082217	5.660401068		2.072329	0.18882
ATG9B	201.598236	47.924146	7.662477803	5.61247475		2.072658	0.18908
ZNF84	57.9940131	285.06604	5.882496648	8.160204421		-2.29732	0.18954
OMA1	698.689777	163.60312	9.450571603	7.362847851		2.094452	0.18994
LOC102159195	0	13.220454	0	3.829895615		-20	0.18999
NTRK3	0	13.220454	0	3.829895615		-20	0.18999
MMP3	0	13.220454	0	3.829895615		-20	0.18999
ADAMTS16	0	13.220454	0	3.829895615		-20	0.18999
LOC102161372	0	13.220454	0	3.829895615		-20	0.18999
LOC102157665	0	13.220454	0	3.829895615		-20	0.18999
IL1B	0	13.220454	0	3.829895615		-20	0.18999
LOC102161112	0	13.220454	0	3.829895615		-20	0.18999
LOC102163782	0	13.220454	0	3.829895615		-20	0.18999
LOC102163797	0	13.220454	0	3.829895615		-20	0.18999
GLDN	0	13.220454	0	3.829895615		-20	0.18999
LOC102166837	0	13.220454	0	3.829895615		-20	0.18999
LOC102162190	0	13.220454	0	3.829895615		-20	0.18999
LOC100516455	0	13.220454	0	3.829895615		-20	0.18999
LOC100624063	0	13.220454	0	3.829895615		-20	0.18999
SEZ6L	0	13.220454	0	3.829895615		-20	0.18999
RDH5	0	13.220454	0	3.829895615		-20	0.18999
VGLL2	0	13.220454	0	3.829895615		-20	0.18999
LOC102166212	0	13.220454	0	3.829895615		-20	0.18999
LOC102158036	0	13.220454	0	3.829895615		-20	0.18999
IL1R2	0	13.220454	0	3.829895615		-20	0.18999
LOC102160085	0	13.220454	0	3.829895615		-20	0.18999
GPR39	0	13.220454	0	3.829895615		-20	0.18999
LOC102164940	0	13.220454	0	3.829895615		-20	0.18999
FGF7	0	13.220454	0	3.829895615		-20	0.18999
CLEC18A	0	13.220454	0	3.829895615		-20	0.18999
LOC102158312	0	13.220454	0	3.829895615		-20	0.18999
LOC100158146	0	13.220454	0	3.829895615		-20	0.18999
LOC102167273	0	13.220454	0	3.829895615		-20	0.18999
LYPD5	0	13.220454	0	3.829895615		-20	0.18999
RIMBP2	0	13.220454	0	3.829895615		-20	0.18999
LRRC34	0	13.220454	0	3.829895615		-20	0.18999
LOC102165714	238.880102	57.013208	7.906169679	5.858309486		2.066919	0.19062
LOC102164154	346.583269	82.627837	8.441214828	6.385911347		2.068502	0.19085
LOC100513188	8.28485901	50.402981	3.214880004	5.683780114		-2.60496	0.19089
KIAA1239	26.2353869	4.1313919	4.767410456	2.359350201		2.666814	0.1909
EXOSC6	1862.71247	419.74941	10.86396358	8.716817448		2.149805	0.1912
C14H1orf198	1025.94171	5776.5121	10.00413858	12.49623266		-2.49325	0.19159
DBR1	898.907203	209.04843	9.813632431	7.714578179		2.104335	0.19181
LRRC27	66.2788721	324.7274	6.072081615	8.347521274		-2.29261	0.19202
SLA-11	1086.69734	6136.7695	10.08706146	12.58349874		-2.49753	0.19225
PPP3CA	191.932567	973.35592	7.591952882	9.92830506		-2.34237	0.19241
PML	2270.05137	13479.905	11.14914462	13.71862977		-2.57001	0.19252
LOC100739807	1117.07516	257.79885	10.12680145	8.015687408		2.115409	0.19261
AP5B1	2301.81	514.77143	11.16917966	9.010588038		2.160765	0.19325

TTC21B	2025.90001	297.46021	10.9850592	8.221394816		2.767794	0.07268
MYO1C	2279.666191	20554.501	11.15523959	14.3272369		-3.17256	0.07274
LOC102159206	3.17207726	36.356248	2.060765874	5.223277669		-3.5187	0.07278
LOC100739011	206.1850219	32.224856	7.694775899	5.054191063		2.677694	0.07278
DTD2	4306.62356	607.3146	12.07267646	9.248673828		2.826041	0.07285
LOC100516678	47.5811589	343.7318	5.602325001	8.429330588		-2.85282	0.07293
LOC102160168	200.8982264	31.398578	7.657484427	5.017858594		2.677694	0.07298
LOC102163743	2996.555651	431.31731	11.54957082	8.755946793		2.796484	0.07299
LOC102167547	15.8603863	119.81036	4.075565686	6.916600414		-2.91725	0.07316
LOC100625160	25.37661808	185.08636	4.721187694	7.539828464		-2.86663	0.07326
LOC100515119	25.37661808	185.08636	4.721187694	7.539828464		-2.86663	0.07326
AMDHD2	634.4154519	5211.3377	9.311556364	12.34774484		-3.03815	0.07337
LOC102164300	823.6827284	126.42059	9.687695383	6.993454622		2.703857	0.07345
PCDHGC3	741.2087197	6150.8162	9.53568114	12.58679668		-3.05283	0.07356
LOC102161817	12.68830904	98.327126	3.774872332	6.634115866		-2.95409	0.07359
LRP1	3352.885664	30988.744	11.71161779	14.91950322		-3.20827	0.0736
L3MBTL1	10.57359087	84.280394	3.532764645	6.414142197		-2.99473	0.07362
LOC102164986	4.229436346	42.966475	2.386655454	5.458331975		-3.34467	0.07382
ACTR1B	384.8787075	3050.6197	8.592003629	11.57535949		-2.98663	0.07386
PLOD3	590.0063703	4813.0715	9.207029871	12.23304186		-3.02816	0.0739
LRR1	951.6231779	145.42499	9.895761841	7.194018019		2.710115	0.07396
RAD51AP1	3699.699444	527.99188	11.85358226	9.047101766		2.80882	0.07402
LOC100524518	29.60605442	214.0061	4.935745167	7.74823377		-2.85369	0.07407
LOC102163107	262.2250535	41.313919	8.040153	5.403060391		2.666106	0.07407
LOC100515297	373.2477576	2949.8138	8.547849862	11.52689717		-2.98242	0.0741
RFX1	293.9458261	2286.3123	8.204306183	11.15943761		-2.9594	0.07423
BRCA2	1275.175058	192.52286	10.31761053	7.59636019		2.727594	0.07429
DTNA	20.08982264	147.90383	4.398475058	7.218237038		-2.88012	0.0744
C18H7orf41	204.0703037	1550.0982	7.67997478	10.59907433		-2.92522	0.07457
LOC102164114	190.3246356	1438.5506	7.579878842	10.49140283		-2.91808	0.07481
ID4	158.603863	1186.5357	7.31835176	10.21375522		-2.90326	0.07483
ENO3	228.3895627	1743.4474	7.84165594	10.76855435		-2.93237	0.07506
LOC100623650	189.2672765	29.746021	7.571883648	4.942327829		2.669656	0.0751
ADCY6	327.7813168	2558.9841	8.360984509	11.32191914		-2.96477	0.07514
ADK	6018.487921	838.67255	12.55542505	9.71368301		2.843222	0.07527
MYCBPAP	355.2726531	56.186929	8.476837939	5.837613535		2.66062	0.07556
MAP3K9	158.603863	24.788351	7.31835176	4.688647628		2.677694	0.07558
EPB41L5	1138.775736	173.51846	10.15453427	7.447235822		2.714323	0.0756
LOC102164436	392.2802211	61.970878	8.619413822	5.976612875		2.662222	0.07566
SGOL2	4577.307486	648.62852	12.16059864	9.343471165		2.819035	0.07569
HNMT	65.55626337	470.97867	6.056502534	8.882577857		-2.84486	0.0758
LOC100739491	328.8386759	2560.6367	8.365616765	11.32285015		-2.96105	0.07592
LRRC46	10.57359087	83.454116	3.532764645	6.400095824		-2.98052	0.07614
C1H9orf40	3651.060926	524.68677	11.83449512	9.038059606		2.798787	0.07616
LOC102161736	31.7207726	3.3051135	5.032134908	2.106051269		3.262656	0.07624
LOC100622811	375.3624757	59.492043	8.555978985	5.918673475		2.657516	0.07647
LOC100625119	68.72834063	492.46191	6.123673244	8.946794917		-2.84104	0.07686
SIGLEC15	22.20454082	1.6525567	4.536335244	1.407383614		3.748083	0.07697
LOC102162589	22.20454082	1.6525567	4.536335244	1.407383614		3.748083	0.07697
LOC100521004	86.7034451	625.49273	6.45456161	9.291153952		-2.85083	0.07708
LOC100736879	127.9404495	19.830681	7.010561107	4.380638094		2.689666	0.07709
LOC102159626	1.057359087	22.309516	1.04079362	4.542847146		-4.39912	0.07716
CDH12	1.057359087	22.309516	1.04079362	4.542847146		-4.39912	0.07716
LOC100513566	1.057359087	22.309516	1.04079362	4.542847146		-4.39912	0.07716
LOC100738882	637.5875292	99.979683	9.318740569	6.657921243		2.672917	0.07736
PANK3	1629.390352	245.40468	10.6710017	7.944885826		2.731098	0.0775
LOC100624767	6446.718351	900.64343	12.65457301	9.81641319		2.839537	0.07756
LOC102161615	796.1913922	123.94176	9.638782322	6.965111899		2.683453	0.07765
SPSB2	56.04003159	398.26618	5.833902876	8.641207045		-2.8292	0.07786
LOC100517135	2457.302517	362.73621	11.26344675	8.506748724		2.760083	0.07796
LOC100515156	253.7661808	40.48764	7.993029968	5.374609695		2.647946	0.07798
CUEDC1	319.3224441	2463.1358	8.323381077	11.26686607		-2.94741	0.07806
LOC102159624	218.8733309	1648.4254	7.780528816	10.68774777		-2.91292	0.07812
LOC100623976	157.5465039	1164.2262	7.308762255	10.18639436		-2.88552	0.07815
LOC102163857	2.114718173	28.919743	1.639101631	4.903025878		-3.77352	0.07818
FKBP10	1288.920727	10941.578	10.33306669	13.41766507		-3.08559	0.0783
DCP2	1856.722556	278.45581	10.85931934	8.126476366		2.737238	0.07833
LOC100516635	3.17207726	35.52997	2.060765874	5.191008663		-3.48554	0.07849
HDDC2	2879.188793	422.22825	11.49194767	8.725292112		2.769567	0.07867
LOC100522069	17.97510447	131.37826	4.246035924	7.048522415		-2.86965	0.07867
LOC102159762	4.229436346	42.140197	2.386655454	5.430960858		-3.31666	0.07872
KDELC2	267.5118489	2035.1236	8.068841943	10.99160945		-2.92744	0.07879
SIPA1	171.292172	1268.3373	7.428713345	10.30985977		-2.88841	0.0789
PLXNA3	304.5194169	2333.4101	8.255120262	11.18884233		-2.93783	0.07909
LOC100623803	53.92531342	380.91433	5.779399292	8.57710524		-2.82043	0.07938
LOC100737524	6.344154519	55.360651	2.876596413	5.816616368		-3.12536	0.0794
ZNRF1	111.0227041	802.3163	6.807647348	9.649824338		-2.85332	0.07949
PPP4R4	51.81059524	365.21504	5.722755497	8.516547233		-2.81743	0.0798
LOC100523364	7.401513606	61.970878	3.070649265	5.976612875		-3.0657	0.07985
SSFA2	7.401513606	61.970878	3.070649265	5.976612875		-3.0657	0.07985
LOC100520306	616.4403475	97.500848	9.270155951	6.622064238		2.660475	0.07987
WNT11	78.24457241	11.567897	6.308240221	3.651671381		2.757864	0.0803
RSPO2	68.72834063	9.9153405	6.123673244	3.448285225		2.793171	0.08033
CDK2AP2	392.2802211	3043.1832	8.619413822	11.57183949		-2.95562	0.08036
ARL6IP5	670.3656609	5394.7715	9.390954938	12.39761354		-3.00854	0.08038
IPO11	767.6426969	120.63664	9.586169308	6.926434087		2.669767	0.08039
PARD3B	25.37661808	180.12869	4.721187694	7.500871231		-2.82746	0.08053
KCNT2	1648.422816	250.36235	10.68774555	7.973624744		2.718997	0.08065
C6H1orf174	132.1698858	959.30919	7.057124067	9.907355173		-2.8596	0.08066
MSH6	8185.016689	1137.7853	12.99894589	10.15328008		2.846757	0.08077
LOC100627867	62.38418611	439.58009	5.986051038	8.783260503		-2.81687	0.08087
LOC102161342	9.516231779	75.191332	3.394545939	6.251554969		-2.9821	0.0809
LOC100626534	242.1352308	1816.9861	7.925615148	10.82812548		-2.90766	0.08097
FCHO1	13.74566813	102.45852	3.882219287	6.692908622		-2.89799	0.08106
NUP153	14864.35404	1997.1148	13.8596662	10.96442378		2.895868	0.08107
CCDC99	2415.008154	360.25737	11.23840961	8.49688321		2.744928	0.08138
MTO1	5500.381968	784.13817	12.42557836	9.616802763		2.810352	0.08162
LOC100518170	150.1449903	1093.1663	7.239789351	10.09561629		-2.86408	0.08169
LOC100739280	730.6351288	115.67897	9.514980536	6.866400771		2.659024	0.08232
MSH4	26.43397716	2.4788351	4.777891883	1.798604302		3.414659	0.08241
LOC100521034	26.43397716	2.4788351	4.777891883	1.798604302		3.414659	0.08241
LOC102167407	26.43397716	2.4788351	4.777891883	1.798604302		3.414659	0.08241
LOC100518476	78.24457241	551.95395	6.308240221	9.111015534		-2.81849	0.08242
UNC13D	15.8603863	115.67897	4.075565686	6.866400771		-2.86663	0.08254
LOC100739554	150.1449903	23.962073	7.239789351	4.641665831		2.647532	0.08263
TMEM8A	396.5096575	3052.2723	8.634846101	11.57614055		-2.94446	0.08289
THSD7A	430.3451482	69.407383	8.752698918	6.137654819		2.632333	0.08307
LOC100622776	804.6502649	127.24687	9.654009886	7.002779797		2.660732	0.08324
LOC100739399	249.5367444	40.48764	7.968878398	5.374609695		2.623699	0.0834
LOC100524254	1496.163107	12622.228	10.54801569	13.62379331		-3.07663	0.08345
GPC4	407.0832483	3130.7688	8.672719679	11.61276197		-2.94312	0.08372
LOC100623125	727.4630516	115.67897	9.508711989	6.866400771		2.652747	0.0838
LOC100621472	814.1664967	128.89943	9.670950948	7.021251245		2.659078	0.08382
JARID2	7704.975664	1084.0772	12.91176191	10.08358201		2.829323	0.08402
LOC100627610	1711.864361	261.93024	10.7421952	8.038536289		2.708314	0.08404
MEF2D	192.4393538	1408.8046	7.59573752	10.46127953		-2.872	0.08422
MINK1	597.4078839	4708.1342	9.224985372	12.20124611		-2.97837	0.0843
SLC30A4	10.57359087	80.97528	3.532764645	6.357117027		-2.93702	0.0843
LOC100738694	10.57359087	80.97528	3.532764645	6.357117027		-2.93702	0.0843
RASL11B	187.1525583	1367.4907	7.555759096	10.41836992		-2.86924	0.08431
PLXNB1	122.653654	875.85507	6.950161063	9.776194605		-2.8361	0.08438
ZMIZ2	71.90041789	502.37725	6.187855179	8.975496206		-2.8047	0.08444
ORC6	790.9045968	125.59431	9.629182825	6.98406878		2.654733	0.08448
LOC100621247	790.9045968	125.59431	9.629182825	6.98406878		2.654733	0.08448
LOC100514533	10996.5345	1517.8734	13.42489251	10.56878588		2.856926	0.08466
SEMA4G	88.81816327	624.66645	6.488935315	9.289249932		-2.81416	0.08467
LOC102159595	16.91774539	0.8262784	4.163317207	0.868906686		4.355766	0.08467
LOC102159753	16.91774539	0.8262784	4.163317207	0.868906686		4.355766	0.08467
LOC100739473	16.91774539	0.8262784	4.163317207	0.868906686		4.355766	0.08467
ESR2	16.91774539	0.8262784	4.163317207	0.868906686		4.355766	0.08467
LRRC40	5147.224033	742.82426	12.32985912	9.538817985		2.792702	0.08481
C4H1orf114	1889.500688	288.37115	10.88455266	8.176777292		2.712006	0.08499
LOC100624329	72.95777697	508.98748	6.208629956	8.994318011		-2.8025	0.08503
LOC102160167	41.23700438	285.06604	5.400435611	8.160204421		-2.78928	0.08505
LOC102159493	0	14.873011	0	3.98850389		-20	0.08509
PF4	0	14.873011	0	3.98850389		-20	0.08509
LOC100518346	0	14.873011	0	3.98850389		-20	0.08509
LOC100156142	0	14.873011	0	3.98850389		-20	0.08509
LOC102164457	0	14.873011	0	3.98850389		-20	0.08509
LOC100738840	0	14.873011	0	3.98850389		-20	0.08509
SUPT20H	0	14.873011	0	3.98850389		-20	0.08509
LOC102165347	0	14.873011	0	3.98850389		-20	0.08509
LOC102163229	0	14.873011	0	3.98850389		-20	0.08509
LOC102165597	0	14.873011	0	3.98850389		-20	0.08509
IZUMO2	0	14.873011	0	3.98850389		-20	0.08509
LOC102167383	0	14.873011	0	3.98850389		-20	0.08509
ROBO4	0	14.873011	0	3.98850389		-20	0.08509
LOC100525030	68.72834063	478.41518	6.123673244	8.905131772		-2.79929	0.08517
KIAA0513	438.8040209	3374.5209	8.780716984	11.72089442		-2.94303	0.0853
DAPK3	939.992228	7616.634	9.878038997	12.89512726		-3.01843	0.08564
LOC102166544	106.7932677	754.39215	6.752123267	9.561081988		-2.82049	0.08568
FARP2	2.114718173	28.093465	1.639101631	4.862623207		-3.7317	0.08576
LOC100522818	2.114718173	28.093465	1.639101631	4.862623207		-3.7317	0.08576
LOC100623895	2.114718173	28.093465	1.639101631	4.862623207		-3.7317	0.08576
ISG20	2.114718173	28.093465	1.639101631	4.862623207		-3.7317	0.08576
SHISA2	25.37661808	176.82357	4.721187694	7.474302764		-2.80074	0.08583
LOC100511846	44.40908164	5.7839486	5.504908955	2.76212524		2.940728	0.08593
EML5	197.7261492	32.224856	7.63463791	5.054191063		2.617258	0.086
LOC100739580	21.14718173	148.73011	4.46905122	7.22622053		-2.81416	0.08603
LOC100739118	152.2597085	1092.34	7.259834656	10.09452641		-2.84282	0.08636
C1H6orf163	1.057359087	21.483238	1.04079362	4.490777899		-4.34467	0.08684
DAW1	1.057359087	21.483238	1.04079362	4.490777899		-4.34467	0.08684
RPP40	3977.784884	585.83137	11.95811219	9.196802173		2.763408	0.08697
TMEM222	179.7510447	1298.9096	7.497860176	10.34419558		-2.85323	0.087
LOC100738306	7.401513606	60.318321	3.070649265	5.938246292		-3.0267	0.08733
CACHD1	3358.172459	499.89841	11.71389015	8.968374236		2.747969	0.08742
LOC100624348	46.52379981	318.94345	5.570578288	8.321673128		-2.77726	0.08763
LOC100739564	17.97510447	127.24687	4.246035924	7.002779797		-2.82356	0.08772
LOC100520194	246.3646672	40.48764	7.950495635	5.374609695		2.605242	0.08772
VPS37C	189.2672765	1368.317	7.571883648	10.41924074		-2.85391	0.08779
FOXRED2	198.7835083	1441.0295	7.642293686	10.49388494		-2.85783	0.08785
MEF2BNB	182.923122	1319.5666	7.52295905	10.3669413		-2.85075	0.08786
UNC13A	84.58872693	587.48392	6.419348884	9.20085919		-2.79601	0.08793
SBNO2	502.2455661	3860.3726	8.975118746	11.91489804		-2.94228	0.0884
B3GNT3	5.286795433	47.097867	2.65232482	5.587901017		-3.1552	0.08847
LOC100737174	57270.79757	7243.1562	15.80553726	12.82260194		2.983109	0.08851
LOC100511223	29.60605442	203.26448	4.935745167	7.674294538		-2.77939	0.08855
FAM175A	1024.580955	162.77684	10.00222566	7.35558754		2.654067	0.08877
FAM185A	679.8818927	109.89502	9.411260757	6.793050814		2.629158	0.08883
RNF135	59.21210885	405.70268	5.911981742	8.667830689		-2.77646	0.08885
LOC102166093	23.2618999	161.12428	4.600620625	7.340956379		-2.79213	0.08888
CRISPLD1	76.12985423	11.567897	6.269217479	3.651671381		2.718336	0.0891
GRTP1	76.12985423	11.567897	6.269217479	3.651671381		2.718336	0.0891
LOC100739781	1061.588523	168.56079	10.05336732	7.405658765		2.654884	0.08917
TMEM205	172.3495311	1233.6336	7.437540124	10.26986725		-2.8395	0.08924
TMEM232	34.89284986	4.1313919	5.165624571	2.359350201		3.078232	0.08927
TNS3	181.8657629	1304.6935	7.514641181	10.35060062		-2.84277	0.0895
LOC102164189	44.40908164	302.41788	5.504908955	8.245162313		-2.76762	0.0896
HHATL	4.229436346	40.48764	2.386655454	5.374609695		-3.25894	0.08965
PPIP5K1	64.49890428	441.23265	6.033398867	8.788661735		-2.77419	0.08996
RRBP1	2469.990826	21178.341	11.27087394	14.37037008		-3.10001	0.09
PKIA	24076.0664	3216.7017	14.555372	11.65181487		2.903946	0.09012
LOC100518592	10.57359087	79.322724	3.532764645	6.327736286		-2.90727	0.09028
HSD11B2	66.61362245	9.9153405	6.079242038	3.448285225		2.748083	0.09037
C5H12orf11	4706.305294	692.42128	12.20068571	9.437588292		2.764873	0.09039
PTCHD1	9.516231779	72.712497	3.394545939	6.20383732		-2.93374	0.09044
STIL	2256.404291	346.21064	11.14044911	8.439667339		2.704304	0.0907
LOC100737028	259.0529762	42.966475	8.022661739	5.458331975		2.591964	0.09087
LOC100512339	186.0951992	1331.1345	7.54762873	10.37952399		-2.83854	0.09087
ULK1	724.2909743	5665.7908	9.502416086	12.46831623		-2.96764	0.09105
TNFRSF10B	74.01513606	506.50864	6.229109817	8.987288579		-2.77469	0.0911
PHYHIP	209.3570991	34.703692	7.716696697	5.158001345		2.592805	0.09135
TMEM91	7.401513606	59.492043	3.070649265	5.918673475		-3.0068	0.09135
PCDH15	21.14718173	1.6525567	4.46905122	1.407383614		3.677694	0.09142
LOC100624640	21.14718173	1.6525567	4.46905122	1.407383614		3.677694	0.09142
LOC102166424	21.14718173	1.6525567	4.46905122	1.407383614		3.677694	0.09142
CCDC102A	215.7012537	1552.5771	7.75956369	10.60137809		-2.84756	0.0917
OLFM3	39.1222862	4.9576702	5.326331909	2.57474827		2.980256	0.09173
LOC100270690	1255.085236	199.13309	10.29471865	7.644815895		2.65598	0.09195
ADAR	1099.65345	8834.5684	10.10414458	13.10910722		-3.00611	0.09213
MTF2	5023.51302	739.51914	12.29476806	9.53239322		2.764037	0.09216
LOC102166613	6.344154519	52.881816	2.876596413	5.751726565		-3.05927	0.09227
LOC100516494	42.29436346	285.06604	5.436107306	8.160204421		-2.75276	0.09279
ERI2	480.0410253	79.322724	8.910016128	6.327736286		2.597352	0.09281
C13H3orf17	1866.238788	290.84999	10.86669072	8.189083193		2.681786	0.09287
LOC100154999	28.54869534	193.34914	4.885022527	7.602506906		-2.75971	0.0931
GNS	506.4750025	3838.8893	8.987192949	11.90684901		-2.92213	0.09319
LOC102159269	11.63094995	0	3.658891241	0		20	0.09328
HECW1	11.63094995	0	3.658891241	0		20	0.09328
LOC102159513	11.63094995	0	3.658891241	0		20	0.09328
LOC102161751	11.63094995	0	3.658891241	0		20	0.09328
LOC102161623	11.63094995	0	3.658891241	0		20	0.09328
LOC100628173	11.63094995	0	3.658891241	0		20	0.09328
LOC102164765	11.63094995	0	3.658891241	0		20	0.09328
LOC102164248	11.63094995	0	3.658891241	0		20	0.09328
LOC100520836	11.63094995	0	3.658891241	0		20	0.09328
TPH1	11.63094995	0	3.658891241	0		20	0.09328
LOC102160597	11.63094995	0	3.658891241	0		20	0.09328
LOC100519511	11.63094995	0	3.658891241	0		20	0.09328
LOC102162224	11.63094995	0	3.658891241	0		20	0.09328
LOC102162293	11.63094995	0	3.658891241	0		20	0.09328
LOC100737184	11.63094995	0	3.658891241	0		20	0.09328
LOC102165746	11.63094995	0	3.658891241	0		20	0.09328
LOC100153663	11.63094995	0	3.658891241	0		20	0.09328
CCDC152	11.63094995	0	3.658891241	0		20	0.09328
LOC100739670	11.63094995	0	3.658891241	0		20	0.09328
LOC100511133	11.63094995	0	3.658891241	0		20	0.09328
LOC100620388	11.63094995	0	3.658891241	0		20	0.09328
SERTM1	11.63094995	0	3.658891241	0		20	0.09328
NUDT8	64.49890428	436.27498	6.033398867	8.772396993		-2.75789	0.09355
PKCNT1	43.35172255	5.7839486	5.470918232	2.76212524		2.905963	0.09383
LOC100521704	1550.088421	244.5784	10.59906522	7.940039851		2.663982	0.09395
GALNTL1	207.242381	1477.3857	7.702119902	10.52980702		-2.83366	0.09408
IL6R	2.114718173	27.267186	1.639101631	4.821056378		-3.68863	0.09417
STOM	214.6438946	1532.7464	7.75250706	10.58284422		-2.8361	0.09423
LOC100520534	20.08982264	137.98849	4.398475058	7.118821584		-2.78001	0.09427
LOC100519396	62.38418611	420.57569	5.986051038	8.719647871		-2.75311	0.09435
COMMD4	272.7986443	1976.4579	8.096971493	10.94943124		-2.85701	0.09441
PTPRB	351.0432167	58.665764	8.459608734	5.89883146		2.581058	0.09445
LOC100521322	296.0605442	49.576702	8.214613188	5.660401069		2.578158	0.09447
IKBIP	511.7617979	3866.9828	9.002144971	11.91736566		-2.91766	0.09449
OPA1	5664.272627	832.8886	12.46792967	9.703710853		2.765695	0.09458
DNAJC21	6659.247527	970.05081	12.70136008	9.923402974		2.779227	0.09461
LOC102165576	52.86795433	354.47342	5.751355374	8.473597884		-2.74521	0.09506
LOC100519736	25.37661808	2.4788351	4.721187694	1.798604302		3.355766	0.09521
LOC102168072	125.8257313	20.656959	6.986703669	4.436758793		2.606727	0.09522
GCLM	10363.17641	1474.0806	13.33931786	10.52657809		2.813579	0.09555
COX7A1	47.5811589	318.11717	5.602325001	8.317942438		-2.7411	0.09557
PROKR1	7.401513606	58.665764	3.070649265	5.89883146		-2.98663	0.09559
SLCO4C1	47.5811589	6.610227	5.602325001	2.927939483		2.847619	0.0957
PHGK	4.229436346	39.661362	2.386655454	5.34558663		-3.2292	0.09573
VMO1	4.229436346	39.661362	2.386655454	5.34558663		-3.2292	0.09573
GRAMD2	4.229436346	39.661362	2.386655454	5.34558663		-3.2292	0.09573
C9H11orf52	4.229436346	39.661362	2.386655454	5.34558663		-3.2292	0.09573
BCAR1	748.6102333	5785.6012	9.549996838	12.49850049		-2.95018	0.09596
LOC100525236	588.9490112	97.500848	9.204446459	6.622064238		2.594656	0.09601
TTC21A	59.21210885	396.61362	5.911981742	8.63522336		-2.74377	0.09607
ZKSCAN7	10.57359087	77.670167	3.532764645	6.29774474		-2.8769	0.09672
FBXL8	48.63851798	323.90112	5.633388138	8.343856914		-2.73538	0.09693
PELI2	4329.88546	650.28108	12.0804463	9.347136503		2.735194	0.09694
LOC100737024	120.5389359	19.830681	6.925274755	4.380638094		2.603693	0.09698
DENND1C	120.5389359	19.830681	6.925274755	4.380638094		2.603693	0.09698
LOC102159733	83.53136784	13.220454	6.40141489	3.829895615		2.659546	0.09701
ZMYND10	6.344154519	52.055537	2.876596413	5.729431428		-3.03655	0.09706
LOC100513562	6.344154519	52.055537	2.876596413	5.729431428		-3.03655	0.09706
TTC14	11106.49985	1579.018	13.4392465	10.62572525		2.814305	0.09711
FBXW5	323.5518805	2351.5882	8.342305305	11.20003312		-2.86157	0.09712

LOC100512828	586.052678	111.54758	9.197346157	6.814391229	6.814391229	2.393371	0.02002
ST3GAL2	159.154334	841.97766	7.323319031	9.71935059	9.71935059	-2.40336	0.02006
ERCC6L	1552.58369	289.19743	10.60138424	8.180890933	8.180890933	2.424544	0.02014
LOC102163871	30.6703665	3.3051135	4.985061656	2.106051269	2.106051269	3.214074	0.02022
LOC102162432	30.6703665	3.3051135	4.985061656	2.106051269	2.106051269	3.214074	0.02022
LOC102159194	26.5257224	2.4788351	4.78270852	1.798604302	1.798604302	3.419658	0.02028
LOC102164464	14.09179	83.454116	3.915692025	6.400095824	6.400095824	-2.56613	0.02032
LOC102162673	248.678647	47.097867	7.963928635	5.587901017	5.587901017	2.400549	0.02035
LOC102166090	380.47833	72.712497	8.575457296	6.20383732	6.20383732	2.387539	0.02041
LCN15	58.8539465	9.0890621	5.90337447	3.334720158	3.334720158	2.694936	0.02046
PHF2	410.319768	2227.6465	8.684116598	11.12195208	11.12195208	-2.4407	0.02047
ZNF420	193.969345	36.356248	7.607103495	5.223277669	5.223277669	2.415553	0.02047
MMP3	0	13.220454	0	3.829895615	3.829895615	-20	0.0205
ADAMTS16	0	13.220454	0	3.829895615	3.829895615	-20	0.0205
IL1B	0	13.220454	0	3.829895615	3.829895615	-20	0.0205
LOC102163797	0	13.220454	0	3.829895615	3.829895615	-20	0.0205
LOC102166940	0	13.220454	0	3.829895615	3.829895615	-20	0.0205
LOC100738368	0	13.220454	0	3.829895615	3.829895615	-20	0.0205
LOC100624063	0	13.220454	0	3.829895615	3.829895615	-20	0.0205
PIH1D3	0	13.220454	0	3.829895615	3.829895615	-20	0.0205
LUZP2	0	13.220454	0	3.829895615	3.829895615	-20	0.0205
LOC102162870	0	13.220454	0	3.829895615	3.829895615	-20	0.0205
SOWAHA	0	13.220454	0	3.829895615	3.829895615	-20	0.0205
FGF7	0	13.220454	0	3.829895615	3.829895615	-20	0.0205
LOC102167273	0	13.220454	0	3.829895615	3.829895615	-20	0.0205
LYPD5	0	13.220454	0	3.829895615	3.829895615	-20	0.0205
LOC100155760	0	13.220454	0	3.829895615	3.829895615	-20	0.0205
IKBIP	697.95807	3866.9828	9.449062101	11.91736566	11.91736566	-2.47	0.02054
LOC100623790	1.65785765	22.309516	1.410263837	4.542847146	4.542847146	-3.75027	0.02065
BTC	26.5257224	144.59872	4.78270852	7.185853814	7.185853814	-2.44659	0.0207
LOC100514211	431.042988	82.627837	8.755031058	6.385911347	6.385911347	2.383132	0.02075
LOC102160286	78.7482382	13.220454	6.317380743	3.829895615	3.829895615	2.574476	0.02085
DDX18	14011.3839	2406.9489	13.7744148	11.23358906	11.23358906	2.541322	0.02091
LOC100518781	272.717583	52.055537	8.096544303	5.729431428	5.729431428	2.389284	0.02098
ZBTB44	2704.79475	497.41958	11.40183669	8.961216934	8.961216934	2.442984	0.02108
LOC100514611	151.693975	28.093465	7.254499324	4.862623207	4.862623207	2.432857	0.02116
LOC100622080	238.731501	45.44531	7.905275684	5.537461031	5.537461031	2.393186	0.02119
LOC100521322	259.454722	49.576702	8.024888782	5.660401068	5.660401068	2.387749	0.02131
LOC100525275	847.994187	161.95056	9.729610865	7.348290508	7.348290508	2.388501	0.02134
SNX15	99.4714588	517.25026	6.65064192	9.017505127	9.017505127	-2.37851	0.02145
CELF3	46.4200141	6.610227	5.567424187	2.927939483	2.927939483	2.811975	0.02145
LOC102165737	780.850952	149.55639	9.610749795	7.234160086	7.234160086	2.384358	0.02151
ANKLE1	518.909444	99.979683	9.02211655	6.657921243	6.657921243	2.375776	0.02154
TREX1	111.076462	577.56858	6.808339514	9.176344172	9.176344172	-2.37844	0.0216
LOC100625556	407.004052	78.496445	8.672439671	6.312818447	6.312818447	2.374344	0.0216
LOC100737019	125.997181	656.06503	6.988652665	9.359892345	9.359892345	-2.38045	0.02164
KLHL8	466.686928	90.064343	8.869399294	6.508814353	6.508814353	2.373427	0.0217
LOC102160307	86.2085977	14.873011	6.446398468	3.98850389	3.98850389	2.535135	0.02176
HSD11B2	62.1696618	9.9153405	5.981159942	3.448285225	3.448285225	2.648477	0.02189
ATG16L2	1700.13302	319.76973	10.73228024	8.325394195	8.325394195	2.410542	0.02198
IGDCC4	910.163848	5046.9083	9.831566697	12.30146999	12.30146999	-2.4712	0.02203
WBP1L	1359.44327	7666.2107	10.40986108	12.90448612	12.90448612	-2.4955	0.0221
EDARADD	3924.14905	716.38335	11.93853172	9.486600448	9.486600448	2.453576	0.02215
LOC100737752	212.205779	40.48764	7.736102732	5.374609695	5.374609695	2.38991	0.02215
NUP160	5974.91896	1072.5093	12.54494487	10.06811901	10.06811901	2.477929	0.02217
MTHFD2	6672.0481	1192.3197	12.70413019	10.22076488	10.22076488	2.484359	0.02219
DBT	1815.35412	341.25297	10.82682979	8.41891924	8.41891924	2.411338	0.02224
ZFP36	76.2614518	394.13478	6.271676881	8.626201041	8.626201041	-2.36966	0.02226
LOC100623625	81.2350247	419.74941	6.361681078	8.716817448	8.716817448	-2.36935	0.02226
C9H11orf71	58.0250177	9.0890621	5.883254663	3.334720158	3.334720158	2.674472	0.02227
RAB26	130.141825	23.962073	7.03498407	4.641665831	4.641665831	2.44126	0.02228
PHF11	1141.43499	6380.5216	10.15789636	12.63968474	12.63968474	-2.48283	0.02228
LOC102157769	589.368394	114.02642	9.205471676	6.845821397	6.845821397	2.369802	0.02232
IKBKE	158.325405	823.79954	7.315832521	9.687899712	9.687899712	-2.3794	0.02236
LOC100511078	877.006695	168.56079	9.778088131	7.405658764	7.405658764	2.379319	0.02239
LOC102161657	38.1307259	4.9576702	5.290229968	2.574748269	2.574748269	2.94322	0.02241
TBX10	38.1307259	4.9576702	5.290229968	2.574748269	2.574748269	2.94322	0.02241
ABHD14B	294.269732	1556.7085	8.205889666	10.60520952	10.60520952	-2.40329	0.02251
CNIH3	73.7746653	12.394176	6.224477643	3.74353388	3.74353388	2.573463	0.02253
LOC102160998	73.7746653	12.394176	6.224477643	3.74353388	3.74353388	2.573463	0.02253
LOC100525698	880.322411	169.38707	9.783526081	7.412672017	7.412672017	2.377708	0.02257
TMEM26	22.3810782	122.2892	4.547269558	6.945902605	6.945902605	-2.44995	0.02257
PUSL1	1091.69926	209.04843	10.09368067	7.714578179	7.714578179	2.384666	0.02266
LOC102162175	190.653629	36.356248	7.582357509	5.223277669	5.223277669	2.390679	0.02283
LRRTM4	19.0653629	105.76363	4.326635347	6.738276475	6.738276475	-2.47182	0.02305
TRIM62	121.852537	627.97156	6.940783842	9.29685098	9.29685098	-2.36556	0.02307
PRR5	193.140416	1004.7545	7.600956677	9.974062478	9.974062478	-2.37912	0.02311
DIP2C	567.816244	3063.0139	9.151818856	11.58120714	11.58120714	-2.43146	0.02326
LOC100623959	4.97357294	38.008805	2.578594101	5.285727902	5.285727902	-2.93398	0.02328
LAD1	4.97357294	38.008805	2.578594101	5.285727902	5.285727902	-2.93398	0.02328
LOC100623096	242.047217	1265.8585	7.925092802	10.30703964	10.30703964	-2.38676	0.02329
TGFBR3L	81.2350247	14.046732	6.361681078	3.911378308	3.911378308	2.531867	0.02338
IL15RA	32.3282241	170.21334	5.058672541	7.419651342	7.419651342	-2.39648	0.02344
LOC100621687	1624.70049	309.02811	10.66684578	8.276255224	8.276255224	2.394364	0.02351
DEPDC1B	1133.97463	218.13749	10.14844434	7.775692512	7.775692512	2.378079	0.02354
LOC102164674	29.8414377	3.3051135	4.946798112	2.106051269	2.106051269	3.174545	0.0236
LOC102166164	29.8414377	3.3051135	4.946798112	2.106051269	2.106051269	3.174545	0.0236
RBM47	116.878964	21.483238	6.881162477	4.490777898	4.490777898	2.443732	0.02364
LOC100153192	191.482558	992.36032	7.588583912	9.956173315	9.956173315	-2.37365	0.02366
LOC100626689	72.9457365	373.47782	6.208395063	8.548736477	8.548736477	-2.35613	0.02367
APLP1	50.5646582	260.27769	5.688310694	8.029440115	8.029440115	-2.36385	0.02378
MTR	3269.29528	607.3146	11.67520519	9.248673828	9.248673828	2.428464	0.0238
LOC100520306	498.186223	97.500848	8.963434308	6.622064238	6.622064238	2.353198	0.02382
ADAM5	17.4075053	0.8262784	4.202222212	0.868906686	0.868906686	4.396938	0.02383
LOC100621245	1447.30973	8085.1339	10.50015445	12.98123437	12.98123437	-2.4819	0.02402
STX1A	98.64253	504.02981	6.638689748	8.980224728	8.980224728	-2.35323	0.02402
SMARCA1	363.070825	1915.3133	8.508075323	10.90411771	10.90411771	-2.39926	0.02408
TMEM151A	33.1571529	173.51846	5.094115824	7.447235822	7.447235822	-2.3877	0.0241
SLC2A6	65.4853771	334.64274	6.054965161	8.390782629	8.390782629	-2.35338	0.02411
42619	13959.9903	2446.6103	13.76911366	11.25715814	11.25715814	2.512442	0.02415
LOC102159824	7.46035941	49.576702	3.080718953	5.660401068	5.660401068	-2.73235	0.02417
GPC2	116.050035	593.26787	6.870981559	9.214969574	9.214969574	-2.35394	0.0242
LOC102158126	57.1960888	9.0890621	5.862850292	3.334720158	3.334720158	2.653713	0.02425
LOC102160482	57.1960888	9.0890621	5.862850292	3.334720158	3.334720158	2.653713	0.02425
LOC102166466	43.1042988	222.26888	5.462847376	7.80263838	7.80263838	-2.3664	0.02433
LOC102166035	18.2364341	100.80596	4.265769485	6.669678233	6.669678233	-2.46669	0.0244
LOC102159817	21.5521494	1.6525567	4.495193036	1.407383613	1.407383613	3.70506	0.02449
DMKN	21.5521494	1.6525567	4.495193036	1.407383613	1.407383613	3.70506	0.02449
LOC102164221	21.5521494	1.6525567	4.495193036	1.407383613	1.407383613	3.70506	0.02449
LRGUK	21.5521494	1.6525567	4.495193036	1.407383613	1.407383613	3.70506	0.02449
LOC100739717	278.520085	54.534373	8.12680814	5.795309087	5.795309087	2.352544	0.0245
LOC100739224	0.82892882	17.351846	0.870998931	4.19785327	4.19785327	-4.3877	0.02458
KCNT2	1298.10254	250.36235	10.34329959	7.973624744	7.973624744	2.374315	0.02462
LOC102164620	4216.76093	779.1805	12.04226161	9.607664138	9.607664138	2.436106	0.02462
LOC100622285	1421.61293	7905.8315	10.47432747	12.94888396	12.94888396	-2.47539	0.02464
EPHA7	7117.18288	1287.3417	12.79729328	10.33129957	10.33129957	2.466911	0.02466
LOC100518417	9.94714588	61.1446	3.452482877	5.957557119	5.957557119	-2.61987	0.02472
LOC100621699	9.94714588	61.1446	3.452482877	5.957557119	5.957557119	-2.61987	0.02472
ZNF382	48.9068006	250.36235	5.641164514	7.973624744	7.973624744	-2.35591	0.02478
LOC100157134	6.63143059	45.44531	2.93195353	5.537461031	5.537461031	-2.77674	0.02479
MAP3K5	111.905391	20.656959	6.818970566	4.436758793	4.436758793	2.43758	0.02494
ARPC1B	1154.69785	6348.2967	10.17454855	12.63238109	12.63238109	-2.45885	0.02503
LOC102162967	2.48678647	25.61463	1.801898017	4.734147579	4.734147579	-3.36461	0.02503
FAM98C	300.901163	1567.4501	8.237932504	10.61512389	10.61512389	-2.38106	0.02505
LOC102162766	256.967935	50.402981	8.011047944	5.683780114	5.683780114	2.350007	0.02509
GABRR1	64.6564482	10.741619	6.036864803	3.553559423	3.553559423	2.589583	0.0251
MAP3K14	243.705074	1260.0745	7.934900207	10.30043781	10.30043781	-2.3703	0.02514
CPEB1	147.549331	28.093465	7.214798294	4.862623207	4.862623207	2.392891	0.02515
TRIB3	673.919134	3618.273	9.398570844	11.82148421	11.82148421	-2.42465	0.02516
LOC100517307	15.7496477	88.411786	4.066058842	6.482393107	6.482393107	-2.48892	0.02522
LOC102167622	9.11821706	57.013208	3.338883189	5.858309486	5.858309486	-2.64447	0.02526
RHBDL1	200.600775	1030.3691	7.655357377	10.01034505	10.01034505	-2.36076	0.02529
C1H6orf163	1.65785765	21.483238	1.410263837	4.490777898	4.490777898	-3.69582	0.02531
TCEAL5	1.65785765	21.483238	1.410263837	4.490777898	4.490777898	-3.69582	0.02531
LOC102163805	1.65785765	21.483238	1.410263837	4.490777898	4.490777898	-3.69582	0.02531
LOC102160840	37.3017971	4.9576702	5.259340178	2.574748269	2.574748269	2.911511	0.02541
IKZF1	37.3017971	4.9576702	5.259340178	2.574748269	2.574748269	2.911511	0.02541
HSD17B13	5.80250177	41.313919	2.766065426	5.403060391	5.403060391	-2.83188	0.02552
CCDC38	119.365751	22.309516	6.911281129	4.542847146	4.542847146	2.419658	0.02555
ILK	2985.80162	17074.216	11.5443857	14.05961623	14.05961623	-2.51563	0.02555
FANCM	1198.63108	233.0105	10.22837509	7.870429459	7.870429459	2.362921	0.02555
LOC102166037	824.78418	161.95056	9.689620969	7.348290508	7.348290508	2.348463	0.0256
GRAMD3	263.599366	1361.7068	8.047665794	10.41225942	10.41225942	-2.369	0.02571
NDUFA4L2	70.45895	356.12598	6.159042811	8.480289273	8.480289273	-2.33753	0.02574
LCP1	5144.33228	949.39385	12.32904853	9.892381689	9.892381689	2.437905	0.02583
ADAMTSL1	8.28928824	52.881816	3.215568059	5.751726565	5.751726565	-2.67345	0.0259
KLHL25	1165.47393	227.22655	10.18793834	7.834322836	7.834322836	2.358713	0.02592
TXLNG	7476.93799	1359.2279	12.86842479	10.4096327	10.4096327	2.45966	0.02593
LOC100522963	3006.52484	567.65324	11.55436094	9.151405371	9.151405371	2.405015	0.02606
CCDC36	571.13196	113.20014	9.160204126	6.83542057	6.83542057	2.334948	0.02609
LOC100739113	126.82611	23.962073	6.998038744	4.641665831	4.641665831	2.404027	0.02612
IL17RD	73.7746653	371.82527	6.224477643	8.542355828	8.542355828	-2.33343	0.02616
TMEM151B	41.4464412	211.52726	5.407571694	7.731504113	7.731504113	-2.35152	0.02621
LOC102165243	162.470049	31.398578	7.352882522	5.017858594	5.017858594	2.371403	0.02623
SLC16A1	7334.36223	1336.9184	12.84065249	10.38577442	10.38577442	2.45576	0.02628
LOC102159568	4.97357294	37.182527	2.578594101	5.254840671	5.254840671	-2.90227	0.02638
LOC102158199	106.931818	19.830681	6.753976422	4.380638094	4.380638094	2.430885	0.02641
GRIK5	1870.89236	360.25737	10.87028176	8.49688321	8.49688321	2.376627	0.02643
PDHA1	33997.6868	5805.4318	15.0531914	12.50343616	12.50343616	2.549961	0.02643
BRI3	602.631255	3196.871	9.237523697	11.64289604	11.64289604	-2.40731	0.02644
GRIP1	675.576991	133.8571	9.402110305	7.075287628	7.075287628	2.335427	0.02646
NFE2L2	1543.46547	8523.8877	10.5928919	13.05746511	13.05746511	-2.46534	0.02648
LOC100624199	44.7621565	227.22655	5.516083133	7.834322836	7.834322836	-2.34378	0.0266
CDS1	254.481149	50.402981	7.997073033	5.683780114	5.683780114	2.335978	0.02667
FAM105A	144.233615	728.77752	7.182231603	9.511312908	9.511312908	-2.33707	0.02672
LOC100739107	1012.12209	199.13309	9.984592332	7.644815895	7.644815895	2.345578	0.02683
TAB3	2102.1635	404.05012	11.03834529	8.661956638	8.661956638	2.379269	0.02686
MYD88	533.001234	2806.0414	9.060699264	11.4548346	11.4548346	-2.39633	0.02691
LOC100739776	68.8010924	345.38436	6.125177709	8.436229978	8.436229978	-2.3277	0.02691
LOC100738613	401.201551	80.149002	8.651774833	6.342501448	6.342501448	2.323571	0.02707
URGCP	289.296159	1486.4748	8.181381675	10.5386495	10.5386495	-2.36128	0.02717
FAM177A1	190.653629	966.74569	7.582357509	9.918484174	9.918484174	-2.34218	0.02725
LAMA4	1692.67266	9344.3821	10.72593935	13.19003794	13.19003794	-2.4648	0.0273
SLC30A2	12.4339324	0	3.747809764	0	0	20	0.02733
LOC100622164	12.4339324	0	3.747809764	0	0	20	0.02733
TDRD5	12.4339324	0	3.747809764	0	0	20	0.02733
C6H1orf177	12.4339324	0	3.747809764	0	0	20	0.02733
LOC102157415	12.4339324	0	3.747809764	0	0	20	0.02733
LOC100511546	12.4339324	0	3.747809764	0	0	20	0.02733
LOC102159971	12.4339324	0	3.747809764	0	0	20	0.02733
LOC102163596	12.4339324	0	3.747809764	0	0	20	0.02733
LOC100624651	12.4339324	0	3.747809764	0	0	20	0.02733
IDH1	12.4339324	0	3.747809764	0	0	20	0.02733
PROK1	12.4339324	0	3.747809764	0	0	20	0.02733
QRICH2	12.4339324	0	3.747809764	0	0	20	0.02733
LOC100154433	12.4339324	0	3.747809764	0	0	20	0.02733
DNAJB7	12.4339324	0	3.747809764	0	0	20	0.02733
DCLK3	12.4339324	0	3.747809764	0	0	20	0.02733
LOC100621469	12.4339324	0	3.747809764	0	0	20	0.02733
LOC100623971	12.4339324	0	3.747809764	0	0	20	0.02733
LOC102160857	12.4339324	0	3.747809764	0	0	20	0.02733
LOC102167024	12.4339324	0	3.747809764	0	0	20	0.02733
LOC102160234	12.4339324	0	3.747809764	0	0	20	0.02733
LOC102167050	12.4339324	0	3.747809764	0	0	20	0.02733
GP91-PHOX	12.4339324	0	3.747809764	0	0	20	0.02733
LOC102158719	12.4339324	0	3.747809764	0	0	20	0.02733
KLHDC7B	12.4339324	0	3.747809764	0	0	20	0.02733
LOC102162534	12.4339324	0	3.747809764	0	0	20	0.02733
TTLL8	12.4339324	0	3.747809764	0	0	20	0.02733
LOC100737191	12.4339324	0	3.747809764	0	0	20	0.02733
LOC100158035	12.4339324	0	3.747809764	0	0	20	0.02733
LOC102163344	12.4339324	0	3.747809764	0	0	20	0.02733
LOC100515852	12.4339324	0	3.747809764	0	0	20	0.02733
LOC100517827	12.4339324	0	3.747809764	0	0	20	0.02733
AVP	12.4339324	0	3.747809764	0	0	20	0.02733
LBX2	12.4339324	0	3.747809764	0	0	20	0.02733
LOC100736836	36.4728682	185.91263	5.227774503	7.546220275	7.546220275	-2.34973	0.02743
LOC100157427	1259.14288	247.05723	10.29937161	7.954529215	7.954529215	2.349525	0.02748
CD38	269.401868	53.708094	8.078961306	5.773682393	5.773682393	2.326548	0.02751
CPT1B	237.073644	47.097867	7.895264103	5.587901017	5.587901017	2.331602	0.02753
LOC100739623	6.63143059	44.619032	2.93195353	5.511563931	5.511563931	-2.75027	0.02755
LOC100156127	40.6175124	5.7839486	5.379118827	2.762125239	2.762125239	2.811975	0.02756
MPC2	7146.19539	1312.9563	12.80316151	10.35970162	10.35970162	2.444356	0.02757
LOC102165250	29.0125088	3.3051135	4.907492018	2.106051269	2.106051269	3.133903	0.02759
BRWD3	3281.72921	623.01389	11.68068003	9.285434338	9.285434338	2.39712	0.02772
LOC102162482	63.8275194	318.94345	6.018534465	8.321673128	8.321673128	-2.32105	0.02784
LOC100621976	59.6828753	9.9153405	5.92321754	3.448285225	3.448285225	2.589583	0.02788
HPCAL4	59.6828753	9.9153405	5.92321754	3.448285225	3.448285225	2.589583	0.02788
LOC100624061	1134.80356	223.92144	10.14949762	7.81327737	7.81327737	2.341378	0.02795
ZNF394	59.6828753	298.28649	5.92321754	8.225383356	8.225383356	-2.32131	0.02798
LRFN2	129.312897	24.788351	7.025836058	4.688647628	4.688647628	2.383132	0.02799
LOC100516697	49.7357294	249.53607	5.664930183	7.968874505	7.968874505	-2.32689	0.02802
LOC100518118	58.0250177	290.02371	5.883254663	8.184992878	8.184992878	-2.32142	0.02805
SCN7A	0	12.394176	0	3.74353388	3.74353388	-20	0.02807
C14H10orf90	0	12.394176	0	3.74353388	3.74353388	-20	0.02807
LOC102159171	0	12.394176	0	3.74353388	3.74353388	-20	0.02807
LOC102161946	0	12.394176	0	3.74353388	3.74353388	-20	0.02807
LOC102164222	0	12.394176	0	3.74353388	3.74353388	-20	0.02807
IL2RB	0	12.394176	0	3.74353388	3.74353388	-20	0.02807
LOC102160757	0	12.394176	0	3.74353388	3.74353388	-20	0.02807
SH3TC1	0	12.394176	0	3.74353388	3.74353388	-20	0.02807
LOC102162948	0	12.394176	0	3.74353388	3.74353388	-20	0.02807
MIR199A-1	0	12.394176	0	3.74353388	3.74353388	-20	0.02807
CCL4	0	12.394176	0	3.74353388	3.74353388	-20	0.02807
LOC102165710	0	12.394176	0	3.74353388	3.74353388	-20	0.02807
CXHXorf64	0	12.394176	0	3.74353388	3.74353388	-20	0.02807
LOC100737539	0	12.394176	0	3.74353388	3.74353388	-20	0.02807
LOC100737634	0	12.394176	0	3.74353388	3.74353388	-20	0.02807
LOC102167920	0	12.394176	0	3.74353388	3.74353388	-20	0.02807
LOC102167901	0	12.394176	0	3.74353388	3.74353388	-20	0.02807
ADM5	382.136188	76.843889	8.581713486	6.282511874	6.282511874	2.314084	0.02823
UPK3B	377.991544	76.01761	8.566021848	6.267116453	6.267116453	2.313948	0.02826
LOC100738549	493.21265	99.153405	8.948988129	6.646067655	6.646067655	2.314476	0.02831
LOC102163690	67.1432347	11.567897	6.090498528	3.65167138	3.65167138	2.537115	0.02839
WNT11	67.1432347	11.567897	6.090498528	3.65167138	3.65167138	2.537115	0.02839
LOC102165085	276.033298	55.360651	8.113915583	5.816616368	5.816616368	2.31791	0.02844
LOC102159125	3.31571529	28.919743	2.109599694	4.903025878	4.903025878	-3.12466	0.02858
GAB3	3.31571529	28.919743	2.109599694	4.903025878	4.903025878	-3.12466	0.02858
42440	3.31571529	28.919743	2.109599694	4.903025878	4.903025878	-3.12466	0.02858
LOC102161691	131.799683	657.71758	7.053107893	9.363516252	9.363516252	-2.31912	0.02863
OAF	484.923362	2519.3228	8.924584985	11.29939278	11.29939278	-2.37721	0.02865
PROCR	55.5382312	276.80325	5.821154844	8.117919691	8.117919691	-2.31731	0.02871
PHF19	62.9985906	313.1595	5.999968229	8.29535341	8.29535341	-2.31351	0.0288
LRRC8C	53.8803735	268.54047	5.778218396	8.074358096	8.074358096	-2.31731	0.02883
MDFIC	293.440804	1494.7376	8.201833804	10.54664136	10.54664136	-2.34875	0.02884
C2H11orf35	36.4728682	4.9576702	5.227774503	2.574748269	2.574748269	2.879089	0.02885
LOC102161729	24.8678647	2.4788351	4.693089065	1.798604302	1.798604302	3.326548	0.02889
LOC100154078	24.8678647	2.4788351	4.693089065	1.798604302	1.798604302	3.326548	0.02889
TMEM116	24.8678647	2.4788351	4.693089065	1.798604302	1.798604302	3.326548	0.02889
GPER1	19.8942918	105.76363	4.385036953	6.738276475	6.738276475	-2.41042	0.02892
LOC100739280	574.447675	115.67897	9.16854094	6.86640077	6.86640077	2.312049	0.02893
MTX3	454.252995	91.716899	8.830524699	6.534760414	6.534760414	2.308237	0.02901
LOC100625581	97.8136012	485.0254	6.62663773	8.924887914	8.924887914	-2.30995	0.02908
POLE	1403.3765	276.80325	10.45571404	8.117919691	8.117919691	2.341969	0.02912
GXYLT2	79.5771671	394.13478	6.332299179	8.626201041	8.626201041	-2.30826	0.02916
LOC100622217	43.9332277	219.79005	5.489710792	7.786531327	7.786531327	-2.32274	0.02925
LOC102165864	145.891473	726.29869	7.19860684	9.506404165	9.506404165	-2.31567	0.02943
JUP	2242.25247	12395.828	11.13137628	13.59768342	13.59768342	-2.46683	0.02946
MDGA1	420.266914	85.106672	8.718590801	6.428053129	6.428053129	2.303962	0.0295
LOC100739240	11.6050035	66.928548	3.655924617	6.085946114	6.085946114	-2.52787	0.02958
C18H7orf10	58.0250177	287.54487	5.883254663	8.172651889	8.172651889	-2.30904	0.0296
LOC100522323	2282.04105	443.71149	11.15674109	8.796725856	8.796725856	2.362631	0.02967
C16H5orf49	50.5646582	251.18863	5.688310694	7.978359394	7.978359394	-2.31257	0.02971
SLC5A4	62.1696618	307.37555	5.981159942	8.268544594	8.268544594	-2.30572	0.02982
B4GALNT1	2.48678647	24.788351	1.801898017	4.688647628	4.688647628	-3.31731	0.02988
PKIB	281.8358	57.013208	8.143820931	5.858309486	5.858309486	2.305487	0.0299
LOC102162044	51.3935871	8.2627837	5.711318333	3.211445828	3.211445828	2.636889	0.02991

LOC102164814	5.52323934	38.008805	2.705588563	5.285727902		-2.78275	0.1936		[illegible]
LOC100624806	5.52323934	38.008805	2.705588563	5.285727902		-2.78275	0.1936		[illegible]
FAM132A	77.3253508	17.351846	6.291407421	4.19785327		2.155852	0.19404		[illegible]
LOC102167052	2000.79345	451.14799	10.96707741	8.820651243		2.1489	0.1948		[illegible]
GPER1	20.7121475	105.76363	4.440430524	6.738276475		-2.35229	0.19556		[illegible]
LOC100738242	96.6566885	22.309516	6.609646952	4.542847146		2.11521	0.19563		[illegible]
LOC100738305	96.6566885	22.309516	6.609646952	4.542847146		2.11521	0.19563		[illegible]
LOC100523874	46.9475344	228.87911	5.583384725	7.844731552		-2.28546	0.19579		[illegible]
MAN1A2	157.412321	786.61701	7.307540742	9.621350459		-2.32111	0.1959		[illegible]
MRAS	595.129039	3210.9178	9.219480843	11.64921924		-2.43171	0.196		[illegible]
TOE1	1810.2417	410.66035	10.82276336	8.685310691		2.140165	0.19613		[illegible]
LOC102158195	38.6626754	189.21775	5.309710091	7.571508044		-2.29103	0.19624		[illegible]
CHCHD4	4117.57493	898.16459	12.00792952	9.812441412		2.196743	0.19631		[illegible]
STK10	55.2323934	268.54047	5.813329549	8.074358096		-2.28155	0.19661		[illegible]
TOP1MT	64.8980623	315.63834	6.042164139	8.306692135		-2.28203	0.19667		[illegible]
FER1L4	106.322357	24.788351	6.745806839	4.688647628		2.100711	0.19674		[illegible]
TAF4B	682.120059	161.12428	9.415995345	7.340956379		2.081852	0.19687		[illegible]
WFIKKN1	13.8080984	74.365053	3.888314478	6.2358238		-2.42911	0.19692		[illegible]
F2RL2	13.8080984	74.365053	3.888314478	6.2358238		-2.42911	0.19692		[illegible]
ISG12%28A%29	3579.05909	21800.529	11.80576769	14.41214167		-2.60671	0.19695		[illegible]
LOC100737768	330.013551	79.322724	8.370746468	6.327736286		2.056719	0.19715		[illegible]
GINS4	2283.85947	513.11887	11.15788972	9.005958152		2.154109	0.1972		[illegible]
DRAM1	59.3748229	288.37115	5.915875148	8.176777292		-2.28	0.19729		[illegible]
ZNF227	67.659682	328.85879	6.101391269	8.365704748		-2.2811	0.19734		[illegible]
TAS1R3	6.90404918	43.792754	2.982591923	5.485193456		-2.66518	0.19748		[illegible]
LOC100625447	6.90404918	43.792754	2.982591923	5.485193456		-2.66518	0.19748		[illegible]
RSPH10B	6.90404918	43.792754	2.982591923	5.485193456		-2.66518	0.19748		[illegible]
LOC100624515	38.6626754	7.4365053	5.309710091	3.076645515		2.378245	0.19767		[illegible]
[illegible]	[illegible]	[illegible]	[illegible]	[illegible]		[illegible]	[illegible]		[illegible]

FCHO1	407.338902	102.45852	8.673623206	6.692908622		1.99119	0.24125
ABLIM3	129.796125	603.18321	7.031175985	9.238842286		-2.21635	0.24184
LCAT	34.5202459	158.64545	5.150569664	7.318727602		-2.20029	0.24187
EFCAB6	12.4272885	63.623435	3.747096094	6.013985524		-2.35605	0.24202
ARHGAP18	2210.67655	520.55537	11.11092469	9.026676623		2.086364	0.24246
SLA-DQB1	11.0464787	57.839486	3.590539588	5.878712739		-2.38847	0.24249
AHNAK	8168.87099	49721.301	12.99609758	15.60160544		-2.60566	0.24308
LOC100626663	27.6161967	128.07315	4.838760036	7.012045083		-2.21338	0.24373
LOC100522150	510.899639	128.07315	8.999717179	7.012045083		1.996072	0.24382
LOC102160750	20.7121475	98.327126	4.440430524	6.634115866		-2.24711	0.24405
GPX2	15.1889082	1.6525567	4.016933786	1.407383613		3.200246	0.24407
PRR18	15.1889082	1.6525567	4.016933786	1.407383613		3.200246	0.24407
LOC100739315	15.1889082	1.6525567	4.016933786	1.407383613		3.200246	0.24407
LOC100739348	15.1889082	1.6525567	4.016933786	1.407383613		3.200246	0.24407
LXN	226.452813	1078.2933	7.829423468	10.07587122		-2.25147	0.24435
LOC100738326	722.163544	3700.9008	9.498178141	11.85405053		-2.35748	0.24438
LOC100624942	269.257918	1294.7782	8.078193076	10.33960308		-2.26564	0.24446
ABHD2	519.184498	2601.1243	9.022879597	11.34547417		-2.32482	0.24474
BMPR2	294.112495	1421.1988	8.205121195	10.47390743		-2.27267	0.24477
LOC100158196	30.3778164	5.7839486	4.971673052	2.762125239		2.392892	0.24477
ALOX5AP	46.9475344	213.17982	5.583384725	7.742678745		-2.18295	0.24484
LOC102162570	8.28485901	46.271589	3.214880004	5.56290145		-2.48158	0.24487
NLRP6	48.3283443	10.741619	5.624344958	3.553559423		2.169658	0.24549
SYNGAP1	611.698757	3095.2388	9.259034115	11.59630102		-2.33916	0.24555
PLEKHG2	266.496298	1278.2526	8.063375117	10.3210855		-2.26199	0.24597
LOC100518921	229.214433	1089.8612	7.846834472	10.09125179		-2.24937	0.24601
PSMC3IP	1245.49047	302.41788	10.28365614	8.245162313		2.042099	0.24628
XYLT1	51.0899639	231.35794	5.702933533	7.86020516		-2.17901	0.24629
LOC100739199	266.496298	1277.4264	8.063375117	10.32015335		-2.26105	0.24647
LOC100738016	983.136603	241.27328	9.942714772	7.920491517		2.026724	0.24664
CA5B	56.6132033	256.1463	5.848327568	8.006445558		-2.17776	0.24678
SLC25A6	114797.768	22161.612	16.80874764	14.43584032		2.37296	0.24696
LOC102164693	6.90404918	40.48764	2.982591923	5.374609695		-2.55197	0.24709
PDGFA	240.260911	1142.743	7.914450382	10.15954718		-2.24983	0.24786
ZACN	153.269892	711.42568	7.269312714	9.476595706		-2.21464	0.24843
TMEM161B	1079.79329	264.40908	10.07787491	8.052073912		2.029912	0.2485
CLDN1	120.130456	551.12767	6.920417836	9.108858104		-2.19778	0.24925
LOC102164444	51.0899639	11.567897	5.702933533	3.65167138		2.142913	0.24956
STARD8	20.7121475	97.500848	4.440430524	6.622064238		-2.23494	0.25005
LOC102158625	96.6566885	23.962073	6.609646952	4.641665831		2.012117	0.25006
MMP2	7308.62646	43777.054	12.83558197	15.41792022		-2.5825	0.25011
C2	13.8080984	68.581105	3.888314478	6.120623682		-2.3123	0.25019
ADORA2A	13.8080984	68.581105	3.888314478	6.120623682		-2.3123	0.25019
LOC102162880	541.277456	136.33593	9.082887383	7.101565318		1.989202	0.2503
LOC102166393	22.0929574	103.2848	4.529381037	6.704385035		-2.22497	0.2505
CENPL	386.626754	98.327126	8.598524339	6.634115866		1.97528	0.25077
LOC102164986	168.4588	42.966475	7.404790748	5.458331975		1.971112	0.25102
CDC45	4957.10731	1129.5225	12.27557378	10.14277403		2.133785	0.25109
SH3PXD2A	1692.87286	9127.0709	10.72610988	13.15609428		-2.43068	0.25147
LOC100621607	24.854577	114.85269	4.692347799	6.856147779		-2.2082	0.25168
TRPM6	33.1394361	150.38266	5.093367322	7.242056188		-2.18202	0.25233
LOC100622425	8.28485901	0	3.214880004	0		20	0.25316
ATP6V1G3	8.28485901	0	3.214880004	0		20	0.25316
EDAR	8.28485901	0	3.214880004	0		20	0.25316
SLC16A14	8.28485901	0	3.214880004	0		20	0.25316
LOC100511549	8.28485901	0	3.214880004	0		20	0.25316
LOC102161264	8.28485901	0	3.214880004	0		20	0.25316
LOC102159941	8.28485901	0	3.214880004	0		20	0.25316
LOC102163950	8.28485901	0	3.214880004	0		20	0.25316
NPHS1	8.28485901	0	3.214880004	0		20	0.25316
LOC100624315	8.28485901	0	3.214880004	0		20	0.25316
LOC102162079	8.28485901	0	3.214880004	0		20	0.25316
LOC102166941	8.28485901	0	3.214880004	0		20	0.25316
KLF1	8.28485901	0	3.214880004	0		20	0.25316
TTC36	8.28485901	0	3.214880004	0		20	0.25316
KCNH4	8.28485901	0	3.214880004	0		20	0.25316
QRICH2	8.28485901	0	3.214880004	0		20	0.25316
LOC100154934	8.28485901	0	3.214880004	0		20	0.25316
LOC102160903	8.28485901	0	3.214880004	0		20	0.25316
PEBP4	8.28485901	0	3.214880004	0		20	0.25316
SLC26A5	8.28485901	0	3.214880004	0		20	0.25316
LOC102158914	8.28485901	0	3.214880004	0		20	0.25316
LOC100512533	8.28485901	0	3.214880004	0		20	0.25316
LOC100623257	8.28485901	0	3.214880004	0		20	0.25316
ANKRD34B	8.28485901	0	3.214880004	0		20	0.25316
PRSS48	8.28485901	0	3.214880004	0		20	0.25316
LOC100523216	8.28485901	0	3.214880004	0		20	0.25316
KRT28	8.28485901	0	3.214880004	0		20	0.25316
ARL14EPL	8.28485901	0	3.214880004	0		20	0.25316
LOC100620428	8.28485901	0	3.214880004	0		20	0.25316
LOC100622535	53.8515836	12.394176	5.777461367	3.74353388		2.119326	0.25334
ABCC5	289.970066	1384.8426	8.184726929	10.43654764		-2.25575	0.25337
GOLIM4	1469.18167	7818.246	10.52177872	12.93281377		-2.41183	0.25402
LOC100525876	8474.02996	1883.0884	13.04900275	10.87965095		2.169947	0.25429
BMP1	2047.74099	11152.279	11.00052189	13.44518032		-2.44523	0.25432
SMAD9	288.589256	1375.7535	8.177864267	10.42705455		-2.25313	0.25458
PRKAG2	127.034505	32.224857	7.000388854	5.054191063		1.978975	0.2547
GAS2L2	4.14242951	28.919743	2.362450112	4.903025878		-2.80351	0.25478
SEMA3F	756.68379	3838.8893	9.565452073	11.90684901		-2.34293	0.2552
ACOT7	7153.97576	1605.4589	12.80473116	10.64966833		2.15576	0.2554
LOC100623695	256.830629	1214.6292	8.010279851	10.24748753		-2.24163	0.25541
NOC4L	6886.09865	1550.0982	12.74968063	10.59907433		2.151327	0.25663
SLC38A6	245.784151	1157.616	7.947105933	10.17818678		-2.23569	0.25665
LOC100622436	306.539784	1462.5127	8.26462924	10.51521957		-2.2543	0.25688
LOC100525621	8.28485901	45.44531	3.214880004	5.537461031		-2.45558	0.25715
LOC100155828	574.416892	145.42499	9.168463763	7.194018019		1.981823	0.25741
LOC102159922	117.368836	29.746021	6.88714549	4.942327829		1.980281	0.25742
RIPK4	48.3283443	215.65866	5.624344958	7.75928006		-2.15781	0.25757
MTCH2	21472.9738	4558.5778	14.39030157	12.15468452		2.235866	0.25769
LETM2	367.295416	94.195734	8.524719632	6.572825024		1.963207	0.25789
LOC100154511	198.836616	51.229259	7.642677144	5.706786332		1.956544	0.25789
LOC100511686	393.530803	100.80596	8.623994134	6.669678233		1.964896	0.25825
LOC100623508	59.3748229	264.40908	5.915875148	8.052073912		-2.15485	0.25886
LOC100739662	17.9505279	2.4788351	4.24416613	1.798604302		2.856292	0.25899
LOC100515112	17.9505279	2.4788351	4.24416613	1.798604302		2.856292	0.25899
WNT10B	67.659682	301.59161	6.101391269	8.241228156		-2.15623	0.25909
LOC102164962	120.130456	30.5723	6.920417836	4.980587446		1.974305	0.25991
LOC100738073	2158.20577	517.25026	11.07628502	9.017505127		2.060898	0.26019
BAG2	69.0404918	307.37555	6.130117309	8.268544594		-2.15449	0.26024
LOC102159328	2.76161967	23.135794	1.911353989	4.593102408		-3.06654	0.26069
LOC102159911	2.76161967	23.135794	1.911353989	4.593102408		-3.06654	0.26069
LOC100626249	2.76161967	23.135794	1.911353989	4.593102408		-3.06654	0.26069
LOC100156782	2.76161967	23.135794	1.911353989	4.593102408		-3.06654	0.26069
IGFN1	2.76161967	23.135794	1.911353989	4.593102408		-3.06654	0.26069
REPS2	107.703167	27.267186	6.764250165	4.821056379		1.981823	0.26094
CCND3	4348.17017	1006.4071	12.08652444	9.976431027		2.111194	0.26102
LOC102167053	34.5202459	154.51406	5.150569664	7.280901168		-2.16222	0.26124
HEBP2	288.589256	1364.1856	8.177864267	10.41488138		-2.24095	0.26139
STXBP1	6663.78827	1509.6106	12.70234333	10.56091609		2.142166	0.26192
MELK	3231.09502	760.1761	11.6582539	9.572086457		2.087618	0.2622
MXRA8	1576.88483	8351.1955	10.62377619	13.02793977		-2.40491	0.26231
LOC102165496	19.3313377	90.064343	4.345633235	6.508814353		-2.22001	0.2628
SLC51B	0	11.567897	0	3.65167138		-20	0.26314
LOC100736905	0	11.567897	0	3.65167138		-20	0.26314
LOC100037944	0	11.567897	0	3.65167138		-20	0.26314
TAGLN3	0	11.567897	0	3.65167138		-20	0.26314
LOC102159496	0	11.567897	0	3.65167138		-20	0.26314
CHRNA7	0	11.567897	0	3.65167138		-20	0.26314
LOC102157487	0	11.567897	0	3.65167138		-20	0.26314
LOC102168069	0	11.567897	0	3.65167138		-20	0.26314
LOC102168116	0	11.567897	0	3.65167138		-20	0.26314
LOC102159958	0	11.567897	0	3.65167138		-20	0.26314
LOC102163690	0	11.567897	0	3.65167138		-20	0.26314
LOC102161085	0	11.567897	0	3.65167138		-20	0.26314
WBSCR17	0	11.567897	0	3.65167138		-20	0.26314
PAX3	0	11.567897	0	3.65167138		-20	0.26314
LOC102162067	0	11.567897	0	3.65167138		-20	0.26314
LOC102166667	0	11.567897	0	3.65167138		-20	0.26314
LOC100626943	0	11.567897	0	3.65167138		-20	0.26314
PROX2	0	11.567897	0	3.65167138		-20	0.26314
LOC100516806	0	11.567897	0	3.65167138		-20	0.26314
LOC100624214	0	11.567897	0	3.65167138		-20	0.26314
LOC102166133	0	11.567897	0	3.65167138		-20	0.26314
DTX1	0	11.567897	0	3.65167138		-20	0.26314
C5H12orf56	0	11.567897	0	3.65167138		-20	0.26314
LOC102166257	0	11.567897	0	3.65167138		-20	0.26314
LOC102166392	0	11.567897	0	3.65167138		-20	0.26314
LOC100623470	0	11.567897	0	3.65167138		-20	0.26314
LOC100623436	0	11.567897	0	3.65167138		-20	0.26314
LOC102158059	0	11.567897	0	3.65167138		-20	0.26314
TXLNB	0	11.567897	0	3.65167138		-20	0.26314
ABCB4	0	11.567897	0	3.65167138		-20	0.26314
PLXNA4	0	11.567897	0	3.65167138		-20	0.26314
VNN3	0	11.567897	0	3.65167138		-20	0.26314
CCM2L	0	11.567897	0	3.65167138		-20	0.26314
ESRRG	0	11.567897	0	3.65167138		-20	0.26314
MCHR1	0	11.567897	0	3.65167138		-20	0.26314
CRISPLD1	0	11.567897	0	3.65167138		-20	0.26314
LOC100623303	0	11.567897	0	3.65167138		-20	0.26314
LOC102167618	0	11.567897	0	3.65167138		-20	0.26314
LOC100153068	0	11.567897	0	3.65167138		-20	0.26314
TBX20	0	11.567897	0	3.65167138		-20	0.26314
LOC102165523	0	11.567897	0	3.65167138		-20	0.26314
LOC102167191	0	11.567897	0	3.65167138		-20	0.26314
LOC100155068	0	11.567897	0	3.65167138		-20	0.26314
LOC102165953	0	11.567897	0	3.65167138		-20	0.26314
LOC102167455	0	11.567897	0	3.65167138		-20	0.26314
LOC100737953	0	11.567897	0	3.65167138		-20	0.26314
ALB	0	11.567897	0	3.65167138		-20	0.26314
LOC102164151	665.550341	168.56079	9.380570028	7.405658764		1.981279	0.26315
CD99L2	443.239957	2145.8449	8.795195352	11.06800227		-2.27539	0.2641
LOC100625763	78.7061606	349.51575	6.316619332	8.453335466		-2.15081	0.26411
LRRC8C	60.7556328	268.54047	5.948498827	8.074358096		-2.14405	0.26471
LOC100517370	1899.99433	460.23705	10.89253852	8.849364605		2.045546	0.26499
LOC102165609	9.66566885	50.402981	3.414902536	5.683780114		-2.38257	0.26572
DLX1	200.217426	52.055537	7.652611443	5.729431428		1.943444	0.26632
IER5L	1071.50843	5495.5775	10.06677328	12.42431786		-2.35863	0.26637
LOC102158683	5.52323934	33.877413	2.705588563	5.124221138		-2.61674	0.26656
MGME1	2641.48922	630.4504	11.36768187	9.3025256		2.066897	0.26679
LOC100525885	44.1859147	9.9153405	5.497801224	3.448285225		2.155852	0.2669
LOC102164918	1.38080984	17.351846	1.251452392	4.19785327		-3.6515	0.26693
KRT83	1.38080984	17.351846	1.251452392	4.19785327		-3.6515	0.26693
LOC102167488	538.515836	137.98849	9.075521496	7.118821585		1.964441	0.26756
LOC102161895	27.6161967	123.94176	4.838760036	6.965111899		-2.16608	0.26773
LOC100624284	28.9970066	129.7257	4.906746634	7.030399034		-2.16149	0.26791
LOC102166710	20.7121475	3.3051135	4.440430524	2.106051269		2.647705	0.2683
LOC100627582	20.7121475	3.3051135	4.440430524	2.106051269		2.647705	0.2683
ZWILCH	2325.28376	559.39046	11.18381137	9.13028858		2.055479	0.26846
LOC102157992	20.7121475	95.022013	4.440430524	6.585293272		-2.19778	0.26877
VEGFA	251.30739	1167.5313	7.979038653	10.19048071		-2.21594	0.26878
LOC100739376	405.958092	1941.7542	8.668736424	10.92388765		-2.25796	0.26936
APP	9436.45442	56296.824	13.20418206	15.78079155		-2.57674	0.26956
NPTXR	1639.02128	401.57129	10.67949881	8.653100473		2.029107	0.26963
EGFL8	919.619351	231.35794	9.846460957	7.86020516		1.99091	0.26972
SQSTM1	7329.33861	42939.208	12.83966413	15.39004156		-2.55054	0.26978
LOC100518456	8.28485901	44.619032	3.214880004	5.511563931		-2.42911	0.26994
WFIKKN2	8.28485901	44.619032	3.214880004	5.511563931		-2.42911	0.26994
LOC100621699	12.4272885	61.1446	3.747096094	5.957557119		-2.29871	0.26997
KSR2	46.9475344	10.741619	5.583384725	3.553559423		2.127838	0.27011
LOC100626756	46.9475344	10.741619	5.583384725	3.553559423		2.127838	0.27011
PPID	6194.31292	1418.72	12.59696144	10.47139067		2.126354	0.27015
ZNF713	62.1364426	272.67186	5.98040107	8.096303303		-2.13365	0.27045
SCN8A	249.92658	65.275991	7.971121491	6.05041444		1.93688	0.27054
LRRC73	610.317947	156.16661	9.255779113	7.296150961		1.966475	0.27056
LOC100525590	2025.64803	491.63563	10.98487984	8.944377168		2.042722	0.27065
SNRPG	21181.6229	4551.1413	14.37059362	12.15232962		2.218513	0.27121
SYK	332.77517	86.759229	8.382732826	6.455478947		1.939459	0.2723
ACVR2B	149.127462	38.835083	7.230044098	5.315967693		1.941113	0.27267
RPAP3	3452.02459	817.18931	11.75364489	9.676290877		2.078701	0.27304
SEMA4G	139.461793	624.66645	7.13403395	9.289249932		-2.16322	0.27318
WDR12	3602.53286	851.06672	11.81519629	9.734822598		2.081667	0.27321
TNIP2	2085.02285	506.50864	11.02653925	8.987288579		2.041404	0.27328
GALNT3	91.1334492	23.135794	6.525653118	4.593102408		1.977854	0.27387
LOC102165670	15.1889082	71.886218	4.016933786	6.187574144		-2.2427	0.27406
PKD1	1648.68694	8640.3929	10.68797656	13.07704817		-2.38978	0.2741
LOC100623895	4.14242951	28.093465	2.362450112	4.862623207		-2.76169	0.27422
LOC102162702	4.14242951	28.093465	2.362450112	4.862623207		-2.76169	0.27422
ADCY9	64.8980623	283.41348	6.042164139	8.151846042		-2.12666	0.2746
LOC102161974	23.4737672	4.1313919	4.613164285	2.359350201		2.50635	0.27465
LOC100624267	23.4737672	4.1313919	4.613164285	2.359350201		2.50635	0.27465
MCM4	13498.797	2979.5598	13.72065009	11.54136761		2.17966	0.27507
LRP2BP	40.0434852	175.17101	5.359081339	7.460832769		-2.12912	0.27511
LOC100739112	146.365843	655.23875	7.203258356	9.358076973		-2.16244	0.27543
KCNJ11	52.4707738	12.394176	5.740678649	3.74353388		2.081852	0.27586
FLNB	3458.92864	19074.636	11.75652657	14.21944355		-2.46326	0.27597
CNTLN	102.179928	450.32171	6.689018532	8.818012376		-2.13984	0.27608
HEG1	153.269892	686.63733	7.269312714	9.42550405		-2.16347	0.27664
LOC102165490	34.5202459	151.20894	5.150569664	7.249909307		-2.13103	0.27764
NUP37	885.099105	224.74772	9.791324254	7.818567588		1.977533	0.27796
LOC100523631	37.2818656	162.77684	5.258589232	7.35558754		-2.12635	0.27811
XIAP	139.461793	620.53506	7.13403395	9.279691957		-2.15364	0.27857
LOC100622939	64.8980623	281.76092	6.042164139	8.143438955		-2.11822	0.27922
SFXN1	2932.84009	703.98917	11.51857452	9.46145729		2.058673	0.27932
LOC100737829	23.4737672	104.93735	4.613164285	6.727067559		-2.16041	0.27959
CACNA1C	23.4737672	104.93735	4.613164285	6.727067559		-2.16041	0.27959
SKA1	1309.00772	327.20624	10.3553596	8.358458837		2.000201	0.27996
CARNS1	3030.87759	727.12497	11.56599579	9.508042269		2.05946	0.28064
LOC102166318	99.4183082	25.61463	6.649878514	4.734147579		1.956544	0.28071
DYNC1I1	310.682213	81.801559	8.283932016	6.371586023		1.925239	0.281
SNRPF	9339.79773	2112.7938	13.18933005	11.04561893		2.144239	0.28114
DIP2B	371.437846	1741.7948	8.540855872	10.76718701		-2.22938	0.28117
LOC100739180	318.967072	1480.6908	8.321779634	10.53302874		-2.21479	0.28152
LOC102162762	1732.91634	428.0122	10.75981858	8.744874853		2.017478	0.28164
ZNF389	34.5202459	150.38266	5.150569664	7.242056188		-2.12312	0.28187
ACP2	276.161967	1269.9899	8.11458549	10.3117368		-2.20123	0.28193
LOC100739054	281.685207	74.365053	8.143052576	6.2358238		1.921387	0.28224
LOC102163167	28.9970066	127.24687	4.906746634	7.002779797		-2.13365	0.28257
LOC100515728	28.9970066	5.7839486	4.906746634	2.762125239		2.325777	0.28293
EDARADD	2977.02601	716.38335	11.54014064	9.486600448		2.055068	0.28309
LOC102166815	8.28485901	43.792754	3.214880004	5.485193456		-2.40214	0.28324
LOC102160827	8.28485901	43.792754	3.214880004	5.485193456		-2.40214	0.28324
JAG2	972.090124	247.05723	9.926429619	7.954529215		1.976245	0.28335
BPTF	220.929574	1000.6231	7.79395812	9.968124036		-2.17924	0.28366
AMPH	5.52323934	33.051135	2.705588563	5.089630977		-2.58111	0.28369
LOC102165374	5.52323934	33.051135	2.705588563	5.089630977		-2.58111	0.28369
LOC100739060	104.941548	458.5845	6.727124677	8.84418632		-2.1276	0.28371
PPM1E	396.292423	104.11107	8.634057469	6.715770874		1.928442	0.28405
CCRL2	11.0464787	54.534373	3.590539588	5.795309087		-2.30358	0.28412
GGCX	773.253508	3796.7491	9.596662204	11.89092889		-2.29575	0.28422
LOC100625333	12287.8267	2746.5493	13.58505956	11.42392966		2.161538	0.28425
LOC100524511	219.548764	993.1866	7.784953864	9.957372852		-2.17752	0.28436
LOC100152487	490.187492	2331.7576	8.940130012	11.18782067		-2.25001	0.28458
LHPP	3153.76967	757.69727	11.62331896	9.56738053		2.057384	0.28468
LOC102159626	2.76161967	22.309516	1.911353989	4.542847146		-3.01407	0.28482
LOC100736864	2.76161967	22.309516	1.911353989	4.542847146		-3.01407	0.28482
LOC100623790	2.76161967	22.309516	1.911353989	4.542847146		-3.01407	0.28482
LOC100739227	835.389951	214.0061	9.708031918	7.74823377		1.964798	0.28491
ST5	872.671816	4314.8257	9.770947641	12.07542087		-2.30579	0.28554
SLCO2B1	17.9505279	81.801559	4.24416613	6.371586023		-2.1881	0.28556
TRAPPC8	57.9940131	249.53607	5.882496648	7.968874505		-2.10527	0.28557
LOC100521858	13.8080984	65.275991	3.888314478	6.05041444		-2.24104	0.28586
OAS1	202.979046	912.21132	7.672277146	9.834804937		-2.16804	0.28616
MGST3	8784.71218	2002.0725	13.10094352	10.96799892		2.133501	0.28619
LOC100625214	624.126046	161.95056	9.288003303	7.348290508		1.946284	0.28627
LOC100519176	1760.53254	436.27498	10.78261541	8.772396993		2.012702	0.28627
ZFC3H1	236.118482	1070.0305	7.889464306	10.06478384		-2.18007	0.28641
TP53INP2	334.15598	1545.9668	8.388688866	10.59522655		-2.20992	0.28688

LOC102164887	16.91774539	108.24247	4.163317207	6.771389985		-2.67766	0.12394
MEGF10	34.89284986	215.65866	5.165624571	7.75928006		-2.62775	0.12395
KLK1	31.7207726	4.1313919	5.032134908	2.359350201		2.940728	0.12418
C3H7orf50	218.8733309	1444.3346	7.780528816	10.4971878		-2.72224	0.1242
ZFAT	33.83549077	209.04843	5.122485983	7.714578179		-2.62723	0.12424
ELOVL4	810.9944194	141.2936	9.665326002	7.15272698		2.520996	0.12425
AASDHPPT	9939.175414	1504.6529	13.2790556	10.55617352		2.723695	0.1243
LOC100739715	519.1633115	3631.4934	9.022820836	11.82674448		-2.8063	0.12434
42618	473.6968708	3291.893	8.890862729	11.68513993		-2.79688	0.12435
TDRD7	253.7661808	1689.7393	7.993029968	10.72343848		-2.73523	0.1244
TNFSF13B	14.80302721	95.848291	3.982129041	6.597654688		-2.69486	0.12452
MYO5B	284.4295943	51.229259	8.156991116	5.706786332		2.473032	0.12455
CKS2	12824.70836	1913.6607	13.64675089	10.90287304		2.744519	0.12482
LOC100736687	99.39175414	17.351846	6.649496966	4.19785327		2.518037	0.12491
NPW	1.057359087	19.004403	1.04079362	4.322245637		-4.1678	0.12492
SLC16A9	1.057359087	19.004403	1.04079362	4.322245637		-4.1678	0.12492
PLEKHA2	1.057359087	19.004403	1.04079362	4.322245637		-4.1678	0.12492
INSRR	1.057359087	19.004403	1.04079362	4.322245637		-4.1678	0.12492
LOC102158176	87.76080419	550.3014	6.471850834	9.106697443		-2.64857	0.12496
RNF224	4.229436346	36.356248	2.386655454	5.223277669		-3.10367	0.12497
GPR62	4.229436346	36.356248	2.386655454	5.223277669		-3.10367	0.12497
LOC100736982	3.17207726	30.5723	2.060765874	4.980587447		-3.26873	0.12508
ROBO3	3.17207726	30.5723	2.060765874	4.980587447		-3.26873	0.12508
LOC102167083	5.286795433	42.140197	2.65232482	5.430960858		-2.99473	0.1251
CACNA1G	88.81816327	556.91162	6.488935315	9.123892796		-2.64852	0.12517
LOC102161787	10.57359087	0	3.532764645	0		20	0.12526
LOC102161554	10.57359087	0	3.532764645	0		20	0.12526
LOC102161270	10.57359087	0	3.532764645	0		20	0.12526
SLC14A1	10.57359087	0	3.532764645	0		20	0.12526
LOC102166884	10.57359087	0	3.532764645	0		20	0.12526
LOC102164534	10.57359087	0	3.532764645	0		20	0.12526
IDH1	10.57359087	0	3.532764645	0		20	0.12526
LOC102164202	10.57359087	0	3.532764645	0		20	0.12526
LOC100514422	10.57359087	0	3.532764645	0		20	0.12526
LOC102164330	10.57359087	0	3.532764645	0		20	0.12526
LOC102164342	10.57359087	0	3.532764645	0		20	0.12526
LOC102157705	10.57359087	0	3.532764645	0		20	0.12526
LOC100738386	10.57359087	0	3.532764645	0		20	0.12526
LOC102164165	10.57359087	0	3.532764645	0		20	0.12526
RGS9	10.57359087	0	3.532764645	0		20	0.12526
RHOH	10.57359087	0	3.532764645	0		20	0.12526
LOC102160989	10.57359087	0	3.532764645	0		20	0.12526
LOC102160183	10.57359087	0	3.532764645	0		20	0.12526
LOC100519723	10.57359087	0	3.532764645	0		20	0.12526
LOC102160369	10.57359087	0	3.532764645	0		20	0.12526
LOC100512605	10.57359087	0	3.532764645	0		20	0.12526
ABCA12	10.57359087	0	3.532764645	0		20	0.12526
LOC102158158	10.57359087	0	3.532764645	0		20	0.12526
P2RY14	10.57359087	0	3.532764645	0		20	0.12526
LOC100627396	10.57359087	0	3.532764645	0		20	0.12526
LOC102165335	10.57359087	0	3.532764645	0		20	0.12526
LOC102165450	10.57359087	0	3.532764645	0		20	0.12526
LOC102165535	10.57359087	0	3.532764645	0		20	0.12526
LOC100625692	10.57359087	0	3.532764645	0		20	0.12526
LOC102167228	10.57359087	0	3.532764645	0		20	0.12526
LOC102167231	10.57359087	0	3.532764645	0		20	0.12526
LOC100517891	10.57359087	0	3.532764645	0		20	0.12526
GRXCR1	10.57359087	0	3.532764645	0		20	0.12526
LOC100620960	10.57359087	0	3.532764645	0		20	0.12526
LOC100037970	2.114718173	24.788351	1.639101631	4.688647628		-3.55112	0.12531
TLR6	2.114718173	24.788351	1.639101631	4.688647628		-3.55112	0.12531
LOC102160931	2.114718173	24.788351	1.639101631	4.688647628		-3.55112	0.12531
LOC100624445	1533.170676	259.45141	10.58324328	8.02487043		2.562982	0.12558
LANCL3	27.49133625	3.3051135	4.832451381	2.106051269		3.056205	0.12561
MYOZ1	27.49133625	3.3051135	4.832451381	2.106051269		3.056205	0.12561
CHEK1	1953.999592	326.37996	10.93295259	8.354822188		2.581806	0.12569
MRPS28	2476.334981	408.18152	11.27457325	8.676597164		2.600924	0.12574
LOC100524359	178.6936856	32.224856	7.489395904	5.054191063		2.471243	0.12578
PTGER4	7.401513606	53.708094	3.070649265	5.773682393		-2.85925	0.12591
ACSM3	214.6438946	38.835083	7.75250706	5.315967693		2.466513	0.1262
LOC100738122	543.4825705	96.674569	9.088742057	6.609911087		2.491025	0.12637
LOC100624590	397.5670166	2715.977	8.638678515	11.40778664		-2.7722	0.12639
ZC3H8	1982.548287	331.33763	10.9538678	8.376305833		2.580982	0.1264
FASTKD1	3911.171261	628.79784	11.93375381	9.298745001		2.636933	0.12658
NUBP2	161.7759402	1041.937	7.346743662	10.02643633		-2.6872	0.12666
LOC102164581	13.74566813	89.238064	3.882219287	6.495664213		-2.69868	0.12682
LOC100620860	94.1049587	589.13648	6.571448659	9.20490483		-2.64626	0.12684
LOC100519435	126.8830904	805.62141	6.998761703	9.655747894		-2.6666	0.12699
ANGPTL6	9.516231779	65.275991	3.394545939	6.05041444		-2.77809	0.12722
LOC100738447	1066.655319	162.77684	9.869910619	7.35558754		2.521616	0.12732
LOC100513825	23.2618999	2.4788351	4.600620625	1.798604302		3.230235	0.12744
CRLF3	4024.308683	646.97797	11.97488371	9.339796491		2.636957	0.12746
ASF1B	3978.842243	640.36574	11.95849553	9.325003477		2.635381	0.12768
LOC100513028	6444.603633	1008.0596	12.65409976	9.978795694		2.676511	0.12782
LOC100522988	149.0876312	952.69896	7.229661278	9.897390138		-2.67586	0.128
C12H17orf59	118.4242177	746.95565	6.899951616	9.546808914		-2.65706	0.12826
WDR12	5377.728314	851.06672	12.3930494	9.734822598		2.659653	0.12827
THAP3	136.3993222	866.76601	7.102231077	9.761162271		-2.66781	0.12827
ALOX5AP	34.89284986	213.17982	5.165624571	7.742678745		-2.61107	0.12858
LOC102163227	72.95777697	12.394176	6.208629956	3.74353388		2.557399	0.12864
LOC100515993	38.06492712	232.18422	5.287802017	7.865326367		-2.60874	0.12896
BCKDHB	1589.210707	270.19303	10.63500222	8.083176277		2.556248	0.12897
LOC102165721	60.26946793	9.9153405	5.937096418	3.448285225		2.603699	0.12912
LOC100520817	97.27703596	606.48832	6.618782442	9.246712872		-2.64031	0.12917
KIAA1522	326.7239578	2186.3326	8.356337332	11.09495688		-2.74237	0.12947
LOC100513807	19.03246356	1.6525567	4.324267947	1.407383614		3.525691	0.12951
LOC100516054	19.03246356	1.6525567	4.324267947	1.407383614		3.525691	0.12951
LOC100516234	19.03246356	1.6525567	4.324267947	1.407383614		3.525691	0.12951
DCHS1	1358.706426	10073.986	10.40907948	13.2984902		-2.89033	0.12961
ACER1	98.33439505	17.351846	6.63422144	4.19785327		2.502607	0.12966
LIN9	1316.412063	226.40027	10.36349095	7.829090181		2.539664	0.12973
SFXN1	4381.696055	703.98917	12.09760291	9.46145729		2.637864	0.12982
LOC100738287	141.6861176	25.61463	7.156701167	4.734147579		2.467658	0.12998
STRADB	7490.331769	1165.8788	12.8710065	10.18843898		2.683612	0.13007
LOC100517767	39.1222862	237.96817	5.326331909	7.900674664		-2.60471	0.13007
ABCE1	19593.92123	2877.1013	14.25819215	11.49090165		2.767718	0.13009
LURAP1L	208.2997401	38.008805	7.70942671	5.285727902		2.454255	0.13018
RHPN1	170.965345	687.46361	6.793965376	9.42723658		-2.64423	0.13045
FANCB	649.2184792	115.67897	9.344780748	6.866400771		2.488577	0.13065
LOC102161720	14.80302721	0.8262784	3.982129041	0.868906686		4.163121	0.13074
EVX1	14.80302721	0.8262784	3.982129041	0.868906686		4.163121	0.13074
DBF4	5057.348511	807.27397	12.30445072	9.658700576		2.647251	0.13093
LOC102157928	47.5811589	7.4365053	5.602325001	3.076645515		2.677694	0.13103
LOC100523455	9422.12682	1449.2923	13.20199014	10.50212795		2.700704	0.1312
SLC12A7	96.21967688	596.57298	6.603176434	9.222971117		-2.63229	0.1313
ADAMTS15	14.80302721	94.195734	3.982129041	6.572825024		-2.66977	0.13136
LTBP1	475.811589	3253.0579	8.897275491	11.66802423		-2.77333	0.13155
LOC100623778	2721.642289	451.14799	11.41079173	8.820651243		2.592805	0.13157
FANCM	1351.304913	233.0105	10.40120477	7.87042946		2.535886	0.13181
RGSL1	6.344154519	47.097867	2.876596413	5.587901017		-2.89216	0.1321
HSD17B7	3884.737284	631.27668	11.92397265	9.304412191		2.621473	0.1321
PFDN4	7084.30588	1110.5181	12.79061442	10.11831577		2.673394	0.13212
LOC100739338	84.58872693	520.55537	6.419348884	9.026676623		-2.62151	0.13214
LOC100737502	3062.111915	504.85609	11.58078236	8.98258319		2.600583	0.13236
CAAP1	2574.669376	428.83847	11.3307317	8.747650814		2.585881	0.13243
LOC100739112	105.7359087	655.23875	6.737901807	9.358076973		-2.63156	0.1334
AGFG2	134.284604	841.97766	7.079853843	9.71935059		-2.64849	0.13363
LOC100623328	11699.67829	1783.935	13.51430455	10.80165583		2.713334	0.13366
MAP7D1	1778.477984	13333.654	10.79723837	13.70289278		-2.90636	0.13368
LOC102157979	4.229436346	35.52997	2.386655454	5.191008663		-3.0705	0.1337
FLI1	4.229436346	35.52997	2.386655454	5.191008663		-3.0705	0.1337
SLA-11	863.8623737	6136.7695	9.756326764	12.58349874		-2.82861	0.13378
MURC	39.1222862	5.7839486	5.326331909	2.76212524		2.757864	0.13378
AVEN	59.21210885	358.60481	5.911981742	8.490268521		-2.59843	0.13397
ACSM5	33.83549077	204.09076	5.122485983	7.68011867		-2.5926	0.13401
MEIS3	287.6016715	1887.2198	8.172935846	10.882811		-2.71412	0.13428
ESRP1	301.3473397	55.360651	8.240063075	5.816616368		2.444495	0.13431
QRSL1	3239.748241	534.60211	11.66211123	9.065017825		2.599344	0.13463
CHAC2	768.7000559	137.16221	9.588152542	7.110219252		2.486538	0.13513
LOC102158049	71.90041789	12.394176	6.187855179	3.74353388		2.536338	0.13521
LOC102163471	547.7120068	99.153405	9.099905335	6.646067655		2.465683	0.13531
LOC102161397	3.17207726	29.746021	2.060765874	4.942327829		-3.2292	0.13542
NUP43	2837.951788	472.63123	11.47114263	8.887620397		2.586063	0.13543
LOC102161480	67.67098154	11.567897	6.10162868	3.651671381		2.548411	0.13566
GLI1	23.2618999	141.2936	4.600620625	7.15272698		-2.60266	0.13591
SLC25A15	3768.427785	618.05622	11.88012982	9.273926629		2.608153	0.1362
LOC100738959	1162.037636	8391.6831	10.18368207	13.0349164		-2.8523	0.13632
WASH1	283.3722352	1847.5584	8.151636803	10.85218494		-2.70485	0.1367
LOC102163796	1605.071093	276.80325	10.64932004	8.117919691		2.535704	0.13684
PTBP2	2563.038426	430.49103	11.32420217	8.753186764		2.5738	0.13686
LOC100739542	59.21210885	9.9153405	5.911981742	3.448285225		2.578158	0.13706
LOC100624860	71.90041789	434.62242	6.187855179	8.766934408		-2.59569	0.13724
LOC100737262	76.12985423	461.06333	6.269217479	8.851946794		-2.59843	0.13729
LOC102158399	143.8008358	26.440908	7.177926119	4.778256309		2.443228	0.13733
IGFALS	10.57359087	69.407383	3.532764645	6.137654819		-2.71462	0.13737
NOXO1	9.516231779	63.623435	3.394545939	6.013985524		-2.7411	0.1374
LMF2	1254.027877	9084.1044	10.29350369	13.14928738		-2.85677	0.13752
GOLGA2	209.3570991	1335.2658	7.716696697	10.38399134		-2.67309	0.13771
MSH3	1405.230226	244.5784	10.45761709	7.940039851		2.522438	0.13791
LOC102166360	231.56164	42.966475	7.86146934	5.458331975		2.430113	0.138
C10H1orf101	2.114718173	23.962073	1.639101631	4.641665831		-3.50222	0.13802
EVI2A	2.114718173	23.962073	1.639101631	4.641665831		-3.50222	0.13802
LOC102164456	2.114718173	23.962073	1.639101631	4.641665831		-3.50222	0.13802
RAI2	2.114718173	23.962073	1.639101631	4.641665831		-3.50222	0.13802
ANKRD22	54.9826725	9.0890621	5.806908455	3.334720158		2.596774	0.13809
TTC16	7.401513606	52.055537	3.070649265	5.729431428		-2.81416	0.13819
LOC100738530	7.401513606	52.055537	3.070649265	5.729431428		-2.81416	0.13819
LOC102165440	187.1525583	34.703692	7.555759096	5.158001345		2.431054	0.13823
PTPRF	439.86138	2946.5087	8.78418129	11.52528034		-2.74389	0.13823
LOC100738066	99.39175414	607.3146	6.649496966	9.248673828		-2.61125	0.13826
BUB1	5062.635307	818.84187	12.30595779	9.679201855		2.628232	0.1383
HDAC2	43145.53753	6126.0278	15.39695717	12.58097169		2.816188	0.13835
PTGES3	42722.59389	6070.6672	15.38274539	12.567877		2.815072	0.13846
LGR4	10368.4632	1606.2852	13.34005359	10.65041019		2.690402	0.13854
SKA1	1909.59051	327.20624	10.89980289	8.358458837		2.544991	0.1386
DEK	53032.90235	7439.8105	15.69462729	12.86124405		2.83355	0.13863
CNN2	2620.135817	19993.458	11.35597639	14.28731254		-2.93181	0.13903
LOC102162570	6.344154519	46.271589	2.876596413	5.56290145		-2.86663	0.13915
CSRNP1	86.7034451	525.51304	6.45456161	9.040325464		-2.59957	0.13918
FOXN2	2096.743069	357.77853	11.03462227	8.486949769		2.551011	0.13922
LOC100627624	56.04003159	334.64274	5.833902876	8.390782629		-2.57809	0.1395
LOC102158840	91.99024053	16.525567	6.539007406	4.131389251		2.476781	0.13987
LOC100515118	8304.498266	1307.1724	13.01985101	10.35333695		2.667444	0.13992
LOC102157971	67.67098154	405.70268	6.10162868	8.667830689		-2.58381	0.13998
LOC100624306	1220.192386	214.83238	10.25407478	7.753767487		2.505825	0.14011
LOC100623430	22.20454082	133.8571	4.536335244	7.075287628		-2.59177	0.14012
LOC100737021	22.20454082	133.8571	4.536335244	7.075287628		-2.59177	0.14012
LOC100739791	2520.744062	426.35964	11.30020615	8.739306855		2.563707	0.14022
GEMIN8	176.5789675	1107.213	7.472316908	10.1140195		-2.64855	0.14063
LOC102166318	138.5140403	25.61463	7.124266508	4.734147579		2.434992	0.14064
GALNT12	846.9446283	152.03522	9.727826248	7.257719911		2.477862	0.14072
LOC100624925	228.3895627	1455.0762	7.84165594	10.50787015		-2.67153	0.14073
SYNJ2	209.3570991	1326.1768	7.716696697	10.37414484		-2.66324	0.1408
LOC102158566	256.938258	47.924146	8.010881963	5.61247475		2.422597	0.14085
LOC100739568	46.52379981	7.4365053	5.570578288	3.076645515		2.645272	0.14115
CLCNKA	1.057359087	18.178124	1.04079362	4.261389711		-4.10367	0.14136
LOC100152036	1.057359087	18.178124	1.04079362	4.261389711		-4.10367	0.14136
LOC102166121	1.057359087	18.178124	1.04079362	4.261389711		-4.10367	0.14136
TMEM198	57.09739067	339.60041	5.860401464	8.411936365		-2.57234	0.14143
RND1	242.1352308	1545.9668	7.925615148	10.59522655		-2.67462	0.14149
TBKBP1	366.903603	2409.4277	8.523183995	11.23507346		-2.71522	0.14151
GAA	336.2401895	2194.5954	8.397632665	11.10039648		-2.70639	0.14154
LOC100626221	419.7715574	77.670167	8.716893377	6.29774474		2.434172	0.1419
SCN7A	0	12.394176	0	3.74353388		-20	0.14209
MOGAT3	0	12.394176	0	3.74353388		-20	0.14209
LOC102159171	0	12.394176	0	3.74353388		-20	0.14209
LOC102161356	0	12.394176	0	3.74353388		-20	0.14209
GJD4	0	12.394176	0	3.74353388		-20	0.14209
LOC102166650	0	12.394176	0	3.74353388		-20	0.14209
KCNK5	0	12.394176	0	3.74353388		-20	0.14209
LOC102164226	0	12.394176	0	3.74353388		-20	0.14209
ZPBP2	0	12.394176	0	3.74353388		-20	0.14209
LOC100736773	0	12.394176	0	3.74353388		-20	0.14209
GUCY1A2	0	12.394176	0	3.74353388		-20	0.14209
IL2RB	0	12.394176	0	3.74353388		-20	0.14209
HTRA4	0	12.394176	0	3.74353388		-20	0.14209
LOC102162770	0	12.394176	0	3.74353388		-20	0.14209
SH3TC1	0	12.394176	0	3.74353388		-20	0.14209
MIR199A-1	0	12.394176	0	3.74353388		-20	0.14209
CCL4	0	12.394176	0	3.74353388		-20	0.14209
CBLC	0	12.394176	0	3.74353388		-20	0.14209
LOC100625253	0	12.394176	0	3.74353388		-20	0.14209
CXHXorf64	0	12.394176	0	3.74353388		-20	0.14209
LOC100737634	0	12.394176	0	3.74353388		-20	0.14209
LOC102167920	0	12.394176	0	3.74353388		-20	0.14209
LOC100737946	1121.857991	199.13309	10.13295976	7.644815895		2.494085	0.14219
KHK	209.3570991	1322.0454	7.716696697	10.36964685		-2.65873	0.14223
EFCAB2	461.0085617	85.106672	8.851775777	6.428053129		2.437449	0.14229
PKDREJ	14.80302721	91.716899	3.982129041	6.534760414		-2.6313	0.14237
LOC100627193	26.43397716	3.3051135	4.777891883	2.106051269		2.999622	0.14246
LOC100626459	28.54869534	169.38707	4.885022527	7.412672017		-2.56883	0.14252
FASTKD2	4985.448093	813.05792	12.28379682	9.668987631		2.616293	0.14252
CCNJ	4430.334573	727.95125	12.11352554	9.509678516		2.605502	0.14273
CCDC18	414.4847619	76.843889	8.698651756	6.282511874		2.431317	0.14274
LOC102160096	207.242381	38.835083	7.702119902	5.315967693		2.415887	0.14292
FADD	138.5140403	851.893	7.124266508	9.736220951		-2.62064	0.14299
LOC102166908	4.229436346	34.703692	2.386655454	5.158001345		-3.03655	0.14306
CD81	114.1947813	694.90011	6.84793155	9.442736427		-2.60531	0.14313
DNASE1L2	100.4491132	607.3146	6.664612444	9.248673828		-2.59598	0.14322
NUDT12	1227.5939	217.31121	10.26279241	7.770242414		2.497999	0.14329
LOC733698	9223.343312	1450.9448	13.17123049	10.50377091		2.668297	0.14333
LOC100623666	42.29436346	6.610227	5.436107306	2.927939483		2.677694	0.14336
NEBL	637.5875292	116.50525	9.318740569	6.876581411		2.452228	0.14343
C9H11orf82	1148.291968	204.09076	10.16652963	7.68011867		2.492207	0.14358
PKIB	304.5194169	57.013208	8.255120262	5.858309485		2.417166	0.14393
CYCS	26734.26714	3944.6529	14.70645647	11.94604835		2.76072	0.14397
TC2N	116.3094995	21.483238	6.874176035	4.490777899		2.436686	0.14414
GNB4	78.24457241	466.84728	6.308240221	8.869893856		-2.57689	0.14438
PUS3	2402.319845	410.66035	11.23081295	8.685310691		2.548411	0.14466
HAT1	7935.479945	1263.3796	12.95428356	10.30421398		2.651029	0.14498
CAMK1D	48.63851798	285.89232	5.633388138	8.164365521		-2.5553	0.14536
LOC102159055	375.3624757	70.233662	8.555978985	6.154487245		2.41805	0.14609
CACYBP	22859.04609	3417.4873	14.48054069	11.73914237		2.741757	0.14615
DNAH10	5237.099556	856.85067	12.35482776	9.744582725		2.611652	0.14616
LOC100620733	38.06492712	5.7839486	5.287802017	2.76212524		2.718336	0.14622
TLN2	391.222862	2554.8527	8.61552982	11.31958899		-2.70718	0.14626
LOC100621314	512.819157	95.022013	9.00511687	6.585293272		2.432116	0.14628
LOC100738154	13.74566813	85.106672	3.882219287	6.428053129		-2.6303	0.1463
EFNB3	13.74566813	85.106672	3.882219287	6.428053129		-2.6303	0.1463
MTSS1L	183.9804811	1141.9167	7.531229237	10.15850456		-2.63383	0.14649
LOC100737594	8344.677911	1327.8293	13.02681353	10.37594012		2.651787	0.1465
LOC100621894	329.896035	61.970878	8.370234194	5.976612875		2.412349	0.1465
LOC100522455	24.31925899	143.77244	4.662163277	7.177643143		-2.56362	0.14663
LOC100157139	473.6968708	3133.2476	8.890862729	11.61390343		-2.72562	0.14666
SCNN1A	3.17207726	28.919743	2.060765874	4.903025878		-3.18855	0.14666
LOC102164195	3.17207726	28.919743	2.060765874	4.903025878		-3.18855	0.14666
LOC100523652	708.430588	129.7257	9.470517725	7.030399033		2.449162	0.1471
CCDC87	10.57359087	67.754826	3.532764645	6.103389087		-2.67986	0.14749
GLB1L	285.4869534	1818.6387	8.16232563	10.8294363		-2.67136	0.14769
TSTD1	85.64608601	508.98748	6.437062676	8.994318011		-2.57117	0.14784
42616	969.5982824	175.17101	9.922730497	7.460832769		2.468623	0.1479
LDLRAD4	659.79207	121.46292	9.368052563	6.936201186		2.441495	0.14831
PLEKHO2	233.6763581	1466.6441	7.874528698	10.51928645		-2.64994	0.1484
LOC100515558	1525.769162	269.36675	10.57626624	8.078773924		2.501893	0.14841
LOC102157740	968.5409233	175.17101	9.921157984	7.460832769		2.467049	0.14849
LOC102157632	115.2521404	21.483238	6.861113469	4.490777899		2.42351	0.14866
MAP2K5	1361.878504	242.09956	10.41244124	7.925403489		2.491926	0.14881

WFIKKN2	7.46035941	44.619032	3.080718953	5.511563931	5.511563931	-2.58034	0.04377
RCC1	5192.41015	1034.5005	12.34246645	10.01611256	10.01611256	2.32747	0.04383
LOC102160037	72.9457365	14.046732	6.208395063	3.911378308	3.911378308	2.376589	0.04385
DYRK1B	260.283651	1236.1124	8.029473043	10.27276092	10.27276092	-2.24765	0.04387
CBLB	366.38654	1761.6255	8.521154961	10.78351026	10.78351026	-2.26547	0.0439
CEBPB	251.165434	1190.6671	7.978226717	10.21876559	10.21876559	-2.24506	0.04403
LOC100738454	121.023608	562.69557	6.931016486	9.138772422	9.138772422	-2.21707	0.04408
ASB16	37.3017971	5.7839486	5.259340178	2.762125239	2.762125239	2.689118	0.04408
LOC102166016	37.3017971	5.7839486	5.259340178	2.762125239	2.762125239	2.689118	0.04408
CPA5	37.3017971	5.7839486	5.259340178	2.762125239	2.762125239	2.689118	0.04408
CLPB	3660.54969	741.1717	11.83823866	9.535609179	9.535609179	2.304181	0.04422
RHBDD2	312.506167	1491.4325	8.29235001	10.54344993	10.54344993	-2.25474	0.0443
NOL9	4545.01674	912.21132	12.15038728	9.834804937	9.834804937	2.316846	0.04433
TPP1	2199.9771	11421.646	11.10392842	13.47960925	13.47960925	-2.37621	0.04442
NUCB1	2734.63619	14340.887	11.41766066	13.80794728	13.80794728	-2.39071	0.04445
PCDHA6	26.5257224	3.3051135	4.78270852	2.106051269	2.106051269	3.00462	0.04447
RSPO4	26.5257224	3.3051135	4.78270852	2.106051269	2.106051269	3.00462	0.04447
LOC100737055	26.5257224	3.3051135	4.78270852	2.106051269	2.106051269	3.00462	0.04447
LOC102165900	26.5257224	3.3051135	4.78270852	2.106051269	2.106051269	3.00462	0.04447
GPR182	26.5257224	3.3051135	4.78270852	2.106051269	2.106051269	3.00462	0.04447
ZNF518B	528.027661	114.02642	9.047199346	6.845821397	6.845821397	2.211245	0.04461
LRRC7	13.2628612	69.407383	3.834191515	6.137654819	6.137654819	-2.3877	0.04465
HAGHL	45.5910853	212.35354	5.541982032	7.737102248	7.737102248	-2.21964	0.04465
IZUMO1	10.7760747	58.665764	3.557786824	5.89883146	5.89883146	-2.44469	0.04476
LOC100737169	1055.22639	5288.1816	10.04470338	12.36882879	12.36882879	-2.32522	0.04488
SASS6	916.795279	195.82797	9.842028575	7.620791468	7.620791468	2.227013	0.045
TRMT13	130.141825	27.267186	7.03498407	4.821056379	4.821056379	2.254847	0.0452
LOC100512723	140.9179	654.41247	7.148912757	9.356259313	9.356259313	-2.21535	0.04521
LOC100739855	940.005286	4680.867	9.878059017	12.19286823	12.19286823	-2.31603	0.04522
GLB1L	379.649401	1818.6387	8.572318998	10.8294363	10.8294363	-2.26012	0.04532
MLKL	51.3935871	237.96817	5.711318333	7.900674664	7.900674664	-2.21111	0.04534
LOC100739646	23.2100071	112.37386	4.597531595	6.824944216	6.824944216	-2.27549	0.0454
GALNT4	33.1571529	156.16661	5.094115824	7.296150961	7.296150961	-2.23569	0.04547
LOC100621801	33.1571529	156.16661	5.094115824	7.296150961	7.296150961	-2.23569	0.04547
LOC102157454	40.6175124	6.610227	5.379118827	2.927939483	2.927939483	2.61933	0.04549
LOC100522001	79.5771671	15.699289	6.332299179	4.061714779	4.061714779	2.341655	0.04554
LOC100627275	440.990134	2122.7091	8.787870357	11.05237047	11.05237047	-2.26709	0.04564
LGI2	54.7093024	9.9153405	5.799846344	3.448285225	3.448285225	2.464052	0.04565
GAS2L1	351.465821	1676.5188	8.461339555	10.71211323	10.71211323	-2.25401	0.04568
LOC654299	902.703489	4478.4288	9.819705682	12.12909905	12.12909905	-2.31067	0.04577
PRDM10	285.151515	61.970878	8.160635436	5.976612875	5.976612875	2.202066	0.04587
FASN	55514.1923	10023.583	15.76059501	13.29125459	13.29125459	2.469458	0.04591
GPX2	19.0653629	1.6525567	4.326635347	1.407383613	1.407383613	3.528182	0.04597
LOC102166864	19.0653629	1.6525567	4.326635347	1.407383613	1.407383613	3.528182	0.04597
CTSW	19.0653629	1.6525567	4.326635347	1.407383613	1.407383613	3.528182	0.04597
LOC100623402	19.0653629	1.6525567	4.326635347	1.407383613	1.407383613	3.528182	0.04597
ATOH1	19.0653629	1.6525567	4.326635347	1.407383613	1.407383613	3.528182	0.04597
LOC102160042	19.0653629	1.6525567	4.326635347	1.407383613	1.407383613	3.528182	0.04597
LOC102165743	19.0653629	1.6525567	4.326635347	1.407383613	1.407383613	3.528182	0.04597
CSMD1	19.0653629	1.6525567	4.326635347	1.407383613	1.407383613	3.528182	0.04597
PPP1R32	19.0653629	1.6525567	4.326635347	1.407383613	1.407383613	3.528182	0.04597
CACNA2D4	19.0653629	1.6525567	4.326635347	1.407383613	1.407383613	3.528182	0.04597
ANXA1	3513.00035	18540.86	11.77889862	14.17849838	14.17849838	-2.39993	0.04603
RPIA	3189.71811	653.58619	11.63966544	9.35443936	9.35443936	2.28698	0.04607
IGSF1	125.997181	26.440908	6.988652665	4.77825631	4.77825631	2.252548	0.04626
TMEM141	180.706484	841.15138	7.505466087	9.717935781	9.717935781	-2.21872	0.04628
ATAD2	3750.90293	764.30749	11.87340679	9.579895716	9.579895716	2.295013	0.04643
NPAS2	313.335095	1483.1697	8.296159547	10.53544032	10.53544032	-2.24291	0.04664
TSGA10IP	29.8414377	4.1313919	4.946798112	2.359350201	2.359350201	2.852617	0.04665
ENO2	549.57981	2659.7901	9.104807896	11.37763898	11.37763898	-2.27491	0.0467
HSPD1	77625.0397	13845.947	16.24425307	13.75728027	13.75728027	2.487058	0.04681
ITGA2	470.002643	102.45852	8.879591345	6.692908622	6.692908622	2.197629	0.04682
LOC100736775	124.339324	572.61091	6.969695301	9.163928658	9.163928658	-2.20327	0.04684
LOC100620793	96.9846724	19.830681	6.614484182	4.380638094	4.380638094	2.290023	0.04684
LOC102162699	321.624384	1522.831	8.33371167	10.57348723	10.57348723	-2.24331	0.04684
SDCBP	2944.35518	15360.515	11.5242259	13.90703288	13.90703288	-2.3832	0.04703
RIF1	7432.17583	1473.2543	12.85976302	10.52576972	10.52576972	2.334778	0.04704
PMFBP1	86.2085977	17.351846	6.446398468	4.19785327	4.19785327	2.312743	0.04708
ENPP1	540.461593	2610.2134	9.080715198	11.35050464	11.35050464	-2.2719	0.0471
TMEM109	820.639535	4035.5436	9.682361792	11.97890475	11.97890475	-2.29794	0.04712
LOC100620578	11220.3806	2184.68	13.45396256	11.09386649	11.09386649	2.360628	0.04715
WASF1	431.042988	94.195734	8.755031058	6.572825024	6.572825024	2.194098	0.04727
LOC102161594	5228.88302	1053.5049	12.35256296	10.04235012	10.04235012	2.311306	0.04733
A3GALT2	1.65785765	19.004403	1.410263837	4.322245637	4.322245637	-3.51894	0.04735
LOC102159557	1.65785765	19.004403	1.410263837	4.322245637	4.322245637	-3.51894	0.04735
SLC34A3	1.65785765	19.004403	1.410263837	4.322245637	4.322245637	-3.51894	0.04735
INHA	1.65785765	19.004403	1.410263837	4.322245637	4.322245637	-3.51894	0.04735
LOC100626840	1.65785765	19.004403	1.410263837	4.322245637	4.322245637	-3.51894	0.04735
LOC100154914	1.65785765	19.004403	1.410263837	4.322245637	4.322245637	-3.51894	0.04735
ADAMTS10	1576.62262	7979.3702	10.62353643	12.96223996	12.96223996	-2.33944	0.04739
RALGAPA2	1063.51568	228.05283	10.05598149	7.839536581	7.839536581	2.221401	0.04744
HSPB2	68.8010924	314.81206	6.125177709	8.302922453	8.302922453	-2.19399	0.04745
KLF7	74.6035941	341.25297	6.240382915	8.41891924	8.41891924	-2.19352	0.04745
LOC102164120	161.641121	34.703692	7.34554825	5.158001345	5.158001345	2.219633	0.04749
UNC93B1	621.696618	3015.0898	9.282385633	11.55846366	11.55846366	-2.27792	0.04764
EBF4	48.9068006	224.74772	5.641164514	7.818567588	7.818567588	-2.2002	0.04775
FBXL4	83.7218112	382.56689	6.404661526	8.583334365	8.583334365	-2.19204	0.04778
LGI4	19.0653629	93.369456	4.326635347	6.560248082	6.560248082	-2.292	0.04801
C4H1orf114	1352.81184	288.37115	10.40281152	8.176777292	8.176777292	2.229962	0.04805
LOC100736628	143.404687	659.37014	7.173973754	9.367131078	9.367131078	-2.201	0.04813
NHLRC1	175.732911	38.008805	7.465426908	5.285727902	5.285727902	2.208979	0.04828
LOC100736720	78.7482382	15.699289	6.317380743	4.061714779	4.061714779	2.326548	0.04829
LOC102162069	33.1571529	4.9576702	5.094115824	2.574748269	2.574748269	2.741586	0.04832
LOC102158566	219.666138	47.924146	7.785721452	5.61247475	5.61247475	2.196488	0.04833
LOC102160864	10.7760747	57.839486	3.557786824	5.878712739	5.878712739	-2.42422	0.04838
LOC102159188	29.0125088	136.33593	4.907492018	7.101565318	7.101565318	-2.23242	0.04841
LOC100738006	2063.20384	433.79615	11.01136973	8.76419534	8.76419534	2.249797	0.04862
MMP15	121.023608	553.60651	6.931016486	9.115320738	9.115320738	-2.19357	0.04863
LOC102157854	117.707893	24.788351	6.891272054	4.688647628	4.688647628	2.247477	0.04869
LOC102161195	50.5646582	9.0890621	5.688310694	3.334720158	3.334720158	2.475926	0.04873
LOC100525796	50.5646582	9.0890621	5.688310694	3.334720158	3.334720158	2.475926	0.04873
ADCY9	62.1696618	283.41348	5.981159942	8.151846042	8.151846042	-2.18863	0.04875
LOC102167511	150.036117	32.224857	7.238749771	5.054191063	5.054191063	2.219064	0.04878
FGFR3	222.981854	48.750424	7.807238043	5.636636914	5.636636914	2.19344	0.0488
LOC100511626	849.652044	184.26008	9.732425314	7.533408207	7.533408207	2.205129	0.04884
FKBP7	295.92759	1387.3214	8.213967342	10.43912586	10.43912586	-2.22899	0.04887
LOC100624593	391.254405	1853.3424	8.615645837	10.85669193	10.85669193	-2.24395	0.04894
BMP1	2181.74066	11152.279	11.09192502	13.44518032	13.44518032	-2.35379	0.04899
LOC100516787	82.0639535	16.525567	6.376150635	4.13138925	4.13138925	2.312049	0.04901
RRAGC	1371.8772	6862.2419	10.42298687	12.74467448	12.74467448	-2.32253	0.04902
NHE5	954.926005	206.56959	9.900755138	7.697451307	7.697451307	2.208761	0.0491
LOC100516275	96.1557435	19.830681	6.602227381	4.380638094	4.380638094	2.277639	0.04916
LOC102158540	193.140416	42.140197	7.600956677	5.430960858	5.430960858	2.196381	0.04954
42430	22.3810782	2.4788351	4.547269558	1.798604302	1.798604302	3.174545	0.04983
LOC100624694	22.3810782	2.4788351	4.547269558	1.798604302	1.798604302	3.174545	0.04983
ATHL1	224.639711	1039.4582	7.817877185	10.02300328	10.02300328	-2.21015	0.04994
MPP6	1596.51691	340.42669	10.64161549	8.415432027		2.229512	0.05
A2M	53.0514447	241.27328	5.756261274	7.920491517		-2.1852	0.05016
IRX6	12.4339324	64.449713	3.747809764	6.032314961		-2.37389	0.05019
SNX10	2887.88802	602.35693	11.49634851	9.236867912		2.261374	0.05037
RPUSD2	547.921952	120.63664	9.100457226	6.926434087		2.183302	0.05041
LOC102158759	170.759338	37.182527	7.424244723	5.254840671		2.199268	0.05049
TNFRSF12A	1064.34461	5236.9523	10.05710446	12.35478721		-2.29876	0.05055
LOC100737285	223.810782	1032.848	7.812567422	10.01380833		-2.20628	0.05072
LOC102157752	39.7885835	6.610227	5.350093502	2.927939483		2.589583	0.05075
LOC102166077	39.7885835	6.610227	5.350093502	2.927939483		2.589583	0.05075
BPIFB3	39.7885835	6.610227	5.350093502	2.927939483		2.589583	0.05075
PPP1R3C	145.062544	661.0227	7.190442454	9.37073687		-2.18802	0.05088
LOC100511764	14.9207188	0.8262784	3.99283357	0.868906686		4.174545	0.05092
LOC100739108	14.9207188	0.8262784	3.99283357	0.868906686		4.174545	0.05092
LOC102167469	14.9207188	0.8262784	3.99283357	0.868906686		4.174545	0.05092
CSTF2	2214.06889	467.67356	11.11313585	8.872439593		2.243126	0.051
STAT2	679.721635	3272.0624	9.410921153	11.67642537		-2.26718	0.05113
ZNF436	221.323996	1019.6275	7.79651986	9.99524072		-2.20381	0.05114
MPZL3	19.8942918	95.848291	4.385036953	6.597654688		-2.2684	0.05116
LOC100523131	645.735554	142.11988	9.337032113	7.161080272		2.183835	0.0513
LOC100524247	901.045631	196.65425	9.817056606	7.626835185		2.195939	0.05136
LOC102159626	2.48678647	22.309516	1.801898017	4.542847146		-3.1653	0.05138
RHBDL3	2.48678647	22.309516	1.801898017	4.542847146		-3.1653	0.05138
SGCG	2.48678647	22.309516	1.801898017	4.542847146		-3.1653	0.05138
LOC102160135	102.787174	464.36844	6.697484358	8.862229579		-2.17561	0.05162
LOC100621417	442.647992	2088.8317	8.793271628	11.02917106		-2.23846	0.05167
ITM2B	4739.81501	24916.424	12.21091938	14.60486733		-2.39419	0.05178
TMCC2	435.187632	96.674569	8.768805054	6.609911086		2.170429	0.05219
LOC100737554	25.6967935	3.3051135	4.73859457	2.106051269		2.958817	0.05226
IGIP	0.82892882	14.873011	0.870998931	3.98850389		-4.1653	0.05232
LOC102157810	0.82892882	14.873011	0.870998931	3.98850389		-4.1653	0.05232
LOC102157791	0.82892882	14.873011	0.870998931	3.98850389		-4.1653	0.05232
LOC102158362	213.034708	47.097867	7.741700952	5.587901017		2.177355	0.05234
LOC100511499	10.7760747	0	3.557786824	0		20	0.05253
LOC102168156	10.7760747	0	3.557786824	0		20	0.05253
RD3	10.7760747	0	3.557786824	0		20	0.05253
DGKK	10.7760747	0	3.557786824	0		20	0.05253
CELA1	10.7760747	0	3.557786824	0		20	0.05253
PAX5	10.7760747	0	3.557786824	0		20	0.05253
LOC100624232	10.7760747	0	3.557786824	0		20	0.05253
LOC102162116	10.7760747	0	3.557786824	0		20	0.05253
LOC102166649	10.7760747	0	3.557786824	0		20	0.05253
LOC102164488	10.7760747	0	3.557786824	0		20	0.05253
LOC102164454	10.7760747	0	3.557786824	0		20	0.05253
LOC100524885	10.7760747	0	3.557786824	0		20	0.05253
LOC102164583	10.7760747	0	3.557786824	0		20	0.05253
LOC102157754	10.7760747	0	3.557786824	0		20	0.05253
HEPACAM	10.7760747	0	3.557786824	0		20	0.05253
LOC102164371	10.7760747	0	3.557786824	0		20	0.05253
SPN	10.7760747	0	3.557786824	0		20	0.05253
MYL7	10.7760747	0	3.557786824	0		20	0.05253
LOC100736798	10.7760747	0	3.557786824	0		20	0.05253
LOC100154934	10.7760747	0	3.557786824	0		20	0.05253
BPIFB4	10.7760747	0	3.557786824	0		20	0.05253
LOC102158158	10.7760747	0	3.557786824	0		20	0.05253
LOC102158300	10.7760747	0	3.557786824	0		20	0.05253
LOC102167787	10.7760747	0	3.557786824	0		20	0.05253
DMRTC2	10.7760747	0	3.557786824	0		20	0.05253
C14H10orf131	10.7760747	0	3.557786824	0		20	0.05253
PIK3CG	10.7760747	0	3.557786824	0		20	0.05253
LOC102165295	10.7760747	0	3.557786824	0		20	0.05253
FAM83B	6.63143059	39.661362	2.93195353	5.34558663		-2.58034	0.05255
LOC102165547	99.4714588	447.84288	6.65064192	8.810066691		-2.17064	0.05256
NMB	70.45895	14.046732	6.159042811	3.911378308		2.326548	0.05314
LOC100519601	70.45895	14.046732	6.159042811	3.911378308		2.326548	0.05314
CASKIN1	872.033122	191.69658	9.76989258	7.590187173		2.185558	0.05339
LAMB1	5532.27097	29156.059	12.43391686	14.83155757		-2.39785	0.05341
CALD1	13442.7387	73879.202	13.71464679	16.17290019		-2.45834	0.05343
LOC100739645	1215.20966	264.40908	10.24817623	8.052073912		2.200362	0.05345
LOC100739238	169.930409	770.91772	7.417265267	9.592303268		-2.18163	0.0535
EGR1	3286.70279	688.28988	11.68286417	9.428967032		2.255553	0.05355
LOC100623380	242.876145	1113.8232	7.930004838	10.12259927		-2.19723	0.05356
LRP4	1833.59056	393.3085	10.8412424	8.623181018		2.220938	0.05368
LOC100512447	659.827344	146.25127	9.368129573	7.202136284		2.173639	0.05372
LOC102159477	0	10.741619	0	3.553559423		-20	0.05379
LOC100525593	0	10.741619	0	3.553559423		-20	0.05379
KCNH7	0	10.741619	0	3.553559423		-20	0.05379
LOC100626820	0	10.741619	0	3.553559423		-20	0.05379
ITGB6	0	10.741619	0	3.553559423		-20	0.05379
CLIC6	0	10.741619	0	3.553559423		-20	0.05379
LOC102160362	0	10.741619	0	3.553559423		-20	0.05379
LOC102165848	0	10.741619	0	3.553559423		-20	0.05379
LOC100515186	0	10.741619	0	3.553559423		-20	0.05379
LRRC66	0	10.741619	0	3.553559423		-20	0.05379
CADM2	0	10.741619	0	3.553559423		-20	0.05379
LOC100739342	288.467231	64.449713	8.177256226	6.032314961		2.162162	0.05382
LOC102160260	12.4339324	63.623435	3.747809764	6.013985524		-2.35528	0.05383
ND2	251841.011	43491.988	17.94215944	15.40849522		2.533692	0.05385
LOC100525978	16.5785765	80.97528	4.135746339	6.357117027		-2.28816	0.05389
LOC102164482	29.0125088	4.1313919	4.907492018	2.359350201		2.811975	0.05389
KIF5A	29.0125088	4.1313919	4.907492018	2.359350201		2.811975	0.05389
LOC102161477	53.0514447	9.9153405	5.756261274	3.448285225		2.419658	0.05392
PUS10	2599.52079	550.3014	11.34458486	9.106697443		2.239952	0.05392
AHNAK2	96.9846724	434.62242	6.614484182	8.766934408		-2.16393	0.05394
TXNDC5	2579.6265	13090.728	11.33350564	13.67636794		-2.34331	0.05398
CEP70	1855.97164	398.26618	10.85873606	8.641207046		2.22037	0.05398
LOC100153094	319.137597	1475.7332	8.322548307	10.52819346		-2.20918	0.05406
MCM2	16704.5737	3261.3207	14.0280419	11.67168291		2.356715	0.05416
LPHN1	209.718992	955.1778	7.719176542	9.901135097		-2.18731	0.05423
RALGDS	504.817654	2375.5503	8.982473579	11.21465323		-2.23443	0.05436
LOC100515505	17.4075053	84.280394	4.202222212	6.414142197		-2.27549	0.05453
SLC22A17	610.091614	2892.8006	9.255244873	11.49874979		-2.24537	0.05453
LOC100737129	140.088971	630.4504	7.140461408	9.3025256		-2.17004	0.05455
COL4A5	1254.16931	273.49814	10.29366627	8.100652569		2.197129	0.05458
NCL	18092.2005	3524.0773	14.14316001	11.78343916		2.36005	0.05464
LOC100153768	3234.48027	680.0271	11.65976416	9.411568398		2.24987	0.05469
TMEM88B	115.221107	24.788351	6.860728285	4.688647628		2.216671	0.05491
CHST2	4786.23503	990.70777	12.22497692	9.953771246		2.27236	0.05513
MDM1	622.525547	138.81477	9.28430486	7.127372928		2.164972	0.05528
LOC100627393	401.201551	90.064343	8.651774833	6.508814353		2.155299	0.05531
RNF24	87.8664553	391.65595	6.473567038	8.617121942		-2.1562	0.05538
SLC9A9	111.905391	499.89841	6.818970566	8.968374236		-2.15936	0.05556
MAFF	227.126498	1032.848	7.83369022	10.01380833		-2.18506	0.05559
SPATA5	1406.69221	306.54928	10.45911621	8.264673769		2.198116	0.05574
LOC102161865	14.9207188	73.538775	3.99283357	6.219919206		-2.30119	0.05581
PNPLA4	689.668781	153.68778	9.431850204	7.273215394		2.165901	0.05586
PIP4K2C	359.755109	80.97528	8.494876018	6.357117027		2.151462	0.05599
LOC100622742	111.905391	499.07214	6.818970566	8.965992412		-2.15697	0.0561
MYO1C	3992.12121	20554.501	11.96330115	14.3272369		-2.36423	0.05616
TMBIM1	615.894116	2906.021	9.268879075	11.50532579		-2.23829	0.05634
LOC100738145	30.6703665	139.64104	4.985061656	7.135873883		-2.18681	0.05667
GNA14	30.6703665	139.64104	4.985061656	7.135873883		-2.18681	0.05667
SOHLH2	93.6689571	19.830681	6.564819524	4.380638094		2.239837	0.05687
SERINC4	201.429704	908.90621	7.661277193	9.829574033		-2.17385	0.05691
DBH	18.2364341	1.6525567	4.265769485	1.407383613		3.464052	0.05695
LOC102159728	18.2364341	1.6525567	4.265769485	1.407383613		3.464052	0.05695
LOC102166350	18.2364341	1.6525567	4.265769485	1.407383613		3.464052	0.05695
LOC102158112	18.2364341	1.6525567	4.265769485	1.407383613		3.464052	0.05695
LOC102163239	18.2364341	1.6525567	4.265769485	1.407383613		3.464052	0.05695
C1H14orf39	18.2364341	1.6525567	4.265769485	1.407383613		3.464052	0.05695
SLC7A2	570.303031	128.07315	9.158112373	7.012045083		2.154761	0.05707
FAM26E	72.9457365	14.873011	6.208395063	3.98850389		2.294127	0.05714
LOC102161964	1384.31114	303.24416	10.43599432	8.249085772		2.190616	0.05738
ZNF703	557.04017	2608.5608	9.124225165	11.34959131		-2.2274	0.05746
LOC396710	995.543517	220.61633	9.960788996	7.791920351		2.173945	0.05751
LOC102157824	300.901163	1374.1009	8.237932504	10.4253218		-2.19113	0.05753
TTLL4	754.32523	168.56079	9.560954167	7.405658764		2.161918	0.0576
OSBPL5	590.197322	2768.0325	9.207495925	11.43516629		-2.22959	0.0578
LOC100738030	5.80250177	35.52997	2.766065426	5.191008663		-2.61429	0.05786
SUSD4	5635.05814	1166.7051	12.46047078	10.18946021		2.271991	0.05786
LOC100738939	288.467231	65.275991	8.177256226	6.05041444		2.143783	0.0579
GUF1	2348.35536	504.85609	11.19804923	8.98258319		2.217707	0.05795
LOC100739054	328.255814	74.365053	8.363065106	6.2358238		2.142124	0.0581
FBL	27696.1699	5340.2371	14.75745095	12.38295822		2.374711	0.0581
LOC100739509	48.9068006	9.0890621	5.641164514	3.334720158		2.427832	0.0582
TUBB6	1962.90345	9716.2074	10.93950829	13.24632604		-2.3074	0.05822
LOC102166190	273.546512	61.970878	8.100906771	5.976612875		2.142124	0.05844
MROH1	65.4853771	289.19743	6.054965161	8.180890933		-2.14281	0.05845
LOC102159264	155.838619	34.703692	7.293137032	5.158001345		2.166892	0.05848
SYNGR1	134.286469	596.57298	7.079873746	9.222971117		-2.15138	0.05855
C5H12orf66	184.022199	41.313919	7.531554564	5.403060391		2.15518	0.05857
LOC102161369	1.65785765	18.178124	1.410263837	4.261389712		-3.45481	0.05859
LOC102163811	1.65785765	18.178124	1.410263837	4.261389712		-3.45481	0.05859
LOC102164807	1.65785765	18.178124	1.410263837	4.261389712		-3.45481	0.05859
LOC100152036	1.65785765	18.178124	1.410263837	4.261389712		-3.45481	0.05859
TMOD2	127.655039	28.093465	7.007364151	4.862623207		2.183944	0.05893
LOC100739498	110.247534	23.962073	6.79762954	4.641665831		2.201922	0.05909
TNFAIP1	498.186223	2311.1006	8.963434308	11.17498846		-2.21382	0.0591
N4BP2	2645.11188	567.65324	11.36965834	9.151405371		2.220247	0.05911
FSD1L	751.009514	168.56079	9.554607104	7.405658764		2.155562	0.05912
IGF2BP3	739.404511	166.08195	9.532169874	7.384412099		2.154469	0.05924

MYSM1	189.170948	846.10905	7.571153052	9.726403897		-2.16115	0.28692
LOC102164574	22.0929574	98.327126	4.529381037	6.634115866		-2.154	0.28695
ASAP3	701.451397	3410.0508	9.456254597	11.73600054		-2.28138	0.28699
LOC100622097	469.475344	123.11548	8.877975309	6.955539223		1.931037	0.28718
B3GALTL	624.126046	3008.4796	9.288003303	11.5552983		-2.26913	0.28735
HMGB2	9357.74826	2128.4931	13.19209987	11.05629433		2.136329	0.28789
IQCB1	1093.60139	277.62953	10.09618988	8.122204372		1.977854	0.28795
LOC100152024	162.935561	722.1673	7.356985025	9.498185627		-2.14803	0.28798
ZDHHC8	432.193479	2027.6871	8.758867714	10.98633067		-2.23009	0.28924
RGC32	675.21601	175.17101	9.401340363	7.460832769		1.946585	0.28928
LOC102160274	41.4242951	177.64985	5.406818783	7.480990892		-2.10049	0.29003
LOC102166683	12.4272885	59.492043	3.747096094	5.918673475		-2.25919	0.29003
TIGD3	298.254925	79.322724	8.225231178	6.327736286		1.91074	0.29054
ZBTB38	193.313377	861.80834	7.602241413	9.752896314		-2.15643	0.29066
C12H17orf72	37.2818656	8.2627837	5.258589232	3.211445828		2.173774	0.2907
LOC100737854	6.90404918	38.008805	2.982591923	5.285727902		-2.46082	0.29101
HERC5	625.506856	3002.6956	9.291186486	11.5525229		-2.26316	0.29109
SIRT4	288.589256	76.843889	8.177864267	6.282511874		1.909015	0.29125
C13H3orf26	2290.76352	562.69557	11.16224247	9.138772422		2.025402	0.29134
HHIP	86.9910197	374.3041	6.459284385	8.55191625		-2.10527	0.29136
MROH6	71.8021115	18.178124	6.185908388	4.261389712		1.981823	0.29181
ZFHX4	653.123052	3141.5104	9.353418248	11.61770179		-2.26603	0.29196
ELAC2	3639.81473	873.37624	11.83004561	9.772110386		2.05919	0.29198
FAM188B	463.952105	122.2892	8.8609383	6.945902605		1.923679	0.2922
ATP6V1G2	67.659682	289.19743	6.101391269	8.180890933		-2.09569	0.29221
PCDH9	55.2323934	235.48934	5.813329549	7.885631319		-2.09208	0.29273
CLCN6	528.850167	2504.4497	9.049440637	11.29085388		-2.24356	0.29291
MLLT3	204.359856	911.38504	7.682010377	9.833498988		-2.15695	0.29295
CCNB1	14390.8001	3212.5703	13.81295943	11.64996132		2.163347	0.29304
LOC102164780	22.0929574	97.500848	4.529381037	6.622064238		-2.14183	0.29342
STXBP6	149.127462	653.58619	7.230044098	9.35443936		-2.13183	0.29369
NCF4	98.0374983	25.61463	6.629902968	4.734147579		1.936366	0.29384
LOC404701	4.14242951	27.267186	2.362450112	4.821056379		-2.71862	0.29485
SP140	1309.00772	6587.9175	10.3553596	12.68582574		-2.33135	0.29541
SAE1	19102.1233	4210.7146	14.22152091	12.04019196		2.181596	0.29607
FZD7	223.691193	998.97055	7.81179977	9.965741799		-2.15893	0.29616
SPSB4	24.854577	108.24247	4.692347799	6.771389985		-2.12268	0.29622
SMAD6	325.871121	1488.9536	8.352578112	10.54105171		-2.19193	0.29622
LOC102161449	16.569718	75.191332	4.135019133	6.251554969		-2.18202	0.2964
LOC100623617	327.251931	1494.7376	8.358659688	10.54664136		-2.19142	0.29676
SHD	8.28485901	42.966475	3.214880004	5.458331975		-2.37466	0.29706
LOC100736834	8.28485901	42.966475	3.214880004	5.458331975		-2.37466	0.29706
PRSS36	8.28485901	42.966475	3.214880004	5.458331975		-2.37466	0.29706
TOMM5	5512.19286	1299.7359	12.42867236	10.34511233		2.084408	0.29734
LOC100737435	887.860724	4334.6563	9.795813571	12.08203469		-2.28751	0.29784
PRSS8	28.9970066	124.76803	4.906746634	6.974621475		-2.10527	0.29785
LOC102164764	171.22042	751.91332	7.428112398	9.556339969		-2.13471	0.29789
LOC102162605	19.3313377	85.932951	4.345633235	6.441831208		-2.15227	0.29801
DPYSL3	974.851744	4789.9357	9.930518173	12.22609174		-2.29675	0.29805
LOC100736659	48.3283443	11.567897	5.624344958	3.65167138		2.062743	0.29811
DST	3701.95117	20015.767	11.85445981	14.28892138		-2.43478	0.29844
[illegible]							

F8A2	119.4815768	719.68846	6.912668746	9.493231938		-2.59058	0.14892
LOC100624071	1243.454286	222.26888	10.28129752	7.80263838		2.483976	0.14917
LOC100516384	301.3473397	57.013208	8.240063075	5.858309485		2.402059	0.1493
LOC100626461	38.06492712	221.4426	5.287802017	7.797289318		-2.5404	0.14937
GTF2IRD2	168.1200948	1031.1954	7.40190428	10.0115004		-2.61675	0.14948
LOC100738884	8.458872693	56.186929	3.241668254	5.837613535		-2.7317	0.14979
LOC102164779	33.83549077	4.9576702	5.122485983	2.57474827		2.770803	0.14995
OPRD1	33.83549077	4.9576702	5.122485983	2.57474827		2.770803	0.14995
F8A1	119.4815768	718.03591	6.912668746	9.489920003		-2.58727	0.15
LOC100523628	3210.142187	544.51745	11.64887083	9.091481528		2.559587	0.15011
LOC100524404	26.43397716	154.51406	4.777891883	7.280901168		-2.54727	0.1502
LOC100621053	14.80302721	90.064343	3.982129041	6.508814353		-2.60506	0.15025
TRAM2	425.0583528	2768.8588	8.734907225	11.43559673		-2.70356	0.1503
LOC100156874	13.74566813	84.280394	3.882219287	6.414142197		-2.61622	0.15055
EMC10	1228.651259	8649.482	10.2640335	13.07856481		-2.81554	0.15061
METTL16	698.9143562	128.89943	9.45103459	7.021251245		2.43887	0.15075
LOC100624105	741.2087197	136.33593	9.53568114	7.101565318		2.442714	0.15075
LOC102164702	12.68830904	78.496445	3.774872332	6.312818447		-2.62913	0.15105
TDRD12	7468.127228	1205.5401	12.86672396	10.2366602		2.631067	0.15109
MED14	10940.49447	1725.2692	13.41752218	10.75344178		2.664785	0.15114
GRK5	236.8484354	1479.0383	7.893898725	10.53141878		-2.64262	0.15126
LOC100624979	466.2953572	87.585507	8.868190893	6.468998788		2.41248	0.15185
LOC100525572	134.284604	808.92653	7.079853853	9.661647227		-2.59071	0.15186
CREB3L2	600.5799612	3997.5348	9.232612701	11.96525572		-2.73468	0.152
ATP10A	180.8084038	1108.0393	7.506275077	10.11509477		-2.61548	0.15202
IGFN1	2.114718173	23.135794	1.639101631	4.593102407		-3.45159	0.1521
WNT1	2.114718173	23.135794	1.639101631	4.593102407		-3.45159	0.1521
SERPINB7	2.114718173	23.135794	1.639101631	4.593102407		-3.45159	0.1521
LOC100623534	2.114718173	23.135794	1.639101631	4.593102407		-3.45159	0.1521
LOC102158033	2.114718173	23.135794	1.639101631	4.593102407		-3.45159	0.1521
CD40LG	2.114718173	23.135794	1.639101631	4.593102407		-3.45159	0.1521
CD34	2.114718173	23.135794	1.639101631	4.593102407		-3.45159	0.1521
LOC100521485	839.5431147	5725.2828	9.715178013	12.48338322		-2.76967	0.15226
MN1	327.7813168	2088.0054	8.360984509	11.02860054		-2.67132	0.15228
SPIN3	54.9826725	318.94345	5.806908455	8.321673128		-2.53625	0.1524
LOC102157511	126.8830904	23.962073	6.998681703	4.641665831		2.404675	0.15248
TYW3	942.1069461	171.8659	9.881277568	7.433509508		2.454608	0.15262
[illegible]							

[illegible]							

CCDC3	238.880102	1019.6275	7.906169679	9.99524072		-2.09368	0.33932
LOC102163695	4.14242951	25.61463	2.362450112	4.734147579		-2.62842	0.33964
LOC100736721	4.14242951	25.61463	2.362450112	4.734147579		-2.62842	0.33964
LOC102162739	158.793131	661.84898	7.320061583	9.372536392		-2.05935	0.33982
FIGNL1	948.616357	253.66746	9.891200975	7.992470822		1.902886	0.34009
GATA4	5.52323934	30.5723	2.705588563	4.980587446		-2.46864	0.34019
LOC100621130	5.52323934	30.5723	2.705588563	4.980587446		-2.46864	0.34019
LOC100521704	912.715301	244.5784	9.835600905	7.940039851		1.899868	0.34045
PHLDA2	243.022531	1036.9794	7.930870551	10.01956204		-2.09323	0.34055
ACAD8	961.043646	256.97257	9.909958537	8.011073883		1.902988	0.34072
ARHGAP23	490.187492	2189.6377	8.940130012	11.09713518		-2.15929	0.34087
HR	244.403341	1042.7633	7.93901108	10.02757887		-2.09308	0.34096
LOC102159522	8.28485901	40.48764	3.214880004	5.374609695		-2.28893	0.34156
MIC-1	28.9970066	118.15781	4.906746634	6.89672967		-2.02674	0.34162
TGFBR3	78.7061606	318.11717	6.316619332	8.317942438		-2.01501	0.34171
LOC733643	2413.65559	12085.147	11.23760171	13.56106683		-2.32394	0.34197
LOC100516676	389.388374	1709.57	8.608766278	10.74026139		-2.13435	0.3423
LOC102159841	9.66566885	45.44531	3.414902536	5.537461031		-2.23319	0.34256
LOC100738261	9244.52185	2197.9005	13.17453904	11.10256659		2.072473	0.3429
LYSMD3	1743.96282	453.62683	10.76898058	8.828539005		1.942791	0.3429
SKP2	1082.55491	288.37115	10.08155655	8.176777292		1.908441	0.34291
NCCRP1	153.269892	42.966475	7.269312714	5.458331975		1.834791	0.34313
RAB5B	637.934144	2894.4531	9.319523428	11.49957343		-2.18181	0.34354
LOC100738331	520.565308	2325.9736	9.026704102	11.18423914		-2.15968	0.34449
LOC102157990	404.577282	112.37386	8.663833033	6.824944216		1.848109	0.34475
LOC102158539	12.4272885	55.360651	3.747096094	5.816616368		-2.15535	0.34511
LOC102161409	24.854577	101.63224	4.692347799	6.681340183		-2.03177	0.34655
LOC100157987	382.484325	106.58991	8.583023796	6.749398975		1.84333	0.34656
LOC100518422	3094.39484	783.3119	11.59590773	9.615283673		1.981999	0.34678
LOC100621280	111.845597	454.4531	6.818206315	8.831158705		-2.02262	0.34766
MTL5	73.1829213	19.830681	6.213015177	4.380638094		1.883773	0.34821
EPB42	73.1829213	19.830681	6.213015177	4.380638094		1.883773	0.34821
PTPMT1	2677.39027	684.15849	11.38715048	9.420293943		1.968425	0.34882
ETV7	201.598236	842.80394	7.662477803	9.720764012		-2.06371	0.34893
RASL12	33.1394361	133.03082	5.093367322	7.066420948		-2.00514	0.349
KCNH2	84.2294	23.135794	6.413279271	4.593102408		1.864197	0.34907
LOC100738528	109.083977	442.05893	6.782460686	8.791354786		-2.0188	0.34908
LOC100624765	56.6132033	14.873011	5.848327568	3.98850389		1.928442	0.34909
CKS1B	6099.03704	1490.6062	12.57460229	10.54265097		2.032682	0.34911
TPP1	2305.95243	11421.646	11.17177254	13.47960925		-2.30834	0.34925
LOC100523131	513.661259	142.11988	9.007479378	7.161080272		1.853709	0.34954
LOC100738557	73.1829213	292.50254	6.213015177	8.197229196		-1.99887	0.34982
RUNX1	178.124469	738.69286	7.484818616	9.530782548		-2.05209	0.34985
CENPA	2395.70507	616.40367	11.22683667	9.270070238		1.958503	0.34999
CD247	175.362849	726.29869	7.462402878	9.506404165		-2.05022	0.35024
LOC102165864	175.362849	726.29869	7.462402878	9.506404165		-2.05022	0.35024
LOC100622851	51.0899639	13.220454	5.702933533	3.829895615		1.950268	0.35036
DHODH	672.45439	184.26008	9.395436432	7.533408207		1.867693	0.35072
SNX15	127.034505	517.25026	7.000388854	9.017505127		-2.02564	0.35077
SIPA1L2	98.0374983	27.267186	6.629902968	4.821056379		1.846168	0.35103
SKIL	179.505279	743.65053	7.495897217	9.540419716		-2.0506	0.35116
EXOSC3	1731.53553	453.62683	10.75866923	8.828539005		1.932474	0.35119
LOC100737495	111.845597	451.97427	6.818206315	8.823285292		-2.01473	0.35248
LOC102167775	111.845597	31.398578	6.818206315	5.017858594		1.832737	0.35354
DTX3L	248.54577	1046.8947	7.963160641	10.03327803		-2.07453	0.35357
LOC100627643	1253.77533	334.64274	10.29321336	8.390782629		1.905585	0.35388
LOC100627645	157.412321	44.619032	7.307540742	5.511563931		1.818817	0.35392
NCK2	276.161967	1170.0102	8.11458549	10.1935379		-2.08293	0.35431
KLHDC8B	289.970066	1231.9811	8.184726929	10.26793491		-2.087	0.35459
TNRC18	885.099105	4063.637	9.791324254	11.98891081		-2.19886	0.35462
MYLIP	302.397354	1287.3417	8.245064693	10.33129957		-2.08988	0.35526
LOC102164436	218.167954	61.970878	7.775893057	5.976612875		1.815777	0.3554
MARVELD2	40.0434852	9.9153405	5.359081339	3.448285225		2.013833	0.35543
RTN4R	262.353869	74.365053	8.040858843	6.2358238		1.818817	0.35555
ANXA5	9243.14104	50369.103	13.17432356	15.62028007		-2.44608	0.35573
LOC100522523	2583.4952	665.15409	11.33566681	9.379712118		1.957564	0.35584
DYRK1B	291.350875	1236.1124	8.191557101	10.27276092		-2.08498	0.35616
LOC102164659	9.66566885	44.619032	3.414902536	5.511563931		-2.20672	0.35676
FYN	1016.27604	4702.3502	9.990495494	12.19947304		-2.21009	0.35682
ABCC2	12.4272885	54.534373	3.747096094	5.795309087		-2.13365	0.35694
EML2	1547.88783	409.83407	10.59701695	8.682412026		1.917189	0.35707
CLEC4F	8.28485901	39.661362	3.214880004	5.34558663		-2.25919	0.35736
FAM83B	8.28485901	39.661362	3.214880004	5.34558663		-2.25919	0.35736
LOC100518075	8.28485901	39.661362	3.214880004	5.34558663		-2.25919	0.35736
PSPH	1001.08713	4623.8538	9.96879224	12.17519203		-2.20753	0.35746
LOC100517664	120.130456	484.19913	6.920417836	8.922433141		-2.011	0.35752
TRPV2	9740.23258	2335.0627	13.24988862	11.18986327		2.060495	0.35756
EXOSC2	4494.53602	1124.5649	12.13427743	10.13643348		1.998805	0.35777
MYO1G	309.301403	87.585507	8.277526411	6.468998788		1.820249	0.35804
SH3BGRL2	378.341895	106.58991	8.567354903	6.749398975		1.82762	0.35834
LOC100627610	965.186075	261.93024	9.91615725	8.038536289		1.881624	0.35853
LOC100511337	52.4707738	206.56959	5.740678649	7.697451307		-1.97704	0.35905
LOC100624915	2373.61211	615.57739	11.21347615	9.268138169		1.947072	0.35926
PLD1	127.034505	512.29259	7.000388854	9.003637625		-2.01175	0.35933
SLC6A2	0	9.9153405	0	3.448285225		-20	0.35936
LOC100622878	0	9.9153405	0	3.448285225		-20	0.35936
LOC100520985	0	9.9153405	0	3.448285225		-20	0.35936
LOC102161770	0	9.9153405	0	3.448285225		-20	0.35936
LOC100525342	0	9.9153405	0	3.448285225		-20	0.35936
LOC102161545	0	9.9153405	0	3.448285225		-20	0.35936
LOC102161376	0	9.9153405	0	3.448285225		-20	0.35936
ECSCR	0	9.9153405	0	3.448285225		-20	0.35936
LOC102159904	0	9.9153405	0	3.448285225		-20	0.35936
IL-18RA	0	9.9153405	0	3.448285225		-20	0.35936
LOC100151963	0	9.9153405	0	3.448285225		-20	0.35936
LOC102161036	0	9.9153405	0	3.448285225		-20	0.35936
LOC102163672	0	9.9153405	0	3.448285225		-20	0.35936
LOC102166894	0	9.9153405	0	3.448285225		-20	0.35936
CTXN3	0	9.9153405	0	3.448285225		-20	0.35936
LOC100156382	0	9.9153405	0	3.448285225		-20	0.35936
LOC102164817	0	9.9153405	0	3.448285225		-20	0.35936
LOC102166729	0	9.9153405	0	3.448285225		-20	0.35936
LOC102164595	0	9.9153405	0	3.448285225		-20	0.35936
LOC100516475	0	9.9153405	0	3.448285225		-20	0.35936
ABRA	0	9.9153405	0	3.448285225		-20	0.35936
WEE1B	0	9.9153405	0	3.448285225		-20	0.35936
LOC100512379	0	9.9153405	0	3.448285225		-20	0.35936
LOC102166009	0	9.9153405	0	3.448285225		-20	0.35936
LOC102166102	0	9.9153405	0	3.448285225		-20	0.35936
LOC100736583	0	9.9153405	0	3.448285225		-20	0.35936
LOC100738855	0	9.9153405	0	3.448285225		-20	0.35936
LOC100738944	0	9.9153405	0	3.448285225		-20	0.35936
LOC102158129	0	9.9153405	0	3.448285225		-20	0.35936
LOC102160963	0	9.9153405	0	3.448285225		-20	0.35936
LOC100623721	0	9.9153405	0	3.448285225		-20	0.35936
LOC100623996	0	9.9153405	0	3.448285225		-20	0.35936
SPOCK1	0	9.9153405	0	3.448285225		-20	0.35936
ZNF551	0	9.9153405	0	3.448285225		-20	0.35936
SMOC2	0	9.9153405	0	3.448285225		-20	0.35936
LOC102162353	0	9.9153405	0	3.448285225		-20	0.35936
FMO1	0	9.9153405	0	3.448285225		-20	0.35936
CAV3	0	9.9153405	0	3.448285225		-20	0.35936
LOC102162468	0	9.9153405	0	3.448285225		-20	0.35936
LOC100153182	0	9.9153405	0	3.448285225		-20	0.35936
LHFPL3	0	9.9153405	0	3.448285225		-20	0.35936
LOC100523503	0	9.9153405	0	3.448285225		-20	0.35936
ENO4	0	9.9153405	0	3.448285225		-20	0.35936
LOC102167835	0	9.9153405	0	3.448285225		-20	0.35936
LOC100627942	0	9.9153405	0	3.448285225		-20	0.35936
MMP12	0	9.9153405	0	3.448285225		-20	0.35936
LOC100739827	0	9.9153405	0	3.448285225		-20	0.35936
IFNA1	0	9.9153405	0	3.448285225		-20	0.35936
ALOX12	0	9.9153405	0	3.448285225		-20	0.35936
LOC100737674	0	9.9153405	0	3.448285225		-20	0.35936
LOC100737663	0	9.9153405	0	3.448285225		-20	0.35936
LOC100737350	0	9.9153405	0	3.448285225		-20	0.35936
LOC102165134	0	9.9153405	0	3.448285225		-20	0.35936
LOC102165163	0	9.9153405	0	3.448285225		-20	0.35936
LOC100626430	42.8051049	168.56079	5.453027102	7.405658764		-1.97741	0.35971
TLX3	34.5202459	8.2627837	5.150569664	3.211445828		2.062743	0.35989
MIPOL1	299.635734	85.106672	8.231872692	6.428053129		1.815866	0.36059
PPP1R9A	5.52323934	29.746021	2.705588563	4.942327829		-2.42911	0.36068
MDM4	215.406334	892.38064	7.757598918	9.803131183		-2.0506	0.36073
AKNA	215.406334	892.38064	7.757598918	9.803131183		-2.0506	0.36073
SCML1	854.721288	233.83678	9.740997172	7.875514565		1.869952	0.36138
SH3YL1	26.2353869	104.93735	4.767410456	6.727067559		-1.99994	0.3617
CAV1	1449.85033	6851.5003	10.50268298	12.74241476		-2.24052	0.3617
PCP4L1	661.407911	2949.8138	9.371576093	11.52689717		-2.15701	0.3622
LOC102166498	20.7121475	84.280394	4.440430524	6.414142197		-2.02472	0.36277
LOC100739072	31.7586262	7.4365053	5.033802952	3.076645515		2.094452	0.36282
LOC100624406	31.7586262	7.4365053	5.033802952	3.076645515		2.094452	0.36282
LOC100737661	31.7586262	7.4365053	5.033802952	3.076645515		2.094452	0.36282
OXTR	102.179928	406.52896	6.689018532	8.67075877		-1.99225	0.36301
PDK4	27.6161967	109.89502	4.838760036	6.793050813		-1.99254	0.3634
LOC102167025	4.14242951	24.788351	2.362450112	4.688647628		-2.58111	0.36374
NUDT12	789.823226	217.31121	9.627211433	7.770242414		1.861767	0.36381
LOC100524323	64.8980623	254.49374	6.042164139	7.997144125		-1.97138	0.36425
LOC102161329	157.412321	637.8869	7.307540742	9.319416755		-2.01875	0.36461
LOC100739517	102.179928	405.70268	6.689018532	8.667830689		-1.98931	0.36482
LOC100154593	1190.25808	321.42229	10.21827028	8.33280766		1.888733	0.36489
WDR93	88.3718295	24.788351	6.481748252	4.688647628		1.833924	0.36514
LOC102162392	82.8485901	326.37996	6.389714621	8.354822188		-1.978	0.36529
NUP93	5711.02948	1418.72	12.47978771	10.47139067		2.009161	0.36556
XPNPEP2	107.703167	30.5723	6.764250165	4.980587446		1.816764	0.36583
FCHSD2	385.245944	1647.5991	8.593375974	10.68702487		-2.09651	0.36598
MAGIX	34.5202459	135.50965	5.150569664	7.092859162		-1.97288	0.36649
LOC102163714	24.854577	99.153405	4.692347799	6.646067655		-1.99615	0.36683
KANSL1L	84.2294	331.33763	6.413279271	8.376505833		-1.97591	0.36702
LOC102159369	98.0374983	387.52456	6.629902968	8.601861975		-1.98288	0.36736
F8	2.76161967	19.830681	1.911353989	4.380638094		-2.84415	0.36764
XK	2.76161967	19.830681	1.911353989	4.380638094		-2.84415	0.36764
LOC102161858	2.76161967	19.830681	1.911353989	4.380638094		-2.84415	0.36764
LOC102163912	2.76161967	19.830681	1.911353989	4.380638094		-2.84415	0.36764
LOC102166455	2.76161967	19.830681	1.911353989	4.380638094		-2.84415	0.36764
C13H3orf25	2.76161967	19.830681	1.911353989	4.380638094		-2.84415	0.36764
LOC102158207	2.76161967	19.830681	1.911353989	4.380638094		-2.84415	0.36764
MIR221	2.76161967	19.830681	1.911353989	4.380638094		-2.84415	0.36764
MAK	238.880102	68.581105	7.906169679	6.120623682		1.800404	0.36781
PPM1L	1068.74681	290.84999	10.06305367	8.189083193		1.877573	0.36782
LOC100737624	26.2353869	104.11107	4.767410456	6.715770874		-1.98854	0.36824
PIEZO1	147.746652	594.09415	7.21671339	9.216974124		-2.00757	0.36862
IZUMO1	13.8080984	58.665764	3.888314478	5.89883146		-2.087	0.36864
LOC100738182	59.3748229	231.35794	5.915875148	7.86020516		-1.9622	0.36866
KDELC2	470.856154	2035.1236	8.882203309	10.99160945		-2.11176	0.36908
LOC100517583	46.9475344	182.60752	5.583384725	7.52048134		-1.95962	0.3695
FAM149A	69.0404918	269.36675	6.130117309	8.078773924		-1.96406	0.36968
LOC102165983	11.0464787	48.750424	3.590539588	5.636636914		-2.14183	0.36987
B3GALNT1	44.1859147	171.8659	5.497801224	7.433509508		-1.95962	0.36989
LOC100519435	197.455807	805.62141	7.632673964	9.655747894		-2.02857	0.36997
LOC102166016	26.2353869	5.7839486	4.767410456	2.762125239		2.181387	0.3705
LOC102167581	26.2353869	5.7839486	4.767410456	2.762125239		2.181387	0.3705
PF4	1.38080984	14.873011	1.251452392	3.98850389		-3.42911	0.37057
LOC100738867	1.38080984	14.873011	1.251452392	3.98850389		-3.42911	0.37057
LOC102160501	1.38080984	14.873011	1.251452392	3.98850389		-3.42911	0.37057
NRG3	1.38080984	14.873011	1.251452392	3.98850389		-3.42911	0.37057
LOC102163229	1.38080984	14.873011	1.251452392	3.98850389		-3.42911	0.37057
LOC102163036	1.38080984	14.873011	1.251452392	3.98850389		-3.42911	0.37057
ROBO4	1.38080984	14.873011	1.251452392	3.98850389		-3.42911	0.37057
RCOR1	229.214433	942.78362	7.846834472	9.882312326		-2.04023	0.37079
LOC100738813	20.7121475	83.454116	4.440430524	6.400095824		-2.01051	0.37082
LOC102165174	191.932567	55.360651	7.591952882	5.816616368		1.793667	0.37092
HCFC2	400.434852	1707.9174	8.649022066	10.73886695		-2.0926	0.37101
NOLC1	21932.7834	5080.7857	14.42086707	12.31111982		2.109965	0.37152
IMP4	2508.93147	655.23875	11.29343226	9.358076973		1.93698	0.37181
LOC100625549	418.38538	1788.0664	8.71213276	10.80499121		-2.0955	0.37196
GPR15	55.2323934	14.873011	5.813329549	3.98850389		1.892818	0.37275
SCN1B	52.4707738	203.26448	5.740678649	7.674294538		-1.95377	0.37291
C4H8orf76	1310.38853	354.47342	10.35687947	8.473597884		1.886245	0.37291
LOC100511078	600.652279	168.56079	9.23278612	7.405658764		1.833261	0.37303
LOC102161210	24.854577	98.327126	4.692347799	6.634115866		-1.98408	0.37376
H2AFZ	24150.364	5574.9002	14.55981705	12.44498902		2.115027	0.37395
NME3	4918.44464	1240.2438	12.26427974	10.27757084		1.987578	0.37428
LOC102161987	52.4707738	14.046732	5.740678649	3.911378308		1.90128	0.37444
UPK1A	23.4737672	4.9576702	4.613164285	2.574748269		2.243315	0.37546
LRRC71	23.4737672	4.9576702	4.613164285	2.574748269		2.243315	0.37546
SLC39A4	298.254925	85.932951	8.225231178	6.441831208		1.795263	0.37622
FAM171A1	664.169531	2919.2415	9.377578275	11.51187196		-2.13597	0.37645
NHLH1	6.90404918	33.877413	2.982591923	5.124221138		-2.29481	0.37703
BMP4	169.83961	681.67966	7.416498698	9.415064951		-2.00492	0.37708
MRC2	6601.65183	34358.307	12.68882986	15.06841333		-2.37976	0.3776
LOC102165702	234.737672	959.30919	7.881038517	9.907355173		-2.03095	0.37809
XYLB	330.013551	95.022013	8.370746468	6.585293272		1.796192	0.37835
LOC102168094	17.9505279	72.712497	4.24416613	6.20383732		-2.01818	0.37856
LOC102160349	16.569718	67.754826	4.135019133	6.103389087		-2.03177	0.3787
TSC22D1	494.329921	2121.8829	8.952245962	11.05180905		-2.1018	0.37889
LOC100626982	77.3253508	299.11277	6.291407421	8.229360901		-1.95168	0.37961
CCDC68	92.514259	26.440908	6.547114458	4.77825631		1.806904	0.37996
SMPDL3A	93.8950688	365.21504	6.568261215	8.516547233		-1.95962	0.38025
CD164L2	6.90404918	0	2.982591923	0		20	0.38095
LOC102157540	6.90404918	0	2.982591923	0		20	0.38095
LOC100511499	6.90404918	0	2.982591923	0		20	0.38095
RRH	6.90404918	0	2.982591923	0		20	0.38095
LOC102161274	6.90404918	0	2.982591923	0		20	0.38095
ATP1A3	6.90404918	0	2.982591923	0		20	0.38095
LOC102163698	6.90404918	0	2.982591923	0		20	0.38095
LOC100626338	6.90404918	0	2.982591923	0		20	0.38095
ELAVL3	6.90404918	0	2.982591923	0		20	0.38095
C7H6orf223	6.90404918	0	2.982591923	0		20	0.38095
LOC100522352	6.90404918	0	2.982591923	0		20	0.38095
LOC100514930	6.90404918	0	2.982591923	0		20	0.38095
HEPACAM	6.90404918	0	2.982591923	0		20	0.38095
LOC100526083	6.90404918	0	2.982591923	0		20	0.38095
SEL1L2	6.90404918	0	2.982591923	0		20	0.38095
NRXN2	6.90404918	0	2.982591923	0		20	0.38095
LOC100623527	6.90404918	0	2.982591923	0		20	0.38095
LOC102164919	6.90404918	0	2.982591923	0		20	0.38095
LOC102158197	6.90404918	0	2.982591923	0		20	0.38095
LOC100623167	6.90404918	0	2.982591923	0		20	0.38095
SBSPON	6.90404918	0	2.982591923	0		20	0.38095
LOC102167676	6.90404918	0	2.982591923	0		20	0.38095
LOC102163328	6.90404918	0	2.982591923	0		20	0.38095
LOC102167703	6.90404918	0	2.982591923	0		20	0.38095
LOC100523921	6.90404918	0	2.982591923	0		20	0.38095
LOC100739530	6.90404918	0	2.982591923	0		20	0.38095
LOC102165972	6.90404918	0	2.982591923	0		20	0.38095
SLA-DOA	6.90404918	0	2.982591923	0		20	0.38095
HSPE1	24244.2591	5624.4769	14.56541505	12.45775968		2.107852	0.38112
LOC102163407	46.9475344	180.12869	5.583384725	7.500871231		-1.93991	0.38123
LOC100621768	20.7121475	4.1313919	4.440430524	2.359350201		2.325777	0.3813
LOC102160094	20.7121475	4.1313919	4.440430524	2.359350201		2.325777	0.3813
LOC100525698	599.271469	169.38707	9.229471287	7.412672017		1.822886	0.38134
SMYD1	44.1859147	11.567897	5.497801224	3.65167138		1.93346	0.38137
LOC102158442	5.52323934	28.919743	2.705588563	4.903025878		-2.38847	0.38194
FNIP1	437.716718	1856.6475	8.777145873	10.85926105		-2.08463	0.38207
LOC102160839	34.5202459	133.03082	5.150569664	7.066420948		-1.94625	0.38207
RPP38	2450.93746	646.14969	11.25970647	9.337955638		1.923393	0.38214
LOC102161359	44.1859147	169.38707	5.497801224	7.412672017		-1.93867	0.38232
LOC100512133	879.575865	244.5784	9.782303494	7.940039851		1.846511	0.38243
MMP16	93.8950688	363.56248	6.568261215	8.510022294		-1.95308	0.3843
LOC102158881	252.6882	1030.3691	7.986912605	10.01034505		-2.02773	0.38446
RDH16	41.4242951	10.741619	5.406818783	3.553559423		1.947266	0.38449
LOC100737962	24897.3822	5786.4274	14.60376438	12.49870648		2.105249	0.38566
IGFBP6	1025.94171	4610.6333	10.00413858	12.17106209		-2.16802	0.38584
LOC100156757	428.051049	123.11548	8.745005502	6.955539223		1.797771	0.38636
DEF8	505.3764	2155.7603	8.984066358	11.07465011		-2.09277	0.38636
WDHD1	2544.83253	671.76432	11.3139218	9.393957376		1.921544	0.38639
ANO6	1851.66599	8688.3171	10.85538709	13.08502708		-2.23025	0.38672

SFXN5	17.97510447	101.63224	4.246035924	6.681340184		-2.49929	0.17608
GBA	916.7303281	6015.3065	9.841926475	12.55466236		-2.71407	0.17609
KIAA0556	12.68830904	74.365053	3.774872332	6.2358238		-2.55112	0.17614
TMPO	8849.038196	1469.1229	13.11146797	10.5217211		2.590566	0.17615
SFR1	1430.606844	265.23536	10.48341963	8.056558371		2.431282	0.1762
LOC102162076	198.7835083	39.661362	7.642293686	5.34558663		2.325392	0.17646
OPHN1	21.14718173	118.15781	4.46905122	6.896729669		-2.48218	0.17648
GXYLT2	70.8430588	394.13478	6.166776871	8.626201041		-2.45799	0.17657
LOC100516994	447.2628936	2778.7742	8.80820127	11.44075196		-2.63525	0.1767
USPL1	2423.467026	436.27498	11.24345192	8.772396993		2.473763	0.17702
IRF2	240.0205127	1426.1565	7.913012126	10.4789278		-2.5709	0.1771
LOC102157796	113.1374223	22.309516	6.834628076	4.542847146		2.342345	0.17745
BNC1	8.458872693	52.881816	3.241668254	5.751726565		-2.64423	0.17751
MPG	295.0031852	1775.6722	8.20946889	10.79496183		-2.58956	0.17776
ATG9A	491.6719753	3067.9716	8.944483599	11.58353958		-2.64152	0.17813
SGCE	255.880899	1523.6573	8.004955809	10.5742693		-2.57399	0.17829
PPP3CA	168.1200948	973.35592	7.40190428	9.92830506		-2.53348	0.17866
SLIT2	422.9436346	2606.9083	8.727728654	11.34867741		-2.6238	0.17882
LOC100514885	13.74566813	79.322724	3.882219287	6.327736286		-2.52876	0.17889
RBMX	48482.02884	7268.7708	15.56519221	12.82769417		2.737667	0.1789
LOC100622077	22.20454082	123.11548	4.536335244	6.955539223		-2.47109	0.17898
LOC100520015	566.7444704	110.7213	9.14909794	6.803760479		2.355766	0.17962
LOC100737265	13181.03837	2146.6712	13.68628585	11.06855743		2.618291	0.18011
PTPMT1	3893.196157	684.15849	11.92710984	9.420293943		2.508553	0.18017
LOC100524465	519.6633115	3239.8375	9.022820836	11.66215097		-2.6166	0.1803
LOC102167277	6.344154519	42.140197	2.876596413	5.430960858		-2.7317	0.18071
TBC1D2B	133.2272449	757.69727	7.068533723	9.56738053		-2.50773	0.1808
LOC102160861	1774.248547	327.20624	10.79380531	8.358458837		2.438936	0.18083
LOC100524374	65.64608601	475.93634	6.437062676	8.897652909		-2.47431	0.18116
LOC100524747	38.06492712	207.39587	5.287802017	7.703182885		-2.44585	0.18117
TMEM104	188.2099174	1092.34	7.563843899	10.09452641		-2.53701	0.18117
LOC100513313	77.18721332	14.873011	6.288860784	3.98850389		2.375665	0.18123
FGF2	64.49890428	354.47342	6.033398867	8.473597884		-2.45833	0.1813
RAB11FIP2	4021.136606	706.46801	11.97374637	9.4665211		2.508907	0.18136
ZSWIM3	23.2618999	128.07315	4.600620625	7.012045083		-2.46093	0.18138
LOC102159454	1.057359087	16.525567	1.04079362	4.131389251		-3.96616	0.18142
LOC102159703	1.057359087	16.525567	1.04079362	4.131389251		-3.96616	0.18142
LOC100624444	1.057359087	16.525567	1.04079362	4.131389251		-3.96616	0.18142
LOC102160869	1.057359087	16.525567	1.04079362	4.131389251		-3.96616	0.18142
LOC102160321	1.057359087	16.525567	1.04079362	4.131389251		-3.96616	0.18142
HPGD	1.057359087	16.525567	1.04079362	4.131389251		-3.96616	0.18142
COL13A1	1.057359087	16.525567	1.04079362	4.131389251		-3.96616	0.18142
LOC100739489	1245.569004	234.66306	10.28374703	7.88058181		2.408142	0.18163
PCNA	18461.48965	2951.4663	14.17230949	11.5277049		2.645015	0.18192
ATRNL1	1068.990037	203.26448	10.06338165	7.674294538		2.394818	0.18221
LOC100520746	3417.514786	607.3146	11.73909899	9.248673828		2.492377	0.18236
LZTS2	365.846244	2216.9049	8.519031704	11.11498177		-2.59924	0.18245
LOC102161526	31.7207276	4.9576702	5.032134908	2.57474827		2.677694	0.18245
LOC100624208	106.7932677	598.22554	6.752123267	9.226955308		-2.48587	0.1825
LOC100627990	840.6004738	161.95056	9.716991707	7.348290507		2.375867	0.18258
LOC102161959	39.1222862	6.610227	5.326331909	2.927939483		2.565219	0.18259
SPATA31D1	39.1222862	6.610227	5.326331909	2.927939483		2.565219	0.18259
DHRS11	300.2899806	1793.8503	8.235008887	10.80964784		-2.57863	0.18259
WBP1L	1160.980277	7666.2107	10.18236987	12.90448612		-2.72317	0.18267
LOC102162449	10.57359087	62.797156	3.532764645	5.995420212		-2.57023	0.18268
TMEM161B	1410.517021	264.40908	10.46303081	8.052073912		2.41538	0.18292
ANG1	463.1232799	91.716899	8.858364253	6.534760414		2.336137	0.183
CLDN5	7.401513606	47.097867	3.070649265	5.587901017		-2.66977	0.18302
PKLR	24.31925899	3.3051135	4.662163277	2.106051269		2.879328	0.1831
ZWINT	902.9846599	173.51846	9.820154481	7.447235822		2.379612	0.18328
SEMA6D	1232.880695	233.0105	10.26898719	7.87042946		2.403566	0.18335
LOC102159131	188.2099174	38.008805	7.563843899	5.285727902		2.307937	0.18351
LOC100739730	49.69587707	270.19303	5.663796517	8.083176277		-2.44279	0.18352
ADPRM	1638.906584	304.89672	10.67939792	8.256900823		2.426341	0.18358
DHX29	11021.91112	1824.4226	13.42821767	10.83401482		2.594863	0.18367
CYP2C49	16.91774539	1.6525567	4.163317207	1.407383614		3.355766	0.18371
LOC102161466	16.91774539	1.6525567	4.163317207	1.407383614		3.355766	0.18371
BAALC	16.91774539	1.6525567	4.163317207	1.407383614		3.355766	0.18371
UOX	16.91774539	1.6525567	4.163317207	1.407383614		3.355766	0.18371
LOC102158274	16.91774539	1.6525567	4.163317207	1.407383614		3.355766	0.18371
LOC100625536	16.91774539	1.6525567	4.163317207	1.407383614		3.355766	0.18371
LOC102167102	16.91774539	1.6525567	4.163317207	1.407383614		3.355766	0.18371
LOC100625149	16.91774539	1.6525567	4.163317207	1.407383614		3.355766	0.18371
ACBD3	112.0800632	627.97156	6.821200784	9.29685098		-2.48617	0.18374
LOC100736805	235.7910763	1382.3637	7.887470902	10.43396481		-2.55156	0.18377
LOC100623245	126.8830904	715.55707	6.998681703	9.484937802		-2.49557	0.18387
FAM49B	8990.724313	1507.958	13.13438209	10.55933696		2.575841	0.18396
NUP155	11744.08737	1937.6228	13.51976983	10.9208164		2.599575	0.18418
ARID5B	844.8299102	5430.3015	9.724223768	12.40708223		-2.6843	0.18423
XKR5	1892.672765	349.51575	10.88697133	8.453335466		2.436996	0.18427
LOC100738482	5697.050758	984.09754	12.47625276	9.944122772		2.533342	0.1846
NRTN	2.114718173	21.483238	1.639101631	4.490777899		-3.34467	0.18486
MST4	1720.323234	319.76973	10.74930232	8.325394195		2.427574	0.1849
LOC100521295	20.08982264	110.7213	4.398475058	6.803760479		-2.4624	0.18496
BTG4	135.3419631	27.267186	7.09108585	4.821056378		2.311371	0.18517
MPP5	5558.536718	962.6143	12.44074895	9.912311998		2.529675	0.18531
C2H11orf68	545.5972887	3386.915	9.094334493	11.72618198		-2.63406	0.18533
CEP70	2170.758205	398.26618	11.08464777	8.641207045		2.446394	0.18536
SCD5	850.1167056	164.4294	9.733213158	7.370071807		2.37045	0.18552
ZDHHC12	199.8408674	1155.1372	7.64990905	10.17509685		-2.53114	0.18553
LOC102159255	171.292172	34.703692	7.428713345	5.158001345		2.303298	0.18573
ANXA1	2649.741871	18540.86	11.37218047	14.17849838		-2.80678	0.18579
TIGD3	356.5096575	79.322724	8.634846101	6.327736286		2.32155	0.18583
LOC102157887	3034.620578	546.17	11.56777576	9.095845332		2.474094	0.18608
CCNC	8511.740647	1436.8981	13.05540796	10.48974571		2.566496	0.18612
ZSWIM8	1025.638314	6665.5876	10.00371229	12.70273277		-2.70021	0.18638
LASS4	185.0378402	1062.594	7.539452286	10.05473181		-2.5217	0.1864
COL23A1	3.17207726	26.440908	2.060765874	4.778256309		-3.05927	0.18653
LOC102163326	3.17207726	26.440908	2.060765874	4.778256309		-3.05927	0.18653
GABRP	19.03246356	104.93735	4.324267947	6.727067559		-2.46299	0.1866
LOC100512133	1291.035445	244.5784	10.33542993	7.940039851		2.40016	0.18664
OLA1	14305.01108	2340.0203	13.80433384	11.19292176		2.611928	0.18692
MOB1A	32645.9618	5071.6966	14.99466112	12.30853717		2.686364	0.18696
GABPA	1489.818953	280.10837	10.54188935	8.1348259		2.41108	0.18701
LOC100524777	1129.259504	215.65866	10.14243833	7.75928006		2.388556	0.18706
LOC100623655	53.92531342	291.67627	5.779399292	8.193161944		-2.43533	0.18714
LOC102163212	382.7639893	76.843889	8.584075532	6.282511874		2.316453	0.18731
LOC100514147	4.229436346	31.398578	2.386655454	5.017858594		-2.89216	0.18783
LOC100626596	216.7586127	1254.2906	7.766585971	10.29380564		-2.53271	0.1879
DLD	20028.49582	3213.3966	14.28983849	11.65033222		2.639883	0.18819
DSG2	945.2790234	182.60752	9.886121835	7.520481339		2.371994	0.18834
ADCK4	68.72834063	373.47782	6.123673244	8.548736477		-2.44205	0.18846
LOC102162398	17.97510447	99.153405	4.246035924	6.646067655		-2.46366	0.18854
LOC100516485	41.23700438	221.4426	5.400435611	7.797289318		-2.42492	0.18889
ST8SIA1	134.284604	752.7396	7.079853853	9.557922374		-2.48686	0.18894
TMEM229A	78.24457241	426.35964	6.308240221	8.739306855		-2.44601	0.18965
LOC100625485	3479.768754	24690.85	11.76519026	14.59174733		-2.82691	0.18997
LOC100625124	240.0205127	1393.1053	7.913012126	10.44512386		-2.53707	0.19005
LOC102164748	284.4295943	57.839486	8.156991116	5.878712739		2.297945	0.19023
LOC102165138	57.09739067	10.741619	5.860401464	3.553559423		2.410213	0.19032
LOC100739690	72.95777697	395.78734	6.208629956	8.632222185		-2.43959	0.19057
LOC102165554	34.89284986	5.7839486	5.165624571	2.76212524		2.592805	0.19062
RTKN	74.01513606	401.57129	6.229109817	8.653100473		-2.43976	0.19081
ACADSB	7884.726709	1346.8337	12.945028	10.39642684		2.549489	0.19114
FOXO4	251.6514626	1462.5127	7.981004722	10.51521957		-2.53895	0.19114
ARMC12	64.49890428	12.394176	6.033398867	3.74353388		2.379612	0.19125
MAST3	29.60605442	158.64545	4.935745167	7.318727602		-2.42184	0.19125
PIEZO2	23.2618999	125.59431	4.600620625	6.98406878		-2.43273	0.19148
FBXL18	66.61362245	359.43109	6.079242038	8.493579657		-2.43183	0.19172
OSBPL7	135.3419631	755.21843	7.09108585	9.562659203		-2.48028	0.19172
NCAPG	8197.704998	1398.063	13.00118034	10.45024522		2.551791	0.19176
DUSP3	416.5994801	2506.1023	8.705976106	11.29180515		-2.58871	0.19192
LOC100522128	46.52379981	248.70979	5.570578288	7.964108573		-2.41842	0.19196
DIMT1	5566.995591	973.35592	12.44294235	9.92830506		2.51586	0.19219
DUSP1	259.0529762	1505.4792	8.022661739	10.55696503		-2.5389	0.1923
LOC102160055	125.8257313	25.61463	6.986703669	4.734147579		2.296387	0.19246
DPM3	127.9404495	710.5994	7.010561107	9.474921483		-2.47356	0.19252
ZUFSP	1324.870935	252.84118	10.37272463	7.987782333		2.389548	0.19263
EPHX3	20.08982264	109.06875	4.398475058	6.782261052		-2.4407	0.19275
LOC100626290	72.95777697	394.13478	6.208629956	8.626201041		-2.43356	0.19286
LOC102166933	17.97510447	98.327126	4.246035924	6.634115866		-2.45159	0.19288
LOC102158208	137.4566813	28.093465	7.113290863	4.862623207		2.290671	0.19313
CPEB2	206.1850219	1177.4467	7.694775899	10.20267077		-2.51365	0.19348
LOC100626126	1846.148965	346.21064	10.8510845	8.439667339		2.414797	0.19383
KIF21B	247.4220263	1428.6353	7.956649285	10.48143145		-2.52959	0.19418
AIP	348.9284986	2063.2171	8.450916354	11.01137899		-2.56389	0.19443
LOC100622933	133.2272449	27.267186	7.068533723	4.821056378		2.288651	0.19446
SLC43A2	218.8733309	1252.638	7.780528816	10.29190511		-2.5168	0.1945
ZFYVE21	210.4144582	1200.5825	7.723930233	10.23071996		-2.51243	0.19473
LOC100622379	19.03246356	103.2848	4.324267947	6.704385034		-2.44009	0.19486
LRRC41	610.096193	3756.2615	9.255255683	11.87546581		-2.62219	0.19494
NPNT	28.54869534	152.03522	4.885022527	7.257719911		-2.41291	0.19499
COL27A1	118.4242177	651.93364	6.899951616	9.350792552		-2.46076	0.19508
MYD88	465.2379981	2806.0414	8.864922778	11.4548346		-2.5925	0.19513
LOC100622574	314.0356487	1839.2957	8.29937128	10.84572185		-2.55015	0.19557
CDKN2D	95.16231779	517.25026	6.587399765	9.017505127		-2.4424	0.19587
LOC100627208	2185.561232	407.35524	11.09444803	8.673680921		2.423644	0.19661
LOC100738546	287.6016715	1669.9086	8.172935846	10.70641709		-2.53763	0.197
LOC102161901	20.08982264	2.4788351	4.398475058	1.798604302		3.018731	0.19739
FBXL7	72.95777697	390.82967	6.208629956	8.614082834		-2.42141	0.19753
PLEKHM2	2054.448705	13823.637	11.00523765	13.754954		-2.75031	0.19754
LARP4	3606.651844	652.75991	11.8168444	9.352617108		2.466036	0.19755
LOC100513794	244.249949	50.402981	7.938109026	5.683780113		2.276777	0.19765
LOC100516996	38.06492712	6.610227	5.287802017	2.927939483		2.525691	0.19777
FAM3A	684.111329	4225.5876	9.420194632	12.04527763		-2.62685	0.19815
LOC100739838	2113.660814	395.78734	11.04621056	8.632222185		2.416947	0.19848
LOC102165544	731.6924879	145.42499	9.517064014	7.194018019		2.330962	0.19866
FAM210A	1827.116502	345.38436	10.8361423	8.436229978		2.403294	0.19902
ARHGAP25	12.68830904	71.05994	3.774872332	6.171125545		-2.48554	0.19918
SNRNP40	8794.055523	1508.7843	13.10247697	10.56012674		2.543142	0.19943
HSPA14	7920.676918	1368.317	12.95159015	10.41924074		2.533221	0.19963
LOC100627613	2524.973499	468.19411	11.30262379	8.874980846		2.430148	0.19965
VEZT	8152.238557	1407.1521	12.99315751	10.45958742		2.534418	0.20037
LOC102162037	5.286795433	35.52997	2.65232482	5.191008663		-2.74857	0.20062
NKX3-1	5.286795433	35.52997	2.65232482	5.191008663		-2.74857	0.20062
IL4I1	5.286795433	35.52997	2.65232482	5.191008663		-2.74857	0.20062
ATP6	1078198.577	137839.76	20.04019282	17.07264301		2.967559	0.20083
JAKMIP3	4.229436346	30.5723	2.386655454	4.980587447		-2.85369	0.20106
PDZD2	672.4803791	134.68337	9.395492105	7.084100147		2.31992	0.20106
LOC102165278	30.66341351	4.9576702	4.98474489	2.57474827		2.628784	0.20108
LOC102159071	70.8430588	14.046732	6.166776871	3.911378308		2.334392	0.20108
LOC100622824	3928.089007	710.5994	11.93997913	9.474921483		2.466719	0.20108
ENOPH1	9525.748011	1630.2472	13.21776811	10.67175973		2.546742	0.20136
RBL1	5322.745642	945.26246	12.37822593	9.886096578		2.493384	0.20151
LOC100620908	1598.726939	305.723	10.64360995	8.260792531		2.386627	0.20162
ZNF697	98.33439505	529.64444	6.63422144	9.051601682		-2.42926	0.20193
RBPMS2	1874.69766	355.2997	10.87321159	8.476947458		2.39955	0.20196
LOC102159074	0	10.741619	0	3.553559423		-20	0.20207
AQP1	0	10.741619	0	3.553559423		-20	0.20207
LOC102159294	0	10.741619	0	3.553559423		-20	0.20207
LOC102159385	0	10.741619	0	3.553559423		-20	0.20207
LOC102159477	0	10.741619	0	3.553559423		-20	0.20207
LOC100511523	0	10.741619	0	3.553559423		-20	0.20207
LOC102157512	0	10.741619	0	3.553559423		-20	0.20207
LOC100525593	0	10.741619	0	3.553559423		-20	0.20207
LOC100739070	0	10.741619	0	3.553559423		-20	0.20207
LOC100626820	0	10.741619	0	3.553559423		-20	0.20207
GRIA2	0	10.741619	0	3.553559423		-20	0.20207
ITGB6	0	10.741619	0	3.553559423		-20	0.20207
LOC102157967	0	10.741619	0	3.553559423		-20	0.20207
CLIC6	0	10.741619	0	3.553559423		-20	0.20207
GATA5	0	10.741619	0	3.553559423		-20	0.20207
LOC102160170	0	10.741619	0	3.553559423		-20	0.20207
LOC102162789	0	10.741619	0	3.553559423		-20	0.20207
CHRNE	0	10.741619	0	3.553559423		-20	0.20207
LOC102162708	0	10.741619	0	3.553559423		-20	0.20207
LOC100739196	0	10.741619	0	3.553559423		-20	0.20207
LOC102165848	0	10.741619	0	3.553559423		-20	0.20207
LOC102165869	0	10.741619	0	3.553559423		-20	0.20207
GPR128	0	10.741619	0	3.553559423		-20	0.20207
LOC100515186	0	10.741619	0	3.553559423		-20	0.20207
RAMP1	3.17207726	25.61463	2.060765874	4.734147579		-3.01347	0.20211
LOC102159467	3.17207726	25.61463	2.060765874	4.734147579		-3.01347	0.20211
LOC100625061	3.17207726	25.61463	2.060765874	4.734147579		-3.01347	0.20211
LOC102163693	14.80302721	80.97528	3.982129041	6.357117027		-2.45159	0.20215
ACTN4	4157.535928	29314.704	12.02185998	14.83938609		-2.81782	0.20233
LOC102164718	12.68830904	0.8262784	3.774872332	0.868906686		3.940728	0.20236
LOC102164756	12.68830904	0.8262784	3.774872332	0.868906686		3.940728	0.20236
LOC102166746	12.68830904	0.8262784	3.774872332	0.868906686		3.940728	0.20236
LOC100524880	191.3819947	1070.8568	7.587829971	10.06589642		-2.48424	0.20241
ADNP2	5448.571373	967.57197	12.41192705	9.919715447		2.493437	0.20257
SLC4A7	7395.169451	1288.168	12.85256256	10.33222455		2.521262	0.20258
VPS13A	588.9490112	118.98409	9.204446459	6.906699252		2.307374	0.20273
LOC102161427	85661.9464	12794.921	16.38638368	13.64339632		2.743083	0.20275
tRNA-Asp	1079.563627	211.52726	10.07756831	7.731504113		2.351533	0.20297
QTRTD1	985.4586687	194.17542	9.946114795	7.608627544		2.343435	0.20341
LOC100512723	120.5389359	654.41247	6.925274755	9.356259313		-2.4407	0.20357
GATSL2	206.1850219	1156.7897	7.694775899	10.17715754		-2.48812	0.20371
LOC102159096	2.114718173	20.656959	1.639101631	4.436758793		-3.28809	0.20381
LOC102164787	2.114718173	20.656959	1.639101631	4.436758793		-3.28809	0.20381
LOC102158398	2.114718173	20.656959	1.639101631	4.436758793		-3.28809	0.20381
LOC102163402	2.114718173	20.656959	1.639101631	4.436758793		-3.28809	0.20381
SEZ6	2.114718173	20.656959	1.639101631	4.436758793		-3.28809	0.20381
MKI67	15370.82904	2561.463	13.90800122	11.32331543		2.585155	0.20416
KIAA0825	343.6417031	71.05994	8.428953474	6.171125545		2.273797	0.20419
LOC102167659	100.4491132	20.656959	6.664612444	4.436758793		2.281765	0.20442
SOGA3	119.4815768	24.788351	6.912668746	4.688647628		2.269054	0.2046
CROT	418.7141983	2465.6147	8.713263458	11.26831664		-2.55791	0.20473
MMP11	463.1232799	2746.5493	8.858364253	11.42392966		-2.56815	0.20496
LOC100511049	16.91774539	90.890621	4.163317207	6.521845711		-2.42559	0.20507
C10H9orf3	677.7671745	4133.0444	9.406772987	12.01333817		-2.60834	0.20548
MRPS27	10297.62014	1763.278	13.33016343	10.78486223		2.545979	0.2055
LOC100620272	1.057359087	15.699289	1.04079362	4.061714779		-3.89216	0.20559
LOC102159488	1.057359087	15.699289	1.04079362	4.061714779		-3.89216	0.20559
LOC102161377	1.057359087	15.699289	1.04079362	4.061714779		-3.89216	0.20559
TULP1	1.057359087	15.699289	1.04079362	4.061714779		-3.89216	0.20559
MUC6	1.057359087	15.699289	1.04079362	4.061714779		-3.89216	0.20559
ERBB3	1.057359087	15.699289	1.04079362	4.061714779		-3.89216	0.20559
LOC100621502	1.057359087	15.699289	1.04079362	4.061714779		-3.89216	0.20559
LOC100623810	1.057359087	15.699289	1.04079362	4.061714779		-3.89216	0.20559
NACAD	1.057359087	15.699289	1.04079362	4.061714779		-3.89216	0.20559
LOC100517188	1.057359087	15.699289	1.04079362	4.061714779		-3.89216	0.20559
LOC100517985	1.057359087	15.699289	1.04079362	4.061714779		-3.89216	0.20559
LOC102165025	1.057359087	15.699289	1.04079362	4.061714779		-3.89216	0.20559
LOC100623445	161.7759402	33.877413	7.346743662	5.124221138		2.255601	0.20592
LOC100511313	191.3819947	1064.2465	7.587829971	10.05697165		-2.47531	0.20605
LOC100154692	6663.476964	1174.1416	12.70227594	10.19861885		2.504669	0.20628
NAA35	4857.507644	873.37624	12.24629752	9.772110386		2.475541	0.20643
KIRREL	498.0161298	2962.208	8.962942639	11.53294417		-2.57241	0.20646
TMEM38A	334.1254714	1928.5337	8.388557533	10.91403654		-2.52904	0.20685
POLD4	475.811598	2818.4355	8.897275491	11.46119064		-2.56643	0.2069
PLCD1	502.2455661	2986.9963	8.975118746	11.54496265		-2.57223	0.20692
BAD	262.2250535	1487.3011	8.040153	10.53945068		-2.50382	0.207
LOC100515763	1851.435761	353.64714	10.8552078	8.470240516		2.388262	0.20708
KLB	145.9155539	30.5723	7.198843332	4.980587447		2.254837	0.20714
LOC100627004	3405.753618	23463.001	11.7341819	14.5181614		-2.78434	0.20727
LOC102161401	23.2618999	3.3051135	4.600620625	2.106051269		2.815197	0.20731
LOC102166059	23.2618999	3.3051135	4.600620625	2.106051269		2.815197	0.20731
PLCXD3	23.2618999	3.3051135	4.600620625	2.106051269		2.815197	0.20731
LOC102162465	23.2618999	3.3051135	4.600620625	2.106051269		2.815197	0.20731
LOC102165337	23.2618999	3.3051135	4.600620625	2.106051269		2.815197	0.20731
LOC102165403	23.2618999	3.3051135	4.600620625	2.106051269		2.815197	0.20731
LOC102167484	23.2618999	3.3051135	4.600620625	2.106051269		2.815197	0.20731
SUPT16H	37119.64809	5875.6655	15.17993428	12.52078207		2.659359	0.20744
LOC102161154	2020.613214	384.21944	10.98129128	8.589536711		2.394791	0.20761
LOC100736603	136.3993222	740.34542	7.102231077	9.534002096		-2.44036	0.20784
LOC102166829	103.6211905	21.483238	6.709031281	4.490777899		2.270036	0.20809

LOC102164436	260.283651	61.970878	8.029473043	5.976612875		2.070422	0.07718
SLC35E3	1668.63372	380.91433	10.70531593	8.577105241		2.131129	0.07719
LOC100153224	727.799507	171.03962	9.509378173	7.426597064		2.08921	0.0773
LOC100620270	311.677238	74.365053	8.288530387	6.2358238		2.067356	0.0778
RAD54B	5645.83422	1224.5445	12.46322656	10.25920721		2.204942	0.07785
MPP5	4386.69134	962.6143	12.09924632	9.912311998		2.188103	0.07799
LOC100739180	339.860818	1480.6908	8.41303896	10.53302874		-2.12325	0.07801
LOC102160068	1485.44045	341.25297	10.53764595	8.41891924		2.121977	0.07802
TMEM68	188.166843	802.3163	7.563515426	9.649824338		-2.09216	0.0781
LOC100626780	3619.93217	801.49002	11.82214544	9.648339641		2.175206	0.07819
LOC100737481	600.144468	142.11988	9.231567934	7.161080272		2.078201	0.07846
PROM1	1035.3321	241.27328	10.01727069	7.920491517		2.101354	0.07853
CYTB	233711.508	42779.736	17.83437542	15.3846737		2.449729	0.07856
CD9	363.070825	1583.9756	8.508075323	10.63024495		-2.12523	0.0786
GAREM	493.21265	117.33153	8.948988129	6.886690713		2.071619	0.07862
FAM166B	9.11821706	46.271589	3.338883189	5.56290145		-2.3433	0.07863
HOXD8	3362.96424	747.78193	11.71594665	9.548401802		2.169044	0.07869
EXOSC5	2963.42054	662.67525	11.53353441	9.374333672		2.160889	0.07875
HEY1	107.760747	24.788351	6.765014156	4.688647628		2.120098	0.07888
CD160	36.4728682	6.610227	5.227774503	2.927939483		2.464052	0.0789
FAM89A	1048.59496	244.5784	10.03561698	7.940039851		2.100089	0.07914
LOC100627468	195.627202	832.8886	7.619319115	9.703710853		-2.09002	0.0793
LOC102160319	58.8539465	12.394176	5.90337447	3.74353388		2.247477	0.07938
JDP2	247.849718	1063.4203	7.959130944	10.05585217		-2.10117	0.07946
ASXL3	362.241896	86.759229	8.504786801	6.455478947		2.061864	0.07975
LOC102159873	282.664729	67.754826	8.148042965	6.103389087		2.060696	0.07984
SLC39A8	285.980444	68.581105	8.164808619	6.120623682		2.060033	0.08002
LOC100625242	33.1571529	5.7839486	5.094115824	2.762125239		2.519193	0.08019
LOC102160623	234.586857	56.186929	7.880115246	5.837613535		2.061816	0.08021
DCAF4	2573.824	580.8737	11.3302581	9.184562218		2.147617	0.08027
LOC100513604	241.218288	57.839486	7.920163984	5.878712739		2.060213	0.08052
LRRC27	77.9193094	324.7274	6.302306427	8.347521274		-2.05918	0.08071
CKAP5	2777.74049	625.49273	11.44021539	9.291153952		2.150847	0.08078
NFIL3	368.044398	1598.8486	8.527650579	10.64371971		-2.11908	0.0808
LOC100737262	110.247534	461.06333	6.79762954	8.851946794		-2.06422	0.0808
SNAPIN	276.862227	1189.0146	8.118225916	10.21676353		-2.10253	0.0809
CHEK1	1409.179	326.37996	10.46166259	8.354822188		2.110231	0.08093
LOC100621480	64.6564482	269.36675	6.036864803	8.078773924		-2.05871	0.08094
LOC102160069	564.500529	134.68337	9.143384563	7.084100147		2.067403	0.08122
CATSPERG	879.493482	207.39587	9.782168514	7.703182885		2.084286	0.08153
LOC102163021	72.1168077	299.93905	6.192131177	8.233327508		-2.05626	0.08154
IRF2BPL	1706.76445	7941.3614	10.73789328	12.9553523		-2.21812	0.08159
LOC100736929	29.8414377	4.9576702	4.946798112	2.574748269		2.589583	0.08167
PPGRP-S	29.8414377	4.9576702	4.946798112	2.574748269		2.589583	0.08167
LOC102164320	29.8414377	4.9576702	4.946798112	2.574748269		2.589583	0.08167
GPRC5C	571.960888	2527.5855	9.16229285	11.30411487		-2.14377	0.08168
PLA1A	113.563249	26.440908	6.8400005	4.77825631		2.102652	0.08178
LOC100513899	944.14993	222.26888	9.884399393	7.80263838		2.08671	0.08183
LOC100738411	110.247534	25.61463	6.79762954	4.734147579		2.105706	0.08194
CTDSP2	1109.9357	5054.3448	10.1175596	12.30359378		-2.18705	0.08202
PDE4DIP	23.2100071	100.80596	4.597531595	6.669678233		-2.11876	0.08207
CCDC102A	358.926181	1552.5771	8.491557236	10.60137809		-2.11291	0.08232
LOC100737512	2171.79352	495.76702	11.08533537	8.956425596		2.131153	0.08234
LOC100516994	627.49912	2778.7742	9.295766913	11.44075196		-2.14676	0.08268
ACE	5.80250177	33.051135	2.766065426	5.089630977		-2.50995	0.08271
HAUS6	2199.9771	502.37725	11.10392842	8.975496206		2.130645	0.08279
STOML1	244.534003	1041.1107	7.939779021	10.02529289		-2.09002	0.08279
NUCB2	461.713355	2016.1192	8.853974928	10.97808065		-2.12651	0.08289
LOC102165911	26.5257224	4.1313919	4.78270852	2.359350201		2.682692	0.08335
LOC102168072	90.3532418	20.656959	6.51338402	4.436758793		2.128948	0.08355
LOC100156682	90.3532418	20.656959	6.51338402	4.436758793		2.128948	0.08355
NELL2	182.364341	43.792754	7.518569296	5.485193456		2.05806	0.08377
RBM24	246.191861	59.492043	7.94948743	5.918673475		2.049014	0.08382
LOC100739584	17.4075053	77.670167	4.202222212	6.29774474		-2.15765	0.08396
TXNDC16	87.0375265	19.830681	6.460046706	4.380638094		2.133903	0.08397
LOC102161397	4.97357294	29.746021	2.578594101	4.942327829		-2.58034	0.08417
PPID	6500.45984	1418.72	12.66654798	10.47139067		2.195952	0.08422
HOXD1	30.6703665	129.7257	4.985061656	7.030399034		-2.08055	0.0844
ARID3A	6806.33457	1483.1697	12.7328743	10.53544032		2.198194	0.08451
LOC102166878	42.27537	8.2627837	5.435474249	3.211445828		2.355118	0.08454
LRRC3B	42.27537	8.2627837	5.435474249	3.211445828		2.355118	0.08454
RPS6KL1	12068.3747	2561.463	13.55906332	11.32331543		2.236191	0.08461
LOC102166185	116.050035	27.267186	6.870981559	4.821056379		2.089509	0.08473
LOC100736635	310.01938	75.191332	8.280860669	6.251554969		2.04372	0.08507
RAP1GAP2	23.2100071	3.3051135	4.597531595	2.106051269		2.811975	0.08516
LOC102163588	23.2100071	3.3051135	4.597531595	2.106051269		2.811975	0.08516
LOC100623933	23.2100071	3.3051135	4.597531595	2.106051269		2.811975	0.08516
LOC102160878	23.2100071	3.3051135	4.597531595	2.106051269		2.811975	0.08516
LOC102163118	23.2100071	3.3051135	4.597531595	2.106051269		2.811975	0.08516
LOC100737555	23.2100071	3.3051135	4.597531595	2.106051269		2.811975	0.08516
RND2	679.721635	162.77684	9.410921153	7.35558754		2.062049	0.08521
ATP8B1	113.563249	470.15239	6.8400005	8.880049963		-2.04963	0.08567
LOC102158518	13.2628612	0.8262784	3.834191515	0.868906686		4.00462	0.08575
TRAPPC5	732.77308	3247.274	9.519190167	11.66545762		-2.14779	0.0858
LOC100620141	102.787174	23.962073	6.697484358	4.641665831		2.100836	0.08582
MLC2V	4.14464412	26.440908	2.363071282	4.77825631		-2.67345	0.08583
LOC102160253	4.14464412	26.440908	2.363071282	4.77825631		-2.67345	0.08583
LOC100739222	9.11821706	45.44531	3.338883189	5.537461031		-2.31731	0.08591
LOC100737501	73.7746653	16.525567	6.224477643	4.13138925		2.158426	0.08627
LOC102161480	54.7093024	11.567897	5.799846344	3.65167138		2.24166	0.08634
SPDYC	54.7093024	11.567897	5.799846344	3.65167138		2.24166	0.08634
CDHR4	131.799683	546.17	7.053107893	9.095845332		-2.051	0.08669
LOC100622579	581.908034	2546.5899	9.187124476	11.31491737		-2.1297	0.08679
LOC100513825	19.8942918	2.4788351	4.385036953	1.798604302		3.00462	0.08686
LOC100626745	19.8942918	2.4788351	4.385036953	1.798604302		3.00462	0.08686
GNRH2	19.8942918	2.4788351	4.385036953	1.798604302		3.00462	0.08686
LOC102163439	19.8942918	2.4788351	4.385036953	1.798604302		3.00462	0.08686
SNX24	1099.98855	260.27769	10.10458375	8.029440115		2.079365	0.08694
ANO5	325.769028	79.322724	8.352127436	6.327736286		2.038043	0.08698
JARID2	4876.58827	1084.0772	12.25195227	10.08358201		2.169405	0.08699
CCDC153	254.481149	61.970878	7.997073033	5.976612875		2.037896	0.08718
LOC100736725	121.852537	28.919743	6.940783842	4.903025878		2.07501	0.08738
LOC100737643	1475.49331	6737.4738	10.5279591	12.71820617		-2.19101	0.08745
SLC12A4	946.636717	4231.3715	9.888190287	12.04725057		-2.16024	0.08748
ILDR2	605.94697	146.25127	9.245426661	7.202136284		2.050742	0.08756
LOC102161984	89.524313	20.656959	6.500233417	4.436758793		2.115652	0.08758
LOC100156062	3.31571529	23.135794	2.109599694	4.593102408		-2.80273	0.08759
WNT1	3.31571529	23.135794	2.109599694	4.593102408		-2.80273	0.08759
LOC100737156	954.097076	227.22655	9.899503566	7.834322836		2.070005	0.0877
SCML4	16.5785765	1.6525567	4.135746339	1.407383613		3.326548	0.08776
ASB4	16.5785765	1.6525567	4.135746339	1.407383613		3.326548	0.08776
BATF	16.5785765	1.6525567	4.135746339	1.407383613		3.326548	0.08776
LOR	16.5785765	1.6525567	4.135746339	1.407383613		3.326548	0.08776
LOC102161092	16.5785765	1.6525567	4.135746339	1.407383613		3.326548	0.08776
PROKR2	16.5785765	1.6525567	4.135746339	1.407383613		3.326548	0.08776
LOC102158314	16.5785765	1.6525567	4.135746339	1.407383613		3.326548	0.08776
LOC102167823	16.5785765	1.6525567	4.135746339	1.407383613		3.326548	0.08776
HNF1B	16.5785765	1.6525567	4.135746339	1.407383613		3.326548	0.08776
CRISP2	16.5785765	1.6525567	4.135746339	1.407383613		3.326548	0.08776
IL1R2	0.82892882	13.220454	0.870998931	3.829895615		-3.99538	0.08783
GPR39	0.82892882	13.220454	0.870998931	3.829895615		-3.99538	0.08783
CLEC18A	0.82892882	13.220454	0.870998931	3.829895615		-3.99538	0.08783
SGPP2	35.6439394	6.610227	5.195502704	2.927939483		2.430885	0.08811
ADAM3A	35.6439394	6.610227	5.195502704	2.927939483		2.430885	0.08811
FRMPD1	35.6439394	6.610227	5.195502704	2.927939483		2.430885	0.08811
TCEAL3	12.4339324	57.839486	3.747809764	5.878712739		-2.21777	0.08814
NANP	1351.15398	318.11717	10.40104374	8.317942438		2.086562	0.0883
LOC100625707	934.202784	223.09516	9.869135415	7.807967682		2.066077	0.08872
DYDC1	24.0389359	102.45852	4.646101346	6.692908622		-2.09159	0.08897
PPP1R16B	63.8275194	14.046732	6.018534465	3.911378308		2.183944	0.08898
ITGB1BP2	63.8275194	14.046732	6.018534465	3.911378308		2.183944	0.08898
P2RX1	2.48678647	19.830681	1.801898017	4.380638094		-2.99538	0.08923
LOC100625628	2.48678647	19.830681	1.801898017	4.380638094		-2.99538	0.08923
LOC100739675	2.48678647	19.830681	1.801898017	4.380638094		-2.99538	0.08923
YDJC	730.286294	175.99729	9.514292512	7.467583487		2.052909	0.08938
GDF1	568.645173	137.98849	9.153919747	7.118821585		2.042981	0.08945
MADD	2454.45825	565.17441	11.26177658	9.145102724		2.118637	0.08948
BCORL1	2724.68904	624.66645	11.41240527	9.289249932		2.124934	0.08956
LOC100623894	1119.05391	266.06164	10.12935246	8.061028933		2.072447	0.08971
LOC100524899	1079.26533	256.97257	10.07716999	8.011073883		2.070363	0.08978
LOC102161575	98.64253	23.135794	6.638689748	4.593102408		2.092083	0.08995
LOC100622546	1.65785765	16.525567	1.410263837	4.13138925		-3.31731	0.09003
HTR7	1.65785765	16.525567	1.410263837	4.13138925		-3.31731	0.09003
CD5L	1.65785765	16.525567	1.410263837	4.13138925		-3.31731	0.09003
HOMER1	246.191861	60.318321	7.94948743	5.938246292		2.029115	0.09015
LOC102168050	173.246124	42.140197	7.444982755	5.430960858		2.039554	0.09019
LOC100624419	679.721635	164.4294	9.410921153	7.370071807		2.047476	0.09024
AXDND1	252.823291	61.970878	7.987680649	5.976612875		2.028467	0.09026
ORAI3	1027.04281	4583.3661	10.00568463	12.16250655		-2.15791	0.0903
SLC12A1	32.3282241	5.7839486	5.058672541	2.762125239		2.482668	0.09042
EHD3	476.634074	2051.6492	8.899761949	11.00327137		-2.10583	0.09065
LOC100519729	318.308668	1346.8337	8.318807908	10.39642684		-2.08107	0.09071
LOC102164101	44.7621565	9.0890621	5.516083133	3.334720158		2.300076	0.09091
RRAD	14.9207188	66.928548	3.99283357	6.085946114		-2.1653	0.091
TTLL7	14.9207188	66.928548	3.99283357	6.085946114		-2.1653	0.091
WSB1	4971.91508	23916.627	12.27987608	14.54578667		-2.26614	0.09141
LOC100519176	1865.08985	436.27498	10.86580274	8.772396993		2.095935	0.09168
LOC100515167	235.415786	57.839486	7.88518256	5.878712739		2.025084	0.0917
SIM2	363.070825	89.238064	8.508075323	6.495664213		2.02452	0.09191
LOC102158971	659.827344	160.298	9.368129573	7.333584776		2.041332	0.092
MIR21	35.6439394	147.07755	5.195502704	7.210209122		-2.04485	0.09201
HSD17B6	14.09179	63.623435	3.915692025	6.013985524		-2.1747	0.09203
LOC100737122	185.680057	45.44531	7.544423999	5.537461031		2.030616	0.09206
GPRIN1	9.94714588	47.924146	3.452482877	5.61247475		-2.2684	0.09207
DUSP23	73.7746653	299.93905	6.224477643	8.233327508		-2.02347	0.09213
LOC102167327	407.004052	99.979683	8.672439671	6.657921243		2.025336	0.09233
ANKS1A	2469.37897	571.78463	11.27051666	9.161848978		2.110605	0.09263
SCN11A	29.0125088	4.9576702	4.907492018	2.574748269		2.548941	0.09315
LOC102165229	29.0125088	4.9576702	4.907492018	2.574748269		2.548941	0.09315
ZNF555	5.80250177	32.224857	2.766065426	5.054191063		-2.47343	0.09317
GNAO1	324.940099	80.149002	8.348463041	6.342501448		2.019417	0.09323
LOC102161004	54.7093024	222.26888	5.799846344	7.80263838		-2.02245	0.09337
RAD54L2	1362.75899	323.90112	10.41337299	8.343856914		2.072905	0.09348
LOC102159752	66.3143059	14.873011	6.072841239	3.98850389		2.156623	0.09354
PDXP	214.692565	52.881816	7.752832639	5.751726565		2.021429	0.09354
SSBP2	321.624384	1353.444	8.33371167	10.403485		-2.07319	0.09373
NMNAT2	1060.19997	254.49374	10.05148082	7.997144125		2.058634	0.09373
LOC102160944	9.11821706	44.619032	3.338883189	5.511563931		-2.29084	0.09385
CDKN1B	361.412967	1526.9624	8.501490766	10.57739336		-2.07894	0.09415
ROM1	87.8664553	356.12598	6.473567038	8.480289273		-2.019	0.09417
THUMPD3	2043.30955	478.41518	10.99739795	8.905131772		2.094573	0.09428
LOC100627255	1752.35553	413.13919	10.77590285	8.693971907		2.084596	0.09456
LETM2	381.307259	94.195734	8.578588783	6.572825024		2.01722	0.09474
LOC100736728	75.432523	304.89672	6.256114748	8.256900824		-2.01506	0.09505
LOC100521307	617.551974	151.20894	9.272751012	7.249909307		2.030017	0.09516
PHF1	257.796864	1072.5093	8.015676326	10.06811901		-2.05668	0.09518
LOC100736937	230.442213	57.013208	7.854508213	5.858309486		2.015037	0.09519
PCDHB15	600.144468	147.07755	9.231567934	7.210209122		2.028733	0.09523
CCNE2	496.528365	122.2892	8.958634969	6.945902605		2.021579	0.09548
SLC25A19	1694.33052	400.74501	10.72735085	8.650136295		2.079959	0.09557
GNL3	9804.57013	2143.3661	13.2593858	11.06633551		2.193576	0.09566
PWWP2A	3002.3802	693.24755	11.5523714	9.439306379		2.114664	0.09584
PHF6	2461.91861	573.43719	11.26615324	9.166005344		2.102076	0.09584
RHBDF2	448.450494	1905.3979	8.812018403	10.89663357		-2.08707	0.09606
LOC102166311	499.844081	2133.4508	8.968217733	11.05964916		-2.09364	0.09619
LOC100737729	1005.49066	242.92584	9.975118073	7.930298794		2.049312	0.09621
LOC102161560	25.6967935	4.1313919	4.73859457	2.359350201		2.636889	0.09641
LOC100739018	25.6967935	4.1313919	4.73859457	2.359350201		2.636889	0.09641
LOC100515242	25.6967935	4.1313919	4.73859457	2.359350201		2.636889	0.09641
NT5DC1	2174.2803	509.81376	11.0869856	8.996653565		2.092496	0.09656
LOC100525291	180.706484	44.619032	7.505466087	5.511563931		2.017917	0.09661
DUSP3	583.565892	2506.1023	9.191221843	11.29180515		-2.10248	0.09666
SLCO2B1	19.0653629	81.801559	4.326635347	6.371586023		-2.10117	0.09667
SLC22A15	299.243305	1247.6803	8.229988267	10.28618848		-2.05986	0.09707
LOC100626101	167.443622	41.313919	7.396121996	5.403060391		2.018976	0.09743
TPST2	1127.3432	271.84558	10.13999023	8.091940885		2.052067	0.09744
C3H16orf71	18.2364341	78.496445	4.265769485	6.312818447		-2.1058	0.09763
ATP6V1F	802.403101	3491.0261	9.64998022	11.76984864		-2.12125	0.09764
HDDC2	1782.19697	422.22825	10.80025036	8.725292112		2.077562	0.09767
CACHD1	123.510395	499.89841	6.96012238	8.968374236		-2.017	0.0977
MAP3K2	1734.1191	411.48663	10.76081898	8.688203544		2.075286	0.09793
KLHL30	22.3810782	94.195734	4.547269558	6.572825024		-2.07338	0.09793
CHPF	2327.63214	10653.207	11.18526703	13.3791356		-2.19435	0.09827
NOL6	7532.47622	1674.8663	12.87910002	10.71069131		2.169078	0.0984
MAGIX	33.1571529	135.50965	5.094115824	7.092859162		-2.031	0.09841
MAP7D1	2883.84338	13333.654	11.49427728	13.70289278		-2.20901	0.09852
LOC100623601	513.935871	127.24687	9.008248962	7.002779797		2.013958	0.09869
RUNX2	21.5521494	90.890621	4.495193036	6.521845711		-2.0763	0.09871
GDPD5	485.752291	2060.7383	8.927043958	11.00964548		-2.08487	0.0988
LOC100738061	43.9332277	9.0890621	5.489710792	3.334720158		2.273109	0.09937
LOC100521777	77.9193094	18.178124	6.302306427	4.261389172		2.099778	0.09941
PQLC3	200.600775	820.49442	7.655357377	9.682106971		-2.03217	0.09943
WBSCR16	7600.44838	1693.0444	12.89205862	10.72625596		2.166465	0.09973
PITRM1	8161.6332	1812.0285	12.99481891	10.82418586		2.171252	0.09975
CHCHD4	3909.22833	898.16459	11.93303714	9.812441412		2.121832	0.09984
LOC102158495	121.852537	29.746021	6.940783842	4.942327829		2.034368	0.09993
LOC100626719	863.743834	3755.4352	9.756129012	11.8751485		-2.1203	0.09996
EEF1E1	4322.03489	989.05521	12.07782876	9.95136517		2.127588	0.10019
LOC102168081	22.3810782	3.3051135	4.547269558	2.106051269		2.759508	0.10023
SLC34A2	22.3810782	3.3051135	4.547269558	2.106051269		2.759508	0.10023
LOC102166710	22.3810782	3.3051135	4.547269558	2.106051269		2.759508	0.10023
YBX2	244.534003	61.1446	7.939779021	5.957557119		1.999738	0.10024
LOC100623342	129.312897	521.38165	7.025836058	9.028960416		-2.01147	0.10028
TMEM79	35.6439394	144.59872	5.195502704	7.185853814		-2.02033	0.10035
HRK	102.787174	24.788351	6.697484358	4.688647628		2.051926	0.10041
DNM1	1036.16103	4540.3997	10.01842419	12.14892129		-2.13157	0.10048
LOC100520276	59.6828753	238.79445	5.92321754	7.905654454		-2.00038	0.10056
LOC100624528	10416.3196	2289.6174	13.3466965	11.16152077		2.185667	0.10064
NUP107	4252.40487	975.00848	12.05440247	9.93074987		2.124792	0.1009
NEU3	156.667548	38.835083	7.300741934	5.315967693		2.012274	0.10091
NAA25	4097.39518	941.13107	12.00084338	9.879783965		2.122239	0.10094
SPATA5L1	818.152749	200.78564	9.677988689	7.65667973		2.026714	0.10099
CPAMD8	41.4464412	166.90823	5.407571694	7.391529145		-2.00973	0.10106
LOC100737595	1010.46424	246.23095	9.982229594	7.949715578		2.036934	0.10109
LOC100620795	316.650811	79.322724	8.31129789	6.327736286		1.997087	0.10112
SLC1A3	1472.17759	353.64714	10.52471564	8.470240516		2.057569	0.10114
LOC102160113	359.755109	90.064343	8.494876018	6.508814353		1.997987	0.10137
RLN3	12.4339324	56.186929	3.747809764	5.837613535		-2.17595	0.10145
SHROOM1	188.166843	765.13377	7.563515426	9.581452508		-2.0237	0.10145
LOC102163693	19.0653629	80.97528	4.326635347	6.357117027		-2.08653	0.10162
BLOC1S1	544.606237	2309.448	9.091716326	11.17395694		-2.08426	0.10191
PMEL	31.4992953	5.7839486	5.022336531	2.762125239		2.445193	0.10192
LOC100515728	31.4992953	5.7839486	5.022336531	2.762125239		2.445193	0.10192
LOC102161798	198.113989	49.576702	7.637450771	5.660401068		1.998597	0.10214
SCN4A	40.6175124	8.2627837	5.379118827	3.211445828		2.297402	0.10226
LOC102159863	40.6175124	8.2627837	5.379118827	3.211445828		2.297402	0.10226
ZNF750	40.6175124	8.2627837	5.379118827	3.211445828		2.297402	0.10226
RASIP1	248.678647	1019.6275	7.963928635	9.99524072		-2.03569	0.10228
TRIM13	1287.32646	311.50695	10.3312825	8.287744447		2.047042	0.10229
PHLPP1	1652.05515	395.78734	10.69091914	8.632222186		2.061464	0.1023
TBC1D1	1492.90081	359.43109	10.54486865	8.493579657		2.054331	0.10277
PA2G4	9470.51181	2098.7471	13.20937901	11.03599984		2.173914	0.10289
INPP5A	617.551974	2629.2178	9.272751012	11.36096654		-2.09	0.10292
LOC102159750	3.31571529	22.309516	2.109599694	4.542847146		-2.75027	0.10297
LOC102159263	9.11821706	0	3.338883189	0		20	0.10309
GP1BA	9.11821706	0	3.338883189	0		20	0.10309
KCNJ13	9.11821706	0	3.338883189	0		20	0.10309
LOC102168095	9.11821706	0	3.338883189	0		20	0.10309
LOC102161258	9.11821706	0	3.338883189	0		20	0.10309
LOC102163765	9.11821706	0	3.338883189	0		20	0.10309
LOC102163635	9.11821706	0	3.338883189	0		20	0.10309
LOC102161080	9.11821706	0	3.338883189	0		20	0.10309
LOC102162014	9.11821706	0	3.338883189	0		20	0.10309
RXFP1	9.11821706	0	3.338883189	0		20	0.10309
LOC100152084	9.11821706	0	3.338883189	0		20	0.10309
LOC102166698	9.11821706	0	3.338883189	0		20	0.10309
LOC102164430	9.11821706	0	3.338883189	0		20	0.10309

RARRES2	19.3313377	76.843889	4.345633235	6.282511874	-1.99099	0.38743
PBX3	274.781157	1122.9123	8.107380078	10.13431376	-2.03089	0.38746
P2RY11	17.9505279	3.3051135	4.24416613	2.106051269	2.441255	0.38805
LOC100739453	17.9505279	3.3051135	4.24416613	2.106051269	2.441255	0.38805
LOC100737554	17.9505279	3.3051135	4.24416613	2.106051269	2.441255	0.38805
LOC100620684	17.9505279	3.3051135	4.24416613	2.106051269	2.441255	0.38805
IGF2R	2127.82796	10073.986	11.05584365	13.2984902	-2.24318	0.38817
LOC100154449	121.511266	473.45751	6.936770608	8.890135073	-1.96214	0.38836
SRSF7	14354.8991	3446.4071	13.80935606	11.75129595	2.058378	0.38842
SDPR	4.14242951	23.962073	2.362450112	4.641665831	-2.5322	0.3889
ALDH1A2	4.14242951	23.962073	2.362450112	4.641665831	-2.5322	0.3889
MOCOS	1130.88326	312.33322	10.14450945	8.291553945	1.856292	0.3892
LOC100627735	1792.29117	483.37285	10.80839403	8.919974184	1.890597	0.38949
PAPLN	37.2818656	142.11988	5.258589232	7.161080272	-1.93056	0.3895
ADCY6	595.129039	2558.9841	9.219480843	11.32191914	-2.1043	0.38995
LOC100620893	468.094534	134.68337	8.873734881	7.084100147	1.797228	0.39056
MCTP2	146.365843	42.966475	7.203258356	5.458331975	1.768296	0.39066
LOC102159423	1436.04223	392.48223	10.48888674	8.620154661	1.871399	0.39145
ANXA8	3920.11912	1014.6698	11.93704976	9.988215791	1.949887	0.39188
CCDC73	168.4588	49.576702	7.404790748	5.660401068	1.764662	0.39208
STARD13	273.400348	1111.3444	8.100138498	10.11938784	-2.02322	0.39213
LOC100153667	35.9010557	9.0890621	5.205590187	3.334720158	1.981823	0.39243
GATM	92.514259	354.47342	6.547114458	8.473597884	-1.93793	0.39319
LOC102162484	35.9010557	136.33593	5.205590187	7.101565318	-1.92507	0.39355
LOC100515496	334.15598	97.500848	8.388688866	6.622064238	1.777035	0.39364
DDAH1	3475.49836	16977.542	11.76341919	14.05142493	-2.28834	0.39371
INPP4A	196.074997	779.1805	7.62260094	9.607664138	-1.99055	0.39384
LOC733698	5716.55272	1450.9448	12.48118205	10.50377091	1.978153	0.39389
CACNB2	12.4272885	52.055537	3.747096094	5.729431428	-2.06654	0.39392
LOC100155029	241.641721	71.05994	7.922683827	6.171125545	1.765761	0.39404
PRRC1	225.072003	901.4697	7.820638529	9.817734691	-2.00189	0.39435
CHPF2	1488.513	6815.144	10.540625	12.7347401	-2.19487	0.39488
LOC100525868	15.1889082	2.4788351	4.016933786	1.798604302	2.615284	0.39545
LOC102168002	15.1889082	2.4788351	4.016933786	1.798604302	2.615284	0.39545
LOC102157793	15.1889082	2.4788351	4.016933786	1.798604302	2.615284	0.39545
LOC100623661	15.1889082	2.4788351	4.016933786	1.798604302	2.615284	0.39545
LOC102162711	15.1889082	2.4788351	4.016933786	1.798604302	2.615284	0.39545
GNRH2	15.1889082	2.4788351	4.016933786	1.798604302	2.615284	0.39545
LOC100515753	15.1889082	2.4788351	4.016933786	1.798604302	2.615284	0.39545
UROC1	15.1889082	2.4788351	4.016933786	1.798604302	2.615284	0.39545
SGOL1	1275.86829	351.99459	10.318394	8.463502248	1.857854	0.39546
LOC102161528	73.1829213	277.62953	6.213015177	8.122204372	-1.92358	0.39552
G3BP1	18879.8129	4487.5178	14.20463326	12.13202341	2.072855	0.39558
LOC102167463	6.90404918	33.051135	2.982591923	5.089630977	-2.25919	0.396
LOC100512370	172.60123	679.20082	7.439633355	9.409816938	-1.9764	0.39604
NAP1L5	516.422879	148.73011	9.015200036	7.226220529	1.795856	0.39611
CADM1	30.3778164	115.67897	4.971673052	6.86640077	-1.92904	0.39623
PSAP	10003.9673	52725.649	13.28842883	15.68624469	-2.39793	0.39644
COQ10B	2192.72602	588.3102	11.09916764	9.202883429	1.898077	0.39673
SERPINH1	10485.8699	55440.8	13.35629651	15.75868647	-2.4025	0.39676
DENND2A	193.313377	765.13377	7.602241413	9.581452508	-1.98477	0.39677
EPB41L2	1817.14574	8427.2131	10.82825214	13.04101108	-2.21338	0.39703
LOC100525368	53.8515836	14.873011	5.777461367	3.98850389	1.856292	0.39713
LOC100739392	11.0464787	47.097867	3.590539588	5.587901017	-2.09208	0.39715
CLGN	63.5172524	239.62073	6.011613095	7.910617116	-1.91553	0.39775
TTC9	178.124469	700.68406	7.484818616	9.454677778	-1.97588	0.39804
WNT4	2.76161967	19.004403	1.911353989	4.322245637	-2.78275	0.3985
LOC100517730	2.76161967	19.004403	1.911353989	4.322245637	-2.78275	0.3985
PUS1	3855.22106	1003.9282	11.91297204	9.972876744	1.941157	0.39855
KIAA1875	93.8950688	357.77854	6.568261215	8.486949769	-1.92995	0.39863
LOC102158835	80.0869705	23.135794	6.341398207	4.593102408	1.791441	0.39908
GTF2A2	5621.27684	1434.4193	12.45693878	10.48725646	1.970431	0.39973
INPP5B	893.383964	3916.5595	9.804750511	11.93573946	-2.13223	0.40012
DDIT4L	55.2323934	207.39587	5.813329549	7.703182885	-1.9088	0.40023
LOC100737474	907.192062	3981.0092	9.826853617	11.95928084	-2.13365	0.40027
LOC102166402	15.1889082	61.1446	4.016933786	5.957557119	-2.00921	0.4007
LOC102160596	15.1889082	61.1446	4.016933786	5.957557119	-2.00921	0.4007
LOC100514687	896.145583	252.84118	9.809198306	7.987782332	1.825502	0.40112
LOC100517308	615.841187	176.82357	9.268755287	7.474302765	1.800248	0.40122
LOC100525538	9.66566885	42.140197	3.414902536	5.430960858	-2.12426	0.40153
LOC102166534	9.66566885	42.140197	3.414902536	5.430960858	-2.12426	0.40153
RIBC1	9.66566885	42.140197	3.414902536	5.430960858	-2.12426	0.40153
SESN3	218.167954	865.11346	7.775893057	9.758412211	-1.98745	0.40187
LOC100736660	28.9970066	109.89502	4.906746634	6.793050813	-1.92215	0.40207
LOC102159881	12.4272885	1.6525567	3.747096094	1.407383613	2.91074	0.40241
LOC102159962	12.4272885	1.6525567	3.747096094	1.407383613	2.91074	0.40241
LOC102166686	12.4272885	1.6525567	3.747096094	1.407383613	2.91074	0.40241
GRIP2	12.4272885	1.6525567	3.747096094	1.407383613	2.91074	0.40241
CTBS	12.4272885	1.6525567	3.747096094	1.407383613	2.91074	0.40241
SORL1	12.4272885	1.6525567	3.747096094	1.407383613	2.91074	0.40241
SPIB	12.4272885	1.6525567	3.747096094	1.407383613	2.91074	0.40241
CYTH4	12.4272885	1.6525567	3.747096094	1.407383613	2.91074	0.40241
LOC100517350	1061.84276	297.46021	10.05371247	8.221394816	1.835802	0.40271
CCDC77	642.076574	184.26008	9.328846725	7.533408207	1.801002	0.40279
LOC100622995	81.4677803	307.37555	6.365758672	8.268544594	-1.9157	0.40308
TENC1	1652.82937	7562.9259	10.69159468	12.88491952	-2.19401	0.40336
LOC100522418	30.3778164	7.4365053	4.971673052	3.076645515	2.030321	0.40341
TCF4	208.502285	822.97326	7.71082217	9.686453706	-1.98078	0.40357
LOC102167655	133.938554	515.5977	7.076158798	9.012897421	-1.94467	0.40367
LOC100154848	5.52323934	28.093465	2.705588563	4.862623207	-2.34665	0.40391
SEC14L2	102.179928	388.35083	6.689018532	8.604926909	-1.92625	0.40393
CD151	4968.15379	24674.325	12.27878448	14.59078145	-2.31223	0.40421

C6H1orf167	84.2294	308.20183	6.413279271	8.27240506		-1.87148	0.43141		SLC16A8
PMCH	80.0869705	23.962073	6.341398207	4.641665831		1.740815	0.43165		LOC102164817
LOC100157694	1260.67938	358.60481	10.30112962	8.490268521		1.813735	0.43263		LOC102166729
CBLN4	19.3313377	72.712497	4.345633235	6.20383732		-1.91126	0.43282		LOC100516475
SLC16A7	30.3778164	110.7213	4.971673052	6.803760479		-1.86584	0.43321		LOC100518952
CXXC5	1325.57744	5788.9063	10.37349318	12.49932428		-2.12667	0.4335		ABRA
LOC100518497	1256.53695	357.77854	10.29638508	8.486949769		1.812314	0.43364		WEE1B
LOC100621115	4475.20468	1188.1883	12.12806029	10.21576146		1.91319	0.43517		LOC100512379
DAAM1	95.2758787	348.68947	6.58910248	8.449930555		-1.87176	0.43537		FAM19A3
TCTEX1D4	6.90404918	31.398578	2.982591923	5.017858594		-2.18518	0.43543		SERPINB9
GIPC2	6.90404918	31.398578	2.982591923	5.017858594		-2.18518	0.43543		LOC100738855
SV2A	6.90404918	31.398578	2.982591923	5.017858594		-2.18518	0.43543		LOC100738944
PCSK1N	5162.84798	1360.0542	12.33423081	10.4105088		1.924503	0.43565		LOC102166422
MYCBP	1789.52955	500.72469	10.80617061	8.970752135		1.837491	0.4359		MIR24-1
CDCA4	4259.79834	1135.3065	12.05690806	10.15013629		1.907703	0.43646		LOC102160963
LOC100738208	31.7586262	8.2627837	5.033802952	3.211445828		1.942449	0.43652		LOC100623996
LRRC43	31.7586262	8.2627837	5.033802952	3.211445828		1.942449	0.43652		LOC102160574
LOC102158861	440.478338	132.20454	8.786198839	7.057499439		1.736299	0.43679		NKG7
LOC102162175	118.749646	36.356248	6.90387758	5.223277669		1.707648	0.43711		ZNF551
LOC100620662	2163.72901	599.8781	11.07997072	9.230928526		1.850779	0.43766		SMOC2
RPF2	2075.35718	576.7423	11.01983893	9.174282325		1.847361	0.4377		FMO1
GOLGA2	339.67922	1335.2658	8.41227014	10.38399134		-1.97488	0.43776		LOC102158533
OVOL1	92.514259	28.093465	6.547114458	4.862623207		1.719441	0.43776		CAV3
LOC100626307	234.737672	71.886218	7.881038517	6.187574144		1.707262	0.43777		LOC102162468
RASSF2	48.3283443	173.51846	5.624344958	7.447235822		-1.84415	0.43863		LOC102158380
LOC100627526	20.7121475	76.843889	4.440430524	6.282511874		-1.89145	0.4388		LOC102167835
LOC100627821	33847.7915	8030.5995	15.04681671	12.97147161		2.075482	0.43884		PRSS54
CECR5	807.773754	236.31561	9.65959237	7.890663216		1.773236	0.43887		MMP12
BTBD19	432.193479	1726.0955	8.758867714	10.75413216		-1.99776	0.43897		LOC102165719
LOC100738718	24.854577	90.890621	4.692347799	6.521845711		-1.87062	0.43927		IFNA1
LOC100513117	24.854577	90.890621	4.692347799	6.521845711		-1.87062	0.43927		LOC100737681
STAT2	784.299987	3272.0624	9.617100062	11.67642537		-2.06072	0.43939		LOC100737674
TEFM	592.36742	175.99729	9.212781904	7.467583487		1.750939	0.43969		LOC100737663
F2R	2930.07847	799.83746	11.51721588	9.645365656		1.873161	0.43979		PPP1R3C
SUSD4	4373.02475	1166.7051	12.09474567	10.18946021		1.906192	0.43983		LOC100620150
LOC100153054	16.569718	62.797156	4.135019133	5.995420212		-1.92215	0.44046		LOC100519166
AARS2	2363.94644	653.58619	11.20759179	9.35443936		1.854748	0.44053		HEATR1

[illegible]

LOC102166329	0	8.2627837	0	3.211445828		-20	0.47745	
LOC102158073	0	8.2627837	0	3.211445828		-20	0.47745	
PRLR	0	8.2627837	0	3.211445828		-20	0.47745	
SCG3	0	8.2627837	0	3.211445828		-20	0.47745	
LOC102160123	0	8.2627837	0	3.211445828		-20	0.47745	
LOC102162824	0	8.2627837	0	3.211445828		-20	0.47745	
LOC102160030	0	8.2627837	0	3.211445828		-20	0.47745	
PNPLA1	0	8.2627837	0	3.211445828		-20	0.47745	
LOC102160419	0	8.2627837	0	3.211445828		-20	0.47745	
LOC102160243	0	8.2627837	0	3.211445828		-20	0.47745	
CD96	0	8.2627837	0	3.211445828		-20	0.47745	
C4H1orf110	0	8.2627837	0	3.211445828		-20	0.47745	
TNFRSF11B	0	8.2627837	0	3.211445828		-20	0.47745	
LOC102158594	0	8.2627837	0	3.211445828		-20	0.47745	
LOC102162594	0	8.2627837	0	3.211445828		-20	0.47745	
LOC100157354	0	8.2627837	0	3.211445828		-20	0.47745	
LOC100739324	0	8.2627837	0	3.211445828		-20	0.47745	
LOC100625823	0	8.2627837	0	3.211445828		-20	0.47745	
LOC102165395	0	8.2627837	0	3.211445828		-20	0.47745	
LOC100513340	0	8.2627837	0	3.211445828		-20	0.47745	
LOC102163351	0	8.2627837	0	3.211445828		-20	0.47745	
LOC100739145	0	8.2627837	0	3.211445828		-20	0.47745	
LOC102165882	0	8.2627837	0	3.211445828		-20	0.47745	
LOC102167149	0	8.2627837	0	3.211445828		-20	0.47745	
GRM1	0	8.2627837	0	3.211445828		-20	0.47745	
LOC102167338	0	8.2627837	0	3.211445828		-20	0.47745	
LOC102167382	0	8.2627837	0	3.211445828		-20	0.47745	
SELP	0	8.2627837	0	3.211445828		-20	0.47745	
MIR210	0	8.2627837	0	3.211445828		-20	0.47745	
LRRC3B	0	8.2627837	0	3.211445828		-20	0.47745	
LOC100737348	0	8.2627837	0	3.211445828		-20	0.47745	
LOC100737369	0	8.2627837	0	3.211445828		-20	0.47745	
C4H8orf46	0	8.2627837	0	3.211445828		-20	0.47745	
LOC100525679	93.8950688	29.746021	6.568261215	4.942327829		1.658353	0.47745	
TAB3	1376.66741	404.05012	10.42801192	8.661956638		1.768574	0.4775	
LOC102159436	27.6161967	95.848291	4.838760036	6.597654688		-1.79524	0.47764	
LOC102166486	59.3748229	204.09076	5.915875148	7.68011867		-1.78129	0.47816	
FAIM	59.3748229	204.09076	5.915875148	7.68011867		-1.78129	0.47816	
LOC100737169	1270.34505	5288.1816	10.31213992	12.36882879		-2.05755	0.4783	
WNT2	8.28485901	33.877413	3.214880004	5.124221138		-2.03177	0.47926	
LOC102167245	8.28485901	33.877413	3.214880004	5.124221138		-2.03177	0.47926	
LOC100515601	8.28485901	33.877413	3.214880004	5.124221138		-2.03177	0.47926	
LOC102163933	280.304397	1040.2845	8.135988291	10.02414854		-1.89191	0.47954	
MYO5A	1053.55791	4315.6519	10.0424226	12.07569705		-2.03431	0.47987	
WIPI1	702.832206	2790.3421	9.459087721	11.44674321		-1.98919	0.47992	
TMEM88B	78.7061606	24.788351	6.316619332	4.688647628		1.666814	0.48001	
ZIC5	35.9010557	123.11548	5.205590187	6.955539223		-1.77791	0.48012	
EIF2C4	88.3718295	306.54928	6.481748252	8.264673769		-1.79446	0.4805	
LOC100737524	15.1889082	55.360651	4.016933786	5.816616368		-1.86584	0.48055	
BAMBI	1346.28959	396.61362	10.39584427	8.63522336		1.763183	0.48058	
LOC100624767	3213.14449	900.64343	11.65021907	9.81641319		1.834958	0.48105	
FBXO4	1027.32252	307.37555	10.0060771	8.268544594		1.740815	0.48131	
LOC102163748	103.560738	33.051135	6.708197415	5.089630977		1.647705	0.48133	
GPRC5C	642.076574	2527.5855	9.328846725	11.30411487		-1.97694	0.48146	
TYW3	556.466364	171.8659	9.122740949	7.433509508		1.695011	0.48186	
NUP85	5357.54216	1458.3813	12.38762484	10.51114118		1.877203	0.4821	
CRYGS	37.2818656	10.741619	5.258589232	3.553559423		1.795263	0.4823	
MAP3K12	706.974636	2801.0837	9.467553865	11.45228432		-1.98625	0.48235	
LOC102160822	469.475344	146.25127	8.877975309	7.202136284		1.6826	0.48283	
LOC100517485	41.4242951	141.2936	5.406818783	7.152726981		-1.77015	0.48292	
SGK1	4207.32757	1162.5737	12.03903129	10.18434684		1.855582	0.48308	
LOC100623679	88.3718295	28.093465	6.481748252	4.862623207		1.653352	0.48309	
C7H15orf38	7300.3416	1953.3221	12.83394586	10.93245253		1.902034	0.48344	
FAM21C	2027.02884	8704.8426	10.98586245	13.08776823		-2.10245	0.48362	
LOC100739011	100.799118	32.224857	6.669581252	5.054191063		1.645237	0.48374	
SYDE2	1016.27604	304.89672	9.990495494	8.256900824		1.7369	0.4839	
IGF1R	842.294	3376.1734	9.719891879	11.72160055		-2.00299	0.48419	
GLG1	4948.82245	22763.143	12.27316106	14.47447552		-2.20154	0.48424	
DYNC1LI2	292.731685	1084.0772	8.198355089	10.08358201		-1.88882	0.4844	
GABARAPL1	1753.62849	7439.8105	10.77694988	12.86124405		-2.08492	0.48447	
ITGAM	11.0464787	42.140197	3.590539588	5.430960858		-1.93161	0.48462	
LOC100622019	20.7121475	4.9576702	4.440430524	2.574748269		2.062743	0.48483	
CCDC64B	20.7121475	4.9576702	4.440430524	2.574748269		2.062743	0.48483	
LOC100737458	20.7121475	4.9576702	4.440430524	2.574748269		2.062743	0.48483	
EPSTI1	548.181505	2124.3617	9.101139229	11.05349266		-1.9543	0.48502	
LOC102162013	91.1334492	314.81206	6.525653118	8.302922453		-1.78844	0.48529	
LOC102159434	92.514259	319.76973	6.547114458	8.325394195		-1.78929	0.48532	
SH2B3	214.025525	775.04911	7.748364114	9.600004147		-1.8565	0.48535	
KLF16	6536.75376	1763.278	12.67457933	10.78486223		1.890314	0.4856	
ATP5H	6052.08951	1640.1626	12.56345597	10.68050244		1.883595	0.48575	
LOC100523069	443.239957	138.81477	8.795195352	7.127372928		1.674927	0.48605	
SLC25A19	1354.57445	400.74501	10.40468863	8.650136295		1.757083	0.48622	
LOC100621109	38.6626754	131.37826	5.309710091	7.048522415		-1.76471	0.48671	
MRM1	1708.06177	499.07214	10.73898882	8.965992412		1.77504	0.48714	
LOC100737102	694.547347	213.17982	9.442004915	7.742678745		1.704002	0.48725	
LOC102162802	110.464787	35.52997	6.800444207	5.191008663		1.636478	0.48728	
CSGALNACT2	179.505279	641.19202	7.495897217	9.32686092		-1.83673	0.48757	
EDEM1	85.6102098	294.1551	6.436465198	8.205329462		-1.78072	0.48779	
LOC100620524	128.415315	449.49543	7.015864542	8.815368674		-1.80749	0.48819	
FGFR1OP2	1691.49205	494.94074	10.72493334	8.954023947		1.772969	0.48823	
NKAIN1	3249.04554	915.51644	11.66624422	9.840016943		1.827358	0.4887	
LOC100522945	26.2353869	90.064343	4.767410456	6.508814353		-1.77944	0.48876	
LOC100627148	26.2353869	90.064343	4.767410456	6.508814353		-1.77944	0.48876	
MDP1	747.018121	228.87911	9.54692941	7.844731552		1.706557	0.48955	
LOC100623428	18907.4291	4807.2876	14.20674189	12.23130747		1.975658	0.48974	
LOC102166356	19.3313377	67.754826	4.345633235	6.103389087		-1.80938	0.49006	
VPS37C	365.914607	1368.317	8.519300528	10.41924074		-1.90282	0.49033	
ORC6	397.673233	125.59431	8.639062934	6.98406878		1.662812	0.49038	
PIGF	669.69277	206.56959	9.38950824	7.697451307		1.696871	0.4909	
LOC100518837	503.99559	1927.7074	8.980126979	10.91341861		-1.9354	0.4915	
CCDC173	92.514259	29.746021	6.547114458	4.942327829		1.636979	0.49193	
LOC100738323	45.5667246	153.68778	5.541227504	7.273215394		-1.75395	0.4921	
LOC100739571	34.5202459	9.9153405	5.150569664	3.448285225		1.799709	0.49231	
LOC100622843	515.042069	161.12428	9.011344871	7.340956379		1.676516	0.4925	
LOC100523107	2112.63905	9021.3073	11.04551331	13.1392807		-2.09429	0.49264	
ROCK1	829.866711	3293.5456	9.698473246	11.68586378		-1.98869	0.49278	
ITPR2	41.4242951	139.64104	5.406818783	7.135873883		-1.75317	0.4928	
PASK	1405.66441	417.27058	10.45806247	8.708292708		1.752197	0.49297	
LOC102167298	230.595243	74.365053	7.855461808	6.2358238		1.632666	0.49323	
CNOT6	2022.88641	588.3102	10.98291261	9.202883429		1.781766	0.49355	
PLXNA1	1104.64787	4478.4288	10.11067627	12.12909905		-2.01941	0.49376	
KIF22	6133.55729	1671.5611	12.58274352	10.70784324		1.875528	0.49422	
PCYT1B	60.7556328	204.91704	5.948498827	7.685919384		-1.75395	0.49485	
LOC100521521	16.569718	58.665764	4.135019133	5.89883146		-1.82397	0.49489	
LOC102165559	55.2323934	185.91263	5.813329549	7.546220275		-1.75104	0.49511	
LEPROTL1	4903.25573	1355.9228	12.25981849	10.40612294		1.854465	0.49595	
NEDD9	1161.26107	4715.5707	10.18271845	12.20352257		-2.02174	0.49602	
GALNT5	98.0374983	335.46902	6.629902968	8.394329862		-1.77477	0.49641	
CA8	11.0464787	1.6525567	3.590539588	1.407383613		2.740815	0.49645	
LOC102163954	11.0464787	1.6525567	3.590539588	1.407383613		2.740815	0.49645	
GRIFIN	11.0464787	1.6525567	3.590539588	1.407383613		2.740815	0.49645	
LOR	11.0464787	1.6525567	3.590539588	1.407383613		2.740815	0.49645	
BSPRY	11.0464787	1.6525567	3.590539588	1.407383613		2.740815	0.49645	
LOC102166844	11.0464787	1.6525567	3.590539588	1.407383613		2.740815	0.49645	
LOC100738162	11.0464787	1.6525567	3.590539588	1.407383613		2.740815	0.49645	
UOX	11.0464787	1.6525567	3.590539588	1.407383613		2.740815	0.49645	
LOC100516952	11.0464787	1.6525567	3.590539588	1.407383613		2.740815	0.49645	
LOC100738819	11.0464787	1.6525567	3.590539588	1.407383613		2.740815	0.49645	
IL12A	11.0464787	1.6525567	3.590539588	1.407383613		2.740815	0.49645	
LOC102162851	11.0464787	1.6525567	3.590539588	1.407383613		2.740815	0.49645	
FGD2	11.0464787	1.6525567	3.590539588	1.407383613		2.740815	0.49645	
CDKL3	11.0464787	1.6525567	3.590539588	1.407383613		2.740815	0.49645	
RDH12	11.0464787	1.6525567	3.590539588	1.407383613		2.740815	0.49645	
LOC100521334	11.0464787	1.6525567	3.590539588	1.407383613		2.740815	0.49645	
LOC100737298	11.0464787	1.6525567	3.590539588	1.407383613		2.740815	0.49645	
LOC102167815	11.0464787	1.6525567	3.590539588	1.407383613		2.740815	0.49645	
LOC102163348	11.0464787	1.6525567	3.590539588	1.407383613		2.740815	0.49645	
LOC100739685	11.0464787	1.6525567	3.590539588	1.407383613		2.740815	0.49645	
LOC102159171	1.38080984	12.394176	1.251452392	3.74353388		-3.16608	0.49666	
LOC102159548	1.38080984	12.394176	1.251452392	3.74353388		-3.16608	0.49666	
LOC102157579	1.38080984	12.394176	1.251452392	3.74353388		-3.16608	0.49666	
LOC102157643	1.38080984	12.394176	1.251452392	3.74353388		-3.16608	0.49666	
LOC102158800	1.38080984	12.394176	1.251452392	3.74353388		-3.16608	0.49666	
LOC100737992	1.38080984	12.394176	1.251452392	3.74353388		-3.16608	0.49666	
LOC100620825	1.38080984	12.394176	1.251452392	3.74353388		-3.16608	0.49666	
LOC100518654	1016.27604	308.20183	9.990495494	8.27240506		1.721345	0.49693	
LOC102165847	111.845597	36.356248	6.818206315	5.223277669		1.621233	0.49725	
LOC100736756	6.90404918	28.919743	2.982591923	4.903025878		-2.06654	0.49742	
PLA2G7	5.52323934	24.788351	2.705588563	4.688647628		-2.16608	0.49747	
LOC100511329	5.52323934	24.788351	2.705588563	4.688647628		-2.16608	0.49747	
LOC100523295	5.52323934	24.788351	2.705588563	4.688647628		-2.16608	0.49747	
LOC102165660	5.52323934	24.788351	2.705588563	4.688647628		-2.16608	0.49747	
BCKDHB	883.718295	270.19303	9.789074346	8.083176277		1.709596	0.49758	
LOC100519752	1824.04979	535.42838	10.83372011	9.067241769		1.76838	0.49763	
LOC100620605	41.4242951	138.81477	5.406818783	7.127372928		-1.74461	0.49776	
DYDC2	8.28485901	33.051135	3.214880004	5.089630977		-1.99615	0.49785	
C10H9orf24	8.28485901	33.051135	3.214880004	5.089630977		-1.99615	0.49785	
CEP76	106.322357	364.38876	6.745806839	8.513288452		-1.77703	0.49837	
SSUH2	2.76161967	16.525567	1.911353989	4.13138925		-2.58111	0.49852	
MKL2	2.76161967	16.525567	1.911353989	4.13138925		-2.58111	0.49852	
LOC102166797	2.76161967	16.525567	1.911353989	4.13138925		-2.58111	0.49852	
LOC100520680	2.76161967	16.525567	1.911353989	4.13138925		-2.58111	0.49852	
LOC102158804	2.76161967	16.525567	1.911353989	4.13138925		-2.58111	0.49852	
LOC102162885	2.76161967	16.525567	1.911353989	4.13138925		-2.58111	0.49852	
LOC102158950	2.76161967	16.525567	1.911353989	4.13138925		-2.58111	0.49852	
HPGD	2.76161967	16.525567	1.911353989	4.13138925		-2.58111	0.49852	
FAM211B	2.76161967	16.525567	1.911353989	4.13138925		-2.58111	0.49852	
LOC102165331	2.76161967	16.525567	1.911353989	4.13138925		-2.58111	0.49852	
LOC102165745	2.76161967	16.525567	1.911353989	4.13138925		-2.58111	0.49852	
NGDN	2218.96141	644.49713	11.11631888	9.334266871		1.783639	0.49872	
USP20	429.431859	1610.4165	8.749641054	10.65411376		-1.90693	0.49884	
LOC102162713	220.929574	789.92212	7.79395812	9.627391839		-1.83812	0.49902	
ELOVL4	446.001577	141.2936	8.804136111	7.152726981		1.658353	0.49941	
LOC102167139	48.3283443	14.873011	5.624344958	3.98850389		1.700173	0.49941	
LOC100620831	70.4213016	237.14189	6.158282521	7.895677624		-1.75167	0.49945	
TRNAE-UUC	11.0464787	41.313919	3.590539588	5.403060391		-1.90304	0.49977	
MYO1F	11.0464787	41.313919	3.590539588	5.403060391		-1.90304	0.49977	
COL4A3BP	212.644715	757.69727	7.739069817	9.56738053		-1.83318	0.49983	
THSD1	37.2818656	124.76803	5.258589232	6.974621475		-1.7427	0.49984	
LOC100623430	40.0434852	133.8571	5.359081339	7.075287628		-1.74105	0.50004	
LOC100624559	110.464787	378.43549	6.800444207	8.567710832		-1.77646	0.50038	
NUP107	3438.21649	975.00848	11.74786422	9.93074987		1.818174	0.50121	
LOC100153559	64.8980623	20.656959	6.042164139	4.436758793		1.651547	0.50138	
HS1BP3	660.027102	2549.8951	9.368565612	11.31678783		-1.94984	0.5017	
LOC102161427	52925.0602	12794.921	15.69169064	13.64339632		2.04838	0.50193	
LOC100152318	48.3283443	161.12428	5.624344958	7.340956379		-1.73723	0.50201	
ANKRD52	455.667246	1710.3962	8.834999507	10.7409581		-1.90828	0.50218	
LOC100511081	146.365843	47.924146	7.203258356	5.61247475		1.610754	0.5023	
CCDC114	86.9910197	294.1551	6.459284385	8.205329462		-1.75764	0.50233	
tRNA-Met	382.484325	122.2892	8.583023796	6.945902605		1.645104	0.50234	
RENBP	829.866711	3260.4945	9.698473246	11.67131746		-1.97414	0.50262	
LOC100622784	4105.14764	1153.4846	12.00356979	10.17303322		1.831435	0.50298	
HSD17B7	2162.3482	631.27668	11.07905018	9.304412191		1.776255	0.50324	
LOC100518938	715.259495	222.26888	9.484338548	7.80263838		1.686161	0.50356	
LOC100515735	133.938554	461.88961	7.076158798	8.854524369		-1.78598	0.5036	
COQ3	1451.23114	433.79615	10.50405538	8.76419534		1.742188	0.50373	
LOC100622769	556.466364	175.17101	9.122740949	7.460832769		1.66753	0.50389	
FAM71E1	110.464787	376.78294	6.800444207	8.561413734		-1.77015	0.50424	
INSC	30.3778164	101.63224	4.971673052	6.681340183		-1.74227	0.50439	
LOC102162000	22.0929574	75.191332	4.529381037	6.251554969		-1.76698	0.50454	
LOC100515685	487.425872	154.51406	8.931995812	7.280901168		1.657445	0.50469	
LOC100627047	3561.10857	1010.5384	11.79851577	9.982335443		1.817202	0.50477	
PHC3	56.6132033	188.39147	5.848327568	7.565227535		-1.73452	0.50516	
CHRNB4	38.6626754	11.567897	5.309710091	3.65167138		1.740815	0.50518	
DPYSL5	156.031511	51.229259	7.294910283	5.706786332		1.606797	0.50533	
CEP85	1581.02726	470.97867	10.62755875	8.882577857		1.747129	0.5056	
HOXB4	207.121475	732.08264	7.70128203	9.517832026		-1.82153	0.5056	
LOC102161010	45.5667246	151.20894	5.541227504	7.249909307		-1.73049	0.50573	
LOC102168176	916.857731	281.76092	9.842126741	8.143438955		1.702226	0.50604	
LOC100737297	27.6161967	92.543178	4.838760036	6.547560532		-1.74461	0.50625	
LOC100524282	21170.5764	5412.9496	14.36984107	12.40246575		1.967574	0.50664	
LOC100513919	457.048056	1707.0911	8.839355155	10.73816922		-1.90112	0.50709	
RIT1	389.388374	1436.8981	8.608766278	10.48974571		-1.88368	0.50716	
PRDM10	17.9505279	61.970878	4.24416613	5.976612875		-1.78756	0.50776	
MYLK2	17.9505279	61.970878	4.24416613	5.976612875		-1.78756	0.50776	
LOC102165483	122.892075	419.74941	6.9529401	8.716817448		-1.77214	0.50791	
TRIM4	230.595243	818.84187	7.855461808	9.679201855		-1.82822	0.50797	
KIF13A	200.217426	703.98917	7.652611443	9.46145729		-1.81399	0.50832	
ZNF407	60.7556328	201.61192	5.948498827	7.662575262		-1.73049	0.5087	
LOC100519100	56.6132033	187.56519	5.848327568	7.558919565		-1.72818	0.50887	
LOC100738041	122.892075	40.48764	6.9529401	5.374609695		1.601838	0.50898	
LOC100624562	24.854577	83.454116	4.692347799	6.400095824		-1.74747	0.50909	
LOC102162530	73.1829213	243.75212	6.213015177	7.935177544		-1.73584	0.5099	
LOC100739796	67.659682	224.74772	6.101391269	7.818567588		-1.73194	0.51016	
LOC100524308	10541.1023	2815.9567	13.36387498	11.45992167		1.904329	0.51048	
NDUFB3	5717.93353	1584.8019	12.48153042	10.63099686		1.851191	0.51094	
LOC102160117	296.874115	96.674569	8.218558948	6.609911086		1.618643	0.51182	
LOC100524518	682.120059	214.0061	9.415995345	7.74823377		1.672374	0.51183	
GLDC	360.391367	116.50525	8.497418233	6.876581411		1.629169	0.51189	
C4H1orf85	1884.80543	7824.8562	10.88096511	12.93403288		-2.05365	0.51197	
CPNE8	530.230977	168.56079	9.053195465	7.405658764		1.653352	0.51245	
LOC100524404	46.9475344	154.51406	5.583384725	7.280901168		-1.71862	0.51266	
HOOK3	41.4242951	136.33593	5.406818783	7.101565318		-1.71862	0.5127	
LOC102160208	352.106508	114.02642	8.463959601	6.845821397		1.626644	0.51278	
ATL1	49.7091541	163.60312	5.664174303	7.362847851		-1.71862	0.51298	
ZSCAN20	49.7091541	163.60312	5.664174303	7.362847851		-1.71862	0.51298	
LOC100625510	317.586262	103.2848	8.315540247	6.704385035		1.620521	0.51306	
PA2G4	7693.87241	2098.7471	12.90968169	11.03599984		1.874182	0.51334	
POMT2	182.266898	632.92923	7.517802419	9.308177987		-1.79599	0.51406	
TPST1	724.925164	2784.5581	9.503677017	11.4437507		-1.94154	0.51426	
ZSCAN2	95.2758787	318.94345	6.58910248	8.321673128		-1.74312	0.51445	
CNOT4	70.4213016	233.0105	6.158282521	7.870429459		-1.72631	0.51449	
TAX1BP3	1869.61652	7736.4444	10.86929812	12.91764142		-2.04893	0.5146	
LOC102159148	11.0464787	40.48764	3.590539588	5.374609695		-1.8739	0.51502	
LOC102167000	12.4272885	44.619032	3.747096094	5.511563931		-1.84415	0.51507	
LOC102158225	12.4272885	44.619032	3.747096094	5.511563931		-1.84415	0.51507	
LOC102167722	9.66566885	36.356248	3.414902536	5.223277669		-1.91126	0.51547	
OASL	9.66566885	36.356248	3.414902536	5.223277669		-1.91126	0.51547	
LOC100525269	13.8080984	48.750424	3.888314478	5.636636914		-1.8199	0.51549	
LOC100626637	191.932567	667.63292	7.591952882	9.385070587		-1.79846	0.51554	
SHISA2	53.8515836	176.82357	5.777461367	7.474302765		-1.71525	0.51572	
LOC102157942	35.9010557	10.741619	5.205590187	3.553559423		1.740815	0.5158	
LOC100621940	35.9010557	10.741619	5.205590187	3.553559423		1.740815	0.5158	
LOC102158270	35.9010557	10.741619	5.205590187	3.553559423		1.740815	0.5158	
FAM222A	35.9010557	10.741619	5.205590187	3.553559423		1.740815	0.5158	
AKT3	984.517413	3866.1565	9.944737552	11.91705744		-1.97341	0.51616	
LOC100621250	15.1889082	52.881816	4.016933786	5.751726565		-1.79975	0.5162	
MAP3K11	1730.15472	517.25026	10.75751896	9.017505127		1.741967	0.51646	
LOC100736857	8.28485901	32.224857	3.214880004	5.054191063		-1.95962	0.51658	
LOC102158582	8.28485901	32.224857	3.214880004	5.054191063		-1.95962	0.51658	
MDM1	430.812669	138.81477	8.75426176	7.127372928		1.6339	0.51671	
ACTG2	4961.24974	22116.993	12.27677863	14.43293287		-2.15638	0.51699	
LOC102166878	28.9970066	8.2627837	4.906746634	3.211445828		1.811204	0.51706	
LOC102162044	28.9970066	8.2627837	4.906746634	3.211445828		1.811204	0.51706	
FGF21	92.514259	30.5723	6.547114458	4.980587446		1.597451	0.51734	
CDKN2AIPNL	5325.78354	1489.7799	12.37904894	10.54185156		1.837895	0.5174	
KRAB	124.272885	41.313919	6.968930373	5.403060391		1.588812	0.51763	
NDUFC1	2079.49961	615.57739	11.0227143	9.268138169		1.756224	0.51767	
EMP3	2370.85049	9964.9172	11.21179736	13.28278687		-2.07145	0.51779	
LOC100525262	2919.03199	846.93533	11.51176846	9.72781043		1.785166	0.5179	
LOC100154692	4131.38303	1174.1416	12.01275827	10.19861885		1.815018	0.51814	
TTC40	6.90404918	28.093465	2.982591923	4.862623207		-2.02472	0.51858	
LOC102158208	6.90404918	28.093465	2.982591923	4.862623207		-2.02472	0.51858	
LOC100521239	64.8980623	213.17982	6.042164139	7.742678745		-1.71582	0.51865	
LOC100152601	73.1829213	23.962073	6.213015177	4.641665831		1.610754	0.51867	
MTMR7	191.932567	665.15409	7.591952882	9.379712118		-1.79309	0.51889	
AKAP17B	260.973059	85.932951	8.033274644	6.441831208		1.602618	0.51908	

CCT2	54291.15966	9091.5409	15.72845625	13.1504678		2.578121	0.2777	
CGNL1	48.63851798	225.574	5.633388138	7.823838478		-2.21343	0.278	
LOC100620348	193.4967128	962.6143	7.603601962	9.912311998		-2.31465	0.27836	
CX3CL1	59.21210885	275.97698	5.911981742	8.113622246		-2.22058	0.27839	
ALKBH4	86.7034451	410.66035	6.45456161	8.685310691		-2.24378	0.27842	
ESCO2	1029.86775	223.09516	10.00964355	7.807967682		2.206728	0.27843	
ARSG	23.2618999	108.24247	4.600620625	6.771389985		-2.21823	0.27848	
LOC100622678	421.8862755	95.848291	8.724125929	6.597654688		2.13803	0.27856	
LRRC8E	163.8906584	805.62141	7.365365858	9.655747894		-2.29737	0.27882	
C6H19orf47	98.33439505	468.49984	6.63422144	8.874980846		-2.25228	0.27888	
LOC102162802	152.2597085	35.52997	7.259834656	5.191008663		2.099426	0.27903	
PRKCG	216.7586127	50.402981	7.766585971	5.683780113		2.104508	0.27907	
SH3BP5	731.6924879	4019.8443	9.517064014	11.97328275		-2.45783	0.27955	
LOC100512042	6167.575552	1193.146	12.59072167	10.22176348		2.369933	0.27965	
TGFB1	572.0312658	3081.192	9.162470047	11.58974104		-2.42932	0.27975	
LOC100627792	12989.65638	2393.7284	13.66518671	11.22564635		2.440032	0.28013	
LOC100624220	43.35172255	199.95937	5.470918232	7.650760007		-2.20555	0.28015	
BDP1	4123.700438	820.49442	12.01007363	9.682106971		2.329374	0.28107	
LOC100520070	86.7034451	409.00779	6.45456161	8.679507525		-2.23797	0.28114	
KLHL35	4.229436346	26.440908	2.386655454	4.778256309		-2.64423	0.28137	
FBXL4	81.41664967	382.56689	6.364863912	8.583334366		-2.23232	0.28171	
LOC100738703	21.14718173	98.327126	4.46905122	6.634115866		-2.21712	0.28171	
PRKRA	3549.554454	713.90451	11.79382862	9.48160675		2.313835	0.2819	
DNAH9	15.8603863	75.191332	4.075565686	6.251554969		-2.24514	0.28194	
LRRC16B	15.8603863	75.191332	4.075565686	6.251554969		-2.24514	0.28194	
LOC100626218	109.965345	524.68677	6.793965376	9.038059606		-2.25441	0.28214	
LOC100737274	82.47400875	387.52456	6.383255151	8.601861976		-2.23228	0.28214	
METTL18	1520.482366	323.07484	10.5712619	8.340183224		2.234589	0.28222	
C12H17orf104	137.4566813	32.224856	7.113290863	5.054191063		2.092731	0.28274	
LOC102164492	627.0139383	140.46732	9.294652769	7.144325041		2.158263	0.283	
LOC102163981	472.6395117	107.41619	8.887645627	6.760436381		2.137529	0.28304	
LOC100144621	11092.75418	2072.3062	13.43746004	11.01771745		2.420309	0.28314	
RPA2	5221.239169	1025.4115	12.35045282	10.00339347		2.348189	0.28331	
DAPK2	31.7207726	145.42499	5.032134908	7.194018019		-2.19678	0.28334	
LOC100518260	62.38418611	14.046732	5.986051038	3.911378308		2.150946	0.28342	
LOC100621762	126.8830904	29.746021	6.998681703	4.942327829		2.092731	0.28342	
C5H12orf57	575.2033431	3083.6709	9.170434221	11.59090085		-2.4225	0.28349	
LOC102159988	95.16231779	449.49543	6.587399765	8.815368674		-2.23984	0.28354	
C17H8orf48	64.49890428	299.11277	6.033398867	8.2293609		-2.21334	0.28379	
CITED1	328.8386759	76.01761	8.365616765	6.267116453		2.112974	0.28411	
CWC15	13287.83164	2455.6993	13.69792665	11.26250558		2.4359	0.28415	
LOC100519345	172.3495311	40.48764	7.437540124	5.374609695		2.089784	0.28422	
TRIOBP	692.5702017	3761.2191	9.437898106	11.87736817		-2.44117	0.2848	
CTSF	540.3104932	2876.275	9.080312545	11.49048741		-2.41234	0.28502	
ZNF619	821.5680103	181.78124	9.683991156	7.513974209		2.176177	0.28511	
RDH10	719.0041789	160.298	9.49186147	7.333584777		2.165244	0.28515	
LOC100737817	9.516231779	47.924146	3.394545939	5.61247475		-2.33229	0.28531	
LOC100737324	109.965345	522.20793	6.793965376	9.0312406		-2.24758	0.28538	
C1H9orf16	631.2433747	3399.3092	9.304336204	11.73145023		-2.42897	0.28559	
ZNF394	64.49890428	298.28649	6.033398867	8.225383356		-2.20935	0.28565	
FKBP7	274.9133625	1387.3214	8.108071518	10.43912586		-2.33525	0.2861	
RTDR1	186.0951992	43.792754	7.54762873	5.485193456		2.087277	0.28611	
LOC102161090	19.03246356	88.411786	4.324267947	6.482393107		-2.21578	0.28612	
EFNA5	531.8516205	120.63664	9.057590042	6.926434087		2.140356	0.28616	
LOC100623685	12.68830904	61.1446	3.774872332	5.957557118		-2.26873	0.28638	
ACOT6	145.9155539	703.98917	7.198843332	9.46145729		-2.27042	0.28647	
LOC100737905	540.3104932	2870.4911	9.080312545	11.48758436		-2.40944	0.28649	
KLF12	39.1222862	178.47613	5.326331909	7.487648157		-2.18967	0.28659	
CNR1	298.1752624	69.407383	8.224847079	6.137654819		2.103	0.28673	
SRSF1	41561.61362	7148.9605	15.34299876	12.80371955		2.539446	0.28681	
LOC102161156	38.06492712	173.51846	5.287802017	7.447235822		-2.18855	0.28697	
LOC100525245	877.6080419	4842.8175	9.779075894	12.24192881		-2.4642	0.28698	
IRG1	0	9.0890621	0	3.334720158		-20	0.28698	
ATP8B4	0	9.0890621	0	3.334720158		-20	0.28698	
HS3ST1	0	9.0890621	0	3.334720158		-20	0.28698	
SEMA5A	0	9.0890621	0	3.334720158		-20	0.28698	
LOC102159482	0	9.0890621	0	3.334720158		-20	0.28698	
LOC102161645	0	9.0890621	0	3.334720158		-20	0.28698	
LOC100518337	0	9.0890621	0	3.334720158		-20	0.28698	
LOC102157601	0	9.0890621	0	3.334720158		-20	0.28698	
LOC102159993	0	9.0890621	0	3.334720158		-20	0.28698	
LOC100511840	0	9.0890621	0	3.334720158		-20	0.28698	
LOC102166969	0	9.0890621	0	3.334720158		-20	0.28698	
LOC100626606	0	9.0890621	0	3.334720158		-20	0.28698	
LOC100512393	0	9.0890621	0	3.334720158		-20	0.28698	
LOC102157999	0	9.0890621	0	3.334720158		-20	0.28698	
LOC100738609	0	9.0890621	0	3.334720158		-20	0.28698	
DRC1	0	9.0890621	0	3.334720158		-20	0.28698	
LOC100738916	0	9.0890621	0	3.334720158		-20	0.28698	
LOC102166502	0	9.0890621	0	3.334720158		-20	0.28698	
LOC102158104	0	9.0890621	0	3.334720158		-20	0.28698	
PLEK	0	9.0890621	0	3.334720158		-20	0.28698	
LOC102162688	0	9.0890621	0	3.334720158		-20	0.28698	
LOC102160353	0	9.0890621	0	3.334720158		-20	0.28698	
LOC102160333	0	9.0890621	0	3.334720158		-20	0.28698	
C15H2orf62	0	9.0890621	0	3.334720158		-20	0.28698	
LOC100524382	0	9.0890621	0	3.334720158		-20	0.28698	
LOC102167546	0	9.0890621	0	3.334720158		-20	0.28698	
LOC100737217	0	9.0890621	0	3.334720158		-20	0.28698	
LOC100157925	0	9.0890621	0	3.334720158		-20	0.28698	
LOC100153667	0	9.0890621	0	3.334720158		-20	0.28698	
LOC100620796	0	9.0890621	0	3.334720158		-20	0.28698	
IFNB1	0	9.0890621	0	3.334720158		-20	0.28698	
LOC100737597	0	9.0890621	0	3.334720158		-20	0.28698	
LOC102165185	0	9.0890621	0	3.334720158		-20	0.28698	
GCNT3	0	9.0890621	0	3.334720158		-20	0.28698	
TOX	4156.478569	832.8886	12.02149311	9.703710853		2.319166	0.28785	
METTL5	4341.516409	867.59229	12.08431559	9.762535337		2.32311	0.28798	
LOC100739867	520.2206706	2748.2019	9.025750489	11.42479713		-2.40129	0.28843	
LOC100517064	167.0627357	810.57908	7.392856063	9.664587872		-2.27856	0.28879	
SLC12A9	453.6070481	2368.9401	8.828476241	11.21063488		-2.38473	0.28896	
LOC100739008	2890.819743	594.09415	11.49776191	9.216974124		2.282715	0.28909	
CADM3	220.9880491	52.055537	7.7943382	5.729431428		2.085845	0.28919	
LOC100624116	3566.472199	722.99358	11.80068647	9.499833085		2.302443	0.2892	
WDYHV1	5573.339745	1097.2977	12.44458522	10.10105341		2.344587	0.28946	
LOC100739807	1184.242177	257.79885	10.21096616	8.015687407		2.199646	0.28971	
VPS9D1	107.8506268	508.1612	6.766205905	8.99197867		-2.23625	0.29002	
MTA3	2252.174854	470.97867	11.13774356	8.882577857		2.257585	0.29012	
FKBP3	10271.18617	1943.4067	13.32645563	10.92511432		2.401943	0.29019	
LOC102162222	78.24457241	18.178124	6.308240221	4.261389711		2.105787	0.29055	
LSM6	5745.689276	1130.3488	12.48851533	10.14382809		2.345712	0.29059	
LOC102161469	7.401513606	38.835083	3.070649265	5.315967693		-2.39147	0.29066	
LOC102160398	7.401513606	38.835083	3.070649265	5.315967693		-2.39147	0.29066	
MED30	1320.641499	285.89232	10.36811518	8.164365521		2.207695	0.29069	
LOC102165472	98.33439505	23.135794	6.63422144	4.593102407		2.087569	0.29074	
LOC100515984	35.95020894	7.4365053	5.207510617	3.076645515		2.273303	0.29109	
LOC100626746	42.29436346	9.0890621	5.436107306	3.334720158		2.218262	0.29132	
NAT6	65.55626337	300.76533	6.056502534	8.23728324		-2.19783	0.29147	
SPOCK1	45.46644072	9.9153405	5.538117235	3.448285225		2.197068	0.29172	
PLLP	29.60605442	5.7839486	4.935745167	2.76212524		2.355766	0.29203	
LOC102158192	29.60605442	5.7839486	4.935745167	2.76212524		2.355766	0.29203	
MTERFD3	830.026883	185.08636	9.698751337	7.539828464		2.16496	0.29213	
ATP1A4	16.91774539	78.496445	4.163317207	6.312818447		-2.21409	0.29219	
LOC100519878	1440.123076	310.68067	10.49297784	8.283924863		2.212688	0.29237	
CYBRD1	738.0366424	3986.7931	9.529502087	11.96137486		-2.43346	0.29248	
PQLC2	234.7337172	1160.0948	7.881014313	10.1812701		-2.30515	0.2927	
F7	26.43397716	4.9576702	4.777891883	2.57474827		2.414659	0.29316	
LOC102166663	26.43397716	4.9576702	4.777891883	2.57474827		2.414659	0.29316	
FTL	18230.98537	127138.63	14.15418405	16.95605423		-2.80194	0.29326	
WDR3	8225.196334	1584.8019	13.00600979	10.63099686		2.375748	0.29335	
MAGOHB	6045.979257	1189.0146	12.56199891	10.21676353		2.34621	0.29337	
RASIP1	208.2997401	1019.6275	7.70942671	9.995240719		-2.29131	0.29339	
SH3BGR	171.292172	826.27837	7.428713345	9.692229055		-2.27017	0.2941	
HTR1D	488.499898	112.37386	8.935164749	6.824944216		2.120052	0.2942	
C3H16orf54	28.54869534	128.89943	4.885022527	7.021245125		-2.17475	0.29423	
LOC102163505	23.2618999	4.1313919	4.600620625	2.359350201		2.493269	0.2949	
HES3	23.2618999	4.1313919	4.600620625	2.359350201		2.493269	0.2949	
ANXA2	15403.60717	105787.59	13.91107427	16.69082456		-2.77983	0.29499	
LOC100737961	20.08982264	91.716899	4.398475058	6.534760414		-2.19072	0.29522	
SLA-DYB	61.32682702	14.046732	5.961781364	3.911378308		2.126284	0.29538	
LOC100524724	367.9609621	85.932951	8.52732437	6.441831208		2.098269	0.29584	
CD276	602.6946793	3188.6082	9.237675276	11.63916352		-2.40343	0.29592	
CWC22	4798.295535	960.96175	12.22860694	9.909835714		2.319971	0.29596	
GPR123	15.8603863	73.538775	4.075565686	6.219919206		-2.21308	0.29606	
C13H21orf2	128.9978086	608.96716	7.022343493	9.252587761		-2.23902	0.2962	
CCNB1	17472.85891	3212.5703	14.09291063	11.64996132		2.443316	0.29628	
MFNG	64.49890428	14.873011	6.033398867	3.98850389		2.116578	0.2963	
LOC102160988	166.0053766	39.661362	7.38375074	5.34558663		2.065424	0.29658	
PLAT	781.388365	4215.6723	9.611741106	12.04188917		-2.43165	0.29684	
RHBDD2	298.1752624	1491.4325	8.224847079	10.54344993		-2.32247	0.29685	
UBE3D	378.534553	88.411786	8.568087425	6.482393107		2.098114	0.29691	
GDF11	97.27703596	451.14799	6.618782442	8.820651243		-2.21343	0.29696	
C14H10orf58	1328.043013	7476.993	10.37617208	12.8684354		-2.49316	0.297	
LOC100623232	2417.122872	507.33492	11.23967184	8.98963553		2.25228	0.29746	
ATP2B4	3015.588115	18131.853	11.55870201	14.14631828		-2.58802	0.29747	
LOC100737036	153.3170676	730.43008	7.269753823	9.51457615		-2.25223	0.29761	
LOC100622572	1.057359087	13.220454	1.04079362	3.829895615		-3.64423	0.29773	
SAG	1.057359087	13.220454	1.04079362	3.829895615		-3.64423	0.29773	
LOC102163797	1.057359087	13.220454	1.04079362	3.829895615		-3.64423	0.29773	
LOC100522360	1.057359087	13.220454	1.04079362	3.829895615		-3.64423	0.29773	
LOC100626947	1.057359087	13.220454	1.04079362	3.829895615		-3.64423	0.29773	
LOC100738368	1.057359087	13.220454	1.04079362	3.829895615		-3.64423	0.29773	
LOC102164940	1.057359087	13.220454	1.04079362	3.829895615		-3.64423	0.29773	
LOC102160420	1.057359087	13.220454	1.04079362	3.829895615		-3.64423	0.29773	
LOC100155760	1.057359087	13.220454	1.04079362	3.829895615		-3.64423	0.29773	
LOC100737676	1.057359087	13.220454	1.04079362	3.829895615		-3.64423	0.29773	
LOC100738689	13.74566813	64.449713	3.882219287	6.032314961		-2.2292	0.29787	
ZCCHC12	13.74566813	64.449713	3.882219287	6.032314961		-2.2292	0.29787	
TMEM143	117.3668586	549.47512	6.887121389	9.104533542		-2.22703	0.29795	
LOC100626689	81.41664967	373.47782	6.364863912	8.548736477		-2.19763	0.29814	
ACOX3	200.8982264	974.1822	7.657484427	9.929527983		-2.27773	0.29828	
GAS2L1	333.0681123	1676.5188	8.38399847	10.71211323		-2.33158	0.29838	
LOC102166835	76.12985423	347.86319	6.269217479	8.446517589		-2.19199	0.29864	
LOC100154511	10.57359087	51.229259	3.532764645	5.706786332		-2.2765	0.29874	
LOC100738222	271.7412852	64.449713	8.091389289	6.032314961		2.075988	0.29899	
ERF	916.7303281	4994.8528	9.841926475	12.28651524		-2.44587	0.29911	
WDR54	100.4491132	465.19472	6.664612444	8.864788865		-2.21137	0.2992	
CCDC160	861.7476556	193.34914	9.752794838	7.602506906		2.156057	0.29923	
MMP-13	2.114718173	17.351846	1.639101631	4.19785327		-3.03655	0.29938	
LOC102163388	2.114718173	17.351846	1.639101631	4.19785327		-3.03655	0.29938	
FAM81A	80.35929058	367.69388	6.346235194	8.526279641		-2.19397	0.29946	
ADAT2	1141.947813	252.0149	10.15854382	7.983078556		2.179916	0.29957	
INTS8	5462.317041	1089.0349	12.41556143	10.0901586		2.326463	0.29974	
TRNAQ-CUG	3.17207726	21.483238	2.060765874	4.490777899		-2.75971	0.29985	
LOC102160773	3.17207726	21.483238	2.060765874	4.490777899		-2.75971	0.29985	
CCDC113	3.17207726	21.483238	2.060765874	4.490777899		-2.75971	0.29985	
DIRAS3	3431.130236	705.64173	11.74488858	9.464835137		2.281676	0.29987	
KIF2A	6587.347109	1297.257	12.68570085	10.34236034		2.344233	0.2999	
PPP1R9B	1527.88388	8666.8338	10.57826312	13.08145578		-2.50397	0.29995	
CREBRF	120.5389359	563.52185	6.925274755	9.140885611		-2.22497	0.30009	
OSCP1	4.229436346	25.61463	2.386655454	4.734147579		-2.59843	0.30045	
LOC102162918	4.229436346	25.61463	2.386655454	4.734147579		-2.59843	0.30045	
LOC102158272	4.229436346	25.61463	2.386655454	4.734147579		-2.59843	0.30045	
NOP58	18815.70495	3458.8013	14.19972639	11.75647345		2.443593	0.30076	
MAP6	267.5118489	63.623435	8.068841943	6.013985524		2.071973	0.30078	
tRNA-Phe	16.91774539	2.4788351	4.163317207	1.798604302		2.770803	0.3008	
TSPAN8	16.91774539	2.4788351	4.163317207	1.798604302		2.770803	0.3008	
LOC102166647	16.91774539	2.4788351	4.163317207	1.798604302		2.770803	0.3008	
LOC102166151	16.91774539	2.4788351	4.163317207	1.798604302		2.770803	0.3008	
LOC102158014	16.91774539	2.4788351	4.163317207	1.798604302		2.770803	0.3008	
YAE1D1	4397.556441	890.72808	12.10281441	9.800460045		2.303645	0.301	
LOC100737737	98.33439505	453.62683	6.63422144	8.828539005		-2.20574	0.3011	
TSPAN12	1964.573183	419.74941	10.94073436	8.716817448		2.226616	0.3013	
LOC102165792	5.286795433	29.746021	2.65232482	4.942327829		-2.49223	0.30136	
LOC102167974	45.46644072	203.26448	5.538117235	7.674294538		-2.16048	0.30154	
CITED4	123.7110131	29.746021	6.962445064	4.942327829		2.056205	0.30189	
JAKMIP2	1218.077668	268.54047	10.25157433	8.074358096		2.181395	0.30215	
LOC100620095	7.401513606	0	3.070649265	0		20	0.30239	
SLC6A3	7.401513606	0	3.070649265	0		20	0.30239	
LOC102159173	7.401513606	0	3.070649265	0		20	0.30239	
LOC102159152	7.401513606	0	3.070649265	0		20	0.30239	
ASZ1	7.401513606	0	3.070649265	0		20	0.30239	
LOC102159400	7.401513606	0	3.070649265	0		20	0.30239	
LOC102159466	7.401513606	0	3.070649265	0		20	0.30239	
LOC102159461	7.401513606	0	3.070649265	0		20	0.30239	
LOC102161699	7.401513606	0	3.070649265	0		20	0.30239	
LOC102159669	7.401513606	0	3.070649265	0		20	0.30239	
KRT5	7.401513606	0	3.070649265	0		20	0.30239	
LOC102157507	7.401513606	0	3.070649265	0		20	0.30239	
LOC102161433	7.401513606	0	3.070649265	0		20	0.30239	
LOC100156006	7.401513606	0	3.070649265	0		20	0.30239	
LOC102161207	7.401513606	0	3.070649265	0		20	0.30239	
LOC102161139	7.401513606	0	3.070649265	0		20	0.30239	
MIP	7.401513606	0	3.070649265	0		20	0.30239	
LOC100626209	7.401513606	0	3.070649265	0		20	0.30239	
LOC102166893	7.401513606	0	3.070649265	0		20	0.30239	
LOC102166657	7.401513606	0	3.070649265	0		20	0.30239	
LOC100624651	7.401513606	0	3.070649265	0		20	0.30239	
LOC102164208	7.401513606	0	3.070649265	0		20	0.30239	
LOC100516477	7.401513606	0	3.070649265	0		20	0.30239	
LOC100624099	7.401513606	0	3.070649265	0		20	0.30239	
TPH2	7.401513606	0	3.070649265	0		20	0.30239	
LOC100621648	7.401513606	0	3.070649265	0		20	0.30239	
UNC80	7.401513606	0	3.070649265	0		20	0.30239	
LOC100623855	7.401513606	0	3.070649265	0		20	0.30239	
MORN5	7.401513606	0	3.070649265	0		20	0.30239	
LOC102162858	7.401513606	0	3.070649265	0		20	0.30239	
LOC102162659	7.401513606	0	3.070649265	0		20	0.30239	
LOC102158667	7.401513606	0	3.070649265	0		20	0.30239	
LOC102160246	7.401513606	0	3.070649265	0		20	0.30239	
IKZF3	7.401513606	0	3.070649265	0		20	0.30239	
LOC100623116	7.401513606	0	3.070649265	0		20	0.30239	
LOC102158579	7.401513606	0	3.070649265	0		20	0.30239	
LOC102158259	7.401513606	0	3.070649265	0		20	0.30239	
CD79B	7.401513606	0	3.070649265	0		20	0.30239	
LOC102158307	7.401513606	0	3.070649265	0		20	0.30239	
LOC102162525	7.401513606	0	3.070649265	0		20	0.30239	
LOC102167694	7.401513606	0	3.070649265	0		20	0.30239	
IGSF10	7.401513606	0	3.070649265	0		20	0.30239	
LOC102165912	7.401513606	0	3.070649265	0		20	0.30239	
LOC100153591	7.401513606	0	3.070649265	0		20	0.30239	
LOC102167312	7.401513606	0	3.070649265	0		20	0.30239	
LOC100620892	7.401513606	0	3.070649265	0		20	0.30239	
TCF24	7.401513606	0	3.070649265	0		20	0.30239	
VBP1	8632.279583	1674.04	13.07569299	10.70997982		2.366408	0.30239	
ATAD2	3726.133421	764.30749	11.86385075	9.579895716		2.285454	0.30246	
LOC102162228	6.344154519	33.877413	2.876596413	5.124221138		-2.41682	0.30257	
LOC100625908	6.344154519	33.877413	2.876596413	5.124221138		-2.41682	0.30257	
LOC100621604	1137.718377	252.0149	10.15319527	7.983078556		2.174563	0.30259	
LOC100739030	28.54869534	127.24687	4.885022527	7.002779797		-2.15613	0.30275	
LOC102159179	192.4393538	46.271589	7.59573752	5.56290145		2.056205	0.30282	
PRR14L	3072.685506	638.71318	11.58575384	9.3212814		2.26626	0.30294	
LOC100157987	456.7791254	106.58991	8.838507868	6.749398975		2.099426	0.30326	
ZBTB47	412.3700438	2095.442	8.691290033	11.03372717		-2.34524	0.30358	
LOC102162807	33.83549077	150.38266	5.122485983	7.242056187		-2.15203	0.3036	
MANEA	2853.812175	596.57298	11.47918011	9.222971117		2.25812	0.30363	
LOC102159264	143.8008358	34.703692	7.177926119	5.158001345		2.050911	0.30376	
ACP2	259.0529762	1269.9899	8.022661739	10.3117368		-2.2935	0.30377	
LIAS	1933.909769	414.79174	10.91805058	8.699717297		2.221061	0.30382	
ORC3	2215.167286	470.97867	11.11385107	8.882577857		2.233682	0.3039	
THUMPD2	3487.170268	719.68846	11.76825475	9.493231938		2.276612	0.30404	
ILK	2874.959356	17074.216	11.48982757	14.05961623		-2.57021	0.30411	
MXRA7	251.6514626	1230.3285	7.981004722	10.26599998		-2.28954	0.30419	
LOC100515460	957.9673324	214.83238	9.90533786	7.753767487		2.156765	0.30421	
LOC102161846	13.74566813	1.6525567	3.882219287	1.407383614		3.056205	0.30497	
HYDIN	13.74566813	1.6525567	3.882219287	1.407383614		3.056205	0.30497	
SVOPL	13.74566813	1.6525567	3.882219287	1.407383614		3.056205	0.30497	

PLCL2	381.307259	103.2848	8.578588783	6.704385035		1.884326	0.15186
MEFV	31.4992953	6.610227	5.022336531	2.927939483		2.252548	0.15215
LOC102163889	31.4992953	6.610227	5.022336531	2.927939483		2.252548	0.15215
CD40LG	4.14464412	23.135794	2.363071282	4.593102408		-2.48081	0.15217
NUP93	5838.97463	1418.72	12.51174639	10.47139067		2.041125	0.1528
LOC100518639	480.778718	129.7257	8.912226855	7.030399034		1.889909	0.15289
LOC100739312	236.244715	894.0332	7.890232137	9.805797385		-1.92005	0.15297
LOC102159144	125.997181	33.877413	6.988652665	5.124221138		1.894996	0.15309
LOC100155846	493.21265	133.03082	8.948988129	7.066420948		1.890449	0.15312
LOC100739633	94.4978859	347.86319	6.57739689	8.446517589		-1.88017	0.15334
ANKRD10	352.29475	1355.0965	8.464728501	10.40524416		-1.94354	0.15337
CBX2	2138.63637	545.34373	11.06314991	9.09366508		1.971453	0.15338
LOC102166317	261.941508	994.83916	8.038598095	9.959768938		-1.92522	0.15339
LOC100737533	33.9860818	126.42059	5.128709195	6.993454623		-1.89522	0.1534
LOC102162614	72.9457365	267.71419	6.208395063	8.06992871		-1.8758	0.15345
FANCB	426.898344	115.67897	8.741124286	6.86640077		1.883766	0.15391
POFUT2	1627.18728	6740.779	10.66905094	12.71891361		-2.05054	0.15393
CDC25C	3043.82664	764.30749	11.57214437	9.579895716		1.993661	0.15418
ZNF643	188.166843	704.81545	7.563515426	9.463147202		-1.90523	0.15425
C4BPB	2.48678647	17.351846	1.801898017	4.19785327		-2.80273	0.15443
GABRE	37.3017971	137.98849	5.259340178	7.118821585		-1.88723	0.15455
CSGALNACT1	330.742601	1265.8585	8.373920477	10.30703964		-1.93634	0.15505
PSMB8	564.500529	2214.426	9.143384563	11.11336845		-1.97189	0.15509
NARS2	1662.00229	429.66475	10.69957444	8.750421444		1.951639	0.15532
LOC102165702	253.65222	959.30919	7.992384484	9.907355173		-1.91914	0.15557
TMEM177	624.183404	167.73451	9.288135672	7.398611253		1.89579	0.15559
SLC26A8	48.0778718	11.567897	5.617000784	3.65167138		2.055246	0.15595
KIFC2	1225.98573	321.42229	10.26090276	8.33280766		1.9314	0.15607
LOC102159590	6.63143059	31.398578	2.93195353	5.017858594		-2.24331	0.15615
TC2N	82.0639535	21.483238	6.376150635	4.490777898		1.933537	0.15616
LOC100625160	50.5646582	185.08636	5.688310694	7.539828464		-1.872	0.15645
C6H19orf33	177.390768	661.0227	7.478897148	9.37073687		-1.89777	0.15657
HOXD10	14.9207188	59.492043	3.99283357	5.918673475		-1.99538	0.15658
LOC100738833	25.6967935	4.9576702	4.73859457	2.574748269		2.373854	0.15663
LOC102167026	25.6967935	4.9576702	4.73859457	2.574748269		2.373854	0.15663
EARS2	4147.95983	1029.5429	12.01853397	10.00918878		2.010398	0.15678
RAB3IL1	781.679881	3108.4592	9.61227855	11.60244799		-1.99155	0.1569
SLC9A3R1	2749.55691	696.55267	11.42550804	9.446158339		1.980895	0.15753
LOC102166996	508.962298	137.98849	8.99424678	7.118821585		1.883011	0.15769
CENPL	359.755109	98.327126	8.494876018	6.634115866		1.871354	0.15774
KRAS	1696.8173	439.58009	10.72946551	8.783260503		1.948633	0.15775
SACS	1380.99542	361.08365	10.43253712	8.500179216		1.935304	0.15795
SYNJ2	347.321177	1326.1768	8.444274378	10.37414484		-1.93293	0.15852
LOC100524872	9550.08898	2281.3546	13.22144952	11.15630723		2.065623	0.15877
POLD1	7689.9727	1856.6475	12.90895036	10.85926105		2.050279	0.15878
BCAT1	9980.30304	2381.3343	13.28501245	11.21816014		2.067313	0.15963
SKIDA1	324.940099	89.238064	8.348463041	6.495664213		1.864443	0.16011
LOC102162229	11.6050035	47.924146	3.655924617	5.61247475		-2.04601	0.16033
LOC102158062	416.951198	114.02642	8.707190687	6.845821397		1.87051	0.16046
MMS22L	475.805145	129.7257	8.897255992	7.030399034		1.874907	0.16061
TAP1	1446.4808	5905.4115	10.49932849	12.52806616		-2.02949	0.16083
LOC100517317	203.916491	56.186929	7.678892279	5.837613535		1.859672	0.16119
TBCC	373.846899	1426.9827	8.550157659	10.47976283		-1.93245	0.16136
G2E3	1325.45719	348.68947	10.3733624	8.449930555		1.926475	0.1615
LOC100515119	685.524137	185.08636	9.423166634	7.539828464		1.889009	0.16171
MINPP1	1774.73661	461.06333	10.79420189	8.851946794		1.944568	0.16172
ATRAID	715.365575	2815.1304	9.4845522	11.45949843		-1.97645	0.16174
DUSP1	393.741191	1505.4792	8.624763262	10.55696503		-1.9349	0.16189
CDCA2	1614.75335	421.40197	10.65799127	8.722472752		1.938045	0.16198
GP5	19.8942918	3.3051135	4.385036953	2.106051269		2.589583	0.16236
LOC102161014	19.8942918	3.3051135	4.385036953	2.106051269		2.589583	0.16236
NAT9	198.942918	738.69286	7.643444369	9.530782548		-1.89262	0.16241
LOC100626307	260.283651	71.886218	8.029473043	6.187574144		1.856298	0.16269
VPS37C	359.755109	1368.317	8.494876018	10.41924074		-1.92732	0.1628
PARP8	663.971988	2599.4718	9.377149757	11.34455765		-1.96902	0.16292
LOC100621053	24.0389359	90.064343	4.646101346	6.508814353		-1.90558	0.16336
CCIN	28.18358	5.7839486	4.867084967	2.762125239		2.284728	0.16344
LOC102163435	28.18358	5.7839486	4.867084967	2.762125239		2.284728	0.16344
FAM222A	44.7621565	10.741619	5.516083133	3.553559423		2.059068	0.16348
CD276	807.376674	3188.6082	9.658883883	11.63916352		-1.98161	0.16372
LOC100627689	5263.69803	1302.2147	12.36213507	10.34785908		2.015109	0.16385
CTNND2	845.5074	227.22655	9.72537887	7.834322836		1.895686	0.16408
PYGB	3522.9475	889.07553	11.78297672	9.797783952		1.986405	0.16425
LOC100737737	124.339324	453.62683	6.969695301	8.828539005		-1.86722	0.16438
LRRIQ1	144.233615	39.661362	7.182231603	5.34558663		1.862601	0.16465
COX7A1	87.8664553	318.11717	6.473567038	8.317942438		-1.85617	0.16479
IKBKAP	4982.69116	1237.765	12.28299895	10.27468681		2.009188	0.16509
LOC100623931	228.784355	850.24044	7.844136766	9.733422888		-1.89388	0.1659
SNX17	1553.41262	6325.1609	10.6021538	12.62711454		-2.02566	0.16608
LOC100622368	3.31571529	19.830681	2.109599694	4.380638094		-2.58034	0.16612
LOC102165817	3.31571529	19.830681	2.109599694	4.380638094		-2.58034	0.16612
LOC102168023	69.6300212	18.178124	6.142209624	4.261389712		1.937506	0.1662
RN18S	212769.45	44093.519	17.69893828	15.42831172		2.270652	0.16661
ZFP36L1	2369.07858	9849.2382	11.21071918	13.2659429		-2.05569	0.16699
LOC102161664	14.09179	1.6525567	3.915692025	1.407383613		3.092083	0.167
ATP2B3	14.09179	1.6525567	3.915692025	1.407383613		3.092083	0.167
NUGGC	14.09179	1.6525567	3.915692025	1.407383613		3.092083	0.167
LOC100524449	14.09179	1.6525567	3.915692025	1.407383613		3.092083	0.167
SMC1B	14.09179	1.6525567	3.915692025	1.407383613		3.092083	0.167
LOC100515551	14.09179	1.6525567	3.915692025	1.407383613		3.092083	0.167
A4GALT	3827.16438	965.09314	11.90243706	9.916018472		1.987536	0.16702
LOC100621810	66.3143059	238.79445	6.072841239	7.905654454		-1.84838	0.16714
LOC102161387	123.510395	33.877413	6.96012238	5.124221138		1.866237	0.16722
KNOP1	5032.42689	1252.638	12.29732524	10.29190511		2.006285	0.16729
LOC100620170	77.9193094	20.656959	6.302306427	4.436758793		1.915353	0.16752
LOC102165479	77.9193094	20.656959	6.302306427	4.436758793		1.915353	0.16752
LOC102159609	38.9596547	9.0890621	5.320472212	3.334720158		2.099778	0.16804
LOC102163409	38.9596547	9.0890621	5.320472212	3.334720158		2.099778	0.16804
OPA1	3272.611	832.8886	11.67666718	9.703710853		1.974247	0.16849
CADM1	418.609056	115.67897	8.712902004	6.86640077		1.855477	0.16867
FOXP4	1490.41403	6033.4847	10.5424651	12.55901486		-2.01728	0.16918
SUSD3	30.6703665	6.610227	4.985061656	2.927939483		2.214074	0.16928
LOC100623365	19.8942918	75.191332	4.385036953	6.251554969		-1.91821	0.1693
LOC100739401	72.1168077	19.004403	6.192131177	4.322245637		1.924002	0.16933
NEFL	402.859408	111.54758	8.657709339	6.814391229		1.852617	0.16954
ZKSCAN5	218.837209	808.10025	7.780291786	9.660174654		-1.88468	0.16957
TCOF1	8840.5259	2146.6712	13.11007966	11.06855743		2.042031	0.1699
ITGB3	596.828753	2304.4904	9.223588476	11.17085793		-1.94906	0.16994
POLE2	864.572763	233.83678	9.757511292	7.875514565		1.886486	0.17004
CLUH	15670.0705	3700.0745	13.93581611	11.85372848		2.082385	0.1702
VWA8	2050.76991	534.60211	11.00265324	9.065017825		1.939628	0.17022
LOC102165736	103.616103	373.47782	6.708961125	8.548736477		-1.84977	0.1704
LOC100623501	1359.44327	361.08365	10.40986108	8.500179216		1.912611	0.17046
LOC100512793	1.65785765	14.046732	1.410263837	3.911378308		-3.08284	0.17048
LOC100623155	1.65785765	14.046732	1.410263837	3.911378308		-3.08284	0.17048
LOC100524114	1.65785765	14.046732	1.410263837	3.911378308		-3.08284	0.17048
MIR421	1.65785765	14.046732	1.410263837	3.911378308		-3.08284	0.17048
LOC102167251	509.791227	140.46732	8.996589935	7.144325041		1.859672	0.1705
TRIM2	851.309902	230.53167	9.735234283	7.855065709		1.884719	0.17056
LOC102157658	22.3810782	4.1313919	4.547269558	2.359350201		2.43758	0.17097
LOC102165445	22.3810782	4.1313919	4.547269558	2.359350201		2.43758	0.17097
PRSS56	18.2364341	69.407383	4.265769485	6.137654819		-1.92827	0.171
LOC102162560	119.365751	431.31731	6.911281129	8.755946793		-1.85336	0.171
LOC102166204	174.903982	48.750424	7.45864433	5.636636914		1.843076	0.17101
LOC102159564	58.0250177	14.873011	5.883254663	3.98850389		1.963978	0.1713
LOC102166485	945.807788	255.32002	9.886927762	8.001802338		1.88924	0.17138
SUMF1	1037.81889	4109.0823	10.02072843	12.00495158		-1.98526	0.17221
ST6GALNAC1	41.4464412	9.9153405	5.407571694	3.448285225		2.063514	0.17236
RBM27	1165.47393	312.33322	10.18793834	8.291553945		1.899759	0.17245
LOC102166394	242.047217	67.754826	7.925092802	6.103389087		1.836893	0.17268
LOC100739772	4412.38813	1112.1707	12.10767091	10.12045911		1.988181	0.17275
GBP2	151.693975	550.3014	7.254499324	9.106697443		-1.85906	0.17332
OXCT1	3134.17988	803.96886	11.61433251	9.652789156		1.962876	0.17363
LIPH	33.1571529	7.4365053	5.094115824	3.076645515		2.156623	0.17437
LOC100511504	267.74401	990.70777	8.070088789	9.953771246		-1.88761	0.17456
GRPR	68.8010924	18.178124	6.125177709	4.261389712		1.920228	0.17473
LOC102164750	4.14464412	22.309516	2.363071282	4.542847146		-2.42834	0.17496
NAGLU	430.21406	1625.2896	8.752260407	10.66736843		-1.91757	0.17499
TNK2	1365.24577	5464.1789	10.41600132	12.416053		-2.00084	0.17506
LOC100513372	145.062544	40.48764	7.190442454	5.374609695		1.841122	0.17524
ATAD5	1071.80497	289.19743	10.06717211	8.180890933		1.889916	0.1753
EBNA1BP2	9634.63972	2344.1517	13.23416474	11.19546556		2.039165	0.17563
TTC27	1568.33333	416.4443	10.6159361	8.705439898		1.913037	0.17565
MRAP2	14.9207188	57.839486	3.99283357	5.878712739		-1.95474	0.17607
FAM175A	589.368394	162.77684	9.205471676	7.35558754		1.856274	0.17611
LOC100512700	5392.182	1350.9651	12.396921	10.40084224		1.996879	0.17619
DLX2	65.4853771	233.0105	6.054965161	7.870429459		-1.83115	0.17626
TMEM164	1808.72269	477.5889	10.82155293	8.902643123		1.92113	0.17639
LOC100521112	2064.8617	542.03861	11.01252796	9.084910972		1.929578	0.17661
B3GALNT2	205.574348	57.839486	7.690517306	5.878712739		1.829534	0.17677
SMG1	6017.19433	1500.5215	12.55511498	10.55220944		2.003627	0.17686
COMMD4	519.738372	1976.4579	9.02441491	10.94943124		-1.92706	0.17706
RSL1D1	10433.7271	2533.3695	13.34910525	11.30741116		2.042125	0.17741
ND3	111538.176	24105.845	16.76719099	14.55715524		2.210083	0.17745
BNC2	333.229387	1240.2438	8.384694778	10.27757084		-1.89604	0.17762
LOC102165187	62.9985906	16.525567	5.999968229	4.13138925		1.93062	0.17777
TSLP	24.8678647	4.9576702	4.693089065	2.574748269		2.326548	0.17777
LOC102167152	24.8678647	4.9576702	4.693089065	2.574748269		2.326548	0.17777
UHRF2	499.015152	138.81477	8.965828003	7.127372928		1.845923	0.17783
RBM28	872.033122	237.96817	9.76989258	7.900674664		1.873614	0.17788
LOC100738805	79.5771671	21.483238	6.332299179	4.490777898		1.889143	0.178
LOC102166048	116.050035	32.224857	6.870981559	5.054191063		1.848501	0.17809
CCNJL	116.050035	32.224857	6.870981559	5.054191063		1.848501	0.17809
CEP97	184.851128	52.055537	7.538003632	5.729431428		1.82824	0.17816
SRBD1	1569.99119	418.09686	10.61745938	8.711139889		1.908847	0.17825
LOC100739719	238.731501	875.0288	7.905275684	9.774834483		-1.87394	0.17844
PLEKHB1	54.7093024	14.046732	5.799846344	3.911378308		1.961552	0.17844
PLD3	711.220931	2742.4179	9.476181024	11.4217587		-1.94708	0.17844
LRR1	523.054088	145.42499	9.03357191	7.194018019		1.846685	0.17855
LOC102158861	474.147287	132.20454	8.892230982	7.057499439		1.842564	0.17856
LOC100152601	87.8664553	23.962073	6.473567038	4.641665831		1.87456	0.1786
LOC100622637	1417.46829	5660.0068	10.47011818	12.46684295		-1.99749	0.17879
LOC100158117	35.6439394	8.2627837	5.195502704	3.211445828		2.108957	0.17889
XIRP1	34.8150106	124.76803	5.162492464	6.974621475		-1.84147	0.17968
FRMD6	770.074877	2974.6021	9.590727153	11.53896592		-1.94963	0.18038
LOC100518827	666.458774	184.26008	9.382534921	7.533408207		1.854772	0.18051
LOC102162398	27.3546512	99.153405	4.825513503	6.646067655		-1.85788	0.1807
ASIC3	16.5785765	2.4788351	4.135746339	1.798604302		2.741586	0.18072
TMPRSS9	239.56043	67.754826	7.910255542	6.103389087		1.821994	0.18075
ZBTB47	552.066597	2095.442	9.1113094	11.03372717		-1.92434	0.18118
SARS2	969.017795	264.40908	9.921867403	8.052073912		1.873751	0.18144
LOC102165033	218.837209	61.970878	7.780291786	5.976612875		1.820196	0.18166
ASTN2	4.97357294	24.788351	2.578594101	4.688647628		-2.31731	0.18197
LOC100524727	4.97357294	24.788351	2.578594101	4.688647628		-2.31731	0.18197
ADAM-9	2015.9549	8169.4143	10.97796311	12.99619351		-2.01877	0.18203
LOC100511492	80.4060959	284.23976	6.347064926	8.156031285		-1.82173	0.18249
PLSCR1	190.653629	689.11616	7.582357509	9.43069541		-1.85379	0.18274
DBF4B	285.980444	80.97528	8.164808619	6.357117027		1.820363	0.18274
GOLGA2	359.755109	1335.2658	8.494876018	10.38399134		-1.89204	0.1829
LOC102165942	38.1307259	9.0890621	5.290229968	3.334720158		2.068751	0.18295
METTL13	3372.08245	870.8974	11.71985187	9.768014573		1.953065	0.18302
tRNA-Met	435.187632	122.2892	8.768805054	6.945902605		1.831341	0.18315
NCALD	14.09179	54.534373	3.915692025	5.795309087		-1.95231	0.1833
LOC100158196	27.3546512	5.7839486	4.825513503	2.762125239		2.24166	0.18334
LOC102158449	8.28928824	35.52997	3.215568059	5.191008663		-2.09972	0.18342
KPNA3	1992.74489	527.99188	10.96126511	9.047101766		1.916169	0.18349
LOC102165352	101.129317	28.093465	6.674253245	4.862623207		1.847895	0.18415
HS6ST1	4169.51198	17511.318	12.02600879	14.0960824		-2.07034	0.18423
LOC100739524	380.47833	107.41619	8.575457296	6.76043638		1.824603	0.18436
CA4	2.48678647	16.525567	1.801898017	4.13138925		-2.73235	0.18455
LOC102166462	2.48678647	16.525567	1.801898017	4.13138925		-2.73235	0.18455
LOC102165440	123.510395	34.703692	6.96012238	5.158001345		1.831471	0.18457
C2H11orf80	137.602185	38.835083	7.114806188	5.315967693		1.825071	0.1848
GAN	799.087386	220.61633	9.64401377	7.791920351		1.856814	0.18507
LOC102166970	252.823291	71.886218	7.987680649	6.187574144		1.814342	0.18522
LOC100518563	332.400458	94.195734	8.381112277	6.572825024		1.819189	0.18524
TMEM198	96.1557435	339.60041	6.602227381	8.411936365		-1.82039	0.18545
LOC100516485	62.9985906	221.4426	5.999968229	7.797289318		-1.81354	0.18589
CLDN5	165.785765	47.097867	7.381852349	5.587901017		1.815587	0.1862
LOC100626019	75.432523	265.23536	6.256114748	8.05655837		-1.81401	0.18628
LOC102159723	70.45895	19.004403	6.159042811	4.322245637		1.890449	0.18638
NUDT11	70.45895	19.004403	6.159042811	4.322245637		1.890449	0.18638
LOC102159332	10.7760747	0.8262784	3.557786824	0.868906686		3.70506	0.18649
LOC102161845	10.7760747	0.8262784	3.557786824	0.868906686		3.70506	0.18649
LOC102157407	10.7760747	0.8262784	3.557786824	0.868906686		3.70506	0.18649
LOC100518000	10.7760747	0.8262784	3.557786824	0.868906686		3.70506	0.18649
LOC102166959	10.7760747	0.8262784	3.557786824	0.868906686		3.70506	0.18649
LOC102162152	10.7760747	0.8262784	3.557786824	0.868906686		3.70506	0.18649
LOC100624772	10.7760747	0.8262784	3.557786824	0.868906686		3.70506	0.18649
LOC102160761	10.7760747	0.8262784	3.557786824	0.868906686		3.70506	0.18649
LOC100627180	10.7760747	0.8262784	3.557786824	0.868906686		3.70506	0.18649
LOC102158982	10.7760747	0.8262784	3.557786824	0.868906686		3.70506	0.18649
ZNF594	10.7760747	0.8262784	3.557786824	0.868906686		3.70506	0.18649
CALCRL	14.9207188	57.013208	3.99283357	5.858309486		-1.93398	0.18658
LOC102162804	40.6175124	9.9153405	5.379118827	3.448285225		2.034368	0.18665
LOC100627541	40.6175124	9.9153405	5.379118827	3.448285225		2.034368	0.18665
RPP14	1129.82999	308.20183	10.14316633	8.27240506		1.874158	0.18686
RRP1B	7200.07576	1798.808	12.81399673	10.81362731		2.000971	0.18698
CD81	193.140416	694.90011	7.600956677	9.442736426		-1.84716	0.18701
LOC102164574	27.3546512	98.327126	4.825513503	6.634115866		-1.8458	0.18709
DFFB	310.848309	88.411786	8.284700625	6.482393107		1.8139	0.18737
LOC102165164	62.1696618	16.525567	5.981159942	4.13138925		1.911511	0.18755
LOC100737239	65.4853771	229.70539	6.054965161	7.849907883		-1.81054	0.18759
LOC100736819	5.80250177	27.267186	2.766065426	4.821056379		-2.23242	0.18771
GRM4	29.8414377	6.610227	4.946798112	2.927939483		2.174545	0.18806
LOC102164054	89.524313	24.788351	6.500233417	4.688647628		1.852617	0.18816
SYCP2	823.955251	228.05283	9.688172053	7.839536581		1.853198	0.18834
LOC100739805	605.118041	169.38707	9.243454975	7.412672017		1.836893	0.18837
AGL	1200.28894	327.20624	10.23036748	8.358458837		1.87511	0.18861
C14H12orf65	736.917724	204.91704	9.527316158	7.685919384		1.846464	0.1887
PGM1	1124.02749	4384.233	10.13574453	12.0984378		-1.96365	0.18897
PHF8	2941.8684	770.09144	11.52300731	9.590758147		1.933631	0.18943
RAD1	644.077696	180.12869	9.333329125	7.500871231		1.838207	0.18946
TMCC3	1104.13319	302.41788	10.11000454	8.245162313		1.868299	0.18965
PSORS1C2	53.8803735	14.046732	5.778218396	3.911378308		1.939525	0.18966
C1H9orf135	19.0653629	3.3051135	4.326635347	2.106051269		2.528182	0.18984
TMEM81	19.0653629	3.3051135	4.326635347	2.106051269		2.528182	0.18984
LOC102158389	19.0653629	3.3051135	4.326635347	2.106051269		2.528182	0.18984
LOC100737157	19.0653629	3.3051135	4.326635347	2.106051269		2.528182	0.18984
LOC102159294	0.82892882	10.741619	0.870998931	3.553559423		-3.69582	0.18991
GPR128	0.82892882	10.741619	0.870998931	3.553559423		-3.69582	0.18991
LOC102165138	0.82892882	10.741619	0.870998931	3.553559423		-3.69582	0.18991
LOC100739807	934.202784	257.79885	9.869135415	8.015687408		1.85749	0.19015
LOC100513063	1731.63231	465.19472	10.75874981	8.864788865		1.896226	0.19021
FGF18	30.6703665	109.06875	4.985061656	6.782261052		-1.83032	0.19026
C17H8orf48	85.3796688	299.11277	6.43261988	8.229360901		-1.80873	0.19047
LOC102159839	92.0110994	25.61463	6.539330984	4.734147579		1.84484	0.19049
FAM117A	1682.72551	452.80055	10.71744125	8.82591454		1.893852	0.19052
LOC102165490	43.1042988	151.20894	5.462847376	7.249909307		-1.81064	0.19095
LOC100516675	21.5521494	78.496445	4.495193036	6.312818447		-1.8648	0.19106
GALNS	300.901163	1097.2977	8.237932504	10.10105341		-1.86659	0.19115
LOC100737449	2117.91314	564.34813	11.04910874	9.142995708		1.907986	0.19137
LOC100519130	9.94714588	40.48764	3.452482877	5.374609695		-2.02513	0.19141
LPAR6	61.340733	214.0061	5.962103212	7.74823377		-1.80273	0.19197
LOC100621672	1892.4445	507.33492	10.88679742	8.98963553		1.899241	0.1921
ART4	32.3282241	7.4365053	5.058672541	3.076645515		2.120098	0.19214
LOC100739568	32.3282241	7.4365053	5.058672541	3.076645515		2.120098	0.19214
KCNN4	6.63143059	29.746021	2.93195353	4.942327829		-2.1653	0.19257
ZSWIM6	1671.94944	451.14799	10.70817813	8.820651243		1.889859	0.19282
ABCC3	1225.98573	4783.3255	10.26090276	12.22409983		-1.96407	0.19284
RRP12	8664.79299	2158.2391	13.08111606	11.0763073		2.005311	0.19307
LOC102158683	119.365751	33.877413	6.911281129	5.124221138		1.816993	0.19327
PRDM2	1310.53647	357.77854	10.35704221	8.486949769		1.873019	0.19346
SEPX1	565.329458	2117.7515	9.145497764	11.04899865		-1.90537	0.1938
CAND2	426.069415	1574.0603	8.738326772	10.62119134		-1.88533	0.19388
TIMP-4	3.31571529	19.004403	2.109599694	4.322245637		-2.51894	0.19394
WNT4	3.31571529	19.004403	2.109599694	4.322245637		-2.51894	0.19394

GCNT2	1373.90579	417.27058	10.42511705	8.708292708		1.719228	0.51926
LOC102159661	48.3283443	157.81917	5.624344958	7.311241242		-1.70733	0.51928
NTHL1	2403.98992	706.46801	11.23181513	9.4665211		1.766735	0.51935
IGJ	19.3313377	65.275991	4.345633235	6.05041444		-1.75561	0.51939
ANKRD32	863.006147	269.36675	9.754897767	8.078773924		1.679799	0.52048
CLOCK	40.0434852	130.55198	5.359081339	7.039489182		-1.70498	0.52062
MRPL50	1311.76934	399.91873	10.35839774	8.647166015		1.713735	0.52062
KIRREL2	22.0929574	5.7839486	4.529381037	2.762125239		1.93346	0.52066
LOC100525890	27.6161967	90.890621	4.838760036	6.521845711		-1.71862	0.52067
LOC100737264	694.547347	2640.7857	9.442004915	11.36729771		-1.92682	0.52083
LOC100516136	115.988026	389.17711	6.870217066	8.607985345		-1.74645	0.52084
LOC102159500	8.28485901	0.8262784	3.214880004	0.868906686		3.325777	0.52125
LOC100522669	8.28485901	0.8262784	3.214880004	0.868906686		3.325777	0.52125
LOC100738252	8.28485901	0.8262784	3.214880004	0.868906686		3.325777	0.52125
SIGLEC5	8.28485901	0.8262784	3.214880004	0.868906686		3.325777	0.52125
LOC100736827	8.28485901	0.8262784	3.214880004	0.868906686		3.325777	0.52125
LOC100522888	8.28485901	0.8262784	3.214880004	0.868906686		3.325777	0.52125
LOC102158760	8.28485901	0.8262784	3.214880004	0.868906686		3.325777	0.52125
LOC102165574	8.28485901	0.8262784	3.214880004	0.868906686		3.325777	0.52125
LOC102163410	8.28485901	0.8262784	3.214880004	0.868906686		3.325777	0.52125
LOC100620444	8.28485901	0.8262784	3.214880004	0.868906686		3.325777	0.52125
POMT1	672.45439	2548.2425	9.395436432	11.3158529		-1.92199	0.52161
LOC102159538	33.1394361	108.24247	5.093367322	6.771389985		-1.70765	0.52164
C10H1orf101	5.52323934	23.962073	2.705588563	4.641665831		-2.11717	0.5218
LOC100155322	5.52323934	23.962073	2.705588563	4.641665831		-2.11717	0.5218
CYB5B	6840.53193	1891.3512	12.74010369	10.88596414		1.854691	0.52185
LOC100524027	342.440839	1231.9811	8.423917793	10.26793491		-1.84705	0.52191
LOC102162088	407.338902	132.20454	8.673623206	7.057499439		1.623458	0.52197
SLC40A1	694.547347	2637.4806	9.442004915	11.36549164		-1.92502	0.52202
SAT2	2974.26439	865.11346	11.53880216	9.758412211		1.781572	0.52246
MRPL55	2218.96141	656.89131	11.11631888	9.361705436		1.756158	0.52252
[illegible]							

LOC102157804	0	7.4365053	0	3.076645515	-20	0.54219	LOC102162308	0	8.2627837	0	3.211445828	-20	0.34039	AP1AR	1836.07734	510.64003	10.84319665	8.998985343	1.846248	0.22696
NKX2-8	0	7.4365053	0	3.076645515	-20	0.54219	CD96	0	8.2627837	0	3.211445828	-20	0.34039	FRMPD3	114.392178	386.69828	6.850401618	8.598790517	-1.75722	0.22714
LOC102166041	0	7.4365053	0	3.076645515	-20	0.54219	C4H1orf110	0	8.2627837	0	3.211445828	-20	0.34039	RPRD1A	5159.253	1363.3593	12.33322608	10.41400792	1.919996	0.22718
PIFO	0	7.4365053	0	3.076645515	-20	0.54219	LOC100157354	0	8.2627837	0	3.211445828	-20	0.34039	PCDHA1	23.2100071	4.9576702	4.597531595	2.574748269	2.227013	0.22739
LOC100738868	0	7.4365053	0	3.076645515	-20	0.54219	LOC100739324	0	8.2627837	0	3.211445828	-20	0.34039	PLCD4	23.2100071	4.9576702	4.597531595	2.574748269	2.227013	0.22739
LOC100738812	0	7.4365053	0	3.076645515	-20	0.54219	LOC100625823	0	8.2627837	0	3.211445828	-20	0.34039	RSPH9	23.2100071	4.9576702	4.597531595	2.574748269	2.227013	0.22739
CPS1	0	7.4365053	0	3.076645515	-20	0.54219	KRT14	0	8.2627837	0	3.211445828	-20	0.34039	RARS2	260.283651	77.670167	8.029473043	6.29774474	1.744652	0.2274
LOC102160922	0	7.4365053	0	3.076645515	-20	0.54219	LOC102167149	0	8.2627837	0	3.211445828	-20	0.34039	CENPA	2236.44997	616.40367	11.12763971	9.270070238	1.859263	0.2275
SEC16B	0	7.4365053	0	3.076645515	-20	0.54219	GRM1	0	8.2627837	0	3.211445828	-20	0.34039	AMER1	2214.06889	610.61972	11.11313585	9.256491105	1.858354	0.22765
LOC102160838	0	7.4365053	0	3.076645515	-20	0.54219	SELP	0	8.2627837	0	3.211445828	-20	0.34039	ARL4D	268.572939	80.149002	8.074531868	6.342501448	1.744557	0.22784
TMEM236	0	7.4365053	0	3.076645515	-20	0.54219	MIR210	0	8.2627837	0	3.211445828	-20	0.34039	GFM2	1269.09003	359.43109	10.31071505	8.493579657	1.820007	0.22789
LOC102160463	0	7.4365053	0	3.076645515	-20	0.54219	LOC100620402	0	8.2627837	0	3.211445828	-20	0.34039	LOC100738106	155.00969	46.271589	7.28549183	5.56290145	1.74416	0.22814
LOC102160272	0	7.4365053	0	3.076645515	-20	0.54219	LOC100522828	12510.67271	2461.4833	13.61098706	11.26589821	2.34556	0.34053	LOC100737327	87.8664553	25.61463	6.473567038	4.734147579	1.778344	0.22827
LOC102162949	0	7.4365053	0	3.076645515	-20	0.54219	PIGX	957.9673324	223.92144	9.90533786	7.81327737	2.096984	0.34079	TBX18	129.312897	437.92754	7.025836058	8.777838973	-1.75983	0.22874
ZNF704	0	7.4365053	0	3.076645515	-20	0.54219	RIOK1	4138.503465	878.33391	12.01524201	9.780267293	2.236268	0.34103	LOC100157816	114.392178	33.877413	6.850401618	5.124221138	1.755593	0.22888
LOC102158739	0	7.4365053	0	3.076645515	-20	0.54219	LOC102166198	72.95777697	18.178124	6.208629956	4.261389711	2.004858	0.34138	CENPB	1569.16226	5977.2977	10.61669794	12.54551903	-1.9295	0.22901
TSGA13	0	7.4365053	0	3.076645515	-20	0.54219	DCUN1D5	6466.808173	1331.9607	12.65906118	10.38041857	2.279502	0.34145	LOC100510918	12.4339324	46.271589	3.747809764	5.56290145	-1.89584	0.22901
SLC45A2	0	7.4365053	0	3.076645515	-20	0.54219	MAP4K1	10.57359087	47.924146	3.532764645	5.61247475	-2.18029	0.34165	LOC102165367	25.6967935	5.7839486	4.73859457	2.762125239	2.151462	0.22931
LOC102158545	0	7.4365053	0	3.076645515	-20	0.54219	KCNRG	4.229436346	23.962073	2.386655454	4.641665831	-2.50222	0.34166	IFFO2	87.0375265	291.67627	6.460046706	8.193161944	-1.74466	0.22955
M1AP	0	7.4365053	0	3.076645515	-20	0.54219	C1H18orf54	2235.257109	494.94074	11.12687035	8.954023947	2.175113	0.34171	HEBP2	385.451903	1364.1856	8.59414506	10.41488138	-1.82342	0.22958
LOC100737245	0	7.4365053	0	3.076645515	-20	0.54219	C5H12orf39	428.2304301	104.93735	8.745608549	6.727067559	2.028859	0.34179	ATP6V1H	851.309902	3136.5527	9.735234283	11.61542398	-1.88142	0.22973
LOC102167845	0	7.4365053	0	3.076645515	-20	0.54219	CCL5	45.46644072	191.69658	5.538117235	7.590187173	-2.07595	0.342	LOC102157786	90.3532418	26.440908	6.51338402	4.77825631	1.772805	0.22976
LOC102163313	0	7.4365053	0	3.076645515	-20	0.54219	LOC102159737	69.78569971	17.351846	6.145386028	4.19785327	2.007842	0.34206	SYDE2	1066.00247	304.89672	10.0593478	8.256900824	1.805818	0.22996
LOC100517479	0	7.4365053	0	3.076645515	-20	0.54219	PRAM1	84.58872693	365.21504	6.419348884	8.516547233	-2.11021	0.34244	HIVEP1	11929.1147	3029.1365	13.54232029	11.56516707	1.977508	0.23038
LOC102167718	0	7.4365053	0	3.076645515	-20	0.54219	ZBTB26	1784.822138	401.57129	10.80237268	8.653100473	2.152052	0.34262	ADAT2	872.862051	252.0149	9.771261742	7.983078557	1.792245	0.23077
ERC2	0	7.4365053	0	3.076645515	-20	0.54219	LOC102164964	27.49133625	5.7839486	4.832451381	2.76212524	2.24885	0.34279	LOC102157458	28.18358	6.610227	4.867084967	2.927939483	2.092083	0.23085
LOC100515984	0	7.4365053	0	3.076645515	-20	0.54219	SLA-DOB	27.49133625	5.7839486	4.832451381	2.76212524	2.24885	0.34279	FBP1	28.18358	6.610227	4.867084967	2.927939483	2.092083	0.23085
MANSC4	0	7.4365053	0	3.076645515	-20	0.54219	FAM91A1	8335.161679	1689.7393	13.02516754	10.72343848	2.30241	0.34284	SYK	290.125088	86.759229	8.185495362	6.455478947	1.741586	0.23086
LOC100511104	0	7.4365053	0	3.076645515	-20	0.54219	LOC100511081	189.2672765	47.924146	7.571883648	5.61247475	1.9816	0.34314	GALNT10	441.819063	1571.5815	8.79057352	10.61891904	-1.83069	0.23121
MYBPC3	0	7.4365053	0	3.076645515	-20	0.54219	NAA15	14411.80435	2815.9567	13.81506345	11.45992167	2.355554	0.34318	PELI2	2356.64465	650.28108	11.20313057	9.347136503	1.857599	0.23142
MIR224	0	7.4365053	0	3.076645515	-20	0.54219	LOC100622354	15.8603863	2.4788351	4.075565686	1.798604302	2.677694	0.3434	LOC100516297	723.654863	210.70098	9.501150225	7.72588417	1.780105	0.23155
LOC100737904	0	7.4365053	0	3.076645515	-20	0.54219	LOC102159786	15.8603863	2.4788351	4.075565686	1.798604302	2.677694	0.3434	ALKBH8	1989.42918	553.60651	10.95886382	9.115320738	1.845422	0.23159
DNAJB13	0	7.4365053	0	3.076645515	-20	0.54219	LOC100738873	15.8603863	2.4788351	4.075565686	1.798604302	2.677694	0.3434	LOC100628087	119.365751	35.52997	6.911281129	5.191008663	1.748281	0.23172
LOC100737379	0	7.4365053	0	3.076645515	-20	0.54219	LOC100623661	15.8603863	2.4788351	4.075565686	1.798604302	2.677694	0.3434	HMCN1	62.9985906	209.87471	5.999968229	7.72024225	-1.73614	0.2319
LOC100737367	0	7.4365053	0	3.076645515	-20	0.54219	LOC102158827	15.8603863	2.4788351	4.075565686	1.798604302	2.677694	0.3434	LOC102164504	4.97357294	23.135794	2.578594101	4.593102408	-2.21777	0.23214
LOC100737489	0	7.4365053	0	3.076645515	-20	0.54219	HSBP1P2	15.8603863	2.4788351	4.075565686	1.798604302	2.677694	0.3434	LOC100154278	24.8678647	85.106672	4.693089065	6.428053129	-1.77499	0.23259
WDR43	5575.71012	1585.6282	12.44519856	10.63174838	1.814101	0.54223	LOC102167642	15.8603863	2.4788351	4.075565686	1.798604302	2.677694	0.3434	NOC4L	5874.61857	1550.0982	12.52052503	10.59907433	1.922136	0.23276
FAM136A	7862.33121	2189.6377	12.94092491	11.09713518	1.844265	0.54225	E2F7	1094.366655	254.49374	10.09719815	7.997144125	2.104394	0.34372	MCAM	6991.1857	1828.554	12.77152779	10.83727631	1.934834	0.23286
SH3D19	1576.88483	6258.2324	10.62377619	12.61177002	-1.98868	0.54239	LOC100736845	146.972913	655.23875	7.209189299	9.358076973	-2.15647	0.34377	LOC102158993	106.931818	358.60481	6.753976422	8.490268521	-1.7457	0.23295
MSH2	3737.85223	1089.8612	11.86837974	10.09125179	1.778325	0.54292	RAB37	25.37661808	106.58991	4.721187694	6.749398975	-2.0705	0.34448	ATIC	9287.31854	2394.5547	13.18120173	11.22614405	1.955505	0.23309
NXN	2445.41422	10046.719	11.25645298	13.29458036	-2.03857	0.54298	LOC100623591	120.5389359	529.64444	6.925274755	9.051601682	-2.13552	0.34482	LOC102158094	33.1571529	8.2627837	5.094115824	3.211445828	2.00462	0.23336
ARHGAP21	1695.63448	6763.9148	10.72846007	12.72385604	-1.99603	0.5431	LOC100517259	81.41664967	349.51575	6.364863912	8.453335466	-2.10196	0.34513	LOC102165339	33.1571529	8.2627837	5.094115824	3.211445828	2.00462	0.23336
CITED2	1496.79786	5911.1955	10.54862722	12.52947825	-1.98157	0.54311	TANC1	558.2855977	2752.3333	9.127441369	11.42696353	-2.30158	0.34514	LOC100523728	971.504581	280.10837	9.925561236	8.13498259	1.794236	0.23402
RC3H2	66.2788721	211.52726	6.072081615	7.731504113	-1.67422	0.54318	MBF1	1231.823336	6480.5013	10.26775036	12.6621123	-2.39531	0.34537	LOC102166685	135.115398	40.48764	7.088686472	5.374609695	1.738639	0.23406
TMEM154	69.0404918	220.61633	6.130117309	7.791920351	-1.67602	0.54319	GGCX	751.7823106	3796.7491	9.556088917	11.89092889	-2.33638	0.34537	SCIN	5.80250177	25.61463	2.766065426	4.734147579	-2.14222	0.23419
SLC25A13	408.719711	1455.0762	8.678493492	10.50787015	-1.83191	0.54382	LOC102159934	41.23700438	172.69218	5.400435611	7.440388989	-2.06619	0.34542	NT5M	159.154334	539.55978	7.323319031	9.078310354	-1.76136	0.23427
SCRN3	77.3253508	247.88351	6.291407421	7.959326845	-1.68065	0.54383	ITGAE	149.0876312	38.008805	7.229661278	5.285727902	1.971755	0.34567	IFT20	571.13196	2051.6492	9.160204126	11.00327137	-1.84489	0.23432
LOC100738146	335.53679	1176.6204	8.394620418	10.20165885	-1.81011	0.54394	LOC100736902	9908.512	1991.3309	13.2745983	10.96024155	2.314935	0.34589	STAB1	186.508985	56.186929	7.55081592	5.837613535	1.730939	0.23466
LOC102166404	1480.22814	456.93194	10.53257815	8.838989383	1.695768	0.544	CYTH1	133.2272449	588.3102	7.068533723	9.202883429	-2.14269	0.34596	LOC100622776	427.727273	127.24687	8.743916385	7.002779797	1.749061	0.23468
TSTD2	483.283443	1741.7948	8.919707868	10.76718701	-1.84963	0.54444	ISOC2	75.07249515	320.59601	6.249303021	8.329105689	-2.0944	0.34597	LRBA	1700.96195	478.41518	10.73298306	8.905131772	1.830016	0.2349
LOC100156195	67.659682	215.65866	6.101391269	7.75928006	-1.67238	0.54475	LOC102165942	39.1222862	9.0890621	5.326331909	3.334720158	2.105787	0.34601	TJP2	10088.0638	2594.5141	13.30050468	11.3418046	1.959113	0.23504
NSUN3	1692.87286	518.90282	10.72610988	9.022098163	1.705937	0.54517	LOC100521084	1578.637116	359.43109	10.62537746	8.493579657	2.134892	0.34612	LOC100736736	205.574348	61.970878	7.690517306	5.976612875	1.729998	0.23518
MDFIC	419.76619	1494.7376	8.716874974	10.54664136	-1.83223	0.54549	GFM1	8877.586891	1797.9817	13.11611436	10.81296483	2.303789	0.34613	LOC100627365	85.3796688	284.23976	6.43261988	8.156031285	-1.73514	0.23535
DOCK4	44.1859147	139.64104	5.497801224	7.135873883	-1.66006	0.54555	ABHD2	530.7942615	2601.1243	9.054724399	11.34547417	-2.29291	0.34662	SYT2	38.1307259	9.9153405	5.290229968	3.448285225	1.94322	0.23551
ICAM3	1455.37357	450.32171	10.50816475	8.818012376	1.692362	0.54561	LOC102159747	155.4317857	39.661362	7.289389877	5.34558663	1.970475	0.34667	CD302	26.5257224	90.064343	4.78270852	6.508814353	-1.76356	0.23571
LOC102164603	11.0464787	38.835083	3.590539588	5.315967693	-1.81377	0.54567	PCDH1	433.5172255	2089.658	8.76326956	11.02974136	-2.26911	0.34669	LOC102162762	1511.96617	428.0122	10.56316402	8.744874853	1.820702	0.23581
LOC100624568	5.52323934	23.135794	2.705588563	4.593102408	-2.06654	0.54626	EFHC2	57.09739067	14.046732	5.860401464	3.911378308	2.02319	0.34678	LOC100516384	188.995772	57.013208	7.569823503	5.858309486	1.728986	0.23585
LOC100625687	5.52323934	23.135794	2.705588563	4.593102408	-2.06654	0.54626	MAK16	11102.27041	2216.0786	13.43869706	11.1144442	2.324774	0.34681	LOC100624675	5543.87597	22451.636	12.43693948	14.45459721	-2.01785	0.23626
LOC100737823	5.52323934	23.135794	2.705588563	4.593102408	-2.06654	0.54626	LOC100738030	7.401513606	35.52997	3.070649265	5.191008663	-2.26314	0.34685	LOC102164415	40.6175124	10.741619	5.379118827	3.553559423	1.91889	0.23653
LOC102165147	5.52323934	23.135794	2.705588563	4.593102408	-2.06654	0.54626	CRY2	199.8408674	906.42737	7.64990905	9.825638371	-2.18134	0.3472	LOC100621448	2533.20649	699.85778	11.30731836	9.45297791	1.855831	0.23653
ADAM11	452.905626	149.55639	8.82624856	7.234160086	1.598521	0.54629	NECAB3	142.7434767	632.10295	7.167352675	9.306296318	-2.14673	0.34731	PITPNM3	68.8010924	19.830681	6.125177709	4.380638094	1.794697	0.23659
ACAT1	23308.07	6100.4132	14.50860383	12.57492773	1.933851	0.54642	LOC100625300	230.5042809	1055.9838	7.854895061	10.04573749	-2.19572	0.34771	MRE11A	2450.3136	678.37454	11.25933935	9.40806335	1.852812	0.23701
LOC100625820	45.5667246	143.77244	5.541227504	7.177643143	-1.65773	0.54693	LOC100525984	23.2618999	97.500848	4.600620625	6.622064238	-2.06745	0.34772	GHDC	246.191861	846.93533	7.94948743	9.72781043	-1.78247	0.23706
NDUFB8	10349.1697	2845.7027	13.3373668	11.47507612	1.862658	0.54735	SNPH	23.2618999	97.500848	4.600620625	6.622064238	-2.06745	0.34772	LOC100737297	27.3546512	92.543178	4.825513503	6.547560532	-1.75834	0.2372
EMILIN3	66.2788721	22.309516	6.072081615	4.542847146	1.57089	0.54758	NKAIN1	4292.877891	915.51644	12.06806545	9.840016943	2.229287	0.34809	LOC102160497	7.46035941	30.5723	3.080718953	4.980587446	-2.03491	0.23726
NUF2	3773.75328	1103.9079	11.88216664	10.10971041	1.77338	0.54771	LOC102167743	467.3527163	114.85269	8.871451622	6.856147778	2.024727	0.34815	LOC102159943	2080.61135	580.8737	11.02348502	9.184562218	1.840711	0.23737
LYL1	428.051049	1522.831	8.745005502	10.57348723	-1.8309	0.54771	LOC102165483	97.27703596	419.74941	6.618782442	8.716817448	-2.10936	0.34852	LOC100736666	480.778718	142.94616	8.912226855	7.169385476	1.749901	0.23767
LOC100621912	563.370413	2047.5178	9.140498547	11.00036472	-1.86172	0.5478	SH2D5	428.2304301	105.76363	8.745608549	6.738276475	2.017544	0.34853	MAP3K4	2132.83386	594.92043	11.05923214	9.218975892	1.842003	0.23771
GATA2	1409.80684	437.92754	10.46230476	8.777838973	1.686733	0.54798	LOC100736826	7040.954157	1452.5974	12.78176012	10.50541201	2.277136	0.34874	IMP3	3692.87791	1002.2757	11.85092047	9.970502347	1.881466	0.23776
MPHOSPH10	3463.07107	1018.8012	11.75825281	9.994072271	1.765179	0.54823	DCBLD1	6911.956349	1427.809	12.7550871	10.48059738	2.275291	0.34876	C2H11orf68	922.597781	3386.915	9.851120896	11.72618198	-1.8762	0.23779
UST	414.242951	1469.1229	8.697811867	10.5217211	-1.82641	0.54825	LOC102164531	3.17207726	19.830681	2.060765874	4.380638094	-2.64423	0.34884	GNPDA1	1061.85782	3927.3011	10.05373291	11.9396898	-1.88695	0.23784
LOC100621702	4241.84782	1232.8073	12.05081722	10.26890141	1.782746	0.54856	FAM169B	3.17207726	19.830681	2.060765874	4.380638094	-2.64423	0.34884	LOC100736834	142.575758	42.966475	7.165668365	5.458331975	1.730445	0.23791
DUSP19	365.914607	122.2892	8.519300528	6.945902605	1.58121	0.54859	C17H20orf196	3.17207726	19.830681	2.060765874	4.380638094	-2.64423	0.34884	IGFALS	229.613284	69.407383	7.849331809	6.137654819	1.726045	0.23821
FAM101A	19.3313377	62.797156	4.345633235	5.995420212	-1.69976	0.54891	GNAI2	5826.048567	34687.992	12.50854962	15.08219031	-2.57385	0.34894	LOC100524940	174.903982	52.881816	7.45864433	5.751726565	1.72572	0.23826
TNFRSF11A	34.5202459	10.741619	5.150569664	3.553559423	1.684231	0.54918	C13H3orf26	2542.948603	562.69557	11.31285381	9.138772422	2.176076	0.34927	LOC102164532	406.175124	121.46292	8.669505612	6.936201185	1.741586	0.2383
LOC102165302	9.66566885	34.703692	3.414902536	5.158001345	-1.84415	0.54927	MSH2	5169.428574	1089.8612	12.33606815	10.09125179	2.24586	0.34927	LOC100623685	17.4075053	61.1446	4.202222212	5.957557119	-1.81252	0.23833
LOC100622996	9.66566885	34.703692	3.414902536	5.158001345	-1.84415	0.54927	RSPH3	46.52379981	194.17542	5.570578288	7.608627544	-2.06132	0.34953	WDR91	2003.52097	561.04301	10.96904179	9.134536737	1.836354	0.23863
LOC102165171	69.0404918	218.96377	6.130117309	7.781122098	-1.66518	0.54938	RSAD1	154.3744266	684.98477	7.279605257	9.422032737	-2.14964	0.34981	LOC100516934	279.349014	84.280394	8.131080184	6.414142197	1.7288	0.23866
LOC102164532	38.6626754	121.46292	5.309710091	6.936201185	-1.6515	0.55054	RASGRP4	15.8603863	67.754826	4.075565686	6.103389087	-2.0949	0.34988	LOC102161610	9.94714588	0.8262784	3.452482877	0.868906686	3.589583	0.23882
ABCG1	16.569718	54.534373	4.135019133	5.795309087	-1.71862	0.55064	PLCG2	291.8311079	73.538775	8.193925011	6.219919206	1.988557	0.35054	SYT7	9.94714588	0.8262784	3.452482877	0.868906686	3.589583	0.23882
AFF4	966.566885	3655.4555	9.918217584	11.8362301	-1.91911	0.55087	LTA4H	18946.81747	3661.2395	14.20974406	11.83851041	2.371551	0.35065	LOC100525750	9.94714588	0.8262784	3.452482877	0.868906686	3.589583	0.23882
NEK3	1012.13361	321.42229	9.984608731	8.33280766	1.654858	0.55102	ZFYVE1	486.3851798	2354.0671	8.928918573	11.20155244	-2.27498	0.35078	ACPP	9.94714588	0.8262784	3.452482877	0.868906686	3.589583	0.23882
KIFAP3	1030.08414	3914.0806	10.00994635	11.93482631	-1.92591	0.55126	MOAP1	61.32682702	257.79885	5.961781364	8.015687407	-2.07166	0.35084	SERPIND1	9.94714588	0.8262784	3.452482877	0.868906686	3.589583	0.23882
LOC102163999	15.1889082	50.402981	4.016933786	5.683780114	-1.73049	0.55216	LOC100627865	19.03246356	80.149002	4.324267947	6.342501448	-2.07422	0.35085	LOC100736827	9.94714588	0.8262784	3.452482877	0.868906686	3.589583	0.23882
LOC100624199	71.8021115	227.22655	6.185908388	7.834322836	-1.66203	0.55224	LOC100624621	47.5811589	198.30681	5.602325001	7.63884719	-2.05927	0.35093	LOC102158079	9.94714588	0.8262784	3.452482877	0.868906686	3.589583	0.23882
LOC100627669	452.905626	150.38266	8.82624856	7.242056188	1.590572	0.55226	FLVCR2	56.04003159	234.66306	5.833902876	7.88058181	-2.06606	0.35117	LOC102160692	9.94714588	0.8262784	3.452482877	0.868906686	3.589583	0.23882
LOC102164507	33.1394361	104.11107	5.093367322	6.715770874	-1.6515	0.55238	TANGO2	838.4857557	4241.2869	9.713362036	12.05062647	-2.33864	0.35129	LOC102164966	9.94714588	0.8262784	3.452482877	0.868906686	3.589583	0.23882
LOC100737328	88.3718295	281.76092	6.481748252	8.143438955	-1.67281	0.55294	SCAF1	978.0571551	5010.552	9.935249273	12.29104175	-2.35698	0.35134	LOC102163468	9.94714588	0.8262784	3.452482877	0.868906686	3.589583	0.23882
LOC100519853	523.326928	172.69218	9.03432283	7.44038899	1.59951	0.55337	LOC102163142	20.08982264	84.280394	4.398475058	6.414142197	-2.06873	0.35141	LOC102165829	9.94714588	0.8262784	3.452482877	0.868906686	3.589583	0.23882
GTPBP2	294.112495	1010.5384	8.205121195	9.982335443	-1.78068	0.55341	LOC100523107	1685.430384	9021.3073	10.71975705	13.1392807	-2.42022	0.35247	LOC100511007	9.94714588	0.8262784	3.452482877	0.868906686	3.589583	0.23882
ISY1	2104.35419	641.19202	11.03984725	9.32686092	1.714549	0.55382	MECR	186.0951992	833.71488	7.54762873	9.705139674	-2.16351	0.35258	LOC100620444	9.94714588	0.8262784	3.452482877	0.868906686	3.589583	0.23882
PION	107.703167	346.21064	6.764250165	8.439667339	-1.68459	0.55419	SYNE1	536.0810569	2608.5608	9.068996028	11.34959131	-2.28273	0.35279	LOC100739598	318.308668	95.848291	8.318807908	6.597654688	1.731602	0.23914
LOC102157907	8.28485901	30.5723	3.214880004	4.980587446	-1.88368	0.55425	LOC102161161	10.57359087	47.097867	3.532764645	5.587901017	-2.1552	0.35305	LY6G5B	36.4728682	121.46292	5.227774503	6.936201185	-1.73562	0.23927
ATIC	8562.40179	2394.5547	13.0639683	11.22614405	1.838258	0.55435	DCUN1D4	3382.491718	737.04031	11.72429714	9.5275558	2.198271	0.35311	LOC100738202	107.760747	32.224857	6.765014156	5.054191063	1.741586	0.23934
LOC102168008	84.2294	28.919743	6.413279271	4.903025878	1.542269	0.55435	CNTN3	858.5755783	204.91704	9.747480684	7.685919384	2.066905	0.35345	LOC102166933	326.597957	98.327126	8.355782547	6.634115866	1.731854	0.2395
KMT2A	631.030095	2297.0539	9.303849446	11.1661969	-1.864	0.55464	LOC100512389	47.5811589	11.567897	5.602325001	3.651671381	2.040264	0.35361	KIF20B	4339.44239	1170.0102	12.08362638	10.1935379	1.890989	0.23951
LOC102159784	124.272885	42.966475	6.968930373	5.458331975	1.532228	0.55476	LOC102165122	47.5811589	11.567897	5.602325001	3.651671381	2.040264	0.35361	CYCS	15617.019	3944.6529	13.93092386	11.94604835	1.985149	0.24
LOC100154844	154.650702	507.33492	7.28216827	8.98963553	-1.71393	0.55484	LOC100519752	2399.147767	535.42838	11.22890751	9.067241769	2.163756	0.35363	LOC100521603	4637.85677	1246.8541	12.17955359	10.28523351	1.895166	0.24006
LOC100515569	53.8515836	168.56079	5.777461367	7.405568764	-1.64621	0.55488	DDHD1	3274.641091	715.55707	11.67756158	9.484937802	2.194198	0.3537	CARD10	130.141825	435.4487	7.03498407	8.769668286	-1.74242	0.24036
NEBL	37.2818656	116.50525	5.258589232	6.876581411	-1.64385	0.55496	GMPPA	402.853812	1912.8344	8.657689347	10.90225031	-2.24738	0.3538	RSG1	113.563249	378.43549	6.8400005	8.567710832	-1.73655	0.24043
ORC5	789.823226	255.32002	9.627211433	8.001802338	1.629223	0.55512	MYLIP	279.1427989	1287.3417	8.130018598	10.33129957	-2.20532	0.35384	LOC102161040	50.5646582	14.046732	5.688310694	3.911378308	1.847895	0.24045
LOC100623777	1031.46495	3903.339	10.01187709	11.93086262	-1.92001	0.55523	LOC100627329	328.8386759	1536.0515	8.365616765	10.58594978	-2.22377	0.35391	CACNA2D1	7861.56096	2059.0857	12.94078358	11.00848864	1.932812	0.24115
ZNF316	202.979046	677.54826	7.672277146	9.406307627	-1.73899	0.5554	MTX2	5882.088599	1235.2862	12.52235805	10.27179701	2.251483	0.35411	LOC100627386	96.9846724	28.919743	6.614484182	4.903025878	1.745702	0.24157
PHF19	98.0374983	313.1595	6.629902968	8.29535341	-1.67549	0.55545	POMC	29.60605442	122.2892	4.935745167	6.945902605	-2.04633	0.35413	LAMP2	1191.99965	4418.1105	10.2203779	12.10954028	-1.89005	0.24209
TIMP-4	4.14242951	19.004403	2.362450112	4.322245637	-2.19778	0.55546	LOC100738145	33.83549077	139.64104	5.122485983	7.135873883	-2.04511	0.35435	LOC102166678	55.5382312	15.699289	5.821154844	4.061714779	1.822782	0.24238
LOC100624626	4.14242951	19.004403	2.362450112	4.322245637	-2.19778	0.55546	LOC100739217	33.83549077	139.64104	5.122485983	7.135873883	-2.04511	0.35435	LOC102166461	55.5382312	15.699289	5.821154844	4.061714779	1.822782	0.24238
AOAH	4.14242951	19.004403	2.362450112	4.322245637	-2.19778	0.55546	C3H16orf96	9.516231779	42.966475	3.394545939	5.458331975	-2.17475	0.3551	RNF169	137.602185	460.23705	7.114806188	8.849364605	-1.74187	0.24245
LOC100739621	4.14242951	19.004403	2.362450112	4.322245637	-2.19778	0.55546	LOC102159337	393.3375802	98.327126	8.623287395	6.634115866	2.000107	0.35525	LOC102167193	153.351832	514.77143	7.2700788	9.010588038	-1.74709	0.24253
LAPTM5	38.6626754	12.394176	5.309710091	3.74353388	1.641279	0.55572	C2H5orf30	3010.301319	662.67525	11.55617136	9.374333673	2.183534	0.35527	LOC100622027	289.296159	87.585507	8.181381675	6.468998788	1.723783	0.24257
LOC102162076	114.607216	39.661362	6.853087646	5.34558663	1.530892	0.55589	LOC100516837	1120.800632	263.5828	10.13160059	8.047575471	2.088202	0.35536	LOC100520985	0.82892882	9.9153405	0.870998931	3.448285225	-3.58034	0.24265
CSF1R	12.4272885	2.4788351	3.747096094	1.798604302	2.325777	0.55595	LOC102166748	108.9079859	28.093465	6.780152406	4.862623207	1.954803	0.35537	LOC100525342	0.82892882	9.9153405	0.870998931	3.448285225	-3.58034	0.24265
LOC102157946	12.4272885	2.4788351	3.747096094	1.798604302	2.325777	0.55595	CFTR	32.77813168	7.4365053	5.078017628	3.076645515	2.140037	0.35538	LOC102163918	0.82892882	9.9153405	0.870998931	3.448285225	-3.58034	0.24265
LOC100737481	45.5667246	142.11988	5.541227504	7.161080272	-1.64106	0.55611	CD72	32.77813168	7.4365053	5.078017628	3.076645515	2.140037	0.35538	AMBP	0.82892882	9.9153405	0.870998931	3.448285225	-3.58034	0.24265
NFE2	131.176954	45.44531	7.04632663	5.537461031	1.529311	0.55669	TMEM109	804.6502649	4035.5436	9.654009886	11.97890475	-2.32633	0.35538	SERPINB9	0.82892882	9.9153405	0.870998931	3.448285225	-3.58034	0.24265
ZNF354C	26.2353869	82.627837	4.767410456	6.385911347	-1.65511	0.5567	LOC102159144	131.1125267	33.877413	7.045623457	5.124221138	1.95241	0.35564	LOC100738944	0.82892882	9.9153405	0.870998931	3.448285225	-3.58034	0.24265
LOC100623570	27.6161967	86.759229	4.838760036	6.455478947	-1.6515	0.55686	CDKN2AIPNL	7178.410839	1489.7799	12.80964974	10.54185156	2.268565	0.35592	LOC100739571	0.82892882	9.9153405	0.870998931	3.448285225	-3.58034	0.24265
ZSCAN31	27.6161967	86.759229	4.838760036	6.455478947	-1.6515	0.55686	PRPH	5.286795433	27.267186	2.65232482	4.821056378	-2.3667	0.35592	LOC100737663	0.82892882	9.9153405	0.870998931	3.448285225	-3.58034	0.24265
PWWP2A	2279.71704	693.24755	11.15527175	9.439306379	1.717412	0.55732	PLIN1	5.286795433	27.267186	2.65232482	4.821056378	-2.3667	0.35592	ARID4B	678.063778	2437.5212	9.407403269	11.2517908	-1.84592	0.24267
LOC100152887	27.6161967	8.2627837	4.838760036	3.211445828	1.740815	0.5574	MRS2	6160.174038	1292.2994	12.58898957	10.33684055	2.253031	0.35618	EXT1	1390.11364	5192.3333	10.44202456	12.3424451	-1.90118	0.2427
LOC102167379	27.6161967	8.2627837	4.838760036	3.211445828	1.740815	0.5574	EPHA5	96.21967688	24.788351	6.603176434	4.688647628	1.95667	0.35634	ZWILCH	1987.77132	559.39046	10.95766168	9.13028858	1.829224	0.24354
LOC100155975	30.3778164	95.022013	4.971673052	6.585293272	-1.64524	0.55752	NAV3	90.93288145	386.69828	6.522509054	8.598790517	-2.08833	0.35636	DVL3	1190.34179	4404.89	10.21837166	12.10521776	-1.88773	0.2437
EZH2	4007.11014	1177.4467	11.96870644	10.20267077	1.7669	0.55788	LOC100625234	445.1481754	110.7213	8.80137913	6.803760479	2.007353	0.35637	LOC100625619	563.6716	167.73451	9.141268263	7.398611253	1.748675	0.244
FASTKD5	840.91319	271.84558	9.717527674	8.091940885	1.629169	0.55916	SMNDC1	5405.219651	1144.3955	12.40040441	10.16163018	2.239767	0.35639	MIOX	12.4339324	45.44531	3.747809764	5.537461031	-1.86985	0.24403
KPNA2	12598.5089	3454.6699	13.62107989	11.75474968	1.866633	0.55932	FAM63A	389.1081439	1835.9905	8.607730306	10.84312848	-2.23832	0.35652	KLHL38	12.4339324	45.44531	3.747809764	5.537461031	-1.86985	0.24403
ITGA4	158.793131	518.90282	7.320061583	9.022098163	-1.70832	0.5596	SLC31A1	3633.085821	790.7484	11.82737677	9.62889824	2.199905	0.35673	CLSPN	2342.55286	654.41247	11.19448162	9.356259313	1.839809	0.24436
LOC100737174	27683.8564	7243.1562	14.75680942	12.82260194	1.934355	0.55972	LOC100526053	44.40908164	10.741619	5.504908955	3.553559423	2.047643	0.35688	WDR36	4344.41597	1177.4467	12.08527857	10.20267077	1.883501	0.24535
FZD4	951.377977	3559.6072	9.895390449	11.79790758	-1.90363	0.55977	C17H20orf112	401.7964529	1899.614	8.653907169	10.89224983	-2.24117	0.35699	TEKT3	748.522728	220.61633	9.549828415	7.791920351	1.762507	0.24538
TMEM68	238.880102	802.3163	7.906169679	9.649824338	-1.74788	0.56004	R3HDM4	656.6199928	3231.5747	9.36111035	11.658468	-2.29911	0.357	CTSC	1605.63513	6028.527	10.64982661	12.55782912	-1.90866	0.2455
LOC100622666	1198.54294	380.08805	10.22826908	8.573980563	1.656876	0.56084	SNX31	12.68830904	1.6525567	3.774872332	1.407383614	2.940728	0.35714	LOC100627519	815.665962	239.62073	9.673602289	7.910617116	1.767226	0.24567
AMY2	11.0464787	38.008805	3.590539588	5.285727902	-1.78275	0.56101	IL-5	12.68830904	1.6525567	3.774872332	1.407383614	2.940728	0.35714	MRPL50	1394.25828	399.91873	10.44631649	8.647166015	1.801719	0.24572
BNIPL	11.0464787	38.008805	3.590539588	5.285727902	-1.78275	0.56101	RAP1GAP	12.68830904	1.6525567	3.774872332	1.407383614	2.940728	0.35714	LOC102161165	181.535412	55.360651	7.512032568	5.816616368	1.713318	0.24597
ATP5G2	15509.2561	4205.7569	13.92093489	12.03849274	1.882692	0.56122	LOC100623402	12.68830904	1.6525567	3.774872332	1.407383614	2.940728	0.35714	CHAF1A	4511.03066	1221.2394	12.13956116	10.25531122	1.885111	0.24626
IRF8	42.8051049	14.046732	5.453027102	3.911378308	1.607548	0.56153	RPL3L	12.68830904	1.6525567	3.774872332	1.407383614	2.940728	0.35714	LDLRAP1	959.899578	3502.594	9.908241855	11.7746199	-1.86747	0.24632
LOC102161256	16.569718	53.708094	4.135019133	5.773682393	-1.69659	0.56181	LOC102160303	12.68830904	1.6525567	3.774872332	1.407383614	2.940728	0.35714	PMPCA	4713.28929	1273.295	12.20282458	10.31548355	1.888168	0.2464
LOC100518740	16.569718	53.708094	4.135019133	5.773682393	-1.69659	0.56181	LOC102158479	12.68830904	1.6525567	3.774872332	1.407383614	2.940728	0.35714	SIGLEC10	559.526956	1983.8944	9.130639944	10.95484652	-1.82606	0.24657
TMEM254	545.419885	1946.7118	9.093866176	10.92756454	-1.8356	0.56206	LOC100627380	12.68830904	1.6525567	3.774872332	1.407383614	2.940728	0.35714	C9H11orf54	2661.69045	740.34542	11.378669	9.534002096	1.846072	0.24658
NUP43	1509.22515	472.63123	10.56054793	8.887620397	1.675021	0.5623	LOC100521145	12.68830904	1.6525567	3.774872332	1.407383614	2.940728	0.35714	TTC21B	1021.24031	297.46021	9.997518673	8.221394816	1.779554	0.2469
ARL6IP5	1401.52198	5394.7715	10.45380767	12.39761354	-1.94457	0.56263	LOC102167150	12.68830904	1.6525567	3.774872332	1.407383614	2.940728	0.35714	LOC100627666	493.21265	147.90383	8.948988129	7.218237038	1.73755	0.24711
DERA	2677.39027	809.7528	11.38715048	9.663118299	1.725274	0.56268	LOC102161575	89.87552236	23.135794	6.505819847	4.593102407	1.957802	0.3573	FAM169B	67.9721635	19.830681	6.107942317	4.380638094	1.77721	0.24713
LOC100620861	2268.67056	693.24755	11.14826719	9.439306379	1.710405	0.56278	SMURF1	168.1200948	742.82426	7.40190428	9.538817985	-2.14353	0.3575	CSTB	779.193094	2809.3465	9.607687418	11.45653228	-1.85018	0.24722
ZMIZ2	154.650702	502.37725	7.28216827	8.975496206	-1.69976	0.5632	HAUS2	2745.961548	609.79344	11.423621	9.254540753	2.170919	0.35764	MRPS5	3773.28401	1032.0217	11.88198727	10.01265483	1.870347	0.2475
CHRNB1	331.394361	1135.3065	8.376752095	10.15013629	-1.77646	0.56397	LOC100519542	100.4491132	428.83847	6.664612444	8.747650814	-2.09397	0.35773	LOC102159638	243.705074	74.365053	7.934900207	6.2358238	1.71244	0.2477
C2CD2	796.727275	2925.0254	9.639751796	11.5147266	-1.87629	0.56406	LOC102161449	17.97510447	75.191332	4.246035924	6.251554969	-2.06457	0.35777	LOC102161938	421.924771	127.24687	8.724257253	7.002779797	1.729356	0.24782
LOC100620494	28.9970066	90.064343	4.906746634	6.508814353	-1.63505	0.56406	MTIF2	4226.264269	911.38504	12.04550859	9.833498988	2.21325	0.35783	LOC100523107	2358.3025	9021.3073	11.20414469	13.1392807	-1.93559	0.24795
LOC102164417	85.6102098	29.746021	6.436465198	4.942327829	1.525086	0.56417	BMS1	11952.38711	2400.3387	13.54513186	11.22962317	2.315989	0.35796	LOC100524253	93.6689571	28.093465	6.564819524	4.862623207	1.737336	0.2482
PIH1D1	5093.80748	1482.3434	12.31481192	10.53463691	1.780865	0.56428	LOC100620243	8.458872693	38.835083	3.241668254	5.315967693	-2.19882	0.35801	NLK	111.905391	33.877413	6.818970566	5.124221138	1.723884	0.24851
CLDN22	33.1394361	102.45852	5.093367322	6.692908622	-1.62842	0.56467	GIPC1	614.3256293	3002.6956	9.265206274	11.5525229	-2.28918	0.3581	LPAR5	127.655039	38.835083	7.007364151	5.315967693	1.716818	0.24878
ZC3H12C	205.740666	680.0271	7.691678382	9.411568398	-1.72477	0.56475	USP33	4806.754408	1027.8903	12.23114749	10.00687345	2.225377	0.35826	NR4A2	151.693975	505.68236	7.254499324	8.984937802	-1.73707	0.24895
MAP3K9	71.8021115	24.788351	6.185908388	4.688647628	1.534364	0.56475	MAGOH	4470.514218	960.96175	12.12654775	9.909835714	2.21789	0.35846	LOC100739700	59.6828753	195.0017	5.92321754	7.614722326	-1.7081	0.24982
RBFA	2203.7725	675.89571	11.10641408	9.402789759	1.705103	0.56503	GFRA3	88.81816327	375.95666	6.488935315	8.558254848	-2.08164	0.35879	MPHOSPH10	3712.7722	1018.8012	11.85866961	9.994072271	1.865624	0.25025
ZC2HC1A	142.223413	458.5845	7.162123543	8.84418632	-1.68903	0.56503	RPP30	6439.316837	1350.9651	12.65291595	10.40084223	2.252917	0.35926	LOC100627329	440.161205	1536.0515	8.785162119	10.58594978	-1.80312	0.25036
SIX4	615.841187	2211.9472	9.268755287	11.11175331	-1.84469	0.56515	MAGI1	6662.419604	1394.7579	12.70204703	10.446833	2.256032	0.35945	LOC100153260	3123.40381	865.11346	11.60936521	9.758412211	1.852158	0.25085
STXBP2	3805.51191	1128.6963	11.89425388	10.14171921	1.753433	0.56544	SLC5A1	13.74566813	58.665764	3.882219287	5.89883146	-2.09354	0.35946	TMEM33	3004.03806	833.71488	11.55316755	9.705139674	1.849277	0.25088
RTN4RL1	321.728692	109.89502	8.334178034	6.793050813	1.549719	0.56561	LOC102163485	21.14718173	4.1313919	4.46905122	2.359350201	2.355766	0.35947	LOC102158714	132.628612	40.48764	7.062085132	5.374609695	1.711839	0.25095
LOC102163972	194.694187	640.36574	7.61245709	9.325003477	-1.71769	0.56567	LOC100515533	21.14718173	4.1313919	4.46905122	2.359350201	2.355766	0.35947	CACNA1S	270.230797	82.627837	8.083377186	6.385911347	1.709492	0.25106
SLC2A3	18255.6868	4918.8351	14.15613735	12.26439426	1.891957	0.5657	THOC1	10030.1083	2041.7339	13.29219339	10.99628554	2.29647	0.35951	CEBPE	12.4339324	1.6525567	3.747809764	1.407383613	2.911511	0.2511
HIF1A	2172.01387	8635.4353	11.08548167	13.07622024	-1.99124	0.56571	LOC102160517	322.4945214	81.801559	8.337597469	6.371586023	1.979074	0.35968	LOC100736994	12.4339324	1.6525567	3.747809764	1.407383613	2.911511	0.2511
KIF20B	3953.25856	1170.0102	11.94919149	10.1935379	1.756521	0.56571	CFP	2.114718173	15.699289	1.639101631	4.061714779	-2.89216	0.35987	LOC102159151	12.4339324	1.6525567	3.747809764	1.407383613	2.911511	0.2511
LOC100739398	1303.48449	413.13919	10.34926407	8.693971907	1.657674	0.56598	LOC102166108	2.114718173	15.699289	1.639101631	4.061714779	-2.89216	0.35987	BSPRY	12.4339324	1.6525567	3.747809764	1.407383613	2.911511	0.2511
CCDC78	9.66566885	33.877413	3.414902536	5.124221138	-1.80938	0.56617	LOC102160771	2.114718173	15.699289	1.639101631	4.061714779	-2.89216	0.35987	PDCL2	12.4339324	1.6525567	3.747809764	1.407383613	2.911511	0.2511
BARD1	334.15598	114.02642	8.388688866	6.845821397	1.551154	0.56626	LOC100738332	13348.10111	2666.4003	13.70445498	11.38121864	2.323669	0.36021	LOC102158392	12.4339324	1.6525567	3.747809764	1.407383613	2.911511	0.2511
P2RY6	16.569718	4.1313919	4.135019133	2.359350201	2.003849	0.56639	LOC100517114	609.0388339	149.55639	9.252757274	7.234160086	2.025845	0.36041	LOC102165310	12.4339324	1.6525567	3.747809764	1.407383613	2.911511	0.2511
LOC102163984	16.569718	4.1313919	4.135019133	2.359350201	2.003849	0.56639	BANP	125.8257313	542.86489	6.986703669	9.087104484	-2.10917	0.36041	LOC100737298	12.4339324	1.6525567	3.747809764	1.407383613	2.911511	0.2511
KCND2	16.569718	4.1313919	4.135019133	2.359350201	2.003849	0.56639	UBA6	4526.55425	974.1822	12.14451621	9.929527983	2.21615	0.36047	LOC102167156	12.4339324	1.6525567	3.747809764	1.407383613	2.911511	0.2511
LOC102162886	16.569718	4.1313919	4.135019133	2.359350201	2.003849	0.56639	LOC100524650	6329.351492	1331.1345	12.62806989	10.37952399	2.249401	0.3605	CRLF3	2298.61963	646.97597	11.16717953	9.339796491	1.828984	0.25157
LOC100625507	16.569718	4.1313919	4.135019133	2.359350201	2.003849	0.56639	LOC100525885	41.23700438	9.9153405	5.400435611	3.448285225	2.056205	0.36083	LOC102164508	80.4060959	23.962073	6.347064926	4.641665831	1.746552	0.25177
LOC102167180	16.569718	4.1313919	4.135019133	2.359350201	2.003849	0.56639	LOC100522999	121.596295	523.03421	6.937771569	9.033517185	-2.10481	0.36099	LOC102165321	462.542284	139.64104	8.856557135	7.135873883	1.727862	0.25178
SULT4A1	46.9475344	15.699289	5.583384725	4.061714779	1.58035	0.56678	DHRS1	376.4198348	1760.7992	8.560026434	10.7828338	-2.22582	0.36119	IGJ	19.0653629	65.275991	4.326635347	6.05041444	-1.7756	0.25209
LOC100627628	46.9475344	15.699289	5.583384725	4.061714779	1.58035	0.56678	GPBP1L1	8048.617367	1666.6035	12.97470449	10.70356057	2.27183	0.36148	LOC102159034	26.5257224	88.411786	4.78270852	6.482393107	-1.73685	0.25215
LOC102160271	40.0434852	123.11548	5.359081339	6.955539223	-1.62037	0.56716	ABLIM1	537.138416	133.03082	9.07183349	7.066420949	2.013533	0.36158	LOC100739468	253.65222	860.15579	7.992384484	9.750130438	-1.76175	0.25271
LOC102161555	13.8080984	45.44531	3.888314478	5.537461031	-1.71862	0.56719	tRNA-Leu	6883.407654	1441.0295	12.74911681	10.49388494	2.256023	0.36168	LOC100627811	137.602185	42.140197	7.114806188	5.430960858	1.707234	0.25305
SOS2	127.034505	405.70268	7.000388854	8.667830689	-1.6752	0.56734	LOC102167511	123.7110131	32.224856	6.962445064	5.054191063	1.940728	0.36192	USP53	604.289112	2137.5821	9.241480591	11.06243891	-1.82267	0.25319
ADAM8	314.824643	1070.0305	8.302979933	10.06478384	-1.76503	0.56752	UBXN2A	2090.39894	475.11006	11.03025255	8.895151314	2.137445	0.36207	LOC100626090	37.3017971	9.9153405	5.259340178	3.448285225	1.911511	0.25384
MAP6	182.266898	63.623435	7.517802419	6.013985524	1.518422	0.5681	LOC100517200	11.63094995	50.402981	3.658891241	5.683780113	-2.11554	0.36238	LOC102167126	37.3017971	9.9153405	5.259340178	3.448285225	1.911511	0.25384
DYRK4	35.9010557	11.567897	5.205590187	3.65167138	1.6339	0.56818	LOC102165332	572.0312658	141.2936	9.162470047	7.15272698	2.017398	0.36238	LOC100519984	70617.2754	16714.785	16.10775397	14.0289235	2.078896	0.25403
F2RL1	35.9010557	11.567897	5.205590187	3.65167138	1.6339	0.56818	LOC100520903	197.7261492	51.229259	7.63463791	5.706786332	1.948464	0.36251	LOC100525039	2732.97833	764.30749	11.41678609	9.579895716	1.838249	0.25409
SEC31A	2370.85049	9468.3239	11.21179736	13.2090457	-1.9977	0.56823	PKN3	228.3895627	1025.4115	7.84165594	10.00339347	-2.16663	0.36255	LOC102160382	44.7621565	12.394176	5.516083133	3.74353388	1.852617	0.2541
SPTBN5	2.76161967	14.873011	1.911353989	3.98850389	-2.42911	0.56863	LOC102162080	152.2597085	39.661362	7.259834656	5.34558663	1.940728	0.3627	LOC100737009	44.7621565	12.394176	5.516083133	3.74353388	1.852617	0.2541
PLCB2	2.76161967	14.873011	1.911353989	3.98850389	-2.42911	0.56863	ANGPT4	29.60605442	120.63664	4.935745167	6.926434087	-2.0267	0.36357	LOC102161035	29.8414377	7.4365053	4.946798112	3.076645515	2.00462	0.25449
PRDM6	2.76161967	14.873011	1.911353989	3.98850389	-2.42911	0.56863	LOC100736619	6657.132809	1399.7156	12.70090193	10.45194831	2.249767	0.3637	TRNAK-UUU	29.8414377	7.4365053	4.946798112	3.076645515	2.00462	0.25449
AS3MT	2.76161967	14.873011	1.911353989	3.98850389	-2.42911	0.56863	LOC102158835	4.229436346	23.135794	2.386655454	4.593102407	-2.45159	0.36377	LOC102167232	49.7357294	14.046732	5.664930183	3.911378308	1.824048	0.25465
LOC100153418	2.76161967	14.873011	1.911353989	3.98850389	-2.42911	0.56863	ZNF862	95.16231779	401.57129	6.587399765	8.653100473	-2.07719	0.36395	LOC100524759	616.723045	2180.5486	9.270816343	11.09113691	-1.822	0.25481
LOC100739704	219.548764	725.47241	7.784953864	9.504764199	-1.72438	0.56892	WDR25	48.63851798	199.13309	5.633388138	7.644815895	-2.03356	0.36402	RFWD3	3372.08245	933.69456	11.71985187	9.868351187	1.852617	0.25488
LOC100621373	44.1859147	135.50965	5.497801224	7.092859162	-1.61674	0.56914	DNAJC12	171.292172	44.619032	7.428713345	5.511563931	1.940728	0.36417	B3GAT3	670.603418	2381.3343	9.391465763	11.21816014	-1.82824	0.25489
NKRF	1499.55948	472.63123	10.55128479	8.887620397	1.665752	0.56931	GINS4	2264.863163	513.11887	11.14584502	9.005958152	2.142059	0.36421	GALNT18	27.3546512	6.610227	4.825513503	2.927939483	2.049014	0.25493
LOC102166171	182.266898	594.09415	7.517802419	9.216974124	-1.70464	0.56953	PER1	446.2055345	2107.0098	8.804794233	11.04166589	-2.23942	0.36461	LOC102160456	27.3546512	6.610227	4.825513503	2.927939483	2.049014	0.25493
LOC102160253	75.944541	26.440908	6.265747069	4.77825631	1.522175	0.5696	MMP16	86.7034451	363.56248	6.45456161	8.510022294	-2.06804	0.36472	LOC100737411	14.9207188	2.4788351	3.99283357	1.798604302	2.589583	0.25531
LOC100622980	220.929574	76.843889	7.79395812	6.282511874	1.523584	0.56973	C9H11orf54	3355.000382	740.34542	11.71252716	9.534002096	2.180042	0.36477	SLC25A13	5380.57699	1455.0762	12.39381328	10.50787015	1.886666	0.25534
RASL11B	396.292423	1367.4907	8.634057469	10.41836992	-1.78689	0.56978	LOC102159054	20.08982264	82.627837	4.398475058	6.385911347	-2.04016	0.36487	LOC100627810	717.852361	214.0061	9.489551688	7.74823377	1.746035	0.25555
LOC102164747	45.5667246	139.64104	5.541227504	7.135873883	-1.61567	0.56986	LOC102166027	532.9089796	2552.3739	9.060450003	11.31818909	-2.25988	0.36492	LOC102167400	22.3810782	4.9576702	4.547269558	2.574748269	2.174545	0.25604
PIBF1	110.464787	38.835083	6.800444207	5.315967693	1.508154	0.57034	LOC100627623	21.14718173	86.759229	4.46905122	6.455478947	-2.03655	0.36492	DRAP1	3609.1561	14027.728	11.8178455	13.77609658	-1.95855	0.25606
PRDM2	113.226407	357.77854	6.835752397	8.486949769	-1.65986	0.57068	PTPRN	31.7207726	128.89943	5.032134908	7.021251245	-2.02275	0.36519	LOC102162453	59.6828753	17.351846	5.92321754	4.19785327	1.782228	0.25637
CDH12	5.52323934	22.309516	2.705588563	4.542847146	-2.01407	0.57074	MCUR1	2176.045	494.94074	11.08815551	8.954023947	2.136381	0.36537	LOC102163272	59.6828753	17.351846	5.92321754	4.19785327	1.782228	0.25637
CYP11A1	5.52323934	22.309516	2.705588563	4.542847146	-2.01407	0.57074	C16H5orf22	868.0918101	209.87471	9.76336478	7.72024225	2.048319	0.36575	LOC100621768	19.8942918	4.1313919	4.385036953	2.359350201	2.267655	0.25649
LOC102162744	31.7586262	97.500848	5.033802952	6.622064238	-1.61827	0.57075	SCRN3	60.26946793	247.88351	5.937096418	7.959326845	-2.04016	0.36603	LOC100626394	17.4075053	3.3051135	4.202222212	2.106051269	2.396938	0.25649
LOC100620865	33.1394361	101.63224	5.093367322	6.681340183	-1.61674	0.57081	KIFAP3	791.9619558	3914.0806	9.631107841	11.93482631	-2.30517	0.36609	PIPOX	17.4075053	3.3051135	4.202222212	2.106051269	2.396938	0.25649
TRIM13	961.043646	311.50695	9.909958537	8.287744447	1.625338	0.57083	LOC100623912	1999.466033	457.75822	10.96612042	8.841590192	2.126957	0.36611	P2RY11	17.4075053	3.3051135	4.202222212	2.106051269	2.396938	0.25649
LOC100620566	12.4272885	41.313919	3.747096094	5.403060391	-1.73312	0.57116	LOC102165677	49.69587707	12.394176	5.663796517	3.74353388	2.003464	0.36624	LOC100521848	17.4075053	3.3051135	4.202222212	2.106051269	2.396938	0.25649
ZBTB43	35.9010557	109.89502	5.205590187	6.793050813	-1.61403	0.57121	NUDT15	1756.273443	405.70268	10.77912299	8.667830689	2.114023	0.3663	WDFY4	17.4075053	3.3051135	4.202222212	2.106051269	2.396938	0.25649
LOC102167293	27.6161967	85.106672	4.838760036	6.428053129	-1.62376	0.57131	TENM1	15.8603863	66.10227	4.075565686	6.068289661	-2.05927	0.36647	SLA-6	1081.75212	3934.7376	10.08048728	11.94241832	-1.8629	0.25659
PXK	834.009141	3048.9672	9.705648181	11.57457801	-1.87019	0.57137	IMP3	4633.347517	1002.2757	12.17815052	9.970502347	2.208776	0.36706	LOC100737288	62.1696618	18.178124	5.981159942	4.261389712	1.774007	0.25689
ERO1L	247.164961	821.3207	7.95515562	9.683557338	-1.73247	0.57146	NR2C1	2308.214886	523.86049	11.17318672	9.035790183	2.139523	0.36712	LOC102168077	241.218288	74.365053	7.920163984	6.2358238	1.697643	0.25753
CHRM4	20.7121475	5.7839486	4.440430524	2.762125239	1.840351	0.57174	LOC100522562	85.64608601	22.309516	6.437062676	4.542847146	1.940728	0.36719	LSMD1	1778.88126	509.81376	10.79756528	8.996653565	1.802928	0.25756
ZC3H6	244.403341	810.57908	7.93901108	9.664587872	-1.72969	0.57241	SUN2	1224.421822	6264.8426	10.25906273	12.61329282	-2.35518	0.36754	LOC100623194	1606.46406	462.71589	10.65057077	8.857097348	1.79569	0.25762
LOC100523457	259.592249	90.064343	8.025650364	6.508814353	1.527219	0.57271	LOC100627067	130.0551676	556.91162	7.034030432	9.123892796	-2.09832	0.36766	ZFP1	1468.03295	424.70708	10.52065104	8.733717284	1.789344	0.25769
ELL3	40.0434852	13.220454	5.359081339	3.829895615	1.598796	0.57275	LOC102164187	6.344154519	30.5723	2.876596413	4.980587447	-2.26873	0.36768	IFT43	227.126498	762.65494	7.83369022	9.576777083	-1.74753	0.25777
PMPCB	3878.69483	1155.9634	11.92172746	10.17612756	1.746475	0.57277	LOC100626252	311.9209305	1425.3302	8.28965435	10.47809228	-2.19204	0.36771	NTHL1	2506.68076	706.46801	11.29213798	9.4665211	1.827082	0.25789
LOC100620325	8.28485901	29.746021	3.214880004	4.942327829	-1.84415	0.57308	LOC102158195	46.52379981	189.21775	5.570578288	7.571508044	-2.02401	0.36788	LOC100518058	896.072058	265.23536	9.809080066	8.05655837	1.756342	0.25791
ZNF697	164.316371	529.64444	7.369085785	9.051601682	-1.68855	0.57309	TTC27	1800.682524	416.4443	10.8151291	8.705439898	2.112348	0.36899	LTBR	1474.66438	5448.4796	10.52714892	12.41190275	-1.88547	0.25792
C1H9orf114	3286.32741	989.88149	11.68269944	9.952568709	1.731149	0.57311	CACNB3	564.6297522	2700.2777	9.143714198	11.39942626	-2.25773	0.3699	LOC100521704	823.126322	244.5784	9.68672168	7.940039851	1.750817	0.25809
AGPAT9	162.935561	524.68677	7.356985025	9.038059606	-1.68715	0.57343	ZFAND4	46.52379981	11.567897	5.570578288	3.651671381	2.007842	0.36994	IFRD2	10001.8552	2626.7389	13.28812424	11.35960624	1.928923	0.25866
RIC3	672.45439	223.09516	9.395436432	7.807967682	1.591777	0.57386	LOC102161733	486.3851798	122.2892	8.928918573	6.945902605	1.991802	0.36998	G6PD	1643.76586	6104.5446	10.68366651	12.57590427	-1.89288	0.2588
ZUFSP	767.730269	252.84118	9.586333665	7.987782332	1.602368	0.57403	HSPE1	29428.4181	5624.4769	14.8449714	12.45775968	2.387419	0.37	STXBP1	5575.37527	1509.6106	12.44511194	10.56091609	1.884892	0.25889
LOC102162418	154.650702	495.76702	7.28216827	8.956425596	-1.68065	0.57435	RBM12B	2660.315462	599.8781	11.37792382	9.230928526	2.148856	0.37024	CASP8	57.1960888	185.08636	5.862850292	7.539828464	-1.69421	0.25896
SLC16A5	3302.89713	995.66544	11.68995305	9.96096549	1.729999	0.57445	LOC100739350	214.6438946	949.39385	7.75250706	9.892381689	-2.14506	0.37039	LOC102161148	9.94714588	37.182527	3.452482877	5.254840671	-1.90227	0.25933
FAM214B	411.481331	1416.2411	8.688185014	10.46886952	-1.78317	0.57467	ADAMTS9	54.9826725	223.92144	5.806908455	7.81327737	-2.02594	0.37062	PTGER1	9.11821706	34.703692	3.338883189	5.158001345	-1.92827	0.25937
SFRP5	22.0929574	68.581105	4.529381037	6.120623682	-1.63422	0.57476	SSBP4	231.56164	1029.5429	7.86146934	10.00918878	-2.15254	0.37086	LOC102166908	9.11821706	34.703692	3.338883189	5.158001345	-1.92827	0.25937
LOC100521612	22.0929574	68.581105	4.529381037	6.120623682	-1.63422	0.57476	XRCC4	895.5831463	217.31121	9.80829357	7.770242414	2.043065	0.37087	SLC35D2	96.9846724	315.63834	6.614484182	8.306692135	-1.70244	0.25948
LOC100623926	443.239957	1533.5727	8.795195352	10.58362124	-1.79074	0.57522	FLOT2	813.1091376	4003.3187	9.669078402	11.96734109	-2.29968	0.37094	LOC102166840	89.524313	290.84999	6.500233417	8.189083193	-1.69992	0.25951
LOC100739531	95.2758787	296.63394	6.58910248	8.217395218	-1.6385	0.57533	LOC102165013	33.83549077	136.33593	5.122485983	7.101565318	-2.01056	0.37111	MFRP	8.28928824	32.224857	3.215568059	5.054191063	-1.95885	0.25951
DIP2C	840.91319	3063.0139	9.717527674	11.58120714	-1.86492	0.57533	LOC100623012	1631.505071	8513.9723	10.67287176	13.05578613	-2.38363	0.37112	LOC102158668	8.28928824	32.224857	3.215568059	5.054191063	-1.95885	0.25951
LOC100625807	24.854577	7.4365053	4.692347799	3.076645515	1.740815	0.57561	CBR1	34.89284986	8.2627837	5.165624571	3.211445828	2.078232	0.37146	LOC100624905	32.3282241	105.76363	5.058672541	6.738276475	-1.70998	0.25968
VPS8	150.508272	480.89401	7.243252754	8.912572065	-1.67588	0.57565	LOC102164574	24.31925899	98.327126	4.662163277	6.634115866	-2.01549	0.37147	LOC100737946	663.143059	199.13309	9.375350227	7.644815895	1.735587	0.25984
ACSL4	1902.75595	7405.933	10.89463283	12.85466058	-1.96059	0.57572	LOC100737034	24.31925899	98.327126	4.662163277	6.634115866	-2.01549	0.37147	LOC100626592	1768.93411	508.1612	10.78947994	8.99197867	1.799522	0.25988
LYPD4	568.893652	190.8703	9.154548913	7.58398763	1.575566	0.57606	LOC100738799	74.01513606	304.89672	6.229109817	8.256900823	-2.04243	0.37162	COQ6	3165.67918	883.29158	11.628755	9.788378341	1.841553	0.25994
NAALAD2	55.2323934	19.004403	5.813329549	4.322245637	1.539181	0.57608	LOC100736995	50.75323616	205.74331	5.693577173	7.691696868	-2.01927	0.372	LOC100517984	175.732911	582.52625	7.465426908	9.188653752	-1.72894	0.26006
LOC100626442	55.2323934	19.004403	5.813329549	4.322245637	1.539181	0.57608	TULP4	8.458872693	38.008805	3.241668254	5.285727902	-2.1678	0.37204	LOC100623120	125.997181	38.835083	6.988652665	5.315967693	1.697959	0.26091
SLC9C2	55.2323934	19.004403	5.813329549	4.322245637	1.539181	0.57608	LOC100523874	945.2790234	228.87911	9.886121835	7.844731552	2.046154	0.37207	PNPLA2	1101.64641	3995.0559	10.10675451	11.96436106	-1.85855	0.26094
LOC102160658	15.1889082	48.750424	4.016933786	5.636636914	-1.6824	0.5761	LOC100739313	307.6914942	1396.4104	8.270021921	10.44854012	-2.18217	0.37219	LOC100625722	110.247534	359.43109	6.79762954	8.493579657	-1.70497	0.26095
ACKR3	1176.44998	4399.1061	10.20145006	12.10332258	-1.90277	0.57643	BMX	17.97510447	3.3051135	4.246035924	2.106051269	2.443228	0.37221	LOC102159960	4.97357294	22.309516	2.578594101	4.542847146	-2.1653	0.26102
C16H5orf51	115.988026	364.38876	6.870217066	8.513288452	-1.6515	0.57654	LOC100522569	17.97510447	3.3051135	4.246035924	2.106051269	2.443228	0.37221	LOC102160302	4.97357294	22.309516	2.578594101	4.542847146	-2.1653	0.26102
LOC102163753	34.5202459	104.93735	5.150569664	6.727067559	-1.60401	0.57687	PIPOX	17.97510447	3.3051135	4.246035924	2.106051269	2.443228	0.37221	LOC102166431	3.31571529	17.351846	2.109599694	4.19785327	-2.3877	0.26119
LOC102159752	44.1859147	14.873011	5.497801224	3.98850389	1.57089	0.57698	LOC102160699	17.97510447	3.3051135	4.246035924	2.106051269	2.443228	0.37221	CYYR1	3.31571529	17.351846	2.109599694	4.19785327	-2.3877	0.26119
LOC100523505	44.1859147	14.873011	5.497801224	3.98850389	1.57089	0.57698	LOC102160163	17.97510447	3.3051135	4.246035924	2.106051269	2.443228	0.37221	LOC100623323	826.442037	246.23095	9.692514443	7.949715578	1.746901	0.26119
TRIM8	1393.23712	5279.0925	10.44526023	12.36634749	-1.92185	0.57701	LOC102167188	17.97510447	3.3051135	4.246035924	2.106051269	2.443228	0.37221	MPPE1	878.664553	261.10397	9.780809668	8.033995371	1.750688	0.26122
LOC100522227	40.0434852	121.46292	5.359081339	6.936201185	-1.60088	0.57747	LGI1	17.97510447	3.3051135	4.246035924	2.106051269	2.443228	0.37221	MLF1	460.884426	140.46732	8.851388091	7.144325041	1.71417	0.26144
ADAP1	513.661259	173.51846	9.007479378	7.447235822	1.565728	0.57751	LOC100737969	270.6839262	70.233662	8.085785403	6.154487245	1.946375	0.37252	VPS28	1970.36381	7368.7505	10.94497833	12.84740007	-1.90296	0.26208
LOC100625055	516.422879	1803.7657	9.015200036	10.81759583	-1.80439	0.57761	LOC100626042	35.95020894	144.59872	5.207510617	7.185853813	-2.00798	0.3726	ALDOC	701.273785	2478.8351	9.455889772	11.27602848	-1.82161	0.26233
MCEE	1853.0468	580.8737	10.85646195	9.184562218	1.673603	0.57807	ZNF516	105.7359087	443.71149	6.737901807	8.796725856	-2.06916	0.37277	PLEKHN1	522.225159	158.64545	9.031288103	7.318727602	1.718866	0.26253
LOC102164238	5908.48529	1722.7904	12.52881676	10.75136865	1.778041	0.57813	LOC102165714	217.8159718	57.013208	7.773574237	5.858309485	1.933742	0.3732	CS	4845.9179	1326.1768	12.24285193	10.37414484	1.869497	0.26286
DNAJC1	465.332915	1610.4165	8.865216451	10.65411376	-1.7911	0.57841	LOC102165784	250.5941035	65.275991	7.974954301	6.05041444	1.940728	0.37327	LOC100155802	60.5118041	195.0017	5.942791385	7.614722326	-1.6882	0.26298
SNTB2	259.592249	858.50323	8.025650364	9.747359248	-1.72558	0.57868	LOC102164981	11.63094995	49.576702	3.658891241	5.660401069	-2.09169	0.37334	LOC102167129	202.258633	62.797156	7.667172819	5.995420212	1.68743	0.26303
SIDT2	816.058613	2953.9452	9.674295766	11.52891565	-1.8559	0.57881	UGP2	1590.268066	8256.9998	10.63596118	13.01157666	-2.37635	0.37352	LOC102166472	87.0375265	26.440908	6.460046706	4.77825631	1.718866	0.26314
LOC100737345	671.07358	223.92144	9.392475381	7.81327737	1.583478	0.57996	SLC16A5	225.2174854	995.66544	7.821566636	9.96096549	-2.14434	0.37359	LOC102165781	87.0375265	26.440908	6.460046706	4.77825631	1.718866	0.26314
SLC2A12	476.379393	161.95056	8.898992482	7.348290508	1.556558	0.58005	PLK4	2423.467026	551.95395	11.24345192	9.111015534	2.134453	0.37369	LOC100738357	964.044222	285.89232	9.914451243	8.164365521	1.753627	0.26345
LOC100621492	13.8080984	44.619032	3.888314478	5.511563931	-1.69214	0.58008	LOC102161066	16.91774539	69.407383	4.163317207	6.137654819	-2.03655	0.37374	RNF135	124.339324	405.70268	6.969695301	8.667830689	-1.70614	0.26365
GCNT1	1902.75595	596.57298	10.89463283	9.222971117	1.67332	0.5802	LOC100622373	194.5540719	850.24044	7.611423767	9.733422888	-2.1277	0.37394	LOC100512626	557.04017	1942.5805	9.124225165	10.92450111	-1.80212	0.2638
PRKAR2A	222.310384	725.47241	7.802906526	9.504764199	-1.70635	0.58035	SNTB1	511.7617979	128.89943	9.002144971	7.021251245	1.989227	0.37406	ZBTB7B	167.443622	551.95395	7.396121996	9.111015534	-1.72087	0.26383
LOC100153751	2925.93604	893.20692	11.51517551	9.8044649	1.711832	0.58043	LOC100520452	157.5465039	677.54826	7.308762255	9.406307627	-2.10455	0.37441	RPS6KA4	503.988725	1747.5788	8.980107366	10.77196706	-1.79389	0.26433
CRY	9968.0662	2819.2618	13.28324266	11.46161338	1.821996	0.58062	LOC100739419	69.78569971	18.178124	6.145386028	4.261389711	1.940728	0.37446	LOC100737543	1479.63795	430.49103	10.5320032	8.753186764	1.781189	0.26438
LOC100626361	399.054043	1360.8805	8.644051094	10.41138438	-1.76988	0.58065	C-FLIP	255.880899	1141.0904	8.004955809	10.15746117	-2.15687	0.37451	NR4A1	261.112579	80.97528	8.034042783	6.357117027	1.689118	0.26447
ELF4	455.667246	1569.9289	8.834999507	10.61740218	-1.78465	0.58089	PHLDA3	1687.545102	8791.6019	10.721565	13.10207443	-2.3812	0.37486	LOC100738744	92.0110994	28.093465	6.539330984	4.862623207	1.711573	0.2645
LOC100511880	51.0899639	154.51406	5.702933533	7.280901168	-1.59663	0.58109	LOC100620851	253.7661808	1130.3488	7.993029968	10.14382809	-2.1552	0.37491	SLC35G1	320.795455	99.153405	8.330000138	6.646067655	1.693919	0.26523
LOC100518118	93.8950688	290.02371	6.568261215	8.184992878	-1.62705	0.58113	LOC102160331	15.8603863	65.275991	4.075565686	6.05041444	-2.04112	0.37495	CSNK1G1	520.567301	158.64545	9.026709615	7.318727602	1.714279	0.26576
LOC102160688	2590.39925	797.35863	11.33951559	9.640893151	1.699874	0.5812	NGDN	2858.041611	644.49713	11.4813159	9.334266871	2.148781	0.37506	ZFC3H1	3853.6901	1070.0305	11.91239916	10.06478384	1.848589	0.26594
C17H20orf108	2463.36475	761.00238	11.26700009	9.573651694	1.694657	0.58175	LOC102159445	3.17207726	19.004403	2.060765874	4.322245637	-2.58283	0.37541	FAM129B	4396.63848	17118.009	12.10251329	14.06331157	-1.96104	0.26641
LOC100627417	1306.24611	419.74941	10.35231506	8.716817448	1.637827	0.5818	AOAH	3.17207726	19.004403	2.060765874	4.322245637	-2.58283	0.37541	DOK1	295.92759	997.31799	8.213967342	9.963355621	-1.75281	0.26643
KIF11	4241.84782	1266.6847	12.05081722	10.3079803	1.743635	0.58193	LOC100517730	3.17207726	19.004403	2.060765874	4.322245637	-2.58283	0.37541	CYP1A1	38.1307259	123.11548	5.290229968	6.955539223	-1.69099	0.2665
LOC100738679	1270.34505	409.00779	10.31213992	8.679507525	1.63502	0.58207	LOC102163558	5.286795433	26.440908	2.65232482	4.778256309	-2.32231	0.37557	SLITRK5	143.404687	44.619032	7.173973754	5.511563931	1.684361	0.26696
LOC100738842	1270.34505	409.00779	10.31213992	8.679507525	1.63502	0.58207	LOC102159332	9.516231779	0.8262784	3.394545939	0.868906686	3.525691	0.37578	GAMT	215.521494	715.55707	7.758366438	9.484937802	-1.73124	0.26703
LOC100513362	231.976052	757.69727	7.864037858	9.56738053	-1.70765	0.58221	LOC102159567	9.516231779	0.8262784	3.394545939	0.868906686	3.525691	0.37578	NDUFAF6	649.880198	196.65425	9.346248212	7.626835185	1.724512	0.26718
LOC102162144	6.90404918	25.61463	2.982591923	4.734147579	-1.89145	0.58239	DIO1	9.516231779	0.8262784	3.394545939	0.868906686	3.525691	0.37578	PTPRM	53.0514447	170.21334	5.756261274	7.419651342	-1.68188	0.26739
SYP	6.90404918	25.61463	2.982591923	4.734147579	-1.89145	0.58239	LOC100621599	9.516231779	0.8262784	3.394545939	0.868906686	3.525691	0.37578	AKAP3	168.272551	552.78023	7.403204238	9.113169742	-1.71591	0.26754
LOC100624174	6.90404918	25.61463	2.982591923	4.734147579	-1.89145	0.58239	PLET	9.516231779	0.8262784	3.394545939	0.868906686	3.525691	0.37578	LOC100737766	1114.90927	329.68507	10.12400401	8.369314102	1.757766	0.26756
LOC102158272	6.90404918	25.61463	2.982591923	4.734147579	-1.89145	0.58239	UCP1	9.516231779	0.8262784	3.394545939	0.868906686	3.525691	0.37578	GPR137	626.670191	2191.2902	9.293862884	11.0982231	-1.806	0.26757
LOC100623063	85.6102098	262.75652	6.436465198	8.043062959	-1.61787	0.58268	LOC102162427	9.516231779	0.8262784	3.394545939	0.868906686	3.525691	0.37578	SLC45A3	67.9721635	218.13749	6.107942317	7.775692512	-1.68222	0.26761
SLC5A4	99.4183082	307.37555	6.649878514	8.268544594	-1.62842	0.58269	LOC102165389	9.516231779	0.8262784	3.394545939	0.868906686	3.525691	0.37578	LOC102162367	127.655039	39.661362	7.007364151	5.34558663	1.686444	0.26793
LOC100523147	73.1829213	223.09516	6.213015177	7.807967682	-1.60808	0.5828	LOC102167692	9.516231779	0.8262784	3.394545939	0.868906686	3.525691	0.37578	ZNF385C	140.088971	456.93194	7.140461408	8.838989383	-1.70564	0.26795
LOC102164161	129.796125	46.271589	7.031175985	5.56290145	1.488049	0.58282	LOC100625977	9.516231779	0.8262784	3.394545939	0.868906686	3.525691	0.37578	LOC100513915	522.225159	159.47173	9.031288103	7.326175314	1.711371	0.26802
AHDC1	724.925164	2587.0776	9.503677017	11.33766515	-1.83542	0.58291	LOC102163303	9.516231779	0.8262784	3.394545939	0.868906686	3.525691	0.37578	ZFP28	122.681466	398.26618	6.950485513	8.641207046	-1.69882	0.26834
LOC100736638	450.144007	1545.1406	8.817444209	10.59445576	-1.77928	0.5833	LOC102165973	9.516231779	0.8262784	3.394545939	0.868906686	3.525691	0.37578	ZYX	3848.71653	14850.701	11.9105365	13.85834057	-1.94808	0.26836
ATP6AP1	189.170948	608.14088	7.571153052	9.250632122	-1.68472	0.58332	LACTB2	3637.315258	807.27397	11.82905484	9.658700576	2.171744	0.37581	LOC102162002	53.8803735	15.699289	5.778218396	4.061714779	1.779061	0.26897
LOC102161093	78.7061606	240.44701	6.316619332	7.915562765	-1.61117	0.58342	ZIC5	486.3851798	123.11548	8.928918573	6.955539223	1.982087	0.37591	LOC102159275	426.898344	131.37826	8.741124286	7.048522415	1.700166	0.26898
LOC100737050	78.7061606	240.44701	6.316619332	7.915562765	-1.61117	0.58342	CBX3	26150.60493	5066.739	14.67461187	12.3071265	2.367715	0.37593	ATN1	2629.36223	9926.0821	11.36104577	13.27715401	-1.91651	0.26941
LOC102162825	56.6132033	171.03962	5.848327568	7.426597064	-1.59512	0.58349	C4H8orf59	3181.593491	713.07823	11.63598718	9.479938335	2.157617	0.37632	DHRS13	232.928999	72.712497	7.869926909	6.20383732	1.679615	0.26946
GIT2	809.154564	2911.805	9.662053366	11.5081934	-1.84743	0.5835	AGO3	84.58872693	22.309516	6.419348884	4.542847146	1.922806	0.37658	LOC100514845	199.771847	659.37014	7.649413169	9.367131078	-1.72274	0.26979
LOC102160403	136.700174	48.750424	7.105386569	5.636636914	1.487528	0.58353	TESC	1.057359087	11.567897	1.04079362	3.651671381	-3.45159	0.37691	POLR3D	14058.6329	3661.2395	13.7792713	11.83851041	1.941052	0.26985

LOC100511504	298.254925	990.70777	8.225231178	9.953771246		-1.73191	0.58388		CHRNA7
GLI1	46.9475344	141.2936	5.583384725	7.152726981		-1.58957	0.58392		LOC102159958
TIPIN	1064.60438	347.03692	10.05745621	8.44309653		1.617156	0.58393		LOC102164427
LOC100738592	451.524816	154.51406	8.821853101	7.280901168		1.547067	0.58402		KCNT1
CCDC116	30.3778164	91.716899	4.971673052	6.534760414		-1.59417	0.58404		LOC102157764
LOC102168023	4.14242951	18.178124	2.362450112	4.261389712		-2.13365	0.58426		LOC100623470
LOC102168067	4.14242951	18.178124	2.362450112	4.261389712		-2.13365	0.58426		PLXNA4
LOC102164807	4.14242951	18.178124	2.362450112	4.261389712		-2.13365	0.58426		MCHR1
LOC102166957	4.14242951	18.178124	2.362450112	4.261389712		-2.13365	0.58426		LOC102167618
LOC102160733	4.14242951	18.178124	2.362450112	4.261389712		-2.13365	0.58426		LOC102165953
IL20RB	4.14242951	18.178124	2.362450112	4.261389712		-2.13365	0.58426		ELFN2
FHIT	4.14242951	18.178124	2.362450112	4.261389712		-2.13365	0.58426		BDNF
IZUMO4	80.0869705	244.5784	6.341398207	7.940039851		-1.61066	0.58428		LOC100624715
UTP3	4065.10416	1219.5869	11.98943146	10.25335927		1.7369	0.58434		LOC100512344
STAT1	3086.10998	12381.781	11.59204117	13.59604779		-2.00436	0.58439		RALGAPA2
CENPM	1256.53695	405.70268	10.29638508	8.667830689		1.630958	0.58448		SHOX2
TIGD7	114.607216	356.12598	6.853087646	8.480289273		-1.63569	0.58489		LOC100621366
KIAA0947	156.031511	494.11447	7.294910283	8.951618292		-1.66301	0.58497		ID1
LOC100511067	12.4272885	40.48764	3.747096094	5.374609695		-1.70397	0.58512		NHLH1
LIPT2	106.322357	38.008805	6.745806839	5.285727902		1.484039	0.58525		LOC102160047
LOC100520439	74.5637311	26.440908	6.239622035	4.77825631		1.495702	0.5853		LOC102158159
LOC100620738	5541.18987	1631.8998	12.43624042	10.67322054		1.763643	0.58544		LOC102158569
TRMT10C	1431.8998	459.41077	10.48472201	8.846777786		1.640074	0.5864		RAPGEFL1
EZH1	517.803688	1790.5452	9.019044926	10.80698875		-1.78992	0.58668		LOC102167293
ESD	13032.0832	3643.0613	13.6698908	11.83133153		1.838845	0.58674		LOC100516136
MDH2	25214.9684	6771.3513	14.62205001	12.72544109		1.896765	0.58683		CALCRL
LOC100153723	1172.30755	380.91433	10.19636551	8.577105241		1.621813	0.58683		SWAP70
TPM4	21805.7489	101928.05	14.41248708	16.63720572		-2.22477	0.58689		LOC102164485

SFR1	778.776747	265.23536	9.606917325	8.05655837	1.553937	0.6108
ZNF667	172.60123	532.94955	7.439633355	9.060559625	-1.62656	0.61095
CSRNP1	1614.1667	525.51304	10.65746735	9.040325464	1.618991	0.61096
TMPO	4841.11928	1469.1229	12.2414229	10.5217211	1.720386	0.611
LOC100739502	138.080984	419.74941	7.119781365	8.716817448	-1.60401	0.61138
LOC100738089	15.1889082	46.271589	4.016933786	5.56290145	-1.60711	0.61164
DZIP3	111.845597	335.46902	6.818206315	8.394329862	-1.58467	0.61165
LOC100736860	42.8051049	123.94176	5.453027102	6.965111899	-1.53381	0.6118
POP1	2464.74556	781.65934	11.26780822	9.612240686	1.656827	0.61187
LOC100511545	17.9505279	53.708094	4.24416613	5.773682393	-1.58111	0.61191
LOC100626942	17.9505279	53.708094	4.24416613	5.773682393	-1.58111	0.61191
MRPS22	1862.71247	601.53065	10.86396358	9.234890832	1.630695	0.61207
AARS	5793.87807	23762.113	12.50056259	14.53643625	-2.03606	0.61225
LOC102159737	4.14242951	17.351846	2.362450112	4.19785327	-2.06654	0.6128
LY6G5C	4.14242951	17.351846	2.362450112	4.19785327	-2.06654	0.6128
KLHL8	248.54577	90.064343	7.963160641	6.508814353	1.464484	0.61296
LOC100626253	248.54577	90.064343	7.963160641	6.508814353	1.464484	0.61296
LOC102158006	20.7121475	61.1446	4.440430524	5.957557119	-1.56175	0.61304
LOC102166835	115.988026	347.86319	6.870217066	8.446517589	-1.58454	0.61338
LGALS9	167.07799	513.11887	7.392987005	9.005958152	-1.61877	0.61342
SLC35E2B	956.901216	3374.5209	9.903733075	11.72089442	-1.81824	0.61381
ART3	35.9010557	12.394176	5.205590187	3.74353388	1.534364	0.61397
CNIH3	35.9010557	12.394176	5.205590187	3.74353388	1.534364	0.61397
CCNC	4714.08478	1436.8981	12.203068	10.48974571	1.71402	0.61415
AGA	220.929574	691.595	7.79395812	9.435868156	-1.64634	0.61428
CHIC1	73.1829213	214.0061	6.213015177	7.74823377	-1.54807	0.61455
MESDC1	2611.1114	827.10465	11.35100071	9.693669287	1.658522	0.61471
LOC100524374	156.031511	475.93634	7.294910283	8.897652909	-1.60893	0.61508
LOC100738575	27.6161967	9.0890621	4.838760036	3.334720158	1.603311	0.61537
LOC102165942	27.6161967	9.0890621	4.838760036	3.334720158	1.603311	0.61537
CDT1	2696.72161	853.54556	11.39752576	9.739013597	1.659667	0.61614
NSUN6	789.823226	270.19303	9.627211433	8.083176277	1.547539	0.61626
LONRF2	9.66566885	31.398578	3.414902536	5.017858594	-1.69976	0.61636
SIN3B	1024.5609	3623.2307	10.00219745	11.82345906	-1.82227	0.61637
MPST	448.763197	158.64545	8.813021802	7.318727602	1.500148	0.61649
SEL1L	2395.70507	9094.0198	11.22683667	13.15086106	-1.92447	0.6166
LOC100626840	52.4707738	19.004403	5.740678649	4.322245637	1.46518	0.61702
LOC102160736	172.60123	63.623435	7.439633355	6.013985524	1.439813	0.61704
LOC102166723	16.569718	49.576702	4.135019133	5.660401068	-1.58111	0.61709
PLOD3	1332.48149	4813.0715	10.38098208	12.23304186	-1.85284	0.61713
LOC100517855	4700.27668	1436.8981	12.19883687	10.48974571	1.709788	0.61733
LOC102159370	23.4737672	7.4365053	4.613164285	3.076645515	1.658353	0.61758
STAP2	23.4737672	7.4365053	4.613164285	3.076645515	1.658353	0.61758
FGD6	51.0899639	147.07755	5.702933533	7.210209122	-1.52547	0.61768
LMLN	107.703167	319.76973	6.764250165	8.325394195	-1.56997	0.61775
LOC100520853	144.985033	53.708094	7.189676653	5.773682393	1.432693	0.61777
SRCRB4D	247.164961	90.064343	7.95515562	6.508814353	1.456446	0.61789
PPA1	9005.64175	2642.4382	13.13677356	11.3681999	1.768959	0.61791
SLC10A4	22.0929574	64.449713	4.529381037	6.032314961	-1.54459	0.61811
LOC102162699	461.190485	1522.831	8.85234375	10.57348723	-1.72332	0.61817
LOC100736896	150.508272	456.10566	7.243252754	8.836383876	-1.59953	0.61825
DNAJC16	157.412321	478.41518	7.307540742	8.905131772	-1.60371	0.61837
PTGER3	48.3283443	138.81477	5.624344958	7.127372928	-1.52222	0.61854
PRIM2	2659.43974	844.4565	11.37744901	9.723586711	1.655027	0.61873
BAZ2B	236.118482	738.69286	7.889464306	9.530782548	-1.64546	0.61878
PCCA	2068.45313	667.63292	11.01503386	9.385070587	1.631425	0.6189
LOC102164787	5.52323934	20.656959	2.705588563	4.436758793	-1.90304	0.61919
RELT	332.77517	119.81036	8.382732826	6.916600414	1.473795	0.61921
LOC100512637	11167.99	3236.5324	13.4472111	11.66067891	1.786849	0.61934
TRMT11	735.971642	253.66746	9.525465297	7.992470822	1.536712	0.61958
CATSPER1	156.031511	57.839486	7.294910283	5.878712739	1.431711	0.61969
CHCHD2	22725.3683	6304.504	14.47207954	12.62239597	1.849849	0.6201
YARS2	432.193479	153.68778	8.758867714	7.273215394	1.491675	0.62024
NFATC4	1289.67639	4628.8114	10.3339116	12.17673772	-1.84363	0.62033
ANO4	44.1859147	126.42059	5.497801224	6.993454623	-1.51657	0.62046
LOC100153934	411.481331	1341.8761	8.688185014	10.39111046	-1.70535	0.62076
LOC102162058	53.8515836	154.51406	5.777461367	7.280901168	-1.52068	0.6209
FAM84B	157.412321	476.76262	7.307540742	8.900150173	-1.59872	0.62106
C4H1orf226	27.6161967	79.322724	4.838760036	6.327736286	-1.52222	0.6213
LOC100738954	42.8051049	122.2892	5.453027102	6.945902605	-1.51444	0.6213
LOC102164786	19.3313377	5.7839486	4.345633235	2.762125239	1.740815	0.62134
LOC102166621	11.0464787	34.703692	3.590539588	5.158001345	-1.6515	0.62165
LOC102164577	11.0464787	34.703692	3.590539588	5.158001345	-1.6515	0.62165
FURIN	2339.09186	8809.78	11.19234945	13.10505403	-1.91316	0.62187
LOC102158185	160.173941	59.492043	7.332474693	5.918673475	1.428871	0.62194
LOC100627774	238.880102	87.585507	7.906169679	6.468998788	1.447523	0.62227
ZMYM4	167.07799	507.33492	7.392987005	8.98963553	-1.60242	0.62229
ZZEF1	310.682213	988.22893	8.283932016	9.950160626	-1.66941	0.62261
ZNF10	23.4737672	67.754826	4.613164285	6.103389087	-1.52927	0.62283
CCDC58	2205.15331	711.42568	11.10731733	9.476595706	1.632094	0.62291
PVRL1	222.310384	81.801559	7.802906526	6.371586023	1.442375	0.62293
LOC100623976	361.772177	1164.2262	8.502920002	10.18639436	-1.68622	0.62306
LMNB2	9274.89967	2728.3712	13.1792715	11.41435289	1.765292	0.62314
PTGR2	2286.62109	736.21403	11.15963239	9.525939716	1.63502	0.62326
ALPK2	30.3778164	86.759229	4.971673052	6.455478947	-1.514	0.62326
LOC102163920	99.4183082	291.67627	6.649878514	8.193161944	-1.55278	0.62329
B3GNT7	40.0434852	114.02642	5.359081339	6.845821397	-1.50973	0.62334
HSD17B1	46.9475344	133.8571	5.583384725	7.075287628	-1.51157	0.62343
VSIG8	6.90404918	23.962073	2.982591923	4.641665831	-1.79524	0.62432
RAB26	6.90404918	23.962073	2.982591923	4.641665831	-1.79524	0.62432
LOC100739113	6.90404918	23.962073	2.982591923	4.641665831	-1.79524	0.62432
TRIM3	657.265482	2218.5574	9.362525737	11.11605632	-1.75507	0.62436
EIF2S1	10750.9854	3136.5527	13.39231546	11.61542398	1.777217	0.62444
RFXANK	6652.74179	2002.0725	12.69995017	10.96799892	1.732455	0.62495
NLN	20337.9481	5703.7996	14.31195744	12.47796049	1.834179	0.62502
FANCM	668.311961	233.0105	9.386534985	7.870429459	1.520127	0.62533
DOK2	33.1394361	94.195734	5.093367322	6.572825024	-1.50711	0.62536
LOC100519089	1209.58942	406.52896	10.24149393	8.67075877	1.573087	0.62538
PALLD	4254.2751	16771.798	12.05503669	14.03383579	-1.97905	0.62549
LOC100738992	353.487318	1132.0014	8.469590204	10.14593389	-1.67915	0.62552
LOC100516480	10398.8789	3043.1832	13.3442791	11.57183949	1.772775	0.62559
LOC102167737	71.8021115	206.56959	6.185908388	7.697451307	-1.52453	0.62592
PITX2	1424.99575	474.28379	10.47775397	8.892645374	1.587135	0.62614
LOC100737447	244.403301	759.34982	7.93901108	9.57051952	-1.6355	0.62636
LOC102160241	45.5667246	16.525567	5.541227504	4.13138925	1.463281	0.62654
LOC100738884	19.3313377	56.186929	4.345633235	5.837613535	-1.53929	0.62675
LOC100736959	49.7091541	18.178124	5.664174303	4.261389712	1.451308	0.62676
LOC100520391	96.6566885	281.76092	6.609646952	8.143438955	-1.54353	0.62689
LOC100521724	285.827636	898.16459	8.164040225	9.812441412	-1.65183	0.62708
HABP2	15.1889082	4.1313919	4.016933786	2.359350201	1.878318	0.62721
LOC102157994	15.1889082	4.1313919	4.016933786	2.359350201	1.878318	0.62721
LOC102166382	31.7586262	90.064343	5.033802952	6.508814353	-1.50381	0.6274
LOC102163031	31.7586262	90.064343	5.033802952	6.508814353	-1.50381	0.6274
LYRM9	1291.0572	432.96987	10.33545422	8.761451061	1.576214	0.62745
RASSF7	1001.08713	341.25297	9.96879224	8.41891924	1.552654	0.62766
MTERFD3	521.946118	185.08636	9.030518495	7.539828464	1.495702	0.62773
SCAMC-1	412.862141	1335.2658	8.69300647	10.38399134	-1.69339	0.62777
LAT	115.988026	341.25297	6.870217066	8.41891924	-1.55687	0.62793
LOC100512841	723.544354	2451.5679	9.500930199	11.26007738	-1.76055	0.62795
PSMG4	4946.06083	1521.1785	12.27235592	10.57192182	1.701091	0.62798
DNAH2	57.9940131	21.483238	5.882496648	4.490777898	1.432693	0.62803
LOC100623471	334.15598	121.46292	8.388688866	6.936201185	1.460006	0.62837
EVC2	312.063023	985.7501	8.290309306	9.946540946	-1.65938	0.62847
KDM5A	124.272885	366.8676	6.968930373	8.523042794	-1.56175	0.62862
LOC100626088	140.842603	52.881816	7.148147109	5.751726565	1.41324	0.6287
LOC102161446	8.28485901	27.267186	3.214880004	4.821056379	-1.71862	0.62875
ATG9A	890.622344	3067.9716	9.800288961	11.58353958	-1.7844	0.62879
ACSS3	3048.82812	968.39825	11.57451222	9.92094567	1.654582	0.62889
HGSNAT	843.67481	2891.148	9.722252217	11.49792568	-1.77688	0.62933
LOC100739483	44.1859147	124.76803	5.497801224	6.974621475	-1.49759	0.62962
FRS3	425.289429	152.8615	8.735689471	7.265488457	1.47622	0.62969
LOC100737737	151.889082	453.62683	7.256341575	8.828539005	-1.57849	0.62992
LOC100518209	3562.48938	1121.2598	11.79907491	10.13219091	1.667765	0.63
LOC100515119	520.565308	185.08636	9.026704102	7.539828464	1.491881	0.63016
LOC100523731	139.461793	413.96546	7.13403395	8.696847462	-1.56964	0.63017
LOC100623684	269.257918	838.67255	8.078193076	9.71368301	-1.63912	0.63033
AUTS2	447.382387	1451.7711	8.808585798	10.50459169	-1.69823	0.63042
METTL16	354.868128	128.89943	8.475198918	7.021251246	1.461037	0.63055
ALDOC	733.210023	2478.8351	9.520048999	11.27602848	-1.75736	0.63073
LOC100737015	3073.68269	977.48731	11.58622182	9.934409336	1.652818	0.63082
RAD1	505.3764	180.12869	8.984066358	7.500871231	1.48833	0.63089
LOC102160451	23.4737672	66.928548	4.613164285	6.085946114	-1.51157	0.6309
NARS2	1275.86829	429.66475	10.318394	8.750421444	1.570196	0.63101
SCAND3	115.988026	43.792754	6.870217066	5.485193456	1.405212	0.63101
HOXC10	20.7121475	59.492043	4.440430524	5.918673475	-1.52222	0.63107
LOC100157053	1246.87128	420.57569	10.28525341	8.719647871	1.567875	0.63115
LOC102163892	28.9970066	9.9153405	4.906746634	3.448285225	1.54817	0.63135
JPH1	28.9970066	9.9153405	4.906746634	3.448285225	1.54817	0.63135
LOC102167735	28.9970066	9.9153405	4.906746634	3.448285225	1.54817	0.63135
FHOD1	552.323934	1821.1175	9.111980519	10.8314003	-1.72124	0.63173
LOC102159744	1.38080984	9.9153405	1.251452392	3.448285225	-2.84415	0.63221
LOC102159722	1.38080984	9.9153405	1.251452392	3.448285225	-2.84415	0.63221
LOC102161180	1.38080984	9.9153405	1.251452392	3.448285225	-2.84415	0.63221
LOC102164319	1.38080984	9.9153405	1.251452392	3.448285225	-2.84415	0.63221
LOC102166157	1.38080984	9.9153405	1.251452392	3.448285225	-2.84415	0.63221
LOC100623553	1.38080984	9.9153405	1.251452392	3.448285225	-2.84415	0.63221
LOC100521923	1.38080984	9.9153405	1.251452392	3.448285225	-2.84415	0.63221
HAO2	17.9505279	52.055537	4.24416613	5.729431428	-1.53603	0.63223
LOC100514848	2919.03199	11069.651	11.51176846	13.43445249	-1.92305	0.6323
MREG	74.5637311	28.093465	6.239622035	4.862623207	1.408239	0.63235
SHQ1	1125.36002	382.56689	10.13745231	8.583334365	1.556603	0.63245
RRP1B	5891.91557	1798.808	12.52476588	10.81362731	1.711696	0.63249
LOC100739801	171.22042	513.94515	7.428112398	9.008274952	-1.58576	0.6325
C7H14orf142	4519.39059	1403.847	12.14223172	10.45619725	1.686743	0.63259
IMMP1L	1336.62392	449.49543	10.38545684	8.815368674	1.572215	0.63266
LOC102160006	9.66566885	30.5723	3.414902536	4.980587446	-1.66128	0.63276
LOC100627897	9.66566885	30.5723	3.414902536	4.980587446	-1.66128	0.63276
LOC102167990	9.66566885	30.5723	3.414902536	4.980587446	-1.66128	0.63276
EREG	120.130456	45.44531	6.920417836	5.537461031	1.402399	0.63286
NOTCH4	120.130456	45.44531	6.920417836	5.537461031	1.402399	0.63286
EXOC4	216.787144	661.84898	7.766774985	9.372536392	-1.61022	0.63288
NDUFA4L2	121.511266	356.12598	6.936770608	8.480289273	-1.5513	0.63296
MATN3	167.07799	62.797156	7.392987005	5.995420212	1.411751	0.63305
ZNF782	45.5667246	128.07315	5.541227504	7.012045083	-1.49092	0.63307
HUS1	1244.10966	420.57569	10.2820571	8.719647871	1.564676	0.6333
LOC100620274	1067.366	364.38876	10.06119026	8.513288452	1.550505	0.63333
RPL26L1	6234.35641	1897.9614	12.60625631	10.89099488	1.71579	0.63341
NCAPD2	4396.49852	1369.1433	12.10246738	10.42011103	1.683082	0.63343
LOC100624297	532.992597	1748.405	9.06067593	10.77264864	-1.71385	0.63348
LOC100155517	212.644715	79.322724	7.739069817	6.327736286	1.422639	0.63354
EPB41	5648.89304	1731.0532	12.46400784	10.75826752	1.706318	0.63362
LOC100622348	168.4588	504.02981	7.404790748	8.980224728	-1.58111	0.63406
RNF152	185.028518	69.407383	7.539379992	6.137654819	1.414587	0.63407
SNRNP35	1669.39909	554.43279	10.70597712	9.117468532	1.590244	0.63464
SPNS3	113.226407	42.966475	6.835752397	5.458331975	1.397927	0.6349
P4HA2	2189.9644	8081.0025	11.09735033	12.98049708	-1.88363	0.63513
C1H6orf165	24.854577	8.2627837	4.692347799	3.211445828	1.588812	0.63524
LOC102161541	24.854577	8.2627837	4.692347799	3.211445828	1.588812	0.63524
LSMEM1	24.854577	8.2627837	4.692347799	3.211445828	1.588812	0.63524
LOC102162308	24.854577	8.2627837	4.692347799	3.211445828	1.588812	0.63524
RAPGEF3	24.854577	8.2627837	4.692347799	3.211445828	1.588812	0.63524
AASDHPPT	4854.92738	1504.6529	12.24553113	10.55617352	1.690019	0.63527
LOC102161729	11.0464787	2.4788351	3.590539588	1.798604302	2.155852	0.6353
LOC102159739	11.0464787	2.4788351	3.590539588	1.798604302	2.155852	0.6353
LOC102157687	11.0464787	2.4788351	3.590539588	1.798604302	2.155852	0.6353
LOC100513825	11.0464787	2.4788351	3.590539588	1.798604302	2.155852	0.6353
DPF3	11.0464787	2.4788351	3.590539588	1.798604302	2.155852	0.6353
RAX2	11.0464787	2.4788351	3.590539588	1.798604302	2.155852	0.6353
PLEKHG4	11.0464787	2.4788351	3.590539588	1.798604302	2.155852	0.6353
LOC102162538	11.0464787	2.4788351	3.590539588	1.798604302	2.155852	0.6353
LOC102163165	11.0464787	2.4788351	3.590539588	1.798604302	2.155852	0.6353
TNFSF9	1946.94187	641.19202	10.92773491	9.32686092	1.602381	0.63634
LOC100522069	46.9475344	131.37826	5.583384725	7.048522415	-1.48461	0.63638
LOC102162394	11.0464787	33.877413	3.590539588	5.124221138	-1.61674	0.63646
RYBP	4366.1207	1364.1856	12.09246669	10.41488138	1.678312	0.6366
EHHADH	222.310384	676.72199	7.802906526	9.404549765	-1.60599	0.63662
LOC102163346	2067.07232	678.37454	11.01407092	9.40806335	1.607435	0.63678
LOC102166967	41.4242951	115.67897	5.406818783	6.86640077	-1.48158	0.63679
SPATA5L1	563.370413	200.78564	9.140498547	7.65667973	1.488428	0.63691
LMNB1	16145.8094	4639.5531	13.9789615	12.18008104	1.799102	0.63691
RCSD1	74.5637311	211.52726	6.239622035	7.731504113	-1.5043	0.63712
LOC102157971	138.080984	405.70268	7.119781365	8.667830689	-1.55491	0.63731
LOC100620250	2.76161967	13.220454	1.911353989	3.829895615	-2.25919	0.63817
LOC102161819	2.76161967	13.220454	1.911353989	3.829895615	-2.25919	0.63817
LOC102164264	2.76161967	13.220454	1.911353989	3.829895615	-2.25919	0.63817
LOC100626947	2.76161967	13.220454	1.911353989	3.829895615	-2.25919	0.63817
LOC100738368	2.76161967	13.220454	1.911353989	3.829895615	-2.25919	0.63817
STK31	2.76161967	13.220454	1.911353989	3.829895615	-2.25919	0.63817
LOC102158046	2.76161967	13.220454	1.911353989	3.829895615	-2.25919	0.63817
LOC102160484	2.76161967	13.220454	1.911353989	3.829895615	-2.25919	0.63817
LOC100623261	2.76161967	13.220454	1.911353989	3.829895615	-2.25919	0.63817
LOC100739363	2.76161967	13.220454	1.911353989	3.829895615	-2.25919	0.63817
LOC100739148	2.76161967	13.220454	1.911353989	3.829895615	-2.25919	0.63817
JMJD1C	802.250515	2708.5405	9.64970619	11.4038325	-1.75539	0.63822
PI4K2B	102.179928	294.1551	6.689018532	8.205329462	-1.52547	0.63835
LOC102162288	231.976052	86.759229	7.864037858	6.455478947	1.418887	0.63886
ZFP92	106.322357	306.54928	6.745806839	8.264673769	-1.52767	0.63894
CD97	1742.58201	6278.8893	10.76783851	12.61652342	-1.84928	0.63901
XRN1	265.115489	815.53675	8.055908671	9.673374014	-1.62113	0.63911
LOC102159202	6.90404918	0.8262784	2.982591923	0.868906686	3.062743	0.63926
LOC102159411	6.90404918	0.8262784	2.982591923	0.868906686	3.062743	0.63926
LOC102161720	6.90404918	0.8262784	2.982591923	0.868906686	3.062743	0.63926
ACPP	6.90404918	0.8262784	2.982591923	0.868906686	3.062743	0.63926
TGFA	6.90404918	0.8262784	2.982591923	0.868906686	3.062743	0.63926
IL17F	6.90404918	0.8262784	2.982591923	0.868906686	3.062743	0.63926
LOC102160892	6.90404918	0.8262784	2.982591923	0.868906686	3.062743	0.63926
LOC102160076	6.90404918	0.8262784	2.982591923	0.868906686	3.062743	0.63926
LOC102162368	6.90404918	0.8262784	2.982591923	0.868906686	3.062743	0.63926
LOC102167692	6.90404918	0.8262784	2.982591923	0.868906686	3.062743	0.63926
LOC102165697	6.90404918	0.8262784	2.982591923	0.868906686	3.062743	0.63926
MIR425	6.90404918	0.8262784	2.982591923	0.868906686	3.062743	0.63926
LOC102165100	6.90404918	0.8262784	2.982591923	0.868906686	3.062743	0.63926
WRN	1770.19821	588.3102	10.79050995	9.202883429	1.589262	0.63936
TMED1	528.850167	1721.1378	9.049440637	10.74998491	-1.70243	0.63938
SLC2A5	161.554751	478.41518	7.34478191	8.905131772	-1.56624	0.63956
RU2B	1705.30015	568.47952	10.73665573	9.153500149	1.584845	0.64
LOC100626371	205.740666	619.70878	7.691678382	9.27777274	-1.59076	0.64017
NFE2L2	2312.85648	8523.8877	11.17608366	13.05746511	-1.88184	0.64023
DUSP12	660.027102	233.83678	9.368565612	7.875514565	1.497023	0.64034
LOC100739028	99.4183082	38.008805	6.649878514	5.285727902	1.387178	0.64036
GFOD1	99.4183082	38.008805	6.649878514	5.285727902	1.387178	0.64036
LOC100524738	115.988026	335.46902	6.870217066	8.394329862	-1.5322	0.64053
LOC100737646	34.5202459	95.848291	5.150569664	6.597654688	-1.47331	0.6407
ARRB2	3195.19396	1022.9326	11.64213925	9.999905072	1.643192	0.64086
LOC102159454	4.14242951	16.525567	2.362450112	4.13138925	-1.99615	0.64088
LOC102168017	4.14242951	16.525567	2.362450112	4.13138925	-1.99615	0.64088
LOC100738049	4.14242951	16.525567	2.362450112	4.13138925	-1.99615	0.64088
HTR7	4.14242951	16.525567	2.362450112	4.13138925	-1.99615	0.64088
LOC100514132	4.14242951	16.525567	2.362450112	4.13138925	-1.99615	0.64088
LOC100153808	4.14242951	16.525567	2.362450112	4.13138925	-1.99615	0.64088
TUBGCP5	1110.17111	380.91433	10.11786528	8.577105241	1.543244	0.64098
RASSF8	218.167954	659.37014	7.775893057	9.367131078	-1.59565	0.641
SUSD3	20.7121475	6.610227	4.440430524	2.927939483	1.647705	0.64119
LOC102160128	20.7121475	6.610227	4.440430524	2.927939483	1.647705	0.64119
LOC100627527	20.7121475	6.610227	4.440430524	2.927939483	1.647705	0.64119
PREI2	151.889082	57.839486	7.256341575	5.878712739	1.392892	0.64178
USP49	84.2294	32.224857	6.413279271	5.054191063	1.38615	0.6418
LOC100737533	45.5667246	126.42059	5.541227504	6.993454623	-1.47218	0.64188
CHAF1B	1893.09029	627.97156	10.88728939	9.29685098	1.591972	0.64199
LOC100153027	17.9505279	51.229259	4.24416613	5.706786332	-1.51294	0.64225
ADAMTS10	2181.67954	7979.3702	11.09188461	12.96223996	-1.87084	0.64256
PBXIP1	1775.72145	6381.3479	10.7950018	12.63987153	-1.84546	0.64266
LOC100522645	1886.18624	626.31901	10.88202109	9.293055462	1.590503	0.6428
LOC100624471	5.52323934	19.830681	2.705588563	4.380638094	-1.84415	0.64289
LOC102164531	5.52323934	19.830681	2.705588563	4.380638094	-1.84415	0.64289
LOC102164099	5.52323934	19.830681	2.705588563	4.380638094	-1.84415	0.64289
PVR	218.167954	657.71758	7.775893057	9.363516252	-1.59203	0.6429

BCAS4	341.526985	1496.3901	8.420073841	10.54823444	-2.13141	0.4065
DNAJC24	2536.604449	595.74671	11.30925149	9.220974888	2.090128	0.40656
ADAM15	499.0734889	2256.5662	8.965996313	11.1405526	-2.1768	0.40661
LOC100738528	109.965345	442.05893	6.793965376	8.791354786	-2.00719	0.40676
FASTK	548.7693659	2500.3184	9.102682709	11.28847297	-2.18784	0.4069
DFNA5	166.0053766	686.63733	7.38375074	9.42550405	-2.04832	0.40704
SDF4	2053.391346	10464.816	11.00449532	13.35339712	-2.34947	0.4073
LOC102165079	468.4100754	123.11548	8.874704998	6.955539223	1.92776	0.40742
CNIH4	1471.843849	359.43109	10.52438877	8.493579657	2.033838	0.40764
LOC100625251	9.516231779	39.661362	3.394545939	5.34558663	-2.05927	0.40774
NCALD	13.74566813	54.534373	3.882219287	5.795309087	-1.98819	0.40841
LEPRE1	1975.146774	10016.973	10.94847439	13.29030296	-2.34241	0.40864
DCAF16	164.9480175	45.44531	7.374587584	5.537461031	1.859808	0.40874
PLTP	539.2531342	2447.4365	9.077491726	11.25764509	-2.18224	0.40885
LOC102158189	66.61362245	18.178124	6.079242038	4.261389711	1.873614	0.40922
CARD6	278.0854398	1193.9722	8.124563049	10.2227614	-2.10217	0.40926
LOC100627476	44.40908164	170.21334	5.504908955	7.419651341	-1.93842	0.40944
CDKN2A	19.03246356	73.538775	4.324267947	6.219919206	-1.95004	0.40948
SAP25	45.46644072	174.34474	5.538117235	7.454050314	-1.93907	0.40957
LOC100738470	358.4447303	95.848291	8.489626143	6.597654688	1.902926	0.4096
RELA	692.5702017	3207.6126	9.437898106	11.64773391	-2.21147	0.40965
MSLN	7.401513606	32.224856	3.070649265	5.054191063	-2.12228	0.41022
LOC100623921	7.401513606	32.224856	3.070649265	5.054191063	-2.12228	0.41022
ZNFX1	1547.973703	7672.821	10.59709694	12.90572939	-2.30938	0.41041
ZNF554	410.2553256	109.06875	8.683890551	6.782261052	1.911284	0.4109
EBF1	109.965345	439.58009	6.793965376	8.783260503	-1.99908	0.41098
CEP55	1746.757211	423.05453	10.77128907	8.728105973	2.045764	0.41107
ALG8	1495.105748	366.04132	10.54699644	8.519798669	2.030169	0.41109
SPAG1	1885.271251	454.4531	10.88132144	8.831158705	2.052569	0.41165
CEBPZ	13442.20607	2821.7406	13.71458963	11.46288086	2.252113	0.41182
EXOSC8	2323.017913	551.95395	11.18240547	9.111015534	2.07338	0.41185
GTF2A1	3987.301115	912.21132	11.96155862	9.834804937	2.127973	0.41217
RNF180	109.965345	30.5723	6.793965376	4.980587447	1.846752	0.41233
NDUFAF5	1707.634925	414.79174	10.73862846	8.699717297	2.04154	0.41237
LOC102157898	162.8332993	667.63292	7.356084806	9.385070587	-2.03566	0.41267
LRFN1	370.0756803	99.153405	8.535569642	6.646067655	1.900086	0.41279
LOC102158651	22.20454082	4.9576702	4.536335244	2.57474827	2.163121	0.41315
PLEKHG5	386.9934257	1698.8283	8.599888397	10.73117334	-2.13416	0.41321
LOC102166702	12.68830904	50.402981	3.774872332	5.683780113	-1.99001	0.41326
OBSCN	89.87552236	353.64714	6.505819847	8.470240516	-1.97631	0.4133
ARHGEF17	777.1589286	3618.273	9.603921026	11.82148421	-2.21902	0.41342
PNMA2	16.91774539	3.3051135	4.163317207	2.106051269	2.355766	0.4135
VSIG2	16.91774539	3.3051135	4.163317207	2.106051269	2.355766	0.4135
LOC102160878	16.91774539	3.3051135	4.163317207	2.106051269	2.355766	0.4135
LOC102158276	16.91774539	3.3051135	4.163317207	2.106051269	2.355766	0.4135
INPP4A	188.2099174	779.1805	7.563843899	9.607664138	-2.04961	0.41363
KIAA1211L	10.57359087	42.966475	3.532764645	5.458331975	-2.02275	0.41372
CBL	3883.679925	891.55436	11.92358002	9.801796232	2.12303	0.41373
FANCD2OS	71.90041789	19.830681	6.187855179	4.380638094	1.858266	0.41388
DNAAF2	2700.495107	636.23435	11.39954235	9.315680218	2.085594	0.41395
CD97	1292.092804	6278.8893	10.3366101	12.61652342	-2.2808	0.41405
LOC100738034	16987.53108	3515.8145	14.05227349	11.78005351	2.272545	0.41409
RTN1	46.52379981	12.394176	5.570578288	3.74353388	1.908307	0.41441
LOC100624585	34.89284986	132.20454	5.165624571	7.057499439	-1.92177	0.41461
LOC102161664	11.63094995	1.6525567	3.658891241	1.407383614	2.815197	0.41463
LOC102163537	11.63094995	1.6525567	3.658891241	1.407383614	2.815197	0.41463
LOC100738560	11.63094995	1.6525567	3.658891241	1.407383614	2.815197	0.41463
LOC100519199	11.63094995	1.6525567	3.658891241	1.407383614	2.815197	0.41463
SLC26A10	11.63094995	1.6525567	3.658891241	1.407383614	2.815197	0.41463
LOC102165743	11.63094995	1.6525567	3.658891241	1.407383614	2.815197	0.41463
LOC100739759	11.63094995	1.6525567	3.658891241	1.407383614	2.815197	0.41463
GAR1	5074.266256	1144.3955	12.30926779	10.16163018	2.148613	0.41496
LOC100517953	2247.945418	537.08094	11.13503293	9.071679398	2.065396	0.41499
LOC102157550	103.6211905	28.919743	6.709031281	4.903025878	1.841192	0.41552
CLSPN	2778.73968	654.41247	11.44073407	9.356259313	2.086159	0.41555
LOC100154243	128.9978086	518.07654	7.022343493	9.019803473	-2.00582	0.41562
ZBTB10	5481.349505	1230.3285	12.42057859	10.26599998	2.155488	0.41568
TRADD	280.2001579	1193.9722	8.135453595	10.2227614	-2.09124	0.41569
PARL	7565.404264	1659.9932	12.88539214	10.69783049	2.18824	0.4158
LACC1	297.1179033	1271.6424	8.219739208	10.31361139	-2.09759	0.41599
HVCN1	195.611431	809.7528	7.619203392	9.663118299	-2.04949	0.41599
LOC102167404	32.77813168	8.2627837	5.078017628	3.211445828	1.988034	0.41606
LOC102161574	8.458872693	35.52997	3.241668254	5.191008663	-2.0705	0.41617
CSTB	617.4977066	2809.3465	9.272624435	11.45653228	-2.18573	0.41621
PAK1IP1	6947.906558	1534.3989	12.76257027	10.58439784	2.178905	0.41626
LOC102160601	5.286795433	24.788351	2.65232482	4.688647628	-2.2292	0.41687
LOC102160363	5.286795433	24.788351	2.65232482	4.688647628	-2.2292	0.41687
LOC100523295	5.286795433	24.788351	2.65232482	4.688647628	-2.2292	0.41687
LOC102167440	5.286795433	24.788351	2.65232482	4.688647628	-2.2292	0.41687
LOC102167980	5.286795433	24.788351	2.65232482	4.688647628	-2.2292	0.41687
DOCK10	512.819157	135.50965	9.00511687	7.092859161	1.920055	0.41693
PSMG1	7051.527748	1556.7085	12.78392472	10.60520952	2.179437	0.41695
LOC100625039	52.86795433	201.61192	5.751355374	7.662575262	-1.93112	0.41704
GTPBP8	2139.037432	513.94515	11.06342032	9.008274952	2.057275	0.41706
LOC100516665	229.4469218	960.13547	7.848290687	9.908595977	-2.06508	0.41731
E2F8	2293.411859	548.64884	11.16390867	9.10236639	2.063541	0.41761
C1H9orf85	413.4274028	110.7213	8.69497559	6.803760479	1.900701	0.41774
LOC100622542	278.0854398	76.01761	8.124563049	6.267116453	1.871123	0.41781
LOC100513233	25.37661808	95.848291	4.721187694	6.597654688	-1.91725	0.4179
LOC100622139	938.9348689	4417.2842	9.876416981	12.1092705	-2.23406	0.41819
LOC100523202	166.0053766	46.271589	7.38375074	5.56290145	1.843031	0.41828
LOC100511759	29.60605442	111.54758	4.935745167	6.814391228	-1.9137	0.41829
LOC100736696	5248.730506	28593.363	12.35802765	14.80344315	-2.44564	0.41832
INSR	59.21210885	226.40027	5.911981742	7.829090181	-1.93491	0.41843
LOC100127151	328.8386759	89.238064	8.365616765	6.495664213	1.881649	0.41855
LOC102160596	15.8603863	61.1446	4.075565686	5.957557118	-1.9468	0.41913
LOC100737943	3458.621572	805.62141	11.75639852	9.655747894	2.102023	0.42002
ABHD17C	4887.113698	1111.3444	12.25506213	10.11938784	2.136677	0.4206
MOK	27.49133625	103.2848	4.832451381	6.704385034	-1.90958	0.42071
ERGIC3	621.7271429	2814.3041	9.282456353	11.45907507	-2.17842	0.42081
CACFD1	242.1352308	1013.0173	7.925615148	9.985866528	-2.06477	0.42083
MGME1	2653.971307	630.4504	11.37448055	9.3025256	2.073698	0.42083
CBX2	2270.149959	545.34373	11.14920725	9.09366508	2.05755	0.42096
SCNN1D	74.01513606	285.06604	6.229109817	8.160204421	-1.9454	0.421
LOC102165155	43.35172255	11.567897	5.470918232	3.651671381	1.905963	0.42126
LOC102157556	174.4642493	48.750424	7.455033302	5.636636914	1.839445	0.42137
LOC102168187	1.057359087	10.741619	1.04079362	3.553559423	-3.34467	0.42158
LOC100156099	1.057359087	10.741619	1.04079362	3.553559423	-3.34467	0.42158
LOC102164415	1.057359087	10.741619	1.04079362	3.553559423	-3.34467	0.42158
LOC100154886	1.057359087	10.741619	1.04079362	3.553559423	-3.34467	0.42158
ZSCAN9	1.057359087	10.741619	1.04079362	3.553559423	-3.34467	0.42158
CCKAR	1.057359087	10.741619	1.04079362	3.553559423	-3.34467	0.42158
LRRC66	1.057359087	10.741619	1.04079362	3.553559423	-3.34467	0.42158
LOC100521282	102.5638314	402.39757	6.694376434	8.656058573	-1.9721	0.4216
LOC100522139	3450.162699	805.62141	11.75286677	9.655747894	2.09849	0.42228
MRPL22	1149.349327	290.02371	10.16785632	8.184992878	1.986575	0.4226
STX5	560.4003159	2508.5811	9.132886065	11.29323087	-2.16234	0.42265
PSMD14	17962.41616	3733.952	14.13277412	11.86687397	2.266206	0.42282
NOTCH3	300.2899806	1274.9475	8.235008887	10.31735329	-2.08601	0.42301
ZNF496	3253.493909	763.48122	11.6682175	9.578337242	2.091325	0.42306
LOC100626673	528.6795433	140.46732	9.048975981	7.144325041	1.912159	0.42348
LOC102160068	1367.165299	341.25297	10.41802683	8.41891924	2.002274	0.42361
LOC102167622	14.80302721	57.013208	3.982129041	5.858309485	-1.9454	0.42362
LOC100737985	310.8635715	85.106672	8.284771232	6.428053129	1.868937	0.42371
LOC100522714	622.784502	2809.3465	9.284903899	11.45653228	-2.17343	0.42375
LOC102161719	478.9836662	128.07315	8.906841502	7.012045083	1.903008	0.42385
TBC1D24	61.32682702	233.0105	5.961781364	7.87042946	-1.9258	0.42414
LOC100739363	48.63851798	13.220454	5.633388138	3.829895615	1.879328	0.42418
MTMR2	14330.3877	3030.7891	13.80689069	11.56595367	2.241312	0.42433
TRAPPC12	211.4718173	870.8974	7.731127682	9.768014573	-2.04204	0.42471
LOC102165946	462.0659208	123.94176	8.855073776	6.965111899	1.898436	0.42478
TBPL1	1850.378402	452.80055	10.85438408	8.82591454	2.030873	0.42489
ARRDC3	618.5550656	2784.5581	9.275088704	11.4437507	-2.17047	0.42494
TAF7	13937.05012	2955.5977	13.76674113	11.52972225	2.237403	0.42512
EXOSC9	6154.887243	1383.19	12.58775109	10.43482627	2.153733	0.42526
LSM3	5577.569182	1262.5534	12.44567942	10.30327087	2.143292	0.42548
GPT	85.64608601	330.51135	6.437062676	8.372914449	-1.94824	0.42561
GBX1	155.4317857	43.792754	7.289389877	5.485193456	1.827517	0.42567
LOC100515752	1894.787483	463.54217	10.88858153	8.859665746	2.031264	0.42624
UBE3A	14411.80435	3053.0986	13.81506345	11.57653092	2.238905	0.4265
ENOX2	778.2162877	202.4382	9.605880024	7.6684468	1.94269	0.42655
LOC100519188	246.3646672	1024.5852	7.950495635	10.00223161	-2.05617	0.42657
BFSP1	461.0085617	123.94176	8.851775777	6.965111899	1.895131	0.4267
SLC4A5	287.6016715	79.322724	8.172935846	6.327736286	1.858266	0.42678
LOC102164780	357.3873713	97.500848	8.485375991	6.622064238	1.874002	0.42694
SLC16A2	179.7510447	728.77752	7.497860176	9.511312908	-2.01948	0.42701
SV2A	7.401513606	31.398578	3.070649265	5.017858594	-2.08481	0.42704
LOC100620352	7.401513606	31.398578	3.070649265	5.017858594	-2.08481	0.42704
EYA4	7.401513606	31.398578	3.070649265	5.017858594	-2.08481	0.42704
UBE2N	18923.55557	3935.5639	14.2079718	11.94272118	2.265541	0.42715
LOC102167148	706.3158698	185.08636	9.466210821	7.539828464	1.932115	0.42747
LOC100524883	29.60605442	7.4365053	4.935745167	3.076645515	1.993196	0.42749
ODF2L	855.403501	221.4426	9.742146883	7.797289318	1.949672	0.42785
LOC100739056	212.5291764	871.72368	7.738289401	9.769381136	-2.03621	0.42815
CLCA2	2.114718173	14.046732	1.639101631	3.911378308	-2.7317	0.42836
LOC100739130	2.114718173	14.046732	1.639101631	3.911378308	-2.7317	0.42836
LOC102167315	2.114718173	14.046732	1.639101631	3.911378308	-2.7317	0.42836
TGFBR3L	2.114718173	14.046732	1.639101631	3.911378308	-2.7317	0.42836
DARS	18443.51455	3847.1521	14.1709042	11.90995011	2.261251	0.4284
LOC102162906	24.31925899	5.7839486	4.662163277	2.76212524	2.071973	0.42846
LOC102165367	24.31925899	5.7839486	4.662163277	2.76212524	2.071973	0.42846
CCDC142	29.60605442	109.89502	4.935745167	6.793050814	-1.89216	0.4287
LOC100622807	56.04003159	210.70098	5.833902876	7.72588417	-1.91067	0.42893
HEXIM2	225.2174854	927.08433	7.821566636	9.858112096	-2.04138	0.42895
LOC100739277	40.17964529	10.741619	5.363859498	3.553559423	1.903253	0.42925
LOC100512841	552.9988023	2451.5679	9.113739047	11.26007738	-2.14836	0.42959
XYLT2	341.526985	1453.4237	8.420073841	10.50623185	-2.08938	0.4296
LOC102161673	93.04759962	26.440908	6.555319217	4.778256309	1.815197	0.43001
TMUB1	379.5919121	1628.5947	8.572101093	10.67029745	-2.10111	0.43019
TMEM168	4349.975282	1007.2333	12.08712311	9.977613845	2.110609	0.43041
LOC100627260	22.20454082	82.627837	4.536335244	6.385911347	-1.89577	0.43061
SENP6	13330.126	2851.4867	13.70251102	11.47800442	2.224904	0.43091
NXPH4	154.3744266	43.792754	7.279605257	5.485193456	1.81767	0.43103
LOC102158008	10866.47933	2359.0248	13.40772973	11.20458627	2.203622	0.43123
ALDH3A2	2063.964937	10229.326	11.01190157	13.32056453	-2.30922	0.43142
LOC102164577	8.458872693	34.703692	3.241668254	5.158001345	-2.03655	0.43148
LOC100623544	19.03246356	4.1313919	4.324267947	2.359350201	2.203763	0.43184
KIF5A	19.03246356	4.1313919	4.324267947	2.359350201	2.203763	0.43184
LOC100625424	323.5518805	89.238064	8.342305305	6.495664213	1.858266	0.43185
SHROOM3	6687.796223	1503.8266	12.70753088	10.55538158	2.152893	0.43188
SHC3	27.49133625	101.63224	4.832451381	6.681340184	-1.88631	0.43191
ASPHD2	41.23700438	152.8615	5.400435611	7.265488457	-1.89021	0.43197
PPP1R16A	507.5323616	2226.8202	8.990195776	11.12141709	-2.13341	0.43202
LOC102160530	81.41664967	23.135794	6.364863912	4.593102407	1.815197	0.43209
LOC100737167	81.41664967	23.135794	6.364863912	4.593102407	1.815197	0.43209
LOC102157806	211.4718173	59.492043	7.731127682	5.918673475	1.829697	0.43221
LOC100520993	64.49890428	242.92584	6.033398867	7.930298794	-1.91317	0.43223
TUBAL3	3.17207726	17.351846	2.060765874	4.19785327	-2.45159	0.43227
LOC102162453	3.17207726	17.351846	2.060765874	4.19785327	-2.45159	0.43227
LOC100627387	3.17207726	17.351846	2.060765874	4.19785327	-2.45159	0.43227
GRK7	3.17207726	17.351846	2.060765874	4.19785327	-2.45159	0.43227
CCDC17	3.17207726	17.351846	2.060765874	4.19785327	-2.45159	0.43227
C4BPB	3.17207726	17.351846	2.060765874	4.19785327	-2.45159	0.43227
LOC102161870	65.55626337	247.05723	6.056502534	7.954529215	-1.91404	0.43237
LOC100738672	2325.132631	564.34813	11.18371764	9.142995708	2.042656	0.43268
LOC100621094	444.0908164	1924.4023	8.797955923	10.91094422	-2.11548	0.43285
CDK7	2029.072087	497.41958	10.98731524	8.961216934	2.028285	0.43294
CNIH	8504.339133	1882.2621	13.05415305	10.87901811	2.175732	0.43322
LOC100737243	1904.303715	469.32612	10.89580527	8.877517631	2.020601	0.43373
HAUS1	3181.593491	756.04471	11.63598718	9.564234696	2.073206	0.43391
SCARF1	38.06492712	140.46732	5.287802017	7.144325041	-1.8837	0.43403
NFKB2	770.8147741	3495.9838	9.59211085	11.77189539	-2.18124	0.43435
LOC100518497	1421.090612	357.77853	10.47379768	8.486949769	1.98986	0.43436
MRPL42	3191.109723	758.52355	11.64029453	9.568950879	2.072792	0.43441
LOC102157987	84.58872693	322.24856	6.419348884	8.336500155	-1.92964	0.4346
LOC100626458	195.611431	55.360651	7.619203392	5.816616368	1.821058	0.43479
ARHGEF3	52.86795433	196.65425	5.751355374	7.626835184	-1.8952	0.43499
LOC100523069	514.9338752	138.81477	9.011042364	7.127372928	1.891226	0.43505
LOC102158919	12.68830904	48.750424	3.774872332	5.636636914	-1.94191	0.43542
NDUFA4L2	93.04759962	356.12598	6.555319217	8.480289273	-1.93635	0.43546
LOC100621823	2435.097976	590.78904	11.25035644	9.208939157	2.043265	0.43549
MAK	244.249949	68.581105	7.938109026	6.120623682	1.832475	0.43556
KCNE3	9.516231779	38.008805	3.394545939	5.285727902	-1.99787	0.43562
QSER1	9710.785851	2134.277	13.24552089	11.06020754	2.185841	0.43573
NUF2	4773.976276	1103.9079	12.22127785	10.10971041	2.112572	0.43574
ATR	3287.3294	780.83306	11.68313911	9.610716783	2.07383	0.4358
LOC100157694	1422.147971	358.60481	10.47486996	8.490268521	1.987605	0.4359
BAK	453.6070481	1961.5849	8.828476241	10.93853932	-2.1125	0.43596
NHE5	785.6178013	206.56959	9.619519025	7.697451307	1.9272	0.43701
IGSF8	118.4242177	459.41077	6.899951616	8.846777786	-1.95582	0.43729
RYBP	5986.767148	1364.1856	12.5478024	10.41488138	2.133737	0.43733
ARL2BP	10513.3214	2302.0115	13.36006812	11.16930593	2.191252	0.4377
LOC102166949	53.92531342	199.95937	5.779399292	7.650760007	-1.89067	0.4378
LOC100625662	61.32682702	17.351846	5.961781364	4.19785327	1.821429	0.43801
LOC100518455	2213.052568	542.03861	11.11247376	9.084910972	2.02957	0.43808
KIF1C	3297.902991	16884.172	11.68777064	14.04346928	-2.35605	0.43812
LOC100736829	33.83549077	123.94176	5.122485983	6.965111899	-1.87305	0.43816
JAK3	5.286795433	23.962073	2.65232482	4.641665831	-2.18029	0.4384
TMEM47	9055.223217	2005.3776	13.1446938	10.97037744	2.174876	0.43844
LOC102161585	37.00756803	9.9153405	5.24821481	3.448285225	1.900086	0.43861
SIN3B	800.4208285	3623.2307	9.646416195	11.82345906	-2.17845	0.43863
LOC100511033	8806.743832	1954.9746	13.10455679	10.93367194	2.171459	0.43884
LOC100624963	367.9609621	101.63224	8.52732437	6.681340184	1.856195	0.4391
LOC102166720	31.7207726	8.2627837	5.032134908	3.211445828	1.940728	0.43922
LOC100626475	13.74566813	2.4788351	3.882219287	1.798604302	2.471243	0.43928
SLC19A3	13.74566813	2.4788351	3.882219287	1.798604302	2.471243	0.43928
LOC102157946	13.74566813	2.4788351	3.882219287	1.798604302	2.471243	0.43928
LOC100736782	13.74566813	2.4788351	3.882219287	1.798604302	2.471243	0.43928
LOC102162538	13.74566813	2.4788351	3.882219287	1.798604302	2.471243	0.43928
PRX	113.1374223	436.27498	6.834628076	8.772396993	-1.94716	0.43943
SYPL2	13.74566813	52.055537	3.882219287	5.729431428	-1.92107	0.43946
FAM168A	117.3668586	453.62683	6.887121389	8.828539005	-1.95048	0.4396
DEF6	206.1850219	831.23604	7.694775899	9.700848959	-2.01132	0.43965
LOC102163855	41.23700438	151.20894	5.400435611	7.249909307	-1.87453	0.43967
SQSTM1	7810.711573	42939.208	12.93142297	15.39004156	-2.45877	0.43982
LOC100522223	2217.282005	544.51745	11.11522707	9.091481528	2.025742	0.4408
LOC102166967	31.7207726	115.67897	5.032134908	6.866400771	-1.86663	0.44101
LOC100621800	1121.857991	289.19743	10.13295976	8.180890933	1.955763	0.44113
ZNF425	6.344154519	27.267186	2.876596413	4.821056378	-2.10367	0.44131
AKNAD1	16.91774539	62.797156	4.163317207	5.995420212	-1.89216	0.44146
LOC100739186	411.3126847	113.20014	8.687595036	6.83542057	1.86136	0.4415
LOC102160570	26.43397716	6.610227	4.777891883	2.927939483	1.999622	0.44151
LOC100738411	88.81816327	25.61463	6.488935315	4.734147579	1.793887	0.44154
LOC100624174	88.81816327	25.61463	6.488935315	4.734147579	1.793887	0.44154
LOC102167139	52.86795433	14.873011	5.751355374	3.98850389	1.829697	0.44161
PRICKLE1	513.8765161	2230.1253	9.008082659	11.12355584	-2.11763	0.44172
LOC100520671	1117.628555	288.37115	10.12751535	8.176777292	1.954442	0.44174
RET	80.35929058	23.135794	6.346235194	4.593102407	1.796338	0.44189
LOC100526084	2861.213688	690.76872	11.48291567	9.434145967	2.050353	0.44202
PTPRD	279.1427989	78.496445	8.130018598	6.312818447	1.830304	0.44209
LOC102167584	25.37661808	92.543178	4.721187694	6.547560532	-1.86663	0.44211
GPR114	25.37661808	92.543178	4.721187694	6.547560532	-1.86663	0.44211
TBC1D16	666.1362245	2953.9452	9.381837569	11.52891565	-2.14785	0.44243
LOC100739296	2065.022296	510.64003	11.01264011	8.998985343	2.015779	0.44256
NHP2L1	26042.7543	5376.5934	14.66864981	12.39274495	2.276118	0.4431
LOC100523888	11.63094995	44.619032	3.658891241	5.511563931	-1.93969	0.4432
LOC100156892	235.7910763	956.00408	7.887470902	9.902381259	-2.01951	0.44362
FBXL16	7.401513606	30.5723	3.070649265	4.980587447	-2.04633	0.44419
LOC102160497	7.401513606	30.5723	3.070649265	4.980587447	-2.04633	0.44419
ACCSL	51.81059524	190.04403	5.722755497	7.577761331	-1.87501	0.44447
TBC1D22A	266.4544898	1089.8612	8.063149612	10.09125179	-2.03218	0.44459
ZNF334	85.64608601	24.788351	6.437062676	4.688647628	1.788725	0.44466
LOC100737955	381.7066303	1608.764	8.580095085	10.65263347	-2.07542	0.44475
LOC100625963	327.7813168	91.716899	8.360984509	6.534760414	1.837474	0.44488
CTNNA2	111.0227041	32.224856	6.807647348	5.054191063	1.784609	0.44537
SNX32	80.35929058	300.76533	6.346235194	8.23728324	-1.9041	0.44542
CNOT2	7510.421592	1697.1758	12.87487026	10.72977008	2.145758	0.44543

LRRC8B	326.597957	104.93735	8.355782547	6.727067559	1.637987	0.30655
LOC100622534	119.365751	372.65155	6.911281129	8.54554968	-1.64244	0.308
USP39	1803.74912	539.55978	10.81758259	9.078310354	1.741144	0.30803
EID3	1.65785765	11.567897	1.410263837	3.65167138	-2.80273	0.3085
LOC100514233	1.65785765	11.567897	1.410263837	3.65167138	-2.80273	0.3085
LOC100738493	1.65785765	11.567897	1.410263837	3.65167138	-2.80273	0.3085
GPIBB	1.65785765	11.567897	1.410263837	3.65167138	-2.80273	0.3085
CDH22	1.65785765	11.567897	1.410263837	3.65167138	-2.80273	0.3085
LOC100623321	1.65785765	11.567897	1.410263837	3.65167138	-2.80273	0.3085
LOC102165806	1.65785765	11.567897	1.410263837	3.65167138	-2.80273	0.3085
GKAP1	625.012333	196.65425	9.29004727	7.626835185	1.668223	0.30868
TACC2	3497.25071	1011.3647	11.77241797	9.983513431	1.789918	0.30879
LOC100523888	13.2628612	44.619032	3.834191515	5.511563931	-1.75027	0.30884
C17H20orf96	562.013742	177.64985	9.137026327	7.480990892	1.661569	0.309
IFIT5	2015.9549	7249.7664	10.97796311	12.82391779	-1.84647	0.30909
UNC5A	48.9068006	150.38266	5.641164514	7.242056188	-1.62053	0.31003
TNIP2	1684.38337	506.50864	10.71886108	8.987288579	1.733562	0.31017
LOC100517736	1138.11928	3959.526	10.1537031	11.95147632	-1.79868	0.31024
NIPAL1	226.297569	73.538775	7.828438443	6.219919206	1.621644	0.31035
MST1	169.930409	55.360651	7.417265267	5.816616368	1.618011	0.31076
KLHL40	19.8942918	63.623435	4.385036953	6.013985524	-1.6772	0.31125
RAD51	1885.81307	564.34813	10.88173579	9.142995708	1.740529	0.31129
CGREF1	125.168252	390.00339	6.979205122	8.611037311	-1.63962	0.31175
CCDC175	19.0653629	61.1446	4.326635347	5.957557119	-1.68127	0.31237
LOC100154262	2914.51374	853.54556	11.50953441	9.739013597	1.771715	0.31252
LOC102165357	1307.22076	399.09245	10.35339029	8.644189607	1.711708	0.31312
LOC102161277	35.6439394	109.89502	5.195502704	6.793050813	-1.6244	0.31334
C5H12orf29	1289.81325	394.13478	10.33406458	8.626201041	1.710401	0.31341
LOC102163107	126.82611	41.313919	6.998038744	5.403060391	1.618152	0.31343
TBC1D8	330.742601	1073.3356	8.373920477	10.06922902	-1.69832	0.31344
LOC100623424	348.150106	112.37386	8.4477036	6.824944216	1.631403	0.31353
C5H12orf23	1186.19715	4124.7816	10.21334381	12.01045175	-1.79797	0.31357
STARD8	31.4992953	97.500848	5.022336531	6.622064238	-1.6301	0.31386
SLA-DOB2	42.27537	129.7257	5.435474249	7.030399034	-1.61758	0.31395
IOCF3	23.2100071	5.7839486	4.597531595	2.762125239	2.00462	0.31404
LOC102160986	23.2100071	5.7839486	4.597531595	2.762125239	2.00462	0.31404
LOC102164964	23.2100071	5.7839486	4.597531595	2.762125239	2.00462	0.31404
LOC100737243	150.036117	469.32612	7.238749771	8.877517631	-1.64528	0.31456
LOC100736861	227.955427	74.365053	7.838922949	6.2358238	1.616055	0.31456
GNL2	6274.16227	1767.4094	12.61543705	10.78823662	1.827787	0.31487
IFNGR1	426.069415	1398.8893	8.738326772	10.45109701	-1.71512	0.31504
LOC102165688	310.848309	100.80596	8.284700625	6.669678233	1.62463	0.31518
KCNAB3	232.928999	76.01761	7.869926909	6.267116453	1.615485	0.31533
APRIL	48.0778718	147.07755	5.617000784	7.210209122	-1.61313	0.31537
LOC100524738	1086.72569	335.46902	10.08709906	8.394329862	1.695736	0.31557
LOC100626361	415.293341	1360.8805	8.70145667	10.41138438	-1.71234	0.3157
TTC9	2359.96036	700.68406	11.2051581	9.454677778	1.751927	0.3158
CCDC58	2398.09109	711.42568	11.22827222	9.476595706	1.753102	0.31583
LOC100521647	92.0110994	29.746021	6.539330984	4.942327829	1.629111	0.31651
LOC100737389	174.903982	549.47512	7.45864433	9.104533542	-1.65149	0.31655
SLC5A1	179.048626	58.665764	7.492242779	5.89883146	1.609761	0.31673
SIPA1	388.767618	1268.3373	8.606470427	10.30985977	-1.70596	0.31682
GEMIN4	5750.27925	1630.2472	12.48966717	10.67175973	1.818541	0.3171
LOC100739531	953.268147	296.63394	9.898250907	8.217395218	1.684198	0.31765
LOC102161441	87.0375265	28.093465	6.460046706	4.862623207	1.631403	0.31774
FGB	15.7496477	51.229259	4.066058842	5.706786332	-1.70165	0.31872
TESK2	198.942918	65.275991	7.643444369	6.05041444	1.60773	0.3189
REV3L	237.902572	756.04471	7.900278578	9.564234696	-1.6681	0.31892
ZNF664	4022.79158	1164.2262	11.97433986	10.18639436	1.788826	0.31903
VTN	5.80250177	23.135794	2.766065426	4.593102408	-1.99538	0.31945
LOC102167819	95.3268147	292.50254	6.589865555	8.197229196	-1.6175	0.31946
NCOA3	3148.27167	923.77922	11.6208025	9.85296517	1.76894	0.31998
LOC102161982	79.5771671	25.61463	6.332299179	4.734147579	1.635387	0.32019
DIXDC1	3070.35236	902.29598	11.58465832	9.819054981	1.766732	0.32024
EVI5L	537.145878	1777.3248	9.071853494	10.79630311	-1.72632	0.32033
ZNF585A	1460.57259	446.19032	10.51330577	8.804745152	1.710803	0.32053
PCSK4	118.536822	38.835083	6.90131128	5.315967693	1.609903	0.32054
MYBBP1A	33922.2542	8774.25	15.04998695	13.09922452	1.950884	0.32056
LOC102161648	20.7232206	4.9576702	4.441166102	2.574748269	2.063514	0.32085
LOC100522173	20.7232206	4.9576702	4.441166102	2.574748269	2.063514	0.32085
LOC102160515	20.7232206	4.9576702	4.441166102	2.574748269	2.063514	0.32085
LOC102167889	20.7232206	4.9576702	4.441166102	2.574748269	2.063514	0.32085
LOC100511183	113.563249	37.182527	6.8400005	5.254840671	1.610799	0.32102
LOC102162253	311.677238	101.63224	8.288530387	6.681340183	1.616695	0.32129
TMEM161B	841.362756	264.40908	9.718297841	8.052073912	1.669956	0.32243
MAP3K12	828.099895	2801.0837	9.695402126	11.45228432	-1.75811	0.32244
ATL1	53.8803735	163.60312	5.778218396	7.362847851	-1.60237	0.32246
DPH5	2231.47639	668.4592	11.1244292	9.386852329	1.739087	0.32319
FIGNL1	804.889888	253.66746	9.65443892	7.992470822	1.665853	0.32339
PLK1	4684.27678	1349.3126	12.19391856	10.3990777	1.795602	0.32339
LOC100620662	1990.25811	599.8781	10.95946452	9.230928526	1.730214	0.32343
LOC102165677	41.4464412	12.394176	5.407571694	3.74353388	1.741586	0.32353
LOC100620825	41.4464412	12.394176	5.407571694	3.74353388	1.741586	0.32353
IMPACT	2975.85448	878.33391	11.53957298	9.780267293	1.760463	0.32361
SEL1L	2526.57505	9094.0198	11.30353822	13.15086106	-1.84774	0.32361
LOC100524777	678.892707	215.65866	9.409163283	7.75928006	1.654434	0.32376
CCNB1	11711.1064	3212.5703	13.51571295	11.64996132	1.866077	0.32377
RCAN1	639.933052	2130.9719	9.324029859	11.05797272	-1.73552	0.32404
LOC100517487	746.86487	236.31561	9.546633806	7.890663216	1.660132	0.32407
ERI2	241.218288	79.322724	7.920163984	6.327736286	1.604533	0.3241
LOC102166720	29.8414377	8.2627837	4.946798112	3.211445828	1.852617	0.32427
LOC100156932	29.8414377	8.2627837	4.946798112	3.211445828	1.852617	0.32427
LOC102158655	29.8414377	8.2627837	4.946798112	3.211445828	1.852617	0.32427
NUP54	2907.05338	860.15579	11.50583804	9.750130438	1.756888	0.32516
ABCA1	465.02907	1520.3522	8.86427614	10.57113847	-1.70901	0.32523
LOC100623151	666.458774	2221.8625	9.382534921	11.11820302	-1.73718	0.32532
L3MBTL1	256.139007	84.280394	8.006404665	6.414142197	1.603658	0.32602
LOC100739192	4.97357294	20.656959	2.578594101	4.436758793	-2.05427	0.32616
LOC102166815	13.2628612	43.792754	3.834191515	5.485193456	-1.7233	0.3263
LOC100739495	13.2628612	43.792754	3.834191515	5.485193456	-1.7233	0.3263
LRRN3	130.141825	42.966475	7.03498407	5.458331975	1.598801	0.32634
TFAP2A	97.8136012	298.28649	6.62663773	8.225383356	-1.60859	0.32677
RGMA	2471.86575	738.69286	11.2719682	9.530782548	1.742554	0.32687
LRRC4C	38.9596547	11.567897	5.320472212	3.65167138	1.751854	0.32737
TMPO	5107.85941	1469.1229	12.31878552	10.5217211	1.797764	0.32742
LOC100511462	194.798274	64.449713	7.613224233	6.032314961	1.595735	0.32746
MRPS28	1321.31255	408.18152	10.3688475	8.676597164	1.694689	0.32797
LOC102161772	1141.43499	355.2997	10.15789636	8.476947458	1.68374	0.32838
SPAG5	3570.19644	1047.721	11.80219178	10.03441517	1.768749	0.32859
LOC102157859	18.2364341	4.1313919	4.265769485	2.359350201	2.142124	0.32905
KIAA1984	18.2364341	4.1313919	4.265769485	2.359350201	2.142124	0.32905
HCN4	18.2364341	4.1313919	4.265769485	2.359350201	2.142124	0.32905
ZBED3	12.4339324	41.313919	3.747809764	5.403060391	-1.73235	0.3296
LOC100737962	21703.0145	5786.4274	14.40567429	12.49870648	1.907151	0.32969
UBXN7	3347.21459	986.57638	11.70917628	9.947748515	1.762458	0.32975
LOC100522034	1425.75758	439.58009	10.47852451	8.783260503	1.697531	0.33024
VASH1	3913.37298	1144.3955	11.93456551	10.16163018	1.773827	0.33037
LOC100270690	620.867689	199.13309	9.280463849	7.644815895	1.640553	0.33049
PSD3	578.592319	1905.3979	9.178894665	10.89663357	-1.71947	0.33059
SIRT4	232.100071	76.843889	7.864805631	6.282511874	1.594745	0.33068
BBS12	373.846899	122.2892	8.550157659	6.945902605	1.612151	0.33086
LOC100524051	48.0778718	14.873011	5.617000784	3.98850389	1.692676	0.33091
LOC100523276	100.300388	304.89672	6.662495885	8.256900824	-1.60399	0.33093
GMPS	5596.92742	1606.2852	12.45067707	10.65041019	1.800907	0.33096
LOC100627009	174.075053	539.55978	7.451829714	9.078310354	-1.63207	0.33117
FN3K	270.230797	89.238064	8.083377186	6.495664213	1.598461	0.33123
LOC100625894	144.233615	47.924146	7.182231603	5.61247475	1.589583	0.33159
GNPNAT1	2473.52361	741.99798	11.27293509	9.537214474	1.737081	0.33167
LOC100622435	96.1557435	291.67627	6.602227381	8.193161944	-1.60092	0.33198
MBD2	279.349014	884.11786	8.131080184	9.78972576	-1.66217	0.33225
KDM2B	1254.16931	390.00339	10.29366627	8.611037311	1.685174	0.33283
VIMP	970.675652	3283.6302	9.924331009	11.68151526	-1.75823	0.33299
TNIP1	2301.10641	8176.0245	11.16873881	12.99736025	-1.82907	0.33306
FUT2	73.7746653	23.962073	6.224477643	4.641665831	1.622373	0.33331
ADSS	4342.75811	1265.8585	12.08472805	10.30703964	1.778495	0.33339
TFE3	1435.70472	4963.4542	10.48854787	12.27741939	-1.78959	0.33361
LOC100520085	136.773256	418.92313	7.106152054	8.713981461	-1.6149	0.33373
RELL2	28.18358	85.932951	4.867084967	6.441831208	-1.60836	0.33394
IL20RB	4.14464412	18.178124	2.363071282	4.261389712	-2.13288	0.33423
LOC102164936	4.14464412	18.178124	2.363071282	4.261389712	-2.13288	0.33423
PPP4R1	684.695208	2268.1341	9.42142363	11.14792617	-1.72797	0.33463
LOC102158986	477.463002	155.34033	8.902263561	7.288546214	1.619956	0.33466
F12	57.1960888	18.178124	5.862850292	4.261389712	1.653713	0.33474
LOC102162214	57.1960888	18.178124	5.862850292	4.261389712	1.653713	0.33474
LOC100628140	149.207188	457.75822	7.230810045	8.841590191	-1.61727	0.33557
LOC102165638	246.191861	81.801559	7.94948743	6.371586023	1.589583	0.33576
LOC100739860	2980.82805	10725.093	11.54198135	13.38883708	-1.84721	0.3358
ZBTB6	284.322587	94.195734	8.156450147	6.572825024	1.593795	0.33614
LOC102166452	183.19327	61.1446	7.525076539	5.957557119	1.58307	0.33623
ESYT3	165.785765	55.360651	7.381852349	5.816616368	1.582387	0.33629
LOC102164279	163.298978	54.534373	7.360179698	5.795309087	1.582278	0.33636
CEP89	302.559021	957.65663	8.245833235	9.90487036	-1.66229	0.3367
LOC102166745	68.8010924	22.309516	6.125177709	4.542847146	1.624772	0.33672
LOC100737124	508.133369	165.25567	8.991899814	7.37725977	1.620507	0.33703
GULP1	329.913672	1048.5473	8.370311088	10.03555141	-1.66823	0.33704
BAIAP2L1	319.966526	105.76363	8.326279034	6.738276475	1.597077	0.33718
FZD2	694.642354	2297.8802	9.442201964	11.16671554	-1.72596	0.33724
CEP78	1271.57682	396.61362	10.31353703	8.63522336	1.680812	0.33736
LOC100622863	24.8678647	6.610227	4.693089065	2.927939483	1.911511	0.33756
MPP3	24.8678647	6.610227	4.693089065	2.927939483	1.911511	0.33756
LOC102160570	24.8678647	6.610227	4.693089065	2.927939483	1.911511	0.33756
SCPEP1	1998.54739	7017.5822	10.96545776	12.77696391	-1.81202	0.33774
TECTA	102.787174	310.68067	6.697484358	8.283924863	-1.59577	0.33783
LOC100623546	104.445032	34.703692	6.720347312	5.158001345	1.589583	0.33792
PLIN1	82.8928824	27.267186	6.39047651	4.821056379	1.604082	0.3381
PELI1	1862.60307	570.13208	10.86387889	9.157680603	1.707952	0.33829
TMEM144	1337.06219	416.4443	10.38592946	8.705439898	1.682871	0.33871
CCDC151	101.958245	33.877413	6.685915561	5.124221138	1.589583	0.33872
LOC102159006	15.7496477	3.3051135	4.066058842	2.106051269	2.252548	0.3389
ADARB2	15.7496477	3.3051135	4.066058842	2.106051269	2.252548	0.3389
LOC102163201	15.7496477	3.3051135	4.066058842	2.106051269	2.252548	0.3389
LOC100622925	15.7496477	3.3051135	4.066058842	2.106051269	2.252548	0.3389
LOC100737336	15.7496477	3.3051135	4.066058842	2.106051269	2.252548	0.3389
HSPA12B	378.820472	1210.4978	8.569173859	10.24257609	-1.67601	0.33911
CFH	1608.95085	496.5933	10.65280093	8.958823254	1.695984	0.33916
UBXN11	405.346195	1299.7359	8.666565574	10.34511233	-1.68099	0.33927
PHLDB3	230.442213	718.03591	7.854508213	9.489920003	-1.63965	0.33939
TBC1D16	882.809197	2953.9452	9.787591134	11.52891565	-1.74247	0.33968
LOC100626244	125.997181	42.140197	6.988652665	5.430960858	1.580122	0.33992
UTP18	5683.13602	1640.9888	12.47272536	10.68122861	1.792122	0.33997
LOC102158823	52.2225159	16.525567	5.733964805	4.13138925	1.659972	0.34041
LOC102158883	52.2225159	16.525567	5.733964805	4.13138925	1.659972	0.34041
SLC48A1	30.6703665	92.543178	4.985061656	6.547560532	-1.59328	0.34046
LOC100525621	14.09179	45.44531	3.915692025	5.537461031	-1.68928	0.3405
LOC100620367	3032.22164	10879.607	11.5666352	13.40947146	-1.84318	0.34056
RDH14	137.602185	418.92313	7.114806188	8.713981461	-1.60618	0.34062
LOC100512115	898.558845	285.89232	9.813073848	8.164365521	1.652141	0.34083
LOC102159182	411.977625	135.50965	8.68991981	7.092859162	1.60417	0.34093
LOC100738332	9485.43253	2666.4003	13.21164993	11.38121864	1.83082	0.34116
NHP2L1	19904.2389	5376.5934	14.28086057	12.39274495	1.888311	0.34119
LOC100512939	553.724454	180.12869	9.115627516	7.500871231	1.62014	0.3414
LOC100622736	214.692565	71.886218	7.752832639	6.187574144	1.578485	0.3415
LOC102158988	94.4978859	31.398578	6.57739689	5.017858594	1.589583	0.34155
FKBP4	17632.9739	4795.7197	14.10607001	12.22783241	1.878457	0.34166
LOC102165223	275.204369	91.716899	8.109592332	6.534760414	1.585244	0.3418
LOC102166173	1511.96617	469.32612	10.56316402	8.877517631	1.687763	0.34226
LOC102166858	29.0125088	87.585507	4.907492018	6.468998788	-1.59402	0.34245
HSPG2	4718.26286	17333.668	12.20434582	14.08137256	-1.87725	0.34256
EPHA1	91.1821706	273.49814	6.526415834	8.100652569	-1.58471	0.34261
GATA4	92.0110994	30.5723	6.539330984	4.980587446	1.589583	0.34266
MESP2	121.023608	366.04132	6.931016486	8.51979867	-1.59672	0.34277
PALM	348.150106	1103.9079	8.4477036	10.10971041	-1.66484	0.34297
LOC102160363	6.63143059	24.788351	2.93195353	4.688647628	-1.90227	0.34315
CELSR1	1572.47798	487.50424	10.61974127	8.932227272	1.689553	0.34323
UNC13D	348.979035	115.67897	8.451124691	6.86640077	1.593014	0.34333
SERINC3	1397.574	4785.8043	10.44974087	12.22484712	-1.77584	0.34342
LOC100512321	28.18358	85.106672	4.867084967	6.428053129	-1.59442	0.3436
LOC100524724	256.967935	85.932951	8.011047944	6.441831208	1.580305	0.34381
SLC35F3	3.31571529	15.699289	2.109599694	4.061714779	-2.24331	0.34393
MIR24-2	3.31571529	15.699289	2.109599694	4.061714779	-2.24331	0.34393
LOC102166362	3.31571529	15.699289	2.109599694	4.061714779	-2.24331	0.34393
LOC100739447	3.31571529	15.699289	2.109599694	4.061714779	-2.24331	0.34393
TFDP2	1487.09831	462.71589	10.53925412	8.857097348	1.684301	0.34418
DRAM1	96.1557435	288.37115	6.602227381	8.176777292	-1.58448	0.34428
ALDH6A1	2655.05902	801.49002	11.37507149	9.648339641	1.727987	0.34441
DAPK2	48.9068006	145.42499	5.641164514	7.194018019	-1.57217	0.34448
PSMC3IP	949.952432	302.41788	9.893229367	8.245162313	1.651312	0.34475
S100A11	1927.25952	6715.1643	10.91308351	12.71342181	-1.80087	0.34478
MOB1B	1238.41966	389.17711	10.27544905	8.607985345	1.670002	0.34492
LOC100516649	132.628612	44.619032	7.062085132	5.511563931	1.571661	0.34515
DHX37	4217.58985	1242.7227	12.04254512	10.28044911	1.762914	0.34532
LOC102160839	44.7621565	133.03082	5.516083133	7.066420948	-1.57141	0.34582
PHF7	127.655039	42.966475	7.007364151	5.458331975	1.570967	0.34616
LOC102164364	22.3810782	5.7839486	4.547269558	2.762125239	1.952153	0.34623
ZBTB39	2359.96036	718.03591	11.2051581	9.489920003	1.716635	0.34642
TTC40	84.55074	28.093465	6.418708429	4.862623207	1.589583	0.34661
LOC100739395	540.461593	176.82357	9.080715198	7.474302765	1.611881	0.34693
ZNF84	95.3268147	285.06604	6.589865555	8.160204421	-1.58034	0.34714
SLC18B1	699.615927	226.40027	9.452479975	7.829090182	1.627687	0.34721
LOC100515732	1294.78682	4399.9323	10.33961268	12.10359347	-1.76477	0.34747
ADRB2	38.1307259	11.567897	5.290229968	3.65167138	1.720827	0.34754
LOC102161413	38.1307259	11.567897	5.290229968	3.65167138	1.720827	0.34754
LOC100739452	38.1307259	11.567897	5.290229968	3.65167138	1.720827	0.34754
ENPP4	38.1307259	11.567897	5.290229968	3.65167138	1.720827	0.34754
EMILIN2	2550.61399	9008.9131	11.31719438	13.13729747	-1.82051	0.34791
NLGN3	40.6175124	120.63664	5.379118827	6.926434087	-1.57049	0.34797
NFKBIE	513.935871	1656.6881	9.008248962	10.6949569	-1.68864	0.34812
NUP153	6948.91033	1997.1148	12.76277865	10.96442378	1.798869	0.34818
LOC100623420	1035.3321	3470.3692	10.01727069	11.76128908	-1.745	0.34856
MMS19	8419.43006	2395.381	13.0396782	11.22664158	1.813467	0.3486
NLRX1	529.685518	1709.57	9.05171337	10.74026139	-1.69043	0.34863
KBTBD11	38.9596547	115.67897	5.320472212	6.86640077	-1.57007	0.34912
NTAN1	703.760571	2306.1429	9.460989403	11.17189167	-1.71233	0.34943
CNOT6	1907.36522	588.3102	10.89812159	9.202883429	1.696932	0.34944
LOC100620893	406.175124	134.68337	8.669505612	7.084100147	1.59253	0.34961
LOC100621861	205.574348	69.407383	7.690517306	6.137654819	1.566499	0.34981
CEP68	3572.68323	1065.8991	11.80319604	10.05920803	1.744937	0.34983
CORO2A	79.5771671	26.440908	6.332299179	4.77825631	1.589583	0.34985
ZNF510	198.113989	66.928548	7.637450771	6.085946114	1.565637	0.34994
LOC100738592	468.344785	154.51406	8.87450432	7.280901168	1.599833	0.34995
DCAF12L2	29.0125088	8.2627837	4.907492018	3.211445828	1.811975	0.35001
HUNK	29.0125088	8.2627837	4.907492018	3.211445828	1.811975	0.35001
LOC102167517	15.7496477	49.576702	4.066058842	5.660401068	-1.65434	0.35009
RMI1	411.148697	136.33593	8.687021122	7.101565318	1.592494	0.35015
LOC100622782	13.2628612	2.4788351	3.834191515	1.798604302	2.419658	0.35058
LOC102157509	13.2628612	2.4788351	3.834191515	1.798604302	2.419658	0.35058
LOC102166647	13.2628612	2.4788351	3.834191515	1.798604302	2.419658	0.35058
LOC100524744	13.2628612	2.4788351	3.834191515	1.798604302	2.419658	0.35058
LOC100524718	13.2628612	2.4788351	3.834191515	1.798604302	2.419658	0.35058
LOC102166301	13.2628612	2.4788351	3.834191515	1.798604302	2.419658	0.35058
LOC102162244	13.2628612	2.4788351	3.834191515	1.798604302	2.419658	0.35058
GPX8	324.11117	1017.1487	8.344789314	9.991732532	-1.64997	0.35062
LOC100738073	1662.83122	517.25026	10.70029338	9.017505127	1.684707	0.35105
RAPGEF6	2248.05497	688.28988	11.13510321	9.428967032	1.707589	0.35111
LOC100738807	77.0903806	25.61463	6.287072939	4.734147579	1.589583	0.35168
LOC100738248	125.168252	375.95666	6.979205122	8.558254848	-1.5867	0.35171
LOC100156526	2412.18288	736.21403	11.23672154	9.525939716	1.712142	0.35188
LOC100736687	53.8803735	17.351846	5.778218396	4.19785327	1.634671	0.35222
LOC102164599	129.312897	43.792754	7.025836058	5.485193456	1.562102	0.35226
LOC100625214	490.725864	161.95056	8.941710429	7.348290508	1.599364	0.35248
AHDC1	786.653454	2587.0776	9.621417212	11.33766515	-1.71752	0.35268
USP28	4406.58563	1303.041	12.10577288	10.34877351	1.757779	0.35307
FIGNL2	1201.94679	380.91433	10.23235712	8.577105241	1.657835	0.35347
LOC100522040	74.6035941	24.788351	6.240382915	4.688647628	1.589583	0.35369
LOC100739570	523.883017	172.69218	9.035852107	7.44038899	1.601042	0.35424
KIAA0391	737.746653	239.62073	9.528935879	7.910617116	1.622373	0.35454
LOC102157491	2.48678647	13.220454	1.801898017	3.829895615	-2.41042	0.35542

LOC102165140	133.938554	51.229259	7.076158798	5.706786332	1.386531	0.64304
LOC102164954	33.1394361	91.716899	5.093367322	6.534760414	-1.46864	0.6431
LOC102160490	173.982039	66.10227	7.451063037	6.068289661	1.396167	0.64316
VSIG10L	13.8080984	40.48764	3.888314478	5.374609695	-1.55197	0.64321
LOC102163743	1264.82181	431.31731	10.30585862	8.755946793	1.552113	0.64329
TSTD3	662.788721	235.48934	9.374580306	7.885631319	1.492887	0.64335
LOC100624813	98.0374983	279.28209	6.629902968	8.130735747	-1.51032	0.64416
LOC100739617	67.659682	189.21775	6.101391269	7.571508044	-1.48368	0.64419
NR3C1	615.841187	2017.7718	9.268755287	10.97926211	-1.71213	0.64434
LOC100738669	1285.53396	4485.039	10.32927382	12.13122645	-1.80275	0.64439
HAUS2	1829.57303	609.79344	10.83807962	9.254540753	1.585114	0.6446
LOC100156062	6.90404918	23.135794	2.982591923	4.593102408	-1.74461	0.64483
LOC102166888	6.90404918	23.135794	2.982591923	4.593102408	-1.74461	0.64483
LOC102164504	6.90404918	23.135794	2.982591923	4.593102408	-1.74461	0.64483
TTC23L	6.90404918	23.135794	2.982591923	4.593102408	-1.74461	0.64483
LOC102158033	6.90404918	23.135794	2.982591923	4.593102408	-1.74461	0.64483
LOC102160136	6.90404918	23.135794	2.982591923	4.593102408	-1.74461	0.64483
LOC100524336	6.90404918	23.135794	2.982591923	4.593102408	-1.74461	0.64483
LOC102167427	6.90404918	23.135794	2.982591923	4.593102408	-1.74461	0.64483
LOC100624862	64.8980623	180.95496	6.042164139	7.507437596	-1.47938	0.6451
LOC102164731	244.403341	741.1717	7.93901108	9.535609179	-1.60054	0.64513
LOC102162789	30.3778164	10.741619	4.971673052	3.553559423	1.499807	0.64535
LOC102167289	30.3778164	10.741619	4.971673052	3.553559423	1.499807	0.64535
STAP1	56.6132033	21.483238	5.848327568	4.490777898	1.397927	0.64568
DNAAF2	1912.42162	636.23435	10.90193909	9.315680218	1.58777	0.64572
LOC102164793	26.2353869	72.712497	4.767410456	6.20383732	-1.47069	0.64588
EPAS1	1311.76934	4575.1033	10.35839774	12.15990392	-1.80229	0.64611
LOC100737441	252.6882	766.78633	7.986912605	9.584561062	-1.60147	0.64664
LOC780439	457.048056	1455.9025	8.839355155	10.50868861	-1.6715	0.64669
FBXL6	3291.85065	1057.6363	11.68512136	10.04799134	1.638055	0.6468
LOC102161673	8.28485901	26.440908	3.214880004	4.77825631	-1.67422	0.64683
LOC100737356	60.7556328	168.56079	5.948498827	7.405658764	-1.47218	0.64685
LOC100623844	310.682213	115.67897	8.283932016	6.86640077	1.425313	0.64689
CHRNA3	77.3253508	29.746021	6.291407421	4.942327829	1.378245	0.6469
LOC100519796	1625.21318	5764.1179	10.66730067	12.4931344	-1.82647	0.64742
LOC100514675	104.941548	40.48764	6.727124677	5.374609695	1.374033	0.64743
LOC100517984	196.074997	582.52625	7.62260094	9.188653752	-1.57092	0.64776
CYP1A1	331.394361	123.11548	8.376752095	6.955539223	1.428537	0.64785
TMEM107	689.024108	245.40468	9.430502957	7.944885826	1.489392	0.64797
ZSWIM8	1859.95085	6665.5876	10.86182424	12.70273277	-1.84147	0.64837
CD164	4161.76085	15964.524	12.02332496	13.96267232	-1.9396	0.64841
STIL	994.183082	346.21064	9.958818149	8.439667339	1.521861	0.64866
LSM7	4222.51648	1336.9184	12.04422896	10.38577442	1.659192	0.64872
LOC102160915	30.3778164	83.454116	4.971673052	6.400095824	-1.45797	0.64879
LOC100514291	303.778164	932.86828	8.251615733	9.867075268	-1.61866	0.64884
TXNRD2	1922.08729	641.19202	10.90920853	9.32686092	1.583845	0.64887
LOC102167747	9.66566885	29.746021	3.414902536	4.942327829	-1.62176	0.64891
EDA	328.632741	122.2892	8.364715736	6.945902605	1.426179	0.64892
DDX31	3160.67371	1020.4538	11.62647277	9.996408222	1.631021	0.64913
LOC100524049	56.6132033	156.16661	5.848327568	7.296150961	-1.46388	0.64916
LOC102161119	89.7526393	34.703692	6.503867696	5.158001345	1.370865	0.64916
LOC100627522	342.440839	127.24687	8.423917793	7.002779797	1.428225	0.64958
STAM	306.539784	941.13107	8.26462924	9.879783965	-1.61832	0.64958
FGFR1	15911.0717	4627.1589	13.95783407	12.17622268	1.781832	0.64984
LOC100622285	2180.29873	7905.8315	11.09097165	12.94888396	-1.85839	0.64991
TUBA8	24.854577	68.581105	4.692347799	6.120623682	-1.4643	0.65002
TMEM206	555.085554	200.78564	9.119163049	7.65667973	1.467054	0.65011
LOC100524810	16.569718	4.9576702	4.135019133	2.574748269	1.740815	0.65018
WNT8B	16.569718	4.9576702	4.135019133	2.574748269	1.740815	0.65018
LOC102163332	16.569718	4.9576702	4.135019133	2.574748269	1.740815	0.65018
LOC100737763	16.569718	4.9576702	4.135019133	2.574748269	1.740815	0.65018
CALU	7637.2592	30741.687	12.89902816	14.90795564	-2.00907	0.65034
LCN15	26.2353869	9.0890621	4.767410456	3.334720158	1.529311	0.65038
RPUSD2	323.109502	120.63664	8.340337505	6.926434087	1.421355	0.6511
DLG5	1819.90736	6487.1115	10.83044181	12.6635829	-1.83371	0.65134
AMMECR1	82.8485901	231.35794	6.389714621	7.86020516	-1.48158	0.65168
TTC21B	842.294	297.46021	9.719891879	8.221394816	1.501627	0.65196
POLA2	5474.911	1709.57	12.41888328	10.74026139	1.679202	0.65223
PDXP	136.700174	52.881816	7.105386569	5.751726565	1.370171	0.65239
PAAF1	2765.7621	903.94854	11.43398288	9.821691943	1.613365	0.6527
CLSPN	1956.60754	654.41247	10.93487585	9.356259313	1.580082	0.65276
JMJD8	502.61478	1601.3275	8.976176814	10.64595332	-1.67174	0.65286
IDS	795.346465	2635.0017	9.637252429	11.3641356	-1.72815	0.65302
PPAN	5168.37122	1621.1582	12.33577309	10.66369878	1.672685	0.65302
LOC100739265	26.2353869	71.886218	4.767410456	6.187574144	-1.4542	0.65304
LOC102162952	12.4272885	36.356248	3.747096094	5.223277669	-1.54869	0.65324
POLE3	4912.9214	1546.7931	12.26265906	10.59599693	1.667301	0.65344
GTSE1	4272.22563	1357.5754	12.06110978	10.40787888	1.653956	0.65346
C14H1orf124	776.015128	275.97698	9.601798876	8.113622247	1.491537	0.65367
TIMM8B	6122.51081	1900.4403	12.58014332	10.89287689	1.68779	0.65375
CTSO	84.2294	234.66306	6.413279271	7.88058181	-1.47819	0.65388
LOC102166590	57.9940131	22.309516	5.882496648	4.542847146	1.378245	0.65396
LOC100739778	42.8051049	116.50525	5.453027102	6.876581411	-1.44454	0.65396
TAPBPL	763.587839	2517.6702	9.578538444	11.29844651	-1.72122	0.65405
LOC100627123	1868.23571	6652.3672	10.86823279	12.69986894	-1.83219	0.65411
ID2	603.413898	1948.3644	9.239393025	10.92878809	-1.69104	0.65461
LOC102159182	49.7091541	135.50965	5.664174303	7.092859162	-1.44681	0.65465
LOC100737543	1246.87128	430.49103	10.28525341	8.753186764	1.534257	0.65472
LOC102159288	38.6626754	104.93735	5.309710091	6.727067559	-1.44051	0.65524
LOC100625251	13.8080984	39.661362	3.888314478	5.34558663	-1.52222	0.65544
WDR7	158.793131	459.41077	7.320061583	8.846777786	-1.53264	0.65572
LOC100511813	95.2758787	37.182527	6.58910248	5.254840671	1.357486	0.65584

CTXN1	220.9880491	889.07553	7.7943382	9.797783952	-2.00834	0.44556
SETMAR	514.9338752	140.46732	9.011042364	7.144325041	1.874153	0.44564
LOC100623612	131.1125267	38.008805	7.045623457	5.285727902	1.7864	0.44568
LOC100521958	82.47400875	309.02811	6.383255151	8.276255224	-1.90573	0.44571
SPSB4	390.1655029	108.24247	8.611635334	6.771389985	1.84982	0.44585
KIAA1598	1803.854602	451.97427	10.8176669	8.823285292	1.996771	0.44594
LOC100155473	244.249949	989.88149	7.938109026	9.952568709	-2.0189	0.44619
LOC100515066	2067.137014	513.11887	11.01411605	9.005958152	2.010269	0.44633
ZBBX	21.14718173	4.9576702	4.46905122	2.57474827	2.092731	0.44663
LOC102165109	21.14718173	4.9576702	4.46905122	2.57474827	2.092731	0.44663
SLA-8	519.1633115	2240.8669	9.022820836	11.13048494	-2.1098	0.44681
ALKBH7	192.4393538	764.30749	7.59573752	9.579895716	-1.98975	0.44697
LOC100513486	139.5713994	40.48764	7.135159284	5.374609695	1.78545	0.44707
HNRNPA1	146346.9565	26954.853	17.15904308	14.71831095	2.440776	0.44736
MTL5	68.72834063	19.830681	6.123673244	4.380638094	1.793171	0.44744
LOC100739334	3792.747044	902.29598	11.88940777	9.819054981	2.07157	0.44761
LOC100624771	323.5518805	90.890621	8.342305305	6.521845711	1.831794	0.44768
LOC100738457	192.4393538	55.360651	7.59573752	5.816616368	1.797471	0.44774
LOC100737380	653.4479155	175.99729	9.354134569	7.467583487	1.892519	0.44788
LOC100738992	277.0280807	1132.0014	8.119086791	10.14593389	-2.03077	0.44788
C12H17orf70	348.9284986	1453.4237	8.450916354	10.50623185	-2.05845	0.44803
DENND2D	39.1222862	10.741619	5.326331909	3.553559423	1.864779	0.44826
CCDC59	3527.349913	844.4565	11.78477793	9.723586711	2.06249	0.44865
RAN	82138.82592	15748.866	16.32579427	13.94305191	2.382816	0.44874
LOC100525350	1294.207522	332.99018	10.33896755	8.383661891	1.958517	0.44875
LOC100155489	360.5594485	1504.6529	8.498089068	10.55617352	-2.06112	0.44877
PDPN	5613.519391	1299.7359	12.45494682	10.34511233	2.110687	0.44884
ESYT2	2005.810187	9710.4234	10.97068845	13.24546705	-2.27535	0.44934
CD101	33.83549077	9.0890621	5.122485983	3.334720158	1.896334	0.44966
LOC100620580	33.83549077	9.0890621	5.122485983	3.334720158	1.896334	0.44966
LOC102158853	64.49890428	237.14189	6.033398867	7.895677624	-1.8784	0.44973
LOC100738407	7295.777697	1659.167	12.83304379	10.69711263	2.136603	0.4498
LPP	9.516231779	37.182527	3.394545939	5.254840671	-1.96616	0.44989
EYA2	9.516231779	37.182527	3.394545939	5.254840671	-1.96616	0.44989
LOC100621532	1686.487743	426.35964	10.72066131	8.739306855	1.983879	0.44991
LOC100625542	13.74566813	51.229259	3.882219287	5.706786332	-1.89799	0.45004
LOC100516295	2355.796045	11553.024	11.2026112	13.4961078	-2.29398	0.45023
LOC102163519	16.91774539	61.970878	4.163317207	5.976612875	-1.87305	0.45033
SEMA4F	468.4100754	1994.636	8.874704998	10.96263288	-2.09028	0.45055
LINS	2078.767964	518.07654	11.02220686	9.019803473	2.004492	0.4506
WDR13	309.8062124	1273.295	8.27987153	10.31548355	-2.03913	0.45068
DPYD	236.8484354	951.87268	7.893898725	9.896139655	-2.0068	0.45076
HAGHL	58.15474976	212.35354	5.886422108	7.737102248	-1.8685	0.45115
LOC100738707	1484.532158	379.26177	10.53676412	8.570849103	1.968742	0.45124
LOC102159419	8.458872693	0.8262784	3.241668254	0.868906686	3.355766	0.45126
LOC102159773	8.458872693	0.8262784	3.241668254	0.868906686	3.355766	0.45126
LOC100622032	8.458872693	0.8262784	3.241668254	0.868906686	3.355766	0.45126
MBP	8.458872693	0.8262784	3.241668254	0.868906686	3.355766	0.45126
LOC100623741	8.458872693	0.8262784	3.241668254	0.868906686	3.355766	0.45126
LOC100157899	8.458872693	0.8262784	3.241668254	0.868906686	3.355766	0.45126
LOC102165100	8.458872693	0.8262784	3.241668254	0.868906686	3.355766	0.45126
RP2	209.3570991	60.318321	7.716696697	5.938246292	1.795298	0.45163
KIAA1841	227.3322036	65.275991	7.834990539	6.05041444	1.800178	0.45168
IREB2	3068.456069	744.47681	11.58376731	9.54201967	2.043214	0.45188
LOC100517757	177.6363265	696.55267	7.480881679	9.446158339	-1.97131	0.45195
DNAJC19	7270.401079	1658.3407	12.82801766	10.69639441	2.132295	0.4526
PPP2R1B	18447.74398	3937.2164	14.17123498	11.94332669	2.228196	0.45267
LOC100738634	28.54869534	7.4365053	4.885022527	3.076645515	1.940728	0.45284
LOC102160332	28.54869534	7.4365053	4.885022527	3.076645515	1.940728	0.45284
LOC102158714	138.5140403	40.48764	7.124266508	5.374609695	1.774479	0.45295
LOC100523137	103.6211905	390.00339	6.709031281	8.611037311	-1.91217	0.45309
SNRPG	21546.86347	4551.1413	14.39525721	12.15232962	2.243178	0.45329
STYK1	17.97510447	65.275991	4.246035924	6.05041444	-1.86055	0.45361
LOC102166770	84.58872693	24.788351	6.419348884	4.688647628	1.770803	0.45395
TCTN1	327.7813168	1346.8337	8.360984509	10.39642684	-2.03877	0.45463
PRDM5	1980.433569	497.41958	10.95232888	8.961216934	1.993281	0.45474
PIGQ	619.6124247	2688.7098	9.27754877	11.39323482	-2.11748	0.45495
LOC100622510	3138.241769	762.65494	11.61620043	9.576777083	2.040854	0.45511
ATN1	2059.735501	9926.0821	11.00894363	13.27715401	-2.26877	0.4552
LOC100738488	115.2521404	33.877413	6.861113469	5.124221138	1.766398	0.45522
LOC100514810	6404.423987	1478.212	12.64507835	10.53061313	2.115216	0.45533
LOC102164618	366.903603	103.2848	8.523183995	6.704385034	1.828773	0.4555
BACE1	555.1135205	2383.8131	9.119235603	11.21966049	-2.10242	0.45564
EIF3M	21353.36675	4523.8741	14.3822435	12.14366193	2.238833	0.45581
BAX	660.8494291	2879.5801	9.370359231	11.49214367	-2.12346	0.45615
SLC22A17	664.0215064	2892.8006	9.377257187	11.49874979	-2.12316	0.45666
EFR3B	1997.351315	502.37725	10.96459452	8.975496206	1.991245	0.4567
LOC102158883	57.09739067	16.525567	5.860401464	4.131389251	1.788725	0.45674
LOC102165187	57.09739067	16.525567	5.860401464	4.131389251	1.788725	0.45674
LOC102165234	57.09739067	16.525567	5.860401464	4.131389251	1.788725	0.45674
LOC102157721	280.2001579	80.149002	8.135453595	6.342501448	1.805701	0.45674
GTF2A1L	15.8603863	3.3051135	4.075565686	2.106051269	2.262656	0.45678
LOC102157414	15.8603863	3.3051135	4.075565686	2.106051269	2.262656	0.45678
LOC100626394	15.8603863	3.3051135	4.075565686	2.106051269	2.262656	0.45678

LOC100626947	2.48678647	13.220454	1.801898017	3.829895615	-2.41042	0.35542
RDH5	2.48678647	13.220454	1.801898017	3.829895615	-2.41042	0.35542
LOC102166212	2.48678647	13.220454	1.801898017	3.829895615	-2.41042	0.35542
RHOB	536.316949	1721.9641	9.069629536	10.75067695	-1.6829	0.35566
LOC100523649	2444.5111	747.78193	11.2559203	9.548401802	1.708856	0.3557
UTP14A	11235.3013	3163.8199	13.45587959	11.62790768	1.828299	0.35588
ARHGAP11A	3307.42601	995.66544	11.6919293	9.96096549	1.731976	0.35607
SCAPER	739.404511	240.44701	9.532169874	7.915562765	1.620645	0.3561
NUMBL	1206.09144	4052.0691	10.23731925	11.98479907	-1.74832	0.35651
LOC102157577	19.8942918	4.9576702	4.385036953	2.574748269	2.00462	0.35672
LOC102160552	19.8942918	4.9576702	4.385036953	2.574748269	2.00462	0.35672
AMH	19.8942918	4.9576702	4.385036953	2.574748269	2.00462	0.35672
DNAJC21	3215.41491	970.05081	11.65123781	9.923402974	1.728873	0.35688
LOC100621800	896.900987	289.19743	9.810412555	8.180890933	1.632894	0.3569
LOC100627422	2581.28436	788.26957	11.33443216	9.624374311	1.711328	0.35726
DCBLD1	4837.62862	1427.809	12.2403825	10.48059738	1.760497	0.35728
CDCP1	148.378259	446.19032	7.222826382	8.804745152	-1.58838	0.3576
LOC100621992	347.321177	1087.3823	8.444274378	10.08796973	-1.64652	0.35762
BIRC3	1138.11928	3807.4907	10.1537031	11.89500367	-1.74219	0.35763
LOC606743	63.8275194	187.56519	6.018534465	7.558919565	-1.55514	0.35765
CASP10	21.5521494	65.275991	4.495193036	6.05041444	-1.59872	0.35767
LRRC6	21.5521494	65.275991	4.495193036	6.05041444	-1.59872	0.35767
LOC100620831	80.4060959	237.14189	6.347064926	7.895677624	-1.56037	0.3577
ADAT3	233.757928	79.322724	7.875030072	6.327736286	1.559209	0.35772
LOC102161197	906.848133	292.50254	9.82630717	8.197229196	1.632412	0.35796
CEP135	909.33492	293.32882	9.830253612	8.201285014	1.632293	0.35822
LOC102157709	26.5257224	7.4365053	4.78270852	3.076645515	1.834695	0.35832
LOC100737379	26.5257224	7.4365053	4.78270852	3.076645515	1.834695	0.35832
LOC100737969	206.403277	70.233662	7.69629488	6.154487245	1.555231	0.35836
RARRES2	25.6967935	76.843889	4.73859457	6.282511874	-1.58034	0.35841
LOC102160025	201.429704	68.581105	7.661277193	6.120623682	1.554393	0.35861
LOC102157714	336.545102	113.20014	8.398936476	6.83542057	1.571924	0.35863
LOC100514086	447.621565	1418.72	8.80935516	10.47139067	-1.66424	0.3592
LOC100155489	473.318358	1504.6529	8.889711898	10.55617352	-1.66855	0.35928
LOC100624626	58.0250177	19.004403	5.883254663	4.322245637	1.610341	0.35986
LOC100154916	33.1571529	9.9153405	5.094115824	3.448285225	1.741586	0.35997
MFSD12	1062.68675	3533.1663	10.05485763	11.78715422	-1.73325	0.3604
RFC4	3683.75969	1106.3867	11.84735482	10.11294344	1.735323	0.36068
SETD7	1569.16226	5331.1481	10.61669794	12.38050112	-1.76445	0.36154
MBLAC2	381.307259	128.07315	8.578588783	7.012045083	1.573986	0.36183
CLHC1	220.495067	75.191332	7.791130759	6.251554969	1.552108	0.36191
LOC102159586	126.82611	377.60922	6.998038744	8.564565719	-1.57404	0.36197
GTF2H3	1245.05109	396.61362	10.28314751	8.63522336	1.650399	0.36209
LOC102166906	4.97357294	19.830681	2.578594101	4.380638094	-1.99538	0.36209
LOC102166568	13.2628612	42.140197	3.834191515	5.430960858	-1.66781	0.36282
LOC100737277	1763.13161	551.95395	10.78474248	9.111015534	1.67552	0.36297
PLA2G12A	472.48943	1497.2164	8.887188408	10.54903031	-1.66393	0.36298
GSTZ1	263.599366	809.7528	8.047665794	9.663118299	-1.61913	0.36319
SNPH	33.1571529	97.500848	5.094115824	6.622064238	-1.55609	0.36361
PCDHA13	10.7760747	1.6525567	3.557786824	1.407383613	2.70506	0.3638
LOC102168084	10.7760747	1.6525567	3.557786824	1.407383613	2.70506	0.3638
LOC102157995	10.7760747	1.6525567	3.557786824	1.407383613	2.70506	0.3638
TMC1	10.7760747	1.6525567	3.557786824	1.407383613	2.70506	0.3638
LOC102164988	10.7760747	1.6525567	3.557786824	1.407383613	2.70506	0.3638
LOC102158902	10.7760747	1.6525567	3.557786824	1.407383613	2.70506	0.3638
LOC100517136	10.7760747	1.6525567	3.557786824	1.407383613	2.70506	0.3638
LOC102158441	10.7760747	1.6525567	3.557786824	1.407383613	2.70506	0.3638
LOC102163191	10.7760747	1.6525567	3.557786824	1.407383613	2.70506	0.3638
LOC102165959	10.7760747	1.6525567	3.557786824	1.407383613	2.70506	0.3638
LOC102165069	10.7760747	1.6525567	3.557786824	1.407383613	2.70506	0.3638
ASAH2B	10.7760747	1.6525567	3.557786824	1.407383613	2.70506	0.3638
NPAS3	7.46035941	26.440908	3.080718953	4.77825631	-1.82545	0.36403
LOC100739867	840.533827	2748.2019	9.716877454	11.42479713	-1.70911	0.36439
FZD6	429.385131	1351.7914	8.749484425	10.4017237	-1.65453	0.36449
LOC100156975	1261.62967	402.39757	10.30221584	8.656058572	1.648595	0.36449
LOC100625808	4750.59109	1411.2835	12.21419497	10.46381397	1.751099	0.36459
LOC102167139	46.4200141	14.873011	5.567424187	3.98850389	1.64205	0.36459
NPM1	141295.892	35005.283	17.1083702	15.09532627	2.013075	0.3648
LOC102160209	73.7746653	24.788351	6.224477643	4.688647628	1.573463	0.36487
BRCA2	581.079105	192.52286	9.18507142	7.59636019	1.593705	0.36549
LOC100153723	1189.51286	380.91433	10.21736749	8.577105241	1.642832	0.36572
FAM83D	1366.90363	434.62242	10.41775088	8.766934408	1.653077	0.36578
USP25	1119.05391	3714.9476	10.12935246	11.85951443	-1.73106	0.36597
LOC102160425	146.720402	50.402981	7.206725282	5.683780114	1.541489	0.36611
NEK1	3994.608	1199.7562	11.96419934	10.22972754	1.735313	0.36651
R3HDM4	981.451727	3231.5747	9.940242712	11.658468	-1.71925	0.36672
AGPAT2	359.755109	1119.6072	8.494876018	10.13006494	-1.63791	0.36682
STYXL1	53.0514447	17.351846	5.756261274	4.19785327	1.612303	0.36735
LOC102167874	53.0514447	17.351846	5.756261274	4.19785327	1.612303	0.36735
FAM76A	619.209831	1984.7206	9.276612585	10.95544696	-1.68044	0.36738
ACTB	133392.884	577885.05	17.025333	19.14042551	-2.1151	0.36739
WDR75	3076.15486	937.82595	11.58738134	9.874713913	1.713736	0.3674
PHACTR3	875.348838	285.06604	9.77536145	8.160204421	1.618562	0.36761
TBC1D30	460.884426	154.51406	8.851388091	7.280901168	1.576667	0.368
COLEC11	24.0389359	6.610227	4.646101346	2.927939483	1.862601	0.36821
LOC102159074	1.65785765	10.741619	1.410263837	3.553559423	-2.69582	0.36839
LOC100622882	1.65785765	10.741619	1.410263837	3.553559423	-2.69582	0.36839
LOC102159385	1.65785765	10.741619	1.410263837	3.553559423	-2.69582	0.36839
LOC102157878	1.65785765	10.741619	1.410263837	3.553559423	-2.69582	0.36839
GRIA2	1.65785765	10.741619	1.410263837	3.553559423	-2.69582	0.36839
RFX4	1.65785765	10.741619	1.410263837	3.553559423	-2.69582	0.36839
LOC100739690	133.457541	395.78734	7.071006856	8.632222186	-1.56834	0.3685
LOC100516884	251.165434	85.932951	7.978226717	6.441831208	1.547355	0.36859
LOC102158746	80.4060959	27.267186	6.347064926	4.821056379	1.560139	0.36872
LOC102163984	17.4075053	4.1313919	4.202222212	2.359350201	2.07501	0.36952
KREMEN2	17.4075053	4.1313919	4.202222212	2.359350201	2.07501	0.36952
LOC100623087	17.4075053	4.1313919	4.202222212	2.359350201	2.07501	0.36952
LOC100519345	117.707893	40.48764	6.891272054	5.374609695	1.539658	0.36965
LOC102158451	43.9332277	14.046732	5.489710792	3.911378308	1.645078	0.36976
LOC100521043	43.9332277	14.046732	5.489710792	3.911378308	1.645078	0.36976
CFB	776.706308	2516.8439	9.60308163	11.29797314	-1.69617	0.36982
ETV6	392.912262	1225.3708	8.621730518	10.26017957	-1.64094	0.36994
MTFP1	2185.05638	680.0271	11.09411489	9.411568398	1.684006	0.37009
LOC100515297	902.703489	2949.8138	9.819705682	11.52689717	-1.7083	0.37012
LOC102159655	68.8010924	23.135794	6.125177709	4.593102408	1.572305	0.3702
LOC100152318	55.5382312	161.12428	5.821154844	7.340956379	-1.53662	0.37025
ADAR	2546.46935	8834.5684	11.31484907	13.10910722	-1.79466	0.37067
MCM3	8791.6191	2536.6746	13.10207726	11.30929137	1.79319	0.37078
TRUB1	206.403277	71.05994	7.69629488	6.171125545	1.538358	0.37114
CIC	1525.22904	5133.6675	10.57575576	12.32605515	-1.75096	0.37122
FKBP10	3118.43023	10941.578	11.60706683	13.41766507	-1.81093	0.3713
LOC100154269	2398.92002	743.65053	11.22877061	9.540419716	1.689688	0.37151
LOC102159703	50.5646582	16.525567	5.688310694	4.13138925	1.61343	0.37172
LOC102160817	50.5646582	16.525567	5.688310694	4.13138925	1.61343	0.37172
LOC102165334	50.5646582	16.525567	5.688310694	4.13138925	1.61343	0.37172
RNF145	1048.59496	3451.3648	10.03561698	11.75336918	-1.71871	0.37192

LOC102162761	0	5.7839486	0	2.762125239		-20	0.67347		LOC100519853
BCL2A1	0	5.7839486	0	2.762125239		-20	0.67347		FBXW4
LOC102158608	0	5.7839486	0	2.762125239		-20	0.67347		CA8
CCIN	0	5.7839486	0	2.762125239		-20	0.67347		LOC102159340
LOC102158456	0	5.7839486	0	2.762125239		-20	0.67347		LOC102159405
CYP19A1	0	5.7839486	0	2.762125239		-20	0.67347		LOC102161092
LOC102158576	0	5.7839486	0	2.762125239		-20	0.67347		IL1RAPL2
TRNAM-CAU	0	5.7839486	0	2.762125239		-20	0.67347		LOC100738112
LOC102162608	0	5.7839486	0	2.762125239		-20	0.67347		PAGE2B
LOC100625891	0	5.7839486	0	2.762125239		-20	0.67347		LOC102158040
LOC102165348	0	5.7839486	0	2.762125239		-20	0.67347		GPR50
LOC100153213	0	5.7839486	0	2.762125239		-20	0.67347		SLC24A3
LOC102167502	0	5.7839486	0	2.762125239		-20	0.67347		PEX5L
LOC102163245	0	5.7839486	0	2.762125239		-20	0.67347		SHC4
LOC102165525	0	5.7839486	0	2.762125239		-20	0.67347		LOC100518740
LOC100155545	0	5.7839486	0	2.762125239		-20	0.67347		RUFY3
LOC102167728	0	5.7839486	0	2.762125239		-20	0.67347		LOC100624079
LOC100521167	0	5.7839486	0	2.762125239		-20	0.67347		DHDDS
LOC102167154	0	5.7839486	0	2.762125239		-20	0.67347		LOC102166268
LOC102165988	0	5.7839486	0	2.762125239		-20	0.67347		PPM1D
LOC100739562	0	5.7839486	0	2.762125239		-20	0.67347		LOC102158880
LOC102165978	0	5.7839486	0	2.762125239		-20	0.67347		LOC100627889
LOC102167472	0	5.7839486	0	2.762125239		-20	0.67347		SPOPL
LYPD1	0	5.7839486	0	2.762125239		-20	0.67347		SRSF7
LOC102163014	0	5.7839486	0	2.762125239		-20	0.67347		ABCB10
LOC100620761	0	5.7839486	0	2.762125239		-20	0.67347		LOC102163583
LOC100737835	0	5.7839486	0	2.762125239		-20	0.67347		CAV2
LOC100521937	0	5.7839486	0	2.762125239		-20	0.67347		CLSTN1
COL19A1	0	5.7839486	0	2.762125239		-20	0.67347		LOC100739072
LOC102167979	0	5.7839486	0	2.762125239		-20	0.67347		LOC100623982
RAB42	178.124469	70.233662	7.484818616	6.154487245		1.342651	0.67361		LOC102162975
TUBE1	483.283443	180.95496	8.919707868	7.507437596		1.417239	0.67365		RBBP9
HOXA4	56.6132033	150.38266	5.848327568	7.242056188		-1.40943	0.67368		LOC102166458
FAM98C	505.3764	1567.4501	8.984066358	10.61512389		-1.63299	0.67375		PLXDC1
LOC102166983	179.505279	510.64003	7.495897217	8.998985343		-1.50828	0.6741		LOC102166426
LOC100523614	214.025525	617.22994	7.748364114	9.271999722		-1.52803	0.67417		CCDC60
LOC102167663	248.54577	96.674569	7.963160641	6.609911086		1.362303	0.67443		C5H12orf29
LOC100625271	147.746652	413.96546	7.21671339	8.696847462		-1.48638	0.67449		CCDC62
MRPS24	2623.53869	879.16019	11.35784815	9.781622305		1.577316	0.67455		LGMN
LOC100626673	368.676226	140.46732	8.530118456	7.144325041		1.39212	0.6748		LOC102164851
RFK	1012.13361	361.90993	9.984608731	8.50346771		1.483697	0.67502		ZC3H3
FAM217B	55.2323934	146.25127	5.813329549	7.202136284		-1.40486	0.67514		LOC100524954
LOC100736628	227.833623	659.37014	7.838155235	9.367131078		-1.53311	0.67522		PIWIL4
HOXA2	98.0374983	266.88791	6.629902968	8.065485684		-1.44483	0.67522		SRRM5
LOC414432	3373.31843	1112.1707	11.72038041	10.12045911		1.60079	0.67532		ND1
DHRS4	5103.47315	1636.8575	12.31754635	10.67759409		1.640551	0.67569		LOC102158252
LOC100519232	1600.3586	555.25907	10.6450807	9.119613134		1.527162	0.67571		WDR34
DLGAP2	1361.4785	477.5889	10.41201775	8.902643123		1.511333	0.67573		FSD2
RAB3D	1496.79786	5090.701	10.54862722	12.313932		-1.76598	0.67588		LOC102162036
RASIP1	341.060029	1019.6275	8.418105721	9.99524072		-1.57994	0.67594		LOC102166157
LOC102159090	1.38080984	9.0890621	1.251452392	3.334720158		-2.71862	0.676		LOC102164752
LOC102159482	1.38080984	9.0890621	1.251452392	3.334720158		-2.71862	0.676		LOC100626975
LOC102159609	1.38080984	9.0890621	1.251452392	3.334720158		-2.71862	0.676		LOC100623063
LOC102163881	1.38080984	9.0890621	1.251452392	3.334720158		-2.71862	0.676		LOC102161982
LOC102161195	1.38080984	9.0890621	1.251452392	3.334720158		-2.71862	0.676		LOC100628051
LOC100738609	1.38080984	9.0890621	1.251452392	3.334720158		-2.71862	0.676		MDK
LOC102158101	1.38080984	9.0890621	1.251452392	3.334720158		-2.71862	0.676		LOC100739034
LOC102160482	1.38080984	9.0890621	1.251452392	3.334720158		-2.71862	0.676		LOC100526036
LOC102160353	1.38080984	9.0890621	1.251452392	3.334720158		-2.71862	0.676		LOC102167889
LOC102160333	1.38080984	9.0890621	1.251452392	3.334720158		-2.71862	0.676		LOC100627469
LOC100627488	1.38080984	9.0890621	1.251452392	3.334720158		-2.71862	0.676		LOC102163968
FER1L5	1.38080984	9.0890621	1.251452392	3.334720158		-2.71862	0.676		LOC100738571
LOC100739509	1.38080984	9.0890621	1.251452392	3.334720158		-2.71862	0.676		LOC100521048
GFRA2	27.6161967	72.712497	4.838760036	6.20383732		-1.39669	0.67605		EXOC7
LOC100623573	2162.3482	735.38775	11.07905018	9.524321819		1.556022	0.67614		MRPL19
LOC100738489	48.3283443	127.24687	5.624344958	7.002779797		-1.39669	0.67626		LOC100517015
MGST2	190.551757	75.191332	7.581590451	6.251554969		1.341545	0.67628		DEPDC7
CNNM2	45.5667246	119.81036	5.541227504	6.916600414		-1.3947	0.67631		LOC102158199
SLC5A1	22.0929574	58.665764	4.529381037	5.89883146		-1.40893	0.67633		LOC100623365
LOC100738240	60.7556328	161.12428	5.948498827	7.340956379		-1.40708	0.67633		LOC100737852
FHL3	863.006147	2794.4735	9.754897767	11.44887693		-1.69513	0.67654		LOC100524619
SLC44A1	11964.7172	3629.8409	13.54661925	11.82608799		1.720808	0.67657		NMD3
INTS12	448.763197	169.38707	8.813021802	7.412672017		1.405631	0.6767		LOC102158599
PCOLCE	2575.21034	9169.2111	11.33103468	13.16273923		-1.83211	0.67692		SPATA5
RRAGC	1973.17726	6862.2419	10.94703582	12.74467448		-1.79816	0.67729		LOC100738417
LOC100620451	129.796125	358.60481	7.031175985	8.490268521		-1.46615	0.67769		PCNP
DTX3	400.434852	1210.4978	8.649022066	10.24257609		-1.59596	0.67784		LOC100523762
USP45	466.713725	175.99729	8.869481953	7.467583487		1.406985	0.67794		LOC102163346
IQSEC1	520.565308	1608.764	9.026704102	10.65263347		-1.6278	0.67833		CTR1
LOC100737115	52.4707738	137.98849	5.740678649	7.118821585		-1.39496	0.67838		RSBN1
LOC100739066	34.5202459	13.220454	5.150569664	3.829895615		1.384671	0.67859		RAB5C
SLC35A5	158.793131	444.53776	7.320061583	8.79940391		-1.48516	0.67869		PLSCR1
LPIN1	886.479915	2868.8385	9.793570658	11.48675384		-1.69431	0.67878		PSMG2
LOC100620231	318.967072	123.11548	8.321779634	6.955539223		1.373395	0.6788		SORT1
KIAA1109	512.280449	1579.8442	9.003603499	10.62647952		-1.62478	0.67884		LOC100739861
[illegible]	[illegible]	[illegible]	[illegible]	[illegible]	[illegible]	[illegible]	[illegible]	[illegible]	[illegible]

PTPN20B	4.14242951	0	2.362450112	0	20	0.6958
LOC100621389	4.14242951	0	2.362450112	0	20	0.6958
LOC100623971	4.14242951	0	2.362450112	0	20	0.6958
LOC102160165	4.14242951	0	2.362450112	0	20	0.6958
ESPNL	4.14242951	0	2.362450112	0	20	0.6958
LOC102167081	4.14242951	0	2.362450112	0	20	0.6958
CLDN18	4.14242951	0	2.362450112	0	20	0.6958
LOC102158259	4.14242951	0	2.362450112	0	20	0.6958
LOC102162630	4.14242951	0	2.362450112	0	20	0.6958
OSBP2	4.14242951	0	2.362450112	0	20	0.6958
LOC100521383	4.14242951	0	2.362450112	0	20	0.6958
LOC100739206	4.14242951	0	2.362450112	0	20	0.6958
LOC100155636	4.14242951	0	2.362450112	0	20	0.6958
LOC100625692	4.14242951	0	2.362450112	0	20	0.6958
LOC102163454	4.14242951	0	2.362450112	0	20	0.6958
TSACC	4.14242951	0	2.362450112	0	20	0.6958
CYP7A1	4.14242951	0	2.362450112	0	20	0.6958
DMRTC2	4.14242951	0	2.362450112	0	20	0.6958
LOC102167956	4.14242951	0	2.362450112	0	20	0.6958
LOC100737384	4.14242951	0	2.362450112	0	20	0.6958
LOC102165114	4.14242951	0	2.362450112	0	20	0.6958
LOC102165241	4.14242951	0	2.362450112	0	20	0.6958
LOC100738703	245.784151	98.327126	7.947105933	6.634115866	1.321731	0.69595
DRAM2	1448.46952	517.25026	10.50130928	9.017505127	1.485595	0.69608
NCAPG	4214.23162	1398.063	12.04139619	10.45024522	1.59184	0.69642
NIPAL1	28.9970066	73.538775	4.906746634	6.219919206	-1.3426	0.69658
TRIM25	299.635734	860.15579	8.231872692	9.750130438	-1.52139	0.69659
LOC102160364	593.748229	1809.5496	9.216135261	10.82221201	-1.60771	0.6968
ROM1	132.557744	356.12598	7.061319821	8.480289273	-1.42577	0.69686
PAP7	680.739249	2097.9208	9.413076234	11.035432	-1.62379	0.69756
FANCI	1778.48307	627.14528	10.79724249	9.294954469	1.503776	0.69758
NR2F2	921.00016	2915.9364	9.848623191	11.5102382	-1.66269	0.69763
GFPT2	1864.09328	655.23875	10.86503207	9.358076973	1.508381	0.69766
LOC100626494	3202.09801	1084.0772	11.64525223	10.08358201	1.56255	0.69771
LOC100624495	432.193479	167.73451	8.758867714	7.398611253	1.365498	0.69784
LMO4	2442.6526	8430.5182	11.25482348	13.04157672	-1.78717	0.69788
GCKR	284.446826	113.20014	8.157078211	6.83542057	1.329283	0.69797
EMG1	5987.19145	1942.5805	12.54790463	10.92450111	1.623905	0.69817
LOC100738361	150.508272	407.35524	7.243252754	8.673680921	-1.43644	0.69819
DNAJC13	1619.68994	5386.5087	10.66239239	12.39540258	-1.73363	0.69851
NDC80	2112.63905	737.04031	11.04551331	9.5275558	1.519231	0.69876
LOC102166739	8.28485901	23.962073	3.214880004	4.641665831	-1.5322	0.6988
LOC102163224	8.28485901	23.962073	3.214880004	4.641665831	-1.5322	0.6988
MTUS1	506.75721	195.0017	8.987995009	7.614722326	1.377808	0.69913
LOC100524253	67.659682	28.093465	6.101391269	4.862623207	1.268062	0.69914
LOC102167175	67.659682	28.093465	6.101391269	4.862623207	1.268062	0.69914
LOC100511061	38.6626754	97.500848	5.309710091	6.622064238	-1.33447	0.69918
PRKG1	98.0374983	256.97257	6.629902968	8.011073883	-1.39021	0.6992
KCTD14	314.824643	124.76803	8.302979933	6.974621475	1.3353	0.69921
LOC100621529	561.989603	214.83238	9.13696447	7.753767487	1.387332	0.69924
KLC3	292.731685	116.50525	8.198355089	6.876581411	1.329184	0.69926
USH1G	99.4183082	41.313919	6.649878514	5.403060391	1.266884	0.69952
LOC102160164	15.1889082	4.9576702	4.016933786	2.574748269	1.615284	0.69958
LOC102167771	70.4213016	180.95496	6.158282521	7.507437596	-1.36155	0.69959
LOC100510914	1291.0572	4201.6255	10.33545422	12.03707519	-1.70239	0.69959
LOC100737635	274.781157	109.89502	8.107380078	6.793050813	1.322157	0.70026
LOC100627548	23.4737672	59.492043	4.613164285	5.918673475	-1.34165	0.70029
LOC100623232	1411.18765	507.33492	10.46371609	8.98963553	1.475899	0.70046
MOGAT1	77.3253508	32.224857	6.291407421	5.054191063	1.262768	0.70048
LOC100524777	563.370413	215.65866	9.140498547	7.75928006	1.385334	0.70051
PPIH	4680.94534	1548.4457	12.19289238	10.59753645	1.595979	0.70061
CRY2	316.205452	906.42737	8.309273759	9.825638371	-1.51933	0.70068
RSPO3	56.6132033	143.77244	5.848327568	7.177643143	-1.34458	0.70072
LOC100511626	71.8021115	184.26008	6.185908388	7.533408207	-1.35965	0.70095
PHGK	15.1889082	39.661362	4.016933786	5.34558663	-1.38472	0.70114
LOC100513639	15.1889082	39.661362	4.016933786	5.34558663	-1.38472	0.70114
ALG13	1568.59997	560.21674	10.61618121	9.132414222	1.48542	0.70115
CRLF1	1333.8623	4342.9191	10.38247521	12.08478153	-1.70306	0.7014
JPH4	484.664252	187.56519	8.923815489	7.558919565	1.369594	0.7014
KIRREL	938.950688	2962.208	9.876441262	11.53294417	-1.65755	0.70146
LOC100521892	60.7556328	154.51406	5.948498827	7.280901168	-1.34665	0.70156
LOC102158524	41.4242951	104.11107	5.406818783	6.715770874	-1.32957	0.70159
LOC100620498	2945.26738	1007.2333	11.52467265	9.977613844	1.548001	0.70173
LOC100627437	1367.00174	493.28819	10.41785435	8.94920862	1.470512	0.7018
FBXO33	869.910197	323.90112	9.766380153	8.343856914	1.425313	0.7018
PDGFD	109.083977	286.7186	6.782460686	8.168514654	-1.3942	0.70185
CREG1	824.343472	2567.2469	9.688850821	11.32656819	-1.6389	0.70234
LOC102163072	33.1394361	13.220454	5.093367322	3.829895615	1.325777	0.70244
ZNF585A	1226.15913	446.19032	10.26110663	8.804745152	1.458415	0.7025
CHRD	1226.15913	446.19032	10.26110663	8.804745152	1.458415	0.7025
LOC100520931	1760.53254	624.66645	10.78261541	9.289249932	1.494854	0.70252
LOC102167299	42.8051049	107.41619	5.453027102	6.76043638	-1.32736	0.70278
LOC100524210	60186.7391	16870.952	15.877182	14.04233926	1.834904	0.70282
LOC102163212	187.790138	76.843889	7.560639591	6.282511874	1.289119	0.70283
LOC100627329	515.042069	1536.0515	9.011344871	10.58594978	-1.57646	0.70306
PRX	161.554751	436.27498	7.34478191	8.772396993	-1.43321	0.70308
ADRA2B	27.6161967	10.741619	4.838760036	3.553559423	1.362303	0.70313
KIF15	1086.69734	399.09245	10.08706146	8.644189607	1.445155	0.70318
PFDN2	5578.47174	1828.554	12.44591282	10.83727631	1.609167	0.70341
LOC102157821	30.3778164	76.01761	4.971673052	6.267116453	-1.32332	0.70342
CDV3	327.251931	936.99967	8.358659688	9.87344361	-1.51765	0.70346
AIFM3	6.90404918	20.656959	2.982591923	4.436758793	-1.58111	0.70352
LOC100520139	6.90404918	20.656959	2.982591923	4.436758793	-1.58111	0.70352
LOC102162065	48.3283443	121.46292	5.624344958	6.936201185	-1.32957	0.70357
LY6G5B	48.3283443	121.46292	5.624344958	6.936201185	-1.32957	0.70357
C13H3orf33	556.466364	214.0061	9.122740949	7.74823377	1.378643	0.70362
LOC102162283	38.6626754	96.674569	5.309710091	6.609911086	-1.32219	0.70388
LOC102161743	2.76161967	11.567897	1.911353989	3.65167138	-2.06654	0.70402
LOC102159642	2.76161967	11.567897	1.911353989	3.65167138	-2.06654	0.70402
LOC102157764	2.76161967	11.567897	1.911353989	3.65167138	-2.06654	0.70402
ACTRT3	2.76161967	11.567897	1.911353989	3.65167138	-2.06654	0.70402
LOC102158109	2.76161967	11.567897	1.911353989	3.65167138	-2.06654	0.70402
LOC102160900	2.76161967	11.567897	1.911353989	3.65167138	-2.06654	0.70402
BMP8B	2.76161967	11.567897	1.911353989	3.65167138	-2.06654	0.70402
LOC102160015	2.76161967	11.567897	1.911353989	3.65167138	-2.06654	0.70402
GPR33	2.76161967	11.567897	1.911353989	3.65167138	-2.06654	0.70402
LOC102162434	2.76161967	11.567897	1.911353989	3.65167138	-2.06654	0.70402
LOC100737230	2.76161967	11.567897	1.911353989	3.65167138	-2.06654	0.70402
LOC100515384	2.76161967	11.567897	1.911353989	3.65167138	-2.06654	0.70402
LOC102158189	44.1859147	18.178124	5.497801224	4.261389712	1.281383	0.70404
CACYBP	10902.8745	3417.4873	13.41255324	11.73914237	1.673701	0.7041
LOC102166252	31.7586262	79.322724	5.033802952	6.327736286	-1.32059	0.70421
LOC100511354	310.682213	884.11786	8.283932016	9.78972576	-1.5088	0.70452
LOC100523528	212.644715	585.83137	7.739069817	9.196802174	-1.46204	0.70454
LOC102161725	95.2758787	247.05723	6.58910248	7.954529215	-1.37466	0.70459
GLCE	182.266898	495.76702	7.517802419	8.956425596	-1.44361	0.70461
LOC100513945	532.992597	1590.5859	9.06067593	10.63624928	-1.57737	0.70474
KIF1B	2247.95841	7632.3333	11.13504127	12.89809747	-1.76351	0.70477
LOC100623965	1645.92532	588.3102	10.68555943	9.202883429	1.48425	0.70486
LOC102158554	53.8515836	135.50965	5.777461367	7.092859162	-1.33133	0.70487
LOC100620270	180.886089	74.365053	7.506891393	6.2358238	1.282385	0.70493
DVL3	1357.33607	4404.89	10.40762475	12.10521776	-1.69833	0.70495
LOC100627260	33.1394361	82.627837	5.093367322	6.385911347	-1.31808	0.70504
TFAP2A	792.584846	298.28649	9.632240666	8.225383356	1.409867	0.70543
KIAA2026	389.388374	1128.6963	8.608766278	10.14171921	-1.53538	0.70548
LOC100623194	171.22042	462.71589	7.428112398	8.857097348	-1.43427	0.70552
GPR68	144.985033	386.69828	7.189676653	8.598790517	-1.4153	0.70559
RBM34	2133.3512	749.43448	11.05958187	9.551582312	1.509247	0.70566
USH1C	22.0929574	8.2627837	4.529381037	3.211445828	1.418887	0.70576
CNBD2	22.0929574	8.2627837	4.529381037	3.211445828	1.418887	0.70576
LOC100524620	22.0929574	8.2627837	4.529381037	3.211445828	1.418887	0.70576
LOC102166690	22.0929574	8.2627837	4.529381037	3.211445828	1.418887	0.70576
LOC100517177	22.0929574	8.2627837	4.529381037	3.211445828	1.418887	0.70576
LOC100737951	22.0929574	8.2627837	4.529381037	3.211445828	1.418887	0.70576
VDAC2	15869.6474	4860.1694	13.95407336	12.24708769	1.707192	0.70587
LOC100515833	74.5637311	190.04403	6.239622035	7.577761331	-1.34979	0.70612
CAMTA2	1053.55791	3334.0332	10.0424226	11.70348542	-1.662	0.70668
LOC102163125	11.0464787	29.746021	3.590539588	4.942327829	-1.42911	0.70677
UMPS	3440.97811	1170.8365	11.74902221	10.19455552	1.555279	0.70711
INO80D	63.5172524	160.298	6.011613095	7.333584776	-1.33554	0.70712
CNN2	5466.62614	19993.458	12.41669888	14.28731254	-1.87081	0.70721
WEE1	2717.43376	940.30479	11.40855996	9.878518122	1.531044	0.70736
WDR66	55.2323934	23.135794	5.813329549	4.593102408	1.255388	0.70743
HNRNPA1	98722.38	26954.853	16.59110417	14.71831095	1.872832	0.70758
LOC102157481	70.4213016	178.47613	6.158282521	7.487648157	-1.34165	0.70761
MON2	607.556328	1826.9015	9.249246994	10.8359726	-1.58831	0.70764
LOC100621254	86.9910197	223.09516	6.459284385	7.807967682	-1.35872	0.70773
LOC100738530	20.7121475	52.055537	4.440430524	5.729431428	-1.32957	0.70781
PHYKPL	5590.89903	1840.9482	12.44912259	10.84701678	1.602631	0.7079
ANGPTL6	26.2353869	65.275991	4.767410456	6.05041444	-1.31504	0.70792
RBL2	216.787144	594.92043	7.766774985	9.218975892	-1.45642	0.70806
LOC100736873	44.1859147	109.89502	5.497801224	6.793050813	-1.31447	0.70807
CCDC142	44.1859147	109.89502	5.497801224	6.793050813	-1.31447	0.70807
SLC30A3	9.66566885	2.4788351	3.414902536	1.798604302	1.963207	0.70811
LOC102157465	9.66566885	2.4788351	3.414902536	1.798604302	1.963207	0.70811
LOC102166151	9.66566885	2.4788351	3.414902536	1.798604302	1.963207	0.70811
LOC100621276	9.66566885	2.4788351	3.414902536	1.798604302	1.963207	0.70811
LOC100625054	9.66566885	2.4788351	3.414902536	1.798604302	1.963207	0.70811
LOC100627489	9.66566885	2.4788351	3.414902536	1.798604302	1.963207	0.70811
LOC100737370	9.66566885	2.4788351	3.414902536	1.798604302	1.963207	0.70811
URB2	2489.60013	867.59229	11.2822777	9.762535337	1.520825	0.70821
DAGLA	853.340479	320.59601	9.738667328	8.329105689	1.412365	0.70821
LOC102166204	19.3313377	48.750424	4.345633235	5.636636914	-1.33447	0.70825
LOC100511223	523.326928	203.26448	9.03432283	7.674294538	1.364354	0.70829
CRLS1	2290.76352	803.14258	11.16224247	9.651307509	1.512101	0.70838
SLA-1	2369.46968	8040.5148	11.21095722	12.97325158	-1.76272	0.70866
LOC100736967	113.226407	294.98138	6.835752397	8.209362603	-1.38141	0.70878
PFKP	7264.44055	2351.5882	12.82683456	11.20003312	1.627216	0.70878
DNAJC6	230.595243	94.195734	7.855461808	6.572825024	1.291629	0.70884
LOC102163365	17.9505279	45.44531	4.24416613	5.537461031	-1.34011	0.70897
REXO4	4135.52546	1392.2791	12.01420375	10.44426852	1.570622	0.70913
LOC102166542	30.3778164	75.191332	4.971673052	6.251554969	-1.30755	0.7092
LOC100624847	167.07799	447.84288	7.392987005	8.810066691	-1.42247	0.7094
LOC100739529	632.410905	242.92584	9.306997895	7.930298794	1.380346	0.70957
TBC1D12	165.69718	443.71149	7.381085891	8.796725856	-1.42107	0.70959
PTCD2	1020.41847	379.26177	9.996358335	8.570849103	1.427895	0.70962
LOC100520338	40.0434852	16.525567	5.359081339	4.13138925	1.276868	0.70962
KIF18B	1567.21916	565.17441	10.61491148	9.145102724	1.471439	0.70962
UBE3C	361.772177	1035.3268	8.502920002	10.01726331	-1.51693	0.70964
CLCN3	3733.7098	1266.6847	11.86678043	10.3079803	1.559552	0.70964
LOC102159973	173.982039	467.67356	7.451063037	8.872439593	-1.42656	0.70966
TPRN	1092.22058	404.05012	10.09436881	8.661956638	1.434658	0.70968
SPR	13819.1448	4287.5585	13.75448512	12.06627708	1.68844	0.70972
LOC102166195	34.5202459	14.046732	5.150569664	3.911378308	1.297208	0.7098
TEX15	146.365843	61.1446	7.203258356	5.957557119	1.259282	0.70988
DHRS13	175.362849	72.712497	7.462402878	6.20383732	1.270068	0.7099
DNA2	436.335908	171.8659	8.772597998	7.433509508	1.344156	0.70995
RAD9B	16.569718	42.140197	4.135019133	5.430960858	-1.34665	0.71003
EBF4	581.320941	224.74772	9.18567069	7.818567588	1.371028	0.71018
LOC102163558	9.66566885	26.440908	3.414902536	4.77825631	-1.45183	0.71031
TRABD2B	5.52323934	17.351846	2.705588563	4.19785327	-1.6515	0.71031
ACTL10	5.52323934	17.351846	2.705588563	4.19785327	-1.6515	0.71031
CDH23	5.52323934	17.351846	2.705588563	4.19785327	-1.6515	0.71031
LOC102163272	5.52323934	17.351846	2.705588563	4.19785327	-1.6515	0.71031
GRK7	5.52323934	17.351846	2.705588563	4.19785327	-1.6515	0.71031
C4BPB	5.52323934	17.351846	2.705588563	4.19785327	-1.6515	0.71031
LOC102162715	33.1394361	81.801559	5.093367322	6.371586023	-1.30358	0.71037
LOC100523728	735.971642	280.10837	9.525465297	8.13498259	1.393665	0.71047
PTPRM	67.659682	170.21334	6.101391269	7.419651342	-1.33098	0.71066
ZNF45	151.889082	403.22385	7.256341575	8.659010619	-1.40856	0.71068
C4H8orf82	2009.07831	713.07823	10.97303599	9.479938335	1.494402	0.71106
LOC102166748	66.2788721	28.093465	6.072081615	4.862623207	1.238314	0.71147
CTNS	440.478338	1279.0789	8.786198839	10.32201704	-1.53796	0.71163
ABR	1467.80086	4752.7532	10.52042309	12.21485129	-1.69511	0.71165
NIN	368.676226	1053.5049	8.530118456	10.04235012	-1.51477	0.71173
CUX2	69.0404918	173.51846	6.130117309	7.447235822	-1.32957	0.71179
EPHB1	16.569718	5.7839486	4.135019133	2.762125239	1.518422	0.7118
MURC	16.569718	5.7839486	4.135019133	2.762125239	1.518422	0.7118
ASB16	16.569718	5.7839486	4.135019133	2.762125239	1.518422	0.7118
LOC102160278	16.569718	5.7839486	4.135019133	2.762125239	1.518422	0.7118
PARVB	3518.30346	1201.4088	11.7810742	10.2317117	1.550153	0.71181
SREK1	532.992597	1574.0603	9.06067593	10.62119134	-1.5623	0.71185
LOC102167943	99.4183082	42.140197	6.649878514	5.430960858	1.238314	0.71211
EPHX3	44.1859147	109.06875	5.497801224	6.782261052	-1.30358	0.71216
MRPS28	1100.50544	408.18152	10.1052609	8.676597164	1.430884	0.71233
TLL2	37.2818656	91.716899	5.258589232	6.534760414	-1.29871	0.71241
AAAS	5187.70255	1725.2692	12.34115812	10.75344178	1.588274	0.71241
SH2B2	180.886089	75.191332	7.506891393	6.251554969	1.266443	0.7126
CNDP2	16058.8184	4947.7549	13.97116796	12.27284987	1.69852	0.71271
ELK4	59.3748229	147.90383	5.915875148	7.218237038	-1.31674	0.71272
TFE3	1529.9373	4963.4542	10.58019948	12.27741939	-1.69787	0.71296
LOC100739646	45.5667246	112.37386	5.541227504	6.824944216	-1.30225	0.71307
RPA3	847.817239	320.59601	9.729310147	8.329105689	1.402997	0.71319
LOC100736703	93.8950688	239.62073	6.568261215	7.910617116	-1.35163	0.71341
APC10	606.175518	234.66306	9.24596981	7.88058181	1.369145	0.71346
ATP6V0D1	2027.02884	6737.4738	10.98586245	12.71820617	-1.73284	0.71368
BEST1	56.6132033	140.46732	5.848327568	7.144325041	-1.31102	0.71378
OSBPL11	99.4183082	254.49374	6.649878514	7.997144125	-1.35605	0.71384
CDAN1	2497.88499	875.85507	11.28706879	9.776194605	1.511943	0.71393
LOC102162646	131.176934	55.360651	7.04632663	5.816616368	1.244581	0.714
LOC100736673	1191.63889	440.40637	10.21994157	8.785963646	1.43604	0.71403
LOC102158986	389.388374	155.34033	8.608766278	7.288546214	1.325777	0.7141
AFAP1L1	218.167954	90.064343	7.775893057	6.508814353	1.276411	0.71425
MGAT4A	92.514259	235.48934	6.547114458	7.885631319	-1.34791	0.71434
LOC102167065	26.2353869	64.449713	4.767410456	6.032314961	-1.29666	0.71442
SGCE	519.184498	1523.6573	9.022879597	10.5742693	-1.55322	0.71447
LOC102158906	27.6161967	67.754826	4.838760036	6.103389087	-1.29481	0.71448
LOC100739618	23.4737672	9.0890621	4.613164285	3.334720158	1.368846	0.71471
TROVE2	62.1364426	154.51406	5.98040107	7.280901168	-1.31423	0.71482
YPEL1	73.1829213	183.4338	6.213015177	7.526959252	-1.32568	0.71506
CYP20A1	53.8515836	133.03082	5.777461367	7.066420948	-1.3047	0.71509
ZNF397	53.8515836	133.03082	5.777461367	7.066420948	-1.3047	0.71509
LOC102157729	8.28485901	23.135794	3.214880004	4.593102408	-1.48158	0.71518
LOC100737167	8.28485901	23.135794	3.214880004	4.593102408	-1.48158	0.71518
NDC1	1378.04822	504.85609	10.42945718	8.98258319	1.448682	0.71533
SLC30A4	194.694187	80.97528	7.61245709	6.357117027	1.265656	0.71539
LOC100513297	153.269892	64.449713	7.269312714	6.032314961	1.249828	0.71546
LOC102160503	124.272885	322.24857	6.968930373	8.336500155	-1.37466	0.71557
AKNAD1	149.127462	62.797156	7.230044098	5.995420212	1.247775	0.71566
TENM3	1496.79786	4824.6394	10.54862722	12.2365044	-1.68854	0.71616
LOC100738125	788.442416	300.76533	9.624690226	8.23728324	1.390367	0.71617
PLXNA3	769.111079	2333.4101	9.588922741	11.18884233	-1.60118	0.71618
KCTD11	417.00457	1196.4511	8.707374906	10.22575101	-1.52063	0.71629
LOC100524522	4077.53145	1384.8426	11.99383406	10.43654764	1.557974	0.7163
C8H4orf29	219.548764	90.890621	7.784953864	6.521845711	1.272338	0.71645
CACNA2D1	6244.02208	2059.0857	12.60849096	11.00848864	1.600472	0.7165
WDR3	4712.70397	1584.8019	12.20264544	10.63099686	1.572253	0.71651
RHOJ	133.938554	348.68947	7.076158798	8.449930555	-1.38037	0.71657
LOC102166858	35.9010557	87.585507	5.205590187	6.468998788	-1.28667	0.71659
EHD4	1107.40949	3470.3692	10.11427525	11.76128908	-1.6479	0.71676
LOC102164705	56.6132033	139.64104	5.848327568	7.135873883	-1.30251	0.71699
FPGS	1331.10068	489.98307	10.37948741	8.939529481	1.441816	0.71726
LOC102160509	1074.27005	401.57129	10.07048332	8.653100473	1.419629	0.71738
LRRC16B	179.505279	75.191332	7.495897217	6.251554969	1.255388	0.71755
LOC102161652	1.38080984	8.2627837	1.251452392	3.211445828	-2.58111	0.71774
LOC102157539	1.38080984	8.2627837	1.251452392	3.211445828	-2.58111	0.71774
LOC100626257	1.38080984	8.2627837	1.251452392	3.211445828	-2.58111	0.71774
LOC102166457	1.38080984	8.2627837	1.251452392	3.211445828	-2.58111	0.71774
LOC102166493	1.38080984	8.2627837	1.251452392	3.211445828	-2.58111	0.71774
LOC102166591	1.38080984	8.2627837	1.251452392	3.211445828	-2.58111	0.71774
LOC100623954	1.38080984	8.2627837	1.251452392	3.211445828	-2.58111	0.71774
LOC102162813	1.38080984	8.2627837	1.251452392	3.211445828	-2.58111	0.71774
LOC102158842	1.38080984	8.2627837	1.251452392	3.211445828	-2.58111	0.71774
LOC102162961	1.38080984	8.2627837	1.251452392	3.211445828	-2.58111	0.71774
KLHDC10	1.38080984	8.2627837	1.251452392	3.211445828	-2.58111	0.71774
CBR1	1.38080984	8.2627837	1.251452392	3.211445828	-2.58111	0.71774
LOC102161299	57.9940131	142.94616	5.882496648	7.169385476	-1.3015	0.71793

PAH	5.286795433	0	2.65232482	0	20	0.50612
LOC102161474	5.286795433	0	2.65232482	0	20	0.50612
LOC100511549	5.286795433	0	2.65232482	0	20	0.50612
LOC102157576	5.286795433	0	2.65232482	0	20	0.50612
FAM83A	5.286795433	0	2.65232482	0	20	0.50612
LOC102163831	5.286795433	0	2.65232482	0	20	0.50612
LOC102161063	5.286795433	0	2.65232482	0	20	0.50612
LOC100624257	5.286795433	0	2.65232482	0	20	0.50612
PVALB1	5.286795433	0	2.65232482	0	20	0.50612
LOC102157910	5.286795433	0	2.65232482	0	20	0.50612
LOC100154934	5.286795433	0	2.65232482	0	20	0.50612
SEL1L2	5.286795433	0	2.65232482	0	20	0.50612
C5H12orf60	5.286795433	0	2.65232482	0	20	0.50612
DNMT3L	5.286795433	0	2.65232482	0	20	0.50612
MOV10L1	5.286795433	0	2.65232482	0	20	0.50612
PSTPIP2	5.286795433	0	2.65232482	0	20	0.50612
LOC102158141	5.286795433	0	2.65232482	0	20	0.50612
LOC102160606	5.286795433	0	2.65232482	0	20	0.50612
LOC100621104	5.286795433	0	2.65232482	0	20	0.50612
LOC102160747	5.286795433	0	2.65232482	0	20	0.50612
LOC102160177	5.286795433	0	2.65232482	0	20	0.50612
LOC102158928	5.286795433	0	2.65232482	0	20	0.50612
CCR8	5.286795433	0	2.65232482	0	20	0.50612
LOC102158429	5.286795433	0	2.65232482	0	20	0.50612
LOC102162285	5.286795433	0	2.65232482	0	20	0.50612
LOC102158197	5.286795433	0	2.65232482	0	20	0.50612
GIMAP4	5.286795433	0	2.65232482	0	20	0.50612
LOC102167761	5.286795433	0	2.65232482	0	20	0.50612
LOC100513689	5.286795433	0	2.65232482	0	20	0.50612
ITLN2	5.286795433	0	2.65232482	0	20	0.50612
GRB7	5.286795433	0	2.65232482	0	20	0.50612
ALOXE3	5.286795433	0	2.65232482	0	20	0.50612
SIT1	5.286795433	0	2.65232482	0	20	0.50612
LOC100155946	5.286795433	0	2.65232482	0	20	0.50612
PPP6C	6228.902379	1515.3945	12.60499384	10.56642945	2.039285	0.50641
LOC100515222	35.95020894	10.741619	5.207510617	3.553559423	1.742789	0.50659
BUB1B	1749.929288	468.49984	10.77390511	8.874980846	1.901176	0.50697
LOC100624175	376.4198348	112.37386	8.560026434	6.824944216	1.744036	0.50723
LOC100626244	134.284604	42.140197	7.079853853	5.430960858	1.672025	0.50734
LOC100739021	21.14718173	70.233662	4.46905122	6.154487245	-1.7317	0.50756
LOC100514456	63.44154519	214.83238	6.009919183	7.753767487	-1.75971	0.50759
YTHDF2	11290.48033	2631.6966	13.46294702	11.36232556	2.101042	0.50766
LOC100738762	1882.099174	501.55097	10.87889327	8.97312612	1.907874	0.50773
LOC102167158	277.0280807	84.280394	8.119086791	6.414142197	1.716763	0.50779
HBQ1	82.47400875	283.41348	6.383255151	8.151846042	-1.7809	0.50824
LOC100516794	194.5540719	60.318321	7.611423767	5.938246292	1.689503	0.50854
LOC100736842	9.516231779	33.877413	3.394545939	5.124221138	-1.83186	0.5086
RPAP2	2615.90638	680.85338	11.3536466	9.413317734	1.941895	0.50881
LOC100627742	28.54869534	94.195734	4.885022527	6.572825024	-1.72224	0.50882
NCAM1	544.5399296	2183.8537	9.091540985	11.09332099	-2.00377	0.50892
FITM2	78.24457241	267.71419	6.308240221	8.069928711	-1.77463	0.50894
TMOD3	2718.470212	705.64173	11.40910991	9.464835137	1.945787	0.509
LOC100624267	16.91774539	4.1313919	4.163317207	2.359350201	2.033838	0.50937
ADAM28	16.91774539	4.1313919	4.163317207	2.359350201	2.033838	0.50937
CLIC5	16.91774539	4.1313919	4.163317207	2.359350201	2.033838	0.50937
LOC102164858	16.91774539	4.1313919	4.163317207	2.359350201	2.033838	0.50937
LOC100511730	150.1449903	47.097867	7.239789351	5.587901017	1.672623	0.50964
ACVR2A	1316.412063	381.06365	10.36349095	8.500179217	1.866206	0.50978
TEN1	59.21210885	199.13309	5.911981742	7.644815895	-1.74977	0.50985
KLC4	472.6395117	1869.0417	8.887645627	10.86885471	-1.98349	0.50994
MED20	2939.458261	759.34982	11.5218253	9.57051952	1.952714	0.51004
LOC100515712	33.83549077	111.54758	5.122485983	6.814391228	-1.72105	0.51022
RNF219	2077.710605	551.12767	11.02147321	9.108858104	1.914536	0.51037
C2H11orf49	140.6287585	500.72469	7.145970432	8.970752134	-1.83213	0.51047
SPDYA	52.86795433	16.525567	5.751355374	4.131389251	1.677694	0.51101
SLC35E3	1392.541913	380.91433	10.44454068	8.57710524	1.870182	0.5111
LOC102157907	8.458872693	30.5723	3.241668254	4.980587447	-1.85369	0.51137
LOC100738813	25.37661808	83.454116	4.721187694	6.400095824	-1.71748	0.51139
ZNF777	305.576776	1160.0948	8.260104603	10.1812701	-1.92464	0.5114
LOC100519476	344.6990622	1322.0454	8.43337288	10.36964685	-1.93936	0.51175
LOC102159642	38.06492712	11.567897	5.287802017	3.651671381	1.718336	0.51212
NOTCH2	1978.318851	8880.0137	10.95078832	13.11650864	-2.16629	0.51218
ANKS6	120.5389359	423.05453	6.925274755	8.728105973	-1.81134	0.51227
FAH	253.7661808	946.08874	7.993029968	9.887355792	-1.89848	0.51282
LOC102168067	4.229436346	18.178124	2.386655454	4.261389711	-2.10367	0.51323
NRL	4.229436346	18.178124	2.386655454	4.261389711	-2.10367	0.51323
IL20RB	4.229436346	18.178124	2.386655454	4.261389711	-2.10367	0.51323
TMPRSS7	4.229436346	18.178124	2.386655454	4.261389711	-2.10367	0.51323
LOC100524250	4.229436346	18.178124	2.386655454	4.261389711	-2.10367	0.51323
LOC102165772	4.229436346	18.178124	2.386655454	4.261389711	-2.10367	0.51323
MIR7	85.64608601	27.267186	6.437062676	4.821056378	1.651221	0.51353
KDM6A	3893.196157	988.22893	11.92710984	9.950160626	1.978038	0.51354
NUDCD1	3395.180027	870.8974	11.72969722	9.768014573	1.962913	0.5137
GPD1	32.77813168	107.41619	5.078017628	6.760436381	-1.71241	0.51389
CYP4V2	119.4815768	418.09686	6.912668746	8.711139889	-1.80705	0.51394
ALG1	108.9079859	378.43549	6.780152406	8.567710832	-1.79694	0.51401
LOC102167667	1798.567806	484.19913	10.81343475	8.922433141	1.893176	0.51409
GATA2	124.7683722	437.92754	6.974625354	8.777838973	-1.81144	0.51412
PDXP	15.8603863	52.881816	4.075565686	5.751726565	-1.73734	0.5142
LOC100626396	56.04003159	186.73891	5.833902876	7.552583893	-1.73649	0.51428
LOC100736679	37.00756803	121.46292	5.24821481	6.936201186	-1.71462	0.5143
SLC16A13	103.6211905	358.60481	6.709031281	8.490268521	-1.79108	0.51432
TMEM132A	1686.487743	7439.8105	10.72066131	12.86124405	-2.14124	0.51466
C14H1orf124	979.1145142	275.97698	9.93680651	8.113622246	1.82693	0.51467
TCEANC	105.7359087	366.04132	6.737901807	8.519798669	-1.79154	0.51514
LOC100739434	13083.76134	3039.0519	13.67559999	11.56988022	2.106084	0.51517
SLC35A2	345.7564213	1320.3928	8.437778789	10.36784372	-1.93314	0.51526
TGM1	7.401513606	27.267186	3.070649265	4.821056378	-1.88127	0.51531
NAGS	7.401513606	27.267186	3.070649265	4.821056378	-1.88127	0.51531
ARHGAP39	44.40908164	146.25127	5.504908955	7.202136284	-1.71952	0.51558
ZMYM5	3339.139995	859.32951	11.70569286	9.748745508	1.958193	0.51564
LOC102162713	215.7012537	789.92212	7.75956369	9.627391839	-1.87268	0.51573
RABEP2	292.888467	1101.4291	8.199124934	10.10647012	-1.91095	0.51577
LOC102166677	19.03246356	4.9576702	4.324267947	2.57474827	1.940728	0.51589
LOC102166558	19.03246356	4.9576702	4.324267947	2.57474827	1.940728	0.51589
LOC102167152	19.03246356	4.9576702	4.324267947	2.57474827	1.940728	0.51589
HSBP1P	19.03246356	4.9576702	4.324267947	2.57474827	1.940728	0.51589
EVL	304.5194169	1148.5269	8.255120262	10.16682456	-1.91518	0.51615
LOC102160327	30.66341351	9.0890621	4.98474489	3.334720158	1.754315	0.51644
CANT1	140.6287585	496.5933	7.145970432	8.958823254	-1.82017	0.51649
CARKD	765.5279787	3135.7264	9.582194642	11.61504399	-2.03427	0.51664
LOC100627645	140.6287585	44.619032	7.145970432	5.511563931	1.656161	0.51669
LOC100739813	192.4393538	60.318321	7.59573752	5.938246292	1.673736	0.51676
LOC100738367	448.3202527	133.8571	8.811600281	7.075287628	1.743836	0.51691
PDP1	2465.76139	650.28108	11.26840245	9.347136503	1.922898	0.51711
GABRG3	40.17964529	12.394176	5.363859498	3.74353388	1.696803	0.51724
LOC100623659	68.72834063	230.53167	6.123673244	7.855065709	-1.74599	0.51729
LOC100737712	100.4491132	345.38436	6.664612444	8.436229978	-1.78174	0.51733
LOC102161441	87.76080419	28.093465	6.471850834	4.862623207	1.643342	0.51734
PCM1	5778.467408	1429.4616	12.49672084	10.48226504	2.015215	0.5174
COQ10B	2210.93785	588.3102	11.11109513	9.202883429	1.91001	0.51775
LAT	99.39175414	341.25297	6.649496966	8.41891924	-1.77964	0.51783
PMS1	2497.482162	658.54386	11.2868362	9.365324797	1.923123	0.51788
LOC102159170	69.78569971	22.309516	6.145386028	4.542847146	1.645272	0.51821
LOC100519042	494.8440525	147.07755	8.953742641	7.210209122	1.750397	0.51869
PARG	3619.340153	928.73689	11.82190954	9.860678689	1.962385	0.51871
LOC100513372	126.8830904	40.48764	6.998681703	5.374609695	1.647946	0.51872
CHST12	150.1449903	531.29699	7.239789351	9.056087606	-1.82316	0.51878
SPATC1L	1.057359087	9.0890621	1.04079362	3.334720158	-3.10367	0.51882
LOC102157555	1.057359087	9.0890621	1.04079362	3.334720158	-3.10367	0.51882
LOC102161429	1.057359087	9.0890621	1.04079362	3.334720158	-3.10367	0.51882
LOC102163786	1.057359087	9.0890621	1.04079362	3.334720158	-3.10367	0.51882
LOC100626680	1.057359087	9.0890621	1.04079362	3.334720158	-3.10367	0.51882
CAPN3	1.057359087	9.0890621	1.04079362	3.334720158	-3.10367	0.51882
LCN15	1.057359087	9.0890621	1.04079362	3.334720158	-3.10367	0.51882
LOC102160344	1.057359087	9.0890621	1.04079362	3.334720158	-3.10367	0.51882
LOC102162382	1.057359087	9.0890621	1.04079362	3.334720158	-3.10367	0.51882
LOC100627488	1.057359087	9.0890621	1.04079362	3.334720158	-3.10367	0.51882
C8H4orf26	1.057359087	9.0890621	1.04079362	3.334720158	-3.10367	0.51882
LOC102165917	1.057359087	9.0890621	1.04079362	3.334720158	-3.10367	0.51882
LOC100738361	117.3668586	407.35524	6.887121389	8.673680921	-1.79526	0.51882
DTX2	87.76080419	298.28649	6.471850834	8.225383356	-1.76505	0.51886
LOC100626397	137.4566813	482.54657	7.113290863	8.917511028	-1.81169	0.51943
FUBP1	14284.92126	3310.8974	13.80230645	11.69344228	2.109199	0.51958
NPM3	11530.50084	2715.977	13.49329267	11.40778664	2.085912	0.51992
LOC100153608	1178.955382	329.68507	10.20451659	8.369314102	1.838349	0.51994
FGFR1OP2	1829.23122	494.94074	10.83781021	8.954023947	1.88591	0.51997
HIGD1A	6816.741002	1669.9086	12.7350893	10.70641709	2.029324	0.51999
LOC102167649	49.69587707	15.699289	5.663796517	4.061714779	1.662427	0.52032
LOC100519844	10.57359087	36.356248	3.532764645	5.223277669	-1.78174	0.52042
ALK	10.57359087	36.356248	3.532764645	5.223277669	-1.78174	0.52042
FER	82.47400875	278.45581	6.383255151	8.126476366	-1.75544	0.52062
LOC102166070	244.249949	76.01761	7.938109026	6.267116453	1.683953	0.52062
ZNF414	267.5118489	991.53405	8.068841943	9.95497278	-1.89006	0.52067
RAB26	6.344154519	23.962073	2.876596413	4.641665831	-1.91725	0.52086
LOC100739113	6.344154519	23.962073	2.876596413	4.641665831	-1.91725	0.52086
EIF4E3	84.58872693	285.89232	6.419348884	8.164365521	-1.75693	0.52106
TMEM164	1757.330802	477.5889	10.7799908	8.902643123	1.879545	0.52126
LOC100620492	413.4274028	124.76803	8.69497559	6.974621476	1.728386	0.52145
ISCA2	485.3278207	1895.4826	8.925785314	10.88911041	-1.96553	0.52147
LOC100156244	21.14718173	5.7839486	4.46905122	2.76212524	1.870339	0.52149
WDR65	32.77813168	9.9153405	5.078017628	3.448285225	1.724999	0.52154
FOXJ1	32.77813168	9.9153405	5.078017628	3.448285225	1.724999	0.52154
TCP11	32.77813168	9.9153405	5.078017628	3.448285225	1.724999	0.52154
RAB33B	2268.035241	604.83577	11.1478633	9.242782946	1.906828	0.52165
ZNF180	76.12985423	255.32002	6.269217479	8.001802338	-1.74577	0.52169
YOD1	123.7110131	39.661362	6.962445064	5.34558663	1.641168	0.52172
CCNB2	4228.378987	1075.8144	12.04623013	10.07255395	1.974675	0.52186
LOC100738183	42.29436346	13.220454	5.436107306	3.829895615	1.677694	0.52201
TPRKB	1884.213892	509.81376	10.8805125	8.996653565	1.885921	0.52205
DDR1	777.1589286	3165.4724	9.603921026	11.62866081	-2.02614	0.52221
P2RX4	265.3971307	980.79243	8.057434734	9.939274229	-1.8858	0.52236
LOC100739201	60.26946793	199.13309	5.937096418	7.644815895	-1.72423	0.5226
SCFD2	326.7239578	1229.5022	8.356337332	10.26503154	-1.91193	0.5226
ZFYVE19	106.7932677	366.04132	6.752123267	8.519798669	-1.77719	0.52272
LOC100626512	150.1449903	47.924146	7.239789351	5.61247475	1.647532	0.5229
C18H7orf49	137.4566813	480.06773	7.113290863	8.910096228	-1.80426	0.52314
ECE2	1031.982468	292.50254	10.01260005	8.197229196	1.818897	0.52317
LOC100514971	9.516231779	33.051135	3.394545939	5.089630977	-1.79624	0.52355
SIRPA	94.1049587	318.94345	6.571448659	8.321673128	-1.76096	0.52422
ZNF316	189.2267765	677.54826	7.571883648	9.406307627	-1.8399	0.52429
PLEKHN1	48.63851798	158.64545	5.633388138	7.318727602	-1.70563	0.52439
ZNF526	126.8830904	439.58009	6.998681703	8.783260503	-1.79263	0.5244
SCCPDH	8397.545866	2034.2197	13.03592384	10.99102387	2.045437	0.52442
SPTBN5	3.17207726	14.873011	2.060765874	3.98850389	-2.2292	0.52455
LOC102167861	3.17207726	14.873011	2.060765874	3.98850389	-2.2292	0.52455
LOC102163343	3.17207726	14.873011	2.060765874	3.98850389	-2.2292	0.52455
ALOX12B	3.17207726	14.873011	2.060765874	3.98850389	-2.2292	0.52455
OVOL3	3.17207726	14.873011	2.060765874	3.98850389	-2.2292	0.52455
LOC100625210	3.17207726	14.873011	2.060765874	3.98850389	-2.2292	0.52455
MTHFR	372.1903985	1412.936	8.543768058	10.46550112	-1.92458	0.52482
LOC100739819	26.43397716	85.106672	4.777891883	6.428053129	-1.66868	0.52483
TRIP6	1149.349327	4835.381	10.16785632	12.2397122	-2.07281	0.52495
TOR2A	75.07249515	250.36235	6.249303021	7.973624744	-1.73766	0.52496
HOOK2	84.58872693	284.23976	6.419348884	8.156031284	-1.74857	0.52509
DAZAP2	1181.0701	4979.9797	10.20709988	12.28221383	-2.07605	0.52515
NIPAL3	315.0930078	1177.4467	8.304205312	10.20267077	-1.90181	0.52545
LOC100516260	27.49133625	88.411786	4.832451381	6.482393107	-1.68526	0.52545
ZNF532	4582.594281	1163.3999	12.16226363	10.18537096	1.977817	0.52566
LOC100627047	3933.375802	1010.5384	11.94191905	9.982335443	1.960644	0.52596
LOC100625555	1483.474798	410.66035	10.53573689	8.685310691	1.852963	0.52643
SLC30A10	23.2618999	6.610227	4.600620625	2.927939483	1.815197	0.52646
LOC102160128	23.2618999	6.610227	4.600620625	2.927939483	1.815197	0.52646
LOC100737487	23.2618999	6.610227	4.600620625	2.927939483	1.815197	0.52646
LOC100521043	44.40908164	14.046732	5.504908955	3.911378308	1.66062	0.52647
CTAGE5	2141.15215	576.7423	11.06484524	9.174282325	1.892389	0.52693
LOC100738636	741.2087197	2986.9963	9.53568114	11.54496265	-2.01074	0.52721
RERG	289.7163897	90.064343	8.183468598	6.508814353	1.685613	0.52751
GPR155	178.6936856	633.75551	7.489395904	9.310057206	-1.82644	0.52756
CD2AP	375.3624757	114.85269	8.555978985	6.856147778	1.7085	0.5277
LOC102162367	122.653654	39.661362	6.950161063	5.34558663	1.628784	0.52787
GPC1	1449.639308	6204.5243	10.50247313	12.59933739	-2.09763	0.52818
SP3	7514.651028	1842.6008	12.87568236	10.84831056	2.027963	0.52818
LOC102161772	104.6785496	355.2997	6.723538761	8.476947458	-1.76307	0.52857
DIRC2	107.8506268	366.8676	6.766205905	8.523042794	-1.76622	0.52859
LOC100152528	68.72834063	22.309516	6.123673244	4.542847146	1.623246	0.5287
AIFM3	5.286795433	20.656959	2.65232482	4.436758793	-1.96616	0.5287
LOC100626871	5.286795433	20.656959	2.65232482	4.436758793	-1.96616	0.5287
LOC102160082	5.286795433	20.656959	2.65232482	4.436758793	-1.96616	0.5287
LOC100521310	5.286795433	20.656959	2.65232482	4.436758793	-1.96616	0.5287
MTR	2259.576368	607.3146	11.14247494	9.248673828	1.895536	0.52872
NFXL1	2155.955178	581.69997	11.07478048	9.186609435	1.88998	0.52897
LOC100627112	2720.58493	721.34102	11.41023134	9.496536287	1.915163	0.52925
LOC100526028	170.2348129	54.534373	7.419832229	5.795309087	1.642288	0.52943
PTRH2	3962.981856	1021.2801	11.95273464	9.997574781	1.956208	0.52947
LOC100736644	444.0908164	134.68337	8.797955923	7.084100147	1.721283	0.52958
IFT43	212.5291764	762.65494	7.738289401	9.576777083	-1.84337	0.52979
PIGA	639.7022474	189.21775	9.323510241	7.571508044	1.757353	0.52995
PSME1	1929.680333	8457.7854	10.9148936	13.04623481	-2.13192	0.53003
IGF1R	832.1416011	3376.1734	9.702417907	11.72160055	-2.02049	0.53027
TMUB2	149.0876312	518.90282	7.229661278	9.022098163	-1.7993	0.5303
HAUS4	1199.045204	338.77413	10.22887304	8.408432212	1.82349	0.53039
LAMP-1	4372.179823	20613.167	12.09446696	14.33134851	-2.23714	0.5305
LOC100626367	20.08982264	64.449713	4.398475058	6.032314961	-1.68171	0.53059
RNF151	20.08982264	64.449713	4.398475058	6.032314961	-1.68171	0.53059
LOC102161711	46.52379981	14.873011	5.570578288	3.98850389	1.645272	0.53066
LOC100514169	46.52379981	14.873011	5.570578288	3.98850389	1.645272	0.53066
AS3MT	46.52379981	14.873011	5.570578288	3.98850389	1.645272	0.53066
PDCD7	1868.353506	510.64003	10.8683237	8.998985343	1.871389	0.5308
NFKBIL1	366.903603	1379.8849	8.523183995	10.43137734	-1.91107	0.53094
KDR	25.37661808	7.4365053	4.721187694	3.076645515	1.770803	0.53095
MRPL51	2958.490724	780.83306	11.53113322	9.610716783	1.921775	0.53097
LOC100736623	26.43397716	84.280394	4.777891883	6.414142197	-1.6728	0.53104
LCA5	161.7759402	52.055537	7.346743662	5.729431428	1.635874	0.53119
LOC100515137	216.7586127	777.52795	7.766585971	9.60460502	-1.84281	0.53133
LOC100739304	24023.19845	5422.0387	14.55220068	12.40488574	2.147521	0.53143
RU2B	2096.743069	568.47952	11.03462227	9.153500149	1.88297	0.53152
LOC100622849	581.5474976	173.51846	9.186231873	7.447235822	1.744808	0.53159
LOC102158493	17.97510447	57.839486	4.246035924	5.878712739	-1.68605	0.53167
LOC100520849	1859.894633	508.98748	10.86178065	8.994318011	1.869519	0.53168
POLR3D	15718.70018	3661.2395	13.94028608	11.83851041	2.102078	0.53182
CLC-5	72.95777697	240.44701	6.208629956	7.915562765	-1.72059	0.53185
LOC102166342	172.3495311	55.360651	7.437540124	5.816616368	1.638405	0.53189
NR4A2	145.9155539	505.68236	7.198843332	8.984937802	-1.7931	0.53212
HSPB8	98.33439505	330.51335	6.63422144	8.372914449	-1.74893	0.53222
LOC100525886	7.401513606	0.8262784	3.070649265	0.868906686	3.163121	0.53223
LOC100628214	7.401513606	0.8262784	3.070649265	0.868906686	3.163121	0.53223
LOC100622805	7.401513606	0.8262784	3.070649265	0.868906686	3.163121	0.53223
LOC102161896	7.401513606	0.8262784	3.070649265	0.868906686	3.163121	0.53223
LOC100628019	7.401513606	0.8262784	3.070649265	0.868906686	3.163121	0.53223
CELF5	7.401513606	0.8262784	3.070649265	0.868906686	3.163121	0.53223
LOC102157921	7.401513606	0.8262784	3.070649265	0.868906686	3.163121	0.53223
LOC100621278	7.401513606	0.8262784	3.070649265	0.868906686	3.163121	0.53223
LOC102160791	7.401513606	0.8262784	3.070649265	0.868906686	3.163121	0.53223
LOC100623209	7.401513606	0.8262784	3.070649265	0.868906686	3.163121	0.53223
TRNAI-AAU	7.401513606	0.8262784	3.070649265	0.868906686	3.163121	0.53223
LOC102167136	7.401513606	0.8262784	3.070649265	0.868906686	3.163121	0.53223
APRT	379.5919121	1429.4616	8.572101093	10.48226504	-1.91295	0.53229
LOC102167808	70.8430588	23.135794	6.166776871	4.593102407	1.6145	0.53234
FDX1L	547.7120068	2132.6245	9.099905335	11.05909057	-1.96114	0.53237
HSPA9	73081.48799	15244.836	16.15723813	13.89612764	2.261185	0.53246
CCDC173	90.93288145	29.746021	6.522509054	4.942327829	1.612105	0.53259
FOS	42.29436346	135.50965	5.436107306	7.092859161	-1.67986	0.53268
LOC102158554	42.29436346	135.50965	5.436107306	7.092859161	-1.67986	0.53268
IDI1	24306.57068	5488.1409	14.5691181	12.42236467	2.146957	0.53272
SGSM3	560.4003159	2185.5063	9.132886065	11.09441179	-1.96344	0.53276
MRPS35	3414.212491	893.20692	11.73775963	9.8044649	1.934487	0.533

LOC102159330	14.09179	3.3051135	3.915692025	2.106051269	2.092083	0.43434
LOC100524097	14.09179	3.3051135	3.915692025	2.106051269	2.092083	0.43434
LOC102165811	14.09179	3.3051135	3.915692025	2.106051269	2.092083	0.43434
LOC100739688	14.09179	3.3051135	3.915692025	2.106051269	2.092083	0.43434
LOC100736852	99.4714588	36.356248	6.65064192	5.223277669	1.452079	0.43476
LOC102160085	38.9596547	13.220454	5.320472212	3.829895615	1.559209	0.43479
METTL23	3110.96988	997.31799	11.60361238	9.963355621	1.641239	0.43479
ALDH2	14845.2863	4356.1396	13.85781448	12.08916561	1.768883	0.43494
GPAT	2388.97287	777.52795	11.22277853	9.60460502	1.619424	0.43503
LOC100626871	58.0250177	20.656959	5.883254663	4.436758793	1.490047	0.43518
LOC102165320	203.087562	578.39486	7.673044449	9.178403076	-1.50995	0.43592
ZNF569	328.255814	118.15781	8.363065106	6.89672967	1.474106	0.43601
FTL	33179.534	127138.63	15.01804949	16.95605423	-1.93804	0.43612
MTERFD3	523.883017	185.08636	9.035852107	7.539828464	1.501046	0.43721
CNPY3	526.369803	1578.1917	9.042671155	10.62497059	-1.58412	0.43733
FABP5	4820.22111	1510.4369	12.23518288	10.561705	1.674133	0.43766
LOC100739815	315.821882	114.02642	8.307528171	6.845821397	1.469743	0.43787
LOC100622112	8.28928824	26.440908	3.215568059	4.77825631	-1.67345	0.43837
LOC102163326	8.28928824	26.440908	3.215568059	4.77825631	-1.67345	0.43837
MYLK4	6.63143059	22.309516	2.93195353	4.542847146	-1.75027	0.43849
LOC102164187	9.94714588	30.5723	3.452482877	4.980587446	-1.61987	0.43879
FBXL16	83.7218112	30.5723	6.404661526	4.980587446	1.453378	0.43882
LOC102165088	4.97357294	18.178124	2.578594101	4.261389712	-1.86985	0.43909
PLOD2	2336.75035	7652.164	11.19090516	12.9018406	-1.71136	0.4392
LOC102163706	3.31571529	14.046732	2.109599694	3.911378308	-2.08284	0.4393
LOC102157701	3.31571529	14.046732	2.109599694	3.911378308	-2.08284	0.4393
FADS6	3.31571529	14.046732	2.109599694	3.911378308	-2.08284	0.4393
LOC102162677	3.31571529	14.046732	2.109599694	3.911378308	-2.08284	0.4393
MIR199A-2	3.31571529	14.046732	2.109599694	3.911378308	-2.08284	0.4393
STAG1	451.766209	1340.2235	8.82262248	10.38933397	-1.56883	0.43937
AGPAT9	185.680057	524.68677	7.544423999	9.038059606	-1.49864	0.43984
BCDIN3D	673.090205	235.48934	9.396797851	7.885631319	1.515138	0.43998
ZNF367	94.4978859	34.703692	6.57739989	5.158001345	1.445193	0.44073
LOC100739573	588.539465	207.39587	9.203444583	7.703182885	1.504752	0.44075
LOC100739112	229.613284	655.23875	7.849331809	9.358076973	-1.51281	0.44088
ZNF391	13.2628612	38.835083	3.834191515	5.315967693	-1.54997	0.44089
LOC102158450	55.5382312	19.830681	5.821154844	4.380638094	1.485747	0.44128
LOC100521067	369.702255	1082.4247	8.534117082	10.08138313	-1.54983	0.44134
LOC100738110	704.5895	246.23095	9.462685282	7.949715578	1.516771	0.4414
LOC102162518	1661.17336	5319.5802	10.69885515	12.37736785	-1.67911	0.44141
PRDX5	499.015152	1486.4748	8.965828003	10.5386495	-1.57474	0.44143
DICER1	12176.9644	3629.8409	13.57198538	11.82608799	1.746176	0.44144
LOC100624422	1855.14271	615.57739	10.85809192	9.268138169	1.591518	0.44172
TSPAN9	724.483792	2205.337	9.502799573	11.10743744	-1.60597	0.44203
PARD3B	66.3143059	180.12869	6.072841239	7.500871231	-1.44164	0.44272
PTTG1IP	4169.51198	14097.962	12.02600879	13.78330129	-1.75754	0.4429
ACSM3	105.273961	38.835083	6.731644339	5.315967693	1.438716	0.44305
GRIA3	474.976216	169.38707	8.894745675	7.412672017	1.487532	0.44307
HEATR2	1081.75212	370.17271	10.08048728	8.535946834	1.547099	0.44308
PLCE1	511.449084	181.78124	9.001264859	7.513974209	1.492387	0.44309
TMEM40	86.2085977	235.48934	6.446398468	7.885631319	-1.44976	0.44329
SUPT16H	20255.7047	5875.6655	14.30611188	12.52078207	1.785504	0.44335
NDC1	1501.1901	504.85609	10.55285168	8.98258319	1.572163	0.44379
LOC102162977	52.2225159	141.2936	5.733964805	7.152726981	-1.43595	0.44399
NTF4	26.5257224	72.712497	4.78270852	6.20383732	-1.45481	0.44425
LOC102166124	99.4714588	272.67186	6.65064192	8.096303303	-1.45481	0.44438
NIPSNAP1	3833.79581	1223.7183	11.90493405	10.2582342	1.647502	0.44494
PAQR6	252.823291	92.543178	7.987680649	6.547560532	1.449931	0.44503
LOC100738250	2140.29422	706.46801	11.06426733	9.4665211	1.599113	0.44537
TMEM231	1786.34162	595.74671	10.80359969	9.220974888	1.584237	0.44552
NOP14	7033.46107	2169.807	12.78022418	11.08401575	1.696668	0.44582
LOC100154071	28.18358	9.0890621	4.867084967	3.334720158	1.632652	0.44615
LOC102164129	28.18358	9.0890621	4.867084967	3.334720158	1.632652	0.44615
MEIS2	751.009514	262.75652	9.554607104	8.043062959	1.515105	0.4468
MED30	820.639535	285.89232	9.682361792	8.164365521	1.521277	0.44717
SLC39A4	233.757928	85.932951	7.875030072	6.441831208	1.443732	0.44727
LOC100624365	34.8150106	94.195734	5.162492464	6.572825024	-1.43595	0.4476
PRADC1	410.319768	1202.235	8.684116598	10.23270276	-1.5509	0.44761
LOC102163719	24.0389359	7.4365053	4.646101346	3.076645515	1.692676	0.4482
LOC102165474	32.3282241	87.585507	5.058672541	6.468998788	-1.4379	0.44824
LOC102167625	155.838619	57.839486	7.293137032	5.878712739	1.429926	0.44882
LOC102161849	100.300388	37.182527	6.662495885	5.254840671	1.43163	0.44884
METTL21B	472.48943	169.38707	8.887188408	7.412672017	1.479958	0.44905
SLC3A1	1515.28189	511.46631	10.56632227	9.001313359	1.566875	0.44917
PSD	57.1960888	20.656959	5.862850292	4.436758793	1.469289	0.44927
FAM210A	999.688161	345.38436	9.96677675	8.436229978	1.533275	0.44981
DCUN1D5	4176.97234	1331.9607	12.02858723	10.38041857	1.648906	0.45026
LOC102164618	281.8358	103.2848	8.143820931	6.704385035	1.448227	0.45037
ARFIP2	1267.43217	3964.4836	10.30883066	11.95328111	-1.64522	0.4504
TRMU	1742.40839	584.17881	10.76769484	9.192733716	1.576601	0.45057
LOC100737556	476.634074	171.03962	8.899761949	7.426597064	1.478552	0.45069
YAE1D1	2724.68904	890.72808	11.41240527	9.800460045	1.613035	0.45075
LOC102158264	256.139007	94.195734	8.006404665	6.572825024	1.443193	0.45076
SSH2	2753.70155	899.81715	11.42768031	9.815090479	1.613668	0.45097
SLC7A1	1736.60589	582.52625	10.76288518	9.188653752	1.575875	0.45098
LOC100738240	447.621565	161.12428	8.80935516	7.340956379	1.474106	0.45098
LOC100738528	159.154334	442.05893	7.323319031	8.791354786	-1.47381	0.45107
LOC102162671	14.09179	40.48764	3.915692025	5.374609695	-1.52263	0.45121
CCDC19	14.09179	40.48764	3.915692025	5.374609695	-1.52263	0.45121
UHMK1	527.198732	188.39147	9.044937027	7.565227535	1.484613	0.45127
LOC102161409	276.862227	101.63224	8.118225916	6.681340183	1.44581	0.45159
LOC102167728	19.8942918	5.7839486	4.385036953	2.762125239	1.782228	0.45159
LOC102167154	19.8942918	5.7839486	4.385036953	2.762125239	1.782228	0.45159
TEN1	73.7746653	199.13309	6.224477643	7.644815895	-1.43254	0.45181
CCDC166	9.11821706	28.093465	3.338883189	4.862623207	-1.62341	0.45188
LOC100737397	9.11821706	28.093465	3.338883189	4.862623207	-1.62341	0.45188
LOC102163793	97.8136012	36.356248	6.62663773	5.223277669	1.427832	0.45196
LOC102164606	2122.88672	704.81545	11.0524911	9.463147202	1.59071	0.45239
LOC102167549	1037.81889	358.60481	10.02072843	8.490268521	1.533088	0.4525
LOC102161333	373.017971	135.50965	8.546963779	7.092859162	1.46085	0.45256
ELK4	408.66191	147.90383	8.678289948	7.218237038	1.466248	0.45269
CENPT	2736.29405	895.68576	11.41853471	9.808458669	1.611159	0.4528
LOC100622330	13739.4953	4100.8196	13.74614638	12.00204831	1.744345	0.45291
LOC102166702	135.115398	50.402981	7.088686472	5.683780114	1.422611	0.4531
CLDN19	108.589676	40.48764	6.775968083	5.374609695	1.423333	0.45344
BAD	503.988725	1487.3011	8.980107366	10.53945068	-1.56123	0.45349
ZNF10	24.8678647	67.754826	4.693089065	6.103389087	-1.44604	0.45361
LOC102159177	7.46035941	23.962073	3.080718953	4.641665831	-1.68344	0.45375
NR5A2	7.46035941	23.962073	3.080718953	4.641665831	-1.68344	0.45375
LOC102160230	7.46035941	23.962073	3.080718953	4.641665831	-1.68344	0.45375
LOC100737683	1594.85906	538.7335	10.64011753	9.07610342	1.565785	0.45383
LOC102166929	69.6300212	25.61463	6.142209624	4.734147579	1.442741	0.45391
FURIN	2697.33439	8809.78	11.39785343	13.10505403	-1.70757	0.45403
MICAL3	5685.6228	1786.4138	12.4733564	10.80365798	1.670252	0.45424
LOC102165280	42.27537	14.873011	5.435474249	3.98850389	1.507121	0.45467
LOC100624599	600.973397	214.0061	9.233555921	7.74823377	1.489649	0.45469
DOCK8	46.4200141	16.525567	5.567424187	4.13138925	1.490047	0.45474
RNF222	84.55074	31.398578	6.418708429	5.017858594	1.429118	0.45488
LOC100523796	276.033298	101.63224	8.113915583	6.681340183	1.441484	0.45488
tRNA-Val	38.1307259	13.220454	5.290229968	3.829895615	1.528182	0.45509
NT5C3A	1461.40152	496.5933	10.51412376	8.958823254	1.557216	0.45518
PLCD3	586.052678	1742.6211	9.197346157	10.76787084	-1.57216	0.45521
RIC8A	1361.10113	4259.465	10.4116181	12.05679519	-1.6459	0.45535
CORO1B	2332.60571	7541.4427	11.18834511	12.88081612	-1.6929	0.45578
FNIP1	622.525547	1856.6475	9.28430486	10.85926105	-1.57649	0.45596
LOC102165430	33.9860818	11.567897	5.128709195	3.65167138	1.554817	0.45618
FOXRED1	2053.2567	684.98477	11.00440075	9.422032737	1.58377	0.45619
LOC100738871	455.081924	164.4294	8.833149183	7.370071807	1.468658	0.45635
LOC102159481	15.7496477	4.1313919	4.066058842	2.359350201	1.93062	0.45659
LOC102166682	15.7496477	4.1313919	4.066058842	2.359350201	1.93062	0.45659
LOC102167444	15.7496477	4.1313919	4.066058842	2.359350201	1.93062	0.45659
LOC102165044	15.7496477	4.1313919	4.066058842	2.359350201	1.93062	0.45659
LOC102165173	15.7496477	4.1313919	4.066058842	2.359350201	1.93062	0.45659
MGAT5	524.711945	188.39147	9.038128706	7.565227535	1.477792	0.45668
GNAQ	392.912262	142.94616	8.621730518	7.169385476	1.458735	0.45683
ZFYVE21	412.806554	1200.5825	8.692812686	10.23071996	-1.5402	0.45691
RAB40B	291.782946	107.41619	8.193687711	6.76043638	1.441684	0.45693
GPR137B	22.3810782	61.1446	4.547269558	5.957557119	-1.44995	0.45721
LOC100517625	99.4714588	37.182527	6.65064192	5.254840671	1.419658	0.4573
GTPBP4	7640.23697	2365.635	12.89959049	11.20862149	1.69139	0.45745
LOC100624904	57.1960888	152.8615	5.862850292	7.265488457	-1.41824	0.45766
LOC102159537	7.46035941	0.8262784	3.080718953	0.868906686	3.174545	0.45817
LOC102159773	7.46035941	0.8262784	3.080718953	0.868906686	3.174545	0.45817
LOC102164271	7.46035941	0.8262784	3.080718953	0.868906686	3.174545	0.45817
LOC100518676	7.46035941	0.8262784	3.080718953	0.868906686	3.174545	0.45817
SP5	7.46035941	0.8262784	3.080718953	0.868906686	3.174545	0.45817
LOC100156741	7.46035941	0.8262784	3.080718953	0.868906686	3.174545	0.45817
LOC100623820	7.46035941	0.8262784	3.080718953	0.868906686	3.174545	0.45817
LOC102160311	7.46035941	0.8262784	3.080718953	0.868906686	3.174545	0.45817
LOC102162597	7.46035941	0.8262784	3.080718953	0.868906686	3.174545	0.45817
IFITM5	7.46035941	0.8262784	3.080718953	0.868906686	3.174545	0.45817
LOC102165315	7.46035941	0.8262784	3.080718953	0.868906686	3.174545	0.45817
LOC100621254	82.8928824	223.09516	6.39047651	7.807967682	-1.42834	0.45817
LOC102165721	29.8414377	9.9153405	4.946798112	3.448285225	1.589583	0.45819
CLSTN1	2304.42213	7430.7214	11.17081522	12.8594807	-1.6891	0.45825
LOC102164523	758.469874	267.71419	9.568848927	8.06992871	1.502398	0.45828
NLE1	3869.43975	1246.8541	11.91828178	10.28523351	1.633832	0.45842
BASP1	5306.80233	17992.212	12.37389893	14.13516509	-1.76146	0.45866
FGF19	36.4728682	97.500848	5.227774503	6.622064238	-1.41859	0.45882
FAM228B	82.0639535	30.5723	6.376150635	4.980587446	1.424524	0.45896
LOC100739814	817.32382	287.54487	9.676528037	8.172651889	1.507121	0.45906
LOC100519831	484.923362	1421.1988	8.924584985	10.47390743	-1.55128	0.45906
SRRM5	56.36716	150.38266	5.842153193	7.242056188	-1.41571	0.45933
LOC102160428	249.507576	92.543178	7.968710424	6.547560532	1.430885	0.45944
LOC100626506	154.180761	57.839486	7.277805898	5.878712739	1.414496	0.4601
HAS1	114.392178	42.966475	6.850401618	5.458331975	1.412705	0.46046
LOC102165622	71.2878788	26.440908	6.175681852	4.77825631	1.430885	0.46047
LOC100512753	160.812192	60.318321	7.338176502	5.938246292	1.414709	0.46051
TNFSF10	472.48943	171.03962	8.887188408	7.426597064	1.465951	0.46061
CYP27B1	288.467231	106.58991	8.177256226	6.749398975	1.436337	0.46069
CHPT1	337.374031	966.74569	8.402475042	9.918484174	-1.51879	0.4607
LOC100521295	300.072234	110.7213	8.233965854	6.803760479	1.438377	0.46072
LOC102159762	14.9207188	42.140197	3.99283357	5.430960858	-1.49788	0.46077
NDUFC1	1826.1302	615.57739	10.83536373	9.268138169	1.568777	0.46104
TTC37	5510.71882	1743.4474	12.42828658	10.76855435	1.660298	0.46122
RNF183	25.6967935	8.2627837	4.73859457	3.211445828	1.636889	0.46148
C1H9orf100	1430.73115	489.1568	10.48354489	8.937099516	1.548384	0.4615
TUFM	27956.4535	8065.3032	14.77094534	12.97769192	1.793381	0.46151
LOC100512339	456.739782	1331.1345	8.838383871	10.37952399	-1.54321	0.46153
ID1	983.109585	3001.043	9.942675165	11.55172895	-1.61004	0.46156
LOC100156848	585.22375	209.87471	9.195307606	7.72024225	1.47946	0.46165
CAPN15	5741.16103	1812.8547	12.48737807	10.82484322	1.663079	0.46182
LOC102167175	75.432523	28.093465	6.256114748	4.862623207	1.424952	0.46185
EXOSC3	1320.48362	453.62683	10.36794282	8.828539005	1.541489	0.46187
NT5C	3081.95737	1008.8859	11.59009922	9.979976575	1.611084	0.46195
LOC100511730	125.168252	47.097867	6.979205122	5.587901017	1.410135	0.46198
TCEA3	3711.94327	1202.235	11.85834756	10.23270276	1.626456	0.46206
UBN2	522.225159	188.39147	9.031288103	7.565227535	1.470938	0.4621
ANKMY1	0.82892882	7.4365053	0.870998931	3.076645515	-3.1653	0.46225
LOC100524883	0.82892882	7.4365053	0.870998931	3.076645515	-3.1653	0.46225
LOC100738704	0.82892882	7.4365053	0.870998931	3.076645515	-3.1653	0.46225
LOC102162949	0.82892882	7.4365053	0.870998931	3.076645515	-3.1653	0.46225
AMFR	1797.11769	5689.7529	10.81227174	12.47440381	-1.66268	0.46226
LOC100738149	640.761981	228.87911	9.325894513	7.844731552	1.485203	0.4623
LOC100626336	11.6050035	2.4788351	3.655924617	1.798604302	2.227013	0.46231
LOC102166760	11.6050035	2.4788351	3.655924617	1.798604302	2.227013	0.46231
DLL4	11.6050035	2.4788351	3.655924617	1.798604302	2.227013	0.46231
COLEC10	11.6050035	2.4788351	3.655924617	1.798604302	2.227013	0.46231
LOC102160267	11.6050035	2.4788351	3.655924617	1.798604302	2.227013	0.46231
LOC102162496	11.6050035	2.4788351	3.655924617	1.798604302	2.227013	0.46231
LOC102165505	11.6050035	2.4788351	3.655924617	1.798604302	2.227013	0.46231
INPP4B	237.073644	665.15409	7.895264103	9.379712118	-1.48835	0.46248
C5H12orf35	3171.48168	1036.9794	11.63139612	10.01956204	1.61277	0.46259
LOC100621894	164.956836	61.970878	7.374664246	5.976612875	1.412426	0.4626
DROSHA	4888.19327	1559.1873	12.25538072	10.60750351	1.648507	0.46292
LOC100519022	79.5771671	29.746021	6.332299179	4.942327829	1.419658	0.46329
NR3C1	677.234849	2017.7718	9.405641104	10.97926211	-1.57503	0.46344
BMP4	242.876145	681.67966	7.930004838	9.415064951	-1.48887	0.46356
LOC100738929	149.207188	56.186929	7.230810045	5.837613535	1.409011	0.46375
LOC102158256	127673.275	33951.778	16.96210834	15.05124201	1.910898	0.46399
SBK1	203.087562	564.34813	7.673044449	9.142995708	-1.47448	0.46417
AAMDC	173.246124	477.5889	7.444982755	8.902643123	-1.46295	0.46418
CHRM3	39.7885835	14.046732	5.350093502	3.911378308	1.50212	0.46454
LMO4	2607.81008	8430.5182	11.3491762	13.04157672	-1.69278	0.46477
RECK	509.791227	1491.4325	8.996589935	10.54344993	-1.54872	0.4648
C1H9orf80	3544.49965	1153.4846	11.79177324	10.17303322	1.619583	0.46487
ROR2	1213.5518	3742.2147	10.2462083	11.8700621	-1.62466	0.46508
CHD3	6962.17319	23875.314	12.76552919	14.54329248	-1.77791	0.46519
LOC100513474	235.415786	658.54386	7.88518256	9.365324797	-1.48407	0.46547
LOC100511732	901.87456	317.29089	9.818381752	8.314202076	1.507121	0.46555
LOC102167796	30.6703665	81.801559	4.985061656	6.371586023	-1.41528	0.46564
LOC100620494	241.218288	90.064343	7.920163984	6.508814353	1.421311	0.4657
SLIT1	68.8010924	25.61463	6.125177709	4.734147579	1.425463	0.46572
LOC102166318	68.8010924	25.61463	6.125177709	4.734147579	1.425463	0.46572
LOC100627583	140.088971	52.881816	7.140461408	5.751726565	1.4055	0.46573
KIAA1377	79.5771671	212.35354	6.332299179	7.737102248	-1.41604	0.46612
LRRC8E	285.151515	805.62141	8.160635436	9.655747894	-1.49837	0.46615
CLEC3B	35.6439394	12.394176	5.195502704	3.74353388	1.523994	0.46628
LOC102157643	35.6439394	12.394176	5.195502704	3.74353388	1.523994	0.46628
TRIM24	1622.21371	552.78023	10.66463724	9.113169742	1.553186	0.46631
LOC102167299	289.296159	107.41619	8.181381675	6.76043638	1.429336	0.46633
EIF2C3	126.82611	47.924146	6.998038744	5.61247475	1.404027	0.46636
SH3GL3	118.536822	320.59601	6.90131128	8.329105689	-1.43542	0.4664
LOC100738364	21.5521494	6.610227	4.495193036	2.927939483	1.70506	0.46656
LOC100519434	21.5521494	6.610227	4.495193036	2.927939483	1.70506	0.46656
LOC102166404	1325.45719	456.93194	10.3733624	8.838989383	1.536439	0.46659
TGM1	72.9457365	27.267186	6.208395063	4.821056379	1.419658	0.46676
ELP4	853.796688	301.59161	9.739437508	8.241228156	1.501296	0.46692
LOC100736905	2.48678647	11.567897	1.801898017	3.65167138	-2.21777	0.46696
LOC102161743	2.48678647	11.567897	1.801898017	3.65167138	-2.21777	0.46696
LOC100624399	8.28928824	25.61463	3.215568059	4.734147579	-1.62765	0.46705
LRRC37A	8.28928824	25.61463	3.215568059	4.734147579	-1.62765	0.46705
DIS3	2223.18711	744.47681	11.11906244	9.54201967	1.578331	0.46706
TIGD3	211.37685	79.322724	7.730482704	6.327736286	1.414011	0.4671
LOC100627613	1360.2722	468.49984	10.41073986	8.874980846	1.537775	0.46726
LOC102164300	342.347604	126.42059	8.423526086	6.993454623	1.43723	0.4675
MXRA7	426.069415	1230.3285	8.738326772	10.26599998	-1.52988	0.46767
LOC100625751	99.4714588	266.88791	6.65064192	8.065485684	-1.42388	0.46784
LOC102158984	77.0903806	28.919743	6.287072939	4.903025878	1.414496	0.46788
SPRY2	655.6827	1941.7542	9.359052637	10.92388765	-1.56629	0.46845
PRDM15	1013.77995	355.2997	9.986951206	8.476947458	1.512636	0.46868
SHB	150.036117	409.00779	7.238749771	8.679507525	-1.44682	0.46903
C3H16orf5	773.390592	2311.9269	9.596917616	11.17550395	-1.57983	0.46931
PYCARD	17.4075053	47.924146	4.202222212	5.61247475	-1.46104	0.46932
TMEM104	381.307259	1092.34	8.578588783	10.0945264	-1.5184	0.46939
LOC100626961	2501.70719	833.71488	11.28927379	9.705139674	1.585287	0.46966
LOC100620106	29.8414377	79.322724	4.946798112	6.327736286	-1.41042	0.46992
ANKRD23	104.445032	280.10837	6.720347312	8.13498259	-1.42324	0.47025
SLC46A3	22.3810782	60.318321	4.547269558	5.938246292	-1.43032	0.47043
GRAMD2	14.09179	39.661362	3.915692025	5.34558663	-1.49288	0.47055
LOC102160023	58.0250177	21.483238	5.883254663	4.490777898	1.433464	0.4706
PDZRN3	2009.32347	6363.996	10.97321194	12.6359439	-1.66322	0.47062
ARHGAP26	203.916491	76.843889	7.678892279	6.282511874	1.407976	0.47067
MAGI1	4316.23239	1394.7579	12.07589104	10.446833	1.629758	0.47075
LOC100737167	62.1696618	23.135794	5.981159942	4.593102408	1.426084	0.47086
ZNF565	48.9068006	128.89943	5.641164514	7.021251246	-1.39814	0.47101
ZNF507	3120.91702	1027.8903	11.60821648	10.00687345	1.602284	0.47102
TSPAN17	906.019204	2730.85	9.824989287	11.41566257	-1.59174	0.47112
ST5	[illegible]	[illegible]	[illegible]	[illegible]	[illegible]	[illegible]
[illegible]	[illegible]	[illegible]	[illegible]	[illegible]	[illegible]	[illegible]
[illegible]	[illegible]	[illegible]	[illegible]	[illegible]	[illegible]	[illegible]
DAW1	[illegible]	[illegible]	[illegible]	[illegible]	[illegible]	[illegible]
[illegible]	[illegible]	[illegible]	[illegible]	[illegible]	[illegible]	[illegible]
[illegible]	[illegible]	[illegible]	[illegible]	[illegible]	[illegible]	[illegible]
[illegible]	[illegible]	[illegible]	[illegible]	[illegible]	[illegible]	[illegible]
[illegible]	[illegible]	[illegible]	[illegible]	[illegible]	[illegible]	[illegible]
[illegible]	[illegible]	[illegible]	[illegible]	[illegible]	[illegible]	[illegible]

MAP3K3	479.141013	1388.1477	8.907314364	10.43998425		-1.53464	0.71817
LOC100739082	92.514259	39.661362	6.547114458	5.34558663		1.221942	0.71831
PATL1	850.578859	2596.1666	9.733996324	11.34272287		-1.60987	0.71834
FAM221B	92.514259	233.83678	6.547114458	7.875514565		-1.33775	0.71836
DNAJC11	4929.49111	1655.8619	12.26751564	10.69423761		1.573856	0.71837
NUDT5	4236.32458	1438.5506	12.04893793	10.49140283		1.558197	0.71859
ALG1	144.985033	378.43549	7.189676653	8.567710832		-1.38414	0.71866
FKBP10	3184.14748	10941.578	11.63714446	13.41766507		-1.78084	0.71873
ICK	1244.10966	461.06333	10.2820571	8.851946794		1.432077	0.71887
PDE4DIP	41.4242951	100.80596	5.406818783	6.669678233		-1.28303	0.71893
DCLK2	71.8021115	178.47613	6.185908388	7.487648157		-1.31363	0.71911
RHOQ	2642.87003	8922.9801	11.36843554	13.12347159		-1.75542	0.71952
CASQ2	42.8051049	104.11107	5.453027102	6.715770874		-1.28227	0.71957
RAN	54990.7517	15748.866	15.74692762	13.94305191		1.803941	0.71961
NPM1	128876.505	35005.283	16.97564095	15.09532627		1.880345	0.71969
IRF7	1427.75737	4557.7515	10.48054523	12.15442305		-1.67457	0.71997
LOC102167315	4.14242951	14.046732	2.362450112	3.911378308		-1.76169	0.72015
LOC102165256	4.14242951	14.046732	2.362450112	3.911378308		-1.76169	0.72015
SLC30A1	593.748229	232.18422	9.216135261	7.865326367		1.354581	0.72023
LOC100521751	385.245944	1090.6875	8.593375974	10.09234416		-1.50139	0.72029
MLF1IP	2227.24627	793.22724	11.12169297	9.633408027		1.489455	0.72043
SATB1	100.799118	255.32002	6.669581252	8.001802338		-1.34082	0.72043
ST6GAL1	292.731685	808.10025	8.198355089	9.660174654		-1.46496	0.72069
LOC102164606	258.211439	704.81545	8.017985577	9.463147202		-1.44869	0.72082
LOC100621201	26.2353869	63.623435	4.767410456	6.013985524		-1.27804	0.72083
LOC102160529	26.2353869	63.623435	4.767410456	6.013985524		-1.27804	0.72083
HNMT	178.124469	470.97867	7.484818616	8.882577857		-1.40278	0.72088
LOC100737059	357.629748	1004.7545	8.486351351	9.974062478		-1.4903	0.7209
MRTO4	6187.40887	2051.6492	12.5953528	11.00327137		1.592551	0.72101
LOC102167496	74.5637311	185.08636	6.239622035	7.539828464		-1.31165	0.72103
LOC100737809	24.854577	60.318321	4.692347799	5.938246292		-1.27908	0.72121
HEATR4	48.3283443	20.656959	5.624344958	4.436758793		1.226242	0.7213
F12	42.8051049	18.178124	5.453027102	4.261389712		1.23558	0.72152
LOC100738476	42.8051049	18.178124	5.453027102	4.261389712		1.23558	0.72152
LOC102166206	84.2294	36.356248	6.413279271	5.223277669		1.212121	0.72165
LOC100620357	53.8515836	23.135794	5.777461367	4.593102408		1.218862	0.72166
LOC100049687	17.9505279	6.610227	4.24416613	2.927939483		1.441255	0.72177
LOC100738676	17.9505279	6.610227	4.24416613	2.927939483		1.441255	0.72177
LOC100738739	17.9505279	6.610227	4.24416613	2.927939483		1.441255	0.72177
LOC100625863	17.9505279	6.610227	4.24416613	2.927939483		1.441255	0.72177
LOC100524915	1244.10966	462.71589	10.2820571	8.857097348		1.426915	0.72182
LOC102166906	6.90404918	19.830681	2.982591923	4.380638094		-1.52222	0.72187
LOC102162966	6.90404918	19.830681	2.982591923	4.380638094		-1.52222	0.72187
LOC100620793	6.90404918	19.830681	2.982591923	4.380638094		-1.52222	0.72187
IDNK	363.152987	147.07755	8.508400868	7.210209122		1.304	0.72197
INTS9	2402.60911	852.71928	11.23098658	9.73761795		1.494459	0.72211
CDC42SE2	5142.13583	1729.4006	12.32843254	10.75689038		1.572096	0.72218
THSD4	48.3283443	117.33153	5.624344958	6.886690713		-1.27965	0.72227
LOC100513918	2299.04838	818.84187	11.16744849	9.679201855		1.48938	0.72243
MIR24-1	24.854577	9.9153405	4.692347799	3.448285225		1.325777	0.72253
SLC1A1	22.0929574	53.708094	4.529381037	5.773682393		-1.28155	0.72254
NUDT2	974.851744	369.34643	9.930518173	8.532731629		1.400208	0.7227
LOC100624866	117.368836	50.402981	6.88714549	5.683780114		1.219468	0.72272
LOC100513028	2873.46527	1008.0596	11.48907788	9.978795694		1.511211	0.72312
LOC100514431	96.6566885	242.92584	6.609646952	7.930298794		-1.32957	0.7232
PARL	4914.30221	1659.9932	12.2630644	10.69783049		1.565809	0.72325
LOC100738417	611.698757	1797.9817	9.259034115	10.81296483		-1.55549	0.72333
LOC100521985	89.7526393	38.835083	6.503867696	5.315967693		1.208594	0.72352
GPCPD1	444.620767	1268.3373	8.799672657	10.30985977		-1.51229	0.72378
AGMO	11.0464787	3.3051135	3.590539588	2.106051269		1.740815	0.72409
LOC102164985	11.0464787	3.3051135	3.590539588	2.106051269		1.740815	0.72409
LOC100739179	11.0464787	3.3051135	3.590539588	2.106051269		1.740815	0.72409
NCMAP	11.0464787	3.3051135	3.590539588	2.106051269		1.740815	0.72409
LOC102165250	11.0464787	3.3051135	3.590539588	2.106051269		1.740815	0.72409
LOC100156909	71.8021115	176.82357	6.185908388	7.474302765		-1.30021	0.72418
ATG14	211.263905	563.52185	7.729715254	9.140885611		-1.41543	0.72425
SLC35F2	3090.25241	1079.9458	11.59397574	10.07807851		1.516766	0.72428
TAOK1	165.69718	432.96987	7.381085891	8.761451061		-1.38572	0.72434
TMEFF2	52.4707738	127.24687	5.740678649	7.002779797		-1.27804	0.72438
LOC100738652	9.66566885	25.61463	3.414902536	4.734147579		-1.40603	0.72465
LOC102165890	9.66566885	25.61463	3.414902536	4.734147579		-1.40603	0.72465
LOC102167138	9.66566885	25.61463	3.414902536	4.734147579		-1.40603	0.72465
LOC102161009	225.072003	94.195734	7.820638529	6.572825024		1.256653	0.72482
LOC100626042	59.3748229	144.59872	5.915875148	7.185853814		-1.28413	0.72492
ENDOV	481.902633	1382.3637	8.915588519	10.43396481		-1.52032	0.72493
ASH1L	1158.49945	3600.9211	10.17928642	11.81455088		-1.63611	0.72521
LOC100738591	113.226407	286.7186	6.835752397	8.168514654		-1.34043	0.72528
FSTL3	46.9475344	113.20014	5.583384725	6.83542057		-1.26975	0.72534
ALKBH8	1502.3211	553.60651	10.55393748	9.115320738		1.44026	0.72535
LOC100524893	2417.79802	860.98206	11.24007459	9.751514039		1.489639	0.72536
LOC100624914	132.557744	339.60041	7.061319821	8.411936365		-1.35722	0.72544
LOC102164895	143.604223	370.17271	7.175965874	8.535946834		-1.3661	0.72552
NLE1	3601.15205	1246.8541	11.81464337	10.28523351		1.530166	0.72568
LOC100520212	1097.74382	413.96546	10.10163933	8.696847462		1.406959	0.72575
ADAM17	532.992597	1541.0092	9.06067593	10.59059562		-1.53169	0.72581
SLC16A2	2018.74398	728.77752	10.97995672	9.511312908		1.469908	0.72596
LOC100623249	1699.77691	621.36134	10.7319782	9.281608625		1.451841	0.72607
CCDC150	393.530803	159.47173	8.623994134	7.326175314		1.303176	0.72615
LOC100738805	49.7091541	21.483238	5.664174303	4.490777898		1.2103	0.72629
RYR3	28.9970066	69.407383	4.906746634	6.137654819		-1.25919	0.72632
CNIH4	941.712308	359.43109	9.880673754	8.493579657		1.389571	0.72659
MPV17L2	2862.41879	1008.8859	11.48352297	9.979976575		1.504472	0.72692
SRM	8507.1694	2777.1216	13.05463304	11.43989404		1.615089	0.72693
LOC100511765	2577.97196	915.51644	11.33258037	9.840016943		1.493579	0.72696
CACNA1G	209.883095	556.91162	7.720299639	9.123892796		-1.40786	0.72704
TBPL1	1206.8278	452.80055	10.23819906	8.82591454		1.414272	0.72716
CCNA2	4099.6244	1408.8046	12.00162789	10.46127953		1.54102	0.72716
LOC102161154	1010.7528	384.21944	9.982641126	8.589536711		1.395428	0.72739
CENPT	2515.83552	895.68576	11.29739522	9.808458669		1.489973	0.7276
PRMT6	1962.13078	711.42568	10.93894057	9.476595706		1.463636	0.72789
ANKRD12	1082.55491	3330.7281	10.08155655	11.70205496		-1.6214	0.72789
C1H6orf162	741.494882	288.37115	9.536237241	8.176777292		1.36251	0.72793
LOC102164594	164.316371	70.233662	7.369085785	6.154487245		1.226242	0.72805
RIOK2	2684.29432	951.87268	11.39086451	9.896139655		1.495702	0.72818
LENEP	38.6626754	16.525567	5.309710091	4.13138925		1.226242	0.72821
NCEH1	955.520406	365.21504	9.901651937	8.516547233		1.387541	0.72852
LOC100739756	647.599813	254.49374	9.341184798	7.997144125		1.347472	0.72858
MERTK	71.8021115	31.398578	6.185908388	5.017858594		1.193327	0.72859
RLN3	23.4737672	56.186929	4.613164285	5.837613535		-1.25919	0.7287
TLR1	16.569718	40.48764	4.135019133	5.374609695		-1.28893	0.72883
LOC102163312	16.569718	40.48764	4.135019133	5.374609695		-1.28893	0.72883
TRMT6	3026.73516	1065.0728	11.5640233	10.05809027		1.50681	0.72896
NTN4	615.841187	1795.5029	9.268755287	10.81097555		-1.54376	0.729
LOC100627925	46.9475344	112.37386	5.583384725	6.824944216		-1.25919	0.72907
RABEP1	262.353869	707.29429	8.040858843	9.468205094		-1.4308	0.72923
LOC102161324	151.889082	65.275991	7.256341575	6.05041444		1.218394	0.72923
EXOC6B	103.560738	258.62513	6.708197415	8.020286225		-1.32039	0.7294
SGMS2	91.1334492	225.574	6.525653118	7.823838478		-1.30755	0.72941
LOC100738871	67.659682	164.4294	6.101391269	7.370071807		-1.2811	0.72949
LOC100625686	129.796125	56.186929	7.031175985	5.837613535		1.207941	0.72973
LOC100737277	1488.513	551.95395	10.540625	9.111015534		1.431252	0.7299
PAXIP1	1679.06476	617.22994	10.71430113	9.271999722		1.443778	0.72994
C9H1orf21	469.475344	1332.787	8.877975309	10.38131259		-1.50532	0.73
FAM78A	19.3313377	7.4365053	4.345633235	3.076645515		1.378245	0.73012
CLIP1	886.479915	2668.0529	9.793570658	11.38211216		-1.58963	0.73026
LOC100153260	2415.0364	865.11346	11.23842648	9.758412211		1.481084	0.73031
LOC102158714	92.514259	40.48764	6.547114458	5.374609695		1.192194	0.73038
RGL1	943.093118	2853.9655	9.882785352	11.47925759		-1.5975	0.73044
RIOK1	2453.69908	878.33391	11.26133046	9.780267293		1.482117	0.73067
CRIM1	3458.92864	11776.946	11.75652657	13.5238003		-1.76757	0.73069
ARHGEF15	161.554751	69.407383	7.34478191	6.137654819		1.218862	0.73079
LOC102161808	33.1394361	14.046732	5.093367322	3.911378308		1.238314	0.7309
LOC102163572	33.1394361	14.046732	5.093367322	3.911378308		1.238314	0.7309
AGO3	51.0899639	22.309516	5.702933533	4.542847146		1.195381	0.73095
LOC102165638	34.5202459	81.801559	5.150569664	6.371586023		-1.24469	0.73099
BMP2	34.5202459	81.801559	5.150569664	6.371586023		-1.24469	0.73099
LOC102166125	8.28485901	22.309516	3.214880004	4.542847146		-1.42911	0.731
LRBA	183.647708	478.41518	7.528631546	8.905131772		-1.38132	0.73112
LOC100622546	5.52323934	16.525567	2.705588563	4.13138925		-1.58111	0.73116
EPX	5.52323934	16.525567	2.705588563	4.13138925		-1.58111	0.73116
LOC102160321	5.52323934	16.525567	2.705588563	4.13138925		-1.58111	0.73116
LOC100523190	20.7121475	49.576702	4.440430524	5.660401068		-1.25919	0.73124
ASAP1	962.424456	2914.2838	9.912027735	11.50942063		-1.5984	0.73127
SRSF2	8355.28032	2744.0705	13.02864517	11.42262747		1.606371	0.73132
LOC100739141	296.874115	806.44769	8.218558948	9.65722499		-1.44173	0.73134
CTSD	1971.79645	6375.5639	10.94602639	12.63856351		-1.69304	0.73144
IMMP2L	30.3778164	71.886218	4.971673052	6.187574144		-1.2427	0.73161
C2H19orf57	135.319364	58.665764	7.090846698	5.89883146		1.205778	0.73163
SUSD2	71.8021115	174.34474	6.185908388	7.454050313		-1.27984	0.73169
C5H12orf11	1895.8519	692.42128	10.88939133	9.437588292		1.453124	0.73185
LOC100620128	28.9970066	68.581105	4.906746634	6.120623682		-1.24191	0.73204
OCIAD2	336.9176	924.6055	8.400527683	9.854253624		-1.45644	0.73211
RRS1	2919.03199	1033.6742	11.51176846	10.01496091		1.497708	0.73212
LOC100510901	270.638728	727.95125	8.08554537	9.509678516		-1.42747	0.73227
LOC100738140	677.977629	266.88791	9.407220232	8.065485684		1.345004	0.7323
LOC100623803	149.127462	380.91433	7.230044098	8.577105241		-1.35292	0.73245
LOC100738161	11.0464787	28.093465	3.590539588	4.862623207		-1.34665	0.7326
LOC102166179	11.0464787	28.093465	3.590539588	4.862623207		-1.34665	0.7326
LOC100627924	27.6161967	65.275991	4.838760036	6.05041444		-1.24104	0.73262
BBIP1	2047.74099	744.47681	11.00052189	9.54201967		1.459734	0.73268
STOM	535.754216	1532.7464	9.068117809	10.58284422		-1.51648	0.73281
TRMT61B	263.734679	110.7213	8.048403381	6.803760479		1.252154	0.7331
APOO	1157.11864	438.75382	10.17756734	8.780552285		1.399053	0.73311
ACAD9	4624.33214	1586.4545	12.17534125	10.6324995		1.543439	0.73318
ACOT13	882.337485	341.25297	9.786820924	8.41891924		1.370489	0.73322
C1H6orf57	498.472351	200.78564	8.964261007	7.65667973		1.311857	0.73332
LOC100521511	153.269892	391.65595	7.269312714	8.617121942		-1.35351	0.73348
ENPP5	70.4213016	170.21334	6.158282521	7.419651342		-1.27326	0.73348
LOC102160025	158.793131	68.581105	7.320061583	6.120623682		1.211265	0.73357
SCN2A	164.316371	422.22825	7.369085785	8.725292112		-1.36155	0.73359
LOC102161798	113.226407	49.576702	6.835752397	5.660401068		1.191476	0.73369
LOC100521322	113.226407	49.576702	6.835752397	5.660401068		1.191476	0.73369
LOC100739649	113.226407	49.576702	6.835752397	5.660401068		1.191476	0.73369
LOC100524077	3514.16103	1230.3285	11.77937507	10.26599998		1.514137	0.73378
LOC102161970	776.015128	303.24416	9.601798876	8.249085772		1.355605	0.73403
LOC100519689	122.892075	53.708094	6.9529401	5.773682393		1.19418	0.73424
LOC102159477	2.76161967	10.741619	1.911353989	3.553559423		-1.95962	0.73465
LOC102161636	2.76161967	10.741619	1.911353989	3.553559423		-1.95962	0.73465
LOC102164153	2.76161967	10.741619	1.911353989	3.553559423		-1.95962	0.73465
GATA5	2.76161967	10.741619	1.911353989	3.553559423		-1.95962	0.73465
ZSCAN9	2.76161967	10.741619	1.911353989	3.553559423		-1.95962	0.73465
LOC102160199	2.76161967	10.741619	1.911353989	3.553559423		-1.95962	0.73465
KIAA1456	2.76161967	10.741619	1.911353989	3.553559423		-1.95962	0.73465
LOC100739277	2.76161967	10.741619	1.911353989	3.553559423		-1.95962	0.73465
LOC100739693	2.76161967	10.741619	1.911353989	3.553559423		-1.95962	0.73465
LOC100515222	2.76161967	10.741619	1.911353989	3.553559423		-1.95962	0.73465
GABRR1	2.76161967	10.741619	1.911353989	3.553559423		-1.95962	0.73465
THUMPD3	1267.58343	478.41518	10.30900269	8.905131772		1.405746	0.73468
LOC100739573	515.042069	207.39587	9.011344871	7.703182885		1.312303	0.73473
SYT5	93.8950688	230.53167	6.568261215	7.855065709		-1.29584	0.73486
LOC102159000	379.722705	156.16661	8.572596799	7.296150961		1.28186	0.73494
LOC100625894	109.083977	47.924146	6.782460686	5.61247475		1.186615	0.73497
LOC100738744	63.5172524	28.093465	6.011613095	4.862623207		1.176914	0.73499
LOC100515534	273.400348	114.85269	8.100138498	6.856147779		1.25123	0.735
SLC25A33	2154.06334	782.48562	11.07351456	9.613762982		1.460925	0.73502
LOC102158116	13.8080984	33.877413	3.888314478	5.124221138		-1.29481	0.73506
TRIOBP	1222.01671	3761.2191	10.25622839	11.87736817		-1.62194	0.73508
TYW5	364.533797	150.38266	8.513860991	7.242056188		1.277414	0.73519
LOC100622494	0	4.9576702	0	2.574748269		-20	0.73528
SPATA21	0	4.9576702	0	2.574748269		-20	0.73528
LOC100620069	0	4.9576702	0	2.574748269		-20	0.73528
LOC100622073	0	4.9576702	0	2.574748269		-20	0.73528
SPPL2C	0	4.9576702	0	2.574748269		-20	0.73528
PCDHA5	0	4.9576702	0	2.574748269		-20	0.73528
LOC100736929	0	4.9576702	0	2.574748269		-20	0.73528
LOC100622673	0	4.9576702	0	2.574748269		-20	0.73528
LOC102161912	0	4.9576702	0	2.574748269		-20	0.73528
SLC5A12	0	4.9576702	0	2.574748269		-20	0.73528
SLC1A7	0	4.9576702	0	2.574748269		-20	0.73528
LOC102161883	0	4.9576702	0	2.574748269		-20	0.73528
LOC102159507	0	4.9576702	0	2.574748269		-20	0.73528
LOC102161657	0	4.9576702	0	2.574748269		-20	0.73528
LOC102157450	0	4.9576702	0	2.574748269		-20	0.73528
ZBBX	0	4.9576702	0	2.574748269		-20	0.73528
LOC102157577	0	4.9576702	0	2.574748269		-20	0.73528
HSPB9	0	4.9576702	0	2.574748269		-20	0.73528
LOC102161032	0	4.9576702	0	2.574748269		-20	0.73528
LOC102163731	0	4.9576702	0	2.574748269		-20	0.73528
LOC102163513	0	4.9576702	0	2.574748269		-20	0.73528
LOC102166839	0	4.9576702	0	2.574748269		-20	0.73528
ANGPTL3	0	4.9576702	0	2.574748269		-20	0.73528
LOC102164798	0	4.9576702	0	2.574748269		-20	0.73528
LOC102166677	0	4.9576702	0	2.574748269		-20	0.73528
LOC102166624	0	4.9576702	0	2.574748269		-20	0.73528
LOC100624550	0	4.9576702	0	2.574748269		-20	0.73528
LOC102157864	0	4.9576702	0	2.574748269		-20	0.73528
LOC102157863	0	4.9576702	0	2.574748269		-20	0.73528
ADCY10	0	4.9576702	0	2.574748269		-20	0.73528
GRIA1	0	4.9576702	0	2.574748269		-20	0.73528
FAM167A	0	4.9576702	0	2.574748269		-20	0.73528
LOC102157751	0	4.9576702	0	2.574748269		-20	0.73528
ONECUT2	0	4.9576702	0	2.574748269		-20	0.73528
LOC102164305	0	4.9576702	0	2.574748269		-20	0.73528
LOC100624077	0	4.9576702	0	2.574748269		-20	0.73528
LOC100152300	0	4.9576702	0	2.574748269		-20	0.73528
LOC102166095	0	4.9576702	0	2.574748269		-20	0.73528
LOC102166026	0	4.9576702	0	2.574748269		-20	0.73528
HTR4	0	4.9576702	0	2.574748269		-20	0.73528
LOC100736708	0	4.9576702	0	2.574748269		-20	0.73528
LOC102166291	0	4.9576702	0	2.574748269		-20	0.73528
LOC102166348	0	4.9576702	0	2.574748269		-20	0.73528
LOC102158069	0	4.9576702	0	2.574748269		-20	0.73528
OPRD1	0	4.9576702	0	2.574748269		-20	0.73528
ARL11	0	4.9576702	0	2.574748269		-20	0.73528
LOC102160552	0	4.9576702	0	2.574748269		-20	0.73528
LOC102160840	0	4.9576702	0	2.574748269		-20	0.73528
LIME1	0	4.9576702	0	2.574748269		-20	0.73528
LOC102164843	0	4.9576702	0	2.574748269		-20	0.73528
OLFM3	0	4.9576702	0	2.574748269		-20	0.73528
LOC102167026	0	4.9576167	0	2.574748269		-20	0.73528
IKZF1	0	4.9576702	0	2.574748269		-20	0.73528
DCDC2B	0	4.9576702	0	2.574748269		-20	0.73528
LOC102162578	0	4.9576702	0	2.574748269		-20	0.73528
LOC100524169	0	4.9576702	0	2.574748269		-20	0.73528
LOC102162631	0	4.9576702	0	2.574748269		-20	0.73528
LOC100521253	0	4.9576702	0	2.574748269		-20	0.73528
C18H7orf31	0	4.9576702	0	2.574748269		-20	0.73528
LOC100155447	0	4.9576702	0	2.574748269		-20	0.73528
LOC102163287	0	4.9576702	0	2.574748269		-20	0.73528
TBX10	0	4.9576702	0	2.574748269		-20	0.73528
LOC102163253	0	4.9576167	0	2.574748269		-20	0.73528
C12H17orf66	0	4.9576702	0	2.574748269		-20	0.73528
LOC102165659	0	4.9576702	0	2.574748269		-20	0.73528
LOC100523304	0	4.9576702	0	2.574748269		-20	0.73528
LOC100625482	0	4.9576702	0	2.574748269		-20	0.73528
SYT14	0	4.9576702	0	2.574748269		-20	0.73528
LOC102165994	0	4.9576702	0	2.574748269		-20	0.73528
LOC102167400	0	4.9576702	0	2.574748269		-20	0.73528
LOC100737772	0	4.9576702	0	2.574748269		-20	0.73528
PPP1R3A	0	4.9576702	0	2.574748269		-20	0.73528
LOC102165042	0	4.9576702	0	2.574748269		-20	0.73528
LOC102167988	0	4.9576702	0	2.574748269		-20	0.73528
LOC100737391	0	4.9576702	0	2.574748269		-20	0.73528
LOC102165109	0	4.9576702	0	2.574748269		-20	0.73528
LOC102165233	0	4.9576702	0	2.574748269		-20	0.73528
LMOD2	0	4.9576702	0	2.574748269		-20	0.73528
LOC102165229	0	4.9576702	0	2.574748269		-20	0.73528

C16H5orf54	506.4750025	152.8615	8.987192949	7.265488457		1.728266	0.53309
DSN1	685.1686881	202.4382	9.422419483	7.6684468		1.758978	0.53326
LOC102166896	7.401513606	26.440908	3.070649265	4.778256309		-1.83688	0.53348
LOC100518101	3315.878095	870.07113	11.69561027	9.766646714		1.930186	0.53371
LOC100738511	393.3375802	1483.1697	8.623287395	10.53544032		-1.91484	0.53375
LRCH2	1973.032056	538.7335	10.9469297	9.07610342		1.872771	0.53376
LOC100624425	32.77813168	104.11107	5.078017628	6.715770874		-1.66732	0.5342
RFX7	4086.69287	1055.9838	11.99707109	10.04573749		1.952346	0.53429
MCAM	7402.570965	1828.554	12.85400558	10.83727631		2.017323	0.53435
LOC102164166	39.1222862	12.394176	5.326331909	3.74353388		1.658328	0.53471
LOC102158800	39.1222862	12.394176	5.326331909	3.74353388		1.658328	0.53471
SERPINB1	482.1557435	1849.211	8.916344501	10.85347409		-1.93934	0.53499
LOC102166878	27.49133625	8.2627837	4.832451381	3.211445828		1.734277	0.53508
LOC102167243	27.49133625	8.2627837	4.832451381	3.211445828		1.734277	0.53508
LRRC43	27.49133625	8.2627837	4.832451381	3.211445828		1.734277	0.53508
LOC100522856	890.2963509	3612.489	9.799761389	11.8191768		-2.02064	0.53509
LOC102164565	1523.654444	6495.3743	10.57426658	12.66541904		-2.09188	0.5351
LOC100153150	856.4608601	249.53607	9.743927009	7.968874505		1.779139	0.53534
TIMM23	9610.336738	2330.105	13.23052138	11.18679828		2.044192	0.53536
HMCN1	64.49890428	209.87471	6.033398867	7.72024225		-1.70218	0.53548
TCTE1	52.86795433	170.21334	5.751355374	7.419651341		-1.68688	0.53553
CKAP4	3144.585923	14307.01	11.61911306	13.80453541		-2.18578	0.53566
LOC100512457	59.21210885	191.69658	5.911981742	7.590187173		-1.69486	0.53569
LOC100620599	228.3895627	72.712497	7.84165594	6.20383732		1.651221	0.53573
LOC100521612	214.6438946	68.581105	7.75250706	6.120623682		1.646062	0.53594
LOC100521808	622.784502	185.91263	9.284903899	7.546220276		1.744108	0.53619
LOC100738540	105.7359087	34.703692	6.737901807	5.158001345		1.607304	0.53624
STK39	6273.311461	1572.4077	12.61524143	10.61967687		1.996252	0.5364
FAM199X	4623.831286	1186.5357	12.17518502	10.21375522		1.962333	0.53669
EIF4EBP1	2520.744062	11227.471	11.30020615	13.4548738		-2.15511	0.53674
UQCRB	2659.258103	712.25196	11.37735051	9.478267989		1.900346	0.53712
MIB1	17584.93897	4083.4677	14.10213475	11.99593236		2.106474	0.53715
DET1	1473.958567	413.13919	10.52645871	8.693971907		1.834996	0.53732
LOC100512680	13.74566813	44.619032	3.882219287	5.511563931		-1.69868	0.53754
LOC100627386	87.76080419	28.919743	6.471850834	4.903025878		1.601522	0.53755
SLC25A23	105.7359087	354.47342	6.737901807	8.473597884		-1.74521	0.53773
LOC102167038	51.81059524	166.08195	5.722755497	7.3844121		-1.68058	0.53779
SAT2	241.0778717	865.11346	7.919327399	9.758412211		-1.84339	0.53786
LOC102161819	41.23700438	13.220454	5.400435611	3.829895615		1.641168	0.53856
LOC100627491	864.9197328	3483.5896	9.758089489	11.76677304		-2.00994	0.53883
LOC102163779	29.60605442	9.0890621	4.935745167	3.334720158		1.703689	0.53891
LOC102164310	29.60605442	9.0890621	4.935745167	3.334720158		1.703689	0.53891
LOC100620723	67.67098154	22.309516	6.10162868	4.542847146		1.600878	0.53917
LOC100523432	158.603863	547.82256	7.31835176	9.100195977		-1.78828	0.5394
LOC100626166	265.3971307	958.48291	8.057434724	9.906113301		-1.8526	0.53944
PYGO1	1141.947813	327.20624	10.15854382	8.358458837		1.803225	0.53966
LOC100516434	1342.84604	380.08805	10.39215215	8.573980563		1.820888	0.53973
LOC102159607	4.229436346	17.351846	2.386655454	4.19785327		-2.03655	0.5399
LOC102162905	4.229436346	17.351846	2.386655454	4.19785327		-2.03655	0.5399
LOC102167874	4.229436346	17.351846	2.386655454	4.19785327		-2.03655	0.5399
DDX46	17123.93041	3995.8822	14.0638105	11.96465934		2.099428	0.54013
SEZ6L2	12.68830904	41.313919	3.774872332	5.403060391		-1.70313	0.54023
KRR1	5128.19157	1310.4775	12.32451574	10.35697734		1.968358	0.54028
OVCH1	9.516231779	1.6525567	3.394545939	1.407383614		2.525691	0.54034
ATP6V0A4	9.516231779	1.6525567	3.394545939	1.407383614		2.525691	0.54034
LOC102163685	9.516231779	1.6525567	3.394545939	1.407383614		2.525691	0.54034
LOC100524515	9.516231779	1.6525567	3.394545939	1.407383614		2.525691	0.54034
LOC100156191	9.516231779	1.6525567	3.394545939	1.407383614		2.525691	0.54034
LOC100520411	9.516231779	1.6525567	3.394545939	1.407383614		2.525691	0.54034
LOC100738383	9.516231779	1.6525567	3.394545939	1.407383614		2.525691	0.54034
LOC100623599	9.516231779	1.6525567	3.394545939	1.407383614		2.525691	0.54034
LOC102158113	9.516231779	1.6525567	3.394545939	1.407383614		2.525691	0.54034
LOC102160539	9.516231779	1.6525567	3.394545939	1.407383614		2.525691	0.54034
LOC102167823	9.516231779	1.6525567	3.394545939	1.407383614		2.525691	0.54034
LOC100517427	9.516231779	1.6525567	3.394545939	1.407383614		2.525691	0.54034
ALOX15B	9.516231779	1.6525567	3.394545939	1.407383614		2.525691	0.54034
SYTL3	9.516231779	1.6525567	3.394545939	1.407383614		2.525691	0.54034
LOC102163081	9.516231779	1.6525567	3.394545939	1.407383614		2.525691	0.54034
PPP1R9A	89.87552236	29.746021	6.505819847	4.942327829		1.595232	0.54064
LOC100157748	122.653654	413.96546	6.950161063	8.696847462		-1.75492	0.54099
LOC102159655	6.344154519	23.135794	2.876596413	4.593102407		-1.86663	0.54121
CDIPT	1125.030068	4627.9852	10.13702964	12.17648022		-2.04042	0.54133
LDHB	36027.39616	7971.9337	15.13684681	12.96089496		2.176093	0.54144
LOC102159004	0	5.7839486	0	2.76212524		-20	0.54154
LOC102159285	0	5.7839486	0	2.76212524		-20	0.54154
MMD2	0	5.7839486	0	2.76212524		-20	0.54154
LOC102157405	0	5.7839486	0	2.76212524		-20	0.54154
LOC102161505	0	5.7839486	0	2.76212524		-20	0.54154
ZFR2	0	5.7839486	0	2.76212524		-20	0.54154
OPTC	0	5.7839486	0	2.76212524		-20	0.54154
KIRREL2	0	5.7839486	0	2.76212524		-20	0.54154
LOC102164743	0	5.7839486	0	2.76212524		-20	0.54154
NT5C1B	0	5.7839486	0	2.76212524		-20	0.54154
LOC100738507	0	5.7839486	0	2.76212524		-20	0.54154
LOC100621988	0	5.7839486	0	2.76212524		-20	0.54154
NUPR1L	0	5.7839486	0	2.76212524		-20	0.54154
CPA5	0	5.7839486	0	2.76212524		-20	0.54154
NR1I2	0	5.7839486	0	2.76212524		-20	0.54154
TACR2	0	5.7839486	0	2.76212524		-20	0.54154
NOD2	0	5.7839486	0	2.76212524		-20	0.54154
BCL2A1	0	5.7839486	0	2.76212524		-20	0.54154
LOC102158608	0	5.7839486	0	2.76212524		-20	0.54154
LOC102162911	0	5.7839486	0	2.76212524		-20	0.54154
LOC102165348	0	5.7839486	0	2.76212524		-20	0.54154
LOC102163245	0	5.7839486	0	2.76212524		-20	0.54154
LOC102167581	0	5.7839486	0	2.76212524		-20	0.54154
LOC100737053	0	5.7839486	0	2.76212524		-20	0.54154
ITM2A	0	5.7839486	0	2.76212524		-20	0.54154
CCDC13	0	5.7839486	0	2.76212524		-20	0.54154
MIR339	0	5.7839486	0	2.76212524		-20	0.54154
MIR29C	0	5.7839486	0	2.76212524		-20	0.54154
LOC100620761	0	5.7839486	0	2.76212524		-20	0.54154
LOC100737835	0	5.7839486	0	2.76212524		-20	0.54154
LOC100620314	0	5.7839486	0	2.76212524		-20	0.54154
CCND2	187.1525583	653.58619	7.555759096	9.35443936		-1.80416	0.54156
SNX21	283.3722352	1026.2377	8.151636803	10.00455439		-1.85659	0.54172
TXLNB	2.114718173	11.567897	1.639101631	3.651671381		-2.45159	0.54194
SMYD1	2.114718173	11.567897	1.639101631	3.651671381		-2.45159	0.54194
BMP8B	2.114718173	11.567897	1.639101631	3.651671381		-2.45159	0.54194
LOC102158467	2.114718173	11.567897	1.639101631	3.651671381		-2.45159	0.54194
LOC100739452	2.114718173	11.567897	1.639101631	3.651671381		-2.45159	0.54194
KCTD19	2.114718173	11.567897	1.639101631	3.651671381		-2.45159	0.54194
PLEKHB1	43.35172255	14.046732	5.470918232	3.911378308		1.625855	0.54218
ITGB1BP2	43.35172255	14.046732	5.470918232	3.911378308		1.625855	0.54218
HSPA4L	170.2348129	589.13648	7.419832229	9.20490483		-1.79108	0.54226
SENP2	7305.293929	1821.1175	12.83492408	10.8314003		2.004118	0.54236
SERINC5	1595.554862	447.0166	10.64074641	8.807408375		1.835658	0.5424
SERINC4	253.7661808	908.90621	7.993029968	9.829574033		-1.84063	0.54259
TAGLN2	4207.231805	19479.513	12.03899846	14.24974402		-2.21101	0.54288
COMTD1	118.4242177	397.4399	6.899951616	8.638218305		-1.74677	0.54301
PEX14	418.7141983	1568.2763	8.713263458	10.61588372		-1.90514	0.54314
C5H12orf41	5038.316048	1293.1257	12.29901222	10.33776199		1.962079	0.54317
LOC102162530	75.07249515	243.75212	6.249303021	7.935177544		-1.69906	0.54318
TMEM9	1115.513836	4574.2771	10.12478541	12.1596434		-2.03584	0.54323
MAP3K5	62.38418611	20.656959	5.986051038	4.436758793		1.594552	0.54333
MRPL3	8502.224415	2097.9208	13.0537943	11.035432		2.01888	0.54359
EPB41L3	468.4100754	1771.5408	8.874704998	10.79160314		-1.91916	0.5436
UBALD1	372.1903985	1377.406	8.543768058	10.42878522		-1.88784	0.54402
QRFPR	11.63094995	2.4788351	3.658891241	1.798604302		2.230235	0.54416
LOC100738337	11.63094995	2.4788351	3.658891241	1.798604302		2.230235	0.54416
LOC102166207	11.63094995	2.4788351	3.658891241	1.798604302		2.230235	0.54416
LOC102158757	11.63094995	2.4788351	3.658891241	1.798604302		2.230235	0.54416
LOC102165866	11.63094995	2.4788351	3.658891241	1.798604302		2.230235	0.54416
LOC102165921	11.63094995	2.4788351	3.658891241	1.798604302		2.230235	0.54416
LOC100523747	11.63094995	2.4788351	3.658891241	1.798604302		2.230235	0.54416
LOC100625259	1354.47699	385.04572	10.40458491	8.592627913		1.814634	0.54421
LOC100627669	47.5811589	150.38266	5.602325001	7.242056187		-1.66018	0.54454
NUDT4	7363.448679	1839.2957	12.84636181	10.84572185		2.001228	0.54491
C12H17orf49	356.3300122	1311.3038	8.481113281	10.357886		-1.87972	0.54525
PSMA1	18036.4313	4214.0197	14.13870628	12.04132366		2.097645	0.54528
LOC102164728	158.603863	52.055537	7.31835176	5.729431428		1.607304	0.54542
LOC102160855	79.30193149	26.440908	6.327362784	4.778256309		1.584584	0.54547
TMEM261	190.3246356	61.970878	7.579878842	5.976612875		1.6188	0.54575
PTPN21	965.368846	3892.5974	9.916430135	11.926888		-2.01158	0.54592
ADD1	2460.474594	10811.026	11.26530713	13.40034929		-2.13549	0.54611
TBC1D20	1825.001783	7801.7204	10.83447246	12.92976149		-2.09589	0.54622
FUCA2	498.0161298	1887.2198	8.962942639	10.882811		-1.922	0.5464
CCSAP	414.4847619	128.89943	8.698651756	7.021251245		1.685073	0.54651
DTNB	2320.903195	634.58179	11.18109211	9.311933979		1.870808	0.54654
FRMD1	13.74566813	3.3051135	3.882219287	2.106051269		2.056205	0.54671
LOC102164985	13.74566813	3.3051135	3.882219287	2.106051269		2.056205	0.54671
LOC102167606	13.74566813	3.3051135	3.882219287	2.106051269		2.056205	0.54671
LOC100739361	385.9360666	120.63664	8.5959514	6.926434087		1.677694	0.54675
GANC	39.1222862	122.2892	5.326331909	6.945902605		-1.64423	0.54701
NARFL	475.811589	1793.8503	8.897275491	10.80964784		-1.9146	0.54708
NR2F2	742.2660788	2915.9364	9.537734957	11.5102382		-1.97395	0.54721
LOC100739740	2545.063321	691.595	11.31405258	9.435868156		1.879702	0.54743
LOC100516731	900.8699418	265.23536	9.816775588	8.056558371		1.764046	0.54776
PHLPP1	1389.36984	395.78734	10.44125298	8.632222185		1.811633	0.54781
RNF149	2951.089211	793.22724	11.5275206	9.633408027		1.895441	0.54793
LOC102161119	10.57359087	34.703692	3.532764645	5.158001345		-1.71462	0.54801
LOC102165302	10.57359087	34.703692	3.532764645	5.158001345		-1.71462	0.54801
LOC100525046	319.3224481	1158.4423	8.323381077	10.17921528		-1.8591	0.54841
UPF0639	81.41664967	27.267186	6.364863912	4.821056378		1.578158	0.54844
PAXIP1	2246.888059	617.22994	11.13435448	9.271999722		1.864048	0.54854
NAGPA	254.8235399	905.6011	7.999005211	9.824324094		-1.82938	0.54854
GDPD3	13.74566813	43.792754	3.882219287	5.485193456		-1.67172	0.54865
SMN1	7435.349097	1863.2577	12.86037878	10.86438561		1.996573	0.54877
ITGB7	15.8603863	4.1313919	4.075565686	2.359350201		1.940728	0.54888
HIBADH	851.1740647	252.0149	9.735004335	7.983078556		1.755945	0.54892
HAUS8	1551.14578	438.75382	10.60004835	8.780552285		1.821851	0.54907
LOC102165430	35.95020894	11.567897	5.207510617	3.651671381		1.635874	0.54909
CD63	7534.740851	36469.448	12.87953364	15.15444032		-2.27506	0.54915
LOC102161649	74.01513606	237.96817	6.229109817	7.900674664		-1.68488	0.54915
ITGAL	217.8159718	762.65494	7.773574237	9.576777083		-1.80792	0.54919
CDK3	28.54869534	88.411786	4.885022527	6.482393107		-1.63081	0.54928
LOC102160623	17.97510447	56.186929	4.246035924	5.837613535		-1.64423	0.54929
LOC102158295	17.97510447	56.186929	4.246035924	5.837613535		-1.64423	0.54929
NXT2	3347.598868	892.38064	11.70934185	9.803131183		1.907396	0.54929
DCST2	31.7207726	98.327126	5.032134908	6.634115866		-1.63216	0.54932
C1H14orf166	22034.30601	5093.1799	14.42752931	12.31463419		2.113113	0.54942
LOC102167237	527.6221842	2002.0725	9.04609316	10.96799892		-1.92392	0.54945
GALNT4	49.69587707	156.16661	5.663796517	7.296150961		-1.65189	0.54951
LOC100621801	49.69587707	156.16661	5.663796517	7.296150961		-1.65189	0.54951
WBP11	12549.795	3025.8314	13.61549113	11.5635926		2.05226	0.54972
FAM129C	34.89284986	108.24247	5.165624571	6.771389985		-1.63326	0.54992
MED6	3641.544694	965.09314	11.83073096	9.916018471		1.91581	0.54993
LOC100621093	75.07249515	241.27328	6.249303021	7.920491517		-1.68431	0.55003
LOC100155978	2167.586127	598.22554	11.08253903	9.226955308		1.857328	0.55025
LOC100511920	24.31925899	75.191332	4.662163277	6.251554969		-1.62847	0.5506
PKP4	7644.706196	1915.3133	12.90043405	10.90411771		1.996881	0.55062
LOC102162576	17.97510447	4.9576702	4.246035924	2.57474827		1.858266	0.55093
LOC102167582	17.97510447	4.9576702	4.246035924	2.57474827		1.858266	0.55093
LOC102165042	17.97510447	4.9576702	4.246035924	2.57474827		1.858266	0.55093
OGFOD2	223.1027673	780.83306	7.808016653	9.610716783		-1.80731	0.55102
LOC100622580	4089.864947	1075.8144	11.99819019	10.07255395		1.926624	0.55131
LOC102159839	7.401513606	25.61463	3.070649265	4.734147579		-1.79108	0.55168
LOC102163695	7.401513606	25.61463	3.070649265	4.734147579		-1.79108	0.55168
MUC1	7.401513606	25.61463	3.070649265	4.734147579		-1.79108	0.55168
LOC102162967	7.401513606	25.61463	3.070649265	4.734147579		-1.79108	0.55168
LOC100517057	7.401513606	25.61463	3.070649265	4.734147579		-1.79108	0.55168
GJD3	2368.484354	650.28108	11.21035742	9.347136503		1.864829	0.55179
LOC100511883	5.286795433	19.830681	2.65232482	4.380638094		-1.90727	0.55183
HSP70	38.06492712	12.394176	5.287802017	3.74353388		1.6188	0.55214
ZNF507	3888.96672	1027.8903	11.9255421	10.00687345		1.919701	0.55217
LOC414417	7673.254891	1925.2286	12.90581097	10.91156322		1.994809	0.55229
BCL2L1	348.9284986	1269.1636	8.450916354	10.31079859		-1.86287	0.55246
ZNF628	375.3624757	1374.1009	8.555978985	10.4253218		-1.87213	0.55269
LOC100736758	5933.899194	27996.79	12.53500781	14.77302534		-2.23821	0.55285
LOC102162751	20.08982264	5.7839486	4.398475058	2.76212524		1.796338	0.55298
TRNAM-CAU	20.08982264	5.7839486	4.398475058	2.76212524		1.796338	0.55298
LOC100156509	326.7239578	104.11107	8.356337332	6.715770874		1.649949	0.55336
CLNS1A	12282.28315	2977.081	13.5844086	11.54016727		2.044608	0.55337
FAM133B	5572.282386	1434.4193	12.44431154	10.48725646		1.957802	0.55342
LOC100738657	9.516231779	31.398578	3.394545939	5.017858594		-1.72224	0.55356
CPOX	5695.993399	1464.1653	12.47598502	10.5168477		1.959869	0.55366
LOC100738746	77.18721332	247.05723	6.288860784	7.954529215		-1.67841	0.55397
GNL2	6977.512612	1767.4094	12.76870386	10.78823662		1.981077	0.55448
LOC100624365	30.66341351	94.195734	4.98474489	6.572825024		-1.61914	0.55465
FAM83D	1524.711803	434.62242	10.57526676	8.766934408		1.810702	0.55478
ACTR6	2570.439939	703.98917	11.32836074	9.46145729		1.86839	0.55536
DNAJC27	378.534553	119.81036	8.568087425	6.916600414		1.659672	0.55579
SPAG5	3953.465625	1047.721	11.94926704	10.03441517		1.915863	0.55586
LOC100737091	59.21210885	185.91263	5.911981742	7.546220276		-1.65066	0.55586
C6H1orf167	95.16231779	308.20183	6.587399765	8.27240506		-1.69541	0.55596
LOC102157660	3.17207726	14.046732	2.060765874	3.911378308		-2.14673	0.55627
LOC102163798	3.17207726	14.046732	2.060765874	3.911378308		-2.14673	0.55627
LOC100736598	3.17207726	14.046732	2.060765874	3.911378308		-2.14673	0.55627
LOC102167870	3.17207726	14.046732	2.060765874	3.911378308		-2.14673	0.55627
LOC102165895	3.17207726	14.046732	2.060765874	3.911378308		-2.14673	0.55627
NOVA2	3.17207726	14.046732	2.060765874	3.911378308		-2.14673	0.55627
LOC102165256	3.17207726	14.046732	2.060765874	3.911378308		-2.14673	0.55627
LOC102165903	256.938258	83.454116	8.010881963	6.400095824		1.622367	0.55654
STAMBPL1	344.6990622	109.89502	8.43337288	6.793050814		1.649211	0.55656
PMAIP1	154.3744266	518.90282	7.279605257	9.022098163		-1.74903	0.55694
VWA5B2	24.31925899	7.4365053	4.662163277	3.076645515		1.709403	0.55712
LOC100624406	24.31925899	7.4365053	4.662163277	3.076645515		1.709403	0.55712
LOC102162727	24.31925899	7.4365053	4.662163277	3.076645515		1.709403	0.55712
MRPL44	3150.930078	851.06672	11.62201982	9.734822598		1.888434	0.55716
LOC100739798	63.44154519	21.483238	6.009919183	4.490777899		1.562216	0.55735
SYT17	42.29436346	14.046732	5.436107306	3.911378308		1.590231	0.55784
LOC102165030	253.7661808	82.627837	7.993029968	6.385911347		1.6188	0.55787
LOC100512753	181.8657629	60.318321	7.514641181	5.938246292		1.592206	0.55789
ZBTB24	1208.561436	351.99459	10.24026833	8.463502248		1.779666	0.55789
HIVEP2	491.6719753	1832.6854	8.944483599	10.84053045		-1.89819	0.5579
NEIL2	259.0529762	84.280394	8.022661739	6.414142197		1.619978	0.5582
MDN1	12142.71175	2960.5554	13.56792183	11.53213936		2.036151	0.55831
LOC100739743	1700.233411	482.54657	10.73236538	8.917511028		1.816993	0.55845
LOC102165761	1915.934665	538.7335	10.90458545	9.07610342		1.830405	0.55846
IFITM2	1698.118693	7100.21	10.73057092	12.79384917		-2.06392	0.55865
LOC100525054	53.92531342	167.73451	5.779399292	7.398611253		-1.63714	0.55866
GSG2	1015.064723	299.93905	9.988776589	8.233327508		1.75583	0.55876
LOC100516841	3827.639893	1020.4538	11.90261626	9.996408222		1.907244	0.5591
CA11	75.07249515	237.96817	6.249303021	7.900674664		-1.66441	0.55916
GTF2F2	3315.878095	894.0332	11.69561027	9.805797385		1.890991	0.55919
LOC100152887	26.43397716	8.2627837	4.777891883	3.211445828		1.677694	0.55921
LOC102158761	26.43397716	8.2627837	4.777891883	3.211445828		1.677694	0.55921
PCDH19	32.77813168	99.979683	5.078017628	6.657921243		-1.6089	0.55965
MAP3K8	13.74566813	42.966475	3.882219287	5.458331975		-1.64423	0.55976
GNB2	1906.418433	8045.4725	10.89740565	12.97414074		-2.07731	0.55979
FSD1	109.965345	357.77853	6.793965376	8.486949769		-1.70202	0.56029
ATP6V0A1	483.2131026	1792.1978	8.919498308	10.80831891		-1.891	0.56041
LOC102159978	16.91774539	52.055537	4.163317207	5.729431428		-1.62151	0.56045
PLXNB3	16.91774539	52.055537	4.163317207	5.729431428		-1.62151	0.56045
POC1B	381.7066303	121.46292	8.580095085	6.936201186		1.651948	0.56053
NPHP3	80.35929058	255.32002	6.346235194	8.001802338		-1.66777	0.56066
VAMP1	66.61362245	209.04843	6.079242038	7.714578179		-1.64995	0.56069
LOC100622481	4237.895219	1122.9123	12.04947259	10.13431376		1.916103	0.56075
LOC100517085	119.4815768	40.48764	6.912668746	5.374609695		1.561235	0.5609
QPCT	868.0918101	260.27769	9.76336478	8.029440115		1.737796	0.56099
LOC100623298	690.4554835	210.70098	9.433492562	7.72588417		1.712351	0.56125
LOC102161099	28.54869534	9.0890621	4.885022527	3.334720158		1.651221	0.56129
LOC102158687	28.54869534	9.0890621	4.885022527	3.334720158		1.651221	0.56129
LOC100622074	6.344154519	22.309516	2.876596413	4.542847146		-1.81416	0.56159
APOM	6.344154519	22.309516	2.876596413	4.542847146		-1.81416	0.56159
LOC100736864	6.344154519	22.309516	2.876596413	4.542847146		-1.81416	0.56159
UBE2D2	2923.597874	798.18491	11.51402256	9.642385527		1.87295	0.56166

FAM13C	136.773256	370.17271	7.106152054	8.535946834		-1.43641	0.47238
LOC102161705	3061.23415	1010.5384	11.58036888	9.982335443		1.598989	0.47256
TMEM209	980.622798	345.38436	9.939024946	8.436229978		1.505496	0.47259
LOC100626030	2136.97851	720.51474	11.06203163	9.494885058		1.568473	0.47301
OSGIN2	1162.15821	405.70268	10.18383163	8.667830689		1.518312	0.4732
SLC4A1	27.3546512	9.0890621	4.825513503	3.334720158		1.589583	0.47321
ABCD1	586.881607	1718.659	9.199381831	10.74790681		-1.55014	0.47387
LOC102158662	347.321177	128.89943	8.444274378	7.021251246		1.430025	0.47394
ATP6	556443.341	137839.76	19.08587786	17.07264301		2.013243	0.47415
LOC102166034	17.4075053	4.9576702	4.202222212	2.574748269		1.811975	0.47415
LOC102160345	17.4075053	4.9576702	4.202222212	2.574748269		1.811975	0.47415
LOC100738442	92.8400282	247.05723	6.552131542	7.954529215		-1.41203	0.47416
LOC100739565	97.8136012	37.182527	6.62663773	5.254840671		1.39541	0.47431
JMJD8	549.57981	1601.3275	9.104807896	10.64595332		-1.54287	0.47515
IPPK	319.137597	118.98409	8.322548307	6.906699252		1.42341	0.47527
LOC100523964	398.714764	1139.4379	8.642827052	10.15537214		-1.51489	0.47537
LOC100524364	37.3017971	13.220454	5.259340178	3.829895615		1.496473	0.47554
ITPKB	30.6703665	80.97528	4.985061656	6.357117027		-1.40064	0.47571
LOC102159157	87.0375265	33.051135	6.460046706	5.089630977		1.396938	0.47572
LOC100738207	585.22375	212.35354	9.195307606	7.737102248		1.46252	0.47579
LOC102160481	657.340557	237.14189	9.362690268	7.895677624		1.47089	0.47592
NYAP1	232.928999	645.32341	7.869928909	9.336112433		-1.47013	0.47592
ARL6IP5	1726.65874	5394.7715	10.75460256	12.39761354		-1.64358	0.47609
PIGL	230.442213	637.8869	7.854508213	9.319416755		-1.4689	0.47623
FAM101A	164.956836	62.797156	7.374664246	5.995420212		1.393317	0.47675
LOC102165668	21.5521494	57.839486	4.495193036	5.878712739		-1.42422	0.47693
ALDH5A1	1088.38355	382.56689	10.08929627	8.583334365		1.508403	0.47724
FANCD2	7510.92407	2361.5036	12.87496677	11.2061008		1.669285	0.47728
LOC100621691	1631.33193	5074.1755	10.67271874	12.30924199		-1.63712	0.47731
LOC100625801	24.8678647	66.10227	4.693089065	6.068289661		-1.41042	0.47778
LOC102157550	76.2614518	28.919743	6.271676881	4.903025878		1.398899	0.47855
LOC100624086	76.2614518	28.919743	6.271676881	4.903025878		1.398899	0.47855
DMTN	164.956836	447.84288	7.374664246	8.810066691		-1.4409	0.47867
LOC102162905	4.97357294	17.351846	2.578594101	4.19785327		-1.80273	0.47925
LOC100627387	4.97357294	17.351846	2.578594101	4.19785327		-1.80273	0.47925
LHX8	4.97357294	17.351846	2.578594101	4.19785327		-1.80273	0.47925
SEZ6L2	14.9207188	41.313919	3.99283357	5.403060391		-1.46931	0.4793
CDH23	47.248943	17.351846	5.592425431	4.19785327		1.445193	0.4796
MST1R	193.969345	530.47071	7.607103495	9.053846388		-1.45144	0.47963
LOC100626246	237.073644	655.23875	7.895264103	9.358076973		-1.46669	0.47975
ZNF385D	881.15134	313.98578	9.784882372	8.299142895		1.488691	0.47981
PPA1	8443.469	2642.4382	13.04379099	11.3681999		1.675966	0.48026
TIRAP	213.863637	587.48392	7.747277532	9.20085919		-1.45786	0.4804
ATG9A	1018.75352	3067.9716	9.994004777	11.58353958		-1.59048	0.48121
LOC100625140	169.10148	458.5845	7.410251883	8.84418632		-1.4393	0.48128
BCL3	618.380902	1805.4182	9.27468309	10.81891624		-1.54577	0.48141
LOC102161992	43.1042988	15.699289	5.462847376	4.061714779		1.457133	0.48143
KIAA1875	133.457541	357.77854	7.071006856	8.486949769		-1.42269	0.48168
PGCP	384.622974	1090.6875	8.591047195	10.09234416		-1.50372	0.482
MTF1	157.496477	60.318321	7.308306958	5.938246292		1.384651	0.48232
ZNF385A	1605.63513	554.43279	10.64982661	9.117468532		1.53406	0.48255
SPNS3	111.905391	42.966475	6.818970566	5.458331975		1.380996	0.48286
IRGC	4.97357294	0	2.578594101	0		20	0.48295
ATP6V0D2	4.97357294	0	2.578594101	0		20	0.48295
SMIM18	4.97357294	0	2.578594101	0		20	0.48295
LOC102159173	4.97357294	0	2.578594101	0		20	0.48295
LOC102159220	4.97357294	0	2.578594101	0		20	0.48295
LOC102159250	4.97357294	0	2.578594101	0		20	0.48295
LOC102161795	4.97357294	0	2.578594101	0		20	0.48295
LOC102159669	4.97357294	0	2.578594101	0		20	0.48295
LOC102161623	4.97357294	0	2.578594101	0		20	0.48295
LOC102168057	4.97357294	0	2.578594101	0		20	0.48295
ECT2L	4.97357294	0	2.578594101	0		20	0.48295
LOC102161552	4.97357294	0	2.578594101	0		20	0.48295
LOC100518475	4.97357294	0	2.578594101	0		20	0.48295
GPRC6A	4.97357294	0	2.578594101	0		20	0.48295
LOC102159941	4.97357294	0	2.578594101	0		20	0.48295
LOC102163950	4.97357294	0	2.578594101	0		20	0.48295
ATP1A3	4.97357294	0	2.578594101	0		20	0.48295
LOC100513824	4.97357294	0	2.578594101	0		20	0.48295
LOC102163639	4.97357294	0	2.578594101	0		20	0.48295
LOC100626338	4.97357294	0	2.578594101	0		20	0.48295
LOC100626209	4.97357294	0	2.578594101	0		20	0.48295
LOC102164700	4.97357294	0	2.578594101	0		20	0.48295
LOC102162079	4.97357294	0	2.578594101	0		20	0.48295
MSLNL	4.97357294	0	2.578594101	0		20	0.48295
LOC100516371	4.97357294	0	2.578594101	0		20	0.48295
LOC100626755	4.97357294	0	2.578594101	0		20	0.48295
LOC102166731	4.97357294	0	2.578594101	0		20	0.48295
TTC36	4.97357294	0	2.578594101	0		20	0.48295
ASB18	4.97357294	0	2.578594101	0		20	0.48295
PPEF2	4.97357294	0	2.578594101	0		20	0.48295
LOC102164033	4.97357294	0	2.578594101	0		20	0.48295
CNFN	4.97357294	0	2.578594101	0		20	0.48295
C5H12orf60	4.97357294	0	2.578594101	0		20	0.48295
CC2D2B	4.97357294	0	2.578594101	0		20	0.48295
DNMT3L	4.97357294	0	2.578594101	0		20	0.48295
LOC102158141	4.97357294	0	2.578594101	0		20	0.48295
LOC102158037	4.97357294	0	2.578594101	0		20	0.48295
OPRK1	4.97357294	0	2.578594101	0		20	0.48295
LRRTM3	4.97357294	0	2.578594101	0		20	0.48295
LOC100621571	4.97357294	0	2.578594101	0		20	0.48295
PEBP4	4.97357294	0	2.578594101	0		20	0.48295
HTR3B	4.97357294	0	2.578594101	0		20	0.48295
LOC102160747	4.97357294	0	2.578594101	0		20	0.48295
GPR22	4.97357294	0	2.578594101	0		20	0.48295
LOC102164866	4.97357294	0	2.578594101	0		20	0.48295
LOC102164859	4.97357294	0	2.578594101	0		20	0.48295
LOC102158429	4.97357294	0	2.578594101	0		20	0.48295
CD8B	4.97357294	0	2.578594101	0		20	0.48295
LOC102162255	4.97357294	0	2.578594101	0		20	0.48295
LOC100623167	4.97357294	0	2.578594101	0		20	0.48295
LOC102165396	4.97357294	0	2.578594101	0		20	0.48295
KPNA7	4.97357294	0	2.578594101	0		20	0.48295
LOC102165522	4.97357294	0	2.578594101	0		20	0.48295
LOC102163328	4.97357294	0	2.578594101	0		20	0.48295
LOC100517491	4.97357294	0	2.578594101	0		20	0.48295
LOC100513415	4.97357294	0	2.578594101	0		20	0.48295
LOC102167341	4.97357294	0	2.578594101	0		20	0.48295
FCGR2B	4.97357294	0	2.578594101	0		20	0.48295
SLA-DOA	4.97357294	0	2.578594101	0		20	0.48295
LOC100620428	4.97357294	0	2.578594101	0		20	0.48295
LOC100737333	4.97357294	0	2.578594101	0		20	0.48295
LOC102165197	4.97357294	0	2.578594101	0		20	0.48295
LOC102165114	4.97357294	0	2.578594101	0		20	0.48295
GORAB	114.392178	304.07044	6.850401618	8.252998589		-1.41042	0.48378
LOC100737943	2384.82823	805.62141	11.22027446	9.655747894		1.565711	0.48379
MSANTD2	138.431114	370.99899	7.123408719	8.53915489		-1.42225	0.48383
LOC100738746	93.6689571	247.05723	6.564819524	7.954529215		-1.3992	0.48403
LOC100627067	203.916491	556.91162	7.678892279	9.123892796		-1.44947	0.48413
KLF16	5477.56167	1763.278	12.41958146	10.78486223		1.635274	0.48437
LOC102166829	57.1960888	21.483238	5.862850292	4.490777898		1.412705	0.4844
P2RX6	27.3546512	71.886218	4.825513503	6.187574144		-1.39393	0.48446
LOC102159569	11.6050035	33.051135	3.655924617	5.089630977		-1.50995	0.48463
TACC3	7965.17707	2509.4074	12.95967183	11.2937058		1.66636	0.48478
CD34	7.46035941	23.135794	3.080718953	4.593102408		-1.63281	0.48478
RASA1	1676.09408	5191.507	10.71174791	12.34221554		-1.63105	0.48481
LOC102157740	474.147287	175.17101	8.892230982	7.460832769		1.436571	0.4849
RGS2	75.432523	197.48053	6.256114748	7.632853688		-1.38845	0.48491
LOC100625158	192.311487	73.538775	7.594783559	6.219919206		1.386868	0.48497
UNC13B	1352.81184	472.63123	10.40281152	8.887620397		1.517174	0.48498
LOC102158426	179.048626	68.581105	7.492242779	6.120623682		1.384468	0.48498
LOC100511509	13.2628612	3.3051135	3.834191515	2.106051269		2.00462	0.48505
LOC100516763	13.2628612	3.3051135	3.834191515	2.106051269		2.00462	0.48505
CENPO	1586.56977	549.47512	10.63260428	9.104533542		1.529785	0.48546
C9H11orf70	50.5646582	131.37826	5.688310694	7.048522415		-1.37753	0.4858
LOC102159255	90.3532418	34.703692	6.51338402	5.158001345		1.380487	0.48591
MAP4K5	118.536822	314.81206	6.90131128	8.302922453		-1.40916	0.48633
LOC102159726	0	4.9576702	0	2.574748269		-20	0.48645
LOC100624077	0	4.9576702	0	2.574748269		-20	0.48645
LOC102160263	0	4.9576702	0	2.574748269		-20	0.48645
C17H20orf106	0	4.9576702	0	2.574748269		-20	0.48645
LOC102163287	0	4.9576702	0	2.574748269		-20	0.48645
PPP1R3A	0	4.9576702	0	2.574748269		-20	0.48645
HSBP1P	0	4.9576702	0	2.574748269		-20	0.48645
LOC100521565	0	4.9576702	0	2.574748269		-20	0.48645
LOC100737537	77.0903806	201.61192	6.287072939	7.662575262		-1.38696	0.48662
MCM8	1228.47252	432.14359	10.26382377	8.758701552		1.507283	0.48672
KCTD1	85.3796688	223.92144	6.43261988	7.81327737		-1.39103	0.48681
LOC102163243	48.9068006	18.178124	5.641164514	4.261389712		1.427832	0.48687
IREB2	2187.54317	744.47681	11.09575512	9.54201967		1.555013	0.48689
LOC100520965	1729.14553	596.57298	10.75667768	9.222971117		1.535289	0.48692
WDTC1	1838.56413	5717.8463	10.84514826	12.48150842		-1.63689	0.48699
LOC100516848	562.842671	206.56959	9.139148854	7.697451307		1.446104	0.48705
LOC102164265	71.2878788	185.91263	6.175681852	7.546220275		-1.3829	0.48746
LOC102164888	510.620155	188.39147	8.99892929	7.565227535		1.438517	0.48754
LOC102163980	20796.9953	6211.9608	14.34415685	12.60106524		1.743254	0.48758
LOC100622780	569.474102	209.04843	9.156017583	7.714578179		1.445793	0.48801
RNF13	451.766209	1286.5154	8.82262248	10.330374		-1.50982	0.48836
LOC102157505	19.0653629	5.7839486	4.326635347	2.762125239		1.720827	0.48848
TRNAA-UGC	19.0653629	5.7839486	4.326635347	2.762125239		1.720827	0.48848
LOC100156244	19.0653629	5.7839486	4.326635347	2.762125239		1.720827	0.48848
UMPS	3533.72358	1170.8365	11.78738168	10.19455552		1.59365	0.4886
LOC100518964	81.2350247	212.35354	6.361681078	7.737102248		-1.38629	0.48868
LOC100737149	344.834391	129.7257	8.433937531	7.030399034		1.410439	0.48916
LOC102163372	143.404687	55.360651	7.173973754	5.816616368		1.373159	0.48933
MMP17	60.5118041	156.99289	5.942791385	7.303715831		-1.37541	0.48939
INPP5J	959.070649	342.90552	9.906996763	8.42586848		1.483826	0.4897
LOC100525841	3.31571529	13.220454	2.109599694	3.829895615		-1.99538	0.48979
LOC100622572	3.31571529	13.220454	2.109599694	3.829895615		-1.99538	0.48979
LOC100522360	3.31571529	13.220454	2.109599694	3.829895615		-1.99538	0.48979
LOC102164940	3.31571529	13.220454	2.109599694	3.829895615		-1.99538	0.48979
LOC102158312	3.31571529	13.220454	2.109599694	3.829895615		-1.99538	0.48979
LOC100626489	24.8678647	65.275991	4.693089065	6.05041444		-1.39227	0.48991
TRIB1	62.9985906	23.962073	5.999968229	4.641665831		1.394567	0.48993
LMNB2	8669.76657	2728.3712	13.08194383	11.41435289		1.667953	0.49006
DHFR	2382.34144	808.92653	11.21876993	9.661647227		1.5583	0.4904
ABCE1	9167.95279	2877.1013	13.16254125	11.49090165		1.671984	0.4907
LOC102158305	58.8539465	22.309516	5.90337447	4.542847146		1.39948	0.49075
LOC102166967	44.7621565	115.67897	5.516083133	6.86640077		-1.36978	0.49095
RBM12B	1733.29017	599.8781	10.76012959	9.230928526		1.530772	0.4911
LOC100737971	894.414201	321.42229	9.806411388	8.33280766		1.476473	0.49123
PLXNB1	314.992953	875.85507	8.303748575	9.776194605		-1.47537	0.49127
LOC102161995	115.221107	44.619032	6.860728285	5.511563931		1.368674	0.49135
LOC102164114	4387.52026	1438.5506	12.09951885	10.49140283		1.60879	0.49157
LOC102163089	54.7093024	20.656959	5.799846344	4.436758793		1.405158	0.49197
LOC100521858	169.10148	65.275991	7.410251883	6.05041444		1.373265	0.49198
LTA4H	11829.6432	3661.2395	13.5302409	11.83851041		1.692003	0.49198
FCGBP	40.6175124	14.873011	5.379118827	3.98850389		1.449405	0.49199
LOC100738012	87.0375265	227.22655	6.460046706	7.834322836		-1.38442	0.49232
LOC102159097	30.6703665	10.741619	4.985061656	3.553559423		1.513634	0.49309
LOC102161601	30.6703665	10.741619	4.985061656	3.553559423		1.513634	0.49309
LOC102160199	30.6703665	10.741619	4.985061656	3.553559423		1.513634	0.49309
LOC102160236	30.6703665	10.741619	4.985061656	3.553559423		1.513634	0.49309
LOC100518100	519.738372	192.52286	9.02441491	7.59636019		1.432756	0.49329
LOC100519891	5.80250177	19.004403	2.766065426	4.322245637		-1.71159	0.49361
HIVEP3	5.80250177	19.004403	2.766065426	4.322245637		-1.71159	0.49361
CDC20	9046.92918	2847.3553	13.14337192	11.47591339		1.667806	0.49363
ELOVL4	375.504757	141.2936	8.556524283	7.152726981		1.410135	0.49365
LOC100739435	77.9193094	202.4382	6.302306427	7.6684468		-1.37743	0.49387
LENG9	149.207188	397.4399	7.230810045	8.638218305		-1.41342	0.49441
PLIN3	384.622974	1079.1196	8.591047195	10.07697529		-1.48834	0.49451
ZNF33B	159.983263	61.970878	7.330766893	5.976612875		1.368259	0.49454
LOC100738161	72.9457365	28.093465	6.208395063	4.862623207		1.376589	0.49468
LOC100623893	746.86487	271.84558	9.546633806	8.091940885		1.45806	0.49482
LOC100525755	901.045631	2662.2689	9.817056606	11.37898239		-1.56298	0.4953
NBEAL1	116.878964	45.44531	6.881162477	5.537461031		1.362812	0.49542
LOC102161137	27.3546512	71.05994	4.825513503	6.171125545		-1.37725	0.49563
LOC100736930	98.64253	257.79885	6.638689748	8.015687408		-1.38596	0.49581
TLR6	8.28928824	24.788351	3.215568059	4.688647628		-1.58034	0.49598
LOC102160664	36.4728682	13.220454	5.227774503	3.829895615		1.464052	0.49607
SNX3	1156.35571	3467.8903	10.17661662	11.76025851		-1.58447	0.49622
PPTC7	508.962298	189.21775	8.99424678	7.571508044		1.427511	0.49634
ELMSAN1	1104.96212	394.13478	10.11108626	8.626201041		1.487236	0.49658
LOC100736923	59.6828753	153.68778	5.92321754	7.273215394		-1.36461	0.49685
NFATC2IP	1993.57382	687.46361	10.9618648	9.42723658		1.536002	0.49689
LOC102162506	168.272551	65.275991	7.403204238	6.05041444		1.366175	0.49706
PDCD2L	602.631255	222.26888	9.237523697	7.80263838		1.43897	0.49709
LOC100524893	2531.54863	860.98206	11.30637425	9.751514039		1.555965	0.49714
CCDC71L	353.952608	985.7501	8.471482603	9.946540946		-1.47767	0.49737
SH2B3	2262.14676	775.04911	11.14411443	9.600004147		1.545333	0.49811
LOC102159024	9.11821706	1.6525567	3.338883189	1.407383613		2.464052	0.49824
LOC100622589	9.11821706	1.6525567	3.338883189	1.407383613		2.464052	0.49824
LOC100511295	9.11821706	1.6525567	3.338883189	1.407383613		2.464052	0.49824
LOC102163882	9.11821706	1.6525567	3.338883189	1.407383613		2.464052	0.49824
OVGP1	9.11821706	1.6525567	3.338883189	1.407383613		2.464052	0.49824
LOC100524679	9.11821706	1.6525567	3.338883189	1.407383613		2.464052	0.49824
LOC102166186	9.11821706	1.6525567	3.338883189	1.407383613		2.464052	0.49824
LOC102160785	9.11821706	1.6525567	3.338883189	1.407383613		2.464052	0.49824
LOC102163348	9.11821706	1.6525567	3.338883189	1.407383613		2.464052	0.49824
KLHL33	9.11821706	1.6525567	3.338883189	1.407383613		2.464052	0.49824
LOC102163081	9.11821706	1.6525567	3.338883189	1.407383613		2.464052	0.49824
ACTR3B	746.86487	272.67186	9.546633806	8.096303303		1.453681	0.49849
DCAKD	604.289112	1736.8371	9.241480591	10.76307717		-1.52315	0.49857
LOC102163415	5767.68676	1870.6942	12.49402721	10.87012906		1.624419	0.49866
MCC	557.04017	206.56959	9.124225165	7.697451307		1.431153	0.49872
MAK16	6903.31924	2216.0786	12.75328346	11.1144442		1.639281	0.49903
LOC102162264	696.300212	255.32002	9.44563611	8.001802338		1.447403	0.49919
FOXN3	1161.32928	413.96546	10.18280312	8.696847462		1.488195	0.49926
LOC102158524	40.6175124	104.11107	5.379118827	6.715770874		-1.35795	0.49955
TMOD3	2044.96741	705.64173	10.99856745	9.464835137		1.53507	0.49964
METTL21A	4332.81096	1430.2879	12.08142051	10.48309814		1.598998	0.49965
RNASEK	873.69098	2567.2469	9.772629606	11.32656819		-1.55503	0.49968
LOC100518510	159.154334	423.05453	7.323319031	8.728105973		-1.41042	0.50015
TMEFF1	664.800917	244.5784	9.378947046	7.940039851		1.442625	0.50021
LIPA	642.419838	1850.8636	9.329616608	10.85476209		-1.52661	0.50025
LOC102167015	26.5257224	9.0890621	4.78270852	3.334720158		1.545189	0.50043
LOC102162817	20.7232206	6.610227	4.441166102	2.927939483		1.648477	0.50048
TLE3	2524.08827	860.98206	11.3021181	9.751514039		1.551707	0.50074
LOC102166743	163.298978	63.623435	7.360179698	6.013985524		1.359886	0.50101
RCC2	21055.6211	6344.1653	14.36198633	12.63144204		1.730703	0.50101
HGSNAT	978.136012	2891.148	9.935365468	11.49792568		-1.56354	0.50115
LOC100737090	43.9332277	112.37386	5.489710792	6.824944216		-1.35492	0.50127
LOC100623683	66.3143059	25.61463	6.072841239	4.734147579		1.372352	0.50133
ACSL3	6928.18711	2227.6465	12.7584704	11.12195208		1.636958	0.50152
LRTM2	32.3282241	11.567897	5.058672541	3.65167138		1.482668	0.50163
LOC100515384	32.3282241	11.567897	5.058672541	3.65167138		1.482668	0.50163
TTF1	600.144468	222.26888	9.231567934	7.80263838		1.433004	0.50174
LOC102157410	14.9207188	4.1313919	3.99283357	2.359350201		1.852617	0.50184
LOC100156557	14.9207188	4.1313919	3.99283357	2.359350201		1.852617	0.50184
LOC102162850	14.9207188	4.1313919	3.99283357	2.359350201		1.852617	0.50184
CD80	14.9207188	4.1313919	3.99283357	2.359350201		1.852617	0.50184
LOC100524370	14.9207188	4.1313919	3.99283357	2.359350201		1.852617	0.50184
LOC100519232	1582.42512	555.25907	10.62883293	9.119613134		1.510904	0.50187
GALK2	884.467055	2596.1666	9.79029482	11.34272287		-1.5535	0.50191
LOC102163244	3218.73062	1083.2509	11.65272428	10.08248299		1.571124	0.50194
LOC100522143	452.595138	1274.1212	8.825261365	10.31641872		-1.49321	0.50207
LOC100154048	2495.07576	852.71928	11.28544601	9.73761795		1.548941	0.50234
PLCL1	1.65785765	9.0890621	1.410263837	3.334720158		-2.45481	0.50248
REP15	1.65785765	9.0890621	1.410263837	3.334720158		-2.45481	0.50248
LOC102157837	1.65785765	9.0890621	1.410263837	3.334720158		-2.45481	0.50248
LOC102160344	1.65785765	9.0890621	1.410263837	3.334720158		-2.45481	0.50248
LOC100524382	1.65785765	9.0890621	1.410263837	3.334720158		-2.45481	0.50248
CA2	62.1696618	23.962073	5.981159942	4.641665831		1.375458	0.50252
LOC102163224	62.1696618	23.962073	5.981159942	4.641665831		1.375458	0.50252
NAT10	6388.55444	2065.6959	12.64149962	11.01311043		1.628862	0.50265
LOC102164512	573.618746	213.17982	9.166461249	7.742678745		1.428021	0.50306

ARHGAP10	231.976052	612.27227	7.864037858	9.260383917		-1.4002	0.7353	
LOC100623642	52.4707738	23.135794	5.740678649	4.593102408		1.181387	0.73533	
ENOX1	55.2323934	131.37826	5.813329549	7.048522415		-1.25014	0.73534	
KIF18A	851.959669	331.33763	9.736333717	8.376505833		1.362483	0.73551	
EXTL1	289.970066	121.46292	8.184726929	6.936201185		1.255388	0.73552	
SLC26A8	27.6161967	11.567897	4.838760036	3.65167138		1.255388	0.73558	
LOC100154485	41.4242951	97.500848	5.406818783	6.622064238		-1.23494	0.73563	
LOC100523138	737.352452	290.02371	9.52816584	8.184992878		1.346184	0.73617	
GLIS2	1379.42903	4285.9059	10.430901	12.06572104		-1.63553	0.73625	
DEPTOR	220.929574	94.195734	7.79395812	6.572825024		1.229853	0.73638	
CTXN1	327.251931	889.07553	8.358659688	9.797783952		-1.4419	0.73657	
MASTL	1252.39452	474.28379	10.29162488	8.892645374		1.400867	0.73663	
RCL1	2503.40823	901.4697	11.29025403	9.817734691		1.473543	0.73682	
MGLL	352.106508	962.6143	8.463959601	9.912311998		-1.45095	0.73684	
SLC8B1	171.22042	438.75382	7.428112398	8.780552285		-1.35756	0.7371	
LOC100623062	354.868128	147.07755	8.475198918	7.210209122		1.270706	0.73716	
CIC	1629.35561	5133.6675	10.67097096	12.32605515		-1.65569	0.73717	
BEST4	20.7121475	8.2627837	4.440430524	3.211445828		1.325777	0.73725	
LOC102158867	20.7121475	8.2627837	4.440430524	3.211445828		1.325777	0.73725	
LOC100623307	1590.69293	592.44159	10.63634632	9.212962234		1.424911	0.7373	
ERF	1589.31212	4994.8528	10.63509423	12.28651524		-1.65204	0.73733	
PIAS3	1001.08713	3014.2635	9.96879224	11.55806837		-1.59024	0.73734	
LOC102158089	16.569718	39.661362	4.135019133	5.34558663		-1.25919	0.73792	
NAALADL1	100.799118	44.619032	6.669581252	5.511563931		1.175752	0.73796	
LOC102162214	41.4242951	18.178124	5.406818783	4.261389712		1.188274	0.73818	
LOC102165363	41.4242951	18.178124	5.406818783	4.261389712		1.188274	0.73818	
LOC100737288	41.4242951	18.178124	5.406818783	4.261389712		1.188274	0.73818	
LOC100525437	1060.46195	407.35524	10.05183695	8.673680921		1.380334	0.73822	
LOC100511572	63.5172524	151.20894	6.011613095	7.249909307		-1.25132	0.73834	
PGAP1	2090.54609	764.30749	11.03035408	9.579895716		1.451655	0.73847	
C14H10orf107	27.6161967	64.449713	4.838760036	6.032314961		-1.22266	0.7385	
LOC102168183	9.66566885	24.788351	3.414902536	4.688647628		-1.35872	0.73853	
LOC102157854	9.66566885	24.788351	3.414902536	4.688647628		-1.35872	0.73853	
PTCD3	6931.66538	2325.1473	12.75919441	11.18372676		1.57588	0.73854	
ERBB2IP	867.148577	2571.3783	9.761798159	11.32888711		-1.56819	0.73855	
SPAG5	2936.98252	1047.721	11.5206101	10.03441517		1.48708	0.73865	
RPF1	3302.89713	1168.3576	11.68995305	10.19150049		1.49925	0.73871	
DUS4L	875.433436	341.25297	9.775500714	8.41891924		1.359156	0.73873	
CAAP1	1119.83678	428.83847	10.13036048	8.747650814		1.384782	0.73891	
LOC100517951	111.845597	275.97698	6.818206315	8.113622247		-1.30304	0.73897	
DCAF4	1553.41107	580.8737	10.60215236	9.184562218		1.419143	0.73908	
POLR3H	1576.88483	589.13648	10.62377619	9.20490483		1.420404	0.73926	
LOC100154278	197.455807	85.106672	7.632673964	6.428053129		1.214186	0.73926	
LOC100624624	171.22042	74.365053	7.428112398	6.2358238		1.203158	0.73931	
SORCS1	6.90404918	19.004403	2.982591923	4.322245637		-1.46082	0.7395	
LOC102160898	6.90404918	19.004403	2.982591923	4.322245637		-1.46082	0.7395	
LOC102160101	6.90404918	19.004403	2.982591923	4.322245637		-1.46082	0.7395	
LOC100737964	6.90404918	19.004403	2.982591923	4.322245637		-1.46082	0.7395	
LOC100154462	6103.17947	2068.1748	12.57558167	11.01483979		1.561203	0.73958	
LOC100738745	113.226407	279.28209	6.835752397	8.130735747		-1.30251	0.73966	
SUZ12	3849.69782	1348.4863	11.9109042	10.39819462		1.513404	0.73975	
PYCR2	5063.42967	1739.316	12.30618409	10.76513355		1.541595	0.73985	
LOC102164038	74.5637311	178.47613	6.239622035	7.487648157		-1.25919	0.74021	
LOC100625870	35.9010557	83.454116	5.205590187	6.400095824		-1.21696	0.74028	
NEK2	2354.28077	855.19811	11.20168334	9.741800849		1.460956	0.74028	
DHX57	234.737672	614.75111	7.881038517	9.26620351		-1.38895	0.74038	
LOC100737685	48.3283443	21.483238	5.624344958	4.490777898		1.169658	0.74062	
LOC100156657	339.67922	919.64783	8.41227014	9.846505582		-1.43691	0.74064	
LOC102161594	2946.64819	1053.5049	11.52534863	10.04235012		1.483878	0.74068	
LOC100737643	2100.21176	6737.4738	11.03700585	12.71820617		-1.68167	0.7407	
LOC102165668	24.854577	57.839486	4.692347799	5.878712739		-1.21854	0.74073	
LOC100514721	687.643298	1987.1995	9.427613082	10.9572468		-1.531	0.74108	
[illegible]								

ZDHHC23	150.508272	67.754826	7.243252754	6.103389087	[illegible]
WWP1	3700.57036	1327.0031	11.85392174	10.37504276	[illegible]
RIMS1	1139.16811	446.19032	10.15503085	8.804745152	[illegible]
IRAK1BP1	98.0374983	231.35794	6.629902968	7.86020516	[illegible]
ENDOG	417.00457	175.99729	8.707374906	7.467583487	[illegible]
LOC100520401	233.356862	102.45852	7.872563229	6.692908622	[illegible]
LOC102166949	85.6102098	199.95937	6.436465198	7.650760008	[illegible]
CYP2C32	1.38080984	7.4365053	1.251452392	3.076645515	[illegible]
LOC102157543	1.38080984	7.4365053	1.251452392	3.076645515	[illegible]
LOC102166832	1.38080984	7.4365053	1.251452392	3.076645515	[illegible]
LOC102157941	1.38080984	7.4365053	1.251452392	3.076645515	[illegible]
RHAG	1.38080984	7.4365053	1.251452392	3.076645515	[illegible]
LOC102162664	1.38080984	7.4365053	1.251452392	3.076645515	[illegible]
LOC102164856	1.38080984	7.4365053	1.251452392	3.076645515	[illegible]
LOC102167693	1.38080984	7.4365053	1.251452392	3.076645515	[illegible]
LOC102165441	1.38080984	7.4365053	1.251452392	3.076645515	[illegible]
FER1L6	1.38080984	7.4365053	1.251452392	3.076645515	[illegible]
LOC102167244	1.38080984	7.4365053	1.251452392	3.076645515	[illegible]
LOC102165886	1.38080984	7.4365053	1.251452392	3.076645515	[illegible]
LOC100739568	1.38080984	7.4365053	1.251452392	3.076645515	[illegible]
LOC100739626	1.38080984	7.4365053	1.251452392	3.076645515	[illegible]
LOC102167923	1.38080984	7.4365053	1.251452392	3.076645515	[illegible]
CCT5	28582.7636	8878.3611	14.80290827	13.11624016	[illegible]
CELF3	16.569718	6.610227	4.135019133	2.927939483	[illegible]
LOC102165672	16.569718	6.610227	4.135019133	2.927939483	[illegible]
DCUN1D2	294.112495	127.24687	8.205121195	7.002779797	[illegible]
PRTG	60.7556328	28.093465	5.948498827	4.862623207	[illegible]
LOC102165796	62.1364426	142.11988	5.98040107	7.161080272	[illegible]
LOC100511457	146.365843	66.10227	7.203258356	6.068289661	[illegible]
GRID1	146.365843	66.10227	7.203258356	6.068289661	[illegible]
LAMP-1	5992.71469	20613.167	12.54923469	14.33134851	[illegible]
LOC100738786	22.0929574	49.576702	4.529381037	5.660401068	[illegible]
CTNND2	548.181505	227.22655	9.101139229	7.834322836	[illegible]
FABP7	73.1829213	33.877413	6.213015177	5.124221138	[illegible]
CREM	2630.44274	969.22453	11.36163829	9.922174845	[illegible]
TBL1X	3217.28692	10451.595	11.65207724	13.35157355	[illegible]
LOC100512389	26.2353869	11.567897	4.767410456	3.65167138	[illegible]
RHOV	26.2353869	11.567897	4.767410456	3.65167138	[illegible]
ENY2	3599.77124	1296.4308	11.81409023	10.34144184	[illegible]
MOV10	3030.87759	9789.7461	11.56599579	13.2572031	[illegible]
NOP16	4210.08919	1498.869	12.03997772	10.55062075	[illegible]
LUZP1	1321.43501	3949.6106	10.36898111	11.94785994	[illegible]
LOC100153608	818.820233	329.68507	9.679163785	8.369314102	[illegible]
PAK1IP1	4316.41155	1534.3989	12.07595091	10.58439784	[illegible]
LOC100155807	697.308967	284.23976	9.447721688	8.156031285	[illegible]
PRKCA	211.263905	529.64444	7.729715254	9.051601682	[illegible]
LOC100624707	34.5202459	76.843889	5.150569664	6.282511874	[illegible]
LOC100157421	78.7061606	181.78124	6.316619332	7.513974209	[illegible]
ADAP2	28.9970066	64.449713	4.906746634	6.032314961	[illegible]
TMEM184A	773.253508	313.1595	9.596662204	8.29535341	[illegible]
FAM161B	102.179928	240.44701	6.689018532	7.915562765	[illegible]
LOC100511796	980.374983	390.00339	9.938660685	8.611037311	[illegible]
LOC102165327	980.374983	390.00339	9.938660685	8.611037311	[illegible]
FAM211A	173.982039	428.0122	7.451063037	8.744874853	[illegible]
SNRNP40	4232.18215	1508.7843	12.04752685	10.56012674	[illegible]
LOC102165153	66.2788721	151.20894	6.072081615	7.249909307	[illegible]
SCNN1A	62.1364426	28.919743	5.98040107	4.903025878	[illegible]
ZNF536	62.1364426	28.919743	5.98040107	4.903025878	[illegible]
LOC100522213	182.266898	81.801559	7.517802419	6.371586023	[illegible]
GBE1	306.539784	793.22724	8.26462924	9.633408027	[illegible]
MTHFR	519.184498	1412.936	9.022879597	10.46550112	[illegible]
ELL	38.6626754	85.932951	5.309710091	6.441831208	[illegible]
ZNF132	8.28485901	20.656959	3.214880004	4.436758793	[illegible]
SGCA	8.28485901	20.656959	3.214880004	4.436758793	[illegible]
CHD2	1420.85332	4256.9862	10.47355693	12.05595555	[illegible]
[illegible]	[illegible]	[illegible]	[illegible]	[illegible]	[illegible]

LOC102165769	66.2788721	147.07755	6.072081615	7.210209122		-1.14996	0.77198		ACAD9
ZNF283	220.929574	542.03861	7.79395812	9.084910972		-1.29481	0.77201		PALB2
MAP7D1	4103.76683	13333.654	12.00308456	13.70289278		-1.70005	0.77208		CSNK2A2
TXLNG	3714.37846	1359.2279	11.85929346	10.4096327		1.450333	0.77214		FAM131A
NRIP1	325.871121	829.58349	8.352578112	9.697981376		-1.34809	0.77227		RNGTT
LOC100623650	62.1364426	29.746021	5.98040107	4.942327829		1.062743	0.7723		SH3PXD2B
HSPA8	77981.2355	23069.692	16.25085789	14.49377367		1.757128	0.7723		CAMLG
DDX25	6.90404918	17.351846	2.982591923	4.19785327		-1.32957	0.77236		FAM214A
LOC100157583	6.90404918	17.351846	2.982591923	4.19785327		-1.32957	0.77236		CELF3
LOC100517371	6.90404918	17.351846	2.982591923	4.19785327		-1.32957	0.77236		LOC100157318
LOC102165708	6.90404918	17.351846	2.982591923	4.19785327		-1.32957	0.77236		SLTM
FBXO10	51.0899639	111.54758	5.702933533	6.814391229		-1.12655	0.77253		TMEM128
LPCAT4	5775.92754	2046.6915	12.49608668	10.99978268		1.496759	0.77264		DVL1
LOC100514515	38.6626754	83.454116	5.309710091	6.400095824		-1.11004	0.77267		IER5
LOC100521282	168.4588	402.39757	7.404790748	8.656058572		-1.25623	0.77268		KCTD3
ETV6	465.332915	1225.3708	8.865216451	10.26017957		-1.39688	0.77285		ACAD8
PLEKHO2	548.181505	1466.6441	9.101139229	10.51928646		-1.41979	0.77298		ZNF638
LOC102160652	277.542777	123.94176	8.121755095	6.965111899		1.163048	0.77311		LOC100154648
ATP5G1	14872.7027	4927.9242	13.86047622	12.26705708		1.593615	0.77343		LOC102166692
CINP	2053.26423	787.44329	11.00440604	9.622863177		1.382671	0.77344		LOC100736755
LOC100515933	3289.08903	1217.108	11.68391091	10.25042639		1.434231	0.7736		LOC100739749
LOC100627183	658.646292	1792.1978	9.365548835	10.80831891		-1.44415	0.77362		BMP3
AS-A	655.884672	1783.935	9.359496291	10.80165583		-1.44355	0.77362		LOC100628117
TMEM128	2515.83552	950.22013	11.29739522	9.893635432		1.404704	0.77366		BTAF1
LOC100516270	5804.92455	2059.0857	12.50331011	11.00848864		1.495273	0.7737		PIF1
LOC100518510	176.743659	423.05453	7.473654282	8.728105973		-1.25919	0.77381		THPO
LOC102160446	33.1394361	71.05994	5.093367322	6.171125545		-1.10049	0.7739		LOC100738290
NDUFA7	8054.26377	2790.3421	12.97571612	11.44674321		1.529311	0.77399		APH1B
LOC100514339	128.415315	60.318321	7.015864542	5.938246292		1.090149	0.77432		IZUMO4
NUDCD2	2642.87003	995.66544	11.36843554	9.96096549		1.408373	0.77453		FBXO16
LOC102158039	41.4242951	19.830681	5.406818783	4.380638094		1.062743	0.77457		SPPL2A
[illegible]	[illegible]	[illegible]	[illegible]	[illegible]	[illegible]	[illegible]	[illegible]	[illegible]	[illegible]

LOC100738329	244.403341	585.00509	7.93901108	9.194769379		-1.25919	0.78835
TERT	1035.60738	428.83847	10.01765385	8.747650814		1.271971	0.78842
LOC100511614	30.3778164	14.873011	4.971673052	3.98850389		1.030321	0.78844
LOC100512173	30.3778164	14.873011	4.971673052	3.98850389		1.030321	0.78844
SPATS2L	1383.57146	3938.869	10.43522379	11.94393195		-1.50938	0.78867
SLC16A4	9.66566885	21.483238	3.414902536	4.490777898		-1.15227	0.78878
LOC102160023	9.66566885	21.483238	3.414902536	4.490777898		-1.15227	0.78878
PARP4	175.362849	405.70268	7.462402878	8.667830689		-1.21008	0.78898
S1PR5	331.394361	150.38266	8.376752095	7.242056188		1.139911	0.78899
NBEAL1	22.0929574	45.44531	4.529381037	5.537461031		-1.04054	0.78908
LOC100516841	2652.53569	1020.4538	11.37370024	9.996408222		1.378161	0.78911
SCAI	28.9970066	59.492043	4.906746634	5.918673475		-1.03679	0.78919
SOCS2	52.4707738	109.89502	5.740678649	6.793050813		-1.06654	0.78924
TIMM44	5309.21382	1936.7965	12.37455424	10.92020136		1.454826	0.78927
LHX2	66.2788721	33.051135	6.072081615	5.089630977		1.003849	0.78933
C3H9orf89	1469.18167	592.44159	10.52177872	9.212962234		1.310268	0.78933
CCDC136	98.0374983	214.83238	6.629902968	7.753767487		-1.13181	0.78938
LOC100524934	3660.52688	1374.1009	11.83822967	10.4253218		1.413563	0.78941
LOC100736778	845.05562	2287.1385	9.724608699	11.15995869		-1.43643	0.78948
LOC102162847	17.9505279	37.182527	4.24416613	5.254840671		-1.0506	0.78951
LOC100517625	17.9505279	37.182527	4.24416613	5.254840671		-1.0506	0.78951
REV3L	309.301403	756.04471	8.277526411	9.564234696		-1.28946	0.78956
TIMM22	1832.33465	726.29869	10.84025444	9.506404165		1.335048	0.78959
LOC102166270	16.569718	7.4365053	4.135019133	3.076645515		1.155852	0.78972
LOC100625094	16.569718	7.4365053	4.135019133	3.076645515		1.155852	0.78972
LRP3	1835.09627	5360.0678	10.84242599	12.38830466		-1.5464	0.78974
ZNF436	405.958092	1019.6275	8.668736424	9.99524072		-1.32864	0.78974
GLRB	11.0464787	23.962073	3.590539588	4.641665831		-1.11717	0.78982
LOC100623339	11.0464787	23.962073	3.590539588	4.641665831		-1.11717	0.78982
LOC100511840	2.76161967	9.0890621	1.911353989	3.334720158		-1.71862	0.7899
LOC102162001	2.76161967	9.0890621	1.911353989	3.334720158		-1.71862	0.7899
LOC102166792	2.76161967	9.0890621	1.911353989	3.334720158		-1.71862	0.7899
UBD	2.76161967	9.0890621	1.911353989	3.334720158		-1.71862	0.7899
LOC100514528	2.76161967	9.0890621	1.911353989	3.334720158		-1.71862	0.7899
ANKRD22	2.76161967	9.0890621	1.911353989	3.334720158		-1.71862	0.7899
ANKRD31	2.76161967	9.0890621	1.911353989	3.334720158		-1.71862	0.7899
LOC102166502	2.76161967	9.0890621	1.911353989	3.334720158		-1.71862	0.7899
CD101	2.76161967	9.0890621	1.911353989	3.334720158		-1.71862	0.7899
LOC102160792	2.76161967	9.0890621	1.911353989	3.334720158		-1.71862	0.7899
LOC102164997	2.76161967	9.0890621	1.911353989	3.334720158		-1.71862	0.7899
LOC102158892	2.76161967	9.0890621	1.911353989	3.334720158		-1.71862	0.7899
LOC102167048	2.76161967	9.0890621	1.911353989	3.334720158		-1.71862	0.7899
LOC102160344	2.76161967	9.0890621	1.911353989	3.334720158		-1.71862	0.7899
LOC100155809	2.76161967	9.0890621	1.911353989	3.334720158		-1.71862	0.7899
LOC100512457	88.3718295	191.69658	6.481748252	7.590187173		-1.11717	0.78997
FBXO2	67.659682	143.77244	6.101391269	7.177643143		-1.08742	0.78999
LOC102160444	67.659682	143.77244	6.101391269	7.177643143		-1.08742	0.78999
ANK2	701.451397	1860.7789	9.456254597	10.86246603		-1.40749	0.79014
STC2	642.076574	1688.0867	9.328846725	10.72202768		-1.39457	0.79014
LOC100737151	10963.6301	3795.0966	13.42056952	11.89030098		1.530517	0.79026
ABI2	243.022531	113.20014	7.930870551	6.83542057		1.102214	0.79042
FAM20A	66.2788721	140.46732	6.072081615	7.144325041		-1.08361	0.79044
LOC102158547	31.7586262	15.699289	5.033802952	4.061714779		1.016449	0.79047
LOC100627889	31.7586262	15.699289	5.033802952	4.061714779		1.016449	0.79047
SHMT2	197.455807	93.369456	7.632673964	6.560248082		1.080507	0.7905
VWCE	88.3718295	43.792754	6.481748252	5.485193456		1.012894	0.79056
CKAP5	260.973059	625.49273	8.033274644	9.291153952		-1.26109	0.79073
LOC102164829	120.130456	58.665764	6.920417836	5.89883146		1.034011	0.79077
LOC100526084	1731.53553	690.76872	10.75866923	9.434145968		1.325777	0.79082
GAS2L3	545.419885	238.79445	9.093866176	7.905654454		1.191598	0.79084
LOC102167104	12.4272885	26.440908	3.747096094	4.77825631		-1.08926	0.791
NLK	67.659682	33.877413	6.101391269	5.124221138		0.997973	0.7911
RASGRP1	67.659682	33.877413	6.101391269	5.124221138		0.997973	0.7911
LOC100625158	35.9010557	73.538775	5.205590187	6.219919206		-1.03448	0.79113
LOC102162051	53.8515836	112.37386	5.777461367	6.824944216		-1.06125	0.79114
LMNA	24451.3806	90316.357	14.57768731	16.46271565		-1.88507	0.79116
CENPK	584.082561	254.49374	9.192496406	7.997144125		1.198542	0.79123
SDHD	9552.44244	3344.7748	13.22180497	11.70812465		1.513961	0.79127
LOC102161806	42.8051049	21.483238	5.453027102	4.490777898		0.994571	0.79129
LOC100738431	19.3313377	39.661362	4.345633235	5.34558663		-1.03679	0.79136
LOC102159863	17.9505279	8.2627837	4.24416613	3.211445828		1.119326	0.79153
LOC102158655	17.9505279	8.2627837	4.24416613	3.211445828		1.119326	0.79153
LOC102159215	0	4.1313919	0	2.359350201		-20	0.7916
LOC102161973	0	4.1313919	0	2.359350201		-20	0.7916
PCDHA10	0	4.1313919	0	2.359350201		-20	0.7916
ART5	0	4.1313919	0	2.359350201		-20	0.7916
LOC102161735	0	4.1313919	0	2.359350201		-20	0.7916
LOC100525040	0	4.1313919	0	2.359350201		-20	0.7916
ANKDD1B	0	4.1313919	0	2.359350201		-20	0.7916
LOC102159741	0	4.1313919	0	2.359350201		-20	0.7916
LOC102157564	0	4.1313919	0	2.359350201		-20	0.7916
LOC102163996	0	4.1313919	0	2.359350201		-20	0.7916
LOC102168178	0	4.1313919	0	2.359350201		-20	0.7916
LOC102168083	0	4.1313919	0	2.359350201		-20	0.7916
LOC102157679	0	4.1313919	0	2.359350201		-20	0.7916
LOC100626136	0	4.1313919	0	2.359350201		-20	0.7916
LOC100626124	0	4.1313919	0	2.359350201		-20	0.7916
LOC102163817	0	4.1313919	0	2.359350201		-20	0.7916
LOC100739018	0	4.1313919	0	2.359350201		-20	0.7916
LOC102166870	0	4.1313919	0	2.359350201		-20	0.7916
LOC102164627	0	4.1313919	0	2.359350201		-20	0.7916
LOC100522424	0	4.1313919	0	2.359350201		-20	0.7916
LOC102164407	0	4.1313919	0	2.359350201		-20	0.7916
LOC102164586	0	4.1313919	0	2.359350201		-20	0.7916
LOC102157886	0	4.1313919	0	2.359350201		-20	0.7916
LOC102157756	0	4.1313919	0	2.359350201		-20	0.7916
ADAM28	0	4.1313919	0	2.359350201		-20	0.7916
LOC100624121	0	4.1313919	0	2.359350201		-20	0.7916
LOC100520705	0	4.1313919	0	2.359350201		-20	0.7916
POU6F2	0	4.1313919	0	2.359350201		-20	0.7916
LOC102166176	0	4.1313919	0	2.359350201		-20	0.7916
LOC100738766	0	4.1313919	0	2.359350201		-20	0.7916
TLX2	0	4.1313919	0	2.359350201		-20	0.7916
LOC100154768	0	4.1313919	0	2.359350201		-20	0.7916
LOC102166243	0	4.1313919	0	2.359350201		-20	0.7916
BANK1	0	4.1313919	0	2.359350201		-20	0.7916
LOC100621504	0	4.1313919	0	2.359350201		-20	0.7916
LOC102160707	0	4.1313919	0	2.359350201		-20	0.7916
LOC102162882	0	4.1313919	0	2.359350201		-20	0.7916
TMEM232	0	4.1313919	0	2.359350201		-20	0.7916
LOC102160368	0	4.1313919	0	2.359350201		-20	0.7916
IFN-ALPHA-8	0	4.1313919	0	2.359350201		-20	0.7916
LOC102162940	0	4.1313919	0	2.359350201		-20	0.7916
LOC102158756	0	4.1313919	0	2.359350201		-20	0.7916
CD80	0	4.1313919	0	2.359350201		-20	0.7916
LOC100524370	0	4.1313919	0	2.359350201		-20	0.7916
LOC102158543	0	4.1313919	0	2.359350201		-20	0.7916
SFTPB	0	4.1313919	0	2.359350201		-20	0.7916
LOC102162640	0	4.1313919	0	2.359350201		-20	0.7916
ADAM7	0	4.1313919	0	2.359350201		-20	0.7916
LOC102167634	0	4.1313919	0	2.359350201		-20	0.7916
LOC102167613	0	4.1313919	0	2.359350201		-20	0.7916
LOC102163264	0	4.1313919	0	2.359350201		-20	0.7916
LOC102165540	0	4.1313919	0	2.359350201		-20	0.7916
GIMAP8	0	4.1313919	0	2.359350201		-20	0.7916
LOC100627957	0	4.1313919	0	2.359350201		-20	0.7916
LOC102163434	0	4.1313919	0	2.359350201		-20	0.7916
LOC102165727	0	4.1313919	0	2.359350201		-20	0.7916
LOC102167265	0	4.1313919	0	2.359350201		-20	0.7916
TSGA10IP	0	4.1313919	0	2.359350201		-20	0.7916
LOC102167133	0	4.1313919	0	2.359350201		-20	0.7916
LOC102167444	0	4.1313919	0	2.359350201		-20	0.7916
LOC102167479	0	4.1313919	0	2.359350201		-20	0.7916
CCDC67	0	4.1313919	0	2.359350201		-20	0.7916
LOC102167339	0	4.1313919	0	2.359350201		-20	0.7916
HCN4	0	4.1313919	0	2.359350201		-20	0.7916
LRRC15	0	4.1313919	0	2.359350201		-20	0.7916
LOC100620363	0	4.1313919	0	2.359350201		-20	0.7916
LOC102165183	0	4.1313919	0	2.359350201		-20	0.7916
LSM3	3327.7517	1262.5534	11.70076555	10.30327087		1.398203	0.79162
KIF2C	4331.60046	1610.4165	12.08101748	10.65411376		1.427466	0.79166
LOC100626166	385.245944	958.48291	8.593375974	9.906113301		-1.31497	0.79171
LOC102167999	298.254925	137.16221	8.225231178	7.110219252		1.120663	0.79173
LOC102167478	592.36742	1539.3566	9.212781904	10.58904867		-1.37776	0.79186
C15H2orf76	693.166538	298.28649	9.439138011	8.225383356		1.216503	0.79201
LOC100621481	312.063023	759.34982	8.290309306	9.57051952		-1.28293	0.79207
LOC102161969	60.7556328	30.5723	5.948498827	4.980587446		0.990793	0.79214
FBXL16	60.7556328	30.5723	5.948498827	4.980587446		0.990793	0.79214
LOC100515763	834.009141	353.64714	9.705648181	8.470240516		1.237753	0.79215
LURAP1L	75.944541	38.008805	6.265747069	5.285727902		0.998613	0.79225
FKBP1B	726.305974	311.50695	9.506418615	8.287744447		1.221313	0.79226
PRPS2	6096.27543	2210.2946	12.573949	11.11067555		1.463689	0.79227
LOC102161680	13.8080984	28.919743	3.888314478	4.903025878		-1.06654	0.79228
MPL	13.8080984	28.919743	3.888314478	4.903025878		-1.06654	0.79228
LOC102160908	316.205452	770.09144	8.309273759	9.590758147		-1.28417	0.79229
TMEM8B	374.199466	927.08433	8.551513962	9.858112096		-1.30889	0.79229
KDM1B	1315.91177	537.90722	10.36294298	9.073893105		1.290634	0.79229
DNAJC25	233.356862	551.12767	7.872563229	9.108858104		-1.23985	0.7923
GMDS	3396.7922	1288.168	11.73038191	10.33222455		1.398852	0.79239
LOC102157547	1.38080984	6.610227	1.251452392	2.927939483		-2.25919	0.79242
LOC102162055	1.38080984	6.610227	1.251452392	2.927939483		-2.25919	0.79242
KCNH6	1.38080984	6.610227	1.251452392	2.927939483		-2.25919	0.79242
CD160	1.38080984	6.610227	1.251452392	2.927939483		-2.25919	0.79242
SYCE1	1.38080984	6.610227	1.251452392	2.927939483		-2.25919	0.79242
LOC102164971	1.38080984	6.610227	1.251452392	2.927939483		-2.25919	0.79242
LOC102167003	1.38080984	6.610227	1.251452392	2.927939483		-2.25919	0.79242
LOC102158431	1.38080984	6.610227	1.251452392	2.927939483		-2.25919	0.79242
LOC100157318	1.38080984	6.610227	1.251452392	2.927939483		-2.25919	0.79242
LOC100513500	1.38080984	6.610227	1.251452392	2.927939483		-2.25919	0.79242
MIR1839	1.38080984	6.610227	1.251452392	2.927939483		-2.25919	0.79242
MIR186	1.38080984	6.610227	1.251452392	2.927939483		-2.25919	0.79242
LOC102159387	220.929574	104.11107	7.79395812	6.715770874		1.085463	0.79271
LOC100625970	1887.56705	5498.0563	10.88307629	12.42496834		-1.54239	0.79272
GPRC5A	405.958092	182.60752	8.668736424	7.52048134		1.152585	0.79274
C4H1orf52	446.001577	199.13309	8.804136111	7.644815895		1.163316	0.79276
DNAJB5	154.650702	350.34203	7.28216827	8.45673236		-1.17975	0.7928
LOC102164640	276.161967	128.07315	8.11458549	7.012045083		1.108547	0.79302
LOC100517200	24.854577	50.402981	4.692347799	5.683780114		-1.02	0.79303
SSBP4	2660.82055	1029.5429	11.3781976	10.00918878		1.369867	0.79305
GPC2	249.92658	593.26787	7.971121491	9.214969574		-1.24718	0.7931
PIP5KL1	44.1859147	22.309516	5.497801224	4.542847146		0.985927	0.79314
WT1	20.7121475	42.140197	4.440430524	5.430960858		-1.02472	0.79316
LOC102157424	19.3313377	9.0890621	4.345633235	3.334720158		1.088738	0.79322
LOC102163670	19.3313377	9.0890621	4.345633235	3.334720158		1.088738	0.79322
LOC102161099	19.3313377	9.0890621	4.345633235	3.334720158		1.088738	0.79322
HSPA12B	477.760203	1210.4978	8.903159423	10.24257609		-1.34124	0.79349
KCNIP3	15.1889082	31.398578	4.016933786	5.017858594		-1.04768	0.79362
GKAP1	439.097528	196.65425	8.781679457	7.626835185		1.15888	0.79375
TDP1	1395.99874	569.3058	10.44811501	9.15559189		1.294022	0.79377
LOC100739114	53.8515836	111.54758	5.777461367	6.814391229		-1.0506	0.79381
LOC102165730	53.8515836	111.54758	5.777461367	6.814391229		-1.0506	0.79381
NMRK1	69.0404918	145.42499	6.130117309	7.194018019		-1.07476	0.79385
LPAR5	77.3253508	38.835083	6.291407421	5.315967693		0.993581	0.79387
MRPL20	7453.61149	2668.0529	12.86391745	11.38211216		1.482152	0.79387
LOC100621972	98.0374983	48.750424	6.629902968	5.636636914		1.007919	0.79393
C8H4orf21	98.0374983	48.750424	6.629902968	5.636636914		1.007919	0.79393
SNX25	335.53679	818.84187	8.394620418	9.679201855		-1.28711	0.79412
DCUN1D3	167.07799	380.08805	7.392987005	8.573980563		-1.18581	0.79418
LOC100156520	34.5202459	17.351846	5.150569664	4.19785327		0.992354	0.79422
ANKRD26	267.877108	638.71318	8.070803121	9.3212814		-1.2536	0.79425
MMP11	1006.61037	2746.5493	9.97672216	11.42392966		-1.44861	0.7943
NRAS	880.956675	2371.4189	9.784563977	11.21214307		-1.42861	0.79434
POLR1D	8507.1694	3017.5686	13.05463304	11.55964888		1.495293	0.7944
ATL2	3336.03656	1270.8161	11.70435178	10.3126744		1.39238	0.79443
LOC102161802	52.4707738	108.24247	5.740678649	6.771389985		-1.04468	0.79472
ETV2	52.4707738	108.24247	5.740678649	6.771389985		-1.04468	0.79472
GAREM	56.6132033	117.33153	5.848327568	6.886690713		-1.05138	0.79474
APITD1	1721.86987	691.595	10.75059802	9.435868156		1.315977	0.7948
LOC100156943	20.7121475	9.9153405	4.440430524	3.448285225		1.062743	0.79483
PABPC1L2A	26.2353869	52.881816	4.767410456	5.751726565		-1.01126	0.79488
WIPF1	1014.89523	2768.0325	9.988535907	11.43516629		-1.44753	0.79493
LOC100625556	38.6626754	78.496445	5.309710091	6.312818447		-1.02169	0.795
LOC100516765	1545.12621	626.31901	10.59444237	9.293055462		1.302755	0.79508
LOC102163541	33.1394361	66.928548	5.093367322	6.085946114		-1.01407	0.79515
LOC100156883	33.1394361	66.928548	5.093367322	6.085946114		-1.01407	0.79515
LOC100127143	419.76619	189.21775	8.716874974	7.571508044		1.149539	0.79527
NDE1	898.907203	380.91433	9.813632431	8.577105241		1.238706	0.79532
EFCAB2	176.743659	85.106672	7.473654282	6.428053129		1.054314	0.79542
LIMS1	1741.2012	5005.5944	10.76669553	12.28961386		-1.52346	0.79549
FDXACB1	160.173941	77.670167	7.332472693	6.29774474		1.044207	0.79555
TRMT112	8213.0569	2925.8517	13.00387923	11.51513394		1.489063	0.7956
LOC100737214	3701.95117	1401.3681	11.85445981	10.45364939		1.40145	0.79563
LOC100737037	131.176934	64.449713	7.04632663	6.032314961		1.025268	0.79568
PPP1R3D	394.911613	976.66104	8.629034575	9.933190545		-1.30633	0.79568
LOC100738156	165.69718	375.13038	7.381085891	8.55508903		-1.17884	0.79586
L3MBTL3	1600.3586	647.80224	10.6450807	9.341634998		1.30477	0.79596
PTCRA	35.9010557	18.178124	5.205590187	4.261389712		0.981823	0.79596
LOC100620225	144.985033	323.90112	7.189676653	8.343856914		-1.15965	0.79601
MMP17	74.5637311	156.99289	6.239622035	7.303715831		-1.07415	0.79618
SCML2	324.490311	149.55639	8.34647079	7.234160086		1.117486	0.79618
IL17D	125.653695	276.80325	6.984745358	8.117919691		-1.13941	0.79619
PDPN	512.280449	1299.7359	9.003603499	10.34511233		-1.34321	0.79623
TNNT1	905.811252	384.21944	9.824658483	8.589536711		1.23728	0.79625
LOC102161581	412.862141	186.73891	8.69300647	7.552583893		1.144638	0.79636
LOC102160460	22.0929574	10.741619	4.529381037	3.553559423		1.040375	0.79637
GJA10	22.0929574	10.741619	4.529381037	3.553559423		1.040375	0.79637
A2ML1	17.9505279	36.356248	4.24416613	5.223277669		-1.01818	0.79639
LOC100517788	444.620767	199.95937	8.799672657	7.650760008		1.152868	0.79649
MOCS2	586.84418	1508.7843	9.19928998	10.56012674		-1.36234	0.79657
LOC100621572	88.3718295	188.39147	6.481748252	7.565227535		-1.09208	0.79671
PPL	534.373406	1360.0542	9.064401668	10.4105088		-1.34774	0.7968
FAM107A	40.0434852	80.97528	5.359081339	6.357117027		-1.01591	0.79681
CASP2	6455.28598	2345.8043	12.65648877	11.19648182		1.460398	0.79683
LOC100626017	233.356862	545.34373	7.872563229	9.09366508		-1.22463	0.79691
LOC100625067	132.557744	65.275991	7.061319821	6.05041444		1.021997	0.79694
LOC100626310	80.0869705	40.48764	6.341398207	5.374609695		0.984086	0.79694
BZRAP1	432.193479	1075.8144	8.758867714	10.07255395		-1.31568	0.79697
LOC100152989	668.311961	1739.316	9.386534985	10.76513355		-1.37993	0.79711
LOC100739269	56.6132033	116.50525	5.848327568	6.876581411		-1.04118	0.79726
LOC102160361	77.3253508	162.77684	6.291407421	7.35558754		-1.07388	0.79731
MCC	459.809675	206.56959	8.848027198	7.697451307		1.154409	0.79737
LOC100627210	45.5667246	92.543178	5.541227504	6.547560532		-1.02215	0.7974
GPR114	45.5667246	92.543178	5.541227504	6.547560532		-1.02215	0.7974
SEPHS2	8195.10638	2927.5043	13.00072299	11.51594828		1.485091	0.7974
LOC100620855	180.886089	411.48663	7.506891393	8.688203544		-1.18576	0.79747
PTPRF	1079.79329	2946.5087	10.07787491	11.52528034		-1.44825	0.79781
LOC100626030	301.016544	720.51474	8.238483771	9.494885058		-1.25919	0.79782
LOC102166485	578.559321	255.32002	9.178812527	8.001802338		1.180158	0.79784
TESC	23.4737672	11.567897	4.613164285	3.65167138		1.020923	0.79785
LOC102159892	23.4737672	11.567897	4.613164285	3.65167138		1.020923	0.79785
LOC102165905	23.4737672	11.567897	4.613164285	3.65167138		1.020923	0.79785
FBXL3	618.602806	1594.7173	9.275199868	10.63998933		-1.36622	0.79787
LOC100739660	625.506856	274.32442	9.291186486	8.104988763		1.189143	0.79793
LOC102162270	168.4588	380.08805	7.404790748	8.573980563		-1.17394	0.79794
LDLR	2366.70806	6977.9208	11.20927549	12.76878825		-1.55992	0.798
LOC100625542	102.179928	51.229259	6.689018532	5.706786332		0.996072	0.79815
LOC100522040	48.3283443	24.788351	5.624344958	4.688647628		0.963207	0.79825
CGREF1	172.60123	390.00339	7.439633355	8.611037311		-1.17604	0.79833
LOC100624978	647.599813	283.41348	9.341184798	8.151846042		1.192194	0.79834
TIMM13	8018.36272	2872.9699	12.96927188	11.48882924		1.480765	0.79835
LOC100737111	1340.76635	552.78023	10.38991775	9.113169742		1.27828	0.79837
CCSAP	274.781157	128.89943	8.107380078	7.021251246		1.092037	0.79848
LOC100623881	399.054043	181.78124	8.644051094	7.513974209		1.134381	0.79862
NR6A1	66.2788721	33.877413	6.072081615	5.124221138		0.968225	0.79865
CTNNAL1	1799.19522	724.64613	10.81393765	9.503122366		1.312003	0.79867
LOC100738901	44.1859147	89.238064	5.497801224	6.495664213		-1.01407	0.79876
LOC102167660	669.69277	292.50254	9.38950824	8.197229196		1.19505	0.79876

LOC102162746	4.229436346	0	2.386655454	0		20	0.62296
VWA2	4.229436346	0	2.386655454	0		20	0.62296
LOC102158457	4.229436346	0	2.386655454	0		20	0.62296
LOC102158592	4.229436346	0	2.386655454	0		20	0.62296
LOC102162610	4.229436346	0	2.386655454	0		20	0.62296
LOC100623167	4.229436346	0	2.386655454	0		20	0.62296
LOC102165396	4.229436346	0	2.386655454	0		20	0.62296
PGLYRP3	4.229436346	0	2.386655454	0		20	0.62296
SNCA	4.229436346	0	2.386655454	0		20	0.62296
LOC100517491	4.229436346	0	2.386655454	0		20	0.62296
LOC102167787	4.229436346	0	2.386655454	0		20	0.62296
LOC100739779	4.229436346	0	2.386655454	0		20	0.62296
LOC102165859	4.229436346	0	2.386655454	0		20	0.62296
LOC100523909	4.229436346	0	2.386655454	0		20	0.62296
LOC100517924	4.229436346	0	2.386655454	0		20	0.62296
LOC102165954	4.229436346	0	2.386655454	0		20	0.62296
OVOL2	4.229436346	0	2.386655454	0		20	0.62296
GPR144	4.229436346	0	2.386655454	0		20	0.62296
GPR151	4.229436346	0	2.386655454	0		20	0.62296
LOC100515519	4.229436346	0	2.386655454	0		20	0.62296
SLC9A4	4.229436346	0	2.386655454	0		20	0.62296
LOC100737450	1002.376414	320.59601	9.970647215	8.329105689		1.644596	0.62309
BUB3	15660.54543	4009.1027	13.93493896	11.96942346		1.965783	0.62326
LOC100737484	697.8569971	2455.6993	9.448853466	11.26250558		-1.81513	0.62349
ARMCX3	1103.882886	4063.637	10.10967774	11.98891081		-1.88018	0.62359
LOC102160490	24.31925899	66.10227	4.662163277	6.068289661		-1.4426	0.62364
GRPEL2	596.3505248	199.13309	9.222433942	7.644815895		1.582428	0.62367
LOC102157443	54.9826725	153.68778	5.806908455	7.273215395		-1.48295	0.62371
PAPOLA	28762.28187	7031.6289	14.81194068	12.77984838		2.032247	0.62393
IL17D	95.16231779	276.80325	6.587399765	8.117919691		-1.5404	0.62394
DCAKD	509.6470797	1736.8371	8.996182745	10.76307717		-1.76889	0.62409
LOC100626199	35.95020894	98.327126	5.207510617	6.634115866		-1.45159	0.62411
BBS9	232.618999	733.73519	7.868013796	9.521080572		-1.65729	0.62419
LOC102161940	307.6914942	108.24247	8.270021921	6.771389985		1.507218	0.62432
UROD	640.7596065	2232.6042	9.325889176	11.12515782		-1.80087	0.62446
LOC102162180	27.49133625	9.9153405	4.832451381	3.448285225		1.471243	0.62449
LOC100622112	69.78569971	26.440908	6.145386028	4.778256309		1.40016	0.6245
PSRC1	3818.123662	1095.6451	11.89902592	10.09888102		1.801083	0.62458
PSTK	504.3602843	171.03962	8.981168479	7.426597064		1.560124	0.6252
CTIF	561.457675	1928.5337	9.135600727	10.91403654		-1.78026	0.62526
LOC100739849	4043.341147	1155.9634	11.98168898	10.17612756		1.806452	0.62539
LOC100524158	3222.830496	938.65223	11.65456018	9.875983097		1.779666	0.62539
LOC102162017	13.74566813	38.008805	3.882219287	5.285727902		-1.46736	0.62546
HFE2	13.74566813	38.008805	3.882219287	5.285727902		-1.46736	0.62546
SLC38A6	353.1579349	1157.616	8.468249056	10.17818678		-1.71277	0.62577
FIGN	401.7964529	138.81477	8.653907169	7.127372928		1.533304	0.62577
LOC100516466	4043.341147	1156.7897	11.98168898	10.17715754		1.805421	0.626
NAMPT	8610.075042	2317.7108	13.07197765	11.1791072		1.893325	0.62605
MKNK1	546.6546478	1869.868	9.097122603	10.86949202		-1.77423	0.62628
LOC102159741	13.74566813	4.1313919	3.882219287	2.359350201		1.734277	0.62645
LOC100621282	13.74566813	4.1313919	3.882219287	2.359350201		1.734277	0.62645
LOC102167133	13.74566813	4.1313919	3.882219287	2.359350201		1.734277	0.62645
AVIL	67.67098154	190.8703	6.10162868	7.58398763		-1.49598	0.62647
LOC100522550	257.9956171	818.84187	8.016783874	9.679201855		-1.66624	0.62665
C2	25.37661808	68.581105	4.721187694	6.120623682		-1.43431	0.62665
LOC102159380	29.60605442	80.149002	4.935745167	6.342501448		-1.43679	0.62672
LOC100511854	2482.679135	740.34542	11.27826309	9.534002096		1.745627	0.62713
LOC102161361	253.7661808	80.890621	7.993029968	6.521845711		1.481296	0.62715
LOC100513875	2491.138008	742.82426	11.28316825	9.538817985		1.745712	0.62732
ATP5A1	115808.3113	25620.413	16.82139173	14.64506245		2.176373	0.62741
ZNF226	93.04759962	268.54047	6.555319217	8.074358096		-1.5291	0.62745
RGL2	2013.211701	7815.7671	10.97559961	12.93235634		-1.95689	0.62745
CSAD	88.81816327	255.32002	6.488935315	8.001802338		-1.52338	0.62754
ABHD1	17.97510447	48.750424	4.246035924	5.636636914		-1.43941	0.62755
ETFA	16196.62649	4155.3539	13.9834948	12.0211028		1.96265	0.62762
COPS4	7991.519976	2168.1544	12.96443473	11.08291706		1.882002	0.62772
ANXA9	98.33439505	37.182527	6.63422144	5.254840671		1.403071	0.62789
NOLC1	20134.23173	5080.7857	14.29743446	12.31111982		1.986527	0.62794
LOC100627992	2042.817755	619.70878	10.99705084	9.27777274		1.720898	0.62794
MBLAC2	366.903603	128.07315	8.523183995	7.012045083		1.518433	0.62813
LOC100525224	251.6514626	794.87979	7.981004722	9.636406738		-1.65931	0.62816
LOC100520688	4631.232799	1313.7826	12.17749204	10.36060857		1.81767	0.62819
KCNQ5	193.4967125	595.74671	7.603601962	9.220974888		-1.62239	0.62826
PA2G4	7708.147741	2098.7471	12.91235566	11.03599884		1.876856	0.62831
LOC100736736	168.1200948	61.970878	7.40190428	5.976612875		1.43983	0.62832
LOC102165421	21.14718173	57.013208	4.46905122	5.858309485		-1.43083	0.62841
PSME2	1491.933671	5617.8666	10.54393435	12.45606345		-1.91284	0.62846
SCN8A	177.6363265	65.275991	7.480881679	6.05041444		1.444302	0.62848
CHML	1000.261696	322.24856	9.96760338	8.336500155		1.634132	0.62857
IPO7	19701.77186	4984.1111	14.26611099	12.28340995		1.982917	0.62869
LAMB1	6713.172841	29156.059	12.71299396	14.83155757		-2.11873	0.62876
MKL1	761.2985423	2681.2733	9.574212307	11.38924054		-1.81638	0.62881
CAND1	17834.47571	4547.8362	14.12246208	12.15128176		1.971417	0.62891
LOC100512726	265.3971307	95.022013	8.057434734	6.585293272		1.481819	0.62907
LOC100515976	658.7347109	219.79005	9.365742202	7.786531327		1.583572	0.62908
NUDT3	15749.36359	4056.2005	13.94309751	11.98626889		1.957093	0.62913
SLC3A1	1653.709611	511.46631	10.69236234	9.001313359		1.692995	0.62937
LOC102161616	333.0681123	117.33153	8.38399847	6.886690713		1.505226	0.6294
LOC100624340	2264.863163	682.50594	11.14584502	9.416810055		1.73051	0.62948
TXNDC5	3231.289369	13090.728	11.65834064	13.67636794		-2.01836	0.62948
LOC100626623	30.66341351	82.627837	4.98474489	6.385911347		-1.43011	0.62954
CHD1	7439.578533	2034.2974	12.86119909	10.99102387		1.87069	0.62956
USP49	84.58872693	32.224856	6.419348884	5.054191063		1.392291	0.62956
TACC2	3476.596677	1011.3647	11.76387491	9.983513431		1.781372	0.62961
MITD1	1295.264881	409.00779	10.34014484	8.679507525		1.663047	0.62969
ARL6IP6	10266.95673	2736.634	13.3258615	11.41871385		1.907534	0.62972
PPP4R2	9464.421184	2539.1534	13.20845099	11.31069993		1.898167	0.62974
ANKDD1A	23.2618999	8.2627837	4.600620625	3.211445828		1.493269	0.62975
LOC100525180	23.2618999	8.2627837	4.600620625	3.211445828		1.493269	0.62975
LOC102164126	23.2618999	8.2627837	4.600620625	3.211445828		1.493269	0.62975
LOC102158865	23.2618999	8.2627837	4.600620625	3.211445828		1.493269	0.62975
LOC102159091	25674.79334	6371.4325	14.64812123	12.63762848		2.010663	0.62977
FOXK1	1683.315666	6401.1785	10.71794683	12.6443472		-1.92703	0.62985
LOC102160658	130.0551676	48.750424	7.034030432	5.636636914		1.415637	0.62994
PIGY	6940.505044	1909.5293	12.76103278	10.89975668		1.861824	0.63005
LOC102162177	223.1027673	80.97528	7.808016653	6.357117027		1.462155	0.63008
FUK	179.7510447	547.82256	7.497860176	9.100195977		-1.60771	0.6302
PRPF4B	12884.97783	3375.3471	13.65535144	11.72124753		1.932582	0.63025
42622	3979.899602	16427.24	11.95887877	14.00389034		-2.04529	0.6303
LRRK1	530.7942615	1798.808	9.054724399	10.81362731		-1.76082	0.63054
LOC100152318	58.15474976	161.12428	5.886422108	7.340956379		-1.47021	0.63064
FOXP1	919.9024053	3291.893	9.846904461	11.68513993		-1.83936	0.63078
LOC102163189	15.8603863	42.966475	4.075565686	5.458331975		-1.43778	0.63093
LOC102158548	47.5811589	18.178124	5.602325001	4.261389711		1.388187	0.63102
ZNF346	1415.803817	444.53776	10.46842429	8.79940391		1.671243	0.63105
AP5M1	1574.40768	489.98307	10.6215095	8.939529481		1.684005	0.63106
GTF3A	105.7359087	306.54928	6.737901807	8.264673769		-1.53565	0.63111
CNOT7	7476.586101	2047.5178	12.8683569	11.00036472		1.868504	0.63123
KLHDC4	363.7315258	1185.7095	8.510691095	10.21275105		-1.7048	0.63126
LOC100624374	1742.527775	537.90722	10.76779363	9.073893105		1.695752	0.63132
FBXO34	2710.011339	806.44769	11.40461543	9.65722499		1.748646	0.63137
GMCL1	2168.643487	657.71758	11.08324228	9.363516252		1.721253	0.63176
CDH24	117.3668586	342.90552	6.887121389	8.42586848		-1.54679	0.63195
ARFIP2	1091.194577	3964.4836	10.09301418	11.95328111		-1.86122	0.632
WBSCR27	29.60605442	79.322724	4.935745167	6.327736286		-1.42184	0.63212
LOC100625158	27.49133625	73.538775	4.832451381	6.219919206		-1.41953	0.6323
KSR1	435.6319437	1443.5083	8.77027387	10.49636279		-1.7284	0.63234
LOC100736894	6109.420802	1702.9597	12.57705602	10.73467552		1.842991	0.63242
CLC-2	140.6287585	417.27058	7.145970432	8.708292708		-1.56909	0.63249
NAGA	213.5865355	659.37014	7.745415745	9.367131078		-1.62627	0.6325
C14H10orf137	2814.689888	836.19371	11.45927273	9.709417666		1.751067	0.63257
DCAF17	701.0290744	233.83678	9.45538697	7.875514565		1.583972	0.63265
MAP4K2	44.40908164	120.63664	5.504908955	6.926434087		-1.44174	0.63273
SMEK1	11466.00193	3040.7044	13.48520063	11.57066424		1.914885	0.63296
LOC102158390	124.7683722	47.097867	6.974625354	5.587901017		1.405519	0.63299
LOC100524051	39.1222862	14.873011	5.326331909	3.98850389		1.395294	0.633
LOC100738957	35.95020894	96.674569	5.207510617	6.609911087		-1.42714	0.63312
PPIL1	5070.03682	1436.0718	12.30806503	10.48891644		1.819868	0.63316
LOC100621569	727.4630516	242.09956	9.508711989	7.925403489		1.587273	0.63322
AIG1	918.8450462	299.93905	9.84524704	8.233327508		1.615152	0.63324
AZI1	243.1925899	759.34982	7.931875612	9.57051952		-1.64267	0.63331
KLHL29	223.1027673	690.76872	7.808016653	9.434145967		-1.63049	0.63331
LOC100622330	15857.21422	4100.8196	13.9529427	12.00204831		1.951155	0.63332
PSD3	561.457675	1905.3979	9.135600727	10.89663357		-1.76284	0.63334
LOC100628061	177.6363265	537.90722	7.480881679	9.073893105		-1.59843	0.63345
RHEBL1	238.9631536	86.759229	7.906669086	6.455478947		1.461699	0.63345
LOC102161524	16.91774539	45.44531	4.163317207	5.537461031		-1.42559	0.63351
LOC102160371	21.14718173	7.4365053	4.46905122	3.076645515		1.507769	0.63353
ZCCHC11	3025.104347	894.85948	11.56324602	9.80712864		1.757252	0.63395
LOC100737880	23.2618999	61.970878	4.600620625	5.976612875		-1.41362	0.63395
ZNF367	12.68830904	34.703692	3.774872332	5.158001345		-1.45159	0.63399
TBK1	305.576776	109.06875	8.260104603	6.782261052		1.486297	0.63399
GPAA1	1126.087427	4091.7305	10.13838371	11.99884795		-1.86139	0.63408
LOC100520401	285.4869534	102.45852	8.16232563	6.692908622		1.478385	0.63429
MNT	108.9079859	314.81206	6.780152406	8.302922453		-1.53138	0.63434
TMEM126A	3635.20054	1060.1152	11.82821605	10.05136551		1.777814	0.63472
ZNF644	2854.869534	849.41417	11.47971436	9.732021819		1.748885	0.63477
LOC100622851	34.89284986	13.220454	5.165624571	3.829895615		1.40016	0.63522
LOC102162803	34.89284986	13.220454	5.165624571	3.829895615		1.40016	0.63522
LOC102168137	154.3744266	57.839486	7.279605257	5.878712739		1.416307	0.63548
LOC100739649	131.1125267	49.576702	7.045623457	5.660401069		1.403071	0.63556
LOC100738250	2332.534145	706.46801	11.18830086	9.4665211		1.723202	0.63557
LOC100513996	11.63094995	3.3051135	3.658891241	2.106051269		1.815197	0.63562
LOC102157722	11.63094995	3.3051135	3.658891241	2.106051269		1.815197	0.63562
LOC102164006	11.63094995	3.3051135	3.658891241	2.106051269		1.815197	0.63562
LOC102158754	11.63094995	3.3051135	3.658891241	2.106051269		1.815197	0.63562
LOC102163063	11.63094995	3.3051135	3.658891241	2.106051269		1.815197	0.63562
LOC102167311	11.63094995	3.3051135	3.658891241	2.106051269		1.815197	0.63562
LRRC6	175.5216084	65.275991	7.463700987	6.05041444		1.427024	0.6357
ABCA1	458.8938436	1520.3522	8.845157075	10.57113847		-1.72817	0.63584
GNG5	2020.613214	619.70878	10.98129128	9.27777274		1.705131	0.636
LOC102159590	81.41664967	31.398578	6.364863912	5.017858594		1.374625	0.63601
LOC100737128	303.4620578	964.26686	8.25011864	9.914784038		-1.66792	0.63607
LOC100621516	297.1179033	941.95734	8.219739208	9.881048699		-1.66463	0.63618
STARD10	156.4891448	466.021	7.299108582	8.867343618		-1.57433	0.63627
ESF1	6595.805982	1836.8168	12.68755196	10.84377726		1.844341	0.63693
RHCG	57.09739067	156.16661	5.860401464	7.296150961		-1.45159	0.63706
LOC102163748	85.64608601	33.051135	6.437062676	5.089630977		1.373688	0.63716
LOC102166498	31.7207726	84.280394	5.032134908	6.414142197		-1.40977	0.63727
VPS37A	70.8430588	196.65425	6.166776871	7.626835184		-1.47296	0.63732
CHMP1A	1360.821144	5013.8572	10.41132152	12.29199289		-1.88144	0.63743
GLIPR2	1376.681531	5077.4806	10.42802671	12.31018121		-1.88292	0.63751
LOC100621470	257.9956171	804.79513	8.016783874	9.654269283		-1.64128	0.63754
IGDCC4	1369.280017	5046.9083	10.42025502	12.30146999		-1.88198	0.63759
B3GALTL	855.403501	3008.4796	9.742146883	11.5552983		-1.81436	0.63767
PPP2CA	6196.124247	1736.0109	12.59738318	10.76239106		1.83559	0.63778
LOC100622329	56.04003159	152.8615	5.833902876	7.265488457		-1.4477	0.63786
SCUBE3	38.06492712	101.63224	5.287802017	6.681340184		-1.41682	0.63804
LOC102168094	27.49133625	72.712497	4.832451381	6.20383732		-1.40323	0.63804
LOC100737888	2866.500484	856.02439	11.48557801	9.743192457		1.743567	0.63805
LOC102161495	234.7337172	724.64613	7.881014313	9.503122366		-1.62625	0.63821
SUMF1	1136.661018	4109.0823	10.15185504	12.00495158		-1.85401	0.63825
KIAA1324L	296.0605442	106.58991	8.214613188	6.749398975		1.473821	0.63825
PHC1	11216.46519	2997.7379	13.45345909	11.55013973		1.903672	0.63826
CDK5RAP3	573.0886249	1934.3177	9.16512966	10.91835468		-1.75499	0.6383
ND3	107104.1313	24105.845	16.70866807	14.55715524		2.151559	0.6386
CCNG1	50961.5359	12120.677	15.63714945	13.56530174		2.071938	0.63869
ZDHHC1	227.3322036	699.0315	7.834990539	9.451276037		-1.62056	0.6387
LOC102160305	30.66341351	11.567897	4.98474489	3.651671381		1.406392	0.63872
LOC100153731	30.66341351	11.567897	4.98474489	3.651671381		1.406392	0.63872
VGLL4	840.6004738	2946.5087	9.716991707	11.52528034		-1.80951	0.63876
LOC102160814	25.37661808	66.928548	4.721187694	6.085946114		-1.39912	0.63904
CHTF18	427.173071	1398.063	8.742050253	10.45024522		-1.71054	0.63909
TTC7A	119.4815768	345.38436	6.912668746	8.436229978		-1.53141	0.63911
LOC100737496	2888.705025	863.4609	11.49670652	9.755656899		1.74222	0.63933
DTNBP1	263.2824126	820.49442	8.04593661	9.682106971		-1.63988	0.63939
LOC100523457	246.3646672	90.064343	7.950495635	6.508814353		1.451767	0.63944
LOC100627556	5363.982646	1523.6573	12.38935779	10.5742693		1.815766	0.63945
TTLL4	485.3278207	168.56079	8.925785314	7.405658765		1.525691	0.64016
LOC102165481	15.8603863	42.140197	4.075565686	5.430960858		-1.40977	0.64031
LOC100623058	109.965345	314.81206	6.793965376	8.302922453		-1.51744	0.64041
LHFPL2	137.4566813	401.57129	7.113290863	8.653100473		-1.54668	0.64043
AKR1E2	106.7932677	304.89672	6.752123267	8.256900823		-1.5135	0.6405
LOC100627583	20.08982264	52.881816	4.398475058	5.751726565		-1.39631	0.64073
GEM	554.0561614	1855.8212	9.116489943	10.8586192		-1.74395	0.64109
DFNB31	28.54869534	10.741619	4.885022527	3.553559423		1.410213	0.64111
LOC102165559	67.67098154	185.91263	6.10162868	7.546220276		-1.45802	0.64114
FITM1	7.401513606	21.483238	3.070649265	4.490777899		-1.53732	0.64123
C1QL3	8.458872693	23.962073	3.241668254	4.641665831		-1.50222	0.6414
LRRC7	26.43397716	69.407383	4.777891883	6.137654819		-1.3927	0.64144
PET100	262.2250535	813.8842	8.040153	9.670451241		-1.63402	0.64166
LOC102163661	6.344154519	19.004403	2.876596413	4.322245637		-1.58283	0.64167
TIMP-4	6.344154519	19.004403	2.876596413	4.322245637		-1.58283	0.64167
LOC102162476	6.344154519	19.004403	2.876596413	4.322245637		-1.58283	0.64167
RSPO1	6.344154519	19.004403	2.876596413	4.322245637		-1.58283	0.64167
LOC100518122	71.90041789	28.093465	6.187855179	4.862623207		1.355766	0.6418
DDX20	3883.679925	1136.1328	11.92358002	10.15118498		1.773293	0.64182
NPAS3	9.516231779	26.440908	3.394545939	4.778256309		-1.47431	0.642
IFT172	822.6253694	2863.0546	9.685844458	11.48384326		-1.79925	0.64208
SLC25A26	1019.294159	333.81646	9.994769438	8.387226654		1.610443	0.64224
LOC100624226	433.5172255	1413.7623	8.76326956	10.46634396		-1.70538	0.64234
LOC100523337	16.91774539	44.619032	4.163317207	5.511563931		-1.39912	0.64236
LOC102162115	46.52379981	18.178124	5.570578288	4.261389711		1.355766	0.64271
LOC100624086	10.57359087	28.919743	3.532764645	4.903025878		-1.45159	0.6429
LOC102161313	5.286795433	16.525567	2.65232482	4.131389251		-1.64423	0.643
AMY2A	5.286795433	16.525567	2.65232482	4.131389251		-1.64423	0.643
LOC102167657	5.286795433	16.525567	2.65232482	4.131389251		-1.64423	0.643
LOC100521789	5.286795433	16.525567	2.65232482	4.131389251		-1.64423	0.643
LOC102165052	5.286795433	16.525567	2.65232482	4.131389251		-1.64423	0.643
LOC102165118	5.286795433	16.525567	2.65232482	4.131389251		-1.64423	0.643
EDIL3	329.896035	1045.2421	8.370234194	10.03100107		-1.66375	0.64315
LOC100738573	85.64608601	238.79445	6.437062676	7.905654455		-1.47931	0.64322
LOC100737500	106.7932677	41.313919	6.752123267	5.403060391		1.370121	0.64327
SIGIRR	72.95777697	200.78564	6.208629956	7.65667973		-1.46052	0.64331
SPR	16440.87644	4287.5585	14.00508734	12.06627708		1.939059	0.64331
LOC100624184	154.3744266	453.62683	7.279605257	8.828539005		-1.55507	0.64342
SLC35A3	1433.778921	456.93194	10.48661274	8.838989383		1.649771	0.64343
FAM188A	2484.793853	756.04471	11.27949094	9.564234696		1.716583	0.64348
LOC100627250	1008.720569	331.33763	9.97974038	8.376505833		1.606153	0.64381
IGSF6	26.43397716	9.9153405	4.777891883	3.448285225		1.414659	0.64406
LOC102161212	3471.309881	1027.8903	11.76167999	10.00687345		1.755794	0.64411
ZSCAN12	305.576776	110.7213	8.260104603	6.803760479		1.464602	0.64415
LOC102161115	70.8430588	194.17542	6.166776871	7.608627544		-1.45466	0.64429
MCCC2	659.79207	224.74772	9.368052563	7.818567588		1.553705	0.64432
LOC102165097	17.97510447	47.097867	4.246035924	5.587901017		-1.38966	0.64437
LOC102166152	46.52379981	123.94176	5.570578288	6.965111899		-1.41362	0.64437
LOC100524210	72375.17212	16870.952	16.14322719	14.04233926		2.100954	0.64444
ARID2	4702.075858	1358.4016	12.19938889	10.40875606		1.791388	0.64451
LOC100049693	6407.596065	27135.808	12.64579262	14.72796341		-2.08234	0.64453
NAPB	591.0637294	203.26448	9.209608665	7.674294538		1.539956	0.6446
IQCF3	16.91774539	5.7839486	4.163317207	2.76212524		1.548411	0.64462
LOC100515766	16.91774539	5.7839486	4.163317207	2.76212524		1.548411	0.64462
PITPNM1	772.9294923	2663.0952	9.596058328	11.37942992		-1.7847	0.64462
LOC100521669	498.0161298	1640.9888	8.962942639	10.68122861		-1.7203	0.64472
CUL3	7658.451864	2126.8405	12.90302544	11.05517432		1.848341	0.64488
STXBP6	2114.718173	653.58619	11.04693175	9.35443936		1.694016	0.64512
LOC102161064	12.68830904	33.877413	3.774872332	5.124221138		-1.41682	0.64528
LOC102165018	12.68830904	33.877413	3.774872332	5.124221138		-1.41682	0.64528
CDO1	404.9685302	143.77244	8.665224087	7.177643143		1.494023	0.64528
ZNF436	323.5518805	1019.6275	8.342305305	9.995240719		-1.65597	0.64529
ZZZ3	6726.918509	1888.8724	12.71594452	10.88407308		1.832421	0.64529
RRP15	5888.432753	1671.5611	12.52391297	10.70784324		1.816688	0.64531
SNX11	212.5291764	642.01829	7.738289401	9.328715975		-1.59495	0.64544
NOXRED1	40.17964529	15.699289	5.363859498	4.061714779		1.355766	0.64548
C4H1orf194	40.17964529	15.699289	5.363859498	4.061714779		1.355766	0.64548
LOC100157359	302.4046988	109.89502	8.245099618	6.793050814		1.460355	0.64557
LOC102158412	4.229436346	14.046732	2.386655454	3.911378308		-1.7317	0.64564

XCR1	4.14464412	0	2.363071282	0		20	0.60089
LOC102167553	4.14464412	0	2.363071282	0		20	0.60089
LOC100739283	4.14464412	0	2.363071282	0		20	0.60089
LOC102163454	4.14464412	0	2.363071282	0		20	0.60089
LOC100158208	4.14464412	0	2.363071282	0		20	0.60089
LOC102165755	4.14464412	0	2.363071282	0		20	0.60089
GLRA2	4.14464412	0	2.363071282	0		20	0.60089
ITLN2	4.14464412	0	2.363071282	0		20	0.60089
LOC100517924	4.14464412	0	2.363071282	0		20	0.60089
LCN5	4.14464412	0	2.363071282	0		20	0.60089
LOC100624079	130.970754	312.33322	7.044074441	8.291553945		-1.25384	0.60117
LOC100623242	276.033298	116.50525	8.113915583	6.876581411		1.244447	0.6013
P2RY2	18.2364341	42.966475	4.265769485	5.458331975		-1.23639	0.6014
LOC102165084	46.4200141	19.830681	5.567424187	4.380638094		1.227013	0.60159
SUZ12	3735.15328	1348.4863	11.86733793	10.39819462		1.469827	0.60192
LOC100737955	612.578401	1608.764	9.261103887	10.65263347		-1.39299	0.60205
LOC100738481	176.561839	76.01761	7.472177749	6.267116453		1.215768	0.60222
TAF1C	1410.83686	542.03861	10.46335768	9.084910972		1.380084	0.60234
LOC102167310	5.80250177	16.525567	2.766065426	4.13138925		-1.50995	0.60244
LOC102165520	825.613108	328.03251	9.691068432	8.362086342		1.331627	0.60248
GLS	4307.11417	1541.8354	12.07284077	10.59136848		1.482073	0.60256
EIF2AK3	440.990134	1132.0014	8.787870357	10.14593389		-1.36006	0.60275
INTS8	2970.8809	1089.0349	11.53716059	10.0901586		1.447841	0.60276
TMEM185A	366.38654	928.73689	8.521154961	9.860678689		-1.3419	0.603
TRIM47	881.980268	2372.2452	9.786237389	11.21264545		-1.42743	0.60301
LOC102160774	19.8942918	7.4365053	4.385036953	3.076645515		1.419658	0.60305
LOC100519264	19.8942918	7.4365053	4.385036953	3.076645515		1.419658	0.60305
ERC2	19.8942918	7.4365053	4.385036953	3.076645515		1.419658	0.60305
YIF1A	955.754934	2584.5987	9.902005625	11.33628269		-1.43523	0.60308
LOC100739076	404.517266	1032.0217	8.663619532	10.01265483		-1.3512	0.60313
TLL1	423.582629	175.17101	8.729901537	7.460832769		1.273879	0.60322
SNRPA	7582.21195	2620.955	12.88859333	11.35642721		1.532526	0.60324
LOC102163747	1052.73961	412.31291	10.04130269	8.691090609		1.352337	0.60325
LOC100625748	431.871917	178.47613	8.757796397	7.487648157		1.274872	0.60362
HABP2	0	4.1313919	0	2.359350201		-20	0.60378
LOC102159741	0	4.1313919	0	2.359350201		-20	0.60378
LOC100626318	0	4.1313919	0	2.359350201		-20	0.60378
LOC102163581	0	4.1313919	0	2.359350201		-20	0.60378
LOC102164514	0	4.1313919	0	2.359350201		-20	0.60378
ADAM28	0	4.1313919	0	2.359350201		-20	0.60378
POU1F1	0	4.1313919	0	2.359350201		-20	0.60378
BANK1	0	4.1313919	0	2.359350201		-20	0.60378
LOC102161405	23.2100071	53.708094	4.597531595	5.773682393		-1.21039	0.60387
ZNF496	2030.87562	763.48122	10.98859637	9.578337243		1.411437	0.60393
CTDSPL	231.271142	568.47952	7.859666109	9.153500149		-1.29752	0.60404
ALDH3A1	19.0653629	44.619032	4.326635347	5.511563931		-1.22671	0.60407
SGK3	226.297569	96.674569	7.828438443	6.609911086		1.227013	0.60416
LOC100737767	1369.39042	527.99188	10.42037125	9.047101766		1.374946	0.60422
LOC102162802	81.2350247	35.52997	6.361681078	5.191008663		1.193065	0.60435
LOC102157806	136.773256	59.492043	7.106152054	5.918673475		1.201018	0.60438
LOC100522069	57.1960888	131.37826	5.862850292	7.048522415		-1.19974	0.60452
FLOT2	1441.50722	4003.3187	10.49436283	11.96734109		-1.47362	0.60453
LOC102168053	37.3017971	15.699289	5.259340178	4.061714779		1.248546	0.60462
GLCE	1279.8661	495.76702	10.32290396	8.956425596		1.368259	0.60462
LPCAT4	5814.9357	2046.6915	12.5057956	10.99978268		1.50647	0.605
LOC100622841	21.5521494	8.2627837	4.495193036	3.211445828		1.383132	0.60518
LOC100739575	33.9860818	77.670167	5.128709195	6.29774474		-1.19242	0.60526
LOC100738653	421.924771	1076.6407	8.724257253	10.07366055		-1.35148	0.60582
LOC102166500	58.8539465	25.61463	5.90337447	4.734147579		1.200171	0.60599
LOC100624307	290.954017	123.11548	8.189597352	6.955539223		1.240779	0.60629
LOC100525614	75.432523	33.051135	6.256114748	5.089630977		1.190487	0.6064
GCNT1	1556.72833	596.57298	10.60522793	9.222971117		1.383747	0.6065
TBX4	19.8942918	46.271589	4.385036953	5.56290145		-1.21777	0.60662
LOC102162570	19.8942918	46.271589	4.385036953	5.56290145		-1.21777	0.60662
PRX	180.706484	436.27498	7.505466087	8.772396993		-1.27159	0.60681
LOC100520066	1075.94961	422.22825	10.07273504	8.725292112		1.349516	0.60689
RPGR	103.616103	45.44531	6.708961125	5.537461031		1.189045	0.60712
LOC100738712	2354.15786	879.16019	11.20160805	9.781622305		1.421013	0.60713
LOC102166792	23.2100071	9.0890621	4.597531595	3.334720158		1.352544	0.6072
LOC100624388	387.109761	980.79243	8.600320906	9.939274229		-1.34121	0.60721
TBL1X	3551.13108	10451.595	11.79446911	13.35157355		-1.55737	0.60723
PPAN	4525.12245	1621.1582	12.1440599	10.66369878		1.480932	0.60725
LOC100154012	266.086152	113.20014	8.061161368	6.83542057		1.233018	0.60728
LOC100523210	266.086152	113.20014	8.061161368	6.83542057		1.233018	0.60728
GPR155	256.967935	633.75551	8.011047944	9.310057206		-1.30234	0.60731
CILP	7.46035941	19.830681	3.080718953	4.380638094		-1.41042	0.60738
LOC100526008	7.46035941	19.830681	3.080718953	4.380638094		-1.41042	0.60738
LOC102158156	7.46035941	19.830681	3.080718953	4.380638094		-1.41042	0.60738
RHOQ	3062.06307	8922.9801	11.58075936	13.12347159		-1.54302	0.60738
EIF4A2	37260.3506	11732.327	15.18539235	13.51832448		1.667152	0.60785
XKR5	878.664553	349.51575	9.780809668	8.453335466		1.329955	0.60787
CHST7	187.337914	80.97528	7.557179646	6.357117027		1.210089	0.60788
ERGIC1	3068.69451	8939.5057	11.58387937	13.12614072		-1.54257	0.60791
LOC100738973	3500.56642	1275.7738	11.77378474	10.31828724		1.456216	0.60822
CCND3	397.056907	1006.4071	8.636830884	9.976431027		-1.3418	0.60852
POMT2	256.967935	632.92923	8.011047944	9.308177987		-1.30046	0.60859
LOC100037948	105.273961	46.271589	6.731644339	5.56290145		1.18595	0.6091
LOC100154243	213.034708	518.07654	7.741700952	9.019803473		-1.28208	0.60947
DGKD	1898.24701	720.51474	10.89121183	9.494885058		1.397568	0.60997
LOC102158250	145.062544	344.55808	7.190442454	8.432784407		-1.24807	0.61015
PAK1IP1	4255.72058	1534.3989	12.05552668	10.58439784		1.47173	0.61021
PAPLN	62.1696618	142.11988	5.981159942	7.161080272		-1.19283	0.61055
LOC100737774	919.282065	2462.3095	9.845932303	11.26638222		-1.42143	0.61055
LOC102165475	78.7482382	34.703692	6.317380743	5.158001345		1.182159	0.61066
LOC100738290	95.3268147	221.4426	6.589865555	7.797289318		-1.21598	0.61073
FAM83C	26.5257224	10.741619	4.78270852	3.553559423		1.304181	0.611
LOC102160460	26.5257224	10.741619	4.78270852	3.553559423		1.304181	0.611
ACOXL	26.5257224	10.741619	4.78270852	3.553559423		1.304181	0.611
LOC100153743	135.944327	321.42229	7.097445694	8.33280766		-1.24145	0.61105
SLC9B2	71.2878788	31.398578	6.175681852	5.017858594		1.182958	0.61121
USP37	851.309902	340.42669	9.735234283	8.415432027		1.32234	0.61142
TMEM204	21.5521494	49.576702	4.495193036	5.660401068		-1.20183	0.61144
LOC102163384	9.11821706	23.135794	3.338883189	4.593102408		-1.3433	0.6116
LOC102167226	149.207188	65.275991	7.230810045	6.05041444		1.192693	0.61167
LOC100626165	262.770437	112.37386	8.043139069	6.824944216		1.225497	0.61169
PTPN9	1050.25282	2835.7874	10.03789396	11.4700423		-1.43301	0.61174
FHAD1	16.5785765	38.835083	4.135746339	5.315967693		-1.22804	0.61177
WDYHV1	2970.05198	1097.2977	11.53675813	10.10105341		1.436533	0.61189
LOC102157962	198.113989	478.41518	7.637450771	8.905131772		-1.27193	0.61191
LOC100511684	379.649401	956.00408	8.572318998	9.902381259		-1.33235	0.6121
ARMCX2	796.6006	2109.4887	9.639522684	11.04336138		-1.40496	0.61224
PODXL2	1153.03999	452.80055	10.17247751	8.82591454		1.348495	0.61242
NUP85	4022.79158	1458.3813	11.97433986	10.51114118		1.463829	0.61244
DFNB59	63.8275194	28.093465	6.018534465	4.862623207		1.183944	0.61257
CHRNB4	28.18358	11.567897	4.867084967	3.65167138		1.284728	0.61281
LOC102160015	28.18358	11.567897	4.867084967	3.65167138		1.284728	0.61281
LOC100518681	282.664729	697.37895	8.148042965	9.447866256		-1.30285	0.61301
COL17A1	95.3268147	42.140197	6.589865555	5.430960858		1.177685	0.6131
ATP9A	1117.39605	3025.8314	10.12721546	11.5635926		-1.43719	0.6132
RXRB	1037.81889	2796.126	10.02072843	11.44972953		-1.42987	0.6132
LOC102163687	188.166843	81.801559	7.563515426	6.371586023		1.201812	0.6133
LOC102166569	87.0375265	200.78564	6.460046706	7.65667973		-1.20595	0.61331
CCDC171	43.9332277	19.004403	5.489710792	4.322245637		1.208979	0.61342
POLD2	2112.93957	799.01119	11.04571842	9.643876361		1.402964	0.61351
LOC100627082	1283.18182	3506.7254	10.32663376	11.7763201		-1.4504	0.61355
LOC102160651	9.94714588	24.788351	3.452482877	4.688647628		-1.31731	0.61357
LOC102166498	37.3017971	84.280394	5.259340178	6.414142197		-1.17595	0.61405
DNMBP	1376.85078	535.42838	10.42820393	9.067241769		1.362607	0.61408
TUFT1	692.155568	1812.0285	9.437035368	10.82418586		-1.38844	0.61414
WDR33	2053.2567	778.35423	11.00440075	9.60613539		1.399415	0.61421
PPFIBP1	1345.35148	3686.0278	10.39483938	11.84824259		-1.45408	0.61429
C17H20orf112	723.654863	1899.614	9.501150225	10.89224983		-1.39233	0.61452
GK	1182.0525	464.36844	10.20829838	8.862229579		1.347952	0.61455
CD3EAP	2729.66262	1016.3224	11.41503536	9.990561237		1.425365	0.61457
LOC100511884	29.8414377	12.394176	4.946798112	3.74353388		1.267655	0.61458
LOC102164166	29.8414377	12.394176	4.946798112	3.74353388		1.267655	0.61458
RTN1	29.8414377	12.394176	4.946798112	3.74353388		1.267655	0.61458
LOC102165605	29.8414377	12.394176	4.946798112	3.74353388		1.267655	0.61458
DHX34	3995.43693	1451.7711	11.96449861	10.50459169		1.460539	0.61461
PKDREJ	40.6175124	91.716899	5.379118827	6.534760414		-1.17509	0.61461
SPAG4	65.4853771	28.919743	6.054965161	4.903025878		1.179118	0.61463
ZBTB9	1957.10095	744.47681	10.93523943	9.54201967		1.394419	0.61464
LOC767626	96.9846724	42.966475	6.614484182	5.458331975		1.174545	0.615
LOC100524613	7242.35113	2535.022	12.8224416	11.30835157		1.51446	0.61505
THOP1	7122.15645	2496.187	12.79830096	11.28608812		1.512588	0.61535
LOC102166455	45.5910853	19.830681	5.541982032	4.380638094		1.201018	0.61543
LOC102164542	10.7760747	26.440908	3.557786824	4.77825631		-1.29494	0.61547
LOC100624035	43.9332277	99.153405	5.489710792	6.646067655		-1.17435	0.61548
FAM160B2	1371.04827	3757.0878	10.42211553	11.87578304		-1.45434	0.61552
LOC100155406	610.920543	250.36235	9.257200523	7.973624744		1.286967	0.61575
CREB3L4	351.465821	148.73011	8.461339555	7.226220529		1.240688	0.61594
PIK3R6	18.2364341	42.140197	4.265769485	5.430960858		-1.20837	0.61617
LOC102161491	31.4992953	13.220454	5.022336531	3.829895615		1.252548	0.61631
PLEKHM1	520.567301	1333.6133	9.026709615	10.38220606		-1.35718	0.61634
LOC100622441	3387.00317	1246.0278	11.72621951	10.28427789		1.442673	0.61671
WDR27	159.983263	70.233662	7.330766893	6.154487245		1.187686	0.61674
tRNA-Asp	509.791227	211.52726	8.996589935	7.731504113		1.269063	0.61737
CCDC148	47.248943	20.656959	5.592425431	4.436758793		1.193654	0.61739
LOC102167771	431.871917	180.95496	8.757796397	7.507437596		1.254973	0.6175
LOC733646	1181.22357	3197.6973	10.20728718	11.64326876		-1.43675	0.61765
ECSIT	3532.89465	1297.257	11.78704331	10.34236034		1.445386	0.61767
CYP2A19	33.1571529	14.046732	5.094115824	3.911378308		1.239086	0.61801
LOC102167870	33.1571529	14.046732	5.094115824	3.911378308		1.239086	0.61801
LOC100627863	1574.96477	4347.0505	10.62201956	12.08615299		-1.46472	0.61803
LOC102166197	19.0653629	43.792754	4.326635347	5.485193456		-1.19974	0.61826
LOC102165059	19.0653629	43.792754	4.326635347	5.485193456		-1.19974	0.61826
LOC102163371	85.3796688	38.008805	6.43261988	5.285727902		1.167559	0.61851
SPRED2	2366.59179	892.38064	11.20920464	9.803131183		1.40708	0.61854
DNASE1	897.729916	360.25737	9.811743816	8.49688321		1.317254	0.61868
FKBP14	929.229211	2471.3986	9.861442434	11.27169564		-1.41122	0.61888
LOC102157645	75.432523	171.8659	6.256114748	7.433509508		-1.18802	0.61902
TRNAQ-CUG	48.9068006	21.483238	5.641164514	4.490777898		1.186824	0.61928
ARHGAP28	843.849542	2227.6465	9.722550627	11.12195208		-1.40046	0.61972
UTP6	5556.30991	1985.5469	12.44017098	10.95604716		1.484591	0.61976
LOC100514227	29.0125088	65.275991	4.907492018	6.05041444		-1.16988	0.61977
NUDCD1	2302.76427	870.8974	11.16977739	9.768014573		1.402792	0.6199
LOC102159128	216.350423	94.195734	7.763879093	6.572825024		1.199636	0.62003
ATP2A1	216.350423	94.195734	7.763879093	6.572825024		1.199636	0.62003
VRK1	2039.16491	777.52795	10.99447005	9.60460502		1.391012	0.62025
CDK7	1264.94539	497.41958	10.30599945	8.961216934		1.34654	0.62029
TTC8	4469.58422	1620.3319	12.12624766	10.66296373		1.463851	0.62032
LOC100627430	333.229387	142.11988	8.384694778	7.161080272		1.229407	0.62072
PANX1	329.084743	140.46732	8.366692646	7.144325041		1.228225	0.62086
TCTEX1D4	13.2628612	31.398578	3.834191515	5.017858594		-1.24331	0.62086
LOC100738482	2621.07294	984.09754	11.3564921	9.944122772		1.413284	0.62113
KIF14	1172.10536	463.54217	10.19611687	8.859665746		1.33833	0.62116
HINFP	1664.48908	643.67085	10.70173018	9.332418944		1.370684	0.62132
LOC100621650	170.759338	75.191332	7.424244723	6.251554969		1.183326	0.62148
LOC102164200	130.141825	57.839486	7.03498407	5.878712739		1.169958	0.622
PAQR8	111.905391	258.62513	6.818970566	8.020286225		-1.20858	0.62226
ZMYM4	1288.98432	507.33492	10.33313782	8.98963553		1.345224	0.62258
DENND6A	1439.84937	562.69557	10.4927038	9.138772422		1.355491	0.6226
LEMD2	1612.26656	4437.9411	10.65576912	12.11599986		-1.4608	0.62274
TEFM	416.951198	175.99729	8.707190687	7.467583487		1.244325	0.62279
RAB3A	477.463002	199.95937	8.902263561	7.650760008		1.255682	0.62279
STIP1	28942.8788	9359.2551	14.82097065	13.19233214		1.628743	0.62281
SPSB4	249.507576	108.24247	7.968710424	6.771389985		1.204817	0.62282
MFSD2A	429.385131	180.95496	8.749484425	7.507437596		1.246642	0.62287
AP3B2	38.1307259	16.525567	5.290229968	4.13138925		1.206254	0.6229
LOC100515894	38.1307259	16.525567	5.290229968	4.13138925		1.206254	0.6229
LOC100521652	38.1307259	16.525567	5.290229968	4.13138925		1.206254	0.6229
LOC102165579	273.546512	118.15781	8.100906771	6.89672967		1.211071	0.62301
TSSK4	106.931818	246.23095	6.753976422	7.949715578		-1.20332	0.62342
LOC102161016	131.799683	58.665764	7.053107893	5.89883146		1.167756	0.62359
RTDR1	97.8136012	43.792754	6.62663773	5.485193456		1.159343	0.62368
SLC16A3	5955.02467	2126.0143	12.54013401	11.05461398		1.485956	0.62386
LOC100736636	599.31554	247.88351	9.229577203	7.959326845		1.273653	0.62397
RBM12	4404.92777	1603.8063	12.10523013	10.64818348		1.457618	0.62427
THAP9	688.839852	282.5872	9.430117666	8.147648622		1.285472	0.62435
TNK1	64.6564482	28.919743	6.036864803	4.903025878		1.16074	0.62451
DLAT	7141.22182	2521.8016	12.80215722	11.30081103		1.501716	0.62459
TMEM110	664.800917	273.49814	9.378947046	8.100652569		1.281391	0.62499
HBQ1	690.49771	283.41348	9.433580664	8.151846042		1.284728	0.62503
RANBP1	22500.444	7401.8017	14.45772997	12.85385566		1.604005	0.62519
LOC100155828	64.6564482	145.42499	6.036864803	7.194018019		-1.16941	0.62528
LGI3	550.408739	1403.0207	9.106978322	10.45534846		-1.34996	0.6253
PDGFC	1144.75071	3069.6242	10.16207746	11.58431622		-1.42303	0.6255
LOC100627623	38.9596547	86.759229	5.320472212	6.455478947		-1.15504	0.62562
CCDC99	891.098485	360.25737	9.801059179	8.49688321		1.306557	0.62595
XPO5	26156.0201	8531.3242	14.67491057	13.05872307		1.616301	0.62598
LOC102159598	203.916491	485.85168	7.678892279	8.927338518		-1.25254	0.62624
EFNA2	79.5771671	180.12869	6.332299179	7.500871231		-1.1786	0.62641
WDR46	5703.85924	2046.6915	12.47797557	10.99978268		1.478645	0.62659
LCAT	70.45895	158.64545	6.159042811	7.318727602		-1.17095	0.62687
GADD45GIP1	2132.00493	815.53675	11.05867159	9.673374014		1.386389	0.62701
EXTL3	5955.02467	2131.7982	12.54013401	11.05853175		1.482037	0.62706
PLXNB3	116.050035	52.055537	6.870981559	5.729431428		1.156623	0.62747
NAAA	1038.64782	416.4443	10.02187918	8.705439898		1.318511	0.62752
LURAP1L	16.5785765	38.008805	4.135746339	5.285727902		-1.19701	0.62753
LOC100624021	1443.99401	566.82696	10.4968478	9.149307546		1.349084	0.6276
SNRPF	5895.34179	2112.7938	12.52560444	11.04561893		1.480423	0.62761
CYP2C42	1056.05532	423.05453	10.04583517	8.728105973		1.31977	0.62772
FBXL6	2813.38443	1057.6363	11.45860369	10.04799134		1.411463	0.62773
LOC100738582	28.18358	62.797156	4.867084967	5.995420212		-1.15584	0.62774
CHN1	290.125088	125.59431	8.185495362	6.98406878		1.207904	0.62788
PPIH	4226.70807	1548.4457	12.04566005	10.59753645		1.448714	0.62833
MKI67	7236.54863	2561.463	12.82128542	11.32331543		1.498334	0.62838
LOC100737768	179.048626	79.322724	7.492242779	6.327736286		1.174545	0.62846
IL17D	120.194679	276.80325	6.921182554	8.117919691		-1.20349	0.6287
RAD54L	1530.20261	599.05182	10.58044948	9.228943285		1.35297	0.62875
EIF2C4	747.679399	306.54928	9.548231995	8.264673769		1.286329	0.62897
LOC100622454	3234.48027	1206.3664	11.65976416	10.23764787		1.422866	0.62902
ANKRD24	424.411558	180.12869	8.732715419	7.500871231		1.236436	0.62911
LOC102160158	78.7482382	35.52997	6.317380743	5.191008663		1.148211	0.62916
HEATR1	7241.5222	2565.5943	12.82227649	11.32563958		1.497	0.62952
IRF2	561.184814	1426.1565	9.134900672	10.4789278		-1.34558	0.62969
LOC100623098	86.2085977	195.0017	6.446398468	7.614722326		-1.17758	0.62993
LOC102168102	5.80250177	0.8262784	2.766065426	0.868906686		2.811975	0.63023
LOC102160505	5.80250177	0.8262784	2.766065426	0.868906686		2.811975	0.63023
LOC102164839	5.80250177	0.8262784	2.766065426	0.868906686		2.811975	0.63023
LOC102162231	5.80250177	0.8262784	2.766065426	0.868906686		2.811975	0.63023
LOC102158322	5.80250177	0.8262784	2.766065426	0.868906686		2.811975	0.63023
LOC100625846	5.80250177	0.8262784	2.766065426	0.868906686		2.811975	0.63023
LOC100739525	5.80250177	0.8262784	2.766065426	0.868906686		2.811975	0.63023
MIR425	5.80250177	0.8262784	2.766065426	0.868906686		2.811975	0.63023
LOC102163221	62.1696618	138.81477	5.981159942	7.127372928		-1.15888	0.63026
GYLTL1B	921.768852	373.47782	9.849825496	8.548736477		1.303382	0.63047
PHIP	1068.48925	428.83847	10.06270627	8.747650814		1.317066	0.63047
ATXN1L	712.04986	293.32882	9.477859149	8.201285014		1.279459	0.63069
MAB21L3	41.4464412	91.716899	5.407571694	6.534760414		-1.14594	0.63082
LOC100514675	89.524313	40.48764	6.500233417	5.374609695		1.144798	0.63113
LOC102160554	188.166843	83.454116	7.563515426	6.400095824		1.172957	0.63121
ARHGEF3	87.0375265	196.65425	6.460046706	7.626835185		-1.17595	0.63125
SFXN5	231.271142	101.63224	7.859666109	6.681340183		1.186227	0.63126
SH2D4A	67.9721635	152.03522	6.107942317	7.257719911		-1.16139	0.63129
SFXN4	2351.67107	897.33831	11.20008392	9.811115052		1.389962	0.63133
LOC100156375	2090.55849	803.96886	11.03036263	9.652789156		1.378677	0.63148
LOC100737868	2367.42072	903.12226	11.20970966	9.820374065		1.390323	0.63153
SF3A2	3287.53171	1227.8497	11.68322787	10.26309271		1.420871	0.63178
WRB	2708.9394	1024.5852	11.40404487	10.00223161		1.402688	0.63178
ANK2	5126.92477	1860.7789	12.32415938	10.86246603		1.462187	0.6319
FASTKD5	655.6827	271.84558	9.359052637	8.091940885		1.27021	0.63195
NLGN1	121.023608	54.534373	6.931016486	5.795309087		1.150051	0.63202
RNF44	4125.57876	1518.6996	12.01073046	10.5695705		1.44176	0.63206
LOC100622939	122.681466	281.76092	6.950485513	8.143438955		-1.19955	0.63207
LDHB	24207.2084	7971.9337	14.56320869	12.96089496		1.602435	0.63214
LOC102166690	20.7232206	8.2627837	4.441166102	3.211445828		1.326548	0.63227

AKR1B1	8260.00444	2954.7715	13.01210149	11.52931901		1.483096	0.79877	
STX1A	218.167954	504.02981	7.775893057	8.980224728		-1.20807	0.79889	
LOC100154243	223.691193	518.07654	7.81179977	9.019803473		-1.21166	0.79892	
ZSWIM3	62.1364426	128.07315	5.98040107	7.012045083		-1.04346	0.79894	
TACC1	831.247521	2203.6844	9.700868858	11.10635644		-1.40657	0.79897	
VMP1	2649.77408	7883.5219	11.372198	12.94480757		-1.57297	0.79901	
LOC102159131	74.5637311	38.008805	6.239622035	5.285727902		0.97214	0.79906	
LOC102161322	33.1394361	66.10227	5.093367322	6.068289661		-0.99615	0.79917	
C7H15orf39	2571.06791	1007.2333	11.32871302	9.977613844		1.35197	0.79924	
SH3TC1	24.854577	12.394176	4.692347799	3.74353388		1.003849	0.79928	
CTF1	82.8485901	174.34474	6.389714621	7.454050313		-1.07339	0.79952	
RPUSD4	1342.14716	554.43279	10.39140166	9.117468532		1.275458	0.79958	
CEP97	26.2353869	52.055537	4.767410456	5.729431428		-0.98854	0.79978	
WLS	2818.23288	8422.2554	11.46108694	13.0401622		-1.57942	0.79981	
LOC100623534	11.0464787	23.135794	3.590539588	4.593102408		-1.06654	0.79986	
SLA-DMB	129.796125	64.449713	7.031175985	6.032314961		1.010001	0.79992	
HOMER1	30.3778164	60.318321	4.971673052	5.938246292		-0.98958	0.8	
HAUS3	1430.51899	588.3102	10.48333109	9.202883429		1.28189	0.80002	
MEF2B	12.4272885	25.61463	3.747096094	4.734147579		-1.04346	0.80013	
HSD11B1	12.4272885	25.61463	3.747096094	4.734147579		-1.04346	0.80013	
LOC102160055	12.4272885	25.61463	3.747096094	4.734147579		-1.04346	0.80013	
LOC102158169	785.680797	339.60041	9.619634556	8.411936365		1.210105	0.80028	
LOC102161184	8.28485901	18.178124	3.214880004	4.261389712		-1.13365	0.80048	
MGAT5B	8.28485901	18.178124	3.214880004	4.261389712		-1.13365	0.80048	
LOC102162644	8.28485901	18.178124	3.214880004	4.261389712		-1.13365	0.80048	
LOC102165772	8.28485901	18.178124	3.214880004	4.261389712		-1.13365	0.80048	
TMOD2	13.8080984	28.093465	3.888314478	4.862623207		-1.02472	0.80064	
SAG	26.2353869	13.220454	4.767410456	3.829895615		0.988742	0.80066	
WASF2	3461.69026	10533.397	11.75767763	13.36282007		-1.60542	0.80074	
LOC100737561	183.647708	89.238064	7.528631546	6.495664213		1.04121	0.80077	
SLC35G1	205.740666	99.153405	7.691678382	6.646067655		1.053093	0.80082	

[illegible]

LOC102164596	2.76161967	8.2627837	1.911353989	3.211445828		-1.58111	0.8141	
LOC102164029	2.76161967	8.2627837	1.911353989	3.211445828		-1.58111	0.8141	
LOC102166265	2.76161967	8.2627837	1.911353989	3.211445828		-1.58111	0.8141	
LOC102160370	2.76161967	8.2627837	1.911353989	3.211445828		-1.58111	0.8141	
LOC102163349	2.76161967	8.2627837	1.911353989	3.211445828		-1.58111	0.8141	
LOC100521853	2.76161967	8.2627837	1.911353989	3.211445828		-1.58111	0.8141	
LRRC19	2.76161967	8.2627837	1.911353989	3.211445828		-1.58111	0.8141	
LOC100521595	2.76161967	8.2627837	1.911353989	3.211445828		-1.58111	0.8141	
LOC102161655	16.569718	8.2627837	4.135019133	3.211445828		1.003849	0.81411	
SLC47A2	16.569718	8.2627837	4.135019133	3.211445828		1.003849	0.81411	
ZNF750	16.569718	8.2627837	4.135019133	3.211445828		1.003849	0.81411	
LOC102166279	17.9505279	9.0890621	4.24416613	3.334720158		0.981823	0.81411	
ST5AR2	17.9505279	9.0890621	4.24416613	3.334720158		0.981823	0.81411	
LOC102163775	161.554751	81.801559	7.34478191	6.371586023		0.981823	0.81414	
CLDN20	37.2818656	71.05994	5.258589232	6.171125545		-0.93056	0.81415	
ATP5B	91477.2708	28201.707	16.48114147	14.78354603		1.697631	0.81419	
C5	19.3313377	9.9153405	4.345633235	3.448285225		0.963207	0.81426	
LOC100738921	19.3313377	9.9153405	4.345633235	3.448285225		0.963207	0.81426	
LOC100627541	19.3313377	9.9153405	4.345633235	3.448285225		0.963207	0.81426	
LOC102161347	15.1889082	7.4365053	4.016933786	3.076645515		1.030321	0.81428	
LOC102161380	15.1889082	7.4365053	4.016933786	3.076645515		1.030321	0.81428	
LOC100626302	15.1889082	7.4365053	4.016933786	3.076645515		1.030321	0.81428	
LOC100623682	15.1889082	7.4365053	4.016933786	3.076645515		1.030321	0.81428	
LOC102165166	15.1889082	7.4365053	4.016933786	3.076645515		1.030321	0.81428	
LOC102161732	62.1364426	122.2892	5.98040107	6.945902605		-0.97679	0.81431	
STOML1	434.955098	1041.1107	8.76803574	10.02529289		-1.25919	0.81444	
VBP1	4346.78936	1674.04	12.08606633	10.70997982		1.376616	0.81448	
FOXM1	2083.64204	851.893	11.02558396	9.736220951		1.290363	0.81454	
LOC100516133	27.6161967	52.055537	4.838760036	5.729431428		-0.91454	0.81462	
AMOT	417.00457	993.1866	8.707374906	9.957372852		-1.252	0.81463	
ZADH2	1315.91177	3538.124	10.36294298	11.78917659		-1.42692	0.81464	
TRIB2	2304.57162	6558.1714	11.17090876	12.67929787		-1.5088	0.81465	
LOC102162135	13.8080984	6.610227	3.888314478	2.927939483		1.062743	0.81467	
ESAM	13.8080984	6.610227	3.888314478	2.927939483		1.062743	0.81467	
LOC102167390	13.8080984	6.610227	3.888314478	2.927939483		1.062743	0.81467	
LOC100516880	197.455807	433.79615	7.632673964	8.76419534		-1.13549	0.81483	
P2RX7	22.0929574	11.567897	4.529381037	3.65167138		0.93346	0.81487	
LRTM2	22.0929574	11.567897	4.529381037	3.65167138		0.93346	0.81487	
LOC100738304	22.0929574	11.567897	4.529381037	3.65167138		0.93346	0.81487	
PGLYRP2	22.0929574	11.567897	4.529381037	3.65167138		0.93346	0.81487	
LOC102165430	22.0929574	11.567897	4.529381037	3.65167138		0.93346	0.81487	
EXOSC8	1296.58044	551.95395	10.34160826	9.111015534		1.232092	0.81489	
MAN2A2	2199.63007	6226.8338	11.10370093	12.60451473		-1.50124	0.81493	
KIAA0196	1583.78888	4337.1352	10.63007495	12.08285929		-1.45336	0.81494	
MIR365-2	6.90404918	14.873011	2.982591923	3.98850389		-1.10718	0.815	
LOC100738118	6.90404918	14.873011	2.982591923	3.98850389		-1.10718	0.815	
LOC100516639	6.90404918	14.873011	2.982591923	3.98850389		-1.10718	0.815	
LOC100524410	6.90404918	14.873011	2.982591923	3.98850389		-1.10718	0.815	
LOC100155368	6.90404918	14.873011	2.982591923	3.98850389		-1.10718	0.815	
LOC100625210	6.90404918	14.873011	2.982591923	3.98850389		-1.10718	0.815	
EID2	100.799118	206.56959	6.669581252	7.697451307		-1.03514	0.81511	
KIAA1522	851.959669	2186.3326	9.736333717	11.09495688		-1.35966	0.81527	
LOC100738565	23.4737672	12.394176	4.613164285	3.74353388		0.921387	0.8153	
ARMC12	23.4737672	12.394176	4.613164285	3.74353388		0.921387	0.8153	
INPP1	2221.72303	904.77482	11.11811247	9.823008619		1.296048	0.81531	
PUS3	937.569879	410.66035	9.874320351	8.685310691		1.190981	0.81535	
SP8	12.4272885	5.7839486	3.747096094	2.762125239		1.103385	0.81535	
LOC100518860	12.4272885	5.7839486	3.747096094	2.762125239		1.103385	0.81535	
LOC102163435	12.4272885	5.7839486	3.747096094	2.762125239		1.103385	0.81535	
LOC102167532	288.589256	659.37014	8.177864267	9.367131078		-1.19207	0.81538	
LOC100739056	371.437846	871.72368	8.540855872	9.769381136		-1.23075	0.81555	
LOC100519617	73.1829213	145.42499	6.213015177	7.194018019		-0.9907	0.81555	
GADD45GIP1	1982.84292	815.53675	10.95408209	9.673374014		1.281749	0.81558	
LOC100626473	16.569718	31.398578	4.135019133	5.017858594		-0.92215	0.81563	
LOC102167295	16.569718	31.398578	4.135019133	5.017858594		-0.92215	0.81563	

BCL9	904.430442	2291.2699	9.822460003	11.16256122	-1.34107	0.8236
LOC100524897	178.124469	91.716899	7.484818616	6.534760414	0.957626	0.82361
UBL5	1162.64188	508.1612	10.18443141	8.99197867	1.194049	0.82372
LOC100624979	46.9475344	87.585507	5.583384725	6.468998788	-0.89964	0.82381
LOC100622750	69.0404918	38.008805	6.130117309	5.285727902	0.861109	0.82381
NCOA7	505.3764	1201.4088	8.984066358	10.2317117	-1.2493	0.82385
LOC100738751	209.883095	452.80055	7.720299639	8.82591454	-1.10929	0.82386
LOC100620714	220.929574	479.24146	7.79395812	8.907616136	-1.11717	0.82392
LOC100620726	57.9940131	109.89502	5.882496648	6.793050813	-0.92215	0.82393
LOC102163372	30.3778164	55.360651	4.971673052	5.816616368	-0.86584	0.82394
LOC100737304	30.3778164	55.360651	4.971673052	5.816616368	-0.86584	0.82394
NT5C2	2761.61967	7847.1657	11.43182125	12.93813979	-1.50666	0.82395
KCNC3	42.8051049	23.962073	5.453027102	4.641665831	0.83703	0.82408
LOC102164068	154.650702	322.24857	7.28216827	8.336500155	-1.05916	0.82434
LOC100626536	67.659682	129.7257	6.101391269	7.030399034	-0.9391	0.82434
ABHD1	89.7526393	48.750424	6.503867696	5.636636914	0.88054	0.82434
LOC102159045	1.38080984	5.7839486	1.251452392	2.762125239	-2.06654	0.82438
LOC100736921	1.38080984	5.7839486	1.251452392	2.762125239	-2.06654	0.82438
LOC102159282	1.38080984	5.7839486	1.251452392	2.762125239	-2.06654	0.82438
LOC102159336	1.38080984	5.7839486	1.251452392	2.762125239	-2.06654	0.82438
LOC102157505	1.38080984	5.7839486	1.251452392	2.762125239	-2.06654	0.82438
LOC102164317	1.38080984	5.7839486	1.251452392	2.762125239	-2.06654	0.82438
LOC102164107	1.38080984	5.7839486	1.251452392	2.762125239	-2.06654	0.82438
LOC100738817	1.38080984	5.7839486	1.251452392	2.762125239	-2.06654	0.82438
LOC102164933	1.38080984	5.7839486	1.251452392	2.762125239	-2.06654	0.82438
LOC102162906	1.38080984	5.7839486	1.251452392	2.762125239	-2.06654	0.82438
LOC102158455	1.38080984	5.7839486	1.251452392	2.762125239	-2.06654	0.82438
LOC102162494	1.38080984	5.7839486	1.251452392	2.762125239	-2.06654	0.82438
LOC102165367	1.38080984	5.7839486	1.251452392	2.762125239	-2.06654	0.82438
LOC100158021	1.38080984	5.7839486	1.251452392	2.762125239	-2.06654	0.82438
LOC102165704	1.38080984	5.7839486	1.251452392	2.762125239	-2.06654	0.82438
LOC100515766	1.38080984	5.7839486	1.251452392	2.762125239	-2.06654	0.82438
GPR179	1.38080984	5.7839486	1.251452392	2.762125239	-2.06654	0.82438
LOC100620887	1.38080984	5.7839486	1.251452392	2.762125239	-2.06654	0.82438
LOC102165034	1.38080984	5.7839486	1.251452392	2.762125239	-2.06654	0.82438
VANGL1	179.509279	380.08805	7.495897217	8.573980563	-1.08231	0.82439
BCAR1	2096.06933	5785.6012	11.03415884	12.49850049	-1.46478	0.82448
TESK1	871.291006	2193.7691	9.768665705	11.09985344	-1.33219	0.82451
RND2	332.77517	162.77684	8.382732826	7.35558754	1.031652	0.82458
COBLL1	189.170948	402.39757	7.571153052	8.656058572	-1.08893	0.8248
SLC8A3	81.4677803	158.64545	6.365758672	7.318727602	-0.9615	0.82483
EXOSC1	1982.84292	829.58349	10.95408209	9.697981376	1.257111	0.82485
MLH1	1629.35561	693.24755	10.67097096	9.439306379	1.232859	0.82487
BCAS2	3323.60928	1331.9607	11.69896908	10.38041857	1.319199	0.82494
ICA1L	12.4272885	23.135794	3.747096094	4.593102408	-0.89662	0.82497
CHODL	12.4272885	23.135794	3.747096094	4.593102408	-0.89662	0.82497
LOC100737863	12.4272885	23.135794	3.747096094	4.593102408	-0.89662	0.82497
GPS2	1284.15315	3365.4318	10.32772458	11.71700453	-1.38997	0.82498
SZT2	1349.05121	3553.8233	10.39879842	11.79556212	-1.39743	0.82498
AKTIP	865.767767	2175.591	9.759501694	11.08785459	-1.32936	0.82505
DZANK1	394.911613	910.55877	8.629034575	9.832191855	-1.20522	0.82516
LOC100626559	69.0404918	132.20454	6.130117309	7.057499439	-0.93726	0.82523
LRP10	2779.5702	7880.2168	11.44116504	12.94420268	-1.50337	0.8253
LOC100736740	120.130456	242.92584	6.920417836	7.930298794	-1.01591	0.82532
ATG4C	720.782734	329.68507	9.495420823	8.369314102	1.128476	0.82535
HOXD9	1286.91477	559.39046	10.33082141	9.13028858	1.201989	0.82539
L3MBTL1	161.554751	84.280394	7.34478191	6.414142197	0.938754	0.82547
LOC102164279	100.799118	54.534373	6.669581252	5.795309087	0.886245	0.82558
MRPS23	1582.40807	675.89571	10.6288174	9.402789759	1.227249	0.82558
LOC100626461	465.332915	221.4426	8.865216451	7.797289318	1.07133	0.82559
LOC100621245	2847.22988	8085.1339	11.47584988	12.98123437	-1.50571	0.82567
PCDHB8	4.14242951	9.9153405	2.362450112	3.448285225	-1.25919	0.82573
LOC100626090	4.14242951	9.9153405	2.362450112	3.448285225	-1.25919	0.82573
LOC102162804	4.14242951	9.9153405	2.362450112	3.448285225	-1.25919	0.82573
LOC102160376	4.14242951	9.9153405	2.362450112	3.448285225	-1.25919	0.82573
LOC102163390	4.14242951	9.9153405	2.362450112	3.448285225	-1.25919	0.82573
SPAG11B	2.76161967	0	1.911353989	0	20	0.82581
ZC2HC1B	2.76161967	0	1.911353989	0	20	0.82581
CNPY1	2.76161967	0	1.911353989	0	20	0.82581
BEND4	2.76161967	0	1.911353989	0	20	0.82581
LOC100622164	2.76161967	0	1.911353989	0	20	0.82581
LOC100622121	2.76161967	0	1.911353989	0	20	0.82581
LOC102159036	2.76161967	0	1.911353989	0	20	0.82581
LOC100622751	2.76161967	0	1.911353989	0	20	0.82581
LOC102159152	2.76161967	0	1.911353989	0	20	0.82581
LOC102159250	2.76161967	0	1.911353989	0	20	0.82581
LOC100622606	2.76161967	0	1.911353989	0	20	0.82581
P2RX2	2.76161967	0	1.911353989	0	20	0.82581
GDF3	2.76161967	0	1.911353989	0	20	0.82581
LOC102159360	2.76161967	0	1.911353989	0	20	0.82581
LOC102161829	2.76161967	0	1.911353989	0	20	0.82581
IGFBPL1	2.76161967	0	1.911353989	0	20	0.82581
LOC102159535	2.76161967	0	1.911353989	0	20	0.82581
OPALIN	2.76161967	0	1.911353989	0	20	0.82581
CHRNA2	2.76161967	0	1.911353989	0	20	0.82581
CNTNAP5	2.76161967	0	1.911353989	0	20	0.82581
LOC102157497	2.76161967	0	1.911353989	0	20	0.82581
ZAR1	2.76161967	0	1.911353989	0	20	0.82581
LOC102168035	2.76161967	0	1.911353989	0	20	0.82581
LOC102157524	2.76161967	0	1.911353989	0	20	0.82581
LOC102168156	2.76161967	0	1.911353989	0	20	0.82581
LOC102168171	2.76161967	0	1.911353989	0	20	0.82581
LOC102157620	2.76161967	0	1.911353989	0	20	0.82581
LOC100511763	2.76161967	0	1.911353989	0	20	0.82581
LOC102161302	2.76161967	0	1.911353989	0	20	0.82581
LOC100626482	2.76161967	0	1.911353989	0	20	0.82581
LOC102166893	2.76161967	0	1.911353989	0	20	0.82581
C7H6orf132	2.76161967	0	1.911353989	0	20	0.82581
LOC102162186	2.76161967	0	1.911353989	0	20	0.82581
LOC100152084	2.76161967	0	1.911353989	0	20	0.82581
LOC100514964	2.76161967	0	1.911353989	0	20	0.82581
LOC100738047	2.76161967	0	1.911353989	0	20	0.82581
LOC100626776	2.76161967	0	1.911353989	0	20	0.82581
LOC102164571	2.76161967	0	1.911353989	0	20	0.82581
GRIK1	2.76161967	0	1.911353989	0	20	0.82581
LOC100516605	2.76161967	0	1.911353989	0	20	0.82581
LOC100514422	2.76161967	0	1.911353989	0	20	0.82581
ASB11	2.76161967	0	1.911353989	0	20	0.82581
SLA-DRB1	2.76161967	0	1.911353989	0	20	0.82581
LOC100738386	2.76161967	0	1.911353989	0	20	0.82581
LOC100522152	2.76161967	0	1.911353989	0	20	0.82581
LOC100624099	2.76161967	0	1.911353989	0	20	0.82581
LOC100152324	2.76161967	0	1.911353989	0	20	0.82581
LOC102164157	2.76161967	0	1.911353989	0	20	0.82581
TPH2	2.76161967	0	1.911353989	0	20	0.82581
LOC100621867	2.76161967	0	1.911353989	0	20	0.82581
LOC100621903	2.76161967	0	1.911353989	0	20	0.82581
GALNTL5	2.76161967	0	1.911353989	0	20	0.82581
LOC102166593	2.76161967	0	1.911353989	0	20	0.82581
LUZP4	2.76161967	0	1.911353989	0	20	0.82581
DNMT3L	2.76161967	0	1.911353989	0	20	0.82581
PDE6A	2.76161967	0	1.911353989	0	20	0.82581
MOV10L1	2.76161967	0	1.911353989	0	20	0.82581
PSTPIP2	2.76161967	0	1.911353989	0	20	0.82581
LOC100623554	2.76161967	0	1.911353989	0	20	0.82581
DCLK3	2.76161967	0	1.911353989	0	20	0.82581
NLRP5	2.76161967	0	1.911353989	0	20	0.82581
LOC102160990	2.76161967	0	1.911353989	0	20	0.82581
ABCC4	2.76161967	0	1.911353989	0	20	0.82581
MMEL1	2.76161967	0	1.911353989	0	20	0.82581
LOC100623946	2.76161967	0	1.911353989	0	20	0.82581
LOC102160609	2.76161967	0	1.911353989	0	20	0.82581
LOC102160848	2.76161967	0	1.911353989	0	20	0.82581
HTR3B	2.76161967	0	1.911353989	0	20	0.82581
LOC102158818	2.76161967	0	1.911353989	0	20	0.82581
LOC102160177	2.76161967	0	1.911353989	0	20	0.82581
LOC102162879	2.76161967	0	1.911353989	0	20	0.82581
ATOH7	2.76161967	0	1.911353989	0	20	0.82581
GPR55	2.76161967	0	1.911353989	0	20	0.82581
TMEM215	2.76161967	0	1.911353989	0	20	0.82581
GPR22	2.76161967	0	1.911353989	0	20	0.82581
GPR18	2.76161967	0	1.911353989	0	20	0.82581
LOC102162746	2.76161967	0	1.911353989	0	20	0.82581
APOBEC4	2.76161967	0	1.911353989	0	20	0.82581
LOC102158972	2.76161967	0	1.911353989	0	20	0.82581
LOC102158974	2.76161967	0	1.911353989	0	20	0.82581
LOC102167066	2.76161967	0	1.911353989	0	20	0.82581
LOC102158700	2.76161967	0	1.911353989	0	20	0.82581
LOC102167039	2.76161967	0	1.911353989	0	20	0.82581
LOC102162318	2.76161967	0	1.911353989	0	20	0.82581
ABCA12	2.76161967	0	1.911353989	0	20	0.82581
LOC102158457	2.76161967	0	1.911353989	0	20	0.82581
LOC102162224	2.76161967	0	1.911353989	0	20	0.82581
LOC100524342	2.76161967	0	1.911353989	0	20	0.82581
LOC102158579	2.76161967	0	1.911353989	0	20	0.82581
CALY	2.76161967	0	1.911353989	0	20	0.82581
LOC102158300	2.76161967	0	1.911353989	0	20	0.82581
LOC102162525	2.76161967	0	1.911353989	0	20	0.82581
LOC102165335	2.76161967	0	1.911353989	0	20	0.82581
LOC102163106	2.76161967	0	1.911353989	0	20	0.82581
CKMT2	2.76161967	0	1.911353989	0	20	0.82581
LOC100158035	2.76161967	0	1.911353989	0	20	0.82581
LOC100523557	2.76161967	0	1.911353989	0	20	0.82581
LOC102167553	2.76161967	0	1.911353989	0	20	0.82581
LOC102165522	2.76161967	0	1.911353989	0	20	0.82581
LOC102165602	2.76161967	0	1.911353989	0	20	0.82581
LOC100737086	2.76161967	0	1.911353989	0	20	0.82581
LOC100157853	2.76161967	0	1.911353989	0	20	0.82581
WNT3A	2.76161967	0	1.911353989	0	20	0.82581
LOC102167793	2.76161967	0	1.911353989	0	20	0.82581
LOC102167787	2.76161967	0	1.911353989	0	20	0.82581
LOC102165782	2.76161967	0	1.911353989	0	20	0.82581
LOC100515852	2.76161967	0	1.911353989	0	20	0.82581
LOC102165912	2.76161967	0	1.911353989	0	20	0.82581
LOC102167163	2.76161967	0	1.911353989	0	20	0.82581
LOC102167179	2.76161967	0	1.911353989	0	20	0.82581
LOC100517924	2.76161967	0	1.911353989	0	20	0.82581
LOC100517827	2.76161967	0	1.911353989	0	20	0.82581
LOC102167341	2.76161967	0	1.911353989	0	20	0.82581
HAO1	2.76161967	0	1.911353989	0	20	0.82581
LRIT3	2.76161967	0	1.911353989	0	20	0.82581
SELV	2.76161967	0	1.911353989	0	20	0.82581
GRXCR1	2.76161967	0	1.911353989	0	20	0.82581
LOC100521617	2.76161967	0	1.911353989	0	20	0.82581
SLC9A4	2.76161967	0	1.911353989	0	20	0.82581
SIT1	2.76161967	0	1.911353989	0	20	0.82581
LOC100155946	2.76161967	0	1.911353989	0	20	0.82581
LOC102165162	2.76161967	0	1.911353989	0	20	0.82581
HOXD13	2.76161967	0	1.911353989	0	20	0.82581
SCML4	5.52323934	1.6525567	2.705588563	1.407383613	1.740815	0.82584
LOC100037947	5.52323934	1.6525567	2.705588563	1.407383613	1.740815	0.82584
CEACAM16	5.52323934	1.6525567	2.705588563	1.407383613	1.740815	0.82584
KMO	5.52323934	1.6525567	2.705588563	1.407383613	1.740815	0.82584
SLC12A8	5.52323934	1.6525567	2.705588563	1.407383613	1.740815	0.82584
LOC102163685	5.52323934	1.6525567	2.705588563	1.407383613	1.740815	0.82584
SIGLEC15	5.52323934	1.6525567	2.705588563	1.407383613	1.740815	0.82584
KIF6	5.52323934	1.6525567	2.705588563	1.407383613	1.740815	0.82584
LOC100156191	5.52323934	1.6525567	2.705588563	1.407383613	1.740815	0.82584
ZP2	5.52323934	1.6525567	2.705588563	1.407383613	1.740815	0.82584
LOC102166700	5.52323934	1.6525567	2.705588563	1.407383613	1.740815	0.82584
LOC102164221	5.52323934	1.6525567	2.705588563	1.407383613	1.740815	0.82584
MYO7B	5.52323934	1.6525567	2.705588563	1.407383613	1.740815	0.82584
LOC100516519	5.52323934	1.6525567	2.705588563	1.407383613	1.740815	0.82584
GPA33	5.52323934	1.6525567	2.705588563	1.407383613	1.740815	0.82584
UCP3	5.52323934	1.6525567	2.705588563	1.407383613	1.740815	0.82584
LOC102158676	5.52323934	1.6525567	2.705588563	1.407383613	1.740815	0.82584
LOC100512568	5.52323934	1.6525567	2.705588563	1.407383613	1.740815	0.82584
C15H2orf54	5.52323934	1.6525567	2.705588563	1.407383613	1.740815	0.82584
LOC102162612	5.52323934	1.6525567	2.705588563	1.407383613	1.740815	0.82584
LOC102165310	5.52323934	1.6525567	2.705588563	1.407383613	1.740815	0.82584
LHFPL4	5.52323934	1.6525567	2.705588563	1.407383613	1.740815	0.82584
GIMAP6	5.52323934	1.6525567	2.705588563	1.407383613	1.740815	0.82584
LOC100515857	5.52323934	1.6525567	2.705588563	1.407383613	1.740815	0.82584
SYTL3	5.52323934	1.6525567	2.705588563	1.407383613	1.740815	0.82584
LOC100515958	5.52323934	1.6525567	2.705588563	1.407383613	1.740815	0.82584
LOC102167102	5.52323934	1.6525567	2.705588563	1.407383613	1.740815	0.82584
LOC102167150	5.52323934	1.6525567	2.705588563	1.407383613	1.740815	0.82584
LOC102165970	5.52323934	1.6525567	2.705588563	1.407383613	1.740815	0.82584
LOC102165959	5.52323934	1.6525567	2.705588563	1.407383613	1.740815	0.82584
LOC102167987	5.52323934	1.6525567	2.705588563	1.407383613	1.740815	0.82584
LOC102165226	5.52323934	1.6525567	2.705588563	1.407383613	1.740815	0.82584
TN-X	208.502285	106.58991	7.71082217	6.749398975	0.967992	0.8259
GMPPB	784.299987	1945.8856	9.617100062	10.92695237	-1.31095	0.82592
ISCA2	766.349459	1895.4826	9.583739935	10.88911041	-1.30649	0.82616
LOC100526168	153.269892	317.29089	7.269312714	8.314202076	-1.04973	0.82619
CDK7	1129.50245	497.41958	10.1427484	8.961216934	1.183152	0.82621
LOC100517722	470.856154	1103.9079	8.882203309	10.10971041	-1.22926	0.82624
LOC100525875	46.9475344	86.759229	5.583384725	6.455478947	-0.88597	0.82634
LOC102165085	102.179928	55.360651	6.689018532	5.816616368	0.884179	0.82635
MMS22L	258.211439	129.7257	8.017985577	7.030399034	0.993089	0.82635
LOC102165507	23.4737672	42.140197	4.613164285	5.430960858	-0.84415	0.82636
TRMT10B	898.907203	404.05012	9.813632431	8.661956638	1.153638	0.82637
LOC102159805	24.854577	44.619032	4.692347799	5.511563931	-0.84415	0.82646
NSF	3043.30488	1231.9811	11.57189713	10.26793491	1.304659	0.82647
MTA3	1063.22357	470.97867	10.05558555	8.882577857	1.174711	0.82648
NHSL1	575.797702	269.36675	9.171921605	8.078773924	1.09599	0.82648
EYA2	20.7121475	37.182527	4.440430524	5.254840671	-0.84415	0.8265
MRPS36	1431.8998	618.05622	10.48472201	9.273926629	1.212121	0.82652
CDK5	2178.91792	907.25365	11.0900581	9.826951452	1.264034	0.82654
MAST4	62.1364426	117.33153	5.98040107	6.886690713	-0.91708	0.82655
LOC102166531	42.8051049	78.496445	5.453027102	6.312818447	-0.87484	0.8267
LOC100623143	1296.58044	3387.7413	10.34160826	11.7265338	-1.38561	0.82672
LOC100737556	349.344888	171.03962	8.452632037	7.426597064	1.030321	0.82677
PROCA1	11.0464787	20.656959	3.590539588	4.436758793	-0.90304	0.82694
LOC100516794	111.845597	60.318321	6.818206315	5.938246292	0.89084	0.82699
LOC100739580	77.3253508	148.73011	6.291407421	7.226220529	-0.94368	0.827
LOC100514932	69.0404918	131.37826	6.130117309	7.048522415	-0.92821	0.82703
LOC102162863	150.508272	79.322724	7.243252754	6.327736286	0.924037	0.82705
RP9	429.431859	206.56959	8.749641054	7.697451307	1.055801	0.82714
NINL	853.340479	2130.1456	9.738667328	11.05741347	-1.31976	0.82716
LOC100522315	28.9970066	52.055537	4.906746634	5.729431428	-0.84415	0.82723
LOC100623746	28.9970066	52.055537	4.906746634	5.729431428	-0.84415	0.82723
SLC35E3	840.91319	380.91433	9.717527674	8.577105241	1.14249	0.82723
DMP1	143.604223	76.01761	7.175965874	6.267116453	0.917693	0.82726
GJA9	17.9505279	32.224857	4.24416613	5.054191063	-0.84415	0.82728
RAB3B	6.90404918	14.046732	2.982591923	3.911378308	-1.02472	0.82731
LOC100739429	6.90404918	14.046732	2.982591923	3.911378308	-1.02472	0.82731
LOC102165718	6.90404918	14.046732	2.982591923	3.911378308	-1.02472	0.82731
CPOX	3667.43092	1464.1653	11.8409474	10.5168477	1.324691	0.82731
MFSD7	6894.38351	2615.9973	12.75141508	11.35369673	1.39806	0.82733
JMY	631.030095	1523.6573	9.303849446	10.5742693	-1.27176	0.82733
ARAP3	1619.68994	4325.5673	10.66239239	12.07900712	-1.41717	0.82734
LZTS2	885.099105	2216.9049	9.791324254	11.11498177	-1.32464	0.82739
SPATA17	49.7091541	28.093465	5.664174303	4.862623207	0.823277	0.82739
LOC100625878	85.6102098	166.08195	6.436465198	7.384412099	-0.95604	0.8274
LOC100626072	44.1859147	80.97528	5.497801224	6.357117027	-0.8739	0.82742
IRGQ	832.628331	2071.4799	9.703260498	11.01714238	-1.31492	0.82744
ENOSF1	1360.09769	590.78904	10.4105549	9.208939157	1.202995	0.82745
ABCA3	581.320941	1390.6265	9.18567069	10.44255634	-1.25833	0.82745
PIH1D2	172.60123	90.064343	7.439633355	6.508814353	0.938415	0.82755
FAM120C	30.3778164	54.534373	4.971673052	5.795309087	-0.84415	0.82759
SGK3	186.409328	96.674569	7.550048951	6.609911086	0.947266	0.82769
G2E3	762.207029	348.68947	9.575930649	8.449930555	1.12824	0.82775
VMAC	151.889082	80.149002	7.256341575	6.342501448	0.922262	0.82775
IFRD2	6919.23809	2626.7389	12.75660596	11.35960624	1.39734	0.82779
LOC100737430	1581.02726	678.37454	10.62755875	9.40806335	1.220708	0.82785
RNF208	104.941548	57.013208	6.727124677	5.858309486	0.880218	0.82786

LOC102160994	40.17964529	97.500848	5.363859498	6.622064238	-1.27895	0.68541
SLC35B2	659.79207	2095.442	9.368052563	11.03372717	-1.66717	0.68542
NME5	15.8603863	38.008805	4.075565686	5.285727902	-1.26091	0.68547
LOC102163372	134.284604	55.360651	7.079853853	5.816616368	1.278361	0.68571
LOC102164825	31.7207726	76.01761	5.032134908	6.267116453	-1.26091	0.68591
EXOSC10	5970.906762	1781.4562	12.54397593	10.79965089	1.744893	0.68594
LOC100153418	5.286795433	14.873011	2.65232482	3.98850389	-1.49223	0.68596
LOC102160374	53.92531342	23.135794	5.779399292	4.593102407	1.220836	0.6861
C1H9orf46	155.4317857	423.05453	7.289389877	8.728105973	-1.44456	0.68612
CATSPER1	24.31925899	57.839486	4.662163277	5.878712739	-1.24996	0.68617
LOC102162452	14.80302721	35.52997	3.982129041	5.191008663	-1.26314	0.68619
UMPS	3770.542503	1170.8365	11.88093897	10.19455552	1.687233	0.68639
LOC100622990	990.7454641	346.21064	9.953826084	8.439667339	1.516864	0.68645
LOC102161805	34.89284986	14.873011	5.165624571	3.98850389	1.230235	0.68652
IFRD1	6785.073259	2004.5513	12.72836129	10.96978318	1.759085	0.68669
LOC102165864	253.7661808	726.29869	7.993029968	9.506404165	-1.51706	0.68669
LOC100738903	8.458872693	21.483238	3.241668254	4.490777899	-1.34467	0.68686
XKR6	8.458872693	21.483238	3.241668254	4.490777899	-1.34467	0.68686
MRE11A	2071.366451	678.37454	11.01706342	9.40806335	1.610429	0.687
TESK1	689.3981244	2193.7691	9.431284735	11.09985344	-1.67	0.68701
LOC100523690	68.72834063	172.69218	6.123673244	7.440388989	-1.32923	0.68716
LOC100154839	169.1774539	463.54217	7.410896102	8.859665746	-1.45416	0.6872
LHX2	13.74566813	33.051135	3.882219287	5.089630977	-1.26572	0.68723
LOC102165352	65.55626337	28.093465	6.056502534	4.862623207	1.222499	0.68724
TMEM180	26.43397716	62.797156	4.777891883	5.995420212	-1.24831	0.68726
LOC100621557	14427.66474	4006.6238	13.81665018	11.96853138	1.848379	0.68731
LOC100626018	725.3483334	261.10397	9.504517774	8.033995371	1.47405	0.68762
PURA	354.215294	1048.5473	8.472549889	10.03555141	-1.56569	0.68773
LOC100739395	472.6395117	176.82357	8.887645627	7.474302764	1.41843	0.68793
ENPP4	27.49133625	11.567897	4.832451381	3.651671381	1.24885	0.68803
TMSB4X	26656.02257	121337.33	14.70222803	16.88867579	-2.18649	0.68806
RBM28	654.5052746	237.96817	9.356463579	7.900674664	1.459636	0.68814
DCLK1	4242.124655	1307.1724	12.05091135	10.35333695	1.698338	0.68822
CRK	10840.04536	3086.1497	13.40421626	11.59205974	1.812491	0.68837
LOC100158017	65.55626337	163.60312	6.056502534	7.362847851	-1.31939	0.6885
STX18	98.33439505	254.49374	6.63422144	7.997144125	-1.37186	0.68854
HDLBP	7270.401079	29288.263	12.82801766	14.83808428	-2.01022	0.68864
LOC100621130	12.68830904	30.5723	3.774872332	4.980587447	-1.26873	0.68868
LOC100524171	12.68830904	30.5723	3.774872332	4.980587447	-1.26873	0.68868
ATP2B1	3598.192972	1125.3911	11.81345774	10.13749218	1.676846	0.6887
LOC396903	6926.759376	27752.212	12.75817311	14.76036711	-2.00235	0.68918
MAN1B1	743.3234379	2375.5503	9.539785855	11.21465323	-1.6762	0.68918
SLC18A1	49.69587707	21.483238	5.663796517	4.490777899	1.209915	0.68921
C3H2orf44	857.5182192	304.89672	9.745704941	8.256900823	1.491847	0.68935
RALGPS2	1097.538732	381.74061	10.10137002	8.580223165	1.523607	0.68945
ANKRD9	153.3170676	413.96546	7.269753823	8.696847462	-1.43299	0.68952
PRKG1	99.39175414	256.97257	6.649496966	8.011073883	-1.37042	0.68953
POM121C	5450.686091	1646.7728	12.41248678	10.68630161	1.726796	0.68955
IRAK4	396.5096575	1184.0569	8.634846101	10.21074062	-1.57831	0.6896
LOC102157627	16.91774539	6.610227	4.163317207	2.927939483	1.355766	0.68961
LOC100156563	16.91774539	6.610227	4.163317207	2.927939483	1.355766	0.68961
PNLDC1	16.91774539	6.610227	4.163317207	2.927939483	1.355766	0.68961
LOC102167952	16.91774539	6.610227	4.163317207	2.927939483	1.355766	0.68961
ZC3HAV1	501.188207	1534.3989	8.97208434	10.58439784	-1.61425	0.68972
LOC100623893	104.6785496	271.84558	6.723538761	8.091940885	-1.37682	0.68981
FMNL3	527.6221842	1623.637	9.04609316	10.66590169	-1.62165	0.68993
LOC102161808	32.77813168	14.046732	5.078017628	3.911378308	1.222499	0.68994
LOC102161197	818.395933	292.50254	9.678416922	8.197229196	1.48435	0.69
LOC100739243	210.4144582	586.65764	7.723930233	9.198832108	-1.47928	0.69007
SKIV2L2	12088.78644	3418.3136	13.56150114	11.73949104	1.822313	0.6901
NCAPH	2441.442131	791.57468	11.25410867	9.630403069	1.624936	0.69016
LOC102159153	272.7986443	107.41619	8.096971493	6.760436381	1.344625	0.6902
MRPL28	2154.897818	7679.4312	11.07407309	12.90697159	-1.83338	0.69021
IPO5	50197.06528	12666.847	15.61534414	13.62888378	1.986546	0.69022
LOC102161331	51.81059524	126.42059	5.722755497	6.993454622	-1.28691	0.69035
LOC100625867	252.7088217	718.03591	7.987029874	9.489920003	-1.50658	0.69037
LOC100517940	80.35929058	203.26448	6.346235194	7.674294538	-1.33882	0.6905
PIK3CB	288.6590306	113.20014	8.178211834	6.83542057	1.350491	0.6906
LOC100738161	11.63094995	28.093465	3.658891241	4.862623207	-1.27227	0.69065
KLHL21	177.6363265	485.85168	7.480881679	8.927338518	-1.45159	0.69076
SCNN1G	0	4.1313919	0	2.359350201	-20	0.69099
LOC102159291	0	4.1313919	0	2.359350201	-20	0.69099
LOC100525040	0	4.1313919	0	2.359350201	-20	0.69099
LOC102168178	0	4.1313919	0	2.359350201	-20	0.69099
LOC102157679	0	4.1313919	0	2.359350201	-20	0.69099
LOC102163737	0	4.1313919	0	2.359350201	-20	0.69099
LOC102163817	0	4.1313919	0	2.359350201	-20	0.69099
LOC100739018	0	4.1313919	0	2.359350201	-20	0.69099
LOC102157980	0	4.1313919	0	2.359350201	-20	0.69099
LOC102166039	0	4.1313919	0	2.359350201	-20	0.69099
LOC100621600	0	4.1313919	0	2.359350201	-20	0.69099
KREMEN2	0	4.1313919	0	2.359350201	-20	0.69099
TLX2	0	4.1313919	0	2.359350201	-20	0.69099
TLX1	0	4.1313919	0	2.359350201	-20	0.69099
LOC100519735	0	4.1313919	0	2.359350201	-20	0.69099
TMPRSS6	0	4.1313919	0	2.359350201	-20	0.69099
IFN-ALPHA-8	0	4.1313919	0	2.359350201	-20	0.69099
LOC102162958	0	4.1313919	0	2.359350201	-20	0.69099
LOC100623087	0	4.1313919	0	2.359350201	-20	0.69099
CD80	0	4.1313919	0	2.359350201	-20	0.69099
HAPLN2	0	4.1313919	0	2.359350201	-20	0.69099
LOC102165336	0	4.1313919	0	2.359350201	-20	0.69099
LOC102165445	0	4.1313919	0	2.359350201	-20	0.69099
LOC102167813	0	4.1313919	0	2.359350201	-20	0.69099
LOC100523291	0	4.1313919	0	2.359350201	-20	0.69099
LOC102167479	0	4.1313919	0	2.359350201	-20	0.69099
LOC102167339	0	4.1313919	0	2.359350201	-20	0.69099
LOC100737810	0	4.1313919	0	2.359350201	-20	0.69099
LOC102165173	0	4.1313919	0	2.359350201	-20	0.69099
LOC102165183	0	4.1313919	0	2.359350201	-20	0.69099
MSANTD1	0	4.1313919	0	2.359350201	-20	0.69099
LOC100738156	140.6287585	375.13038	7.145970432	8.55508903	-1.4155	0.69116
LOC100737635	279.1427989	109.89502	8.130018598	6.793050814	1.344877	0.69121
ADAL	412.3700438	156.99289	8.691290033	7.303715831	1.39324	0.69127
LOC100624234	7.401513606	19.004403	3.070649265	4.322245637	-1.36044	0.69143
FAIM2	7.401513606	19.004403	3.070649265	4.322245637	-1.36044	0.69143
LOC100519891	7.401513606	19.004403	3.070649265	4.322245637	-1.36044	0.69143
NARF	1109.169682	3682.7227	10.11656448	11.84694875	-1.73129	0.69157
EOGT	186.0951992	510.64003	7.54762873	8.998985343	-1.45627	0.69162
TMEM86B	22.20454082	52.055537	4.536335244	5.729431428	-1.2292	0.69175
CTPS1	7999.978849	2346.6306	12.9659608	11.19698969	1.769405	0.69183
LOC102162927	1563.834089	528.81816	10.61179399	9.049353478	1.564244	0.69187
REL	59.21210885	145.42499	5.911981742	7.194018019	-1.29631	0.69203
LOC100519617	59.21210885	145.42499	5.911981742	7.194018019	-1.29631	0.69203
OPLAH	879.72276	2848.1815	9.782544139	11.47633184	-1.69492	0.69205
KIAA2013	1573.350321	5412.9496	10.62054089	12.40246575	-1.78258	0.69207
LOC100511482	1315.354704	451.97427	10.36233257	8.823285292	1.541139	0.69221
FAM171A1	899.8125827	2919.2415	9.815083169	11.51187196	-1.6979	0.69224
PIAS2	5021.398302	1533.5727	12.29416073	10.58362124	1.711193	0.69237
ZNF596	1301.609036	447.84288	10.34718842	8.810066691	1.539232	0.69246
LOC102167310	38.06492712	16.525567	5.287802017	4.131389251	1.203763	0.69248
LOC733651	12804.61854	3614.9679	13.64448932	11.82016615	1.82461	0.69257
LOC100622725	1826.059143	609.79344	10.83530762	9.254540753	1.582341	0.69278
SH3GLB2	1026.695673	3374.5209	10.00519739	11.72089442	-1.71667	0.69278
CCDC66	1245.569004	430.49103	10.28374703	8.753186764	1.53275	0.69282
SLC20A1	24847.93853	6649.8883	14.60089661	12.69933133	1.901724	0.69294
LOC102162338	25.37661808	10.741619	4.721187694	3.553559423	1.240288	0.69315
LOC100513639	16.91774539	39.661362	4.163317207	5.34558663	-1.2292	0.69318
ZNF280D	1215.96295	421.40197	10.24906953	8.722472752	1.52883	0.69318
LOC102161121	10.57359087	25.61463	3.532764645	4.734147579	-1.2765	0.69325
LOC102166500	10.57359087	25.61463	3.532764645	4.734147579	-1.2765	0.69325
NRAP	10.57359087	25.61463	3.532764645	4.734147579	-1.2765	0.69325
LOC102165890	10.57359087	25.61463	3.532764645	4.734147579	-1.2765	0.69325
CIDEC	10.57359087	25.61463	3.532764645	4.734147579	-1.2765	0.69325
LRRC37A	10.57359087	25.61463	3.532764645	4.734147579	-1.2765	0.69325
LOC100739355	754.9543878	2399.5124	9.562155379	11.22912667	-1.66828	0.69337
LOC102158539	132.1698858	55.360651	7.057124067	5.816616368	1.255461	0.69342
POMT1	797.2487513	2548.2425	9.640694581	11.3158529	-1.6764	0.69346
PCDH12	5.286795433	0.8262784	2.65232482	0.868906686	2.677694	0.6935
LOC102159619	5.286795433	0.8262784	2.65232482	0.868906686	2.677694	0.6935
LOC100520307	5.286795433	0.8262784	2.65232482	0.868906686	2.677694	0.6935
FA2H	5.286795433	0.8262784	2.65232482	0.868906686	2.677694	0.6935
LOC102160550	5.286795433	0.8262784	2.65232482	0.868906686	2.677694	0.6935
SOX10	5.286795433	0.8262784	2.65232482	0.868906686	2.677694	0.6935
LOC100152671	5.286795433	0.8262784	2.65232482	0.868906686	2.677694	0.6935
LOC102160180	5.286795433	0.8262784	2.65232482	0.868906686	2.677694	0.6935
LOC102160076	5.286795433	0.8262784	2.65232482	0.868906686	2.677694	0.6935
RTL1	5.286795433	0.8262784	2.65232482	0.868906686	2.677694	0.6935
LOC100625374	5.286795433	0.8262784	2.65232482	0.868906686	2.677694	0.6935
LOC102167924	5.286795433	0.8262784	2.65232482	0.868906686	2.677694	0.6935
TAF2	4679.871317	1440.2032	12.19256139	10.49305805	1.700196	0.69351
OGFOD1	1962.458465	651.93364	10.93918136	9.350792552	1.589865	0.69361
PPFIA3	393.3375802	1165.0525	8.623287395	10.18741704	-1.56656	0.69365
METTL8	1967.74526	653.58619	10.94306073	9.35443936	1.590094	0.69367
LOC100737729	662.9641473	242.92584	9.374961531	7.930298794	1.448415	0.6938
LOC102159631	30.66341351	13.220454	4.98474489	3.829895615	1.213747	0.69383
SOWAHA	30.66341351	13.220454	4.98474489	3.829895615	1.213747	0.69383
SRSF10	7536.855569	2229.299	12.87993844	11.12302145	1.757373	0.69434
ROD1	9668.491488	2801.0837	13.23922431	11.45228433	1.787306	0.6945
TMEM87B	128.9978086	338.77413	7.022343493	8.408432212	-1.39298	0.69454
LOC100624686	20.08982264	8.2627837	4.398475058	3.211445828	1.281765	0.69455
PCDH8	20.08982264	8.2627837	4.398475058	3.211445828	1.281765	0.69455
LOC100626976	163.8906584	67.754826	7.365365858	6.103389087	1.274338	0.69465
EVI2B	2.114718173	8.2627837	1.639101631	3.211445828	-1.96616	0.69475
LOC102159687	2.114718173	8.2627837	1.639101631	3.211445828	-1.96616	0.69475
LOC102161354	2.114718173	8.2627837	1.639101631	3.211445828	-1.96616	0.69475
LOC100511868	2.114718173	8.2627837	1.639101631	3.211445828	-1.96616	0.69475
LOC102164396	2.114718173	8.2627837	1.639101631	3.211445828	-1.96616	0.69475
LOC102164084	2.114718173	8.2627837	1.639101631	3.211445828	-1.96616	0.69475
DNAI2	2.114718173	8.2627837	1.639101631	3.211445828	-1.96616	0.69475
CPA4	2.114718173	8.2627837	1.639101631	3.211445828	-1.96616	0.69475
LOC102166591	2.114718173	8.2627837	1.639101631	3.211445828	-1.96616	0.69475
LOC102158594	2.114718173	8.2627837	1.639101631	3.211445828	-1.96616	0.69475
LOC100737348	2.114718173	8.2627837	1.639101631	3.211445828	-1.96616	0.69475
ZNF239	1257.199954	435.4487	10.2971455	8.769668286	1.529639	0.69481
LOC102166437	4.229436346	12.394176	2.386655454	3.74353388	-1.55112	0.69518
LOC100512852	4.229436346	12.394176	2.386655454	3.74353388	-1.55112	0.69518
LOC102158901	4.229436346	12.394176	2.386655454	3.74353388	-1.55112	0.69518
LOC102165710	4.229436346	12.394176	2.386655454	3.74353388	-1.55112	0.69518
LOC100737691	4.229436346	12.394176	2.386655454	3.74353388	-1.55112	0.69518
LOC102167901	4.229436346	12.394176	2.386655454	3.74353388	-1.55112	0.69518
TCEAL4	123.7110131	323.07484	6.962445064	8.340183224	-1.38489	0.6952
LOC100038006	1710.807002	576.7423	10.74130434	9.174282325	1.568678	0.69527
CCDC171	43.35172255	19.004403	5.470918232	4.322245637	1.189756	0.69532
LOC100737964	43.35172255	19.004403	5.470918232	4.322245637	1.189756	0.69532
ACAP2	10858.02046	3119.2009	13.40660635	11.60742319	1.799513	0.69544
CENPK	695.742279	254.49374	9.4444813	7.997144125	1.450923	0.69545
USP37	957.9673324	340.42669	9.90533786	8.415432027	1.492632	0.6955
EXOC4	236.8484354	661.84898	7.893898725	9.372536392	-1.48254	0.69552
ITGA4	1522.597085	518.90282	10.57326572	9.022098163	1.552998	0.69553
UTP14A	11024.02584	3163.8199	13.42849442	11.62790768	1.800912	0.69567
LOC100627242	104.6785496	268.54047	6.723538761	8.074358096	-1.35917	0.69573
PON2	273.8560034	776.70167	8.102532181	9.603073025	-1.50394	0.69595
AMIGO3	285.4869534	813.05792	8.16232563	9.668987631	-1.50993	0.69613
DPH2	44.40908164	105.76363	5.504908955	6.738276475	-1.25192	0.69628
LOC100510952	91.99024053	39.661362	6.539007406	5.34558663	1.213747	0.69669
PRDM8	9.516231779	23.135794	3.394545939	4.593102407	-1.28166	0.69669
KCNH2	9.516231779	23.135794	3.394545939	4.593102407	-1.28166	0.69669
LOC100513218	71.90041789	177.64985	6.187855179	7.480990892	-1.30496	0.69676
LOC102159561	8.458872693	2.4788351	3.241668254	1.798604302	1.770803	0.69679
TRNAS-AGA	8.458872693	2.4788351	3.241668254	1.798604302	1.770803	0.69679
LOC102163500	8.458872693	2.4788351	3.241668254	1.798604302	1.770803	0.69679
LOC102166423	8.458872693	2.4788351	3.241668254	1.798604302	1.770803	0.69679
LOC102160337	8.458872693	2.4788351	3.241668254	1.798604302	1.770803	0.69679
C9H1orf105	8.458872693	2.4788351	3.241668254	1.798604302	1.770803	0.69679
LOC102167749	8.458872693	2.4788351	3.241668254	1.798604302	1.770803	0.69679
GCET2	8.458872693	2.4788351	3.241668254	1.798604302	1.770803	0.69679
LOC100521408	8.458872693	2.4788351	3.241668254	1.798604302	1.770803	0.69679
LOC100523833	585.776934	218.13749	9.19666835	7.775692512	1.425114	0.69689
ZBTB7C	240.0205127	96.674569	7.913012126	6.609911087	1.311949	0.69696
BMP2	34.89284986	81.801559	5.165624571	6.371586023	-1.2292	0.69699
GORASP1	340.4696259	986.57638	8.415613445	9.947748515	-1.5349	0.69709
SRRD	330.9533941	129.7257	8.374836893	7.030399033	1.351164	0.69711
POLR2L	1127.144786	3714.1213	10.13973652	11.85919359	-1.72035	0.69711
ACSF2	2224.683518	7865.3438	11.12003275	12.94147753	-1.82191	0.69723
LOC100623062	379.5919121	147.07755	8.572101093	7.210209122	1.367872	0.69738
TIAF1	392.2802211	1153.4846	8.619413822	10.17303322	-1.55604	0.69749
WLS	2368.484354	8422.2554	11.21035742	13.0401622	-1.83024	0.69761
LOC100737407	35.95020894	84.280394	5.207510617	6.414142197	-1.2292	0.69762
SSU72	793.0193149	2515.1914	9.633030292	11.29702593	-1.66524	0.69763
LOC102163553	6.344154519	16.525567	2.876596413	4.131389251	-1.3812	0.6977
LOC102158804	6.344154519	16.525567	2.876596413	4.131389251	-1.3812	0.6977
LOC102165892	6.344154519	16.525567	2.876596413	4.131389251	-1.3812	0.6977
LOC102167202	120.5389359	312.33322	6.925274755	8.291553945	-1.37359	0.69772
LOC100620714	177.6363265	479.24146	7.480881679	8.907616136	-1.43183	0.69776
LOC102167486	201.9555855	82.627837	7.665020235	6.385911347	1.289338	0.69778
TMEM108	41.23700438	18.178124	5.400435611	4.261389711	1.181736	0.69783
POLR1B	7471.299306	2221.8625	12.86733653	11.11820302	1.74959	0.69783
MARK1	1126.087427	395.78734	10.13838371	8.632222185	1.508521	0.69788
LOC100524077	3912.22862	1230.3285	11.93414368	10.26599998	1.668947	0.69793
LOC100622083	11.63094995	4.1313919	3.658891241	2.359350201	1.493269	0.69815
LOC102168083	11.63094995	4.1313919	3.658891241	2.359350201	1.493269	0.69815
LOC102162379	11.63094995	4.1313919	3.658891241	2.359350201	1.493269	0.69815
LOC102158759	85.64608601	37.182527	6.437062676	5.254840671	1.203763	0.6982
ABCC10	272.7986443	770.09144	8.096971493	9.590758147	-1.49719	0.6982
LOC102157824	459.9512027	1374.1009	8.848470222	10.4253218	-1.57894	0.69826
LOC100738565	28.54869534	12.394176	4.885022527	3.74353388	1.203763	0.69828
LOC100739831	28.54869534	12.394176	4.885022527	3.74353388	1.203763	0.69828
LOC102162460	48.63851798	21.483238	5.633388138	4.490777899	1.178888	0.69828
LOC102165184	63.44154519	154.51406	6.009919183	7.280901168	-1.28424	0.69834
TSGA14	93.04759962	234.66306	6.555319217	7.88058181	-1.33455	0.69836
ZNF827	131.1125267	342.07925	7.045623457	8.422398044	-1.38353	0.69856
FRY	74.01513606	182.60752	6.229109817	7.520481339	-1.30285	0.69861
LOC100511905	549.826725	206.56959	9.105454748	7.697451307	1.412349	0.6988
ZNF662	831.084242	300.76533	9.700585787	8.23728324	1.466356	0.69889
OSGEP	1392.541917	480.89401	10.44454068	8.912572065	1.53393	0.69897
LOC100625070	1945.540719	651.93364	10.92669681	9.350792552	1.577374	0.69901
LOC100524687	33.83549077	14.873011	5.122485983	3.98850389	1.185841	0.69903
LOC102160161	23.2618999	9.9153405	4.600620625	3.448285225	1.230235	0.69911
ENO4	23.2618999	9.9153405	4.600620625	3.448285225	1.230235	0.69911
SIRT4	186.0951992	76.843889	7.54762873	6.282511874	1.276038	0.69922
LOC100517759	19.03246356	43.792754	4.324267947	5.485193456	-1.20223	0.69935
LOC100620395	511.7617979	1543.488	9.002144971	10.59291295	-1.59265	0.69941
LOC100518605	260.1103353	728.77752	8.028515753	9.511312908	-1.48635	0.69942
LOC100628087	81.41664967	35.52997	6.364863912	5.191008663	1.196287	0.69944
MAPK6	11188.97385	3223.3119	13.44991904	11.65477561	1.795462	0.6995
SH3YL1	44.40908164	104.93735	5.504908955	6.727067559	-1.2406	0.69962
PEPD	391.222862	1145.2218	8.61552982	10.16267055	-1.54956	0.69976
WDR24	604.8093975	1855.8212	9.242720148	10.8586192	-1.61751	0.69989
MPLKIP	1601.899016	546.99628	10.64646782	9.098022295	1.55018	0.69991
LOC100519357	2862.271047	927.91061	11.48344853	9.859395963	1.625103	0.69997
LOC100624061	599.5226021	223.92144	9.230074736	7.81327737	1.420821	0.7
GMPR	1434.83628	494.94074	10.48767554	8.954023947	1.535558	0.70002
LOC100521167	14.80302721	5.7839486	3.982129041	2.76212524	1.355766	0.70031
LOC100627444	2194.020105	728.77752	11.10001844	9.511312908	1.590026	0.70039
LOC100621126	2000.523392	670.11176	10.96688276	9.390409227	1.577904	0.70046
LOC100514543	145.9155539	383.39316	7.198843332	8.586438871	-1.39369	0.70055
LOC100625999	450.4349709	1336.9184	8.818374373	10.38577442	-1.56952	0.70059
LOC100622995	119.4815768	307.37555	6.912668746	8.268544593	-1.36321	0.70074
RIC8B	2061.850219	689.11616	11.01042336	9.43069541	1.58112	0.70079
KRAB	95.16231779	41.313919	6.587399765	5.403060391	1.203763	0.70086
E2F3	1427.434767	493.28819	10.48021944	8.94920862	1.532922	0.70091
LOC100739002	161.7759402	67.754826	7.346743662	6.103389087	1.255601	0.70091
BAG4	233.6763581	95.022013	7.874528698	6.585293272	1.298178	0.70097
IRX3	169.1774539	451.14799	7.410896102	8.820651243	-1.41506	0.70097
LMBR1L	492.7293343	1475.7332	8.947576553	10.52819346	-1.58256	0.70099
TECPR1	543.4825705	1645.1202	9.088742057	10.684854	-1.59789	0.70101

LOC102163947	9.94714588	3.3051135	3.452482877	2.106051269	1.589583	0.68161
LOC102161111	9.94714588	3.3051135	3.452482877	2.106051269	1.589583	0.68161
LOC102166826	9.94714588	3.3051135	3.452482877	2.106051269	1.589583	0.68161
LOC102162006	9.94714588	3.3051135	3.452482877	2.106051269	1.589583	0.68161
LCA5L	9.94714588	3.3051135	3.452482877	2.106051269	1.589583	0.68161
LOC100624770	9.94714588	3.3051135	3.452482877	2.106051269	1.589583	0.68161
CNDP1	9.94714588	3.3051135	3.452482877	2.106051269	1.589583	0.68161
LOC102160997	9.94714588	3.3051135	3.452482877	2.106051269	1.589583	0.68161
LOC102160163	9.94714588	3.3051135	3.452482877	2.106051269	1.589583	0.68161
LOC102162704	9.94714588	3.3051135	3.452482877	2.106051269	1.589583	0.68161
LOC102163417	9.94714588	3.3051135	3.452482877	2.106051269	1.589583	0.68161
LOC100511115	9.94714588	3.3051135	3.452482877	2.106051269	1.589583	0.68161
LOC102167368	9.94714588	3.3051135	3.452482877	2.106051269	1.589583	0.68161
LOC100620901	9.94714588	3.3051135	3.452482877	2.106051269	1.589583	0.68161
C2H19orf52	1642.93693	674.24315	10.68293923	9.399263292	1.284936	0.68168
LOC100738768	429.385131	1012.191	8.749484425	9.984690459	-1.23714	0.68177
AHR	1095.84391	2768.0325	10.09914251	11.43516629	-1.33682	0.68183
DIAPH2	551.237668	243.75212	9.109145486	7.935177544	1.17726	0.68189
LOC100523455	3734.32435	1449.2923	11.86701781	10.50212795	1.365499	0.68198
DDX25	36.4728682	17.351846	5.227774503	4.19785327	1.071735	0.68209
DHX29	4778.77467	1824.4226	12.22272689	10.83401482	1.389201	0.6822
GLMN	610.920543	268.54047	9.257200523	8.074358096	1.185845	0.68268
CD59	1738.26374	711.42568	10.76426101	9.476595706	1.288862	0.68279
EPHX2	3879.38689	1503.0004	11.92198479	10.5545892	1.367983	0.68286
MKL2	34.8150106	16.525567	5.162492464	4.13138925	1.07501	0.6829
PHTF2	421.095842	989.88149	8.721426809	9.952568709	-1.23311	0.68293
LOC100627731	462.542284	1094.8188	8.856557135	10.0977936	-1.24304	0.68297
LOC100620601	1672.77837	686.63733	10.70889279	9.42550405	1.284626	0.68307
LOC102160517	172.417195	81.801559	7.438103147	6.371586023	1.075703	0.68307
CPA4	19.0653629	8.2627837	4.326635347	3.211445828	1.206254	0.68315
PRLR	19.0653629	8.2627837	4.326635347	3.211445828	1.206254	0.68315
LOC100513340	19.0653629	8.2627837	4.326635347	3.211445828	1.206254	0.68315
LOC102165720	19.0653629	8.2627837	4.326635347	3.211445828	1.206254	0.68315
LOC100153559	9.11821706	20.656959	3.338883189	4.436758793	-1.1798	0.68348
COX17	7126.3011	2652.3536	12.79914016	11.37360122	1.42588	0.6835
ELOVL6	368.873327	167.73451	8.530887454	7.398611253	1.136946	0.68355
GTSE1	3474.86963	1357.5754	11.76315826	10.40787888	1.355927	0.68375
BAZ2A	9205.25459	3369.5632	13.16839862	11.71877396	1.449896	0.68376
ADCY5	24.8678647	51.229259	4.693089065	5.706786332	-1.04269	0.68384
C4H1orf194	33.1571529	15.699289	5.094115824	4.061714779	1.078621	0.68388
LOC100526167	1262.4586	3215.0491	10.30316267	11.65107374	-1.3486	0.68388
ESRRA	4656.92213	1783.935	12.18547081	10.80165583	1.384314	0.6839
LOC102159722	3.31571529	9.9153405	2.109599694	3.448285225	-1.58034	0.68459
LOC100151963	3.31571529	9.9153405	2.109599694	3.448285225	-1.58034	0.68459
LOC100516475	3.31571529	9.9153405	2.109599694	3.448285225	-1.58034	0.68459
LOC102160963	3.31571529	9.9153405	2.109599694	3.448285225	-1.58034	0.68459
FMO1	3.31571529	9.9153405	2.109599694	3.448285225	-1.58034	0.68459
LOC100627942	3.31571529	9.9153405	2.109599694	3.448285225	-1.58034	0.68459
C4H1orf56	31.4992953	14.873011	5.022336531	3.98850389	1.082623	0.68505
LOC100739094	31.4992953	14.873011	5.022336531	3.98850389	1.082623	0.68505
LOC102166802	31.4992953	14.873011	5.022336531	3.98850389	1.082623	0.68505
LOC102162356	31.4992953	14.873011	5.022336531	3.98850389	1.082623	0.68505
LOC102163229	31.4992953	14.873011	5.022336531	3.98850389	1.082623	0.68505
LOC102166314	186.508985	88.411786	7.55081592	6.482393107	1.076935	0.68536
MTERF	667.287703	292.50254	9.384325518	8.197229196	1.18986	0.68545
SAMD8	1095.84391	464.36844	10.09914251	8.862229579	1.2387	0.68555
CD55	387.109761	901.4697	8.600320906	9.817734691	-1.21954	0.68564
LOC102161090	43.1042988	88.411786	5.462847376	6.482393107	-1.03641	0.68579
LOC100739263	1029.5296	2577.1622	10.00917023	11.33212734	-1.3238	0.68591
CEP19	741.891297	323.07484	9.537007315	8.340183224	1.199339	0.68601
LOC102163312	82.8928824	40.48764	6.39047651	5.374609695	1.033767	0.68615
LOC100525248	29.8414377	14.046732	4.946798112	3.911378308	1.087082	0.68644
LOC100737848	164.956836	361.08365	7.374664246	8.500179216	-1.13024	0.68645
LOC100623573	1793.80197	735.38775	10.80960896	9.524321819	1.286444	0.68648
UNKL	190.653629	421.40197	7.582357509	8.722472752	-1.14424	0.6866
PTCD2	880.322411	379.26177	9.783526081	8.570849103	1.214838	0.68687
LOC100518152	439.332277	1031.1954	8.782448788	10.0115004	-1.23093	0.68688
NCAPH	1940.52238	791.57468	10.92297262	9.630403069	1.293648	0.6869
PPP1R21	2369.90751	953.52524	11.21122367	9.898639538	1.313488	0.68694
LOC102162243	17.4075053	7.4365053	4.202222212	3.076645515	1.227013	0.68695
KRT25	17.4075053	7.4365053	4.202222212	3.076645515	1.227013	0.68695
ALG8	847.165258	366.04132	9.728201579	8.51979867	1.210637	0.68712
RPA2	2561.39007	1025.4115	11.32327439	10.00339347	1.320724	0.68726
LOC100624780	193.969345	428.83847	7.607103495	8.747650814	-1.14461	0.68733
CDK1	2807.58193	1117.1284	11.45562617	10.1268701	1.329533	0.68746
BCAS3	436.016561	1022.1063	8.771544143	9.998740397	-1.22909	0.68749
PLEKHH2	525.540874	234.66306	9.040401719	7.88058181	1.163212	0.68756
PDE4B	45.5910853	93.369456	5.541982032	6.560248082	-1.0342	0.68758
PAK2	2352.5	947.74129	11.20059214	9.889870929	1.31163	0.6877
QRSL1	1271.57682	534.60211	10.31353703	9.065017825	1.250081	0.68776
ANXA4	888.611699	2195.4216	9.79703195	11.10093931	-1.30487	0.68792
RBM19	9327.93605	3424.9239	13.18749684	11.74227737	1.445486	0.68794
LOC100623064	336.545102	773.39656	8.398936476	9.596928726	-1.20041	0.68809
LOC100620250	28.18358	13.220454	4.867084967	3.829895615	1.092083	0.6881
CALCOCO2	2728.83369	7315.0424	11.41459734	12.83684773	-1.42258	0.68813
LOC102162056	36.4728682	74.365053	5.227774503	6.2358238	-1.0278	0.68815
C1H6orf57	444.30585	200.78564	8.798652753	7.65667973	1.145897	0.68833
ARF5	648.22234	1563.3187	9.342568834	10.61131873	-1.27005	0.68849
ERLEC1	1042.79246	2605.2557	10.02761917	11.34776292	-1.32097	0.68855
VAV3	97.8136012	206.56959	6.62663773	7.697451307	-1.07852	0.68872
HEXIM1	1592.37227	4102.4721	10.63786766	12.00262943	-1.36532	0.68891
LOC100522374	1085.06783	461.88961	10.08489849	8.854524369	1.232165	0.68907
LOC102157465	8.28928824	2.4788351	3.215568059	1.798604302	1.741586	0.68953
KRT222	8.28928824	2.4788351	3.215568059	1.798604302	1.741586	0.68953
LOC102161209	8.28928824	2.4788351	3.215568059	1.798604302	1.741586	0.68953
TRNAE-CUC	8.28928824	2.4788351	3.215568059	1.798604302	1.741586	0.68953
LOC102160485	8.28928824	2.4788351	3.215568059	1.798604302	1.741586	0.68953
LOC102158819	8.28928824	2.4788351	3.215568059	1.798604302	1.741586	0.68953
LOC102167583	8.28928824	2.4788351	3.215568059	1.798604302	1.741586	0.68953
LOC102165606	8.28928824	2.4788351	3.215568059	1.798604302	1.741586	0.68953
LOC102165851	8.28928824	2.4788351	3.215568059	1.798604302	1.741586	0.68953
LOC102167166	8.28928824	2.4788351	3.215568059	1.798604302	1.741586	0.68953
RGAG4	221.323996	492.46191	7.79651986	8.946794917	-1.15385	0.68959
LOC100518476	246.191861	551.95395	7.94948743	9.111015534	-1.16476	0.68963
SMCR7L	2640.96723	1057.6363	11.36739686	10.04799134	1.320223	0.68966
LOC100621421	65.4853771	32.224857	6.054965161	5.054191063	1.022999	0.68988
PMCH	48.9068006	23.962073	5.641164514	4.641665831	1.029282	0.69004
LOC102158995	26.5257224	12.394176	4.78270852	3.74353388	1.09773	0.69006
VDAC2	13534.7498	4860.1694	13.72448719	12.24708769	1.47759	0.69016
MYOM3	7.46035941	17.351846	3.080718953	4.19785327	-1.21777	0.69021
GNAZ	1642.108	680.0271	10.6822116	9.411568398	1.271885	0.69023
LOC100623302	1037.81889	443.71149	10.02072843	8.796725856	1.225861	0.69023
CYB5R1	1248.36681	3154.7308	10.28698139	11.62375844	-1.33748	0.69024
LOC100739517	942.492072	405.70268	9.881866586	8.667830689	1.216058	0.69034
LOC100626663	62.1696618	128.07315	5.981159942	7.012045083	-1.04269	0.69038
REXO1	2881.35659	1148.5269	11.49303311	10.16682456	1.326964	0.69095
LOC100513338	2872.23837	1145.2218	11.48846197	10.16267055	1.326548	0.69102
ITGB4	532.172305	1260.9008	9.058458033	10.30138278	-1.24449	0.69105
COIL	1477.98009	617.22994	10.53038692	9.271999722	1.259747	0.69124
BTBD2	3126.71952	8434.6496	11.61089543	13.04228346	-1.43168	0.69142
RFX2	15.7496477	6.610227	4.066058842	2.927939483	1.252548	0.69158
LOC102158808	15.7496477	6.610227	4.066058842	2.927939483	1.252548	0.69158
LOC102163385	15.7496477	6.610227	4.066058842	2.927939483	1.252548	0.69158
LOC100523329	42.27537	20.656959	5.435474249	4.436758793	1.033189	0.69182
DNAJA4	216.350423	479.24146	7.763879093	8.907616136	-1.14738	0.69183
PAX9	1183.71036	502.37725	10.21031867	8.975496206	1.236473	0.69185
C1H9orf40	1240.07752	524.68677	10.27737752	9.038059606	1.240902	0.69201
MSH3	546.264095	244.5784	9.096093396	7.940039851	1.159302	0.69206
SPPL3	764.272375	1858.3001	9.579829512	10.8605439	-1.28182	0.69212
PES1	9007.1406	3328.2493	13.13701363	11.70098118	1.436306	0.6922
GJC2	2.48678647	8.2627837	1.801898017	3.211445828	-1.73235	0.69232
LOC100511868	2.48678647	8.2627837	1.801898017	3.211445828	-1.73235	0.69232
LOC100154249	2.48678647	8.2627837	1.801898017	3.211445828	-1.73235	0.69232
TLX3	2.48678647	8.2627837	1.801898017	3.211445828	-1.73235	0.69232
LOC102160243	2.48678647	8.2627837	1.801898017	3.211445828	-1.73235	0.69232
LOC102162308	2.48678647	8.2627837	1.801898017	3.211445828	-1.73235	0.69232
SLCO1A2	2.48678647	8.2627837	1.801898017	3.211445828	-1.73235	0.69232
LOC102167149	2.48678647	8.2627837	1.801898017	3.211445828	-1.73235	0.69232
LOC100737951	2.48678647	8.2627837	1.801898017	3.211445828	-1.73235	0.69232
LRRC19	2.48678647	8.2627837	1.801898017	3.211445828	-1.73235	0.69232
RHOV	24.8678647	11.567897	4.693089065	3.65167138	1.104156	0.69237
NSUN6	607.604828	270.19303	9.249361968	8.083176277	1.169143	0.69239
STXBP6	1569.16226	653.58619	10.61669794	9.35443936	1.263545	0.69267
BAG3	1725.00088	4451.9879	10.75321749	12.12055797	-1.36785	0.69272
RARRES1	202.258633	445.36404	7.667172819	8.802077003	-1.13878	0.69274
KIAA0196	1683.55444	4337.1352	10.71815134	12.08285929	-1.36523	0.69275
LOC100519617	70.45895	145.42499	6.159042811	7.194018019	-1.04542	0.69281
DDX28	863.743834	375.13038	9.756129012	8.55508903	1.203211	0.69288
HAUS8	1022.06924	438.75382	9.998688073	8.780552285	1.220009	0.69292
BAMBI	181.535412	396.61362	7.512032568	8.63522336	-1.12748	0.69293
LOC100625867	315.821882	718.03591	8.307528171	9.489920003	-1.18494	0.693
LOC102159133	135.944327	66.10227	7.097445694	6.068289661	1.040244	0.69316
LOC100739251	415.293341	963.44058	8.70145667	9.913548547	-1.21406	0.69316
TAMM41	2330.94785	945.26246	11.18731981	9.886096578	1.30213	0.69342
LOC100738530	25.6967935	52.055537	4.73859457	5.729431428	-1.01846	0.69353
SELH	6012.22076	2285.486	12.55392221	11.15891636	1.395397	0.69362
LOC100521041	1742.40839	721.34102	10.76769484	9.496536287	1.272329	0.6938
LOC102168094	150.036117	72.712497	7.238749771	6.20383732	1.045035	0.69388
LOC100152503	445.134778	1036.9794	8.801335807	10.01956204	-1.22007	0.69402
C14H1orf131	1492.07188	624.66645	10.54406791	9.289249932	1.256159	0.69415
LOC102161930	23.2100071	47.097867	4.597531595	5.587901017	-1.02091	0.69419
HPCA	23.2100071	47.097867	4.597531595	5.587901017	-1.02091	0.69419
ZNF169	182.364341	87.585507	7.518569296	6.468998788	1.05806	0.69426
PLAGL2	1428.24436	599.8781	10.48103688	9.230928526	1.251502	0.69431
LOC102166198	37.3017971	18.178124	5.259340178	4.261389712	1.037042	0.69432
SLC27A6	37.3017971	18.178124	5.259340178	4.261389712	1.037042	0.69432
LOC102165363	37.3017971	18.178124	5.259340178	4.261389712	1.037042	0.69432
ADORA2A	33.9860818	68.581105	5.128709195	6.120623682	-1.01287	0.69437
TIFA	257.796864	121.46292	8.015676326	6.936201185	1.085719	0.69438
LRRK2	495.699437	1163.3999	8.9562293	10.18537096	-1.23081	0.69455
PIAS3	1201.94679	3014.2635	10.23235712	11.55806837	-1.32643	0.69464
LOC102159905	2113.7685	864.28718	11.04628403	9.757035212	1.290235	0.69479
TMEM2	387.109761	891.55436	8.600320906	9.801796232	-1.20358	0.69481
LOC102167397	67.9721635	139.64104	6.107942317	7.135873883	-1.03871	0.69484
LOC100620765	67.9721635	139.64104	6.107942317	7.135873883	-1.03871	0.69484
OGFOD1	1560.87298	651.93364	10.60906141	9.350792552	1.259556	0.69488
MAGI3	789.969169	1919.4447	9.627477651	10.90722467	-1.28082	0.69489
LOC100621578	98.64253	206.56959	6.638689748	7.697451307	-1.06635	0.69507
CPNE9	23.2100071	10.741619	4.597531595	3.553559423	1.111536	0.6951
LOC100520070	187.337914	409.00779	7.557179646	8.679507525	-1.12649	0.69513
LOC100622420	36.4728682	73.538775	5.227774503	6.219919206	-1.01168	0.69516
LOC100153642	62.9985906	128.89943	5.999968229	7.021251246	-1.03285	0.69531
MEIS1	599.31554	267.71419	9.229577203	8.06992871	1.162622	0.69543
CEP44	95.3268147	47.097867	6.589865555	5.587901017	1.01722	0.69562
LOC102161527	95.3268147	47.097867	6.589865555	5.587901017	1.01722	0.69562
RHOG	233.757928	518.07654	7.875030072	9.019803473	-1.14815	0.69573
LOC100621888	11.6050035	24.788351	3.655924617	4.688647628	-1.09492	0.69577
CLMN	144.233615	309.02811	7.182231603	8.276255224	-1.09933	0.69579
LOC100157694	819.810607	358.60481	9.680905563	8.490268521	1.192896	0.69597
LOC102160467	917.624208	398.26618	9.843330992	8.641207046	1.20417	0.696
FLNB	6733.38883	19074.636	12.71733131	14.21944355	-1.50225	0.69608
LOC100622224	447.621565	204.09076	8.80935516	7.68011867	1.133069	0.69609
LOC100627522	270.230797	127.24687	8.083377186	7.002779797	1.086562	0.69611
LOC100623836	377.991544	174.34474	8.566021848	7.454050313	1.116411	0.69644
LOC100520338	33.9860818	16.525567	5.128709195	4.13138925	1.040244	0.69677
LOC102159915	167.443622	361.90993	7.396121996	8.50346771	-1.11196	0.69682
LOC100626969	147.549331	71.886218	7.214798294	6.187574144	1.03741	0.69708
LOC102168042	273.546512	128.89943	8.100906771	7.021251246	1.08554	0.69722
LOC100624112	366.38654	837.84627	8.521154961	9.712262629	-1.19332	0.69724
LOC102164743	14.09179	5.7839486	3.915692025	2.762125239	1.284728	0.69725
LOC100158021	14.09179	5.7839486	3.915692025	2.762125239	1.284728	0.69725
THOC3	2388.14394	970.87709	11.22227806	9.924630058	1.298529	0.69739
GFRA2	149.207188	72.712497	7.230810045	6.20383732	1.037042	0.69762
LOC100739482	66.3143059	33.051135	6.072841239	5.089630977	1.00462	0.6977
MSH2	2703.13689	1089.8612	11.40095247	10.09125179	1.31049	0.6977
TMEM63C	62.9985906	31.398578	5.999968229	5.017858594	1.00462	0.69771
LOC100154628	174.075053	376.78294	7.451829714	8.561413734	-1.11402	0.69775
LOC102158359	61.340733	30.5723	5.962103212	4.980587446	1.00462	0.69776
SALL2	6124.95508	2335.0627	12.58071907	11.18986327	1.391238	0.6978
ANXA9	74.6035941	37.182527	6.240382915	5.254840671	1.00462	0.69819
LOC100155367	237.902572	113.20014	7.900278578	6.83542057	1.071495	0.69828
SLC2A4	21.5521494	9.9153405	4.495193036	3.448285225	1.120098	0.69832
LOC102163625	21.5521494	9.9153405	4.495193036	3.448285225	1.120098	0.69832
LOC102164761	21.5521494	9.9153405	4.495193036	3.448285225	1.120098	0.69832
LOC102162180	21.5521494	9.9153405	4.495193036	3.448285225	1.120098	0.69832
SDR42E1	390.425476	180.12869	8.612593847	7.500871231	1.116019	0.69834
LOC102157574	10.7760747	23.135794	3.557786824	4.593102408	-1.10229	0.69842
LOC100628061	242.876145	537.90722	7.930004838	9.073893105	-1.14714	0.69843
MAML3	384.622974	177.64985	8.591047195	7.480990892	1.114408	0.69845
ECHS1	1572.47798	4005.7975	10.61974127	11.9682339	-1.34905	0.69845
EHD4	1376.02185	3470.3692	10.42733573	11.76128908	-1.33459	0.69859
BCAR3	1108.27784	2749.0281	10.11540504	11.42523067	-1.3106	0.69904
ENSA	768.41702	1854.1687	9.587621932	10.85733464	-1.27081	0.69908
HCK	6.63143059	1.6525567	2.93195353	1.407383613	2.00462	0.69912
OVCH1	6.63143059	1.6525567	2.93195353	1.407383613	2.00462	0.69912
LOC102159176	6.63143059	1.6525567	2.93195353	1.407383613	2.00462	0.69912
DCT	6.63143059	1.6525567	2.93195353	1.407383613	2.00462	0.69912
GDF7	6.63143059	1.6525567	2.93195353	1.407383613	2.00462	0.69912
LOC102159372	6.63143059	1.6525567	2.93195353	1.407383613	2.00462	0.69912
WDR96	6.63143059	1.6525567	2.93195353	1.407383613	2.00462	0.69912
PLCH2	6.63143059	1.6525567	2.93195353	1.407383613	2.00462	0.69912
LOC102159780	6.63143059	1.6525567	2.93195353	1.407383613	2.00462	0.69912
IL25	6.63143059	1.6525567	2.93195353	1.407383613	2.00462	0.69912
LOC102161189	6.63143059	1.6525567	2.93195353	1.407383613	2.00462	0.69912
LOC100626296	6.63143059	1.6525567	2.93195353	1.407383613	2.00462	0.69912
PAX2	6.63143059	1.6525567	2.93195353	1.407383613	2.00462	0.69912
LOC102166992	6.63143059	1.6525567	2.93195353	1.407383613	2.00462	0.69912
KLLN	6.63143059	1.6525567	2.93195353	1.407383613	2.00462	0.69912
SVOPL	6.63143059	1.6525567	2.93195353	1.407383613	2.00462	0.69912
LOC100738112	6.63143059	1.6525567	2.93195353	1.407383613	2.00462	0.69912
LOC102164385	6.63143059	1.6525567	2.93195353	1.407383613	2.00462	0.69912
LOC100738383	6.63143059	1.6525567	2.93195353	1.407383613	2.00462	0.69912
LOC102166545	6.63143059	1.6525567	2.93195353	1.407383613	2.00462	0.69912
CRHBP	6.63143059	1.6525567	2.93195353	1.407383613	2.00462	0.69912
HPSE2	6.63143059	1.6525567	2.93195353	1.407383613	2.00462	0.69912
IL28B	6.63143059	1.6525567	2.93195353	1.407383613	2.00462	0.69912
LOC102160393	6.63143059	1.6525567	2.93195353	1.407383613	2.00462	0.69912
LOC102162947	6.63143059	1.6525567	2.93195353	1.407383613	2.00462	0.69912
CDKL3	6.63143059	1.6525567	2.93195353	1.407383613	2.00462	0.69912
C15H2orf54	6.63143059	1.6525567	2.93195353	1.407383613	2.00462	0.69912
LOC100627319	6.63143059	1.6525567	2.93195353	1.407383613	2.00462	0.69912
SIDT1	6.63143059	1.6525567	2.93195353	1.407383613	2.00462	0.69912
LOC102165764	6.63143059	1.6525567	2.93195353	1.407383613	2.00462	0.69912
LOC102165801	6.63143059	1.6525567	2.93195353	1.407383613	2.00462	0.69912
FRMPD2	6.63143059	1.6525567	2.93195353	1.407383613	2.00462	0.69912
LOC100153710	6.63143059	1.6525567	2.93195353	1.407383613	2.00462	0.69912
LOC100620719	6.63143059	1.6525567	2.93195353	1.407383613	2.00462	0.69912
LOC102167989	6.63143059	1.6525567	2.93195353	1.407383613	2.00462	0.69912
LOC100737315	6.63143059	1.6525567	2.93195353	1.407383613	2.00462	0.69912
LOC102165239	6.63143059	1.6525567	2.93195353	1.407383613	2.00462	0.69912
ALKBH4	188.995772	410.66035	7.569823503	8.685310691	-1.11959	0.69919
LOC102160397	141.746829	69.407383	7.157314885	6.137654819	1.030155	0.69932
LOC100510914	1645.42372	4201.6255	10.68511995	12.03707519	-1.35249	0.69933
42620	4692.56607	12918.862	12.19646875	13.65730308	-1.46103	0.69954
NEK3	725.312721	321.42229	9.504447038	8.33280766	1.174133	0.69982
LOC100738736	7630.28982	2872.1436	12.8977112	11.4884144	1.40961	0.69987
LOC100515976	105.273961	219.79005	6.731644339	7.786531327	-1.06198	0.6999
HNRNPA0	20062.5643	7087.8159	14.29229031	12.79132894	1.501093	0.69995
ZSCAN20	351.465821	163.60312	8.461339555	7.362847851	1.103184	0.69997
FUCT1	132.628612	280.93465	7.062085132	8.139216969	-1.08284	0.70005
TMA16	2975.85448	1194.7985	11.53957298	10.22375862	1.316537	0.70007
JAKMIP1	48.0778718	23.962073	5.617000784	4.641665831	1.00462	0.70007
THUMPD1	5707.17495	2191.2902	12.47881384	11.0982231	1.380996	0.70011
KLHL24	950.781361	2328.4525	9.894486391	11.18577517	-1.29219	0.70015
LOC100519113	89.524313	44.619032	6.500233417	5.511563931	1.00462	0.70024
IRF8	5.80250177	14.046732	2.766065426	3.911378308	-1.27549	0.70025
LOC102160904	5.80250177	14.046732	2.766065426	3.911378308	-1.27549	0.70025

OMC	8976.64474	3339.8172	13.13212129	11.70598532	1.426407	0.82794
LOC102160338	64.8980623	36.356248	6.042164139	5.223277669	0.835972	0.82797
SLC25A34	51.0899639	28.919743	5.702933533	4.903025878	0.820985	0.82804
MPP6	741.494882	340.42669	9.536237271	8.415432027	1.123093	0.82825
LOC100626019	131.176934	265.23536	7.04632663	8.05655837	-1.01576	0.82834
CCDC86	1467.80086	634.58179	10.52042309	9.311933979	1.209778	0.82841
ZNF37A	33.1394361	59.492043	5.093367322	5.918673475	-0.84415	0.82844
GLT8D1	1205.44699	3112.5906	10.23654881	11.60436356	-1.36855	0.82848
ZNF643	316.205452	704.81545	8.309273759	9.463147202	-1.15638	0.82852
TMEM216	588.22499	275.97698	9.202674809	8.113622247	1.09182	0.82863
LOC102166236	55.2323934	102.45852	5.813329549	6.692908622	-0.89145	0.82867
LOC102158238	2383.27778	989.05521	11.21933661	9.95136517	1.268824	0.82878
N4BP1	1444.32709	3798.4017	10.49718031	11.89155653	-1.39499	0.8288
GLT8D2	944.473928	2373.8978	9.884893865	11.21364969	-1.32967	0.82883
LOC100620687	817.439423	2022.7295	9.676731829	10.98280072	-1.30712	0.82883
LOC102159419	4.14242951	0.8262784	2.362450112	0.868906686	2.325777	0.82895
LOC102157421	4.14242951	0.8262784	2.362450112	0.868906686	2.325777	0.82895
LOC100513950	4.14242951	0.8262784	2.362450112	0.868906686	2.325777	0.82895
LOC102166971	4.14242951	0.8262784	2.362450112	0.868906686	2.325777	0.82895
LOC100738463	4.14242951	0.8262784	2.362450112	0.868906686	2.325777	0.82895
LOC100736578	4.14242951	0.8262784	2.362450112	0.868906686	2.325777	0.82895
C12H17orf100	4.14242951	0.8262784	2.362450112	0.868906686	2.325777	0.82895
TRIM42	4.14242951	0.8262784	2.362450112	0.868906686	2.325777	0.82895
LOC102166555	4.14242951	0.8262784	2.362450112	0.868906686	2.325777	0.82895
LOC102164903	4.14242951	0.8262784	2.362450112	0.868906686	2.325777	0.82895
ADAM5	4.14242951	0.8262784	2.362450112	0.868906686	2.325777	0.82895
LOC102163359	4.14242951	0.8262784	2.362450112	0.868906686	2.325777	0.82895
LOC100739133	4.14242951	0.8262784	2.362450112	0.868906686	2.325777	0.82895
NIT1	3116.4878	1265.0322	11.6061682	10.30609837	1.300747	0.829
LOC100738753	88.3718295	171.03962	6.481748252	7.426597064	-0.95267	0.82902
LOC102159308	15.1889082	27.267186	4.016933786	4.821056379	-0.84415	0.82904
LOC100524051	26.2353869	14.873011	4.767410456	3.98850389	0.818817	0.82918
LOC102165280	26.2353869	14.873011	4.767410456	3.98850389	0.818817	0.82918
LOC102164619	27.6161967	15.699289	4.838760036	4.061714779	0.814815	0.82922
ZC2HC1C	24.854577	14.046732	4.692347799	3.911378308	0.823277	0.82923
C5H12orf39	202.979046	104.93735	7.672277146	6.727067559	0.951802	0.82923
LOC100517819	680.739249	315.63834	9.413076234	8.306692135	1.10883	0.82925
SLC2A11	243.022531	524.68677	7.930870551	9.038059606	-1.11037	0.82932
LOC102158883	28.9970066	16.525567	4.906746634	4.13138925	0.811204	0.82933
TMEM221	23.4737672	13.220454	4.613164285	3.829895615	0.828278	0.82936
ATF7IP2	67.659682	38.008805	6.101391269	5.285727902	0.831963	0.82937
LOC100738586	81.4677803	156.16661	6.365758672	7.296150961	-0.93878	0.82944
LOC102159356	30.3778164	17.351846	4.971673052	4.19785327	0.807929	0.8295
HSD17B12	7986.60409	3006.827	12.96354711	11.55450587	1.40934	0.82952
LOC100737831	140.842603	285.89232	7.148147109	8.164365521	-1.02139	0.82956
ANAPC4	2018.74398	851.06672	10.97995672	9.734822598	1.246114	0.8296
LOC100156118	22.0929574	12.394176	4.529381037	3.74353388	0.833924	0.82962
DNAH1	22.0929574	12.394176	4.529381037	3.74353388	0.833924	0.82962
LOC102160998	22.0929574	12.394176	4.529381037	3.74353388	0.833924	0.82962
LOC102165605	22.0929574	12.394176	4.529381037	3.74353388	0.833924	0.82962
LOC100521170	9.66566885	18.178124	3.414902536	4.261389712	-0.91126	0.82967
LOC100737927	9.66566885	18.178124	3.414902536	4.261389712	-0.91126	0.82967
LOC100737746	1010.7528	452.80055	9.982641126	8.82591454	1.158483	0.82972
SLC25A35	117.368836	63.623435	6.88714549	6.013985524	0.883419	0.82987
WDR90	1899.99433	805.62141	10.89253852	9.655747894	1.237821	0.82988
MST1R	245.784151	530.47071	7.947105933	9.053846388	-1.10988	0.82988
YAP1	1719.10825	4592.4552	10.74828364	12.16536404	-1.41761	0.8299
PEX7	776.015128	356.12598	9.601798876	8.480289273	1.123697	0.82993
TASP1	534.373406	253.66746	9.064401668	7.992470822	1.07491	0.82995
HSPA14	3388.50734	1368.317	11.72685988	10.41924074	1.308247	0.82995
LOC100624234	33.1394361	19.004403	5.093367322	4.322245637	0.802215	0.82997
LOC100737971	693.166538	321.42229	9.439138011	8.33280766	1.108732	0.83002
GNAO1	44.1859147	80.149002	5.497801224	6.342501448	-0.8591	0.83003
LOC100624586	8311.0944	3122.506	13.02099632	11.60895057	1.412334	0.83013
NAF1	1644.54451	706.46801	10.68434934	9.4665211	1.218992	0.83016
LOC100737149	255.44982	129.7257	8.002532746	7.030399034	0.977576	0.83016
LOC100625160	95.2758787	185.08636	6.58910248	7.539828464	-0.95802	0.83019
POLR3E	2967.36034	1212.1504	11.53545052	10.24454267	1.291611	0.83019
HRH1	49.7091541	90.890621	5.664174303	6.521845711	-0.87062	0.83022
CSNK1G1	82.8485901	158.64545	6.389714621	7.318727602	-0.93726	0.83023
LOC100623064	345.202459	773.39656	8.435472161	9.596928726	-1.16377	0.83025
LOC100515532	8740.52626	3272.0624	13.09366948	11.67642537	1.41752	0.83033
ST3GAL1	800.869705	1969.8476	9.647224023	10.94460053	-1.29844	0.83035
MFN2	339.67922	759.34982	8.41227014	9.57051952	-1.16059	0.83038
LOC100623601	249.92658	127.24687	7.971121491	7.002779797	0.973874	0.83039
DNAJC21	2325.28376	970.05081	11.18381137	9.923402974	1.261275	0.83041
NUFIP1	1369.76336	598.22554	10.42076382	9.226955308	1.195165	0.83043
LOC100620396	550.943125	261.10397	9.108375801	8.033995371	1.077279	0.83044
EPHA5	13.8080984	24.788351	3.888314478	4.688647628	-0.84415	0.83046
LOC102162411	13.8080984	24.788351	3.888314478	4.688647628	-0.84415	0.83046
LOC102162669	56.6132033	32.224857	5.848327568	5.054191063	0.812965	0.83055
LOC100739342	118.749646	64.449713	6.90387758	6.032314961	0.881677	0.83055
SLC5A7	19.3313377	10.741619	4.345633235	3.553559423	0.84773	0.83056
LOC102162338	19.3313377	10.741619	4.345633235	3.553559423	0.84773	0.83056
EMX2	45.5667246	82.627837	5.541227504	6.385911347	-0.85865	0.83066
TMEM106A	291.350875	639.53946	8.191557101	9.323143639	-1.13428	0.83069
RALGPS1	23.4737672	41.313919	4.613164285	5.403060391	-0.81558	0.83079
LOC102165111	23.4737672	41.313919	4.613164285	5.403060391	-0.81558	0.83079
LOC100524911	66.2788721	123.94176	6.072081615	6.965111899	-0.90304	0.83082
SPATA6L	28.9970066	51.229259	4.906746634	5.706786332	-0.82106	0.83093
TMEM37	37.2818656	21.483238	5.258589232	4.490777898	0.795263	0.83094
LOC100621997	37.2818656	21.483238	5.258589232	4.490777898	0.795263	0.83094
LOC102164826	447.382387	216.48493	8.808585798	7.764771649	1.047242	0.83121
LOC100522725	17115.1379	6094.6293	14.06306959	12.57355945	1.489663	0.83123
ZDHHC14	588.22499	277.62953	9.202674809	8.122204372	1.083207	0.83125
LOC102158168	111.845597	61.1446	6.818206315	5.957557119	0.871211	0.83138
SBSN	20.7121475	36.356248	4.440430524	5.223277669	-0.81173	0.83139
LOC100737242	20.7121475	36.356248	4.440430524	5.223277669	-0.81173	0.83139
HNRNPAB	14907.223	5365.8517	13.86382068	12.38986032	1.474132	0.83143
LOC100623187	2038.07532	5521.1921	10.99369935	12.43102536	-1.43777	0.8315
LOC100737291	531.611787	1246.0278	9.056940546	10.28427789	-1.22889	0.8315
DFFB	167.07799	88.411786	7.392987005	6.482393107	0.918211	0.83153
CBX1	9742.9942	3624.0569	13.25029756	11.82378794	1.42676	0.83153
FUCT1	139.461793	280.93465	7.13403395	8.139216969	-1.01036	0.83162
LOC100739748	505.3764	242.09956	8.984066358	7.925403489	1.061758	0.83167
HGF	225.072003	478.41518	7.820638529	8.905131772	-1.08788	0.83168
GATSL3	519.184498	1212.9767	9.022879597	10.24552496	-1.22423	0.83172
LOC100513021	103.560738	57.013208	6.708197415	5.858309486	0.861109	0.83172
LOC100511482	1003.84875	451.97427	9.972762649	8.823285292	1.151229	0.8318
LOC100626988	295.493305	148.73011	8.211855717	7.226220529	0.990429	0.8318
NUP210L	459.809675	222.26888	8.848027198	7.80263838	1.048731	0.83183
GOLGB1	2497.88499	6904.3821	11.28706879	12.75350553	-1.46681	0.83184
LOC100620138	149.127462	302.41788	7.230044098	8.245162313	-1.02	0.83186
LOC100739201	407.338902	199.13309	8.673623206	7.644815895	1.032497	0.83188
RMDN1	1840.61951	785.79073	10.84675931	9.619836152	1.227974	0.83193
LOC100737713	73.1829213	41.313919	6.213015177	5.403060391	0.824879	0.83204
LOC100623685	34.5202459	61.1446	5.150569664	5.957557119	-0.82478	0.83207
LOC100625613	266.496298	576.7423	8.063375117	9.174282325	-1.11381	0.8321
TLR9	5208.4147	2038.4287	12.34690557	10.99394939	1.353387	0.83214
LOC100518738	191.932567	399.91873	7.591952882	8.647166015	-1.05911	0.83229
LOC100156373	16.569718	9.0890621	4.135019133	3.334720158	0.866346	0.83232
C15H2orf62	16.569718	9.0890621	4.135019133	3.334720158	0.866346	0.83232
GLI2	1123.97921	501.55097	10.13568262	8.97312612	1.164147	0.83233
LOC100739659	349.344888	779.1805	8.452632037	9.607664138	-1.15731	0.83234
LOC102164769	12.4272885	22.309516	3.747096094	4.542847146	-0.84415	0.83237
CLC-5	121.511266	240.44701	6.936770608	7.915562765	-0.98463	0.83243
LAMA3	35.9010557	63.623435	5.205590187	6.013985524	-0.82553	0.83245
LOC100628140	216.787144	457.75822	7.766774985	8.841590191	-1.07831	0.83247
OBFC1	370.057036	830.40976	8.535497154	9.69941588	-1.16608	0.83251
ASB7	1099.12463	491.63563	10.10345126	8.944377168	1.160694	0.83252
CDC40	1825.4306	780.83306	10.83481122	9.610716783	1.225151	0.83255
EIF4EBP3	371.437846	833.71488	8.540855872	9.705139674	-1.16643	0.83257
MED27	2178.91792	917.99527	11.0900581	9.843913627	1.247053	0.8326
DYNC1I2	3300.13551	9365.8653	11.68874665	13.19335061	-1.50489	0.83266
LOC102165481	74.5637311	42.140197	6.239622035	5.430960858	0.823277	0.83268
LOC102159980	17.9505279	31.398578	4.24416613	5.017858594	-0.80667	0.83272
LOC102162823	17.9505279	31.398578	4.24416613	5.017858594	-0.80667	0.83272
KIFC1	2506.16985	1043.5896	11.29184402	10.02872051	1.26393	0.83275
LOC100517121	1364.24012	3531.5138	10.414939	11.78647947	-1.37219	0.83291
ERC1	2185.82197	5944.2466	11.09462006	12.53752094	-1.44332	0.83296
CCDC138	643.457384	302.41788	9.331941149	8.245162313	1.089301	0.83298
C9H11orf42	45.5667246	81.801559	5.541227504	6.371586023	-0.84415	0.83314
LOC102157541	5.52323934	11.567897	2.705588563	3.65167138	-1.06654	0.83321
LOC102163801	5.52323934	11.567897	2.705588563	3.65167138	-1.06654	0.83321
LOC100738493	5.52323934	11.567897	2.705588563	3.65167138	-1.06654	0.83321
LOC102160237	5.52323934	11.567897	2.705588563	3.65167138	-1.06654	0.83321
LOC100739730	135.319364	270.19303	7.090846698	8.083176277	-0.99762	0.83331
CDC14A	67.659682	125.59431	6.101391269	6.98406878	-0.8924	0.83332
PITRM1	4570.48056	1812.0285	12.15844577	10.82418586	1.33474	0.83337
RBM17	6053.47032	2345.8043	12.56378503	11.19648182	1.36768	0.83338
LOC102161377	8.28485901	15.699289	3.214880004	4.061714779	-0.92215	0.83343
LOC100621502	8.28485901	15.699289	3.214880004	4.061714779	-0.92215	0.83343
NACAD	8.28485901	15.699289	3.214880004	4.061714779	-0.92215	0.83343
LOC100739447	8.28485901	15.699289	3.214880004	4.061714779	-0.92215	0.83343
LOC102167723	8.28485901	15.699289	3.214880004	4.061714779	-0.92215	0.83343
TTC31	555.085554	1300.5622	9.119163049	10.34602849	-1.22835	0.83346
LIN7B	129.796125	257.79885	7.031175985	8.015687408	-0.99	0.8335
MOK	56.6132033	103.2848	5.848327568	6.704385035	-0.86742	0.83355
ANKDD1A	15.1889082	8.2627837	4.016933786	3.211445828	0.878318	0.83363
DCAF12L2	15.1889082	8.2627837	4.016933786	3.211445828	0.878318	0.83363
ST8SIA2	15.1889082	8.2627837	4.016933786	3.211445828	0.878318	0.83363
GSG2	636.553334	299.93905	9.316402225	8.233327508	1.085612	0.83366
HFM1	64.8980623	119.81036	6.042164139	6.916600414	-0.88451	0.83372
BAK	4979.20027	1961.5849	12.28198804	10.93853932	1.343894	0.83373
GAB3	16.569718	28.919743	4.135019133	4.903025878	-0.80351	0.83377
LOC102164480	16.569718	28.919743	4.135019133	4.903025878	-0.80351	0.83377
LOC100625861	2953.55224	1215.4555	11.52872379	10.24846781	1.280954	0.83385
TMEM104	474.998584	1092.34	8.89481347	10.0945264	-1.20143	0.83388
SLA-7	951.377977	2363.1561	9.895390449	11.20710961	-1.31262	0.83392
CHST6	863.006147	395.78734	9.754897767	8.632222186	1.124645	0.83398
MRPL18	4254.2751	1698.8283	12.05503669	10.73117334	1.324373	0.83406
COQ6	2082.26123	883.29158	11.02462804	9.788378341	1.237189	0.83407
TMEM62	574.416892	273.49814	9.168463763	8.100652569	1.070567	0.83407
CEP85L	62.1364426	114.02642	5.98040107	6.845821397	-0.87586	0.83422
MST1	99.4183082	55.360651	6.649878514	5.816616368	0.844651	0.83425
C14H1orf131	1423.61494	624.66645	10.47635631	9.289249932	1.188401	0.83432
LOC100620629	53.8515836	97.500848	5.777461367	6.622064238	-0.85643	0.83437
RAD54B	2974.26439	1224.5445	11.53880216	10.25920721	1.280288	0.83439
LYPLAL1	919.619351	419.74941	9.846460957	8.716817448	1.131509	0.83444
LOC100523337	78.7061606	44.619032	6.316619332	5.511563931	0.818817	0.83454
LOC100525950	1528.55649	666.80665	10.57889767	9.383286641	1.196829	0.83454
LOC102161965	118.749646	65.275991	6.90387758	6.05041444	0.863299	0.8346
LOC100738939	118.749646	65.275991	6.90387758	6.05041444	0.863299	0.8346
LOC100623713	745.637311	347.03692	9.544263795	8.44309653	1.103385	0.83461
LOC100525112	70.4213016	130.55198	6.158282521	7.039489182	-0.89054	0.83469
LOC100626268	303.778164	153.68778	8.251615733	7.273215394	0.983016	0.83474
LOC100622298	342.440839	756.87099	8.423917793	9.565808471	-1.14419	0.83474
CCDC66	944.473928	430.49103	9.884893865	8.753186764	1.133528	0.8349
LOC100525731	962.424456	437.92754	9.912027735	8.777838973	1.135981	0.8349
LOC100516938	202.979046	422.22825	7.672277146	8.725292112	-1.05669	0.8349
LOC102158722	11.0464787	19.830681	3.590539588	4.380638094	-0.84415	0.83492
LOC102165046	11.0464787	19.830681	3.590539588	4.380638094	-0.84415	0.83492
LOC102167526	110.464787	61.1446	6.800444207	5.957557119	0.85329	0.83493
LOC100515167	33.1394361	57.839486	5.093367322	5.878712739	-0.80351	0.83493
DDX39A	11203.891	4147.0911	13.451841	12.01823188	1.433828	0.83497
GABBR1	838.15157	2047.5178	9.712787608	11.00036472	-1.28859	0.83501
DCUN1D5	3255.94959	1331.9607	11.66930568	10.38041857	1.289527	0.83503
ENKUR	470.856154	228.87911	8.882203309	7.844731552	1.040701	0.83503
LOC102160626	23.4737672	40.48764	4.613164285	5.374609695	-0.78643	0.83506
HAUS8	963.805265	438.75382	9.91409397	8.780552285	1.13533	0.83516
LOC100625801	120.130456	66.10227	6.920417836	6.068289661	0.86183	0.83521
LOC102164485	13.8080984	7.4365053	3.888314478	3.076645515	0.892818	0.83531
LOC102164472	13.8080984	7.4365053	3.888314478	3.076645515	0.892818	0.83531
GAL3ST1	13.8080984	7.4365053	3.888314478	3.076645515	0.892818	0.83531
LOC100521442	71.8021115	133.03082	6.185908388	7.066420948	-0.88966	0.83535
C3H16orf91	1887.56705	809.7528	10.88307629	9.663118299	1.220974	0.8354
LOC102167165	22.0929574	38.008805	4.529381037	5.285727902	-0.78275	0.83549
LOC102165909	64.8980623	118.98409	6.042164139	6.906699252	-0.87452	0.83551
LOC100513197	1228.92075	547.82256	10.26434965	9.100195978	1.165611	0.83559
LRRK1	747.018121	1798.808	9.54692941	10.81362731	-1.26783	0.83565
ZNF71	146.365843	292.50254	7.203258356	8.197229196	-0.99887	0.83572
BACH2	69.0404918	39.661362	6.130117309	5.34558663	0.799709	0.83581
LOC102161921	2.76161967	7.4365053	1.911353989	3.076645515	-1.42911	0.83583
P2X3R	2.76161967	7.4365053	1.911353989	3.076645515	-1.42911	0.83583
C3H2orf74	2.76161967	7.4365053	1.911353989	3.076645515	-1.42911	0.83583
LOC102161304	2.76161967	7.4365053	1.911353989	3.076645515	-1.42911	0.83583
AGAP2	2.76161967	7.4365053	1.911353989	3.076645515	-1.42911	0.83583
LOC100738157	2.76161967	7.4365053	1.911353989	3.076645515	-1.42911	0.83583
LOC100156825	2.76161967	7.4365053	1.911353989	3.076645515	-1.42911	0.83583
LOC102164040	2.76161967	7.4365053	1.911353989	3.076645515	-1.42911	0.83583
LOC102162727	2.76161967	7.4365053	1.911353989	3.076645515	-1.42911	0.83583
LOC102160250	2.76161967	7.4365053	1.911353989	3.076645515	-1.42911	0.83583
SYNE4	2.76161967	7.4365053	1.911353989	3.076645515	-1.42911	0.83583
KRT25	2.76161967	7.4365053	1.911353989	3.076645515	-1.42911	0.83583
LOC102165894	2.76161967	7.4365053	1.911353989	3.076645515	-1.42911	0.83583
LOC102167981	2.76161967	7.4365053	1.911353989	3.076645515	-1.42911	0.83583
BRI3	1255.15614	3196.871	10.29480009	11.64289604	-1.34879	0.83589
LOC100154339	1003.84875	456.10566	9.972762649	8.836383876	1.138102	0.83596
DUSP3	1007.99118	2506.1023	9.978697848	11.29180515	-1.31396	0.83604
LOC102159873	38.6626754	67.754826	5.309710091	6.103389087	-0.80938	0.83606
LOC102163571	20.7121475	35.52997	4.440430524	5.191008663	-0.77856	0.83608
LOC100515435	3712.99765	1505.4792	11.85875719	10.55696503	1.302362	0.83613
PAIP2	5538.42825	2173.1121	12.43552136	11.08621063	1.349714	0.83624
LOC100521766	118.749646	231.35794	6.90387758	7.86020516	-0.9622	0.83626
LFNG	791.204036	367.69388	9.629728241	8.526279641	1.105545	0.83633
LOC100520194	70.4213016	40.48764	6.158282521	5.374609695	0.79853	0.83636
ZBTB40	222.310384	465.19472	7.802906526	8.864788865	-1.06526	0.83637
C10H1orf53	220.929574	115.67897	7.79395812	6.86640077	0.93346	0.83654
ZNF575	179.505279	366.04132	7.495897217	8.51979867	-1.02798	0.83671
HECTD4	1278.62991	3256.3631	10.3215109	11.66948881	-1.34866	0.83673
PFKFB4	421.147	947.74129	8.721601651	9.889870929	-1.17017	0.83675
REXO2	3440.97811	9716.2074	11.74902221	13.24632604	-1.49757	0.83675
MAP3K14	544.039075	1260.0745	9.090215854	10.30043781	-1.21173	0.83688
LOC100738789	71.8021115	41.313919	6.185908388	5.403060391	0.797398	0.83691
LOC100624208	278.923587	598.22554	8.128889246	9.226955308	-1.10082	0.83692
LOC100737714	277.542777	594.92043	8.121755095	9.218975892	-1.09999	0.83692
TWIST2	385.245944	857.6795	8.593375974	9.745971655	-1.15465	0.83693
CD274	13.8080984	23.962073	3.888314478	4.641665831	-0.79524	0.83698
LHX9	13.8080984	23.962073	3.888314478	4.641665831	-0.79524	0.83698
CDK3	49.7091541	88.411786	5.664174303	6.482393107	-0.83073	0.83704
C4	42.8051049	75.191332	5.453027102	6.251554969	-0.81278	0.83716
LOC100152415	492.949111	1127.87	8.948218607	10.14066361	-1.19409	0.83725
INTS8	2600.06492	1089.0349	11.34488669	10.0901586	1.255497	0.83728
SMIM20	3215.90611	1322.8717	11.65145811	10.37054757	1.281552	0.83732
LOC100514616	647.599813	307.37555	9.341184798	8.268544594	1.0751	0.83744
LOC102157458	12.4272885	6.610227	3.747096094	2.927939483	0.91074	0.83746
DNALI1	12.4272885	6.610227	3.747096094	2.927939483	0.91074	0.83746
AGER	12.4272885	6.610227	3.747096094	2.927939483	0.91074	0.83746
LOC100516996	12.4272885	6.610227	3.747096094	2.927939483	0.91074	0.83746
LOC100737005	57.9940131	33.877413	5.882496648	5.124221138	0.77558	0.83748
SLC6A15	56.6132033	101.63224	5.848327568	6.681340183	-0.84415	0.83756
TLE3	2007.6975	860.98206	10.9720446	9.751514039	1.221487	0.83785
LRRC6	117.368836	65.275991	6.88714549	6.05041444	0.846425	0.83787
LOC102159690	77.3253508	142.94616	6.291407421	7.169385476	-0.88646	0.8379
ROBO3	17.9505279	30.5723	4.24416613	4.980587446	-0.7682	0.83792
LOC102167365	59.3748229	34.703692	5.915875148	5.158001345	0.774762	0.83794
EXPH5	151.889082	82.627837	7.256341575	6.385911347	0.878318	0.83796
LOC102160377	74.5637311	42.966475	6.239622035	5.458331975	0.795263	0.83797
LOC100625079	31.7586262	54.534373	5.033802952	5.795309087	-0.78002	0.83798
LOC100521079	31.7586262	54.534373	5.033802952	5.795309087	-0.78002	0.83798
LOC100624425	57.9940131	104.11107	5.882496648	6.715770874	-0.84415	0.83809
TAF13	3052.97055	1263.3796	11.57647043	10.30421398	1.272925	0.83811
LOC100738321	106.322357	203.26448	6.745806839	7.674294538	-0.93491	0.83813
RAB39A	27.6161967	47.097867	4.838760036	5.587901017	-0.77015	0.83815
TMOD3	324.490311	705.64173	8.34647079	9.464835137	-1.12076	0.83818
HOXD10	34.5202459	59.492043	5.150569664	5.918673475	-0.78525	0.83818
ACAA1	4388.21366	1761.6255	12.09974678	10.78351026	1.316727	0.8382

FAM107A	34.89284986	80.97528	5.165624571	6.357117027	-1.21455	0.70116
RMND5A	5675.903577	1736.0109	12.47088852	10.76239106	1.709074	0.70118
LOC100513913	8.458872693	20.656959	3.241668254	4.436758793	-1.28809	0.70122
LOC100153765	8.458872693	20.656959	3.241668254	4.436758793	-1.28809	0.70122
PROCA1	8.458872693	20.656959	3.241668254	4.436758793	-1.28809	0.70122
GAP43	935.7627916	336.2953	9.871539963	8.397868394	1.476414	0.70133
HSF2	2895.049179	939.47851	11.49987039	9.877251166	1.623656	0.70137
YIPF2	1267.773545	4198.3204	10.30921888	12.03594015	-1.72752	0.70151
LOC102167560	240.0205127	97.500848	7.913012126	6.622064238	1.299671	0.70163
LOC102158407	11.63094995	27.267186	3.658891241	4.821056378	-1.2292	0.70169
PDZK1	119.4815768	51.229259	6.912668746	5.706786332	1.221748	0.70171
LOC100524712	75.07249515	33.051135	6.249303021	5.089630977	1.183585	0.70174
METTL10	1668.512639	569.3058	10.7052113	9.15559189	1.551287	0.70186
ZNF200	280.2001579	112.37386	8.135453595	6.824944216	1.318151	0.70187
LOC100737596	185.0378402	76.843889	7.539452286	6.282511874	1.267818	0.70198
PDK1	5115.503261	1580.6705	12.32094246	10.62723339	1.694339	0.70207
LOC100625332	946.3363825	3034.0942	9.887732982	11.56752557	-1.68084	0.70229
LOC100517307	38.06492712	88.411786	5.287802017	6.482393107	-1.21578	0.70276
CCDC40	199.8408674	82.627837	7.64990905	6.385911347	1.274152	0.70294
MAPK10	44.40908164	104.11107	5.504908955	6.715770874	-1.2292	0.70295
ASH1L	1105.997605	3600.9211	10.11243638	11.81455088	-1.70302	0.703
LOC102159107	17.97510447	7.4365053	4.246035924	3.076645515	1.273303	0.70305
C3H2orf74	17.97510447	7.4365053	4.246035924	3.076645515	1.273303	0.70305
LOC102163574	17.97510447	7.4365053	4.246035924	3.076645515	1.273303	0.70305
DNAJB13	17.97510447	7.4365053	4.246035924	3.076645515	1.273303	0.70305
DPY19L3	8083.510217	2405.2963	12.98094466	11.23259861	1.748767	0.70322
LOC100512382	26.43397716	11.567897	4.777891883	3.651671381	1.192267	0.70337
LOC102160900	26.43397716	11.567897	4.777891883	3.651671381	1.192267	0.70337
PFKFB4	332.0107532	947.74129	8.379424954	9.889870929	-1.51326	0.70361
KIF26B	285.4869534	801.49002	8.16232563	9.648339641	-1.48926	0.70365
ETV5	8820.4895	2606.9083	13.10680656	11.34867741	1.758519	0.7037
LOC102165334	37.00756803	16.525567	5.24821481	4.131389251	1.163121	0.70374
ALMS1	3087.488533	999.79683	11.59268526	9.966933409	1.626727	0.70386
PAPOLG	1737.240979	592.44159	10.76341239	9.212962234	1.552053	0.70393
LOC102159805	102.5638314	44.619032	6.694376434	5.511563931	1.200791	0.70394
ADAMTSL1	23.2618999	52.881816	4.600620625	5.751726565	-1.1848	0.70397
UHRF2	58.15474976	138.81477	5.886422108	7.127372928	-1.25519	0.70397
RAPGEF1	1025.638314	3306.766	10.00371229	11.69164148	-1.6889	0.70402
MAN2C1	1001.319055	3220.0068	9.9691261	11.653296	-1.68516	0.70404
LOC100621769	145.9155539	61.970878	7.198843332	5.976612875	1.235471	0.70404
RWDD4	6643.387141	2012.8141	12.69792042	10.97571481	1.722705	0.70417
B3GAT2	59.21210885	26.440908	5.911981742	4.778256309	1.163121	0.7042
NOL9	2790.370629	912.21132	11.44675798	9.834804937	1.613017	0.7042
LOC100622764	632.3007338	1934.3177	9.306746941	10.91835468	-1.61314	0.70427
LOC102160806	112.0800632	284.23976	6.821200784	8.156031284	-1.34258	0.70441
LOC102165096	748.6102333	275.97698	9.549996838	8.113622246	1.439667	0.70445
LOC102157770	532.9089796	1598.8486	9.060450003	10.64371971	-1.58507	0.70472
LOC102164274	59.21210885	141.2936	5.911981742	7.15272698	-1.25473	0.70475
LOC100526148	186.0951992	77.670167	7.54762873	6.29774474	1.260608	0.70482
ORAI3	1379.853608	4583.3661	10.43134466	12.16250655	-1.73189	0.70498
KDM4B	42.29436346	98.327126	5.436107306	6.634115866	-1.21712	0.70505
LOC100625560	4923.063907	1532.7464	12.26563377	10.58284422	1.683437	0.70509
CCNB1IP1	2417.122872	801.49002	11.23967184	9.648339641	1.592534	0.70511
LRFN2	10.57359087	24.788351	3.532764645	4.688647628	-1.2292	0.70512
LOC100522498	9330.13658	2749.8544	13.1878371	11.42566408	1.762543	0.70516
BMP4	247.4220263	681.67966	7.956649285	9.415064951	-1.46212	0.70526
FAIM	536.0810569	204.09076	9.068996028	7.68011867	1.39324	0.70535
NDC80	2203.536336	737.04031	11.10625954	9.5275558	1.580005	0.70536
KDELC1	1193.758409	422.22825	10.22250321	8.725292112	1.499416	0.70539
LOC100625892	976.999796	351.99459	9.933690354	8.463502248	1.472805	0.70541
LOC100510960	431.4025073	1262.5534	8.756231078	10.30327087	-1.54924	0.70564
TAF11	3817.066303	1216.2818	11.89862644	10.24944743	1.649987	0.70571
UTP11L	6289.171847	1918.6184	12.61888372	10.90660382	1.712802	0.70573
LOC100623683	57.09739067	25.61463	5.860401464	4.734147579	1.156457	0.70573
ELAC1	602.6946793	227.22655	9.237675276	7.834322837	1.407296	0.70596
CAND2	526.5648251	1574.0603	9.043204567	10.62119135	-1.57981	0.706
GALR3	21.14718173	9.0890621	4.46905122	3.334720158	1.218262	0.70608
LOC102158672	739.0940015	2292.0962	9.531564713	11.16308117	-1.63284	0.70609
LOC100626319	296.0605442	830.40976	8.214613188	9.69941588	-1.48793	0.7062
LOC102160361	67.67098154	162.77684	6.10162868	7.35558754	-1.26629	0.70629
LOC100515340	366.903603	1053.5049	8.523183995	10.04235012	-1.52172	0.70631
ATP6V1E2	5.286795433	14.046732	2.65232482	3.911378308	-1.40977	0.70634
TSSK6	5.286795433	14.046732	2.65232482	3.911378308	-1.40977	0.70634
CENPB	1758.388161	5977.2977	10.78085809	12.54551903	-1.76524	0.70635
LOC100512939	466.2953572	180.12869	8.868190893	7.500871231	1.372216	0.70642
HUS1	1186.356895	420.57569	10.21353793	8.719647871	1.496101	0.7065
INTS4	3399.409463	1095.6451	11.73149276	10.09888102	1.633504	0.70652
ARHGEF10	315.0930078	889.07553	8.304205312	9.797783952	-1.49653	0.70666
LOC100522493	922.0171235	334.64274	9.850213602	8.390782629	1.462172	0.70668
CCDC151	76.12985423	33.877413	6.269217479	5.124221138	1.168139	0.70678
XPOT	9468.65062	2793.6472	13.20909548	11.44845044	1.761009	0.70687
LOC102163333	109.965345	47.924146	6.793965376	5.61247475	1.198224	0.70688
SKAP1	47.5811589	21.483238	5.602325001	4.490777899	1.147179	0.70713
RPL36AL	15561.15368	4405.7163	13.92575411	12.1054883	1.820501	0.70716
TMOD4	7.401513606	18.178124	3.070649265	4.261389711	-1.29631	0.7072
ASIC4	7.401513606	18.178124	3.070649265	4.261389711	-1.29631	0.7072
DLEC1	7.401513606	18.178124	3.070649265	4.261389711	-1.29631	0.7072
LOC102165088	7.401513606	18.178124	3.070649265	4.261389711	-1.29631	0.7072
TTBK1	34.89284986	15.699289	5.165624571	4.061714779	1.152232	0.70723
NEK5	34.89284986	15.699289	5.165624571	4.061714779	1.152232	0.70723
HSD17B6	149.0876312	63.623435	7.229661278	6.013985524	1.22853	0.70725
LOC102158312	29.60605442	13.220454	4.935745167	3.829895615	1.163121	0.70737
LOC102158777	46.52379981	108.24247	5.570578288	6.771389985	-1.21823	0.70745
LOC102160428	40.17964529	92.543178	5.363859498	6.547560532	-1.20366	0.7075
CHST2	347.8711395	990.70777	8.446550445	9.953771246	-1.50991	0.70752
SPIN2B	185.0378402	77.670167	7.539452286	6.29774474	1.252388	0.70752
NXPH2	2988.096779	975.83476	11.54549389	9.931970724	1.614518	0.70767
LOC102158271	74.01513606	33.051135	6.229109817	5.089630977	1.163121	0.70773
LOC100739409	74.01513606	33.051135	6.229109817	5.089630977	1.163121	0.70773
LOC100513290	538.1957751	205.74331	9.074665382	7.691696868	1.387286	0.708
LOC102168147	103.6211905	45.44531	6.709031281	5.537461031	1.189116	0.70803
PLXNA1	1357.649067	4478.4288	10.40795715	12.12909905	-1.72188	0.70804
SLC5A3	566.7444704	1701.3072	9.14909794	10.73327567	-1.58587	0.70809
LOC102157962	180.8084038	478.41518	7.506275077	8.905131772	-1.4038	0.70831
C7H14orf169	3062.111915	998.97055	11.58078236	9.965741799	1.616013	0.70846
LOC100155725	28.54869534	64.449713	4.885022527	6.032314961	-1.17475	0.70858
C14H1orf131	1827.116502	624.66645	10.8361423	9.289249932	1.548411	0.70863
LOC102159205	79.30193149	192.52286	6.327362784	7.59636019	-1.2796	0.70866
STAG2	10676.1547	3125.8111	13.38223962	11.61047634	1.77209	0.70869
LOC102160004	210.4144582	87.585507	7.723930233	6.468998788	1.26447	0.70871
LOC100626090	3.17207726	9.9153405	2.060765874	3.448285225	-1.64423	0.70876
LOC100626400	3.17207726	9.9153405	2.060765874	3.448285225	-1.64423	0.70876
LOC102164595	3.17207726	9.9153405	2.060765874	3.448285225	-1.64423	0.70876
LOC102166102	3.17207726	9.9153405	2.060765874	3.448285225	-1.64423	0.70876
LOC102160600	3.17207726	9.9153405	2.060765874	3.448285225	-1.64423	0.70876
LOC102167859	3.17207726	9.9153405	2.060765874	3.448285225	-1.64423	0.70876
LOC100627942	3.17207726	9.9153405	2.060765874	3.448285225	-1.64423	0.70876
LOC100521923	3.17207726	9.9153405	2.060765874	3.448285225	-1.64423	0.70876
GIGYF1	478.9836662	1409.6309	8.906841502	10.46212483	-1.55727	0.70882
MAP2	231.56164	628.79784	7.86146934	9.298745001	-1.4412	0.70889
MTCH2	16108.86568	4558.5778	13.97565685	12.15468452	1.821199	0.70896
LOC102159311	5385.129828	1673.2137	12.39503329	10.70926798	1.686359	0.70921
PRMT10	1051.014932	378.43549	10.03893947	8.567710832	1.473664	0.70926
NDUFA3	777.1589286	2409.4277	9.603921026	11.23507346	-1.63241	0.70929
TWISTNB	2979.637906	975.83476	11.54140541	9.931970724	1.610428	0.70943
LOC102159750	9.516231779	22.309516	3.394545939	4.542847146	-1.2292	0.7095
LOC102167010	9.516231779	22.309516	3.394545939	4.542847146	-1.2292	0.7095
ZFYVE16	2199.3069	740.34542	11.10348905	9.534002096	1.570778	0.70951
LOC100620687	664.0215064	2022.7295	9.377257187	10.98280072	-1.607	0.70956
LOC100523020	850.1167056	312.33322	9.733213158	8.291553945	1.444575	0.70957
NDUFA5	5993.111303	1845.9059	12.54933016	10.85089463	1.698976	0.7096
LOC100522201	696.7996381	2133.4508	9.446669039	11.05964916	-1.61437	0.70964
SSH1	1144.062532	3693.4643	10.16121067	11.85114948	-1.69081	0.70996
HYAL3	314.0356487	879.98647	8.29937128	9.782976046	-1.48655	0.71003
SLC15A2	169.1774539	71.886218	7.410896102	6.187574144	1.23475	0.71004
ZDHHC23	158.603863	67.754826	7.31835176	6.103389087	1.227032	0.71015
LOC100526020	3430.072877	1110.5181	11.74444405	10.11831577	1.627006	0.71017
LOC102167226	152.2597085	65.275991	7.259834656	6.05041444	1.22191	0.71032
LOC100511541	148.0302721	63.623435	7.219461601	6.013985524	1.218262	0.71049
PDDC1	2372.71379	8261.1312	11.21293028	13.01229825	-1.7998	0.71062
APBA1	70.8430588	169.38707	6.166776871	7.412672017	-1.25763	0.71068
ADARB1	74.01513606	177.64985	6.229109817	7.480990892	-1.26314	0.71079
LOC102160104	74.01513606	177.64985	6.229109817	7.480990892	-1.26314	0.71079
TGFBR1	14623.27617	4181.7948	13.8360776	12.03025152	1.806072	0.7108
IQSEC1	541.3678523	1608.764	9.083127858	10.65263347	-1.57127	0.71084
TNFRSF19	803.5929058	297.46021	9.652115207	8.221394816	1.433768	0.71094
MOB3C	230.5042809	623.01389	7.854895061	9.285434338	-1.43447	0.71095
C14H10orf84	11719.76812	3415.0085	13.5167795	11.73809585	1.778983	0.71097
TMEM183A	5867.285571	1814.5073	12.51872336	10.82615702	1.693115	0.71118
HPCA	21.14718173	47.097867	4.46905122	5.587901017	-1.1552	0.7113
GPR64	485.3278207	188.39147	8.925785314	7.565227535	1.365226	0.71133
GYLTL1B	1031.982468	373.47782	10.01260005	8.548736477	1.466324	0.71137
LOC100622470	1355.534349	478.41518	10.40570986	8.905131772	1.502527	0.71147
LOC100739863	75.07249515	180.12869	6.249303021	7.500871231	-1.26267	0.7115
MAP7D3	48.63851798	112.37386	5.633388138	6.824944216	-1.20814	0.71165
LOC100623030	335.1828304	134.68337	8.393102235	7.084100147	1.315376	0.71169
GPX1	6497.471587	24994.921	12.66588473	14.60940505	-1.94368	0.71173
LOC100625686	128.9978086	56.186929	7.022343493	5.837613535	1.19904	0.71191
PPHLN1	7111.797216	2165.6756	12.79620132	11.08126746	1.715397	0.71205
TIGD2	1144.062532	410.66035	10.16121067	8.685310691	1.478148	0.71222
PYGM	20.08982264	44.619032	4.398475058	5.511563931	-1.15119	0.71229
ARHGAP32	27.49133625	12.394176	4.832451381	3.74353388	1.149315	0.71238
LOC100739483	307.6914942	124.76803	8.270021921	6.974621476	1.302236	0.71244
USF2	1607.185812	5355.1101	10.65121839	12.38696991	-1.73638	0.71256
SMARCD3	957.9673324	3019.2212	9.90533786	11.56043849	-1.65613	0.71276
TDP1	1637.849225	569.3058	10.67846742	9.15559189	1.524527	0.71287
LOC100514246	11486.09176	3361.3004	13.48772597	11.71523292	1.772797	0.71288
LOC100622468	1170.496509	419.74941	10.19413694	8.716817448	1.47952	0.71295
SLC25A5	18943.6454	5317.9276	14.20950252	12.37691968	1.832778	0.71301
LOC102168106	15.8603863	6.610227	4.075565686	2.927939483	1.262656	0.71329
LOC102157752	15.8603863	6.610227	4.075565686	2.927939483	1.262656	0.71329
LOC100738971	15.8603863	6.610227	4.075565686	2.927939483	1.262656	0.71329
CD160	15.8603863	6.610227	4.075565686	2.927939483	1.262656	0.71329
LOC102162817	15.8603863	6.610227	4.075565686	2.927939483	1.262656	0.71329
LOC102167390	15.8603863	6.610227	4.075565686	2.927939483	1.262656	0.71329
MIR1839	15.8603863	6.610227	4.075565686	2.927939483	1.262656	0.71329
GCNT4	15.8603863	6.610227	4.075565686	2.927939483	1.262656	0.71329
PARP1	15161.47194	4337.1352	13.88821736	12.08285929	1.805596	0.71329
LIMK1	760.2411832	2334.2364	9.572209803	11.18935289	-1.61842	0.71332
LOC102161904	2121.062328	720.51474	11.05125132	9.494885058	1.557687	0.71332
NRP1	677.7671745	2054.9543	9.406772987	11.00559249	-1.60024	0.71333
LOC100513472	153.3170676	66.10227	7.269753823	6.068289661	1.213747	0.71336
RBM26	7320.096956	2227.6465	12.83784412	11.12195208	1.716342	0.71336
ST3GAL3	83.53136784	201.61192	6.40141489	7.662575262	-1.27119	0.71339
RIBC1	19.03246356	42.140197	4.324267947	5.430960858	-1.14673	0.7135
RNPC3	1073.219473	388.35083	10.06907306	8.604926909	1.466513	0.71354
LOC100526222	12.68830904	4.9576702	3.774872332	2.57474827	1.355766	0.71364
LOC102165102	12.68830904	4.9576702	3.774872332	2.57474827	1.355766	0.71364
LRRC8B	253.7661808	104.93735	7.993029968	6.727067559	1.273972	0.71372
MTHFD2	3687.011135	1192.3197	11.8486273	10.22076488	1.628681	0.71377
LOC100511492	114.1947813	284.23976	6.84793155	8.156031284	-1.31561	0.71386
PRCP	4560.38974	1447.6397	12.15525773	10.50048311	1.655455	0.71398
BTF3	55650.92344	14343.366	15.76414393	13.80819661	1.956022	0.71404
PAK4	401.7964529	1147.7007	8.653907169	10.16578718	-1.51421	0.71426
ETV3L	19.03246356	8.2627837	4.324267947	3.211445828	1.203763	0.71427
ITPA	901.9273008	332.16391	9.818466024	8.380088299	1.441116	0.71438
FAM73A	1048.900214	380.91433	10.0360365	8.57710524	1.461339	0.71443
LOC100626323	2982.809983	984.09754	11.54293995	9.944122772	1.599799	0.71446
ECHDC2	144.8581949	369.34643	7.188422634	8.532731628	-1.35033	0.71446
NKIRAS1	468.4100754	183.4338	8.874704998	7.526959252	1.352513	0.71448
LOC100621480	108.9079859	269.36675	6.780152406	8.078773924	-1.30646	0.71448
LOC100736569	94.1049587	42.140197	6.571448659	5.430960858	1.159074	0.71472
LOC102157815	380.6492712	152.03522	8.576103626	7.257719911	1.324057	0.71476
CCDC3	361.6168076	1019.6275	8.502301987	9.995240719	-1.49551	0.71488
LOC100738489	54.9826725	127.24687	5.806908455	7.002779797	-1.21058	0.71495
LOC102168128	53.92531342	24.788351	5.779399292	4.688647628	1.1213	0.71502
COX2	610010.5469	131541.04	19.21847703	17.0051644	2.213321	0.71509
PITPNM3	8.458872693	19.830681	3.241668254	4.380638094	-1.2292	0.71512
LOC102158020	8.458872693	19.830681	3.241668254	4.380638094	-1.2292	0.71512
BCL2L11	8.458872693	19.830681	3.241668254	4.380638094	-1.2292	0.71512
LOC102158547	6.344154519	15.699289	2.876596413	4.061714779	-1.3072	0.71513
LOC100155163	80.35929058	36.356248	6.346235194	5.223277669	1.144261	0.71517
LOC100626268	384.8787075	153.68778	8.592003629	7.273215395	1.324401	0.71519
LOC102159436	42.29436346	95.848291	5.436107306	6.597654688	-1.18029	0.71527
TSEN15	3396.237386	1108.8656	11.73014632	10.11616923	1.614853	0.71528
LOC100625017	60.26946793	140.46732	5.937096418	7.144325041	-1.22074	0.71529
LOC102163516	226.2748445	604.83577	7.828294201	9.242782946	-1.41847	0.71537
LOC100514541	243.1925899	655.23875	7.931875612	9.358076973	-1.42992	0.71542
LOC102159936	50.75323616	116.50525	5.693577173	6.876581411	-1.19882	0.71559
LOC102164617	13.74566813	30.5723	3.882219287	4.980587447	-1.15325	0.71565
LOC102167836	13.74566813	30.5723	3.882219287	4.980587447	-1.15325	0.71565
RARRES1	1243.454286	445.36404	10.28129752	8.802077003	1.481296	0.71568
DMTF1	2480.564417	833.71488	11.27703419	9.705139674	1.573042	0.71569
PKD2L2	929.4186371	342.07925	9.861736185	8.422398044	1.441998	0.71571
PKMYT1	213.5865355	566.82696	7.745415745	9.149307546	-1.40809	0.71574
SMARCA5	27183.64476	7435.6791	14.73050435	12.8604428	1.870202	0.71576
LOC102160023	46.52379981	21.483238	5.570578288	4.490777899	1.114757	0.71576
RBBP4	38159.03208	10157.44	15.21977476	13.31039125	1.909488	0.71585
PTPN2	2555.636912	856.85067	11.32003157	9.744582725	1.576567	0.71587
LOC102167831	209.3570991	88.411786	7.716696567	6.482393107	1.243655	0.71588
ATRAID	904.042019	2815.1304	9.821840965	11.45949843	-1.63874	0.71595
RASL11A	22.20454082	9.9153405	4.536335244	3.448285225	1.163121	0.71598
CBLL1	1636.791866	571.78463	10.67753631	9.161848978	1.517327	0.71601
LOC100516601	548.7693659	212.35354	9.102682709	7.737102248	1.369732	0.71609
NCAPG2	3390.950591	1108.8656	11.72789944	10.11616923	1.612605	0.71624
IL17RB	103.6211905	46.271589	6.709031281	5.56290145	1.163121	0.71626
LOC100522864	1070.047396	389.17711	10.06480561	8.607985345	1.459176	0.71649
LOC102159499	9.516231779	3.3051135	3.394545939	2.106051269	1.525691	0.71649
LCA5L	9.516231779	3.3051135	3.394545939	2.106051269	1.525691	0.71649
DPEP2	9.516231779	3.3051135	3.394545939	2.106051269	1.525691	0.71649
LOC100738931	9.516231779	3.3051135	3.394545939	2.106051269	1.525691	0.71649
RGS3	9.516231779	3.3051135	3.394545939	2.106051269	1.525691	0.71649
NAT8	9.516231779	3.3051135	3.394545939	2.106051269	1.525691	0.71649
LOC100153697	9.516231779	3.3051135	3.394545939	2.106051269	1.525691	0.71649
MIR92A-1	9.516231779	3.3051135	3.394545939	2.106051269	1.525691	0.71649
LOC102167951	9.516231779	3.3051135	3.394545939	2.106051269	1.525691	0.71649
CD274	10.57359087	23.962073	3.532764645	4.641665831	-1.18029	0.71664
LOC100515824	10.57359087	23.962073	3.532764645	4.641665831	-1.18029	0.71664
LOC102167454	10.57359087	23.962073	3.532764645	4.641665831	-1.18029	0.71664
LOC102167353	10.57359087	23.962073	3.532764645	4.641665831	-1.18029	0.71664
LOC100737125	9985.699214	2972.9496	13.2857922	11.53816447	1.747968	0.71674
LOC102165807	16.91774539	37.182527	4.163317207	5.254840671	-1.13609	0.71676
TAF8	94.1049587	228.05283	6.571448659	7.839536581	-1.27703	0.71681
LOC100738549	236.8484354	99.153405	7.893898725	6.646067655	1.25623	0.71693
DLL1	372.1903985	149.55639	8.543768058	7.234160086	1.315351	0.71693
PBLD	949.5084597	349.51575	9.892555657	8.453335466	1.441823	0.71699
LOC102162907	99.39175414	44.619032	6.649496966	5.511563931	1.155467	0.71739
LOC102166542	33.83549077	75.191332	5.122485983	6.251554969	-1.15203	0.71745
LOC100621868	71.90041789	169.38707	6.187855179	7.412672017	-1.23625	0.71752
NDUFS4	6610.609009	2041.7339	12.6907857	10.99628554	1.694988	0.71757
LOC100625384	179.7510447	466.021	7.497860176	8.867343618	-1.37439	0.71772
CSTF1	3919.630134	1268.3373	11.93686983	10.30985977	1.627779	0.71789
BICD1	465.2379981	183.4338	8.864922778	7.526959252	1.342709	0.71793
WDR91	212.5291764	561.04301	7.738289401	9.134536737	-1.40045	0.718
EFNA2	76.12985423	180.12869	6.269217479	7.500871231	-1.24249	0.718
DEDD2	154.3744266	393.3085	7.279605257	8.623181018	-1.34923	0.71801
LOC102159884	195.611431	83.454116	7.619203392	6.400095824	1.228936	0.71807
ADRB2	25.37661808	11.567897	4.721187694	3.651671381	1.133373	0.71808
LOC102165520	131.1125267	328.03251	7.045623457	8.362086342	-1.32303	0.71816
EBNA1BP2	7682.771123	2344.1517	12.90759883	11.19546556	1.712561	0.71818
NTAN1	758.1264651	2306.1429	9.568196438	11.17189167	-1.60497	0.71821
FAM207A	506.4750025	1473.2543	8.987192949	10.52576972	-1.54044	0.71822
LOC102163033	74.01513606	33.877413	6.229109817	5.124221138	1.127497	0.71825
LOC100525600	3106.520996	1027.064	11.60154842	10.00571439	1.596774	0.71829
LOC100620170	44.40908164	20.656959	5.504908955	4.436758793	1.104227	0.71834
ANXA13	4.229436346	11.567897	2.386655454	3.651671381	-1.45159	0.71843
LOC102161290	4.229436346	11.567897	2.386655454	3.651671381	-1.45159	0.71843

LOC102165256	5.80250177	14.046732	2.766065426	3.911378308	-1.27549	0.70025
WNT2	16.5785765	33.877413	4.135746339	5.124221138	-1.031	0.7003
ADAP2	130.970754	64.449713	7.044074441	6.032314961	1.022999	0.70035
HECA	573.618746	1351.7914	9.166461249	10.4017237	-1.23671	0.70035
NUDCD2	2445.34003	995.66544	11.25640923	9.96096549	1.296302	0.7004
HEXB	1182.88143	2942.3773	10.20930888	11.52325676	-1.31468	0.70085
FAAH	347.321177	787.44329	8.444274378	9.622863177	-1.18091	0.70091
EXOSC1	2007.66561	829.58349	10.9720217	9.697981376	1.27506	0.70125
IDE	5708.00388	2194.5954	12.47902333	11.10039648	1.379031	0.70148
LOC100511695	882.809197	386.69828	9.787591134	8.598790517	1.190893	0.70164
GALR1	1.65785765	6.610227	1.410263837	2.927939483	-1.99538	0.70169
SPATA18	1.65785765	6.610227	1.410263837	2.927939483	-1.99538	0.70169
CYP2B22	1.65785765	6.610227	1.410263837	2.927939483	-1.99538	0.70169
WDR87	1.65785765	6.610227	1.410263837	2.927939483	-1.99538	0.70169
LOC100518382	1.65785765	6.610227	1.410263837	2.927939483	-1.99538	0.70169
LOC102157547	1.65785765	6.610227	1.410263837	2.927939483	-1.99538	0.70169
KCNH6	1.65785765	6.610227	1.410263837	2.927939483	-1.99538	0.70169
LOC102160131	1.65785765	6.610227	1.410263837	2.927939483	-1.99538	0.70169
LOC102162866	1.65785765	6.610227	1.410263837	2.927939483	-1.99538	0.70169
LOC102158630	1.65785765	6.610227	1.410263837	2.927939483	-1.99538	0.70169
LOC100519510	1.65785765	6.610227	1.410263837	2.927939483	-1.99538	0.70169
HDGFL1	1.65785765	6.610227	1.410263837	2.927939483	-1.99538	0.70169
LOC102158431	1.65785765	6.610227	1.410263837	2.927939483	-1.99538	0.70169
PNLDC1	1.65785765	6.610227	1.410263837	2.927939483	-1.99538	0.70169
LOC102165215	1.65785765	6.610227	1.410263837	2.927939483	-1.99538	0.70169
GJA9	15.7496477	32.224857	4.066058842	5.054191063	-1.03285	0.70177
LOC100738976	19.8942918	9.0890621	4.385036953	3.334720158	1.130151	0.70213
LOC102165752	19.8942918	9.0890621	4.385036953	3.334720158	1.130151	0.70213
SAMD4A	399.543693	913.86388	8.645815816	9.837413293	-1.19363	0.70232
RAB4B	612.578401	1446.8134	9.261103887	10.49965999	-1.23991	0.70266
LOC102158889	234.586857	112.37386	7.880115246	6.824944216	1.061816	0.70266
LOC100624867	399.543693	185.08636	8.645815816	7.539828464	1.110155	0.70278
LOC100524919	393.741191	182.60752	8.624763262	7.52048134	1.108501	0.70292
LOC100627437	1144.75071	493.28819	10.16207746	8.94920862	1.214531	0.70333
TROVE2	329.084743	154.51406	8.366692646	7.280901168	1.090721	0.70338
ABCD4	322.453312	724.64613	8.337413677	9.503122366	-1.16819	0.70344
TTC7B	360.584038	817.18931	8.498187183	9.676290877	-1.18033	0.70346
ERGIC3	1138.11928	2814.3041	10.1537031	11.45907507	-1.30613	0.70352
GALC	310.848309	696.55267	8.284700625	9.446158339	-1.16402	0.70354
ADCK3	1270.74789	543.69117	10.31259698	9.089294666	1.224819	0.70356
LOC100621444	54.7093024	109.89502	5.799846344	6.793050813	-1.00627	0.7036
LOC100521751	471.660501	1090.6875	8.884660496	10.09234416	-1.20942	0.70366
LOC100518497	809.863461	357.77854	9.663315193	8.486949769	1.178612	0.70373
LOC100739564	62.9985906	127.24687	5.999968229	7.002779797	-1.01424	0.70374
KXD1	1515.28189	3826.4951	10.56632227	11.90218483	-1.33644	0.70386
PCDHA5	12.4339324	4.9576702	3.747809764	2.574748269	1.326548	0.70422
LOC100624550	12.4339324	4.9576702	3.747809764	2.574748269	1.326548	0.70422
LOC102158223	12.4339324	4.9576702	3.747809764	2.574748269	1.326548	0.70422
LOC100627940	12.4339324	4.9576702	3.747809764	2.574748269	1.326548	0.70422
LOC100626299	23.2100071	46.271589	4.597531595	5.56290145	-0.99538	0.70434
AHCYL2	1065.17354	461.88961	10.05822657	8.854524369	1.205468	0.70436
LOC100522714	1137.29035	2809.3465	10.15265288	11.45653228	-1.30463	0.70437
SPINT1	27.3546512	13.220454	4.825513503	3.829895615	1.049014	0.70443
WWP1	3314.05744	1327.0031	11.69481815	10.37504276	1.320427	0.70462
GABARAPL1	2817.52907	7439.8105	11.46072673	12.86124405	-1.40084	0.70465
LOC100738447	347.321177	162.77684	8.444274378	7.35558754	1.093375	0.70466
POLA2	4350.21847	1709.57	12.08720374	10.74026139	1.347454	0.70471
LOC100625740	852.96776	2060.7383	9.738037793	11.00964548	-1.2726	0.70477
LOC100525350	748.522728	332.99018	9.549828415	8.383661891	1.168566	0.70493
TIPIN	782.50881	347.03692	9.613805686	8.44309653	1.173018	0.70497
LOC102161064	67.1432347	33.877413	6.090498528	5.124221138	0.986918	0.70507
LOC100621915	65.4853771	33.051135	6.054965161	5.089630977	0.986473	0.70518
RPGRIP1L	114.392178	57.013208	6.850401618	5.858309486	1.00462	0.70521
LOC100624499	720.339148	1717.0065	9.494533912	10.74651974	-1.25315	0.7053
FAM162B	63.8275194	32.224857	6.018534465	5.054191063	0.986005	0.70533
MYO19	2392.28859	980.79243	11.22477865	9.939274229	1.286372	0.70538
LOC102163532	14.09179	28.919743	3.915692025	4.903025878	-1.0372	0.70543
SERBP1	63830.0062	21074.23	15.96196977	14.36326075	1.598755	0.70545
PRIM2	2035.84919	844.4565	10.99212345	9.723586711	1.269536	0.70566
TMEM219	599.31554	1407.9783	9.229577203	10.46043372	-1.23224	0.70567
UBL4A	847.165258	2043.3864	9.728201579	10.99745219	-1.27025	0.70571
LOC100621130	60.5118041	30.5723	5.942791385	4.980587446	0.984992	0.70576
LOC100624950	110.247534	228.87911	6.79762954	7.844731552	-1.05384	0.70577
LOC100514955	324.11117	726.29869	8.344789314	9.506404165	-1.16407	0.70596
LOC100737519	301.730092	142.94616	8.241888278	7.169385476	1.077787	0.70597
IFT122	511.449084	1186.5357	9.001264859	10.21375522	-1.21409	0.70604
TASP1	557.869098	253.66746	9.126366596	7.992470822	1.136988	0.70606
TTL	622.525547	280.93465	9.28430486	8.139216969	1.147898	0.70611
SPARCL1	198.942918	96.674569	7.643444369	6.609911086	1.041146	0.70614
ALDH18A1	19500.5506	6942.3909	14.25130122	12.76142468	1.49001	0.70615
LOC100739002	136.773256	67.754826	7.106152054	6.103389087	1.013391	0.70615
LOC100627033	90.3532418	45.44531	6.51338402	5.537461031	0.991445	0.70615
LRCH2	1254.16931	538.7335	10.29366627	9.07610342	1.219088	0.70623
TMEM214	2537.35113	6637.4942	11.30967594	12.69664031	-1.38732	0.70628
LOC100623829	575.276604	261.10397	9.170617638	8.033995371	1.139631	0.7063
LOC100622246	3411.87104	1365.8381	11.73677019	10.4166267	1.320777	0.7063
KIF1C	6066.10113	16884.172	12.56679164	14.04346928	-1.47683	0.70636
DDX52	1705.93552	717.20963	10.73719284	9.48826118	1.250096	0.70654
ITPR3	9424.0918	3520.7721	13.20229095	11.78208585	1.420462	0.70655
LOC100625901	67.9721635	137.16221	6.107942317	7.110219252	-1.01287	0.70658
KIAA1147	60.5118041	121.46292	5.942791385	6.936201185	-1.00523	0.7066
LOC102164932	18.2364341	8.2627837	4.265769485	3.211445828	1.142124	0.70664
GFAP	20.7232206	41.313919	4.441166102	5.403060391	-0.99538	0.70668
SENP5	2878.8698	1166.7051	11.49178788	10.18946021	1.303063	0.7067
ELANE	74.6035941	151.20894	6.240382915	7.249909307	-1.01923	0.70673
LOC100626038	867.05955	382.56689	9.761650206	8.583334365	1.180419	0.70675
SHROOM2	522.225159	238.79445	9.031288103	7.905654454	1.128903	0.70689
LOC100737060	647.393411	291.67627	9.34072562	8.193161944	1.150275	0.707
LOC100739114	231.271142	111.54758	7.859666109	6.814391229	1.051926	0.70706
LOC100739300	232.100071	506.50864	7.864805631	8.987288579	-1.12584	0.70707
LOC102161507	25.6967935	12.394176	4.73859457	3.74353388	1.051926	0.70713
LOC102158027	25.6967935	12.394176	4.73859457	3.74353388	1.051926	0.70713
LOC102162524	25.6967935	12.394176	4.73859457	3.74353388	1.051926	0.70713
P2RY1	290.125088	643.67085	8.185495362	9.332418944	-1.14965	0.70721
LOC102158661	53.8803735	27.267186	5.778218396	4.821056379	0.982594	0.70724
LOC102161315	124.339324	61.970878	6.969695301	5.976612875	1.00462	0.70741
LOC102158489	124.339324	61.970878	6.969695301	5.976612875	1.00462	0.70741
ERO1LB	62.1696618	124.76803	5.981159942	6.974621475	-1.00497	0.70745
BEST1	69.6300212	140.46732	6.142209624	7.144325041	-1.01245	0.70752
POP1	1869.2345	781.65934	10.86900346	9.612240686	1.257836	0.70754
ANXA2	33918.9385	105787.59	15.04984593	16.69082456	-1.64101	0.70769
SLC38A2	3057.0895	8093.3967	11.57841492	12.98270783	-1.40459	0.70771
SNRNP200	8397.87791	3165.4724	13.03598088	11.62866081	1.407604	0.70773
LOC100625074	473.318358	218.13749	8.889711898	7.775692512	1.117573	0.70776
CCDC68	52.2225159	26.440908	5.733964805	4.77825631	0.9819	0.70777
LOC100153027	101.958245	51.229259	6.685915561	5.706786332	0.992939	0.70778
TBC1D17	1364.41684	3403.4406	10.41512574	11.73320205	-1.31871	0.70779
TMEM102	231.271142	504.02981	7.859666109	8.980224728	-1.12392	0.70786
LOC100623533	145.062544	305.723	7.190442454	8.260792531	-1.07555	0.70791
LOC100517777	1269.91896	546.17	10.31165632	9.095845332	1.217314	0.70808
LOC100526129	50.5646582	25.61463	5.688310694	4.734147579	0.981161	0.70837
LOC100737307	50.5646582	25.61463	5.688310694	4.734147579	0.981161	0.70837
DDX27	2613.61258	1068.3779	11.35238148	10.0625561	1.290623	0.7084
TMEM8B	407.832981	927.08433	8.675367775	9.858112096	-1.18472	0.70844
TMEM184B	1032.01639	2518.4965	10.01264742	11.29891972	-1.2871	0.70852
LOC100620408	106.931818	53.708094	6.753976422	5.773682393	0.99348	0.70859
EIF2C1	5291.05268	2059.912	12.36961171	11.00906718	1.360972	0.70866
LOC100627463	1581.5962	670.11176	10.62807748	9.390409227	1.238908	0.70866
LOC102160255	111.076462	229.70539	6.808339514	7.849907883	-1.04823	0.70874
PPRC1	10262.9678	3821.5375	13.32530092	11.90031493	1.425223	0.70896
LOC100739454	553.724454	252.84118	9.115627516	7.987782332	1.130937	0.70902
RSC1A1	2192.51674	908.07993	11.09903	9.828263338	1.271697	0.70906
KLF4	1249.19574	3090.2811	10.28793827	11.59398914	-1.30674	0.70924
LOC100626534	762.614518	1816.9861	9.576700721	10.82812548	-1.25252	0.70929
LOC100736940	1058.54211	461.88961	10.04922521	8.854524369	1.196459	0.70935
LOC100738745	615.894116	279.28209	9.268879075	8.130735747	1.140959	0.70942
SLC30A4	164.127907	80.97528	7.36744015	6.357117027	1.019267	0.70943
GPR180	1245.05109	537.08094	10.28314751	9.071679398	1.212994	0.70945
LOC100625244	290.954017	138.81477	8.189597352	7.127372928	1.06763	0.7095
LOC102159202	4.97357294	0.8262784	2.578594101	0.868906686	2.589583	0.70955
LOC102168123	4.97357294	0.8262784	2.578594101	0.868906686	2.589583	0.70955
LOC102168075	4.97357294	0.8262784	2.578594101	0.868906686	2.589583	0.70955
LOC102161181	4.97357294	0.8262784	2.578594101	0.868906686	2.589583	0.70955
LOC102163843	4.97357294	0.8262784	2.578594101	0.868906686	2.589583	0.70955
INMT	4.97357294	0.8262784	2.578594101	0.868906686	2.589583	0.70955
LOC100626635	4.97357294	0.8262784	2.578594101	0.868906686	2.589583	0.70955
LOC100736578	4.97357294	0.8262784	2.578594101	0.868906686	2.589583	0.70955
LOC100152671	4.97357294	0.8262784	2.578594101	0.868906686	2.589583	0.70955
LOC102160180	4.97357294	0.8262784	2.578594101	0.868906686	2.589583	0.70955
LOC100625009	4.97357294	0.8262784	2.578594101	0.868906686	2.589583	0.70955
TMPRSS2	4.97357294	0.8262784	2.578594101	0.868906686	2.589583	0.70955
LOC102164904	4.97357294	0.8262784	2.578594101	0.868906686	2.589583	0.70955
LOC102167814	4.97357294	0.8262784	2.578594101	0.868906686	2.589583	0.70955
LOC100739734	4.97357294	0.8262784	2.578594101	0.868906686	2.589583	0.70955
LOC100153579	4.97357294	0.8262784	2.578594101	0.868906686	2.589583	0.70955
NRSN2	8.28928824	18.178124	3.215568059	4.261389712	-1.13288	0.70966
LOC102161184	8.28928824	18.178124	3.215568059	4.261389712	-1.13288	0.70966
UFC1	959.070649	2324.3211	9.906996763	11.18321421	-1.2771	0.70968
SETD4	307.532594	146.25127	8.269279095	7.202136284	1.07229	0.70975
GLRB	47.248943	23.962073	5.592425431	4.641665831	0.979529	0.70984
DIAPH3	607.604828	275.97698	9.249361968	8.113622247	1.138585	0.70998
CLIP2	998.030304	2425.127	9.96438463	11.24443937	-1.2809	0.71001
LOC102164953	135.944327	67.754826	7.097445694	6.103389087	1.00462	0.71002
KCNJ8	115.221107	57.839486	6.860728285	5.878712739	0.994278	0.71004
LOC100736659	24.0389359	11.567897	4.646101346	3.65167138	1.055246	0.71026
LOC100623470	24.0389359	11.567897	4.646101346	3.65167138	1.055246	0.71026
LOC100517560	24.0389359	11.567897	4.646101346	3.65167138	1.055246	0.71026
LOC100737758	2830.79193	7431.5477	11.46749955	12.8596411	-1.39246	0.71032
LOC100520026	12.4339324	25.61463	3.747809764	4.734147579	-1.04269	0.71034
TICAM2	192.311487	94.195734	7.594783559	6.572825024	1.029711	0.71038
LOC102160098	2563.87685	1051.8524	11.32467384	10.04008744	1.285395	0.71048
ZRANB3	620.867689	281.76092	9.280463849	8.143438955	1.139814	0.71051
SYMPK	8614.22834	3250.5791	13.07267332	11.66692481	1.406025	0.71052
LOC100624707	155.00969	76.843889	7.28549183	6.282511874	1.012356	0.71069
CDK2AP2	1233.44609	3043.1832	10.26964812	11.57183949	-1.30289	0.71071
LOC100739767	139.260042	69.407383	7.131960259	6.137654819	1.00462	0.71076
IKZF4	68.8010924	137.98849	6.125177709	7.118821585	-1.00404	0.71084
CEP85L	235.415786	114.02642	7.88518256	6.845821397	1.045843	0.71087
CNTF	48.9068006	96.674569	5.641164514	6.609911086	-0.9831	0.71107
LOC100515432	183.19327	90.064343	7.525076539	6.508814353	1.024339	0.71108
LOC100515232	312.506167	148.73011	8.29235001	7.226220529	1.071188	0.71109
LOC100737713	81.2350247	41.313919	6.361681078	5.403060391	0.975474	0.71136
LOC100623904	77.9193094	39.661362	6.302306427	5.34558663	0.974247	0.71136
LOC100513639	77.9193094	39.661362	6.302306427	5.34558663	0.974247	0.71136
RBL2	271.059725	594.92043	8.087779591	9.218975892	-1.13409	0.71146
LOC100737242	71.2878788	36.356248	6.175681852	5.223277669	0.971453	0.71168
LOC100738959	3174.79739	8391.6831	11.63290316	13.0349164	-1.4023	0.7117
LOC102157450	0.82892882	4.9576702	0.870998931	2.574748269	-2.58034	0.71185
LOC102163738	0.82892882	4.9576702	0.870998931	2.574748269	-2.58034	0.71185
RAB25	0.82892882	4.9576702	0.870998931	2.574748269	-2.58034	0.71185
LOC100738604	0.82892882	4.9576702	0.870998931	2.574748269	-2.58034	0.71185
PILRA	0.82892882	4.9576702	0.870998931	2.574748269	-2.58034	0.71185
CCR7	0.82892882	4.9576702	0.870998931	2.574748269	-2.58034	0.71185
LOC102158372	0.82892882	4.9576702	0.870998931	2.574748269	-2.58034	0.71185
LOC102167884	0.82892882	4.9576702	0.870998931	2.574748269	-2.58034	0.71185
LOC102167172	0.82892882	4.9576702	0.870998931	2.574748269	-2.58034	0.71185
LOC102167417	0.82892882	4.9576702	0.870998931	2.574748269	-2.58034	0.71185
AKR1C1	0.82892882	4.9576702	0.870998931	2.574748269	-2.58034	0.71185
LOC100737686	0.82892882	4.9576702	0.870998931	2.574748269	-2.58034	0.71185
C7H15orf39	2442.85324	1007.2333	11.25494194	9.977613844	1.278169	0.71193
LOC100738156	176.561839	375.13038	7.472177749	8.55508903	-1.08722	0.71198
LOC102166561	16.5785765	7.4365053	4.135746339	3.076645515	1.156623	0.71201
LOC102166270	16.5785765	7.4365053	4.135746339	3.076645515	1.156623	0.71201
LOC100737245	16.5785765	7.4365053	4.135746339	3.076645515	1.156623	0.71201
NPR3	52.2225159	103.2848	5.733964805	6.704385035	-0.98388	0.71211
LOC102158547	31.4992953	15.699289	5.022336531	4.061714779	1.00462	0.71216
LOC100620622	174.075053	369.34643	7.451829714	8.532731629	-1.08527	0.71221
RNF2	3304.93922	1334.4396	11.69084449	10.38309898	1.30839	0.71225
RUFY3	2514.97005	1035.3268	11.29689903	10.01726331	1.280455	0.71236
LOC100520931	1459.74366	624.66645	10.51248731	9.289249932	1.224557	0.71237
C1H18orf25	1099.15962	480.06773	10.10349714	8.910096229	1.195091	0.71245
NKIRAS2	720.339148	1702.1334	9.494533912	10.73397576	-1.2406	0.71249
LOC100517973	991.398873	436.27498	9.954776287	8.772396993	1.184228	0.71256
LOC102161167	10.7760747	4.1313919	3.557786824	2.359350201	1.383132	0.71286
C8H4orf36	10.7760747	4.1313919	3.557786824	2.359350201	1.383132	0.71286
SRFBP1	1194.48644	518.90282	10.22338204	9.022098163	1.202854	0.71294
PRKAR1A	6872.64888	19162.222	12.74686044	14.22605251	-1.47933	0.7131
SPAG11B	3.31571529	0	2.109599694	0	20	0.71313
LOC102159146	3.31571529	0	2.109599694	0	20	0.71313
LOC102159152	3.31571529	0	2.109599694	0	20	0.71313
LOC102159210	3.31571529	0	2.109599694	0	20	0.71313
LOC102159204	3.31571529	0	2.109599694	0	20	0.71313
LOC102159258	3.31571529	0	2.109599694	0	20	0.71313
P2RX2	3.31571529	0	2.109599694	0	20	0.71313
LOC102159400	3.31571529	0	2.109599694	0	20	0.71313
LOC102161687	3.31571529	0	2.109599694	0	20	0.71313
LOC102161637	3.31571529	0	2.109599694	0	20	0.71313
LOC102157576	3.31571529	0	2.109599694	0	20	0.71313
LOC102168169	3.31571529	0	2.109599694	0	20	0.71313
LOC102157691	3.31571529	0	2.109599694	0	20	0.71313
LOC102161264	3.31571529	0	2.109599694	0	20	0.71313
SERPINB11	3.31571529	0	2.109599694	0	20	0.71313
LOC100156006	3.31571529	0	2.109599694	0	20	0.71313
LOC102161013	3.31571529	0	2.109599694	0	20	0.71313
LOC102166893	3.31571529	0	2.109599694	0	20	0.71313
LOC100624315	3.31571529	0	2.109599694	0	20	0.71313
ADRA1A	3.31571529	0	2.109599694	0	20	0.71313
LOC100738107	3.31571529	0	2.109599694	0	20	0.71313
LOC102164230	3.31571529	0	2.109599694	0	20	0.71313
RAC2	3.31571529	0	2.109599694	0	20	0.71313
CREB3L3	3.31571529	0	2.109599694	0	20	0.71313
LOC102166434	3.31571529	0	2.109599694	0	20	0.71313
LOC102160903	3.31571529	0	2.109599694	0	20	0.71313
LY75	3.31571529	0	2.109599694	0	20	0.71313
LOC100621138	3.31571529	0	2.109599694	0	20	0.71313
LOC100627724	3.31571529	0	2.109599694	0	20	0.71313
ABCA12	3.31571529	0	2.109599694	0	20	0.71313
CCR1	3.31571529	0	2.109599694	0	20	0.71313
TMEM130	3.31571529	0	2.109599694	0	20	0.71313
FXYD3	3.31571529	0	2.109599694	0	20	0.71313
LOC100623228	3.31571529	0	2.109599694	0	20	0.71313
LOC102167637	3.31571529	0	2.109599694	0	20	0.71313
LOC102165335	3.31571529	0	2.109599694	0	20	0.71313
LOC100523413	3.31571529	0	2.109599694	0	20	0.71313
LOC102167688	3.31571529	0	2.109599694	0	20	0.71313
LOC100523434	3.31571529	0	2.109599694	0	20	0.71313
LOC100157853	3.31571529	0	2.109599694	0	20	0.71313
LOC100155636	3.31571529	0	2.109599694	0	20	0.71313
TSACC	3.31571529	0	2.109599694	0	20	0.71313
LOC100739733	3.31571529	0	2.109599694	0	20	0.71313
LOC100513690	3.31571529	0	2.109599694	0	20	0.71313
LOC102165837	3.31571529	0	2.109599694	0	20	0.71313
LOC100625468	3.31571529	0	2.109599694	0	20	0.71313
LOC100517804	3.31571529	0	2.109599694	0	20	0.71313
LOC102167312	3.31571529	0	2.109599694	0	20	0.71313
LRRC18	3.31571529	0	2.109599694	0	20	0.71313
LOC102167956	3.31571529	0	2.109599694	0	20	0.71313
TCF24	3.31571529	0	2.109599694	0	20	0.71313
LOC100621529	461.713355	214.83238	8.853974928	7.753767487	1.103786	0.7139
LOC102158880	307.532594	147.07755	8.269279095	7.210209122	1.064162	0.71392
ZNF219	116.050035	238.79445	6.870981559	7.905654454	-1.04102	0.714
EEFSEC	1317.99683	569.3058	10.36522538	9.15559189	1.211071	0.71411
LOC100627544	7.46035941	16.525567	3.080718953	4.13138925	-1.14738	0.71491
TCAIM	241.218288	117.33153	7.920163984	6.886690713	1.039749	0.71495
LOC100521892	77.0903806	154.51406	6.287072939	7.280901168	-1.00312	0.71497
LOC100513919	724.483792	1707.0911	9.502799573	10.73816922	-1.23651	0.71515
LOC100154542	54.7093024	28.093465	5.799846344	4.862623207	0.961552	0.71516

IL9R	9.66566885	17.351846	3.414902536	4.19785327		-0.84415	0.83831	
FAM71F2	9.66566885	17.351846	3.414902536	4.19785327		-0.84415	0.83831	
LOC102162453	9.66566885	17.351846	3.414902536	4.19785327		-0.84415	0.83831	
EPS8L1	35.9010557	61.970878	5.205590187	5.976612875		-0.78756	0.83835	
CEPT1	1259.29857	563.52185	10.29954984	9.140885611		1.160077	0.83836	
PRRG2	26.2353869	44.619032	4.767410456	5.511563931		-0.76615	0.83838	
ST6GALNAC2	1708.06177	4467.6872	10.73898882	12.12563533		-1.38717	0.83845	
PRKG2	1133.64488	512.29259	10.14802511	9.003637625		1.145929	0.83849	
LOC100736562	53.8515836	95.848291	5.777461367	6.597654688		-0.83176	0.83853	
MRPS26	7079.41203	2734.9814	12.7896176	11.41784271		1.372099	0.83859	
LOC102167077	6.90404918	13.220454	2.982591923	3.829895615		-0.93726	0.83861	
LOC100155760	6.90404918	13.220454	2.982591923	3.829895615		-0.93726	0.83861	
LOC102167640	59.3748229	106.58991	5.915875148	6.749398975		-0.84415	0.83864	
MYO19	2311.47567	980.79243	11.17522247	9.939274229		1.236794	0.83866	
LOC102163415	4679.56453	1870.6942	12.19246683	10.87012906		1.322801	0.83878	
LOC102166332	48.3283443	85.106672	5.624344958	6.428053129		-0.8164	0.83881	
ESPL1	3519.68427	1441.0295	11.78164014	10.49388494		1.288346	0.83881	
CDK8	120.130456	66.928548	6.920417836	6.085946114		0.843908	0.83899	
LOC100156231	69.0404918	125.59431	6.130117309	6.98406878		-0.86326	0.83903	
SCN3B	40.0434852	69.407383	5.359081339	6.137654819		-0.79352	0.83905	
LOC102160397	40.0434852	69.407383	5.359081339	6.137654819		-0.79352	0.83905	
LOC100620143	95.2758787	179.30241	6.58910248	7.494274844		-0.91221	0.8391	
NTN3	100.799118	57.013208	6.669581252	5.858309486		0.822115	0.83912	
LOC100623579	30.3778164	18.178124	4.971673052	4.261389712		0.740815	0.83914	
BTF3	42502.7076	14343.366	15.37530107	13.80819661		1.567171	0.83923	
LOC102158305	37.2818656	22.309516	5.258589232	4.542847146		0.740815	0.83924	
LOC102165474	49.7091541	87.585507	5.664174303	6.468998788		-0.81718	0.83924	
LOC102165352	16.569718	28.093465	4.135019133	4.862623207		-0.76169	0.83925	
LOC100625662	28.9970066	17.351846	4.906746634	4.19785327		0.740815	0.83929	
LOC102162033	12.4272885	21.483238	3.747096094	4.490777898		-0.7897	0.83933	
TMTC1	118.749646	228.87911	6.90387758	7.844731552		-0.94666	0.83933	
LOC100624657	140.842603	276.80325	7.148147109	8.117919691		-0.97478	0.83936	
RNFT1	434.955098	975.00848	8.76803574	9.93074987		-1.16455	0.83941	
LOC100736861	42.8051049	74.365053	5.453027102	6.2358238		-0.79684	0.83964	
KIF17	51.0899639	90.064343	5.702933533	6.508814353		-0.81792	0.83968	
LOC100739512	22.0929574	37.182527	4.529381037	5.254840671		-0.75104	0.83979	
PLIN2	4856.30819	1939.2753	12.24594131	10.92204568		1.324342	0.83982	
LOC100738287	42.8051049	25.61463	5.453027102	4.734147579		0.740815	0.83996	
LOC100522598	11709.2674	4357.7921	13.5154864	12.08971269		1.425982	0.83999	
TP53	10208.3271	3840.5419	13.31760016	11.90746976		1.410365	0.84003	
CD300LG	80.0869705	46.271589	6.341398207	5.56290145		0.791441	0.84003	
FAXDC2	103.560738	58.665764	6.708197415	5.89883146		0.819886	0.84019	
ASPRV1	11.0464787	5.7839486	3.590539588	2.762125239		0.93346	0.84021	
MMD2	11.0464787	5.7839486	3.590539588	2.762125239		0.93346	0.84021	
LOC100624577	11.0464787	5.7839486	3.590539588	2.762125239		0.93346	0.84021	
CCDC13	11.0464787	5.7839486	3.590539588	2.762125239		0.93346	0.84021	
ZNF518B	63.5172524	114.02642	6.011613095	6.845821397		-0.84415	0.84023	
LOC100518575	5009.57808	1997.1148	12.29076135	10.96442378		1.326772	0.84028	
LOC102164741	132.557744	73.538775	7.061319821	6.219919206		0.850044	0.84029	
LOC100738454	266.496298	562.69557	8.063375117	9.138772422		-1.07824	0.84029	
UHRF2	75.944541	138.81477	6.265747069	7.127372928		-0.87014	0.84031	
LOC100523883	132.557744	257.79885	7.061319821	8.015687408		-0.95962	0.84036	
RAB5A	204.359856	418.09686	7.682010377	8.711139889		-1.03273	0.84037	
LOC102166185	45.5667246	27.267186	5.541227504	4.821056379		0.740815	0.84045	
BTG4	45.5667246	27.267186	5.541227504	4.821056379		0.740815	0.84045	
LOC100155954	265.115489	137.98849	8.055908671	7.118821585		0.942073	0.84045	
STX6	622.745236	1450.1185	9.284813081	10.50294966		-1.21946	0.8405	
ADAMTS5	151.889082	299.93905	7.256341575	8.233327508		-0.98165	0.84053	
CUL7	1042.51143	2571.3783	10.02723068	11.32888711		-1.30248	0.84055	
LOC100624499	724.925164	1717.0065	9.503677017	10.74651974		-1.24399	0.84057	
LOC100521958	156.031511	309.02811	7.294910283	8.276255224		-0.9859	0.84059	
LOC100622890	7257.5365	21872.415	12.82546298	14.41689084		-1.59156	0.84062	
METTL13	2020.12479	870.8974	10.98094269	9.768014573		1.21387	0.84063	
LOC102163742	23.4737672	14.046732	4.613164285	3.911378308		0.740815	0.84076	
LOC100049692	23.4737672	14.046732	4.613164285	3.911378308		0.740815	0.84076	
LOC100737219	23.4737672	14.046732	4.613164285	3.911378308		0.740815	0.84076	
LOC100736563	84.2294	155.34033	6.413279271	7.288546214		-0.88304	0.84087	
FOXP1	1303.48449	3291.893	10.34926407	11.68513993		-1.33654	0.84087	
SCARF2	1534.07973	3943.0004	10.58409787	11.94544398		-1.36192	0.84087	
TFEC	0	3.3051135	0	2.106051269		-20	0.84087	
GP5	0	3.3051135	0	2.106051269		-20	0.84087	
NRSN1	0	3.3051135	0	2.106051269		-20	0.84087	
GTF2A1L	0	3.3051135	0	2.106051269		-20	0.84087	
PCDHA6	0	3.3051135	0	2.106051269		-20	0.84087	
LOC102159135	0	3.3051135	0	2.106051269		-20	0.84087	
LOC100736942	0	3.3051135	0	2.106051269		-20	0.84087	
LOC102159330	0	3.3051135	0	2.106051269		-20	0.84087	
LOC102159352	0	3.3051135	0	2.106051269		-20	0.84087	
LOC102161839	0	3.3051135	0	2.106051269		-20	0.84087	
TSSK2	0	3.3051135	0	2.106051269		-20	0.84087	
PTGDR	0	3.3051135	0	2.106051269		-20	0.84087	
OSM	0	3.3051135	0	2.106051269		-20	0.84087	
PGR	0	3.3051135	0	2.106051269		-20	0.84087	
LOC102168010	0	3.3051135	0	2.106051269		-20	0.84087	
LOC102161483	0	3.3051135	0	2.106051269		-20	0.84087	
LOC102163979	0	3.3051135	0	2.106051269		-20	0.84087	
RLN	0	3.3051135	0	2.106051269		-20	0.84087	
LOC102161339	0	3.3051135	0	2.106051269		-20	0.84087	
FAM71D	0	3.3051135	0	2.106051269		-20	0.84087	
LOC102161285	0	3.3051135	0	2.106051269		-20	0.84087	
LOC102163871	0	3.3051135	0	2.106051269		-20	0.84087	
LOC102163947	0	3.3051135	0	2.106051269		-20	0.84087	
[illegible]								

CCDC51	1208.20861	550.3014	10.23984744	9.106697443		1.134576	0.84453
LOC100512753	104.941548	60.318321	6.727124677	5.938246292		0.798918	0.84453
LOC102162495	3305.65875	1376.5798	11.69115845	10.42792014		1.26385	0.84457
LOC102161342	44.1859147	75.191332	5.497801224	6.251554969		-0.76698	0.84465
MYO1E	2774.04696	7518.3069	11.43829647	12.87638397		-1.43842	0.84472
EML4	1963.51159	5131.1887	10.93995496	12.3253585		-1.38586	0.84474
LOC102166814	106.322357	198.30681	6.745806839	7.63884719		-0.89929	0.84474
LOC100621570	1205.44699	549.47512	10.23654881	9.104533542		1.133442	0.84478
CREB1	71.8021115	128.07315	6.185908388	7.012045083		-0.83487	0.84481
HIBADH	509.518829	252.0149	8.995820363	7.983078557		1.015626	0.84483
LOC100737579	1737.05877	765.96005	10.76326115	9.583007622		1.181306	0.84484
CCP110	977.613364	454.4531	9.934595174	8.831158705		1.105133	0.84484
HCN3	55.2323934	95.848291	5.813329549	6.597654688		-0.79524	0.84487
PTPRS	2359.80401	6285.4996	11.20506256	12.61804121		-1.41336	0.8449
LOC102164987	987.279033	458.5845	9.948774622	8.84418632		1.10627	0.8449
TTC13	422.52781	928.73689	8.726312893	9.860678689		-1.13622	0.84491
MRPL39	2056.02585	893.20692	11.00634421	9.8044649		1.202792	0.84493
CD302	162.935561	90.064343	7.356985025	6.508814353		0.855274	0.84494
LOC102160096	64.8980623	38.835083	6.042164139	5.315967693		0.740815	0.84495
LOC100737767	1152.97621	527.99188	10.17239777	9.047101766		1.126775	0.84499
MYL6	8886.8921	27044.091	13.11762559	14.72307914		-1.60556	0.84503
LRRC57	1839.2387	807.27397	10.8456772	9.658700576		1.187978	0.84507
DMXL2	1081.1741	498.24586	10.0797169	8.963606649		1.117669	0.84514
ZNF75D	3866.26754	1590.5859	11.91709886	10.63624928		1.281383	0.84518
LOC100625133	3374.69924	1404.6732	11.72097066	10.45704554		1.264524	0.8452
LOC100516601	113.226407	212.35354	6.835752397	7.737102248		-0.90726	0.84522
LOC100738967	544.039075	1230.3285	9.090215854	10.26599998		-1.17726	0.8453
RPL9	115406.705	36666.929	16.81638002	15.16223117		1.654176	0.84533
CCDC40	48.3283443	82.627837	5.624344958	6.385911347		-0.77376	0.84533
LOC100624925	632.410905	1455.0762	9.306997895	10.50787015		-1.20216	0.84536
PPTC7	102.179928	189.21775	6.689018532	7.571508044		-0.88894	0.84539
LOC100516289	1036.98819	480.06773	10.01957431	8.910096229		1.11109	0.84542
ACAP2	1255.15614	3119.2009	10.29480009	11.60742319		-1.31331	0.84544
TMEM25	226.452813	121.46292	7.829423468	6.936201185		0.898694	0.84548
AK3L1	2147.1593	930.38945	11.06888526	9.863240725		1.206523	0.84551
LOC102158891	26.2353869	42.966475	4.767410456	5.458331975		-0.7117	0.84554
ENOPH1	3968.44747	1630.2472	11.95472249	10.67175973		1.283484	0.84555
LOC100152693	96.6566885	56.186929	6.609646952	5.837613535		0.782635	0.84563
ANKFY1	1350.43202	3381.1311	10.40027323	11.72371687		-1.32408	0.84565
ZFAT	111.845597	209.04843	6.818206315	7.714578179		-0.90233	0.84574
LOC100736962	480.521823	239.62073	8.911457373	7.910617116		1.003849	0.84576
LONRF1	222.310384	451.14799	7.802906526	8.820651243		-1.02103	0.84585
DFNB31	5.52323934	10.741619	2.705588563	3.553559423		-0.95962	0.84586
FAM83C	5.52323934	10.741619	2.705588563	3.553559423		-0.95962	0.84586
LOC100154858	5.52323934	10.741619	2.705588563	3.553559423		-0.95962	0.84586
RFX4	5.52323934	10.741619	2.705588563	3.553559423		-0.95962	0.84586
LOC102160638	5.52323934	10.741619	2.705588563	3.553559423		-0.95962	0.84586
LOC100517695	5.52323934	10.741619	2.705588563	3.553559423		-0.95962	0.84586
LOC102165362	5.52323934	10.741619	2.705588563	3.553559423		-0.95962	0.84586
ACOXL	5.52323934	10.741619	2.705588563	3.553559423		-0.95962	0.84586
LOC100627110	506.75721	1134.4802	8.987995009	10.14908684		-1.16266	0.84592
C12H17orf53	589.6058	288.37115	9.206051714	8.176777292		1.031824	0.84594
FAM13C	186.409328	370.17271	7.550048951	8.535946834		-0.98972	0.84595
LOC102157882	120.130456	68.581105	6.920417836	6.120623682		0.808719	0.846
LOC100627787	69.0404918	41.313919	6.130117309	5.403060391		0.740815	0.84601
POPDC2	16.569718	9.9153405	4.135019133	3.448285225		0.740815	0.84603
LOC102160846	16.569718	9.9153405	4.135019133	3.448285225		0.740815	0.84603
LOC100738559	98.0374983	57.013208	6.629902968	5.858309486		0.782037	0.84606
LOC100621056	6858.48245	2694.4938	12.74388401	11.39633386		1.347875	0.84606
CLMN	158.793131	309.02811	7.320061583	8.276255224		-0.96059	0.84617
PMPCA	3023.97354	1273.295	11.56270681	10.31548355		1.247879	0.84618
ELOVL6	323.109502	167.73451	8.340337505	7.398611253		0.945844	0.84623
LGALS7	9.66566885	16.525567	3.414902536	4.13138925		-0.77376	0.84629
LIG1	5031.67104	2029.3397	12.29710858	10.9875054		1.310027	0.84636
LOC100627528	15.1889082	24.788351	4.016933786	4.688647628		-0.70664	0.84641
NUDCD1	1992.50859	870.8974	10.96109411	9.768014573		1.194011	0.84642
LOC102164508	38.6626754	23.962073	5.309710091	4.641665831		0.690189	0.84643
LOC100520008	37.2818656	23.135794	5.258589232	4.593102408		0.688347	0.84646
LEPRE1	3612.19853	10016.973	11.81906081	13.29030296		-1.4715	0.84648
PLA1A	42.8051049	26.440908	5.453027102	4.77825631		0.695011	0.84654
TEKT1	42.8051049	26.440908	5.453027102	4.77825631		0.695011	0.84654
LIN9	450.144007	226.40027	8.817444209	7.829090182		0.991511	0.84657
TUSC3	639.314954	1467.4704	9.322637893	10.52009846		-1.19873	0.84661
NEURL2	149.127462	287.54487	7.230044098	8.172651889		-0.94724	0.84662
ATP11C	1529.9373	3872.7667	10.58019948	11.91952136		-1.33989	0.84666
COX6B	16977.0569	6213.6134	14.05138374	12.60144892		1.450082	0.84667
TFRC	1324.19663	3299.3295	10.37199073	11.68839437		-1.31706	0.84667
RSG1	190.551757	378.43549	7.581590451	8.567710832		-0.98986	0.84669
LOC767626	71.8021115	42.966475	6.185908388	5.458331975		0.740815	0.84671
LGI4	168.4588	93.369456	7.404790748	6.560248082		0.851373	0.84672
CUL5	3885.59888	1602.98	11.9242925	10.64744047		1.277381	0.84673
LOC100738946	1975.93888	865.11346	10.94905255	9.758412211		1.191577	0.84685
LOC100624078	33.1394361	54.534373	5.093367322	5.795309087		-0.71862	0.84691
LOC100153768	1516.1292	680.0271	10.56712824	9.411568398		1.156729	0.84691
CCDC42B	23.4737672	38.008805	4.613164285	5.285727902		-0.69528	0.84692
DACH1	151.889082	85.106672	7.256341575	6.428053129		0.835674	0.84698
LOC100625070	1447.08871	651.93364	10.49993427	9.350792552		1.150356	0.84698
LOC102158020	31.7586262	19.830681	5.033802952	4.380638094		0.679414	0.84702
CCT6B	48.3283443	29.746021	5.624344958	4.942327829		0.700173	0.84702
SCMH1	227.833623	461.88961	7.838155235	8.854524369		-1.01957	0.84702
LOC100518781	88.3718295	52.055537	6.481748252	5.729431428		0.763535	0.84703
LOC100624306	423.90862	214.83238	8.7310088	7.753767487		0.980542	0.84719
LOC100524171	49.7091541	30.5723	5.664174303	4.980587446		0.701286	0.84719
LOC100516792	2127.82796	926.25805	11.05584365	9.856827085		1.199895	0.84724
LOC102159723	30.3778164	19.004403	4.971673052	4.322245637		0.676684	0.8473
PPP1R3E	243.022531	495.76702	7.930870551	8.956425596		-1.02857	0.8475
LOC102159646	17.9505279	28.919743	4.24416613	4.903025878		-0.68803	0.84754
LOC100737999	383.865134	196.65425	8.58820917	7.626835185		0.964938	0.84755
BAG4	171.22042	95.022013	7.428112398	6.585293272		0.849521	0.84757
LOC100512626	824.343472	1942.5805	9.688850821	10.92450111		-1.23666	0.84763
FAM8A1	788.442416	1848.3847	9.624690226	10.85282966		-1.22919	0.84766
LOC102167711	144.985033	81.801559	7.189676653	6.371586023		0.825704	0.84768
ACO	147.746652	283.41348	7.21671339	8.151846042		-0.93978	0.84772
TMEM150C	66.2788721	115.67897	6.072081615	6.86640077		-0.80351	0.84779
C2H11orf95	201.598236	401.57129	7.662477803	8.653100473		-0.99417	0.8478
SKAP2	182.266898	358.60481	7.517802419	8.490268521		-0.97634	0.8478
DHX35	925.14259	435.4487	9.855090519	8.769668286		1.087173	0.84783
LOC100511730	28.9970066	47.097867	4.906746634	5.587901017		-0.69976	0.84784
LOC102166114	22.0929574	35.52997	4.529381037	5.191008663		-0.68545	0.84784
LOC100737226	22.0929574	35.52997	4.529381037	5.191008663		-0.68545	0.84784
LOC100626397	237.499292	482.54657	7.897841171	8.917511028		-1.02274	0.84786
PKP2	1195.78132	549.47512	10.22494384	9.104533542		1.121828	0.84788
MTF2	1658.35261	739.51914	10.69640478	9.53239322		1.165091	0.84796
MASP2	85.6102098	153.68778	6.436465198	7.273215394		-0.84415	0.84796
LOC100518101	1982.84292	870.07113	10.95408209	9.766646714		1.188365	0.84798
SLC31A2	89.7526393	161.95056	6.503867696	7.348290508		-0.85153	0.84805
ABHD14B	676.59682	1556.7085	9.404283293	10.60520952		-1.20213	0.84805
LOC100627387	27.6161967	17.351846	4.838760036	4.19785327		0.670426	0.84809
DCTN6	1582.40807	708.94684	10.6288174	9.471567197		1.158372	0.84812
LOC733653	2079.49961	908.90621	11.0227143	9.829574033		1.194033	0.84814
ZNF367	56.6132033	34.703692	5.848327568	5.158001345		0.706049	0.84821
LOC100525232	446.001577	225.574	8.804136111	7.823838478		0.983448	0.84827
LOC102159291	8.28485901	4.1313919	3.214880004	2.359350201		1.003849	0.84831
LOC100511392	8.28485901	4.1313919	3.214880004	2.359350201		1.003849	0.84831
LOC102161560	8.28485901	4.1313919	3.214880004	2.359350201		1.003849	0.84831
LOC100621282	8.28485901	4.1313919	3.214880004	2.359350201		1.003849	0.84831
C13H3orf35	8.28485901	4.1313919	3.214880004	2.359350201		1.003849	0.84831
LOC102160141	8.28485901	4.1313919	3.214880004	2.359350201		1.003849	0.84831
LOC102162958	8.28485901	4.1313919	3.214880004	2.359350201		1.003849	0.84831
FNDC1	8.28485901	4.1313919	3.214880004	2.359350201		1.003849	0.84831
LOC102163019	8.28485901	4.1313919	3.214880004	2.359350201		1.003849	0.84831
PHTF2	451.524816	989.88149	8.821853101	9.952568709		-1.13245	0.84833
LOC100525517	62.1364426	107.41619	5.98040107	6.76043638		-0.7897	0.84836
LOC102161772	737.352452	355.2997	9.52816584	8.476947458		1.053318	0.84853
CNTN3	400.434852	204.91704	8.649022066	7.685919384		0.966528	0.84864
VAC14	454.286436	995.65544	8.830630669	9.96096549		-1.13206	0.84865
LOC102160340	157.412321	303.24416	7.307540742	8.249085772		-0.94593	0.84865
LOC102161277	63.5172524	109.89502	6.011613095	6.793050813		-0.79091	0.84869
HAGHL	114.607216	212.35354	6.853087646	7.737102248		-0.88977	0.8487
TNFRSF21	640.695764	313.1595	9.325745649	8.29535341		1.032742	0.84878
SLC7A1	281.685207	582.52625	8.143052576	9.188653752		-1.04824	0.8488
ATG2B	515.042069	1145.2218	9.011344871	10.16267055		-1.15286	0.8488
LOC100738572	1129.50245	523.03421	10.1427484	9.033517185		1.11071	0.84887
ST3GAL6	56.6132033	96.674569	5.848327568	6.609911086		-0.772	0.84887
LOC102164123	6.90404918	12.394176	2.982591923	3.74353388		-0.84415	0.8489
LOC102166384	6.90404918	12.394176	2.982591923	3.74353388		-0.84415	0.8489
LOC102158901	6.90404918	12.394176	2.982591923	3.74353388		-0.84415	0.8489
VWA3A	6.90404918	12.394176	2.982591923	3.74353388		-0.84415	0.8489
LOC102160319	6.90404918	12.394176	2.982591923	3.74353388		-0.84415	0.8489
LOC100516934	149.127462	84.280394	7.230044098	6.414142197		0.823277	0.84893
LOC102158759	60.7556328	37.182527	5.948498827	5.254840671		0.708393	0.84894
ECE2	593.748229	292.50254	9.216135261	8.197229196		1.021402	0.84895
LOC102159106	20.7121475	33.051135	4.440430524	5.089630977		-0.67422	0.84897
LOC100624002	20.7121475	33.051135	4.440430524	5.089630977		-0.67422	0.84897
LOC100737090	64.8980623	112.37386	6.042164139	6.824944216		-0.79206	0.84903
LOC100623533	158.793131	305.723	7.320061583	8.260792531		-0.94508	0.8491
CCDC175	37.2818656	61.1446	5.258589232	5.957557119		-0.71375	0.84914
LOC102167559	45.5667246	76.01761	5.541227504	6.267116453		-0.73835	0.84922
LOC100623438	113.226407	209.04843	6.835752397	7.714578179		-0.88463	0.84922
PLBD1	11.0464787	18.178124	3.590539588	4.261389712		-0.71862	0.84925
LOC102168015	11.0464787	18.178124	3.590539588	4.261389712		-0.71862	0.84925
LOC100520170	11.0464787	18.178124	3.590539588	4.261389712		-0.71862	0.84925
LOC100739841	11.0464787	18.178124	3.590539588	4.261389712		-0.71862	0.84925
LRFN3	120.130456	69.407383	6.920417836	6.137654819		0.791441	0.84926
SLC35F3	24.854577	15.699289	4.692347799	4.061714779		0.662812	0.84926
KLF8	35.9010557	58.665764	5.205590187	5.89883146		-0.70849	0.84926
NCAPH2	4899.1133	1992.1572	12.25859939	10.96083975		1.298189	0.84928
SLC35F5	676.59682	1550.9245	9.404283293	10.59984266		-1.19676	0.84932
LOC100155280	100.799118	183.4338	6.669581252	7.526959252		-0.86378	0.84932
NEK11	16.569718	26.440908	4.135019133	4.77825631		-0.67422	0.84933
LOC100627440	93.8950688	169.38707	6.568261215	7.412672017		-0.8512	0.84938
S100A13	680.739249	331.33763	9.413076234	8.376505833		1.0388	0.84943
IL4R	1469.18167	3674.4599	10.52177872	11.84370907		-1.32252	0.84951
WRB	2363.94644	1024.5852	11.20759179	10.00223161		1.206157	0.84952
CHM	425.289429	922.12666	8.735689471	9.850384804		-1.11652	0.84958
LOC100627009	263.734679	539.55978	8.048403381	9.078310354		-1.0327	0.84961
LOC102159884	49.7091541	83.454116	5.664174303	6.400095824		-0.74747	0.84963
LOC102164408	49.7091541	83.454116	5.664174303	6.400095824		-0.74747	0.84963
LOC102165903	49.7091541	83.454116	5.664174303	6.400095824		-0.74747	0.84963
LRRC46	49.7091541	83.454116	5.664174303	6.400095824		-0.74747	0.84963
LOC100624412	121.511266	70.233662	6.936770608	6.154487245		0.790856	0.84966
LOC102161163	247.164961	501.55097	7.95515562	8.97312612		-1.02092	0.84966
LOC100622542	132.557744	76.01761	7.061319821	6.267116453		0.802215	0.8498
FAM172A	95.2758787	171.8659	6.58910248	7.433509508		-0.8511	0.84981
LOC100623125	211.263905	115.67897	7.729715254	6.86640077		0.86892	0.84981
SNRPD3	13349.6695	5008.0732	13.70462447	12.29032798		1.414476	0.84986
WDR5B	393.530803	202.4382	8.623994134	7.6684468		0.958995	0.84986
LOC100156697	719.401924	348.68947	9.492568225	8.449930555		1.044855	0.84988
TIMM9	2466.12637	1065.8991	11.2686159	10.05920803		1.210176	0.84996
LOC102157436	13.8080984	8.2627837	3.888314478	3.211445828		0.740815	0.85011
LOC102161368	13.8080984	8.2627837	3.888314478	3.211445828		0.740815	0.85011
LOC100511868	13.8080984	8.2627837	3.888314478	3.211445828		0.740815	0.85011
LOC102158865	13.8080984	8.2627837	3.888314478	3.211445828		0.740815	0.85011
LOC102167243	13.8080984	8.2627837	3.888314478	3.211445828		0.740815	0.85011
LOC100626489	111.845597	65.275991	6.818206315	6.05041444		0.776884	0.85012
SMCR7L	2444.03341	1057.6363	11.25563846	10.04799134		1.20842	0.85015
TUBB1	80.0869705	141.2936	6.341398207	7.152726981		-0.81906	0.85019
LOC102165614	113.226407	208.22215	6.835752397	7.708891783		-0.87891	0.85021
TMEM209	711.117065	345.38436	9.475970616	8.436229978		1.041884	0.85024
MAATS1	67.659682	41.313919	6.101391269	5.403060391		0.711668	0.85025
LOC100624184	226.452813	453.62683	7.829423468	8.828539005		-1.0023	0.85027
LOC100152387	19.3313377	30.5723	4.345633235	4.980587446		-0.66128	0.85033
LOC100627208	853.340479	407.35524	9.738667328	8.673680921		1.066834	0.85038
ZNF614	30.3778164	48.750424	4.971673052	5.636636914		-0.6824	0.8504
LOC100737804	75.9445541	133.03082	6.265747069	7.066420948		-0.80874	0.8504
LOC100152436	100.799118	182.60752	6.669581252	7.52048134		-0.85726	0.85041
LOC100737851	162.935561	313.1595	7.356985025	8.29535341		-0.9426	0.85041
CREBBP	559.227984	1249.3329	9.129870238	10.28809654		-1.15965	0.85045
MRPL46	1982.84292	875.0288	10.95408209	9.774834483		1.180168	0.85049
LOC100623970	639.314954	313.98578	9.322637893	8.299142895		1.025828	0.85051
KMT2D	1679.06476	4249.5497	10.71430113	12.0534337		-1.33965	0.85053
PKNOX2	753.92217	364.38876	9.560184105	8.513288452		1.048937	0.85054
LOC102161371	23.4737672	37.182527	4.613164285	5.254840671		-0.66358	0.85054
LOC102157695	23.4737672	37.182527	4.613164285	5.254840671		-0.66358	0.85054
ANXA9	23.4737672	37.182527	4.613164285	5.254840671		-0.66358	0.85054
LOC100525758	299.635734	620.53506	8.231872692	9.279691957		-1.0503	0.85063
LOC100621766	7773.95938	3050.6197	12.92461942	11.57535949		1.349547	0.85066
KBTBD7	491.568302	247.88351	8.944179978	7.959326845		0.98773	0.85067
SMURF2	421.147	908.90621	8.721601651	9.829574033		-1.10981	0.85067
PLEKHA8	922.38097	2182.2012	9.85078219	11.09222936		-1.24235	0.85071
PLCD3	753.92217	1742.6211	9.560184105	10.76787084		-1.20877	0.85075
FTH1	47481.9078	166579.37	15.53512067	17.3458589		-1.81076	0.85081
IGDCC3	28.9970066	46.271589	4.906746634	5.56290145		-0.67422	0.85088
ADK	1890.32867	838.67255	10.88518437	9.71368301		1.172458	0.85093
LOC100736845	314.824643	655.23875	8.302979933	9.358076973		-1.05747	0.85095
LOC102157443	288.589256	153.68778	8.177864267	7.273215394		0.909015	0.85099
LOC102164106	71.8021115	43.792754	6.185908388	5.485193456		0.713334	0.85108
TRIP11	597.890659	1343.5286	9.22614882	10.39288476		-1.16807	0.85112
LOC100739021	42.8051049	70.233662	5.453027102	6.154487245		-0.71438	0.85128
SNRPC	7003.46749	2775.4691	12.77405966	11.4390356		1.335338	0.85129
OTUB2	84.2294	148.73011	6.413279271	7.226220529		-0.8203	0.85134
LOC102159272	40.0434852	65.275991	5.359081339	6.05041444		-0.70498	0.85136
LOC100517424	660.027102	323.90112	9.368565612	8.343856914		1.026972	0.85139
LOC102161791	8.28485901	14.046732	3.214880004	3.911378308		-0.76169	0.85141
LOC102159942	8.28485901	14.046732	3.214880004	3.911378308		-0.76169	0.85141
42614	8.28485901	14.046732	3.214880004	3.911378308		-0.76169	0.85141
LOC100516365	8.28485901	14.046732	3.214880004	3.911378308		-0.76169	0.85141
PPAPDC3	8.28485901	14.046732	3.214880004	3.911378308		-0.76169	0.85141
LOC404703	8.28485901	14.046732	3.214880004	3.911378308		-0.76169	0.85141
LOC100523481	8.28485901	14.046732	3.214880004	3.911378308		-0.76169	0.85141
LOC102167376	8.28485901	14.046732	3.214880004	3.911378308		-0.76169	0.85141
WDR18	4817.64552	1970.6739	12.23441196	10.94520526		1.289639	0.85144
RPL24	79852.2328	26294.657	16.28506319	14.6825369		1.602563	0.85144
LOC100627865	48.3283443	80.149002	5.624344958	6.342501448		-0.72981	0.85149
LOC102166268	15.1889082	23.962073	4.016933786	4.641665831		-0.65773	0.85151
LOC102160063	15.1889082	23.962073	4.016933786	4.641665831		-0.65773	0.85151
LOC396858	22.0929574	34.703692	4.529381037	5.158001345		-0.6515	0.8516
HDHD1	149.127462	85.106672	7.230044098	6.428053129		0.809202	0.85166
ZMYND11	1338.00473	3291.893	10.38694534	11.68513993		-1.29883	0.85172
LOC100621981	7641.40163	22559.052	12.89981036	14.46148278		-1.5618	0.85172
LOC102160255	124.272885	229.70539	6.968930373	7.849907883		-0.88627	0.85178
MYO9B	2855.51474	7624.8968	11.48004026	12.89669129		-1.41697	0.85179
LOC100626126	709.736256	346.21064	9.473170485	8.439637339		1.035633	0.85181
RNF113A	1206.8278	559.39046	10.23819906	9.13028858		1.109292	0.85183
SDAD1	4795.55256	1964.0637	12.22778215	10.94036036		1.287855	0.85184
LOC100623628	12.4272885	19.830681	3.747096094	4.380638094		-0.67422	0.85184
LOC100623424	202.979046	112.37386	7.672277146	6.824944216		0.853024	0.85191
CENPP	625.506856	309.02811	9.291186486	8.276255224		1.017288	0.85192
LOC100738280	17.9505279	28.093465	4.24416613	4.862623207		-0.64621	0.85196
LOC100625641	17.9505279	28.093465	4.24416613	4.862623207		-0.64621	0.85196
LOC102165773	20.7121475	13.220454	4.440430524	3.829895615		0.647705	0.85197
LOC100737676	20.7121475	13.220454	4.440430524	3.829895615		0.647705	0.85197
ESYT3	34.5202459	55.360651	5.150569664	5.816616368		-0.68142	0.85206
ATP8A1	26.2353869	41.313919	4.767410456	5.403060391		-0.65511	0.85217
SAMD15	26.2353869	41.313919	4.767410456	5.403060391		-0.65511	0.85217
LOC102166660	55.2323934	92.543178	5.813329549	6.547560532		-0.74461	0.85221
HSD17B6	107.703167	63.623435	6.764250165	6.013985524		0.759431	0.85227
LOC100739186	204.359856	113.20014	7.682010377	6.83542057		0.852236	0.85227
LOC100626282	947.235547	2237.5618	9.889101667	11.12835645		-1.24013	0.85231
MUC13	476.379393	242.09956	8.898992482	7.925403489		0.976511	0.85232
GAN	120.130456	220.61633	6.920417836	7.791920351		-0.87694	0.85233
DFNB59	44.1859147	28.093465	5.497801224	4.862623207		0.653352	0.85234
PCDHA1	1.38080984	4.9576702	1.251452392	2.574748269		-1.84415	0.85235
LOC100736907	1.38080984	4.9576702	1.251452392	2.574748269		-1.84415	0.85235
LOC102159552	1.38080984	4.9576702	1.251452392	2.574748269		-1.84415	0.85235
LOC102168150	1.38080984	4.9576702	1.251452392	2.574748269		-1.84415	0.85235
LOC102168173	1.38080984	4.9576702	1.251452392	2.574748269		-1.84415	0.85235

TRA2A	7470.241947	2335.889	12.86713237	11.19037347		1.677183	0.73333
LOC102158442	13.74566813	28.919743	3.882219287	4.903025878		-1.07308	0.73342
CDKN2AIP	1754.158725	626.31901	10.77738579	9.293055462		1.48581	0.73349
LOC102161009	215.7012537	94.195734	7.75956369	6.572825024		1.195301	0.73353
LOC100522104	97.27703596	45.44531	6.618782442	5.537461031		1.097968	0.73371
HMGCR	14429.77945	4264.4227	13.81686161	12.058473		1.758627	0.73371
LOC100620664	66.61362245	149.55639	6.079242038	7.234160086		-1.1668	0.73371
MYT1L	65.55626337	31.398578	6.056502534	5.017858594		1.062034	0.73372
PSD	9.516231779	20.656959	3.394545939	4.436758793		-1.11817	0.73384
LOC102167575	30.66341351	14.873011	4.98474489	3.98850389		1.043822	0.73399
ANKRD46	2128.463841	746.95565	11.05627452	9.546808914		1.510718	0.73407
NOP2	7025.093771	2211.1209	12.77850712	11.11121454		1.66774	0.73408
LOC100738527	6133.740061	22616.892	12.5827865	14.46517683		-1.88256	0.73413
LOC100737304	26.43397716	55.360651	4.777891883	5.816616368		-1.06647	0.73415
DCTN1	3911.171261	13770.755	11.93375381	13.74942484		-1.81594	0.73432
P2RY1	1804.911961	643.67085	10.81851185	9.332418944		1.487533	0.73439
LOC100511579	225.2174854	577.56858	7.821566636	9.176344172		-1.35867	0.73441
LOC102163404	7266.171643	2281.3546	12.82717827	11.15630723		1.671305	0.73442
VAMP2	729.5777697	2141.7135	9.512894045	11.06522327		-1.55363	0.73452
LOC100518286	31.7207726	66.928548	5.032134908	6.085946114		-1.07719	0.73456
LOC100520821	31.7207726	66.928548	5.032134908	6.085946114		-1.07719	0.73456
HAL	3.17207726	0	2.060765874	0		20	0.73463
A4GNT	3.17207726	0	2.060765874	0		20	0.73463
SLC22A16	3.17207726	0	2.060765874	0		20	0.73463
LOC100628233	3.17207726	0	2.060765874	0		20	0.73463
TDRD9	3.17207726	0	2.060765874	0		20	0.73463
LOC100736882	3.17207726	0	2.060765874	0		20	0.73463
DPPA4	3.17207726	0	2.060765874	0		20	0.73463
LOC102159250	3.17207726	0	2.060765874	0		20	0.73463
LOC102159263	3.17207726	0	2.060765874	0		20	0.73463
LOC102159350	3.17207726	0	2.060765874	0		20	0.73463
IL31RA	3.17207726	0	2.060765874	0		20	0.73463
LOC102159559	3.17207726	0	2.060765874	0		20	0.73463
LOC102161625	3.17207726	0	2.060765874	0		20	0.73463
LOC102168057	3.17207726	0	2.060765874	0		20	0.73463
LOC102168043	3.17207726	0	2.060765874	0		20	0.73463
LOC102157517	3.17207726	0	2.060765874	0		20	0.73463
LOC100628186	3.17207726	0	2.060765874	0		20	0.73463
LOC102161357	3.17207726	0	2.060765874	0		20	0.73463
LOC102168171	3.17207726	0	2.060765874	0		20	0.73463
LOC102157634	3.17207726	0	2.060765874	0		20	0.73463
LOC102161258	3.17207726	0	2.060765874	0		20	0.73463
LOC102161228	3.17207726	0	2.060765874	0		20	0.73463
LOC102161101	3.17207726	0	2.060765874	0		20	0.73463
LOC102161179	3.17207726	0	2.060765874	0		20	0.73463
NMU	3.17207726	0	2.060765874	0		20	0.73463
C7H6orf223	3.17207726	0	2.060765874	0		20	0.73463
LOC102166833	3.17207726	0	2.060765874	0		20	0.73463
LOC102166947	3.17207726	0	2.060765874	0		20	0.73463
LOC102166668	3.17207726	0	2.060765874	0		20	0.73463
LOC102166698	3.17207726	0	2.060765874	0		20	0.73463
LOC100524824	3.17207726	0	2.060765874	0		20	0.73463
LOC102157880	3.17207726	0	2.060765874	0		20	0.73463
ASB10	3.17207726	0	2.060765874	0		20	0.73463
SP6	3.17207726	0	2.060765874	0		20	0.73463
LOC102164061	3.17207726	0	2.060765874	0		20	0.73463
LOC102164157	3.17207726	0	2.060765874	0		20	0.73463
LOC102157965	3.17207726	0	2.060765874	0		20	0.73463
GALNTL5	3.17207726	0	2.060765874	0		20	0.73463
LOC102166491	3.17207726	0	2.060765874	0		20	0.73463
NYAP2	3.17207726	0	2.060765874	0		20	0.73463
LOC100623946	3.17207726	0	2.060765874	0		20	0.73463
LOC102160572	3.17207726	0	2.060765874	0		20	0.73463
HTR3B	3.17207726	0	2.060765874	0		20	0.73463
LOC100625090	3.17207726	0	2.060765874	0		20	0.73463
LOC102158834	3.17207726	0	2.060765874	0		20	0.73463
GPR18	3.17207726	0	2.060765874	0		20	0.73463
LOC102162730	3.17207726	0	2.060765874	0		20	0.73463
LOC102158768	3.17207726	0	2.060765874	0		20	0.73463
LOC102162318	3.17207726	0	2.060765874	0		20	0.73463
CDHR1	3.17207726	0	2.060765874	0		20	0.73463
CDHR3	3.17207726	0	2.060765874	0		20	0.73463
LOC100524342	3.17207726	0	2.060765874	0		20	0.73463
LOC100519458	3.17207726	0	2.060765874	0		20	0.73463
LOC102162534	3.17207726	0	2.060765874	0		20	0.73463
LOC102167670	3.17207726	0	2.060765874	0		20	0.73463
LOC102165592	3.17207726	0	2.060765874	0		20	0.73463
LOC102167703	3.17207726	0	2.060765874	0		20	0.73463
LOC102163454	3.17207726	0	2.060765874	0		20	0.73463
IGSF9B	3.17207726	0	2.060765874	0		20	0.73463
LOC102167179	3.17207726	0	2.060765874	0		20	0.73463
SYT10	3.17207726	0	2.060765874	0		20	0.73463
LOC100515722	3.17207726	0	2.060765874	0		20	0.73463
LGR6	3.17207726	0	2.060765874	0		20	0.73463
LOC100515380	3.17207726	0	2.060765874	0		20	0.73463
SELV	3.17207726	0	2.060765874	0		20	0.73463
C3H16orf11	3.17207726	0	2.060765874	0		20	0.73463
LCN8	3.17207726	0	2.060765874	0		20	0.73463
C9H11orf53	3.17207726	0	2.060765874	0		20	0.73463
PCNX	10060.77171	3071.2767	13.29659674	11.58509244		1.711831	0.73469
LOC102167514	58.15474976	28.093465	5.886422108	4.862623207		1.049662	0.73477
ZCWPW1	48.63851798	105.76363	5.633388138	6.738276475		-1.12067	0.73478
LOC100738718	42.29436346	90.890621	5.436107306	6.521845711		-1.10367	0.73492
ARL9	562.5150341	224.74772	9.13831029	7.818567588		1.323585	0.73503
LOC100525267	229.4469218	99.979683	7.848290687	6.657921243		1.198454	0.7352
NAV1	747.5528742	2197.0742	9.547960415	11.10202437		-1.55534	0.73531
PRMT6	2012.154342	711.42568	10.97524207	9.476595168		1.499956	0.73538
LOC102157454	14.80302721	6.610227	3.982129041	2.927939483		1.163121	0.73543
SUSD3	14.80302721	6.610227	3.982129041	2.927939483		1.163121	0.73543
TMEM71	14.80302721	6.610227	3.982129041	2.927939483		1.163121	0.73543
LOC102166846	14.80302721	6.610227	3.982129041	2.927939483		1.163121	0.73543
BCL2L14	14.80302721	6.610227	3.982129041	2.927939483		1.163121	0.73543
LOC102167396	14.80302721	6.610227	3.982129041	2.927939483		1.163121	0.73543
LOC102160274	78.24457241	177.64985	6.308240221	7.480990892		-1.18297	0.73567
LOC102167095	38.06492712	80.97528	5.287802017	6.357117027		-1.08902	0.73572
LOC102159921	3.17207726	9.0890621	2.060765874	3.334720158		-1.5187	0.7358
PTCH2	3.17207726	9.0890621	2.060765874	3.334720158		-1.5187	0.7358
LOC102161100	3.17207726	9.0890621	2.060765874	3.334720158		-1.5187	0.7358
LOC100154071	3.17207726	9.0890621	2.060765874	3.334720158		-1.5187	0.7358
FAM71F1	3.17207726	9.0890621	2.060765874	3.334720158		-1.5187	0.7358
GPR137C	3.17207726	9.0890621	2.060765874	3.334720158		-1.5187	0.7358
MATN2	3.17207726	9.0890621	2.060765874	3.334720158		-1.5187	0.7358
LOC102167048	3.17207726	9.0890621	2.060765874	3.334720158		-1.5187	0.7358
LOC102165845	3.17207726	9.0890621	2.060765874	3.334720158		-1.5187	0.7358
MTFP1	1912.762588	680.0271	10.90219615	9.411568398		1.491994	0.73582
LOC100738466	428.2304301	175.99729	8.745608549	7.467583487		1.282834	0.73588
SEPHS1	7617.21486	2387.1182	12.89523726	11.22165854		1.673994	0.73594
LOC100512926	975.9424369	370.17271	9.932129748	8.535946834		1.398598	0.73596
MUS81	393.3375802	1072.5093	8.623287395	10.06811901		-1.44715	0.73602
LOC102165320	1598.726939	578.39486	10.64360995	9.178403076		1.466797	0.73608
ENKD1	145.9155539	354.47342	7.198843332	8.473597884		-1.28054	0.73613
LOC100523704	84.58872693	193.34914	6.419348884	7.602506906		-1.19267	0.73624
LOC100626826	269.6265671	703.16289	8.080159664	9.459765397		-1.3829	0.73628
LOC100514512	14822.05968	4386.7119	13.85555565	12.09925308		1.756534	0.7363
LOC102158659	121.596295	289.19743	6.937771569	8.180890933		-1.24996	0.73632
C2H19orf24	464.180639	1288.9943	8.861647242	10.33314893		-1.47349	0.73645
JAG1	3928.089007	1307.9987	11.93997913	10.35424791		1.586467	0.73672
LOC100517438	1609.30053	582.52625	10.65311425	9.188653752		1.466039	0.73676
POU2F1	253.7661808	109.89502	7.993029968	6.793050814		1.207374	0.73676
LRRC8D	507.5323616	1422.8514	8.990195776	10.47558283		-1.48721	0.73682
EPX	7.401513606	16.525567	3.070649265	4.131389251		-1.15881	0.7369
NEU3	81.41664967	38.835083	6.364863912	5.315967693		1.067963	0.73705
MRPL46	2520.744062	875.0288	11.30020615	9.774834483		1.526447	0.73708
DIS3L2	201.9555855	508.1612	7.665020235	8.99197867		-1.33125	0.73719
HSD17B8	308.7488533	816.36303	8.274955131	9.674833183		-1.40278	0.73721
LOC100739745	1233.938054	458.5845	10.27022296	8.84418632		1.42801	0.73727
MAFK	465.2379981	1289.8205	8.864922778	10.33407272		-1.47113	0.73736
ABCC1	1054.187009	3206.7864	10.04328299	11.64736235		-1.605	0.73741
LOC102160522	117.3668586	54.534373	6.887121389	5.795309087		1.105787	0.73743
LOC100737089	970.6556415	369.34643	9.924301297	8.532731628		1.393985	0.73745
USP35	684.111329	269.36675	9.420194632	8.078773924		1.344659	0.73746
JMJD1C	8725.327182	2708.5405	13.09115885	11.4038325		1.687694	0.73757
LOC100736873	50.75323616	109.89502	5.693577173	6.793050814		-1.11455	0.73762
TOMM20L	142.7434767	65.275991	7.167352675	6.05041444		1.1288	0.73763
LOC102159366	20.08982264	41.313919	4.398475058	5.403060391		-1.04016	0.73785
LOC100627897	14.80302721	30.5723	3.982129041	4.980587447		-1.04633	0.73793
DOLK	522.3353888	1465.8178	9.031592009	10.51847399		-1.48866	0.73794
LOC100524897	207.242381	91.716899	7.702119902	6.534760414		1.17606	0.738
C1H15orf48	23.2618999	47.924146	4.600620625	5.61247475		-1.04278	0.73806
LOC102163391	86.7034451	41.313919	6.45456161	5.403060391		1.069461	0.73831
TRAK1	2145.381587	757.69727	11.06769087	9.56738053		1.501541	0.73836
SGPP1	2564.095785	890.72808	11.32479698	9.800460045		1.525393	0.73849
GPR75	10.57359087	22.309516	3.532764645	4.542847146		-1.07719	0.73855
TMEM206	492.7293343	200.78564	8.947576553	7.65667973		1.295139	0.73856
ZNF74	86.7034451	197.48053	6.45456161	7.632853689		-1.18755	0.73863
LOC102165586	654.5052746	259.45141	9.356463579	8.02487043		1.33494	0.73874
LOC100626038	1006.60585	382.56689	9.976715689	8.583334366		1.395715	0.73876
MRPS9	5719.255299	1846.7322	12.48186382	10.85153993		1.630853	0.73881
LOC100625079	26.43397716	54.534373	4.777891883	5.795309087		-1.04477	0.73898
LOC100739372	834.2563193	2463.9621	9.706075182	11.26734975		-1.56242	0.73908
LOC100154858	22.20454082	10.741619	4.536335244	3.553559423		1.047643	0.73912
DMXL1	2642.340357	916.34271	11.36814648	9.841317007		1.527857	0.73917
POLR3E	3596.078253	1212.1504	11.81260983	10.24454267		1.568856	0.73929
DGAT	315.0930078	831.23604	8.304205312	9.700848959		-1.39948	0.73934
CDR2L	931.5333553	2783.7318	9.865011519	11.44332269		-1.57934	0.73943
IKBKB	386.9934257	1045.2421	8.599888397	10.03100107		-1.43346	0.73958
MAP4K5	132.1698858	314.81206	7.057124067	8.302922453		-1.2521	0.73958
ARF5	555.1135205	1563.3187	9.119235603	10.61131873		-1.49376	0.73961
PRPF4	4054.972097	1352.6177	11.98583201	10.40260462		1.583938	0.73965
LOC100622965	22.20454082	45.44531	4.536335244	5.537461031		-1.03328	0.73976
IDNK	347.8711395	147.07755	8.446550445	7.210209122		1.241976	0.73989
CNTROB	312.9782896	823.79954	8.294520996	9.687899712		-1.39623	0.74
PDE6D	3376.147563	1146.0481	11.7215895	10.16371018		1.55871	0.74003
LOC100621926	526.5648251	1472.4281	9.043204567	10.52496091		-1.48351	0.7401
SLC16A1	3999.989425	1336.9184	11.9661411	10.38577442		1.581085	0.7401
ATF7	1257.199954	3879.377	10.2971455	11.92198109		-1.62561	0.74013
LOC100620632	759.1838242	297.46021	9.570204516	8.221394816		1.351753	0.74032
LOC100739778	53.92531342	116.50525	5.779399292	6.876581411		-1.11136	0.74036
DNAH12	4.229436346	10.741619	2.386655454	3.553559423		-1.34467	0.74044
LOC102160236	4.229436346	10.741619	2.386655454	3.553559423		-1.34467	0.74044
LOC100516662	19.03246356	9.0890621	4.324267947	3.334720158		1.066259	0.74048
LOC100155531	19.03246356	9.0890621	4.324267947	3.334720158		1.066259	0.74048
C9H11orf71	19.03246356	9.0890621	4.324267947	3.334720158		1.066259	0.74048
LOC102162237	58.15474976	126.42059	5.886422108	6.993454622		-1.12026	0.7407
SCAF8	6354.72811	2039.255	12.63384169	10.99453378		1.639788	0.74091
ANGPTL3	11.63094995	4.9576702	3.658891241	2.57474827		1.230235	0.74094
LOC102166775	11.63094995	4.9576702	3.658891241	2.57474827		1.230235	0.74094
ANKRD53	11.63094995	4.9576702	3.658891241	2.57474827		1.230235	0.74094
LOC100521253	11.63094995	4.9576702	3.658891241	2.57474827		1.230235	0.74094
GNG10	16297.076	4817.2029	13.99241401	12.23427944		1.758346	0.74099
CAMK1G	7.401513606	2.4788351	3.070649265	1.798604302		1.578158	0.74103
LOXHD1	7.401513606	2.4788351	3.070649265	1.798604302		1.578158	0.74103
CXCR6	7.401513606	2.4788351	3.070649265	1.798604302		1.578158	0.74103
LOC102166625	7.401513606	2.4788351	3.070649265	1.798604302		1.578158	0.74103
LOC100626684	7.401513606	2.4788351	3.070649265	1.798604302		1.578158	0.74103
KCNK4	7.401513606	2.4788351	3.070649265	1.798604302		1.578158	0.74103
DLL4	7.401513606	2.4788351	3.070649265	1.798604302		1.578158	0.74103
LOC102160695	7.401513606	2.4788351	3.070649265	1.798604302		1.578158	0.74103
LOC102160576	7.401513606	2.4788351	3.070649265	1.798604302		1.578158	0.74103
LOC102162329	7.401513606	2.4788351	3.070649265	1.798604302		1.578158	0.74103
LOC102162496	7.401513606	2.4788351	3.070649265	1.798604302		1.578158	0.74103
LOC100153492	7.401513606	2.4788351	3.070649265	1.798604302		1.578158	0.74103
LOC102163480	7.401513606	2.4788351	3.070649265	1.798604302		1.578158	0.74103
GLRA1	7.401513606	2.4788351	3.070649265	1.798604302		1.578158	0.74103
LOC100519597	725.3483334	285.89232	9.504517774	8.164365521		1.343202	0.74108
SNAPC3	1593.440143	581.69997	10.63883422	9.186609435		1.453798	0.74115
POP1	2208.823132	781.65934	11.10971519	9.612240686		1.498666	0.74119
ADM5	169.1774539	76.843889	7.410896102	6.282511874		1.138535	0.74125
LOC102165643	169.1774539	76.843889	7.410896102	6.282511874		1.138535	0.74125
LOC100155534	149.0876312	358.60481	7.229661278	8.490268521		-1.26623	0.74127
PRMT2	113.1374223	263.5828	6.834628076	8.047574471		-1.22018	0.74132
ZFYVE26	1077.448909	3260.4945	10.07474212	11.67131746		-1.59747	0.74133
DNAAF1	8.458872693	18.178124	3.241668254	4.261389711		-1.10367	0.74134
LOC102161393	8.458872693	18.178124	3.241668254	4.261389711		-1.10367	0.74134
LOC102163646	8.458872693	18.178124	3.241668254	4.261389711		-1.10367	0.74134
LOC102162214	8.458872693	18.178124	3.241668254	4.261389711		-1.10367	0.74134
HNRNPH1	64546.48544	17043.644	15.97807327	14.05703084		1.921105	0.74144
ALPK2	41.23700438	86.759229	5.400435611	6.455478947		-1.07308	0.7415
LOC102167686	41.23700438	86.759229	5.400435611	6.455478947		-1.07308	0.7415
KCTD13	41.23700438	86.759229	5.400435611	6.455478947		-1.07308	0.7415
LOC102166665	4572.02069	1512.9157	12.15893173	10.56406916		1.5955	0.7415
PEX11A	810.9944194	316.46462	9.665326002	8.310451992		1.357648	0.74162
GBP2	218.8733309	550.3014	7.780528816	9.106697443		-1.33013	0.74162
AAK1	699.9717153	2016.1192	9.453212421	10.97808065		-1.52621	0.74164
SLC35C2	704.2011517	2029.3397	9.46189102	10.9875054		-1.52695	0.74171
DNAJC4	357.3873713	951.87268	8.485375991	9.896139655		-1.41328	0.74172
NOTCH4	95.16231779	45.44531	6.587399765	5.537461031		1.066259	0.74182
LOC102164205	13.74566813	28.093465	3.882219287	4.862623207		-1.03126	0.74191
STEAP2	75.07249515	166.90823	6.249303021	7.391529145		-1.1527	0.74193
LOC102164870	66.61362245	146.25127	6.079242038	7.202136284		-1.13456	0.74199
NR3C1	701.0290744	2017.7718	9.45538697	10.97926211		-1.52522	0.74204
LOC100620598	320.3798032	137.16221	8.328135457	7.110219252		1.2239	0.74205
MICALL1	803.5929058	2349.1094	9.652115207	11.19851221		-1.54758	0.74208
LOC100624624	162.8332993	74.365053	7.356084806	6.2358238		1.130699	0.74209
CCR4	67.67098154	148.73011	6.10162868	7.22622053		-1.13609	0.74219
NOL3	282.3148761	730.43008	8.146262546	9.51457615		-1.37144	0.74226
LOC100623571	182.923122	449.49543	7.52295905	8.815368674		-1.29707	0.74236
CTNND2	560.4003159	227.22655	9.132886065	7.834322837		1.302326	0.74254
ARL4D	176.5789675	80.149002	7.472316908	6.342501448		1.139557	0.74266
FAM155A	31.7207726	65.275991	5.032134908	6.05041444		-1.04112	0.74272
AASS	2202.478977	781.65934	11.10556742	9.612240686		1.494516	0.74273
SIRT7	348.9284986	924.6055	8.450916354	9.854253624		-1.40591	0.74276
LOC100155322	11.63094995	23.962073	3.658891241	4.641665831		-1.04278	0.74278
RC3H1	6172.862347	1992.1572	12.5919576	10.96083975		1.631608	0.74288
LOC102167002	659.79207	263.5828	9.368052563	8.047574471		1.323755	0.74325
LOC100519693	296.0605442	128.07315	8.214613188	7.012045083		1.208924	0.7433
C2H19orf25	653.4479155	1860.7789	9.354134569	10.86246603		-1.50976	0.74332
ITCH	6043.864539	1955.8009	12.56149429	10.93428126		1.627712	0.74338
LOC100155029	154.3744266	71.05994	7.279605257	6.171125545		1.119325	0.74339
LOC102165076	591.0637294	238.79445	9.209608665	7.905654455		1.307544	0.74341
LOC100737121	41.23700438	20.656959	5.400435611	4.436758793		0.997312	0.74341
LOC100737156	559.3429568	227.22655	9.130166286	7.834322837		1.299602	0.74342
ART4	15.8603863	7.4365053	4.075565686	3.076645515		1.092731	0.74347
CPS1	15.8603863	7.4365053	4.075565686	3.076645515		1.092731	0.74347
GAL3ST1	15.8603863	7.4365053	4.075565686	3.076645515		1.092731	0.74347
COL4A4	15.8603863	7.4365053	4.075565686	3.076645515		1.092731	0.74347
LOC100524873	15016.61375	4486.6916	13.87436797	12.13175781		1.742836	0.74353
LOC100624081	117.3668586	55.360651	6.887121389	5.816616368		1.084092	0.74358
LOC100737790	786.6751604	309.02811	9.62145697	8.276255224		1.34803	0.74358
GCKR	257.9956171	113.20014	8.016783874	6.83542057		1.188471	0.74364
LOC102167251	64.49890428	140.46732	6.033398867	7.144235041		-1.12289	0.74371
LOC100517951	693.6275608	275.97698	9.440095844	8.113622246		1.329613	0.74373
EGR1	268.569208	688.28988	8.074511901	9.428967032		-1.35772	0.74384
SNIP1	473.6968708	195.82797	8.890862729	7.620791467		1.274377	0.74389
CALU	8231.540489	30741.687	13.00712199	14.90795564		-1.90096	0.74393
RBM12	4854.335566	1603.8063	12.24535529	10.64818348		1.597774	0.74394
LOC102164453	436.6893028	1184.8832	8.773763314	10.21174619		-1.44007	0.744
LNX1	166.0053766	76.01761	7.38375074	6.267116453		1.126824	0.744
ZGPAT	873.3786055	2567.2469	9.772114291	11.32656819		-1.55554	0.74403
BRAF	919.9024053	356.12598	9.846904461	8.480289273		1.369093	0.74404
LOC100737360	617.4977066	248.70979	9.272624435	7.964108573		1.31197	0.74407
ZNHIT3	702.0864335	279.28209	9.457558247	8.130735747		1.329926	0.74424
FAM117A	1200.102563	452.80055	10.23014363	8.82591454		1.40621	0.74426
FRRS1L	5.286795433	12.394176	2.65232482	3.74353388		-1.2292	0.74427
LOC102159548	5.286795433	12.394176	2.65232482	3.74353388		-1.2292	0.74427
LOC100152103	5.286795433	12.394176	2.65232482	3.74353388		-1.2292	0.74427
DNAH1	5.286795433	12.394176	2.65232482	3.74353388		-1.2292	0.74427

FAM109A	925.913496	420.57569	9.856290896	8.719647871		1.138512	0.73409
LOC102161361	178.219697	90.890621	7.485585395	6.521845711		0.971453	0.73418
MIR7	14.09179	27.267186	3.915692025	4.821056379		-0.95231	0.7342
IPO5	36009.497	12666.847	15.13612989	13.62888378		1.50732	0.73436
LOC102161806	10.7760747	21.483238	3.557786824	4.490777898		-0.99538	0.73436
LOC100625963	179.877555	91.716899	7.498869583	6.534760414		0.971756	0.7344
ASH2L	3653.08933	1501.3478	11.83529619	10.55300313		1.282859	0.73448
LOC100626340	661.485201	308.20183	9.371744417	8.27240506		1.101833	0.73449
KLF5	28.18358	52.881816	4.867084967	5.751726565		-0.90792	0.7345
KCNN2	28.18358	52.881816	4.867084967	5.751726565		-0.90792	0.7345
ZSCAN16	28.18358	52.881816	4.867084967	5.751726565		-0.90792	0.7345
LOC100627242	133.457541	268.54047	7.071006856	8.074358096		-1.00876	0.7346
XKR9	38.9596547	20.656959	5.320472212	4.436758793		0.915353	0.73486
KIAA1328	105.273961	208.22215	6.731644339	7.708891783		-0.98397	0.73488
PISD	1793.80197	776.70167	10.80960896	9.603073025		1.207588	0.73491
LOC102164492	283.493658	140.46732	8.15225268	7.144325041		1.013082	0.73496
LOC100524404	79.5771671	154.51406	6.332299179	7.280901168		-0.95731	0.73514
MECR	382.136188	833.71488	8.581713486	9.705139674		-1.12547	0.73521
LOC102162422	31.4992953	16.525567	5.022336531	4.13138925		0.93062	0.73528
LOC100739157	221.323996	111.54758	7.79651986	6.814391229		0.988501	0.7353
GPR161	542.94838	256.97257	9.087325937	8.011073883		1.079201	0.73534
FAM91A1	4144.64412	1689.7393	12.01738056	10.72343848		1.294448	0.73534
LOC102167693	15.7496477	7.4365053	4.066058842	3.076645515		1.082623	0.73536
COL4A4	15.7496477	7.4365053	4.066058842	3.076645515		1.082623	0.73536
DNAJB13	15.7496477	7.4365053	4.066058842	3.076645515		1.082623	0.73536
PPARGC-1	15.7496477	7.4365053	4.066058842	3.076645515		1.082623	0.73536
C7H6orf141	820.639535	376.78294	9.682361792	8.561413734		1.123015	0.73546
LOC100736562	188.166843	95.848291	7.563515426	6.597654688		0.973188	0.7355
LOC102157559	11.6050035	4.9576702	3.655924617	2.574748269		1.227013	0.73556
LOC102166775	11.6050035	4.9576702	3.655924617	2.574748269		1.227013	0.73556
LOC100152300	11.6050035	4.9576702	3.655924617	2.574748269		1.227013	0.73556
LOC102164164	11.6050035	4.9576702	3.655924617	2.574748269		1.227013	0.73556
LOC102166291	11.6050035	4.9576702	3.655924617	2.574748269		1.227013	0.73556
LOC102164944	11.6050035	4.9576702	3.655924617	2.574748269		1.227013	0.73556
LOC102158474	11.6050035	4.9576702	3.655924617	2.574748269		1.227013	0.73556
TMEM125	11.6050035	4.9576702	3.655924617	2.574748269		1.227013	0.73556
LOC102165659	11.6050035	4.9576702	3.655924617	2.574748269		1.227013	0.73556
GPR108	285.151515	607.3146	8.160635436	9.248673828		-1.09072	0.73567
LOC102161744	57.1960888	30.5723	5.862850292	4.980587446		0.903691	0.73591
LOC100514500	57.1960888	30.5723	5.862850292	4.980587446		0.903691	0.73591
LOC100515931	566.987315	267.71419	9.149714901	8.06992871		1.082623	0.73593
MTA3	1043.62139	470.97867	10.02876443	8.882577857		1.147865	0.73595
ABCB7	3486.47463	1440.2032	11.76796701	10.49305805		1.275497	0.736
LOC100737507	212.205779	441.23265	7.736102732	8.788661735		-1.05608	0.7361
LOC102160736	33.9860818	63.623435	5.128709195	6.013985524		-0.90461	0.73616
LOC100515558	570.303031	269.36675	9.158112373	8.078773924		1.082157	0.73647
NCAPD3	805.718817	370.99899	9.655922097	8.53915489		1.118861	0.7366
LOC102161180	19.8942918	9.9153405	4.385036953	3.448285225		1.00462	0.73666
LOC102162479	19.8942918	9.9153405	4.385036953	3.448285225		1.00462	0.73666
CCPG1	929.229211	2172.2858	9.861442434	11.08566222		-1.22511	0.7367
LOC100737890	1145.57963	513.94515	10.16312084	9.008274952		1.156391	0.73675
SPAG8	33.1571529	61.970878	5.094115824	5.976612875		-0.90227	0.73681
FANCD2OS	37.3017971	19.830681	5.259340178	4.380638094		0.911511	0.73685
LOC100622855	502.330867	239.62073	8.975363265	7.910617116		1.067885	0.73688
RNF19B	592.684109	1336.0921	9.213551687	10.38488316		-1.17268	0.73705
FOXN2	774.219521	357.77854	9.59846109	8.486949769		1.113676	0.73707
LOC100511081	90.3532418	47.924146	6.51338402	5.61247475		0.914824	0.7372
AKIP1	731.115222	1675.6925	9.515926912	10.71140244		-1.19659	0.73723
LOC102167481	67.9721635	36.356248	6.107942317	5.223277669		0.902741	0.73732
TOMM40	8324.93218	3239.0112	13.02339609	11.66178309		1.361885	0.73735
VMP1	3077.81272	7883.5219	11.5881584	12.94480757		-1.35693	0.73735
LOC102157581	7.46035941	2.4788351	3.080718953	1.798604302		1.589583	0.73745
LOC102157606	7.46035941	2.4788351	3.080718953	1.798604302		1.589583	0.73745
LOC102157659	7.46035941	2.4788351	3.080718953	1.798604302		1.589583	0.73745
LOC102163799	7.46035941	2.4788351	3.080718953	1.798604302		1.589583	0.73745
LOC100624553	7.46035941	2.4788351	3.080718953	1.798604302		1.589583	0.73745
LOC102164474	7.46035941	2.4788351	3.080718953	1.798604302		1.589583	0.73745
LOC100516782	7.46035941	2.4788351	3.080718953	1.798604302		1.589583	0.73745
DPP6	7.46035941	2.4788351	3.080718953	1.798604302		1.589583	0.73745
LOC102158108	7.46035941	2.4788351	3.080718953	1.798604302		1.589583	0.73745
LOC100519797	7.46035941	2.4788351	3.080718953	1.798604302		1.589583	0.73745
LOC100519305	7.46035941	2.4788351	3.080718953	1.798604302		1.589583	0.73745
LOC102163110	7.46035941	2.4788351	3.080718953	1.798604302		1.589583	0.73745
LOC100627770	7.46035941	2.4788351	3.080718953	1.798604302		1.589583	0.73745
GCET2	7.46035941	2.4788351	3.080718953	1.798604302		1.589583	0.73745
LOC102163038	7.46035941	2.4788351	3.080718953	1.798604302		1.589583	0.73745
LOC100620987	7.46035941	2.4788351	3.080718953	1.798604302		1.589583	0.73745
SNIP1	403.688337	195.82797	8.660667462	7.620791468		1.043655	0.7375
IDH3A	7201.73362	2830.8297	12.81432883	11.46751879		1.347119	0.73751
NRAP	13.2628612	25.61463	3.834191515	4.734147579		-0.94958	0.73762
LOC100739678	204.745419	104.11107	7.684716501	6.715770874		0.975708	0.73775
LOC102162758	20.7232206	38.835083	4.441166102	5.315967693		-0.90611	0.73786
SULT4A1	7.46035941	15.699289	3.080718953	4.061714779		-1.07338	0.73791
TULP1	7.46035941	15.699289	3.080718953	4.061714779		-1.07338	0.73791
LOC102165025	7.46035941	15.699289	3.080718953	4.061714779		-1.07338	0.73791
LOC100738679	893.585272	409.00779	9.805075196	8.679507525		1.127477	0.73792
LOC102157541	4.97357294	11.567897	2.578594101	3.65167138		-1.21777	0.73797
LOC102166257	4.97357294	11.567897	2.578594101	3.65167138		-1.21777	0.73797
PLXNA4	4.97357294	11.567897	2.578594101	3.65167138		-1.21777	0.73797
CRISPLD1	4.97357294	11.567897	2.578594101	3.65167138		-1.21777	0.73797
LOC100737230	4.97357294	11.567897	2.578594101	3.65167138		-1.21777	0.73797
BCAN	4.97357294	11.567897	2.578594101	3.65167138		-1.21777	0.73797
LOC100737953	4.97357294	11.567897	2.578594101	3.65167138		-1.21777	0.73797
LOC100626473	16.5785765	31.398578	4.135746339	5.017858594		-0.92138	0.73815
RPAP3	1886.642	817.18931	10.88236946	9.676290877		1.207078	0.73823
LOC100523337	83.7218112	44.619032	6.404661526	5.511563931		0.907944	0.73833
TYW5	303.387949	150.38266	8.249767434	7.242056188		1.012526	0.73836
C12H17orf49	583.565892	1311.3038	9.191221843	10.357886		-1.16803	0.73849
LOC100738842	892.756343	409.00779	9.803737765	8.679507525		1.126138	0.73857
LOC100738348	1924.77273	832.8886	10.91122174	9.703710853		1.208493	0.73864
ZCWPW2	24.0389359	12.394176	4.646101346	3.74353388		0.955711	0.73879
INHBA	1090.0414	2573.0309	10.09149013	11.32981363		-1.23909	0.73895
IMMP1L	988.083158	449.49543	9.949948011	8.815368674		1.136326	0.73897
SCN2A	923.42671	422.22825	9.852415134	8.725292112		1.128974	0.739
NPW	35.6439394	19.004403	5.195502704	4.322245637		0.907323	0.73904
LOC100514431	508.133369	242.92584	8.991899814	7.930298794		1.064691	0.73906
LOC100626837	171.588267	88.411786	7.431190575	6.482393107		0.95664	0.7391
IFN-DELTA-1	9.94714588	19.830681	3.452482877	4.380638094		-0.99538	0.73929
ITPRIP	571.960888	1281.5578	9.16229285	10.32480808		-1.16391	0.73938
LOC100626975	78.7482382	42.140197	6.317380743	5.430960858		0.902051	0.73947
LOC100623881	371.360113	181.78124	8.54055473	7.513974209		1.030616	0.73958
LOC733634	112.73432	59.492043	6.829523851	5.918673475		0.922158	0.73961
LOC102159107	2.48678647	7.4365053	1.801898017	3.076645515		-1.58034	0.73967
LOC102160250	2.48678647	7.4365053	1.801898017	3.076645515		-1.58034	0.73967
LOC100625807	2.48678647	7.4365053	1.801898017	3.076645515		-1.58034	0.73967
MANSC4	2.48678647	7.4365053	1.801898017	3.076645515		-1.58034	0.73967
MIR224	2.48678647	7.4365053	1.801898017	3.076645515		-1.58034	0.73967
LOC100737489	2.48678647	7.4365053	1.801898017	3.076645515		-1.58034	0.73967
LOC102161329	300.072234	637.8869	8.233965854	9.319416755		-1.08799	0.7397
EXOC7	1233.44609	2936.5933	10.26964812	11.52041897		-1.25145	0.73984
LOC100626561	107.760747	57.013208	6.765014156	5.858309486		0.918464	0.73986
SAT2	397.885835	865.11346	8.639832083	9.758412211		-1.12053	0.73988
RBAK	202.258633	416.4443	7.667172819	8.705439898		-1.04192	0.74001
LOC102159912	29.8414377	55.360651	4.946798112	5.816616368		-0.89154	0.74003
LOC102166872	345.663319	742.82426	8.437391382	9.538817985		-1.10365	0.74009
KIF21B	3438.39676	1428.6353	11.74793984	10.48143145		1.267098	0.74011
AMPH	61.340733	33.051135	5.962103212	5.089630977		0.892146	0.74019
LOC100511067	75.432523	40.48764	6.256114748	5.374609695		0.897705	0.74039
APON	293.440804	146.25127	8.201833804	7.202136284		1.00462	0.74044
VASH2	1304.73397	582.52625	10.35064527	9.188653752		1.163361	0.74055
LOC100519868	218.008281	451.14799	7.774841608	8.820651243		-1.04922	0.74056
LOC100512133	510.620155	244.5784	8.99892929	7.940039851		1.061953	0.74066
CENPK	533.001234	254.49374	9.060699264	7.997144125		1.066509	0.74067
LOC100738242	41.4464412	22.309516	5.407571694	4.542847146		0.893589	0.74074
MTRR	5227.22516	2109.4887	12.35210556	11.04336138		1.309152	0.74085
LOC100156117	721.997005	337.12158	9.497845861	8.401398268		1.098724	0.74089
LOC102162096	189.824701	97.500848	7.576104118	6.622064238		0.961181	0.74101
SAE1	10985.7937	4210.7146	13.4234828	12.04019196		1.383502	0.74109
STAG3	35.6439394	66.10227	5.195502704	6.068289661		-0.89104	0.74122
CDKN1A	8087.0296	22121.951	12.98157256	14.43325621		-1.4518	0.74127
LOC102159828	28.18358	14.873011	4.867084967	3.98850389		0.922158	0.74134
LOC100513313	28.18358	14.873011	4.867084967	3.98850389		0.922158	0.74134
ZNF580	398.714764	865.11346	8.642827052	9.758412211		-1.11753	0.74143
LOC100738621	48.9068006	26.440908	5.641164514	4.77825631		0.887263	0.74147
LOC100517350	629.985906	297.46021	9.30146397	8.221394816		1.082623	0.74159
LOC100525262	1952.12738	846.93533	10.93157033	9.72781043		1.204723	0.74159
NRDE2	1541.80761	680.85338	10.59134245	9.413317734		1.179207	0.74171
RFK	778.364165	361.90993	9.606153789	8.50346771		1.104815	0.74193
MAP3K8	23.2100071	42.966475	4.597531595	5.458331975		-0.88846	0.74199
PRMT3	236.244715	119.81036	7.890232137	6.916600414		0.979529	0.74203
FOSL1	673.919134	1524.4836	9.398570844	10.57505095		-1.17767	0.74207
LOC100738979	28.18358	52.055537	4.867084967	5.729431428		-0.8852	0.74207
LOC100517569	215.521494	444.53776	7.758366438	8.79940391		-1.04447	0.74209
PEAK1	512.278013	1132.8276	9.003596652	10.14698564		-1.14493	0.7424
ZNF346	971.504581	444.53776	9.925561236	8.79940391		1.127915	0.74242
CARNS1	1653.713	727.12497	10.6923653	9.508042269		1.185434	0.74242
LOC100156103	203.087562	104.11107	7.673044449	6.715770874		0.963978	0.74249
FAM178B	150.865046	302.41788	7.246646039	8.245162313		-1.00328	0.74253
WDPCP	39.7885835	21.483238	5.350093502	4.490777898		0.889143	0.74262
SLC25A47	227.955427	471.80495	7.838922949	8.88510133		-1.04944	0.74275
LOC102159292	128.483968	67.754826	7.016629669	6.103389087		0.923193	0.74297
LOC100738318	184.022199	95.022013	7.531554564	6.585293272		0.953546	0.74304
LOC100623125	227.126498	115.67897	7.83369022	6.86640077		0.973369	0.74305
LOC100524658	14.09179	6.610227	3.915692025	2.927939483		1.092083	0.74327
LOC102162191	14.09179	6.610227	3.915692025	2.927939483		1.092083	0.74327
GFRA3	184.851128	375.95666	7.538003632	8.558254848		-1.0242	0.74329
LOC102162364	33.1571529	61.1446	5.094115824	5.957557119		-0.8829	0.74336
SEMA4G	295.92759	624.66645	8.213967342	9.289249932		-1.07784	0.74364
TRPV4	22.3810782	41.313919	4.547269558	5.403060391		-0.88435	0.74367
ENPEP	40.6175124	75.191332	5.379118827	6.251554969		-0.88846	0.74368
HDAC9	75.432523	143.77244	6.256114748	7.177643143		-0.93053	0.74371
LOC100626096	233.757928	118.98409	7.875030072	6.906699252		0.974247	0.74388
CCR4	77.9193094	148.73011	6.302306427	7.226220529		-0.93264	0.744
LOC100154848	14.9207188	28.093465	3.99283357	4.862623207		-0.91292	0.74406
LOC102166181	32.3282241	17.351846	5.058672541	4.19785327		0.897705	0.7441
LOC102165420	32.3282241	17.351846	5.058672541	4.19785327		0.897705	0.7441
LOC100523297	18.2364341	33.877413	4.265769485	5.124221138		-0.8935	0.74426
LOC100739477	454.252995	220.61633	8.830524699	7.791920351		1.041956	0.74427
LOC102165733	79.5771671	42.966475	6.332299179	5.458331975		0.889143	0.74428
RNF103	441.819063	962.6143	8.79057352	9.912311998		-1.1235	0.74435
CLASP2	3658.89183	1521.1785	11.83758529	10.57192182		1.266217	0.74441
CUL7	1096.67283	2571.3783	10.1002324	11.32888711		-1.22941	0.74442
NFATC1	121.852537	239.62073	6.940783842	7.910617116		-0.97562	0.74443
DNAJC28	96.9846724	187.56519	6.614484182	7.558919565		-0.95156	0.74468
BUB1	1874.20807	818.84187	10.87283497	9.679201855		1.194624	0.74476
AMPD3	130.141825	256.97257	7.03498407	8.011073883		-0.98153	0.74488
LOC102159509	9.11821706	18.178124	3.338883189	4.261389712		-0.99538	0.74495
CLCNKA	9.11821706	18.178124	3.338883189	4.261389712		-0.99538	0.74495
RC3H2	433.529775	211.52726	8.763311226	7.731504113		1.035287	0.74506
SMNDC1	2689.0451	1144.3955	11.39341465	10.16163018		1.232508	0.74513
TIAM2	470.002643	1027.8903	8.879591345	10.00687345		-1.12895	0.74523
TMCC1	1453.94116	647.80224	10.50674509	9.341634998		1.166344	0.74524
RPP38	1449.79651	646.14969	10.50262947	9.337955638		1.16591	0.7453
CACYBP	8732.76516	3417.4873	13.09238802	11.73914237		1.353503	0.74554
NECAB3	300.072234	632.10295	8.233965854	9.306296318		-1.07485	0.74574
ZCCHC11	2059.88813	894.85948	11.00905048	9.80712864		1.202833	0.74583
NAPSA	37.3017971	68.581105	5.259340178	6.120623682		-0.87857	0.74589
STK32C	30.6703665	56.186929	4.985061656	5.837613535		-0.87339	0.74605
LOC100627112	1630.503	721.34102	10.67198592	9.496536287		1.176564	0.74641
SDS	51.3935871	28.093465	5.711318333	4.862623207		0.871354	0.74647
LOC100515663	1853.48485	812.23164	10.85680277	9.667522535		1.190277	0.74648
RYBP	3244.42742	1364.1856	11.66419278	10.41488138		1.249924	0.74652
LOC100739331	70.45895	133.03082	6.159042811	7.066420948		-0.91691	0.74657
TOMM20L	122.681466	65.275991	6.950485513	6.05041444		0.910293	0.7466
LOC102157970	712.04986	335.46902	9.477859149	8.394329862		1.085799	0.74662
CXHXorf56	1739.09267	765.96005	10.76494843	9.583007622		1.182994	0.74671
ATG10	115.221107	224.74772	6.860728285	7.818567588		-0.9639	0.74675
RAPGEFL1	162.470049	85.106672	7.352882522	6.428053129		0.93283	0.74694
LOC102166332	162.470049	85.106672	7.352882522	6.428053129		0.93283	0.74694
LOC100627551	369.702255	790.7484	8.534117082	9.62889824		-1.09685	0.74697
LOC102161793	9.94714588	4.1313919	3.452482877	2.359350201		1.267655	0.74707
LOC102166243	9.94714588	4.1313919	3.452482877	2.359350201		1.267655	0.74707
LOC102162490	9.94714588	4.1313919	3.452482877	2.359350201		1.267655	0.74707
WDR45	644.906625	1443.5083	9.335181807	10.49636279		-1.16242	0.74709
KIAA0247	525.540874	1156.7897	9.040401719	10.17715754		-1.13825	0.7471
LOC100627283	2809.23978	1194.7985	11.45647752	10.22375862		1.233412	0.74724
LOC100737852	271.888654	566.82696	8.092168603	9.149307546		-1.05989	0.74724
PPP1R14A	57.1960888	106.58991	5.862850292	6.749398975		-0.89808	0.74736
FAM208B	7612.88232	3015.0898	12.89441656	11.55846366		1.336242	0.74762
LOC102168137	107.760747	57.839486	6.765014156	5.878712739		0.897705	0.74773
LOC100738647	380.47833	814.71047	8.575457296	9.671913368		-1.09847	0.74779
C9H11orf65	42.27537	23.135794	5.435474249	4.593102408		0.869691	0.74781
GOT2	5818.25141	2349.1094	12.50661786	11.19851221		1.308472	0.74782
RNF25	254.481149	527.1656	7.997073033	9.044846533		-1.0507	0.74786
RIPK4	111.076462	215.65866	6.808339514	7.75928006		-0.9572	0.74788
LOC102165939	313.335095	660.19642	8.296159547	9.368935101		-1.07519	0.7479
SNRK	2752.04369	1173.3153	11.42681179	10.19760409		1.229913	0.74798
DNAJC27	233.757928	119.81036	7.875030072	6.916600414		0.964263	0.74817
CCDC86	1415.81043	634.58179	10.46843102	9.311933979		1.15775	0.74827
LOC100517695	20.7232206	10.741619	4.441166102	3.553559423		0.948037	0.74829
LOC102167663	185.680057	96.674569	7.544423999	6.609911086		0.941611	0.74833
LOC102162720	239.56043	493.28819	7.910255542	8.94920862		-1.04204	0.74854
FEM1C	392.912262	194.17542	8.621730518	7.608627545		1.016847	0.74874
LOC102159631	24.8678647	13.220454	4.693089065	3.829895615		0.911511	0.74887
LOC100739066	24.8678647	13.220454	4.693089065	3.829895615		0.911511	0.74887
CD200	193.969345	100.80596	7.607103495	6.669678233		0.944248	0.74894
MCCC1	1658.68658	735.38775	10.69669511	9.524321819		1.173464	0.74907
LOC102160123	16.5785765	8.2627837	4.135746339	3.211445828		1.00462	0.74914
LOC102160574	4.14464412	9.9153405	2.363071282	3.448285225		-1.25841	0.74927
LOC102160376	4.14464412	9.9153405	2.363071282	3.448285225		-1.25841	0.74927
LOC102165134	4.14464412	9.9153405	2.363071282	3.448285225		-1.25841	0.74927
TAF1D	5019.16403	2051.6492	12.29351879	11.00327137		1.290663	0.74933
METTL9	956.583862	2203.6844	9.903255029	11.10635644		-1.20395	0.74939
PLEKHM3	143.404687	76.01761	7.173973754	6.267116453		0.915687	0.74942
LOC102163263	48.9068006	90.064343	5.641164514	6.508814353		-0.88092	0.74961
LOC100737194	19.8942918	36.356248	4.385036953	5.223277669		-0.86985	0.74968
LOC102157907	16.5785765	30.5723	4.135746339	4.980587446		-0.8829	0.74968
APOM	40.6175124	22.309516	5.379118827	4.542847146		0.864443	0.74974
LOC100154753	40.6175124	22.309516	5.379118827	4.542847146		0.864443	0.74974
C3H9orf89	284.322587	592.44159	8.156450147	9.212962234		-1.05914	0.74992
TMEM53	67.1432347	125.59431	6.090498528	6.98406878		-0.90346	0.74996
LOC100155308	250.336505	128.07315	7.973476417	7.012045083		0.966901	0.74998
LOC102165699	164.956836	328.85879	7.374664246	8.365704748		-0.99538	0.74998
PITX3	29.0125088	15.699289	4.907492018	4.061714779		0.885976	0.75023
LOC100517188	29.0125088	15.699289	4.907492018	4.061714779		0.885976	0.75023
RHBDD1	198.942918	402.39757	7.643444369	8.656058572		-1.01627	0.75027
LOC100521958	155.838619	309.02811	7.293137032	8.276255224		-0.98769	0.75048
LOC100623469	75.432523	41.313919	6.256114748	5.403060391		0.868559	0.75056
LOC100737591	747.693799	1685.6079	9.548231995	10.71990888		-1.17275	0.75056
LOC102161848	288.467231	146.25127	8.177256226	7.202136284		0.979958	0.75063
LOC100738639	23.2100071	42.140197	4.597531595	5.430960858		-0.86045	0.75063
LOC100627439	464.200141	227.22655	8.861707724	7.834322836		1.030616	0.75068
LOC102162624	10.7760747	20.656959	3.557786824	4.436758793		-0.9388	0.75075
ARID4A	472.48943	1025.4115	8.887188408	10.00339347		-1.11785	0.75078
LOC100738644	89.524313	48.750424	6.500233417	5.636636914		0.876865	0.75087
LOC100621972	89.524313	48.750424	6.500233417	5.636636914		0.876865	0.75087
LOC100515974	818.981678	384.21944	9.679447863	8.589536711		1.091901	0.75121
BACE1	1031.18746	2383.8131	10.01148929	11.21966049		-1.20896	0.75127
CARF	92.0110994	175.17101	6.539330984	7.460832769		-0.92888	0.75134
LOC102159973	1012.95102	467.67356	9.985772251	8.872439593		1.114991	0.75139
ZNF862	198.942918	401.57129	7.643444369	8.653100473		-1.0133	0.75151
LOC102161367	44.7621565	81.801559	5.516083133	6.371586023		-0.86985	0.75156
B3GNT3	86.2085977	47.097867	6.446398468	5.587901017		0.87217	0.75172
LOC102164004	38.9596547	21.483238	5.320472212	4.490777898		0.858769	0.75183
LOC102162460	38.9596547	21.483238	5.320472212	4.490777898		0.858769	0.75183
LOC100510904	1479.63795	3517.467	10.5320032	11.78073128		-1.24929	0.75193
LOC102157599	2442.85324	6038.4423	11.25494194	12.56019963		-1.30561	0.75202

LOC102162183	1.38080984	4.9576702	1.251452392	2.574748269	-1.84415	0.85235
LOC102157772	1.38080984	4.9576702	1.251452392	2.574748269	-1.84415	0.85235
NLRP14	1.38080984	4.9576702	1.251452392	2.574748269	-1.84415	0.85235
GATA1	1.38080984	4.9576702	1.251452392	2.574748269	-1.84415	0.85235
LOC102166558	1.38080984	4.9576702	1.251452392	2.574748269	-1.84415	0.85235
LOC100625680	1.38080984	4.9576702	1.251452392	2.574748269	-1.84415	0.85235
LOC100620741	1.38080984	4.9576702	1.251452392	2.574748269	-1.84415	0.85235
LOC102165102	1.38080984	4.9576702	1.251452392	2.574748269	-1.84415	0.85235
CACNA1E	1.38080984	4.9576702	1.251452392	2.574748269	-1.84415	0.85235
FBXO40	46.9475344	29.746021	5.583384725	4.942327829	0.658353	0.85238
NTF3	280.304397	150.38266	8.135988291	7.242056188	0.898356	0.85241
DENND2C	48.3283443	30.5723	5.624344958	4.980587446	0.660644	0.85243
LOC102165223	161.554751	91.716899	7.34478191	6.534760414	0.816764	0.85244
EDEM3	1239.96723	3017.5686	10.27724931	11.55964888	-1.28308	0.85257
LOC100624029	57.9940131	97.500848	5.882496648	6.622064238	-0.74951	0.8526
PRRG4	38.6626754	24.788351	5.309710091	4.688647628	0.641279	0.8526
ELP4	607.556328	301.59161	9.249246994	8.241228156	1.010422	0.8526
LOC100518399	4628.47457	1904.5716	12.17663275	10.89600814	1.28107	0.85263
SERPINI1	121.511266	71.05994	6.936770608	6.171125545	0.773982	0.85271
MAP2	305.158974	628.79784	8.25813716	9.298745001	-1.04304	0.85277
LOC102158962	80.0869705	48.750424	6.341398207	5.636636914	0.716153	0.85278
PLEKHA6	80.0869705	139.64104	6.341398207	7.135873883	-0.80208	0.8528
LOC100739217	80.0869705	139.64104	6.341398207	7.135873883	-0.80208	0.8528
LOC100621608	412.862141	213.17982	8.69300647	7.742678745	0.953589	0.85281
WNT3	12.4272885	7.4365053	3.747096094	3.076645515	0.740815	0.85284
LOC100519264	12.4272885	7.4365053	3.747096094	3.076645515	0.740815	0.85284
LRWD1	1126.74083	527.1656	10.13921983	9.044846533	1.095828	0.85296
LOC102165515	1060.46195	499.07214	10.05183695	8.965992412	1.087373	0.85299
ZNF391	24.854577	38.835083	4.692347799	5.315967693	-0.64385	0.85301
FBXO44	687.643298	1561.6661	9.427613082	10.60979385	-1.18335	0.85305
LOC102166414	56.6132033	35.52997	5.848327568	5.191008663	0.672102	0.85306
LOC102157815	283.066016	152.03522	8.150082438	7.257719911	0.896733	0.85308
LRRC49	357.629748	187.56519	8.486351351	7.558919565	0.931075	0.85314
MTO1	1745.34363	784.13817	10.77012175	9.616802763	1.154331	0.85317
LOC102157796	34.5202459	22.309516	5.150569664	4.542847146	0.629784	0.85318
LOC100737009	19.3313377	12.394176	4.345633235	3.74353388	0.641279	0.85321
YPEL5	1019.03766	2420.9956	9.994406701	11.24198055	-1.24839	0.85321
ACADM	9470.97466	3676.1125	13.20944951	11.84435759	1.365332	0.85324
LOC100623894	527.469357	266.06164	9.045676011	8.061028933	0.987327	0.85328
SLMO2	3421.64677	1447.6397	11.74089669	10.50048311	1.240988	0.85338
LOC102163325	45.5667246	74.365053	5.541227504	6.2358238	-0.70664	0.8534
LOC100621967	1375.2866	632.10295	10.42656521	9.306296318	1.121501	0.8534
LOC102158248	46.9475344	76.843889	5.583384725	6.282511874	-0.71088	0.85341
FIGN	255.44982	138.81477	8.002532746	7.127372928	0.879879	0.85342
ITGAE	60.7556328	38.008805	5.948498827	5.285727902	0.676684	0.85354
LOC100738684	4417.21067	1828.554	12.10924649	10.83727631	1.272432	0.85356
LOC102166108	9.66566885	15.699289	3.414902536	4.061714779	-0.69976	0.8536
LOC102162642	9.66566885	15.699289	3.414902536	4.061714779	-0.69976	0.8536
LOC102163062	9.66566885	15.699289	3.414902536	4.061714779	-0.69976	0.8536
FBLN1	2780.95101	1198.9299	11.4418813	10.22873443	1.213831	0.85361
SYNGR3	111.845597	202.4382	6.818206315	7.6684468	-0.85597	0.85367
LOC100738535	4415.82986	1828.554	12.10879554	10.83727631	1.271981	0.85369
LOC100737568	1226.15913	570.13208	10.26110663	9.157680603	1.104778	0.8537
LOC102165033	38.6626754	61.970878	5.309710091	5.976612875	-0.68065	0.85381
RWDD2B	367.295416	192.52286	8.524719632	7.59636019	0.931911	0.85385
RPL32	100300.646	32639.648	16.61398575	14.99438209	1.619633	0.85389
CCDC11	63.5172524	39.661362	6.011613095	5.34558663	0.679414	0.8539
COX7A2	10995.3887	4222.2825	13.42474219	12.04414903	1.380804	0.85391
AIRE	16.569718	25.61463	4.135019133	4.734147579	-0.62842	0.85392
ACYP1	1674.92233	756.87099	10.71073957	9.565808471	1.145975	0.854
LOC102162277	23.4737672	36.356248	4.613164285	5.223277669	-0.63115	0.854
LOC733634	37.2818656	59.492043	5.258589232	5.918673475	-0.67422	0.85402
LOC100625722	186.409328	359.43109	7.550048951	8.493579657	-0.94724	0.85404
LOC102167939	56.6132033	94.195734	5.848327568	6.572825024	-0.73452	0.85412
LOC102164004	13.8080984	21.483238	3.888314478	4.490777898	-0.6377	0.85416
SLC25A2	13.8080984	21.483238	3.888314478	4.490777898	-0.6377	0.85416
LOC102158767	13.8080984	21.483238	3.888314478	4.490777898	-0.6377	0.85416
LOC100515851	2647.01246	1147.7007	11.37069419	10.16578718	1.205619	0.85417
NECAB3	307.920593	632.10295	8.271092238	9.306296318	-1.0376	0.85419
TXNDC16	30.3778164	19.830681	4.971673052	4.380638094	0.615284	0.85423
TRPM2	30.3778164	19.830681	4.971673052	4.380638094	0.615284	0.85423
USHBP1	6.90404918	3.3051135	2.982591923	2.106051269	1.062743	0.85426
LOC102164789	6.90404918	3.3051135	2.982591923	2.106051269	1.062743	0.85426
LOC102164139	6.90404918	3.3051135	2.982591923	2.106051269	1.062743	0.85426
LOC102166092	6.90404918	3.3051135	2.982591923	2.106051269	1.062743	0.85426
PIPOX	6.90404918	3.3051135	2.982591923	2.106051269	1.062743	0.85426
LOC102158067	6.90404918	3.3051135	2.982591923	2.106051269	1.062743	0.85426
LOC100621028	6.90404918	3.3051135	2.982591923	2.106051269	1.062743	0.85426
LOC102160217	6.90404918	3.3051135	2.982591923	2.106051269	1.062743	0.85426
CLDN23	6.90404918	3.3051135	2.982591923	2.106051269	1.062743	0.85426
LOC100627350	6.90404918	3.3051135	2.982591923	2.106051269	1.062743	0.85426
LOC102165621	6.90404918	3.3051135	2.982591923	2.106051269	1.062743	0.85426
LOC100153359	6.90404918	3.3051135	2.982591923	2.106051269	1.062743	0.85426
LOC100625521	6.90404918	3.3051135	2.982591923	2.106051269	1.062743	0.85426
LOC100628191	157.412321	296.63394	7.307540742	8.217395218	-0.91414	0.85441
LOC102157609	27.6161967	42.966475	4.838760036	5.458331975	-0.6377	0.8545
LOC100737659	27.6161967	42.966475	4.838760036	5.458331975	-0.6377	0.8545
TAF12	1129.50245	530.47071	10.1427484	9.053846388	1.090342	0.85456
LOC102166853	144.985033	270.19303	7.189676653	8.083176277	-0.89809	0.85457
PIGX	433.574288	223.92144	8.76345901	7.81327737	0.953287	0.85458
LOC100625309	34.5202459	54.534373	5.150569664	5.795309087	-0.65972	0.8546
RIPK2	439.097528	940.30479	8.781679457	9.878518122	-1.09859	0.85464
UMODL1	17.9505279	11.567897	4.24416613	3.65167138	0.6339	0.85467
SPDYC	17.9505279	11.567897	4.24416613	3.65167138	0.6339	0.85467
LOC102164559	28.9970066	19.004403	4.906746634	4.322245637	0.60957	0.85471
LOC100625795	104.941548	62.797156	6.727124677	5.995420212	0.740815	0.8548
LOC102162328	125.653695	229.70539	6.984745358	7.849907883	-0.87033	0.85481
ZKSCAN5	383.865134	808.10025	8.58820917	9.660174654	-1.07393	0.85482
TCIRG1	1535.46054	3802.5331	10.585395	11.89312443	-1.30829	0.85484
RFWD3	2105.735	933.69456	11.04079314	9.868351187	1.173301	0.8549
LOC100739495	70.4213016	43.792754	6.158282521	5.485193456	0.68532	0.8549
LOC100620496	8040.45567	3173.7352	12.97324097	11.63242056	1.341095	0.85491
SLC25A26	675.21601	333.81646	9.401340363	8.387226654	1.016294	0.85495
MUSTN1	2.76161967	6.610227	1.911353989	2.927939483	-1.25919	0.855
TDRD6	2.76161967	6.610227	1.911353989	2.927939483	-1.25919	0.855
LOC102161597	2.76161967	6.610227	1.911353989	2.927939483	-1.25919	0.855
LOC102161606	2.76161967	6.610227	1.911353989	2.927939483	-1.25919	0.855
LPPR5	2.76161967	6.610227	1.911353989	2.927939483	-1.25919	0.855
RAB9B	2.76161967	6.610227	1.911353989	2.927939483	-1.25919	0.855
LOC100624378	2.76161967	6.610227	1.911353989	2.927939483	-1.25919	0.855
LOC102164751	2.76161967	6.610227	1.911353989	2.927939483	-1.25919	0.855
LOC102164506	2.76161967	6.610227	1.911353989	2.927939483	-1.25919	0.855
ANKRD61	2.76161967	6.610227	1.911353989	2.927939483	-1.25919	0.855
FAM3B	2.76161967	6.610227	1.911353989	2.927939483	-1.25919	0.855
LOC102158003	2.76161967	6.610227	1.911353989	2.927939483	-1.25919	0.855
NEFH	2.76161967	6.610227	1.911353989	2.927939483	-1.25919	0.855
LOC102160958	2.76161967	6.610227	1.911353989	2.927939483	-1.25919	0.855
LOC102160992	2.76161967	6.610227	1.911353989	2.927939483	-1.25919	0.855
LOC102160131	2.76161967	6.610227	1.911353989	2.927939483	-1.25919	0.855
LOC102162788	2.76161967	6.610227	1.911353989	2.927939483	-1.25919	0.855
LOC102162866	2.76161967	6.610227	1.911353989	2.927939483	-1.25919	0.855
CAPSL	2.76161967	6.610227	1.911353989	2.927939483	-1.25919	0.855
LOC102158630	2.76161967	6.610227	1.911353989	2.927939483	-1.25919	0.855
SFTPC	2.76161967	6.610227	1.911353989	2.927939483	-1.25919	0.855
LOC100519434	2.76161967	6.610227	1.911353989	2.927939483	-1.25919	0.855
LOC102167860	2.76161967	6.610227	1.911353989	2.927939483	-1.25919	0.855
LOC102167952	2.76161967	6.610227	1.911353989	2.927939483	-1.25919	0.855
LOC100737487	2.76161967	6.610227	1.911353989	2.927939483	-1.25919	0.855
KLHL21	244.403341	485.85168	7.93901108	8.927338518	-0.99125	0.85507
LOC100620614	255.44982	510.64003	8.002532746	8.998985343	-0.99927	0.85508
METTL1	1489.89381	682.50594	10.54196179	9.416810055	1.126296	0.85511
GPC3	157.412321	295.80766	7.307540742	8.213384501	-0.91011	0.85511
RCC2	17066.8096	6344.1653	14.0589903	12.63144204	1.427691	0.85511
LOC100625822	379.722705	199.13309	8.572596799	7.644815895	0.931213	0.85514
LOC102158294	1119.83678	527.1656	10.13036048	9.044846533	1.08696	0.85515
IKZF2	22.0929574	33.877413	4.529381037	5.124221138	-0.61674	0.85517
C1H9orf41	4083.05468	1707.9174	11.99578647	10.73886695	1.257411	0.85522
STAR	27.6161967	18.178124	4.838760036	4.261389712	0.603311	0.85527
SIAH2	494.329921	1071.683	8.952245962	10.06700814	-1.11633	0.85529
CPSF4	1469.18167	674.24315	10.52177872	9.399263292	1.123672	0.85531
CTRB2	73.1829213	45.44531	6.213015177	5.537461031	0.687376	0.85533
LOC100524650	3105.44132	1331.1345	11.60104709	10.37952399	1.222142	0.8554
HINT3	321.728692	171.8659	8.334178034	7.433509508	0.904561	0.85555
LOC100626448	11.0464787	17.351846	3.590539588	4.19785327	-0.6515	0.85557
LOC100516210	11.0464787	17.351846	3.590539588	4.19785327	-0.6515	0.85557
STYXL1	11.0464787	17.351846	3.590539588	4.19785327	-0.6515	0.85557
LOC102160184	11.0464787	17.351846	3.590539588	4.19785327	-0.6515	0.85557
PLCXD2	11.0464787	17.351846	3.590539588	4.19785327	-0.6515	0.85557
LOC100525275	301.016544	161.95056	8.238483771	7.348290508	0.894289	0.85561
UQCC	1503.70191	689.11616	10.55526199	9.43069541	1.1257	0.85563
VEZF1	694.547347	1566.6238	9.442004915	10.61436366	-1.17351	0.85565
PITPNM2	646.219003	1445.1609	9.338110157	10.49801233	-1.16114	0.85565
LOC100736949	56.6132033	93.369456	5.848327568	6.560248082	-0.72181	0.8558
TRIM46	296.874115	603.18321	8.218558948	9.238842286	-1.02274	0.85582
LOC100523652	234.737672	129.7257	7.881038517	7.030399034	0.855585	0.85583
ZYG11B	186.409328	356.95226	7.550048951	8.483623365	-0.93726	0.85584
ORAI3	1821.28817	4583.3661	10.83153541	12.16250655	-1.33145	0.85585
APOBEC3F	541.277456	274.32442	9.082887383	8.104988763	0.980485	0.85586
MCPH1	40.0434852	63.623435	5.359081339	6.013985524	-0.66799	0.85587
TMEM143	273.400348	549.47512	8.100138498	9.104533542	-1.00704	0.85596
C6H1orf174	448.763197	959.30919	8.813021802	9.907355173	-1.09604	0.85598
PRDX2	25702.3943	9273.3222	14.64967127	13.17902613	1.470745	0.85603
AASDH	593.748229	298.28649	9.216135261	8.225383356	0.993153	0.85607
HEXA	3494.82969	9426.1837	11.77141919	13.20261112	-1.43145	0.85607
GPR137B	38.6626754	61.1446	5.309710091	5.957557119	-0.66128	0.85608
EIF2B3	3507.25698	1489.7799	11.77653872	10.54185156	1.235244	0.85614
TRIM52	110.464787	197.48053	6.800444207	7.632853688	-0.83812	0.85617
LOC102168106	11.0464787	6.610227	3.590539588	2.927939483	0.740815	0.85618
TMEM71	11.0464787	6.610227	3.590539588	2.927939483	0.740815	0.85618
LOC100626845	11.0464787	6.610227	3.590539588	2.927939483	0.740815	0.85618
RFX2	11.0464787	6.610227	3.590539588	2.927939483	0.740815	0.85618
SLC30A10	11.0464787	6.610227	3.590539588	2.927939483	0.740815	0.85618
MFAP5	11.0464787	6.610227	3.590539588	2.927939483	0.740815	0.85618
FAM222B	187.790138	359.43109	7.560639591	8.493579657	-0.93659	0.8562
LOC102161249	4.14242951	8.2627837	2.362450112	3.211445828	-0.99615	0.85621
LOC102163910	4.14242951	8.2627837	2.362450112	3.211445828	-0.99615	0.85621
LOC102166720	4.14242951	8.2627837	2.362450112	3.211445828	-0.99615	0.85621
LOC100624001	4.14242951	8.2627837	2.362450112	3.211445828	-0.99615	0.85621
LOC102166012	4.14242951	8.2627837	2.362450112	3.211445828	-0.99615	0.85621
HUNK	4.14242951	8.2627837	2.362450112	3.211445828	-0.99615	0.85621
LOC102166370	4.14242951	8.2627837	2.362450112	3.211445828	-0.99615	0.85621
LOC102162759	4.14242951	8.2627837	2.362450112	3.211445828	-0.99615	0.85621
HRH4	4.14242951	8.2627837	2.362450112	3.211445828	-0.99615	0.85621
LOC100739457	4.14242951	8.2627837	2.362450112	3.211445828	-0.99615	0.85621
LOC102167940	4.14242951	8.2627837	2.362450112	3.211445828	-0.99615	0.85621
LOC102165958	24.854577	38.008805	4.692347799	5.285727902	-0.61282	0.85621
TUFM	22087.4341	8065.3032	14.43100353	12.97769192	1.453425	0.85624
LOC102167808	15.1889082	23.135794	4.016933786	4.593102408	-0.60711	0.85627
LOC102165055	15.1889082	23.135794	4.016933786	4.593102408	-0.60711	0.85627
DPY19L4	214.025525	416.4443	7.748364114	8.705439898	-0.96034	0.85629
ZNF473	267.877108	536.25466	8.070803121	9.06946229	-1.00135	0.85634
LOC100152072	37.2818656	58.665764	5.258589232	5.89883146	-0.65404	0.85635
GAPDHS	62.1364426	103.2848	5.98040107	6.704385035	-0.73312	0.85638
FAM57A	379.722705	794.05352	8.572596799	9.634908162	-1.06429	0.85639
SEC23A	2170.63306	5559.2009	11.08456464	12.44092129	-1.35676	0.85643
LOC102158440	20.7121475	31.398578	4.440430524	5.017858594	-0.60022	0.85653
GPX8	473.617774	1017.1487	8.890622318	9.991732528	-1.10274	0.85657
LOC100512680	28.9970066	44.619032	4.906746634	5.511563931	-0.62176	0.85664
LOC100152872	28.9970066	44.619032	4.906746634	5.511563931	-0.62176	0.85664
LOC102158840	24.854577	16.525567	4.692347799	4.13138925	0.588812	0.85667
LOC102165052	24.854577	16.525567	4.692347799	4.13138925	0.588812	0.85667
LOC100739814	568.893652	287.54487	9.154548913	8.172651889	0.984372	0.85668
LOC100739544	985.898223	471.80495	9.946757499	8.88510133	1.063248	0.85673
THUMPD2	1572.7424	719.68846	10.6199837	9.493231938	1.127838	0.85673
ZNF23	144.985033	267.71419	7.189676653	8.06992871	-0.88479	0.85682
LOC100739017	255.44982	507.33492	8.002532746	8.98963553	-0.9899	0.85686
PPP4R1	970.709315	2268.1341	9.924380988	11.14792617	-1.22439	0.85694
TYMS	13005.8478	4957.6702	13.66698375	12.27573757	1.391426	0.85696
LOC102163320	100.799118	61.1446	6.669581252	5.957557119	0.721186	0.85703
LOC100739490	2253.48165	998.14427	11.13858005	9.964549203	1.174835	0.85707
NCL	8968.35988	3524.0773	13.13078931	11.78343916	1.347599	0.85707
CCDC102A	691.785728	1552.5771	9.436265399	10.60137809	-1.16627	0.8571
LOC100738335	67.659682	113.20014	6.101391269	6.83542057	-0.74251	0.85712
LOC100739020	389.388374	204.91704	8.608766278	7.685919384	0.92617	0.85712
C12H17orf47	278.923587	152.03522	8.128889246	7.257719911	0.875464	0.85714
OXCT2	5.52323934	9.9153405	2.705588563	3.448285225	-0.84415	0.85717
LOC102163918	5.52323934	9.9153405	2.705588563	3.448285225	-0.84415	0.85717
FSD2	5.52323934	9.9153405	2.705588563	3.448285225	-0.84415	0.85717
LOC102162075	5.52323934	9.9153405	2.705588563	3.448285225	-0.84415	0.85717
FAM19A3	5.52323934	9.9153405	2.705588563	3.448285225	-0.84415	0.85717
LOC102160161	5.52323934	9.9153405	2.705588563	3.448285225	-0.84415	0.85717
LOC102167027	5.52323934	9.9153405	2.705588563	3.448285225	-0.84415	0.85717
FGF14	5.52323934	9.9153405	2.705588563	3.448285225	-0.84415	0.85717
LOC100512993	5.52323934	9.9153405	2.705588563	3.448285225	-0.84415	0.85717
LOC102158380	5.52323934	9.9153405	2.705588563	3.448285225	-0.84415	0.85717
LOC102165330	5.52323934	9.9153405	2.705588563	3.448285225	-0.84415	0.85717
SLITRK6	5.52323934	9.9153405	2.705588563	3.448285225	-0.84415	0.85717
LOC102165531	5.52323934	9.9153405	2.705588563	3.448285225	-0.84415	0.85717
PRSS54	5.52323934	9.9153405	2.705588563	3.448285225	-0.84415	0.85717
USP13	82.8485901	142.11988	6.389714621	7.161080272	-0.77856	0.85717
ACVR1B	162.935561	304.89672	7.356985025	8.256900824	-0.90402	0.85726
LOC102166317	465.332915	994.83916	8.865216451	9.959768938	-1.0962	0.85728
LOC102160924	23.4737672	35.52997	4.613164285	5.191008663	-0.59799	0.85729
LOC102158717	52.4707738	33.877413	5.740678649	5.124221138	0.63119	0.8573
LOC102162978	69.0404918	115.67887	6.130117309	6.86640077	-0.74461	0.85732
ADRBK2	397.673233	833.71488	8.639062934	9.705139674	-1.06797	0.85732
CTNNBL1	1957.98835	879.16019	10.9358931	9.781622305	1.155174	0.85735
STAT6	1154.35702	2748.2019	10.17412302	11.42479713	-1.2514	0.85735
LOC100621191	328.632741	672.59059	8.364715736	9.395728184	-1.03325	0.85738
LOC102163995	12.4272885	19.004403	3.747096094	4.322245637	-0.61282	0.85739
LOC102158370	12.4272885	19.004403	3.747096094	4.322245637	-0.61282	0.85739
LOC102160428	56.6132033	92.543178	5.848327568	6.547560532	-0.70899	0.85746
LOC102165842	23.4737672	15.699289	4.613164285	4.061714779	0.58035	0.85754
EMC8	2638.7276	1153.4846	11.36617334	10.17303322	1.193844	0.85757
MRPL16	2676.00946	1168.3576	11.38640652	10.19150049	1.195601	0.85759
EFNA1	642.076574	321.42229	9.328846725	8.33280766	0.998275	0.8576
TBC1D9	219.548764	426.35964	7.784953864	8.739306855	-0.95753	0.85766
LOC100623659	443.29397	230.53167	8.795195352	7.855065709	0.943123	0.85768
MCF2L2	142.223413	83.454116	7.162123543	6.400095824	0.769104	0.85769
DNAJC17	1325.57744	618.05622	10.37349318	9.273926629	1.100811	0.85773
SPRY3	40.0434852	26.440908	5.359081339	4.77825631	0.598796	0.85781
STAT5A	80.0869705	136.33593	6.341398207	7.101565318	-0.76753	0.85786
LOC100620601	1488.513	686.63733	10.540625	9.42550405	1.116252	0.85786
E2F4	5358.92297	2202.8581	12.38799655	11.10581565	1.282566	0.85796
LOC100522905	38.6626754	25.61463	5.309710091	4.734147579	0.593973	0.85801
LOC100623683	38.6626754	25.61463	5.309710091	4.734147579	0.593973	0.85801
LOC100521645	38.6626754	25.61463	5.309710091	4.734147579	0.593973	0.85801
CKB	7004.8483	2815.1304	12.77434403	11.45949843	1.315152	0.85801
UBAP1	389.388374	812.23164	8.608766278	9.667522535	-1.06068	0.85803
CCDC170	31.7586262	48.750424	5.033802952	5.636636914	-0.61827	0.85803
KRI1	3272.51931	1404.6732	11.67662677	10.45704554	1.220167	0.85803
MED18	976.232554	469.32612	9.932558114	8.877517631	1.056634	0.85809
LOC102161290	6.90404918	11.567897	2.982591923	3.65167138	-0.74461	0.85813
LOC102164286	6.90404918	11.567897	2.982591923	3.65167138	-0.74461	0.85813
FCER1G	6.90404918	11.567897	2.982591923	3.65167138	-0.74461	0.85813
LOC102160560	6.90404918	11.567897	2.982591923	3.65167138	-0.74461	0.85813
TMEM182	6.90404918	11.567897	2.982591923	3.65167138	-0.74461	0.85813
LOC102158363	6.90404918	11.567897	2.982591923	3.65167138	-0.74461	0.85813
LOC100517560	6.90404918	11.567897	2.982591923	3.65167138	-0.74461	0.85813
LOC100739722	6.90404918	11.567897	2.982591923	3.65167138	-0.74461	0.85813
LOC100624381	19.3313377	28.919743	4.345633235	4.903025878	-0.58111	0.85813
SPAG4	19.3313377	28.919743	4.345633235	4.903025878	-0.58111	0.85813
NSUN5	1394.61793	647.80224	10.44668833	9.341634998	1.106245	0.85815
LOC100739488	98.0374983	171.03962	6.629902968	7.426597064	-0.80293	0.85816
LOC102163508	16.569718	24.788351	4.135019133	4.688647628	-0.58111	0.85821
LOC102162252	16.569718	24.788351	4.135019133	4.688647628	-0.58111	0.85821
KLHL15	421.147	220.61633	8.721601651	7.791920351	0.932784	0.85828

LOC102160979	5.286795433	12.394176	2.65232482	3.74353388	-1.2292	0.74427
LOC102162953	5.286795433	12.394176	2.65232482	3.74353388	-1.2292	0.74427
LOC102165305	5.286795433	12.394176	2.65232482	3.74353388	-1.2292	0.74427
LOC100620825	5.286795433	12.394176	2.65232482	3.74353388	-1.2292	0.74427
LOC102159114	635.472811	255.32002	9.313955076	8.001802338	1.315524	0.74428
PARP16	2321.960554	822.14698	11.18174894	9.685006249	1.497875	0.74431
NR1H3	780.3310059	2263.1765	9.609790056	11.14477068	-1.53619	0.74431
IMMP2L	34.89284986	71.886218	5.165624571	6.187574144	-1.04278	0.74432
KRAS	1160.980277	439.58009	10.18236987	8.783260503	1.401146	0.74441
VAT1	7699.688868	28524.782	12.91077179	14.79997881	-1.88934	0.74471
LOC102157933	26.43397716	13.220454	4.777891883	3.829895615	0.999622	0.74474
CLEC18A	26.43397716	13.220454	4.777891883	3.829895615	0.999622	0.74474
ZFX	4791.95138	1587.2808	12.22669859	10.63325024	1.594056	0.74481
CCDC15	382.7639893	161.95056	8.584075532	7.348290507	1.240902	0.74485
PPM1B	5331.204514	1749.2313	12.3805164	10.77332989	1.60774	0.74496
TET1	275.9707216	120.63664	8.113589668	6.926434087	1.193847	0.74496
LOC100628113	58.15474976	28.919743	5.886422108	4.903025878	1.007842	0.74497
BGLAP	33.83549077	69.407383	5.122485983	6.137654819	-1.03655	0.74502
LOC100516259	1205.389359	455.27938	10.23647989	8.833773656	1.404675	0.74509
TBC1D9	175.5216084	426.35964	7.463700987	8.739306855	-1.28042	0.74512
RAB11FIP4	68.72834063	33.877413	6.123673244	5.124221138	1.020581	0.74519
AHCYL2	1224.421822	461.88961	10.25906273	8.854524369	1.406481	0.74525
CCDC132	2087.226837	747.78193	11.02806276	9.548401802	1.480898	0.74526
LOC100737677	132.1698858	61.970878	7.057124067	5.976612875	1.092731	0.74529
HERC2	87.76080419	196.65425	6.471850834	7.626835184	-1.16401	0.74529
LOC100623370	57.09739067	122.2892	5.860401464	6.945902605	-1.0988	0.7453
FBXO15	52.86795433	26.440908	5.751355374	4.778256309	0.999622	0.74538
CCDC28A	1117.628555	425.53336	10.12751535	8.736514777	1.393097	0.74543
AGK	2327.24735	825.45209	11.18502862	9.690787384	1.495368	0.74546
LOC100737829	49.69587707	104.93735	5.663796517	6.727067559	-1.07833	0.74564
SLC2A5	1272.002981	478.41518	10.31402008	8.905131772	1.410767	0.74567
DR1	3624.626949	1233.6336	11.82401477	10.26986725	1.554919	0.74567
FRRS1	83.53136784	185.91263	6.40141489	7.546220276	-1.15423	0.74578
LOC100628115	1044.670778	400.74501	10.03021298	8.650136295	1.382292	0.74589
TBL1XR1	6571.486723	2119.404	12.68222361	11.05012347	1.632561	0.74603
TPGS2	4162.822724	1399.7156	12.02369293	10.45194831	1.572428	0.74609
LOC780436	2458.359876	8066.9557	11.26406714	12.97798745	-1.71433	0.74623
SETD7	1692.831898	5331.1481	10.72607499	12.38050112	-1.65501	0.74655
LOC102167559	37.00756803	76.01761	5.24821481	6.267116453	-1.03851	0.7466
SCIN	12.68830904	25.61463	3.774872332	4.734147579	-1.01347	0.74661
LOC100152699	348.9284986	149.55639	8.450916354	7.234160086	1.222242	0.74663
USP25	1223.364463	3714.9476	10.25781736	11.85951443	-1.60249	0.74675
TTC13	353.1579349	928.73689	8.468249056	9.860678689	-1.39496	0.74681
ATP5G3	40288.55328	11135.754	15.29811819	13.44304113	1.855171	0.7469
LOC100522374	189.2672765	461.88961	7.571883648	8.854524369	-1.28712	0.7469
LOC100738761	557.2282386	1545.9668	9.124711296	10.59522655	-1.47217	0.74692
RAD18	1203.274641	456.10566	10.23394873	8.836383876	1.399526	0.74696
ATP13A3	9641.000151	3010.9584	13.23511674	11.55648612	1.67896	0.74731
SRFBP1	1387.255122	518.90282	10.439057	9.022098163	1.418697	0.74732
MESDC1	2324.075272	827.10465	11.18306171	9.693669287	1.490515	0.74735
RGP1	322.4945214	837.84627	8.337597469	9.712262629	-1.37741	0.7474
LOC102163742	6.344154519	14.046732	2.876596413	3.911378308	-1.14673	0.74764
LOC100520091	6.344154519	14.046732	2.876596413	3.911378308	-1.14673	0.74764
LOC100623155	6.344154519	14.046732	2.876596413	3.911378308	-1.14673	0.74764
LOC100523481	6.344154519	14.046732	2.876596413	3.911378308	-1.14673	0.74764
BDH1	6.344154519	14.046732	2.876596413	3.911378308	-1.14673	0.74764
EPG5	6.344154519	14.046732	2.876596413	3.911378308	-1.14673	0.74764
LOC100623242	54.9826725	116.50525	5.806908455	6.876581411	-1.08335	0.7477
GPRASP2	2211.995209	791.57468	11.11178461	9.630403069	1.482551	0.74781
LOC102164657	341.526985	147.07755	8.420073841	7.210209122	1.215423	0.74785
STARD9	377.4771939	998.14427	8.564062559	9.964549203	-1.40286	0.7479
PRUNE2	819.4532921	2372.2452	9.680277394	11.21264545	-1.53352	0.74793
CENPE	5383.01511	1773.1934	12.39446674	10.79294756	1.602065	0.74796
PRR3	2331.476786	830.40976	11.18764701	9.69941588	1.489349	0.748
LOC100627551	306.6341351	790.7484	8.265071784	9.62889824	-1.3667	0.74801
SMC6	8531.8347	2696.9726	13.05880767	11.39765998	1.661514	0.74812
LOC100624129	253.7661808	639.53946	7.993029968	9.323143639	-1.33353	0.74815
LOC100522861	308.7488533	796.53235	8.274955131	9.63939923	-1.3673	0.74819
LOC100519820	359.5020894	944.43618	8.493863811	9.884836263	-1.39345	0.74821
LOC102158524	49.69587707	104.11107	5.663796517	6.715770874	-1.06693	0.74828
DFFA	526.5648251	1446.8134	9.043204567	10.49965999	-1.4582	0.74834
KDM1A	10072.40266	3139.0315	13.29826347	11.61656333	1.682016	0.74843
SERPING1	1862.009351	5902.1064	10.8634192	12.52725863	-1.66437	0.74859
LOC100518322	1163.094995	443.71149	10.18499308	8.796725856	1.390275	0.74864
TTC19	559.3429568	1546.7931	9.130166286	10.59599693	-1.46748	0.74864
EFNA1	137.4566813	321.42229	7.113290863	8.33280766	-1.22549	0.74875
TMEM249	102.5638314	49.576702	6.694376434	5.660401069	1.048788	0.74876
LOC100625519	2200.364259	7099.3838	11.10418217	12.79368129	-1.68995	0.74882
LOC102157843	10.57359087	21.483238	3.532764645	4.490777899	-1.02275	0.74889
LOC100739335	2600.045994	917.99527	11.3448762	9.843913627	1.501979	0.74894
TBC1D1	919.9024053	359.43109	9.846904461	8.493579657	1.355766	0.74898
PYGB	2509.113112	889.07553	11.29353666	9.797783952	1.4968	0.74906
LOC102163687	177.6363265	81.801559	7.480881679	6.371586023	1.118726	0.74909
LOC102159053	45.46644072	23.135794	5.538117235	4.593102407	0.974675	0.7491
LOC102165354	45.46644072	23.135794	5.538117235	4.593102407	0.974675	0.7491
CHAF1B	1707.634925	627.97156	10.73862846	9.29685098	1.443228	0.74913
LOC100622293	12.68830904	5.7839486	3.774872332	2.76212524	1.133373	0.74918
LOC102159045	12.68830904	5.7839486	3.774872332	2.76212524	1.133373	0.74918
LOC102159457	12.68830904	5.7839486	3.774872332	2.76212524	1.133373	0.74918
LOC102159796	12.68830904	5.7839486	3.774872332	2.76212524	1.133373	0.74918
CCIN	12.68830904	5.7839486	3.774872332	2.76212524	1.133373	0.74918
LOC102158576	12.68830904	5.7839486	3.774872332	2.76212524	1.133373	0.74918
LOC100627739	12.68830904	5.7839486	3.774872332	2.76212524	1.133373	0.74918
MAF1	1765.789675	5557.5483	10.78691459	12.44049244	-1.65414	0.74924
MERTK	15.8603863	31.398578	4.075565686	5.017858594	-0.98527	0.74932
PLK1	3973.555447	1349.3126	11.95657779	10.3990777	1.558206	0.74946
LOC100620087	980.1718733	380.91433	9.938362067	8.57710524	1.363568	0.74947
LOC100739617	85.64608601	189.21775	6.437062676	7.571508044	-1.14359	0.74948
NDUFB3	4743.312862	1584.8019	12.21198344	10.63099686	1.581592	0.74956
LOC396710	98.33439505	220.61633	6.63422144	7.79192035	-1.16577	0.74959
FBXO46	44.40908164	91.716899	5.504908955	6.534760414	-1.04633	0.74961
TELO2	1268.830904	3846.3258	10.31042068	11.9096403	-1.59998	0.74966
CUL4B	7571.748419	2425.127	12.88660129	11.24443937	1.642566	0.74969
LOC100415928	1890.558047	689.11616	10.88535933	9.43069541	1.455993	0.74971
YPEL5	837.4283966	2420.9956	9.71154377	11.24198055	-1.53156	0.74978
MNAT1	107.8506268	52.055537	6.766205905	5.729431428	1.050911	0.7498
ATG13	1003.433773	2961.3817	9.972166728	11.53254182	-1.56133	0.7498
LOC100623494	59.21210885	125.59431	5.911981742	6.98406878	-1.08481	0.74985
LOC100155807	707.3732289	284.23976	9.46836588	8.156031284	1.315363	0.74986
AP3M1	8359.480938	2654.8324	13.02937022	11.3749484	1.654793	0.74993
GDF1	64.49890428	137.98849	6.033398867	7.118821584	-1.0972	0.74993
LOC100626866	33.83549077	17.351846	5.122485983	4.19785327	0.963448	0.75001
LOC102161987	27.49133625	14.046732	4.832451381	3.911378308	0.968742	0.75009
LOC102158751	27.49133625	14.046732	4.832451381	3.911378308	0.968742	0.75009
LOC102158661	13.74566813	27.267186	3.882219287	4.821056378	-0.98819	0.75013
LOC102164684	16.91774539	8.2627837	4.163317207	3.211445828	1.033838	0.75025
LRRC19	16.91774539	8.2627837	4.163317207	3.211445828	1.033838	0.75025
LOC100519550	105.7359087	238.79445	6.737901807	7.905654455	-1.1753	0.75025
LOC100736985	2951.089211	1032.0217	11.5275206	10.01265483	1.515774	0.75037
ZNF420	72.95777697	36.356248	6.208629956	5.223277669	1.004858	0.7504
LOC100516350	105.7359087	51.229259	6.737901807	5.706786332	1.045425	0.75061
LOC102161960	22.20454082	43.792754	4.536335244	5.485193456	-0.97984	0.75067
LOC102166395	7.401513606	15.699289	3.070649265	4.061714779	-1.08481	0.75072
LOC102166362	7.401513606	15.699289	3.070649265	4.061714779	-1.08481	0.75072
LOC100521960	5570.167468	1837.6431	12.44376402	10.84442575	1.599864	0.75082
EGR2	24.31925899	12.394176	4.662163277	3.74353388	0.972437	0.751
NT5DC1	1350.247554	509.81376	10.40007629	8.996653565	1.405182	0.75102
FBXO28	6455.177224	2102.0522	12.65646447	11.03826893	1.618658	0.75106
GSPT1	23554.78837	6849.8477	14.523794	12.7420668	1.781877	0.75112
LOC102166466	99.39175414	222.26888	6.649496966	7.80263838	-1.16111	0.75123
AATF	4082.463433	1387.3214	11.99557759	10.43912586	1.557138	0.75142
USP39	1435.89364	539.55978	10.48873756	9.078310354	1.412094	0.75171
KIAA0391	582.6048567	239.62073	9.188848079	7.910617116	1.281765	0.75171
TFAP2A	743.3234379	298.28649	9.539785855	8.225383356	1.317291	0.75173
LOC100736562	46.52379981	95.848291	5.570578288	6.597654688	-1.04278	0.75174
HMGN3	7572.805778	2433.3898	12.88680271	11.24934448	1.63786	0.75175
EDEM2	576.2607022	1588.107	9.173079205	10.63400058	-1.46251	0.75184
LOC100739587	860.6902965	340.42669	9.751025627	8.415432027	1.33815	0.75189
KIF11	3689.125853	1266.6847	11.84945431	10.3079803	1.542221	0.75194
TTC8	4839.532539	1620.3319	12.24095006	10.66296373	1.578578	0.75195
LOC100519283	3808.60743	1303.8673	11.89542662	10.34968735	1.546467	0.75197
C7H6orf201	8.458872693	3.3051135	3.241668254	2.106051269	1.355766	0.75199
LOC100516763	8.458872693	3.3051135	3.241668254	2.106051269	1.355766	0.75199
LOC102160758	8.458872693	3.3051135	3.241668254	2.106051269	1.355766	0.75199
ACSL6	8.458872693	3.3051135	3.241668254	2.106051269	1.355766	0.75199
LOC100523928	8.458872693	3.3051135	3.241668254	2.106051269	1.355766	0.75199
TNFAIP1	806.7649831	2311.1006	9.657791796	11.17498846	-1.51836	0.75209
DTWD2	43.35172255	22.309516	5.470918232	4.542847146	0.95843	0.7521
LOC102157990	250.5941035	112.37386	7.974954301	6.824944216	1.157046	0.75215
LOC100520008	11.63094995	23.135794	3.658891241	4.593102407	-0.99216	0.75218
LOC102162929	11.63094995	23.135794	3.658891241	4.593102407	-0.99216	0.75218
LOC100520599	222.0454082	545.34373	7.801193638	9.09366508	-1.29631	0.75219
LOC102163920	724.2909743	291.67627	9.502416806	8.193161944	1.312201	0.75219
CDK15	19.03246356	37.182527	4.324267947	5.254840671	-0.96616	0.75255
USP45	412.3700438	175.99729	8.691290033	7.467583487	1.228386	0.7526
PDCD10	4492.718759	1516.2208	12.13369412	10.56721535	1.567109	0.7526
LOC100626000	1594.497505	594.09415	10.63979064	9.216974124	1.424338	0.7527
SLC16A11	21.14718173	10.741619	4.46905122	3.553559423	0.977254	0.75283
FAM222A	21.14718173	10.741619	4.46905122	3.553559423	0.977254	0.75283
LOC102163345	21.14718173	10.741619	4.46905122	3.553559423	0.977254	0.75283
KIAA1715	1157.8082	445.36404	10.17842608	8.802077003	1.378339	0.75284
LOC102165733	86.7034451	42.966475	6.45456161	5.458331975	1.012878	0.75294
LOC100622814	1252.970518	478.41518	10.29228771	8.905131772	1.389017	0.75308
LOC102166222	5855.654621	1930.1863	12.5158611	10.91527161	1.60109	0.7531
GTPBP10	4707.362653	1583.1494	12.20100973	10.62949265	1.572122	0.75311
LOC100624689	475.811589	1278.2526	8.897275491	10.3210855	-1.42571	0.75312
LOC100156608	2842.181225	1002.2757	11.47329034	9.970502347	1.503719	0.75316
LOC100625289	88.81816327	195.0017	6.488935315	7.614722326	-1.13456	0.75319
TCAIM	262.2250535	117.33153	8.040153	6.886690713	1.160215	0.75319
SLC12A4	1391.484558	4231.3715	10.44344561	12.04725057	-1.6045	0.75325
LOC100625644	31856.11456	9060.9686	14.95932798	13.14560878	1.813833	0.75328
ARHGAP26	163.8906584	76.843889	7.365365858	6.282511874	1.092731	0.75336
LOC100626489	32.77813168	65.275991	5.078017628	6.05041444	-0.99382	0.75343
LOC100626837	191.3819947	88.411786	7.587829971	6.482393107	1.114144	0.75344
LOC100156103	229.4469218	104.11107	7.848290687	6.715770874	1.140037	0.75345
OXNAD1	1601.899016	597.39926	10.64646782	9.224964588	1.423016	0.75345
LOC100628179	1024.580955	3007.6533	10.00222566	11.55490214	-1.5536	0.75355
MAPKBP1	584.7195749	1607.9377	9.1940663	10.65189276	-1.45939	0.75361
SUGT1	6140.084216	2016.9455	12.58427767	10.9786715	1.606086	0.75363
SPTY2D1	2940.51562	1034.5005	11.52234398	10.01611256	1.507135	0.75366
ATF6	339.4122668	874.20252	8.411139215	9.773473077	-1.36493	0.75382
SEC62	927.3039189	2689.5361	9.858453399	11.39367795	-1.53624	0.75383
LOC100625244	65.55626337	138.81477	6.056502534	7.127372928	-1.08236	0.754
LOC100524802	138.5140403	66.10227	7.124266508	6.068289661	1.067261	0.75405
LOC100737279	49.69587707	25.61463	5.663796517	4.734147579	0.956158	0.75417
NDUFA4	15487.13854	4690.7823	13.91887614	12.19592036	1.72317	0.7543
TMED6	58.15474976	29.746021	5.886422108	4.942327829	0.9672	0.75432
ADAMTS14	546.6546478	1488.9536	9.097122603	10.54105171	-1.4456	0.75434
MAD2L2	612.2109111	1689.7393	9.260239557	10.72343848	-1.4647	0.75441
LOC100516938	177.6363265	422.22825	7.480881679	8.725292112	-1.2491	0.75442
LOC102162548	34.89284986	18.178124	5.165624571	4.261389711	0.940728	0.75448
TNXB	25.37661808	49.576702	4.721187694	5.660401069	-0.96616	0.75469
STIP1	32924.04724	9359.2551	15.00689789	13.19233214	1.814676	0.75475
EXOSC1	2298.698654	829.58349	11.16722911	9.697981376	1.470358	0.75475
LOC100623758	38.06492712	19.830681	5.287802017	4.380638094	0.940728	0.75478
RB1	315.0930078	802.3163	8.304205312	9.649824338	-1.34839	0.75486
PAQR6	45.46644072	92.543178	5.538117235	6.547560532	-1.02532	0.75489
XPO5	29748.7979	8531.3242	14.86059225	13.05872307	1.80199	0.75496
NARG2	1508.851417	567.65324	10.56019087	9.151405371	1.410369	0.75502
LOC100513474	264.3397716	658.54386	8.051697126	9.365324797	-1.31689	0.75505
LOC100519380	291.8311079	129.7257	8.193925011	7.030399033	1.169669	0.75509
MFSD3	382.7639893	997.31799	8.584075532	9.963355621	-1.3816	0.75509
RNF152	34.89284986	69.407383	5.165624571	6.137654819	-0.99216	0.7551
KPNA4	474.7542299	200.78564	8.894072673	7.65667973	1.241525	0.75511
STIM2	1202.217281	462.71589	10.23268148	8.857097348	1.377499	0.75514
LOC100621733	176.5789675	82.627837	7.472316908	6.385911347	1.095614	0.75549
UBE2B	7733.524359	2495.3607	12.91709686	11.28561068	1.631878	0.7555
LOC100622391	277.0280807	123.94176	8.119086791	6.965111899	1.16037	0.75559
LOC100524801	259.0529762	642.84457	8.022661739	9.330568647	-1.31122	0.75565
BCAS2	3872.048975	1331.9607	11.91925403	10.38041857	1.539546	0.75571
AMPD1	13.74566813	6.610227	3.882219287	2.927939483	1.056205	0.75589
LOC102167724	213.5865355	517.25026	7.745415745	9.017505127	-1.27604	0.75595
ELP4	745.438156	301.59161	9.543878925	8.241228155	1.305492	0.75601
CAMK1	337.2975486	863.4609	8.402148913	9.755656899	-1.35611	0.7561
ZDHHC24	360.5594485	930.38945	8.498089068	9.863240725	-1.3676	0.75616
SFXN4	2499.596881	897.33831	11.28805679	9.811115052	1.477972	0.75633
LOC100514500	15.8603863	30.5723	4.075565686	4.980587447	-0.9468	0.75637
LOC102167234	443.0334573	189.21775	8.794524575	7.571508044	1.227368	0.75639
ATXN1L	128.9978086	293.32882	7.022343493	8.201285014	-1.18517	0.75648
LOC100627009	222.0454082	539.55978	7.801193638	9.078310354	-1.28093	0.75651
LOC102163371	75.07249515	38.008805	6.249303021	5.285727902	0.981951	0.75656
SYS1	959.0246915	2776.2953	9.906927702	11.43946488	-1.53352	0.75656
LOC102162583	24.31925899	47.097867	4.662163277	5.587901017	-0.95356	0.7566
LOC102165529	24.31925899	47.097867	4.662163277	5.587901017	-0.95356	0.7566
PSMA5	14711.0367	4491.6492	13.84470939	12.13335071	1.711582	0.7566
FKBP9	6538.708591	23319.228	12.67501064	14.50929429	-1.83444	0.75672
SLC25A25	195.611431	467.67356	7.619203392	8.872439593	-1.25751	0.75677
LOC100520083	47.5811589	96.674569	5.602325001	6.609911087	-1.02275	0.75677
RPP14	762.3559014	308.20183	9.576212035	8.27240506	1.306589	0.75678
GPT2	5414.735882	1808.7234	12.40294167	10.82155346	1.581919	0.7568
LOC100523623	11130.8191	3484.4159	13.44240175	11.7671151	1.675571	0.7569
LOC102165838	156.4891448	74.365053	7.299108582	6.2358238	1.073366	0.75695
LOC100513987	4391.212287	1496.3901	12.10073207	10.54823444	1.553133	0.7571
SLC39A14	11163.59724	3495.1575	13.44664359	11.77155447	1.675373	0.75715
LOC102164277	579.4327794	1579.018	9.180985186	10.62572525	-1.44631	0.75722
LOC102160739	53.92531342	110.7213	5.779399292	6.803760479	-1.0379	0.75736
XRN2	12964.27976	4008.2764	13.66236572	11.96912616	1.693488	0.75755
ND4L	42728.93805	11937.244	15.3829596	13.54330298	1.839744	0.75762
MAST2	1661.111125	5106.4003	10.69880113	12.31837343	-1.62016	0.75767
BRD7	8234.712566	2651.5273	13.00767776	11.37315188	1.634895	0.7577
LOC100514291	362.6741667	932.86828	8.506502639	9.867075268	-1.363	0.75781
RABEPK	979.1145142	386.69828	9.93680651	8.598790517	1.340269	0.75783
CYP24A1	152.2597085	72.712497	7.259834656	6.20383732	1.066259	0.75784
TIMM9	3014.530756	1065.8991	11.55819623	10.05920803	1.499863	0.75791
CLDN15	22.20454082	11.567897	4.536335244	3.651671381	0.940728	0.75794
FBXO33	803.5929058	323.90112	9.652115207	8.343856914	1.310911	0.75797
NFIL3	586.834293	1598.8486	9.199265715	10.64371971	-1.44601	0.75799
LOC100621245	2514.399908	8085.1339	11.29657207	12.98123437	-1.68506	0.75804
CCDC68	13.74566813	26.440908	3.882219287	4.778256309	-0.94379	0.75804
LOC100626842	326.7239578	144.59872	8.356373332	7.185853813	1.176018	0.75828
SNX14	10061.82907	3186.1294	13.29674834	11.63804188	1.659016	0.75847
LOC102159723	35.95020894	19.004403	5.207510617	4.322245637	0.919666	0.75861
LOC102167000	23.2618999	44.619032	4.600620625	5.511563931	-0.93969	0.75872
FEZ1	23.2618999	44.619032	4.600620625	5.511563931	-0.93969	0.75872
LOC100624533	121.596295	59.492043	6.937771569	5.918673475	1.031331	0.75892
EFCAB11	299.2326215	133.8571	8.22993693	7.075287628	1.160574	0.75899
TMEM170A	735.9219243	299.93905	9.525367966	8.233327508	1.294883	0.75901
NDUFS7	1108.112323	3244.7952	10.11518976	11.66435624	-1.55002	0.75901
LOC100623407	3839.270843	1329.4819	11.90699235	10.37773317	1.529968	0.75911
ENPEP	38.06492712	75.191332	5.287802017	6.251554969	-0.9821	0.75918
COL24A1	16.91774539	32.224856	4.163317207	5.054191063	-0.92964	0.75918
LOC100738644	25.37661808	48.750424	4.721187694	5.636636914	-0.94191	0.7593
MRPS17	1741.470416	651.10736	10.76691845	9.348965686	1.419339	0.75931
LOC100512907	42.29436346	84.280394	5.436107306	6.414142197	-0.99473	0.75932
HS2ST1	6131.625343	2033.4711	12.5822891	10.99043805	1.592325	0.75933
PRDM11	127.9404495	288.37115	7.010561107	8.176777292	-1.17245	0.75934
LOC102163062	29.60605442	15.699289	4.935745167	4.061714779	0.915193	0.75936
MEMO1	7044.126235	2307.7955	12.78240984	11.17292467	1.609905	0.75963
LOC102157658	9.516231779	4.1313919	3.394545939	2.359350201	1.203763	0.75979
KCND2	9.516231779	4.1313919	3.394545939	2.359350201	1.203763	0.75979
LOC102164514	9.516231779	4.1313919	3.394545939	2.359350201	1.203763	0.75979
IGSF5	9.516231779	4.1313919	3.394545939	2.359350201	1.203763	0.75979
LOC100522889	9.516231779	4.1313919	3.394545939	2.359350201	1.203763	0.75979
LOC102163490	9.516231779	4.1313919	3.394545939	2.359350201	1.203763	0.75979
LOC102167444	9.516231779	4.1313919	3.394545939	2.359350201	1.203763	0.75979
LOC100621242	275.9707216	124.76803	8.113589668	6.974621476	1.145267	0.75996
SDHB	9897.938409	3146.468	13.2730581	11.61997601	1.653395	0.75997

GP9	19.0653629	34.703692	4.326635347	5.158001345	-0.86414	0.75206
LOC102164577	19.0653629	34.703692	4.326635347	5.158001345	-0.86414	0.75206
ZNF536	52.2225159	28.919743	5.733964805	4.903025878	0.852617	0.75234
RSBN1	303.387949	153.68778	8.249767434	7.273215394	0.981161	0.75239
LOC100522432	209.718992	109.06875	7.719176542	6.782261052	0.94322	0.75247
LOC100517085	22.3810782	40.48764	4.547269558	5.374609695	-0.8552	0.75251
RAB1B	828.099895	1877.3045	9.695402126	10.87521522	-1.18079	0.75252
LOC102161553	12.4339324	5.7839486	3.747809764	2.762125239	1.104156	0.75266
SYT6	12.4339324	5.7839486	3.747809764	2.762125239	1.104156	0.75266
LOC100516122	12.4339324	5.7839486	3.747809764	2.762125239	1.104156	0.75266
LOC102160777	12.4339324	5.7839486	3.747809764	2.762125239	1.104156	0.75266
LOC100519860	12.4339324	5.7839486	3.747809764	2.762125239	1.104156	0.75266
LOC102158455	12.4339324	5.7839486	3.747809764	2.762125239	1.104156	0.75266
SLC29A2	12.4339324	5.7839486	3.747809764	2.762125239	1.104156	0.75266
SCG5	213.034708	110.7213	7.741700952	6.803760479	0.944156	0.75272
LOC102160446	132.628612	71.05994	7.062085132	6.171125545	0.900284	0.75279
BHLHE22	15.7496477	28.919743	4.066058842	4.903025878	-0.87674	0.75282
FANCL	175.732911	92.543178	7.465426908	6.547560532	0.925186	0.75286
LOC102162274	180.706484	361.08365	7.505466087	8.500179216	-0.99868	0.75293
LOC100738402	179.048626	94.195734	7.492242779	6.572825024	0.926618	0.753
LOC100627742	179.048626	94.195734	7.492242779	6.572825024	0.926618	0.753
GNRHR2	435.187632	214.83238	8.768805054	7.753767487	1.018426	0.75305
LOC100736984	455.081924	223.92144	8.833149183	7.81327737	1.023134	0.75313
LOC100737247	445.963707	219.79005	8.804013881	7.786531327	1.0208	0.75318
LOC100623494	243.705074	125.59431	7.934900207	6.98406878	0.956365	0.75327
LOC102160979	23.2100071	12.394176	4.597531595	3.74353388	0.905085	0.7533
LOC100157232	50.5646582	92.543178	5.688310694	6.547560532	-0.872	0.75335
MTAP	4999.26974	2054.128	12.28779021	11.00501256	1.283191	0.75341
WFDC3	66.3143059	123.11548	6.072841239	6.955539223	-0.89262	0.75342
LOC100154692	2733.80726	1174.1416	11.41722344	10.19861885	1.219305	0.75345
MFSD11	1280.69503	3002.6956	10.32383731	11.5525229	-1.22933	0.75367
KIF27	431.042988	213.17982	8.755031058	7.742678745	1.015761	0.75378
LOC102166191	27.3546512	14.873011	4.825513503	3.98850389	0.879089	0.75379
LOC100620514	27.3546512	14.873011	4.825513503	3.98850389	0.879089	0.75379
USE1	597.657682	1316.2614	9.225587482	10.363326	-1.13905	0.754
LOC102158789	19.0653629	9.9153405	4.326635347	3.448285225	0.94322	0.75402
LOC100512993	19.0653629	9.9153405	4.326635347	3.448285225	0.94322	0.75402
LOC102161118	58.0250177	32.224857	5.883254663	5.054191063	0.848501	0.75411
LOC100623921	58.0250177	32.224857	5.883254663	5.054191063	0.848501	0.75411
LOC102160063	43.1042988	23.962073	5.462847376	4.641665831	0.847079	0.75433
LOC102165515	1081.75212	499.07214	10.08048728	8.965992412	1.11605	0.75451
BCL2L15	21.5521494	38.835083	4.495193036	5.315967693	-0.84953	0.75454
LOC100521985	21.5521494	38.835083	4.495193036	5.315967693	-0.84953	0.75454
DHX38	1337.89112	607.3146	10.38682293	9.248673828	1.139445	0.75456
LOC100520452	324.11117	677.54826	8.344789314	9.406307627	-1.06383	0.75475
LOC100737992	5.80250177	12.394176	2.766065426	3.74353388	-1.09492	0.75486
CDKN3	565.329458	274.32442	9.145497764	8.104988763	1.043209	0.75488
RIPK1	1665.31801	3980.1829	10.70244804	11.95898144	-1.25704	0.75492
FAM171B	3186.4024	1355.9228	11.63816545	10.40612294	1.232653	0.75512
TBR1	5.80250177	1.6525567	2.766065426	1.407383613	1.811975	0.75519
LOC102159881	5.80250177	1.6525567	2.766065426	1.407383613	1.811975	0.75519
LOC102161384	5.80250177	1.6525567	2.766065426	1.407383613	1.811975	0.75519
KMO	5.80250177	1.6525567	2.766065426	1.407383613	1.811975	0.75519
LOC102163712	5.80250177	1.6525567	2.766065426	1.407383613	1.811975	0.75519
LOC102166759	5.80250177	1.6525567	2.766065426	1.407383613	1.811975	0.75519
KCNC2	5.80250177	1.6525567	2.766065426	1.407383613	1.811975	0.75519
LOC102164587	5.80250177	1.6525567	2.766065426	1.407383613	1.811975	0.75519
LOC100516805	5.80250177	1.6525567	2.766065426	1.407383613	1.811975	0.75519
LOC100512372	5.80250177	1.6525567	2.766065426	1.407383613	1.811975	0.75519
LOC102166011	5.80250177	1.6525567	2.766065426	1.407383613	1.811975	0.75519
LOC100516952	5.80250177	1.6525567	2.766065426	1.407383613	1.811975	0.75519
FOXD4	5.80250177	1.6525567	2.766065426	1.407383613	1.811975	0.75519
LOC102160575	5.80250177	1.6525567	2.766065426	1.407383613	1.811975	0.75519
VTCN1	5.80250177	1.6525567	2.766065426	1.407383613	1.811975	0.75519
LOC102158912	5.80250177	1.6525567	2.766065426	1.407383613	1.811975	0.75519
SH3RF3	5.80250177	1.6525567	2.766065426	1.407383613	1.811975	0.75519
LOC102158738	5.80250177	1.6525567	2.766065426	1.407383613	1.811975	0.75519
LOC102162306	5.80250177	1.6525567	2.766065426	1.407383613	1.811975	0.75519
LOC100521334	5.80250177	1.6525567	2.766065426	1.407383613	1.811975	0.75519
LOC100523828	5.80250177	1.6525567	2.766065426	1.407383613	1.811975	0.75519
LOC102167279	5.80250177	1.6525567	2.766065426	1.407383613	1.811975	0.75519
LOC102167150	5.80250177	1.6525567	2.766065426	1.407383613	1.811975	0.75519
LOC102167177	5.80250177	1.6525567	2.766065426	1.407383613	1.811975	0.75519
LOC100739685	5.80250177	1.6525567	2.766065426	1.407383613	1.811975	0.75519
LOC102167987	5.80250177	1.6525567	2.766065426	1.407383613	1.811975	0.75519
MIR1271	5.80250177	1.6525567	2.766065426	1.407383613	1.811975	0.75519
LOC100626842	282.664729	144.59872	8.148042965	7.185853814	0.967037	0.75525
PREI2	106.102889	57.839486	6.742853591	5.878712739	0.875337	0.75546
CYP2J34	178.219697	94.195734	7.485585395	6.572825024	0.919923	0.75547
PIK3R4	2819.18693	1211.3241	11.46157508	10.24355971	1.218694	0.75559
STBD1	505.646582	1095.6451	8.984835918	10.09888102	-1.11558	0.7556
LOC102167616	12.4339324	23.135794	3.747809764	4.593102408	-0.89584	0.75573
CEP112	158.325405	311.50695	7.315832521	8.287744447	-0.97637	0.75575
OSGEPL1	864.572763	406.52896	9.757511292	8.67075877	1.088629	0.75575
SRRD	251.165434	129.7257	7.978226717	7.030399034	0.953174	0.75589
TRAF2	1704.27766	4075.2049	10.73579095	11.99301087	-1.25771	0.75593
LOC100627270	33.1571529	59.492043	5.094115824	5.918673475	-0.84338	0.75594
LOC100625011	1415.81043	641.19202	10.46843102	9.32686092	1.1428	0.75604
COMMD5	251.994362	514.77143	7.982961427	9.010588038	-1.03054	0.75605
OCIAD2	432.700846	924.6055	8.760556447	9.854253624	-1.09547	0.7562
NAGS	14.9207188	27.267186	3.99283357	4.821056379	-0.86985	0.75629
LYPD3	14.9207188	27.267186	3.99283357	4.821056379	-0.86985	0.75629
LOC100514871	9.94714588	19.004403	3.452482877	4.322245637	-0.93398	0.75635
LOC102165502	9.94714588	19.004403	3.452482877	4.322245637	-0.93398	0.75635
DPH2	201.429704	105.76363	7.661277193	6.738276475	0.929433	0.75643
DENND2C	54.7093024	30.5723	5.799846344	4.980587446	0.839561	0.75651
LOC102165211	443.476921	219.79005	8.795964699	7.786531327	1.012733	0.75655
CDK5R1	35.6439394	19.830681	5.195502704	4.380638094	0.845923	0.75657
CELF2	213.863637	430.49103	7.747277532	8.753186764	-1.00929	0.75672
LOC102164472	14.9207188	7.4365053	3.99283357	3.076645515	1.00462	0.7569
LOC102165886	14.9207188	7.4365053	3.99283357	3.076645515	1.00462	0.7569
LOC100624767	2042.48062	900.64343	10.99681285	9.81641319	1.181294	0.75693
LOC102166671	62.1696618	34.703692	5.981159942	5.158001345	0.841122	0.75701
LOC100738540	62.1696618	34.703692	5.981159942	5.158001345	0.841122	0.75701
LOC100627685	402.030479	852.71928	8.654745137	9.73761795	-1.08477	0.75701
TMEM185B	1380.16649	627.14528	10.43167152	9.294954469	1.137971	0.75706
LOC100736585	143.404687	279.28209	7.173973754	8.130735747	-0.96163	0.75707
LOC100622563	1.65785765	5.7839486	1.410263837	2.762125239	-1.80273	0.75712
ASGR1	1.65785765	5.7839486	1.410263837	2.762125239	-1.80273	0.75712
LOC100518349	1.65785765	5.7839486	1.410263837	2.762125239	-1.80273	0.75712
KIRREL2	1.65785765	5.7839486	1.410263837	2.762125239	-1.80273	0.75712
LOC102160097	1.65785765	5.7839486	1.410263837	2.762125239	-1.80273	0.75712
LOC102167586	1.65785765	5.7839486	1.410263837	2.762125239	-1.80273	0.75712
MIR339	1.65785765	5.7839486	1.410263837	2.762125239	-1.80273	0.75712
ZSCAN26	784.995596	372.65155	9.618377419	8.54554968	1.074857	0.7572
42618	1400.88971	3291.893	10.45315714	11.68513993	-1.23257	0.75729
LOC102164821	2949.32875	1265.8585	11.52666001	10.30703964	1.220271	0.75736
OGG1	801.574172	380.08805	9.648490919	8.573980563	1.076502	0.75753
CDK5RAP2	3168.99489	1353.444	11.6302648	10.403485	1.22739	0.75768
LOC102161987	25.6967935	14.046732	4.73859457	3.911378308	0.871354	0.75772
LOC102164093	25.6967935	14.046732	4.73859457	3.911378308	0.871354	0.75772
LOC102158751	25.6967935	14.046732	4.73859457	3.911378308	0.871354	0.75772
HDHD1	159.154334	85.106672	7.323319031	6.428053129	0.903082	0.75784
LOC102162623	113.563249	61.970878	6.8400005	5.976612875	0.873834	0.75785
ZMYND10	94.4978859	52.055537	6.57739689	5.729431428	0.86023	0.75785
ADAMTS15	177.390768	94.195734	7.478897148	6.572825024	0.913197	0.75791
LOC102161587	1779.71018	794.05352	10.79823702	9.634908162	1.164334	0.75792
LOC100623437	507.30444	1095.6451	8.989549022	10.09888102	-1.11086	0.75794
LOC102167000	80.4060959	44.619032	6.347064926	5.511563931	0.849646	0.758
ALKBH1	150.865046	80.97528	7.246646039	6.357117027	0.897705	0.75801
LOC102163680	21.5521494	11.567897	4.495193036	3.65167138	0.897705	0.75826
GPR33	21.5521494	11.567897	4.495193036	3.65167138	0.897705	0.75826
LOC102158363	21.5521494	11.567897	4.495193036	3.65167138	0.897705	0.75826
LOC100736912	140.9179	76.01761	7.148912757	6.267116453	0.890449	0.75842
TBKBP1	1051.08175	2409.4277	10.03903109	11.23507346	-1.19682	0.75842
DLD	8042.26745	3213.3966	12.97356598	11.65033222	1.323503	0.75847
SLMO2	3403.58175	1447.6397	11.73326186	10.50048311	1.233351	0.7586
CHDH	137.602185	74.365053	7.114806188	6.2358238	0.887807	0.75861
RGS7	137.602185	74.365053	7.114806188	6.2358238	0.887807	0.75861
ZNF75D	3767.4815	1590.5859	11.8797676	10.63624928	1.244042	0.75868
HAUS4	706.247358	338.77413	9.466071071	8.408432212	1.05985	0.7587
LOC100523528	285.151515	585.83137	8.160635436	9.196802174	-1.03876	0.75888
ZSCAN31	48.0778718	86.759229	5.617000784	6.455478947	-0.85164	0.7589
METAP1D	231.271142	466.84728	7.859666109	8.869893856	-1.01337	0.7589
TUT1	1949.64059	865.11346	10.92973227	9.758412211	1.172247	0.75901
LOC102162484	73.7746653	136.33593	6.224477643	7.101565318	-0.88597	0.75904
SPTBN5	7.46035941	14.873011	3.080718953	3.98850389	-0.99538	0.7591
LIN7C	3527.09214	1497.2164	11.78467253	10.54903031	1.236197	0.75912
NICN1	471.660501	1010.5384	8.884660496	9.982335443	-1.0993	0.75917
FHOD1	812.350247	1821.1175	9.667732933	10.8314003	-1.16465	0.75923
CBFA2T2	306.703665	156.99289	8.265397816	7.303715831	0.966146	0.75929
CDC25B	2126.20243	5150.1931	11.05474162	12.3306909	-1.27635	0.7593
ALDH1B1	733.602009	351.16831	9.520819031	8.460121275	1.062835	0.75931
NFATC2	71.2878788	131.37826	6.175681852	7.048522415	-0.882	0.75931
LOC102164793	40.6175124	72.712497	5.379118827	6.20383732	-0.8401	0.75941
LOC100739392	26.5257224	47.097867	4.78270852	5.587901017	-0.82827	0.75953
LOC102165421	103.616103	57.013208	6.708961125	5.858309486	0.86188	0.75956
COQ10B	1282.35289	588.3102	10.32570221	9.202883429	1.124144	0.75991
VANGL2	396.227978	199.13309	8.633823429	7.644815895	0.992598	0.75995
LOC102158955	620.867689	1359.2279	9.280463849	10.4096327	-1.13043	0.76016
ARAP1	1792.97305	4279.2957	10.8089425	12.06349475	-1.25502	0.7603
LOC102162252	43.9332277	24.788351	5.489710792	4.688647628	0.82565	0.76035
AP3M2	183.19327	97.500848	7.525076538	6.622064238	0.90988	0.7604
LOC100156373	17.4075053	9.0890621	4.202222212	3.334720158	0.937506	0.76054
LOC100524650	3100.1938	1331.1345	11.59860797	10.37952399	1.219702	0.76057
LOC102162033	11.6050035	21.483238	3.655924617	4.490777898	-0.88846	0.76058
LOC102165104	16.5785765	29.746021	4.135746339	4.942327829	-0.84338	0.76065
MLF1IP	1770.59197	793.22724	10.79083065	9.633408027	1.158426	0.76067
LOC102167550	38.1307259	21.483238	5.290229968	4.490777898	0.827743	0.76067
LOC102164382	49.7357294	28.093465	5.664930183	4.862623207	0.824048	0.76076
LOC100626072	150.036117	80.97528	7.238749771	6.357117027	0.889756	0.76078
LOC100626607	5299.34197	2187.9851	12.37186973	11.09604644	1.27621	0.76081
MTHFR	644.077696	1412.936	9.333329125	10.46550112	-1.13339	0.76084
LOC100739351	275.204369	561.86929	8.109592332	9.136656134	-1.02973	0.76086
LOC100518839	33.9860818	60.318321	5.128709195	5.938246292	-0.82765	0.7609
PPCS	678.892707	1495.5639	9.409163283	10.54743812	-1.13943	0.76098
RRP8	2723.86011	1181.5781	11.41196645	10.20771972	1.204938	0.76099
LOC100524214	324.940099	166.08195	8.348463041	7.384412099	0.968278	0.76103
GTF3C4	4055.11981	1707.9174	11.98588455	10.73886695	1.247506	0.76107
LOC100736660	208.061135	109.89502	7.707781074	6.793050813	0.920881	0.76109
SPIN2B	143.404687	77.670167	7.173973754	6.29774474	0.88466	0.76113
MRPS24	1977.82417	879.16019	10.95042771	9.781622305	1.169716	0.76113
ABCB9	452.595138	225.574	8.825261365	7.823838478	1.00462	0.76115
LOC100621303	87.0375265	161.95056	6.460046706	7.348290508	-0.89584	0.76125
MAP7D3	213.034708	112.37386	7.741700952	6.824944216	0.922782	0.76129
BMX	8.28928824	3.3051135	3.215568059	2.106051269	1.326548	0.76138
LOC100628107	8.28928824	3.3051135	3.215568059	2.106051269	1.326548	0.76138
LOC100626139	8.28928824	3.3051135	3.215568059	2.106051269	1.326548	0.76138
LOC102161160	8.28928824	3.3051135	3.215568059	2.106051269	1.326548	0.76138
KCNB2	8.28928824	3.3051135	3.215568059	2.106051269	1.326548	0.76138
PHEX	8.28928824	3.3051135	3.215568059	2.106051269	1.326548	0.76138
LOC102160699	8.28928824	3.3051135	3.215568059	2.106051269	1.326548	0.76138
LOC102165300	8.28928824	3.3051135	3.215568059	2.106051269	1.326548	0.76138
PGLYRP1	8.28928824	3.3051135	3.215568059	2.106051269	1.326548	0.76138
NCMAP	8.28928824	3.3051135	3.215568059	2.106051269	1.326548	0.76138
LOC102166171	1293.12897	594.09415	10.33776568	9.216974124	1.122103	0.76138
LOC100515534	218.008281	114.85269	7.774841608	6.856147779	0.924598	0.76151
SLC29A3	194.798274	385.872	7.613224233	8.595712505	-0.98614	0.76155
PAM	31180.9866	11394.379	14.92842523	13.47616126	1.452344	0.76157
TCF7L2	3756.70543	1592.2384	11.87563626	10.63774646	1.238412	0.76159
MYOF	5801.67284	15124.199	12.50250187	13.88466653	-1.38232	0.76161
SLC25A4	5986.52396	2452.3942	12.54774381	11.26056335	1.287528	0.76161
LOC102162641	28.18358	15.699289	4.867084967	4.061714779	0.844156	0.7617
MIR1306	28.18358	15.699289	4.867084967	4.061714779	0.844156	0.7617
LOC102159201	29.0125088	51.229259	4.907492018	5.706786332	-0.82029	0.76198
PROKR1	33.1571529	58.665764	5.094115824	5.89883146	-0.8232	0.76198
BMPR1B	301.730092	155.34033	8.241888278	7.288546214	0.957826	0.76202
TRAF5	558.698027	274.32442	9.128504852	8.104988763	1.026186	0.76207
DTD2	1322.9704	607.3146	10.37065516	9.248673828	1.123265	0.76207
LOC100622964	24.0389359	13.220454	4.646101346	3.829895615	0.862601	0.76208
LOC100522132	253.65222	513.11887	7.992384484	9.005958152	-1.01644	0.76224
KCNC3	42.27537	23.962073	5.435474249	4.641665831	0.819065	0.76232
PIANP	232.928999	467.67356	7.869926909	8.872439593	-1.00561	0.76242
LOC100624238	92.0110994	51.229259	6.539330984	5.706786332	0.84484	0.76242
ALG11	580.250177	284.23976	9.183015439	8.156031285	1.029567	0.76247
LOC100625505	3272.611	1403.0207	11.67666718	10.45534846	1.221906	0.76251
LNPEP	1775.56554	797.35863	10.7948752	9.640893151	1.154978	0.76258
LOC100524292	238.731501	480.06773	7.905275684	8.910096229	-1.00785	0.76272
EBF2	642.419838	312.33322	9.329616608	8.291553945	1.04043	0.76275
LOC100623800	125.168252	68.581105	6.979205122	6.120623682	0.867986	0.76278
FBXO46	170.759338	91.716899	7.424244723	6.534760414	0.896705	0.76278
SLC36A1	707.076287	1558.361	9.467760991	10.60673925	-1.14009	0.76294
TYW1	1579.93834	716.38335	10.62656538	9.486600448	1.141065	0.76307
LOC100525237	249.507576	130.55198	7.968710424	7.039489182	0.934459	0.76321
KCNF1	3.31571529	8.2627837	2.109599694	3.211445828	-1.31731	0.76334
LOC102164327	3.31571529	8.2627837	2.109599694	3.211445828	-1.31731	0.76334
LOC102166493	3.31571529	8.2627837	2.109599694	3.211445828	-1.31731	0.76334
LOC102158858	3.31571529	8.2627837	2.109599694	3.211445828	-1.31731	0.76334
LOC102163351	3.31571529	8.2627837	2.109599694	3.211445828	-1.31731	0.76334
GRM1	3.31571529	8.2627837	2.109599694	3.211445828	-1.31731	0.76334
GIT2	1260.80074	2911.805	10.30126839	11.5081934	-1.20757	0.76361
LOC100523801	5805.81748	2389.5971	12.50353197	11.22315526	1.280732	0.76363
SHAS2	41.4464412	73.538775	5.407571694	6.219919206	-0.82726	0.76365
GPR85	41.4464412	73.538775	5.407571694	6.219919206	-0.82726	0.76365
ZNF784	92.0110994	171.03962	6.539330984	7.426597064	-0.89445	0.76374
LOC102167038	89.524313	166.08195	6.500233417	7.384412099	-0.89154	0.76375
NET1	4631.22534	11839.743	12.17748972	13.53147197	-1.35417	0.76376
MIB1	10336.7424	4083.4677	13.33563354	11.99593236	1.339915	0.76378
LOC102159629	10.7760747	4.9576702	3.557786824	2.574748269	1.120098	0.76392
CKMT1B	10.7760747	4.9576702	3.557786824	2.574748269	1.120098	0.76392
LOC102162294	10.7760747	4.9576702	3.557786824	2.574748269	1.120098	0.76392
LRRC71	10.7760747	4.9576702	3.557786824	2.574748269	1.120098	0.76392
LOC102165102	10.7760747	4.9576702	3.557786824	2.574748269	1.120098	0.76392
LOC102158717	59.6828753	33.877413	5.92321754	5.124221138	0.816993	0.76395
LOC100526148	142.575758	77.670167	7.165668365	6.29774474	0.876296	0.76397
TMEM245	4745.61752	1983.0681	12.21268409	10.95424582	1.258862	0.76398
HACE1	886.953841	1988.852	9.794340872	10.95844545	-1.16501	0.76403
HIC1	211.37685	419.74941	7.730482704	8.716817448	-0.98971	0.76405
ADAMTS8	232.100071	122.2892	7.864805631	6.945902605	0.92445	0.76414
PAPD5	991.398873	466.84728	9.954776287	8.869893856	1.086515	0.76416
ZMIZ1	2408.033523	5850.8772	11.23424157	12.51468377	-1.28079	0.76428
DYNC1I2	3726.86399	9365.8653	11.86413351	13.19335061	-1.32945	0.7643
ZFPL1	973.162439	2197.9005	9.928018548	11.10256659	-1.17537	0.76436
LOC100153411	518.080515	1109.6919	9.019814523	10.1172429	-1.09891	0.76446
MATN3	35.6439394	62.797156	5.195502704	5.995420212	-0.81704	0.76453
MFSD4	18.2364341	32.224857	4.265769485	5.054191063	-0.82135	0.76459
NPTN	1598.17477	3757.0878	10.6431119	11.87578304	-1.23319	0.76462
LOC102164068	165.785765	322.24857	7.381852349	8.336500155	-0.95885	0.76464
LOC100522150	243.705074	128.07315	7.934900207	7.012045083	0.928168	0.76471
LOC100511545	96.1557435	53.708094	6.602227381	5.773682393	0.840234	0.76499
LOC100621510	461.713355	978.31359	8.853974928	9.935627098	-1.0833	0.7651
FAM186B	110.247534	61.1446	6.79762954	5.957557119	0.850449	0.76515
LPP	65.4853771	37.182527	6.054965161	5.254840671	0.816548	0.76516
LOC102162188	83.7218112	47.097867	6.404661526	5.587901017	0.829942	0.7652
CECR5	472.48943	236.31561	8.887188408	7.890663216	0.999567	0.76546
LOC100525571	161.641121	87.585507	7.34554825	6.468998788	0.88403	0.76549
ZNF451	2872.23837	1248.5066	11.48846197	10.28714283	1.201972	0.76558
PPFIA2	20.7232206	36.356248	4.441166102	5.223277669	-0.81096	0.76561
LOC100620396	526.369803	261.10397	9.042671155	8.033995371	1.011452	0.76561
LOC100522612	463.371212	232.18422	8.859134728	7.865326367	0.996898	0.76566
IL1RAP	2385.65715	1051.8524	11.22077562	10.04008744	1.181454	0.7657
CREM	2183.39852	969.22453	11.09302037	9.922174845	1.171673	0.76571
LOC100736879	34.8150106	19.830681	5.162492464	4.380638094	0.811975	0.76579
LOC102161858	34.8150106	19.830681	5.162492464	4.380638094	0.811975	0.76579
LOC102167660	595.170895	292.50254	9.219582135	8.197229196	1.024855	0.76586
LOC100737616	518.909444	257.79885	9.02211655	8.015687408	1.009237	0.76587
LOC102168187	4.97357294	10.741619	2.578594101	3.553559423	-1.11086	0.76592

SGOL2	1395.99874	648.62852	10.44811501	9.343471165		1.105833	0.8583		KPNA3	1377.73889	527.99188	10.42913354	9.047101766		1.383715	0.76006		LOC100624918	4.97357294	10.741619	2.578594101	3.553559423		-1.11086	0.76592
LOC102159672	93.8950688	162.77684	6.568261215	7.35558754		-0.79377	0.85832		LOC102164403	482.1557435	205.74331	8.916344501	7.691696868		1.228654	0.76006		LOC102160170	4.97357294	10.741619	2.578594101	3.553559423		-1.11086	0.76592
SMCHD1	927.90421	2147.4975	9.859386021	11.06911237		-1.21061	0.85835		LOC100154462	6238.418611	2068.1748	12.60719589	11.01483979		1.592822	0.76007		LOC100625061	44.7621565	25.61463	5.516083133	4.734147579		0.805312	0.76593
SLC4A5	133.938554	79.322724	7.076158798	6.327736286		0.755765	0.8584		GPC3	131.1125267	295.80766	7.045623457	8.213384501		-1.17385	0.76008		DNALI1	13.2628612	6.610227	3.834191515	2.927939483		1.00462	0.76595
LOC100622845	15.1889082	9.9153405	4.016933786	3.448285225		0.615284	0.85841		CHUK	2004.752828	740.34542	10.96992812	9.534002096		1.437154	0.76013		LOC102157975	13.2628612	6.610227	3.834191515	2.927939483		1.00462	0.76595
LOC102164761	15.1889082	9.9153405	4.016933786	3.448285225		0.615284	0.85841		LOC100620709	104.6785496	229.70539	6.723538761	7.849907883		-1.13382	0.76019		ANKRD61	13.2628612	6.610227	3.834191515	2.927939483		1.00462	0.76595
LOC100517731	15.1889082	9.9153405	4.016933786	3.448285225		0.615284	0.85841		RCC1	2903.508052	1034.5005	11.50407811	10.01611256		1.488863	0.76019		CAPSL	13.2628612	6.610227	3.834191515	2.927939483		1.00462	0.76595
LOC100738806	292.731685	159.47173	8.198355089	7.326175314		0.876278	0.85852		TRAM1	17415.76151	5270.8297	14.08818877	12.36408806		1.724292	0.76035		MFAP5	13.2628612	6.610227	3.834191515	2.927939483		1.00462	0.76595
SLC30A6	167.07799	96.674569	7.392987005	6.609911086		0.789313	0.85852		ANKRD13C	3693.355289	1286.5154	11.8511069	10.330374		1.521463	0.7604		LOC102167387	13.2628612	6.610227	3.834191515	2.927939483		1.00462	0.76595
LOC102160066	22.0929574	14.873011	4.529381037	3.98850389		0.57089	0.85854		MAP3K13	443.0334573	190.8703	8.794524575	7.58398763		1.214823	0.76042		CBX7	92.8400282	52.055537	6.552131542	5.729431428		0.834695	0.76596
QIL1	4252.89429	1786.4138	12.05456847	10.80365798		1.251379	0.85868		LOC100151973	26.43397716	14.046732	4.777891883	3.911378308		0.912159	0.76053		LOC102163972	1391.7715	640.36574	10.44374287	9.325003477		1.119954	0.76649
C15H4orf47	70.4213016	44.619032	6.158282521	5.511563931		0.658353	0.8587		CLDN22	50.75323616	102.45852	5.693577173	6.692908622		-1.01347	0.7607		GYPC	242.876145	128.07315	7.930004838	7.012045083		0.923253	0.7665
C16H5orf22	397.673233	209.87471	8.639062934	7.72024225		0.922055	0.85874		ICA	0	3.3051135	0	2.106051269		-20	0.76076		AGPS	3305.76815	1423.6776	11.69120618	10.4764198		1.215363	0.76656
ERCC6	336.9176	688.28988	8.400527683	9.428967032		-1.03062	0.85877		HPN	0	3.3051135	0	2.106051269		-20	0.76076		DUSP28	534.659091	1144.3955	9.06517131	10.16163018		-1.09789	0.76661
LOC102159053	34.5202459	23.135794	5.150569664	4.593102408		0.577316	0.85883		SLC6A7	0	3.3051135	0	2.106051269		-20	0.76076		PCBP4	1932.23309	4599.0654	10.91679988	12.16743866		-1.25107	0.76663
LOC100517015	34.5202459	23.135794	5.150569664	4.593102408		0.577316	0.85883		PCDHA6	0	3.3051135	0	2.106051269		-20	0.76076		HSPA14	3166.50811	1368.317	11.6291326	10.41924074		1.21049	0.76667
B3GALNT2	93.8950688	57.839486	6.568261215	5.878712739		0.698995	0.85884		AQP9	0	3.3051135	0	2.106051269		-20	0.76076		WBP2	3294.99207	8176.8508	11.68649706	12.99750602		-1.31127	0.7667
ATP2B1	521.946118	1125.3911	9.030518495	10.13749218		-1.10845	0.85895		USHBP1	0	3.3051135	0	2.106051269		-20	0.76076		LOC100625107	402.030479	204.09076	8.654745137	7.68011867		0.978094	0.76683
CXCR4	215.406334	121.46292	7.757598918	6.936201185		0.826545	0.85896		LOC102161822	0	3.3051135	0	2.106051269		-20	0.76076		PABPC1	99118.3352	33807.18	16.59687889	15.04508472		1.551822	0.76684
ATG4D	839.53238	410.66035	9.715159588	8.685310691		1.03164	0.85896		LOC100525229	0	3.3051135	0	2.106051269		-20	0.76076		PHYHIPL	22.3810782	12.394176	4.547269558	3.74353388		0.852617	0.76691
LOC100623098	110.464787	195.0017	6.800444207	7.614722326		-0.8199	0.85899		LOC100511475	0	3.3051135	0	2.106051269		-20	0.76076		LOC102166786	23.2100071	40.48764	4.597531595	5.374609695		-0.80273	0.76694
LOC102166563	13.8080984	20.656959	3.888314478	4.436758793		-0.58111	0.85908		LOC100628107	0	3.3051135	0	2.106051269		-20	0.76076		PPM1J	48.9068006	86.759229	5.641164514	6.455478947		-0.82698	0.76694
LOC100037971	8.28485901	13.220454	3.214880004	3.829895615		-0.67422	0.85914		RAP1GAP2	0	3.3051135	0	2.106051269		-20	0.76076		UXT	962.386364	456.10566	9.911970694	8.836383876		1.077248	0.76694
PIH1D3	8.28485901	13.220454	3.214880004	3.829895615		-0.67422	0.85914		LOC102163911	0	3.3051135	0	2.106051269		-20	0.76076		LOC100521998	117.707893	65.275991	6.891272054	6.05041444		0.850587	0.76697
LOC102160664	8.28485901	13.220454	3.214880004	3.829895615		-0.67422	0.85914		LOC100626139	0	3.3051135	0	2.106051269		-20	0.76076		LOC100739706	499.844081	249.53607	8.968217733	7.968874505		1.00223	0.76707
ZMAT1	8.28485901	13.220454	3.214880004	3.829895615		-0.67422	0.85914		MROH7	0	3.3051135	0	2.106051269		-20	0.76076		SOCS7	456.739782	229.70539	8.838383871	7.849907883		0.991588	0.76724
HOXD11	8.28485901	13.220454	3.214880004	3.829895615		-0.67422	0.85914		LOC102163556	0	3.3051135	0	2.106051269		-20	0.76076		ALG1	193.140416	378.43549	7.600956677	8.567710832		-0.9704	0.76728
LOC100512313	412.862141	217.31121	8.69300647	7.770242414		0.925898	0.85914		BEST2	0	3.3051135	0	2.106051269		-20	0.76076		LOC100624029	180.706484	97.500848	7.505466087	6.622064238		0.890162	0.76731
RMND5B	57.9940131	94.195734	5.882496648	6.572825024		-0.69976	0.85915		ADRA2C	0	3.3051135	0	2.106051269		-20	0.76076		LOC100737754	179.048626	96.674569	7.492242779	6.609911086		0.889143	0.76735
TMEM131	2147.1593	5450.9584	11.06888526	12.41255884		-1.34408	0.85916		LOC102162006	0	3.3051135	0	2.106051269		-20	0.76076		LOC100738470	177.390768	95.848291	7.478897148	6.597654688		0.888106	0.76738
FANK1	33.1394361	22.309516	5.093367322	4.542847146		0.57089	0.8592		SLC25A48	0	3.3051135	0	2.106051269		-20	0.76076		RBPMS	2060.71706	4924.6191	11.00963064	12.26608935		-1.25687	0.76743
LOC102167010	33.1394361	22.309516	5.093367322	4.542847146		0.57089	0.8592		LOC102162173	0	3.3051135	0	2.106051269		-20	0.76076		LOC100157828	69.6300212	39.661362	6.142209624	5.34558663		0.811975	0.76743
LOC100625443	122.892075	219.79005	6.9529401	7.786531327		-0.83873	0.85921		LOC102164789	0	3.3051135	0	2.106051269		-20	0.76076		LOC100626018	524.711945	261.10397	9.038128706	8.033995371		1.006901	0.76744
ANKS1A	1210.97023	571.78463	10.24313854	9.161848978		1.08262	0.85923		LOC102166766	0	3.3051135	0	2.106051269		-20	0.76076		KLC2	5349.90663	2225.9939	12.38556764	11.12088191		1.265064	0.76752
ZNF77	160.173941	93.369456	7.332474693	6.560248082		0.778617	0.85926		SARDH	0	3.3051135	0	2.106051269		-20	0.76076		SPATA17	48.9068006	28.093465	5.641164514	4.862623207		0.799801	0.76756
WDFY2	24.854577	37.182527	4.692347799	5.254840671		-0.58111	0.85926		LOC100626847	0	3.3051135	0	2.106051269		-20	0.76076		SLC10A7	170.759338	92.543178	7.424244723	6.547560532		0.883766	0.76757
LOC100624467	341.060029	696.55267	8.418105721	9.446158339		-1.03021	0.85929		RORB	0	3.3051135	0	2.106051269		-20	0.76076		LOC102161149	715.365575	1566.6238	9.4845522	10.61436366		-1.13091	0.76778
LOC100517759	28.9970066	43.792754	4.906746634	5.485193456		-0.59479	0.85935		LOC100738225	0	3.3051135	0	2.106051269		-20	0.76076		SERGEF	861.257048	412.31291	9.751974205	8.691090609		1.062704	0.76782
LOC100522576	925.14259	448.66916	9.855090519	8.812720118		1.044024	0.85936		TNF	0	3.3051135	0	2.106051269		-20	0.76076		LOC100737045	98.64253	55.360651	6.638689748	5.816616368		0.833349	0.76788
ZSCAN16	34.5202459	52.881816	5.150569664	5.751726565		-0.61533	0.85943		TDH	0	3.3051135	0	2.106051269		-20	0.76076		PLIN2	4607.1864	1939.2753	12.16998336	10.92204568		1.248368	0.76797
MEF2C	225.072003	435.4487	7.820638529	8.769668286		-0.95212	0.85947		LOC102157734	0	3.3051135	0	2.106051269		-20	0.76076		LOC102161372	6.63143059	13.220454	2.93195353	3.829895615		-0.99538	0.76798
POLR2K	2673.24784	1172.489	11.38491746	10.19658861		1.189019	0.85948		LOC100514392	0	3.3051135	0	2.106051269		-20	0.76076		VGLL2	6.63143059	13.220454	2.93195353	3.829895615		-0.99538	0.76798
TMBIM1	1220.6359	2906.021	10.25459864	11.50532579		-1.25141	0.85949		ACAN	0	3.3051135	0	2.106051269		-20	0.76076		LOC102164240	15.7496477	8.2627837	4.066058842	3.211445828		0.93062	0.76799
LOC102163597	167.07799	310.68067	7.392987005	8.283924863		-0.89491	0.8596		AARD	0	3.3051135	0	2.106051269		-20	0.76076		LOC102166012	15.7496477	8.2627837	4.066058842	3.211445828		0.93062	0.76799
CENPQ	748.398931	370.99899	9.549590109	8.53915489		1.012392	0.85961		ZFP42	0	3.3051135	0	2.106051269		-20	0.76076		FAM46C	47.248943	83.454116	5.592425431	6.400095824		-0.8207	0.7681
PRSS53	31.7586262	21.483238	5.033802952	4.490777898		0.563937	0.85962		LOC100736655	0	3.3051135	0	2.106051269		-20	0.76076		NUDT1	1316.33897	609.79344	10.36341091	9.254540753		1.110139	0.7682
CCDC9	1557.5535	3809.1433	10.60599196	11.89562954		-1.29018	0.85976		LOC100624770	0	3.3051135	0	2.106051269		-20	0.76076		LOC102165983	86.2085977	48.750424	6.446398468	5.636636914		0.822417	0.76821
ATP10D	429.431859	225.574	8.749641054	7.823838478		0.928828	0.85982		AGMO	0	3.3051135	0	2.106051269		-20	0.76076		GPR176	232.928999	462.71589	7.869926909	8.857097348		-0.99024	0.76853
CEP70	809.154564	398.26618	9.662053366	8.641207046		1.022682	0.85987		LOC100520018	0	3.3051135	0	2.106051269		-20	0.76076		LOC102167303	91.1821706	167.73451	6.526415834	7.398611253		-0.87936	0.76857
TMCC1	1387.71389	647.80224	10.43953368	9.341634998		1.099085	0.85989		LOC100624962	0	3.3051135	0	2.106051269		-20	0.76076		LOC100738629	33.1571529	19.004403	5.094115824	4.322245637		0.802986	0.76866
LOC100523842	255.44982	501.55097	8.002532746	8.97312612		-0.97336	0.85992		LOC102158038	0	3.3051135	0	2.106051269		-20	0.76076		DNASE1L1	430.21406	900.64343	8.752260407	9.81641319		-1.0659	0.76882
RBM28	455.667246	237.96817	8.834999507	7.900674664		0.937212	0.85995		IL20RA	0	3.3051135	0	2.106051269		-20	0.76076		C16H5orf51	751.838443	364.38876	9.55619649	8.513288452		1.044944	0.76883
UTP11L	4584.28866	1918.6184	12.16279684	10.90660382		1.25663	0.85997		LOC102162742	0	3.3051135	0	2.106051269		-20	0.76076		LOC100520853	95.3268147	53.708094	6.589865555	5.773682393		0.827743	0.76885
LOC102166896	17.9505279	26.440908	4.24416613	4.77825631		-0.55875	0.86		LOC100627069	0	3.3051135	0	2.106051269		-20	0.76076		LOC100738619	498.186223	249.53607	8.963434308	7.968874505		0.997437	0.76897
POLR2I	3712.99765	1583.1494	11.85875719	10.62949265		1.229787	0.86001		NOX5	0	3.3051135	0	2.106051269		-20	0.76076		LOC102165013	258.625793	136.33593	8.020289907	7.101565318		0.9237	0.76897
NUDT6	457.048056	965.91942	8.839355155	9.91725185		-1.07956	0.86002		LOC100152874	0	3.3051135	0	2.106051269		-20	0.76076		VMAC	145.891473	80.149002	7.19860684	6.342501448		0.864139	0.76906
BLMH	6489.80623	2636.6543	12.66418197	11.36503977		1.299467	0.86005		LOC102158635	0	3.3051135	0	2.106051269		-20	0.76076		LOC102167010	12.4339324	22.309516	3.747809764	4.542847146		-0.84338	0.76909
ASTN1	30.3778164	20.656959	4.971673052	4.436758793		0.55639	0.8601		LAMC3	0	3.3051135	0	2.106051269		-20	0.76076		LOC102167621	12.4339324	22.309516	3.747809764	4.542847146		-0.84338	0.76909
LOC100737500	27.6161967	41.313919	4.838760036	5.403060391		-0.58111	0.86017		CLDN23	0	3.3051135	0	2.106051269		-20	0.76076		SLMAP	1430.73115	659.37014	10.48354489	9.367131078		1.117592	0.76914
LOC100524687	9.66566885	14.873011	3.414902536	3.98850389		-0.62176	0.86021		LOC102162536	0	3.3051135	0	2.106051269		-20	0.76076		PRSS53	37.3017971	21.483238	5.259340178	4.490777898		0.796034	0.76914
LOC100738840	9.66566885	14.873011	3.414902536	3.98850389		-0.62176	0.86021		LOC100623270	0	3.3051135	0	2.106051269		-20	0.76076		HERC3	4104.02661	1745.0999	12.00317586	10.7699204		1.23373	0.76918
LOC102163343	9.66566885	14.873011	3.414902536	3.98850389		-0.62176	0.86021		LOC100524164	0	3.3051135	0	2.106051269		-20	0.76076		ST7L	285.980444	577.56858	8.164808619	9.176344172		-1.01408	0.76919
FAM26E	9.66566885	14.873011	3.414902536	3.98850389		-0.62176	0.86021		ZDHHC19	0	3.3051135	0	2.106051269		-20	0.76076		LOC100738607	2723.03119	6635.8416	11.4115275	12.69628113		-1.28507	0.7692
LOC100739805	98.0374983	169.38707	6.629902968	7.412672017		-0.78892	0.86021		P2RY11	0	3.3051135	0	2.106051269		-20	0.76076		LOC100620172	58.8539465	104.93735	5.90337447	6.727067559		-0.83432	0.76922
LOC100523995	70.4213016	116.50525	6.158282521	6.876581411		-0.72631	0.86023		FAM205A	0	3.3051135	0	2.106051269		-20	0.76076		LOC100519655	260.283651	521.38165	8.029473043	9.028960416		-1.00226	0.76923
NEK7	1607.26265	3938.869	10.65128732	11.94393195		-1.29318	0.86026		LOC100524097	0	3.3051135	0	2.106051269		-20	0.76076		KIAA1407	256.139007	512.29259	8.006404665	9.003637625		-1.00004	0.76931
LOC102159457	9.66566885	5.7839486	3.414902536	2.762125239		0.740815	0.86028		LOC102165300	0	3.3051135	0	2.106051269		-20	0.76076		LOC100737447	1666.97586	759.34982	10.7038827	9.57051952		1.134397	0.76934
PMEL	9.66566885	5.7839486	3.414902536	2.762125239		0.740815	0.86028		LOC100739453	0	3.3051135	0	2.106051269		-20	0.76076		LOC100624806	66.3143059	38.008805	6.072841239	5.285727902		0.802986	0.76937
MIR339	9.66566885	5.7839486	3.414902536	2.762125239		0.740815	0.86028		PGLYRP1	0	3.3051135	0	2.106051269		-20	0.76076		LOC100622476	1081.75212	509.81376	10.08048728	8.996653565		1.085328	0.7694
LOC100624963	175.362849	101.63224	7.462402878	6.681340183		0.786985	0.86031		RSPO4	0	3.3051135	0	2.106051269		-20	0.76076		LOC100620086	67.1432347	120.63664	6.090498528	6.926434087		-0.84535	0.76951
LOC100739862	451.524816	236.31561	8.821853101	7.890663216		0.93409	0.86034		LOC102163201	0	3.3051135	0	2.106051269		-20	0.76076		DDX59	307.532594	624.66645	8.269279095	9.289249932		-1.02235	0.76952
LOC100623546	23.4737672	34.703692	4.613164285	5.158001345		-0.56404	0.86041		LOC102167554	0	3.3051135	0	2.106051269		-20	0.76076		LOC100520855	73.7746653	42.140197	6.224477643	5.430960858		0.807928	0.76961
LOC100524779	393.530803	209.04843	8.623994134	7.714578179		0.912639	0.86045		LOC100153359	0	3.3051135	0	2.106051269		-20	0.76076		TMOD1	437.674419	916.34271	8.777006768	9.841317007		-1.06603	0.76967
LOC102163333	31.7586262	47.924146	5.033802952	5.61247475		-0.5936	0.86052		LOC102165811	0	3.3051135	0	2.106051269		-20	0.76076		LOC100516365	24.8678647	14.046732	4.693089065	3.911378308		0.824048	0.76979
TZP	109.083977	190.8703	6.782460686	7.58398763		-0.80715	0.86054		LOC100513717	0	3.3051135	0	2.106051269		-20	0.76076		GDPD2	24.8678647	14.046732	4.693089065	3.911378308		0.824048	0.76979
ZNF662	162.935561	300.76533	7.356985025	8.23728324		-0.88434	0.86056		LOC100511115	0	3.3051135	0	2.106051269		-20	0.76076		LOC100523481	24.8678647	14.046732	4.693089065	3.911378308		0.824048	0.76979
FAM210A	689.024108	345.38436	9.430502957	8.436229978		0.996352	0.86056		LRIT1	0	3.3051135	0	2.106051269		-20	0.76076		ZFYVE28	137.602185	76.01761	7.114806188	6.267116453		0.856098	0.76992
LOC100521082	846.436429	415.61802	9.726961338	8.702581435		1.026144	0.86063		LOC100737508	0	3.3051135	0	2.106051269		-20	0.76076		LOC100620865	57.1960888	101.63224	5.862850292	6.681340183		-0.82937	0.76998
RHOC	6166.69673	17426.211	12.59051611	14.08905407		-1.49869	0.86064		LOC100737392	0	3.3051135	0	2.106051269		-20	0.76076		LOC100738877	41.4464412	23.962073	5.407571694	4.641665831		0.790496	0.77
LOC100626246	324.490311	655.23875	8.34647079	9.358076973		-1.01385	0.86066		LOC100519177	102.5638314	51.229259	6.694376434	5.706786332		1.001482	0.76079		LOC100156311	1095.84391	516.42398	10.09914251	9.015203113		1.085414	0.77005
UNKL	219.548764	421.40197	7.784953864	8.722472752		-0.94066	0.86066		LOC102166743	32.77813168	63.623435	5.078017628	6.013985524		-0.95682	0.7608		LOC102163390	18.2364341	9.9153405	4.265769485	3.448285225		0.879089	0.77011
LOC100626193	55.2323934	88.411786	5.813329549	6.482393107		-0.67872	0.86066		MCPH1	32.77813168	63.623435	5.078017628	6.013985524		-0.95682	0.7608		SH2B2	135.944327	75.191332	7.097445694	6.251554969		0.854378	0.77012
BARX1	55.2323934	88.411786	5.813329549	6.482393107		-0.67872	0.86066		VIMP	1123.972709	3283.6302	10.13567429	11.68151526		-1.54668	0.76087		UPF2	1962.90345	884.11786	10.93950829	9.78972576		1.150679	0.77033
LOC100739549	15.1889082	22.309516	4.016933786	4.542847146		-0.55464	0.86067		LOC100737435	1442.237794	4334.6563	10.49509331	12.08203469		-1.58761	0.76092		LOC102165838	134.286469	74.365053	7.079873746	6.2358238		0.852617	0.77033
LOC100524724	53.8515836	85.932951	5.777461367	6.441831208		-0.67422	0.86067		LOC100739516	44.40908164	88.411786	5.504908955	6.482393107		-0.99338	0.76094		PCDH19	56.36716	99.979683	5.842153193	6.657921243		-0.82678	0.77039
[illegible]	[illegible]	[illegible]	[illegible]	[illegible]		[illegible]	[illegible]		[illegible]	[illegible]	[illegible]	[illegible]	[illegible]		[illegible]	[illegible]		[illegible]	[illegible]	[illegible]	[illegible]	[illegible]		[illegible]	[illegible]

CALR3	52.4707738	35.52997	5.740678649	5.191008663	0.562478	0.86526
TRIM32	185.028518	340.42669	7.539379992	8.415432027	-0.8796	0.86533
LOC102159761	42.8051049	64.449713	5.453027102	6.032314961	-0.59039	0.86533
LOC100155725	42.8051049	64.449713	5.453027102	6.032314961	-0.59039	0.86533
LOC100517114	265.115489	149.55639	8.055908671	7.234160086	0.825931	0.86535
LOC102159794	8.28485901	4.9576702	3.214880004	2.574748269	0.740815	0.86536
LOC102162131	8.28485901	4.9576702	3.214880004	2.574748269	0.740815	0.86536
TMEM255B	8.28485901	4.9576702	3.214880004	2.574748269	0.740815	0.86536
LOC102158648	8.28485901	4.9576702	3.214880004	2.574748269	0.740815	0.86536
FLT3	8.28485901	4.9576702	3.214880004	2.574748269	0.740815	0.86536
LOC102165590	8.28485901	4.9576702	3.214880004	2.574748269	0.740815	0.86536
SLC4A10	8.28485901	4.9576702	3.214880004	2.574748269	0.740815	0.86536
AMH	8.28485901	4.9576702	3.214880004	2.574748269	0.740815	0.86536
ZNF621	132.557744	80.97528	7.061319821	6.357117027	0.711067	0.86537
LOC102162557	92.514259	154.51406	6.547114458	7.280901168	-0.73999	0.86538
LOC100513987	3440.97811	1496.3901	11.74902221	10.54823444	1.201332	0.86543
LOC100738357	158.793131	285.89232	7.320061583	8.164365521	-0.84832	0.86546
RND3	613.079567	1318.7403	9.262281789	10.36603833	-1.10501	0.86548
CERKL	52.4707738	80.97528	5.740678649	6.357117027	-0.62597	0.86555
C7H6orf226	1140.54892	551.12767	10.15677698	9.108858104	1.04927	0.86556
LOC100521274	85.6102098	141.2936	6.436465198	7.152726981	-0.72284	0.86557
CCT2	24646.0748	9091.5409	14.58912881	13.1504678	1.438761	0.86562
LOC102167659	28.9970066	20.656959	4.906746634	4.436758793	0.489276	0.86565
CCDC148	28.9970066	20.656959	4.906746634	4.436758793	0.489276	0.86565
LOC100156357	74.5637311	120.63664	6.239622035	6.926434087	-0.69412	0.86567
RCC1	2289.38271	1034.5005	11.16137297	10.01611256	1.146024	0.86573
LOC102167575	20.7121475	14.873011	4.440430524	3.98850389	0.47778	0.86578
LIX1	20.7121475	14.873011	4.440430524	3.98850389	0.47778	0.86578
SEC16A	528.850167	1115.4758	9.049440637	10.12473627	-1.07673	0.86579
CD63	12217.4054	36469.448	13.5767684	15.15444032	-1.57775	0.86579
LOC102168167	16.569718	23.135794	4.135019133	4.593102408	-0.48158	0.86581
LOC102161929	379.722705	767.61261	8.572596799	9.58611283	-1.01543	0.86581
FOXN2	704.213016	357.77854	9.461915293	8.486949769	0.976945	0.86584
LOC102159510	49.7091541	76.01761	5.664174303	6.267116453	-0.61282	0.86585
GYG1	1238.58642	2896.1057	10.27564314	11.5003966	-1.22542	0.86586
CCNB1IP1	1726.0123	801.49002	10.75406264	9.648339641	1.106686	0.86587
LOC102165446	30.3778164	43.792754	4.971673052	5.485193456	-0.52767	0.86593
RPL10	43066.078	15234.921	15.39429782	13.89518906	1.49917	0.86599
LOC102161951	8.28485901	12.394176	3.214880004	3.74353388	-0.58111	0.866
LOC100624977	8.28485901	12.394176	3.214880004	3.74353388	-0.58111	0.866
LOC100739831	8.28485901	12.394176	3.214880004	3.74353388	-0.58111	0.866
GRM2	8.28485901	12.394176	3.214880004	3.74353388	-0.58111	0.866
AK5	8.28485901	12.394176	3.214880004	3.74353388	-0.58111	0.866
RYK	3569.39343	9389.0011	11.80186734	13.19690962	-1.39529	0.86603
LOC100157234	1097.74382	2529.2381	10.10163933	11.30505743	-1.20416	0.86604
IFT74	285.827636	556.08534	8.164040225	9.121754552	-0.96016	0.86605
TDRD3	285.827636	160.298	8.164040225	7.333584776	0.834389	0.86606
TSSK6	9.66566885	14.046732	3.414902536	3.911378308	-0.53929	0.86609
LOC100624261	9.66566885	14.046732	3.414902536	3.911378308	-0.53929	0.86609
LOC100625286	706.974636	1544.3143	9.467553865	10.59368456	-1.12724	0.86612
LOC100621516	455.667246	941.95734	8.834999507	9.8810487	-1.04768	0.86612
LOC102166267	23.4737672	33.051135	4.613164285	5.089630977	-0.49365	0.86613
LOC100525984	162.935561	97.500848	7.356985025	6.622064238	0.740815	0.86614
LOC100519022	42.8051049	29.746021	5.453027102	4.942327829	0.525086	0.86615
LOC102157512	15.1889082	10.741619	4.016933786	3.553559423	0.499807	0.86617
LOC102159851	6.90404918	10.741619	2.982591923	3.553559423	-0.6377	0.86627
LOC100739070	6.90404918	10.741619	2.982591923	3.553559423	-0.6377	0.86627
LOC102157878	6.90404918	10.741619	2.982591923	3.553559423	-0.6377	0.86627
LOC100526053	6.90404918	10.741619	2.982591923	3.553559423	-0.6377	0.86627
LOC100625842	6.90404918	10.741619	2.982591923	3.553559423	-0.6377	0.86627
SMARCC2	1622.45156	3908.2967	10.66484862	11.93269337	-1.26836	0.86632
APEX1	12135.9377	4744.4904	13.56711683	12.21234147	1.354961	0.86633
RNPS1	23530.3804	8724.6733	14.52229833	13.09105075	1.431352	0.86635
RAB22A	1448.46952	3443.102	10.50130928	11.74991214	-1.24918	0.8664
LOC100525721	650.361433	1404.6732	9.347314489	10.45704554	-1.11092	0.86641
LOC100623323	462.571295	246.23095	8.856647425	7.949715578	0.909664	0.86641
LOC102163591	197.455807	115.67897	7.632673964	6.86640077	0.771403	0.86641
LOC100515277	196.074997	361.90993	7.62260094	8.50346771	-0.88423	0.86642
LOC102159127	11.0464787	15.699289	3.590539588	4.061714779	-0.50711	0.86644
CRIPT	893.383964	443.71149	9.804750511	8.796725856	1.009658	0.8665
EDN1	100.799118	169.38707	6.669581252	7.412672017	-0.74884	0.86651
ZBTB41	64.8980623	102.45852	6.042164139	6.692908622	-0.65879	0.86654
OGT	3130.2959	8106.6171	11.61254413	12.98506224	-1.3728	0.86654
C3H16orf93	60.7556328	95.022013	5.948498827	6.585293272	-0.64524	0.86657
NFATC2	227.833623	131.37826	7.838155235	7.048522415	0.794254	0.86659
LOC100623698	40.0434852	28.093465	5.359081339	4.862623207	0.511333	0.86662
LOC100623469	28.9970066	41.313919	4.906746634	5.403060391	-0.51072	0.86677
LOC100623775	28.9970066	41.313919	4.906746634	5.403060391	-0.51072	0.86677
LOC100627743	219.548764	127.24687	7.784953864	7.002779797	0.786911	0.86679
LOC100739508	890.622344	442.88521	9.800288961	8.79404282	1.007881	0.86681
PRPF38A	700.070587	356.95226	9.453415898	8.483623365	0.971769	0.86681
LSAMP	109.083977	185.08636	6.782460686	7.539828464	-0.76276	0.86682
LOC100621861	110.464787	69.407383	6.800444207	6.137654819	0.670426	0.86682
ATAT1	368.676226	739.51914	8.530118456	9.53239322	-1.00423	0.86684
ZBTB18	226.452813	425.53336	7.829423468	8.736514776	-0.91006	0.86685
HS3ST3B1	142.223413	86.759229	7.162123543	6.455478947	0.71307	0.86689
PRPH	38.6626754	27.267186	5.309710091	4.821056379	0.503776	0.86689
ADRA2A	11.0464787	7.4365053	3.590539588	3.076645515	0.57089	0.8669
PRRT4	11.0464787	7.4365053	3.590539588	3.076645515	0.57089	0.8669
LOC102163059	11.0464787	7.4365053	3.590539588	3.076645515	0.57089	0.8669
LOC100738149	425.289429	228.87911	8.735689471	7.844731552	0.893859	0.86693
CACNA1S	53.8515836	82.627837	5.777461367	6.385911347	-0.61764	0.86696
LOC102165156	12.4272885	17.351846	3.747096094	4.19785327	-0.48158	0.86696
SVCT1	26.2353869	19.004403	4.767410456	4.322245637	0.46518	0.86698
C5H12orf29	781.538367	394.13478	9.612017677	8.626201041	0.987628	0.86698
LOC100737574	330.013551	651.93364	8.370746468	9.350792552	-0.9822	0.86701
HOXA10	110.464787	187.56519	6.800444207	7.558919565	-0.76381	0.86703
CYP2A19	19.3313377	14.046732	4.345633235	3.911378308	0.460707	0.86705
LOC102166381	19.3313377	14.046732	4.345633235	3.911378308	0.460707	0.86705
LOC102167232	19.3313377	14.046732	4.345633235	3.911378308	0.460707	0.86705
LOC100625273	370.057036	741.99798	8.535497154	9.537214474	-1.00367	0.86707
LOC100736939	5.52323934	9.0890621	2.705588563	3.334720158	-0.71862	0.86708
SLC39A2	5.52323934	9.0890621	2.705588563	3.334720158	-0.71862	0.86708
LOC100626320	5.52323934	9.0890621	2.705588563	3.334720158	-0.71862	0.86708
FAM71F1	5.52323934	9.0890621	2.705588563	3.334720158	-0.71862	0.86708
LOC100738976	5.52323934	9.0890621	2.705588563	3.334720158	-0.71862	0.86708
LOC102166447	5.52323934	9.0890621	2.705588563	3.334720158	-0.71862	0.86708
PABPC5	5.52323934	9.0890621	2.705588563	3.334720158	-0.71862	0.86708
LOC102162382	5.52323934	9.0890621	2.705588563	3.334720158	-0.71862	0.86708
LOC100625576	5.52323934	9.0890621	2.705588563	3.334720158	-0.71862	0.86708
LYPD6B	5.52323934	9.0890621	2.705588563	3.334720158	-0.71862	0.86708
LOC100737597	5.52323934	9.0890621	2.705588563	3.334720158	-0.71862	0.86708
LOC100737947	240.260911	137.98849	7.914450382	7.118821585	0.800054	0.86712
IGSF1	37.2818656	26.440908	5.258589232	4.77825631	0.495702	0.8672
LOC100622067	850.578859	425.53336	9.733996324	8.736514776	0.999173	0.86724
ZKSCAN7	51.0899639	77.670167	5.702933533	6.29774474	-0.60432	0.86726
C8H4orf46	214.025525	398.26618	7.748364114	8.641207046	-0.89595	0.86732
LOC100625644	24458.2846	9060.9686	14.57809459	13.14560878	1.432586	0.86739
LOC100736826	3312.5628	1452.5974	11.69416755	10.50541201	1.189313	0.8674
LOC102159255	24.854577	34.703692	4.692347799	5.158001345	-0.48158	0.86746
LOC100154345	24.854577	34.703692	4.692347799	5.158001345	-0.48158	0.86746
C14H10orf116	24.854577	34.703692	4.692347799	5.158001345	-0.48158	0.86746
YIPF2	1735.67796	4198.3204	10.76211454	12.03594015	-1.27431	0.86747
CHCHD1	2119.5431	969.22453	11.05021809	9.922174845	1.12885	0.8675
LOC102163666	289.970066	561.86929	8.184726929	9.136656134	-0.95433	0.86753
DUSP28	544.039075	1144.3955	9.090215854	10.16163018	-1.0728	0.86754
RRP8	2637.34679	1181.5781	11.36541849	10.20771972	1.158372	0.86755
C16H5orf42	885.099105	1977.2841	9.791324254	10.95003394	-1.15961	0.86759
LOC102165893	13.8080984	19.004403	3.888314478	4.322245637	-0.46082	0.86761
C13H3orf17	555.085554	290.84999	9.119163049	8.189083193	0.932435	0.86762
GGH	3341.5598	1464.9916	11.70673766	10.51766108	1.189629	0.86763
LOC102161315	96.6566885	61.970878	6.609646952	5.976612875	0.641279	0.86768
LOC100621894	96.6566885	61.970878	6.609646952	5.976612875	0.641279	0.86768
DNAJB12	1017.65685	2310.2743	9.992452423	11.17447279	-1.18281	0.8678
ATG2A	1179.2116	2724.2398	10.20482983	11.41216746	-1.20803	0.86781
LOC100512042	2663.58217	1193.146	11.37969361	10.22176348	1.158597	0.86785
NDUFA8	6837.77031	2815.1304	12.73952122	11.45949843	1.280324	0.8679
LOC100623373	46.9475344	70.233662	5.583384725	6.154487245	-0.58111	0.86791
LASS4	2343.23429	1062.594	11.19490105	10.05473181	1.140911	0.86794
LOC100737936	1056.31952	518.07654	10.04619571	9.019803473	1.027809	0.86804
RPL26	88848.2089	29922.845	16.43907131	14.86900794	1.570095	0.86806
LOC100739772	2463.36475	1112.1707	11.26700009	10.12045911	1.147252	0.86806
FAM69A	393.530803	792.40096	8.623994134	9.63190633	-1.00975	0.86806
CES2	34.5202459	49.576702	5.150569664	5.660401068	-0.52222	0.86807
TNXB	34.5202459	49.576702	5.150569664	5.660401068	-0.52222	0.86807
NDUFB4	5941.62472	2477.1826	12.53688456	11.27506675	1.262157	0.86808
KIAA1147	75.944541	121.46292	6.265747069	6.936201185	-0.6775	0.8681
C3H16orf96	30.3778164	42.966475	4.971673052	5.458331975	-0.50019	0.86814
ULK2	1824.04979	4427.1995	10.83372011	12.11250451	-1.27925	0.86819
LOC102167753	45.5667246	67.754826	5.541227504	6.103389087	-0.57234	0.86819
ZNF577	176.743659	318.94345	7.473654282	8.321673128	-0.85164	0.86824
FAM227B	78.7061606	126.42059	6.316619332	6.993454623	-0.68368	0.86825
TUSC1	265.115489	151.20894	8.055908671	7.249909307	0.810077	0.86827
LOC100515340	506.75721	1053.5049	8.987995009	10.04235012	-1.05583	0.86828
CCT6A	13987.6036	5428.6489	13.77196434	12.4066432	1.365484	0.8683
E2F5	741.494882	377.60922	9.536237271	8.564565719	0.973543	0.86837
ST6GALNAC1	13.8080984	9.9153405	3.888314478	3.448285225	0.47778	0.86842
HIGD1B	17.9505279	13.220454	4.24416613	3.829895615	0.441255	0.86846
tRNA-Val	17.9505279	13.220454	4.24416613	3.829895615	0.441255	0.86846
LOC100627651	56.6132033	38.835083	5.848327568	5.315967693	0.543778	0.86847
RMDN2	70.4213016	47.097867	6.158282521	5.587901017	0.58035	0.86848
SET	35745.0242	12889.116	15.12549517	13.65397766	1.471589	0.86848
PXMP4	165.69718	99.979683	7.381085891	6.657921243	0.728842	0.86852
IL15RA	102.179928	170.21334	6.689018532	7.419651342	-0.73623	0.86853
MAGEL2	631.030095	1347.66	9.303849446	10.397311	-1.09468	0.86853
RBBP7	14596.5408	5648.4389	13.83343772	12.46389188	1.369702	0.86855
LOC100511262	73.1829213	48.750424	6.213015177	5.636636914	0.586092	0.86858
RAB32	335.53679	660.19642	8.394620418	9.368935101	-0.97642	0.86858
MYO6	798.108085	1754.189	9.642246841	10.77741066	-1.13615	0.86865
LOC100518219	7050.41502	2900.2371	12.78369708	11.50245248	1.281537	0.86866
ATP2C2	4.14242951	7.4365053	2.362450112	3.076645515	-0.84415	0.8687
LOC100738634	4.14242951	7.4365053	2.362450112	3.076645515	-0.84415	0.8687
LOC102160774	4.14242951	7.4365053	2.362450112	3.076645515	-0.84415	0.8687
LOC102160332	4.14242951	7.4365053	2.362450112	3.076645515	-0.84415	0.8687
KRT26	4.14242951	7.4365053	2.362450112	3.076645515	-0.84415	0.8687
LOC102165293	4.14242951	7.4365053	2.362450112	3.076645515	-0.84415	0.8687
LOC100622742	262.353869	499.07214	8.040858843	8.965992412	-0.92773	0.86871
TIE-1	23.4737672	32.224857	4.613164285	5.054191063	-0.45712	0.86871
LOC102161527	33.1394361	47.097867	5.093367322	5.587901017	-0.50711	0.86872
LOC100155163	26.2353869	36.356248	4.767410456	5.223277669	-0.47069	0.86873
ACVR2A	197.455807	361.08365	7.632673964	8.500179216	-0.8708	0.86874
LOC100628087	51.0899639	35.52997	5.702933533	5.191008663	0.524003	0.86878
LOC100739561	51.0899639	35.52997	5.702933533	5.191008663	0.524003	0.86878
KIAA0391	444.620767	239.62073	8.799627657	7.910617116	0.891823	0.86879
PTPN9	1226.15913	2835.7874	10.26110663	11.4700423	-1.2096	0.86889
DUT	13117.6934	5124.5785	13.67933642	12.32349912	1.356009	0.8689
LOC102161205	577.178511	1217.108	9.17537118	10.25042639	-1.07637	0.86893
LOC102165045	128.415315	220.61633	7.015864542	7.791920351	-0.78072	0.86898
ZC3HC1	2009.07831	927.08433	10.97303599	9.858112096	1.115761	0.86898
LOC102166685	28.9970066	40.48764	4.906746634	5.374609695	-0.48158	0.869
USP38	218.167954	404.05012	7.775893057	8.661956638	-0.8891	0.86902
TRAF3	532.992597	1112.1707	9.06067593	10.12045911	-1.06119	0.86903
ANXA2	32547.0686	105787.59	14.99028432	16.69082456	-1.70057	0.86904
LOC102167598	20.7121475	28.093465	4.440430524	4.862623207	-0.43976	0.86906
PPP3CC	1436.04223	684.98477	10.48888674	9.422032737	1.067954	0.8691
LOC102167621	16.569718	22.309516	4.135019133	4.542847146	-0.42911	0.86911
LOC100513566	16.569718	22.309516	4.135019133	4.542847146	-0.42911	0.86911
COL18A1	1695.63448	4068.5947	10.72846007	11.99066941	-1.26271	0.86918
NCKIPSD	399.054043	801.49002	8.644051094	9.648339641	-1.0061	0.86919
TLE1	816.058613	1794.6766	9.674295766	10.81031185	-1.13698	0.86927
LOC100620528	147.746652	258.62513	7.21671339	8.020286225	-0.80774	0.86928
DPYSL2	1299.34206	3021.7	10.34467546	11.56162209	-1.21758	0.86929
NAT9	371.437846	738.69286	8.540855872	9.530782548	-0.99185	0.86929
C1H9orf57	30.3778164	22.309516	4.971673052	4.542847146	0.445359	0.86929
LOC100154753	30.3778164	22.309516	4.971673052	4.542847146	0.445359	0.86929
RAB9A	756.68379	1648.4254	9.565452073	10.68774777	-1.12333	0.86931
LOC100526125	654.503862	338.77413	9.35646047	8.408432212	0.950078	0.86932
JMJD6	1908.27919	885.77041	10.89881237	9.792416828	1.107268	0.86932
ASAP2	937.569879	2096.2682	9.874320351	11.03429567	-1.16083	0.86939
LOC102167899	35.9010557	51.229259	5.205590187	5.706786332	-0.51294	0.8694
ZBED5	1231.68237	2844.8764	10.26758539	11.47465731	-1.20774	0.86945
DEDD	894.764774	1988.852	9.806976122	10.95844545	-1.15236	0.86945
LOC102157639	93.8950688	153.68778	6.568261215	7.273215394	-0.71088	0.86946
LOC100623372	941.712308	469.32612	9.880673754	8.877517631	1.004696	0.86947
LOC100517977	140.842603	244.5784	7.148147109	7.940039851	-0.79621	0.8695
INTS2	149.127462	261.10397	7.230044098	8.033995371	-0.80808	0.86951
LTA4H	9076.06305	3661.2395	13.14800986	11.83851041	1.309734	0.86952
LOC102160098	508.13802	1051.8524	8.991912992	10.04008744	-1.04964	0.86953
LOC102167310	22.0929574	16.525567	4.529381037	4.13138925	0.418887	0.86955
RUNDC3A	82.8485901	133.03082	6.389714621	7.066420948	-0.68321	0.86958
PTPN4	443.239957	239.62073	8.795195352	7.910617116	0.887335	0.86959
SLC9A1	466.713725	955.1778	8.869481953	9.901135097	-1.03323	0.86963
LOC100524580	91.1334492	59.492043	6.525653118	5.918673475	0.615284	0.8697
PTK2	142.223413	247.05723	7.162123543	7.954529215	-0.79669	0.86972
IPO13	890.622344	1976.4579	9.800288961	10.94943124	-1.15003	0.86975
ARL10	1536.84135	729.6038	10.58669096	9.512945452	1.074783	0.86981
LOC102164845	28.9970066	21.483238	4.906746634	4.490777898	0.432693	0.86985
UTP18	3762.7068	1640.9888	11.87793853	10.68122861	1.197205	0.86985
LOC100621211	17.9505279	23.962073	4.24416613	4.641665831	-0.41673	0.86992
CACNA1D	17.9505279	23.962073	4.24416613	4.641665831	-0.41673	0.86992
PTPRJ	658.646292	1407.1521	9.365548835	10.45958742	-1.0952	0.86993
UGCG	665.550341	1423.6776	9.380570028	10.4764198	-1.097	0.86994
LOC100625114	27.6161967	38.008805	4.838760036	5.285727902	-0.46082	0.86994
LOC102167424	27.6161967	38.008805	4.838760036	5.285727902	-0.46082	0.86994
TNK2	2214.81898	5464.1789	11.11362431	12.416053	-1.30282	0.86999
LOC100511270	16.569718	12.394176	4.135019133	3.74353388	0.418887	0.87004
LOC102166437	16.569718	12.394176	4.135019133	3.74353388	0.418887	0.87004
LOC102158877	16.569718	12.394176	4.135019133	3.74353388	0.418887	0.87004
LOC102165512	16.569718	12.394176	4.135019133	3.74353388	0.418887	0.87004
CNTD2	22.0929574	29.746021	4.529381037	4.942327829	-0.42911	0.8701
ZMYND8	798.108085	1745.0999	9.642246841	10.7699204	-1.12865	0.87014
NAGPA	446.001577	905.6011	8.804136111	9.824324094	-1.02183	0.87015
LOC102160586	44.1859147	64.449713	5.497801224	6.032314961	-0.54459	0.87015
LOC100622567	370.057036	732.90892	8.535497154	9.519457213	-0.98589	0.87021
UFSP1	367.295416	203.26448	8.524719632	7.674294538	0.853583	0.87021
FAAH2	40.0434852	28.919743	5.359081339	4.903025878	0.469513	0.87024
LOC102167448	1072.88924	528.81816	10.06862949	9.049353478	1.020658	0.87026
NOXO1	98.0374983	63.623435	6.629902968	6.013985524	0.623775	0.87028
LOC102166070	51.0899639	76.01761	5.702933533	6.267116453	-0.57329	0.87029
RAB40B	69.0404918	107.41619	6.130117309	6.76043638	-0.6377	0.87029
LOC100621428	342.440839	670.93804	8.423917793	9.392184392	-0.97032	0.87031
LOC102164356	66.2788721	102.45852	6.072081615	6.692908622	-0.62842	0.87034
FRYL	791.204036	1726.9218	9.629728241	10.75482221	-1.12608	0.87034
USP32	389.388374	775.87539	8.608766278	9.601539402	-0.99462	0.87039
LOC100511275	27.6161967	20.656959	4.838760036	4.436758793	0.418887	0.87045
LOC102160861	626.887665	327.20624	9.294362661	8.358458837	0.938007	0.87047
LOC102158766	330.013551	185.08636	8.370746468	7.539828464	0.834327	0.8705
HMBS	2144.39768	987.40265	11.06702938	9.948955075	1.118862	0.8705
LOC100626333	212.644715	125.59431	7.739069817	6.98406878	0.759674	0.8705
LOC100738318	62.1364426	95.022013	5.98040107	6.585293272	-0.61282	0.87051
MGAT3	95.2758787	155.34033	6.58910248	7.288546214	-0.70525	0.87053
LOC100525546	984.517413	489.98307	9.944737552	8.939529481	1.006685	0.87053
FAXC	143.604223	248.70979	7.175965874	7.964108573	-0.79237	0.8706
RARS2	122.892075	77.670167	6.9529401	6.29774474	0.661959	0.87062
PPP5C	5096.5691	2167.3282	12.31559371	11.0823674	1.233609	0.87068
SLC25A15	1274.48748	618.05622	10.31683302	9.273926629	1.044107	0.8707
LOC100524598	2597.3033	1174.9678	11.34335413	10.1996329	1.144393	0.87071
LOC100522130	3801.36948	1659.9932	11.89268301	10.69783049	1.195342	0.87072
LOC102166825	19.3313377	25.61463	4.345633235	4.734147579	-0.40603	0.87076
CCDC59	1801.95684	844.4565	10.81614914	9.723586711	1.093469	0.87077
CASP3	305.158974	587.48392	8.25813716	9.20085919	-0.94499	0.87078
UPF0639	37.2818656	27.267186	5.258589232	4.821056379	0.451308	0.87083
KIRREL3	9.66566885	6.610227	3.414902536	2.927939483	0.54817	0.87085
LOC100156563	9.66566885	6.610227	3.414902536	2.927939483	0.54817	0.87085
LOC102160570	9.66566885	6.610227	3.414902536	2.927939483	0.54817	0.87085
LOC102160222	9.66566885	6.610227	3.414902536	2.927939483	0.54817	0.87085
LOC102158784	9.66566885	6.610227	3.414902536	2.927939483	0.54817	0.87085

[illegible]

[illegible]

LOC102167872	9.66566885	6.610227	3.414902536	2.927939483	0.54817	0.87085	HARBI1	43.35172255	81.801559	5.470918232	6.371586023	-0.91604	0.7752	PRICKLE3	1731.63231	813.8842	10.75874981	9.670451241	1.089237	0.7923
LOC102165802	9.66566885	6.610227	3.414902536	2.927939483	0.54817	0.87085	LOC100157816	19.03246356	33.877413	4.324267947	5.124221138	-0.83186	0.77531	ACAD11	1007.14852	494.94074	9.977492478	8.954023947	1.024949	0.7923
CD276	1369.76336	3188.6082	10.42076382	11.63916352	-1.219	0.87088	CCHCR1	328.8386759	799.83746	8.365616765	9.645365656	-1.28233	0.77537	SYNE1	1186.19715	2608.5608	10.21334381	11.34959131	-1.13691	0.7923
PDXDC1	657.265482	1398.8893	9.362525737	10.45109701	-1.08973	0.8709	LOC100515652	142.7434767	311.50695	7.167352675	8.287744447	-1.12584	0.77542	ZNF37A	100.300388	59.492043	6.662495885	5.918673475	0.753559	0.79233
SDCCAG8	256.830629	482.54657	8.010279851	8.917511028	-0.90985	0.87092	TNPO1	14757.56077	4644.5107	13.84926442	12.1816215	1.667856	0.77547	LOC100626252	680.550564	1425.3302	9.412676884	10.47809228	-1.06652	0.79233
LOC102159564	11.0464787	14.873011	3.590539588	3.98850389	-0.42911	0.87094	LOC100515619	1292.092804	513.94515	10.3366101	9.008274952	1.330023	0.77553	LOC102164997	15.7496477	9.0890621	4.066058842	3.334720158	0.793116	0.79236
LOC102166802	11.0464787	14.873011	3.590539588	3.98850389	-0.42911	0.87094	LOC100739598	196.6687901	95.848291	7.626941292	6.597654688	1.036943	0.77556	LOC100625986	15.7496477	9.0890621	4.066058842	3.334720158	0.793116	0.79236
LOC102162924	11.0464787	14.873011	3.590539588	3.98850389	-0.42911	0.87094	TTC18	177.6363265	87.585507	7.480881679	6.468998788	1.020163	0.77577	SETD8	2241.42354	1032.0217	11.13084308	10.01265483	1.118942	0.79241
LOC102160330	394.911613	786.61701	8.629034575	9.621350459	-0.99413	0.87095	LOC102158185	115.2521404	59.492043	6.861113469	5.918673475	0.954025	0.77581	LOC102162254	53.8803735	33.051135	5.778218396	5.089630977	0.70506	0.79243
LOC102159788	12.4272885	16.525567	3.747096094	4.13138925	-0.41119	0.87096	TMCC3	713.7173834	302.41788	9.481229068	8.245162313	1.238809	0.77582	LOC100623509	249.507576	137.98849	7.968710424	7.118821585	0.854536	0.79251
LOC100624652	12.4272885	16.525567	3.747096094	4.13138925	-0.41119	0.87096	TRAPPC9	907.2140963	2499.4921	9.826888619	11.28799632	-1.46212	0.77588	C15H4orf47	73.7746653	44.619032	6.224477643	5.511563931	0.725466	0.79253
RAPGEF2	788.442416	1716.1802	9.624690226	10.74582571	-1.12212	0.87099	LOC100739086	1725.610029	666.80665	10.75372656	9.383286641	1.371766	0.77592	LOC102166623	62.9985906	106.58991	5.999968229	6.749398975	-0.75868	0.7926
FKBPL	372.818656	206.56959	8.546194759	7.697451307	0.851846	0.871	RHOG	224.1601264	518.07654	7.814807553	9.019803473	-1.20864	0.77596	TP53BP1	67.9721635	41.313919	6.107942317	5.403060391	0.718316	0.79275
ARHGAP11A	2160.96739	995.66544	11.07812905	9.96096549	1.117944	0.87101	LOC102164424	77.18721332	155.34033	6.288860784	7.288546214	-1.009	0.77602	LOC100522989	41.4464412	25.61463	5.407571694	4.734147579	0.69428	0.7928
LOC102159075	12.4272885	9.0890621	3.747096094	3.334720158	0.451308	0.87101	CYP2A19	7.401513606	14.046732	3.070649265	3.911378308	-0.92434	0.77602	ZNF691	486.58122	254.49374	8.929498748	7.997144125	0.93505	0.79284
LOC100516662	12.4272885	9.0890621	3.747096094	3.334720158	0.451308	0.87101	KCNA7	7.401513606	14.046732	3.070649265	3.911378308	-0.92434	0.77602	LOC100625121	2825.81836	1278.2526	11.46496347	10.3210855	1.144496	0.79286
LOC102158126	12.4272885	9.0890621	3.747096094	3.334720158	0.451308	0.87101	LOC100515214	7.401513606	14.046732	3.070649265	3.911378308	-0.92434	0.77602	LOC100524762	305.874736	594.92043	8.261506068	9.218975892	-0.95976	0.79288
LOC100519531	12.4272885	9.0890621	3.747096094	3.334720158	0.451308	0.87101	LOC102164763	8.458872693	15.699289	3.241668254	4.061714779	-0.89216	0.77607	ABL1	4458.80814	10922.574	12.12276593	13.41515731	-1.29258	0.79289
LOC102167708	12.4272885	9.0890621	3.747096094	3.334720158	0.451308	0.87101	LOC102165637	8.458872693	15.699289	3.241668254	4.061714779	-0.89216	0.77607	LOC100626218	272.717583	524.68677	8.096544303	9.038059606	-0.94405	0.793
MSS51	158.793131	278.45581	7.320061583	8.126476366	-0.8103	0.87105	LOC102163128	2382.230022	891.55436	11.21870249	9.801796232	1.417918	0.7762	PFDN2	4164.53841	1828.554	12.02428727	10.83727631	1.187453	0.79318
POMC	205.740666	122.2892	7.691678382	6.945902605	0.75053	0.87106	SLC17A7	125.8257313	64.449713	6.986703669	6.032314961	0.965181	0.77626	TMOD4	10.7760747	18.178124	3.557786824	4.261389712	-0.75437	0.79319
LACTB	637.934144	332.99018	9.319523428	8.383661891	0.937928	0.87106	LOC100623269	142.7434767	310.68067	7.167352675	8.283924863	-1.12201	0.77631	LOC102162644	10.7760747	18.178124	3.557786824	4.261389712	-0.75437	0.79319
LOC100525030	956.901216	478.41518	9.903733075	8.905131772	1.000107	0.87106	ALG6	849.0593465	353.64714	9.731419756	8.470240516	1.263555	0.77645	LOC100739355	1099.98855	2399.5124	10.10458375	11.22912667	-1.12525	0.79324
LRRC10B	26.2353869	19.830681	4.767410456	4.380638094	0.40378	0.8711	LOC102160184	9.516231779	17.351846	3.394545939	4.19785327	-0.86663	0.77647	MRI1	2096.361	971.70337	11.03435948	9.925856099	1.109299	0.79327
LOC100157828	28.9970066	39.661362	4.906746634	5.34558663	-0.45183	0.8711	INCA1	9.516231779	17.351846	3.394545939	4.19785327	-0.86663	0.77647	ATG16L1	1870.06343	875.0288	10.86964275	9.774834483	1.095685	0.79341
MYT1L	23.4737672	31.398578	4.613164285	5.017858594	-0.41965	0.87111	LOC102165156	9.516231779	17.351846	3.394545939	4.19785327	-0.86663	0.77647	LOC102162501	77.9193094	47.097867	6.302306427	5.587901017	0.726319	0.79346
LOC100625723	2025.64803	939.47851	10.98487984	9.877251166	1.108451	0.87113	LOC102167662	500.1308479	220.61633	8.969043538	7.79192035	1.180766	0.7766	PITPNA	1613.92442	3636.4511	10.65725093	11.82871214	-1.17196	0.7935
LOC100738621	35.9010557	26.440908	5.205590187	4.77825631	0.441255	0.87117	SLC16A7	230.5042809	110.7213	7.854895061	6.803760479	1.057861	0.77662	LOC100516316	123.510395	220.61633	6.96012238	7.791920351	-0.83691	0.79351
TMOD4	13.8080984	18.178124	3.888314478	4.261389712	-0.39669	0.87118	LOC100525830	33.83549077	61.970878	5.122485983	5.976612875	-0.87305	0.77662	LOC100623304	157.496477	287.54487	7.308306958	8.172651889	-0.86847	0.79365
LOC102159509	13.8080984	18.178124	3.888314478	4.261389712	-0.39669	0.87118	RNF170	1073.219473	436.27498	10.06907306	8.772396993	1.298635	0.77667	LOC102159053	37.3017971	23.135794	5.259340178	4.593102408	0.689118	0.79369
CREB3L4	256.830629	148.73011	8.010279851	7.226220529	0.788121	0.87119	CNPPD1	1014.007364	2825.872	9.987274479	11.46499086	-1.47863	0.77668	LOC102166888	37.3017971	23.135794	5.259340178	4.593102408	0.689118	0.79369
BICD1	110.464787	183.4338	6.800444207	7.526959252	-0.73167	0.87122	MAST4	60.26946793	117.33153	5.937096418	6.886690713	-0.96109	0.7768	WDR74	2445.34003	1120.4335	11.25640923	10.13112832	1.125978	0.79376
LOC102159343	9.66566885	13.220454	3.414902536	3.829895615	-0.45183	0.87122	RASA2	1629.390352	634.58179	10.6710017	9.311933979	1.360454	0.7769	SLC20A2	2795.14799	1267.511	11.44922501	10.30892034	1.140926	0.79393
LOC100523339	9.66566885	13.220454	3.414902536	3.829895615	-0.45183	0.87122	PKN2	6358.957547	2167.3282	12.63480142	11.0823674	1.552873	0.77704	LOC100625234	195.627202	110.7213	7.619319115	6.803760479	0.821174	0.79394
RPL22L1	8451.93701	3443.9283	13.04523698	11.75025822	1.295227	0.87124	UGDH	13915.90294	4414.8053	13.76455057	12.10846086	1.656313	0.7771	LOC100512871	532.172305	276.80325	9.058458033	8.117919691	0.943033	0.79397
CHML	614.460377	322.24857	9.265522169	8.336500155	0.931146	0.87125	LOC100521666	10.57359087	19.004403	3.532764645	4.322245637	-0.84587	0.77711	LOC100620216	9.11821706	15.699289	3.338883189	4.061714779	-0.78388	0.794
BPNT1	2667.7246	1205.5401	11.38193472	10.2366602	1.14593	0.87126	HO2	891.35371	2443.3051	9.801471867	11.25520869	-1.45476	0.77715	ZPBP	15.7496477	25.61463	4.066058842	4.734147579	-0.70165	0.79405
NCOA3	455.657246	923.77922	8.834999507	9.85296517	-1.01957	0.87128	LOC102166880	1276.232417	3650.4978	10.31880536	11.83427266	-1.5162	0.77719	LOC100521645	15.7496477	25.61463	4.066058842	4.734147579	-0.70165	0.79405
C10H9orf72	766.349459	392.48223	9.583739935	8.620154661	0.965375	0.87128	MFSD2A	399.6817347	180.95496	8.646312935	7.507437596	1.143221	0.77721	EPC2	316.650811	616.40367	8.31129789	9.270070238	-0.96098	0.79408
POM121C	3761.32599	1646.7728	11.87740914	10.68630161	1.1916	0.87136	DYRK1A	4692.559626	1646.7728	12.19646677	10.68630161	1.510734	0.77725	TRPC1	75.432523	128.89943	6.256114748	7.021251246	-0.77299	0.79419
TNFRSF19	168.4588	297.46021	7.404790748	8.221394816	-0.8203	0.87139	LOC100739655	205.1276628	99.979683	7.687394321	6.657921243	1.036815	0.77728	LOC102165784	110.247534	65.275991	6.79762954	6.05041444	0.756122	0.79425
RFN19	1618.30913	3833.9316	10.66116271	11.90498515	-1.24434	0.87147	LOC100739678	214.6438946	104.11107	7.75250706	6.715770874	1.043822	0.77733	LOC102161310	13.2628612	7.4365053	3.834191515	3.076645515	0.834695	0.79427
PLDN	69.0404918	106.58991	6.130117309	6.749398975	-0.62656	0.87147	LOC102158048	94.1049587	193.34914	6.571448659	7.602506906	-1.03887	0.77735	LOC102167845	13.2628612	7.4365053	3.834191515	3.076645515	0.834695	0.79427
MGAT5	113.226407	188.39147	6.835752397	7.565227535	-0.73452	0.87153	HEG1	288.6590306	686.63733	8.178211834	9.42550405	-1.25018	0.77744	LOC100739626	13.2628612	7.4365053	3.834191515	3.076645515	0.834695	0.79427
TAF1D	4787.2677	2051.6492	12.2252881	11.00327137	1.222418	0.87155	LOC100623775	23.2618999	41.313919	4.600620625	5.403060391	-0.82866	0.77747	LOC102168047	7.46035941	3.3051135	3.080718953	2.106051269	1.174545	0.79429
LOC100622293	2.76161967	5.7839486	1.911353989	2.762125239	-1.06654	0.87155	LOC102159394	19.03246356	10.741619	4.324267947	3.553559423	0.825251	0.77754	RLN	7.46035941	3.3051135	3.080718953	2.106051269	1.174545	0.79429
LOC102159183	2.76161967	5.7839486	1.911353989	2.762125239	-1.06654	0.87155	LOC102166185	48.63851798	27.267186	5.633388138	4.821056378	0.834933	0.77756	MROH7	7.46035941	3.3051135	3.080718953	2.106051269	1.174545	0.79429
LOC100622563	2.76161967	5.7839486	1.911353989	2.762125239	-1.06654	0.87155	LOC100738426	620.6697838	1626.1158	9.280004648	10.66810125	-1.38953	0.77759	LOC102162087	7.46035941	3.3051135	3.080718953	2.106051269	1.174545	0.79429
LOC102157444	2.76161967	5.7839486	1.911353989	2.762125239	-1.06654	0.87155	GINS3	776.1015695	327.20624	9.601959365	8.358458837	1.246045	0.77761	LOC100738225	7.46035941	3.3051135	3.080718953	2.106051269	1.174545	0.79429
ASGR1	2.76161967	5.7839486	1.911353989	2.762125239	-1.06654	0.87155	SH3RF1	1873.640301	720.51474	10.87239809	9.494885058	1.378744	0.77769	TNF	7.46035941	3.3051135	3.080718953	2.106051269	1.174545	0.79429
LOC102159796	2.76161967	5.7839486	1.911353989	2.762125239	-1.06654	0.87155	ARMC5	164.9480175	364.38876	7.374587584	8.513288452	-1.14347	0.77769	LOC102164139	7.46035941	3.3051135	3.080718953	2.106051269	1.174545	0.79429
LOC102157596	2.76161967	5.7839486	1.911353989	2.762125239	-1.06654	0.87155	LOC102159097	5.286795433	10.741619	2.65232482	3.553559423	-1.02275	0.77769	LOC102166059	7.46035941	3.3051135	3.080718953	2.106051269	1.174545	0.79429
LOC102168098	2.76161967	5.7839486	1.911353989	2.762125239	-1.06654	0.87155	LOC102157404	5.286795433	10.741619	2.65232482	3.553559423	-1.02275	0.77769	CSF3	7.46035941	3.3051135	3.080718953	2.106051269	1.174545	0.79429
LOC102161293	2.76161967	5.7839486	1.911353989	2.762125239	-1.06654	0.87155	LOC102157610	5.286795433	10.741619	2.65232482	3.553559423	-1.02275	0.77769	TRNAW-CCA	7.46035941	3.3051135	3.080718953	2.106051269	1.174545	0.79429
SLC12A1	2.76161967	5.7839486	1.911353989	2.762125239	-1.06654	0.87155	LOC102164291	5.286795433	10.741619	2.65232482	3.553559423	-1.02275	0.77769	LHFPL5	7.46035941	3.3051135	3.080718953	2.106051269	1.174545	0.79429
MOS	2.76161967	5.7839486	1.911353989	2.762125239	-1.06654	0.87155	LOC102166023	5.286795433	10.741619	2.65232482	3.553559423	-1.02275	0.77769	LOC102165654	7.46035941	3.3051135	3.080718953	2.106051269	1.174545	0.79429
ORM1	2.76161967	5.7839486	1.911353989	2.762125239	-1.06654	0.87155	CYP4F55	46.52379981	87.585507	5.570578288	6.468998788	-0.91272	0.77778	LRIT1	7.46035941	3.3051135	3.080718953	2.106051269	1.174545	0.79429
TRNAA-UGC	2.76161967	5.7839486	1.911353989	2.762125239	-1.06654	0.87155	ZNF518A	2159.127255	818.84187	11.07690059	9.679201855	1.398792	0.77793	KIAA0101	307.532594	167.73451	8.269279095	7.398611253	0.87456	0.79433
LOC102166608	2.76161967	5.7839486	1.911353989	2.762125239	-1.06654	0.87155	LOC102161984	11.63094995	20.656959	3.658891241	4.436758793	-0.82866	0.77793	PRKCD	1192.82858	580.04742	10.22137998	9.182512091	1.040144	0.79442
LOC100154127	2.76161967	5.7839486	1.911353989	2.762125239	-1.06654	0.87155	LOC102161347	13.74566813	7.4365053	3.882219287	3.076645515	0.88628	0.77795	DCLRE1B	1192.82858	580.04742	10.22137998	9.182512091	1.040144	0.79442
[truncated: 1,667,626 more chars]
