# Supplementary material for: Common microRNA–mRNA interactions exist among distinct porcine iPSC lines independent of their metastable pluripotent states
Source: Cell Death Dis. 2017 Aug 31;8(8):e3027–. doi: 10.1038/cddis.2017.426 (PMC5596602; doi:10.1038/cddis.2017.426)
Supplement: Supplementary Table 6 [file cddis2017426x7.pdf]

piPS-L Vs PEFs

AccID	piPS-L	PEFs	Log2FC	FDR	Style	liPSCs	PEFs	AccID	KeggID	E-Value	Blast_AccID	Blast_Symbol
CCK	1006.61037	0	20	0	High	729	0	NM_214237.2	ssc:397468	2E-50	NM_001174138.1	CCK
LOC100518318	2441.27179	0	20	0	High	1768	0	XP_005656383.1	ssc:100518318	2E-55	NM_152453.2	TMCO5A
DARC	1654.210183	0	20	0	High	1198	0	NM_001244095.1	ssc:100154447	3E-96	NM_002036.3	DARC
KRT5	1668.018282	0	20	0	High	1208	0	XP_005652613.1	ssc:100511564	0	NM_000424.3	KRT5
GPX6	1299.342055	0	20	0	High	941	0	NM_001137607.1	ssc:100154091	7E-104	NM_182701.1	GPX6
XDH	901.6688227	0	20	0	High	653	0	NM_001285974.1	ssc:100515259	2E-76	NM_000379.3	XDH
PRKCZ	2528.262809	0	20	0	High	1831	0	NM_001204374.1	ssc:100533200			
DIRAS2	1036.988187	0	20	0	High	751	0	XP_005657251.1	ssc:100739440	5E-114	NM_017594.3	DIRAS2
POU2F3	1030.084137	0	20	0	High	746	0	XP_003130003.2	ssc:397514	1E-157	NM_014352.3	POU2F3
IBSP	4230.801337	0	20	0	High	3064	0	XP_003129385.1	ssc:397137	2E-93	NM_004967.3	IBSP
RAC2	1122.598396	0	20	0	High	813	0	XP_003355390.1	ssc:100621675	2E-109	NM_002872.4	RAC2
TRIML2	2685.675131	0	20	0	High	1945	0	XP_005674640.1	ssc:100737283	1E-17	NM_173553.1	TRIML2
VRTN	2573.829534	0	20	0	High	1864	0	NM_001195113.1	ssc:100157734	0	NM_018228.2	VRTN
BPIFB6	2134.732006	0	20	0	High	1546	0	XP_003483988.1	ssc:100736799	0	NM_174897.2	BPIFB6
TCL1A	17978.14406	0	20	0	High	13020	0	XP_005656518.1	ssc:100156364	8E-27	NR_049726.1	TCL1A
PKP1	1083.935721	0	20	0	High	785	0	XP_005674635.1	ssc:100623590			
CALB2	817.4394228	0	20	0	High	592	0	NM_001194980.1	ssc:100127479	1E-154	NR_027910.2	CALB2
PNCK	1168.165121	0	20	0	High	846	0	NM_001195366.1	ssc:100337678	0	NM_198452.1	PNCK
CCR1	2275.574609	0	20	0	High	1648	0	NM_001001621.1	ssc:414374	3E-172	NM_001295.2	CCR1
CRYBA4	987.2790326	0	20	0	High	715	0	XP_005670834.1	ssc:100152041	3E-110	NM_001886.2	CRYBA4
TMEM171	1239.967232	0	20	0	High	898	0	NM_001244323.1	ssc:100514277	1E-139	NM_173490	TMEM171
SNCA	1273.106669	0	20	0	High	922	0	NM_001037145.1	ssc:641350	9E-71	NM_007308.2	SNCA
LMO1	875.4334359	0	20	0	High	634	0	NM_001113055.1	ssc:100127356	3E-86	NR_073006.1	LMO1
LOC100737072	2039.456127	0	20	0	High	1477	0	XP_003483032.1	ssc:100737072	0	NM_000422.2	KRT17
KRT36	1601.739409	0	20	0	High	1160	0	XP_003131480.2	ssc:100514315	0	NM_003771.4	KRT36
UTF1	1198.542937	0	20	0	High	868	0	XP_001925647.3	ssc:100158138	3E-29	NM_003577.2	UTF1
LOC100523609	1297.961246	0	20	0	High	940	0	XR_303888.1	ssc:100523609	0	NM_032803.5	SLC7A3
ST14	1615.547508	0	20	0	High	1170	0	XP_005667570.1	ssc:100517843	0	NM_021978.3	ST14
LOC100521510	4598.096753	0	20	0	High	3330	0	XP_003130576.2	ssc:100521510	2E-52	NM_178820.3	FBXO27
KRT6A	112193.5608	1.652556743	16.05093054	0	High	81252	2	XP_005652608.1	ssc:100737483	0	NM_173086.4	KRT6C
LOC102164968	33008.25912	1.652556743	14.28583962	0	High	23905	2	XP_005652633.1	ssc:102164968	2E-47	NM_005555.3	KRT6B
HENMT1	48853.05199	4.95767023	13.26649876	0	High	35380	6	XP_005663670.1	ssc:100153448	3E-126	NM_144584.2	HENMT1
LOC100519925	4896.351678	0.826278372	12.53279166	0	High	3546	1	XR_307139.1	ssc:100519925			
SLC52A3	7405.283149	1.652556743	12.12963937	0	High	5363	2	XP_003134400.2	ssc:100516303	0	NM_033409.3	SLC52A3
LOC102164762	2756.096432	0.826278372	11.70371084	0	High	1996	1	XP_005663149.1	ssc:102164762	5E-126	NM_144584.2	HENMT1
LOC100152743	2735.384285	0.826278372	11.692828	0	High	1981	1	XP_001926795.1	ssc:100152743	3E-126	NM_144584.2	HENMT1
LOC102160289	2177.537111	0.826278372	11.36378178	0	High	1577	1	XP_005670228.1	ssc:102160289	8E-74	NR_046079.1	SEMA5B
OTX2	1931.75296	0.826278372	11.19099508	0	High	1399	1	XP_005660050.1	ssc:100512643	2E-174	NR_073036.1	OTX2
EMSP1	1854.427609	0.826278372	11.13205843	0	High	1343	1	NM_213802.1	ssc:396720	2E-111	NM_004917.3	KLK4
CDH1	4965.392169	3.305113487	10.55299214	0	High	3596	4	NM_001163060.1	ssc:100048953			
FOXD4	2089.165281	1.652556743	10.30401111	0	High	1513	2	XP_003353582.1	ssc:100155087	3E-134	NM_012184.4	FOXD4L1
KRT18	4650.567527	4.131391859	10.13656316	0	High	3368	5	XP_005652636.1	ssc:100126286	0	NM_199187.1	KRT18
LOC102159782	1353.193639	1.652556743	9.677452775	0	High	980	2	XR_304869.1	ssc:102159782			
ERBB4	1303.484485	1.652556743	9.623457886	0	High	944	2	XP_001926521.1				
LOC102158452	2606.96897	3.305113487	9.623457886	0	High	1888	4	XR_298845.1	ssc:102158452			
LOC100511106	2604.20735	3.305113487	9.621928797	0	High	1886	4	XP_003133370.3	ssc:100511106	2E-85	NR_048571.1	GPM6A
FAM43B	5131.08935	6.610226974	9.600349623	0	High	3716	8	XP_003356225.1	ssc:100624917	5E-164	NM_207334.2	FAM43B
NOXA1	2326.664573	3.305113487	9.459347712	0	High	1685	4	XP_005652790.1	ssc:100516894	3E-114	NM_006647.1	NOXA1
SOX21	2307.333236	3.305113487	9.447310854	0	High	1671	4	XP_005653879.1	ssc:100517115	8E-155	NM_007084.2	SOX21
LOC100622539	2537.928478	4.131391859	9.262807793	0	High	1838	5	XP_005674221.1	ssc:100622539	4E-129	NR_048571.1	GPM6A
ANO9	2490.980944	4.131391859	9.235870365	0	High	1804	5	XP_005655100.1	ssc:100627366	0	NM_001012302.2	ANO9
MFNG	8947.647736	14.87301069	9.232667932	0	High	6480	18	XP_005663880.1	ssc:102161832	1E-129	NR_029413.1	MFNG
LOC396757	3612.19853	6.610226974	9.093961662	0	High	2616	8	NM_213819.1	ssc:396757	2E-29	NM_002638.3	PI3
LCP2	2467.507176	4.95767023	8.959176254	0	High	1787	6	XP_003134116.3	ssc:100511843	0	NM_005565.3	LCP2
KIT	5454.198851	12.39417558	8.781561178	0	High	3950	15	NM_001044525.1	ssc:396810	7E-53	NM_001093772.1	KIT
RAB3C	2071.214754	4.95767023	8.706599121	0	High	1500	6	XP_003483878.1	ssc:100523703	2E-128	NM_138453.2	RAB3C
FOS	53500.8579	135.509653	8.62502259	0	High	38746	164	NM_001123113.1	ssc:100144486	0	NM_005252.3	FOS
DOCK8	6331.013097	16.52556743	8.58159276	0	High	4585	20	XP_003121977.2	ssc:100153522	0	NM_203447.3	DOCK8
SOX2	4460.015769	14.04673232	8.310670444	0	High	3230	17	NM_001123197.1	ssc:407739	1E-158	NM_003106.3	SOX2
LOC100738213	161.5547508	387901.3393	-11.22945087	0	Low	117	469456	XP_005668985.1	ssc:100738213	0	NM_000088.3	COL1A1
TAGLN	38.6626754	120386.2799	-11.60444189	0	Low	28	145697	NM_001244150.1	ssc:397021	1E-116	NM_003186.3	TAGLN
THBS1	62.13644261	211976.7586	-11.73617883	0	Low	45	256544	NM_001244536.1	ssc:492313	0	NM_003246.2	THBS1
ACTA2	16.56971803	109145.589	-12.68541715	0	Low	12	132093	NM_001164650.1	ssc:733615	0	NM_001613.2	ACTA2
ANGPTL2	2.761619671	18276.4513	-12.69218371	0	Low	2	22119	NM_001109946.1	ssc:100126164	0	NM_012098.2	ANGPTL2
TNC	2.761619671	18393.78283	-12.70141595	0	Low	2	22261	NM_214230.1	ssc:397460	0	NM_002160.3	TNC
LOC102160657	2.761619671	22024.45	-12.96130373	0	Low	2	26655	XP_005660435.1	ssc:102160657	0	NM_002160.3	TNC
POSTN	5.523239343	55326.77349	-13.29017553	0	Low	4	66959	NM_001206351.1	ssc:100152401	0	NM_006475.2	POSTN
ITGA8	1.380809836	16505.73675	-13.54516527	0	Low	1	19976	XP_003130794.1	ssc:100512676	0	NM_003638.1	ITGA8
MGP	1.380809836	26806.94921	-14.24480477	0	Low	1	32443	NM_214116.1	ssc:397206	2E-42	NM_001190839.1	MGP
ASPN	0	15025.04591	-20	0	Low	0	18184	NM_001243889.1	ssc:100511749	0	NM_017680.4	ASPN
LUM	0	13427.84982	-20	0	Low	0	16251	NM_001243339.1	ssc:100152607	4E-177	NM_002345.3	LUM
LOC100621401	0	8792.428154	-20	0	Low	0	10641	XP_003359182.1	ssc:100621401	5E-98	NM_181724.2	TMEM119
FMOD	0	59302.82502	-20	0	Low	0	71771	XP_003130153.1	ssc:100526237	0	NR_103757.1	FMOD
COL15A1	0	16447.07099	-20	0	Low	0	19905	XP_003122085.4	ssc:100512644	6E-145	NM_001855.4	COL15A1
COL8A2	0	16593.32226	-20	0	Low	0	20082	XP_003356361.2	ssc:100525585			
PDZD4	1763.29416	4.131391859	8.737429551	1.11E-16	High	1277	5	XP_005674070.1	ssc:100525676	0	NM_032512.2	PDZD4
LOC100512967	1861.331659	4.95767023	8.552457117	1.11E-16	High	1348	6	XR_303930.1	ssc:100512967			
CP	47742.88088	213.1798199	7.807070938	1.11E-16	High	34576	258	NM_001267694.2	ssc:406870	0	NR_046371.1	CP
MXRA5	2.761619671	14656.52576	-12.3737409	1.11E-16	Low	2	17738	XP_005658001.1	ssc:100519997	0	NM_015419.3	MXRA5
LOC733603	1.380809836	10361.53078	-12.87343489	1.11E-16	Low	1	12540	NM_001044552.1	ssc:733603	2E-51	NM_199161.3	SAA1
FAM198B	1.380809836	11644.74109	-13.0418763	1.11E-16	Low	1	14093	XP_005666715.1	ssc:100515440	0	NM_016613.6	FAM198B
LOC100620394	0	8025.641825	-20	1.11E-16	Low	0	9713	XP_005674263.1	ssc:100620394	1E-77	NM_001855.4	COL15A1
LOC100520836	735.9716424	0	20	2.22E-16	High	533	0	XP_005655636.1	ssc:100520836	2E-152	NM_007210.3	GALNT6
tRNA-Ala	1106.028678	1.652556743	9.386473269	2.22E-16	High	801	2	tRNA-Ala				
BATF3	2856.89555	9.089062089	8.296101147	2.22E-16	High	2069	11	XP_003482796.1	ssc:100524332	8E-49	NM_018664.2	BATF3
LINGO1	3011.546252	10.74161883	8.131149173	2.22E-16	High	2181	13	XP_005666192.1	ssc:100155465	0	NM_032808.5	LINGO1
LAMA4	1.380809836	9344.382106	-12.72436891	2.22E-16	Low	1	11309	XP_003121419.3	ssc:100154581	0	NM_002290.4	LAMA4
ELN	0	7319.173817	-20	2.22E-16	Low	0	8858	ELN				
LOC100738461	0	7119.214451	-20	2.22E-16	Low	0	8616	XP_005656330.1	ssc:100738461	0	NM_018689.1	KIAA1199
LOC100153689	726.3059736	0	20	3.33E-16	High	526	0	LOC100153689				
LOC100523909	709.7362556	0	20	3.33E-16	High	514	0	XP_005665511.1	ssc:100523909	0	NM_001099772.1	CYP4B1
LTBP2	27.61619671	56402.58793	-10.996031	3.33E-16	Low	20	68261	XP_005656461.1	ssc:100514300	0	NM_000428.2	LTBP2
LOC102166564	8.284859014	21479.10627	-11.3401692	3.33E-16	Low	6	25995	XP_005661871.1	ssc:102166564	0	NM_002609.3	PDGFRB
MMP1	2.761619671	11619.95274	-12.03880193	3.33E-16	Low	2	14063	NM_001166229.1	ssc:397320	0	NM_002421.3	MMP1
IRG6	0	6658.151119	-20	3.33E-16	Low	0	8058	NM_213817.1	ssc:396752	1E-177	NM_080657.4	RSAD2

DRD2	2948.028999	11.5678972	7.993480269	4.44E-16	High	2135	14	NM_001244253.1	ssc:100624857	0	NM_016574.3	DRD2
COL8A1	9.66566885	22202.92613	-11.16559263	4.44E-16	Low	7	26871	XP_005670312.1	ssc:397430	0	NM_020351.3	COL8A1
KCNK5	3093.014032	12.39417558	7.963207257	5.55E-16	High	2240	15	XP_001928289.1	ssc:100154866	0	NM_003740.3	KCNK5
VCAM1	0	6099.58694	-20	5.55E-16	Low	0	7382	NM_213891.1	ssc:396925	4E-134	NM_080682.2	VCAM1
NRARP	1306.246105	3.305113487	8.626511209	6.66E-16	High	946	4	XP_003353772.1	ssc:100620133	8E-63	NM_001004354.2	NRARP
NPTX1	6434.573835	31.39857813	7.679001562	7.77E-16	High	4660	38	XP_003131182.2	ssc:100525071	0	NM_002522.3	NPTX1
LOC102160189	11.04647869	22683.82014	-11.00386136	7.77E-16	Low	8	27453	XP_005656326.1	ssc:102160189	6E-102	NM_018689.1	KIAA1199
LOC102157779	654.5038621	0	20	8.88E-16	High	474	0	XR_300762.1	ssc:102157779			
LRAT	973.4709342	1.652556743	9.202294283	8.88E-16	High	705	2	NM_001244920.1	ssc:100513994	1E-111	NM_004744.3	LRAT
EGR2	2919.031993	12.39417558	7.879683902	8.88E-16	High	2114	15	NM_001097488.1	ssc:100038004	0	NM_001136179.1	EGR2
PTF-BETA	1.380809836	7819.072232	-12.46726707	8.88E-16	Low	1	9463	NM_214336.1	ssc:397609	8E-63	NM_002825.5	PTN
RAX	958.282026	1.652556743	9.179606689	9.99E-16	High	694	2	XP_003121760.1	ssc:100157455	2E-117	NM_013435.2	RAX
CITED4	5965.09849	29.74602138	7.647705432	9.99E-16	High	4320	36	XP_003128154.1	ssc:100525468	2E-40	NM_133467.2	CITED4
LOC100627277	0	5187.375618	-20	1.55E-15	Low	0	6278	XP_005674513.1	ssc:100627277	3E-60	NM_181724.2	TMEM119
LOC100522691	1904.136763	7.436505346	8.000296576	1.67E-15	High	1379	9	XP_005653653.1	ssc:100522691	4E-144	NM_024873.5	TNIP3
PVRL4	1477.466524	4.95767023	8.219247417	1.78E-15	High	1070	6	XP_005663234.1	ssc:100154054	0	NM_030916.2	PVRL4
LOC100156689	15.18890819	25125.47273	-10.69191686	1.78E-15	Low	11	30408	XP_005659493.1	ssc:100156689	0	NM_080645.2	COL12A1
LOC100514847	593.7482294	0	20	2.11E-15	High	430	0	XP_005653440.1	ssc:100514847	1E-133	NM_005268.3	GJB5
GRM3	585.4633704	0	20	2.44E-15	High	424	0	XP_005656765.1	ssc:100521916	0	NM_000840.2	GRM3
PRELP	0	4799.851061	-20	2.55E-15	Low	0	5809	XP_005656706.1	ssc:100511032	2E-172	NM_201348.1	PRELP
WDR72	575.7977015	0	20	2.89E-15	High	417	0	XP_003353403.2	ssc:100153251	0	NR_102336.1	WDR72
DUOXA1	729.0675933	0.826278372	9.785208956	2.89E-15	High	528	1	NM_001243551.1	ssc:100653516	1E-164	NM_144565.3	DUOXA1
HOXB13	861.6253375	1.652556743	9.026217055	3.11E-15	High	624	2	XP_003131599.1	ssc:100522893	7E-148	NM_006361.5	HOXB13
IFIT2	0	4632.116552	-20	3.22E-15	Low	0	5606	XP_005671321.1	ssc:100155467	0	NM_001547.4	IFIT2
CLDN5	8011.458667	47.09786719	7.410259402	3.55E-15	High	5802	57	NM_001161636.1	ssc:396997	1E-113	NM_003277.3	CLDN5
C1QL4	1742.582013	7.436505346	7.87238603	4.11E-15	High	1262	9	XP_003126157.1	ssc:100512604	1E-131	NM_001008223.1	C1QL4
NHigh210	13085.93481	80.14900206	7.351116716	4.33E-15	High	9477	97	XP_005669873.1	ssc:100515926	0	NM_024923.3	NHigh210
FBN1	15.18890819	21241.96438	-10.4496833	4.77E-15	Low	11	25708	NM_001001771.1	ssc:414836	0	NM_000138.4	FBN1
HSPA2	17157.94302	108.2424667	7.308466174	5.44E-15	High	12426	131	XP_003356782.1	ssc:100621324	0	NM_021979.3	HSPA2
CDA	532.9925966	0	20	6.00E-15	High	386	0	NM_001244385.1	ssc:100515954	2E-70	NM_001785.2	CDA
COLL11A1	22.09295737	27569.60415	-10.28527629	6.44E-15	Low	16	33366	XP_001929407.4	ssc:397175	0	NM_080630.3	COL11A1
LOC102163958	1027.322518	3.305113487	8.279973647	8.22E-15	High	744	4	XR_304068.1	ssc:102163958			
FOLR1	1500.940291	6.610226974	7.826951061	8.33E-15	High	1087	8	NM_213830.1	ssc:396784	9E-105	NM_016730.1	FOLR1
VWC2	3395.411386	19.83068092	7.419708355	8.33E-15	High	2459	24	XP_003130481.1	ssc:100515158	3E-169	NM_198570.3	VWC2
LOC102164256	0	3954.568287	-20	8.33E-15	Low	0	4786	XP_005660342.1	ssc:102164256	4E-52	NM_001855.4	COL15A1
LOXL4	8.284859014	12123.15627	-10.51500059	9.55E-15	Low	6	14672	XP_005671434.1	ssc:100157207	0	NM_032211.6	LOXL4
TPD52L1	505.3763999	0	20	1.03E-14	High	366	0	XP_005659275.1	ssc:100515246	4E-95	NM_003287.2	TPD52L1
VIT	1230.301564	4.95767023	7.955133957	1.10E-14	High	891	6	XP_005662691.1	ssc:100524557	0	NM_053276.3	VIT
LOC100738383	759.4454097	1.652556743	8.844102645	1.11E-14	High	550	2	XP_005657003.1	ssc:100738383	6E-162	NM_004382.4	CRHR1
ARL4D	11884.63026	80.14900206	7.212196747	1.14E-14	High	8607	97	XP_005668837.1	ssc:100516931	2E-104	NM_001661.3	ARL4D
SYBU	2580.733583	14.87301069	7.438940688	1.23E-14	High	1869	18	XP_005659855.1	ssc:100157483	0	NM_017786.5	SYBU
CORO1A	3904.930215	24.78835115	7.299490645	1.39E-14	High	2828	30	XP_005658185.1	ssc:100621483	9E-156	NM_007074.3	CORO1A
LOC100739233	0	3576.132793	-20	1.55E-14	Low	0	4328	XR_307534.1	ssc:100739233	1E-67	NM_002508.2	NID1
EIF2S3	0	3510.030523	-20	1.73E-14	Low	0	4248	XP_005658005.1	ssc:100624149	0	NM_001415.3	EIF2S3
NPW	3008.784632	19.00440255	7.306703361	1.95E-14	High	2179	23	NM_213786.1	ssc:396680	5E-28	NM_001099456.2	NPW
LOC100628118	0	3442.275697	-20	1.97E-14	Low	0	4166	XP_005662044.1	ssc:100628118			
ACTL8	469.4753442	0	20	2.11E-14	High	340	0	XP_003356215.1	ssc:100622307	3E-54	NM_030812.2	ACTL8
VSNL1	693.1665375	1.652556743	8.71235839	2.71E-14	High	502	2	XP_003125391.1	ssc:100517707	8E-111	NM_003385.4	VSNL1
MX2	0	3252.231671	-20	2.78E-14	Low	0	3936	NM_001097416.1	ssc:396893	8E-160	NM_002463.1	MX2
PROK1	455.6672458	0	20	2.84E-14	High	330	0	NM_001172586.1	ssc:100359362	7E-49	NM_032414.2	PROK1
COL4A1	52.47077376	47585.37143	-9.824788352	2.86E-14	Low	38	57590	XP_003131139.3	ssc:100515336	0	NM_001845.4	COL4A1
LOC102163104	4.142429507	6692.854811	-10.65792886	2.99E-14	Low	3	8100	XP_005673058.1	ssc:102163104	0	NM_198596.2	SULF2
TTC22	452.9056261	0	20	3.01E-14	High	328	0	XP_003356503.1	ssc:100620707	0	NM_017904.3	TTC22
LOC100519889	1518.890819	8.262783717	7.52217455	3.21E-14	High	1100	10	XR_297275.1	ssc:100519889			
LEFTY2	996.9447014	4.131391859	7.914741768	3.32E-14	High	722	5	NM_001195346.1	ssc:100337677	1E-176	NM_003240.3	LEFTY2
LOC102164134	0	3124.984802	-20	3.52E-14	Low	0	3782	XP_005660341.1	ssc:102164134	4E-116	NM_001855.4	COL15A1
COL4A2	49.70915409	43357.305	-9.768547816	3.69E-14	Low	36	52473	XP_005668604.1	ssc:100153454	0	NM_001846.2	COL4A2
DUOXA2	1284.154123	6.610226974	7.601901742	3.97E-14	High	930	8	NM_001243254.1	ssc:100155365	6E-148	NM_207581.3	DUOXA2
TRIM29	769.1110785	2.478835115	8.277385853	4.16E-14	High	557	3	XP_003129991.3	ssc:100520758	0	NM_058193.1	TRIM29
LOC102165673	9.66566885	10474.73092	-10.08175599	4.45E-14	Low	7	12677	XP_005661865.1	ssc:102165673	5E-87	NM_002609.3	PDGFRB
LOC100738003	0	2990.301427	-20	4.62E-14	Low	0	3619	XP_005658279.1	ssc:100738003	0	NM_004820.3	CYP7B1
COL3A1	564.7512228	487725.6819	-9.754238737	4.87E-14	Low	409	590268	NM_001243297.1	ssc:100152001	0	NM_000090.3	COL3A1
LOC100738704	1353.193639	7.436505346	7.507527774	5.02E-14	High	980	9	XP_003482495.2	ssc:100738704	1E-125	NM_024873.5	TNIP3
PEX5L	644.8381933	1.652556743	8.608093576	5.55E-14	High	467	2	XP_005670067.1	ssc:100524828	0	NM_016559.2	PEX5L
KIAA1244	1521.652439	9.089062089	7.387291726	6.05E-14	High	1102	11	XP_003353246.3	ssc:100621590			
KLK15	419.7661901	0	20	6.35E-14	High	304	0	XP_003356062.1	ssc:100620705	9E-112	NR_102274.1	KLK15
LOX	12.42728852	11478.65914	-9.851226858	7.80E-14	Low	9	13892	NM_001206403.1	ssc:100525278	0	NM_002317.5	LOX
LOC100157228	4.142429507	5598.862247	-10.40044082	8.86E-14	Low	3	6776	XP_005671626.1	ssc:100157228	0	NM_021641	ADAM12
PAPP-A	0	2684.57843	-20	8.88E-14	Low	0	3249	XP_001926788.4	ssc:397219	0	NM_002581.3	PAPPA
DUSP9	1543.745396	9.915340461	7.282556808	9.66E-14	High	1118	12	XP_003135519.2	ssc:100519006	9E-65	NM_001395	DUSP9
LOC100511328	401.8156622	0	20	9.75E-14	High	291	0	XP_003126562.2	ssc:100511328	2E-13	NM_199286.2	DPPA3
LOC102161103	2.761619671	4513.958745	-10.67466287	1.07E-13	Low	2	5463	XP_005656329.1	ssc:102161103	0	NM_018689.1	KIAA1199
DPPA4	393.5308032	0	20	1.19E-13	High	285	0	XP_005654122.1	ssc:100620290	2E-77	NM_018189.3	DPPA4
LOC100737363	596.509849	1.652556743	8.495702338	1.20E-13	High	432	2	XR_135271.2	ssc:100737363			
LOC102160384	966.566885	4.95767023	7.607063447	1.21E-13	High	700	6	XR_307351.1	ssc:102160384			
FRMPD4	589.6057999	1.652556743	8.478907096	1.34E-13	High	427	2	XP_003465.1	ssc:100526196	0	NM_014728.3	FRMPD4
NID2	33.13943606	23899.27562	-9.494202124	1.38E-13	Low	24	28924	XP_005660021.1	ssc:100156994	0	NM_007361.3	NID2
COL1A2	534.3734064	385650.557	-9.495230267	1.43E-13	Low	387	466732	NM_001243655.1	ssc:100626716	0	NM_000089.3	COL1A2
LOC102166965	0	2473.877445	-20	1.46E-13	Low	0	2994	XP_005671014.1	ssc:102166965	3E-150	NM_002508.2	NID1
LOC100738989	244.4033409	170068.7459	-9.442638314	1.49E-13	Low	177	205825	XP_005674338.1	ssc:100738989	0	NM_000089.3	COL1A2
XCR1	383.8651343	0	20	1.52E-13	High	278	0	NM_001001622.1	ssc:414375	2E-149	NM_005283.2	XCR1
LOC100737120	4.142429507	5022.946222	-10.24384097	1.71E-13	Low	3	6079	XP_003480633.2	ssc:100737120	0	NM_002160.3	TNC
LOC100736872	2.761619671	4152.875096	-10.55438012	1.77E-13	Low	2	5026	XP_003482733.1	ssc:100736872	8E-178	NM_006379.3	SEMA3C
FOXA3	570.2744622	1.652556743	8.430812808	1.86E-13	High	413	2	XP_003127277.1	ssc:100512416	2E-144	NM_004497.2	FOXA3
INHBE	0	2371.418927	-20	1.89E-13	Low	0	2870	XP_003126368.1	ssc:100518554	9E-164	NM_031479.3	INHBE
LOC100153335	0	2349.935689	-20	2.00E-13	Low	0	2844	XP_005666216.1	ssc:100153335	0	NM_022369.3	STRA6
E4	2292.144327	18.17812418	6.978350744	2.03E-13	High	1660	22	NM_213947.1	ssc:396987	1E-18	NM_080736.1	WFDC2
LOC100626687	0	2323.494781	-20	2.14E-13	Low	0	2812	XP_005674224.1	ssc:100626687	0	NM_173817.1	PIEZO2
NID1	0	2274.744357	-20	2.43E-13	Low	0	2753	XP_003361217.1		4E-154	NM_002508.2	NID1
SLC6A17	0	2253.26112	-20	2.58E-13	Low	0	2727	XP_001929358.2	ssc:100156430	0	NM_001010898.2	SLC6A17
STMN2	5.523239343	5444.348191	-9.945028975	2.70E-13	Low	4	6589	XP_005663069.1	ssc:100156768	6E-98	NM_007029.3	STMN2
LRRN4CL	5.523239343	5439.390521	-9.943714646	2.71E-13	Low	4	6583	XP_003353868.1	ssc:100620756	1E-67	NM_203422.3	LRRN4CL
CCL2	74.56373113	45810.52549	-9.262989343	2.90E-13	Low	54	55442	NM_214214.1	ssc:397422	7E-36	NM_002982.3	CCL2
LOC100516162	356.2489376	0	20	3.16E-13	High	258	0	XR_299606.1	ssc:100516162			
CAMSAP3	2024.267219	16.52556743	6.936556129	3.20E-13	High	1466	20	XP_005654804.1	ssc:100511741	0	NM_020902.1	CAMSAP3
DSE	2.761619671	3738.083354	-10.40256838	3.36E-13	Low	2	4524	XP_003121429.1	ssc:100520877	0	NM_013352.2	DSE
LOC100511351	0	2149.976323	-20	3.42E-13	Low	0	2602	XP_005671015.1	ssc:100511351	1E-144	NM_002508.2	NID1
LOC100153940	2.761619671	3705.858497	-10.39007743	3.54E-13	Low	2	4485	XP_005659162.1	ssc:100153940	0	NM_003247.3	THBS2
ADAM19	26.23538688	16526.39371	-9.299042163	3.62E-13	Low	19	20001	XP_003134154.4	ssc:100521528	0	NM_033274.4	ADAM19
KCNAB1	2078.118803	17.35184581	6.904045185	3.65E-13	High	1505	21	NM_001105294.1	ssc:100125830			

LOC102163946	350.7256983	0	20	3.68E-13	High	254	0	XR_308621.1	ssc:102163946			
LOC100620960	350.7256983	0	20	3.68E-13	High	254	0	XP_003361910.1	ssc:100620960	6E-36	NM_018967.3	SNTG1
LOC100621417	0	2088.831724	-20	4.08E-13	Low	0	2528	XP_003361726.1	ssc:100621417	5E-68	NM_015696.4	GPX7
LOC100524999	1012.13361	6.610226974	7.258484224	4.19E-13	High	733	8	XP_005656631.1	ssc:100524999	6E-33	NM_016619.2	PLAC8
MOBP	342.4408393	0	20	4.64E-13	High	248	0	XR_306740.1	ssc:100152154			
SLC8A1	0	2005.377608	-20	5.22E-13	Low	0	2427	HighI0001834F10		0	NM_021097.2	SLC8A1
ALPL	1143.310544	8.262783717	7.112373699	5.34E-13	High	828	10	XP_005658515.1	ssc:100170147	0	NM_001177520.1	ALPL
LOC100155138	5082.761005	49.5767023	6.679806275	5.39E-13	High	3681	60	XP_005654160.1	ssc:100155138	0	NM_080386.3	TUBA3D
NFE2L3	2936.982521	27.26718627	6.751025035	5.43E-13	High	2127	33	XP_003360238.1	ssc:100622324	0	NM_004289.6	NFE2L3
LOC100738735	664.169531	3.305113487	7.65070792	6.13E-13	High	481	4	XP_005655617.1	ssc:100738735	0	NM_012284.1	KCNH3
DNAJC12	4542.86436	44.61903207	6.669799203	6.15E-13	High	3290	54	XP_001925277.1	ssc:100153566	8E-98	NM_201262.1	DNAJC12
SEMA3C	0	1944.233009	-20	6.30E-13	Low	0	2353	XP_005667747.1	ssc:100624508	0	NM_006379.3	SEMA3C
DAAM2	23.47376721	13680.691	-9.186875994	6.31E-13	Low	17	16557	XP_003356671.1		0	NM_015345.3	DAAM2
C12H17orf67	0	1926.054885	-20	6.67E-13	Low	0	2331	XP_005657027.1	ssc:100525534	1E-34	NM_001085430.2	C17orf67
SULF2	1.380809836	2634.175449	-10.89762108	6.90E-13	Low	1	3188	XP_005673059.1	ssc:100152351	0	NM_198596.2	SULF2
LOC100152082	16.56971803	9950.044152	-9.230010065	7.37E-13	Low	12	12042	XP_005666212.1	ssc:100152082	0	NM_201526.1	ISLR
MMP23B	5.523239343	4607.328201	-9.70420001	7.45E-13	Low	4	5576	XP_005653406.1	ssc:100525702	8E-126	NM_006983.1	MMP23B
LOC100621838	5.523239343	4604.849366	-9.703423602	7.47E-13	Low	4	5573	XP_005658329.1	ssc:100621838	0	NM_201526.1	ISLR
LOC100525680	2.761619671	3270.409795	-10.20974106	7.56E-13	Low	2	3958	XP_005661152.1	ssc:100525680	6E-52	NM_199161.3	SAA1
SDC2	15.18890819	9156.816916	-9.235684197	7.98E-13	Low	11	11082	XP_001926974.2	ssc:100152754	7E-100	NM_002998.3	SDC2
DDX60	0	1853.342388	-20	8.43E-13	Low	0	2243	XP_005657343.1	ssc:100158037	8E-21	NM_017631.5	DDX60
DUOX2	867.1485768	5.783948602	7.228080663	8.54E-13	High	628	7	NM_213999.2	ssc:397060	0	NM_014080.4	DUOX2
AWN	0	1846.732161	-20	8.61E-13	Low	0	2235	NM_213829.1	ssc:396783			
PTGFR	0	1842.600769	-20	8.73E-13	Low	0	2230	NM_214059.1	ssc:397126	6E-177	NM_001039585.1	PTGFR
PLN	320.3478819	0	20	8.87E-13	High	232	0	NM_214213.1	ssc:397421	3E-14	NM_002667.3	PLN
LOC100512873	318.9670721	0	20	9.24E-13	High	231	0	XP_003134513.1	ssc:100512873	7E-35	NM_003064.3	SLPI
LOC100738123	423.9086196	219619.0073	-9.017033903	1.02E-12	Low	307	265793	XR_300525.1	ssc:100738123			
LOC100157992	31.75862622	16447.89727	-9.016538936	1.06E-12	Low	23	19906	XP_005663151.1	ssc:100157992	9E-94	NM_022716	PRRX1
LOC102165390	313.4438327	0	20	1.10E-12	High	227	0	XP_005665019.1	ssc:102165390	7E-66	NM_005427.3	TP73
MFAP2	23.47376721	12445.40483	-9.050348052	1.12E-12	Low	17	15062	XP_005665105.1	ssc:100523794	2E-80	NM_017459.2	MFAP2
OCLN	469.4753442	1.652556743	8.150205772	1.25E-12	High	340	2	NM_001163647.2	ssc:397236	0	NM_002538.3	OCLN
F7	755.3029801	4.95767023	7.251249358	1.37E-12	High	547	6	NM_001044591.1	ssc:733661	0	NR_051961.1	F7
GABRA5	463.9521048	1.652556743	8.133132259	1.41E-12	High	336	2	XP_005654528.1	ssc:100520472	0	NM_001165037.1	GABRA5
NPY	0	1688.086713	-20	1.48E-12	Low	0	2043	NM_001256367.1	ssc:397304	4E-47	NM_000905.3	NPY
SPOCK3	0	1679.82393	-20	1.53E-12	Low	0	2033	XP_001927799.2	ssc:100154805	3E-105	NM_016950.2	SPOCK3
MYBPC2	2078.118803	20.65695929	6.652506418	1.54E-12	High	1505	25	XP_003127419.2	ssc:100511817	0	NM_004533.3	MYBPC2
LOC100626400	1159.880262	9.915340461	6.870097853	1.63E-12	High	840	12	XP_003361608.1	ssc:100626400	7E-47	NM_005764.3	PDZK1IP1
GREM1	0	1662.472084	-20	1.63E-12	Low	0	2012	XP_005659828.1	ssc:100156280	5E-107	NM_013372.6	GREM1
COL1A1	265.1154885	124736.6356	-8.878048579	1.64E-12	Low	192	150962	XP_005668984.1	ssc:397571	3E-121	NM_000088.3	COL1A1
CRHR1	1017.656849	8.262783717	6.94440755	1.68E-12	High	737	10	NM_001144110.1	ssc:397426	0	NM_004382.4	CRHR1
CCL11	0	1648.425352	-20	1.72E-12	Low	0	1995	NM_001256774.1	ssc:100038010	1E-27	NM_002986.2	CCL11
FAP	1.380809836	2238.388109	-10.66272984	1.85E-12	Low	1	2709	XP_005671941.1	ssc:100736572	0	NM_004460.2	FAP
NTRK2	450.1440065	1.652556743	8.08954299	1.89E-12	High	326	2	XP_003130695.4	ssc:100519209	0	NM_006180.3	NTRK2
tRNA-Cys	3075.063504	33.05113487	6.539772584	1.89E-12	High	2227	40	tRNA-Cys				
KCNE4	0	1620.331887	-20	1.90E-12	Low	0	1961	XP_005672306.1	ssc:397167	1E-76	NM_080671.3	KCNE4
SRPX2	0	1613.72166	-20	1.95E-12	Low	0	1953	XP_001924554.3	ssc:100153130	0	NM_014467.2	SRPX2
LOC102167439	0	1613.72166	-20	1.95E-12	Low	0	1953	XP_005662978.1	ssc:102167439	7E-54	NM_138455.3	CTHRC1
AVPR2	294.112495	0	20	2.03E-12	High	213	0	NM_214232.1	ssc:397462	2E-152	NR_027419.1	AVPR2
ARG1	515.0420687	2.478835115	7.698884156	2.14E-12	High	373	3	NM_214048.2	ssc:397115	9E-171	NM_001244438.1	ARG1
DUSP2	2823.756114	30.57229975	6.529246598	2.19E-12	High	2045	37	XP_005662400.1	ssc:100622812	9E-117	NM_004418.3	DUSP2
CHI3L1	5.523239343	3854.588604	-9.44684653	2.20E-12	Low	4	4665	NM_001001540.1	ssc:396865	0	NM_001276.2	CHI3L1
NKX2-5	291.3508753	0	20	2.22E-12	High	211	0	XP_003134089.1	ssc:100521411	3E-157	NM_004387.3	NKX2-5
LOC102167666	441.8591474	1.652556743	8.062742931	2.26E-12	High	320	2	XR_300674.1	ssc:102167666			
LOC100624590	2.761619671	2715.977008	-9.941740905	2.34E-12	Low	2	3287	XP_003360576.1	ssc:100624590	0	NM_024005.1	DDX3X
POU5F1	705.5938261	4.95767023	7.153031816	2.67E-12	High	511	6	NM_001113060.1	ssc:100127461	1E-176	NM_203289.5	POU5F1
TDGF1	433.5742884	1.652556743	8.035435585	2.72E-12	High	314	2	XP_003358458.2	ssc:100623389	3E-75	NM_003212.3	TDGF1
AIF1	967.9476949	8.262783717	6.872157375	2.74E-12	High	701	10	NM_001129950.1	ssc:397271	1E-74	NM_032955.1	AIF1
LOC100737939	2.761619671	2620.128117	-9.889907326	2.90E-12	Low	2	3171	XP_003481552.1	ssc:100737939	5E-125	NM_080630.3	COL11A1
PPP1R1A	2003.555072	21.48323766	6.543206922	3.06E-12	High	1451	26	XP_005674358.1	ssc:100621808	8E-39	NM_006741.3	PPP1R1A
CREB3L1	24.85457704	10908.52706	-8.777729045	3.39E-12	Low	18	13202	XP_003122877.1	ssc:100516663	0	NM_052854.3	CREB3L1
GBX2	622.7452359	4.131391859	7.235870365	3.42E-12	High	451	5	XP_003133803.1	ssc:100514608	0	NM_001485.2	GBX2
CAMKV	2613.873019	29.74602138	6.45734853	3.67E-12	High	1893	36	XP_003358517.1	ssc:100623981	0	NM_024046.3	CAMKV
MYLK	0	1434.419253	-20	3.98E-12	Low	0	1736	XP_005670216.1	ssc:396848	0	NM_053032.2	MYLK
POF1B	273.4003475	0	20	4.10E-12	High	198	0	XP_005673805.1	ssc:100523581	0	NM_024921.3	POF1B
CHGA	2006.316169	22.30951604	6.490746322	4.14E-12	High	1453	27	NM_001164005.2	ssc:397540	1E-117	NM_001275.3	CHGA
NT5E	12.42728852	5935.983823	-8.899831797	4.31E-12	Low	9	7184	XP_003353298.1	ssc:100157995	0	NM_002526.3	NT5E
CSF1	4.142429507	2943.20356	-9.472694464	4.41E-12	Low	3	3562	NM_001244523.1	ssc:100513084	1E-32	NM_172212.2	CSF1
CARP	8.284859014	4422.241845	-9.060085064	4.43E-12	Low	6	5352	NM_213922.1	ssc:396959	7E-172	NM_014391.2	ANKRD1
COL5A3	0	1383.189994	-20	4.96E-12	Low	0	1674	NM_001105288.1	ssc:397531			
GADL1	266.4962983	0	20	5.24E-12	High	193	0	XP_005669383.1	ssc:100623700	0	NM_207359.2	GADL1
CXCL10	2.761619671	2351.588246	-9.73390511	5.60E-12	Low	2	2846	NM_001008691.1	ssc:494019	3E-22	NM_001565.3	CXCL10
LOC100513671	0	1343.528632	-20	5.92E-12	Low	0	1626	XP_003129107.4	ssc:100513671	9E-114	NM_182502.3	TMPRSS11B
LOC102158737	262.3538688	0	20	6.09E-12	High	190	0	XR_305607.1	ssc:102158737			
CCN2	125.6536951	46688.85939	-8.537481437	6.18E-12	Low	91	56505	NM_213833.1	ssc:100152141	0	NM_001901.2	CTGF
LOC100739307	1.380809836	1824.422645	-10.36770962	6.41E-12	Low	1	2208	XP_003483519.1	ssc:100739307	2E-121	NM_002508.2	NID1
LOC102164667	258.2114393	0	20	7.09E-12	High	187	0	XP_005653489.1	ssc:102164667	2E-40	NM_015978.2	TNNI3K
LOC100521530	0	1300.562157	-20	7.21E-12	Low	0	1574	XP_005673187.1	ssc:100521530	3E-62	NM_002825.5	PTN
LOC100517050	1578.265642	18.17812418	6.439992906	8.00E-12	High	1143	22	XR_305793.1	ssc:100517050	2E-32	NM_017898.3	2-Mar
LOC102164776	1.380809836	1751.710148	-10.30903371	8.20E-12	Low	1	2120	XR_305411.1	ssc:102164776			
TENM4	2.761619671	2188.811407	-9.630417292	8.64E-12	Low	2	2649	XR_305203.1	ssc:100514784	0	NM_001098816.2	TENM4
IFIT3	24.85457704	9279.106115	-8.54433047	9.04E-12	Low	18	11230	NM_001204395.1	ssc:100154248	0	NM_001549.4	IFIT3
KCNH3	382.4843245	1.652556743	7.854557002	9.17E-12	High	277	2	XP_003126164.1	ssc:100514837	0	NM_012284.1	KCNH3
EPCAM	381.1035147	1.652556743	7.849339293	9.49E-12	High	276	2	NM_214419.1	ssc:403163	6E-136	NM_002354.2	EPCAM
LOC100623720	71.80211146	24788.35115	-8.431420326	9.70E-12	Low	52	30000	XP_005659104.1	ssc:100623720	2E-177	NM_001848.2	COL6A1
EGR3	499.8531605	3.305113487	7.240660723	9.83E-12	High	362	4	XP_005670471.1	ssc:100516296	0	NM_004430.2	EGR3
DPT	0	1233.633609	-20	9.92E-12	Low	0	1493	XP_003125697.1	ssc:100516366	3E-108	NM_001937.4	DPT
ERG	0	1231.981052	-20	1.00E-11	Low	0	1491	NM_001244456.1	ssc:100625364			
LOC102166492	314.8246425	0.826278372	8.57370485	1.05E-11	High	228	1	XP_005661126.1	ssc:102166492	1E-51	NM_001252010.1	LUZP2
TGM1	2172.013872	27.26718627	6.315723672	1.06E-11	High	1573	33	XP_001927930.2	ssc:100156489	0	NM_000359.2	TGM1
GBP1	8.284859014	3798.401675	-8.840699614	1.11E-11	Low	6	4597	NM_001128473.1	ssc:100151938	0	NM_002053.2	GBP1
ADAMTS2	0	1203.887588	-20	1.15E-11	Low	0	1457	XP_003123699.3	ssc:100521434	0	NM_021599.2	ADAMTS2
FN1	2339.091862	881757.1804	-8.558289088	1.15E-11	Low	1694	1067143	XP_005672231.1	ssc:397620	0	NM_212482.1	FN1
LOC100037970	1978.700495	24.78835115	6.318747135	1.16E-11	High	1433	30	XP_003480802.2	ssc:100037970	2E-160	NR_040546.1	PDE4C
ITGB5	19.3313377	7134.91374	-8.527810704	1.20E-11	Low	14	8635	NM_001246669.1	ssc:100134977	0	NM_002213.3	ITGB5
FIBIN	0	1193.972247	-20	1.21E-11	Low	0	1445	XP_005658260.1	ssc:100620302	4E-116	NM_203371.1	FIBIN
KLHDC8A	488.8066819	3.305113487	7.208420386	1.22E-11	High	354	4	XP_003357440.1	ssc:100625797	0	NM_018203.2	KLHDC8A
VEGFC	1.380809836	1636.031176	-10.21046988	1.24E-11	Low	1	1980	VEGFC		0	NM_005429.3	VEGFC
LOC100623930	370.057036	1.652556743	7.806904027	1.26E-11	High	268	2	XP_005658231.1	ssc:100623930	6E-121	NM_001012302.2	ANO9
ACTC1	15.18890819	5781.469767	-8.572274322	1.30E-11	Low	11	6997	NM_001170517.1	ssc:100152267	0	NM_005159.4	ACTC1
ITGA11	11.04647869	4512.306188	-8.674134605	1.34E-11	Low	8	5461	XP_005659927.1	ssc:100520350	0	NM_012211.3	ITGA11

LOC100628220	0	1165.878783	-20	1.40E-11	Low	0	1411	XP_003354556.3	ssc:100628220	6E-32	NM_001278939.1	ELN
LOC100737327	1996.651022	25.61462952	6.284470363	1.40E-11	High	1446	31	XR_135454.2	ssc:100737327			
PROC	592.3674195	4.95767023	6.900686173	1.47E-11	High	429	6	NM_213918.1	ssc:396954	0	NM_000312.3	PROC
SALL4	361.772177	1.652556743	7.774237838	1.57E-11	High	262	2	NM_001114673.1	ssc:100136902			
LUZP2	1136.406495	13.22045395	6.425563457	1.63E-11	High	823	16	XP_003122961.4	ssc:100520176	6E-67	NM_001252010.1	LUZP2
LOC100621284	0	1129.522534	-20	1.69E-11	Low	0	1367	XP_003361912.2	ssc:100621284	5E-96	NM_003728.3	UNC5C
CLDN10	356.2489376	1.652556743	7.752042092	1.82E-11	High	258	2	NM_001243444.1	ssc:100153752	2E-124	NM_182848.3	CLDN10
SERPINF1	86.99101965	26974.68372	-8.276523854	1.84E-11	Low	63	32646	NM_001078662.1	ssc:780402	2E-16	NM_002615.5	SERPINF1
SAMD5	632.4109048	5.783948602	6.772663702	1.86E-11	High	458	7	XP_005659216.1	ssc:100522528	1E-80	NM_001030060.2	SAMD5
KDR	737.3524523	7.436505346	6.631585766	1.96E-11	High	534	9	XP_003129035.3	ssc:397311	0	NM_002253.2	KDR
LOC100523870	2.761619671	1878.957017	-9.410201703	2.18E-11	Low	2	2274	XR_299536.1	ssc:100523870			
KRT8	30781.01286	442.0589289	6.121658193	2.21E-11	High	22292	535	NM_001159615.1	ssc:100152077	0	NR_045962.1	KRT8
LOC100626686	229.2144327	0	20	2.21E-11	High	166	0	XP_005658179.1	ssc:100626686	9E-71	NM_006152.3	LRMP
LOC102159390	8.284859014	3391.872716	-8.67738917	2.21E-11	Low	6	4105	XR_302916.1	ssc:102159390			
LOC100622513	0	1071.683048	-20	2.32E-11	Low	0	1297	XP_005674372.1	ssc:100622513	6E-111	NM_005576.2	LOXL1
LOC100525856	1.380809836	1469.949223	-10.05603596	2.37E-11	Low	1	1779	XP_005661151.1	ssc:100525856	9E-43	NM_199161.3	SAA1
LOC100624640	345.2024589	1.652556743	7.706599121	2.46E-11	High	250	2	XP_005669304.1	ssc:100624640	8E-150	NM_207386.3	SHISA6
VGF	6.904049179	2927.504271	-8.728012819	2.63E-11	Low	5	3543	XP_005658665.1	ssc:102163755	4E-50	NM_003378.3	VGF
TRPC6	0	1048.547254	-20	2.65E-11	Low	0	1269	XP_003357304.2	ssc:100271728	0	NM_004621.5	TRPC6
LOC102166331	342.4408393	1.652556743	7.695011147	2.66E-11	High	248	2	XR_302282.1	ssc:102166331			
PLAC9	5.523239343	2550.721334	-8.851174931	2.69E-11	Low	4	3087	NM_001190209.1	ssc:100155282	1E-31	NM_001012973.1	PLAC9
EDIL3	0	1045.24214	-20	2.70E-11	Low	0	1265	XP_005661599.1	ssc:100516065	0	NM_005711.4	EDIL3
PDGFRB	44.18591474	13038.67271	-8.204994749	2.78E-11	Low	32	15780	XP_005661873.1	ssc:100126842	0	NM_002609.3	PDGFRB
BPIFB1	283.0660163	0.826278372	8.420294936	2.92E-11	High	205	1	NM_001101032.1	ssc:100113426	3E-173	NM_033197.2	BPIFB1
DAB2	40.04348524	11665.39805	-8.186452275	3.12E-11	Low	29	14118	XP_005672491.1	ssc:100519746	0	NM_001343.3	DAB2
MATK	854.7212883	9.915340461	6.429647935	3.21E-11	High	619	12	XP_005661404.1	ssc:100521543	0	NM_139355.2	MATK
ITGB8	0	1013.843562	-20	3.24E-11	Low	0	1227	NM_001097424.1	ssc:100037284	0	NM_002214.2	ITGB8
GLIS1	2.761619671	1754.188983	-9.31107382	3.30E-11	Low	2	2123	XP_003128030.4	ssc:100515552	0	NM_147193.2	GLIS1
LOC100739460	1056.319524	13.22045395	6.320130774	3.32E-11	High	765	16	XP_003481348.1	ssc:100739460	6E-112	NM_001307.5	CLDN7
FLRT2	17.95052786	5674.053579	-8.304209694	3.32E-11	Low	13	6867	XP_005656501.1	ssc:100151812	0	NM_013231.4	FLRT2
SLC16A6	332.7751704	1.652556743	7.653704172	3.50E-11	High	241	2	XP_005674608.1	ssc:100739042	0	NM_004694.4	SLC16A6
CLDN7	1197.162128	15.69928906	6.252775506	3.68E-11	High	867	19	NM_001160076.1	ssc:100127486	6E-112	NM_001307.5	CLDN7
LOC102165477	216.7871442	0	20	3.75E-11	High	157	0	XR_304464.1	ssc:102165477			
ELMOD1	1952.465108	28.09346464	6.1189184	3.87E-11	High	1414	34	XP_003357322.2	ssc:100626413	0	NM_018712.3	ELMOD1
SEMA5B	215.4063344	0	20	3.99E-11	High	156	0	XP_005670227.1	ssc:102160172			
CERS3	215.4063344	0	20	3.99E-11	High	156	0	XP_005659847.1	ssc:100152265	0	NM_178842.3	CERS3
GRIK2	481.9026327	4.131391859	6.865969967	4.11E-11	High	349	5	XP_003121367.3	ssc:100516526	0	NM_175768.3	GRIK2
ATP2A3	1888.947855	27.26718627	6.114273232	4.13E-11	High	1368	33	XP_005669187.1	ssc:396820	5E-159	NM_174958.2	ATP2A3
ROBO2	1367.001737	19.00440255	6.168537595	4.65E-11	High	990	23	XP_005670349.1	ssc:100739586	0	NM_002942.4	ROBO2
LOC780430	209.883095	0	20	5.10E-11	High	152	0	NM_001078682.1	ssc:780430	5E-114	NM_017594.3	DIRAS2
MSRB3	41.42429507	11075.43529	-8.062670539	5.18E-11	Low	30	13404	XP_005664013.1	ssc:100511693	5E-95	NM_198080.3	MSRB3
LOC102157888	208.5022852	0	20	5.42E-11	High	151	0	XP_005673916.1	ssc:102157888	3E-15	NM_153448.3	ESX1
GPR37	1933.13377	28.91974301	6.062742931	5.48E-11	High	1400	35	XP_003134766.2	ssc:100523220	0	NM_005302.3	GPR37
LOC100736763	15.18890819	4545.357323	-8.225231733	5.60E-11	Low	11	5501	XR_309518.1	ssc:100736763			
COL6A3	81.46778031	20982.51297	-8.008742157	5.68E-11	Low	59	25394	XP_005672367.1	ssc:100101551	0	NM_057167.3	COL6A3
CCR8	207.1214754	0	20	5.78E-11	High	150	0	XP_003358425.1	ssc:100622393	1E-163	NM_005201.3	CCR8
TMPRSS5	205.7406655	0	20	6.15E-11	High	149	0	XP_005667414.1	ssc:100515912	0	NR_110047.1	TMPRSS5
LOC102159987	1.380809836	1254.290568	-9.827141239	6.18E-11	Low	1	1518	XP_005668226.1	ssc:102159987	7E-93	NM_032812.8	PLXDC2
ENPEP	4674.041294	75.19133183	5.957960314	6.22E-11	High	3385	91	NM_214017.1	ssc:397080	0	NM_001977.3	ENPEP
CDH8	694.5473474	8.262783717	6.393301331	6.92E-11	High	503	10	XP_003355837.2	ssc:100625758	2E-105	NM_001796.4	CDH8
HighTI	202.9790459	0	20	6.99E-11	High	147	0	NM_213871.1	ssc:396887	8E-11	NM_001633.3	AMBP
APBB1IP	0	892.3806415	-20	7.00E-11	Low	0	1080	XP_003130799.3	ssc:100514179	0	NM_019043.3	APBB1IP
LOC100739007	6.904049179	2482.966507	-8.490406345	7.14E-11	Low	5	3005	XP_005663642.1	ssc:100739007	2E-87	NM_172212.2	CSF1
LOC100516994	8.284859014	2778.774164	-8.389755727	7.40E-11	Low	6	3363	NM_001243219.1	ssc:100516994	0	NM_052966.3	FAM129A
PHACTR1	1.380809836	1215.455485	-9.781766695	7.47E-11	Low	1	1471	XP_005665632.1	ssc:100153737	0	NM_030948.2	PHACTR1
PEG3	0	882.465301	-20	7.49E-11	Low	0	1068	XP_005653381.1	ssc:100522552	0	NM_006210.2	PEG3
CDH2	0	880.8127443	-20	7.58E-11	Low	0	1066	HighI00028F4AC1		0	NM_001792.3	CDH2
LOC102164249	306.5397835	1.652556743	7.535230703	7.68E-11	High	222	2	XR_300352.1	ssc:102164249			
LOC102166491	200.2174262	0	20	7.95E-11	High	145	0	XR_307709.1	ssc:102166491			
INPP5D	305.1589737	1.652556743	7.528717396	8.02E-11	High	221	2	XP_003133787.3	ssc:100526019	0	NM_005541.3	INPP5D
LOC100515546	2.761619671	1513.741977	-9.098388952	8.05E-11	Low	2	1832	XP_003126463.1	ssc:100515546	4E-118	NM_005086.4	SSPN
ENTPD3	22.09295737	5839.309253	-8.046067358	8.06E-11	Low	16	7067	XP_005669433.1	ssc:100623360	0	NM_001248.2	ENTPD3
ATP12A	198.8366163	0	20	8.49E-11	High	144	0	XP_005653908.1	ssc:100521921	0	NM_001676.5	ATP12A
LOC100739808	198.8366163	0	20	8.49E-11	High	144	0	XP_005659867.1	ssc:100739808	4E-164	NM_005503.3	APBA2
LOC100513415	198.8366163	0	20	8.49E-11	High	144	0	XP_003129043.2	ssc:100513415	1E-89	NM_152620.2	TRIM60
LOC100737477	84.22939998	20208.29014	-7.906407653	8.72E-11	Low	61	24457	XP_005654501.1	ssc:100737477	0	NM_000138.4	FBN1
LOC100158194	4.142429507	1785.587561	-8.75170602	9.09E-11	Low	3	2161	XP_001926938.4	ssc:100158194	0	NM_152380.2	TBX15
BEST2	396.2924229	3.305113487	6.905721763	9.21E-11	High	287	4	XP_003123378.1	ssc:100522788	0	NM_017682.2	BEST2
FOXH1	3232.475825	53.70809416	5.911356242	9.62E-11	High	2341	65	XP_001925315.1	ssc:100154027	4E-154	NM_003923.2	FOXH1
KCND3	196.0749967	0	20	9.68E-11	High	142	0	XP_001928554.1	ssc:100037946	0	NM_172198.2	KCND3
EVPL	759.4454097	9.915340461	6.259140144	1.01E-10	High	550	12	XP_003131232.3	ssc:100519916	0	NM_001988.2	EVPL
ISM1	0	838.6725473	-20	1.02E-10	Low	0	1015	XP_001925035.2	ssc:100154170	0	NM_080826.1	ISM1
LOC100739059	0	832.0623203	-20	1.07E-10	Low	0	1007	XP_003484027.2	ssc:100739059	5E-146	NM_198596.2	SULF2
CTHRC1	24.85457704	6120.243899	-7.943933784	1.13E-10	Low	18	7407	XP_003125583.1	ssc:100152510	4E-73	NM_138455.3	CTHRC1
LOC100517681	929.2850195	13.22045395	6.135277531	1.15E-10	High	673	16	XR_309472.1	ssc:100517681			
DNMT3B	12390.00666	214.0060983	5.855381244	1.18E-10	High	8973	259	XP_001928639.1		0	NM_175850.2	DNMT3B
GUCY1B3	0	804.7951341	-20	1.30E-10	Low	0	974	NM_001018032.1	ssc:444999			
CNN1	27.61619671	6544.124704	-7.888541784	1.32E-10	Low	20	7920	NM_213878.1	ssc:396911	8E-177	NM_001299.4	CNN1
LOC100626688	8.284859014	2512.712528	-8.244552764	1.36E-10	Low	6	3041	XP_005658385.1	ssc:100626688	0	NM_199512.2	CCDC80
C1QTNF5	13.80809836	3612.489041	-8.031334755	1.43E-10	Low	10	4372	XP_003129988.2	ssc:100520210	1E-119	NM_015645.4	C1QTNF5
PLXDC2	2.761619671	1360.880478	-8.944810004	1.53E-10	Low	2	1647	XP_005668225.1	ssc:100522568	0	NM_032812.8	PLXDC2
ETNK2	375.5802753	3.305113487	6.828277677	1.54E-10	High	272	4	XP_005656723.1	ssc:100622861	0	NM_018208.2	ETNK2
DPP10	1604.501029	26.4409079	5.92320919	1.54E-10	High	1162	32	XP_001925671.4	ssc:100157195	0	NM_020868.3	DPP10
C18H7orf58	0	775.8753911	-20	1.63E-10	Low	0	939	XP_005673279.1	ssc:100511847	0	NM_024913.4	CPED1
CLEC14A	0	775.0491127	-20	1.64E-10	Low	0	938	XP_005666250.1	ssc:100157365	0	NM_175060.2	CLEC14A
HSD17B2	185.028518	0	20	1.67E-10	High	134	0	NM_001167649.1	ssc:100312973	1E-25	NM_002153.2	HSD17B2
GAS7	24.85457704	5731.893065	-7.849356332	1.68E-10	Low	18	6937	XP_005669293.1	ssc:100518690	5E-179	NM_201433.1	GAS7
MMP19	15.18890819	3729.82057	-7.939944248	1.85E-10	Low	11	4514	XP_005663940.1	ssc:100518427	0	NR_073606.1	MMP19
MCSF	27.61619671	6173.125715	-7.804342716	1.88E-10	Low	20	7471	MCSF		2E-175	NM_172212.2	CSF1
LOC100514524	182.2668983	0	20	1.92E-10	High	132	0	XP_005652643.1	ssc:100514524			
FXYD3	182.2668983	0	20	1.92E-10	High	132	0	NM_214208.2	ssc:397413	1E-33	NM_021910.2	FXYD3
LIN28A	182.2668983	0	20	1.92E-10	High	132	0	NM_001123133.1	ssc:100142662	1E-105	NM_024674.4	LIN28A
EGFR	2.761619671	1304.693549	-8.88398062	1.97E-10	Low	2	1579	NM_214007.1	ssc:397070	0	NM_201284.1	EGFR
LOC102161608	998.3255112	15.69928906	5.99073916	2.13E-10	High	723	19	XP_005671034.1	ssc:102161608	0	NM_024554.3	PGBD5
SVEP1	22.09295737	4956.843952	-7.80969148	2.17E-10	Low	16	5999	XP_003122123.2	ssc:100155613	0	NM_153366.3	SVEP1
LOC100737434	82.84859014	17070.08488	-7.68677737	2.19E-10	Low	60	20659	XP_003480673.1	ssc:100737434	0	NM_001278074.1	COL5A1
LOC102161270	179.5052786	0	20	2.21E-10	High	130	0	XR_297327.1	ssc:102161270			
LOC100738350	657.2654818	9.089062089	6.176200981	2.22E-10	High	476	11	XP_005653900.1	ssc:100738350	1E-76	NM_024719.3	GRTP1
GBX1	2441.27179	43.7927537	5.800796941	2.26E-10	High	1768	53	XP_003360124.1	ssc:100625616	7E-162	NM_001098834.2	GBX1
LOC102159296	0	732.9089157	-20	2.29E-10	Low	0	887	XP_005666934.1	ssc:102159296			

LOC100736874	1369.763357	23.13579441	5.887656224	2.30E-10	High	992	28	XP_003483456.2	ssc:100736874	0	NM_198827.3	GPR133
LOC100152528	1311.769344	22.30951604	5.877711037	2.58E-10	High	950	27	XP_005665716.1	ssc:100152528	1E-68	NM_003524.2	HIST1H2BH
LOC100524089	269.257918	1.652556743	7.34814515	2.63E-10	High	195	2	XP_005664591.1	ssc:100524089	0	NM_004646.3	NPHS1
SLC15A2	3812.415956	71.88621834	5.728846513	2.94E-10	High	2761	87	NM_001097514.1	ssc:100048951	0	NM_021082.3	SLC15A2
GRAP2	306.5397835	2.478835115	6.950268202	3.16E-10	High	222	3	XP_005663838.1	ssc:100516438	9E-162	NM_004810.2	GRAP2
LOC102163185	306.5397835	2.478835115	6.950268202	3.16E-10	High	222	3	XR_302805.1	ssc:102163185			
CSPG4	66.27887212	12812.27243	-7.594761613	3.29E-10	Low	48	15506	XP_003128533.3	ssc:733676	0	NM_001897.4	CSPG4
LOC100518132	74.56373113	14235.12379	-7.57676527	3.50E-10	Low	54	17228	XP_003127801.2	ssc:100518132	0	NM_001856.3	COL16A1
GLT1D1	260.973059	1.652556743	7.30305726	3.53E-10	High	189	2	XP_003359123.2	ssc:100623172	1E-67	NM_144669.1	GLT1D1
GPR124	179.5052786	34432.67231	-7.583608073	3.55E-10	Low	130	41672	XP_003133414.1	ssc:100524344	0	NM_032777.9	GPR124
LOC100736865	169.8396098	0	20	3.71E-10	High	123	0	XP_005654951.1	ssc:100736865			
LOC100155551	169.8396098	0	20	3.71E-10	High	123	0	XP_005666029.1	ssc:100155551	1E-46	NM_001159726.1	TSPO2
LOC100519956	169.8396098	0	20	3.71E-10	High	123	0	XP_003481627.2	ssc:100519956	2E-141	NM_007210.3	GALNT6
NDST3	341.0600294	3.305113487	6.689182068	3.85E-10	High	247	4	XP_005666957.1	ssc:100525060	0	NM_004784.2	NDST3
PARM1	3244.903114	62.79715625	5.691332364	3.94E-10	High	2350	76	NM_001243716.1	ssc:100511208	3E-95	NM_015393.3	PARM1
ARSJ	0	666.806646	-20	4.04E-10	Low	0	807	XP_003129285.2	ssc:100512548	0	NM_024590.3	ARSJ
INPP4B	0	665.1540892	-20	4.10E-10	Low	0	805	XP_003129226.3	ssc:100511393	0	NM_003866.2	INPP4B
NOL4	255.4498196	1.652556743	7.272196297	4.32E-10	High	185	2	XP_005665391.1	ssc:100518847	2E-155	NR_036752.1	NOL4
LOC102164335	0	656.8913055	-20	4.42E-10	Low	0	795	XR_299884.1	ssc:102164335			
LOC100523750	254.0690098	1.652556743	7.264376792	4.55E-10	High	184	2	XP_005669193.1	ssc:100523750	0	NM_198501.2	SMTNL2
ACVRL1	2.761619671	1132.827648	-8.68019802	4.62E-10	Low	2	1371	XP_003481626.1	ssc:100153279	0	NM_001077401.1	ACVRL1
LOC100622870	294.112495	2.478835115	6.890561956	4.67E-10	High	213	3	XR_297931.1	ssc:100622870	1E-36	NM_003980.4	MAP7
LOC100515628	171.2204196	31142.43183	-7.506883028	4.86E-10	Low	124	37690	XP_003133256.2	ssc:100515628	0	NM_002775.4	HTRA1
LOC102163398	289.9700655	2.478835115	6.870097853	5.34E-10	High	210	3	XP_005666709.1	ssc:102163398	1E-34	NM_152620.2	TRIM60
LOC100739671	0	632.1029544	-20	5.57E-10	Low	0	765	XP_005672506.1	ssc:100739671	8E-55	NM_007036.4	ESM1
NXPH3	248.5457704	1.652556743	7.232667932	5.59E-10	High	180	2	XP_003483055.1	ssc:100736722	2E-142	NM_007225.2	NXPH3
CHST15	2.761619671	1094.818843	-8.630961808	5.67E-10	Low	2	1325	XP_005671601.1	ssc:100152393	0	NM_015892.4	CHST15
ANGPT2	0	629.6241193	-20	5.70E-10	Low	0	762	NM_213808.1	ssc:396730	0	NM_001147.2	ANGPT2
ARHGAP4	441.8591474	5.783948602	6.255388009	5.82E-10	High	320	7	XP_003135532.1	ssc:100523659	0	NM_001666.4	ARHGAP4
MYBPH	0	625.4927274	-20	5.93E-10	Low	0	757	NM_001033014.1	ssc:613132	0	NM_004997.2	MYBPH
ADM	26.23538688	4836.20731	-7.526218214	6.24E-10	Low	19	5853	NM_214107.1	ssc:397195	9E-91	NM_001124.1	ADM
LOC100738990	6.904049179	1729.400632	-7.968613665	6.34E-10	Low	5	2093	XP_003484353.1	ssc:100738990	8E-150	NM_033093.2	TRIM5
DPP4	1.380809836	851.8930013	-9.269013781	6.35E-10	Low	1	1031	NM_214257.1	ssc:397492			
COLQ	0	618.0562221	-20	6.37E-10	Low	0	748	XP_003358361.2	ssc:100624545	1E-37	NM_080544.2	COLQ
CCDC85C	2075.357183	41.31391859	5.65058794	6.39E-10	High	1503	50	XP_005666495.1	ssc:100512614	3E-83	NM_001144995.1	CCDC85C
CNTN5	473.6177737	6.610226974	6.162879602	6.62E-10	High	343	8	XP_003129849.3	ssc:100519379	0	NM_175566.2	CNTN5
LOC100738803	0	610.6197167	-20	6.85E-10	Low	0	739	XP_003481541.2	ssc:100738803	9E-168	NM_001010898.2	SLC6A17
LOC102165808	158.7931311	0	20	6.91E-10	High	115	0	XR_308897.1	ssc:102165808			
CLSTN2	1633.498036	32.2248565	5.663646976	7.11E-10	High	1183	39	XP_001926251.2	ssc:100157330	0	NM_022131.2	CLSTN2
PNP	35485.43197	722.9935753	5.617100231	7.47E-10	High	25699	875	XP_005656368.1	ssc:100157138	3E-155	NM_000270.3	PNP
LOC733579	9.66566885	2098.747064	-7.762443023	7.64E-10	Low	7	2540	NM_001044532.1	ssc:733579	8E-156	NM_033093.2	TRIM5
LOXL1	81.46778031	13617.89384	-7.385058276	7.77E-10	Low	59	16481	XP_005666227.1	ssc:100154899	0	NM_005576.2	LOXL1
EGR1	33577.15278	688.2898837	5.608319784	7.82E-10	High	24317	833	XP_003124022.1	ssc:100520726	0	NM_001964.2	EGR1
CLMP	24.85457704	4428.025794	-7.477008263	7.97E-10	Low	18	5359	XP_003130027.3	ssc:100512427	2E-147	NM_024769.2	CLMP
CYP7B1	0	594.0941493	-20	8.07E-10	Low	0	719	XP_005663118.1	ssc:100286867	0	NM_004820.3	CYP7B1
LOC100622713	0	587.4839223	-20	8.63E-10	Low	0	711	XP_005656599.1	ssc:100622713	0	NM_024582.4	FAT4
THBS4	0	585.8313656	-20	8.77E-10	Low	0	709	XP_003123802.3	ssc:100513005	0	NM_003248.4	THBS4
SCN4A	531.6117868	8.262783717	6.007601377	8.95E-10	High	385	10	XP_003131332.1	ssc:100511715	0	NM_000334.4	SCN4A
CCL20	2.761619671	1005.580778	-8.508298617	9.46E-10	Low	2	1217	NM_001024589.1	ssc:553951	5E-39	NM_004591.2	CCL20
GH1	153.2698918	0	20	9.58E-10	High	111	0	NM_213869.1	ssc:396884	2E-72	NM_022562.2	GH1
SORBS2	5.523239343	1409.630902	-7.995587095	9.66E-10	Low	4	1706	XP_005671790.1	ssc:396719	0	NM_021069.4	SORBS2
FAM65B	490.1874917	7.436505346	6.042565049	9.86E-10	High	355	9	XP_005665694.1	ssc:100154661	0	NM_015864.3	FAM65B
PLA2R1	2.761619671	996.4917163	-8.495199356	9.99E-10	Low	2	1206	XP_005671924.1	ssc:100622105	0	NM_007366.4	PLA2R1
IFNGR1	5.523239343	1398.889283	-7.984551422	1.01E-09	Low	4	1693	NM_001177907.1	ssc:100152238	2E-142	NM_000416.2	IFNGR1
GBP7	0	568.4795197	-20	1.05E-09	Low	0	688	XP_005663773.1	ssc:100155195	0	NM_052941.4	GBP4
SLC26A7	2.761619671	986.5763758	-8.480772285	1.06E-09	Low	2	1194	XP_005663034.1	ssc:100626316	0	NM_134266.1	SLC26A7
LOC100525452	0	563.5218495	-20	1.11E-09	Low	0	682	XP_005655056.1	ssc:100525452	5E-136	NM_207317.1	ZNF474
LOC102162332	4.142429507	1172.489009	-8.144881537	1.15E-09	Low	3	1419	XP_005663147.1	ssc:102162332	2E-144	NM_003068.4	SNAI2
LOC100513259	302.397354	3.305113487	6.515601896	1.20E-09	High	219	4	XP_005654370.1	ssc:100513259	2E-70	NM_005364.4	MAGEA8
CKMT1B	372.8186556	4.95767023	6.232667932	1.23E-09	High	270	6	XP_005659734.1	ssc:100519994	0	NM_001015001.1	CKMT1A
VDR	4.142429507	1154.310885	-8.122338969	1.26E-09	Low	3	1397	NM_001097414.1	ssc:396628	0	NM_001017536.1	VDR
LOC100525045	2577.971963	56.18692928	5.519858207	1.29E-09	High	1867	68	XP_005655695.1	ssc:100525045	0	NM_001982.3	ERBB3
CDH10	147.7466524	0	20	1.34E-09	High	107	0	XP_005657658.1	ssc:100523754	0	NM_006727.3	CDH10
CCDC80	66.27887212	10149.17724	-7.25859801	1.34E-09	Low	48	12283	XP_003132728.1	ssc:100516578	0	NM_199512.2	CCDC80
LOC102161596	2.761619671	947.7412924	-8.42283484	1.35E-09	Low	2	1147	XP_005668184.1	ssc:102161596			
MASP1	23.47376721	3815.753521	-7.34477478	1.45E-09	Low	17	4618	NM_001184947.1	ssc:100152125	0	NR_033519.1	MASP1
PPP1R16B	747.0181211	14.04673232	5.732836779	1.46E-09	High	541	17	XP_003134487.3	ssc:100517865	0	NM_015568.2	PPP1R16B
TJP3	146.3658426	0	20	1.46E-09	High	106	0	XP_005661408.1	ssc:100626455	0	NM_014428.1	TJP3
RGS4	3075.063504	68.58110485	5.486661248	1.49E-09	High	2227	83	NM_001185184.1	ssc:100157437	1E-113	NM_005613.5	RGS4
AGT	0	533.7758281	-20	1.53E-09	Low	0	646	AGT		2E-75	NM_000029.3	AGT
LOC100514326	4.142429507	1101.42907	-8.054683728	1.67E-09	Low	3	1333	XP_005672251.1	ssc:100514326	1E-132	NM_022648.4	TNS1
C1H15orf48	2154.063344	47.92414556	5.490164155	1.69E-09	High	1560	58	XP_003353424.1	ssc:100152534	1E-35	NM_197955.2	C15orf48
LOC102161294	0	524.686766	-20	1.70E-09	Low	0	635	XP_005670314.1	ssc:102161294			
ZBP1	2.761619671	911.385044	-8.366402239	1.71E-09	Low	2	1103	NM_001123216.1	ssc:100144524	1E-113	NM_030776.2	ZBP1
LOC102158099	4.142429507	1097.297678	-8.049262094	1.71E-09	Low	3	1328	XP_005660601.1	ssc:102158099	1E-41	NM_001278074.1	COL5A1
LOC100152144	0	521.3816526	-20	1.76E-09	Low	0	631	XP_005660642.1	ssc:100152144	1E-131	NM_138325.2	PCSK6
EHF	219.5487639	1.652556743	7.053697791	1.78E-09	High	159	2	XP_005661076.1	ssc:100526094	3E-175	NM_012153.5	EHF
STOX1	560.6087933	9.915340461	5.821188253	1.82E-09	High	406	12	XP_005657443.1	ssc:100157127	0	NM_152709.4	STOX1
KAZALD1	2134.732006	47.92414556	5.477158445	1.84E-09	High	1546	58	NM_001244551.1	ssc:100624902	6E-146	NM_030929.4	KAZALD1
ATP8A2	1785.387118	39.66136184	5.492358895	1.87E-09	High	1293	48	XP_003482910.1	ssc:100625134	0	NM_016529.4	ATP8A2
FAM151A	142.2234131	0	20	1.90E-09	High	103	0	XP_003128023.2	ssc:100513740	4E-17	NM_176782.2	FAM151A
IL1A	0	507.3349202	-20	2.07E-09	Low	0	614	NM_214029.1	ssc:397094	2E-105	NM_000575.3	IL1A
GRTP1	618.6028064	11.5678972	5.740814836	2.11E-09	High	448	14	XP_003131151.3	ssc:100518272	2E-72	NM_024719.3	GRTP1
NR5A2	1119.836777	23.96207278	5.546391945	2.11E-09	High	811	29	NM_001267893.1	ssc:100523688			
KCNA6	0	503.2035284	-20	2.18E-09	Low	0	609	HighI00025DE6BB		0	NM_002235.3	KCNA6
AIM1	20.71214754	3200.176134	-7.271530354	2.18E-09	Low	15	3873	XP_003121377.3	ssc:100521168	0	NM_001624.2	AIM1
COL5A1	751.1605506	110737.8274	-7.203811109	2.42E-09	Low	544	134020	NM_001014971.1	ssc:397533			
EFCAB4B	280.3043967	3.305113487	6.406150753	2.44E-09	High	203	4	XP_003355652.2	ssc:100625935	0	NM_032680.3	EFCAB4B
IL33	11.04647869	1905.397925	-7.430361961	2.44E-09	Low	8	2306	NM_001285978.1	ssc:100518643	3E-68	NM_033439.3	IL33
ADRB3	138.0809836	0	20	2.49E-09	High	100	0	NM_001099927.1	ssc:397356	2E-163	NM_000025.2	ADRB3
LOC102159289	138.0809836	0	20	2.49E-09	High	100	0	XR_304016.1	ssc:102159289			
LOC102159469	0	486.6779609	-20	2.66E-09	Low	0	589	XP_005659451.1	ssc:102159469			
LOC102159598	0	485.8516826	-20	2.68E-09	Low	0	588	XP_005659452.1	ssc:102159598	2E-88	NM_002290.4	LAMA4
CD14	2.761619671	841.1513824	-8.25070701	2.76E-09	Low	2	1018	NM_001097445.2	ssc:100037938	1E-144	NM_001174105.1	CD14
LOC102163523	0	482.5465691	-20	2.79E-09	Low	0	584	XP_005667043.1	ssc:102163523	0	NM_003728.3	UNC5C
USP18	102.1799278	13783.14952	-7.075649956	2.83E-09	Low	74	16681	NM_213826.1	ssc:396777	2E-151	NM_017414.3	USP18
ISLR2	0	480.8940123	-20	2.85E-09	Low	0	582	XP_005666221.1	ssc:100157309	0	NM_020851.2	ISLR2
PLCH2	208.5022852	1.652556743	6.979219575	2.88E-09	High	151	2	XP_003356173.1	ssc:100623940	0	NM_014638.2	PLCH2
CACNG6	274.7811573	3.305113487	6.377439457	2.93E-09	High	199	4	XP_005664874.1	ssc:100519779	3E-112	NR_102308.1	CACNG6
CHL1	2391.562635	57.01320765	5.390513594	3.01E-09	High	1732	69	XP_003132376.3	ssc:100511780	0	NR_045572.1	CHL1

PHOSPHO1	466.7137245	8.262783717	5.819766178	3.06E-09	High	338	10	XP_005668920.1	ssc:100621753	2E-153	NM_178500.3	PHOSPHO1
LOC100519663	0	473.457507	-20	3.13E-09	Low	0	573	XP_005665337.1	ssc:100519663	0	NM_173817.1	PIEZO2
SNX22	205.7406655	1.652556743	6.959983357	3.25E-09	High	149	2	XP_003362155.2	ssc:100624664	1E-38	NR_073534.1	SNX22
LOC102157807	133.9385541	0	20	3.28E-09	High	97	0	XP_005665620.1	ssc:102157807	2E-159	NM_145655.3	GCNT2
TRIM44	9.66566885	1645.120238	-7.411107748	3.31E-09	Low	7	1991	XP_005661051.1	ssc:100524974	9E-137	NM_017583.4	TRIM44
LOC102165705	0	467.6735584	-20	3.37E-09	Low	0	566	XR_301879.1	ssc:102165705			
LOC100625207	5.523239343	1140.264153	-7.689637716	3.45E-09	Low	4	1380	XP_003484218.1	ssc:100625207	0	NM_021906.2	USP9X
LOC100737066	204.3598557	1.652556743	6.950268202	3.46E-09	High	148	2	XP_003481468.1	ssc:100737066	0	NM_030916.2	PVRL4
CRABP1	0	463.5421665	-20	3.55E-09	Low	0	561	NM_001128492.1	ssc:100169745	3E-78	NM_004378.2	CRABP1
KRT2	132.5577442	0	20	3.61E-09	High	96	0	NM_001098583.1	ssc:100049665	1E-166	NM_000423.2	KRT2
KIF26B	2.761619671	801.4900206	-8.181026101	3.68E-09	Low	2	970	XP_005668023.1	ssc:100516102	0	NM_018012.3	KIF26B
TFAP2C	1213.731846	28.09346464	5.43307135	3.72E-09	High	879	34	NM_001123201.1	ssc:100144525	0	NM_003222.3	TFAP2C
PDZD3	519.1844982	9.915340461	5.710441187	3.76E-09	High	376	12	XP_005667485.1	ssc:100519043	0	NR_033122.1	PDZD3
COL16A1	33.13943606	4378.449092	-7.045727075	4.03E-09	Low	24	5299	XP_005665218.1	ssc:100737666	6E-118	NM_001856.3	COL16A1
GDNF	0	453.6268261	-20	4.04E-09	Low	0	549	XP_005672481.1	ssc:100322894	2E-73	NM_199234.1	GDNF
KIAA1598	0	451.9742693	-20	4.13E-09	Low	0	547	XP_005671545.1	ssc:100156189	0	NM_018330.6	KIAA1598
LOC100623179	200.2174262	1.652556743	6.920723926	4.18E-09	High	145	2	XP_005674463.1	ssc:100623179	0	NM_130806.3	RXFP2
LOC100621648	129.7961246	0	20	4.37E-09	High	94	0	XR_305874.1	ssc:100621648			
LOC102159043	2.761619671	772.5702776	-8.128007719	4.59E-09	Low	2	935	XR_303323.1	ssc:102159043			
BNC2	6.904049179	1240.243836	-7.488965331	4.67E-09	Low	5	1501	XP_005660148.1	ssc:100513064	0	NM_017637.5	BNC2
LOC102166634	197.4558065	1.652556743	6.900686173	4.76E-09	High	143	2	XP_005669381.1	ssc:102166634	5E-95	NM_207359.2	GADL1
PRSS55	353.4873179	5.783948602	5.933459914	4.76E-09	High	256	7	XP_005670526.1	ssc:100513823	8E-120	NM_198464.3	PRSS55
LOC100158001	196.0749967	1.652556743	6.890561956	5.07E-09	High	142	2	NM_001244603.1	ssc:100158001	0	NR_046371.1	CP
KCNK4	227.8336229	2.478835115	6.52217455	5.08E-09	High	165	3	XP_003122639.3	ssc:100524785	1E-165	NM_033311.1	KCNK4
TRIM50	258.2114393	3.305113487	6.287709296	5.23E-09	High	187	4	NM_214186.1	ssc:397383	0	NM_178125.3	TRIM50
ST8SIA1	2.761619671	752.7395966	-8.090492408	5.36E-09	Low	2	911	XP_003355598.1	ssc:100621251	6E-92	NM_003034.3	ST8SIA1
LOC100739699	38.6626754	4813.071515	-6.959872576	5.38E-09	Low	28	5825	XP_005663972.1	ssc:100739699	0	NM_002332.2	LRP1
POU3F3	194.6941868	1.652556743	6.880366189	5.41E-09	High	141	2	XP_003356366.2	ssc:397513	5E-120	NM_002699.3	POU3F1
SERPINB7	977.6133637	23.13579441	5.401065464	5.72E-09	High	708	28	XP_005652752.1	ssc:100152588	2E-178	NM_003784.3	SERPINB7
LOC100516568	193.313377	1.652556743	6.870097853	5.78E-09	High	140	2	XP_005667956.1	ssc:100516568	1E-177	NM_015198.3	COBL
GALNTL2	22.09295737	2873.796177	-7.023227381	5.80E-09	Low	16	3478	NM_001206447.1	ssc:100157572	0	NM_054110.4	GALNT15
WSCD2	0	426.3596398	-20	5.84E-09	Low	0	516	WSCD2				
LOC100525793	125.6536951	0	20	5.85E-09	High	91	0	XP_005652520.1	ssc:100525793	0	NM_033125.3	SLC22A16
LOC100625479	23.47376721	3020.047449	-7.007378013	5.90E-09	Low	17	3655	XP_005658348.1	ssc:100625479			
NIPAL4	16.56971803	2246.650893	-7.083083099	5.99E-09	Low	12	2719	XP_003134161.1	ssc:100523099			
LOC100626567	0	423.0545263	-20	6.11E-09	Low	0	512	LOC100626567				
LOC100512657	2.761619671	730.4300806	-8.047087723	6.42E-09	Low	2	884	XP_003360629.2	ssc:100512657	1E-140	NM_007191.4	WIF1
CRYBB1	124.2728852	0	20	6.46E-09	High	90	0	NM_001078681.1	ssc:780429	2E-115	NM_001887.3	CRYBB1
LOC100524147	124.2728852	0	20	6.46E-09	High	90	0	XP_005664870.1	ssc:100524147	0	NM_032803.5	SLC7A3
COL6A2	596.509849	73816.40462	-6.951251701	6.53E-09	Low	432	89336	XP_005657250.1	ssc:100101552	0	NM_058175.2	COL6A2
THSD7B	2259.004891	58.66576439	5.26702475	6.68E-09	High	1636	71	XP_003359479.2	ssc:100624965	0	NM_001080427.1	THSD7B
MAP1LC3A	33.13943606	4021.496835	-6.923039722	6.73E-09	Low	24	4867	NM_001170827.1	ssc:100322893	5E-66	NM_181509.2	MAP1LC3A
NRK	0	415.618021	-20	6.79E-09	Low	0	503	XP_003361569.1	ssc:100620665			
MYL9	260.973059	30984.61266	-6.891507242	6.86E-09	Low	189	37499	NM_001244472.1	ssc:100157760	9E-99	NM_181526.2	MYL9
TRIM63	0	414.7917426	-20	6.87E-09	Low	0	502	NM_001184756.1	ssc:100431101	0	NM_032588.3	TRIM63
LOC100525778	189.1709475	1.652556743	6.838846919	7.05E-09	High	137	2	LOC100525778				
LOC100515181	0	409.007794	-20	7.47E-09	Low	0	495	XP_005673866.1	ssc:100515181	0	NM_198465	NRK
LOC100737080	1067.366003	26.4409079	5.33513944	7.52E-09	High	773	32	XR_302312.1	ssc:100737080			
P4HA3	85.61020982	9791.398705	-6.837588292	7.64E-09	Low	62	11850	XP_003357239.1	ssc:100624543	0	NR_110031.1	P4HA3
CLDN4	1063.223574	26.4409079	5.329529472	7.80E-09	High	770	32	NM_001161637.1	ssc:733578	4E-96	NM_001305.4	CLDN4
LOC100514340	0	405.7026805	-20	7.84E-09	Low	0	491	NM_001244954.1	ssc:100514340			
EIF1AY	0	405.7026805	-20	7.84E-09	Low	0	491	NM_001244349.1	ssc:100625415	2E-79	NM_001412.3	EIF1AX
COL11A2	245.7841508	3.305113487	6.216548267	8.25E-09	High	178	4	XP_003128381.1	ssc:100520915	0	NM_080681.2	COL11A2
CGN	1168.165121	29.74602138	5.295403688	8.63E-09	High	846	36	XP_005663506.1	ssc:100157963	0	NM_020770.2	CGN
MYL1	0	399.0924535	-20	8.64E-09	Low	0	483	NM_214374.2	ssc:397666	3E-81	NM_079422.2	MYL1
APOA2	120.1304557	0	20	8.76E-09	High	87	0	NM_001186294.1	ssc:100153243	3E-34	NM_001643.1	APOA2
NXF2B	120.1304557	0	20	8.76E-09	High	87	0	XP_005657925.1	ssc:100153087	0	NM_001099686.2	NXF2B
GDF10	2.761619671	688.2898837	-7.961357849	9.15E-09	Low	2	833	XP_003133153.1	ssc:100519926	0	NM_004962.3	GDF10
SMOC1	4.142429507	825.4520934	-7.638563531	9.38E-09	Low	3	999	XP_005656414.1	ssc:100157351	0	NM_022137.5	SMOC1
LOC102158081	1097.743819	28.09346464	5.288163045	9.57E-09	High	795	34	XP_005666535.1	ssc:102158081	3E-107	NM_178557.3	NAT8L
CDHR1	118.7496459	0	20	9.72E-09	High	86	0	XP_005671212.1	ssc:100512562	0	NM_033100.3	CDHR1
LOC100739473	151.8890819	0.826278372	7.52217455	9.74E-09	High	110	1	XP_005673241.1	ssc:100739473			
LOC100514506	0	385.8719996	-20	1.06E-08	Low	0	467	XR_115703.3	ssc:100514506			
LOC100156358	392.1499933	43927.43708	-6.807572942	1.07E-08	Low	284	53163	NM_001252605.1	ssc:100156358	1E-53	NM_019554.2	S100A4
EDNRA	0	385.0457212	-20	1.07E-08	Low	0	466	NM_214229.1	ssc:397457	2E-163	NR_045958.1	EDNRA
LOC100624487	1.380809836	529.6444363	-8.58336571	1.09E-08	Low	1	641	XP_005673051.1	ssc:100624487	1E-60	NM_030777.3	SLC2A10
COL14A1	207.1214754	22555.74699	-6.766874108	1.10E-08	Low	150	27298	XP_005662938.1	ssc:100158059	0	NM_021110.2	COL14A1
IGF2	0	382.5668861	-20	1.11E-08	Low	0	463	NM_213883.2	ssc:396916			
LOC102166950	0	382.5668861	-20	1.11E-08	Low	0	463	XR_299386.1	ssc:102166950			
CDKN2B	4.142429507	799.8374638	-7.5930859	1.13E-08	Low	3	968	NM_214124.1	ssc:397227	4E-59	NM_078487.2	CDKN2B
FLT1	16.56971803	2003.725051	-6.917991695	1.19E-08	Low	12	2425	XP_001925775.4	ssc:396763	0	NM_002019.4	FLT1
SLC30A2	115.9880262	0	20	1.20E-08	High	84	0	NM_001139475.1	ssc:100170651	2E-179	NM_032513.3	SLC30A2
MYOG	0	376.7829375	-20	1.22E-08	Low	0	456	NM_001012406.1	ssc:497618	3E-118	NM_002479.5	MYOG
LOC100513699	147.7466524	0.826278372	7.482281823	1.25E-08	High	107	1	XP_005674426.1	ssc:100513699	2E-178	NM_001008783.1	SLC35D3
PREX2	289.9700655	4.95767023	5.870097853	1.28E-08	High	210	6	XP_001925866.1		0	NM_025170.4	PREX2
LOC100156127	317.5862622	5.783948602	5.777949965	1.29E-08	High	230	7	XP_001927974.1	ssc:100156127	1E-67	NM_138720.2	HIST1H2BD
LOC100624693	23.47376721	2649.04846	-6.818281033	1.30E-08	Low	17	3206	XP_005662674.1	ssc:100624693	1E-99	NM_000104.3	CYP1B1
LOC100739422	23.47376721	2648.222181	-6.817830964	1.30E-08	Low	17	3205	XP_005662673.1	ssc:100739422	3E-100	NM_000104.3	CYP1B1
LOC100626112	114.6072164	0	20	1.33E-08	High	83	0	XP_003358836.2	ssc:100626112	8E-38	NM_015341.4	NCAPH
KIAA1199	0	370.1727105	-20	1.35E-08	Low	0	448	XP_005656328.1	ssc:102160984	2E-59	NM_018689.1	KIAA1199
LOC102166778	2.761619671	644.4971299	-7.866515478	1.35E-08	Low	2	780	XP_005662309.1	ssc:102166778	9E-150	NM_001393.3	ECM2
DHX58	82.84859014	8611.47319	-6.699639108	1.36E-08	Low	60	10422	NM_001199132.1	ssc:100524520	0	NM_024119.2	DHX58
LOC100624329	1.380809836	508.987477	-8.525971705	1.38E-08	Low	1	616	XR_131258.2	ssc:100624329			
ABI3BP	0	366.0413187	-20	1.44E-08	Low	0	443	XP_005670307.1	ssc:100154641	0	NM_015429.3	ABI3BP
LOC100622618	8.284859014	1144.395545	-7.109892924	1.54E-08	Low	6	1385	XP_003357081.2	ssc:100622618	0	NM_024582.4	FAT4
SLC7A11	0	361.9099268	-20	1.54E-08	Low	0	438	XP_003360599.1	ssc:100049683			
NOX4	16.56971803	1915.313266	-6.852887514	1.56E-08	Low	12	2318	XP_005667271.1	ssc:100523323	0	NR_026571.1	NOX4
EMILIN1	270.6387278	27857.9753	-6.685578297	1.63E-08	Low	196	33715	XP_005662732.1	ssc:100523665	0	NM_007046.3	EMILIN1
LOC100519739	111.8455967	0	20	1.66E-08	High	81	0	XP_003132687.1	ssc:100519739	8E-38	NM_015341.4	NCAPH
REG3G	111.8455967	0	20	1.66E-08	High	81	0	NM_001144847.1	ssc:100240745	7E-71	NM_198448.3	REG3G
BNC1	1854.427609	52.88181579	5.132058426	1.68E-08	High	1343	64	XP_003128498.1	ssc:100156444	0	NM_001717.3	BNC1
LOC100737503	2.761619671	617.2299437	-7.804149597	1.75E-08	Low	2	747	XR_135144.2	ssc:100737503			
LOC100521443	26.23538688	2772.163937	-6.723354701	1.77E-08	Low	19	3355	XP_005662679.1	ssc:100521443	1E-123	NM_006449.4	CDC42EP3
FGFR4	1568.599973	44.61903207	5.135674453	1.79E-08	High	1136	54	XP_003123730.1	ssc:100127133	0	NM_213647.1	FGFR4
PTGS1	15.18890819	1743.447364	-6.842780829	1.80E-08	Low	11	2110	XP_001926164.1	ssc:397541	0	NM_080591.2	PTGS1
LOC100513632	15.18890819	1742.621086	-6.842096925	1.80E-08	Low	11	2109	XP_005672612.1	ssc:100513632	0	NM_003062.3	SLIT3
PIK3R6	1485.751383	42.14019696	5.139851857	1.80E-08	High	1076	51	XP_003132032.2	ssc:100034246	0	NM_001010855.2	PIK3R6
DES	481.9026327	49798.14491	-6.691206507	1.82E-08	Low	349	60268	NM_001001535.1	ssc:396725	0	NM_001927.3	DES
LOC102167854	110.4647869	0	20	1.85E-08	High	80	0	XR_297828.1	ssc:102167854			
TGFBI	4.142429507	735.3877508	-7.471884189	1.87E-08	Low	3	890	XP_005661717.1	ssc:100519300	0	NM_000358.2	TGFBI

KANK3	542.6582654	13.22045395	5.359200338	1.87E-08	High	393	16	XP_003123210.1	ssc:100513584	0	NM_198471.2	KANK3
MN1	19.3313377	2088.005445	-6.755040191	1.99E-08	Low	14	2527	XP_005657395.1	ssc:100156493	0	NM_002430.2	MN1
GMPR	17043.3358	494.9407447	5.105808111	2.00E-08	High	12343	599	XP_005665652.1	ssc:100154573	0	NM_006877.3	GMPR
LOC100516145	0	346.2106378	-20	2.00E-08	Low	0	419	XR_304856.1	ssc:100516145			
LRGUK	168.4588	1.652556743	6.671552174	2.03E-08	High	122	2	XP_003134708.2	ssc:100526023	0	NM_144648.1	LRGUK
GNG11	8.284859014	1091.513729	-7.041637414	2.04E-08	Low	6	1321	XP_005667670.1	ssc:100522202	3E-35	NM_004126.3	GNG11
FGF10	1.380809836	475.9363421	-8.429110165	2.05E-08	Low	1	576	XP_003133972.1	ssc:100525086	2E-104	NM_004465.1	FGF10
ASB9	0	344.558081	-20	2.06E-08	Low	0	417	NM_001243703.1	ssc:100158057	2E-114	NM_024087.2	ASB9
EPHA8	109.083977	0	20	2.07E-08	High	79	0	XP_003127734.2	ssc:100518378	0	NM_020526.3	EPHA8
LOC100525427	139.4617934	0.826278372	7.399026319	2.10E-08	High	101	1	LOC100525427	ssc:100525427			
IFIT1	71.80211146	6919.255085	-6.590446648	2.16E-08	Low	52	8374	NM_001244363.1	ssc:100153038	0	NM_001548.4	IFIT1
LOC102165099	12.42728852	1430.287861	-6.846650172	2.30E-08	Low	9	1731	XP_005665219.1	ssc:102165099	1E-55	NM_001856.3	COL16A1
LOC102161422	77.3253508	7343.962168	-6.569473343	2.34E-08	Low	56	8888	XP_005663980.1	ssc:102161422	0	NM_002332.2	LRP1
DPPA2	165.6971803	1.652556743	6.647705432	2.35E-08	High	120	2	XP_003358870.1	ssc:100620381	3E-97	NM_138815.3	DPPA2
NRGN	191.9325672	2.478835115	6.274793408	2.44E-08	High	139	3	XP_003482685.1	ssc:100738830			
ESM1	0	334.6427406	-20	2.45E-08	Low	0	405	XP_005674289.1	ssc:100519340	2E-71	NM_007036.4	ESM1
CYP2D25	1855.808419	55.36065091	5.067043068	2.51E-08	High	1344	67	NM_214394.1	ssc:397687	0	NM_001025161.2	CYP2D6
LOC100739427	0	332.1639054	-20	2.56E-08	Low	0	402	XP_005670555.1	ssc:100739427	2E-81	NM_016950.2	SPOCK3
TFPI2	13.80809836	1517.873369	-6.78039298	2.62E-08	Low	10	1837	XP_005667666.1	ssc:100520144	3E-85	NM_006528.3	TFPI2
LOC100625802	472.2369638	11.5678972	5.351312429	2.63E-08	High	342	14	XP_005668756.1	ssc:100625802	2E-127	NM_014834.4	LRRC37A
RAPGEF4	1296.580436	38.0088051	5.092234228	2.63E-08	High	939	46	XP_001925789.1		0	NM_007023.3	RAPGEF4
LOC102157467	0	330.5113487	-20	2.64E-08	Low	0	400	XR_298763.1	ssc:102157467			
TNS1	19.3313377	1972.326473	-6.672813093	2.80E-08	Low	14	2387	XP_005672258.1	ssc:100514801	0	NM_022648.4	TNS1
PPP1R14C	3972.589897	122.289199	5.021710976	2.88E-08	High	2877	148	XP_005659204.1	ssc:100521302	2E-41	NM_030949.2	PPP1R14C
LOC100739698	20.71214754	2080.56894	-6.650357136	2.88E-08	Low	15	2518	XP_003481292.1	ssc:100739698	1E-123	NM_006449.4	CDC42EP3
BGN	1946.941868	198659.6301	-6.672945111	2.95E-08	Low	1410	240427	XP_003135523.1	ssc:397396	0	NM_001711.4	BGN
SIRPA	0	318.9434515	-20	3.25E-08	Low	0	386	NM_001011508.2	ssc:494566			
LOC102162482	0	318.9434515	-20	3.25E-08	Low	0	386	XR_305617.1	ssc:102162482			
LOC100627431	103.5607377	0	20	3.27E-08	High	75	0	XP_005658254.1	ssc:100627431	0	NM_033086.2	FGD3
CA12	0	318.1171731	-20	3.30E-08	Low	0	385	XP_005659573.1	ssc:100152749	3E-153	NM_206925.1	CA12
LIPG	56.61320327	5103.921502	-6.494323775	3.33E-08	Low	41	6177	NM_001243029.1	ssc:100155736	0	NM_006033.2	LIPG
DKK2	11.04647869	1229.502217	-6.798343975	3.36E-08	Low	8	1488	XP_003129317.1	ssc:100519672	5E-140	NM_014421.2	DKK2
RBP4	11.04647869	1226.197104	-6.79446054	3.41E-08	Low	8	1484	NM_214057.1	ssc:397124	4E-106	NM_006744.3	RBP4
LOC100736640	0	313.9857813	-20	3.56E-08	Low	0	380	XR_305303.1	ssc:100736640	2E-33	NM_004621.5	TRPC6
EPB41L3	17.95052786	1771.540829	-6.624834636	3.68E-08	Low	13	2144	XP_005665352.1	ssc:100520913	0	NM_012307.3	EPB41L3
LOC100737030	102.1799278	0	20	3.68E-08	High	74	0	XP_003483031.1	ssc:100737030	2E-171	NM_000422.2	KRT17
SEMA6A	5.523239343	765.9600506	-7.115610692	3.71E-08	Low	4	927	XP_005661650.1	ssc:100522008	0	NM_020796.3	SEMA6A
LOC100622872	258.2114393	4.95767023	5.702746795	3.72E-08	High	187	6	XP_005654533.1	ssc:100622872	0	NM_153181.2	NETO1
LOC102158591	0	311.5069461	-20	3.74E-08	Low	0	377	XP_005654691.1	ssc:102158591	8E-52	NR_030691.1	ANO1
FAM124A	429.4318589	10.74161883	5.321145888	3.78E-08	High	311	13	XP_001925756.4	ssc:100152644	0	NM_145019.3	FAM124A
LOC100156932	354.8681278	8.262783717	5.42451129	3.89E-08	High	257	10	XP_003356615.1	ssc:100156932	2E-68	NM_003518.3	HIST1H2BG
ANGPTL1	0	309.028111	-20	3.91E-08	Low	0	374	NM_001109947.1	ssc:100126165	0	NM_004673.3	ANGPTL1
KREMEN2	231.9760524	4.131391859	5.811204164	3.94E-08	High	168	5	XP_005662277.1	ssc:100737079	3E-96	NM_172229.2	KREMEN2
LOC100511417	549.5623146	14.87301069	5.207514455	3.94E-08	High	398	18	XP_003133982.1	ssc:100511417	0	NM_002202.2	ISL1
LSR	893.3839637	26.4409079	5.078436738	3.95E-08	High	647	32	XP_005664540.1	ssc:100516738	1E-115	NM_205835.3	LSR
GHR	0	306.5492759	-20	4.10E-08	Low	0	371	NM_214254.2	ssc:397488	0	NM_001242462.1	GHR
IFIH1	27.61619671	2520.975312	-6.512323534	4.11E-08	Low	20	3051	NM_001100194.1	ssc:100101927	0	NM_022168.3	IFIH1
LOC102158635	205.7406655	3.305113487	5.959983357	4.19E-08	High	149	4	XP_005654948.1	ssc:102158635	1E-51	NR_040546.1	PDE4C
S1PR3	8.284859014	961.7880247	-6.859098006	4.33E-08	Low	6	1164	XP_001925305.1	ssc:100154607	0	NM_005226.3	S1PR3
LOC100628185	5.523239343	741.1716994	-7.068149339	4.51E-08	Low	4	897	XP_005658364.1	ssc:100628185			
ZIC4	4.142429507	631.276676	-7.251651491	4.63E-08	Low	3	764	ZIC4	ssc:100519623	9E-12	NR_040762.1	ZIC4
PACSIN1	347.9640786	8.262783717	5.396166665	4.66E-08	High	252	10	XP_001926343.3	ssc:100157037	0	NM_020804.4	PACSIN1
LOC100737139	1169.545931	36.35624836	5.007601377	4.68E-08	High	847	44	XP_005663921.1	ssc:100737139	1E-43	NM_006741.3	PPP1R1A
LOC100514082	99.41830817	0	20	4.68E-08	High	72	0	XP_003121229.3	ssc:100514082	1E-137	NM_003980.4	MAP7
LOC100155774	153.2698918	1.652556743	6.535230703	4.74E-08	High	111	2	XP_001928002.3	ssc:100155774	0	NM_130806.3	RXFP2
KCNK2	0	296.6339355	-20	4.98E-08	Low	0	359	XP_003357633.2	ssc:100627197	8E-110	NM_014217.3	KCNK2
TLR4	0	296.6339355	-20	4.98E-08	Low	0	359	NM_001113039.1	ssc:399541	0	NM_138557.2	TLR4
LPCAT2	3590.105573	117.3315288	4.935363625	5.03E-08	High	2600	142	XP_005653311.1	ssc:100517894	0	NM_017839.4	LPCAT2
LOC100739542	392.1499933	9.915340461	5.305599455	5.07E-08	High	284	12	XP_003481349.1	ssc:100739542	0	NM_001042.2	SLC2A4
AEBP1	483.2834425	41982.37779	-6.440770502	5.17E-08	Low	350	50809	XP_003134934.1	ssc:733628	0	NM_001129.4	AEBP1
NANOG	98.03749834	0	20	5.29E-08	High	71	0	NM_001129971.1	ssc:100170132	5E-121	NM_024865.2	NANOG
CACNA2D2	3145.484806	103.2847965	4.928582583	5.30E-08	High	2278	125	XP_005669658.1	ssc:100517557	0	NM_006030.2	CACNA2D2
LOC100736658	0	293.328822	-20	5.32E-08	Low	0	355	XP_005659684.1	ssc:100736658	2E-54	NM_002009.3	FGF7
LOC100737285	9.66566885	1032.847965	-6.739542621	5.34E-08	Low	7	1250	XP_005661241.1	ssc:100737285	0	NM_001080421.2	UNC13A
ABAT	294.112495	6.610226974	5.475524456	5.58E-08	High	213	8	NM_214263.1	ssc:397500	0	NM_020686.5	ABAT
LOC100623608	4.142429507	611.4459951	-7.205604124	5.59E-08	Low	3	740	XP_003360896.1	ssc:100623608	2E-129	NM_001017961.3	FAM78B
OSR1	2.761619671	507.3349202	-7.521280009	5.60E-08	Low	2	614	XP_005674600.1	ssc:100520131	1E-117	NM_145260.2	OSR1
ERBB3	550.9431245	15.69928906	5.133132259	5.74E-08	High	399	19	HighI00025DF8BD		0	NM_001982.3	ERBB3
LOC100737813	124.2728852	0.826278372	7.232667932	5.88E-08	High	90	1	XP_005652749.1	ssc:100737813	9E-47	NM_153181.2	NETO1
LIN7A	1021.799278	32.2248565	4.986794078	5.88E-08	High	740	39	XP_005664343.1	ssc:100511198	2E-133	NM_004664.2	LIN7A
PAX9	2.761619671	502.37725	-7.507112677	5.94E-08	Low	2	608	XP_005666257.1	ssc:100156821	0	NM_006194.3	PAX9
UNC5C	0	287.5448734	-20	5.98E-08	Low	0	348	XP_005667044.1	ssc:100622952	1E-157	NM_003728.3	UNC5C
LOC100738686	96.6566885	0	20	5.99E-08	High	70	0	XR_307991.1	ssc:100738686	5E-71	NR_048571.1	GPM6A
ETNPPL	149.1274623	1.652556743	6.495702338	6.06E-08	High	108	2	XP_005667008.1	ssc:100519324	2E-175	NR_027475.1	ETNPPL
LOC102159197	197.4558065	3.305113487	5.900686173	6.07E-08	High	143	4	XR_302293.1	ssc:102159197			
LOC100152679	0	286.718595	-20	6.08E-08	Low	0	347	XP_005672752.1	ssc:100152679	0	NM_182734.2	PLCB1
MBNL3	2.761619671	496.5933014	-7.490406345	6.36E-08	Low	2	601	NM_001243933.1	ssc:100512149	0	NM_133486.2	MBNL3
ALX1	0	284.2397599	-20	6.40E-08	Low	0	344	XP_003126788.1	ssc:100525700	0	NM_006982.2	ALX1
PTPRU	17.95052786	1613.72166	-6.49022168	6.41E-08	Low	13	1953	XP_005653451.1	ssc:100515955	0	NM_133178.3	PTPRU
LOC100622410	48.32834425	3927.301101	-6.344524833	6.42E-08	Low	35	4753	LOC100622410				
LOC100157370	6.904049179	799.0111855	-6.854629148	6.43E-08	Low	5	967	XP_001925219.2	ssc:100157370	6E-42	NM_138325.2	PCSK6
LOC100739187	6.904049179	798.1849071	-6.853136448	6.47E-08	Low	5	966	XP_003480678.1	ssc:100739187	6E-42	NM_138325.2	PCSK6
LRFN2	796.7272752	24.78835115	5.006351749	6.72E-08	High	577	30	XP_001928805.1	ssc:100153249	0	NM_020737.1	LRFN2
LOC102157546	0	281.7609248	-20	6.73E-08	Low	0	341	XR_298764.1	ssc:102157546			
LOC100622219	95.27587867	0	20	6.78E-08	High	69	0	XP_003357960.2	ssc:100622219	0	NM_001676.5	ATP12A
KIFC3	26.23538688	2210.294644	-6.396580827	6.85E-08	Low	19	2675	XP_005664439.1	ssc:100514463	3E-11	NM_005550.3	KIFC3
LOC102161219	13.80809836	1290.646817	-6.546435807	6.89E-08	Low	10	1562	XP_005674129.1	ssc:102161219	4E-40	NM_002962.1	S100A5
IL8	6.904049179	789.095845	-6.836613992	6.93E-08	Low	5	955	NM_213867.1	ssc:396880	5E-35	NM_000584.3	IL8
LOC100620730	6.904049179	789.095845	-6.836613992	6.93E-08	Low	5	955	XP_003362006.1	ssc:100620730	5E-35	NM_000584.3	IL8
WIF1	0	280.108368	-20	6.97E-08	Low	0	339	XP_003481699.1	ssc:100511326	1E-67	NM_007191.4	WIF1
LOC100736668	0	279.2820896	-20	7.09E-08	Low	0	338	XP_005669299.1	ssc:100736668	6E-46	NM_201433.1	GAS7
LEPREL1	1.380809836	385.8719996	-8.126463904	7.09E-08	Low	1	467	XP_005670122.1	ssc:100520219	0	NM_018192.3	LEPREL1
LOC100520559	9.66566885	983.2712624	-6.6685761	7.15E-08	Low	7	1190	XP_005658373.1	ssc:100520559	0	NM_024408.3	NOTCH2
HMGA2	30.37781639	2496.186961	-6.360564014	7.26E-08	Low	22	3021	XP_005664014.1	ssc:100513206	1E-51	NM_003484.1	HMGA2
LOC100628052	13.80809836	1274.947528	-6.528779416	7.41E-08	Low	10	1543	XP_003360600.1	ssc:100628052	1E-50	NM_020130.4	C8orf4
LOC100517809	763.5878392	23.96207278	4.993969511	7.56E-08	High	553	29	XP_005661493.1	ssc:100517809			
MAMLD1	5.523239343	679.2008216	-6.942179148	7.57E-08	Low	4	822	XP_005657994.1	ssc:100517073	0	NM_005491.4	MAMLD1
ISG15	305.1589737	24180.21027	-6.308121964	8.02E-08	Low	221	29264	NM_001128469.1	ssc:100145895	3E-52	NM_005101.3	ISG15
SLC34A2	190.5517573	3.305113487	5.849339293	8.36E-08	High	138	4	NM_001256772.1	ssc:641347	0	NM_006424.2	SLC34A2
LOC100739780	190.5517573	3.305113487	5.849339293	8.36E-08	High	138	4	XP_005659865.1	ssc:100739780	3E-161	NM_005503.3	APBA2

SCRN1	28.99700655	2325.973616	-6.325786948	8.61E-08	Low	21	2815	XP_003134860.1	ssc:100513510	0	NM_014766.4	SCRN1
DLK1	92.51425899	0	20	8.75E-08	High	67	0	NM_001126101.1	ssc:497066			
LOC100624633	92.51425899	0	20	8.75E-08	High	67	0	XP_005666519.1	ssc:100624633	0	NM_003836.5	DLK1
LOC102160402	92.51425899	0	20	8.75E-08	High	67	0	XR_303203.1	ssc:102160402			
LOC102167761	92.51425899	0	20	8.75E-08	High	67	0	XR_297827.1	ssc:102167761			
LOC102165800	92.51425899	0	20	8.75E-08	High	67	0	XR_301414.1	ssc:102165800			
LOC102157763	8.284859014	854.3718364	-6.688243133	8.75E-08	Low	6	1034	XP_005666811.1	ssc:102157763	6E-33	NM_002089.3	CXCL2
PAX1	1.380809836	370.9989889	-8.069756799	8.94E-08	Low	1	449	XP_005672808.1	ssc:100739820	0	NM_006192.4	PAX1
PRRX2	179.5052786	13710.43702	-6.255104478	9.03E-08	Low	130	16593	XP_005654631.1	ssc:100521241	5E-97	NM_016307.3	PRRX2
POU3F2	434.9550983	12.39417558	5.133132259	9.04E-08	High	315	15	XP_001926276.1	ssc:100157279	0	NM_005604.3	POU3F2
CELF4	929.2850195	30.57229975	4.925824165	9.08E-08	High	673	37	XP_005665413.1	ssc:100521138	0	NM_020180.3	CELF4
SLC2A4	365.9146065	9.915340461	5.205700885	9.54E-08	High	265	12	NM_001128433.1	ssc:396754	0	NM_001042.2	SLC2A4
NCAM1	27.61619671	2183.853736	-6.305217779	9.66E-08	Low	20	2643	XP_005667401.1	ssc:100515564	0	NM_181351.4	NCAM1
USP43	117.368836	0.826278372	7.150205772	9.73E-08	High	85	1	XP_005669291.1	ssc:100515447	0	NM_153210.4	USP43
PDE1C	0	264.409079	-20	9.77E-08	Low	0	320	XP_003134849.3	ssc:100525902	0	NM_005020.2	PDE1C
KHDRBS3	0	264.409079	-20	9.77E-08	Low	0	320	XR_302764.1	ssc:100155706	1E-169	NM_006558.1	KHDRBS3
SGCD	0	263.5828006	-20	9.95E-08	Low	0	319	NM_001144123.1	ssc:100240724			
CGA	91.13344916	0	20	9.97E-08	High	66	0	NM_214446.1	ssc:406869	6E-48	NM_001252383.1	CGA
ATP6V1B1	91.13344916	0	20	9.97E-08	High	66	0	XP_005662542.1	ssc:100523368	0	NM_001692.3	ATP6V1B1
GRM8	91.13344916	0	20	9.97E-08	High	66	0	XP_005673251.1	ssc:100524120	0	NR_028041.1	GRM8
HS3ST2	1324.196632	45.44531045	4.864842128	1.01E-07	High	959	55	XP_003124592.1	ssc:100522426	0	NM_006043.1	HS3ST2
LOC100738076	0	262.7565222	-20	1.01E-07	Low	0	318	XP_005665361.1	ssc:100738076	2E-34	NM_032048.2	EMILIN2
TMEM30B	0	262.7565222	-20	1.01E-07	Low	0	318	XP_003480534.1	ssc:100738730	0	NM_001017970.2	TMEM30B
COL5A2	1072.889242	86391.53516	-6.331316906	1.02E-07	Low	777	104555	NM_001105289.1	ssc:397532			
LRRC32	20.71214754	1681.476486	-6.343107647	1.03E-07	Low	15	2035	XP_003129719.1	ssc:100522452	0	NM_005512.2	LRRC32
PCDH17	42.80510491	3234.053547	-6.239416894	1.03E-07	Low	31	3914	XP_005668514.1	ssc:100157362	0	NM_014459.2	PCDH17
NPY1R	0	261.9302438	-20	1.03E-07	Low	0	317	NM_214288.1	ssc:397547	0	NM_000909.5	NPY1R
BEND6	15.18890819	1298.9096	-6.418139047	1.04E-07	Low	11	1572	XP_005665923.1	ssc:100153820	6E-127	NM_152731.2	BEND6
LOC102160493	6.904049179	732.0826374	-6.728419958	1.08E-07	Low	5	886	XP_005672259.1	ssc:102160493	4E-89	NM_022648.4	TNS1
SYT6	251.3073901	5.783948602	5.441254554	1.09E-07	High	182	7	XR_298361.1	ssc:100152816	0	np_001240701	np_001240701
FAT4	2.761619671	452.8005477	-7.357217247	1.10E-07	Low	2	548	XP_005666936.1	ssc:102159549	0	NM_024582.4	FAT4
PRR5L	2.761619671	451.9742693	-7.354582187	1.11E-07	Low	2	547	XP_005652862.1	ssc:100523837	0	NM_024841.4	PRR5L
ABCA5	1264.82181	43.7927537	4.85209817	1.12E-07	High	916	53	XP_003483003.1	ssc:100520049	2E-129	NM_172232	ABCA5
ZEB2	5.523239343	635.4080679	-6.846024952	1.12E-07	Low	4	769	ZEB2	ssc:100739669	0	NR_033258.1	ZEB2
LOC100517243	1.380809836	354.4734215	-8.004019001	1.17E-07	Low	1	429	XP_003133148.1	ssc:100517243	2E-25	NM_006829.2	ADIRF
PRR16	0	256.1462952	-20	1.18E-07	Low	0	310	XP_005655053.1	ssc:100524363	2E-98	NM_016644.1	PRR16
LOC100515261	991.4214621	33.87741324	4.871102865	1.18E-07	High	718	41	XP_003126103.4	ssc:100515261	0	NM_025045	BAIAP2L2
LOC100737566	114.6072164	0.826278372	7.115854268	1.20E-07	High	83	1	LOC100737566				
MYL4	0	254.4937385	-20	1.22E-07	Low	0	308	XP_003131354.1	ssc:100516998	4E-91	NM_002476.2	MYL4
PLAUR	33.13943606	2475.530002	-6.223044572	1.22E-07	Low	24	2996	XP_005655990.1	ssc:100521017	7E-119	NM_002659.3	PLAUR
ZBED2	0	253.6674601	-20	1.24E-07	Low	0	307	ZBED2	ssc:100517972	1E-97	NM_024508.4	ZBED2
LAD1	1094.9822	38.0088051	4.848429936	1.25E-07	High	793	46	XP_005668054.1	ssc:100622691	2E-129	NM_005558.3	LAD1
LOC100623668	0	252.8411817	-20	1.27E-07	Low	0	306	XP_003482903.1	ssc:100623668	2E-123	NM_000231.2	SGCG
TAC4	88.37182949	0	20	1.30E-07	High	64	0	NM_001287414.1	ssc:100511101	2E-15	NM_170685.2	TAC4
LOC100738537	88.37182949	0	20	1.30E-07	High	64	0	XP_005670069.1	ssc:100738537	2E-116	NM_016559.2	PEX5L
CFTR	288.5892557	7.436505346	5.278248967	1.33E-07	High	209	9	NM_001104950.1	ssc:403154			
OLFML2B	4.142429507	526.3393228	-6.989372225	1.36E-07	Low	3	637	XP_005663213.1	ssc:100156452	0	NM_015441.1	OLFML2B
LOC100739643	0	249.5360683	-20	1.37E-07	Low	0	302	XR_309652.1	ssc:100739643			
OSBPL3	4.142429507	523.8604877	-6.982561693	1.39E-07	Low	3	634	XP_003484108.1	ssc:100523342	0	NR_104112.1	OSBPL3
GAS1	53.85158359	3811.622129	-6.145272522	1.42E-07	Low	39	4613	XP_003130684.3	ssc:100516459	2E-100	NM_002048.2	GAS1
LOC100620584	2423.321262	88.41178578	4.776603165	1.43E-07	High	1755	107	XP_003359538.1	ssc:100620584	4E-157	NM_013964.3	NRG1
SFN	1031.464947	36.35624836	4.82634765	1.49E-07	High	747	44	NM_001044564.1	ssc:733625	6E-141	NM_006142.3	SFN
LOC100624681	86.99101965	0	20	1.49E-07	High	63	0	XP_003360222.2	ssc:100624681			
HPDL	3729.567366	137.1622097	4.765053329	1.50E-07	High	2701	166	XP_003128096.1	ssc:100514057	5E-173	NM_032756.2	HPDL
LOC100519542	2.761619671	428.8384749	-7.278775892	1.51E-07	Low	2	519	XP_005661991.1	ssc:100519542	2E-130	NM_133457.3	COL26A1
MMP14	745.6373113	54838.44297	-6.200569746	1.58E-07	Low	540	66368	NM_214239.1	ssc:397471	0	NM_004995.3	MMP14
ADAMTSL3	11.04647869	946.915014	-6.421576493	1.58E-07	Low	8	1146	XP_005666168.1	ssc:100157238	0	NM_207517.2	ADAMTSL3
LRMP	1956.607537	71.88621834	4.766495383	1.58E-07	High	1417	87	XP_005655711.1	ssc:100518428	0	NM_006152.3	LRMP
SP100	5.523239343	598.2255411	-6.759031051	1.60E-07	Low	4	724	XP_005657638.1	ssc:100516940	1E-80	NM_003113.3	SP100
LOC100736853	1.380809836	335.4690189	-7.924521081	1.61E-07	Low	1	406	XP_003482717.1	ssc:100736853	5E-166	NM_024788.2	C7orf63
AFF2	2.761619671	423.0545263	-7.259185164	1.63E-07	Low	2	512	XP_005654357.1	ssc:100512515	0	NM_002025.3	AFF2
AVIL	5149.039877	190.8703039	4.753638877	1.64E-07	High	3729	231	XP_005658919.1	ssc:100624986	0	NM_006576.3	AVIL
LOC102162371	1405.664413	51.22925905	4.778140372	1.65E-07	High	1018	62	XP_005662335.1	ssc:102162371	0	NM_153214.2	FBLN7
LOC102160802	85.61020982	0	20	1.71E-07	High	62	0	XP_005653469.1	ssc:102160802	3E-46	NR_036752.1	NOL4
LOC100511905	5549.47473	206.5695929	4.747651414	1.72E-07	High	4019	250	XR_308633.1	ssc:100511905	8E-149	NM_001160179	NAT1
FAM180A	0	239.6207278	-20	1.73E-07	Low	0	290	XP_003484061.1	ssc:100737678	5E-78	NM_205855.3	FAM180A
RNASEL	5.523239343	589.9627574	-6.738965428	1.74E-07	Low	4	714	NM_001097512.1	ssc:100048946	0	NM_021133.3	RNASEL
CRYAB	16.56971803	1274.947528	-6.26574501	1.76E-07	Low	12	1543	CRYAB		1E-95	NM_001885.1	CRYAB
NPFFR2	0	237.9681711	-20	1.81E-07	Low	0	288	XP_005656581.1	ssc:100524634	5E-135	NM_053036.2	NPFFR2
KCNJ3	153.2698918	2.478835115	5.950268202	1.83E-07	High	111	3	XP_003483696.1	ssc:396586	0	NM_002239.3	KCNJ3
LOC102166606	109.083977	0.826278372	7.044595584	1.84E-07	High	79	1	XR_301715.1	ssc:102166606			
MCF2L	1495.417052	55.36065091	4.755543173	1.85E-07	High	1083	67	XP_005668614.1	ssc:100518447	0	NM_024979.4	MCF2L
GATA3	22.09295737	1609.590268	-6.186963126	1.85E-07	Low	16	1948	NM_001044567.1	ssc:733631	0	NM_002051.2	GATA3
SLIT3	86.99101965	5827.741356	-6.065926565	1.87E-07	Low	63	7053	XP_003134124.3	ssc:100513444	0	NM_003062.3	SLIT3
ZNF423	8.284859014	751.0870399	-6.502359147	1.88E-07	Low	6	909	ZNF423	ssc:100520077	0	NM_015069.3	ZNF423
LOC100628107	173.9820393	3.305113487	5.71809476	1.88E-07	High	126	4	XP_005659245.1	ssc:100628107	7E-88	NM_001244438.1	ARG1
MFSD6L	236.1184819	5.783948602	5.351312429	1.90E-07	High	171	7	XP_005674701.1	ssc:100514186	0	NM_152599.3	MFSD6L
TNN	0	235.4893359	-20	1.92E-07	Low	0	285	XP_005656793.1	ssc:100520506	0	NM_022093.1	TNN
LOC100625326	0	235.4893359	-20	1.92E-07	Low	0	285	XP_005665351.1	ssc:100625326	6E-36	NM_012307.3	EPB41L3
LOC102163367	876.8142457	31.39857813	4.803500104	1.93E-07	High	635	38	LOC102163367				
FBLN7	1529.937298	57.01320765	4.746032545	1.94E-07	High	1108	69	XP_005662337.1	ssc:100517582	0	NM_153214.2	FBLN7
LOC100523969	84.22939998	0	20	1.96E-07	High	61	0	LOC100523969				
HTR1B	15.18890819	1159.268556	-6.254052839	2.04E-07	Low	11	1403	NM_214298.1	ssc:397563	0	NM_000863.1	HTR1B
BHLHE41	8.284859014	738.6928643	-6.478353684	2.07E-07	Low	6	894	XP_003355589.1	ssc:100626912	0	NM_030762.2	BHLHE41
FAM78B	4.142429507	488.3305177	-6.881236983	2.11E-07	Low	3	591	XP_001925744.3	ssc:100153010	1E-152	NM_001017961.3	FAM78B
tRNA-Asn	908.5728719	33.05113487	4.780830515	2.13E-07	High	658	40	tRNA-Asn				
RASL11A	334.1559802	9.915340461	5.074715573	2.17E-07	High	242	12	XP_003482916.1	ssc:100736950	6E-130	NM_206827.1	RASL11A
LOC100620398	0	229.7053873	-20	2.22E-07	Low	0	278	XP_005674448.1	ssc:100620398	0	NM_006080.2	SEMA3A
LOC100515404	8.284859014	729.6038022	-6.460492291	2.23E-07	Low	6	883	XP_003130868.1	ssc:100515404	0	NM_032817.5	ITIH5
LOC100518586	2.761619671	400.7450103	-7.181026101	2.25E-07	Low	2	485	XP_003134207.3	ssc:100518586	2E-96	NM_001145161.2	UBE2QL1
ESRRB	128.4153147	1.652556743	6.279973647	2.27E-07	High	93	2	XP_003482338.2	ssc:100151940	0	NM_004452.3	ESRRB
TMEM255A	16.56971803	1215.455485	-6.196804194	2.33E-07	Low	12	1471	XP_003360475.1	ssc:100152537	1E-128	NM_017938.3	TMEM255A
ABCB1	38.6626754	2546.589942	-6.041481388	2.41E-07	Low	28	3082	XP_003130253.2	ssc:396910	0	NM_000927.4	ABCB1
SULT1C4	2.761619671	395.7873401	-7.16306701	2.42E-07	Low	2	479	XP_005662396.1	ssc:100623441	4E-147	NM_006588.2	SULT1C4
ROBO1	147.7466524	9523.684513	-6.010322482	2.42E-07	Low	107	11526	XP_003132784.3	ssc:100517310	0	NM_133631.3	ROBO1
LOC100738183	410.1005212	13.22045395	4.955133957	2.42E-07	High	297	16	XP_003480280.2	ssc:100738183	1E-53	NM_025218.2	ULBP1
LOC102157759	208.5022852	4.95767023	5.394257075	2.55E-07	High	151	6	XR_298275.1	ssc:102157759			
BACE2	117.368836	7459.64114	-5.989984931	2.58E-07	Low	85	9028	XP_005670420.1	ssc:100517374	0	NM_138992.2	BACE2
LOC100522330	15148.86471	576.7423035	4.715139022	2.58E-07	High	10971	698	XP_005666317.1	ssc:100522330	7E-161	NR_049767.1	SLC7A8
TGFB2	8.284859014	710.5993997	-6.422415513	2.60E-07	Low	6	860	XP_005653819.1	ssc:397084	0	NM_003238.3	TGFB2

CADPS2	2.761619671	389.1771131	-7.138768413	2.67E-07	Low	2	471	XP_005673275.1	ssc:100525671	0	NM_017954.10	CADPS2
SARDH	167.0779901	3.305113487	5.659678073	2.69E-07	High	121	4	NM_001253922.1	ssc:100154338	0	NM_007101.3	SARDH
GPR160	345.2024589	10.74161883	5.006159403	2.74E-07	High	250	13	XP_005674626.1	ssc:100626150	3E-55	NM_014373.2	GPR160
LOC100737466	12.42728852	936.9996735	-6.236465087	2.84E-07	Low	9	1134	XP_003484332.1	ssc:100737466	0	NM_014314.3	DDX58
LOC100512333	103.5607377	0.826278372	6.969633527	2.89E-07	High	75	1	XR_297265.1	ssc:100512333			
MYOD1	0	218.1374901	-20	2.99E-07	Low	0	264	NM_001002824.1	ssc:407604	6E-155	NM_002478.4	MYOD1
LOC102164230	80.08697047	0	20	3.01E-07	High	58	0	XR_304295.1	ssc:102164230			
MYHC	63.51725244	3943.00039	-5.956001535	3.01E-07	Low	46	4772	XP_003132042.1	ssc:396711	0	NM_002470.3	MYH3
ADRA1B	0	217.3112118	-20	3.06E-07	Low	0	263	XP_005672634.1	ssc:100519931	0	NM_000679.3	ADRA1B
LOC102164379	595.1290392	21.48323766	4.791919177	3.09E-07	High	431	26	XR_306462.1	ssc:102164379			
LOC100739280	2905.223894	115.678972	4.650450809	3.10E-07	High	2104	140	XP_005671858.1	ssc:100739280	2E-73	NM_013964.3	NRG1
LOC100626974	0	216.4849334	-20	3.13E-07	Low	0	262	XP_003361236.1	ssc:100626974	0	NM_001039523.2	CHRNA1
LOC100152729	11002.29277	431.31731	4.672910772	3.15E-07	High	7968	522	XP_001929591.1	ssc:100152729	6E-47	NM_005978.3	S100A2
PHYHD1	5688.936523	226.4002739	4.651211375	3.21E-07	High	4120	274	XP_003122259.1	ssc:100157556	1E-147	NM_174933.3	PHYHD1
ARHGAP27	1065.985193	41.31391859	4.689415684	3.24E-07	High	772	50	XP_005668771.1	ssc:100628007	0	NM_199282.2	ARHGAP27
LOC100621379	10956.72605	431.31731	4.666923347	3.28E-07	High	7935	522	XP_003360610.1	ssc:100621379	6E-47	NM_005978.3	S100A2
NES	372.8186556	23472.08971	-5.976328426	3.31E-07	Low	270	28407	XP_005663322.1	ssc:100153933	0	NM_006617.1	NES
LOC100738892	1582.408072	62.79715625	4.655278651	3.32E-07	High	1146	76	XP_005652485.1	ssc:100738892	2E-112	NM_007115.3	TNFAIP6
PDZRN3	104.9415475	6363.996019	-5.922275058	3.38E-07	Low	76	7702	XP_003132374.3	ssc:100511410	0	NM_015009.1	PDZRN3
PDGFRL	9.66566885	755.2184318	-6.287880597	3.41E-07	Low	7	914	XP_005672702.1	ssc:100736738	9E-141	NM_006207.2	PDGFRL
RGS14	182.2668983	4.131391859	5.463280861	3.41E-07	High	132	5	XP_005661511.1	ssc:100524790	0	NM_006480.4	RGS14
KCNMA1	0	213.1798199	-20	3.42E-07	Low	0	258	NM_214219.1	ssc:397434	5E-44	NM_002247.3	KCNMA1
LOC100156724	78.70616064	0	20	3.48E-07	High	57	0	XP_001925377.2	ssc:100156724	0	NM_002112.3	HDC
GDPD4	78.70616064	0	20	3.48E-07	High	57	0	XP_005667211.1	ssc:100511652	0	NM_182833.1	GDPD4
LOC100621385	78.70616064	0	20	3.48E-07	High	57	0	XP_005674345.1	ssc:100621385	0	NM_198182.2	GRHL1
CCR2	78.70616064	0	20	3.48E-07	High	57	0	NM_001001619.1	ssc:414372	4E-169	NM_001123396.1	CCR2
LOC100511133	78.70616064	0	20	3.48E-07	High	57	0	XP_005655746.1	ssc:100511133			
PRDM1	335.5367901	10.74161883	4.965187622	3.54E-07	High	243	13	XP_005659399.1	ssc:100154284	0	NM_182907.2	PRDM1
RTN1	372.8186556	12.39417558	4.910739838	3.63E-07	High	270	15	NM_001244641.1	ssc:100152394	0	NM_206857.1	RTN1
IQCA1	100.799118	0.826278372	6.930639395	3.65E-07	High	73	1	XP_005672363.1	ssc:100515512	7E-131	NR_073043.1	IQCA1
MFI2	2.761619671	367.6938754	-7.05684669	3.72E-07	Low	2	445	XP_001926353.5	ssc:100156949	0	NM_033316.3	MFI2
PHLDB2	41.42429507	2522.627869	-5.928306442	3.74E-07	Low	30	3053	XP_005674250.1	ssc:100623057			
RASGRP2	256.8306294	7.436505346	5.110048646	3.78E-07	High	186	9	NM_001244158.1	ssc:100626093	0	NM_153819.1	RASGRP2
LOC100523291	179.5052786	4.131391859	5.441254554	3.90E-07	High	130	5	XP_005654313.1	ssc:100523291	3E-15	NM_080736.1	WFDC2
CD36	0	208.2221497	-20	3.92E-07	Low	0	252	NM_001044622.1	ssc:733702	0	NM_001127444.1	CD36
LGR5	0	208.2221497	-20	3.92E-07	Low	0	252	XP_005664040.1	ssc:100151994	0	NM_003667.3	LGR5
LOC102160246	77.3253508	0	20	4.04E-07	High	56	0	XP_005658525.1	ssc:102160246	1E-120	NM_024938.2	PCNXL2
CRYM	788.4424162	30.57229975	4.688708406	4.05E-07	High	571	37	XP_003124605.2	ssc:100525333	2E-168	NM_001888.3	CRYM
LOC100621578	0	206.5695929	-20	4.10E-07	Low	0	250	XP_003356977.2	ssc:100621578	3E-97	NM_000857.2	GUCY1B3
PTHLH	0	205.7433146	-20	4.20E-07	Low	0	249	NM_213916.2	ssc:396951	1E-73	NM_198966.1	PTHLH
LOC100621672	5.523239343	507.3349202	-6.521280009	4.23E-07	Low	4	614	XP_003361780.1	ssc:100621672	2E-55	NM_006200.3	PCSK5
TIMP-3	127.0345049	7434.02651	-5.870851515	4.23E-07	Low	92	8997	XP_003126121.3	ssc:396775	2E-124	NM_000362.4	TIMP3
CHRM1	118.7496459	1.652556743	6.167079591	4.48E-07	High	86	2	NM_214034.1	ssc:397099	0	NM_000738.2	CHRM1
ANPEP	70.42130162	4080.1626	-5.856470943	4.48E-07	Low	51	4938	NM_214277.1	ssc:397520	0	NM_001150.2	ANPEP
TSTD1	12518.42197	508.987477	4.620278741	4.58E-07	High	9066	616	XP_005663867.1	ssc:100153230	4E-162	NM_003312.5	TST
LOC100626014	2.761619671	353.6471431	-7.00065215	4.67E-07	Low	2	428	XP_005653020.1	ssc:100626014	0	NM_171825.2	CAMK2A
PAPSS2	42.80510491	2503.623466	-5.870090932	4.69E-07	Low	31	3030	XP_005671332.1	ssc:100156262	0	NM_004670.3	PAPSS2
LOC102166772	75.94454097	0	20	4.70E-07	High	55	0	XR_307715.1	ssc:102166772			
LOC102160606	75.94454097	0	20	4.70E-07	High	55	0	XR_306517.1	ssc:102160606	3E-44	NM_198393.3	TEX14
LOC100514098	4.142429507	424.7070831	-6.679847212	4.78E-07	Low	3	514	XP_005653093.1	ssc:100514098	0	NM_001098814.1	SRL
IL1RL1	0	200.7856443	-20	4.83E-07	Low	0	243	XP_005662414.1	ssc:100127134	0	NR_104167.1	IL1RL1
LOC100517161	0	199.959366	-20	4.95E-07	Low	0	242	XP_005653357.1	ssc:100517161	1E-155	NM_014650.2	ZNF432
LOC100622715	0	199.1330876	-20	5.07E-07	Low	0	241	XP_005667638.1	ssc:100622715	0	NM_024788.2	C7orf63
LOC102158748	0	199.1330876	-20	5.07E-07	Low	0	241	XR_307291.1	ssc:102158748			
C1QTNF3	0	199.1330876	-20	5.07E-07	Low	0	241	NM_001145386.1	ssc:100270814	4E-167	NM_181435.5	C1QTNF3
FBLN5	103.5607377	5856.661099	-5.821529362	5.11E-07	Low	75	7088	XP_005656504.1	ssc:100155248	0	NM_006329.3	FBLN5
LOC102168112	487.425872	18.17812418	4.744907591	5.20E-07	High	353	22	XP_005652759.1	ssc:102168112	2E-24	NM_006914.3	RORB
CHRDL1	28.99700655	1716.180178	-5.887153242	5.22E-07	Low	21	2077	XP_005673875.1	ssc:100521058	0	NM_145234.3	CHRDL1
SAMD9	74.56373113	4197.494128	-5.814910443	5.29E-07	Low	54	5080	XP_005667663.1	ssc:100519098	0	NM_017654.3	SAMD9
ITGA9	0	197.4805308	-20	5.32E-07	Low	0	239	XP_005669412.1	ssc:100626707	0	NM_002207.2	ITGA9
CHRNG	12.42728852	841.9776608	-6.082198499	5.33E-07	Low	9	1019	XP_003133779.1	ssc:100522221	0	NM_005199.4	CHRNG
LOC100525180	265.1154885	8.262783717	5.003849242	5.38E-07	High	192	10	XR_135488.2	ssc:100525180			
LOC100621315	74.56373113	0	20	5.48E-07	High	54	0	XR_298397.1	ssc:100621315			
LOC100521229	74.56373113	0	20	5.48E-07	High	54	0	XP_003133773.1	ssc:100521229	0	NM_001631.3	ALPI
LOC100049650	140.8426032	7884.348223	-5.806835793	5.55E-07	Low	102	9542	XP_005657045.1	ssc:100049650	0	NM_015194.1	MYO1D
LOC100525848	1400.141173	58.66576439	4.576909654	5.56E-07	High	1014	71	XP_003135321.1	ssc:100525848	3E-18	NM_194324.2	TMSB15B
LOC100626977	2.761619671	342.9055243	-6.95615269	5.59E-07	Low	2	415	XP_003361612.2	ssc:100626977	5E-108	NR_033519.1	MASP1
PRICKLE2	5.523239343	481.7202907	-6.446537237	5.72E-07	Low	4	583	XP_005669761.1	ssc:100520050	0	NM_198859.3	PRICKLE2
LOC100739844	9.66566885	689.9424404	-6.157462629	5.79E-07	Low	7	835	XP_003482731.1	ssc:100739844	3E-154	NM_006080.2	SEMA3A
LOC102158049	353.4873179	12.39417558	4.833924241	5.84E-07	High	256	15	LOC102158049				
PENK	0	194.1754174	-20	5.86E-07	Low	0	235	XP_003125669.1	ssc:100152093	5E-25	NM_006211.3	PENK
PPFIA4	17.95052786	1108.039296	-5.947838968	5.93E-07	Low	13	1341	XP_005653788.1	ssc:100512361	0	NR_038265.1	PPFIA4
DFNA5	9.66566885	686.6373269	-6.150534909	5.96E-07	Low	7	831	XP_005673376.1	ssc:100522585	0	NM_004403.2	DFNA5
ANO1	2.761619671	338.7741324	-6.938665263	6.00E-07	Low	2	410	XP_005654695.1	ssc:100738137	0	NR_030691.1	ANO1
NR2E1	189.1709475	4.95767023	5.253884418	6.01E-07	High	137	6	XP_005659417.1	ssc:100155153	0	NM_003269.4	NR2E1
TRIM72	0	193.349139	-20	6.01E-07	Low	0	234	XP_003124542.2	ssc:100511188	0	NM_001008274.3	TRIM72
ANG1	2145.778485	91.71689926	4.548169758	6.01E-07	High	1554	111	NM_001044573.2	ssc:733639	2E-48	NM_001145.4	ANG
LOC100515260	258.2114393	14486.31241	-5.809993667	6.04E-07	Low	187	17532	XR_303102.1	ssc:100515260			
SLC28A3	73.18292129	0	20	6.40E-07	High	53	0	NM_001244637.1	ssc:100621750			
TBX5	2.761619671	332.9901838	-6.913821192	6.63E-07	Low	2	403	HighI00025DF922		0	NM_181486.2	TBX5
LOC102165620	11.04647869	741.1716994	-6.068149339	6.71E-07	Low	8	897	XP_005671013.1	ssc:102165620	3E-18	NM_080647.1	TBX1
WDR17	0	189.2177471	-20	6.80E-07	Low	0	229	XP_005671730.1	ssc:100157060	0	NM_181265.3	WDR17
HSPB7	0	189.2177471	-20	6.80E-07	Low	0	229	NM_001243388.1	ssc:100627988	7E-80	NM_014424.4	HSPB7
ADCY2	0	189.2177471	-20	6.80E-07	Low	0	229	XP_003134201.4	ssc:100517503	0	NM_020546.2	ADCY2
LOC100513619	272.0195376	14958.11736	-5.781074522	6.88E-07	Low	197	18103	XP_005668657.1	ssc:100513619	0	NM_020954.3	RNF213
LOC102163961	9.66566885	669.2854811	-6.11360834	6.92E-07	Low	7	810	XP_005656794.1	ssc:102163961	0	NM_022093.1	TNN
LOC100127164	2.761619671	330.5113487	-6.903041354	6.92E-07	Low	2	400	XP_003353536.1	ssc:100127164	1E-156	NM_000956.3	PTGER2
CPXM2	8.284859014	598.2255411	-6.17406855	7.15E-07	Low	6	724	XP_005671590.1	ssc:100153578	0	NM_198148.2	CPXM2
LOC100516621	2114.019859	92.54317763	4.513718482	7.44E-07	High	1531	112	XP_005656332.1	ssc:100516621	3E-55	NM_138573.3	NRG4
ZBTB8B	71.80211146	0	20	7.50E-07	High	52	0	ZBTB8B	ssc:102163469	2E-150	NM_001145720.1	ZBTB8B
LOC102165956	71.80211146	0	20	7.50E-07	High	52	0	XP_005659863.1	ssc:102165956	1E-100	NM_005503.3	APBA2
SLC13A5	682.1200589	28.09346464	4.601719226	7.50E-07	High	494	34	XP_003131921.2	ssc:100520976	0	NM_177550.4	SLC13A5
IGFBP7	608.9371376	34102.98724	-5.807461005	7.53E-07	Low	441	41273	NM_001163801.1	ssc:100302573	8E-111	NM_001553.2	IGFBP7
LOC100737747	6.904049179	523.8604877	-6.245596099	7.74E-07	Low	5	634	XP_003483564.1	ssc:100737747	0	NR_045675.1	ARHGAP22
BATF2	4.142429507	390.8296698	-6.559919036	7.77E-07	Low	3	473	XP_005660776.1	ssc:100515875	3E-90	NM_138456.3	BATF2
LOC102165113	183.6477082	4.95767023	5.211134771	7.78E-07	High	133	6	XR_306174.1	ssc:102165113			
MSLN	767.7302687	32.2248565	4.57435369	7.89E-07	High	556	39	XP_003124743.1	ssc:100524016	2E-124	NM_013404.4	MSLN
SYNJ2	23.47376721	1326.176787	-5.820079905	7.90E-07	Low	17	1605	XP_005654412.1	ssc:100516167	0	NM_003898.3	SYNJ2
LOC100738589	41.42429507	2205.336974	-5.734378158	8.26E-07	Low	30	2669	XP_005660660.1	ssc:100738589	1E-84	NM_053056.2	CCND1
LOC100517484	374.1994655	14.04673232	4.735501036	8.40E-07	High	271	17	XP_003129305.1	ssc:100517484	4E-57	NR_045756.2	COL25A1

LOC100739751	2.761619671	318.9434515	-6.851642201	8.52E-07	Low	2	386	XP_003481268.2	ssc:100739751	4E-171	NM_022055.1	KCNK12
LOC100622637	109.083977	5660.006846	-5.697292688	8.53E-07	Low	79	6850	LOC100622637		2E-68	NM_201279.1	NRP2
LOC100737392	146.3658426	3.305113487	5.468735291	8.54E-07	High	106	4	XP_003483393.2	ssc:100737392	0	NM_004540.3	NCAM2
LOC102159094	70.42130162	0	20	8.81E-07	High	51	0	XR_305155.1	ssc:102159094			
GIMAP4	70.42130162	0	20	8.81E-07	High	51	0	XP_003134607.2	ssc:100518347	3E-76	NM_018326.2	GIMAP4
DIRAS3	16274.22472	705.6417295	4.527509127	8.93E-07	High	11786	854	NM_001044598.2	ssc:733668	5E-70	NM_004675.2	DIRAS3
tRNA-Tyr	509.5188294	20.65695929	4.624435652	9.17E-07	High	369	25	tRNA-Tyr				
LOC100623058	2.761619671	314.8120596	-6.832832351	9.19E-07	Low	2	381	XP_003361152.2	ssc:100623058	8E-156	NM_005711.4	EDIL3
TSPAN1	109.083977	1.652556743	6.044595584	9.28E-07	High	79	2	NM_001243860.1	ssc:100511571	4E-98	NM_005727.3	TSPAN1
IRX2	1.380809836	246.2309548	-7.478353684	9.76E-07	Low	1	298	XP_005674585.1	ssc:100620587	2E-100	NM_033267.4	IRX2
CASQ1	0	177.6498499	-20	9.77E-07	Low	0	215	HighI0002330337		0	NM_001231.4	CASQ1
LOC102160748	13.80809836	820.4944231	-5.892906976	9.99E-07	Low	10	993	XR_308984.1	ssc:102160748			
DMRTB1	314.8246425	11.5678972	4.766349928	1.02E-06	High	228	14	XP_003128031.3	ssc:100515733	8E-93	NM_033067.1	DMRTB1
LOC100626670	1025.941708	45.44531045	4.496673523	1.02E-06	High	743	55	XP_005664518.1	ssc:100626670	0	NM_018092.4	NETO2
CELA1	69.04049179	0	20	1.04E-06	High	50	0	NM_213823.1	ssc:396766	5E-135	NM_001971.5	CELA1
LOC100519780	69.04049179	0	20	1.04E-06	High	50	0	XP_003127524.2	ssc:100519780	0	NM_033405.3	HELZ2
LOC100514323	1441.565469	65.27599137	4.464940085	1.08E-06	High	1044	79	XP_003132557.2	ssc:100514323			
LOC100523405	0	174.3447364	-20	1.09E-06	Low	0	211	XP_003133273.1	ssc:100523405	4E-16	NM_021641	ADAM12
PROM1	1.380809836	241.2732845	-7.449009723	1.10E-06	Low	1	292	XP_005666595.1	ssc:100511336	0	NM_006017.2	PROM1
PODXL	702.8322064	30.57229975	4.522883317	1.13E-06	High	509	37	XP_005657812.1	ssc:100512201	2E-100	NM_005397.3	PODXL
TMEM246	2.761619671	303.2441624	-6.778821417	1.14E-06	Low	2	367	XP_005660360.1	ssc:100621832	0	NM_032342.1	TMEM246
C1H14orf37	45.56672458	2280.528306	-5.645243596	1.15E-06	Low	33	2760	NM_001244542.1	ssc:100514619	0	NM_001001872.2	C14orf37
LOC100623862	2.761619671	300.7653273	-6.766979804	1.20E-06	Low	2	364	XP_005674074.1	ssc:100623862	5E-164	XP_003960890.2	LOC100509091
FHL2	160.1739409	7891.784728	-5.622640246	1.20E-06	Low	116	9551	XP_005662407.1	ssc:100513532	4E-120	NM_201557.3	FHL2
LOC102161109	165.6971803	8165.282869	-5.622881909	1.20E-06	Low	120	9882	XR_305383.1	ssc:102161109			
LOC100737693	0	171.0396229	-20	1.21E-06	Low	0	207	XP_003482794.2	ssc:100737693	5E-64	NM_014217.3	KCNK2
ZBTB32	67.65968195	0	20	1.22E-06	High	49	0	ZBTB32	ssc:100627119	0	NM_014383.1	ZBTB32
SLC46A2	4.142429507	361.0836484	-6.445712133	1.23E-06	Low	3	437	XP_005660391.1	ssc:100157182	0	NM_033051.3	SLC46A2
CRISPLD2	16.56971803	915.5164359	-5.787964829	1.24E-06	Low	12	1108	XP_005664373.1	ssc:100625354	0	NM_031476.3	CRISPLD2
RCVRN	0	170.2133446	-20	1.25E-06	Low	0	206	XR_306608.1	ssc:100516153			
TCF21	0	170.2133446	-20	1.25E-06	Low	0	206	XP_005652728.1	ssc:100152962	6E-84	NM_198392.2	TCF21
LOC100511846	190.5517573	5.783948602	5.041984371	1.26E-06	High	138	7	XP_003121317.2	ssc:100511846	2E-14	NM_030568.3	KHDC1
HOXB9	5.523239343	420.5756912	-6.25070701	1.26E-06	Low	4	509	XP_003131603.2	ssc:100523635	1E-145	NM_024017.4	HOXB9
SLCO4C1	207.1214754	6.610226974	4.969633527	1.27E-06	High	150	8	XP_003480930.1	ssc:100737557	0	NM_180991.4	SLCO4C1
GPR56	24.85457704	1284.862868	-5.691959027	1.28E-06	Low	18	1555	NM_001243056.1	ssc:100515029	6E-109	NM_201525.2	GPR56
KIAA0319	273.4003475	9.915340461	4.785208956	1.28E-06	High	198	12	XP_005665689.1	ssc:100738226	0	NM_014809.3	KIAA0319
ARMC2	0	169.3870662	-20	1.28E-06	Low	0	205	XP_005659424.1	ssc:100523341	0	NM_032131.5	ARMC2
tRNA-Thr	760.8262195	33.87741324	4.48917134	1.28E-06	High	551	41	tRNA-Thr				
PRR19	1271.725859	58.66576439	4.438125063	1.31E-06	High	921	71	XP_005655937.1	ssc:100513285	1E-112	NM_199285.2	PRR19
SLITRK2	11.04647869	656.0650272	-5.892180361	1.37E-06	Low	8	794	XP_005674008.1	ssc:100511615	0	NM_032539.4	SLITRK2
BEX1	3534.873179	166.0819527	4.41169124	1.40E-06	High	2560	201	NM_001244771.1	ssc:100513130	2E-49	NM_018476.3	BEX1
IL20RA	138.0809836	3.305113487	5.384671026	1.41E-06	High	100	4	XP_005659241.1	ssc:100622457	3E-135	NM_014432.3	IL20RA
EPHA3	8.284859014	531.296993	-6.00289759	1.43E-06	Low	6	643	NM_001195335.1	ssc:100337658	6E-153	NM_182644.2	EPHA3
SLITRK5	970.7093145	44.61903207	4.443308213	1.43E-06	High	703	54	XP_003357919.1	ssc:100155909	0	NM_015567.1	SLITRK5
LOC100515962	5.523239343	411.4866291	-6.219187096	1.43E-06	Low	4	498	XP_003482718.1	ssc:100515962	8E-173	NM_012395.3	CDK14
KIAA1210	0	166.0819527	-20	1.44E-06	Low	0	201	XP_005673928.1	ssc:100518589	0	NM_020721.1	KIAA1210
PRRX1	48.32834425	2323.494781	-5.587283026	1.44E-06	Low	35	2812	XP_003481450.1	ssc:100738910	9E-143	NM_022716	PRRX1
LOC100516442	12.42728852	709.7731213	-5.835774484	1.45E-06	Low	9	859	XP_003126812.2	ssc:100516442	0	NM_133642.3	LARGE
RASL10A	66.27887212	0	20	1.45E-06	High	48	0	XP_003132992.1	ssc:100513761	2E-108	NM_006477.4	RASL10A
LOC100737239	1.380809836	229.7053873	-7.378126237	1.46E-06	Low	1	278	XP_005661240.1	ssc:100737239	0	NM_001080421.2	UNC13A
NR4A1	1717.727436	80.97528043	4.406875762	1.47E-06	High	1244	98	HighI00025E09A7				
DDR2	276.1619671	13338.61175	-5.593950066	1.48E-06	Low	200	16143	XP_005655438.1	ssc:100154199	0	NM_006182.2	DDR2
TNFAIP6	5042.71752	237.9681711	4.405360885	1.51E-06	High	3652	288	NM_001159607.1	ssc:100286870	4E-149	NM_007115.3	TNFAIP6
LOC100153981	201.598236	6.610226974	4.930639395	1.60E-06	High	146	8	XP_003480454.1	ssc:100153981	0	NM_020168.5	PAK6
LOC102165358	2.761619671	285.8923166	-6.693813391	1.60E-06	Low	2	346	XR_298944.1	ssc:102165358			
LOC102161654	0	162.7768392	-20	1.61E-06	Low	0	197	XP_005672858.1	ssc:102161654	1E-145	NM_006065.3	SIRPB1
LOC102159728	102.1799278	1.652556743	5.950268202	1.61E-06	High	74	2	XR_307548.1	ssc:102159728			
LOC100524545	13.80809836	752.7395966	-5.768564313	1.65E-06	Low	10	911	XP_005652536.1	ssc:100524545	0	NM_138325.2	PCSK6
PPP1R17	64.89806228	0	20	1.72E-06	High	47	0	XP_003134851.1	ssc:100511608	2E-79	NM_006658.4	PPP1R17
LOC102161046	34.52024589	1641.815125	-5.571705131	1.72E-06	Low	25	1987	XR_308191.1	ssc:102161046			
TPM2	1140.548924	57079.30992	-5.645167655	1.76E-06	Low	826	69080	NM_001129947.1	ssc:396693	8E-150	NM_213674.1	TPM2
PCSK6	0	160.2980041	-20	1.76E-06	Low	0	194	XP_005660641.1	ssc:102164742	9E-132	NM_138325.2	PCSK6
LOC102163369	0	160.2980041	-20	1.76E-06	Low	0	194	XP_005658421.1	ssc:102163369	7E-54	NM_001286057.1	ADCY7
LMCD1	5.523239343	394.9610617	-6.160051972	1.82E-06	Low	4	478	NM_001008692.1	ssc:494020	2E-62	NM_014583.3	LMCD1
LOC100627808	4.142429507	334.6427406	-6.336000761	1.91E-06	Low	3	405	XP_005658038.1	ssc:100627808			
ZNF503	41.42429507	1911.181874	-5.527844119	1.91E-06	Low	30	2313	ZNF503	ssc:100521339	2E-174	NM_032772.4	ZNF503
TLR3	22.09295737	1075.81444	-5.605698897	1.98E-06	Low	16	1302	NM_001097444.1	ssc:100037937	0	NM_003265.2	TLR3
PSAT1	610.3179474	28975.92994	-5.569150137	2.00E-06	Low	442	35068	XP_005660231.1	ssc:100154160	4E-173	NM_058179.3	PSAT1
LOC102159412	0	156.1666123	-20	2.04E-06	Low	0	189	XP_005666935.1	ssc:102159412	9E-139	NM_024582.4	FAT4
LOC102159293	63.51725244	0	20	2.05E-06	High	46	0	XR_298801.1	ssc:102159293			
MNX1	63.51725244	0	20	2.05E-06	High	46	0	XP_003134570.1	ssc:100511165	2E-145	NM_005515.3	MNX1
LOC100621416	26.23538688	1238.591279	-5.561042318	2.09E-06	Low	19	1499	XP_005658770.1	ssc:100621416	1E-156	NR_046095.1	DACT1
MEGF10	1.380809836	215.6588655	-7.28709116	2.09E-06	Low	1	261	XP_005661666.1	ssc:100515713	0	NM_032446.2	MEGF10
ITGBL1	0	155.3403339	-20	2.10E-06	Low	0	188	XP_005668583.1	ssc:100525532	2E-175	NM_004791.2	ITGBL1
LOC100625270	0	155.3403339	-20	2.10E-06	Low	0	188	XP_005658030.1	ssc:100625270	4E-65	NM_003034.3	ST8SIA1
COL9A2	33860.21879	1578.19169	4.423246942	2.16E-06	High	24522	1910	XP_003356382.1	ssc:100621911	0	NM_001852.3	COL9A2
LOC102163032	81.46778031	0.826278372	6.623457886	2.19E-06	High	59	1	XR_302802.1	ssc:102163032			
PLA2G4A	82.84859014	3654.629238	-5.463104095	2.21E-06	Low	60	4423	XP_005667902.1	ssc:100520687	0	NM_024420.2	PLA2G4A
SERTAD4	63.51725244	2809.346464	-5.466942339	2.22E-06	Low	46	3400	XP_005667938.1	ssc:100511654	0	NM_019605.3	SERTAD4
LOC102163538	4.142429507	325.5536785	-6.296274483	2.24E-06	Low	3	394	XP_005668658.1	ssc:102163538	2E-59	NM_020954.3	RNF213
LOC100737186	4.142429507	324.7274001	-6.292608165	2.27E-06	Low	3	393	XR_305624.1	ssc:100737186			
MAPK4	0	152.8614988	-20	2.30E-06	Low	0	185	XP_005659518.1	ssc:100523414	0	NM_002747.3	MAPK4
LOC100622976	2.761619671	266.8879141	-6.594575519	2.39E-06	Low	2	323	XP_003356553.1	ssc:100622976	4E-104	NM_001452.1	FOXF2
NEURL1B	238.8801016	9.089062089	4.716011445	2.40E-06	High	173	11	XP_005674629.1	ssc:100522651	0	NR_015355.3	NEURL1B
SSC5D	20.71214754	981.6187056	-5.566613689	2.44E-06	Low	15	1188	XP_005664918.1	ssc:100621391	0	NM_182571.2	SSC5D
NLRP3	0	151.208942	-20	2.45E-06	Low	0	183	NM_001256770.1	ssc:100514823	0	NM_183395.2	NLRP3
LOC100621009	0	151.208942	-20	2.45E-06	Low	0	183	XP_005668267.1	ssc:100621009	0	NM_001029880.2	SFMBT2
LOC100620561	0	151.208942	-20	2.45E-06	Low	0	183	XP_005674328.1	ssc:100620561	0	NM_138557.2	TLR4
LOC102164132	62.13644261	0	20	2.45E-06	High	45	0	XR_307148.1	ssc:102164132			
LOC100157629	62.13644261	0	20	2.45E-06	High	45	0	LOC100157629				
PHYH	86.99101965	3767.003097	-5.436406934	2.46E-06	Low	63	4559	NM_001113447.1	ssc:100134962	4E-118	NM_006214.3	PHYH
SLPI	144.9850328	4.131391859	5.133132259	2.47E-06	High	105	5	NM_213870.1	ssc:396886			
SYNPR	0	150.3826637	-20	2.52E-06	Low	0	182	XP_003358538.2	ssc:100623290	4E-117	NM_144642.4	SYNPR
DDX58	125.6536951	5427.822624	-5.432848574	2.53E-06	Low	91	6569	NM_213804.2	ssc:396723	0	NM_014314.3	DDX58
TMEM229A	6.904049179	426.3596398	-5.948484324	2.56E-06	Low	5	516	XP_003134771.1	ssc:100524656	9E-164	NM_001136002.1	TMEM229A
UNC13A	11.04647869	587.4839223	-5.732890913	2.60E-06	Low	8	711	XP_005654971.1	ssc:100627038	0	NM_001080421.2	UNC13A
CD44	563.370413	25303.94886	-5.489134936	2.71E-06	Low	408	30624	K7GLL1		0	NM_001202557.1	CD44
BOK	28.99700655	1291.473095	-5.479969804	2.77E-06	Low	21	1563	XP_005657656.1	ssc:100627765	2E-49	NM_032515.4	BOK
GRIA4	0	147.9038285	-20	2.77E-06	Low	0	179	XP_003129869.2	ssc:100524886	0	NR_046356.1	GRIA4
TRIM9	2.761619671	259.4514087	-6.553805913	2.81E-06	Low	2	314	XP_001928246.2	ssc:100153738			

TDRKH	509.5188294	23.96207278	4.410310847	2.84E-06	High	369	29	NM_001244274.1	ssc:100622748	0	NM_006862.3	TDRKH
LOC102163288	6.904049179	418.0968561	-5.920250644	2.87E-06	Low	5	506	XP_005668656.1	ssc:102163288	2E-135	NM_020954.3	RNF213
SLA-DMA	95.27587867	1.652556743	5.849339293	2.90E-06	High	69	2	NM_001113705.1	ssc:445528	9E-105	NM_006120.3	HLA-DMA
LOC102166360	864.3869572	42.96647533	4.330393965	2.91E-06	High	626	52	LOC102166360				
LOC100627578	1973.177255	100.8059614	4.2908677	2.93E-06	High	1429	122	XP_003360596.1	ssc:100627578	2E-41	NM_001127688.2	BEX4
MCF2	60.75563277	0	20	2.93E-06	High	44	0	XP_001925660.1		0	NM_005369.4	MCF2
LOC102159104	60.75563277	0	20	2.93E-06	High	44	0	XR_300771.1	ssc:102159104			
CLDN3	60.75563277	0	20	2.93E-06	High	44	0	NM_001160075.1	ssc:431781			
LOC102158037	60.75563277	0	20	2.93E-06	High	44	0	XR_305151.1	ssc:102158037			
SOWAHB	60.75563277	0	20	2.93E-06	High	44	0	XP_005666837.1	ssc:100523320	0	NM_001029870.1	SOWAHB
CDH18	1.380809836	203.2644794	-7.201699669	2.94E-06	Low	1	246	XP_003359779.3	ssc:100625674	0	NM_004934.3	CDH18
OMD	0	146.2512718	-20	2.95E-06	Low	0	177	XP_003124829.1	ssc:100511925	0	NM_005014.2	OMD
TPMT	2692.57918	137.9884881	4.286368952	2.99E-06	High	1950	167	NM_001243675.1	ssc:100157630	1E-118	NM_000367.2	TPMT
BMP6	110.4647869	2.478835115	5.47778043	3.07E-06	High	80	3	NM_001168001.1	ssc:100155536	0	NM_001718.4	BMP6
SEMA3E	231.9760524	9.089062089	4.67370064	3.09E-06	High	168	11	XP_003130268.3	ssc:100524100	0	NM_012431.2	SEMA3E
LOC100624220	1.380809836	199.959366	-7.178048401	3.22E-06	Low	1	242	XP_005667639.1	ssc:100624220	2E-85	NM_012395.3	CDK14
PPAP2B	96.6566885	3982.661752	-5.364719578	3.29E-06	Low	70	4820	XP_005665445.1	ssc:100512419	1E-178	NM_177414.1	PPAP2B
LOC100739745	8.284859014	458.5844963	-5.790566624	3.36E-06	Low	6	555	XP_003484078.1	ssc:100739745	0	NM_001127500.1	MET
RPRML	109.083977	2.478835115	5.459633084	3.41E-06	High	79	3	XP_005668755.1	ssc:100738728	2E-52	NM_203400.4	RPRML
LOC100510930	2080.880422	108.2424667	4.264855536	3.42E-06	High	1507	131	XP_003133048.1	ssc:100510930	0	NM_080386.3	TUBA3D
LOC100620918	139.4617934	4.131391859	5.077098224	3.44E-06	High	101	5	XP_005655043.1	ssc:100620918	0	NR_003149.1	GPR98
LOC102162556	124.2728852	3.305113487	5.232667932	3.45E-06	High	90	4	XR_299180.1	ssc:102162556			
B4GALNT2	363.1529868	16.52556743	4.457805731	3.45E-06	High	263	20	NM_001244330.1	ssc:100621328	0	NM_153446.2	B4GALNT2
LOC100158117	214.0255245	8.262783717	4.695011147	3.47E-06	High	155	10	XP_005665718.1	ssc:100158117	2E-52	NM_003539.3	HIST1H4D
OPRK1	59.37482294	0	20	3.52E-06	High	43	0	XP_003355107.2	ssc:100152118	0	NM_001282904.1	OPRK1
MPP3	183.6477082	6.610226974	4.796097272	3.56E-06	High	133	8	XP_003483023.1	ssc:100512807	0	NR_003562.2	MPP3
PODNL1	23.47376721	1022.932624	-5.445517922	3.60E-06	Low	17	1238	XP_003123408.2	ssc:100513269	0	NM_024825.3	PODNL1
LOC102161846	92.51425899	1.652556743	5.806904027	3.70E-06	High	67	2	XR_300659.1	ssc:102161846			
LOC102163456	4.142429507	298.2864922	-6.17007769	3.71E-06	Low	3	361	XP_005673399.1	ssc:102163456			
LOC100152091	93.89506883	3790.138891	-5.335057515	3.71E-06	Low	68	4587	XP_005670217.1	ssc:100152091	2E-147	NM_053032.2	MYLK
LOC100516232	0	140.4673232	-20	3.71E-06	Low	0	170	XP_003133753.2	ssc:100516232			
LOC100524008	165.6971803	6728.384781	-5.343639255	3.75E-06	Low	120	8143	XP_005660013.1	ssc:100524008	1E-35	NM_053064.4	GNG2
LOC102158176	11.04647869	550.3013956	-5.638563531	3.80E-06	Low	8	666	XP_005658880.1	ssc:102158176	6E-71	NM_001999.3	FBN2
TRANK1	13.80809836	651.1073569	-5.559308888	3.84E-06	Low	10	788	XP_005652448.1	ssc:102161776	0	NM_014831.2	TRANK1
KCNK12	11.04647869	548.6488388	-5.634224595	3.87E-06	Low	8	664	XP_005674663.1	ssc:100521189	6E-109	NM_022055.1	KCNK12
PTGS2	93.89506883	3762.045426	-5.324324067	3.88E-06	Low	68	4553	NM_214321.1	ssc:397590	0	NM_000963	PTGS2
LOC100511782	760.8262195	38.83508347	4.292134493	3.89E-06	High	551	47	XP_003132905.1	ssc:100511782	0	NM_007197.3	FZD10
KCNQ5	12.42728852	595.746706	-5.583115612	4.01E-06	Low	9	721	XP_003480326.1	ssc:100154391	0	NM_019842.3	KCNQ5
ENPP6	2.761619671	242.9258413	-6.458857509	4.09E-06	Low	2	294	XP_005671763.1	ssc:100152050	1E-169	NM_153343.3	ENPP6
FOXS1	48.32834425	1942.580452	-5.328960971	4.09E-06	Low	35	2351	XP_003134430.1	ssc:100524116	2E-154	NM_004118.3	FOXS1
CCL5	1.380809836	191.6965822	-7.117166159	4.10E-06	Low	1	232	NM_001129946.1	ssc:396613	3E-37	NM_002985.2	CCL5
ARHGAP24	0	137.9884881	-20	4.10E-06	Low	0	167	XP_003129392.2	ssc:100522450	0	NM_031305.2	ARHGAP24
GABRE	0	137.9884881	-20	4.10E-06	Low	0	167	XP_003135510.1	ssc:100516716	0	NM_021990.2	GABRE
PCNXL2	106.3223574	2.478835115	5.422638876	4.22E-06	High	77	3	XP_005671027.1	ssc:100158027	0	NM_024938.2	PCNXL2
KLF7	5.523239343	341.2529675	-5.949183135	4.23E-06	Low	4	413	NM_001097487.1	ssc:100038003			
LOC102158418	0	137.1622097	-20	4.24E-06	Low	0	166	XP_005658586.1	ssc:102158418	5E-74	NM_133631.3	ROBO1
TRIM34	2.761619671	240.4470062	-6.444060507	4.34E-06	Low	2	291	XP_005667139.1	ssc:100738479	1E-146	NM_001003819.3	TRIM6-TRIM34
PLAU	104.9415475	4118.997683	-5.29463544	4.38E-06	Low	76	4985	NM_213945.1	ssc:396985	0	NM_002658.3	PLAU
LOC102159188	0	136.3359313	-20	4.39E-06	Low	0	165	XR_300828.1	ssc:102159188			
LOC100627356	52.47077376	2076.437548	-5.306452606	4.40E-06	Low	38	2513	XR_297923.1	ssc:100627356			
LOC100520702	1.380809836	189.2177471	-7.098388952	4.42E-06	Low	1	229	XR_308834.1	ssc:100520702			
PLLP	164.3163705	5.783948602	4.828277677	4.47E-06	High	119	7	XP_003127021.2	ssc:100516614	1E-84	NM_015993.2	PLLP
LOC100620286	4.142429507	288.3711517	-6.121305889	4.50E-06	Low	3	349	XP_005668185.1	ssc:100620286	9E-135	NM_173576.2	MKX
SNAI2	1.380809836	188.3914688	-7.092075178	4.53E-06	Low	1	228	NM_001128439.1	ssc:641345	2E-144	NM_003068.4	SNAI2
PTER	1.380809836	188.3914688	-7.092075178	4.53E-06	Low	1	228	NM_001244248.1	ssc:100625702	0	NM_030664.4	PTER
SYT7	74.56373113	0.826278372	6.495702338	4.53E-06	High	54	1	XP_003122697.4	ssc:100521541	0	NM_004200.3	SYT7
AHNAK2	8.284859014	434.6224235	-5.713141652	4.58E-06	Low	6	526	XP_005656542.1	ssc:100522557	6E-135	NM_138420.2	AHNAK2
ZC3H12D	4.142429507	287.5448734	-6.117166159	4.58E-06	Low	3	348	ZC3H12D	ssc:100156984	0	NM_207360.2	ZC3H12D
RFTN1	4.142429507	287.5448734	-6.117166159	4.58E-06	Low	3	348	XP_005669346.1	ssc:100155924	3E-76	NM_015150.1	RFTN1
SIGIRR	3740.613845	200.7856443	4.219547011	4.64E-06	High	2709	243	XP_005655097.1	ssc:100626800	0	NM_021805.2	SIGIRR
RNF43	0	134.6833746	-20	4.70E-06	Low	0	163	XP_005669052.1	ssc:100517305	0	NM_017763.4	RNF43
GPNMB	0	134.6833746	-20	4.70E-06	Low	0	163	NM_001098584.1	ssc:100049669			
LOC100513943	89.75263932	1.652556743	5.763182649	4.75E-06	High	65	2	XP_005658103.1	ssc:100513943	1E-118	NM_015464.2	SOSTDC1
LOC102164569	205.7406655	8.262783717	4.638055262	4.85E-06	High	149	10	XP_005671855.1	ssc:102164569	1E-33	NM_013964.3	NRG1
LOC100736608	0	133.8570962	-20	4.87E-06	Low	0	162	XP_005667737.1	ssc:100736608	8E-88	NM_006080.2	SEMA3A
KCNQ4	262.3538688	11.5678972	4.503315522	4.95E-06	High	190	14	XP_003128158.3	ssc:100526228	0	NM_172163.2	KCNQ4
ECEL1	13.80809836	623.0138923	-5.495677782	4.96E-06	Low	10	754	XP_003133775.1	ssc:100521587	0	NM_004826.2	ECEL1
LOC100620852	2.761619671	233.8367792	-6.403843407	5.09E-06	Low	2	283	XP_005658148.1	ssc:100620852	0	NM_001080826.1	SGK223
CLDN11	8.284859014	426.3596398	-5.685449919	5.11E-06	Low	6	516	NM_001161641.1	ssc:100302016	5E-101	NM_005602.5	CLDN11
SDS	548.1815048	28.09346464	4.286347192	5.12E-06	High	397	34	XP_001928337.1	ssc:100155595	1E-138	NM_006843.2	SDS
TFAP2D	56.61320327	0	20	5.14E-06	High	41	0	XP_003356687.1	ssc:100155785	0	NM_172238.3	TFAP2D
LOC100513452	56.61320327	0	20	5.14E-06	High	41	0	LOC100513452	ssc:100513452	3E-72	NM_171999.3	SALL3
LOC102163860	0	132.2045395	-20	5.22E-06	Low	0	160	XP_005673333.1	ssc:102163860	7E-65	NM_005020.2	PDE1C
LOC100624585	0	132.2045395	-20	5.22E-06	Low	0	160	XP_005660189.1	ssc:100624585	5E-159	NM_152629.3	GLIS3
BCL6B	103.5607377	2.478835115	5.384671026	5.25E-06	High	75	3	XP_005669250.1	ssc:100517187	0	NM_181844.3	BCL6B
CABLES1	16.56971803	712.2519564	-5.425766722	5.32E-06	Low	12	862	XP_005665371.1	ssc:100526114	0	NR_023359.1	CABLES1
TMEM140	8.284859014	423.0545263	-5.674222663	5.35E-06	Low	6	512	XP_003134698.1	ssc:100523705	5E-54	NM_018295.4	TMEM140
LOC100525572	19.3313377	808.9265259	-5.386995291	5.35E-06	Low	14	979	XP_005663306.1	ssc:100525572	2E-14	NM_002348.3	LY9
MYOM1	13.80809836	614.7511086	-5.47641588	5.36E-06	Low	10	744	XP_005665357.1	ssc:100522496	0	NM_019856.1	MYOM1
FOLR2	19.3313377	806.4476908	-5.382567579	5.44E-06	Low	14	976	NM_213853.1	ssc:396853	3E-106	NM_001113536.1	FOLR2
ADAM3A	173.9820393	6.610226974	4.71809476	5.64E-06	High	126	8	NM_001097513.1	ssc:100048948	1E-103	NM_014237.2	ADAM18
RAB27B	1222.016705	66.92854811	4.190498478	5.73E-06	High	885	81	XP_001925846.1	ssc:100154771	2E-121	NM_004163.4	RAB27B
CCRN4L	4103.766832	225.5739955	4.185276096	5.80E-06	High	2972	273	XP_005653649.1	ssc:100513746	0	NM_012118.3	CCRN4L
C8H4orf19	370.057036	18.17812418	4.347472408	5.80E-06	High	268	22	XP_003482412.1	ssc:100738623	2E-85	NM_018302.2	C4orf19
tRNA-Pro	158.7931311	5.783948602	4.778949965	5.97E-06	High	115	7	tRNA-Pro				
LOC100736999	86.99101965	1.652556743	5.71809476	6.14E-06	High	63	2	XP_003482726.1	ssc:100736999	1E-118	NM_015464.2	SOSTDC1
WNT11	255.4498196	11.5678972	4.464841375	6.22E-06	High	185	14	XP_005667195.1	ssc:100521912	0	NM_004626.2	WNT11
PALM2	0	128.0731476	-20	6.23E-06	Low	0	155	NM_001204195.1	ssc:100152369	0	NM_053016.5	PALM2
DPEP1	0	128.0731476	-20	6.23E-06	Low	0	155	NM_214108.1	ssc:397196	0	NM_004413.3	DPEP1
CGNL1	2.761619671	225.5739955	-6.351942305	6.24E-06	Low	2	273	XP_005659624.1	ssc:102161202	7E-178	NM_032866.4	CGNL1
LOC100522769	2.761619671	225.5739955	-6.351942305	6.24E-06	Low	2	273	XP_003134501.1	ssc:100522769	1E-90	NM_003881.2	WISP2
LOC100518491	55.23239343	0	20	6.25E-06	High	40	0	XP_005664662.1	ssc:100518491	1E-68	NM_178820.3	FBXO27
GCS	1107.409488	61.14459951	4.178819897	6.30E-06	High	802	74	Y17718	ssc:397342	4E-143	NM_173849.2	GSC
LOC100510899	2.761619671	224.7477171	-6.346648005	6.37E-06	Low	2	272	XP_003125029.3	ssc:100510899	4E-46	NM_032181.2	EVA1A
FAR2	19.3313377	783.3118964	-5.340573491	6.44E-06	Low	14	948	XP_003355581.1	ssc:100512348	0	NM_018099.4	FAR2
NTNG2	0	127.2468692	-20	6.46E-06	Low	0	154	XP_003122308.3	ssc:100512264	0	NM_014917.2	NTNG1
LOC102160132	0	127.2468692	-20	6.46E-06	Low	0	154	XR_308188.1	ssc:102160132			
ADAMTS9	2.761619671	223.9214387	-6.341334205	6.51E-06	Low	2	271	XP_003132346.3	ssc:100520218	0	NM_182920.1	ADAMTS9
AQP3	2107.115809	118.1578072	4.156482746	6.58E-06	High	1526	143	NM_001110172.1	ssc:100126235	8E-161	NM_004925.4	AQP3
ANGPT4	2149.920914	120.6366423	4.155543506	6.62E-06	High	1557	146	XP_003134398.2	ssc:100515978	0	NM_015985.2	ANGPT4

LOC100154987	13.80809836	589.9627574	-5.417037333	6.79E-06	Low	10	714	XP_003480443.2	ssc:100154987	0	NM_002373.5	MAP1A
CBWD2	8179.917467	451.147991	4.180413621	6.84E-06	High	5924	546	XP_005660199.1	ssc:100156293	0	NM_172003.3	CBWD2
ARHGEF6	67.65968195	2463.962104	-5.186539862	6.85E-06	Low	49	2982	XP_005657985.1	ssc:100622228	0	NM_004840.2	ARHGEF6
LOC100522935	156.0315114	5.783948602	4.753638877	6.91E-06	High	113	7	XR_304390.1	ssc:100522935			
UNC45B	12.42728852	542.0386119	-5.446812167	6.92E-06	Low	9	656	HighI00005FE9DD		0	NM_173167.2	UNC45B
TMPRSS11F	0	125.5943125	-20	6.95E-06	Low	0	152	XP_003129105.3	ssc:100513296	0	NM_207407.2	TMPRSS11F
PIEZO2	0	125.5943125	-20	6.95E-06	Low	0	152	XP_005674226.1	ssc:100620725	0	NM_173817.1	PIEZO2
LOC102159707	17.95052786	726.2986887	-5.338464801	6.96E-06	Low	13	879	XR_304052.1	ssc:102159707			
LOC100525814	20.71214754	817.1893096	-5.302121279	7.07E-06	Low	15	989	XP_005662638.1	ssc:100525814	9E-139	NM_016932.4	SIX2
LOC102164037	16.56971803	677.5482648	-5.353702763	7.10E-06	Low	12	820	XP_005664371.1	ssc:102164037			
LOC102159872	70.42130162	0.826278372	6.413240178	7.18E-06	High	51	1	XP_005668770.1	ssc:102159872	2E-86	NM_001006607.2	LRRC37A2
LHFPL2	8.284859014	401.5712887	-5.599035167	7.21E-06	Low	6	486	NM_001243213.1	ssc:100523234	4E-118	NM_005779.2	LHFPL2
LOC100524114	291.3508753	14.04673232	4.374451184	7.37E-06	High	211	17	XP_005657692.1	ssc:100524114	7E-165	NR_015355.3	NEURL1B
LOC100737399	2.761619671	218.9637685	-6.309033713	7.39E-06	Low	2	265	XP_005665066.1	ssc:100737399	2E-100	NM_152942.2	TNFRSF8
LOC102163792	0	123.9417558	-20	7.49E-06	Low	0	150	XP_005667045.1	ssc:102163792	6E-59	NM_003728.3	UNC5C
FAM83F	386.626754	19.83068092	4.285135352	7.49E-06	High	280	24	XP_005663834.1	ssc:100155438	0	NM_138435.2	FAM83F
KNDC1	2.761619671	218.1374901	-6.303579283	7.55E-06	Low	2	264	XP_001925624.3	ssc:100152900			
KIF1A	102.1799278	3651.324125	-5.159236014	7.56E-06	Low	74	4419	XP_003133880.3	ssc:100517246	0	NM_004321.6	KIF1A
LOC100628233	53.85158359	0	20	7.62E-06	High	39	0	XP_003356094.2	ssc:100628233	0	NM_032803.5	SLC7A3
LOC102166884	53.85158359	0	20	7.62E-06	High	39	0	XR_300733.1	ssc:102166884			
LOC100621290	53.85158359	0	20	7.62E-06	High	39	0	XP_005668080.1	ssc:100621290	0	NM_006180.3	NTRK2
LOC100623445	619.9836162	33.87741324	4.193834466	7.67E-06	High	449	41	XP_005656036.1	ssc:100623445	1E-48	NM_178545.3	TMEM52
TMEM45A	28.99700655	1079.119553	-5.217806923	7.85E-06	Low	21	1306	XP_005654125.1	ssc:100522901	3E-111	NM_018004.1	TMEM45A
TPM1	3149.627235	128639.9746	-5.352014123	7.95E-06	Low	2281	155686	NM_001097483.2	ssc:100037999	1E-149	NM_001018020.1	TPM1
MLXIPL	84.22939998	1.652556743	5.671552174	7.98E-06	High	61	2	XP_003481050.2	ssc:100170769	0	NM_032994.2	MLXIPL
LOC100737919	1.380809836	170.2133446	-6.945685691	8.04E-06	Low	1	206	XR_308589.1	ssc:100737919			
TMEM26	0	122.289199	-20	8.07E-06	Low	0	148	NM_001244582.1	ssc:100157089	6E-172	NM_178505.6	TMEM26
LOC100515339	6609.936684	373.477824	4.145541964	8.10E-06	High	4787	452	XP_003131485.1	ssc:100515339	0	NM_002276.4	KRT19
RNASE1	247.1649606	11.5678972	4.417275691	8.22E-06	High	179	14	NM_001167655.1	ssc:100312980	2E-51	NM_198235.2	RNASE1
XIRP2	0	121.4629206	-20	8.38E-06	Low	0	147	NM_214396.1	ssc:397689	0	NM_152381.5	XIRP2
LOC100522432	1891.709475	109.0687451	4.116380895	8.39E-06	High	1370	132	XP_005662460.1	ssc:100522432	1E-88	NM_022912.2	REEP1
LOC100515458	69.04049179	0.826278372	6.384671026	8.42E-06	High	50	1	XP_005673467.1	ssc:100515458	0	NM_014728.3	FRMPD4
GARNL3	151.8890819	5.783948602	4.714819628	8.65E-06	High	110	7	XP_005660494.1	ssc:100156351	0	NR_104591.1	GARNL3
ADM2	0	120.6366423	-20	8.70E-06	Low	0	146	NM_001206384.1	ssc:100517471	3E-47	NM_024866.5	ADM2
LOC102164566	0	120.6366423	-20	8.70E-06	Low	0	146	XR_300984.1	ssc:102164566			
PLEKHF1	62.13644261	2174.764674	-5.129275841	8.72E-06	Low	45	2632	XP_005653317.1	ssc:100522869	7E-133	NM_024310.4	PLEKHF1
HS3ST5	191.9325672	8.262783717	4.537827814	8.73E-06	High	139	10	XP_005659453.1	ssc:100155403	0	NM_153612.3	HS3ST5
FN3K	1540.983777	89.23806415	4.110048646	8.84E-06	High	1116	108	XP_005656937.1	ssc:100523990	7E-167	NM_022158.3	FN3K
LOC102162560	9.66566885	431.31731	-5.479736238	8.85E-06	Low	7	522	XP_005667735.1	ssc:102162560	6E-67	NM_152754.2	SEMA3D
GUCY1A2	258.2114393	12.39417558	4.3808187	8.90E-06	High	187	15	XP_003130141.3	ssc:100522091	0	NM_001256424.1	GUCY1A2
STEAP2	1.380809836	166.9082311	-6.917396647	8.98E-06	Low	1	202	XP_005667635.1	ssc:100623287	0	NM_152999.3	STEAP2
CCDC42	0	119.8103639	-20	9.04E-06	Low	0	145	NM_001244077.1	ssc:100514007	8E-156	NM_144681.2	CCDC42
LOC100514839	851.9596687	31767.09828	-5.220604369	9.07E-06	Low	617	38446	XP_005663981.1	ssc:100514839	0	NM_002332.2	LRP1
LOC100157489	110.4647869	3.305113487	5.062742931	9.17E-06	High	80	4	XP_003359120.2	ssc:100157489	0	NM_198827.3	GPR133
LOC100738690	96.6566885	2.478835115	5.285135352	9.26E-06	High	70	3	XR_302950.1	ssc:100738690			
LOC100622674	52.47077376	0	20	9.33E-06	High	38	0	XP_003360468.1	ssc:100622674	0	NM_003604.2	IRS4
LOC102159414	52.47077376	0	20	9.33E-06	High	38	0	XR_305368.1	ssc:102159414			
LOC100626938	52.47077376	0	20	9.33E-06	High	38	0	XP_005658251.1	ssc:100626938	8E-37	NM_032414.2	PROK1
LOC100512117	52.47077376	0	20	9.33E-06	High	38	0	LOC100512117				
UBASH3A	52.47077376	0	20	9.33E-06	High	38	0	XP_003483414.1	ssc:100627915	0	NM_018961.3	UBASH3A
CSF2RB	52.47077376	0	20	9.33E-06	High	38	0	XP_001924814.3	ssc:100153254	0	NM_000395.2	CSF2RB
KRT13	52.47077376	0	20	9.33E-06	High	38	0	XP_003131484.1	ssc:100515166	7E-175	NM_153490.2	KRT13
LIPC	52.47077376	0	20	9.33E-06	High	38	0	NM_001143714.1	ssc:100233189	2E-162	NM_000236.2	LIPC
LOC102166489	0	118.9840855	-20	9.40E-06	Low	0	144	XP_005670814.1	ssc:102166489	5E-55	NM_014653.2	WSCD2
GDAP1	0	118.9840855	-20	9.40E-06	Low	0	144	XP_005663078.1	ssc:100157012	0	NR_046346.1	GDAP1
LOC100737142	5.523239343	296.6339355	-5.747025198	9.43E-06	Low	4	359	XR_303908.1	ssc:100737142			
TNFRSF1B	77.3253508	2657.311243	-5.102881933	9.50E-06	Low	56	3216	NM_001097441.2	ssc:100037306			
CYBRD1	115.9880262	3986.743144	-5.103180968	9.54E-06	Low	84	4825	NM_001128452.1	ssc:100144591	5E-137	NM_024843.3	CYBRD1
FAM212A	28.99700655	1042.763305	-5.168363936	9.56E-06	Low	21	1262	XP_003132261.3	ssc:100512437	4E-110	NM_203370.1	FAM212A
LOC100625277	806.3929441	46.27158882	4.123284473	9.74E-06	High	584	56	XP_005658718.1	ssc:100625277	5E-61	NR_109837.1	HighP1
CHRNA1	0	118.1578072	-20	9.77E-06	Low	0	143	XP_001924357.4	ssc:396587	0	NM_001039523.2	CHRNA1
ADCY4	1148.833783	66.92854811	4.101404551	9.78E-06	High	832	81	XP_001927591.3	ssc:100152067	0	NM_139247.3	ADCY4
LOC100521067	30.37781639	1082.424667	-5.155104642	9.83E-06	Low	22	1310	XP_005661132.1	ssc:100521067	0	NM_182964.5	NAV2
FZD3	1118.455967	65.27599137	4.098812186	9.99E-06	High	810	79	XR_307344.1	ssc:100153078	0	NM_145866.1	FZD3
LOC102160653	40.04348524	1390.6265	-5.11802363	1.01E-05	Low	29	1683	XP_005670311.1	ssc:102160653	0	NM_182909.3	FILIP1L
FILIP1L	40.04348524	1389.800221	-5.117166159	1.01E-05	Low	29	1682	XP_005670309.1	ssc:100157058	0	NM_182909.3	FILIP1L
FAM65C	0	117.3315288	-20	1.02E-05	Low	0	142	XP_005673066.1	ssc:100153156	0	NM_080829.2	FAM65C
PTH1R	2.761619671	206.5695929	-6.224969449	1.03E-05	Low	2	250	NM_214382.1	ssc:397675	0	NM_001184744.1	PTH1R
LOC100525496	81.46778031	1.652556743	5.623457886	1.04E-05	High	59	2	XP_003121308.1	ssc:100525496			
LOC100621915	582.7017507	33.05113487	4.13998593	1.07E-05	High	422	40	XP_003356617.1	ssc:100621915	2E-68	NM_003520.3	HIST1H2BN
CDH13	16.56971803	629.6241193	-5.247869851	1.08E-05	Low	12	762	NM_001109945.1	ssc:100126163	1E-51	NM_001257.4	CDH13
LOC102166397	9.66566885	415.618021	-5.426244832	1.09E-05	Low	7	503	XR_306138.1	ssc:102166397			
LOC100621905	0	115.678972	-20	1.10E-05	Low	0	140	LOC100621905				
KBTBD11	0	115.678972	-20	1.10E-05	Low	0	140	XP_005657582.1	ssc:100625139	0	NM_014867.2	KBTBD11
LOC102165781	474.9985835	26.4409079	4.167079591	1.10E-05	High	344	32	XR_299236.1	ssc:102165781			
ARHGEF26	1763.29416	104.9373532	4.070672959	1.11E-05	High	1277	127	XP_005669998.1	ssc:100626582	0	NM_015595.3	ARHGEF26
MYLPF	19.3313377	713.0782348	-5.205046991	1.11E-05	Low	14	863	NM_001006592.1	ssc:474162	8E-93	NM_013292.3	MYLPF
IL17RD	8.284859014	371.8252673	-5.488003854	1.12E-05	Low	6	450	XP_003132314.3	ssc:100513377	0	NM_017563.3	IL17RD
THEMIS2	394.911613	21.48323766	4.200246455	1.12E-05	High	286	26	XP_005653435.1	ssc:100514466	0	NM_004848.3	THEMIS2
FBXO32	27.61619671	970.0508084	-5.134473762	1.13E-05	Low	20	1174	NM_001044588.1	ssc:733657	1E-145	NM_148177.2	FBXO32
LOC102164089	5.523239343	287.5448734	-5.70212866	1.13E-05	Low	4	348	XP_005656795.1	ssc:102164089	3E-117	NM_022093.1	TNN
LOC102167554	107.7031672	3.305113487	5.026217055	1.13E-05	High	78	4	XR_305889.1	ssc:102167554			
GABRA1	133.9385541	4.95767023	4.755765178	1.14E-05	High	97	6	XP_005672614.1	ssc:100516706	0	NM_001127648.1	GABRA1
SEMA3A	0	114.8526937	-20	1.14E-05	Low	0	139	XP_005667738.1	ssc:102163327	2E-152	NM_006080.2	SEMA3A
LOC100739707	0	114.8526937	-20	1.14E-05	Low	0	139	XP_003483969.1	ssc:100739707	2E-90	NM_006065.3	SIRPB1
LOC100622474	51.08996392	0	20	1.15E-05	High	37	0	XP_003361735.2	ssc:100622474	4E-41	NM_173803.3	MPV17L
SLC6A1	51.08996392	0	20	1.15E-05	High	37	0	XP_005669835.1	ssc:100156668	0	NM_003042.3	SLC6A1
CLDN8	51.08996392	0	20	1.15E-05	High	37	0	NM_001161646.1	ssc:100302021	3E-114	NM_199328.2	CLDN8
LOC100628123	51.08996392	0	20	1.15E-05	High	37	0	XP_003355108.1	ssc:100628123	2E-156	NM_005285.3	NPBWR1
ASB18	51.08996392	0	20	1.15E-05	High	37	0	XP_003483831.1	ssc:100621336	3E-178	NM_212556.2	ASB18
LOC102160070	51.08996392	0	20	1.15E-05	High	37	0	XR_306367.1	ssc:102160070			
LOC100037976	2522.73957	151.208942	4.060375916	1.18E-05	High	1827	183	XP_001925729.3	ssc:100037976	2E-161	NM_033012.3	TNFSF11
ARPP21	0	114.0264153	-20	1.19E-05	Low	0	138	XP_005669408.1	ssc:100512986	0	NM_198399.1	ARPP21
CALD1	1986.985354	73879.20177	-5.216515135	1.19E-05	Low	1439	89412	XP_005673207.1	ssc:100524227	0	NM_033157.3	CALD1
LOC102158695	1.380809836	158.6454474	-6.844147665	1.19E-05	Low	1	192	XP_005669287.1	ssc:102158695	4E-59	NM_004822.2	NTN1
LOC102157425	80.08697047	1.652556743	5.598795831	1.20E-05	High	58	2	XR_305501.1	ssc:102157425			
LOC100155404	80.08697047	1.652556743	5.598795831	1.20E-05	High	58	2	XP_003355257.1	ssc:100155404	1E-68	NM_001161334.1	HIST2H2BF
TSPAN7	9.66566885	409.007794	-5.403114957	1.20E-05	Low	7	495	XP_003135059.2	ssc:100526026	7E-130	NM_004615.3	TSPAN7
NMB	274.7811573	14.04673232	4.289976615	1.21E-05	High	199	17	NM_001123145.1	ssc:100141313	2E-18	NM_205858.1	NMB
PDE8B	13.80809836	532.9495498	-5.270412419	1.22E-05	Low	10	645	XP_005661559.1	ssc:100522306	0	NM_003719.3	PDE8B

FBN2	403.196472	13763.31884	-5.093201573	1.24E-05	Low	292	16657	XP_003123945.2	ssc:100048956			
LOC100737019	17.95052786	656.0650272	-5.191740643	1.25E-05	Low	13	794	LOC100737019				
CTSC	182.2668983	6028.527	-5.047681059	1.26E-05	Low	132	7296	XP_003129789.1	ssc:100522387	0	NM_148170.3	CTSC
LOC100626363	4.142429507	240.4470062	-5.859098006	1.27E-05	Low	3	291	XR_300111.1	ssc:100626363			
16S	13826.04889	564649.7195	-5.351895332	1.28E-05	Low	10013	683365	16S				
FBP1	157.4123213	6.610226974	4.57370485	1.30E-05	High	114	8	NM_213979.1	ssc:397038	7E-176	NM_001127628.1	FBP1
LOC100523785	157.4123213	6.610226974	4.57370485	1.30E-05	High	114	8	XP_003355254.1	ssc:100523785	1E-62	NM_003517.2	HIST2H2AC
IL7	2.761619671	197.4805308	-6.160051972	1.33E-05	Low	2	239	NM_214135.2	ssc:397253	8E-63	NM_001199888.1	IL7
PSP-I	0	111.5475802	-20	1.34E-05	Low	0	135	NM_213837.1	ssc:396818			
LOC102164681	4.142429507	237.9681711	-5.844147665	1.34E-05	Low	3	288	XR_299526.1	ssc:102164681			
LOC100521565	131.1769344	4.95767023	4.725707944	1.35E-05	High	95	6	XP_003129134.1	ssc:100521565			
GJA1	980.3749834	34378.13793	-5.132013923	1.35E-05	Low	710	41606	NM_001244212.1	ssc:100518636	0	NM_000165.3	GJA1
PTGIS	71.80211146	2316.058276	-5.011501475	1.38E-05	Low	52	2803	XP_005654305.1	ssc:100126284	0	NM_000961.3	PTGIS
MPP7	283.0660163	14.87301069	4.250369934	1.39E-05	High	205	18	XP_003130810.1	ssc:100516752	0	NM_173496.3	MPP7
GVIN1	2.761619671	195.8279741	-6.147928413	1.39E-05	Low	2	237	XP_003482569.2	ssc:100512797	1E-80	NM_017920.3	URGCP
SLC5A5	104.9415475	3.305113487	4.98874235	1.40E-05	High	76	4	NM_214410.1	ssc:399542	0	NM_000453.2	SLC5A5
GALNT12	2485.457704	152.0352204	4.031034071	1.41E-05	High	1800	184	XP_003122084.1	ssc:100512452	0	NM_024642.4	GALNT12
TBX18	11.04647869	437.927537	-5.309033713	1.41E-05	Low	8	530	XP_001926986.1	ssc:100152747	0	NM_001080508.2	TBX18
SGIP1	49.70915409	0	20	1.41E-05	High	36	0	XP_005658837.1	ssc:100620397	9E-180	NM_032291.2	SGIP1
ACVR1C	49.70915409	0	20	1.41E-05	High	36	0	XP_003133466.3	ssc:100156756	0	NM_145259.2	ACVR1C
S100A7	49.70915409	0	20	1.41E-05	High	36	0	XP_005663471.1	ssc:100144623	6E-27	NM_002963.3	S100A7
CD70	6616.840733	398.2661752	4.054337693	1.42E-05	High	4792	482	NM_001044531.1	ssc:733577	8E-66	NM_001252	CD70
MSMP	122.8920754	3930.606214	-4.999288043	1.45E-05	Low	89	4757	XP_003122034.1	ssc:100512517	1E-73	NM_001044264.2	MSMP
CMKLR1	0	109.8950234	-20	1.46E-05	Low	0	133	NM_001123100.1	ssc:780421	0	NM_004072.2	CMKLR1
SHC4	0	109.8950234	-20	1.46E-05	Low	0	133	XP_003121568.1	ssc:100526147	0	NM_203349.3	SHC4
CCDC88B	255.4498196	13.22045395	4.272196297	1.49E-05	High	185	16	XP_003122643.3	ssc:100525503	9E-165	NM_032251.5	CCDC88B
FLNC	636.5533343	21473.32232	-5.076120213	1.50E-05	Low	461	25988	XP_005657826.1	ssc:100518997	0	NM_001458.4	FLNC
LOC100511379	662.7887212	39.66136184	4.062742931	1.51E-05	High	480	48	LOC100511379				
LOC100737860	1.380809836	152.0352204	-6.78274712	1.52E-05	Low	1	184	XP_005668944.1	ssc:100737860	0	NM_017957.2	EPN3
RUSC2	128.4153147	4077.683764	-4.988860722	1.52E-05	Low	93	4935	XP_005660277.1	ssc:100158062	0	NR_052015.1	RUSC2
AARD	103.5607377	3.305113487	4.969633527	1.55E-05	High	75	4	XP_003125560.1	ssc:100522252	4E-36	NM_001025357.2	AARD
LAMA2	0	108.2424667	-20	1.59E-05	Low	0	131	XP_005659257.1	ssc:100154420	0	NM_001079823.1	LAMA2
KLK1	115.9880262	4.131391859	4.811204164	1.59E-05	High	84	5	NM_001001911.1	ssc:431673	5E-107	NM_002257.3	KLK1
LOC100153192	30.37781639	992.3603244	-5.029773981	1.62E-05	Low	22	1201	XP_005656320.1	ssc:100153192	0	NM_014272.3	ADAMTS7
LOC100154071	190.5517573	9.089062089	4.389907674	1.63E-05	High	138	11	XP_001928646.4	ssc:100154071	2E-68	NM_003509.2	HIST1H2AI
LOC100737780	63.51725244	0.826278372	6.264376792	1.63E-05	High	46	1	XP_003480480.1	ssc:100737780	1E-73	NM_153181.2	NETO1
LAMB1	865.767767	29156.05862	-5.073671807	1.65E-05	Low	627	35286	XP_005667793.1	ssc:396707	0	NM_002291.2	LAMB1
GLIPR1	0	107.4161883	-20	1.65E-05	Low	0	130	XP_005664057.1	ssc:100523551	4E-121	NM_006851.2	GLIPR1
ADAMTS4	23.47376721	781.6593397	-5.057418696	1.70E-05	Low	17	946	XP_003481462.1	ssc:100157276	0	NM_005099.4	ADAMTS4
LOC574051	251.3073901	13.22045395	4.248609476	1.71E-05	High	182	16	NM_001025216.1	ssc:574051	5E-41	NM_005322.2	HIST1H1B
LOC102166598	1.380809836	148.7301069	-6.75103826	1.71E-05	Low	1	180	XP_005670367.1	ssc:102166598	6E-53	NR_072999.1	JAM2
CXCL11	0	106.58991	-20	1.73E-05	Low	0	129	NM_001128491.1	ssc:100169744	5E-40	NM_005409.4	CXCL11
LOC102159413	0	106.58991	-20	1.73E-05	Low	0	129	XP_005667232.1	ssc:102159413	0	NM_001098816.2	TENM4
LOC100738449	0	106.58991	-20	1.73E-05	Low	0	129	XP_005667233.1	ssc:100738449	0	NM_001098816.2	TENM4
LOC102165979	0	106.58991	-20	1.73E-05	Low	0	129	XR_303700.1	ssc:102165979			
C13H3orf56	48.32834425	0	20	1.75E-05	High	35	0	XP_005669882.1	ssc:102162402			
IL23RA	48.32834425	0	20	1.75E-05	High	35	0	NM_001137621.1	ssc:733613	0	NM_144701.2	IL23R
LOC100738968	0	105.7636316	-20	1.80E-05	Low	0	128	XP_003481531.1	ssc:100738968	7E-123	NM_002506.2	NGF
LOC100525666	26.23538688	852.7192796	-5.022484906	1.81E-05	Low	19	1032	XR_297813.1	ssc:100525666	1E-18	NM_024563.3	NPR3
DENND3	2449.556649	154.5140555	3.986710671	1.83E-05	High	1774	187	XP_005662889.1	ssc:102164758	3E-104	NM_014957.2	DENND3
HS3ST1	187.7901377	9.089062089	4.368846059	1.84E-05	High	136	11	XP_003361717.1	ssc:100628223	2E-158	NM_005114.2	HS3ST1
ZNF211	8.284859014	340.4266892	-5.36072319	1.85E-05	Low	6	412	ZNF211	ssc:100514110	1E-56	NR_049754.1	ZNF211
FOXL1	13.80809836	493.2881879	-5.15884419	1.89E-05	Low	10	597	XP_005664360.1	ssc:100511634	4E-124	NM_005250.2	FOXL1
CAMK2B	113.2264065	4.131391859	4.776438746	1.93E-05	High	82	5	XP_005673407.1	ssc:100516166	7E-169	NM_172084.2	CAMK2B
DLC1	52.47077376	1607.111433	-4.93681209	1.94E-05	Low	38	1945	XP_005674578.1	ssc:100525491	0	NM_182643.2	DLC1
WNT5A	11.04647869	413.9654642	-5.227851957	1.95E-05	Low	8	501	XP_005669717.1	ssc:100627056	0	NM_003392.4	WNT5A
CPNE5	186.4093278	9.089062089	4.358198815	1.95E-05	High	135	11	XP_001927623.2	ssc:100152591	0	NM_020939.1	CPNE5
LOC102165933	0	104.1110748	-20	1.96E-05	Low	0	126	XR_303906.1	ssc:102165933			
LOC100518848	480.5218228	28.91974301	4.054475315	1.97E-05	High	348	35	Z84015	ssc:100518848	7E-44	NM_001024.3	RPS21
IER3	219.5487639	6745.736627	-4.941362676	1.99E-05	Low	159	8164	XP_001927586.2	ssc:100154770	3E-60	NM_052815.1	IER3
YPEL2	35.90105573	1114.649523	-4.956420079	2.01E-05	Low	26	1349	YPEL2	ssc:100524105	2E-68	NM_001005404.3	YPEL2
ID3	157.4123213	4783.325494	-4.925393591	2.01E-05	Low	114	5789	NM_001243602.1	ssc:100626978			
VSTM4	89.75263932	2702.756554	-4.912333395	2.03E-05	Low	65	3271	XP_005671270.1	ssc:100157009	3E-160	NM_144984.3	VSTM4
CMPK2	111.8455967	3366.258086	-4.911565426	2.05E-05	Low	81	4074	XP_005662825.1	ssc:100623872	1E-176	NR_046236.1	CMPK2
DCHS1	325.8711212	10073.98591	-4.950189302	2.09E-05	Low	236	12192	XP_003357178.1	ssc:100623166	0	NM_024542.1	DCHS1
LOC100626144	330.0135507	19.00440255	4.118119688	2.10E-05	High	239	23	XP_005656792.1	ssc:100626144	0	NM_207108.2	ASTN1
TMEM8C	2.761619671	181.7812418	-6.040544877	2.11E-05	Low	2	220	XP_003353750.1	ssc:100627299	5E-117	NM_001080483.2	TMEM8C
SLC36A4	26.23538688	829.5834852	-4.982801204	2.12E-05	Low	19	1004	XP_005667282.1	ssc:100511947	0	NM_152313.3	SLC36A4
AMPD3	5.523239343	256.9725736	-5.539955934	2.13E-05	Low	4	311	XP_005661192.1	ssc:100620914	0	NM_001172431.1	AMPD3
PDZK1IP1	439.0975278	26.4409079	4.053697791	2.14E-05	High	318	32	NM_001001769.1	ssc:414756	8E-50	NM_005764.3	PDZK1IP1
LOC100628148	0	102.4585181	-20	2.14E-05	Low	0	124	LOC100628148				
LOC100623513	0	102.4585181	-20	2.14E-05	Low	0	124	XP_005663610.1	ssc:100623513	1E-122	NM_002506.2	NGF
VASN	298.2549245	9142.770183	-4.938013492	2.15E-05	Low	216	11065	XP_005662231.1	ssc:102158247	0	NM_138440.2	VASN
LOC100738012	3552.823707	227.2265522	3.966762769	2.16E-05	High	2573	275	XP_003483394.2	ssc:100738012	0	NM_006988.3	ADAMTS1
FRMD4A	2.761619671	180.9549634	-6.033972223	2.17E-05	Low	2	219	XP_005668182.1	ssc:100737056	3E-143	NM_018027.3	FRMD4A
JAM3	187.7901377	5652.570341	-4.911713837	2.18E-05	Low	136	6841	XP_003130135.2	ssc:100520043	1E-151	NM_032801.4	JAM3
RNASE6	46.94753442	0	20	2.19E-05	High	34	0	NM_001167656.1	ssc:100312981	6E-51	NM_005615.4	RNASE6
LOC102168166	46.94753442	0	20	2.19E-05	High	34	0	XP_005654921.1	ssc:102168166	1E-162	NM_005498.4	AP1M2
LOC100524561	46.94753442	0	20	2.19E-05	High	34	0	XP_003126210.1	ssc:100524561	8E-24	NM_002704.3	PPBP
LOC102162598	46.94753442	0	20	2.19E-05	High	34	0	XP_005668111.1	ssc:102162598	1E-42	NM_012144.3	DNAI1
RHPN2	1032.845757	66.10226974	3.965781201	2.19E-05	High	748	80	XP_003355886.2	ssc:100620673			
NTRK1	8.284859014	328.0325136	-5.30721786	2.28E-05	Low	6	397	XP_001929561.2	ssc:100154471	0	NM_002529.3	NTRK1
PRAP1	60.75563277	0.826278372	6.200246455	2.30E-05	High	44	1	XP_001927920.1	ssc:100154502	1E-19	NM_145202.4	PRAP1
LOC100627133	17.95052786	588.3102007	-5.034478877	2.33E-05	Low	13	712	XP_005657517.1	ssc:100627133	0	NM_203380.1	ACSL5
LOC102166035	0	100.8059614	-20	2.34E-05	Low	0	122	XR_306634.1	ssc:102166035			
LOC102161934	24.85457744	776.7016694	-4.965777109	2.35E-05	Low	18	940	XP_005670750.1	ssc:102161934	1E-40	NM_016817.2	OAS2
LOC100515533	110.4647869	4.131391859	4.740814836	2.36E-05	High	80	5	XP_005661750.1	ssc:100515533	3E-22	NM_006527.2	SLBP
PDE5	9.66566885	361.0836484	-5.223319711	2.44E-05	Low	7	437	XP_003482498.1	ssc:397616	0	NM_033437.3	PDE5A
SYNPO2L	4.142429507	214.0060983	-5.691030951	2.45E-05	Low	3	259	XP_003359286.2	ssc:100152222	0	NM_024875.4	SYNPO2L
PCDH19	0	99.97968298	-20	2.45E-05	Low	0	121	XP_005670816.1	ssc:100515639	0	NM_020766.2	PCDH19
LOC100739139	73.18292129	1.652556743	5.468735291	2.45E-05	High	53	2	XP_005667923.1	ssc:100739139			
CD248	251.3073901	7462.946253	-4.892220294	2.49E-05	Low	182	9032	XP_003122563.1	ssc:100523587	0	NM_020404.2	CD248
PEA15	658.6462916	20362.80419	-4.952889615	2.51E-05	Low	477	24644	XP_001928995.1	ssc:100512480	5E-71	NM_003768.3	PEA15
RASSF10	121.5112655	4.95767023	4.615283954	2.52E-05	High	88	6	XP_005661179.1	ssc:100625955	0	NM_001080521.2	RASSF10
AFGF	0	99.15340461	-20	2.57E-05	Low	0	120	XP_003124058.1	ssc:397497	8E-88	NM_033137.2	FGF1
RPS6KA2	226.4528131	6651.540892	-4.876406227	2.60E-05	Low	164	8050	XP_003121145.2	ssc:100515286	0	NM_021135.4	RPS6KA2
LOC100739291	2.761619671	175.1710148	-5.987105618	2.60E-05	Low	2	212	XP_005670218.1	ssc:100739291	0	NM_053032.2	MYLK
TINAGL1	51.08996392	1483.995956	-4.860303433	2.64E-05	Low	37	1796	XP_005665217.1	ssc:100517950	0	NM_022164.2	TINAGL1
SDK1	0	98.32712624	-20	2.69E-05	Low	0	119	XP_005661878.1	ssc:100512953	0	NM_152744.3	SDK1
GGT5	17.95052786	573.43719	-4.997537298	2.70E-05	Low	13	694	XP_005670952.1	ssc:100157523	0	NM_004121.2	GGT5

FSHR	45.56672458	0	20	2.74E-05	High	33	0	NM_214386.2	ssc:397679			
LOC102162836	45.56672458	0	20	2.74E-05	High	33	0	XR_304093.1	ssc:102162836			
WAP-3	45.56672458	0	20	2.74E-05	High	33	0	NM_001206553.1	ssc:100607966			
LOC102158429	45.56672458	0	20	2.74E-05	High	33	0	XR_301975.1	ssc:102158429			
LOC100517891	45.56672458	0	20	2.74E-05	High	33	0	XP_003125724.1	ssc:100517891	3E-67	NM_001639.3	APCS
LOC100625208	45.56672458	0	20	2.74E-05	High	33	0	XP_005658068.1	ssc:100625208	2E-143	NM_002538.3	OCLN
OBSCN	9.66566885	353.6471431	-5.193297228	2.74E-05	Low	7	428	XP_005661481.1	ssc:100516544			
LOC102161923	59.37482294	0.826278372	6.167079591	2.75E-05	High	43	1	XR_299940.1	ssc:102161923			
CELF5	59.37482294	0.826278372	6.167079591	2.75E-05	High	43	1	XP_005654782.1	ssc:100518716	0	NR_033342.1	CELF5
KIAA1211	17.95052786	570.1320765	-4.989197997	2.79E-05	Low	13	690	XP_005666754.1	ssc:100522154	2E-94	NM_020722.1	KIAA1211
LOC102157883	84.22939998	2.478835115	5.086589673	2.80E-05	High	61	3	XP_005661699.1	ssc:102157883			
AR	0	97.50084786	-20	2.81E-05	Low	0	118	NM_214314.2	ssc:397582	0	NM_001011645.2	AR
FGF19	0	97.50084786	-20	2.81E-05	Low	0	118	XP_003122468.1	ssc:100518950	2E-93	NM_005117.2	FGF19
GULO	6.904049179	280.108368	-5.342398532	2.83E-05	Low	5	339	NM_001129948.1	ssc:396759			
ROR2	131.1769344	3742.214746	-4.83430638	2.83E-05	Low	95	4529	XP_005657255.1	ssc:100154208	0	NM_004560.3	ROR2
CHAC1	175.3628491	5014.683438	-4.837743582	2.90E-05	Low	127	6069	XP_005659810.1	ssc:100524723	3E-138	NM_024111.3	CHAC1
LOC102165476	0	96.67456949	-20	2.95E-05	Low	0	117	XR_298251.1	ssc:102165476			
LOC100623420	122.8920754	3470.369161	-4.819625345	2.98E-05	Low	89	4200	XP_003357434.2	ssc:100623420	0	NM_001684.4	ATP2B4
LOC100621773	430.8126688	12664.3686	-4.877570666	2.99E-05	Low	312	15327	XP_003361781.1	ssc:100621773			
EBF2	8.284859014	312.3332245	-5.236465087	3.01E-05	Low	6	378	XP_005670500.1	ssc:100155094	0	NM_022659.3	EBF2
ID4	41.42429507	1186.535742	-4.840134602	3.02E-05	Low	30	1436	NM_001123130.1	ssc:100144508	1E-86	NM_001546.3	ID4
LOC100622925	95.27587867	3.305113487	4.849339293	3.04E-05	High	69	4	XR_299376.1	ssc:100622925			
SEMA3D	225.0720032	6419.35667	-4.833970206	3.07E-05	Low	163	7769	XP_005667736.1	ssc:100523915	0	NM_152754.2	SEMA3D
LOC100518411	1.380809836	133.8570962	-6.599035167	3.07E-05	Low	1	162	XP_005660658.1	ssc:100518411	0	NR_030691.1	ANO1
LOC100622678	0	95.84829112	-20	3.09E-05	Low	0	116	XP_005674454.1	ssc:100622678	0	NM_017718.1	DOCK10
ZNF804A	0	95.84829112	-20	3.09E-05	Low	0	116	ZNF804A	ssc:100524346	0	NM_194250.1	ZNF804A
LOC102158140	0	95.84829112	-20	3.09E-05	Low	0	116	XP_005661136.1	ssc:102158140	0	NM_182964.5	NAV2
LOC102159096	338.2984098	20.65695929	4.033596585	3.09E-05	High	245	25	XR_305609.1	ssc:102159096			
LOC102167733	2.761619671	169.3870662	-5.938665263	3.13E-05	Low	2	205	XP_005666981.1	ssc:102167733	6E-54	NM_024590.3	ARSJ
PDE3B	820.2010424	54.53437253	3.910739838	3.19E-05	High	594	66	XP_005661171.1	ssc:100516060	0	NM_000922.3	PDE3B
MICAL2	49.70915409	1398.063005	-4.813774016	3.19E-05	Low	36	1692	XP_005661181.1	ssc:100519075	0	NM_014632.3	MICAL2
CCDC69	2.761619671	168.5607878	-5.931610506	3.22E-05	Low	2	204	XP_003134191.3	ssc:100515590	3E-130	NM_015621.2	CCDC69
ANGPT1	12.42728852	413.9654642	-5.057926956	3.22E-05	Low	9	501	NM_213959.1	ssc:397009			
LOC102161276	0	95.02201275	-20	3.24E-05	Low	0	115	XR_304701.1	ssc:102161276			
LOC102162312	0	95.02201275	-20	3.24E-05	Low	0	115	XR_306270.1	ssc:102162312			
SLC2A13	0	95.02201275	-20	3.24E-05	Low	0	115	XP_003126653.3	ssc:100520492	1E-161	NM_052885.3	SLC2A13
ENTPD8	70.42130162	1.652556743	5.413240178	3.32E-05	High	51	2	XP_005652794.1	ssc:100516719	0	NM_198585.2	ENTPD8
LOC100512920	70.42130162	1.652556743	5.413240178	3.32E-05	High	51	2	XR_306463.1	ssc:100512920			
SERPINE1	352.1065081	10062.41801	-4.836821325	3.35E-05	Low	255	12178	NM_213910.1	ssc:396945			
SWAP70	115.9880262	3205.960082	-4.788708678	3.35E-05	Low	84	3880	XP_003123029.2	ssc:100521312	0	NM_015055.2	SWAP70
SPEG	64.89806228	1794.676623	-4.7894047	3.36E-05	Low	47	2172	XP_005672303.1	ssc:100737722	0	NM_005876.4	SPEG
MAOB	415.6237606	26.4409079	3.974434513	3.38E-05	High	301	32	NM_001001864.1	ssc:414909	0	NM_000898.4	MAOB
LOC100517858	46.94753442	1310.477498	-4.802899378	3.38E-05	Low	34	1586	XR_131003.3	ssc:100517858			
LOC100513982	0	94.19573438	-20	3.39E-05	Low	0	114	XP_005662499.1	ssc:100513982	3E-40	NM_016026.3	RDH11
KLHL30	0	94.19573438	-20	3.39E-05	Low	0	114	XP_003133822.1	ssc:100519226	0	NM_198582.3	KLHL30
LOC100511475	93.89506883	3.305113487	4.828277677	3.41E-05	High	68	4	XP_005671063.1	ssc:100511475	8E-24	NM_021910.2	FXYD3
ATP6V0D2	44.18591474	0	20	3.44E-05	High	32	0	XP_003125629.1	ssc:100521494	0	NM_152565.1	ATP6V0D2
LOC100624460	44.18591474	0	20	3.44E-05	High	32	0	XP_003354138.3	ssc:100624460	0	NM_024690.2	MUC16
LOC102164033	44.18591474	0	20	3.44E-05	High	32	0	LOC102164033				
TNS4	44.18591474	0	20	3.44E-05	High	32	0	XP_003131518.1	ssc:100521862	0	NM_032865.5	TNS4
LOC100515669	44.18591474	0	20	3.44E-05	High	32	0	XP_003127928.1	ssc:100515669	0	NM_024421.2	DSC1
GRB7	44.18591474	0	20	3.44E-05	High	32	0	XP_005653981.1	ssc:100513230	0	NM_005310.3	GRB7
KCTD12	2.761619671	166.0819527	-5.910236855	3.49E-05	Low	2	201	XP_005668528.1	ssc:100524761	2E-166	NM_138444.3	KCTD12
ACSL5	24.85457704	723.8198536	-4.864047222	3.52E-05	Low	18	876	NM_001195321.1	ssc:100157521	0	NM_203380.1	ACSL5
SLIT2	95.27587867	2606.908263	-4.774084997	3.52E-05	Low	69	3155	XP_005666614.1	ssc:100515495	0	NM_004787	SLIT2
LOC102163771	0	93.36945601	-20	3.56E-05	Low	0	113	XR_309073.1	ssc:102163771			
LOC102157709	150.5082721	7.436505346	4.339074159	3.56E-05	High	109	9	XR_298258.1	ssc:102157709			
LOC102165155	207.1214754	11.5678972	4.162278605	3.58E-05	High	150	14	XP_005668615.1	ssc:102165155	2E-162	NM_024979.4	MCF2L
SOX11	71.80211146	1957.453463	-4.768807929	3.62E-05	Low	52	2369	XP_003354985.1	ssc:100514639	0	NM_003108.3	SOX11
THY1	1473.324095	44528.14145	-4.917570669	3.62E-05	Low	1067	53890	NM_001146129.1	ssc:100271931	2E-67	NM_006288.3	THY1
LOC102161162	81.46778031	2.478835115	5.038495385	3.64E-05	High	59	3	XP_005670001.1	ssc:102161162	7E-89	NM_172160.2	KCNAB1
RANBP3L	2.761619671	164.429396	-5.895809784	3.69E-05	Low	2	199	XP_003133942.4	ssc:100517433	0	NM_145000.3	RANBP3L
LOC102157822	2.761619671	164.429396	-5.895809784	3.69E-05	Low	2	199	XR_306600.1	ssc:102157822			
SCG2	0	92.54317763	-20	3.74E-05	Low	0	112	NM_001012299.1	ssc:497237	0	NM_003469.4	SCG2
LOC100624621	4.142429507	198.3068092	-5.581113259	3.74E-05	Low	3	240	LOC100624621				
TRIM24	8280.716585	552.7802307	3.904977693	3.75E-05	High	5997	669	XP_003360173.2	ssc:100620590			
LOC100525624	2.761619671	163.6031176	-5.888541784	3.80E-05	Low	2	198	XP_005654339.1	ssc:100525624	3E-117	NM_021963.3	NAP1L2
ADAM22	238.8801016	14.04673232	4.087980223	3.87E-05	High	173	17	XP_005667627.1	ssc:100513676	0	NM_021723.3	ADAM22
LOC102164270	13.80809836	434.6224235	-4.976176058	3.88E-05	Low	10	526	XP_005674366.1	ssc:102164270	4E-105	NM_133337.2	MYOF
LOC100518277	8.284859014	298.2864922	-5.17007769	3.90E-05	Low	6	361	XP_003133337.3	ssc:100518277	4E-31	NM_016815.3	GYPC
PTPRE	2.761619671	162.7768392	-5.881236983	3.91E-05	Low	2	197	XP_005671631.1	ssc:100155079	0	NM_130435.3	PTPRE
B3GNT9	2.761619671	162.7768392	-5.881236983	3.91E-05	Low	2	197	XP_005659126.1	ssc:100620432	1E-97	NM_033309.2	B3GNT9
IRX4	1.380809836	128.0731476	-6.535309569	3.92E-05	Low	1	155	XP_003134213.1	ssc:100519749	0	NM_016358.2	IRX4
SLC7A2	1.380809836	128.0731476	-6.535309569	3.92E-05	Low	1	155	NM_001110420.1	ssc:100037298	0	NM_003046.5	SLC7A2
PIK3CD	1056.319524	72.71249671	3.860699155	3.96E-05	High	765	88	XP_005665035.1	ssc:100518131	0	NM_005026.3	PIK3CD
LOC100519977	5.523239343	229.7053873	-5.378126237	4.00E-05	Low	4	278	XR_299307.1	ssc:100519977			
TIMP2	2250.720032	68241.50444	-4.922190937	4.05E-05	Low	1630	82589	NM_001145985.1	ssc:396988	7E-116	NM_003255.4	TIMP2
LOC102161722	0	90.89062089	-20	4.12E-05	Low	0	110	XP_005658702.1	ssc:102161722	7E-105	NM_153343.3	ENPP6
LOC100626465	0	90.89062089	-20	4.12E-05	Low	0	110	XP_005663840.1	ssc:100626465	3E-60	NM_033016.2	PDGFB
LOC102162178	0	90.89062089	-20	4.12E-05	Low	0	110	XP_005658008.1	ssc:102162178	0	NM_004654.3	USP9Y
LOC102166299	0	90.89062089	-20	4.12E-05	Low	0	110	XP_005653880.1	ssc:102166299	5E-87	NM_005708.3	GPC6
CLU	4364.739891	136815.1728	-4.970188742	4.12E-05	Low	3161	165580	NM_213971.1	ssc:397025	2E-177	NR_045494.1	CLU
LOC100738391	2.761619671	161.1242825	-5.866515478	4.13E-05	Low	2	195	XP_003480603.1	ssc:100738391	6E-180	NM_032342.1	TMEM246
MAN1C1	164.3163705	4399.106051	-4.742662256	4.19E-05	Low	119	5324	XP_005665172.1	ssc:100524150	0	NM_020379	MAN1C1
LOC102162237	1.380809836	126.4205909	-6.516573007	4.21E-05	Low	1	153	XP_005671728.1	ssc:102162237	5E-57	NM_005429.3	VEGFC
CDCA7	11873.58378	795.706072	3.899376028	4.27E-05	High	8599	963	XP_001928667.1	ssc:100154583	0	NM_145810.2	CDCA7
ARHGAP22	11.04647869	360.2573701	-5.027369489	4.27E-05	Low	8	436	XP_005671266.1	ssc:100156289	2E-142	NR_045675.1	ARHGAP22
PTGIR	0	90.06434252	-20	4.33E-05	Low	0	109	XP_003356000.1	ssc:100622305	0	NM_000960.3	PTGIR
LOC100514834	0	90.06434252	-20	4.33E-05	Low	0	109	XR_303007.1	ssc:100514834			
LOC102167305	0	90.06434252	-20	4.33E-05	Low	0	109	XR_297258.1	ssc:102167305			
LOC102159210	42.80510491	0	20	4.35E-05	High	31	0	XR_298894.1	ssc:102159210			
LOC102157580	42.80510491	0	20	4.35E-05	High	31	0	XR_298273.1	ssc:102157580			
LOC100155246	42.80510491	0	20	4.35E-05	High	31	0	LOC100155246				
SOX7	42.80510491	0	20	4.35E-05	High	31	0	XP_003359100.1	ssc:100155811	0	NM_031439.3	SOX7
TSPAN33	1875.139757	131.3782611	3.835199645	4.43E-05	High	1358	159	XP_003134729.1	ssc:100515287	3E-158	NM_178562.3	TSPAN33
LOC100738887	1.380809836	124.7680341	-6.497589903	4.53E-05	Low	1	151	XP_005653802.1	ssc:100738887	0	NR_038265.1	PPFIA2
LOC102157630	0	89.23806415	-20	4.55E-05	Low	0	108	XR_305358.1	ssc:102157630			
PCPA1	0	89.23806415	-20	4.55E-05	Low	0	108	NM_214244.1	ssc:397476	0	NM_001868.2	CPA1
IGF2BP2	230.5952426	6145.858529	-4.736179904	4.55E-05	Low	167	7438	XP_003132621.2	ssc:100516156	0	NM_006548.4	IGF2BP2
LOC102162895	13.80809836	422.228248	-4.93443655	4.57E-05	Low	10	511	XP_005661674.1	ssc:102162895	1E-103	NM_001999.3	FBN2

CILP2	8.284859014	289.1974301	-5.125433775	4.64E-05	Low	6	350	XP_003123618.1	ssc:100518061	0	NM_153221.2	CILP2
LOC102165157	793.9656555	55.36065091	3.842143792	4.71E-05	High	575	67	XR_306510.1	ssc:102165157			
IRK1	2.761619671	156.9928906	-5.829040772	4.77E-05	Low	2	190	NM_214151.1	ssc:397293	0	NM_000891.2	KCNJ2
PRSS16	122.8920754	5.783948602	4.409193345	4.87E-05	High	89	7	XP_003356607.1	ssc:100628128	0	NM_005865.3	PRSS16
SLC15A3	38.6626754	1022.106346	-4.724460027	4.88E-05	Low	28	1237	XP_003122726.3	ssc:100513135	0	NR_027391.1	SLC15A3
LOC100739389	243.0225311	14.87301069	4.030321453	4.89E-05	High	176	18	XP_005655045.1	ssc:100739389	1E-77	NR_003149.1	GPR98
RHCG	2.761619671	156.1666123	-5.821427588	4.91E-05	Low	2	189	NM_001044577.1	ssc:733644			
MYO16	209.883095	12.39417558	4.081851754	4.95E-05	High	152	15	XP_001925002.1		0	NM_015011.1	MYO16
ITGB2	834.0091408	58.66576439	3.829472456	4.98E-05	High	604	71	NM_213908.1	ssc:396943	0	NM_001127491.1	ITGB2
OCA2	0	87.5855074	-20	5.03E-05	Low	0	106	NM_214094.2	ssc:397171	7E-11	NM_000275.2	OCA2
PLSCR4	22.09295737	610.6197167	-4.788615718	5.14E-05	Low	16	739	XP_005669981.1	ssc:100518400	8E-152	NM_020353.2	PLSCR4
3-Mar	34.52024589	910.5587656	-4.721237483	5.16E-05	Low	25	1102	NM_001244831.1	ssc:100513526	2E-146	NM_178450.4	3-Mar
ATOH8	8.284859014	283.4134815	-5.096287429	5.20E-05	Low	6	343	XP_005662472.1	ssc:100523367	2E-126	NM_032827.6	ATOH8
ACACB	75.94454097	1940.927895	-4.675656448	5.21E-05	Low	55	2349	NM_001206399.1	ssc:100154993	0	NM_001093.3	ACACB
LOC100736679	1.380809836	121.4629206	-6.458857509	5.24E-05	Low	1	147	XP_003482978.1	ssc:100736679	1E-73	NM_004791.2	ITGBL1
LRP1	1110.171108	30988.74405	-4.802890327	5.26E-05	Low	804	37504	XP_005663982.1	ssc:100627964	0	NM_002332.2	LRP1
LOC100523833	5.523239343	218.1374901	-5.303579283	5.33E-05	Low	4	264	XP_003135475.3	ssc:100523833	4E-143	NM_033642.2	FGF13
ARNT2	73.18292129	1853.342388	-4.662478614	5.50E-05	Low	53	2243	XP_001926107.3	ssc:100157219	0	NM_014862.3	ARNT2
LOC102157775	41.42429507	0	20	5.54E-05	High	30	0	XP_005659066.1	ssc:102157775	2E-14	NM_004360.3	CDH1
SP6	41.42429507	0	20	5.54E-05	High	30	0	XP_003131582.1	ssc:100519513	0	NM_199262.2	SP6
ALOXE3	41.42429507	0	20	5.54E-05	High	30	0	XP_005657096.1	ssc:100511043	0	NM_021628.2	ALOXE3
TOX	31.75862622	832.8885987	-4.712903131	5.54E-05	Low	23	1008	XP_005663138.1	ssc:100155888	0	NM_014729.2	TOX
PEAK1	44.18591474	1132.827648	-4.68019802	5.57E-05	Low	32	1371	NM_001244188.1	ssc:100152646	0	NM_024776.3	PEAK1
F5	0	85.93295066	-20	5.57E-05	Low	0	104	NM_214120.1	ssc:397217	0	NM_000130.4	F5
LOC100623096	49.70915409	1265.858465	-4.670460744	5.62E-05	Low	36	1532	XR_305640.1	ssc:100623096			
HEPH	2.761619671	152.0352204	-5.78274712	5.69E-05	Low	2	184	XP_005673751.1	ssc:100512938	0	NM_138737.4	HEPH
ATP8B1	16.56971803	470.1523935	-4.826507505	5.70E-05	Low	12	569	XP_003121763.2	ssc:100153006	0	NM_005603.4	ATP8B1
GPX3	33.13943606	849.4141661	-4.679847212	6.18E-05	Low	24	1028	NM_001115155.1	ssc:396598	6E-108	NM_002084.3	GPX3
LOC100156874	0	84.28039392	-20	6.18E-05	Low	0	102	XR_307539.1	ssc:100156874			
LOC100736623	0	84.28039392	-20	6.18E-05	Low	0	102	XP_005672862.1	ssc:100736623	5E-138	NM_080792.2	SIRPA
LOC100512907	0	84.28039392	-20	6.18E-05	Low	0	102	XR_304317.1	ssc:100512907			
LOC102163142	0	84.28039392	-20	6.18E-05	Low	0	102	XP_005652456.1	ssc:102163142	2E-110	NM_014831.2	TRANK1
LOC100525036	5.523239343	212.3535415	-5.264809713	6.19E-05	Low	4	257	XP_003123849.4	ssc:100525036	0	NM_022350.3	ERAP2
FOXF2	11.04647869	337.1215757	-4.931610506	6.20E-05	Low	8	408	XP_005665585.1	ssc:100158066	6E-118	NM_001452.1	FOXF2
LOC100519756	129.7961246	6.610226974	4.295403688	6.22E-05	High	94	8	XP_005657976.1	ssc:100519756	9E-24	NM_001163438.1	SMIM10
LY6H	64.89806228	1.652556743	5.295403688	6.23E-05	High	47	2	XP_005662880.1	ssc:100516078	3E-63	NM_002347.4	LY6H
ATCAY	64.89806228	1.652556743	5.295403688	6.23E-05	High	47	2	XP_005661400.1	ssc:100626830	0	NM_033064.4	ATCAY
KLHL41	11.04647869	336.2952973	-4.928070148	6.28E-05	Low	8	407	XP_003359596.1	ssc:100627572	0	NM_006063.2	KLHL41
RBP5	1.380809836	117.3315288	-6.408932283	6.33E-05	Low	1	142	NM_001145223.1	ssc:100270724	3E-69	NM_031491.2	RBP5
LOC102159968	2.761619671	148.7301069	-5.75103826	6.43E-05	Low	2	180	XR_302917.1	ssc:102159968			
LOC100620787	62.13644261	1531.093823	-4.622979234	6.52E-05	Low	45	1853	XR_299787.1	ssc:100620787			
LOC102164464	0	83.45411554	-20	6.52E-05	Low	0	101	XR_303994.1	ssc:102164464			
SLAIN1	1371.144167	100.8059614	3.765727406	6.62E-05	High	993	122	XP_005668535.1	ssc:100526183	6E-92	NM_144595.3	SLAIN1
ZNF613	11.04647869	332.9901838	-4.913821192	6.64E-05	Low	8	403	ZNF613	ssc:100625971	0	NM_024840.3	ZNF613
ACTN2	12.42728852	363.5624836	-4.870619876	6.68E-05	Low	9	440	NM_001243666.1	ssc:100157406	0	NM_001278344.1	ACTN2
MADCAM1	96.6566885	4.131391859	4.548169758	6.81E-05	High	70	5	NM_001037998.1	ssc:492312	3E-60	NM_130762.2	MADCAM1
LOC100523068	2.761619671	147.0775502	-5.734918595	6.83E-05	Low	2	178	LOC100523068	ssc:100523068			
RGS7BP	0	82.62783717	-20	6.88E-05	Low	0	100	NM_001243809.1	ssc:100511050			
EMILIN2	357.6297475	9008.913087	-4.654814408	6.93E-05	Low	259	10903	XP_005665358.1	ssc:100522875	0	NM_032048.2	EMILIN2
PPIF	3083.348363	226.4002739	3.767550289	6.93E-05	High	2233	274	XP_001929518.1	ssc:100152612	9E-101	NM_005729.3	PPIF
LOC102167710	15.18890819	422.228248	-4.796933026	7.00E-05	Low	11	511	XP_005672881.1	ssc:102167710	4E-63	NR_046000	IRF4
VPAC1	52.47077376	0.826278372	5.98874235	7.04E-05	High	38	1	NM_214036.1	ssc:100155402	0	NM_004624.3	VIPR1
SPP1	57.9940131	1412.109737	-4.605804423	7.04E-05	Low	42	1709	NM_214023.1	ssc:397087	2E-96	NM_001251830.1	SPP1
LOC100627899	2.761619671	146.2512718	-5.726790714	7.05E-05	Low	2	177	XP_005658520.1	ssc:100627899	0	NM_004274.4	AKAP6
LOC102161206	40.04348524	0	20	7.09E-05	High	29	0	XR_307765.1	ssc:102161206			
LOC100737191	40.04348524	0	20	7.09E-05	High	29	0	XP_003483873.1	ssc:100737191	3E-124	NM_001190787.1	MCIDAS
LOC100737075	40.04348524	0	20	7.09E-05	High	29	0	XP_003483725.1	ssc:100737075	5E-41	NM_001145250.1	SP9
LOC102162614	8.284859014	267.7141924	-5.014072666	7.14E-05	Low	6	324	XR_301641.1	ssc:102162614			
LOC100737584	20.71214754	544.517447	-4.716429223	7.15E-05	Low	15	659	XR_303847.1	ssc:100737584	2E-30	NM_021913.4	AXL
MDGA2	0	81.8015588	-20	7.26E-05	Low	0	99	XP_003353527.1	ssc:100621082			
NKX2-2	0	81.8015588	-20	7.26E-05	Low	0	99	XP_003359963.1	ssc:100626520	7E-149	NM_002509.3	NKX2-2
LOC100739353	0	81.8015588	-20	7.26E-05	Low	0	99	XP_005660124.1	ssc:100739353	3E-75	NM_002169.2	IFNA5
ANKRD35	33.13943606	825.4520934	-4.638563531	7.27E-05	Low	24	999	XP_003125839.1	ssc:100511691	0	NM_144698.4	ANKRD35
ADAMTS12	2.761619671	145.4249934	-5.718616782	7.27E-05	Low	2	176	XP_005672463.1	ssc:100511353	0	NM_030955.2	ADAMTS12
GLIS3	6.904049179	236.3156143	-5.097128406	7.31E-05	Low	5	286	XP_003121968.3	ssc:100523225	2E-180	NM_152629.3	GLIS3
LOC100517249	63.51725244	1.652556743	5.264376792	7.34E-05	High	46	2	XP_003134406.1	ssc:100517249	2E-86	NM_004609.3	TCF15
AKAP6	8.284859014	266.0616357	-5.005139541	7.39E-05	Low	6	322	XP_003482294.1	ssc:100155181	0	NM_004274.4	AKAP6
LGI2	168.4588	9.915340461	4.086589673	7.43E-05	High	122	12	XP_005674273.1	ssc:100623207	2E-162	NM_018176.3	LGI2
HOXC4	27.61619671	694.9001106	-4.653219059	7.53E-05	Low	20	841	XP_005663910.1	ssc:100154759	1E-153	NM_153633.2	HOXC4
LOC102163193	95.27587867	4.131391859	4.527411198	7.62E-05	High	69	5	XP_005665735.1	ssc:102163193			
MFSD6	1634.878846	122.289199	3.740814836	7.62E-05	High	1184	148	NM_001097457.1	ssc:100037960	0	NM_017694.3	MFSD6
LOC100515328	15.18890819	415.618021	-4.774168135	7.65E-05	Low	11	503	XP_003361055.1	ssc:100515328	0	NM_004787	SLIT2
LOC100525978	0	80.97528043	-20	7.66E-05	Low	0	98	XR_301768.1	ssc:100525978			
LOC100625107	5.523239343	204.0907578	-5.207552395	7.71E-05	Low	4	247	XR_304842.1	ssc:100625107			
PODN	5.523239343	203.2644794	-5.201699669	7.88E-05	Low	4	246	XP_005665471.1	ssc:100622214			
NKAIN3	84.22939998	3.305113487	4.671552174	7.98E-05	High	61	4	XP_001925563.2	ssc:100157871	2E-87	NM_173688.2	NKAIN3
LOC102163118	84.22939998	3.305113487	4.671552174	7.98E-05	High	61	4	XR_299182.1	ssc:102163118			
CXCL2	89.75263932	2125.187972	-4.565492278	8.01E-05	Low	65	2572	XP_005652611.1	ssc:396594	9E-35	NM_002089.3	CXCL2
LOC100622435	9.66566885	291.6762652	-4.915354615	8.07E-05	Low	7	353	XP_005658189.1	ssc:100622435	3E-116	NM_153498.2	CAMK1D
FNDC5	0	80.14900206	-20	8.09E-05	Low	0	97	XP_005665249.1	ssc:100622587	4E-77	NM_153756.2	FNDC5
LOC102163368	0	80.14900206	-20	8.09E-05	Low	0	97	XR_306051.1	ssc:102163368			
LOC102157645	4.142429507	171.8659013	-5.374662381	8.27E-05	Low	3	208	XR_301276.1	ssc:102157645			
QPCT	8.284859014	260.2776871	-4.973430682	8.35E-05	Low	6	315	XP_003481293.1	ssc:397424	6E-175	NM_012413.3	QPCT
LOC102160320	114.6072164	5.783948602	4.308499345	8.46E-05	High	83	7	XP_005666992.1	ssc:102160320	2E-69	NR_045756.2	COL25A1
ANO5	0	79.32272369	-20	8.55E-05	Low	0	96	XP_005661130.1	ssc:100520542	0	NM_213599.2	ANO5
C3	187.7901377	4449.509032	-4.566452952	8.58E-05	Low	136	5385	NM_214009.1	ssc:397072			
LOC102158609	51.08996392	0.826278372	5.950268202	8.58E-05	High	37	1	XP_005658284.1	ssc:102158609	7E-113	NM_178500.3	PHOSPHO1
LOC102162369	74.56373113	1743.447364	-4.547324945	8.64E-05	Low	54	2110	XR_308396.1	ssc:102162369			
CEBPE	62.13644261	1.652556743	5.232667932	8.67E-05	High	45	2	XP_001924449.1	ssc:100157564	8E-156	NM_001805.3	CEBPE
LOC100154755	62.13644261	1.652556743	5.232667932	8.67E-05	High	45	2	XP_001927693.1	ssc:100154755	1E-49	NM_003087.2	SNCG
EXOC3L1	62.13644261	1.652556743	5.232667932	8.67E-05	High	45	2	XP_005664474.1	ssc:100522868	0	NM_178516.3	EXOC3L1
LOC100625994	62.13644261	1.652556743	5.232667932	8.67E-05	High	45	2	XP_005658381.1	ssc:100625994	2E-106	NM_138435.2	FAM83F
SORD	8440.890526	620.5350572	3.76581109	8.72E-05	High	6113	751	NM_001244162.1	ssc:100158181	0	NR_034039.1	SORD
LOC100153281	59.37482294	1390.6265	-4.54973987	8.74E-05	Low	43	1683	XP_005670481.1	ssc:100153281	8E-86	NM_003844.3	TNFRSF10A
LOC102158647	124.2728852	6.610226974	4.232667932	8.77E-05	High	90	8	XR_300173.1	ssc:102158647			
LOC100623939	19.3313377	494.1144663	-4.675831916	8.87E-05	Low	14	598	XP_005664537.1	ssc:100623939	2E-43	NM_021902.3	FXYD1
LOC100522644	4.142429507	169.3870662	-5.353702763	8.95E-05	Low	3	205	XR_307034.1	ssc:100522644	1E-65	NM_207015.2	NAALADL2
LOC100621868	4.142429507	169.3870662	-5.353702763	8.95E-05	Low	3	205	XP_003361364.2	ssc:100621868	3E-81	NR_045563.1	GULP1
LOC102159468	0	78.49644531	-20	9.04E-05	Low	0	95	XR_307030.1	ssc:102159468			
NTS	0	78.49644531	-20	9.04E-05	Low	0	95	XP_003481806.1	ssc:100739079	1E-82	NM_006183.4	NTS

LOC102164702	0	78.49644531	-20	9.04E-05	Low	0	95	XP_005661990.1	ssc:102164702	2E-44	NM_133457.3	COL26A1
LOC100622236	38.6626754	0	20	9.12E-05	High	28	0	XP_005664698.1	ssc:100622236			
LOC102157622	38.6626754	0	20	9.12E-05	High	28	0	XR_298343.1	ssc:102157622			
LOC100511891	38.6626754	0	20	9.12E-05	High	28	0	XR_305437.1	ssc:100511891			
PTAFR	38.6626754	0	20	9.12E-05	High	28	0	XP_005653452.1	ssc:100523973	8E-168	NM_001164723.2	PTAFR
LOC102166434	38.6626754	0	20	9.12E-05	High	28	0	XP_005657111.1	ssc:102166434	4E-68	NM_207386.3	SHISA6
SLC6A20	38.6626754	0	20	9.12E-05	High	28	0	XP_003358454.2	ssc:100621879	0	NM_022405.3	SLC6A20
AVP	38.6626754	0	20	9.12E-05	High	28	0	NM_213952.2	ssc:396995	4E-79	NM_000490.4	AVP
LOC100737912	8.284859014	256.1462952	-4.950347068	9.12E-05	Low	6	310	XP_005664976.1	ssc:100737912	0	NM_001144989.1	ZNF814
ESRP1	731.8292129	55.36065091	3.724574195	9.22E-05	High	530	67	XP_001925909.1		0	NM_017697.3	ESRP1
LOC102160929	113.2264065	5.783948602	4.291011919	9.30E-05	High	82	7	XR_298164.1	ssc:102160929			
IFI44L	370.057036	8855.22531	-4.580709463	9.36E-05	Low	268	10717	XP_003127967.2	ssc:100511267	2E-158	NM_006820.2	IFI44L
HOXB8	78.70616064	1809.549634	-4.523010304	9.48E-05	Low	57	2190	X06668		9E-144	NM_024016.3	HOXB8
HLF	153.2698918	9.089062089	4.075799084	9.49E-05	High	111	11	XP_003483076.1	ssc:100736723	3E-79	NM_002126.4	HLF
NETO2	153.2698918	9.089062089	4.075799084	9.49E-05	High	111	11	XP_003360626.1	ssc:100624026	0	NM_018092.4	NETO2
LOC100622492	0	77.67016694	-20	9.56E-05	Low	0	94	XP_005657142.1	ssc:100622492	0	YC006_HUMAN	LOC100132731
C1QTNF7	0	77.67016694	-20	9.56E-05	Low	0	94	NM_001143702.1	ssc:100233173	2E-145	NM_031911.4	C1QTNF7
LOC102166831	2.761619671	137.9884881	-5.642889456	9.68E-05	Low	2	167	XR_299766.1	ssc:102166831			
HSPG2	706.9746359	17333.66768	-4.615774684	9.69E-05	Low	512	20978	XP_005656099.1	ssc:100517288	0	NM_005529.5	HSPG2
LOC102167451	747.0181211	57.01320765	3.711775163	9.83E-05	High	541	69	XP_005666318.1	ssc:102167451	2E-16	NR_049767.1	SLC7A8
GPM6B	46.94753442	1083.250945	-4.528174293	9.93E-05	Low	34	1311	XP_005673502.1	ssc:100155237	1E-155	NM_005278.3	GPM6B
LOC102165939	27.61619671	660.196419	-4.579308762	1.00E-04	Low	20	799	XP_005656101.1	ssc:102165939	0	NM_005529.5	HSPG2
FSCN2	13.80809836	366.867597	-4.731672935	1.00E-04	Low	10	444	XP_003357978.1	ssc:100625228	0	NM_012418.3	FSCN2
SLC1A6	1027.322518	79.32272369	3.695011147	1.01E-04	High	744	96	XP_005652965.1	ssc:100518296	0	NR_073589.1	SLC1A6
LOC102161033	42.80510491	987.4026542	-4.527783756	1.02E-04	Low	31	1195	XR_299987.1	ssc:102161033			
PAQR5	111.8455967	5.783948602	4.273309917	1.02E-04	High	81	7	XP_005659931.1	ssc:100521597	1E-165	NM_017705.3	PAQR5
C14H10orf82	60.75563277	1.652556743	5.200246455	1.03E-04	High	44	2	XP_005671539.1	ssc:100154177	9E-70	NM_144661.2	C10orf82
LOC100738560	60.75563277	1.652556743	5.200246455	1.03E-04	High	44	2	XP_005662804.1	ssc:100738560	3E-107	NM_198182.2	GRHL1
ACAP1	372.8186556	27.26718627	3.773236314	1.03E-04	High	270	33	XP_005669280.1	ssc:100623422	0	NM_014716.3	ACAP1
LOC100516238	121.5112655	6.610226974	4.200246455	1.05E-04	High	88	8	XP_005673868.1	ssc:100516238	1E-92	NM_018015.5	CXorf57
LOC100624711	1.380809836	106.58991	-6.270412419	1.07E-04	Low	1	129	XP_003360906.1	ssc:100624711	0	NM_152999.3	STEAP2
LOC102163074	6.904049179	220.6163253	-4.997953001	1.07E-04	Low	5	267	XP_005654413.1	ssc:102163074			
DOCK10	2.761619671	135.509653	-5.616737168	1.07E-04	Low	2	164	XP_005672312.1	ssc:100512141	0	NM_017718.1	DOCK10
SSTR1	0	76.0176102	-20	1.07E-04	Low	0	92	NM_001190231.1	ssc:397004	0	NM_001049.2	SSTR1
CCDC110	0	76.0176102	-20	1.07E-04	Low	0	92	XP_005671780.1	ssc:100517861	0	NM_152775.3	CCDC110
LEPREL2	436.3359081	10254.11459	-4.554619911	1.08E-04	Low	316	12410	XP_005652654.1	ssc:100156539	0	NM_014262.4	LEPREL2
LOC100516650	26.23538688	621.3613355	-4.565846502	1.09E-04	Low	19	752	XP_005658305.1	ssc:100516650	0	NM_004735.3	LRRFIP1
LOC100515976	6.904049179	219.7900469	-4.992539504	1.09E-04	Low	5	266	XR_135434.2	ssc:100515976			
GAS6	88.37182949	1974.805308	-4.481980067	1.11E-04	Low	64	2390	XP_005653903.1	ssc:100519331	0	NM_001143946.1	GAS6
LOC100621566	5.523239343	190.8703039	-5.110934205	1.11E-04	Low	4	231	XP_003361361.3	ssc:100621566	4E-167	NM_030672.2	ARHGAP28
TNNI1	12.42728852	331.3376271	-4.736718589	1.12E-04	Low	9	401	NM_213912.3	ssc:396947	1E-103	NM_003281.3	TNNI1
SRPX	44.18591474	999.7968298	-4.499976496	1.13E-04	Low	32	1210	XP_005673612.1	ssc:100156108	0	NM_006307.4	SRPX
KCNIP4	179.5052786	11.5678972	3.955827727	1.13E-04	High	130	14	XP_003482404.2	ssc:100516499	4E-148	NM_147183.3	KCNIP4
FAM185A	1400.141173	109.8950234	3.671374338	1.13E-04	High	1014	133	XP_005667762.1	ssc:100512804	4E-116	NR_026879.1	FAM185A
MYOT	0	75.19133183	-20	1.14E-04	Low	0	91	NM_001099941.2	ssc:100101550	0	NM_006790.2	MYOT
LOC100156578	0	75.19133183	-20	1.14E-04	Low	0	91	XP_005674329.1	ssc:100156578	2E-117	NM_002506.2	NGF
LOC102164159	0	75.19133183	-20	1.14E-04	Low	0	91	XP_005666956.1	ssc:102164159	5E-52	NM_016599.4	MYOZ2
LOC100736635	0	75.19133183	-20	1.14E-04	Low	0	91	XR_300401.1	ssc:100736635			
LOC100623148	5.523239343	190.0440255	-5.104675215	1.14E-04	Low	4	230	XP_003361739.2	ssc:100623148	2E-122	NM_014331.3	SLC7A11
FRMPD1	120.1304557	6.610226974	4.183758332	1.14E-04	High	87	8	XP_005660309.1	ssc:100153190	0	NM_014907.2	FRMPD1
BICC1	28.99700655	672.5905946	-4.535752725	1.16E-04	Low	21	814	XP_001929158.3	ssc:100154253	0	NM_001080512.1	BICC1
VCAN	374.1994655	8627.998758	-4.527146566	1.16E-04	Low	271	10442	NM_001206429.1	ssc:397328	0	NM_004385.4	VCAN
ARHGAP8	792.5848457	61.97087788	3.676903072	1.17E-04	High	574	75	NM_001204383.1	ssc:100533202	0	NM_181334.4	PRR5-ARHGAP8
SNED1	34.52024589	786.6170099	-4.510146737	1.18E-04	Low	25	952	XP_003133861.2	ssc:100512566	0	NM_001080437.1	SNED1
LOC102162351	2.761619671	133.0308178	-5.590102042	1.18E-04	Low	2	161	XP_005657866.1	ssc:102162351	0	NR_027121.1	SHROOM4
LOC100525734	37.28186556	0	20	1.18E-04	High	27	0	XR_298103.1	ssc:100525734	2E-35	NM_005498.4	AP1M2
LOC102164369	37.28186556	0	20	1.18E-04	High	27	0	XR_305196.1	ssc:102164369			
LOC100521071	37.28186556	0	20	1.18E-04	High	27	0	XR_298107.1	ssc:100521071			
MET	5.523239343	188.3914688	-5.092075178	1.20E-04	Low	4	228	NM_001038008.1	ssc:654328	0	NM_001127500.1	MET
LOC102166540	0	74.36505346	-20	1.20E-04	Low	0	90	XR_304431.1	ssc:102166540			
LOC102167766	0	74.36505346	-20	1.20E-04	Low	0	90	XP_005655621.1	ssc:102167766	6E-117	NM_020983.2	ADCY6
PBX1	194.6941868	4349.529349	-4.481577585	1.21E-04	Low	141	5264	XP_005655431.1	ssc:100154639	0	NM_002585.3	PBX1
LOC100156557	89.75263932	4.131391859	4.441254554	1.21E-04	High	65	5	XP_001928622.1	ssc:100156557	4E-74	NM_003537.3	HIST1H3B
LOC100623335	1.380809836	104.1110748	-6.236465087	1.21E-04	Low	1	126	XP_005658115.1	ssc:100623335	1E-135	NM_022137.5	SMOC1
CCDC147	59.37482294	1.652556743	5.167079591	1.22E-04	High	43	2	XP_005671580.1	ssc:100152602	0	NM_001008723.1	CCDC147
LOC100155350	110.4647869	2431.737248	-4.460328896	1.22E-04	Low	80	2943	XP_003134499.4	ssc:100155350	2E-111	NM_175913.3	JPH2
LOC102164964	109.083977	5.783948602	4.237240662	1.24E-04	High	79	7	XR_308814.1	ssc:102164964			
SDK2	8.284859014	242.0995629	-4.868979518	1.25E-04	Low	6	293	XP_005668701.1	ssc:100518574	0	NM_019064.3	SDK2
PLXNB3	664.169531	52.05553742	3.673427996	1.25E-04	High	481	63	XP_005674065.1	ssc:100525146	0	NM_005393.2	PLXNB3
SEMA7A	1.380809836	103.2847965	-6.224969449	1.26E-04	Low	1	125	XP_005658058.1	ssc:100623369	0	NM_003612.3	SEMA7A
LOC100626467	0	73.53877508	-20	1.28E-04	Low	0	89	XP_005664123.1	ssc:100626467	3E-154	NM_004963.3	GUCY2C
GPR123	0	73.53877508	-20	1.28E-04	Low	0	89	XP_005658819.1	ssc:100623440	2E-139	NM_032422.2	GPR123
FUT4	11.04647869	295.8076571	-4.743000941	1.28E-04	Low	8	358	XP_005667293.1	ssc:100513419	5E-170	NM_002033.3	FUT4
KLF6	287.2084458	6422.661783	-4.483001247	1.30E-04	Low	208	7773	NM_001134353.2	ssc:100174961	1E-139	NR_027653.1	KLF6
ELTD1	2.761619671	130.5519827	-5.562965912	1.31E-04	Low	2	158	XP_005665418.1	ssc:100525348	0	NM_022159.3	ELTD1
DRP2	1054.938715	84.28039392	3.645818322	1.33E-04	High	764	102	XP_005673821.1	ssc:100517071	0	NM_001939.2	DRP2
BMP5	78.70616064	3.305113487	4.57370485	1.34E-04	High	57	4	NM_001204901.1	ssc:100151964			
PTCHD1	0	72.71249671	-20	1.35E-04	Low	0	88	XP_001926295.3	ssc:100151829	0	NM_173495.2	PTCHD1
MYF5	0	72.71249671	-20	1.35E-04	Low	0	88	Y17154		3E-143	NM_005593.2	MYF5
SHOX	0	72.71249671	-20	1.35E-04	Low	0	88	XP_003134962.1	ssc:100520816	3E-138	NM_006883.2	SHOX
LOC100739096	55.23239343	1198.103639	-4.439094254	1.36E-04	Low	40	1450	XP_003482089.2	ssc:100739096	7E-87	NR_023359.1	CABLES1
LOC100524566	243.0225311	17.35184581	3.807929032	1.36E-04	High	176	21	LOC100524566				
THBD	787.0616064	62.79715625	3.647705432	1.38E-04	High	570	76	NM_001130732.1	ssc:100157642	0	NM_000361.2	THBD
PLCH1	1222.016705	98.32712624	3.635530718	1.39E-04	High	885	119	XP_005669995.1	ssc:100627163	0	NM_014996.2	PLCH1
LOC100156937	615.8411867	48.75042393	3.659071687	1.39E-04	High	446	59	XP_001927752.1	ssc:100156937	2E-68	NM_003518.3	HIST1H2BG
LOC595121	3552.823707	282.5872031	3.652198063	1.40E-04	High	2573	342	NM_001031791.1	ssc:595121	3E-115	NM_212460.3	ARL4A
LOC100519237	4.142429507	156.1666123	-5.236465087	1.40E-04	Low	3	189	XP_005652760.1	ssc:100519237	1E-127	NM_006200.3	PCSK5
LOC102160583	1.380809836	100.8059614	-6.189922501	1.44E-04	Low	1	122	XR_302321.1	ssc:102160583			
LOC100626969	0	71.88621834	-20	1.44E-04	Low	0	87	XP_003360867.2	ssc:100626969			
DNAH6	0	71.88621834	-20	1.44E-04	Low	0	87	XP_003125020.3	ssc:100524798	0	NM_173645.1	DNAH6
LOC100738727	0	71.88621834	-20	1.44E-04	Low	0	87	XP_003483006.1	ssc:100738727	0	NM_172386.1	ABCA9
LOC102167011	0	71.88621834	-20	1.44E-04	Low	0	87	XR_302256.1	ssc:102167011			
ZNF25	15.18890819	370.9989889	-4.61032518	1.44E-04	Low	11	449	ZNF25		0	NM_145011.2	ZNF25
LOC100624881	46.94753442	1013.017284	-4.431465586	1.45E-04	Low	34	1226	LOC100624881				
LOC102161702	164.3163705	10.74161883	3.935192881	1.45E-04	High	119	13	XR_298374.1	ssc:102161702			
MKRN3	57.9940131	1.652556743	5.133132259	1.45E-04	High	42	2	XP_001925048.1	ssc:100155343	2E-152	NM_005664.3	MKRN3
NRP1	96.6566885	2054.95431	-4.410092939	1.47E-04	Low	70	2487	XP_005668239.1	ssc:100286859	0	NR_045259.1	NRP1
LOC100525821	385.2459442	29.74602138	3.695011147	1.49E-04	High	279	36	XR_304334.1	ssc:100525821	9E-75	NM_003537.3	HIST1H3B
CNNM1	19.3313377	450.3217126	-4.541942661	1.49E-04	Low	14	545	XP_005671437.1	ssc:100154357	2E-24	NM_020348.2	CNNM1
EFHD1	689.024108	55.36065091	3.637621651	1.50E-04	High	499	67	XP_001925420.4	ssc:100153398	1E-92	NM_025202.3	EFHD1

LOC102161782	67.65968195	2.478835115	4.77056218	1.52E-04	High	49	3	XP_005663571.1	ssc:102161782	1E-74	NM_001005464.2	HIST2H3A
PTPRR	31.75862622	695.726389	-4.453299631	1.52E-04	Low	23	842	XP_005664036.1	ssc:100154962	0	NR_073474.1	PTPRR
ARHGAP25	0	71.05993997	-20	1.53E-04	Low	0	86	XP_003125117.3	ssc:100514401	0	NM_014882.2	ARHGAP25
CDKL1	0	71.05993997	-20	1.53E-04	Low	0	86	XP_001925107.3	ssc:100152969			
FILIP1	0	71.05993997	-20	1.53E-04	Low	0	86	XP_005659495.1	ssc:100514614	0	NM_015687.2	FILIP1
IL11RA	245.7841508	5303.880868	-4.431584579	1.53E-04	Low	178	6419	XP_003130718.3	ssc:100524166	9E-153	NR_052010.1	IL11RA
C-ERBA-B1	52.47077376	1115.475802	-4.410001342	1.54E-04	Low	38	1350	XP_005669358.1	ssc:396776	0	NM_001252634.1	THRB
LOC102161562	35.90105573	0	20	1.54E-04	High	26	0	XP_005668602.1	ssc:102161562	2E-91	NM_015011.1	MYO16
SASH3	35.90105573	0	20	1.54E-04	High	26	0	XP_003135424.2	ssc:100525094	0	NM_018990.3	SASH3
LOC102157889	35.90105573	0	20	1.54E-04	High	26	0	XP_005652891.1	ssc:102157889	1E-138	NM_007134.1	ZNF135
LOC102164008	35.90105573	0	20	1.54E-04	High	26	0	XR_300594.1	ssc:102164008			
LCN12	35.90105573	0	20	1.54E-04	High	26	0	XP_003122373.1	ssc:100511618	1E-44	NM_178536.3	LCN12
LOC100623014	35.90105573	0	20	1.54E-04	High	26	0	XR_299375.1	ssc:100623014	6E-49	NR_038257.1	TRPM2
LRG1	35.90105573	0	20	1.54E-04	High	26	0	XP_003123119.1	ssc:100524848	3E-132	NM_052972.2	LRG1
LOC102167688	35.90105573	0	20	1.54E-04	High	26	0	XR_298736.1	ssc:102167688			
HSD17B3	35.90105573	0	20	1.54E-04	High	26	0	NM_001244790.1	ssc:100513224	1E-150	NM_000197.1	HSD17B3
SR-PSOX	85.61020982	1803.765685	-4.397085268	1.55E-04	Low	62	2183	NM_213811.1	ssc:396735	8E-62	NM_022059.3	CXCL16
ARHGAP31	140.8426032	2977.907252	-4.402143118	1.57E-04	Low	102	3604	XP_003132708.3	ssc:100511412	0	NM_020754	ARHGAP31
DPF1	1314.530964	107.4161883	3.613264786	1.57E-04	High	952	130	XP_005664622.1	ssc:100625938	0	NM_004647.2	DPF1
ZNF568	11.04647869	285.0660382	-4.689637716	1.57E-04	Low	8	345	ZNF568	ssc:100511135	0	NM_198539.3	ZNF568
TMPRSS2	46.94753442	0.826278372	5.828277677	1.59E-04	High	34	1	XP_005670425.1	ssc:100739292	6E-121	NM_005656.3	TMPRSS2
IGDCC4	236.1184819	5046.908295	-4.417816996	1.60E-04	Low	171	6108	XP_001926606.1		0	NM_020962.1	IGDCC4
LOC102164461	0	70.2336616	-20	1.62E-04	Low	0	85	XP_005674472.1	ssc:102164461	9E-17	NR_027382.1	RDH13
LOC100621791	0	70.2336616	-20	1.62E-04	Low	0	85	XP_003359328.1	ssc:100621791	2E-64	NM_007021.3	C10orf10
LOC100624226	67.65968195	1413.762294	-4.385099364	1.64E-04	Low	49	1711	XP_005670489.1	ssc:100624226	5E-80	NR_027140.1	TNFRSF10B
LACC1	60.75563277	1271.642414	-4.387531062	1.64E-04	Low	44	1539	XP_005668502.1	ssc:100153856	0	NM_153218.2	LACC1
LOC100153773	13.80809836	335.4690189	-4.602592986	1.65E-04	Low	10	406	XP_005656291.1	ssc:100153773	2E-36	NM_005822.3	RCAN2
NEXN	388.0075638	8418.95033	-4.439483688	1.65E-04	Low	281	10189	XP_005665432.1	ssc:100510913	0	NM_144573.3	NEXN
SEMA3B	182.2668983	3839.715593	-4.396874974	1.66E-04	Low	132	4647	XP_005669648.1	ssc:100153200	0	NM_004636.2	SEMA3B
LOC102159472	33.13943606	712.2519564	-4.425766722	1.66E-04	Low	24	862	XR_307747.1	ssc:102159472			
XIRP1	2.761619671	124.7680341	-5.497589903	1.67E-04	Low	2	151	NM_001143928.1	ssc:100134978	0	NM_194293.2	XIRP1
EBF1	19.3313377	439.5800938	-4.507112677	1.70E-04	Low	14	532	XP_005672639.1	ssc:100520467	0	NM_024007.3	EBF1
HKDC1	95.27587867	4.95767023	4.264376792	1.70E-04	High	69	6	XP_001928917.1	ssc:100153520	0	NM_025130.3	HKDC1
HES7	95.27587867	4.95767023	4.264376792	1.70E-04	High	69	6	XP_003483152.1	ssc:100620745	4E-85	NM_032580.3	HES7
LOC102165278	95.27587867	4.95767023	4.264376792	1.70E-04	High	69	6	XP_005668616.1	ssc:102165278	8E-112	NM_024979.4	MCF2L
SCN9A	302.397354	23.13579441	3.708246974	1.71E-04	High	219	28	XP_003133500.3	ssc:100516701	0	NM_002977.3	SCN9A
LOC100519616	57.9940131	1206.366423	-4.378620395	1.71E-04	Low	42	1460	XR_299064.1	ssc:100519616	2E-29	NM_014656.2	KIAA0040
LOC102162793	0	69.40738323	-20	1.73E-04	Low	0	84	XR_298475.1	ssc:102162793			
LIFR	7281.010264	582.5262521	3.64374367	1.73E-04	High	5273	705	XP_005672485.1	ssc:397451	0	NM_002310.5	LIFR
LOC100520029	628.2684753	13820.33205	-4.459267276	1.74E-04	Low	455	16726	XR_304045.1	ssc:100520029	4E-29	NM_006868.3	RAB31
DNAH3	216.7871442	15.69928906	3.787508072	1.74E-04	High	157	19	XP_005662161.1	ssc:100511869	4E-30	NM_001888.3	CRYM
LOC100627460	24.85457704	544.517447	-4.453394817	1.74E-04	Low	18	659	LOC100627460				
LOC100736977	23.47376721	516.4239823	-4.459434702	1.76E-04	Low	17	625	XP_005665750.1	ssc:100736977	5E-46	NM_002127.5	HLA-G
LOC100620664	4.142429507	149.5563853	-5.17406855	1.77E-04	Low	3	181	XR_131350.3	ssc:100620664			
LOC100513741	19.3313377	434.6224235	-4.490749231	1.81E-04	Low	14	526	XR_303907.1	ssc:100513741	0	NR_049754.1	ZNF211
LOC102161964	12.42728852	303.2441624	-4.608896415	1.83E-04	Low	9	367	XP_005656083.1	ssc:102161964	0	NM_020765.2	UBR4
PDLIM3	0	68.58110485	-20	1.84E-04	Low	0	83	NM_001001637.1	ssc:414421	0	NR_047562.1	PDLIM3
LOC100623800	0	68.58110485	-20	1.84E-04	Low	0	83	XP_005658194.1	ssc:100623800	2E-136	NM_022659.3	EBF2
SELENBP1	12666.16862	1003.101943	3.658440054	1.85E-04	High	9173	1214	XP_001929678.1	ssc:100152724	0	NM_003944.3	SELENBP1
AKAP5	403.196472	32.2248565	3.645237176	1.85E-04	High	292	39	XP_005666363.1	ssc:100153460	8E-172	NM_004857.3	AKAP5
LOC100522856	175.3628491	3612.489041	-4.364578163	1.87E-04	Low	127	4372	XR_301863.1	ssc:100522856	6E-64	NM_198709.2	ARSB
RASAL2	92.51425899	1876.478182	-4.342207964	1.92E-04	Low	67	2271	XP_005667843.1	ssc:100522392	0	NM_170692.2	RASAL2
GATA-6	2297.667567	191.6965822	3.583273559	1.93E-04	High	1664	232	NM_214328.2	ssc:397600	0	NM_005257.4	GATA6
AKR1CL1	325.8711212	6820.927959	-4.387594614	1.94E-04	Low	236	8255	NM_001038626.2	ssc:654406	6E-140	NM_003739.5	AKR1C3
NLRC5	8.284859014	223.0951604	-4.75103826	1.95E-04	Low	6	270	NM_001278781.1	ssc:100135667	2E-30	NM_032206.4	NLRC5
H2AFY2	20.71214754	454.4531045	-4.455582377	1.96E-04	Low	15	550	XR_299447.1	ssc:100152359	5E-64	NM_018649.2	H2AFY2
RGS16	0	67.75482648	-20	1.96E-04	Low	0	82	NM_001113017.1	ssc:397544	3E-65	NM_002928.3	RGS16
TNMD	0	67.75482648	-20	1.96E-04	Low	0	82	NM_001099934.1	ssc:100048961	0	NM_022144.2	TNMD
LOC102158004	0	67.75482648	-20	1.96E-04	Low	0	82	XP_005658330.1	ssc:102158004	1E-45	NM_022369.3	STRA6
LOC100739264	0	67.75482648	-20	1.96E-04	Low	0	82	XP_003481977.1	ssc:100739264	1E-81	NM_021170.3	HES4
AMOTL2	238.8801016	4927.924209	-4.366621478	1.96E-04	Low	173	5964	XP_005669919.1	ssc:100523696	0	NM_016201.3	AMOTL2
COL4A5	11.04647869	273.498141	-4.629872571	1.97E-04	Low	8	331	XP_003135355.3	ssc:100519180	0	NM_033381.1	COL4A5
CDH15	5.523239343	171.8659013	-4.959624882	1.98E-04	Low	4	208	XP_005655832.1	ssc:100187727	0	NM_001793.4	CDH3
LOC100622470	22.09295737	478.4151772	-4.436604702	2.01E-04	Low	16	579	XP_005658278.1	ssc:100622470	0	NM_016608.1	ARMCX1
ADRA1A	34.52024589	0	20	2.02E-04	High	25	0	NM_001123072.1	ssc:100144471	0	NM_033304.2	ADRA1A
PPEF2	34.52024589	0	20	2.02E-04	High	25	0	XP_003129165.2	ssc:100511942	0	NM_152934.1	PPEF2
MORC1	34.52024589	0	20	2.02E-04	High	25	0	XP_005654123.1	ssc:100620480	0	NM_014429.3	MORC1
LOC102163381	34.52024589	0	20	2.02E-04	High	25	0	XR_307961.1	ssc:102163381			
TNFSF10	5.523239343	171.0396229	-4.952672121	2.03E-04	Low	4	207	NM_001024696.1	ssc:406191	1E-85	NR_033994.1	TNFSF10
LOC100522595	4.142429507	145.4249934	-5.133654282	2.05E-04	Low	3	176	XR_309484.1	ssc:100522595			
KCNN1	397.6732327	32.2248565	3.625337619	2.06E-04	High	288	39	KCNN1		0	NM_002248.4	KCNN1
MAGI2	2.761619671	119.8103639	-5.439094254	2.08E-04	Low	2	145	XP_005667758.1	ssc:100515047	0	NM_012301.3	MAGI2
TRIM55	0	66.92854811	-20	2.09E-04	Low	0	81	NM_001244691.1	ssc:100152010	0	NM_184087.1	TRIM55
LRFN5	0	66.92854811	-20	2.09E-04	Low	0	81	XP_005659971.1	ssc:102163172	0	NM_152447.3	LRFN5
NOR-1	276.1619671	21.48323766	3.684231308	2.09E-04	High	200	26	NM_214247.1	ssc:397479	0	NM_173200.2	NR4A3
TNK1	359.0105573	28.91974301	3.633896632	2.12E-04	High	260	35	XP_003358294.1	ssc:100621720	0	NM_003985.4	TNK1
CADM3	621.3644261	52.05553742	3.577316104	2.14E-04	High	450	63	XP_003481475.1	ssc:100157273	0	NM_021189.3	CADM3
SLC16A10	508.1380196	42.14019696	3.59195145	2.14E-04	High	368	51	XP_005654457.1	ssc:100513770	4E-105	NM_018593.4	SLC16A10
LOC102163300	19.3313377	421.4019696	-4.446183659	2.15E-04	Low	14	510	XP_005671664.1	ssc:102163300	9E-77	NR_033258.1	ZEB2
GULP1	52.47077376	1048.547254	-4.320734004	2.17E-04	Low	38	1269	XP_003133585.4	ssc:100511723	5E-92	NR_045563.1	GULP1
LOC102162054	28.99700655	599.8780979	-4.370693479	2.19E-04	Low	21	726	XR_301502.1	ssc:102162054			
DKK1	5.523239343	168.5607878	-4.931610506	2.19E-04	Low	4	204	NM_001145384.1	ssc:100157640	9E-133	NM_012242.2	DKK1
LOC100622865	6.904049179	193.349139	-4.807621789	2.20E-04	Low	5	234	XP_005669276.1	ssc:100622865	1E-78	NM_001365.3	DLG4
LOC100738804	23.47376721	495.767023	-4.400541013	2.21E-04	Low	17	600	XP_003481936.1	ssc:100738804	3E-174	NM_032040.4	CCDC8
TENM1	0	66.10226974	-20	2.23E-04	Low	0	80	XP_005673947.1	ssc:100520416	0	NM_014253.3	TENM1
PRRT1	2.761619671	118.1578072	-5.419056501	2.24E-04	Low	2	143	NM_001123170.1	ssc:100144520	9E-106	NM_030651.3	PRRT1
TPST2	3221.429347	271.8455843	3.566841654	2.25E-04	High	2333	329	NM_001243490.1	ssc:100154448	0	NM_003595.3	TPST2
LOC100523100	73.18292129	3.305113487	4.468735291	2.32E-04	High	53	4	LOC100523100	ssc:100523100			
MAP2K6	327.2519311	26.4409079	3.629558085	2.33E-04	High	237	32	XR_306438.1	ssc:100625946	0	NM_031988.1	MAP2K6
LRRC37A	317.5862622	25.61462952	3.632108577	2.36E-04	High	230	31	XP_005668769.1	ssc:102159591	0	NM_014834.4	LRRC37A
OSAP	307.9205934	24.78835115	3.63482414	2.39E-04	High	223	30	NM_001195363.1	ssc:100359351	6E-85	NM_032623.3	MGARP
LOC100737121	262.3538688	20.65695929	3.666814255	2.40E-04	High	190	25	XP_005662278.1	ssc:100737121			
FRZB	4.142429507	141.2936016	-5.092075178	2.40E-04	Low	3	171	NM_001243330.1	ssc:100152419	3E-171	NM_001463.3	FRZB
CASZ1	144.9850328	9.915340461	3.870097853	2.41E-04	High	105	12	XP_005665059.1	ssc:100510911	0	NM_017766.4	CASZ1
CD200	1169.545931	100.8059614	3.536295658	2.42E-04	High	847	122	XP_005670288.1	ssc:100626182	1E-116	NM_005944.5	CD200
LOC100037967	1746.724442	150.3826637	3.537941866	2.43E-04	High	1265	182	XP_003359834.1	ssc:100037967	0	NM_006203.4	PDE4D
C7H15orf27	316.2054524	25.61462952	3.625822314	2.44E-04	High	229	31	XP_001928867.3	ssc:100152648	0	NM_152335.2	C15orf27
CMAH	5.523239343	165.2556743	-4.903041354	2.44E-04	Low	4	200	NM_001113015.1	ssc:396918			
NNAT	414.2429507	8396.640814	-4.341263222	2.46E-04	Low	300	10162	NM_001122990.1	ssc:449004	2E-41	NM_181689.1	NNAT
LOC100511764	44.18591474	0.826278372	5.740814836	2.46E-04	High	32	1	XR_304933.1	ssc:100511764			

MAST1	44.18591474	0.826278372	5.740814836	2.46E-04	High	32	1	XP_005652946.1	ssc:100626936	0	NM_014975.2	MAST1
LOC102167117	44.18591474	0.826278372	5.740814836	2.46E-04	High	32	1	XR_309083.1	ssc:102167117			
ITGB3	118.7496459	2304.490379	-4.278452628	2.49E-04	Low	86	2789	NM_214002.1	ssc:397063	0	NM_000212.2	ITGB3
A4GALT	11727.21793	965.0931382	3.603048812	2.51E-04	High	8493	1168	XP_005663821.1	ssc:100524987	2E-170	NM_017436.4	A4GALT
GDAP1L1	5.523239343	164.429396	-4.895809784	2.51E-04	Low	4	199	XP_005673013.1	ssc:100625236	0	NR_046353.1	GDAP1L1
ZNF521	93.89506883	1812.028469	-4.270412419	2.53E-04	Low	68	2193	ZNF521	ssc:100739769	0	NM_015461.2	ZNF521
IGFBP4	125.6536951	2432.563526	-4.27495248	2.53E-04	Low	91	2944	NM_001123129.1	ssc:100144490	1E-134	NM_001552.2	IGFBP4
TMEM100	0	64.44971299	-20	2.54E-04	Low	0	78	XP_005669010.1	ssc:100523465	2E-63	NM_018286.2	TMEM100
LOC100739211	0	64.44971299	-20	2.54E-04	Low	0	78	XP_003482272.2	ssc:100739211	3E-102	NM_207517.2	ADAMTSL3
LOC100521743	0	64.44971299	-20	2.54E-04	Low	0	78	XP_003357786.2	ssc:100521743	0	NM_001081.3	CUBN
FCGBP	197.4558065	14.87301069	3.730761171	2.55E-04	High	143	18	XP_005674577.1	ssc:100625830	2E-104	NM_003890.2	FCGBP
LOC102165618	4.142429507	139.6410448	-5.075102099	2.56E-04	Low	3	169	XP_005660020.1	ssc:102165618			
PIP5K1B	143.6042229	9.915340461	3.856292054	2.59E-04	High	104	12	XP_003480579.2	ssc:100737867	3E-157	NM_003558.3	PIP5K1B
LOC102164445	8.284859014	211.5272632	-4.674222663	2.61E-04	Low	6	256	XR_301561.1	ssc:102164445			
HIGD1A	20549.21198	1669.908589	3.621242033	2.66E-04	High	14882	2021	XP_005669484.1	ssc:100524219	2E-47	NM_014056.3	HIGD1A
TEF1	38.6626754	755.2184318	-4.287880597	2.66E-04	Low	28	914	NM_001142669.1	ssc:100216478	0	NM_021961.5	TEAD1
LOC100736595	89.75263932	4.95767023	4.178220148	2.68E-04	High	65	6	XR_135365.2	ssc:100736595			
LOC102161514	33.13943606	0	20	2.68E-04	High	24	0	XR_299126.1	ssc:102161514			
LOC102157576	33.13943606	0	20	2.68E-04	High	24	0	XR_303177.1	ssc:102157576			
ZNF488	33.13943606	0	20	2.68E-04	High	24	0	ZNF488	ssc:100519569	5E-122	NM_153034.2	ZNF488
HNF4A	33.13943606	0	20	2.68E-04	High	24	0	NM_001044571.1	ssc:733636	0	NM_178850.2	HNF4A
LOC102160260	0	63.62343462	-20	2.72E-04	Low	0	77	XR_302319.1	ssc:102160260			
F13A1	0	63.62343462	-20	2.72E-04	Low	0	77	XP_001927665.3	ssc:100153504	0	NM_000129.3	F13A1
LOC102165879	0	63.62343462	-20	2.72E-04	Low	0	77	XR_301495.1	ssc:102165879			
GPD1L	6355.867674	538.7334984	3.560445409	2.72E-04	High	4603	652	XP_003483187.1	ssc:100624347	0	NM_015141.3	GPD1L
LOC396897	1.380809836	89.23806415	-6.014072666	2.75E-04	Low	1	108	NM_001113286.1	ssc:396897	7E-107	NM_173600.2	MUC19
RET	284.4468262	23.13579441	3.619960441	2.76E-04	High	206	28	XP_001926404.3	ssc:100153852	0	NM_020975.4	RET
MND1	2201.010878	192.5228606	3.515064605	2.83E-04	High	1594	233	XP_005653639.1	ssc:100517105	4E-87	NR_045605.1	MND1
RAB3IP	857.482908	75.19133183	3.511469654	2.84E-04	High	621	91	XP_005664031.1	ssc:100152578	2E-61	NR_103520.1	RAB3IP
LOC102164246	1494.036242	131.3782611	3.507416665	2.85E-04	High	1082	159	XR_306461.1	ssc:102164246			
LOC100737785	196.0749967	3757.914035	-4.260454583	2.85E-04	Low	142	4548	XP_003482952.2	ssc:100737785			
LOC100623840	930.6658293	81.8015588	3.508062997	2.86E-04	High	674	99	XP_005658230.1	ssc:100623840	1E-58	NM_021805.2	SIGIRR
ZNF180	11.04647869	255.3200169	-4.530648192	2.87E-04	Low	8	309	ZNF180	ssc:100625792	0	NM_013256.4	ZNF180
SYT11	16.56971803	351.168308	-4.405541694	2.88E-04	Low	12	425	XP_001928839.3	ssc:100154741	0	NM_152280.4	SYT11
KCNS3	3715.759268	322.248565	3.527411198	2.90E-04	High	2691	390	NM_001044596.1	ssc:733666	0	NM_002252.4	KCNS3
KITLG	0	62.79715625	-20	2.90E-04	Low	0	76	NM_214269.2	ssc:397509	3E-78	NM_003994.5	KITLG
BCO2	0	62.79715625	-20	2.90E-04	Low	0	76	XP_005667395.1	ssc:100517547	0	NM_031938.5	BCO2
DSP	2993.595724	261.1039655	3.519183129	2.91E-04	High	2168	316	XP_003128216.1	ssc:100156744	0	NM_004415.2	DSP
LOC102165895	185.028518	14.04673232	3.719441185	2.94E-04	High	134	17	XP_005655387.1	ssc:102165895	2E-35	NM_014957.2	DENND3
ADAMTS1	4458.63496	385.8719996	3.530407822	2.97E-04	High	3229	467	NM_001144843.1	ssc:497061			
TOM1L1	559.2279835	48.75042393	3.519949885	3.00E-04	High	405	59	XP_005669004.1	ssc:100522701	0	NM_005486.2	TOM1L1
RNF128	140.8426032	9.915340461	3.828277677	3.00E-04	High	102	12	XP_003135339.3	ssc:100515872	0	NM_194463.1	RNF128
LOC100515100	219.5487639	17.35184581	3.661380369	3.01E-04	High	159	21	XR_307393.1	ssc:100515100			
LOC100737132	20.71214754	418.9231345	-4.338136505	3.06E-04	Low	15	507	XP_005672443.1	ssc:100737132	0	NM_007118.2	TRIO
MRVI1	0	61.97087788	-20	3.11E-04	Low	0	75	XP_005661186.1	ssc:100739522	0	NM_130385.3	MRVI1
SLC35G2	0	61.97087788	-20	3.11E-04	Low	0	75	XP_005654084.1	ssc:100620415	0	NM_025246.2	SLC35G2
LOC102163560	0	61.97087788	-20	3.11E-04	Low	0	75	XR_297410.1	ssc:102163560			
LOC100626132	240.2609114	4564.361725	-4.247739272	3.12E-04	Low	174	5524	XP_003361657.1	ssc:100626132	7E-45	NM_144617.2	HSPB6
LOC100624137	2.761619671	110.7213018	-5.325274354	3.16E-04	Low	2	134	XR_299148.1	ssc:100624137			
RNF24	19.3313377	391.6559482	-4.340573491	3.21E-04	Low	14	474	XP_005672824.1	ssc:100519342	2E-83	NM_007219.3	RNF24
LOC396679	1322.815873	118.1578072	3.484826345	3.21E-04	High	958	143	NM_001244426.1	ssc:396679	0	NR_028435.1	AMT
LOC100510895	9.66566885	226.4002739	-4.549862325	3.24E-04	Low	7	274	XP_003124046.2	ssc:100510895	0	NM_032101.2	PCDHGB7
LOC100737762	34.52024589	653.586192	-4.242862859	3.29E-04	Low	25	791	XP_005672304.1	ssc:100737762			
LOC100524517	723.5443539	64.44971299	3.488835619	3.29E-04	High	524	78	XP_005653846.1	ssc:100524517	2E-175	NM_003240.3	LEFTY2
LOC494560	390.7691835	7495.17111	-4.26157281	3.31E-04	Low	283	9071	XP_003127107.1	ssc:494560	2E-76	NM_144617.2	HSPB6
KCTD7	38.6626754	725.4724104	-4.229907371	3.32E-04	Low	28	878	XP_005662070.1	ssc:100622993	4E-169	NM_153033.4	KCTD7
TMEM200A	16.56971803	342.0792459	-4.367709621	3.32E-04	Low	12	414	XP_005659253.1	ssc:100157644	0	NM_052913.2	TMEM200A
PCDH7	254.0690098	4780.020038	-4.233724452	3.33E-04	Low	184	5785	NM_001244484.1	ssc:100520035	0	NM_032457.3	PCDH7
GBP6	0	61.14459951	-20	3.33E-04	Low	0	74	XP_005663763.1	ssc:100523310	0	NM_198460.2	GBP6
LOC102159630	0	61.14459951	-20	3.33E-04	Low	0	74	XR_305716.1	ssc:102159630			
MED13L	52.47077376	970.0508084	-4.208474344	3.34E-04	Low	38	1174	XP_005657377.1	ssc:100151978	0	NM_015335.4	MED13L
LOC100513356	51.08996392	945.2624573	-4.209603135	3.34E-04	Low	37	1144	XP_003127281.1	ssc:100513356	3E-174	NM_032040.4	CCDC8
LOC100737260	60.75563277	2.478835115	4.615283954	3.35E-04	High	44	3	XR_299072.1	ssc:100737260	6E-79	NM_207108.2	ASTN1
NPAS1	1426.37656	128.0731476	3.47731497	3.36E-04	High	1033	155	XP_003127301.2	ssc:100517221	0	NM_002517.2	NPAS1
TP53I3	1966.273206	175.9972932	3.481838647	3.37E-04	High	1424	213	XP_003125411.1	ssc:100521786	4E-174	NM_147184.3	TP53I3
TTC39B	12.42728852	271.0193059	-4.446812167	3.38E-04	Low	9	328	XP_003121951.2	ssc:100515182	0	NM_152574.2	TTC39B
MME	4951.584071	434.6224235	3.51005562	3.42E-04	High	3586	526	XP_003132549.1	ssc:100511536	0	NM_007289.2	MME
LOC100623083	22.09295737	433.7961452	-4.295358776	3.44E-04	Low	16	525	XR_299921.1	ssc:100623083			
LOC100736916	62.13644261	1134.480204	-4.190447978	3.49E-04	Low	45	1373	XP_005667953.1	ssc:100736916	0	NM_025179.3	PLXNA2
PLXNA2	106.3223574	1935.970225	-4.186539862	3.51E-04	Low	77	2343	XP_005667952.1	ssc:100514595	0	NM_025179.3	PLXNA2
LOC100625965	1.380809836	85.10667229	-5.945685691	3.52E-04	Low	1	103	XR_302183.1	ssc:100625965			
KCNJ14	189.1709475	14.87301069	3.668921918	3.54E-04	High	137	18	XP_003127320.1	ssc:100521454	0	np_733838	np_733838
VSIG2	69.04049179	3.305113487	4.384671026	3.56E-04	High	50	4	XP_005667517.1	ssc:100522338	5E-119	NM_014312.3	VSIG2
LOC100620517	2.761619671	108.2424667	-5.292608165	3.57E-04	Low	2	131	XP_005657265.1	ssc:100620517	6E-84	NM_001495.4	GFRA2
LOC100621447	0	60.31832114	-20	3.57E-04	Low	0	73	XR_309679.1	ssc:100621447			
GFI1	31.75862622	0	20	3.58E-04	High	23	0	XP_005663730.1	ssc:102164883	0	NM_005263.3	GFI1
KCNK13	31.75862622	0	20	3.58E-04	High	23	0	NM_001244596.1	ssc:100623906	0	NM_022054.3	KCNK13
LOC102163722	31.75862622	0	20	3.58E-04	High	23	0	XP_005673901.1	ssc:102163722	4E-143	NM_001256761.1	HTR2C
ELAVL2	31.75862622	0	20	3.58E-04	High	23	0	XP_005654549.1	ssc:100153303	0	NM_004432.3	ELAVL2
C22orf41	31.75862622	0	20	3.58E-04	High	23	0	NM_001206362.1	ssc:100517654	2E-39	NM_001123225.1	SYCE3
SDR9C7	31.75862622	0	20	3.58E-04	High	23	0	XP_003355526.2	ssc:100626104	2E-172	NM_148897.2	SDR9C7
CRIP3	31.75862622	0	20	3.58E-04	High	23	0	XP_001926813.1	ssc:100154285	2E-98	NM_206922.2	CRIP3
LOC100737042	31.75862622	0	20	3.58E-04	High	23	0	XP_003483238.2	ssc:100737042	5E-81	NM_018398.2	CACNA2D3
KRT15	31.75862622	0	20	3.58E-04	High	23	0	XP_003131486.1	ssc:100515506	0	NM_002275.3	KRT15
GSDMA	31.75862622	0	20	3.58E-04	High	23	0	XP_003131545.1	ssc:100512434	0	NM_178171.4	GSDMA
RAD51C	760.8262195	68.58110485	3.471683913	3.58E-04	High	551	83	XP_005669031.1	ssc:100518577	2E-168	NR_103873.1	RAD51C
CMTM8	370.057036	32.2248565	3.521501808	3.63E-04	High	268	39	XP_003358400.2	ssc:100624671	8E-79	NM_178868.3	CMTM8
SLC25A53	248.5457704	20.65695929	3.588811743	3.64E-04	High	180	25	XP_003135335.1	ssc:100514813	1E-164	NM_001012755.4	SLC25A53
IL1R1	115.9880262	2099.573343	-4.178048401	3.65E-04	Low	84	2541	XP_005662423.1	ssc:100626904	0	NM_001288706.1	IL1R1
PHLDA1	231.9760524	4271.859182	-4.202816306	3.68E-04	Low	168	5170	XP_003126437.1	ssc:100523904	2E-134	NM_007350.3	PHLDA1
PLA2G16	5088.284245	451.147991	3.495506599	3.74E-04	High	3685	546	NM_001244514.1	ssc:100512584	8E-82	NM_007069.3	PLA2G16
PRSS23	2676.009462	241.2732845	3.471343133	3.74E-04	High	1938	292	XP_003357279.1	ssc:100520683	0	NM_007173.4	PRSS23
SNX18	40.04348524	732.0826374	-4.192367057	3.79E-04	Low	29	886	XP_003134014.3	ssc:100518876	0	NM_052870.2	SNX18
P14ARF	16.56971803	333.8164622	-4.332434146	3.79E-04	Low	12	404	NM_213735.1	ssc:396553	1E-32	NM_078487.2	CDKN2B
LOC102158372	85.61020982	4.95767023	4.110048646	3.80E-04	High	62	6	XP_005668759.1	ssc:102158372	5E-167	NM_001006607.2	LRRC37A2
ZNF814	34.52024589	636.2343462	-4.20404361	3.81E-04	Low	25	770	ZNF814	ssc:100737218	0	NM_005773.2	ZNF256
RBM24	0	59.49204276	-20	3.83E-04	Low	0	72	XP_005656270.1	ssc:100156013	5E-130	NM_153020.2	RBM24
LOC100512714	0	59.49204276	-20	3.83E-04	Low	0	72	XP_005663639.1	ssc:100512714	1E-126	NM_006492.2	ALX3
LIPM	0	59.49204276	-20	3.83E-04	Low	0	72	XP_001928475.1	ssc:100153843	0	NM_001128215.1	LIPM
PARVA	600.6522785	11429.08244	-4.250035727	3.87E-04	Low	435	13832	XP_005652864.1	ssc:100518536	2E-168	NM_018222.4	PARVA

MOGAT2	41.42429507	0.826278372	5.647705432	3.88E-04	High	30	1	NM_001167651.1	ssc:100312975	1E-164	NM_025098.2	MOGAT2
FAM167B	41.42429507	0.826278372	5.647705432	3.88E-04	High	30	1	XP_005665233.1	ssc:100519719	6E-33	NM_032648.2	FAM167B
MAP4K2	1318.673393	120.6366423	3.4503472	3.89E-04	High	955	146	XP_005660785.1	ssc:100523716	0	NM_004579.3	MAP4K2
PARP3	417.0045704	7787.673654	-4.223057328	3.90E-04	Low	302	9425	XP_005669672.1	ssc:100620838	0	NM_005485.4	PARP3
LOC102161128	1.380809836	83.45411554	-5.917396647	3.90E-04	Low	1	101	XP_005672168.1	ssc:102161128	2E-43	NM_201279.1	NRP2
WBP5	52.47077376	941.9573438	-4.166075759	3.92E-04	Low	38	1140	XP_003135329.2	ssc:100513331	4E-37	NM_016303.2	WBP5
ATP1B2	280.3043967	23.96207278	3.548169758	3.93E-04	High	203	29	XP_003131993.1	ssc:100522212	1E-174	NM_001678.3	ATP1B2
LOC100515214	178.1244688	14.04673232	3.66457925	3.94E-04	High	129	17	LOC100515214				
LOC100626745	59.37482294	2.478835115	4.58211709	3.96E-04	High	43	3	XP_005658833.1	ssc:100626745	1E-17	NM_203297.1	TRIM7
LOC100517092	186.4093278	14.87301069	3.647705432	3.97E-04	High	135	18	XR_303452.1	ssc:100517092	2E-55	NM_198320.3	CPM
EPB41L4B	127.0345049	9.089062089	3.804945174	4.04E-04	High	92	11	XP_005674634.1	ssc:100510946	0	NM_019114.4	EPB41L4B
ELMO1	3929.784793	354.4734215	3.470700945	4.06E-04	High	2846	429	XP_005673304.1	ssc:100522109	0	NR_038121.1	ELMO1
LOC102161796	57.9940131	1031.195408	-4.15226996	4.07E-04	Low	42	1248	XP_005657344.1	ssc:102161796			
SLC4A8	227.8336229	19.00440255	3.583575094	4.10E-04	High	165	23	XP_003481628.2	ssc:100521009	0	NR_047672.1	SLC4A8
PDLIM4	136.7001737	2430.084691	-4.151919613	4.11E-04	Low	99	2941	NM_001285969.1	ssc:100520235	7E-168	NM_003687.3	PDLIM4
LOC102164137	0	58.66576439	-20	4.11E-04	Low	0	71	XR_308197.1	ssc:102164137			
LOC102160155	0	58.66576439	-20	4.11E-04	Low	0	71	XP_005666779.1	ssc:102160155	0	NM_015236.4	LPHN3
SHROOM4	0	58.66576439	-20	4.11E-04	Low	0	71	XP_005674526.1	ssc:100513967	0	NR_027121.1	SHROOM4
IGSF11	510.8996392	46.27158882	3.464841375	4.12E-04	High	370	56	XP_005670253.1	ssc:100511781	0	NM_152538.2	IGSF11
AKR1C4	374.1994655	6869.678383	-4.198363155	4.18E-04	Low	271	8314	NM_001123075.1	ssc:100144511	6E-140	NM_003739.5	AKR1C3
LOC100739564	4.142429507	127.2468692	-4.941009204	4.20E-04	Low	3	154	XP_005660414.1	ssc:100739564	8E-118	NR_074078.1	RGS3
POPDC3	2.761619671	104.9373532	-5.247869851	4.20E-04	Low	2	127	NM_001144111.1	ssc:100151910	1E-156	NR_024539.1	POPDC3
LOC102161649	11.04647869	237.9681711	-4.429110165	4.21E-04	Low	8	288	XP_005659186.1	ssc:102161649	3E-174	NM_012454.3	TIAM2
LOC102160252	75.94454097	4.131391859	4.200246455	4.23E-04	High	55	5	XR_307479.1	ssc:102160252			
ARL4C	193.313377	3445.58081	-4.155733815	4.26E-04	Low	140	4170	NM_001244315.1	ssc:100514255	1E-111	NM_005737.3	ARL4C
PRDM16	5.523239343	148.7301069	-4.75103826	4.30E-04	Low	4	180	XP_005674579.1	ssc:100624476	0	NM_199454.2	PRDM16
LOC100737053	92.51425899	5.783948602	3.999549105	4.33E-04	High	67	7	XP_005666581.1	ssc:100737053	2E-107	NM_181876.2	PPP2R2C
AMACR	16.56971803	325.5536785	-4.296274483	4.34E-04	Low	12	394	XP_003133921.1	ssc:100512020	0	NM_203382.2	AMACR
LOC100512652	82.84859014	1448.465986	-4.127904849	4.37E-04	Low	60	1753	XR_130502.3	ssc:100512652			
DKK3	538.5158359	9967.395998	-4.210155972	4.39E-04	Low	390	12063	NM_001039749.1	ssc:664653	4E-151	NM_015881.5	DKK3
HSPB8	3617.72177	330.5113487	3.452309743	4.42E-04	High	2620	400	XP_001929620.1	ssc:100155338	1E-107	NM_014365.2	HSPB8
LOC102158649	0	57.83948602	-20	4.42E-04	Low	0	70	XR_300644.1	ssc:102158649			
CASP1	0	57.83948602	-20	4.42E-04	Low	0	70	NM_214162.1	ssc:397319	1E-174	NM_033295.3	CASP1
LOC100739130	175.3628491	14.04673232	3.642036682	4.43E-04	High	127	17	XP_005654941.1	ssc:100739130	2E-75	NM_001199209.1	CYP4F3
LOC100620529	26.23538688	480.8940123	-4.196132993	4.48E-04	Low	19	582	XP_005658322.1	ssc:100620529	0	NM_004408	DNM1
LOC100737127	40.04348524	708.9468429	-4.146038006	4.51E-04	Low	29	858	XP_005665344.1	ssc:100737127	2E-65	NM_006868.3	RAB31
ADAMTS7	259.5922491	4623.853768	-4.154776559	4.54E-04	Low	188	5596	XP_005656319.1	ssc:100154398	0	NM_014272.3	ADAMTS7
FSCN1	915.4769211	17303.09538	-4.240362821	4.54E-04	Low	663	20941	NM_001146300.1	ssc:100286741	4E-113	NM_003088.3	FSCN1
LOC100738694	1.380809836	80.97528043	-5.873895008	4.56E-04	Low	1	98	XP_005672166.1	ssc:100738694	2E-176	NM_205863.3	PARD3B
SYT3	291.3508753	25.61462952	3.507717714	4.61E-04	High	211	31	XP_003127416.2	ssc:100511086	0	NM_032298.2	SYT3
LOC100522999	28.99700655	523.0342093	-4.17292943	4.64E-04	Low	21	633	XR_309499.1	ssc:100522999			
LOC102166076	443.2399573	40.48764021	3.452534479	4.65E-04	High	321	49	XP_005672353.1	ssc:102166076	5E-105	NM_205862.1	UGT1A6
C6H19orf81	443.2399573	40.48764021	3.452534479	4.65E-04	High	321	49	XP_005664847.1	ssc:100510910	2E-88	NM_001195076.1	C19orf81
LOC100620994	46.94753442	819.6681448	-4.125918633	4.67E-04	Low	34	992	XP_005658809.1	ssc:100620994	0	NM_001286057.1	ADCY7
LOC102163089	240.2609114	20.65695929	3.539902142	4.73E-04	High	174	25	XR_306702.1	ssc:102163089			
CLIC6	140.8426032	10.74161883	3.71280046	4.73E-04	High	102	13	XP_003358996.1	ssc:100622868	0	NM_053277.1	CLIC6
LOC100621934	0	57.01320765	-20	4.76E-04	Low	0	69	XP_003361045.1	ssc:100621934	7E-52	NM_022369.3	STRA6
FAT3	0	57.01320765	-20	4.76E-04	Low	0	69	XP_003357289.2	ssc:100624134	0	NM_001008781.2	FAT3
CDRT1	0	57.01320765	-20	4.76E-04	Low	0	69	XP_005654045.1	ssc:100523091	2E-146	NR_051988.1	FBXW10
SLC2A2	30.37781639	0	20	4.81E-04	High	22	0	NM_001097417.1	ssc:397429	0	NM_001278659.1	SLC2A2
HAVCR1	30.37781639	0	20	4.81E-04	High	22	0	NM_001164736.1	ssc:100303721	2E-33	NM_012206.2	HAVCR1
LOC100626505	30.37781639	0	20	4.81E-04	High	22	0	XR_135242.2	ssc:100626505			
LOC100516860	30.37781639	0	20	4.81E-04	High	22	0	XP_003127932.3	ssc:100516860	0	NM_024421.2	DSC1
LOC102166196	30.37781639	0	20	4.81E-04	High	22	0	XP_005658392.1	ssc:102166196	3E-69	NM_014369.3	PTPN18
BCL2L14	99.41830817	6.610226974	3.910739838	4.82E-04	High	72	8	XP_003126516.1	ssc:100514901	2E-132	NM_138724.1	BCL2L14
SLITRK3	82.84859014	4.95767023	4.062742931	4.84E-04	High	60	6	XP_005657154.1	ssc:100153567	0	NM_014926.2	SLITRK3
LOC102159404	40.04348524	0.826278372	5.598795831	4.91E-04	High	29	1	XR_298033.1	ssc:102159404			
LOC102164629	40.04348524	0.826278372	5.598795831	4.91E-04	High	29	1	XR_303379.1	ssc:102164629			
LOC100739170	1034.226567	97.50084786	3.406993695	4.92E-04	High	749	118	XR_301936.1	ssc:100739170	2E-73	NM_022140.3	EPB41L4A
RAB33A	205.7406655	17.35184581	3.567665934	4.95E-04	High	149	21	NM_001123177.1	ssc:100144495	1E-140	NM_004794.2	RAB33A
LOC100628155	1445.707898	136.3359313	3.406538349	4.97E-04	High	1047	165	XP_005659044.1	ssc:100628155	1E-92	NM_005729.3	PPIF
BTC	5.523239343	144.5987151	-4.710396276	4.99E-04	Low	4	175	XP_005666828.1	ssc:100505411	8E-68	NM_001729.2	BTC
LOC100523701	53.85158359	923.7792196	-4.100487418	5.01E-04	Low	39	1118	XP_005672121.1	ssc:100523701	0	NM_001159.3	AOX1
CDC6	1478.847334	139.6410448	3.404678165	5.03E-04	High	1071	169	XP_005668909.1	ssc:100522031	0	NM_001254.3	CDC6
LOC100625519	399.0540425	7099.38377	-4.153037759	5.05E-04	Low	289	8592	XP_003354492.1	ssc:100625519	0	NM_003088.3	FSCN1
ANTXR2	383.8651343	6816.796567	-4.150422495	5.05E-04	Low	278	8250	XP_003129419.3	ssc:100514416	0	NM_058172.5	ANTXR2
METRNL	186.4093278	3214.222866	-4.107924007	5.09E-04	Low	135	3890	XP_005668624.1	ssc:100512305	1E-118	NM_001004431.1	METRNL
GBP5	0	56.18692928	-20	5.12E-04	Low	0	68	XP_001929321.1		0	NM_052942.3	GBP5
LOC102160100	131.1769344	9.915340461	3.725707944	5.13E-04	High	95	12	XR_298050.1	ssc:102160100			
LOC102167381	5.523239343	143.7724367	-4.70212866	5.15E-04	Low	4	174	XP_005659063.1	ssc:102167381	1E-21	NR_103804.1	PDLIM7
PLEKHH1	254.0690098	22.30951604	3.50948929	5.15E-04	High	184	27	XP_005656409.1	ssc:100152619	1E-143	NM_020715.2	PLEKHH1
LOC100519459	461.1904851	42.96647533	3.42407941	5.28E-04	High	334	52	XR_135471.2	ssc:100519459			
VAV3	9.66566885	206.5695929	-4.417614526	5.31E-04	Low	7	250	XP_005663675.1	ssc:100737105	0	NM_006113.4	VAV3
LOC100620419	171.2204196	2921.720322	-4.092891413	5.32E-04	Low	124	3536	XP_005657649.1	ssc:100620419	4E-113	NM_005737.3	ARL4C
LOC100523671	26.23538688	466.0210017	-4.150809003	5.32E-04	Low	19	564	LOC100523671				
WBSCR27	834.0091408	79.32272369	3.394257075	5.34E-04	High	604	96	XP_005662042.1	ssc:100526161	3E-92	NM_152559.2	WBSCR27
TGFB3	27.61619671	487.5042393	-4.141828213	5.35E-04	Low	20	590	NM_214198.1	ssc:397400	0	NM_003239.2	TGFB3
ATP1A4	1.380809836	78.49644531	-5.829040772	5.36E-04	Low	1	95	XP_005663264.1	ssc:100157711	0	NM_144699.3	ATP1A4
EPN3	1.380809836	78.49644531	-5.829040772	5.36E-04	Low	1	95	XP_005668947.1	ssc:100512679	0	NM_017957.2	EPN3
LOC100627414	1933.13377	183.4337985	3.397610082	5.38E-04	High	1400	222	XP_005666537.1	ssc:100627414	5E-25	NM_022965.3	FGFR3
UNC5CL	48.32834425	1.652556743	4.870097853	5.41E-04	High	35	2	XP_005674216.1	ssc:100156294	0	NM_173561.2	UNC5CL
C2H19orf38	2.761619671	99.97968298	-5.178048401	5.42E-04	Low	2	121	XP_005661306.1	ssc:100627549	3E-98	NM_001136482.1	C19orf38
SULF1	244.4033409	4198.320407	-4.102476363	5.47E-04	Low	177	5081	XP_005663089.1	ssc:100152427	0	NM_015170.2	SULF1
LOC102159912	0	55.36065091	-20	5.52E-04	Low	0	67	XR_306121.1	ssc:102159912			
LOC100738457	0	55.36065091	-20	5.52E-04	Low	0	67	XP_003480959.1	ssc:100738457	2E-116	NM_012159.4	FBXL21
LOC100621708	0	55.36065091	-20	5.52E-04	Low	0	67	XP_003361674.1	ssc:100621708	0	NM_001145030.1	TOPAZ1
FRMD7	0	55.36065091	-20	5.52E-04	Low	0	67	XP_003360499.2	ssc:100626525	0	NM_194277.2	FRMD7
ZNF311	56.61320327	2.478835115	4.51340434	5.56E-04	High	41	3	ZNF311	ssc:100524874	0	NM_001010877.2	ZNF311
SH2D2A	617.2219966	58.66576439	3.395198738	5.58E-04	High	447	71	XP_005663320.1	ssc:100153265	3E-154	NM_003975.3	SH2D2A
LOC100736904	64.89806228	3.305113487	4.295403688	5.58E-04	High	47	4	XP_003483712.2	ssc:100736904	6E-44	NM_004490.2	GRB14
AHCYL2	4955.7265	461.8896098	3.423476571	5.60E-04	High	3589	559	XP_005657816.1	ssc:100514931	0	NM_015328.3	AHCYL2
ALDH1L2	434.9550983	7625.723093	-4.131935765	5.60E-04	Low	315	9229	XP_001926622.3	ssc:100151976	0	NR_027752.1	ALDH1L2
LOC102163778	2.761619671	99.15340461	-5.166075759	5.66E-04	Low	2	120	XP_005674396.1	ssc:102163778	5E-37	NM_152629.3	GLIS3
ANKRD63	2.761619671	99.15340461	-5.166075759	5.66E-04	Low	2	120	XP_005674617.1	ssc:100627919			
ZKSCAN2	1.380809836	77.67016694	-5.813774016	5.66E-04	Low	1	94	ZKSCAN2	ssc:100520899	0	NM_001012981.4	ZKSCAN2
LOC102160712	1.380809836	77.67016694	-5.813774016	5.66E-04	Low	1	94	XR_302696.1	ssc:102160712			
SMIM5	113.2264065	8.262783717	3.776438746	5.71E-04	High	82	10	XP_005653944.1	ssc:100625566	1E-22	NR_028439.1	SMIM5
FUCA2	113.2264065	1887.219801	-4.058980095	5.75E-04	Low	82	2284	XP_005654434.1	ssc:100154940	0	NM_032020.4	FUCA2
ZPBP2	153.2698918	12.39417558	3.628340107	5.76E-04	High	111	15	NM_001164513.1	ssc:100302699	8E-63	NM_199321.2	ZPBP2
LOC102166411	5.523239343	140.4673232	-4.6685761	5.82E-04	Low	4	170	XP_005672450.1	ssc:102166411			

BVES	5.523239343	140.4673232	-4.6685761	5.82E-04	Low	4	170	NM_001144112.1	ssc:100153106	0	NM_147147.3	BVES
MECOM	140.8426032	2351.588246	-4.061479768	5.83E-04	Low	102	2846	XP_005670049.1	ssc:100518275	0	NM_005241.3	MECOM
YBX2	637.9341441	61.14459951	3.383110512	5.90E-04	High	462	74	YBX2	ssc:100519387	8E-131	NM_015982.3	YBX2
LOC100515686	520.5653081	49.5767023	3.392344954	5.94E-04	High	377	60	XP_003131634.1	ssc:100515686	5E-44	NM_153229.2	TMEM92
LOC100738497	0	54.53437253	-20	5.95E-04	Low	0	66	XP_005667234.1	ssc:100738497	2E-87	NM_001098816.2	TENM4
TGM5	0	54.53437253	-20	5.95E-04	Low	0	66	XP_001926111.1	ssc:100158178	0	NM_201631.3	TGM5
LOC102160949	0	54.53437253	-20	5.95E-04	Low	0	66	XR_300993.1	ssc:102160949	4E-128	NM_025137.3	SPG11
NCALD	0	54.53437253	-20	5.95E-04	Low	0	66	NM_001244466.1	ssc:100156785	1E-112	NM_032041.2	NCALD
LOC100736869	796.7272752	76.84388857	3.374083534	5.97E-04	High	577	93	XR_302263.1	ssc:100736869	2E-37	NM_194320.2	ZNF169
CBLN2	5.523239343	139.6410448	-4.6600646	6.01E-04	Low	4	169	NM_001244702.1	ssc:100517134	6E-112	NM_182511.3	CBLN2
LOC102165321	5.523239343	139.6410448	-4.6600646	6.01E-04	Low	4	169	XR_300382.1	ssc:102165321			
SEPP1	40.04348524	672.5905946	-4.070089153	6.01E-04	Low	29	814	NM_001134823.1	ssc:100037964	4E-139	NM_005410.2	SEPP1
MALL	88.37182949	5.783948602	3.933459914	6.06E-04	High	64	7	NM_001243633.1	ssc:100626835			
LOC100623028	273.4003475	4631.290274	-4.082327201	6.06E-04	Low	198	5605	XP_003361790.1	ssc:100623028	2E-109	NM_015927.4	TGFB1I1
LOC100626712	585.4633704	56.18692928	3.381272449	6.07E-04	High	424	68	XP_005661466.1	ssc:100626712	5E-70	NM_138393.1	REEP6
ZNF134	38.6626754	647.8022434	-4.066540086	6.16E-04	Low	28	784	ZNF134	ssc:100512237	0	NM_003435.3	ZNF134
FRMD5	111.8455967	8.262783717	3.758736744	6.26E-04	High	81	10	XP_005659740.1	ssc:100152168	0	NR_104455.1	FRMD5
SIX1	622.7452359	10886.21755	-4.127716882	6.27E-04	Low	451	13175	NM_001199718.1	ssc:100156847	2E-167	NM_005982.3	SIX1
SEMA3G	255.4498196	23.13579441	3.464841375	6.30E-04	High	185	28	XP_001928286.1	ssc:100157653	0	NM_020163.1	SEMA3G
LOC100626722	17.95052786	324.7274001	-4.177130948	6.31E-04	Low	13	393	XR_131435.2	ssc:100626722			
LOC100624013	1.380809836	76.0176102	-5.78274712	6.32E-04	Low	1	92	XP_005669325.1	ssc:100624013	3E-102	NM_001102608.1	COL6A6
LOC102157928	103.5607377	7.436505346	3.799708525	6.41E-04	High	75	9	XR_309125.1	ssc:102157928			
LOC102159216	0	53.70809416	-20	6.43E-04	Low	0	65	XR_307086.1	ssc:102159216			
ASPA	0	53.70809416	-20	6.43E-04	Low	0	65	NM_001123077.1	ssc:100142661	1E-174	NM_001128085.1	ASPA
TMPRSS11A	0	53.70809416	-20	6.43E-04	Low	0	65	XP_005666787.1	ssc:100525933	0	NM_182606.3	TMPRSS11A
SGSM1	0	53.70809416	-20	6.43E-04	Low	0	65	XP_005670818.1	ssc:100152606	0	NM_133454.2	SGSM1
SPINK2	0	53.70809416	-20	6.43E-04	Low	0	65	XP_003129067.1	ssc:100520393	9E-24	NR_073419.1	SPINK2
ZNF286A	2.761619671	96.67456949	-5.129549883	6.45E-04	Low	2	117	ZNF286A	ssc:100627421	0	NM_020652.2	ZNF286A
LOC100736584	3587.343953	343.7318026	3.383560833	6.50E-04	High	2598	416	XP_003480860.1	ssc:100736584	0	NM_006087	TUBB4A
IFI44	519.1844982	8936.20059	-4.105342357	6.50E-04	Low	376	10815	NM_001246205.1	ssc:100525523	4E-151	NM_006417.4	IFI44
LOC100511509	63.51725244	3.305113487	4.264376792	6.51E-04	High	46	4	XR_303488.1	ssc:100511509			
LOC102165318	19.3313377	343.7318026	-4.15226996	6.51E-04	Low	14	416	XR_306421.1	ssc:102165318			
LOC100628186	28.99700655	0	20	6.53E-04	High	21	0	XP_003354623.2	ssc:100628186	0	NM_170664.2	OTOA
PPBP	28.99700655	0	20	6.53E-04	High	21	0	NM_213862.2	ssc:396870	8E-24	NM_002704.3	PPBP
PGLYRP3	28.99700655	0	20	6.53E-04	High	21	0	NM_001244361.1	ssc:100626786	1E-151	NM_052891.1	PGLYRP3
RNASE4	1876.520567	182.6075202	3.361242018	6.54E-04	High	1359	221	NM_213936.2	ssc:396976	1E-66	NM_194431.2	RNASE4
LOC100521376	2136.112816	207.3958713	3.364528764	6.55E-04	High	1547	251	XP_005664247.1	ssc:100521376	0	NM_016575.1	NT5DC3
RAP1GAP	46.94753442	1.652556743	4.828277677	6.62E-04	High	34	2	XP_005658516.1	ssc:100627043	1E-125	NM_002885.2	RAP1GAP
LOC100739759	46.94753442	1.652556743	4.828277677	6.62E-04	High	34	2	XP_003483318.2	ssc:100739759			
C1H14orf39	46.94753442	1.652556743	4.828277677	6.62E-04	High	34	2	XP_005660080.1	ssc:100157646	0	NM_174978.2	C14orf39
LOC102165266	46.94753442	1.652556743	4.828277677	6.62E-04	High	34	2	XP_005666958.1	ssc:102165266			
LOC100737062	2489.600134	241.2732845	3.367173959	6.62E-04	High	1803	292	XP_005661334.1	ssc:100737062	0	NM_006087	TUBB4A
QRFPR	55.23239343	2.478835115	4.47778043	6.63E-04	High	40	3	XP_003129267.3	ssc:100522335	3E-175	NM_198179.2	QRFPR
LOC100624264	2.761619671	95.84829112	-5.117166159	6.75E-04	Low	2	116	XP_003361331.2	ssc:100624264			
LOC100513233	2.761619671	95.84829112	-5.117166159	6.75E-04	Low	2	116	XP_005657050.1	ssc:100513233	0	NM_144610.1	SGK494
TRABD2A	8.284859014	176.8235716	-4.41568965	6.82E-04	Low	6	214	XP_005655281.1	ssc:100524432	2E-19	NM_001277053.1	TRABD2A
LOC100625416	75.94454097	1223.718269	-4.010181376	6.84E-04	Low	55	1481	XR_309344.1	ssc:100625416			
LOC100737535	16.56971803	299.1127706	-4.17406855	6.86E-04	Low	12	362	LOC100737535				
LOC102168145	157.4123213	13.22045395	3.57370485	6.92E-04	High	114	16	XR_301204.1	ssc:102168145			
AXL	544.0390753	9277.453558	-4.091946693	6.93E-04	Low	394	11228	NM_001128458.1	ssc:100144875			
C7H15orf59	0	52.88181579	-20	6.95E-04	Low	0	64	XP_001928647.1	ssc:100156505	5E-139	NM_001039614.1	C15orf59
ADAMTSL1	0	52.88181579	-20	6.95E-04	Low	0	64	XP_005660146.1	ssc:102160697			
SLC38A4	0	52.88181579	-20	6.95E-04	Low	0	64	XP_005664217.1	ssc:100286742	0	NM_018018.4	SLC38A4
PID1	0	52.88181579	-20	6.95E-04	Low	0	64	NM_001173520.1	ssc:100302508	3E-23	NM_017933.4	PID1
LOC100627583	0	52.88181579	-20	6.95E-04	Low	0	64	XP_005658592.1	ssc:100627583	1E-64	NR_073474.1	PTPRR
LOC100515572	0	52.88181579	-20	6.95E-04	Low	0	64	XP_003130869.4	ssc:100515572	3E-92	NM_001029880.2	SFMBT2
ABCA13	526.0885474	51.22925905	3.360265713	6.97E-04	High	381	62	XP_005674688.1	ssc:100514786	0	NM_152701.3	ABCA13
PPFIA2	379.7227048	36.35624836	3.384676026	7.03E-04	High	275	44	XP_003126797.4	ssc:100512281	0	NR_038265.1	PPFIA2
ABLIM1	1349.05121	133.0308178	3.34211271	7.03E-04	High	977	161	XP_005671534.1	ssc:100154343	0	NM_006720.3	ABLIM1
LOC100521998	661.4079113	65.27599137	3.340915934	7.29E-04	High	479	79	XP_003121205.4	ssc:100521998	4E-55	NM_139165.2	RAET1E
LYSMD2	70.42130162	4.131391859	4.091312083	7.30E-04	High	51	5	XP_005659663.1	ssc:100155966	1E-60	NM_153374.2	LYSMD2
MPPED2	1679.06476	166.0819527	3.337690658	7.34E-04	High	1216	201	XP_005661121.1	ssc:100515427	3E-175	NM_001584.2	MPPED2
CYP4V2	24.85457704	418.0968561	-4.072253737	7.37E-04	Low	18	506	XP_005671802.1	ssc:100113469	1E-124	NM_207352.3	CYP4V2
SLITRK4	156.0315114	13.22045395	3.560993799	7.39E-04	High	113	16	XP_005674002.1	ssc:100525800	0	NM_173078.4	SLITRK4
LOC100524058	376.9610852	36.35624836	3.374140358	7.43E-04	High	273	44	XP_003121439.3	ssc:100524058	1E-68	NM_198920.1	UBE3D
LOC100737021	5.523239343	133.8570962	-4.599035167	7.50E-04	Low	4	162	XR_135112.2	ssc:100737021			
LOC100514491	613.0795671	10365.66217	-4.079594148	7.51E-04	Low	444	12545	XP_005669732.1	ssc:100514491	0	NM_001457.3	FLNB
LOC100736817	0	52.05553742	-20	7.51E-04	Low	0	63	XP_003484258.1	ssc:100736817	4E-31	NM_001257.4	CDH13
LRRC31	0	52.05553742	-20	7.51E-04	Low	0	63	XP_005670050.1	ssc:100625984	2E-99	NM_024727.3	LRRC31
KCNF1	109.083977	8.262783717	3.722667489	7.52E-04	High	79	10	XP_005655334.1	ssc:100621537	0	NM_002236.4	KCNF1
LOC100737137	45.56672458	729.6038022	-4.001060672	7.53E-04	Low	33	883	LOC100737137				
LOC102158261	62.13644261	3.305113487	4.232667932	7.61E-04	High	45	4	XR_304928.1	ssc:102158261			
TMEM177	1687.349619	167.7345095	3.330507489	7.64E-04	High	1222	203	XP_003133358.1	ssc:100523095	5E-140	NM_030577.2	TMEM177
UNC5B	274.7811573	4459.424372	-4.020502515	7.68E-04	Low	199	5397	NM_001044549.1	ssc:733600	0	NM_170744.4	UNC5B
LOC100623035	24.85457704	414.7917426	-4.060803716	7.69E-04	Low	18	502	XP_005664949.1	ssc:100623035	0	NM_001267779.1	ZNF850
NOTCH2	531.6117868	8880.031661	-4.062116899	7.72E-04	Low	385	10747	XP_003481525.2	ssc:100153369	0	NM_024408.3	NOTCH2
LOC100738863	75.94454097	1196.451082	-3.977671337	7.73E-04	Low	55	1448	XP_005656100.1	ssc:100738863	1E-161	NM_005529.5	HSPG2
LOC102162528	22.09295737	373.477824	-4.079364126	7.73E-04	Low	16	452	XP_005670322.1	ssc:102162528	1E-29	NM_080927.3	DCBLD2
LOC100628061	33.13943606	537.90722	-4.020736356	7.74E-04	Low	24	651	XP_005659043.1	ssc:100628061	0	NM_020338.3	ZMIZ1
LOC100739847	272.0195376	25.61462952	3.408670345	7.76E-04	High	197	31	XP_005668534.1	ssc:100739847	4E-65	NM_144595.3	SLAIN1
CRABP2	921.0001604	15784.39574	-4.099153814	7.84E-04	Low	667	19103	NM_001164509.1	ssc:100155151	4E-76	NM_001878.3	CRABP2
SYNM	182.2668983	2902.71592	-3.993278906	7.85E-04	Low	132	3513	XP_003353468.2	ssc:100623833	0	NM_145728.2	SYNM
LOC100622846	1.380809836	72.71249671	-5.718616782	7.94E-04	Low	1	88	XP_005674362.1	ssc:100622846	0	NM_152753.2	SCUBE3
LOC100739371	1.380809836	72.71249671	-5.718616782	7.94E-04	Low	1	88	XP_003481077.1	ssc:100739371			
VCL	2587.637632	46834.28439	-4.177857517	7.95E-04	Low	1874	56681	NM_213934.1	ssc:396974	0	NM_014000.2	VCL
LOC102157528	37.28186556	0.826278372	5.495702338	7.98E-04	High	27	1	XP_005672489.1	ssc:102157528			
LOC102161349	37.28186556	0.826278372	5.495702338	7.98E-04	High	27	1	XP_005664545.1	ssc:102161349	6E-114	NM_024916.1	CD22
LOC100156741	37.28186556	0.826278372	5.495702338	7.98E-04	High	27	1	NM_001244642.1	ssc:100156741	1E-74	NM_001005464.2	HIST2H3A
LOC100737408	37.28186556	0.826278372	5.495702338	7.98E-04	High	27	1	XP_005658762.1	ssc:100737408	0	NR_104591.1	GARNL3
LOC100154508	169.8396098	14.87301069	3.51340434	7.99E-04	High	123	18	XP_005665717.1	ssc:100154508	1E-68	NM_021065.3	HIST1H2AD
CASP4	244.4033409	3914.906925	-4.001642097	8.04E-04	Low	177	4738	XP_005667345.1	ssc:100522887	1E-170	NM_033307.2	CASP4
SLC44A5	2513.073901	249.5360683	3.332132832	8.05E-04	High	1820	302	XP_005653486.1	ssc:100513866	0	NM_152697.4	SLC44A5
LOC100738373	4.142429507	112.3738586	-4.761685504	8.07E-04	Low	3	136	XP_003481727.1	ssc:100738373	2E-46	NM_004447.5	EPS8
LOC100516350	0	51.22925905	-20	8.14E-04	Low	0	62	XP_003121685.1	ssc:100516350	3E-73	NM_198500.1	FMN1
LOC100626805	0	51.22925905	-20	8.14E-04	Low	0	62	XP_003355736.2	ssc:100626805	4E-39	NM_003994.5	KITLG
LOC102166113	0	51.22925905	-20	8.14E-04	Low	0	62	XP_005669278.1	ssc:102166113	1E-81	NM_001365.3	DLG4
UCMA	0	51.22925905	-20	8.14E-04	Low	0	62	XP_005658512.1	ssc:100626016	2E-56	NM_145314.1	UCMA
LOC100737841	0	51.22925905	-20	8.14E-04	Low	0	62	XP_003481563.1	ssc:100737841	7E-136	NM_004120.4	GBP2
SULT2B1	92.51425899	6.610226974	3.806904027	8.15E-04	High	67	8	NM_001243697.1	ssc:100626471			
LOC100736741	967.9476949	97.50084786	3.311442421	8.22E-04	High	701	118	XP_005660763.1	ssc:100736741	2E-59	NM_002419.3	MAP3K11

LOC102158761	107.7031672	8.262783717	3.70428896	8.25E-04	High	78	10	XP_005672766.1	ssc:102158761	1E-43	NM_080676.5	MACROD2
KRT14	107.7031672	8.262783717	3.70428896	8.25E-04	High	78	10	XP_005653960.1	ssc:100737113	0	NM_000526.4	KRT14
LOC102165647	16.56971803	288.3711517	-4.121305889	8.34E-04	Low	12	349	XP_005666496.1	ssc:102165647	4E-172	NM_032425.4	HHIPL1
LOC100626318	69.04049179	4.131391859	4.062742931	8.41E-04	High	50	5	NM_001244165.1	ssc:100626318	0	NM_032803.5	SLC7A3
SPINT1	153.2698918	13.22045395	3.535230703	8.43E-04	High	111	16	NM_001244422.2	ssc:100157387	0	NM_181642.2	SPINT1
MID1	12.42728852	228.0528306	-4.197784619	8.53E-04	Low	9	276	XP_005673451.1	ssc:100524415	0	NM_033291.1	MID1
UNC-6	19.3313377	326.3799568	-4.077539085	8.59E-04	Low	14	395	NM_001044548.1	ssc:733599	0	NM_004822.2	NTN1
LOC102157895	0	50.40298068	-20	8.82E-04	Low	0	61	XP_005655003.1	ssc:102157895	4E-60	NM_021599.2	ADAMTS2
HOXD1	5.523239343	129.7257044	-4.553805913	8.84E-04	Low	4	157	XP_001925052.2	ssc:100157662	3E-131	NM_024501.2	HOXD1
ZNFX1	473.6177737	7672.82096	-4.017961963	8.86E-04	Low	343	9286	ZNFX1	ssc:100624243	0	NM_021035.2	ZNFX1
LOC102159499	60.75563277	3.305113487	4.200246455	8.91E-04	High	44	4	XR_298674.1	ssc:102159499			
LOC100520832	60.75563277	3.305113487	4.200246455	8.91E-04	High	44	4	NM_001285972.1	ssc:100520832	9E-73	NM_198448.3	REG3G
LOC100155159	1.380809836	71.05993997	-5.685449919	8.93E-04	Low	1	86	XP_001926734.4	ssc:100155159	0	NM_032709.2	PYROXD2
LOC100622412	27.61619671	0	20	8.95E-04	High	20	0	XP_003355255.1	ssc:100622412	1E-74	NM_001005464.2	HIST2H3A
TDRD1	27.61619671	0	20	8.95E-04	High	20	0	XP_005671525.1	ssc:100157555	0	NM_198795.1	TDRD1
LOC102159671	27.61619671	0	20	8.95E-04	High	20	0	XR_305057.1	ssc:102159671			
LOC102161389	27.61619671	0	20	8.95E-04	High	20	0	XR_299016.1	ssc:102161389			
PRDM14	27.61619671	0	20	8.95E-04	High	20	0	XP_003125648.1	ssc:100519142	0	NM_024504.3	PRDM14
MIP	27.61619671	0	20	8.95E-04	High	20	0	XP_003126321.1	ssc:100522373	1E-139	NM_012064.3	MIP
FAM179A	27.61619671	0	20	8.95E-04	High	20	0	XP_003125325.3	ssc:100518963	0	NM_199280.2	FAM179A
P2RY14	27.61619671	0	20	8.95E-04	High	20	0	XP_003132536.1	ssc:100524220	2E-163	NM_014879.3	P2RY14
MARVELD3	27.61619671	0	20	8.95E-04	High	20	0	NM_001243842.1	ssc:100511453	2E-140	NM_052858.5	MARVELD3
IGLON5	69.04049179	1059.288873	-3.939509521	8.96E-04	Low	50	1282	XP_003127424.2	ssc:100512905	7E-175	NM_001101372.1	IGLON5
RNF213	776.0151277	12863.50169	-4.051054838	8.96E-04	Low	562	15568	XP_005668644.1	ssc:100626814	0	NM_020954.3	RNF213
LOC100620141	251.3073901	23.96207278	3.390628481	9.00E-04	High	182	29	XP_003484302.2	ssc:100620141	0	NM_030632.1	ASXL3
LOC102163243	197.4558065	18.17812418	3.441254554	9.05E-04	High	143	22	XR_306704.1	ssc:102163243			
KBTBD8	785.6807965	80.14900206	3.293186836	9.15E-04	High	569	97	XP_003132353.3	ssc:100521640	0	NM_032505.2	KBTBD8
TRPC3	26.23538688	421.4019696	-4.005611087	9.15E-04	Low	19	510	NM_001145749.1	ssc:397210	0	NM_003305.2	TRPC3
C1QTNF1	138.0809836	2121.05658	-3.941196555	9.17E-04	Low	100	2567	XP_005658522.1	ssc:100217377	2E-99	NR_049769.1	C1QTNF1
LOC102158142	8.284859014	166.9082311	-4.332434146	9.25E-04	Low	6	202	XP_005661569.1	ssc:102158142			
SRSF12	847.8172391	86.75922903	3.288664164	9.32E-04	High	614	105	NM_001195598.1	ssc:100152352			
PIP4K2C	791.2040359	80.97528043	3.288496321	9.37E-04	High	573	98	XP_005674442.1	ssc:100516802	2E-155	NM_024779.4	PIP4K2C
LOC102157511	249.9265803	23.96207278	3.382679728	9.37E-04	High	181	29	XR_305505.1	ssc:102157511			
LOC102159425	128.4153147	10.74161883	3.579533929	9.38E-04	High	93	13	XR_300992.1	ssc:102159425			
DENND2D	128.4153147	10.74161883	3.579533929	9.38E-04	High	93	13	XP_005663617.1	ssc:100154003	0	NM_024901.4	DENND2D
LOC102166308	1.380809836	70.2336616	-5.6685761	9.47E-04	Low	1	85	XR_299392.1	ssc:102166308			
LOC100627126	1.380809836	70.2336616	-5.6685761	9.47E-04	Low	1	85	XR_305631.1	ssc:100627126			
1-Mar	52.47077376	2.478835115	4.403779849	9.48E-04	High	38	3	XP_005666735.1	ssc:100738764	2E-70	NM_017923.3	1-Mar
KCP	9.66566885	185.0863553	-4.259185164	9.52E-04	Low	7	224	XP_005657822.1	ssc:100517738	0	NM_199349.2	KCP
LOC100520518	0	49.5767023	-20	9.57E-04	Low	0	60	XR_308015.1	ssc:100520518			
OMG	67.65968195	4.131391859	4.033596585	9.70E-04	High	49	5	XP_003131799.1	ssc:100525125	0	NM_002544.4	OMG
IRAK3	8.284859014	165.2556743	-4.318078853	9.74E-04	Low	6	200	XP_005664015.1	ssc:100514645	0	NM_007199.2	IRAK3
FOXC2	49.70915409	753.565875	-3.922150177	9.94E-04	Low	36	912	XP_005674616.1	ssc:100526107	1E-167	NM_005251.2	FOXC2
LOC100620268	104.9415475	8.262783717	3.666814255	9.96E-04	High	76	10	XP_005658181.1	ssc:100620268	0	NM_005026.3	PIK3CD
FNDC3A	15.18890819	259.4514087	-4.094374294	0.001001	Low	11	314	XP_003357889.1		1E-91	NR_103528.1	FNDC3A
HYDIN	44.18591474	1.652556743	4.740814836	0.001003	High	32	2	XP_003126939.3	ssc:100513208			
LOC100624271	44.18591474	671.7643162	-3.926296706	0.001004	Low	32	813	XR_300555.1	ssc:100624271			
CACNA1I	89.75263932	6.610226974	3.763182649	0.001014	High	65	8	XP_003481582.1	ssc:100154640	0	NM_021096.3	CACNA1I
LOC100520306	941.712308	97.50084786	3.271799716	0.001014	High	682	118	XP_005663986.1	ssc:100520306	1E-135	NM_024779.4	PIP4K2C
LOC100520093	127.0345049	10.74161883	3.563937074	0.001015	High	92	13	LOC100520093				
TRIO	69.04049179	1034.500521	-3.905347821	0.001018	Low	50	1252	XP_003133886.3	ssc:100518519	0	NM_007118.2	TRIO
ZIC2	44.18591474	669.2854811	-3.920963262	0.001024	Low	32	810	ZIC2	ssc:100622978	0	NM_007129.3	ZIC2
LOC100157899	35.90105573	0.826278372	5.441254554	0.001026	High	26	1	XP_005658300.1	ssc:100157899	0	NM_001115.2	ADCY8
TSPAN18	110.4647869	1655.035579	-3.905203775	0.001027	Low	80	2003	XP_003122898.2	ssc:100520775	4E-95	NM_130783.4	TSPAN18
LOC100515900	74.56373113	4.95767023	3.910739838	0.001034	High	54	6	LOC100515900				
F3	53.85158359	808.1002476	-3.9074736	0.001036	Low	39	978	NM_213785.1	ssc:396677			
LOC102161728	0	48.75042393	-20	0.00104	Low	0	59	XR_308080.1	ssc:102161728			
GUCA1B	0	48.75042393	-20	0.00104	Low	0	59	XP_005666050.1	ssc:102167816	2E-23	NM_002098.5	GUCA1B
FAM133A	0	48.75042393	-20	0.00104	Low	0	59	XP_005673830.1	ssc:100519754	6E-46	NM_173698.2	FAM133A
PNOC	0	48.75042393	-20	0.00104	Low	0	59	NM_001244476.1	ssc:397257	3E-75	NM_006228.4	PNOC
LOC102158919	0	48.75042393	-20	0.00104	Low	0	59	XR_303076.1	ssc:102158919			
LOC100737190	0	48.75042393	-20	0.00104	Low	0	59	XP_005672396.1	ssc:100737190	2E-130	NM_022817.2	PER2
ZNF583	11.04647869	200.7856443	-4.183997667	0.001041	Low	8	243	ZNF583	ssc:100620770	0	NM_152478.2	ZNF583
ITPKA	59.37482294	3.305113487	4.167079591	0.001047	High	43	4	XP_003121630.1	ssc:100516235	0	NM_002220.2	ITPKA
ACSL6	59.37482294	3.305113487	4.167079591	0.001047	High	43	4	XR_301990.1	ssc:100522126	0	NM_015256.3	ACSL6
LOC100737760	646.2190031	66.92854811	3.271329553	0.001049	High	468	81	XP_003483086.2	ssc:100737760	2E-72	NR_103873.1	RAD51C
ABCA9	19.3313377	313.9857813	-4.02168585	0.001056	Low	14	380	XP_003131306.1	ssc:100520216	0	NM_172386.1	ABCA9
LRFN1	951.3779768	99.15340461	3.262284413	0.001066	High	689	120	XP_005657556.1	ssc:100515802	0	NM_020862.1	LRFN1
LOC100155449	63.51725244	943.6099005	-3.892970143	0.001073	Low	46	1142	XP_005663033.1	ssc:100155449	0	NM_175636.2	RUNX1T1
LOC100620317	81.46778031	5.783948602	3.816102963	0.001087	High	59	7	XP_003357253.1	ssc:100620317	0	NM_004626.2	WNT11
OAS2	563.370413	8866.793207	-3.976256726	0.001089	Low	408	10731	NM_001031796.1	ssc:595128			
DEPDC1B	2105.734999	218.1374901	3.271014244	0.001091	High	1525	264	XP_005654276.1	ssc:100524052	0	NM_018369.2	DEPDC1B
ZBTB4	117.368836	1736.837137	-3.887341182	0.001104	Low	85	2102	ZBTB4	ssc:100739718	0	NM_020899.3	ZBTB4
PARP12	281.6852065	4273.511739	-3.923266382	0.001117	Low	204	5172	XP_003134663.1	ssc:100515805	0	NM_022750.2	PARP12
GNB3	24.85457704	386.698278	-3.959624882	0.001119	Low	18	468	NM_001243493.1	ssc:100154522	0	NM_002075.2	GNB3
LOC100739452	132.5577442	11.5678972	3.518422415	0.001128	High	96	14	XR_135364.2	ssc:100739452			
LOC102164820	0	47.92414556	-20	0.001131	Low	0	58	XR_299650.1	ssc:102164820			
IFNE	0	47.92414556	-20	0.001131	Low	0	58	NM_001105310.1	ssc:100125969	2E-80	NM_176891.4	IFNE
LOC102160618	0	47.92414556	-20	0.001131	Low	0	58	XP_005672260.1	ssc:102160618			
LOC100627982	0	47.92414556	-20	0.001131	Low	0	58	XP_005672751.1	ssc:100627982	1E-32	NM_182734.2	PLCB1
TTNLOC100620	0	47.92414556	-20	0.001131	Low	0	58	XP_003483740.1	ssc:100620261	0	NM_133437.3	TTN
CACNA1A	0	47.92414556	-20	0.001131	Low	0	58	XP_003480824.2	ssc:100525387	0	NM_023035.2	CACNA1A
LOC100739107	1909.660003	199.1330876	3.261510941	0.001131	High	1383	241	XP_005667610.1	ssc:100739107	0	NM_003582.2	DYRK3
TMEM117	26.23538688	404.8764021	-3.947895589	0.001133	Low	19	490	XP_003126671.4	ssc:100524623	0	NM_032256.2	TMEM117
ACTA1	1.380809836	67.75482648	-5.616737168	0.001136	Low	1	82	NM_001167795.1	ssc:100154254	0	NM_001100.3	ACTA1
UBA7	288.5892557	4366.881195	-3.919513788	0.001139	Low	209	5285	XR_306784.1	ssc:100512631	0	NM_003335.2	UBA7
LOC102166134	51.08996392	2.478835115	4.365305701	0.001139	High	37	3	XR_303848.1	ssc:102166134			
KLF12	9.66566885	178.4761283	-4.206717744	0.001152	Low	7	216	NM_001134347.2	ssc:100174955			
LOC100739776	22.09295737	345.3843594	-3.966544296	0.001174	Low	16	418	XP_005670275.1	ssc:100739776	3E-55	NM_033254.2	BOC
PTPN14	33.13943606	497.4195798	-3.90784234	0.001177	Low	24	602	XP_003130446.3	ssc:100521917			
LOC100737799	197.4558065	19.00440255	3.377124217	0.001198	High	143	23	XP_003483087.1	ssc:100737799			
RNF39	1.380809836	66.92854811	-5.599035167	0.001208	Low	1	81	NM_001128446.1	ssc:100141418	9E-167	NM_170770.1	RNF39
TTLL7	1.380809836	66.92854811	-5.599035167	0.001208	Low	1	81	XP_003482112.1	ssc:100522876	0	NM_024686.4	TTLL7
LOC102161930	0	47.09786719	-20	0.001231	Low	0	57	XR_306838.1	ssc:102161930			
RGSL1	0	47.09786719	-20	0.001231	Low	0	57	XP_005667865.1	ssc:100513105	0	NM_181572.2	RGSL1
LOC100621405	0	47.09786719	-20	0.001231	Low	0	57	XP_005658046.1	ssc:100621405	1E-123	NM_006492.2	ALX3
LOC102158175	0	47.09786719	-20	0.001231	Low	0	57	XP_005658681.1	ssc:102158175	8E-65	NM_133178.3	PTPRU
CCL19	0	47.09786719	-20	0.001231	Low	0	57	NM_001170516.1	ssc:494464	3E-32	NM_006274.2	CCL19
ICA	57.9940131	3.305113487	4.133132259	0.001232	High	42	4	NM_213847.1	ssc:396845	0	NM_001063.3	TF
LOC100622779	26.23538688	0	20	0.001239	High	19	0	XP_003354428.1	ssc:100622779	5E-146	NM_001003750.1	OR8I2

NMUR1	26.23538688	0	20	0.001239	High	19	0	HighI00019DC5D0		3E-170	NM_006056.4	NMUR1
FAM83A	26.23538688	0	20	0.001239	High	19	0	XP_001928788.1	ssc:100152159	0	NM_207006.2	FAM83A
LOC102157666	26.23538688	0	20	0.001239	High	19	0	XR_298274.1	ssc:102157666			
MSLNL	26.23538688	0	20	0.001239	High	19	0	XP_005662300.1	ssc:100519473	1E-40	NM_013404.4	MSLN
KCNG3	26.23538688	0	20	0.001239	High	19	0	XP_003354899.1	ssc:100514638	0	NM_172344.2	KCNG3
LOC100156285	26.23538688	0	20	0.001239	High	19	0	XP_005654361.1	ssc:100156285	1E-66	NM_005366.4	MAGEA11
LOC100621683	26.23538688	0	20	0.001239	High	19	0	XP_005667909.1	ssc:100621683	2E-33	NM_018664.2	BATF3
ABCG8	26.23538688	0	20	0.001239	High	19	0	XP_005655297.1	ssc:100048963	4E-166	NM_022437.2	ABCG8
NXNL2	26.23538688	0	20	0.001239	High	19	0	XP_005670441.1	ssc:100739001	8E-47	NM_145283.2	NXNL2
LOC100623337	26.23538688	0	20	0.001239	High	19	0	XP_003354433.1	ssc:100623337	1E-135	NM_001005201.1	OR8H3
TMIGD2	26.23538688	0	20	0.001239	High	19	0	XP_005661396.1	ssc:102164394	7E-69	NM_144615.2	TMIGD2
SDCBP2	404.5772819	42.14019696	3.263146349	0.001239	High	293	51	NM_001244863.1	ssc:100513633	2E-142	NM_080489.4	SDCBP2
GNG8	2.761619671	85.10667229	-4.945685691	0.001243	Low	2	103	XP_005664677.1	ssc:100622520	2E-34	NM_033258.1	GNG8
LOC100624937	2.761619671	85.10667229	-4.945685691	0.001243	Low	2	103	XP_005658007.1	ssc:100624937	9E-145	NM_005089.3	ZRSR2
LOC102166014	4.142429507	103.2847965	-4.640006948	0.001248	Low	3	125	XP_005664109.1	ssc:102166014			
NPR3	4.142429507	103.2847965	-4.640006948	0.001248	Low	3	125	XP_005654272.1	ssc:102163867	2E-22	NM_024563.3	NPR3
SOX15	240.2609114	23.96207278	3.325777337	0.001255	High	174	29	XP_003131994.1	ssc:100522397	5E-106	NM_006942.1	SOX15
FOXJ1	115.9880262	9.915340461	3.548169758	0.001265	High	84	12	XP_003358007.1	ssc:100623071	0	NM_001454.3	FOXJ1
LOC102158789	115.9880262	9.915340461	3.548169758	0.001265	High	84	12	XR_307821.1	ssc:102158789			
GEM	128.4153147	1855.821223	-3.853168565	0.001266	Low	93	2246	Z80109.1	ssc:404772	6E-173	NM_181702.2	GEM
LOC100523704	11.04647869	193.349139	-4.129549883	0.001269	Low	8	234	XP_003134227.1	ssc:100523704	0	NM_001164440.1	ANKRD33B
LOC100621888	247.1649606	24.78835115	3.317740018	0.001273	High	179	30	XP_005672495.1	ssc:100621888	3E-129	NM_021072.3	HCN1
LOC102167192	15.18890819	247.8835115	-4.028572236	0.001274	Low	11	300	XP_005656807.1	ssc:102167192	0	NM_014810.4	CEP350
STEAP3	705.5938261	75.19133183	3.230199677	0.001281	High	511	91	XP_005671689.1	ssc:100620912	0	NM_182915.2	STEAP3
LOC102164189	19.3313377	302.4178841	-3.96753008	0.001289	Low	14	366	XR_298722.1	ssc:102164189			
ZFP57	93.89506883	7.436505346	3.658352676	0.0013	High	68	9	ZFP57	ssc:100514715	6E-172	NM_001109809.2	ZFP57
LOC100623836	9.66566885	174.3447364	-4.17292943	0.001302	Low	7	211	XR_297840.1	ssc:100623836			
LOC100521038	129.7961246	11.5678972	3.488048766	0.001314	High	94	14	XP_003131310.3	ssc:100521038	2E-155	NM_007168	ABCA8
HOXB5	24.85457704	375.1303808	-3.91580865	0.001315	Low	18	454	XP_003131598.1	ssc:100522700	5E-157	NM_002147.3	HOXB5
LOC100626218	35.90105573	524.686766	-3.869358227	0.001318	Low	26	635	XP_003361078.2	ssc:100626218	0	NM_018489.2	ASH1L
SLC38A1	48.32834425	694.9001106	-3.845864137	0.001328	Low	35	841	XP_003355677.1	ssc:100154364	0	NM_030674.3	SLC38A1
LOC100624348	20.71214754	318.9434515	-3.944751606	0.00133	Low	15	386	XP_003358918.2	ssc:100624348	0	NM_153605.3	CRYBG3
DMPK	34.52024589	504.8560851	-3.870357544	0.001331	Low	25	611	XP_005659031.1	ssc:100627008	0	NM_004409	DMPK
LOC102167359	5.523239343	119.8103639	-4.439094254	0.001336	Low	4	145	XR_303838.1	ssc:102167359			
IGFBP2	108753.9635	10062.41801	3.434019047	0.00134	High	78761	12178	NM_214003.1	ssc:397064			
TBX4	0	46.27158882	-20	0.001342	Low	0	56	NM_001246249.1	ssc:100620126	0	NM_018488.2	TBX4
LOC102159229	0	46.27158882	-20	0.001342	Low	0	56	XP_005673115.1	ssc:102159229	9E-52	NR_003259.1	GNAS
LOC102167982	0	46.27158882	-20	0.001342	Low	0	56	XR_303713.1	ssc:102167982			
WFDC1	463.9521048	6949.827385	-3.904929361	0.001353	Low	336	8411	NM_001244987.1	ssc:100514648	1E-89	NM_021197.3	WFDC1
SHC3	4.142429507	101.6322397	-4.616737168	0.001355	Low	3	123	XP_001928162.3	ssc:100155002	6E-74	NM_016848.5	SHC3
SYNPO2	24.85457704	372.6515456	-3.906243786	0.001362	Low	18	451	XP_005666955.1	ssc:100525414	0	NM_133477.2	SYNPO2
LOC100737382	53.85158359	767.6126073	-3.833317731	0.001365	Low	39	929	XP_005672448.1	ssc:100737382	0	NM_007118.2	TRIO
LOC100153492	49.70915409	2.478835115	4.325777337	0.001373	High	36	3	XP_005670514.1	ssc:100153492	1E-29	NM_213725.1	RPLP1
CDH11	2971.502767	49085.06667	-4.046019546	0.001378	Low	2152	59405	NM_001244482.1	ssc:100525579	0	NM_001797.2	CDH11
LOC100520275	5.523239343	118.9840855	-4.429110165	0.001385	Low	4	144	XR_307525.1	ssc:100520275			
KCNG1	842.2939998	90.89062089	3.212120555	0.001389	High	610	110	NM_001113450.1	ssc:100134965	0	NM_172318.2	KCNG1
DBNDD2	2844.468262	302.4178841	3.23354362	0.001417	High	2060	366	NM_001243672.1	ssc:100157614	5E-62	NM_018478.3	DBNDD2
CTTNBP2	53.85158359	761.8286587	-3.822405885	0.001422	Low	39	922	HighI0000680B24		0	NM_033427.2	CTTNBP2
PRUNE2	167.0779901	2372.245205	-3.827659542	0.001444	Low	121	2871	XP_005660227.1	ssc:100153374	2E-65	NM_138818.2	PRUNE2
ARHGAP28	157.4123213	2227.64649	-3.822899931	0.001457	Low	114	2696	XP_005656168.1	ssc:100520745	0	NM_030672.2	ARHGAP28
LOC100739736	1.380809836	64.44971299	-5.544587383	0.001459	Low	1	78	XR_305107.1	ssc:100739736	6E-37	NM_003998.3	NFKB1
REEP3	321.7286917	4670.951635	-3.859800001	0.001463	Low	233	5653	XP_001925694.2	ssc:100158143	2E-137	NM_001001330.2	REEP3
LOC102161524	0	45.44531045	-20	0.001465	Low	0	55	XR_300626.1	ssc:102161524			
KLHL38	0	45.44531045	-20	0.001465	Low	0	55	XP_005662925.1	ssc:102160009	1E-64	NM_001081675.2	KLHL38
CCL3L1	0	45.44531045	-20	0.001465	Low	0	55	NM_001009579.1	ssc:494459	3E-39	NM_001001437.3	CCL3L3
SERPINB8	56.61320327	794.8797936	-3.811526243	0.00147	Low	41	962	XP_005652751.1	ssc:100156248	0	NM_198833.1	SERPINB8
SYT1	12.42728852	204.9170362	-4.043456473	0.001499	Low	9	248	XP_005652680.1	ssc:100513599	0	NM_005639.2	SYT1
LOC100624650	327.2519311	4731.269957	-3.85375387	0.001503	Low	237	5726	XP_003360420.1	ssc:100624650	2E-109	NM_001024921.2	RPL9
EXO5	20.71214754	311.5069461	-3.910715282	0.001507	Low	15	377	XP_003128160.1	ssc:100511388	4E-178	NM_022774.1	EXO5
LOC102163252	63.51725244	4.131391859	3.942448697	0.001507	High	46	5	LOC102163252				
LOC100626459	9.66566885	169.3870662	-4.131310341	0.001513	Low	7	205	XP_005661870.1	ssc:100626459	0	NM_171825.2	CAMK2A
SLC16A11	120.1304557	10.74161883	3.483318614	0.001518	High	87	13	XP_005669249.1	ssc:100516827	1E-173	NM_153357.1	SLC16A11
LOC102166137	15.18890819	239.6207278	-3.979662635	0.001523	Low	11	290	XP_005655468.1	ssc:102166137	4E-20	NM_016954.2	TBX22
LOC100524679	41.42429507	1.652556743	4.647705432	0.001548	High	30	2	XP_005655748.1	ssc:100524679	0	NM_173498.1	OVOS2
LOC100625548	41.42429507	1.652556743	4.647705432	0.001548	High	30	2	XP_003361336.1	ssc:100625548	1E-137	NR_103801.1	GABRB3
LEPR	190.5517573	19.00440255	3.325777337	0.001553	High	138	23	NM_001024587.1	ssc:396836	8E-121	NM_002303.5	LEPR
LOC102166743	1.380809836	63.62343462	-5.525971705	0.001556	Low	1	77	XP_005654240.1	ssc:102166743			
KLHL29	49.70915409	690.7687188	-3.796619294	0.001584	Low	36	836	XP_005662760.1	ssc:100522799	0	NM_052920.1	KLHL29
TSHZ3	124.2728852	1718.659013	-3.789699881	0.001599	Low	90	2080	XP_005655858.1	ssc:100627966	0	NM_020856.2	TSHZ3
LOC102162177	2.761619671	80.97528043	-4.873895008	0.0016	Low	2	98	XR_305968.1	ssc:102162177			
LOC100522267	2.761619671	80.97528043	-4.873895008	0.0016	Low	2	98	LOC100522267				
LOC102160052	0	44.61903207	-20	0.001601	Low	0	54	XR_302532.1	ssc:102160052			
LOC102162300	0	44.61903207	-20	0.001601	Low	0	54	XR_304485.1	ssc:102162300			
LOC100513736	0	44.61903207	-20	0.001601	Low	0	54	XP_005664671.1	ssc:100513736	9E-99	NM_018215.3	PNMAL1
CSRP3	0	44.61903207	-20	0.001601	Low	0	54	NM_001172368.1	ssc:100337687	3E-110	NM_003476.4	CSRP3
C6H1orf233	3079.205934	332.1639054	3.21259114	0.001615	High	2230	402	XP_005653419.1	ssc:100511201	5E-72	NM_001242659.1	C1orf233
LOC100738475	13.80809836	218.9637685	-3.987105618	0.001634	Low	10	265	XP_005658467.1	ssc:100738475			
ARX	111.8455967	9.915340461	3.495702338	0.001639	High	81	12	XP_005673568.1	ssc:100627139	3E-141	NM_139058.2	ARX
LOC102159586	26.23538688	377.6092159	-3.847308005	0.001639	Low	19	457	XR_299222.1	ssc:102159586			
JAM2	6.904049179	132.2045395	-4.259185164	0.001648	Low	5	160	XP_005674369.1	ssc:100621244	3E-111	NR_072999.1	JAM2
C1QTNF4	118.7496459	10.74161883	3.466639873	0.001649	High	86	13	XP_003122853.1	ssc:100511621	2E-157	NM_031909.2	C1QTNF4
GRB14	252.6881999	26.4409079	3.256514674	0.001655	High	183	32	XP_005671947.1	ssc:100514075	0	NM_004490.2	GRB14
DENND6A	5284.359241	562.6955711	3.231302034	0.001664	High	3827	681	XP_005669730.1	ssc:100152812	0	NM_152678.2	DENND6A
ENO3	127.0345049	1743.447364	-3.778650491	0.00167	Low	92	2110	NM_001044527.1	ssc:692156	0	NM_053013.3	ENO3
LOC102160994	4.142429507	97.50084786	-4.556865712	0.001675	Low	3	118	XP_005658556.1	ssc:102160994	4E-145	NM_025137.3	SPG11
SNPH	4.142429507	97.50084786	-4.556865712	0.001675	Low	3	118	XP_005672870.1	ssc:100513829	0	NM_014723.2	SNPH
IGFBP3	1317.292583	145.4249934	3.179228674	0.001677	High	954	176	NM_001005156.1	ssc:448812			
ZNRF2	2742.288334	298.2864922	3.200609717	0.001681	High	1986	361	ZNRF2	ssc:100514021	1E-97	NM_147128.3	ZNRF2
NAPSA	621.3644261	68.58110485	3.179556596	0.001684	High	450	83	XP_005664836.1	ssc:100525581	0	NM_004851.1	NAPSA
GNS	273.4003475	3838.889315	-3.811601983	0.001688	Low	198	4646	XP_003355555.2	ssc:100623722	0	NM_002076.3	GNS
LOC100525748	12.42728852	199.959366	-4.0081234	0.001703	Low	9	242	XP_005664110.1	ssc:100525748	2E-71	NM_004447.5	EPS8
LOC100625867	52.47077376	718.035905	-3.774470017	0.001704	Low	38	869	LOC100625867				
LOC102160758	55.23239343	3.305113487	4.062742931	0.001721	High	40	4	LOC102160758				
LOC102159424	24.85457704	0	20	0.001734	High	18	0	XR_307055.1	ssc:102159424			
LOC102159402	24.85457704	0	20	0.001734	High	18	0	XR_297885.1	ssc:102159402			
LOC100516308	24.85457704	0	20	0.001734	High	18	0	XP_003121549.3	ssc:100516308			
LOC102166765	24.85457704	0	20	0.001734	High	18	0	XP_005669382.1	ssc:102166765	2E-47	NM_207359.2	GADL1
LOC100156977	24.85457704	0	20	0.001734	High	18	0	XP_001927348.1	ssc:100156977	4E-24	NM_022359.5	PDE4DIP
TCL1B	24.85457704	0	20	0.001734	High	18	0	XP_005656517.1	ssc:100525932	1E-35	NM_199206.2	TCL1B
ARGFX	24.85457704	0	20	0.001734	High	18	0	XP_005654117.1	ssc:100152745	2E-64	NM_001012659.1	ARGFX

LOC100739411	24.85457704	0	20	0.001734	High	18	0	XP_005670427.1	ssc:100739411	2E-109	NM_005656.3	TMPRSS2
WNT8A	24.85457704	0	20	0.001734	High	18	0	XP_005661739.1	ssc:100739710	0	NM_058244.2	WNT8A
LOC100739117	24.85457704	0	20	0.001734	High	18	0	XP_005669518.1	ssc:100739117	4E-169	NM_001123396.1	CCR2
LOC100513450	24.85457704	0	20	0.001734	High	18	0	XP_005673677.1	ssc:100513450	8E-19	NM_007003.3	PAGE4
LOC102165082	24.85457704	0	20	0.001734	High	18	0	XR_301689.1	ssc:102165082			
DEFB123	24.85457704	0	20	0.001734	High	18	0	XP_003134421.1	ssc:100521232	6E-17	NM_153324.2	DEFB123
LOC100737333	24.85457704	0	20	0.001734	High	18	0	XP_005662275.1	ssc:100737333	3E-118	NR_073012.1	PRSS21
FBXL7	27.61619671	390.8296698	-3.822953442	0.001745	Low	20	473	XP_003133898.3	ssc:100521047	0	NM_012304.4	FBXL7
LOC100626660	11.04647869	181.7812418	-4.040544877	0.00175	Low	8	220	LOC100626660				
ZNF660	0	43.7927537	-20	0.001752	Low	0	53	ZNF660	ssc:100513047	1E-93	NM_173658.2	ZNF660
CADPS	0	43.7927537	-20	0.001752	Low	0	53	XP_005669756.1	ssc:100518990	0	NM_183394.2	CADPS
LMOD3	0	43.7927537	-20	0.001752	Low	0	53	XP_003132362.1	ssc:100524108	0	NM_198271.3	LMOD3
NR2F1	133.9385541	1822.770088	-3.766489397	0.001758	Low	97	2206	XP_005661606.1	ssc:100621341	0	NM_005654.4	NR2F1
GPR19	559.2279835	61.97087788	3.173774243	0.001762	High	405	75	XP_005659112.1	ssc:100625907	0	NM_006143.2	GPR19
NPFF	15.18890819	233.0105008	-3.939304898	0.001762	Low	11	282	XP_003126243.1	ssc:100518250	2E-33	NM_003717.2	NPFF
RSPH9	69.04049179	4.95767023	3.799708525	0.00177	High	50	6	XP_005666103.1	ssc:100157153	2E-147	NM_152732.4	RSPH9
RELB	71.80211146	970.0508084	-3.755962139	0.001772	Low	52	1174	XP_005659055.1	ssc:100622609	0	NM_006509.3	RELB
KCNN3	2.761619671	79.32272369	-4.844147665	0.001775	Low	2	96	NM_213985.1	ssc:397045	7E-150	NM_170782.2	KCNN3
LOC100737389	40.04348524	549.4751172	-3.778414699	0.00178	Low	29	665	XR_303554.1	ssc:100737389	2E-71	NM_052885.3	SLC2A13
PRR15L	82.84859014	6.610226974	3.647705432	0.001784	High	60	8	XP_003358119.1	ssc:100626612	9E-46	NM_024320.3	PRR15L
DNM1	324.4903114	4540.399653	-3.806571983	0.00179	Low	235	5495	XP_005660537.1	ssc:100153921	0	NM_004408	DNM1
KIAA1033	11.04647869	180.9549634	-4.033972223	0.001792	Low	8	219	XP_005664243.1	ssc:100737665	5E-129	NM_015275.1	KIAA1033
TMEM55A	9.66566885	163.6031176	-4.081186862	0.001812	Low	7	198	XP_001928458.1	ssc:100156639	3E-147	NM_018710.2	TMEM55A
NAV3	27.61619671	386.698278	-3.807621789	0.001845	Low	20	468	XP_005655801.1	ssc:100622883	0	NM_014903.5	NAV3
LOC102164565	459.8096753	6495.37428	-3.820303931	0.001857	Low	333	7861	XP_005659016.1	ssc:102164565	0	NM_021035.2	ZNFX1
DLG4	104.9415475	1406.325789	-3.744272972	0.001864	Low	76	1702	XP_005655337.1	ssc:100626077	0	NM_001365.3	DLG4
LOC100516675	2.761619671	78.49644531	-4.829040772	0.001871	Low	2	95	XP_005664681.1	ssc:100516675	5E-12	NM_152796.2	HIF3A
SNTB1	6.904049179	128.899426	-4.222659288	0.001876	Low	5	156	XP_005662942.1	ssc:100154009	0	NM_021021.3	SNTB1
DAPK2	8.284859014	145.4249934	-4.133654282	0.001893	Low	6	176	NM_001243563.1	ssc:100155578	6E-105	NM_014326.3	DAPK2
LOC102159666	0	42.96647533	-20	0.00192	Low	0	52	XP_005655857.1	ssc:102159666			
DMKN	40.04348524	1.652556743	4.598795831	0.001935	High	29	2	XP_005664543.1	ssc:100515381	6E-70	NR_033746.1	DMKN
GYPC	6.904049179	128.0731476	-4.213381474	0.001939	Low	5	155	XP_003483649.2	ssc:100739383	5E-31	NM_016815.3	GYPC
COL7A1	566.1320327	8015.726484	-3.82362282	0.00194	Low	410	9701	XP_005669576.1	ssc:100523209	0	NM_000094.3	COL7A1
LOC100514810	14084.26032	1478.212007	3.252158696	0.001963	High	10200	1789	NM_001244997.1	ssc:100514810	1E-46	NR_109934.1	KIAA0101
BZW2	23070.57074	2376.376597	3.279218304	0.001965	High	16708	2876	NM_001245007.1	ssc:100514864	0	NR_027624.1	BZW2
LOXL3	93.89506883	1244.375228	-3.728228377	0.001965	Low	68	1506	XP_003361946.1	ssc:100626748	0	NM_032603	LOXL3
AMCF-II	687.6432982	9804.619159	-3.833729299	0.001972	Low	498	11866	NM_213876.1	ssc:396900	4E-33	NM_002994.4	CXCL5
LOC100739584	2.761619671	77.67016694	-4.813774016	0.001973	Low	2	94	XR_306259.1	ssc:100739584	4E-105	NM_015843.1	LMO7
KIF5C	6391.76873	691.5949971	3.208215871	0.001988	High	4629	837	XP_005671654.1	ssc:100622102	0	NM_004522.2	KIF5C
LOC100739030	6.904049179	127.2468692	-4.20404361	0.002004	Low	5	154	XP_003483340.1	ssc:100739030	7E-76	NM_005578.3	LPP
LOC100625312	46.94753442	2.478835115	4.243315177	0.002016	High	34	3	XP_005674423.1	ssc:100625312	0	NM_182907.2	PRDM1
CAPNS2	46.94753442	2.478835115	4.243315177	0.002016	High	34	3	XP_003127026.1	ssc:100518072	2E-119	NM_032330.1	CAPNS2
DIAPH2	16.56971803	243.7521197	-3.878793807	0.002022	Low	12	295	XP_003135273.2	ssc:100155257	0	NM_007309.3	DIAPH2
LOC100518476	41.42429507	551.9539523	-3.735998861	0.00206	Low	30	668	LOC100518476				
UBE2L6	370.057036	5069.217811	-3.775943602	0.002073	Low	268	6135	NM_001246215.1	ssc:100514753	2E-66	NM_198183.2	UBE2L6
CTSK	450.1440065	6223.528696	-3.789274294	0.002074	Low	326	7532	NM_214302.1	ssc:397569	0	NM_000396.3	CTSK
SPON1	2.761619671	76.84388857	-4.798343975	0.002081	Low	2	93	VSGP				
SCN4B	0	42.14019696	-20	0.002107	Low	0	51	XP_003129950.1	ssc:100512800	5E-89	NR_024527.1	SCN4B
LOC102158540	0	42.14019696	-20	0.002107	Low	0	51	XP_005664039.1	ssc:102158540	5E-57	NR_073474.1	PTPRR
SPMI	0	42.14019696	-20	0.002107	Low	0	51	NM_001031776.1	ssc:396758			
LOC102167277	0	42.14019696	-20	0.002107	Low	0	51	LOC102167277				
DCTPP1	3170.339383	353.6471431	3.164254776	0.002111	High	2296	428	XP_003354588.1	ssc:100621901	2E-74	NM_024096.1	DCTPP1
LOC100738192	56.61320327	741.9979778	-3.712204794	0.002119	Low	41	898	XP_005661482.1	ssc:100738192	0	NM_052843.3	OBSCN
LOC100622576	24.85457704	342.0792459	-3.78274712	0.002136	Low	18	414	XP_005658397.1	ssc:100622576	4E-178	NM_022774.1	EXO5
LOC100739399	361.772177	40.48764021	3.159527994	0.002137	High	262	49	XP_005659354.1	ssc:100739399	2E-79	NM_080743.4	SRSF12
LOC100154916	107.7031672	9.915340461	3.441254554	0.002139	High	78	12	XP_001927985.1	ssc:100154916	2E-52	NM_003539.3	HIST1H4D
ROR1	27.61619671	375.9566591	-3.766979804	0.002139	Low	20	455	XP_003127992.3	ssc:100520620	0	NM_005012.3	ROR1
HAUS1	6934.426995	756.0447101	3.197233216	0.002159	High	5022	915	NM_001185166.1	ssc:100152640	2E-141	NR_026978.1	HAUS1
SIPA1L1	392.1499933	5339.410838	-3.76720308	0.002173	Low	284	6462	XP_005656430.1	ssc:100157762	0	NM_015556.2	SIPA1L1
LOC100037974	4.142429507	92.54317763	-4.481577585	0.002181	Low	3	112	XP_005674384.1	ssc:100037974	0	NM_018058.6	CRTAC1
KLB	277.542777	30.57229975	3.182413162	0.002185	High	201	37	XP_003482415.1	ssc:100525825	0	NM_175737.3	KLB
VIM	30631.8854	533350.2948	-4.121977307	0.002186	Low	22184	645485	VIM		0	NM_003380.3	VIM
C6H19orf68	13.80809836	206.5695929	-3.903041354	0.002211	Low	10	250	XP_005664703.1	ssc:102165016	0	NM_199341.3	C19orf68
EPB41L4A	948.6163572	109.0687451	3.120587006	0.002229	High	687	132	XP_005661637.1	ssc:100518651	0	NM_022140.3	EPB41L4A
LOC100515642	133.9385541	13.22045395	3.340727678	0.002229	High	97	16	XP_005674021.1	ssc:100515642	3E-120	NR_033262.1	PRRG3
LOC100152707	5.523239343	108.2424667	-4.292608165	0.002245	Low	4	131	XP_003126250.1	ssc:100152707	2E-130	NR_003084.2	HOXC5
WDR63	386.626754	43.7927537	3.142177399	0.002264	High	280	53	XP_005652563.1	ssc:100157444	0	NM_145172.4	WDR63
WISP1	78.70616064	1014.66984	-3.688389995	0.002268	Low	57	1228	XP_005662899.1	ssc:100739115	1E-135	NR_037944.1	WISP1
LOC102158578	120.1304557	11.5678972	3.37640341	0.002277	High	87	14	XP_005662319.1	ssc:102158578	3E-58	NM_006648.3	WNK2
LOC102165965	248.5457704	27.26718627	3.188273813	0.002281	High	180	33	XR_302277.1	ssc:102165965			
MAB21L3	4.142429507	91.71689926	-4.468638529	0.002282	Low	3	111	XR_303162.1	ssc:100154970			
MLKL	16.56971803	237.9681711	-3.844147665	0.002291	Low	12	288	XP_005664404.1	ssc:100736792	2E-90	NM_152649.2	MLKL
ZNF350	9.66566885	156.1666123	-4.014072666	0.002301	Low	7	189	ZNF350	ssc:100737585	0	NM_021632.3	ZNF350
ZBED3	0	41.31391859	-20	0.002315	Low	0	50	ZBED3	ssc:100521776	1E-57	NM_032367.2	ZBED3
HSD17B13	0	41.31391859	-20	0.002315	Low	0	50	NM_001243622.1	ssc:100626886	9E-149	NM_178135.4	HSD17B13
LOC102167347	0	41.31391859	-20	0.002315	Low	0	50	XR_309153.1	ssc:102167347			
LOC100620105	2.761619671	75.19133183	-4.766979804	0.002319	Low	2	91	XP_005658075.1	ssc:100620105	0	NM_152280.4	SYT11
LOC100515588	1.380809836	58.66576439	-5.408932283	0.002323	Low	1	71	XP_005672167.1	ssc:100515588	3E-157	NM_205863.3	PARD3B
LOC102167736	900.2880129	104.1110748	3.112263067	0.002323	High	652	126	XP_005656849.1	ssc:102167736			
LOC102164190	66.27887212	4.95767023	3.740814836	0.00234	High	48	6	XR_298858.1	ssc:102164190			
SYT2	106.3223574	9.915340461	3.422638876	0.00234	High	77	12	XP_005668073.1	ssc:100511582	0	NM_177402.4	SYT2
LOC100156401	3283.565789	370.9989889	3.145776201	0.002351	High	2378	449	XP_005673029.1	ssc:100156401	5E-62	NM_018478.3	DBNDD2
LOC102157983	19.3313377	269.3667492	-3.800558396	0.002363	Low	14	326	XP_005662850.1	ssc:102157983	4E-71	NM_012293.1	PXDN
LOC100737024	186.4093278	19.83068092	3.232667932	0.002367	High	135	24	XP_003480864.1	ssc:100737024	0	NM_024898.2	DENND1C
DENND1C	186.4093278	19.83068092	3.232667932	0.002367	High	135	24	XP_003123166.1	ssc:100518598	0	NM_024898.2	DENND1C
ESPN	59.37482294	4.131391859	3.845151496	0.002386	High	43	5	XP_003127578.1	ssc:100515838	0	NM_031475.2	ESPN
LOC100517234	22.09295737	301.5916057	-3.770937818	0.002394	Low	16	365	XP_005667982.1	ssc:100517234	1E-83	NM_021958.3	HLX
ITGA10	154.6507016	1992.157154	-3.687246177	0.002405	Low	112	2411	XP_003125838.2	ssc:100511507	0	NM_003637.3	ITGA10
BOC	202.9790459	2637.480563	-3.699757755	0.002406	Low	147	3192	XP_005670285.1	ssc:100626085	0	NM_033254.2	BOC
STX3	84.22939998	1073.335605	-3.671633994	0.002413	Low	61	1299	XP_005660928.1	ssc:100520886	4E-130	NM_004177.4	STX3
DTL	3233.856635	366.867597	3.13992433	0.002417	High	2342	444	XR_305730.1	ssc:100525066	0	NM_016448.3	DTL
LOC100737431	22.09295737	300.7653273	-3.766979804	0.002429	Low	16	364	XP_005669345.1	ssc:100737431	6E-58	NM_015150.1	RFTN1
OPRL1	52.47077376	3.305113487	3.98874235	0.002429	High	38	4	NM_214176.1	ssc:397364	2E-12	NM_182647.2	OPRL1
LAMC3	52.47077376	3.305113487	3.98874235	0.002429	High	38	4	XP_003353735.3	ssc:100157958	0	NM_006059.3	LAMC3
HRC	9.66566885	154.5140555	-3.998724702	0.00243	Low	7	187	XP_003127335.2	ssc:100525465	1E-162	NM_002152.2	HRC
LOC102165764	38.6626754	1.652556743	4.548169758	0.002432	High	28	2	XR_303865.1	ssc:102165764			
LOC100519278	93.89506883	1194.798526	-3.66957416	0.002439	Low	68	1446	LOC100519278		0	NM_080424.2	SP110
ARSI	51.08996392	653.586192	-3.677265683	0.002443	Low	37	791	XP_003124150.1	ssc:100517463	0	NM_001012301.2	ARSI
ACN9	479.141013	55.36065091	3.113517498	0.002448	High	347	67	XP_005667689.1	ssc:102158000	5E-28	NM_020186.2	ACN9
PTI	23.47376721	0	20	0.002454	High	17	0	NM_001190215.1	ssc:100156830			

LOC102164656	23.47376721	0	20	0.002454	High	17	0	XR_308524.1	ssc:102164656			
LOC100736733	23.47376721	0	20	0.002454	High	17	0	XP_005663243.1	ssc:100736733	5E-155	NM_080878.2	ITLN2
RGS9	23.47376721	0	20	0.002454	High	17	0	XP_005656992.1	ssc:102166057	3E-81	NM_003835.3	RGS9
OPRM1	23.47376721	0	20	0.002454	High	17	0	NM_001001538.1	ssc:396802	0	NR_104351.1	OPRM1
DNAJC22	23.47376721	0	20	0.002454	High	17	0	XP_005655614.1	ssc:100513730	1E-157	NM_024902.2	DNAJC22
WNT9A	6.904049179	122.289199	-4.146710435	0.002454	Low	5	148	XP_003123659.2	ssc:100512949	0	NM_003395.2	WNT9A
LOC100737468	6.904049179	122.289199	-4.146710435	0.002454	Low	5	148	LOC100737468		2E-55	NM_152456.2	IL34
LOC102160150	41.42429507	533.7758281	-3.687684923	0.002456	Low	30	646	XP_005665346.1	ssc:102160150	1E-40	NM_015210.3	SOGA2
TAL1	4576.003796	514.7714256	3.15208437	0.002466	High	3314	623	XR_298705.1	ssc:100521731	4E-145	NM_003189	TAL1
LOC100739471	51.08996392	649.4548002	-3.6681173	0.002525	Low	37	786	XP_003483758.1	ssc:100739471	2E-37	NM_144629.2	RFTN2
LOC100518295	0	40.48764021	-20	0.002548	Low	0	49	XP_005652880.1	ssc:100518295	4E-23	NM_021030.2	ZNF14
LOC102166786	0	40.48764021	-20	0.002548	Low	0	49	XR_298591.1	ssc:102166786			
MYH2	0	40.48764021	-20	0.002548	Low	0	49	NM_214136.1	ssc:397256	0	NM_017534.5	MYH2
PPP2R2B	0	40.48764021	-20	0.002548	Low	0	49	NM_214025.2	ssc:397089	2E-52	NR_073527.1	PPP2R2B
PLD5	0	40.48764021	-20	0.002548	Low	0	49	XP_005668008.1	ssc:100622421	0	NM_152666.2	PLD5
CLDN19	0	40.48764021	-20	0.002548	Low	0	49	NM_001160084.1	ssc:100294682	1E-121	NM_148960.2	CLDN19
LOC100517085	0	40.48764021	-20	0.002548	Low	0	49	XP_003124008.3	ssc:100517085	4E-117	NM_012159.4	FBXL21
SHAS2	2.761619671	73.53877508	-4.734918595	0.00259	Low	2	89	NM_214053.1	ssc:397120	0	NM_005328.2	HAS2
LOC100623769	62.13644261	782.485618	-3.654552683	0.002592	Low	45	947	XP_005654767.1	ssc:100623769	1E-104	NM_205843.2	NFIC
LRRK2	92.51425899	1163.399947	-3.652527592	0.002595	Low	67	1408	NM_001113437.1	ssc:780403			
ZNF567	16.56971803	232.1842225	-3.808648983	0.002601	Low	12	281	ZNF567	ssc:100525521	0	NM_152603.2	ZNF567
LOC100739118	86.99101965	1092.340007	-3.650411702	0.002609	Low	63	1322	XP_005671292.1	ssc:100739118	3E-146	NM_178145.1	RASSF4
LOC102165757	5.523239343	104.9373532	-4.247869851	0.002625	Low	4	127	XR_309548.1	ssc:102165757			
CHAD	15.18890819	215.658655	-3.827659542	0.002632	Low	11	261	XP_005668942.1	ssc:100624069	0	NM_001267.2	CHAD
RGAG4	38.6626754	492.4619096	-3.670998762	0.002659	Low	28	596	XP_005657889.1	ssc:102167195	0	NM_001024455.3	RGAG4
MYPN	1.380809836	57.01320765	-5.367709621	0.002671	Low	1	69	XP_003359264.1	ssc:100624901	0	NR_045663.3	MYPN
MIR214	27.61619671	360.2573701	-3.705441394	0.002672	Low	20	436	NR_038497.1	ssc:100316560			
ZNF385C	35.90105573	456.9319396	-3.669881116	0.002731	Low	26	553	ZNF385C	ssc:100622424	0	NM_001242704.1	ZNF385C
MAPK10	5.523239343	104.1110748	-4.236465087	0.002732	Low	4	126	XP_005667090.1	ssc:100521736	0	NM_138982.2	MAPK10
PAOX	2118.162288	247.0572331	3.099895929	0.002733	High	1534	299	XP_005674486.1	ssc:100626281	6E-144	NR_109766.1	PAOX
MSI1	822.9626621	97.50084786	3.077340307	0.002773	High	596	118	XP_005670771.1	ssc:100627974			
LOC102167902	9.66566885	150.3826637	-3.959624882	0.002791	Low	7	182	XP_005666644.1	ssc:102167902	7E-62	NM_199039.3	KLHL5
LOC100736715	57.9940131	4.131391859	3.811204164	0.002793	High	42	5	XP_003480861.1	ssc:100736715	2E-32	NM_174881.2	CRB3
LOC100517699	57.9940131	4.131391859	3.811204164	0.002793	High	42	5	XP_003123163.1	ssc:100517699	2E-32	NM_174881.2	CRB3
DCX	0	39.66136184	-20	0.002808	Low	0	48	XP_005673890.1	ssc:100523160	0	NM_178153.2	DCX
LOC102159458	0	39.66136184	-20	0.002808	Low	0	48	XR_299573.1	ssc:102159458			
LOC102159747	0	39.66136184	-20	0.002808	Low	0	48	XP_005666777.1	ssc:102159747	3E-154	NM_015236.4	LPHN3
LOC100154648	0	39.66136184	-20	0.002808	Low	0	48	XP_005660358.1	ssc:100154648	0	NM_207299.1	LPPR1
ZSCAN4	0	39.66136184	-20	0.002808	Low	0	48	ZSCAN4	ssc:102159852	1E-103	NM_152677.2	ZSCAN4
LOC100510952	0	39.66136184	-20	0.002808	Low	0	48	XP_005666778.1	ssc:100510952			
LOC100625959	0	39.66136184	-20	0.002808	Low	0	48	XP_003360914.2	ssc:100625959	7E-96	NM_006558.1	KHDRBS3
CLVS2	0	39.66136184	-20	0.002808	Low	0	48	XP_001926095.1	ssc:100155458	0	NM_001010852.3	CLVS2
LOC102165051	0	39.66136184	-20	0.002808	Low	0	48	XR_301799.1	ssc:102165051			
TCP11	103.5607377	9.915340461	3.384671026	0.002809	High	75	12	XP_005665956.1	ssc:100154604	0	NM_018679.5	TCP11
CELSR1	4248.751865	487.5042393	3.12355242	0.002821	High	3077	590	HighI0000088500		0	NM_014246.1	CELSR1
ABCG4	26.23538688	339.6004108	-3.694252234	0.002855	Low	19	411	XP_003129978.1	ssc:100518504	0	NM_022169.4	ABCG4
IL13RA2	142.2234131	14.87301069	3.257390362	0.002867	High	103	18	NM_001243634.1	ssc:100156991	2E-168	NM_000640.2	IL13RA2
DLL1	9.66566885	149.5563853	-3.951676129	0.00287	Low	7	181	XP_005659153.1	ssc:100620481	0	NM_005618.3	DLL1
NAALADL2	2.761619671	71.88621834	-4.70212866	0.002897	Low	2	87	XP_003132587.3	ssc:100522279	0	NM_207015.2	NAALADL2
KANK4	2.761619671	71.88621834	-4.70212866	0.002897	Low	2	87	XP_003128002.2	ssc:100523255	0	NM_181712.4	KANK4
LOC102167620	2.761619671	71.88621834	-4.70212866	0.002897	Low	2	87	XP_005658643.1	ssc:102167620			
RAP1GAP2	51.08996392	3.305113487	3.950268202	0.002898	High	37	4	XP_003358244.2	ssc:100624704	0	NM_015085.4	RAP1GAP2
LOC100620269	631.0300949	8327.23343	-3.72205655	0.002921	Low	457	10078	XP_005658558.1	ssc:100620269	4E-78	NM_016201.3	AMOTL2
LOC100626812	266.4962983	30.57229975	3.123818508	0.002924	High	193	37	XP_003357377.3	ssc:100626812	0	NM_003105.5	SORL1
PGM5	5.523239343	102.4585181	-4.213981473	0.00296	Low	4	124	XP_003121981.3	ssc:100511977	0	NM_021965.3	PGM5
XRCC2	926.5233998	110.7213018	3.064894602	0.002961	High	671	134	XRCC2	ssc:100514260			
LOC100736946	77.3253508	946.915014	-3.614221571	0.002974	Low	56	1146	XR_304087.1	ssc:100736946	5E-71	NM_001792.3	CDH2
LOC100621070	53.85158359	661.8489758	-3.619441377	0.00299	Low	39	801	XP_005666583.1	ssc:100621070			
LOC100154114	30.37781639	0.826278372	5.200246455	0.003001	High	22	1	XR_297331.1	ssc:100154114			
LOC100154530	128.4153147	1579.017968	-3.620138416	0.003001	Low	93	1911	XP_003360037.2	ssc:100154530	0	NM_198951.1	TGM2
C17H20orf96	1492.655432	177.6498499	3.070772794	0.003002	High	1081	215	XP_001925791.3	ssc:100154679	3E-114	NM_153269.2	C20orf96
LOC100158017	11.04647869	163.6031176	-3.888541784	0.003007	Low	8	198	XP_005671108.1	ssc:100158017	0	NM_014767.2	SPOCK2
LOC100627840	6.904049179	117.3315288	-4.087004188	0.003025	Low	5	142	XP_003355471.1	ssc:100627840	2E-130	NR_003084.2	HOXC5
LOC102159203	2.761619671	71.05993997	-4.685449919	0.003067	Low	2	86	XR_303394.1	ssc:102159203			
EGR4	37.28186556	1.652556743	4.495702338	0.003071	High	27	2	NM_001285968.1	ssc:100516906	0	NM_001965.3	EGR4
LOC102166513	1.380809836	55.36065091	-5.325274354	0.00308	Low	1	67	XR_298946.1	ssc:102166513			
ZNF256	5.523239343	101.6322397	-4.201699669	0.003083	Low	4	123	ZNF256	ssc:100516085	0	NM_005773.2	ZNF256
SOX13	277.542777	32.2248565	3.106464308	0.003093	High	201	39	XP_005656725.1	ssc:100524269	0	NM_005686.2	SOX13
STEAP4	0	38.83508347	-20	0.0031	Low	0	47	NM_001166489.1	ssc:100310802	0	NM_024636.3	STEAP4
LOC100624603	0	38.83508347	-20	0.0031	Low	0	47	XP_003356537.2	ssc:100624603	3E-53	NM_057160.2	ARTN
LOC100514680	0	38.83508347	-20	0.0031	Low	0	47	XP_005669411.1	ssc:100514680	2E-141	NM_002207.2	ITGA9
LOC100519903	0	38.83508347	-20	0.0031	Low	0	47	HighI00025DF217	ssc:100519903	0	NM_001080397.1	SLC45A1
LOC102162758	0	38.83508347	-20	0.0031	Low	0	47	XP_005658708.1	ssc:102162758	9E-20	NR_110311.1	LONRF3
GSC2	82.84859014	7.436505346	3.47778043	0.003102	High	60	9	XP_001927461.3	ssc:100152323	9E-62	NM_005315.1	GSC2
LOC100739480	11.04647869	161.9505609	-3.873895008	0.003166	Low	8	196	XP_003482190.2	ssc:100739480	8E-138	NR_028077.1	ZSCAN12
FZD5	211.2639049	23.96207278	3.140221684	0.003171	High	153	29	XP_005672190.1	ssc:100519058	0	NM_030804.1	FZD5
LOC100737938	8.284859014	131.3782611	-3.987105618	0.003176	Low	6	159	XP_003481433.1	ssc:100737938			
LOC102165489	664.169531	80.14900206	3.050795078	0.003189	High	481	97	XP_005670721.1	ssc:102165489	2E-64	NM_014365.2	HSPB8
FAM102B	16.56971803	223.0951604	-3.75103826	0.003194	Low	12	270	XP_005663673.1	ssc:100519836	2E-98	NM_001010883.2	FAM102B
PSD3	156.0315114	1905.397925	-3.610182999	0.003196	Low	113	2306	XP_005672728.1	ssc:100623956	0	NM_206909.2	PSD3
LOC102164936	165.6971803	18.17812418	3.188273813	0.003202	High	120	22	XP_005664341.1	ssc:102164936	6E-110	NR_038265.1	PPFIA2
LOC102159147	6.904049179	115.678972	-4.066540086	0.003248	Low	5	140	XP_005659213.1	ssc:102159147			
PRMT8	2.761619671	70.2336616	-4.6685761	0.003248	Low	2	85	XP_005664132.1	ssc:100526051	0	NR_046301.1	PRMT8
LOC100739592	2118.162288	252.8411817	3.066509761	0.003257	High	1534	306	XR_302158.1	ssc:100739592			
PROX1	197.4558065	22.30951604	3.145798671	0.003264	High	143	27	NM_001128490.1	ssc:100169702	0	NM_002763.4	PROX1
LOC100519735	56.61320327	4.131391859	3.776438746	0.003278	High	41	5	XP_005669305.1	ssc:100519735	0	NM_004662.2	DNAH9
LCN2	5614.372792	651.1073569	3.108157517	0.003298	High	4066	788	NM_001244410.1	ssc:100153501	2E-66	NM_005564.3	LCN2
LOC100739717	1.380809836	54.53437253	-5.303579283	0.003312	Low	1	66	XR_135312.2	ssc:100739717			
FOXA1	301.0165442	35.52996998	3.082734406	0.003312	High	218	43	XP_001929311.1	ssc:100156502	0	NM_004496.3	FOXA1
ARSB	41.42429507	504.0298068	-3.604960001	0.003312	Low	30	610	XP_005661568.1	ssc:102158016	2E-145	NM_198709.2	ARSB
LOC100127144	626.8876654	76.0176102	3.043801367	0.003315	High	454	92	XP_005654136.1	ssc:100127144	0	NM_002606.2	PDE9A
TMEM35	1422.234131	171.8659013	3.04880374	0.003341	High	1030	208	XP_003135283.1	ssc:100516714	2E-77	NM_021637.2	TMEM35
LOC102161288	466.7137245	5902.932688	-3.660822093	0.003374	Low	338	7144	XR_306392.1	ssc:102161288			
LOC100624304	20.71214754	266.0616357	-3.683211446	0.003421	Low	15	322	XP_005662404.1	ssc:100624304	0	NM_006267.4	RANBP2
LOC100623959	0	38.0088051	-20	0.003427	Low	0	46	XP_005674164.1	ssc:100623959	1E-33	NM_153343.3	ENPP6
LOC102167421	0	38.0088051	-20	0.003427	Low	0	46	XR_306488.1	ssc:102167421			
KLHL4	2.761619671	69.40738323	-4.651502587	0.003442	Low	2	84	XP_003135270.3	ssc:100524840	0	NM_057162.2	KLHL4
DIO3	2.761619671	69.40738323	-4.651502587	0.003442	Low	2	84	NM_001001625.2	ssc:414378	1E-154	NM_001362.3	DIO3
LOC100519177	423.9086196	51.22925905	3.048713371	0.003446	High	307	62	XP_005657769.1	ssc:100519177	6E-91	NM_130759.3	GIMAP1
LOC100514136	2800.282347	38936.71571	-3.797486995	0.003462	Low	2028	47123	XP_003361338.1	ssc:100514136	6E-156	NM_003118.3	SPARC

SLC6A7	49.70915409	3.305113487	3.910739838	0.003468	High	36	4	XP_003124145.2	ssc:100516251	0	NM_014228.3	SLC6A7
LOC102160475	476.3793933	57.83948602	3.041984371	0.003469	High	345	70	XR_299423.1	ssc:102160475			
KIAA0101	1379.429026	167.7345095	3.039819787	0.003485	High	999	203	XP_005659557.1	ssc:100623233	2E-40	NR_109934.1	KIAA0101
CDO1	9.66566885	143.7724367	-3.894773738	0.003504	Low	7	174	NM_001167643.1	ssc:100312964	8E-116	NM_001801.2	CDO1
LOC100525873	22.09295737	0	20	0.003514	High	16	0	XP_005664152.1	ssc:100525873	7E-108	NM_032680.3	EFCAB4B
CKM	22.09295737	0	20	0.003514	High	16	0	NM_001129949.1	ssc:397264	0	NM_001824.4	CKM
LOC102161637	22.09295737	0	20	0.003514	High	16	0	XR_305387.1	ssc:102161637			
CIDE-B	22.09295737	0	20	0.003514	High	16	0	NM_001112688.1	ssc:100127160	2E-110	NM_014430.2	CIDEB
ZAN	22.09295737	0	20	0.003514	High	16	0	NM_214383.1	ssc:397676	0	NM_173059.1	ZAN
LOC102164198	22.09295737	0	20	0.003514	High	16	0	XR_306376.1	ssc:102164198			
S1PR4	22.09295737	0	20	0.003514	High	16	0	XP_005654771.1	ssc:100517997	6E-176	NM_003775.3	S1PR4
HMGCLL1	22.09295737	0	20	0.003514	High	16	0	XP_003356654.1	ssc:100627473	0	NR_109869.1	HMGCLL1
ZNF541	22.09295737	0	20	0.003514	High	16	0	ZNF541	ssc:100519367	0	np_001094889	np_001094889
CD8B	22.09295737	0	20	0.003514	High	16	0	NM_213762.2	ssc:396636	3E-58	NM_172213.3	CD8B
TNFRSF18	22.09295737	0	20	0.003514	High	16	0	XP_003356166.1	ssc:100622025	1E-43	NM_148902.1	TNFRSF18
OVOL2	22.09295737	0	20	0.003514	High	16	0	XP_003483950.1	ssc:100739162	1E-50	NM_021220.2	OVOL2
PNAS-5	8696.340345	1003.101943	3.115940172	0.00358	High	6298	1214	XP_003128900.1	ssc:414404	1E-152	NM_017816.2	LYAR
LOC100523690	12.42728852	172.6921797	-3.796619294	0.003615	Low	9	209	XR_306191.1	ssc:100523690			
ZNF462	45.56672458	542.0386119	-3.572343049	0.003645	Low	33	656	ZNF462	ssc:100158126	0	NM_021224.4	ZNF462
LOC100624418	2.761619671	68.58110485	-4.634224595	0.003649	Low	2	83	XR_131274.2	ssc:100624418			
DLK2	42.80510491	2.478835115	4.110048646	0.003684	High	31	3	NM_001128466.1	ssc:100145892	0	NM_206539.2	DLK2
SPATA6	12.42728852	171.8659013	-3.789699881	0.003704	Low	9	208	NM_001177917.1	ssc:100415810	0	NM_019073.3	SPATA6
LOC102161426	0	37.18252673	-20	0.003795	Low	0	45	XP_005664948.1	ssc:102161426	0	NM_020813.2	ZNF471
LPP	0	37.18252673	-20	0.003795	Low	0	45	LPP		1E-118	NM_005578.3	LPP
LOC100738788	0	37.18252673	-20	0.003795	Low	0	45	XP_003482722.2	ssc:100738788	5E-47	NM_000446.5	PON1
IL21R	0	37.18252673	-20	0.003795	Low	0	45	XP_003124575.1	ssc:100518723	0	NM_181079.4	IL21R
GUCY2C	0	37.18252673	-20	0.003795	Low	0	45	NM_214105.1	ssc:397193	1E-118	NM_004963.3	GUCY2C
LOC102167801	0	37.18252673	-20	0.003795	Low	0	45	XR_302359.1	ssc:102167801			
LOC102164981	404.5772819	49.5767023	3.028681095	0.003835	High	293	60	XR_305141.1	ssc:102164981			
ARHGEF10L	93.89506883	1095.645121	-3.544587383	0.003846	Low	68	1326	XP_003482027.1	ssc:100525994	0	NM_018125.3	ARHGEF10L
LOC102167148	13.80809836	185.0863553	-3.744611991	0.003878	Low	10	224	XP_005667759.1	ssc:102167148	1E-61	NM_012301.3	MAGI2
LOC100738613	646.2190031	80.14900206	3.011266714	0.003884	High	468	97	XP_003483004.1	ssc:100738613	0	NM_172232	ABCA5
HSP70	117.368836	12.39417558	3.243315177	0.003895	High	85	15	NM_213766.1	ssc:396648			
GPR4	35.90105573	1.652556743	4.441254554	0.003897	High	26	2	NM_001123118.1	ssc:100144489	1E-51	NM_005282.2	GPR4
CXHXorf57	35.90105573	1.652556743	4.441254554	0.003897	High	26	2	XP_003360455.3	ssc:100627173			
BMPR1B	11.04647869	155.3403339	-3.813774016	0.003909	Low	8	188	NM_001039745.1	ssc:396691	1E-64	NM_001256794.1	BMPR1B
ZBTB7C	5.523239343	96.67456949	-4.129549883	0.003953	Low	4	117	ZBTB7C	ssc:100520594	0	NM_001039360.2	ZBTB7C
DUSP27	5.523239343	96.67456949	-4.129549883	0.003953	Low	4	117	XP_003355120.1	ssc:100153962	0	NM_001080426.1	DUSP27
GRIA3	12.42728852	169.3870662	-3.768740262	0.003986	Low	9	205	XP_003135428.1	ssc:100152391	0	NM_181894.1	GRIA3
S1PR2	144.9850328	1693.870662	-3.546347841	0.00399	Low	105	2050	NM_001243855.1	ssc:100511551	2E-173	NM_004230.3	S1PR2
INMT	28.99700655	0.826278372	5.133132259	0.003994	High	21	1	NM_001244733.1	ssc:100512997	1E-78	NM_006774.4	INMT
WNT2B	66.27887212	765.9600506	-3.530648192	0.004042	Low	48	927	XP_003125897.1	ssc:100520560	0	NM_024494.2	WNT2B
MED30	2332.187813	285.8923166	3.028140221	0.004057	High	1689	346	XP_003135209.1	ssc:100519234	7E-102	NM_080651.3	MED30
PLAG1	13.80809836	183.4337985	-3.731672935	0.004058	Low	10	222	XP_005663144.1	ssc:100154905	0	NM_002655.2	PLAG1
TACSTD2	73.18292129	6.610226974	3.468735291	0.004149	High	53	8	XP_003128015.1	ssc:100510966	1E-145	NM_002353.2	TACSTD2
RORB	48.32834425	3.305113487	3.870097853	0.004162	High	35	4	XP_005674570.1	ssc:100739111	5E-83	NM_006914.3	RORB
MYCN	247.1649606	29.74602138	3.054705612	0.004162	High	179	36	XP_003125394.1	ssc:100518190	0	NM_005378.4	MYCN
INSRR	165.6971803	19.00440255	3.124143476	0.004173	High	120	23	XP_001929559.1	ssc:100155702	0	NM_014215.2	INSRR
CSRNP3	60.75563277	4.95767023	3.615283954	0.004181	High	44	6	XP_005671951.1	ssc:100515450	0	NM_024969.3	CSRNP3
SLA-DQA1	0	36.35624836	-20	0.00421	Low	0	44	NM_001130224.1	ssc:100153387	3E-124	NM_002122.3	HLA-DQA1
LOC102166264	0	36.35624836	-20	0.00421	Low	0	44	XP_005655572.1	ssc:102166264			
LOC100737194	0	36.35624836	-20	0.00421	Low	0	44	XP_005672676.1	ssc:100737194	0	NM_003966.2	SEMA5A
LOC100620938	154.6507016	1791.37151	-3.533979283	0.004213	Low	112	2168	XP_005668470.1	ssc:100620938	0	NR_103528.1	FNDC3A
LOC100738029	296.8741147	36.35624836	3.029576067	0.00425	High	215	44	XP_005674356.1	ssc:100738029	0	NM_007168	ABCA8
LOC102161705	88.37182949	1010.538449	-3.515393852	0.004262	Low	64	1223	XP_005656081.1	ssc:102161705	0	NM_020765.2	UBR4
LOC100519166	183.6477082	21.48323766	3.095657554	0.004289	High	133	26	XR_305826.1	ssc:100519166			
ANK1	196.0749967	23.13579441	3.083207034	0.004291	High	142	28	XP_001926385.2	ssc:100155697	0	NM_020481.2	ANK1
LOC100624442	4483.489537	543.6911686	3.043762732	0.004372	High	3247	658	XR_300029.1	ssc:100624442			
REC8	73.18292129	827.9309285	-3.499931502	0.004498	Low	53	1002	HighI0001C95632		0	NM_005132.2	REC8
TIAM1	632.4109048	80.14900206	2.980105782	0.00453	High	458	97	XP_001927581.1				
LOC102165794	41.42429507	2.478835115	4.062742931	0.004538	High	30	3	XR_300521.1	ssc:102165794			
LOC100514384	53.85158359	4.131391859	3.70428896	0.004544	High	39	5	XP_003135498.2	ssc:100514384	5E-59	NM_005364.4	MAGEA8
CHST11	22.09295737	266.0616357	-3.590102042	0.00456	Low	16	322	XP_001926288.4	ssc:100157186	6E-171	NM_018413.5	CHST11
LOC102164824	9.66566885	136.3359313	-3.818152456	0.004574	Low	7	165	XR_306661.1	ssc:102164824			
LOC102165013	9.66566885	136.3359313	-3.818152456	0.004574	Low	7	165	XR_297707.1	ssc:102165013			
LOC100623866	120.1304557	1360.880478	-3.501866508	0.004577	Low	87	1647	XP_005674350.1	ssc:100623866	0	NM_015210.3	SOGA2
FJX1	135.3193639	1535.225215	-3.504010106	0.004607	Low	98	1858	XP_003353946.1	ssc:100626682	0	NM_014344.3	FJX1
LOC100736644	1061.842764	134.6833746	2.97892647	0.004613	High	769	163	XP_003483288.2	ssc:100736644	3E-53	NM_052883.1	TXNRD3
GAL3ST4	111.8455967	1262.553352	-3.496763989	0.004629	Low	81	1528	XP_005661948.1	ssc:100511123	0	NM_024637.4	GAL3ST4
STYK1	2.761619671	65.27599137	-4.562965912	0.004637	Low	2	79	XP_003126549.1	ssc:100523437	0	NM_018423.2	STYK1
COL4A6	2.761619671	65.27599137	-4.562965912	0.004637	Low	2	79	XP_001925433.3	ssc:100152680	0	NM_033641.3	COL4A6
TNNT2	77.3253508	868.4185687	-3.489377196	0.004669	Low	56	1051	NM_001257353.1	ssc:100622450	2E-108	NM_001276347.1	TNNT2
LOC102161574	0	35.52996998	-20	0.004678	Low	0	43	XR_297074.1	ssc:102161574			
GALNT9	0	35.52996998	-20	0.004678	Low	0	43	XP_001927211.4	ssc:100156842	0	NM_021808.3	GALNT9
ADCYAP1	0	35.52996998	-20	0.004678	Low	0	43	NM_001001544.1	ssc:414283	9E-71	NM_001117.4	ADCYAP1
PON1	0	35.52996998	-20	0.004678	Low	0	43	NM_001097515.2	ssc:100048952			
LOC100627616	0	35.52996998	-20	0.004678	Low	0	43	XP_005674315.1	ssc:100627616	2E-140	NM_002247.3	KCNMA1
FLI1	0	35.52996998	-20	0.004678	Low	0	43	XP_005667560.1	ssc:100515914	1E-121	NM_002017.4	FLI1
LOC102157627	71.80211146	6.610226974	3.441254554	0.004705	High	52	8	XR_298952.1	ssc:102157627			
ATP8B3	120.1304557	13.22045395	3.183758332	0.004726	High	87	16	XP_003123063.3	ssc:100513137	0	NR_047593	ATP8B3
SLC9A9	44.18591474	499.8984149	-3.499976496	0.00475	Low	32	605	XP_005669980.1	ssc:100517680	0	NM_173653.3	SLC9A9
LOC100516316	17.95052786	220.6163253	-3.619441377	0.004755	Low	13	267	XP_003124070.4	ssc:100516316	0	NM_032053.2	PCDHGA4
RAB27A	1511.98677	191.6965822	2.979548996	0.004808	High	1095	232	NM_001032357.1	ssc:606749	1E-123	NM_183236.2	RAB27A
POLR3G	2474.411226	309.8543894	2.997422973	0.004819	High	1792	375	XP_005652997.1	ssc:100519356	9E-94	NM_006467.2	POLR3G
ARSE	1.380809836	50.40298068	-5.189922501	0.004823	Low	1	61	NM_001244120.1	ssc:100627778	0	NM_001282631.1	ARSE
LOC102159829	1.380809836	50.40298068	-5.189922501	0.004823	Low	1	61	XR_306120.1	ssc:102159829			
GRAMD1C	1009.37199	128.899426	2.969140213	0.004824	High	731	156	XP_001925196.1		0	NM_017577.4	GRAMD1C
LOC100736899	254.0690098	31.39857813	3.016449279	0.004858	High	184	38	XP_005669279.1	ssc:100736899	4E-122	NM_015982.3	YBX2
LOC102164998	6.904049179	106.58991	-3.948484324	0.00488	Low	5	129	XR_307847.1	ssc:102164998			
SEMA4D	4124.478979	508.987477	3.018509818	0.004885	High	2987	616	XP_005670454.1	ssc:100152598	0	NM_182635.1	SEMA4D
GABRA2	5.523239343	92.54317763	-4.066540086	0.004902	Low	4	112	XP_005666686.1	ssc:100622617	0	NM_001286827.1	GABRA2
PTPRD	4.142429507	78.49644531	-4.244078271	0.004926	Low	3	95	XP_005660164.1	ssc:100155429	0	NM_130393.3	PTPRD
LOC100738222	2.761619671	64.44971299	-4.544587383	0.00493	Low	2	78	XP_005674414.1	ssc:100738222	3E-134	NM_012413.3	QPCT
LOC100518095	516.4228786	6110.328559	-3.564625136	0.004936	Low	374	7395	XP_005670428.1	ssc:100518095	1E-28	NM_002462.4	MX1
OBSL1	570.2744622	6779.61404	-3.571474811	0.004947	Low	413	8205	XP_005672301.1	ssc:100624019	6E-162	NM_015311.2	OBSL1
FAT1	1777.102259	22497.90751	-3.66219222	0.004964	Low	1287	27228	NM_001190170.1	ssc:100154043	0	NM_005245.3	FAT1
LOC100515353	34.52024589	1.652556743	4.384671026	0.004973	High	25	2	XP_003135337.2	ssc:100515353	1E-174	NM_018015.5	CXorf57
LOC102165291	34.52024589	1.652556743	4.384671026	0.004973	High	25	2	XP_005662692.1	ssc:102165291	3E-45	NM_053276.3	VIT
GRIP1	9.66566885	133.8570962	-3.791680245	0.005011	Low	7	162	XP_005664019.1	ssc:100514286	0	NM_021150.3	GRIP1
LOC102163494	673.8351998	86.75922903	2.957306656	0.005051	High	488	105	XP_005658979.1	ssc:102163494	4E-37	NR_109766.1	PAOX

TNNI3	539.8966458	69.40738323	2.959522211	0.005067	High	391	84	NM_001098599.1	ssc:100049696	2E-96	NM_000363.4	TNNI3
GIP	20.71214754	0	20	0.005099	High	15	0	HighI000003415C		2E-49	NM_004123.2	GIP
LOC102161699	20.71214754	0	20	0.005099	High	15	0	XP_005653909.1	ssc:102161699	2E-144	NM_001676.5	ATP12A
MDH1B	20.71214754	0	20	0.005099	High	15	0	XP_003359673.2	ssc:100621831	0	NR_104261.1	MDH1B
USH2A	20.71214754	0	20	0.005099	High	15	0	XP_003130538.3	ssc:100513222			
LOC102161179	20.71214754	0	20	0.005099	High	15	0	XR_309353.1	ssc:102161179			
LOC102161013	20.71214754	0	20	0.005099	High	15	0	XP_005652810.1	ssc:102161013			
LOC100522379	20.71214754	0	20	0.005099	High	15	0	LOC100522379	ssc:100522379			
LOC102166200	20.71214754	0	20	0.005099	High	15	0	XR_306717.1	ssc:102166200			
LOC102158025	20.71214754	0	20	0.005099	High	15	0	XR_309275.1	ssc:102158025			
LOC102162201	20.71214754	0	20	0.005099	High	15	0	XP_005663353.1	ssc:102162201			
CDH9	20.71214754	0	20	0.005099	High	15	0	XP_003359781.2	ssc:100626823	0	NM_016279.3	CDH9
LOC102167461	20.71214754	0	20	0.005099	High	15	0	XP_005669384.1	ssc:102167461			
LOC100523792	20.71214754	0	20	0.005099	High	15	0	XP_003127471.1	ssc:100523792	0	NM_032803.5	SLC7A3
LOC100737801	20.71214754	0	20	0.005099	High	15	0	XP_005673249.1	ssc:100737801	1E-98	NR_028041.1	GRM8
LOC100514709	118.7496459	13.22045395	3.167079591	0.005109	High	86	16	XP_003125309.3	ssc:100514709	6E-47	NM_003352.4	SUMO1
LOC100516615	5.523239343	91.71689926	-4.05360103	0.005122	Low	4	111	XP_005653378.1	ssc:100516615	0	NM_198480.3	ZNF615
NHSL2	17.95052786	217.3112118	-3.597664435	0.005131	Low	13	263	XP_005657890.1	ssc:100151779	0	NM_001013627.2	NHSL2
TRIL	11.04647869	147.0775502	-3.734918595	0.00514	Low	8	178	XP_005673340.1	ssc:100515456	0	NM_014817.3	TRIL
LOC100627762	20.71214754	245.4046764	-3.566613689	0.005161	Low	15	297	XR_131068.3	ssc:100627762			
LOC100522650	4.142429507	77.67016694	-4.228811515	0.005186	Low	3	94	XP_003133905.2	ssc:100522650	1E-12	NM_004934.3	CDH18
LOC100516594	4.142429507	77.67016694	-4.228811515	0.005186	Low	3	94	XP_003123294.2	ssc:100516594	2E-82	NM_022377.3	ICAM4
LOC100520600	10897.35122	1307.998662	3.058544537	0.005203	High	7892	1583	XP_005661030.1	ssc:100520600	8E-90	NM_006034.3	TP53I11
PRICKLE1	198.8366163	2230.125325	-3.487469424	0.005207	Low	144	2699	XP_005664204.1	ssc:100157209	0	NM_153026.2	PRICKLE1
GP9	0	34.70369161	-20	0.005208	Low	0	42	NM_001141989.1	ssc:100216312	5E-45	NM_000174.3	GP9
PTGER1	0	34.70369161	-20	0.005208	Low	0	42	XP_003123421.2	ssc:100515879	9E-74	NM_000955.2	PTGER1
LOC100738540	0	34.70369161	-20	0.005208	Low	0	42	XR_302001.1	ssc:100738540	2E-71	NM_012159.4	FBXL21
LOC102167614	0	34.70369161	-20	0.005208	Low	0	42	XR_299563.1	ssc:102167614			
LOC102157611	1.380809836	49.5767023	-5.166075759	0.005214	Low	1	60	XP_005674347.1	ssc:102157611	2E-13	NR_046000	IRF4
LOC102162488	1.380809836	49.5767023	-5.166075759	0.005214	Low	1	60	XR_299752.1	ssc:102162488			
ELFN2	106.3223574	11.5678972	3.200246455	0.005255	High	77	14	XP_005663863.1	ssc:100511563	0	NM_052906	ELFN2
ITGA5	2089.165281	26341.75449	-3.656352901	0.005334	Low	1513	31880	XP_001925287.3	ssc:100155091	0	NM_002205.2	ITGA5
LOC102160731	70.42130162	6.610226974	3.413240178	0.005344	High	51	8	XR_299209.1	ssc:102160731	8E-61	NM_015011.1	MYO16
ZNF432	5.523239343	90.89062089	-4.040544877	0.005354	Low	4	110	ZNF432	ssc:100624217	0	NM_014650.2	ZNF432
SPOCD1	27.61619671	0.826278372	5.062742931	0.005359	High	20	1	XP_003127817.1	ssc:100521137	0	NM_144569.5	SPOCD1
GGT1	38.6626754	428.8384749	-3.47142097	0.005428	Low	28	519	NM_214030.1	ssc:397095	0	NM_013430.2	GGT1
LOC100516424	19.3313377	228.879109	-3.565572408	0.005431	Low	14	277	XP_005661613.1	ssc:100516424			
PLEKHG1	4.142429507	76.84388857	-4.213381474	0.005462	Low	3	93	XP_005658150.1	ssc:100621167			
LOC100738797	6.904049179	104.1110748	-3.914536992	0.005479	Low	5	126	XP_005657730.1	ssc:100738797	6E-113	NM_032883.2	TOX2
TBX3	66.27887212	721.3410185	-3.444060507	0.005509	Low	48	873	XP_001928037.1	ssc:100152741	0	NM_016569.3	TBX3
LOC100523082	80.08697047	869.2448471	-3.440123158	0.005569	Low	58	1052	XP_005667841.1	ssc:100523082			
FEZ1	346.5832688	44.61903207	2.957470888	0.005585	High	251	54	XP_003130092.1	ssc:100511148	2E-48	NM_022549.3	FEZ1
SPIC	40.04348524	2.478835115	4.013833331	0.005612	High	29	3	XP_003481784.1	ssc:100738994	2E-91	NM_152323.1	SPIC
LOC102162264	22.09295737	255.3200169	-3.530648192	0.005616	Low	16	309	XP_005657676.1	ssc:102162264	8E-32	NM_001014279.2	ANXA2R
MYCL	457.0480556	59.49204276	2.941577242	0.005642	High	331	72	XP_003127875.2	ssc:100518432	0	NM_005376.4	MYCL
LOC102164425	1.380809836	48.75042393	-5.141828213	0.005643	Low	1	59	XR_305664.1	ssc:102164425			
LOC102157816	1.380809836	48.75042393	-5.141828213	0.005643	Low	1	59	XP_005657894.1	ssc:102157816	1E-57	NM_002637.3	PHKA1
ACBD3	57.9940131	627.9715625	-3.436723349	0.005708	Low	42	760	XP_003357690.2	ssc:100621148	9E-80	NM_022735.3	ACBD3
LOC100739594	30.37781639	338.7741324	-3.479233645	0.005726	Low	22	410	LOC100739594		2E-65	NM_022658.3	HOXC8
FAM184A	8.284859014	116.5052504	-3.813774016	0.005779	Low	6	141	XP_005659294.1	ssc:100626155	0	NM_024581.5	FAM184A
LOC100154674	241.6417213	30.57229975	2.982572582	0.005792	High	175	37	XP_001926885.2	ssc:100154674	1E-100	NM_139057.2	ADAMTS17
LOC100037951	0	33.87741324	-20	0.005809	Low	0	41	NM_001097448.1	ssc:100037951			
LOC102157549	0	33.87741324	-20	0.005809	Low	0	41	XR_304924.1	ssc:102157549			
LOC102161387	0	33.87741324	-20	0.005809	Low	0	41	XP_005656386.1	ssc:102161387			
LOC102161064	0	33.87741324	-20	0.005809	Low	0	41	XR_298198.1	ssc:102161064			
COLGALT2	0	33.87741324	-20	0.005809	Low	0	41	XP_003482786.1	ssc:100624924			
LOC102166942	0	33.87741324	-20	0.005809	Low	0	41	XR_298158.1	ssc:102166942			
LOC100625760	0	33.87741324	-20	0.005809	Low	0	41	XP_003356564.2	ssc:100625760	0	NM_000129.3	F13A1
COX17	22411.92444	2652.353573	3.078921521	0.005822	High	16231	3210	NM_001190922.1	ssc:100156799	9E-28	NM_005694.1	COX17
CA14	9.66566885	129.7257044	-3.746450991	0.005852	Low	7	157	XP_005658313.1	ssc:100153371	3E-167	NM_012113.1	CA14
PIF1	411.481331	53.70809416	2.937615544	0.005861	High	298	65	XP_001926830.2	ssc:100153538	0	NM_025049.3	PIF1
RYR2	27.61619671	309.028111	-3.484151529	0.005864	Low	20	374	XP_001924803.4	ssc:396856	0	NM_001035.2	RYR2
MAP1A	557.8471736	6397.873432	-3.519650608	0.005938	Low	404	7743	XP_005659735.1	ssc:100520470	0	NM_002373.5	MAP1A
EFNB2	30.37781639	336.2952973	-3.468638529	0.005942	Low	22	407	NM_001114286.1	ssc:100135673	0	NM_004093.3	EFNB2
LOC102163830	2.761619671	61.97087788	-4.488003854	0.005947	Low	2	75	XP_005656594.1	ssc:102163830			
HTR2A	2.761619671	61.97087788	-4.488003854	0.005947	Low	2	75	NM_214217.1	ssc:397432	0	NM_001165947.2	HTR2A
TNFRSF10B	46.94753442	506.5086419	-3.431465586	0.005983	Low	34	613	XP_005670476.1	ssc:100156086	5E-89	NM_003844.3	TNFRSF10A
LOC100622807	17.95052786	210.7009848	-3.553098883	0.005991	Low	13	255	LOC100622807				
LOC102157722	45.56672458	3.305113487	3.785208956	0.006049	High	33	4	XP_005658236.1	ssc:102157722			
LOC102160764	4.142429507	75.19133183	-4.182017303	0.006065	Low	3	91	XR_299169.1	ssc:102160764			
LOC100739138	342.4408393	44.61903207	2.940123644	0.006069	High	248	54	XP_005666063.1	ssc:100739138	5E-128	NM_001164446	C6orf132
SAMD4A	85.61020982	913.8638791	-3.416124524	0.006074	Low	62	1106	XP_003121863.3	ssc:100156574	0	NM_015589.5	SAMD4A
S100A14	1046.653855	137.9884881	2.923164582	0.006086	High	758	167	NM_001190168.1	ssc:100153930	3E-55	NM_020672.2	S100A14
ATF5	466.7137245	5278.266239	-3.499454325	0.006088	Low	338	6388	XP_005664813.1	ssc:100523675	4E-98	NM_012068.5	ATF5
EPS8	365.9146065	4086.772827	-3.481383139	0.006102	Low	265	4946	XP_005664108.1	ssc:100525921	0	NM_004447.5	EPS8
LOC102162629	1.380809836	47.92414556	-5.117166159	0.006112	Low	1	58	XP_005666567.1	ssc:102162629			
LOC102159034	5.523239343	88.41178578	-4.00065215	0.006124	Low	4	107	XP_005660990.1	ssc:102159034	4E-98	NM_032645.4	RAPSN
LOC100158075	1633.498036	214.0060983	2.932240907	0.006191	High	1183	259	XP_005663242.1	ssc:100158075	6E-54	NM_001113207.1	TSTD1
RXFP4	8.284859014	114.8526937	-3.793163736	0.006199	Low	6	139	NM_001005844.1	ssc:450213	4E-167	NM_181885.2	RXFP4
LOC100737060	26.23538688	291.6762652	-3.474782024	0.006208	Low	19	353	XP_005672507.1	ssc:100737060	0	NM_001897.4	CSPG4
LOC102165247	132.5577442	15.69928906	3.077849823	0.006302	High	96	19	XR_302925.1	ssc:102165247			
CYP2C91	24.85457704	276.8032545	-3.477277448	0.006326	Low	18	335	XR_307738.1	ssc:100516879			
SGCB	75.94454097	803.1425773	-3.402637954	0.00636	Low	55	972	NM_001114287.1	ssc:100135674	3E-179	NM_000232.4	SGCB
ITGB7	51.08996392	4.131391859	3.628340107	0.006361	High	37	5	XP_005652618.1	ssc:100626600	0	NR_104181.1	ITGB7
OTOG	33.13943606	1.652556743	4.325777337	0.00638	High	24	2	XP_003123004.2	ssc:100514091	0	xp_001717583	xp_001717583
LOC100516234	33.13943606	1.652556743	4.325777337	0.00638	High	24	2	XP_003134134.3	ssc:100516234	0	NM_001127648.1	GABRA1
LOC100521567	33.13943606	1.652556743	4.325777337	0.00638	High	24	2	LOC100521567	ssc:100521567			
LRRN1	35.90105573	386.698278	-3.429110165	0.006434	Low	26	468	XP_005657135.1	ssc:100627698	0	NM_020873.5	LRRN1
CD40	46.94753442	499.0721365	-3.410127062	0.00645	Low	34	604	NM_214194.1	ssc:397395	3E-115	NM_152854.2	CD40
LOC100622016	0	33.05113487	-20	0.006492	Low	0	40	XP_003360831.2	ssc:100622016	6E-68	NM_183394.2	CADPS
LOC102162837	0	33.05113487	-20	0.006492	Low	0	40	XR_304257.1	ssc:102162837			
MYO18B	0	33.05113487	-20	0.006492	Low	0	40	XP_001924463.4	ssc:100154025	0	NM_032608.5	MYO18B
LOC102167726	0	33.05113487	-20	0.006492	Low	0	40	XP_005654659.1	ssc:102167726	1E-49	NM_152572.2	AK8
ACE	0	33.05113487	-20	0.006492	Low	0	40	NM_001083941.1	ssc:613133	0	NM_152831.1	ACE
PARD3B	15.18890819	180.128685	-3.56793787	0.00654	Low	11	218	XP_001925471.1		2E-134	NM_205863.3	PARD3B
RPIA	5128.32773	653.586192	2.972039052	0.006597	High	3714	791	NM_001097495.1	ssc:100038014	1E-160	NM_144563.2	RPIA
B2R	1.380809836	47.09786719	-5.092075178	0.006627	Low	1	57	NM_214146.1	ssc:397277	4E-173	NM_000623.3	BDKRB2
C1H15orf52	110.4647869	1162.573669	-3.395663682	0.006638	Low	80	1407	XP_003353441.1	ssc:100153607			
LAMC2	9.66566885	126.4205909	-3.709218084	0.006644	Low	7	153	XP_003130413.2	ssc:100514975	0	NM_018891.2	LAMC2
MX1	4136.906268	52119.16085	-3.655189681	0.006645	Low	2996	63077	NM_214061.1	ssc:397128	0	NM_002462.4	MX1

LOC100524899	1951.084298	256.9725736	2.924589816	0.006655	High	1413	311	XP_005657087.1	ssc:100524899	4E-74	NM_032356.3	LSMD1
LOC102167038	13.80809836	166.0819527	-3.58830876	0.006669	Low	10	201	XP_005656806.1	ssc:102167038	3E-64	NM_145034.4	TOR1AIP2
EPHB3	138.0809836	1459.207604	-3.401598602	0.00667	Low	100	1766	XP_005670109.1	ssc:100337659	0	NM_004443.3	EPHB3
STK32C	421.1469999	56.18692928	2.906017427	0.00676	High	305	68	XP_003483625.1	ssc:100737116			
ITM2A	62.13644261	5.783948602	3.42531301	0.006776	High	45	7	NM_001031798.1	ssc:595131	3E-129	NM_004867.4	ITM2A
LOC102159565	6.904049179	99.15340461	-3.844147665	0.00695	Low	5	120	XP_005660430.1	ssc:102159565	5E-61	NM_032888.2	COL27A1
GIPR	38.6626754	2.478835115	3.963207257	0.006969	High	28	3	XP_005659028.1	ssc:100626628	2E-170	NM_000164.2	GIPR
SLC19A3	38.6626754	2.478835115	3.963207257	0.006969	High	28	3	XP_005672321.1	ssc:100513826	0	NM_025243.3	SLC19A3
RIPPLY3	38.6626754	2.478835115	3.963207257	0.006969	High	28	3	XP_001928108.2	ssc:100153272	9E-38	NM_018962.2	RIPPLY3
CD55	86.99101965	901.4697036	-3.373340627	0.007072	Low	63	1091	NM_213815.1	ssc:396743			
LOC100519345	305.1589737	40.48764021	2.914007551	0.007089	High	221	49	XR_115668.2	ssc:100519345			
SH2D3C	35.90105573	379.2617726	-3.401095789	0.007095	Low	26	459	XP_003353720.1	ssc:100151897	0	NM_170600.2	SH2D3C
LOC100737455	84.22939998	871.7236822	-3.37147511	0.007111	Low	61	1055	XP_005660119.1	ssc:100737455	0	NR_033258.1	ZEB2
LOC100621670	4.142429507	72.71249671	-4.133654282	0.007122	Low	3	88	XP_003361253.1	ssc:100621670	4E-51	NM_001048.3	SST
FAM166B	1.380809836	46.27158882	-5.066540086	0.007194	Low	1	56	NM_001243630.1	ssc:100156839	6E-130	NM_001287239.1	FAM166B
CHST1	2.761619671	59.49204276	-4.429110165	0.007215	Low	2	72	XP_005661027.1	ssc:100519885	0	NM_003654.5	CHST1
LOC100622805	26.23538688	0.826278372	4.98874235	0.007247	High	19	1	LOC100622805				
MFRP	0	32.2248565	-20	0.007269	Low	0	39	XP_003129989.1	ssc:100520397	0	NM_031433.3	MFRP
ADAMDEC1	0	32.2248565	-20	0.007269	Low	0	39	XP_003132859.1	ssc:100517917	0	NM_014479.3	ADAMDEC1
CXCL9	0	32.2248565	-20	0.007269	Low	0	39	NM_001114289.2	ssc:100135681	2E-28	NM_002416.1	CXCL9
LOC102163603	0	32.2248565	-20	0.007269	Low	0	39	LOC102163603				
LOC102166048	0	32.2248565	-20	0.007269	Low	0	39	XP_005674403.1	ssc:102166048	5E-58	NR_073474.1	PTPRR
CTNNA2	0	32.2248565	-20	0.007269	Low	0	39	XP_005662473.1	ssc:100525337	0	NM_004389.3	CTNNA2
LOC102158668	0	32.2248565	-20	0.007269	Low	0	39	LOC102158668				
LOC100739358	0	32.2248565	-20	0.007269	Low	0	39	XP_003482517.1	ssc:100739358	0	NM_178833.4	SLC9B2
LOC102161149	150.5082721	1566.623793	-3.379744088	0.0073	Low	109	1896	XP_005667984.1	ssc:102161149	0	NM_198551.2	MIA3
LOC100621092	35.90105573	376.7829375	-3.39163546	0.007333	Low	26	456	XP_005674141.1	ssc:100621092	7E-85	NM_153367.3	ZCCHC24
LOC100738154	5.523239343	85.10667229	-3.945685691	0.00736	Low	4	103	XR_303634.1	ssc:100738154			
LOC100512321	5.523239343	85.10667229	-3.945685691	0.00736	Low	4	103	XP_005652738.1	ssc:100512321	0	NM_014702.4	KIAA0408
LOC100526189	12.42728852	149.5563853	-3.589106049	0.007409	Low	9	181	XP_005657470.1	ssc:100526189	3E-44	NM_199440.1	HSPD1
FGFR3	361.772177	48.75042393	2.891594788	0.007461	High	262	59	XP_005666536.1	ssc:100514115	5E-165	NM_022965.3	FGFR3
DSCC1	923.7617801	125.5943125	2.878749723	0.007488	High	669	152	XP_001926458.1	ssc:100156441	7E-179	NM_024094.2	DSCC1
LOC100517087	186.4093278	23.96207278	2.959649438	0.00749	High	135	29	XP_005655161.1	ssc:100517087	2E-65	NM_001192.2	TNFRSF17
SLC22A16	19.3313377	0	20	0.007499	High	14	0	XP_003353330.2	ssc:100620810	3E-48	NM_033125.3	SLC22A16
TCTEX1D1	19.3313377	0	20	0.007499	High	14	0	NM_001243355.1	ssc:100628189	4E-83	NM_152665.2	TCTEX1D1
SMIM18	19.3313377	0	20	0.007499	High	14	0	XP_005671874.1	ssc:102167879	9E-50	NM_001206847.1	SMIM18
LOC102159232	19.3313377	0	20	0.007499	High	14	0	XP_005673921.1	ssc:102159232	2E-19	NM_153448.3	ESX1
LOC102157403	19.3313377	0	20	0.007499	High	14	0	XP_005673914.1	ssc:102157403	2E-19	NM_153448.3	ESX1
LOC102159763	19.3313377	0	20	0.007499	High	14	0	XP_005662371.1	ssc:102159763	0	NM_144707.2	PROM2
FAM71A	19.3313377	0	20	0.007499	High	14	0	XP_003130457.1	ssc:100524701	0	NM_153606.3	FAM71A
LCK	19.3313377	0	20	0.007499	High	14	0	NM_001143713.1	ssc:100233188	0	NM_005356.3	LCK
LTF	19.3313377	0	20	0.007499	High	14	0	NM_214362.1	ssc:397649	0	NM_002343	LTF
SPN	19.3313377	0	20	0.007499	High	14	0	XP_005655154.1	ssc:100623653	3E-68	NM_003123.4	SPN
TRIM15	19.3313377	0	20	0.007499	High	14	0	NM_001123208.1	ssc:100144461	0	NM_052812.1	TRIM15
LOC100623474	19.3313377	0	20	0.007499	High	14	0	XP_005655616.1	ssc:100623474	1E-157	NM_024902.2	DNAJC22
LOC100519721	19.3313377	0	20	0.007499	High	14	0	XP_005656204.1	ssc:100519721	1E-111	np_001013696	np_001013696
CCNO	19.3313377	0	20	0.007499	High	14	0	XP_005672504.1	ssc:100517790	1E-174	NM_021147.3	CCNO
LOC102158406	19.3313377	0	20	0.007499	High	14	0	XR_298395.1	ssc:102158406			
LOC102167637	19.3313377	0	20	0.007499	High	14	0	XP_005663590.1	ssc:102167637			
BIN2	19.3313377	0	20	0.007499	High	14	0	XP_005655633.1	ssc:100519772	1E-167	NM_016293.2	BIN2
LOC100513607	19.3313377	0	20	0.007499	High	14	0	XP_003129044.2	ssc:100513607	1E-104	NM_152620.2	TRIM60
LOC100153882	19.3313377	0	20	0.007499	High	14	0	XP_005659670.1	ssc:100153882	1E-158	NM_002112.3	HDC
LOC100515722	19.3313377	0	20	0.007499	High	14	0	XR_302485.1	ssc:100515722	1E-77	NM_001039753.2	EML6
CXHXorf48	19.3313377	0	20	0.007499	High	14	0	XR_297879.1	ssc:102168080			
LOC100515519	19.3313377	0	20	0.007499	High	14	0	XP_003135338.1	ssc:100515519			
LOC100525236	6.904049179	97.50084786	-3.819900118	0.007539	Low	5	118	XP_005666775.1	ssc:100525236	1E-154	NM_015236.4	LPHN3
LOC100621366	30.37781639	320.5960082	-3.399666387	0.007556	Low	22	388	XP_005670111.1	ssc:100621366	0	NM_004443.3	EPHB3
CAPRIN2	2341.853481	312.3332245	2.906492866	0.007581	High	1696	378	XP_005664074.1	ssc:100624034	0	NR_038177	CAPRIN2
LOC100736917	26.23538688	280.108368	-3.416399114	0.007602	Low	19	339	XP_005669333.1	ssc:100736917	0	NM_001281726.1	BTD
LOC100739014	26.23538688	280.108368	-3.416399114	0.007602	Low	19	339	XP_003480267.1	ssc:100739014			
NRAP	197.4558065	25.61462952	2.946489863	0.007614	High	143	31	NM_001044616.1	ssc:733691			
LOC100620844	755.3029801	103.2822895	2.870427574	0.007704	High	547	125	XP_005660357.1	ssc:100620844	5E-67	NM_001018116.2	MURC
C3H2orf40	2.761619671	58.66576439	-4.408932283	0.007705	Low	2	71	NM_001244729.1	ssc:100512958	5E-60	NM_032411.2	C2orf40
LOC102159377	5.523239343	84.28039392	-3.931610506	0.007712	Low	4	102	XP_005659568.1	ssc:102159377	0	NM_003922.3	HERC1
GLS2	122.8920754	14.87301069	3.046623266	0.007722	High	89	18	XP_005663945.1	ssc:100738224	0	NM_138566.1	GLS2
LOC100737630	122.8920754	14.87301069	3.046623266	0.007722	High	89	18	XP_005655692.1	ssc:100737630	0	NM_138566.1	GLS2
RAB20	128.4153147	15.69928906	3.032046134	0.007778	High	93	19	XP_003482979.1	ssc:100737446	6E-112	NM_017817.1	RAB20
LAMB3	60.75563277	5.783948602	3.392891533	0.007816	High	44	7	XP_005656816.1	ssc:100513489	0	NM_001127641.1	LAMB3
AIF1L	24.85457704	265.2353573	-3.41568965	0.007829	Low	18	321	XP_005660585.1	ssc:100624075	3E-69	NR_033701.1	AIF1L
LOC100154173	122.8920754	1251.811733	-3.348553811	0.007932	Low	89	1515	XP_005660024.1	ssc:100154173	0	NM_006832.2	FERMT2
LOC102166571	4.142429507	71.05993997	-4.100487418	0.007944	Low	3	86	XR_309215.1	ssc:102166571			
LOC100736836	16.56971803	185.9126336	-3.488003854	0.007982	Low	12	225	XP_005664407.1	ssc:100736836	2E-72	NM_152649.2	MLKL
CHSY3	12.42728852	147.0775502	-3.564993593	0.008041	Low	9	178	XP_005661677.1	ssc:100518720	0	NM_175856.4	CHSY3
LOC100621248	249.9265803	2599.471757	-3.378642232	0.008062	Low	181	3146	XP_003362128.1	ssc:100621248	6E-105	NM_002076.3	GNS
C3H16orf54	937.5698785	128.899426	2.862680382	0.008108	High	679	156	XP_005655153.1	ssc:100623246	3E-82	NM_175900.3	C16orf54
EDN3	0	31.39857813	-20	0.008156	Low	0	38	NM_001098582.2	ssc:100049663	5E-59	NM_207034.1	EDN3
LOC102164680	0	31.39857813	-20	0.008156	Low	0	38	XR_305540.1	ssc:102164680			
LOC100626991	0	31.39857813	-20	0.008156	Low	0	38	XP_005667732.1	ssc:100626991			
LOC100738453	0	31.39857813	-20	0.008156	Low	0	38	XP_005656961.1	ssc:100738453	7E-59	NM_006456.2	ST6GALNAC2
SLFNL1	0	31.39857813	-20	0.008156	Low	0	38	XP_005656255.1	ssc:100526058	1E-138	NM_144990.3	SLFNL1
ABCC9	0	31.39857813	-20	0.008156	Low	0	38	XP_005655715.1	ssc:100127449	0	NM_020298.2	ABCC9
LMX1B	0	31.39857813	-20	0.008156	Low	0	38	NM_001258424.1	ssc:100157562			
LOC100625402	0	31.39857813	-20	0.008156	Low	0	38	XP_003358402.1	ssc:100625402	1E-62	NM_198399.1	ARPP21
SLC9B2	0	31.39857813	-20	0.008156	Low	0	38	XP_003129331.2	ssc:100522336	0	NM_178833.4	SLC9B2
HMHA1	1333.862301	182.6075202	2.868791656	0.008219	High	966	221	XP_005661429.1	ssc:100623239	0	NR_047652.1	HMHA1
PTPRC	31.75862622	1.652556743	4.264376792	0.008231	High	23	2	XP_003130644.3	ssc:100522631	0	NR_052021.1	PTPRC
TM4SF5	31.75862622	1.652556743	4.264376792	0.008231	High	23	2	XP_003131959.3	ssc:100513308	1E-78	NM_003963.2	TM4SF5
ATP2B3	31.75862622	1.652556743	4.264376792	0.008231	High	23	2	XP_005674059.1	ssc:100520061	0	NM_021949.3	ATP2B3
RNF182	31.75862622	1.652556743	4.264376792	0.008231	High	23	2	XP_005665645.1	ssc:100154575	4E-136	NM_152737.3	RNF182
KIAA1958	450.1440065	61.97087788	2.8607243	0.008253	High	326	75	XP_005660389.1	ssc:100513712	0	NM_133465.3	KIAA1958
DSTN	2444.033409	28625.58791	-3.549969412	0.008272	Low	1770	34644	NM_001004031.1	ssc:445516	3E-92	NM_006870.3	DSTN
LOC100519396	41.42429507	420.5756912	-3.343816414	0.008338	Low	30	509	XP_005674154.1	ssc:100519396	0	NM_015690.4	STK36
TRPC4	23.47376721	248.7097899	-3.405341999	0.008363	Low	17	301	NM_001145868.1	ssc:100157635	0	NM_016179.2	TRPC4
LOC102168045	4.142429507	70.2336616	-4.083613599	0.008396	Low	3	85	XP_005668048.1	ssc:102168045			
LOC100624776	52.47077376	527.1656012	-3.328670264	0.008434	Low	38	638	LOC100624776				
LOC100736922	15.18890819	171.0396229	-3.493240503	0.008441	Low	11	207	XP_005660194.1	ssc:100736922	1E-52	NM_003383.3	VLDLR
PFAS	2774.04696	373.477824	2.892897722	0.008444	High	2009	452	XP_003358324.2	ssc:100620093	0	NM_012393.2	PFAS
C14H10orf54	24.85457704	261.1039655	-3.393040911	0.008463	Low	18	316	XP_005671104.1	ssc:100154373	1E-141	NM_022153.1	C10orf54
LOC100627892	4330.219645	574.2634684	2.914655513	0.008467	High	3136	695	XR_301599.1	ssc:100627892			
TMEM198	33.13943606	339.6004108	-3.357217247	0.008476	Low	24	411	XP_003359699.1	ssc:100623329	4E-148	NM_001005209.1	TMEM198

LOC100626623	5.523239343	82.62783717	-3.903041354	0.008478	Low	4	100	XR_300167.1	ssc:100626623			
LOC100519366	5.523239343	82.62783717	-3.903041354	0.008478	Low	4	100	XR_298538.1	ssc:100519366	2E-35	NM_001863.4	COX6B1
LOC102160944	1.380809836	44.61903207	-5.014072666	0.008502	Low	1	54	XP_005658725.1	ssc:102160944			
TNNC1	44.18591474	445.3640424	-3.333326627	0.008529	Low	32	539	NM_001130243.1	ssc:100156435	8E-87	NM_003280.2	TNNC1
CCDC38	171.2204196	22.30951604	2.940123644	0.008629	High	124	27	XP_005664300.1	ssc:100157054	0	NM_182496.2	CCDC38
NRIP3	313.4438327	42.96647533	2.866923605	0.008673	High	227	52	XP_005667099.1	ssc:100519788	8E-122	NM_020645.2	NRIP3
LOC100519519	127.0345049	1271.642414	-3.323400724	0.008702	Low	92	1539	XP_005657624.1	ssc:100519519	0	NM_133437.3	TTN
LOC100737124	1193.019698	165.2556743	2.851846149	0.008788	High	864	200	XP_005662410.1	ssc:100737124	6E-136	NM_006236.1	POU3F3
CCDC153	446.0015769	61.97087788	2.8473865	0.008789	High	323	75	XP_005667484.1	ssc:100522389	2E-67	NM_001145018.1	CCDC153
LOC102159735	2.761619671	57.01320765	-4.367709621	0.008805	Low	2	69	XR_302589.1	ssc:102159735			
LOC100516384	2.761619671	57.01320765	-4.367709621	0.008805	Low	2	69	XP_003129759.3	ssc:100516384	5E-130	NM_001364.3	DLG2
LOC102165421	2.761619671	57.01320765	-4.367709621	0.008805	Low	2	69	XP_005673291.1	ssc:102165421	1E-51	NM_152556.2	C7orf60
RAPGEF5	422.5278097	58.66576439	2.848455559	0.008813	High	306	71	XP_005656743.1	ssc:100518983	0	NM_012294.3	RAPGEF5
LOC100520570	546.800695	5802.126726	-3.407494825	0.008848	Low	396	7022	XR_304053.1	ssc:100520570	0	NM_015210.3	SOGA2
LOC100155273	871.2910064	121.4629206	2.842638686	0.00888	High	631	147	LOC100155273	ssc:100155273			
TRHDE	8.284859014	106.58991	-3.685449919	0.008906	Low	6	129	XP_003355569.2	ssc:100620702			
CCNA1	48.32834425	4.131391859	3.548169758	0.008994	High	35	5	XP_005668399.1	ssc:100156017	0	NM_003914.3	CCNA1
LOC100623000	15.18890819	168.5607878	-3.472178887	0.009066	Low	11	204	XR_303409.1	ssc:100623000			
ELMO3	1909.660003	262.7565222	2.861517322	0.009094	High	1383	318	XR_303768.1	ssc:100522492	0	NM_024712.3	ELMO3
PDSS1	1484.370573	205.7433146	2.850933849	0.009136	High	1075	249	XP_005668199.1	ssc:100514361	0	NM_014317.3	PDSS1
LOC100736982	0	30.57229975	-20	0.009171	Low	0	37	XP_005669300.1	ssc:100736982	0	NM_003802.2	MYH13
LOC102164187	0	30.57229975	-20	0.009171	Low	0	37	XP_005655457.1	ssc:102164187			
RNF180	0	30.57229975	-20	0.009171	Low	0	37	XP_003483883.1	ssc:100526254	0	NM_178532.3	RNF180
INHBB	0	30.57229975	-20	0.009171	Low	0	37	NM_001164842.1	ssc:397490			
LOC102158359	0	30.57229975	-20	0.009171	Low	0	37	XP_005656512.1	ssc:102158359			
KLHL34	0	30.57229975	-20	0.009171	Low	0	37	XP_005658889.1	ssc:102163579	8E-44	NM_153270.1	KLHL34
MIR145	0	30.57229975	-20	0.009171	Low	0	37	NR_038484.1	ssc:100316550			
LOC100737745	0	30.57229975	-20	0.009171	Low	0	37	XP_003482971.1	ssc:100737745	3E-75	NM_005708.3	GPC6
IER2	6879.194602	906.4273738	2.923976332	0.009185	High	4982	1097	XP_003123390.1	ssc:100525209	2E-87	NM_004907.2	IER2
ARHGAP9	11.04647869	130.5519827	-3.562965912	0.009226	Low	8	158	XP_005674439.1	ssc:100516267	0	NM_032496.2	ARHGAP9
LOC100622512	1.380809836	43.7927537	-4.987105618	0.009259	Low	1	53	XP_005658491.1	ssc:100622512	7E-164	NM_013992.3	PAX8
LOC100738599	1.380809836	43.7927537	-4.987105618	0.009259	Low	1	53	XP_003481459.1	ssc:100738599	1E-69	NM_015441.1	OLFML2B
SPARC	2270.05137	25894.73789	-3.511862105	0.009282	Low	1644	31339	NM_001031794.1	ssc:595124	2E-89	NM_003118.3	SPARC
SYNPO	196.0749967	1963.237411	-3.323757165	0.009316	Low	142	2376	XP_005658874.1	ssc:100624154	2E-159	NM_007286.5	SYNPO
PPA2	2191.345209	301.5916057	2.861148595	0.009381	High	1587	365	XP_005667016.1	ssc:100521105	1E-98	NM_176869.2	PPA2
TOX2	4.142429507	68.58110485	-4.049262094	0.009392	Low	3	83	XP_005657727.1	ssc:102166192	3E-48	NM_032883.2	TOX2
LOC100513133	4118.95574	48519.89227	-3.558225818	0.009464	Low	2983	58721	XR_115737.2	ssc:100513133			
TMEM38A	193.313377	1928.53372	-3.318490993	0.009465	Low	140	2334	XP_005661247.1	ssc:100514568	5E-130	NM_024074.1	TMEM38A
CHDH	527.4693573	74.36505346	2.826390568	0.009533	High	382	90	XP_005669704.1	ssc:100151982	0	NM_018397.4	CHDH
CPEB2	120.1304557	1177.44668	-3.292987872	0.009618	Low	87	1425	NM_001185049.1	ssc:100462674			
SLC29A4	291.3508753	40.48764021	2.847204181	0.009686	High	211	49	XP_003354487.1	ssc:100624843	5E-124	NM_153247.2	SLC29A4
ZNF70	5.523239343	80.14900206	-3.859098006	0.009795	Low	4	97	ZNF70	ssc:100157520	0	NM_021916.2	ZNF70
MYB	313.4438327	43.7927537	2.839442869	0.0098	High	227	53	XP_005652721.1	ssc:100152439	0	NM_005375.2	MYB
ZNF827	34.52024589	342.0792459	-3.308815932	0.009879	Low	25	414	ZNF827	ssc:100523980	0	NM_178835.3	ZNF827
LOC100156509	8.284859014	104.1110748	-3.651502587	0.009969	Low	6	126	XP_001927312.2	ssc:100156509	9E-98	NR_104579.1	RGS20
LOC100738923	71.80211146	693.2475539	-3.271272446	0.010083	Low	52	839	XP_005662241.1	ssc:100738923	0	NM_004380.2	CREBBP
LOC102157484	2.761619671	55.36065091	-4.325274354	0.01009	Low	2	67	XR_307133.1	ssc:102157484			
LOC100737045	2.761619671	55.36065091	-4.325274354	0.01009	Low	2	67	XP_003484149.1	ssc:100737045	6E-117	NM_004615.3	TSPAN7
TIMM8A	4645.044287	629.6241193	2.883129639	0.01013	High	3364	762	XP_003135287.1	ssc:100517443	9E-49	NM_032696.1	TIMM8A
RASGRF2	167.0779901	22.30951604	2.904790571	0.010136	High	121	27	XP_005661584.1	ssc:100515363	0	NM_006909.2	RASGRF2
B3GNT3	334.1559802	47.09786719	2.826788059	0.01019	High	242	57	XP_003123547.1	ssc:100518473	9E-159	NM_014256.3	B3GNT3
UGP2	784.2999867	8256.999769	-3.396140184	0.010203	Low	568	9993	NM_213980.1	ssc:397040			
LRRC70	144.9850328	19.00440255	2.931498398	0.010206	High	105	23	XR_308429.1	ssc:100525544	0	NM_181506.4	LRRC70
HTRA3	103.5607377	999.7968298	-3.271157806	0.010228	Low	75	1210	NM_001195343.1	ssc:100337673			
LOC102158232	1743.962823	244.578398	2.834000394	0.010233	High	1263	296	XR_300389.1	ssc:102158232			
LOC102159568	266.4962983	37.18252673	2.841418777	0.010252	High	193	45	XP_005661607.1	ssc:102159568	7E-67	NM_024717.4	MCTP1
NYX	5.523239343	79.32272369	-3.844147665	0.010286	Low	4	96	XP_003360321.1	ssc:100620950	0	NM_022567.2	NYX
PIM2	2351.51915	327.2062352	2.845320936	0.010312	High	1703	396	XP_003360359.1	ssc:100621339	3E-161	NM_006875.3	PIM2
LOC100626135	92.51425899	889.9018064	-3.265898508	0.010335	Low	67	1077	XP_005652614.1	ssc:100626135	5E-130	NM_198316.1	TENC1
KCNN4	0	29.74602138	-20	0.010335	Low	0	36	NM_214018.2	ssc:397081	0	NM_002250.2	KCNN4
LOC100621628	0	29.74602138	-20	0.010335	Low	0	36	XP_003360430.1	ssc:100621628	3E-141	NM_144657.4	HDX
LOC100621630	0	29.74602138	-20	0.010335	Low	0	36	XP_005658328.1	ssc:100621630			
LOC100624808	0	29.74602138	-20	0.010335	Low	0	36	XP_003360907.2	ssc:100624808	1E-179	NM_024788.2	C7orf63
UNCX	0	29.74602138	-20	0.010335	Low	0	36	XP_003124306.1	ssc:100523430	9E-115	NM_001080461.1	UNCX
CSMD2	0	29.74602138	-20	0.010335	Low	0	36	XP_005656143.1	ssc:100736699	0	NM_052896.4	CSMD2
BLNK	57.9940131	5.783948602	3.325777337	0.010452	High	42	7	XP_001928268.1	ssc:100152350	0	NR_047683.1	BLNK
ANKLE1	698.6897769	99.97968298	2.804945174	0.010503	High	506	121	XP_005658082.1	ssc:100620785	8E-174	NR_103530.1	ANKLE1
LOC100518964	20.71214754	212.3535415	-3.357919117	0.010579	Low	15	257	XP_003125468.1	ssc:100518964	2E-172	NM_145175.2	FAM84A
LOC102164131	198.8366163	27.26718627	2.866345718	0.01059	High	144	33	XR_307100.1	ssc:102164131			
LOC100519123	46.94753442	451.9742693	-3.267119345	0.010602	Low	34	547	XP_003122102.3	ssc:100519123	0	NM_005502.3	ABCA1
MDGA1	593.7482294	85.10667229	2.802507159	0.010609	High	430	103	XP_001924602.1	ssc:397529	0	NM_153487.3	MDGA1
XKR5	35.90105573	349.5157512	-3.283259299	0.010655	Low	26	423	XP_003133364.3	ssc:100524909	0	NM_207411.4	XKR5
LOC102161872	30.37781639	1.652556743	4.200246455	0.010679	High	22	2	XR_305191.1	ssc:102161872			
ARHGAP30	30.37781639	1.652556743	4.200246455	0.010679	High	22	2	XP_001928095.4	ssc:100158101	0	NM_181720.2	ARHGAP30
ARHGEF16	30.37781639	1.652556743	4.200246455	0.010679	High	22	2	XP_005665015.1	ssc:100625324	0	NM_014448.3	ARHGEF16
LOC100626715	30.37781639	1.652556743	4.200246455	0.010679	High	22	2	XP_005674325.1	ssc:100626715			
RPL3L	30.37781639	1.652556743	4.200246455	0.010679	High	22	2	XP_005658938.1	ssc:100622679	2E-52	NM_005061.2	RPL3L
SPRP	30.37781639	1.652556743	4.200246455	0.010679	High	22	2	NM_214238.2	ssc:397469	4E-18	NM_003125.2	SPRR1B
RAB6B	4651.948337	635.4080679	2.872079761	0.010717	High	3369	769	XP_005669909.1	ssc:100522830	7E-121	NM_016577.3	RAB6B
LOC100622083	46.94753442	4.131391859	3.506349583	0.010736	High	34	5	XP_003354876.2	ssc:100622083	1E-173	NM_138735.2	NRXN1
GNAT1	22.09295737	223.9214387	-3.341334205	0.010762	Low	16	271	XP_001928011.1	ssc:100157237	0	NM_144499.2	GNAT1
PDLIM1	835.3899506	8727.152162	-3.384989258	0.010813	Low	605	10562	XP_003483582.1	ssc:100155592	1E-136	NM_020992.3	PDLIM1
LOC102159729	2.761619671	54.53437253	-4.303579283	0.010814	Low	2	66	XP_005671271.1	ssc:102159729	1E-33	NR_045675.1	ARHGAP22
ZWINT	1216.493465	173.5184581	2.809567527	0.010829	High	881	210	ZWINT	ssc:100623641	7E-102	NM_032997.2	ZWINT
LOC102164486	41.42429507	3.305113487	3.647705432	0.010851	High	30	4	XP_005670537.1	ssc:102164486			
LOC100517279	17.95052786	186.738912	-3.378924408	0.010859	Low	13	226	XR_130597.3	ssc:100517279			
LOC102167215	35.90105573	2.478835115	3.856292054	0.01088	High	26	3	XR_297157.1	ssc:102167215			
LOC100515829	52.47077376	500.7246933	-3.254431634	0.010901	Low	38	606	XP_005663562.1	ssc:100515829	5E-65	NM_016274.4	PLEKHO1
LOC102157672	44.18591474	423.8808047	-3.262000179	0.010906	Low	32	513	XR_305312.1	ssc:102157672			
EML5	230.5952426	32.2248565	2.83911691	0.010997	High	167	39	XP_003356819.2	ssc:100153276	0	NM_183387.2	EML5
TLR7	84.22939998	9.915340461	3.086589673	0.011002	High	61	12	NM_001097434.1	ssc:100037296			
LOC733663	15.18890819	161.9505609	-3.414463389	0.011009	Low	11	196	NM_001044593.1	ssc:733663			
RAPSN	6.904049179	90.06432252	-3.705441394	0.011011	Low	5	109	XP_003122858.3	ssc:100512645	3E-136	NM_032645.4	RAPSN
SST	1.380809836	42.14019696	-4.931610506	0.011018	Low	1	51	NM_001009583.1	ssc:494469	2E-61	NM_001048.3	SST
LIMS2	102.1799278	971.7033652	-3.249404143	0.01102	Low	74	1176	XP_005671891.1	ssc:100517009	0	NM_017980.4	LIMS2
SCUBE3	8.284859014	101.6322397	-3.616737168	0.011181	Low	6	123	XP_005665957.1	ssc:100152993	0	NM_152753.2	SCUBE3
SLC6A3	17.95052786	0	20	0.011186	High	13	0	XP_005674695.1	ssc:100521346	0	NM_001044.4	SLC6A3
PTCHD4	17.95052786	0	20	0.011186	High	13	0	XP_001925843.4	ssc:100157414	1E-175	NM_207499.2	PTCHD4
LOC102161687	17.95052786	0	20	0.011186	High	13	0	XR_301223.1	ssc:102161687			
PLA2G4F	17.95052786	0	20	0.011186	High	13	0	XP_005659772.1	ssc:100513834	1E-167	NR_033151.1	PLA2G4F

LOC100154181	17.95052786	0	20	0.011186	High	13	0	XP_001926704.1	ssc:100154181	3E-69	NM_175065.2	HIST2H2AB
LOC100624606	17.95052786	0	20	0.011186	High	13	0	XR_305119.1	ssc:100624606			
LOC102164094	17.95052786	0	20	0.011186	High	13	0	XR_300596.1	ssc:102164094			
GFI1B	17.95052786	0	20	0.011186	High	13	0	XP_003122315.3	ssc:100514698	0	NM_004188.4	GFI1B
LOC102165799	17.95052786	0	20	0.011186	High	13	0	LOC102165799				
LOC102167231	17.95052786	0	20	0.011186	High	13	0	XR_298105.1	ssc:102167231			
GABRR2	17.95052786	0	20	0.011186	High	13	0	XP_003121344.1	ssc:100522289	0	NM_002043.3	GABRR2
PSORS1C2	110.4647869	14.04673232	2.97528009	0.011345	High	80	17	NM_001123171.1	ssc:396560	1E-33	NM_014069.2	PSORS1C2
PN-1	1952.465108	21228.74393	-3.44265034	0.011367	Low	1414	25692	NM_214287.1	ssc:397542	0	NR_073116.1	SERPINE2
PTGER4	2.761619671	53.70809416	-4.281552977	0.011597	Low	2	65	XP_005659149.1	ssc:100625072	0	NM_000958.2	PTGER4
TMEM132C	2.761619671	53.70809416	-4.281552977	0.011597	Low	2	65	XP_003359125.2	ssc:100623362	0	NM_001136103.2	TMEM132C
LOC102157560	0	28.91974301	-20	0.011673	Low	0	35	XP_005658851.1	ssc:102157560			
11-Mar	0	28.91974301	-20	0.011673	Low	0	35	XP_003133899.3	ssc:100521230	1E-81	NM_001102562.1	11-Mar
LOC102164195	0	28.91974301	-20	0.011673	Low	0	35	XR_305910.1	ssc:102164195			
IDO1	0	28.91974301	-20	0.011673	Low	0	35	NM_001246240.1	ssc:100519877	2E-175	NM_002164.5	IDO1
BHLHE22	0	28.91974301	-20	0.011673	Low	0	35	XP_003481442.1	ssc:100739687	1E-138	NM_152414.4	BHLHE22
LOC102158984	0	28.91974301	-20	0.011673	Low	0	35	XP_005657897.1	ssc:102158984			
CCL8	0	28.91974301	-20	0.011673	Low	0	35	NM_001164515.1	ssc:100302703	4E-31	NM_005623.2	CCL8
LOC100627386	0	28.91974301	-20	0.011673	Low	0	35	XR_307946.1	ssc:100627386			
LOC102163138	0	28.91974301	-20	0.011673	Low	0	35	XP_005662123.1	ssc:102163138	9E-94	NM_001012981.4	ZKSCAN2
LDLRAD4	836.7707605	121.4629206	2.784316475	0.0117	High	606	147	XP_005665323.1	ssc:100622189	4E-102	NM_181483.3	LDLRAD4
PKC	4.142429507	65.27599137	-3.978003411	0.011823	Low	3	79	NM_001080206.2	ssc:791125	0	NM_006255.3	PRKCH
LOC100516787	125.6536951	16.52556743	2.926681381	0.011856	High	91	20	XR_115797.3	ssc:100516787			
LIF	24.85457704	243.7521197	-3.293831307	0.01186	Low	18	295	NM_214402.2	ssc:399503	3E-105	NM_002309.4	LIF
TTC28	198.8366163	1893.830028	-3.251651491	0.012029	Low	144	2292	XP_005657394.1	ssc:100516229	0	NM_015281.1	TTC28
LOC102158553	1.380809836	41.31391859	-4.903041354	0.01204	Low	1	50	XR_299267.1	ssc:102158553			
APRIL	13.80809836	147.0775502	-3.4129905	0.012052	Low	10	178	NM_001112690.1	ssc:100127162			
C10H1orf115	303.7781639	43.7927537	2.794254095	0.012062	High	220	53	XP_003130555.1	ssc:100516689	4E-39	NM_024709.4	C1orf115
TMEM173	151.8890819	20.65695929	2.87831836	0.012097	High	110	25	NM_001142838.1	ssc:100217389	4E-165	NM_198282.2	TMEM173
LOC102157898	71.80211146	667.6329244	-3.216956928	0.012161	Low	52	808	XP_005655626.1	ssc:102157898	0	NM_198900.2	FMNL3
LOC102166840	30.37781639	290.8499868	-3.259185164	0.01222	Low	22	352	XP_005671817.1	ssc:102166840	1E-42	NR_027878.1	ADAM9
LOC100511616	22.09295737	218.1374901	-3.303579283	0.012222	Low	16	264	XP_005658573.1	ssc:100511616	5E-75	NM_014397.5	NEK6
PARP8	270.6387278	2599.471757	-3.263778275	0.012308	Low	196	3146	XP_003133980.2	ssc:100511108	0	NM_024615.3	PARP8
KCNN2	2.761619671	52.88181579	-4.259185164	0.012447	Low	2	64	XP_005661539.1	ssc:100519705	0	NR_103458.1	KCNN2
LOC100738205	2.761619671	52.88181579	-4.259185164	0.012447	Low	2	64	XR_309627.1	ssc:100738205			
PPGRP-S	51.08996392	4.95767023	3.365305701	0.01246	High	37	6	NM_213737.1	ssc:396556	2E-37	NM_020393.2	PGLYRP4
ZNF711	1383.571455	200.7856443	2.784669126	0.012472	High	1002	243	ZNF711	ssc:100152061	0	NM_021998.4	ZNF711
ADRA1D	544.0390753	80.14900206	2.762953813	0.012766	High	394	97	NM_001123073.1	ssc:552898	0	NM_000678.3	ADRA1D
NUAK1	361.772177	3493.504956	-3.271521824	0.012841	Low	262	4228	XP_003126141.3	ssc:100523669	0	NM_014840.2	NUAK1
RBP1	689.024108	101.6322397	2.761196336	0.01289	High	499	123	NM_001031789.1	ssc:100156666	2E-74	NM_002899.3	RBP1
LOC396904	12685.49996	150749.5313	-3.570901231	0.012945	Low	9187	182444	LOC396904		0	NM_005964.3	MYH10
HOMER2	2085.022852	300.7653273	2.79335303	0.01297	High	1510	364	XP_005666167.1	ssc:100156419	0	NM_199332.2	HOMER2
FHOD3	135.3193639	1252.638012	-3.210529358	0.013006	Low	98	1516	XP_003356465.2	ssc:100622006	0	NM_025135.3	FHOD3
SETBP1	9.66566885	109.8950234	-3.507112677	0.013041	Low	7	133	XP_005659496.1	ssc:100737551	0	NM_015559.2	SETBP1
LOC100621279	8.284859014	98.32712624	-3.569040426	0.013069	Low	6	119	XR_299836.1	ssc:100621279			
LOC100737092	52.47077376	482.5465691	-3.201082209	0.013082	Low	38	584	XP_003480928.1	ssc:100737092	0	NM_022350.3	ERAP2
STK36	66.27887212	606.4883248	-3.193858916	0.013184	Low	48	734	XP_005672268.1	ssc:100622459	0	NM_015690.4	STK36
LOC100626511	0	28.09346464	-20	0.013214	Low	0	34	XP_003357514.2	ssc:100626511	2E-84	NM_178425.2	HDAC9
ISG20	0	28.09346464	-20	0.013214	Low	0	34	NM_001005351.2	ssc:448855	2E-76	NM_002201.4	ISG20
BMP3	0	28.09346464	-20	0.013214	Low	0	34	NM_001206388.1	ssc:100512974	0	NM_001201.2	BMP3
ARHGAP42	182.2668983	1698.002054	-3.219713723	0.013222	Low	132	2055	XP_003129850.3	ssc:100519555	0	NM_152432.2	ARHGAP42
KLHL40	4.142429507	63.62343462	-3.941009204	0.013305	Low	3	77	XP_005669475.1	ssc:100523695	0	NM_152393.3	KLHL40
SYPL2	2.761619671	52.05553742	-4.236465087	0.013368	Low	2	63	XP_003125917.1	ssc:100514960	2E-137	NM_001040709.1	SYPL2
WASF3	13.80809836	143.7724367	-3.380200565	0.013434	Low	10	174	XP_005656890.1	ssc:100155782	3E-130	NM_006646.5	WASF3
LOC102162901	15.18890819	155.3403339	-3.354342397	0.013446	Low	11	188	XP_005673768.1	ssc:102162901	0	NM_181303.1	NLGN3
SNAP91	9.66566885	109.0687451	-3.496224361	0.013513	Low	7	132	XP_005659462.1	ssc:100521763	0	NR_026669.1	SNAP91
SLC22A17	305.1589737	2892.800579	-3.244833951	0.013522	Low	221	3501	XP_001928953.3	ssc:100152841	0	NR_110290.1	SLC22A17
CYP26A1	23.47376721	0.826278372	4.828277677	0.013595	High	17	1	XP_003483578.1	ssc:100124374	4E-120	NM_057157.2	CYP26A1
SAMD12	23.47376721	0.826278372	4.828277677	0.013595	High	17	1	XP_003481395.1	ssc:100738445	2E-30	NR_109794.1	SAMD12
VAV1	23.47376721	0.826278372	4.828277677	0.013595	High	17	1	NM_001267833.1	ssc:100519821	0	NM_005428.3	VAV1
SLC7A9	23.47376721	0.826278372	4.828277677	0.013595	High	17	1	NM_001110171.1	ssc:100037973	0	NM_014270.4	SLC7A9
PTGDS	8.284859014	97.50084786	-3.556865712	0.013597	Low	6	118	NM_214228.1	ssc:397456			
DAPK1	1271.725859	187.5651904	2.761323695	0.013758	High	921	227	XP_003482851.1	ssc:100516103	0	NM_004938	DAPK1
SLC4A11	169.8396098	23.96207278	2.825348346	0.013776	High	123	29	XP_001924597.3	ssc:100157069	9E-165	NM_032034.3	SLC4A11
LOC100738338	12.42728852	131.3782611	-3.402143118	0.013843	Low	9	159	XP_003480496.2	ssc:100738338	9E-51	NR_104583.1	CALML4
LOC100737078	22.09295737	212.3535415	-3.264809713	0.013913	Low	16	257	XP_005672707.1	ssc:100737078	5E-128	NM_006197.3	PCM1
CDH24	37.28186556	342.9055243	-3.201265188	0.013927	Low	27	415	NM_001243615.1	ssc:100156315	0	NM_144985.3	CDH24
HCK	28.99700655	1.652556743	4.133132259	0.013937	High	21	2	XP_003134439.4	ssc:100526085	0	NM_002110.3	HCK
CACNA1F	28.99700655	1.652556743	4.133132259	0.013937	High	21	2	XP_003135132.1	ssc:100513061	0	NM_005183.3	CACNA1F
GCNT3	75.94454097	9.089062089	3.062742931	0.013937	High	55	11	XP_005659605.1	ssc:100522112	0	NM_004751.2	GCNT3
NT5C	7245.109208	1008.885892	2.844244426	0.013977	High	5247	1221	UMPH2		2E-99	NR_045513.1	NT5C
LOC100739719	96.6566885	875.0287957	-3.178389021	0.014016	Low	70	1059	XP_005669968.1	ssc:100739719	5E-140	NM_003270.3	TSPAN6
APOA1	146.3658426	1337.744684	-3.19215198	0.014016	Low	106	1619	NM_214398.1	ssc:397691	3E-122	NM_000039.1	APOA1
LOC100737413	45.56672458	414.7917426	-3.186334598	0.01403	Low	33	502	XP_003481951.1	ssc:100737413	0	NM_021632.3	ZNF350
ICA1	4.142429507	62.79715625	-3.922150177	0.014126	Low	3	76	XP_005667694.1	ssc:100511342	2E-142	NM_022308.1	ICA1
KLF9	77.3253508	697.3789457	-3.17292943	0.014141	Low	56	844	U57346.1		1E-141	NM_001206.2	KLF9
LOC100627081	132.5577442	18.17812418	2.866345718	0.014168	High	96	22	XP_003361707.1	ssc:100627081	1E-170	NM_018593.4	SLC16A10
LOC100621406	1713.585006	252.0149034	2.765436804	0.01425	High	1241	305	XP_005658086.1	ssc:100621406	5E-147	NM_018290.3	PGM2
BDKRB1	2.761619671	51.22925905	-4.213381474	0.01437	Low	2	62	NM_001113064.1	ssc:100127469	8E-141	NM_000710.3	BDKRB1
AZGP1	2.761619671	51.22925905	-4.213381474	0.01437	Low	2	62	XP_003124355.2	ssc:100519648	2E-114	NM_001185.3	AZGP1
FAM154B	127.0345049	17.35184581	2.872059369	0.014401	High	92	21	XP_003356701.2	ssc:100513023	0	NM_001008226.1	FAM154B
LOC102164007	1.380809836	39.66136184	-4.844147665	0.01443	Low	1	48	XR_300276.1	ssc:102164007			
ELOVL2	106.3223574	14.04673232	2.920138536	0.014441	High	77	17	XP_005665625.1	ssc:100153368	3E-156	NM_017770.3	ELOVL2
LOC100524954	106.3223574	14.04673232	2.920138536	0.014441	High	77	17	XR_299308.1	ssc:100524954			
CDHR4	60.75563277	546.1700037	-3.168260007	0.014441	Low	44	661	XP_005669638.1	ssc:100513757	0	NM_001007540.2	CDHR4
USP44	1433.280609	212.3535415	2.754781015	0.014492	High	1038	257	XP_005664303.1	ssc:100516911	0	NM_032147.3	USP44
ICOSLG	204.3598557	1877.304461	-3.199478918	0.014494	Low	148	2272	XP_005657224.1	ssc:100621467	4E-84	NM_015259.5	ICOSLG
LOC102159265	24.85457704	233.8367792	-3.233918405	0.014497	Low	18	283	XR_300558.1	ssc:102159265			
LOC100736682	180.8860885	1653.383022	-3.192267614	0.014518	Low	131	2001	XP_003480471.2	ssc:100736682	0	NM_175610.2	TJP1
LOC100624658	5.523239343	73.53877508	-3.734918595	0.014634	Low	4	89	XP_005658285.1	ssc:100624658	5E-28	NM_175913.3	JPH2
LOC100736744	121.5112655	16.52556743	2.87831836	0.014661	High	88	20	XP_003481688.2	ssc:100736744	3E-63	NM_024779.4	PIP4K2C
ADM5	510.8996392	76.84388857	2.733037486	0.014664	High	370	93	NM_001099939.1	ssc:100101476			
VWF	49.70915409	4.95767023	3.325777337	0.014685	High	36	6	NM_001246221.1	ssc:399543	0	NM_000552.3	VWF
CCR7	49.70915409	4.95767023	3.325777337	0.014685	High	36	6	NM_001001532.2	ssc:396663	0	NM_001838.3	CCR7
LPAR3	49.70915409	4.95767023	3.325777337	0.014685	High	36	6	NM_001162402.1	ssc:100113360	5E-175	NM_012152.2	LPAR3
LOC100518639	12.42728852	129.7257044	-3.383880911	0.014697	Low	9	157	XP_005659502.1	ssc:100518639	0	NM_020964.2	EPG5
LOC102167807	20.71214754	198.3068092	-3.259185164	0.014722	Low	15	240	XP_005673813.1	ssc:102167807			
LOC100155572	267.8771081	39.66136184	2.755765178	0.014786	High	194	48	XR_304540.1	ssc:100155572			
LOC100739077	143.6042229	1297.257044	-3.175294289	0.01481	Low	104	1570	XP_005674296.1	ssc:100739077	3E-85	NM_020992.3	PDLIM1
HHIPL1	77.3253508	690.7687188	-3.159189374	0.014819	Low	56	836	XP_005666497.1	ssc:100625046	0	NM_032425.4	HHIPL1

RAVER2	537.1350261	80.97528043	2.729731337	0.014863	High	389	98	XP_003356487.2	ssc:100623447	0	NM_018716.1	RAVER2
WSCD1	0	27.26718627	-20	0.014996	Low	0	33	XP_005669205.1	ssc:100522103	0	NM_015253.1	WSCD1
LOC102161240	0	27.26718627	-20	0.014996	Low	0	33	XP_005658006.1	ssc:102161240			
CNTN4	0	27.26718627	-20	0.014996	Low	0	33	XP_005669790.1	ssc:100153462	0	NM_175613.2	CNTN4
TM4SF18	0	27.26718627	-20	0.014996	Low	0	33	XP_003132527.1	ssc:100522214	2E-101	NM_138786.3	TM4SF18
LOC100522814	0	27.26718627	-20	0.014996	Low	0	33	XP_005656616.1	ssc:100522814	7E-107	NM_031305.2	ARHGAP24
LOC102160144	0	27.26718627	-20	0.014996	Low	0	33	XR_303501.1	ssc:102160144			
LOC102158661	0	27.26718627	-20	0.014996	Low	0	33	XR_309221.1	ssc:102158661			
LOC102158724	0	27.26718627	-20	0.014996	Low	0	33	XR_304233.1	ssc:102158724			
FAM26F	0	27.26718627	-20	0.014996	Low	0	33	NM_001258434.1	ssc:100158219			
LOC100737032	0	27.26718627	-20	0.014996	Low	0	33	XP_003483161.1	ssc:100737032	0	NM_017534.5	MYH2
LOC100737008	0	27.26718627	-20	0.014996	Low	0	33	XP_005661612.1	ssc:100737008	3E-108	NM_022350.3	ERAP2
LOC100737091	19.3313377	185.9126336	-3.265611433	0.015052	Low	14	225	XP_005657970.1	ssc:100737091	4E-139	NM_033381.1	COL4A5
LOC100622791	484.6642523	4600.717974	-3.246801439	0.015105	Low	351	5568	XR_304346.1	ssc:100622791	6E-16	NM_002116.7	HLA-A
LOC100513486	272.0195376	40.48764021	2.748156811	0.015199	High	197	49	XR_297518.1	ssc:100513486	2E-33	NM_152770.2	C4orf22
MPC2	9417.12308	1312.956333	2.842467451	0.015235	High	6820	1589	XP_005663197.1	ssc:100154777	8E-69	NR_026550.2	MPC2
CDON	4110.670881	592.4415925	2.794629034	0.015253	High	2977	717	XP_005658056.1	ssc:100622989			
ASXL3	573.0360818	86.75922903	2.723536845	0.015277	High	415	105	XP_005665392.1	ssc:100518495	0	NM_030632.1	ASXL3
LOC102163869	8.284859014	95.02201275	-3.519712714	0.015333	Low	6	115	XR_303904.1	ssc:102163869			
PHF11	662.7887212	6380.521586	-3.267053407	0.015343	Low	480	7722	NM_001244327.1	ssc:100518446	2E-96	NM_016119.1	PHF11
KAL1	5.523239343	72.71249671	-3.718616782	0.015413	Low	4	88	XP_001925105.2	ssc:100154011	0	NM_000216.2	KAL1
LOC102160564	2863.799599	418.9231345	2.773173078	0.015427	High	2074	507	XR_304333.1	ssc:102160564			
LOC100622375	447.3823868	67.75482648	2.723112834	0.015454	High	324	82	XP_005658968.1	ssc:100622375	0	NM_033517.1	SHANK3
CMYA5	2.761619671	50.40298068	-4.189922501	0.015458	Low	2	61	NM_001197307.1	ssc:100505410			
BCAR3	298.2549245	2749.028143	-3.204303813	0.015481	Low	216	3327	XP_005663717.1	ssc:100153143	0	NM_003567.3	BCAR3
LOC102158822	12.42728852	128.0731476	-3.365384568	0.015611	Low	9	155	XR_299778.1	ssc:102158822			
LOC100513211	100.799118	889.9018064	-3.14216314	0.015912	Low	73	1077	XR_304085.1	ssc:100513211	3E-51	NM_015461.2	ZNF521
SLC41A2	93.89506883	827.9309285	-3.140389116	0.015924	Low	68	1002	XP_001926561.1		0	NM_032148.3	SLC41A2
LOC100624689	143.6042229	1278.252641	-3.154002927	0.015931	Low	104	1547	XP_005658671.1	ssc:100624689	0	NM_001431.3	EPB41L2
LOC102162364	4.142429507	61.14459951	-3.883676029	0.015948	Low	3	74	XP_005660293.1	ssc:102162364	1E-47	NM_021111.2	RECK
PLXNB1	99.41830817	875.855074	-3.139108712	0.01606	Low	72	1060	XP_005669596.1	ssc:100522643	0	NM_002673	PLXNB1
LOC102166921	742.8756916	113.2001369	2.714245116	0.016095	High	538	137	XR_300637.1	ssc:102166921			
LOC100736995	22.09295737	205.7433146	-3.219187096	0.016185	Low	16	249	XP_005655802.1	ssc:100736995	0	NM_014903.5	NAV3
CAMK1D	31.75862622	285.8923166	-3.170251435	0.016208	Low	23	346	NM_001243935.1	ssc:100511150			
LOC102159363	573.0360818	87.5855074	2.709861908	0.016258	High	415	106	XR_299269.1	ssc:102159363			
LOC102167481	243.0225311	36.35624836	2.740814836	0.016279	High	176	44	XP_005654940.1	ssc:102167481	3E-67	NM_001199209.1	CYP4F3
LOC102168047	38.6626754	3.305113487	3.548169758	0.016281	High	28	4	XR_306030.1	ssc:102168047			
PSRC1	7704.918883	1095.645121	2.813999183	0.016551	High	5580	1326	XP_005663654.1	ssc:100156693	7E-146	NM_032636.7	PSRC1
LOC100620668	2.761619671	49.5767023	-4.166075759	0.016642	Low	2	60	XP_005658903.1	ssc:100620668	1E-110	NM_001164440.1	ANKRD33B
LOC102166028	646.2190031	99.15340461	2.70428896	0.016719	High	468	120	XR_305096.1	ssc:102166028			
LOC100513915	16.56971803	159.4717257	-3.2666797	0.016725	Low	12	193	XP_003123933.1	ssc:100513915	2E-37	NM_032446.2	MEGF10
BTG2	7499.178218	1069.204213	2.810195082	0.016733	High	5431	1294	NM_001097505.2	ssc:100048932	1E-84	NM_006763.2	BTG2
LOC102158304	133.9385541	19.00440255	2.817165722	0.016906	High	97	23	XR_297416.1	ssc:102158304			
LOC100737501	118.7496459	16.52556743	2.845151496	0.016924	High	86	20	XP_005660647.1	ssc:100737501			
TFF1	16.56971803	0	20	0.016936	High	12	0	XP_003359021.1	ssc:780401	1E-18	NM_003225.2	TFF1
KLHL6	16.56971803	0	20	0.016936	High	12	0	XP_003358750.2	ssc:100625137	0	NM_130446.2	KLHL6
EPHA6	16.56971803	0	20	0.016936	High	12	0	XP_001924986.4	ssc:100156650	0	NM_173655.3	EPHA6
TARSL2	16.56971803	0	20	0.016936	High	12	0	XP_005652533.1	ssc:100523715	0	NM_152334.2	TARSL2
LOC102161998	16.56971803	0	20	0.016936	High	12	0	XP_005657563.1	ssc:102161998	5E-39	NM_017579.2	DMBT1
PGA	16.56971803	0	20	0.016936	High	12	0	NM_213873.2	ssc:396892	0	NM_014224.2	PGA5
LOC102159894	16.56971803	0	20	0.016936	High	12	0	XP_005652898.1	ssc:102159894	1E-133	NM_024733.3	ZNF665
RXFP2	16.56971803	0	20	0.016936	High	12	0	RXFP2		6E-71	NM_130806.3	RXFP2
LOC100514940	16.56971803	0	20	0.016936	High	12	0	XP_005654359.1	ssc:100514940	4E-67	NM_005364.4	MAGEA8
ADAM18	16.56971803	0	20	0.016936	High	12	0	XP_005654290.1	ssc:100519521	0	NM_014237.2	ADAM18
LOC100621138	16.56971803	0	20	0.016936	High	12	0	LOC100621138				
THEM5	16.56971803	0	20	0.016936	High	12	0	XP_003355217.1	ssc:100622268	2E-107	NM_182578.3	THEM5
LOC102160414	16.56971803	0	20	0.016936	High	12	0	XR_299094.1	ssc:102160414			
LOC102158704	16.56971803	0	20	0.016936	High	12	0	XR_308019.1	ssc:102158704			
ZSCAN10	16.56971803	0	20	0.016936	High	12	0	ZSCAN10	ssc:100628188	0	NM_032805.1	ZSCAN10
CACNB4	16.56971803	0	20	0.016936	High	12	0	NM_213750.1	ssc:396585	0	NM_001145798.1	CACNB4
LOC102163198	16.56971803	0	20	0.016936	High	12	0	XR_305725.1	ssc:102163198			
PAPPA2	16.56971803	0	20	0.016936	High	12	0	XP_003130378.3	ssc:100521390	0	NM_021936.2	PAPPA2
LOC102163242	16.56971803	0	20	0.016936	High	12	0	XR_306618.1	ssc:102163242			
LOC100157667	16.56971803	0	20	0.016936	High	12	0	XR_299681.1	ssc:100157667	4E-22	NM_182540.4	DDX26B
KRTDAP	16.56971803	0	20	0.016936	High	12	0	XP_003127087.1	ssc:100515206	3E-37	NM_207392.2	KRTDAP
FCGR2B	16.56971803	0	20	0.016936	High	12	0	NM_001033013.2	ssc:613131	4E-104	NM_004001.4	FCGR2B
LCN8	16.56971803	0	20	0.016936	High	12	0	XP_005652780.1	ssc:100303612	1E-59	NM_178469.3	LCN8
LOC102162060	4.142429507	60.31832114	-3.864047222	0.016959	Low	3	73	XR_302109.1	ssc:102162060			
MLC2V	0	26.4409079	-20	0.017059	Low	0	32	NM_213791.2	ssc:396690	6E-91	NM_000432.3	MYL2
ZBTB16	0	26.4409079	-20	0.017059	Low	0	32	ZBTB16	ssc:100625290	0	NM_006006.4	ZBTB16
LOC102160855	0	26.4409079	-20	0.017059	Low	0	32	XR_305249.1	ssc:102160855			
LOC102160410	0	26.4409079	-20	0.017059	Low	0	32	XR_298807.1	ssc:102160410			
LOC102158399	0	26.4409079	-20	0.017059	Low	0	32	XR_297559.1	ssc:102158399			
LOC102162226	0	26.4409079	-20	0.017059	Low	0	32	XP_005658705.1	ssc:102162226			
LOC102163370	0	26.4409079	-20	0.017059	Low	0	32	XR_306619.1	ssc:102163370			
LOC100738691	69.04049179	599.0518195	-3.117166159	0.017082	Low	50	725	XP_005665024.1	ssc:100738691	2E-107	NM_012102.3	RERE
RNF207	637.9341441	98.32712624	2.697746114	0.017217	High	462	119	XP_003481985.1	ssc:100515143	0	NM_207396.2	RNF207
MEG3	13.80809836	136.3359313	-3.303579283	0.017261	Low	10	165	NR_021488.1	ssc:100144590			
LOC100623747	48.32834425	4.95767023	3.285135352	0.017342	High	35	6	XP_005658336.1	ssc:100623747	6E-93	NM_183239.1	GSTO2
LOC100517709	48.32834425	4.95767023	3.285135352	0.017342	High	35	6	XP_005658255.1	ssc:100517709	4E-55	NR_034020.1	LOC100130301
HFE2	1.380809836	38.0088051	-4.78274712	0.017381	Low	1	46	XP_005663578.1	ssc:100155778	4E-155	NM_213653.3	HFE2
LOC100624749	421.1469999	3857.067439	-3.195108577	0.017413	Low	305	4668	XP_005659064.1	ssc:100624749	7E-55	NR_103804.1	PDLIM7
LOC100625694	11.04647869	114.0264153	-3.367709621	0.017532	Low	8	138	XP_005653361.1	ssc:100625694	0	NM_024840.3	ZNF613
CHADL	255.4498196	38.83508347	2.717607445	0.017636	High	185	47	XP_005653164.1	ssc:102162247	3E-146	NM_138481.1	CHADL
TRIB3	397.6732327	3618.27299	-3.185645897	0.017726	Low	288	4379	XP_003134414.1	ssc:100518810	2E-154	NM_021158.3	TRIB3
LOC102162975	13.80809836	135.509653	-3.294809074	0.017758	Low	10	164	XR_301352.1	ssc:102162975			
ALOX12B	107.7031672	14.87301069	2.856292054	0.017881	High	78	18	XP_003132016.1	ssc:100526246	0	NM_001139.2	ALOX12B
ARHGDIB	2.761619671	48.75042393	-4.141828213	0.017931	Low	2	59	NM_001244240.1	ssc:100155466	1E-100	NM_001175	ARHGDIB
CNKSR2	2.761619671	48.75042393	-4.141828213	0.017931	Low	2	59	XP_005673548.1	ssc:100523478	0	NM_014927.3	CNKSR2
LOC102163693	6.904049179	80.97528043	-3.551966913	0.018049	Low	5	98	XR_299347.1	ssc:102163693			
TRAM2	309.3014032	2768.858824	-3.162206213	0.018053	Low	224	3351	XP_001928862.4	ssc:100157796	0	NM_012288.3	TRAM2
PDZD2	13.80809836	134.6833746	-3.285985223	0.018272	Low	10	163	XP_003359788.2	ssc:100525132	7E-29	NM_178140.2	PDZD2
MAFB	38.6626754	335.4690189	-3.117166159	0.01828	Low	28	406	XP_003134488.2	ssc:100518227	5E-109	NM_005461.4	MAFB
BAALC	27.61619671	1.652556743	4.062742931	0.018293	High	20	2	NM_001129969.1	ssc:100170128	3E-69	NM_024812.2	BAALC
LOC102164563	27.61619671	1.652556743	4.062742931	0.018293	High	20	2	XR_306377.1	ssc:102164563			
LOC100152383	27.61619671	1.652556743	4.062742931	0.018293	High	20	2	LOC100152383				
LOC102165191	27.61619671	1.652556743	4.062742931	0.018293	High	20	2	XR_305142.1	ssc:102165191			
ZNF185	33.13943606	290.0237085	-3.129549883	0.018295	Low	24	351	ZNF185	ssc:100152632	0	NM_007150.3	ZNF185
LOC102165339	67.65968195	8.262783717	3.033596585	0.01833	High	49	10	XP_005672875.1	ssc:102165339	9E-117	NM_018354.1	TMEM74B
LOC100737119	67.65968195	8.262783717	3.033596585	0.01833	High	49	10	XP_005672876.1	ssc:100737119	1E-116	NM_018354.1	TMEM74B

LOC100622991	34.52024589	300.7653273	-3.123123614	0.018461	Low	25	364	XP_003360998.2	ssc:100622991	5E-77	NR_073131.1	NEDD9
NRN1	167.0779901	24.78835115	2.752787478	0.018536	High	121	30	NM_001243601.1	ssc:100155948	2E-78	NM_016588.2	NRN1
LOC100518152	120.1304557	1031.195408	-3.101643887	0.018582	Low	87	1248	XP_005654120.1	ssc:100518152	0	NM_015480.2	PVRL3
SPARCL1	618.6028064	96.67456949	2.677805039	0.018826	High	448	117	NM_001097422.1	ssc:100037275	0	NM_004684.4	SPARCL1
CHD5	15.18890819	144.5987151	-3.250964657	0.018854	Low	11	175	XP_005656076.1	ssc:100738053	0	NM_015557.2	CHD5
FAM50B	22.09295737	0.826278372	4.740814836	0.01886	High	16	1	XP_003128207.1	ssc:100156092	3E-116	NM_012135.1	FAM50B
LOC102161067	22.09295737	0.826278372	4.740814836	0.01886	High	16	1	XR_299124.1	ssc:102161067			
C15H2orf66	22.09295737	0.826278372	4.740814836	0.01886	High	16	1	XP_005654241.1	ssc:100151853	6E-33	NM_213608.2	C2orf66
SCARA3	2732.622665	27463.84052	-3.329175221	0.018881	Low	1979	33238	XP_003359089.1	ssc:100628076	0	NM_182826.1	SCARA3
LOC102158952	86.99101965	11.5678972	2.910739838	0.018895	High	63	14	XR_308706.1	ssc:102158952			
NTM	6.904049179	80.14900206	-3.537169911	0.018913	Low	5	97	XP_003482697.1	ssc:100519556	0	NM_016522.2	NTM
LOC102161148	1.380809836	37.18252673	-4.75103826	0.019113	Low	1	45	XP_005657636.1	ssc:102161148			
MYH1	1.380809836	37.18252673	-4.75103826	0.019113	Low	1	45	NM_001104951.1	ssc:100125538			
LOC100624445	1688.730429	259.4514087	2.702402776	0.019203	High	1223	314	XP_005658616.1	ssc:100624445	1E-40	NR_037669.1	GGCT
GCK	484.6642523	76.0176102	2.6725801	0.019267	High	351	92	XP_003134931.1	ssc:100514142	0	NM_033508.1	GCK
CLDN9	537.1350261	84.28039392	2.672015839	0.019278	High	389	102	NM_001161647.1	ssc:100302022	9E-95	NM_020982.3	CLDN9
KIAA1755	2.761619671	47.92414556	-4.117166159	0.019335	Low	2	58	XP_003134484.3	ssc:100516472	0	NM_001029864.1	KIAA1755
LOC102165555	62.13644261	7.436505346	3.062742931	0.019378	High	45	9	XR_303667.1	ssc:102165555			
TBC1D30	16.56971803	154.5140555	-3.221117123	0.019395	Low	12	187	XP_003126403.2	ssc:100512468			
RAB3A	1289.676387	199.959366	2.689230339	0.0194	High	934	242	NM_001123179.1	ssc:100144498	1E-129	NM_002866.4	RAB3A
HIF3A	0	25.61462952	-20	0.019455	Low	0	31	XP_003356031.2	ssc:100621574	0	NM_152796.2	HIF3A
LOC102157470	0	25.61462952	-20	0.019455	Low	0	31	XR_304922.1	ssc:102157470			
LOC100624399	0	25.61462952	-20	0.019455	Low	0	31	XP_003356177.2	ssc:100624399	7E-59	NM_199454.2	PRDM16
GPRIN3	0	25.61462952	-20	0.019455	Low	0	31	XP_005667050.1	ssc:100517107	0	NM_198281.2	GPRIN3
TRPA1	0	25.61462952	-20	0.019455	Low	0	31	XP_001926150.1	ssc:100152934	0	NM_007332.2	TRPA1
LOC100625061	0	25.61462952	-20	0.019455	Low	0	31	XP_005674243.1	ssc:100625061	2E-52	NM_001868.2	CPA1
LOC102162967	0	25.61462952	-20	0.019455	Low	0	31	XP_005658709.1	ssc:102162967	1E-70	NM_020721.1	KIAA1210
LOC100737228	0	25.61462952	-20	0.019455	Low	0	31	XP_005654379.1	ssc:100737228	3E-130	NM_022138.2	SMOC2
LOC102163531	24.85457704	219.7900469	-3.144542598	0.019468	Low	18	266	XP_005658011.1	ssc:102163531	4E-102	NM_198240.1	CLIP1
LOC100620996	175.3628491	1507.958028	-3.104181226	0.019509	Low	127	1825	XR_302439.1	ssc:100620996	4E-27	NM_006759.3	UGP2
LOC100738612	7285.152693	1062.593986	2.777368758	0.019512	High	5276	1286	XP_003482797.2	ssc:100738612	2E-92	NM_004024.4	ATF3
IL6ST	388.0075638	3457.148707	-3.15542598	0.019543	Low	281	4184	NM_001097432.1	ssc:100037294	0	NM_175767.2	IL6ST
LOC100514841	270.6387278	2366.461257	-3.128291097	0.019651	Low	196	2864	XP_005664153.1	ssc:100514841	1E-70	NM_001080509.2	TSPAN11
LOC100623234	219.5487639	1897.96142	-3.11183735	0.019856	Low	159	2297	XP_003359698.1	ssc:100623234	3E-119	NM_005876.4	SPEG
LOC100520605	15.18890819	142.9461583	-3.234381773	0.01989	Low	11	173	XP_005661698.1	ssc:100520605			
LOC100736655	37.28186556	3.305113487	3.495702338	0.020038	High	27	4	XR_299091.1	ssc:100736655	8E-25	NM_001127641.1	LAMB3
LOC102165654	37.28186556	3.305113487	3.495702338	0.020038	High	27	4	XR_306028.1	ssc:102165654			
SYT17	100.799118	14.04673232	2.843176554	0.020057	High	73	17	XP_005655160.1	ssc:100513981	0	NM_016524.2	SYT17
SLC39A8	5.523239343	68.58110485	-3.634224595	0.020101	Low	4	83	XP_005667018.1	ssc:100524695	0	NM_022154.5	SLC39A8
LOC100627741	144.9850328	21.48323766	2.754620636	0.020171	High	105	26	XP_005658625.1	ssc:100627741	0	NM_198060.3	NRAP
SMAD3	196.0749967	1681.476486	-3.100251837	0.020188	Low	142	2035	NM_214137.1	ssc:397260			
TP53INP1	95.27587867	799.8374638	-3.069523944	0.020243	Low	69	968	XP_003355067.1	ssc:100157451	2E-118	NM_033285.3	TP53INP1
LOC102166259	273.4003475	2373.071484	-3.117668578	0.020421	Low	198	2872	XR_297673.1	ssc:102166259			
PCDH11X	474.9985835	75.19133183	2.659284951	0.020447	High	344	91	XP_005673836.1	ssc:414736	0	NM_032969.3	PCDH11X
MAML2	62.13644261	519.7290958	-3.064248274	0.020451	Low	45	629	XP_005667324.1	ssc:100739729	6E-16	NM_018717.4	MAML3
FABP5	10437.54155	1510.436864	2.788744155	0.020522	High	7559	1828	NM_001039746.2	ssc:574074	5E-66	NM_001444.2	FABP5
LOC100157017	720.7827343	114.0264153	2.660196376	0.020563	High	522	138	XP_005660626.1	ssc:100157017	1E-48	NM_012203.1	GRHPR
TET1	12.42728852	120.6366143	-3.279084721	0.02062	Low	9	146	XP_003359270.2	ssc:100625859	0	NM_030625.2	TET1
SGPP2	56.61320327	6.610226974	3.098366841	0.020666	High	41	8	XP_003133728.2	ssc:100524832	6E-179	NM_152386.2	SGPP2
KIAA1324L	672.45439	106.58991	2.657365543	0.020729	High	487	129	XP_003130265.3	ssc:100523199	0	NR_030672.1	KIAA1324L
ULBP1	75.94454097	9.915340461	2.937212049	0.020757	High	55	12	NM_001004035.1	ssc:445523	1E-53	NM_025218.2	ULBP1
CALN1	11.04647869	109.8950234	-3.314467599	0.020787	Low	8	133	XP_005662055.1	ssc:100737793	2E-45	NM_031468.3	CALN1
LOC100512171	114.6072164	960.1354679	-3.066540086	0.020798	Low	83	1162	XP_005664844.1	ssc:100512171			
BCAN	85.61020982	11.5678972	2.887656224	0.020806	High	62	14	XP_003481480.1	ssc:100738802	0	NM_198427.1	BCAN
TEC	41.42429507	348.6894729	-3.073393757	0.02081	Low	30	422	XP_003129008.1	ssc:404277	0	NM_003215.2	TEC
ARHGEF10	106.3223574	889.075528	-3.063860986	0.020823	Low	77	1076	XP_005657576.1	ssc:100624966	0	NM_014629.2	ARHGEF10
MYOF	1586.550501	15124.19932	-3.252893418	0.02085	Low	1149	18304	XP_005671355.1	ssc:100154898	0	NM_133337.2	MYOF
LOC100624393	2.761619671	47.09786719	-4.092075178	0.020867	Low	2	57	XP_003362153.1	ssc:100624393	0	NM_001145030.1	TOPAZ1
LOC100524378	2.761619671	47.09786719	-4.092075178	0.020867	Low	2	57	XP_005664828.1	ssc:100524378	3E-13	XP_003960829.2	LOC100287477
NTNG1	57.9940131	483.3728475	-3.059160556	0.020879	Low	42	585	XP_005663676.1	ssc:100152257	0	NM_014917.2	NTNG1
LOC100525578	24.85457704	216.4849334	-3.122683164	0.020905	Low	18	262	XP_003126925.3	ssc:100525578	4E-121	NM_017990.3	PDPR
MAP4K5	37.28186556	314.8120596	-3.077944849	0.020985	Low	27	381	XP_005660007.1	ssc:102161529	2E-90	NM_198794.2	MAP4K5
LOC102162398	9.66566885	99.15340461	-3.358720837	0.021024	Low	7	120	XR_306562.1	ssc:102162398			
PTPN21	440.4783376	3892.597409	-3.143590167	0.021029	Low	319	4711	XP_005666425.1	ssc:100152076	0	NM_007039.3	PTPN21
LOC102159206	1.380809836	36.35624836	-4.718616782	0.021046	Low	1	44	XP_005654696.1	ssc:102159206	4E-127	NR_030691.1	ANO1
CTSA	1637.640465	15585.26265	-3.250491907	0.021228	Low	1186	18862	NM_001243629.1	ssc:100156798	0	NM_001167594.1	CTSA
LOC100521307	955.5204063	151.208942	2.659743226	0.02123	High	692	183	XP_003484160.1	ssc:100521307	1E-55	NM_015871.4	ZNF593
NAP1L3	115.9880262	967.5719733	-3.060393102	0.02126	Low	84	1171	XP_003360439.1	ssc:100519582	0	NM_004538.5	NAP1L3
LOC100625305	20.71214754	183.4337985	-3.146710435	0.021273	Low	15	222	XP_005674204.1	ssc:100625305	1E-47	NM_002397.4	MEF2C
LOC100739516	8.284859014	88.41178578	-3.41568965	0.021351	Low	6	107	XP_005656580.1	ssc:100739516	6E-142	NM_003759.3	SLC4A4
FAM169A	858.8637178	136.3359313	2.655263392	0.021385	High	622	165	XP_005661542.1	ssc:100518001	0	NR_046462.1	FAM169A
OTUD1	996.9447014	157.819169	2.659241035	0.021402	High	722	191	XP_005674574.1	ssc:100738624			
TLN1	5331.306776	54472.40166	-3.352964371	0.021413	Low	3861	65925	XP_003353615.1	ssc:100157220	0	NM_006289.3	TLN1
ZBED6	31.75862622	269.3667492	-3.084351362	0.021509	Low	23	326	ZBED6	ssc:100622313	0	NM_001174108.1	ZBED6
LOC102158959	11.04647869	109.0687451	-3.303579283	0.021518	Low	8	132	XR_309520.1	ssc:102158959			
LOC100738662	277.542777	43.7927537	2.663946073	0.021557	High	201	53	XP_003483783.1	ssc:100738662	8E-67	NM_018000.2	MREG
HDDC2	2745.049953	422.228248	2.700737397	0.021646	High	1988	511	XP_005659270.1	ssc:100514692	1E-75	NM_016063.2	HDDC2
EPM2A	22.09295737	193.349139	-3.129549883	0.021688	Low	16	234	XP_001927667.1	ssc:100153341	6E-177	NM_005670.3	EPM2A
LOC100525245	545.4198851	4842.817537	-3.150407437	0.021756	Low	395	5861	XP_005653847.1	ssc:100525245	3E-100	NM_018212.4	ENAH
SOX9	51.08996392	423.0545263	-3.049731798	0.021783	Low	37	512	NM_213843.1	ssc:396840	0	NM_000346.3	SOX9
ZC3HAV1	182.2668983	1534.398936	-3.073549144	0.021789	Low	132	1857	NM_001177489.1	ssc:100415778	0	NM_024625.3	ZC3HAV1
CRSP-2	4.142429507	57.01320765	-3.78274712	0.021808	Low	3	69	NM_213747.1	ssc:396574	5E-38	NM_001741.2	CALCA
LOC100739537	56.61320327	467.6735584	-3.046291402	0.021823	Low	41	566	XP_005669380.1	ssc:100739537	1E-59	NM_003242.5	TGFBR2
LOC100621698	9.66566885	98.32712624	-3.346648005	0.02184	Low	7	119	XP_005674142.1	ssc:100621698	3E-170	NM_170662.3	CBLB
LOC100737211	42.80510491	356.1259782	-3.056532913	0.021858	Low	31	431	XP_005672441.1	ssc:100737211	2E-175	NM_007118.2	TRIO
LOC100519468	31.75862622	2.478835115	3.679414291	0.021904	High	23	3	NM_001260482.1	ssc:100519468	1E-98	NM_172014.2	TNFSF14
HOMER3	1295.199626	12073.57957	-3.22060709	0.021932	Low	938	14612	XP_003480800.1	ssc:100526210	1E-38	NR_027297.1	HOMER3
LOC100523560	44.18591474	366.867597	-3.05360103	0.021947	Low	32	444	XR_297529.1	ssc:100523560			
JHDM1D	2129.208767	331.3376271	2.68394346	0.022048	High	1542	401	XP_003134662.1	ssc:100515632	0	NM_030647.1	KDM7A
EVA1B	151.8890819	1266.684744	-3.059947432	0.022098	Low	110	1533	XP_005658947.1	ssc:100625314	9E-43	NM_018166.1	EVA1B
LOC100511492	1815.764934	284.2397599	2.675397166	0.022147	High	1315	344	XP_003354012.3	ssc:100511492	0	NM_024888.2	LPPR3
HRK	0	24.78835115	-20	0.022246	Low	0	30	XP_003359152.1	ssc:100155596	5E-28	NR_073189.1	HRK
TCEAL7	0	24.78835115	-20	0.022246	Low	0	30	XP_005657951.1	ssc:100524125	2E-48	NM_152278.3	TCEAL7
LOC102163993	0	24.78835115	-20	0.022246	Low	0	30	XP_005654329.1	ssc:102163993	5E-40	NM_001008749.2	RAB19
TNFSF15	0	24.78835115	-20	0.022246	Low	0	30	NM_001244555.1	ssc:100624969	6E-126	NM_005118.3	TNFSF15
LOC102160363	0	24.78835115	-20	0.022246	Low	0	30	XR_298209.1	ssc:102160363			
LOC102162241	0	24.78835115	-20	0.022246	Low	0	30	XP_005672464.1	ssc:102162241	1E-76	NM_030955.2	ADAMTS12
SOGA3	0	24.78835115	-20	0.022246	Low	0	30	XP_005652741.1	ssc:100512995	1E-173	NM_001012279.2	SOGA3
LOC102167440	0	24.78835115	-20	0.022246	Low	0	30	LOC102167440				
LOC100737220	167.0779901	25.61462952	2.705481763	0.022284	High	121	31	XP_005665604.1	ssc:100737220	2E-78	NM_016588.2	NRN1

LOC100513317	80.08697047	657.7175839	-3.037828789	0.022294	Low	58	796	XP_003133863.1	ssc:100513317	8E-55	NM_016139.2	CHCHD2
HAUS4	2174.775491	338.7741324	2.68247085	0.022311	High	1575	410	NM_001244156.1	ssc:100626075	2E-153	NM_017815.2	HAUS4
HAPLN2	41.42429507	4.131391859	3.325777337	0.022356	High	30	5	XP_003355190.2	ssc:100628124	0	NM_021817.2	HAPLN2
LOC102160991	38.6626754	321.4222866	-3.055456587	0.022395	Low	28	389	XP_005658344.1	ssc:102160991	0	NM_022359.5	PDE4DIP
LOC100520821	5.523239343	66.92854811	-3.599035167	0.022418	Low	4	81	XP_005654355.1	ssc:100520821	9E-53	NM_006520.2	DYNLT3
LOC100038019	201.598236	1693.870662	-3.070768799	0.022423	Low	146	2050	XP_005669379.1	ssc:100038019	2E-177	NM_003242.5	TGFBR2
NEDD4	34.52024589	288.3711517	-3.0624122	0.022529	Low	25	349	XP_001925710.1		0	NR_104302.1	NEDD4
LOC102162209	2.761619671	46.27158882	-4.066540086	0.022538	Low	2	56	XR_304023.1	ssc:102162209			
LOC100737550	2.761619671	46.27158882	-4.066540086	0.022538	Low	2	56	XP_003480275.1	ssc:100737550	0	NM_182961.3	SYNE1
LOC102164546	132.5577442	19.83068092	2.740814836	0.022655	High	96	24	XP_005653488.1	ssc:102164546			
GCOM1	324.4903114	52.05553742	2.640051859	0.023073	High	235	63	XP_003121532.4	ssc:100154487	0	NR_104371	GCOM1
HCN1	200.2174262	31.39857813	2.672796413	0.023186	High	145	38	XP_005672496.1	ssc:100510937	7E-33	NM_021072.3	HCN1
LOC102166276	113.2264065	927.0843331	-3.03349012	0.023194	Low	82	1122	XP_005669378.1	ssc:102166276	3E-54	NM_003242.5	TGFBR2
PCNA	20629.29895	2951.466344	2.805190999	0.023291	High	14940	3572	XP_003359931.1	ssc:692192	1E-152	NM_182649.1	PCNA
PDXK	4411.687425	674.2431513	2.70998971	0.023466	High	3195	816	NM_213943.1	ssc:396983			
IL16	51.08996392	416.4442994	-3.027011722	0.02347	Low	37	504	NM_213751.1	ssc:396589	0	NM_172217.3	IL16
APC	156.0315114	1285.689146	-3.042632546	0.02352	Low	113	1556	NM_001206430.1	ssc:100517932			
COL17A1	263.7346786	42.14019696	2.645818322	0.023546	High	191	51	XP_001929475.1	ssc:414914	0	NM_130778.1	COL17A1
ZNF582	19.3313377	168.5607878	-3.124255584	0.023832	Low	14	204	ZNF582	ssc:100521903	0	NM_144690.1	ZNF582
LOC100627071	55.23239343	6.610226974	3.062742931	0.023846	High	40	8	XP_005658072.1	ssc:100627071	0	NM_016524.2	SYT17
LOC102165346	55.23239343	6.610226974	3.062742931	0.023846	High	40	8	XR_309473.1	ssc:102165346			
SCUBE2	1307.626914	209.048428	2.645041877	0.023878	High	947	253	XP_005667098.1	ssc:100519613	0	NM_020974.2	SCUBE2
LOC102166805	22.09295737	189.2177471	-3.098388952	0.023976	Low	16	229	XR_301473.1	ssc:102166805			
GADD45B	327.2519311	2772.163937	-3.082538966	0.023999	Low	237	3355	XP_005654758.1	ssc:100621090	8E-89	NM_015675.3	GADD45B
LIAS	2651.154885	414.7917426	2.676161878	0.024109	High	1920	502	NM_001243462.1	ssc:100627627	0	NM_194451.2	LIAS
LOC102158450	131.1769344	19.83068092	2.725707944	0.024138	High	95	24	XP_005666618.1	ssc:102158450	0	NM_018176.3	LGI2
LSDP5	26.23538688	1.652556743	3.98874235	0.024148	High	19	2	NM_001123135.1	ssc:100142670	8E-151	NM_001013706.2	PLIN5
LOC102158611	26.23538688	1.652556743	3.98874235	0.024148	High	19	2	XR_306498.1	ssc:102158611			
LOC102162275	26.23538688	1.652556743	3.98874235	0.024148	High	19	2	XP_005659301.1	ssc:102162275			
ALOX15B	26.23538688	1.652556743	3.98874235	0.024148	High	19	2	XP_003132015.2	ssc:100525835	0	NM_001141.2	ALOX15B
ADAMTS17	1383.571455	221.4426036	2.643392439	0.024315	High	1002	268	XP_003121693.2	ssc:100155073	0	NM_139057.2	ADAMTS17
LOC102166958	45.56672458	4.95767023	3.200246455	0.024334	High	33	6	XP_005658825.1	ssc:102166958	4E-48	NM_138994.3	CNTNAP4
LOC100621169	45.56672458	4.95767023	3.200246455	0.024334	High	33	6	XP_005658484.1	ssc:100621169	0	NM_020340.4	KIAA1244
SH3BGR	102.1799278	826.2783717	-3.015516083	0.024367	Low	74	1000	NM_001244236.1	ssc:100626262	7E-61	NM_007341.2	SH3BGR
SLC35D2	38.6626754	315.638338	-3.02925907	0.024389	Low	28	382	NM_001244362.1	ssc:100514312	6E-90	NR_104627.1	SLC35D2
LOC100620506	40.04348524	326.3799568	-3.026913012	0.024391	Low	29	395	XP_003356780.3	ssc:100620506	0	NM_144767.3	AKAP13
LOC100156854	825.7242818	133.8570962	2.624966507	0.024424	High	598	162	XP_005655493.1	ssc:100156854	0	NM_003051.3	SLC16A1
LOC100521848	35.90105573	3.305113487	3.441254554	0.024738	High	26	4	XR_304625.1	ssc:100521848			
NFIX	278.9235868	2329.27873	-3.061941444	0.024767	Low	202	2819	XP_005652941.1	ssc:100523536	0	NM_002501	NFIX
LOC102164768	218.167954	1801.28685	-3.045516835	0.024799	Low	158	2180	XP_005664907.1	ssc:102164768	2E-57	NM_182571.2	SSC5D
PEAR1	302.397354	2534.195766	-3.067010556	0.024813	Low	219	3067	XP_005663619.1	ssc:100156908	0	NM_207369.1	PEAR1
LOC100623829	31.75862622	261.1039655	-3.039403956	0.024885	Low	23	316	XP_003358850.1	ssc:100623829	0	NM_015642.4	ZBTB20
FGF11	404.5772819	3438.144305	-3.087142882	0.024917	Low	293	4161	XP_005669270.1	ssc:100739797	4E-129	NM_004112.2	FGF11
LOC100514227	5.523239343	65.27599137	-3.562965912	0.025043	Low	4	79	XP_005663077.1	ssc:100514227			
LOC102164214	167.0779901	1361.706757	-3.026822454	0.025082	Low	121	1648	XR_302272.1	ssc:102164214			
LOC100739101	651.7422425	106.58991	2.61223063	0.025313	High	472	129	XP_003480812.1	ssc:100739101	5E-67	NM_001199209.1	CYP4F3
LOC100625781	30.37781639	249.5360683	-3.038158285	0.025353	Low	22	302	XP_003361074.1	ssc:100625781	3E-133	NM_145260.2	OSR1
IFFO2	35.90105573	291.6762652	-3.022269819	0.025393	Low	26	353	XP_005656079.1	ssc:100737107	0	NM_001136265.1	IFFO2
EVI2A	0	23.96207278	-20	0.025505	Low	0	29	XP_003131800.3	ssc:100525302	2E-103	NM_014210.3	EVI2A
LOC102164456	0	23.96207278	-20	0.025505	Low	0	29	XR_302902.1	ssc:102164456			
KCNRG	0	23.96207278	-20	0.025505	Low	0	29	XP_001928247.2	ssc:100152625	3E-129	NM_199464.2	KCNRG
LOC100738421	0	23.96207278	-20	0.025505	Low	0	29	HighI00025DF5A1				
RAI2	0	23.96207278	-20	0.025505	Low	0	29	XP_005673527.1	ssc:100154649	0	NR_033349.1	RAI2
C1QL3	0	23.96207278	-20	0.025505	Low	0	29	XP_003130781.1	ssc:100524890	1E-147	NM_001010908.1	C1QL3
LOC102165701	0	23.96207278	-20	0.025505	Low	0	29	XP_005660454.1	ssc:102165701			
LOC100524214	19.3313377	166.0819527	-3.102881933	0.025513	Low	14	201	XP_005668499.1	ssc:100524214	5E-33	NM_001010897.1	SERP2
LOC102166908	1.380809836	34.70369161	-4.651502587	0.02562	Low	1	42	XR_298559.1	ssc:102166908			
MYBPHL	1.380809836	34.70369161	-4.651502587	0.02562	Low	1	42	XP_005663653.1	ssc:100157465	9E-77	NM_001265613.1	MYBPHL
RAB3IL1	370.057036	3108.459234	-3.070380107	0.025774	Low	268	3762	XP_005660868.1	ssc:100739680	2E-164	NM_013401.3	RAB3IL1
LOC102161631	191.9325672	30.57229975	2.650302543	0.025803	High	139	37	XR_304474.1	ssc:102161631			
LOC100517767	28.99700655	237.9681711	-3.036792743	0.025884	Low	21	288	XR_304403.1	ssc:100517767			
LOC102164058	13.80809836	124.7680341	-3.175661808	0.025973	Low	10	151	XR_300563.1	ssc:102164058			
WASF1	573.0360818	94.19573438	2.604892348	0.025978	High	415	114	XP_003121401.2	ssc:100154562	0	NM_003931.2	WASF1
LOC100620251	15.18890819	0	20	0.026027	High	11	0	LOC100620251				
GNAL	15.18890819	0	20	0.026027	High	11	0	XP_003482083.2	ssc:100736677	3E-103	NM_182978.3	GNAL
LOC102164431	15.18890819	0	20	0.026027	High	11	0	XR_299666.1	ssc:102164431			
NLRP12	15.18890819	0	20	0.026027	High	11	0	XP_005664869.1	ssc:100523066	0	NM_144687.3	NLRP12
LOC100621822	15.18890819	0	20	0.026027	High	11	0	XP_005664661.1	ssc:100621822	1E-70	NM_178820.3	FBXO27
LOC102166344	15.18890819	0	20	0.026027	High	11	0	XP_005655042.1	ssc:102166344	5E-141	NR_003149.1	GPR98
HES2	15.18890819	0	20	0.026027	High	11	0	XP_003127577.1	ssc:100515666	1E-58	NM_019089.4	HES2
LOC102162972	15.18890819	0	20	0.026027	High	11	0	XR_307095.1	ssc:102162972			
CD79B	15.18890819	0	20	0.026027	High	11	0	NM_001243912.1	ssc:100511898	1E-88	NM_021602.2	CD79B
LOC102162510	15.18890819	0	20	0.026027	High	11	0	XR_297843.1	ssc:102162510			
LOC100739323	15.18890819	0	20	0.026027	High	11	0	XP_005654371.1	ssc:100739323	1E-57	NM_001164415.2	HSFX2
LOC102165746	15.18890819	0	20	0.026027	High	11	0	XR_301509.1	ssc:102165746			
LOC102167219	15.18890819	0	20	0.026027	High	11	0	XP_005674304.1	ssc:102167219	1E-69	NM_006180.3	NTRK2
LOC100625116	15.18890819	0	20	0.026027	High	11	0	XP_005658067.1	ssc:100625116	0	NM_144724.1	MARVELD2
OTOL1	15.18890819	0	20	0.026027	High	11	0	XP_003358706.1	ssc:100623105	0	NM_001080440.1	OTOL1
CACNA2D3	15.18890819	0	20	0.026027	High	11	0	XP_005669714.1	ssc:100153986	0	NM_018398.2	CACNA2D3
SLCO2A1	49.70915409	396.6136184	-2.996150758	0.026034	Low	36	480	NM_001123195.2	ssc:100144510			
LRRN4	49.70915409	5.783948602	3.103384916	0.026047	High	36	7	XP_003359940.1	ssc:100152719	3E-140	NM_152611.4	LRRN4
TLL1	20.71214754	175.1710148	-3.080215023	0.026323	Low	15	212	XP_003129040.3	ssc:100512480	0	NM_012464.4	TLL1
LOC100621387	2.761619671	44.61903207	-4.014072666	0.026361	Low	2	54	LOC100621387				
LOC100739607	2.761619671	44.61903207	-4.014072666	0.026361	Low	2	54	XP_005669288.1	ssc:100739607	2E-66	NM_144681.2	CCDC42
ZMYND12	20.71214754	0.826278372	4.647705432	0.026386	High	15	1	ZMYND12	ssc:100524028	2E-179	NM_032257.4	ZMYND12
AOX1	20.71214754	0.826278372	4.647705432	0.026386	High	15	1	XP_005672134.1	ssc:100525616	0	NM_001159.3	AOX1
LOC100621509	20.71214754	0.826278372	4.647705432	0.026386	High	15	1	XP_003360671.1	ssc:100621509	7E-29	NM_145306.2	C10orf35
LOC100152671	20.71214754	0.826278372	4.647705432	0.026386	High	15	1	XP_005663923.1	ssc:100152671	0	NM_005337.4	NCKAP1L
LOC102158743	20.71214754	0.826278372	4.647705432	0.026386	High	15	1	XP_005658587.1	ssc:102158743	2E-31	NM_019114.4	EPB41L4B
LOC100625374	20.71214754	0.826278372	4.647705432	0.026386	High	15	1	XP_003360022.2	ssc:100625374	0	NM_080834.2	CNBD2
LOC100518827	1130.883255	184.2600769	2.617634578	0.02639	High	819	223	XR_301937.1	ssc:100518827			
LOC102162827	35.90105573	289.1974301	-3.009956558	0.026422	Low	26	350	XR_309498.1	ssc:102162827			
GSTO1	18606.41254	2722.587235	2.772749739	0.026464	High	13475	3295	NM_214050.1	ssc:397117	4E-116	NM_004832.2	GSTO1
LOC102164245	5.523239343	64.44971299	-3.544587383	0.026486	Low	4	78	XP_005658386.1	ssc:102164245	2E-49	NM_001430.4	EPAS1
APH1B	5.523239343	64.44971299	-3.544587383	0.026486	Low	4	78	XP_003353379.1	ssc:100624829	1E-76	NM_031301.3	APH1B
LOC102157563	63.51725244	8.262783717	2.942448697	0.026549	High	46	10	XP_005659473.1	ssc:102157563			
LOC102158094	63.51725244	8.262783717	2.942448697	0.026549	High	46	10	XR_307083.1	ssc:102158094			
ARHGEF40	1014.895229	8967.599168	-3.143390993	0.026586	Low	735	10853	XP_005659058.1	ssc:100623088			
LOC100521211	12.42728852	114.0264153	-3.197784619	0.026658	Low	9	138	XP_003130444.3	ssc:100521211	0	NM_005401.4	PTPN14
HOXA3	51.08996392	404.8764021	-2.986369737	0.026792	Low	37	490	XP_005673358.1	ssc:100519809	0	NM_153632.2	HOXA3

KIF12	312.0630229	2577.98852	-3.046336515	0.026794	Low	226	3120	XP_005660422.1	ssc:100156816	0	NM_138424.1	KIF12
MFSD2A	1107.409488	180.9549634	2.613486203	0.026799	High	802	219	XP_005656144.1	ssc:100518612	0	NR_109896.1	MFSD2A
LRRC8D	176.743659	1422.851356	-3.009054591	0.026876	Low	128	1722	XP_005663753.1	ssc:100152378	0	NM_018103.4	LRRC8D
LOC100157320	37.28186556	298.2864922	-3.000152689	0.027018	Low	27	361	XP_005665766.1	ssc:100157320	9E-168	NM_170770.1	RNF39
DEPDC4	40.04348524	4.131391859	3.276867736	0.027021	High	29	5	XP_005664272.1	ssc:100511197	3E-100	NM_152317.2	DEPDC4
ZFP36	2468.887986	394.1347833	2.647100401	0.027148	High	1788	477	NM_001168419.1	ssc:100316849	1E-127	NM_003407.3	ZFP36
LOC102159432	31.75862622	256.1462952	-3.011747613	0.027198	Low	23	310	XP_005671932.1	ssc:102159432	7E-15	NM_016839.2	RBMS1
PEX3	2638.727596	420.5756912	2.649405053	0.027303	High	1911	509	NM_001244185.1	ssc:100153726	0	NM_003630.2	PEX3
PYCARD	291.3508753	47.92414556	2.60393303	0.027356	High	211	58	XP_003354571.1	ssc:100522011	3E-72	NM_145183.1	PYCARD
LRIG1	265.1154885	2159.891664	-3.026266089	0.027584	Low	192	2614	XP_005669774.1	ssc:100521990	0	NM_015541.2	LRIG1
PPP1R12A	272.0195376	2218.557428	-3.027839717	0.027601	Low	197	2685	XP_005652681.1	ssc:397190	2E-21	NM_002480.2	PPP1R12A
LOC100521647	185.028518	29.74602138	2.636979025	0.027618	High	134	36	XP_003121203.4	ssc:100521647	3E-57	NM_139165.2	RAET1E
CDKN2A	6.904049179	73.53877508	-3.4129905	0.027805	Low	5	89	XP_005660118.1	ssc:102157875	1E-12	NM_058197.4	CDKN2A
LOC100737490	30.37781639	2.478835115	3.615283954	0.027884	High	22	3	XP_003483716.2	ssc:100737490	0	NM_006920.4	SCN1A
RFC3	4203.18514	660.196419	2.670515787	0.027936	High	3044	799	NM_001190192.1	ssc:100156440	0	NM_181558.2	RFC3
LOC100624195	22.09295737	182.6075202	-3.047087723	0.028231	Low	16	221	XP_003357314.2	ssc:100624195	0	NM_024606.2	DYNC2H1
LOC102160967	1.380809836	33.87741324	-4.616737168	0.028326	Low	1	41	LOC102160967				
LOC102160047	1.380809836	33.87741324	-4.616737168	0.028326	Low	1	41	XP_005661480.1	ssc:102160047			
F2	2.761619671	43.7927537	-3.987105618	0.028545	Low	2	53	NM_001122985.1	ssc:100144442	0	NM_000506.3	F2
CACNG7	80.08697047	623.8401707	-2.961537003	0.028643	Low	58	755	XP_003127454.3	ssc:100519605	3E-163	NM_031896.4	CACNG7
ESCO2	1358.716878	223.0951604	2.606513744	0.028688	High	984	270	XP_003483444.1	ssc:100627165	0	NM_001017420.2	ESCO2
SCIN	160.1739409	25.61462952	2.644599521	0.028781	High	116	31	NM_001244731.1	ssc:100512981	0	NM_033128.3	SCIN
ASS1	425.2894294	71.05993997	2.581336622	0.028788	High	308	86	XP_005660580.1	ssc:414411	0	NM_054012.3	ASS1
LOC100514541	84.22939998	655.2387488	-2.959624882	0.028883	Low	61	793	XP_005670110.1	ssc:100514541	0	NM_004443.3	EPHB3
LOC100625039	24.85457704	201.6119227	-3.0199975	0.029062	Low	18	244	XR_309618.1	ssc:100625039	4E-94	NM_207003.2	BCL2L11
LOC102166021	150.5082721	23.96207278	2.651018166	0.029085	High	109	29	XP_005655388.1	ssc:102166021	1E-31	NM_014957.2	DENND3
LOC100524801	82.84859014	642.8445732	-2.955920913	0.029211	Low	60	778	XP_003125656.1	ssc:100524801	0	NM_025054.4	VCPIP1
ZFPM2	34.52024589	272.6718627	-2.981651188	0.029228	Low	25	330	NM_001195374.1	ssc:100337657			
LOC100520746	3826.224055	607.3146032	2.655405392	0.029238	High	2771	735	XP_005665483.1	ssc:100520746	0	NM_004153.3	ORC1
LOC102164797	127.0345049	19.83068092	2.679414291	0.02924	High	92	24	XR_308378.1	ssc:102164797			
PRDM8	0	23.13579441	-20	0.02932	Low	0	28	XP_005653685.1	ssc:100514243	0	NM_020226.3	PRDM8
SPTLC3	0	23.13579441	-20	0.02932	Low	0	28	XP_003134305.1	ssc:100519280	0	NM_018327.2	SPTLC3
MUSK	0	23.13579441	-20	0.02932	Low	0	28	XP_003122125.3	ssc:100515121	0	NM_005592.3	MUSK
RGS22	0	23.13579441	-20	0.02932	Low	0	28	XP_005663008.1	ssc:100154772	0	NM_015668.4	RGS22
LOC100522946	0	23.13579441	-20	0.02932	Low	0	28	XP_003130837.3	ssc:100522946			
CD40LG	0	23.13579441	-20	0.02932	Low	0	28	NM_214126.1	ssc:397231	9E-134	NM_000074.2	CD40LG
LOC102167616	0	23.13579441	-20	0.02932	Low	0	28	XR_306110.1	ssc:102167616			
LOC102165354	0	23.13579441	-20	0.02932	Low	0	28	XR_298688.1	ssc:102165354			
LOC102165408	0	23.13579441	-20	0.02932	Low	0	28	XR_299918.1	ssc:102165408			
LOC100516485	27.61619671	221.4426036	-3.003346259	0.029329	Low	20	268	XP_005661823.1	ssc:100516485	0	NM_032096.1	PCDHGB2
LOC100623625	2600.064921	419.7494128	2.630947434	0.02964	High	1883	508	XP_003355955.1	ssc:100623625	1E-127	NM_003407.3	ZFP36
LOC102164420	103.5607377	15.69928906	2.721706013	0.02971	High	75	19	XP_005666407.1	ssc:102164420	7E-87	NM_012074.4	DPF3
SH2D4B	131.1769344	20.65695929	2.666814255	0.029924	High	95	25	XP_005671206.1	ssc:100153283	2E-167	NM_207372.2	SH2D4B
CCNE2	729.0675933	122.289199	2.57575559	0.030008	High	528	148	NM_001243931.1	ssc:100512048	0	NM_057749.2	CCNE2
LOC102164708	22.09295737	180.128685	-3.027369489	0.030043	Low	16	218	XP_005662798.1	ssc:102164708	1E-164	NR_052013.2	NBAS
LOC100154002	1266.202619	209.8747064	2.592908073	0.030109	High	917	254	XP_001928249.1	ssc:100154002	0	NM_032435.2	KIAA1804
LOC100626399	4.142429507	52.88181579	-3.674222663	0.030252	Low	3	64	XP_005662181.1	ssc:100626399	3E-103	NM_032167.3	SNX29
GFRA4	4.142429507	52.88181579	-3.674222663	0.030252	Low	3	64	XP_005672831.1	ssc:100738405	2E-80	NM_145763.1	GFRA4
LOC102165762	4.142429507	52.88181579	-3.674222663	0.030252	Low	3	64	XP_005664614.1	ssc:102165762	4E-137	NM_032689.4	ZNF607
LOC100523914	26.23538688	209.8747064	-2.999942337	0.030256	Low	19	254	XP_003130144.1	ssc:100523914	0	NM_001174108.1	ZBED6
LOC100622080	272.0195376	45.44531045	2.581506942	0.030405	High	197	55	XP_005656566.1	ssc:100622080	3E-37	NM_014317.3	PDSS1
LOC100521295	12.42728852	110.7213018	-3.155349353	0.030415	Low	9	134	XR_306847.1	ssc:100521295	2E-51	NM_015520.1	MAGI1
SLC17A7	382.4843245	64.44971299	2.569154783	0.030498	High	277	78	XP_003127359.1	ssc:100514772	0	NM_020309.3	SLC17A7
EID1	457.0480556	3748.824973	-3.036020701	0.03053	Low	331	4537	XP_001928796.1	ssc:100155122	6E-81	NM_014335.2	EID1
HPN	34.52024589	3.305113487	3.384671026	0.030631	High	25	4	XP_005664551.1	ssc:100624088	8E-152	NM_182983.2	HPN
LOC100512875	34.52024589	3.305113487	3.384671026	0.030631	High	25	4	LOC100512875				
S100A9	34.52024589	3.305113487	3.384671026	0.030631	High	25	4	NM_001177906.1	ssc:100127489	3E-37	NM_002965.3	S100A9
LOC102166369	24.85457704	199.1330876	-3.002149499	0.030746	Low	18	241	XR_309210.1	ssc:102166369			
LOC100512382	80.08697047	11.5678972	2.791440909	0.030775	High	58	14	XP_005673280.1	ssc:100512382	8E-151	NM_012281.2	KCND2
ADGB	2.761619671	42.96647533	-3.959624882	0.030937	Low	2	52	XP_005659219.1	ssc:100155164	0	NM_024694.3	ADGB
PAK7	2.761619671	42.96647533	-3.959624882	0.030937	Low	2	52	XP_001928687.3	ssc:100152316	0	NM_177990.2	PAK7
LOC102162112	2.761619671	42.96647533	-3.959624882	0.030937	Low	2	52	XR_303844.1	ssc:102162112			
ADAMTS6	237.4992917	1876.478182	-2.9820324	0.031171	Low	172	2271	XP_003134060.3	ssc:100513765	0	NM_197941.2	ADAMTS6
FAM110B	88.37182949	676.7219864	-2.936904805	0.031174	Low	64	819	XP_005655419.1	ssc:100156516	0	NM_147189.2	FAM110B
TMEM180	371.4378458	62.79715625	2.564349685	0.031189	High	269	76	XP_005671479.1	ssc:100157822	0	NM_024789.3	TMEM180
BAI3	66.27887212	9.089062089	2.866345718	0.031193	High	48	11	XP_003353288.1		0	NM_001704.2	BAI3
LOC100522323	2732.622665	443.7114856	2.622592455	0.031195	High	1979	537	XP_003127755.1	ssc:100522323	5E-62	NM_004102.3	FABP3
SLC9B1	139.4617934	22.30951604	2.644138817	0.031228	High	101	27	XP_005674707.1	ssc:100522506	0	NR_047515.1	SLC9B1
LOC100626667	1.380809836	33.05113487	-4.581113259	0.031361	Low	1	40	XP_005655752.1	ssc:100626667	2E-39	NM_031491.2	RBP5
LOC100739482	1.380809836	33.05113487	-4.581113259	0.031361	Low	1	40	XP_003482457.2	ssc:100739482	2E-38	NM_003759.3	SLC4A4
LOC102163519	5.523239343	61.97087788	-3.488003854	0.031414	Low	4	75	XP_005655803.1	ssc:102163519	1E-88	NM_014903.5	NAV3
LOC100737880	5.523239343	61.97087788	-3.488003854	0.031414	Low	4	75	XP_005663831.1	ssc:100737880	2E-123	NM_001002034.2	FAM109B
RAB11FIP4	204.3598557	33.87741324	2.592716197	0.03153	High	148	41	XP_005669131.1	ssc:100524645	0	NM_032932.3	RAB11FIP4
LOC102163221	16.56971803	138.8147665	-3.066540086	0.031662	Low	12	168	XR_309034.1	ssc:102163221			
JUNB	20373.84913	3046.488357	2.741499435	0.031671	High	14755	3687	XP_005674540.1	ssc:100523358			
KLHL25	1362.859308	227.2265522	2.584433302	0.031721	High	987	275	XP_001928010.1	ssc:100153363	0	NM_022480.3	KLHL25
LOC100737398	62.13644261	473.457507	-2.929723396	0.031733	Low	45	573	XP_005661710.1	ssc:100737398	0	NM_014423.3	AFF4
SFRP1	994.1830817	8455.306578	-3.088273608	0.031839	Low	720	10233	XP_003359916.2	ssc:100621622	2E-150	NM_003012.4	SFRP1
LOC100738126	52.47077376	6.610226974	2.98874235	0.031874	High	38	8	XR_302948.1	ssc:100738126	5E-13	NM_052958.2	C8orf34
ZNF135	27.61619671	217.3112118	-2.976176058	0.031957	Low	20	263	ZNF135	ssc:102159974	7E-28	NM_007134.1	ZNF135
LOC100520944	49.70915409	379.2617726	-2.931610506	0.032046	Low	36	459	XP_005660084.1	ssc:100520944	1E-89	NM_181054.2	HIF1A
MYO3B	24.85457704	1.652556743	3.910739838	0.032049	High	18	2	XP_003359605.2	ssc:100621555	0	NR_045684.1	MYO3B
LOC100524449	24.85457704	1.652556743	3.910739838	0.032049	High	18	2	XR_304944.1	ssc:100524449			
LOC100517257	24.85457704	1.652556743	3.910739838	0.032049	High	18	2	XP_005673827.1	ssc:100517257	2E-147	NM_024885.3	TAF7L
MIR935	24.85457704	1.652556743	3.910739838	0.032049	High	18	2	NR_038568.1	ssc:100498743			
RIMKLA	24.85457704	1.652556743	3.910739838	0.032049	High	18	2	XP_005665572.1	ssc:100522878	2E-178	NM_173642.3	RIMKLA
LOC102159769	272.0195376	2149.976323	-2.982538591	0.032069	Low	197	2602	XR_303859.1	ssc:102159769			
NIP7	5650.273848	894.8594766	2.658587738	0.032092	High	4092	1083	NM_001048071.1	ssc:595107	1E-104	NM_016101.4	NIP7
VLDLR	999.7063211	8486.705156	-3.085628308	0.032175	Low	724	10271	NM_001199890.1	ssc:733630	0	NM_003383.3	VLDLR
AMDHD1	70.42130162	9.915340461	2.828277677	0.032197	High	51	12	XP_001925019.3	ssc:100151804	0	NM_152435.2	AMDHD1
HIC1	55.23239343	419.7494128	-2.925941756	0.032321	Low	40	508	XP_005669169.1	ssc:100525779	0	NM_006497.3	HIC1
LOC100738919	4.142429507	52.05553742	-3.651502587	0.032355	Low	3	63	XP_005659218.1	ssc:100738919	1E-126	NM_024694.3	ADGB
FAM131B	1003.848751	169.3870662	2.567146291	0.032475	High	727	205	XP_003134617.1	ssc:100520650	0	NM_014690.4	FAM131B
VSTM5	28.99700655	226.4002739	-2.964899824	0.032527	Low	21	274	XP_003129835.1	ssc:100516505	5E-102	NM_001144871.1	VSTM5
MMD	1199.923747	201.6119227	2.573289865	0.032561	High	869	244	NM_001044595.1	ssc:733665	2E-139	NM_012329.2	MMD
LOC100157418	1463.658426	244.578398	2.58121002	0.032645	High	1060	296	XP_005659699.1	ssc:100157418	0	NM_138356.2	SHF
NFASC	38.6626754	4.131391859	3.226241663	0.032733	High	28	5	XP_005667588.1	ssc:100626921			
CX3CL1	35.90105573	275.9769762	-2.942449738	0.032771	Low	26	334	HighI0000E5D135		1E-123	NM_002996.3	CX3CL1
DBI	38394.79829	5612.082701	2.774302695	0.032864	High	27806	6792	NM_214119.1	ssc:397212	5E-41	NR_104221.1	DBI
LOC100739477	1314.530964	220.6163253	2.574936668	0.032874	High	952	267	XR_302512.1	ssc:100739477	6E-50	NM_001281494.1	MSH6

LOC100622387	74.56373113	10.74161883	2.79526262	0.033125	High	54	13	XP_003356618.1	ssc:100622387	2E-69	NM_003509.2	HIST1H2AI
ZNF793	13.80809836	118.1578072	-3.097128406	0.033153	Low	10	143	ZNF793	ssc:100511696	0	NM_001013659.2	ZNF793
LOC100623627	5.523239343	61.14459951	-3.468638529	0.033281	Low	4	74	XR_303903.1	ssc:100623627			
LOC100621170	5.523239343	61.14459951	-3.468638529	0.033281	Low	4	74	XP_005658604.1	ssc:100621170	0	NM_014718.3	CLSTN3
LOC100625901	16.56971803	137.1622097	-3.049262094	0.033402	Low	12	166	XP_005674150.1	ssc:100625901	3E-140	NM_015589.5	SAMD4A
GREB1L	110.4647869	17.35184581	2.670425508	0.033554	High	80	21	XP_005653460.1	ssc:100524319	0	NM_024935.2	GREB1L
LOC100626354	2.761619671	42.14019696	-3.931610506	0.033559	Low	2	51	XP_005673127.1	ssc:100626354	0	NM_001794.3	CDH4
LOC102166568	2.761619671	42.14019696	-3.931610506	0.033559	Low	2	51	XR_308991.1	ssc:102166568			
ADAMTS14	193.313377	1488.953626	-2.945285443	0.033709	Low	140	1802	XP_005657462.1	ssc:100153539	0	NM_139155.2	ADAMTS14
SCN2B	0	22.30951604	-20	0.0338	Low	0	27	XP_003129961.1	ssc:100514594	6E-120	NM_004588.4	SCN2B
LOC102159543	0	22.30951604	-20	0.0338	Low	0	27	XR_298208.1	ssc:102159543			
LOC102157538	0	22.30951604	-20	0.0338	Low	0	27	XP_005674188.1	ssc:102157538			
LOC102159960	0	22.30951604	-20	0.0338	Low	0	27	XR_301995.1	ssc:102159960			
LOC102164750	0	22.30951604	-20	0.0338	Low	0	27	XR_308701.1	ssc:102164750			
LOC100516820	0	22.30951604	-20	0.0338	Low	0	27	XP_003130120.2	ssc:100516820			
LOC100624148	0	22.30951604	-20	0.0338	Low	0	27	XR_301796.1	ssc:100624148			
LOC100736739	0	22.30951604	-20	0.0338	Low	0	27	XP_003480551.1	ssc:100736739	2E-28	NM_002170.3	IFNA8
GPR75	0	22.30951604	-20	0.0338	Low	0	27	NM_001205045.1	ssc:100517153	0	NM_006794.3	GPR75
RHBDL3	0	22.30951604	-20	0.0338	Low	0	27	XP_005674602.1	ssc:100522032	4E-124	NM_138328.2	RHBDL3
SGCG	0	22.30951604	-20	0.0338	Low	0	27	XP_003482902.1	ssc:100272144	3E-38	NM_000231.2	SGCG
LOC100739700	24.85457704	195.0016957	-2.971903212	0.033804	Low	18	236	XP_003482111.1	ssc:100739700	0	NM_024686.4	TTLL7
LOC100155802	24.85457704	195.0016957	-2.971903212	0.033804	Low	18	236	XP_005658316.1	ssc:100155802	6E-32	NM_024579.3	C1orf54
LOC100737465	457.0480556	78.49644531	2.541646635	0.033993	High	331	95	XP_003480713.1	ssc:100737465			
LOC100515974	2321.141334	384.2194429	2.594831939	0.03403	High	1681	465	XP_005671952.1	ssc:100515974	0	NM_024753.4	TTC21B
HOGA1	11.04647869	98.32712624	-3.154002927	0.034247	Low	8	119	NM_001190169.1	ssc:100153961			
LRRC29	11.04647869	98.32712624	-3.154002927	0.034247	Low	8	119	XP_005664476.1	ssc:100524680			
EFCAB4A	27.61619671	214.0060983	-2.954065357	0.034252	Low	20	259	NM_001243838.1	ssc:100511435	2E-86	NM_173584.4	EFCAB4A
NKX3-2	40.04348524	303.2441624	-2.920840422	0.034259	Low	29	367	XP_003356908.1	ssc:100625162	5E-141	NM_001189.3	NKX3-2
LOC100516665	127.0345049	960.1354679	-2.918017561	0.034356	Low	92	1162	XP_005661834.1	ssc:100516665	0	NM_032054.1	PCDHGA5
LDB3	19.3313377	155.3403339	-3.006419093	0.034526	Low	14	188	XP_005657484.1	ssc:100151883	0	NM_007078.2	LDB3
TTLL6	8.284859014	79.32272369	-3.259185164	0.034555	Low	6	96	XP_005654019.1	ssc:100523818	0	NM_173623.3	TTLL6
EXOC6	360.3913671	61.97087788	2.539902142	0.034605	High	261	75	XP_003361613.2	ssc:100627369	0	NM_019053.4	EXOC6
NXPH4	256.8306294	43.7927537	2.552053193	0.034656	High	186	53	XP_003355530.1	ssc:100626804	1E-129	NM_007224.3	NXPH4
GPR143	207.1214754	1589.759587	-2.940259557	0.034736	Low	150	1924	XP_003360273.1	ssc:100624736			
LOC102162973	1.380809836	32.2248565	-4.544587383	0.034771	Low	1	39	XR_301315.1	ssc:102162973			
LOC100737173	1.380809836	32.2248565	-4.544587383	0.034771	Low	1	39	LOC100737173				
TOMM20	25725.86805	3859.546274	2.736716597	0.034818	High	18631	4671	XP_001926970.1	ssc:100152814	8E-81	NM_014765.2	TOMM20
MDFI	363.1529868	2858.096888	-2.976405473	0.03506	Low	263	3459	XP_005666020.1	ssc:100152701	8E-102	NM_005586.3	MDFI
GOLGA3	216.7871442	1663.298362	-2.939695872	0.035124	Low	157	2013	XP_005657358.1	ssc:100155657	0	NM_005895.3	GOLGA3
LOC100738084	5.523239343	60.31832114	-3.449009723	0.035274	Low	4	73	XR_305162.1	ssc:100738084	6E-14	NM_015159.2	FAM168A
NOD1	85.61020982	637.886903	-2.897445891	0.03533	Low	62	772	NM_001114277.1	ssc:100155660	0	NM_006092.2	NOD1
APC2	28.99700655	222.268882	-2.938330104	0.03535	Low	21	269	NM_001206431.1	ssc:100579172	0	NM_005883.2	APC2
B4GALNT4	1383.571455	234.6630576	2.55973451	0.035539	High	1002	284	XP_005658229.1	ssc:100623649	1E-145	NM_178537.4	B4GALNT4
LOC100157933	28.99700655	2.478835115	3.548169758	0.035629	High	21	3	XP_005666085.1	ssc:100157933	5E-175	NM_206539.2	DLK2
LIPI	28.99700655	2.478835115	3.548169758	0.035629	High	21	3	XP_005670350.1	ssc:100154888	0	NM_198996.2	LIPI
PAX6	491.5683015	85.10667229	2.53004774	0.035707	High	356	103	NM_001244178.1	ssc:100514152	0	NM_001604.5	PAX6
THSD7A	6.904049179	69.40738323	-3.329574492	0.035752	Low	5	84	XP_003357499.2	ssc:100512249	0	NM_015204.2	THSD7A
LOC100623494	15.18890819	125.5943125	-3.047681059	0.03576	Low	11	152	XP_005657783.1	ssc:100623494	0	NM_004668.2	MGAM
ABCA4	158.7931311	26.4409079	2.586304887	0.035952	High	115	32	XP_001924499.3	ssc:100155583	0	NM_000350.2	ABCA4
SERPINB9	69.04049179	9.915340461	2.799708525	0.035995	High	50	12	NM_001111256.1	ssc:100126851	2E-32	NM_004155.5	SERPINB9
LOC100738857	78.70616064	582.5262521	-2.887774597	0.036301	Low	57	705	XP_003484265.2	ssc:100738857	0	NM_004612.2	TGFBR1
OTUD7A	22.09295737	172.6921797	-2.966544296	0.036316	Low	16	209	XP_005659864.1	ssc:100525093	0	NM_130901.1	OTUD7A
FABP3	490.1874917	85.10667229	2.525989523	0.036321	High	355	103	NM_001099931.1	ssc:399532			
KLHL42	122.8920754	915.5164359	-2.897193899	0.03658	Low	89	1108	XP_003126455.1	ssc:100513858	0	NM_020782.1	KLHL42
OAZ3	20.71214754	162.7768392	-2.974346388	0.036662	Low	15	197	NM_001122996.1	ssc:100144448			
BST2	111.8455967	829.5834852	-2.890878715	0.036907	Low	81	1004	NM_001161755.1	ssc:100302088			
STEAP1	182.2668983	1372.448375	-2.912627402	0.037062	Low	132	1661	NM_214305.1	ssc:397573	1E-18	NM_012449.2	STEAP1
KRT81	4.142429507	50.40298068	-3.604960001	0.037076	Low	3	61	XP_003126201.1	ssc:100523123	0	NM_002281.3	KRT81
ZNF226	35.90105573	268.5404708	-2.903041354	0.037091	Low	26	325	ZNF226	ssc:100523907	0	NM_016444.1	ZNF226
RECK	197.4558065	1491.432461	-2.917096949	0.037094	Low	143	1805	XP_005660294.1	ssc:100519638	0	NM_021111.2	RECK
LOC100518000	19.3313377	0.826278372	4.548169758	0.037212	High	14	1	XP_003123683.2	ssc:100518000	2E-38	xp_003846745	xp_003846745
LOC102164525	19.3313377	0.826278372	4.548169758	0.037212	High	14	1	XR_300812.1	ssc:102164525			
LOC100514688	19.3313377	0.826278372	4.548169758	0.037212	High	14	1	XP_003133995.3	ssc:100514688	1E-34	NM_001190787.1	MCIDAS
LOC102158079	19.3313377	0.826278372	4.548169758	0.037212	High	14	1	XR_304451.1	ssc:102158079			
LOC102160118	19.3313377	0.826278372	4.548169758	0.037212	High	14	1	XR_306159.1	ssc:102160118			
LOC100627180	19.3313377	0.826278372	4.548169758	0.037212	High	14	1	XP_003354464.1	ssc:100627180	6E-56	NM_001025295.2	IFITM5
LOC100625977	19.3313377	0.826278372	4.548169758	0.037212	High	14	1	XP_005668112.1	ssc:100625977	6E-173	NM_012144.3	DNAI1
DGAT2	19.3313377	0.826278372	4.548169758	0.037212	High	14	1	NM_001160080.1	ssc:100294675	4E-18	NM_032564.4	DGAT2
WNK3	12.42728852	105.7636316	-3.089260162	0.037225	Low	9	128	XP_003360382.2	ssc:100156408	0	NM_020922.4	WNK3
RASSF4	103.5607377	765.1337722	-2.885234857	0.037277	Low	75	926	NM_001243337.1	ssc:100152580	3E-146	NM_178145.1	RASSF4
LOC102157981	153.2698918	25.61462952	2.581034392	0.037312	High	111	31	XP_005662316.1	ssc:102157981	7E-116	NM_006648.3	WNK2
SLCO3A1	95.27587867	702.336616	-2.881979738	0.037389	Low	69	850	XP_005666353.1	ssc:100156054	0	NM_013272.3	SLCO3A1
PGM2	2624.919498	438.7538154	2.580789602	0.037392	High	1901	531	XP_003128958.1	ssc:100522261	0	NM_018290.3	PGM2
RNF17	5.523239343	59.49204276	-3.429110165	0.037402	Low	4	72	XP_005653906.1	ssc:100738776	0	NM_031994.1	RNF17
ASF1B	3891.122117	640.3657381	2.603218232	0.037413	High	2818	775	NM_001137625.1	ssc:100192434	5E-112	NM_018154.2	ASF1B
LDHD	1441.565469	246.2309548	2.549552312	0.037495	High	1044	298	XP_005664401.1	ssc:100523064	0	NM_194436.2	LDHD
LCA5	299.6357344	52.05553742	2.525086145	0.037534	High	217	63	XP_005659474.1	ssc:100152810	0	NM_181714.3	LCA5
TLE6	77.3253508	11.5678972	2.740814836	0.037554	High	56	14	XP_003354048.2	ssc:100623564	7E-152	NM_024760.2	TLE6
LOC100626258	374.1994655	65.27599137	2.519183129	0.037582	High	271	79	XR_304844.1	ssc:100626258			
IFITM1	306.5397835	2355.719638	-2.942021621	0.037649	Low	222	2851	XP_003124278.1	ssc:100127358	7E-46	NM_003641.3	IFITM1
LOC100622645	157.4123213	26.4409079	2.57370485	0.037826	High	114	32	XP_005658846.1	ssc:100622645	3E-85	NM_001024071.1	GCH1
FAM54A	1379.429026	236.3156143	2.545284367	0.037874	High	999	286	NM_001190176.1	ssc:100157026	1E-138	NM_138419.3	MTFR2
LOC100628205	33.13943606	3.305113487	3.325777337	0.038031	High	24	4	XP_005669920.1	ssc:100628205	9E-118	NM_004441.4	EPHB1
LOC100739496	33.13943606	3.305113487	3.325777337	0.038031	High	24	4	XP_003480667.2	ssc:100739496	0	NM_006059.3	LAMC3
NFKBIA	563.370413	4461.076929	-2.985236323	0.038192	Low	408	5399	NM_001005150.1	ssc:406188	9E-174	NM_020529.2	NFKBIA
HHAT	59.37482294	434.6224235	-2.871839398	0.038215	Low	43	526	XP_005667936.1	ssc:100512733	0	NM_018194	HHAT
DDIT3	310.682213	2378.029154	-2.936254849	0.038484	Low	225	2878	NM_001144845.1	ssc:100240743	3E-85	NM_004083.5	DDIT3
LOC100523668	1.380809836	31.39857813	-4.507112677	0.038604	Low	1	38	XP_003125970.4	ssc:100523668	0	NM_004120.4	GBP2
CMTM5	59.37482294	8.262783717	2.845151496	0.03878	High	43	10	NM_001244546.1	ssc:100514659	2E-68	NM_181618.1	CMTM5
LOC102163855	19.3313377	151.208942	-2.96753008	0.03891	Low	14	183	XP_005661678.1	ssc:102163855	0	NM_175856.4	CHSY3
URGCP	198.8366163	1486.474791	-2.902239634	0.03897	Low	144	1799	XP_005673385.1	ssc:100525140	0	NM_017920.3	URGCP
LOC100522672	15.18890819	123.1154774	-3.018922066	0.039033	Low	11	149	XP_003355325.1	ssc:100522672	0	NM_000699.2	AMY2A
TCEAL5	0	21.48323766	-20	0.039071	Low	0	26	XP_005657950.1	ssc:100523940	2E-36	NM_001012979.2	TCEAL5
LOC102161784	0	21.48323766	-20	0.039071	Low	0	26	XP_005663765.1	ssc:102161784	8E-50	NM_198460.2	GBP6
LOC102163527	0	21.48323766	-20	0.039071	Low	0	26	XP_005667704.1	ssc:102163527	0	NM_015204.2	THSD7A
SLC18A1	0	21.48323766	-20	0.039071	Low	0	26	XP_005657256.1	ssc:100154435	4E-21	NM_003053.3	SLC18A1
HEPHL1	0	21.48323766	-20	0.039071	Low	0	26	XP_003357292.1	ssc:100625356	0	NM_001098672.1	HEPHL1
LOC102160773	0	21.48323766	-20	0.039071	Low	0	26	XR_299986.1	ssc:102160773			
LOC102160716	0	21.48323766	-20	0.039071	Low	0	26	XR_302919.1	ssc:102160716			
LOC102158216	0	21.48323766	-20	0.039071	Low	0	26	XR_304391.1	ssc:102158216			

LOC102167375	0	21.48323766	-20	0.039071	Low	0	26	XR_306387.1	ssc:102167375			
MBOAT1	8889.653722	1422.025078	2.644180316	0.039139	High	6438	1721	XP_005665670.1	ssc:100152017	0	NR_073465.1	MBOAT1
MYCBPAP	320.3478819	56.18692928	2.51133299	0.039331	High	232	68	XP_003131625.3	ssc:100513622	0	NM_032133.4	MYCBPAP
PTPRB	334.1559802	58.66576439	2.509930954	0.039379	High	242	71	XP_001926902.5	ssc:100156181	0	NM_002837.4	PTPRB
LOC100737262	63.51725244	461.0633314	-2.85974452	0.03961	Low	46	558	XR_306753.1	ssc:100737262	1E-10	NM_018075.3	ANO10
LOC100737774	323.1095016	2462.309548	-2.92991706	0.039654	Low	234	2980	XP_003482743.1	ssc:100737774	3E-131	NM_005824.2	LRRC17
LOC102161016	5.523239343	58.66576439	-3.408932283	0.039676	Low	4	71	XP_005653904.1	ssc:102161016	2E-143	NM_031994.1	RNF17
LOC102163005	37.28186556	4.131391859	3.173774243	0.039735	High	27	5	XP_005665435.1	ssc:102163005	1E-100	NM_020794.2	LRRC7
LOC102162807	19.3313377	150.3826637	-2.959624882	0.03986	Low	14	182	XR_300403.1	ssc:102162807			
LOC100626701	1876.520567	15834.79872	-3.076966521	0.039925	Low	1359	19164	XP_003356232.1	ssc:100626701	0	NM_005529.5	HSPG2
WWC1	13.80809836	113.2001369	-3.035289152	0.040018	Low	10	137	XP_005658135.1	ssc:100513827	0	NM_015238.2	WWC1
LOC102161587	109.083977	794.0535152	-2.863797036	0.040126	Low	79	961	XP_005656080.1	ssc:102161587	9E-178	NM_020765.2	UBR4
HSD11B2	67.65968195	9.915340461	2.77056218	0.040266	High	49	12	NM_213913.1	ssc:396948	0	NM_000196.3	HSD11B2
TDRD7	226.4528131	1689.73927	-2.899518287	0.040368	Low	164	2045	XP_001924715.1	ssc:100152652	0	NM_014290.2	TDRD7
LOC102159605	13.80809836	0	20	0.040573	High	10	0	XR_307568.1	ssc:102159605			
LOC102168014	13.80809836	0	20	0.040573	High	10	0	XP_005672557.1	ssc:102168014			
LOC102161357	13.80809836	0	20	0.040573	High	10	0	XR_299270.1	ssc:102161357			
LOC102159869	13.80809836	0	20	0.040573	High	10	0	XR_299907.1	ssc:102159869			
SERPINB11	13.80809836	0	20	0.040573	High	10	0	XP_001925217.2	ssc:100156623	0	NM_080475.2	SERPINB11
LOC102163657	13.80809836	0	20	0.040573	High	10	0	XP_005656342.1	ssc:102163657	2E-33	NM_001866.2	COX7B
LOC102163658	13.80809836	0	20	0.040573	High	10	0	XP_005666710.1	ssc:102163658	2E-102	NM_152620.2	TRIM60
LOC102166688	13.80809836	0	20	0.040573	High	10	0	XP_005662944.1	ssc:102166688	1E-19	NR_109794.1	SAMD12
LOC102166698	13.80809836	0	20	0.040573	High	10	0	XR_298125.1	ssc:102166698			
ELOVL3	13.80809836	0	20	0.040573	High	10	0	NM_001167634.1	ssc:100155434	6E-128	NM_152310.2	ELOVL3
DLL3	13.80809836	0	20	0.040573	High	10	0	XP_003481899.1	ssc:100520433	0	NM_203486.2	DLL3
LOC100520863	13.80809836	0	20	0.040573	High	10	0	XR_306494.1	ssc:100520863			
LOC102166129	13.80809836	0	20	0.040573	High	10	0	XP_005653392.1	ssc:102166129	3E-159	NM_153254.2	TTLL10
ANKRD65	13.80809836	0	20	0.040573	High	10	0	XP_005653409.1	ssc:100526225	1E-120	NM_001243536.1	ANKRD65
CREB3L3	13.80809836	0	20	0.040573	High	10	0	XP_005661395.1	ssc:100522979	0	NM_032607.2	CREB3L3
IL12B	13.80809836	0	20	0.040573	High	10	0	NM_214013.1	ssc:397076	3E-153	NM_002187.2	IL12B
LOC100623615	13.80809836	0	20	0.040573	High	10	0	XP_005658569.1	ssc:100623615	6E-25	NM_003695.2	LY6D
LOC102160807	13.80809836	0	20	0.040573	High	10	0	XR_298056.1	ssc:102160807			
LOC102160718	13.80809836	0	20	0.040573	High	10	0	XR_297394.1	ssc:102160718			
LOC102160138	13.80809836	0	20	0.040573	High	10	0	XP_005673576.1	ssc:102160138	3E-135	NM_002365.4	MAGEB3
TMEM130	13.80809836	0	20	0.040573	High	10	0	XP_003481028.1	ssc:100517935	0	NM_152913.2	TMEM130
LOC102163175	13.80809836	0	20	0.040573	High	10	0	XR_307642.1	ssc:102163175			
HSH2D	13.80809836	0	20	0.040573	High	10	0	NM_001243826.1	ssc:100511370	3E-119	NM_032855.2	HSH2D
CWH43	13.80809836	0	20	0.040573	High	10	0	XP_003129016.3	ssc:100521324	0	NM_025087.2	CWH43
LOC100737290	13.80809836	0	20	0.040573	High	10	0	XP_005662280.1	ssc:100737290	2E-95	NM_001135086.1	PRSS41
LGR6	13.80809836	0	20	0.040573	High	10	0	XP_003130662.2	ssc:100511400	0	NM_021636.2	LGR6
ZSWIM5	98.03749834	15.69928906	2.642634442	0.040683	High	71	19	ZSWIM5	ssc:100514968	0	NM_020883.1	ZSWIM5
NFAT5	189.1709475	1397.236727	-2.884814025	0.040818	Low	137	1691	XP_005655828.1	ssc:100516737	0	NM_173215.2	NFAT5
SPAG9	44.18591474	319.7697299	-2.85537492	0.041357	Low	32	387	XP_003131645.2	ssc:100518147	3E-143	NM_172345.1	SPAG9
GUCY1A3	135.3193639	984.0975407	-2.862433018	0.041423	Low	98	1191	NM_001018033.1	ssc:553100	0	NM_001256449.1	GUCY1A3
SLIT1	150.5082721	25.61462952	2.554802851	0.041435	High	109	31	XP_003359360.1		1E-176	NM_003061.2	SLIT1
LOC102159348	34.52024589	252.0149034	-2.867994407	0.04175	Low	25	305	XR_309456.1	ssc:102159348			
SEMA6D	31.75862622	233.0105008	-2.87517456	0.041766	Low	23	282	XP_005654516.1	ssc:100511850	0	NM_153619.1	SEMA6D
LOC100624376	24.85457704	185.9126336	-2.903041354	0.041814	Low	18	225	XP_003358885.3	ssc:100624376	7E-102	NM_015429.3	ABI3BP
LOC102168189	45.56672458	5.783948602	2.977854033	0.041999	High	33	7	XR_307473.1	ssc:102168189			
LOC100152103	80.08697047	12.39417558	2.691905236	0.042076	High	58	15	LOC100152103				
KCNJ8	5.523239343	57.83948602	-3.388468181	0.042106	Low	4	70	XP_003126482.2	ssc:100152631	0	NM_004982.3	KCNJ8
LOC100737241	5.523239343	57.83948602	-3.388468181	0.042106	Low	4	70	XP_005662800.1	ssc:100737241	2E-113	NR_052013.2	NBAS
DACT3	665.5503408	5208.858855	-2.968347647	0.042249	Low	482	6304	XP_003356003.2	ssc:100622612	0	NM_145056.2	DACT3
LOC100621470	111.8455967	804.7951341	-2.847113123	0.042422	Low	81	974	XP_005659623.1	ssc:100621470	0	NM_032866.4	CGNL1
PCDH1	280.3043967	2089.658002	-2.898200568	0.042488	Low	203	2529	XP_005661829.1	ssc:100626940	0	NM_032420.3	PCDH1
EFNA5	15.18890819	120.6366423	-2.989578104	0.042645	Low	11	146	XP_005661627.1	ssc:100513721	2E-86	NM_001962.2	EFNA5
SCN1A	23.47376721	1.652556743	3.828277677	0.042743	High	17	2	HighI00025DFBBD		0	NM_006920.4	SCN1A
SPAG6	23.47376721	1.652556743	3.828277677	0.042743	High	17	2	NM_001159312.1	ssc:100286811	0	NM_172242.2	SPAG6
EML6	23.47376721	1.652556743	3.828277677	0.042743	High	17	2	XP_003125191.1	ssc:100516074	0	NM_001039753.2	EML6
GABRB3	23.47376721	1.652556743	3.828277677	0.042743	High	17	2	XP_005654529.1	ssc:100739031	7E-139	NR_103801.1	GABRB3
LOC100739034	17.95052786	138.8147665	-2.951062868	0.042762	Low	13	168	XP_005673812.1	ssc:100739034	2E-45	NM_007309.3	DIAPH2
LOC102157752	49.70915409	6.610226974	2.910739838	0.042806	High	36	8	XR_309346.1	ssc:102157752			
LOC102162573	70.42130162	502.37725	-2.834687335	0.042819	Low	51	608	XP_005659712.1	ssc:102162573	0	NM_025137.3	SPG11
CEP170	57.9940131	413.9654642	-2.835534534	0.042864	Low	42	501	XP_005668011.1	ssc:100623007	0	NM_014812.2	CEP170
LOC102160497	1.380809836	30.57229975	-4.468638529	0.042921	Low	1	37	XR_302320.1	ssc:102160497			
SERPINB2	2.761619671	39.66136184	-3.844147665	0.043055	Low	2	48	XP_003121745.1	ssc:100519286	0	NM_002575.2	SERPINB2
LOC102165012	2.761619671	39.66136184	-3.844147665	0.043055	Low	2	48	XP_005653389.1	ssc:102165012			
ZFP37	9.66566885	84.28039392	-3.124255584	0.04309	Low	7	102	ZFP37	ssc:100517074	0	NM_003408.2	ZFP37
SETD7	683.5008687	5331.148054	-2.963431172	0.043249	Low	495	6452	XP_005660590.1	ssc:100626704	0	NM_030648.2	SETD7
LOC102166427	74.56373113	530.4707147	-2.830727149	0.043394	Low	54	642	XR_297926.1	ssc:102166427			
ZMYND15	8.284859014	75.19133183	-3.182017303	0.043451	Low	6	91	ZMYND15	ssc:100513818	0	NM_032265.2	ZMYND15
DNAH9	8.284859014	75.19133183	-3.182017303	0.043451	Low	6	91	XP_003132047.4	ssc:100518397	0	NM_004662.2	DNAH9
GSTO2	53.85158359	7.436505346	2.856292054	0.043484	High	39	9	XP_001927323.2	ssc:100152209	2E-111	NM_183239.1	GSTO2
VWA5B2	53.85158359	7.436505346	2.856292054	0.043484	High	39	9	XP_005670091.1	ssc:100626924	0	NM_138345.1	VWA5B2
LOC102167547	15.18890819	119.8103639	-2.979662635	0.043931	Low	11	145	XP_005656878.1	ssc:102167547			
LOC100519128	3122.011039	531.296993	2.554885186	0.043931	High	2261	643	XP_005660774.1	ssc:100519128	3E-102	NM_080668.3	CDCA5
HPX	13.80809836	110.7213018	-3.003346259	0.044039	Low	10	134	NM_213953.2	ssc:396998	0	NM_000613.2	HPX
RINL	13.80809836	110.7213018	-3.003346259	0.044039	Low	10	134	XP_005664658.1	ssc:102163514	2E-129	NM_198445.3	RINL
PDE3A	13.80809836	110.7213018	-3.003346259	0.044039	Low	10	134	NM_213736.1	ssc:396555	2E-79	NM_001244683.1	PDE3A
LOC100152184	635.1725244	4908.919806	-2.950185186	0.044246	Low	460	5941	XP_005655456.1	ssc:100152184	0	NM_016274.4	PLEKHO1
BRI3BP	455.6672458	81.8015588	2.47778043	0.044331	High	330	99	XP_001924308.4	ssc:100155348	2E-90	NM_080626.5	BRI3BP
SMOX	305.1589737	2263.17646	-2.890716155	0.044388	Low	221	2739	NM_001185170.1	ssc:100152781	0	NM_175842.2	SMOX
LOC102158716	259.5922491	46.27158882	2.488048766	0.044489	High	188	56	LOC102158716				
LOC100624745	241.6417213	42.96647533	2.49158623	0.044515	High	175	52	XP_005661895.1	ssc:100624745	4E-130	NM_153247.2	SLC29A4
LOC100514211	459.8096753	82.62783717	2.476337013	0.044599	High	333	100	XP_003122517.1	ssc:100514211			
LOC100738006	2521.35876	433.7961452	2.539112274	0.044635	High	1826	525	XP_003482423.2	ssc:100738006	0	NM_020453.3	ATP10D
ZNF606	81.46778031	575.9160251	-2.82155696	0.044797	Low	59	697	ZNF606		0	NM_025027.3	ZNF606
LOC100737020	11093.42622	102321.3559	-3.205330369	0.044817	Low	8034	123834	XP_005672671.1	ssc:100737020	6E-63	NM_003118.3	SPARC
LOC100736687	104.9415475	17.35184581	2.596424927	0.044903	High	76	21	XR_304519.1	ssc:100736687	2E-17	NM_025152.2	NUBPL
LOC102166544	106.3223574	754.3921534	-2.826869673	0.044966	Low	77	913	XP_005666528.1	ssc:102166544			
TMEM169	317.5862622	57.01320765	2.47778043	0.045018	High	230	69	XP_005672233.1	ssc:100513571	2E-158	NM_138390.3	TMEM169
RABEPK	2234.150314	386.698278	2.530446009	0.045026	High	1618	468	NM_001244400.1	ssc:100152056	0	NM_005833.3	RABEPK
ALDH3A1	249.9265803	44.61903207	2.485773221	0.045209	High	181	54	XP_005669315.1	ssc:100526075	0	NM_001135168.1	ALDH3A1
C14H10orf71	0	20.65695929	-20	0.045292	Low	0	25	XP_005671275.1	ssc:100521158	0	NM_199459.3	C10orf71
LOC102159603	0	20.65695929	-20	0.045292	Low	0	25	XR_307292.1	ssc:102159603			
LOC102163989	0	20.65695929	-20	0.045292	Low	0	25	XR_302943.1	ssc:102163989			
LOC100513913	0	20.65695929	-20	0.045292	Low	0	25	LOC100513913				
PTPRCAP	0	20.65695929	-20	0.045292	Low	0	25	XP_003122509.1	ssc:100511860	9E-55	NM_005608.2	PTPRCAP
NKX6-1	0	20.65695929	-20	0.045292	Low	0	25	XP_003129394.3	ssc:397289	1E-113	NM_006168.2	NKX6-1
COMP	0	20.65695929	-20	0.045292	Low	0	25	XP_003123575.1	ssc:396554	0	NM_000095.2	COMP

LOC100736567	0	20.65695929	-20	0.045292	Low	0	25	XP_005664285.1	ssc:100736567	1E-158	NM_181670.3	ANKS1B
LOC102166377	0	20.65695929	-20	0.045292	Low	0	25	XR_297549.1	ssc:102166377			
LOC102158398	0	20.65695929	-20	0.045292	Low	0	25	XP_005664004.1	ssc:102158398	2E-84	NM_020700.1	PPM1H
LOC100739192	0	20.65695929	-20	0.045292	Low	0	25	XP_003482090.2	ssc:100739192	0	NM_198129.1	LAMA3
CCDC176	0	20.65695929	-20	0.045292	Low	0	25	XP_005656460.1	ssc:100152098	0	NM_025057.2	CCDC176
SEZ6	0	20.65695929	-20	0.045292	Low	0	25	XP_005657062.1	ssc:100518932	0	NM_178860.4	SEZ6
ZMYND10	289.9700655	52.05553742	2.47778043	0.045504	High	210	63	ZMYND10	ssc:100518334	0	NM_015896.2	ZMYND10
IRAK2	8.284859014	74.36505346	-3.166075759	0.045523	Low	6	90	XP_005669832.1	ssc:102161037	9E-87	NM_001570.3	IRAK2
RGS7	8.284859014	74.36505346	-3.166075759	0.045523	Low	6	90	XP_003130574.3	ssc:100521144	1E-54	NM_002924.5	RGS7
TNFAIP3	274.7811573	2016.119227	-2.875225976	0.045525	Low	199	2440	NM_001267890.1	ssc:100622156	0	NM_006290.3	TNFAIP3
LOC100523822	27.61619671	2.478835115	3.47778043	0.045675	High	20	3	LOC100523822				
LOC100525801	4.142429507	47.92414556	-3.532203658	0.04568	Low	3	58	XR_301277.1	ssc:100525801			
LOC100622259	30.37781639	218.9637685	-2.849602095	0.045766	Low	22	265	XP_005674294.1	ssc:100622259	3E-165	NM_003618.3	MAP4K3
ARNTL2	70.42130162	494.9407447	-2.813172015	0.045788	Low	51	599	XP_003355586.2	ssc:100625791	0	NM_020183.4	ARNTL2
LOC102160806	40.04348524	284.2397599	-2.827468923	0.045803	Low	29	344	XP_005665044.1	ssc:102160806	2E-35	NM_006048.4	UBE4B
LOC396903	3268.376881	27752.21167	-3.085956492	0.045843	Low	2367	33587	XP_003483154.2	ssc:396903	0	NM_005964.3	MYH10
LOC780415	741.4948818	5739.32957	-2.952373581	0.045859	Low	537	6946	NM_001078670.1	ssc:780415	4E-177	NM_006084.4	IRF9
CDCP1	63.51725244	446.1903207	-2.812438805	0.045912	Low	46	540	XP_005669509.1	ssc:100512497	0	NM_178181.2	CDCP1
LOC102167078	74.56373113	11.5678972	2.688347416	0.045913	High	54	14	XR_298982.1	ssc:102167078			
RALGAPA2	1288.295577	228.0528306	2.49802365	0.045955	High	933	276	XP_005672795.1	ssc:100154830	0	NM_020343.3	RALGAPA2
LOC102167727	37.28186556	265.2353573	-2.830727149	0.045987	Low	27	321	XR_303912.1	ssc:102167727			
PXDN	2377.754537	19766.23121	-3.055366127	0.046056	Low	1722	23922	XP_005662846.1	ssc:100516076	0	NM_012293.1	PXDN
JAZF1	1350.432019	238.7944494	2.499579809	0.046075	High	978	289	XP_005673344.1	ssc:100517019	2E-119	NM_175061.3	JAZF1
DYNC2H1	23.47376721	172.6921797	-2.879081455	0.046093	Low	17	209	XP_005667360.1	ssc:100624373	0	NM_024606.2	DYNC2H1
LOC100739570	23.47376721	172.6921797	-2.879081455	0.046093	Low	17	209	XP_005669829.1	ssc:100739570	9E-163	NM_018187.2	SETD5
LOC100738110	34.52024589	246.2309548	-2.834497495	0.046264	Low	25	298	XP_005661122.1	ssc:100738110	0	NM_005734.4	HIPK3
FBXO15	151.8890819	26.4409079	2.52217455	0.046403	High	110	32	XP_005659890.1	ssc:100516237	1E-97	NM_152676.2	FBXO15
NPM3	17037.81256	2715.977008	2.649196952	0.046422	High	12339	3287	XP_003359391.1	ssc:100627227	2E-75	NM_006993.2	NPM3
LOC100526005	650.3614326	117.3315288	2.470650966	0.046576	High	471	142	XP_005667541.1	ssc:100526005	7E-109	NM_022549.3	FEZ1
OPHN1	15.18890819	118.1578072	-2.959624882	0.046635	Low	11	143	XP_003484167.1	ssc:100515117			
LOC102164300	701.4513966	126.4205909	2.47211168	0.046656	High	508	153	XR_301709.1	ssc:102164300			
LOC102157933	82.84859014	13.22045395	2.647705432	0.046666	High	60	16	XR_302823.1	ssc:102157933			
PRSS22	140.8426032	998.9705514	-2.826358351	0.046705	Low	102	1209	XP_005655249.1	ssc:100519474	3E-137	NM_022119.3	PRSS22
HPRT1	4440.684432	751.9133183	2.562143792	0.046736	High	3216	910	NM_001032376.2	ssc:397351	5E-119	NM_000194.2	HPRT1
ADAT2	1422.234131	252.0149034	2.496578026	0.047173	High	1030	305	XP_001924271.1	ssc:100154785	2E-99	NM_182503.2	ADAT2
ABHD6	102.1799278	716.3833483	-2.809619981	0.04727	Low	74	867	XP_003132321.1	ssc:100515411	0	NM_020676.5	ABHD6
SLC13A4	19.3313377	144.5987151	-2.903041354	0.047283	Low	14	175	XP_003134691.1	ssc:100522048	0	NM_012450.2	SLC13A4
LOC100738956	31.75862622	3.305113487	3.264376792	0.04733	High	23	4	XR_135238.2	ssc:100738956			
LOC102167627	31.75862622	3.305113487	3.264376792	0.04733	High	23	4	XR_301535.1	ssc:102167627			
LOC100512447	811.9161834	146.2512718	2.472881631	0.047353	High	588	177	XR_309077.1	ssc:100512447	1E-46	NR_037669.1	GGCT
LOC102160623	5.523239343	56.18692928	-3.346648005	0.047483	Low	4	68	XP_005673163.1	ssc:102160623	2E-154	NM_170606.2	KMT2C
FAM114A1	738.7332621	5671.574744	-2.94062392	0.047586	Low	535	6864	XP_005666637.1	ssc:100525173	0	NR_033290.1	FAM114A1
DCP2	1574.123213	278.4558113	2.499028164	0.047685	High	1140	337	XP_003123880.1	ssc:100516785	0	NR_038352.1	DCP2
LOC100623810	95.27587867	15.69928906	2.601411779	0.047689	High	69	19	XP_003361641.1	ssc:100623810	0	NM_147204.2	TRPV4
USP51	1.380809836	29.74602138	-4.429110165	0.047786	Low	1	36	XP_003135158.1	ssc:100519179	0	NM_201286.3	USP51
LOC100152800	1.380809836	29.74602138	-4.429110165	0.047786	Low	1	36	XP_005674266.1	ssc:100152800	1E-101	NM_006192.4	PAX1
LOC102160261	1.380809836	29.74602138	-4.429110165	0.047786	Low	1	36	XR_302345.1	ssc:102160261			
ELK3	777.3959375	5977.297741	-2.942771934	0.047937	Low	563	7234	XP_003355727.1	ssc:100622445	0	NM_005230.2	ELK3
SMG1	209.883095	1500.521523	-2.837806138	0.048379	Low	152	1816	XP_005658674.1	ssc:100625964	0	NM_015092.4	SMG1
NFIC	822.9626621	6331.771163	-2.943710234	0.048563	Low	596	7663	XP_005654783.1	ssc:100737330	5E-165	NM_205843.2	NFIC
IRX6	6.904049179	64.44971299	-3.222659288	0.048864	Low	5	78	XP_005653312.1	ssc:100518430	0	NM_024335.2	IRX6
LOC102158240	4.142429507	47.09786719	-3.507112677	0.049028	Low	3	57	XR_307528.1	ssc:102158240			
LOC102165529	4.142429507	47.09786719	-3.507112677	0.049028	Low	3	57	LOC102165529				
ZFYVE26	440.4783376	3260.494455	-2.887947791	0.049036	Low	319	3946	ZFYVE26	ssc:100155869	0	NM_015346.3	ZFYVE26
MAP4K1	263.7346786	47.92414556	2.460262669	0.049404	High	191	58	XP_003127163.3	ssc:100516374	0	NM_007181.5	MAP4K1
LOC100515885	20.71214754	152.0352204	-2.875856524	0.049414	Low	15	184	XP_005661868.1	ssc:100515885	4E-116	NM_000112.3	SLC26A2
FLRT3	2818.232875	490.8093528	2.521556182	0.049616	High	2041	594	XP_005672767.1	ssc:100158214	0	NM_198391.2	FLRT3
ARHGEF3	27.61619671	196.6542525	-2.832074832	0.049787	Low	20	238	XP_003358533.1	ssc:100627663	0	NM_019555.2	ARHGEF3
STXBP5L	52.47077376	7.436505346	2.818817348	0.049918	High	38	9	XP_005670233.1	ssc:100620808	0	NM_014980.2	STXBP5L
EGFLAM	24.85457704	178.4761283	-2.844147665	0.049964	Low	18	216	XP_005672488.1	ssc:100518875	0	NM_182801.2	EGFLAM

piPS-F Vs PEFs

AccID	piPS-F	PEFs	Log2FC	FDR	Style	piPS-F	PEFs	AccID	KeggID	E-Value	Blast_AccID	Blast_Symbol
ACTL8	7079.019085	0	20	0	High	6695	0	XP_003356215.1	ssc:100622307	3E-54	NM_030812.2	ACTL8
LIN28A	5810.188181	0	20	0	High	5495	0	NM_001123133.1	ssc:100142662	1E-105	NM_024674.4	LIN28A
GABRA5	13109.13796	1.652556743	12.95358539	0	High	12398	2	XP_005654528.1	ssc:100520472	0	NM_001165037.1	GABRA5
OTX2	6196.124247	0.826278372	12.87245055	0	High	5860	1	XP_005660050.1	ssc:100512643	2E-174	NR_073036.1	OTX2
POSTN	1.057359087	55326.77349	-15.67522476	0	Low	1	66959	NM_001206351.1	ssc:100152401	0	NM_006475.2	POSTN
COL3A1	7.401513606	487725.6819	-16.00789008	0	Low	7	590268	NM_001243297.1	ssc:100152001	0	NM_000090.3	COL3A1
MGP	0	26806.94921	-20	0	Low	0	32443	NM_214116.1	ssc:397206	2E-42	NM_001190839.1	MGP
AEBP1	0	41982.37779	-20	0	Low	0	50809	XP_003134934.1	ssc:733628	0	NM_001129.4	AEBP1
LOC100738123	0	219619.0073	-20	0	Low	0	265793	XR_300525.1	ssc:100738123			
LOC100738213	0	387901.3393	-20	0	Low	0	469456	XP_005668985.1	ssc:100738213	0	NM_000088.3	COL1A1
COL1A1	0	124736.6356	-20	0	Low	0	150962	XP_005668984.1	ssc:397571	3E-121	NM_000088.3	COL1A1
CCL2	0	45810.52549	-20	0	Low	0	55442	NM_214214.1	ssc:397422	7E-36	NM_002982.3	CCL2
COL4A2	3.17207726	43357.305	-13.73855955	1.11E-16	Low	3	52473	XP_005668604.1	ssc:100153454	0	NM_001846.2	COL4A2
COL4A1	5.286795433	47585.37143	-13.13583701	3.33E-16	Low	5	57590	XP_003131139.3	ssc:100515336	0	NM_001845.4	COL4A1
ERAS	3455.449495	0	20	4.44E-16	High	3268	0	XP_005673659.1	ssc:100621986	2E-101	NM_181532.3	ERAS
LOC100513556	0	13236.15324	-20	7.77E-16	Low	0	16019	XP_003357187.1	ssc:100513556	6E-115	NM_145040.2	PRKCDBP
MFAP2	0	12445.40483	-20	9.99E-16	Low	0	15062	XP_005665105.1	ssc:100523794	2E-80	NM_017459.2	MFAP2
CSPG4	0	12812.27243	-20	9.99E-16	Low	0	15506	XP_003128533.3	ssc:733676	0	NM_001897.4	CSPG4
LOC100156358	7.401513606	43927.43708	-12.53501438	1.33E-15	Low	7	53163	NM_001252605.1	ssc:100156358	1E-53	NM_019554.2	S100A4
HSD17B2	2799.886861	0	20	1.55E-15	High	2648	0	NM_001167649.1	ssc:100312973	1E-25	NM_002153.2	HSD17B2
MMP1	0	11619.95274	-20	1.55E-15	Low	0	14063	NM_001166229.1	ssc:397320	0	NM_002421.3	MMP1
COL1A2	87.76080419	385650.557	-12.10142983	1.78E-15	Low	83	466732	NM_001243655.1	ssc:100626716	0	NM_000089.3	COL1A2
PXDN	2.114718173	19766.23121	-13.19028479	1.89E-15	Low	2	23922	XP_005662846.1	ssc:100516076	0	NM_012293.1	PXDN
SOX21	5971.964121	3.305113487	10.81928997	2.22E-15	High	5648	4	XP_005653879.1	ssc:100517115	8E-155	NM_007084.2	SOX21
FMOD	12.68830904	59302.82502	-12.1903853	2.22E-15	Low	12	71771	XP_003130153.1	ssc:100526237	0	NR_103757.1	FMOD
DES	10.57359087	49798.14491	-12.20141088	2.55E-15	Low	10	60268	NM_001001535.1	ssc:396725	0	NM_001927.3	DES

LOC733603	0	10361.53078	-20	2.78E-15	Low	0	12540	NM_001044552.1	ssc:733603	2E-51	NM_199161.3	SAA1
LOC100623720	4.229436346	24788.35115	-12.51690928	3.33E-15	Low	4	30000	XP_005659104.1	ssc:100623720	2E-177	NM_001848.2	COL6A1
PRRX2	1.057359087	13710.43702	-13.66252153	3.44E-15	Low	1	16593	XP_005654631.1	ssc:100521241	5E-97	NM_016307.3	PRRX2
COL6A3	3.17207726	20982.51297	-12.69147194	3.55E-15	Low	3	25394	XP_005672367.1	ssc:100101551	0	NM_057167.3	COL6A3
LOC100738989	44.40908164	170068.7459	-11.90297568	3.66E-15	Low	42	205825	XP_005674338.1	ssc:100738989	0	NM_000089.3	COL1A2
LUM	1.057359087	13427.84982	-13.63247528	3.77E-15	Low	1	16251	NM_001243339.1	ssc:100152607	4E-177	NM_002345.3	LUM
VRTN	2408.663999	0	20	4.00E-15	High	2278	0	NM_001195113.1	ssc:100157734	0	NM_018228.2	VRTN
ST8SIA6	2379.057945	0	20	4.44E-15	High	2250	0	XP_003130770.1	ssc:100522567	1E-166	NM_001004470.1	ST8SIA6
ST14	2351.566609	0	20	4.77E-15	High	2224	0	XP_005667570.1	ssc:100517843	0	NM_021978.3	ST14
DCN	0	8943.637096	-20	5.77E-15	Low	0	10824	NM_213920.1	ssc:396957	0	NM_133507.2	DCN
LOC100621401	0	8792.428154	-20	6.22E-15	Low	0	10641	XP_003359182.1	ssc:100621401	5E-98	NM_181724.2	TMEM119
PTGES	0	7529.048523	-20	1.39E-14	Low	0	9112	NM_001038631.1	ssc:654407	1E-73	NM_198797.1	PTGES
BACE2	0	7459.64114	-20	1.45E-14	Low	0	9028	XP_005670420.1	ssc:100517374	0	NM_138992.2	BACE2
ELN	0	7319.173817	-20	1.61E-14	Low	0	8858	ELN				
LOC102160657	6.344154519	22024.45	-11.76139046	2.38E-14	Low	6	26655	XP_005660435.1	ssc:102160657	0	NM_002160.3	TNC
LOC100622870	3240.8056	2.478835115	10.35247446	4.66E-14	High	3065	3	XR_297931.1	ssc:100622870	1E-36	NM_003980.4	MAP7
ENTPD3	0	5839.309253	-20	5.07E-14	Low	0	7067	XP_005669433.1	ssc:100623360	0	NM_001248.2	ENTPD3
COL8A2	5.286795433	16593.32226	-11.61592164	5.40E-14	Low	5	20082	XP_003356361.2	ssc:100525585			
GAS7	0	5731.893065	-20	5.57E-14	Low	0	6937	XP_005669293.1	ssc:100518690	5E-179	NM_201433.1	GAS7
LOC100157992	5.286795433	16447.89727	-11.60322203	5.65E-14	Low	5	19906	XP_005663151.1	ssc:100157992	9E-94	NM_022716	PRRX1
SALL4	2628.594689	1.652556743	10.63537618	5.90E-14	High	2486	2	NM_001114673.1	ssc:100136902			
TNC	6.344154519	18393.78283	-11.50150268	5.94E-14	Low	6	22261	NM_214230.1	ssc:397460	0	NM_002160.3	TNC
LOC100152082	2.114718173	9950.044152	-12.2000218	6.00E-14	Low	2	12042	XP_005666212.1	ssc:100152082	0	NM_201526.1	ISLR
AMCF-II	2.114718173	9804.619159	-12.17878047	6.46E-14	Low	2	11866	NM_213876.1	ssc:396900	4E-33	NM_002994.4	CXCL5
CDH1	3527.349913	3.305113487	10.05966917	6.97E-14	High	3336	4	NM_001163060.1	ssc:100048953			
LOC102159782	2541.891244	1.652556743	10.58698678	7.33E-14	High	2404	2	XR_304869.1	ssc:102159782			
TMEM171	1541.629548	0	20	7.41E-14	High	1458	0	NM_001244323.1	ssc:100514277	1E-139	NM_173490	TMEM171
LOC100511106	3464.965727	3.305113487	10.03392556	7.84E-14	High	3277	4	XP_003133370.3	ssc:100511106	2E-85	NR_048571.1	GPM6A
LOC100628205	3404.696259	3.305113487	10.00861057	8.77E-14	High	3220	4	XP_005669920.1	ssc:100628205	9E-118	NM_004441.4	EPHB1
LOC100627277	0	5187.375618	-20	9.28E-14	Low	0	6278	XP_005674513.1	ssc:100627277	3E-60	NM_181724.2	TMEM119
LOC100623527	1486.646876	0	20	9.36E-14	High	1406	0	LOC100623527		7E-28	NM_001029887.1	HELT
CRMP1	0	5056.823635	-20	1.06E-13	Low	0	6120	XP_003128882.3	ssc:100520574			
SVEP1	0	4956.843952	-20	1.17E-13	Low	0	5999	XP_003122123.2	ssc:100155613	0	NM_153366.3	SVEP1
AHNAK	25.37661808	49721.30102	-10.93614852	1.26E-13	Low	24	60175	XP_005674623.1	ssc:100515251	0	NM_024060.3	AHNAK
PRELP	0	4799.851061	-20	1.38E-13	Low	0	5809	XP_005656706.1	ssc:100511032	2E-172	NM_201348.1	PRELP
LOC100622539	3575.988431	4.131391859	9.757498449	1.46E-13	High	3382	5	XP_005674221.1	ssc:100622539	4E-129	NR_048571.1	GPM6A
LEFTY2	3472.36724	4.131391859	9.715075916	1.77E-13	High	3284	5	NM_001195346.1	ssc:100337677	1E-176	NM_003240.3	LEFTY2
LOC100736763	0	4545.357323	-20	1.82E-13	Low	0	5501	XR_309518.1	ssc:100736763			
FAM198B	5.286795433	11644.74109	-11.10499744	3.28E-13	Low	5	14093	XP_005666715.1	ssc:100515440	0	NM_016613.6	FAM198B
TMEM163	2003.695469	1.652556743	10.24374773	3.44E-13	High	1895	2	XP_005671679.1	ssc:100512373	1E-98	NM_030923.4	TMEM163
GPR124	21.14718173	34432.67231	-10.66909703	3.49E-13	Low	20	41672	XP_003133414.1	ssc:100524344	0	NM_032777.9	GPR124
CASP4	0	3914.906925	-20	3.90E-13	Low	0	4738	XP_005667345.1	ssc:100522887	1E-170	NM_033307.2	CASP4
LOC100736983	1190.586331	0	20	3.96E-13	High	1126	0	XP_003483672.1	ssc:100736983	2E-61	NM_001029887.1	HELT
CHI3L1	0	3854.588604	-20	4.22E-13	Low	0	4665	NM_001001540.1	ssc:396865	0	NM_001276.2	CHI3L1
LOC100513452	1169.43915	0	20	4.44E-13	High	1106	0	LOC100513452	ssc:100513452	3E-72	NM_171999.3	SALL3
SIX1	5.286795433	10886.21755	-11.00782165	4.63E-13	Low	5	13175	NM_001199718.1	ssc:100156847	2E-167	NM_005982.3	SIX1
IRG6	2.114718173	6658.151119	-11.62044049	4.65E-13	Low	2	8058	NM_213817.1	ssc:396752	1E-177	NM_080657.4	RSAD2
LTBP2	39.1222862	56402.58793	-10.49355496	5.40E-13	Low	37	68261	XP_005656461.1	ssc:100514300	0	NM_000428.2	LTBP2
C1QTNF5	0	3612.489041	-20	5.88E-13	Low	0	4372	XP_003129988.2	ssc:100520210	1E-119	NM_015645.4	C1QTNF5
ADM	1.057359087	4836.20731	-12.15919496	7.01E-13	Low	1	5853	NM_214107.1	ssc:397195	9E-91	NM_001124.1	ADM
LOC100628118	0	3442.275697	-20	7.51E-13	Low	0	4166	XP_005662044.1	ssc:100628118			
SLC6A1	1067.932677	0	20	8.00E-13	High	1010	0	XP_005669835.1	ssc:100156668	0	NM_003042.3	SLC6A1
CMPK2	0	3366.258086	-20	8.42E-13	Low	0	4074	XP_005662825.1	ssc:100623872	1E-176	NR_046236.1	CMPK2
MCF2	1057.359087	0	20	8.54E-13	High	1000	0	XP_001925660.1		0	NM_005369.4	MCF2
IGFBP6	1.057359087	4610.633314	-12.09028381	8.94E-13	Low	1	5580	NM_001100190.1	ssc:100101923	1E-100	NM_002178.2	IGFBP6
LOC100525680	0	3270.409795	-20	9.75E-13	Low	0	3958	XP_005661152.1	ssc:100525680	6E-52	NM_199161.3	SAA1
MX2	0	3252.231671	-20	1.00E-12	Low	0	3936	NM_001097416.1	ssc:396893	8E-160	NM_002463.1	MX2
EHBP1L1	3.17207726	6902.729517	-11.08752333	1.03E-12	Low	3	8354	XP_005660818.1	ssc:100518056	0	NM_001099409.1	EHBP1L1
OBSL1	3.17207726	6779.61404	-11.06155952	1.13E-12	Low	3	8205	XP_005672301.1	ssc:100624019	6E-162	NM_015311.2	OBSL1
CRLF1	1.057359087	4342.919122	-12.00398396	1.21E-12	Low	1	5256	XP_003480798.1	ssc:100738750			
LOC102166006	973.8277187	0	20	1.45E-12	High	921	0	XR_309180.1	ssc:102166006			
MCSF	3.17207726	6173.125715	-10.92635755	1.82E-12	Low	3	7471	MCSF		2E-175	NM_172212.2	CSF1
LOC100737434	13.74566813	17070.08488	-10.27827748	1.82E-12	Low	13	20659	XP_003480673.1	ssc:100737434	0	NM_001278074.1	COL5A1
LOC100737120	2.114718173	5022.946222	-11.2138527	1.96E-12	Low	2	6079	XP_003480633.2	ssc:100737120	0	NM_002160.3	TNC
NPYR5	919.9024053	0	20	2.10E-12	High	870	0	XR_304867.1	ssc:100517770	0	NM_006174.2	NPY5R
LOC100156689	22.20454082	25125.47273	-10.14408029	2.18E-12	Low	21	30408	XP_005659493.1	ssc:100156689	0	NM_080645.2	COL12A1
HOXB6	0	2758.943483	-20	2.32E-12	Low	0	3339	XP_003358123.1	ssc:100627849	2E-124	NM_156037.1	HOXB6
KIF1A	1.057359087	3651.324125	-11.75373862	2.93E-12	Low	1	4419	XP_003133880.3	ssc:100517246	0	NM_004321.6	KIF1A
LOC100621838	2.114718173	4604.849366	-11.08847284	3.05E-12	Low	2	5573	XP_005658329.1	ssc:100621838	0	NM_201526.1	ISLR
CDC42EP5	2.114718173	4564.361725	-11.07573201	3.19E-12	Low	2	5524	XP_003127481.2	ssc:100525753	2E-45	NM_145057.3	CDC42EP5
TIMP-3	5.286795433	7434.02651	-10.45753461	3.24E-12	Low	5	8997	XP_003126121.3	ssc:396775	2E-124	NM_000362.4	TIMP3
INSM1	842.715192	0	20	3.70E-12	High	797	0	XP_005672796.1	ssc:100511966	3E-158	NM_002196.2	INSM1
SERPINH1	57.09739067	55440.79991	-9.923307541	3.93E-12	Low	54	67097	NM_001244132.1	ssc:396773	1E-122	NM_001235.3	SERPINH1
PDLIM4	0	2430.084691	-20	4.43E-12	Low	0	2941	NM_001285969.1	ssc:100520235	7E-168	NM_003687.3	PDLIM4
ADAM33	0	2406.948897	-20	4.65E-12	Low	0	2913	XP_005672830.1	ssc:100738366	4E-124	NM_153202.2	ADAM33
LOC100152170	0	2376.376597	-20	4.96E-12	Low	0	2876	XP_005659694.1	ssc:100152170	0	NM_021199.3	SQRDL
LOC102166259	0	2373.071484	-20	4.99E-12	Low	0	2872	XR_297673.1	ssc:102166259			
MAB21L2	1.057359087	3284.456528	-11.60097355	5.03E-12	Low	1	3975	XP_003129198.1	ssc:100519500	0	NM_006439.4	MAB21L2
FABP7	11260.87427	33.87741324	8.376779405	5.05E-12	High	10650	41	NM_001025229.1	ssc:574075	5E-71	NM_001446.3	FABP7
CXCL10	0	2351.588246	-20	5.23E-12	Low	0	2846	NM_001008691.1	ssc:494019	3E-22	NM_001565.3	CXCL10
LOC100153335	0	2349.935689	-20	5.25E-12	Low	0	2844	XP_005666216.1	ssc:100153335	0	NM_022369.3	STRA6
SLC2A1	3.17207726	5001.462984	-10.62270654	5.31E-12	Low	3	6053	XP_005665564.1	ssc:397404	6E-76	NM_006516.2	SLC2A1
THY1	48.63851798	44528.14145	-9.838402411	5.34E-12	Low	46	53890	NM_001146129.1	ssc:100271931	2E-67	NM_006288.3	THY1
PRRX1	0	2323.494781	-20	5.56E-12	Low	0	2812	XP_003481450.1	ssc:100738910	9E-143	NM_022716	PRRX1
C1QTNF6	0	2309.448049	-20	5.73E-12	Low	0	2795	NM_001142830.1	ssc:100217379	2E-104	NM_182486.1	C1QTNF6
LOC102161109	7.401513606	8165.282869	-10.10746682	6.51E-12	Low	7	9882	XR_305383.1	ssc:102161109			
LOC100622285	7.401513606	7905.831461	-10.06088115	7.68E-12	Low	7	9568	XP_005657028.1	ssc:100622285	1E-165	NM_005082.4	TRIM25
KLHDC8A	1697.061334	3.305113487	9.004123181	8.03E-12	High	1605	4	XP_003357440.1	ssc:100625797	0	NM_018203.2	KLHDC8A
POU5F1	2148.553664	4.95767023	8.759487785	8.29E-12	High	2032	6	NM_001113060.1	ssc:100127461	1E-176	NM_203289.5	POU5F1
ASPN	16.91774539	15025.04591	-9.79461637	8.67E-12	Low	16	18184	NM_001243889.1	ssc:100511749	0	NM_017680.4	ASPN
LOC100622125	738.0366424	0	20	8.72E-12	High	698	0	XP_005669371.1	ssc:100622125	0	NM_005442.3	EOMES
EOMES	738.0366424	0	20	8.72E-12	High	698	0	XP_005669373.1	ssc:100524218	0	NM_005442.3	EOMES
LPPR5	2583.128248	6.610226974	8.610203748	8.84E-12	High	2443	8	NM_001245001.1	ssc:100514836	0	NM_001037317.1	LPPR5
ACTA2	131.1125267	109145.589	-9.701232581	9.23E-12	Low	124	132093	NM_001164650.1	ssc:733615	0	NM_001613.2	ACTA2
AKR1CL1	6.344154519	6820.927959	-10.0703244	9.34E-12	Low	6	8255	NM_001038626.2	ssc:654406	6E-140	NM_003739.5	AKR1C3
C3	3.17207726	4449.509032	-10.45400253	9.63E-12	Low	3	5385	NM_214009.1	ssc:397072			
PLA2G4A	2.114718173	3654.629238	-10.75504393	9.88E-12	Low	2	4423	XP_005667902.1	ssc:100520687	0	NM_024420.2	PLA2G4A
LOC100519925	963.2541279	0.826278372	10.18707284	9.89E-12	High	911	1	XR_307139.1	ssc:100519925			
SERTAD4	1.057359087	2809.346464	-11.37555343	1.11E-11	Low	1	3400	XP_005667938.1	ssc:100511654	0	NM_019605.3	SERTAD4
SLA-3	6.344154519	6546.603539	-10.01110299	1.15E-11	Low	6	7923	NM_001097427.1	ssc:100037288	2E-147	NM_002116.7	HLA-A

PARP12	3.17207726	4273.511739	-10.39577846	1.18E-11	Low	3	5172	XP_003134663.1	ssc:100515805	0	NM_022750.2	PARP12
LOC100513601	5.286795433	5755.855137	-10.08842106	1.19E-11	Low	5	6966	NM_001246242.1	ssc:100513601	3E-145	NM_002116.7	HLA-A
PDGFRA	2.114718173	3512.509358	-10.69782094	1.21E-11	Low	2	4251	PDGFRA		0	NM_006206.4	PDGFRA
MAP7D2	1586.03863	3.305113487	8.906512385	1.24E-11	High	1500	4	XP_005673545.1	ssc:100739061	0	NM_152780.3	MAP7D2
KCNJ3	1366.10794	2.478835115	9.106193453	1.26E-11	High	1292	3	XP_003483696.1	ssc:396586	0	NM_002239.3	KCNJ3
HOXB3	1.057359087	2740.765359	-11.3398977	1.26E-11	Low	1	3317	XP_003131595.1	ssc:100522160	0	NM_002146.4	HOXB3
SOX11	0	1957.453463	-20	1.33E-11	Low	0	2369	XP_003354985.1	ssc:100514639	0	NM_003108.3	SOX11
C12H17orf67	0	1926.054885	-20	1.44E-11	Low	0	2331	XP_005657027.1	ssc:100525534	1E-34	NM_001085430.2	C17orf67
COL5A1	144.8581949	110737.8274	-9.578291104	1.45E-11	Low	137	134020	NM_001014971.1	ssc:397533			
LOC102165113	1962.458465	4.95767023	8.628784094	1.49E-11	High	1856	6	XR_306174.1	ssc:102165113			
LOC102161974	1726.667388	4.131391859	8.70714658	1.64E-11	High	1633	5	XR_307170.1	ssc:102161974			
NFE2L3	7627.788451	27.26718627	8.127955188	1.67E-11	High	7214	33	XP_003360238.1	ssc:100622324	0	NM_004289.6	NFE2L3
AWN	0	1846.732161	-20	1.79E-11	Low	0	2235	NM_213829.1	ssc:396783			
PLAC9	1.057359087	2550.721334	-11.23622417	1.82E-11	Low	1	3087	NM_001190209.1	ssc:100155282	1E-31	NM_001012973.1	PLAC9
LOC100153981	2307.157527	6.610226974	8.447200986	1.84E-11	High	2182	8	XP_003480454.1	ssc:100153981	0	NM_020168.5	PAK6
ZNF275	0	1793.024067	-20	2.08E-11	Low	0	2170	ZNF275	ssc:100623368	9E-140	NM_020636.2	ZNF275
LOC102164776	0	1751.710148	-20	2.34E-11	Low	0	2120	XR_305411.1	ssc:102164776			
INPP5D	1042.556059	1.652556743	9.301209436	2.36E-11	High	986	2	XP_003133787.3	ssc:100526019	0	NM_005541.3	INPP5D
DAAM2	19.03246356	13680.691	-9.48946307	2.38E-11	Low	18	16557	XP_003356671.1		0	NM_015345.3	DAAM2
TSHZ3	0	1718.659013	-20	2.58E-11	Low	0	2080	XP_005655858.1	ssc:100627966	0	NM_020856.2	TSHZ3
tRNA-Ala	1025.638314	1.652556743	9.277606536	2.62E-11	High	970	2	tRNA-Ala				
LOC100514841	1.057359087	2366.461257	-11.12805018	2.67E-11	Low	1	2864	XP_005664153.1	ssc:100514841	1E-70	NM_001080509.2	TSPAN11
LOC100620394	10.57359087	8025.641825	-9.568007552	2.84E-11	Low	10	9713	XP_005674263.1	ssc:100620394	1E-77	NM_001855.4	COL15A1
LOC100627044	0	1683.955322	-20	2.86E-11	Low	0	2038	XP_005658552.1	ssc:100627044	1E-66	NR_037944.1	WISP1
NFIX	1.057359087	2329.27873	-11.10520216	2.89E-11	Low	1	2819	XP_005652941.1	ssc:100523536	0	NM_002501	NFIX
LOC100739233	3.17207726	3576.132793	-10.13875668	2.93E-11	Low	3	4328	XR_307534.1	ssc:100739233	1E-67	NM_002508.2	NID1
GM2A	3.17207726	3566.217452	-10.13475105	2.97E-11	Low	3	4316	XP_003134190.1	ssc:100515283	3E-63	NM_001167607.1	GM2A
NECD	0	1658.340692	-20	3.09E-11	Low	0	2007	NM_001123144.1	ssc:100144475	3E-11	NM_002487.2	NDN
LOC102157759	1749.929288	4.95767023	8.4634186	3.12E-11	High	1655	6	XR_298275.1	ssc:102157759			
CCL11	0	1648.425352	-20	3.19E-11	Low	0	1995	NM_001256774.1	ssc:100038010	1E-27	NM_002986.2	CCL11
TPD52L1	598.465243	0	20	3.36E-11	High	566	0	XP_005659275.1	ssc:100515246	4E-95	NM_003287.2	TPD52L1
SLC6A17	1.057359087	2253.26112	-11.05733339	3.42E-11	Low	1	2727	XP_001929358.2	ssc:100156430	0	NM_001010898.2	SLC6A17
KCNE4	0	1620.331887	-20	3.48E-11	Low	0	1961	XP_005672306.1	ssc:397167	1E-76	NM_080671.3	KCNE4
TAC3	593.1784476	0	20	3.56E-11	High	561	0	NM_001007196.1	ssc:492314	2E-40	NR_033654.1	TAC3
CRABP2	24.31925899	15784.39574	-9.342184046	3.57E-11	Low	23	19103	NM_001164509.1	ssc:100155151	4E-76	NM_001878.3	CRABP2
PHF2	1.057359087	2227.64649	-11.04083918	3.63E-11	Low	1	2696	XP_005662327.1	ssc:100514572	0	NM_024517.1	PHF2
LOC100525528	0	1597.196093	-20	3.74E-11	Low	0	1933	XP_005666809.1	ssc:100525528	1E-33	NM_002089.3	CXCL2
KIFC3	1.057359087	2210.294644	-11.02955758	3.78E-11	Low	1	2675	XP_005664439.1	ssc:100514463	3E-11	NM_005550.3	KIFC3
LOC102159390	3.17207726	3391.872716	-10.06243841	3.83E-11	Low	3	4105	XR_302916.1	ssc:102159390			
LOC100518132	22.20454082	14235.12379	-9.324384586	3.95E-11	Low	21	17228	XP_003127801.2	ssc:100518132	0	NM_001856.3	COL16A1
PLEKHF1	1.057359087	2174.764674	-11.00617817	4.10E-11	Low	1	2632	XP_005653317.1	ssc:100522869	7E-133	NM_024310.4	PLEKHF1
ACTG2	35.95020894	22116.99318	-9.264939392	4.20E-11	Low	34	26767	XP_003125076.4	ssc:100520667	5E-117	NM_001615.3	ACTG2
AIF1	2388.574177	8.262783717	8.17530606	4.21E-11	High	2259	10	NM_001129950.1	ssc:397271	1E-74	NM_032955.1	AIF1
LOC102166564	34.89284986	21479.10627	-9.265786816	4.21E-11	Low	33	25995	XP_005661871.1	ssc:102166564	0	NM_002609.3	PDGFRB
PPP1R26	0	1550.924504	-20	4.34E-11	Low	0	1877	XP_005660604.1	ssc:100519641	0	NM_014811.3	PPP1R26
LOC100525396	0	1536.877771	-20	4.55E-11	Low	0	1860	XP_005652609.1	ssc:100525396	1E-33	NM_002089.3	CXCL2
CXCL2	1.057359087	2125.187972	-10.97290933	4.61E-11	Low	1	2572	XP_005652611.1	ssc:396594	9E-35	NM_002089.3	CXCL2
MVP	14.80302721	9626.143031	-9.344921813	4.64E-11	Low	14	11650	XP_003354597.1	ssc:100622738	0	NM_017458.3	MVP
PAPP-A	2.114718173	2684.57843	-10.31001443	4.75E-11	Low	2	3249	XP_001926788.4	ssc:397219	0	NM_002581.3	PAPPA
LOC733579	1.057359087	2098.747064	-10.95484718	4.91E-11	Low	1	2540	NM_001044532.1	ssc:733579	8E-156	NM_033093.2	TRIM5
LOC100515546	0	1513.741977	-20	4.91E-11	Low	0	1832	XP_003126463.1	ssc:100515546	4E-118	NM_005086.4	SSPN
SQRDL	0	1509.610585	-20	4.98E-11	Low	0	1827	NM_001244238.1	ssc:100154990			
TINAGL1	0	1483.995956	-20	5.43E-11	Low	0	1796	XP_005665217.1	ssc:100517950	0	NM_022164.2	TINAGL1
TAGLN	203.0129446	120386.2799	-9.211883546	5.64E-11	Low	192	145697	NM_001244150.1	ssc:397021	1E-116	NM_003186.3	TAGLN
TNS1	1.057359087	1972.326473	-10.86521725	6.74E-11	Low	1	2387	XP_005672258.1	ssc:100514801	0	NM_022648.4	TNS1
LOC100624156	0	1416.241129	-20	6.89E-11	Low	0	1714	XR_300518.1	ssc:100624156			
LOC100525721	0	1404.673232	-20	7.18E-11	Low	0	1700	XP_003359988.2	ssc:100525721	1E-130	NM_177991.1	DUSP15
LOC100627123	10.57359087	6652.367171	-9.297258579	7.38E-11	Low	10	8051	XP_005666705.1	ssc:100627123	1E-115	NM_006206.4	PDGFRA
LOC102164419	1.057359087	1927.707441	-10.83220499	7.57E-11	Low	1	2333	XP_005665565.1	ssc:102164419	1E-92	NM_006516.2	SLC2A1
CSF1	3.17207726	2943.20356	-9.857743701	7.88E-11	Low	3	3562	NM_001244523.1	ssc:100513084	1E-32	NM_172212.2	CSF1
ACKR3	6.344154519	4399.106051	-9.437566756	8.70E-11	Low	6	5324	XP_003133807.1	ssc:100515346	0	NM_020311.2	ACKR3
WFDC1	11.63094995	6949.827385	-9.222864402	8.73E-11	Low	11	8411	NM_001244987.1	ssc:100514648	1E-89	NM_021197.3	WFDC1
LOC100513671	0	1343.528632	-20	9.00E-11	Low	0	1626	XP_003129107.4	ssc:100513671	9E-114	NM_182502.3	TMPRSS11B
ODF4	511.7617979	0	20	9.18E-11	High	484	0	NM_001202461.1	ssc:100529212	1E-36	NM_153007.4	ODF4
LOC102165697	678.8245336	0.826278372	9.682195086	9.42E-11	High	642	1	XR_300413.1	ssc:102165697			
SLIT3	9.516231779	5827.741356	-9.258330724	9.48E-11	Low	9	7053	XP_003134124.3	ssc:100513444	0	NM_003062.3	SLIT3
KCNK13	507.5323616	0	20	9.69E-11	High	480	0	NM_001244596.1	ssc:100623906	0	NM_022054.3	KCNK13
BOK	0	1291.473095	-20	1.10E-10	Low	0	1563	XP_005657656.1	ssc:100627765	2E-49	NM_032515.4	BOK
LOC102161219	0	1290.646817	-20	1.10E-10	Low	0	1562	XP_005674129.1	ssc:102161219	4E-40	NM_002962.1	S100A5
CLDN10	812.0517785	1.652556743	8.9407281	1.18E-10	High	768	2	NM_001243444.1	ssc:100153752	2E-124	NM_182848.3	CLDN10
UROC1	965.368846	2.478835115	8.605274148	1.18E-10	High	913	3	NM_001244219.1	ssc:100157217	0	NM_144639.2	UROC1
FAP	2.114718173	2238.388109	-10.04777908	1.20E-10	Low	2	2709	XP_005671941.1	ssc:100736572	0	NM_004460.2	FAP
EPAS1	7.401513606	4575.103344	-9.271768285	1.24E-10	Low	7	5537	NM_001097420.1	ssc:100037272	0	NM_001430.4	EPAS1
LRAT	802.5355467	1.652556743	8.923721675	1.27E-10	High	759	2	NM_001244920.1	ssc:100513994	1E-111	NM_004744.3	LRAT
IER5L	9.516231779	5495.57745	-9.173664955	1.28E-10	Low	9	6651	XP_005674645.1	ssc:102161937	1E-42	NM_203434.2	IER5L
LOC100049650	14.80302721	7884.348223	-9.05695545	1.28E-10	Low	14	9542	XP_005657455.1	ssc:100049650	0	NM_015194.1	MYO1D
LOC100738990	1.057359087	1729.400632	-10.675591	1.31E-10	Low	1	2093	XP_003484353.1	ssc:100738990	8E-150	NM_033093.2	TRIM5
KIAA1244	2139.037432	9.089062089	7.878614585	1.37E-10	High	2023	11	XP_003353246.3	ssc:100621590			
DPT	0	1233.609609	-20	1.39E-10	Low	0	1493	XP_003125697.1	ssc:100516366	3E-108	NM_001937.4	DPT
C1QL4	1831.345938	7.436505346	7.944063813	1.41E-10	High	1732	9	XP_003126157.1	ssc:100512604	1E-131	NM_001008223.1	C1QL4
LOC100738662	8616.419196	43.7927537	7.62025246	1.41E-10	High	8149	53	XP_003483783.1	ssc:100738662	8E-67	NM_018000.2	MREG
RBP4	0	1226.197104	-20	1.43E-10	Low	0	1484	NM_214057.1	ssc:397124	4E-106	NM_006744.3	RBP4
LOC100625416	0	1223.718269	-20	1.45E-10	Low	0	1481	XR_309344.1	ssc:100625416			
LOC102164256	6.344154519	3954.568287	-9.283876582	1.50E-10	Low	6	4786	XP_005660342.1	ssc:102164256	4E-52	NM_001855.4	COL15A1
HSPA12B	0	1210.497815	-20	1.53E-10	Low	0	1465	XP_005672829.1	ssc:100524916	0	NM_052970.4	HSPA12B
RFX4	2391.746254	10.74161883	7.798709095	1.56E-10	High	2262	13	XP_005655591.1	ssc:100521450	0	NM_213594.2	RFX4
C1QTNF1	2.114718173	2121.05658	-9.970101981	1.57E-10	Low	2	2567	XP_005658522.1	ssc:100217377	2E-99	NR_049769.1	C1QTNF1
LOC100153689	470.5247935	0	20	1.57E-10	High	445	0	LOC100153689				
HOXC6	0	1198.929917	-20	1.60E-10	Low	0	1451	XP_003355472.1	ssc:100627932	8E-140	NM_153693.4	HOXC6
ARAP3	7.401513606	4325.567276	-9.190853301	1.65E-10	Low	7	5235	XP_003124050.1	ssc:100511744	0	NM_022481.5	ARAP3
TRIM44	1.057359087	1645.120238	-10.60351191	1.69E-10	Low	1	1991	XP_005661051.1	ssc:100524974	9E-137	NM_017583.4	TRIM44
GLI3	2.114718173	2078.916383	-9.941150608	1.74E-10	Low	2	2516	XP_005673412.1	ssc:100516888	0	NM_000168.5	GLI3
ENPP2	4402.843236	22.30951604	7.62463245	1.76E-10	High	4164	27	XP_005662941.1	ssc:503658	0	NR_045555.1	ENPP2
VSNL1	761.2985423	1.652556743	8.847618696	1.78E-10	High	720	2	XP_003125391.1	ssc:100517707	8E-111	NM_003385.4	VSNL1
OCLN	760.2411832	1.652556743	8.84561356	1.80E-10	High	719	2	NM_001163647.2	ssc:397236	0	NM_002538.3	OCLN
LOC100628220	0	1165.878783	-20	1.85E-10	Low	0	1411	XP_003354556.3	ssc:100628220	6E-32	NM_001278939.1	ELN
LOC100739007	3.17207726	2482.966507	-9.612421176	1.87E-10	Low	3	3005	XP_005663642.1	ssc:100739007	2E-87	NM_172212.2	CSF1
GALNTL2	4.229436346	2873.796177	-9.408276618	2.01E-10	Low	4	3478	NM_001206447.1	ssc:100157572	0	NM_054110.4	GALNT15
LOC100623776	0	1146.048102	-20	2.02E-10	Low	0	1387	XP_003362087.1	ssc:100623776			
LOC100625207	0	1140.264153	-20	2.07E-10	Low	0	1380	XP_003484218.1	ssc:100625207	0	NM_021906.2	USP9X

EPHB1	1442.237794	5.783948602	7.962038606	2.09E-10	High	1364	7	XP_003132484.3	ssc:100524585	0	NM_004441.4	EPHB1
HOXA5	3.17207726	2425.127021	-9.578416688	2.11E-10	Low	3	2935	NM_001195232.1	ssc:100499568	3E-155	NM_019102.3	HOXA5
ACTC1	11.63094995	5781.469767	-8.957323558	2.23E-10	Low	11	6997	NM_001170517.1	ssc:100152267	0	NM_005159.4	ACTC1
LOC102165673	23.2618999	10474.73092	-8.814728535	2.33E-10	Low	22	12677	XP_005661865.1	ssc:102165673	5E-87	NM_002609.3	PDGFRB
LOC100627141	1.057359087	1538.530328	-10.50687176	2.38E-10	Low	1	1862	XP_005674171.1	ssc:100627141	0	NM_001099409.1	EHBP1L1
LOC100514326	0	1101.42907	-20	2.47E-10	Low	0	1333	XP_005672251.1	ssc:100514326	1E-132	NM_022648.4	TNS1
GNG11	0	1091.513729	-20	2.58E-10	Low	0	1321	XP_005667670.1	ssc:100522202	3E-35	NM_004126.3	GNG11
LOC102164134	5.286795433	3124.984802	-9.207239954	2.66E-10	Low	5	3782	XP_005660341.1	ssc:102164134	4E-116	NM_001855.4	COL15A1
ZNF503	2.114718173	1911.181874	-9.819783951	2.67E-10	Low	2	2313	ZNF503	ssc:100521339	2E-174	NM_032772.4	ZNF503
IL33	2.114718173	1905.397925	-9.815411198	2.71E-10	Low	2	2306	NM_001285978.1	ssc:100518643	3E-68	NM_033439.3	IL33
LOC102160289	572.0312658	0.826278372	9.435250383	2.82E-10	High	541	1	XP_005670228.1	ssc:102160289	8E-74	NR_046079.1	SEMA5B
LOXL4	28.54869534	12123.15627	-8.730124822	2.93E-10	Low	27	14672	XP_005671434.1	ssc:100157207	0	NM_032211.6	LOXL4
MMP23B	9.516231779	4607.328201	-8.919324245	3.13E-10	Low	9	5576	XP_005653406.1	ssc:100525702	8E-126	NM_006983.1	MMP23B
SAMD9	8.458872693	4197.494128	-8.954847182	3.18E-10	Low	8	5080	XP_005667663.1	ssc:100519098	0	NM_017654.3	SAMD9
LOC100739307	2.114718173	1824.422645	-9.752758858	3.38E-10	Low	2	2208	XP_003483519.1	ssc:100739307	2E-121	NM_002508.2	NID1
PLAU	8.458872693	4118.997683	-8.92761219	3.50E-10	Low	8	4985	NM_213945.1	ssc:396985	0	NM_002658.3	PLAU
BCL3	2.114718173	1805.418242	-9.737651965	3.56E-10	Low	2	2185	XR_297404.1	ssc:100738193	0	NM_005178.4	BCL3
WISP1	0	1014.66984	-20	3.74E-10	Low	0	1228	XP_005662899.1	ssc:100739115	1E-135	NR_037944.1	WISP1
ZNF12	0	1012.191005	-20	3.78E-10	Low	0	1225	ZNF12	ssc:100519656	0	NM_016265.3	ZNF12
CCL20	0	1005.580778	-20	3.91E-10	Low	0	1217	NM_001024589.1	ssc:553951	5E-39	NM_004591.2	CCL20
ELOVL2	2573.612017	14.04673232	7.517416211	4.05E-10	High	2434	17	XP_005665625.1	ssc:100153368	3E-156	NM_017770.3	ELOVL2
COL5A3	1.057359087	1383.189994	-10.35331821	4.08E-10	Low	1	1674	NM_001105288.1	ssc:397531			
LOC100514082	404.9685302	0	20	4.10E-10	High	383	0	XP_003121229.3	ssc:100514082	1E-137	NM_003980.4	MAP7
IFFO1	2.114718173	1750.88387	-9.693402273	4.16E-10	Low	2	2119	XP_005652668.1	ssc:100515778	0	NR_036467.1	IFFO1
LOC102161033	0	987.4026542	-20	4.29E-10	Low	0	1195	XR_299987.1	ssc:102161033			
SLC26A7	0	986.5763758	-20	4.31E-10	Low	0	1194	XP_005663034.1	ssc:100626316	0	NM_134266.1	SLC26A7
EVC2	0	985.7500975	-20	4.33E-10	Low	0	1193	XP_005658650.1	ssc:100628058	0	NM_147127.4	EVC2
FBXO32	0	970.0508084	-20	4.69E-10	Low	0	1174	NM_001044588.1	ssc:733657	1E-145	NM_148177.2	FBXO32
S1PR3	0	961.7880247	-20	4.90E-10	Low	0	1164	XP_001925305.1	ssc:100154607	0	NM_005226.3	S1PR3
LOC100517050	3121.324024	18.17812418	7.423810987	4.94E-10	High	2952	22	XR_305793.1	ssc:100517050	2E-32	NM_017898.3	2-Mar
P4HA3	25.37661808	9791.398705	-8.591871339	4.95E-10	Low	24	11850	XP_003357239.1	ssc:100624543	0	NR_110031.1	P4HA3
SEMA3B	8.458872693	3839.715593	-8.82631833	4.99E-10	Low	8	4647	XP_005669648.1	ssc:100153200	0	NM_004636.2	SEMA3B
ADAMTSL3	0	946.915014	-20	5.30E-10	Low	0	1146	XP_005666168.1	ssc:100157238	0	NM_207517.2	ADAMTSL3
NLRP12	388.0507848	0	20	5.39E-10	High	367	0	XP_005664869.1	ssc:100523066	0	NM_144687.3	NLRP12
LOC100739662	758.1264651	2.478835115	8.256632407	5.59E-10	High	717	3	XP_003481686.1	ssc:100739662	0	NM_005379.3	MYO1A
LOC102166331	636.5301701	1.652556743	8.589385276	5.62E-10	High	602	2	XR_302282.1	ssc:102166331			
LMO3	379.5919121	0	20	6.20E-10	High	359	0	NM_001112685.2	ssc:100127154	1E-82	NR_045014.1	LMO3
OAS1	0	912.2113224	-20	6.41E-10	Low	0	1104	NM_214303.1	ssc:397570	5E-155	NM_016816.2	OAS1
PEG3	0	882.465301	-20	7.58E-10	Low	0	1068	XP_005653381.1	ssc:100522552	0	NM_006210.2	PEG3
ROBO2	3033.563219	19.00440255	7.318535896	7.65E-10	High	2869	23	XP_005670349.1	ssc:100739586	0	NM_002942.4	ROBO2
GIMAP8	944.2216643	4.131391859	7.836353869	7.97E-10	High	893	5	XP_005657771.1	ssc:100736760	0	NM_175571.2	GIMAP8
NSUN7	2430.86854	14.87301069	7.352631349	8.00E-10	High	2299	18	XP_005666674.1	ssc:100620833	0	NM_024677.4	NSUN7
ZSWIM5	2541.891244	15.69928906	7.339059267	8.10E-10	High	2404	19	ZSWIM5	ssc:100514968	0	NM_020883.1	ZSWIM5
FAM111A	1.057359087	1205.140144	-10.15499857	8.19E-10	Low	1	1459	XP_005660932.1	ssc:100525029	6E-166	NM_198847.2	FAM111A
LOC100513263	0	868.4185687	-20	8.22E-10	Low	0	1051	XP_005660693.1	ssc:100513263			
TNNT2	0	868.4185687	-20	8.22E-10	Low	0	1051	NM_001257353.1	ssc:100622450	2E-108	NM_001276347.1	TNNT2
TRIM25	0	860.155785	-20	8.63E-10	Low	0	1041	XP_005657024.1	ssc:100737114	1E-114	NM_005082.4	TRIM25
LOC102166965	5.286795433	2473.877445	-8.870164812	8.71E-10	Low	5	2994	XP_005671014.1	ssc:102166965	3E-150	NM_002508.2	NID1
TWIST2	0	857.6769499	-20	8.75E-10	Low	0	1038	XP_003133824.1	ssc:100519927	1E-90	NM_057179.2	TWIST2
LOC100519889	1486.646876	8.262783717	7.491218383	8.89E-10	High	1406	10	XR_297275.1	ssc:100519889			
LOC102157763	0	854.3718364	-20	8.93E-10	Low	0	1034	XP_005666811.1	ssc:102157763	6E-33	NM_002089.3	CXCL2
LOC100621315	358.4447303	0	20	8.93E-10	High	339	0	XR_298397.1	ssc:100621315			
NHigh210	11747.25945	80.14900206	7.195423953	9.04E-10	High	11110	97	XP_005669873.1	ssc:100515926	0	NM_024923.3	NHigh210
VPAC1	474.7542299	0.826278372	9.166337234	9.29E-10	High	449	1	NM_214036.1	ssc:100155402	0	NM_004624.3	VIPR1
LOC100519601	2249.002777	14.04673232	7.322907076	9.63E-10	High	2127	17	XR_135210.2	ssc:100519601			
MYO5C	695.742279	2.478835115	8.132746872	9.67E-10	High	658	3	XP_005659654.1	ssc:100155557	0	NM_018728.3	MYO5C
LOC100525856	2.114718173	1469.949223	-9.441085196	1.01E-09	Low	2	1779	XP_005661151.1	ssc:100525856	9E-43	NM_199161.3	SAA1
LOC100517681	2113.660814	13.22045395	7.320828356	1.03E-09	High	1999	16	XR_309472.1	ssc:100517681			
C1R	20.08982264	6800.270999	-8.402983603	1.06E-09	Low	19	8230	XP_005658602.1	ssc:445464			
COL6A2	222.0454082	73816.40462	-8.376942922	1.09E-09	Low	210	89336	XP_005657250.1	ssc:100101552	0	NM_058175.2	COL6A2
TRIM4	0	818.8418664	-20	1.11E-09	Low	0	991	XP_003124381.1	ssc:100525393	0	NM_033091.2	TRIM4
PTGS1	3.17207726	1743.447364	-9.102299184	1.12E-09	Low	3	2110	XP_001926164.1	ssc:397541	0	NM_080591.2	PTGS1
TSPO	7.401513606	2946.508674	-8.636970466	1.16E-09	Low	7	3566	NM_213753.1	ssc:396592	4E-77	NR_046308.1	TSPO
LOC102165099	2.114718173	1430.287861	-9.40162441	1.16E-09	Low	2	1731	XP_005665219.1	ssc:102165099	1E-55	NM_001856.3	COL16A1
LOC102159856	672.4803791	2.478835115	8.083686054	1.20E-09	High	636	3	LOC102159856				
PTGIS	5.286795433	2316.058276	-8.775062335	1.22E-09	Low	5	2803	XP_005654305.1	ssc:100126284	0	NM_000961.3	PTGIS
MATN4	0	803.1425773	-20	1.22E-09	Low	0	972	XP_003360059.1	ssc:100510939	0	NM_030592.3	MATN4
GREB1L	2590.529762	17.35184581	7.22201421	1.26E-09	High	2450	21	XP_005653460.1	ssc:100524319	0	NM_024935.2	GREB1L
ZSCAN10	339.4122668	0	20	1.26E-09	High	321	0	ZSCAN10	ssc:100628188	0	NM_032805.1	ZSCAN10
HOXB2	0	796.5323503	-20	1.27E-09	Low	0	964	XP_003131594.1	ssc:100521989	9E-154	NM_002145.3	HOXB2
COL16A1	12.68830904	4378.449092	-8.430776312	1.31E-09	Low	12	5299	XP_005665218.1	ssc:100737666	6E-118	NM_001856.3	COL16A1
LOC102158099	1.057359087	1097.297678	-10.01927383	1.32E-09	Low	1	1328	XP_005660601.1	ssc:102158099	1E-41	NM_001278074.1	COL5A1
IL8	0	789.095845	-20	1.33E-09	Low	0	955	NM_213867.1	ssc:396880	5E-35	NM_000584.3	IL8
LOC100620730	0	789.095845	-20	1.33E-09	Low	0	955	XP_003362006.1	ssc:100620730	5E-35	NM_000584.3	IL8
LRRC32	3.17207726	1681.476486	-9.050084979	1.35E-09	Low	3	2035	XP_003129719.1	ssc:100522452	0	NM_005512.2	LRRC32
CDC42EP1	12.68830904	4337.135173	-8.417098782	1.38E-09	Low	12	5249	XP_003481667.1	ssc:100738372	3E-133	NM_152243.2	CDC42EP1
SYNPO	4.229436346	1963.237411	-8.858553522	1.39E-09	Low	4	2376	XP_005658874.1	ssc:100624154	2E-159	NM_007286.5	SYNPO
LOC100623769	0	782.485618	-20	1.39E-09	Low	0	947	XP_005654767.1	ssc:100623769	1E-104	NM_205843.2	NFIC
DAB1	550.8840841	1.652556743	8.380905162	1.41E-09	High	521	2	NM_001097442.1	ssc:100037307			
CLEC14A	0	775.0491127	-20	1.46E-09	Low	0	938	XP_005666250.1	ssc:100157365	0	NM_175060.2	CLEC14A
FXYD5	5.286795433	2213.599758	-8.709785172	1.53E-09	Low	5	2679	XP_005664552.1	ssc:100624274	1E-28	NR_028406.1	FXYD5
IQCJ	328.8386759	0	20	1.54E-09	High	311	0	XP_005652468.1	ssc:100155582	4E-34	NM_001197114.1	IQCJ-SCHIP1
PRKCZ	328.8386759	0	20	1.54E-09	High	311	0	NM_001204374.1	ssc:100533200			
GADD45B	7.401513606	2772.163937	-8.54897653	1.58E-09	Low	7	3355	XP_005654758.1	ssc:100621090	8E-89	NM_015675.3	GADD45B
LOC100513362	0	757.6972669	-20	1.64E-09	Low	0	917	XP_005664972.1	ssc:100513362	8E-156	NM_133460.1	ZNF418
TRPC6	1.057359087	1048.547254	-9.953710755	1.66E-09	Low	1	1269	XP_003357304.2	ssc:100271728	0	NM_004621.5	TRPC6
CLU	435.6319437	136815.1728	-8.294902772	1.71E-09	Low	412	165580	NM_213971.1	ssc:397025	2E-177	NR_045494.1	CLU
LOC100520893	322.4945214	0	20	1.75E-09	High	305	0	XP_005661555.1	ssc:100520893	0	NM_014979.1	SV2C
NID2	81.41664967	23899.27562	-8.197427321	1.75E-09	Low	77	28924	XP_005660021.1	ssc:100156994	0	NM_007361.3	NID2
LGALS1	268.569208	82013.91234	-8.254430944	1.75E-09	Low	254	99257	NM_001001867.1	ssc:414915	4E-68	NM_002305.3	LGALS1
LOC100515902	3.17207726	1595.543536	-8.974404349	1.76E-09	Low	3	1931	XR_304348.1	ssc:100515902			
EMSP1	429.2877891	0.826278372	9.021101516	1.76E-09	High	406	1	NM_213802.1	ssc:396720	2E-111	NM_004917.3	KLK4
LOC100511351	5.286795433	2149.976323	-8.667711553	1.77E-09	Low	5	2602	XP_005671015.1	ssc:100511351	1E-144	NM_002508.2	NID1
EMILIN1	95.16231779	27857.9753	-8.193484282	1.80E-09	Low	90	33715	XP_005662732.1	ssc:100523665	0	NM_007046.3	EMILIN1
DNMT3B	28611.07952	214.0060983	7.062778215	1.85E-09	High	27059	259	XP_001928639.1		0	NM_175850.2	DNMT3B
ONECUT1	319.3224441	0	20	1.86E-09	High	302	0	XP_005659647.1	ssc:100152052	0	NR_073510.1	ONECUT1
PODNL1	1.057359087	1022.932624	-9.91803	1.88E-09	Low	1	1238	XP_003123408.2	ssc:100513269	0	NM_024825.3	PODNL1
HOXB4	0	732.0826374	-20	1.95E-09	Low	0	886	XP_003131596.1	ssc:100522341	6E-140	NM_024015.4	HOXB4
LOC102160493	0	732.0826374	-20	1.95E-09	Low	0	886	XP_005672259.1	ssc:102160493	4E-89	NM_022648.4	TNS1
LOC100737137	0	729.6038022	-20	1.98E-09	Low	0	883	LOC100737137				

LOC100620681	0	727.1249671	-20	2.02E-09	Low	0	880	XR_304725.1	ssc:100620681	1E-146	NM_153717.2	EVC
ACSL5	0	723.8198536	-20	2.06E-09	Low	0	876	NM_001195321.1	ssc:100157521	0	NM_203380.1	ACSL5
SLC35F1	2290.239782	16.52556743	7.114655032	2.10E-09	High	2166	20	XP_005654451.1	ssc:100522582	0	NM_001029858.3	SLC35F1
ALDH3B1	3.17207726	1522.004761	-8.906329246	2.24E-09	Low	3	1842	XP_005660674.1	ssc:100739347	0	NM_001161473.1	ALDH3B1
LOC100516442	0	709.7731213	-20	2.28E-09	Low	0	859	XP_003126812.2	ssc:100516442	0	NM_133642.3	LARGE
FNDC1	796.1913922	4.131391859	7.590343559	2.37E-09	High	753	5	XP_005659175.1	ssc:100154276	3E-87	NM_032532.2	FNDC1
DRD2	1654.76697	11.5678972	7.160357619	2.44E-09	High	1565	14	NM_001244253.1	ssc:100624857	0	NM_016574.3	DRD2
LOC100623748	0	697.3789457	-20	2.49E-09	Low	0	844	XP_005658500.1	ssc:100623748	0	NM_002501	NFIX
HOXC4	0	694.9001106	-20	2.54E-09	Low	0	841	XP_005663910.1	ssc:100154759	1E-153	NM_153633.2	HOXC4
GDF10	0	688.2898837	-20	2.66E-09	Low	0	833	XP_003133153.1	ssc:100519926	0	NM_004962.3	GDF10
GBP1	12.68830904	3798.401675	-8.225748851	2.70E-09	Low	12	4597	NM_001128473.1	ssc:100151938	0	NM_002053.2	GBP1
VASN	33.83549077	9142.770183	-8.077950231	2.78E-09	Low	32	11065	XP_005662231.1	ssc:102158247	0	NM_138440.2	VASN
SLA-1	29.60605442	8040.514835	-8.085251834	2.80E-09	Low	28	9731	NM_001097427.1	ssc:100037288	2E-147	NM_002116.7	HLA-A
IRF7	15.8603863	4557.751498	-8.166750547	2.80E-09	Low	15	5516	NM_001097428.1	ssc:100037289	7E-160	NM_004031.2	IRF7
VWA5A	7.401513606	2446.610259	-8.368748254	2.97E-09	Low	7	2961	XP_005653769.1	ssc:100515913	0	NM_198315.2	VWA5A
NPY	4.229436346	1688.086713	-8.640707889	2.99E-09	Low	4	2043	NM_001256367.1	ssc:397304	4E-47	NM_000905.3	NPY
MGAT5B	2348.394531	18.17812418	7.013327658	3.06E-09	High	2221	22	XP_005668662.1	ssc:100516218	0	NM_198955.1	MGAT5B
CNN1	24.31925899	6544.124704	-8.07195716	3.13E-09	Low	23	7920	NM_213878.1	ssc:396911	8E-177	NM_001299.4	CNN1
LOC100739808	293.9458261	0	20	3.14E-09	High	278	0	XP_005659867.1	ssc:100739808	4E-164	NM_005503.3	APBA2
S100A16	11.63094995	3415.00851	-8.197776431	3.22E-09	Low	11	4133	NM_001190208.1	ssc:100155146	2E-51	NM_080388.1	S100A16
LOC102164335	0	656.8913055	-20	3.37E-09	Low	0	795	XR_299884.1	ssc:102164335			
LOC100621630	3628.856385	29.74602138	6.930674435	3.38E-09	High	3432	36	XP_005658328.1	ssc:100621630			
ARSI	0	653.586192	-20	3.45E-09	Low	0	791	XP_003124150.1	ssc:100517463	0	NM_001012301.2	ARSI
SLAIN1	11985.16525	100.8059614	6.893525032	3.51E-09	High	11335	122	XP_005668535.1	ssc:100526183	6E-92	NM_144595.3	SLAIN1
ZNF134	0	647.8022434	-20	3.61E-09	Low	0	784	ZNF134	ssc:100512237	0	NM_003435.3	ZNF134
MASP1	13.74566813	3815.753521	-8.11684714	3.70E-09	Low	13	4618	NM_001184947.1	ssc:100152125	0	NR_033519.1	MASP1
LOC100153751	1.057359087	893.2069198	-9.722385209	3.73E-09	Low	1	1081	XP_005670536.1	ssc:100153751	5E-84	NM_021973.2	HAND2
LOC102164249	472.6395117	1.652556743	8.15989662	3.75E-09	High	447	2	XR_300352.1	ssc:102164249			
DPYSL5	6025.889434	51.22925905	6.878062366	3.76E-09	High	5699	62	XP_003125368.1	ssc:100511810	0	NM_020134.3	DPYSL5
ACVRL1	2.114718173	1132.827648	-9.065247257	3.77E-09	Low	2	1371	XP_003481626.1	ssc:100153279	0	NM_001077401.1	ACVRL1
ZNF814	0	636.2343462	-20	3.96E-09	Low	0	770	ZNF814	ssc:100737218	0	NM_005773.2	ZNF256
CYGB	2.114718173	1117.954637	-9.046180525	4.03E-09	Low	2	1353	XP_003131227.2	ssc:100518861	2E-97	NM_134268.4	CYGB
LOC100626670	5299.483742	45.44531045	6.865576585	4.04E-09	High	5012	55	XP_005664518.1	ssc:100626670	0	NM_018092.4	NETO2
CDH13	0	629.6241193	-20	4.17E-09	Low	0	762	NM_001109945.1	ssc:100126163	1E-51	NM_001257.4	CDH13
LYSMD2	728.5204106	4.131391859	7.462197677	4.17E-09	High	689	5	XP_005659663.1	ssc:100155966	1E-60	NM_153374.2	LYSMD2
SYT6	901.9273008	5.783948602	7.284812608	4.23E-09	High	853	7	XR_298361.1	ssc:100152816	0	np_001240701	np_001240701
NFIB	10.57359087	2991.959384	-8.144478767	4.26E-09	Low	10	3621	XP_005660156.1	ssc:100155995	0	NM_005596.3	NFIB
MYBPH	0	625.4927274	-20	4.31E-09	Low	0	757	NM_001033014.1	ssc:613132	0	NM_004997.2	MYBPH
ITGB5	28.54869534	7134.91374	-7.965327361	4.31E-09	Low	27	8635	NM_001246669.1	ssc:100134977	0	NM_002213.3	ITGB5
ECEL1	0	623.0138923	-20	4.40E-09	Low	0	754	XP_003133775.1	ssc:100521587	0	NM_004826.2	ECEL1
VGF	10.57359087	2927.504271	-8.113062056	4.75E-09	Low	10	3543	XP_005658665.1	ssc:102163755	4E-50	NM_003378.3	VGF
LOC100738803	0	610.6197167	-20	4.87E-09	Low	0	739	XP_003481541.2	ssc:100738803	9E-168	NM_001010898.2	SLC6A17
LTBP3	25.37661808	6236.74915	-7.941150608	4.87E-09	Low	24	7548	XP_005660760.1	ssc:100517877	4E-63	NM_021070.4	LTBP3
TLR3	2.114718173	1075.81444	-8.990748134	4.90E-09	Low	2	1302	NM_001097444.1	ssc:100037937	0	NM_003265.2	TLR3
NRCAM	3505.145372	30.57229975	6.841105389	4.98E-09	High	3315	37	XP_005667799.1	ssc:100520970	0	NM_005010.4	NRCAM
CHRNG	1.057359087	841.9776608	-9.637172737	5.02E-09	Low	1	1019	XP_003133779.1	ssc:100522221	0	NM_005199.4	CHRNG
LOC100737260	535.0236978	2.478835115	7.753796673	5.16E-09	High	506	3	XR_299072.1	ssc:100737260	6E-79	NM_207108.2	ASTN1
CAMKV	3389.893232	29.74602138	6.832399308	5.23E-09	High	3206	36	XP_003358517.1	ssc:100623981	0	NM_024046.3	CAMKV
IGLON5	2.114718173	1059.288873	-8.968414947	5.30E-09	Low	2	1282	XP_003127424.2	ssc:100512905	7E-175	NM_001101372.1	IGLON5
BGN	768.7000559	198659.6301	-8.013662241	5.38E-09	Low	727	240427	XP_003135523.1	ssc:397396	0	NM_001711.4	BGN
SP100	0	598.2255411	-20	5.40E-09	Low	0	724	XP_005657638.1	ssc:100516940	1E-80	NM_003113.3	SP100
LOC100627133	0	588.3102007	-20	5.87E-09	Low	0	712	XP_005657517.1	ssc:100627133	0	NM_203380.1	ACSL5
CHL1	6229.959738	57.01320765	6.771782857	5.97E-09	High	5892	69	XP_003132376.3	ssc:100511780	0	NR_045572.1	CHL1
LOC102161796	2.114718173	1031.195408	-8.92963662	6.07E-09	Low	2	1248	XP_005657344.1	ssc:102161796			
UTF1	264.3397716	0	20	6.14E-09	High	250	0	XP_001925647.3	ssc:100158138	3E-29	NM_003577.2	UTF1
GUCY1B3	1.057359087	804.7951341	-9.572012363	6.31E-09	Low	1	974	NM_001018032.1	ssc:444999			
SDC4	8.458872693	2324.32106	-8.102128238	6.35E-09	Low	8	2813	NM_214284.1	ssc:397528	7E-59	NM_002999.3	SDC4
ZNF606	0	575.9160251	-20	6.53E-09	Low	0	697	ZNF606		0	NM_025027.3	ZNF606
NR0B1	255.880899	0	20	7.53E-09	High	242	0	NM_214387.1	ssc:397680	0	NM_000475.4	NR0B1
SRPX2	5.286795433	1613.72166	-8.25378254	7.57E-09	Low	5	1953	XP_001924554.3	ssc:100153130	0	NM_014467.2	SRPX2
FIBIN	3.17207726	1193.972247	-8.556125677	7.63E-09	Low	3	1445	XP_005658260.1	ssc:100620302	4E-116	NM_203371.1	FIBIN
HOXB8	6.344154519	1809.549634	-8.155987055	7.87E-09	Low	6	2190	X06668		9E-144	NM_024016.3	HOXB8
PPP1R3D	2.114718173	976.6610354	-8.851248721	7.98E-09	Low	2	1182	XP_003134554.1	ssc:100521997	2E-147	NM_006242.3	PPP1R3D
ID3	21.14718173	4783.325494	-7.821404747	8.00E-09	Low	20	5789	NM_001243602.1	ssc:100626978			
LOC100525868	498.0161298	2.478835115	7.650386348	8.12E-09	High	471	3	LOC100525868				
KCNK12	0	548.6488388	-20	8.34E-09	Low	0	664	XP_005674663.1	ssc:100521189	6E-109	NM_022055.1	KCNK12
LOC102165782	251.6514626	0	20	8.36E-09	High	238	0	XR_305670.1	ssc:102165782			
LOC100620576	0	547.8225605	-20	8.40E-09	Low	0	663	XP_005666577.1	ssc:100620576	1E-148	NM_153717.2	EVC
COL15A1	77.18721332	16447.07099	-7.735253093	8.84E-09	Low	73	19905	XP_003122085.4	ssc:100512644	6E-145	NM_001855.4	COL15A1
CLEC11A	2.114718173	949.3938491	-8.810397483	9.20E-09	Low	2	1149	XP_005664849.1	ssc:100620310	2E-132	NM_002975.2	CLEC11A
GLIS1	6.344154519	1754.188983	-8.111160556	9.21E-09	Low	6	2123	XP_003128030.4	ssc:100515552	0	NM_147193.2	GLIS1
ZNF473	0	536.2546633	-20	9.36E-09	Low	0	649	ZNF473	ssc:100524202	0	NM_015428.1	ZNF473
CARP	20.08982264	4422.241845	-7.782169288	9.40E-09	Low	19	5352	NM_213922.1	ssc:396959	7E-172	NM_014391.2	ANKRD1
ZNF667	0	532.9495498	-20	9.65E-09	Low	0	645	ZNF667	ssc:100521730	0	NR_030740.1	ZNF667
RUNX1	1.057359087	738.6928643	-9.448365422	9.72E-09	Low	1	894	NM_001246252.1	ssc:100512633	0	NM_001754.4	RUNX1
SYBU	1629.390352	14.87301069	6.775491744	1.03E-08	High	1541	18	XP_005655398.1	ssc:100157483	0	NM_017786.5	SYBU
LOC100515404	1.057359087	729.6038022	-9.430504028	1.03E-08	Low	1	883	XP_003130868.1	ssc:100515404	0	NM_032817.5	ITIH5
NETO2	1088.0225	9.089062089	6.903361247	1.04E-08	High	1029	11	XP_003360626.1	ssc:100624026	0	NM_018092.4	NETO2
LUZP4	242.1352308	0	20	1.07E-08	High	229	0	XP_005673902.1	ssc:100153362	1E-51	NM_016383.3	LUZP4
LOC102165344	37.00756803	7593.498236	-7.68080053	1.10E-08	Low	35	9190	XR_309235.1	ssc:102165344			
NID1	9.516231779	2274.744357	-7.901098294	1.12E-08	Low	9	2753	XP_003361217.1		4E-154	NM_002508.2	NID1
NBL1	39.1222862	7981.849071	-7.672588509	1.12E-08	Low	37	9660	XP_005656092.1	ssc:100623582	8E-88	NM_001204089.1	MINOS1-NBL1
LOC100736977	0	516.4239823	-20	1.13E-08	Low	0	625	XP_005665750.1	ssc:100736977	5E-46	NM_002127.5	HLA-G
ZBP1	2.114718173	911.385044	-8.751451476	1.13E-08	Low	2	1103	NM_001123216.1	ssc:100144524	1E-113	NM_030776.2	ZBP1
HOXA9	0	513.9451472	-20	1.16E-08	Low	0	622	XP_003134886.1	ssc:100519066	1E-159	NM_152739.3	HOXA9
LGALS9	0	513.1188688	-20	1.17E-08	Low	0	621	NM_213932.1	ssc:396972	3E-162	NR_024043.1	LGALS9
IL1A	0	507.3349202	-20	1.24E-08	Low	0	614	NM_214029.1	ssc:397094	2E-105	NM_000575.3	IL1A
OSR1	0	507.3349202	-20	1.24E-08	Low	0	614	XP_005674600.1	ssc:100520131	1E-117	NM_145260.2	OSR1
HOXB7	2.114718173	892.3806415	-8.721049998	1.26E-08	Low	2	1080	XP_003131597.1	ssc:100522513	7E-118	NM_004502.3	HOXB7
KCNA6	0	503.2035284	-20	1.29E-08	Low	0	609	HighI00025DE6BB		0	NM_002235.3	KCNA6
CDH10	234.7337172	0	20	1.29E-08	High	222	0	XP_005657658.1	ssc:100523754	0	NM_006727.3	CDH10
ECM1	3.17207726	1061.767708	-8.386824544	1.38E-08	Low	3	1285	XP_005655446.1	ssc:100620701	0	NM_022664.2	ECM1
TARSL2	231.56164	0	20	1.41E-08	High	219	0	XP_005652533.1	ssc:100523715	0	NM_152334.2	TARSL2
LOC102166536	231.56164	0	20	1.41E-08	High	219	0	XR_303556.1	ssc:102166536			
LOC100623939	0	494.1144663	-20	1.41E-08	Low	0	598	XP_005664537.1	ssc:100623939	2E-43	NM_021902.3	FXYD1
FOXL1	0	493.2881879	-20	1.42E-08	Low	0	597	XP_005664360.1	ssc:100511634	4E-124	NM_005250.2	FOXL1
DKK2	4.229436346	1229.502217	-8.183393212	1.48E-08	Low	4	1488	XP_003129317.1	ssc:100519672	5E-140	NM_014421.2	DKK2
MAMLD1	1.057359087	679.2008216	-9.327228984	1.48E-08	Low	1	822	XP_005657994.1	ssc:100517073	0	NM_005491.4	MAMLD1
NDRG1	15.8603863	3261.320733	-7.683884609	1.52E-08	Low	15	3947	XP_005662896.1	ssc:100156071	0	NM_006096.3	NDRG1
DPP4	2.114718173	851.8930013	-8.654063018	1.59E-08	Low	2	1031	NM_214257.1	ssc:397492			

LOC100737092	0	482.5465691	-20	1.59E-08	Low	0	584	XP_003480928.1	ssc:100737092	0	NM_022350.3	ERAP2
ISLR2	0	480.8940123	-20	1.62E-08	Low	0	582	XP_005666221.1	ssc:100157309	0	NM_020851.2	ISLR2
LOC100513632	7.401513606	1742.621086	-7.879222859	1.65E-08	Low	7	2109	XP_005672612.1	ssc:100513632	0	NM_003062.3	SLIT3
LOC100512163	0	478.4151772	-20	1.66E-08	Low	0	579	XR_298279.1	ssc:100512163			
LOC100738561	0	478.4151772	-20	1.66E-08	Low	0	579	XR_298278.1	ssc:100738561			
GRIK1	224.1601264	0	20	1.73E-08	High	212	0	XP_005657195.1	ssc:100624513	0	NM_175611.2	GRIK1
LOC100521659	2.114718173	837.0199906	-8.62865286	1.74E-08	Low	2	1013	XP_005662529.1	ssc:100521659	0	NM_019885.3	CYP26B1
SLC7A3	8938.913718	96.67456949	6.530819277	1.74E-08	High	8454	117	NM_001130973.1	ssc:100171387	0	NM_032803.5	SLC7A3
LOC494560	40.17964529	7495.17111	-7.543352776	1.76E-08	Low	38	9071	XP_003127107.1	ssc:494560	2E-76	NM_144617.2	HSPB6
LOC102165604	223.1027673	0	20	1.78E-08	High	211	0	XR_299597.1	ssc:102165604			
SCMH1	0	461.8896098	-20	1.98E-08	Low	0	559	XP_003128156.3	ssc:100525880			
LOC100515735	0	461.8896098	-20	1.98E-08	Low	0	559	XR_304349.1	ssc:100515735			
VDR	4.229436346	1154.310885	-8.092350706	2.04E-08	Low	4	1397	NM_001097414.1	ssc:396628	0	NM_001017536.1	VDR
FOLR2	2.114718173	806.4476908	-8.574971738	2.09E-08	Low	2	976	NM_213853.1	ssc:396853	3E-106	NM_001113536.1	FOLR2
CHGA	2074.538528	22.30951604	6.538987423	2.18E-08	High	1962	27	NM_001164005.2	ssc:397540	1E-117	NM_001275.3	CHGA
MDFI	14.80302721	2858.096888	-7.593018777	2.19E-08	Low	14	3459	XP_005666020.1	ssc:100152701	8E-102	NM_005586.3	MDFI
SPEG	8.458872693	1794.676623	-7.729042789	2.34E-08	Low	8	2172	XP_005672303.1	ssc:100737722	0	NM_005876.4	SPEG
UGT1A10	0	445.3640424	-20	2.38E-08	Low	0	539	XP_003133790.3	ssc:100511841	3E-128	NM_019075.2	UGT1A10
GLCCI1	13417.88681	152.0352204	6.463608092	2.47E-08	High	12690	184	XP_005667693.1	ssc:100511149	0	NM_138426.3	GLCCI1
PDGFRB	75.07249515	13038.67271	-7.440296866	2.48E-08	Low	71	15780	XP_005661873.1	ssc:100126842	0	NM_002609.3	PDGFRB
CALB2	211.4718173	0	20	2.49E-08	High	200	0	NM_001194980.1	ssc:100127479	1E-154	NR_027910.2	CALB2
ZNF333	0	440.4063721	-20	2.51E-08	Low	0	533	ZNF333	ssc:100516595	0	NM_032433.2	ZNF333
LOC100622872	611.153552	4.95767023	6.945728781	2.61E-08	High	578	6	XP_005654533.1	ssc:100622872	0	NM_153181.2	NETO1
LOC100512967	611.153552	4.95767023	6.945728781	2.61E-08	High	578	6	XR_303930.1	ssc:100512967			
POU2F3	209.3570991	0	20	2.65E-08	High	198	0	XP_003130003.2	ssc:397514	1E-157	NM_014352.3	POU2F3
LOC100521657	609.0388339	4.95767023	6.9407281	2.67E-08	High	576	6	XP_003123900.2	ssc:100521657	0	NM_173800.4	AQPEP
LOC100737813	279.1427989	0.826278372	8.400159719	2.67E-08	High	264	1	XP_005652749.1	ssc:100737813	9E-47	NM_153181.2	NETO1
AHNAK2	0	434.6224235	-20	2.69E-08	Low	0	526	XP_005656542.1	ssc:100522557	6E-135	NM_138420.2	AHNAK2
LOC100513741	0	434.6224235	-20	2.69E-08	Low	0	526	XR_303907.1	ssc:100513741	0	NR_049754.1	ZNF211
CRISPLD2	3.17207726	915.5164359	-8.173014066	2.91E-08	Low	3	1108	XP_005664373.1	ssc:100625354	0	NM_031476.3	CRISPLD2
IFI35	2.114718173	755.2184318	-8.480284756	2.91E-08	Low	2	914	XP_003358072.1	ssc:100624758	6E-118	NM_005533.4	IFI35
WSCD2	0	426.3596398	-20	2.96E-08	Low	0	516	WSCD2				
CLDN11	0	426.3596398	-20	2.96E-08	Low	0	516	NM_001161641.1	ssc:100302016	5E-101	NM_005602.5	CLDN11
CEBPD	188.2099174	32373.5866	-7.426330747	2.97E-08	Low	178	39180	XP_005663148.1	ssc:100153946	3E-115	NM_005195.3	CEBPD
LOC100626567	0	423.0545263	-20	3.08E-08	Low	0	512	LOC100626567				
CD86	0	423.0545263	-20	3.08E-08	Low	0	512	NM_214222.1	ssc:397441	2E-99	NM_176892.1	CD86
SOWAHB	204.0703037	0	20	3.11E-08	High	193	0	XP_005666837.1	ssc:100523320	0	NM_001029870.1	SOWAHB
ZNF181	0	422.228248	-20	3.11E-08	Low	0	511	NM_001244818.1	ssc:100513473	0	NM_001145665.1	ZNF181
HOXB9	0	420.5756912	-20	3.17E-08	Low	0	509	XP_003131603.2	ssc:100523635	1E-145	NM_024017.4	HOXB9
LOC100623028	27.49133625	4631.290274	-7.39629334	3.20E-08	Low	26	5605	XP_003361790.1	ssc:100623028	2E-109	NM_015927.4	TGFB1I1
COL9A1	2745.961548	32.2248565	6.412993679	3.23E-08	High	2597	39	XP_003121321.2	ssc:100155319	0	NM_078485.3	COL9A1
BHLHE41	2.114718173	738.6928643	-8.448365422	3.25E-08	Low	2	894	XP_003355589.1	ssc:100626912	0	NM_030762.2	BHLHE41
RBAK	0	416.4442994	-20	3.33E-08	Low	0	504	XP_005653115.1	ssc:100620178	0	NM_021163.3	RBAK
NRK	0	415.618021	-20	3.36E-08	Low	0	503	XP_003361569.1	ssc:100620665			
TRIM63	0	414.7917426	-20	3.39E-08	Low	0	502	NM_001184756.1	ssc:100431101	0	NM_032588.3	TRIM63
LOC100623035	0	414.7917426	-20	3.39E-08	Low	0	502	XP_005664949.1	ssc:100623035	0	NM_001267779.1	ZNF850
LOC100737413	0	414.7917426	-20	3.39E-08	Low	0	502	XP_003481951.1	ssc:100737413	0	NM_021632.3	ZNF350
LOC100519278	5.286795433	1194.798526	-7.820158143	3.44E-08	Low	5	1446	LOC100519278		0	NM_080424.2	SP110
EHF	332.0107532	1.652556743	7.650386348	3.48E-08	High	314	2	XP_005661076.1	ssc:100526094	3E-175	NM_012153.5	EHF
RAB3C	582.6048567	4.95767023	6.876711607	3.53E-08	High	551	6	XP_003483878.1	ssc:100523703	2E-128	NM_138453.2	RAB3C
FUT9	199.8408674	0	20	3.54E-08	High	189	0	XP_005659365.1	ssc:102160171	0	NM_006581.3	FUT9
GBP7	1.057359087	568.4795197	-9.070499155	3.63E-08	Low	1	688	XP_005663773.1	ssc:100155195	0	NM_052941.4	GBP4
LOC100515181	0	409.007794	-20	3.64E-08	Low	0	495	XP_005673866.1	ssc:100515181	0	NM_198465	NRK
LOC102161103	27.49133625	4513.958745	-7.35927239	3.64E-08	Low	26	5463	XP_005656329.1	ssc:102161103	0	NM_018689.1	KIAA1199
TBX3	2.114718173	721.3410185	-8.414072244	3.67E-08	Low	2	873	XP_001928037.1	ssc:100152741	0	NM_016569.3	TBX3
HMGCLL1	197.7261492	0	20	3.78E-08	High	187	0	XP_003356654.1	ssc:100627473	0	NR_109869.1	HMGCLL1
LOC100514340	0	405.7026805	-20	3.79E-08	Low	0	491	NM_001244954.1	ssc:100514340			
EIF1AY	0	405.7026805	-20	3.79E-08	Low	0	491	NM_001244349.1	ssc:100625415	2E-79	NM_001412.3	EIF1AX
LRRN4CL	33.83549077	5439.390521	-7.328763883	3.84E-08	Low	32	6583	XP_003353868.1	ssc:100620756	1E-67	NM_203422.3	LRRN4CL
LOC100521069	0	404.0501238	-20	3.87E-08	Low	0	489	ZNF564	ssc:100521069	1E-177	NM_152601.3	ZNF709
SERPINE1	63.44154519	10062.41801	-7.309333403	3.90E-08	Low	60	12178	NM_213910.1	ssc:396945			
LOC100518586	0	400.7450103	-20	4.03E-08	Low	0	485	XP_003134207.3	ssc:100518586	2E-96	NM_001145161.2	UBE2QL1
LOC100518738	0	399.9187319	-20	4.08E-08	Low	0	484	XP_005664996.1	ssc:100518738	0	NM_014480.2	ZNF544
MYL1	0	399.0924535	-20	4.12E-08	Low	0	483	NM_214374.2	ssc:397666	3E-81	NM_079422.2	MYL1
MXRA5	93.04759962	14656.52576	-7.299358513	4.14E-08	Low	88	17738	XP_005658001.1	ssc:100519997	0	NM_015419.3	MXRA5
ZFP28	0	398.2661752	-20	4.16E-08	Low	0	482	ZFP28	ssc:100624090	0	NM_020828.1	ZFP28
COL8A1	139.5713994	22202.92613	-7.313602673	4.16E-08	Low	132	26871	XP_005670312.1	ssc:397430	0	NM_020351.3	COL8A1
PTGS2	23.2618999	3762.045426	-7.337404526	4.17E-08	Low	22	4553	NM_214321.1	ssc:397590	0	NM_000963	PTGS2
ZNF268	0	397.4398968	-20	4.20E-08	Low	0	481	ZNF268	ssc:100154415	0	NM_152943.2	ZNF268
SOX2	1237.110131	14.04673232	6.460595572	4.33E-08	High	1170	17	NM_001123197.1	ssc:407739	1E-158	NM_003106.3	SOX2
PARP14	16.91774539	2802.736237	-7.372154855	4.34E-08	Low	16	3392	XP_005670231.1	ssc:100153948	0	NM_017554.2	PARP14
ZBTB4	9.516231779	1736.837137	-7.511856353	4.36E-08	Low	9	2102	ZBTB4	ssc:100739718	0	NM_020899.3	ZBTB4
THBS1	1211.733513	211976.7586	-7.450689831	4.42E-08	Low	1146	256544	NM_001244536.1	ssc:492313	0	NM_003246.2	THBS1
PDZD4	499.0734889	4.131391859	6.916480554	4.57E-08	High	472	5	XP_005674070.1	ssc:100525676	0	NM_032512.2	PDZD4
UNC45B	1.057359087	542.0386119	-9.001786405	4.61E-08	Low	1	656	HighI00005FE9DD		0	NM_173167.2	UNC45B
CYYR1	1470.786489	17.35184581	6.405354881	4.62E-08	High	1391	21	XP_001924644.3	ssc:100154451	3E-40	NM_052954.2	CYYR1
LOC102165956	191.3819947	0	20	4.63E-08	High	181	0	XP_005659863.1	ssc:102165956	1E-100	NM_005503.3	APBA2
CCNA1	498.0161298	4.131391859	6.913420754	4.63E-08	High	471	5	XP_005668399.1	ssc:100156017	0	NM_003914.3	CCNA1
RIPK3	2.114718173	686.6373269	-8.342939068	4.70E-08	Low	2	831	XP_001927459.1	ssc:100153263	3E-155	NM_006871.3	RIPK3
NNAT	54.9826725	8396.640814	-7.254691431	4.72E-08	Low	52	10162	NM_001122990.1	ssc:449004	2E-41	NM_181689.1	NNAT
LOC102159197	435.6319437	3.305113487	7.042266126	4.79E-08	High	412	4	XR_302293.1	ssc:102159197			
GPR68	0	386.698278	-20	4.82E-08	Low	0	468	XP_005666454.1	ssc:100153615	0	NM_003485.3	GPR68
LMNA	549.826725	90316.35742	-7.359866459	4.86E-08	Low	520	109305	NM_001111257.1	ssc:100126859	0	NR_047545.1	LMNA
LOC100514506	0	385.8719996	-20	4.87E-08	Low	0	467	XR_115703.3	ssc:100514506			
EDNRA	0	385.0457212	-20	4.93E-08	Low	0	466	NM_214229.1	ssc:397457	2E-163	NR_045958.1	EDNRA
IGF2	0	382.5668861	-20	5.09E-08	Low	0	463	NM_213883.2	ssc:396916			
LOC102166950	0	382.5668861	-20	5.09E-08	Low	0	463	XR_299386.1	ssc:102166950			
RNF128	899.8125827	9.915340461	6.50381842	5.31E-08	High	851	12	XP_003135339.3	ssc:100515872	0	NM_194463.1	RNF128
SCARA3	179.7510447	27463.84052	-7.255389419	5.35E-08	Low	170	33238	XP_003359089.1	ssc:100628076	0	NM_182826.1	SCARA3
LOC102161294	1.057359087	524.686766	-8.954847182	5.42E-08	Low	1	635	XP_005670314.1	ssc:102161294			
PARP10	12.68830904	2094.615672	-7.367041932	5.45E-08	Low	12	2535	XP_005655371.1	ssc:100516730	0	NM_032789.3	PARP10
MYOG	0	376.7829375	-20	5.49E-08	Low	0	456	NM_001012406.1	ssc:497618	3E-118	NM_002479.5	MYOG
HOXB5	0	375.1303808	-20	5.61E-08	Low	0	454	XP_003131598.1	ssc:100522700	5E-157	NM_002147.3	HOXB5
PDE1A	1.057359087	518.9028174	-8.93885515	5.73E-08	Low	1	628	XP_005672048.1	ssc:100152819	0	NM_005019.4	PDE1A
LOC100515339	0	373.477824	-20	5.74E-08	Low	0	452	XP_003131485.1	ssc:100515339	0	NM_002276.4	KRT19
FAM65B	712.6600243	7.436505346	6.582445379	5.79E-08	High	674	9	XP_005665694.1	ssc:100154661	0	NM_015864.3	FAM65B
PAX1	0	370.9989889	-20	5.93E-08	Low	0	449	XP_005672808.1	ssc:100739820	0	NM_006192.4	PAX1
LOC102164895	0	370.1727105	-20	6.00E-08	Low	0	448	XP_005666576.1	ssc:102164895	1E-114	NM_001313.4	CRMP1
SLA-5	1.057359087	513.1188688	-8.922683859	6.06E-08	Low	1	621	NM_001114056.1	ssc:100135029	5E-138	NM_002116.7	HLA-A
SNED1	3.17207726	786.6170099	-7.954089663	6.23E-08	Low	3	952	XP_003133861.2	ssc:100512566	0	NM_001080437.1	SNED1

LOC100523560	0	366.867597	-20	6.27E-08	Low	0	444	XR_297529.1	ssc:100523560			
CHRM4	587.8916521	5.783948602	6.66735175	6.34E-08	High	556	7	XP_003122876.1	ssc:100516483	0	NM_000741.2	CHRM4
LOC100739847	1983.605646	25.61462952	6.275013401	6.47E-08	High	1876	31	XP_005668534.1	ssc:100739847	4E-65	NM_144595.3	SLAIN1
LOC100738461	49.69587707	7119.214451	-7.162448084	6.54E-08	Low	47	8616	XP_005656330.1	ssc:100738461	0	NM_018689.1	KIAA1199
LOC102167050	180.8084038	0	20	6.59E-08	High	171	0	XR_300973.1	ssc:102167050			
WNT5B	6.344154519	1177.44668	-7.536018104	6.86E-08	Low	6	1425	XP_005664181.1	ssc:100516268	2E-84	NM_032642.2	WNT5B
LOC100620451	0	358.6048133	-20	7.03E-08	Low	0	434	NM_001244338.1	ssc:100620451	2E-52	NR_037939.1	HOXA10
LRRC4	238.9631536	0.826278372	8.175944562	7.06E-08	High	226	1	XP_005673248.1	ssc:100521592	0	NM_022143.4	LRRC4
LOC100517243	0	354.4734215	-20	7.45E-08	Low	0	429	XP_003133148.1	ssc:100517243	2E-25	NM_006829.2	ADIRF
ISG12%28A%29	153.3170676	21800.52856	-7.151700994	7.46E-08	Low	145	26384	ISG12%28A%29		3E-19	NM_005532	IFI27
FRMD5	740.1513606	8.262783717	6.485048616	7.47E-08	High	700	10	XP_005659740.1	ssc:100152168	0	NR_104455.1	FRMD5
LOC102165913	61.32682702	8517.277456	-7.117730227	7.61E-08	Low	58	10308	XR_301647.1	ssc:102165913			
UNC5B	31.7207726	4459.424372	-7.135285777	7.66E-08	Low	30	5397	NM_001044549.1	ssc:733600	0	NM_170744.4	UNC5B
LOC100622791	32.77813168	4600.717974	-7.132981586	7.67E-08	Low	31	5568	XR_304346.1	ssc:100622791	6E-16	NM_002116.7	HLA-A
LOC100623474	175.5216084	0	20	7.92E-08	High	166	0	XP_005655616.1	ssc:100623474	1E-157	NM_024902.2	DNAJC22
LOC102167067	175.5216084	0	20	7.92E-08	High	166	0	XR_303490.1	ssc:102167067			
COLQ	2.114718173	618.0562221	-8.191128861	7.96E-08	Low	2	748	XP_003358361.2	ssc:100624545	1E-37	NM_080544.2	COLQ
LOC100626144	1461.270258	19.00440255	6.264745544	8.00E-08	High	1382	23	XP_005656792.1	ssc:100626144	0	NM_207108.2	ASTN1
RHOJ	0	348.6894729	-20	8.08E-08	Low	0	422	NM_001168417.1	ssc:100155474			
LOC100526171	2.114718173	613.9248302	-8.181452801	8.23E-08	Low	2	743	XR_303843.1	ssc:100526171	0	NM_001267779.1	ZNF850
LOC100620529	1.057359087	480.8940123	-8.829109744	8.38E-08	Low	1	582	XP_005658322.1	ssc:100620529	0	NM_004408	DNM1
TSPAN18	10.57359087	1655.035579	-7.290253012	8.43E-08	Low	10	2003	XP_003122898.2	ssc:100520775	4E-95	NM_130783.4	TSPAN18
KRT18	451.49233	4.131391859	6.771929764	8.57E-08	High	427	5	XP_005652636.1	ssc:100126286	0	NM_199187.1	KRT18
LOC100622576	0	342.0792459	-20	8.89E-08	Low	0	414	XP_005658397.1	ssc:100622576	4E-178	NM_022774.1	EXO5
ACPT	2.114718173	604.0094897	-8.157961997	8.93E-08	Low	2	731	XP_005664855.1	ssc:100620403	0	NM_080791.1	ACPT
BTBD11	4416.588905	61.97087788	6.155198337	8.97E-08	High	4177	75	HighI00025E0D7E		0	NM_152322.2	BTBD11
ALPL	717.9468198	8.262783717	6.441105269	9.05E-08	High	679	10	XP_005658515.1	ssc:100170147	0	NM_001177520.1	ALPL
ZNF211	0	340.4266892	-20	9.11E-08	Low	0	412	ZNF211	ssc:100514110	1E-56	NR_049754.1	ZNF211
LOC102162054	2.114718173	599.8780979	-8.148060139	9.24E-08	Low	2	726	XR_301502.1	ssc:102162054			
LOC100739594	0	338.7741324	-20	9.33E-08	Low	0	410	LOC100739594		2E-65	NM_022658.3	HOXC8
C8H4orf19	1370.337376	18.17812418	6.236183983	9.34E-08	High	1296	22	XP_003482412.1	ssc:100738623	2E-85	NM_018302.2	C4orf19
EFHB	170.2348129	0	20	9.56E-08	High	161	0	XP_003132117.2	ssc:100519922	0	NM_144715.3	EFHB
FOXF2	0	337.1215757	-20	9.56E-08	Low	0	408	XP_005665585.1	ssc:100158066	6E-118	NM_001452.1	FOXF2
LOC100523671	1.057359087	466.0210017	-8.783785753	9.81E-08	Low	1	564	LOC100523671				
CREB3L1	82.47400875	10908.52706	-7.047301065	9.87E-08	Low	78	13202	XP_003122877.1	ssc:100516663	0	NM_052854.3	CREB3L1
RNF182	280.2001579	1.652556743	7.405614149	1.01E-07	High	265	2	XP_005665645.1	ssc:100154575	4E-136	NM_152737.3	RNF182
ZNF613	0	332.9901838	-20	1.02E-07	Low	0	403	ZNF613	ssc:100625971	0	NM_024840.3	ZNF613
TNNI1	0	331.3376271	-20	1.04E-07	Low	0	401	NM_213912.3	ssc:396947	1E-103	NM_003281.3	TNNI1
LOC102157467	0	330.5113487	-20	1.06E-07	Low	0	400	XR_298763.1	ssc:102157467			
STOX1	803.5929058	9.915340461	6.340658707	1.08E-07	High	760	12	XP_005657443.1	ssc:100157127	0	NM_152709.4	STOX1
LOC100737756	0	328.8587919	-20	1.08E-07	Low	0	398	XP_005664973.1	ssc:100737756	0	NM_001267779.1	ZNF850
FOXS1	13.74566813	1942.580452	-7.142853506	1.12E-07	Low	13	2351	XP_003134430.1	ssc:100524116	2E-154	NM_004118.3	FOXS1
FAM124A	851.1740647	10.74161883	6.308170854	1.13E-07	High	805	13	XP_001925756.4	ssc:100152644	0	NM_145019.3	FAM124A
USP44	14892.90273	212.3535415	6.132012993	1.13E-07	High	14085	257	XP_005664303.1	ssc:100516911	0	NM_032147.3	USP44
METRNL	24.31925899	3214.222866	-7.046226885	1.13E-07	Low	23	3890	XP_005668624.1	ssc:100512305	1E-118	NM_001004431.1	METRNL
LOC100626302	638.6448883	7.436505346	6.424245337	1.15E-07	High	604	9	LOC100626302				
LOC100626722	0	324.7274001	-20	1.15E-07	Low	0	393	XR_131435.2	ssc:100626722			
DAB2	91.99024053	11665.39805	-6.986539011	1.23E-07	Low	87	14118	XP_005672491.1	ssc:100519746	0	NM_001343.3	DAB2
LOC100626132	35.95020894	4564.361725	-6.988269164	1.25E-07	Low	34	5524	XP_003361657.1	ssc:100626132	7E-45	NM_144617.2	HSPB6
ZNF577	0	318.9434515	-20	1.26E-07	Low	0	386	ZNF577	ssc:100624128	9E-180	NR_024181.1	ZNF577
LOC100739751	0	318.9434515	-20	1.26E-07	Low	0	386	XP_003481268.2	ssc:100739751	4E-171	NM_022055.1	KCNK12
HR	6.344154519	1042.763305	-7.360768095	1.26E-07	Low	6	1262	NM_001083930.1	ssc:397617	0	NM_018411.4	HR
C1H15orf52	7.401513606	1162.573669	-7.295286092	1.26E-07	Low	7	1407	XP_003353441.1	ssc:100153607			
DNM1	35.95020894	4540.399653	-6.980675325	1.28E-07	Low	34	5495	XP_005660537.1	ssc:100153921	0	NM_004408	DNM1
CXCL12	2.114718173	556.9116225	-8.040839180	1.34E-07	Low	2	674	NM_001009580.1	ssc:494460	9E-40	NM_199168.3	CXCL12
PDLIM2	25.37661808	3231.574712	-6.992593721	1.34E-07	Low	24	3911	XP_005670470.1	ssc:100737811	6E-141	NM_198042.3	PDLIM2
LOC100736640	0	313.9857813	-20	1.36E-07	Low	0	380	XR_305303.1	ssc:100736640	2E-33	NM_004621.5	TRPC6
PREX2	469.4674344	4.95767023	6.565218965	1.37E-07	High	444	6	XP_001925866.1		0	NM_025170.4	PREX2
HHAT	1.057359087	434.6224235	-8.68315339	1.39E-07	Low	1	526	XP_005667936.1	ssc:100512733	0	NM_018194	HHAT
EHD2	157.5465039	19790.19328	-6.972864152	1.40E-07	Low	149	23951	XP_003356007.1	ssc:100624668	0	NM_014601.3	EHD2
LOC100514323	4332.000178	65.27599137	6.052337029	1.40E-07	High	4097	79	XP_003132557.2	ssc:100514323			
EXO5	0	311.5069461	-20	1.42E-07	Low	0	377	XP_003128160.1	ssc:100511388	4E-178	NM_022774.1	EXO5
LOC100621379	1.057359087	431.31731	-8.672140397	1.44E-07	Low	1	522	XP_003360610.1	ssc:100621379	6E-47	NM_005978.3	S100A2
LOC100152729	1.057359087	431.31731	-8.672140397	1.44E-07	Low	1	522	XP_001929591.1	ssc:100152729	6E-47	NM_005978.3	S100A2
ZNF329	0	309.8543894	-20	1.46E-07	Low	0	375	ZNF329	ssc:100516617	0	NM_024620.3	ZNF329
RIN3	6.344154519	1010.538449	-7.315480589	1.48E-07	Low	6	1223	XP_005656507.1	ssc:100155709	0	NM_024832.3	RIN3
LOC100523750	262.2250535	1.652556743	7.30996191	1.52E-07	High	248	2	XP_005669193.1	ssc:100523750	0	NM_198501.2	SMTNL2
EIF2S3	28.54869534	3510.030523	-6.941914949	1.54E-07	Low	27	4248	XP_005658005.1	ssc:100624149	0	NM_001415.3	EIF2S3
LOC100737013	0	305.7229975	-20	1.56E-07	Low	0	370	XP_005653310.1	ssc:100737013	1E-140	NM_001266.4	CES1
EPCAM	261.1676944	1.652556743	7.304132831	1.56E-07	High	247	2	NM_214419.1	ssc:403163	6E-136	NM_002354.2	EPCAM
LOC100514098	1.057359087	424.7070831	-8.64985895	1.56E-07	Low	1	514	XP_005653093.1	ssc:100514098	0	NM_001098814.1	SRL
PRSS22	6.344154519	998.9705514	-7.298870429	1.56E-07	Low	6	1209	XP_005655249.1	ssc:100519474	3E-137	NM_022119.3	PRSS22
FAM64A	8.458872693	1222.89199	-7.175615861	1.61E-07	Low	8	1480	XP_005669202.1	ssc:100521819	2E-95	NM_019013.2	FAM64A
LOC102160150	2.114718173	533.7758281	-7.979624755	1.66E-07	Low	2	646	XP_005665346.1	ssc:102160150	1E-40	NM_015210.3	SOGA2
LOC100623862	0	300.7653273	-20	1.69E-07	Low	0	364	XP_005674074.1	ssc:100623862	5E-164	XP_003960890.2	LOC100509091
NFATC4	39.1222862	4628.811438	-6.886507303	1.75E-07	Low	37	5602	XP_001927389.2	ssc:100154469	0	NM_004554.4	NFATC4
LOC102163456	0	298.2864922	-20	1.76E-07	Low	0	361	XP_005673399.1	ssc:102163456			
TLR4	0	296.6339355	-20	1.81E-07	Low	0	359	NM_001113039.1	ssc:399541	0	NM_138557.2	TLR4
LOC100519459	2750.190984	42.96647533	6.000176565	1.83E-07	High	2601	52	XR_135471.2	ssc:100519459			
tRNA-Cys	2132.693278	33.05113487	6.011832873	1.85E-07	High	2017	40	tRNA-Cys				
LOC100739671	3.17207726	632.1029544	-7.638587838	1.86E-07	Low	3	765	XP_005672506.1	ssc:100739671	8E-55	NM_007036.4	ESM1
LOC100620918	397.5670166	4.131391859	6.588426356	1.90E-07	High	376	5	XP_005655043.1	ssc:100620918	0	NR_003149.1	GPR98
LOC100736658	0	293.328822	-20	1.91E-07	Low	0	355	XP_005659684.1	ssc:100736658	2E-54	NM_002009.3	FGF7
KIAA1549L	1073.219473	15.69928906	6.095102098	1.93E-07	High	1015	19	XP_005661089.1	ssc:100513003	0	NM_012194.2	KIAA1549L
ZNF71	0	292.5025436	-20	1.94E-07	Low	0	354	ZNF71	ssc:100739752	0	NM_021216.4	ZNF71
LOC100153940	31.7207726	3705.858497	-6.868236075	1.94E-07	Low	30	4485	XP_005659162.1	ssc:100153940	0	NM_003247.3	THBS2
HOXA3	1.057359087	404.8764021	-8.58087234	1.98E-07	Low	1	490	XP_005673358.1	ssc:100519809	0	NM_153632.2	HOXA3
BEND4	151.2023494	0	20	1.98E-07	High	143	0	XP_005666678.1	ssc:100514664	0	NM_207406.3	BEND4
CD14	5.286795433	841.1513824	-7.313828152	2.00E-07	Low	5	1018	NM_001097445.2	ssc:100037938	1E-144	NM_001174105.1	CD14
LOC100626715	250.5941035	1.652556743	7.244508848	2.01E-07	High	237	2	XP_005674325.1	ssc:100626715			
ETS1	5.286795433	840.325104	-7.31241027	2.01E-07	Low	5	1017	NM_001162886.1	ssc:100302363			
IER3	59.21210885	6745.736627	-6.831939946	2.06E-07	Low	56	8164	XP_001927586.2	ssc:100154770	3E-60	NM_052815.1	IER3
OLIG2	150.1449903	0	20	2.07E-07	High	142	0	XP_003358977.2	ssc:100152482	3E-92	NM_005806.3	OLIG2
LOC102165288	150.1449903	0	20	2.07E-07	High	142	0	XP_005672232.1	ssc:102165288	1E-24	NM_018000.2	MREG
LOC102165647	0	288.3711517	-20	2.08E-07	Low	0	349	XP_005666496.1	ssc:102165647	4E-172	NM_032425.4	HHIPL1
LOC780415	50.75323616	5739.32957	-6.821238595	2.15E-07	Low	48	6946	NM_001078670.1	ssc:780415	4E-177	NM_006084.4	IRF9
LOC100622121	149.0876312	0	20	2.16E-07	High	141	0	XP_005666622.1	ssc:100622121	5E-168	NM_152692.4	C1GALT1C1
LOC102161556	149.0876312	0	20	2.16E-07	High	141	0	XR_299329.1	ssc:102161556			
CACNB4	149.0876312	0	20	2.16E-07	High	141	0	NM_213750.1	ssc:396585	0	NM_001145798.1	CACNB4
ANPEP	35.95020894	4080.1626	-6.826482681	2.19E-07	Low	34	4938	NM_214277.1	ssc:397520	0	NM_001150.2	ANPEP

ZNF568	0	285.0660382	-20	2.20E-07	Low	0	345	ZNF568	ssc:100511135	0	NM_198539.3	ZNF568
LOC102161608	1049.957573	15.69928906	6.063487993	2.22E-07	High	993	19	XP_005671034.1	ssc:102161608	0	NM_024554.3	PGBD5
CD248	66.61362245	7462.946253	-6.807784248	2.25E-07	Low	63	9032	XP_003122563.1	ssc:100523587	0	NM_020404.2	CD248
LOC100623866	10.57359087	1360.880478	-7.007931146	2.25E-07	Low	10	1647	XP_005674350.1	ssc:100623866	0	NM_015210.3	SOGA2
BAI3	666.1362245	9.089062089	6.195541999	2.28E-07	High	630	11	XP_003353288.1		0	NM_001704.2	BAI3
RYR1	0	282.5872031	-20	2.30E-07	Low	0	342	NM_001001534.1	ssc:396718	0	NM_001042723.1	RYR1
LOC102157546	0	281.7609248	-20	2.33E-07	Low	0	341	XR_298764.1	ssc:102157546			
LOC100520391	0	281.7609248	-20	2.33E-07	Low	0	341	XP_005658326.1	ssc:100520391	1E-82	NM_001313.4	CRMP1
SLC24A4	244.249949	1.652556743	7.207514641	2.36E-07	High	231	2	XP_005656506.1	ssc:100156914	0	NM_153648.3	SLC24A4
LOC102167451	3474.481958	57.01320765	5.929357907	2.41E-07	High	3286	69	XP_005666318.1	ssc:102167451	2E-16	NR_049767.1	SLC7A8
SEC14L2	1.057359087	388.3508347	-8.520751347	2.43E-07	Low	1	470	NM_001198918.1	ssc:100152451	0	NM_033382.2	SEC14L2
LOC100736668	0	279.2820896	-20	2.44E-07	Low	0	338	XP_005669299.1	ssc:100736668	6E-46	NM_201433.1	GAS7
ERG	9.516231779	1231.981052	-7.016373942	2.44E-07	Low	9	1491	NM_001244456.1	ssc:100625364			
CPXM2	3.17207726	598.2255411	-7.559117787	2.45E-07	Low	3	724	XP_005671590.1	ssc:100153578	0	NM_198148.2	CPXM2
NEK6	61.32682702	6726.732224	-6.777243709	2.49E-07	Low	58	8141	XP_005660482.1	ssc:100514624	0	NM_014397.5	NEK6
LOC100739179	334.1254714	3.305113487	6.659546347	2.51E-07	High	316	4	XP_003482145.1	ssc:100739179	2E-126	NM_020883.1	ZSWIM5
CYP2C91	0	276.8032545	-20	2.55E-07	Low	0	335	XR_307738.1	ssc:100516879			
KLF9	4.229436346	697.3789457	-7.365333589	2.55E-07	Low	4	844	U57346.1		1E-141	NM_001206.2	KLF9
XK	1258.257313	19.83068092	5.987548957	2.60E-07	High	1190	24	XP_003135062.1	ssc:100511169	0	NM_021083.2	XK
PHACTR1	9.516231779	1215.455485	-6.996890931	2.61E-07	Low	9	1471	XP_005665632.1	ssc:100153737	0	NM_030948.2	PHACTR1
AKR1C4	63.44154519	6869.678383	-6.758670838	2.66E-07	Low	60	8314	NM_001123075.1	ssc:100144511	6E-140	NM_003739.5	AKR1C3
ZNF624	0	272.6718627	-20	2.75E-07	Low	0	330	ZNF624	ssc:100626993	0	NM_020787.3	ZNF624
LOC102157983	0	269.3667492	-20	2.92E-07	Low	0	326	XP_005662850.1	ssc:102157983	4E-71	NM_012293.1	PXDN
LOC100624511	0	267.7141924	-20	3.01E-07	Low	0	324	XP_003358225.2	ssc:100624511	3E-176	NM_015077.3	SARM1
TRH	4.229436346	673.416873	-7.31489065	3.04E-07	Low	4	815	NM_001260479.1	ssc:100513309	2E-70	NM_007117.4	TRH
HOXA2	0	266.8879141	-20	3.05E-07	Low	0	323	XP_003134891.1	ssc:100519993	0	NM_006735.3	HOXA2
LOC100622976	0	266.8879141	-20	3.05E-07	Low	0	323	XP_003356553.1	ssc:100622976	4E-104	NM_001452.1	FOXF2
CRYAB	10.57359087	1274.947528	-6.913828652	3.12E-07	Low	10	1543	CRYAB		1E-95	NM_001885.1	CRYAB
LOC100738589	20.08982264	2205.336974	-6.778390477	3.12E-07	Low	19	2669	XP_005660660.1	ssc:100738589	1E-84	NM_053056.2	CCND1
RARG	41.23700438	4338.78773	-6.717208662	3.13E-07	Low	39	5251	XP_005652619.1	ssc:397221	0	NM_001243732.1	RARG
LOC102167727	0	265.2353573	-20	3.15E-07	Low	0	321	XR_303912.1	ssc:102167727			
TMEM132E	0	264.409079	-20	3.20E-07	Low	0	320	XP_005669107.1	ssc:100519732	0	NM_207313.1	TMEM132E
KHDRBS3	0	264.409079	-20	3.20E-07	Low	0	320	XR_302764.1	ssc:100155706	1E-169	NM_006558.1	KHDRBS3
PPP1R1A	1307.95319	21.48323766	5.927955666	3.20E-07	High	1237	26	XP_005674358.1	ssc:100621808	8E-39	NM_006741.3	PPP1R1A
SLC24A2	139.5713994	0	20	3.24E-07	High	132	0	NM_001244595.1	ssc:100625506			
TMEM30B	0	262.7565222	-20	3.30E-07	Low	0	318	XP_003480534.1	ssc:100738730	0	NM_001017970.2	TMEM30B
LOXL1	130.0551676	13617.89384	-6.710236057	3.38E-07	Low	123	16481	XP_005666227.1	ssc:100154899	0	NM_005576.2	LOXL1
ETNK2	318.2650851	3.305113487	6.589385276	3.39E-07	High	301	4	XP_005656723.1	ssc:100622861	0	NM_018208.2	ETNK2
LOC100524543	273.8560034	2.478835115	6.787611386	3.44E-07	High	259	3	XP_005674240.1	ssc:100524543	4E-146	NM_199141.1	CARM1
PROK2	229.4469218	1.652556743	7.117316832	3.47E-07	High	217	2	XP_005669784.1	ssc:100526076	4E-42	NM_021935.3	PROK2
LOC102157620	137.4566813	0	20	3.55E-07	High	130	0	XR_304039.1	ssc:102157620			
LOC100157667	137.4566813	0	20	3.55E-07	High	130	0	XR_299681.1	ssc:100157667	4E-22	NM_182540.4	DDX26B
LOC100513356	7.401513606	945.2624573	-6.996750815	3.56E-07	Low	7	1144	XP_003127281.1	ssc:100513356	3E-174	NM_032040.4	CCDC8
ZBTB7B	3.17207726	551.9539523	-7.442976193	3.66E-07	Low	3	668	ZBTB7B	ssc:100622022	7E-110	NR_049765.1	ZBTB7B
IGFBP4	23.2618999	2432.563526	-6.708364738	3.73E-07	Low	22	2944	NM_001123129.1	ssc:100144490	1E-134	NM_001552.2	IGFBP4
LOC100737912	0	256.1462952	-20	3.74E-07	Low	0	310	XP_005664976.1	ssc:100737912	0	NM_001144989.1	ZNF814
FRMPD4	226.2748445	1.652556743	7.097232586	3.78E-07	High	214	2	XP_005673465.1	ssc:100526196	0	NM_014728.3	FRMPD4
LOC102166778	4.229436346	644.4971299	-7.251564715	3.78E-07	Low	4	780	XP_005662309.1	ssc:102166778	9E-150	NM_001393.3	ECM2
LOC102164903	181.8657629	0.826278372	7.782030354	3.82E-07	High	172	1	LOC102164903				
MYL4	0	254.4937385	-20	3.86E-07	Low	0	308	XP_003131354.1	ssc:100516998	4E-91	NM_002476.2	MYL4
LOC100521619	0	254.4937385	-20	3.86E-07	Low	0	308	LOC100521619				
LOC100626014	1.057359087	353.6471431	-8.385701387	3.88E-07	Low	1	428	XP_005653020.1	ssc:100626014	0	NM_171825.2	CAMK2A
TRAF3IP2	21.14718173	2211.947201	-6.70870773	3.88E-07	Low	20	2677	XP_005659446.1	ssc:100157000	0	NR_028338.2	TRAF3IP2
ZBED2	0	253.6674601	-20	3.93E-07	Low	0	307	ZBED2	ssc:100517972	1E-97	NM_024508.4	ZBED2
FAM46A	26.43397716	2706.061667	-6.677657402	3.96E-07	Low	25	3275	XP_003121442.1	ssc:100156873	0	NM_017633.2	FAM46A
LOC100623668	0	252.8411817	-20	3.99E-07	Low	0	306	XP_003482903.1	ssc:100623668	2E-123	NM_000231.2	SGCG
SYT11	1.057359087	351.168308	-8.375553432	4.01E-07	Low	1	425	XP_001928839.3	ssc:100154741	0	NM_152280.4	SYT11
ZNF287	0	252.0149034	-20	4.05E-07	Low	0	305	ZNF287	ssc:100524396	0	NM_020653.2	ZNF287
LOC102157666	134.284604	0	20	4.10E-07	High	127	0	XR_298274.1	ssc:102157666			
ZNF382	0	250.3623466	-20	4.19E-07	Low	0	303	ZNF382	ssc:100525701	0	NM_032825.4	ZNF382
SLC44A5	0	249.5360683	-20	4.26E-07	Low	0	302	XP_005653486.1	ssc:100513866	0	NM_152697.4	SLC44A5
LOC100625781	0	249.5360683	-20	4.26E-07	Low	0	302	XP_003361074.1	ssc:100625781	3E-133	NM_145260.2	OSR1
LOC100522001	945.2790234	15.69928906	5.911969107	4.27E-07	High	894	19	XP_005659664.1	ssc:100522001	4E-120	NM_207381.2	TNFAIP8L3
LOC100621773	126.8830904	12664.3686	-6.641131526	4.28E-07	Low	120	15327	XP_003361781.1	ssc:100621773			
LOC100516145	1.057359087	346.2106378	-8.355040834	4.31E-07	Low	1	419	XR_304856.1	ssc:100516145			
POU4F1	304.5194169	3.305113487	6.525690601	4.46E-07	High	288	4	HighI00025E0FFA		3E-78	NM_006237.3	POU4F1
LOC102165318	1.057359087	343.7318026	-8.344674119	4.46E-07	Low	1	416	XR_306421.1	ssc:102165318			
LOC100625507	345.7564213	4.131391859	6.38698433	4.51E-07	High	327	5	LOC100625507				
LOC102158406	132.1698858	0	20	4.51E-07	High	125	0	XR_298395.1	ssc:102158406			
LOC100626977	1.057359087	342.9055243	-8.341201927	4.52E-07	Low	1	415	XP_003361612.2	ssc:100626977	5E-108	NR_033519.1	MASP1
IRX2	0	246.2309548	-20	4.55E-07	Low	0	298	XP_005674585.1	ssc:100620587	2E-100	NM_033267.4	IRX2
LOC100625479	30.66341351	3020.047449	-6.621909096	4.60E-07	Low	29	3655	XP_005658348.1	ssc:100625479			
IGSF11	2532.375012	46.27158882	5.774220618	4.83E-07	High	2395	56	XP_005670253.1	ssc:100511781	0	NM_152538.2	IGSF11
BCL6	15.8603863	1634.378619	-6.687170516	4.90E-07	Low	15	1978	XP_005657169.1	ssc:100156549	0	NM_138931.1	BCL6
FAM211A	2.114718173	428.0121966	-7.661042688	4.98E-07	Low	2	518	XP_003358347.1	ssc:100626706	0	NM_207387.3	FAM211A
ABI3	2.114718173	425.5333614	-7.652663023	5.12E-07	Low	2	515	XP_005668917.1	ssc:100621521	4E-139	NM_016428.2	ABI3
LOC100511574	255.880899	2.478835115	6.689666336	5.22E-07	High	242	3	XP_003356976.3	ssc:100511574	3E-20	NM_152605.3	ZNF781
LOC102167956	128.9978086	0	20	5.23E-07	High	122	0	XP_005658719.1	ssc:102167956	2E-102	NM_005379.3	MYO1A
AFF2	2.114718173	423.0545263	-7.644234401	5.27E-07	Low	2	512	XP_005654357.1	ssc:100512515	0	NM_002025.3	AFF2
LOC100739427	1.057359087	332.1639054	-8.295286092	5.29E-07	Low	1	402	XP_005670555.1	ssc:100739427	2E-81	NM_016950.2	SPOCK3
LOC102164681	0	237.9681711	-20	5.38E-07	Low	0	288	XR_299526.1	ssc:102164681			
TNFAIP6	0	237.9681711	-20	5.38E-07	Low	0	288	NM_001159607.1	ssc:100286870	4E-149	NM_007115.3	TNFAIP6
LOC100739844	5.286795433	689.9424404	-7.027938693	5.38E-07	Low	5	835	XP_003482731.1	ssc:100739844	3E-154	NM_006080.2	SEMA3A
LOC102161709	127.9404495	0	20	5.50E-07	High	121	0	XR_299330.1	ssc:102161709			
ZNF227	1.057359087	328.8587919	-8.280859021	5.56E-07	Low	1	398	ZNF227	ssc:100627091	0	NM_182490	ZNF227
MYCN	1618.816762	29.74602138	5.766099165	5.56E-07	High	1531	36	XP_003125394.1	ssc:100518190	0	NM_005378.4	MYCN
UNC-6	1.057359087	326.3799568	-8.269943244	5.77E-07	Low	1	395	NM_001044548.1	ssc:733599	0	NM_004822.2	NTN1
CRIP3	126.8830904	0	20	5.78E-07	High	120	0	XP_001926813.1	ssc:100154285	2E-98	NM_206922.2	CRIP3
RAB3IP	3971.440729	75.19133183	5.722952307	5.88E-07	High	3756	91	XP_005664031.1	ssc:100152578	2E-61	NR_103520.1	RAB3IP
CD40	3.17207726	499.0721365	-7.297676639	6.04E-07	Low	3	604	NM_214194.1	ssc:397395	3E-115	NM_152854.2	CD40
LOC102159768	0	232.1842225	-20	6.08E-07	Low	0	281	XP_005664648.1	ssc:102159768	0	NM_198539.3	ZNF568
ZNF567	0	232.1842225	-20	6.08E-07	Low	0	281	ZNF567	ssc:100525521	0	NM_152603.2	ZNF567
LOC102163653	168.1200948	0.826278372	7.668648555	6.17E-07	High	159	1	XR_304536.1	ssc:102163653			
ANKRD34B	124.7683722	0	20	6.40E-07	High	118	0	XP_005661580.1	ssc:100513200	0	NM_001004441.2	ANKRD34B
SYCP2	0	228.0528306	-20	6.64E-07	Low	0	276	XP_005673123.1	ssc:100134968	0	NM_014258.2	SYCP2
LOC100522457	440.9187391	6.610226974	6.059669173	6.79E-07	High	417	8	LOC100522457				
BST2	7.401513606	829.5834852	-6.808423033	6.82E-07	Low	7	1004	NM_001161755.1	ssc:100302088			
GAS6	21.14718173	1974.805308	-6.545101209	6.83E-07	Low	20	2390	XP_005653903.1	ssc:100519331	0	NM_001143946.1	GAS6
EDNRB	400.7390938	5.783948602	6.114464715	6.90E-07	High	379	7	NM_001038002.1	ssc:414911	0	NR_047024.1	EDNRB
LOC102159598	3.17207726	485.8516826	-7.258944245	6.91E-07	Low	3	588	XP_005659452.1	ssc:102159598	2E-88	NM_002290.4	LAMA4

LOC102165620	6.344154519	741.1716994	-6.868236075	6.93E-07	Low	6	897	XP_005671013.1	ssc:102165620	3E-18	NM_080647.1	TBX1
LOC100522769	0	225.5739955	-20	7.01E-07	Low	0	273	XP_003134501.1	ssc:100522769	1E-90	NM_003881.2	WISP2
TRIM71	122.653654	0	20	7.09E-07	High	116	0	XP_005674691.1	ssc:100623572	0	NM_001039111.1	TRIM71
LOC100737965	2.114718173	398.2661752	-7.557123737	7.11E-07	Low	2	482	XP_005666131.1	ssc:100737965	0	np_004339	np_004339
LOC100510899	0	224.7477171	-20	7.13E-07	Low	0	272	XP_003125029.3	ssc:100510899	4E-46	NM_032181.2	EVA1A
LOC100523822	242.1352308	2.478835115	6.610006887	7.33E-07	High	229	3	LOC100523822				
LOC102159108	203.0129446	1.652556743	6.9407281	7.36E-07	High	192	2	XP_005671338.1	ssc:102159108	1E-58	NM_152429.4	FGFBP3
NLRC5	0	223.0951604	-20	7.40E-07	Low	0	270	NM_001278781.1	ssc:100135667	2E-30	NM_032206.4	NLRC5
LOC100512381	746.4955151	13.22045395	5.819289973	7.42E-07	High	706	16	XP_003121319.4	ssc:100512381	0	NM_001704.2	BAI3
LOC100624693	29.60605442	2649.04846	-6.483438189	7.50E-07	Low	28	3206	XP_005662674.1	ssc:100624693	1E-99	NM_000104.3	CYP1B1
PODXL	1576.522398	30.57229975	5.688376776	7.70E-07	High	1491	37	XP_005657812.1	ssc:100512201	2E-100	NM_005397.3	PODXL
F3	7.401513606	808.1002476	-6.770570134	7.78E-07	Low	7	978	NM_213785.1	ssc:396677			
POU3F2	701.0290744	12.39417558	5.821740064	7.83E-07	High	663	15	XP_001926276.1	ssc:100157279	0	NM_005604.3	POU3F2
GHR	1.057359087	306.5492759	-8.179509777	7.87E-07	Low	1	371	NM_214254.2	ssc:397488	0	NM_001242462.1	GHR
FAM43B	430.3451482	6.610226974	6.024650584	7.89E-07	High	407	8	XP_003356225.1	ssc:100624917	5E-164	NM_207334.2	FAM43B
LOC100625443	0	219.7900469	-20	7.96E-07	Low	0	266	XP_003483626.1	ssc:100625443	0	NM_138499.3	PWWP2B
LOC100737399	0	218.9637685	-20	8.11E-07	Low	0	265	XP_005665066.1	ssc:100737399	2E-100	NM_152942.2	TNFRSF8
LOC102163958	275.9707216	3.305113487	6.383671596	8.17E-07	High	261	4	XR_304068.1	ssc:102163958			
MYOD1	0	218.1374901	-20	8.26E-07	Low	0	264	NM_001002824.1	ssc:407604	6E-155	NM_002478.4	MYOD1
DHX58	99.39175414	8611.47319	-6.436990089	8.39E-07	Low	94	10422	NM_001199132.1	ssc:100524520	0	NM_024119.2	DHX58
ZNF135	0	217.3112118	-20	8.42E-07	Low	0	263	ZNF135	ssc:102159974	7E-28	NM_007134.1	ZNF135
LOC100512171	9.516231779	960.1354679	-6.656703753	8.47E-07	Low	9	1162	XP_005664844.1	ssc:100512171			
LOC100522421	0	216.4849334	-20	8.58E-07	Low	0	262	XR_301560.1	ssc:100522421	1E-91	NM_001161473.1	ALDH3B1
LOC100626974	0	216.4849334	-20	8.58E-07	Low	0	262	XP_003361236.1	ssc:100626974	0	NM_001039523.2	CHRNA1
LOC100737584	4.229436346	544.517447	-7.008369056	8.74E-07	Low	4	659	XR_303847.1	ssc:100737584	2E-30	NM_021913.4	AXL
LOC100739422	30.66341351	2648.222181	-6.432362047	8.87E-07	Low	29	3205	XP_005662673.1	ssc:100739422	3E-100	NM_000104.3	CYP1B1
FAM181B	195.611431	1.652556743	6.88714706	9.23E-07	High	185	2	XP_003129763.2	ssc:100517667	5E-168	NM_175885.3	FAM181B
KCNK2	1.057359087	296.6339355	-8.132074435	9.25E-07	Low	1	359	XP_003357633.2	ssc:100627197	8E-110	NM_014217.3	KCNK2
CNPY1	117.3668586	0	20	9.26E-07	High	111	0	XP_005673147.1	ssc:102167342	3E-36	NM_001103176.1	CNPY1
FAM71E1	2.114718173	376.7829375	-7.477124415	9.36E-07	Low	2	456	XP_005664843.1	ssc:100511996	1E-60	NM_138411.1	FAM71E1
DPP6	232.618999	2.478835115	6.552162812	9.37E-07	High	220	3	XP_005673153.1	ssc:100621864	0	NM_130797.2	DPP6
LOC100525036	0	212.3535415	-20	9.43E-07	Low	0	257	XP_003123849.4	ssc:100525036	0	NM_022350.3	ERAP2
CCDC141	1.057359087	294.9813787	-8.124014665	9.51E-07	Low	1	357	XP_003133560.3	ssc:100520053	0	NM_173648.3	CCDC141
AGT	4.229436346	533.7758281	-6.979624755	9.65E-07	Low	4	646	AGT		2E-75	NM_000029.3	AGT
ARSD	10.57359087	1014.66984	-6.584401151	9.73E-07	Low	10	1228	XP_005658943.1	ssc:100624032	3E-148	NM_009589.2	ARSD
ARHGEF10L	11.63094995	1095.645121	-6.557667842	9.75E-07	Low	11	1326	XP_003482027.1	ssc:100525994	0	NM_018125.3	ARHGEF10L
LOC102166633	116.3094995	0	20	9.77E-07	High	110	0	XR_306669.1	ssc:102166633			
LOC100525818	11.63094995	1093.992564	-6.555490189	9.82E-07	Low	11	1324	XP_003482028.1	ssc:100525818	0	NM_018125.3	ARHGEF10L
EPHA3	4.229436346	531.296993	-6.972909328	9.87E-07	Low	4	643	NM_001195335.1	ssc:100337658	6E-153	NM_182644.2	EPHA3
LOC100628224	2.114718173	371.8252673	-7.458015592	9.99E-07	Low	2	450	XP_005655269.1	ssc:100628224	4E-33	NM_006634.2	VAMP5
LOC102159087	53.92531342	4474.297383	-6.374554681	1.01E-06	Low	51	5415	XP_005654763.1	ssc:102159087			
ISM1	8.458872693	838.6725473	-6.631498413	1.06E-06	Low	8	1015	XP_001925035.2	ssc:100154170	0	NM_080826.1	ISM1
LOC100515919	60093.94633	1142.742988	5.716646794	1.06E-06	High	56834	1383	XP_005656853.1	ssc:100515919	4E-155	NM_145918.2	CTSL
CLMP	53.92531342	4428.025794	-6.35955716	1.06E-06	Low	51	5359	XP_003130027.3	ssc:100527427	2E-147	NM_024769.2	CLMP
PLA2R1	10.57359087	996.4917163	-6.558320498	1.06E-06	Low	10	1206	XP_005671924.1	ssc:100622105	0	NM_007366.4	PLA2R1
LOC100621578	0	206.5695929	-20	1.08E-06	Low	0	250	XP_003356977.2	ssc:100621578	3E-97	NM_000857.2	GUCY1B3
TPM2	638.6448883	57079.30992	-6.481810124	1.08E-06	Low	604	69080	NM_001129947.1	ssc:396693	8E-150	NM_213674.1	TPM2
LOC102164037	6.344154519	677.5482648	-6.738752	1.08E-06	Low	6	820	XP_005664371.1	ssc:102164037			
SPRN	22.20454082	1881.435852	-6.404835554	1.09E-06	Low	21	2277	XP_005671641.1	ssc:100154535	9E-23	NM_001012508.3	SPRN
PDGFRL	7.401513606	755.2184318	-6.672929834	1.09E-06	Low	7	914	XP_005672702.1	ssc:100736738	9E-141	NM_006207.2	PDGFRL
SLC16A10	2025.90001	42.14019696	5.587222103	1.10E-06	High	1916	51	XP_005654457.1	ssc:100513770	4E-105	NM_018593.4	SLC16A10
AMDHD1	552.9988023	9.915340461	5.801470235	1.11E-06	High	523	12	XP_001925019.3	ssc:100151804	0	NM_152435.2	AMDHD1
PCP4L1	35.95020894	2949.813787	-6.358479918	1.11E-06	Low	34	3570	XP_003125705.2	ssc:100523600	1E-23	NM_001102566.1	PCP4L1
ZSCAN22	0	204.9170362	-20	1.12E-06	Low	0	248	ZSCAN22	ssc:100516809	2E-19	NM_181846.2	ZSCAN22
DPYSL3	59.21210885	4789.935721	-6.337970249	1.14E-06	Low	56	5797	XP_005655082.1	ssc:100525453	0	NM_001387.2	DPYSL3
SLC46A2	2.114718173	361.0836484	-7.41572387	1.15E-06	Low	2	437	XP_005660391.1	ssc:100157182	0	NM_033051.3	SLC46A2
SCN1B	0	203.2644794	-20	1.17E-06	Low	0	246	XP_005664550.1	ssc:100736963	2E-82	NM_199037.3	SCN1B
ZNF175	0	203.2644794	-20	1.17E-06	Low	0	246	ZNF175	ssc:100514293	0	NM_007147.2	ZNF175
CDH18	0	203.2644794	-20	1.17E-06	Low	0	246	XP_003359779.3	ssc:100625674	0	NM_004934.3	CDH18
RNASEL	5.286795433	589.9627574	-6.80208657	1.17E-06	Low	5	714	NM_001097512.1	ssc:100048946	0	NM_021133.3	RNASEL
DNAH11	692.5702017	13.22045395	5.711116696	1.18E-06	High	655	16	XP_003361782.1		0	np_003768	np_003768
CARD10	3.17207726	435.4487019	-7.100931052	1.19E-06	Low	3	527	XP_005663881.1	ssc:102161954	0	NM_014550.3	CARD10
LOC102164377	187.1525583	1.652556743	6.823371149	1.21E-06	High	177	2	XP_005668663.1	ssc:102164377	6E-94	NM_198955.1	MGAT5B
LOC100737436	3.17207726	432.9698668	-7.092694902	1.22E-06	Low	3	524	XP_005663032.1	ssc:100737436	2E-50	NM_080388.1	S100A16
FLYWCH2	1.057359087	280.108368	-8.049375864	1.23E-06	Low	1	339	NM_001243398.1	ssc:100627929	7E-49	NM_138439.2	FLYWCH2
GULO	1.057359087	280.108368	-8.049375864	1.23E-06	Low	1	339	NM_001129948.1	ssc:396759			
ZNF583	0	200.7856443	-20	1.24E-06	Low	0	243	ZNF583	ssc:100620770	0	NM_152478.2	ZNF583
IL1RL1	0	200.7856443	-20	1.24E-06	Low	0	243	XP_005662414.1	ssc:100127134	0	NR_104167.1	IL1RL1
LOC100736585	1.057359087	279.2820896	-8.045113837	1.25E-06	Low	1	338	XP_003484319.1	ssc:100736585	4E-117	NM_033382.2	SEC14L2
SAMD5	363.7315258	5.783948602	5.974675432	1.25E-06	High	344	7	XP_005659216.1	ssc:100522528	1E-80	NM_001030060.2	SAMD5
LOC100738326	46.52379981	3700.900827	-6.313763733	1.25E-06	Low	44	4479	XP_005658395.1	ssc:100738326	9E-47	NM_002305.3	LGALS1
LOC100517161	0	199.959366	-20	1.27E-06	Low	0	242	XP_005653357.1	ssc:100517161	1E-155	NM_014650.2	ZNF432
NR2F1	22.20454082	1822.770088	-6.359134054	1.27E-06	Low	21	2206	XP_005661606.1	ssc:100621341	0	NM_005654.4	NR2F1
KLF13	4.229436346	503.2035284	-6.894532819	1.29E-06	Low	4	609	NM_001011505.1	ssc:494563	5E-132	NM_015995.2	KLF13
LOC102158748	0	199.1330876	-20	1.29E-06	Low	0	241	XR_307291.1	ssc:102158748			
LOC100521334	185.0378402	1.652556743	6.806976711	1.29E-06	High	175	2	LOC100521334				
LOC100737762	6.344154519	653.586192	-6.686805785	1.29E-06	Low	6	791	XP_005672304.1	ssc:100737762			
LOC100154530	19.03246356	1579.017968	-6.374421462	1.30E-06	Low	18	1911	XP_003360037.2	ssc:100154530	0	NM_198951.1	TGM2
LOC100739780	255.880899	3.305113487	6.274628837	1.30E-06	High	242	4	XP_005659865.1	ssc:100739780	3E-161	NM_005503.3	APBA2
SPARC	312.9782896	25894.73789	-6.37045256	1.32E-06	Low	296	31339	NM_001031794.1	ssc:595124	2E-89	NM_003118.3	SPARC
LOC102160189	275.9707216	22683.82014	-6.361004596	1.32E-06	Low	261	27453	XP_005656326.1	ssc:102160189	6E-102	NM_018689.1	KIAA1199
CAR	21975.0939	451.9742693	5.603484876	1.34E-06	High	20783	547	XP_001924589.4	ssc:397333	0	NM_001338.4	CXADR
DOCK3	183.9804811	1.652556743	6.798709095	1.34E-06	High	174	2	XP_003132287.4	ssc:100519389	0	NM_004947.4	DOCK3
LOC100622474	109.965345	0	20	1.37E-06	High	104	0	XP_003361735.2	ssc:100622474	4E-41	NM_173803.3	MPV17L
LOC100738804	4.229436346	495.767023	-6.873053091	1.39E-06	Low	4	600	XP_003481936.1	ssc:100738804	3E-174	NM_032040.4	CCDC8
LOC102166124	1.057359087	272.6718627	-8.010556615	1.40E-06	Low	1	330	XP_005672718.1	ssc:102166124	1E-89	NR_024040.1	CSGALNACT1
MPZL2	0	195.8279741	-20	1.40E-06	Low	0	237	NM_001244821.1	ssc:100513488	1E-113	NM_144765.2	MPZL2
GPR88	566.7444704	10.74161883	5.721415072	1.41E-06	High	536	13	XP_005663693.1	ssc:100623311	7E-89	NM_022049.2	GPR88
NTRK2	181.8657629	1.652556743	6.782030354	1.44E-06	High	172	2	XP_003130695.4	ssc:100519209	0	NM_006180.3	NTRK2
LOC102159424	108.9079859	0	20	1.45E-06	High	103	0	XR_307055.1	ssc:102159424			
LOC102159869	108.9079859	0	20	1.45E-06	High	103	0	XR_299907.1	ssc:102159869			
PENK	0	194.1754174	-20	1.46E-06	Low	0	235	XP_003125669.1	ssc:100152093	5E-25	NM_006211.3	PENK
HPSE	1095.424014	23.13579441	5.565218965	1.47E-06	High	1036	28	NM_001146130.2	ssc:100271932	0	NM_006665.5	HPSE
PDE3B	2485.851213	54.53437253	5.510430304	1.48E-06	High	2351	66	XP_005661171.1	ssc:100516060	0	NM_000922.3	PDE3B
RRAS	91.99024053	7079.553089	-6.26603367	1.50E-06	Low	87	8568	XP_003356046.1	ssc:100516558	7E-100	NM_006270.3	RRAS
SPOCK3	21.14718173	1679.82393	-6.311700806	1.53E-06	Low	20	2033	XP_001927799.2	ssc:100154805	3E-105	NM_016950.2	SPOCK3
LOC102162047	1.057359087	267.7141924	-7.984084404	1.53E-06	Low	1	324	XP_005668699.1	ssc:102162047			
IFIH1	32.77813168	2520.975312	-6.265104555	1.56E-06	Low	31	3051	NM_001100194.1	ssc:100101927	0	NM_022168.3	IFIH1
LOC102159425	556.1708795	10.74161883	5.69424487	1.58E-06	High	526	13	XR_300992.1	ssc:102159425			
LOC102159043	8.458872693	772.5702776	-6.513056956	1.59E-06	Low	8	935	XR_303323.1	ssc:102159043			

CLIC2	0	190.8703039	-20	1.59E-06	Low	0	231	NM_001244428.1	ssc:100516055	7E-135	NM_001289.5	CLIC2
LOC102166696	0	190.0440255	-20	1.62E-06	Low	0	230	XR_303880.1	ssc:102166696			
LOC100623508	1.057359087	264.409079	-7.966162496	1.63E-06	Low	1	320	XP_005658780.1	ssc:100623508	5E-149	NM_001754.4	RUNX1
LOC102159370	415.542121	7.436505346	5.8042261	1.65E-06	High	393	9	XP_005658623.1	ssc:102159370	6E-89	NM_005559.3	LAMA1
HSPB7	0	189.2177471	-20	1.66E-06	Low	0	229	NM_001243388.1	ssc:100627988	7E-80	NM_014424.4	HSPB7
ADCY2	0	189.2177471	-20	1.66E-06	Low	0	229	XP_003134201.4	ssc:100517503	0	NM_020546.2	ADCY2
LOC100520570	77.18721332	5802.126726	-6.232076123	1.66E-06	Low	73	7022	XR_304053.1	ssc:100520570	0	NM_015210.3	SOGA2
LOC100625132	2108.374019	47.09786719	5.484325279	1.68E-06	High	1994	57	XP_003357570.3	ssc:100625132	4E-101	NM_014141.5	CNTNAP2
ESM1	2.114718173	334.6427406	-7.306012499	1.68E-06	Low	2	405	XP_005674289.1	ssc:100519340	2E-71	NM_007036.4	ESM1
SNX22	176.5789675	1.652556743	6.739469892	1.72E-06	High	167	2	XP_003362155.2	ssc:100624664	1E-38	NR_073534.1	SNX22
DIRAS2	105.7359087	0	20	1.73E-06	High	100	0	XP_005657251.1	ssc:100739440	5E-114	NM_017594.3	DIRAS2
LOC100626788	0	187.5651904	-20	1.73E-06	Low	0	227	XR_303915.1	ssc:100626788	8E-19	NM_181846.2	ZSCAN22
HOXA10	0	187.5651904	-20	1.73E-06	Low	0	227	NM_001257354.1	ssc:100737679	7E-51	NR_037939.1	HOXA10
CSGALNACT1	15.8603863	1265.858465	-6.318544387	1.75E-06	Low	15	1532	XP_003134251.2	ssc:100517735	6E-180	NR_024040.1	CSGALNACT1
LOC100524748	0	185.9126336	-20	1.81E-06	Low	0	225	XP_005664829.1	ssc:100524748	0	NM_024729.3	MYH14
NTRK1	2.114718173	328.0325136	-7.277229598	1.85E-06	Low	2	397	XP_001929561.2	ssc:100154471	0	NM_002529.3	NTRK1
AK5	609.0388339	12.39417558	5.618800005	1.86E-06	High	576	15	XP_005653478.1	ssc:733697	0	NM_174858.2	AK5
PTPRU	21.14718173	1613.72166	-6.25378254	1.87E-06	Low	20	1953	XP_005653451.1	ssc:100515955	0	NM_133178.3	PTPRU
LOC100626135	10.57359087	889.9018064	-6.39510884	1.87E-06	Low	10	1077	XP_005652614.1	ssc:100626135	5E-130	NM_198316.1	TENC1
HERC6	58.15474976	4262.77012	-6.195750418	1.87E-06	Low	55	5159	XP_003360755.1	ssc:100626657			
LOC100737711	0	184.2600769	-20	1.89E-06	Low	0	223	XP_003484104.1	ssc:100737711	1E-159	NM_152739.3	HOXA9
EPSTI1	28.54869534	2124.361694	-6.217460793	1.90E-06	Low	27	2571	XP_005656905.1	ssc:100625050	1E-113	NM_033255.3	EPSTI1
LOC100738115	1.057359087	256.1462952	-7.920358806	1.91E-06	Low	1	310	XP_003482229.2	ssc:100738115	9E-133	NM_007162.2	TFEB
LOC102159817	173.4068902	1.652556743	6.713317604	1.92E-06	High	164	2	XR_297940.1	ssc:102159817			
LOC102157989	2.114718173	325.5536785	-7.26628622	1.92E-06	Low	2	394	XP_005654078.1	ssc:102157989	1E-54	NM_020876.1	ARHGAP23
SLC26A5	103.6211905	0	20	1.95E-06	High	98	0	NM_001135963.1	ssc:100190993	0	NM_206885.2	SLC26A5
TSPAN33	5761.549663	131.3782611	5.45465842	1.96E-06	High	5449	159	XP_003134729.1	ssc:100515287	3E-158	NM_178562.3	TSPAN33
HTR1B	14.80302721	1159.268556	-6.291178772	2.01E-06	Low	14	1403	NM_214298.1	ssc:397563	0	NM_000863.1	HTR1B
TMEM8C	0	181.7812418	-20	2.02E-06	Low	0	220	XP_003353750.1	ssc:100627299	5E-117	NM_001080483.2	TMEM8C
NR6A1	1484.532158	33.87741324	5.453540815	2.04E-06	High	1404	41	NM_001097499.1	ssc:100038028	2E-55	NM_033335.2	NR6A1
LY96	9.516231779	801.4900206	-6.396150336	2.07E-06	Low	9	970	NM_001104956.1	ssc:100125555	1E-60	NM_015364.4	LY96
ADAMTS2	15.8603863	1203.887588	-6.246128967	2.24E-06	Low	15	1457	XP_003123699.3	ssc:100521434	0	NM_021599.2	ADAMTS2
LOC100519519	16.91774539	1271.642414	-6.232011917	2.26E-06	Low	16	1539	XP_005657624.1	ssc:100519519	0	NM_133437.3	TTN
UBE2L6	71.90041789	5069.217811	-6.139619188	2.28E-06	Low	68	6135	NM_001246215.1	ssc:100514753	2E-66	NM_198183.2	UBE2L6
LOC100624847	4.229436346	447.8428775	-6.726383442	2.30E-06	Low	4	542	XP_003362157.1	ssc:100624847	2E-126	NM_020731.4	AHRR
ST3GAL1	27.49133625	1969.847638	-6.162963203	2.31E-06	Low	26	2384	NM_001004047.1	ssc:445537	9E-177	NM_173344.2	ST3GAL1
NANOG	100.4491132	0	20	2.35E-06	High	95	0	NM_001129971.1	ssc:100170132	5E-121	NM_024865.2	NANOG
LOC100622075	37.00756803	2604.429428	-6.137003203	2.36E-06	Low	35	3152	XP_005655081.1	ssc:100622075	0	NM_001387.2	DPYSL3
FGFBP3	167.0627357	1.652556743	6.659546347	2.40E-06	High	158	2	NM_001258356.1	ssc:100153311	1E-25	NM_152429.4	FGFBP3
LOC100621092	3.17207726	376.7829375	-6.892161914	2.43E-06	Low	3	456	XP_005674141.1	ssc:100621092	7E-85	NM_153367.3	ZCCHC24
ZNF418	6.344154519	573.43719	-6.498063753	2.47E-06	Low	6	694	ZNF418	ssc:100514656	0	NM_133460.1	ZNF418
GREM1	23.2618999	1662.472084	-6.159217372	2.48E-06	Low	22	2012	XP_005659828.1	ssc:100156280	5E-107	NM_013372.6	GREM1
LOC100738686	99.39175414	0	20	2.50E-06	High	94	0	XR_307991.1	ssc:100738686	5E-71	NR_048571.1	GPM6A
BRINP2	904.042019	20.65695929	5.451690019	2.53E-06	High	855	25	XP_003130380.3	ssc:100522028	0	NM_021165.2	BRINP2
ADAP1	0	173.5184581	-20	2.53E-06	Low	0	210	NM_214226.1	ssc:397453	2E-76	NM_006869.3	ADAP1
TMEM151A	0	173.5184581	-20	2.53E-06	Low	0	210	XP_003122547.1	ssc:100520884	0	NM_153266.3	TMEM151A
A2M	1.057359087	241.2732845	-7.83405896	2.56E-06	Low	1	292	XP_001925235.3	ssc:403166	0	NM_000014.4	A2M
TENC1	108.9079859	7562.925936	-6.117762839	2.56E-06	Low	103	9153	XP_005652637.1	ssc:100156301	0	NM_198316.1	TENC1
COL2A1	1097.538732	25.61462952	5.421160017	2.58E-06	High	1038	31	XP_001925994.5	ssc:397323	0	NM_033150.2	COL2A1
WNT2B	9.516231779	765.9600506	-6.330734928	2.59E-06	Low	9	927	XP_003125897.1	ssc:100520560	0	NM_024494.2	WNT2B
LOC100736962	1.057359087	239.6207278	-7.824143491	2.64E-06	Low	1	290	XP_005653313.1	ssc:100736962	0	NM_001266.4	CES1
FAM180A	1.057359087	239.6207278	-7.824143491	2.64E-06	Low	1	290	XP_003484061.1	ssc:100737678	5E-78	NM_205855.3	FAM180A
PCOLCE	132.1698858	9169.211091	-6.116332202	2.65E-06	Low	125	11097	XP_005661969.1	ssc:100627524	0	NM_002593.3	PCOLCE
KIAA1199	3.17207726	370.1727105	-6.866626822	2.65E-06	Low	3	448	XP_005656328.1	ssc:102160984	2E-59	NM_018689.1	KIAA1199
EPB41L1	107.8506268	7434.02651	-6.107037366	2.65E-06	Low	102	8997	XP_005672964.1	ssc:100156952	0	NM_177996.2	EPB41L1
LOC100512533	98.33439505	0	20	2.67E-06	High	93	0	XP_005658249.1	ssc:100512533	8E-138	NR_036437.1	KCNC4
LOC396583	5.286795433	499.0721365	-6.560711045	2.68E-06	Low	5	604	XP_005658936.1	ssc:396583	4E-108	NM_005985.3	SNAI1
LOC100624942	17.97510447	1294.778208	-6.170561024	2.69E-06	Low	17	1567	XP_005661896.1	ssc:100624942	1E-127	NM_001080495.2	TNRC18
TNFSF10	0	171.0396229	-20	2.72E-06	Low	0	207	NM_001024696.1	ssc:406191	1E-85	NR_033994.1	TNFSF10
LOC100737693	0	171.0396229	-20	2.72E-06	Low	0	207	XP_003482794.2	ssc:100737693	5E-64	NM_014217.3	KCNK2
CRELD1	17.97510447	1290.646817	-6.165950298	2.74E-06	Low	17	1562	XP_005669822.1	ssc:100152256	0	NM_015513.4	CRELD1
SYNE3	8.458872693	690.7687188	-6.351593533	2.76E-06	Low	8	836	XP_003128757.2	ssc:100525411	0	NM_152592.3	SYNE3
RCVRN	0	170.2133446	-20	2.78E-06	Low	0	206	XR_306608.1	ssc:100516153			
LOC100737919	0	170.2133446	-20	2.78E-06	Low	0	206	XR_308589.1	ssc:100737919			
TCF21	0	170.2133446	-20	2.78E-06	Low	0	206	XP_005652728.1	ssc:100152962	6E-84	NM_198392.2	TCF21
ABI3BP	3.17207726	366.0413187	-6.850434789	2.80E-06	Low	3	443	XP_005670307.1	ssc:100154641	0	NM_015429.3	ABI3BP
LOC100736904	225.2174854	3.305113487	6.090475219	2.82E-06	High	213	4	XP_003483712.2	ssc:100736904	6E-44	NM_004490.2	GRB14
LOC100739541	2.114718173	299.9390489	-7.148060139	2.88E-06	Low	2	363	XP_003481236.1	ssc:100739541	4E-49	NM_012476.2	VAX2
DKK1	0	168.5607878	-20	2.92E-06	Low	0	204	NM_001145384.1	ssc:100157640	9E-133	NM_012242.2	DKK1
ZNF582	0	168.5607878	-20	2.92E-06	Low	0	204	ZNF582	ssc:100521903	0	NM_144690.1	ZNF582
CCDC69	0	168.5607878	-20	2.92E-06	Low	0	204	XP_003134191.3	ssc:100515590	3E-130	NM_015621.2	CCDC69
LOC100736876	975.9424369	23.13579441	5.398597515	2.98E-06	High	923	28	XP_003483522.2	ssc:100736876	2E-51	NM_020808.3	SIPA1L2
ALPK1	7.401513606	614.7511086	-6.37603829	3.01E-06	Low	7	744	XP_003129293.3	ssc:100514116	0	NM_025144.3	ALPK1
LOC102158081	1166.267072	28.09346464	5.375519834	3.02E-06	High	1103	34	XP_005666535.1	ssc:102158081	3E-107	NM_178557.3	NAT8L
LOC102164919	96.21967688	0	20	3.03E-06	High	91	0	XR_301479.1	ssc:102164919			
LOC100521071	96.21967688	0	20	3.03E-06	High	91	0	XR_298107.1	ssc:100521071			
CYLC1	96.21967688	0	20	3.03E-06	High	91	0	XP_003135262.4	ssc:100522114	1E-105	NM_021118.2	CYLC1
FN1	10314.53789	881757.1804	-6.417630327	3.07E-06	Low	9755	1067143	XP_005672231.1	ssc:397620	0	NM_212482.1	FN1
LYL1	22.20454082	1522.831039	-6.099757334	3.10E-06	Low	21	1843	XP_005652937.1	ssc:100523949	4E-123	NM_005583.4	LYL1
MFSD6	4967.472989	122.289199	5.344143232	3.12E-06	High	4698	148	NM_001097457.1	ssc:100037960	0	NM_017694.3	MFSD6
SLC16A12	374.3051166	7.436505346	5.653446148	3.13E-06	High	354	9	XP_001928846.2	ssc:100157079	0	NM_213606.3	SLC16A12
CES1	0	165.2556743	-20	3.21E-06	Low	0	200	NM_214246.1	ssc:397478			
LOC100738970	127.9404495	0.826278372	7.274628837	3.21E-06	High	121	1	XP_003482513.1	ssc:100738970	3E-115	NM_025212.2	CXXC4
ATP12A	95.16231779	0	20	3.24E-06	High	90	0	XP_005653908.1	ssc:100521921	0	NM_001676.5	ATP12A
CACNA2D3	95.16231779	0	20	3.24E-06	High	90	0	XP_005669714.1	ssc:100153986	0	NM_018398.2	CACNA2D3
LOC100523701	12.68830904	923.7792196	-6.185976373	3.24E-06	Low	12	1118	XP_005672121.1	ssc:100523701	0	NM_001159.3	AOX1
KIF12	39.1222862	2577.98852	-6.042111349	3.25E-06	Low	37	3120	XP_005660422.1	ssc:100156816	0	NM_138424.1	KIF12
LOC100620398	1.057359087	229.7053873	-7.763175473	3.25E-06	Low	1	278	XP_005674448.1	ssc:100620398	0	NM_006080.2	SEMA3A
LOC100153543	463.1232799	9.915340461	5.545590158	3.28E-06	High	438	12	XP_005670048.1	ssc:100153543	6E-89	NM_001409.3	MEGF6
LOC100521749	1.057359087	228.879109	-7.757976567	3.31E-06	Low	1	277	XP_005669167.1	ssc:100521749	1E-29	NR_046393.1	ABR
COL18A1	62.38418611	4068.594702	-6.027206398	3.34E-06	Low	59	4924	HighI00025DF767		2E-122	NM_130445.2	COL18A1
SSC5D	13.74566813	981.6187056	-6.158113803	3.35E-06	Low	13	1188	XP_005664918.1	ssc:100621391	0	NM_182571.2	SSC5D
PARP9	37.00756803	2427.605856	-6.035570064	3.35E-06	Low	35	2938	XP_005670226.1	ssc:100519390	0	NM_031458.2	PARP9
LOC100624297	26.43397716	1748.405035	-6.047502123	3.48E-06	Low	25	2116	XP_003360691.1	ssc:100624297	6E-60	NM_031477.4	YPEL3
FBLN2	30.66341351	2011.161557	-6.035366858	3.48E-06	Low	29	2434	XP_003483279.1	ssc:100738595	3E-141	NM_001998.2	FBLN2
ITGA8	245.3073081	16505.73675	-6.072233512	3.49E-06	Low	232	19976	XP_003130794.1	ssc:100512676	0	NM_003638.1	ITGA8
PHLDA2	14.80302721	1036.979357	-6.130351128	3.49E-06	Low	14	1255	NM_001174057.1	ssc:100379631			
PVR	8.458872693	657.7175839	-6.280859021	3.52E-06	Low	8	796	XP_005653340.1	ssc:100525346	8E-107	NM_006505.3	PVR
SELM	96.21967688	6239.227985	-6.018891762	3.54E-06	Low	91	7551	NM_001161648.1	ssc:100302025	2E-54	NM_080430.2	SELM
TEC	3.17207726	348.6894729	-6.780371089	3.55E-06	Low	3	422	XP_003129008.1	ssc:404277	0	NM_003215.2	TEC

TIMP2	948.4511007	68241.50444	-6.168932248	3.57E-06	Low	897	82589	NM_001145985.1	ssc:396988	7E-116	NM_003255.4	TIMP2
LOC102162518	82.47400875	5319.580157	-6.011229043	3.58E-06	Low	78	6438	XP_005657243.1	ssc:102162518	5E-47	NM_130445.2	COL18A1
TFEB	4.229436346	408.1815156	-6.592601632	3.63E-06	Low	4	494	XP_001927183.2	ssc:100157940	0	NM_007162.2	TFEB
LOC100736654	186.0951992	2.478835115	6.230234717	3.63E-06	High	176	3	XP_005667803.1	ssc:100736654			
PWWP2B	2.114718173	285.8923166	-7.078862628	3.64E-06	Low	2	346	XP_005654212.1	ssc:100154915	0	NM_138499.3	PWWP2B
GNAT1	1.057359087	223.9214387	-7.726383442	3.68E-06	Low	1	271	XP_001928011.1	ssc:100157237	0	NM_144499.2	GNAT1
LCN2	8.458872693	651.1073569	-6.26628622	3.70E-06	Low	8	788	NM_001244410.1	ssc:100153501	2E-66	NM_005564.3	LCN2
FBXO43	155.4317857	1.652556743	6.555437944	3.71E-06	High	147	2	XP_001925369.1	ssc:100153144	0	NR_036491.1	FBXO43
PARP4	4.229436346	405.7026805	-6.583813615	3.74E-06	Low	4	491	XP_005658099.1	ssc:100626565			
CALML4	4.229436346	405.7026805	-6.583813615	3.74E-06	Low	4	491	NM_001244619.1	ssc:100519528	2E-78	NR_104583.1	CALML4
LOC100525848	2296.583936	58.66576439	5.290826868	3.76E-06	High	2172	71	XP_003135321.1	ssc:100525848	3E-18	NM_194324.2	TMSB15B
IL4R	58.15474976	3674.459919	-5.981491377	3.90E-06	Low	55	4447	NM_214340.1	ssc:397614			
LOC606745	5.286795433	461.8896098	-6.449010779	3.92E-06	Low	5	559	XP_005658202.1	ssc:606745	2E-70	NM_000676.2	ADORA2B
ABCC8	212.5291764	3.305113487	6.00681729	4.00E-06	High	201	4	XP_005661160.1	ssc:100158245	0	NM_001287174.1	ABCC8
WIF1	2.114718173	280.108368	-7.049375864	4.02E-06	Low	2	339	XP_003481699.1	ssc:100511326	1E-67	NM_007191.4	WIF1
LOC100737517	21.14718173	1380.711159	-6.028802324	4.04E-06	Low	20	1671	XP_003483749.1	ssc:100737517	0	NM_014585.5	SLC40A1
NOR-1	864.9197328	21.48323766	5.331282914	4.12E-06	High	818	26	NM_214247.1	ssc:397479	0	NM_173200.2	NR4A3
SNAP25	3026.161706	78.49644531	5.268717948	4.13E-06	High	2862	95	XP_003483940.1	ssc:100620588	4E-117	NM_130811.2	SNAP25
LOC100513699	122.653654	0.826278372	7.213746594	4.13E-06	High	116	1	XP_005674426.1	ssc:100513699	2E-178	NM_001008783.1	SLC35D3
LOC100522153	28.54869534	1814.507304	-5.990009238	4.14E-06	Low	27	2196	XP_005653617.1	ssc:100522153	0	NM_198595.2	AFAP1
LOC100511616	1.057359087	218.1374901	-7.68862852	4.18E-06	Low	1	264	XP_005658573.1	ssc:100511616	5E-75	NM_014397.5	NEK6
LOC100739750	12.68830904	875.855074	-6.109120449	4.21E-06	Low	12	1060	XP_003481092.1	ssc:100739750	1E-37	NM_014015.3	DEXI
ARHGAP23	34.89284986	2189.637685	-5.971616926	4.21E-06	Low	33	2650	XP_005654077.1	ssc:100514368	0	NM_020876.1	ARHGAP23
RHPN2	2538.719167	66.10226974	5.263257193	4.21E-06	High	2401	80	XP_003355886.2	ssc:100620673			
ZNF350	0	156.1666123	-20	4.23E-06	Low	0	189	ZNF350	ssc:100737585	0	NM_021632.3	ZNF350
GGT5	7.401513606	573.43719	-6.275671331	4.24E-06	Low	7	694	XP_005670952.1	ssc:100157523	0	NM_004121.2	GGT5
CCN2	685.1686881	46688.85939	-6.090475311	4.26E-06	Low	648	56505	NM_213833.1	ssc:100152141	0	NM_001901.2	CTGF
TCIRG1	61.32682702	3802.533067	-5.954298674	4.28E-06	Low	58	4602	XP_005660671.1	ssc:100521769	0	NM_006053.3	TCIRG1
MGAT3	0	155.3403339	-20	4.34E-06	Low	0	188	XP_005674708.1	ssc:100625969	3E-132	NM_002409.4	MGAT3
ITGBL1	0	155.3403339	-20	4.34E-06	Low	0	188	XP_005668583.1	ssc:100525532	2E-175	NM_004791.2	ITGBL1
PPL	21.14718173	1360.0542	-6.007054926	4.35E-06	Low	20	1646	XP_003124688.3	ssc:100512773	0	NM_002705.4	PPL
DEXI	12.68830904	870.0711254	-6.099561621	4.35E-06	Low	12	1053	XP_003124646.1	ssc:100519361	1E-37	NM_014015.3	DEXI
LGALS3	172.3495311	10919.26868	-5.985395051	4.36E-06	Low	163	13215	NM_001097501.2	ssc:100038033	4E-56	NM_006498.2	LGALS2
MYL9	467.3527163	30984.61266	-6.050896344	4.40E-06	Low	442	37499	NM_001244472.1	ssc:100157760	9E-99	NM_181526.2	MYL9
LOC102167053	0	154.5140555	-20	4.45E-06	Low	0	187	XP_005671638.1	ssc:102167053	0	NM_173572.3	TTC40
BPIFB6	89.87552236	0	20	4.54E-06	High	85	0	XP_003483988.1	ssc:100736799	0	NM_174897.2	BPIFB6
LOC100736923	0	153.6877771	-20	4.57E-06	Low	0	186	XP_003484321.1	ssc:100736923	0	NM_052970.4	HSPA12B
LOC102163933	15.8603863	1040.28447	-6.035406373	4.61E-06	Low	15	1259	XP_005669573.1	ssc:102163933	3E-29	NM_016479.4	SHISA5
LOC100519934	116921.7104	2704.409111	5.434085617	4.61E-06	High	110579	3273	XP_003121374.2	ssc:100519934	4E-13	NM_013230.2	CD24
HSPG2	271.7412852	17333.66768	-5.995199372	4.65E-06	Low	257	20978	XP_005656099.1	ssc:100517288	0	NM_005529.5	HSPG2
TNNC1	5.286795433	445.3640424	-6.396447769	4.69E-06	Low	5	539	NM_001130243.1	ssc:100156435	8E-87	NM_003280.2	TNNC1
BAHCC1	54.9826725	3346.427406	-5.927500875	4.70E-06	Low	52	4050	XP_005656945.1	ssc:100625456	0	NM_001080519.2	BAHCC1
HS3ST5	378.534553	8.262783717	5.517653282	4.72E-06	High	358	10	XP_005659453.1	ssc:100155403	0	NM_153612.3	HS3ST5
LOC100738767	0	152.0352204	-20	4.81E-06	Low	0	184	XR_306551.1	ssc:100738767			
RASGRF2	870.2065282	22.30951604	5.285626717	4.90E-06	High	823	27	XP_005661584.1	ssc:100515363	0	NM_006909.2	RASGRF2
LOC100522330	22978.52767	576.7423035	5.316215705	4.91E-06	High	21732	698	XP_005666317.1	ssc:100522330	7E-161	NR_049767.1	SLC7A8
MYOM1	8.458872693	614.7511086	-6.183393212	4.91E-06	Low	8	744	XP_005665357.1	ssc:100522496	0	NM_019856.1	MYOM1
KCNK6	0	151.208942	-20	4.94E-06	Low	0	183	XP_003127153.1	ssc:100514651	1E-149	NM_004823.1	KCNK6
NLRP3	0	151.208942	-20	4.94E-06	Low	0	183	NM_001256770.1	ssc:100514823	0	NM_183395.2	NLRP3
LOC100621009	0	151.208942	-20	4.94E-06	Low	0	183	XP_005668267.1	ssc:100621009	0	NM_001029880.2	SFMBT2
LOC100620561	0	151.208942	-20	4.94E-06	Low	0	183	XP_005674328.1	ssc:100620561	0	NM_138557.2	TLR4
LOC102161872	148.0302721	1.652556743	6.485048616	4.97E-06	High	140	2	XR_305191.1	ssc:102161872			
NTF3	0	150.3826637	-20	5.08E-06	Low	0	182	NM_001123152.1	ssc:100144493	4E-143	NM_002527.4	NTF3
GYG2	8.458872693	609.7934383	-6.171711407	5.11E-06	Low	8	738	XP_005658942.1	ssc:102163050	3E-26	NM_003918.2	GYG2
STX11	6.344154519	494.1144663	-6.283273574	5.14E-06	Low	6	598	XP_001926274.1	ssc:100152253	2E-143	NM_003764.3	STX11
SH2D3C	4.229436346	379.2617726	-6.486584744	5.20E-06	Low	4	459	XP_003353720.1	ssc:100151897	0	NM_170600.2	SH2D3C
ANXA8	15.8603863	1014.66984	-5.999438651	5.21E-06	Low	15	1228	NM_001243599.1	ssc:100155930	0	NM_001271703.1	ANXA8
LOC102157888	87.76080419	0	20	5.22E-06	High	83	0	XP_005673916.1	ssc:102157888	3E-15	NM_153448.3	ESX1
NXNL2	87.76080419	0	20	5.22E-06	High	83	0	XP_005670441.1	ssc:100739001	8E-47	NM_145283.2	NXNL2
LMO1	87.76080419	0	20	5.22E-06	High	83	0	NM_001113055.1	ssc:100127356	3E-86	NR_073006.1	LMO1
CCNJL	1210.676154	32.2248565	5.231495263	5.31E-06	High	1145	39	XP_003359878.2	ssc:100626618	5E-174	NM_024565.5	CCNJL
LOC102160711	7.401513606	546.9962821	-6.207566886	5.35E-06	Low	7	662	XP_005652444.1	ssc:102160711			
LOC102159968	0	148.7301069	-20	5.35E-06	Low	0	180	XR_302917.1	ssc:102159968			
PRDM16	0	148.7301069	-20	5.35E-06	Low	0	180	XP_005674579.1	ssc:100624476	0	NM_199454.2	PRDM16
ARID5A	7.401513606	546.1700037	-6.20538594	5.39E-06	Low	7	661	XP_005662382.1	ssc:100524615	0	NM_212481.1	ARID5A
LOC100157228	94.1049587	5598.862247	-5.894719129	5.41E-06	Low	89	6776	XP_005671626.1	ssc:100157228	0	NM_021641	ADAM12
LOC100518095	102.5638314	6110.328559	-5.896655991	5.43E-06	Low	97	7395	XP_005670428.1	ssc:100518095	1E-28	NM_002462.4	MX1
KIT	510.7044388	12.39417558	5.364754382	5.45E-06	High	483	15	NM_001044525.1	ssc:396810	7E-53	NM_001093772.1	KIT
LOC100621301	0	147.9038285	-20	5.50E-06	Low	0	179	XR_309146.1	ssc:100621301			
LOC100523068	0	147.0775502	-20	5.65E-06	Low	0	178	LOC100523068	ssc:100523068			
DPYSL4	0	147.0775502	-20	5.65E-06	Low	0	178	XP_003483624.1	ssc:100512868	0	NM_006426.2	DPYSL4
CEP170B	50.75323616	2975.428417	-5.873453784	5.66E-06	Low	48	3601	XP_005656532.1	ssc:100521503	0	NM_015005.2	CEP170B
SLC25A53	790.9045968	20.65695929	5.258803869	5.72E-06	High	748	25	XP_003135335.1	ssc:100514813	1E-164	NM_001012755.4	SLC25A53
ICOSLG	31.7207726	1877.304461	-5.887090925	5.74E-06	Low	30	2272	XP_005657224.1	ssc:100621467	4E-84	NM_015259.5	ICOSLG
FAM217B	0	146.2512718	-20	5.81E-06	Low	0	177	XP_005673124.1	ssc:102162581	1E-112	NM_022106.2	FAM217B
WIPF3	12094.07323	319.7697299	5.241123033	5.82E-06	High	11438	387	XP_005673342.1	ssc:100514743	2E-138	NM_001080529.2	WIPF3
TRIM9	2.114718173	259.4514087	-6.93885515	5.85E-06	Low	2	314	XP_001928246.2	ssc:100153738			
PODN	1.057359087	203.2644794	-7.586748906	5.90E-06	Low	1	246	XP_005665471.1	ssc:100622214			
IGFBP3	0	145.4249934	-20	5.97E-06	Low	0	176	NM_001005156.1	ssc:448812			
LOC100522595	0	145.4249934	-20	5.97E-06	Low	0	176	XR_309484.1	ssc:100522595			
LOC100737264	45.46644072	2640.785676	-5.860021339	5.97E-06	Low	43	3196	XP_003483748.1	ssc:100737264	0	NM_014585.5	SLC40A1
SLC40A1	45.46644072	2637.480563	-5.858214582	6.01E-06	Low	43	3192	XP_003359638.1	ssc:100512076	0	NM_014585.5	SLC40A1
ELOVL3	85.64608601	0	20	6.02E-06	High	81	0	NM_001167634.1	ssc:100155434	6E-128	NM_152310.2	ELOVL3
TRPC3	5.286795433	421.4019696	-6.316659743	6.15E-06	Low	5	510	NM_001145749.1	ssc:397210	0	NM_003305.2	TRPC3
KCNH3	142.7434767	1.652556743	6.432581196	6.17E-06	High	135	2	XP_003126164.1	ssc:100514837	0	NM_012284.1	KCNH3
NOL4	142.7434767	1.652556743	6.432581196	6.17E-06	High	135	2	XP_005665391.1	ssc:100518847	2E-155	NR_036752.1	NOL4
LOC102163597	3.17207726	310.6806678	-6.613860752	6.24E-06	Low	3	376	XP_005662469.1	ssc:102163597	4E-32	NM_006634.2	VAMP5
LOC100517809	892.4110691	23.96207278	5.218883793	6.27E-06	High	844	29	XP_005661493.1	ssc:100517809			
RSPO3	0	143.7724367	-20	6.31E-06	Low	0	174	XP_001926731.3	ssc:100155208	3E-120	NM_032784.4	RSPO3
AFAP1	27.49133625	1600.501206	-5.863402921	6.45E-06	Low	26	1937	NM_001253821.1	ssc:100522448	0	NM_198595.2	AFAP1
C1QTNF3	1.057359087	199.1330876	-7.557123737	6.52E-06	Low	1	241	NM_001145386.1	ssc:100270814	4E-167	NM_181435.5	C1QTNF3
PCDHB10	358.4447303	8.262783717	5.438978967	6.56E-06	High	339	10	XP_003480986.1	ssc:100739340	0	NM_019119.4	PCDHB9
PDK2	15.8603863	967.5719733	-5.930869166	6.58E-06	Low	15	1171	XP_005668938.1	ssc:100516037	0	NM_002611.4	PDK2
TMEM45A	17.97510447	1079.119553	-5.907710741	6.61E-06	Low	17	1306	XP_005654125.1	ssc:100522901	3E-111	NM_018004.1	TMEM45A
PPFIBP2	0	142.1198799	-20	6.67E-06	Low	0	172	XP_005653690.1	ssc:100738007	7E-154	NM_003621.3	PPFIBP2
NYNRIN	62.38418611	3536.471431	-5.824986433	6.67E-06	Low	59	4280	XP_005666290.1	ssc:100155701	4E-64	NM_025081.2	NYNRIN
MREG	1022.466237	28.09346464	5.185674537	6.75E-06	High	967	34	XP_003359689.1	ssc:100627946	8E-103	NM_018000.2	MREG
JAM3	99.39175414	5652.570341	-5.829637064	6.80E-06	Low	94	6841	XP_003130135.2	ssc:100520043	1E-151	NM_032801.4	JAM3
LOC100626048	0	141.2936016	-20	6.86E-06	Low	0	171	XR_303884.1	ssc:100626048	2E-139	NM_021632.3	ZNF350
DNAJC22	83.53136784	0	20	6.97E-06	High	79	0	XP_005655614.1	ssc:100513730	1E-157	NM_024902.2	DNAJC22

LOC100739615	193.4967128	3.305113487	5.871465438	7.03E-06	High	183	4	XR_303107.1	ssc:100739615			
DDX58	96.21967688	5427.822624	-5.817897811	7.05E-06	Low	91	6569	NM_213804.2	ssc:396723	0	NM_014314.3	DDX58
LOC100516232	0	140.4673232	-20	7.06E-06	Low	0	170	XP_003133753.2	ssc:100516232			
ALDH1L1	843.7725511	23.13579441	5.188655613	7.24E-06	High	798	28	NM_001244647.1	ssc:100622210	0	NR_072979.1	ALDH1L1
LOC102166594	7.401513606	513.9451472	-6.117650249	7.26E-06	Low	7	622	XP_005658920.1	ssc:102166594	7E-36	NM_017912.3	HERC6
LRRC4B	1.057359087	194.1754174	-7.520751347	7.36E-06	Low	1	235	XP_003356057.2		0	NM_001080457.1	LRRC4B
LOC100521600	5.286795433	404.8764021	-6.258944245	7.47E-06	Low	5	490	XP_005652546.1	ssc:100521600			
LOC102165837	82.47400875	0	20	7.51E-06	High	78	0	XR_309178.1	ssc:102165837			
GABRE	0	137.9884881	-20	7.69E-06	Low	0	167	XP_003135510.1	ssc:100516716	0	NM_021990.2	GABRE
FAM184B	137.4566813	1.652556743	6.378133412	7.72E-06	High	130	2	XP_005674598.1	ssc:100627969	2E-35	NM_015688.1	FAM184B
LOC100737490	163.8906584	2.478835115	6.046927504	7.77E-06	High	155	3	XP_003483716.2	ssc:100737490	0	NM_006920.4	SCN1A
LOC102163451	268.569208	5.783948602	5.537095364	7.89E-06	High	254	7	XR_301982.1	ssc:102163451			
PLD5	1411.574381	40.48764021	5.123679782	7.94E-06	High	1335	49	XP_005668008.1	ssc:100622421	0	NM_152666.2	PLD5
ENPP6	2.114718173	242.9258413	-6.843906746	8.06E-06	Low	2	294	XP_005671763.1	ssc:100152050	1E-169	NM_153343.3	ENPP6
LTBR	99.39175414	5448.479583	-5.776583721	8.15E-06	Low	94	6594	XP_005653218.1	ssc:100520196	1E-144	NM_002342.2	LTBR
SDK2	2.114718173	242.0995629	-6.838991255	8.19E-06	Low	2	293	XP_005668701.1	ssc:100518574	0	NM_019064.3	SDK2
LOC100737780	108.9079859	0.826278372	7.042266126	8.36E-06	High	103	1	XP_003480480.1	ssc:100737780	1E-73	NM_153181.2	NETO1
LOC100737019	10.57359087	656.0650272	-5.955301503	8.37E-06	Low	10	794	LOC100737019				
TNFAIP3	37.00756803	2016.119227	-5.767616816	8.38E-06	Low	35	2440	NM_001267890.1	ssc:100622156	0	NM_006290.3	TNFAIP3
LOC100623510	10.57359087	655.2387488	-5.953483362	8.43E-06	Low	10	793	XP_003362027.1	ssc:100623510	4E-46	NM_006272.2	S100B
NPHS2	135.3419631	1.652556743	6.355765599	8.46E-06	High	128	2	XP_005667854.1	ssc:100523986	0	NM_014625.2	NPHS2
PARP3	141.6861176	7787.673654	-5.780422113	8.49E-06	Low	134	9425	XP_005669672.1	ssc:100620838	0	NM_005485.4	PARP3
FOXC2	12.68830904	753.565875	-5.892161914	8.82E-06	Low	12	912	XP_005674616.1	ssc:100526107	1E-167	NM_005251.2	FOXC2
LOC100627460	8.458872693	544.517447	-6.008369056	8.89E-06	Low	8	659	LOC100627460				
SCARA5	0	133.8570962	-20	8.90E-06	Low	0	162	XP_005670506.1	ssc:100524281	0	NM_173833.5	SCARA5
LOC100620286	3.17207726	288.3711517	-6.506355126	8.98E-06	Low	3	349	XP_005668185.1	ssc:100620286	9E-135	NM_173576.2	MKX
HYAL1	1.057359087	185.9126336	-7.458015592	9.09E-06	Low	1	225	NM_214441.1	ssc:404698	4E-132	NR_047690.1	HYAL1
SOCS3	1.057359087	185.9126336	-7.458015592	9.09E-06	Low	1	225	NM_001123196.1	ssc:493186	1E-23	NM_003955.4	SOCS3
LOC100521595	339.4122668	8.262783717	5.360266991	9.11E-06	High	321	10	XP_005659460.1	ssc:100521595	5E-44	NR_103525.1	RIPPLY2
ZNF792	0	133.0308178	-20	9.17E-06	Low	0	161	ZNF792	ssc:100514843	0	NM_175872.4	ZNF792
LOC102162351	0	133.0308178	-20	9.17E-06	Low	0	161	XP_005657866.1	ssc:102162351	0	NR_027121.1	SHROOM4
LOC100739500	7.401513606	489.9830744	-6.048767773	9.17E-06	Low	7	593	XP_003481823.2	ssc:100739500	0	NR_104293.1	ACSF3
ADAMTS19	468.4100754	12.39417558	5.240037892	9.19E-06	High	443	15	XP_003123951.4	ssc:100518181	0	NM_133638.3	ADAMTS19
MYLK	26.43397716	1434.419253	-5.761929443	9.23E-06	Low	25	1736	XP_005670216.1	ssc:396848	0	NM_053032.2	MYLK
KCP	1.057359087	185.0863553	-7.451589323	9.29E-06	Low	1	224	XP_005657822.1	ssc:100517738	0	NM_199349.2	KCP
FZD5	835.3136784	23.96207278	5.123493447	9.35E-06	High	790	29	XP_005672190.1	ssc:100519058	0	NM_030804.1	FZD5
ACOT12	79.30193149	0	20	9.44E-06	High	75	0	XP_003480920.1	ssc:100738062	0	NM_130767.2	ACOT12
LOC100736853	4.229436346	335.4690189	-6.309570318	9.46E-06	Low	4	406	XP_003482717.1	ssc:100736853	5E-166	NM_024788.2	C7orf63
SERPINF1	474.7542299	26974.68372	-5.82828138	9.50E-06	Low	449	32646	NM_001078662.1	ssc:780402	2E-16	NM_002615.5	SERPINF1
AIPL1	0	131.3782611	-20	9.73E-06	Low	0	159	XP_003131940.1	ssc:100524458	0	NM_014336	AIPL1
NT5E	112.0800632	5935.983823	-5.726885581	9.82E-06	Low	106	7184	XP_003353298.1	ssc:100157995	0	NM_002526.3	NT5E
LOC100626812	1040.441341	30.57229975	5.088826739	9.84E-06	High	984	37	XP_003357377.3	ssc:100626812	0	NM_003105.5	SORL1
ELTD1	0	130.5519827	-20	1.00E-05	Low	0	158	XP_005665418.1	ssc:100525348	0	NM_022159.3	ELTD1
NXF2B	78.24457241	0	20	1.02E-05	High	74	0	XP_005657925.1	ssc:100153087	0	NM_001099686.2	NXF2B
APOE	57.09739067	2928.330549	-5.680509787	1.09E-05	Low	54	3544	NM_214308.1	ssc:397576	7E-118	NM_000041.2	APOE
LOC102163538	4.229436346	325.5536785	-6.26628622	1.10E-05	Low	4	394	XP_005668658.1	ssc:102163538	2E-59	NM_020954.3	RNF213
TMEM140	6.344154519	423.0545263	-6.0592719	1.10E-05	Low	6	512	XP_003134698.1	ssc:100523705	5E-54	NM_018295.4	TMEM140
IRX4	0	128.0731476	-20	1.10E-05	Low	0	155	XP_003134213.1	ssc:100519749	0	NM_016358.2	IRX4
LOC100516179	2.114718173	227.2265522	-6.747522209	1.11E-05	Low	2	275	XP_003123076.3	ssc:100516179	1E-154	NM_021217.2	ZNF77
PTPLAD2	51.81059524	2645.743346	-5.674282149	1.12E-05	Low	49	3202	XP_003121932.1	ssc:100523711	6E-75	NM_001010915.3	PTPLAD2
LOC102157535	428.2304301	11.5678972	5.210188775	1.12E-05	High	405	14	LOC102157535				
LOC100621484	3.17207726	275.1506978	-6.438650267	1.13E-05	Low	3	333	XP_005658487.1	ssc:100621484	0	NM_138931.1	BCL6
NDST3	178.6936856	3.305113487	5.756645036	1.13E-05	High	169	4	XP_005666957.1	ssc:100525060	0	NM_004784.2	NDST3
NTNG2	0	127.2468692	-20	1.13E-05	Low	0	154	XP_003122308.3	ssc:100512264	0	NM_014917.2	NTNG1
LOC100625548	127.9404495	1.652556743	6.274628837	1.18E-05	High	121	2	XP_003361336.1	ssc:100625548	1E-137	NR_103801.1	GABRB3
ANKRD6	324.6092396	8.262783717	5.29593235	1.19E-05	High	307	10	XP_005674610.1	ssc:102167335	0	NM_014942.4	ANKRD6
HPRT1	26233.07894	751.9133183	5.124676979	1.19E-05	High	24810	910	NM_001032376.2	ssc:397351	5E-119	NM_000194.2	HPRT1
ANKS1B	76.12985423	0	20	1.20E-05	High	72	0	XP_005664274.1	ssc:100621174	3E-165	NM_181670.3	ANKS1B
TMPRSS11F	0	125.5943125	-20	1.21E-05	Low	0	152	XP_003129105.3	ssc:100513296	0	NM_207407.2	TMPRSS11F
FAM162B	1057.359087	32.2248565	5.036147665	1.21E-05	High	1000	39	XP_005654448.1	ssc:100152527	1E-66	NM_001085480.2	FAM162B
BEX1	5428.48155	166.0819527	5.030581489	1.24E-05	High	5134	201	NM_001244771.1	ssc:100513130	2E-49	NM_018476.3	BEX1
SRPX	19.03246356	999.7968298	-5.715100731	1.24E-05	Low	18	1210	XP_005673612.1	ssc:100156108	0	NM_006307.4	SRPX
LOC100523405	1.057359087	174.3447364	-7.365333589	1.24E-05	Low	1	211	XP_003133273.1	ssc:100523405	4E-16	NM_021641	ADAM12
HOMER3	232.618999	12073.57957	-5.697740726	1.24E-05	Low	220	14612	XP_003480800.1	ssc:100526210	1E-38	NR_027297.1	HOMER3
C2H19orf66	15.8603863	848.5878878	-5.741564271	1.25E-05	Low	15	1027	NM_001244321.1	ssc:100512458	7E-153	NM_018381.2	C19orf66
TRNAG-UCC	101.5064723	0.826278372	6.9407281	1.26E-05	High	96	1	TRNAG-UCC				
LOC100521298	101.5064723	0.826278372	6.9407281	1.26E-05	High	96	1	LOC100521298				
LOC102161895	0	123.9417558	-20	1.29E-05	Low	0	150	XR_297140.1	ssc:102161895			
OTUD7A	1.057359087	172.6921797	-7.351593533	1.30E-05	Low	1	209	XP_005659864.1	ssc:100525093	0	NM_130901.1	OTUD7A
GABRB3	125.8257313	1.652556743	6.250583363	1.30E-05	High	119	2	XP_005654529.1	ssc:100739031	7E-139	NR_103801.1	GABRB3
AFF3	614.3256293	18.17812418	5.078728334	1.31E-05	High	581	22	XP_005662434.1	ssc:100522606	0	NM_002285.2	AFF3
MX1	943.1643052	52119.16085	-5.788160926	1.32E-05	Low	892	63077	NM_214061.1	ssc:397128	0	NM_002462.4	MX1
LOC100517194	1.057359087	171.8659013	-7.344674119	1.33E-05	Low	1	208	XR_307362.1	ssc:100517194			
CDH15	1.057359087	171.8659013	-7.344674119	1.33E-05	Low	1	208	XP_005655832.1	ssc:100187727	0	NM_001793.4	CDH3
OSTF1	71.90041789	3543.081658	-5.62286075	1.34E-05	Low	68	4288	NM_214005.1	ssc:397066	1E-123	NM_012383.4	OSTF1
LOC100524949	0	122.289199	-20	1.37E-05	Low	0	148	XP_003130654.3	ssc:100524949	2E-136	NM_004433.4	ELF3
CXCL14	9.516231779	545.3437253	-5.840631614	1.37E-05	Low	9	660	NM_001244128.1	ssc:494467	6E-44	NM_004887.4	CXCL14
LOC100627693	17.97510447	927.9106115	-5.689913772	1.39E-05	Low	17	1123	XP_005667597.1	ssc:100627693	2E-106	NM_177403.4	RAB7B
SEMA3E	340.4696259	9.089062089	5.227250859	1.39E-05	High	322	11	XP_003130268.3	ssc:100524100	0	NM_012431.2	SEMA3E
PLAUR	50.75323616	2475.530002	-5.608093809	1.40E-05	Low	48	2996	XP_005655990.1	ssc:100521017	7E-119	NM_002659.3	PLAUR
LOC100738076	3.17207726	262.7565222	-6.372154855	1.41E-05	Low	3	318	XP_005665361.1	ssc:100738076	2E-34	NM_032048.2	EMILIN2
LOC100520093	385.9360666	10.74161883	5.167078535	1.45E-05	High	365	13	LOC100520093				
LOC100623829	3.17207726	261.1039655	-6.363052648	1.45E-05	Low	3	316	XP_003358850.1	ssc:100623829	0	NM_015642.4	ZBTB20
LOC102164566	0	120.6366423	-20	1.46E-05	Low	0	146	XR_300984.1	ssc:102164566			
EFEMP2	220.9880491	11063.04112	-5.645635861	1.47E-05	Low	209	13389	XP_005660736.1	ssc:100526031	0	NR_037718.1	EFEMP2
JHigh	247.4220263	12395.82813	-5.646736908	1.50E-05	Low	234	15002	NM_214323.1	ssc:397592	0	NM_021991.2	JHigh
CCDC42	0	119.8103639	-20	1.51E-05	Low	0	145	NM_001244077.1	ssc:100514007	8E-156	NM_144681.2	CCDC42
LOC100625208	72.95777697	0	20	1.53E-05	High	69	0	XP_005658068.1	ssc:100625208	2E-143	NM_002538.3	OCLN
LAMA1	429.2877891	12.39417558	5.114210921	1.55E-05	High	406	15	XP_005658620.1	ssc:100625589			
LOC100524517	1997.351315	64.44971299	4.953770367	1.55E-05	High	1889	78	XP_005653846.1	ssc:100524517	2E-175	NM_003240.3	LEFTY2
LOC102166489	0	118.9840855	-20	1.56E-05	Low	0	144	XP_005670814.1	ssc:102166489	5E-55	NM_014653.2	WSCD2
GDAP1	0	118.9840855	-20	1.56E-05	Low	0	144	XP_005663078.1	ssc:100157012	0	NR_046346.1	GDAP1
COL5A2	1571.235603	86391.53516	-5.78091853	1.57E-05	Low	1486	104555	NM_001105289.1	ssc:397532			
HTRA3	20.08982264	999.7968298	-5.637098219	1.57E-05	Low	19	1210	NM_001195343.1	ssc:100337673			
AXL	189.2672765	9277.453558	-5.615231972	1.58E-05	Low	179	11228	NM_001128458.1	ssc:100144875			
SH2D6	34.89284986	1673.213703	-5.583546474	1.58E-05	Low	33	2025	XP_005655277.1	ssc:100520186	1E-60	NM_198482.1	SH2D6
LOC102160907	121.596295	1.652556743	6.20125565	1.59E-05	High	115	2	XP_005669665.1	ssc:102160907	0	NM_004947.4	DOCK3
KRT6A	121.596295	1.652556743	6.20125565	1.59E-05	High	115	2	XP_005652608.1	ssc:100737483	0	NM_173086.4	KRT6C
LOC100153163	40.17964529	1916.139544	-5.575593992	1.59E-05	Low	38	2319	XP_003128354.1	ssc:100153163	4E-77	NM_005931	MICB
CMAH	1.057359087	165.2556743	-7.288090591	1.60E-05	Low	1	200	NM_001113015.1	ssc:396918			

MYLPF	13.74566813	713.0782348	-5.697011432	1.61E-05	Low	13	863	NM_001006592.1	ssc:474162	8E-93	NM_013292.3	MYLPF
CHRNA1	0	118.1578072	-20	1.62E-05	Low	0	143	XP_001924357.4	ssc:396587	0	NM_001039523.2	CHRNA1
ASB9	5.286795433	344.558081	-6.026209879	1.64E-05	Low	5	417	NM_001243703.1	ssc:100158057	2E-114	NM_024087.2	ASB9
RAPGEF5	1801.739884	58.66576439	4.9407281	1.64E-05	High	1704	71	XP_005656743.1	ssc:100518983	0	NM_012294.3	RAPGEF5
LOC100737535	4.229436346	299.1127706	-6.144080288	1.65E-05	Low	4	362	LOC100737535				
LOC100623234	40.17964529	1897.96142	-5.561842029	1.67E-05	Low	38	2297	XP_003359698.1	ssc:100623234	3E-119	NM_005876.4	SPEG
RBP5	0	117.3315288	-20	1.67E-05	Low	0	142	NM_001145223.1	ssc:100270724	3E-69	NM_031491.2	RBP5
LOC100627840	0	117.3315288	-20	1.67E-05	Low	0	142	XP_003355471.1	ssc:100627840	2E-130	NR_003084.2	HOXC5
MFAP3L	281.257517	7.436505346	5.241123033	1.74E-05	High	266	9	XP_005670543.1	ssc:100519447	0	NM_021647.6	MFAP3L
LOC100511337	2.114718173	206.5695929	-6.610018685	1.77E-05	Low	2	250	XP_005667019.1	ssc:100511337			
LOC102167737	2.114718173	206.5695929	-6.610018685	1.77E-05	Low	2	250	LOC102167737				
PCDH17	69.78569971	3234.053547	-5.534268322	1.80E-05	Low	66	3914	XP_005668514.1	ssc:100157362	0	NM_014459.2	PCDH17
CCDC38	701.0290744	22.30951604	4.973743157	1.81E-05	High	663	27	XP_005664300.1	ssc:100157054	0	NM_182496.2	CCDC38
LOC100622219	70.8430588	0	20	1.81E-05	High	67	0	XP_003357960.2	ssc:100622219	0	NM_001676.5	ATP12A
LOC102163689	70.8430588	0	20	1.81E-05	High	67	0	XR_305017.1	ssc:102163689			
LOC100157853	70.8430588	0	20	1.81E-05	High	67	0	LOC100157853				
RELB	20.08982264	970.0508084	-5.59352358	1.82E-05	Low	19	1174	XP_005659055.1	ssc:100622609	0	NM_006509.3	RELB
SEMA3A	0	114.8526937	-20	1.85E-05	Low	0	139	XP_005667738.1	ssc:102163327	2E-152	NM_006080.2	SEMA3A
RXFP4	0	114.8526937	-20	1.85E-05	Low	0	139	NM_001005844.1	ssc:450213	4E-167	NM_181885.2	RXFP4
LOC100623937	187.1525583	4.131391859	5.501443054	1.85E-05	High	177	5	XP_005663636.1	ssc:100623937	0	NR_036437.1	KCNC4
LOC102164968	118.4242177	1.652556743	6.163120521	1.86E-05	High	112	2	XP_005652633.1	ssc:102164968	2E-47	NM_005555.3	KRT6B
COL14A1	455.7217663	22555.74699	-5.629197977	1.86E-05	Low	431	27298	XP_005662938.1	ssc:100158059	0	NM_021110.2	COL14A1
LOC102161702	370.0756803	10.74161883	5.106536993	1.86E-05	High	350	13	XR_298374.1	ssc:102161702			
SPINT2	2.114718173	203.2644794	-6.586748906	1.91E-05	Low	2	246	XP_005664623.1	ssc:100515207	3E-74	NM_021102.3	SPINT2
ENO2	58.15474976	2659.790079	-5.515271549	1.91E-05	Low	55	3219	XP_005652652.1	ssc:100157750	0	NM_001975.2	ENO2
LOC100625694	0	114.0264153	-20	1.92E-05	Low	0	138	XP_005653361.1	ssc:100625694	0	NM_024840.3	ZNF613
LOC102167708	322.4945214	9.089062089	5.148999413	1.92E-05	High	305	11	XR_308346.1	ssc:102167708			
LOC100512657	14.80302721	730.4300806	-5.624782038	1.94E-05	Low	14	884	XP_003360629.2	ssc:100512657	1E-140	NM_007191.4	WIF1
FOXH1	1605.071093	53.70809416	4.901353862	1.95E-05	High	1518	65	XP_001925315.1	ssc:100154027	4E-154	NM_003923.2	FOXH1
LOC102158695	1.057359087	158.6454474	-7.229196901	1.95E-05	Low	1	192	XP_005669287.1	ssc:102158695	4E-59	NM_004822.2	NTN1
CLDN1	10.57359087	551.1276739	-5.703849257	1.96E-05	Low	10	667	NM_001244539.1	ssc:100625166	1E-109	NM_021101.4	CLDN1
CCDC85C	1238.16749	41.31391859	4.90543477	1.97E-05	High	1171	50	XP_005666495.1	ssc:100512614	3E-83	NM_001144995.1	CCDC85C
FSTL3	0	113.2001369	-20	1.98E-05	Low	0	137	XP_005659092.1	ssc:102164292	2E-37	NM_005860.2	FSTL3
LOC102163288	7.401513606	418.0968561	-5.819873053	1.98E-05	Low	7	506	XP_005668656.1	ssc:102163288	2E-135	NM_020954.3	RNF213
CCDC85A	1116.571195	37.18252673	4.908306622	1.99E-05	High	1056	45	XP_005662596.1	ssc:100511688	0	NM_001080433.1	CCDC85A
LOC100739238	15.8603863	770.9177208	-5.603077076	2.00E-05	Low	15	933	XP_005660681.1	ssc:100739238	0	NM_002335.2	LRP5
LOC100511825	433.5172255	13.22045395	5.035245699	2.02E-05	High	410	16	XP_005653655.1	ssc:100511825			
IL16	7.401513606	416.4442994	-5.814159402	2.02E-05	Low	7	504	NM_213751.1	ssc:396589	0	NM_172217.3	IL16
CAMSAP3	524.4501069	16.52556743	4.988033815	2.05E-05	High	496	20	XP_005654804.1	ssc:100511741	0	NM_020902.1	CAMSAP3
LOC102165358	4.229436346	285.8923166	-6.078862628	2.06E-05	Low	4	346	XR_298944.1	ssc:102165358			
JUNB	67.67098154	3046.488357	-5.492466102	2.08E-05	Low	64	3687	XP_005674540.1	ssc:100523358			
KCNJ14	476.868948	14.87301069	5.002824221	2.09E-05	High	451	18	XP_003127320.1	ssc:100521454	0	np_733838	np_733838
SIPA1L2	823.6827284	27.26718627	4.916850998	2.10E-05	High	779	33	XP_001928528.3	ssc:100158048	0	NM_020808.3	SIPA1L2
LOC100738401	89.87552236	4055.374248	-5.495762998	2.10E-05	Low	85	4908	XP_003483277.2	ssc:100738401			
VSTM4	60.26946793	2702.756554	-5.486860431	2.11E-05	Low	57	3271	XP_005671270.1	ssc:100157009	3E-160	NM_144984.3	VSTM4
LOC100512258	30.66341351	1392.279056	-5.504786282	2.12E-05	Low	29	1685	XR_308149.1	ssc:100512258			
CKMT1B	205.1276628	4.95767023	5.370715941	2.12E-05	High	194	6	XP_005659734.1	ssc:100519994	0	NM_001015001.1	CKMT1A
PSP-I	0	111.5475802	-20	2.13E-05	Low	0	135	NM_213837.1	ssc:396818			
PTK2B	28.54869534	1297.257044	-5.505895742	2.15E-05	Low	27	1570	XP_005657321.1	ssc:100157507	0	NM_173176.2	PTK2B
TRIM34	3.17207726	240.4470062	-6.244147243	2.17E-05	Low	3	291	XP_005667139.1	ssc:100738479	1E-146	NM_001003819.3	TRIM6-TRIM34
LOC100624137	0	110.7213018	-20	2.20E-05	Low	0	134	XR_299148.1	ssc:100624137			
PDE3A	0	110.7213018	-20	2.20E-05	Low	0	134	NM_213736.1	ssc:396555	2E-79	NM_001244683.1	PDE3A
LOC100525798	746.4955151	24.78835115	4.912399377	2.22E-05	High	706	30	XP_003135234.1	ssc:100525798	0	NM_021963.3	NAP1L2
MT-2B	49.69587707	2206.163253	-5.472269576	2.22E-05	Low	47	2670	XP_003355856.1	ssc:396827	2E-14	NM_005953.3	MT2A
LOC100738863	26.43397716	1196.451082	-5.500224098	2.24E-05	Low	25	1448	XP_005656100.1	ssc:100738863	1E-161	NM_005529.5	HSPG2
WNT8B	203.0129446	4.95767023	5.355765599	2.26E-05	High	192	6	XP_003125637.4	ssc:100513655	0	NM_003393.3	WNT8B
EN1	0	109.8950234	-20	2.28E-05	Low	0	133	XP_003133330.1	ssc:100516112	1E-122	NM_001426.3	EN1
CMKLR1	0	109.8950234	-20	2.28E-05	Low	0	133	NM_001123100.1	ssc:780421	0	NM_004072.2	CMKLR1
OSR2	7.401513606	404.8764021	-5.773517418	2.32E-05	Low	7	490	XP_005655406.1	ssc:100155180	0	NM_053001.3	OSR2
LOC780439	32.77813168	1455.902491	-5.473036299	2.33E-05	Low	31	1762	NM_001078688.1	ssc:780439	1E-70	NM_003102.2	SOD3
MMP19	84.58872693	3729.82057	-5.462497009	2.33E-05	Low	80	4514	XP_005663940.1	ssc:100518427	0	NR_073606.1	MMP19
ADAM22	445.1481754	14.04673232	4.985979181	2.35E-05	High	421	17	XP_005667627.1	ssc:100513676	0	NM_021723.3	ADAM22
GUCY2C	1083.793064	37.18252673	4.865320697	2.37E-05	High	1025	45	NM_214105.1	ssc:397193	1E-118	NM_004963.3	GUCY2C
TNN	3.17207726	235.4893359	-6.214090009	2.39E-05	Low	3	285	XP_005656793.1	ssc:100520506	0	NM_022093.1	TNN
LOC100628185	15.8603863	741.1716994	-5.54630798	2.42E-05	Low	15	897	XP_005658364.1	ssc:100628185			
LOC100620844	2983.867342	103.2847965	4.852483587	2.43E-05	High	2822	125	XP_005660357.1	ssc:100620844	5E-67	NM_001018116.2	MURC
ANKRD35	17.97510447	825.4520934	-5.521112427	2.45E-05	Low	17	999	XP_003125839.1	ssc:100511691	0	NM_144698.4	ANKRD35
LOC100152707	0	108.2424667	-20	2.45E-05	Low	0	131	XP_003126250.1	ssc:100152707	2E-130	NR_003084.2	HOXC5
LOC100620517	0	108.2424667	-20	2.45E-05	Low	0	131	XP_005657265.1	ssc:100620517	6E-84	NM_001495.4	GFRA2
ARHGEF19	14.80302721	695.726389	-5.554555902	2.46E-05	Low	14	842	XP_003127681.1	ssc:100521628	0	NM_153213.3	ARHGEF19
TNFRSF1B	61.32682702	2657.311243	-5.437305097	2.49E-05	Low	58	3216	NM_001097441.2	ssc:100037306			
SCG3	286.5443125	8.262783717	5.115986546	2.50E-05	High	271	10	XP_001925946.1	ssc:100154760	0	NM_013243.3	SCG3
NRG3	462.0659208	14.87301069	4.957330067	2.52E-05	High	437	18	XP_001927043.2	ssc:100158106			
STXBP5L	264.3397716	7.436505346	5.151624882	2.52E-05	High	250	9	XP_005670233.1	ssc:100620808	0	NM_014980.2	STXBP5L
LOC102158596	66.61362245	0	20	2.57E-05	High	63	0	XR_298346.1	ssc:102158596			
PDLIM1	196.6687901	8727.152162	-5.471671016	2.59E-05	Low	186	10562	XP_003483582.1	ssc:100155592	1E-136	NM_020992.3	PDLIM1
FRAS1	2664.544898	93.36945601	4.834794655	2.59E-05	High	2520	113	XP_003129179.3	ssc:100514970	0	NM_206841.1	FRAS1
MYO7A	133.2272449	2.478835115	5.748083022	2.63E-05	High	126	3	NM_001099928.1	ssc:397373	0	NM_001127180.1	MYO7A
CXCL11	0	106.58991	-20	2.64E-05	Low	0	129	NM_001128491.1	ssc:100169744	5E-40	NM_005409.4	CXCL11
LOC102165979	0	106.58991	-20	2.64E-05	Low	0	129	XR_303700.1	ssc:102165979			
C-JUN	62.38418611	2668.879141	-5.418909801	2.66E-05	Low	59	3230	NM_213880.1	ssc:396913	9E-166	NM_002228.3	JUN
LOC100738921	326.7239578	9.915340461	5.042266126	2.66E-05	High	309	12	XP_003484203.1	ssc:100738921	0	NM_144967.3	ARHGAP36
LOC100737466	21.14718173	936.9996735	-5.469511231	2.70E-05	Low	20	1134	XP_003484332.1	ssc:100737466	0	NM_014314.3	DDX58
CTBS	111.0227041	1.652556743	6.070011117	2.70E-05	High	105	2	XP_003356466.3	ssc:100523254	2E-89	NM_004388.2	CTBS
GLDC	3314.820736	116.5052504	4.830463975	2.70E-05	High	3135	141	XP_001925488.2	ssc:100155425	0	NM_000170.2	GLDC
LOC100738968	0	105.7636316	-20	2.74E-05	Low	0	128	XP_003481531.1	ssc:100738968	7E-123	NM_002506.2	NGF
CEBPB	27.49133625	1190.667134	-5.436649303	2.74E-05	Low	26	1441	NM_001199889.1	ssc:397360	1E-132	NM_005194.3	CEBPB
PTER	2.114718173	188.3914688	-6.477124415	2.75E-05	Low	2	228	NM_001244248.1	ssc:100625702	0	NM_030664.4	PTER
ANGPTL1	5.286795433	309.028111	-5.869200766	2.77E-05	Low	5	374	NM_001109947.1	ssc:100126165	0	NM_004673.3	ANGPTL1
ATP6V1G3	65.55626337	0	20	2.82E-05	High	62	0	XP_003130645.3	ssc:100522819	1E-48	NM_133326.1	ATP6V1G3
GAD2	65.55626337	0	20	2.82E-05	High	62	0	NM_213895.2	ssc:396929	0	NM_001134366.1	GAD2
GGN	4.229436346	267.7141924	-5.984084404	2.82E-05	Low	4	324	XP_005664642.1	ssc:100515664	3E-177	NM_182477.1	GGN
PLEKHH1	650.2758382	22.30951604	4.865320697	2.83E-05	High	615	27	XP_005656409.1	ssc:100152619	1E-143	NM_020715.2	PLEKHH1
LEPREL2	233.6763581	10254.11459	-5.455547336	2.84E-05	Low	221	12410	XP_005652654.1	ssc:100156539	0	NM_014262.4	LEPREL2
POPDC3	0	104.9373532	-20	2.84E-05	Low	0	127	NM_001144111.1	ssc:100151910	1E-156	NR_024539.1	POPDC3
FOXC1	13.74566813	633.7555111	-5.52687745	2.85E-05	Low	13	767	XP_005665586.1	ssc:102159047	6E-112	NM_001453.2	FOXC1
PPARG	17.97510447	797.3586287	-5.471156692	2.90E-05	Low	17	965	NM_214379.1	ssc:397671	0	NM_138712.3	PPARG
SPOCD1	87.76080419	0.826278372	6.730805031	2.94E-05	High	83	1	XP_003127817.1	ssc:100521137	0	NM_144569.5	SPOCD1
LOC102167736	0	104.1110748	-20	2.95E-05	Low	0	126	XP_005656849.1	ssc:102167736			
FLNC	475.811589	21473.32232	-5.496011199	2.95E-05	Low	450	25988	XP_005657826.1	ssc:100518997	0	NM_001458.4	FLNC

SP140	154.3744266	6587.917458	-5.415316796	2.99E-05	Low	146	7973	XP_005657635.1	ssc:100516764	4E-178	NM_007237.4	SP140
LOC100627814	108.9079859	1.652556743	6.042266126	3.02E-05	High	103	2	XP_003361818.3	ssc:100627814	8E-141	NM_003914.3	CCNA1
LOC100152976	130.0551676	2.478835115	5.713317604	3.03E-05	High	123	3	LOC100152976				
PAX6	2363.197558	85.10667229	4.795324188	3.03E-05	High	2235	103	NM_001244178.1	ssc:100514152	0	NM_001604.5	PAX6
LOC102161685	24430.2817	813.8841962	4.907703154	3.04E-05	High	23105	985	XP_005670278.1	ssc:102161685	3E-31	NM_014590.3	ERVW-1
CHD5	1.057359087	144.5987151	-7.095445513	3.04E-05	Low	1	175	XP_005656076.1	ssc:100738053	0	NM_015557.2	CHD5
LRRC70	555.1135205	19.00440255	4.868377256	3.05E-05	High	525	23	XR_308429.1	ssc:100525544	0	NM_181506.4	LRRC70
LOC100523418	1485.589517	53.70809416	4.789752201	3.09E-05	High	1405	65	XP_003480572.2	ssc:100523418	0	NM_134428.2	RFX3
MSC	9.516231779	461.0633314	-5.598430711	3.10E-05	Low	9	558	XP_003125643.1	ssc:100155518	6E-93	NM_005098.3	MSC
RIC3	3.17207726	223.0951604	-6.136087497	3.10E-05	Low	3	270	XP_005667118.1	ssc:100520142	1E-140	NR_045405.1	RIC3
ZIC1	37.00756803	1539.356607	-5.378363343	3.14E-05	Low	35	1863	ZIC1	ssc:100153570	0	NM_003412.3	ZIC1
LOC100622513	25.37661808	1071.683048	-5.400234664	3.18E-05	Low	24	1297	XP_005674372.1	ssc:100622513	6E-111	NM_005576.2	LOXL1
LOC100628148	0	102.4585181	-20	3.18E-05	Low	0	124	LOC100628148				
LOC100623513	0	102.4585181	-20	3.18E-05	Low	0	124	XP_005663610.1	ssc:100623513	1E-122	NM_002506.2	NGF
LOC102162044	274.9133625	8.262783717	5.056205317	3.19E-05	High	260	10	XP_005668029.1	ssc:102162044	5E-79	NM_016002.2	SCCPDH
LOC102160748	19.03246356	820.4944231	-5.429959307	3.23E-05	Low	18	993	XR_308984.1	ssc:102160748			
LOC100737503	13.74566813	617.2299437	-5.488759115	3.24E-05	Low	13	747	XR_135144.2	ssc:100737503			
CAPG	556.1708795	24799.09277	-5.478615324	3.27E-05	Low	526	30013	XP_005655276.1	ssc:100519653	0	NM_001747.3	CAPG
RBMS3	1.057359087	142.1198799	-7.070499155	3.30E-05	Low	1	172	XR_299279.1	ssc:100739397	1E-56	NM_014483.3	RBMS3
LOC102166397	8.458872693	415.618021	-5.618648991	3.30E-05	Low	8	503	XR_306138.1	ssc:102166397			
ZNF256	0	101.6322397	-20	3.30E-05	Low	0	123	ZNF256	ssc:100516085	0	NM_005773.2	ZNF256
LOC100624765	440.9187391	14.87301069	4.889744171	3.32E-05	High	417	18	XP_005654553.1	ssc:100624765	3E-167	NM_134428.2	RFX3
LOC100512853	63.44154519	0	20	3.39E-05	High	60	0	LOC100512853				
PROX1	630.1860156	22.30951604	4.820046618	3.41E-05	High	596	27	NM_001128490.1	ssc:100169702	0	NM_002763.4	PROX1
LHFPL3	312.9782896	9.915340461	4.980256464	3.43E-05	High	296	12	XP_005667775.1	ssc:100738576	5E-42	NM_199000.2	LHFPL3
LOC100621722	61.32682702	2484.619064	-5.340362558	3.46E-05	Low	58	3007	XP_005669870.1	ssc:100621722			
TGFB3	10.57359087	487.5042393	-5.52687745	3.55E-05	Low	10	590	NM_214198.1	ssc:397400	0	NM_003239.2	TGFB3
DTX3L	25.37661808	1046.894697	-5.366472709	3.56E-05	Low	24	1267	XP_005670232.1	ssc:100520459	0	NM_138287.3	DTX3L
FABP3	2295.526577	85.10667229	4.753409083	3.61E-05	High	2171	103	NM_001099931.1	ssc:399532			
PLXND1	189.2672765	7831.466407	-5.370785569	3.62E-05	Low	179	9478	XP_005654079.1	ssc:102159849	0	NM_015103.2	PLXND1
LOC100624035	0	99.15340461	-20	3.71E-05	Low	0	120	XP_005653366.1	ssc:100624035	0	NM_144689.3	ZNF420
ANKRD63	0	99.15340461	-20	3.71E-05	Low	0	120	XP_005674617.1	ssc:100627919			
SEMA5B	62.38418611	0	20	3.73E-05	High	59	0	XP_005670227.1	ssc:102160172			
LOC100156194	62.38418611	0	20	3.73E-05	High	59	0	LOC100156194				
SLC24A5	5030.914534	184.2600769	4.771005256	3.73E-05	High	4758	223	XP_003121571.1	ssc:100511672	0	NM_205850.2	SLC24A5
LOC100154753	620.6697838	22.30951604	4.79809479	3.73E-05	High	587	27	XR_299439.1	ssc:100154753	1E-62	NM_000984.5	RPL23A
SPATA20	6.344154519	328.0325136	-5.692267097	3.77E-05	Low	6	397	NM_001159313.1	ssc:100286812	0	NM_022827.3	SPATA20
C16H5orf49	4.229436346	251.188625	-5.892161914	3.83E-05	Low	4	304	XP_003134195.3	ssc:100516406	2E-65	NM_001089584.2	C5orf49
LOC102157883	124.7683722	2.478835115	5.653446148	3.85E-05	High	118	3	XP_005661699.1	ssc:102157883			
KCNMA1	3.17207726	213.1798199	-6.070499155	3.86E-05	Low	3	258	NM_214219.1	ssc:397434	5E-44	NM_002247.3	KCNMA1
LOC100621279	0	98.32712624	-20	3.86E-05	Low	0	119	XR_299836.1	ssc:100621279			
SDK1	0	98.32712624	-20	3.86E-05	Low	0	119	XP_005661878.1	ssc:100512953	0	NM_152744.3	SDK1
ZC3H12D	5.286795433	287.5448734	-5.765249802	3.92E-05	Low	5	348	ZC3H12D	ssc:100156984	0	NM_207360.2	ZC3H12D
LOC102163961	15.8603863	669.2854811	-5.399121903	3.97E-05	Low	15	810	XP_005656794.1	ssc:102163961	0	NM_022093.1	TNN
VAMP8	75.07249515	2961.381684	-5.301842203	3.98E-05	Low	71	3584	XP_003354791.1	ssc:100620246	6E-40	NM_003761.4	VAMP8
LOC102164445	3.17207726	211.5272632	-6.0592719	4.01E-05	Low	3	256	XR_301561.1	ssc:102164445			
STARD8	0	97.50084786	-20	4.02E-05	Low	0	118	XP_005673758.1	ssc:100519288	0	NM_014725.4	STARD8
LOC102159188	1.057359087	136.3359313	-7.010556615	4.02E-05	Low	1	165	XR_300828.1	ssc:102159188			
APOA1	33.83549077	1337.744684	-5.305121671	4.07E-05	Low	32	1619	NM_214398.1	ssc:397691	3E-122	NM_000039.1	APOA1
LOC102162014	61.32682702	0	20	4.10E-05	High	58	0	XP_005661582.1	ssc:102162014	8E-65	NM_130767.2	ACOT12
LOC100154928	61.32682702	0	20	4.10E-05	High	58	0	LOC100154928				
LOC100621385	61.32682702	0	20	4.10E-05	High	58	0	XP_005674345.1	ssc:100621385	0	NM_198182.2	GRHL1
MAGIX	1.057359087	135.509653	-7.001786405	4.14E-05	Low	1	164	XP_005673668.1	ssc:100514936	3E-85	NM_024859.3	MAGIX
TSPAN13	6370.588497	235.4893359	4.757693016	4.14E-05	High	6025	285	XP_005667721.1	ssc:100624728	5E-53	NM_014399.3	TSPAN13
ZNF286A	0	96.67456949	-20	4.18E-05	Low	0	117	ZNF286A	ssc:100627421	0	NM_020652.2	ZNF286A
PDE4A	34.89284986	1369.96954	-5.295068573	4.19E-05	Low	33	1658	NM_001123159.1	ssc:396825	0	NM_006202.2	PDE4A
SMTN	81.41664967	3178.692896	-5.286965974	4.21E-05	Low	77	3847	NM_001244360.1	ssc:414369	0	NM_134270.2	SMTN
C6H19orf33	15.8603863	661.0226974	-5.381199995	4.21E-05	Low	15	800	XP_003127155.1	ssc:100515030			
LOC102161934	19.03246356	776.7016694	-5.350826346	4.22E-05	Low	18	940	XP_005670750.1	ssc:102161934	1E-40	NM_016817.2	OAS2
LOC100738735	142.7434767	3.305113487	5.432581196	4.22E-05	High	135	4	XP_005655617.1	ssc:100738735	0	NM_012284.1	KCNH3
BEX5	142.7434767	3.305113487	5.432581196	4.22E-05	High	135	4	XP_005657928.1	ssc:100155526	3E-36	NM_001159560.1	BEX5
LOC100627489	122.653654	2.478835115	5.628784094	4.25E-05	High	116	3	LOC100627489				
GPNMB	1.057359087	134.6833746	-6.992962555	4.26E-05	Low	1	163	NM_001098584.1	ssc:100049669			
CD36	3.17207726	208.2221497	-6.036551824	4.32E-05	Low	3	252	NM_001044622.1	ssc:733702	0	NM_001127444.1	CD36
tRNA-Tyr	563.5723931	20.65695929	4.769901132	4.36E-05	High	533	25	tRNA-Tyr				
ICAM-1	169.1774539	6696.986203	-5.306902776	4.39E-05	Low	160	8105	NM_213816.1	ssc:396750	9E-150	NM_000201.2	ICAM1
BATF2	8.458872693	390.8296698	-5.529930774	4.44E-05	Low	8	473	XP_005660776.1	ssc:100515875	3E-90	NM_138456.3	BATF2
HHIPL1	16.91774539	690.7687188	-5.351593533	4.48E-05	Low	16	836	XP_005666497.1	ssc:100625046	0	NM_032425.4	HHIPL1
LOC100627331	81.41664967	0.826278372	6.62255214	4.51E-05	High	77	1	XP_003361708.1	ssc:100627331	8E-152	NM_207108.2	ASTN1
LOC102159258	60.26946793	0	20	4.53E-05	High	57	0	XR_299710.1	ssc:102159258			
LOC102165390	60.26946793	0	20	4.53E-05	High	57	0	XP_005665019.1	ssc:102165390	7E-66	NM_005427.3	TP73
LRIT3	60.26946793	0	20	4.53E-05	High	57	0	XP_003129299.2	ssc:100516451	0	NM_198506.4	LRIT3
AREG	0	95.02201275	-20	4.54E-05	Low	0	115	NM_214376.1	ssc:397668	8E-101	XP_001125684.1	AREGB
LOC102157992	0	95.02201275	-20	4.54E-05	Low	0	115	XP_005664985.1	ssc:102157992	0	NM_021089.2	ZNF8
LOC102162312	0	95.02201275	-20	4.54E-05	Low	0	115	XR_306270.1	ssc:102162312			
LOC102159707	17.97510447	726.2986887	-5.336490915	4.56E-05	Low	17	879	XR_304052.1	ssc:102159707			
LOC100522644	2.114718173	169.3870662	-6.3237145	4.57E-05	Low	2	205	XR_307034.1	ssc:100522644	1E-65	NM_207015.2	NAALADL2
TMEM74	457.8364845	16.52556743	4.792060719	4.57E-05	High	433	20	XP_005662957.1	ssc:100525987	2E-140	NM_153015.1	TMEM74
AHDC1	67.67098154	2587.077582	-5.256642194	4.60E-05	Low	64	3131	XP_005656140.1	ssc:100512968	0	NM_001029882.2	AHDC1
LOC100624675	541.3678523	22451.63592	-5.37406754	4.63E-05	Low	512	27172	XR_299377.1	ssc:100624675			
LOC100739178	20.08982264	799.8374638	-5.315170125	4.63E-05	Low	19	968	XP_005654927.1	ssc:100739178	0	NM_006202.2	PDE4A
TPMT	3601.365049	137.9884881	4.705924026	4.64E-05	High	3406	167	NM_001243675.1	ssc:100157630	1E-118	NM_000367.2	TPMT
GRB14	699.9717153	26.4409079	4.726453006	4.65E-05	High	662	32	XP_005671947.1	ssc:100514075	0	NM_004490.2	GRB14
LOC100514871	515.9912342	19.00440255	4.762940981	4.70E-05	High	488	23	NM_001245009.1	ssc:100514871	6E-180	NM_014519.2	ZNF232
LOC100513982	0	94.19573438	-20	4.72E-05	Low	0	114	XP_005662499.1	ssc:100513982	3E-40	NM_016026.3	RDH11
LOC100517213	0	94.19573438	-20	4.72E-05	Low	0	114	XP_005652879.1	ssc:100517213	0	NM_152601.3	ZNF709
KLHL30	0	94.19573438	-20	4.72E-05	Low	0	114	XP_003133822.1	ssc:100519226	0	NM_198582.3	KLHL30
LOC100620869	0	94.19573438	-20	4.72E-05	Low	0	114	XP_005664934.1	ssc:100620869	6E-84	NM_198539.3	ZNF568
LOC100516787	454.6644072	16.52556743	4.782030354	4.77E-05	High	430	20	XR_115797.3	ssc:100516787			
LOC102161205	31.7207726	1217.108042	-5.26188552	4.77E-05	Low	30	1473	XP_005660659.1	ssc:102161205	3E-166	NM_001876.3	CPT1A
LOC100155098	860.6902965	33.05113487	4.702722489	4.77E-05	High	814	40	XP_003360438.1	ssc:100155098	3E-63	NM_005008.3	NHP2L1
ERBB4	100.4491132	1.652556743	5.925621208	4.81E-05	High	95	2	XP_001926521.1				
RAB33A	473.6968708	17.35184581	4.770803099	4.82E-05	High	448	21	NM_001123177.1	ssc:100144495	1E-140	NM_004794.2	RAB33A
KRBA2	354.215294	12.39417558	4.836892289	4.84E-05	High	335	15	XP_005674593.1	ssc:100621261	5E-61	NM_213597.2	KRBA2
LOC100514231	2914.081643	113.2001369	4.686093689	4.90E-05	High	2756	137	NM_001244939.1	ssc:100514231	3E-103	NR_024537.1	GSTM3
ZNF77	0	93.36945601	-20	4.92E-05	Low	0	113	ZNF77	ssc:100516355	6E-166	NM_021217.2	ZNF77
LOC100627927	0	93.36945601	-20	4.92E-05	Low	0	113	XP_005674341.1	ssc:100627927	4E-68	NM_001080509.2	TSPAN11
LOC102161634	876.5506828	33.87741324	4.693441886	4.92E-05	High	829	41	XR_299036.1	ssc:102161634			
LOC100523918	9.516231779	418.0968561	-5.457302974	4.97E-05	Low	9	506	XP_005668531.1	ssc:100523918	0	NM_015843.1	LMO7
ELAVL2	59.21210885	0	20	5.00E-05	High	56	0	XP_005654549.1	ssc:100153303	0	NM_004432.3	ELAVL2

SLFN14	59.21210885	0	20	5.00E-05	High	56	0	XP_003358199.2	ssc:100625890	0	NM_001129820.1	SLFN14
NELL1	59.21210885	0	20	5.00E-05	High	56	0	XP_005661131.1	ssc:100520718	0	NM_201551	NELL1
SIGLEC10	52.86795433	1983.894371	-5.229797899	5.02E-05	Low	50	2401	XP_005653353.1	ssc:100513863	0	NM_033130.4	SIGLEC10
LOC100037974	0	92.54317763	-20	5.13E-05	Low	0	112	XP_005674384.1	ssc:100037974	0	NM_018058.6	CRTAC1
PADI1	0	92.54317763	-20	5.13E-05	Low	0	112	XP_003127695.3	ssc:100524567	0	NM_013358.2	PADI1
JOSD2	51.81059524	1932.665111	-5.221200703	5.17E-05	Low	49	2339	NM_001243416.1	ssc:100627842	2E-101	NM_138334.3	JOSD2
LOC780424	137.4566813	3.305113487	5.378133412	5.25E-05	High	130	4	NM_001078676.1	ssc:780424	2E-77	NM_006688.3	C1QL1
LOC102167439	43.35172255	1613.72166	-5.21815863	5.27E-05	Low	41	1953	XP_005662978.1	ssc:102167439	7E-54	NM_138455.3	CTHRC1
LOC100511614	407.0832483	14.87301069	4.774555233	5.32E-05	High	385	18	XP_005673974.1	ssc:100511614	0	NM_144967.3	ARHGAP36
LOC100622715	3.17207726	199.1330876	-5.972161236	5.34E-05	Low	3	241	XP_005667638.1	ssc:100622715	0	NM_024788.2	C7orf63
OSMR	67.67098154	2507.754858	-5.211715202	5.35E-05	Low	64	3035	XP_003133952.2	ssc:100519398	0	NM_003999.2	OSMR
LOC100516615	0	91.71689926	-20	5.35E-05	Low	0	111	XP_005653378.1	ssc:100516615	0	NM_198480.3	ZNF615
LOC102164954	0	91.71689926	-20	5.35E-05	Low	0	111	XR_301606.1	ssc:102164954			
LOC100521431	0	91.71689926	-20	5.35E-05	Low	0	111	XP_005652913.1	ssc:100521431	0	NM_153358.2	ZNF791
DPEP1	1.057359087	128.0731476	-6.920358806	5.40E-05	Low	1	155	NM_214108.1	ssc:397196	0	NM_004413.3	DPEP1
EMID1	90.93288145	3378.652262	-5.215501994	5.41E-05	Low	86	4089	XP_005657415.1	ssc:100152849	3E-117	NM_133455.3	EMID1
DLX2	4.229436346	233.0105008	-5.783785753	5.49E-05	Low	4	282	XP_003133525.1	ssc:100523029	4E-141	NM_004405.3	DLX2
LOC100625116	58.15474976	0	20	5.53E-05	High	55	0	XP_005658067.1	ssc:100625116	0	NM_144724.1	MARVELD2
GPRC5C	68.72834063	2527.585539	-5.200710979	5.56E-05	Low	65	3059	XP_005674317.1	ssc:100620956	0	NM_022036.2	GPRC5C
LOC102159568	938.9348689	37.18252673	4.658328369	5.57E-05	High	888	45	XP_005661607.1	ssc:102159568	7E-67	NM_024717.4	MCTP1
LOC102165939	16.91774539	660.196419	-5.286286094	5.57E-05	Low	16	799	XP_005656101.1	ssc:102165939	0	NM_005529.5	HSPG2
LOC100626465	0	90.89062089	-20	5.59E-05	Low	0	110	XP_005663840.1	ssc:100626465	3E-60	NM_033016.2	PDGFB
LOC102162178	0	90.89062089	-20	5.59E-05	Low	0	110	XP_005658008.1	ssc:102162178	0	NM_004654.3	USP9Y
ZNF432	0	90.89062089	-20	5.59E-05	Low	0	110	ZNF432	ssc:100624217	0	NM_014650.2	ZNF432
LMX1A	78.24457241	0.826278372	6.565218965	5.65E-05	High	74	1	XP_003125689.1	ssc:100513081	0	NM_177399.2	LMX1A
LOC100738381	1300.551676	52.05553742	4.642928276	5.67E-05	High	1230	63	XP_005664214.1	ssc:100738381	9E-154	NM_006159.2	NELL2
LOC100624327	5.286795433	266.0616357	-5.653223184	5.69E-05	Low	5	322	XP_003360220.2	ssc:100624327	9E-170	NM_030636.2	EEPD1
LOC100622991	6.344154519	300.7653273	-5.56706654	5.71E-05	Low	6	364	XP_003360998.2	ssc:100622991	5E-77	NR_073131.1	NEDD9
MAFB	7.401513606	335.4690189	-5.502215396	5.73E-05	Low	7	406	XP_003134488.2	ssc:100518227	5E-109	NM_005461.4	MAFB
LOC100522842	596.3505248	23.13579441	4.68796203	5.75E-05	High	564	28	XR_309010.1	ssc:100522842			
LOC100153177	97.27703596	1.652556743	5.879327555	5.78E-05	High	92	2	LOC100153177				
RIMBP2	362.6741667	13.22045395	4.777830365	5.80E-05	High	343	16	XP_005670600.1	ssc:100153193	0	NM_015347.4	RIMBP2
PTGIR	0	90.06434252	-20	5.83E-05	Low	0	109	XP_003356000.1	ssc:100622305	0	NM_000960.3	PTGIR
SALL1	0	90.06434252	-20	5.83E-05	Low	0	109	XP_003127035.1	ssc:100519899	0	NM_002968.2	SALL1
LOC102160134	4230.493705	167.7345095	4.656574595	5.88E-05	High	4001	203	LOC102160134				
LOC102167673	1.057359087	125.5943125	-6.892161914	5.93E-05	Low	1	152	XP_005661696.1	ssc:102167673			
CD99L2	59.21210885	2145.844931	-5.179509777	5.94E-05	Low	56	2597	XP_003360528.1	ssc:100517448	3E-68	NM_134446.3	CD99L2
LOC100620963	9.516231779	402.397567	-5.402087361	5.97E-05	Low	9	487	XP_003355420.2	ssc:100620963	0	NM_152772.2	TCP11L2
RAB3IL1	85.64608601	3108.459234	-5.181668531	6.02E-05	Low	81	3762	XP_005660868.1	ssc:100739680	2E-164	NM_013401.3	RAB3IL1
LPAR3	171.292172	4.95767023	5.110653101	6.09E-05	High	162	6	NM_001162402.1	ssc:100113360	5E-175	NM_012152.2	LPAR3
LOC100623149	340.4696259	12.39417558	4.779791882	6.10E-05	High	322	15	XP_003361879.1	ssc:100623149			
PRSS8	1.057359087	124.7680341	-6.88263914	6.11E-05	Low	1	151	XP_003124520.1	ssc:100522730	2E-139	NM_002773.3	PRSS8
XIRP1	1.057359087	124.7680341	-6.88263914	6.11E-05	Low	1	151	NM_001143928.1	ssc:100134978	0	NM_194293.2	XIRP1
LOC100624356	57.09739067	0	20	6.12E-05	High	54	0	XP_005658199.1	ssc:100624356	8E-109	NM_144575.2	CAPN13
LOC100152658	57.09739067	0	20	6.12E-05	High	54	0	XP_003360532.1	ssc:100152658	1E-61	NM_005008.3	NHP2L1
LOC100737042	57.09739067	0	20	6.12E-05	High	54	0	XP_003483238.2	ssc:100737042	5E-81	NM_018398.2	CACNA2D3
KCND1	12.68830904	504.8560851	-5.31430047	6.16E-05	Low	12	611	XP_005673658.1	ssc:100620758	0	NM_004979.5	KCND1
C14H10orf54	5.286795433	261.1039655	-5.626087054	6.22E-05	Low	5	316	XP_005671104.1	ssc:100154373	1E-141	NM_022153.1	C10orf54
SLC8A3	2.114718173	158.6454474	-6.229196901	6.24E-05	Low	2	192	XP_005656415.1	ssc:100152101	0	NR_104122.1	SLC8A3
LOC100622258	499.0734889	19368.79131	-5.278337848	6.26E-05	Low	472	23441	XP_003354392.1	ssc:100622258	2E-43	NM_001387.2	DPYSL3
FST	6456.234583	255.3200169	4.660314446	6.31E-05	High	6106	309	NM_001003662.1	ssc:445002	0	NM_013409.2	FST
LOC100153517	0	88.41178578	-20	6.36E-05	Low	0	107	XP_005658136.1	ssc:100153517	0	NM_024832.3	RIN3
ADAMTSL2	1.057359087	123.1154774	-6.863402921	6.51E-05	Low	1	149	XP_005654665.1	ssc:100628047	0	NM_014694.3	ADAMTSL2
WFDC3	1.057359087	123.1154774	-6.863402921	6.51E-05	Low	1	149	XP_005673032.1	ssc:100155625	5E-46	NM_181530.1	WFDC3
DGKB	169.1774539	4.95767023	5.092731193	6.54E-05	High	160	6	XP_003357509.2	ssc:100625799	0	NM_145695.2	DGKB
REPS2	677.7671745	27.26718627	4.635552026	6.66E-05	High	641	33	XP_003484126.1	ssc:100738275	5E-162	NM_004726.2	REPS2
LOC100737468	1.057359087	122.289199	-6.853687766	6.72E-05	Low	1	148	LOC100737468		2E-55	NM_152456.2	IL34
CD2	56.04003159	0	20	6.79E-05	High	53	0	NM_213776.1	ssc:396662	2E-113	NM_001767.3	CD2
LOC102157986	56.04003159	0	20	6.79E-05	High	53	0	XR_303229.1	ssc:102157986			
CRYBB1	56.04003159	0	20	6.79E-05	High	53	0	NM_001078681.1	ssc:780429	2E-115	NM_001887.3	CRYBB1
CAPN13	56.04003159	0	20	6.79E-05	High	53	0	XP_003125312.3	ssc:100515606			
LOC100525452	14.80302721	563.5218495	-5.250507408	6.81E-05	Low	14	682	XP_005655056.1	ssc:100525452	5E-136	NM_207317.1	ZNF474
LOC102167155	13.74566813	528.8181579	-5.265722778	6.82E-05	Low	13	640	XP_005669150.1	ssc:102167155	0	NM_015077.3	SARM1
LOC102161111	131.1125267	3.305113487	5.30996191	6.89E-05	High	124	4	XR_305751.1	ssc:102161111			
LOC100522115	0	86.75922903	-20	6.94E-05	Low	0	105	XR_299665.1	ssc:100522115			
INPP4B	17.97510447	665.1540892	-5.209616533	6.96E-05	Low	17	805	XP_003129226.3	ssc:100511393	0	NM_003866.2	INPP4B
FOXA1	863.8623737	35.52996998	4.603693113	7.03E-05	High	817	43	XP_001929311.1	ssc:100156502	0	NM_004496.3	FOXA1
LOC100627143	6489.012714	261.1039655	4.635302719	7.03E-05	High	6137	316	XR_300070.1	ssc:100627143			
LOC102163074	4.229436346	220.6163253	-5.704930332	7.12E-05	Low	4	267	XP_005654413.1	ssc:102163074			
LOC100518676	75.07249515	0.826278372	6.505512719	7.14E-05	High	71	1	LOC100518676				
CD82	19.03246356	694.9001106	-5.19027139	7.21E-05	Low	18	841	NM_001145218.2	ssc:100270689			
CCDC158	148.0302721	4.131391859	5.163120521	7.26E-05	High	140	5	XP_003129142.3	ssc:100522937	0	NM_178555.3	CCDC158
LDHC	166.0053766	4.95767023	5.065423847	7.29E-05	High	157	6	NM_001195775.1	ssc:100502559	6E-156	NM_017448.3	LDHC
FAM212A	29.60605442	1042.763305	-5.138375674	7.32E-05	Low	28	1262	XP_003132261.3	ssc:100512437	4E-110	NM_203370.1	FAM212A
LOC100518541	111.0227041	2.478835115	5.485048616	7.54E-05	High	105	3	XP_005674179.1	ssc:100518541	5E-101	NM_133638.3	ADAMTS19
SH3PXD2A	254.8235399	9127.070894	-5.162581477	7.79E-05	Low	241	11046	XP_005671487.1	ssc:100153803	0	NM_014631.2	SH3PXD2A
ERICH2	362.6741667	14.04673232	4.690367524	7.83E-05	High	343	17	XP_005672010.1	ssc:100155783	1E-44	XP_209489.5	ERICH2
AQP3	1.057359087	118.1578072	-6.804105738	7.90E-05	Low	1	143	NM_001110172.1	ssc:100126235	8E-161	NM_004925.4	AQP3
HOXA1	1.057359087	118.1578072	-6.804105738	7.90E-05	Low	1	143	XP_005673359.1	ssc:100520162	6E-165	NM_153620.2	HOXA1
PRTG	676.7098154	28.09346464	4.590230853	7.92E-05	High	640	34	XP_005654495.1	ssc:100154460	0	NM_173814.4	PRTG
ZFP37	0	84.28039392	-20	7.94E-05	Low	0	102	ZFP37	ssc:100517074	0	NM_003408.2	ZFP37
MYHC	114.1947813	3943.00039	-5.109725226	7.95E-05	Low	108	4772	XP_003132042.1	ssc:396711	0	NM_002470.3	MYH3
SLC16A3	62.38418611	2126.01425	-5.090827093	8.00E-05	Low	59	2573	XP_003357973.1	ssc:100158243	4E-16	NM_004207.3	SLC16A3
TMEM2	22845.30042	891.5543631	4.679430835	8.06E-05	High	21606	1079	XP_005674542.1	ssc:100516588	0	NM_013390.2	TMEM2
FAM65C	1.057359087	117.3315288	-6.79398152	8.16E-05	Low	1	142	XP_005673066.1	ssc:100153156	0	NM_080829.2	FAM65C
ZNF500	2.114718173	149.5563853	-6.144080288	8.24E-05	Low	2	181	ZNF500	ssc:100625066	2E-22	NM_021646.1	ZNF500
LOC102164464	0	83.45411554	-20	8.31E-05	Low	0	101	XR_303994.1	ssc:102164464			
BCL9L	148.0302721	5106.400337	-5.108342507	8.34E-05	Low	140	6180	XP_005667470.1	ssc:100515846	0	NM_182557.2	BCL9L
LOC100155138	1159.922918	49.5767023	4.548222814	8.38E-05	High	1097	60	XP_005654160.1	ssc:100155138	0	NM_080386.3	TUBA3D
DPPA5	53.92531342	0	20	8.41E-05	High	51	0	NM_001160273.1	ssc:100301558	5E-45	NM_001025290.2	DPPA5
GNAL	53.92531342	0	20	8.41E-05	High	51	0	XP_003482083.2	ssc:100736677	3E-103	NM_182978.3	GNAL
SYT13	53.92531342	0	20	8.41E-05	High	51	0	XP_003122894.3	ssc:100520062	0	NM_020826.2	SYT13
IFIT1	198.7835083	6919.255085	-5.121346752	8.47E-05	Low	188	8374	NM_001244363.1	ssc:100153038	0	NM_001548.4	IFIT1
LOC100738918	90.93288145	1.652556743	5.782030354	8.48E-05	High	86	2	LOC100738918				
LOC102166090	1690.717179	72.71249671	4.539288204	8.62E-05	High	1599	88	XP_005665407.1	ssc:102166090			
IL10RA	5.286795433	243.7521197	-5.52687745	8.63E-05	Low	5	295	XP_005656694.1	ssc:100525654	0	NR_026691.1	IL10RA
ZNF354C	0	82.62783717	-20	8.71E-05	Low	0	100	ZNF879	ssc:102159167	3E-35	NM_001136116.1	ZNF879
EMX2	0	82.62783717	-20	8.71E-05	Low	0	100	XP_005671546.1	ssc:100152562	6E-112	NM_004098.3	EMX2
GPRIN3	610.096193	25.61462952	4.573996797	8.75E-05	High	577	31	XP_005667050.1	ssc:100517107	0	NM_198281.2	GPRIN3
SMOX	67.67098154	2263.17646	-5.063667951	8.79E-05	Low	64	2739	NM_001185170.1	ssc:100152781	0	NM_175842.2	SMOX

LOC102167598	664.0215064	28.09346464	4.562923507	8.85E-05	High	628	34	XP_005671016.1	ssc:102167598	8E-20	NM_004485.3	GNG4
1-Mar	107.8506268	2.478835115	5.44322844	8.89E-05	High	102	3	XP_005666735.1	ssc:100738764	2E-70	NM_017923.3	1-Mar
SLC9A9	13.74566813	499.8984149	-5.184586015	8.92E-05	Low	13	605	XP_005669980.1	ssc:100517680	0	NM_173653.3	SLC9A9
GARNL3	177.6363265	5.783948602	4.9407281	8.96E-05	High	168	7	XP_005660494.1	ssc:100156351	0	NR_104591.1	GARNL3
NPTXR	10.57359087	401.5712887	-5.247118809	9.00E-05	Low	10	486	XP_005655541.1	ssc:100521449	2E-170	NM_058178.1	NPTXR
LOC100514469	0	81.8015588	-20	9.12E-05	Low	0	99	XP_005664974.1	ssc:100514469	2E-85	NR_049754.1	ZNF211
NKX2-2	0	81.8015588	-20	9.12E-05	Low	0	99	XP_003359963.1	ssc:100626520	7E-149	NM_002509.3	NKX2-2
LOC102165471	0	81.8015588	-20	9.12E-05	Low	0	99	XR_303832.1	ssc:102165471			
OMD	2.114718173	146.2512718	-6.111839951	9.16E-05	Low	2	177	XP_003124829.1	ssc:100511925	0	NM_005014.2	OMD
PCDHB8	264.3397716	9.915340461	4.736587383	9.19E-05	High	250	12	XP_005655077.1	ssc:102166091	3E-33	NM_019120.3	PCDHB8
LOC100157629	52.86795433	0	20	9.37E-05	High	50	0	LOC100157629				
GGT1	11.63094995	428.8384749	-5.20439351	9.52E-05	Low	11	519	NM_214030.1	ssc:397095	0	NM_013430.2	GGT1
SLCO2A1	10.57359087	396.6136184	-5.229196901	9.55E-05	Low	10	480	NM_001123195.2	ssc:100144510			
LOC100525978	0	80.97528043	-20	9.56E-05	Low	0	98	XR_301768.1	ssc:100525978			
IFNAR2	66.61362245	2186.332572	-5.036551824	9.61E-05	Low	63	2646	NM_001204775.2	ssc:100533555			
LOC100156010	88.81816327	1.652556743	5.748083022	9.69E-05	High	84	2	XP_001924141.1	ssc:100156010	1E-68	NM_001025.4	RPS23
SV2C	88.81816327	1.652556743	5.748083022	9.69E-05	High	84	2	XP_005661560.1	ssc:100517268	6E-92	NM_014979.1	SV2C
LMCD1	10.57359087	394.9610617	-5.223173114	9.74E-05	Low	10	478	NM_001008692.1	ssc:494020	2E-62	NM_014583.3	LMCD1
STAC2	10.57359087	394.9610617	-5.223173114	9.74E-05	Low	10	478	XP_005653995.1	ssc:100514486	0	NM_198993.3	STAC2
ADAMTS5	7.401513606	299.9390489	-5.340705217	9.78E-05	Low	7	363	XP_003132795.3	ssc:100522952	0	NM_007038.3	ADAMTS5
THBS4	16.91774539	585.8313656	-5.113876218	9.88E-05	Low	16	709	XP_003123802.3	ssc:100513005	0	NM_003248.4	THBS4
SEMA3F	116.3094995	3838.889315	-5.044648126	9.91E-05	Low	110	4646	XP_001925178.2	ssc:100153225	0	NM_004186.3	SEMA3F
LOC100155449	28.54869534	943.6099005	-5.046693834	1.00E-04	Low	27	1142	XP_005663033.1	ssc:100155449	0	NM_175636.2	RUNX1T1
LOC102163368	0	80.14900206	-20	1.00E-04	Low	0	97	XR_306051.1	ssc:102163368			
ETV7	25.37661808	842.8039392	-5.053625337	1.01E-04	Low	24	1020	XP_005665991.1	ssc:100156210	2E-137	NM_016135.3	ETV7
LOC102159469	13.74566813	486.6779609	-5.145918506	1.01E-04	Low	13	589	XP_005659451.1	ssc:102159469			
RIN1	23.2618999	776.7016694	-5.061319729	1.02E-04	Low	22	940	XP_003122546.1	ssc:100520715	0	NM_004292.2	RIN1
IL17RC	53.92531342	1746.752478	-5.01756872	1.02E-04	Low	51	2114	XP_003483265.1	ssc:100152641	0	NR_037807.1	IL17RC
LOC100037976	3462.851009	151.208942	4.517344952	1.02E-04	High	3275	183	XP_001925729.3	ssc:100037976	2E-161	NM_033012.3	TNFSF11
LOC100738817	173.4068902	5.783948602	4.905962682	1.03E-04	High	164	7	XR_135305.2	ssc:100738817			
LOC100737142	7.401513606	296.6339355	-5.324719513	1.03E-04	Low	7	359	XR_303908.1	ssc:100737142			
C6H16orf74	61.32682702	1981.415535	-5.013869349	1.03E-04	Low	58	2398	XP_005664362.1	ssc:100513354	8E-27	NM_206967.2	C16orf74
PAPOLB	1.057359087	111.5475802	-6.721049998	1.03E-04	Low	1	135	XP_005674606.1	ssc:100511746	0	NM_020144.4	PAPOLB
SATB1	5934.956553	255.3200169	4.538858915	1.04E-04	High	5613	309	XP_005669348.1	ssc:100155111	0	NM_002971.4	SATB1
GRM8	51.81059524	0	20	1.05E-04	High	49	0	XP_005673251.1	ssc:100524120	0	NR_028041.1	GRM8
ACACB	60.26946793	1940.927895	-5.009175385	1.05E-04	Low	57	2349	NM_001206399.1	ssc:100154993	0	NM_001093.3	ACACB
SLC1A6	0	79.32272369	-20	1.05E-04	Low	0	96	XP_005652965.1	ssc:100518296	0	NR_073589.1	SLC1A6
ANO5	0	79.32272369	-20	1.05E-04	Low	0	96	XP_005661130.1	ssc:100520542	0	NM_213599.2	ANO5
PDE1C	6.344154519	264.409079	-5.381199995	1.05E-04	Low	6	320	XP_003134849.3	ssc:100525902	0	NM_005020.2	PDE1C
LOC100737020	2659.258103	102321.3559	-5.265939667	1.06E-04	Low	2515	123834	XP_005672671.1	ssc:100737020	6E-63	NM_003118.3	SPARC
CACNG6	121.596295	3.305113487	5.20125565	1.06E-04	High	115	4	XP_005664874.1	ssc:100519779	3E-112	NR_102308.1	CACNG6
PLXNA2	60.26946793	1935.970225	-5.005485625	1.06E-04	Low	57	2343	XP_005667952.1	ssc:100514595	0	NM_025179.3	PLXNA2
LOC100158196	172.3495311	5.783948602	4.897138831	1.07E-04	High	163	7	XP_001927439.1	ssc:100158196	4E-47	NM_018947.5	CYCS
LOC100737216	223.1027673	8.262783717	4.754936693	1.07E-04	High	211	10	XP_003481711.1	ssc:100737216			
LOC100515628	869.1491692	31142.43183	-5.163133992	1.08E-04	Low	822	37690	XP_003133256.2	ssc:100515628	0	NM_002775.4	HTRA1
C7H14orf50	1221.249745	54.53437253	4.485048616	1.08E-04	High	1155	66	XP_001926490.2	ssc:100157071	1E-166	NM_172365.1	PPP1R36
LOC102164962	694.6849199	30.57229975	4.506061794	1.08E-04	High	657	37	LOC102164962				
PGF	42.29436346	1355.922808	-5.002665829	1.09E-04	Low	40	1641	XP_003356799.1	ssc:100156404	2E-83	NM_002632.5	PGF
CLGN	5507.783482	239.6207278	4.522647231	1.10E-04	High	5209	290	NM_001243207.1	ssc:100624991	0	NM_004362.2	CLGN
HHEX	746.4955151	33.05113487	4.497361878	1.10E-04	High	706	40	NM_001244579.1	ssc:397232	2E-146	NM_002729.4	HHEX
NTS	0	78.49644531	-20	1.10E-04	Low	0	95	XP_003481806.1	ssc:100739079	1E-82	NM_006183.4	NTS
LOC100738143	0	78.49644531	-20	1.10E-04	Low	0	95	XR_300479.1	ssc:100738143			
LOC100516675	0	78.49644531	-20	1.10E-04	Low	0	95	XP_005664681.1	ssc:100516675	5E-12	NM_152796.2	HIF3A
PEAR1	79.30193149	2534.195766	-4.998028162	1.11E-04	Low	75	3067	XP_005663319.1	ssc:100156908	0	NM_207369.1	PEAR1
CACNA1E	154.3744266	4.95767023	4.960667863	1.11E-04	High	146	6	XP_005667863.1	ssc:100511769	1E-89	NM_001205294.1	CACNA1E
FAM20A	2.114718173	140.4673232	-6.053625337	1.11E-04	Low	2	170	XP_005656990.1	ssc:100522273	0	NR_027751.1	FAM20A
PIK3AP1	709.4879471	31.39857813	4.498007042	1.11E-04	High	671	38	NM_001244503.1	ssc:100155165	0	NM_152309.2	PIK3AP1
LOC100623904	888.1816327	39.66136184	4.485048616	1.11E-04	High	840	48	LOC100623904				
PCDH11X	1673.799434	75.19133183	4.476416499	1.11E-04	High	1583	91	XP_005673836.1	ssc:414736	0	NM_032969.3	PCDH11X
SLC35G1	2214.109927	99.15340461	4.480920731	1.12E-04	High	2094	120	XP_003359346.1	ssc:100156133	1E-148	NM_153226.2	SLC35G1
LOC102165992	2325.132631	104.1110748	4.481117565	1.12E-04	High	2199	126	XP_005668550.1	ssc:102165992			
LOC100156657	28.54869534	919.6478277	-5.009584776	1.13E-04	Low	27	1113	NM_001190191.1	ssc:100156657	8E-12	NM_016210.4	C3orf18
FCHSD1	46.52379981	1475.733172	-4.987319147	1.13E-04	Low	44	1786	XP_005661826.1	ssc:100511554	0	NM_033449.2	FCHSD1
LOC100627081	425.0583528	18.17812418	4.547385672	1.16E-04	High	402	22	XP_003361707.1	ssc:100627081	1E-170	NM_018593.4	SLC16A10
LOC100522650	0	77.67016694	-20	1.16E-04	Low	0	94	XP_003133905.2	ssc:100522650	1E-12	NM_004934.3	CDH18
LOC100516594	0	77.67016694	-20	1.16E-04	Low	0	94	XP_003123294.2	ssc:100516594	2E-82	NM_022377.3	ICAM4
ZKSCAN2	0	77.67016694	-20	1.16E-04	Low	0	94	ZKSCAN2	ssc:100520899	0	NM_001012981.4	ZKSCAN2
LOC102160712	0	77.67016694	-20	1.16E-04	Low	0	94	XR_302696.1	ssc:102160712			
TXNIP	83.53136784	2644.917068	-4.984760614	1.16E-04	Low	79	3201	NM_001044614.2	ssc:733688	0	NM_006472.4	TXNIP
SULT1A1	5.286795433	228.879109	-5.436048472	1.16E-04	Low	5	277	NM_213765.1	ssc:396640	5E-70	NM_001017391.1	SULT1A4
USP18	408.1406074	13783.14952	-5.077695522	1.16E-04	Low	386	16681	NM_213826.1	ssc:396777	2E-151	NM_017414.3	USP18
LOC102157594	68.72834063	0.826278372	6.378133412	1.17E-04	High	65	1	XR_300709.1	ssc:102157594			
CYR61	538.1957751	18432.61792	-5.09798611	1.17E-04	Low	509	22308	XP_001927775.1	ssc:100153791	0	NM_001554.4	CYR61
CDX4	50.75323616	0	20	1.17E-04	High	48	0	XP_003360417.1	ssc:100623433	3E-127	NM_005193.1	CDX4
LOC100625970	170.2348129	5498.056285	-5.013323674	1.18E-04	Low	161	6654	XP_005652631.1	ssc:100625970	0	NR_026554.1	CALCOCO1
VGLL3	206.1850219	6709.380378	-5.024168099	1.18E-04	Low	195	8120	NM_001143929.1	ssc:100233225	4E-150	NM_016206.2	VGLL3
LOC100736898	22.20454082	720.5147401	-5.020101303	1.19E-04	Low	21	872	XP_003482725.1	ssc:100736898	9E-155	NM_005924.4	MEOX2
ISOC1	11746.20209	502.37725	4.547279421	1.19E-04	High	11109	608	XP_003123949.1	ssc:100517640	2E-163	NM_016048.2	ISOC1
LOC102162275	85.64608601	1.652556743	5.695615602	1.19E-04	High	81	2	XP_005659301.1	ssc:102162275			
LOC100738512	6.344154519	257.798852	-5.344674119	1.19E-04	Low	6	312	XP_003483066.1	ssc:100738512	0	NM_022827.3	SPATA20
LOC100519287	235.7910763	9.089062089	4.697233881	1.19E-04	High	223	11	XP_005673936.1	ssc:100519287	2E-109	NM_033495.3	KLHL13
NRSN1	118.4242177	3.305113487	5.163120521	1.23E-04	High	112	4	XP_005665686.1	ssc:100154240	2E-105	NM_080723.4	NRSN1
LOC100738248	10.57359087	375.9566591	-5.152029041	1.23E-04	Low	10	455	XP_005673317.1	ssc:100738248	7E-146	NM_030636.2	EEPD1
GLIPR1	1.057359087	107.4161883	-6.666602214	1.23E-04	Low	1	130	XP_005664057.1	ssc:100523551	4E-121	NM_006851.2	GLIPR1
LOC102162524	301.3473397	12.39417558	4.603693113	1.24E-04	High	285	15	XR_300241.1	ssc:102162524			
LOC102161674	10.57359087	375.1303808	-5.148854793	1.24E-04	Low	10	454	XP_005668059.1	ssc:102161674	5E-126	NM_020443.4	NAV1
ZNF84	7.401513606	285.0660382	-5.26733203	1.24E-04	Low	7	345	ZNF84	ssc:100620909	0	NM_152943.2	ZNF268
MTTP	216.7586127	8.262783717	4.713317604	1.27E-04	High	205	10	NM_214185.1	ssc:397381	0	NM_000253.2	MTTP
SCN1A	84.58872693	1.652556743	5.677693694	1.27E-04	High	80	2	HighI00025DFBBD		0	NM_006920.4	SCN1A
LOC102160003	1617.759402	74.36505346	4.44322844	1.27E-04	High	1530	90	LOC102160003				
GDPD5	66.61362245	2060.738259	-4.951200227	1.28E-04	Low	63	2494	XP_005667191.1	ssc:100627912	0	NM_030792.6	GDPD5
LOC102164998	1.057359087	106.58991	-6.655461656	1.28E-04	Low	1	129	XR_307847.1	ssc:102164998			
SSTR1	0	76.0176102	-20	1.28E-04	Low	0	92	NM_001190231.1	ssc:397004	0	NM_001049.2	SSTR1
LOC102164235	0	76.0176102	-20	1.28E-04	Low	0	92	XR_304726.1	ssc:102164235			
LOC100514465	0	76.0176102	-20	1.28E-04	Low	0	92	XP_005653370.1	ssc:100514465	2E-71	NM_003779.3	B4GALT3
ZNF709	0	76.0176102	-20	1.28E-04	Low	0	92	ZNF709	ssc:100624296	0	NM_152601.3	ZNF709
LOC102165199	0	76.0176102	-20	1.28E-04	Low	0	92	XP_005658191.1	ssc:102165199	4E-64	NM_012236.3	SCMH1
LOC100737124	3.17207726	165.2556743	-5.70312809	1.29E-04	Low	3	200	XP_005662410.1	ssc:100737124	6E-136	NM_006236.1	POU3F3
LOC102165157	1200.102563	55.36065091	4.438152991	1.30E-04	High	1135	67	XR_306510.1	ssc:102165157			
TLE2	19.03246356	613.9248302	-5.0115278	1.30E-04	Low	18	743	XP_003354045.2	ssc:100622728	2E-97	NM_003260.4	TLE2

DDX60	60.26946793	1853.342388	-4.942558292	1.31E-04	Low	57	2243	XP_005657343.1	ssc:100158037	8E-21	NM_017631.5	DDX60
LOC102159119	5.286795433	223.0951604	-5.399121903	1.31E-04	Low	5	270	XR_297475.1	ssc:102159119			
LOC102160857	49.69587707	0	20	1.32E-04	High	47	0	XP_005657197.1	ssc:102160857	0	NM_175611.2	GRIK1
GPER1	1.057359087	105.7636316	-6.644234401	1.33E-04	Low	1	128	XP_005655115.1	ssc:100521003	3E-169	NM_001505.2	GPER1
LOC100620105	0	75.19133183	-20	1.35E-04	Low	0	91	XP_005658075.1	ssc:100620105	0	NM_152280.4	SYT11
LOC102159708	0	75.19133183	-20	1.35E-04	Low	0	91	XR_298415.1	ssc:102159708			
ZMYND15	0	75.19133183	-20	1.35E-04	Low	0	91	ZMYND15	ssc:100513818	0	NM_032265.2	ZMYND15
LOC100156578	0	75.19133183	-20	1.35E-04	Low	0	91	XP_005674329.1	ssc:100156578	2E-117	NM_002506.2	NGF
LOC102164159	0	75.19133183	-20	1.35E-04	Low	0	91	XP_005666956.1	ssc:102164159	5E-52	NM_016599.4	MYOZ2
GPR19	1334.387167	61.97087788	4.428443104	1.35E-04	High	1262	75	XP_005659112.1	ssc:100625907	0	NM_006143.2	GPR19
TBX2	114.1947813	3531.513761	-4.950718091	1.35E-04	Low	108	4274	NM_001246248.1	ssc:100622658	0	NM_005994.3	TBX2
LOC100738350	230.5042809	9.089062089	4.664518305	1.36E-04	High	218	11	XP_005653900.1	ssc:100738350	1E-76	NM_024719.3	GRTP1
CLMN	8.458872693	309.028111	-5.191128861	1.36E-04	Low	8	374	NM_001243882.1	ssc:100511700	0	NM_024734.3	CLMN
SLC16A6	83.53136784	1.652556743	5.659546347	1.37E-04	High	79	2	XP_005674608.1	ssc:100739042	0	NM_004694.4	SLC16A6
LOC100517136	83.53136784	1.652556743	5.659546347	1.37E-04	High	79	2	LOC100517136				
LOC102164974	164.9480175	5.783948602	4.833812896	1.37E-04	High	156	7	XR_303780.1	ssc:102164974			
FAM105A	23.2618999	728.7775239	-4.969437628	1.38E-04	Low	22	882	XP_003133888.1	ssc:100518874	0	NM_019018.2	FAM105A
GATA-6	4.229436346	191.6965822	-5.502215396	1.38E-04	Low	4	232	NM_214328.2	ssc:397600	0	NM_005257.4	GATA6
LOC100626903	5.286795433	220.6163253	-5.383002238	1.38E-04	Low	5	267	XP_003354462.1	ssc:100626903	0	NM_007183.2	PKP3
OAZ3	3.17207726	162.7768392	-5.68132372	1.39E-04	Low	3	197	NM_001122996.1	ssc:100144448			
LOC100736608	2.114718173	133.8570962	-5.984084404	1.39E-04	Low	2	162	XP_005667737.1	ssc:100736608	8E-88	NM_006080.2	SEMA3A
PRR5	32.77813168	1004.7545	-4.937965604	1.39E-04	Low	31	1216	NM_001204382.1	ssc:100152201	0	NM_181333.3	PRR5
TMSB15A	20.08982264	635.4080679	-4.983146675	1.40E-04	Low	19	769	XP_003135306.1	ssc:100522845	2E-18	NM_194324.2	TMSB15B
MTMR8	5.286795433	219.7900469	-5.377588741	1.41E-04	Low	5	266	XP_003135196.3	ssc:100514746	0	NM_017677.3	MTMR8
F2RL2	0	74.36505346	-20	1.42E-04	Low	0	90	XP_003123759.1	ssc:100519355	0	NM_004101.3	F2RL2
NFKBIA	144.8581949	4461.076929	-4.944678819	1.43E-04	Low	137	5399	NM_001005150.1	ssc:406188	9E-174	NM_020529.2	NFKBIA
LOC100621070	21.14718173	661.8489758	-4.967964738	1.44E-04	Low	20	801	XP_005666583.1	ssc:100621070			
LOC100626467	1562.77673	73.53877508	4.409462722	1.46E-04	High	1478	89	XP_005664123.1	ssc:100626467	3E-154	NM_004963.3	GUCY2C
GABRB2	82.47400875	1.652556743	5.641167818	1.47E-04	High	78	2	XP_003483903.1	ssc:100517063	0	NM_021911.2	GABRB2
LOC100514136	1140.890454	38936.71571	-5.092899019	1.47E-04	Low	1079	47123	XP_003361338.1	ssc:100514136	6E-156	NM_003118.3	SPARC
CHRNA6	48.63851798	0	20	1.48E-04	High	46	0	XP_005657708.1	ssc:100622106	0	NM_004198.3	CHRNA6
LOC102167207	48.63851798	0	20	1.48E-04	High	46	0	XP_005661561.1	ssc:102167207	1E-60	NM_014979.1	SV2C
GABRR2	48.63851798	0	20	1.48E-04	High	46	0	XP_003121344.1	ssc:100522289	0	NM_002043.3	GABRR2
LOC100624658	0	73.53877508	-20	1.49E-04	Low	0	89	XP_005658285.1	ssc:100624658	5E-28	NM_175913.3	JPH2
LOC100517751	0	73.53877508	-20	1.49E-04	Low	0	89	XP_005652884.1	ssc:100517751	0	NM_152601.3	ZNF709
ITGA5	790.9045968	26341.75449	-5.05770395	1.49E-04	Low	748	31880	XP_001925287.3	ssc:100155091	0	NM_002205.2	ITGA5
LOC100738338	2.114718173	131.3782611	-5.957117356	1.51E-04	Low	2	159	XP_003480496.2	ssc:100738338	9E-51	NR_104583.1	CALML4
LOC100737408	65.55626337	0.826278372	6.30996191	1.52E-04	High	62	1	XP_005658762.1	ssc:100737408	0	NR_104591.1	GARNL3
PPFIA4	37.00756803	1108.039296	-4.904044906	1.53E-04	Low	35	1341	XP_005653788.1	ssc:100512361	0	NR_038265.1	PPFIA2
EFHD2	128.9978086	3902.51275	-4.918984883	1.53E-04	Low	122	4723	XP_003127676.1	ssc:100520743	5E-109	NM_024329.5	EFHD2
LIF	6.344154519	243.7521197	-5.263843044	1.55E-04	Low	6	295	NM_214402.2	ssc:399503	3E-105	NM_002309.4	LIF
MAMDC2	2.114718173	130.5519827	-5.948015149	1.56E-04	Low	2	158	NM_001244983.1	ssc:100514623	9E-69	NM_153267.4	MAMDC2
CBLN4	0	72.71249671	-20	1.57E-04	Low	0	88	NM_001123092.1	ssc:100141408	1E-113	NM_080617.5	CBLN4
MYF5	0	72.71249671	-20	1.57E-04	Low	0	88	Y17154		3E-143	NM_005593.2	MYF5
LOC100621670	0	72.71249671	-20	1.57E-04	Low	0	88	XP_003361253.1	ssc:100621670	4E-51	NM_001048.3	SST
LOC100739371	0	72.71249671	-20	1.57E-04	Low	0	88	XP_003481077.1	ssc:100739371			
SHOX	0	72.71249671	-20	1.57E-04	Low	0	88	XP_003134962.1	ssc:100520816	3E-138	NM_006883.2	SHOX
FRMPD1	176.5789675	6.610226974	4.739469892	1.59E-04	High	167	8	XP_005660309.1	ssc:100153190	0	NM_014907.2	FRMPD1
INSC	1.057359087	101.6322397	-6.586748906	1.59E-04	Low	1	123	XP_003123009.2	ssc:100515187	0	NM_001278316.1	INSC
LOC100624487	16.91774539	529.6444363	-4.968414947	1.60E-04	Low	16	641	XP_005673051.1	ssc:100624487	1E-60	NM_030777.3	SLC2A10
KRCC1	40.17964529	1189.014577	-4.887157764	1.60E-04	Low	38	1439	NM_001243946.1	ssc:100515537	6E-23	NM_016618.1	KRCC1
STRBP	11176.28555	504.0298068	4.470787923	1.62E-04	High	10570	610	XP_001927197.1		0	NR_033234.1	STRBP
MSX1	13.74566813	440.4063721	-5.001786405	1.63E-04	Low	13	533	NM_001162887.1	ssc:100302364	1E-140	NM_002448.3	MSX1
FLT3LG	40.17964529	1183.230628	-4.880122665	1.64E-04	Low	38	1432	XP_005664789.1	ssc:100322867	1E-66	NM_001459.3	FLT3LG
CHRND	3.17207726	156.9928906	-5.629127508	1.64E-04	Low	3	190	XP_005672338.1	ssc:100522042	0	NR_046334.1	CHRND
NAALADL2	0	71.88621834	-20	1.65E-04	Low	0	87	XP_003132587.3	ssc:100522279	0	NM_207015.2	NAALADL2
LOC102160281	47.5811589	0	20	1.67E-04	High	45	0	XR_305285.1	ssc:102160281			
LOC100517446	47.5811589	0	20	1.67E-04	High	45	0	LOC100517446				
C14H10orf131	47.5811589	0	20	1.67E-04	High	45	0	XP_005671396.1	ssc:100158005	4E-50	NM_001130446.2	C10orf131
LOC100155368	334.1254714	14.87301069	4.489621346	1.67E-04	High	316	18	XP_005663084.1	ssc:100155368	3E-125	NM_020647.2	JPH1
CD151	760.2411832	24674.32474	-5.020409613	1.67E-04	Low	719	29862	NM_001243865.1	ssc:100511619	1E-121	NM_139030.3	CD151
XAF1	97.27703596	2859.749445	-4.877645671	1.69E-04	Low	92	3461	XP_005669195.1	ssc:102159947			
TPTE2	317.207726	14.04673232	4.497121448	1.70E-04	High	300	17	XP_005668431.1	ssc:100625564	0	NR_073487.1	TPTE2
S1PR2	58.15474976	1693.870662	-4.864282882	1.70E-04	Low	55	2050	NM_001243855.1	ssc:100511551	2E-173	NM_004230.3	S1PR2
NIPAL4	77.18721332	2246.650893	-4.863270278	1.73E-04	Low	73	2719	XP_003134161.1	ssc:100523099			
CAPN6	756.0117469	36.35624836	4.378133412	1.74E-04	High	715	44	XP_003135366.1	ssc:100522971	0	NM_014289.3	CAPN6
LOC100738726	40.17964529	1168.357618	-4.861873292	1.74E-04	Low	38	1414	XP_005668623.1	ssc:100738726	0	NM_006437.3	PARP4
LOC102159203	0	71.05993997	-20	1.74E-04	Low	0	86	XR_303394.1	ssc:102159203			
KCNE1L	0	71.05993997	-20	1.74E-04	Low	0	86	XP_005673883.1	ssc:100521707	5E-49	NM_012282.2	KCNE1L
LOC102158716	954.7952552	46.27158882	4.366992855	1.75E-04	High	903	56	LOC102158716				
ZNF311	95.16231779	2.478835115	5.262656195	1.80E-04	High	90	3	ZNF311	ssc:100524874	0	NM_001010877.2	ZNF311
ANGPT2	21.14718173	629.6241193	-4.895953493	1.82E-04	Low	20	762	NM_213808.1	ssc:396730	0	NM_001147.2	ANGPT2
TGFA	63.44154519	0.826278372	6.262656195	1.82E-04	High	60	1	NM_214251.1	ssc:397484			
LOC100620408	1098.596091	53.70809416	4.354377725	1.82E-04	High	1039	65	XP_005666757.1	ssc:100620408	7E-35	NM_006937.3	SUMO2
STOX2	4724.280399	224.7477171	4.393716228	1.82E-04	High	4468	272	XP_005671756.1	ssc:100156897	0	NM_020225.1	STOX2
CA12	9.516231779	318.1171731	-5.063024035	1.83E-04	Low	9	385	XP_005659573.1	ssc:100152749	3E-153	NM_206925.1	CA12
SAMD14	19.03246356	571.7846332	-4.908937627	1.83E-04	Low	18	692	XP_005668983.1	ssc:102159512	4E-174	NM_174920.3	SAMD14
LOC102162158	28.54869534	831.236042	-4.863761488	1.83E-04	Low	27	1006	XP_005653293.1	ssc:102162158	4E-160	NM_000512.4	GALNS
LOC102168045	0	70.2336616	-20	1.84E-04	Low	0	85	XP_005668048.1	ssc:102168045			
LOC102164461	0	70.2336616	-20	1.84E-04	Low	0	85	XP_005674472.1	ssc:102164461	9E-17	NR_027382.1	RDH13
LOC100621791	0	70.2336616	-20	1.84E-04	Low	0	85	XP_003359328.1	ssc:100621791	2E-64	NM_007021.3	C10orf10
LRRC29	1.057359087	98.32712624	-6.539052164	1.86E-04	Low	1	119	XP_005664476.1	ssc:100524680			
LOC100620684	109.965345	3.305113487	5.056205317	1.87E-04	High	104	4	LOC100620684				
TEX15	1244.511645	61.14459951	4.347210839	1.87E-04	High	1177	74	XP_005671870.1	ssc:100513440	0	NM_031271.3	TEX15
UNC93B1	104.6785496	3015.089778	-4.848163216	1.88E-04	Low	99	3649	XP_003122484.3	ssc:100522238	0	NM_030930.2	UNC93B1
RASSF10	140.6287585	4.95767023	4.826085534	1.88E-04	High	133	6	XP_005661179.1	ssc:100625955	0	NM_001080521.2	RASSF10
LOC102161206	46.52379981	0	20	1.88E-04	High	44	0	XR_307765.1	ssc:102161206			
LOC102159265	6.344154519	233.8367792	-5.203930143	1.88E-04	Low	6	283	XR_300558.1	ssc:102159265			
C6H19orf68	5.286795433	206.5695929	-5.288090591	1.88E-04	Low	5	250	XP_005664703.1	ssc:102165016	0	NM_199341.3	C19orf68
RBM20	326.7239578	14.87301069	4.457303626	1.90E-04	High	309	18	XP_001925009.4	ssc:100157563	0	NM_001134363.1	RBM20
LOC100738887	2.114718173	124.7680341	-5.88263914	1.92E-04	Low	2	151	XP_005653802.1	ssc:100738887	0	NR_038265.1	PPFIA2
PTGDS	1.057359087	97.50084786	-6.52687745	1.93E-04	Low	1	118	NM_214228.1	ssc:397456			
DIO3	0	69.40738323	-20	1.94E-04	Low	0	84	NM_001001625.2	ssc:414378	1E-154	NM_001362.3	DIO3
LOC102162793	0	69.40738323	-20	1.94E-04	Low	0	84	XR_298475.1	ssc:102162793			
PRSS56	0	69.40738323	-20	1.94E-04	Low	0	84	XP_003483809.1	ssc:100737499	0	NM_001195129.1	PRSS56
LAMA4	312.9782896	9344.382106	-4.899964779	1.97E-04	Low	296	11309	XP_003121419.3	ssc:100154581	0	NM_002290.4	LAMA4
OAF	88.81816327	2519.322755	-4.826037413	1.98E-04	Low	84	3049	XP_003130002.2	ssc:100522940	1E-132	NM_178507.2	OAF
HOOK1	1229.708618	61.14459951	4.329947615	2.00E-04	High	1163	74	XP_005653494.1	ssc:100525467	0	NM_015888.4	HOOK1
DUSP27	1.057359087	96.67456949	-6.51459912	2.01E-04	Low	1	117	XP_003355120.1	ssc:100153962	0	NM_001080426.1	DUSP27
CEL	0	68.58110485	-20	2.05E-04	Low	0	83	XP_003353742.1	ssc:100625953	0	NM_001807.4	CEL

CD19	0	68.58110485	-20	2.05E-04	Low	0	83	NM_214377.1	ssc:397669	0	NM_001770.5	CD19
NAPSA	0	68.58110485	-20	2.05E-04	Low	0	83	XP_005664836.1	ssc:100525581	0	NM_004851.1	NAPSA
RAB27A	3927.031648	191.6965822	4.356542704	2.05E-04	High	3714	232	NM_001032357.1	ssc:606749	1E-123	NM_183236.2	RAB27A
ABCA3	49.69587707	1390.6265	-4.80646501	2.06E-04	Low	47	1683	XP_005655233.1	ssc:100518064	0	NM_001089.2	ABCA3
LRP5	94.1049587	2646.569625	-4.813709053	2.07E-04	Low	89	3203	XP_005660680.1	ssc:100524299	0	NM_002335.2	LRP5
OSBPL5	98.33439505	2768.032545	-4.81502097	2.08E-04	Low	93	3350	XP_003122456.2	ssc:100515995	0	NM_145638.2	OSBPL5
LOC100514555	6.344154519	228.879109	-5.173014066	2.08E-04	Low	6	277	XP_005657585.1	ssc:100514555	0	NM_017677.3	MTMR8
UBA7	153.3170676	4366.881195	-4.832013067	2.11E-04	Low	145	5285	XR_306784.1	ssc:100512631	0	NM_003335.2	UBA7
LOC100525496	77.18721332	1.652556743	5.545590158	2.12E-04	High	73	2	XP_003121308.1	ssc:100525496			
LOC102166992	77.18721332	1.652556743	5.545590158	2.12E-04	High	73	2	XP_005668368.1	ssc:102166992			
LOC100626713	77.18721332	1.652556743	5.545590158	2.12E-04	High	73	2	XP_005674123.1	ssc:100626713	0	NM_207499.2	PTCHD4
C2H19orf71	67.67098154	1883.088409	-4.79841961	2.12E-04	Low	64	2279	XP_003123092.1	ssc:100519760	1E-88	NM_001135580.1	C19orf71
KLK15	45.46644072	0	20	2.13E-04	High	43	0	XP_003356062.1	ssc:100620705	9E-112	NR_102274.1	KLK15
LOC102159429	45.46644072	0	20	2.13E-04	High	43	0	XR_301667.1	ssc:102159429			
LOC102159508	45.46644072	0	20	2.13E-04	High	43	0	XR_306155.1	ssc:102159508			
LOC100626112	45.46644072	0	20	2.13E-04	High	43	0	XP_003358836.2	ssc:100626112	8E-38	NM_015341.4	NCAPH
SASH3	45.46644072	0	20	2.13E-04	High	43	0	XP_003135424.2	ssc:100525094	0	NM_018990.3	SASH3
LOC100519739	45.46644072	0	20	2.13E-04	High	43	0	XP_003132687.1	ssc:100519739	8E-38	NM_015341.4	NCAPH
HSF4	12.68830904	388.3508347	-4.935788847	2.14E-04	Low	12	470	XP_003126988.1	ssc:100523672	0	NM_001538.3	HSF4
ZNF10	0	67.75482648	-20	2.17E-04	Low	0	82	ZNF10	ssc:100627773	0	NM_015394.4	ZNF10
TNMD	0	67.75482648	-20	2.17E-04	Low	0	82	NM_001099934.1	ssc:100048961	0	NM_022144.2	TNMD
LOC102166578	0	67.75482648	-20	2.17E-04	Low	0	82	XP_005654560.1	ssc:102166578	1E-89	NM_153267.4	MAMDC2
LOC102158004	0	67.75482648	-20	2.17E-04	Low	0	82	XP_005658330.1	ssc:102158004	1E-45	NM_022369.3	STRA6
LOC102167753	0	67.75482648	-20	2.17E-04	Low	0	82	XR_302371.1	ssc:102167753			
LOC100739264	0	67.75482648	-20	2.17E-04	Low	0	82	XP_003481977.1	ssc:100739264	1E-81	NM_021170.3	HES4
LOC102161276	1.057359087	95.02201275	-6.489724452	2.17E-04	Low	1	115	XR_304701.1	ssc:102161276			
XIRP2	2.114718173	121.4629206	-5.843906746	2.18E-04	Low	2	147	NM_214396.1	ssc:397689	0	NM_152381.5	XIRP2
LOC100737604	4222.034833	208.2221497	4.341743032	2.21E-04	High	3993	252	XP_003481799.2	ssc:100737604	2E-154	NM_018351.3	FGD6
LOC102168130	121.596295	4.131391859	4.879327555	2.22E-04	High	115	5	XR_304864.1	ssc:102168130			
UNC5D	136.3993222	4.95767023	4.782030354	2.23E-04	High	129	6	XP_005671842.1	ssc:100156454	0	NM_080872.2	UNC5D
ADAMTS10	275.9707216	7979.370236	-4.853687766	2.23E-04	Low	261	9657	NM_001246250.1	ssc:100624519	0	NM_030957.3	ADAMTS10
SLC29A2	151.2023494	5.783948602	4.708282014	2.24E-04	High	143	7	XP_003122535.1	ssc:100518596	0	NM_001532.2	SLC29A2
SMPDL3B	2.114718173	120.6366423	-5.83405896	2.25E-04	Low	2	146	XP_003127785.1	ssc:100514109	0	NM_014474.2	SMPDL3B
LOC100736916	41.23700438	1134.480204	-4.781948092	2.26E-04	Low	39	1373	XP_005667953.1	ssc:100736916	0	NM_025179.3	PLXNA2
FLT1	72.95777697	2003.725051	-4.779478976	2.27E-04	Low	69	2425	XP_001925775.4	ssc:396763	0	NM_002019.4	FLT1
LOC100513619	503.3029252	14958.11736	-4.893357815	2.28E-04	Low	476	18103	XP_005668657.1	ssc:100513619	0	NM_020954.3	RNF213
LOC100516245	234.7337172	6696.159924	-4.834236993	2.29E-04	Low	222	8104	XR_298106.1	ssc:100516245			
LOC100625598	0	66.92854811	-20	2.29E-04	Low	0	81	XP_005653364.1	ssc:100625598			
LOC102157659	90.93288145	2.478835115	5.197067853	2.32E-04	High	86	3	XR_309375.1	ssc:102157659			
LOC100510883	90.93288145	2.478835115	5.197067853	2.32E-04	High	86	3	LOC100510883				
CA14	2569.38258	129.7257044	4.307885415	2.33E-04	High	2430	157	XP_005658313.1	ssc:100153371	3E-167	NM_012113.1	CA14
HES1	33.83549077	927.9106115	-4.777376613	2.35E-04	Low	32	1123	NM_001195231.1	ssc:100499567	6E-162	NM_005524.3	HES1
ELMOD1	559.3429568	28.09346464	4.31542667	2.38E-04	High	529	34	XP_003357322.2	ssc:100626413	0	NM_018712.3	ELMOD1
LOC100621672	10534.46858	507.3349202	4.376035262	2.39E-04	High	9963	614	XP_003361780.1	ssc:100621672	2E-55	NM_006200.3	PCSK5
CXCR5	100.4491132	2744.896751	-4.77221515	2.40E-04	Low	95	3322	XP_003129963.1	ssc:100515679	1E-177	NM_032966.2	CXCR5
PDZRN4	60.26946793	0.826278372	6.188655613	2.41E-04	High	57	1	XP_003481760.1	ssc:100156795	0	NM_013377.3	PDZRN4
LOC102167098	60.26946793	0.826278372	6.188655613	2.41E-04	High	57	1	XR_300692.1	ssc:102167098			
LOC102158029	4.229436346	170.2133446	-5.330734928	2.41E-04	Low	4	206	XR_303678.1	ssc:102158029			
IL15RA	4.229436346	170.2133446	-5.330734928	2.41E-04	Low	4	206	XP_005668268.1	ssc:733692	1E-54	NR_046362.1	IL15RA
DBX1	44.40908164	0	20	2.41E-04	High	42	0	XP_003122964.1	ssc:100520887	2E-156	NM_001029865.2	DBX1
LOC100512242	44.40908164	0	20	2.41E-04	High	42	0	XR_305005.1	ssc:100512242	4E-65	NM_054013.3	MGAT4B
EPHA1	8.458872693	273.498141	-5.014921808	2.42E-04	Low	8	331	XP_003134614.1	ssc:100519989	0	NM_005232.4	EPHA1
LOC100511457	0	66.10226974	-20	2.42E-04	Low	0	80	XP_003127417.4	ssc:100511457	8E-89	NM_024729.3	MYH14
LOC102164768	66.61362245	1801.28685	-4.757066897	2.43E-04	Low	63	2180	XP_005664907.1	ssc:102164768	2E-57	NM_182571.2	SSC5D
LOC100622080	886.0669145	45.44531045	4.285212319	2.43E-04	High	838	55	XP_005658656.1	ssc:100622080	3E-37	NM_014317.3	PDSS1
SDC2	320.3798032	9156.816916	-4.836991069	2.44E-04	Low	303	11082	XP_001926974.2	ssc:100152754	7E-100	NM_002998.3	SDC2
LOC100620614	17.97510447	510.6400337	-4.828234587	2.45E-04	Low	17	618	XP_005669253.1	ssc:100620614	0	NM_000697.2	ALOX12
ZNF385C	15.8603863	456.9319396	-4.848479475	2.45E-04	Low	15	553	ZNF385C	ssc:100622424	0	NM_001242704.1	ZNF385C
ARHGEF9	1.057359087	92.54317763	-6.451589323	2.46E-04	Low	1	112	XP_005657868.1	ssc:100512575	0	NM_015185.2	ARHGEF9
LOC102165355	75.07249515	1.652556743	5.505512719	2.47E-04	High	71	2	XR_298728.1	ssc:102165355			
PPAT	3701.814162	187.5651904	4.302768458	2.54E-04	High	3501	227	XP_003129071.2	ssc:100521102	0	NM_002703.4	PPAT
HSD3B7	63.44154519	1697.175776	-4.741564271	2.55E-04	Low	60	2054	XP_005655142.1	ssc:100525281	0	NM_025193.3	HSD3B7
ASTN1	415.542121	20.65695929	4.330294912	2.55E-04	High	393	25	XP_003482773.1	ssc:100737305	0	NM_207108.2	ASTN1
LOC100622865	5.286795433	193.349139	-5.192671025	2.57E-04	Low	5	234	XP_005669276.1	ssc:100622865	1E-78	NM_001365.3	DLG4
TRIM72	5.286795433	193.349139	-5.192671025	2.57E-04	Low	5	234	XP_003124542.2	ssc:100511188	0	NM_001008274.3	TRIM72
PALMD	8.458872693	269.3667492	-4.992962555	2.60E-04	Low	8	326	NM_001038645.2	ssc:654413	0	NM_017734.4	PALMD
LOC100523100	103.6211905	3.305113487	4.970475443	2.60E-04	High	98	4	LOC100523100	ssc:100523100			
TIAM1	1542.686907	80.14900206	4.266616925	2.60E-04	High	1459	97	XP_001927581.1				
NFIC	228.3895627	6331.771163	-4.793040489	2.61E-04	Low	216	7663	XP_005654783.1	ssc:100737330	5E-165	NM_205843.2	NFIC
LOC100624958	5.286795433	192.5228606	-5.186492451	2.62E-04	Low	5	233	XP_005656941.1	ssc:100624958			
GAL3ST4	47.5811589	1262.553352	-4.729810132	2.65E-04	Low	45	1528	XP_005661948.1	ssc:100511123	0	NM_024637.4	GAL3ST4
ADRA1B	6.344154519	217.3112118	-5.098190889	2.65E-04	Low	6	263	XP_005672634.1	ssc:100519931	0	NM_000679.3	ADRA1B
NHSL2	6.344154519	217.3112118	-5.098190889	2.65E-04	Low	6	263	XP_005657890.1	ssc:100151779	0	NM_001013627.2	NHSL2
FRMD3	74.01513606	1.652556743	5.485048616	2.67E-04	High	70	2	XP_005668097.1	ssc:100519727	3E-179	NM_174938.5	FRMD3
CDH7	74.01513606	1.652556743	5.485048616	2.67E-04	High	70	2	XP_001926047.1	ssc:100156376	0	NM_033646.1	CDH7
AKAP5	623.8418611	32.2248565	4.274934525	2.69E-04	High	590	39	XP_005666363.1	ssc:100153460	8E-172	NM_004857.3	AKAP5
GALNT18	160.7185812	6.610226974	4.603693113	2.70E-04	High	152	8	XP_005661184.1	ssc:100519410	0	NM_198516.2	GALNT18
LOC102164245	0	64.44971299	-20	2.72E-04	Low	0	78	XP_005658386.1	ssc:102164245	2E-49	NM_001430.4	EPAS1
TMEM100	0	64.44971299	-20	2.72E-04	Low	0	78	XP_005669010.1	ssc:100523465	2E-63	NM_018286.2	TMEM100
LOC100739211	0	64.44971299	-20	2.72E-04	Low	0	78	XP_003482272.2	ssc:100739211	3E-102	NM_207517.2	ADAMTSL3
LOC102167757	0	64.44971299	-20	2.72E-04	Low	0	78	XR_303389.1	ssc:102167757			
LOC100737830	0	64.44971299	-20	2.72E-04	Low	0	78	LOC100737830				
C1H6orf118	43.35172255	0	20	2.74E-04	High	41	0	XR_300598.1	ssc:100157121			
C6H1orf173	43.35172255	0	20	2.74E-04	High	41	0	XP_005653487.1	ssc:100514238	0	NM_001002912.4	C1orf173
TEX11	43.35172255	0	20	2.74E-04	High	41	0	XP_003484173.1	ssc:100739555			
LOC100511509	102.5638314	3.305113487	4.955678441	2.75E-04	High	97	4	XR_303488.1	ssc:100511509			
LOC100627550	102.5638314	3.305113487	4.955678441	2.75E-04	High	97	4	XP_003360923.1	ssc:100627550	0	NM_005068.2	SIM1
FZD3	1240.282209	65.27599137	4.247972149	2.78E-04	High	1173	79	XR_307344.1	ssc:100153078	0	NM_145866.1	FZD3
LOC100514834	1.057359087	90.06434252	-6.412418726	2.78E-04	Low	1	109	XR_303007.1	ssc:100514834			
RAPSN	1.057359087	90.06434252	-6.412418726	2.78E-04	Low	1	109	XP_003122858.3	ssc:100512645	3E-136	NM_032645.4	RAPSN
SEMA3C	74.01513606	1944.233009	-4.715236989	2.80E-04	Low	70	2353	XP_005667747.1	ssc:100624508	0	NM_006379.3	SEMA3C
LOC102166137	7.401513606	239.6207278	-5.016788569	2.82E-04	Low	7	290	XP_005655468.1	ssc:102166137	4E-20	NM_016954.2	TBX22
LOC102157822	4.229436346	164.429396	-5.280859021	2.83E-04	Low	4	199	XR_306600.1	ssc:102157822			
LOC396679	2256.404291	118.1578072	4.255238723	2.83E-04	High	2134	143	NM_001244426.1	ssc:396679	0	NR_028435.1	AMT
LOC100520702	5.286795433	189.2177471	-5.161510094	2.84E-04	Low	5	229	XR_308834.1	ssc:100520702			
LIMS2	37.00756803	971.7033652	-4.714623729	2.85E-04	Low	35	1176	XP_005671891.1	ssc:100517009	0	NM_017980.4	LIMS2
EFCAB6	0	63.62343462	-20	2.88E-04	Low	0	77	XP_005663806.1	ssc:100524434	0	NM_198856.2	EFCAB6
LOC102160260	0	63.62343462	-20	2.88E-04	Low	0	77	XR_302319.1	ssc:102160260			
F13A1	0	63.62343462	-20	2.88E-04	Low	0	77	XP_001927665.3	ssc:100153504	0	NM_000129.3	F13A1
LOC102165879	0	63.62343462	-20	2.88E-04	Low	0	77	XR_301495.1	ssc:102165879			

LOC100155089	0	63.62343462	-20	2.88E-04	Low	0	77	XP_005671240.1	ssc:100155089	5E-21	NM_001137548.1	FAM25C
DRAM1	9.516231779	288.3711517	-4.921392626	2.89E-04	Low	9	349	NM_001190286.1	ssc:100462750	3E-132	NM_018370.2	DRAM1
SLC15A3	39.1222862	1022.106346	-4.70741082	2.90E-04	Low	37	1237	XP_003122726.3	ssc:100513135	0	NR_027391.1	SLC15A3
LOC100155118	19.03246356	518.0765391	-4.766631032	2.91E-04	Low	18	627	XP_001929233.1	ssc:100155118	3E-92	NM_002475.4	MYL6B
LOC100739439	2.114718173	114.0264153	-5.752758858	2.92E-04	Low	2	138	XP_005669377.1	ssc:100739439	5E-88	NM_014483.3	RBMS3
THSD1	2369.541713	124.7680341	4.247287793	2.95E-04	High	2241	151	XP_005668435.1	ssc:100513373	0	NM_199263.2	THSD1
TUBB6	352.1005758	9716.207373	-4.78633379	2.96E-04	Low	333	11759	XP_005656166.1	ssc:100623583	0	NM_032525.1	TUBB6
SERPINI1	1334.387167	71.05993997	4.23099704	2.98E-04	High	1262	86	XP_001926075.4	ssc:100154352	0	NM_005025.4	SERPINI1
LOC100737939	100.4491132	2620.128717	-4.705100955	2.99E-04	Low	95	3171	XP_003481552.1	ssc:100737939	5E-125	NM_080630.3	COL11A1
GNB3	13.74566813	386.698278	-4.814159402	3.01E-04	Low	13	468	NM_001243493.1	ssc:100154522	0	NM_002075.2	GNB3
LOC100738892	0	62.79715625	-20	3.05E-04	Low	0	76	XP_005652485.1	ssc:100738892	2E-112	NM_007115.3	TNFAIP6
ST6GALNAC2	169.1774539	4467.687156	-4.722918947	3.08E-04	Low	160	5407	NM_213784.1	ssc:396676	3E-165	NM_175040.3	ST6GALNAC4
PINK1	93.04759962	2406.122618	-4.692597422	3.09E-04	Low	88	2912	XP_005665148.1	ssc:100515613	0	NM_032409.2	PINK1
LOC100622713	22.20454082	587.4839223	-4.725622728	3.12E-04	Low	21	711	XP_005656599.1	ssc:100622713	0	NM_024582.4	FAT4
LOC100621972	909.3288145	48.75042393	4.221315399	3.12E-04	High	860	59	LOC100621972				
LOC100739329	6.344154519	209.8747064	-5.047956587	3.12E-04	Low	6	254	XP_005656527.1	ssc:100739329	2E-135	NM_206918.2	DEGS2
LOC100739389	299.2326215	14.87301069	4.330498841	3.13E-04	High	283	18	XP_005655045.1	ssc:100739389	1E-77	NR_003149.1	GPR98
SOX7	42.29436346	0	20	3.13E-04	High	40	0	XP_003359100.1	ssc:100155811	0	NM_031439.3	SOX7
LOC102160632	9.516231779	283.4134815	-4.896374165	3.14E-04	Low	9	343	XP_005665348.1	ssc:102160632	1E-80	NM_002845.3	PTPRM
WIPI1	107.8506268	2790.342061	-4.693335523	3.14E-04	Low	102	3377	NM_001190294.1	ssc:100462758	0	NM_017983.5	WIPI1
SCUBE2	6.344154519	209.048428	-5.042265475	3.18E-04	Low	6	253	XP_005667098.1	ssc:100519613	0	NM_020974.2	SCUBE2
LOC102163369	4.229436346	160.2980041	-5.244147243	3.19E-04	Low	4	194	XP_005658421.1	ssc:102163369	7E-54	NM_001286057.1	ADCY7
CDC42EP4	62.38418611	1591.412144	-4.672983339	3.19E-04	Low	59	1926	XP_003358042.1	ssc:100517117	3E-143	NM_012121.4	CDC42EP4
SCP2	9.516231779	281.7609248	-4.887937328	3.23E-04	Low	9	341	NM_001168415.1	ssc:397439	5E-177	NM_002979.4	SCP2
C10H1orf95	57.09739067	0.826278372	6.110653101	3.23E-04	High	54	1	XP_005667995.1	ssc:100621258	4E-65	NM_001003665.3	C1orf95
TMCO4	19.03246356	506.5086419	-4.734052663	3.23E-04	Low	18	613	XP_005656093.1	ssc:100623693	0	NM_181719.4	TMCO4
ARHGAP8	0	61.97087788	-20	3.24E-04	Low	0	75	NM_001204383.1	ssc:100533202	0	NM_181334.4	PRR5-ARHGAP8
MRVI1	0	61.97087788	-20	3.24E-04	Low	0	75	XP_005661186.1	ssc:100739522	0	NM_130385.3	MRVI1
LOC102163830	0	61.97087788	-20	3.24E-04	Low	0	75	XP_005656594.1	ssc:102163830			
LOC102163560	0	61.97087788	-20	3.24E-04	Low	0	75	XR_297410.1	ssc:102163560			
LOC100627089	0	61.97087788	-20	3.24E-04	Low	0	75	XP_005655754.1	ssc:100627089	0	NM_203416.3	CD163
ZNF33B	0	61.97087788	-20	3.24E-04	Low	0	75	ZNF33B	ssc:100738050	0	NM_006955.1	ZNF33B
HPDL	2567.267862	137.1622097	4.226278874	3.25E-04	High	2428	166	XP_003128096.1	ssc:100514057	5E-173	NM_032756.2	HPDL
FBXO24	4.229436346	159.4717257	-5.236691438	3.26E-04	Low	4	193	XP_005661970.1	ssc:100627436	0	NM_033506.2	FBXO24
WT1	781.388365	42.14019696	4.212770811	3.27E-04	High	739	51	NM_001001264.1	ssc:397338	0	NM_024426.4	WT1
LOC102159137	99.39175414	3.305113487	4.910354451	3.28E-04	High	94	4	XP_005668096.1	ssc:102159137			
LOC102165469	99.39175414	3.305113487	4.910354451	3.28E-04	High	94	4	XR_303733.1	ssc:102165469			
LOC100526008	382.7639893	19.83068092	4.270648986	3.29E-04	High	362	24	LOC100526008				
LOC100525875	1.057359087	86.75922903	-6.358479918	3.30E-04	Low	1	105	XR_115968.2	ssc:100525875			
ZIC4	24.31925899	631.276676	-4.698101273	3.30E-04	Low	23	764	ZIC4	ssc:100519623	9E-12	NR_040762.1	ZIC4
LOC102159296	28.54869534	732.9089157	-4.682137193	3.32E-04	Low	27	887	XP_005666934.1	ssc:102159296			
RINL	2.114718173	110.7213018	-5.710323591	3.34E-04	Low	2	134	XP_005664658.1	ssc:102163514	2E-129	NM_198445.3	RINL
FAT4	16.91774539	452.8005477	-4.742266484	3.34E-04	Low	16	548	XP_005666936.1	ssc:102159549	0	NM_024582.4	FAT4
RNF43	3.17207726	134.6833746	-5.408000054	3.34E-04	Low	3	163	XP_005669052.1	ssc:100517305	0	NM_017763.4	RNF43
LOC102159987	49.69587707	1254.290568	-4.657601625	3.35E-04	Low	47	1518	XP_005668226.1	ssc:102159987	7E-93	NM_032812.8	PLXDC2
ADAMTS4	30.66341351	781.6593397	-4.671949779	3.38E-04	Low	29	946	XP_003481462.1	ssc:100157276	0	NM_005099.4	ADAMTS4
FOXA3	70.8430588	1.652556743	5.42185479	3.39E-04	High	67	2	XP_003127277.1	ssc:100512416	2E-144	NM_004497.2	FOXA3
LOC100157134	834.2563193	45.44531045	4.198287376	3.43E-04	High	789	55	ZNF704	ssc:100157134	0	NM_032651.1	ZNF704
LOC100623679	524.4501069	28.09346464	4.222499068	3.44E-04	High	496	34	XP_003360371.1	ssc:100623679	2E-56	NM_033022.3	RPS24
LOC102159630	0	61.14459951	-20	3.44E-04	Low	0	74	XR_305716.1	ssc:102159630			
LOC102166402	0	61.14459951	-20	3.44E-04	Low	0	74	XR_300488.1	ssc:102166402			
LOC100623627	0	61.14459951	-20	3.44E-04	Low	0	74	XR_303903.1	ssc:100623627			
PKHD1L1	251.6514626	12.39417558	4.343692767	3.45E-04	High	238	15	XP_003125570.3	ssc:100525460	0	NM_177531.4	PKHD1L1
CALN1	2.114718173	109.8950234	-5.699516836	3.46E-04	Low	2	133	XP_005662055.1	ssc:100737793	2E-45	NM_031468.3	CALN1
KIF5C	13650.50581	691.5949971	4.302883107	3.49E-04	High	12910	837	XP_005671654.1	ssc:100622102	0	NM_004522.2	KIF5C
GPR146	4.229436346	156.9928906	-5.214090009	3.51E-04	Low	4	190	XP_003481022.2	ssc:100738733	9E-117	NM_138445.2	GPR146
LOC100627578	1847.206324	100.8059614	4.195692155	3.52E-04	High	1747	122	XP_003360596.1	ssc:100627578	2E-41	NM_001127688.2	BEX4
LOC102166486	6.344154519	204.0907578	-5.007639132	3.55E-04	Low	6	247	XR_300446.1	ssc:102166486			
DUSP23	10.57359087	299.9390489	-4.826132044	3.56E-04	Low	10	363	XP_005663272.1	ssc:100620368	2E-80	NM_017823.3	DUSP23
LOC102158959	2.114718173	109.0687451	-5.68862852	3.58E-04	Low	2	132	XR_309520.1	ssc:102158959			
FADS3	215.7012537	5576.552731	-4.692267097	3.59E-04	Low	204	6749	NM_001206434.1	ssc:100518057	0	NM_021727.4	FADS3
MSMP	154.3744266	3930.606514	-4.670246152	3.60E-04	Low	146	4757	XP_003122034.1	ssc:100512517	1E-73	NM_001044264.2	MSMP
LOC100626701	581.5474976	15834.79872	-4.76705769	3.60E-04	Low	550	19164	XP_003356232.1	ssc:100626701	0	NM_005529.5	HSPG2
SMPD1	134.284604	3395.177829	-4.660121329	3.63E-04	Low	127	4109	XP_005656664.1	ssc:100518980	2E-70	NR_027400.1	SMPD1
LOC102163860	3.17207726	132.2045395	-5.381199995	3.64E-04	Low	3	160	XP_005673333.1	ssc:102163860	7E-65	NM_005020.2	PDE1C
tRNA-Asn	605.8667566	33.05113487	4.196228833	3.65E-04	High	573	40	tRNA-Asn				
LOC102159863	179.7510447	8.262783717	4.44322844	3.65E-04	High	170	10	XR_304873.1	ssc:102159863			
LOC102164483	0	60.31832114	-20	3.66E-04	Low	0	73	XP_005669520.1	ssc:102164483	1E-109	NR_033815.1	ALS2CL
LOC100621447	0	60.31832114	-20	3.66E-04	Low	0	73	XR_309679.1	ssc:100621447			
LOC100153113	0	60.31832114	-20	3.66E-04	Low	0	73	XP_005671238.1	ssc:100153113	5E-21	NM_001137548.1	FAM25C
HOXC11	0	60.31832114	-20	3.66E-04	Low	0	73	XP_003355473.2	ssc:100628033	6E-106	NM_014212.3	HOXC11
SCN9A	432.4598664	23.13579441	4.22436771	3.67E-04	High	409	28	XP_003133500.3	ssc:100516701	0	NM_002977.3	SCN9A
TMEM158	40.17964529	997.3179947	-4.633516848	3.68E-04	Low	38	1207	XP_003358446.1	ssc:100620777	5E-108	NM_015444.2	TMEM158
LOC102162854	97.27703596	3.305113487	4.879327555	3.69E-04	High	92	4	XR_301570.1	ssc:102162854			
LOC102167838	83.53136784	2.478835115	5.074583847	3.70E-04	High	79	3	XR_307942.1	ssc:102167838			
ABCA5	793.0193149	43.7927537	4.17859193	3.73E-04	High	750	53	XP_003483003.1	ssc:100520049	2E-129	NM_172232	ABCA5
TACC1	88.81816327	2203.684417	-4.632919088	3.74E-04	Low	84	2667	XP_003359532.2	ssc:100523215	0	NM_006283.2	TACC1
ENOX1	3.17207726	131.3782611	-5.372154855	3.75E-04	Low	3	159	XP_005668503.1	ssc:100525605	0	NM_017993.3	ENOX1
DAGLA	11.63094995	320.5960082	-4.784715624	3.75E-04	Low	11	388	XP_003122693.1	ssc:100520824	0	NM_006133.2	DAGLA
DLG4	57.09739067	1406.325789	-4.62236222	3.76E-04	Low	54	1702	XP_005655337.1	ssc:100626077	0	NM_001365.3	DLG4
SSX2IP	7269.34372	383.3931645	4.244928602	3.77E-04	High	6875	464	XR_298669.1	ssc:100522497	0	NM_014021.3	SSX2IP
LOC100152428	12.68830904	342.0792459	-4.752758858	3.89E-04	Low	12	414	XP_005670351.1	ssc:100152428	1E-170	NM_019902.1	ABCC1
LOC100512714	0	59.49204276	-20	3.89E-04	Low	0	72	XP_005663639.1	ssc:100512714	1E-126	NM_006492.2	ALX3
LOC102158670	0	59.49204276	-20	3.89E-04	Low	0	72	XR_303842.1	ssc:102158670			
ZNF37A	0	59.49204276	-20	3.89E-04	Low	0	72	ZNF37A	ssc:100152623	0	NM_003421.2	ZNF37A
LOC102162416	0	59.49204276	-20	3.89E-04	Low	0	72	XR_308922.1	ssc:102162416			
HOXC10	0	59.49204276	-20	3.89E-04	Low	0	72	XP_003355474.2	ssc:100628125	2E-158	NM_017409.3	HOXC10
HOXD10	0	59.49204276	-20	3.89E-04	Low	0	72	XP_003133545.2	ssc:100515344	0	NM_002148.3	HOXD10
COL7A1	309.8062124	8015.726484	-4.693395302	3.89E-04	Low	293	9701	XP_005669576.1	ssc:100523209	0	NM_000094.3	COL7A1
SLC9C2	357.3873713	19.00440255	4.233083079	3.90E-04	High	338	23	XP_005656772.1	ssc:100624136	0	NM_178527.3	SLC9C2
LOC100519456	31.7207726	780.8330613	-4.621514324	3.96E-04	Low	30	945	XP_005673355.1	ssc:100519456	6E-134	NM_024014.3	HOXA6
CYP26A1	54.9826725	0.826278372	6.056205317	3.96E-04	High	52	1	XP_003483578.1	ssc:100124374	4E-120	NM_057157.2	CYP26A1
PHYH	151.2023494	3767.003097	-4.638864758	3.97E-04	Low	143	4559	NM_001113447.1	ssc:100134962	4E-118	NM_006214.3	PHYH
RBMS2	63.44154519	1542.66172	-4.603850017	4.00E-04	Low	60	1867	XP_003126323.4	ssc:100522738	0	NM_002898.3	RBMS2
LOC100038019	69.78569971	1693.870662	-4.601248476	4.06E-04	Low	66	2050	XP_005669379.1	ssc:100038019	2E-177	NM_003242.5	TGFBR2
LOC100519796	228.3895627	5764.117921	-4.657531223	4.08E-04	Low	216	6976	XP_005654011.1	ssc:100519796	2E-93	NM_016429.2	COPZ2
FSBP	216.7586127	10.74161883	4.334805981	4.10E-04	High	205	13	XP_003355069.1	ssc:100624535	1E-171	NM_001256141.1	FSBP
CR2	40.17964529	0	20	4.10E-04	High	38	0	XP_005667612.1	ssc:100623585	0	NM_001877.4	CR2
NMUR1	40.17964529	0	20	4.10E-04	High	38	0	HighI00019DC5D0		3E-170	NM_006056.4	NMUR1
LOC100739622	40.17964529	0	20	4.10E-04	High	38	0	XP_003482905.1	ssc:100739622	2E-152	NM_199138.2	AMER2

SHANK1	0	58.66576439	-20	4.15E-04	Low	0	71	XP_005664848.1	ssc:100512353	0	NM_016148.2	SHANK1
LOC100517530	0	58.66576439	-20	4.15E-04	Low	0	71	XP_003127023.2	ssc:100517530	3E-53	XP_003403927.4	LOC100653057
C3H2orf40	0	58.66576439	-20	4.15E-04	Low	0	71	NM_001244729.1	ssc:100512958	5E-60	NM_032411.2	C2orf40
SHROOM4	0	58.66576439	-20	4.15E-04	Low	0	71	XP_005674526.1	ssc:100513967	0	NR_027121.1	SHROOM4
LOC100521521	0	58.66576439	-20	4.15E-04	Low	0	71	XR_307615.1	ssc:100521521			
CTF1	5.286795433	174.3447364	-5.043405495	4.15E-04	Low	5	211	XP_005662095.1	ssc:100736818	2E-61	NM_001330.3	CTF1
LOC102158432	95.16231779	3.305113487	4.847618696	4.16E-04	High	90	4	XP_005662318.1	ssc:102158432	3E-83	NM_006648.3	WNK2
LOC100038017	95.16231779	3.305113487	4.847618696	4.16E-04	High	90	4	XP_005673792.1	ssc:100038017	0	NM_005296.2	LPAR4
LOC100521603	51.81059524	1246.854063	-4.588901647	4.19E-04	Low	49	1509	XP_003122968.4	ssc:100521603	6E-134	NM_006410.4	HTATIP2
LOC100622618	47.5811589	1144.395545	-4.588051565	4.21E-04	Low	45	1385	XP_003357081.2	ssc:100622618	0	NM_024582.4	FAT4
LOC100621406	4599.512027	252.0149034	4.189899852	4.24E-04	High	4350	305	XP_005658086.1	ssc:100621406	5E-147	NM_018290.3	PGM2
GALNT5	12.68830904	335.4690189	-4.724607817	4.26E-04	Low	12	406	XP_005671911.1	ssc:100627600	0	NM_014568.1	GALNT5
SLA-7	98.33439505	2363.156143	-4.586875021	4.40E-04	Low	93	2860	NM_213768.1	ssc:396650	4E-132	NM_002116.7	HLA-A
IFI27L2	140.6287585	3417.487345	-4.602972436	4.41E-04	Low	133	4136	XP_005666469.1	ssc:100152306	3E-31	NM_032036.2	IFI27L2
LOC100154532	80.35929058	1920.270936	-4.578701241	4.41E-04	Low	76	2324	XP_003360024.3	ssc:100154532	4E-162	NM_183006.2	DLGAP4
SLA-DQB1	0	57.83948602	-20	4.42E-04	Low	0	70	NM_001113694.1	ssc:100037921	2E-113	xp_003846522	xp_003846522
LOC102158649	0	57.83948602	-20	4.42E-04	Low	0	70	XR_300644.1	ssc:102158649			
CASP1	0	57.83948602	-20	4.42E-04	Low	0	70	NM_214162.1	ssc:397319	1E-174	NM_033295.3	CASP1
HOXA11	0	57.83948602	-20	4.42E-04	Low	0	70	XP_003134898.2	ssc:100521236	3E-149	NM_005523.5	HOXA11
LOC102158933	120.5389359	4.95767023	4.603693113	4.43E-04	High	114	6	LOC102158933				
NPTX2	3.17207726	126.4205909	-5.316659743	4.47E-04	Low	3	153	XP_005661911.1	ssc:100521613	5E-166	NM_002523.2	NPTX2
ANO4	3.17207726	126.4205909	-5.316659743	4.47E-04	Low	3	153	XP_003126736.3	ssc:100525519	0	NM_178826.3	ANO4
FAM108B1	12456.7474	660.196419	4.23788829	4.47E-04	High	11781	799	NM_001243558.1	ssc:100155547	7E-173	NM_016014.2	ABHD17B
EXO1	5101.757593	280.9346464	4.18268789	4.47E-04	High	4825	340	XP_003130603.3	ssc:100512301	0	NM_130398.3	EXO1
LOC100623151	93.04759962	2221.862542	-4.577656822	4.50E-04	Low	88	2689	XP_005659139.1	ssc:100623151	1E-123	NM_032714.2	INF2
LOC100156854	2361.08284	133.8570962	4.140683132	4.55E-04	High	2233	162	XP_005655493.1	ssc:100156854	0	NM_003051.3	SLC16A1
FGFR4	779.2736468	44.61903207	4.126398906	4.56E-04	High	737	54	XP_003123730.1	ssc:100127133	0	NM_213647.1	FGFR4
LOC100737127	29.60605442	708.9468429	-4.581713316	4.57E-04	Low	28	858	XP_005665344.1	ssc:100737127	2E-65	NM_006868.3	RAB31
LOC100738006	7995.749413	433.7961452	4.204144121	4.57E-04	High	7562	525	XP_003482423.2	ssc:100738006	0	NM_020453.3	ATP10D
LOC102166276	39.1222862	927.0843331	-4.566637996	4.58E-04	Low	37	1122	XP_005669378.1	ssc:102166276	3E-54	NM_003242.5	TGFBR2
SOX9	16.91774539	423.0545263	-4.644234401	4.59E-04	Low	16	512	NM_213843.1	ssc:396840	0	NM_000346.3	SOX9
LOC100739077	54.9826725	1297.257044	-4.560343526	4.60E-04	Low	52	1570	XP_005674296.1	ssc:100739077	3E-85	NM_020992.3	PDLIM1
LOC102164501	172.3495311	8.262783717	4.382565659	4.61E-04	High	163	10	XR_302668.1	ssc:102164501			
GLS2	279.1427989	14.87301069	4.230234717	4.62E-04	High	264	18	XP_005663945.1	ssc:100738224	0	NM_138566.1	GLS2
LOC100737630	279.1427989	14.87301069	4.230234717	4.62E-04	High	264	18	XP_005655692.1	ssc:100737630	0	NM_138566.1	GLS2
HOMER2	5430.596269	300.7653273	4.174400446	4.70E-04	High	5136	364	XP_005666167.1	ssc:100156419	0	NM_199332.2	HOMER2
LOC100621934	0	57.01320765	-20	4.71E-04	Low	0	69	XP_003361045.1	ssc:100621934	7E-52	NM_022369.3	STRA6
RNF208	0	57.01320765	-20	4.71E-04	Low	0	69	XP_005652807.1	ssc:100515466	3E-111	NM_031297.4	RNF208
LOC102164644	39.1222862	0	20	4.71E-04	High	37	0	XR_306541.1	ssc:102164644			
LOC100621683	39.1222862	0	20	4.71E-04	High	37	0	XP_005667909.1	ssc:100621683	2E-33	NM_018664.2	BATF3
CXHXorf48	39.1222862	0	20	4.71E-04	High	37	0	XR_297879.1	ssc:102168080			
LOC100515867	66.61362245	1.652556743	5.333045523	4.73E-04	High	63	2	LOC100515867				
LOC396877	3.17207726	124.7680341	-5.297676639	4.75E-04	Low	3	151	Z49058	ssc:396877	8E-121	NM_001928.2	CFD
NEDD9	194.5540719	4715.570667	-4.599189292	4.76E-04	Low	184	5707	XP_005665630.1	ssc:100154178	0	NR_073131.1	NEDD9
LOC100511504	42.29436346	990.7077677	-4.549222249	4.80E-04	Low	40	1199	XP_005655379.1	ssc:100511504	0	NM_032450.2	MROH1
LOC102164478	2947.917133	167.7345095	4.135444528	4.83E-04	High	2788	203	XR_299466.1	ssc:102164478			
NEIL1	8.458872693	235.4893359	-4.79905251	4.85E-04	Low	8	285	XP_005666203.1	ssc:100155341	1E-180	NR_046311.1	NEIL1
LOC100155264	52.86795433	0.826278372	5.999621789	4.88E-04	High	50	1	LOC100155264				
AGTR1	12.68830904	325.5536785	-4.68132372	4.89E-04	Low	12	394	AGTR1		0	NM_032049.3	AGTR1
NFKBIZ	78.24457241	1825.248923	-4.543958738	4.93E-04	Low	74	2209	XP_005657172.1	ssc:100520981	0	NM_031419.3	NFKBIZ
PLCH1	1697.061334	98.32712624	4.109305418	4.94E-04	High	1605	119	XP_005669995.1	ssc:100627163	0	NM_014996.2	PLCH1
LOC100517234	11.63094995	301.5916057	-4.696555436	4.98E-04	Low	11	365	XP_005667982.1	ssc:100517234	1E-83	NM_021958.3	HLX
RALGDS	101.5064723	2375.550319	-4.548618141	5.00E-04	Low	96	2875	XP_005660599.1	ssc:100513970	0	NM_006266.3	RALGDS
LOC100513500	143.8008358	6.610226974	4.44322844	5.01E-04	High	136	8	XP_005672139.1	ssc:100513500	0	xp_003846271	xp_003846271
GBP5	0	56.18692928	-20	5.02E-04	Low	0	68	XP_001929321.1		0	NM_052942.3	GBP5
LOC102162114	20.08982264	483.3728475	-4.588599702	5.06E-04	Low	19	585	XP_005655914.1	ssc:102162114	2E-96	NM_003573.2	LTBP4
TLR7	195.611431	9.915340461	4.302184559	5.06E-04	High	185	12	NM_001097434.1	ssc:100037296			
LPHN1	41.23700438	955.1777977	-4.533757864	5.07E-04	Low	39	1156	XP_005661305.1	ssc:100514215	0	NM_014921.4	LPHN1
SLC9B1	394.3949393	22.30951604	4.143909917	5.10E-04	High	373	27	XP_005674707.1	ssc:100522506	0	NR_047515.1	SLC9B1
MMP14	2011.096983	54838.44297	-4.769133049	5.11E-04	Low	1902	66368	NM_214239.1	ssc:397471	0	NM_004995.3	MMP14
MAT1A	2.114718173	100.8059614	-5.574971738	5.12E-04	Low	2	122	NM_001243187.1	ssc:100156922	0	NM_000429.2	MAT1A
LOC102160583	2.114718173	100.8059614	-5.574971738	5.12E-04	Low	2	122	XR_302321.1	ssc:102160583			
EVA1B	54.9826725	1266.684744	-4.525936664	5.14E-04	Low	52	1533	XP_005658947.1	ssc:100625314	9E-43	NM_018166.1	EVA1B
LOC102162606	65.55626337	1.652556743	5.30996191	5.16E-04	High	62	2	XP_005669713.1	ssc:102162606	1E-45	NM_018398.2	CACNA2D3
LOC100739059	35.95020894	832.0623203	-4.532619258	5.17E-04	Low	34	1007	XP_003484027.2	ssc:100739059	5E-146	NM_198596.2	SULF2
LOC100739699	201.9555855	4813.071515	-4.574847907	5.20E-04	Low	191	5825	XP_005663972.1	ssc:100739699	0	NM_002332.2	LRP1
CPVL	233.6763581	12.39417558	4.236777563	5.22E-04	High	221	15	XP_005673343.1	ssc:100515288	0	NM_031311.3	CPVL
LOC100623143	144.8581949	3387.741324	-4.547610512	5.31E-04	Low	137	4100	XP_003361153.1	ssc:100623143	1E-74	NR_037150.1	CDKN1A
LOC100510930	1847.206324	108.2424667	4.093006491	5.33E-04	High	1747	131	XP_003133048.1	ssc:100510930	0	NM_080386.3	TUBA3D
XRCC2	1888.443329	110.7213018	4.092192774	5.36E-04	High	1786	134	XRCC2	ssc:100514260			
LOC100621708	0	55.36065091	-20	5.37E-04	Low	0	67	XP_003361674.1	ssc:100621708	0	NM_001145030.1	TOPAZ1
ZNF366	0	55.36065091	-20	5.37E-04	Low	0	67	ZNF366	ssc:100519576	0	NM_152625.1	ZNF366
LOC100155273	3.17207726	121.4629206	-5.258944245	5.37E-04	Low	3	147	LOC100155273	ssc:100155273			
CCDC64	403.9111711	23.13579441	4.125839505	5.38E-04	High	382	28	XR_307437.1	ssc:100153008	0	NM_207311.2	CCDC64
LOC102165829	51.81059524	0.826278372	5.970475443	5.43E-04	High	49	1	XP_005658842.1	ssc:102165829	2E-149	np_003768	np_003768
LOC102159268	38.06492712	0	20	5.43E-04	High	36	0	XP_005670045.1	ssc:102159268			
LOC100624237	38.06492712	0	20	5.43E-04	High	36	0	LOC100624237				
LOC102166085	38.06492712	0	20	5.43E-04	High	36	0	LOC102166085				
LOC102160802	38.06492712	0	20	5.43E-04	High	36	0	XP_005653469.1	ssc:102160802	3E-46	NR_036752.1	NOL4
LOC100157657	38.06492712	0	20	5.43E-04	High	36	0	LOC100157657				
LOC100521899	38.06492712	0	20	5.43E-04	High	36	0	LOC100521899				
LOC100736628	28.54869534	659.3701406	-4.529591835	5.45E-04	Low	27	798	XP_005670352.1	ssc:100736628	2E-152	NM_000392.3	ABCC2
AK1	103.6211905	2376.376597	-4.519372517	5.52E-04	Low	98	2876	XP_005660509.1	ssc:100521423	3E-105	NM_000476.2	AK1
TOM1L1	820.5106512	48.75042393	4.073035392	5.58E-04	High	776	59	XP_005669004.1	ssc:100522701	0	NM_005486.2	TOM1L1
DCC	64.49890428	1.652556743	5.286502937	5.63E-04	High	61	2	XP_005659521.1	ssc:100152102	0	NM_005215.3	DCC
LOC102159871	89.87552236	3.305113487	4.765156535	5.68E-04	High	85	4	XR_306365.1	ssc:102159871			
GALNS	48.63851798	1097.297678	-4.495711876	5.68E-04	Low	46	1328	NM_213955.1	ssc:397000	9E-159	NM_000512.4	GALNS
AMOTL2	210.4144582	4927.924209	-4.549674323	5.70E-04	Low	199	5964	XP_005669919.1	ssc:100523696	0	NM_016201.3	AMOTL2
RARRES2	1.057359087	76.84388857	-6.183393212	5.71E-04	Low	1	93	NM_001123186.1	ssc:100141314	6E-41	NM_002889.3	RARRES2
PLEKHG1	1.057359087	76.84388857	-6.183393212	5.71E-04	Low	1	93	XP_005658150.1	ssc:100621167			
ZBTB20	0	54.53437253	-20	5.74E-04	Low	0	66	ZBTB20	ssc:100624908	0	NM_015642.4	ZBTB20
LOC100738497	0	54.53437253	-20	5.74E-04	Low	0	66	XP_005667234.1	ssc:100738497	2E-87	NM_001098816.2	TENM4
LOC100625309	0	54.53437253	-20	5.74E-04	Low	0	66	LOC100625309				
LOC100620540	2.114718173	98.32712624	-5.539052164	5.74E-04	Low	2	119	XP_005653412.1	ssc:100620540	5E-166	NM_080601.1	PTPN11
PTX3	91.99024053	2081.395218	-4.499926312	5.79E-04	Low	87	2519	NM_001244783.1	ssc:100513178	0	NM_002852.3	PTX3
ARHGEF40	373.2477576	8967.599168	-4.586516296	5.80E-04	Low	353	10853	XP_005669058.1	ssc:100623088			
CDC6	2360.025481	139.6410448	4.079007475	5.84E-04	High	2232	169	XP_005668909.1	ssc:100522031	0	NM_001254.3	CDC6
LOC102163736	56.04003159	1255.943125	-4.486169555	5.85E-04	Low	53	1520	XP_005655920.1	ssc:102163736	8E-42	NM_000660.5	TGFB1
LOC100511417	267.5118489	14.87301069	4.168834172	5.85E-04	High	253	18	XP_003133982.1	ssc:100511417	0	NM_002202.2	ISL1
LOC102162148	152.2597085	7.436505346	4.355765599	5.89E-04	High	144	9	XP_005662169.1	ssc:102162148	0	NM_024847.3	TMC7

RND2	2754.420421	162.7768392	4.080781437	5.96E-04	High	2605	197	XP_003358080.1	ssc:100625457	1E-123	NM_005440.4	RND2
BCAM	72.95777697	1631.899784	-4.483346882	5.97E-04	Low	69	1975	XP_005653341.1	ssc:100738241	0	NM_005581.4	BCAM
LOC100624013	1.057359087	76.0176102	-6.167796357	6.00E-04	Low	1	92	XP_005669325.1	ssc:100624013	3E-102	NM_001102608.1	COL6A6
SLC4A8	330.9533941	19.00440255	4.12222249	6.00E-04	High	313	23	XP_003481628.2	ssc:100521009	0	NR_047672.1	SLC4A8
PLEKHA6	4.229436346	139.6410448	-5.045113837	6.01E-04	Low	4	169	XP_005656727.1	ssc:100512182	0	NM_138480.1	PLEKHA6
SLC41A2	37.00756803	827.9309285	-4.483618177	6.03E-04	Low	35	1002	XP_001926561.1		0	NM_032148.3	SLC41A2
BATF3	176.5789675	9.089062089	4.280038273	6.08E-04	High	167	11	XP_003482796.1	ssc:100524332	8E-49	NM_018664.2	BATF3
PRRT1	3.17207726	118.1578072	-5.219143237	6.09E-04	Low	3	143	NM_001123170.1	ssc:100144520	9E-106	NM_030651.3	PRRT1
ASPA	0	53.70809416	-20	6.14E-04	Low	0	65	NM_001123077.1	ssc:100142661	1E-174	NM_001128085.1	ASPA
TMPRSS11A	0	53.70809416	-20	6.14E-04	Low	0	65	XP_005666787.1	ssc:100525933	0	NM_182606.3	TMPRSS11A
SPINK2	0	53.70809416	-20	6.14E-04	Low	0	65	XP_003129067.1	ssc:100520393	9E-24	NR_073419.1	SPINK2
TMEM132C	0	53.70809416	-20	6.14E-04	Low	0	65	XP_003359125.2	ssc:100623362	0	NM_001136103.2	TMEM132C
LOC100153559	355.2726531	20.65695929	4.104226832	6.16E-04	High	336	25	XP_005660159.1	ssc:100153559	6E-54	NM_203403.1	LURAP1L
FRMD4B	751.7823106	45.44531045	4.048111635	6.18E-04	High	711	55	XP_005669778.1	ssc:100524460	0	NM_015123.1	FRMD4B
LOC102164089	11.63094995	287.5448734	-4.627746278	6.21E-04	Low	11	348	XP_005656795.1	ssc:102164089	3E-117	NM_022093.1	TNN
SULT1C4	16.91774539	395.7873401	-4.548116247	6.25E-04	Low	16	479	XP_005662396.1	ssc:100623441	4E-147	NM_006588.2	SULT1C4
LOC102159415	37.00756803	0	20	6.29E-04	High	35	0	XP_005657627.1	ssc:102159415	5E-53	NM_020864.1	NYAP2
LOC102161632	37.00756803	0	20	6.29E-04	High	35	0	XR_298642.1	ssc:102161632			
MYOT	1.057359087	75.19133183	-6.152029041	6.30E-04	Low	1	91	NM_001099941.2	ssc:100101550	0	NM_006790.2	MYOT
TMEM176B	4.229436346	137.9884881	-5.027938693	6.34E-04	Low	4	167	XP_005658758.1	ssc:100517619	3E-71	NM_014020.3	TMEM176B
C13H21orf63	4.229436346	137.9884881	-5.027938693	6.34E-04	Low	4	167	XP_003358978.2	ssc:100628171	0	NR_104472.1	EVA1C
SDC3	224.1601264	5130.36241	-4.516459163	6.44E-04	Low	212	6209	XP_005665222.1	ssc:100516859	4E-171	NM_014654.3	SDC3
IFI30	191.3819947	4342.919122	-4.504138074	6.47E-04	Low	181	5256	NM_001131046.1	ssc:100174943	2E-89	NM_006332.4	IFI30
RNF24	16.91774539	391.6559482	-4.53297765	6.56E-04	Low	16	474	XP_005672824.1	ssc:100519342	2E-83	NM_007219.3	RNF24
GFRA4	0	52.88181579	-20	6.57E-04	Low	0	64	XP_005672831.1	ssc:100738405	2E-80	NM_145763.1	GFRA4
PID1	0	52.88181579	-20	6.57E-04	Low	0	64	NM_001173520.1	ssc:100302508	3E-23	NM_017933.4	PID1
LOC102165762	0	52.88181579	-20	6.57E-04	Low	0	64	XP_005664614.1	ssc:102165762	4E-137	NM_032689.4	ZNF607
LOC100515572	0	52.88181579	-20	6.57E-04	Low	0	64	XP_003130869.4	ssc:100515572	3E-92	NM_001029880.2	SFMBT2
LOC102157481	6.344154519	178.4761283	-4.814159402	6.58E-04	Low	6	216	XR_306640.1	ssc:102157481			
LZTS1	6.344154519	178.4761283	-4.814159402	6.58E-04	Low	6	216	XP_001925540.1	ssc:100155722	0	NM_021020	LZTS1
HGF	21.14718173	478.4151772	-4.499725844	6.59E-04	Low	20	579	XP_005667745.1	ssc:100525120	0	NM_001010934.1	HGF
LOC102166540	1.057359087	74.36505346	-6.136087497	6.62E-04	Low	1	90	XR_304431.1	ssc:102166540			
LOC100525112	2153.840459	130.5519827	4.044215116	6.63E-04	High	2037	158	XP_005653492.1	ssc:100525112	0	NM_000775.2	CYP2J2
HSPA2	1776.363265	108.2424667	4.036588115	6.65E-04	High	1680	131	XP_003356782.1	ssc:100621324	0	NM_021979.3	HSPA2
LOC102163869	2.114718173	95.02201275	-5.489724452	6.69E-04	Low	2	115	XR_303904.1	ssc:102163869			
LOC102159147	3.17207726	115.678972	-5.188554917	6.70E-04	Low	3	140	XP_005659213.1	ssc:102159147			
PION	14.80302721	346.2106378	-4.547685912	6.76E-04	Low	14	419	XP_003130279.1	ssc:100512622	5E-125	NM_017439.3	GSAP
LOC100623503	49.69587707	0.826278372	5.910354451	6.77E-04	High	47	1	LOC100623503				
C15H2orf66	49.69587707	0.826278372	5.910354451	6.77E-04	High	47	1	XP_005654241.1	ssc:100151853	6E-33	NM_213608.2	C2orf66
LOC100737186	13.74566813	324.7274001	-4.562180185	6.77E-04	Low	13	393	XR_305624.1	ssc:100737186			
LOC100515210	20.08982264	452.8005477	-4.49433897	6.84E-04	Low	19	548	XP_003128028.1	ssc:100515210	1E-80	NM_078626.2	CDKN2C
PER2	5.286795433	156.1666123	-4.88454873	6.87E-04	Low	5	189	XP_005672407.1	ssc:100154609	0	NM_022817.2	PER2
MGLL	44.40908164	962.6143031	-4.438031218	6.87E-04	Low	42	1165	NM_001143718.1	ssc:100233193	8E-167	NM_007283.6	MGLL
LOC102159369	16.91774539	387.5245563	-4.517678513	6.89E-04	Low	16	469	XP_005668982.1	ssc:102159369	2E-66	NM_005501.2	ITGA3
LOC100155350	111.0227041	2431.737248	-4.45306071	6.91E-04	Low	105	2943	XP_003134499.4	ssc:100155350	2E-111	NM_175913.3	JPH2
ZHX3	22.20454082	494.9407447	-4.478329171	6.93E-04	Low	21	599	NM_001258405.1	ssc:100156548	1E-177	NM_015035.3	ZHX3
WNT10B	12.68830904	301.5916057	-4.571024554	6.96E-04	Low	12	365	NM_001287016.1	ssc:100126276	0	NM_003394.3	WNT10B
LOC102162336	2.114718173	94.19573438	-5.477124415	6.96E-04	Low	2	114	XR_297593.1	ssc:102162336			
LOC102164225	135.3419631	6.610226974	4.355765599	6.98E-04	High	128	8	XR_303299.1	ssc:102164225			
DLX1	0	52.05553742	-20	7.04E-04	Low	0	63	XP_003359610.1	ssc:100522648	5E-146	NM_178120.4	DLX1
LOC100627144	0	52.05553742	-20	7.04E-04	Low	0	63	XP_005658517.1	ssc:100627144	6E-26	NM_198797.1	PTGES
LOC102165534	0	52.05553742	-20	7.04E-04	Low	0	63	XR_308137.1	ssc:102165534			
LRRC31	0	52.05553742	-20	7.04E-04	Low	0	63	XP_005670050.1	ssc:100625984	2E-99	NM_024727.3	LRRC31
LOC100620473	0	52.05553742	-20	7.04E-04	Low	0	63	XP_005664910.1	ssc:100620473	1E-95	NM_016535.3	ZNF581
LOC102164797	334.1254714	19.83068092	4.074583847	7.04E-04	High	316	24	XR_308378.1	ssc:102164797			
LOC102160069	2192.962746	134.6833746	4.025237624	7.17E-04	High	2074	163	XR_305815.1	ssc:102160069			
DFNB59	459.9512027	28.09346464	4.033174349	7.18E-04	High	435	34	XP_003133557.3	ssc:100518993	0	NM_001042702.3	DFNB59
LGI4	2.114718173	93.36945601	-5.464413363	7.24E-04	Low	2	113	XP_003481877.1	ssc:100737096	0	NM_139284.2	LGI4
JDP2	49.69587707	1063.420264	-4.419441887	7.26E-04	Low	47	1287	XP_003356802.2	ssc:100624820	6E-48	NM_130469.3	JDP2
LOC100622674	35.95020894	0	20	7.29E-04	High	34	0	XP_003360468.1	ssc:100622674	0	NM_003604.2	IRS4
LOC100738821	35.95020894	0	20	7.29E-04	High	34	0	LOC100738821				
LOC102162482	13.74566813	318.9434515	-4.53625172	7.35E-04	Low	13	386	XR_305617.1	ssc:102162482			
MRC2	1392.541917	34358.30725	-4.6248663	7.38E-04	Low	1317	41582	XP_005653951.1	ssc:100516106	0	NM_006039.4	MRC2
TMEM40	9.516231779	235.4893359	-4.629127508	7.39E-04	Low	9	285	XP_003132425.1	ssc:100526186	1E-82	NM_018306.3	TMEM40
SCML2	2428.753822	149.5563853	4.021454854	7.41E-04	High	2297	181	XP_005673528.1	ssc:100519636	0	NR_033717.1	SCML2
LOC100628093	12.68830904	297.4602138	-4.551124996	7.42E-04	Low	12	360	XP_005653150.1	ssc:100628093	0	NM_052963.2	TOP1MT
ACN9	883.9521964	55.36065091	3.997035541	7.45E-04	High	836	67	XP_005667689.1	ssc:102158000	5E-28	NM_020186.2	ACN9
LOC100520228	6.344154519	173.5184581	-4.773517418	7.49E-04	Low	6	210	XP_003135261.1	ssc:100520228	1E-27	NM_001288583.1	SMIM1
ZNRF2	4957.956757	298.2864922	4.054975136	7.52E-04	High	4689	361	ZNRF2	ssc:100514021	1E-97	NM_147128.3	ZNRF2
ADA	23.2618999	507.3349202	-4.446897627	7.54E-04	Low	22	614	XP_005673015.1	ssc:100625920	0	NM_000022.2	ADA
LOC102166660	2.114718173	92.54317763	-5.451589323	7.54E-04	Low	2	112	XP_005665958.1	ssc:102166660			
CPNE4	280.2001579	16.52556743	4.083686054	7.55E-04	High	265	20	XP_001924956.3	ssc:100157532	0	NM_153429.1	CPNE4
FGB	0	51.22925905	-20	7.55E-04	Low	0	62	NM_001244113.1	ssc:100514354	0	NM_005141.4	FGB
LOC102166113	0	51.22925905	-20	7.55E-04	Low	0	62	XP_005669278.1	ssc:102166113	1E-81	NM_001365.3	DLG4
AZGP1	0	51.22925905	-20	7.55E-04	Low	0	62	XP_003124355.2	ssc:100519648	2E-114	NM_001185.3	AZGP1
LOC100739734	48.63851798	0.826278372	5.879327555	7.57E-04	High	46	1	XP_005674428.1	ssc:100739734	0	NM_057157.2	CYP26A1
LOC100517801	90.93288145	1935.970225	-4.412110885	7.68E-04	Low	86	2343	XP_005660415.1	ssc:100517801	2E-111	np_066929	np_066929
PLCB4	1168.381791	73.53877508	3.989862823	7.69E-04	High	1105	89	XP_001926310.1		0	NM_182797.2	PLCB4
APOBEC3F	11.63094995	274.3244194	-4.559842213	7.71E-04	Low	11	332	NM_001097446.1	ssc:100037939			
CGN	478.9836662	29.74602138	4.009207838	7.75E-04	High	453	36	XP_005663506.1	ssc:100157963	0	NM_020770.2	CGN
LOC100737860	5.286795433	152.0352204	-4.845868262	7.76E-04	Low	5	184	XP_005668944.1	ssc:100737860	0	NM_017957.2	EPN3
KIAA1958	978.0571551	61.97087788	3.980256464	7.94E-04	High	925	75	XP_005660389.1	ssc:100513712	0	NM_133465.3	KIAA1958
RPS6KA6	1305.838472	82.62783717	3.982204736	7.99E-04	High	1235	100	XP_005657901.1	ssc:100522294	0	NM_014496.4	RPS6KA6
TRIM62	29.60605442	627.9715625	-4.406735087	8.03E-04	Low	28	760	XP_005665260.1	ssc:100519905	0	NM_018207.2	TRIM62
LOC102162660	60.26946793	1.652556743	5.188655613	8.07E-04	High	57	2	XR_302177.1	ssc:102162660			
LOC100737862	60.26946793	1.652556743	5.188655613	8.07E-04	High	57	2	LOC100737862				
SLC9A3R2	633.3580929	14609.42789	-4.52773446	8.10E-04	Low	599	17681	NM_001137639.1	ssc:100192448			
LOC102157895	0	50.40298068	-20	8.11E-04	Low	0	61	XP_005655003.1	ssc:102157895	4E-60	NM_021599.2	ADAMTS2
MYH14	0	50.40298068	-20	8.11E-04	Low	0	61	XP_005664827.1	ssc:100525110	0	NM_024729.3	MYH14
KRT81	0	50.40298068	-20	8.11E-04	Low	0	61	XP_003126201.1	ssc:100523123	0	NM_002281.3	KRT81
LOC100512321	1340.731322	85.10667229	3.977604102	8.15E-04	High	1268	103	XP_005652738.1	ssc:100512321	0	NM_014702.4	KIAA0408
ARHGAP6	11.63094995	271.0193059	-4.542354787	8.15E-04	Low	11	328	XP_005673452.1	ssc:100524780	0	NR_109776.1	ARHGAP6
LOC100627393	1420.033253	90.06434252	3.978824864	8.15E-04	High	1343	109	XP_003360381.2	ssc:100627393	0	NM_020922.4	WNK3
HOXA4	5.286795433	150.3826637	-4.830100946	8.16E-04	Low	5	182	XP_003134889.1	ssc:100519635	6E-126	NM_002141.4	HOXA4
LOC102161722	2.114718173	90.89062089	-5.425594114	8.17E-04	Low	2	110	XP_005658702.1	ssc:102161722	7E-105	NM_153343.3	ENPP6
SYNPO2	16.91774539	372.6515456	-4.461218024	8.25E-04	Low	16	451	XP_005666955.1	ssc:100525414	0	NM_133477.2	SYNPO2
SIM1	131.1125267	6.610226974	4.30996191	8.29E-04	High	124	8	NM_001172585.2	ssc:100154026	0	NM_005068.2	SIM1
RSPH4A	71.90041789	2.478835115	4.85826594	8.30E-04	High	68	3	XP_005658999.1	ssc:100524228	0	NM_001161664.1	RSPH4A
LOC102159423	17.97510447	392.4822266	-4.448555263	8.33E-04	Low	17	475	XP_005659148.1	ssc:102159423	6E-51	NM_002228.3	JUN
TOR4A	14.80302721	330.5113487	-4.480735668	8.36E-04	Low	14	400	XP_005652797.1	ssc:100514387	0	NM_017723.2	TOR4A

MAB21L1	11.63094995	269.3667492	-4.533530936	8.38E-04	Low	11	326	XP_001927813.1	ssc:100155671	0	NM_005584.4	MAB21L1
LOC100738242	360.5594485	22.30951604	4.014506026	8.39E-04	High	341	27	XP_003482539.1	ssc:100738242	1E-85	NM_005342.2	HMGB3
LOC100738305	360.5594485	22.30951604	4.014506026	8.39E-04	High	341	27	XP_003482541.1	ssc:100738305	1E-85	NM_005342.2	HMGB3
GPR160	190.3246356	10.74161883	4.147178977	8.40E-04	High	180	13	XP_005674626.1	ssc:100626150	3E-55	NM_014373.2	GPR160
MXRA8	376.4198348	8351.195503	-4.471568184	8.46E-04	Low	356	10107	XP_005653399.1	ssc:100621355	0	NM_032348.3	MXRA8
PROSER2	1384.083044	88.41178578	3.968547995	8.47E-04	High	1309	107	XP_003357817.1	ssc:100627913	1E-100	NM_153256.3	PROSER2
TEP1	254.8235399	5533.586255	-4.440644317	8.49E-04	Low	241	6697	XP_005656370.1	ssc:100152287	0	NM_007110.4	TEP1
MYRF	17.97510447	390.8296698	-4.442467933	8.49E-04	Low	17	473	XP_005660869.1	ssc:100520659	0	NM_013279.2	MYRF
KCNV1	34.89284986	0	20	8.50E-04	High	33	0	XP_001924676.2	ssc:100155464	0	NM_014379.2	KCNV1
FAM19A4	34.89284986	0	20	8.50E-04	High	33	0	XP_005669775.1	ssc:100736627	9E-77	NM_182522.4	FAM19A4
GPR55	34.89284986	0	20	8.50E-04	High	33	0	XP_005657645.1	ssc:100517685	1E-140	NM_005683.3	GPR55
LOC102164859	34.89284986	0	20	8.50E-04	High	33	0	XR_305979.1	ssc:102164859			
LOC102163452	43.35172255	897.3383117	-4.371490784	8.53E-04	Low	41	1086	XP_005661777.1	ssc:102163452			
PRMT8	1.057359087	70.2336616	-6.053625337	8.54E-04	Low	1	85	XP_005664132.1	ssc:100526051	0	NR_046301.1	PRMT8
LOC102164150	6.344154519	168.5607878	-4.731697242	8.54E-04	Low	6	204	XP_005674291.1	ssc:102164150	1E-108	NM_002518.3	NPAS2
LOC102163734	106.7932677	4.95767023	4.429014581	8.56E-04	High	101	6	XP_005654591.1	ssc:102163734	6E-20	NM_199160.3	LHX6
PDSS1	3289.444118	205.7433146	3.998926347	8.61E-04	High	3111	249	XP_005668199.1	ssc:100514361	0	NM_014317.3	PDSS1
SEMA3G	371.1330394	23.13579441	4.003737897	8.61E-04	High	351	28	XP_001928286.1	ssc:100157653	0	NM_020163.1	SEMA3G
NEURL2	12.68830904	287.5448734	-4.502215396	8.66E-04	Low	12	348	XP_005673033.1	ssc:100157993	7E-150	NM_080749.3	NEURL2
LOC102157611	0	49.5767023	-20	8.72E-04	Low	0	60	XP_005674347.1	ssc:102157611	2E-13	NR_046000	IRF4
LOC100515686	0	49.5767023	-20	8.72E-04	Low	0	60	XP_003131634.1	ssc:100515686	5E-44	NM_153229.2	TMEM92
PDGFD	12.68830904	286.718595	-4.498063753	8.78E-04	Low	12	347	XP_005667362.1	ssc:100524161	1E-92	NM_033135.3	PDGFD
FAM98C	76.12985423	1567.450071	-4.363813363	8.82E-04	Low	72	1897	XP_003127161.1	ssc:100516018	2E-142	NM_174905.3	FAM98C
PCPA1	2.114718173	89.23806415	-5.399121903	8.87E-04	Low	2	108	NM_214244.1	ssc:397476	0	NM_001868.2	CPA1
LOC100628155	2135.865355	136.3359313	3.969582963	8.93E-04	High	2020	165	XP_005659044.1	ssc:100628155	1E-92	NM_005729.3	PPIF
LOC102166317	48.63851798	994.8391596	-4.354292121	8.96E-04	Low	46	1204	XP_005658614.1	ssc:102166317	9E-86	NM_001135602.1	GLB1
IFI44L	402.853812	8855.22531	-4.458200703	8.99E-04	Low	381	10717	XP_003127967.2	ssc:100511267	2E-158	NM_006820.2	IFI44L
LOC100524058	564.6297522	36.35624836	3.957029912	9.05E-04	High	534	44	XP_003121439.3	ssc:100524058	1E-68	NM_198920.1	UBE3D
LOC396904	5837.679517	150749.5313	-4.690614695	9.12E-04	Low	5521	182444	LOC396904		0	NM_005964.3	MYH10
LOC100737139	562.5150341	36.35624836	3.951616416	9.24E-04	High	532	44	XP_005663921.1	ssc:100737139	1E-43	NM_006741.3	PPP1R1A
IFT27	24.31925899	505.6823635	-4.378060287	9.26E-04	Low	23	612	XP_003126110.3	ssc:100517032	1E-90	NR_033531.2	IFT27
NPAS2	72.95777697	1483.169677	-4.345478073	9.32E-04	Low	69	1795	XP_003124946.3	ssc:100521893	0	NM_002518.3	NPAS2
LOC100621949	0	48.75042393	-20	9.38E-04	Low	0	59	XP_005667121.1	ssc:100621949	2E-98	NM_003621.3	PPFIBP2
ZNF614	0	48.75042393	-20	9.38E-04	Low	0	59	ZNF614	ssc:100516977	0	NM_025040.3	ZNF614
TMEM150C	1786.936856	115.678972	3.949290113	9.42E-04	High	1690	140	XP_005653678.1	ssc:100622888	2E-142	NM_001080506.1	TMEM150C
LOC100624418	1.057359087	68.58110485	-6.019273832	9.49E-04	Low	1	83	XR_131274.2	ssc:100624418			
SRSF12	1330.157731	86.75922903	3.938436288	9.50E-04	High	1258	105	NM_001195598.1	ssc:100152352			
SOCS1	15.8603863	341.2529675	-4.427341777	9.52E-04	Low	15	413	NM_001204768.1	ssc:100307052	2E-96	NM_003745.1	SOCS1
LOC100622291	46.52379981	0.826278372	5.815197218	9.55E-04	High	44	1	LOC100622291				
ZAP70	93.04759962	4.131391859	4.493269123	9.58E-04	High	88	5	ZAP70	ssc:100524930	0	NM_207519.1	ZAP70
SCARF2	189.2672765	3943.00039	-4.380796951	9.64E-04	Low	179	4772	XP_005654177.1	ssc:100155943	2E-147	NM_182895	SCARF2
POLR3G	4931.52278	309.8543894	3.992370887	9.69E-04	High	4664	375	XP_005652997.1	ssc:100519356	9E-94	NM_006467.2	POLR3G
IGSF1	411.3126847	26.4409079	3.959391944	9.71E-04	High	389	32	XP_001926045.1	ssc:100157227	0	NM_205833.3	IGSF1
LOC100524679	58.15474976	1.652556743	5.137125313	9.74E-04	High	55	2	XP_005655748.1	ssc:100524679	0	NM_173498.1	OVOS2
SIDT1	58.15474976	1.652556743	5.137125313	9.74E-04	High	55	2	XP_005670270.1	ssc:100625203	0	NM_017699.2	SIDT1
LOC102164866	33.83549077	0	20	9.93E-04	High	32	0	XP_005659276.1	ssc:102164866	1E-23	NM_003287.2	TPD52L1
CPPED1	11.63094995	259.4514087	-4.479423531	9.95E-04	Low	11	314	NM_001243581.1	ssc:100627178	3E-158	NM_018340.2	CPPED1
ELF4	78.24457241	1569.928906	-4.326564738	9.96E-04	Low	74	1900	XP_005673959.1	ssc:100523880	0	NM_001421.3	ELF4
B3GALT4	24.31925899	497.4195798	-4.354292121	9.99E-04	Low	23	602	XP_001927603.1	ssc:100155431	1E-158	NM_003782.3	B3GALT4
LSR	409.1979665	26.4409079	3.951955355	9.99E-04	High	387	32	XP_005664540.1	ssc:100516738	1E-115	NM_205835.3	LSR
LOC102167381	5.286795433	143.7724367	-4.765249802	1.00E-03	Low	5	174	XP_005659063.1	ssc:102167381	1E-21	NR_103804.1	PDLIM7
MFGE8	818.395933	18321.07034	-4.484561004	0.001002	Low	774	22173	NM_001122984.1	ssc:397545	9E-141	NM_005928.2	MFGE8
ITFG3	126.8830904	2577.162241	-4.344211643	0.001004	Low	120	3119	XP_005655180.1	ssc:100738032	0	NR_104317.1	ITFG3
LOC100623747	103.6211905	4.95767023	4.385512943	0.001006	High	98	6	XP_005658336.1	ssc:100623747	6E-93	NM_183239.1	GSTO2
PPM1J	2.114718173	86.75922903	-5.358479918	0.001006	Low	2	105	XP_003125899.2	ssc:100520904	4E-40	NM_005167.5	PPM1J
LOC102164820	0	47.92414556	-20	0.001011	Low	0	58	XR_299650.1	ssc:102164820			
IFNE	0	47.92414556	-20	0.001011	Low	0	58	NM_001105310.1	ssc:100125969	2E-80	NM_176891.4	IFNE
LOC102160618	0	47.92414556	-20	0.001011	Low	0	58	XP_005672260.1	ssc:102160618			
KIAA1755	0	47.92414556	-20	0.001011	Low	0	58	XP_003134484.3	ssc:100516472	0	NM_001029864.1	KIAA1755
TTNLOC100620	0	47.92414556	-20	0.001011	Low	0	58	XP_003483740.1	ssc:100620261	0	NM_133437.3	TTN
CACNA1A	0	47.92414556	-20	0.001011	Low	0	58	XP_003480824.2	ssc:100525387	0	NM_023035.2	CACNA1A
TRIM36	372.1903985	23.96207278	3.957216223	0.001014	High	352	29	XP_003480940.1	ssc:100519890	0	NM_018700.3	TRIM36
LOC102162418	24.31925899	495.767023	-4.349491135	0.001014	Low	23	600	XP_005662716.1	ssc:102162418	4E-58	NM_005253.3	FOSL2
LOC100155572	601.6373203	39.66136184	3.923087941	0.001017	High	569	48	XR_304540.1	ssc:100155572			
MFSD12	172.3495311	3533.166318	-4.357552384	0.001018	Low	163	4276	XP_005654766.1	ssc:100519240	2E-176	NM_174983.4	MFSD12
NELL2	661.9067882	43.7927537	3.917863992	0.001025	High	626	53	XP_005664213.1	ssc:100525817	0	NM_006159.2	NELL2
LOC100737847	4.229436346	123.9417558	-4.873053091	0.001031	Low	4	150	XP_005671815.1	ssc:100737847	2E-156	NM_021623.1	PLEKHA2
MAP1LC3A	195.611431	4021.496835	-4.36167	0.001032	Low	185	4867	NM_001170827.1	ssc:100322893	5E-66	NM_181509.2	MAP1LC3A
ATP8	164794.7004	8846.136248	4.219478575	0.001034	High	155855	10706	HighI000181E8D6	ssc:808505			
LOC102163871	80.35929058	3.305113487	4.603693113	0.001034	High	76	4	XR_298108.1	ssc:102163871			
LOC102159288	3.17207726	104.9373532	-5.047956587	0.001039	Low	3	127	XR_303800.1	ssc:102159288			
LOC102161477	171.292172	9.915340461	4.110653101	0.001046	High	162	12	XR_305189.1	ssc:102161477			
TRPM4	6.344154519	161.1242825	-4.666602214	0.001048	Low	6	195	XP_005664768.1	ssc:102167071	1E-163	NM_017636.3	TRPM4
MYEF2	8570.952756	532.9495498	4.007384709	0.00105	High	8106	645	XP_005654508.1	ssc:100153072	0	NM_016132.3	MYEF2
CSDC2	13.74566813	294.9813787	-4.423574947	0.001051	Low	13	357	NM_001243420.1	ssc:100153407	3E-82	NM_014460.3	CSDC2
AKAP17B	1293.150163	85.93295066	3.91153457	0.001053	High	1223	104	XP_003135380.2	ssc:100513257	1E-81	NR_027383.1	AKAP17A
TRIM55	1.057359087	66.92854811	-5.984084404	0.001057	Low	1	81	NM_001244691.1	ssc:100152010	0	NM_184087.1	TRIM55
RNF39	1.057359087	66.92854811	-5.984084404	0.001057	Low	1	81	NM_001128446.1	ssc:100141418	9E-167	NM_170770.1	RNF39
PSMB8	111.0227041	2214.426036	-4.318006168	0.001069	Low	105	2680	NM_213935.1	ssc:396975			
LOC102157456	45.46644072	0.826278372	5.782030354	0.001075	High	43	1	XR_297431.1	ssc:102157456			
LOC102164296	45.46644072	0.826278372	5.782030354	0.001075	High	43	1	XR_301127.1	ssc:102164296			
LOC100739677	45.46644072	0.826278372	5.782030354	0.001075	High	43	1	XP_005654455.1	ssc:100739677	2E-100	NM_001161664.1	RSPH4A
CADM2	181.8657629	10.74161883	4.081596636	0.001075	High	172	13	XP_005670344.1	ssc:100626294	2E-170	NM_153184.3	CADM2
LOC100625123	38.06492712	751.0870399	-4.302445884	0.001075	Low	36	909	XP_005659108.1	ssc:100625123	1E-43	NM_019009.3	TOLLIP
TMEM61	0	47.09786719	-20	0.00109	Low	0	57	NM_001243365.1	ssc:100628096	3E-67	NM_182532.1	TMEM61
LOC100624393	0	47.09786719	-20	0.00109	Low	0	57	XP_003362153.1	ssc:100624393	0	NM_001145030.1	TOPAZ1
MEOX2	0	47.09786719	-20	0.00109	Low	0	57	NM_214121.1	ssc:397222	4E-73	NM_005924.4	MEOX2
LOC102160923	0	47.09786719	-20	0.00109	Low	0	57	XR_309605.1	ssc:102160923			
LOC100621405	0	47.09786719	-20	0.00109	Low	0	57	XP_005658046.1	ssc:100621405	1E-123	NM_006492.2	ALX3
LOC100524378	0	47.09786719	-20	0.00109	Low	0	57	XP_005664828.1	ssc:100524378	3E-13	XP_003960829.2	LOC100287477
B2R	0	47.09786719	-20	0.00109	Low	0	57	NM_214146.1	ssc:397277	4E-173	NM_000623.3	BDKRB2
LOC100738669	219.93069	4485.039002	-4.349999688	0.0011	Low	208	5428	XP_005665628.1	ssc:100738669	0	NR_073131.1	NEDD9
FCGRT	953.7378961	21103.97589	-4.467778169	0.001105	Low	902	25541	NM_214197.2	ssc:397399	1E-148	NM_004107.4	FCGRT
ARNT2	94.1049587	1853.342388	-4.299714875	0.001108	Low	89	2243	XP_001926107.3	ssc:100157219	0	NM_014862.3	ARNT2
NPR3	3.17207726	103.2847965	-5.025056185	0.001116	Low	3	125	XP_005654272.1	ssc:102163867	2E-22	NM_024563.3	NPR3
GVIN1	8.458872693	195.8279741	-4.53297765	0.001128	Low	8	237	XP_003482569.2	ssc:100512797	1E-80	NM_017920.3	URGCP
FSCN2	17.97510447	366.867597	-4.351187426	0.001135	Low	17	444	XP_003357978.1	ssc:100626228	0	NM_012418.3	FSCN2
GPR37	434.5745846	28.91974301	3.909477166	0.001143	High	411	35	XP_003134766.2	ssc:100523220	0	NM_005302.3	GPR37
LOC100627770	67.67098154	2.478835115	4.770803099	0.001143	High	64	3	XP_005658481.1	ssc:100627770	2E-73	NM_130811.2	SNAP25
ADAMTSL5	2.114718173	84.28039392	-5.316659743	0.001145	Low	2	102	XP_005661472.1	ssc:100512586	0	NM_213604.2	ADAMTSL5

SHPK	7.401513606	175.9972932	-4.571589099	0.001161	Low	7	213	XP_005669175.1	ssc:100515408	0	NM_013276.2	SHPK
ADM2	4.229436346	120.6366423	-4.83405896	0.001164	Low	4	146	NM_001206384.1	ssc:100517471	3E-47	NM_024866.5	ADM2
ECT2L	32.77813168	0	20	0.001165	High	31	0	XP_001926401.2	ssc:100153588	0	NM_001195037.2	ECT2L
LOC102168070	32.77813168	0	20	0.001165	High	31	0	XR_297365.1	ssc:102168070			
LOC102160718	32.77813168	0	20	0.001165	High	31	0	XR_297394.1	ssc:102160718			
LOC100521810	32.77813168	0	20	0.001165	High	31	0	XP_003129328.1	ssc:100521810	3E-115	NM_025212.2	CXXC4
C1H15orf43	32.77813168	0	20	0.001165	High	31	0	XP_003121579.3	ssc:100516583	2E-123	NM_152448.2	C15orf43
RBP1	1505.679339	101.6322397	3.888984525	0.001169	High	1424	123	NM_001031789.1	ssc:100156666	2E-74	NM_002899.3	RBP1
PIGL	32.77813168	637.886903	-4.282495128	0.00117	Low	31	772	XP_003132072.1	ssc:100523821	4E-101	NM_004278.3	PIGL
LOC102167710	21.14718173	422.228248	-4.319485787	0.00117	Low	20	511	XP_005672881.1	ssc:102167710	4E-63	NR_046000	IRF4
LOC100523351	0	46.27158882	-20	0.001177	Low	0	56	XP_003122145.3	ssc:100523351	2E-99	NM_198188.2	ASTN2
LOC102158339	1.057359087	65.27599137	-5.948015149	0.00118	Low	1	79	XR_308249.1	ssc:102158339			
LOC100627924	1.057359087	65.27599137	-5.948015149	0.00118	Low	1	79	XR_299871.1	ssc:100627924			
LOC100738148	56.04003159	1.652556743	5.083686054	0.001182	High	53	2	XP_005668045.1	ssc:100738148	0	NR_047564.1	CRB1
MAL2	431.4025073	28.91974301	3.898907924	0.001189	High	408	35	NM_001161639.1	ssc:100153186	2E-88	NM_052886.2	MAL2
COLL11A1	1245.569004	27569.60415	-4.468201699	0.001192	Low	1178	33366	XP_001929407.4	ssc:397175	0	NM_080630.3	COL11A1
S100A14	5.286795433	137.9884881	-4.706010598	0.001202	Low	5	167	NM_001190168.1	ssc:100153930	3E-55	NM_020672.2	S100A14
KDELR3	286.5443125	5807.910675	-4.341189099	0.001204	Low	271	7029	NM_001244970.1	ssc:100514523	1E-119	NM_016657.2	KDELR3
IQCA1	44.40908164	0.826278372	5.748083022	0.001213	High	42	1	XP_005672363.1	ssc:100515512	7E-131	NR_073043.1	IQCA1
FGF10	24.31925899	475.9363421	-4.290597446	0.001222	Low	23	576	XP_003133972.1	ssc:100525086	2E-104	NM_004465.1	FGF10
LOC100520275	4.229436346	118.9840855	-4.814159402	0.001238	Low	4	144	XR_307525.1	ssc:100520275			
NKX3-2	14.80302721	303.2441624	-4.356515732	0.001239	Low	14	367	XP_003356908.1	ssc:100625162	5E-141	NM_001189.3	NKX3-2
LOC100520244	21.14718173	416.4442994	-4.299586229	0.001246	Low	20	504	XP_003126314.1	ssc:100520244	3E-92	NM_002475.4	MYL6B
LOC100627628	244.249949	15.69928906	3.959587127	0.001248	High	231	19	LOC100627628				
CACNA1S	2.114718173	82.62783717	-5.288090591	0.00125	Low	2	100	HighI0000DA8508		0	NM_000069.2	CACNA1S
CHCHD6	41.23700438	784.1381748	-4.249096459	0.001265	Low	39	949	XP_005669885.1	ssc:100624760	8E-32	NM_032343.2	CHCHD6
KLF4	158.603863	3090.28111	-4.284238265	0.001268	Low	150	3740	NM_001031782.2	ssc:595111	0	NM_004235.4	KLF4
MEG3	5.286795433	136.3359313	-4.68862852	0.001269	Low	5	165	NR_021488.1	ssc:100144590			
SEC24D	217.8159718	4304.910317	-4.304801531	0.001269	Low	206	5210	XP_003129278.3	ssc:100525592	0	NM_014822.2	SEC24D
ME3	43.35172255	822.9732582	-4.246684328	0.00127	Low	41	996	NM_001244258.1	ssc:100520040	0	NM_006680.2	ME3
LOC100627863	219.93069	4347.050514	-4.304916017	0.001272	Low	208	5261	XR_300176.1	ssc:100627863			
LOC100524524	24.31925899	471.8049503	-4.27801938	0.001272	Low	23	571	XP_003132006.3	ssc:100524524	2E-51	NM_203411.1	TMEM88
PRSS33	0	45.44531045	-20	0.001272	Low	0	55	XP_003481124.1	ssc:100737240	2E-123	NM_152891.2	PRSS33
LOC100739222	0	45.44531045	-20	0.001272	Low	0	55	XR_135291.2	ssc:100739222			
EREG	0	45.44531045	-20	0.001272	Low	0	55	XP_005666827.1	ssc:100620969	4E-76	NM_001432.2	EREG
CCL3L1	0	45.44531045	-20	0.001272	Low	0	55	NM_001009579.1	ssc:494459	3E-39	NM_001001437.3	CCL3L3
LOC100515260	688.3407654	14486.31241	-4.395423631	0.001274	Low	651	17532	XR_303102.1	ssc:100515260			
LOC100627728	243.1925899	15.69928906	3.953328137	0.001278	High	230	19	LOC100627728				
LOC102158067	77.18721332	3.305113487	4.545590158	0.001279	High	73	4	XP_005674378.1	ssc:102158067	1E-96	NM_019053.4	EXOC6
LOC100626168	20.08982264	394.9610617	-4.297173695	0.001281	Low	19	478	XP_005663469.1	ssc:100626168	2E-46	NM_002962.1	S100A5
LOC100625903	48.63851798	920.4741061	-4.242205662	0.001282	Low	46	1114	XP_003361387.1	ssc:100625903	0	NM_003930.3	SKAP2
PQLC3	43.35172255	820.4944231	-4.242332304	0.001288	Low	41	993	XR_298268.1	ssc:100525694	6E-84	NR_104231.1	PQLC3
LOC102168044	33.83549077	642.8445732	-4.247860746	0.001299	Low	32	778	XP_005657691.1	ssc:102168044	4E-167	NM_005990.3	STK10
LOC102162560	22.20454082	431.31731	-4.279822975	0.001303	Low	21	522	XP_005667735.1	ssc:102162560	6E-67	NM_152754.2	SEMA3D
LOC102162575	54.9826725	1.652556743	5.056205317	0.001304	High	52	2	XR_301394.1	ssc:102162575			
LOC100739353	2.114718173	81.8015588	-5.273591021	0.001307	Low	2	99	XP_005660124.1	ssc:100739353	3E-75	NM_002169.2	IFNA5
LOC102166028	1438.008358	99.15340461	3.85826594	0.001311	High	1360	120	XR_305096.1	ssc:102166028			
DSP	3907.999184	263.1039655	3.903733892	0.001313	High	3696	316	XP_003128216.1	ssc:100156744	0	NM_004415.2	DSP
LOC102165736	19.03246356	373.477824	-4.294488362	0.001322	Low	18	452	XP_005658637.1	ssc:102165736	2E-14	NR_073150.1	RAP2C
SIRT2	204.0703037	3982.661752	-4.286594795	0.001327	Low	193	4820	NM_001114271.1	ssc:100125964			
AFGF	3.17207726	99.15340461	-4.966162496	0.001338	Low	3	120	XP_003124050.1	ssc:397497	8E-88	NM_033137.2	FGF1
LRRN4	108.9079859	5.783948602	4.234911204	0.001348	High	103	7	XP_003359940.1	ssc:100152719	3E-140	NM_152611.4	LRRN4
ITM2C	1080.620986	23078.7812	-4.416634526	0.001352	Low	1022	27931	NM_001123131.1	ssc:100144481	7E-138	NM_030926.5	ITM2C
TOP1MT	15.8603863	315.638338	-4.314772633	0.001358	Low	15	382	XP_005662884.1	ssc:100517528	0	NM_052963.2	TOP1MT
PTHLH	9.516231779	205.7433146	-4.434311331	0.001365	Low	9	249	NM_213916.2	ssc:396951	1E-73	NM_198966.1	PTHLH
LOC102158647	119.4815768	6.610226974	4.175944562	0.001365	High	113	8	XR_300173.1	ssc:102158647			
SOX13	466.2953572	32.2248565	3.854998226	0.001367	High	441	39	XP_005656725.1	ssc:100524269	0	NM_005686.2	SOX13
OPALIN	31.7207726	0	20	0.001372	High	30	0	NM_214210.1	ssc:397416	3E-63	NR_104297.1	OPALIN
TJP3	31.7207726	0	20	0.001372	High	30	0	XP_005661408.1	ssc:100626455	0	NM_014428.1	TJP3
TMEM215	31.7207726	0	20	0.001372	High	30	0	XP_003130731.1	ssc:100512303	9E-127	NM_212558.2	TMEM215
LOC100736881	0	44.61903207	-20	0.001376	Low	0	54	XP_005665115.1	ssc:100736881	3E-22	NM_018125.3	ARHGEF10L
LOC102163702	0	44.61903207	-20	0.001376	Low	0	54	XR_300485.1	ssc:102163702			
LOC102160052	0	44.61903207	-20	0.001376	Low	0	54	XR_302532.1	ssc:102160052			
LOC102162300	0	44.61903207	-20	0.001376	Low	0	54	XR_304485.1	ssc:102162300			
EDN1	7.401513606	169.3870662	-4.516359578	0.00138	Low	7	205	NM_213882.1	ssc:396915	1E-80	NM_001955.4	EDN1
SULF2	138.5140403	2634.175449	-4.249247313	0.001383	Low	131	3188	XP_005673059.1	ssc:100152351	0	NM_198596.2	SULF2
HOGA1	3.17207726	98.32712624	-4.954089663	0.001389	Low	3	119	NM_001190169.1	ssc:100153961			
SYT1	9.516231779	204.9170362	-4.42850571	0.00139	Low	9	248	XP_005652680.1	ssc:100513599	0	NM_005639.2	SYT1
LOC100624471	295.0031852	19.83068092	3.89492441	0.001393	High	279	24	XP_005658875.1	ssc:100624471	3E-91	NM_182797.2	PLCB4
LOC102161978	140.6287585	8.262783717	4.08911994	0.001395	High	133	10	XR_301702.1	ssc:102161978			
LOC100738135	140.6287585	8.262783717	4.08911994	0.001395	High	133	10	XP_005659503.1	ssc:100738135	3E-150	NR_046357.1	RNF165
FAM101A	1.057359087	62.79715625	-5.892161914	0.001398	Low	1	76	XP_005670625.1	ssc:100158166	3E-69	NM_001204299.1	ZNF664-FAM101A
CD207	1.057359087	62.79715625	-5.892161914	0.001398	Low	1	76	NM_001129957.1	ssc:100037935	1E-147	NM_015717.4	CD207
LOC102158551	1.057359087	62.79715625	-5.892161914	0.001398	Low	1	76	XP_005656640.1	ssc:102158551			
BCO2	1.057359087	62.79715625	-5.892161914	0.001398	Low	1	76	XP_005667395.1	ssc:100517547	0	NM_031938.5	BCO2
TMEFF1	3609.823922	244.578398	3.883559577	0.001399	High	3414	296	XP_005660355.1	ssc:102166244	0	NM_001198812.1	MSANTD3-TMEFF1
IRF1	142.7434767	2710.193059	-4.246898903	0.001401	Low	135	3280	NM_001097413.1	ssc:396611	1E-173	NM_002198.2	IRF1
LOC100621905	4.229436346	115.678972	-4.773517418	0.001404	Low	4	140	LOC100621905				
FRMPD3	20.08982264	386.698278	-4.266671607	0.00141	Low	19	468	XP_003135350.1	ssc:100517925	0	NM_032428.1	FRMPD3
B3GALNT1	2498.539522	171.8659013	3.861729795	0.001412	High	2363	208	NM_214351.1	ssc:397634	0	NM_033169.2	B3GALNT1
LOC102166068	107.8506268	5.783948602	4.220836019	0.00142	High	102	7	XR_300918.1	ssc:102166068			
ARPC1B	322.4945214	6348.29673	-4.299023007	0.00142	Low	305	7683	NM_001134356.1	ssc:100174964			
HEPH	2197.192182	152.0352204	3.853183582	0.001426	High	2078	184	XP_005673751.1	ssc:100512938	0	NM_138737.4	HEPH
LOC100521170	270.6839262	18.17812418	3.896333981	0.001449	High	256	22	LOC100521170				
RHBDL2	14.80302721	292.5025436	-4.304485029	0.001459	Low	14	354	NM_001244943.1	ssc:100514296	9E-110	NM_017821.3	RHBDL2
CD44	1196.930486	25303.94886	-4.401951272	0.001461	Low	1132	30624	K7GLL1		0	NM_001202557.1	CD44
IGFBP5	23.2618999	438.7538154	-4.237370833	0.001466	Low	22	531	NM_214099.1	ssc:397182	1E-91	NM_000599.3	IGFBP5
LOC100514709	204.0703037	13.22045395	3.948222637	0.001474	High	193	16	XP_003125309.3	ssc:100514709	6E-47	NM_003352.4	SUMO1
TBX18	23.2618999	437.927537	-4.234651332	0.001479	Low	22	530	XP_001926986.1	ssc:100152747	0	NM_001080508.2	TBX18
LOC100523504	75.07249515	3.305113487	4.505512719	0.00148	High	71	4	XP_005667494.1	ssc:100523504	0	NM_014619.3	GRIK4
MYLK2	1.057359087	61.97087788	-5.873053091	0.001481	Low	1	75	XP_003134431.2	ssc:100524288	9E-91	NM_033118.3	MYLK2
HTR2A	1.057359087	61.97087788	-5.873053091	0.001481	Low	1	75	NM_214217.1	ssc:397432	0	NM_001165947.2	HTR2A
EPS8L1	1.057359087	61.97087788	-5.873053091	0.001481	Low	1	75	XP_003356116.1	ssc:100627336	6E-42	NM_139204.1	EPS8L1
STAT1	612.2109111	12381.7814	-4.338046326	0.001485	Low	579	14985	NM_213769.1	ssc:396655			
LOC100520085	22.20454082	418.9231345	-4.237758915	0.001487	Low	21	507	XP_003128374.1	ssc:100520085	2E-121	NM_148954.2	PSMB9
LOC100622512	0	43.7927537	-20	0.001491	Low	0	53	XP_005658491.1	ssc:100622512	7E-164	NM_013992.3	PAX8
ZNF660	0	43.7927537	-20	0.001491	Low	0	53	ZNF660	ssc:100513047	1E-93	NM_173658.2	ZNF660
CADPS	0	43.7927537	-20	0.001491	Low	0	53	XP_005669756.1	ssc:100518990	0	NM_183394.2	CADPS
EXOC6	872.3212464	61.97087788	3.815197218	0.001495	High	825	75	XP_003361613.2	ssc:100627369	0	NM_019053.4	EXOC6
ARPP21	4.229436346	114.0264153	-4.752758858	0.001496	Low	4	138	XP_005669408.1	ssc:100512986	0	NM_198399.1	ARPP21

TSHZ2	2.114718173	79.32272369	-5.229196901	0.001497	Low	2	96	NM_001114674.1	ssc:100136903	0	NM_173485.5	TSHZ2
NYX	2.114718173	79.32272369	-5.229196901	0.001497	Low	2	96	XP_003360321.1	ssc:100620950	0	NM_022567.2	NYX
LOC100627666	6.344154519	147.9038285	-4.543087677	0.001539	Low	6	179	XP_003359071.1	ssc:100627666	0	NM_021020	LZTS1
LOC100522323	6607.436932	443.7114856	3.896396946	0.001541	High	6249	537	XP_003127755.1	ssc:100522323	5E-62	NM_004102.3	FABP3
LOC102165468	5675.903577	384.2194429	3.884847649	0.001552	High	5368	465	XP_005664059.1	ssc:102165468	1E-79	NM_015936.1	YARS2
STX3	59.21210885	1073.335605	-4.180065194	0.001562	Low	56	1299	XP_005660928.1	ssc:100520886	4E-130	NM_004177.4	STX3
PAICS	60846.786	3678.591311	4.047955752	0.001565	High	57546	4452	XP_005666753.1	ssc:100521281	0	NM_006452.3	PAICS
MTHFD2L	1.057359087	61.14459951	-5.853687766	0.00157	Low	1	74	XP_003129156.4	ssc:100525706	4E-135	NM_001144978.1	MTHFD2L
TNFRSF21	4573.078049	313.1595029	3.868195978	0.001573	High	4325	379	XP_003128478.2	ssc:100513161	1E-172	NM_014452.4	TNFRSF21
CHSY3	6.344154519	147.0775502	-4.535005331	0.001578	Low	6	178	XP_005661677.1	ssc:100518720	0	NM_175856.4	CHSY3
PSMB9	20.08982264	376.7829375	-4.229196901	0.001587	Low	19	456	NM_001037961.1	ssc:654294	2E-121	NM_148954.2	PSMB9
LOC100739537	25.37661808	467.6735584	-4.203930143	0.001587	Low	24	566	XP_005669380.1	ssc:100739537	1E-59	NM_003242.5	TGFBR2
CAMTA2	178.6936856	3334.03323	-4.221707923	0.001587	Low	169	4035	XP_005669220.1	ssc:100511531	0	NM_015099.3	CAMTA2
TRIM50	74.01513606	3.305113487	4.485048616	0.001594	High	70	4	NM_214186.1	ssc:397383	0	NM_178125.3	TRIM50
LOC100737157	74.01513606	3.305113487	4.485048616	0.001594	High	70	4	XR_304834.1	ssc:100737157			
LOC100737319	25.37661808	466.84728	-4.201378957	0.001599	Low	24	565	XP_005667722.1	ssc:100737319	0	NM_001621.4	AHR
PPM1E	1455.983462	104.1110748	3.80579852	0.001611	High	1377	126	XP_005669053.1	ssc:100518751	0	NR_048561.1	PPM1E
NES	1137.718377	23472.08971	-4.366730898	0.001613	Low	1076	28407	XP_005663322.1	ssc:100153933	0	NM_006617.1	NES
LOC102159666	0	42.96647533	-20	0.001618	Low	0	52	XP_005655857.1	ssc:102159666			
HAS1	0	42.96647533	-20	0.001618	Low	0	52	NM_001136212.1	ssc:100191062	0	NM_001523.2	HAS1
C1RL	0	42.96647533	-20	0.001618	Low	0	52	XP_005658603.1	ssc:100513152	5E-117	NM_016546.2	C1RL
LOC100626234	30.66341351	0	20	0.001623	High	29	0	XP_005667351.1	ssc:100626234	6E-49	NM_018947.5	CYCS
PDIA2	30.66341351	0	20	0.001623	High	29	0	XP_005655182.1	ssc:100738360	0	NM_006849.2	PDIA2
LOC102166344	30.66341351	0	20	0.001623	High	29	0	XP_005655042.1	ssc:102166344	5E-141	NR_003149.1	GPR98
LOC100623257	30.66341351	0	20	0.001623	High	29	0	XP_005667272.1	ssc:100623257	2E-120	NM_001143975.1	UBTFL1
LOC102165082	30.66341351	0	20	0.001623	High	29	0	XR_301689.1	ssc:102165082			
LOC100623608	33.83549077	611.4459951	-4.175615861	0.001632	Low	32	740	XP_003360896.1	ssc:100623608	2E-129	NM_001017961.3	FAM78B
LIN28B	285.4869534	19.83068092	3.847618696	0.001662	High	270	24	XP_005659396.1	ssc:100738513	7E-94	NM_001004317.3	LIN28B
LOC102157733	628.0712974	45.44531045	3.788725007	0.001669	High	594	55	XR_301024.1	ssc:102157733			
IFIT3	477.9263071	9279.106115	-4.279125746	0.001677	Low	452	11230	NM_001204395.1	ssc:100154248	0	NM_001549.4	IFIT3
RMND5B	3.17207726	94.19573438	-4.892161914	0.001679	Low	3	114	NM_001243800.1	ssc:100510951	4E-18	NM_022762	RMND5B
EGFR	72.95777697	1304.693549	-4.1605054	0.001682	Low	69	1579	NM_214007.1	ssc:397070	0	NM_201284.1	EGFR
GPR56	71.90041789	1284.862868	-4.159470425	0.001685	Low	68	1555	NM_001243056.1	ssc:100515029	6E-109	NM_201525.2	GPR56
WNK3	1466.557053	105.7636316	3.793517672	0.001692	High	1387	128	XP_003360382.2	ssc:100156408	0	NM_020922.4	WNK3
LMTK3	6.344154519	144.5987151	-4.510483012	0.001703	Low	6	175	XP_005664728.1	ssc:100623034	0	NM_001080434.1	LMTK3
NRROS	197.7261492	13.22045395	3.902660059	0.001745	High	187	16	XP_003358810.1	ssc:100620479	0	NM_198565.1	NRROS
LOC102163771	3.17207726	93.36945601	-4.879450862	0.001745	Low	3	113	XR_309073.1	ssc:102163771			
LOC102163119	103.6211905	5.783948602	4.163120521	0.001753	High	98	7	XP_005657145.1	ssc:102163119	4E-37	NM_024768.2	EFCC1
SCN4B	0	42.14019696	-20	0.001757	Low	0	51	XP_003129950.1	ssc:100512800	5E-89	NR_024527.1	SCN4B
SST	0	42.14019696	-20	0.001757	Low	0	51	NM_001009583.1	ssc:494469	2E-61	NM_001048.3	SST
LOC102165507	0	42.14019696	-20	0.001757	Low	0	51	XR_304363.1	ssc:102165507			
SPMI	0	42.14019696	-20	0.001757	Low	0	51	NM_001031776.1	ssc:396758			
TMEM151B	10.57359087	211.5272632	-4.322306306	0.00176	Low	10	256	XP_001929612.1	ssc:100157535	0	NM_001137560.1	TMEM151B
PDE2A	4.229436346	109.8950234	-4.699516836	0.001762	Low	4	133	XP_005653749.1	ssc:100737701	0	NM_002599.4	PDE2A
LOC100512333	41.23700438	0.826278372	5.641167818	0.001768	High	39	1	XR_297265.1	ssc:100512333			
RNF17	1.057359087	59.49204276	-5.814159402	0.001768	Low	1	72	XP_005653906.1	ssc:100738776	0	NM_031994.1	RNF17
LOC100522117	51.81059524	1.652556743	4.970475443	0.001771	High	49	2	LOC100522117				
FAM129B	861.7476556	17118.00903	-4.312105635	0.001777	Low	815	20717	XP_005660503.1	ssc:100739641	0	NM_022833.3	FAM129B
LOC100625740	115.2521404	2060.738259	-4.160295826	0.001779	Low	109	2494	XP_003359703.3	ssc:100625740	0	NM_015470.2	RAB11FIP5
LOC102161422	388.0507848	7343.962168	-4.242241249	0.001787	Low	367	8888	XP_005663980.1	ssc:102161422	0	NM_002332.2	LRP1
LOC100737519	6.344154519	142.9461583	-4.493900128	0.001792	Low	6	173	XP_003480458.2	ssc:100737519	2E-145	NR_051953.1	MEIS2
ZNF704	123.7110131	7.436505346	4.056205317	0.0018	High	117	9	ZNF704	ssc:102167304	8E-180	NM_032651.1	ZNF704
ANO9	82.47400875	4.131391859	4.319239723	0.001811	High	78	5	XP_005655100.1	ssc:100627366	0	NM_001012302.2	ANO9
CTSA	794.076674	15585.26265	-4.294760341	0.001836	Low	751	18862	NM_001243629.1	ssc:100156798	0	NM_001167594.1	CTSA
PAPLN	6.344154519	142.1198799	-4.485536655	0.001839	Low	6	172	XP_003482334.1	ssc:100523451	0	NM_173462.3	PAPLN
NUDT22	90.93288145	1602.153763	-4.139066734	0.00184	Low	86	1939	XP_005660810.1	ssc:100521244	4E-141	NM_032344.3	NUDT22
LOC100510895	11.63094995	226.4002739	-4.282834865	0.001841	Low	11	274	XP_003124046.2	ssc:100510895	0	NM_032101.2	PCDHGB7
KCNK10	71.90041789	3.305113487	4.44322844	0.001853	High	68	4	XP_003482344.1	ssc:100155276	0	NM_138318.2	KCNK10
BTBD17	8.458872693	175.1710148	-4.372154855	0.001861	Low	8	212	XP_005668698.1	ssc:100623910			
LOC102167052	25.37661808	451.147991	-4.152029041	0.001867	Low	24	546	XR_301611.1	ssc:102167052			
LOC102161016	1.057359087	58.66576439	-5.79398152	0.001879	Low	1	71	XP_005653904.1	ssc:102161016	2E-143	NM_031994.1	RNF17
LAMA2	4.229436346	108.2424667	-4.677657402	0.001884	Low	4	131	XP_005659257.1	ssc:100154420	0	NM_001079823.1	LAMA2
LOC100622295	27.49133625	484.1991258	-4.138551537	0.001908	Low	26	586	XP_005674228.1	ssc:100622295	4E-116	NM_000599.3	IGFBP5
LOC102167347	0	41.31391859	-20	0.001911	Low	0	50	XR_309153.1	ssc:102167347			
LOC102167351	0	41.31391859	-20	0.001911	Low	0	50	XP_005652885.1	ssc:102167351	7E-152	NM_152601.3	ZNF709
LOC100620566	0	41.31391859	-20	0.001911	Low	0	50	XP_003361906.2	ssc:100620566	4E-110	NM_031916.4	ROPN1L
TCP11L2	113.1374223	1988.852041	-4.135787841	0.001917	Low	107	2407	XP_003126140.3	ssc:100523311	0	NM_152772.2	TCP11L2
EMILIN2	477.9263071	9008.913087	-4.23649297	0.001923	Low	452	10903	XP_005665358.1	ssc:100522875	0	NM_032048.2	EMILIN2
RBCK1	95.16231779	1661.645806	-4.126078671	0.001929	Low	90	2011	XP_005654297.1	ssc:100519985	3E-89	NM_031229.2	RBCK1
LOC102159120	29.60605442	0	20	0.001929	High	28	0	XR_303642.1	ssc:102159120			
LOC102161287	29.60605442	0	20	0.001929	High	28	0	XR_306164.1	ssc:102161287			
PNLIPRP2	29.60605442	0	20	0.001929	High	28	0	NM_001190291.1	ssc:100462755	0	NR_103727.1	PNLIPRP2
LOC100738781	29.60605442	0	20	0.001929	High	28	0	XP_003483797.1	ssc:100738781	8E-179	NM_020864.1	NYAP2
LOC100627398	29.60605442	0	20	0.001929	High	28	0	XP_003361503.1	ssc:100627398	7E-25	NM_001867.2	COX7C
LOC100623228	29.60605442	0	20	0.001929	High	28	0	LOC100623228				
LOC102167676	29.60605442	0	20	0.001929	High	28	0	XR_309448.1	ssc:102167676			
DYNC1I1	1101.768168	81.8015588	3.751548541	0.001929	High	1042	99	XP_003130207.2	ssc:100523985			
FSD1L	2313.501681	168.5607878	3.77873728	0.001931	High	2188	204	XP_005660371.1	ssc:100521765	0	NM_207647.2	FSD1L
LOC100520265	13.74566813	257.798852	-4.229196901	0.001932	Low	13	312	XP_003131064.2	ssc:100520265	0	NM_203487.2	PCDH9
LOC100152091	210.4144582	3790.138891	-4.170944972	0.001933	Low	199	4587	XP_005670217.1	ssc:100152091	2E-147	NM_053032.2	MYLK
LOC102161873	81.41664967	4.131391859	4.300624045	0.001936	High	77	5	XR_305513.1	ssc:102161873			
LOC102164231	65.55626337	1134.480204	-4.113154001	0.001939	Low	62	1373	XR_304489.1	ssc:102164231			
E2F5	5354.466414	377.6092159	3.825776934	0.001939	High	5064	457	XP_005663053.1	ssc:100157957	5E-167	NM_001951.3	E2F5
PDE10A	308.7488533	22.30951604	3.790702656	0.001949	High	292	27	XP_005659167.1	ssc:100154378	0	NR_045597.1	PDE10A
LOC100521376	2862.271047	207.3958713	3.786701217	0.001955	High	2707	251	XP_005664247.1	ssc:100521376	0	NR_016575.1	NT5DC3
RUNX2	3.17207726	90.89062089	-4.840631614	0.001964	Low	3	110	XP_005666129.1	ssc:100155806	6E-45	NR_103533.1	RUNX2
LOC100738501	1176.840663	87.5855074	3.748083022	0.001967	High	1113	106	XP_003480583.1	ssc:100738501	1E-179	NM_013390.2	TMEM2
LOC102166805	9.516231779	189.2177471	-4.313513187	0.001986	Low	9	229	XR_301473.1	ssc:102166805			
LONRF3	68.72834063	1183.230628	-4.105682365	0.001991	Low	65	1432	XP_003360480.1	ssc:100517799	0	NR_110311.1	LONRF3
LOC100621853	488.499898	36.35624836	3.748083022	0.001996	High	462	44	XP_003482633.1	ssc:100621853	4E-121	NM_022337.2	RAB38
LOC100154020	90.93288145	4.95767023	4.197067853	0.002005	High	86	6	LOC100154020				
LOC100624372	40.17964529	0.826278372	5.603693113	0.002015	High	38	1	LOC100624372				
LOC100621908	40.17964529	0.826278372	5.603693113	0.002015	High	38	1	LOC100621908				
LOC102166444	40.17964529	0.826278372	5.603693113	0.002015	High	38	1	XR_301131.1	ssc:102166444			
PPP1R14A	4.229436346	106.58991	-4.655461656	0.002016	Low	4	129	NM_214337.1	ssc:397610	4E-64	NM_033256.2	PPP1R14A
KBTBD8	1070.047396	80.14900206	3.738846332	0.00202	High	1012	97	XP_003132353.3	ssc:100521640	0	NM_032505.2	KBTBD8
JPH1	151.2023494	9.915340461	3.930674435	0.002037	High	143	12	XP_005663079.1	ssc:100626574	0	NM_020647.2	JPH1
LOC102167305	3.17207726	90.06434252	-4.827456225	0.002045	Low	3	109	XR_297258.1	ssc:102167305			
TRIM24	7903.759172	552.7802307	3.837761063	0.002047	High	7475	669	XP_003360173.2	ssc:100620590			
LOC102163367	421.8862755	31.39857813	3.748083022	0.002053	High	399	38	LOC102163367				

ZNF784	8.458872693	171.0396229	-4.337721358	0.00207	Low	8	207	ZNF784	ssc:100628161	2E-146	NM_203374.1	ZNF784
MIEN1	115.2521404	1992.157154	-4.111466012	0.002077	Low	109	2411	XP_003131565.3	ssc:100516461	1E-57	NM_032339.3	MIEN1
LOC102162058	7.401513606	154.5140555	-4.383773939	0.002079	Low	7	187	XP_005672640.1	ssc:102162058			
LOC100518295	0	40.48764021	-20	0.002082	Low	0	49	XP_005652880.1	ssc:100518295	4E-23	NM_021030.2	ZNF14
TLR1	0	40.48764021	-20	0.002082	Low	0	49	NM_001031775.1	ssc:396607	0	NM_003263.3	TLR1
CRB2	60.26946793	2.478835115	4.603693113	0.002084	High	57	3	XP_003122203.3	ssc:100511431	0	NR_104603.1	CRB2
LOC102161865	2.114718173	73.53877508	-5.119967832	0.002084	Low	2	89	XR_303108.1	ssc:102161865			
DSCC1	1683.315666	125.5943125	3.744462706	0.002088	High	1592	152	XP_001926458.1	ssc:100156441	7E-179	NM_024094.2	DSCC1
BTBD19	100.4491132	1726.095519	-4.10297557	0.00209	Low	95	2089	XP_005656243.1	ssc:100520914	2E-104	NM_001136537.1	BTBD19
LOC100514339	798.3061104	60.31832114	3.726273875	0.002091	High	755	73	XP_005655078.1	ssc:100514339	2E-132	NM_018936.3	PCDHB2
LOC100620584	1174.725945	88.41178578	3.731941714	0.002092	High	1111	107	XP_003359538.1	ssc:100620584	4E-157	NM_013964.3	NRG1
STOML1	61.32682702	1041.110748	-4.085461424	0.002109	Low	58	1260	XP_005666226.1	ssc:100156113	0	NM_004809.4	STOML1
LOC102164569	130.0551676	8.262783717	3.97635201	0.00211	High	123	10	XP_005671855.1	ssc:102164569	1E-33	NM_013964.3	NRG1
LRRC3B	130.0551676	8.262783717	3.97635201	0.00211	High	123	10	XP_005669369.1	ssc:100523139	5E-154	NM_052953.2	LRRC3B
FAM169A	1828.173861	136.3359313	3.745165539	0.002111	High	1729	165	XP_005661542.1	ssc:100518001	0	NR_046462.1	FAM169A
LOC100738653	63.44154519	1076.640718	-4.084965174	0.002115	Low	60	1303	XR_306378.1	ssc:100738653			
LOC102161421	5.286795433	121.4629206	-4.521978651	0.002121	Low	5	147	XR_303328.1	ssc:102161421			
LOC102159735	1.057359087	57.01320765	-5.752758858	0.002125	Low	1	69	XR_302589.1	ssc:102159735			
SADC	1.057359087	57.01320765	-5.752758858	0.002125	Low	1	69	NM_001123193.1	ssc:100144439	0	NM_052998.2	ADC
ARHGEF26	1394.656635	104.9373532	3.732309762	0.00213	High	1319	127	XP_005669998.1	ssc:100626582	0	NM_015595.3	ARHGEF26
SLC22A4	89.87552236	4.95767023	4.180194035	0.00213	High	85	6	NM_001145752.1	ssc:100271724	0	NM_003059.2	SLC22A4
PROM1	3296.845632	241.2732845	3.772346253	0.002134	High	3118	292	XP_005666595.1	ssc:100511336	0	NM_006017.2	PROM1
ZNF423	44.40908164	751.0870399	-4.080053462	0.002146	Low	42	909	ZNF423	ssc:100520077	0	NM_015069.3	ZNF423
LRCH1	42.29436346	714.7307915	-4.078862628	0.00216	Low	40	865	XP_005668473.1	ssc:100521036	0	NM_015116.2	LRCH1
LOC102167733	8.458872693	169.3870662	-4.3237145	0.002161	Low	8	205	XP_005666981.1	ssc:102167733	6E-54	NM_024590.3	ARSJ
LOC100738956	69.78569971	3.305113487	4.400159719	0.002161	High	66	4	XR_135238.2	ssc:100738956			
CNDP1	69.78569971	3.305113487	4.400159719	0.002161	High	66	4	XP_005659878.1	ssc:100516053	0	NM_032649.5	CNDP1
LOC100627365	15.8603863	284.2397599	-4.16360856	0.002176	Low	15	344	XP_005658208.1	ssc:100627365	0	NM_152727.5	CPNE2
LOC100738544	201.9555855	3536.471431	-4.130200654	0.00218	Low	191	4280	XP_003481990.1	ssc:100738544	3E-92	NM_018948.3	ERRFI1
LOC100738308	134.284604	2308.621771	-4.103666019	0.002185	Low	127	2794	XP_003483753.2	ssc:100738308	7E-142	NM_139266.2	STAT1
LOC100737363	49.69587707	1.652556743	4.910354451	0.002189	High	47	2	XR_135271.2	ssc:100737363			
PIK3CD	2.114718173	72.71249671	-5.103666019	0.002189	Low	2	88	XP_005665035.1	ssc:100518131	0	NM_005026.3	PIK3CD
FGF14	149.0876312	9.915340461	3.910354451	0.002194	High	141	12	XP_005668578.1	ssc:100156844	4E-145	NM_175929.2	FGF14
LOC100737414	27.49133625	469.3261151	-4.093541802	0.002196	Low	26	568	XP_005665350.1	ssc:100737414	0	NM_002845.3	PTPRM
LOC100513133	2399.147767	48519.89227	-4.337982424	0.002205	Low	2269	58721	XR_115737.2	ssc:100513133			
BMP1	601.6373203	11152.27918	-4.212300711	0.002212	Low	569	13497	XP_003359075.1	ssc:100156461	0	NR_033404.1	BMP1
ARHGEF37	3.17207726	88.41178578	-4.800738886	0.002217	Low	3	107	XP_005661860.1	ssc:100514827	0	NM_001001669.2	ARHGEF37
LOC102162762	5957.161094	428.0121966	3.79890116	0.00222	High	5634	518	XR_300561.1	ssc:102162762			
BEND6	77.18721332	1298.9096	-4.072795344	0.002229	Low	73	1572	XP_005665923.1	ssc:100153820	6E-127	NM_152731.2	BEND6
LOC100621171	198.7835083	14.04673232	3.82289161	0.002237	High	188	17	XP_005674343.1	ssc:100621171	7E-60	NM_007280.1	OIP5
LOC100737686	88.81816327	4.95767023	4.163120521	0.002265	High	84	6	LOC100737686				
DCX	0	39.66136184	-20	0.002271	Low	0	48	XP_005673890.1	ssc:100523160	0	NM_178153.2	DCX
CLEC4F	0	39.66136184	-20	0.002271	Low	0	48	XP_005658763.1	ssc:100524617	0	NM_173535.2	CLEC4F
LOC102159458	0	39.66136184	-20	0.002271	Low	0	48	XR_299573.1	ssc:102159458			
LOC100511379	0	39.66136184	-20	0.002271	Low	0	48	LOC100511379				
BACH2	0	39.66136184	-20	0.002271	Low	0	48	XP_001924369.1		0	NM_021813.2	BACH2
SERPINB2	0	39.66136184	-20	0.002271	Low	0	48	XP_003121745.1	ssc:100519286	0	NM_002575.2	SERPINB2
ZSCAN4	0	39.66136184	-20	0.002271	Low	0	48	ZSCAN4	ssc:102159852	1E-103	NM_152677.2	ZSCAN4
LOC100625959	0	39.66136184	-20	0.002271	Low	0	48	XP_003360914.2	ssc:100625959	7E-96	NM_006558.1	KHDRBS3
LAG3	0	39.66136184	-20	0.002271	Low	0	48	NM_001105306.1	ssc:100125962	0	NM_002286.5	LAG3
LOC102165012	0	39.66136184	-20	0.002271	Low	0	48	XP_005653389.1	ssc:102165012			
CDCA7	11368.7249	795.706072	3.836691034	0.002273	High	10752	963	XP_001928667.1	ssc:100154583	0	NM_145810.2	CDCA7
PRKAB2	9683.294515	684.1584918	3.823095491	0.002295	High	9158	828	NM_001243683.1	ssc:100157793	3E-158	NR_103871.1	PRKAB2
DNA2	2287.067704	171.8659013	3.734143831	0.002296	High	2163	208	XP_003483536.1	ssc:100153906	0	NR_102264.1	DNA2
KANK4	2.114718173	71.88621834	-5.087177897	0.0023	Low	2	87	XP_003128002.2	ssc:100523255	0	NM_181712.4	KANK4
LOC102167011	2.114718173	71.88621834	-5.087177897	0.0023	Low	2	87	XR_302256.1	ssc:102167011			
LOC100737896	2.114718173	71.88621834	-5.087177897	0.0023	Low	2	87	XR_302944.1	ssc:100737896	1E-20	NM_172060.3	EYA1
LOC102159402	28.54869534	0	20	0.002302	High	27	0	XR_297885.1	ssc:102159402			
GLB1L2	28.54869534	0	20	0.002302	High	27	0	XP_005667581.1	ssc:100520582	0	NM_138342.3	GLB1L2
LOC102158142	8.458872693	166.9082311	-4.302445884	0.002307	Low	8	202	XP_005661569.1	ssc:102158142			
ATP2A3	360.5594485	27.26718627	3.724999409	0.002319	High	341	33	XP_005669187.1	ssc:396820	5E-159	NM_174958.2	ATP2A3
LOC100625764	257.9956171	19.00440255	3.762940981	0.002322	High	244	23	XP_005668032.1	ssc:100625764	7E-54	NM_016309.2	LCMT1
CPT1A	349.9858577	6218.571026	-4.151214568	0.002327	Low	331	7526	NM_001129805.1	ssc:399527	1E-155	NM_001876.3	CPT1A
LOC100624495	2224.683518	167.7345095	3.729348671	0.002327	High	2104	203	XP_005658667.1	ssc:100624495	2E-140	NM_182609.2	ZNF677
APCDD1	207.242381	14.87301069	3.800550442	0.002334	High	196	18	XP_005658356.1	ssc:100626308	6E-112	NM_153000.4	APCDD1
HELLS	7534.740851	540.3860551	3.801495542	0.002338	High	7126	654	XP_005657492.1	ssc:100154547	0	NM_018063	HELLS
GPR63	267.5118489	19.83068092	3.753796673	0.002352	High	253	24	XP_005659366.1	ssc:100152431	0	NM_030784.3	GPR63
LOC100739471	39.1222862	649.4548002	-4.053166537	0.002355	Low	37	786	XP_003483758.1	ssc:100739471	2E-37	NM_144629.2	RFTN2
LOC100519689	693.6275608	53.70809416	3.690949791	0.002387	High	656	65	XR_297787.1	ssc:100519689			
LOC100037968	8.458872693	165.2556743	-4.288090591	0.002411	Low	8	200	XP_003482606.1	ssc:100037968			
FRMD7	1.057359087	55.36065091	-5.710323591	0.002411	Low	1	67	XP_003360499.2	ssc:100626525	0	NM_194277.2	FRMD7
LOC100157806	1.057359087	55.36065091	-5.710323591	0.002411	Low	1	67	XR_135278.2	ssc:100157806			
LOC102166984	2.114718173	71.05993997	-5.070499155	0.002418	Low	2	86	XR_304405.1	ssc:102166984			
LOC100738304	166.0053766	11.5678972	3.843031426	0.002425	High	157	14	XP_003482411.1	ssc:100738304	3E-75	NM_022551.2	RPS18
SPRY4	98.33439505	1631.073506	-4.051981864	0.002447	Low	93	1974	XP_003124057.1	ssc:100512891	7E-161	NM_030964.3	SPRY4
LOC100736719	64.49890428	1060.115151	-4.038802518	0.002447	Low	61	1283	XP_003482045.1	ssc:100736719			
PTPRZ1	501.188207	38.83508347	3.689919996	0.002454	High	474	47	XP_003134783.1	ssc:100511294	0	NM_002851.2	PTPRZ1
KIAA0319	145.9155539	9.915340461	3.879327555	0.002456	High	138	12	XP_005665689.1	ssc:100738226	0	NM_014809.3	KIAA0319
LOC100627920	59.21210885	970.8770868	-4.03532452	0.002465	Low	56	1175	XP_003360105.1	ssc:100627920	0	NM_015077.3	SARM1
RHOC	936.8201507	17426.21086	-4.217342987	0.002466	Low	886	21090	XP_003125894.1	ssc:100520019	4E-112	NM_175744.4	RHOC
NEFM	23.2618999	390.8296698	-4.070499155	0.002469	Low	22	473	XP_005670496.1	ssc:100625407	0	NM_005382.2	NEFM
FAM171A2	22.20454082	374.3041024	-4.075284218	0.00247	Low	21	453	XP_005668832.1	ssc:100514069	0	NM_198475.2	FAM171A2
RIN2	20.08982264	341.2529675	-4.086304859	0.002476	Low	19	413	XP_003359960.2	ssc:100625676	0	NM_018993.3	RIN2
STEAP4	0	38.83508347	-20	0.002481	Low	0	47	NM_001166489.1	ssc:100310802	0	NM_024636.3	STEAP4
LOC100624603	0	38.83508347	-20	0.002481	Low	0	47	XP_003356537.2	ssc:100624603	3E-53	NM_057160.2	ARTN
HighK3A	0	38.83508347	-20	0.002481	Low	0	47	XP_003355356.1	ssc:397558	8E-142	NM_006953.3	HighK3A
LOC102162758	0	38.83508347	-20	0.002481	Low	0	47	XP_005658708.1	ssc:102162758	9E-20	NR_110311.1	LONRF3
PCNXL2	58.15474976	2.478835115	4.552162812	0.002499	High	55	3	XP_005671027.1	ssc:100158027	0	NM_024938.2	PCNXL2
RMI1	1772.133829	136.3359313	3.700249819	0.0025	High	1676	165	XP_005668084.1	ssc:100517963	0	NM_024945.2	RMI1
LOC100154987	35.95020894	589.9627574	-4.036551824	0.002502	Low	34	714	XP_003480443.2	ssc:100154987	0	NM_002373.5	MAP1A
LOC100624199	12.68830904	227.2265522	-4.162559708	0.002505	Low	12	275	XP_005672073.1	ssc:100624199	8E-71	NM_014585.5	SLC40A1
F5	3.17207726	85.93295066	-4.759711618	0.002509	Low	3	104	NM_214120.1	ssc:397217	0	NM_000130.4	F5
SLC39A4	3.17207726	85.93295066	-4.759711618	0.002509	Low	3	104	ZIP4	ssc:100156896	0	NM_130849.3	SLC39A4
LOC102163982	3.17207726	85.93295066	-4.759711618	0.002509	Low	3	104	XR_302179.1	ssc:102163982			
PGM2	5978.308275	438.7538154	3.768253716	0.002511	High	5654	531	XP_003128958.1	ssc:100522261	0	NM_018290.3	PGM2
FAM78B	29.60605442	488.3305177	-4.043893799	0.002522	Low	28	591	XP_001925744.3	ssc:100153010	1E-152	NM_001017961.3	FAM78B
LOC100624428	135.3419631	9.089062089	3.896333981	0.00253	High	128	11	XP_003356823.3	ssc:100624428	7E-106	NM_182398.1	RPS6KA5
LOC102160000	67.67098154	3.305113487	4.355765599	0.00253	High	64	4	XR_308365.1	ssc:102160000			
LOC100626559	6.344154519	132.2045395	-4.381199995	0.002531	Low	6	160	XP_005660597.1	ssc:100626559	2E-138	NM_152572.2	AK8
LOC102157981	334.1254714	25.61462952	3.705350037	0.002542	High	316	31	XP_005662316.1	ssc:102157981	7E-116	NM_006648.3	WNK2

DACT3	301.3473397	5208.858855	-4.111468111	0.002542	Low	285	6304	XP_003356003.2	ssc:100622612	0	NM_145056.2	DACT3
LOC102166308	2.114718173	70.2336616	-5.053625337	0.002544	Low	2	85	XR_299392.1	ssc:102166308			
LOC102161560	77.18721332	4.131391859	4.223662063	0.00255	High	73	5	XP_005658071.1	ssc:102161560	2E-47	NM_006095.2	ATP8A1
LOC100739312	54.9826725	894.0331982	-4.023279466	0.002555	Low	52	1082	LOC100739312				
ZDHHC8	122.653654	2027.687124	-4.047172939	0.00257	Low	116	2454	ZDHHC8	ssc:100157140	0	NM_013373.3	ZDHHC8
LOC102159729	1.057359087	54.53437253	-5.68862852	0.002571	Low	1	66	XP_005671271.1	ssc:102159729	1E-33	NR_045675.1	ARHGAP22
TMEM176A	1.057359087	54.53437253	-5.68862852	0.002571	Low	1	66	XP_005657767.1	ssc:100523103	7E-43	NM_018487.2	TMEM176A
INHBE	142.7434767	2371.418927	-4.054253825	0.002582	Low	135	2870	XP_003126368.1	ssc:100518554	9E-164	NM_031479.3	INHBE
MIR21	7.401513606	147.0775502	-4.31261291	0.002585	Low	7	178	NR_038508.1	ssc:100316568			
CILP2	16.91774539	289.1974301	-4.095445513	0.002598	Low	16	350	XP_003123618.1	ssc:100518061	0	NM_153221.2	CILP2
LOC595121	3749.395321	282.5872031	3.729889905	0.002601	High	3546	342	NM_001031791.1	ssc:595121	3E-115	NM_212460.3	ARL4A
TP53I3	2289.182422	175.9972932	3.701207289	0.002612	High	2165	213	XP_003125411.1	ssc:100521786	4E-174	NM_147184.3	TP53I3
GNG8	3.17207726	85.10667229	-4.745772427	0.002617	Low	3	103	XP_005664677.1	ssc:100622520	2E-34	NM_033258.1	GNG8
KBTBD11	5.286795433	115.678972	-4.451589323	0.002628	Low	5	140	XP_005657582.1	ssc:100625139	0	NM_014867.2	KBTBD11
SKP2	3821.295739	288.3711517	3.728063164	0.002632	High	3614	349	NM_001171755.1	ssc:100329128	0	NM_032637.3	SKP2
LOC102159432	14.80302721	256.1462952	-4.113003884	0.002645	Low	14	310	XP_005671932.1	ssc:102159432	7E-15	NM_016839.2	RBMS1
LOC100736872	245.3073081	4152.875096	-4.081448362	0.00266	Low	232	5026	XP_003482733.1	ssc:100736872	8E-178	NM_006379.3	SEMA3C
GLTPD1	30.66341351	499.0721365	-4.024658145	0.00266	Low	29	604	XP_005653398.1	ssc:100628234	7E-102	NM_001029885.1	GLTPD1
TLL1	2270.149959	175.1710148	3.695951621	0.002661	High	2147	212	XP_003129040.3	ssc:100512480	0	NM_012464.4	TLL1
C2H19orf38	4.229436346	99.97968298	-4.563097638	0.002669	Low	4	121	XP_005661306.1	ssc:100627549	3E-98	NM_001136482.1	C19orf38
SLC12A5	6.344154519	130.5519827	-4.363052648	0.002674	Low	6	158	XP_005673037.1	ssc:100155991	0	NM_020708.4	SLC12A5
FBLN5	340.4696259	5856.661099	-4.104480411	0.00268	Low	322	7088	XP_005656504.1	ssc:100155248	0	NM_006329.3	FBLN5
IGFBP7	1792.223652	34102.98724	-4.250075529	0.002682	Low	1695	41273	NM_001163801.1	ssc:100302573	8E-111	NM_001553.2	IGFBP7
DEPDC1B	2845.353302	218.1374901	3.705298156	0.00269	High	2691	264	XP_005654276.1	ssc:100524052	0	NM_018369.2	DEPDC1B
LOC100622097	1573.350321	123.1154774	3.67575589	0.002696	High	1488	149	XP_003357757.1	ssc:100622097	0	NM_024945.2	RMI1
TAPBP	384.8787075	6649.888336	-4.11085435	0.00271	Low	364	8048	XP_005665941.1	ssc:100155428	0	NM_172209.2	TAPBP
ATF7IP2	0	38.0088051	-20	0.002715	Low	0	46	XP_003354662.1	ssc:100520369	0	NR_045816.1	ATF7IP2
MAGEL2	83.53136784	1347.660024	-4.011994719	0.002719	Low	79	1631	XP_005659854.1	ssc:100154924	2E-67	NM_019066.4	MAGEL2
LOC100621449	30.66341351	496.5933014	-4.017474586	0.00272	Low	29	601	XP_005659046.1	ssc:100621449	3E-35	NM_144999.2	LRRC45
LHFPL4	47.5811589	1.652556743	4.847618696	0.002724	High	45	2	XP_003132402.3	ssc:100157883	4E-142	NM_198560.2	LHFPL4
LOC102160475	728.5204106	57.83948602	3.654842755	0.002728	High	689	70	XR_299423.1	ssc:102160475			
PLEKHA4	107.8506268	1750.057591	-4.020295933	0.002739	Low	102	2118	XP_005664744.1	ssc:100522871	0	NM_020904.2	PLEKHA4
LOC102163525	66.61362245	3.305113487	4.333045523	0.002742	High	63	4	XR_305221.1	ssc:102163525			
LOC100623420	208.2997401	3470.369161	-4.058356194	0.002755	Low	197	4200	XP_003357434.2	ssc:100623420	0	NM_001684.4	ATP2B4
LOC102167066	27.49133625	0	20	0.002761	High	26	0	XR_297493.1	ssc:102167066			
LOC102162598	27.49133625	0	20	0.002761	High	26	0	XP_005668111.1	ssc:102162598	1E-42	NM_012144.3	DNAI1
LOC102163342	27.49133625	0	20	0.002761	High	26	0	XP_005661676.1	ssc:102163342	3E-66	NM_133638.3	ADAMTS19
LOC100739170	1231.823336	97.50084786	3.659236789	0.002777	High	1165	118	XR_301936.1	ssc:100739170	2E-73	NM_022140.3	EPB41L4A
STK10	15.8603863	268.5404708	-4.081639713	0.002802	Low	15	325	XP_003359866.1		2E-68	NM_005990.3	STK10
SERINC2	47.5811589	757.6972669	-3.993159228	0.002808	Low	45	917	NM_001244148.1	ssc:100626004	5E-148	NM_178865.4	SERINC2
SLC39A8	2.114718173	68.58110485	-5.019273832	0.002818	Low	2	83	XP_005667018.1	ssc:100524695	0	NM_022154.5	SLC39A8
LOC102161965	817.3385739	65.27599137	3.646309455	0.002822	High	773	79	XP_005656559.1	ssc:102161965	9E-27	NR_046382.1	ZNF195
ATF5	311.9209305	5278.266239	-4.080811854	0.002825	Low	295	6388	XP_005664813.1	ssc:100523675	4E-98	NM_012068.5	ATF5
BRIX1	15353.9113	1099.776513	3.803323932	0.002831	High	14521	1331	NM_001244824.1	ssc:100513502	0	NM_018321.3	BRIX1
LOC102165686	7.401513606	143.7724367	-4.279822975	0.002857	Low	7	174	XP_005665993.1	ssc:102165686			
LOC100157017	1438.008358	114.0264153	3.656632079	0.002862	High	1360	138	XP_005660626.1	ssc:100157017	1E-48	NM_012203.1	GRHPR
EHHADH	9191.62254	676.7219864	3.763684387	0.002871	High	8693	819	XP_003358782.2	ssc:448846	6E-105	NM_001025266.1	C3orf70
DEF8	133.2272449	2155.760272	-4.016235706	0.002873	Low	126	2609	XP_005653273.1	ssc:100523845	0	NM_207514.2	DEF8
AIM1	194.5540719	3200.176134	-4.039908231	0.002877	Low	184	3873	XP_003121377.3	ssc:100521168	0	NM_001624.2	AIM1
LOC100622302	1033.039828	82.62783717	3.644124161	0.002887	High	977	100	XP_003362135.2	ssc:100622302	4E-141	NR_037936.1	LIPT1
ARAP2	5.286795433	113.2001369	-4.420338189	0.002888	Low	5	137	XP_003128954.1	ssc:100521564	0	NM_139182.1	ARAP2
PCDH9	13.74566813	235.4893359	-4.098612792	0.002891	Low	13	285	XP_001925538.1	ssc:100154773	0	NM_203487.2	PCDH9
LOC102162356	198.7835083	14.87301069	3.740429449	0.002905	High	188	18	XR_299995.1	ssc:102162356			
CHRNA3	375.3624757	29.74602138	3.657515812	0.002905	High	355	36	XP_001925795.3	ssc:100151989	0	NR_046313.1	CHRNA3
FBN1	1168.381791	21241.96438	-4.184333504	0.00292	Low	1105	25708	NM_001001771.1	ssc:414836	0	NM_000138.4	FBN1
MCF2L	688.3407654	55.36065091	3.636190142	0.002924	High	651	67	XP_005668614.1	ssc:100518447	0	NM_024979.4	MCF2L
LOC100621201	789.8472377	63.62343462	3.633943491	0.002952	High	747	77	XP_003360777.3	ssc:100621201	9E-53	NM_001042367.1	C15orf60
ANXA5	2637.053562	50369.10326	-4.255540267	0.002956	Low	2494	60959	XP_003129266.2	ssc:100521982	0	NM_001154.3	ANXA5
LOC102161426	0	37.18252673	-20	0.002975	Low	0	45	XP_005664948.1	ssc:102161426	0	NM_020813.2	ZNF471
LOC102161148	0	37.18252673	-20	0.002975	Low	0	45	XP_005657636.1	ssc:102161148			
LOC102167801	0	37.18252673	-20	0.002975	Low	0	45	XR_302359.1	ssc:102167801			
LOC100511183	0	37.18252673	-20	0.002975	Low	0	45	XP_005654914.1	ssc:100511183	0	NM_003259.3	ICAM5
LOC102162744	4.229436346	97.50084786	-4.52687745	0.002977	Low	4	118	XR_298718.1	ssc:102162744			
PBXIP1	377.4771939	6381.347865	-4.079399793	0.002979	Low	357	7723	XP_005663400.1	ssc:100151902	4E-180	NM_020524.2	PBXIP1
LOC100522480	197.7261492	14.87301069	3.732735058	0.002987	High	187	18	XP_005661609.1	ssc:100522480	3E-175	NM_024717.4	MCTP1
TNIP1	477.9263071	8176.024488	-4.09653943	0.003003	Low	452	9895	NM_001145987.1	ssc:100271903	0	NM_006058.4	TNIP1
LOC102162373	56.04003159	2.478835115	4.498723553	0.003013	High	53	3	XR_309104.1	ssc:102162373			
LOC102162699	96.21967688	1522.831039	-3.984280116	0.003017	Low	91	1843	XP_005672219.1	ssc:102162699			
LOC102161046	103.6211905	1641.815125	-3.985900714	0.003033	Low	98	1987	XR_308191.1	ssc:102161046			
IL12A	46.52379981	1.652556743	4.815197218	0.003047	High	44	2	NM_213993.1	ssc:397053			
FAM227B	6.344154519	126.4205909	-4.316659743	0.003078	Low	6	153	XP_005659683.1	ssc:102164442	5E-65	NM_152647.2	FAM227B
LOC100521274	7.401513606	141.2936016	-4.254731994	0.003083	Low	7	171	XP_005665093.1	ssc:100521274	8E-123	NM_182623.2	FAM131C
TENM4	137.4566813	2188.811407	-3.993098716	0.003107	Low	130	2649	XR_305203.1	ssc:100514784	0	NM_001098816.2	TENM4
LOC100736817	1.057359087	52.05553742	-5.621514324	0.003133	Low	1	63	XP_003484258.1	ssc:100736817	4E-31	NM_001257.4	CDH13
AMZ1	1.057359087	52.05553742	-5.621514324	0.003133	Low	1	63	NM_001195325.1	ssc:100337670	0	NM_133463.2	AMZ1
SPNS2	1.057359087	52.05553742	-5.621514324	0.003133	Low	1	63	XP_005669203.1	ssc:100523398	0	NM_001124758.1	SPNS2
LOC100623746	1.057359087	52.05553742	-5.621514324	0.003133	Low	1	63	LOC100623746				
CBX7	1.057359087	52.05553742	-5.621514324	0.003133	Low	1	63	XP_003126060.3	ssc:100520132	4E-137	NM_175709.3	CBX7
GSTO2	111.0227041	7.436505346	3.900086115	0.003162	High	105	9	XP_001923723.2	ssc:100152209	2E-111	NM_183239.1	GSTO2
GPD1L	7106.510421	538.7334984	3.721497633	0.003166	High	6721	652	XP_003483187.1	ssc:100624347	0	NM_015141.3	GPD1L
MYB	537.138416	43.7927537	3.616529831	0.003176	High	508	53	XP_005652721.1	ssc:100152439	0	NM_005375.2	MYB
LOC100517224	101.5064723	6.610226974	3.9407281	0.0032	High	96	8	XR_130739.2	ssc:100517224			
MPZL3	4.229436346	95.84829112	-4.502215396	0.003205	Low	4	116	XR_297544.1	ssc:100513300	2E-123	NR_104405.1	MPZL3
ZFP92	19.03246356	306.5492759	-4.009584776	0.003206	Low	18	371	ZFP92	ssc:100623680	8E-170	NM_001136273.1	ZFP92
LOC102165900	64.49890428	3.305113487	4.286502937	0.003228	High	61	4	XR_298690.1	ssc:102165900			
DDIT3	150.1449903	2378.029154	-3.985338158	0.003237	Low	142	2878	NM_001144845.1	ssc:100240743	3E-85	NM_004083.5	DDIT3
SP8	91.99024053	5.783948602	3.991354173	0.003249	High	87	7	XP_005667734.1	ssc:100627196	0	NM_198956.2	SP8
MSI1	1195.873127	97.50084786	3.616505764	0.003255	High	1131	118	XP_005670771.1	ssc:100627974			
ITGA10	126.8830904	1992.157154	-3.972759741	0.003264	Low	120	2411	XP_003125838.2	ssc:100511507	0	NM_003637.3	ITGA10
SLA-DQA1	0	36.35624836	-20	0.003266	Low	0	44	NM_001130224.1	ssc:100153387	3E-124	NM_002122.3	HLA-DQA1
LOC102166264	0	36.35624836	-20	0.003266	Low	0	44	XP_005655572.1	ssc:102166264			
PUS10	7221.762561	550.3013956	3.714057095	0.003275	High	6830	666	XP_005662583.1	ssc:100523369	0	NM_144709.2	PUS10
LOC102160955	376.4198348	30.57229975	3.622045665	0.003287	High	356	37	XR_308120.1	ssc:102160955			
LOC100739788	2739.617393	218.1374901	3.65066478	0.003302	High	2591	264	XP_005668726.1	ssc:100739788	8E-87	NM_015462.3	NOL11
LOC100737556	2126.349123	171.0396229	3.635976008	0.003316	High	2011	207	XP_005674253.1	ssc:100737556	0	NM_019005.3	MIOS
LOC100155975	4.229436346	95.02201275	-4.489724452	0.003328	Low	4	115	XR_304375.1	ssc:100155975	1E-39	NM_002120.3	HLA-DOB
GC	26.43397716	0	20	0.003329	High	25	0	XP_003357019.1	ssc:448964	0	NM_001204307.1	GC
GPRC6A	26.43397716	0	20	0.003329	High	25	0	XP_003480314.1	ssc:100152099	0	NM_148963.3	GPRC6A
LOC100523434	26.43397716	0	20	0.003329	High	25	0	LOC100523434				
LOC100739530	26.43397716	0	20	0.003329	High	25	0	XR_309490.1	ssc:100739530			

MMRN1	26.43397716	0	20	0.003329	High	25	0	XP_005667049.1	ssc:100516922	0	NM_007351.2	MMRN1
BDKRB1	1.057359087	51.22925905	-5.598430711	0.003352	Low	1	62	NM_001113064.1	ssc:100127469	8E-141	NM_000710.3	BDKRB1
FGF9	1.057359087	51.22925905	-5.598430711	0.003352	Low	1	62	NM_213801.1	ssc:396717	6E-120	NM_002010.2	FGF9
DEPTOR	1147.234609	94.19573438	3.606354912	0.003366	High	1085	114	XP_001926120.1	ssc:100157658	0	NM_022783.3	DEPTOR
MAML2	33.83549077	519.7290958	-3.941150608	0.003389	Low	32	629	XP_005667324.1	ssc:100739729	6E-16	NM_018717.4	MAML3
LHFPL1	45.46644072	1.652556743	4.782030354	0.003415	High	43	2	XP_003360469.1	ssc:100152966	2E-110	NM_178175.3	LHFPL1
LOC100153771	12.68830904	211.5272632	-4.0592719	0.003436	Low	12	256	XP_003121623.1	ssc:100153771	4E-79	NM_139265.3	EHD4
LOC102160986	90.93288145	5.783948602	3.974675432	0.003446	High	86	7	XR_299123.1	ssc:102160986			
CA5B	15.8603863	256.1462952	-4.01346821	0.003453	Low	15	310	XP_005673507.1	ssc:100156418	2E-175	NM_007220.3	CA5B
LOC100624568	286.5443125	23.13579441	3.630559719	0.003459	High	271	28	XP_005662968.1	ssc:100624568	0	NM_014677.4	RIMS2
NAGLU	105.7359087	1625.289557	-3.942159453	0.003487	Low	100	1967	XP_003131436.1	ssc:100519685	0	NM_000263.3	NAGLU
LOC102168075	35.95020894	0.826278372	5.44322844	0.003489	High	34	1	XR_297615.1	ssc:102168075			
LOC100737566	35.95020894	0.826278372	5.44322844	0.003489	High	34	1	LOC100737566				
LOC102159538	5.286795433	108.2424667	-4.355729307	0.003509	Low	5	131	XP_005653321.1	ssc:102159538	1E-11	NR_045692.1	C19orf12
LOC102162175	438.8040209	36.35624836	3.593301507	0.003533	High	415	44	XP_005668030.1	ssc:102162175	6E-45	NM_022366.2	TFB2M
LTBP4	241.0778717	3829.800253	-3.98969801	0.003536	Low	228	4635	XP_005674700.1	ssc:100627785	0	NM_003573.2	LTBP4
RGS4	822.6253694	68.58110485	3.584352513	0.003555	High	778	83	NM_001185184.1	ssc:100157437	1E-113	NM_005613.5	RGS4
DLX4	8.458872693	151.208942	-4.159934239	0.00356	Low	8	183	XP_005674532.1	ssc:100517238	8E-46	NM_138281.2	DLX4
LOC102165490	8.458872693	151.208942	-4.159934239	0.00356	Low	8	183	XR_307778.1	ssc:102165490			
HighK1B	681.9966108	57.01320765	3.580396493	0.00359	High	645	69	NM_001123212.1	ssc:100144488	3E-147	NM_006952.3	HighK1B
IRF5	0	35.52996998	-20	0.003591	Low	0	43	XP_005657821.1	ssc:100515633	0	NM_032643.3	IRF5
LOC102166114	0	35.52996998	-20	0.003591	Low	0	43	XR_306716.1	ssc:102166114			
ADCYAP1	0	35.52996998	-20	0.003591	Low	0	43	NM_001001544.1	ssc:414283	9E-71	NM_001117.4	ADCYAP1
PON1	0	35.52996998	-20	0.003591	Low	0	43	NM_001097515.2	ssc:100048952			
LOC100627616	0	35.52996998	-20	0.003591	Low	0	43	XP_005674315.1	ssc:100627616	2E-140	NM_002247.3	KCNMA1
LOC100736946	12557.19651	946.915014	3.729135648	0.003597	High	11876	1146	XR_304087.1	ssc:100736946	5E-71	NM_001792.3	CDH2
IL-6	7.401513606	136.3359313	-4.203201693	0.003602	Low	7	165	NM_001252429.1	ssc:100628202	2E-68	NM_000600.3	IL6
ADCY9	17.97510447	283.4134815	-3.978836326	0.00361	Low	17	343	XP_005653094.1	ssc:100625423	0	NM_001116.3	ADCY9
ATOH8	17.97510447	283.4134815	-3.978836326	0.00361	Low	17	343	XP_005662472.1	ssc:100523367	2E-126	NM_032827.6	ATOH8
LOC100522418	107.8506268	7.436505346	3.85826594	0.003668	High	102	9	XR_130344.2	ssc:100522418			
MND1	2357.910763	192.5228606	3.614407449	0.00368	High	2230	233	XP_005653639.1	ssc:100517105	4E-87	NR_045605.1	MND1
CCDC159	9.516231779	164.429396	-4.11093402	0.003689	Low	9	199	XR_298080.1	ssc:100515600			
PAFAH2	117.3668586	1786.41384	-3.927969342	0.003704	Low	111	2162	XP_005665185.1	ssc:100623755	0	NM_000437.3	PAFAH2
EPN3	3.17207726	78.49644531	-4.629127508	0.003708	Low	3	95	XP_005668947.1	ssc:100512679	0	NM_017957.2	EPN3
SCG2	4.229436346	92.54317763	-4.451589323	0.003729	Low	4	112	NM_001012299.1	ssc:497237	0	NM_003469.4	SCG2
DTL	4607.970899	366.867597	3.650800214	0.003735	High	4358	444	XR_305730.1	ssc:100525066	0	NM_016448.3	DTL
LOC102159413	5.286795433	106.58991	-4.333533561	0.00375	Low	5	129	XP_005667232.1	ssc:102159413	0	NM_001098816.2	TENM4
PLSCR3	407.0832483	6569.739334	-4.01244037	0.003752	Low	385	7951	XP_005669265.1	ssc:102163020	1E-150	NM_020360.3	PLSCR3
MZT2B	34.89284986	522.2079309	-3.90362103	0.003788	Low	33	632	XP_005654159.1	ssc:100153925	4E-68	NM_001085365.1	MZT2A
LOC102160306	7.401513606	134.6833746	-4.185607633	0.003798	Low	7	163	XP_005653420.1	ssc:102160306			
LOC100738836	21.14718173	324.7274001	-3.940691808	0.003801	Low	20	393	XP_003483784.2	ssc:100738836	5E-51	NM_000599.3	IGFBP5
LOC102159151	44.40908164	1.652556743	4.748083022	0.003835	High	42	2	XR_307681.1	ssc:102159151			
FSTL4	44.40908164	1.652556743	4.748083022	0.003835	High	42	2	XP_003123983.4	ssc:100525565	0	NM_015082.1	FSTL4
LOC102167316	44.40908164	1.652556743	4.748083022	0.003835	High	42	2	LOC102167316				
ETV4	822.6253694	13773.23418	-4.065487993	0.00385	Low	778	16669	XP_005668843.1	ssc:100516755	0	NM_001986.2	ETV4
ZNF8	31.7207726	474.2837854	-3.902250732	0.003857	Low	30	574	ZNF8	ssc:100517831	0	NM_021089.2	ZNF8
LOC102158885	3.17207726	77.67016694	-4.613860752	0.003879	Low	3	94	XP_005664477.1	ssc:102158885	3E-33	NM_013241.2	FHOD1
C1QTNF7	3.17207726	77.67016694	-4.613860752	0.003879	Low	3	94	NM_001143702.1	ssc:100233173	2E-145	NM_031911.4	C1QTNF7
LOC102163435	88.81816327	5.783948602	3.9407281	0.003884	High	84	7	XR_299697.1	ssc:102163435			
BHLHE40	282.3148761	4428.852072	-3.971555754	0.003887	Low	267	5360	NM_001245010.1	ssc:100514873	0	NM_003670.2	BHLHE40
PPA2	3724.018703	301.5916057	3.626192139	0.0039	High	3522	365	XP_005667016.1	ssc:100521105	1E-98	NM_176869.2	PPA2
GLRX	132.1698858	1995.462268	-3.91625759	0.003921	Low	125	2415	NM_214233.1	ssc:397463	1E-47	NM_002064.2	GLRX
PTGER1	0	34.70369161	-20	0.003956	Low	0	42	XP_003123421.2	ssc:100515879	9E-74	NM_000955.2	PTGER1
LOC102167614	0	34.70369161	-20	0.003956	Low	0	42	XR_299563.1	ssc:102167614			
LOC102165475	0	34.70369161	-20	0.003956	Low	0	42	XP_005655128.1	ssc:102165475	6E-27	NM_006869.3	ADAP1
C14H10orf116	0	34.70369161	-20	0.003956	Low	0	42	NM_001243503.1	ssc:100154721	9E-25	NM_006829.2	ADIRF
LOC100513464	62.38418611	920.4741061	-3.883124869	0.003964	Low	59	1114	XP_005661965.1	ssc:100513464	9E-79	NM_145030.2	PPP1R35
KLHL41	22.20454082	336.2952973	-3.920801962	0.003972	Low	21	407	XP_003359596.1	ssc:100627572	0	NM_006063.2	KLHL41
SNX18	49.69587707	732.0826374	-3.880808438	0.003972	Low	47	886	XP_003134014.3	ssc:100518876	0	NM_052870.2	SNX18
LIPT2	447.2628936	38.0088051	3.556717496	0.004011	High	423	46	XP_003129688.1	ssc:100515269	9E-103	NM_001144869.1	LIPT2
SORD	7892.128222	620.5350572	3.668829773	0.004012	High	7464	751	NM_001244162.1	ssc:100158181	0	NR_034039.1	SORD
CNKSR2	570.9739067	48.75042393	3.549938147	0.004025	High	540	59	XP_005673548.1	ssc:100523478	0	NM_014927.3	CNKSR2
ZNHIT2	62.38418611	917.1689926	-3.877935313	0.004028	Low	59	1110	ZNHIT2	ssc:100514699	0	NM_014205.2	ZNHIT2
GPR83	34.89284986	0.826278372	5.400159719	0.004032	High	33	1	HighI000263A7E0		0	NM_016540.3	GPR83
PTPRO	25.37661808	0	20	0.004036	High	24	0	XP_003126501.1	ssc:100524563	0	NM_030671.2	PTPRO
CDA	25.37661808	0	20	0.004036	High	24	0	NM_001244385.1	ssc:100515954	2E-70	NM_001785.2	CDA
IGFBPL1	25.37661808	0	20	0.004036	High	24	0	XP_001924387.1	ssc:100155070	3E-101	NM_001007563.2	IGFBPL1
LOC102157691	25.37661808	0	20	0.004036	High	24	0	XR_301930.1	ssc:102157691			
LOC100624756	25.37661808	0	20	0.004036	High	24	0	LOC100624756				
FOXB2	25.37661808	0	20	0.004036	High	24	0	XP_001925193.2	ssc:100156565	4E-161	NM_001013735.1	FOXB2
SBSPON	25.37661808	0	20	0.004036	High	24	0	XP_003125646.3	ssc:100517890	2E-100	NM_153225.3	SBSPON
CKMT2	25.37661808	0	20	0.004036	High	24	0	NM_001044551.2	ssc:733602	0	NM_001825.2	CKMT2
LOC100622742	33.83549077	499.0721365	-3.88263914	0.004056	Low	32	604	XP_005658868.1	ssc:100622742	8E-48	NM_032525.1	TUBB6
LOC100627808	22.20454082	334.6427406	-3.913695076	0.004059	Low	21	405	XP_005658038.1	ssc:100627808			
GRIN3B	3.17207726	76.84388857	-4.598430711	0.004061	Low	3	93	XP_003354014.1	ssc:100623799	0	NM_138690.1	GRIN3B
LOC100523276	20.08982264	304.8967192	-3.923783893	0.004078	Low	19	369	XP_005654017.1	ssc:100523276	0	NR_024573.1	GHDC
ARHGDIB	1.057359087	48.75042393	-5.52687745	0.004129	Low	1	59	NM_001244240.1	ssc:100155466	1E-100	NM_001175	ARHGDIB
LOC102160403	1.057359087	48.75042393	-5.52687745	0.004129	Low	1	59	XP_005654496.1	ssc:102160403	2E-32	NM_001311.4	CRIP1
LOC100737190	1.057359087	48.75042393	-5.52687745	0.004129	Low	1	59	XP_005672396.1	ssc:100737190	2E-130	NM_022817.2	PER2
HUNK	114.1947813	8.262783717	3.788725007	0.004133	High	108	10	XP_005657200.1	ssc:100739691	8E-100	NM_014586.1	HUNK
MROH1	19.03246356	289.1974301	-3.925520511	0.004144	Low	18	350	XP_005674638.1	ssc:100515480			
LOC100623471	1427.434767	121.4629206	3.554836946	0.004214	High	1350	147	XR_300240.1	ssc:100623471			
SORBS3	762.3559014	12447.88367	-4.029292004	0.004224	Low	721	15065	XP_005670469.1	ssc:100153623	0	NM_005775.4	SORBS3
NYAP1	44.40908164	645.3234083	-3.861095716	0.004232	Low	42	781	XP_005661966.1	ssc:100514396	0	NM_173564.3	NYAP1
OSAP	293.9458261	24.78835115	3.567816076	0.004236	High	278	30	NM_001195363.1	ssc:100359351	6E-85	NM_032623.3	MGARP
ITGB3	154.3744266	2304.490379	-3.899942061	0.004247	Low	146	2789	NM_214002.1	ssc:397063	0	NM_000212.2	ITGB3
LOC100623304	19.03246356	287.5448734	-3.917252895	0.004249	Low	18	348	XP_003361419.2	ssc:100623304	5E-98	NM_022744.3	C16orf58
RFTN1	19.03246356	287.5448734	-3.917252895	0.004249	Low	18	348	XP_005669346.1	ssc:100155924	3E-76	NM_015150.1	RFTN1
SEMA3D	409.1979665	6419.35667	-3.971557841	0.004271	Low	387	7769	XP_005667736.1	ssc:100523915	0	NM_152754.2	SEMA3D
BLM	3691.240571	304.0704408	3.601628285	0.004282	High	3491	368	NM_001123084.1	ssc:100141304	1E-107	NM_001287248.1	BLM
MPST	9.516231779	158.6454474	-4.0592719	0.00431	Low	9	192	XP_001926486.3	ssc:100154490			
MEOX1	9.516231779	158.6454474	-4.0592719	0.00431	Low	9	192	XP_005668827.1	ssc:100516574	7E-88	NM_013999.3	MEOX1
PTPRC	43.35172255	1.652556743	4.713317604	0.004315	High	41	2	XP_003130644.3	ssc:100522631	0	NR_052021.1	PTPRC
LOC100521953	43.35172255	1.652556743	4.713317604	0.004315	High	41	2	LOC100521953				
LOC100037951	0	33.87741324	-20	0.004365	Low	0	41	NM_001097448.1	ssc:100037951			
LOC102157549	0	33.87741324	-20	0.004365	Low	0	41	XR_304924.1	ssc:102157549			
LOC102158116	0	33.87741324	-20	0.004365	Low	0	41	XR_303914.1	ssc:102158116			
LOC100625760	0	33.87741324	-20	0.004365	Low	0	41	XP_003356564.2	ssc:100625760	0	NM_000129.3	F13A1
LOC100739000	148.0302721	11.5678972	3.677693694	0.00439	High	140	14	LOC100739000				
TBC1D10C	12.68830904	199.959366	-3.978135137	0.004393	Low	12	242	XP_005660691.1	ssc:100512518	0	NR_046266.1	TBC1D10C

OTUD1	9.516231779	157.819169	-4.051738227	0.004408	Low	9	191	XP_005674574.1	ssc:100738624			
TMEM102	34.89284986	504.0298068	-3.852505714	0.00443	Low	33	610	XP_005669269.1	ssc:100623827	0	NM_178518.2	TMEM102
PYCARD	1.057359087	47.92414556	-5.502215396	0.004434	Low	1	58	XP_003354571.1	ssc:100522011	3E-72	NM_145183.1	PYCARD
MOV10	614.3256293	9789.746148	-3.994195973	0.00444	Low	581	11848	XP_005655499.1	ssc:100521086	0	NM_020963.4	MOV10
LOC100515348	51.81059524	2.478835115	4.385512943	0.004452	High	49	3	XP_003133934.1	ssc:100515348	2E-56	NM_033022.3	RPS24
LOC102158083	3.17207726	75.19133183	-4.56706654	0.004454	Low	3	91	XP_005656768.1	ssc:102158083			
LOC100624749	254.8235399	3857.067439	-3.91993391	0.004455	Low	241	4668	XP_005659064.1	ssc:100624749	7E-55	NR_103804.1	PDLIM7
ITGA3	372.1903985	5752.550024	-3.950088881	0.004456	Low	352	6962	XP_005668937.1	ssc:100517053	0	NM_005501.2	ITGA3
N4BP2	7045.183594	567.6532414	3.633555489	0.004468	High	6663	687	XP_005666666.1	ssc:100512116	0	NM_018177.4	N4BP2
RRAGD	1838.747452	156.9928906	3.549952208	0.004485	High	1739	190	NM_001243623.1	ssc:100156654	0	NM_021244.4	RRAGD
LOC100737472	391.222862	33.87741324	3.529595055	0.004502	High	370	41	XP_003482505.1	ssc:100737472			
SLC35F6	103.6211905	1502.17408	-3.857661041	0.004517	Low	98	1818	XP_003125375.1	ssc:100513653	0	NM_017877.3	SLC35F6
LOC102164731	51.81059524	741.1716994	-3.838488732	0.004523	Low	49	897	XP_005668251.1	ssc:102164731	5E-53	NM_001191055.1	ERVV-2
NRSN2	217.8159718	18.17812418	3.582834508	0.004558	High	206	22	XP_001928139.1	ssc:100157494	2E-93	NM_024958.2	NRSN2
TAF4B	1883.156533	161.1242825	3.546907087	0.004558	High	1781	195	XP_003127923.1	ssc:100037274	0	NM_005640.1	TAF4B
C3H16orf58	10.57359087	170.2133446	-4.008806833	0.004584	Low	10	206	XP_005662084.1	ssc:102165338	3E-90	NM_022744.3	C16orf58
PKD1	549.826725	8640.392933	-3.974047985	0.004587	Low	520	10457	NM_001246202.1	ssc:100516429	0	NM_001009944.2	PKD1
GCS	2.114718173	61.14459951	-4.853687766	0.004592	Low	2	74	Y17718	ssc:397342	4E-143	NM_173849.2	GSC
GBP6	2.114718173	61.14459951	-4.853687766	0.004592	Low	2	74	XP_005663763.1	ssc:100523310	0	NM_198460.2	GBP6
MMP9	2.114718173	61.14459951	-4.853687766	0.004592	Low	2	74	NM_001038004.1	ssc:654325	0	NM_004994.2	MMP9
LOC102160223	2.114718173	61.14459951	-4.853687766	0.004592	Low	2	74	XP_005662742.1	ssc:102160223	1E-23	NM_175630.1	DNMT3A
LOC102167526	2.114718173	61.14459951	-4.853687766	0.004592	Low	2	74	XR_297123.1	ssc:102167526	1E-41	NM_003566.3	EEA1
DUS4L	4109.95477	341.2529675	3.59020902	0.004597	High	3887	413	XP_003357562.2	ssc:100623288	4E-169	NR_073005.1	DUS4L
LOC102159412	9.516231779	156.1666123	-4.036551824	0.004614	Low	9	189	XP_005666935.1	ssc:102159412	9E-139	NM_024582.4	FAT4
LOC102159410	77.18721332	4.95767023	3.960627657	0.004627	High	73	6	XR_304801.1	ssc:102159410			
tRNA-Thr	389.1081439	33.87741324	3.521775551	0.004631	High	368	41	tRNA-Thr				
IL7	12.68830904	197.4805308	-3.960138708	0.004637	Low	12	239	NM_214135.2	ssc:397253	8E-63	NM_001199888.1	IL7
LOC102163843	33.83549077	0.826278372	5.355765599	0.004673	High	32	1	XR_300466.1	ssc:102163843			
LOC100515458	33.83549077	0.826278372	5.355765599	0.004673	High	32	1	XP_005673467.1	ssc:100515458	0	NM_014728.3	FRMPD4
SLC5A7	137.4566813	10.74161883	3.677693694	0.004681	High	130	13	XP_003124900.1	ssc:100512044	0	NM_021815.2	SLC5A7
RXRG	297.1179033	25.61462952	3.535995609	0.0047	High	281	31	NM_001130213.1	ssc:445465	0	NR_033824.1	RXRG
LOC100520252	52.86795433	749.4344832	-3.825336952	0.00471	Low	50	907	XP_005665214.1	ssc:100520252	9E-58	NM_178865.4	SERINC2
LDB3	9.516231779	155.3403339	-4.028898251	0.00472	Low	9	188	XP_005657484.1	ssc:100151883	0	NM_007078.2	LDB3
CALD1	4163.880083	73879.20177	-4.149167853	0.004758	Low	3938	89412	XP_005673207.1	ssc:100524227	0	NM_033157.3	CALD1
LOC102158175	1.057359087	47.09786719	-5.477124415	0.004767	Low	1	57	XP_005658681.1	ssc:102158175	8E-65	NM_133178.3	PTPRU
CCL19	1.057359087	47.09786719	-5.477124415	0.004767	Low	1	57	NM_001170516.1	ssc:494464	3E-32	NM_006274.2	CCL19
NPEPL1	175.5216084	2566.420623	-3.870037084	0.004783	Low	166	3106	NM_001123148.1	ssc:100144530	0	NM_024663.3	NPEPL1
CXCR4	1392.541917	121.4629206	3.519132885	0.004803	High	1317	147	NM_213773.1	ssc:396659	0	NM_003467.2	CXCR4
CAST	427.173071	6543.298426	-3.937125463	0.00481	Low	404	7919	NM_214067.1	ssc:397135			
LOC100622016	0	33.05113487	-20	0.004827	Low	0	40	XP_003360831.2	ssc:100622016	6E-68	NM_183394.2	CADPS
LOC102166930	0	33.05113487	-20	0.004827	Low	0	40	XR_301654.1	ssc:102166930			
LOC100626667	0	33.05113487	-20	0.004827	Low	0	40	XP_005655752.1	ssc:100626667	2E-39	NM_031491.2	RBP5
AMPH	0	33.05113487	-20	0.004827	Low	0	40	NM_001244203.1	ssc:100626324			
LOC102162837	0	33.05113487	-20	0.004827	Low	0	40	XR_304257.1	ssc:102162837			
MYO18B	0	33.05113487	-20	0.004827	Low	0	40	XP_001924463.4	ssc:100154025	0	NM_032608.5	MYO18B
LOC102167726	0	33.05113487	-20	0.004827	Low	0	40	XP_005654659.1	ssc:102167726	1E-49	NM_152572.2	AK8
RUSC2	273.8560034	4077.683764	-3.896260482	0.004879	Low	259	4935	XP_005660277.1	ssc:100158062	0	NR_052015.1	RUSC2
LOC100737894	19.03246356	278.4558113	-3.870914181	0.004889	Low	18	337	XP_005653002.1	ssc:100737894	3E-50	NM_000943.4	PPIC
LOC100517487	2767.10873	236.3156143	3.549592472	0.004892	High	2617	286	XP_003130303.1	ssc:100517487			
RNF213	810.9944194	12863.50169	-3.987447628	0.004902	Low	767	15568	XP_005668644.1	ssc:100626814	0	NM_020954.3	RNF213
GHDC	60.26946793	846.935331	-3.812752581	0.004913	Low	57	1025	NM_001246239.1	ssc:100523395	0	NR_024573.1	GHDC
LOC102163494	4.229436346	86.75922903	-4.358479918	0.004914	Low	4	105	XP_005658979.1	ssc:102163494	4E-37	NR_109766.1	PAOX
LOC102157411	24.31925899	0	20	0.004921	High	23	0	XP_005653886.1	ssc:102157411			
LOC780430	24.31925899	0	20	0.004921	High	23	0	NM_001078682.1	ssc:780430	5E-114	NM_017594.3	DIRAS2
LRRTM3	24.31925899	0	20	0.004921	High	23	0	XP_001924493.1	ssc:100154787	0	NM_178011.3	LRRTM3
HS1BP3	175.5216084	2549.895055	-3.860717316	0.004923	Low	166	3086	XP_003354961.2	ssc:100625429	3E-164	NM_022460.3	HS1BP3
ZNF618	46.52379981	652.7599137	-3.810511625	0.004933	Low	44	790	ZNF618	ssc:100157179	0	NM_133374.2	ZNF618
AS-A	124.7683722	1783.935005	-3.837738879	0.004949	Low	118	2159	NM_213933.1	ssc:396973	9E-35	NM_001085428.2	ARSA
LOC100738335	6.344154519	113.2001369	-4.157303983	0.004961	Low	6	137	XP_005664917.1	ssc:100738335	6E-141	NM_203374.1	ZNF784
LRP10	512.819157	7880.216831	-3.941713266	0.00498	Low	485	9537	NM_001143715.1	ssc:100233190			
CDKL2	275.9707216	23.96207278	3.525690601	0.004981	High	261	29	XP_005666830.1	ssc:100737771	0	NM_003948.3	CDKL2
RHBDF1	332.0107532	4967.585571	-3.903242946	0.005008	Low	314	6012	XP_005655169.1	ssc:100517883	0	NM_022450.3	RHBDF1
C1QTNF2	87.76080419	1236.112444	-3.816089429	0.00502	Low	83	1496	NM_001142829.1	ssc:100217378	8E-140	NM_031908.4	C1QTNF2
LOC100512370	48.63851798	679.2008216	-3.803667028	0.005033	Low	46	822	XR_309610.1	ssc:100512370			
ZNF362	11.63094995	180.128685	-3.952987107	0.005036	Low	11	218	ZNF362	ssc:100739793	2E-64	NM_152493.2	ZNF362
DNASE1L1	64.49890428	900.6434252	-3.803609483	0.00507	Low	61	1090	NM_001038632.1	ssc:654408	4E-30	NM_006730.2	DNASE1L1
TSPAN2	656.6199928	58.66576439	3.484467938	0.005091	High	621	71	XP_005663612.1	ssc:100156161	5E-114	NM_005725.4	TSPAN2
PPIP5K2	14118.91588	1125.391142	3.649130897	0.005107	High	13353	1362	XP_003354281.3	ssc:100625306	0	NR_074081.1	PPIP5K2
TRIM26	271.7412852	4000.013598	-3.87969923	0.005126	Low	257	4841	NM_001123209.1	ssc:100144460	0	NM_003449.4	TRIM26
TBX4	1.057359087	46.27158882	-5.451589323	0.005129	Low	1	56	NM_001246249.1	ssc:100620126	0	NM_018488.2	TBX4
FAM166B	1.057359087	46.27158882	-5.451589323	0.005129	Low	1	56	NM_001243630.1	ssc:100156839	6E-130	NM_001287239.1	FAM166B
LOC100627548	2.114718173	59.49204276	-4.814159402	0.005151	Low	2	72	XP_003360457.1	ssc:100627548	2E-18	NM_024657.4	MORC4
SH2D4A	9.516231779	152.0352204	-3.997871355	0.005179	Low	9	184	XP_005654286.1	ssc:100518097	1E-124	NM_022071.3	SH2D4A
PUS7	2201.421618	191.6965822	3.521538957	0.005181	High	2082	232	XP_005658694.1	ssc:100627719	2E-159	NM_019042.3	PUS7
CECR2	4694.674344	396.6136184	3.565218965	0.005238	High	4440	480	XP_005674672.1	ssc:100737051	2E-140	NM_031413	CECR2
LOC100624822	2255.346932	196.6542525	3.519616086	0.005246	High	2133	238	XP_003482510.1	ssc:100624822	0	NM_024751.3	GSTCD
LOC102163625	125.8257313	9.915340461	3.665620862	0.005259	High	119	12	XR_300001.1	ssc:102163625			
LRRC1	1735.126261	152.8614988	3.504743662	0.005268	High	1641	185	XP_001927723.2	ssc:100154780	0	NM_025168.1	LRRC1
FAM43A	97.27703596	1359.227921	-3.804544313	0.005271	Low	92	1645	XP_003132648.1	ssc:100523468	0	NM_153690.4	FAM43A
LOC102167780	75.07249515	4.95767023	3.920550218	0.005314	High	71	6	XR_305599.1	ssc:102167780			
MFRP	0	32.2248565	-20	0.005347	Low	0	39	XP_003129989.1	ssc:100520397	0	NM_031433.3	MFRP
LOC100514309	0	32.2248565	-20	0.005347	Low	0	39	XP_005653770.1	ssc:100514309	1E-93	NM_001004462.1	OR10G4
LOC100736857	0	32.2248565	-20	0.005347	Low	0	39	XP_003483252.2	ssc:100736857	2E-119	NM_198178.2	MITF
LOC102158668	0	32.2248565	-20	0.005347	Low	0	39	LOC102158668				
PPAPDC1A	66.61362245	4.131391859	4.011117428	0.005353	High	63	5	XP_001924214.1	ssc:100152806	6E-159	NM_001030059.1	PPAPDC1A
BAIAP2L1	1183.184818	105.7636316	3.48375992	0.005354	High	1119	128	XP_003354495.1	ssc:100521439	3E-73	NM_018842.4	BAIAP2L1
ZFPM2	19.03246356	272.6718627	-3.840631614	0.005356	Low	18	330	NM_001195374.1	ssc:100337657			
LOC100627705	5678.018295	477.5888989	3.57154629	0.005372	High	5370	578	XP_003359943.1	ssc:100627705	7E-61	NM_004102.3	FABP3
MAOB	298.1752624	26.4409079	3.495316952	0.005384	High	282	32	NM_001001864.1	ssc:414909	0	NM_000898.4	MAOB
LOC102157527	3.17207726	71.88621834	-4.502215396	0.005386	Low	3	87	XP_005672099.1	ssc:102157527			
RASGRP3	3.17207726	71.88621834	-4.502215396	0.005386	Low	3	87	XP_003125298.4	ssc:100511446	0	NM_170672.2	RASGRP3
LOC102167620	3.17207726	71.88621834	-4.502215396	0.005386	Low	3	87	XP_005658643.1	ssc:102167620			
UCP2	2796.714784	242.9258413	3.525145261	0.005386	High	2645	294	NM_214289.1	ssc:397549	1E-171	NM_003355.2	UCP2
MICAL2	100.4491132	1398.063005	-3.798892646	0.005388	Low	95	1692	XP_005661181.1	ssc:100519075	0	NM_014632.3	MICAL2
RPS6KA4	124.7683722	1747.578756	-3.808033299	0.005422	Low	118	2115	XP_005660815.1	ssc:100626270	0	NM_003942.2	RPS6KA4
HEY2	9.516231779	150.3826637	-3.98210404	0.005427	Low	9	182	NM_001243329.1	ssc:100152404	5E-166	NM_012259.2	HEY2
PPFIA2	403.9111711	36.35624836	3.473762909	0.005441	High	382	44	XP_003126797.4	ssc:100512281	0	NR_038265.1	PPFIA2
LOC102165667	17.97510447	257.798852	-3.842173778	0.00546	Low	17	312	XP_005671808.1	ssc:102165667	2E-71	NM_021623.1	PLEKHA2
B3GAT3	168.1200948	2381.334267	-3.824206066	0.005461	Low	159	2882	XP_003353876.1	ssc:397642	1E-176	NM_012200.3	B3GAT3
STC1	2.114718173	58.66576439	-4.79398152	0.005461	Low	2	71	NM_001103212.1	ssc:100125345	2E-138	NM_003155.2	STC1

PIEZO1	43.35172255	594.0941493	-3.776530357	0.005483	Low	41	719	XP_005653296.1	ssc:102162867	0	NM_001142864.2	PIEZO1
CTNNA3	41.23700438	1.652556743	4.641167818	0.005498	High	39	2	XP_005671093.1	ssc:100153974	0	NM_013266.2	CTNNA3
TTC39B	19.03246356	271.0193059	-3.831861404	0.005499	Low	18	328	XP_003121951.2	ssc:100515182	0	NM_152574.2	TTC39B
LOC100157320	21.14718173	298.2864922	-3.818161333	0.005509	Low	20	361	XP_005665766.1	ssc:100157320	9E-168	NM_170770.1	RNF39
NPR2	391.222862	5780.643489	-3.885167514	0.005525	Low	370	6996	NM_001244322.1	ssc:100512698	0	NM_003995.3	NPR2
MIOX	1.057359087	45.44531045	-5.425594114	0.005525	Low	1	55	NM_214102.2	ssc:397189	2E-150	NM_017584.5	MIOX
CBLN3	1.057359087	45.44531045	-5.425594114	0.005525	Low	1	55	NM_001112680.1	ssc:100127149	1E-89	NM_001039771.2	CBLN3
LOC102159841	1.057359087	45.44531045	-5.425594114	0.005525	Low	1	55	XP_005662541.1	ssc:102159841	4E-32	NM_012476.2	VAX2
LOC100525621	1.057359087	45.44531045	-5.425594114	0.005525	Low	1	55	LOC100525621	ssc:100525621			
PHC2	1.057359087	45.44531045	-5.425594114	0.005525	Low	1	55	XP_005665263.1	ssc:100622975	0	NM_198040.2	PHC2
AMPD3	17.97510447	256.9725736	-3.83754233	0.005537	Low	17	311	XP_005661192.1	ssc:100620914	0	NM_001172431.1	AMPD3
PLEKHH3	85.64608601	1179.099236	-3.783154017	0.005537	Low	81	1427	XP_005668856.1	ssc:100518749	0	NR_073574.1	PLEKHH3
C1H9orf116	4.229436346	84.28039392	-4.316659743	0.005555	Low	4	102	XP_003353756.1	ssc:100518594	6E-63	NM_144654.2	C9orf116
LOC100515505	4.229436346	84.28039392	-4.316659743	0.005555	Low	4	102	XP_005668821.1	ssc:100515505	0	NM_005374.4	MPP2
HS3ST3A1	8.458872693	136.3359313	-4.010556615	0.005567	Low	8	165	XP_003132059.1	ssc:100521333	0	NM_006042.1	HS3ST3A1
BBC3	42.29436346	577.5685818	-3.771454951	0.005575	Low	40	699	XP_005664684.1	ssc:100517403	3E-74	NM_014417.4	BBC3
CNOT6	7016.634898	588.3102007	3.576130339	0.005586	High	6636	712	XP_003123675.2	ssc:100516183	0	NM_015455.3	CNOT6
SYN1	6.344154519	109.8950234	-4.114554336	0.005629	Low	6	133	NM_001141988.1	ssc:100216310	0	NM_133499.2	SYN1
tRNA-Pro	82.47400875	5.783948602	3.833812896	0.005631	High	78	7	tRNA-Pro				
CENPL	1086.965141	98.32712624	3.466572385	0.005644	High	1028	119	XP_003357589.1	ssc:100623697	0	NM_033319.3	CENPL
LOC100155159	3.17207726	71.05993997	-4.485536655	0.005654	Low	3	86	XP_001926734.4	ssc:100155159	0	NM_032709.2	PYROXD2
DMPK	37.00756803	504.8560851	-3.769979954	0.005656	Low	35	611	XP_005659031.1	ssc:100627008	0	NM_004409	DMPK
NEFL	1234.995413	111.5475802	3.468774561	0.005696	High	1168	135	NM_001244331.1	ssc:100521224	0	NM_006158.4	NEFL
CD200	1112.341759	100.8059614	3.463947251	0.005716	High	1052	122	XP_005670288.1	ssc:100626182	1E-116	NM_005944.5	CD200
HMOX1	653.4479155	9883.115604	-3.918821764	0.005716	Low	618	11961	NM_001004027.1	ssc:445512			
LOC100622843	1803.854602	161.1242825	3.484837217	0.005721	High	1706	195	XP_003361322.1	ssc:100622843	6E-130	NR_104590.1	TEX30
ATAD5	3315.878095	289.1974301	3.519264332	0.005734	High	3136	350	XP_005669120.1	ssc:100522895	0	NM_024857.3	ATAD5
ARHGEF6	175.5216084	2463.962104	-3.811259512	0.005734	Low	166	2982	XP_005657985.1	ssc:100622228	0	NM_004840.2	ARHGEF6
TRAPPC5	228.3895627	3247.274001	-3.829660496	0.005743	Low	216	3930	XP_003123197.1	ssc:100524729	2E-105	NM_174894.2	TRAPPC5
WNT9A	7.401513606	122.289199	-4.046332844	0.005752	Low	7	148	XP_003123659.2	ssc:100512949	0	NM_003395.2	WNT9A
MICALL2	152.2597085	2120.230302	-3.799614854	0.005774	Low	144	2566	XP_005655130.1	ssc:100523596	0	NM_182924.3	MICALL2
NDUFAF4	2671.946412	235.4893359	3.504157439	0.005776	High	2527	285	XP_001925512.1	ssc:100157673	2E-79	NM_014165.3	NDUFAF4
LOC102160753	2.114718173	57.83948602	-4.773517418	0.005793	Low	2	70	XR_309570.1	ssc:102160753			
MRAP2	2.114718173	57.83948602	-4.773517418	0.005793	Low	2	70	XP_003353296.2	ssc:100515980	3E-111	NM_138409.2	MRAP2
KLF6	436.6893028	6422.661783	-3.87849223	0.005806	Low	413	7773	NM_001134353.2	ssc:100174961	1E-139	NR_027653.1	KLF6
LOC100627559	77.18721332	1048.547254	-3.763886196	0.005807	Low	73	1269	LOC100627559		6E-100	NM_016596.3	HDAC7
VIM	27757.79074	533350.2948	-4.264118728	0.005852	Low	26252	645485	VIM		0	NM_003380.3	VIM
PCDH7	330.9533941	4780.02038	-3.852316798	0.005859	Low	313	5785	NM_001244484.1	ssc:100520035	0	NM_032457.3	PCDH7
IL6	23.2618999	320.5960082	-3.784715624	0.00591	Low	22	388	NM_214399.1	ssc:399500	2E-68	NM_000600.3	IL6
CAMK4	89.87552236	6.610226974	3.765156535	0.005914	High	85	8	XP_003123873.2	ssc:100515364	0	NM_001744.4	CAMK4
LOC100736998	0	31.39857813	-20	0.005936	Low	0	38	XP_003482500.1	ssc:100736998	3E-45	NM_025144.3	ALPK1
LOC102164680	0	31.39857813	-20	0.005936	Low	0	38	XR_305540.1	ssc:102164680			
LOC100626991	0	31.39857813	-20	0.005936	Low	0	38	XP_005667732.1	ssc:100626991			
LMX1B	0	31.39857813	-20	0.005936	Low	0	38	NM_001258424.1	ssc:100157562			
LOC100625402	0	31.39857813	-20	0.005936	Low	0	38	XP_003358402.1	ssc:100625402	1E-62	NM_198399.1	ARPP21
LOC102167414	0	31.39857813	-20	0.005936	Low	0	38	XP_005656946.1	ssc:102167414			
WFIKKN2	1.057359087	44.61903207	-5.399121903	0.005958	Low	1	54	XP_005668980.1	ssc:100512012	0	NM_175575.5	WFIKKN2
LOC102158225	1.057359087	44.61903207	-5.399121903	0.005958	Low	1	54	XP_005667764.1	ssc:102158225	2E-102	NM_017439.3	GSAP
MIR214	26.43397716	360.2573701	-3.768562536	0.005999	Low	25	436	NR_038497.1	ssc:100316560			
GLIS2	300.2899806	4285.905914	-3.835171936	0.006029	Low	284	5187	XP_005662237.1	ssc:100624334	0	NM_032575.2	GLIS2
CST3	1002.376414	15387.78212	-3.940289028	0.006034	Low	948	18623	NM_001044602.1	ssc:733672	6E-47	NM_001288614.1	CST3
KLHL6	23.2618999	0	20	0.006035	High	22	0	XP_003358750.2	ssc:100625137	0	NM_130446.2	KLHL6
PTCHD4	23.2618999	0	20	0.006035	High	22	0	XP_001925843.4	ssc:100157414	1E-175	NM_207499.2	PTCHD4
LOC102161637	23.2618999	0	20	0.006035	High	22	0	XR_305387.1	ssc:102161637			
LOC100511763	23.2618999	0	20	0.006035	High	22	0	LOC100511763				
LOC102164021	23.2618999	0	20	0.006035	High	22	0	XR_308287.1	ssc:102164021			
LOC100158105	23.2618999	0	20	0.006035	High	22	0	XP_001926048.1	ssc:100158105	2E-63	NM_007179.2	INSL6
HAO1	23.2618999	0	20	0.006035	High	22	0	NM_001243431.1	ssc:100627803	0	NM_017545.2	HAO1
LRRC18	23.2618999	0	20	0.006035	High	22	0	XP_005671269.1	ssc:100152341	4E-121	NM_001006939.3	LRRC18
SGSH	168.1200948	2326.799895	-3.790783064	0.006052	Low	159	2816	XP_003482989.1	ssc:100737146	0	NM_000199.3	SGSH
LOC100515974	4411.302109	384.2194429	3.521202136	0.00611	High	4172	465	XP_005671952.1	ssc:100515974	0	NM_024753.4	TTC21B
OLFM1	19.03246356	264.409079	-3.796237494	0.006118	Low	18	320	XP_005652767.1	ssc:100620523	0	NM_058199.2	OLFM1
LOC100625482	72.95777697	4.95767023	3.879327555	0.006119	High	69	6	XP_005658672.1	ssc:100625482	2E-57	NM_152666.2	PLD5
RPS6KA2	456.7791254	6651.540892	-3.864119966	0.006144	Low	432	8050	XP_003121145.2	ssc:100515286	0	NM_021135.4	RPS6KA2
UMODL1	138.5140403	11.5678972	3.581833679	0.006145	High	131	14	XP_005670432.1	ssc:100620228	0	NM_173568.3	UMODL1
CDRT1	2.114718173	57.01320765	-4.752758858	0.00615	Low	2	69	XP_005654045.1	ssc:100523091	2E-146	NR_051988.1	FBXW10
GDNF	33.83549077	453.6268261	-3.74489674	0.006163	Low	32	549	XP_005672481.1	ssc:100322894	2E-73	NM_199234.1	GDNF
LOC100525679	324.6092396	29.74602138	3.447935443	0.006184	High	307	36	XP_003122846.1	ssc:100525679	4E-23	NM_001000.3	RPL39
WDR66	255.880899	23.13579441	3.467273914	0.006201	High	242	28	XP_003132921.2	ssc:100524906	0	NM_144668.5	WDR66
YARS2	1690.717179	153.6877771	3.459561012	0.006215	High	1599	186	YARS2	ssc:100624597	0	NM_015936.1	YARS2
RYR3	3.17207726	69.40738323	-4.451589323	0.006239	Low	3	84	XP_003361898.1	ssc:396888	9E-99	NM_001243996.1	RYR3
LOC100519123	33.83549077	451.9742693	-3.739631424	0.006262	Low	32	547	XP_003122102.3	ssc:100519123	0	NM_005502.3	ABCA1
RHBDL1	77.18721332	1030.36913	-3.738655592	0.00627	Low	73	1247	XP_005662298.1	ssc:102164926	0	np_003952	np_003952
MEIS2	19.03246356	262.7565222	-3.787192355	0.006286	Low	18	318	XP_005659823.1	ssc:100512756	5E-179	NR_051953.1	MEIS2
TAF5	2025.90001	183.4337985	3.465231579	0.006305	High	1916	222	XP_001929370.2	ssc:100152210	0	NM_006951.3	TAF5
RPF1	14114.68645	1168.357618	3.594643245	0.006324	High	13349	1414	XP_003361191.1	ssc:100620426	2E-166	NM_025065.6	RPF1
LOC102166581	31.7207726	0.826278372	5.262656195	0.006344	High	30	1	XP_005655044.1	ssc:102166581	9E-165	NR_003149.1	GPR98
LOC100739369	611.153552	57.01320765	3.422166825	0.006366	High	578	69	XP_003480987.2	ssc:100739369	0	NM_020957.2	PCDHB16
SR-PSOX	133.2272449	1803.765685	-3.759050892	0.006374	Low	126	2183	NM_213811.1	ssc:396735	8E-62	NM_022059.3	CXCL16
PPP1R13B	17.97510447	248.7097899	-3.790391236	0.006375	Low	17	301	XP_005653591.1	ssc:100521849	0	NM_015316.2	PPP1R13B
CCDC73	530.7942615	49.5767023	3.420418558	0.006414	High	502	60	XP_003480760.2	ssc:100516315	0	NM_001008391.3	CCDC73
TAGAP	1.057359087	43.7927537	-5.372154855	0.006432	Low	1	53	NM_001243543.1	ssc:100155114	0	NM_152133.2	TAGAP
LOC102160827	1.057359087	43.7927537	-5.372154855	0.006432	Low	1	53	XP_005671031.1	ssc:102160827	0	NM_018662.2	DISC1
TMEM81	56.04003159	3.305113487	4.083686054	0.006473	High	53	4	XP_003357451.2	ssc:100626729	6E-69	NM_203376.1	TMEM81
SYCP3	161.7759402	14.04673232	3.525690601	0.006474	High	153	17	XP_005664257.1	ssc:100523378	4E-114	NM_153694.4	SYCP3
LOC100156943	120.5389359	9.915340461	3.603693113	0.006522	High	114	12	XP_005671346.1	ssc:100156943	2E-176	NR_019053.4	EXOC6
SOAT1	12542.39348	1050.19981	3.578076941	0.006522	High	11862	1271	XP_003357604.2	ssc:100628166	0	NR_045530.1	SOAT1
TUBA8	3.17207726	68.58110485	-4.434311331	0.006558	Low	3	83	XP_005664192.1	ssc:100518253	0	NM_018943.2	TUBA8
ARSJ	50.75323616	666.806646	-3.715696763	0.006571	Low	48	807	XP_003129285.2	ssc:100512548	0	NM_024590.3	ARSJ
ITPKB	4.229436346	80.97528043	-4.258944245	0.006573	Low	4	98	XP_005667996.1	ssc:102164283	4E-48	NM_002221.3	ITPKB
GABRA1	71.90041789	4.95767023	3.85826594	0.006574	High	68	6	XP_005672614.1	ssc:100516706	0	NM_001127648.1	GABRA1
MESP2	27.49133625	366.0413187	-3.734957571	0.006578	Low	26	443	XP_005653586.1	ssc:100153433	7E-118	NM_001039958.1	MESP2
LOC102161478	5.286795433	93.36945601	-4.142485268	0.006593	Low	5	113	XR_305386.1	ssc:102161478			
RTKN2	606.9241157	57.01320765	3.412148069	0.006597	High	574	69	XP_005671080.1	ssc:100157770	7E-157	NM_145307.3	RTKN2
LOC100624161	0	30.57229975	-20	0.006604	Low	0	37	NM_001286804.1	ssc:100624161	7E-31	NM_015714.3	G0S2
XPNPEP2	0	30.57229975	-20	0.006604	Low	0	37	NM_001004048.1	ssc:445538	0	NM_003399.5	XPNPEP2
INHBB	0	30.57229975	-20	0.006604	Low	0	37	NM_001164842.1	ssc:397490			
LOC102158359	0	30.57229975	-20	0.006604	Low	0	37	XP_005656512.1	ssc:102158359			
MIR145	0	30.57229975	-20	0.006604	Low	0	37	NR_038484.1	ssc:100316550			
LOC100620819	337.2975486	31.39857813	3.4252507	0.006625	High	319	38	XP_005658436.1	ssc:100620819	3E-22	NM_001134664.1	SAMD13

NPM2	169.1774539	14.87301069	3.507768693	0.006643	High	160	18	NM_001195362.1	ssc:100154488	4E-59	NM_182795.1	NPM2
LOC100736869	817.3385739	76.84388857	3.410931392	0.00672	High	773	93	XR_302263.1	ssc:100736869	2E-37	NM_194320.2	ZNF169
GPR126	161.7759402	2179.722345	-3.752075407	0.00676	Low	153	2638	XP_003121222.2	ssc:100511292	0	NM_198569.2	GPR126
C5H12orf35	12281.22579	1036.979357	3.565995484	0.006796	High	11615	1255	NM_001243821.1	ssc:100511327	0	NM_018169.3	KIAA1551
ART5	63.44154519	4.131391859	3.9407281	0.006798	High	60	5	HighI0001C9836A		1E-109	NM_053017.3	ART5
ABCD1	128.9978086	1718.659013	-3.735864876	0.006802	Low	122	2080	XP_003135521.2	ssc:100519529	0	NM_000033.3	ABCD1
PPAP2B	287.6016715	3982.661752	-3.791588991	0.006822	Low	272	4820	XP_005665445.1	ssc:100512419	1E-178	NM_177414.1	PPAP2B
C7H15orf60	2302.928091	210.7009848	3.450200401	0.006857	High	2178	255	XR_298777.1	ssc:100154080			
FNDC5	4.229436346	80.14900206	-4.244147243	0.006862	Low	4	97	XP_005665249.1	ssc:100622587	4E-77	NM_153756.2	FNDC5
STRIP2	411.3126847	38.83508347	3.404803093	0.006895	High	389	47	XP_005657814.1	ssc:100517565	0	NM_020704.2	STRIP2
HRC	10.57359087	154.5140555	-3.869200766	0.006942	Low	10	187	XP_003127335.2	ssc:100525465	1E-162	NM_002152.2	HRC
EFHD1	2.114718173	55.36065091	-4.710323591	0.006945	Low	2	67	XP_001925420.4	ssc:100153398	1E-92	NM_025202.3	EFHD1
LOC102162112	1.057359087	42.96647533	-5.344674119	0.006952	Low	1	52	XR_303844.1	ssc:102162112			
NHigh54	10013.19055	860.155785	3.541159956	0.007062	High	9470	1041	XP_003129166.3	ssc:100512118	0	NR_103782.1	NHigh54
G2E3	3876.278411	348.6894729	3.474657481	0.007067	High	3666	422	XP_005656363.1	ssc:100153402	0	NM_017769.3	G2E3
LOC102162556	54.9826725	3.305113487	4.056205317	0.007097	High	52	4	XR_299180.1	ssc:102162556			
CCDC114	22.20454082	294.1551003	-3.727650409	0.007108	Low	21	356	XP_005664716.1	ssc:100622853	0	NM_144577.3	CCDC114
LOC100622845	118.4242177	9.915340461	3.578158021	0.007122	High	112	12	XP_005658628.1	ssc:100622845	3E-90	NM_014988	LIMCH1
SKAP2	27.49133625	358.6048133	-3.705345915	0.007187	Low	26	434	XP_005673360.1	ssc:100520346	5E-123	NM_003930.3	SKAP2
TIMP1	687.2834063	9904.598842	-3.849121511	0.007196	Low	650	11987	NM_213857.1	ssc:396862	6E-104	NM_003254.2	TIMP1
LOC100037967	1606.128453	150.3826637	3.416877114	0.007222	High	1519	182	XP_003359834.1	ssc:100037967	0	NM_006203.4	PDE4D
LOC100628067	56.04003159	720.5147401	-3.684498271	0.007231	Low	53	872	LOC100628067				
ADCY4	3.17207726	66.92854811	-4.399121903	0.007258	Low	3	81	XP_001927591.3	ssc:100152067	0	NM_139247.3	ADCY4
HSP70.2	750.7249515	10857.2978	-3.854236842	0.00726	Low	710	13140	NM_213766.1	ssc:396648			
EPHA2	340.4696259	4693.261151	-3.784992737	0.007262	Low	322	5680	XP_005665094.1	ssc:100522873	0	NM_004431.3	EPHA2
UBASH3B	2914.081643	266.8879141	3.448735417	0.007275	High	2756	323	XP_005667496.1	ssc:100521108	0	NM_032873.4	UBASH3B
DAPP1	223.1027673	20.65695929	3.433008598	0.007295	High	211	25	XP_005667023.1	ssc:100525936	3E-117	NM_014395.2	DAPP1
KCNN4	0	29.74602138	-20	0.007363	Low	0	36	NM_214018.2	ssc:397081	0	NM_002250.2	KCNN4
LOC100152800	0	29.74602138	-20	0.007363	Low	0	36	XP_005674266.1	ssc:100152800	1E-101	NM_006192.4	PAX1
LOC102160261	0	29.74602138	-20	0.007363	Low	0	36	XR_302345.1	ssc:102160261			
LOC102165740	0	29.74602138	-20	0.007363	Low	0	36	XR_306715.1	ssc:102165740			
UNCX	0	29.74602138	-20	0.007363	Low	0	36	XP_003124306.1	ssc:100523430	9E-115	NM_001080461.1	UNCX
CSMD2	0	29.74602138	-20	0.007363	Low	0	36	XP_005656143.1	ssc:100736699	0	NM_052896.4	CSMD2
LOC102159364	30.66341351	0.826278372	5.213746594	0.00743	High	29	1	XR_305572.1	ssc:102159364			
GDF3	22.20454082	0	20	0.00745	High	21	0	XP_003126560.2	ssc:100510963	9E-173	NM_020634.1	GDF3
LOC100626482	22.20454082	0	20	0.00745	High	21	0	XR_131024.3	ssc:100626482			
LOC100519009	22.20454082	0	20	0.00745	High	21	0	XP_005660942.1	ssc:100519009	4E-138	NM_001005469.1	OR5B3
LOC100620123	4.229436346	78.49644531	-4.214090009	0.007484	Low	4	95	XP_003357074.1	ssc:100620123	0	NM_032961.1	PCDH10
LOC102161798	514.9338752	49.5767023	3.376652966	0.007491	High	487	60	XP_005668027.1	ssc:102161798	9E-67	NM_022366.2	TFB2M
LOC100626354	1.057359087	42.14019696	-5.316659743	0.007522	Low	1	51	XP_005673127.1	ssc:100626354	0	NM_001794.3	CDH4
LOC100520855	1.057359087	42.14019696	-5.316659743	0.007522	Low	1	51	XR_299041.1	ssc:100520855			
LOC102166568	1.057359087	42.14019696	-5.316659743	0.007522	Low	1	51	XR_308991.1	ssc:102166568			
RAVER2	841.6578329	80.97528043	3.377680376	0.007601	High	796	98	XP_003356487.2	ssc:100623447	0	NM_018716.1	RAVER2
PROCR	21.14718173	276.8032545	-3.710323591	0.007602	Low	20	335	NM_001163406.1	ssc:654289	9E-94	NM_006404.4	PROCR
KLF2	10.57359087	151.208942	-3.838006144	0.007609	Low	10	183	NM_001134351.2	ssc:100174959			
RNF144B	6118.937034	546.1700037	3.485859066	0.007629	High	5787	661	XP_005665667.1	ssc:100155665	1E-169	NM_182757.3	RNF144B
PEX11G	9.516231779	138.8147665	-3.866626822	0.00763	Low	9	168	XP_005654798.1	ssc:100511182	1E-101	NM_080662.3	PEX11G
ACER3	4772.918917	431.31731	3.468050306	0.007645	High	4514	522	XP_005667218.1	ssc:100524160	6E-97	NM_018367.5	ACER3
LOC100523056	259.0529762	3474.500553	-3.745486547	0.007659	Low	245	4205	XR_302076.1	ssc:100523056			
AFAP1L1	5.286795433	90.06434252	-4.090490631	0.007668	Low	5	109	NM_001244817.1	ssc:100513463	0	NM_152406.2	AFAP1L1
LOC100625094	93.04759962	7.436505346	3.645272216	0.007681	High	88	9	XR_300137.1	ssc:100625094			
LOC100623777	289.7163897	3903.339028	-3.751995567	0.007716	Low	274	4724	XP_003354931.2	ssc:100623777	2E-84	NM_005253.3	FOSL2
LOC100154430	630.1860156	8889.929001	-3.818322252	0.007725	Low	596	10759	XP_005669581.1	ssc:100154430	8E-45	xp_003120356	xp_003120356
CNNM1	4980.161298	450.3217126	3.467164524	0.007757	High	4710	545	XP_005671437.1	ssc:100154357	2E-24	NM_020348.2	CNNM1
IFIT2	341.526985	4632.116552	-3.761600075	0.007808	Low	323	5606	XP_005671321.1	ssc:100155467	0	NM_001547.4	IFIT2
CCNE2	1277.289777	122.289199	3.384716973	0.007818	High	1208	148	NM_001243931.1	ssc:100512048	0	NM_057749.2	CCNE2
LOC100739584	4.229436346	77.67016694	-4.198823252	0.007821	Low	4	94	XR_306259.1	ssc:100739584	4E-105	NM_015843.1	LMO7
TFAM	4473.686295	407.3552373	3.457104733	0.007838	High	4231	493	NM_001130211.1	ssc:397279			
MITF	2.114718173	53.70809416	-4.666602214	0.007865	Low	2	65	NM_001038001.1	ssc:414902			
PTK7	2569.38258	39515.93685	-3.942940984	0.007881	Low	2430	47824	XP_005666076.1	ssc:100153873	0	NR_072998.1	PTK7
LINGO1	123.7110131	10.74161883	3.525690601	0.007935	High	117	13	XP_005666192.1	ssc:100155465	0	NM_032808.5	LINGO1
PPIF	2409.721358	226.4002739	3.411918726	0.008	High	2279	274	XP_001929518.1	ssc:100152612	9E-101	NM_005729.3	PPIF
LOC100737177	159.6612221	2069.001043	-3.695848508	0.008	Low	151	2504	XP_005653161.1	ssc:100737177	0	NM_000262.2	NAGA
DBH	38.06492712	1.652556743	4.525690601	0.008041	High	36	2	XP_001927246.3	ssc:733609	0	NM_000787.3	DBH
ASB4	38.06492712	1.652556743	4.525690601	0.008041	High	36	2	XP_003130204.2	ssc:100523325	0	NM_145872.2	ASB4
CCDC146	38.06492712	1.652556743	4.525690601	0.008041	High	36	2	XP_003482737.1	ssc:100737427	0	NM_020879.2	CCDC146
LOC100153710	38.06492712	1.652556743	4.525690601	0.008041	High	36	2	LOC100153710				
LOC102161288	432.4598664	5902.932688	-3.770793733	0.008071	Low	409	7144	XR_306392.1	ssc:102161288			
LOC100519871	131.1125267	11.5678972	3.502606988	0.008077	High	124	14	XP_003132737.1	ssc:100519871	3E-59	NM_005175.2	ATP5G1
TRIM3	171.292172	2218.557428	-3.695090771	0.008135	Low	162	2685	XP_003482568.1	ssc:100739484	0	NM_033278.3	TRIM3
LOC102167735	115.2521404	9.915340461	3.538987423	0.008143	High	109	12	XR_305104.1	ssc:102167735			
HSD17B13	1.057359087	41.31391859	-5.288090591	0.008148	Low	1	50	NM_001243622.1	ssc:100626886	9E-149	NM_178135.4	HSD17B13
TRPV4	1.057359087	41.31391859	-5.288090591	0.008148	Low	1	50	NM_001130729.1	ssc:100126845	0	NM_147204.2	TRPV4
LOC102159125	0	28.91974301	-20	0.008229	Low	0	35	XR_304469.1	ssc:102159125			
ZNF536	0	28.91974301	-20	0.008229	Low	0	35	ZNF536	ssc:100738519	0	NM_014717.1	ZNF536
CCL8	0	28.91974301	-20	0.008229	Low	0	35	NM_001164515.1	ssc:100302703	4E-31	NM_005623.2	CCL8
LOC102163138	0	28.91974301	-20	0.008229	Low	0	35	XP_005662123.1	ssc:102163138	9E-94	NM_001012981.4	ZKSCAN2
MMP15	44.40908164	553.6065091	-3.639934263	0.008265	Low	42	670	XP_003127010.4	ssc:100514105	0	NM_002428.2	MMP15
LOC102159034	5.286795433	88.41178578	-4.063773292	0.008283	Low	5	107	XP_005660990.1	ssc:102159034	4E-98	NM_032645.4	RAPSN
FMO5	5.286795433	88.41178578	-4.063773292	0.008283	Low	5	107	XP_005653132.1	ssc:100152950	0	NM_001461.3	FMO5
ATG9B	487.4425389	47.92414556	3.346407545	0.008342	High	461	58	NM_001190280.1	ssc:100462720	0	NR_073169.1	ATG9B
LOC100621534	45.46644072	2.478835115	4.197067853	0.008364	High	43	3	XR_302292.1	ssc:100621534	1E-15	NM_014930.1	ZNF510
NRGN	45.46644072	2.478835115	4.197067853	0.008364	High	43	3	XP_003482685.1	ssc:100738830			
LOC102165005	45.46644072	2.478835115	4.197067853	0.008364	High	43	3	LOC102165005				
LOC100623841	3516.776322	328.0325136	3.422342855	0.008413	High	3326	397	XP_005674239.1	ssc:100623841	4E-122	NM_014878.4	KIAA0020
PNPLA2	302.4046988	3995.055927	-3.723663238	0.008502	Low	286	4835	NM_001098605.1	ssc:100049704	0	NM_020376.3	PNPLA2
CDKN2B	64.49890428	799.8374638	-3.6323603	0.008514	Low	61	968	NM_214124.1	ssc:397227	4E-59	NM_078487.2	CDKN2B
LRRN3	435.6319437	42.96647533	3.341826408	0.008526	High	412	52	XP_003134818.1	ssc:100520161	0	NM_018334.4	LRRN3
LOC102164131	280.2001579	27.26718627	3.361220029	0.008555	High	265	33	XR_307100.1	ssc:102164131			
LOC100739477	2313.501681	220.6163253	3.39046669	0.008591	High	2188	267	XR_302512.1	ssc:100739477	6E-50	NM_001281494.1	MSH6
PLOD1	1172.611227	16785.01884	-3.839377477	0.008632	Low	1109	20314	XP_003127625.1	ssc:100525583	0	NM_000302.3	PLOD1
LOC100626165	1148.291968	112.3738586	3.353111146	0.008635	High	1086	136	XP_003361992.1	ssc:100626165	1E-75	NM_001757.3	CBR1
LAMB2	763.4132605	10629.22497	-3.799431065	0.008637	Low	722	12864	XP_005669592.1	ssc:101101688	2E-125	NM_002292.3	LAMB2
WASF1	956.9099733	94.19573438	3.344649567	0.008686	High	905	114	XP_003121401.2	ssc:100154562	0	NM_003931.2	WASF1
DDX18	28491.59795	2406.948897	3.565259121	0.008708	High	26946	2913	XP_005654221.1	ssc:100153560	0	NM_006773.3	DDX18
tRNA-Lys	12005.25507	1060.941429	3.500249138	0.008709	High	11354	1284	tRNA-Lys				
CSF3R	29.60605442	0.826278372	5.163120521	0.008734	High	28	1	XP_003361917.1	ssc:100622336	0	NM_172313.2	CSF3R
LOC100738517	29.60605442	0.826278372	5.163120521	0.008734	High	28	1	LOC100738517				
LOC102166078	29.60605442	0.826278372	5.163120521	0.008734	High	28	1	XR_302444.1	ssc:102166078			
PTPRM	12.68830904	170.2133446	-3.745772427	0.008756	Low	12	206	XP_005665347.1	ssc:102160512	3E-57	NM_002845.3	PTPRM

TMEM26	8.458872693	122.289199	-3.853687766	0.008817	Low	8	148	NM_001244582.1	ssc:100157089	6E-172	NM_178505.6	TMEM26
LOC102159522	1.057359087	40.48764021	-5.258944245	0.008838	Low	1	49	XR_301182.1	ssc:102159522			
LOC102166786	1.057359087	40.48764021	-5.258944245	0.008838	Low	1	49	XR_298591.1	ssc:102166786			
MYH2	1.057359087	40.48764021	-5.258944245	0.008838	Low	1	49	NM_214136.1	ssc:397256	0	NM_017534.5	MYH2
GSDMD	238.9631536	3087.802275	-3.691720287	0.00885	Low	226	3737	XP_005653149.1	ssc:100515607	2E-168	NM_024736.6	GSDMD
FLRT2	425.0583528	5674.053579	-3.738646958	0.008864	Low	402	6867	XP_005656501.1	ssc:100151812	0	NM_013231.4	FLRT2
IFFO2	23.2618999	291.6762652	-3.648327155	0.008867	Low	22	353	XP_005656079.1	ssc:100737107	0	NM_001136265.1	IFFO2
DUOX2	75.07249515	5.783948602	3.698157797	0.008921	High	71	7	NM_213999.2	ssc:397060	0	NM_014080.4	DUOX2
LOC102164273	19.03246356	241.2732845	-3.664133958	0.009049	Low	18	292	XP_005664904.1	ssc:102164273	2E-53	NM_001142864.2	PIEZO1
NMB	151.2023494	14.04673232	3.428174095	0.009061	High	143	17	NM_001123145.1	ssc:100141313	2E-18	NM_205858.1	NMB
PKDCC	402.853812	5333.62689	-3.726788593	0.009062	Low	381	6455	XP_005662648.1	ssc:100516968	0	NM_138370.2	PKDCC
NHSL1	2818.919325	269.3667492	3.38749851	0.009091	High	2666	326	XP_005659234.1	ssc:100156257	0	NM_020464.1	NHSL1
TRIM47	187.1525583	2372.245205	-3.663966466	0.009109	Low	177	2871	XP_003131238.1	ssc:100520974	0	NM_033452.2	TRIM47
APBB1IP	72.95777697	892.3806415	-3.612525541	0.009122	Low	69	1080	XP_003130799.3	ssc:100514179	0	NM_019043.3	APBB1IP
MPP7	158.603863	14.87301069	3.414659288	0.009158	High	150	18	XP_003130810.1	ssc:100516752	0	NM_173496.3	MPP7
ETNPPL	37.00756803	1.652556743	4.485048616	0.009172	High	35	2	XP_005667008.1	ssc:100519324	2E-175	NR_027475.1	ETNPPL
LOC100624854	185.0378402	2339.19407	-3.660119323	0.009193	Low	175	2831	XP_003356402.2	ssc:100624854	0	NM_002845.3	PTPRM
FHL2	584.7195749	7891.784728	-3.754534808	0.009202	Low	553	9551	XP_005662407.1	ssc:100513532	4E-120	NM_201557.3	FHL2
LOC100516062	0	28.09346464	-20	0.009218	Low	0	34	LOC100516062				
LOC102162702	0	28.09346464	-20	0.009218	Low	0	34	XP_005673125.1	ssc:102162702	5E-116	NM_014258.2	SYCP2
ZNF215	0	28.09346464	-20	0.009218	Low	0	34	ZNF215	ssc:100738243	3E-103	NM_013250.2	ZNF215
SPP1	114.1947813	1412.109737	-3.62828358	0.009223	Low	108	1709	NM_214023.1	ssc:397087	2E-96	NM_001251830.1	SPP1
LOC100620998	44.40908164	539.5597767	-3.60285616	0.009229	Low	42	653	LOC100620998				
LOC102161687	21.14718173	0	20	0.009259	High	20	0	XR_301223.1	ssc:102161687			
LOC102157412	21.14718173	0	20	0.009259	High	20	0	XP_005664273.1	ssc:102157412	5E-104	NM_181670.3	ANKS1B
LOC102163618	21.14718173	0	20	0.009259	High	20	0	XR_305016.1	ssc:102163618			
MYO3A	21.14718173	0	20	0.009259	High	20	0	XP_003130805.3	ssc:100515334	0	NM_017433.4	MYO3A
SHE	21.14718173	0	20	0.009259	High	20	0	XP_001929477.1	ssc:100152691	0	NM_001010846.2	SHE
GP91-PHOX	21.14718173	0	20	0.009259	High	20	0	NM_214043.1	ssc:397108	0	NM_000397.3	CYBB
LOC102167163	21.14718173	0	20	0.009259	High	20	0	XR_308072.1	ssc:102167163			
LOC100620631	21.14718173	0	20	0.009259	High	20	0	XR_309688.1	ssc:100620631			
ITGB4	102.5638314	1260.900795	-3.619860805	0.009302	Low	97	1526	XP_003131243.2	ssc:100521988	0	NM_001005731.1	ITGB4
CHPF2	510.7044388	6815.14401	-3.738183639	0.009323	Low	483	8248	XP_005657748.1	ssc:100517065	0	NM_019015.2	CHPF2
RHOB	138.5140403	1721.964127	-3.635950962	0.009325	Low	131	2084	NM_001123189.1	ssc:100144503	1E-113	NM_004040.2	RHOB
GPAT	8533.945188	777.5279478	3.456246406	0.009363	High	8071	941	XP_005671520.1	ssc:397629	0	NM_020918.5	GPAM
STAT2	255.880899	3272.062352	-3.676655878	0.009415	Low	242	3960	NM_213889.1	ssc:396923	0	NM_198332.1	STAT2
LOC102163118	51.81059524	3.305113487	3.970475443	0.009426	High	49	4	XR_299182.1	ssc:102163118			
LOC100738582	3.17207726	62.79715625	-4.307199413	0.009432	Low	3	76	XR_307370.1	ssc:100738582			
THBD	3.17207726	62.79715625	-4.307199413	0.009432	Low	3	76	NM_001130732.1	ssc:100157642	0	NM_000361.2	THBD
LOC100153054	3.17207726	62.79715625	-4.307199413	0.009432	Low	3	76	XR_301385.1	ssc:100153054			
LOC102157994	59.21210885	4.131391859	3.841192426	0.009475	High	56	5	XR_298358.1	ssc:102157994			
LOC102160252	59.21210885	4.131391859	3.841192426	0.009475	High	56	5	XR_307479.1	ssc:102160252			
LOC102163264	59.21210885	4.131391859	3.841192426	0.009475	High	56	5	XP_005652457.1	ssc:102163264	6E-48	NM_002207.2	ITGA9
ERAP1	322.4945214	4171.05322	-3.693065163	0.009482	Low	305	5048	XP_005661611.1	ssc:100523953	0	NM_016442.3	ERAP1
NPAS1	1289.978086	128.0731476	3.332306626	0.009491	High	1220	155	XP_003127301.2	ssc:100517221	0	NM_002517.2	NPAS1
GJA5	2.114718173	51.22925905	-4.598430711	0.009532	Low	2	62	XP_005653125.1	ssc:100157795	0	NM_181703.3	GJA5
LOC102165035	2.114718173	51.22925905	-4.598430711	0.009532	Low	2	62	XP_005657654.1	ssc:102165035	5E-83	NM_014808.3	FARP2
SLC18B1	2329.362068	226.4002739	3.362987296	0.009533	High	2203	274	XP_005652731.1	ssc:100155211	0	NM_052831.2	SLC18B1
LOC102159592	1.057359087	39.66136184	-5.229196901	0.009597	Low	1	48	XP_005669324.1	ssc:102159592	3E-92	NM_001102608.1	COL6A6
FBXO41	498.0161298	50.40298068	3.304611511	0.009642	High	471	61	XP_003125058.1	ssc:100517089	0	NM_001080410.2	FBXO41
DARS2	3160.44631	304.0704408	3.377650825	0.009696	High	2989	368	XP_005656786.1	ssc:100624574	0	NM_018122.4	DARS2
LOC100625965	5.286795433	85.10667229	-4.008806833	0.009697	Low	5	103	XR_302183.1	ssc:100625965			
HLF	103.6211905	9.089062089	3.511043825	0.009725	High	98	11	XP_003483076.1	ssc:100736723	3E-79	NM_002126.4	HLF
LOC100620831	19.03246356	237.1418927	-3.639216326	0.009734	Low	18	287	XP_005653414.1	ssc:100620831	2E-127	NM_198317.2	KLHL17
LOC100627685	70.8430588	852.7192796	-3.589372546	0.009749	Low	67	1032	XP_005655915.1	ssc:100627685	0	NM_003573.2	LTBP4
NUS1	24657.6139	2146.67121	3.521860052	0.009875	High	23320	2598	XP_001928107.4	ssc:100154658	8E-153	NM_138459.3	NUS1
TRIB3	284.4295943	3618.27299	-3.669157773	0.009881	Low	269	4379	XP_003134414.1	ssc:100518810	2E-154	NM_021158.3	TRIB3
GPX3	70.8430588	849.4141661	-3.58376976	0.009913	Low	67	1028	NM_001115155.1	ssc:396598	6E-108	NM_002084.3	GPX3
PPP1R1B	24.31925899	295.8076571	-3.604488222	0.009966	Low	23	358	XP_005653983.1	ssc:100736966	2E-69	NM_181505.3	PPP1R1B
LOC100516621	914.6156099	92.54317763	3.304967	0.009969	High	865	112	XP_005656332.1	ssc:100516621	3E-55	NM_138573.3	NRG4
LOC100624264	6.344154519	95.84829112	-3.917252895	0.009986	Low	6	116	XP_003361331.2	ssc:100624264			
B3GNTL1	90.93288145	1093.992564	-3.588657053	0.01004	Low	86	1324	XP_005656939.1	ssc:100511953	0	NM_001009905.1	B3GNTL1
ZNF793	8.458872693	118.1578072	-3.804105738	0.010173	Low	8	143	ZNF793	ssc:100511696	0	NM_001013659.2	ZNF793
LIMCH1	2326.18999	228.879109	3.345311242	0.010177	High	2200	277	XP_005666676.1	ssc:100515040	0	NM_014988	LIMCH1
CEACAM1	2.114718173	50.40298068	-4.574971738	0.010179	Low	2	61	XP_005655946.1	ssc:100512285	2E-115	NM_001712.4	CEACAM1
LOC100513188	2.114718173	50.40298068	-4.574971738	0.010179	Low	2	61	XR_308963.1	ssc:100513188			
FBLIM1	541.3678523	7099.38377	-3.713012679	0.010226	Low	512	8592	XP_005656078.1	ssc:100521094	8E-150	NM_017556.2	FBLIM1
LOC102167091	340.4696259	34.70369161	3.294365055	0.010283	High	322	42	XR_300336.1	ssc:102167091			
LOC102167744	6.344154519	95.02201275	-3.904761951	0.010349	Low	6	115	XP_005659025.1	ssc:102167744	5E-20	NM_198086	AJUBA
LOC102161240	0	27.26718627	-20	0.010351	Low	0	33	XP_005658006.1	ssc:102161240			
TM4SF18	0	27.26718627	-20	0.010351	Low	0	33	XP_003132527.1	ssc:100522214	2E-101	NM_138786.3	TM4SF18
LOC100522814	0	27.26718627	-20	0.010351	Low	0	33	XP_005656616.1	ssc:100522814	7E-107	NM_031305.2	ARHGAP24
LOC404701	0	27.26718627	-20	0.010351	Low	0	33	XP_001925654.1	ssc:404701	0	NM_152860.1	SP7
LOC102158724	0	27.26718627	-20	0.010351	Low	0	33	XR_304233.1	ssc:102158724			
FAM26F	0	27.26718627	-20	0.010351	Low	0	33	NM_001258434.1	ssc:100158219			
LOC100737008	0	27.26718627	-20	0.010351	Low	0	33	XP_005661612.1	ssc:100737008	3E-108	NM_022350.3	ERAP2
LOC100620373	0	27.26718627	-20	0.010351	Low	0	33	XP_005664906.1	ssc:100620373	1E-95	NM_016535.3	ZNF581
GPRASP1	882.8948373	90.06434252	3.293213662	0.010355	High	835	109	XP_005657934.1	ssc:100523039	0	NM_014710.4	GPRASP1
LOC102161569	50.75323616	3.305113487	3.9407281	0.010388	High	48	4	XR_301805.1	ssc:102161569			
KCTD7	61.32682702	725.4724104	-3.564330535	0.010395	Low	58	878	XP_005662070.1	ssc:100622993	4E-169	NM_153033.4	KCTD7
LOC100738277	159.6612221	1947.538122	-3.608565705	0.010409	Low	151	2357	XP_005661995.1	ssc:100738277	2E-137	NM_032831.3	ORAI2
CCND3	84.58872693	1006.407057	-3.572604724	0.010437	Low	80	1218	NM_001078678.1	ssc:780426	7E-61	NM_001760.4	CCND3
LOC100517092	154.3744266	14.87301069	3.375665157	0.010455	High	146	18	XR_303452.1	ssc:100517092	2E-55	NM_198320.3	CPM
LOC100521440	159.6612221	1945.059287	-3.606728266	0.010466	Low	151	2354	XP_005662009.1	ssc:100521440	2E-137	NM_032831.3	ORAI2
ACE2	35.95020894	1.652556743	4.44322844	0.010487	High	34	2	NM_001123070.1	ssc:100144303			
LOC100623751	161.7759402	15.69928906	3.365225928	0.0105	High	153	19	XR_309643.1	ssc:100623751			
LOC102161128	5.286795433	83.45411554	-3.980517789	0.010511	Low	5	101	XP_005672168.1	ssc:102161128	2E-43	NM_201279.1	NRP2
LOC100518417	3.17207726	61.14459951	-4.268725266	0.010513	Low	3	74	XP_005654939.1	ssc:100518417	0	NM_152921.1	EMR2
LOC100625759	12.68830904	162.7768392	-3.68132372	0.010559	Low	12	197	XP_005665016.1	ssc:100625759	3E-29	NM_001288583.1	SMIM1
RPF2	6088.27362	576.7423035	3.400034447	0.010563	High	5758	698	XP_005652746.1	ssc:100525264	5E-135	NM_032194	RPF2
tRNA-Arg	4123.700438	398.2661752	3.372134672	0.010583	High	3900	482	tRNA-Arg				
REC8	8906.135586	827.9309285	3.427217257	0.010584	High	8423	1002	HighI0001C95632		0	NM_005132.2	REC8
KCTD17	124.7683722	1498.868966	-3.586550092	0.01061	Low	118	1814	XP_005663865.1	ssc:100512279	7E-104	NM_024681.3	KCTD17
KCTD11	100.4491132	1196.451082	-3.57422468	0.010628	Low	95	1448	XP_003358298.1	ssc:100622059	5E-122	NM_001002914.2	KCTD11
LOC100521530	108.9079859	1300.562157	-3.577953699	0.010641	Low	103	1574	XP_005673187.1	ssc:100521530	3E-62	NM_002825.5	PTN
ITGA9	1976.204133	197.4805308	3.322949645	0.010664	High	1869	239	XP_005669412.1	ssc:100626707	0	NM_002207.2	ITGA9
ANO1	28.54869534	338.7741324	-3.568826998	0.010679	Low	27	410	XP_005654695.1	ssc:100738137	0	NR_030691.1	ANO1
PALM2	9.516231779	128.0731476	-3.750433805	0.010687	Low	9	155	NM_001204195.1	ssc:100152369	0	NM_053016.5	PALM2
PDGFA	96.21967688	1142.742988	-3.570025202	0.010695	Low	91	1383	HighI0001C965DD		9E-90	NM_033023.4	PDGFA
XYLT1	19.03246356	231.3579441	-3.603592416	0.010799	Low	18	280	XYLT1	ssc:100514160	0	NM_022166.3	XYLT1

AMMECR1	2325.132631	231.3579441	3.329114471	0.010805	High	2199	280	XP_003135359.4	ssc:100520351			
LOC100738786	2.114718173	49.5767023	-4.551124996	0.010878	Low	2	60	XP_005655922.1	ssc:100738786	2E-124	NM_001712.4	CEACAM1
ABAT	79.30193149	6.610226974	3.58458429	0.01088	High	75	8	NM_214263.1	ssc:397500	0	NM_020686.5	ABAT
LOC102163221	10.57359087	138.8147665	-3.714623729	0.010887	Low	10	168	XR_309034.1	ssc:102163221			
LOC100518021	31.7207726	372.6515456	-3.554327428	0.010953	Low	30	451	XP_003128185.1	ssc:100518021			
COL19A1	71.90041789	5.783948602	3.635873518	0.010969	High	68	7	XP_003121320.2	ssc:100512751	0	NM_001858.4	COL19A1
PDE8A	71.90041789	841.9776608	-3.549709896	0.010982	Low	68	1019	XP_003128509.4	ssc:100514527	0	NM_173457.1	PDE8A
MEGF6	35.95020894	420.5756912	-3.548293406	0.010984	Low	34	509	XP_005665018.1	ssc:102165253	0	NM_001409.3	MEGF6
E4F1	124.7683722	1483.995956	-3.572162986	0.011075	Low	118	1796	XP_005655232.1	ssc:100517704	0	NM_004424	E4F1
LOC100518848	281.257517	28.91974301	3.281765018	0.011094	High	266	35	Z84015	ssc:100518848	7E-44	NM_001024.3	RPS21
EPB41L4A	1059.473805	109.0687451	3.280038273	0.011121	High	1002	132	XP_005661637.1	ssc:100518651	0	NM_022140.3	EPB41L4A
PLCD3	145.9155539	1742.621086	-3.578053324	0.011202	Low	138	2109	XP_005668783.1	ssc:100521111	0	NM_133373.3	PLCD3
SEPP1	7073.732289	672.5905946	3.39467111	0.011231	High	6690	814	NM_001134823.1	ssc:100037964	4E-139	NM_005410.2	SEPP1
FGF11	279.1427989	3438.144305	-3.622554854	0.011319	Low	264	4161	XP_005669270.1	ssc:100739797	4E-129	NM_004112.2	FGF11
LOC100623959	1.057359087	38.0088051	-5.167796357	0.011363	Low	1	46	XP_005674164.1	ssc:100623959	1E-33	NM_153343.3	ENPP6
FOXP2	1.057359087	38.0088051	-5.167796357	0.011363	Low	1	46	NM_001113049.1	ssc:100127352			
LOC102167165	1.057359087	38.0088051	-5.167796357	0.011363	Low	1	46	XP_005672197.1	ssc:102167165			
LAD1	1.057359087	38.0088051	-5.167796357	0.011363	Low	1	46	XP_005668054.1	ssc:100622691	2E-129	NM_005558.3	LAD1
LDLRAP1	284.4295943	3502.594018	-3.622280289	0.01138	Low	269	4239	XP_003127756.1	ssc:100125967	1E-169	NM_015627.2	LDLRAP1
SLCO2B1	5.286795433	81.8015588	-3.951662926	0.011407	Low	5	99	XP_005667189.1	ssc:102160472	0	NM_007256.4	SLCO2B1
WDR43	17412.58944	1585.628195	3.457004336	0.011446	High	16468	1919	XP_005662713.1	ssc:100519832	4E-167	NM_015131.1	WDR43
LOC100620491	49.69587707	3.305113487	3.910354451	0.011464	High	47	4	XP_005658078.1	ssc:100620491	0	NM_004296.5	RGS6
SLC2A11	45.46644072	524.686766	-3.528452813	0.011484	Low	43	635	XP_005670960.1	ssc:100125552	0	NR_104248.1	SLC2A11
BCAS3	87.76080419	1022.106346	-3.541824754	0.011487	Low	83	1237	XP_005669071.1	ssc:100620226	0	NM_017679.3	BCAS3
TNFRSF1A	464.180639	5869.055274	-3.660370041	0.011507	Low	439	7103	NM_213969.1	ssc:397020	2E-103	NM_001065.3	TNFRSF1A
ZIC3	647.103761	67.75482648	3.255601437	0.011509	High	612	82	ZIC3	ssc:100156863	0	NM_003413.3	ZIC3
MIOS	4936.809575	480.067734	3.362269119	0.011511	High	4669	581	NM_001195353.1	ssc:100337662	0	NM_019005.3	MIOS
LOC102159210	20.08982264	0	20	0.011588	High	19	0	XR_298894.1	ssc:102159210			
LOC102159797	20.08982264	0	20	0.011588	High	19	0	XP_005659519.1	ssc:102159797			
SUN3	20.08982264	0	20	0.011588	High	19	0	XP_003360598.1	ssc:100627860			
LOC100626755	20.08982264	0	20	0.011588	High	19	0	XP_005668037.1	ssc:100626755			
LOC102164033	20.08982264	0	20	0.011588	High	19	0	LOC102164033				
LOC100621043	20.08982264	0	20	0.011588	High	19	0	XP_003353592.2	ssc:100621043	1E-62	NM_004293.4	GDA
LOC102167745	20.08982264	0	20	0.011588	High	19	0	XP_005659138.1	ssc:102167745	1E-18	NM_004102.3	FABP3
LOC102165751	20.08982264	0	20	0.011588	High	19	0	XR_309050.1	ssc:102165751			
DEFB123	20.08982264	0	20	0.011588	High	19	0	XP_003134421.1	ssc:100521232	6E-17	NM_153324.2	DEFB123
HOXD13	20.08982264	0	20	0.011588	High	19	0	XP_003483728.1	ssc:100152793	1E-161	NM_000523.3	HOXD13
LOC100520029	1040.441341	13820.33205	-3.731524743	0.011606	Low	984	16726	XR_304045.1	ssc:100520029	4E-29	NM_006868.3	RAB31
MAP3K15	15771.56814	1447.639707	3.445551621	0.011619	High	14916	1752	XP_003484135.1	ssc:100739216	0	NM_001001671.3	MAP3K15
CCDC170	2.114718173	48.75042393	-4.52687745	0.011635	Low	2	59	XP_005659194.1	ssc:100516653	6E-168	NM_025059.3	CCDC170
DPP10	0	26.4409079	-20	0.011654	Low	0	32	XP_001925671.4	ssc:100157195	0	NM_020868.3	DPP10
MLC2V	0	26.4409079	-20	0.011654	Low	0	32	NM_213791.2	ssc:396690	6E-91	NM_000432.3	MYL2
CLDN4	0	26.4409079	-20	0.011654	Low	0	32	NM_001161637.1	ssc:733578	4E-96	NM_001305.4	CLDN4
ANO2	0	26.4409079	-20	0.011654	Low	0	32	XP_005655769.1	ssc:100521792	1E-129	np_065106	np_065106
PDZK1IP1	0	26.4409079	-20	0.011654	Low	0	32	NM_001001769.1	ssc:414756	8E-50	NM_005764.3	PDZK1IP1
LOC102162791	0	26.4409079	-20	0.011654	Low	0	32	XP_005655114.1	ssc:102162791			
LOC102160410	0	26.4409079	-20	0.011654	Low	0	32	XR_298807.1	ssc:102160410			
LOC102162226	0	26.4409079	-20	0.011654	Low	0	32	XP_005658705.1	ssc:102162226			
LOC102163370	0	26.4409079	-20	0.011654	Low	0	32	XR_306619.1	ssc:102163370			
ZNF575	31.7207726	366.0413187	-3.528506694	0.011811	Low	30	443	ZNF575	ssc:100519430	2E-112	NM_174945.2	ZNF575
PIK3R3	3591.848817	356.9522566	3.3309236	0.011916	High	3397	432	XP_005665526.1	ssc:100511937	0	NM_003629.3	PIK3R3
APLP1	22.20454082	260.2776871	-3.551124996	0.011939	Low	21	315	XP_005664574.1	ssc:100524259	1E-73	NM_005166.3	APLP1
ZNF396	1289.978086	133.8570962	3.268581029	0.011964	High	1220	162	ZNF396	ssc:100620188	4E-97	NM_145756.2	ZNF396
LOC100738354	6.344154519	91.71689926	-3.853687766	0.011966	Low	6	111	XR_307304.1	ssc:100738354			
LOC100515261	322.4945214	33.87741324	3.250879027	0.011979	High	305	41	XP_003126103.4	ssc:100515261	0	NM_025045	BAIAP2L2
HCRTR1	34.89284986	1.652556743	4.400159719	0.012022	High	33	2	NM_001043346.1	ssc:387287	0	NM_001525.2	HCRTR1
FLNA	590.0063703	7493.518553	-3.666840853	0.012041	Low	558	9069	XP_005659106.1	ssc:733585	1E-44	NM_001456.3	FLNA
DYDC1	7.401513606	102.4585181	-3.791075789	0.01205	Low	7	124	XP_003133137.1	ssc:100524960	3E-69	NM_138812.3	DYDC1
LOC100515204	735.9219243	77.67016694	3.244120243	0.012099	High	696	94	XR_130605.1	ssc:100515204			
SFMBT1	775.0442105	81.8015588	3.244078367	0.01216	High	733	99	XP_005669712.1	ssc:100152413	0	NM_016329.3	SFMBT1
TIMM8A	6497.471587	629.6241193	3.367315707	0.012163	High	6145	762	XP_003135287.1	ssc:100517443	9E-49	NM_032696.1	TIMM8A
LOC102164238	18776.58266	1722.790405	3.446115417	0.012197	High	17758	2085	XR_305265.1	ssc:102164238			
LOC100513950	27.49133625	0.826278372	5.056205317	0.012212	High	26	1	XP_003358699.1	ssc:100513950	2E-32	NM_001030.4	RPS27
LOC100522050	27.49133625	0.826278372	5.056205317	0.012212	High	26	1	LOC100522050				
ABCG4	29.60605442	339.6004108	-3.519874062	0.012241	Low	28	411	XP_003129978.1	ssc:100518504	0	NM_022169.4	ABCG4
CERKL	765.5279787	80.97528043	3.240901642	0.012283	High	724	98	XP_005654239.1	ssc:100521824	0	NR_027690.1	CERKL
C9H11orf88	56.04003159	4.131391859	3.761757959	0.012283	High	53	5	XP_005653753.1	ssc:100513750	1E-53	NM_207430.2	C11orf88
LOC100738016	2367.426995	241.2732845	3.294579853	0.012323	High	2239	292	XP_005674168.1	ssc:100738016	4E-31	NM_032390.4	NIFK
LENG9	34.89284986	397.4398968	-3.509733365	0.012332	Low	33	481	XP_003127477.1	ssc:100525050	5E-117	NM_198988.1	LENG9
LOC100155621	1994.179237	204.9170362	3.28268325	0.012388	High	1886	248	XP_001927807.1	ssc:100155621	1E-32	NM_006304.1	SHFM1
MYH1	1.057359087	37.18252673	-5.136087497	0.012389	Low	1	45	NM_001104951.1	ssc:100125538			
LOC100738788	1.057359087	37.18252673	-5.136087497	0.012389	Low	1	45	XP_003482722.2	ssc:100738788	5E-47	NM_000446.5	PON1
ZNF70	5.286795433	80.14900206	-3.922219148	0.012395	Low	5	97	ZNF70	ssc:100157520	0	NM_021916.2	ZNF70
LOC102159969	2.114718173	47.92414556	-4.502215396	0.012455	Low	2	58	XP_005652856.1	ssc:102159969			
LOC100623625	37.00756803	419.7494128	-3.503636071	0.012484	Low	35	508	XP_003355955.1	ssc:100623625	1E-127	NM_003407.3	ZFP36
EGFLAM	14.80302721	178.4761283	-3.591766981	0.012507	Low	14	216	XP_005672488.1	ssc:100518875	0	NM_182801.2	EGFLAM
TPM1	8558.264447	128639.9746	-3.909876958	0.012511	Low	8094	155686	NM_001097483.2	ssc:100037999	1E-149	NM_001018020.1	TPM1
AMD1	32603.66743	2919.241487	3.481368791	0.012533	High	30835	3533	XP_005652745.1	ssc:100155925	0	NR_109768.1	AMD1
DLGAP4	367.9609621	4495.780621	-3.610947018	0.012563	Low	348	5441	XP_005672968.1	ssc:100153319	0	NM_183006.2	DLGAP4
EMP3	779.2736468	9964.917163	-3.676655878	0.012628	Low	737	12060	NM_001244530.1	ssc:100625126	2E-77	NM_001425.2	EMP3
LOC102161059	1365.050581	142.9461583	3.255410657	0.012675	High	1291	173	XR_297740.1	ssc:102161059			
ZNF711	1943.426001	200.7856443	3.274874147	0.012676	High	1838	243	ZNF711	ssc:100152061	0	NM_021998.4	ZNF711
CDH8	90.93288145	8.262783717	3.460102259	0.012689	High	86	10	XP_003355837.2	ssc:100625758	2E-105	NM_001796.4	CDH8
TMEM79	11.63094995	144.5987151	-3.636013894	0.012732	Low	11	175	XP_001928013.1	ssc:100152302	1E-174	NR_026678.1	TMEM79
PMM1	100.4491132	1146.87438	-3.513170645	0.012737	Low	95	1388	NM_001184895.1	ssc:100151790	2E-148	NM_002676.2	PMM1
LOC100625270	12.68830904	155.3403339	-3.613860752	0.012818	Low	12	188	XP_005658030.1	ssc:100625270	4E-65	NM_003034.3	ST8SIA1
LOC100737431	26.43397716	300.7653273	-3.508172851	0.012937	Low	25	364	XP_005669345.1	ssc:100737431	6E-58	NM_015150.1	RFTN1
LOC100624077	62.38418611	4.95767023	3.653446148	0.012973	High	59	6	XR_299856.1	ssc:100624077			
KIAA1875	31.7207726	357.778535	-3.49556702	0.012999	Low	30	433	XP_003125510.3	ssc:100515310			
LOC102164625	11.63094995	143.7724367	-3.627746278	0.013036	Low	11	174	XR_297300.1	ssc:102164625			
LOC100737543	4294.99261	430.4910317	3.318600561	0.013061	High	4062	521	XR_135187.2	ssc:100737543			
LOC100738027	152.2597085	1757.494097	-3.528913717	0.013073	Low	144	2127	XP_005654694.1	ssc:100738027	7E-35	NM_138565.2	CTTN
HS6ST1	1333.329808	17511.31753	-3.715182045	0.013099	Low	1261	21193	XP_003133447.3	ssc:100156717			
LOC100515685	12.68830904	154.5140555	-3.60616636	0.013102	Low	12	187	XP_005653962.1	ssc:100515685	5E-32	NM_006014.4	LAGE3
NT5DC3	161.7759402	16.52556743	3.291225347	0.013105	High	153	20	XP_003481780.1	ssc:100738320	0	NM_016575.1	NT5DC3
LOC102161907	0	25.61462952	-20	0.013156	Low	0	31	XP_005654262.1	ssc:102161907	6E-147	XP_005275995.1	LOC728763
HIF3A	0	25.61462952	-20	0.013156	Low	0	31	XP_003356031.2	ssc:100621574	0	NM_152796.2	HIF3A
LOC102157470	0	25.61462952	-20	0.013156	Low	0	31	XR_304922.1	ssc:102157470			
AMICA1	0	25.61462952	-20	0.013156	Low	0	31	NM_001244730.1	ssc:100512978	7E-132	NR_104479.1	AMICA1
LOC100624399	0	25.61462952	-20	0.013156	Low	0	31	XP_003356177.2	ssc:100624399	7E-59	NM_199454.2	PRDM16

AIRE	0	25.61462952	-20	0.013156	Low	0	31	XP_003359037.1	ssc:100621659	0	NM_000659.1	AIRE
LOC102160901	0	25.61462952	-20	0.013156	Low	0	31	XR_306021.1	ssc:102160901	3E-51	NM_001029880.2	SFMBT2
LOC100623023	0	25.61462952	-20	0.013156	Low	0	31	XP_005665919.1	ssc:100623023	1E-72	NM_138569.2	MLIP
LOC100623327	0	25.61462952	-20	0.013156	Low	0	31	XR_297216.1	ssc:100623327	9E-65	NM_032784.4	RSPO3
LOC100737228	0	25.61462952	-20	0.013156	Low	0	31	XP_005654379.1	ssc:100737228	3E-130	NM_022138.2	SMOC2
ZPBP	0	25.61462952	-20	0.013156	Low	0	31	NM_214106.1	ssc:397194	9E-163	NM_007009.2	ZPBP
LOC100737327	0	25.61462952	-20	0.013156	Low	0	31	XR_135454.2	ssc:100737327			
LOC102165794	41.23700438	2.478835115	4.056205317	0.013173	High	39	3	XR_300521.1	ssc:102165794			
NAGK	344.6990622	4147.091148	-3.588690477	0.013205	Low	326	5019	XP_005662538.1	ssc:100523729	0	NM_017567.4	NAGK
LOC100626982	26.43397716	299.1127706	-3.500224098	0.013238	Low	25	362	XR_297141.1	ssc:100626982			
LOC100628219	71.90041789	805.6214124	-3.486029968	0.013248	Low	68	975	XP_005655102.1	ssc:100628219	2E-127	NM_020223.3	FAM20C
EXTL1	9.516231779	121.4629206	-3.673981744	0.013293	Low	9	147	XP_005665190.1	ssc:100623848	0	NM_004455.2	EXTL1
TMEM101	125.8257313	1432.766697	-3.509304821	0.013365	Low	119	1734	NM_001244685.1	ssc:100512625	9E-147	NM_032376.2	TMEM101
NASP	41747.70882	3732.299405	3.483560393	0.013372	High	39483	4517	XP_005658844.1	ssc:100622547	0	NM_172164.1	NASP
RGS14	54.9826725	4.131391859	3.734277222	0.013421	High	52	5	XP_005661511.1	ssc:100524790	0	NM_006480.4	RGS14
ITGA1	51.81059524	575.9160251	-3.474539403	0.01345	Low	49	697	HighI00028F495E		0	NM_181501.1	ITGA1
C3H16orf71	5.286795433	78.49644531	-3.892161914	0.013485	Low	5	95	XP_005662220.1	ssc:100525811	2E-106	NM_139170.2	C16orf71
PRAF2	326.7239578	3897.555079	-3.576425258	0.013513	Low	309	4717	NM_001243880.1	ssc:100511673	5E-83	NM_007213.2	PRAF2
LOC102167722	1.057359087	36.35624836	-5.103666019	0.013526	Low	1	44	XP_005654070.1	ssc:102167722			
RORA	21.14718173	241.2732845	-3.512130865	0.013562	Low	20	292	XP_005659598.1	ssc:100156637	0	NM_134262.2	RORA
CHN1	1177.898022	125.5943125	3.229371603	0.013563	High	1114	152	XP_005657605.1	ssc:100513051	6E-105	NR_038133.1	CHN1
KIAA0020	13403.08378	1276.600084	3.392186421	0.013577	High	12676	1545	XP_005660193.1	ssc:100152831	0	NM_014878.4	KIAA0020
LOC100737938	10.57359087	131.3782611	-3.635189261	0.01366	Low	10	159	XP_003481433.1	ssc:100737938			
LOC102163664	8.458872693	109.8950234	-3.699516836	0.013704	Low	8	133	XP_005657760.1	ssc:102163664			
TBC1D4	655.5626337	71.05993997	3.20562525	0.013714	High	620	86	XP_005668526.1	ssc:100523015	0	NM_014832.3	TBC1D4
LOC102159565	7.401513606	99.15340461	-3.743770074	0.013774	Low	7	120	XP_005660430.1	ssc:102159565	5E-61	NM_032888.2	COL27A1
MLXIPL	33.83549077	1.652556743	4.355765599	0.013817	High	32	2	XP_003481050.2	ssc:100170769	0	NM_032994.2	MLXIPL
C8H4orf48	22.20454082	251.188625	-3.499844491	0.013843	Low	21	304	XP_003128861.3	ssc:100515739	1E-31	NM_001168243.1	C4orf48
LDB2	138.5140403	14.04673232	3.30172576	0.013863	High	131	17	XP_005666597.1	ssc:100513100	4E-44	NM_001290.3	LDB2
LOC100524931	622.784502	67.75482648	3.200337418	0.01392	High	589	82	XP_005662588.1	ssc:100524931	0	NM_144709.2	PUS10
LOC100514966	3.17207726	57.01320765	-4.167796357	0.013926	Low	3	69	XP_005656048.1	ssc:100514966	1E-95	NM_172130.2	KCNAB2
LOC100155040	5118.675338	514.7714256	3.313766625	0.013928	High	4841	623	XP_001925316.3	ssc:100155040	1E-101	NM_014673.3	EMC2
LOC102158317	47.5811589	3.305113487	3.847618696	0.014018	High	45	4	XR_298831.1	ssc:102158317			
LOC102164141	5.286795433	77.67016694	-3.876895158	0.014073	Low	5	94	XP_005662176.1	ssc:102164141			
ARHGAP27	378.534553	41.31391859	3.195725187	0.01414	High	358	50	XP_005668771.1	ssc:100628007	0	NM_199282.2	ARHGAP27
LOC100624332	3920.687493	400.7450103	3.290350191	0.014163	High	3708	485	XP_005674282.1	ssc:100624332	0	NM_203292.1	RBBP8
LOC100736959	173.4068902	18.17812418	3.253885985	0.014182	High	164	22	XR_301972.1	ssc:100736959	1E-50	NM_173666.2	DTWD2
LOC102159229	2.114718173	46.27158882	-4.451589323	0.01431	Low	2	56	XP_005673115.1	ssc:102159229	9E-52	NR_003259.1	GNAS
LOC102162209	2.114718173	46.27158882	-4.451589323	0.01431	Low	2	56	XR_304023.1	ssc:102162209			
ELANE	12.68830904	151.208942	-3.574971738	0.014319	Low	12	183	XP_003123034.2	ssc:100522182	3E-92	NM_001972.2	ELANE
BICC1	61.32682702	672.5905946	-3.45513839	0.014326	Low	58	814	XP_001929158.3	ssc:100154253	0	NM_001080512.1	BICC1
YPEL2	100.4491132	1114.649523	-3.472053425	0.014374	Low	95	1349	YPEL2	ssc:100524105	2E-68	NM_001005404.3	YPEL2
LOC100624284	10.57359087	129.7257044	-3.616927055	0.014385	Low	10	157	LOC100624284				
LOC100516279	4270.673351	436.2749803	3.291153911	0.014438	High	4039	528	XR_130813.2	ssc:100516279			
WDHD1	6720.574354	671.7643162	3.322557467	0.014514	High	6356	813	XP_005660040.1	ssc:100152808	0	NM_007086.3	WDHD1
LOC100625846	26.43397716	0.826278372	4.999621789	0.014529	High	25	1	XP_005658893.1	ssc:100625846			
PAQR5	67.67098154	5.783948602	3.548410677	0.014588	High	64	7	XP_005659931.1	ssc:100521597	1E-165	NM_017705.3	PAQR5
SERTAD1	187.1525583	2126.01425	-3.505864592	0.014609	Low	177	2573	XP_003355962.1	ssc:100624989	1E-81	NM_013376.3	SERTAD1
LOC102161863	19.03246356	0	20	0.014613	High	18	0	XR_303022.1	ssc:102161863			
ACSBG1	19.03246356	0	20	0.014613	High	18	0	XP_005656303.1	ssc:100156446	0	NM_015162.4	ACSBG1
LOC100525247	19.03246356	0	20	0.014613	High	18	0	XR_297588.1	ssc:100525247			
LOC102161224	19.03246356	0	20	0.014613	High	18	0	XP_005664286.1	ssc:102161224	1E-90	xp_003846405	xp_003846405
ASB11	19.03246356	0	20	0.014613	High	18	0	NM_001243489.1	ssc:100154431	2E-177	NM_080873.2	ASB11
LOC102166082	19.03246356	0	20	0.014613	High	18	0	LOC102166082				
LOC102166417	19.03246356	0	20	0.014613	High	18	0	XR_309080.1	ssc:102166417			
CALY	19.03246356	0	20	0.014613	High	18	0	XP_001926990.2	ssc:100158118	3E-74	NM_015722.3	CALY
LOC100625927	19.03246356	0	20	0.014613	High	18	0	XP_003361286.2	ssc:100625927	6E-29	NM_016945.2	TAS2R16
LOC100737086	19.03246356	0	20	0.014613	High	18	0	XP_003483715.1	ssc:100737086	5E-129	NM_173512.2	SLC38A11
LOC100737534	19.03246356	0	20	0.014613	High	18	0	XP_003483254.1	ssc:100737534	0	NM_014461	CNTN6
TRPM6	12.68830904	150.3826637	-3.56706654	0.014643	Low	12	182	XP_005652756.1	ssc:100157775	0	NM_017662.4	TRPM6
GJA1	2571.497299	34378.13793	-3.740810861	0.014683	Low	2432	41606	NM_001244212.1	ssc:100518636	0	NM_000165.3	GJA1
LOC102160528	7.401513606	97.50084786	-3.719522528	0.014744	Low	7	118	XR_306481.1	ssc:102160528			
CDKN1A	1703.405488	22121.95085	-3.698984808	0.014772	Low	1611	26773	XP_001929593.1	ssc:100152215	1E-74	NR_037150.1	CDKN1A
TSPAN4	230.5042809	2638.306841	-3.516746914	0.014825	Low	218	3193	XP_005652829.1	ssc:100513456	2E-113	NM_003271.4	TSPAN4
PAOX	22.20454082	247.0572331	-3.475918652	0.014826	Low	21	299	XP_005674486.1	ssc:100626281	6E-144	NR_109766.1	PAOX
LOC102161333	1250.855799	135.509653	3.206447953	0.014874	High	1183	164	XP_005659625.1	ssc:102161333	1E-109	NM_019092.1	FAM63B
LOC100620234	0	24.78835115	-20	0.014894	Low	0	30	XP_005673289.1	ssc:100620234			
ASTN2	0	24.78835115	-20	0.014894	Low	0	30	XP_005660442.1	ssc:102162976	4E-94	NM_198188.2	ASTN2
LOC100511329	0	24.78835115	-20	0.014894	Low	0	30	ZNF829	ssc:100511329	0	NM_001171979.1	ZNF829
HSPB3	0	24.78835115	-20	0.014894	Low	0	30	XP_003134015.1	ssc:100519061	2E-75	NM_006308.2	HSPB3
LOC100737673	0	24.78835115	-20	0.014894	Low	0	30	XP_003483458.1	ssc:100737673	0	NM_001136103.2	TMEM132C
EFCAB4A	19.03246356	214.0060983	-3.491117687	0.014929	Low	18	259	NM_001243838.1	ssc:100511435	2E-86	NM_173584.4	EFCAB4A
LOC100738036	583.6622158	64.44971299	3.178887837	0.014933	High	552	78	XP_003482696.1	ssc:100738036	0	NM_014155.4	ZBTB44
IGDCC3	418.7141983	46.27158882	3.177767297	0.014935	High	396	56	XP_001926550.1	ssc:100155852	0	NM_004884.3	IGDCC3
LOC100623570	6.344154519	86.75922903	-3.773517418	0.014989	Low	6	105	XP_005658162.1	ssc:100623570	5E-52	NM_207336.1	ZNF467
TMEM141	77.18721332	841.1513824	-3.445931688	0.015	Low	73	1018	XP_005652781.1	ssc:100512883	1E-43	NM_032928.3	TMEM141
LOC100523138	26.43397716	290.0237085	-3.455705431	0.015047	Low	25	351	XP_003131998.1	ssc:100523138	2E-51	NM_203411.1	TMEM88
DYRK4	115.2521404	11.5678972	3.316595002	0.015064	High	109	14	XP_005655765.1	ssc:100524805	0	NR_104115.1	DYRK4
CHCHD4	9053.108499	898.1645901	3.333361494	0.015145	High	8562	1087	NM_001243499.1	ssc:100154463	4E-68	NM_144636.2	CHCHD4
LOC100739435	17.97510447	202.4382011	-3.493409499	0.015159	Low	17	245	XP_003482147.1	ssc:100739435	4E-128	NM_024587.2	TMEM53
LOC100153192	90.93288145	992.3603244	-3.447990082	0.015207	Low	86	1201	XP_005656320.1	ssc:100153192	0	NM_014272.3	ADAMTS7
LOC102158833	142.7434767	14.87301069	3.262656195	0.015249	High	135	18	XP_005671681.1	ssc:102158833	6E-78	NM_207481.3	NCKAP5
AGPAT3	41.23700438	444.537764	-3.430294544	0.015335	Low	39	538	NM_001143700.1	ssc:100233171			
OSBPL3	48.63851798	523.8604877	-3.429011475	0.015341	Low	46	634	XP_003484108.1	ssc:100523342	0	NR_104112.1	OSBPL3
LOC100522668	156.4891448	16.52556743	3.24329087	0.015366	High	148	20	LOC100522668				
LOC100738613	723.2336152	80.14900206	3.173705272	0.015453	High	684	97	XP_003483004.1	ssc:100738613	0	NM_172232	ABCA5
CHD7	8902.963509	887.4229712	3.326591839	0.01546	High	8420	1074	XP_003355099.1	ssc:100157083	0	NM_017780.3	CHD7
LOC102160467	37.00756803	398.2661752	-3.42784072	0.015547	Low	35	482	XP_005655635.1	ssc:102160467	4E-35	NM_001033873.1	SMAGP
PTPN6	13.74566813	158.6454474	-3.528757183	0.015575	Low	13	192	XP_005652650.1	ssc:100171389	0	NM_080549.3	PTPN6
PLXNC1	2136.922714	228.879109	3.22287704	0.015638	High	2021	277	XP_005655774.1	ssc:100519487	0	NR_037687.1	PLXNC1
LOC102164447	3.17207726	55.36065091	-4.125361091	0.015648	Low	3	67	XP_005661275.1	ssc:102164447			
RAPGEF4	341.526985	38.0088051	3.167593998	0.015656	High	323	46	XP_001925789.1		0	NM_007023.3	RAPGEF4
RNASE1	114.1947813	11.5678972	3.303298179	0.015733	High	108	14	NM_001167655.1	ssc:100312980	2E-51	NM_198235.2	RNASE1
CYP2D25	494.8440525	55.36065091	3.160041128	0.015833	High	468	67	NM_214394.1	ssc:397687	0	NM_001025161.2	CYP2D6
LOC100516519	32.77813168	1.652556743	4.30996191	0.015925	High	31	2	LOC100516519	ssc:100516519			
CRHBP	32.77813168	1.652556743	4.30996191	0.015925	High	31	2	NM_001195332.1	ssc:100337682	2E-180	NM_001882.3	CRHBP
LOC102160710	32.77813168	1.652556743	4.30996191	0.015925	High	31	2	XR_302566.1	ssc:102160710			
VWA3B	32.77813168	1.652556743	4.30996191	0.015925	High	31	2	XP_005655264.1	ssc:100623963	0	NM_144992.4	VWA3B
LOC102158296	32.77813168	1.652556743	4.30996191	0.015925	High	31	2	XP_005662317.1	ssc:102158296	2E-43	NM_006648.3	WNK2
LOC100739139	32.77813168	1.652556743	4.30996191	0.015925	High	31	2	XP_005667923.1	ssc:100739139			

PWWP2A	6813.621954	693.2475539	3.296979378	0.016021	High	6444	839	XP_005674590.1	ssc:100518878	2E-21	NM_052927.2	PWWP2A
LOC102166621	310.8635715	34.70369161	3.163120521	0.016057	High	294	42	XR_304529.1	ssc:102166621			
OMA1	1497.220467	163.6031176	3.194014529	0.016084	High	1416	198	XP_005665443.1	ssc:100626843	0	NM_145243.3	OMA1
TMEM169	507.5323616	57.01320765	3.154131738	0.016162	High	480	69	XP_005672233.1	ssc:100513571	2E-158	NM_138390.3	TMEM169
SLC38A3	1.057359087	34.70369161	-5.036551824	0.016194	Low	1	42	XP_005669647.1	ssc:100156039	0	NM_006841.4	SLC38A3
LOC100624593	168.1200948	1853.342388	-3.462565351	0.016219	Low	159	2243	XP_005658463.1	ssc:100624593	4E-129	NM_001135602.1	GLB1
ZFP36	37.00756803	394.1347833	-3.41279684	0.016233	Low	35	477	NM_001168419.1	ssc:100316849	1E-127	NM_003407.3	ZFP36
LOC100521355	6434.030042	658.5438623	3.288371233	0.016281	High	6085	797	XR_298026.1	ssc:100521355			
LOC100621139	1005.548491	12318.98424	-3.614828744	0.016345	Low	951	14909	XR_297411.1	ssc:100621139			
LIX1	140.6287585	14.87301069	3.241123033	0.016367	High	133	18	XP_003123859.2	ssc:100511371	1E-164	NM_153234.4	LIX1
LOC100620861	6781.901181	693.2475539	3.290247237	0.016404	High	6414	839	XR_302487.1	ssc:100620861	4E-80	NM_012120.2	CD2AP
RNF207	880.7801191	98.32712624	3.163120521	0.016407	High	833	119	XP_003481985.1	ssc:100515143	0	NM_207396.2	RNF207
LOC102160944	2.114718173	44.61903207	-4.399121903	0.016499	Low	2	54	XP_005658725.1	ssc:102160944			
LOC100739623	2.114718173	44.61903207	-4.399121903	0.016499	Low	2	54	XP_005668532.1	ssc:100739623	7E-63	NM_015843.1	LMO7
DMTN	42.29436346	447.8428775	-3.404455347	0.016502	Low	40	542	XP_005657288.1	ssc:100157264	0	NM_001978.2	DMTN
HENMT1	59.21210885	4.95767023	3.578158021	0.016517	High	56	6	XP_005663670.1	ssc:100153448	3E-126	NM_144584.2	HENMT1
LOC100737474	348.9284986	3981.009195	-3.512130865	0.016631	Low	330	4818	XP_003483083.1	ssc:100737474	2E-178	np_060419	np_060419
LOC100156520	160.7185812	17.35184581	3.21137569	0.016671	High	152	21	LOC100156520				
LOC100521743	4.229436346	64.44971299	-3.92963662	0.016671	Low	4	78	XP_003357786.2	ssc:100521743	0	NM_001081.3	CUBN
SUMF2	96.21967688	1027.064016	-3.416050342	0.016819	Low	91	1243	XP_003124494.1	ssc:100518241	2E-154	NM_015411.2	SUMF2
VSIG8	0	23.96207278	-20	0.016909	Low	0	29	XP_003125715.1	ssc:100514765	0	NM_001013661.1	VSIG8
LOC102157475	0	23.96207278	-20	0.016909	Low	0	29	XR_305628.1	ssc:102157475			
NKAPL	0	23.96207278	-20	0.016909	Low	0	29	XP_001929056.1	ssc:100154907	2E-118	NM_001007531.2	NKAPL
KCNC3	0	23.96207278	-20	0.016909	Low	0	29	XR_303881.1	ssc:100511263	0	NM_004977.2	KCNC3
LOC100739318	0	23.96207278	-20	0.016909	Low	0	29	XP_005665583.1	ssc:100739318			
LHX9	0	23.96207278	-20	0.016909	Low	0	29	XP_005668044.1	ssc:397597	0	NM_020204.2	LHX9
SDPR	0	23.96207278	-20	0.016909	Low	0	29	XP_005652497.1	ssc:100517010	0	NM_004657.5	SDPR
ALDH1A2	0	23.96207278	-20	0.016909	Low	0	29	XP_005659619.1	ssc:100737077	6E-148	NM_170697.2	ALDH1A2
JAKMIP1	0	23.96207278	-20	0.016909	Low	0	29	XP_003128880.2	ssc:100520203	0	NM_144720.3	JAKMIP1
CTSD	546.6546478	6375.563916	-3.543851358	0.017042	Low	517	7716	NM_001037721.1	ssc:494568	2E-42	NM_001909.4	CTSD
LOC100737316	560.4003159	63.62343462	3.138827608	0.017089	High	530	77	XP_003480992.1	ssc:100737316	0	NM_018931.2	PCDHB11
EPB41L4B	91.99024053	9.089062089	3.339277476	0.017124	High	87	11	XP_005674634.1	ssc:100510946	0	NM_019114.4	EPB41L4B
LOC100511785	4397.556441	463.5421665	3.245929608	0.017182	High	4159	561	XP_003133427.2	ssc:100511785	3E-122	NM_031243.2	HNRNPA2B1
LOC100512875	45.46644072	3.305113487	3.782030354	0.017238	High	43	4	LOC100512875				
GNL3	22115.72265	2143.366096	3.36712219	0.017254	High	20916	2594	XP_005669697.1	ssc:100155235	0	NM_206826.1	GNL3
LOC100624503	64.49890428	676.7219864	-3.391216705	0.017298	Low	61	819	XP_005656249.1	ssc:100624503	1E-107	NR_048549.1	SLC6A9
LOC100739107	1810.198756	199.1330876	3.184343249	0.017318	High	1712	241	XP_005667610.1	ssc:100739107	0	NM_003582.2	DYRK3
MST1R	50.75323616	530.4707147	-3.385701387	0.017386	Low	48	642	XP_005669639.1	ssc:100626292	0	NM_002447.2	MST1R
SNX10	5779.524767	602.356933	3.262260339	0.01741	High	5466	729	XP_005673364.1	ssc:100520876	2E-93	NR_037670.1	SNX10
ATP6AP1	58.15474976	608.1408816	-3.386436643	0.017427	Low	55	736	XP_005674116.1	ssc:102159115	1E-35	NM_001183.5	ATP6AP1
CACNA2D2	914.6156099	103.2847965	3.146537637	0.017477	High	865	125	XP_005669658.1	ssc:100157557	0	NM_006030.2	CACNA2D2
BMPER	48.63851798	507.3349202	-3.38276729	0.017525	Low	46	614	XP_003134846.1	ssc:100525197	0	NM_133468.4	BMPER
LOC102160736	4.229436346	63.62343462	-3.911020941	0.017543	Low	4	77	XP_005658937.1	ssc:102160736	1E-12	NM_001311.4	CRIP1
LOC100518118	27.49133625	290.0237085	-3.399121903	0.017547	Low	26	351	XP_005662185.1	ssc:100518118	5E-44	NM_003498.5	SNN
SGSM1	3.17207726	53.70809416	-4.081639713	0.017627	Low	3	65	XP_005670818.1	ssc:100152606	0	NM_133454.2	SGSM1
PLEKHG6	3.17207726	53.70809416	-4.081639713	0.017627	Low	3	65	XP_005653219.1	ssc:100520565	0	NM_018173.3	PLEKHG6
LOC100511112	234.7337172	2581.293633	-3.458997273	0.017646	Low	222	3124	XR_300888.1	ssc:100511112	6E-73	NM_015832.4	MBD2
ZNF580	82.47400875	865.1134552	-3.390877909	0.017729	Low	78	1047	ZNF580	ssc:100627967			
WDR63	2.114718173	43.7927537	-4.372154855	0.017741	Low	2	53	XP_005652563.1	ssc:100157444	0	NM_145172.4	WDR63
MBNL2	81.41664967	853.545558	-3.390072399	0.017743	Low	77	1033	XP_005653881.1	ssc:100519915	0	NM_207304.2	MBNL2
MBOAT1	133.2272449	1422.025078	-3.415985859	0.01775	Low	126	1721	XP_005665670.1	ssc:100152017	0	NR_073465.1	MBOAT1
LOC102166942	1.057359087	33.87741324	-5.001786405	0.017759	Low	1	41	XR_298158.1	ssc:102166942			
ATP10D	2051.276628	225.5739955	3.184849395	0.01777	High	1940	273	XP_005666689.1	ssc:100520087	0	NM_020453.3	ATP10D
ABCB9	21.14718173	225.5739955	-3.415063447	0.017882	Low	20	273	XP_005670660.1	ssc:100155346	0	NM_203445.1	ABCB9
LOC102165085	481.0983844	55.36065091	3.119399144	0.018149	High	455	67	XP_005661556.1	ssc:102165085	3E-151	NM_006633.3	IQGAP2
THAP8	33.83549077	351.168308	-3.375553432	0.018204	Low	32	425	XP_005664583.1	ssc:100522440	6E-66	NM_152658.2	THAP8
LOC100739646	9.516231779	112.3738586	-3.561772241	0.018211	Low	9	136	XP_003482943.1	ssc:100739646	3E-90	NM_017993.3	ENOX1
LOC100526069	1923.336178	213.1798199	3.173468171	0.018245	High	1819	258	XP_005668580.1	ssc:100526069	6E-130	NR_104590.1	TEX30
UBALD2	318.2650851	3526.556091	-3.469959185	0.018396	Low	301	4268	XP_005656979.1	ssc:100519560	5E-81	NM_182565.3	UBALD2
TRAF2	364.7888849	4075.204929	-3.481738934	0.018396	Low	345	4932	XP_005652776.1	ssc:100513840	0	NM_021138.3	TRAF2
GLT8D2	218.8733309	2373.897762	-3.439089721	0.018404	Low	207	2873	XP_003126693.1	ssc:100514961	0	NM_031302.3	GLT8D2
BCAS1	31.7207726	1.652556743	4.262656195	0.018404	High	30	2	NM_001110175.1	ssc:100126238	1E-170	NM_003657.2	BCAS1
LOC102157584	31.7207726	1.652556743	4.262656195	0.018404	High	30	2	XP_005666982.1	ssc:102157584	2E-69	NM_005184.2	CALM3
GPR4	31.7207726	1.652556743	4.262656195	0.018404	High	30	2	NM_001123118.1	ssc:100144489	1E-51	NM_005282.2	GPR4
SP4	1664.283202	185.9126336	3.162204234	0.018409	High	1574	225	XP_003482730.1	ssc:100620437	3E-78	NM_003112.3	SP4
EEF1E1	9643.11487	989.055211	3.285376271	0.018426	High	9120	1197	NM_001243545.1	ssc:100155141	2E-93	NM_004280.4	EEF1E1
TGFBR3	30.66341351	318.1171731	-3.374968041	0.018458	Low	29	385	NM_214272.1	ssc:397512	0	NR_036634.1	TGFBR3
LOC100623211	13.74566813	152.0352204	-3.467356639	0.018507	Low	13	184	XP_003361418.1	ssc:100623211	0	NM_003041.3	SLC5A2
C8H4orf21	421.8862755	48.75042393	3.113367486	0.018513	High	399	59	XP_005656600.1	ssc:102167653	0	NM_138698.2	C4orf21
EGF	17.97510447	0	20	0.018572	High	17	0	NM_214020.1	ssc:397083	7E-156	NM_001963.4	EGF
LOC100511328	17.97510447	0	20	0.018572	High	17	0	XP_003126562.2	ssc:100511328	2E-13	NM_199286.2	DPPA3
RRH	17.97510447	0	20	0.018572	High	17	0	XP_003129314.1	ssc:100518979	2E-180	NM_006583.2	RRH
PAQR9	17.97510447	0	20	0.018572	High	17	0	XP_005669976.1	ssc:100516760	0	NM_198504.2	PAQR9
LOC100626259	17.97510447	0	20	0.018572	High	17	0	XP_003357039.1	ssc:100626259	0	NM_199348.1	DCHS2
LOC102164431	17.97510447	0	20	0.018572	High	17	0	XR_299666.1	ssc:102164431			
LOC102166773	17.97510447	0	20	0.018572	High	17	0	XR_301586.1	ssc:102166773			
KCND3	17.97510447	0	20	0.018572	High	17	0	XP_001928554.1	ssc:100037946	0	NM_172198.2	KCND3
CPB2	17.97510447	0	20	0.018572	High	17	0	XP_001929181.1	ssc:100155038	0	NM_016413.3	CPB2
DNAJB7	17.97510447	0	20	0.018572	High	17	0	XP_003481581.2	ssc:100516609	2E-91	NM_145174.1	DNAJB7
FOXB1	17.97510447	0	20	0.018572	High	17	0	XP_005658514.1	ssc:100626570	6E-132	NM_012182.2	FOXB1
LOC102162630	17.97510447	0	20	0.018572	High	17	0	XP_005666706.1	ssc:102162630	9E-23	NR_049749.1	ZNF267
LHX1	17.97510447	0	20	0.018572	High	17	0	XP_003131754.1	ssc:100513681	0	NM_005568.3	LHX1
LOC100737801	17.97510447	0	20	0.018572	High	17	0	XP_005673249.1	ssc:100737801	1E-98	NR_028041.1	GRM8
PLBD2	470.5247935	5323.711549	-3.500089754	0.018629	Low	445	6443	XP_001925814.4	ssc:100156824	0	NM_173542.3	PLBD2
C7H15orf59	3.17207726	52.88181579	-4.0592719	0.018726	Low	3	64	XP_001928647.1	ssc:100156505	5E-139	NM_001039614.1	C15orf59
TNFRSF12A	464.180639	5236.95232	-3.495969216	0.018788	Low	439	6338	NM_001142839.1	ssc:100217390	2E-51	NM_016639.2	TNFRSF12A
LOC100514839	2520.744062	31767.09828	-3.655611765	0.018878	Low	2384	38446	XP_005663981.1	ssc:100514839	0	NM_002332.2	LRP1
LOC100738371	1034.097187	118.1578072	3.129584917	0.018891	High	978	143	XP_003481457.1	ssc:100738371	2E-43	NM_016371.2	HSD17B7
ID2	182.923122	1948.364401	-3.412954176	0.019084	Low	173	2358	NM_001037965.1	ssc:654298	1E-72	NM_002166.4	ID2
LOC100511757	2.114718173	42.96647533	-4.344674119	0.019095	Low	2	52	XP_003127204.1	ssc:100511757	1E-180	NM_198540.2	B3GNT8
SHD	2.114718173	42.96647533	-4.344674119	0.019095	Low	2	52	XP_003123110.1	ssc:100523303	1E-162	NM_020209.3	SHD
PRSS36	2.114718173	42.96647533	-4.344674119	0.019095	Low	2	52	XP_005662090.1	ssc:100511374	0	NM_173502.4	PRSS36
LOC100155517	685.1686881	79.32272369	3.110653101	0.019103	High	648	96	LOC100155517				
LYPD6	306.6341351	35.52996998	3.109409935	0.019137	High	290	43	XP_005671651.1	ssc:100521871	1E-85	NM_194317.3	LYPD6
LOC100620901	44.40908164	3.305113487	3.748083022	0.019157	High	42	4	LOC100620901				
LOC100624862	16.91774539	180.9549634	-3.41902146	0.019168	Low	16	219	XR_299220.1	ssc:100624862			
TLN1	4192.428778	54472.40166	-3.699667296	0.019213	Low	3965	65925	XP_003353615.1	ssc:100157220	0	NM_006289.3	TLN1
TAPBPL	233.6763581	2517.670199	-3.429505626	0.019214	Low	221	3047	XP_005653216.1	ssc:100520908	1E-176	NM_018009.4	TAPBPL
MFI2	35.95020894	367.6938754	-3.354433085	0.019218	Low	34	445	XP_001926353.5	ssc:100156949	0	NM_033316.3	MFI2
LOC100622310	0	23.13579441	-20	0.019255	Low	0	28	XP_005653694.1	ssc:100622310	1E-82	NM_013250.2	ZNF215

SPTLC3	0	23.13579441	-20	0.019255	Low	0	28	XP_003134305.1	ssc:100519280	0	NM_018327.2	SPTLC3
LOC100626249	0	23.13579441	-20	0.019255	Low	0	28	XP_005658575.1	ssc:100626249	8E-103	NM_052896.4	CSMD2
MUSK	0	23.13579441	-20	0.019255	Low	0	28	XP_003122125.3	ssc:100515121	0	NM_005592.3	MUSK
LOC102164504	0	23.13579441	-20	0.019255	Low	0	28	XR_309207.1	ssc:102164504			
VTN	0	23.13579441	-20	0.019255	Low	0	28	NM_214104.1	ssc:397192	0	NM_000638.3	VTN
MAP3K7CL	0	23.13579441	-20	0.019255	Low	0	28	XP_003358952.2	ssc:100624256			
LOC102167616	0	23.13579441	-20	0.019255	Low	0	28	XR_306110.1	ssc:102167616			
LOC100737823	0	23.13579441	-20	0.019255	Low	0	28	XP_005652871.1	ssc:100737823	1E-125	NM_152601.3	ZNF709
LOC102165147	0	23.13579441	-20	0.019255	Low	0	28	XP_005666890.1	ssc:102165147			
URGCP	141.6861176	1486.474791	-3.391124682	0.019299	Low	134	1799	XP_005673385.1	ssc:100525140	0	NM_017920.3	URGCP
LOC100517788	1778.477984	199.959366	3.152864352	0.019302	High	1682	242	XP_005654270.1	ssc:100517788	0	NM_020726.4	NLN
LOC100515242	50.75323616	4.131391859	3.618800005	0.01934	High	48	5	XR_308152.1	ssc:100515242			
LRRC27	31.7207726	324.7274001	-3.355729307	0.019402	Low	30	393	XP_005654210.1	ssc:100737235	1E-150	NR_026559.1	LRRC27
ASB6	295.0031852	3210.917753	-3.444183275	0.019467	Low	279	3886	NM_001099937.2	ssc:100101474	0	NM_177999.2	ASB6
STMN2	485.3278207	5444.348191	-3.487727869	0.019479	Low	459	6589	XP_005663069.1	ssc:100156768	6E-98	NM_007029.3	STMN2
LOC100739776	33.83549077	345.3843594	-3.351593533	0.019484	Low	32	418	XP_005670275.1	ssc:100739776	3E-55	NM_033254.2	BOC
LOC100152601	209.3570991	23.96207278	3.127141224	0.019496	High	198	29	LOC100152601				
DYDC2	1.057359087	33.05113487	-4.966162496	0.019506	Low	1	40	XP_003133138.1	ssc:100525130	7E-70	NR_070309.1	DYDC2
HMGA2	232.618999	2496.186961	-3.423685156	0.019521	Low	220	3021	XP_005664014.1	ssc:100513206	1E-51	NM_003484.1	HMGA2
OLFML2B	51.81059524	526.3393228	-3.344674119	0.019558	Low	49	637	XP_005663213.1	ssc:100156452	0	NM_015441.1	OLFML2B
IFI44	773.9868514	8936.20059	-3.529280607	0.01958	Low	732	10815	NM_001246205.1	ssc:100525523	4E-151	NM_006417.4	IFI44
GUF1	4683.043394	504.8560851	3.213502311	0.019664	High	4429	611	XP_003128999.1	ssc:100516681	0	NM_021927.2	GUF1
LOC102163337	181.8657629	20.65695929	3.138174164	0.019717	High	172	25	XR_301446.1	ssc:102163337	3E-16	NR_033730.1	ZNF630
GAS1	347.8711395	3811.622129	-3.453780204	0.019726	Low	329	4613	XP_003130684.3	ssc:100516459	2E-100	NM_002048.2	GAS1
LOC100522267	6.344154519	80.97528043	-3.673981744	0.01973	Low	6	98	LOC100522267				
SLA-DOB2	11.63094995	129.7257044	-3.479423531	0.019778	Low	11	157	SLA-DOB2				
DHFR	7680.656405	808.9265259	3.247149037	0.0199	High	7264	979	NM_001244064.1	ssc:100525912	6E-13	NM_000791.3	DHFR
LOC100516420	3.17207726	52.05553742	-4.036551824	0.019907	Low	3	63	XP_005654932.1	ssc:100516420	0	NM_152921.1	EMR2
LOC102160060	5.286795433	71.05993997	-3.748571061	0.020038	Low	5	86	XR_297538.1	ssc:102160060			
SASS6	1726.667388	195.8279741	3.140331426	0.020052	High	1633	237	XP_003361047.1	ssc:100622404	0	NM_194292.1	SASS6
ENG	252.7088217	2706.887946	-3.421087234	0.020056	Low	239	3276	NM_214031.1	ssc:397096	0	NM_001278138.1	ENG
MBTD1	9130.295713	955.1777977	3.256820379	0.020158	High	8635	1156	XP_003358155.1	ssc:100518331	0	NM_017643.2	MBTD1
LOC100627892	5335.433951	574.2634684	3.215820923	0.020191	High	5046	695	XR_301599.1	ssc:100627892			
LOC102165360	13.74566813	148.7301069	-3.435647779	0.020215	Low	13	180	XP_005657079.1	ssc:102165360			
LOC100620269	729.5777697	8327.23343	-3.51270359	0.020226	Low	690	10078	XP_005658558.1	ssc:100620269	4E-78	NM_016201.3	AMOTL2
TMEM200A	33.83549077	342.0792459	-3.337721358	0.020263	Low	32	414	XP_005659253.1	ssc:100157644	0	NM_052913.2	TMEM200A
CAMK2N2	441.9760982	52.05553742	3.085844808	0.020277	High	418	63	XP_003358761.1	ssc:100627130	1E-40	NM_033259.2	CAMK2N2
tRNA-His	3285.214682	362.7362052	3.178994996	0.02032	High	3107	439	tRNA-His				
LOC100737169	476.868948	5288.181579	-3.471106967	0.020357	Low	451	6400	XP_005666111.1	ssc:100737169	7E-57	NM_003376.5	VEGFA
GAMT	70.8430588	715.5570699	-3.336368425	0.020401	Low	67	866	XP_005661457.1	ssc:100625146	4E-122	NM_138924.2	GAMT
CUX1	261.1676944	2791.16834	-3.417820782	0.020408	Low	247	3378	XP_003481047.2	ssc:100521258	6E-150	NM_181552.3	CUX1
FAM46B	77.18721332	780.8330613	-3.338580361	0.020448	Low	73	945	XP_003127772.2	ssc:100511570	0	NM_052943.3	FAM46B
LOC100737799	167.0627357	19.00440255	3.135984391	0.020492	High	158	23	XP_003483087.1	ssc:100737799			
YBX2	4.229436346	61.14459951	-3.853687766	0.020496	Low	4	74	YBX2	ssc:100519387	8E-131	NM_015982.3	YBX2
LOC102167822	4.229436346	61.14459951	-3.853687766	0.020496	Low	4	74	XP_005657220.1	ssc:102167822			
LOC102164444	107.8506268	11.5678972	3.220836019	0.020534	High	102	14	XR_307603.1	ssc:102164444			
LOC102165489	6.344154519	80.14900206	-3.659184742	0.020542	Low	6	97	XP_005670721.1	ssc:102165489	2E-64	NM_014365.2	HSPB8
LOC100738639	2.114718173	42.14019696	-4.316659743	0.020572	Low	2	51	XP_005667014.1	ssc:100738639	4E-22	NM_198278.1	NPNT
LOC100737149	1118.685914	129.7257044	3.108268762	0.020619	High	1058	157	LOC100737149				
MLKL	23.2618999	237.9681711	-3.354727784	0.02063	Low	22	288	XP_005664404.1	ssc:100736792	2E-90	NM_152649.2	MLKL
LOC102164270	43.35172255	434.6224235	-3.325601385	0.020646	Low	41	526	XP_005674366.1	ssc:102164270	4E-105	NM_133337.2	MYOF
TAP1	530.7942615	5905.411523	-3.47581292	0.020663	Low	502	7147	NM_001044581.1	ssc:733649	0	NM_000593.5	TAP1
AGRN	175.5216084	1829.380315	-3.381634476	0.020713	Low	166	2214	XP_005674514.1	ssc:100623869	0	NM_198576.3	AGRN
ARHGAP24	12.68830904	137.9884881	-3.442976193	0.020726	Low	12	167	XP_003129392.2	ssc:100522450	0	NM_031305.2	ARHGAP24
LOC100737115	12.68830904	137.9884881	-3.442976193	0.020726	Low	12	167	XP_005669410.1	ssc:100737115			
LOC102157421	24.31925899	0.826278372	4.879327555	0.020831	High	23	1	XR_304865.1	ssc:102157421			
LOC100621509	24.31925899	0.826278372	4.879327555	0.020831	High	23	1	XP_003360671.1	ssc:100621509	7E-29	NM_145306.2	C10orf35
LOC100624812	1225.479181	14376.41739	-3.552286319	0.020832	Low	1159	17399	XP_003354541.1	ssc:100624812	1E-100	NM_001540.3	HSPB1
LOC102167134	273.8560034	32.2248565	3.087171668	0.020855	High	259	39	XR_297610.1	ssc:102167134			
DDX21	64471.41294	6108.676002	3.399727973	0.020921	High	60974	7393	XP_005657444.1	ssc:100153111	0	NM_004728.3	DDX21
LOC102163704	34.89284986	349.5157512	-3.324354135	0.020977	Low	33	423	XR_300756.1	ssc:102163704			
LAPTM4B	47883.56359	4616.417263	3.374684989	0.021048	High	45286	5587	XP_005655413.1	ssc:100522993	6E-119	NM_018407.4	LAPTM4B
LOC100625581	48.63851798	485.0254042	-3.317889138	0.021082	Low	46	587	XP_005654702.1	ssc:100625581	1E-45	NM_014578.3	RHOD
PTH1R	20.08982264	206.5695929	-3.362091172	0.021083	Low	19	250	NM_214382.1	ssc:397675	0	NM_001184744.1	PTH1R
WDR93	212.5291764	24.78835115	3.099926695	0.021107	High	201	30	XP_003128523.1	ssc:100156276	0	NM_020212.1	WDR93
LOC100516238	68.72834063	6.610226974	3.378133412	0.021164	High	65	8	XP_005673868.1	ssc:100516238	1E-92	NM_018015.5	CXorf57
LOC100737841	3.17207726	51.22925905	-4.01346821	0.021176	Low	3	62	XP_003481563.1	ssc:100737841	7E-136	NM_004120.4	GBP2
LOC100522521	1104.940245	128.899426	3.099650607	0.021201	High	1045	156	XP_005670905.1	ssc:100522521	1E-26	NM_032747.3	USMG5
ZER1	203.0129446	2118.577745	-3.383452447	0.021267	Low	192	2564	XP_005663085.1	ssc:100514508	0	NM_006336.3	ZER1
SORL1	30.66341351	1.652556743	4.213746594	0.02133	High	29	2	XP_003357379.2	ssc:100524036	4E-168	NM_003105.5	SORL1
LOC100512568	30.66341351	1.652556743	4.213746594	0.02133	High	29	2	XP_003134119.1	ssc:100512568	2E-121	NM_001436.3	FBL
LOC102163191	30.66341351	1.652556743	4.213746594	0.02133	High	29	2	XR_303596.1	ssc:102163191			
LOC102168050	354.215294	42.14019696	3.071357543	0.021377	High	335	51	XR_306144.1	ssc:102168050			
LOC102167215	37.00756803	2.478835115	3.900086115	0.021381	High	35	3	XR_297157.1	ssc:102167215			
KCNN3	6.344154519	79.32272369	-3.644234401	0.021395	Low	6	96	NM_213985.1	ssc:397045	7E-150	NM_170782.2	KCNN3
LAMTOR4	255.880899	2699.45144	-3.399121903	0.021441	Low	242	3267	XP_003124385.1	ssc:100526100	3E-51	NM_001008395.2	LAMTOR4
CXCL9	1.057359087	32.2248565	-4.92963662	0.021458	Low	1	39	NM_001114289.2	ssc:100135681	2E-28	NM_002416.1	CXCL9
KCNN1	1.057359087	32.2248565	-4.92963662	0.021458	Low	1	39	KCNN1		0	NM_002248.4	KCNN1
ICAM5	1.057359087	32.2248565	-4.92963662	0.021458	Low	1	39	XP_003123292.3	ssc:100515878	0	NM_003259.3	ICAM5
LOC102166231	1.057359087	32.2248565	-4.92963662	0.021458	Low	1	39	XR_299598.1	ssc:102166231			
ZNF555	1.057359087	32.2248565	-4.92963662	0.021458	Low	1	39	ZNF555	ssc:100517633	0	NM_152791.4	ZNF555
LOC102158582	1.057359087	32.2248565	-4.92963662	0.021458	Low	1	39	XR_302731.1	ssc:102158582			
LOC100515384	106.7932677	11.5678972	3.20662216	0.021485	High	101	14	XP_005656224.1	ssc:100515384	0	NM_032110.2	DMRTA2
SPRY1	158.603863	1629.420949	-3.360859547	0.021534	Low	150	1972	NM_001267835.1	ssc:100623283	4E-164	NM_199327.2	SPRY1
ZFP112	25.37661808	255.3200169	-3.330734928	0.021618	Low	24	309	ZFP112	ssc:100524627	0	NM_013380.3	ZNF112
SCUBE1	421.8862755	50.40298068	3.065273198	0.0217	High	399	61	XP_003126031.4	ssc:100524621			
RCN3	1903.246356	22778.01587	-3.581107863	0.021718	Low	1800	27567	XP_003127376.1	ssc:100518493	2E-180	NM_020650.2	RCN3
LOC100739855	431.4025073	4680.866976	-3.4396693	0.021732	Low	408	5665	XP_003484031.1	ssc:100739855	2E-132	NM_005194.3	CEBPB
PGAM2	20.08982264	204.9170362	-3.350503198	0.021774	Low	19	248	NM_001134968.1	ssc:100188980	1E-144	NM_000290.3	PGAM2
SCN2B	0	22.30951604	-20	0.021995	Low	0	27	XP_003129961.1	ssc:100514594	6E-120	NM_004588.4	SCN2B
LOC102157538	0	22.30951604	-20	0.021995	Low	0	27	XP_005674188.1	ssc:102157538			
LOC102164750	0	22.30951604	-20	0.021995	Low	0	27	XR_308701.1	ssc:102164750			
MYLK4	0	22.30951604	-20	0.021995	Low	0	27	XP_005665587.1	ssc:100157526	2E-180	NM_001012418.3	MYLK4
LOC100516820	0	22.30951604	-20	0.021995	Low	0	27	XP_003130120.2	ssc:100516820			
LOC100623732	0	22.30951604	-20	0.021995	Low	0	27	XR_135360.2	ssc:100623732			
LOC100623790	0	22.30951604	-20	0.021995	Low	0	27	XP_005669531.1	ssc:100623790	4E-109	NR_033815.1	ALS2CL
LOC100739181	0	22.30951604	-20	0.021995	Low	0	27	XP_003482873.1	ssc:100739181	2E-75	NM_030664.4	PTER
CYP11A1	0	22.30951604	-20	0.021995	Low	0	27	NM_214427.1	ssc:403329			
SGCG	0	22.30951604	-20	0.021995	Low	0	27	XP_003482902.1	ssc:100272144	3E-38	NM_000231.2	SGCG
IFT140	141.6861176	1438.550645	-3.343845698	0.022115	Low	134	1741	XP_003124763.1	ssc:100513727	0	NM_014714.3	IFT140

SYNPO2L	21.14718173	214.0060983	-3.339114594	0.02212	Low	20	259	XP_003359286.2	ssc:100152222	0	NM_024875.4	SYNPO2L
PALM	109.965345	1103.907905	-3.327498975	0.022154	Low	104	1336	NM_001038636.1	ssc:654410	1E-90	NM_002579.2	PALM
CELF4	256.938258	30.57229975	3.071124737	0.022159	High	243	37	XP_005665413.1	ssc:100521138	0	NM_020180.3	CELF4
LOC100512652	142.7434767	1448.465986	-3.343029084	0.022199	Low	135	1753	XR_130502.3	ssc:100512652			
LOC100738449	9.516231779	106.58991	-3.485536655	0.022462	Low	9	129	XP_005667233.1	ssc:100738449	0	NM_001098816.2	TENM4
PHYHIPL	112.0800632	12.39417558	3.176795458	0.022521	High	106	15	NM_001244581.1	ssc:100525186			
HAUS3	5351.294337	588.3102007	3.185238927	0.022576	High	5061	712	XP_003482380.1	ssc:100739653	0	NM_024511.5	HAUS3
LOC102165474	7.401513606	87.5855074	-3.564799933	0.022591	Low	7	106	XR_298096.1	ssc:102165474			
PLAG1	17.97510447	183.4337985	-3.351187426	0.022595	Low	17	222	XP_005663144.1	ssc:100154905	0	NM_002655.2	PLAG1
LOC102162199	67.67098154	6.610226974	3.355765599	0.022695	High	64	8	XR_302735.1	ssc:102162199			
FAM136A	21417.86566	2189.637685	3.290050644	0.022775	High	20256	2650	XP_005662544.1	ssc:100525693	2E-69	NM_032822.2	FAM136A
LOC733634	4.229436346	59.49204276	-3.814159402	0.022792	Low	4	72	NM_001044569.1	ssc:733634	7E-146	NM_001353.5	AKR1C1
LOC100624577	61.32682702	5.783948602	3.406391672	0.022855	High	58	7	XP_005668626.1	ssc:100624577	8E-98	NM_007135.2	ZNF79
LOC102157639	14.80302721	153.6877771	-3.37603829	0.022943	Low	14	186	XP_005669317.1	ssc:102157639	1E-154	NM_018242.2	SLC47A1
CHODL	195.611431	23.13579441	3.079792138	0.022958	High	185	28	XP_005654133.1	ssc:100520642	3E-141	NM_024944.2	CHODL
GPRC5A	17.97510447	182.6075202	-3.344674119	0.023005	Low	17	221	XP_003362205.1	ssc:100624186	3E-157	NM_003979.3	GPRC5A
PDLIM3	5.286795433	68.58110485	-3.697345737	0.023012	Low	5	83	NM_001001637.1	ssc:414421	0	NR_047562.1	PDLIM3
LOC102164151	1434.83628	168.5607878	3.089545263	0.023065	High	1357	204	XR_303157.1	ssc:102164151			
LOC102166840	29.60605442	290.8499868	-3.296311097	0.023143	Low	28	352	XP_005671817.1	ssc:102166840	1E-42	NR_027878.1	ADAM9
USP20	159.6612221	1610.416547	-3.334348037	0.023268	Low	151	1949	XR_298042.1	ssc:100157102			
LOC100736715	48.63851798	4.131391859	3.55739946	0.023375	High	46	5	XP_003480861.1	ssc:100736715	2E-32	NM_174881.2	CRB3
LOC100517699	48.63851798	4.131391859	3.55739946	0.023375	High	46	5	XP_003123163.1	ssc:100517699	2E-32	NM_174881.2	CRB3
HSD17B1	12.68830904	133.8570962	-3.399121903	0.023383	Low	12	162	NM_001128472.1	ssc:100147712	8E-120	NM_000413.2	HSD17B1
LOC100620994	83.53136784	819.6681448	-3.294649963	0.023383	Low	79	992	XP_005658809.1	ssc:100620994	0	NM_001286057.1	ADCY7
ISG15	2047.047192	24180.21027	-3.562210523	0.023458	Low	1936	29264	NM_001128469.1	ssc:100145895	3E-52	NM_005101.3	ISG15
FASTKD5	2355.796045	271.8455843	3.115355343	0.023502	High	2228	329	XP_005672840.1	ssc:102167168	0	NM_021826.4	FASTKD5
LOC102162280	130.0551676	14.87301069	3.128355103	0.023546	High	123	18	LOC102162280				
LOC100736899	1.057359087	31.39857813	-4.892161914	0.023644	Low	1	38	XP_005669279.1	ssc:100736899	4E-122	NM_015982.3	YBX2
EDN3	1.057359087	31.39857813	-4.892161914	0.023644	Low	1	38	NM_001098582.2	ssc:100049663	5E-59	NM_207034.1	EDN3
LOC102163868	1.057359087	31.39857813	-4.892161914	0.023644	Low	1	38	XP_005664750.1	ssc:102163868	6E-143	NM_001080434.1	LMTK3
NPTX1	1.057359087	31.39857813	-4.892161914	0.023644	Low	1	38	XP_003131182.2	ssc:100525071	0	NM_002522.3	NPTX1
ABCC9	1.057359087	31.39857813	-4.892161914	0.023644	Low	1	38	XP_005655715.1	ssc:100127449	0	NM_020298.2	ABCC9
LOC100737344	39.1222862	379.2617726	-3.277131379	0.023753	Low	37	459	XP_005658147.1	ssc:100737344	8E-57	NM_182642.2	CTDSP1
OPRL1	42.29436346	3.305113487	3.677693694	0.023765	High	40	4	NM_214176.1	ssc:397364	2E-12	NM_182647.2	OPRL1
LOC100519977	23.2618999	229.7053873	-3.303743855	0.023776	Low	22	278	XR_299307.1	ssc:100519977			
SCML1	2006.867546	233.8367792	3.101371633	0.023786	High	1898	283	XP_005673526.1	ssc:100521306	4E-65	NM_006746.5	SCML1
PRDX6	52679.74441	5168.371215	3.349466746	0.023789	High	49822	6255	NM_214408.1	ssc:399538	1E-121	NM_004905.2	PRDX6
LOC100622296	16.91774539	0	20	0.023798	High	16	0	XP_003361258.1	ssc:100622296	8E-21	NM_002413.4	MGST2
ATP6V0D2	16.91774539	0	20	0.023798	High	16	0	XP_003125629.1	ssc:100521494	0	NM_152565.1	ATP6V0D2
DLGAP1	16.91774539	0	20	0.023798	High	16	0	XP_003356412.2	ssc:100626809	0	NM_004746.3	DLGAP1
LOC102159146	16.91774539	0	20	0.023798	High	16	0	XR_300364.1	ssc:102159146			
EYS	16.91774539	0	20	0.023798	High	16	0	XP_005658648.1	ssc:102158510	0	NM_198283.1	EYS
LOC102162153	16.91774539	0	20	0.023798	High	16	0	XP_005673765.1	ssc:102162153	7E-121	NM_031276.2	TEX11
LOC100516399	16.91774539	0	20	0.023798	High	16	0	LOC100516399				
LOC100738107	16.91774539	0	20	0.023798	High	16	0	LOC100738107				
PPEF2	16.91774539	0	20	0.023798	High	16	0	XP_003129165.2	ssc:100511942	0	NM_152934.1	PPEF2
LOC100522181	16.91774539	0	20	0.023798	High	16	0	LOC100522181				
LOC102157915	16.91774539	0	20	0.023798	High	16	0	XR_307025.1	ssc:102157915			
LOC100514051	16.91774539	0	20	0.023798	High	16	0	XR_303619.1	ssc:100514051			
RFX6	16.91774539	0	20	0.023798	High	16	0	XP_005654453.1	ssc:102161990	1E-138	NM_173560.3	RFX6
LOC102162801	16.91774539	0	20	0.023798	High	16	0	LOC102162801				
LOC102158914	16.91774539	0	20	0.023798	High	16	0	XR_308116.1	ssc:102158914			
LOC102158923	16.91774539	0	20	0.023798	High	16	0	XR_303372.1	ssc:102158923			
LOC102158700	16.91774539	0	20	0.023798	High	16	0	XR_306944.1	ssc:102158700			
LOC100627657	16.91774539	0	20	0.023798	High	16	0	XP_005667309.1	ssc:100627657	0	NM_018039.2	KDM4D
LOC102167553	16.91774539	0	20	0.023798	High	16	0	LOC102167553				
LOC100155636	16.91774539	0	20	0.023798	High	16	0	LOC100155636				
ZNF703	253.7661808	2608.56082	-3.36168235	0.02384	Low	240	3157	ZNF703	ssc:100525011	0	NM_025069.1	ZNF703
B3GNT8	2.114718173	40.48764021	-4.258944245	0.023948	Low	2	49	XP_003481910.1	ssc:100738168	1E-180	NM_198540.2	B3GNT8
PPP2R2B	2.114718173	40.48764021	-4.258944245	0.023948	Low	2	49	NM_214025.2	ssc:397089	2E-52	NR_073527.1	PPP2R2B
THSD7B	4.229436346	58.66576439	-3.79398152	0.024052	Low	4	71	XP_003359473.2	ssc:100624965	0	NM_001080427.1	THSD7B
LOC102160349	5.286795433	67.75482648	-3.67985831	0.024116	Low	5	82	XR_302077.1	ssc:102160349			
TMOD2	232.618999	28.09346464	3.049662472	0.024123	High	220	34	XP_005659661.1	ssc:100157165	0	NM_014548.3	TMOD2
ATG2A	265.3971307	2724.239792	-3.359629018	0.02424	Low	251	3297	NM_001190278.1	ssc:100462717	0	NM_015104.2	ATG2A
LOC100738129	35.95020894	347.0369161	-3.271017077	0.024295	Low	34	420	XP_005656050.1	ssc:100738129	0	NM_172130.2	KCNAB2
LOC100524893	7879.439913	860.9820633	3.194037996	0.024318	High	7452	1042	XP_003131325.1	ssc:100524893	0	NM_015462.3	NOL11
ZNF467	86.7034451	843.6302175	-3.282449547	0.024319	Low	82	1021	ZNF467	ssc:100623053	0	NM_207336.1	ZNF467
C1H9orf41	16252.66652	1707.917394	3.250366332	0.024329	High	15371	2067	XP_003122012.3	ssc:100518712	0	NM_152420.1	C9orf41
LOC102160850	66.61362245	6.610226974	3.333045523	0.024352	High	63	8	XR_304878.1	ssc:102160850			
LOC102160131	66.61362245	6.610226974	3.333045523	0.024352	High	63	8	LOC102160131				
POLE2	1996.293955	233.8367792	3.093750406	0.024403	High	1888	283	XP_003480516.1	ssc:100521651	0	NM_002692.3	POLE2
LOC102166369	20.08982264	199.1330876	-3.309196224	0.02441	Low	19	241	XR_309210.1	ssc:102166369			
LOC100739472	355.2726531	3702.553384	-3.381521989	0.024463	Low	336	4481	XR_308243.1	ssc:100739472			
LOC102165153	14.80302721	151.208942	-3.352579317	0.024468	Low	14	183	XP_005657655.1	ssc:102165153	2E-42	NM_014808.3	FARP2
LOC100621998	23.2618999	228.0528306	-3.293327239	0.024471	Low	22	276	XP_003361869.1	ssc:100621998	5E-44	NM_003498.5	SNN
OXTR	42.29436346	406.5289589	-3.264820811	0.024508	Low	40	492	NM_214027.1	ssc:397092	4E-176	NM_000916.3	OXTR
PNPLA4	1285.748649	153.6877771	3.064534302	0.024619	High	1216	186	NM_001160082.1	ssc:100294680	4E-113	NM_004650.2	PNPLA4
LOC100623437	112.0800632	1095.645121	-3.289179006	0.024783	Low	106	1326	XP_003361057.2	ssc:100623437	0	NM_024423.2	DSC3
CHST9	29.60605442	1.652556743	4.163120521	0.024792	High	28	2	XP_005653467.1	ssc:100515146	0	NM_031422.5	CHST9
TMIGD1	29.60605442	1.652556743	4.163120521	0.024792	High	28	2	XP_005669166.1	ssc:100521401	9E-87	NM_206832.1	TMIGD1
SELRC1	2128.463841	249.5360683	3.092492317	0.024874	High	2013	302	NM_001243594.1	ssc:100627075			
CTSZ	256.938258	2615.997325	-3.347867437	0.024879	Low	243	3166	NM_001123104.1	ssc:100141405	2E-149	NM_001336.3	CTSZ
NUCB1	1272.002981	14340.88742	-3.494960344	0.024924	Low	1203	17356	NM_001244488.1	ssc:100512538	0	NM_006184.5	NUCB1
TERF1	4855.392926	546.9962821	3.149985119	0.024966	High	4592	662	XP_005663086.1	ssc:100516798	0	NM_017489.2	TERF1
LOC100526059	975.9424369	118.1578072	3.0460811	0.024972	High	923	143	LOC100526059				
LOC102161198	23.2618999	0.826278372	4.815197218	0.025112	High	22	1	XR_306691.1	ssc:102161198			
LOC102164762	23.2618999	0.826278372	4.815197218	0.025112	High	22	1	XP_005663149.1	ssc:102164762	5E-126	NM_144584.2	HENMT1
LOC100152743	23.2618999	0.826278372	4.815197218	0.025112	High	22	1	XP_001926795.1	ssc:100152743	3E-126	NM_144584.2	HENMT1
SESN1	4877.597466	550.3013956	3.147876809	0.025186	High	4613	666	XP_005659430.1	ssc:100523876	0	NM_014454.2	SESN1
KLHL1	0	21.48323766	-20	0.025207	Low	0	26	XP_003131065.1	ssc:100520632	0	NM_020866.2	KLHL1
TCEAL5	0	21.48323766	-20	0.025207	Low	0	26	XP_005657950.1	ssc:100523940	2E-36	NM_001012979.2	TCEAL5
LOC102161784	0	21.48323766	-20	0.025207	Low	0	26	XP_005663765.1	ssc:102161784	8E-50	NM_198460.2	GBP6
LOC100626194	0	21.48323766	-20	0.025207	Low	0	26	XP_005658513.1	ssc:100626194	5E-90	NM_033119.4	NKD1
LOC102163805	0	21.48323766	-20	0.025207	Low	0	26	XP_005668659.1	ssc:102163805	0	NM_173628.3	DNAH17
LOC100156469	0	21.48323766	-20	0.025207	Low	0	26	XP_005666287.1	ssc:100156469	2E-46	NM_001836.3	CMA1
LOC102164355	0	21.48323766	-20	0.025207	Low	0	26	XP_005662885.1	ssc:102164355			
HEPHL1	0	21.48323766	-20	0.025207	Low	0	26	XP_003357292.1	ssc:100625356	0	NM_001098672.1	HEPHL1
LOC102166296	0	21.48323766	-20	0.025207	Low	0	26	XR_303339.1	ssc:102166296			
LOC100623643	0	21.48323766	-20	0.025207	Low	0	26	XP_005659567.1	ssc:100623643	2E-50	NM_014326.3	DAPK2
LOC102167375	0	21.48323766	-20	0.025207	Low	0	26	XR_306387.1	ssc:102167375			

LOC100737944	0	21.48323766	-20	0.025207	Low	0	26	XP_005653624.1	ssc:100737944	0	NM_018986.3	SH3TC1
LOC100737685	0	21.48323766	-20	0.025207	Low	0	26	XP_005672342.1	ssc:100737685	0	NM_001631.3	ALPI
SYNGR1	62.38418611	596.5729844	-3.257446378	0.025245	Low	59	722	NM_001244066.1	ssc:100626046	9E-111	NM_145738.2	SYNGR1
EPHA4	37.00756803	353.6471431	-3.25641837	0.025249	Low	35	428	NM_001134967.1	ssc:100188979	1E-144	NM_004438.3	EPHA4
LOC102160451	5.286795433	66.92854811	-3.662156309	0.025283	Low	5	81	XP_005674387.1	ssc:102160451	3E-22	NM_014905.4	GLS
LOC102166411	13.74566813	140.4673232	-3.353185619	0.025359	Low	13	170	XP_005672450.1	ssc:102166411			
LOC102160864	4.229436346	57.83948602	-3.773517418	0.025394	Low	4	70	XP_005658760.1	ssc:102160864	0	xp_003846818	xp_003846818
LOC102166014	9.516231779	103.2847965	-3.440093684	0.025412	Low	9	125	XP_005664109.1	ssc:102166014			
KCNJ8	468.4100754	57.83948602	3.017645471	0.025417	High	443	70	XP_003126482.2	ssc:100152631	0	NM_004982.3	KCNJ8
HAP1	204.0703037	2039.255021	-3.320904043	0.025481	Low	193	2468	XP_003483029.1	ssc:100736934	0	NM_177977.2	HAP1
METTL13	7899.529736	870.8974038	3.181192094	0.025491	High	7471	1054	NM_001244726.1	ssc:100512915	0	NM_015935.4	METTL13
PRRT2	31.7207726	303.2441624	-3.256980058	0.025576	Low	30	367	XP_005674631.1	ssc:100623439	2E-62	NM_145239.2	PRRT2
LOC102160629	2693.093593	313.9857813	3.100493235	0.025581	High	2547	380	LOC102160629				
LOC102161728	3.17207726	48.75042393	-3.941914949	0.025594	Low	3	59	XR_308080.1	ssc:102161728			
GUCA1B	3.17207726	48.75042393	-3.941914949	0.025594	Low	3	59	XP_005666050.1	ssc:102167816	2E-23	NM_002098.5	GUCA1B
LOC100739553	3.17207726	48.75042393	-3.941914949	0.025594	Low	3	59	XR_308078.1	ssc:100739553			
LRP3	508.5897206	5360.067797	-3.39767704	0.025642	Low	481	6487	XP_005653323.1	ssc:100524868	0	NM_002333.3	LRP3
NPL	108.9079859	12.39417558	3.135375531	0.025662	High	103	15	NM_214071.1	ssc:397141	1E-173	NM_030769.2	NPL
NFASC	47.5811589	4.131391859	3.525690601	0.025741	High	45	5	XP_005667588.1	ssc:100626921			
FAM114A1	537.138416	5671.574744	-3.400383551	0.025817	Low	508	6864	XP_005666637.1	ssc:100525173	0	NR_033290.1	FAM114A1
LOC100153934	137.4566813	1341.876076	-3.287202505	0.025842	Low	130	1624	XP_005670434.1	ssc:100153934	2E-104	NR_004858.1	POFUT2
LOC100522777	2.114718173	39.66136184	-4.229196901	0.025878	Low	2	48	XP_003121930.1	ssc:100522777	4E-68	NM_021268.2	IFNA17
SDR42E2	2.114718173	39.66136184	-4.229196901	0.025878	Low	2	48	XP_003481083.1	ssc:100737047	1E-101	NM_145168.2	SDR42E1
LOC102165051	2.114718173	39.66136184	-4.229196901	0.025878	Low	2	48	XR_301799.1	ssc:102165051			
NOX1	1045.728137	127.2468692	3.038805769	0.025899	High	989	154	XP_003484187.1	ssc:100739747	2E-161	NM_013955.2	NOX1
CEP78	3433.244954	396.6136184	3.113766673	0.02594	High	3247	480	XP_005660230.1	ssc:100154982	0	NM_032171.1	CEP78
SCG5	905.0993781	110.7213018	3.031143395	0.025963	High	856	134	NM_001105287.1	ssc:397110	1E-77	NM_003020.3	SCG5
ADAMTS7	444.0908164	4623.853768	-3.38016913	0.026018	Low	420	5596	XP_005656319.1	ssc:100154398	0	NM_014272.3	ADAMTS7
LOC102160006	1.057359087	30.57229975	-4.853687766	0.026096	Low	1	37	XR_309093.1	ssc:102160006			
NOS3	203.0129446	24.78835115	3.033837504	0.026103	High	192	30	NM_214295.1	ssc:397557			
KCNAB2	31.7207726	301.5916057	-3.249096459	0.02614	Low	30	365	XP_005656045.1	ssc:397248	5E-116	NM_172130.2	KCNAB2
ELMO3	27.49133625	262.7565222	-3.256677638	0.026141	Low	26	318	XR_303768.1	ssc:100522492	0	NM_024712.3	ELMO3
CGREF1	41.23700438	390.0033915	-3.241475231	0.026174	Low	39	472	XP_005662728.1	ssc:100522927	1E-92	NM_006569.5	CGREF1
LOC100737528	14104.11286	1514.568255	3.219137423	0.026176	High	13339	1833	XP_005665225.1	ssc:100737528	8E-34	NM_004102.3	FABP3
LOC102159908	372.1903985	46.27158882	3.007842296	0.026192	High	352	56	XR_305574.1	ssc:102159908			
ATG4A	43.35172255	409.007794	-3.237967111	0.026395	Low	41	495	XP_005657968.1	ssc:100156732	1E-133	NM_178271.1	ATG4A
LOC100514431	2042.817755	242.9258413	3.071972633	0.026474	High	1932	294	XR_308097.1	ssc:100514431	0	NM_014900.4	COBLL1
LOX	1046.785496	11478.65914	-3.454916381	0.026505	Low	990	13892	NM_001206403.1	ssc:100525278	0	NM_002317.5	LOX
ANXA4	220.9880491	2195.421634	-3.312457781	0.026576	Low	209	2657	NM_001167639.1	ssc:100312960			
CARHSP1	218.8733309	2171.459561	-3.310497004	0.026667	Low	207	2628	XP_005662205.1	ssc:100521547	2E-72	NM_014316.3	CARHSP1
RNASET2	450.4349709	4665.993965	-3.372793697	0.026676	Low	426	5647	XP_001928120.2	ssc:100157985	9E-91	NM_003730.4	RNASET2
ZCWPW2	107.8506268	12.39417558	3.121300346	0.026815	High	102	15	ZCWPW2	ssc:100524764	5E-68	NM_001040432.1	ZCWPW2
LOC100624791	1147.234609	12617.27074	-3.459167515	0.026867	Low	1085	15270	XR_130867.2	ssc:100624791			
FGD6	1205.389359	147.0775502	3.034850277	0.026934	High	1140	178	XP_005664312.1	ssc:100517825	0	NM_018351.3	FGD6
LOC102166675	576.2607022	71.88621834	3.002934523	0.027016	High	545	87	XR_300568.1	ssc:102166675			
CDHR4	58.15474976	546.1700037	-3.231381149	0.027034	Low	55	661	XP_005669638.1	ssc:100513757	0	NM_001007540.2	CDHR4
TNFSF12	44.40908164	416.4442994	-3.229196901	0.027035	Low	42	504	NM_001112691.1	ssc:100127163	2E-63	NR_037146.1	TNFSF12
SLC48A1	8.458872693	92.54317763	-3.451589323	0.027119	Low	8	112	XP_005658370.1	ssc:100621096	5E-38	NM_017842.2	SLC48A1
LOC102158177	8.458872693	92.54317763	-3.451589323	0.027119	Low	8	112	XR_300505.1	ssc:102158177			
LOC100628231	6094.617775	689.9424404	3.142987832	0.02722	High	5764	835	XP_003355740.2	ssc:100628231	0	NM_182767.5	SLC6A15
LOC100626204	895.5831463	110.7213018	3.015894568	0.027295	High	847	134	XP_005666256.1	ssc:100626204	2E-107	NM_005499.2	UBA2
LOC102162629	3.17207726	47.92414556	-3.917252895	0.027301	Low	3	58	XP_005666567.1	ssc:102162629			
LOC100626096	964.311487	118.9840855	3.018730612	0.027355	High	912	144	XR_309358.1	ssc:100626096			
LOC100518209	10168.62234	1121.25975	3.180931797	0.027387	High	9617	1357	XP_005667788.1	ssc:100518209	7E-161	NM_019042.3	PUS7
PNAS-5	9038.305472	1003.101943	3.171584086	0.0274	High	8548	1214	XP_003128900.1	ssc:414404	1E-152	NM_017816.2	LYAR
LOC102160130	52.86795433	4.95767023	3.414659288	0.027426	High	50	6	XR_308075.1	ssc:102160130			
RABAC1	969.5982824	10491.25649	-3.435656519	0.027431	Low	917	12697	NM_001031795.1	ssc:595125	6E-102	NM_006423.2	RABAC1
IRAK2	6.344154519	74.36505346	-3.551124996	0.027472	Low	6	90	XP_005669832.1	ssc:102161037	9E-87	NM_001570.3	IRAK2
CYP7B1	63.44154519	594.0941493	-3.227191766	0.027505	Low	60	719	XP_005663118.1	ssc:100286867	0	NM_004820.3	CYP7B1
LOC100627454	859.6329374	106.58991	3.011649886	0.027517	High	813	129	XP_005657202.1	ssc:100627454	3E-133	NM_014586.1	HUNK
LOC100524744	34.89284986	2.478835115	3.815197218	0.027566	High	33	3	XP_005664051.1	ssc:100524744	4E-81	NM_032606.3	CAPS2
LOC100627470	17.97510447	174.3447364	-3.277870748	0.027627	Low	17	211	XP_005655812.1	ssc:100627470	3E-116	NM_152342.2	CDYL2
DMRTA1	88.81816327	9.915340461	3.163120521	0.02769	High	84	12	XP_003121928.1	ssc:100517627	0	NM_022160.2	DMRTA1
SMAD6	154.3744266	1488.953626	-3.269793138	0.027767	Low	146	1802	XP_003480494.1	ssc:100152069	0	NR_027654.1	SMAD6
TUFT1	186.0951992	1812.028469	-3.283492817	0.027772	Low	176	2193	XP_001927905.3	ssc:397240	2E-177	NM_020127.2	TUFT1
TNNT1	41.23700438	384.2194429	-3.219919088	0.027784	Low	39	465	NM_213748.2	ssc:396579	1E-105	NM_003283.4	TNNT1
TRIM21	168.1200948	1627.768392	-3.27533136	0.027815	Low	159	1970	NM_001163649.2	ssc:100302538	0	NM_003141.3	TRIM21
LOC100622637	545.5972887	5660.006846	-3.374895418	0.027901	Low	516	6850	LOC100622637		2E-68	NM_201279.1	NRP2
LOC100737748	897.6978645	111.5475802	3.008570746	0.027989	High	849	135	XP_003483718.1	ssc:100737748	0	NM_024753.4	TTC21B
LOC100519903	2.114718173	38.83508347	-4.198823252	0.027993	Low	2	47	HighI00025DF217	ssc:100519903	0	NM_001080397.1	SLC45A1
LOC102158861	1068.990037	132.2045395	3.015404786	0.028157	High	1011	160	XP_005666989.1	ssc:102158861	1E-41	NM_152400.2	C4orf32
CCDC112	1221.249745	150.3826637	3.021648095	0.028248	High	1155	182	XP_005661642.1	ssc:100520234	4E-67	NM_152549.2	CCDC112
PRMT3	964.311487	119.8103639	3.008746523	0.028296	High	912	145	XR_301721.1	ssc:100521429	5E-138	NM_005788.3	PRMT3
LOC102158578	100.4491132	11.5678972	3.118266286	0.028347	High	95	14	XP_005662319.1	ssc:102158578	3E-58	NM_006648.3	WNK2
LOC100736638	160.7185812	1545.140555	-3.265129442	0.028364	Low	152	1870	XR_298726.1	ssc:100736638			
ADAM19	1496.163107	16526.39371	-3.465432576	0.028457	Low	1415	20001	XP_003134154.4	ssc:100521528	0	NM_033274.4	ADAM19
PHLDB1	244.249949	2399.512392	-3.296311097	0.028478	Low	231	2904	XP_005656698.1	ssc:100623285	0	NM_015157.3	PHLDB1
CHPF	993.9175414	10653.20705	-3.422017829	0.028734	Low	940	12893	XP_003359697.1	ssc:100623047			
LOC100626660	19.03246356	181.7812418	-3.255669113	0.02876	Low	18	220	LOC100626660				
LOC100523964	120.5389359	1139.437875	-3.240751128	0.028763	Low	114	1379	XP_005663939.1	ssc:100523964	7E-63	NM_001005209.1	TMEM198
LOC100624808	1.057359087	29.74602138	-4.814159402	0.028852	Low	1	36	XP_003360907.2	ssc:100624808	1E-179	NM_024788.2	C7orf63
LOC100737709	1.057359087	29.74602138	-4.814159402	0.028852	Low	1	36	XP_005657702.1	ssc:100737709	3E-26	NM_022071.3	SH2D4A
PLEKHA7	871.2638873	109.0687451	2.997872007	0.028883	High	824	132	XP_003123007.3	ssc:100514820	0	NM_175058.4	PLEKHA7
LOC102166061	28.54869534	1.652556743	4.110653101	0.028901	High	27	2	XR_306180.1	ssc:102166061			
LOC102158314	28.54869534	1.652556743	4.110653101	0.028901	High	27	2	XR_298473.1	ssc:102158314			
PANK1	2690.978875	321.4222866	3.065589195	0.028909	High	2545	389	XP_005671326.1	ssc:100154650	0	NM_148978.2	PANK1
C14H10orf71	0	20.65695929	-20	0.028983	Low	0	25	XP_005671275.1	ssc:100521158	0	NM_199459.3	C10orf71
LOC102159603	0	20.65695929	-20	0.028983	Low	0	25	XR_307292.1	ssc:102159603			
LOC102163989	0	20.65695929	-20	0.028983	Low	0	25	XR_302943.1	ssc:102163989			
PTPRCAP	0	20.65695929	-20	0.028983	Low	0	25	XP_003122509.1	ssc:100511860	9E-55	NM_005608.2	PTPRCAP
LOC100520139	0	20.65695929	-20	0.028983	Low	0	25	ZNF544	ssc:100520139	0	NM_014480.2	ZNF544
LOC100624548	0	20.65695929	-20	0.028983	Low	0	25	LOC100624548				
NKX6-1	0	20.65695929	-20	0.028983	Low	0	25	XP_003129394.3	ssc:397289	1E-113	NM_006168.2	NKX6-1
COMP	0	20.65695929	-20	0.028983	Low	0	25	XP_003123575.1	ssc:396554	0	NM_000095.2	COMP
LOC102166377	0	20.65695929	-20	0.028983	Low	0	25	XR_297549.1	ssc:102166377			
LOC102160315	0	20.65695929	-20	0.028983	Low	0	25	XR_304641.1	ssc:102160315			
ZNF132	0	20.65695929	-20	0.028983	Low	0	25	ZNF132	ssc:102161626	2E-20	NM_017652.3	ZNF586
TMEM173	0	20.65695929	-20	0.028983	Low	0	25	NM_001142838.1	ssc:100217389	4E-165	NM_198282.2	TMEM173
LOC100739192	0	20.65695929	-20	0.028983	Low	0	25	XP_003482090.2	ssc:100739192	0	NM_198129.1	LAMA3
SGCA	0	20.65695929	-20	0.028983	Low	0	25	NM_001144122.1	ssc:100240723	0	NM_001135697.1	SGCA

LOC100525890	8.458872693	90.89062089	-3.425594114	0.029068	Low	8	110	XP_005669487.1	ssc:100525890	2E-45	NM_001129908.2	FAM198A
PM20D2	3855.13123	452.8005477	3.089835362	0.029084	High	3646	548	XP_001928624.1	ssc:100157591	0	NM_001010853.2	PM20D2
SLC9B2	248.4793853	31.39857813	2.984355033	0.029147	High	235	38	XP_003129331.2	ssc:100522336	0	NM_178833.4	SLC9B2
MAPRE3	83.53136784	775.8753911	-3.215435	0.029212	Low	79	939	XP_003125359.3	ssc:100524857	2E-165	NM_012326.2	MAPRE3
MAP3K14	133.2272449	1260.074517	-3.241548005	0.029221	Low	126	1525	XP_003131369.1	ssc:100520402	0	NM_003954.3	MAP3K14
PTPN18	76.12985423	704.8154511	-3.210711331	0.02928	Low	72	853	XP_003359490.1	ssc:100521585	0	NM_014369.3	PTPN18
RFC3	5728.771531	660.196419	3.117258585	0.029332	High	5418	799	NM_001190192.1	ssc:100156440	0	NM_181558.2	RFC3
LOC102158232	2011.096983	244.578398	3.039613764	0.029519	High	1902	296	XR_300389.1	ssc:102158232			
NRARP	40.17964529	3.305113487	3.603693113	0.029658	High	38	4	XP_003353772.1	ssc:100620133	8E-63	NM_001004354.2	NRARP
PNP	6291.286565	722.9935753	3.121300346	0.029662	High	5950	875	XP_005656368.1	ssc:100157138	3E-155	NM_000270.3	PNP
LOC102162960	2502.768958	301.5916057	3.052856936	0.02969	High	2367	365	XP_005667315.1	ssc:102162960	0	NM_015036.2	ENDOD1
ABCC3	472.6395117	4783.325494	-3.339201821	0.029745	Low	447	5789	XP_003131623.3	ssc:100513231	0	NM_020038.1	ABCC3
16S	41120.69488	564649.7195	-3.77941962	0.029764	Low	38890	683365	16S				
OLFML2A	31.7207726	291.6762652	-3.200868178	0.029844	Low	30	353	NM_001258360.1	ssc:100154062	0	NM_182487.3	OLFML2A
WDR17	20.08982264	189.2177471	-3.235510675	0.02985	Low	19	229	XP_005671730.1	ssc:100157060	0	NM_181265.3	WDR17
NCKIPSD	86.7034451	801.4900206	-3.208523333	0.029921	Low	82	970	XP_003132242.1	ssc:100524278	0	NM_184231.2	NCKIPSD
LOC100153854	4.229436346	55.36065091	-3.710323591	0.029979	Low	4	67	XP_005663685.1	ssc:100153854	0	NM_000699.2	AMY2A
LOC100625063	166.0053766	20.65695929	3.006530158	0.030002	High	157	25	XP_003361279.2	ssc:100625063	5E-57	NM_003628.3	PKP4
GSTZ1	87.76080419	809.7528043	-3.205832908	0.030194	Low	83	980	NM_001243638.1	ssc:100626791	3E-108	NM_145871.2	GSTZ1
LOC102167612	63.44154519	6.610226974	3.262656195	0.030196	High	60	8	XP_005657085.1	ssc:102167612	8E-60	NM_014519.2	ZNF232
TRIM13	2579.956171	311.5069461	3.050010318	0.030208	High	2440	377	XP_005674321.1	ssc:100153839	0	NM_213590	TRIM13
TMEM170B	3107.578355	371.8252673	3.063094052	0.030226	High	2939	450	XP_003482180.1	ssc:100622777	7E-52	NM_001100829.2	TMEM170B
LOC102159321	2.114718173	38.0088051	-4.167796357	0.030312	Low	2	46	XP_005665447.1	ssc:102159321			
NHLRC1	2.114718173	38.0088051	-4.167796357	0.030312	Low	2	46	NM_001258302.1	ssc:100152396			
LOC100625114	2.114718173	38.0088051	-4.167796357	0.030312	Low	2	46	XP_003359959.1	ssc:100625114			
LOC102159404	22.20454082	0.826278372	4.748083022	0.03041	High	21	1	XR_298033.1	ssc:102159404			
LOC100518808	22.20454082	0.826278372	4.748083022	0.03041	High	21	1	LOC100518808				
ACHE	7.401513606	80.97528043	-3.451589323	0.030584	Low	7	98	ACHE		0	NM_015831.2	ACHE
LOC100626258	508.5897206	65.27599137	2.961877935	0.03062	High	481	79	XR_304844.1	ssc:100626258			
ART3	104.6785496	12.39417558	3.078231624	0.03064	High	99	15	NM_001244205.1	ssc:100522086	4E-156	NM_001179.5	ART3
KLHL40	5.286795433	63.62343462	-3.589092847	0.030661	Low	5	77	XP_005669475.1	ssc:100523695	0	NM_152393.3	KLHL40
LOC100737628	93.04759962	856.8506715	-3.203002961	0.030676	Low	88	1037	XP_003484102.2	ssc:100737628	3E-44	NM_003930.3	SKAP2
FGG	15.8603863	0	20	0.030754	High	15	0	NM_001244524.1	ssc:403164	0	NM_021870.2	FGG
STMND1	15.8603863	0	20	0.030754	High	15	0	XP_005656271.1	ssc:100524942	5E-91	NM_001190766.1	STMND1
C6H1orf177	15.8603863	0	20	0.030754	High	15	0	XP_003356502.2	ssc:100620608	0	NM_152607.2	C1orf177
LOC102168169	15.8603863	0	20	0.030754	High	15	0	XP_005655709.1	ssc:102168169	2E-25	NM_001906.4	CTRB1
LOC100525793	15.8603863	0	20	0.030754	High	15	0	XP_005652520.1	ssc:100525793	0	NM_033125.3	SLC22A16
LOC100520278	15.8603863	0	20	0.030754	High	15	0	XP_003133459.2	ssc:100520278	1E-99	NM_052917.2	GALNT13
DLL3	15.8603863	0	20	0.030754	High	15	0	XP_003481899.1	ssc:100520433	0	NM_203486.2	DLL3
LOC100516636	15.8603863	0	20	0.030754	High	15	0	LOC100516636				
LOC100516605	15.8603863	0	20	0.030754	High	15	0	LOC100516605				
LOC102157755	15.8603863	0	20	0.030754	High	15	0	LOC102157755				
LOC100621571	15.8603863	0	20	0.030754	High	15	0	XP_003481213.2	ssc:100621571	3E-78	NM_001135649.1	FOXI3
LOC100623971	15.8603863	0	20	0.030754	High	15	0	XP_003356400.2	ssc:100623971	2E-70	NM_182978.3	GNAL
LOC102158492	15.8603863	0	20	0.030754	High	15	0	XR_298132.1	ssc:102158492			
LOC100627431	15.8603863	0	20	0.030754	High	15	0	XP_005658254.1	ssc:100627431	0	NM_033086.2	FGD3
LOC100625468	15.8603863	0	20	0.030754	High	15	0	LOC100625468				
PPP1R17	15.8603863	0	20	0.030754	High	15	0	XP_003134851.1	ssc:100511608	2E-79	NM_006658.4	PPP1R17
SYNDIG1L	15.8603863	0	20	0.030754	High	15	0	XP_003128712.2	ssc:100514113	8E-107	NM_001105579.1	SYNDIG1L
LOC100737710	3437.47439	410.6603507	3.065331398	0.03077	High	3251	497	XP_003484062.1	ssc:100737710	3E-110	NM_015135.2	NHigh205
SLA-6	396.5096575	3934.737606	-3.310839521	0.030803	Low	375	4762	NM_001113704.1	ssc:100135037	5E-121	xp_003846540	xp_003846540
PHF16	5723.484736	665.9803676	3.103342243	0.030806	High	5413	806	XP_005673639.1	ssc:100521831	0	NM_014735.4	JADE3
LOC102165713	251.6514626	32.2248565	2.965181144	0.030853	High	238	39	LOC102165713				
LOC100621575	67.67098154	616.4036653	-3.187266221	0.030886	Low	64	746	XP_005664922.1	ssc:100621575	3E-87	NM_152600.2	ZNF579
LOC100511684	103.6211905	956.0040761	-3.205697706	0.030938	Low	98	1157	XP_003124393.1	ssc:100511684	9E-79	NM_145030.2	PPP1R35
ANGPTL2	1675.914152	18276.4513	-3.446965818	0.031015	Low	1585	22119	NM_001109946.1	ssc:100126164	0	NM_012098.2	ANGPTL2
PMCH	189.2672765	23.96207278	2.981600381	0.031045	High	179	29	NM_001244440.1	ssc:396962	1E-86	NM_002674.2	PMCH
LOC404703	116.3094995	14.04673232	3.049662472	0.031067	High	110	17	NM_214444.1	ssc:404703	6E-20	NM_001081551.2	DEFB103A
LOC100510918	3.17207726	46.27158882	-3.866626822	0.03113	Low	3	56	NR_045041.1	ssc:100510918			
SH3RF2	45.46644072	4.131391859	3.460102259	0.031327	High	43	5	XP_005661839.1	ssc:102168153	0	NM_152550.3	SH3RF2
HCN4	45.46644072	4.131391859	3.460102259	0.031327	High	43	5	XP_003128545.2	ssc:100156534	9E-38	NM_005477.2	HCN4
LOC100620089	1565.948807	195.0016957	3.005478475	0.031397	High	1481	236	XP_005656556.1	ssc:100620089	0	NM_007153.3	ZNF208
STAP1	170.2348129	21.48323766	2.986242759	0.031561	High	161	26	XP_003129092.2	ssc:100525760	8E-40	NM_012108.2	STAP1
CEP152	3570.701635	428.0121966	3.060483776	0.031603	High	3377	518	XP_005659693.1	ssc:100152345	0	NM_014985.3	CEP152
TGM5	4.229436346	54.53437253	-3.68862852	0.031717	Low	4	66	XP_001926111.1	ssc:100158178	0	NM_201631.3	TGM5
LOC102160662	4.229436346	54.53437253	-3.68862852	0.031717	Low	4	66	XP_005673316.1	ssc:102160662	6E-84	NM_030636.2	EEPD1
HEATR4	163.8906584	20.65695929	2.988033815	0.031781	High	155	25	XP_005666406.1	ssc:102164275	6E-35	NM_203309.2	HEATR4
C3H9orf89	65.55626337	592.4415925	-3.175867399	0.031787	Low	62	717	NM_001244279.1	ssc:100514222			
HSPB2	34.89284986	314.8120596	-3.173487469	0.031857	Low	33	381	XP_003129916.2	ssc:100520143	1E-90	NM_001541.3	HSPB2
LOC102160307	121.596295	14.87301069	3.031330649	0.031887	High	115	18	XP_005663712.1	ssc:102160307			
LOC102164620	6707.886045	779.1805045	3.105828694	0.031888	High	6344	943	XP_005672589.1	ssc:102164620	2E-15	NM_022132.4	MCCC2
LOC100525472	6502.758382	756.8709885	3.102932506	0.031943	High	6150	916	XP_005666589.1	ssc:100525472	7E-108	NM_016045.2	SLMO2
C7H14orf79	25.37661808	231.3579441	-3.188554917	0.031956	Low	24	280	XP_005656534.1	ssc:102163919	7E-32	NM_174891.3	C14orf79
TRABD2A	19.03246356	176.8235716	-3.215776386	0.032029	Low	18	214	XP_005655281.1	ssc:100524432	2E-19	NM_001277053.1	TRABD2A
SLC1A1	413.4274028	53.70809416	2.944422584	0.032092	High	391	65	NM_001164649.1	ssc:397003			
PXDC1	498.0161298	4962.627901	-3.316839911	0.032142	Low	471	6006	XP_001928887.1	ssc:100154877	3E-125	NM_183373.3	PXDC1
JAK1	219.93069	2083.874054	-3.244147243	0.032202	Low	208	2522	NM_214114.1	ssc:397202	0	NM_002227.2	JAK1
LOC100522672	12.68830904	123.1154774	-3.278440421	0.032385	Low	12	149	XP_003355325.1	ssc:100522672	0	NM_000699.2	AMY2A
PLXDC2	146.972913	1360.880478	-3.210918168	0.032435	Low	139	1647	XP_005668225.1	ssc:100522568	0	NM_032812.8	PLXDC2
LOC100621280	50.75323616	454.4531045	-3.162559708	0.032512	Low	48	550	LOC100621280				
CAMK2N1	84.58872693	765.1337722	-3.177174689	0.032532	Low	80	926	XP_003127723.2	ssc:100515264	4E-40	NM_018584.5	CAMK2N1
NENF	146.972913	1359.227921	-3.209165197	0.032594	Low	139	1645	XP_005667924.1	ssc:100525942	4E-60	NR_026598.1	NENF
HOMER1	463.1232799	60.31832114	2.9407281	0.032608	High	438	73	NM_001243811.1	ssc:100511070			
LOC102167488	1082.735705	137.9884881	2.972061307	0.032673	High	1024	167	XR_298482.1	ssc:102167488			
LOC100627784	72.95777697	656.0650272	-3.168705141	0.03274	Low	69	794	XP_003355880.2	ssc:100627784	1E-36	NR_045692.1	C19orf12
LOC102165476	9.516231779	96.67456949	-3.344674119	0.032783	Low	9	117	XR_298251.1	ssc:102165476			
TLDC1	57.09739067	510.6400337	-3.160809927	0.032821	Low	54	618	XP_005655807.1	ssc:100514962	0	NM_020947.3	TLDC1
IL21R	2.114718173	37.18252673	-4.136087497	0.032858	Low	2	45	XP_003124575.1	ssc:100518723	0	NM_181079.4	IL21R
STK36	67.67098154	606.4883248	-3.163870654	0.032942	Low	64	734	XP_005672268.1	ssc:100622459	0	NM_015690.4	STK36
NOSIP	175.5216084	1632.726063	-3.217562201	0.033011	Low	166	1976	XP_003127375.1	ssc:100518311	5E-169	NM_015953.4	NOSIP
LOC100737465	603.7520384	78.49644531	2.943256926	0.033012	High	571	95	XP_003480713.1	ssc:100737465			
PIK3IP1	133.2272449	1222.065712	-3.197360814	0.033057	Low	126	1479	XP_001926569.1	ssc:100155147	2E-129	NM_052880.4	PIK3IP1
ND2	470238.2492	43491.98837	3.43457032	0.033074	High	444729	52636	HighI00021402B2	ssc:808502	4E-89	YP_003024027.1	ND2
PGR	39.1222862	3.305113487	3.565218965	0.033207	High	37	4	NM_001166488.1	ssc:396805	6E-136	NR_073143.2	PGR
LOC100514211	635.472811	82.62783717	2.94313059	0.033208	High	601	100	XP_003122517.1	ssc:100514211			
PHLDB2	265.3971307	2522.627869	-3.248702721	0.033219	Low	251	3053	XP_005674250.1	ssc:100623057			
LOC100155756	10.57359087	104.9373532	-3.310990993	0.03324	Low	10	127	XR_298749.1	ssc:100155756	1E-17	NM_001195543.1	GS1-259H13.2
KLHL38	3.17207726	45.44531045	-3.840631614	0.033278	Low	3	55	XP_005662925.1	ssc:102160009	1E-64	NM_001081675.2	KLHL38
LOC100739358	247.4220263	32.2248565	2.9407281	0.033313	High	234	39	XP_003482517.1	ssc:100739358	0	NM_178833.4	SLC9B2
LOC100513647	166.0053766	1536.051493	-3.209924707	0.033331	Low	157	1859	NM_001244869.1	ssc:100513647			

LOC102165406	1178.955382	150.3826637	2.970798954	0.033337	High	1115	182	XP_005667968.1	ssc:102165406	7E-13	NM_025081.2	NYNRIN
LOC100622368	0	19.83068092	-20	0.03344	Low	0	24	XP_003354393.1	ssc:100622368			
LOC102159118	0	19.83068092	-20	0.03344	Low	0	24	XR_303283.1	ssc:102159118			
LOC102161858	0	19.83068092	-20	0.03344	Low	0	24	XR_302139.1	ssc:102161858			
SNX20	0	19.83068092	-20	0.03344	Low	0	24	XP_005659094.1	ssc:100621814	1E-122	NM_182854.2	SNX20
FAM83F	0	19.83068092	-20	0.03344	Low	0	24	XP_005663834.1	ssc:100155438	0	NM_138435.2	FAM83F
LOC102164546	0	19.83068092	-20	0.03344	Low	0	24	XP_005653488.1	ssc:102164546			
LOC100738697	0	19.83068092	-20	0.03344	Low	0	24	XP_003480533.1	ssc:100738697			
LOC102158147	0	19.83068092	-20	0.03344	Low	0	24	XP_005661879.1	ssc:102158147			
LOC100623646	0	19.83068092	-20	0.03344	Low	0	24	XP_005672273.1	ssc:100623646	1E-79	NM_025216.2	WNT10A
LOC102158584	0	19.83068092	-20	0.03344	Low	0	24	XP_005674229.1	ssc:102158584	0	NM_000784.3	CYP27A1
LOC100517727	0	19.83068092	-20	0.03344	Low	0	24	XR_305775.1	ssc:100517727			
LOC102165393	0	19.83068092	-20	0.03344	Low	0	24	XR_304072.1	ssc:102165393			
MIR221	0	19.83068092	-20	0.03344	Low	0	24	NR_035371.1	ssc:100316576			
LOC100620821	0	19.83068092	-20	0.03344	Low	0	24	XP_005658964.1	ssc:100620821	7E-15	NM_002704.3	PPBP
LOC100620445	0	19.83068092	-20	0.03344	Low	0	24	XP_005672042.1	ssc:100620445	0	NM_133437.3	TTN
LOC100623686	32.77813168	292.5025436	-3.15764364	0.033446	Low	31	354	LOC100623686				
PGAP1	6506.987819	764.3074938	3.089764772	0.033466	High	6154	925	XP_005672106.1	ssc:100620753	0	NM_024989.3	PGAP1
LOC102166858	8.458872693	87.5855074	-3.372154855	0.033475	Low	8	106	XP_005664919.1	ssc:102166858			
ZBTB44	4131.101951	497.4195798	3.053991463	0.033576	High	3907	602	ZBTB44	ssc:100518028	0	NM_014155.4	ZBTB44
DENND5B	259.0529762	33.87741324	2.934851534	0.033721	High	245	41	XP_005653187.1	ssc:102165384	0	NM_144973.3	DENND5B
LOC102160785	27.49133625	1.652556743	4.056205317	0.033789	High	26	2	XP_005661331.1	ssc:102160785	3E-82	NR_104345.1	FLJ22184
CORO1B	746.4955151	7541.442699	-3.336635044	0.033895	Low	706	9127	XP_003122508.3	ssc:100511677	0	NM_020441.2	CORO1B
LOC102159647	1034.097187	133.0308178	2.958539376	0.033905	High	978	161	LOC102159647				
LOC102164364	56.04003159	5.783948602	3.276331132	0.033908	High	53	7	LOC102164364				
AHCY	45523.53811	4814.724072	3.241087663	0.033945	High	43054	5827	NM_001011727.1	ssc:497050	0	NM_001161766.1	AHCY
C4H1orf85	773.9868514	7824.85618	-3.337683273	0.034132	Low	732	9470	XP_005663352.1	ssc:100153484	3E-157	NM_144580.2	C1orf85
ARHGAP18	4317.19715	520.5553742	3.051971433	0.034208	High	4083	630	XP_003121255.3	ssc:100525547	0	NM_033515.2	ARHGAP18
LOC100627437	4078.233997	493.2881879	3.047441909	0.034235	High	3857	597	XP_005658693.1	ssc:100627437	0	NR_103464.1	HSPA14
RAB11FIP5	166.0053766	1525.309874	-3.19980049	0.034283	Low	157	1846	XP_005662524.1	ssc:100622743	0	NM_015470.2	RAB11FIP5
LOC100626687	247.4220263	2323.494781	-3.23125056	0.034308	Low	234	2812	XP_005674224.1	ssc:100626687	0	NM_173817.1	PIEZO2
ACOX2	54.9826725	485.8516826	-3.143467027	0.034349	Low	52	588	XP_005669744.1	ssc:100516877	0	NM_003500.3	ACOX2
LOC100627468	93.04759962	832.8885987	-3.162082706	0.03435	Low	88	1008	XP_003355687.3	ssc:100627468	4E-88	NM_016596.3	HDAC7
3-Mar	101.5064723	910.5587656	-3.165180409	0.034499	Low	96	1102	NM_001244831.1	ssc:100513526	2E-146	NM_178450.4	3-Mar
LOC100521366	85.64608601	763.4812155	-3.156133439	0.034527	Low	81	924	XP_003124801.3	ssc:100521366	4E-78	NM_080861.3	SPSB3
KLHL23	2103.087223	263.5828006	2.996180546	0.034632	High	1989	319	XP_003483721.2	ssc:100520221	0	NM_001199290.1	PHOSPHO2-KLHL23
RANBP3L	17.97510447	164.429396	-3.19339618	0.034676	Low	17	199	XP_003133942.4	ssc:100517433	0	NM_145000.3	RANBP3L
LOC100627251	3237.633523	397.4398968	3.026131083	0.034726	High	3062	481	XP_005656907.1	ssc:100627251	0	NM_005845.3	ABCC4
CDK8	507.5323616	66.92854811	2.922806192	0.034737	High	480	81	XR_299175.1	ssc:100524102			
VRK1	6574.6588	777.5279478	3.079949588	0.034738	High	6218	941	NM_001243688.1	ssc:100157930	0	NM_003384.2	VRK1
LOC102163775	622.784502	81.8015588	2.928532803	0.03475	High	589	99	XP_005673806.1	ssc:102163775	2E-60	NM_198450.5	APOOL
MSN	1828.173861	19515.04258	-3.416111427	0.034754	Low	1729	23618	NM_001009578.1	ssc:494458	0	NM_002444.2	MSN
PHYHD1	25.37661808	226.4002739	-3.157303983	0.03476	Low	24	274	XP_003122259.1	ssc:100157556	1E-147	NM_174933.3	PHYHD1
IL17RD	42.29436346	371.8252673	-3.136087497	0.034921	Low	40	450	XP_003132314.3	ssc:100513377	0	NM_017563.3	IL17RD
SLC5A4	34.89284986	307.3755543	-3.138999093	0.034976	Low	33	372	NM_214182.1	ssc:397376	0	NM_014227.2	SLC5A4
PAQR4	34.89284986	307.3755543	-3.138999093	0.034976	Low	33	372	XP_003354688.1	ssc:100627080	8E-148	NM_152341.4	PAQR4
NDUFAF6	1543.744266	196.6542525	2.97270049	0.03499	High	1460	238	XP_001925076.3	ssc:100152224	0	NM_152416.3	NDUFAF6
FBXO5	2276.494113	285.0660382	2.997445652	0.035134	High	2153	345	XP_005659190.1	ssc:100514379	0	NM_012177.3	FBXO5
COBLL1	3270.411655	402.397567	3.022778755	0.035221	High	3093	487	XP_003483713.1	ssc:100737004	0	NM_014900.4	COBLL1
LOC102162332	130.0551676	1172.489009	-3.172378769	0.035267	Low	123	1419	XP_005663147.1	ssc:102162332	2E-144	NM_003068.4	SNAI2
CNTNAP1	76.12985423	672.5905946	-3.143194383	0.035272	Low	72	814	XP_003131443.3	ssc:100521039	0	NM_003632.2	CNTNAP1
CMTM8	244.249949	32.2248565	2.922112422	0.035301	High	231	39	XP_003358400.2	ssc:100624671	8E-79	NM_178868.3	CMTM8
LOC100738280	1.057359087	28.09346464	-4.731697242	0.035452	Low	1	34	XR_303920.1	ssc:100738280	2E-21	NM_003433.3	ZNF132
SDS	1.057359087	28.09346464	-4.731697242	0.035452	Low	1	34	XP_001928337.1	ssc:100155595	1E-138	NM_006843.2	SDS
CCDC166	1.057359087	28.09346464	-4.731697242	0.035452	Low	1	34	XP_005653153.1	ssc:100516437	2E-138	NM_001162914.1	CCDC166
MRPL47	4514.9233	546.9962821	3.045098547	0.035452	High	4270	662	XP_005670063.1	ssc:100523924	1E-121	NM_177988.1	MRPL47
SLC6A15	775.0442105	101.6322397	2.930920482	0.03546	High	733	123	XP_005655797.1	ssc:100739274	2E-162	NM_182767.5	SLC6A15
HCLS1	3.17207726	44.61903207	-3.814159402	0.035599	Low	3	54	XP_005654114.1	ssc:100521992	0	NM_005335.4	HCLS1
LOC100621492	3.17207726	44.61903207	-3.814159402	0.035599	Low	3	54	XP_003356130.2	ssc:100621492	1E-154	NM_001101401.2	SBK2
LOC100739607	3.17207726	44.61903207	-3.814159402	0.035599	Low	3	54	XP_005669288.1	ssc:100739607	2E-66	NM_144681.2	CCDC42
ARHGEF15	6.344154519	69.40738323	-3.451589323	0.035662	Low	6	84	XP_005657103.1	ssc:100627972	0	NM_173728.3	ARHGEF15
GCK	574.145984	76.0176102	2.917012031	0.035768	High	543	92	XP_003134931.1	ssc:100514142	0	NM_033508.1	GCK
LOC100627397	6518.618769	775.8753911	3.070669431	0.035802	High	6165	939	XP_005658589.1	ssc:100627397	1E-172	NR_049768.1	LARP7
LOC733662	8.458872693	85.93295066	-3.344674119	0.035967	Low	8	104	NM_001044592.1	ssc:733662	0	NM_000504.3	F10
STAC	225.2174854	2075.61127	-3.204145529	0.036202	Low	213	2512	XP_005669409.1	ssc:100625500	2E-169	NM_003149.1	STAC
SKIDA1	674.5950972	89.23806415	2.918290711	0.036269	High	638	108	XP_003130830.1	ssc:100521330	0	NM_207371.3	SKIDA1
PGM5	10.57359087	102.4585181	-3.276502616	0.036392	Low	10	124	XP_003121981.3	ssc:100511977	0	NM_021965.3	PGM5
LOC100522989	194.5540719	25.61462952	2.925131245	0.0364	High	184	31	XP_003125153.1	ssc:100522989	4E-64	NM_002622.4	PFDN1
LOC100739654	19.03246356	171.0396229	-3.167796357	0.036404	Low	18	207	XP_003482652.1	ssc:100739654	2E-149	NM_032427.2	MAML2
TMTC1	1793.281011	228.879109	2.969943888	0.036502	High	1696	277	XP_003355582.1	ssc:100624475	9E-158	NM_175861.3	TMTC1
LOC102166396	200.8982264	1835.164264	-3.19137247	0.036556	Low	190	2221	XP_005657983.1	ssc:102166396	2E-96	NM_152695.5	ZNF449
LOC100153723	3062.111915	380.9143294	3.006988546	0.036574	High	2896	461	XP_001928444.1	ssc:100153723	6E-118	NM_013375.3	ABT1
TOMM20	35464.88112	3859.546274	3.199887951	0.036624	High	33541	4671	XP_001926970.1	ssc:100152814	8E-81	NM_014765.2	TOMM20
SORCS1	146.972913	19.00440255	2.951144716	0.036641	High	139	23	XP_003359410.1	ssc:100155686	0	NM_052918.4	SORCS1
LOC100520559	111.0227041	983.2712624	-3.146734741	0.036815	Low	105	1190	XP_005658373.1	ssc:100520559	0	NM_024408.3	NOTCH2
LOC102166971	21.14718173	0.826278372	4.677693694	0.036996	High	20	1	XR_308939.1	ssc:102166971			
LOC102167388	21.14718173	0.826278372	4.677693694	0.036996	High	20	1	XR_307536.1	ssc:102167388			
LOC100739082	296.0605442	39.66136184	2.900086115	0.037073	High	280	48	XP_005667364.1	ssc:100739082			
LOC100737457	167.0627357	1505.479193	-3.171760896	0.037099	Low	158	1822	XP_005662698.1	ssc:100737457	0	NM_206943.2	LTBP1
CCNB3	4922.006548	599.0518195	3.038493867	0.037117	High	4655	725	XP_005657865.1	ssc:100515638	0	NM_033671.1	CCNB3
LOC100625751	30.66341351	266.8879141	-3.12164376	0.037119	Low	29	323	XP_005662464.1	ssc:100625751	4E-91	NM_003896.3	ST3GAL5
EVI5L	195.611431	1777.324778	-3.18364475	0.037128	Low	185	2151	XP_005661327.1	ssc:100519944	0	NM_145245	EVI5L
BRINP3	38.06492712	3.305113487	3.525690601	0.037235	High	36	4	XP_005667971.1	ssc:100512672	0	NM_199051.1	BRINP3
LOC102160217	38.06492712	3.305113487	3.525690601	0.037235	High	36	4	XR_308366.1	ssc:102160217			
C7H15orf29	3737.764371	461.8896098	3.016555626	0.037241	High	3535	559	NM_001190181.1	ssc:100154146	2E-171	NM_024713.2	KATNBL1
LOC100523505	117.3668586	14.87301069	2.980256464	0.037261	High	111	18	XP_005667682.1	ssc:100523505	1E-89	NM_004411.4	DYNC1I1
TREX1	66.61362245	577.5685818	-3.116103123	0.037477	Low	63	699	XP_005669584.1	ssc:100155669	5E-124	NM_033629	TREX1
LOC100737760	499.0734889	66.92854811	2.898558646	0.037494	High	472	81	XP_003483086.2	ssc:100737760	2E-72	NR_103873.1	RAD51C
RASA4	10.57359087	101.6322397	-3.264820811	0.037519	Low	10	123	XP_005662006.1	ssc:100624152	0	NM_006989.5	RASA4
CENPQ	2954.261288	370.9989889	2.99331027	0.037985	High	2794	449	XP_005666157.1	ssc:100513550	3E-100	NM_018132.3	CENPQ
TAS1R3	3.17207726	43.7927537	-3.787192355	0.038111	Low	3	53	NM_001113288.1	ssc:100127440	0	NM_152228.1	TAS1R3
LOC100738599	3.17207726	43.7927537	-3.787192355	0.038111	Low	3	53	XP_003481459.1	ssc:100738599	1E-69	NM_015441.1	OLFML2B
HMHA1	1402.058149	182.6075202	2.9407281	0.038177	High	1326	221	XP_005661429.1	ssc:100623239	0	NR_047652.1	HMHA1
LOC100620307	82.47400875	9.915340461	3.056205317	0.038292	High	78	12	XR_309604.1	ssc:100620307			
PCDHB4	533.9663387	71.88621834	2.892961681	0.038381	High	505	87	XP_003124099.2	ssc:100521779	0	NM_018938.3	PCDHB4
LOC100623307	4830.016307	592.4415925	3.027283226	0.038392	High	4568	717	XR_135499.1	ssc:100623307			
LOC102165223	685.1686881	91.71689926	2.901199736	0.038416	High	648	111	XP_005666635.1	ssc:102165223	1E-62	NM_015173.3	TBC1D1
LOC102162469	34.89284986	299.9390489	-3.103666019	0.038455	Low	33	363	XP_005653429.1	ssc:102162469	3E-17	NM_013943.2	CLIC4
CCDC58	5853.539903	711.4256781	3.040524399	0.038617	High	5536	861	XP_003358841.2	ssc:100627794	8E-80	NM_001017928.2	CCDC58
LOC100627210	9.516231779	92.54317763	-3.281664321	0.038652	Low	9	112	XR_300528.1	ssc:100627210			

C5AR2	0	19.00440255	-20	0.038719	Low	0	23	XP_005664690.1	ssc:100515612	9E-144	NM_018485.2	C5AR2
WDR16	0	19.00440255	-20	0.038719	Low	0	23	XP_003132040.1	ssc:100516337	0	NM_145054.4	WDR16
A3GALT2	0	19.00440255	-20	0.038719	Low	0	23	XP_005665265.1	ssc:100739829	1E-44	NM_020469.2	ABO
LOC102159557	0	19.00440255	-20	0.038719	Low	0	23	XP_005658027.1	ssc:102159557	8E-47	NM_021599.2	ADAMTS2
LOC102163875	0	19.00440255	-20	0.038719	Low	0	23	LOC102163875				
SLC34A3	0	19.00440255	-20	0.038719	Low	0	23	XP_005652809.1	ssc:641343	6E-59	NM_080877.2	SLC34A3
INHA	0	19.00440255	-20	0.038719	Low	0	23	NM_214189.1	ssc:397386	3E-155	NM_002191.3	INHA
LOC100626840	0	19.00440255	-20	0.038719	Low	0	23	XP_003354913.2	ssc:100626840	7E-155	NM_170672.2	RASGRP3
LOC100621844	0	19.00440255	-20	0.038719	Low	0	23	XP_003355443.2	ssc:100621844	0	NM_002281.3	KRT81
WNT4	0	19.00440255	-20	0.038719	Low	0	23	NM_001170828.1	ssc:100327037	0	NM_030761.4	WNT4
LOC100154914	0	19.00440255	-20	0.038719	Low	0	23	XP_005660120.1	ssc:100154914	5E-27	NM_002177.1	IFNW1
LOC102160975	0	19.00440255	-20	0.038719	Low	0	23	XR_303919.1	ssc:102160975			
LOC102160101	0	19.00440255	-20	0.038719	Low	0	23	XP_005665000.1	ssc:102160101	2E-21	NM_052860.2	ZNF300
LOC102165502	0	19.00440255	-20	0.038719	Low	0	23	XP_005664197.1	ssc:102165502			
HIVEP3	0	19.00440255	-20	0.038719	Low	0	23	XP_005674673.1	ssc:100626195	0	NR_038261.1	HIVEP3
LOC102165893	0	19.00440255	-20	0.038719	Low	0	23	XP_005654340.1	ssc:102165893			
KIF19	0	19.00440255	-20	0.038719	Low	0	23	XP_003358037.1	ssc:100623453	0	NM_153209.3	KIF19
LOC100621187	2.114718173	35.52996998	-4.070499155	0.038736	Low	2	43	XP_003358355.3	ssc:100621187	8E-70	NR_073067.1	SLC5A10
LOC102158449	2.114718173	35.52996998	-4.070499155	0.038736	Low	2	43	XP_005656318.1	ssc:102158449			
POLK	80.35929058	694.0738323	-3.110552405	0.038791	Low	76	840	XP_005661549.1	ssc:100518650	0	NM_016218.2	POLK
LOC100739565	274.9133625	37.18252673	2.886280316	0.03886	High	260	45	XR_135127.2	ssc:100739565			
LOC100524940	390.1655029	52.88181579	2.883242605	0.038879	High	369	64	XP_005653491.1	ssc:100524940	0	NM_000775.2	CYP2J2
TNRC18	431.4025073	4063.637032	-3.235665078	0.038898	Low	408	4918	XP_005661897.1	ssc:100515017	6E-137	NM_001080495.2	TNRC18
LOC100518088	21104.88737	2394.554721	3.13974782	0.039072	High	19960	2898	XR_299059.1	ssc:100518088			
EME2	29.60605442	254.4937385	-3.103666019	0.039114	Low	28	308	XP_005655193.1	ssc:100521548			
ZFPL1	242.1352308	2197.900469	-3.182241143	0.039115	Low	229	2660	ZFPL1	ssc:100515072	2E-154	NM_006782.3	ZFPL1
COCH	48.63851798	4.95767023	3.294365055	0.039167	High	46	6	XP_003361379.1	ssc:100624179	0	NM_004086.2	COCH
LOC396709	389.1081439	3634.798557	-3.223632331	0.03917	Low	368	4399	XP_005668908.1	ssc:396709	0	NM_001145302.2	RARA
LOC100736819	1.057359087	27.26718627	-4.68862852	0.039403	Low	1	33	XR_297386.1	ssc:100736819	3E-14	NM_001266.4	CES1
LOC100737032	1.057359087	27.26718627	-4.68862852	0.039403	Low	1	33	XP_003483161.1	ssc:100737032	0	NM_017534.5	MYH2
LYPD3	1.057359087	27.26718627	-4.68862852	0.039403	Low	1	33	XP_003481923.1	ssc:100736591	3E-144	NM_014400.2	LYPD3
LOC100737558	1.057359087	27.26718627	-4.68862852	0.039403	Low	1	33	XP_003484333.1	ssc:100737558	1E-151	NM_002286.5	LAG3
NKRF	3783.230812	472.6312286	3.000831935	0.039433	High	3578	572	XP_005673926.1	ssc:100155370	0	NM_017544.3	NKRF
SUV39H2	1850.378402	239.6207278	2.948995716	0.039455	High	1750	290	NM_001039747.1	ssc:664651	0	NR_034181.1	SUV39H2
TMSB10	13628.30127	162224.059	-3.573310147	0.039553	Low	12889	196331	NM_001097482.1	ssc:100037998	5E-15	NM_021103.3	TMSB10
AAMDC	56.04003159	477.5888989	-3.091239629	0.039601	Low	53	578	XP_005667226.1	ssc:100737883	4E-37	NM_024684.2	AAMDC
SUMO1	26.43397716	1.652556743	3.999621789	0.039618	High	25	2	NM_001112676.1	ssc:100127139			
LOC102163428	26.43397716	1.652556743	3.999621789	0.039618	High	25	2	XR_304425.1	ssc:102163428			
CDC42SE2	14903.47633	1729.400632	3.10730486	0.039671	High	14095	2093	XP_005661685.1	ssc:100523052	5E-45	NM_020240.2	CDC42SE2
MAN2B1	530.7942615	5042.776903	-3.247993722	0.039682	Low	502	6103	XP_005652912.1	ssc:100518647	0	NM_001173498.1	MAN2B1
LOC100512447	1101.768168	146.2512718	2.913299611	0.039863	High	1042	177	XR_309077.1	ssc:100512447	1E-46	NR_037669.1	GGCT
ETV2	11.63094995	108.2424667	-3.218225784	0.039887	Low	11	131	XP_005664588.1	ssc:100622447	6E-52	NM_014209.2	ETV2
LOC100516109	131.1125267	1146.048102	-3.127790163	0.039916	Low	124	1387	XP_005669527.1	ssc:100516109	1E-19	NM_002507.3	NGFR
UCMA	4.229436346	51.22925905	-3.598430711	0.039947	Low	4	62	XP_005658512.1	ssc:100626016	2E-56	NM_145314.1	UCMA
CAPN12	4.229436346	51.22925905	-3.598430711	0.039947	Low	4	62	XP_003481895.1	ssc:100517656	0	NM_144691.3	CAPN12
HIPK4	87.76080419	754.3921534	-3.103666019	0.039966	Low	83	913	XP_003355967.1	ssc:100625431	0	NM_144685.3	HIPK4
LOC102161383	14.80302721	0	20	0.040087	High	14	0	XR_298180.1	ssc:102161383			
LOC102164700	14.80302721	0	20	0.040087	High	14	0	XR_308377.1	ssc:102164700			
LOC100522152	14.80302721	0	20	0.040087	High	14	0	XP_005666362.1	ssc:100522152	7E-14	NM_025059.3	CCDC170
LOC100512349	14.80302721	0	20	0.040087	High	14	0	LOC100512349				
CC2D2B	14.80302721	0	20	0.040087	High	14	0	XR_307749.1	ssc:100524048			
LOC102158037	14.80302721	0	20	0.040087	High	14	0	XR_305151.1	ssc:102158037			
LOC102158071	14.80302721	0	20	0.040087	High	14	0	XR_297450.1	ssc:102158071			
ABCA6	14.80302721	0	20	0.040087	High	14	0	XP_005668717.1	ssc:100520861	0	NM_172346.1	ABCA6
LOC100623122	14.80302721	0	20	0.040087	High	14	0	XP_005674431.1	ssc:100623122			
WNT8A	14.80302721	0	20	0.040087	High	14	0	XP_005661739.1	ssc:100739710	0	NM_058244.2	WNT8A
LOC102163470	14.80302721	0	20	0.040087	High	14	0	XR_304024.1	ssc:102163470			
LOC100513690	14.80302721	0	20	0.040087	High	14	0	XP_003133486.4	ssc:100513690	3E-74	NM_173512.2	SLC38A11
LOC100513764	14.80302721	0	20	0.040087	High	14	0	XP_003133695.1	ssc:100513764	9E-21	NM_003284.3	TNP1
LOC100517804	14.80302721	0	20	0.040087	High	14	0	XP_005654729.1	ssc:100517804	3E-136	NM_001005323.1	OR5AK2
SLA-DOA	14.80302721	0	20	0.040087	High	14	0	NM_001185143.1	ssc:100157996	4E-143	NM_006748.3	SLA
NUMBL	433.5172255	4052.069135	-3.224497565	0.040177	Low	410	4904	XP_005655913.1	ssc:100627312	0	NM_004756.3	NUMBL
NHigh160	8959.003541	1072.509327	3.062348085	0.040238	High	8473	1298	XP_003122865.3	ssc:100513973	0	NM_015231.1	NHigh160
CD109	283.3722352	2577.162241	-3.185013048	0.040291	Low	268	3119	XP_003480363.1	ssc:100155478	0	NM_133493.3	CD109
C4	7.401513606	75.19133183	-3.344674119	0.040385	Low	7	91	NM_001123089.2	ssc:445467	0	NM_007293.2	C4A
FAM54A	1814.428193	236.3156143	2.9407281	0.040385	High	1716	286	NM_001190176.1	ssc:100157026	1E-138	NM_138419.3	MTFR2
JMJD8	180.8084038	1601.327484	-3.146734741	0.040406	Low	171	1938	XP_003124757.2	ssc:100511444	3E-113	NM_001005920.2	JMJD8
C10H9orf72	3092.775328	392.4822266	2.978202805	0.040457	High	2925	475	XP_005668139.1	ssc:100515791	0	NM_145005.5	C9orf72
CTSH	739.0940015	7132.434905	-3.270564905	0.040728	Low	699	8632	NM_213929.2	ssc:396969	0	NM_148979.2	CTSH
LOC102159784	3.17207726	42.96647533	-3.759711618	0.040829	Low	3	52	XP_005666937.1	ssc:102159784			
NCCRP1	3.17207726	42.96647533	-3.759711618	0.040829	Low	3	52	XP_003355939.2	ssc:100628094	9E-111	NM_001001414.1	NCCRP1
SUSD2	20.08982264	174.3447364	-3.117406076	0.040892	Low	19	211	XP_003359219.2	ssc:100625804	0	NM_019601.3	SUSD2
CLIP2	268.569208	2425.127021	-3.174694502	0.040924	Low	254	2935	XP_003124472.4	ssc:100512954	0	NM_032421.2	CLIP2
LOC102164112	31.7207726	2.478835115	3.677693694	0.040975	High	30	3	XR_303993.1	ssc:102164112			
LOC102165555	64.49890428	7.436505346	3.116577935	0.041178	High	61	9	XR_303667.1	ssc:102165555			
NARS2	3388.835872	429.6647533	2.979506428	0.041226	High	3205	520	XP_003129750.1	ssc:100514245	0	NM_024678.5	NARS2
LOC102160340	35.95020894	303.2441624	-3.076407812	0.041262	Low	34	367	XR_306930.1	ssc:102160340			
LOC102167200	85.64608601	728.7775239	-3.089019243	0.041446	Low	81	882	XP_005668864.1	ssc:102167200	0	NM_003632.2	CNTNAP1
SVCT1	142.7434767	19.00440255	2.90901924	0.041621	High	135	23	XP_005661775.1	ssc:396578	0	NM_152685.3	SLC23A1
LEPR	142.7434767	19.00440255	2.90901924	0.041621	High	135	23	NM_001024587.1	ssc:396836	8E-121	NM_002303.5	LEPR
C2H2orf81	8.458872693	82.62783717	-3.288090591	0.041626	Low	8	100	NM_001244876.1	ssc:100513788	0	NM_001145054.1	C2orf81
LOC102165339	69.78569971	8.262783717	3.078231624	0.04174	High	66	10	XP_005672875.1	ssc:102165339	9E-117	NM_018354.1	TMEM74B
LOC100737119	69.78569971	8.262783717	3.078231624	0.04174	High	66	10	XP_005672876.1	ssc:100737119	1E-116	NM_018354.1	TMEM74B
LOC102162757	37.00756803	3.305113487	3.485048616	0.041815	High	35	4	XP_005658630.1	ssc:102162757	3E-48	NM_018150.2	RNF220
LOC100627350	37.00756803	3.305113487	3.485048616	0.041815	High	35	4	LOC100627350				
FBLN1	138.5140403	1198.929917	-3.113643203	0.041911	Low	131	1451	XP_005663793.1	ssc:100519144	0	NM_006487.2	FBLN1
NFATC2	14.80302721	131.3782611	-3.149762434	0.042034	Low	14	159	NM_001113452.1	ssc:100134969	0	NM_173091.3	NFATC2
CBLN2	15.8603863	139.6410448	-3.138223241	0.042054	Low	15	169	NM_001244702.1	ssc:100517134	6E-112	NM_182511.3	CBLN2
LOC102165965	199.8408674	27.26718627	2.873613904	0.042059	High	189	33	XR_302277.1	ssc:102165965			
LOC102162056	7.401513606	74.36505346	-3.328732575	0.042063	Low	7	90	XR_307766.1	ssc:102162056			
BCHE	891.35371	120.6366423	2.885329861	0.042071	High	843	146	XP_005670020.1	ssc:100624138	0	NM_000055.2	BCHE
GP9	2.114718173	34.70369161	-4.036551824	0.042127	Low	2	42	NM_001141989.1	ssc:100216312	5E-45	NM_000174.3	GP9
P4HA1	904.042019	8770.118638	-3.278134624	0.042141	Low	855	10614	NM_001097435.1	ssc:100037299	0	NM_001142596.1	P4HA1
LOC100622731	2523.91614	326.3799568	2.951039607	0.04217	High	2387	395	XR_131266.2	ssc:100622731			
LOC100622890	2125.291764	21872.41478	-3.363379691	0.04217	Low	2010	26471	XP_003357726.1	ssc:100622890	1E-100	NM_004078.2	CSRP1
LOC100737616	30.66341351	257.798852	-3.071655625	0.042387	Low	29	312	XR_302346.1	ssc:100737616	2E-68	NM_018271.4	THNSL2
IRF6	10.57359087	98.32712624	-3.217124069	0.042448	Low	10	119	NM_214278.1	ssc:397581	0	NM_006147.3	IRF6
PSIP1	35906.85722	4022.323114	3.158158502	0.042654	High	33959	4868	NM_001243670.1	ssc:100157597	0	NM_033222.3	PSIP1
PLIN3	125.8257313	1079.119553	-3.100355819	0.042685	Low	119	1306	NM_001031778.1	ssc:595103	9E-82	NM_005817.4	PLIN3
BCL7A	5674.846218	705.6417295	3.007573512	0.042902	High	5367	854	XP_003483472.1	ssc:100736875	3E-101	NM_020993.3	BCL7A

LOC100621169	47.5811589	4.95767023	3.262656195	0.042913	High	45	6	XP_005658484.1	ssc:100621169	0	NM_020340.4	KIAA1244
VRK3	79.30193149	666.806646	-3.071840574	0.042993	Low	75	807	XP_005656825.1	ssc:100523004	0	NM_016440.3	VRK3
TMEM229B	43.35172255	361.0836484	-3.058171866	0.043012	Low	41	437	XP_005656401.1	ssc:100153043	9E-94	NM_182526.2	TMEM229B
PIP4K2C	587.8916521	80.97528043	2.859996828	0.043045	High	556	98	XP_005674442.1	ssc:100516802	2E-155	NM_024779.4	PIP4K2C
ITGA11	487.4425389	4512.306188	-3.210560902	0.043054	Low	461	5461	XP_005659927.1	ssc:100520350	0	NM_012211.3	ITGA11
IL10RB	8.458872693	81.8015588	-3.273591021	0.043197	Low	8	99	NM_213771.1	ssc:396657			
LRRN1	3000.785088	386.698278	2.956059754	0.043281	High	2838	468	XP_005657135.1	ssc:100627698	0	NM_020873.5	LRRN1
SNAPIN	138.5140403	1189.014577	-3.101662276	0.043295	Low	131	1439	NM_001243486.1	ssc:100154273	3E-73	NR_052020.1	SNAPIN
ELK3	635.472811	5977.297741	-3.233591103	0.043339	Low	601	7234	XP_003355727.1	ssc:100622445	0	NM_005230.2	ELK3
LOC100516210	130.0551676	17.35184581	2.905962682	0.043461	High	123	21	XP_003129368.2	ssc:100516210	1E-66	NM_207491.2	CCSER1
LOC100624905	11.63094995	105.7636316	-3.184802782	0.043482	Low	11	128	XP_003353632.2	ssc:100624905	7E-65	NM_003028.2	SHB
LOC100514038	214.6438946	1882.262131	-3.132450515	0.043622	Low	203	2278	XP_003124405.1	ssc:100514038	9E-165	NM_030935.3	TSC22D4
LOC100739094	113.1374223	14.87301069	2.927307584	0.043657	High	107	18	XP_005664116.1	ssc:100739094	7E-140	NM_024829.5	PLBD1
ASIC1	3.17207726	42.14019696	-3.731697242	0.043775	Low	3	51	XP_003126177.3	ssc:100517467	0	NR_046389.1	ASIC1
LOC102162331	3.17207726	42.14019696	-3.731697242	0.043775	Low	3	51	XP_005673433.1	ssc:102162331	4E-92	NM_032534.2	KRBA1
COL17A1	3.17207726	42.14019696	-3.731697242	0.043775	Low	3	51	XP_001929475.1	ssc:414914	0	NM_130778.1	COL17A1
TRIO	121.596295	1034.500521	-3.088763197	0.043777	Low	115	1252	XP_003133886.3	ssc:100518519	0	NM_007118.2	TRIO
LOC100624637	414.4847619	3780.223551	-3.189080585	0.043804	Low	392	4575	XP_003357240.2	ssc:100624637	0	NM_014786.3	ARHGEF17
TOX3	1.057359087	26.4409079	-4.644234401	0.043873	Low	1	32	XP_005653316.1	ssc:100519713	0	NM_001146188.1	TOX3
LOC100627275	241.0778717	2122.709137	-3.138335562	0.04408	Low	228	2569	XP_005655098.1	ssc:100627275	0	NM_025092.4	ATHL1
LOC100622410	430.3451482	3927.301101	-3.189972103	0.04412	Low	407	4753	LOC100622410				
LOC100738798	157.5465039	21.48323766	2.874494402	0.044379	High	149	26	XP_005673180.1	ssc:100738798	3E-71	NM_001195278.1	TMEM178B
SYN2	174.4642493	23.96207278	2.864106818	0.044484	High	165	29	XP_005669854.1	ssc:100626060	0	NM_133625.3	SYN2
LOC100514227	6.344154519	65.27599137	-3.363052648	0.044705	Low	6	79	XP_005663077.1	ssc:100514227			
C3H16orf5	262.2250535	2311.926884	-3.140218338	0.044726	Low	248	2798	NM_001244099.1	ssc:100512098	8E-72	NM_013399.2	CDIP1
RENBP	362.6741667	3260.494455	-3.168344875	0.044826	Low	343	3946	NM_213900.1	ssc:396934	0	NM_002910.5	RENBP
ARG2	10615.88523	1286.515425	3.044684019	0.044853	High	10040	1557	XP_001928714.1	ssc:100155893	0	NM_001172.3	ARG2
LOC100520518	4.229436346	49.5767023	-3.551124996	0.044975	Low	4	60	XR_308015.1	ssc:100520518			
LOC102167220	4.229436346	49.5767023	-3.551124996	0.044975	Low	4	60	XR_303341.1	ssc:102167220			
E4	0	18.17812418	-20	0.044993	Low	0	22	NM_213947.1	ssc:396987	1E-18	NM_080736.1	WFDC2
LOC102159509	0	18.17812418	-20	0.044993	Low	0	22	XP_005668686.1	ssc:102159509	3E-22	NM_006678.4	CD300C
LOC100156073	0	18.17812418	-20	0.044993	Low	0	22	XP_005663279.1	ssc:100156073	2E-128	NM_002432.1	MNDA
LOC102163811	0	18.17812418	-20	0.044993	Low	0	22	XP_005670535.1	ssc:102163811			
METTL11B	0	18.17812418	-20	0.044993	Low	0	22	NM_001243649.1	ssc:100157210	5E-156	NM_001136107.1	METTL11B
LOC102164807	0	18.17812418	-20	0.044993	Low	0	22	XP_005654422.1	ssc:102164807			
LOC100738476	0	18.17812418	-20	0.044993	Low	0	22	XP_003481153.2	ssc:100738476	7E-132	NM_031948.3	PRSS27
DISP2	0	18.17812418	-20	0.044993	Low	0	22	XP_001924685.3	ssc:100153589	0	NM_033510.1	DISP2
LOC100512345	0	18.17812418	-20	0.044993	Low	0	22	LOC100512345				
LOC102166285	0	18.17812418	-20	0.044993	Low	0	22	XP_005671602.1	ssc:102166285	4E-26	NM_198148.2	CPXM2
HTR3C	0	18.17812418	-20	0.044993	Low	0	22	XP_003358754.1		0	NM_130770.2	HTR3C
LOC100517129	0	18.17812418	-20	0.044993	Low	0	22	XR_299517.1	ssc:100517129	2E-100	NM_003113.3	SP100
LOC102162278	0	18.17812418	-20	0.044993	Low	0	22	XR_307435.1	ssc:102162278			
LOC102165168	0	18.17812418	-20	0.044993	Low	0	22	XR_301714.1	ssc:102165168			
TMED3	141.6861176	1205.540144	-3.088909378	0.045007	Low	134	1459	NM_001204363.1	ssc:100155219	4E-68	NM_007364.2	TMED3
RNASEH2C	697.8569971	6540.819591	-3.228468084	0.045083	Low	660	7916	XP_005660749.1	ssc:100517142	1E-64	NM_032193.3	RNASEH2C
LOC102168042	14.80302721	128.899426	-3.122281698	0.045127	Low	14	156	XP_005657623.1	ssc:102168042	0	NM_133437.3	TTN
LOC102163531	26.43397716	219.7900469	-3.055660646	0.045146	Low	25	266	XP_005658011.1	ssc:102163531	4E-102	NM_198240.1	CLIP1
FAM171B	11206.94896	1355.922808	3.04704661	0.045167	High	10599	1641	XP_003133578.1	ssc:100525259	0	NM_177454.3	FAM171B
SPARCL1	10.57359087	96.67456949	-3.192671025	0.045191	Low	10	117	NM_001097422.1	ssc:100037275	0	NM_004684.4	SPARCL1
ENDOD1	2526.030858	331.3376271	2.930498311	0.045192	High	2389	401	XP_005667307.1	ssc:100526236	0	NM_015036.2	ENDOD1
LOC100738463	20.08982264	0.826278372	4.603693113	0.045215	High	19	1	XP_003482674.2	ssc:100738463	1E-144	NM_014619.3	GRIK4
LOC102158743	20.08982264	0.826278372	4.603693113	0.045215	High	19	1	XP_005658587.1	ssc:102158743	2E-31	NM_019114.4	EPB41L4B
KIF18A	2524.973499	331.3376271	2.929894294	0.045279	High	2388	401	XP_005654745.1	ssc:100518110	0	NM_031217.3	KIF18A
WDR36	9645.229588	1177.44668	3.03415385	0.045287	High	9122	1425	XP_003123877.2	ssc:100516248	0	NM_139281.2	WDR36
ROM1	43.35172255	356.1259782	-3.038226455	0.04534	Low	41	431	XP_005660852.1	ssc:100622038	1E-138	NM_000327.3	ROM1
LOC100627730	2357.910763	310.6806678	2.924004742	0.045418	High	2230	376	XP_003357326.2	ssc:100627730	1E-42	NM_004398.3	DDX10
NIP7	7205.902175	894.8594766	3.009446013	0.045488	High	6815	1083	NM_001048071.1	ssc:595107	1E-104	NM_016101.4	NIP7
LOC100737804	969.5982624	133.0308178	2.865626465	0.045508	High	917	161	XP_003480932.1	ssc:100737804	2E-141	NR_074081.1	PPIP5K2
KIF21A	1942.368642	258.6251304	2.908882663	0.04562	High	1837	313	XP_003126654.4	ssc:100520674	0	NM_017641.3	KIF21A
PRIM1	4100.438538	525.5130444	2.963979732	0.045654	High	3878	636	NM_001243669.1	ssc:100157576	0	NM_000946.2	PRIM1
MTFR1	11026.14055	1339.397241	3.041272091	0.045868	High	10428	1621	XP_005663114.1	ssc:100522611	4E-145	NM_014637.3	MTFR1
BHLHE22	207.242381	28.91974301	2.841192426	0.045906	High	196	35	XP_003481442.1	ssc:100739667	1E-138	NM_152414.4	BHLHE22
CD55	107.8506268	901.4697036	-3.063244445	0.045946	Low	102	1091	NM_213815.1	ssc:396743			
LOC100739425	76.12985423	627.9715625	-3.044165008	0.046075	Low	72	760	ZFP30	ssc:100739425	0	NM_014898.2	ZFP30
LOC102165933	11.63094995	104.1110748	-3.162082706	0.046086	Low	11	126	XR_303906.1	ssc:102165933			
GOLGA3	193.4967128	1663.298362	-3.103666019	0.046101	Low	183	2013	XP_005657358.1	ssc:100155657	0	NM_005895.3	GOLGA3
LOC100620483	3026.161706	394.9610617	2.937706748	0.046137	High	2862	478	XP_005659991.1	ssc:100620483	0	NM_018353.4	MIS18BP1
PLA2G4B	14.80302721	128.0731476	-3.113003884	0.046215	Low	14	155	NM_001204400.1	ssc:100152927	0	NM_001114633.1	PLA2G4B
DIS3	5910.637294	744.4768129	2.989014871	0.046218	High	5590	901	XP_003131067.3	ssc:100521470	0	NM_014953.4	DIS3
SAC3D1	71.90041789	591.6153142	-3.040587337	0.046231	Low	68	716	XP_003122615.2	ssc:100519293	1E-156	NM_013299.3	SAC3D1
NLRX1	198.7835083	1709.569951	-3.104363479	0.046285	Low	188	2069	NM_001204769.1	ssc:100518678	0	NM_170722.1	NLRX1
CD9	185.0378402	1583.975639	-3.097657911	0.046406	Low	175	1917	NM_214006.1	ssc:397067	1E-112	NM_001769.3	CD9
LOC102167359	13.74566813	119.8103639	-3.123703773	0.046524	Low	13	145	XR_303838.1	ssc:102167359			
KLHL32	25.37661808	1.652556743	3.9407281	0.046587	High	24	2	XR_300774.1	ssc:100621727	8E-75	NR_104421.1	KLHL32
PIM2	2474.220263	327.2062352	2.918701794	0.046772	High	2340	396	XP_003360359.1	ssc:100621339	3E-161	NM_006875.3	PIM2
LOC102165240	30.66341351	2.478835115	3.628784094	0.04695	High	29	3	XR_301921.1	ssc:102165240			
LOC100515371	58.15474976	474.2837854	-3.027781614	0.046995	Low	55	574	XP_003124976.3	ssc:100515371	3E-102	NM_018271.4	THNSL2
LOC102163588	35.95020894	3.305113487	3.44322844	0.047025	High	34	4	XR_301263.1	ssc:102163588			
LOC102160325	35.95020894	3.305113487	3.44322844	0.047025	High	34	4	XR_299539.1	ssc:102160325			
LOC102158261	35.95020894	3.305113487	3.44322844	0.047025	High	34	4	XR_304928.1	ssc:102158261			
LOC102167356	26.43397716	217.3112118	-3.0392972	0.047109	Low	25	263	XP_005663852.1	ssc:102167356	0	NM_138632.2	TRIOBP
LOC100737253	21.14718173	175.9972932	-3.057015926	0.047178	Low	20	213	XP_003480897.1	ssc:100737253	9E-75	NM_213604.2	ADAMTSL5
LOC102165603	57.09739067	6.610226974	3.110653101	0.047201	High	54	8	XP_005667495.1	ssc:102165603	0	NM_003105.5	SORL1
CCDC30	545.5972887	76.84388857	2.827834044	0.047265	High	516	93	XP_005665576.1	ssc:100522685	0	NM_001080850.2	CCDC30
KIAA1211	69.78569971	570.1320765	-3.030292833	0.04737	Low	66	690	XP_005666754.1	ssc:100522154	2E-94	NM_020722.1	KIAA1211
TMBIM1	329.896035	2906.021033	-3.138961802	0.047441	Low	312	3517	XP_003361310.1	ssc:100152389	1E-139	NM_022152.4	TMBIM1
LOC102160135	57.09739067	464.3684449	-3.023773219	0.047442	Low	54	562	XR_302088.1	ssc:102160135			
SLC35G2	437.7466618	61.97087788	2.820433866	0.047489	High	414	75	XP_005654084.1	ssc:100620415	0	NM_025246.2	SLC35G2
LOC100621400	2727.986443	360.2573701	2.920736625	0.047584	High	2580	436	XR_131009.3	ssc:100621400			
P2RX6	7.401513606	71.88621834	-3.279822975	0.047602	Low	7	87	XP_005670980.1	ssc:100515342	0	NM_005446.3	P2RX6
LOC102165670	7.401513606	71.88621834	-3.279822975	0.047602	Low	7	87	XR_308289.1	ssc:102165670			
LOC100624700	4008.448297	518.9028174	2.949507592	0.047657	High	3791	628	XP_003357005.1	ssc:100624700	0	NR_024010.1	UGT2A3
NOL11	1981.490928	266.0616357	2.896753959	0.047711	High	1874	322	XP_003131324.3	ssc:100524704	0	NM_015462.3	NOL11
PNOC	4.229436346	48.75042393	-3.52687745	0.04776	Low	4	59	NM_001244476.1	ssc:397257	3E-75	NM_006228.4	PNOC
LOC100520605	16.91774539	142.9461583	-3.078862628	0.047809	Low	16	173	XP_005661698.1	ssc:100520605			
MARVELD2	78.24457241	9.915340461	2.980256464	0.047842	High	74	12	NM_001243948.1	ssc:100516404			
LOC100739291	21.14718173	175.1710148	-3.05022676	0.048012	Low	20	212	XP_005670218.1	ssc:100739291	0	NM_053032.2	MYLK
NOD1	78.24457241	637.886903	-3.027238072	0.048343	Low	74	772	NM_001114277.1	ssc:100135660	0	NM_006092.2	NOD1
TRMT10C	3513.604245	459.4107747	2.935095104	0.048345	High	3323	556	XP_005657178.1	ssc:100522163	0	NM_017819.3	TRMT10C
PHLDB3	87.76080419	718.035905	-3.032407336	0.048379	Low	83	869	XP_005655987.1	ssc:100520495	0	NM_198850.3	PHLDB3

RBBP8	5687.534527	724.646132	2.97245484	0.048388	High	5379	877	XP_005665370.1	ssc:100621518	0	NM_203292.1	RBBP8
RLN3	5.286795433	56.18692928	-3.409769147	0.048429	Low	5	68	NM_001045679.1	ssc:503836	4E-56	NM_080864.2	RLN3
WDTC1	625.9565793	5717.846332	-3.191337358	0.048503	Low	592	6920	NM_001135964.1	ssc:100190994	0	NM_015023.4	WDTC1
KLF7	42.29436346	341.2529675	-3.012304277	0.048545	Low	40	413	NM_001097487.1	ssc:100038003			
LOC100738207	1554.317857	212.3535415	2.87174149	0.049032	High	1470	257	XP_005662646.1	ssc:100738207			
LOC102160529	6.344154519	63.62343462	-3.326058441	0.049044	Low	6	77	LOC102160529				
LOC100621254	27.49133625	223.0951604	-3.02061028	0.049138	Low	26	270	XP_005653384.1	ssc:100621254	1E-62	NM_017891.4	C1orf159
NUDT19	1923.336178	260.2776871	2.885487408	0.049176	High	1819	315	XP_005655869.1	ssc:100524317			
HDAC10	153.3170676	1279.905198	-3.06144674	0.049205	Low	145	1549	XR_297361.1	ssc:100518786	0	NM_032019.5	HDAC10
LOC100518411	15.8603863	133.8570962	-3.077193808	0.049209	Low	15	162	XP_005660658.1	ssc:100518411	0	NR_030691.1	ANO1
LOC102157728	684.111329	6259.058666	-3.193642672	0.049358	Low	647	7575	XR_300556.1	ssc:102157728			
EGFL7	145.9155539	1213.802928	-3.056328625	0.049402	Low	138	1469	XR_297247.1	ssc:100511434	7E-86	NR_046367.1	EGFL7
LOC100158003	563.5723931	80.14900206	2.81384448	0.049551	High	533	97	XP_001928233.1	ssc:100158003	0	NM_032704.3	TUBA1C
SYDE1	412.3700438	3643.887619	-3.143467027	0.049564	Low	390	4410	XP_003123474.1	ssc:100517807	0	NM_033025.4	SYDE1
DGKA	160.7185812	1341.876076	-3.061642805	0.049646	Low	152	1624	NM_214032.2	ssc:397097	0	NM_201554.1	DGKA
LOC100516840	248.4793853	35.52996998	2.806017791	0.049752	High	235	43	LOC100516840				
SYT5	28.54869534	230.5316657	-3.01346821	0.049784	Low	27	279	XP_005656025.1	ssc:100626439	0	NM_003180.2	SYT5
LOC102161004	27.49133625	222.268882	-3.015257045	0.049822	Low	26	269	XP_005672261.1	ssc:102161004	3E-65	NM_022572.4	PNKD
LOC102158597	16.91774539	141.2936016	-3.062086916	0.049904	Low	16	171	XR_304480.1	ssc:102158597			
ARHGEF2	1712.92172	16653.64058	-3.281306466	0.049932	Low	1620	20155	NM_001128463.1	ssc:100145887	0	NM_004723.3	ARHGEF2

piPS-LF Vs PEFS

AccID	piPS-LF	PEFs	Log2FC	FDR	Style	piPS-LF	PEFs	AccID	KeggID	E-Value	Blast_AccID	Blast_Symbol
SLC6A1	1663.660149	0	20	0	High	2007	0	XP_005669835.1	ssc:100156668	0	NM_003042.3	SLC6A1
LOC100736983	3771.626147	0	20	0	High	4550	0	XP_003483672.1	ssc:100736983	2E-61	NM_001029887.1	HELT
NLRP12	925.0845671	0	20	0	High	1116	0	XP_005664869.1	ssc:100523066	0	NM_144687.3	NLRP12
LOC100514082	933.3738554	0	20	0	High	1126	0	XP_003121229.3	ssc:100514082	1E-137	NM_003980.4	MAP7
C1QL2	2182.569593	0	20	0	High	2633	0	XP_005671685.1	ssc:100738874	1E-130	NM_182528.3	C1QL2
TRIM71	859.5991901	0	20	0	High	1037	0	XP_005674691.1	ssc:100623572	0	NM_001039111.1	TRIM71
LOC100623527	2132.833863	0	20	0	High	2573	0	LOC100623527		7E-28	NM_001029887.1	HELT
VRTN	852.1388307	0	20	0	High	1028	0	NM_001195113.1	ssc:100157734	0	NM_018228.2	VRTN
BPIFB6	5035.742603	0	20	0	High	6075	0	XP_003483988.1	ssc:100736799	0	NM_174897.2	BPIFB6
NXNL2	2489.273257	0	20	0	High	3003	0	XP_005670441.1	ssc:100739001	8E-47	NM_145283.2	NXNL2
SNCA	2962.591616	0	20	0	High	3574	0	NM_001037145.1	ssc:641350	9E-71	NM_007308.2	SNCA
LOC102165604	877.0066954	0	20	0	High	1058	0	XR_299597.1	ssc:102165604			
UTF1	2461.918606	0	20	0	High	2970	0	XP_001925647.3	ssc:100158138	3E-29	NM_003577.2	UTF1
ACTL8	5309.289115	0	20	0	High	6405	0	XP_003356215.1	ssc:100622307	3E-54	NM_030812.2	ACTL8
LIN28A	10943.51833	0	20	0	High	13202	0	NM_001123133.1	ssc:100142662	1E-105	NM_024674.4	LIN28A
ST14	1661.173363	0	20	0	High	2004	0	XP_005667570.1	ssc:100517843	0	NM_021978.3	ST14
HSD17B2	3343.069946	0	20	0	High	4033	0	NM_001167649.1	ssc:100312973	1E-25	NM_002153.2	HSD17B2
GABRR2	1272.405744	0	20	0	High	1535	0	XP_003121344.1	ssc:100522289	0	NM_002043.3	GABRR2
INPP5D	9428.23644	1.652556743	12.47807241	0	High	11374	2	XP_003133787.3	ssc:100526019	0	NM_005541.3	INPP5D
GABRA5	3870.268677	1.652556743	11.1935182	0	High	4669	2	XP_005654528.1	ssc:100520472	0	NM_001165037.1	GABRA5
OTX2	1613.92442	0.826278372	10.93165749	0	High	1947	1	XP_005660050.1	ssc:100512643	2E-174	NR_073036.1	OTX2
KRT6A	2853.173011	1.652556743	10.75365171	0	High	3442	2	XP_005652608.1	ssc:100737483	0	NM_173086.4	KRT6C
SALL4	2776.911559	1.652556743	10.7145657	0	High	3350	2	NM_001114673.1	ssc:100136902			
SOX21	5280.276606	3.305113487	10.64169798	0	High	6370	4	XP_005653879.1	ssc:100517115	8E-155	NM_007084.2	SOX21
HENMT1	6600.760222	4.95767023	10.37875417	0	High	7963	6	XP_005663670.1	ssc:100153448	3E-126	NM_144584.2	HENMT1
CDH1	3384.516387	3.305113487	10.00003418	0	High	4083	4	NM_001163060.1	ssc:100048953			
LOC100511106	3238.624914	3.305113487	9.936465865	0	High	3907	4	XP_003133370.3	ssc:100511106	2E-85	NR_048571.1	GPM6A
AIF1	7267.218997	8.262783717	9.780559762	0	High	8767	10	NM_001129950.1	ssc:397271	1E-74	NM_032955.1	AIF1
ESRRB	1307.220755	1.652556743	9.627587269	0	High	1577	2	XP_003482338.2	ssc:100151940	0	NM_004452.3	ESRRB
LOC100622539	3264.321707	4.131391859	9.625939625	0	High	3938	5	XP_005674221.1	ssc:100622539	4E-129	NR_048571.1	GPM6A
LOC100524089	1293.128965	1.652556743	9.611950638	0	High	1560	2	XP_005664591.1	ssc:100524089	0	NM_004646.3	NPHS1
NTRK2	1223.498944	1.652556743	9.53209733	0	High	1476	2	XP_003130695.4	ssc:100519209	0	NM_006180.3	NTRK2
TDH	2137.807436	3.305113487	9.337216382	0	High	2579	4	NM_214004.1	ssc:397065			
NUTM1	1051.081748	1.652556743	9.312959354	0	High	1268	2	XP_003356776.2	ssc:100154145	0	NM_175741.2	NUTM1
MAP7D2	2070.664201	3.305113487	9.291178086	0	High	2498	4	XP_005673545.1	ssc:100739061	0	NM_152780.3	MAP7D2
LOC100519889	4690.079284	8.262783717	9.148768691	0	High	5658	10	XR_297275.1	ssc:100519889			
ANO9	2135.32065	4.131391859	9.013609108	0	High	2576	5	XP_005655100.1	ssc:100627366	0	NM_001012302.2	ANO9
LOC100521657	2499.220403	4.95767023	8.97760011	0	High	3015	6	XP_003123900.2	ssc:100521657	0	NM_173800.4	AQPEP
NPTX1	13338.2937	31.39857813	8.730659178	0	High	16091	38	XP_003131182.2	ssc:100525071	0	NM_002522.3	NPTX1
NRARP	1315.510043	3.305113487	8.636706737	0	High	1587	4	XP_003353772.1	ssc:100620133	8E-63	NM_001004354.2	NRARP
FAM43B	2572.16614	6.610226974	8.604068306	0	High	3103	8	XP_003356225.1	ssc:100624917	5E-164	NM_207334.2	FAM43B
SAMD5	2104.650283	5.783948602	8.507310082	0	High	2539	7	XP_005659216.1	ssc:100522528	1E-80	NM_001030060.2	SAMD5
SOX2	5045.689749	14.04673232	8.488673134	0	High	6087	17	NM_001123197.1	ssc:407739	1E-158	NM_003106.3	SOX2
C1QL4	2426.274667	7.436505346	8.349902353	0	High	2927	9	XP_003126157.1	ssc:100512604	1E-131	NM_001008223.1	C1QL4
ALPL	2573.823997	8.262783717	8.283069783	0	High	3105	10	XP_005658515.1	ssc:100170147	0	NM_001177520.1	ALPL
NHigh210	21174.9868	80.14900206	8.045460797	0	High	25545	97	XP_005669873.1	ssc:100515926	0	NM_024923.3	NHigh210
ELMOD1	6216.137248	28.09346464	7.789639991	0	High	7499	34	XP_003357322.2	ssc:100626413	0	NM_018712.3	ELMOD1
PODXL	6663.758813	30.57229975	7.767967294	0	High	8039	37	XP_005657812.1	ssc:100512201	2E-100	NM_005397.3	PODXL
COL6A2	363.8997536	73816.40462	-7.664256585	0	Low	439	89336	XP_005657250.1	ssc:100101552	0	NM_058175.2	COL6A2
PDGFRB	58.85394648	13038.67271	-7.791442141	0	Low	71	15780	XP_005661873.1	ssc:100126842	0	NM_002609.3	PDGFRB
MCSF	25.69679353	6173.125715	-7.908269012	0	Low	31	7471	MCSF		2E-175	NM_172212.2	CSF1
SERPINF1	103.616103	26974.68372	-8.024214005	0	Low	125	32646	NM_001078662.1	ssc:780402	2E-16	NM_002615.5	SERPINF1
LOC102159390	12.43393235	3391.872716	-8.091655587	0	Low	15	4105	XR_302916.1	ssc:102159390			
LOC100624590	9.947145883	2715.977008	-8.092972916	0	Low	12	3287	XP_003360576.1	ssc:100624590	0	NM_024005.1	DDX3X
CRABP2	56.36716001	15784.39574	-8.129428436	0	Low	68	19103	NM_001164509.1	ssc:100155151	4E-76	NM_001878.3	CRABP2
LOC100627123	23.21000706	6652.367171	-8.162977027	0	Low	28	8051	XP_005666705.1	ssc:100627123	1E-115	NM_006206.4	PDGFRA
IGFBP4	8.289288236	2432.563526	-8.197013537	0	Low	10	2944	NM_001123129.1	ssc:100144490	1E-134	NM_001552.2	IGFBP4
ITGB5	23.21000706	7134.91374	-8.264005216	0	Low	28	8635	NM_001246669.1	ssc:100134977	0	NM_002213.3	ITGB5
PDLIM4	7.460359412	2430.084691	-8.347545743	0	Low	9	2941	NM_001285969.1	ssc:100520235	7E-168	NM_003687.3	PDLIM4
LOC100515628	95.32681471	31142.43183	-8.351783799	0	Low	115	37690	XP_003133256.2	ssc:100515628	0	NM_002775.4	HTRA1
DHX58	25.69679353	8611.47319	-8.388527905	0	Low	31	10422	NM_001199132.1	ssc:100524520	0	NM_024119.2	DHX58
LOC100515260	43.10429883	14486.31241	-8.392642921	0	Low	52	17532	XR_303102.1	ssc:100515260			
BAHCC1	9.947145883	3346.427406	-8.394123368	0	Low	12	4050	XP_005656945.1	ssc:100625456	0	NM_001080519.2	BAHCC1
NPY	4.973572942	1688.086713	-8.406890664	0	Low	6	2043	NM_001256367.1	ssc:397304	4E-47	NM_000905.3	NPY
GBP1	10.77607471	3798.401675	-8.461416908	0	Low	13	4597	NM_001128473.1	ssc:100151938	0	NM_002053.2	GBP1
LOC100157228	15.74964765	5598.862247	-8.473670322	0	Low	19	6776	XP_005671626.1	ssc:100157228	0	NM_021641	ADAM12
IFIT3	25.69679353	9279.106115	-8.496253673	0	Low	31	11230	NM_001204395.1	ssc:100154248	0	NM_001549.4	IFIT3
LRRN4CL	14.92071882	5439.390521	-8.509984157	0	Low	18	6583	XP_003353868.1	ssc:100620756	1E-67	NM_203422.3	LRRN4CL
RPS6KA2	17.4075053	6651.540892	-8.577835321	0	Low	21	8050	XP_003121145.2	ssc:100515286	0	NM_021135.4	RPS6KA2
SLC6A17	5.802501765	2253.26112	-8.601123739	0	Low	7	2727	XP_001929358.2	ssc:100156430	0	NM_001010898.2	SLC6A17

LOC102167439	4.144644118	1613.72166	-8.604927815	0	Low	5	1953	XP_005662978.1	ssc:102167439	7E-54	NM_138455.3	CTHRC1
CSF1	7.460359412	2943.20356	-8.623926475	0	Low	9	3562	NM_001244523.1	ssc:100513084	1E-32	NM_172212.2	CSF1
GREM1	4.144644118	1662.472084	-8.647866171	0	Low	5	2012	XP_005659828.1	ssc:100156280	5E-107	NM_013372.6	GREM1
LOC100623720	61.34073295	24788.35115	-8.65860119	0	Low	74	30000	XP_005659104.1	ssc:100623720	2E-177	NM_001848.2	COL6A1
PCDH7	11.60500353	4780.02038	-8.686125998	0	Low	14	5785	NM_001244484.1	ssc:100520035	0	NM_032457.3	PCDH7
HOXA5	5.802501765	2425.127021	-8.707169542	0	Low	7	2935	NM_001195232.1	ssc:100499568	3E-155	NM_019102.3	HOXA5
LOC100525721	3.315715294	1404.673232	-8.726698707	0	Low	4	1700	XP_003359988.2	ssc:100525721	1E-130	NM_177991.1	DUSP15
TBX2	7.460359412	3531.513761	-8.886825867	0	Low	9	4274	NM_001246248.1	ssc:100622658	0	NM_005994.3	TBX2
THBS1	442.6479918	211976.7586	-8.903530503	0	Low	534	256544	NM_001244536.1	ssc:492313	0	NM_003246.2	THBS1
VEGFC	3.315715294	1636.031176	-8.946664391	0	Low	4	1980	VEGFC		0	NM_005429.3	VEGFC
COL1A1	247.8497183	124736.6356	-8.975203917	0	Low	299	150962	XP_005668984.1	ssc:397571	3E-121	NM_000088.3	COL1A1
GPR124	65.48537706	34432.67231	-9.038389649	0	Low	79	41672	XP_003133414.1	ssc:100524344	0	NM_032777.9	GPR124
BGN	370.5311842	198659.6301	-9.066488049	0	Low	447	240427	XP_003135523.1	ssc:397396	0	NM_001711.4	BGN
ISG15	44.76215647	24180.21027	-9.077331533	0	Low	54	29264	NM_001128469.1	ssc:100145895	3E-52	NM_005101.3	ISG15
NES	42.27537	23472.08971	-9.116913193	0	Low	51	28407	XP_005663322.1	ssc:100153933	0	NM_006617.1	NES
LOC100624156	2.486786471	1416.241129	-9.153568569	0	Low	3	1714	XR_300518.1	ssc:100624156			
ACTC1	9.947145883	5781.469767	-9.182937951	0	Low	12	6997	NM_001170517.1	ssc:100152267	0	NM_005159.4	ACTC1
DES	85.37966883	49798.14491	-9.187983718	0	Low	103	60268	NM_001001535.1	ssc:396725	0	NM_001927.3	DES
THY1	76.26145177	44528.14145	-9.189547686	0	Low	92	53890	NM_001146129.1	ssc:100271931	2E-67	NM_006288.3	THY1
SEMA3C	3.315715294	1944.233009	-9.195665281	0	Low	4	2353	XP_005667747.1	ssc:100624508	0	NM_006379.3	SEMA3C
ITGA8	26.52572236	16505.73675	-9.281359782	0	Low	32	19976	XP_003130794.1	ssc:100512676	0	NM_003638.1	ITGA8
SAMD9	6.631430589	4197.494128	-9.305992457	0	Low	8	5080	XP_005667663.1	ssc:100519098	0	NM_017654.3	SAMD9
IGLON5	1.657857647	1059.288873	-9.319560222	0	Low	2	1282	XP_003127424.2	ssc:100512905	7E-175	NM_001101372.1	IGLON5
CXCL2	3.315715294	2125.187972	-9.324054603	0	Low	4	2572	XP_005652611.1	ssc:396594	9E-35	NM_002089.3	CXCL2
CNN1	9.947145883	6544.124704	-9.36170189	0	Low	12	7920	NM_213878.1	ssc:396911	8E-177	NM_001299.4	CNN1
HOXB3	4.144644118	2740.765359	-9.369114878	0	Low	5	3317	XP_003131595.1	ssc:100522160	0	NM_002146.4	HOXB3
CARP	6.631430589	4422.241845	-9.381242076	0	Low	8	5352	NM_213922.1	ssc:396959	7E-172	NM_014391.2	ANKRD1
LOXL1	19.89429177	13617.89384	-9.418933337	0	Low	24	16481	XP_005666227.1	ssc:100154899	0	NM_005576.2	LOXL1
EPAS1	6.631430589	4575.103344	-9.430268482	0	Low	8	5537	NM_001097420.1	ssc:100037272	0	NM_001430.4	EPAS1
LOC100622618	1.657857647	1144.395545	-9.431049937	0	Low	2	1385	XP_003357081.2	ssc:100622618	0	NM_024582.4	FAT4
LOC102165913	11.60500353	8517.277456	-9.519501575	0	Low	14	10308	XR_301647.1	ssc:102165913			
LOC100157992	22.38107824	16447.89727	-9.521407902	0	Low	27	19906	XP_005663151.1	ssc:100157992	9E-94	NM_022716	PRRX1
CTHRC1	8.289288236	6120.243899	-9.528125203	0	Low	10	7407	XP_003125583.1	ssc:100152510	4E-73	NM_138455.3	CTHRC1
DKK2	1.657857647	1229.502217	-9.534538487	0	Low	2	1488	XP_003129317.1	ssc:100519672	5E-140	NM_014421.2	DKK2
ARNT2	2.486786471	1853.342388	-9.54163108	0	Low	3	2243	XP_001926107.3	ssc:100157219	0	NM_014862.3	ARNT2
LOC100525680	4.144644118	3270.409795	-9.624007478	0	Low	5	3958	XP_005661152.1	ssc:100525680	6E-52	NM_199161.3	SAA1
COL4A2	54.70930236	43357.305	-9.63027321	0	Low	66	52473	XP_005668604.1	ssc:100153454	0	NM_001846.2	COL4A2
MX1	65.48537706	52119.16085	-9.636425353	0	Low	79	63077	NM_214061.1	ssc:397128	0	NM_002462.4	MX1
LOC100152082	12.43393235	9950.044152	-9.644276482	0	Low	15	12042	XP_005666212.1	ssc:100152082	0	NM_201526.1	ISLR
LTBP2	69.63002118	56402.58793	-9.661836182	0	Low	84	68261	XP_005656461.1	ssc:100514300	0	NM_000428.2	LTBP2
LOC100736872	4.973572942	4152.875096	-9.705612131	0	Low	6	5026	XP_003482733.1	ssc:100736872	8E-178	NM_006379.3	SEMA3C
SVEP1	5.802501765	4956.843952	-9.73853107	0	Low	7	5999	XP_003122123.2	ssc:100155613	0	NM_153366.3	SVEP1
PRRX2	15.74964765	13710.43702	-9.76573929	0	Low	19	16593	XP_005654631.1	ssc:100521241	5E-97	NM_016307.3	PRRX2
FAP	2.486786471	2238.388109	-9.813961853	0	Low	3	2709	XP_005671941.1	ssc:100736572	0	NM_004460.2	FAP
LOC100621838	4.973572942	4604.849366	-9.854655613	0	Low	6	5573	XP_005658329.1	ssc:100621838	0	NM_201526.1	ISLR
LOC100525396	1.657857647	1536.877771	-9.856466582	0	Low	2	1860	XP_005652609.1	ssc:100525396	1E-33	NM_002089.3	CXCL2
IFIT2	4.973572942	4632.116552	-9.863173204	0	Low	6	5606	XP_005671321.1	ssc:100155467	0	NM_001547.4	IFIT2
LOC100738123	233.7579283	219619.0073	-9.875771908	0	Low	282	265793	XR_300525.1	ssc:100738123			
LOC100525528	1.657857647	1597.196093	-9.912005598	0	Low	2	1933	XP_005666809.1	ssc:100525528	1E-33	NM_002089.3	CXCL2
SRPX2	1.657857647	1613.72166	-9.92685591	0	Low	2	1953	XP_001924554.3	ssc:100153130	0	NM_014467.2	SRPX2
LOC102161109	8.289288236	8165.282869	-9.944038921	0	Low	10	9882	XR_305383.1	ssc:102161109			
LOC100627920	0.828928824	970.8770868	-10.19382472	0	Low	1	1175	XP_003360105.1	ssc:100627920	0	NM_015077.3	SARM1
ST3GAL1	1.657857647	1969.847638	-10.2145482	0	Low	2	2384	NM_001004047.1	ssc:445537	9E-177	NM_173344.2	ST3GAL1
HR	0.828928824	1042.763305	-10.29687587	0	Low	1	1262	NM_001083930.1	ssc:397617	0	NM_018411.4	HR
FAM198B	9.11821706	11644.74109	-10.31863919	0	Low	11	14093	XP_005666715.1	ssc:100515440	0	NM_016613.6	FAM198B
ACKR3	3.315715294	4399.106051	-10.37367453	0	Low	4	5324	XP_003133807.1	ssc:100515346	0	NM_020311.2	ACKR3
AEBP1	30.67036647	41982.37779	-10.41872276	0	Low	37	50809	XP_003134934.1	ssc:733628	0	NM_001129.4	AEBP1
COL8A2	11.60500353	16593.32261	-10.48164009	0	Low	14	20082	XP_003356361.2	ssc:100525585			
LOXL4	8.289288236	12123.15627	-10.5142295	0	Low	10	14672	XP_005671434.1	ssc:100157207	0	NM_032211.6	LOXL4
LOC100518095	4.144644118	6110.328559	-10.52578601	0	Low	5	7395	XP_005670428.1	ssc:100518095	1E-28	NM_002462.4	MX1
KIF12	1.657857647	2577.98852	-10.60270999	0	Low	2	3120	XP_005660422.1	ssc:100156816	0	NM_138424.1	KIF12
LOC100049650	4.973572942	7884.348223	-10.63049315	0	Low	6	9542	XP_005657045.1	ssc:100049650	0	NM_015194.1	MYO1D
HOXB6	1.657857647	2758.943483	-10.70058005	0	Low	2	3339	XP_003358123.1	ssc:100627849	2E-124	NM_156037.1	HOXB6
ELN	4.144644118	7319.173817	-10.78621686	0	Low	5	8858	ELN				
ACTA2	59.6828753	109145.589	-10.83664916	0	Low	72	132093	NM_001164650.1	ssc:733615	0	NM_001613.2	ACTA2
LOC100738989	90.35324177	170068.7459	-10.87825405	0	Low	109	205825	XP_005674338.1	ssc:100738989	0	NM_000089.3	COL1A2
CCL11	0.828928824	1648.425352	-10.95755271	0	Low	1	1995	NM_001256774.1	ssc:100038010	1E-27	NM_002986.2	CCL11
TSHZ3	0.828928824	1718.659013	-11.01774749	0	Low	1	2080	XP_005655858.1	ssc:100627966	0	NM_020856.2	TSHZ3
LOC100628118	1.657857647	3442.275697	-11.0198268	0	Low	2	4166	XP_005662044.1	ssc:100628118			
COL1A2	173.2461241	385650.557	-11.1202554	0	Low	209	466732	NM_001243655.1	ssc:100626716	0	NM_000089.3	COL1A2
MMP19	1.657857647	3729.82057	-11.13557038	0	Low	2	4514	XP_005663940.1	ssc:100518427	0	NR_073606.1	MMP19
ASPN	6.631430589	15025.04591	-11.14576164	0	Low	8	18184	NM_001243889.1	ssc:100511749	0	NM_017680.4	ASPN
IL33	0.828928824	1905.397925	-11.16655647	0	Low	1	2306	NM_001285978.1	ssc:100518643	3E-68	NM_033439.3	IL33
C12H17orf67	0.828928824	1926.054885	-11.18211296	0	Low	1	2331	XP_005657027.1	ssc:100525534	1E-34	NM_001085430.2	C17orf67
LOC100736763	1.657857647	4545.357323	-11.42085786	0	Low	2	5501	XR_309518.1	ssc:100736763			
LOC100738213	130.9707541	387901.3393	-11.53222935	0	Low	158	469456	XP_005668985.1	ssc:100738213	0	NM_000088.3	COL1A1
CSPG4	3.315715294	12812.27243	-11.91591863	0	Low	4	15506	XP_003128533.3	ssc:733676	0	NM_001897.4	CSPG4
IRG6	1.657857647	6658.151119	-11.97158577	0	Low	2	8058	NM_213817.1	ssc:396752	1E-177	NM_080657.4	RSAD2
MGP	4.973572942	26806.94921	-12.39603679	0	Low	6	32443	NM_214116.1	ssc:397206	2E-42	NM_001190839.1	MGP
PRELP	0.828928824	4799.851061	-12.49945379	0	Low	1	5809	XP_005656706.1	ssc:100511032	2E-172	NM_201348.1	PRELP
LOC100737120	0.828928824	5022.946222	-12.56499798	0	Low	1	6079	XP_003480633.2	ssc:100737120	0	NM_002160.3	TNC
LOC100627277	0.828928824	5187.375618	-12.61146899	0	Low	1	6278	XP_005674513.1	ssc:100627277	3E-60	NM_181724.2	TMEM119
LOC102165673	1.657857647	10474.73092	-12.62530543	0	Low	2	12677	XP_005661865.1	ssc:102165673	5E-87	NM_002609.3	PDGFRB
COL3A1	56.36716001	487725.6819	-13.07892744	0	Low	68	590268	NM_001243297.1	ssc:100152001	0	NM_000090.3	COL3A1
LOC100621401	0.828928824	8792.428154	-13.37272579	0	Low	1	10641	XP_003359182.1	ssc:100621401	5E-98	NM_181724.2	TMEM119
LOC102166564	1.657857647	21479.10627	-13.66132621	0	Low	2	25995	XP_005661871.1	ssc:102166564	0	NM_002609.3	PDGFRB
POSTN	3.315715294	55326.77349	-14.02637004	0	Low	4	66959	NM_001206351.1	ssc:100152401	0	NM_006475.2	POSTN
LOC102160657	0.828928824	22024.45	-14.69749824	0	Low	1	26655	XP_005660435.1	ssc:102160657	0	NM_002160.3	TNC
COL5A1	1.657857647	110737.8274	-16.02746846	0	Low	2	134020	NM_001014971.1	ssc:397533			
IL8	0	789.095845	-20	0	Low	0	955	NM_213867.1	ssc:396880	5E-35	NM_000584.3	IL8
CXCL10	0	2351.588246	-20	0	Low	0	2846	NM_001008691.1	ssc:494019	3E-22	NM_001565.3	CXCL10
GAS7	0	5731.893065	-20	0	Low	0	6937	XP_005669293.1	ssc:100518690	5E-179	NM_201433.1	GAS7
LOC100628220	0	1165.878783	-20	0	Low	0	1411	XP_003354556.3	ssc:100628220	6E-32	NM_001278939.1	ELN
ISM1	0	838.6725473	-20	0	Low	0	1015	XP_001925035.2	ssc:100154170	0	NM_080826.1	ISM1
HOXB2	0	796.5323503	-20	0	Low	0	964	XP_003131594.1	ssc:100521989	9E-154	NM_002145.3	HOXB2
DPT	0	1233.633609	-20	0	Low	0	1493	XP_003125697.1	ssc:100516366	3E-108	NM_001937.4	DPT
SERTAD4	0	2809.346464	-20	0	Low	0	3400	XP_005667938.1	ssc:100511654	0	NM_019605.3	SERTAD4
LOC100622513	0	1071.683048	-20	0	Low	0	1297	XP_005674372.1	ssc:100622513	6E-111	NM_005576.2	LOXL1
DDX60	0	1853.342388	-20	0	Low	0	2243	XP_005657343.1	ssc:100158037	8E-21	NM_017631.5	DDX60
VCAM1	0	6099.58694	-20	0	Low	0	7382	NM_213891.1	ssc:396925	4E-134	NM_080682.2	VCAM1

MMP1	0	11619.95274	-20	0	Low	0	14063	NM_001166229.1	ssc:397320	0	NM_002421.3	MMP1
ZBP1	0	911.385044	-20	0	Low	0	1103	NM_001123216.1	ssc:100144524	1E-113	NM_030776.2	ZBP1
BACE2	0	7459.64114	-20	0	Low	0	9028	XP_005670420.1	ssc:100517374	0	NM_138992.2	BACE2
LOC102161103	0	4513.958745	-20	0	Low	0	5463	XP_005656329.1	ssc:102161103	0	NM_018689.1	KIAA1199
LUM	0	13427.84982	-20	0	Low	0	16251	NM_001243339.1	ssc:100152607	4E-177	NM_002345.3	LUM
TMEM98	0	5284.050187	-20	0	Low	0	6395	XP_003131776.1	ssc:100520269	9E-127	NM_015544.2	TMEM98
MX2	0	3252.231671	-20	0	Low	0	3936	NM_001097416.1	ssc:396893	8E-160	NM_002463.1	MX2
LOC102164776	0	1751.710148	-20	0	Low	0	2120	XR_305411.1	ssc:102164776			
LOC100626687	0	2323.494781	-20	0	Low	0	2812	XP_005674224.1	ssc:100626687	0	NM_173817.1	PIEZO2
KCNE4	0	1620.331887	-20	0	Low	0	1961	XP_005672306.1	ssc:397167	1E-76	NM_080671.3	KCNE4
CHI3L1	0	3854.588604	-20	0	Low	0	4665	NM_001001540.1	ssc:396865	0	NM_001276.2	CHI3L1
TNC	0	18393.78283	-20	0	Low	0	22261	NM_214230.1	ssc:397460	0	NM_002160.3	TNC
LOC102157763	0	854.3718364	-20	0	Low	0	1034	XP_005666811.1	ssc:102157763	6E-33	NM_002089.3	CXCL2
LOC100516442	0	709.7731213	-20	0	Low	0	859	XP_003126812.2	ssc:100516442	0	NM_133642.3	LARGE
LOC100738461	0	7119.214451	-20	0	Low	0	8616	XP_005656330.1	ssc:100738461	0	NM_018689.1	KIAA1199
LOC100738990	0	1729.400632	-20	0	Low	0	2093	XP_003484353.1	ssc:100738990	8E-150	NM_033093.2	TRIM5
PEG3	0	882.465301	-20	0	Low	0	1068	XP_005653381.1	ssc:100522552	0	NM_006210.2	PEG3
LOC102166259	0	2373.071484	-20	0	Low	0	2872	XR_297673.1	ssc:102166259			
NECD	0	1658.340692	-20	0	Low	0	2007	NM_001123144.1	ssc:100144475	3E-11	NM_002487.2	NDN
LOC102158099	0	1097.297678	-20	0	Low	0	1328	XP_005660601.1	ssc:102158099	1E-41	NM_001278074.1	COL5A1
LOC100623769	0	782.485618	-20	0	Low	0	947	XP_005654767.1	ssc:100623769	1E-104	NM_205843.2	NFIC
FOXS1	0	1942.580452	-20	0	Low	0	2351	XP_003134430.1	ssc:100524116	2E-154	NM_004118.3	FOXS1
AMCF-II	0	9804.619159	-20	0	Low	0	11866	NM_213876.1	ssc:396900	4E-33	NM_002994.4	CXCL5
LOC102160189	0	22683.82014	-20	0	Low	0	27453	XP_005656326.1	ssc:102160189	6E-102	NM_018689.1	KIAA1199
MXRA5	0	14656.52576	-20	0	Low	0	17738	XP_005658001.1	ssc:100519997	0	NM_015419.3	MXRA5
LOC100627044	0	1683.955322	-20	0	Low	0	2038	XP_005658552.1	ssc:100627044	1E-66	NR_037944.1	WISP1
FMOD	0	59302.82502	-20	0	Low	0	71771	XP_003130153.1	ssc:100526237	0	NR_103757.1	FMOD
CCL2	0	45810.52549	-20	0	Low	0	55442	NM_214214.1	ssc:397422	7E-36	NM_002982.3	CCL2
EIF2S3	0	3510.030523	-20	0	Low	0	4248	XP_005658005.1	ssc:100624149	0	NM_001415.3	EIF2S3
TRIM4	0	818.8418664	-20	0	Low	0	991	XP_003124381.1	ssc:100525393	0	NM_033091.2	TRIM4
LOC100737137	0	729.6038022	-20	0	Low	0	883	LOC100737137				
TSPAN18	0	1655.035579	-20	0	Low	0	2003	XP_003122898.2	ssc:100520775	4E-95	NM_130783.4	TSPAN18
LOC100153335	0	2349.935689	-20	0	Low	0	2844	XP_005666216.1	ssc:100153335	0	NM_022369.3	STRA6
LOC100513671	0	1343.528632	-20	0	Low	0	1626	XP_003129107.4	ssc:100513671	9E-114	NM_182502.3	TMPRSS11B
LOC733603	0	10361.53078	-20	0	Low	0	12540	NM_001044552.1	ssc:733603	2E-51	NM_199161.3	SAA1
LOC733579	0	2098.747064	-20	0	Low	0	2540	NM_001044532.1	ssc:733579	8E-156	NM_033093.2	TRIM5
LOC100625300	0	1055.983759	-20	0	Low	0	1278	XP_005672791.1	ssc:100625300	0	NM_018993.3	RIN2
LOC100625207	0	1140.264153	-20	0	Low	0	1380	XP_003484218.1	ssc:100625207	0	NM_021906.2	USP9X
LOC100620730	0	789.095845	-20	0	Low	0	955	XP_003362006.1	ssc:100620730	5E-35	NM_000584.3	IL8
OAS1	0	912.2113224	-20	0	Low	0	1104	NM_214303.1	ssc:397570	5E-155	NM_016816.2	OAS1
CCL20	0	1005.580778	-20	0	Low	0	1217	NM_001024589.1	ssc:553951	5E-39	NM_004591.2	CCL20
AWN	0	1846.732161	-20	0	Low	0	2235	NM_213829.1	ssc:396783			
COL6A3	0	20982.51297	-20	0	Low	0	25394	XP_005672367.1	ssc:100101551	0	NM_057167.3	COL6A3
SLC8A1	0	2005.377608	-20	0	Low	0	2427	HighI0001834F10		0	NM_021097.2	SLC8A1
LOC100737434	0	17070.08488	-20	0	Low	0	20659	XP_003480673.1	ssc:100737434	0	NM_001278074.1	COL5A1
POF1B	675.5769912	0	20	1.11E-16	High	815	0	XP_005673805.1	ssc:100523581	0	NM_024921.3	POF1B
LOC102164762	816.4948912	0.826278372	9.948600239	1.11E-16	High	985	1	XP_005663149.1	ssc:102164762	5E-126	NM_144584.2	HENMT1
LOC100152743	814.8370336	0.826278372	9.945667931	1.11E-16	High	983	1	XP_001926795.1	ssc:100152743	3E-126	NM_144584.2	HENMT1
LOC102164968	1026.213884	1.652556743	9.278415924	1.11E-16	High	1238	2	XP_005652633.1	ssc:102164968	2E-47	NM_005555.3	KRT6B
PCNXL2	1153.039994	2.478835115	8.861564528	1.11E-16	High	1391	3	XP_005671027.1	ssc:100158027	0	NM_024938.2	PCNXL2
LOC100620918	1448.138655	4.131391859	8.453356122	1.11E-16	High	1747	5	XP_005655043.1	ssc:100620918	0	NR_003149.1	GPR98
GREB1L	3600.037881	17.35184581	7.696779139	1.11E-16	High	4343	21	XP_005653460.1	ssc:100524319	0	NM_024935.2	GREB1L
HSPA2	19714.41421	108.2424667	7.508840411	1.11E-16	High	23783	131	XP_003356782.1	ssc:100621324	0	NM_021979.3	HSPA2
SDC2	48.90680059	9156.816916	-7.548667278	1.11E-16	Low	59	11082	XP_001926974.2	ssc:100152754	7E-100	NM_002998.3	SDC2
COL5A2	450.93728	86391.53516	-7.581819365	1.11E-16	Low	544	104555	NM_001105289.1	ssc:397532			
IFIT1	35.64393941	6919.255085	-7.600816124	1.11E-16	Low	43	8374	NM_001244363.1	ssc:100153038	0	NM_001548.4	IFIT1
C1R	33.98608177	6800.270999	-7.644504386	1.11E-16	Low	41	8230	XP_005658602.1	ssc:445464			
ARAP3	19.06536294	4325.567276	-7.825791541	1.11E-16	Low	23	5235	XP_003124050.1	ssc:100511744	0	NM_022481.5	ARAP3
MAB21L2	13.26286118	3284.456528	-7.952118821	1.11E-16	Low	16	3975	XP_003129198.1	ssc:100519500	0	NM_006439.4	MAB21L2
LOC100525856	4.144644118	1469.949223	-8.470302376	1.11E-16	Low	5	1779	XP_005661151.1	ssc:100525856	9E-43	NM_199161.3	SAA1
EVC2	1.657857647	985.7500975	-9.215758003	1.11E-16	Low	2	1193	XP_005658650.1	ssc:100628058	0	NM_147127.4	EVC2
LOC102164335	0	656.8913055	-20	1.11E-16	Low	0	795	XR_299884.1	ssc:102164335			
COL27A1	0	651.9336353	-20	1.11E-16	Low	0	789	XP_005660432.1	ssc:100520119	0	NM_032888.2	COL27A1
ZNF814	0	636.2343462	-20	1.11E-16	Low	0	770	ZNF814	ssc:100737218	0	NM_005773.2	ZNF256
GDF10	0	688.2898837	-20	1.11E-16	Low	0	833	XP_003133153.1	ssc:100519926	0	NM_004962.3	GDF10
ZNF134	0	647.8022434	-20	1.11E-16	Low	0	784	ZNF134	ssc:100512237	0	NM_003435.3	ZNF134
LOC100739844	0	689.9424404	-20	1.11E-16	Low	0	835	XP_003482731.1	ssc:100739844	3E-154	NM_006080.2	SEMA3A
LOC100624681	630.8148348	0	20	2.22E-16	High	761	0	XP_003360222.2	ssc:100624681			
SLC26A5	612.5784006	0	20	2.22E-16	High	739	0	NM_001135963.1	ssc:100190993	0	NM_206885.2	SLC26A5
LOC100155551	610.0916142	0	20	2.22E-16	High	736	0	XP_005666029.1	ssc:100155551	1E-46	NM_001159726.1	TSPO2
LOC100523750	911.821706	1.652556743	9.107908133	2.22E-16	High	1100	2	XP_005669193.1	ssc:100523750	0	NM_198501.2	SMTNL2
GPR150	911.821706	1.652556743	9.107908133	2.22E-16	High	1100	2	XP_003123816.1	ssc:100516597	7E-139	NM_199243.1	GPR150
EMILIN1	159.983263	27857.9753	-7.444025616	2.22E-16	Low	193	33715	XP_005662732.1	ssc:100523665	0	NM_007046.3	EMILIN1
LOC102165344	40.61751236	7593.498236	-7.546518978	2.22E-16	Low	49	9190	XR_309235.1	ssc:102165344			
SERPINE1	52.22251589	10062.41801	-7.59008935	2.22E-16	Low	63	12178	NM_213910.1	ssc:396945			
CDC42EP5	23.21000706	4564.361725	-7.619522358	2.22E-16	Low	28	5524	XP_003127481.2	ssc:100525753	2E-45	NM_145057.3	CDC42EP5
PTGS2	17.4075053	3762.045426	-7.755663997	2.22E-16	Low	21	4553	NM_214321.1	ssc:397590	0	NM_000963	PTGS2
C1QTNF6	9.947145883	2309.448049	-7.859049743	2.22E-16	Low	12	2795	NM_001142830.1	ssc:100217379	2E-104	NM_182486.1	C1QTNF6
VGF	12.43393235	2927.504271	-7.87924483	2.22E-16	Low	15	3543	XP_005658665.1	ssc:102163755	4E-50	NM_003378.3	VGF
TENM4	8.289288236	2188.811407	-8.044683709	2.22E-16	Low	10	2649	XR_305203.1	ssc:100514784	0	NM_001098816.2	TENM4
ERG	3.315715294	1231.981052	-8.537444218	2.22E-16	Low	4	1491	NM_001244456.1	ssc:100625364			
BST2	0.828928824	829.5834852	-9.96692323	2.22E-16	Low	1	1004	NM_001161755.1	ssc:100302088			
COLQ	0	618.0562221	-20	2.22E-16	Low	0	748	XP_003358361.2	ssc:100624545	1E-37	NM_080544.2	COLQ
ECEL1	0	623.0138923	-20	2.22E-16	Low	0	754	XP_003133775.1	ssc:100521587	0	NM_004826.2	ECEL1
LOC100737503	0	617.2299437	-20	2.22E-16	Low	0	747	XR_135144.2	ssc:100737503			
BMP6	1001.346019	2.478835115	8.658062563	3.33E-16	High	1208	3	NM_001168001.1	ssc:100155536	0	NM_001718.4	BMP6
FAM65B	1832.761629	7.436505346	7.945178633	3.33E-16	High	2211	9	XP_005665694.1	ssc:100154661	0	NM_015864.3	FAM65B
CAMKV	5327.525549	29.74602138	7.48462508	3.33E-16	High	6427	36	XP_003358517.1	ssc:100623981	0	NM_024046.3	CAMKV
HOXB7	1.657857647	892.3806415	-9.072195273	3.33E-16	Low	2	1080	XP_003131597.1	ssc:100522513	7E-118	NM_004502.3	HOXB7
RUNX1	0.828928824	738.6928643	-9.799510697	3.33E-16	Low	1	894	NM_001246252.1	ssc:100512633	0	NM_001754.4	RUNX1
SP100	0	598.2255411	-20	3.33E-16	Low	0	724	XP_005657638.1	ssc:100516940	1E-80	NM_003113.3	SP100
LOC100513699	736.0887954	0.826278372	9.799036191	4.44E-16	High	888	1	XP_005674426.1	ssc:100513699	2E-178	NM_001008783.1	SLC35D3
LOC102160252	1254.16931	4.131391859	8.245888502	4.44E-16	High	1513	5	XR_307479.1	ssc:102160252			
TNNT2	1.657857647	868.4185687	-9.03292663	4.44E-16	Low	2	1051	NM_001257353.1	ssc:100622450	2E-108	NM_001276347.1	TNNT2
ZNF418	0	573.43719	-20	4.44E-16	Low	0	694	ZNF418	ssc:100514656	0	NM_133460.1	ZNF418
COL4A1	289.2961594	47585.37143	-7.361827151	5.55E-16	Low	349	57590	XP_003131139.3	ssc:100515336	0	NM_001845.4	COL4A1
LOC100513556	79.57716707	13236.15324	-7.377913644	5.55E-16	Low	96	16019	XP_003357187.1	ssc:100513556	6E-115	NM_145040.2	PRKCDBP
LOC100152170	11.60500353	2376.376597	-7.677872714	5.55E-16	Low	14	2876	XP_005659694.1	ssc:100152170	0	NM_021199.3	SQRDL
LOC100737566	696.3002118	0.826278372	9.718865842	6.66E-16	High	840	1	LOC100737566				
LOC100738626	1094.186047	3.305113487	8.370942539	6.66E-16	High	1320	4	XP_005659282.1	ssc:100738626	2E-43	NM_006073.3	TRDN

LOC100153981	1575.793694	6.610226974	7.897163141	6.66E-16	High	1901	8	XP_003480454.1	ssc:100153981	0	NM_020168.5	PAK6
ZSWIM5	2973.36769	15.69928906	7.565254841	6.66E-16	High	3587	19	ZSWIM5	ssc:100514968	0	NM_020883.1	ZSWIM5
VSTM4	14.09179	2702.756554	-7.583432878	7.77E-16	Low	17	3271	XP_005671270.1	ssc:100157009	3E-160	NM_144984.3	VSTM4
LOC100514326	3.315715294	1101.42907	-8.375840741	7.77E-16	Low	4	1333	XP_005672251.1	ssc:100514326	1E-132	NM_022648.4	TNS1
KCNK12	0	548.6488388	-20	7.77E-16	Low	0	664	XP_005674663.1	ssc:100521189	6E-109	NM_022055.1	KCNK12
FIBIN	4.144644118	1193.972247	-8.170305358	8.88E-16	Low	5	1445	XP_005658260.1	ssc:100620302	4E-116	NM_203371.1	FIBIN
LOC102166331	795.7716707	1.652556743	8.91151092	9.99E-16	High	960	2	XR_302282.1	ssc:102166331			
GABRB2	793.2848842	1.652556743	8.906995439	9.99E-16	High	957	2	XP_003483903.1	ssc:100517063	0	NM_021911.2	GABRB2
MATN4	1.657857647	803.1425773	-8.920192179	9.99E-16	Low	2	972	XP_003360059.1	ssc:100510939	0	NM_030592.3	MATN4
EPHA3	0	531.296993	-20	9.99E-16	Low	0	643	NM_001195335.1	ssc:100337658	6E-153	NM_182644.2	EPHA3
ZNF667	0	532.9495498	-20	9.99E-16	Low	0	645	ZNF667	ssc:100521730	0	NR_030740.1	ZNF667
ZNF473	0	536.2546633	-20	9.99E-16	Low	0	649	ZNF473	ssc:100524202	0	NM_015428.1	ZNF473
LOC100622870	912.6506348	2.478835115	8.524256577	1.11E-15	High	1101	3	XR_297931.1	ssc:100622870	1E-36	NM_003980.4	MAP7
LOC102161294	0	524.686766	-20	1.22E-15	Low	0	635	XP_005670314.1	ssc:102161294			
COL14A1	150.0361171	22555.74699	-7.232041423	1.33E-15	Low	181	27298	XP_005662938.1	ssc:100158059	0	NM_021110.2	COL14A1
PDE1A	0	518.9028174	-20	1.33E-15	Low	0	628	XP_005672048.1	ssc:100152819	0	NM_005019.4	PDE1A
FOLR1	1461.401516	6.610226974	7.788437084	1.44E-15	High	1763	8	NM_213830.1	ssc:396784	9E-105	NM_016730.1	FOLR1
WISP1	3.315715294	1014.66984	-8.257474521	1.44E-15	Low	4	1228	XP_005662899.1	ssc:100739115	1E-135	NR_037944.1	WISP1
CLEC14A	1.657857647	775.0491127	-8.868823788	1.44E-15	Low	2	938	XP_005666250.1	ssc:100157365	0	NM_175060.2	CLEC14A
LOC100511846	1328.772904	5.783948602	7.843824112	1.67E-15	High	1603	7	XP_003121317.2	ssc:100511846	2E-14	NM_030568.3	KHDC1
CHGA	3634.023963	22.30951604	7.347764905	1.67E-15	High	4384	27	NM_001164005.2	ssc:397540	1E-117	NM_001275.3	CHGA
KCNA6	0	503.2035284	-20	1.78E-15	Low	0	609	HighI00025DE6BB		0	NM_002235.3	KCNA6
PLN	497.3572942	0	20	1.89E-15	High	600	0	NM_214213.1	ssc:397421	3E-14	NM_002667.3	PLN
MFAP2	85.37966883	12445.40483	-7.187504878	1.89E-15	Low	103	15062	XP_005665105.1	ssc:100523794	2E-80	NM_017459.2	MFAP2
C1QTNF1	11.60500353	2121.05658	-7.513892334	1.89E-15	Low	14	2567	XP_005658522.1	ssc:100217377	2E-99	NR_049769.1	C1QTNF1
KCNH3	741.0623683	1.652556743	8.808751346	2.00E-15	High	894	2	XP_003126164.1	ssc:100514837	0	NM_012284.1	KCNH3
GIMAP8	1080.923186	4.131391859	8.031420384	2.00E-15	High	1304	5	XP_005657771.1	ssc:100736760	0	NM_175571.2	GIMAP8
DRD2	2058.230269	11.5678972	7.475133948	2.33E-15	High	2483	14	NM_001244253.1	ssc:100624857	0	NM_016574.3	DRD2
SQRDL	7.460359412	1509.610585	-7.660715593	2.55E-15	Low	9	1827	NM_001244238.1	ssc:100154990			
LOC100515404	1.657857647	729.6038022	-8.781649303	2.55E-15	Low	2	883	XP_003130868.1	ssc:100515404	0	NM_032817.5	ITIH5
LOC100738803	0.828928824	610.6197167	-9.52481023	2.66E-15	Low	1	739	XP_003481541.2	ssc:100738803	9E-168	NM_001010898.2	SLC6A17
ACSL5	1.657857647	723.8198536	-8.770166735	2.89E-15	Low	2	876	NM_001195321.1	ssc:100157521	0	NM_203380.1	ACSL5
ST8SIA6	474.9762159	0	20	3.00E-15	High	573	0	XP_003130770.1	ssc:100522567	1E-166	NM_001004470.1	ST8SIA6
LOC100512163	0	478.4151772	-20	3.11E-15	Low	0	579	XR_298279.1	ssc:100512163			
LOC100738561	0	478.4151772	-20	3.11E-15	Low	0	579	XR_298278.1	ssc:100738561			
JPH1	1770.591967	9.915340461	7.480353755	3.33E-15	High	2136	12	XP_005663079.1	ssc:100626574	0	NM_020647.2	JPH1
LOC100739422	16.57857647	2648.221781	-7.319560222	3.33E-15	Low	20	3205	XP_005662673.1	ssc:100739422	3E-100	NM_000104.3	CYP1B1
LOC100624693	16.57857647	2649.04846	-7.320010291	3.33E-15	Low	20	3206	XP_005662674.1	ssc:100624693	1E-99	NM_000104.3	CYP1B1
LOC100519663	0	473.457507	-20	3.33E-15	Low	0	573	XP_005665337.1	ssc:100519663	0	NM_173817.1	PIEZO2
LOC102160246	466.6869277	0	20	3.44E-15	High	563	0	XP_005658525.1	ssc:102160246	1E-120	NM_024938.2	PCNXL2
ID3	33.15715294	4783.325494	-7.172550022	3.55E-15	Low	40	5789	NM_001243602.1	ssc:100626978			
THBS4	0.828928824	585.8313656	-9.465021493	3.89E-15	Low	1	709	XP_003123802.3	ssc:100513005	0	NM_003248.4	THBS4
PTGFR	10.77607471	1842.600769	-7.417767952	4.55E-15	Low	13	2230	NM_214059.1	ssc:397126	6E-177	NM_001039585.1	PTGFR
ZNF606	0.828928824	575.9160251	-9.440394522	4.77E-15	Low	1	697	ZNF606		0	NM_025027.3	ZNF606
TPD52L1	445.1347783	0	20	5.55E-15	High	537	0	XP_005659275.1	ssc:100515246	4E-95	NM_003287.2	TPD52L1
SLC26A7	4.144644118	986.5763758	-7.895038702	5.77E-15	Low	5	1194	XP_005663034.1	ssc:100626316	0	NM_134266.1	SLC26A7
DAB2	89.52431295	11665.39805	-7.025740279	5.88E-15	Low	108	14118	XP_005672491.1	ssc:100519746	0	NM_001343.3	DAB2
TINAGL1	8.289288236	1483.995956	-7.484023216	6.22E-15	Low	10	1796	XP_005665217.1	ssc:100517950	0	NM_022164.2	TINAGL1
KIAA1244	1562.530832	9.089062089	7.425537514	6.33E-15	High	1885	11	XP_003353246.3	ssc:100621590			
LOC100739007	16.57857647	2482.966507	-7.226600856	6.33E-15	Low	20	3005	XP_005663642.1	ssc:100739007	2E-87	NM_172212.2	CSF1
CACNB4	434.3587036	0	20	6.99E-15	High	524	0	NM_213750.1	ssc:396585	0	NM_001145798.1	CACNB4
COL8A1	174.075053	22202.92613	-6.99489655	6.99E-15	Low	210	26871	XP_005670312.1	ssc:397430	0	NM_020351.3	COL8A1
MYO5C	754.3252295	2.478835115	8.249380559	7.22E-15	High	910	3	XP_005659654.1	ssc:100155557	0	NM_018728.3	MYO5C
LOC100513741	0	434.6224235	-20	7.66E-15	Low	0	526	XR_303907.1	ssc:100513741	0	NR_049754.1	ZNF211
PNCK	430.2140595	0	20	7.77E-15	High	519	0	NM_001195366.1	ssc:100337678	0	NM_198452.1	PNCK
FRMPD4	647.3934112	1.652556743	8.613799062	7.77E-15	High	781	2	XP_005673465.1	ssc:100526196	0	NM_014728.3	FRMPD4
VDR	5.802501765	1154.310885	-7.636141059	7.77E-15	Low	7	1397	NM_001097414.1	ssc:396628	0	NM_001017536.1	VDR
LOC100620576	0.828928824	547.8225605	-9.368244736	7.77E-15	Low	1	663	XP_005666577.1	ssc:100620576	1E-148	NM_153717.2	EVC
KCNJ3	746.8648701	2.478835115	8.235041119	7.99E-15	High	901	3	XP_003483696.1	ssc:396586	0	NM_002239.3	KCNJ3
LRAT	639.104123	1.652556743	8.595207374	8.66E-15	High	771	2	NM_001244920.1	ssc:100513994	1E-111	NM_004744.3	LRAT
WSCD2	0	426.3596398	-20	9.33E-15	Low	0	516	WSCD2				
DPPA4	421.0958424	0	20	9.55E-15	High	508	0	XP_005654122.1	ssc:100620290	2E-77	NM_018189.3	DPPA4
DDAH1	137.6021847	16977.5417	-6.94698039	9.66E-15	Low	166	20547	XP_003125985.3	ssc:100153814			
LOC102167710	0	422.228248	-20	1.02E-14	Low	0	511	XP_005672881.1	ssc:102167710	4E-63	NR_046000	IRF4
TRPC3	0	421.4019696	-20	1.04E-14	Low	0	510	NM_001145749.1	ssc:397210	0	NM_003305.2	TRPC3
NFE2L3	3566.051799	27.26718627	7.031018014	1.07E-14	High	4302	33	XP_003360238.1	ssc:100622324	0	NM_004289.6	NFE2L3
TNS1	13.26286118	1972.326473	-7.216362527	1.09E-14	Low	16	2387	XP_005672258.1	ssc:100514801	0	NM_022648.4	TNS1
LOC100625479	22.38107824	3020.047449	-7.076147864	1.11E-14	Low	27	3655	XP_005658348.1	ssc:100625479			
LOC102157946	721.1680765	2.478835115	8.184529414	1.12E-14	High	870	3	XR_303677.1	ssc:102157946			
ITGB8	4.973572942	1013.843562	-7.671336709	1.15E-14	Low	6	1227	NM_001097424.1	ssc:100037284	0	NM_002214.2	ITGB8
LOC100737785	29.01250883	3757.914035	-7.017113198	1.19E-14	Low	35	4548	XP_003482952.2	ssc:100737785			
NRK	0	415.618021	-20	1.19E-14	Low	0	503	XP_003361569.1	ssc:100620665			
MYBPH	1.657857647	625.4927274	-8.559529166	1.20E-14	Low	2	757	NM_001033014.1	ssc:613132	0	NM_004997.2	MYBPH
BEND4	411.1486965	0	20	1.21E-14	High	496	0	XP_005666678.1	ssc:100514664	0	NM_207406.3	BEND4
TRIM63	0	414.7917426	-20	1.21E-14	Low	0	502	NM_001184756.1	ssc:100431101	0	NM_032588.3	TRIM63
LOC100623035	0	414.7917426	-20	1.21E-14	Low	0	502	XP_005664949.1	ssc:100623035	0	NM_001267779.1	ZNF850
CMPK2	25.69679353	3366.258086	-7.03341363	1.23E-14	Low	31	4074	XP_005662825.1	ssc:100623872	1E-176	NR_046236.1	CMPK2
TPM2	467.5158565	57079.30992	-6.931808786	1.27E-14	Low	564	69080	NM_001129947.1	ssc:396693	8E-150	NM_213674.1	TPM2
MCF2	407.8329812	0	20	1.31E-14	High	492	0	XP_001925660.1		0	NM_005369.4	MCF2
LOC100515181	0	409.007794	-20	1.40E-14	Low	0	495	XP_005673866.1	ssc:100515181	0	NM_198465	NRK
THEM5	402.0304794	0	20	1.51E-14	High	485	0	XP_003355217.1	ssc:100622268	2E-107	NM_182578.3	THEM5
EIF1AY	0	405.7026805	-20	1.51E-14	Low	0	491	NM_001244349.1	ssc:100625415	2E-79	NM_001412.3	EIF1AX
LOC100521069	0	404.0501238	-20	1.58E-14	Low	0	489	ZNF564	ssc:100521069	1E-177	NM_152601.3	ZNF709
SLC24A4	601.8023259	1.652556743	8.508446062	1.59E-14	High	726	2	XP_005656506.1	ssc:100156914	0	NM_153648.3	SLC24A4
LOC100521383	399.543693	0	20	1.60E-14	High	482	0	LOC100521383				
OSR1	0.828928824	507.3349202	-9.257474521	1.65E-14	Low	1	614	XP_005674600.1	ssc:100520131	1E-117	NM_145260.2	OSR1
LOC100518586	0	400.7450103	-20	1.71E-14	Low	0	485	XP_003134207.3	ssc:100518586	2E-96	NM_001145161.2	UBE2QL1
LOC100518738	0	399.9187319	-20	1.74E-14	Low	0	484	XP_005664996.1	ssc:100518738	0	NM_014480.2	ZNF544
LOC100519221	395.3990489	0	20	1.78E-14	High	477	0	XP_005657564.1	ssc:100519221	5E-175	NM_017579.2	DMBT1
MYL1	0	399.0924535	-20	1.78E-14	Low	0	483	NM_214374.2	ssc:397666	3E-81	NM_079422.2	MYL1
TFAP2E	586.0526783	1.652556743	8.470186729	2.05E-14	High	707	2	XP_003362031.1	ssc:100623903	4E-145	NM_178548.3	TFAP2E
LOC100627133	1.657857647	588.3102007	-8.471113107	2.19E-14	Low	2	712	XP_005657517.1	ssc:100627133	0	NM_203380.1	ACSL5
SLC52A3	578.5923189	1.652556743	8.451703551	2.33E-14	High	698	2	XP_003134400.2	ssc:100516303	0	NM_033409.3	SLC52A3
VIT	929.2292113	4.95767023	7.550228386	2.48E-14	High	1121	6	XP_005662691.1	ssc:100524557	0	NM_053276.3	VIT
EDNRA	0	385.0457212	-20	2.53E-14	Low	0	466	NM_214229.1	ssc:397457	2E-163	NR_045958.1	EDNRA
PODNL1	5.802501765	1022.932624	-7.461820353	2.59E-14	Low	7	1238	XP_003123408.2	ssc:100513269	0	NM_024825.3	PODNL1
LOC102161998	380.47833	0	20	2.60E-14	High	459	0	XP_005657563.1	ssc:102161998	5E-39	NM_017579.2	DMBT1
FAM184B	571.9608883	1.652556743	8.435072876	2.61E-14	High	690	2	XP_005674598.1	ssc:100627969	2E-35	NM_015688.1	FAM184B
IGF2	0	382.5668861	-20	2.69E-14	Low	0	463	NM_213883.2	ssc:396916			
LOC102166950	0	382.5668861	-20	2.69E-14	Low	0	463	XR_299386.1	ssc:102166950			

LOC100521510	377.1626147	0	20	2.82E-14	High	455	0	XP_003130576.2	ssc:100521510	2E-52	NM_178820.3	FBXO27
ID4	7.460359412	1186.535742	-7.313294708	2.82E-14	Low	9	1436	NM_001123130.1	ssc:100144508	1E-86	NM_001546.3	ID4
TMEM163	566.1583865	1.652556743	8.420362093	2.90E-14	High	683	2	XP_005671679.1	ssc:100512373	1E-98	NM_030923.4	TMEM163
LOC102159856	654.0248418	2.478835115	8.043539314	2.93E-14	High	789	3	LOC102159856				
PDGFRA	29.84143765	3512.509358	-6.879041218	2.99E-14	Low	36	4251	PDGFRA		0	NM_006206.4	PDGFRA
LOC100524999	1080.094257	6.610226974	7.352241693	3.05E-14	High	1303	8	XP_005656631.1	ssc:100524999	6E-33	NM_016619.2	PLAC8
GBP7	1.657857647	568.4795197	-8.42164443	3.06E-14	Low	2	688	XP_005663773.1	ssc:100155195	0	NM_052941.4	GBP4
FGF10	0.828928824	475.9363421	-9.165304677	3.10E-14	Low	1	576	XP_003133972.1	ssc:100525086	2E-104	NM_004465.1	FGF10
LOC100523100	736.9177242	3.305113487	7.800659933	3.11E-14	High	889	4	LOC100523100	ssc:100523100			
CDH8	1245.880022	8.262783717	7.236321523	3.14E-14	High	1503	10	XP_003355837.2	ssc:100625758	2E-105	NM_001796.4	CDH8
LOC102162332	7.460359412	1172.489009	-7.296113548	3.18E-14	Low	9	1419	XP_005663147.1	ssc:102162332	2E-144	NM_003068.4	SNAI2
RAX	559.5269559	1.652556743	8.403364016	3.25E-14	High	675	2	XP_003121760.1	ssc:100157455	2E-117	NM_013435.2	RAX
HOXB5	0	375.1303808	-20	3.25E-14	Low	0	454	XP_003131598.1	ssc:100522700	5E-157	NM_002147.3	HOXB5
ODF4	368.0443977	0	20	3.59E-14	High	444	0	NM_001202461.1	ssc:100529212	1E-36	NM_153007.4	ODF4
PAX1	0	370.9989889	-20	3.63E-14	Low	0	449	XP_005672808.1	ssc:100739820	0	NM_006192.4	PAX1
LOC102159296	3.315715294	732.9089157	-7.78816997	3.64E-14	Low	4	887	XP_005666934.1	ssc:102159296			
PTGS1	13.26286118	1743.447364	-7.038406959	3.69E-14	Low	16	2110	XP_001926164.1	ssc:397541	0	NM_080591.2	PTGS1
KIAA1199	0	370.1727105	-20	3.71E-14	Low	0	448	XP_005656328.1	ssc:102160984	2E-59	NM_018689.1	KIAA1199
LOC100623028	41.44644118	4631.290274	-6.804022144	3.75E-14	Low	50	5605	XP_003361790.1	ssc:100623028	2E-109	NM_015927.4	TGFB1I1
ITGA3	52.22251589	5752.550024	-6.783385851	3.76E-14	Low	63	6962	XP_005668937.1	ssc:100517053	0	NM_005501.2	ITGA3
LOC100512657	3.315715294	730.4300806	-7.783282235	3.76E-14	Low	4	884	XP_003360629.2	ssc:100512657	1E-140	NM_007191.4	WIF1
TMEM171	365.5576112	0	20	3.83E-14	High	441	0	NM_001244323.1	ssc:100514277	1E-139	NM_173490	TMEM171
LOC100511574	635.7884077	2.478835115	8.002740591	3.89E-14	High	767	3	XP_003356976.3	ssc:100511574	3E-20	NM_152605.3	ZNF781
ATP12A	363.8997536	0	20	4.01E-14	High	439	0	XP_005653908.1	ssc:100521921	0	NM_001676.5	ATP12A
LOC100523560	0	366.867597	-20	4.05E-14	Low	0	444	XR_297529.1	ssc:100523560			
LOC102161270	359.7551094	0	20	4.49E-14	High	434	0	XR_297327.1	ssc:102161270			
TRPC6	6.631430589	1048.547254	-7.30485603	4.53E-14	Low	8	1269	XP_003357304.2	ssc:100271728	0	NM_004621.5	TRPC6
LOC102157888	358.9261806	0	20	4.59E-14	High	433	0	XP_005673916.1	ssc:102157888	3E-15	NM_153448.3	ESX1
LOC102159782	540.461593	1.652556743	8.353348479	4.59E-14	High	652	2	XR_304869.1	ssc:102159782			
HOXC6	8.289288236	1198.929917	-7.176283385	5.11E-14	Low	10	1451	XP_003355472.1	ssc:100627932	8E-140	NM_153693.4	HOXC6
SYBU	1826.959127	14.87301069	6.940603831	5.18E-14	High	2204	18	XP_005655398.1	ssc:100157483	0	NM_017786.5	SYBU
CR2	353.9526077	0	20	5.27E-14	High	427	0	XP_005667612.1	ssc:100623585	0	NM_001877.4	CR2
NDST3	697.1291407	3.305113487	7.720582315	5.38E-14	High	841	4	XP_005666957.1	ssc:100525060	0	NM_004784.2	NDST3
LOC100622674	352.29475	0	20	5.51E-14	High	425	0	XP_003360468.1	ssc:100622674	0	NM_003604.2	IRS4
LOC100517243	0	354.4734215	-20	5.67E-14	Low	0	429	XP_003133148.1	ssc:100517243	2E-25	NM_006829.2	ADIRF
AGT	1.657857647	533.7758281	-8.33077003	5.70E-14	Low	2	646	AGT		2E-75	NM_000029.3	AGT
HOXB8	14.92071882	1809.549634	-6.922169829	6.35E-14	Low	18	2190	X06668		9E-144	NM_024016.3	HOXB8
VSNL1	520.5673012	1.652556743	8.299241073	6.61E-14	High	628	2	XP_003125391.1	ssc:100517707	8E-111	NM_003385.4	VSNL1
LOC102166344	345.6633194	0	20	6.64E-14	High	417	0	XP_005655042.1	ssc:102166344	5E-141	NR_003149.1	GPR98
LOC100738735	678.0637777	3.305113487	7.680577357	7.08E-14	High	818	4	XP_005655617.1	ssc:100738735	0	NM_012284.1	KCNH3
HTRA3	6.631430589	999.7968298	-7.236171008	7.25E-14	Low	8	1210	NM_001195343.1	ssc:100337673			
PLEKHF1	19.06536294	2174.764674	-6.833761493	7.42E-14	Low	23	2632	XP_005653317.1	ssc:100522869	7E-133	NM_024310.4	PLEKHF1
IER3	66.31430589	6745.736627	-6.668512048	7.47E-14	Low	80	8164	XP_001927586.2	ssc:100154770	3E-60	NM_052815.1	IER3
EHBP1L1	67.97216354	6902.729517	-6.666079099	7.53E-14	Low	82	8354	XP_005660818.1	ssc:100518056	0	NM_001099409.1	EHBP1L1
SELM	61.34073295	6239.227985	-6.668378312	7.69E-14	Low	74	7551	NM_001161648.1	ssc:100302025	2E-54	NM_080430.2	SELM
LOC100622576	0	342.0792459	-20	8.02E-14	Low	0	414	XP_005658397.1	ssc:100622576	4E-178	NM_022774.1	EXO5
RIN2	0	341.2529675	-20	8.22E-14	Low	0	413	XP_003359960.2	ssc:100625676	0	NM_018993.3	RIN2
ZNF211	0	340.4266892	-20	8.39E-14	Low	0	412	ZNF211	ssc:100514110	1E-56	NR_049754.1	ZNF211
DIRAS2	337.3740312	0	20	8.42E-14	High	407	0	XP_005657251.1	ssc:100739440	5E-114	NM_017594.3	DIRAS2
LOC100739594	0	338.7741324	-20	8.82E-14	Low	0	410	LOC100739594		2E-65	NM_022658.3	HOXC8
GPR56	9.947145883	1284.862868	-7.01311604	9.05E-14	Low	12	1555	NM_001243056.1	ssc:100515029	6E-109	NM_201525.2	GPR56
UNC5B	43.93322765	4459.424372	-6.665401193	9.36E-14	Low	53	5397	NM_001044549.1	ssc:733600	0	NM_170744.4	UNC5B
LOC100627012	420.2669136	0.826278372	8.990462261	9.53E-14	High	507	1	XP_005665356.1	ssc:100627012	0	NM_004746.3	DLGAP1
LOC100736853	0	335.4690189	-20	9.70E-14	Low	0	406	XP_003482717.1	ssc:100736853	5E-166	NM_024788.2	C7orf63
BARHL2	332.4004583	0	20	9.74E-14	High	401	0	XP_003125961.2	ssc:100521190	0	NM_020063.1	BARHL2
CREB3L1	111.9053912	10908.52706	-6.607032961	1.02E-13	Low	135	13202	XP_003122877.1	ssc:100516663	0	NM_052854.3	CREB3L1
ZNF613	0	332.9901838	-20	1.04E-13	Low	0	403	ZNF613	ssc:100625971	0	NM_024840.3	ZNF613
LOC100628205	649.8801977	3.305113487	7.619330168	1.08E-13	High	784	4	XP_005669120.1	ssc:100628205	9E-118	NM_004441.4	EPHB1
HOXB4	4.144644118	732.0826374	-7.464614469	1.09E-13	Low	5	886	XP_003131596.1	ssc:100522341	6E-140	NM_024015.4	HOXB4
LOC102160493	4.144644118	732.0826374	-7.464614469	1.09E-13	Low	5	886	XP_005672259.1	ssc:102160493	4E-89	NM_022648.4	TNS1
TNNI1	0	331.3376271	-20	1.09E-13	Low	0	401	NM_213912.3	ssc:396947	1E-103	NM_003281.3	TNNI1
LOC102157467	0	330.5113487	-20	1.12E-13	Low	0	400	XR_298763.1	ssc:102157467			
ARSI	3.315715294	653.586192	-7.62291356	1.12E-13	Low	4	791	XP_003124150.1	ssc:100517463	0	NM_001012301.2	ARSI
LOC102165808	326.5979565	0	20	1.16E-13	High	394	0	XR_308897.1	ssc:102165808			
ZNF227	0	328.8587919	-20	1.18E-13	Low	0	398	ZNF227	ssc:100627091	0	NM_182490	ZNF227
LOC100737756	0	328.8587919	-20	1.18E-13	Low	0	398	XP_005664973.1	ssc:100737756	0	NM_001267779.1	ZNF850
LOC100737413	0.828928824	414.7917426	-8.96692323	1.19E-13	Low	1	502	XP_003481951.1	ssc:100737413	0	NM_021632.3	ZNF350
FOXL1	1.657857647	493.2881879	-8.216966797	1.23E-13	Low	2	597	XP_005664360.1	ssc:100511634	4E-124	NM_005250.2	FOXL1
RBM46	2115.426358	19.83068092	6.737070437	1.25E-13	High	2552	24	XP_005666859.1	ssc:100513804	0	NM_144979.4	RBM46
ZNF503	17.4075053	1911.181874	-6.778611804	1.27E-13	Low	21	2313	ZNF503	ssc:100521339	2E-174	NM_032772.4	ZNF503
UNC-6	0	326.3799568	-20	1.27E-13	Low	0	395	NM_001044548.1	ssc:733599	0	NM_004822.2	NTN1
LOC100626722	0	324.7274001	-20	1.33E-13	Low	0	393	XR_131435.2	ssc:100626722			
ZNF584	0	324.7274001	-20	1.33E-13	Low	0	393	ZNF584	ssc:100519782	3E-154	NM_173548.1	ZNF584
COL5A3	11.60500353	1383.189994	-6.897108566	1.33E-13	Low	14	1674	NM_001105288.1	ssc:397531			
LOC100513452	319.9665259	0	20	1.41E-13	High	386	0	LOC100513452	ssc:100513452	3E-72	NM_171999.3	SALL3
ANGPTL2	193.9693447	18276.4513	-6.558013498	1.43E-13	Low	234	22119	NM_001109946.1	ssc:100126164	0	NM_012098.2	ANGPTL2
PLAC9	24.86786471	2550.721334	-6.680478848	1.44E-13	Low	30	3087	NM_001190209.1	ssc:100155282	1E-31	NM_001012973.1	PLAC9
SLC16A12	993.0567307	7.436505346	7.061107516	1.48E-13	High	1198	9	XP_001928846.2	ssc:100157079	0	NM_213606.3	SLC16A12
MMP23B	48.07787177	4607.328201	-6.582413526	1.56E-13	Low	58	5576	XP_005653464.1	ssc:100525702	8E-126	NM_006983.1	MMP23B
DPP4	5.802501765	851.8930013	-7.197853371	1.57E-13	Low	7	1031	NM_214257.1	ssc:397492			
ZNF577	0	318.9434515	-20	1.59E-13	Low	0	386	ZNF577	ssc:100624128	9E-180	NR_024181.1	ZNF577
LOC100739751	0	318.9434515	-20	1.59E-13	Low	0	386	XP_003481268.2	ssc:100739751	4E-171	NM_022055.1	KCNK12
LOC100622121	315.8218818	0	20	1.60E-13	High	381	0	XP_005666622.1	ssc:100622121	5E-168	NM_152692.4	C1GALT1C1
GPX6	315.8218818	0	20	1.60E-13	High	381	0	NM_001137607.1	ssc:100154091	7E-104	NM_182701.1	GPX6
NMUR1	314.1640241	0	20	1.69E-13	High	379	0	HighI00019DC5D0		3E-170	NM_006056.4	NMUR1
CACNG7	3.315715294	623.8401707	-7.55571251	1.77E-13	Low	4	755	XP_003127454.3	ssc:100519605	3E-163	NM_031896.4	CACNG7
CD14	5.802501765	841.1513824	-7.1795466	1.77E-13	Low	7	1018	NM_001097445.2	ssc:100037938	1E-144	NM_001174105.1	CD14
GBX1	4117.289467	43.7927537	6.554858901	1.85E-13	High	4967	53	XP_003360124.1	ssc:100625616	7E-162	NM_001098834.2	GBX1
APOA1	11.60500353	1337.744684	-6.848912024	1.85E-13	Low	14	1619	NM_214398.1	ssc:397691	3E-122	NM_000039.1	APOA1
HOMER3	131.799683	12073.57957	-6.517362759	1.87E-13	Low	159	14612	XP_003480800.1	ssc:100526210	1E-38	NR_027297.1	HOMER3
PDZD4	685.5241371	4.131391859	7.374435749	1.88E-13	High	827	5	XP_005674070.1	ssc:100525676	0	NM_032512.2	PDZD4
PSTPIP2	309.1904512	0	20	1.97E-13	High	373	0	XP_005659501.1	ssc:100519694	1E-178	NM_024430.3	PSTPIP2
LOC100153689	309.1904512	0	20	1.97E-13	High	373	0	LOC100153689				
EXO5	0	311.5069461	-20	1.99E-13	Low	0	377	XP_003128160.1	ssc:100511388	4E-178	NM_022774.1	EXO5
PYGM	4145.473047	44.61903207	6.537733712	2.05E-13	High	5001	54	XP_005660791.1	ssc:733659	0	NM_005609.2	PYGM
CRYBB1	307.5325936	0	20	2.08E-13	High	371	0	NM_001078681.1	ssc:780429	2E-115	NM_001887.3	CRYBB1
LOC102158081	2713.912968	28.09346464	6.59399609	2.08E-13	High	3274	34	XP_005666535.1	ssc:102158081	3E-107	NM_178557.3	NAT8L
ZNF329	0	309.8543894	-20	2.10E-13	Low	0	375	ZNF329	ssc:100516617	0	NM_024620.3	ZNF329
ELAVL2	305.8747359	0	20	2.19E-13	High	369	0	XP_005654549.1	ssc:100153303	0	NM_004432.3	ELAVL2
MAMLD1	4.144644118	679.2008216	-7.356446164	2.27E-13	Low	5	822	XP_005657994.1	ssc:100517073	0	NM_005491.4	MAMLD1

PLAU	44.76215647	4118.997683	-6.523869963	2.44E-13	Low	54	4985	NM_213945.1	ssc:396985	0	NM_002658.3	PLAU
TDRD1	300.901163	0	20	2.57E-13	High	363	0	XP_005671525.1	ssc:100157555	0	NM_198795.1	TDRD1
OSBPL3	2.486786471	523.8604877	-7.718756205	2.86E-13	Low	3	634	XP_003484108.1	ssc:100523342	0	NR_104112.1	OSBPL3
TAGLN	1317.99683	120386.2799	-6.513180272	2.96E-13	Low	1590	145697	NM_001244150.1	ssc:397021	1E-116	NM_003186.3	TAGLN
LOC494560	85.37966883	7495.17111	-6.455925037	2.99E-13	Low	103	9071	XP_003127107.1	ssc:494560	2E-76	NM_144617.2	HSPB6
LOC102163456	0	298.2864922	-20	3.04E-13	Low	0	361	XP_005673399.1	ssc:102163456			
PTX3	21.55214941	2081.395218	-6.593575364	3.18E-13	Low	26	2519	NM_001244783.1	ssc:100513178	0	NM_002852.3	PTX3
LOC100515380	294.2697324	0	20	3.19E-13	High	355	0	XP_003127015.4	ssc:100515380	0	NM_032269.5	CCDC135
LOC100622713	3.315715294	587.4839223	-7.469085425	3.19E-13	Low	4	711	XP_005656599.1	ssc:100622713	0	NM_024582.4	FAT4
KCNK2	0	296.6339355	-20	3.20E-13	Low	0	359	XP_003357633.2	ssc:100627197	8E-110	NM_014217.3	KCNK2
TLR4	0	296.6339355	-20	3.20E-13	Low	0	359	NM_001113039.1	ssc:399541	0	NM_138557.2	TLR4
LOC102162293	293.4408036	0	20	3.28E-13	High	354	0	XP_005654264.1	ssc:102162293			
NPAS1	10984.13584	128.0731476	6.422309537	3.31E-13	High	13251	155	XP_003127301.2	ssc:100517221	0	NM_002517.2	NPAS1
LOC100736658	0	293.328822	-20	3.57E-13	Low	0	355	XP_005659684.1	ssc:100736658	2E-54	NM_002009.3	FGF7
ADAMTS4	5.802501765	781.6593397	-7.073721127	3.64E-13	Low	7	946	XP_003481462.1	ssc:100157276	0	NM_005099.4	ADAMTS4
LOC102165782	287.6383018	0	20	3.98E-13	High	347	0	XR_305670.1	ssc:102165782			
CPVL	1288.155392	12.39417558	6.699500517	4.05E-13	High	1554	15	XP_005673343.1	ssc:100515288	0	NM_031311.3	CPVL
CD248	87.8664553	7462.946253	-6.408288992	4.12E-13	Low	106	9032	XP_003122563.1	ssc:100523587	0	NM_020404.2	CD248
BEND6	12.43393235	1298.9096	-6.706874582	4.13E-13	Low	15	1572	XP_005665923.1	ssc:100153820	6E-127	NM_152731.2	BEND6
LINGO1	1153.039994	10.74161883	6.746087311	4.21E-13	High	1391	13	XP_005666192.1	ssc:100155465	0	NM_032808.5	LINGO1
PDZRN4	360.5840383	0.826278372	8.769491915	4.26E-13	High	435	1	XP_003481760.1	ssc:100156795	0	NM_013377.3	PDZRN4
DAB1	427.727273	1.652556743	8.01584758	4.54E-13	High	516	2	NM_001097442.1	ssc:100037307			
LOC102165358	0	285.8923166	-20	4.58E-13	Low	0	346	XR_298944.1	ssc:102165358			
ZNF568	0	285.0660382	-20	4.71E-13	Low	0	345	ZNF568	ssc:100511135	0	NM_198539.3	ZNF568
HOXC4	4.973572942	694.9001106	-7.126379165	4.79E-13	Low	6	841	XP_005663910.1	ssc:100154759	1E-153	NM_153633.2	HOXC4
LOC100620451	0.828928824	358.6048133	-8.756930908	4.90E-13	Low	1	434	NM_001244338.1	ssc:100620451	2E-52	NR_037939.1	HOXA10
SPEG	19.06536294	1794.676623	-6.556626107	4.94E-13	Low	23	2172	XP_005672303.1	ssc:100737722	0	NM_005876.4	SPEG
VWA5A	27.35465118	2446.610259	-6.482854331	4.97E-13	Low	33	2961	XP_005653769.1	ssc:100515913	0	NM_198315.2	VWA5A
FN1	9553.404692	881757.1804	-6.528222629	4.97E-13	Low	11525	1067143	XP_005672231.1	ssc:397620	0	NM_212482.1	FN1
LOC100623939	2.486786471	494.1144663	-7.634418849	5.07E-13	Low	3	598	XP_005664537.1	ssc:100623939	2E-43	NM_021902.3	FXYD1
LOC100158194	19.06536294	1785.587561	-6.549301076	5.19E-13	Low	23	2161	XP_001926938.4	ssc:100158194	0	NM_152380.2	TBX15
LOC102157546	0	281.7609248	-20	5.27E-13	Low	0	341	XR_298764.1	ssc:102157546			
LOC100515669	278.5200847	0	20	5.44E-13	High	336	0	XP_003127928.1	ssc:100515669	0	NM_024421.2	DSC1
ELOVL2	1374.36399	14.04673232	6.612385775	5.54E-13	High	1658	17	XP_005665625.1	ssc:100153368	3E-156	NM_017770.3	ELOVL2
LOC100626132	54.70930236	4564.361725	-6.382483161	5.73E-13	Low	66	5524	XP_003361657.1	ssc:100626132	7E-45	NM_144617.2	HSPB6
LOC100736668	0	279.2820896	-20	5.74E-13	Low	0	338	XP_005669299.1	ssc:100736668	6E-46	NM_201433.1	GAS7
LOC100523909	276.8622271	0	20	5.76E-13	High	334	0	XP_005665511.1	ssc:100523909	0	NM_001099772.1	CYP4B1
HOXB9	1.657857647	420.5756912	-7.986901522	5.85E-13	Low	2	509	XP_003131603.2	ssc:100523635	1E-145	NM_024017.4	HOXB9
RHOJ	0.828928824	348.6894729	-8.716478864	6.43E-13	Low	1	422	NM_001168417.1	ssc:100155474			
CXCL14	3.315715294	545.3437253	-7.36170189	6.60E-13	Low	4	660	NM_001244128.1	ssc:494467	6E-44	NM_004887.4	CXCL14
SR-PSOX	19.89429177	1803.765685	-6.50251359	6.67E-13	Low	24	2183	NM_213811.1	ssc:396735	8E-62	NM_022059.3	CXCL16
HGF	2.486786471	478.4151772	-7.587836713	6.94E-13	Low	3	579	XP_005667745.1	ssc:100525120	0	NM_001010934.1	HGF
PTPRB	4745.617515	58.66576439	6.337933183	6.97E-13	High	5725	71	XP_001926902.5	ssc:100156181	0	NM_002837.4	PTPRB
NOX4	21.55214941	1915.313266	-6.473604809	7.20E-13	Low	26	2318	XP_005667271.1	ssc:100523323	0	NR_026571.1	NOX4
DMRTB1	1152.211065	11.5678972	6.63813457	7.27E-13	High	1390	14	XP_003128031.3	ssc:100515733	8E-93	NM_033067.1	DMRTB1
LOC102166405	270.2307965	0	20	7.28E-13	High	326	0	XR_300687.1	ssc:102166405			
DAAM2	172.4171953	13680.691	-6.310093628	7.75E-13	Low	208	16557	XP_003356671.1		0	NM_015345.3	DAAM2
ID2	22.38107824	1948.364401	-6.443840176	8.35E-13	Low	27	2358	NM_001037965.1	ssc:654298	1E-72	NM_002166.4	ID2
ZNF226	0	268.5404708	-20	8.38E-13	Low	0	325	ZNF226	ssc:100523907	0	NM_016444.1	ZNF226
LOC100737260	464.2001412	2.478835115	7.548940841	8.52E-13	High	560	3	XR_299072.1	ssc:100737260	6E-79	NM_207108.2	ASTN1
PTGES	96.15574354	7529.048523	-6.290950712	9.03E-13	Low	116	9112	NM_001038631.1	ssc:654407	1E-73	NM_198797.1	PTGES
LOC102159043	6.631430589	772.5702776	-6.86420223	9.13E-13	Low	8	935	XR_303323.1	ssc:102159043			
LOC102167727	0	265.2353573	-20	9.44E-13	Low	0	321	XR_303912.1	ssc:102167727			
OLFM1	0	264.409079	-20	9.73E-13	Low	0	320	XP_005652767.1	ssc:100620523	0	NM_058199.2	OLFM1
TMEM132E	0	264.409079	-20	9.73E-13	Low	0	320	XP_005669107.1	ssc:100519732	0	NM_207313.1	TMEM132E
LOC100738589	26.52572236	2205.336974	-6.377463266	1.06E-12	Low	32	2669	XP_005660660.1	ssc:100738589	1E-84	NM_053056.2	CCND1
EPSTI1	25.69679353	2124.361694	-6.36929726	1.16E-12	Low	31	2571	XP_005656905.1	ssc:100625050	1E-113	NM_033255.3	EPSTI1
GGT5	4.144644118	573.43719	-7.112243433	1.19E-12	Low	5	694	XP_005670952.1	ssc:100157523	0	NM_004121.2	GGT5
TPM1	1618.897992	128639.9746	-6.312183133	1.25E-12	Low	1953	155686	NM_001097483.2	ssc:100037999	1E-149	NM_001018020.1	TPM1
CAMSAP3	1440.678295	16.52556743	6.445904596	1.25E-12	High	1738	20	XP_005654804.1	ssc:100511741	0	NM_020902.1	CAMSAP3
POU2F3	255.3100777	0	20	1.26E-12	High	308	0	XP_003130003.2	ssc:397514	1E-157	NM_014352.3	POU2F3
FRMD3	384.6229742	1.652556743	7.862601319	1.28E-12	High	464	2	XP_005668097.1	ssc:100519727	3E-179	NM_174938.5	FRMD3
TRIML2	254.4811488	0	20	1.30E-12	High	307	0	XP_005674640.1	ssc:100737283	1E-17	NM_173553.1	TRIML2
CEBPD	424.4115577	32373.5866	-6.253209453	1.30E-12	Low	512	39180	XP_005663148.1	ssc:100153946	3E-115	NM_005195.3	CEBPD
DNMT3B	16000.81308	214.0060983	6.224349499	1.31E-12	High	19303	259	XP_001928639.1		0	NM_175850.2	DNMT3B
LOC100737912	0	256.1462952	-20	1.32E-12	Low	0	310	XP_005664976.1	ssc:100737912	0	NM_001144989.1	ZNF814
ZFP112	0	255.3200169	-20	1.36E-12	Low	0	309	ZFP112	ssc:100524627	0	NM_013380.3	ZNF112
LOC100621315	252.8232912	0	20	1.39E-12	High	305	0	XR_298397.1	ssc:100621315			
BATF3	906.0192042	9.089062089	6.639266391	1.39E-12	High	1093	11	XP_003482796.1	ssc:100524332	8E-49	NM_018664.2	BATF3
MYL4	0	254.4937385	-20	1.41E-12	Low	0	308	XP_003131354.1	ssc:100516998	4E-91	NM_002476.2	MYL4
LOC100521619	0	254.4937385	-20	1.41E-12	Low	0	308	LOC100521619				
NR0B1	249.5075759	0	20	1.57E-12	High	301	0	NM_214387.1	ssc:397680	0	NM_000475.4	NR0B1
CCDC129	374.6758283	1.652556743	7.824799287	1.65E-12	High	452	2	XP_005673334.1	ssc:100512640	0	NR_047565.1	CCDC129
LOC100621169	605.1180412	4.95767023	6.931410477	1.69E-12	High	730	6	XP_005658484.1	ssc:100621169	0	NM_020340.4	KIAA1244
LOC100736640	0.828928824	313.9857813	-8.565235284	1.77E-12	Low	1	380	XR_305303.1	ssc:100736640	2E-33	NM_004621.5	TRPC6
SLIT2	33.98608177	2606.908263	-6.261251961	1.80E-12	Low	41	3155	XP_005666614.1	ssc:100515495	0	NM_004787	SLIT2
LOC100736898	6.631430589	720.5147401	-6.763564	1.81E-12	Low	8	872	XP_003482725.1	ssc:100736898	9E-155	NM_005924.4	MEOX2
NTN4	22.38107824	1795.502902	-6.325964633	1.86E-12	Low	27	2173	XP_003481795.1	ssc:100516554	0	NM_021229.3	NTN4
POU3F2	1102.475335	12.39417558	6.474940259	1.87E-12	High	1330	15	XP_001926276.1	ssc:100157279	0	NM_005604.3	POU3F2
IRX2	0	246.2309548	-20	1.93E-12	Low	0	298	XP_005674585.1	ssc:100620587	2E-100	NM_033267.4	IRX2
LOC100519459	3153.245245	42.96647533	6.197482137	2.16E-12	High	3804	52	XR_135471.2	ssc:100519459			
LOC100155368	1249.195737	14.87301069	6.392159024	2.21E-12	High	1507	18	XP_005663084.1	ssc:100155368	3E-125	NM_020647.2	JPH1
DIRAS3	6.631430589	705.6417295	-6.733471935	2.21E-12	Low	8	854	NM_001044598.2	ssc:733668	5E-70	NM_004675.2	DIRAS3
SDK2	0	242.0995629	-20	2.27E-12	Low	0	293	XP_005668701.1	ssc:100518574	0	NM_019064.3	SDK2
LOC100626567	2.486786471	423.0545263	-7.410417175	2.30E-12	Low	3	512	LOC100626567				
LOC102166778	5.802501765	644.4971299	-6.795355067	2.40E-12	Low	7	780	XP_005662309.1	ssc:102166778	9E-150	NM_001393.3	ECM2
EPHB1	637.4462654	5.783948602	6.78410519	2.44E-12	High	769	7	XP_003132484.3	ssc:100524585	0	NM_004441.4	EPHB1
EGR4	358.9261806	1.652556743	7.762843539	2.50E-12	High	433	2	NM_001285968.1	ssc:100516906	0	NM_001965.3	EGR4
LOC100623930	358.9261806	1.652556743	7.762843539	2.50E-12	High	433	2	XP_005658231.1	ssc:100623930	6E-121	NM_001012302.2	ANO9
FAM180A	0	239.6207278	-20	2.51E-12	Low	0	290	XP_003484061.1	ssc:100737678	5E-78	NM_205855.3	FAM180A
KLHDC8A	467.5158565	3.305113487	7.144171677	2.71E-12	High	564	4	XP_003357440.1	ssc:100625797	0	NM_018203.2	KLHDC8A
PRTG	2085.58492	28.09346464	6.21407369	2.79E-12	High	2516	34	XP_005654495.1	ssc:100154460	0	NM_173814.4	PRTG
ZYG11A	234.5868571	0	20	2.85E-12	High	283	0	ZYG11A	ssc:100516616	7E-148	NM_001004339.2	ZYG11A
RIPK3	6.631430589	686.6373269	-6.694084342	2.89E-12	Low	8	831	XP_001927459.2	ssc:100153263	3E-155	NM_006871.3	RIPK3
MKRN3	353.1236789	1.652556743	7.739329945	2.93E-12	High	426	2	XP_001925048.1	ssc:100155343	2E-152	NM_005664.3	MKRN3
LOC100737142	0.828928824	296.6339355	-8.483219709	3.07E-12	Low	1	359	XR_303908.1	ssc:100737142			
UBE2L6	72.94573648	5069.217811	-6.118795686	3.10E-12	Low	88	6135	NM_001246215.1	ssc:100514753	2E-66	NM_198183.2	UBE2L6
LOC100620475	293.4408036	0.826278372	8.472225874	3.15E-12	High	354	1	XP_005653629.1	ssc:100620475	7E-25	NM_001039127	ZNF718
LOC100627141	19.89429177	1538.530328	-6.273054533	3.17E-12	Low	24	1862	XP_005674171.1	ssc:100627141	0	NM_001099409.1	EHBP1L1
RGS4	4719.091793	68.58110485	6.104554277	3.24E-12	High	5693	83	NM_001185184.1	ssc:100157437	1E-113	NM_005613.5	RGS4

KIT	1041.963531	12.39417558	6.393498663	3.25E-12	High	1257	15	NM_001044525.1	ssc:396810	7E-53	NM_001093772.1	KIT
NFIB	42.27537	2991.953984	-6.145126795	3.35E-12	Low	51	3621	XP_005660156.1	ssc:100155995	0	NM_005596.3	NFIB
LOC102159087	64.65644824	4474.297383	-6.112723079	3.36E-12	Low	78	5415	XP_005654763.1	ssc:102159087			
ARL14EPL	230.442213	0	20	3.38E-12	High	278	0	XP_003123901.2	ssc:100521841	5E-77	NM_001195581.1	ARL14EPL
NDRG1	46.42001412	3261.320733	-6.134565557	3.39E-12	Low	56	3947	XP_005662896.1	ssc:100156071	0	NM_006096.3	NDRG1
ZNF567	0	232.1842225	-20	3.39E-12	Low	0	281	ZNF567	ssc:100525521	0	NM_152603.2	ZNF567
LRRC32	22.38107824	1681.476486	-6.231305253	3.54E-12	Low	27	2035	XP_003129719.1	ssc:100522452	0	NM_005512.2	LRRC32
LOC100620398	0	229.7053873	-20	3.76E-12	Low	0	278	XP_005674448.1	ssc:100620398	0	NM_006080.2	SEMA3A
OCLN	344.0054618	1.652556743	7.701587851	3.78E-12	High	415	2	NM_001163647.2	ssc:397236	0	NM_002538.3	OCLN
LOC100516145	1.657857647	346.2106378	-7.706186109	3.86E-12	Low	2	419	XR_304856.1	ssc:100516145			
VASN	135.9443271	9142.770183	-6.071543502	3.92E-12	Low	164	11065	XP_005662231.1	ssc:102158247	0	NM_138440.2	VASN
LOC100622219	226.2975688	0	20	4.02E-12	High	273	0	XP_003357960.2	ssc:100622219	0	NM_001676.5	ATP12A
EPHA8	226.2975688	0	20	4.02E-12	High	273	0	XP_003127734.2	ssc:100518378	0	NM_020526.3	EPHA8
NEK6	100.3003877	6726.732224	-6.067506742	4.11E-12	Low	121	8141	XP_005660482.1	ssc:100514624	0	NM_014397.5	NEK6
LOC102159901	225.46864	0	20	4.17E-12	High	272	0	XR_303649.1	ssc:102159901			
LOC100516179	0	227.2265522	-20	4.17E-12	Low	0	275	XP_003123076.3	ssc:100516179	1E-154	NM_021217.2	ZNF77
PARP3	116.8789641	7787.673654	-6.058105226	4.32E-12	Low	141	9425	XP_005669672.1	ssc:100620838	0	NM_005485.4	PARP3
LOC100522769	0	225.5739955	-20	4.47E-12	Low	0	273	XP_003134501.1	ssc:100522769	1E-90	NM_003881.2	WISP2
LOC100621385	223.8107824	0	20	4.47E-12	High	270	0	XP_005674345.1	ssc:100621385	0	NM_198182.2	GRHL1
LOC102165697	282.6647288	0.826278372	8.418248253	4.51E-12	High	341	1	XR_300413.1	ssc:102165697			
BEX1	10922.79511	166.0819527	6.039302963	4.56E-12	High	13177	201	NM_001244771.1	ssc:100513130	2E-49	NM_018476.3	BEX1
NR6A1	2341.723927	33.87741324	6.111103472	4.67E-12	High	2825	41	NM_001097499.1	ssc:100038028	2E-55	NM_033335.2	NR6A1
ZNF618	6.631430589	652.7599137	-6.621088519	4.72E-12	Low	8	790	ZNF618	ssc:100157179	0	NM_133374.2	ZNF618
TIMP-3	113.5632488	7434.02651	-6.0325759	5.15E-12	Low	137	8997	XP_003126121.3	ssc:396775	2E-124	NM_000362.4	TIMP3
GULO	0.828928824	280.108368	-8.400521139	5.33E-12	Low	1	339	NM_001129948.1	ssc:396759			
CCK	219.6661383	0	20	5.35E-12	High	265	0	NM_214237.2	ssc:397468	2E-50	NM_001174138.1	CCK
VPAC1	277.6911559	0.826278372	8.39263761	5.36E-12	High	335	1	NM_214036.1	ssc:100155402	0	NM_004624.3	VIPR1
SIPA1L2	1894.931291	27.26718627	6.118835893	5.52E-12	High	2286	33	XP_001928528.3	ssc:100158048	0	NM_020808.3	SIPA1L2
LOC100514506	2.486786471	385.8719996	-7.277695915	5.59E-12	Low	3	467	XR_115703.3	ssc:100514506			
IFI44L	136.7732559	8855.22531	-6.016670951	5.69E-12	Low	165	10717	XP_003127967.2	ssc:100511267	2E-158	NM_006820.2	IFI44L
WNT3A	217.1793518	0	20	5.97E-12	High	262	0	WNT3A		0	NM_033131.3	WNT3A
ADM	74.60359412	4836.20731	-6.018487143	6.08E-12	Low	90	5853	NM_214107.1	ssc:397195	9E-91	NM_001124.1	ADM
MYOD1	0	218.1374901	-20	6.16E-12	Low	0	264	NM_001002824.1	ssc:407604	6E-155	NM_002478.4	MYOD1
ZNF135	0	217.3112118	-20	6.38E-12	Low	0	263	ZNF135	ssc:102159974	7E-28	NM_007134.1	ZNF135
PPP1R1A	1513.624032	21.48323766	6.138651656	6.55E-12	High	1826	26	XP_005674358.1	ssc:100621808	8E-39	NM_006741.3	PPP1R1A
LOC100738970	271.0597253	0.826278372	8.35776715	6.76E-12	High	327	1	XP_003482513.1	ssc:100738970	3E-115	NM_025212.2	CXXC4
LOC102167155	4.973572942	528.8181579	-6.73234527	6.86E-12	Low	6	640	XP_005669150.1	ssc:102167155	0	NM_015077.3	SARM1
ADAMTS19	964.8731507	12.39417558	6.282605072	6.89E-12	High	1164	15	XP_003123951.4	ssc:100518181	0	NM_133638.3	ADAMTS19
MYOG	2.486786471	376.7829375	-7.243307189	7.04E-12	Low	3	456	NM_001012406.1	ssc:497618	3E-118	NM_002479.5	MYOG
MDFI	43.93322765	2858.096888	-6.023598519	7.44E-12	Low	53	3459	XP_005666020.1	ssc:100152701	8E-102	NM_005586.3	MDFI
LOC100514098	3.315715294	424.7070831	-7.001004225	7.49E-12	Low	4	514	XP_005653093.1	ssc:100514098	0	NM_001098814.1	SRL
EHD2	312.5061665	19790.19328	-5.984757234	7.65E-12	Low	377	23951	XP_003356007.1	ssc:100624668	0	NM_014601.3	EHD2
ISG12%28A%29	344.0054618	21800.52856	-5.985787833	7.73E-12	Low	415	26384	ISG12%28A%29		3E-19	NM_005532	IFI27
LOC102163961	7.460359412	669.2854811	-6.487232772	7.74E-12	Low	9	810	XP_005656794.1	ssc:102163961	0	NM_022093.1	TNN
LOC100156358	683.0373506	43927.43708	-6.007014047	7.83E-12	Low	824	53163	NM_001252605.1	ssc:100156358	1E-53	NM_019554.2	S100A4
ACACB	29.01250883	1940.927895	-6.063927657	8.02E-12	Low	35	2349	NM_001206399.1	ssc:100154993	0	NM_001093.3	ACACB
LOC100514231	7020.198207	113.2001369	5.954564156	8.16E-12	High	8469	137	NM_001244939.1	ssc:100514231	3E-103	NR_024537.1	GSTM3
CSF2RB	209.7189924	0	20	8.33E-12	High	253	0	XP_001924814.3	ssc:100153254	0	NM_000395.2	CSF2RB
HOXA2	0.828928824	266.8879141	-8.33077003	8.47E-12	Low	1	323	XP_003134891.1	ssc:100519993	0	NM_006735.3	HOXA2
NT5E	95.32681471	5935.983823	-5.960461259	8.57E-12	Low	115	7184	XP_003353298.1	ssc:100157995	0	NM_002526.3	NT5E
SNX22	315.8218818	1.652556743	7.578267512	8.63E-12	High	381	2	XP_003362155.2	ssc:100624664	1E-38	NR_073534.1	SNX22
LOC102161219	18.23643412	1290.646817	-6.145126795	8.67E-12	Low	22	1562	XP_005674129.1	ssc:102161219	4E-40	NM_002962.1	S100A5
PLA2R1	13.26286118	996.4917163	-6.231393868	8.95E-12	Low	16	1206	XP_005671924.1	ssc:100622105	0	NM_007366.4	PLA2R1
ATP6V0A4	314.1640241	1.652556743	7.570674363	9.08E-12	High	379	2	XP_005657806.1	ssc:100519402	0	NM_130841.2	ATP6V0A4
PAH	206.4032771	0	20	9.70E-12	High	249	0	XP_003126719.3	ssc:100521900	2E-171	NM_000277.1	PAH
PTF-BETA	127.6550388	7819.072232	-5.936675045	9.81E-12	Low	154	9463	NM_214336.1	ssc:397609	8E-63	NM_002825.5	PTN
LOC780415	93.66895707	5739.32957	-5.937167409	1.01E-11	Low	113	6946	NM_001078670.1	ssc:780415	4E-177	NM_006084.4	IRF9
PKP1	205.5743483	0	20	1.01E-11	High	248	0	XP_005674635.1	ssc:100623590			
PTHLH	0	205.7433146	-20	1.07E-11	Low	0	249	NM_213916.2	ssc:396951	1E-73	NM_198966.1	PTHLH
STXBP5L	640.7619806	7.436505346	6.429019927	1.08E-11	High	773	9	XP_005670233.1	ssc:100620808	0	NM_014980.2	STXBP5L
PLEKHH1	1483.782594	22.30951604	6.055476694	1.09E-11	High	1790	27	XP_005656409.1	ssc:100152619	1E-143	NM_020715.2	PLEKHH1
LOC102165155	872.8620513	11.5678972	6.237555123	1.10E-11	High	1053	14	XP_005668615.1	ssc:102165155	2E-162	NM_024979.4	MCF2L
ADAM33	38.13072589	2406.948897	-5.980107706	1.10E-11	Low	46	2913	XP_005672830.1	ssc:100738366	4E-124	NM_153202.2	ADAM33
ZSCAN22	0	204.9170362	-20	1.12E-11	Low	0	248	ZSCAN22	ssc:100516809	2E-19	NM_181846.2	ZSCAN22
C1H15orf52	16.57857647	1162.573669	-6.131858194	1.12E-11	Low	20	1407	XP_003353441.1	ssc:100153607			
ANGPTL1	1.657857647	309.028111	-7.542274136	1.15E-11	Low	2	374	NM_001109947.1	ssc:100126165	0	NM_004673.3	ANGPTL1
LOC100738686	202.258633	0	20	1.18E-11	High	244	0	XR_307991.1	ssc:100738686	5E-71	NR_048571.1	GPM6A
PRPH	1753.184462	27.26718627	6.006668153	1.18E-11	High	2115	33	XP_005655608.1	ssc:100152434	0	NM_006262.3	PRPH
PODN	0	203.2644794	-20	1.21E-11	Low	0	246	XP_005665471.1	ssc:100622214			
ZNF175	0	203.2644794	-20	1.21E-11	Low	0	246	ZNF175	ssc:100514293	0	NM_007147.2	ZNF175
CDH18	0	203.2644794	-20	1.21E-11	Low	0	246	XP_003359779.3	ssc:100625674	0	NM_004934.3	CDH18
POU3F3	305.0458071	1.652556743	7.52818228	1.21E-11	High	368	2	XP_003356366.2	ssc:397513	5E-120	NM_002699.3	POU3F1
LOC100737363	305.0458071	1.652556743	7.52818228	1.21E-11	High	368	2	XR_135271.2	ssc:100737363			
LOC100511549	201.4297041	0	20	1.22E-11	High	243	0	XP_003123129.1	ssc:100511549	2E-146	NM_181710.3	ZNRF4
LOC102166770	1599.83263	24.78835115	6.012114861	1.27E-11	High	1930	30	XP_005660166.1	ssc:102166770	8E-32	NM_000170.2	GLDC
RGS14	442.6479918	4.131391859	6.743388161	1.35E-11	High	534	5	XP_005661511.1	ssc:100524790	0	NM_006480.4	RGS14
ZNF583	0	200.7856443	-20	1.36E-11	Low	0	243	ZNF583	ssc:100620770	0	NM_152478.2	ZNF583
SOWAHB	198.9429177	0	20	1.38E-11	High	240	0	XP_005666837.1	ssc:100523320	0	NM_001029870.1	SOWAHB
GNG11	15.74964765	1091.513729	-6.114866913	1.38E-11	Low	19	1321	XP_005667670.1	ssc:100522202	3E-35	NM_004126.3	GNG11
LOC100517161	0	199.959366	-20	1.41E-11	Low	0	242	XP_005653357.1	ssc:100517161	1E-155	NM_014650.2	ZNF432
MYO7A	347.3211771	2.478835115	7.130464257	1.42E-11	High	419	3	NM_001099928.1	ssc:397373	0	NM_001127180.1	MYO7A
LOC102158748	0	199.1330876	-20	1.47E-11	Low	0	241	XR_307291.1	ssc:102158748			
LOC102166965	40.61751236	2473.877445	-5.928528338	1.49E-11	Low	49	2994	XP_005671014.1	ssc:102166965	3E-150	NM_002508.2	NID1
PRRX1	38.13072589	2323.494781	-5.929198599	1.56E-11	Low	46	2812	XP_003481450.1	ssc:100738910	9E-143	NM_022716	PRRX1
FABP7	2069.835273	33.87741324	5.933048426	1.56E-11	High	2497	41	NM_001025229.1	ssc:574075	5E-71	NM_001446.3	FABP7
LOC100625781	0.828928824	249.5360683	-8.233784415	1.61E-11	Low	1	302	XP_003361074.1	ssc:100625781	3E-133	NM_145260.2	OSR1
LOC100157489	388.7676183	3.305113487	6.878064437	1.62E-11	High	469	4	XP_003359120.2	ssc:100157489	0	NM_198827.3	GPR133
LOC102166884	194.7982735	0	20	1.68E-11	High	235	0	XR_300733.1	ssc:102166884			
LOC102165082	194.7982735	0	20	1.68E-11	High	235	0	XR_301689.1	ssc:102165082			
ISLR2	4.973572942	480.8940123	-6.595292518	1.72E-11	Low	6	582	XP_005666221.1	ssc:100157309	0	NM_020851.2	ISLR2
LOC100739179	384.6229742	3.305113487	6.862601319	1.80E-11	High	464	4	XP_003482145.1	ssc:100739179	2E-126	NM_020883.1	ZSWIM5
LOC102159369	3.315715294	387.5245563	-6.868823788	1.81E-11	Low	4	469	XP_005668982.1	ssc:102159369	2E-66	NM_005501.2	ITGA3
LOC100626259	193.1404159	0	20	1.83E-11	High	233	0	XP_003357039.1	ssc:100626259	0	NM_199348.1	DCHS2
CLU	2240.59461	136815.1728	-5.932202777	1.83E-11	Low	2703	165580	NM_213971.1	ssc:397025	2E-177	NR_045494.1	CLU
PENK	0	194.1754174	-20	1.86E-11	Low	0	235	XP_003125669.1	ssc:100152093	5E-25	NM_006211.3	PENK
COL15A1	285.1515153	16447.07099	-5.849958172	1.88E-11	Low	344	19905	XP_003122085.4	ssc:100512644	6E-145	NM_001855.4	COL15A1
TIMP2	1152.211065	68241.50444	-5.88817253	2.02E-11	Low	1390	82589	NM_001145985.1	ssc:396988	7E-116	NM_003255.4	TIMP2
LOC102161974	423.5826289	4.131391859	6.67987171	2.06E-11	High	511	5	XR_307170.1	ssc:102161974			
LOC100622879	3.315715294	380.088051	-6.840869727	2.18E-11	Low	4	460	XR_300016.1	ssc:100622879			
LOC100626686	188.9957718	0	20	2.24E-11	High	228	0	XP_005658179.1	ssc:100626686	9E-71	NM_006152.3	LRMP

SOX9	4.144644118	423.0545263	-6.673451581	2.27E-11	Low	5	512	NM_213843.1	ssc:396840	0	NM_000346.3	SOX9
LOC102166696	0	190.0440255	-20	2.28E-11	Low	0	230	XR_303880.1	ssc:102166696			
TRIM34	0.828928824	240.4470062	-8.180255019	2.30E-11	Low	1	291	XP_005667139.1	ssc:100738479	1E-146	NM_001003819.3	TRIM6-TRIM34
KCNJ5	285.1515153	1.652556743	7.430885079	2.31E-11	High	344	2	NM_214225.1	ssc:397448	0	NM_000890.3	KCNJ5
ADAMTSL3	14.09179	946.915014	-6.070308163	2.32E-11	Low	17	1146	XP_005666168.1	ssc:100157238	0	NM_207517.2	ADAMTSL3
IL1A	5.802501765	507.3349202	-6.450119599	2.44E-11	Low	7	614	NM_214029.1	ssc:397094	2E-105	NM_000575.3	IL1A
SNAI2	0	188.3914688	-20	2.48E-11	Low	0	228	NM_001128439.1	ssc:641345	2E-144	NM_003068.4	SNAI2
LOC100626788	0	187.5651904	-20	2.58E-11	Low	0	227	XR_303915.1	ssc:100626788	8E-19	NM_181846.2	ZSCAN22
ATOH8	1.657857647	283.4134815	-7.417444442	2.64E-11	Low	2	343	XP_005662472.1	ssc:100523367	2E-126	NM_032827.6	ATOH8
MTL5	1225.98573	19.83068092	5.950064161	2.64E-11	High	1479	24	XP_003122474.1	ssc:100520538	0	NM_004923.3	MTL5
DLGAP1	185.6800565	0	20	2.65E-11	High	224	0	XP_003356412.2	ssc:100626809	0	NM_004746.3	DLGAP1
LOC102164021	185.6800565	0	20	2.65E-11	High	224	0	XR_308287.1	ssc:102164021			
LOC100622872	454.2529953	4.95767023	6.517689907	2.74E-11	High	548	6	XP_005654533.1	ssc:100622872	0	NM_153181.2	NETO1
LOC100516860	184.8511277	0	20	2.77E-11	High	223	0	XP_003127932.3	ssc:100516860	0	NM_024421.2	DSC1
LOC100524748	0	185.9126336	-20	2.81E-11	Low	0	225	XP_005664829.1	ssc:100524748	0	NM_024729.3	MYH14
ATP2A3	1602.319416	27.26718627	5.876852127	2.84E-11	High	1933	33	XP_005669187.1	ssc:396820	5E-159	NM_174958.2	ATP2A3
UNC45B	6.631430589	542.0386119	-6.35293168	2.86E-11	Low	8	656	HighI00005FE9DD		0	NM_173167.2	UNC45B
LOC102159197	366.38654	3.305113487	6.792522884	2.87E-11	High	442	4	XR_302293.1	ssc:102159197			
LOC100520893	184.0221988	0	20	2.89E-11	High	222	0	XP_005661555.1	ssc:100520893	0	NM_014979.1	SV2C
CHST15	17.4075053	1094.818843	-5.974838897	2.95E-11	Low	21	1325	XP_005671601.1	ssc:100152393	0	NM_015892.4	CHST15
ABI3BP	3.315715294	366.0413187	-6.786542564	3.13E-11	Low	4	443	XP_005670307.1	ssc:100154641	0	NM_015429.3	ABI3BP
PXDN	359.7551094	19766.23121	-5.779878834	3.15E-11	Low	434	23922	XP_005662846.1	ssc:100516076	0	NM_012293.1	PXDN
LOC100513704	181.5354124	0	20	3.28E-11	High	219	0	XP_003135242.2	ssc:100513704	0	NM_001008537.2	KIAA2022
LOC100520029	258.625793	13820.33205	-5.739782304	3.89E-11	Low	312	16726	XR_304045.1	ssc:100520029	4E-29	NM_006868.3	RAB31
LOC102165278	436.0165612	4.95767023	6.458576813	4.07E-11	High	526	6	XP_005668616.1	ssc:102165278	8E-112	NM_024979.4	MCF2L
FBXO32	15.74964765	970.0508084	-5.944668855	4.33E-11	Low	19	1174	NM_001044588.1	ssc:733657	1E-145	NM_148177.2	FBXO32
RNF128	676.4059201	9.915340461	6.092083166	4.36E-11	High	816	12	XP_003135339.3	ssc:100515872	0	NM_194463.1	RNF128
LOC102166536	175.7329106	0	20	4.46E-11	High	212	0	XR_303556.1	ssc:102166536			
LOC102167761	175.7329106	0	20	4.46E-11	High	212	0	XR_297827.1	ssc:102167761			
DPP10	1487.09831	26.4409079	5.813584499	4.50E-11	High	1794	32	XP_001925671.4	ssc:100157195	0	NM_020868.3	DPP10
LOC100736874	1322.970402	23.13579441	5.837510339	4.52E-11	High	1596	28	XP_003483456.2	ssc:100736874	0	NM_198827.3	GPR133
ADAM22	875.3488377	14.04673232	5.961551602	4.55E-11	High	1056	17	XP_005667627.1	ssc:100513676	0	NM_021723.3	ADAM22
ELAVL3	174.9039818	0	20	4.67E-11	High	211	0	XP_005654836.1	ssc:100515074	8E-154	NM_032281.2	ELAVL3
ICAM-2	469.1737142	5.783948602	6.341923645	4.78E-11	High	566	7	NM_001001631.1	ssc:414387	7E-84	NM_001099789.1	ICAM2
LOC102163958	347.3211771	3.305113487	6.715426758	4.79E-11	High	419	4	XR_304068.1	ssc:102163958			
MCF2L	2915.342673	55.36065091	5.718660754	4.82E-11	High	3517	67	XP_005668614.1	ssc:100518447	0	NM_024979.4	MCF2L
BATF2	4.144644118	390.8296698	-6.559147954	4.85E-11	Low	5	473	XP_005660776.1	ssc:100515875	3E-90	NM_138456.3	BATF2
GABRB3	263.5993659	1.652556743	7.31750328	4.89E-11	High	318	2	XP_005654529.1	ssc:100739031	7E-139	NR_103801.1	GABRB3
HNMT	5.802501765	470.9786719	-6.342842863	4.99E-11	Low	7	570	NM_001244561.1	ssc:100624677	5E-155	NM_006895.2	HNMT
LOC102161224	173.2461241	0	20	5.11E-11	High	209	0	XP_005664286.1	ssc:102161224	1E-90	xp_003846405	xp_003846405
LOC100523405	0	174.3447364	-20	5.15E-11	Low	0	211	XP_003133273.1	ssc:100523405	4E-16	NM_021641	ADAM12
LOC100625548	261.1125794	1.652556743	7.303828343	5.35E-11	High	315	2	XP_003361336.1	ssc:100625548	1E-137	NR_103801.1	GABRB3
RASGRF2	1258.313954	22.30951604	5.817688898	5.43E-11	High	1518	27	XP_005661584.1	ssc:100515363	0	NM_006909.2	RASGRF2
TMEM30B	1.657857647	262.7565222	-7.308262631	5.43E-11	Low	2	318	XP_003480534.1	ssc:100738730	0	NM_001017970.2	TMEM30B
CCDC158	381.3072589	4.131391859	6.52818228	5.68E-11	High	460	5	XP_003129142.3	ssc:100522937	0	NM_178555.3	CCDC158
CDH15	0	171.8659013	-20	5.89E-11	Low	0	208	XP_005655832.1	ssc:100187727	0	NM_001793.4	CDH3
LOC102157883	299.2433053	2.478835115	6.915512851	5.95E-11	High	361	3	XP_005661699.1	ssc:102157883			
NYNRIN	68.80109236	3536.471431	-5.683735326	5.97E-11	Low	83	4280	XP_005666290.1	ssc:100155701	4E-64	NM_025081.2	NYNRIN
SLC6A20	169.9304088	0	20	6.13E-11	High	205	0	XP_003358454.2	ssc:100621879	0	NM_022405.3	SLC6A20
LOC100737693	0	171.0396229	-20	6.16E-11	Low	0	207	XP_003482794.2	ssc:100737693	5E-64	NM_014217.3	KCNK2
ZIC3	3446.686049	67.75482648	5.668742363	6.24E-11	High	4158	82	ZIC3	ssc:100156863	0	NM_003413.3	ZIC3
SMPD1	66.31430589	3395.177829	-5.678023196	6.28E-11	Low	80	4109	XP_005656664.1	ssc:100518980	2E-70	NR_027400.1	SMPD1
LOC102162630	169.10148	0	20	6.42E-11	High	204	0	XP_005666706.1	ssc:102162630	9E-23	NR_049749.1	ZNF267
RCVRN	0	170.2133446	-20	6.45E-11	Low	0	206	XR_306608.1	ssc:100516153			
LOC100737919	0	170.2133446	-20	6.45E-11	Low	0	206	XR_308589.1	ssc:100737919			
KIAA2022	255.3100777	1.652556743	7.271406865	6.63E-11	High	308	2	XP_003484179.1	ssc:100738677	0	NM_001008537.2	KIAA2022
GRIN2B	168.2725512	0	20	6.72E-11	High	203	0	XP_003355615.1	ssc:100626022	0	NM_000834.3	GRIN2B
LOC100522644	0	169.3870662	-20	6.75E-11	Low	0	205	XR_307034.1	ssc:100522644	1E-65	NM_207015.2	NAALADL2
ARMC2	0	169.3870662	-20	6.75E-11	Low	0	205	XP_005659424.1	ssc:100523341	0	NM_032131.5	ARMC2
PREX2	413.635483	4.95767023	6.382553829	6.75E-11	High	499	6	XP_001925866.1		0	NM_025170.4	PREX2
FOXF2	3.315715294	337.1215757	-6.667805018	6.89E-11	Low	4	408	XP_005665585.1	ssc:100158066	6E-118	NM_001452.1	FOXF2
LOC102167717	167.4436224	0	20	7.04E-11	High	202	0	XR_309273.1	ssc:102167717			
ZNF582	0	168.5607878	-20	7.06E-11	Low	0	204	ZNF582	ssc:100521903	0	NM_144690.1	ZNF582
CCDC69	0	168.5607878	-20	7.06E-11	Low	0	204	XP_003134191.3	ssc:100515590	3E-130	NM_015621.2	CCDC69
LOC100738003	58.85394648	2990.301427	-5.667007949	7.07E-11	Low	71	3619	XP_005658279.1	ssc:100738003	0	NM_004820.3	CYP7B1
PPP1R26	29.01250883	1550.924504	-5.740309594	7.08E-11	Low	35	1877	XP_005660604.1	ssc:100519641	0	NM_014811.3	PPP1R26
INSM1	166.6146935	0	20	7.38E-11	High	201	0	XP_005672796.1	ssc:100511966	3E-158	NM_002196.2	INSM1
LOC100624495	0	167.7345095	-20	7.40E-11	Low	0	203	XP_005658667.1	ssc:100624495	2E-140	NM_182609.2	ZNF677
C8H4orf19	1022.06924	18.17812418	5.81314579	7.62E-11	High	1233	22	XP_003482412.1	ssc:100738623	2E-85	NM_018302.2	C4orf19
OTOF	251.1654336	1.652556743	7.247794308	7.75E-11	High	303	2	XP_005662739.1	ssc:102159615	0	NM_194323.2	OTOF
LEFTY2	368.8733265	4.131391859	6.480353755	7.81E-11	High	445	5	NM_001195346.1	ssc:100337677	1E-176	NM_003240.3	LEFTY2
LOC100521530	24.03893588	1300.562157	-5.757618506	7.83E-11	Low	29	1574	XP_005673187.1	ssc:100521530	3E-62	NM_002825.5	PTN
LOC100512875	328.2558141	3.305113487	6.633976944	8.23E-11	High	396	4	LOC100512875				
LOC100519287	591.0262512	9.089062089	6.022946972	8.84E-11	High	713	11	XP_005673936.1	ssc:100519287	2E-109	NM_033495.3	KLHL13
SLC44A5	1.657857647	249.5360683	-7.233784415	8.87E-11	Low	2	302	XP_005653486.1	ssc:100513866	0	NM_152697.4	SLC44A5
LOC100521810	163.2989782	0	20	8.91E-11	High	197	0	XP_003129328.1	ssc:100521810	3E-115	NM_025212.2	CXXC4
GDAP1L1	0	164.429396	-20	8.91E-11	Low	0	199	XP_005673013.1	ssc:100625236	0	NR_046353.1	GDAP1L1
PVRL4	401.2015506	4.95767023	6.338521061	9.05E-11	High	484	6	XP_005663234.1	ssc:100154054	0	NM_030916.2	PVRL4
SCARA3	555.3823118	27463.84648	-5.627908345	9.66E-11	Low	670	33238	XP_003359089.1	ssc:100628076	0	NM_182826.1	SCARA3
PTPRU	31.4992953	1613.72166	-5.678928396	9.84E-11	Low	38	1953	XP_005653451.1	ssc:100515955	0	NM_133178.3	PTPRU
PWWP2B	2.486786471	285.8923166	-6.845045403	9.91E-11	Low	3	346	XP_005654212.1	ssc:100154915	0	NM_138499.3	PWWP2B
HAPLN1	11.60500353	699.8577809	-5.914242913	1.02E-10	Low	14	847	NM_001004028.1	ssc:445513	0	NM_001884.3	HAPLN1
MSMP	81.23502471	3930.606214	-5.596506142	1.03E-10	Low	98	4757	XP_003122034.1	ssc:100512517	1E-73	NM_001044264.2	MSMP
PRKCZ	160.8121918	0	20	1.03E-10	High	194	0	NM_001204374.1	ssc:100533200			
LOC102163185	281.8358	2.478835115	6.82904876	1.05E-10	High	340	3	XR_302805.1	ssc:102163185			
LOC100623083	5.802501765	433.7961452	-6.224198366	1.10E-10	Low	7	525	XR_299921.1	ssc:100623083			
LOC102157644	242.0472165	1.652556743	7.194444883	1.10E-10	High	292	2	XR_301138.1	ssc:102157644			
FAM124A	649.0512689	10.74161883	5.917049103	1.14E-10	High	783	13	XP_001925756.4	ssc:100152644	0	NM_145019.3	FAM124A
GM2A	74.60359412	3566.217452	-5.579005729	1.18E-10	Low	90	4316	XP_003134190.1	ssc:100515283	3E-63	NM_001167607.1	GM2A
LOC100523609	158.3254053	0	20	1.19E-10	High	191	0	XR_303888.1	ssc:100523609	0	NM_032803.5	SLC7A3
FZD5	1232.617161	23.96207278	5.684828261	1.20E-10	High	1487	29	XP_005672190.1	ssc:100519058	0	NM_030804.1	FZD5
GGT1	5.802501765	428.8384749	-6.207615482	1.23E-10	Low	7	519	NM_214030.1	ssc:397095	0	NM_013430.2	GGT1
SLC4A11	1230.130374	23.96207278	5.681914706	1.23E-10	High	1484	29	XP_001924597.3	ssc:100157069	9E-165	NM_032034.3	SLC4A11
SPOCD1	199.7718465	0.826278372	7.917509661	1.24E-10	High	241	1	XP_003127817.1	ssc:100521137	0	NM_144569.5	SPOCD1
LOC102158695	0	158.6454474	-20	1.25E-10	Low	0	192	XP_005669287.1	ssc:102158695	4E-59	NM_004822.2	NTN1
LOC100626014	4.144644118	353.6471431	-6.414918567	1.26E-10	Low	5	428	XP_005653020.1	ssc:100626014	0	NM_171825.2	CAMK2A
LOC100517050	969.8467236	18.17812418	5.73748152	1.26E-10	High	1170	22	XR_305793.1	ssc:100517050	2E-32	NM_017898.3	2-Mar
KLHL32	237.9025724	1.652556743	7.169527251	1.30E-10	High	287	2	XR_300774.1	ssc:100621727	8E-75	NR_104421.1	KLHL32
LOC100624237	156.6675477	0	20	1.31E-10	High	189	0	LOC100624237				
CRIP3	156.6675477	0	20	1.31E-10	High	189	0	XP_001926813.1	ssc:100154285	2E-98	NM_206922.2	CRIP3

LOC102161288	125.9971812	5902.932688	-5.549968527	1.33E-10	Low	152	7144	XR_306392.1	ssc:102161288			
DPYSL5	2437.050741	51.22925905	5.572024454	1.34E-10	High	2940	62	XP_003125368.1	ssc:100511810	0	NM_020134.3	DPYSL5
GALNTL2	60.51180412	2873.796177	-5.569597334	1.34E-10	Low	73	3478	NM_001206447.1	ssc:100157572	0	NM_054110.4	GALNT15
CDA	155.8386188	0	20	1.38E-10	High	188	0	NM_001244385.1	ssc:100515954	2E-70	NM_001785.2	CDA
NPFFR2	1.657857647	237.9681711	-7.165304677	1.39E-10	Low	2	288	XP_005656581.1	ssc:100524634	5E-135	NM_053036.2	NPFFR2
LOC100738804	7.460359412	495.767023	-6.054273365	1.40E-10	Low	9	600	XP_003481936.1	ssc:100738804	3E-174	NM_032040.4	CCDC8
CYP17A1	273.5465118	2.478835115	6.785980038	1.40E-10	High	330	3	NM_214428.1	ssc:403330	0	NM_000102.3	CYP17A1
LOC100739233	76.26145177	3576.132793	-5.551302503	1.41E-10	Low	92	4328	XR_307534.1	ssc:100739233	1E-67	NM_002508.2	NID1
SMYD1	670.6034183	11.5678972	5.857261295	1.41E-10	High	809	14	NM_001160089.1	ssc:100294702			
SLIT3	125.1682524	5827.741356	-5.540996261	1.41E-10	Low	151	7053	XP_003134124.3	ssc:100513444	0	NM_003062.3	SLIT3
ZNF350	0	156.1666123	-20	1.44E-10	Low	0	189	ZNF350	ssc:100737585	0	NM_021632.3	ZNF350
MNX1	155.00969	0	20	1.45E-10	High	187	0	XP_003134570.1	ssc:100511165	2E-145	NM_005515.3	MNX1
KIFC3	46.42001412	2210.294644	-5.57334793	1.49E-10	Low	56	2675	XP_005664439.1	ssc:100514463	3E-11	NM_005550.3	KIFC3
GLDC	5362.34056	116.5052504	5.524395969	1.49E-10	High	6469	141	XP_001925488.2	ssc:100155425	0	NM_000170.2	GLDC
ITGBL1	0	155.3403339	-20	1.52E-10	Low	0	188	XP_005668583.1	ssc:100525532	2E-175	NM_004791.2	ITGBL1
LOC100626812	1492.071882	30.57229975	5.60894815	1.52E-10	High	1800	37	XP_003357377.3	ssc:100626812	0	NM_003105.5	SORL1
KIAA0319	591.0262512	9.915340461	5.89741609	1.60E-10	High	713	12	XP_005665689.1	ssc:100738226	0	NM_014809.3	KIAA0319
LOC102163032	193.9693447	0.826278372	7.874985044	1.64E-10	High	234	1	XR_302802.1	ssc:102163032			
IER5L	120.1946794	5495.57745	-5.514826141	1.69E-10	Low	145	6651	XP_005674645.1	ssc:102161937	1E-42	NM_203434.2	IER5L
GPC4	67.97216354	3130.76875	-5.525429096	1.73E-10	Low	82	3789	XP_001925506.4	ssc:100156029	0	NM_001448.2	GPC4
LY96	14.92071882	801.4900206	-5.747295611	1.79E-10	Low	18	970	NM_001104956.1	ssc:100125555	1E-60	NM_015364.4	LY96
IGFBP7	730.2862936	34102.98724	-5.545292153	1.81E-10	Low	881	41273	NM_001163801.1	ssc:100302573	8E-111	NM_001553.2	IGFBP7
TRIM50	301.7300918	3.305113487	6.512414965	1.84E-10	High	364	4	NM_214186.1	ssc:397383	0	NM_178125.3	TRIM50
LOC100738767	0	152.0352204	-20	1.85E-10	Low	0	184	XR_306551.1	ssc:100738767			
DSP	11807.26216	261.1039655	5.498906297	1.86E-10	High	14244	316	XP_003128216.1	ssc:100156744	0	NM_004415.2	DSP
RBM20	791.6270265	14.87301069	5.734052246	1.86E-10	High	955	18	XP_001925009.4	ssc:100157563	0	NM_001134363.1	RBM20
10-Mar	931.7159977	18.17812418	5.679615026	1.86E-10	High	1124	22	XP_005653950.1	ssc:100515922	6E-102	NM_152598	10-Mar
LOC102161978	511.4490842	8.262783717	5.951818909	1.88E-10	High	617	10	XR_301702.1	ssc:102161978			
HLF	546.2640948	9.089062089	5.909323361	1.88E-10	High	659	11	XP_003483076.1	ssc:100736723	3E-79	NM_002126.4	HLF
CCDC85C	1921.457013	41.31391859	5.539428986	1.89E-10	High	2318	50	XP_005666495.1	ssc:100512614	3E-83	NM_001144995.1	CCDC85C
LOC100622976	2.486786471	266.8879141	-6.74580753	1.90E-10	Low	3	323	XP_003356553.1	ssc:100622976	4E-104	NM_001452.1	FOXF2
NLRP3	0	151.208942	-20	1.95E-10	Low	0	183	NM_001256770.1	ssc:100514823	0	NM_183395.2	NLRP3
LOC100621009	0	151.208942	-20	1.95E-10	Low	0	183	XP_005668267.1	ssc:100621009	0	NM_001029880.2	SFMBT2
LOC100620561	0	151.208942	-20	1.95E-10	Low	0	183	XP_005674328.1	ssc:100620561	0	NM_138557.2	TLR4
LOC100739307	38.95965471	1824.422645	-5.549315281	1.96E-10	Low	47	2208	XP_003483519.1	ssc:100739307	2E-121	NM_002508.2	NID1
CD2	150.0361171	0	20	1.97E-10	High	181	0	NM_213776.1	ssc:396662	2E-113	NM_001767.3	CD2
PXDC1	110.2475335	4962.627901	-5.492286	1.97E-10	Low	133	6006	XP_001928887.1	ssc:100154877	3E-125	NM_183373.3	PXDC1
LOC102164134	68.80109236	3124.984802	-5.505273892	1.98E-10	Low	83	3782	XP_005660341.1	ssc:102164134	4E-116	NM_001855.4	COL15A1
LOC100623508	2.486786471	264.409079	-6.73234527	2.08E-10	Low	3	320	XP_005658780.1	ssc:100623508	5E-149	NM_001754.4	RUNX1
LOC100153773	4.144644118	335.4690189	-6.338787498	2.08E-10	Low	5	406	XP_005656291.1	ssc:100153773	2E-36	NM_005822.3	RCAN2
VGLL3	150.0361171	6709.380378	-5.482797801	2.09E-10	Low	181	8120	NM_001143929.1	ssc:100233225	4E-150	NM_016206.2	VGLL3
ATP8B3	712.8787883	13.22045395	5.752813174	2.09E-10	High	860	16	XP_003123063.3	ssc:100513137	0	NR_047593	ATP8B3
LOC100519796	129.3128965	5764.117921	-5.478161782	2.15E-10	Low	156	6976	XP_005654011.1	ssc:100519796	2E-93	NM_016429.2	COPZ2
LOC102162275	225.46864	1.652556743	7.092083166	2.16E-10	High	272	2	XP_005659301.1	ssc:102162275			
LOC102164644	147.5493306	0	20	2.30E-10	High	178	0	XR_306541.1	ssc:102164644			
LOC102164249	223.8107824	1.652556743	7.081435921	2.31E-10	High	270	2	XR_300352.1	ssc:102164249			
LOC100623234	41.44644118	1897.96142	-5.517058627	2.33E-10	Low	50	2297	XP_003359698.1	ssc:100623234	3E-119	NM_005876.4	SPEG
LOC100739780	294.2697324	3.305113487	6.476295539	2.33E-10	High	355	4	XP_005659865.1	ssc:100739780	3E-161	NM_005503.3	APBA2
LOC102158150	363.0708247	4.95767023	6.194444883	2.35E-10	High	438	6	XP_005672846.1	ssc:102158150	2E-81	NM_032034.3	SLC4A11
LOC100620394	181.5354124	8025.641825	-5.466293862	2.35E-10	Low	219	9713	XP_005674263.1	ssc:100620394	1E-77	NM_001855.4	COL15A1
HPDL	6015.536748	137.1622097	5.454738448	2.37E-10	High	7257	166	XP_003128096.1	ssc:100514057	5E-173	NM_032756.2	HPDL
LOC100510899	1.657857647	224.7477171	-7.082842517	2.38E-10	Low	2	272	XP_003125029.3	ssc:100510899	4E-46	NM_032181.2	EVA1A
PPP1R1B	3.315715294	295.8076571	-6.479195453	2.39E-10	Low	4	358	XP_005653983.1	ssc:100736966	2E-69	NM_181505.3	PPP1R1B
HOXA10	0.828928824	187.5651904	-7.821928163	2.40E-10	Low	1	227	NM_001257354.1	ssc:100737679	7E-51	NR_037939.1	HOXA10
LOC102157580	146.7204018	0	20	2.42E-10	High	177	0	XR_298273.1	ssc:102157580			
LOC100626144	940.005286	19.00440255	5.628263293	2.44E-10	High	1134	23	XP_005656792.1	ssc:100626144	0	NM_207108.2	ASTN1
LOC100628185	14.09179	741.1716994	-5.716881009	2.46E-10	Low	17	897	XP_005658364.1	ssc:100628185			
LOC102167451	2524.917197	57.01320765	5.468796094	2.55E-10	High	3046	69	XP_005666318.1	ssc:102167451	2E-16	NR_049767.1	SLC7A8
LOC102161206	145.891473	0	20	2.55E-10	High	176	0	XR_307765.1	ssc:102161206			
LOC100514847	145.891473	0	20	2.55E-10	High	176	0	XP_005653440.1	ssc:100514847	1E-133	NM_005268.3	GJB5
C22orf41	145.891473	0	20	2.55E-10	High	176	0	NM_001206362.1	ssc:100517654	2E-39	NM_001123225.1	SYCE3
ZNF541	145.891473	0	20	2.55E-10	High	176	0	ZNF541	ssc:100519367	0	np_001094889	np_001094889
ERAS	145.891473	0	20	2.55E-10	High	176	0	XP_005673659.1	ssc:100621986	2E-101	NM_181532.3	ERAS
LOC102162054	10.77607471	599.8780979	-5.798765696	2.63E-10	Low	13	726	XR_301502.1	ssc:102162054			
FGFR4	1988.600248	44.61903207	5.477950264	2.72E-10	High	2399	54	XP_003123730.1	ssc:100127133	0	NM_213647.1	FGFR4
PHLDA2	21.55214941	1036.979357	-5.588411606	2.80E-10	Low	26	1255	NM_001174057.1	ssc:100379631			
IGFBP3	0	145.4249934	-20	2.80E-10	Low	0	176	NM_001005156.1	ssc:448812			
FAM179A	144.2336153	0	20	2.84E-10	High	174	0	XP_003125325.3	ssc:100518963	0	NM_199280.2	FAM179A
NID1	51.39358706	2274.744357	-5.46797226	2.85E-10	Low	62	2753	XP_003361217.1		4E-154	NM_002508.2	NID1
ALOX12B	755.9830871	14.87301069	5.667585337	2.89E-10	High	912	18	XP_003132016.1	ssc:100526246	0	NM_001139.2	ALOX12B
ZNF180	2.486786471	255.3200169	-6.681880203	2.89E-10	Low	3	309	ZNF180	ssc:100625792	0	NM_013256.4	ZNF180
NOV	0	144.5987151	-20	2.95E-10	Low	0	175	XP_001927085.1	ssc:100154395	3E-156	NM_002514.3	NOV
LOC102157759	353.9526077	4.95767023	6.157750083	2.99E-10	High	427	6	XR_298275.1	ssc:102157759			
GPRIN3	1186.197147	25.61462952	5.533231971	3.05E-10	High	1431	31	XP_005667050.1	ssc:100517107	0	NM_198281.2	GPRIN3
EFEMP2	256.9679353	11063.04112	-5.428015862	3.16E-10	Low	310	13389	XP_005660736.1	ssc:100526031	0	NR_037718.1	EFEMP2
TRIM29	249.5075759	2.478835115	6.6532775	3.35E-10	High	301	3	XP_003129991.3	ssc:100520758	0	NM_058193.1	TRIM29
INHBE	54.70930236	2371.418927	-5.437820578	3.38E-10	Low	66	2870	XP_003126368.1	ssc:100518554	9E-164	NM_031479.3	INHBE
LOC100626974	1.657857647	216.4849334	-7.028802677	3.39E-10	Low	2	262	XP_003361236.1	ssc:100626974	0	NM_001039523.2	CHRNA1
SLA-3	154.1807612	6546.603539	-5.408051951	3.44E-10	Low	186	7923	NM_001097427.1	ssc:100037288	2E-147	NM_002116.7	HLA-A
PPFIBP2	0	142.1198799	-20	3.46E-10	Low	0	172	XP_005653690.1	ssc:100738007	7E-154	NM_003621.3	PPFIBP2
LOC100736977	9.11821706	516.4239823	-5.823660437	3.49E-10	Low	11	625	XP_005665750.1	ssc:100736977	5E-46	NM_002127.5	HLA-G
SPOCK3	38.13072589	1679.82393	-5.461212219	3.58E-10	Low	46	2033	XP_001927799.2	ssc:100154805	3E-105	NM_016950.2	SPOCK3
LOC100519925	178.2196971	0.826278372	7.752813174	3.63E-10	High	215	1	XR_307139.1	ssc:100519925			
LOC100520570	137.6021847	5802.126726	-5.398006525	3.67E-10	Low	166	7022	XR_304053.1	ssc:100520570	0	NM_015210.3	SOGA2
LOC100516232	0	140.4673232	-20	3.86E-10	Low	0	170	XP_003133753.2	ssc:100516232			
GRB14	1190.341791	26.4409079	5.492460358	3.86E-10	High	1436	32	XP_005671947.1	ssc:100514075	0	NM_004490.2	GRB14
SPRY4	37.30179706	1631.073506	-5.450432854	3.91E-10	Low	45	1974	XP_003124057.1	ssc:100512891	7E-161	NM_030964.3	SPRY4
LOC102157497	139.2600424	0	20	3.93E-10	High	168	0	XP_005664035.1	ssc:102157497	6E-66	NM_002837.4	PTPRB
LOC102166521	139.2600424	0	20	3.93E-10	High	168	0	XR_300688.1	ssc:102166521			
LOC100513263	18.23643412	868.4185687	-5.573495011	3.97E-10	Low	22	1051	XP_005660693.1	ssc:100513263			
EVPL	536.3169489	9.915340461	5.757279726	4.06E-10	High	647	12	XP_003131232.3	ssc:100519916	0	NM_001988.2	EVPL
MEIS3	43.93322765	1887.219801	-5.424806156	4.14E-10	Low	53	2284	XP_005664696.1	ssc:100518492	0	NM_020160.2	MEIS3
GABRQ	138.4311135	0	20	4.16E-10	High	167	0	XP_001927219.1	ssc:100153018	0	NM_018558.3	GABRQ
LOC100511351	50.56465824	2149.976323	-5.410047585	4.22E-10	Low	61	2602	XP_005671015.1	ssc:100511351	1E-144	NM_002508.2	NID1
C12H17orf72	469.1737142	8.262783717	5.827350472	4.29E-10	High	566	10	XP_005668743.1	ssc:100511153	1E-45	NR_015354.2	C17orf72
PCOLCE	220.4950671	9169.211091	-5.377979326	4.32E-10	Low	266	11097	XP_005661969.1	ssc:100627524	0	NM_002593.3	PCOLCE
PHACTR1	27.35465118	1215.455485	-5.473567088	4.46E-10	Low	33	1471	XP_005665632.1	ssc:100153737	0	NM_030948.2	PHACTR1
HOXA3	6.631430589	404.8764021	-5.932017615	4.65E-10	Low	8	490	XP_005673358.1	ssc:100519809	0	NM_153632.2	HOXA3
SYNPO	46.42001412	1963.237411	-5.402343874	4.65E-10	Low	56	2376	XP_005658874.1	ssc:100624154	2E-159	NM_007286.5	SYNPO
PKHD1L1	623.3544753	12.39417558	5.65231858	4.70E-10	High	752	15	XP_003125570.3	ssc:100525460	0	NM_177531.4	PKHD1L1

LOC100620419	70.45895001	2921.720322	-5.373891299	4.71E-10	Low	85	3536	XP_005657649.1	ssc:100620419	4E-113	NM_005737.3	ARL4C
SNED1	16.57857647	786.6170099	-5.568269344	4.83E-10	Low	20	952	XP_003133861.2	ssc:100512566	0	NM_001080437.1	SNED1
PLEKHG4	239.56043	2.478835115	6.594583506	4.92E-10	High	289	3	XP_005664484.1	ssc:100524497	0	NM_015432.3	PLEKHG4
CCN2	1097.501762	46688.85939	-5.410783182	4.98E-10	Low	1324	56505	NM_213833.1	ssc:100152141	0	NM_001901.2	CTGF
LOC100522153	43.10429883	1814.507304	-5.395602297	5.09E-10	Low	52	2196	XP_005653617.1	ssc:100522153	0	NM_198595.2	AFAP1
LOC102158049	617.5519736	12.39417558	5.638826344	5.13E-10	High	745	15	LOC102158049				
LOC100517194	0.828928824	171.8659013	-7.695819394	5.43E-10	Low	1	208	XR_307362.1	ssc:100517194			
LOC100521789	770.0748771	16.52556743	5.542227016	5.46E-10	High	929	20	XP_005663683.1	ssc:100521789	0	NM_000699.2	AMY2A
MYLPF	14.92071882	713.0782348	-5.578671423	5.48E-10	Low	18	863	NM_001006592.1	ssc:474162	8E-93	NM_013292.3	MYLPF
LIN28B	893.5852718	19.83068092	5.493799286	5.59E-10	High	1078	24	XP_005659396.1	ssc:100738513	7E-94	NM_001004317.3	LIN28B
GPNMB	0	134.6833746	-20	5.69E-10	Low	0	163	NM_001098584.1	ssc:100049669			
NOXA1	267.74401	3.305113487	6.340010679	5.70E-10	High	323	4	XP_005652790.1	ssc:100516894	3E-114	NM_006647.1	NOXA1
AMY2A	765.1013042	16.52556743	5.532879067	5.81E-10	High	923	20	XP_001929171.1	ssc:100152255	0	NM_000699.2	AMY2A
LOC102167854	133.4575406	0	20	5.83E-10	High	161	0	XR_297828.1	ssc:102167854			
LOC100739427	4.973572942	332.1639054	-6.061468866	5.89E-10	Low	6	402	XP_005670555.1	ssc:100739427	2E-81	NM_016950.2	SPOCK3
SLC15A3	23.21000706	1022.106346	-5.460654539	5.91E-10	Low	28	1237	XP_003122726.3	ssc:100513135	0	NR_027391.1	SLC15A3
ERBB4	202.258633	1.652556743	6.935357662	6.00E-10	High	244	2	XP_001926521.1				
SLC5A7	545.4351659	10.74161883	5.66612438	6.03E-10	High	658	13	XP_003124900.1	ssc:100512044	0	NM_021815.2	SLC5A7
ABCC8	266.0861524	3.305113487	6.331049811	6.05E-10	High	321	4	XP_005661160.1	ssc:100158245	0	NM_001287174.1	ABCC8
LOC100624511	3.315715294	267.7141924	-6.335229679	6.13E-10	Low	4	324	XP_003358225.2	ssc:100624511	3E-176	NM_015077.3	SARM1
TFAP2D	132.6286118	0	20	6.18E-10	High	160	0	XP_003356687.1	ssc:100155785	0	NM_172238.3	TFAP2D
TMEM174	132.6286118	0	20	6.18E-10	High	160	0	XP_005661535.1	ssc:100514450	3E-109	NM_153217.2	TMEM174
LOC100515546	36.47286824	1513.741977	-5.375151845	6.56E-10	Low	44	1832	XP_003126463.1	ssc:100515546	4E-118	NM_005086.4	SSPN
HOXA9	9.947145883	513.9451472	-5.691187945	6.58E-10	Low	12	622	XP_003134886.1	ssc:100519066	1E-159	NM_152739.3	HOXA9
LOC100627460	10.77607471	544.517447	-5.659074613	6.62E-10	Low	13	659	LOC100627460				
ARL4C	87.03752648	3445.58081	-5.306965826	7.00E-10	Low	105	4170	NM_001244315.1	ssc:100514255	1E-111	NM_005737.3	ARL4C
MPP7	688.8398524	14.87301069	5.53339999	7.04E-10	High	831	18	XP_003130810.1	ssc:100516752	0	NM_173496.3	MPP7
GPR68	6.631430589	386.698278	-5.865744395	7.19E-10	Low	8	468	XP_005666454.1	ssc:100153615	0	NM_003485.3	GPR68
LOC100624329	9.947145883	508.987477	-5.677203716	7.21E-10	Low	12	616	XR_131258.2	ssc:100624329			
SLITRK3	322.4533124	4.95767023	6.023284169	7.25E-10	High	389	6	XP_005657154.1	ssc:100153567	0	NM_014926.2	SLITRK3
LOC100622715	1.657857647	199.1330876	-6.908269012	7.41E-10	Low	2	241	XP_005667638.1	ssc:100622715	0	NM_024788.2	C7orf63
C1QTNF3	1.657857647	199.1330876	-6.908269012	7.41E-10	Low	2	241	NM_001145386.1	ssc:100270814	4E-167	NM_181435.5	C1QTNF3
PPP1R14C	4759.709305	122.289199	5.282504574	7.51E-10	High	5742	148	XP_005659204.1	ssc:100521302	2E-41	NM_030949.2	PPP1R14C
ELTD1	0	130.5519827	-20	7.58E-10	Low	0	158	XP_005665418.1	ssc:100525348	0	NM_022159.3	ELTD1
ELK3	153.3518324	5977.297741	-5.284576098	7.83E-10	Low	185	7234	XP_003355727.1	ssc:100622445	0	NM_005230.2	ELK3
LOC100525496	196.4561312	1.652556743	6.893363573	7.87E-10	High	237	2	XP_003121308.1	ssc:100525496			
FAM111A	29.01250883	1205.540144	-5.376860827	7.96E-10	Low	35	1459	XP_005660932.1	ssc:100525029	6E-166	NM_198847.2	FAM111A
LOC102163734	319.1375971	4.95767023	6.008372459	7.99E-10	High	385	6	XP_005654591.1	ssc:102163734	6E-20	NM_199160.3	LHX6
GFI1	128.4839677	0	20	8.28E-10	High	155	0	XP_005663730.1	ssc:102164883	0	NM_005263.3	GFI1
LOC100739473	162.4700494	0.826278372	7.619330168	8.60E-10	High	196	1	XP_005673241.1	ssc:100739473			
LOC100737813	162.4700494	0.826278372	7.619330168	8.60E-10	High	196	1	XP_005652749.1	ssc:100737813	9E-47	NM_153181.2	NETO1
LOC100511383	13.26286118	618.8825004	-5.544201584	8.73E-10	Low	16	749	XR_303665.1	ssc:100511383	9E-55	NM_024560.2	ACSS3
SORL1	193.9693447	1.652556743	6.874985044	8.87E-10	High	234	2	XP_003357379.2	ssc:100524036	4E-168	NM_003105.5	SORL1
PCDH17	83.72181118	3234.053547	-5.271596234	8.91E-10	Low	101	3914	XP_005668514.1	ssc:100157362	0	NM_014459.2	PCDH17
IRX4	0	128.0731476	-20	9.04E-10	Low	0	155	XP_003134213.1	ssc:100519749	0	NM_016358.2	IRX4
PTPN20B	126.82611	0	20	9.33E-10	High	153	0	XR_307653.1	ssc:100153108			
LOC100157667	126.82611	0	20	9.33E-10	High	153	0	XR_299681.1	ssc:100157667	4E-22	NM_182540.4	DDX26B
LRRC4B	1.657857647	194.1754174	-6.871896622	9.37E-10	Low	2	235	XP_003356057.2		0	NM_001080457.1	LRRC4B
LOC100739480	0.828928824	161.9505609	-7.61008952	9.43E-10	Low	1	196	XP_003482190.2	ssc:100739480	8E-138	NR_028077.1	ZSCAN12
HHAT	8.289288236	434.6224235	-5.71237057	9.57E-10	Low	10	526	XP_005667936.1	ssc:100512733	0	NM_018194	HHAT
LOC100521600	7.460359412	404.8764021	-5.762092613	9.58E-10	Low	9	490	XP_005652546.1	ssc:100521600			
NTNG2	0	127.2468692	-20	9.59E-10	Low	0	154	XP_003122308.3	ssc:100512264	0	NM_014917.2	NTNG1
UROC1	222.9818535	2.478835115	6.491120186	9.65E-10	High	269	3	NM_001244219.1	ssc:100157217	0	NM_144639.2	UROC1
LOC102164256	103.616103	3954.568287	-5.254200073	9.69E-10	Low	125	4786	XP_005660342.1	ssc:102164256	4E-52	NM_001855.4	COL15A1
ENTPD3	153.3518324	5839.309253	-5.250880409	9.79E-10	Low	185	7067	XP_005669433.1	ssc:100623360	0	NM_001248.2	ENTPD3
FAM151A	125.9971812	0	20	9.91E-10	High	152	0	XP_003128023.2	ssc:100513740	4E-17	NM_176782.2	FAM151A
LOC102164341	125.9971812	0	20	9.91E-10	High	152	0	XR_307308.1	ssc:102164341			
SLA-DRB1	125.9971812	0	20	9.91E-10	High	152	0	NM_001113695.1	ssc:100153386	2E-115	xp_003846520	xp_003846520
LOC100737780	159.983263	0.826278372	7.597077362	9.92E-10	High	193	1	XP_003480480.1	ssc:100737780	1E-73	NM_153181.2	NETO1
NPTX2	0	126.4205909	-20	1.02E-09	Low	0	153	XP_005661911.1	ssc:100521613	5E-166	NM_002523.2	NPTX2
CAMK2B	281.0068712	4.131391859	6.087833693	1.04E-09	High	339	5	XP_005673407.1	ssc:100516166	7E-169	NM_172084.2	CAMK2B
LOC100515458	159.1543341	0.826278372	7.589582825	1.04E-09	High	192	1	XP_005673467.1	ssc:100515458	0	NM_014728.3	FRMPD4
LOC100626448	748.5227277	17.35184581	5.430885079	1.05E-09	High	903	21	XP_003358860.2	ssc:100626448	0	NM_018338.3	WDR52
LOC102159297	125.1682524	0	20	1.05E-09	High	151	0	XP_005657231.1	ssc:102159297	5E-68	NR_038257.1	TRPM2
PLCXD2	746.8648701	17.35184581	5.427686197	1.07E-09	High	901	21	XP_003358867.1	ssc:100518335	2E-163	NM_153268.3	PLCXD2
TMPRSS11F	0	125.5943125	-20	1.08E-09	Low	0	152	XP_003129105.3	ssc:100513296	0	NM_207407.2	TMPRSS11F
PIEZO2	0	125.5943125	-20	1.08E-09	Low	0	152	XP_005674226.1	ssc:100620725	0	NM_173817.1	PIEZO2
ESR2	158.3254053	0.826278372	7.582049152	1.09E-09	High	191	1	NM_001001533.1	ssc:396697	0	NR_073505.1	ESR2
ZAP70	279.3490136	4.131391859	6.079297011	1.09E-09	High	337	5	ZAP70	ssc:100524930	0	NM_207519.1	ZAP70
CLIC2	1.657857647	190.8703039	-6.847128717	1.10E-09	Low	2	231	NM_001244428.1	ssc:100516055	7E-135	NM_001289.5	CLIC2
ABAT	366.38654	6.610226974	5.792522884	1.11E-09	High	442	8	NM_214263.1	ssc:397500	0	NM_020686.5	ABAT
LOC100511763	124.3393235	0	20	1.12E-09	High	150	0	LOC100511763				
SHE	124.3393235	0	20	1.12E-09	High	150	0	XP_001929477.1	ssc:100152691	0	NM_001010846.2	SHE
IFI44	237.0736436	8936.20059	-5.236254384	1.12E-09	Low	286	10815	NM_001246205.1	ssc:100525523	4E-151	NM_006417.4	IFI44
LOC102165792	1182.052502	29.74602138	5.312453589	1.13E-09	High	1426	36	XP_005658778.1	ssc:102165792	4E-57	NM_139072.3	DNER
LOC102160134	6249.294401	167.7345095	5.219441864	1.15E-09	High	7539	203	LOC102160134				
LOC102164058	0	124.7680341	-20	1.15E-09	Low	0	151	XR_300563.1	ssc:102164058			
CRYAB	32.32822412	1274.947528	-5.301499803	1.18E-09	Low	39	1543	CRYAB		1E-95	NM_001885.1	CRYAB
LOC100625443	2.486786471	219.7900469	-6.46569961	1.18E-09	Low	3	266	XP_003483626.1	ssc:100625443	0	NM_138499.3	PWWP2B
LOC100739808	123.5103947	0	20	1.19E-09	High	149	0	XP_005659867.1	ssc:100739808	4E-164	NM_005503.3	APBA2
JAM3	151.6939747	5652.570341	-5.219671352	1.20E-09	Low	183	6841	XP_003130135.2	ssc:100520043	1E-151	NM_032801.4	JAM3
HERC6	114.3921777	4262.77012	-5.21973095	1.21E-09	Low	138	5159	XP_003360755.1	ssc:100626657			
RNF213	342.3476041	12863.50169	-5.231677699	1.23E-09	Low	413	15568	XP_005668644.1	ssc:100626814	0	NM_020954.3	RNF213
LOC102165620	17.4075053	741.1716994	-5.412026428	1.24E-09	Low	21	897	XP_005671013.1	ssc:102165620	3E-18	NM_080647.1	TBX1
PDE10A	907.6770618	22.30951604	5.346447977	1.25E-09	High	1095	27	XP_005659167.1	ssc:100154378	0	NR_045597.1	PDE10A
LOC102163451	332.4004583	5.783948602	5.844723829	1.28E-09	High	401	7	XR_301982.1	ssc:102163451			
CYP7B1	13.26286118	594.0941493	-5.485227636	1.29E-09	Low	16	719	XP_005663118.1	ssc:100286867	0	NM_004820.3	CYP7B1
LOC100623776	29.01250883	1146.048102	-5.303848731	1.29E-09	Low	35	1387	XP_003362087.1	ssc:100623776			
GPR63	818.1527489	19.83068092	5.366564098	1.30E-09	High	987	24	XP_005659366.1	ssc:100152431	0	NM_030784.3	GPR63
LOC100737127	16.57857647	708.9468429	-5.418285418	1.30E-09	Low	20	858	XP_005665344.1	ssc:100737127	2E-65	NM_006868.3	RAB31
LOC102159412	0.828928824	156.1666123	-7.5576221	1.32E-09	Low	1	189	XP_005666935.1	ssc:102159412	9E-139	NM_024582.4	FAT4
VWCE	1654.541932	43.7927537	5.239595875	1.34E-09	High	1996	53	XP_003122698.3	ssc:100522060	0	NM_152718.2	VWCE
FBLN1	30.67036647	1198.929917	-5.288758114	1.35E-09	Low	37	1451	XP_005663793.1	ssc:100519144	0	NM_006487.2	FBLN1
LOC100154530	41.44644118	1579.017968	-5.251635549	1.35E-09	Low	50	1911	XP_003360037.2	ssc:100154530	0	NM_198951.1	TGM2
GLIS1	46.42001412	1754.188983	-5.23991341	1.36E-09	Low	56	2123	XP_003128030.4	ssc:100515552	0	NM_147193.2	GLIS1
LOC100736715	272.717583	4.131391859	6.044636003	1.37E-09	High	329	5	XP_003480861.1	ssc:100736715	2E-32	NM_174881.2	CRB3
LOC100517699	272.717583	4.131391859	6.044636003	1.37E-09	High	329	5	XP_003123163.1	ssc:100517699	2E-32	NM_174881.2	CRB3
RAPGEF4	1446.480797	38.0088051	5.250069689	1.38E-09	High	1745	46	XP_001925789.1		0	NM_007023.3	RAPGEF4
PLA2G4A	99.47145883	3654.629238	-5.199298607	1.40E-09	Low	120	4423	XP_005667902.1	ssc:100520687	0	NM_024420.2	PLA2G4A
PPAT	6843.636368	187.5651904	5.189299092	1.41E-09	High	8256	227	XP_003129071.2	ssc:100521102	0	NM_002703.4	PPAT

TNNC1	9.11821706	445.3640424	-5.61008952	1.42E-09	Low	11	539	NM_001130243.1	ssc:100156435	8E-87	NM_003280.2	TNNC1
MYOM1	14.09179	614.7511086	-5.447075646	1.46E-09	Low	17	744	XP_005665357.1	ssc:100522496	0	NM_019856.1	MYOM1
LOC100515328	8.289288236	415.618021	-5.647866171	1.46E-09	Low	10	503	XP_003361055.1	ssc:100515328	0	NM_004787	SLIT2
LOC100623866	35.64393941	1360.880478	-5.254739761	1.48E-09	Low	43	1647	XP_005674350.1	ssc:100623866	0	NM_015210.3	SOGA2
LOC102160883	120.1946794	0	20	1.53E-09	High	145	0	XR_297325.1	ssc:102160883			
MYHC	108.5896759	3943.00039	-5.182335002	1.55E-09	Low	131	4772	XP_003132042.1	ssc:396711	0	NM_002470.3	MYH3
LOC102164566	0	120.6366423	-20	1.56E-09	Low	0	146	XR_300984.1	ssc:102164566			
KCNMA1	2.486786471	213.1798199	-6.42164443	1.57E-09	Low	3	258	NM_214219.1	ssc:397434	5E-44	NM_002247.3	KCNMA1
LOC100622791	127.6550388	4600.717974	-5.171536631	1.65E-09	Low	154	5568	XR_304346.1	ssc:100622791	6E-16	NM_002116.7	HLA-A
LOC100513356	24.03893588	945.2624573	-5.297270017	1.66E-09	Low	29	1144	XP_003127281.1	ssc:100513356	3E-174	NM_032040.4	CCDC8
CCDC42	0	119.8103639	-20	1.66E-09	Low	0	145	NM_001244077.1	ssc:100514007	8E-156	NM_144681.2	CCDC42
CA14	4646.146056	129.7257044	5.162498233	1.67E-09	High	5605	157	XP_005658313.1	ssc:100153371	3E-167	NM_012113.1	CA14
TSPAN8	209.7189924	2.478835115	6.402651398	1.71E-09	High	253	3	XP_005664038.1	ssc:100521553	4E-55	NM_004616.2	TSPAN8
LOC102160758	237.9025724	3.305113487	6.169527251	1.73E-09	High	287	4	LOC102160758				
CD79B	118.5368218	0	20	1.73E-09	High	143	0	NM_001243912.1	ssc:100511898	1E-88	NM_021602.2	CD79B
LOC102166489	0	118.9840855	-20	1.77E-09	Low	0	144	XP_005670814.1	ssc:102166489	5E-55	NM_014653.2	WSCD2
FAM83A	117.707893	0	20	1.85E-09	High	142	0	XP_001928788.1	ssc:100152159	0	NM_207006.2	FAM83A
LOC102160402	117.707893	0	20	1.85E-09	High	142	0	XR_303203.1	ssc:102160402			
HNF4A	117.707893	0	20	1.85E-09	High	142	0	NM_001044571.1	ssc:733636	0	NM_178850.2	HNF4A
ACVR2B	1430.73115	38.83508347	5.203248222	1.85E-09	High	1726	47	NM_001005350.1	ssc:448845	0	NM_001106.3	ACVR2B
NFIX	64.65644824	2329.27873	-5.170945219	1.86E-09	Low	78	2819	XP_005652941.1	ssc:100523536	0	NM_002501	NFIX
HOXA1	0	118.1578072	-20	1.89E-09	Low	0	143	XP_005673359.1	ssc:100520162	6E-165	NM_153620.2	HOXA1
CHRNA1	0	118.1578072	-20	1.89E-09	Low	0	143	XP_001924357.4	ssc:396587	0	NM_001039523.2	CHRNA1
ALDH3B1	41.44644118	1522.004761	-5.198580832	1.92E-09	Low	50	1842	XP_005660674.1	ssc:100739347	0	NM_001161473.1	ALDH3B1
CD36	2.486786471	208.2221497	-6.387697098	1.95E-09	Low	3	252	NM_001044622.1	ssc:733702	0	NM_001127444.1	CD36
CACNA1I	344.8343906	6.610226974	5.705060042	1.97E-09	High	416	8	XP_003481582.1	ssc:100154640	0	NM_021096.3	CACNA1I
PFAS	13290.21583	373.477824	5.153198141	1.99E-09	High	16033	452	XP_003358324.2	ssc:100620093	0	NM_012393.2	PFAS
RBP5	0	117.3315288	-20	2.01E-09	Low	0	142	NM_001145223.1	ssc:100270724	3E-69	NM_031491.2	RBP5
LOC102161872	177.3907683	1.652556743	6.746087311	2.04E-09	High	214	2	XR_305191.1	ssc:102161872			
REEP3	132.6286118	4670.951635	-5.138252562	2.06E-09	Low	160	5653	XP_001925694.2	ssc:100158143	2E-137	NM_001001330.2	REEP3
LOC102159968	0.828928824	148.7301069	-7.487232772	2.07E-09	Low	1	180	XR_302917.1	ssc:102159968			
CRYM	1135.632488	30.57229975	5.215127137	2.08E-09	High	1370	37	XP_003124605.2	ssc:100525333	2E-168	NM_001888.3	CRYM
LOC100627331	147.5493306	0.826278372	7.480353755	2.10E-09	High	178	1	XP_003361708.1	ssc:100627331	8E-152	NM_207108.2	ASTN1
LOC100621648	116.0500353	0	20	2.10E-09	High	140	0	XR_305874.1	ssc:100621648			
LOC102167737	2.486786471	206.5695929	-6.37620146	2.10E-09	Low	3	250	LOC102167737				
AMY2	1380.166491	38.0088051	5.18236483	2.15E-09	High	1665	46	NM_214195.1	ssc:397397	0	NM_000699.2	AMY2A
LOC100736904	232.1000706	3.305113487	6.133903341	2.18E-09	High	280	4	XP_003483712.2	ssc:100736904	6E-44	NM_004490.2	GRB14
LOC102160150	12.43393235	533.7758281	-5.423879435	2.21E-09	Low	15	646	XP_005665346.1	ssc:102160150	1E-40	NM_015210.3	SOGA2
TMEM200A	6.631430589	342.0792459	-5.688866633	2.28E-09	Low	8	414	XP_005659253.1	ssc:100157644	0	NM_052913.2	TMEM200A
LOC100621905	0	115.678972	-20	2.29E-09	Low	0	140	LOC100621905				
SLC16A6	174.9039818	1.652556743	6.725719513	2.32E-09	High	211	2	XP_005674608.1	ssc:100739042	0	NM_004694.4	SLC16A6
LOC100153940	106.9318182	3705.858497	-5.11504469	2.43E-09	Low	129	4485	XP_005659162.1	ssc:100153940	0	NM_003247.3	THBS2
DPPA2	174.075053	1.652556743	6.718865842	2.43E-09	High	210	2	XP_003358870.1	ssc:100620381	3E-97	NM_138815.3	DPPA2
SEMA3A	0	114.8526937	-20	2.44E-09	Low	0	139	XP_005667738.1	ssc:102163327	2E-152	NM_006080.2	SEMA3A
MYL9	870.3752648	30984.61266	-5.153770566	2.58E-09	Low	1050	37499	NM_001244472.1	ssc:100157760	9E-99	NM_181526.2	MYL9
LOC100625694	0	114.0264153	-20	2.61E-09	Low	0	138	XP_005653361.1	ssc:100625694	0	NM_024840.3	ZNF613
SV2C	172.4171953	1.652556743	6.705060042	2.65E-09	High	208	2	XP_005661560.1	ssc:100517268	6E-92	NM_014979.1	SV2C
LOC100518938	7590.501238	222.268882	5.093817269	2.71E-09	High	9157	269	XP_003133764.3	ssc:100518938	0	NM_139072.3	DNER
ZNF467	22.38107824	843.6302175	-5.236259324	2.71E-09	Low	27	1021	ZNF467	ssc:100623053	0	NM_207336.1	ZNF467
IL1R1	60.51180412	2099.573343	-5.116735777	2.71E-09	Low	73	2541	XP_005662423.1	ssc:100626904	0	NM_001288706.1	IL1R1
NETO2	411.9776253	9.089062089	5.502290707	2.75E-09	High	497	11	XP_003360626.1	ssc:100624026	0	NM_018092.4	NETO2
STX11	11.60500353	494.1144663	-5.412026428	2.79E-09	Low	14	598	XP_001926274.1	ssc:100152253	2E-143	NM_003764.3	STX11
LOC100737762	16.57857647	653.586192	-5.300985465	2.80E-09	Low	20	791	XP_005672304.1	ssc:100737762			
ZFP57	358.0972518	7.436505346	5.589582825	2.83E-09	High	432	9	ZFP57	ssc:100514715	6E-172	NM_001109809.2	ZFP57
KCND2	251.9943624	4.131391859	5.930619743	2.88E-09	High	304	5	XP_003360199.1	ssc:100038027	0	NM_012281.2	KCND2
NT5DC3	645.7355536	16.52556743	5.288171748	2.91E-09	High	779	20	XP_003481780.1	ssc:100738320	0	NM_016575.1	NT5DC3
LOC102161753	111.9053912	0	20	2.92E-09	High	135	0	XP_005657561.1	ssc:102161753			
CDHR1	111.9053912	0	20	2.92E-09	High	135	0	XP_005671212.1	ssc:100512562	0	NM_033100.3	CDHR1
EPS8L3	111.9053912	0	20	2.92E-09	High	135	0	XP_003125912.1	ssc:100514048	0	NM_139053.2	EPS8L3
IFITM1	68.80109236	2355.719638	-5.097592568	2.95E-09	Low	83	2851	XP_003124278.1	ssc:100127358	7E-46	NM_003641.3	IFITM1
F3	21.55214941	808.1002476	-5.228630612	3.00E-09	Low	26	978	NM_213785.1	ssc:396677			
LOC100738565	510.6201553	12.39417558	5.364516269	3.12E-09	High	616	15	XP_003482036.1	ssc:100738565	0	NM_020816.3	KIF17
LOC102157775	111.0764624	0	20	3.13E-09	High	134	0	XP_005659066.1	ssc:102157775	2E-14	NM_004360.3	CDH1
LOC100737374	218.0082806	7370.403076	-5.079288681	3.13E-09	Low	263	8920	XP_005662697.1	ssc:100737374	0	NM_206943.2	LTBP1
LOC100620538	196.4561312	2.478835115	6.308401073	3.14E-09	High	237	3	XP_005653264.1	ssc:100620538	0	NM_020227.2	PRDM9
POU4F1	222.9818535	3.305113487	6.076082687	3.17E-09	High	269	4	HighI00025E0FFA		3E-78	NM_006237.3	POU4F1
PSP-I	0	111.5475802	-20	3.18E-09	Low	0	135	NM_213837.1	ssc:396818			
LOC100521376	6945.594613	207.3958713	5.065639129	3.24E-09	High	8379	251	XP_005664247.1	ssc:100521376	0	NM_016575.1	NT5DC3
RBP4	34.81501059	1226.197104	-5.13833763	3.24E-09	Low	42	1484	NM_214057.1	ssc:397124	4E-106	NM_006744.3	RBP4
LOC102167733	1.657857647	169.3870662	-6.674859775	3.32E-09	Low	2	205	XP_005666981.1	ssc:102167733	6E-54	NM_024590.3	ARSJ
CX3CL1	4.973572942	275.9769762	-5.794121467	3.35E-09	Low	6	334	HighI0000E5D135		1E-123	NM_002996.3	CX3CL1
LOC100624137	0	110.7213018	-20	3.40E-09	Low	0	134	XR_299148.1	ssc:100624137			
RBP1	3382.0296	101.6322397	5.056459256	3.41E-09	High	4080	123	NM_001031789.1	ssc:100156666	2E-74	NM_002899.3	RBP1
AKAP5	1130.658915	32.2248565	5.132846034	3.41E-09	High	1364	39	XP_005666363.1	ssc:100153460	8E-172	NM_004857.3	AKAP5
CLMP	132.6286118	4428.025794	-5.061199681	3.43E-09	Low	160	5359	XP_003130027.3	ssc:100512427	2E-147	NM_024769.2	CLMP
TRPC4	4.144644118	248.7097899	-5.907071258	3.47E-09	Low	5	301	NM_001145868.1	ssc:100157635	0	NM_016179.2	TRPC4
LOC100738546	48.90680059	1669.908589	-5.093590233	3.47E-09	Low	59	2021	XR_305317.1	ssc:100738546	2E-62	NM_033135.3	PDGFD
LOC100623862	5.802501765	300.7653273	-5.695819394	3.50E-09	Low	7	364	XP_005674074.1	ssc:100623862	5E-164	XP_003960890.2	LOC100509091
SPRN	55.53823118	1881.435852	-5.082209061	3.53E-09	Low	67	2277	XP_005671641.1	ssc:100154535	9E-23	NM_001012508.3	SPRN
AK5	503.1597959	12.39417558	5.343282435	3.58E-09	High	607	15	XP_005653478.1	ssc:733697	0	NM_174858.2	AK5
SLC6A3	109.4186047	0	20	3.59E-09	High	132	0	XP_005674695.1	ssc:100521346	0	NM_001044.4	SLC6A3
ALOXE3	109.4186047	0	20	3.59E-09	High	132	0	XP_005657096.1	ssc:100511043	0	NM_021628.2	ALOXE3
CMKLR1	0	109.8950234	-20	3.64E-09	Low	0	133	NM_001123100.1	ssc:780421	0	NM_004072.2	CMKLR1
CRLF1	130.9707541	4342.919122	-5.051348488	3.66E-09	Low	158	5256	XP_003480798.1	ssc:100738750			
IRF7	137.6021847	4557.751498	-5.049746986	3.69E-09	Low	166	5516	NM_001097428.1	ssc:100037289	7E-160	NM_004031.2	IRF7
LOC100626670	1536.00511	45.44531045	5.078907777	3.79E-09	High	1853	55	XP_005664518.1	ssc:100626670	0	NM_018092.4	NETO2
LOC100514841	71.28787883	2366.461257	-5.052930698	3.92E-09	Low	86	2864	XP_005664153.1	ssc:100514841	1E-70	NM_001080509.2	TSPAN11
LOC102161875	107.7607471	0	20	4.12E-09	High	130	0	XP_005657562.1	ssc:102161875	2E-104	NM_017579.2	DMBT1
LOC102166597	107.7607471	0	20	4.12E-09	High	130	0	XR_300690.1	ssc:102166597			
ZSCAN10	107.7607471	0	20	4.12E-09	High	130	0	ZSCAN10	ssc:100628188	0	NM_032805.1	ZSCAN10
IQCA1P1	107.7607471	0	20	4.12E-09	High	130	0	XP_005657764.1	ssc:100739366	4E-145	NR_073043.1	IQCA1
LOC100620517	0	108.2424667	-20	4.17E-09	Low	0	131	XP_005657265.1	ssc:100620517	6E-84	NM_001495.4	GFRA2
ST8SIA1	20.72322059	752.7395966	-5.18283073	4.27E-09	Low	25	911	XP_003355598.1	ssc:100621251	6E-92	NM_003034.3	ST8SIA1
MAPRE3	21.55214941	775.8753911	-5.169921305	4.40E-09	Low	26	939	XP_003125359.3	ssc:100524857	2E-165	NM_012326.2	MAPRE3
IFIH1	77.0903806	2520.975312	-5.031287329	4.43E-09	Low	93	3051	NM_001100194.1	ssc:100101927	0	NM_022168.3	IFIH1
ARID5A	14.09179	546.1700037	-5.276423296	4.45E-09	Low	17	661	XP_005662382.1	ssc:100524615	0	NM_212481.1	ARID5A
CCDC85A	1252.511452	37.18252673	5.074055173	4.46E-09	High	1511	45	XP_005662596.1	ssc:100511688	0	NM_001080433.1	CCDC85A
GLS2	566.1583865	14.87301069	5.250437091	4.51E-09	High	683	18	XP_005663945.1	ssc:100738224	0	NM_138566.1	GLS2
LOC100737630	565.3294577	14.87301069	5.248323252	4.58E-09	High	682	18	XP_005655692.1	ssc:100737630	0	NM_138566.1	GLS2
CACNA2D2	3328.978156	103.2847965	5.010379594	4.61E-09	High	4016	125	XP_005669658.1	ssc:100517557	0	NM_006030.2	CACNA2D2

HMHA1	5879.592146	182.6075202	5.008897997	4.66E-09	High	7093	221	XP_005661429.1	ssc:100623239	0	NR_047652.1	HMHA1
LOC100523440	106.1028894	0	20	4.74E-09	High	128	0	XP_003127469.1	ssc:100523440	2E-168	NM_032803.5	SLC7A3
PTPRE	1.657857647	162.7768392	-6.617431495	4.79E-09	Low	2	197	XP_005671631.1	ssc:100155079	0	NM_130435.3	PTPRE
CXCL11	0	106.58991	-20	4.79E-09	Low	0	129	NM_001128491.1	ssc:100169744	5E-40	NM_005409.4	CXCL11
LOC102159413	0	106.58991	-20	4.79E-09	Low	0	129	XP_005667232.1	ssc:102159413	0	NM_001098816.2	TENM4
LOC102164998	0	106.58991	-20	4.79E-09	Low	0	129	XR_307847.1	ssc:102164998			
PARP14	87.03752648	2802.736237	-5.009054613	5.00E-09	Low	105	3392	XP_005670231.1	ssc:100153948	0	NM_017554.2	PARP14
SLA-5	13.26286118	513.1188688	-5.273829134	5.11E-09	Low	16	621	NM_001114056.1	ssc:100135029	5E-138	NM_002116.7	HLA-A
SGCD	4.973572942	263.5828006	-5.727829789	5.14E-09	Low	6	319	NM_001144123.1	ssc:100240724			
ANK1	806.5477454	23.13579441	5.123561397	5.17E-09	High	973	28	XP_001926385.2	ssc:100155697	0	NM_020481.2	ANK1
M1AP	335.7161736	7.436505346	5.496473421	5.18E-09	High	405	9	XP_005662495.1	ssc:100518008	0	NM_138804.4	M1AP
LOC100518541	185.6800565	2.478835115	6.227012746	5.30E-09	High	224	3	XP_005674179.1	ssc:100518541	5E-101	NM_133638.3	ADAMTS19
ZNF311	185.6800565	2.478835115	6.227012746	5.30E-09	High	224	3	ZNF311	ssc:100524874	0	NM_001010877.2	ZNF311
LOC102164089	5.802501765	287.5448734	-5.630968249	5.32E-09	Low	7	348	XP_005656795.1	ssc:102164089	3E-117	NM_022093.1	TNN
WT1	1379.337562	42.14019696	5.032634701	5.37E-09	High	1664	51	NM_001001264.1	ssc:397338	0	NM_024426.4	WT1
LOC100736608	0.828928824	133.8570962	-7.335229679	5.42E-09	Low	1	162	XP_005667737.1	ssc:100736608	8E-88	NM_006080.2	SEMA3A
CKMT2	104.4450318	0	20	5.46E-09	High	126	0	NM_001044551.2	ssc:733602	0	NM_001825.2	CKMT2
C2CD4D	104.4450318	0	20	5.46E-09	High	126	0	XP_005663496.1	ssc:100622378	7E-134	NM_001136003.1	C2CD4D
LOC102165956	104.4450318	0	20	5.46E-09	High	126	0	XP_005659863.1	ssc:102165956	1E-100	NM_005503.3	APBA2
POPDC3	0	104.9373532	-20	5.52E-09	Low	0	127	NM_001144111.1	ssc:100151910	1E-156	NR_024539.1	POPDC3
FOXH1	1727.487668	53.70809416	5.007392074	5.56E-09	High	2084	65	XP_001925315.1	ssc:100154027	4E-154	NM_003923.2	FOXH1
MIR214	8.289288236	360.2573701	-5.441635906	5.57E-09	Low	10	436	NR_038497.1	ssc:100316560			
FOLR2	23.21000706	806.4476908	-5.118762091	5.58E-09	Low	28	976	NM_213853.1	ssc:396853	3E-106	NM_001113536.1	FOLR2
WNT5B	35.64393941	1177.44668	-5.045861125	5.82E-09	Low	43	1425	XP_005664181.1	ssc:100516268	2E-84	NM_032642.2	WNT5B
JAM2	0.828928824	132.2045395	-7.317307771	6.06E-09	Low	1	160	XP_005674369.1	ssc:100621244	3E-111	NR_072999.1	JAM2
ITGA1	15.74964765	575.9160251	-5.192467008	6.22E-09	Low	19	697	HighI00028F495E		0	NM_181501.1	ITGA1
LRP2	102.7871741	0	20	6.30E-09	High	124	0	LRP2		0	NM_004525.2	LRP2
FAT4	11.60500353	452.8005477	-5.286056837	6.33E-09	Low	14	548	XP_005666936.1	ssc:102159549	0	NM_024582.4	FAT4
FOXA3	156.6675477	1.652556743	6.566862749	6.40E-09	High	189	2	XP_003127277.1	ssc:100512416	2E-144	NM_004497.2	FOXA3
LOC102165113	254.4811488	4.95767023	5.681752669	6.66E-09	High	307	6	XR_306174.1	ssc:102165113			
ECM1	32.32822412	1061.767708	-5.037530101	6.67E-09	Low	39	1285	XP_005655446.1	ssc:100620701	0	NM_022664.2	ECM1
LOC102163342	101.9582453	0	20	6.78E-09	High	123	0	XP_005661676.1	ssc:102163342	3E-66	NM_133638.3	ADAMTS19
FAM71E1	9.11821706	376.7829375	-5.368838071	6.80E-09	Low	11	456	XP_005664843.1	ssc:100511996	1E-60	NM_138411.1	FAM71E1
LOC100515902	50.56465824	1595.543536	-4.979774787	7.19E-09	Low	61	1931	XR_304348.1	ssc:100515902			
TNFAIP3	64.65644824	2016.119227	-4.962642889	7.28E-09	Low	78	2440	NM_001267890.1	ssc:100622156	0	NM_006290.3	TNFAIP3
SOX7	101.1293165	0	20	7.29E-09	High	122	0	XP_003359100.1	ssc:100155811	0	NM_031439.3	SOX7
ZBED2	4.973572942	253.6674601	-5.67251202	7.33E-09	Low	6	307	ZBED2	ssc:100517972	1E-97	NM_024508.4	ZBED2
RNASEL	16.57857647	589.9627574	-5.153231845	7.33E-09	Low	20	714	NM_001097512.1	ssc:100048946	0	NM_021133.3	RNASEL
ZNF256	0	101.6322397	-20	7.35E-09	Low	0	123	ZNF256	ssc:100516085	0	NM_005773.2	ZNF256
PSTPIP1	179.0486259	2.478835115	6.174545326	7.40E-09	High	216	3	NM_001244186.1	ssc:100152628	0	NM_003978.3	PSTPIP1
KCNQ4	441.819063	11.5678972	5.255257125	7.42E-09	High	533	14	XP_003128158.3	ssc:100526228	0	NM_172163.2	KCNQ4
TMEM158	30.67036647	997.3179947	-5.023136271	7.68E-09	Low	37	1207	XP_003358446.1	ssc:100620777	5E-108	NM_015444.2	TMEM158
ADAMTS5	6.631430589	299.9390489	-5.499205414	7.77E-09	Low	8	363	XP_003132795.3	ssc:100522952	0	NM_007038.3	ADAMTS5
TEX15	1887.470931	61.14459951	4.948085535	7.78E-09	High	2277	74	XP_005671870.1	ssc:100513440	0	NM_031271.3	TEX15
NIPAL4	72.94573648	2246.650893	-4.944808493	7.90E-09	Low	88	2719	XP_003134161.1	ssc:100523099			
LGALS9	14.09179	513.1188688	-5.186366293	7.97E-09	Low	17	621	NM_213932.1	ssc:396972	3E-162	NR_024043.1	LGALS9
APBB1	125.1682524	3802.533067	-4.925020205	8.38E-09	Low	151	4602	XP_005656659.1	ssc:100519163	0	NR_047512.2	APBB1
IL12RB2	176.5618394	2.478835115	6.154367444	8.42E-09	High	213	3	NM_214097.2	ssc:397178	0	NR_047584.1	IL12RB2
RAB33A	600.1444683	17.35184581	5.112148789	8.45E-09	High	724	21	NM_001123177.1	ssc:100144495	1E-140	NM_004794.2	RAB33A
KCNK5	456.7397818	12.39417558	5.203638237	8.88E-09	High	551	15	XP_001928289.1	ssc:100154866	0	NM_003740.3	KCNK5
TDRKH	784.9795596	23.96207278	5.033859945	8.88E-09	High	947	29	NM_001244274.1	ssc:100622748	0	NM_006862.3	TDRKH
LOC100624487	14.92071882	529.6444363	-5.149635221	9.06E-09	Low	18	641	XP_005673051.1	ssc:100624487	1E-60	NM_030777.3	SLC2A10
ANO4	0.828928824	126.4205909	-7.252767518	9.10E-09	Low	1	153	XP_003126736.3	ssc:100525519	0	NM_178826.3	ANO4
FAM19A4	98.64253001	0	20	9.12E-09	High	119	0	XP_005669775.1	ssc:100736627	9E-77	NM_182522.4	FAM19A4
CITED4	944.9788589	29.74602138	4.989513432	9.38E-09	High	1140	36	XP_003128154.1	ssc:100525468	2E-40	NM_133467.2	CITED4
C6H16orf74	65.48537706	1981.415535	-4.919214871	9.63E-09	Low	79	2398	XP_005664362.1	ssc:100513354	8E-27	NM_206967.2	C16orf74
LPPR5	290.9540171	6.610226974	5.459947545	9.64E-09	High	351	8	NM_001245001.1	ssc:100514836	0	NM_001037317.1	LPPR5
LOC102157983	5.802501765	269.3667492	-5.536752908	9.74E-09	Low	7	326	XP_005662850.1	ssc:102157983	4E-71	NM_012293.1	PXDN
LOC102165469	197.28506	3.305113487	5.899438088	9.83E-09	High	238	4	XR_303733.1	ssc:102165469			
LOC100621279	0	98.32712624	-20	9.87E-09	Low	0	119	XR_299836.1	ssc:100621279			
SDK1	0	98.32712624	-20	9.87E-09	Low	0	119	XP_005661878.1	ssc:100512953	0	NM_152744.3	SDK1
FLT4	266.0861524	5.783948602	5.523694889	1.02E-08	High	321	7	HighI0001C97D6A		0	NM_182925.4	FLT4
RSPO2	379.6494012	9.915340461	5.258861612	1.06E-08	High	458	12	XP_003481408.1	ssc:100154008	3E-131	NM_178565.4	RSPO2
LOC102167117	123.5103947	0.826278372	7.223788845	1.06E-08	High	149	1	XR_309083.1	ssc:102167117			
PTGDS	0	97.50084786	-20	1.06E-08	Low	0	118	NM_214228.1	ssc:397456			
LOC100627081	608.4337565	18.17812418	5.064824959	1.07E-08	High	734	22	XP_003361707.1	ssc:100627081	1E-170	NM_018593.4	SLC16A10
LOC100153213	263.5993659	5.783948602	5.510148358	1.11E-08	High	318	7	XP_005673981.1	ssc:100153213	2E-167	NM_147175.3	HS6ST2
MPZL2	3.315715294	195.8279741	-5.884122925	1.12E-08	Low	4	237	NM_001244821.1	ssc:100513488	1E-113	NM_144765.2	MPZL2
LOC100519458	96.15574354	0	20	1.14E-08	High	116	0	XP_005657966.1	ssc:100519458	1E-103	NM_031274.3	TEX13A
LOC102165476	0	96.67456949	-20	1.15E-08	Low	0	117	XR_298251.1	ssc:102165476			
ZNF286A	0	96.67456949	-20	1.15E-08	Low	0	117	ZNF286A	ssc:100627421	0	NM_020652.2	ZNF286A
ASTN1	671.4323471	20.65695929	5.022542232	1.16E-08	High	810	25	XP_003482773.1	ssc:100737305	0	NM_207108.2	ASTN1
TBX3	22.38107824	721.3410185	-5.010330017	1.18E-08	Low	27	873	XP_001928037.1	ssc:100152741	0	NM_016569.3	TBX3
NFKBIA	152.5229035	4461.076929	-4.870294221	1.20E-08	Low	184	5399	NM_001005150.1	ssc:406188	9E-174	NM_020529.2	NFKBIA
LOC100523068	1.657857647	147.0775502	-6.471113107	1.21E-08	Low	2	178	LOC100523068	ssc:100523068			
DPYSL4	1.657857647	147.0775502	-6.471113107	1.21E-08	Low	2	178	XP_003483624.1	ssc:100512868	0	NM_006426.2	DPYSL4
NID2	795.7716707	23899.27562	-4.908468541	1.27E-08	Low	960	28924	XP_005660021.1	ssc:100156994	0	NM_007361.3	NID2
AMDHD1	372.1890418	9.915340461	5.230229458	1.27E-08	High	449	12	XP_001925019.3	ssc:100151804	0	NM_152435.2	AMDHD1
LOC100511782	1166.302885	38.83508347	4.908438086	1.28E-08	High	1407	47	XP_003132905.1	ssc:100511782	0	NM_007197.3	FZD10
LOC102163689	94.49788589	0	20	1.34E-08	High	114	0	XR_305017.1	ssc:102163689			
LOC100522595	1.657857647	145.4249934	-6.454811294	1.34E-08	Low	2	176	XR_309484.1	ssc:100522595			
AREG	0	95.02201275	-20	1.34E-08	Low	0	115	NM_214376.1	ssc:397668	8E-101	XP_001125684.1	AREGB
LOC102157992	0	95.02201275	-20	1.34E-08	Low	0	115	XP_005664985.1	ssc:102157992	0	NM_021089.2	ZNF8
LOC102162312	0	95.02201275	-20	1.34E-08	Low	0	115	XR_306270.1	ssc:102162312			
LOC100515735	13.26286118	461.8896098	-5.122084149	1.36E-08	Low	16	559	XR_304349.1	ssc:100515735			
STOX1	368.8733265	9.915340461	5.217319349	1.38E-08	High	445	12	XP_005657443.1	ssc:100157127	0	NM_152709.4	STOX1
LOC100623579	590.1973224	18.17812418	5.020922137	1.42E-08	High	712	22	LOC100623579				
TSPAN33	3750.902927	131.3782611	4.835439446	1.43E-08	High	4525	159	XP_003134729.1	ssc:100515287	3E-158	NM_178562.3	TSPAN33
LOC102165266	143.4046865	1.652556743	6.439248552	1.43E-08	High	173	2	XP_005666958.1	ssc:102165266			
LOC100524773	93.66895707	0	20	1.45E-08	High	113	0	XP_005652515.1	ssc:100524773	0	NM_004525.2	LRP2
LOC100513982	0	94.19573438	-20	1.45E-08	Low	0	114	XP_005662499.1	ssc:100513982	3E-40	NM_016026.3	RDH11
WIF1	6.631430589	280.108368	-5.400521139	1.46E-08	Low	8	339	XP_003481699.1	ssc:100511326	1E-67	NM_007191.4	WIF1
RSPO3	1.657857647	143.7724367	-6.438323172	1.48E-08	Low	2	174	XP_001926731.1	ssc:100155208	3E-120	NM_032784.4	RSPO3
PRSS33	1324.62826	45.44531045	4.865312304	1.53E-08	High	1598	55	XP_003481124.1	ssc:100737240	2E-123	NM_152891.2	PRSS33
GATA3	55.53823118	1609.590268	-4.857068447	1.53E-08	Low	67	1948	NM_001044567.1	ssc:733631	0	NM_002051.2	GATA3
PRR16	5.802501765	256.1462952	-5.464149159	1.55E-08	Low	7	310	XP_005655053.1	ssc:100524363	2E-98	NM_016644.1	PRR16
TWIST2	28.18358	857.6769499	-4.927507563	1.55E-08	Low	34	1038	XP_003133824.1	ssc:100519927	1E-90	NM_057179.2	TWIST2
CACNA1E	232.1000706	4.95767023	5.548940841	1.56E-08	High	280	6	XP_005667863.1	ssc:100511769	1E-89	NM_001205294.1	CACNA1E
ZNF77	0	93.36945601	-20	1.56E-08	Low	0	113	ZNF77	ssc:100516355	6E-166	NM_021217.2	ZNF77
FNDC7	92.84002824	0	20	1.56E-08	High	112	0	XP_001925018.4	ssc:100155482	0	NM_173532.1	FNDC7

LOC100520275	0.828928824	118.9840855	-7.165304677	1.57E-08	Low	1	144	XR_307525.1	ssc:100520275			
DDX58	190.6536294	5427.822624	-4.831347675	1.57E-08	Low	230	6569	NM_213804.2	ssc:396723	0	NM_014314.3	DDX58
LOC102163193	208.8900635	4.131391859	5.659972153	1.63E-08	High	252	5	XP_005665735.1	ssc:102163193			
LOC102160653	48.07787177	1390.6265	-4.854218142	1.67E-08	Low	58	1683	XP_005670311.1	ssc:102160653	0	NM_182909.3	FILIP1L
FILIP1L	48.07787177	1389.800221	-4.853360671	1.68E-08	Low	58	1682	XP_005670309.1	ssc:100157058	0	NM_182909.3	FILIP1L
SLC41A2	27.35465118	827.9309285	-4.91965235	1.68E-08	Low	33	1002	XP_001926561.1		0	NM_032148.3	SLC41A2
CYP2C49	140.9179	1.652556743	6.41401126	1.68E-08	High	170	2	NM_214420.1	ssc:403215	0	NM_001128925.1	CYP2C18
HSPA12A	4141.328403	147.9038285	4.807362309	1.73E-08	High	4996	179	XP_001925646.5	ssc:100157399	0	NM_025015.2	HSPA12A
LOC100626048	1.657857647	141.2936016	-6.413232191	1.74E-08	Low	2	171	XR_303884.1	ssc:100626048	2E-139	NM_021632.3	ZNF350
SYT5	4.973572942	230.5316657	-5.534538487	1.77E-08	Low	6	279	XP_005656025.1	ssc:100626439	0	NM_003180.2	SYT5
LOC100152091	135.1153982	3790.138891	-4.809986714	1.77E-08	Low	163	4587	XP_005670217.1	ssc:100152091	2E-147	NM_053032.2	MYLK
LOC100513632	61.34073295	1742.621086	-4.82826969	1.78E-08	Low	74	2109	XP_005672612.1	ssc:100513632	0	NM_003062.3	SLIT3
LOC100627840	0.828928824	117.3315288	-7.145126795	1.78E-08	Low	1	142	XP_003355471.1	ssc:100627840	2E-130	NR_003084.2	HOXC5
SOCS3	3.315715294	185.9126336	-5.809160867	1.80E-08	Low	4	225	NM_001123196.1	ssc:493186	1E-23	NM_003955.4	SOCS3
ARSJ	21.55214941	666.806646	-4.951364821	1.82E-08	Low	26	807	XP_003129285.2	ssc:100512548	0	NM_024590.3	ARSJ
LOC100516615	0	91.71689926	-20	1.83E-08	Low	0	111	XP_005653378.1	ssc:100516615	0	NM_198480.3	ZNF615
LOC102157807	91.1821706	0	20	1.84E-08	High	110	0	XP_005665620.1	ssc:102157807	2E-159	NM_145655.3	GCNT2
LOC100522330	16423.56678	576.7423035	4.831696821	1.84E-08	High	19813	698	XP_005666317.1	ssc:100522330	7E-161	NR_049767.1	SLC7A8
PDE8B	16.57857647	532.9495498	-5.006606931	1.89E-08	Low	20	645	XP_005661559.1	ssc:100522306	0	NM_003719.3	PDE8B
LOC100737711	3.315715294	184.2600769	-5.796279576	1.95E-08	Low	4	223	XP_003484104.1	ssc:100737711	1E-159	NM_152739.3	HOXA9
LOC100626465	0	90.89062089	-20	1.98E-08	Low	0	110	XP_005663840.1	ssc:100626465	3E-60	NM_033016.2	PDGFB
LOC102162178	0	90.89062089	-20	1.98E-08	Low	0	110	XP_005658008.1	ssc:102162178	0	NM_004654.3	USP9Y
ZNF432	0	90.89062089	-20	1.98E-08	Low	0	110	ZNF432	ssc:100624217	0	NM_014650.2	ZNF432
VENTX	90.35324177	0	20	1.99E-08	High	109	0	XP_005674523.1	ssc:102160445	8E-14	NM_014468.3	VENTX
TJP3	90.35324177	0	20	1.99E-08	High	109	0	XP_005661408.1	ssc:100626455	0	NM_014428.1	TJP3
CALB2	90.35324177	0	20	1.99E-08	High	109	0	NM_001194980.1	ssc:100127479	1E-154	NR_027910.2	CALB2
SLC1A1	1508.650459	53.70809416	4.811975246	1.99E-08	High	1820	65	NM_001164649.1	ssc:397003			
METRNL	116.0500353	3214.222866	-4.791651099	2.00E-08	Low	140	3890	XP_005668624.1	ssc:100512305	1E-118	NM_001004431.1	METRNL
TLR3	37.30179706	1075.81444	-4.850040312	2.01E-08	Low	45	1302	NM_001097444.1	ssc:100037937	0	NM_003265.2	TLR3
LOC100514169	482.4365753	14.87301069	5.019570666	2.02E-08	High	582	18	XP_005665756.1	ssc:100514169	1E-113	NM_206814.5	MOG
LOC102162114	14.92071882	483.3728475	-5.017747489	2.12E-08	Low	18	585	XP_005655914.1	ssc:102162114	2E-96	NM_003573.2	LTBP4
PTGIR	0	90.06434252	-20	2.15E-08	Low	0	109	XP_003356000.1	ssc:100622305	0	NM_000960.3	PTGIR
LOC102167305	0	90.06434252	-20	2.15E-08	Low	0	109	XR_297258.1	ssc:102167305			
LOC100623608	19.89429177	611.4459951	-4.941798635	2.16E-08	Low	24	740	XP_003360896.1	ssc:100623608	2E-129	NM_001017961.3	FAM78B
LOC100739707	0.828928824	114.8526937	-7.114320748	2.16E-08	Low	1	139	XP_003483969.1	ssc:100739707	2E-90	NM_006065.3	SIRPB1
FAM212A	36.47286824	1042.763305	-4.837444252	2.21E-08	Low	44	1262	XP_003132261.3	ssc:100512437	4E-110	NM_203370.1	FAM212A
SPINT2	4.144644118	203.2644794	-5.615966086	2.23E-08	Low	5	246	XP_005664263.1	ssc:100515207	3E-74	NM_021102.3	SPINT2
LOC102166581	113.5632488	0.826278372	7.102652407	2.26E-08	High	137	1	XP_005655044.1	ssc:102166581	9E-165	NR_003149.1	GPR98
LMX1A	113.5632488	0.826278372	7.102652407	2.26E-08	High	137	1	XP_003125689.1	ssc:100513081	0	NM_177399.2	LMX1A
CASP4	143.4046865	3914.906925	-4.770813931	2.29E-08	Low	173	4738	XP_005667345.1	ssc:100522887	1E-170	NM_033307.2	CASP4
BRINP2	624.1834042	20.65695929	4.917270189	2.29E-08	High	753	25	XP_003130380.3	ssc:100522028	0	NM_021165.2	BRINP2
LOC100525045	1550.925829	56.18692928	4.786751326	2.30E-08	High	1871	68	XP_005655695.1	ssc:100525045	0	NM_001982.3	ERBB3
LOC100738946	29.84143765	865.1134552	-4.857500401	2.30E-08	Low	36	1047	XP_005672098.1	ssc:100738946	0	NM_020760.1	HECW2
GNAT1	4.973572942	223.9214387	-5.492566216	2.31E-08	Low	6	271	XP_001928011.1	ssc:100157237	0	NM_144499.2	GNAT1
C17H20orf174	135.9443271	1.652556743	6.362172329	2.33E-08	High	164	2	NM_001123087.1	ssc:100144512	0	NM_178457.2	ZNF831
LOC102161556	88.69538413	0	20	2.34E-08	High	107	0	XR_299329.1	ssc:102161556			
LOC102159119	4.973572942	223.0951604	-5.487232772	2.39E-08	Low	6	270	XR_297475.1	ssc:102159119			
IGDCC3	1284.839677	46.27158882	4.795317902	2.39E-08	High	1550	56	XP_001926550.1	ssc:100155852	0	NM_004884.3	IGDCC3
IL10RA	5.802501765	243.7521197	-5.392595898	2.44E-08	Low	7	295	XP_005656694.1	ssc:100525654	0	NR_026691.1	IL10RA
FBXO41	1383.482207	50.40298068	4.778651226	2.54E-08	High	1669	61	XP_003125058.1	ssc:100517089	0	NM_001080410.2	FBXO41
LOC100621773	460.8844259	12664.3686	-4.780226324	2.55E-08	Low	556	15327	XP_003361781.1	ssc:100621773			
EHF	134.2864694	1.652556743	6.344470327	2.60E-08	High	162	2	XP_005661076.1	ssc:100526094	3E-175	NM_012153.5	EHF
CRISPLD2	32.32822412	915.5164359	-4.823719623	2.66E-08	Low	39	1108	XP_005664373.1	ssc:100625354	0	NM_031476.3	CRISPLD2
LOC100738350	322.4533124	9.089062089	5.148815051	2.69E-08	High	389	11	XP_005653900.1	ssc:100738350	1E-76	NM_024719.3	GRTP1
LOC100519542	13.26286118	428.8384749	-5.014970404	2.71E-08	Low	16	519	XP_005661991.1	ssc:100519542	2E-130	NM_133457.3	COL26A1
LOC100517809	696.3002118	23.96207278	4.860884847	2.72E-08	High	840	29	XP_005661493.1	ssc:100517809			
TMEM45B	133.4575406	1.652556743	6.335537202	2.75E-08	High	161	2	XP_003130121.3	ssc:100516991	6E-134	NM_138788.3	TMEM45B
KCNG3	87.03752648	0	20	2.77E-08	High	105	0	XP_003354899.1	ssc:100514638	0	NM_172344.2	KCNG3
SPRED3	100.3003877	2686.230986	-4.743184279	2.77E-08	Low	121	3251	XP_005664646.1	ssc:100515836	5E-160	NR_073032.1	SPRED3
SIX1	401.2015506	10886.21755	-4.76203178	2.79E-08	Low	484	13175	NM_001199718.1	ssc:100156847	2E-167	NM_005982.3	SIX1
STK17B	100.3003877	2682.925873	-4.741408108	2.81E-08	Low	121	3247	XP_001928316.3	ssc:100153033	0	NM_004226.3	STK17B
LOC100625270	2.486786471	155.3403339	-5.965006027	2.87E-08	Low	3	188	XP_005658030.1	ssc:100625270	4E-65	NM_003034.3	ST8SIA1
OSR2	12.43393235	404.8764021	-5.025127019	2.91E-08	Low	15	490	XP_005655406.1	ssc:100155180	0	NM_053001.3	OSR2
LOC100522115	0	86.75922903	-20	2.99E-08	Low	0	105	XR_299665.1	ssc:100522115			
TPH1	86.20859765	0	20	3.01E-08	High	104	0	XP_005661154.1	ssc:100511002	0	NM_004179.2	TPH1
TCL1A	86.20859765	0	20	3.01E-08	High	104	0	XP_005656518.1	ssc:100156364	8E-27	NR_049726.1	TCL1A
ADRA1B	4.973572942	217.3112118	-5.449336164	3.03E-08	Low	6	263	XP_005672634.1	ssc:100519931	0	NM_000679.3	ADRA1B
SUSD2	3.315715294	174.3447364	-5.716478864	3.23E-08	Low	4	211	XP_003359219.2	ssc:100625804	0	NM_019601.3	SUSD2
F5	0	85.93295066	-20	3.25E-08	Low	0	104	NM_214120.1	ssc:397217	0	NM_000130.4	F5
GARNL3	234.5868571	5.783948602	5.341923645	3.26E-08	High	283	7	XP_005660494.1	ssc:100156351	0	NR_104591.1	GARNL3
LOC102166947	85.37966883	0	20	3.28E-08	High	103	0	XR_305099.1	ssc:102166947			
LOC102160384	213.8636365	4.95767023	5.430885079	3.30E-08	High	258	6	XR_307351.1	ssc:102160384			
OMG	193.1404159	4.131391859	5.546878374	3.34E-08	High	233	5	XP_003131799.1	ssc:100525125	0	NM_002544.4	OMG
LOC100737938	1.657857647	131.3782611	-6.308262631	3.34E-08	Low	2	159	XP_003481433.1	ssc:100737938			
APBB1IP	32.32822412	892.3806415	-4.786793054	3.38E-08	Low	39	1080	XP_003130799.3	ssc:100514179	0	NM_019043.3	APBB1IP
LOC102158959	0.828928824	109.0687451	-7.039773795	3.42E-08	Low	1	132	XR_309520.1	ssc:102158959			
KLHL41	9.947145883	336.2952973	-5.079302159	3.46E-08	Low	12	407	XP_003359596.1	ssc:100627572	0	NM_006063.2	KLHL41
AFAP1	60.51180412	1600.501206	-4.725163355	3.48E-08	Low	73	1937	NM_001253821.1	ssc:100522448	0	NM_198595.2	AFAP1
CCDC141	8.289288235	294.9813787	-5.153231845	3.53E-08	Low	10	357	XP_003133065.1	ssc:100520053	0	NM_173648.3	CCDC141
LOC100737661	272.717583	7.436505346	5.196639097	3.55E-08	High	329	9	XP_003481215.1	ssc:100737661	9E-130	NM_198274.3	SMYD1
ATP8A2	1065.173538	39.66136184	4.747210468	3.57E-08	High	1285	48	XP_003482910.1	ssc:100625134	0	NM_016529.4	ATP8A2
ACSS3	35.64393941	968.3982517	-4.763871775	3.62E-08	Low	43	1172	XP_003481811.1	ssc:100512104	4E-108	NM_024560.2	ACSS3
LOC100152707	0.828928824	108.2424667	-7.028802677	3.66E-08	Low	1	131	XP_003126250.1	ssc:100152707	2E-130	NR_003084.2	HOXC5
SEMA3B	148.3782594	3839.715593	-4.693647828	3.78E-08	Low	179	4647	XP_005669648.1	ssc:100153200	0	NM_004636.2	SEMA3B
LOC100739215	330.7426006	9.915340461	5.05990276	3.78E-08	High	399	12	XP_005659950.1	ssc:100739215			
LOC100623748	24.86786471	697.3789457	-4.809588269	3.79E-08	Low	30	844	XP_005658500.1	ssc:100623748	0	NM_002501	NFIX
HERC5	116.0500353	3002.695603	-4.693439363	3.79E-08	Low	140	3634	XP_005667057.1	ssc:100518083	0	NM_016323.3	HERC5
SLC7A3	2492.588973	96.67456949	4.688364757	3.80E-08	High	3007	117	NM_001130973.1	ssc:100171387	0	NM_032803.5	SLC7A3
TMSB15A	22.38107824	635.4080679	-4.827331961	3.83E-08	Low	27	769	XP_003135306.1	ssc:100522845	2E-18	NM_194324.2	TMSB15B
HS3ST5	290.1250883	8.262783717	5.133903341	3.86E-08	High	350	10	XP_005659453.1	ssc:100155403	0	NM_153612.3	HS3ST5
LOC102161274	83.72181118	0	20	3.89E-08	High	101	0	XP_005654500.1	ssc:102161274			
GLIPR1	0.828928824	107.4161883	-7.017747489	3.91E-08	Low	1	130	XP_005664057.1	ssc:100523551	4E-121	NM_006851.2	GLIPR1
PDGFRL	27.35465118	755.2184318	-4.787035911	3.95E-08	Low	33	914	XP_005672702.1	ssc:100736738	9E-141	NM_006207.2	PDGFRL
LOC100737466	34.81501059	936.9996735	-4.750267178	4.02E-08	Low	42	1134	XP_003484332.1	ssc:100737466	0	NM_014314.3	DDX58
LOC102161846	127.6550388	1.652556743	6.271406865	4.10E-08	High	154	2	XR_300659.1	ssc:102161846			
LOC102163653	106.1028894	0.826278372	7.004620324	4.15E-08	High	128	1	XR_304536.1	ssc:102163653			
LOC100512381	406.1751236	13.22045339	4.941258263	4.19E-08	High	490	16	XP_003121319.4	ssc:100512381	0	NM_001704.2	BAI3
LOC100737582	0	83.45411554	-20	4.20E-08	Low	0	101	LOC100737582				
ITM2A	227.9554265	5.783948602	5.300553211	4.23E-08	High	275	7	NM_001031798.1	ssc:595131	3E-129	NM_004867.4	ITM2A
LOC100623553	326.5979565	9.915340461	5.041709643	4.25E-08	High	394	12	XP_003357619.1	ssc:100623553	2E-103	NM_033345.2	RGS8

LOC102163946	82.89288236	0	20	4.25E-08	High	100	0	XR_308621.1	ssc:102163946			
LOC100626442	543.7773083	19.00440255	4.838610373	4.28E-08	High	656	23	LOC100626442				
KIRREL	116.0500353	2962.207963	-4.673854062	4.30E-08	Low	140	3585	XP_003125739.2	ssc:100512834	0	NM_018240.6	KIRREL
ODF3B	126.82611	1.652556743	6.262008167	4.35E-08	High	153	2	NM_001206375.1	ssc:100517096	6E-121	NM_001014440.3	ODF3B
LOC102165647	8.289288236	288.3711517	-5.120534807	4.35E-08	Low	10	349	XP_005666496.1	ssc:102165647	4E-172	NM_032425.4	HHIPL1
LOC100620681	26.52572236	727.1249671	-4.776739389	4.35E-08	Low	32	880	XR_304725.1	ssc:100620681	1E-146	NM_153717.2	EVC
PDLIM2	126.82611	3231.574712	-4.671313654	4.36E-08	Low	153	3911	XP_005670470.1	ssc:100737811	6E-141	NM_198042.3	PDLIM2
DKK1	3.315715294	168.5607878	-5.667805018	4.38E-08	Low	4	204	NM_001145384.1	ssc:100157640	9E-133	NM_012242.2	DKK1
CLDN11	14.09179	426.3596398	-4.91914409	4.43E-08	Low	17	516	NM_001161641.1	ssc:100302016	5E-101	NM_005602.5	CLDN11
LOC100621301	2.486786471	147.9038285	-5.894232952	4.46E-08	Low	3	179	XR_309146.1	ssc:100621301			
LOC100157017	2884.672306	114.0264153	4.660967458	4.48E-08	High	3480	138	XP_005660626.1	ssc:100157017	1E-48	NM_012203.1	GRHPR
EMX2	0	82.62783717	-20	4.58E-08	Low	0	100	XP_005671546.1	ssc:100152562	6E-112	NM_004098.3	EMX2
HighK1B	1460.572587	57.01320765	4.679094076	4.59E-08	High	1762	69	NM_001123212.1	ssc:100144488	3E-147	NM_006952.3	HighK1B
LOC780430	82.06395354	0	20	4.64E-08	High	99	0	NM_001078682.1	ssc:780430	5E-114	NM_017594.3	DIRAS2
TWIST1	247.0207894	6279.715625	-4.667994858	4.68E-08	Low	298	7600	XP_003130240.2	ssc:100516456	2E-78	NM_000474.3	TWIST1
LOC100511379	1034.503172	39.66136184	4.705060042	4.69E-08	High	1248	48	LOC100511379				
LOC102162237	1.657857647	126.4205909	-6.252767518	4.72E-08	Low	2	153	XP_005671728.1	ssc:102162237	5E-57	NM_005429.3	VEGFC
LOC100737186	9.947145883	324.7274001	-5.028802677	4.77E-08	Low	12	393	XR_305624.1	ssc:100737186			
OMD	2.486786471	146.2512718	-5.878022725	4.93E-08	Low	3	177	XP_003124829.1	ssc:100511925	0	NM_005014.2	OMD
LOC102159425	340.6897465	10.74161883	4.98717519	4.94E-08	High	411	13	XR_300992.1	ssc:102159425			
LOC100514469	0	81.8015588	-20	5.00E-08	Low	0	99	XP_005664974.1	ssc:100514469	2E-85	NR_049754.1	ZNF211
NKX2-2	0	81.8015588	-20	5.00E-08	Low	0	99	XP_003359963.1	ssc:100626520	7E-149	NM_002509.3	NKX2-2
CGN	788.3113112	29.74602138	4.727996834	5.09E-08	High	951	36	XP_005663506.1	ssc:100157963	0	NM_020770.2	CGN
LOC100152487	92.84002824	2331.757565	-4.650527026	5.09E-08	Low	112	2822	XP_005659871.1	ssc:100152487	0	NM_005786.5	TSHZ1
LOC100737020	3759.192215	102321.3559	-4.766540792	5.11E-08	Low	4535	123834	XP_005672671.1	ssc:100737020	6E-63	NM_003118.3	SPARC
P4HA3	385.451903	9791.398705	-4.666892208	5.12E-08	Low	465	11850	XP_003357239.1	ssc:100624543	0	NR_110031.1	P4HA3
NPHS2	124.3393235	1.652556743	6.233439015	5.19E-08	High	150	2	XP_005667854.1	ssc:100523986	0	NM_014625.2	NPHS2
LOC102164431	80.40609589	0	20	5.54E-08	High	97	0	XR_299666.1	ssc:102164431			
PPP1R17	80.40609589	0	20	5.54E-08	High	97	0	XP_003134851.1	ssc:100511608	2E-79	NM_006658.4	PPP1R17
LOC100739471	24.03893588	649.4548002	-4.755784183	5.55E-08	Low	29	786	XP_003483758.1	ssc:100739471	2E-37	NM_144629.2	RFTN2
NR2F1	72.94573648	1822.770088	-4.643165133	5.58E-08	Low	88	2206	XP_005661606.1	ssc:100621341	0	NM_005654.4	NR2F1
NNAT	335.7161736	8396.640814	-4.644498327	5.75E-08	Low	405	10162	NM_001122990.1	ssc:449004	2E-41	NM_181689.1	NNAT
LXN	42.27537	1078.293275	-4.672788425	5.78E-08	Low	51	1305	XP_003358692.1	ssc:100620551	1E-113	NM_020169.3	LXN
LOC100512967	200.6007753	4.95767023	5.338521061	5.91E-08	High	242	6	XR_303930.1	ssc:100512967			
LOC102163368	0	80.14900206	-20	5.97E-08	Low	0	97	XR_306051.1	ssc:102163368			
ARGFX	79.57716707	0	20	6.07E-08	High	96	0	XP_005654117.1	ssc:100152745	2E-64	NM_001012659.1	ARGFX
LOC100627431	79.57716707	0	20	6.07E-08	High	96	0	XP_005658254.1	ssc:100627431	0	NM_033086.2	FGD3
LOC100524424	121.8525371	1.652556743	6.204292669	6.21E-08	High	147	2	XP_005652950.1	ssc:100524424	0	NM_022904.1	RASAL3
TGFB3	17.4075053	487.5042393	-4.807633397	6.22E-08	Low	21	590	NM_214198.1	ssc:397400	0	NM_003239.2	TGFB3
DPYD	37.30179706	951.8726842	-4.673451581	6.24E-08	Low	45	1152	NM_214044.2	ssc:397109	0	NM_001160301.1	DPYD
SERPINB8	30.67036647	794.8797936	-4.695819394	6.33E-08	Low	37	962	XP_005652715.1	ssc:100156248	0	NM_198833.1	SERPINB8
CELF5	101.1293165	0.826278372	6.935357662	6.35E-08	High	122	1	XP_005654782.1	ssc:100518716	0	NR_033342.1	CELF5
SLC15A1	198.9429177	4.95767023	5.326548419	6.37E-08	High	240	6	NM_214347.1	ssc:397624	0	NM_005073.3	SLC15A1
LOC100514323	1603.977274	65.27599137	4.618957427	6.44E-08	High	1935	79	XP_003132557.2	ssc:100514323			
LOC100622354	140.9179	2.478835115	5.82904876	6.51E-08	High	170	3	XP_005657472.1	ssc:100622354	8E-106	NM_001165973.1	NRG3
CWH43	78.74823824	0	20	6.64E-08	High	95	0	XP_003129016.3	ssc:100521324	0	NM_025087.2	CWH43
CACNA2D3	78.74823824	0	20	6.64E-08	High	95	0	XP_005669714.1	ssc:100153986	0	NM_018398.2	CACNA2D3
COLL11A1	1085.06783	27569.60415	-4.667221416	6.66E-08	Low	1309	33366	XP_001929407.4	ssc:397175	0	NM_080630.3	COL11A1
C1QTNF5	148.3782594	3612.489041	-4.605641584	6.66E-08	Low	179	4372	XP_003129988.2	ssc:100520210	1E-119	NM_015645.4	C1QTNF5
LOC100511616	5.802501765	218.1374901	-5.232418873	6.70E-08	Low	7	264	XP_005658573.1	ssc:100511616	5E-75	NM_014397.5	NEK6
SMOX	92.84002824	2263.17646	-4.607458304	6.71E-08	Low	112	2739	NM_001185170.1	ssc:100152781	0	NM_175842.2	SMOX
LOC100512568	120.1946794	1.652556743	6.184529414	7.02E-08	High	145	2	XP_003134119.1	ssc:100512568	2E-121	NM_001436.3	FBL
GAS6	81.23502471	1974.805308	-4.603464734	7.03E-08	Low	98	2390	XP_005653903.1	ssc:100519331	0	NM_001143946.1	GAS6
LOC100737024	533.0012336	19.83068092	4.748332751	7.16E-08	High	643	24	XP_003480864.1	ssc:100737024	0	NM_024898.2	DENND1C
LOC100738422	77.91930942	0	20	7.28E-08	High	94	0	XP_003481781.2	ssc:100738422	2E-76	NM_000277.1	PAH
DENND1C	531.3433759	19.83068092	4.74383837	7.37E-08	High	641	24	XP_003123166.1	ssc:100518598	0	NM_024898.2	DENND1C
LOC102166594	19.06536294	513.9451472	-4.75258849	7.68E-08	Low	23	622	XP_005658920.1	ssc:102166594	7E-36	NM_017912.3	HERC6
AOAH	509.7912265	19.00440255	4.745500969	7.75E-08	High	615	23	XP_005673309.1	ssc:100522290	0	NM_001637.3	AOAH
LOC100522650	0	77.67016694	-20	7.83E-08	Low	0	94	XP_003133905.2	ssc:100522650	1E-12	NM_004934.3	CDH18
ZKSCAN2	0	77.67016694	-20	7.83E-08	Low	0	94	ZKSCAN2	ssc:100520899	0	NM_001012981.4	ZKSCAN2
LOC102160712	0	77.67016694	-20	7.83E-08	Low	0	94	XR_302696.1	ssc:102160712			
GDAP1	1.657857647	118.9840855	-6.165304677	8.09E-08	Low	2	144	XP_005663078.1	ssc:100157012	0	NR_046346.1	GDAP1
FZD3	1563.359761	65.27599137	4.581953537	8.17E-08	High	1886	79	XR_307344.1	ssc:100153078	0	NM_145866.1	FZD3
SIM1	230.442213	6.610226974	5.123561397	8.18E-08	High	278	8	NM_001172585.2	ssc:100154026	0	NM_005068.2	SIM1
SH2D3C	13.26286118	379.2617726	-4.837730019	8.37E-08	Low	16	459	XP_003353720.1	ssc:100151897	0	NM_170600.2	SH2D3C
MASP1	160.8121918	3815.753521	-4.568519291	8.49E-08	Low	194	4618	NM_001184947.1	ssc:100152125	0	NR_033519.1	MASP1
CD82	27.35465118	694.9001106	-4.666947547	8.49E-08	Low	33	841	NM_001145218.2	ssc:100270689			
LOC100737474	168.2725512	3981.009195	-4.564262437	8.75E-08	Low	203	4818	XP_003483083.1	ssc:100737474	2E-178	np_060419	np_060419
PGA	76.26145177	0	20	8.77E-08	High	92	0	NM_213873.2	ssc:396892	0	NM_014224.2	PGA5
KIAA1958	1475.493306	61.97087788	4.57346316	8.77E-08	High	1780	75	XP_005660389.1	ssc:100513712	0	NM_133465.3	KIAA1958
LOC102165788	337.3740312	11.5678972	4.866150387	8.96E-08	High	407	14	XR_299889.1	ssc:102165788			
SERPINB7	593.5130377	23.13579441	4.68108118	8.98E-08	High	716	28	XP_005652752.1	ssc:100152588	2E-178	NM_003784.3	SERPINB7
C3	188.9957718	4449.509032	-4.557220291	9.25E-08	Low	228	5385	NM_214009.1	ssc:397072			
LOC102164235	0	76.0176102	-20	9.43E-08	Low	0	92	XR_304726.1	ssc:102164235			
ZNF709	0	76.0176102	-20	9.43E-08	Low	0	92	ZNF709	ssc:100624296	0	NM_152601.3	ZNF709
LOC102166686	116.0500353	1.652556743	6.133903341	9.58E-08	High	140	2	XP_005652513.1	ssc:102166686	7E-19	NM_001145798.1	CACNB4
ETV1	244.534003	5750.897467	-4.555680122	9.64E-08	Low	295	6960	XP_005667715.1	ssc:100513560	0	NM_004956.4	ETV1
LTBP1	139.2600424	3253.057949	-4.54594325	9.76E-08	Low	168	3937	XP_003354915.3	ssc:100627243	0	NM_206943.2	LTBP1
DUSP27	0.828928824	96.67456949	-6.865744395	9.91E-08	Low	1	117	XP_003355120.1	ssc:100153962	0	NM_001080426.1	DUSP27
MAFB	11.60500353	335.4690189	-4.853360671	1.00E-07	Low	14	406	XP_003134488.2	ssc:100518227	5E-109	NM_005461.4	MAFB
LOC100737535	9.947145883	299.1127706	-4.910263062	1.01E-07	Low	12	362	LOC100737535				
LOC102160003	1734.119099	74.36505346	4.543434364	1.01E-07	High	2092	90	LOC102160003				
LOC100625609	296.7565188	9.915340461	4.903473601	1.02E-07	High	358	12	XP_005671448.1	ssc:100625609	3E-129	NM_024895.4	PDZD7
OTX1	1041.963531	43.7927537	4.572468804	1.03E-07	High	1257	53	XP_003125144.1	ssc:100520485	7E-150	NM_014562.3	OTX1
MAGEL2	57.19608883	1347.660024	-4.558396286	1.04E-07	Low	69	1631	XP_005659854.1	ssc:100154924	2E-67	NM_019066.4	MAGEL2
C1H15orf43	74.60359412	0	20	1.06E-07	High	90	0	XP_003121579.3	ssc:100516583	2E-123	NM_152448.2	C15orf43
LOC102158432	151.6939747	3.305113487	5.520320163	1.07E-07	High	183	4	XP_005662318.1	ssc:102158432	3E-83	NM_006648.3	WNK2
EFCC1	114.3921777	1.652556743	6.113144781	1.09E-07	High	138	2	XP_003483282.1	ssc:100738889	0	NM_024768.2	EFCC1
OTOG	114.3921777	1.652556743	6.113144781	1.09E-07	High	138	2	XP_003123004.2	ssc:100514091	0	xp_001717583	xp_001717583
RXFP4	1.657857647	114.8526937	-6.114320748	1.11E-07	Low	2	139	NM_001005844.1	ssc:450213	4E-167	NM_181885.2	RXFP4
WNT2B	31.4992953	765.9600506	-4.603877691	1.11E-07	Low	38	927	XP_003125897.1	ssc:100520560	0	NM_024494.2	WNT2B
LOC100523457	2064.032771	90.06434252	4.518366027	1.15E-07	High	2490	109	XP_005667217.1	ssc:100523457	1E-74	NM_001522.2	GUCY2F
LOC100738544	154.1807612	3536.471431	-4.519615946	1.16E-07	Low	186	4280	XP_003481990.1	ssc:100738544	3E-92	NM_018948.3	ERRFI1
LOC100037976	3.315715294	151.208942	-5.511079514	1.16E-07	Low	4	183	XP_001925729.3	ssc:100037976	2E-161	NM_033012.3	TNFSF11
DCC	113.5632488	1.652556743	6.102652407	1.16E-07	High	137	2	XP_005659521.1	ssc:100152102	0	NM_005215.3	DCC
NLRC5	6.631430589	223.0951604	-5.072195273	1.16E-07	Low	8	270	NM_001278781.1	ssc:100135667	2E-30	NM_032206.4	NLRC5
NSUN7	398.7147642	14.87301069	4.744588407	1.16E-07	High	481	18	XP_005666674.1	ssc:100620833	0	NM_024677.4	NSUN7
ESRRG	327.4268853	11.5678972	4.822974245	1.18E-07	High	395	14	XP_005667974.1	ssc:100622056	0	NM_206595.2	ESRRG
PPARG	33.15715294	797.3586287	-4.587836713	1.18E-07	Low	40	965	NM_214379.1	ssc:397671	0	NM_138712.3	PPARG
NR2E1	185.6800565	4.95767023	5.227012746	1.19E-07	High	224	6	XP_005659417.1	ssc:100155153	0	NM_003269.4	NR2E1

ACTG2	930.8870689	22116.99318	-4.570405296	1.19E-07	Low	1123	26767	XP_003125076.4	ssc:100520667	5E-117	NM_001615.3	ACTG2
LOC100738758	256.1390065	8.262783717	4.954155257	1.20E-07	High	309	10	XP_005660163.1	ssc:100738758	6E-56	NM_130393.3	PTPRD
HOXA4	3.315715294	150.3826637	-5.503174316	1.22E-07	Low	4	182	XP_003134889.1	ssc:100519635	6E-126	NM_002141.4	HOXA4
LOC100737139	860.4281189	36.35624836	4.564779434	1.22E-07	High	1038	44	XP_005663921.1	ssc:100737139	1E-43	NM_006741.3	PPP1R1A
LOC102166137	7.460359412	239.6207278	-5.005363764	1.22E-07	Low	9	290	XP_005655468.1	ssc:102166137	4E-20	NM_016954.2	TBX22
PARP12	187.3379141	4273.511739	-4.511707273	1.24E-07	Low	226	5172	XP_003134663.1	ssc:100515805	0	NM_022750.2	PARP12
LOC100624658	0	73.53877508	-20	1.25E-07	Low	0	89	XP_005658285.1	ssc:100624658	5E-28	NM_175913.3	JPH2
LOC100517751	0	73.53877508	-20	1.25E-07	Low	0	89	XP_005652884.1	ssc:100517751	0	NM_152601.3	ZNF709
PDZD3	289.2961594	9.915340461	4.86674105	1.28E-07	High	349	12	XP_005667485.1	ssc:100519043	0	NR_033122.1	PDZD3
IGFBPL1	72.94573648	0	20	1.29E-07	High	88	0	XP_001924387.1	ssc:100155070	3E-101	NM_001007563.2	IGFBPL1
LOC100624606	72.94573648	0	20	1.29E-07	High	88	0	XR_305119.1	ssc:100624606			
LOC100621379	16.57857647	431.31731	-4.701357577	1.32E-07	Low	20	522	XP_003360610.1	ssc:100621379	6E-47	NM_005978.3	S100A2
ERBB3	409.4908389	15.69928906	4.705060042	1.36E-07	High	494	19	HighI00025DF8BD		0	NM_001982.3	ERBB3
PPFIA4	48.07787177	1108.039296	-4.526492202	1.37E-07	Low	58	1341	XP_005653788.1	ssc:100512361	0	NR_038265.1	PPFIA2
MYF5	0	72.71249671	-20	1.38E-07	Low	0	88	Y17154		3E-143	NM_005593.2	MYF5
LOC100621670	0	72.71249671	-20	1.38E-07	Low	0	88	XP_003361253.1	ssc:100621670	4E-51	NM_001048.3	SST
LOC100739371	0	72.71249671	-20	1.38E-07	Low	0	88	XP_003481077.1	ssc:100739371			
SHOX	0	72.71249671	-20	1.38E-07	Low	0	88	XP_003134962.1	ssc:100520816	3E-138	NM_006883.2	SHOX
PIF1	1223.498944	53.70809416	4.509729517	1.39E-07	High	1476	65	XP_001926830.2	ssc:100153538	0	NM_025049.3	PIF1
LOC102165603	217.1793518	6.610226974	5.038043326	1.40E-07	High	262	8	XP_005667495.1	ssc:102165603	0	NM_003105.5	SORL1
LOC100622474	72.11680765	0	20	1.42E-07	High	87	0	XP_003361735.2	ssc:100622474	4E-41	NM_173803.3	MPV17L
GATA-6	4290.535591	191.6965822	4.484261231	1.42E-07	High	5176	232	NM_214328.2	ssc:397600	0	NM_005257.4	GATA6
TNN	7.460359412	235.4893359	-4.980272783	1.43E-07	Low	9	285	XP_005656793.1	ssc:100520506	0	NM_022093.1	TNN
PM20D2	10258.82312	452.8005477	4.501845723	1.47E-07	High	12376	548	XP_001928624.1	ssc:100157591	0	NM_001010853.2	PM20D2
ACVRL1	49.73572942	1132.827648	-4.509501936	1.50E-07	Low	60	1371	XP_003481626.1	ssc:100153279	0	NM_001077401.1	ACVRL1
YPEL2	48.90680059	1114.649523	-4.510411259	1.50E-07	Low	59	1349	YPEL2	ssc:100524105	2E-68	NM_001005404.3	YPEL2
PTPRN	2.486786471	128.899426	-5.695819394	1.52E-07	Low	3	156	XP_005672297.1	ssc:100155609	0	NM_002846.3	PTPRN
KANK4	0	71.88621834	-20	1.52E-07	Low	0	87	XP_003128002.2	ssc:100523255	0	NM_181712.4	KANK4
LOC102167011	0	71.88621834	-20	1.52E-07	Low	0	87	XR_302256.1	ssc:102167011			
LCN2	27.35465118	651.1073569	-4.573037376	1.54E-07	Low	33	788	NM_001244410.1	ssc:100153501	2E-66	NM_005564.3	LCN2
LOC102164954	0.828928824	91.71689926	-6.789795542	1.57E-07	Low	1	111	XR_301606.1	ssc:102164954			
ETNK2	145.0625441	3.305113487	5.455831436	1.59E-07	High	175	4	XP_005656723.1	ssc:100622861	0	NM_018208.2	ETNK2
GLI3	93.66895707	2078.916383	-4.47211692	1.59E-07	Low	113	2516	XP_005673412.1	ssc:100516888	0	NM_000168.5	GLI3
ALX1	9.947145883	284.2397599	-4.83668193	1.60E-07	Low	12	344	XP_003126788.1	ssc:100525700	0	NM_006982.2	ALX1
OLFML2B	21.55214941	526.3393228	-4.61008952	1.60E-07	Low	26	637	XP_005663213.1	ssc:100156452	0	NM_015441.1	OLFML2B
FOXC2	32.32822412	753.565875	-4.542867471	1.60E-07	Low	39	912	XP_005674616.1	ssc:100526107	1E-167	NM_005251.2	FOXC2
SLC25A53	504.8176556	20.65695929	4.611062552	1.62E-07	High	609	25	XP_003135335.1	ssc:100514813	1E-164	NM_001012755.4	SLC25A53
LOC100738277	87.8664553	1947.538122	-4.470195264	1.63E-07	Low	106	2357	XP_005661995.1	ssc:100738277	2E-137	NM_032831.3	ORAI2
HMOX1	439.3322765	9883.115604	-4.491581517	1.63E-07	Low	530	11961	NM_001004027.1	ssc:445512			
LOC102161608	401.2015506	15.69928906	4.675556048	1.64E-07	High	484	19	XP_005671034.1	ssc:102161608	0	NM_024554.3	PGBD5
HTR4	179.0486259	4.95767023	5.174545326	1.64E-07	High	216	6	NM_001001267.1	ssc:397431	0	NR_104445.1	HTR4
LOC100521440	87.8664553	1945.059287	-4.468357826	1.65E-07	Low	106	2354	XP_005662009.1	ssc:100521440	2E-137	NM_032831.3	ORAI2
ARHGEF40	400.3726218	8967.599168	-4.485306566	1.66E-07	Low	483	10853	XP_005659058.1	ssc:100623088			
ZNF45	15.74964765	403.2238454	-4.6781895	1.66E-07	Low	19	488	ZNF45	ssc:100524260	0	NM_003425.3	ZNF45
LOC102159203	0	71.05993997	-20	1.68E-07	Low	0	86	XR_303394.1	ssc:102159203			
KCNE1L	0	71.05993997	-20	1.68E-07	Low	0	86	XP_005673883.1	ssc:100521707	5E-49	NM_012282.2	KCNE1L
FBLIM1	319.1375971	7099.38377	-4.475443318	1.69E-07	Low	385	8592	XP_005656078.1	ssc:100521094	8E-150	NM_017556.2	FBLIM1
KRCC1	53.05144471	1189.014577	-4.486230552	1.69E-07	Low	64	1439	NM_001243946.1	ssc:100515537	6E-23	NM_016618.1	KRCC1
LOC100737408	90.35324177	0.826278372	6.772804649	1.70E-07	High	109	1	XP_005658762.1	ssc:100737408	0	NR_104591.1	GARNL3
IL18	56.36716001	1258.42196	-4.480617061	1.71E-07	Low	68	1523	NM_213997.1	ssc:397057	2E-84	NM_001562.3	IL18
BCAM	73.7746653	1631.899784	-4.467283183	1.71E-07	Low	89	1975	XP_005653341.1	ssc:100738241	0	NM_005581.4	BCAM
TMEM45A	48.07787177	1079.119553	-4.488337862	1.74E-07	Low	58	1306	XP_005654125.1	ssc:100522901	3E-111	NM_018004.1	TMEM45A
GAS1	174.075053	3811.622129	-4.452623735	1.79E-07	Low	210	4613	XP_003130684.3	ssc:100516459	2E-100	NM_002048.2	GAS1
LOC102157981	600.9733971	25.61462952	4.552261199	1.81E-07	High	725	31	XP_005662316.1	ssc:102157981	7E-116	NM_006648.3	WNK2
LOC100620265	278.5200847	9.915340461	4.811975246	1.81E-07	High	336	12	XP_003354173.1	ssc:100620265	0	NM_173483.3	CYP4F22
CYR61	815.6659624	18432.61792	-4.498138726	1.83E-07	Low	984	22308	XP_001927775.1	ssc:100153791	0	NM_001554.4	CYR61
LOC100519689	1187.026075	53.70809416	4.466068289	1.84E-07	High	1432	65	XR_297787.1	ssc:100519689			
SULT1A1	7.460359412	228.879109	-4.93919684	1.85E-07	Low	9	277	NM_213765.1	ssc:396640	5E-70	NM_001017391.1	SULT1A4
LOC102166397	16.57857647	415.618021	-4.647866171	1.85E-07	Low	20	503	XR_306138.1	ssc:102166397			
LOC102164461	0	70.2336616	-20	1.86E-07	Low	0	85	XP_005674472.1	ssc:102164461	9E-17	NR_027382.1	RDH13
LOC100623777	179.0486259	3903.339028	-4.446285423	1.87E-07	Low	216	4724	XP_003354931.2	ssc:100623777	2E-84	NM_005253.3	FOSL2
STOX2	4884.877557	224.7477171	4.441944034	1.90E-07	High	5893	272	XP_005671756.1	ssc:100156897	0	NM_020225.1	STOX2
KCNJ14	376.3336859	14.87301069	4.66124381	1.97E-07	High	454	18	XP_003127320.1	ssc:100521454	0	np_733838	np_733838
SVCT1	458.3976395	19.00440255	4.592194039	2.04E-07	High	553	23	XP_005661775.1	ssc:396578	0	NM_152685.3	SLC23A1
KLHL4	0	69.40738323	-20	2.05E-07	Low	0	84	XP_003135270.3	ssc:100524840	0	NM_057162.2	KLHL4
LOC102162793	0	69.40738323	-20	2.05E-07	Low	0	84	XR_298475.1	ssc:102162793			
LOC100737121	491.5547924	20.65695929	4.572652429	2.06E-07	High	593	25	XP_005662278.1	ssc:100737121			
CCDC80	463.3712124	10149.17724	-4.45305055	2.11E-07	Low	559	12283	XP_003132728.1	ssc:100516578	0	NM_199512.2	CCDC80
LOC100514461	68.80109236	0	20	2.12E-07	High	83	0	XP_003126631.2	ssc:100514461	3E-145	NM_172364.4	CACNA2D4
DNAH11	339.8608177	13.22045395	4.684100424	2.13E-07	High	410	16	XP_003361782.1		0	np_003768	np_003768
LRP11	14.92071882	374.3041024	-4.648821914	2.20E-07	Low	18	453	XP_001929023.1	ssc:100153355	0	NM_032832.5	LRP11
CYBRD1	186.5089853	3986.793144	-4.417911711	2.25E-07	Low	225	4825	NM_001128452.1	ssc:100144591	5E-137	NM_024843.3	CYBRD1
RAB3C	172.4171953	4.95767023	5.120097542	2.30E-07	High	208	6	XP_003483878.1	ssc:100523703	2E-128	NM_138453.2	RAB3C
LOC102162827	10.77607471	289.1974301	-4.746151069	2.32E-07	Low	13	350	XR_309498.1	ssc:102162827			
LOC100156977	67.97216354	0	20	2.35E-07	High	82	0	XP_001927348.1	ssc:100156977	4E-24	NM_022359.5	PDE4DIP
LOC102158406	67.97216354	0	20	2.35E-07	High	82	0	XR_298395.1	ssc:102158406			
LOC100626166	43.93322765	958.4829112	-4.447368311	2.37E-07	Low	53	1160	XP_003362104.1	ssc:100626166	3E-99	NM_198086	AJUBA
WFDC1	324.11117	6949.827385	-4.422416498	2.38E-07	Low	391	8411	NM_001244987.1	ssc:100514648	1E-89	NM_021197.3	WFDC1
LOC100626688	118.5368218	2512.712528	-4.40583844	2.39E-07	Low	143	3041	XP_005658385.1	ssc:100626688	0	NM_199512.2	CCDC80
SP8	188.166843	5.783948602	5.02381389	2.39E-07	High	227	7	XP_005667734.1	ssc:100627196	0	NM_198956.2	SP8
ADCY2	5.802501765	189.2177471	-5.027228542	2.40E-07	Low	7	229	XP_003134201.4	ssc:100517503	0	NM_020546.2	ADCY2
SLC16A10	913.4795636	42.14019696	4.438103491	2.48E-07	High	1102	51	XP_005654457.1	ssc:100513770	4E-105	NM_018593.4	SLC16A10
LOC100625416	57.19608883	1223.718269	-4.419211144	2.50E-07	Low	69	1481	XR_309344.1	ssc:100625416			
LOC102158004	0	67.75482648	-20	2.52E-07	Low	0	82	XP_005658330.1	ssc:102158004	1E-45	NM_022369.3	STRA6
LOC102158906	0	67.75482648	-20	2.52E-07	Low	0	82	XP_005659109.1	ssc:102158906	9E-74	NM_182609.2	ZNF677
LOC100739264	0	67.75482648	-20	2.52E-07	Low	0	82	XP_003481977.1	ssc:100739264	1E-81	NM_021170.3	HES4
FRAS1	1958.75881	93.36945601	4.390845276	2.57E-07	High	2363	113	XP_003129179.3	ssc:100514970	0	NM_206841.1	FRAS1
LOC100517801	92.01109942	1935.970225	-4.395105048	2.60E-07	Low	111	2343	XP_005660415.1	ssc:100517801	2E-111	np_066929	np_066929
TMEM176B	3.315715294	137.9884881	-5.379083968	2.61E-07	Low	4	167	XP_005657758.1	ssc:100517619	3E-71	NM_014020.3	TMEM176B
CNPY1	67.14323471	0	20	2.61E-07	High	81	0	XP_005673147.1	ssc:102167342	3E-36	NM_001103176.1	CNPY1
DACT3	247.0207894	5208.858855	-4.398262975	2.64E-07	Low	298	6304	XP_003356003.2	ssc:100622612	0	NM_145056.2	DACT3
LOC100737080	591.8551801	26.4409079	4.484400588	2.68E-07	High	714	32	XR_302312.1	ssc:100737080			
LOC100152729	18.23643412	431.31731	-4.563854054	2.69E-07	Low	22	522	XP_001929591.1	ssc:100152729	6E-47	NM_005978.3	S100A2
PCDH9	8.289288236	235.4893359	-4.82826969	2.71E-07	Low	10	285	XP_001925538.1	ssc:100154773	0	NM_203487.2	PCDH9
RNF39	0	66.92854811	-20	2.79E-07	Low	0	81	NM_001128446.1	ssc:100141418	9E-167	NM_170770.1	RNF39
LOC100625598	0	66.92854811	-20	2.79E-07	Low	0	81	XP_005653364.1	ssc:100625598			
LOC100519468	119.3657506	2.478835115	5.589582825	2.84E-07	High	144	3	NM_001260482.1	ssc:100519468	1E-98	NM_172014.2	TNFSF14
CCBE1	1347.009338	64.44971299	4.385442108	2.87E-07	High	1625	78	XP_005659897.1	ssc:100510995	2E-167	NM_133459.3	CCBE1
LOC102162224	66.31430589	0	20	2.90E-07	High	80	0	XP_005658301.1	ssc:102162224	6E-82	NM_020715.2	PLEKHH1
LOC100625903	43.10429883	920.4741061	-4.416473475	2.91E-07	Low	52	1114	XP_003361387.1	ssc:100625903	0	NM_003930.3	SKAP2

LOC100738669	215.5214941	4485.039002	-4.379216868	2.92E-07	Low	260	5428	XP_005665628.1	ssc:100738669	0	NR_073131.1	NEDD9
LOC100521659	38.95965471	837.0199906	-4.425209283	2.93E-07	Low	47	1013	XP_005662529.1	ssc:100521659	0	NM_019885.3	CYP26B1
LTBP3	298.4143765	6236.74915	-4.385405287	2.96E-07	Low	360	7548	XP_005660760.1	ssc:100517877	4E-63	NM_021070.4	LTBP3
MYLK	68.80109236	1434.419253	-4.381891477	2.99E-07	Low	83	1736	XP_005670216.1	ssc:396848	0	NM_053032.2	MYLK
EVI2B	231.2711418	8.262783717	4.806813541	3.01E-07	High	279	10	XP_005669134.1	ssc:100524827	5E-159	NM_006495.3	EVI2B
MSH4	118.5368218	2.478835115	5.57952916	3.02E-07	High	143	3	XP_003356477.3	ssc:100627191	0	NM_002440.3	MSH4
LOC100511457	0	66.10226974	-20	3.10E-07	Low	0	80	XP_003127417.4	ssc:100511457	8E-89	NM_024729.3	MYH14
LOC102165318	14.09179	343.7318026	-4.608356553	3.14E-07	Low	17	416	XR_306421.1	ssc:102165318			
VLDLR	406.1751236	8486.705156	-4.385030768	3.16E-07	Low	490	10271	NM_001199890.1	ssc:733630	0	NM_003383.3	VLDLR
MMP2	1998.547394	43777.05441	-4.453151194	3.17E-07	Low	2411	52981	NM_214192.1	ssc:397391	0	NM_004530.4	MMP2
PADI2	404.5172659	17.35184581	4.543040239	3.19E-07	High	488	21	XP_003356214.1	ssc:100621356	0	NM_007365.2	PADI2
GRIK1	65.48537706	0	20	3.22E-07	High	79	0	XP_005657195.1	ssc:100624513	0	NM_175611.2	GRIK1
ASB10	65.48537706	0	20	3.22E-07	High	79	0	XP_005657749.1	ssc:100626709	0	NM_080871.3	ASB10
SOX13	692.1555677	32.2248565	4.424850493	3.25E-07	High	835	39	XP_005656725.1	ssc:100524269	0	NM_005686.2	SOX13
EPS8	198.9429177	4086.772827	-4.360535604	3.26E-07	Low	240	4946	XP_005664108.1	ssc:100525921	0	NM_004447.5	EPS8
SH3BGR	38.95965471	826.2783717	-4.406575109	3.29E-07	Low	47	1000	NM_001244236.1	ssc:100626262	7E-61	NM_007341.2	SH3BGR
MBP	83.72181118	0.826278372	6.662831807	3.29E-07	High	101	1	NM_001001546.2	ssc:414286	2E-82	NM_002385.2	MBP
AGTR1	13.26286118	325.5536785	-4.617431495	3.33E-07	Low	16	394	AGTR1		0	NM_032049.3	AGTR1
RGS17	9.947145883	261.9302438	-4.718756205	3.33E-07	Low	12	317	XP_005659189.1	ssc:100514020	4E-104	NM_012419.4	RGS17
LOC100737477	949.9524318	20208.29014	-4.410948174	3.33E-07	Low	1146	24457	XP_005654501.1	ssc:100737477	0	NM_000138.4	FBN1
LOC100736876	513.1069418	23.13579441	4.471061001	3.38E-07	High	619	28	XP_003483522.2	ssc:100736876	2E-51	NM_020808.3	SIPA1L2
RXRG	560.3558848	25.61462952	4.45130345	3.42E-07	High	676	31	NM_001130213.1	ssc:445465	0	NR_033824.1	RXRG
LOC102164974	180.7064835	5.783948602	4.965449727	3.42E-07	High	218	7	XR_303780.1	ssc:102164974			
LOC100153446	1058.542108	51.22925905	4.368966824	3.46E-07	High	1277	62	XR_130570.2	ssc:100153446			
LOC102160055	558.6980271	25.61462952	4.447028795	3.51E-07	High	674	31	XP_005663036.1	ssc:102160055			
LOC102161569	132.6286118	3.305113487	5.326548419	3.51E-07	High	160	4	XR_301805.1	ssc:102161569			
DCN	432.7008459	8943.637096	-4.369419803	3.54E-07	Low	522	10824	NM_213920.1	ssc:396957	0	NM_133507.2	DCN
LOC100625208	64.65644824	0	20	3.59E-07	High	78	0	XP_005658068.1	ssc:100625208	2E-143	NM_002538.3	OCLN
TRIM9	9.947145883	259.4514087	-4.705037924	3.62E-07	Low	12	314	XP_001928246.2	ssc:100153738			
LOC100738560	99.47145883	1.652556743	5.91151092	3.70E-07	High	120	2	XP_005662804.1	ssc:100738560	3E-107	NM_198182.2	GRHL1
ADAMTS2	58.85394648	1203.887588	-4.354417718	3.73E-07	Low	71	1457	XP_003123699.3	ssc:100521434	0	NM_021599.2	ADAMTS2
SDC3	252.8232912	5130.36241	-4.34285946	3.77E-07	Low	305	6209	XP_005665222.1	ssc:100516859	4E-171	NM_014654.3	SDC3
LOC102162148	209.7189924	7.436505346	4.817688898	3.81E-07	High	253	9	XP_005662169.1	ssc:102162148	0	NM_024847.3	TMC7
LOC100520265	9.947145883	257.798852	-4.695819394	3.84E-07	Low	12	312	XP_003131064.2	ssc:100520265	0	NM_203487.2	PCDH9
RNF32	0	64.44971299	-20	3.84E-07	Low	0	78	XP_005673141.1	ssc:100511726	4E-149	NM_030936.3	RNF32
TMEM100	0	64.44971299	-20	3.84E-07	Low	0	78	XP_005669010.1	ssc:100523465	2E-63	NM_018286.2	TMEM100
LOC102167757	0	64.44971299	-20	3.84E-07	Low	0	78	XR_303389.1	ssc:102167757			
ARHGEF6	122.6814659	2463.962104	-4.327990852	3.90E-07	Low	148	2982	XP_005657985.1	ssc:100622228	0	NM_004840.2	ARHGEF6
DNHD1	756.8120159	36.35624836	4.379659756	3.94E-07	High	913	44	XP_005656655.1	ssc:100518441	0	NM_173589.3	DNHD1
FAM181B	98.64253001	1.652556743	5.899438088	3.98E-07	High	119	2	XP_003129763.2	ssc:100517667	5E-168	NM_175885.3	FAM181B
LOC102159565	1.657857647	99.15340461	-5.902270271	3.99E-07	Low	2	120	XP_005660430.1	ssc:102159565	5E-61	NM_032888.2	COL27A1
AFGF	1.657857647	99.15340461	-5.902270271	3.99E-07	Low	2	120	XP_003124058.1	ssc:397497	8E-88	NM_033137.2	FGF1
SIRPA	13.26286118	318.9434515	-4.587836713	4.00E-07	Low	16	386	NM_001011508.2	ssc:494566			
LOC102159508	63.82751942	0	20	4.00E-07	High	77	0	XR_306155.1	ssc:102159508			
ZNF488	63.82751942	0	20	4.00E-07	High	77	0	ZNF488	ssc:100519569	5E-122	NM_153034.2	ZNF488
LOC100739323	63.82751942	0	20	4.00E-07	High	77	0	XP_005654371.1	ssc:100739323	1E-57	NM_001164415.2	HSFX2
LOC100737854	786.6534536	38.0088051	4.371322645	4.02E-07	High	949	46	XP_003481550.1	ssc:100737854	0	NM_000699.2	AMY2A
LOC102161654	4.973572942	162.7768392	-5.032468994	4.05E-07	Low	6	197	XP_005657858.1	ssc:102161654	1E-145	NM_006065.3	SIRPB1
RCAN2	16.57857647	380.088051	-4.518941632	4.13E-07	Low	20	460	XP_003482255.1	ssc:100738282	9E-82	NM_005822.3	RCAN2
LOC100626222	130.1418253	3.305113487	5.299241073	4.14E-07	High	157	4	XR_300291.1	ssc:100626222			
LOC102162739	31.4992953	661.8489758	-4.393110595	4.19E-07	Low	38	801	XR_304063.1	ssc:102162739			
KDELR3	289.2961594	5807.910675	-4.327400189	4.25E-07	Low	349	7029	NM_001244970.1	ssc:100514523	1E-119	NM_016657.2	KDELR3
EVA1B	62.99859059	1266.684744	-4.329594144	4.26E-07	Low	76	1533	XP_005658947.1	ssc:100625314	9E-43	NM_018166.1	EVA1B
MOGAT2	81.23502471	0.826278372	6.619330168	4.26E-07	High	98	1	NM_001167651.1	ssc:100312975	1E-164	NM_025098.2	MOGAT2
LOC100739133	81.23502471	0.826278372	6.619330168	4.26E-07	High	98	1	XP_003484318.1	ssc:100739133	1E-122	NM_000341.3	SLC3A1
EFCAB6	0	63.62343462	-20	4.28E-07	Low	0	77	XP_005663806.1	ssc:100524434	0	NM_198856.2	EFCAB6
F13A1	0	63.62343462	-20	4.28E-07	Low	0	77	XP_001927665.3	ssc:100153504	0	NM_000129.3	F13A1
LOC102165879	0	63.62343462	-20	4.28E-07	Low	0	77	XR_301495.1	ssc:102165879			
HOGA1	1.657857647	98.32712624	-5.890197439	4.29E-07	Low	2	119	NM_001190169.1	ssc:100153961			
KRT18	145.0625441	4.131391859	5.133903341	4.33E-07	High	175	5	XP_005652636.1	ssc:100126286	0	NM_199187.1	KRT18
LOC100738183	313.3350953	13.22045395	4.566862749	4.42E-07	High	378	16	XP_003480280.2	ssc:100738183	1E-53	NM_025218.2	ULBP1
LOC102166532	62.99859059	0	20	4.46E-07	High	76	0	LOC102166532				
CCL5	6.631430589	191.6965822	-4.853360671	4.50E-07	Low	8	232	NM_001129946.1	ssc:396613	3E-37	NM_002985.2	CCL5
SLC38A4	1058.542108	52.88181579	4.323163134	4.56E-07	High	1277	64	XP_005664217.1	ssc:100286742	0	NM_018018.4	SLC38A4
ANTXR2	341.5186753	6816.796567	-4.319057553	4.63E-07	Low	412	8250	XP_003129419.3	ssc:100514416	0	NM_058172.5	ANTXR2
RGS3	128.4839677	3.305113487	5.28074473	4.64E-07	High	155	4	XP_003480627.1	ssc:100517994	0	NR_074078.1	RGS3
PRSS35	87.8664553	1734.358302	-4.302945672	4.68E-07	Low	106	2099	XP_005659463.1	ssc:100156168	0	NM_153362.2	PRSS35
CDC42EP1	219.6661383	4337.135173	-4.303358008	4.74E-07	Low	265	5249	XP_003481602.1	ssc:100738372	3E-133	NM_152243.2	CDC42EP1
USP18	680.5505642	13783.14952	-4.340059422	4.77E-07	Low	821	16681	NM_213826.1	ssc:396777	2E-151	NM_017414.3	USP18
BCO2	0	62.79715625	-20	4.77E-07	Low	0	76	XP_005667395.1	ssc:100517547	0	NM_031938.5	BCO2
LOC102161934	38.13072589	776.7016694	-4.348334666	4.80E-07	Low	46	940	XP_005670750.1	ssc:102161934	1E-40	NM_016817.2	OAS2
SYNPO2	16.57857647	372.6515456	-4.490435204	4.93E-07	Low	20	451	XP_005666955.1	ssc:100525414	0	NM_133477.2	SYNPO2
LOC100739759	96.15574354	1.652556743	5.862601319	4.96E-07	High	116	2	XP_003483318.2	ssc:100739759			
DPPA5	62.16966177	0	20	4.99E-07	High	75	0	NM_001160273.1	ssc:100301558	5E-45	NM_001025290.2	DPPA5
LOC100623474	62.16966177	0	20	4.99E-07	High	75	0	XP_005655616.1	ssc:100623474	1E-157	NM_024902.2	DNAJC22
LOC100623014	62.16966177	0	20	4.99E-07	High	75	0	XR_299375.1	ssc:100623014	6E-49	NR_038257.1	TRPM2
FNDC5	0.828928824	80.14900206	-6.595292518	5.00E-07	Low	1	97	XP_005665249.1	ssc:100622587	4E-77	NM_153756.2	FNDC5
LOC100513362	37.30179706	757.6972669	-4.344304503	5.00E-07	Low	45	917	XP_005664972.1	ssc:100513362	8E-156	NM_133460.1	ZNF418
HSPB7	6.631430589	189.2177471	-4.834583884	5.05E-07	Low	8	229	NM_001243388.1	ssc:100627988	7E-80	NM_014424.4	HSPB7
PPARGC1B	4209.300566	215.658655	4.286759007	5.06E-07	High	5078	261	XP_005661866.1	ssc:100329221	0	NM_133263.3	PPARGC1B
CD151	1208.578225	24674.32474	-4.351627844	5.21E-07	Low	1458	29862	NM_001243865.1	ssc:100511619	1E-121	NM_139030.3	CD151
BAI3	232.1000706	9.089062089	4.674471723	5.29E-07	High	280	11	XP_003353288.1		0	NM_001704.2	BAI3
LOC102163560	0	61.97087788	-20	5.33E-07	Low	0	75	XR_297410.1	ssc:102163560			
MYLK2	0	61.97087788	-20	5.33E-07	Low	0	75	XP_003134431.2	ssc:100524288	9E-91	NM_033118.3	MYLK2
HTR2A	0	61.97087788	-20	5.33E-07	Low	0	75	NM_214217.1	ssc:397432	0	NM_001165947.2	HTR2A
ZNF71	12.43393235	292.5025436	-4.55609463	5.53E-07	Low	15	354	ZNF71	ssc:100739752	0	NM_021216.4	ZNF71
LOC102159619	78.74823824	0.826278372	6.574475933	5.56E-07	High	95	1	XR_297436.1	ssc:102159619			
FAM211A	19.89429177	428.0121966	-4.427225463	5.57E-07	Low	24	518	XP_003358347.1	ssc:100626706	0	NM_207387.3	FAM211A
LOC100512873	61.34073295	0	20	5.58E-07	High	74	0	XP_003134513.1	ssc:100512873	7E-35	NM_003064.3	SLPI
LOC100737042	61.34073295	0	20	5.58E-07	High	74	0	XP_003483238.2	ssc:100737042	5E-81	NM_018398.2	CACNA2D3
LOC102167231	61.34073295	0	20	5.58E-07	High	74	0	XR_298105.1	ssc:102167231			
SH3PXD2A	463.3712124	9127.070894	-4.299911615	5.58E-07	Low	559	11046	XP_005671487.1	ssc:100153803	0	NM_014631.2	SH3PXD2A
RNASET2	240.3893588	4665.993965	-4.278739502	5.61E-07	Low	290	5647	XP_001928120.2	ssc:100157985	9E-91	NM_003730.4	RNASET2
FAM78B	23.21000706	488.3305177	-4.395039074	5.61E-07	Low	28	591	XP_001925744.3	ssc:100153010	1E-152	NM_001017961.3	FAM78B
GRTP1	275.2043694	11.5678972	4.572304834	5.62E-07	High	332	14	XP_003131151.3	ssc:100518272	2E-72	NM_024719.3	GRTP1
LOC100521274	4.144644118	141.2936016	-5.091304096	5.74E-07	Low	5	171	XP_005665093.1	ssc:100521274	8E-123	NM_182623.2	FAM131C
LOC100154755	94.49788589	1.652556743	5.837510339	5.76E-07	High	114	2	XP_001927693.1	ssc:100154755	1E-49	NM_003087.2	SNCG
ADAMTS20	1154.697851	59.49204276	4.278674865	5.76E-07	High	1393	72	NM_001257275.1	ssc:100049661	0	NM_175851.1	ADAMTS20
LYPD6	709.563073	35.52996998	4.319822556	5.82E-07	High	856	43	XP_005671651.1	ssc:100521871	1E-85	NM_194317.3	LYPD6
SORBS2	72.94573648	1409.630902	-4.272349988	5.87E-07	Low	88	1706	XP_005671790.1	ssc:396719	0	NM_021069.4	SORBS2

LAMB3	169.9304088	5.783948602	4.876745502	5.90E-07	High	205	7	XP_005656816.1	ssc:100513489	0	NM_001127641.1	LAMB3
LOC100512382	273.5465118	11.5678972	4.563587617	5.93E-07	High	330	14	XP_005673280.1	ssc:100512382	8E-151	NM_012281.2	KCND2
LOC102166402	0	61.14459951	-20	5.96E-07	Low	0	74	XR_300488.1	ssc:102166402			
LOC100623627	0	61.14459951	-20	5.96E-07	Low	0	74	XR_303903.1	ssc:100623627			
ESRP1	1070.97604	55.36065091	4.273921489	6.09E-07	High	1292	67	XP_001925909.1		0	NM_017697.3	ESRP1
CPPED1	10.77607471	259.4514087	-4.589560706	6.12E-07	Low	13	314	NM_001243581.1	ssc:100627178	3E-158	NM_018340.2	CPPED1
HES7	154.1807612	4.95767023	4.958816635	6.19E-07	High	186	6	XP_003483152.1	ssc:100620745	4E-85	NM_032580.3	HES7
LOC100517213	1.657857647	94.19573438	-5.82826969	6.20E-07	Low	2	114	XP_005652879.1	ssc:100517213	0	NM_152601.3	ZNF709
LOC100736832	60.51180412	0	20	6.25E-07	High	73	0	XP_003481096.1	ssc:100736832	0	NM_001134408.1	GRIN2A
LOC100737628	43.93322765	856.8506715	-4.2856594	6.52E-07	Low	53	1037	XP_003484102.2	ssc:100737628	3E-44	NM_003930.3	SKAP2
LOC100627241	8.289288236	213.1798199	-4.684678836	6.56E-07	Low	10	258	XP_005658856.1	ssc:100627241	0	NM_198893.2	ZNF160
CAMK2N1	38.95965471	765.1337722	-4.295659207	6.58E-07	Low	47	926	XP_003127723.2	ssc:100515264	4E-40	NM_018584.5	CAMK2N1
LOC100738306	0	60.31832114	-20	6.67E-07	Low	0	73	XP_005667122.1	ssc:100738306	1E-155	NM_207186.2	OR10A4
LOC100621447	0	60.31832114	-20	6.67E-07	Low	0	73	XR_309679.1	ssc:100621447			
HOXC11	0	60.31832114	-20	6.67E-07	Low	0	73	XP_003355473.2	ssc:100628033	6E-106	NM_014212.3	HOXC11
LOC102158192	167.4436224	5.783948602	4.855476885	6.71E-07	High	202	7	XR_307563.1	ssc:102158192			
CBL	17453.09638	891.5543631	4.29101643	6.75E-07	High	21055	1079	XP_003129980.1	ssc:100518854	0	NM_005188.3	CBL
KIF5C	13427.81801	691.5949971	4.279153646	6.79E-07	High	16199	837	XP_005671654.1	ssc:100622102	0	NM_004522.2	KIF5C
LOC100738873	107.7607471	2.478835115	5.442025637	6.91E-07	High	130	3	XP_005657471.1	ssc:100738873	8E-106	NM_001165973.1	NRG3
LOC102164919	59.6828753	0	20	7.01E-07	High	72	0	XR_301479.1	ssc:102164919			
LOC100623122	59.6828753	0	20	7.01E-07	High	72	0	XP_005674431.1	ssc:100623122			
LOC102167333	59.6828753	0	20	7.01E-07	High	72	0	XR_300673.1	ssc:102167333			
CST3	789.1402401	15387.78212	-4.285359789	7.02E-07	Low	952	18623	NM_001044602.1	ssc:733672	6E-47	NM_001288614.1	CST3
UGT1A10	21.55214941	445.3640424	-4.36908142	7.20E-07	Low	26	539	XP_003133790.3	ssc:100511841	3E-128	NM_019075.2	UGT1A10
LOC100523202	885.2959836	46.27158882	4.257961334	7.29E-07	High	1068	56	XP_005668527.1	ssc:100523202	0	NM_014832.3	TBC1D4
LOC100522856	192.3114871	3612.489041	-4.231476366	7.33E-07	Low	232	4372	XR_301863.1	ssc:100522856	6E-64	NM_198709.2	ARSB
MGAT5B	382.9651165	18.17812418	4.396937747	7.37E-07	High	462	22	XP_005668662.1	ssc:100516218	0	NM_198955.1	MGAT5B
FAM132A	368.0443977	17.35184581	4.406718768	7.44E-07	High	444	21	XP_003127537.2	ssc:100523314	1E-115	NM_001014980.2	FAM132A
GADD45B	148.3782594	2772.163937	-4.223660949	7.54E-07	Low	179	3355	XP_005654758.1	ssc:100621090	8E-89	NM_015675.3	GADD45B
WDR93	498.186223	24.78835115	4.328950909	7.59E-07	High	601	30	XP_003128523.1	ssc:100156276	0	NM_020212.1	WDR93
SERPINH1	2753.701552	55440.79991	-4.33150396	7.60E-07	Low	3322	67097	NM_001244132.1	ssc:396773	1E-122	NM_001235.3	SERPINH1
LOC102159990	135.9443271	4.131391859	5.040244234	7.65E-07	High	164	5	XR_300206.1	ssc:102159990			
LOC100623668	10.77607471	252.8411817	-4.5523278	7.69E-07	Low	13	306	XP_003482903.1	ssc:100623668	2E-123	NM_000231.2	SGCG
KLF2	4.973572942	151.208942	-4.926117013	7.72E-07	Low	6	183	NM_001134351.2	ssc:100174959			
CCDC110	0.828928824	76.0176102	-6.518941632	7.82E-07	Low	1	92	XP_005671780.1	ssc:100517861	0	NM_152775.3	CCDC110
LOC100738449	2.486786471	106.58991	-5.42164443	7.96E-07	Low	3	129	XP_005667233.1	ssc:100738449	0	NM_001098816.2	TENM4
SLC9C2	392.9122624	19.00440255	4.369801167	8.18E-07	High	474	23	XP_005656772.1	ssc:100624136	0	NM_178527.3	SLC9C2
LOC100737092	24.03893588	482.5465691	-4.327223239	8.20E-07	Low	29	584	XP_003480928.1	ssc:100737092	0	NM_022350.3	ERAP2
LOC100515900	149.2071882	4.95767023	4.91151092	8.24E-07	High	180	6	LOC100515900				
ITPKA	120.1946794	3.305113487	5.184529414	8.28E-07	High	145	4	XP_003121630.1	ssc:100516235	0	NM_002220.2	ITPKA
PTPRD	1451.45437	78.49644531	4.208728084	8.31E-07	High	1751	95	XP_005660164.1	ssc:100155429	0	NM_130393.3	PTPRD
PIK3R5	10.77607471	250.3623466	-4.538113941	8.39E-07	Low	13	303	NM_213851.1	ssc:396851	0	NM_014308.3	PIK3R5
THSD7B	0	58.66576439	-20	8.40E-07	Low	0	71	XP_003359473.2	ssc:100624965	0	NM_001080427.1	THSD7B
STC1	0	58.66576439	-20	8.40E-07	Low	0	71	NM_001103212.1	ssc:100125345	2E-138	NM_003155.2	STC1
PROK2	90.35324177	1.652556743	5.772804649	8.46E-07	High	109	2	XP_005669784.1	ssc:100526076	4E-42	NM_021935.3	PROK2
IL12A	90.35324177	1.652556743	5.772804649	8.46E-07	High	109	2	NM_213993.1	ssc:397053			
LOC100514541	33.98608177	655.2387488	-4.269004727	8.54E-07	Low	41	793	XP_005670110.1	ssc:100514541	0	NM_004443.3	EPHB3
MYOT	0.828928824	75.19133183	-6.503174316	8.58E-07	Low	1	91	NM_001099941.2	ssc:100101550	0	NM_006790.2	MYOT
CLDN1	28.18358	551.1276739	-4.289459786	8.81E-07	Low	34	667	NM_001244539.1	ssc:100625166	1E-109	NM_021101.4	CLDN1
GIPC2	605.1180412	31.39857813	4.268445465	8.82E-07	High	730	38	XR_304112.1	ssc:100625104	3E-148	NM_017655.4	GIPC2
ESM1	15.74964765	334.6427406	-4.40923026	8.84E-07	Low	19	405	XP_005674289.1	ssc:100519340	2E-71	NM_007036.4	ESM1
DUSP6	163.2989782	2999.390489	-4.199081689	8.85E-07	Low	197	3630	NM_001267842.1	ssc:100622968	9E-14	NM_022652.2	DUSP6
PTCHD4	58.02501765	0	20	8.85E-07	High	70	0	XP_001925843.4	ssc:100157414	1E-175	NM_207499.2	PTCHD4
LOC102158579	58.02501765	0	20	8.85E-07	High	70	0	XP_005673077.1	ssc:102158579	2E-31	NM_203357.1	CTAGE5
CRABP1	23.21000706	463.5421665	-4.319881714	8.93E-07	Low	28	561	NM_001128492.1	ssc:100169745	3E-78	NM_004378.2	CRABP1
DENND5B	646.5644824	33.87741324	4.254398633	9.06E-07	High	780	41	XP_005653187.1	ssc:102165384	0	NM_144973.3	DENND5B
LOC102161912	147.5493306	4.95767023	4.895391255	9.08E-07	High	178	6	XR_304412.1	ssc:102161912			
KSR2	246.1918606	10.74161883	4.518499727	9.21E-07	High	297	13	XP_005657376.1	ssc:100519054			
LOC102158324	1298.931467	71.05993997	4.192145034	9.41E-07	High	1567	86	XR_300390.1	ssc:102158324			
LOC102158649	0	57.83948602	-20	9.44E-07	Low	0	70	XR_300644.1	ssc:102158649			
CASP1	0	57.83948602	-20	9.44E-07	Low	0	70	NM_214162.1	ssc:397319	1E-174	NM_033295.3	CASP1
TBX20	259.4547218	11.5678972	4.487284249	9.48E-07	High	313	14	XP_003134842.1	ssc:100524471	0	NM_020417.1	TBX20
TENC1	408.66191	7562.925936	-4.209964805	9.56E-07	Low	493	9153	XP_005652637.1	ssc:100156301	0	NM_198316.1	TENC1
SSC5D	53.05144471	981.6187056	-4.209698796	9.57E-07	Low	64	1188	XP_005664918.1	ssc:100621391	0	NM_182571.2	SSC5D
MED12L	399.543693	19.83068092	4.33254716	9.71E-07	High	482	24	XP_005669985.1	ssc:100524586	0	NM_053002.4	MED12L
LOC102167736	2.486786471	104.1110748	-5.387697098	9.74E-07	Low	3	126	XP_005656849.1	ssc:102167736			
RAP1GAP	88.69538413	1.652556743	5.746087311	9.91E-07	High	107	2	XP_005658516.1	ssc:100627043	1E-125	NM_002885.2	RAP1GAP
LOC100522894	88.69538413	1.652556743	5.746087311	9.91E-07	High	107	2	XP_003131662.3	ssc:100522894	5E-85	NM_002126.4	HLF
SKAP2	17.4075053	358.6048133	-4.364613485	9.92E-07	Low	21	434	XP_005673360.1	ssc:100520346	5E-123	NM_003930.3	SKAP2
GIMAP4	57.19608883	0	20	9.97E-07	High	69	0	XP_003134607.2	ssc:100518347	3E-76	NM_018326.2	GIMAP4
PRRT1	3.315715294	118.1578072	-5.155251012	1.01E-06	Low	4	143	NM_001123170.1	ssc:100144520	9E-106	NM_030651.3	PRRT1
PDZD7	369.7022553	18.17812418	4.346088606	1.01E-06	High	446	22	XP_005671449.1	ssc:100625985	0	NM_024895.4	PDZD7
NEDD9	259.4547218	4715.570667	-4.183877676	1.03E-06	Low	313	5707	XP_005665630.1	ssc:100154178	0	NR_073131.1	NEDD9
GPR123	0.828928824	73.53877508	-6.471113107	1.03E-06	Low	1	89	XP_005658819.1	ssc:100623440	2E-139	NM_032422.2	GPR123
LOC102160892	72.94573648	0.826278372	6.464051943	1.06E-06	High	88	1	LOC102160892				
LOC100621934	0	57.01320765	-20	1.06E-06	Low	0	69	XP_003361045.1	ssc:100621934	7E-52	NM_022369.3	STRA6
CDRT1	0	57.01320765	-20	1.06E-06	Low	0	69	XP_005654045.1	ssc:100523091	2E-146	NR_051988.1	FBXW10
GPR4	87.8664553	1.652556743	5.732540779	1.07E-06	High	106	2	NM_001123118.1	ssc:100144489	1E-51	NM_005282.2	GPR4
FCGR1A	283.4936577	13.22045395	4.422472839	1.08E-06	High	342	16	NM_001033011.1	ssc:613130	2E-155	NM_000566.3	FCGR1A
JPH4	3356.332807	187.5651904	4.161421765	1.10E-06	High	4049	227	XP_005666308.1	ssc:100152448	0	NM_032452.2	JPH4
LOC102166068	158.3254053	5.783948602	4.77469423	1.10E-06	High	191	7	XR_300918.1	ssc:102166068			
RAB3IP	1346.18041	75.19133183	4.162161601	1.12E-06	High	1624	91	XP_005664031.1	ssc:100152578	2E-61	NR_103520.1	RAB3IP
LOC100621903	56.36716001	0	20	1.12E-06	High	68	0	XP_003361676.2	ssc:100621903	8E-48	NM_020340.4	KIAA1244
LOC100517279	3323.175654	186.738912	4.153468064	1.15E-06	High	4009	226	XR_130597.3	ssc:100517279			
GAL3ST4	70.45895001	1262.553352	-4.163417568	1.17E-06	Low	85	1528	XP_005661948.1	ssc:100511123	0	NM_024637.4	GAL3ST4
LOC100739554	460.0554971	23.96207278	4.26298329	1.18E-06	High	555	29	XP_003480332.1	ssc:100739554	1E-164	NM_021244.4	RRAGD
LOC100624765	307.5325936	14.87301069	4.3699707	1.20E-06	High	371	18	XP_005654553.1	ssc:100624765	3E-167	NM_134428.2	RFX3
DFNA5	37.30179706	686.6373269	-4.202231246	1.21E-06	Low	45	831	XP_005673376.1	ssc:100522585	0	NM_004403.2	DFNA5
LOC100623844	3.315715294	115.678972	-5.124662693	1.21E-06	Low	4	140	XR_309588.1	ssc:100623844			
DUSP9	224.6397112	9.915340461	4.501806865	1.23E-06	High	271	12	XP_003135519.2	ssc:100519006	9E-65	NM_001395	DUSP9
FXYD5	125.1682524	2213.599758	-4.144453802	1.23E-06	Low	151	2679	XP_005664552.1	ssc:100624274	1E-28	NR_028406.1	FXYD5
SMOC1	45.5910853	825.4520934	-4.17836083	1.24E-06	Low	55	999	XP_005656414.1	ssc:100157351	0	NM_022137.5	SMOC1
NAALADL2	0.828928824	71.88621834	-6.438323172	1.25E-06	Low	1	87	XP_003132587.3	ssc:100522279	0	NM_207015.2	NAALADL2
LOC100737896	0.828928824	71.88621834	-6.438323172	1.25E-06	Low	1	87	XR_302944.1	ssc:100737896	1E-20	NM_172060.3	EYA1
MMP14	2874.72516	54838.44297	-4.253691671	1.26E-06	Low	3468	66368	NM_214239.1	ssc:397471	0	NM_004995.3	MMP14
LOC100511328	55.53823118	0	20	1.27E-06	High	67	0	XP_003126562.2	ssc:100511328	2E-13	NM_199286.2	DPPA3
LOC100522379	55.53823118	0	20	1.27E-06	High	67	0	LOC100522379	ssc:100522379			
LOC100522074	114.3921777	3.305113487	5.113144781	1.27E-06	High	138	4	XR_298326.1	ssc:100522074	5E-175	NM_024759.1	NIPAL2
RPP25	747.6937989	41.31391859	4.177747758	1.28E-06	High	902	50	XP_001927152.1	ssc:100157338	7E-87	NM_017793.2	RPP25
IL7	8.289288236	197.4805308	-4.574318389	1.28E-06	Low	10	239	NM_214135.2	ssc:397253	8E-63	NM_001199888.1	IL7

PPAP2B	225.46864	3982.661752	-4.142734266	1.30E-06	Low	272	4820	XP_005665445.1	ssc:100512419	1E-178	NM_177414.1	PPAP2B
C2H19orf66	47.24894295	848.5878878	-4.166710128	1.31E-06	Low	57	1027	NM_001244321.1	ssc:100512458	7E-153	NM_018381.2	C19orf66
PAQR5	155.00969	5.783948602	4.744159862	1.32E-06	High	187	7	XP_005659931.1	ssc:100521597	1E-165	NM_017705.3	PAQR5
NFASC	127.6550388	4.131391859	4.94947877	1.32E-06	High	154	5	XP_005667588.1	ssc:100626921			
FBLN5	329.9136718	5856.661099	-4.149917944	1.33E-06	Low	398	7088	XP_005656504.1	ssc:100155248	0	NM_006329.3	FBLN5
CHRNA3	549.57981	29.74602138	4.207560383	1.34E-06	High	663	36	XP_001925795.3	ssc:100151989	0	NR_046313.1	CHRNA3
LOC780439	82.89288236	1455.902491	-4.134521695	1.35E-06	Low	100	1762	NM_001078688.1	ssc:780439	1E-70	NM_003102.2	SOD3
LOC100621708	0	55.36065091	-20	1.35E-06	Low	0	67	XP_003361674.1	ssc:100621708	0	NM_001145030.1	TOPAZ1
GPR160	235.4157859	10.74161883	4.453927726	1.37E-06	High	284	13	XP_005674626.1	ssc:100626150	3E-55	NM_014373.2	GPR160
SCN1A	85.37966883	1.652556743	5.691120852	1.37E-06	High	103	2	HighI00025DFBBD		0	NM_006920.4	SCN1A
LOC100737066	85.37966883	1.652556743	5.691120852	1.37E-06	High	103	2	XP_003481468.1	ssc:100737066	0	NM_030916.2	PVRL4
C2H19orf38	2.486786471	99.97968298	-5.329280412	1.38E-06	Low	3	121	XP_005661306.1	ssc:100627549	3E-98	NM_001136482.1	C19orf38
MSI1	1698.47516	97.50084786	4.122681544	1.38E-06	High	2049	118	XP_005670771.1	ssc:100627974			
PLAUR	141.7468288	2475.530002	-4.126349069	1.38E-06	Low	171	2996	XP_005655990.1	ssc:100521017	7E-119	NM_002659.3	PLAUR
LOC100514136	2086.413849	38936.71571	-4.222033943	1.39E-06	Low	2517	47123	XP_003361338.1	ssc:100514136	6E-156	NM_003118.3	SPARC
EMILIN3	424.4115577	22.30951604	4.249732822	1.39E-06	High	512	27	XP_005673002.1	ssc:100519117	1E-120	NM_052846.1	EMILIN3
LOC100623445	615.0651871	33.87741324	4.182343696	1.41E-06	High	742	41	XP_005656036.1	ssc:100623445	1E-48	NM_178545.3	TMEM52
LOC100739847	477.4630024	25.61462952	4.220349015	1.43E-06	High	576	31	XP_005668534.1	ssc:100739847	4E-65	NM_144595.3	SLAIN1
LOC102161548	54.70930236	0	20	1.44E-06	High	66	0	XP_005655403.1	ssc:102161548			
ZADH2	203.0875618	3538.123988	-4.122810817	1.45E-06	Low	245	4282	ZADH2	ssc:100737192	4E-122	NM_175907.4	ZADH2
DOCK3	84.55074001	1.652556743	5.677045666	1.49E-06	High	102	2	XP_003132287.4	ssc:100519389	0	NM_004947.4	DOCK3
KIAA1549L	313.3350953	15.69928906	4.318935235	1.49E-06	High	378	19	XP_005661089.1	ssc:100513003	0	NM_012194.2	KIAA1549L
SERPINB1	106.9318182	1849.210996	-4.112146741	1.51E-06	Low	129	2238	XP_005665588.1	ssc:100155145	2E-175	NR_073112.1	SERPINB1
LOC100738497	0	54.53437253	-20	1.53E-06	Low	0	66	XP_005667234.1	ssc:100738497	2E-87	NM_001098816.2	TENM4
ABCG1	0	54.53437253	-20	1.53E-06	Low	0	66	XP_003483412.1	ssc:100620416	8E-148	NM_207630.1	ABCG1
LOC100522569	111.9053912	3.305113487	5.081435921	1.53E-06	High	135	4	XP_003130932.1	ssc:100522569	4E-160	NM_178540.3	C1QTNF9
GJA1	1870.063426	34378.13793	-4.200332297	1.54E-06	Low	2256	41606	NM_001244212.1	ssc:100518636	0	NM_000165.3	GJA1
LOC102161843	448.4504936	7883.521945	-4.135819661	1.55E-06	Low	541	9541	XR_306493.1	ssc:102161843			
LOC100624847	24.03893588	447.8428775	-4.219547722	1.59E-06	Low	29	542	XP_003362157.1	ssc:100624847	2E-126	NM_020731.4	AHRR
MXRA8	475.8051447	8351.195503	-4.133539963	1.60E-06	Low	574	10107	XP_005653399.1	ssc:100621355	0	NM_032348.3	MXRA8
LOC100518848	524.7119453	28.91974301	4.181398997	1.62E-06	High	633	35	Z84015	ssc:100518848	7E-44	NM_001024.3	RPS21
LOC102159424	53.88037353	0	20	1.63E-06	High	65	0	XR_307055.1	ssc:102159424			
PTAFR	53.88037353	0	20	1.63E-06	High	65	0	XP_005653452.1	ssc:100523973	8E-168	NM_001164723.2	PTAFR
WNT8A	53.88037353	0	20	1.63E-06	High	65	0	XP_005661739.1	ssc:100739710	0	NM_058244.2	WNT8A
LEPREL2	583.5658918	10254.11459	-4.135163552	1.66E-06	Low	704	12410	XP_005652654.1	ssc:100156539	0	NM_014262.4	LEPREL2
MAP4K1	833.0734677	47.92414556	4.119619115	1.69E-06	High	1005	58	XP_003127163.3	ssc:100516374	0	NM_007181.5	MAP4K1
LOC102166831	4.973572942	137.9884881	-4.794121467	1.70E-06	Low	6	167	XR_299766.1	ssc:102166831			
TEX14	575.2766036	32.2248565	4.158009958	1.71E-06	High	694	39	XP_005669032.1	ssc:100737915	0	NM_198393.3	TEX14
ASPA	0	53.70809416	-20	1.73E-06	Low	0	65	NM_001123077.1	ssc:100142661	1E-174	NM_001128085.1	ASPA
TMPRSS11A	0	53.70809416	-20	1.73E-06	Low	0	65	XP_005666787.1	ssc:100525933	0	NM_182606.3	TMPRSS11A
TMEM132C	0	53.70809416	-20	1.73E-06	Low	0	65	XP_003359125.2	ssc:100623362	0	NM_001136103.2	TMEM132C
CAMK4	163.2989782	6.610226974	4.626672144	1.74E-06	High	197	8	XP_003123873.2	ssc:100515364	0	NM_001744.4	CAMK4
OPRL1	110.2475335	3.305113487	5.05990276	1.74E-06	High	133	4	NM_214176.1	ssc:397364	2E-12	NM_182647.2	OPRL1
RHPN2	1129.001058	66.10226974	4.094203217	1.76E-06	High	1362	80	XP_003355886.2	ssc:100620673			
ROR1	19.89429177	375.9566591	-4.24013991	1.76E-06	Low	24	455	XP_003127992.3	ssc:100520620	0	NM_005012.3	ROR1
RYR2	15.74964765	309.028111	-4.294346622	1.78E-06	Low	19	374	XP_001924803.4	ssc:396856	0	NM_001035.2	RYR2
TEC	18.23643412	348.6894729	-4.257047246	1.79E-06	Low	22	422	XP_003129008.1	ssc:404277	0	NM_003215.2	TEC
CELF4	545.4351659	30.57229975	4.157110732	1.79E-06	High	658	37	XP_005665413.1	ssc:100521138	0	NM_020180.3	CELF4
PDLIM3	0.828928824	68.58110485	-6.370419107	1.85E-06	Low	1	83	NM_001001637.1	ssc:414421	0	NR_047562.1	PDLIM3
LOC100623270	109.4186047	3.305113487	5.049014444	1.86E-06	High	132	4	XR_299924.1	ssc:100623270			
C9H11orf88	122.6814659	4.131391859	4.892145595	1.86E-06	High	148	5	XP_005653753.1	ssc:100513750	1E-53	NM_207430.2	C11orf88
PRKCG	864.572763	50.40298068	4.100406429	1.86E-06	High	1043	61	XP_005664873.1	ssc:100518074	0	NM_002739.3	PRKCG
WDR17	8.289288236	189.2177471	-4.512655369	1.86E-06	Low	10	229	XP_005671730.1	ssc:100157060	0	NM_181265.3	WDR17
LOC100520702	8.289288236	189.2177471	-4.512655369	1.86E-06	Low	10	229	XR_308834.1	ssc:100520702			
EPHB3	86.20859765	1459.207604	-4.081209585	1.87E-06	Low	104	1766	XP_005670109.1	ssc:100337659	0	NM_004443.3	EPHB3
DSE	220.4950671	3738.083354	-4.083480454	1.88E-06	Low	266	4524	XP_003121429.1	ssc:100520877	0	NM_013352.2	DSE
CYP2D25	944.1499301	55.36065091	4.092083166	1.89E-06	High	1139	67	NM_214394.1	ssc:397687	0	NM_001025161.2	CYP2D6
MLXIPL	82.06395354	1.652556743	5.633976944	1.91E-06	High	99	2	XP_003481050.2	ssc:100170769	0	NM_032994.2	MLXIPL
RNF207	1651.226217	98.32712624	4.069804493	1.91E-06	High	1992	119	XP_003481985.1	ssc:100515143	0	NM_207396.2	RNF207
ETS1	48.90680059	840.325104	-4.10284059	1.92E-06	Low	59	1017	NM_001162886.1	ssc:100302363			
DNAH3	304.2168783	15.69928906	4.276329064	1.94E-06	High	367	19	XP_005662161.1	ssc:100511869	4E-30	NM_001888.3	CRYM
PID1	0	52.88181579	-20	1.96E-06	Low	0	64	NM_001173520.1	ssc:100302508	3E-23	NM_017933.4	PID1
LOC102165870	0	52.88181579	-20	1.96E-06	Low	0	64	XR_306630.1	ssc:102165870			
LOC102166598	5.802501765	148.7301069	-4.67987785	1.98E-06	Low	7	180	XP_005670367.1	ssc:102166598	6E-53	NR_072999.1	JAM2
LIMS2	57.19608883	971.7033652	-4.086527564	1.99E-06	Low	69	1176	XP_005671891.1	ssc:100517009	0	NM_017980.4	LIMS2
LOC100737468	4.144644118	122.289199	-4.882904946	2.00E-06	Low	5	148	LOC100737468		2E-55	NM_152456.2	IL34
SLCO2A1	21.55214941	396.6136184	-4.201830553	2.01E-06	Low	26	480	NM_001123195.2	ssc:100144510			
GEM	111.0764624	1855.821223	-4.062432698	2.05E-06	Low	134	2246	Z80109.1	ssc:404772	6E-173	NM_181702.2	GEM
LOC100739671	36.47286824	632.1029544	-4.115263995	2.09E-06	Low	44	765	XP_005672506.1	ssc:100739671	8E-55	NM_007036.4	ESM1
TFAP2C	496.5283653	28.09346464	4.143569676	2.10E-06	High	599	34	NM_001123201.1	ssc:100144525	0	NM_003222.3	TFAP2C
DLK2	94.49788589	2.478835115	5.252547838	2.12E-06	High	114	3	NM_001128466.1	ssc:100145892	0	NM_206539.2	DLK2
LOC102158777	3.315715294	108.2424667	-5.028802677	2.13E-06	Low	4	131	XR_299709.1	ssc:102158777			
TNFAIP6	11.60500353	237.9681711	-4.357949755	2.13E-06	Low	14	288	NM_001159607.1	ssc:100286870	4E-149	NM_007115.3	TNFAIP6
PHLDB2	151.6939747	2522.627869	-4.055691712	2.15E-06	Low	183	3053	XP_005674250.1	ssc:100623057			
LOC100625212	2038.335977	123.1154774	4.049307823	2.15E-06	High	2459	149	XP_003361120.2	ssc:100625212	0	NM_025151.4	RAB11FIP1
DGKB	133.4575406	4.95767023	4.750574702	2.16E-06	High	161	6	XP_003357509.2	ssc:100625799	0	NM_145695.2	DGKB
NUAK2	71.28787883	1190.667134	-4.061969541	2.18E-06	Low	86	1441	XP_005667585.1	ssc:100625699	0	NM_030952.1	NUAK2
LOC100737021	4.973572942	133.8570962	-4.750267178	2.21E-06	Low	6	162	XR_135112.2	ssc:100737021			
PRDM11	14.92071882	288.3711517	-4.2725379	2.23E-06	Low	18	349	XP_005661028.1	ssc:100520417	0	NR_046338.1	PRDM11
NDNF	0	52.05553742	-20	2.23E-06	Low	0	63	XP_003482497.1	ssc:100738765	0	NM_024574.3	NDNF
LOC102165534	0	52.05553742	-20	2.23E-06	Low	0	63	XR_308137.1	ssc:102165534			
TRIM55	0.828928824	66.92854811	-6.335229679	2.26E-06	Low	1	81	NM_001244691.1	ssc:100152010	0	NM_184087.1	TRIM55
LOC102162336	2.486786471	94.19573438	-5.243307189	2.28E-06	Low	3	114	XR_297593.1	ssc:102162336			
LOC102161923	66.31430589	0.826278372	6.326548419	2.35E-06	High	80	1	XR_299940.1	ssc:102161923			
LOC102159364	66.31430589	0.826278372	6.326548419	2.35E-06	High	80	1	XR_305572.1	ssc:102159364			
TCTEX1D1	51.39358706	0	20	2.38E-06	High	62	0	NM_001243355.1	ssc:100628189	4E-83	NM_152665.2	TCTEX1D1
LOC102161709	51.39358706	0	20	2.38E-06	High	62	0	XR_299330.1	ssc:102161709			
LOC102163762	51.39358706	0	20	2.38E-06	High	62	0	XR_307146.1	ssc:102163762			
ZAN	51.39358706	0	20	2.38E-06	High	62	0	NM_214383.1	ssc:397676	0	NM_173059.1	ZAN
LOC102165390	51.39358706	0	20	2.38E-06	High	62	0	XP_005665019.1	ssc:102165390	7E-66	NM_005427.3	TP73
CCDC63	51.39358706	0	20	2.38E-06	High	62	0	XP_005670705.1	ssc:100514603	6E-114	NM_152591.2	CCDC63
HOXD13	51.39358706	0	20	2.38E-06	High	62	0	XP_003483728.1	ssc:100152793	1E-161	NM_000523.3	HOXD13
PARM1	1037.818887	62.77915625	4.046711658	2.39E-06	High	1252	76	NM_001243716.1	ssc:100511208	3E-95	NM_015393.3	PARM1
LOC100736922	7.460359412	171.0396229	-4.518941632	2.39E-06	Low	9	207	XP_005660194.1	ssc:100736922	1E-52	NM_003383.3	VLDLR
LOC100156782	411.9776253	23.13579441	4.154367444	2.39E-06	High	497	28	XP_005659972.1	ssc:100156782			
LOC100622410	237.9025724	3927.301101	-4.045095435	2.41E-06	Low	287	4753	LOC100622410				
LOC100517858	79.57716707	1310.477498	-4.041594231	2.41E-06	Low	96	1586	XR_131003.3	ssc:100517858			
ST8SIA4	59.6828753	989.055211	-4.050662111	2.45E-06	Low	72	1197	NM_001037322.1	ssc:641356	0	NM_175052.2	ST8SIA4
ACE2	79.57716707	1.652556743	5.589582825	2.47E-06	High	96	2	NM_001123070.1	ssc:100144303			
GNB3	21.55214941	386.698278	-4.165304677	2.51E-06	Low	26	468	NM_001243493.1	ssc:100154522	0	NM_002075.2	GNB3

KAZALD1	796.6005995	47.92414556	4.05503195	2.51E-06	High	961	58	NM_001244551.1	ssc:100624902	6E-146	NM_030929.4	KAZALD1
PRSS22	60.51180412	998.9705514	-4.045153646	2.52E-06	Low	73	1209	XP_005655249.1	ssc:100519474	3E-137	NM_022119.3	PRSS22
BDKRB1	0	51.22925905	-20	2.53E-06	Low	0	62	NM_001113064.1	ssc:100127469	8E-141	NM_000710.3	BDKRB1
AZGP1	0	51.22925905	-20	2.53E-06	Low	0	62	XP_003124355.2	ssc:100519648	2E-114	NM_001185.3	AZGP1
LOC102167899	0	51.22925905	-20	2.53E-06	Low	0	62	XR_298582.1	ssc:102167899			
AARD	105.2739606	3.305113487	4.993305011	2.58E-06	High	127	4	XP_003125560.1	ssc:100522252	4E-36	NM_001025357.2	AARD
FLRT2	344.0054618	5674.053579	-4.043876398	2.59E-06	Low	415	6867	XP_005656501.1	ssc:100151812	0	NM_013231.4	FLRT2
LOC100512333	65.48537706	0.826278372	6.308401073	2.61E-06	High	79	1	XR_297265.1	ssc:100512333			
F7	130.1418253	4.95767023	4.714278573	2.68E-06	High	157	6	NM_001044591.1	ssc:733661	0	NR_051961.1	F7
VPS13A	1920.628084	118.9840855	4.012737652	2.70E-06	High	2317	144	XP_003480587.2	ssc:100153747	0	NM_033305.2	VPS13A
LOC102159108	78.74823824	1.652556743	5.574475933	2.70E-06	High	95	2	XP_005671338.1	ssc:102159108	1E-58	NM_152429.4	FGFBP3
SH2D2A	958.2417201	58.66576439	4.029798887	2.71E-06	High	1156	71	XP_005663320.1	ssc:100153265	3E-154	NM_003975.3	SH2D2A
KLHL6	50.56465824	0	20	2.72E-06	High	61	0	XP_003358750.2	ssc:100625137	0	NM_130446.2	KLHL6
KCNK13	50.56465824	0	20	2.72E-06	High	61	0	NM_001244596.1	ssc:100623906	0	NM_022054.3	KCNK13
LOC102163618	50.56465824	0	20	2.72E-06	High	61	0	XR_305016.1	ssc:102163618			
LOC102166633	50.56465824	0	20	2.72E-06	High	61	0	XR_306669.1	ssc:102166633			
GFOD1	634.9594789	38.0088051	4.06225895	2.73E-06	High	766	46	XP_001928478.1	ssc:100154602	0	NR_038459.1	GFOD1
MAMDC2	4.973572942	130.5519827	-4.714197923	2.74E-06	Low	6	158	NM_001244983.1	ssc:100514623	9E-69	NM_153267.4	MAMDC2
LOC100622295	28.18358	484.1991258	-4.102673689	2.77E-06	Low	34	586	XP_005674228.1	ssc:100622295	4E-116	NM_000599.3	IGFBP5
LOC102165757	3.315715294	104.9373532	-4.984064362	2.77E-06	Low	4	127	XR_309548.1	ssc:102165757			
NGEF	49.73572942	818.8418664	-4.041230327	2.77E-06	Low	60	991	XP_005672344.1	ssc:100153801	0	NM_019850.2	NGEF
CCNA1	116.8789641	4.131391859	4.822243582	2.81E-06	High	141	5	XP_005668399.1	ssc:100156017	0	NM_003914.3	CCNA1
DDIT3	147.5493306	2378.029154	-4.010497121	2.83E-06	Low	178	2878	NM_001144845.1	ssc:100240743	3E-85	NM_004083.5	DDIT3
LOC102159707	43.93322765	726.2986887	-4.047178576	2.84E-06	Low	53	879	XR_304052.1	ssc:102159707			
PGAM2	9.947145883	204.9170362	-4.364613485	2.88E-06	Low	12	248	NM_001134968.1	ssc:100188980	1E-144	NM_000290.3	PGAM2
MYH14	0	50.40298068	-20	2.89E-06	Low	0	61	XP_005664827.1	ssc:100525110	0	NM_024729.3	MYH14
KRT81	0	50.40298068	-20	2.89E-06	Low	0	61	XP_003126201.1	ssc:100523123	0	NM_002281.3	KRT81
LOC102164702	1.657857647	78.49644531	-5.565235284	2.89E-06	Low	2	95	XP_005661990.1	ssc:102164702	2E-44	NM_133457.3	COL26A1
SAMD12	64.65644824	0.826278372	6.290022543	2.89E-06	High	78	1	XP_003481395.1	ssc:100738445	2E-30	NR_109794.1	SAMD12
LOC102164131	465.8579989	27.26718627	4.094652525	2.90E-06	High	562	33	XR_307100.1	ssc:102164131			
IFITM2	433.5297747	7100.210048	-4.033658719	2.91E-06	Low	523	8593	NM_001246214.1	ssc:100620056	3E-30	NR_049759.1	IFITM3
MAP1A	392.0833336	6397.873432	-4.028360229	2.94E-06	Low	473	7743	XP_005659735.1	ssc:100520470	0	NM_002373.5	MAP1A
FSCN2	20.72322059	366.867597	-4.145939352	2.96E-06	Low	25	444	XP_003357978.1	ssc:100625228	0	NM_012418.3	FSCN2
THSD4	1874.20807	117.3315288	3.997618482	2.96E-06	High	2261	142	XP_003121818.3	ssc:100524417	0	NM_024817.2	THSD4
ANKRD6	178.2196971	8.262783717	4.430885079	2.97E-06	High	215	10	XP_005674610.1	ssc:102167335	0	NM_014942.4	ANKRD6
LOC100157318	153.3518324	6.610226974	4.536001785	2.99E-06	High	185	8	XP_005670140.1	ssc:100157318	2E-83	NM_001647.3	APOD
SPTBN4	128.4839677	4.95767023	4.695782229	2.99E-06	High	155	6	XP_005655898.1	ssc:100626544	0	NM_025213.2	SPTBN4
NEFM	22.38107824	390.8296698	-4.126188547	3.04E-06	Low	27	473	XP_005670496.1	ssc:100625407	0	NM_005382.2	NEFM
TN-X	1696.817302	106.58991	3.992688456	3.06E-06	High	2047	129	NM_001123204.1	ssc:445520	0	NM_032470.3	TNXB
LOC102167808	400.3726218	23.13579441	4.113144781	3.07E-06	High	483	28	XP_005673912.1	ssc:102167808			
LOC102164245	0.828928824	64.44971299	-6.280781895	3.09E-06	Low	1	78	XP_005658386.1	ssc:102164245	2E-49	NM_001430.4	EPAS1
LOC100737830	0.828928824	64.44971299	-6.280781895	3.09E-06	Low	1	78	LOC100737830				
S100A7	49.73572942	0	20	3.10E-06	High	60	0	XP_005663471.1	ssc:100144623	6E-27	NM_002963.3	S100A7
LOC100737386	49.73572942	0	20	3.10E-06	High	60	0	XP_003481085.1	ssc:100737386	3E-169	NM_145865.2	ANKS4B
MN1	130.9707541	2088.005445	-3.994808876	3.11E-06	Low	158	2527	XP_005657395.1	ssc:100156493	0	NM_002430.2	MN1
LOC100516594	1.657857647	77.67016694	-5.549968527	3.16E-06	Low	2	94	XP_003123294.2	ssc:100516594	2E-82	NM_022377.3	ICAM4
LOC102167554	102.7871741	3.305113487	4.958816635	3.16E-06	High	124	4	XR_305889.1	ssc:102167554			
TLX2	115.2211065	4.131391859	4.801633302	3.17E-06	High	139	5	XP_003125043.1	ssc:100514453	1E-108	NM_016170.4	TLX2
WNT10B	16.57857647	301.5916057	-4.185204235	3.18E-06	Low	20	365	NM_001287016.1	ssc:100126276	0	NM_003394.3	WNT10B
LOC100626713	77.0903806	1.652556743	5.543779135	3.22E-06	High	93	2	XP_005674123.1	ssc:100626713	0	NM_207499.2	PTCHD4
ATG9B	774.2195212	47.92414556	4.013918069	3.23E-06	High	934	58	NM_001190280.1	ssc:100462720	0	NR_073169.1	ATG9B
LOC102157611	0	49.5767023	-20	3.30E-06	Low	0	60	XP_005674347.1	ssc:102157611	2E-13	NR_046000	IRF4
LOC100621926	92.84002824	1472.428058	-3.987306375	3.30E-06	Low	112	1782	XP_005654593.1	ssc:100621926	1E-44	NM_003641.3	IFITM1
RANBP3L	7.460359412	164.429396	-4.462079295	3.36E-06	Low	9	199	XP_003133942.4	ssc:100517433	0	NM_145000.3	RANBP3L
LOC100628148	3.315715294	102.4585181	-4.949575986	3.39E-06	Low	4	124	LOC100628148				
LOC100524008	418.6090559	6728.384781	-4.006584785	3.41E-06	Low	505	8143	XP_005660013.1	ssc:100524008	1E-35	NM_053064.4	GNG2
RNF182	76.26145177	1.652556743	5.52818228	3.53E-06	High	92	2	XP_005665645.1	ssc:100154575	4E-136	NM_152737.3	RNF182
WNK4	629.1569771	38.83508347	4.017987548	3.53E-06	High	759	47	XP_003358083.1	ssc:100520693	0	NM_032387.4	WNK4
LOC100525572	50.56465824	808.9265259	-3.999807388	3.55E-06	Low	61	979	XP_005663306.1	ssc:100525572	2E-14	NM_002348.3	LY9
ANKS4B	48.90680059	0	20	3.55E-06	High	59	0	XP_003124606.1	ssc:100525512	0	NM_145865.2	ANKS4B
C6H1orf173	48.90680059	0	20	3.55E-06	High	59	0	XP_005653487.1	ssc:100514238	0	NM_001002912.4	C1orf173
LOC102167461	48.90680059	0	20	3.55E-06	High	59	0	XP_005669384.1	ssc:102167461			
LOC100522128	13.26286118	248.7097899	-4.228999352	3.56E-06	Low	16	301	XP_005655108.1	ssc:100522128	2E-121	NM_002735.2	PRKAR1B
LOC100739389	271.0597253	14.87301069	4.187842148	3.62E-06	High	327	18	XP_005655045.1	ssc:100739389	1E-77	NR_003149.1	GPR98
ARPP21	4.144644118	114.0264153	-4.781976038	3.62E-06	Low	5	138	XP_005669408.1	ssc:100512986	0	NM_198399.1	ARPP21
LOC100519456	48.90680059	780.8330613	-3.996907145	3.66E-06	Low	59	945	XP_005673355.1	ssc:100519456	6E-134	NM_024014.3	HOXA6
PARP9	155.00969	2427.605856	-3.969103896	3.66E-06	Low	187	2938	XP_005670226.1	ssc:100519390	0	NM_031458.2	PARP9
LOC100621949	0	48.75042393	-20	3.77E-06	Low	0	59	XP_005667121.1	ssc:100621949	2E-98	NM_003621.3	PPFIBP2
SEL1L3	0	48.75042393	-20	3.77E-06	Low	0	59	XP_003128937.3	ssc:100518082	0	NM_015187.3	SEL1L3
ZNF614	0	48.75042393	-20	3.77E-06	Low	0	59	ZNF614	ssc:100516977	0	NM_025040.3	ZNF614
SSTR1	1.657857647	76.0176102	-5.518941632	3.77E-06	Low	2	92	NM_001190231.1	ssc:397004	0	NM_001049.2	SSTR1
LOC102161873	112.73432	4.131391859	4.770155071	3.81E-06	High	136	5	XR_305513.1	ssc:102161873			
LOC102160583	3.315715294	100.8059614	-4.926117013	3.89E-06	Low	4	122	XR_302321.1	ssc:102160583			
SPARC	1571.64905	25894.73789	-4.042307952	3.92E-06	Low	1896	31339	NM_001031794.1	ssc:595124	2E-89	NM_003118.3	SPARC
PPM1E	1610.608704	104.1110748	3.951410586	3.95E-06	High	1943	126	XP_005669053.1	ssc:100518751	0	NR_048561.1	PPM1E
SSX2IP	6022.167903	383.3931645	3.973386412	3.97E-06	High	7265	464	XR_298669.1	ssc:100522497	0	NM_014021.3	SSX2IP
THSD1	4.973572942	124.7680341	-4.648821914	4.02E-06	Low	6	151	XP_005668435.1	ssc:100513373	0	NM_199263.2	THSD1
XK	339.8608177	19.83068092	4.099137923	4.03E-06	High	410	24	XP_003135062.1	ssc:100511169	0	NM_021083.2	XK
PARVA	713.7077171	11429.08244	-4.001232402	4.03E-06	Low	861	13832	XP_005652864.1	ssc:100518536	2E-168	NM_018222.4	PARVA
RAPGEF5	915.9663501	58.66576439	3.964703859	4.03E-06	High	1105	71	XP_005656743.1	ssc:100518983	0	NM_012294.3	RAPGEF5
PPP4R4	21.55214941	365.2150403	-4.082842517	4.13E-06	Low	26	442	XP_003482359.1	ssc:100737576			
LOC102164159	1.657857647	75.19133183	-5.503174316	4.13E-06	Low	2	91	XP_005666956.1	ssc:102164159	5E-52	NM_016599.4	MYOZ2
CNKSR2	765.1013042	48.75042393	3.972164113	4.14E-06	High	923	59	XP_005673548.1	ssc:100523478	0	NM_014927.3	CNKSR2
LOC100627072	556.2112406	34.70369161	4.002471858	4.17E-06	High	671	42	XR_309386.1	ssc:100627072			
LOC100620614	31.4992953	510.6400337	-4.01891519	4.18E-06	Low	38	618	XP_005669253.1	ssc:100620614	0	NM_000697.2	ALOX12
LOC100512951	63.82751942	992.3603244	-3.958613571	4.21E-06	Low	77	1201	XR_298143.1	ssc:100512951			
IL4R	237.0736436	3674.459919	-3.954125028	4.21E-06	Low	286	4447	NM_214340.1	ssc:397614			
WNT3	159.1543341	7.436505346	4.419657824	4.23E-06	High	192	9	XP_005668754.1	ssc:100519512			
NKX3-2	17.4075053	303.2441624	-4.122698506	4.30E-06	Low	21	367	XP_003356908.1	ssc:100625162	5E-141	NM_001189.3	NKX3-2
IFNE	0	47.92414556	-20	4.32E-06	Low	0	58	NM_001105310.1	ssc:100125969	2E-80	NM_176891.4	IFNE
LOC102160618	0	47.92414556	-20	4.32E-06	Low	0	58	XP_005672260.1	ssc:102160618			
KIAA1755	0	47.92414556	-20	4.32E-06	Low	0	58	XP_003134484.3	ssc:100516472	0	NM_001029864.1	KIAA1755
OAS2	562.0137424	8866.793207	-3.979735116	4.32E-06	Low	678	10731	NM_001031796.1	ssc:595128			
ARHGEF2	1036.16103	16653.64058	-4.006517459	4.33E-06	Low	1250	20155	NM_001128463.1	ssc:100145887	0	NM_004723.3	ARHGEF2
LOC102166540	1.657857647	74.36505346	-5.487232772	4.52E-06	Low	2	90	XR_304431.1	ssc:102166540			
LOC102162076	623.3544753	39.66136184	3.974246675	4.54E-06	High	752	48	XP_005666211.1	ssc:102162076	3E-65	NM_006465.2	ARID3B
PPIF	3464.093554	226.4002739	3.935530285	4.55E-06	High	4179	274	XP_001929518.1	ssc:100152612	9E-101	NM_005729.3	PPIF
PKD2	528.8565895	8270.220223	-3.966977283	4.60E-06	Low	638	10009	NM_001245979.1	ssc:641309	0	NM_000297.3	PKD2
HYDIN	73.7746653	1.652556743	5.480353755	4.64E-06	High	89	2	XP_003126939.3	ssc:100513208			
LOC102160247	73.7746653	1.652556743	5.480353755	4.64E-06	High	89	2	XP_005669705.1	ssc:102160247			

LOC100620269	533.0012336	8327.23343	-3.965626489	4.65E-06	Low	643	10078	XP_005658558.1	ssc:100620269	4E-78	NM_016201.3	AMOTL2
DNAJC22	47.24894295	0	20	4.67E-06	High	57	0	XP_005655614.1	ssc:100513730	1E-157	NM_024902.2	DNAJC22
SMTN	208.0611347	3178.692896	-3.933354236	4.70E-06	Low	251	3847	NM_001244360.1	ssc:414369	0	NM_134270.2	SMTN
NRG3	262.7704371	14.87301069	4.143034353	4.73E-06	High	317	18	XP_001927043.2	ssc:100158106			
LOC102159630	0.828928824	61.14459951	-6.204833041	4.74E-06	Low	1	74	XR_305716.1	ssc:102159630			
PHYHIP	547.9219524	34.70369161	3.980809363	4.76E-06	High	661	42	XP_001927945.1	ssc:100155255	0	NM_014759.3	PHYHIP
LOC100628052	83.72181118	1274.947528	-3.928690539	4.77E-06	Low	101	1543	XP_003360600.1	ssc:100628052	1E-50	NM_020130.4	C8orf4
LYL1	100.3003877	1522.831039	-3.924356794	4.81E-06	Low	121	1843	XP_005652937.1	ssc:100523949	4E-123	NM_005583.4	LYL1
KRAB	643.2487671	41.31391859	3.960676977	4.82E-06	High	776	50	NM_001123132.1	ssc:100141415	1E-171	NM_001109809.2	ZFP57
SLC36A4	53.88037353	829.5834852	-3.944555417	4.84E-06	Low	65	1004	XP_005667282.1	ssc:100511947	0	NM_152313.3	SLC36A4
LOC100624393	0	47.09786719	-20	4.95E-06	Low	0	57	XP_003362153.1	ssc:100624393	0	NM_001145030.1	TOPAZ1
LOC100621405	0	47.09786719	-20	4.95E-06	Low	0	57	XP_005658046.1	ssc:100621405	1E-123	NM_006492.2	ALX3
LOC100524378	0	47.09786719	-20	4.95E-06	Low	0	57	XP_005664828.1	ssc:100524378	3E-13	XP_003960829.2	LOC100287477
LOC100157899	60.51180412	0.826278372	6.194444883	4.97E-06	High	73	1	XP_005658300.1	ssc:100157899	0	NM_001115.2	ADCY8
GPR37	461.7133547	28.91974301	3.996870825	4.98E-06	High	557	35	XP_003134766.2	ssc:100523220	0	NM_005302.3	GPR37
ARHGAP4	132.6286118	5.783948602	4.519193497	5.01E-06	High	160	7	XP_003135532.1	ssc:100523659	0	NM_001666.4	ARHGAP4
TNFRSF1B	175.7329106	2657.311243	-3.918510912	5.04E-06	Low	212	3216	NM_001097441.2	ssc:100037306			
LOC100736679	4.973572942	121.4629206	-4.61008952	5.05E-06	Low	6	147	XP_003482978.1	ssc:100736679	1E-73	NM_004791.2	ITGBL1
DKK3	640.7619806	9967.395998	-3.959356193	5.07E-06	Low	773	12063	NM_001039749.1	ssc:664653	4E-151	NM_015881.5	DKK3
LOC100155289	72.94573648	1.652556743	5.464051943	5.09E-06	High	88	2	XP_005672733.1	ssc:100155289	3E-174	NM_014915.2	ANKRD26
SMAD9	91.1821706	1375.753489	-3.915326424	5.12E-06	Low	110	1665	NM_001195510.1	ssc:100153584	0	NM_005905.5	SMAD9
IL6	19.06536294	320.5960082	-4.071730562	5.15E-06	Low	23	388	NM_214399.1	ssc:399500	2E-68	NM_000600.3	IL6
LOC100621366	19.06536294	320.5960082	-4.071730562	5.15E-06	Low	23	388	XP_005670111.1	ssc:100621366	0	NM_004443.3	EPHB3
LOC100621416	82.06395354	1238.591279	-3.915807724	5.18E-06	Low	99	1499	XP_005658770.1	ssc:100621416	1E-156	NR_046095.1	DACT1
LOC102162371	780.8509518	51.22925905	3.930007264	5.23E-06	High	942	62	XP_005662335.1	ssc:102162371	0	NM_153214.2	FBLN7
CXCL12	35.64393941	556.9116225	-3.965719702	5.26E-06	Low	43	674	NM_001009580.1	ssc:494460	9E-40	NM_199168.3	CXCL12
PTK2B	86.20859765	1297.257044	-3.911488801	5.28E-06	Low	104	1570	XP_005657321.1	ssc:100157507	0	NM_173176.2	PTK2B
LOC100626977	20.72322059	342.9055243	-4.048491012	5.32E-06	Low	25	415	XP_003361612.2	ssc:100626977	5E-108	NR_033519.1	MASP1
SPATC1	46.42001412	0	20	5.37E-06	High	56	0	XP_005655380.1	ssc:100739839			
KRT5	46.42001412	0	20	5.37E-06	High	56	0	XP_005652613.1	ssc:100511564	0	NM_000424.3	KRT5
MOBP	46.42001412	0	20	5.37E-06	High	56	0	XR_306740.1	ssc:100152154			
LOC102162659	46.42001412	0	20	5.37E-06	High	56	0	XP_005672743.1	ssc:102162659	3E-109	NM_014915.2	ANKRD26
LRIT3	46.42001412	0	20	5.37E-06	High	56	0	XP_003129299.2	ssc:100516451	0	NM_198506.4	LRIT3
LOC100737490	84.55074001	2.478835115	5.092083166	5.39E-06	High	102	3	XP_003483716.2	ssc:100737490	0	NM_006920.4	SCN1A
CBLN4	1.657857647	72.71249671	-5.454811294	5.44E-06	Low	2	88	NM_001123092.1	ssc:100141408	1E-113	NM_080617.5	CBLN4
MGAT3	7.460359412	155.3403339	-4.380043526	5.45E-06	Low	9	188	XP_005674708.1	ssc:100625969	3E-132	NM_002409.4	MGAT3
GHR	18.23643412	306.5492759	-4.071223434	5.48E-06	Low	22	371	NM_214254.2	ssc:397488	0	NM_001242462.1	GHR
TGFA	59.6828753	0.826278372	6.174545326	5.56E-06	High	72	1	NM_214251.1	ssc:397484			
NPY2R	72.11680765	1.652556743	5.44756382	5.59E-06	High	87	2	NM_214150.1	ssc:397291	0	NM_000910.2	NPY2R
LOC102159229	0	46.27158882	-20	5.69E-06	Low	0	56	XP_005673115.1	ssc:102159229	9E-52	NR_003259.1	GNAS
LOC102162209	0	46.27158882	-20	5.69E-06	Low	0	56	XR_304023.1	ssc:102162209			
LOC102167982	0	46.27158882	-20	5.69E-06	Low	0	56	XR_303713.1	ssc:102167982			
KIAA1210	8.289288236	166.0819527	-4.324503272	5.70E-06	Low	10	201	XP_005673928.1	ssc:100518589	0	NM_020721.1	KIAA1210
FLT1	135.1153982	2003.725051	-3.890420553	5.88E-06	Low	163	2425	XP_001925775.4	ssc:396763	0	NM_002019.4	FLT1
SERPINB5	130.1418253	5.783948602	4.491886151	5.88E-06	High	157	7	XP_001924663.2	ssc:100155836	0	NM_002639.4	SERPINB5
LOC100624195	2704.794751	182.6075202	3.888701037	5.90E-06	High	3263	221	XP_003357314.2	ssc:100624195	0	NM_024606.2	DYNC2H1
KCNS3	4821.050038	322.248565	3.903101565	5.90E-06	High	5816	390	NM_001044596.1	ssc:733666	0	NM_002252.4	KCNS3
LOC100512714	0.828928824	59.49204276	-6.165304677	5.91E-06	Low	1	72	XP_005663639.1	ssc:100512714	1E-126	NM_006492.2	ALX3
LOC100512370	44.76215647	679.2008216	-3.923448757	5.95E-06	Low	54	822	XR_309610.1	ssc:100512370			
MT-2B	149.2071882	2206.163253	-3.886150606	6.07E-06	Low	180	2670	XP_003355856.1	ssc:396827	2E-14	NM_005953.3	MT2A
SCML2	2200.806027	149.5563853	3.879270583	6.14E-06	High	2655	181	XP_005673528.1	ssc:100519636	0	NR_033717.1	SCML2
LBX1	71.28787883	1.652556743	5.430885079	6.15E-06	High	86	2	NM_001206336.1	ssc:100155030	3E-145	NM_006562.4	LBX1
LOC102157666	45.5910853	0	20	6.19E-06	High	55	0	XR_298274.1	ssc:102157666			
MIP	45.5910853	0	20	6.19E-06	High	55	0	XP_003126321.1	ssc:100522373	1E-139	NM_012064.3	MIP
SP6	45.5910853	0	20	6.19E-06	High	55	0	XP_003131582.1	ssc:100519513	0	NM_199262.2	SP6
LOC102160572	45.5910853	0	20	6.19E-06	High	55	0	XR_299472.1	ssc:102160572			
PAPPA2	45.5910853	0	20	6.19E-06	High	55	0	XP_003130378.3	ssc:100521390	0	NM_021936.2	PAPPA2
CHRNG	56.36716001	841.9776608	-3.900855171	6.20E-06	Low	68	1019	XP_003133779.1	ssc:100522221	0	NM_005199.4	CHRNG
TMEM27	58.85394648	0.826278372	6.154367444	6.22E-06	High	71	1	XP_005673508.1	ssc:102167302	9E-32	NM_020665.5	TMEM27
CALML4	25.69679353	405.7026805	-3.98076258	6.29E-06	Low	31	491	NM_001244619.1	ssc:100519528	2E-78	NR_104583.1	CALML4
LOC100513601	384.6229742	5755.855137	-3.903513438	6.30E-06	Low	464	6966	NM_001246242.1	ssc:100513601	3E-145	NM_002116.7	HLA-A
POLR3G	4594.752469	309.8543894	3.890324831	6.33E-06	High	5543	375	XP_005652997.1	ssc:100519356	9E-94	NM_006467.2	POLR3G
LOC102159786	82.89288236	2.478835115	5.063514013	6.35E-06	High	100	3	XR_299227.1	ssc:102159786			
SLC9A7	151.6939747	7.436505346	4.350395161	6.36E-06	High	183	9	XP_003135112.2	ssc:100524182	0	NM_032591.2	SLC9A7
MREG	436.84549	28.09346464	3.958816635	6.42E-06	High	527	34	XP_003359689.1	ssc:100627946	8E-103	NM_018000.2	MREG
LOC100627578	1474.664377	100.8059614	3.870733782	6.47E-06	High	1779	122	XP_003360596.1	ssc:100627578	2E-41	NM_001127688.2	BEX4
LOC102157563	162.4700494	8.262783717	4.297402074	6.55E-06	High	196	10	XP_005659473.1	ssc:102157563			
ALOX5	162.4700494	8.262783717	4.297402074	6.55E-06	High	196	10	XP_005671286.1	ssc:100156205	0	NM_001256154.1	ALOX5
LOC102166984	1.657857647	71.05993997	-5.42164443	6.56E-06	Low	2	86	XR_304405.1	ssc:102166984			
SHROOM4	0.828928824	58.66576439	-6.145126795	6.62E-06	Low	1	71	XP_005674526.1	ssc:100513967	0	NR_027121.1	SHROOM4
HSD3B7	116.0500353	1697.175776	-3.870317125	6.64E-06	Low	140	2054	XP_005655142.1	ssc:100525281	0	NM_025193.3	HSD3B7
LDHC	116.8789641	4.95767023	4.559209176	6.67E-06	High	141	6	NM_001195775.1	ssc:100502559	6E-156	NM_017448.3	LDHC
ICOSLG	128.4839677	1877.304461	-3.86900239	6.69E-06	Low	155	2272	XP_005657224.1	ssc:100621467	4E-84	NM_015259.5	ICOSLG
NEURL2	17.4075053	287.5448734	-4.046005749	6.80E-06	Low	21	348	XP_005673033.1	ssc:100157993	7E-150	NM_080749.3	NEURL2
LOC100620869	3.315715294	94.19573438	-4.82826969	6.84E-06	Low	4	114	XP_005664934.1	ssc:100620869	6E-84	NM_198539.3	ZNF568
GYG2	40.61751236	609.7934383	-3.908146838	6.87E-06	Low	49	738	XP_005658942.1	ssc:102163050	3E-26	NM_003918.2	GYG2
MVP	641.5909095	9626.143031	-3.907232254	6.98E-06	Low	774	11650	XP_003354597.1	ssc:100622738	0	NM_017458.3	MVP
TARSL2	44.76215647	0	20	7.15E-06	High	54	0	XP_005652533.1	ssc:100523715	0	NM_152334.2	TARSL2
LOC100738279	44.76215647	0	20	7.15E-06	High	54	0	XP_005664249.1	ssc:100738279	0	NM_017564.9	STAB2
PDE6A	44.76215647	0	20	7.15E-06	High	54	0	XP_005661864.1	ssc:100515715	0	NM_000440.2	PDE6A
LOC102167207	44.76215647	0	20	7.15E-06	High	54	0	XP_005661561.1	ssc:102167207	1E-60	NM_014979.1	SV2C
SH3YL1	4.144644118	104.9373532	-4.662136268	7.27E-06	Low	5	127	NM_001244340.1	ssc:100625380			
LOC100512258	96.15574354	1392.279056	-3.855931557	7.30E-06	Low	116	1685	XR_308149.1	ssc:100512258			
LOC100738662	647.3934112	43.7927537	3.885878608	7.33E-06	High	781	53	XP_003483783.1	ssc:100738662	8E-67	NM_018000.2	MREG
NHSL1	3911.715119	269.3667492	3.860157626	7.42E-06	High	4719	326	XP_005659234.1	ssc:100156257	0	NM_020464.1	NHSL1
ABCA4	406.1751236	26.4409079	3.941258263	7.54E-06	High	490	32	XP_001924499.3	ssc:100155583	0	NM_000350.2	ABCA4
LOC102163702	0	44.61903207	-20	7.57E-06	Low	0	54	XR_300485.1	ssc:102163702			
LOC102162300	0	44.61903207	-20	7.57E-06	Low	0	54	XR_304485.1	ssc:102162300			
LOC100525848	852.1388307	58.66576439	3.860497754	7.59E-06	High	1028	71	XP_003135321.1	ssc:100525848	3E-18	NM_194324.2	TMSB15B
FBLN7	828.9288236	57.01320765	3.861880152	7.59E-06	High	1000	69	XP_005662337.1	ssc:100517582	0	NM_153214.2	FBLN7
LOC102162112	632.4726924	42.96647533	3.879719853	7.68E-06	High	763	52	XR_303844.1	ssc:102162112			
LOC100153163	133.4575406	1916.139544	-3.843749902	7.81E-06	Low	161	2319	XP_003128354.1	ssc:100153163	4E-77	NM_005931	MICB
NKX2-3	57.19608883	0.826278372	6.113144781	7.83E-06	High	69	1	XP_003359369.1	ssc:100152347	3E-139	NM_145285.2	NKX2-3
LOC102160550	57.19608883	0.826278372	6.113144781	7.83E-06	High	69	1	XP_005673466.1	ssc:102160550	3E-73	NM_014728.3	FRMPD4
LOC100516245	456.7397818	6696.159924	-3.873889626	7.85E-06	Low	551	8104	XR_298106.1	ssc:100516245			
SCG2	3.315715294	92.54317763	-4.802734598	7.91E-06	Low	4	112	NM_001012299.1	ssc:497237	0	NM_003469.4	SCG2
SLC46A2	23.21000706	361.0836484	-3.959514223	7.93E-06	Low	28	437	XP_005660391.1	ssc:100157182	0	NM_033051.3	SLC46A2
RYR3	1.657857647	69.40738323	-5.387697098	7.94E-06	Low	2	84	XP_003361898.1	ssc:396888	9E-99	NM_001243996.1	RYR3
PN-1	1401.718641	21228.74393	-3.920750312	8.02E-06	Low	1691	25692	NM_214287.1	ssc:397542	0	NR_073116.1	SERPINE2
LOC100739399	594.3419665	40.48764021	3.875739789	8.13E-06	High	717	49	XP_005659354.1	ssc:100739399	2E-79	NM_080743.4	SRSF12

HAPLN2	102.7871741	4.131391859	4.63688854	8.27E-06	High	124	5	XP_003355190.2	ssc:100628124	0	NM_021817.2	HAPLN2
LOC102159402	43.93322765	0	20	8.27E-06	High	53	0	XR_297885.1	ssc:102159402			
LOC102160786	14.09179	236.3156143	-4.067788171	8.34E-06	Low	17	286	XR_301850.1	ssc:102160786			
LOC100154002	2989.117338	209.8747064	3.832119319	8.46E-06	High	3606	254	XP_001928249.1	ssc:100154002	0	NM_032435.2	KIAA1804
LOC102163119	124.3393235	5.783948602	4.426084093	8.63E-06	High	150	7	XP_005657145.1	ssc:102163119	4E-37	NM_024768.2	EFCC1
CD19	1.657857647	68.58110485	-5.370419107	8.75E-06	Low	2	83	NM_214377.1	ssc:397669	0	NM_001770.5	CD19
LOC100622512	0	43.7927537	-20	8.76E-06	Low	0	53	XP_005658491.1	ssc:100622512	7E-164	NM_013992.3	PAX8
ZNF660	0	43.7927537	-20	8.76E-06	Low	0	53	ZNF660	ssc:100513047	1E-93	NM_173658.2	ZNF660
LOC100738887	5.802501765	124.7680341	-4.426429493	8.77E-06	Low	7	151	XP_005653802.1	ssc:100738887	0	NR_038265.1	PPFIA2
STX3	75.43252295	1073.335605	-3.830770751	8.77E-06	Low	91	1299	XP_005660928.1	ssc:100520886	4E-130	NM_004177.4	STX3
LOC102161046	116.0500353	1641.815125	-3.822472816	8.86E-06	Low	140	1987	XR_308191.1	ssc:102161046			
SORD	8995.535594	620.5350572	3.857624559	9.09E-06	High	10852	751	NM_001244162.1	ssc:100158181	0	NR_034039.1	SORD
RASGEF1B	3.315715294	90.89062089	-4.776739389	9.18E-06	Low	4	110	NM_001195367.1	ssc:100337671	4E-67	NM_152545.1	RASGEF1B
LOC102158317	90.35324177	3.305113487	4.772804649	9.25E-06	High	109	4	XR_298831.1	ssc:102158317			
TNXB	708.7341442	49.5767023	3.837510339	9.26E-06	High	855	60	NM_001123204.1	ssc:445520	0	NM_032470.3	TNXB
ROBO2	297.5854477	19.00440255	3.968898402	9.27E-06	High	359	23	XP_005670349.1	ssc:100739586	0	NM_002942.4	ROBO2
CEP152	6117.494718	428.0121966	3.837217138	9.39E-06	High	7380	518	XP_005659693.1	ssc:100152345	0	NM_014985.3	CEP152
LOC100623103	188.166843	10.74161883	4.130729093	9.42E-06	High	227	13	XP_003358562.1	ssc:100623103	1E-42	NM_014850.3	SRGAP3
LOC100620159	101.1293165	4.131391859	4.613429567	9.46E-06	High	122	5	XP_003357925.3	ssc:100620159	0	NM_005845.3	ABCC4
LOC100737799	296.7565188	19.00440255	3.964874146	9.49E-06	High	358	23	XP_003483087.1	ssc:100737799			
IGSF11	660.6562724	46.27158882	3.835701316	9.65E-06	High	797	56	XP_005670253.1	ssc:100511781	0	NM_152538.2	IGSF11
TNMD	1.657857647	67.75482648	-5.35293168	9.65E-06	Low	2	82	NM_001099934.1	ssc:100048961	0	NM_022144.2	TNMD
LOC102166578	1.657857647	67.75482648	-5.35293168	9.65E-06	Low	2	82	XP_005654560.1	ssc:102166578	1E-89	NM_153267.4	MAMDC2
LOC102158142	9.11821706	166.9082311	-4.19415954	9.66E-06	Low	11	202	XP_005661569.1	ssc:102158142			
LOC100738373	4.973572942	112.3738586	-4.497880016	9.67E-06	Low	6	136	XP_003481727.1	ssc:100738373	2E-46	NM_004447.5	EPS8
LRMP	1008.806378	71.88621834	3.810790281	9.71E-06	High	1217	87	XP_005655711.1	ssc:100518428	0	NM_006152.3	LRMP
LOC100738167	155.00969	8.262783717	4.229586689	9.74E-06	High	187	10	XP_003481896.1	ssc:100738167	0	NM_007181.5	MAP4K1
LOC100525798	372.1890418	24.78835115	3.908301363	9.77E-06	High	449	30	XP_003135234.1	ssc:100525798	0	NM_021963.3	NAP1L2
PTER	10.77607471	188.3914688	-4.127829972	9.78E-06	Low	13	228	NM_001244248.1	ssc:100625702	0	NM_030664.4	PTER
LOC102166369	11.60500353	199.1330876	-4.10091409	9.83E-06	Low	14	241	XR_309210.1	ssc:102166369			
LOC102166065	404.5172659	27.26718627	3.890963543	9.89E-06	High	488	33	XP_005659238.1	ssc:102166065	0	NM_020340.4	KIAA1244
LOC100514834	3.315715294	90.06434252	-4.763564	9.90E-06	Low	4	109	XR_303007.1	ssc:100514834			
LOC102157594	55.53823118	0.826278372	6.070709515	9.90E-06	High	67	1	XR_300709.1	ssc:102157594			
PTGDR	89.52431295	3.305113487	4.759507826	9.98E-06	High	108	4	XP_003121861.1	ssc:100524418	4E-145	NM_001281469.1	PTGDR
MSC	31.4992953	461.0633314	-3.871573474	1.01E-05	Low	38	558	XP_003125643.1	ssc:100155518	6E-93	NM_005098.3	MSC
LOC102159666	0	42.96647533	-20	1.01E-05	Low	0	52	XP_005655857.1	ssc:102159666			
NTS	2.486786471	78.49644531	-4.980272783	1.03E-05	Low	3	95	XP_003481806.1	ssc:100739079	1E-82	NM_006183.4	NTS
ARHGAP27	586.8816071	41.31391859	3.828369685	1.07E-05	High	708	50	XP_005668771.1	ssc:100628007	0	NM_199282.2	ARHGAP27
DAPP1	314.1640241	20.65695929	3.926818173	1.07E-05	High	379	25	XP_005667023.1	ssc:100525936	3E-117	NM_014395.2	DAPP1
FOS	1869.234497	135.509653	3.785980038	1.08E-05	High	2255	164	NM_001123113.1	ssc:100144486	0	NM_005252.3	FOS
JAG2	3432.594259	247.0572331	3.796382138	1.08E-05	High	4141	299	XP_005656541.1	ssc:100522743	0	NM_145159.2	JAG2
HPX	4.973572942	110.7213018	-4.476506365	1.09E-05	Low	6	134	NM_213953.2	ssc:396998	0	NM_000613.2	HPX
LOC102165705	32.32822412	467.6735584	-3.8546357	1.10E-05	Low	39	566	XR_301879.1	ssc:102165705			
LOC100739138	628.3280483	44.61903207	3.81578686	1.11E-05	High	758	54	XP_005666063.1	ssc:100739138	5E-128	NM_001164446	C6orf132
EXOC3L1	66.31430589	1.652556743	5.326548419	1.11E-05	High	80	2	XP_005664474.1	ssc:100522868	0	NM_178516.3	EXOC3L1
STMND1	42.27537	0	20	1.11E-05	High	51	0	XP_005656271.1	ssc:100524942	5E-91	NM_001190766.1	STMND1
LOC102164307	42.27537	0	20	1.11E-05	High	51	0	XP_005673335.1	ssc:102164307	8E-90	NR_047565.1	CCDC129
SLFN14	42.27537	0	20	1.11E-05	High	51	0	XP_003358199.2	ssc:100625890	0	NM_001129820.1	SLFN14
LOC102160718	42.27537	0	20	1.11E-05	High	51	0	XR_297394.1	ssc:102160718			
LOC102162598	42.27537	0	20	1.11E-05	High	51	0	XP_005668111.1	ssc:102162598	1E-42	NM_012144.3	DNAI1
LOC102165105	42.27537	0	20	1.11E-05	High	51	0	XR_305120.1	ssc:102165105			
LOC396583	34.81501059	499.0721365	-3.841466992	1.12E-05	Low	42	604	XP_005658936.1	ssc:396583	4E-108	NM_005985.3	SNAI1
PHLDA1	306.7036647	4271.859182	-3.79994678	1.13E-05	Low	370	5170	XP_003126437.1	ssc:100523904	2E-134	NM_007350.3	PHLDA1
NFKBIZ	132.6286118	1825.248923	-3.782629284	1.13E-05	Low	160	2209	XP_005657172.1	ssc:100520981	0	NM_031419.3	NFKBIZ
LPAR3	109.4186047	4.95767023	4.464051943	1.16E-05	High	132	6	NM_001162402.1	ssc:100113360	5E-175	NM_012152.2	LPAR3
TENM1	1.657857647	66.10226974	-5.317307771	1.18E-05	Low	2	80	XP_005673947.1	ssc:100520416	0	NM_014253.3	TENM1
SCN4B	0	42.14019696	-20	1.18E-05	Low	0	51	XP_003129950.1	ssc:100512800	5E-89	NR_024527.1	SCN4B
SST	0	42.14019696	-20	1.18E-05	Low	0	51	NM_001009583.1	ssc:494469	2E-61	NM_001048.3	SST
PHYH	273.5465118	3767.003097	-3.783559155	1.22E-05	Low	330	4559	NM_001113447.1	ssc:100134962	4E-118	NM_006214.3	PHYH
PACSIN1	150.8650459	8.262783717	4.19048687	1.22E-05	High	182	10	XP_001926343.3	ssc:100157037	0	NM_020804.4	PACSIN1
CLCA1	65.48537706	1.652556743	5.308401073	1.22E-05	High	79	2	NM_214148.1	ssc:397284	0	NM_001285.3	CLCA1
LOC100517850	65.48537706	1.652556743	5.308401073	1.22E-05	High	79	2	XR_306300.1	ssc:100517850			
TAL1	7171.892182	514.7714256	3.800349921	1.23E-05	High	8652	623	XR_298705.1	ssc:100521731	4E-145	NM_003189	TAL1
PHYHD1	3093.56237	226.4002739	3.772321512	1.23E-05	High	3732	274	XP_003122259.1	ssc:100157556	1E-147	NM_174933.3	PHYHD1
LOC100524517	881.9802683	64.44971299	3.774500541	1.24E-05	High	1064	78	XP_005653846.1	ssc:100524517	2E-175	NM_003240.3	LEFTY2
EGR2	203.0875618	12.39417558	4.034367668	1.24E-05	High	245	15	NM_001097488.1	ssc:100038004	0	NM_001136179.1	EGR2
TSPO	215.5214941	2946.508674	-3.77310285	1.25E-05	Low	260	3566	NM_213753.1	ssc:396592	4E-77	NR_046308.1	TSPO
LOC100622032	53.88037353	0.826278372	6.026988137	1.26E-05	High	65	1	XP_005657229.1	ssc:100622032	0	NR_038257.1	TRPM2
LOC100623510	47.24894295	655.2387488	-3.793666717	1.26E-05	Low	57	793	XP_003362027.1	ssc:100623510	4E-46	NM_006272.2	S100B
TUB	80.40609589	1095.645121	-3.768331894	1.26E-05	Low	97	1326	XP_005667117.1	ssc:100520318	0	NM_177972.2	TUB
SLC13A5	402.8594083	28.09346464	3.841969987	1.29E-05	High	486	34	XP_003131921.2	ssc:100520976	0	NM_177550.4	SLC13A5
PAOX	3362.964237	247.0572331	3.766816237	1.29E-05	High	4057	299	XP_005674486.1	ssc:100626281	6E-144	NR_109766.1	PAOX
PEAR1	186.5089853	2534.195766	-3.764210936	1.29E-05	Low	225	3067	XP_005663319.1	ssc:100156908	0	NM_207369.1	PEAR1
LOC102157540	41.44644118	0	20	1.30E-05	High	50	0	LOC102157540				
LOC102161357	41.44644118	0	20	1.30E-05	High	50	0	XR_299270.1	ssc:102161357			
LOC102157836	41.44644118	0	20	1.30E-05	High	50	0	XR_302130.1	ssc:102157836			
LOC102158737	41.44644118	0	20	1.30E-05	High	50	0	XR_305607.1	ssc:102158737			
LOC102163174	41.44644118	0	20	1.30E-05	High	50	0	XR_307554.1	ssc:102163174			
LOC102158339	1.657857647	65.27599137	-5.299160424	1.30E-05	Low	2	79	XR_308249.1	ssc:102158339			
LOC100621769	843.0206136	61.97087788	3.765905598	1.32E-05	High	1017	75	XP_005658187.1	ssc:100621769	5E-121	NM_004734.4	DCLK1
LOC100626660	10.77607471	181.7812418	-4.076299671	1.32E-05	Low	13	220	LOC100626660				
LOC100520252	54.70930236	749.4344832	-3.775944297	1.32E-05	Low	66	907	XP_005665214.1	ssc:100520252	9E-58	NM_178865.4	SERINC2
LOXL3	92.01109942	1244.375228	-3.757469864	1.32E-05	Low	111	1506	XP_003361946.1	ssc:100626748	0	NM_032603	LOXL3
NUDT7	35.64393941	499.8984149	-3.809906253	1.33E-05	Low	43	605	XP_003484264.1	ssc:100519193	8E-95	NM_001243661.1	NUDT7
SGSM1	0.828928824	53.70809416	-6.017747489	1.34E-05	Low	1	65	XP_005670818.1	ssc:100152606	0	NM_133454.2	SGSM1
HS3ST2	625.012333	45.44531045	3.781681324	1.34E-05	High	754	55	XP_003124592.1	ssc:100522426	0	NM_006043.1	HS3ST2
LOC100623570	3.315715294	86.75922903	-4.709625193	1.34E-05	Low	4	105	XP_005658162.1	ssc:100623570	5E-52	NM_207336.1	ZNF467
ENTPD8	64.65644824	1.652556743	5.290022543	1.36E-05	High	78	2	XP_005652794.1	ssc:100516719	0	NM_198585.2	ENTPD8
LOC100525229	86.20859765	3.305113487	4.705060042	1.36E-05	High	104	4	XP_003127903.2	ssc:100525229			
LOC102167069	86.20859765	3.305113487	4.705060042	1.36E-05	High	104	4	XP_005664425.1	ssc:102167069	2E-37	NM_015020	PHLPP2
LOC102167347	0	41.31391859	-20	1.37E-05	Low	0	50	XR_309153.1	ssc:102167347			
LOC102167351	0	41.31391859	-20	1.37E-05	Low	0	50	XP_005652885.1	ssc:102167351	7E-152	NM_152601.3	ZNF709
ZDHHC8	150.8650459	2027.687124	-3.748504569	1.39E-05	Low	182	2454	ZDHHC8	ssc:100157140	0	NM_013373.3	ZDHHC8
DUSP10	9.947145883	169.3870662	-4.089897274	1.45E-05	Low	12	205	XP_003357676.1	ssc:100517416	0	NM_144729.2	DUSP10
IFT27	36.47286824	505.6823635	-3.7933359	1.45E-05	Low	44	612	XP_003126110.3	ssc:100517032	1E-90	NR_033531.2	IFT27
CD27	168.2725512	9.915340461	4.084993741	1.46E-05	High	203	12	NM_001278763.1	ssc:100520023	1E-97	NM_001242.4	CD27
LOC100525748	12.43393235	199.959366	-4.007352317	1.49E-05	Low	15	242	XP_005664110.1	ssc:100525748	2E-71	NM_004447.5	EPS8
FLT3	106.1028894	4.95767023	4.419657824	1.49E-05	High	128	6	XP_005668350.1	ssc:100515445	0	NM_004119.2	FLT3
ARSB	36.47286824	504.0298068	-3.788613489	1.49E-05	Low	44	610	XP_005661568.1	ssc:102158016	2E-145	NM_198709.2	ARSB
TRHDE	4.973572942	106.58991	-4.42164443	1.50E-05	Low	6	129	XP_003355569.2	ssc:100620702			

SMARCD3	225.46864	3019.22117	-3.743177753	1.50E-05	Low	272	3654	XP_003134625.2	ssc:100521999	0	NM_003078.3	SMARCD3
C7H6orf132	40.61751236	0	20	1.52E-05	High	49	0	XP_005666049.1	ssc:102167684	9E-39	NM_001164446	C6orf132
LOC100158041	40.61751236	0	20	1.52E-05	High	49	0	XP_005663092.1	ssc:100158041	1E-120	NM_020361.4	CPA6
HSD17B3	40.61751236	0	20	1.52E-05	High	49	0	NM_001244790.1	ssc:100513224	1E-150	NM_000197.1	HSD17B3
RRAGD	2083.098134	156.9928906	3.729959672	1.52E-05	High	2513	190	NM_001243623.1	ssc:100156654	0	NM_021244.4	RRAGD
CYP4V2	29.84143765	418.0968561	-3.808448249	1.53E-05	Low	36	506	XP_005671802.1	ssc:100113469	1E-124	NM_207352.3	CYP4V2
IGFBP5	31.4992953	438.7538154	-3.800020213	1.54E-05	Low	38	531	NM_214099.1	ssc:397182	1E-91	NM_000599.3	IGFBP5
LOC100738154	3.315715294	85.10667229	-4.681880203	1.57E-05	Low	4	103	XR_303634.1	ssc:100738154			
LOC100518295	0	40.48764021	-20	1.60E-05	Low	0	49	XP_005652880.1	ssc:100518295	4E-23	NM_021030.2	ZNF14
LOC102159522	0	40.48764021	-20	1.60E-05	Low	0	49	XR_301182.1	ssc:102159522			
MYH2	0	40.48764021	-20	1.60E-05	Low	0	49	NM_214136.1	ssc:397256	0	NM_017534.5	MYH2
PPP2R2B	0	40.48764021	-20	1.60E-05	Low	0	49	NM_214025.2	ssc:397089	2E-52	NR_073527.1	PPP2R2B
SRPX	75.43252295	999.7968298	-3.728376368	1.62E-05	Low	91	1210	XP_005673612.1	ssc:100156108	0	NM_006307.4	SRPX
SULF1	314.1640241	4198.320407	-3.740222386	1.62E-05	Low	379	5081	XP_005663089.1	ssc:100152427	0	NM_015170.2	SULF1
MEG3	7.460359412	136.3359313	-4.191776888	1.63E-05	Low	9	165	NR_021488.1	ssc:100144590			
EPB41L5	2286.185695	173.5184581	3.719781548	1.63E-05	High	2758	210	XP_005654234.1	ssc:100523470	2E-161	NM_020909.3	EPB41L5
LOC100622375	896.0720583	67.75482648	3.725219127	1.64E-05	High	1081	82	XP_005658968.1	ssc:100622375	0	NM_033517.1	SHANK3
SATB1	3382.0296	255.3200169	3.727510733	1.64E-05	High	4080	309	XP_005669348.1	ssc:100155111	0	NM_002971.4	SATB1
AMOTL2	368.0443977	4927.924209	-3.743028352	1.65E-05	Low	444	5964	XP_005669919.1	ssc:100523696	0	NM_016201.3	AMOTL2
FNDC1	94.49788589	4.131391859	4.515582244	1.65E-05	High	114	5	XP_005669175.1	ssc:100154276	3E-87	NM_032532.2	FNDC1
IFNAR2	165.7857647	2186.332572	-3.721120832	1.65E-05	Low	200	2646	NM_001204775.2	ssc:100533555			
STEAP1	104.4450318	1372.448375	-3.71593611	1.68E-05	Low	126	1661	NM_214305.1	ssc:397573	1E-18	NM_012449.2	STEAP1
CAP2	745.2070124	56.18692928	3.729334789	1.69E-05	High	899	68	XP_003128236.3	ssc:100523257			
PLBD2	397.8858353	5323.711549	-3.742005959	1.69E-05	Low	480	6443	XP_001925814.4	ssc:100156824	0	NM_173542.3	PLBD2
CHRNA9	104.4450318	4.95767023	4.396937747	1.70E-05	High	126	6	NM_001244806.1	ssc:100513414	0	NM_017581.3	CHRNA9
IRF1	205.5743483	2710.193059	-3.720663465	1.70E-05	Low	248	3280	NM_001097413.1	ssc:396611	1E-173	NM_002198.2	IRF1
H2AFY2	33.15715294	454.4531045	-3.776739389	1.71E-05	Low	40	550	XR_299447.1	ssc:100152359	5E-64	NM_018649.2	H2AFY2
LOC100737939	198.9429177	2620.128717	-3.719211242	1.71E-05	Low	240	3171	XP_003481552.1	ssc:100737939	5E-125	NM_080630.3	COL11A1
LOC102167180	93.66895707	4.131391859	4.502871192	1.78E-05	High	113	5	XP_005664381.1	ssc:102167180	5E-49	NM_002661.3	PLCG2
CYP2C91	19.06536294	276.8032545	-3.859835005	1.80E-05	Low	23	335	XR_307738.1	ssc:100516879			
LOC102160408	103.616103	4.95767023	4.385442108	1.81E-05	High	125	6	XP_005656235.1	ssc:102160408	5E-35	NM_001145636.1	C1orf228
LOC100738797	4.973572942	104.1110748	-4.387697098	1.81E-05	Low	6	126	XP_005657730.1	ssc:100738797	6E-113	NM_032883.2	TOX2
LGALS7	244.534003	16.52556743	3.887263374	1.82E-05	High	295	20	NM_001142843.1	ssc:100217394	9E-55	NM_001042507.3	LGALS7B
FAM49A	62.99859059	827.1046501	-3.714678421	1.82E-05	Low	76	1001	XP_005662796.1	ssc:100518011	0	NM_030797.3	FAM49A
LOC100621509	51.39358706	0.826278372	5.958816635	1.83E-05	High	62	1	XP_003360671.1	ssc:100621509	7E-29	NM_145306.2	C10orf35
LOC100738527	1632.989782	22616.89159	-3.791812993	1.84E-05	Low	1970	27372	XP_005659708.1	ssc:100738527	5E-43	NM_004048.2	B2M
EGLN3	14866.83845	1092.340007	3.766603988	1.85E-05	High	17935	1322	XP_001928542.3	ssc:100152368	4E-140	NM_022073.3	EGLN3
LOC102157898	50.56465824	667.6329244	-3.722853821	1.87E-05	Low	61	808	XP_005655626.1	ssc:102157898	0	NM_198900.2	FMNL3
LOC102159458	0	39.66136184	-20	1.87E-05	Low	0	48	XR_299573.1	ssc:102159458			
SERPINB2	0	39.66136184	-20	1.87E-05	Low	0	48	XP_003121745.1	ssc:100519286	0	NM_002575.2	SERPINB2
ZSCAN4	0	39.66136184	-20	1.87E-05	Low	0	48	ZSCAN4	ssc:102159852	1E-103	NM_152677.2	ZSCAN4
CCDC64	324.11117	23.13579441	3.8082902	1.89E-05	High	391	28	XR_307437.1	ssc:100153008	0	NM_207311.2	CCDC64
LOC102163771	4.144644118	93.36945601	-4.493630543	1.90E-05	Low	5	113	XR_309073.1	ssc:102163771			
MAOB	363.8997536	26.4409079	3.782697454	1.93E-05	High	439	32	NM_001001864.1	ssc:414909	0	NM_000898.4	MAOB
PDE1C	18.23643412	264.409079	-3.857876152	1.93E-05	Low	22	320	XP_003134849.3	ssc:100525902	0	NM_005020.2	PDE1C
LOC100521253	102.7871741	4.95767023	4.373854134	1.93E-05	High	124	6	XP_005661562.1	ssc:100521253	3E-173	NM_006633.3	IQGAP2
GJA5	0.828928824	51.22925905	-5.949575986	1.94E-05	Low	1	62	XP_005653125.1	ssc:100157795	0	NM_181703.3	GJA5
LOC100738836	23.21000706	324.7274001	-3.806410256	1.96E-05	Low	28	393	XP_003483784.2	ssc:100738836	5E-51	NM_000599.3	IGFBP5
LOC100628155	1754.013391	136.3359313	3.685422022	1.97E-05	High	2116	165	XP_005659044.1	ssc:100628155	1E-92	NM_005729.3	PPIF
SCN1B	13.26286118	203.2644794	-3.937894181	1.98E-05	Low	16	246	XP_005664550.1	ssc:100736963	2E-82	NM_199037.3	SCN1B
LOC396858	464.2001412	34.70369161	3.741585919	1.98E-05	High	560	42	XP_005658343.1	ssc:396858	0	NM_002222.5	ITPR1
LOC100624234	271.8886541	19.00440255	3.838610373	1.99E-05	High	328	23	XP_005657762.1	ssc:100624234	1E-26	NM_198285.2	WDR86
LOC100511223	2616.099367	203.2644794	3.685987309	2.03E-05	High	3156	246	XP_005669168.1	ssc:100511223	0	NM_178568.2	RTN4RL1
LOC102165895	211.37685	14.04673232	3.91151092	2.04E-05	High	255	17	XP_005655387.1	ssc:102165895	2E-35	NM_014957.2	DENND3
LOC100037974	4.144644118	92.54317763	-4.480806503	2.04E-05	Low	5	112	XP_005674384.1	ssc:100037974	0	NM_018058.6	CRTAC1
B2M	991.398873	13340.26431	-3.750177822	2.04E-05	Low	1196	16145	NM_213978.1	ssc:397033	5E-43	NM_004048.2	B2M
S1PR3	74.60359412	961.7880247	-3.688401922	2.05E-05	Low	90	1164	XP_001925305.1	ssc:100154607	0	NM_005226.3	S1PR3
LOC100516519	61.34073295	1.652556743	5.21407369	2.06E-05	High	74	2	LOC100516519	ssc:100516519			
RGAG1	61.34073295	1.652556743	5.21407369	2.06E-05	High	74	2	XP_003360466.1		0	NM_020769.2	RGAG1
LOC100738971	121.8525371	6.610226974	4.204292669	2.06E-05	High	147	8	XR_306806.1	ssc:100738971			
C10H1orf95	50.56465824	0.826278372	5.935357662	2.07E-05	High	61	1	XP_005667995.1	ssc:100621258	4E-65	NM_001003665.3	C1orf95
SLC14A1	38.95965471	0	20	2.08E-05	High	47	0	XP_003480366.1	ssc:100157861	3E-165	NM_015865.6	SLC14A1
LOC102164571	38.95965471	0	20	2.08E-05	High	47	0	XP_005672345.1	ssc:102164571			
LOC102158818	38.95965471	0	20	2.08E-05	High	47	0	XR_305484.1	ssc:102158818			
PRSS48	38.95965471	0	20	2.08E-05	High	47	0	XP_005666883.1	ssc:102163040	9E-50	NM_183375.2	PRSS48
LOC100737937	38.95965471	0	20	2.08E-05	High	47	0	XP_005662740.1	ssc:100737937	1E-142	NM_194323.2	OTOF
LOC100513619	1109.935695	14958.11736	-3.752380608	2.08E-05	Low	1339	18103	XP_005668657.1	ssc:100513619	0	NM_020954.3	RNF213
LOC102163860	7.460359412	132.2045395	-4.147382769	2.10E-05	Low	9	160	XP_005673333.1	ssc:102163860	7E-65	NM_005020.2	PDE1C
LOC100737860	9.11821706	152.0352204	-4.059510013	2.10E-05	Low	11	184	XP_005668944.1	ssc:100737860	0	NM_017957.2	EPN3
RGS9BP	359.7551094	26.4409079	3.766171557	2.12E-05	High	434	32	XP_005655862.1	ssc:102160982	1E-64	NM_207391.2	RGS9BP
RAD51C	879.4934818	68.58110485	3.680789834	2.13E-05	High	1061	83	XP_005669031.1	ssc:100518577	2E-168	NR_103873.1	RAD51C
LOC102163494	1105.791051	86.75922903	3.671917758	2.16E-05	High	1334	105	XP_005658979.1	ssc:102163494	4E-37	NR_109766.1	PAOX
TMEM82	71.28787883	2.478835115	4.845922578	2.16E-05	High	86	3	XP_003127685.3	ssc:100522322	4E-108	NM_001013641.2	TMEM82
LOC100152059	71.28787883	2.478835115	4.845922578	2.16E-05	High	86	3	XP_005658714.1	ssc:100152059	5E-116	NM_020481.2	ANK1
LOC102165794	71.28787883	2.478835115	4.845922578	2.16E-05	High	86	3	XR_300521.1	ssc:102165794			
LOC102165247	229.6132841	15.69928906	3.870434977	2.16E-05	High	277	19	XR_302925.1	ssc:102165247			
XAF1	222.9818535	2859.749445	-3.680890539	2.18E-05	Low	269	3461	XP_005669195.1	ssc:102159947			
LOC100517362	479.12086	36.35624836	3.720114388	2.18E-05	High	578	44	XP_005668028.1	ssc:100517362	2E-65	NM_199414.1	PRC1
MYCBPAP	722.8259342	56.18692928	3.685341808	2.19E-05	High	872	68	XP_003131625.3	ssc:100513622	0	NM_032133.4	MYCBPAP
HECW2	38.95965471	512.2925905	-3.716915229	2.19E-05	Low	47	620	NM_001198924.1	ssc:100155879	0	NM_020760.1	HECW2
LOC102167526	1.657857647	61.14459951	-5.204833041	2.19E-05	Low	2	74	XR_297123.1	ssc:102167526	1E-41	NM_003566.3	EEA1
LOC102164190	101.1293165	4.95767023	4.350395161	2.21E-05	High	122	6	XR_298858.1	ssc:102164190			
RAB11FIP4	447.6215647	33.87741324	3.723883917	2.23E-05	High	540	41	XP_005669131.1	ssc:100524645	0	NM_032932.3	RAB11FIP4
MCCC2	2858.975513	224.7477171	3.669119897	2.28E-05	High	3449	272	XP_005672588.1	ssc:100518224	3E-179	NM_022132.4	MCCC2
REN	60.51180412	1.652556743	5.194444883	2.30E-05	High	73	2	XP_003130148.3	ssc:100524822	1E-95	NM_000537.3	REN
LOC102163428	60.51180412	1.652556743	5.194444883	2.30E-05	High	73	2	XR_304425.1	ssc:102163428			
TBC1D4	901.8745601	71.05993997	3.665818411	2.31E-05	High	1088	86	XP_005668526.1	ssc:100523015	0	NM_014832.3	TBC1D4
LOC102162786	218.0082806	14.87301069	3.873614312	2.31E-05	High	263	18	XP_005654228.1	ssc:102162786	2E-33	NM_020909.3	EPB41L5
TDRD7	133.4575406	1689.73927	-3.662347925	2.31E-05	Low	161	2045	XP_001924715.1	ssc:100152652	0	NM_014290.2	TDRD7
LOC102158232	3107.65416	244.578398	3.667457069	2.33E-05	High	3749	296	XR_300389.1	ssc:102158232			
LOC100520903	655.6826995	51.22925905	3.677957898	2.36E-05	High	791	62	XP_005653109.1	ssc:100520903	6E-90	NM_198539.3	ZNF568
LOC102167582	100.3003877	4.95767023	4.338521061	2.36E-05	High	121	6	XR_298951.1	ssc:102167582			
LOC100516621	1166.302855	92.54317763	3.655672016	2.36E-05	High	1407	112	XP_005656332.1	ssc:100516621	3E-55	NM_138573.3	NRG4
LOC100623661	70.45895001	2.478835115	4.82904876	2.38E-05	High	85	3	LOC100623661				
CXCR5	216.350423	2744.896751	-3.665310037	2.38E-05	Low	261	3322	XP_003129963.1	ssc:100515679	1E-177	NM_032966.2	CXCR5
LOC100627550	80.40609589	3.305113487	4.604533167	2.39E-05	High	97	4	XP_003360923.1	ssc:100627550	0	NM_005068.2	SIM1
NOS3	334.8872447	24.78835115	3.755941211	2.40E-05	High	404	30	NM_214295.1	ssc:397557			
LOC100738511	117.707893	1483.169677	-3.655400685	2.41E-05	Low	142	1795	XP_005667673.1	ssc:100738511	2E-39	NM_003919.2	SGCE
LOC102167200	57.19608883	728.7775239	-3.671490064	2.42E-05	Low	69	882	XP_005668864.1	ssc:102167200	0	NM_003632.2	CNTNAP1
PLCG2	927.5713536	73.53877508	3.656881214	2.42E-05	High	1119	89	XP_005664379.1	ssc:100518663	0	NM_002661.3	PLCG2

PAPSS2	198.1139888	2503.623466	-3.659614946	2.43E-05	Low	239	3030	XP_005671332.1	ssc:100156262	0	NM_004670.3	PAPSS2
RRH	38.13072589	0	20	2.45E-05	High	46	0	XP_003129314.1	ssc:100518979	2E-180	NM_006583.2	RRH
SKOR2	38.13072589	0	20	2.45E-05	High	46	0	XP_005652528.1	ssc:100154175	9E-155	xp_003403746	xp_003403746
LOC102165954	38.13072589	0	20	2.45E-05	High	46	0	XP_005659237.1	ssc:102165954	1E-23	NM_020340.4	KIAA1244
LOC102157511	324.11117	23.96207278	3.757664127	2.45E-05	High	391	29	XR_305505.1	ssc:102157511			
FGF12	1058.542108	84.28039392	3.650737792	2.46E-05	High	1277	102	XP_001925448.3	ssc:100152498	1E-77	NM_021032.4	FGF12
COL9A1	422.7537	32.2248565	3.713571542	2.46E-05	High	510	39	XP_003121321.2	ssc:100155319	0	NM_078485.3	COL9A1
ICAM-1	518.9094436	6696.986203	-3.689957299	2.46E-05	Low	626	8105	NM_213816.1	ssc:396750	9E-150	NM_000201.2	ICAM1
P2RX7	177.3907683	11.5678972	3.938732389	2.47E-05	High	214	14	XP_001926839.2	ssc:497623	0	NR_033956.1	P2RX7
LOC102165830	109.4186047	5.783948602	4.241659522	2.48E-05	High	132	7	XP_005669982.1	ssc:102165830	9E-128	NM_053002.4	MED12L
LOC100523418	681.379493	53.70809416	3.665247095	2.50E-05	High	822	65	XP_003480572.2	ssc:100523418	0	NM_134428.2	RFX3
PRMT8	2.486786471	70.2336616	-4.819808111	2.53E-05	Low	3	85	XP_005664132.1	ssc:100526051	0	NR_046301.1	PRMT8
LOC100621791	2.486786471	70.2336616	-4.819808111	2.53E-05	Low	3	85	XP_003359328.1	ssc:100621791	2E-64	NM_007021.3	C10orf10
LOC100511392	89.52431295	4.131391859	4.437579732	2.57E-05	High	108	5	XP_005658878.1	ssc:100511392	0	NM_003948.3	CDKL2
C3H16orf54	7.460359412	128.899426	-4.110856893	2.58E-05	Low	9	156	XP_005655153.1	ssc:100623246	3E-82	NM_175900.3	C16orf54
LOC100625114	0	38.0088051	-20	2.58E-05	Low	0	46	XP_003359959.1	ssc:100625114			
SCMH1	35.64393941	461.8896098	-3.695819394	2.61E-05	Low	43	559	XP_003128156.3	ssc:100525880			
LDLRAD4	1510.308317	121.4629206	3.636255223	2.63E-05	High	1822	147	XP_005665323.1	ssc:100622189	4E-102	NM_181483.3	LDLRAD4
PRAP1	48.90680059	0.826278372	5.887263374	2.69E-05	High	59	1	XP_001927920.1	ssc:100154502	1E-19	NM_145202.4	PRAP1
FSCN1	1315.510043	17303.09538	-3.71733598	2.71E-05	Low	1587	20941	NM_001146300.1	ssc:100286741	4E-113	NM_003088.3	FSCN1
LOC102158670	1.657857647	59.49204276	-5.165304677	2.72E-05	Low	2	72	XR_303842.1	ssc:102158670			
HOXC10	1.657857647	59.49204276	-5.165304677	2.72E-05	Low	2	72	XP_003355474.2	ssc:100628125	2E-158	NM_017409.3	HOXC10
SYT11	26.52572236	351.168308	-3.726698707	2.73E-05	Low	32	425	XP_001928839.2	ssc:100154741	0	NM_152280.4	SYT11
LOC100624297	140.9179	1748.405035	-3.633112652	2.76E-05	Low	170	2116	XP_003360691.1	ssc:100624297	6E-60	NM_031477.4	YPEL3
TSHZ2	3.315715294	79.32272369	-4.580342176	2.77E-05	Low	4	96	NM_001114674.1	ssc:100136903	0	NM_173485.5	TSHZ2
KCNN3	3.315715294	79.32272369	-4.580342176	2.77E-05	Low	4	96	NM_213985.1	ssc:397045	7E-150	NM_170782.2	KCNN3
LOC100512652	116.8789641	1448.465986	-3.631438604	2.77E-05	Low	141	1753	XR_130502.3	ssc:100512652			
LAMA2	5.802501765	108.2424667	-4.221447755	2.82E-05	Low	7	131	XP_005659257.1	ssc:100154420	0	NM_001079823.1	LAMA2
PDE3B	680.5505642	54.53437253	3.641464617	2.86E-05	High	821	66	XP_005661171.1	ssc:100516060	0	NM_000922.3	PDE3B
TMEFF2	7.460359412	127.2468692	-4.092241215	2.87E-05	Low	9	154	XP_003133602.3	ssc:654831	4E-145	NM_016192.2	TMEFF2
LOC102161901	68.80109236	2.478835115	4.794697255	2.87E-05	High	83	3	XR_303259.1	ssc:102161901			
LOC100622802	37.30179706	0	20	2.89E-05	High	45	0	XP_003360329.2	ssc:100622802	0	NM_176819.3	CXorf36
LOC102159633	37.30179706	0	20	2.89E-05	High	45	0	XR_300367.1	ssc:102159633			
PDIA2	37.30179706	0	20	2.89E-05	High	45	0	XP_005655182.1	ssc:100738360	0	NM_006849.2	PDIA2
LOC102158259	37.30179706	0	20	2.89E-05	High	45	0	XR_298393.1	ssc:102158259			
LOC100153517	4.144644118	88.41178578	-4.414918567	2.95E-05	Low	5	107	XP_005658136.1	ssc:100153517	0	NM_024832.3	RIN3
CAPG	1880.839501	24799.09277	-3.720838695	2.95E-05	Low	2269	30013	XP_005655276.1	ssc:100519653	0	NM_001747.3	CAPG
KIF17	1105.791051	90.06434252	3.617978951	2.96E-05	High	1334	109	XP_005665149.1	ssc:100738531	0	NM_020816.3	KIF17
TPMT	1691.843729	137.9884881	3.615976499	2.97E-05	High	2041	167	NM_001243675.1	ssc:100157630	1E-118	NM_000367.2	TPMT
OSMR	203.0875618	2507.754858	-3.626222538	2.98E-05	Low	245	3035	XP_003133952.2	ssc:100519398	0	NM_003999.2	OSMR
LOC102160475	716.1945036	57.83948602	3.63022481	3.00E-05	High	864	70	XR_299423.1	ssc:102160475			
VAMP8	239.56043	2961.381684	-3.627808915	3.02E-05	Low	289	3584	XP_003354791.1	ssc:100620246	6E-40	NM_003761.4	VAMP8
C3H2orf40	1.657857647	58.66576439	-5.145126795	3.03E-05	Low	2	71	NM_001244729.1	ssc:100512958	5E-60	NM_032411.2	C2orf40
LOC102161426	0	37.18252673	-20	3.05E-05	Low	0	45	XP_005664948.1	ssc:102161426	0	NM_020813.2	ZNF471
LOC100738788	0	37.18252673	-20	3.05E-05	Low	0	45	XP_003482722.2	ssc:100738788	5E-47	NM_000446.5	PON1
TOX2	2.486786471	68.58110485	-4.785456606	3.05E-05	Low	3	83	XP_005657727.1	ssc:102166192	3E-48	NM_032883.2	TOX2
SLC25A41	286.809373	21.48323766	3.738808834	3.07E-05	High	346	26	XP_005661337.1	ssc:100517328	1E-83	NM_173637.3	SLC25A41
LOC100523827	77.91930942	3.305113487	4.559209176	3.08E-05	High	94	4	XP_005672650.1	ssc:100523827	1E-68	NM_138379.2	TIMD4
LOC102162369	142.5757577	1743.447364	-3.612142205	3.12E-05	Low	172	2110	XR_308396.1	ssc:102162369			
LOC100623149	181.5354124	12.39417558	3.872516788	3.17E-05	High	219	15	XP_003361879.1	ssc:100623149			
LOC100623245	58.02501765	715.5570699	-3.624319873	3.18E-05	Low	70	866	XR_300092.1	ssc:100623245			
TSPAN1	58.02501765	1.652556743	5.133903341	3.19E-05	High	70	2	NM_001243860.1	ssc:100511571	4E-98	NM_005727.3	TSPAN1
LOC100739139	58.02501765	1.652556743	5.133903341	3.19E-05	High	70	2	XP_005667923.1	ssc:100739139			
ETV4	1075.120684	13773.23418	-3.679296848	3.20E-05	Low	1297	16669	XP_005668843.1	ssc:100516755	0	NM_001986.2	ETV4
TTNLOC100620	0.828928824	47.92414556	-5.853360671	3.24E-05	Low	1	58	XP_003483740.1	ssc:100620261	0	NM_133437.3	TTN
CACNA1A	0.828928824	47.92414556	-5.853360671	3.24E-05	Low	1	58	XP_003480824.2	ssc:100525387	0	NM_023035.2	CACNA1A
LOC102159646	370.5311842	28.91974301	3.679468329	3.26E-05	High	447	35	XP_005661843.1	ssc:102159646			
LOC100737177	169.9304088	2069.001043	-3.605918423	3.29E-05	Low	205	2504	XP_005653161.1	ssc:100737177	0	NM_000262.2	NAGA
NFATC4	374.6758283	4628.811438	-3.626926981	3.31E-05	Low	452	5602	XP_001927389.2	ssc:100154469	0	NM_004554.4	NFATC4
VWF	96.15574354	4.95767023	4.277638819	3.32E-05	High	116	6	NM_001246221.1	ssc:399543	0	NM_000552.3	VWF
LOC102164625	9.11821706	143.7724367	-3.978891553	3.33E-05	Low	11	174	XR_297300.1	ssc:102164625			
EGFL7	100.3003877	1213.802928	-3.597135119	3.40E-05	Low	121	1469	XR_297247.1	ssc:100514434	7E-86	NR_046367.1	EGFL7
TMEM249	608.4337565	49.5767023	3.617365982	3.41E-05	High	734	60	XP_005662873.1	ssc:100154454	4E-93	NR_047684.2	TMEM249
LOC100525247	36.47286824	0	20	3.42E-05	High	44	0	XR_297588.1	ssc:100525247			
LOC100525793	36.47286824	0	20	3.42E-05	High	44	0	XP_005652520.1	ssc:100525793	0	NM_033125.3	SLC22A16
LOC102163735	36.47286824	0	20	3.42E-05	High	44	0	XP_005655873.1	ssc:102163735	2E-72	NM_017579.2	DMBT1
TNP2	36.47286824	0	20	3.42E-05	High	44	0	XP_003354656.1	ssc:406180	7E-17	NM_005425.4	TNP2
RASL10A	36.47286824	0	20	3.42E-05	High	44	0	XP_003132992.1	ssc:100513761	2E-108	NM_006477.4	RASL10A
ABCC4	36.47286824	0	20	3.42E-05	High	44	0	XP_005674687.1	ssc:100738425	1E-22	NM_005845.3	ABCC4
HMGCLL1	36.47286824	0	20	3.42E-05	High	44	0	XP_003356654.1	ssc:100627473	0	NR_109869.1	HMGCLL1
LOC102160749	36.47286824	0	20	3.42E-05	High	44	0	XR_302514.1	ssc:102160749			
LOC100523671	37.30179706	466.0210017	-3.643077932	3.45E-05	Low	45	564	LOC100523671				
LOC100622473	396.2279777	31.39857813	3.657559619	3.47E-05	High	478	38	XP_003361634.1	ssc:100622473	3E-75	NM_006366.2	CAP2
HIST1H2BD	83.72181118	1012.191005	-3.595734227	3.48E-05	Low	101	1225	NM_001243656.1	ssc:100157327	2E-68	NM_138720.2	HIST1H2BD
C18H7orf31	95.32681471	4.95767023	4.265147875	3.56E-05	High	115	6	XP_003484111.1	ssc:100738759	2E-162	NM_138811.3	C7orf31
PLEKHG1	3.315715294	76.84388857	-4.534538487	3.57E-05	Low	4	93	XP_005658150.1	ssc:100621167			
RIMBP2	188.166843	13.22045395	3.831168812	3.58E-05	High	227	16	XP_005670600.1	ssc:100153193	0	NM_015347.4	RIMBP2
SLA-DQA1	0	36.35624836	-20	3.60E-05	Low	0	44	NM_001130224.1	ssc:100153387	3E-124	NM_002122.3	HLA-DQA1
LOC102166264	0	36.35624836	-20	3.60E-05	Low	0	44	XP_005655572.1	ssc:102166264			
PPP1R9A	375.5047571	29.74602138	3.658062563	3.61E-05	High	453	36	XP_005667675.1	ssc:100621071	0	NM_017650.2	PPP1R9A
DTX3L	87.03752648	1046.894697	-3.588334967	3.62E-05	Low	105	1267	XP_005670232.1	ssc:100520459	0	NM_138287.3	DTX3L
LOC100627004	1824.472341	23463.00064	-3.684836344	3.63E-05	Low	2201	28396	XP_005655099.1	ssc:100627004	3E-51	NM_003641.3	IFITM1
CAR	5526.468467	451.9742693	3.612045313	3.69E-05	High	6667	547	XP_001924589.4	ssc:397333	0	NM_001338.4	CXADR
LOC100522421	15.74964765	216.4849334	-3.780875164	3.69E-05	Low	19	262	XR_301560.1	ssc:100522421	1E-91	NM_001161473.1	ALDH3B1
LOX	915.9663501	11478.65914	-3.647515717	3.70E-05	Low	1105	13892	NM_001206403.1	ssc:100525278	0	NM_002317.5	LOX
KCNK6	9.947145883	151.208942	-3.926117013	3.70E-05	Low	12	183	XP_003127153.1	ssc:100514651	1E-149	NM_004823.1	KCNK6
DLX4	9.947145883	151.208942	-3.926117013	3.70E-05	Low	12	183	XP_005674532.1	ssc:100517238	8E-46	NM_138281.2	DLX4
LOC102160506	122.6814659	7.436505346	4.044148689	3.70E-05	High	148	9	XR_303205.1	ssc:102160506			
MEOX2	0.828928824	47.09786719	-5.82826969	3.70E-05	Low	1	57	NM_214121.1	ssc:397222	4E-73	NM_005924.4	MEOX2
SLC35F1	223.8107824	16.52556743	3.759507826	3.80E-05	High	270	20	XP_005654451.1	ssc:100522582	0	NM_001029858.3	SLC35F1
FAM114A1	464.2001412	5671.574744	-3.610930496	3.83E-05	Low	560	6864	XP_005666637.1	ssc:100525173	0	NR_033290.1	FAM114A1
PAX6	1013.779951	85.10667229	3.574328486	3.85E-05	High	1223	103	NM_001244178.1	ssc:100514152	0	NM_001604.5	PAX6
ASS1	848.8231154	71.05993997	3.57835557	3.86E-05	High	1024	86	XP_005660580.1	ssc:414411	0	NM_054012.3	ASS1
THBS3	330.7426006	4000.013598	-3.596224118	3.87E-05	Low	399	4841	XP_005663383.1	ssc:100155108	0	NR_045553.1	THBS3
CXXC5	474.1472871	5788.906272	-3.609883612	3.88E-05	Low	572	7006	XP_003124042.1	ssc:100524854	7E-136	NM_016463.7	CXXC5
LOC102159510	3.315715294	76.0176102	-4.518941632	3.89E-05	Low	4	92	XP_005658451.1	ssc:102159510	2E-26	NM_145177.2	DHRSX
LOC100513288	306.7036647	23.96207278	3.67802079	3.89E-05	High	370	29	XP_005665067.1	ssc:100513288	4E-102	NM_002223.2	ITPR2
KLF6	525.5408742	6422.661783	-3.611296446	3.94E-05	Low	634	7773	NM_001134353.2	ssc:100174961	1E-139	NR_027653.1	KLF6
NRSN1	75.43252295	3.305113487	4.512414965	3.98E-05	High	91	4	XP_005665686.1	ssc:100154240	2E-105	NM_080723.4	NRSN1
LOC102163525	75.43252295	3.305113487	4.512414965	3.98E-05	High	91	4	XR_305221.1	ssc:102163525			

UNC5CL	56.36716001	1.652556743	5.092083166	4.00E-05	High	68	2	XP_005674216.1	ssc:100156294	0	NM_173561.2	UNC5CL
RPL3L	56.36716001	1.652556743	5.092083166	4.00E-05	High	68	2	XP_005658938.1	ssc:100622679	2E-52	NM_005061.2	RPL3L
GVIN1	14.09179	195.8279741	-3.796660083	4.01E-05	Low	17	237	XP_003482569.2	ssc:100512797	1E-80	NM_017920.3	URGCP
PRICKLE1	187.3379141	2230.125325	-3.573409975	4.02E-05	Low	226	2699	XP_005664204.1	ssc:100157209	0	NM_153026.2	PRICKLE1
PRKAB2	8384.615051	684.1584918	3.615342066	4.02E-05	High	10115	828	NM_001243683.1	ssc:100157793	3E-158	NR_103871.1	PRKAB2
LOC102161699	35.64393941	0	20	4.05E-05	High	43	0	XP_005653909.1	ssc:102161699	2E-144	NM_001676.5	ATP12A
TMPRSS5	35.64393941	0	20	4.05E-05	High	43	0	XP_005667414.1	ssc:100515912	0	NR_110047.1	TMPRSS5
LOC100156689	1974.508458	25125.47273	-3.669585288	4.08E-05	Low	2382	30408	XP_005659493.1	ssc:100156689	0	NM_080645.2	COL12A1
CPEB2	99.47145883	1177.44668	-3.565235284	4.11E-05	Low	120	1425	NM_001185049.1	ssc:100462674			
CCDC88C	3038.853067	255.3200169	3.573148367	4.12E-05	High	3666	309	XP_001928768.1	ssc:100152429	0	NM_001080414.3	CCDC88C
LOC102166021	304.2168783	23.96207278	3.666275582	4.16E-05	High	367	29	XP_005655388.1	ssc:102166021	1E-31	NM_014957.2	DENND3
GBP5	1.657857647	56.18692928	-5.082842517	4.24E-05	Low	2	68	XP_001929321.1		0	NM_052942.3	GBP5
IFI30	362.2418959	4342.919122	-3.583639766	4.25E-05	Low	437	5256	NM_001131046.1	ssc:100174943	2E-89	NM_006332.4	IFI30
STEAP2	11.60500353	166.9082311	-3.846236236	4.26E-05	Low	14	202	XP_005667635.1	ssc:100623287	0	NM_152999.3	STEAP2
ADCYAP1	0	35.52996998	-20	4.27E-05	Low	0	43	NM_001001544.1	ssc:414283	9E-71	NM_001117.4	ADCYAP1
LOC100627616	0	35.52996998	-20	4.27E-05	Low	0	43	XP_005674315.1	ssc:100627616	2E-140	NM_002247.3	KCNMA1
FLI1	0	35.52996998	-20	4.27E-05	Low	0	43	XP_005667560.1	ssc:100515914	1E-121	NM_002017.4	FLI1
CCDC60	0	35.52996998	-20	4.27E-05	Low	0	43	XP_005670725.1	ssc:100155334	0	NM_178499.3	CCDC60
ADAMTS6	159.1543341	1876.478182	-3.559529166	4.29E-05	Low	192	2271	XP_003134060.3	ssc:100513765	0	NM_197941.2	ADAMTS6
TLE2	51.39358706	613.9248302	-3.578401766	4.32E-05	Low	62	743	XP_003354045.2	ssc:100622728	2E-97	NM_003260.4	TLE2
LOC100512907	4.144644118	84.28039392	-4.345876923	4.32E-05	Low	5	102	XR_304317.1	ssc:100512907			
COL4A6	770.0748771	65.27599137	3.560374363	4.37E-05	High	929	79	XP_001925433.3	ssc:100152680	0	NM_033641.3	COL4A6
MADCAM1	83.72181118	4.131391859	4.340903712	4.39E-05	High	101	5	NM_001037998.1	ssc:492312	3E-60	NM_130762.2	MADCAM1
LOC100736921	101.9582453	5.783948602	4.139779908	4.40E-05	High	123	7	XP_005659361.1	ssc:100736921	2E-140	NM_001004339.2	ZYG11A
HHEX	402.8594083	33.05113487	3.607504733	4.47E-05	High	486	40	NM_001244579.1	ssc:397232	2E-146	NM_002729.4	HHEX
LOC100626903	16.57857647	220.6163253	-3.734147512	4.48E-05	Low	20	267	XP_003354462.1	ssc:100626903	0	NM_007183.2	PKP3
GUCY1A2	174.075053	12.39417558	3.811975246	4.48E-05	High	210	15	XP_003130141.3	ssc:100522091	0	NM_001256424.1	GUCY1A2
CDKL2	300.901163	23.96207278	3.650465067	4.56E-05	High	363	29	XP_005666830.1	ssc:100737771	0	NM_003948.3	CDKL2
SAMD14	48.07787177	571.7846332	-3.572026908	4.60E-05	Low	58	692	XP_005668983.1	ssc:102159512	4E-174	NM_174920.3	SAMD14
LOC100524687	200.6007753	14.87301069	3.75355856	4.60E-05	High	242	18	XP_003128286.1	ssc:100524687	4E-47	NM_198076.4	COX20
C1H14orf37	194.7982735	2280.528306	-3.549315281	4.66E-05	Low	235	2760	NM_001244542.1	ssc:100514619	0	NM_001001872.2	C14orf37
FXYD6	368.0443977	4360.270968	-3.566466078	4.72E-05	Low	444	5277	XP_005656693.1	ssc:102158732	2E-46	NM_022003.3	FXYD6
RAB27A	2232.305322	191.6965822	3.541637844	4.75E-05	High	2693	232	NM_001032357.1	ssc:606749	1E-123	NM_183236.2	RAB27A
LOC100513259	73.7746653	3.305113487	4.480353755	4.75E-05	High	89	4	XP_005654730.1	ssc:100513259	2E-70	NM_005364.4	MAGEA8
EFHD1	1.657857647	55.36065091	-5.061468866	4.76E-05	Low	2	67	XP_001925420.4	ssc:100153398	1E-92	NM_025202.3	EFHD1
TMED8	818.1527489	70.2336616	3.542135663	4.78E-05	High	987	85	XP_003356809.1	ssc:100627014	8E-149	NM_213601.1	TMED8
CKM	34.81501059	0	20	4.82E-05	High	42	0	NM_001129949.1	ssc:397264	0	NM_001824.4	CKM
LOC100153139	34.81501059	0	20	4.82E-05	High	42	0	XR_130735.2	ssc:100153139			
SIT1	34.81501059	0	20	4.82E-05	High	42	0	XP_005660278.1	ssc:100153191	1E-83	NM_014450.2	SIT1
SLAIN1	1167.131784	100.8059614	3.533314605	4.83E-05	High	1408	122	XP_005668535.1	ssc:100526183	6E-92	NM_144595.3	SLAIN1
LOC102159737	226.2975688	17.35184581	3.705060042	4.85E-05	High	273	21	XP_005663035.1	ssc:102159737			
CCL3L1	0.828928824	45.44531045	-5.776739389	4.86E-05	Low	1	55	NM_001009579.1	ssc:494459	3E-39	NM_001001437.3	CCL3L3
GLCCI1	1758.158035	152.0352204	3.531587274	4.92E-05	High	2121	184	XP_005667693.1	ssc:100511149	0	NM_138426.3	GLCCI1
EFNB2	27.35465118	336.2952973	-3.619870541	4.92E-05	Low	33	407	NM_001114286.1	ssc:100135673	0	NM_004093.3	EFNB2
BCL2L14	109.4186047	6.610226974	4.049014444	4.95E-05	High	132	8	XP_003126516.1	ssc:100514901	2E-132	NM_138724.1	BCL2L14
TMEM8C	13.26286118	181.7812418	-3.776739389	4.97E-05	Low	16	220	XP_003353750.1	ssc:100627299	5E-117	NM_001080483.2	TMEM8C
WNT16	145.0625441	9.915340461	3.870868935	4.98E-05	High	175	12	XP_003134784.1	ssc:100511484	0	NM_057168.1	WNT16
LOC100738354	4.973572942	91.71689926	-4.204833041	5.04E-05	Low	6	111	XR_307304.1	ssc:100738354			
TSHR	54.70930236	1.652556743	5.049014444	5.04E-05	High	66	2	NM_214297.1	ssc:397560			
DUOX1	261.1125794	20.65695929	3.659972153	5.05E-05	High	315	25	NM_214096.2	ssc:397177			
C14H10orf116	0	34.70369161	-20	5.07E-05	Low	0	42	NM_001243503.1	ssc:100154721	9E-25	NM_006829.2	ADIRF
RIMBP3	82.06395354	4.131391859	4.31204885	5.15E-05	High	99	5	XP_001926988.2	ssc:100153093	0	NM_015672.1	RIMBP3
LOC100737660	124.3393235	1431.11414	-3.5247842	5.18E-05	Low	150	1732	XP_005661833.1	ssc:100737660	0	NM_032090.1	PCDHGA10
TCP11L2	172.4171953	1988.852041	-3.527960384	5.20E-05	Low	208	2407	XP_003126140.3	ssc:100523311	0	NM_152772.2	TCP11L2
LOC102159756	242.0472165	19.00440255	3.670882927	5.24E-05	High	292	23	XR_306970.1	ssc:102159756			
RRBP1	1722.514095	21178.34095	-3.620001883	5.25E-05	Low	2078	25631	XP_005672770.1	ssc:100154945	0	NM_004587.2	RRBP1
MYEF2	6280.793696	532.9495498	3.558876007	5.26E-05	High	7577	645	XP_005654508.1	ssc:100153072	0	NM_016132.3	MYEF2
AOX1	44.76215647	0.826278372	5.759507826	5.29E-05	High	54	1	XP_005672134.1	ssc:100525616	0	NM_001159.3	AOX1
CNTNAP1	58.02501765	672.5905946	-3.534981643	5.34E-05	Low	70	814	XP_003131443.3	ssc:100521039	0	NM_003632.2	CNTNAP1
TMEM176A	1.657857647	54.53437253	-5.039773795	5.34E-05	Low	2	66	XP_005657767.1	ssc:100523103	7E-43	NM_018487.2	TMEM176A
GPX3	73.7746653	849.4141661	-3.525270794	5.35E-05	Low	89	1028	NM_001115155.1	ssc:396598	6E-108	NM_002084.3	GPX3
ITPR2	1595.687985	139.6410448	3.514383619	5.41E-05	High	1925	169	XP_005664091.1	ssc:102161905	0	NM_002223.2	ITPR2
CHD7	10572.15822	887.4229712	3.574504212	5.51E-05	High	12754	1074	XP_003355099.1	ssc:100157083	0	NM_017780.3	CHD7
STON2	959.8995777	84.28039392	3.50961452	5.62E-05	High	1158	102	XP_005656499.1	ssc:100157426	0	NM_033104.3	STON2
LOC100512841	213.8636365	2451.567929	-3.518941632	5.63E-05	Low	258	2967	XP_003127846.1	ssc:100512841	1E-159	NM_004468.4	FHL3
FOXE1	53.88037353	1.652556743	5.026988137	5.67E-05	High	65	2	XP_001925267.1	ssc:100157892	4E-153	NM_004473.3	FOXE1
CHPF2	580.2501765	6815.14401	-3.553997181	5.67E-05	Low	700	8248	XP_005657748.1	ssc:100517065	0	NM_019015.2	CHPF2
LANCL3	72.11680765	3.305113487	4.44756382	5.68E-05	High	87	4	XP_005673608.1	ssc:100626898	7E-134	NM_198511.2	LANCL3
LOC100739322	472.4894295	40.48764021	3.544728589	5.69E-05	High	570	49	XP_003484204.1	ssc:100739322			
LOC100519082	1327.943975	16023.19018	-3.592895227	5.70E-05	Low	1602	19392	XP_003124283.1	ssc:100519082	3E-50	NM_003641.3	IFITM1
MSMB	31.4992953	375.1303808	-3.57400065	5.74E-05	Low	38	454	NM_213852.1	ssc:396852	1E-32	NM_138634.2	MSMB
LOC102167673	8.289288236	125.5943125	-3.921379094	5.75E-05	Low	10	152	XP_005661696.1	ssc:102167673			
LOC100154257	33.98608177	0	20	5.75E-05	High	41	0	NM_001243180.1	ssc:100154257	0	NR_049774.1	KIAA1324
LOC100523829	33.98608177	0	20	5.75E-05	High	41	0	XP_003134635.1	ssc:100523829	2E-156	NM_012369.2	OR2F1
PMEPA1	344.8343906	3982.661752	-3.529757389	5.77E-05	Low	416	4820	NM_001123203.1	ssc:100144523	7E-129	NM_199171.2	PMEPA1
LOC100526036	7.460359412	116.5052504	-3.965006027	5.85E-05	Low	9	141	XP_005654805.1	ssc:100526036	0	NM_001079817.1	INSR
FAM134B	59.6828753	683.3322134	-3.517198193	5.87E-05	Low	72	827	XP_005672453.1	ssc:100625174	0	NM_019000.4	FAM134B
B3GALTL	262.7704371	3008.479551	-3.517159671	5.89E-05	Low	317	3641	XP_005668387.1	ssc:100517236	0	NM_194318.3	B3GALTL
LOC102161418	88.69538413	1005.587778	-3.503026142	5.94E-05	Low	107	1217	XP_005653043.1	ssc:102161418	1E-40	NM_003641.3	IFITM1
SPINK2	1.657857647	53.70809416	-5.017747489	6.01E-05	Low	2	65	XP_003129067.1	ssc:100520393	9E-24	NR_073419.1	SPINK2
LYSMD2	80.40609589	4.131391859	4.282605072	6.05E-05	High	97	5	XP_005659663.1	ssc:100155966	1E-60	NM_153374.2	LYSMD2
LOC102161915	80.40609589	4.131391859	4.282605072	6.05E-05	High	97	5	XR_298772.1	ssc:102161915			
LOC100037951	0	33.87741324	-20	6.05E-05	Low	0	41	NM_001097448.1	ssc:100037951			
LOC102157549	0	33.87741324	-20	6.05E-05	Low	0	41	XR_304924.1	ssc:102157549			
LOC102166942	0	33.87741324	-20	6.05E-05	Low	0	41	XR_298158.1	ssc:102166942			
LOC102158116	0	33.87741324	-20	6.05E-05	Low	0	41	XR_303914.1	ssc:102158116			
LOC100625760	0	33.87741324	-20	6.05E-05	Low	0	41	XP_003356564.2	ssc:100625760	0	NM_000129.3	F13A1
LOC100622310	281.8358	23.13579441	3.606656338	6.07E-05	High	340	28	XP_005653694.1	ssc:100622310	1E-82	NM_013250.2	ZNF215
REC8	9729.137603	827.9309285	3.554729611	6.07E-05	High	11737	1002	HighI0001C95632		0	NM_005132.2	REC8
DUOXA1	43.93322765	0.826278372	5.732540779	6.09E-05	High	53	1	NM_001243551.1	ssc:100653516	1E-164	NM_144565.3	DUOXA1
BMPER	43.93322765	507.3349202	-3.529554066	6.10E-05	Low	53	614	XP_003134846.1	ssc:100525197	0	NM_133468.4	BMPER
UBA7	379.6494012	4366.881195	-3.523863644	6.11E-05	Low	458	5285	XR_306784.1	ssc:100512631	0	NM_003335.2	UBA7
HYAL1	14.09179	185.9126336	-3.721698026	6.13E-05	Low	17	225	NM_214441.1	ssc:404698	4E-132	NR_047690.1	HYAL1
SIRT2	348.1501059	3982.661752	-3.515951589	6.27E-05	Low	420	4820	NM_001114271.1	ssc:100125964			
LOC100624711	6.631430589	106.58991	-4.006606931	6.35E-05	Low	8	129	XP_003360906.1	ssc:100624711	0	NM_152999.3	STEAP2
SEMA3F	336.5451024	3838.889315	-3.511817197	6.38E-05	Low	406	4646	XP_001925178.2	ssc:100153225	0	NM_004186.3	SEMA3F
RASAL3	53.05144471	1.652556743	5.004620324	6.39E-05	High	64	2	XP_005652967.1	ssc:102159003	8E-42	NM_022904.1	RASAL3
LOC102159718	114.3921777	7.436505346	3.94321978	6.51E-05	High	138	9	XP_005669030.1	ssc:102159718			
MAP1LC3A	353.1236789	4021.496835	-3.509487115	6.54E-05	Low	426	4867	NM_001170827.1	ssc:100322893	5E-66	NM_181509.2	MAP1LC3A
EPT1	1420.784004	127.2468692	3.480985178	6.54E-05	High	1714	154	NM_001244662.1	ssc:100512162	2E-117	NM_033505.2	EPT1

LOC100738465	705.4184289	62.79715625	3.489708133	6.64E-05	High	851	76	XP_005668987.1	ssc:100738465	0	NM_032133.4	MYCBPAP
LOC100736756	339.8608177	28.91974301	3.554817407	6.68E-05	High	410	35	XP_003482433.1	ssc:100736756			
LOC100739118	97.81360119	1092.340007	-3.481243088	6.70E-05	Low	118	1322	XP_005671292.1	ssc:100739118	3E-146	NM_178145.1	RASSF4
YOD1	454.2529953	39.66136184	3.517689907	6.72E-05	High	548	48	YOD1	ssc:100520400	0	NM_018566.3	YOD1
LOC100516260	4.973572942	88.41178578	-4.151884161	6.74E-05	Low	6	107	XR_302733.1	ssc:100516260			
C7H15orf59	1.657857647	52.88181579	-4.995379676	6.76E-05	Low	2	64	XP_001928647.1	ssc:100156505	5E-139	NM_001039614.1	C15orf59
LOC100155492	1.657857647	52.88181579	-4.995379676	6.76E-05	Low	2	64	XP_005660322.1	ssc:100155492	1E-123	NM_197978.2	HEMGN
LOC100515572	1.657857647	52.88181579	-4.995379676	6.76E-05	Low	2	64	XP_003130869.4	ssc:100515572	3E-92	NM_001029880.2	SFMBT2
LOC100627069	70.45895001	3.305113487	4.41401126	6.81E-05	High	85	4	XP_005673982.1	ssc:100627069	2E-160	NM_147175.3	HS6ST2
FHL3	248.6786471	2794.473453	-3.49022193	6.83E-05	Low	300	3382	NM_213946.2	ssc:396986	1E-159	NM_004468.4	FHL3
LOC102159890	33.15715294	0	20	6.88E-05	High	40	0	XP_005662372.1	ssc:102159890			
LOC102163772	33.15715294	0	20	6.88E-05	High	40	0	XR_309197.1	ssc:102163772			
FUT9	33.15715294	0	20	6.88E-05	High	40	0	XP_005659365.1	ssc:102160171	0	NM_006581.3	FUT9
LOC100738781	33.15715294	0	20	6.88E-05	High	40	0	XP_003483797.1	ssc:100738781	8E-179	NM_020864.1	NYAP2
LOC102165955	33.15715294	0	20	6.88E-05	High	40	0	XP_005659279.1	ssc:102165955			
LOC100739711	66.31430589	741.9979778	-3.484023216	6.92E-05	Low	80	898	XP_003484352.2	ssc:100739711	9E-136	NM_012449.2	STEAP1
C14H10orf54	21.55214941	261.1039655	-3.598720706	6.98E-05	Low	26	316	XP_005671104.1	ssc:100154373	1E-141	NM_022153.1	C10orf54
ARID3B	3608.327169	321.4222866	3.488788284	7.06E-05	High	4353	389	XP_003128540.3	ssc:100521805	0	NM_006465.2	ARID3B
MCOLN2	337.3740312	28.91974301	3.544222292	7.09E-05	High	407	35	XP_003127954.1	ssc:100522324	0	NM_153259.2	MCOLN2
GDNF	39.78858353	453.6268261	-3.511079514	7.12E-05	Low	48	549	XP_005672481.1	ssc:100322894	2E-73	NM_199234.1	GDNF
LOC100626667	0	33.05113487	-20	7.23E-05	Low	0	40	XP_005655752.1	ssc:100626667	2E-39	NM_031491.2	RBP5
RHCG	11.60500353	156.1666123	-3.750267178	7.31E-05	Low	14	189	NM_001044577.1	ssc:733644			
GSC2	112.73432	7.436505346	3.922158164	7.32E-05	High	136	9	XP_001927461.3	ssc:100152323	9E-62	NM_005315.1	GSC2
NRROS	172.4171953	13.22045395	3.705060042	7.32E-05	High	208	16	XP_003358810.1	ssc:100620479	0	NM_198565.1	NRROS
TDRD10	87.03752648	4.95767023	4.133903341	7.35E-05	High	105	6	NM_001246198.1	ssc:100157928	2E-155	NM_182499.3	TDRD10
DENND2D	146.7204018	10.74161883	3.771786156	7.36E-05	High	177	13	XP_005663617.1	ssc:100154003	0	NM_024901.4	DENND2D
C8H4orf21	545.4351659	48.75042393	3.483921049	7.42E-05	High	658	59	XP_005656600.1	ssc:102167653	0	NM_138698.2	C4orf21
BICC1	60.51180412	672.5905946	-3.474440101	7.48E-05	Low	73	814	XP_001929158.3	ssc:100154253	0	NM_001080512.1	BICC1
XIRP2	8.289288236	121.4629206	-3.873123926	7.51E-05	Low	10	147	NM_214396.1	ssc:397689	0	NM_152381.5	XIRP2
ARMC12	163.2989782	12.39417558	3.719781548	7.53E-05	High	197	15	XP_005665973.1	ssc:100157423	5E-24	NM_145028.4	ARMC12
SGCE	138.4311135	1523.657317	-3.460298324	7.57E-05	Low	167	1844	NM_001144124.1	ssc:100240725			
LOC102161685	9310.528547	813.8841962	3.51596763	7.60E-05	High	11232	985	XP_005670278.1	ssc:102161685	3E-31	NM_014590.3	ERVW-1
NFIC	558.6980271	6331.771163	-3.502468485	7.60E-05	Low	674	7663	XP_005654783.1	ssc:100737330	5E-165	NM_205843.2	NFIC
LOC100623746	1.657857647	52.05553742	-4.972659599	7.62E-05	Low	2	63	LOC100623746				
LRRC31	1.657857647	52.05553742	-4.972659599	7.62E-05	Low	2	63	XP_005670050.1	ssc:100625984	2E-99	NM_024727.3	LRRC31
TMEM74	205.5743483	16.52556743	3.63688854	7.63E-05	High	248	20	XP_005662957.1	ssc:100525987	2E-140	NM_153015.1	TMEM74
LOC102163572	179.8775547	14.04673232	3.678708716	7.68E-05	High	217	17	XR_305335.1	ssc:102163572			
TAF4B	1766.447323	161.1242825	3.454604888	7.74E-05	High	2131	195	XP_003127923.1	ssc:100037274	0	NM_005640.1	TAF4B
LOC100622991	25.69679353	300.7653273	-3.548978005	7.79E-05	Low	31	364	XP_003360998.2	ssc:100622991	5E-77	NR_073131.1	NEDD9
LOC102164379	256.1390065	21.48323766	3.575643634	7.81E-05	High	309	26	XR_306462.1	ssc:102164379			
LOC102159691	60.51180412	2.478835115	4.609482382	7.83E-05	High	73	3	XR_302105.1	ssc:102159691			
LOC100515112	60.51180412	2.478835115	4.609482382	7.83E-05	High	73	3	XR_309082.1	ssc:100515112			
DIO3	3.315715294	69.40738323	-4.387697098	7.93E-05	Low	4	84	NM_001001625.2	ssc:414378	1E-154	NM_001362.3	DIO3
MOGAT1	367.2154689	32.2248565	3.510380994	7.94E-05	High	443	39	XP_003133724.1	ssc:100524113	3E-137	NM_058165.2	MOGAT1
LOC100626135	81.23502471	889.9018064	-3.453472366	7.98E-05	Low	98	1077	XP_005652614.1	ssc:100626135	5E-130	NM_198316.1	TENC1
LOC102161276	5.802501765	95.02201275	-4.033514805	8.01E-05	Low	7	115	XR_304701.1	ssc:102161276			
PTPRC	51.39358706	1.652556743	4.958816635	8.14E-05	High	62	2	XP_003130644.3	ssc:100522631	0	NR_052021.1	PTPRC
THBD	688.0109236	62.79715625	3.453660337	8.17E-05	High	830	76	NM_001130732.1	ssc:100157642	0	NM_000361.2	THBD
ITGB2	644.0776959	58.66576439	3.456643993	8.17E-05	High	777	71	NM_213908.1	ssc:396943	0	NM_001127491.1	ITGB2
LOC102162465	68.80109236	3.305113487	4.379659756	8.20E-05	High	83	4	XP_005662749.1	ssc:102162465	2E-46	NM_014971.1	EFR3B
ANXA2R	32.32822412	0	20	8.26E-05	High	39	0	XP_005675675.1	ssc:102162127	4E-11	NM_001014279.2	ANXA2R
LOC102164815	32.32822412	0	20	8.26E-05	High	39	0	XP_005656540.1	ssc:102164815			
SLC18A3	32.32822412	0	20	8.26E-05	High	39	0	XP_005671287.1	ssc:100157804			
LOC102158025	32.32822412	0	20	8.26E-05	High	39	0	XR_309275.1	ssc:102158025			
LOC102163446	32.32822412	0	20	8.26E-05	High	39	0	XP_005670718.1	ssc:102163446	7E-74	NM_152591.2	CCDC63
JAK1	190.6536294	2083.874054	-3.450242185	8.27E-05	Low	230	2522	NM_214114.1	ssc:397202	0	NM_002227.2	JAK1
EPB41L3	162.4700494	1771.540829	-3.446759022	8.29E-05	Low	196	2144	XP_005665352.1	ssc:100520913	0	NM_012307.3	EPB41L3
PIM2	3600.037881	327.2062352	3.459739942	8.40E-05	High	4343	396	XP_003360359.1	ssc:100621339	3E-161	NM_006875.3	PIM2
LOC100739791	4720.74965	426.3596398	3.468873196	8.45E-05	High	5695	516	XR_309134.1	ssc:100739791	4E-33	NM_033054.2	MYO1G
TCF21	13.26286118	170.2133446	-3.681880203	8.46E-05	Low	16	206	XP_005652728.1	ssc:100152962	6E-84	NM_198392.2	TCF21
RAB42	763.4434465	70.2336616	3.442286734	8.51E-05	High	921	85	XP_003356293.1	ssc:100621746	2E-99	NM_152304.1	RAB42
SPMI	0.828928824	42.14019696	-5.667805018	8.56E-05	Low	1	51	NM_001031776.1	ssc:396758			
CXCL9	0	32.2248565	-20	8.68E-05	Low	0	39	NM_001114289.2	ssc:100135681	2E-28	NM_002416.1	CXCL9
LOC102163603	0	32.2248565	-20	8.68E-05	Low	0	39	LOC102163603				
LOC100623513	6.631430589	102.4585181	-3.949575986	8.69E-05	Low	8	124	XP_005663610.1	ssc:100623513	1E-122	NM_002506.2	NGF
CTSA	1356.956484	15585.26265	-3.521736107	8.73E-05	Low	1637	18862	NM_001243629.1	ssc:100156798	0	NM_001167594.1	CTSA
ARID3C	319.1375971	28.09346464	3.505872119	9.02E-05	High	385	34	XP_005668105.1	ssc:100524334	5E-141	NM_001017363.1	ARID3C
LOC100624937	4.973572942	85.10667229	-4.096917702	9.09E-05	Low	6	103	XP_005658007.1	ssc:100624937	9E-145	NM_005089.3	ZRSR2
LOC100625519	637.4462654	7099.38377	-3.477318166	9.12E-05	Low	769	8592	XP_003354492.2	ssc:100625519	0	NM_003088.3	FSCN1
LOC100627927	5.802501765	93.36945601	-4.008203716	9.19E-05	Low	7	113	XP_005674341.1	ssc:100627927	4E-68	NM_001080509.2	TSPAN11
LOC102162660	50.56465824	1.652556743	4.935357662	9.20E-05	High	61	2	XR_302177.1	ssc:102162660			
HOMER2	3265.150636	300.7653273	3.440439386	9.24E-05	High	3939	364	XP_005666167.1	ssc:100156419	0	NM_199332.2	HOMER2
LOC102162761	92.84002824	5.783948602	4.004620324	9.26E-05	High	112	7	XR_306805.1	ssc:102162761			
LRRC43	117.707893	8.262783717	3.832439349	9.27E-05	High	142	10	XP_005654144.1	ssc:100739732	0	NM_152759.4	LRRC43
SNX9	820.6395354	9194.825721	-3.486001665	9.29E-05	Low	990	11128	XP_005654411.1	ssc:100627455	0	NM_016224.4	SNX9
FAM185A	1176.250001	109.8950234	3.419996763	9.29E-05	High	1419	133	XP_005667762.1	ssc:100512804	4E-116	NR_026879.1	FAM185A
LRRC1	1637.134427	152.8614988	3.420875802	9.36E-05	High	1975	185	XP_001927723.2	ssc:100154780	0	NM_025168.1	LRRC1
SHPK	14.09179	175.9972932	-3.642626455	9.55E-05	Low	17	213	XP_005669175.1	ssc:100515408	0	NM_013276.2	SHPK
LOC100620996	140.9179	1507.958028	-3.419669488	9.58E-05	Low	170	1825	XR_302439.1	ssc:100620996	4E-27	NM_006759.3	UGP2
OLFML3	694.6423542	7709.177208	-3.472234608	9.61E-05	Low	838	9330	XP_005655478.1	ssc:100158055	0	NM_020190.3	OLFML3
FMNL3	151.6939747	1623.637	-3.419993435	9.61E-05	Low	183	1965	XP_005674225.1	ssc:100620423	0	NM_198900.2	FMNL3
RNF144B	5998.128968	546.1700037	3.457090555	9.63E-05	High	7236	661	XP_005665667.1	ssc:100155665	1E-169	NM_182757.3	RNF144B
IL1RL1	16.57857647	200.7856443	-3.598264814	9.63E-05	Low	20	243	XP_005662414.1	ssc:100127134	0	NR_104167.1	IL1RL1
LOC102163367	349.8079636	31.39857813	3.477792	9.73E-05	High	422	38	LOC102163367				
TRIB2	595.1708953	6558.171436	-3.461917732	9.79E-05	Low	718	7937	XP_003125426.2	ssc:100525217	2E-161	NR_027303.1	TRIB2
C1H9orf116	4.973572942	84.28039392	-4.082842517	9.81E-05	Low	6	102	XP_003353756.1	ssc:100518594	6E-63	NM_144654.2	C9orf116
SRGAP3	535.48802	49.5767023	3.433120083	9.86E-05	High	646	60	XP_003130506.2	ssc:100521110			
NTF3	11.60500353	150.3826637	-3.695819394	9.89E-05	Low	14	182	NM_001123152.1	ssc:100144493	4E-143	NM_002527.4	NTF3
LOC102167902	11.60500353	150.3826637	-3.695819394	9.89E-05	Low	14	182	XP_005666644.1	ssc:102167902	7E-62	NM_199039.3	KLHL5
LOC100519756	100.3003877	6.610226974	3.923483562	9.89E-05	High	121	8	XP_005657976.1	ssc:100519756	9E-24	NM_001163438.1	SMIM10
SLC5A5	67.14323471	3.305113487	4.344470327	9.90E-05	High	81	4	NM_214410.1	ssc:399542	0	NM_000453.2	SLC5A5
LOC100155163	399.543693	36.35624836	3.458078042	9.90E-05	High	482	44	XP_005656348.1	ssc:100155163	0	NM_194301.2	RALGAPA1
RET	265.2572236	23.13579441	3.519193497	9.95E-05	High	320	28	XP_001926404.3	ssc:100153852	0	NM_020975.4	RET
LOC100621683	31.4992953	0	20	9.96E-05	High	38	0	XP_005667909.1	ssc:100621683	2E-33	NM_018664.2	BATF3
VWA2	31.4992953	0	20	9.96E-05	High	38	0	XP_001927121.3	ssc:100152718	0	np_940898	np_940898
LOC102158596	31.4992953	0	20	9.96E-05	High	38	0	XR_298346.1	ssc:102158596			
UNC5D	83.72181118	4.95767023	4.077869306	9.97E-05	High	101	6	XP_005671842.1	ssc:100156454	0	NM_080872.2	UNC5D
TSPAN13	2519.114695	235.4893359	3.419183175	9.99E-05	High	3039	285	XP_005667721.1	ssc:100624728	5E-53	NM_014399.3	TSPAN13
RAB39A	508.1333689	47.09786719	3.431473574	1.02E-04	High	613	57	XP_003129872.1	ssc:100525827	7E-126	NM_017516.1	RAB39A
SPOCK1	132.6286118	9.915340461	3.741585919	1.02E-04	High	160	12	XP_005661736.1	ssc:100620623	0	NM_004598.3	SPOCK1

LOC102157425	49.73572942	1.652556743	4.91151092	1.04E-04	High	60	2	XR_305501.1	ssc:102157425			
LOC100512920	49.73572942	1.652556743	4.91151092	1.04E-04	High	60	2	XR_306463.1	ssc:100512920			
MAP2	58.85394648	628.7978409	-3.4173852	1.04E-04	Low	71	761	XP_005672207.1	ssc:100153306	0	NM_031847.2	MAP2
LOC102164680	0	31.39857813	-20	1.04E-04	Low	0	38	XR_305540.1	ssc:102164680			
LOC100626991	0	31.39857813	-20	1.04E-04	Low	0	38	XP_005667732.1	ssc:100626991			
ABCC9	0	31.39857813	-20	1.04E-04	Low	0	38	XP_005655715.1	ssc:100127449	0	NM_020298.2	ABCC9
LOC100625402	0	31.39857813	-20	1.04E-04	Low	0	38	XP_003358402.1	ssc:100625402	1E-62	NM_198399.1	ARPP21
LOC102167414	0	31.39857813	-20	1.04E-04	Low	0	38	XP_005656946.1	ssc:102167414			
LOC100620352	0	31.39857813	-20	1.04E-04	Low	0	38	XP_003353285.1	ssc:100620352	0	NM_172105.3	EYA4
EYA4	0	31.39857813	-20	1.04E-04	Low	0	38	XP_005652730.1	ssc:100157256	0	NM_172105.3	EYA4
ZNF185	25.69679353	290.0237085	-3.496510586	1.05E-04	Low	31	351	ZNF185	ssc:100152632	0	NM_007150.3	ZNF185
SRSF12	915.1374213	86.75922903	3.398899263	1.06E-04	High	1104	105	NM_001195598.1	ssc:100152352			
RHOC	1550.0969	17426.21086	-3.490828596	1.09E-04	Low	1870	21090	XP_003125894.1	ssc:100520019	4E-112	NM_175744.4	RHOC
MYL5	40.61751236	0.826278372	5.619330168	1.09E-04	High	49	1	XP_005653614.1	ssc:100625329	1E-89	NM_002477.1	MYL5
LOC100515686	1.657857647	49.5767023	-4.902270271	1.10E-04	Low	2	60	XP_003131634.1	ssc:100515686	5E-44	NM_153229.2	TMEM92
BHLHE40	412.8065542	4428.852072	-3.423395029	1.12E-04	Low	498	5360	NM_001245010.1	ssc:100514873	0	NM_003670.2	BHLHE40
KRBA2	155.00969	12.39417558	3.644624189	1.14E-04	High	187	15	XP_005674593.1	ssc:100621261	5E-61	NM_213597.2	KRBA2
SYDE1	342.3476041	3643.887619	-3.411944645	1.14E-04	Low	413	4410	XP_003123474.1	ssc:100517807	0	NM_033025.4	SYDE1
SKP2	3047.971284	288.3711517	3.401850559	1.15E-04	High	3677	349	NM_001171755.1	ssc:100329128	0	NM_032637.3	SKP2
C14H10orf82	48.90680059	1.652556743	4.887263374	1.18E-04	High	59	2	XP_005671539.1	ssc:100154177	9E-70	NM_144661.2	C10orf82
LOC100736825	48.90680059	1.652556743	4.887263374	1.18E-04	High	59	2	LOC100736825				
LOC100621440	48.90680059	1.652556743	4.887263374	1.18E-04	High	59	2	XP_005658221.1	ssc:100621440	1E-134	NM_024759.1	NIPAL2
CUEDC1	234.5868571	2463.135826	-3.392302093	1.19E-04	Low	283	2981	NM_001243919.1	ssc:100511955	4E-179	np_060419	np_060419
CYP7A1	30.67036647	0	20	1.20E-04	High	37	0	NM_001005352.2	ssc:448985	0	NM_000780.3	CYP7A1
LOC100620374	57.19608883	2.478835115	4.52818228	1.20E-04	High	69	3	XP_003356374.2	ssc:100620374			
LOC100625123	72.11680765	751.0870399	-3.380572664	1.23E-04	Low	87	909	XP_005659108.1	ssc:100625123	1E-43	NM_019009.3	TOLLIP
LOC102160096	413.635483	38.83508347	3.412927478	1.23E-04	High	499	47	XR_297711.1	ssc:102160096			
ARHGDIB	1.657857647	48.75042393	-4.878022725	1.25E-04	Low	2	59	NM_001244240.1	ssc:100155466	1E-100	NM_001175	ARHGDIB
EMP2	1.657857647	48.75042393	-4.878022725	1.25E-04	Low	2	59	XP_003481093.1	ssc:100739825	5E-80	NM_001424.4	EMP2
LOC100511091	49.73572942	523.0342093	-3.394550769	1.25E-04	Low	60	633	XP_005666652.1	ssc:100511091	5E-84	NM_199039.3	KLHL5
STRBP	5349.077699	504.0298068	3.407709202	1.26E-04	High	6453	610	XP_001927197.1		0	NR_033234.1	STRBP
LOC100738968	7.460359412	105.7636316	-3.825454674	1.26E-04	Low	9	128	XP_003481531.1	ssc:100738968	7E-123	NM_002506.2	NGF
LOC100736982	0	30.57229975	-20	1.26E-04	Low	0	37	XP_005669300.1	ssc:100736982	0	NM_003802.2	MYH13
DCTPP1	3711.114343	353.6471431	3.391469941	1.27E-04	High	4477	428	XP_003354588.1	ssc:100621901	2E-74	NM_024096.1	DCTPP1
LOC102161750	39.78858353	0.826278372	5.589582825	1.27E-04	High	48	1	XR_299037.1	ssc:102161750			
LOC102166494	39.78858353	0.826278372	5.589582825	1.27E-04	High	48	1	XR_302283.1	ssc:102166494			
PGM2	4630.396409	438.7538154	3.399652132	1.27E-04	High	5586	531	XP_003128958.1	ssc:100522261	0	NM_018290.3	PGM2
SADC	2.486786471	57.01320765	-4.518941632	1.27E-04	Low	3	69	NM_001123193.1	ssc:100144439	0	NM_052998.2	ADC
LOC100523505	176.5618394	14.87301069	3.569404943	1.29E-04	High	213	18	XP_005667682.1	ssc:100523505	1E-89	NM_004411.4	DYNC1I1
SH2D5	1087.554617	105.7636316	3.362172329	1.29E-04	High	1312	128	XP_005665125.1	ssc:100515781	0	NM_001103161.1	SH2D5
HSP70.2	1001.346019	10857.2978	-3.438652592	1.30E-04	Low	1208	13140	NM_213766.1	ssc:396648			
USH1G	435.1876324	41.31391859	3.396937747	1.31E-04	High	525	50	XP_003131267.1	ssc:100512251	0	NM_173477.3	USH1G
ARAP2	8.289288236	113.2001369	-3.771483664	1.31E-04	Low	10	137	XP_003128954.1	ssc:100521564	0	NM_139182.1	ARAP2
LOC102167148	15.74964765	185.0863553	-3.554807084	1.32E-04	Low	19	224	XP_005667759.1	ssc:102167148	1E-61	NM_012301.3	MAGI2
SNTB1	9.947145883	128.899426	-3.695819394	1.33E-04	Low	12	156	XP_005662942.1	ssc:100154009	0	NM_021021.3	SNTB1
PANK3	2537.351129	245.4046764	3.370088533	1.34E-04	High	3061	297	XP_005672611.1	ssc:100512746	8E-158	NM_024594.3	PANK3
NRIP3	450.1083512	42.96647533	3.388988994	1.34E-04	High	543	52	XP_005667099.1	ssc:100519788	8E-122	NM_020645.2	NRIP3
ITIH2	48.07787177	1.652556743	4.862601319	1.34E-04	High	58	2	NM_213903.1	ssc:396937	0	NM_002216.2	ITIH2
LOC102158304	215.5214941	19.00440255	3.503426181	1.35E-04	High	260	23	XR_297416.1	ssc:102158304			
LOC102167532	63.82751942	659.3701406	-3.368838071	1.35E-04	Low	77	798	LOC102167532				
TIAM1	819.8106065	80.14900206	3.354534193	1.38E-04	High	989	97	XP_001927581.1				
LOC102158822	9.947145883	128.0731476	-3.68654158	1.40E-04	Low	12	155	XR_299778.1	ssc:102158822			
DNAJC12	463.3712124	44.61903207	3.376437295	1.42E-04	High	559	54	XP_001925277.1	ssc:100153566	8E-98	NM_201262.1	DNAJC12
LOC102167199	2.486786471	56.18692928	-4.497880016	1.42E-04	Low	3	68	XR_306384.1	ssc:102167199			
LOC100156509	7.460359412	104.1110748	-3.802734598	1.42E-04	Low	9	126	XP_001927312.2	ssc:100156509	9E-98	NR_104579.1	RGS20
LOC102167158	857.9413324	84.28039392	3.347610035	1.42E-04	High	1035	102	XR_300919.1	ssc:102167158			
LOC100523701	9847.674424	923.7792196	3.414163067	1.43E-04	High	11880	1118	XP_005672121.1	ssc:100523701	0	NM_001159.3	AOX1
GADD45G	1862.603067	182.6075202	3.350502174	1.43E-04	High	2247	221	NM_001185129.1	ssc:100152997	5E-87	NM_006705.3	GADD45G
LOC102157728	594.3419665	6259.058666	-3.39658054	1.43E-04	Low	717	7575	XR_300556.1	ssc:102157728			
LOC102167359	9.11821706	119.8103639	-3.715857147	1.43E-04	Low	11	145	XR_303838.1	ssc:102167359			
LOC102161787	29.84143765	0	20	1.46E-04	High	36	0	XP_005664221.1	ssc:102161787	2E-146	NM_001004134.1	OR10AD1
DAO1	29.84143765	0	20	1.46E-04	High	36	0	NM_214066.2	ssc:397134	4E-180	NM_001917.4	DAO
NPHS1	29.84143765	0	20	1.46E-04	High	36	0	XP_005664590.1	ssc:100622946	0	NM_004646.3	NPHS1
LOC100516162	29.84143765	0	20	1.46E-04	High	36	0	XR_299606.1	ssc:100516162			
SCTR	29.84143765	0	20	1.46E-04	High	36	0	NM_001204902.1	ssc:100521045	4E-119	NM_002980.2	SCTR
LOC100515312	29.84143765	0	20	1.46E-04	High	36	0	XP_003125807.1	ssc:100515312	7E-134	NM_001136003.1	C2CD4D
LOC102165288	29.84143765	0	20	1.46E-04	High	36	0	XP_005672232.1	ssc:102165288	1E-24	NM_018000.2	MREG
LOC100738827	33.15715294	350.3420296	-3.401372035	1.47E-04	Low	40	424	XP_005655814.1	ssc:100738827	8E-95	NM_001243661.1	NUDT7
LIMD1	2060.717055	202.4382011	3.347592966	1.47E-04	High	2486	245	XP_005669512.1	ssc:100738579	0	NM_014240.2	LIMD1
POU5F1	79.57716707	4.95767023	4.004620324	1.48E-04	High	96	6	NM_001113060.1	ssc:100127461	1E-176	NM_203289.5	POU5F1
LOC102161720	38.95965471	0.826278372	5.559209176	1.48E-04	High	47	1	XP_005668731.1	ssc:102161720			
CYGB	110.2475335	1117.954637	-3.342043364	1.48E-04	Low	133	1353	XP_003131227.2	ssc:100518861	2E-97	NM_134268.4	CYGB
LOC100737264	257.7968641	2640.785676	-3.356660598	1.49E-04	Low	311	3196	XP_003483748.1	ssc:100737264	0	NM_014585.5	SLC40A1
SLC40A1	257.7968641	2637.480563	-3.354853842	1.50E-04	Low	311	3192	XP_003359638.1	ssc:100512076	0	NM_014585.5	SLC40A1
LOC100514340	38.95965471	405.7026805	-3.380370038	1.51E-04	Low	47	491	NM_001244954.1	ssc:100514340			
FAM78A	102.7871741	7.436505346	3.788891633	1.52E-04	High	124	9	XP_003353738.1	ssc:100624292	1E-162	NM_033387.3	FAM78A
MDK	2364.933934	25947.61971	-3.455730413	1.52E-04	Low	2853	31403	NM_001195352.1	ssc:100359358	2E-53	NR_073039.1	MDK
TAPBP	634.9594789	6649.888336	-3.388593684	1.52E-04	Low	766	8048	XP_005665941.1	ssc:100155428	0	NR_172209.2	TAPBP
CCDC155	47.24894295	1.652556743	4.837510339	1.53E-04	High	57	2	XP_005664784.1	ssc:100515317	0	NM_144688.4	CCDC155
LOC100624808	0	29.74602138	-20	1.53E-04	Low	0	36	XP_003360907.2	ssc:100624808	1E-179	NM_024788.2	C7orf63
LOC100152800	0	29.74602138	-20	1.53E-04	Low	0	36	XP_005674266.1	ssc:100152800	1E-101	NM_006192.4	PAX1
UNCX	0	29.74602138	-20	1.53E-04	Low	0	36	XP_003124306.1	ssc:100523430	9E-115	NM_001080461.1	UNCX
CSMD2	0	29.74602138	-20	1.53E-04	Low	0	36	XP_005656143.1	ssc:100736699	0	NM_052896.4	CSMD2
LOC102160529	3.315715294	63.62343462	-4.262166216	1.54E-04	Low	4	77	LOC102160529				
SDCBP2	434.3587036	42.14019696	3.365617984	1.54E-04	High	524	51	NM_001244863.1	ssc:100513633	2E-142	NM_080489.4	SDCBP2
PLEKHH3	116.8789641	1179.099236	-3.334597943	1.55E-04	Low	141	1427	XP_005668856.1	ssc:100518749	0	NR_073574.1	PLEKHH3
SYT1	18.23643412	204.9170362	-3.490144367	1.55E-04	Low	22	248	XP_005652680.1	ssc:100513599	0	NM_005639.2	SYT1
LOC100738039	46.42001412	476.7626205	-3.360452262	1.56E-04	Low	56	577	XR_306946.1	ssc:100738039	2E-40	NM_181897.2	PPP2R3A
LOC100621472	1296.44468	128.899426	3.330242903	1.56E-04	High	1564	156	XP_003480569.1	ssc:100621472	0	NM_130393.3	PTPRD
LOC100628224	35.64393941	371.8252673	-3.382896112	1.56E-04	Low	43	450	XP_005655269.1	ssc:100628224	4E-33	NM_006634.2	VAMP5
HRH1	914.3084924	90.89062089	3.330477686	1.56E-04	High	1103	110	XP_005669849.1	ssc:100523996	0	NM_001098213.1	HRH1
KANK3	156.6675477	13.22045395	3.566862749	1.57E-04	High	189	16	XP_003123210.1	ssc:100513584	0	NM_198471.2	KANK3
SRCIN1	94.49788589	6.610226974	3.837510339	1.58E-04	High	114	8	XP_005654075.1	ssc:100513819	0	NM_025248.2	SRCIN1
TCIRG1	370.5311842	3802.533067	-3.359293923	1.58E-04	Low	447	4602	XP_005660671.1	ssc:100521769	0	NM_006053.3	TCIRG1
LOC100153854	561.1848136	55.36065091	3.341543157	1.60E-04	High	677	67	XP_005663685.1	ssc:100153854	0	NM_000699.2	AMY2A
LOC100624979	878.664553	87.5855074	3.326548419	1.60E-04	High	1060	106	XR_131348.2	ssc:100624979			
TMEM61	1.657857647	47.09786719	-4.82826969	1.61E-04	Low	2	57	NM_001243365.1	ssc:100628096	3E-67	NM_182532.1	TMEM61
B2R	1.657857647	47.09786719	-4.82826969	1.61E-04	Low	2	57	NM_214146.1	ssc:397277	4E-173	NM_000623.3	BDKRB2
FLNA	717.8523612	7493.518553	-3.383884225	1.62E-04	Low	866	9069	XP_005659106.1	ssc:733585	1E-44	NM_001456.3	FLNA
FRZB	11.60500353	141.2936016	-3.605877268	1.62E-04	Low	14	171	NM_001243330.1	ssc:100152419	3E-171	NM_001463.3	FRZB

ENPP6	22.38107824	242.9258413	-3.440164518	1.63E-04	Low	27	294	XP_005671763.1	ssc:100152050	1E-169	NM_153343.3	ENPP6
ZIC1	153.3518324	1539.356607	-3.327410174	1.63E-04	Low	185	1863	ZIC1	ssc:100153570	0	NM_003412.3	ZIC1
APC2	2238.107824	222.268882	3.331901654	1.63E-04	High	2700	269	NM_001206431.1	ssc:100579172	0	NM_005883.2	APC2
APOBEC3F	25.69679353	274.3244194	-3.416222797	1.64E-04	Low	31	332	NM_001097446.1	ssc:100037939			
LOC100627126	704.5895001	70.2336616	3.326548419	1.64E-04	High	850	85	XR_305631.1	ssc:100627126			
LEF1	3284.215999	323.9011217	3.341923645	1.66E-04	High	3962	392	NM_001129967.1	ssc:100170126	0	NM_016269.4	LEF1
LOC102160168	327.4268853	31.39857813	3.382401654	1.66E-04	High	395	38	XP_005669789.1	ssc:102160168	2E-61	NM_020872.1	CNTN3
LOC102167659	225.46864	20.65695929	3.448226976	1.66E-04	High	272	25	XR_305951.1	ssc:102167659			
LOC102162329	54.70930236	2.478835115	4.464051943	1.68E-04	High	66	3	XR_302438.1	ssc:102162329			
LOC100738892	3.315715294	62.79715625	-4.243307189	1.70E-04	Low	4	76	XP_005652485.1	ssc:100738892	2E-112	NM_007115.3	TNFAIP6
LOC100153054	3.315715294	62.79715625	-4.243307189	1.70E-04	Low	4	76	XR_301385.1	ssc:100153054			
DENND3	1538.491897	154.5140555	3.31570686	1.71E-04	High	1856	187	XP_005662889.1	ssc:102164758	3E-104	NM_014957.2	DENND3
PRSS8	9.947145883	124.7680341	-3.648821914	1.72E-04	Low	12	151	XP_003124520.1	ssc:100522730	2E-139	NM_002773.3	PRSS8
PINK1	239.56043	2406.122618	-3.328248633	1.72E-04	Low	289	2912	XP_005665148.1	ssc:100515613	0	NM_032409.2	PINK1
LOC102161702	131.799683	10.74161883	3.617063561	1.73E-04	High	159	13	XR_298374.1	ssc:102161702			
GCK	756.8120159	76.0176102	3.315529418	1.73E-04	High	913	92	XP_003134931.1	ssc:100514142	0	NM_033508.1	GCK
LOC102160289	38.13072589	0.826278372	5.52818228	1.73E-04	High	46	1	XP_005670228.1	ssc:102160289	8E-74	NR_046079.1	SEMA5B
LOC100623141	38.13072589	0.826278372	5.52818228	1.73E-04	High	46	1	XP_005658224.1	ssc:100623141	5E-80	NM_001647.3	APOD
TGFBI	73.7746653	735.3877508	-3.317307771	1.75E-04	Low	89	890	XP_005661717.1	ssc:100519300	0	NM_000358.2	TGFBI
ALDH1L1	247.0207894	23.13579441	3.416433923	1.77E-04	High	298	28	NM_001244647.1	ssc:100622210	0	NR_072979.1	ALDH1L1
LOC102161807	562.0137424	56.18692928	3.322298946	1.77E-04	High	678	68	XP_005669129.1	ssc:102161807	0	NM_024857.3	ATAD5
LOC100515054	85.37966883	5.783948602	3.883765929	1.78E-04	High	103	7	XP_005669796.1	ssc:100515054	3E-139	NM_002222.5	ITPR1
LOC102168065	29.01250883	0	20	1.78E-04	High	35	0	XR_309024.1	ssc:102168065			
CAPN9	29.01250883	0	20	1.78E-04	High	35	0	XP_001927273.1	ssc:100154688	0	NM_016452.1	CAPN9
LOC102163405	29.01250883	0	20	1.78E-04	High	35	0	XR_306189.1	ssc:102163405			
LOC100523921	29.01250883	0	20	1.78E-04	High	35	0	LOC100523921				
NANOG	29.01250883	0	20	1.78E-04	High	35	0	NM_001129971.1	ssc:100170132	5E-121	NM_024865.2	NANOG
MFSD6	1206.920367	122.289199	3.302961599	1.81E-04	High	1456	148	NM_001097457.1	ssc:100037960	0	NM_017694.3	MFSD6
LOC102163538	31.4992953	325.5536785	-3.369503982	1.82E-04	Low	38	394	XP_005668658.1	ssc:102163538	2E-59	NM_020954.3	RNF213
TMEM108	199.7718465	18.17812418	3.458078042	1.82E-04	High	241	22	XP_005669897.1	ssc:100521929	0	NM_023943.3	TMEM108
CECR2	3993.779072	396.6136184	3.331948407	1.83E-04	High	4818	480	XP_005674672.1	ssc:100737051	2E-140	NM_031413	CECR2
FBN1	1992.744892	21241.96438	-3.414088252	1.84E-04	Low	2404	25708	NM_001001771.1	ssc:414836	0	NM_000138.4	FBN1
HSP27	653.195913	6690.375976	-3.356499618	1.85E-04	Low	788	8097	NM_001007518.1	ssc:493184	1E-100	NM_001540.3	HSPB1
LOC100152887	107.7607471	8.262783717	3.705060042	1.85E-04	High	130	10	XP_005654153.1	ssc:100152887	0	NM_152759.4	LRRC43
LTBP4	380.47833	3829.800253	-3.331382956	1.86E-04	Low	459	4635	XP_005674700.1	ssc:100627785	0	NM_003573.2	LTBP4
LOC102164195	0	28.91974301	-20	1.86E-04	Low	0	35	XR_305910.1	ssc:102164195			
IDO1	0	28.91974301	-20	1.86E-04	Low	0	35	NM_001246240.1	ssc:100519877	2E-175	NM_002164.5	IDO1
CCL8	0	28.91974301	-20	1.86E-04	Low	0	35	NM_001164515.1	ssc:100302703	4E-31	NM_005623.2	CCL8
LOC102163138	0	28.91974301	-20	1.86E-04	Low	0	35	XP_005662123.1	ssc:102163138	9E-94	NM_001012981.4	ZKSCAN2
LOC100739460	153.3518324	13.22045395	3.536001785	1.86E-04	High	185	16	XP_003481348.1	ssc:100739460	6E-112	NM_001307.5	CLDN7
LOC102163981	1056.055321	107.4161883	3.297402074	1.87E-04	High	1274	130	XP_005672587.1	ssc:102163981	5E-60	NM_022132.4	MCCC2
APBA1	14.92071882	169.3870662	-3.504934774	1.87E-04	Low	18	205	XP_003121990.3	ssc:100513711	0	NM_001163.3	APBA1
TMEM180	621.6966177	62.79715625	3.307439596	1.87E-04	High	750	76	XP_005671479.1	ssc:100157822	0	NM_024789.3	TMEM180
LOC100737457	152.5229035	1505.479193	-3.303124963	1.87E-04	Low	184	1822	XP_005662698.1	ssc:100737457	0	NM_206943.2	LTBP1
RTN4RL1	1080.094257	109.8950234	3.296959257	1.87E-04	High	1303	133	XP_005674394.1	ssc:100625930	0	NM_178568.2	RTN4RL1
ARHGAP8	3.315715294	61.97087788	-4.224198366	1.88E-04	Low	4	75	NM_001204383.1	ssc:100533202	0	NM_181334.4	PRR5-ARHGAP8
LOC100627089	3.315715294	61.97087788	-4.224198366	1.88E-04	Low	4	75	XP_005655754.1	ssc:100627089	0	NM_203416.3	CD163
LOC102164246	1288.984321	131.3782611	3.294436234	1.91E-04	High	1555	159	XR_306461.1	ssc:102164246			
PADI1	6.631430589	92.54317763	-3.802734598	1.92E-04	Low	8	112	XP_003127695.3	ssc:100524567	0	NM_013358.2	PADI1
UCP2	2402.235731	242.9258413	3.305789859	1.93E-04	High	2898	294	NM_214289.1	ssc:397549	1E-171	NM_003355.2	UCP2
3-Mar	92.84002824	910.5587656	-3.293933262	1.95E-04	Low	112	1102	NM_001244831.1	ssc:100513526	2E-146	NM_178450.4	3-Mar
C7H15orf27	266.9150812	25.61462952	3.381340892	1.95E-04	High	322	31	XP_001928867.3	ssc:100152648	0	NM_152335.2	C15orf27
MFAP3L	99.47145883	7.436505346	3.741585919	1.96E-04	High	120	9	XP_005670543.1	ssc:100519447	0	NM_021647.6	MFAP3L
CDCA7	8111.897468	795.706072	3.349731904	1.98E-04	High	9786	963	XP_001928667.1	ssc:100154583	0	NM_145810.2	CDCA7
TRAF1	4.973572942	76.84388857	-3.949575986	2.00E-04	Low	6	93	XP_005660453.1	ssc:100627029	4E-69	NM_005658.4	TRAF1
RGS7BP	805.7188165	82.62783717	3.285576638	2.03E-04	High	972	100	NM_001243809.1	ssc:100511050			
ATP10A	113.5632488	1108.039296	-3.286441115	2.03E-04	Low	137	1341	XP_005659852.1	ssc:100158162	0	NM_024490.3	ATP10A
LOC100736623	5.802501765	84.28039392	-3.860450096	2.03E-04	Low	7	102	XP_005672862.1	ssc:100736623	5E-138	NM_080792.2	SIRPA
LOC100737407	5.802501765	84.28039392	-3.860450096	2.03E-04	Low	7	102	XP_003484314.1	ssc:100737407	5E-63	NR_073112.1	SERPINB1
LOC100624812	1383.482207	14376.41739	-3.377328206	2.03E-04	Low	1669	17399	XP_003354541.1	ssc:100624812	1E-100	NM_001540.3	HSPB1
LOC100737961	6.631430589	91.71689926	-3.789795542	2.06E-04	Low	8	111	XR_302877.1	ssc:100737961			
LOC102159457	83.72181118	5.783948602	3.855476885	2.07E-04	High	101	7	XP_005656855.1	ssc:102159457			
MAP3K9	256.9679353	24.78835115	3.373854134	2.10E-04	High	310	30	XP_001925681.3	ssc:100155753	0	NM_033141.3	MAP3K9
NFE2	1.657857647	45.44531045	-4.776739389	2.10E-04	Low	2	55	NM_001185152.1	ssc:100157948	6E-24	NM_006163.2	NFE2
PCDHB8	121.0236082	9.915340461	3.609482382	2.10E-04	High	146	12	XP_005655077.1	ssc:102166091	3E-33	NM_019120.3	PCDHB8
KAL1	707.0762865	72.71249671	3.281590637	2.11E-04	High	853	88	XP_001925105.2	ssc:100154011	0	NM_000216.2	KAL1
BCL6B	53.05144471	2.478835115	4.419657824	2.12E-04	High	64	3	XP_005669250.1	ssc:100517187	0	NM_181844.3	BCL6B
LOC100739491	261.1125794	2560.636674	-3.293758697	2.13E-04	Low	315	3099	XP_003482499.1	ssc:100739491	5E-109	NM_014822.2	SEC24D
PRR14L	6407.619806	638.7131813	3.326548419	2.13E-04	High	7730	773	XP_005670927.1	ssc:100156356	0	NM_173566.2	PRR14L
SH3BP5	405.3461947	4019.844278	-3.30991311	2.13E-04	Low	489	4865	XP_003132111.2	ssc:100517856	0	NM_004844.4	SH3BP5
LOC100624418	4.144644118	68.58110485	-4.048491012	2.14E-04	Low	5	83	XR_131274.2	ssc:100624418			
WASF3	12.43393235	143.7724367	-3.531432576	2.15E-04	Low	15	174	XP_005656890.1	ssc:100155782	3E-130	NM_006646.5	WASF3
HIC2	1762.302679	181.7812418	3.277186492	2.17E-04	High	2126	220	XP_005670974.1	ssc:100151900	0	NM_015094.2	HIC2
KLK15	28.18358	0	20	2.18E-04	High	34	0	XP_003356062.1	ssc:100620705	9E-112	NR_102274.1	KLK15
EGF	28.18358	0	20	2.18E-04	High	34	0	NM_214020.1	ssc:397083	7E-156	NM_001963.4	EGF
NMU	28.18358	0	20	2.18E-04	High	34	0	XR_304890.1	ssc:100523263	2E-65	NM_006681.2	NMU
LOC100514667	28.18358	0	20	2.18E-04	High	34	0	LOC100514667				
LOC102162884	28.18358	0	20	2.18E-04	High	34	0	XR_306025.1	ssc:102162884			
PGLYRP3	28.18358	0	20	2.18E-04	High	34	0	NM_001244361.1	ssc:100626786	1E-151	NM_052891.1	PGLYRP3
LOC100519278	123.5103947	1194.798526	-3.274062992	2.18E-04	Low	149	1446	LOC100519278		0	NM_080424.2	SP110
FAM155B	3462.435696	352.8208647	3.294779493	2.21E-04	High	4177	427	XP_003135206.1	ssc:100518049	0	NM_015686.2	FAM155B
TBX5	33.15715294	332.9901838	-3.328087609	2.21E-04	Low	40	403	HighI00025DF922		0	NM_181486.2	TBX5
USH1C	105.2739606	8.262783717	3.671376916	2.22E-04	High	127	10	XP_005661158.1	ssc:100514274	0	NM_153676.3	USH1C
DUOX2	82.89288236	5.783948602	3.841121592	2.23E-04	High	100	7	NM_213999.2	ssc:397060	0	NM_014080.4	DUOX2
LOC102163336	82.89288236	5.783948602	3.841121592	2.23E-04	High	100	7	XR_307501.1	ssc:102163336			
LOC100737804	1280.695032	133.0308178	3.267094569	2.23E-04	High	1545	161	XP_003480932.1	ssc:100737804	2E-141	NR_074081.1	PPIP5K2
PDGFD	28.18358	286.718595	-3.346708687	2.23E-04	Low	34	347	XP_005667362.1	ssc:100524161	1E-92	NM_033135.3	PDGFD
ETV5	267.74401	2606.908263	-3.283413611	2.27E-04	Low	323	3155	NM_001243025.1	ssc:100623208	1E-108	NM_004454.2	ETV5
LOC100626511	0	28.09346464	-20	2.28E-04	Low	0	34	XP_003357514.2	ssc:100626511	2E-84	NM_178425.2	HDAC9
LOC100516062	0	28.09346464	-20	2.28E-04	Low	0	34	LOC100516062				
LOC100738280	0	28.09346464	-20	2.28E-04	Low	0	34	XR_303920.1	ssc:100738280	2E-21	NM_003433.3	ZNF132
ISG20	0	28.09346464	-20	2.28E-04	Low	0	34	NM_001005351.2	ssc:448855	2E-76	NM_002201.4	ISG20
BMP3	0	28.09346464	-20	2.28E-04	Low	0	34	NM_001206388.1	ssc:100512974	0	NM_001201.2	BMP3
MOG	44.76215647	1.652556743	4.759507826	2.28E-04	High	54	2	NM_001123139.1	ssc:100141421	4E-112	NM_206814.5	MOG
LOC102160907	44.76215647	1.652556743	4.759507826	2.28E-04	High	54	2	XP_005669665.1	ssc:102160907	0	NM_004947.4	DOCK3
ZNF423	77.91930942	751.0870399	-3.268927308	2.28E-04	Low	94	909	ZNF423	ssc:100520077	0	NM_015069.3	ZNF423
LOC102159216	521.39623	53.70809416	3.279168718	2.28E-04	High	629	65	XR_307086.1	ssc:102159216			
CD2AP	1101.646407	114.8526937	3.261804641	2.29E-04	High	1329	139	XP_001925766.5	ssc:100154585	7E-124	NM_012120.2	CD2AP
STOM	159.1543341	1532.74638	-3.267620646	2.30E-04	Low	192	1855	XP_003122151.2	ssc:100510998	2E-112	NR_073037.1	STOM
SERINC2	78.74823824	757.6972669	-3.266301991	2.31E-04	Low	95	917	NM_001244148.1	ssc:100626004	5E-148	NM_178865.4	SERINC2

CCNB3	5935.130377	599.0518195	3.308527012	2.33E-04	High	7160	725	XP_005657865.1	ssc:100515638	0	NM_033671.1	CCNB3
AK1	245.3629318	2376.376597	-3.27577427	2.33E-04	Low	296	2876	XP_005660509.1	ssc:100521423	3E-105	NM_000476.2	AK1
LOC100626258	627.4991195	65.27599137	3.264989066	2.35E-04	High	757	79	XR_304844.1	ssc:100626258			
LOC100620105	4.973572942	75.19133183	-3.918211815	2.36E-04	Low	6	91	XP_005658075.1	ssc:100620105	0	NM_152280.4	SYT11
LOC100156578	4.973572942	75.19133183	-3.918211815	2.36E-04	Low	6	91	XP_005674329.1	ssc:100156578	2E-117	NM_002506.2	NGF
CYP2C32	96.98467236	7.436505346	3.705060042	2.38E-04	High	117	9	XP_003359352.1	ssc:403106	3E-73	NM_001128925.1	CYP2C18
SEMA3G	237.9025724	23.13579441	3.362172329	2.38E-04	High	287	28	XP_001928286.1	ssc:100157653	0	NM_020163.1	SEMA3G
HCLS1	1.657857647	44.61903207	-4.750267178	2.40E-04	Low	2	54	XP_005654114.1	ssc:100521992	0	NM_005335.4	HCLS1
LOC102160052	1.657857647	44.61903207	-4.750267178	2.40E-04	Low	2	54	XR_302532.1	ssc:102160052			
CSRP3	1.657857647	44.61903207	-4.750267178	2.40E-04	Low	2	54	NM_001172368.1	ssc:100337687	3E-110	NM_003476.4	CSRP3
EFEMP1	8980.614875	898.1645901	3.321762475	2.41E-04	High	10834	1087	NM_001244261.1	ssc:100512046	0	NM_004105.3	EFEMP1
LOC100154987	61.34073295	589.9627574	-3.265706574	2.42E-04	Low	74	714	XP_003480443.2	ssc:100154987	0	NM_002373.5	MAP1A
DNAH1	140.9179	12.39417558	3.507120665	2.42E-04	High	170	15	XP_005669688.1	ssc:100156016	0	NM_015512.4	DNAH1
LOC102165438	834.7313254	87.5855074	3.252547838	2.43E-04	High	1007	106	XP_005668039.1	ssc:102165438	0	NM_018136.4	ASPM
LOC100738469	974.8202966	102.4585181	3.250096359	2.44E-04	High	1176	124	XP_003480448.2	ssc:100738469	0	NM_001164273.1	MGA
MST4	3090.246654	319.7697299	3.272616713	2.45E-04	High	3728	387	NM_001143727.1	ssc:100233205	1E-43	NM_016542.3	MST4
SEMA3D	649.8801977	6419.35667	-3.304183028	2.49E-04	Low	784	7769	XP_005667736.1	ssc:100523915	0	NM_152754.2	SEMA3D
LDB2	155.00969	14.04673232	3.464051943	2.51E-04	High	187	17	XP_005666597.1	ssc:100513100	4E-44	NM_001290.3	LDB2
LOC100623335	8.289288236	104.1110748	-3.650731504	2.51E-04	Low	10	126	XP_005658115.1	ssc:100623335	1E-135	NM_022137.5	SMOC1
LOC100627144	2.486786471	52.05553742	-4.387697098	2.51E-04	Low	3	63	XP_005658517.1	ssc:100627144	6E-26	NM_198797.1	PTGES
CADM3	2.486786471	52.05553742	-4.387697098	2.51E-04	Low	3	63	XP_003481475.1	ssc:100157273	0	NM_021189.3	CADM3
LOC100737760	637.4462654	66.92854811	3.251610109	2.52E-04	High	769	81	XP_003483086.2	ssc:100737760	2E-72	NR_103873.1	RAD51C
HOOK1	583.5658918	61.14459951	3.254598577	2.53E-04	High	704	74	XP_005653494.1	ssc:100525467	0	NM_015888.4	HOOK1
DTL	3544.49965	366.867597	3.272250596	2.53E-04	High	4276	444	XR_305730.1	ssc:100525066	0	NM_016448.3	DTL
ZNF287	24.86786471	252.0149034	-3.341154513	2.55E-04	Low	30	305	ZNF287	ssc:100524396	0	NM_020653.2	ZNF287
ELFN2	132.6286118	11.5678972	3.519193497	2.56E-04	High	160	14	XP_005663863.1	ssc:100511563	0	NM_052906	ELFN2
ACOX2	50.56465824	485.8516826	-3.264314683	2.58E-04	Low	61	588	XP_005669744.1	ssc:100516877	0	NM_003500.3	ACOX2
NR1I2	81.23502471	5.783948602	3.811975246	2.60E-04	High	98	7	NM_001038005.1	ssc:397228	0	NM_033013.2	NR1I2
LOC100739348	43.93322765	1.652556743	4.732540779	2.61E-04	High	53	2	XP_005662906.1	ssc:100739348	0	NM_001115.2	ADCY8
RAB3D	523.0540877	5090.701048	-3.282832301	2.65E-04	Low	631	6161	XP_003123225.1	ssc:100516180	1E-114	NM_004283.3	RAB3D
LOC102166835	35.64393941	347.8631945	-3.286791344	2.66E-04	Low	43	421	XR_306590.1	ssc:102166835			
TMEM140	43.93322765	423.0545263	-3.267459221	2.67E-04	Low	53	512	XP_003134698.1	ssc:100523705	5E-54	NM_018295.4	TMEM140
LOC100624257	27.35465118	0	20	2.67E-04	High	33	0	LOC100624257				
LOC102164438	27.35465118	0	20	2.67E-04	High	33	0	XR_306660.1	ssc:102164438			
ONECUT1	27.35465118	0	20	2.67E-04	High	33	0	XP_005659647.1	ssc:100152052	0	NR_073510.1	ONECUT1
LOC102158768	27.35465118	0	20	2.67E-04	High	33	0	XR_303462.1	ssc:102158768			
LOC102165746	27.35465118	0	20	2.67E-04	High	33	0	XR_301509.1	ssc:102165746			
LHX1	27.35465118	0	20	2.67E-04	High	33	0	XP_003131754.1	ssc:100513681	0	NM_005568.3	LHX1
LOC102165228	51.39358706	2.478835115	4.373854134	2.68E-04	High	62	3	XR_306328.1	ssc:102165228			
PKDCC	547.9219524	5333.62689	-3.283074594	2.68E-04	Low	661	6455	XP_005662648.1	ssc:100516968	0	NM_138370.2	PKDCC
FLYWCH2	28.18358	280.108368	-3.313058298	2.68E-04	Low	34	339	NM_001243398.1	ssc:100627929	7E-49	NM_138439.2	FLYWCH2
GTF2IRD2	109.4186047	1031.195408	-3.236387775	2.69E-04	Low	132	1248	XP_005662053.1	ssc:100620992	0	NM_001003795.2	GTF2IRD2B
PLXDC2	144.2336153	1360.880478	-3.23806102	2.69E-04	Low	174	1647	XP_005668225.1	ssc:100522568	0	NM_032812.8	PLXDC2
ADAM19	1635.476569	16526.39371	-3.336988947	2.70E-04	Low	1973	20001	XP_003134154.4	ssc:100521528	0	NM_033274.4	ADAM19
TNNT1	39.78858353	384.2194429	-3.271504081	2.73E-04	Low	48	465	NM_213748.2	ssc:396579	1E-105	NM_003283.4	TNNT1
YARS2	1444.82294	153.6877771	3.232818367	2.73E-04	High	1743	186	YARS2	ssc:100624597	0	NM_015936.1	YARS2
MSRB3	1114.080339	11075.43529	-3.313438224	2.75E-04	Low	1344	13404	XP_005664013.1	ssc:100511693	5E-95	NM_198080.3	MSRB3
LOC100515642	145.891473	13.22045395	3.464051943	2.75E-04	High	176	16	XP_005674021.1	ssc:100515642	3E-120	NR_033262.1	PRRG3
SLC2A1	517.2515859	5001.462984	-3.273412093	2.79E-04	Low	624	6053	XP_005665564.1	ssc:397404	6E-76	NM_006516.2	SLC2A1
LOC102161240	0	27.26718627	-20	2.79E-04	Low	0	33	XP_005658006.1	ssc:102161240			
TM4SF18	0	27.26718627	-20	2.79E-04	Low	0	33	XP_003132527.1	ssc:100522214	2E-101	NM_138786.3	TM4SF18
LOC102158724	0	27.26718627	-20	2.79E-04	Low	0	33	XR_304233.1	ssc:102158724			
FAM26F	0	27.26718627	-20	2.79E-04	Low	0	33	NM_001258434.1	ssc:100158219			
LOC100737032	0	27.26718627	-20	2.79E-04	Low	0	33	XP_003483161.1	ssc:100737032	0	NM_017534.5	MYH2
LOC100524359	314.1640241	32.2248565	3.285272144	2.80E-04	High	379	39	XP_003135477.1	ssc:100524359	2E-95	NM_014117.2	C16orf72
LOC102167098	35.64393941	0.826278372	5.430885079	2.81E-04	High	43	1	XR_300692.1	ssc:102167098			
LOC100737835	80.40609589	5.783948602	3.797178244	2.81E-04	High	97	7	XP_005674237.1	ssc:100737835	0	NR_104445.1	HTR4
LOC100519934	27641.46055	2704.409111	3.353448531	2.81E-04	High	33346	3273	XP_003121374.2	ssc:100519934	4E-13	NM_013230.2	CD24
LOC100626247	258.625793	2447.436537	-3.242333382	2.84E-04	Low	312	2962	XP_003354456.1	ssc:100626247	1E-50	NM_003641.3	IFITM1
MAN1C1	458.3976395	4399.106051	-3.262538861	2.88E-04	Low	553	5324	XP_005665172.1	ssc:100524150	0	NM_020379	MAN1C1
SLC29A4	386.2808318	40.48764021	3.254096625	2.92E-04	High	466	49	XP_003354487.1	ssc:100624843	5E-124	NM_153247.2	SLC29A4
PON1	0.828928824	35.52996998	-5.42164443	2.95E-04	Low	1	43	NM_001097515.2	ssc:100048952			
MAB21L1	27.35465118	269.3667492	-3.299713711	2.95E-04	Low	33	326	XP_001927813.1	ssc:100155671	0	NM_005584.4	MAB21L1
LOC102166268	238.7315012	23.96207278	3.316564331	2.96E-04	High	288	29	XP_005667680.1	ssc:102166268	2E-170	NM_017650.2	PPP1R9A
ARHGEF19	74.6035912	695.726389	-3.221203002	2.99E-04	Low	90	842	XP_003127681.1	ssc:100521628	0	NM_153213.3	ARHGEF19
CA8	43.10429883	1.652556743	4.705060042	2.99E-04	High	52	2	XP_005663130.1	ssc:100157869	1E-105	NM_004056.4	CA8
LOC100626715	43.10429883	1.652556743	4.705060042	2.99E-04	High	52	2	XP_005674325.1	ssc:100626715			
LOC102165140	481.6076465	51.22925905	3.232818367	3.00E-04	High	581	62	XP_005654338.1	ssc:102165140			
OBSL1	700.4448559	6779.61404	-3.274859763	3.00E-04	Low	845	8205	XP_005672301.1	ssc:100624019	6E-162	NM_015311.2	OBSL1
LOC100625132	444.3058495	47.09786719	3.237819501	3.00E-04	High	536	57	XP_003357570.3	ssc:100625132	4E-101	NM_014141.5	CNTNAP2
LOC100627889	165.7857647	15.69928906	3.400549001	3.01E-04	High	200	19	XP_005660475.1	ssc:100627889	0	NR_104603.1	CRB2
LOC102167380	50.56465824	2.478835115	4.350395161	3.02E-04	High	61	3	XP_005658961.1	ssc:102167380	2E-11	NM_199071.2	C21orf58
PTCHD1	4.973572942	72.71249671	-3.869848794	3.03E-04	Low	6	88	XP_001926295.3	ssc:100151829	0	NM_173495.2	PTCHD1
LOC100627808	34.81501059	334.6427406	-3.264840351	3.04E-04	Low	42	405	XP_005658038.1	ssc:100627808			
AIFM2	297.5854477	2802.736237	-3.235460096	3.04E-04	Low	359	3392	XP_005657460.1	ssc:100153541	2E-174	NM_032797.5	AIFM2
LOC100621484	28.18358	275.1506978	-3.287295201	3.09E-04	Low	34	333	XP_005658487.1	ssc:100621484	0	NM_138931.1	BCL6
LOC100736584	3235.309199	343.7318026	3.234548354	3.09E-04	High	3903	416	XP_003480860.1	ssc:100736584	0	NM_006087	TUBB4A
LOC100158003	741.8912971	80.14900206	3.210451354	3.09E-04	High	895	97	XP_001928233.1	ssc:100158003	0	NM_032704.3	TUBA1C
CNN2	1996.889536	19993.45776	-3.323701567	3.10E-04	Low	2409	24197	Z19539	ssc:397565	5E-179	NM_201277.1	CNN2
GUCY2C	353.9526077	37.18252673	3.250859488	3.11E-04	High	427	45	NM_214105.1	ssc:397193	1E-118	NM_004963.3	GUCY2C
FRMD4B	426.8983442	45.44531045	3.231689233	3.15E-04	High	515	55	XP_005669778.1	ssc:100524460	0	NM_015123.1	FRMD4B
LOC100154430	915.9663501	8889.929001	-3.278805393	3.16E-04	Low	1105	10759	XP_005669581.1	ssc:100154430	8E-45	xp_003120356	xp_003120356
LOC102157895	2.486786471	50.40298068	-4.341154513	3.18E-04	Low	3	61	XP_005655003.1	ssc:102157895	4E-60	NM_021599.2	ADAMTS2
LRFN1	912.6506348	99.15340461	3.202328482	3.19E-04	High	1101	120	XP_005657556.1	ssc:100515802	0	NM_020862.1	LRFN1
LOC100737965	42.27537	398.2661752	-3.23584367	3.23E-04	Low	51	482	XP_005666131.1	ssc:100737965	0	np_004339	np_004339
BCHE	1108.277837	120.6366423	3.199579516	3.24E-04	High	1337	146	XP_005670020.1	ssc:100624138	0	NM_000055.2	BCHE
CLDN7	164.1279071	15.69928906	3.386049431	3.26E-04	High	198	19	NM_001160076.1	ssc:100127486	6E-112	NM_001307.5	CLDN7
LOC102159547	26.52572236	0	20	3.29E-04	High	32	0	XR_298740.1	ssc:102159547			
LOC102161712	26.52572236	0	20	3.29E-04	High	32	0	XP_005667862.1	ssc:102161712	1E-131	NM_001205294.1	CACNA1E
LOC102161639	26.52572236	0	20	3.29E-04	High	32	0	XP_005657560.1	ssc:102161639			
OLIG2	26.52572236	0	20	3.29E-04	High	32	0	XP_003358977.2	ssc:100152482	3E-92	NM_005806.3	OLIG2
LOC102162285	26.52572236	0	20	3.29E-04	High	32	0	XP_005672742.1	ssc:102162285	4E-100	NM_014915.2	ANKRD26
LOC102158347	26.52572236	0	20	3.29E-04	High	32	0	XP_005652694.1	ssc:102158347	7E-56	NM_145027.4	KIF6
LOC102165047	26.52572236	0	20	3.29E-04	High	32	0	XR_307531.1	ssc:102165047			
TRIM72	19.06536294	193.349139	-3.342182439	3.30E-04	Low	23	234	XP_003124542.2	ssc:100511188	0	NM_001008274.3	TRIM72
CACNG1	34.81501059	0.826278372	5.396937747	3.31E-04	High	42	1	NM_001044605.1	ssc:733677	7E-106	NM_000727.3	CACNG1
LOC100738931	57.19608883	3.305113487	4.113144781	3.32E-04	High	69	4	XP_003483233.1	ssc:100738931	5E-49	NM_001145059.1	IQCF5
SLC1A6	727.7995071	79.32272369	3.197734953	3.32E-04	High	878	96	XP_005652965.1	ssc:100518296	0	NR_073589.1	SLC1A6
LOC100739211	4.144644118	64.44971299	-3.9588538	3.40E-04	Low	5	78	XP_003482272.2	ssc:100739211	3E-102	NM_207517.2	ADAMTSL3
LOC102167243	99.47145883	8.262783717	3.589582825	3.43E-04	High	120	10	XR_300255.1	ssc:102167243			

ZNF500	14.09179	149.5563853	-3.407762721	3.44E-04	Low	17	181	ZNF500	ssc:100625066	2E-22	NM_021646.1	ZNF500
PDZK1IP1	0	26.4409079	-20	3.44E-04	Low	0	32	NM_001001769.1	ssc:414756	8E-50	NM_005764.3	PDZK1IP1
LOC102160410	0	26.4409079	-20	3.44E-04	Low	0	32	XR_298807.1	ssc:102160410			
BCL2L10	42.27537	1.652556743	4.677045666	3.44E-04	High	51	2	XP_001924296.3	ssc:100153119	4E-46	NM_020396.2	BCL2L10
C6H19orf68	20.72322059	206.5695929	-3.317307771	3.45E-04	Low	25	250	XP_005664703.1	ssc:102165016	0	NM_199341.3	C19orf68
LOC100739439	9.947145883	114.0264153	-3.518941632	3.45E-04	Low	12	138	XP_005669377.1	ssc:100739439	5E-88	NM_014483.3	RBMS3
DEXI	95.32681471	870.0711254	-3.190179346	3.48E-04	Low	115	1053	XP_003124646.1	ssc:100519361	1E-37	NM_014015.3	DEXI
LOC102159735	3.315715294	57.01320765	-4.103904132	3.50E-04	Low	4	69	XR_302589.1	ssc:102159735			
PLCH1	894.4142007	98.32712624	3.185281711	3.51E-04	High	1079	119	XP_005669995.1	ssc:100627163	0	NM_014996.2	PLCH1
TMEM54	67.97216354	622.1876139	-3.194333726	3.52E-04	Low	82	753	XP_005665253.1	ssc:100621456	2E-106	NM_033504.2	TMEM54
LOC100621139	1271.576815	12318.98424	-3.276192782	3.54E-04	Low	1534	14909	XR_297411.1	ssc:100621139			
LOC100525418	204.7454194	20.65695929	3.309131366	3.55E-04	High	247	25	XP_003130397.1	ssc:100525418			
CMTM8	304.2168783	32.2248565	3.238854358	3.60E-04	High	367	39	XP_003358400.2	ssc:100624671	8E-79	NM_178868.3	CMTM8
LOC100624745	397.8858353	42.96647533	3.211071202	3.61E-04	High	480	52	XP_005661895.1	ssc:100624745	4E-130	NM_153247.2	SLC29A4
LOC100156854	1213.551798	133.8570962	3.18047016	3.62E-04	High	1464	162	XP_005655493.1	ssc:100156854	0	NM_003051.3	SLC16A1
LOC100157320	31.4992953	298.2864922	-3.243307189	3.63E-04	Low	38	361	XP_005665766.1	ssc:100157320	9E-168	NM_170770.1	RNF39
CKAP4	1473.835448	14307.01001	-3.279074834	3.64E-04	Low	1778	17315	NM_001244631.1	ssc:100523493			
LOC102164934	19.06536294	190.8703039	-3.323566761	3.65E-04	Low	23	231	XR_297412.1	ssc:102164934			
INSR	23.21000706	226.4002739	-3.286056837	3.65E-04	Low	28	274	XP_005654806.1	ssc:396755	0	NM_001079817.1	INSR
FLVCR1	993.8856595	109.8950234	3.176953832	3.67E-04	High	1199	133	NM_001142846.1	ssc:100217398	0	NM_014053.3	FLVCR1
DUOXA2	84.55074001	6.610226974	3.677045666	3.69E-04	High	102	8	NM_001243550.1	ssc:100155365	6E-148	NM_207581.3	DUOXA2
LOC100513717	56.36716001	3.305113487	4.092083166	3.69E-04	High	68	4	XP_005661415.1	ssc:100513717	2E-148	NR_047593	ATP8B3
LOC100738475	22.38107824	218.9637685	-3.290340723	3.69E-04	Low	27	265	XP_005658467.1	ssc:100738475			
SALL1	814.0081048	90.06434252	3.176015214	3.71E-04	High	982	109	XP_003127035.1	ssc:100519899	0	NM_002968.2	SALL1
ONECUT2	70.45895001	4.95767023	3.82904876	3.72E-04	High	85	6	XP_001926642.3	ssc:100153973	0	NM_004852.2	ONECUT2
LOC100623430	12.43393235	133.8570962	-3.428339083	3.74E-04	Low	15	162	XP_005672880.1	ssc:100623430	2E-114	NM_014012.5	REM1
ZKSCAN7	5.802501765	77.67016694	-3.742613605	3.77E-04	Low	7	94	ZKSCAN7	ssc:100627632	0	NM_025169.2	ZKSCAN7
LOC100152206	281.0068712	29.74602138	3.239836786	3.78E-04	High	339	36	XR_300938.1	ssc:100152206	6E-94	NM_207322.2	C2CD4A
CLEC11A	105.2739606	949.3938491	-3.172858072	3.82E-04	Low	127	1149	XP_005664849.1	ssc:100620310	2E-132	NM_002975.2	CLEC11A
SLC23A2	825.6131083	91.71689926	3.17020639	3.82E-04	High	996	111	NM_214178.1	ssc:397367			
ANXA5	4985.177945	50369.10326	-3.336822139	3.84E-04	Low	6014	60959	XP_003129266.2	ssc:100521982	0	NM_001154.3	ANXA5
FAM46A	295.92759	2706.061667	-3.192878603	3.85E-04	Low	357	3275	XP_003121442.1	ssc:100156873	0	NM_017633.2	FAM46A
LOC100516098	344.0054618	37.18252673	3.209734754	3.89E-04	High	415	45	XP_003130116.4	ssc:100516098	0	NM_014715.3	ARHGAP32
ARHGAP18	4821.050038	520.5553742	3.21122386	3.89E-04	High	5816	630	XP_003121255.3	ssc:100525547	0	NM_033515.2	ARHGAP18
MDM4	8415.285417	892.3806415	3.237281081	3.89E-04	High	10152	1080	XP_005656732.1	ssc:100512731	0	NM_002393.4	MDM4
P4HA1	926.7424248	8770.118638	-3.242356036	3.90E-04	Low	1118	10614	NM_001097435.1	ssc:100037299	0	NM_001142596.1	P4HA1
IQCA1	33.98608177	0.826278372	5.362172329	3.92E-04	High	41	1	XP_005672363.1	ssc:100515512	7E-131	NR_073043.1	IQCA1
LOC100737062	2184.22745	241.2732845	3.178383012	3.94E-04	High	2635	292	XP_005661334.1	ssc:100737062	0	NM_006087	TUBB4A
CREG2	41.44644118	1.652556743	4.648476514	3.97E-04	High	50	2	XP_003124922.1	ssc:100517088	2E-125	NM_153836.3	CREG2
LOC396679	1057.713179	118.1578072	3.162161601	3.99E-04	High	1276	143	NM_001244426.1	ssc:396679	0	NR_028435.1	AMT
LOC102158578	125.1682524	11.5678972	3.435670142	3.99E-04	High	151	14	XP_005662319.1	ssc:102158578	3E-58	NM_006648.3	WNK2
BTG4	257.7968641	27.26718627	3.240996975	4.00E-04	High	311	33	XP_005653752.1	ssc:100514308	1E-110	NM_017589.3	BTG4
SEMA6A	85.37966883	765.9600506	-3.165304677	4.02E-04	Low	103	927	XP_005661650.1	ssc:100522008	0	NM_020796.3	SEMA6A
SPATS2L	428.5562018	3938.868998	-3.200225115	4.02E-04	Low	517	4767	NM_001177916.1	ssc:100415809	0	NM_015535.2	SPATS2L
SLC22A4	69.63002118	4.95767023	3.811975246	4.06E-04	High	84	6	NM_001145752.1	ssc:100271724	0	NM_003059.2	SLC22A4
CCRN4L	2031.704547	225.5739955	3.171017952	4.06E-04	High	2451	273	XP_005653649.1	ssc:100513746	0	NM_012118.3	CCRN4L
SH3BGRL2	951.6102895	106.58991	3.158299996	4.07E-04	High	1148	129	XP_005659476.1	ssc:100510992	2E-48	NM_031469.2	SH3BGRL2
LOC100628123	25.69679353	0	20	4.08E-04	High	31	0	XP_003355108.1	ssc:100628123	2E-156	NM_005285.3	NPBWR1
LOC100522352	25.69679353	0	20	4.08E-04	High	31	0	XR_308557.1	ssc:100522352	1E-19	NM_138379.2	TIMD4
LOC102164667	25.69679353	0	20	4.08E-04	High	31	0	XP_005653489.1	ssc:102164667	2E-40	NM_015978.2	TNNI3K
ELOVL3	25.69679353	0	20	4.08E-04	High	31	0	NM_001167634.1	ssc:100155434	6E-128	NM_152310.2	ELOVL3
ZBTB32	25.69679353	0	20	4.08E-04	High	31	0	ZBTB32	ssc:100627119	0	NM_014383.1	ZBTB32
FOXB2	25.69679353	0	20	4.08E-04	High	31	0	XP_001925193.2	ssc:100156565	4E-161	NM_001013735.1	FOXB2
LOC102160165	25.69679353	0	20	4.08E-04	High	31	0	XR_300050.1	ssc:102160165			
IKZF3	25.69679353	0	20	4.08E-04	High	31	0	XP_005653980.1	ssc:100516292	0	NR_047561.1	IKZF3
CRMP1	547.0930236	5056.823635	-3.208373399	4.08E-04	Low	660	6120	XP_003128882.3	ssc:100520574			
C14H10orf2	5397.984499	584.1788088	3.207938904	4.09E-04	High	6512	707	XP_003483588.1	ssc:100625408	0	NM_021830.4	C10orf2
PDLIM1	927.5713536	8727.152162	-3.233980781	4.09E-04	Low	1119	10562	XP_003483582.1	ssc:100155592	1E-136	NM_020992.3	PDLIM1
LOC102167245	0.828928824	33.87741324	-5.35293168	4.11E-04	Low	1	41	XR_306916.1	ssc:102167245			
CLDN23	55.53823118	3.305113487	4.070709515	4.12E-04	High	67	4	NM_001159778.1	ssc:100156235	7E-109	NM_194284.2	CLDN23
LOC100739178	89.52431295	799.8374638	-3.159355411	4.14E-04	Low	108	968	XP_005654927.1	ssc:100739178	0	NM_006202.2	PDE4A
LOC100515261	312.5061665	33.87741324	3.205489033	4.19E-04	High	377	41	XP_003126103.4	ssc:100515261	0	NM_025045	BAIAP2L2
PPP1R3G	186.5089853	19.00440255	3.294839559	4.20E-04	High	225	23	XP_003128202.1	ssc:100522260	5E-116	NM_001145115	PPP1R3G
LOC100521355	6085.995423	658.5438623	3.208141809	4.22E-04	High	7342	797	XR_298026.1	ssc:100521355			
MSH5	227.9554265	23.96207278	3.249927138	4.24E-04	High	275	29	NM_001195358.1	ssc:100270818	5E-50	NM_172166.3	MSH5
MCM10	4434.769206	485.0254042	3.192726807	4.24E-04	High	5350	587	XP_003361288.2	ssc:100626098			
LOC102157470	0	25.61462952	-20	4.26E-04	Low	0	31	XR_304922.1	ssc:102157470			
LOC102160901	0	25.61462952	-20	4.26E-04	Low	0	31	XR_306021.1	ssc:102160901	3E-51	NM_001029880.2	SFMBT2
TRPA1	0	25.61462952	-20	4.26E-04	Low	0	31	XP_001926150.1	ssc:100152934	0	NM_007332.2	TRPA1
LOC100623327	0	25.61462952	-20	4.26E-04	Low	0	31	XR_297216.1	ssc:100623327	9E-65	NM_032784.4	RSPO3
LOC100517057	0	25.61462952	-20	4.26E-04	Low	0	31	XP_005670211.1	ssc:100517057	3E-179	NR_028136.1	KALRN
LOC100621406	2262.14676	252.0149034	3.166111571	4.26E-04	High	2729	305	XP_005658086.1	ssc:100621406	5E-147	NM_018290.3	PGM2
CBFA2T3	503.1597959	56.18692928	3.162710189	4.29E-04	High	607	68	XP_005653290.1	ssc:100525577	0	NM_175931.2	CBFA2T3
LOC100739750	98.64253001	875.855074	-3.150410462	4.33E-04	Low	119	1060	XP_003481092.1	ssc:100739750	1E-37	NM_014015.3	DEXI
LOC102159434	34.81501059	319.7697299	-3.199252009	4.34E-04	Low	42	387	XR_302046.1	ssc:102159434			
S1PR2	189.8247006	1693.870662	-3.157584082	4.34E-04	Low	229	2050	NM_001243855.1	ssc:100511551	2E-173	NM_004230.3	S1PR2
FRMD7	3.315715294	55.36065091	-4.061468866	4.34E-04	Low	4	67	XP_003360499.2	ssc:100626525	0	NM_194277.2	FRMD7
LOC100157806	3.315715294	55.36065091	-4.061468866	4.34E-04	Low	4	67	XR_135278.2	ssc:100157806			
LOC102161634	310.8483089	33.87741324	3.197815105	4.37E-04	High	375	41	XR_299036.1	ssc:102161634			
ROBO1	1019.582453	9523.684513	-3.22354138	4.46E-04	Low	1230	11526	XP_003132784.3	ssc:100517310	0	NM_133631.3	ROBO1
AGRN	205.5743483	1829.380315	-3.153622872	4.49E-04	Low	248	2214	XP_005674514.1	ssc:100623869	0	NM_198576.3	AGRN
RIN3	114.3921777	1010.538449	-3.143063907	4.51E-04	Low	138	1223	XP_005656507.1	ssc:100155709	0	NM_024832.3	RIN3
SLC24A5	1631.331925	184.2600769	3.14623493	4.52E-04	High	1968	223	XP_003121571.1	ssc:100511672	0	NM_205850.2	SLC24A5
PION	38.13072589	346.2106378	-3.182624153	4.52E-04	Low	46	419	XP_003130279.1	ssc:100512622	5E-125	NM_017439.3	GSAP
LOC100524931	599.3155395	67.75482648	3.144920157	4.54E-04	High	723	82	XP_005662588.1	ssc:100524931	0	NM_144709.2	PUS10
LOC102163597	33.98608177	310.6806678	-3.192416523	4.56E-04	Low	41	376	XP_005662469.1	ssc:102163597	4E-32	NM_006634.2	VAMP5
LOC102166841	40.61751236	1.652556743	4.619330168	4.58E-04	High	49	2	XR_301690.1	ssc:102166841			
LOC102166076	364.7286824	40.48764021	3.171270194	4.60E-04	High	440	49	XP_005672353.1	ssc:102166076	5E-105	NM_205862.1	UGT1A6
LOC100155273	1066.831396	121.4629206	3.134744318	4.64E-04	High	1287	147	LOC100155273	ssc:100155273			
LOC102157456	33.15715294	0.826278372	5.326548419	4.66E-04	High	40	1	XR_297431.1	ssc:102157456			
DGAT2	33.15715294	0.826278372	5.326548419	4.66E-04	High	40	1	NM_001160080.1	ssc:100294675	4E-18	NM_032564.4	DGAT2
LOC100622678	8.289288236	95.84829112	-3.531432576	4.70E-04	Low	10	116	XP_005674454.1	ssc:100622678	0	NM_017718.1	DOCK10
LOC100624264	8.289288236	95.84829112	-3.531432576	4.70E-04	Low	10	116	XP_003361331.2	ssc:100624264			
RASSF4	87.03752648	765.1337722	-3.136002541	4.71E-04	Low	105	926	NM_001243337.1	ssc:100152580	3E-146	NM_178145.1	RASSF4
HK2	5851.408566	642.8445732	3.186242085	4.74E-04	High	7059	778	NM_001122987.1	ssc:494561	0	NM_000189.4	HK2
IRK1	15.74964765	156.9928906	-3.317307771	4.76E-04	Low	19	190	NM_214151.1	ssc:397293	0	NM_000891.2	KCNJ2
REPS2	251.9943624	27.26718627	3.208153718	4.77E-04	High	304	33	XP_003484126.1	ssc:100738275	5E-162	NM_004726.2	REPS2
LOC100625071	101.9582453	9.089062089	3.487703211	4.78E-04	High	123	11	XP_003362096.3	ssc:100625071	2E-176	NM_181871.3	PKLR
MATN2	101.9582453	9.089062089	3.487703211	4.78E-04	High	123	11	XP_005655412.1	ssc:100155607	2E-38	NM_030583.2	MATN2
LOC100738418	67.97216354	4.95767023	3.777209828	4.85E-04	High	82	6	XP_003480674.2	ssc:100738418	2E-49	NM_031303.2	KATNAL2

IGSF3	1402.54757	160.2980041	3.129221335	4.89E-04	High	1692	194	XP_005663604.1	ssc:100512602	0	NM_001542.3	IGSF3
OAZ3	16.57857647	162.7768392	-3.2955034	5.00E-04	Low	20	197	NM_001122996.1	ssc:100144448			
BCL6	186.5089853	1634.378619	-3.131425195	5.00E-04	Low	225	1978	XP_005657169.1	ssc:100156549	0	NM_138931.1	BCL6
ADRB3	24.86786471	0	20	5.07E-04	High	30	0	NM_001099927.1	ssc:397356	2E-163	NM_000025.2	ADRB3
LOC102159682	24.86786471	0	20	5.07E-04	High	30	0	XR_300801.1	ssc:102159682			
LOC102164429	24.86786471	0	20	5.07E-04	High	30	0	XP_005657765.1	ssc:102164429	4E-24	NR_073043.1	IQCA1
LOC102157713	24.86786471	0	20	5.07E-04	High	30	0	XP_005656361.1	ssc:102157713			
LOC102166554	24.86786471	0	20	5.07E-04	High	30	0	XR_300447.1	ssc:102166554			
LOC102158667	24.86786471	0	20	5.07E-04	High	30	0	XR_297739.1	ssc:102158667			
LOC102167081	24.86786471	0	20	5.07E-04	High	30	0	XR_305435.1	ssc:102167081			
ANKRD34B	24.86786471	0	20	5.07E-04	High	30	0	XP_005661580.1	ssc:100513200	0	NM_001004441.2	ANKRD34B
LOC100521071	24.86786471	0	20	5.07E-04	High	30	0	XR_298107.1	ssc:100521071			
CXHXorf48	24.86786471	0	20	5.07E-04	High	30	0	XR_297879.1	ssc:102168080			
GZMA	24.86786471	0	20	5.07E-04	High	30	0	NM_001143709.1	ssc:100233183	4E-110	NM_006144.3	GZMA
LOC100620529	54.70930236	480.8940123	-3.135860899	5.08E-04	Low	66	582	XP_005658322.1	ssc:100620529	0	NM_004408	DNM1
TNFRSF21	2763.648698	313.1595029	3.141604675	5.09E-04	High	3334	379	XP_003128478.2	ssc:100513161	1E-172	NM_014452.4	TNFRSF21
VIM	49905.65983	533350.2948	-3.417808033	5.11E-04	Low	60205	645485	VIM		0	NM_003380.3	VIM
PRDM16	14.92071882	148.7301069	-3.317307771	5.12E-04	Low	18	180	XP_005674579.1	ssc:100624476	0	NM_199454.2	PRDM16
LOC102160923	2.486786471	47.09786719	-4.243307189	5.19E-04	Low	3	57	XR_309605.1	ssc:102160923			
LOC102158175	2.486786471	47.09786719	-4.243307189	5.19E-04	Low	3	57	XP_005658681.1	ssc:102158175	8E-65	NM_133178.3	PTPRU
LOC102168145	134.2864694	13.22045395	3.344470327	5.20E-04	High	162	16	XR_301204.1	ssc:102168145			
DPEP1	12.43393235	128.0731476	-3.364613485	5.24E-04	Low	15	155	NM_214108.1	ssc:397196	0	NM_004413.3	DPEP1
GAP43	2962.591616	336.2952973	3.139059259	5.25E-04	High	3574	407	NM_001244335.1	ssc:100623423	2E-86	NM_002045.3	GAP43
CDC6	1208.578225	139.6410448	3.113515892	5.26E-04	High	1458	169	XP_005668909.1	ssc:100522031	0	NM_001254.3	CDC6
JOSD2	221.3239959	1932.665111	-3.12635989	5.28E-04	Low	267	2339	NM_001243416.1	ssc:100627842	2E-101	NM_138334.3	JOSD2
LOC100622303	0	24.78835115	-20	5.29E-04	Low	0	30	XP_005659134.1	ssc:100622303			
PLA2G7	0	24.78835115	-20	5.29E-04	Low	0	30	NM_001113013.1	ssc:396593	0	NM_005084.3	PLA2G7
LOC100511329	0	24.78835115	-20	5.29E-04	Low	0	30	ZNF829	ssc:100511329	0	NM_001171979.1	ZNF829
LOC100737673	0	24.78835115	-20	5.29E-04	Low	0	30	XP_003483458.1	ssc:100737673	0	NM_001136103.2	TMEM132C
PLOD1	1797.11769	16785.01884	-3.22341736	5.29E-04	Low	2168	20314	XP_003127625.1	ssc:100525583	0	NM_000302.3	PLOD1
LOC100736946	109.4186047	946.915014	-3.113376885	5.30E-04	Low	132	1146	XR_304087.1	ssc:100736946	5E-71	NM_001792.3	CDH2
SLC12A8	39.78858353	1.652556743	4.589582825	5.30E-04	High	48	2	XP_003358826.2	ssc:100623914	0	NM_024628.5	SLC12A8
LOC102158629	214.6925653	23.13579441	3.21407369	5.30E-04	High	259	28	XR_297342.1	ssc:102158629			
LOC102159922	268.5729388	29.74602138	3.174545326	5.37E-04	High	324	36	XP_005661636.1	ssc:102159922			
HRC	15.74964765	154.5140555	-3.294346622	5.37E-04	Low	19	187	XP_003127335.2	ssc:100525465	1E-162	NM_002152.2	HRC
MDN1	28066.70104	2960.555406	3.244919741	5.38E-04	High	33859	3583	XP_003480335.1	ssc:100525725	0	NM_014611.1	MDN1
LOC102159647	1147.237492	133.0308178	3.108331674	5.39E-04	High	1384	161	LOC102159647				
ARHGEF10L	126.82611	1095.645121	-3.110856893	5.40E-04	Low	153	1326	XP_003482027.1	ssc:100525994	0	NM_018125.3	ARHGEF10L
KIFAP3	442.6479918	3914.080647	-3.144441701	5.53E-04	Low	534	4737	XP_005663163.1	ssc:100157611	0	NM_014970.3	KIFAP3
LOC102164483	4.144644118	60.31832114	-3.86327614	5.53E-04	Low	5	73	XP_005669520.1	ssc:102164483	1E-109	NR_033815.1	ALS2CL
NAGK	468.3447853	4147.071198	-3.146456849	5.54E-04	Low	565	5019	XP_005662538.1	ssc:100523729	0	NM_017567.4	NAGK
LOC102165406	1293.957894	150.3826637	3.105080506	5.54E-04	High	1561	182	XP_005667968.1	ssc:102165406	7E-13	NM_025081.2	NYNRIN
LOC100737877	1.657857647	39.66136184	-4.580342176	5.57E-04	Low	2	48	XP_003484241.1	ssc:100737877			
LOC100523520	1448.138655	13321.25991	-3.201458889	5.61E-04	Low	1747	16122	XR_297837.1	ssc:100523520	4E-51	NM_004394.2	DAP
LOC100736842	300.901163	33.87741324	3.150894058	5.62E-04	High	363	41	XP_005660046.1	ssc:100736842	2E-35	NM_021255.2	PELI2
CNNM1	3963.108706	450.3217126	3.137604595	5.66E-04	High	4781	545	XP_005671437.1	ssc:100154357	2E-24	NM_020348.2	CNNM1
LOC100515962	47.24894295	411.4866291	-3.122491594	5.72E-04	Low	57	498	XP_003482718.1	ssc:100515962	8E-173	NM_012395.3	CDK14
LOC100736655	53.05144471	3.305113487	4.004620324	5.74E-04	High	64	4	XR_299091.1	ssc:100736655	8E-25	NM_001127641.1	LAMB3
PKLR	53.05144471	3.305113487	4.004620324	5.74E-04	High	64	4	HighI00000878B8		4E-112	NM_181871.3	PKLR
LOC102158761	92.84002824	8.262783717	3.490047152	5.77E-04	High	112	10	XP_005672766.1	ssc:102158761	1E-43	NM_080676.5	MACROD2
LOC102167634	59.6828753	4.131391859	3.852617231	5.80E-04	High	72	5	XR_297082.1	ssc:102167634			
LOC100514309	0.828928824	32.2248565	-5.280781895	5.81E-04	Low	1	39	XP_005653770.1	ssc:100514309	1E-93	NM_001004462.1	OR10G4
LOC100522935	72.94573648	5.783948602	3.656697021	5.81E-04	High	88	7	XR_304390.1	ssc:100522935			
ADAMTS12	14.92071882	145.4249934	-3.284886293	6.07E-04	Low	18	176	XP_005672463.1	ssc:100511353	0	NM_030955.2	ADAMTS12
BOK	151.6939747	1291.473095	-3.0897819	6.14E-04	Low	183	1563	XP_005657656.1	ssc:100627765	2E-49	NM_032515.4	BOK
GRID1	4.973572942	66.10226974	-3.73234527	6.14E-04	Low	6	80	XP_005671220.1	ssc:100153116	0	NM_017551.2	GRID1
FBXO43	38.95965471	1.652556743	4.559209176	6.14E-04	High	47	2	XP_001925369.1	ssc:100153144	0	NR_036491.1	FBXO43
SNX31	38.95965471	1.652556743	4.559209176	6.14E-04	High	47	2	XP_001924965.1	ssc:100154375	0	NM_152628.3	SNX31
LOC102160627	38.95965471	1.652556743	4.559209176	6.14E-04	High	47	2	XP_005653116.1	ssc:102160627			
CDH7	38.95965471	1.652556743	4.559209176	6.14E-04	High	47	2	XP_001926047.1	ssc:100156376	0	NM_033646.1	CDH7
LOC100620361	38.95965471	1.652556743	4.559209176	6.14E-04	High	47	2	XP_005674287.1	ssc:100620361	1E-80	NM_006144.3	GZMA
LOC102164420	150.8650459	15.69928906	3.264487451	6.21E-04	High	182	19	XP_005666407.1	ssc:102164420	7E-87	NM_012074.4	DPF3
LOC102167649	150.8650459	15.69928906	3.264487451	6.21E-04	High	182	19	XP_005655820.1	ssc:102167649			
KCTD11	140.9179	1196.451082	-3.085834627	6.23E-04	Low	170	1448	XP_003358298.1	ssc:100622059	5E-122	NM_001002914.2	KCTD11
LOC102159415	24.03893588	0	20	6.34E-04	High	29	0	XP_005657627.1	ssc:102159415	5E-53	NM_020864.1	NYAP2
C17H20orf26	24.03893588	0	20	6.34E-04	High	29	0	XP_003483953.1	ssc:100511790	0	NM_015585.3	C20orf26
AGXT2	24.03893588	0	20	6.34E-04	High	29	0	NM_001244884.1	ssc:100513890	0	NM_031900.3	AGXT2
LOC102158664	24.03893588	0	20	6.34E-04	High	29	0	XR_303461.1	ssc:102158664			
TMEM132D	24.03893588	0	20	6.34E-04	High	29	0	XP_003359122.2	ssc:100623073	0	NM_133448.2	TMEM132D
LOC100523842	58.85394648	501.5509716	-3.091185262	6.35E-04	Low	71	607	XR_298427.1	ssc:100523842	1E-15	NM_207376.1	C12orf75
LOC100738338	13.26286118	131.3782611	-3.308262631	6.36E-04	Low	16	159	XP_003480496.2	ssc:100738338	9E-51	NR_104583.1	CALML4
LOC100514384	58.85394648	4.131391859	3.832439349	6.42E-04	High	71	5	XP_003135498.2	ssc:100514384	5E-59	NM_005364.4	MAGEA8
STEAP4	1.657857647	38.83508347	-4.549968527	6.45E-04	Low	2	47	NM_001166489.1	ssc:100310802	0	NM_024636.3	STEAP4
LMNA	9298.094614	90316.35742	-3.27998029	6.50E-04	Low	11217	109305	NM_001111257.1	ssc:100126859	0	NR_047545.1	LMNA
LOC100511614	143.4046865	14.87301069	3.269323551	6.51E-04	High	173	18	XP_005673974.1	ssc:100511614	0	NM_144967.3	ARHGAP36
TSTD3	2001.03418	235.4893359	3.087012176	6.51E-04	High	2414	285	XP_003121361.2	ssc:100514380	4E-34	NM_001195131.1	TSTD3
LOC102162310	97.81360119	9.089062089	3.427831755	6.52E-04	High	118	11	XR_306133.1	ssc:102162310			
ANO6	977.307083	8688.317079	-3.152192902	6.57E-04	Low	1179	10515	XP_005655770.1	ssc:100155593	0	NM_001204803.1	ANO6
NKAPL	0	23.96207278	-20	6.61E-04	Low	0	29	XP_001929056.1	ssc:100154907	2E-118	NM_001007531.2	NKAPL
LOC100738421	0	23.96207278	-20	6.61E-04	Low	0	29	HighI00025DF5A1				
SCML1	1981.968817	233.8367792	3.083360496	6.64E-04	High	2391	283	XP_005673526.1	ssc:100521306	4E-65	NM_006746.5	SCML1
LOC100516650	73.7746653	621.3613355	-3.074235096	6.69E-04	Low	89	752	XP_005658305.1	ssc:100516650	0	NM_004735.3	LRRFIP1
LOC100626629	4380.059904	506.5086419	3.112291812	6.71E-04	High	5284	613	LOC100626629		3E-159	NM_001286807.1	SLC25A30
LOC102160331	4.973572942	65.27599137	-3.714197923	6.73E-04	Low	6	79	XR_306122.1	ssc:102160331			
LOC100627924	4.973572942	65.27599137	-3.714197923	6.73E-04	Low	6	79	XR_299871.1	ssc:100627924			
SQSTM1	4569.055676	42939.20814	-3.232327659	6.76E-04	Low	5512	51967	XP_005654994.1	ssc:100518718	0	NM_003900.4	SQSTM1
LGALS3	1222.670015	10919.26868	-3.158769241	6.76E-04	Low	1475	13215	NM_001097501.2	ssc:100038033	4E-56	NM_006498.2	LGALS2
SCP2	32.32822412	281.7609248	-3.123605386	6.79E-04	Low	39	341	NM_001168415.1	ssc:397439	5E-177	NM_002979.4	SCP2
LOC100620963	47.24894295	402.397567	-3.090267624	6.80E-04	Low	57	487	XP_003355420.2	ssc:100620963	0	NM_152772.2	TCP11L2
ICK	3968.082279	461.0633314	3.105405103	6.81E-04	High	4787	558	XP_005666529.1	ssc:100511822	0	NM_016513.4	ICK
LOC100738921	103.616103	9.915340461	3.385442108	6.86E-04	High	125	12	XP_003484203.1	ssc:100738921	0	NM_144967.3	ARHGAP36
KCNIP3	0.828928824	31.39857813	-5.243307189	6.93E-04	Low	1	38	XP_003124884.2	ssc:100524248	4E-131	NM_013434.4	KCNIP3
HCN1	0.828928824	31.39857813	-5.243307189	6.93E-04	Low	1	38	XP_005672496.1	ssc:100510937	7E-33	NM_021072.3	HCN1
HTR1B	138.4311135	1159.268556	-3.065974677	6.93E-04	Low	167	1403	NM_214298.1	ssc:397563	0	NM_000863.1	HTR1B
IL-6	14.09179	136.3359313	-3.274239049	6.95E-04	Low	17	165	NM_001252429.1	ssc:100628202	2E-68	NM_000600.3	IL6
FCHSD1	175.7329106	1475.733172	-3.069975586	6.95E-04	Low	212	1786	XP_005661826.1	ssc:100511554	0	NM_033449.2	FCHSD1
LOC102161722	8.289288236	90.89062089	-3.454811294	6.98E-04	Low	10	110	XP_005658702.1	ssc:102161722	7E-105	NM_153343.3	ENPP6
RASSF10	64.65644824	4.95767023	3.705060042	6.99E-04	High	78	6	XP_005661179.1	ssc:100625955	0	NM_001080521.2	RASSF10
CD70	3399.437106	398.2661752	3.093491016	7.02E-04	High	4101	482	NM_001044531.1	ssc:733577	8E-66	NM_001252	CD70
LOC102160423	148.3782594	15.69928906	3.240508588	7.05E-04	High	179	19	XP_005668829.1	ssc:102160423	2E-108	NM_002390.4	ADAM11

BRSK1	12.43393235	123.1154774	-3.307657601	7.05E-04	Low	15	149	XP_005656030.1	ssc:100512610	2E-85	NM_032430.1	BRSK1
LOC102161596	113.5632488	947.7412924	-3.060997269	7.05E-04	Low	137	1147	XP_005668184.1	ssc:102161596			
LOC102158163	1373.535061	164.429396	3.062353591	7.07E-04	High	1657	199	XR_298453.1	ssc:102158163			
MFNG	141.7468288	14.87301069	3.252547838	7.11E-04	High	171	18	XP_005663880.1	ssc:102161832	1E-129	NR_029413.1	MFNG
TLX1	58.02501765	4.131391859	3.811975246	7.11E-04	High	70	5	XP_005671450.1	ssc:100157458	2E-25	NM_005521.3	TLX1
LOC102158355	58.02501765	4.131391859	3.811975246	7.11E-04	High	70	5	XR_304478.1	ssc:102158355			
LDB3	16.57857647	155.3403339	-3.228040432	7.13E-04	Low	20	188	XP_005657484.1	ssc:100151883	0	NM_007078.2	LDB3
LOC102162964	38.13072589	1.652556743	4.52818228	7.14E-04	High	46	2	XR_306026.1	ssc:102162964			
LOC100627814	38.13072589	1.652556743	4.52818228	7.14E-04	High	46	2	XP_003361818.3	ssc:100627814	8E-141	NM_003914.3	CCNA1
LOC100515857	38.13072589	1.652556743	4.52818228	7.14E-04	High	46	2	XP_003131759.3	ssc:100515857	2E-20	NM_002983.2	CCL3
LOC102163041	38.13072589	1.652556743	4.52818228	7.14E-04	High	46	2	XR_305087.1	ssc:102163041			
SYNPO2L	24.03893588	214.0060983	-3.154206968	7.16E-04	Low	29	259	XP_003359286.2	ssc:100152222	0	NM_024875.4	SYNPO2L
MGA	1099.15962	132.2045395	3.05555729	7.19E-04	High	1326	160	XP_005659774.1	ssc:100156980	5E-119	NM_001164273.1	MGA
COL11A2	51.39358706	3.305113487	3.958816635	7.21E-04	High	62	4	XP_003128381.1	ssc:100520915	0	NM_080681.2	COL11A2
ITGA5	2878.869804	26341.75449	-3.193776989	7.25E-04	Low	3473	31880	XP_001925287.3	ssc:100155091	0	NM_002205.2	ITGA5
LOC102157793	44.76215647	2.478835115	4.174545326	7.25E-04	High	54	3	XR_302167.1	ssc:102157793			
LOC102166014	9.947145883	103.2847965	-3.37620146	7.26E-04	Low	12	125	XP_005664109.1	ssc:102166014			
LOC100155159	5.802501765	71.05993997	-3.614289508	7.26E-04	Low	7	86	XP_001926734.4	ssc:100155159	0	NM_032709.2	PYROXD2
CCDC92	34.81501059	299.1127706	-3.10290814	7.27E-04	Low	42	362	XP_005670635.1	ssc:100521520	4E-171	NM_025140.1	CCDC92
ADAM11	1242.564307	149.5563853	3.054559105	7.31E-04	High	1499	181	XP_003131395.2	ssc:100525073	0	NM_002390.4	ADAM11
TMEM150C	959.0706489	115.678972	3.051510456	7.31E-04	High	1157	140	XP_005653678.1	ssc:100622888	2E-142	NM_001080506.1	TMEM150C
NPR2	668.1166318	5780.643489	-3.113058221	7.32E-04	Low	806	6996	NM_001244322.1	ssc:100512698	0	NM_003995.3	NPR2
TNFRSF1A	678.0637777	5869.055274	-3.113635413	7.33E-04	Low	818	7103	NM_213969.1	ssc:397020	2E-103	NM_001065.3	TNFRSF1A
SCAI	496.5283653	59.49204276	3.061107516	7.33E-04	High	599	72	XP_003122214.3	ssc:100515704	0	NM_173690.4	SCAI
LAMA1	121.8525371	12.39417558	3.297402074	7.35E-04	High	147	15	XP_005658620.1	ssc:100625589			
LOC100738036	536.3169489	64.44971299	3.056840007	7.38E-04	High	647	78	XP_003482696.1	ssc:100738036	0	NM_014155.4	ZBTB44
LOC100515685	16.57857647	154.5140555	-3.220346041	7.42E-04	Low	20	187	XP_005653962.1	ssc:100515685	5E-32	NM_006014.4	LAGE3
OLFML2A	33.98608177	291.6762652	-3.101352044	7.43E-04	Low	41	353	NM_001258360.1	ssc:100154062	0	NM_182487.3	OLFML2A
LOC102160753	4.144644118	57.83948602	-3.802734598	7.49E-04	Low	5	70	XR_309570.1	ssc:102160753			
LOC102168189	70.45895001	5.783948602	3.606656338	7.49E-04	High	85	7	XR_307473.1	ssc:102168189			
ATP10D	1880.839501	225.5739955	3.059704077	7.51E-04	High	2269	273	XP_005666689.1	ssc:100520087	0	NM_020453.3	ATP10D
COL16A1	512.278013	4378.449092	-3.09542106	7.52E-04	Low	618	5299	XP_005665218.1	ssc:100737666	6E-118	NM_001856.3	COL16A1
EDA	1009.635307	122.289199	3.045465377	7.57E-04	High	1218	148	XP_005657874.1	ssc:100518887	2E-155	NM_001399.4	EDA
LOC100625680	63.82751942	4.95767023	3.686444364	7.68E-04	High	77	6	XP_005674186.1	ssc:100625680	1E-131	NM_001080433.1	CCDC85A
LOC102161259	121.0236082	12.39417558	3.287554288	7.74E-04	High	146	15	XR_308371.1	ssc:102161259			
PLXND1	901.8745601	7831.466407	-3.11828378	7.74E-04	Low	1088	9478	XP_005654079.1	ssc:102159849	0	NM_015103.2	PLXND1
AS3MT	140.0889712	14.87301069	3.235574759	7.76E-04	High	169	18	XP_003359405.2	ssc:100622012	0	NM_020682.3	AS3MT
PLXDC1	275.2043694	32.2248565	3.094257537	7.83E-04	High	332	39	XP_003131573.2	ssc:100517914	0	NM_020405.4	PLXDC1
MB21D1	23.21000706	204.9170362	-3.142221064	7.86E-04	Low	28	248	XP_003121459.1	ssc:100516408	0	NM_138441.2	MB21D1
BRI3BP	672.2612759	81.8015588	3.038821808	7.89E-04	High	811	99	XP_001924308.4	ssc:100155348	2E-90	NM_080626.5	BRI3BP
SEMA3E	95.32681471	9.089062089	3.390678757	7.90E-04	High	115	11	XP_003130268.3	ssc:100524100	0	NM_012431.2	SEMA3E
LOC102166276	112.73432	927.0843331	-3.039773795	7.91E-04	Low	136	1122	XP_005669378.1	ssc:102166276	3E-54	NM_003242.5	TGFBR2
LRPPRC	42518.24615	4621.374933	3.201688042	7.91E-04	High	51293	5593	XP_003125231.3	ssc:100512225	0	NM_133259.3	LRPPRC
LOC102161605	76.26145177	6.610226974	3.52818228	7.93E-04	High	92	8	XR_307169.1	ssc:102161605			
MAST1	30.67036647	0.826278372	5.21407369	7.93E-04	High	37	1	XP_005652946.1	ssc:100626936	0	NM_014975.2	MAST1
LOC102159645	23.21000706	0	20	7.96E-04	High	28	0	XR_308451.1	ssc:102159645			
LOC102157403	23.21000706	0	20	7.96E-04	High	28	0	XP_005673914.1	ssc:102157403	2E-19	NM_153448.3	ESX1
LOC102161139	23.21000706	0	20	7.96E-04	High	28	0	XR_304057.1	ssc:102161139			
LOC100626203	23.21000706	0	20	7.96E-04	High	28	0	XP_003356186.3	ssc:100626203	1E-136	NM_001242672.1	TTC34
DRD1	23.21000706	0	20	7.96E-04	High	28	0	NM_001123108.1	ssc:100144487	0	NM_000794.3	DRD1
SERPINE3	23.21000706	0	20	7.96E-04	High	28	0	XP_005668451.1	ssc:100157886	2E-167	NM_001101320.1	SERPINE3
LOC100621043	23.21000706	0	20	7.96E-04	High	28	0	XP_003353592.2	ssc:100621043	1E-62	NM_004293.4	GDA
LOC102165901	23.21000706	0	20	7.96E-04	High	28	0	XR_298979.1	ssc:102165901			
LOC100521617	23.21000706	0	20	7.96E-04	High	28	0	XP_003354742.2	ssc:100521617	5E-68	NM_022440.2	MAL
LOC102165269	23.21000706	0	20	7.96E-04	High	28	0	XR_299108.1	ssc:102165269			
HPRT1	6447.40839	751.9133183	3.100081109	7.99E-04	High	7778	910	NM_001032376.2	ssc:397351	5E-119	NM_000194.2	HPRT1
LOC102164161	385.451903	46.27158882	3.058352308	7.99E-04	High	465	56	XR_305455.1	ssc:102164161			
FAM169A	1117.396054	136.3359313	3.034902891	8.06E-04	High	1348	165	XP_005661542.1	ssc:100518001	0	NR_046462.1	FAM169A
MFGE8	2056.572411	18321.07034	-3.155190015	8.09E-04	Low	2481	22173	NM_001122984.1	ssc:397545	9E-141	NM_005928.2	MFGE8
PAPP-A	321.6243836	2684.57843	-3.061246862	8.12E-04	Low	388	3249	XP_001926788.4	ssc:397219	0	NM_002581.3	PAPPA
LOC102161711	139.2600424	14.87301069	3.227012746	8.12E-04	High	168	18	XR_299444.1	ssc:102161711			
LOC100520391	33.15715294	281.7609248	-3.08707951	8.12E-04	Low	40	341	XP_005658326.1	ssc:100520391	1E-82	NM_001313.4	CRMP1
PLCB4	602.6312548	73.53877508	3.034698447	8.15E-04	High	727	89	XP_001926310.1		0	NM_182797.2	PLCB4
NAGA	80.40609589	659.3701406	-3.03571177	8.16E-04	Low	97	798	NM_001123222.1	ssc:100142667	0	NM_000262.2	NAGA
JAKMIP2	2221.529247	268.5404708	3.048341702	8.25E-04	High	2680	325	XP_003124123.3	ssc:100511808	0	NR_073101.1	JAKMIP2
LOC100737306	43.93322765	2.478835115	4.147578278	8.27E-04	High	53	3	XR_297554.1	ssc:100737306	2E-18	NM_006060.4	IKZF1
LOC100626333	1025.384955	125.5943125	3.029322596	8.27E-04	High	1237	152	XP_003361231.1	ssc:100626333			
LOC100154674	260.2836506	30.57229975	3.089787708	8.28E-04	High	314	37	XP_001925686.3	ssc:100154674	1E-100	NM_139057.2	ADAMTS17
LOC100626249	0	23.13579441	-20	8.30E-04	Low	0	28	XP_005658575.1	ssc:100626249	8E-103	NM_052896.4	CSMD2
LOC100624568	0	23.13579441	-20	8.30E-04	Low	0	28	XP_005662968.1	ssc:100624568	0	NM_014677.4	RIMS2
MAP3K7CL	0	23.13579441	-20	8.30E-04	Low	0	28	XP_003358952.2	ssc:100624256			
LOC100522946	0	23.13579441	-20	8.30E-04	Low	0	28	XP_003130837.3	ssc:100522946			
LOC100623534	0	23.13579441	-20	8.30E-04	Low	0	28	XP_003360622.1	ssc:100623534			
LOC100627897	0.828928824	30.57229975	-5.204833041	8.30E-04	Low	1	37	XP_005674235.1	ssc:100627897	2E-115	NM_025140.1	CCDC92
LOC100739315	37.30179706	1.652556743	4.496473421	8.31E-04	High	45	2	XP_003481384.1	ssc:100739315	0	NM_001115.2	ADCY8
PTCRA	164.1279071	18.17812418	3.174545326	8.33E-04	High	198	22	NM_001257090.1	ssc:100126289	1E-51	NM_138296.2	PTCRA
LOC102167974	23.21000706	203.2644794	-3.130539259	8.35E-04	Low	28	246	XR_309124.1	ssc:102167974			
LOC100513611	144.2336153	1179.925515	-3.032216444	8.35E-04	Low	174	1428	XP_003130285.1	ssc:100513611	1E-99	NM_005824.2	LRRC17
TUBA4A	3545.328579	423.8808047	3.064188801	8.37E-04	High	4277	513	XP_005672289.1	ssc:100151951	0	NM_006000.2	TUBA4A
LOC100127144	6.631430589	76.0176102	-3.518941632	8.38E-04	Low	8	92	XP_005654136.1	ssc:100127144	0	NM_002606.2	PDE9A
TMEM145	455.0819242	55.36065091	3.039193473	8.39E-04	High	549	67	XP_005655933.1	ssc:100513091	0	NM_173633.2	TMEM145
LOC100520194	337.3740312	40.48764021	3.058795464	8.40E-04	High	407	49	XP_003126126.3	ssc:100520194	0	NM_152322.2	BTBD11
CTSD	746.8648701	6375.563916	-3.093633808	8.41E-04	Low	901	7716	NM_001037721.1	ssc:494568	2E-42	NM_001909.4	CTSD
LOC100739618	94.49788589	9.089062089	3.37807872	8.42E-04	High	114	11	LOC100739618				
MECOM	284.3225865	2351.588246	-3.048034856	8.50E-04	Low	343	2846	XP_005670049.1	ssc:100518275	0	NM_005241.3	MECOM
AIM1	383.7940453	3200.176134	-3.059747078	8.52E-04	Low	463	3873	XP_003121377.3	ssc:100521168	0	NM_001624.2	AIM1
LOC100524058	304.2168783	36.35624836	3.064824959	8.56E-04	High	367	44	XP_003121439.3	ssc:100524058	1E-68	NM_198920.1	UBE3D
LOC100513808	141.7468288	1155.137164	-3.026675806	8.60E-04	Low	171	1398	XP_003130286.2	ssc:100513808	3E-99	NM_005824.2	LRRC17
LOC102157556	401.2015506	48.75042393	3.040840512	8.62E-04	High	484	59	XP_005668377.1	ssc:102157556	2E-146	NM_005800.4	USPL1
SCN9A	201.4297041	23.13579441	3.122077906	8.62E-04	High	243	28	XP_003133500.3	ssc:100516701	0	NM_002977.3	SCN9A
DDN	355.6104653	42.96647533	3.049014444	8.64E-04	High	429	52	XP_003126149.1	ssc:100525642	0	NM_015086.1	DDN
LOC102164731	91.1821706	741.1716994	-3.022984137	8.67E-04	Low	110	897	XP_005668251.1	ssc:102164731	5E-53	NM_001191055.1	ERVV-2
CADPS	2.486786471	43.7927537	-4.13833763	8.68E-04	Low	3	53	XP_005669756.1	ssc:100518990	0	NM_183394.2	CADPS
MT1A	1.657857647	37.18252673	-4.487232772	8.71E-04	Low	2	45	NM_001001266.2	ssc:397417	6E-12	NM_175617.3	MT1E
LOC102167801	1.657857647	37.18252673	-4.487232772	8.71E-04	Low	2	45	XR_302359.1	ssc:102167801			
ZBTB4	212.2057788	1736.837137	-3.03292663	8.74E-04	Low	256	2102	ZBTB4	ssc:100739718	0	NM_020899.3	ZBTB4
THPO	439.3322765	53.70809416	3.032101061	8.78E-04	High	530	65	XP_005670107.1	ssc:100620258	3E-120	NM_199356.1	THPO
MDGA2	7.460359412	81.8015588	-3.454811294	8.79E-04	Low	9	99	XP_003353527.1	ssc:100621082			
LOC102163580	137.6021847	14.87301069	3.209734754	8.88E-04	High	166	18	XR_306705.1	ssc:102163580			
LOC100620687	247.0207894	2022.729454	-3.033598998	8.94E-04	Low	298	2448	XP_005667681.1	ssc:100620687	0	NM_001018161.1	PON2

ZNF396	1080.094257	133.8570962	3.01239169	9.10E-04	High	1303	162	ZNF396	ssc:100620188	4E-97	NM_145756.2	ZNF396
OCA2	8.289288236	87.5855074	-3.401372035	9.16E-04	Low	10	106	NM_214094.2	ssc:397171	7E-11	NM_000275.2	OCA2
OSBPL10	831.4156101	103.2847965	3.00894193	9.18E-04	High	1003	125	XP_003483186.1	ssc:100526184	0	NM_017784.4	OSBPL10
KHDRBS3	31.4992953	264.409079	-3.069380257	9.19E-04	Low	38	320	XR_302764.1	ssc:100155706	1E-169	NM_006558.1	KHDRBS3
EPB41L4A	877.8356242	109.0687451	3.008713079	9.19E-04	High	1059	132	XP_005661637.1	ssc:100518651	0	NM_022140.3	EPB41L4A
LOC102158723	250.3365047	29.74602138	3.073100062	9.21E-04	High	302	36	XP_005654356.1	ssc:102158723			
TP53I3	1422.441861	175.9972932	3.014744542	9.24E-04	High	1716	213	XP_003125411.1	ssc:100521786	4E-174	NM_147184.3	TP53I3
WDR65	99.47145883	9.915340461	3.326548419	9.27E-04	High	120	12	XR_304209.1	ssc:100520084	0	NR_030778.1	WDR65
COCH	62.16966177	4.95767023	3.648476514	9.28E-04	High	75	6	XP_003361379.1	ssc:100624179	0	NM_004086.2	COCH
ZNF684	11.60500353	112.3738586	-3.275487595	9.29E-04	Low	14	136	ZNF684	ssc:102167697	0	NM_152373.3	ZNF684
CARD14	149.2071882	16.52556743	3.174545326	9.31E-04	High	180	20	XP_003482992.1	ssc:100514004	0	NR_047566.1	CARD14
LOC100522999	64.65644824	523.0342093	-3.016039146	9.32E-04	Low	78	633	XR_309499.1	ssc:100522999			
ATP6V1G2	34.81501059	289.1974301	-3.054273365	9.40E-04	Low	42	350	NM_001145380.1	ssc:100152358	2E-60	NM_138282.2	ATP6V1G2
LOC102158445	748.5227277	93.36945601	3.003023539	9.49E-04	High	903	113	XR_298454.1	ssc:102158445			
LOC100625542	415.2933406	51.22925905	3.019090807	9.53E-04	High	501	62	XP_005652742.1	ssc:100625542	2E-42	NM_015278.3	SASH1
LOC100525036	24.86786471	212.3535415	-3.094113629	9.54E-04	Low	30	257	XP_003123849.4	ssc:100525036	0	NM_022350.3	ERAP2
LOC100154114	29.84143765	0.826278372	5.174545326	9.54E-04	High	36	1	XR_297331.1	ssc:100154114			
LOC100518635	250.3365047	2032.644794	-3.021417537	9.58E-04	Low	302	2460	XR_300638.1	ssc:100518635	7E-41	NM_138785.3	GINM1
MYO3B	36.47286824	1.652556743	4.464051943	9.69E-04	High	44	2	XP_003359605.2	ssc:100621555	0	NR_045684.1	MYO3B
CTBS	36.47286824	1.652556743	4.464051943	9.69E-04	High	44	2	XP_003356466.3	ssc:100523254	2E-89	NM_004388.2	CTBS
LOC100515463	67.97216354	5.783948602	3.554817407	9.72E-04	High	82	7	XP_005659920.1	ssc:100515463	0	NM_032445.2	MEGF11
LOC100736869	615.0651871	76.84388857	3.00073689	9.73E-04	High	742	93	XR_302263.1	ssc:100736869	2E-37	NM_194320.2	ZNF169
TMSB10	17135.61664	162224.059	-3.242917783	9.79E-04	Low	20672	196331	NM_001097482.1	ssc:100037998	5E-15	NM_021103.3	TMSB10
MYO9A	247.8497183	29.74602138	3.058696997	9.93E-04	High	299	36	XP_003362136.1	ssc:100188909	5E-169	NM_006901.3	MYO9A
LOC100737472	279.3490136	33.87741324	3.043673101	9.94E-04	High	337	41	XP_003482505.1	ssc:100737472			
HHIP	3050.458071	374.3041024	3.026743135	9.97E-04	High	3680	453	XP_003129219.3	ssc:100525059	0	NM_022475.2	HHIP
LOC100625253	116.8789641	12.39417558	3.237281081	0.001002	High	141	15	XP_005653813.1	ssc:100625253	0	NM_006060.4	IKZF1
LOC100524712	272.717583	33.05113487	3.044636003	0.001002	High	329	40	XP_005670167.1	ssc:100524712	0	NM_025163.3	PIGZ
DDX21	55173.5025	6108.676002	3.175043939	0.001005	High	66560	7393	XP_005657444.1	ssc:100153111	0	NM_004728.3	DDX21
HAL	22.38107824	0	20	0.001006	High	27	0	XP_001925096.1	ssc:100154617	0	NM_002108.3	HAL
LOC102159232	22.38107824	0	20	0.001006	High	27	0	XP_005673921.1	ssc:102159232	2E-19	NM_153448.3	ESX1
EDAR	22.38107824	0	20	0.001006	High	27	0	XP_003124894.1	ssc:100526213	0	NM_022336.3	EDAR
LOC100738215	22.38107824	0	20	0.001006	High	27	0	LOC100738215		3E-22	NM_080676.5	MACROD2
FAM163B	22.38107824	0	20	0.001006	High	27	0	XP_003353751.1	ssc:100627389	3E-75	NM_001080515.2	FAM163B
LOC102160606	22.38107824	0	20	0.001006	High	27	0	XR_306517.1	ssc:102160606	3E-44	NM_198393.3	TEX14
FAM228A	22.38107824	0	20	0.001006	High	27	0	XP_005662759.1	ssc:100738869	8E-46	NM_198553.1	FAM228B
LOC102165254	22.38107824	0	20	0.001006	High	27	0	XR_304028.1	ssc:102165254			
PRKAG2	266.0861524	32.2248565	3.045647593	0.001012	High	321	39	PRKAG2		2E-107	NM_024429.1	PRKAG2
RNF224	1.657857647	36.35624836	-4.454811294	0.001016	Low	2	44	XP_005652805.1	ssc:100738070	9E-56	NM_001190228.1	RNF224
LOC100627304	431.0429883	53.70809416	3.004620324	0.001016	High	520	65	XP_005658588.1	ssc:100627304	0	NM_002661.3	PLCG2
BTBD11	494.8705077	61.97087788	2.997388755	0.001022	High	597	75	HighI00025E0D7E		0	NM_152322.2	BTBD11
BIRC6	322.4533124	39.66136184	3.023284169	0.001023	High	389	48	XP_003125300.1	ssc:100511750	3E-169	NM_016252.3	BIRC6
LOC102166272	48.90680059	3.305113487	3.887263374	0.001024	High	59	4	XR_306100.1	ssc:102166272			
LOC102162854	48.90680059	3.305113487	3.887263374	0.001024	High	59	4	XR_301570.1	ssc:102162854			
SCARF2	480.7787177	3943.00039	-3.035848913	0.001025	Low	580	4772	XP_005654177.1	ssc:100155943	2E-147	NM_182895	SCARF2
KLB	252.8232912	30.57229975	3.047832391	0.001033	High	305	37	XP_003482415.1	ssc:100525825	0	NM_175737.3	KLB
RORA	29.01250883	241.2732845	-3.055921218	0.001039	Low	35	292	XP_005659598.1	ssc:100156637	0	NM_134262.2	RORA
SHMT2	739.4045107	93.36945601	2.985341262	0.001043	High	892	113	XP_005658600.1	ssc:397181	8E-101	NM_148918.2	SHMT1
SCN2B	0	22.30951604	-20	0.001047	Low	0	27	XP_003129961.1	ssc:100514594	6E-120	NM_004588.4	SCN2B
CDH12	0	22.30951604	-20	0.001047	Low	0	27	XP_005672455.1	ssc:100523408	0	NM_004061.3	CDH12
CYP11A1	0	22.30951604	-20	0.001047	Low	0	27	NM_214427.1	ssc:403329			
OXCT2	97.81360119	9.915340461	3.302300873	0.001049	High	118	12	XP_005674674.1	ssc:102162296	6E-108	NM_022120.1	OXCT2
LOC102163982	8.289288236	85.93295066	-3.373891299	0.001053	Low	10	104	XR_302179.1	ssc:102163982			
QTRTD1	1544.294398	194.1754174	2.991515337	0.001061	High	1863	235	XP_005658142.1	ssc:100514011	0	NM_024638.3	QTRTD1
GUCA1B	3.315715294	48.75042393	-3.878022725	0.001076	Low	4	59	XP_005666050.1	ssc:102167816	2E-23	NM_002098.5	GUCA1B
GBP6	4.973572942	61.14459951	-3.619870541	0.001076	Low	6	74	XP_005663763.1	ssc:100523310	0	NM_198460.2	GBP6
LOC102159805	358.0972518	44.61903207	3.004620324	0.001076	High	432	54	XP_005672734.1	ssc:102159805	7E-58	NM_203327.1	SLC23A2
LOC102168054	54.70930236	4.131391859	3.727086349	0.001081	High	66	5	XR_300361.1	ssc:102168054			
SCUBE1	402.0304794	50.40298068	2.995723924	0.001084	High	485	61	XP_003126031.4	ssc:100524621			
LOC102157733	363.8997536	45.44531045	3.00133774	0.001088	High	439	55	XR_301024.1	ssc:102157733			
LOC102166279	91.1821706	9.089062089	3.326548419	0.001094	High	110	11	XP_005659546.1	ssc:102166279	1E-52	NM_004852.2	ONECUT2
LOC100623058	38.95965471	314.8120596	-3.014438011	0.001096	Low	47	381	XP_003361152.2	ssc:100623058	8E-156	NM_005711.4	EDIL3
LOC100511112	321.6243836	2581.293633	-3.004645572	0.001104	Low	388	3124	XR_300888.1	ssc:100511112	6E-73	NM_015832.4	MBD2
LOC100515730	810.6923895	103.2847965	2.972526695	0.001115	High	978	125	XP_003126464.1	ssc:100515730	7E-64	NM_002223.2	ITPR2
LOC100510895	27.35465118	226.4002739	-3.049017639	0.001122	Low	33	274	XP_003124046.2	ssc:100510895	0	NM_032101.2	PCDHGB7
DLGAP4	552.0665965	4495.780621	-3.025657422	0.001123	Low	666	5441	XP_005672968.1	ssc:100153319	0	NM_183006.2	DLGAP4
NLRP14	60.51180412	4.95767023	3.609482382	0.001125	High	73	6	XP_005674670.1	ssc:100622523	4E-12	NM_176822.3	NLRP14
SLC17A7	507.30444	64.44971299	2.976605948	0.001133	High	612	78	XP_003127359.1	ssc:100514772	0	NM_020309.3	SLC17A7
XBP1	537.1458777	4365.228638	-3.022671367	0.001134	Low	648	5283	NM_001271738.1	ssc:100217386			
LOC100157542	35.64393941	1.652556743	4.430885079	0.001134	High	43	2	XR_309390.1	ssc:100157542	6E-31	NM_018417.4	ADCY10
AKR1CL1	828.0998948	6820.927959	-3.042091308	0.001149	Low	999	8255	NM_001038626.2	ssc:654406	6E-140	NM_003739.5	AKR1C3
THEMIS2	181.5354124	21.48323766	3.078967666	0.001153	High	219	26	XP_005653435.1	ssc:100514466	0	NM_004848.3	THEMIS2
TTBK2	1177.907858	150.3826637	2.969516523	0.001157	High	1421	182	XP_005659750.1	ssc:100511488	0	NM_173500.3	TTBK2
LOC102161200	323.2822412	40.48764021	2.997240794	0.001163	High	390	49	XP_005659078.1	ssc:102161200	2E-178	NM_003035.2	STIL
KCNJ11	114.3921777	12.39417558	3.206254186	0.001174	High	138	15	XP_005661161.1	ssc:100158240	0	NM_001166290.1	KCNJ11
LOC100738143	7.460359412	78.49644531	-3.395310283	0.001185	Low	9	95	XR_300479.1	ssc:100738143			
BNC1	416.1222694	52.88181579	2.976163878	0.001185	High	502	64	XP_003128498.1	ssc:100156444	0	NM_001717.3	BNC1
LOC100516635	1.657857647	35.52996998	-4.42164443	0.001187	Low	2	43	XR_116125.2	ssc:100516635			
EDN1	19.89429177	169.3870662	-3.089897274	0.001191	Low	24	205	NM_213882.1	ssc:396915	1E-80	NM_001955.4	EDN1
LOC100737584	69.63002118	544.517447	-2.967196908	0.001194	Low	84	659	XR_303847.1	ssc:100737584	2E-30	NM_021913.4	AXL
LOC102161631	247.8497183	30.57229975	3.019168633	0.001199	High	299	37	XR_304474.1	ssc:102161631			
TMEM117	51.39358706	404.8764021	-2.977821304	0.001202	Low	62	490	XP_003126671.4	ssc:100524623	0	NM_032256.2	TMEM117
KIAA1239	53.88037353	4.131391859	3.705060042	0.001204	High	65	5	XP_003128959.3	ssc:100522449	0	NM_001144990.1	KIAA1239
AK7	3.315715294	47.92414556	-3.853360671	0.001212	Low	4	58	XP_003482362.1	ssc:100152573	0	NM_152327.3	AK7
LOC100737517	176.5618394	1380.711159	-2.967166073	0.001212	Low	213	1671	XP_003483749.1	ssc:100737517	0	NM_014585.5	SLC40A1
TDRD12	9959.579816	1205.540144	3.046405184	0.001232	High	12015	1459	XP_003127054.2	ssc:100523608	0	NM_001110822.1	TDRD12
LOC102158014	41.44644118	2.478835115	4.063514013	0.001238	High	50	3	XR_307757.1	ssc:102158014			
TOP1MT	39.78858353	315.638338	-2.987846003	0.001248	Low	48	382	XP_005662884.1	ssc:100517528	0	NM_052963.2	TOP1MT
AKAP17B	664.8009165	85.93295066	2.951639033	0.001252	High	802	104	XP_003135380.2	ssc:100513257	1E-81	NR_027383.1	AKAP17A
ARHGAP24	15.74964765	137.9884881	-3.131156455	0.001253	Low	19	167	XP_003129392.2	ssc:100522450	0	NM_031305.2	ARHGAP24
C13H21orf63	15.74964765	137.9884881	-3.131156455	0.001253	Low	19	167	XP_003358978.2	ssc:100628171	0	NR_104472.1	EVA1C
RELB	125.1682524	970.0508084	-2.954191629	0.001256	Low	151	1174	XP_005659055.1	ssc:100622609	0	NM_006509.3	RELB
KBTBD8	620.0387601	80.14900206	2.951601942	0.001258	High	748	97	XP_003132353.3	ssc:100521640	0	NM_032505.2	KBTBD8
GTSF1L	21.55214941	0	20	0.001277	High	26	0	NM_001258404.1	ssc:100156402	1E-57	NM_176791.3	GTSF1L
LOC102161484	21.55214941	0	20	0.001277	High	26	0	XR_300122.1	ssc:102161484			
LOC100518709	21.55214941	0	20	0.001277	High	26	0	XP_005659833.1	ssc:100518709	2E-65	NM_001167902.1	PGPEP1L
ATOH7	21.55214941	0	20	0.001277	High	26	0	XP_005671096.1	ssc:100624800	3E-67	NM_145178.3	ATOH7
LOC102162525	21.55214941	0	20	0.001277	High	26	0	XR_306596.1	ssc:102162525			
LOC102163222	21.55214941	0	20	0.001277	High	26	0	LOC102163222				
MARVELD3	21.55214941	0	20	0.001277	High	26	0	NM_001243842.1	ssc:100511453	2E-140	NM_052858.5	MARVELD3

LOC100739530	21.55214941	0	20	0.001277	High	26	0	XR_309490.1	ssc:100739530			
C1QTNF7	7.460359412	77.67016694	-3.380043526	0.001278	Low	9	94	NM_001143702.1	ssc:100233173	2E-145	NM_031911.4	C1QTNF7
AXL	1129.001058	9277.453558	-3.038682036	0.001281	Low	1362	11228	NM_001128458.1	ssc:100144875			
INSRR	160.8121918	19.00440255	3.08097121	0.001288	High	194	23	XP_001929559.1	ssc:100155702	0	NM_014215.2	INSRR
LOC102158067	47.24894295	3.305113487	3.837510339	0.001302	High	57	4	XP_005674378.1	ssc:102158067	1E-96	NM_019053.4	EXOC6
LONRF2	251.1654336	31.39857813	2.999866794	0.001304	High	303	38	XP_005662433.1	ssc:100522431	0	NM_198461.3	LONRF2
ITM2C	2721.373328	23078.7812	-3.084160247	0.001309	Low	3283	27931	NM_001123131.1	ssc:100144481	7E-138	NM_030926.5	ITM2C
PDSS1	1594.030128	205.7433146	2.95376144	0.001312	High	1923	249	XP_005668199.1	ssc:100514361	0	NM_014317.3	PDSS1
KCP	22.38107824	185.0863553	-3.047847096	0.001324	Low	27	224	XP_005657822.1	ssc:100517738	0	NM_199349.2	KCP
LOC100520605	16.57857647	142.9461583	-3.108079808	0.001324	Low	20	173	XP_005661698.1	ssc:100520605			
KLHL1	0	21.48323766	-20	0.001328	Low	0	26	XP_003131065.1	ssc:100520632	0	NM_020866.2	KLHL1
HEPHL1	0	21.48323766	-20	0.001328	Low	0	26	XP_003357292.1	ssc:100625356	0	NM_001098672.1	HEPHL1
LOC100738798	0	21.48323766	-20	0.001328	Low	0	26	XP_005673180.1	ssc:100738798	3E-71	NM_001195278.1	TMEM178B
SKAP1	0	21.48323766	-20	0.001328	Low	0	26	XP_003131592.3	ssc:100521471	2E-121	NM_003726.3	SKAP1
LOC102167375	0	21.48323766	-20	0.001328	Low	0	26	XR_306387.1	ssc:102167375			
LOC100737944	0	21.48323766	-20	0.001328	Low	0	26	XP_005653624.1	ssc:100737944	0	NM_018986.3	SH3TC1
LOC102165903	640.7619806	83.45411554	2.940733446	0.001329	High	773	101	XP_005667679.1	ssc:102165903	5E-97	NM_017650.2	PPP1R9A
ETNPPL	34.81501059	1.652556743	4.396937747	0.001329	High	42	2	XP_005667008.1	ssc:100519324	2E-175	NR_027475.1	ETNPPL
LOC100626746	88.69538413	9.089062089	3.286655692	0.001336	High	107	11	LOC100626746				
LOC102167744	9.947145883	95.02201275	-3.255907226	0.001337	Low	12	115	XP_005659025.1	ssc:102167744	5E-20	NM_198086	AJUBA
LSR	213.8636365	26.4409079	3.01584758	0.001346	High	258	32	XP_005664540.1	ssc:100516738	1E-115	NM_205835.3	LSR
LOC102159808	100.3003877	10.74161883	3.223043843	0.001359	High	121	13	XR_302422.1	ssc:102159808			
PDZD2	1031.187457	134.6833746	2.93666294	0.001365	High	1244	163	XP_003359788.2	ssc:100525132	7E-29	NM_178140.2	PDZD2
SLC38A3	1.657857647	34.70369161	-4.387697098	0.001391	Low	2	42	XP_005669647.1	ssc:100156039	0	NM_006841.4	SLC38A3
LOC102163557	1.657857647	34.70369161	-4.387697098	0.001391	Low	2	42	XP_005674082.1	ssc:102163557	4E-108	NM_007166.3	PICALM
FA2H	28.18358	0.826278372	5.092083166	0.001391	High	34	1	XP_003126916.2	ssc:100523966	0	NM_024306.4	FA2H
LOC102158893	28.18358	0.826278372	5.092083166	0.001391	High	34	1	XR_299087.1	ssc:102158893			
LOC100519366	8.289288236	82.62783717	-3.317307771	0.001396	Low	10	100	XR_298538.1	ssc:100519366	2E-35	NM_001863.4	COX6B1
LOC100739077	169.10148	1297.257044	-2.939503177	0.001397	Low	204	1570	XP_005674296.1	ssc:100739077	3E-85	NM_020992.3	PDLIM1
LOC100516844	4.144644118	52.88181579	-3.673451581	0.001411	Low	5	64	LOC100516844				
ADA	66.31430589	507.3349202	-2.935546426	0.00142	Low	80	614	XP_005673015.1	ssc:100625920	0	NM_000022.2	ADA
LOC102159172	40.61751236	2.478835115	4.034367668	0.001422	High	49	3	XR_305313.1	ssc:102159172			
LOC100157933	40.61751236	2.478835115	4.034367668	0.001422	High	49	3	XP_005666085.1	ssc:100157933	5E-175	NM_206539.2	DLK2
LOC100521521	4.973572942	58.66576439	-3.560164294	0.001441	Low	6	71	XR_307615.1	ssc:100521521			
LOC102166414	277.6911559	35.52996998	2.966372855	0.00145	High	335	43	XP_005672794.1	ssc:102166414	0	NM_020343.3	RALGAPA2
CORIN	0.828928824	28.09346464	-5.082842517	0.001452	Low	1	34	XP_003482424.1	ssc:100738048	0	NM_006587.3	CORIN
PCDH1	271.0597253	2089.658002	-2.946584172	0.001455	Low	327	2529	XP_005661829.1	ssc:100626940	0	NM_032420.3	PCDH1
LOC102164895	48.07787177	370.1727105	-2.944753603	0.001457	Low	58	448	XP_005666576.1	ssc:102164895	1E-114	NM_001313.4	CRMP1
SEZ6	169.9304088	20.65695929	3.040244234	0.00146	High	205	25	XP_005657062.1	ssc:100518932	0	NM_178860.4	SEZ6
LOC100156757	933.3738554	123.1154774	2.922442916	0.001463	High	1126	149	XP_003481671.1	ssc:100156757	4E-79	NM_001638.2	APOF
LOC102168181	122.6814659	14.04673232	3.126610849	0.001467	High	148	17	LOC102168181				
DUSP2	241.2182877	30.57229975	2.980042302	0.001469	High	291	37	XP_005662400.1	ssc:100622812	9E-117	NM_004418.3	DUSP2
LOC102164486	46.42001412	3.305113487	3.811975246	0.001471	High	56	4	XP_005670537.1	ssc:102164486			
STPG1	46.42001412	3.305113487	3.811975246	0.001471	High	56	4	XP_005665168.1	ssc:100736928	8E-84	NM_178122.4	STPG1
FIGN	1051.910677	138.8147665	2.921779255	0.001481	High	1269	168	XP_003133487.1	ssc:100513886	0	NM_018086.2	FIGN
DDR1	407.0040524	3165.472442	-2.959305772	0.001488	Low	491	3831	NM_001123105.1	ssc:100144462	0	NM_013994.2	DDR1
CD97	789.9691689	6278.889347	-2.990641134	0.001499	Low	953	7599	NM_213925.1	ssc:396964			
GSDMD	397.8858353	3087.802275	-2.956153928	0.001505	Low	480	3737	XP_005653149.1	ssc:100515607	2E-168	NM_024736.6	GSDMD
DYNC1I1	617.5519736	81.8015588	2.91636032	0.001513	High	745	99	XP_003130207.2	ssc:100523985			
AK3L1	7413.110469	930.3894466	2.994172373	0.001517	High	8943	1126	NM_001044597.1	ssc:733667	2E-125	NM_203464.2	AK4
LOC100625463	218.8372094	1669.082311	-2.931125134	0.001518	Low	264	2020	XP_003358926.3	ssc:100625463	6E-60	NM_000313.3	PROS1
PLLP	63.82751942	5.783948602	3.464051943	0.001521	High	77	7	XP_003127021.2	ssc:100516614	1E-84	NM_015993.2	PLLP
WIPI1	361.4129671	2790.342061	-2.948721815	0.001531	Low	436	3377	NM_001190294.1	ssc:100462758	0	NM_017983.5	WIPI1
RUNDC3B	98.64253001	10.74161883	3.19899837	0.001533	High	119	13	XP_003357529.1	ssc:100522092	0	NM_138290.2	RUNDC3B
LOC100156657	121.8525371	919.6478277	-2.915945208	0.001536	Low	147	1113	NM_001190191.1	ssc:100156657	8E-12	NM_016210.4	C3orf18
MYLIP	169.9304088	1287.341703	-2.921379094	0.001539	Low	205	1558	XP_001929111.1	ssc:100155795	0	NM_013262.3	MYLIP
LOC100152184	625.012333	4908.919806	-2.973449036	0.001541	Low	754	5941	XP_005655456.1	ssc:100152184	0	NM_016274.4	PLEKHO1
LOC100523351	3.315715294	46.27158882	-3.802734558	0.001544	Low	4	56	XP_003122145.3	ssc:100523351	2E-99	NM_198188.2	ASTN2
LOC100627892	4485.333865	574.2634684	2.965430683	0.001548	High	5411	695	XR_301599.1	ssc:100627892			
BARX1	664.8009165	88.41178578	2.910611764	0.001552	High	802	107	XP_003124833.2	ssc:100512774	4E-88	NM_021570.3	BARX1
LOC100516198	33.98608177	1.652556743	4.362172329	0.001562	High	41	2	XR_303804.1	ssc:100516198			
LOC102164563	33.98608177	1.652556743	4.362172329	0.001562	High	41	2	XR_306377.1	ssc:102164563			
PITX2	3676.299333	474.2837854	2.954431787	0.001563	High	4435	574	NM_001206435.1	ssc:100515395	1E-171	NM_153427.2	PITX2
UBASH3B	2033.362404	266.8879141	2.929561488	0.001565	High	2453	323	XP_005667496.1	ssc:100521108	0	NM_032873.4	UBASH3B
LOC102165046	162.4700494	19.83068092	3.034367668	0.001565	High	196	24	XP_005660240.1	ssc:102165046	1E-96	NM_006377.3	UNC13B
CHST12	70.45895001	531.296993	-2.914663667	0.001571	Low	85	643	XP_003124312.1	ssc:100524793	2E-180	NM_018641.4	CHST12
LOC102165618	16.57857647	139.6410448	-3.074331017	0.001571	Low	20	169	XP_005660020.1	ssc:102165618			
CTNS	169.9304088	1279.078919	-2.912089332	0.001617	Low	205	1548	XP_005669176.1	ssc:100515235	0	NM_004937.2	CTNS
ARHGEF28	11.60500353	104.1110748	-3.165304677	0.001621	Low	14	126	XP_005661540.1	ssc:102160659			
LOC102165933	11.60500353	104.1110748	-3.165304677	0.001621	Low	14	126	XR_303906.1	ssc:102165933			
EN1	12.43393235	109.8950234	-3.143771516	0.001624	Low	15	133	XP_003133330.1	ssc:100516112	1E-122	NM_001426.3	EN1
LOC100622125	20.72322059	0	20	0.001631	High	25	0	XP_005669371.1	ssc:100622125	0	NM_005442.3	EOMES
LOC102168196	20.72322059	0	20	0.001631	High	25	0	XR_302310.1	ssc:102168196			
SASH3	20.72322059	0	20	0.001631	High	25	0	XP_003135424.2	ssc:100525094	0	NM_018990.3	SASH3
EOMES	20.72322059	0	20	0.001631	High	25	0	XP_005669373.1	ssc:100524218	0	NM_005442.3	EOMES
LOC102162983	20.72322059	0	20	0.001631	High	25	0	XR_308175.1	ssc:102162983			
LOC100623257	20.72322059	0	20	0.001631	High	25	0	XP_005667272.1	ssc:100623257	2E-120	NM_001143975.1	UBTFL1
GLB1L2	20.72322059	0	20	0.001631	High	25	0	XP_005667581.1	ssc:100520582	0	NM_138342.3	GLB1L2
LOC100737451	20.72322059	0	20	0.001631	High	25	0	LOC100737451				
LOC100151957	243.7050741	31.39857813	2.956365156	0.001633	High	294	38	NM_001130532.1	ssc:100151957			
LOC100621946	39.78858353	2.478835115	4.004620324	0.001636	High	48	3	XP_005665150.1	ssc:100621946	6E-48	NM_178122.4	STPG1
RUNDC3A	15.74964765	133.0308178	-3.07836904	0.001637	Low	19	161	XP_005668816.1	ssc:100511099	0	NM_006695.4	RUNDC3A
LOC102167875	61.34073295	461.0633314	-2.910047622	0.001644	Low	74	558	XR_307206.1	ssc:102167875			
LOC102160391	80.40609589	8.262783717	3.282605072	0.001645	High	97	10	XP_005672107.1	ssc:102160391	4E-55	NM_024989.3	PGAP1
LOC100622933	213.8636365	27.26718627	2.97145346	0.001654	High	258	33	XP_005658453.1	ssc:100622933	5E-173	NM_024751.3	GSTCD
LOC102160929	62.99859059	5.783948602	3.445192916	0.001667	High	76	7	XR_298164.1	ssc:102160929			
RASSF2	21.55214941	173.5184581	-3.009185475	0.001667	Low	26	210	XP_003359929.1	ssc:100157105	0	NM_170774.1	RASSF2
NEBL	867.8884783	116.5052504	2.897114699	0.001668	High	1047	141	XP_005668224.1	ssc:100522395	2E-162	NM_213569.2	NEBL
HIPK4	101.1293165	754.3921534	-2.899113388	0.001669	Low	122	913	XP_003355967.1	ssc:100625431	0	NM_144685.3	HIPK4
LOC100622673	57.19608883	4.95767023	3.52818228	0.001673	High	69	6	XP_003360240.2	ssc:100622673	1E-120	NM_138811.3	C7orf31
ARSD	135.9443271	1014.66984	-2.899922516	0.001683	Low	164	1228	XP_005658943.1	ssc:100624032	3E-148	NM_009589.2	ARSD
LOC102159603	0	20.65695929	-20	0.001694	Low	0	25	XR_307292.1	ssc:102159603			
LOC102163989	0	20.65695929	-20	0.001694	Low	0	25	XR_302943.1	ssc:102163989			
PTPRCAP	0	20.65695929	-20	0.001694	Low	0	25	XP_003122509.1	ssc:100511860	9E-55	NM_005608.2	PTPRCAP
LOC100520139	0	20.65695929	-20	0.001694	Low	0	25	ZNF544	ssc:100520139	0	NM_014480.2	ZNF544
ZNF132	0	20.65695929	-20	0.001694	Low	0	25	ZNF132	ssc:102161626	2E-20	NM_017652.3	ZNF586
E2F7	1916.48344	254.4937385	2.912759466	0.001695	High	2312	308	XP_003126804.1	ssc:100154851	0	NM_203394.2	E2F7
LOC102167381	17.4075053	143.7724367	-3.046005749	0.001715	Low	21	174	XP_005659063.1	ssc:102167381	1E-21	NR_103804.1	PDLIM7
EXO1	2115.426358	280.9346464	2.912642002	0.00173	High	2552	340	XP_003130603.3	ssc:100512301	0	NM_130398.3	EXO1
C1QTNF2	165.7857647	1236.112444	-2.898417946	0.001732	Low	200	1496	NM_001142829.1	ssc:100217378	8E-140	NM_031908.4	C1QTNF2

F2RL2	7.460359412	74.36505346	-3.317307771	0.001741	Low	9	90	XP_003123759.1	ssc:100519355	0	NM_004101.3	F2RL2
CBLN3	3.315715294	45.44531045	-3.776739389	0.001747	Low	4	55	NM_001112680.1	ssc:100127149	1E-89	NM_001039771.2	CBLN3
SFRP1	1072.633898	8455.306578	-2.978699314	0.001747	Low	1294	10233	XP_003359916.2	ssc:100621622	2E-150	NM_003012.4	SFRP1
SLC9A3R2	1817.011981	14609.42789	-3.007259845	0.001756	Low	2192	17681	NM_001137639.1	ssc:100192448			
LOC100737008	0.828928824	27.26718627	-5.039773795	0.001762	Low	1	33	XP_005661612.1	ssc:100737008	3E-108	NM_022350.3	ERAP2
LOC102157990	830.5866813	112.3738586	2.885824276	0.001767	High	1002	136	XP_005664433.1	ssc:102157990	1E-146	NM_001361.4	DHODH
LDOC1L	76.26145177	565.1744063	-2.889670234	0.001774	Low	92	684	XP_005663798.1	ssc:100520193	5E-129	NM_032287.2	LDOC1L
SLA-7	313.3350953	2363.156143	-2.914936683	0.001775	Low	378	2860	NM_213768.1	ssc:396650	4E-132	NM_002116.7	HLA-A
GRAMD1B	2568.021496	340.4266892	2.915241226	0.001779	High	3098	412	XP_005667502.1	ssc:100512979	0	NM_020716.2	GRAMD1B
LOC102157928	73.7746653	7.436505346	3.310428754	0.001788	High	89	9	XR_309125.1	ssc:102157928			
CD44	3089.417726	25303.94886	-3.033955686	0.001794	Low	3727	30624	K7GLL1		0	NM_001202557.1	CD44
LOC100739537	62.99859059	467.6735584	-2.892110405	0.001795	Low	76	566	XP_005669380.1	ssc:100739537	1E-59	NM_003242.5	TGFBR2
UGP2	1052.739606	8256.999769	-2.971469033	0.001807	Low	1270	9993	NM_213980.1	ssc:397040			
LOC100625326	30.67036647	235.4893359	-2.940744419	0.001818	Low	37	285	XP_005665351.1	ssc:100625326	6E-36	NM_012307.3	EPB41L3
SEPP1	91.1821706	672.5905946	-2.882904946	0.001818	Low	110	814	NM_001134823.1	ssc:100037964	4E-139	NM_005410.2	SEPP1
FHL2	1008.806378	7891.784728	-2.9677023	0.001821	Low	1217	9551	XP_005662407.1	ssc:100513532	4E-120	NM_201557.3	FHL2
CUX1	369.7022553	2791.16834	-2.916433389	0.001829	Low	446	3378	XP_003481047.2	ssc:100521258	6E-150	NM_181552.3	CUX1
CHRM4	62.16966177	5.783948602	3.426084093	0.001829	High	75	7	XP_003122876.1	ssc:100516483	0	NM_000741.2	CHRM4
TM4SF5	33.15715294	1.652556743	4.326548419	0.00184	High	40	2	XP_003131959.3	ssc:100513308	1E-78	NM_003963.2	TM4SF5
LOC102167610	33.15715294	1.652556743	4.326548419	0.00184	High	40	2	XR_304948.1	ssc:102167610			
ST6GALNAC2	583.5658918	4467.687156	-2.936560698	0.001844	Low	704	5407	NM_213784.1	ssc:396676	3E-165	NM_175040.3	ST6GALNAC4
LOC102163288	56.36716001	418.0968561	-2.890910409	0.001846	Low	68	506	XP_005668656.1	ssc:102163288	2E-135	NM_020954.3	RNF213
EFNB3	9.11821706	85.10667229	-3.222448584	0.001852	Low	11	103	NM_001114285.1	ssc:100135672	2E-157	NM_001406.3	EFNB3
CMAH	20.72322059	165.2556743	-2.995379676	0.001853	Low	25	200	NM_001113015.1	ssc:396918			
SGK1	157.4964765	1162.573669	-2.883930681	0.001858	Low	190	1407	NM_001244459.1	ssc:100625739	0	NM_005627.3	SGK1
LOC100739105	90.35324177	9.915340461	3.187842148	0.00186	High	109	12	XR_135272.2	ssc:100739105			
NOR-1	169.9304088	21.48323766	2.983660706	0.001877	High	205	26	NM_214247.1	ssc:397479	0	NM_173200.2	NR4A3
LOC100515829	67.97216354	500.7246933	-2.881001655	0.00188	Low	82	606	XP_005663562.1	ssc:100515829	5E-65	NM_016274.4	PLEKHO1
LOC102163881	84.55074001	9.089062089	3.217614048	0.001882	High	102	11	LOC102163881				
ADI1	900.2167024	6982.052241	-2.955306914	0.001883	Low	1086	8450	NM_001243698.1	ssc:100626434	2E-90	NM_018269.3	ADI1
LOC102166544	102.7871741	754.3921534	-2.875654415	0.001886	Low	124	913	XP_005666528.1	ssc:102166544			
LOC100739615	44.76215647	3.305113487	3.759507826	0.001887	High	54	4	XR_303107.1	ssc:100739615			
NTRK1	43.93322765	328.0325136	-2.900454418	0.001887	Low	53	397	XP_001929561.2	ssc:100154471	0	NM_002529.3	NTRK1
CD99L2	287.6383018	2145.844931	-2.899218122	0.001895	Low	347	2597	XP_003360528.1	ssc:100517448	3E-68	NM_134446.3	CD99L2
LOC100739698	279.3490136	2080.56894	-2.896837462	0.001907	Low	337	2518	XP_003481292.1	ssc:100739698	1E-123	NM_006449.4	CDC42EP3
KATNAL2	146.7204018	18.17812418	3.012794256	0.001908	High	177	22	XP_003361500.3	ssc:100627107	4E-175	NM_031303.2	KATNAL2
UNG	6071.074704	789.9221234	2.942169592	0.001916	High	7324	956	XP_003132973.1	ssc:100519979	4E-155	NM_080911.2	UNG
COL2A1	198.1139888	25.61462952	2.951290822	0.001917	High	239	31	XP_001925994.5	ssc:397323	0	NM_033150.2	COL2A1
LOC100622016	1.657857647	33.05113487	-4.317307771	0.001922	Low	2	40	XP_003360831.2	ssc:100622016	6E-68	NM_183394.2	CADPS
LOC102167726	1.657857647	33.05113487	-4.317307771	0.001922	Low	2	40	XP_005654659.1	ssc:102167726	1E-49	NM_152572.2	AK8
MEOX1	19.89429177	158.6454474	-2.995379676	0.001926	Low	24	192	XP_005668827.1	ssc:100516574	7E-88	NM_013999.3	MEOX1
DMPK	68.80109236	504.8560851	-2.875368814	0.001932	Low	83	611	XP_005659031.1	ssc:100627008	0	NM_004409	DMPK
DOCK10	16.57857647	135.509653	-3.031003585	0.001953	Low	20	164	XP_005672312.1	ssc:100512141	0	NM_017718.1	DOCK10
APCDD1	123.5103947	14.87301069	3.053863におい	0.00196	High	149	18	XP_005658356.1	ssc:100626308	6E-112	NM_153000.4	APCDD1
GCNT2	3127.548451	417.2705777	2.905977132	0.001961	High	3773	505	XP_005665619.1	ssc:100152566	0	NM_145655.3	GCNT2
IGFBP6	606.7758989	4610.633314	-2.925729244	0.001974	Low	732	5580	NM_001100190.1	ssc:100101923	1E-100	NM_002178.2	IGFBP6
BTBD17	22.38107824	175.1710148	-2.968412628	0.001983	Low	27	212	XP_005668698.1	ssc:100623910			
ZNF793	14.09179	118.1578072	-3.067788171	0.001988	Low	17	143	ZNF793	ssc:100511696	0	NM_001013659.2	ZNF793
EML5	242.8761453	32.2248565	2.91397496	0.001998	High	293	39	XP_003356819.2	ssc:100153276	0	NM_183387.2	EML5
ZNF624	36.47286824	272.6718627	-2.902270271	0.002018	Low	44	330	ZNF624	ssc:100626993	0	NM_020787.3	ZNF624
LOC102163074	29.01250883	220.6163253	-2.92679259	0.00202	Low	35	267	XP_005654413.1	ssc:102163074			
LIPG	671.4323471	5103.921502	-2.926292193	0.002024	Low	810	6177	NM_001243029.1	ssc:100155736	0	NM_006033.2	LIPG
LOC100517788	1463.059374	199.959366	2.871209555	0.002024	High	1765	242	XP_005654270.1	ssc:100517788	0	NM_020726.4	NLN
PAIP2B	1229.301445	168.5607878	2.866497865	0.002024	High	1483	204	XP_005662535.1	ssc:100739314	9E-64	NM_020459.1	PAIP2B
GPR19	451.7662089	61.97087788	2.865914054	0.002034	High	545	75	XP_005659112.1	ssc:100625907	0	NM_006143.2	GPR19
GAREML	1889.957718	256.9725736	2.878667663	0.002035	High	2280	311	XP_005662735.1	ssc:100513275	0	NM_001191033.1	GAREML
DSTN	3532.894646	28625.58791	-3.018382691	0.002038	Low	4262	34644	NM_001004031.1	ssc:445516	3E-92	NM_006870.3	DSTN
LOC100738135	77.91930942	8.262783717	3.237281081	0.002055	High	94	10	XP_005659503.1	ssc:100738135	3E-150	NR_046357.1	RNF165
LOC102161349	26.52572236	0.826278372	5.004620324	0.002059	High	32	1	XP_005664545.1	ssc:102161349	6E-114	NM_024916.1	CD22
SOX3	26.52572236	0.826278372	5.004620324	0.002059	High	32	1	XP_003360512.1	ssc:100623770	3E-166	NM_005634.2	SOX3
CHRND	19.89429177	156.9928906	-2.980272783	0.002078	Low	24	190	XP_005672338.1	ssc:100522042	0	NR_046334.1	CHRND
SPHK1	73.7746653	535.4283849	-2.859496248	0.002081	Low	89	648	XP_005656977.1	ssc:100519210	2E-180	NM_182965.2	SPHK1
LOC100621940	94.49788589	10.74161883	3.13707062	0.002084	High	114	13	XP_005663397.1	ssc:100621940	1E-171	NM_181871.3	PKLR
FAM65C	14.09179	117.3315288	-3.057663954	0.002091	Low	17	142	XP_005673066.1	ssc:100153156	0	NM_080829.2	FAM65C
ZIC5	890.2695565	123.1154774	2.854230082	0.002094	High	1074	149	ZIC5	ssc:100153729			
GNAL	19.89429177	0	20	0.002095	High	24	0	XP_003482083.2	ssc:100736677	3E-103	NM_182978.3	GNAL
LOC102168035	19.89429177	0	20	0.002095	High	24	0	XR_298783.1	ssc:102168035			
LOC100628139	19.89429177	0	20	0.002095	High	24	0	XP_005672714.1	ssc:100628139	0	NM_014915.2	ANKRD26
LOC100739063	19.89429177	0	20	0.002095	High	24	0	XP_005662714.1	ssc:100739063	9E-52	NM_182756.3	SPDYA
PAQR9	19.89429177	0	20	0.002095	High	24	0	XP_005669976.1	ssc:100516760	0	NM_198504.2	PAQR9
LOC102157880	19.89429177	0	20	0.002095	High	24	0	XP_005671678.1	ssc:102157880			
PVALB1	19.89429177	0	20	0.002095	High	24	0	NM_001190157.1	ssc:100157265	6E-52	NM_002854.2	PVALB
LOC100623946	19.89429177	0	20	0.002095	High	24	0	XP_005668209.1	ssc:100623946	1E-49	NM_153714.2	C10orf67
LOC102158173	19.89429177	0	20	0.002095	High	24	0	XR_300144.1	ssc:102158173			
CRYBA4	19.89429177	0	20	0.002095	High	24	0	XP_005670834.1	ssc:100152041	3E-110	NM_001886.2	CRYBA4
SLC9A4	19.89429177	0	20	0.002095	High	24	0	XP_003354759.1	ssc:396786	0	NM_001011552.3	SLC9A4
ARMC4	49.73572942	4.131391859	3.589582825	0.002097	High	60	5	XP_005656871.1	ssc:100516925	0	NM_018076.2	ARMC4
WIPF3	2356.644646	319.7697299	2.881628952	0.002097	High	2843	387	XP_005673342.1	ssc:100514743	2E-138	NM_001080529.2	WIPF3
LOC102163933	143.4046865	1040.28447	-2.858814016	0.002098	Low	173	1259	XP_005669573.1	ssc:102163933	3E-29	NM_016479.4	SHISA5
KPNA5	315.8218818	42.96647533	2.877827794	0.002108	High	381	52	XP_005654450.1	ssc:397220	0	NM_002269.2	KPNA5
NHS	1553.412615	213.1798199	2.865298307	0.00211	High	1874	258	XP_005673517.1	ssc:100152239	0	NM_198270	NHS
LOC102167027	88.69538413	9.915340461	3.16112481	0.002121	High	107	12	XP_005664380.1	ssc:102167027	3E-28	NM_002661.3	PLCG2
FNDC9	88.69538413	9.915340461	3.16112481	0.002121	High	107	12	XP_003134155.1	ssc:100525165	9E-114	NM_001001343.3	FNDC9
LOC100153751	123.5103947	893.2069198	-2.854361963	0.002122	Low	149	1081	XP_005670536.1	ssc:100153751	5E-84	NM_021973.2	HAND2
LOC100738813	9.11821706	83.45411554	-3.19415954	0.002127	Low	11	101	XP_005662821.1	ssc:100738813	5E-109	NM_014746	RNF144A
LOC100627669	1087.554617	150.3826637	2.854377689	0.002128	High	1312	182	XR_301823.1	ssc:100627669	5E-40	NM_031918.3	KLF16
EPHA2	623.3544753	4693.261151	-2.912466038	0.002134	Low	752	5680	XP_005665094.1	ssc:100522873	0	NM_004431.3	EPHA2
LOC102167627	43.93322765	3.305113487	3.732540779	0.002142	High	53	4	XR_301535.1	ssc:102167627			
LOC102163179	43.93322765	3.305113487	3.732540779	0.002142	High	53	4	XP_005672747.1	ssc:102163179	2E-86	NM_014915.2	ANKRD26
MTMR9	1488.756167	204.9170362	2.860995649	0.002144	High	1796	248	XP_003360815.1	ssc:100626566	0	NM_015458.3	MTMR9
LOC100514339	436.84549	60.31832114	2.856454917	0.002146	High	527	73	XP_005655078.1	ssc:100514339	2E-132	NM_018936.3	PCDHB2
LOC100152536	1139.777132	157.819169	2.8524074	0.002162	High	1375	191	XP_003131097.3	ssc:100152536	0	NM_005845.3	ABCC4
LOC102162575	32.32822412	1.652556743	4.290022543	0.002173	High	39	2	XR_301394.1	ssc:102162575			
LOC102167675	32.32822412	1.652556743	4.290022543	0.002173	High	39	2	XR_303101.1	ssc:102167675			
LOC100515353	32.32822412	1.652556743	4.290022543	0.002173	High	39	2	XP_003135337.2	ssc:100515353	1E-174	NM_018015.5	CXorf57
LOC102159118	0	19.83068092	-20	0.002175	Low	0	24	XR_303283.1	ssc:102159118			
LOC100736919	0	19.83068092	-20	0.002175	Low	0	24	XP_005670569.1	ssc:100736919	2E-106	NM_021808.3	GALNT9
LOC102164546	0	19.83068092	-20	0.002175	Low	0	24	XP_005653488.1	ssc:102164546			
LOC102158147	0	19.83068092	-20	0.002175	Low	0	24	XP_005661879.1	ssc:102158147			
LOC102165393	0	19.83068092	-20	0.002175	Low	0	24	XR_304072.1	ssc:102165393			

MIR221	0	19.83068092	-20	0.002175	Low	0	24	NR_035371.1	ssc:100316576			
LOC100620821	0	19.83068092	-20	0.002175	Low	0	24	XP_005658964.1	ssc:100620821	7E-15	NM_002704.3	PPBP
ADAMTS7	616.7230448	4623.853768	-2.906401112	0.002198	Low	744	5596	XP_005656319.1	ssc:100154398	0	NM_014272.3	ADAMTS7
LOC100739082	290.9540171	39.66136184	2.874985044	0.002206	High	351	48	XP_005667364.1	ssc:100739082			
LOC102162476	149.2071882	19.00440255	2.972911465	0.002226	High	180	23	XP_005656392.1	ssc:102162476			
LOC100628093	40.61751236	297.4602138	-2.872522928	0.002242	Low	49	360	XP_005653150.1	ssc:100628093	0	NM_052963.2	TOP1MT
LOC100513927	149.2071882	1073.335605	-2.846712295	0.002244	Low	180	1299	XP_005664452.1	ssc:100513927	0	NR_040072.1	NDRG4
LOC100737118	3.315715294	43.7927537	-3.72330013	0.002245	Low	4	53	XP_005672709.1	ssc:100737118	2E-69	NM_014012.5	REM1
LMOD3	3.315715294	43.7927537	-3.72330013	0.002245	Low	4	53	XP_003132362.1	ssc:100524108	0	NM_198271.3	LMOD3
C2H19orf67	216.350423	28.91974301	2.903243304	0.00225	High	261	35	XP_005661295.1	ssc:100513846	7E-170	xp_003403752	xp_003403752
ADAMDEC1	1.657857647	32.2248565	-4.280781895	0.002269	Low	2	39	XP_003132859.1	ssc:100517917	0	NM_014479.3	ADAMDEC1
LIN7A	1.657857647	32.2248565	-4.280781895	0.002269	Low	2	39	XP_005664343.1	ssc:100511198	2E-133	NM_004664.2	LIN7A
LOC102158582	1.657857647	32.2248565	-4.280781895	0.002269	Low	2	39	XR_302731.1	ssc:102158582			
LOC100623995	84.55074001	605.6620465	-2.840623722	0.00227	Low	102	733	XR_299855.1	ssc:100623995			
ATP2C2	71.28787883	7.436505346	3.260960078	0.002274	High	86	9	XP_005655808.1	ssc:100514770	0	NM_014861.2	ATP2C2
PUS7	1377.679705	191.6965822	2.845343996	0.002303	High	1662	232	XP_005658694.1	ssc:100627719	2E-159	NM_019042.3	PUS7
ZFAND4	98.64253001	11.5678972	3.092083166	0.00231	High	119	14	ZFAND4	ssc:100152192	0	NR_104256.1	ZFAND4
LOC100739621	148.3782594	19.00440255	2.964874146	0.002317	High	179	23	XP_005667077.1	ssc:100739621	0	NM_005935.2	AFF1
PLK2	1333.746477	10222.71602	-2.938222183	0.002318	Low	1609	12372	XP_005672528.1	ssc:397251	0	NM_006622.3	PLK2
LOC100737851	43.10429883	313.1595029	-2.860993996	0.002325	Low	52	379	XP_005658607.1	ssc:100737851	3E-91	NM_007364.2	TMED3
USP49	237.9025724	32.2248565	2.884125032	0.002325	High	287	39	XP_003128438.3	ssc:100520499	0	NM_018561.4	USP49
ELAC2	6565.116283	873.3762389	2.910145378	0.002341	High	7920	1057	NM_001243216.1	ssc:100520804	0	NM_173717.1	ELAC2
MROH6	142.5757577	18.17812418	2.97145346	0.002346	High	172	22	XP_003481376.1	ssc:100737318	0	NM_001100878.1	MROH6
LOC100517681	109.4186047	13.22045395	3.049014444	0.002348	High	132	16	XR_309472.1	ssc:100517681			
LOC100626701	2035.849191	15834.79872	-2.959395928	0.002356	Low	2456	19164	XP_003356232.1	ssc:100626701	0	NM_005529.5	HSPG2
LOC100622645	198.1139888	26.4409079	2.905487132	0.002361	High	239	32	XP_005658846.1	ssc:100622645	3E-85	NM_001024071.1	GCH1
TGFBR3	43.93322765	318.1171731	-2.856173857	0.002366	Low	53	385	NM_214272.1	ssc:397512	0	NR_036634.1	TGFBR3
LRP3	718.6812901	5360.067797	-2.898827216	0.002387	Low	867	6487	XP_005653323.1	ssc:100524868	0	NM_002333.3	LRP3
SERTAD1	294.2697324	2126.01425	-2.852940203	0.00243	Low	355	2573	XP_003355962.1	ssc:100624989	1E-81	NM_013376.3	SERTAD1
PNMA2	43.10429883	3.305113487	3.705060042	0.002435	High	52	4	XP_005670504.1	ssc:100157132	0	NM_007257.5	PNMA2
LOC102158261	43.10429883	3.305113487	3.705060042	0.002435	High	52	4	XR_304928.1	ssc:102158261			
IRF6	11.60500353	98.32712624	-3.082842517	0.002436	Low	14	119	NM_214278.1	ssc:397521	0	NM_006147.3	IRF6
LRRC29	11.60500353	98.32712624	-3.082842517	0.002436	Low	14	119	XP_005664476.1	ssc:100524680			
LOC100623982	70.45895001	7.436505346	3.244086259	0.002467	High	85	9	XP_003358539.2	ssc:100623982	5E-76	NM_032505.2	KBTBD8
LOC100622980	543.7773083	76.84388857	2.823013518	0.002468	High	656	93	XP_003353294.2	ssc:100622980	2E-125	NM_016230.3	CYB5R4
MRAS	440.1612053	3210.917753	-2.86688181	0.00248	Low	531	3886	XP_003358618.1	ssc:100621398	2E-121	NM_012219.4	MRAS
LOC100517940	27.35465118	203.2644794	-2.893500062	0.002482	Low	33	246	XP_005663898.1	ssc:100517940	6E-62	NR_027835.1	APOL3
MARCKS	2181.740664	16901.52409	-2.953601819	0.002484	Low	2632	20455	XP_003121421.2	ssc:100517207	2E-34	NM_002356.5	MARCKS
LRFN5	474.1472871	66.92854811	2.824641658	0.002485	High	572	81	XP_005659971.1	ssc:102163172	0	NM_152447.3	LRFN5
TOM1L1	348.1501059	48.75042393	2.836222793	0.002502	High	420	59	XP_005669004.1	ssc:100522701	0	NM_005486.2	TOM1L1
LOC102166801	382.1361877	53.70809416	2.830875452	0.002507	High	461	65	XP_005659205.1	ssc:102166801	1E-39	NM_025218.2	ULBP1
ZSCAN9	92.01109942	10.74161883	3.098596473	0.002516	High	111	13	ZSCAN9	ssc:100154531	0	NM_006299.4	ZSCAN9
EMSP1	25.69679353	0.826278372	4.958816635	0.002518	High	31	1	NM_213802.1	ssc:396720	2E-111	NM_004917.3	KLK4
LOC102162937	25.69679353	0.826278372	4.958816635	0.002518	High	31	1	XR_308244.1	ssc:102162937			
LOC100625977	25.69679353	0.826278372	4.958816635	0.002518	High	31	1	XP_005668112.1	ssc:100625977	6E-173	NM_012144.3	DNAI1
GPD1L	3939.06977	538.7334984	2.870211291	0.002531	High	4752	652	XP_003483187.1	ssc:100624347	0	NM_015141.3	GPD1L
EMP3	1316.338972	9964.917163	-2.920326765	0.002541	Low	1588	12060	NM_001244530.1	ssc:100625126	2E-77	NM_001425.2	EMP3
LOC102164768	251.9943624	1801.28685	-2.837564582	0.002551	Low	304	2180	XP_005664907.1	ssc:102164768	2E-57	NM_182571.2	SSC5D
EFHC2	113.5632488	14.04673232	3.015189566	0.002556	High	137	17	XP_003360327.2	ssc:100622362	0	NM_025184.3	EFHC2
ATF5	714.5366459	5278.266239	-2.884984214	0.002564	Low	862	6388	XP_005664813.1	ssc:100523675	4E-98	NM_012068.5	ATF5
UCP3	31.4992953	1.652556743	4.252547838	0.002573	High	38	2	NM_214049.1	ssc:397116	6E-166	NM_022803.2	UCP3
LOC100155404	31.4992953	1.652556743	4.252547838	0.002573	High	38	2	XP_003355257.1	ssc:100155404	1E-68	NM_001161334.1	HIST2H2BF
NDRG4	152.5229035	1077.466997	-2.820545874	0.002577	Low	184	1304	XP_003481858.1	ssc:100626374	0	NR_040072.1	NDRG4
ANKDD1A	75.43252295	8.262783717	3.19048687	0.002578	High	91	10	XP_005659548.1	ssc:100515000	0	NM_182703	ANKDD1A
LOC100621092	53.05144471	376.7829375	-2.82826969	0.002581	Low	64	456	XP_005674141.1	ssc:100621092	7E-85	NM_153367.3	ZCCHC24
C7H14orf50	386.2808318	54.53437253	2.82441235	0.002582	High	466	66	XP_001926490.2	ssc:100157071	1E-166	NM_172365.1	PPP1R36
LOC102163527	162.4700494	21.48323766	2.91889045	0.002598	High	196	26	XP_005667704.1	ssc:102163527	0	NM_015204.2	THSD7A
LOC100737316	448.4504936	63.62343462	2.817318567	0.002599	High	541	77	XP_003480992.1	ssc:100737316	0	NM_018931.2	PCDHB11
LOC102162929	173.2461241	23.13579441	2.904624534	0.002622	High	209	28	XR_306934.1	ssc:102162929			
LOC102161907	0.828928824	25.61462952	-4.949575986	0.002622	Low	1	31	XP_005654262.1	ssc:102161907	6E-147	XP_005275995.1	LOC728763
LOC100623023	0.828928824	25.61462952	-4.949575986	0.002622	Low	1	31	XP_005665919.1	ssc:100623023	1E-72	NM_138569.2	MLIP
FAM107A	9.11821706	80.97528043	-3.150657001	0.002627	Low	11	98	NM_001252206.1	ssc:100516512	3E-70	NM_007177.3	FAM107A
LOC100621145	986.4253001	140.4673232	2.811975246	0.002635	High	1190	170	XR_305036.1	ssc:100621145	0	NM_178043.2	LARP1B
ART5	48.07787177	4.131391859	3.540673225	0.002639	High	58	5	HighI0001C9836A		1E-109	NM_053017.3	ART5
TSTD1	3692.877909	508.987477	2.8590435	0.002645	High	4455	616	XP_005663867.1	ssc:100153230	4E-162	NM_003312.5	TST
UNC13A	83.72181118	587.4839223	-2.810873943	0.002647	Low	101	711	XP_005654971.1	ssc:100627038	0	NM_001080421.2	UNC13A
PTGER4	4.973572942	53.70809416	-3.432784988	0.002649	Low	6	65	XP_005659149.1	ssc:100625072	0	NM_000958.2	PTGER4
NBEA	2048.283123	287.5448734	2.832556124	0.002653	High	2471	348	XP_003130974.3	ssc:100521513	0	NM_015678.4	NBEA
LOC102166590	167.4436224	22.30951604	2.907944305	0.002657	High	202	27	XR_305915.1	ssc:102166590			
ANKRD35	117.707893	825.4520934	-2.809973424	0.002663	Low	142	999	XP_003125839.1	ssc:100511691	0	NM_144698.4	ANKRD35
IFI35	107.7607471	755.2184318	-2.809062218	0.002666	Low	130	914	XP_003358072.1	ssc:100624758	6E-118	NM_005533.4	IFI35
LOC100515984	69.63002118	7.436505346	3.227012746	0.002678	High	84	9	XP_003135147.1	ssc:100515984	4E-90	NM_018159.3	NUDT11
LOC100738689	6.631430589	64.44971299	-3.280781895	0.002691	Low	8	78	XP_003484299.2	ssc:100738689			
ZCCHC12	6.631430589	64.44971299	-3.280781895	0.002691	Low	8	78	ZCCHC12	ssc:100517990	0	NM_173798.2	ZCCHC12
DOCK4	977.307083	139.6410448	2.807088891	0.002701	High	1179	169	XP_003134821.3	ssc:100520706	0	NM_014705.3	DOCK4
LOC100620095	19.06536294	0	20	0.002709	High	23	0	XP_005670384.1	ssc:100620095	3E-100	NM_138983.2	OLIG1
TDRD9	19.06536294	0	20	0.002709	High	23	0	XP_003128797.3	ssc:100522382	0	NM_153046.2	TDRD9
LOC102161829	19.06536294	0	20	0.002709	High	23	0	XR_297346.1	ssc:102161829			
LOC102161675	19.06536294	0	20	0.002709	High	23	0	LOC102161675				
LOC102159758	19.06536294	0	20	0.002709	High	23	0	XR_307949.1	ssc:102159758			
LOC102159995	19.06536294	0	20	0.002709	High	23	0	XR_300804.1	ssc:102159995			
USH2A	19.06536294	0	20	0.002709	High	23	0	XP_003130538.3	ssc:100513222			
LOC102166688	19.06536294	0	20	0.002709	High	23	0	XP_005662944.1	ssc:102166688	1E-19	NR_109794.1	SAMD12
LOC102164534	19.06536294	0	20	0.002709	High	23	0	XR_301856.1	ssc:102164534			
NT5C1A	19.06536294	0	20	0.002709	High	23	0	XP_003127863.1	ssc:100516203	0	NM_032526.1	NT5C1A
ZBTB8B	19.06536294	0	20	0.002709	High	23	0	ZBTB8B	ssc:102163469	2E-150	NM_001145720.1	ZBTB8B
CPB2	19.06536294	0	20	0.002709	High	23	0	XP_001929181.1	ssc:100155038	0	NM_016413.3	CPB2
MOV10L1	19.06536294	0	20	0.002709	High	23	0	XP_005653251.1	ssc:100519488	0	NM_018995.2	MOV10L1
LOC100739117	19.06536294	0	20	0.002709	High	23	0	XP_005669518.1	ssc:100739117	4E-169	NM_001123396.1	CCR2
LOC102163079	19.06536294	0	20	0.002709	High	23	0	XR_305289.1	ssc:102163079			
LOC102165193	19.06536294	0	20	0.002709	High	23	0	XR_305541.1	ssc:102165193			
LOC102159708	8.289288236	75.19133183	-3.181246221	0.002713	Low	10	91	XR_298415.1	ssc:102159708			
ZMYND15	8.289288236	75.19133183	-3.181246221	0.002713	Low	10	91	ZMYND15	ssc:100513818	0	NM_032265.2	ZMYND15
LOC100511572	19.89429177	151.208942	-2.926117013	0.002722	Low	24	183	XP_005658396.1	ssc:100511572	0	NM_198494.2	ZFP69
CD40	71.28787883	499.0721365	-2.80751966	0.002726	Low	86	604	NM_214194.1	ssc:397395	3E-115	NM_152854.2	CD40
LOC396904	18071.47728	150749.5313	-3.060367164	0.002751	Low	21801	182444	LOC396904		0	NM_005964.3	MYH10
LOC100515339	53.05144471	373.477824	-2.815558638	0.002753	Low	64	452	XP_003131485.1	ssc:100515339	0	NM_002276.4	KRT19
SYNC	315.8218818	2248.303449	-2.831653729	0.002758	Low	381	2721	XP_005665242.1	ssc:100620771	0	NM_030786.2	SYNC
LOC100516409	869.546336	124.7680341	2.801014548	0.002762	High	1049	151	XR_301021.1	ssc:100516409	5E-109	NM_007280.1	OIP5
BLM	2156.872799	304.0704408	2.826463615	0.002772	High	2602	368	NM_001123084.1	ssc:100141304	1E-107	NM_001287248.1	BLM

FADS3	760.9566601	5576.552731	-2.873487371	0.002774	Low	918	6749	NM_001206434.1	ssc:100518057	0	NM_021727.4	FADS3
SPATA20	46.42001412	328.0325136	-2.821019951	0.002777	Low	56	397	NM_001159313.1	ssc:100286812	0	NM_022827.3	SPATA20
LOC100624220	27.35465118	199.959366	-2.869848794	0.002793	Low	33	242	XP_005667639.1	ssc:100624220	2E-85	NM_012395.3	CDK14
C5AR2	0	19.00440255	-20	0.002809	Low	0	23	XP_005664690.1	ssc:100515612	9E-144	NM_018485.2	C5AR2
WDR16	0	19.00440255	-20	0.002809	Low	0	23	XP_003132040.1	ssc:100516337	0	NM_145054.4	WDR16
LOC102163875	0	19.00440255	-20	0.002809	Low	0	23	LOC102163875				
DLX3	0	19.00440255	-20	0.002809	Low	0	23	XP_003131638.1	ssc:100516335	4E-166	NM_005220.2	DLX3
LOC102160975	0	19.00440255	-20	0.002809	Low	0	23	XR_303919.1	ssc:102160975			
LOC102160101	0	19.00440255	-20	0.002809	Low	0	23	XP_005665000.1	ssc:102160101	2E-21	NM_052860.2	ZNF300
LOC102158370	0	19.00440255	-20	0.002809	Low	0	23	XR_299864.1	ssc:102158370			
RSPO1	0	19.00440255	-20	0.002809	Low	0	23	XP_005658939.1	ssc:100623344	4E-57	NM_173640.1	RSPO1
LOC100627895	0	19.00440255	-20	0.002809	Low	0	23	XP_003360659.2	ssc:100627895	0	NM_007332.2	TRPA1
ZNF8	67.97216354	474.2837854	-2.802734598	0.002809	Low	82	574	ZNF8	ssc:100517831	0	NM_021089.2	ZNF8
MSN	2543.98256	19515.04258	-2.939425926	0.002813	Low	3069	23618	NM_001009578.1	ssc:494458	0	NM_002444.2	MSN
IQGAP2	539.6326642	77.67016694	2.796545206	0.002825	High	651	94	XP_005661563.1	ssc:100519536	0	NM_006633.3	IQGAP2
C9H11orf82	1429.073292	204.0907578	2.807797152	0.002829	High	1724	247	XP_003129764.3	ssc:100517842	0	NM_145018.3	C11orf82
PDE8A	121.0236082	841.9776608	-2.798493453	0.002829	Low	146	1019	XP_003128509.4	ssc:100514527	0	NM_173457.1	PDE8A
ERICH2	111.9053912	14.04673232	2.99397308	0.002836	High	135	17	XP_005672010.1	ssc:100155783	1E-44	XP_209489.5	ERICH2
FUCA1	316.6508106	2244.998336	-2.825749698	0.002846	Low	382	2717	XP_003356247.1	ssc:100621112	0	NM_000147.4	FUCA1
ADAMTS3	62.16966177	433.7961452	-2.802734598	0.002846	Low	75	525	XP_003129152.3	ssc:100524818	0	NM_014243.2	ADAMTS3
PRAF2	540.461593	3897.555079	-2.850305407	0.002853	Low	652	4717	NM_001243880.1	ssc:100511673	5E-83	NM_007213.2	PRAF2
XRCC2	767.5880907	110.7213018	2.793399517	0.002854	High	926	134	XRCC2	ssc:100514260			
LOC100626128	646.5644824	93.36945601	2.791771676	0.002872	High	780	113	XP_003361125.3	ssc:100626128	0	NM_024086.3	METTL16
CHD5	19.06536294	144.5987151	-2.923028831	0.002878	Low	23	175	XP_005656076.1	ssc:100738053	0	NM_015557.2	CHD5
LOC102158821	79.57716707	9.089062089	3.130151206	0.00288	High	96	11	XR_299671.1	ssc:102158821			
RAB39B	20.72322059	155.3403339	-2.906112338	0.002894	Low	25	188	XP_003135565.1	ssc:100516775	1E-123	NM_171998.3	RAB39B
LOC100518132	1885.813074	14235.12379	-2.916196454	0.0029	Low	2275	17228	XP_003127801.2	ssc:100518132	0	NM_001856.3	COL16A1
TXNIP	372.1890418	2644.917068	-2.829115002	0.002902	Low	449	3201	NM_001044614.2	ssc:733688	0	NM_006472.4	TXNIP
LOC100621534	36.47286824	2.478835115	3.879089442	0.002918	High	44	3	XR_302292.1	ssc:100621534	1E-15	NM_014930.1	ZNF510
GNG8	9.947145883	85.10667229	-3.096917702	0.002919	Low	12	103	XP_005664771.1	ssc:100622520	2E-34	NM_033258.1	GNG8
LOC100625965	9.947145883	85.10667229	-3.096917702	0.002919	Low	12	103	XR_302183.1	ssc:100625965			
LOC102159363	605.1180412	87.5855074	2.788452524	0.002925	High	730	106	XR_299269.1	ssc:102159363			
STRIP2	274.3754406	38.83508347	2.820718879	0.002928	High	331	47	XP_005657814.1	ssc:100517565	0	NM_020704.2	STRIP2
TMEM229A	61.34073295	426.3596398	-2.797153565	0.002934	Low	74	516	XP_003134771.1	ssc:100524656	9E-164	NM_001136002.1	TMEM229A
TMEM182	95.32681471	11.5678972	3.042755453	0.002938	High	115	14	XP_005662411.1	ssc:100515134	1E-104	NM_144632.3	TMEM182
COL19A1	58.02501765	5.783948602	3.326548419	0.002942	High	70	7	XP_003121320.2	ssc:100512751	0	NM_001858.4	COL19A1
ZNF774	34.81501059	247.8835115	-2.831880943	0.002944	Low	42	300	ZNF774	ssc:100524056	0	NM_001004309.2	ZNF774
FGF11	480.7787177	3438.144305	-2.838185159	0.002949	Low	580	4161	XP_005669270.1	ssc:100739797	4E-129	NM_004112.2	FGF11
IL7R	47.24894295	4.131391859	3.515582244	0.002966	High	57	5	NM_001146128.2	ssc:100271930	0	NM_002185.3	IL7R
TMEM136	47.24894295	4.131391859	3.515582244	0.002966	High	57	5	XP_005667492.1	ssc:102162685	6E-33	NM_174926.2	TMEM136
LOC102162075	84.55074001	9.915340461	3.092083166	0.002967	High	102	12	XR_304414.1	ssc:102162075			
LOC100737349	474.1472871	3382.783654	-2.834803726	0.002991	Low	572	4094	XP_005656322.1	ssc:100737349	2E-38	NM_007364.2	TMED3
SELENBP1	7339.335804	1003.101943	2.871181276	0.002998	High	8854	1214	XP_001929678.1	ssc:100152724	0	NM_003944.3	SELENBP1
LOC100513945	227.9554265	1590.585866	-2.802734598	0.003	Low	275	1925	XP_005668259.1	ssc:100513945	3E-138	NM_024693.4	ECHDC3
NDUFAF4	1641.279071	235.4893359	2.80108693	0.003004	High	1980	285	XP_001925512.1	ssc:100157673	2E-79	NM_014165.3	NDUFAF4
PCDHB10	73.7746653	8.262783717	3.15842566	0.003007	High	89	10	XP_003480986.1	ssc:100739340	0	NM_019119.4	PCDHB9
PDGFA	164.9568359	1142.742988	-2.792340496	0.003009	Low	199	1383	HighI0001C965DD		9E-90	NM_033023.4	PDGFA
LOC100738512	36.47286824	257.798852	-2.821350276	0.003036	Low	44	312	XP_003483066.1	ssc:100738512	0	NM_022827.3	SPATA20
LOC102164705	961.5574354	139.6410448	2.783649978	0.003048	High	1160	169	XP_005662348.1	ssc:102164705	2E-115	NM_022662.3	ANAPC1
GPR62	2.486786471	36.35624836	-3.869848794	0.00305	Low	3	44	XP_005669678.1	ssc:100625569	1E-134	NM_080865.3	GPR62
SIGLEC15	30.67036647	1.652556743	4.21407369	0.003054	High	37	2	XP_003121465.2	ssc:100518460	3E-144	NM_213602.2	SIGLEC15
VWA3B	30.67036647	1.652556743	4.21407369	0.003054	High	37	2	XP_005655264.1	ssc:100623963	0	NM_144992.4	VWA3B
LOC102159131	266.9150812	38.0088051	2.811975246	0.003087	High	322	46	XP_005656854.1	ssc:102159131	8E-63	NM_004938	DAPK1
HOXA11	5.802501765	57.83948602	-3.317307771	0.003088	Low	7	70	XP_003134898.2	ssc:100521236	3E-149	NM_005523.5	HOXA11
LOC100738709	24.86786471	0.826278372	4.91151092	0.003093	High	30	1	XP_005659932.1	ssc:100738709	1E-179	NR_033672.1	NOX5
LOC102163869	11.60500353	95.02201275	-3.033514805	0.003096	Low	14	115	XR_303904.1	ssc:102163869			
LOC102158104	78.74823824	9.089062089	3.115044314	0.003096	High	95	11	XR_302314.1	ssc:102158104			
LOC102161161	4.144644118	47.09786719	-3.506341595	0.003107	Low	5	57	XP_005669833.1	ssc:102161161	4E-66	NM_001570.3	IRAK2
AS-A	256.1390065	1783.935005	-2.800064176	0.00311	Low	309	2159	NM_213933.1	ssc:396973	9E-35	NM_001085428.2	ARSA
AHCY	37346.55922	4814.724072	2.955450306	0.003114	High	45054	5827	NM_001011727.1	ssc:497050	0	NM_001161766.1	AHCY
PCDHB4	493.21265	71.88621834	2.778422687	0.003119	High	595	87	XP_003124099.2	ssc:100521779	0	NM_018938.3	PCDHB4
GLIS2	599.3155395	4285.905914	-2.838212493	0.003125	Low	723	5187	XP_005662237.1	ssc:100624334	0	NM_032575.2	GLIS2
EGFR	188.9957718	1304.693549	-2.787285117	0.003149	Low	228	1579	NM_214007.1	ssc:397070	0	NM_201284.1	EGFR
LOC100621171	110.2475335	14.04673232	2.972439919	0.00315	High	133	17	XP_005674343.1	ssc:100621171	7E-60	NM_007280.1	OIP5
PLB1	41.44644118	3.305113487	3.648476514	0.00316	High	50	4	XP_005662715.1	ssc:100519306	0	NM_153021.4	PLB1
LOC102158452	41.44644118	3.305113487	3.648476514	0.00316	High	50	4	XR_298845.1	ssc:102158452			
VWA5B2	67.97216354	7.436505346	3.192247328	0.003161	High	82	9	XP_005670091.1	ssc:100626924	0	NM_138345.1	VWA5B2
LOC100738652	184.8511277	25.61462952	2.851323914	0.003164	High	223	31	XP_005667676.1	ssc:100738652	2E-81	NM_017650.2	PPP1R9A
LOC102164231	164.9568359	1134.480204	-2.781870965	0.003176	Low	199	1373	XR_304489.1	ssc:102164231			
XPNPEP2	1.657857647	30.57229975	-4.204833041	0.003186	Low	2	37	NM_001004048.1	ssc:445538	0	NM_003399.5	XPNPEP2
INHBB	1.657857647	30.57229975	-4.204833041	0.003186	Low	2	37	NM_001164842.1	ssc:397490			
LOC102167836	1.657857647	30.57229975	-4.204833041	0.003186	Low	2	37	XR_301484.1	ssc:102167836			
MIR145	1.657857647	30.57229975	-4.204833041	0.003186	Low	2	37	NR_038484.1	ssc:100316550			
ROBO3	1.657857647	30.57229975	-4.204833041	0.003186	Low	2	37	XP_005667520.1	ssc:100524637	0	NM_022370.3	ROBO3
NPL	99.47145883	12.39417558	3.004620324	0.003193	High	120	15	NM_214071.1	ssc:397141	1E-173	NM_030769.2	NPL
LOC100524077	8992.219878	1230.328496	2.869633749	0.003212	High	10848	1489	XR_298206.1	ssc:100524077			
LHX2	232.9289994	33.05113487	2.81711855	0.003212	High	281	40	NM_001170519.1	ssc:100156063	0	NM_004789.3	LHX2
ZNF334	0.828928824	24.78835115	-4.902270271	0.003217	Low	1	30	ZNF334	ssc:102161731	4E-56	NR_073026.1	ZNF334
LOC100517269	1045.279247	152.8614988	2.773591424	0.003241	High	1261	185	XP_003123820.4	ssc:100517269	2E-85	NM_014639.3	TTC37
RBMS3	19.06536294	142.1198799	-2.898082474	0.003253	Low	23	172	XR_299279.1	ssc:100739397	1E-56	NM_014483.3	RBMS3
EPN3	9.11821706	78.49644531	-3.105803665	0.003257	Low	11	95	XP_005668947.1	ssc:100512679	0	NM_017957.2	EPN3
LOC100515214	109.4186047	14.04673232	2.961551602	0.003321	High	132	17	LOC100515214				
LOC102167753	7.460359412	67.75482648	-3.183006679	0.003321	Low	9	82	XR_302371.1	ssc:102167753			
LOC102159690	973.1624389	142.9461583	2.76720879	0.003324	High	1174	173	LOC102159690				
PADI3	46.42001412	4.131391859	3.490047152	0.003337	High	56	5	XP_003127694.1	ssc:100524379	0	NM_016233.2	PADI3
SPO11	1206.920367	176.8235716	2.770947978	0.003347	High	1456	214	NM_001105313.1	ssc:100125974	0	NM_198265.1	SPO11
PIGA	1293.128965	189.2177471	2.77274685	0.00335	High	1560	229	XP_001928352.3	ssc:100156880			
LOC102164681	33.98608177	237.9681711	-2.807752672	0.003359	Low	41	288	XR_299526.1	ssc:102164681			
PNAS-5	7223.285769	1003.101943	2.848187016	0.003385	High	8714	1214	XP_003128900.1	ssc:414404	1E-152	NM_017816.2	LYAR
ACPL2	1906.536294	276.8032545	2.784021185	0.003388	High	2300	335	XP_005659967.1	ssc:100515055	0	NM_152282.4	ACPL2
LOC102161782	35.64393941	2.478835115	3.845922578	0.003389	High	43	3	XP_005663571.1	ssc:102161782	1E-74	NM_001005464.2	HIST2H3A
LOC100739662	35.64393941	2.478835115	3.845922578	0.003389	High	43	3	XP_003481686.1	ssc:100739662	0	NM_005379.3	MYO1A
CITED1	514.7647995	76.0176162	2.759507826	0.003415	High	621	92	NM_001037964.1	ssc:654297	5E-98	NM_004143.3	CITED1
GPR146	21.55214941	156.9928906	-2.864795566	0.003427	Low	26	190	XP_003481022.2	ssc:100738733	9E-117	NM_138445.2	GPR146
FAM214B	207.2322059	1416.241129	-2.772746785	0.003444	Low	250	1714	XP_005654579.1	ssc:100624931	0	NM_025182.2	FAM214B
GPC1	867.0595495	6204.524293	-2.839117616	0.00345	Low	1046	7509	XP_005654255.1	ssc:100525965	0	NM_002081.2	GPC1
LOC100514190	1855.142707	270.1930276	2.77946782	0.003451	High	2238	327	XP_005670266.1	ssc:100514190	0	NM_024638.3	QTRTD1
DCP2	1911.509867	278.4558113	2.779192341	0.003478	High	2306	337	XP_003123880.1	ssc:100516785	0	NR_038352.1	DCP2
TMEM169	387.1097606	57.01320765	2.763374607	0.003483	High	467	69	XP_005672233.1	ssc:100513571	2E-158	NM_138390.3	TMEM169
DNA2	1165.473926	171.8659013	2.761561485	0.003499	High	1406	208	XP_003483536.1	ssc:100153906	0	NR_102264.1	DNA2

LOC100737985	573.6187459	85.10667229	2.752748025	0.003511	High	692	103	XP_005653680.1	ssc:100737985			
MYC	16086.19275	2177.24351	2.885248235	0.003523	High	19406	2635	NM_001005154.1	ssc:448810	0	NM_002467.4	MYC
LOC102161593	18.23643412	0	20	0.003525	High	22	0	XP_005667861.1	ssc:102161593	2E-47	NM_001205294.1	CACNA1E
RGR	18.23643412	0	20	0.003525	High	22	0	XP_005671214.1	ssc:100155692	9E-150	NM_002921.3	RGR
LOC100512242	18.23643412	0	20	0.003525	High	22	0	XR_305005.1	ssc:100512242	4E-65	NM_054013.3	MGAT4B
CCR2	18.23643412	0	20	0.003525	High	22	0	NM_001001619.1	ssc:414372	4E-169	NM_001123396.1	CCR2
OSBP2	18.23643412	0	20	0.003525	High	22	0	XP_001924672.1		0	NM_030758.3	OSBP2
LOC102163106	18.23643412	0	20	0.003525	High	22	0	LOC102163106				
LOC102167670	18.23643412	0	20	0.003525	High	22	0	XP_005659903.1	ssc:102167670			
LOC102165684	18.23643412	0	20	0.003525	High	22	0	XP_005665653.1	ssc:102165684			
LOC102166114	2.486786471	35.52996998	-3.83668193	0.00354	Low	3	43	XR_306716.1	ssc:102166114			
SFN	251.1654336	36.35624836	2.788362689	0.003541	High	303	44	NM_001044564.1	ssc:733625	6E-141	NM_006142.3	SFN
LOC100517371	129.3128965	17.35184581	2.89770512	0.003541	High	156	21	XP_005669525.1	ssc:100517371	1E-130	NM_015175.2	NBEAL2
SLA-1	1116.567125	8040.514835	-2.848217895	0.003543	Low	1347	9731	NM_001097427.1	ssc:100037288	2E-147	NM_002116.7	HLA-A
LOC100523056	498.186223	3474.500553	-2.80204858	0.003593	Low	601	4205	XR_302076.1	ssc:100523056			
LOC100624749	551.2376677	3857.067439	-2.806757991	0.003601	Low	665	4668	XP_005659064.1	ssc:100624749	7E-55	NR_103804.1	PDLIM7
DEF8	314.1640241	2155.760272	-2.778606866	0.003627	Low	379	2609	XP_005653273.1	ssc:100523845	0	NM_207514.2	DEF8
LOC100516568	29.84143765	1.652556743	4.174545326	0.003636	High	36	2	XP_005667956.1	ssc:100516568	1E-177	NM_015198.3	COBL
EDIL3	155.00969	1045.24214	-2.753406885	0.003645	Low	187	1265	XP_005661599.1	ssc:100516065	0	NM_005711.4	EDIL3
LOC100156073	0	18.17812418	-20	0.003651	Low	0	22	XP_005663279.1	ssc:100156073	2E-128	NM_002432.1	MNDA
LOC100520170	0	18.17812418	-20	0.003651	Low	0	22	XP_005652770.1	ssc:100520170	0	NM_024733.3	ZNF665
HTR3C	0	18.17812418	-20	0.003651	Low	0	22	XP_003358754.1		0	NM_130770.2	HTR3C
LOC100512775	0	18.17812418	-20	0.003651	Low	0	22	XP_005662405.1	ssc:100512775	0	NM_032528.2	ST6GAL2
LOC100517129	0	18.17812418	-20	0.003651	Low	0	22	XR_299517.1	ssc:100517129	2E-100	NM_003113.3	SP100
LOC102162278	0	18.17812418	-20	0.003651	Low	0	22	XR_307435.1	ssc:102162278			
LOC102165168	0	18.17812418	-20	0.003651	Low	0	22	XR_301714.1	ssc:102165168			
CNIH2	33.15715294	229.7053873	-2.792392653	0.003669	Low	40	278	XP_003353812.1	ssc:100521359	5E-91	NR_073079.1	CNIH2
ANPEP	583.5658918	4080.1626	-2.805659178	0.003678	Low	704	4938	NM_214277.1	ssc:397520	0	NM_001150.2	ANPEP
LOC100627110	168.2725512	1134.480204	-2.753159669	0.003688	Low	203	1373	XP_005658897.1	ssc:100627110	3E-66	NM_020371.2	AVEN
RBCK1	244.534003	1661.645806	-2.764505898	0.003702	Low	295	2011	XP_005654297.1	ssc:100519985	3E-89	NM_031229.2	RBCK1
WDR66	164.9568359	23.13579441	2.833890023	0.003719	High	199	28	XP_003132921.2	ssc:100524906	0	NM_144668.5	WDR66
LOC100623488	93.66895707	627.1452841	-2.743156789	0.003723	Low	113	759	XP_003358791.1	ssc:100623488	2E-164	NM_004454.2	ETV5
PHF21B	19.89429177	144.5987151	-2.861628287	0.003734	Low	24	175	XP_003126022.1	ssc:100522673	0	NM_138415.4	PHF21B
PERP	232.9289994	33.87741324	2.78149464	0.003786	High	281	41	NM_001244825.1	ssc:100513507	7E-27	NM_022121.4	PERP
SORBS3	1714.224807	12447.88367	-2.860272258	0.003787	Low	2068	15065	XP_005670469.1	ssc:100153623	0	NM_005775.4	SORBS3
LOC102162451	899.3877736	134.6833746	2.739371497	0.00381	High	1085	163	XP_005670055.1	ssc:102162451	4E-31	NM_020390.5	EIF5A2
LOC102160118	24.03893588	0.826278372	4.862601319	0.003813	High	29	1	XR_306159.1	ssc:102160118			
LOC102165574	24.03893588	0.826278372	4.862601319	0.003813	High	29	1	XR_300334.1	ssc:102165574			
TTC39B	39.78858353	271.0193059	-2.76796918	0.003819	Low	48	328	XP_003121951.2	ssc:100515182	0	NM_152574.2	TTC39B
KCTD17	222.1529247	1498.868966	-2.754249226	0.003833	Low	268	1814	XP_005663865.1	ssc:100512279	7E-104	NM_024681.3	KCTD17
AIF1L	38.95965471	265.2353573	-2.767220311	0.003865	Low	47	321	XP_005660585.1	ssc:100624075	3E-69	NR_033701.1	AIF1L
CSRNP3	50.56465824	4.95767023	3.350395161	0.00387	High	61	6	XP_005671951.1	ssc:100515450	0	NM_024969.3	CSRNP3
PIK3CD	483.2655042	72.71249671	2.732540779	0.003927	High	583	88	XP_005665035.1	ssc:100518131	0	NM_005026.3	PIK3CD
PLEKHA7	723.654863	109.0687451	2.730064049	0.00394	High	873	132	XP_003123007.3	ssc:100514820	0	NM_175058.4	PLEKHA7
LOC102166423	34.81501059	2.478835115	3.811975246	0.003945	High	42	3	XR_297383.1	ssc:102166423			
LOC102167189	34.81501059	2.478835115	3.811975246	0.003945	High	42	3	LOC102167189				
PROX1	158.3254053	22.30951604	2.82716165	0.003956	High	191	27	NM_001128490.1	ssc:100169702	0	NM_002763.4	PROX1
LOC102164153	86.20859765	10.74161883	3.004620324	0.003956	High	104	13	XP_005663990.1	ssc:102164153	0	NM_004984.2	KIF5A
LOC100155322	0.828928824	23.96207278	-4.853360671	0.003964	Low	1	29	XP_005665715.1	ssc:100155322	2E-172	NM_001076781.1	ZNF391
RTN4R	493.21265	74.36505346	2.729513086	0.00398	High	595	90	XP_005671012.1	ssc:100152289	0	NM_023004.5	RTN4R
IKZF2	231.2711418	33.87741324	2.771189632	0.003984	High	279	41	XP_005672217.1	ssc:100510935	0	NM_016260.2	IKZF2
PTPLAD2	387.9386894	2645.743346	-2.769772549	0.003987	Low	468	3202	XP_003121932.1	ssc:100523711	6E-75	NM_001010915.3	PTPLAD2
ACPT	91.1821706	604.0094897	-2.727747558	0.004025	Low	110	731	XP_005664855.1	ssc:100620403	0	NM_080791.1	ACPT
LOC100517200	4.973572942	50.40298068	-3.341154513	0.004054	Low	6	61	XP_003134248.2	ssc:100517200	9E-128	NM_014012.5	REM1
LOC102165199	9.11821706	76.0176102	-3.059510013	0.004056	Low	11	92	XP_005658191.1	ssc:102165199	4E-64	NM_012236.3	SCMH1
LOC100526125	2291.159268	338.7741324	2.757682125	0.004057	High	2764	410	XPR1	ssc:100526125			
SEMA4D	3488.13249	508.987477	2.776752773	0.004065	High	4208	616	XP_005670454.1	ssc:100152598	0	NM_182635.1	SEMA4D
E2F5	2561.390065	377.6092159	2.761961092	0.004071	High	3090	457	XP_005663053.1	ssc:100157957	5E-167	NM_001951.3	E2F5
GEMIN5	11113.44874	1554.229617	2.838035021	0.004073	High	13407	1881	XP_005672658.1	ssc:100524914	0	NM_015465.4	GEMIN5
LOC102165146	319.9665259	47.92414556	2.739096366	0.004097	High	386	58	XP_005656500.1	ssc:102165146			
MTTP	70.45895001	8.262783717	3.092083166	0.004117	High	85	10	NM_214185.1	ssc:397381	0	NM_000253.2	MTTP
LOC102164274	933.3738554	141.2936016	2.723758922	0.004143	High	1126	171	XP_005655514.1	ssc:102164274			
ZNF366	5.802501765	55.36065091	-3.254113944	0.004144	Low	7	67	ZNF366	ssc:100519576	0	NM_152625.1	ZNF366
C18H7orf41	232.1000706	1550.098225	-2.739540771	0.004165	Low	280	1876	NM_001244785.1	ssc:100513191	4E-40	NM_152793.2	MTURN
HELLS	3697.022553	540.3860551	2.774301494	0.004183	High	4460	654	XP_005657492.1	ssc:100154547	0	NM_018063	HELLS
DMXL2	3397.779248	498.2458582	2.769662407	0.004194	High	4099	603	XP_005652748.1	ssc:100155516	0	NM_015263.3	DMXL2
REG4	250.3365047	37.18252673	2.751171967	0.004215	High	302	45	NM_001190251.1	ssc:100154580	3E-67	NM_032044.3	REG4
LOC102160087	41.44644118	277.6295329	-2.743840909	0.004239	Low	50	336	XP_005662708.1	ssc:102160087	1E-44	NM_030915.3	LBH
LOC100625662	125.9971812	17.35184581	2.860230415	0.004246	High	152	21	XP_005668986.1	ssc:100625662	7E-109	NR_028000.1	RDM1
SMYD2	104.4450318	686.6373269	-2.716804419	0.004257	Low	126	831	NM_001160091.1	ssc:100294706			
NEIL2	552.8955253	84.28039392	2.713737934	0.004275	High	667	102	XP_005657331.1	ssc:100624483	2E-132	NM_145043.2	NEIL2
MAT1A	13.26286118	100.8059614	-2.926117013	0.004293	Low	16	122	NM_001243181.1	ssc:100156922	0	NM_000429.2	MAT1A
DCX	3.315715294	39.66136184	-3.580342176	0.004314	Low	4	48	XP_005673890.1	ssc:100523160	0	NM_178153.2	DCX
LOC102165012	3.315715294	39.66136184	-3.580342176	0.004314	Low	4	48	XP_005653389.1	ssc:102165012			
C9H11orf52	3.315715294	39.66136184	-3.580342176	0.004314	Low	4	48	XP_003357343.1	ssc:100515398	8E-38	NM_080659.2	C11orf52
FGFBP3	29.01250883	1.652556743	4.133903341	0.004341	High	35	2	NM_001258356.1	ssc:100153311	1E-25	NM_152429.4	FGFBP3
SPAG6	29.01250883	1.652556743	4.133903341	0.004341	High	35	2	NM_001159312.1	ssc:100286811	0	NM_172242.2	SPAG6
LOC100739429	105.2739606	14.04673232	2.90584217	0.004343	High	127	17	LOC100739429				
ANGPT1	62.99859059	413.9654642	-2.716118955	0.004388	Low	76	501	NM_213959.1	ssc:397009			
LOC100521443	411.1486965	2772.163937	-2.753280416	0.004402	Low	496	3355	XP_005662679.1	ssc:100521443	1E-123	NM_006449.4	CDC42EP3
LOC102164478	1101.646407	167.7345095	2.715409796	0.004412	High	1329	203	XR_299466.1	ssc:102164478			
ISOC1	3403.58175	502.37725	2.760210723	0.004413	High	4106	608	XP_003123949.1	ssc:100517640	2E-163	NM_016048.2	ISOC1
MOB3C	95.32681571	623.0138923	-2.708310338	0.004436	Low	115	754	XP_005665513.1	ssc:100738460	3E-125	NM_201403.2	MOB3C
LOC102158225	4.144644118	44.61903207	-3.428339083	0.004438	Low	5	54	XP_005667764.1	ssc:102158225	2E-102	NM_017439.3	GSAP
BEST4	69.63002118	8.262783717	3.075009652	0.00446	High	84	10	XP_003128107.1	ssc:100516679	0	NM_153274.2	BEST4
LOC100738208	69.63002118	8.262783717	3.075009652	0.00446	High	84	10	XP_003481435.1	ssc:100738208	2E-145	NM_030958.2	SLCO5A1
LOC102164565	936.6895707	6495.37428	-2.793769753	0.00447	Low	1130	7861	XP_005659016.1	ssc:102164565	0	NM_021035.2	ZNFX1
KIAA1644	25.69679353	176.8235716	-2.782650352	0.004473	Low	31	214	XP_005663800.1	ssc:100522865	3E-90	NM_001099294.1	KIAA1644
FAM117B	814.0081048	124.7680341	2.705794799	0.004488	High	982	151	XP_003133638.1	ssc:100512564	0	NM_173511.3	FAM117B
USP44	1398.402925	212.3535415	2.719240033	0.004489	High	1687	257	XP_005664303.1	ssc:100516911	0	NM_032147.3	USP44
LOC102160100	79.57716707	9.915340461	3.004620324	0.0045	High	96	12	XR_298050.1	ssc:102160100			
PLAT	618.3809024	4215.672253	-2.769195039	0.004512	Low	746	5102	NM_214054.1	ssc:397121	0	NM_033011.2	PLAT
TRNP1	629.1569771	96.67456949	2.70221168	0.004521	High	759	117	NM_001243828.1	ssc:100511386	2E-66	NM_001013642.2	TRNP1
MYCL	388.7676183	59.49204276	2.708139435	0.004553	High	469	72	XP_003127875.2	ssc:100518432	0	NM_005376.4	MYCL
LOC100516938	64.65644824	422.228248	-2.707156938	0.004571	Low	78	511	XP_005670347.1	ssc:100516938	1E-56	NM_000158.3	GBE1
LOC100622731	2169.306731	326.3799568	2.73260969	0.004575	High	2617	395	XR_131266.2	ssc:100622731			
NPFF	34.81501059	233.0105008	-2.742613605	0.004578	Low	42	282	XP_003126243.1	ssc:100518250	2E-33	NM_003717.2	NPFF
LOC102165992	676.4059201	104.1110748	2.699765743	0.004582	High	816	126	XP_005668550.1	ssc:102165992			
LOC100157370	122.6814659	799.0111855	-2.70329839	0.004593	Low	148	967	XP_001925219.2	ssc:100157370	6E-42	NM_138325.2	PCSK6
PON2	119.3657506	776.7016694	-2.701971621	0.004615	Low	144	940	NM_001123223.1	ssc:100142663	0	NM_001018161.1	PON2

LOC102159132	17.4075053	0	20	0.004618	High	21	0	XR_299161.1	ssc:102159132			
LOC102157412	17.4075053	0	20	0.004618	High	21	0	XP_005664273.1	ssc:102157412	5E-104	NM_181670.3	ANKS1B
LOC102157590	17.4075053	0	20	0.004618	High	21	0	XP_005668554.1	ssc:102157590	6E-148	NM_005845.3	ABCC4
LOC100738052	17.4075053	0	20	0.004618	High	21	0	XR_309068.1	ssc:100738052			
LOC100524824	17.4075053	0	20	0.004618	High	21	0	LOC100524824				
MYCT1	17.4075053	0	20	0.004618	High	21	0	XP_003480274.1	ssc:100515064	6E-76	NM_025107.2	MYCT1
OLIG3	17.4075053	0	20	0.004618	High	21	0	XP_001928671.1	ssc:100153431	4E-156	NM_175747.2	OLIG3
IGSF9B	17.4075053	0	20	0.004618	High	21	0	XP_005667578.1	ssc:100626754	0	np_055802	np_055802
LOC102167179	17.4075053	0	20	0.004618	High	21	0	XR_303627.1	ssc:102167179			
LOC102165997	17.4075053	0	20	0.004618	High	21	0	XR_300634.1	ssc:102165997			
LOC733664	17.4075053	0	20	0.004618	High	21	0	NM_001044594.1	ssc:733664			
IFITM3	1319.654687	9234.487082	-2.806871361	0.004624	Low	1592	11176	NM_001201382.1	ssc:100518544	3E-30	NR_049759.1	IFITM3
LOC100739187	122.6814659	798.1849071	-2.701805689	0.004628	Low	148	966	XP_003480678.1	ssc:100739187	6E-42	NM_138325.2	PCSK6
LOC100737646	621.6966177	95.84829112	2.697386115	0.004631	High	750	116	XP_003482010.2	ssc:100737646	0	NM_018156.2	VPS13D
LOC100739295	702.1027136	108.2424667	2.697415482	0.004643	High	847	131	XP_003484045.1	ssc:100739295	6E-141	NM_198285.2	WDR86
LOC102162418	76.26145177	495.767023	-2.70063641	0.004643	Low	92	600	XP_005662716.1	ssc:102162418	4E-58	NM_005253.3	FOSL2
ACN9	361.4129671	55.36065091	2.706715459	0.004647	High	436	67	XP_005667689.1	ssc:102158000	5E-28	NM_020186.2	ACN9
LOC100737806	3861.979389	571.7846332	2.755796712	0.004672	High	4659	692	XP_003481229.1	ssc:100737806	4E-113	NR_027405.1	MTHFD2
LOC102161831	23.21000706	0.826278372	4.811975246	0.004721	High	28	1	XR_303247.1	ssc:102161831			
LOC102161463	23.21000706	0.826278372	4.811975246	0.004721	High	28	1	XP_005673714.1	ssc:102161463	4E-82	NM_020922.4	WNK3
LOC102164756	23.21000706	0.826278372	4.811975246	0.004721	High	28	1	XR_302603.1	ssc:102164756			
C12H17orf100	23.21000706	0.826278372	4.811975246	0.004721	High	28	1	XP_005674657.1	ssc:102161844			
BARX2	23.21000706	0.826278372	4.811975246	0.004721	High	28	1	XP_003130117.3	ssc:100516284	4E-134	NM_003658.4	BARX2
LOC396634	23.21000706	0.826278372	4.811975246	0.004721	High	28	1	XP_005668049.1	ssc:396634	5E-124	NR_052021.1	PTPRC
LOC100622302	534.6590912	82.62783717	2.693919485	0.004724	High	645	100	XP_003362135.2	ssc:100622302	4E-141	NR_037936.1	LIPT1
LRP10	1136.461417	7880.216831	-2.793686622	0.004735	Low	1371	9537	NM_001143715.1	ssc:100233190			
ITPR1	2989.946267	447.8428775	2.739054991	0.004777	High	3607	542	XP_005669797.1	ssc:397454			
EPHB6	0	17.35184581	-20	0.004779	Low	0	21	XP_005673169.1	ssc:100511727	0	NR_104001.1	EPHB6
CLEC1A	0	17.35184581	-20	0.004779	Low	0	21	XP_003126531.2	ssc:100519486	2E-88	NM_016511.2	CLEC1A
LOC102159826	0	17.35184581	-20	0.004779	Low	0	21	XR_305204.1	ssc:102159826			
LOC100626866	0	17.35184581	-20	0.004779	Low	0	21	XP_003360866.2	ssc:100626866			
POU3F4	0	17.35184581	-20	0.004779	Low	0	21	XP_001925801.1	ssc:100152446	0	NM_000307.4	POU3F4
LOC102164918	0	17.35184581	-20	0.004779	Low	0	21	XR_307310.1	ssc:102164918			
LAMB4	0	17.35184581	-20	0.004779	Low	0	21	XP_003482749.1	ssc:100623759	0	NM_007356.2	LAMB4
KRT83	0	17.35184581	-20	0.004779	Low	0	21	XP_003126205.1	ssc:100523670	0	NM_002282.3	KRT83
LOC100515100	0	17.35184581	-20	0.004779	Low	0	21	XR_307393.1	ssc:100515100			
FABP3	549.57981	85.10667229	2.690984857	0.004789	High	663	103	NM_001099931.1	ssc:399532			
LOC100515533	43.93322765	4.131391859	3.410612684	0.004792	High	53	5	XP_005661750.1	ssc:100515533	3E-22	NM_006527.2	SLBP
LOC102159614	2.486786471	33.87741324	-3.76796918	0.004799	Low	3	41	XR_297053.1	ssc:102159614			
LOC102163714	13.26286118	99.15340461	-2.902270271	0.004807	Low	16	120	XP_005671801.1	ssc:102163714	5E-126	NM_207352.3	CYP4V2
LOC100620580	73.7746653	9.089062089	3.020922137	0.004832	High	89	11	LOC100620580				
LOC102157596	53.88037353	5.783948602	3.219633215	0.004833	High	65	7	LOC102157596				
TRNAM-CAU	53.88037353	5.783948602	3.219633215	0.004833	High	65	7	TRNAM-CAU				
LOC102157941	63.82751942	7.436505346	3.101481864	0.004836	High	77	9	XR_303343.1	ssc:102157941			
SFTPC	58.85394648	6.610226974	3.154367444	0.004837	High	71	8	NM_001044533.1	ssc:733580	3E-78	NM_003018.3	SFTPC
LOC574051	98.64253001	13.22045395	2.899438088	0.004838	High	119	16	NM_001025216.1	ssc:574051	5E-41	NM_005322.2	HIST1H1B
DARS2	1999.376323	304.0704408	2.71707256	0.004874	High	2412	368	XP_005656786.1	ssc:100624574	0	NM_018122.4	DARS2
LOC102157822	24.03893588	164.429396	-2.774023301	0.004888	Low	29	199	XR_306600.1	ssc:102157822			
ZC3H12D	43.93322765	287.5448734	-2.710402717	0.004899	Low	53	348	ZC3H12D	ssc:100156984	0	NM_207360.2	ZC3H12D
LOC102161895	17.4075053	123.9417558	-2.831880943	0.004903	Low	21	150	XR_297140.1	ssc:102161895			
PRDM8	0.828928824	23.13579441	-4.802734598	0.004903	Low	1	28	XP_005653685.1	ssc:100514243	0	NM_020226.3	PRDM8
SPTLC3	0.828928824	23.13579441	-4.802734598	0.004903	Low	1	28	XP_003134305.1	ssc:100519280	0	NM_018327.2	SPTLC3
MUSK	0.828928824	23.13579441	-4.802734598	0.004903	Low	1	28	XP_003122125.3	ssc:100515121	0	NM_005592.3	MUSK
LOC100737823	0.828928824	23.13579441	-4.802734598	0.004903	Low	1	28	XP_005652871.1	ssc:100737823	1E-125	NM_152601.3	ZNF709
MBTD1	6526.985557	955.1777977	2.772575639	0.004952	High	7874	1156	XP_003358155.1	ssc:100518331	0	NM_017643.2	MBTD1
CAMK2N2	337.3740312	52.05553742	2.696225385	0.004959	High	407	63	XP_003358761.1	ssc:100627130	1E-40	NM_033259.2	CAMK2N2
LOC100520746	4074.185168	607.3146032	2.745995586	0.004995	High	4915	735	XP_005665483.1	ssc:100520746	0	NM_004153.3	ORC1
ENOPH1	11354.66703	1630.247227	2.800122729	0.00504	High	13698	1973	NM_001243830.1	ssc:100511394	3E-140	NM_021204.3	ENOPH1
ABLIM1	854.6256171	133.0308178	2.683532064	0.005046	High	1031	161	XP_005671534.1	ssc:100154343	0	NM_006720.3	ABLIM1
LOC102160740	4.973572942	48.75042393	-3.293060224	0.005046	Low	6	59	XR_301212.1	ssc:102160740			
PLEKHG2	197.28506	1278.252641	-2.695819394	0.005052	Low	238	1547	XP_005657550.1	ssc:100514923	0	NM_022835.2	PLEKHG2
LOC100739199	197.28506	1277.426363	-2.694886516	0.005076	Low	238	1546	XP_005655886.1	ssc:100739199	0	NM_022835.2	PLEKHG2
LOC102161478	12.43393235	93.36945601	-2.908668042	0.005083	Low	15	113	XR_305386.1	ssc:102161478			
PPA2	1971.192743	301.5916057	2.70840067	0.005085	High	2378	365	XP_005667016.1	ssc:100521105	1E-98	NM_176869.2	PPA2
ADCK1	639.104123	99.97968298	2.676344137	0.005147	High	771	121	XP_001926766.4	ssc:100154765	0	NM_020421.3	ADCK1
LOC100038019	260.2836506	1693.870662	-2.702167121	0.00515	Low	314	2050	XP_005669379.1	ssc:100038019	2E-177	NM_003242.5	TGFBR2
LOC102167980	167.4436224	24.78835115	2.755941211	0.00516	High	202	30	XR_303537.1	ssc:102167980			
AKR1C4	1007.148521	6869.678383	-2.76996611	0.005165	Low	1215	8314	NM_001123075.1	ssc:100144511	6E-140	NM_003739.5	AKR1C3
CFI	19.89429177	137.9884881	-2.794121467	0.005171	Low	24	167	XP_005656603.1	ssc:100516921	0	NM_000204.3	CFI
RAB6B	4249.08915	635.4080679	2.7413983	0.005178	High	5126	769	XP_005669909.1	ssc:100522830	7E-121	NM_016577.3	RAB6B
LOC606745	72.11680765	461.8896098	-2.679140653	0.005188	Low	87	559	XP_005658202.1	ssc:606745	2E-70	NM_000676.2	ADORA2B
LOC102163537	28.18358	1.652556743	4.092083166	0.005198	High	34	2	XR_299969.1	ssc:102163537			
LOC102166020	28.18358	1.652556743	4.092083166	0.005198	High	34	2	XR_304135.1	ssc:102166020			
GPR37L1	28.18358	1.652556743	4.092083166	0.005198	High	34	2	XP_005668062.1	ssc:100624431	0	NM_004767.3	GPR37L1
LOC102162606	28.18358	1.652556743	4.092083166	0.005198	High	34	2	XP_005669713.1	ssc:102162606	1E-45	NM_018398.2	CACNA2D3
LOC102164986	278.5200847	42.96647533	2.696498029	0.005222	High	336	52	XP_005657837.1	ssc:102164986	0	NM_001199637.1	ADCYAP1R1
MYO1G	558.6980271	87.5855074	2.673304651	0.005225	High	674	106	XP_005673395.1	ssc:100512998	0	NM_033054.2	MYO1G
LOC100738326	556.2112406	3700.900827	-2.73417167	0.005249	Low	671	4479	XP_005658395.1	ssc:100738326	9E-47	NM_002305.3	LGALS1
LOC102162530	37.30179706	243.7521197	-2.708097724	0.005256	Low	45	295	XP_005659850.1	ssc:102162530	8E-142	NM_000693.2	ALDH1A3
LOC100739541	46.42001412	299.9390489	-2.691850492	0.005276	Low	56	363	XP_003481236.1	ssc:100739541	4E-49	NM_012476.2	VAX2
B3GALT4	77.91930942	497.4195798	-2.674410501	0.00528	Low	94	602	XP_001927603.1	ssc:100155431	1E-158	NM_003782.3	B3GALT4
TBC1D20	1142.263919	7801.720386	-2.771896271	0.005308	Low	1378	9442	NM_001244620.1	ssc:100519805	4E-109	NM_144628.2	TBC1D20
CCDC17	121.8525371	17.35184581	2.811975246	0.005349	High	147	21	XP_005658845.1	ssc:100622440	2E-57	NM_152500.2	CCDC17
LOC100737091	1191.17072	185.9126336	2.67968348	0.005364	High	1437	225	XP_005657970.1	ssc:100737091	4E-139	NM_033381.1	COL4A5
LOC100524949	17.4075053	122.289199	-2.812515619	0.005376	Low	21	148	XP_003130654.3	ssc:100524949	2E-136	NM_004433.4	ELF3
GPR15	106.9318182	14.87301069	2.845922578	0.005387	High	129	18	XP_005670337.1	ssc:100153787	2E-130	NM_005290.2	GPR15
LOC100738281	48.07787177	4.95767023	3.277638819	0.00539	High	58	6	XP_003481987.1	ssc:100738281	0	NM_031475.2	ESPN
LOC102165796	20.72322059	142.1198799	-2.777788241	0.005396	Low	25	172	XR_300947.1	ssc:102165796			
LOC102162958	43.10429883	4.131391859	3.383131948	0.00542	High	52	5	XR_298939.1	ssc:102162958			
CAPN2	2569.679353	18084.75472	-2.815113776	0.005444	Low	3100	21887	NM_001100188.1	ssc:397393	0	NM_001748.4	CAPN2
MAP6	404.5172659	63.62343462	2.668571121	0.00548	High	488	77	XP_005667193.1	ssc:100519724	0	NM_207577.1	MAP6
CCSAP	818.1527489	128.899426	2.66612438	0.005489	High	987	156	XP_003133075.3	ssc:100521297	2E-99	NM_145257	CCSAP
SNCG	87.03752648	11.5678972	2.91151092	0.00549	High	105	14	NM_001103211.1	ssc:100125343	1E-49	NM_003087.2	SNCG
LOC100623642	155.8386188	23.13579441	2.751854254	0.005533	High	188	28	XP_003359491.2	ssc:100623642	9E-90	NM_002980.2	SCTR
LOC100623296	1554.241544	242.0995629	2.68253835	0.005538	High	1875	293	XP_005659893.1	ssc:100623296	0	NM_173630.3	RTTN
LOC100738678	1793.801974	278.4558113	2.687500327	0.005557	High	2164	337	XP_003480922.2	ssc:100738678	1E-140	NM_014639.3	TTC37
ANKH	668.1166318	4438.767413	-2.731987237	0.00558	Low	806	5372	XP_005672447.1	ssc:100624458	0	NM_054027.4	ANKH
DYDC2	2.486786471	33.05113487	-3.73234527	0.005605	Low	3	40	XP_003133138.1	ssc:100525130	7E-70	NR_070309.1	DYDC2
LOC102166930	2.486786471	33.05113487	-3.73234527	0.005605	Low	3	40	XR_301654.1	ssc:102166930			
SMAD7	365.5576112	2373.897762	-2.699087108	0.005639	Low	441	2873	NM_001244175.1	ssc:100521305	0	NM_005904.3	SMAD7

LOC100624376	28.18358	185.9126336	-2.721698026	0.005661	Low	34	225	XP_003358885.3	ssc:100624376	7E-102	NM_015429.3	ABI3BP
SERPING1	881.1513395	5902.106409	-2.7437682	0.005683	Low	1063	7143	NM_001123194.1	ssc:100144304	6E-174	NM_001032295.1	SERPING1
SHC3	14.09179	101.6322397	-2.85043134	0.005686	Low	17	123	XP_001928162.3	ssc:100155002	6E-74	NM_016848.5	SHC3
LOC100738987	67.14323471	8.262783717	3.022542232	0.005688	High	81	10	XP_003484181.1	ssc:100738987	5E-38	NM_144969.2	ZDHHC15
HighF0639	179.8775547	27.26718627	2.721777437	0.005711	High	217	33	NM_001174165.1	ssc:100156934	0	NM_001161498.1	PLEKHD1
LGR5	1325.457189	208.2221497	2.670294624	0.005729	High	1599	252	XP_005664040.1	ssc:100151994	0	NM_003667.3	LGR5
LOC100624395	1316.338972	8948.594766	-2.765130101	0.005736	Low	1588	10830	XP_003354986.1	ssc:100624395	5E-64	NM_007278.1	GABARAP
WNK3	665.6298454	105.7636316	2.653876502	0.005764	High	803	128	XP_003360382.2	ssc:100156408	0	NM_020922.4	WNK3
PAICS	26015.1022	3678.591311	2.822124067	0.0058	High	31384	4452	XP_005666753.1	ssc:100521281	0	NM_006452.3	PAICS
GRN	2742.925477	19193.6203	-2.806839527	0.005801	Low	3309	23229	NM_001044578.1	ssc:733645	0	NM_002087.2	GRN
3-Sep	26.52572236	175.1710148	-2.72330013	0.005831	Low	32	212	XP_005653157.1	ssc:100157034	0	NM_145734.1	3-Sep
SLC12A5	19.06536294	130.5519827	-2.775598468	0.005868	Low	23	158	XP_005673037.1	ssc:100155991	0	NM_020708.4	SLC12A5
LOC100519237	984.7674424	156.1666123	2.656697021	0.005872	High	1188	189	XP_005652760.1	ssc:100519237	1E-127	NM_006200.3	PCSK5
PDE4A	215.5214941	1369.96954	-2.668240154	0.005892	Low	260	1658	NM_001123159.1	ssc:396825	0	NM_006202.2	PDE4A
LOC733637	3325.66244	23411.77138	-2.81552045	0.005894	Low	4012	28334	NM_001244251.1	ssc:733637	2E-97	NM_033546.3	MYL12B
GABARAP	1307.220755	8849.441361	-2.759083591	0.005905	Low	1577	10710	NM_001190288.1	ssc:100462752	5E-64	NM_007278.1	GABARAP
HSPB8	52.22251589	330.5113487	-2.661955942	0.005926	Low	63	400	XP_001929620.1	ssc:100155338	1E-107	NM_014365.2	HSPB8
CADPS2	2510.825407	389.1771131	2.689662938	0.005933	High	3029	471	XP_005673275.1	ssc:100525671	0	NM_017954.10	CADPS2
LOC102158625	159.1543341	23.96207278	2.73160183	0.00594	High	192	29	XR_302690.1	ssc:102158625			
C18H7orf58	123.5103947	775.8753911	-2.651192503	0.00596	Low	149	939	XP_005673279.1	ssc:100511847	0	NM_024913.4	CPED1
GRASP	268.5729388	1714.527621	-2.674425294	0.005961	Low	324	2075	XP_003126196.1	ssc:100522015	4E-168	NM_181711.3	GRASP
LOC100626459	25.69679353	169.3870662	-2.720663465	0.006028	Low	31	205	XP_005661870.1	ssc:100626459	0	NM_171825.2	CAMK2A
PLXNC1	1449.796512	228.879109	2.663192732	0.006039	High	1749	277	XP_005655774.1	ssc:100519487	0	NR_037687.1	PLXNC1
LOC102157538	0.828928824	22.30951604	-4.750267178	0.006091	Low	1	27	XP_005674188.1	ssc:102157538			
LOC100516820	0.828928824	22.30951604	-4.750267178	0.006091	Low	1	27	XP_003130120.2	ssc:100516820			
LOC100739181	0.828928824	22.30951604	-4.750267178	0.006091	Low	1	27	XP_003482873.1	ssc:100739181	2E-75	NM_030664.4	PTER
LOC102161519	16.57857647	0	20	0.006093	High	20	0	XR_306581.1	ssc:102161519			
LOC102159917	16.57857647	0	20	0.006093	High	20	0	XP_005659520.1	ssc:102159917	2E-122	NM_005215.3	DCC
LOC100514940	16.57857647	0	20	0.006093	High	20	0	XP_005654359.1	ssc:100514940	4E-67	NM_005364.4	MAGEA8
LOC102166773	16.57857647	0	20	0.006093	High	20	0	XR_301586.1	ssc:102166773			
LOC100520863	16.57857647	0	20	0.006093	High	20	0	XR_306494.1	ssc:100520863			
LOC102166149	16.57857647	0	20	0.006093	High	20	0	XR_300635.1	ssc:102166149			
NPYR5	16.57857647	0	20	0.006093	High	20	0	XR_304867.1	ssc:100517770	0	NM_006174.2	NPY5R
MORN5	16.57857647	0	20	0.006093	High	20	0	XP_005654589.1	ssc:100514270	6E-80	NM_198469.3	MORN5
LOC102158871	16.57857647	0	20	0.006093	High	20	0	XR_308021.1	ssc:102158871			
LOC102160350	16.57857647	0	20	0.006093	High	20	0	XP_005662197.1	ssc:102160350	3E-66	NM_001134408.1	GRIN2A
CLDN18	16.57857647	0	20	0.006093	High	20	0	NM_001160081.1	ssc:100294676	3E-142	NM_016369.3	CLDN18
LOC102158428	16.57857647	0	20	0.006093	High	20	0	XR_308279.1	ssc:102158428			
LOC102165972	16.57857647	0	20	0.006093	High	20	0	XP_005674222.1	ssc:102165972	4E-34	NM_004647.2	DPF1
SPTSSB	16.57857647	0	20	0.006093	High	20	0	XP_005670019.1	ssc:100623291	1E-33	NM_145035.2	SPTSSB
LOC102157933	95.32681471	13.22045395	2.850110375	0.0061	High	115	16	XR_302823.1	ssc:102157933			
RNF43	19.89429177	134.6833746	-2.759145329	0.006106	Low	24	163	XP_005669052.1	ssc:100517305	0	NM_017763.4	RNF43
TPK1	233.7579283	1481.517121	-2.663988096	0.00611	Low	282	1793	XP_005667832.1	ssc:100524699	3E-111	NM_022445.3	TPK1
SHOX2	79.57716707	497.4195798	-2.644036852	0.006125	Low	96	602	NM_001243352.1	ssc:100628206	3E-147	NM_006884.3	SHOX2
LOC102168083	42.27537	4.131391859	3.355117571	0.006139	High	51	5	XR_304450.1	ssc:102168083			
LRCH1	114.3921777	714.7307915	-2.643411541	0.006162	Low	138	865	XP_005668473.1	ssc:100521036	0	NM_015116.2	LRCH1
CCBL1	1354.469698	214.8323766	2.656444779	0.006175	High	1634	260	XP_005660561.1	ssc:100515071	0	NR_109829.1	CCBL1
LOC100516420	5.802501765	52.05553742	-3.165304677	0.006208	Low	7	63	XP_005654932.1	ssc:100516420	0	NM_152921.1	EMR2
BEST2	37.30179706	3.305113487	3.496473421	0.006238	High	45	4	XP_003123378.1	ssc:100522788	0	NM_017682.2	BEST2
BEX5	37.30179706	3.305113487	3.496473421	0.006238	High	45	4	XP_005657928.1	ssc:100155526	3E-36	NM_001159560.1	BEX5
LOC102161009	586.0526783	94.19573438	2.637296715	0.00624	High	707	114	XP_005673391.1	ssc:102161009	5E-45	NM_199122.2	TBRG4
EPCAM	27.35465118	1.652556743	4.049014444	0.006243	High	33	2	NM_214419.1	ssc:403163	6E-136	NM_002354.2	EPCAM
CCDC146	27.35465118	1.652556743	4.049014444	0.006243	High	33	2	XP_003482737.1	ssc:100737427	0	NM_020879.2	CCDC146
LOC100628113	187.3379141	28.91974301	2.69551627	0.006255	High	226	35	LOC100628113				
LOC100622904	109.4186047	15.69928906	2.80108693	0.006258	High	132	19	XP_005658493.1	ssc:100622904	0	NM_030594	CPEB1
F11R	87.03752648	542.0386119	-2.638686163	0.006269	Low	105	656	NM_001128444.1	ssc:100127138	7E-139	NM_144504.1	F11R
GFPT2	4273.128086	655.2387488	2.705199981	0.006284	High	5155	793	XP_003123686.3	ssc:100518539	0	NM_005110.2	GFPT2
LOC102163998	0	16.52556743	-20	0.006299	Low	0	20	XR_304724.1	ssc:102163998			
IL15	0	16.52556743	-20	0.006299	Low	0	20	NM_214390.1	ssc:397683	2E-64	NR_037840.2	IL15
LOC100738049	0	16.52556743	-20	0.006299	Low	0	20	XP_003483274.2	ssc:100738049	0	NM_152536.3	FGD5
KCNA3	0	16.52556743	-20	0.006299	Low	0	20	XP_001924064.2	ssc:100156614	0	NR_109846.1	KCNA3
LOC100520680	0	16.52556743	-20	0.006299	Low	0	20	XP_003129129.2	ssc:100520680	7E-22	NM_002620.2	PF4V1
HPGD	0	16.52556743	-20	0.006299	Low	0	20	NM_001190248.1	ssc:100156186	9E-76	NM_001256307.1	HPGD
FAM211B	0	16.52556743	-20	0.006299	Low	0	20	XP_003359215.1	ssc:100625262	3E-117	NM_207644.2	FAM211B
LOC102165745	0	16.52556743	-20	0.006299	Low	0	20	XR_307647.1	ssc:102165745			
SELE	0	16.52556743	-20	0.006299	Low	0	20	NM_214268.1	ssc:397508	1E-147	NM_000450.2	SELE
LOC102164099	133.4575406	19.83068092	2.750574702	0.006309	High	161	24	XR_301559.1	ssc:102164099			
EFR3B	3241.940629	502.37725	2.690014632	0.006318	High	3911	608	XP_005674566.1	ssc:100621843	0	NM_014971.1	EFR3B
SLC6A15	630.8148348	101.6322397	2.633858463	0.006355	High	761	123	XP_005655797.1	ssc:100739274	2E-162	NM_182767.5	SLC6A15
RTKN2	355.6104653	57.01320765	2.640929705	0.006381	High	429	69	XP_005671080.1	ssc:100157770	7E-157	NM_145307.3	RTKN2
LOC100158075	1342.864694	214.0060983	2.649590134	0.006386	High	1620	259	XP_005663242.1	ssc:100158075	6E-54	NM_001113207.1	TSTD1
LOC100626354	4.144644118	42.14019696	-3.345876923	0.006412	Low	5	51	XP_005673127.1	ssc:100626354	0	NM_001794.3	CDH4
CELSR2	380.47833	61.14459951	2.637517302	0.006415	High	459	74	XP_005674678.1	ssc:100736791	0	NM_001408.2	CELSR2
LOC102162199	56.36716001	6.610226974	3.092083166	0.006424	High	68	8	XR_302735.1	ssc:102162199			
SLCO4C1	56.36716001	6.610226974	3.092083166	0.006424	High	68	8	XP_003480930.1	ssc:100737557	0	NM_180991.4	SLCO4C1
LOC100518740	334.8872447	53.70809416	2.640463994	0.006469	High	404	65	XP_005666933.1	ssc:100518740	0	NM_015693.3	INTU
RAPSN	12.43393235	90.06434252	-2.856673405	0.006479	Low	15	109	XP_003122858.3	ssc:100512645	3E-136	NM_032645.4	RAPSN
LOC100522814	1.657857647	27.26718627	-4.039773795	0.006492	Low	2	33	XP_005656616.1	ssc:100522814	7E-107	NM_031305.2	ARHGAP24
MYH1	3.315715294	37.18252673	-3.487232772	0.006507	Low	4	45	NM_001104951.1	ssc:100125538			
EYA2	3.315715294	37.18252673	-3.487232772	0.006507	Low	4	45	XP_005673053.1	ssc:100624872	0	NM_172113.1	EYA2
LOC102158961	2.486786471	32.2248565	-3.695819394	0.006561	Low	3	39	XR_303232.1	ssc:102158961			
DEPTOR	581.9080342	94.19573438	2.62705753	0.006562	High	702	114	XP_001926120.1	ssc:100157658	0	NM_022783.3	DEPTOR
LOC102164786	51.39358706	5.783948602	3.151461713	0.00658	High	62	7	XR_300353.1	ssc:102164786			
MYH6	51.39358706	5.783948602	3.151461713	0.00658	High	62	7	XP_005674565.1	ssc:100736765	2E-102	NM_002471.3	MYH6
LOC102164531	132.6286118	19.83068092	2.741585919	0.006582	High	160	24	XR_301562.1	ssc:102164531			
TMED3	193.1404159	1205.540144	-2.641957699	0.006592	Low	233	1459	NM_001204363.1	ssc:100155219	4E-68	NM_007364.2	TMED3
ZDHHC23	418.6090559	67.75482648	2.627207897	0.006659	High	505	82	ZDHHC23	ssc:100515099	0	NM_173570.3	ZDHHC23
TLN1	7592.988024	54472.40166	-2.842785838	0.006661	Low	9160	65925	XP_003353615.1	ssc:100157220	0	NM_006289.3	TLN1
MEGF10	1345.351481	215.658655	2.641161612	0.006669	High	1623	261	XP_005661666.1	ssc:100515713	0	NM_032446.2	MEGF10
LOC102161970	1909.85201	303.2441624	2.654909073	0.006669	High	2304	367	XP_005668142.1	ssc:102161970			
ZNFX1	1158.013567	7672.82096	-2.728104936	0.006674	Low	1397	9286	ZNFX1	ssc:100624243	0	NM_021035.2	ZNFX1
ZNF215	180.7064835	28.09346464	2.685341808	0.006677	High	218	34	ZNF215	ssc:100738243	3E-103	NM_013250.2	ZNF215
ANKRD63	14.09179	99.15340461	-2.81480743	0.006714	Low	17	120	XP_005674617.1	ssc:100627919			
LOC102164779	46.42001412	4.95767023	3.227012746	0.00676	High	56	6	XR_305830.1	ssc:102164779			
CTSB	7797.733444	55891.9479	-2.841513712	0.006764	Low	9407	67643	NM_001097458.1	ssc:100037961	2E-169	NM_147783.2	CTSB
SGCB	130.1418253	803.1425773	-2.62557143	0.006793	Low	157	972	NM_001114287.1	ssc:100135674	3E-179	NM_000232.4	SGCB
LOC102164151	1042.79246	168.5607878	2.629111189	0.006804	High	1258	204	XR_303157.1	ssc:102164151			
KCND1	82.06395354	504.8560851	-2.621051625	0.006845	Low	99	611	XP_005673658.1	ssc:100620758	0	NM_004979.5	KCND1
LOC102161185	5.802501765	51.22925905	-3.142221064	0.006883	Low	7	62	XR_304022.1	ssc:102161185			
FOXL2	41.44644118	4.131391859	3.326548419	0.006963	High	50	5	NM_001244665.1	ssc:100622956	1E-113	NM_023067.3	FOXL2

ECE2	1828.616985	292.5025436	2.64423185	0.00698	High	2206	354	XP_005670098.1	ssc:100627224	0	NM_032331.3	ECE2
LOC102159472	116.0500353	712.2519564	-2.617640718	0.007001	Low	140	862	XR_307747.1	ssc:102159472			
LOC100621853	227.9554265	36.35624836	2.648476514	0.007005	High	275	44	XP_003482633.1	ssc:100621853	4E-121	NM_022337.2	RAB38
LOC100627990	996.372446	161.9505609	2.621131661	0.007039	High	1202	196	XP_005674262.1	ssc:100627990	0	NM_012120.2	CD2AP
LOC100622769	27.35465118	175.1710148	-2.678906011	0.007067	Low	33	212	XP_005659243.1	ssc:100622769	0	NM_005923.3	MAP3K5
EFCAB4A	33.98608177	214.0060983	-2.654635959	0.007076	Low	41	259	NM_001243838.1	ssc:100511435	2E-86	NM_173584.4	EFCAB4A
LOC100623841	2054.085625	328.0325136	2.646585599	0.007077	High	2478	397	XP_005674239.1	ssc:100623841	4E-122	NM_014878.4	KIAA0020
MND1	1187.855004	192.5228606	2.625257074	0.007078	High	1433	233	XP_005653639.1	ssc:100517105	4E-87	NR_045605.1	MND1
EGR3	36.47286824	3.305113487	3.464051943	0.007182	High	44	4	XP_005670471.1	ssc:100516296	0	NM_004430.2	EGR3
BPIFB2	36.47286824	3.305113487	3.464051943	0.007182	High	44	4	NM_001101030.1	ssc:100113424	0	NM_025227.2	BPIFB2
LOC100517234	48.90680059	301.5916057	-2.62448928	0.007212	Low	59	365	XP_005667982.1	ssc:100517234	1E-83	NM_021958.3	HLX
ARHGEF26	639.104123	104.9373532	2.606522687	0.007275	High	771	127	XP_005669998.1	ssc:100626582	0	NM_015595.3	ARHGEF26
LOC100622851	92.84002824	13.22045395	2.811975246	0.007281	High	112	16	XP_005659098.1	ssc:100622851	4E-74	NM_001080465.2	C17orf98
LOC100522888	21.55214941	0.826278372	4.705060042	0.007331	High	26	1	XP_005667611.1	ssc:100522888	2E-98	NM_032029.4	FCAMR
DDX31	6628.943802	1020.453789	2.69956817	0.007342	High	7997	1235	XP_003122310.2	ssc:100513393	0	NM_138620.1	DDX31
LGALS1	11382.85061	82013.91234	-2.849006764	0.007351	Low	13732	99257	NM_001001867.1	ssc:414915	4E-68	NM_002305.3	LGALS1
LOC100738337	31.4992953	2.478835115	3.667585337	0.007393	High	38	3	XP_003482698.1	ssc:100738337			
GRAP2	31.4992953	2.478835115	3.667585337	0.007393	High	38	3	XP_005663838.1	ssc:100516438	9E-162	NM_004810.2	GRAP2
LRP5	421.0958424	2646.569625	-2.651903072	0.007402	Low	508	3203	XP_005660680.1	ssc:100524299	0	NM_002335.2	LRP5
LOC100156943	73.7746653	9.915340461	2.895391255	0.00746	High	89	12	XP_005671346.1	ssc:100156943	2E-176	NM_019053.4	EXOC6
NT5C2	1201.117865	7847.165696	-2.707793934	0.007482	Low	1449	9497	XP_005657513.1	ssc:100154612	0	NM_012229.4	NT5C2
LOC102167053	24.03893588	154.5140555	-2.68429314	0.007492	Low	29	187	XP_005671638.1	ssc:102167053	0	NM_173572.3	TTC40
LOC100738148	26.52572236	1.652556743	4.004620324	0.007519	High	32	2	XP_005668045.1	ssc:100738148	0	NR_047564.1	CRB1
LOC102162724	26.52572236	1.652556743	4.004620324	0.007519	High	32	2	XR_306885.1	ssc:102162724			
CXHXorf57	26.52572236	1.652556743	4.004620324	0.007519	High	32	2	XP_003360455.3	ssc:100627173			
CACNA1F	26.52572236	1.652556743	4.004620324	0.007519	High	32	2	XP_003135132.1	ssc:100513061	0	NM_005183.3	CACNA1F
CDC42EP4	257.7968641	1591.412144	-2.626000893	0.007526	Low	311	1926	XP_003358042.1	ssc:100517117	3E-143	NM_012121.4	CDC42EP4
LOC100737964	125.1682524	19.00440255	2.719463108	0.007581	High	151	23	XP_005664434.1	ssc:100737964	0	NM_015020	PHLPP2
LOC100526222	45.5910853	4.95767023	3.201017537	0.007582	High	55	6	XP_005664333.1	ssc:100526222			
ATP2B4	2688.216175	18131.85259	-2.753805274	0.007583	Low	3243	21944	XP_005656734.1	ssc:733701	0	NM_001684.4	ATP2B4
LOC102161784	0.828928824	21.48323766	-4.695819394	0.0076	Low	1	26	XP_005663765.1	ssc:102161784	8E-50	NM_198460.2	GBP6
LOC100626194	0.828928824	21.48323766	-4.695819394	0.0076	Low	1	26	XP_005658513.1	ssc:100626194	5E-90	NM_033119.4	NKD1
LOC102160773	0.828928824	21.48323766	-4.695819394	0.0076	Low	1	26	XR_299986.1	ssc:102160773			
LOC100737685	0.828928824	21.48323766	-4.695819394	0.0076	Low	1	26	XP_005672342.1	ssc:100737685	0	NM_001631.3	ALPI
GFRA1	19.06536294	125.5943125	-2.719745233	0.007626	Low	23	152	XP_001925181.1	ssc:100152565	0	NM_145793.3	GFRA1
SMPDL3B	18.23643412	120.6366423	-2.725772616	0.007722	Low	22	146	XP_003127785.1	ssc:100514109	0	NM_014474.2	SMPDL3B
C1H9orf9	67.14323471	406.5289589	-2.598044178	0.007758	Low	81	492	XP_005660596.1	ssc:100151892	4E-87	NM_018956.3	C9orf9
C2H11orf24	565.3294577	3566.217452	-2.657230896	0.007782	Low	682	4316	XP_005660666.1	ssc:100738698			
SLC4A5	478.2919312	79.32272369	2.592085332	0.007807	High	577	96	XP_005662513.1	ssc:100620628	0	NM_133479.1	SLC4A5
TOX3	1.657857647	26.4409079	-3.995379676	0.007813	Low	2	32	XP_005653316.1	ssc:100519713	0	NM_001146188.1	TOX3
ANO2	1.657857647	26.4409079	-3.995379676	0.007813	Low	2	32	XP_005655769.1	ssc:100521792	1E-129	np_065106	np_065106
LOC102163370	1.657857647	26.4409079	-3.995379676	0.007813	Low	2	32	XR_306619.1	ssc:102163370			
LOC100736778	368.8733265	2287.138533	-2.632346377	0.0079	Low	445	2768	XP_005656248.1	ssc:100736778	2E-63	NR_048549.1	SLC6A9
LOC100736644	812.3502471	134.6833746	2.592530109	0.007929	High	980	163	XP_003483288.2	ssc:100736644	3E-53	NM_052883.1	TXNRD3
CLGN	1463.059374	239.6207278	2.610163702	0.007933	High	1765	290	NM_001243207.1	ssc:100624991	0	NM_004362.2	CLGN
CEP128	1245.880022	204.9170362	2.604053308	0.007943	High	1503	248	XP_003128727.4	ssc:100520033	0	NM_152446.3	CEP128
CDH11	7028.487495	49085.06667	-2.80399801	0.007954	Low	8479	59405	NM_001244482.1	ssc:100525579	0	NM_001797.2	CDH11
UBE3D	531.3433759	88.41178578	2.587333885	0.007966	High	641	107	XP_003480358.1	ssc:100739214	8E-105	NM_198920.1	UBE3D
ARNTL	19.06536294	124.7680341	-2.710222459	0.007972	Low	23	151	NM_001097425.1	ssc:100037286	7E-160	NM_001178.4	ARNTL
RNF152	417.7801271	69.40738323	2.589582825	0.007972	High	504	84	NM_001244578.1	ssc:100155834	1E-113	NM_173557.2	RNF152
LOC102159661	24.86786471	157.819169	-2.665917908	0.00798	Low	30	191	XR_297907.1	ssc:102159661			
SFRP5	9.11821706	68.58110485	-2.910987488	0.008041	Low	11	83	NM_001243406.1	ssc:100153176	7E-164	NM_003015.3	SFRP5
LOC100739444	520.5673012	86.75922903	2.584995556	0.008059	High	628	105	XP_003483998.1	ssc:100739444			
ARRB1	1129.829987	186.738912	2.597011209	0.008097	High	1363	226	XP_005667188.1	ssc:100521141	0	NM_020251.3	ARRB1
TMEM206	1216.867513	200.7856443	2.599444074	0.008097	High	1468	243	XP_005667916.1	ssc:100524887	0	NM_018252.2	TMEM206
SEMA5B	15.74964765	0	20	0.008099	High	19	0	XP_005670227.1	ssc:102160172			
LOC100736890	15.74964765	0	20	0.008099	High	19	0	XP_005672898.1	ssc:100736890	2E-78	NM_182519.2	BPIFB4
SGIP1	15.74964765	0	20	0.008099	High	19	0	XP_005658837.1	ssc:100620397	9E-180	NM_032291.2	SGIP1
LOC102161533	15.74964765	0	20	0.008099	High	19	0	XR_301794.1	ssc:102161533			
LOC102161217	15.74964765	0	20	0.008099	High	19	0	XR_297055.1	ssc:102161217			
LOC102161302	15.74964765	0	20	0.008099	High	19	0	XR_302430.1	ssc:102161302			
PRDM13	15.74964765	0	20	0.008099	High	19	0	XP_003121362.2	ssc:100514742	0	NM_021620.3	PRDM13
C7H6orf223	15.74964765	0	20	0.008099	High	19	0	XP_005674646.1	ssc:102167579			
LOC102162034	15.74964765	0	20	0.008099	High	19	0	XR_304917.1	ssc:102162034			
LOC102164165	15.74964765	0	20	0.008099	High	19	0	XR_306398.1	ssc:102164165			
LOC100621024	15.74964765	0	20	0.008099	High	19	0	XP_005652604.1	ssc:100621024	5E-29	NM_002281.3	KRT81
LOC102158850	15.74964765	0	20	0.008099	High	19	0	XR_303463.1	ssc:102158850			
LOC102158700	15.74964765	0	20	0.008099	High	19	0	XR_306944.1	ssc:102158700			
LMO3	15.74964765	0	20	0.008099	High	19	0	NM_001112685.2	ssc:100127154	1E-82	NR_045014.1	LMO3
LOC100157849	15.74964765	0	20	0.008099	High	19	0	LOC100157849				
LOC100625527	15.74964765	0	20	0.008099	High	19	0	XR_303583.1	ssc:100625527			
LOC100518209	7213.338623	1121.25975	2.68554662	0.008106	High	8702	1357	XP_005667788.1	ssc:100518209	7E-161	NM_019042.3	PUS7
LOC100153417	125.9971812	757.6972669	-2.588230086	0.008133	Low	152	917	XP_005663014.1	ssc:100153417	1E-87	NM_177965.3	C8orf37
LOC100736851	470.002643	78.49644531	2.581969641	0.008202	High	567	95	XP_005666887.1	ssc:100736851	0	NM_006726.4	LRBA
B3GAT2	165.7857647	26.4409079	2.648476514	0.008243	High	200	32	XP_003121311.1	ssc:100526195	2E-19	NM_080742.2	B3GAT2
TRERF1	3402.752821	545.3437253	2.641464617	0.008244	High	4105	660	XP_005666051.1	ssc:100153071	0	NM_033502.2	TRERF1
LOC100736913	4.144644118	40.48764021	-3.288161425	0.00825	Low	5	49	XP_003481708.2	ssc:100736913	0	NM_013381.2	TRHDE
LOC102158401	98808.31577	13836.85761	2.836116125	0.008263	High	119200	16746	XP_005664352.1	ssc:102158401			
PSRC1	7021.856065	1095.645121	2.680071838	0.008277	High	8471	1326	XP_005663654.1	ssc:100156693	7E-146	NM_032636.7	PSRC1
LOC102164789	35.64393941	3.305113487	3.430885079	0.008284	High	43	4	XR_301074.1	ssc:102164789			
ANKRD31	67.97216354	9.089062089	2.90274071	0.008305	High	82	11	XP_003480913.2	ssc:100627989	0	NM_001164443.1	ANKRD31
LOC100519128	3307.426006	531.296993	2.638118428	0.008325	High	3990	643	XP_005660774.1	ssc:100519128	3E-102	NM_080668.3	CDCA5
RAB20	0	15.69928906	-20	0.008363	Low	0	19	XP_003482979.1	ssc:100737446	6E-112	NM_017817.1	RAB20
LOC102163721	0	15.69928906	-20	0.008363	Low	0	19	XP_005652769.1	ssc:102163721	1E-30	NM_001102657.1	ZNF836
LOC100522375	0	15.69928906	-20	0.008363	Low	0	19	XP_005653380.1	ssc:100522375			
LOC102166142	0	15.69928906	-20	0.008363	Low	0	19	XR_299369.1	ssc:102166142			
HAND1	0	15.69928906	-20	0.008363	Low	0	19	NM_001014428.1	ssc:541594	4E-88	NM_004821.2	HAND1
GPHB5	0	15.69928906	-20	0.008363	Low	0	19	XP_003121917.2	ssc:100526029	3E-66	NM_145171.3	GPHB5
LOC102158137	0	15.69928906	-20	0.008363	Low	0	19	XR_307422.1	ssc:102158137			
PSD2	0	15.69928906	-20	0.008363	Low	0	19	XP_005661778.1	ssc:100525214	3E-170	NM_032289.2	PSD2
FOXQ1	0	15.69928906	-20	0.008363	Low	0	19	XP_001928083.1	ssc:100152843	7E-115	NM_033260.3	FOXQ1
LOC102165637	0	15.69928906	-20	0.008363	Low	0	19	LOC102165637				
LOC102165671	0	15.69928906	-20	0.008363	Low	0	19	XR_301958.1	ssc:102165671			
VAT1L	0	15.69928906	-20	0.008363	Low	0	19	XP_003126898.3	ssc:100520197	0	NM_020927.1	VAT1L
IL11RA	837.2181118	5303.880868	-2.663372944	0.008421	Low	1010	6419	XP_003130718.3	ssc:100524166	9E-153	NR_052010.1	IL11RA
HUS1	2592.060431	420.5756912	2.623661982	0.008423	High	3127	509	NM_001136511.1	ssc:100192318			
LOC100739863	29.01250883	180.128685	-2.634280983	0.00843	Low	35	218	XP_005669242.1	ssc:100739863	2E-142	NM_201566.2	SLC16A13
CD63	5330.012336	36469.44849	-2.774477606	0.008477	Low	6430	44137	XP_005663935.1	ssc:100155929	6E-117	NM_001780.5	CD63
TLR2	31.4992953	194.1754174	-2.623969109	0.008479	Low	38	235	NM_213761.1	ssc:396623	0	NM_003264.3	TLR2
MSX1	73.7746653	440.4063721	-2.577637967	0.00849	Low	89	533	NM_001162887.1	ssc:100302364	1E-140	NM_002448.3	MSX1

LOC100626458	331.5715294	55.36065091	2.582387324	0.008506	High	400	67	XP_005658098.1	ssc:100626458	0	NM_017645.4	HAUS6
RASL11B	226.2975688	1367.490705	-2.595238036	0.008538	Low	273	1655	XP_003129018.1	ssc:100521670	4E-140	NM_023940.2	RASL11B
LOC100625892	2151.899226	351.9945864	2.611985372	0.008547	High	2596	426	XP_003358370.2	ssc:100625892	0	NM_015184.5	PLCL2
TCTE1	27.35465118	170.2133446	-2.637486083	0.008587	Low	33	206	XP_005666126.1	ssc:100156326	0	NM_182539.3	TCTE1
FITM1	136.7732559	21.48323766	2.67050282	0.008594	High	165	26	NM_001128470.1	ssc:100147706	3E-102	NM_203402.2	FITM1
LOC102163187	113.5632488	17.35184581	2.710334985	0.008607	High	137	21	XR_302975.1	ssc:102163187			
GALNT9	3.315715294	35.52996998	-3.42164443	0.008631	Low	4	43	XP_001927211.4	ssc:100156842	0	NM_021808.3	GALNT9
LOC102160208	678.0637777	114.0264153	2.5720529	0.008642	High	818	138	XR_307349.1	ssc:102160208			
PPEF1	9506.984678	1474.080615	2.68917241	0.008643	High	11469	1784	XP_005673538.1	ssc:100623891	0	NM_152226.1	PPEF1
GALNT3	145.891473	23.13579441	2.656697021	0.00867	High	176	28	XP_003133495.3	ssc:100515629	0	NM_004482.3	GALNT3
LOC102165893	122.6814659	19.00440255	2.690511734	0.008671	High	148	23	XP_005654340.1	ssc:102165893			
LOC102158418	21.55214941	137.1622097	-2.669979389	0.008678	Low	26	166	XP_005658586.1	ssc:102158418	5E-74	NM_133631.3	ROBO1
GSX2	30.67036647	2.478835115	3.629111189	0.008698	High	37	3	XP_003129034.1	ssc:100524448	2E-133	NM_133267.2	GSX2
RSPH4A	30.67036647	2.478835115	3.629111189	0.008698	High	37	3	XP_005658999.1	ssc:100524228	0	NM_001161664.1	RSPH4A
GBA	949.123503	6015.306546	-2.663970528	0.008707	Low	1145	7280	NM_001005730.1	ssc:449572	0	NM_001171812.1	GBA
FCGRT	3166.508106	21103.97589	-2.736550144	0.008723	Low	3820	25541	NM_214197.2	ssc:397399	1E-148	NM_004107.4	FCGRT
CTSH	1118.224983	7132.434905	-2.673184192	0.008725	Low	1349	8632	NM_213929.2	ssc:396969	0	NM_148979.2	CTSH
DHODH	1102.475335	184.2600769	2.580930955	0.008742	High	1330	223	XP_005674422.1	ssc:100624213	2E-110	NM_001361.4	DHODH
ZSCAN30	272.717583	45.44531045	2.585204385	0.008776	High	329	55	ZSCAN30	ssc:100519372	0	NM_001288711.1	ZSCAN30
LOC100627470	28.18358	174.3447364	-2.629016023	0.008781	Low	34	211	XP_005655812.1	ssc:100627470	3E-116	NM_152342.2	CDYL2
LOC100737097	2146.925653	352.8208647	2.605264447	0.008841	High	2590	427	XP_003481926.2	ssc:100737097	0	NM_002088.4	GRIK5
DDR2	2043.30955	13338.61175	-2.706628837	0.008854	Low	2465	16143	XP_005655438.1	ssc:100154199	0	NM_006182.2	DDR2
LOC100738146	196.4561312	1176.620401	-2.582369858	0.008866	Low	237	1424	XP_003482192.1	ssc:100738146	0	NM_021905.2	GABBR1
LOC100513736	4.973572942	44.61903207	-3.165304677	0.008889	Low	6	54	XP_005664671.1	ssc:100513736	9E-99	NM_018215.3	PNMAL1
SLPI	39.78858353	4.131391859	3.26765473	0.008993	High	48	5	NM_213870.1	ssc:396886			
ABL2	1513.624032	252.0149034	2.586425942	0.009008	High	1826	305	XP_005667849.1	ssc:100627019	0	NM_007314.3	ABL2
LOC100518860	48.90680059	5.783948602	3.079908452	0.009033	High	59	7	XR_306301.1	ssc:100518860			
LOC102159987	209.7189924	1254.290568	-2.580342176	0.009057	Low	253	1518	XP_005668226.1	ssc:102159987	7E-93	NM_032812.8	PLXDC2
SCAND3	261.9415083	43.7927537	2.580480618	0.009069	High	316	53	XP_001929194.1		0	NM_052923.1	SCAND3
LOC100627356	342.3476041	2076.437548	-2.60057666	0.009083	Low	413	2513	XR_297923.1	ssc:100627356			
LOC100736999	25.69679353	1.652556743	3.958816635	0.009085	High	31	2	XP_003482726.1	ssc:100736999	1E-118	NM_015464.2	SOSTDC1
LOC100513943	25.69679353	1.652556743	3.958816635	0.009085	High	31	2	XP_005658103.1	ssc:100513943	1E-118	NM_015464.2	SOSTDC1
LOC102158289	25.69679353	1.652556743	3.958816635	0.009085	High	31	2	XR_301830.1	ssc:102158289			
C1H9orf57	140.0889712	22.30951604	2.650612258	0.009158	High	169	27	XP_003480584.1	ssc:100516173			
PROSER2	520.5673012	88.41178578	2.557774087	0.009187	High	628	107	XP_003357817.1	ssc:100627913	1E-100	NM_153256.3	PROSER2
LOC102167922	20.72322059	0.826278372	4.648476514	0.009196	High	25	1	XR_307817.1	ssc:102167922			
LOC100519396	71.28787883	420.5756912	-2.560636767	0.009232	Low	86	509	XP_005674154.1	ssc:100519396	0	NM_015690.4	STK36
CDC14B	2225.673891	367.6938754	2.597665171	0.00927	High	2685	445	XP_003357743.1	ssc:100626260			
PLEKHA4	290.9540171	1750.057591	-2.588539329	0.009288	Low	351	2118	XP_005664744.1	ssc:100522871	0	NM_020904.2	PLEKHA4
FAM171A1	477.4630024	2919.241487	-2.612132708	0.009316	Low	576	3533	XP_003130784.3	ssc:100525944	0	NM_001010924.1	FAM171A1
SGSH	383.7940453	2326.799895	-2.599942911	0.009364	Low	463	2816	XP_003482989.1	ssc:100737146	0	NM_000199.3	SGSH
RAMP1	1.657857647	25.61462952	-3.949575986	0.009432	Low	2	31	NM_214199.1	ssc:397401			
AMICA1	1.657857647	25.61462952	-3.949575986	0.009432	Low	2	31	NM_001244730.1	ssc:100512978	7E-132	NR_104479.1	AMICA1
LOC102162197	1.657857647	25.61462952	-3.949575986	0.009432	Low	2	31	XR_309102.1	ssc:102162197			
LOC100737228	1.657857647	25.61462952	-3.949575986	0.009432	Low	2	31	XP_005654379.1	ssc:100737228	3E-130	NM_022138.2	SMOC2
IL13RA1	221.3239959	1317.914003	-2.574024453	0.009433	Low	267	1595	NM_214341.1	ssc:397615	0	NM_001560.2	IL13RA1
PBXIP1	1015.437809	6381.347865	-2.651759298	0.009442	Low	1225	7723	XP_005663400.1	ssc:100151902	4E-180	NM_020524.2	PBXIP1
EPB41	11098.52802	1731.053189	2.680646388	0.009479	High	13389	2095	XP_005653450.1	ssc:100515614	0	NM_203343.2	EPB41
C4H1orf85	1235.932876	7824.85618	-2.662463844	0.009488	Low	1491	9470	XP_005663352.1	ssc:100153484	3E-157	NM_144580.2	C1orf85
LOC102166252	465.02907	79.32272369	2.551514784	0.00949	High	561	96	XR_302446.1	ssc:102166252			
ZNRF3	989.7410154	167.7345095	2.560871528	0.009504	High	1194	203	ZNRF3	ssc:100153654	0	NM_032173.3	ZNRF3
PTK7	5843.119278	39515.93685	-2.757623967	0.009521	Low	7049	47824	XP_005666076.1	ssc:100153873	0	NR_072998.1	PTK7
LOC102161567	0.828928824	20.65695929	-4.639235865	0.009524	Low	1	25	XR_307920.1	ssc:102161567			
SPHKAP	0.828928824	20.65695929	-4.639235865	0.009524	Low	1	25	XP_005672324.1	ssc:100515691	9E-147	NM_030623.3	SPHKAP
TRABD2A	29.01250883	176.8235716	-2.607563645	0.00955	Low	35	214	XP_005655281.1	ssc:100524432	2E-19	NM_001277053.1	TRABD2A
LOC102164419	320.7954547	1927.707441	-2.587160512	0.009558	Low	387	2333	XP_005665565.1	ssc:102164419	1E-92	NM_006516.2	SLC2A1
LOC100621028	34.81501059	3.305113487	3.396937747	0.009571	High	42	4	XP_005653388.1	ssc:100621028	4E-93	NM_001205252.1	RNF223
KLRG2	43.93322765	4.95767023	3.147578278	0.009572	High	53	6	XP_003134674.3	ssc:100518525	3E-138	NM_198508.2	KLRG2
HKDC1	43.93322765	4.95767023	3.147578278	0.009572	High	53	6	XP_001928917.1	ssc:100153520	0	NM_025130.3	HKDC1
LOC100738938	785.8245248	133.8570962	2.55351357	0.009579	High	948	162	XP_005662712.1	ssc:100738938	9E-60	NM_015131.1	WDR43
LOC100739070	75.43252295	10.74161883	2.811975246	0.009637	High	91	13	XP_005657066.1	ssc:100739070	4E-141	NM_178860.4	SEZ6
PARP10	348.1501059	2094.615672	-2.58890419	0.009658	Low	420	2535	XP_005655371.1	ssc:100516730	0	NM_032789.3	PARP10
SPATA6	28.18358	171.8659013	-2.608356553	0.009667	Low	34	208	NM_001177917.1	ssc:100415810	0	NM_019073.3	SPATA6
CYP24A1	424.4115577	72.71249671	2.545188706	0.009826	High	512	88	NM_214075.2	ssc:397145	0	NM_001128915.1	CYP24A1
ACP2	214.6925653	1269.989857	-2.564472838	0.009827	Low	259	1537	NM_001244740.1	ssc:100513002	0	NM_001610.2	ACP2
LOC100513961	57.19608883	7.436505346	2.94321978	0.00988	High	69	9	XP_005673226.1	ssc:100513961	0	NM_080385.4	CPA5
LOC102160371	57.19608883	7.436505346	2.94321978	0.00988	High	69	9	XR_305579.1	ssc:102160371			
LOC100525112	762.6145177	130.5519827	2.546329627	0.009893	High	920	158	XP_005653492.1	ssc:100525112	0	NM_000775.2	CYP2J2
LOC100511813	221.3239959	37.18252673	2.57346316	0.009901	High	267	45	XP_003126335.3	ssc:100511813	0	NM_015257.2	TMEM194A
LOC100513317	112.73432	657.7175839	-2.544541455	0.009962	Low	136	796	XP_003133863.1	ssc:100513317	8E-55	NM_016139.2	CHCHD2
LOC100511598	3.315715294	34.70369161	-3.387697098	0.009965	Low	4	42	XP_003133373.3	ssc:100511598	1E-160	NM_181265.3	WDR17
SLC16A13	61.34073295	358.6048133	-2.547477542	0.009981	Low	74	434	XP_003131974.1	ssc:100517000	0	NM_201566.2	SLC16A13
F2	4.973572942	43.7927537	-3.13833763	0.009987	Low	6	53	NM_001122985.1	ssc:100144442	0	NM_000506.3	F2
WDR63	4.973572942	43.7927537	-3.13833763	0.009987	Low	6	53	XP_005652563.1	ssc:100157444	0	NM_145172.4	WDR63
LOC102160827	4.973572942	43.7927537	-3.13833763	0.009987	Low	6	53	XP_005671031.1	ssc:102160827	0	NM_018662.2	DISC1
UBXN2A	2871.409445	475.1100637	2.595425393	0.009994	High	3464	575	XP_003125416.3	ssc:100522609	6E-134	NM_181713.3	UBXN2A
THNSL1	571.9608883	98.32712624	2.540255113	0.010005	High	690	119	XP_003357805.1	ssc:100517488	0	NM_024838.4	THNSL1
AVL9	1016.266738	173.5184581	2.55011807	0.010063	High	1226	210	XP_005673332.1	ssc:100624970	0	NM_015060.1	AVL9
LOC102166805	31.4992953	189.2177471	-2.58665595	0.010092	Low	38	229	XR_301473.1	ssc:102166805			
MVB12B	10.77607471	75.19133183	-2.802734598	0.010095	Low	13	91	XP_005660502.1	ssc:102159800	5E-40	NM_033446.2	MVB12B
LOC100627716	142.5757577	23.13579441	2.623530157	0.010108	High	172	28	XP_005658518.1	ssc:100627716	0	NM_198468.2	MMS22L
BCL9L	828.0998948	5106.400337	-2.624429931	0.010197	Low	999	6180	XP_005667470.1	ssc:100515846	0	NM_182557.2	BCL9L
HS3ST3B1	503.1597959	86.75922903	2.535927513	0.010202	High	607	105	XP_003483166.1	ssc:100737777	0	NM_006041.1	HS3ST3B1
MYO5B	299.2433053	51.22925905	2.546279041	0.010248	High	361	62	XP_003121482.3	ssc:100521829	0	NM_001080467.2	MYO5B
LOC100521408	29.84143765	2.478835115	3.589582825	0.010257	High	36	3	XP_005654225.1	ssc:100521408	0	np_001025167	np_001025167
PLOD3	783.3377383	4813.071515	-2.619251491	0.010297	Low	945	5825	XP_005658666.1	ssc:100624420	0	NM_001084.4	PLOD3
SHROOM3	9475.485383	1503.826637	2.655561587	0.010302	High	11431	1820	XP_005666836.1	ssc:100512481	0	NM_020859.3	SHROOM3
FAM46B	134.2864694	780.8330613	-2.539700192	0.010346	Low	162	945	XP_003127772.2	ssc:100511570	0	NM_052943.3	FAM46B
ABCA3	236.2447147	1390.6265	-2.557381028	0.010357	Low	285	1683	XP_005655233.1	ssc:100518064	0	NM_001089.2	ABCA3
ITGA11	736.9177242	4512.306188	-2.614289508	0.010372	Low	889	5461	XP_005659927.1	ssc:100520350	0	NM_012211.3	ITGA11
LOC100152693	326.5979565	56.18692928	2.539209303	0.010414	High	394	68	XP_001925516.2	ssc:100152693			
KLHL15	1293.128965	220.6163253	2.551254706	0.010422	High	1560	267	XP_003135027.1	ssc:100525794	0	NM_030624.2	KLHL15
LAMB2	1679.409797	10629.24497	-2.662012908	0.010438	Low	2026	12864	XP_005669592.1	ssc:101101688	2E-125	NM_002292.3	LAMB2
ALCAM	644.0776959	3918.212039	-2.604888835	0.010464	Low	777	4742	XP_003358875.2	ssc:397269			
METTL16	745.2070124	128.899426	2.531395411	0.010616	High	899	156	XP_005657078.1	ssc:100512985	0	NM_024086.3	METTL16
CCNG2	155.8386188	904.774817	-2.537505978	0.010641	Low	188	1095	XP_003129148.1	ssc:100523979	9E-176	NM_004354.2	CCNG2
LOC102165740	2.486786471	29.74602138	-3.580342176	0.010662	Low	3	36	XR_306715.1	ssc:102165740			
LOC100153608	1948.811664	329.6850703	2.563434212	0.010688	High	2351	399	XR_299406.1	ssc:100153608	2E-118	NM_032783.4	CBR4
LOC100157742	992.2278019	171.0396229	2.536340804	0.010733	High	1197	207	XP_003482945.2	ssc:100157742	0	NM_178009.3	DGKH
LOC100621630	177.3907683	29.74602138	2.576162309	0.010798	High	214	36	XP_005658328.1	ssc:100621630			

LOC100737635	632.4726924	109.8950234	2.524877136	0.01082	High	763	133	XR_299117.1	ssc:100737635			
LOC100622900	527.1987318	91.71689926	2.523087413	0.010845	High	636	111	XR_131273.3	ssc:100622900			
LOC100622412	14.92071882	0	20	0.010848	High	18	0	XP_003355255.1	ssc:100622412	1E-74	NM_001005464.2	HIST2H3A
LOC100628233	14.92071882	0	20	0.010848	High	18	0	XP_003356094.2	ssc:100628233	0	NM_032803.5	SLC7A3
ATP6V1B1	14.92071882	0	20	0.010848	High	18	0	XP_005662542.1	ssc:100523368	0	NM_001692.3	ATP6V1B1
LOC102161632	14.92071882	0	20	0.010848	High	18	0	XR_298642.1	ssc:102161632			
LOC100518085	14.92071882	0	20	0.010848	High	18	0	XP_003357214.1	ssc:100518085	7E-143	NM_001004137.1	OR52M1
KCNG2	14.92071882	0	20	0.010848	High	18	0	XP_005660638.1	ssc:100523354	0	NM_012283.1	KCNG2
KCND3	14.92071882	0	20	0.010848	High	18	0	XP_001928554.1	ssc:100037946	0	NM_172198.2	KCND3
LOC100512349	14.92071882	0	20	0.010848	High	18	0	LOC100512349				
LOC100152223	14.92071882	0	20	0.010848	High	18	0	XP_001928875.1	ssc:100152223	7E-118	NM_003868.1	FGF16
LOC102162774	14.92071882	0	20	0.010848	High	18	0	XP_005672746.1	ssc:102162774	4E-37	NM_014915.2	ANKRD26
LOC102158834	14.92071882	0	20	0.010848	High	18	0	XR_308005.1	ssc:102158834			
LOC102162746	14.92071882	0	20	0.010848	High	18	0	XR_305165.1	ssc:102162746			
LOC100623116	14.92071882	0	20	0.010848	High	18	0	XP_005658455.1	ssc:100623116	7E-31	NM_021238.2	FAM60A
LOC102162215	14.92071882	0	20	0.010848	High	18	0	XR_304415.1	ssc:102162215			
CDX4	14.92071882	0	20	0.010848	High	18	0	XP_003360417.1	ssc:100623433	3E-127	NM_005193.1	CDX4
LOC100737178	14.92071882	0	20	0.010848	High	18	0	XP_005662594.1	ssc:100737178	5E-43	NM_001080433.1	CCDC85A
LOC102165392	14.92071882	0	20	0.010848	High	18	0	XR_304029.1	ssc:102165392			
LOC100739206	14.92071882	0	20	0.010848	High	18	0	XP_003484182.1	ssc:100739206	7E-118	NM_003868.1	FGF16
SGK2	14.92071882	0	20	0.010848	High	18	0	XP_005657728.1	ssc:100157757	2E-171	NM_170693.2	SGK2
DEFB123	14.92071882	0	20	0.010848	High	18	0	XP_003134421.1	ssc:100521232	6E-17	NM_153324.2	DEFB123
L1CAM	1548.439042	264.409079	2.549970969	0.010871	High	1868	320	XP_005674071.1	ssc:100525324	0	NM_024003.3	L1CAM
TPST1	465.8579989	2784.558113	-2.579486232	0.010871	Low	562	3370	NM_001244291.1	ssc:100517581	0	NM_003596.3	TPST1
POU2F1	631.6437636	109.8950234	2.522985076	0.010918	High	762	133	NM_214264.1	ssc:397501	0	NR_037163.1	POU2F1
PRR2	433.5297747	2582.94619	-2.574814602	0.01092	Low	523	3126	NM_001097494.1	ssc:100038012	0	NM_002856.2	PVRL2
LOC100738919	6.631430589	52.05553742	-2.972659599	0.010939	Low	8	63	XP_005659218.1	ssc:100738919	1E-126	NM_024694.3	ADGB
LOC100624942	222.1529247	1294.778208	-2.54307995	0.01097	Low	268	1567	XP_005661896.1	ssc:100624942	1E-127	NM_001080495.2	TNRC18
SAT1	354.7815365	2097.094507	-2.563389043	0.010991	Low	428	2538	NM_214358.1	ssc:397645	5E-97	NR_027783.1	SAT1
LOC100524515	24.86786471	1.652556743	3.91151092	0.01101	High	30	2	XP_003130392.1	ssc:100524515	3E-82	NM_173509.2	FAM163A
LOC100624640	24.86786471	1.652556743	3.91151092	0.01101	High	30	2	XP_005669304.1	ssc:100624640	8E-150	NM_207386.3	SHISA6
LOC102166061	24.86786471	1.652556743	3.91151092	0.01101	High	30	2	XR_306180.1	ssc:102166061			
LOC100625773	291.7829459	1712.048786	-2.552756345	0.011068	Low	352	2072	XP_003353796.3	ssc:100625773	2E-69	NM_014578.3	RHOD
MYOZ1	33.98608177	3.305113487	3.362172329	0.011077	High	41	4	NM_001025222.2	ssc:574060	1E-138	NM_021245.3	MYOZ1
COL10A1	33.98608177	3.305113487	3.362172329	0.011077	High	41	4	NM_001005153.1	ssc:448809	0	NM_000493.3	COL10A1
MIR92A-1	33.98608177	3.305113487	3.362172329	0.011077	High	41	4	NR_035388.1	ssc:100498723			
TTC13	160.8121918	928.7368898	-2.529893154	0.011084	Low	194	1124	XP_005652476.1	ssc:100157092	0	NM_024525.4	TTC13
LOC100516814	73.7746653	10.74161883	2.779914037	0.011118	High	89	13	LOC100516814				
LOC102159468	11.60500353	78.49644531	-2.757880362	0.011124	Low	14	95	XR_307030.1	ssc:102159468			
LOC102159493	0	14.87301069	-20	0.011188	Low	0	18	XP_005664997.1	ssc:102159493			
PF4	0	14.87301069	-20	0.011188	Low	0	18	XP_003126209.1	ssc:100524373	2E-22	NM_002620.2	PF4V1
LOC100522480	0	14.87301069	-20	0.011188	Low	0	18	XP_005661609.1	ssc:100522480	3E-175	NM_024717.4	MCTP1
LOC100156142	0	14.87301069	-20	0.011188	Low	0	18	XP_005652415.1	ssc:100156142	0	NM_130770.2	HTR3C
SHighT20H	0	14.87301069	-20	0.011188	Low	0	18	XP_001926564.2	ssc:100156694	0	NM_017569.3	SHighT20H
LOC102165347	0	14.87301069	-20	0.011188	Low	0	18	XR_297671.1	ssc:102165347			
IZUMO2	0	14.87301069	-20	0.011188	Low	0	18	XP_005664826.1	ssc:100524938	7E-77	NM_152358.2	IZUMO2
BMP7	0	14.87301069	-20	0.011188	Low	0	18	NM_001105290.1	ssc:492315	0	NM_001719.2	BMP7
ROBO4	0	14.87301069	-20	0.011188	Low	0	18	XP_005667522.1	ssc:100524821	0	NM_019055.5	ROBO4
CABP1	0	14.87301069	-20	0.011188	Low	0	18	XP_003359172.1	ssc:100157460	3E-125	NM_031205.3	CABP1
NCCRP1	4.973572942	42.96647533	-3.110856893	0.011234	Low	6	52	XP_003355939.2	ssc:100628094	9E-111	NM_001001414.1	NCCRP1
C1RL	4.973572942	42.96647533	-3.110856893	0.011234	Low	6	52	XP_005658603.1	ssc:100513152	5E-117	NM_016546.2	C1RL
SYTL1	126.82611	20.65695929	2.618151977	0.011271	High	153	25	XP_005656137.1	ssc:100511935	0	NM_032872.2	SYTL1
LOC100524171	180.7064835	30.57229975	2.563351283	0.011298	High	218	37	XP_003131669.3	ssc:100524171	3E-84	NM_021213.3	PCTP
ZNF575	63.82751942	366.0413187	-2.519756024	0.011319	Low	77	443	ZNF575	ssc:100519430	2E-112	NM_174945.2	ZNF575
FUT1	98.64253001	564.3481279	-2.516303681	0.011326	Low	119	683	NM_214068.2	ssc:397138	9E-178	NM_000148.3	FUT1
MALT1	163.2989782	940.304787	-2.525612699	0.011339	Low	197	1138	XP_003353501.2	ssc:100153830	0	NM_173844.2	MALT1
ARHGEF15	9.947145883	69.40738323	-2.802734598	0.011352	Low	12	84	XP_005657103.1	ssc:100627972	0	NM_173728.3	ARHGEF15
ST6GALNAC6	644.0776959	3871.114172	-2.58744222	0.011403	Low	777	4685	XP_005660516.1	ssc:100523532	4E-173	NR_104629.1	ST6GALNAC6
ZNRF2	1740.75053	298.2864922	2.54493891	0.011433	High	2100	361	ZNRF2	ssc:100514021	1E-97	NM_147128.3	ZNRF2
FAM20A	23.21000706	140.4673232	-2.59741569	0.011472	Low	28	170	XP_005656990.1	ssc:100522273	0	NR_027751.1	FAM20A
BDP1	4963.625796	820.4944231	2.596828923	0.01153	High	5988	993	XP_005672586.1	ssc:100517687	0	NM_018429.2	BDP1
MUC13	1401.718641	242.0995629	2.533524414	0.011563	High	1691	293	NM_001105293.1	ssc:100125829	7E-95	NM_033049.3	MUC13
LOC102161067	19.89429177	0.826278372	4.589582825	0.011587	High	24	1	XR_299124.1	ssc:102161067			
LOC102160796	19.89429177	0.826278372	4.589582825	0.011587	High	24	1	XR_302833.1	ssc:102160796			
LCA5	298.4143765	52.05553742	2.519193497	0.011598	High	360	63	XP_005659474.1	ssc:100512810	0	NM_181714.3	LCA5
WDR3	9844.358709	1584.801917	2.634994697	0.011602	High	11876	1918	XP_005663596.1	ssc:100154556	0	NM_006784.2	WDR3
LOC100627417	2472.694681	419.7494128	2.5584839	0.011607	High	2983	508	XP_005667082.1	ssc:100627417	9E-43	NM_144645.3	C4orf36
TRAM2	467.5158565	2768.858824	-2.566204294	0.011611	Low	564	3351	XP_001928862.4	ssc:100157796	0	NM_012288.3	TRAM2
CD300LG	266.0861524	46.27158882	2.523694889	0.011661	High	321	56	XP_005668826.1	ssc:100515853	4E-56	NM_145273.3	CD300LG
LOC100739715	607.6048277	3631.493444	-2.579357738	0.011675	Low	733	4395	XP_003481902.1	ssc:100739715	7E-63	NM_012268.2	PLD3
OSAP	148.3782594	24.78835115	2.581545506	0.011698	High	179	30	NM_001195363.1	ssc:100359351	6E-85	NM_032623.3	MGARP
LOC100620089	1119.882841	195.0016957	2.521789234	0.011755	High	1351	236	XP_005656556.1	ssc:100620089	0	NM_007153.3	ZNF208
FAM227B	20.72322059	126.4205909	-2.608911329	0.011832	Low	25	153	XP_005659683.1	ssc:102164442	5E-65	NM_152647.2	FAM227B
LOC100621115	7264.73221	1188.188299	2.612146133	0.011915	High	8764	1438	XR_304891.1	ssc:100621115	0	NM_032313.2	NOA1
GTPBP2	176.5618394	1010.538449	-2.516878744	0.011961	Low	213	1223	XP_005666107.1	ssc:100514413	0	NM_019096.4	GTPBP2
DYNC2H1	986.4253001	172.6921797	2.51400705	0.011966	High	1190	209	XP_005667360.1	ssc:100624373	0	NM_024606.2	DYNC2H1
LOC100511883	0.828928824	19.83068092	-4.580342176	0.01199	Low	1	24	XP_005658094.1	ssc:100511883			
LOC100623758	0.828928824	19.83068092	-4.580342176	0.01199	Low	1	24	XP_003357157.1	ssc:100623758	1E-62	NM_152770.2	C4orf22
LOC102158207	0.828928824	19.83068092	-4.580342176	0.01199	Low	1	24	XR_303312.1	ssc:102158207			
LOC100517727	0.828928824	19.83068092	-4.580342176	0.01199	Low	1	24	XR_305775.1	ssc:100517727			
LOC100620445	0.828928824	19.83068092	-4.580342176	0.01199	Low	1	24	XP_005672042.1	ssc:100620445	0	NM_133437.3	TTN
PCDH11X	426.0694153	75.19133183	2.502450233	0.012004	High	514	91	XP_005673836.1	ssc:414736	0	NM_032969.3	PCDH11X
ANXA8	6156.454373	1014.66984	2.601089342	0.012009	High	7427	1228	NM_001243599.1	ssc:100155930	0	NM_001271703.1	ANXA8
COL23A1	156.6675477	26.4409079	2.566862749	0.012024	High	189	32	XP_005655031.1	ssc:100521775	1E-146	NM_173465.3	COL23A1
CEP85	2769.4512	470.9786719	2.555866483	0.012107	High	3341	570	XP_005659051.1	ssc:100621740	0	NM_022778.3	CEP85
FAM129C	17.4075053	108.2424667	-2.636485254	0.01212	Low	21	131	XP_005661238.1	ssc:100517808	0	NM_173544.4	FAM129C
LOXHD1	29.01250883	2.478835115	3.548940841	0.012122	High	35	3	XP_003121475.4	ssc:100520225	0	NM_144612.6	LOXHD1
LOC100511579	101.9582453	577.5685818	-2.502013816	0.012133	Low	123	699	XP_005667860.1	ssc:100511579	5E-98	NM_020950.1	KIAA1614
PIK3IP1	213.0347077	1222.065712	-2.520161464	0.012155	Low	257	1479	XP_001926569.1	ssc:100155147	2E-129	NM_052880.4	PIK3IP1
LOC102161615	702.1027136	123.9417558	2.502019793	0.012171	High	847	150	XP_005673208.1	ssc:102161615	2E-166	NM_015135.2	NHigh205
LOC100152254	702.1027136	123.9417558	2.502019793	0.012171	High	847	150	XP_005669869.1	ssc:100152254	0	NR_103507.1	SLC6A6
YPEL5	413.635483	2420.995629	-2.54916862	0.012228	Low	499	2930	YPEL5	ssc:100620823	5E-68	NM_016061.2	YPEL5
LOC102165314	138.4311135	23.13579441	2.580969695	0.012277	High	167	28	XP_005668205.1	ssc:102165314	1E-61	NM_019590.4	KIAA1217
LOC102165099	248.6786471	1430.287861	-2.523950995	0.012297	Low	300	1731	XP_005665219.1	ssc:102165099	1E-55	NM_001856.3	COL16A1
LOC102158208	164.9568359	28.09346464	2.553782104	0.012333	High	199	34	XR_297644.1	ssc:102158208			
LOC100154532	331.5715294	1920.270936	-2.533917839	0.01248	Low	400	2324	XP_003360024.3	ssc:100154532	4E-162	NM_183006.2	DLGAP4
POLA1	851.3099018	150.3826637	2.50104615	0.012487	High	1027	182	XP_005673567.1	ssc:100157467	0	NM_016937.3	POLA1
TSPAN2	331.5715294	58.66576439	2.498729395	0.012505	High	400	71	XP_005663612.1	ssc:100156161	5E-114	NM_005725.4	TSPAN2
DSCC1	708.7341442	125.5943125	2.496473421	0.012511	High	855	152	XP_001926458.1	ssc:100156441	7E-179	NM_024094.2	DSCC1
LOC100739472	625.012333	3702.553384	-2.566563974	0.012526	Low	754	4481	XR_308243.1	ssc:100739472			

LOC100511825	85.37966883	13.22045395	2.691120852	0.012577	High	103	16	XP_005653655.1	ssc:100511825			
LOC102166534	4.973572942	42.14019696	-3.082842517	0.01265	Low	6	51	XR_309367.1	ssc:102166534			
ZNF554	613.4073295	109.0687451	2.491607665	0.012667	High	740	132	ZNF554	ssc:100517454	0	NM_152303.2	ZNF554
IFT140	251.1654336	1438.550645	-2.51790618	0.012685	Low	303	1741	XP_003124763.1	ssc:100513727	0	NM_014714.3	IFT140
DRP2	473.3183583	84.28039392	2.489541918	0.01271	High	571	102	XP_005673821.1	ssc:100517071	0	NM_001939.2	DRP2
RP2	339.8608177	60.31832114	2.494275865	0.012719	High	410	73	XP_005673641.1	ssc:100622983	0	NM_006915.2	RP2
PCSK6	27.35465118	160.2980041	-2.550898398	0.012796	Low	33	194	XP_005660641.1	ssc:102164742	9E-132	NM_138325.2	PCSK6
PLEK2	97.81360119	549.4751172	-2.489947157	0.012824	Low	118	665	XP_001928470.1	ssc:100154251	0	NM_016445.1	PLEK2
LOC100738956	33.15715294	3.305113487	3.326548419	0.012842	High	40	4	XR_135238.2	ssc:100738956			
LOC102158276	33.15715294	3.305113487	3.326548419	0.012842	High	40	4	XR_300229.1	ssc:102158276			
NEK10	320.7954547	57.01320765	2.492285624	0.01294	High	387	69	XP_003358395.2	ssc:100622627	0	NM_199347.2	NEK10
LOC102159370	54.70930236	7.436505346	2.879089442	0.013059	High	66	9	XP_005658623.1	ssc:102159370	6E-89	NM_005559.3	LAMA1
SLC43A2	220.4950671	1252.638012	-2.506151278	0.013081	Low	266	1516	XP_005657072.1	ssc:100623009	0	NM_152346.2	SLC43A2
ZSCAN23	115.2211065	19.00440255	2.599999441	0.013092	High	139	23	ZSCAN23	ssc:100155318	0	NM_001012455.1	ZSCAN23
RIMS1	2587.915787	446.1903207	2.536059549	0.013147	High	3122	540	XP_005659321.1	ssc:100157596	0	NM_014989.5	RIMS1
CCDC114	52.22251589	294.1551003	-2.493833183	0.01316	Low	63	356	XP_005664716.1	ssc:100622853	0	NM_144577.3	CCDC114
LOC100621058	27.35465118	159.4717257	-2.543442594	0.013236	Low	33	193	XP_003361911.1	ssc:100621058	4E-87	NM_032430.1	BRSK1
LOC102166380	433.5297747	77.67016694	2.480698609	0.013273	High	523	94	XP_005664916.1	ssc:102166380	2E-66	NM_175931.2	CBFA2T3
LOC100737652	6047.864697	1011.364727	2.580122496	0.013304	High	7296	1224	XP_005669310.1	ssc:100737652	1E-147	NM_173717.1	ELAC2
PKC	364.7286824	65.27599137	2.48219929	0.013334	High	440	79	NM_001080206.2	ssc:791125	0	NM_006255.3	PRKCH
TMPRSS6	37.30179706	4.131391859	3.174545326	0.013336	High	45	5	XP_001924784.1				
ESPN	37.30179706	4.131391859	3.174545326	0.013336	High	45	5	XP_003127578.1	ssc:100515838	0	NM_031475.2	ESPN
SLC38A6	204.7454194	1157.615999	-2.499253685	0.013336	Low	247	1401	NM_001267849.1	ssc:100519583	0	NR_033344.1	SLC38A6
LOC100517249	24.03893588	1.652556743	3.862601319	0.013384	High	29	2	XP_003134406.1	ssc:100517249	2E-86	NM_004609.3	TCF15
LOC100512171	170.7593377	960.1354679	-2.491273502	0.013443	Low	206	1162	XP_005664844.1	ssc:100512171			
LOC100524511	176.5618394	993.1866028	-2.491891236	0.013473	Low	213	1202	XP_003129342.2	ssc:100524511	0	NM_005908.3	MANBA
PABPC5	62.99859059	9.089062089	2.793116219	0.013477	High	76	11	XP_005674625.1	ssc:100518351	0	NM_080832.2	PABPC5
LOC100620700	298.4143765	1702.959724	-2.512655369	0.013497	Low	360	2061	XP_005659086.1	ssc:100620700	8E-91	NM_006602.2	TCFL5
LOC100625970	923.4267095	5498.056285	-2.573852309	0.013503	Low	1114	6654	XP_005652631.1	ssc:100625970	0	NR_026554.1	CALCOCO1
LOC100627705	2763.648698	477.5888989	2.532733031	0.013596	High	3334	578	XP_003359943.1	ssc:100627705	7E-61	NM_004102.3	FABP3
ULBP1	67.14323471	9.915340461	2.759507826	0.013655	High	81	12	NM_001004035.1	ssc:445523	1E-53	NM_025218.2	ULBP1
DMRTA1	67.14323471	9.915340461	2.759507826	0.013655	High	81	12	XP_003121928.1	ssc:100517627	0	NM_022160.2	DMRTA1
LOC100620819	179.8775547	31.39857813	2.518244043	0.013712	High	217	38	XP_005658436.1	ssc:100620819	3E-22	NM_001134664.1	SAMD13
GCKR	629.1569771	113.2001369	2.474544317	0.01376	High	759	137	XP_005655314.1	ssc:100625192	0	NM_001486.3	GCKR
RAI2	1.657857647	23.96207278	-3.853360671	0.013872	Low	2	29	XP_005673527.1	ssc:100154649	0	NR_033349.1	RAI2
LOC100739318	1.657857647	23.96207278	-3.853360671	0.013872	Low	2	29	XP_005665583.1	ssc:100739318			
ZIC4	113.5632488	631.276676	-2.474776421	0.013877	Low	137	764	ZIC4	ssc:100519623	9E-12	NR_040762.1	ZIC4
CA11	42.27537	237.9681711	-2.492879335	0.0139	Low	51	288	NM_001004038.1	ssc:445526	0	NM_001217.3	CA11
ARHGAP29	775.0484501	4562.709169	-2.557532293	0.013903	Low	935	5522	XP_005663714.1	ssc:100152734	0	NM_004815.3	ARHGAP29
LOC102158109	75.43252295	11.5678972	2.705060042	0.01397	High	91	14	XR_303197.1	ssc:102158109			
SH2D6	295.0986612	1673.213703	-2.503352437	0.014076	Low	356	2025	XP_005655277.1	ssc:100520186	1E-60	NM_198482.1	SH2D6
LOC102158716	258.625793	46.27158882	2.482667621	0.014094	High	312	56	LOC102158716				
LOC102166675	397.8858353	71.88621834	2.468567424	0.014098	High	480	87	XR_300568.1	ssc:102166675			
LOC100515833	1061.028894	190.0440255	2.481058368	0.014217	High	1280	230	XP_005664305.1	ssc:100515833	0	NM_018351.3	FGD6
LOC100514456	38.13072589	214.8323766	-2.494185533	0.014275	Low	46	260	XP_005662824.1	ssc:100514456	4E-140	NM_014746	RNF144A
LOC100739376	341.5186753	1941.754174	-2.50732419	0.014281	Low	412	2350	XP_003482942.1	ssc:100739376	2E-80	NM_183422.3	TSC22D1
LRRC8D	252.8232912	1422.851356	-2.492583671	0.014336	Low	305	1722	XP_005663753.1	ssc:100152378	0	NM_018103.4	LRRC8D
LOC102161347	53.88037353	7.436505346	2.857063136	0.014351	High	65	9	XP_005664287.1	ssc:102161347			
LOC100524408	410.3197677	74.36505346	2.464051943	0.014369	High	495	90	XP_005672948.1	ssc:100524408	0	NM_194323.2	OTOF
LOC100152144	94.49788589	521.3816526	-2.463985856	0.014466	Low	114	631	XP_005660642.1	ssc:100152144	1E-131	NM_138325.2	PCSK6
TRIM36	139.2600424	23.96207278	2.538956752	0.014495	High	168	29	XP_003480940.1	ssc:100519890	0	NM_018700.3	TRIM36
LOC102158867	58.02501765	8.262783717	2.811975246	0.014502	High	70	10	XR_300174.1	ssc:102158867			
LOC102164640	707.0762865	128.0731476	2.46489785	0.014548	High	853	155	XR_299551.1	ssc:102164640			
SLC5A9	14.09179	0	20	0.014644	High	17	0	HighI0001987D48		0	NM_001135181.1	SLC5A9
LOC102159070	14.09179	0	20	0.014644	High	17	0	XR_306449.1	ssc:102159070			
LOC102159289	14.09179	0	20	0.014644	High	17	0	XR_304016.1	ssc:102159289			
LOC102159341	14.09179	0	20	0.014644	High	17	0	XR_308538.1	ssc:102159341			
LOC100525238	14.09179	0	20	0.014644	High	17	0	LOC100525238				
LOC102164452	14.09179	0	20	0.014644	High	17	0	XR_302297.1	ssc:102164452			
LOC102164248	14.09179	0	20	0.014644	High	17	0	XR_300305.1	ssc:102164248			
LOC102166194	14.09179	0	20	0.014644	High	17	0	XR_306099.1	ssc:102166194			
LOC102160990	14.09179	0	20	0.014644	High	17	0	XR_305648.1	ssc:102160990			
LOC100621290	14.09179	0	20	0.014644	High	17	0	XP_005668080.1	ssc:100621290	0	NM_006180.3	NTRK2
FOXB1	14.09179	0	20	0.014644	High	17	0	XP_005658514.1	ssc:100626570	6E-132	NM_012182.2	FOXB1
LOC102160621	14.09179	0	20	0.014644	High	17	0	XR_302208.1	ssc:102160621			
LOC102162810	14.09179	0	20	0.014644	High	17	0	XR_307235.1	ssc:102162810			
LOC102160074	14.09179	0	20	0.014644	High	17	0	XR_300293.1	ssc:102160074			
LOC100519633	14.09179	0	20	0.014644	High	17	0	XP_003134612.1	ssc:100519633	6E-158	NM_012369.2	OR2F1
C15H2orf82	14.09179	0	20	0.014644	High	17	0	NM_001185185.1	ssc:100157430	2E-38	NM_206895.1	C2orf82
LOC102162647	14.09179	0	20	0.014644	High	17	0	XR_300298.1	ssc:102162647			
LOC100521169	14.09179	0	20	0.014644	High	17	0	LOC100521169				
GSDMA	14.09179	0	20	0.014644	High	17	0	XP_003131545.1	ssc:100512434	0	NM_178171.4	GSDMA
LOC102159404	19.06536294	0.826278372	4.52818228	0.014669	High	23	1	XR_298033.1	ssc:102159404			
LOC102164525	19.06536294	0.826278372	4.52818228	0.014669	High	23	1	XR_300812.1	ssc:102164525			
LOC102166555	19.06536294	0.826278372	4.52818228	0.014669	High	23	1	XR_306752.1	ssc:102166555			
LOC102160240	19.06536294	0.826278372	4.52818228	0.014669	High	23	1	XR_305817.1	ssc:102160240			
PNP	4205.155922	722.9935753	2.540104563	0.014683	High	5073	875	XP_005656368.1	ssc:100157138	3E-155	NM_000270.3	PNP
ENG	472.4894295	2706.887946	-2.518281208	0.014686	Low	570	3276	NM_214031.1	ssc:397096	0	NM_001278138.1	ENG
LOC100525818	196.4561312	1093.992564	-2.477323834	0.014691	Low	237	1324	XP_003482028.1	ssc:100525818	0	NM_018125.3	ARHGEF10L
LOC100622725	3522.118571	609.7934383	2.530050947	0.014704	High	4249	738	XR_298025.1	ssc:100622725			
LOC102164603	217.1793518	38.83508347	2.483454474	0.014767	High	262	47	XP_005658357.1	ssc:102164603			
LOC100623650	169.10148	29.74602138	2.507120665	0.014796	High	204	36	XP_005658282.1	ssc:100623650	6E-103	NM_201567.1	CDC25A
TFR2	70.45895001	10.74161883	2.713571542	0.014868	High	85	13	XP_005661971.1	ssc:100517580	0	NM_003227.3	TFR2
SCUBE2	37.30179706	209.048428	-2.486520154	0.014893	Low	45	253	XP_005667098.1	ssc:100519613	0	NM_020974.2	SCUBE2
ASB15	32.32822412	3.305113487	3.290022543	0.014915	High	39	4	XP_005673268.1	ssc:100513190	0	NM_080928.3	ASB15
LOC102162757	32.32822412	3.305113487	3.290022543	0.014915	High	39	4	XP_005658630.1	ssc:102162757	3E-48	NM_018150.2	RNF220
LOC100620843	32.32822412	3.305113487	3.290022543	0.014915	High	39	4	LOC100620843				
RRAS	1193.657506	7079.553089	-2.568269344	0.014968	Low	1440	8568	XP_003356046.1	ssc:100516558	7E-100	NM_006270.3	RRAS
LOC102164286	74.60359412	11.5678972	2.689118499	0.014974	High	90	14	XP_005668577.1	ssc:102164286			
LOC102160237	74.60359412	11.5678972	2.689118499	0.014974	High	90	14	XR_305314.1	ssc:102160237			
DLGAP2	2725.517972	477.5888989	2.512689226	0.014977	High	3288	578	NM_001244586.1	ssc:100523643	0	NM_004745.4	DLGAP2
LOC102159409	194.7982735	34.70369161	2.488819848	0.015012	High	235	42	XR_304693.1	ssc:102159409			
RBM38	1730.803384	308.2018327	2.489494501	0.015038	High	2088	373	NM_001105312.1	ssc:100125973	1E-129	NM_183425.2	RBM38
FAM111B	164.9568359	911.385044	-2.465972131	0.015071	Low	199	1103	XP_005660934.1	ssc:100517326	0	NM_198947.3	FAM111B
LOC100622585	0	14.04673232	-20	0.015085	Low	0	17	XR_297423.1	ssc:100622585	1E-21	NM_004350.2	RUNX3
FREM1	0	14.04673232	-20	0.015085	Low	0	17	XP_005660151.1	ssc:100514332	0	NM_144966.5	FREM1
LOC100739463	0	14.04673232	-20	0.015085	Low	0	17	XR_298932.1	ssc:100739463			
LOC102165274	0	14.04673232	-20	0.015085	Low	0	17	LOC102165274				
LOC100738992	203.9164906	1132.001369	-2.472825348	0.015105	Low	246	1370	XP_005655396.1	ssc:100738992	0	NM_014112	TRPS1
LOC100621844	0.828928824	19.00440255	-4.518941632	0.015162	Low	1	23	XP_003355443.2	ssc:100621844	0	NM_002281.3	KRT81

LOC102161819	82.89288236	13.22045395	2.648476514	0.015171	High	100	16	XR_308618.1	ssc:102161819			
LOC100739587	1915.654511	340.4266892	2.492421342	0.015174	High	2311	412	XP_005670668.1	ssc:100739587	0	NM_014708.4	KNTC1
LOC100518021	67.97216354	372.6515456	-2.454811294	0.015217	Low	82	451	XP_003128185.1	ssc:100518021			
LOC100739813	330.7426006	60.31832114	2.455040702	0.015249	High	399	73	XP_003482471.2	ssc:100739813	1E-77	NM_033632.3	FBXW7
MRVI1	9.11821706	61.97087788	-2.764766748	0.015286	Low	11	75	XP_005661186.1	ssc:100739522	0	NM_130385.3	MRVI1
GAS2L3	1327.115047	238.7944494	2.474452234	0.015298	High	1601	289	XP_001928851.4	ssc:100154381	8E-170	NM_174942.1	GAS2L3
PKD1L3	15.74964765	95.84829112	-2.605433157	0.015309	Low	19	116	XR_309594.1	ssc:102158206			
ITGA4	2960.933758	518.9028174	2.512515941	0.01532	High	3572	628	XP_003133565.1	ssc:100521477	0	NM_000885.4	ITGA4
MSRA	53.05144471	291.6762652	-2.458904049	0.015406	Low	64	353	XP_003359097.2	ssc:100152960	2E-87	NM_012331.3	MSRA
LOC102164843	40.61751236	4.95767023	3.034367668	0.01546	High	49	6	XR_297171.1	ssc:102164843			
NBL1	1345.351481	7981.849071	-2.568739865	0.015479	Low	1623	9660	XP_005656092.1	ssc:100623582	8E-88	NM_001204089.1	MINOS1-NBL1
CTNNA2	3.315715294	32.2248565	-3.280781895	0.015497	Low	4	39	XP_005662473.1	ssc:100525337	0	NM_004389.3	CTNNA2
LOC102162973	3.315715294	32.2248565	-3.280781895	0.015497	Low	4	39	XR_301315.1	ssc:102162973			
SDC4	411.1486965	2324.32106	-2.499077203	0.01554	Low	496	2813	NM_214284.1	ssc:397528	7E-59	NM_002999.3	SDC4
LOC102160850	48.90680059	6.610226974	2.887263374	0.015703	High	59	8	XR_304878.1	ssc:102160850			
LOC102162222	107.7607471	18.17812418	2.567556519	0.015704	High	130	22	XR_305722.1	ssc:102162222			
STAT1	2054.085625	12381.7814	-2.591650667	0.015747	Low	2478	14985	NM_213769.1	ssc:396655			
GAL3ST1	53.05144471	7.436505346	2.834695323	0.015781	High	64	9	NM_001244429.1	ssc:100155265	0	NM_004861.1	GAL3ST1
TET1	657.3405571	120.6366423	2.445972821	0.015807	High	793	146	XP_003359270.2	ssc:100625859	0	NM_030625.2	TET1
OMA1	896.0720583	163.6031176	2.453414512	0.01582	High	1081	198	XP_005665443.1	ssc:100626843	0	NM_145243.3	OMA1
GNAS	8859.591267	56922.31703	-2.683682341	0.01584	Low	10688	68890	NR_073434.1	ssc:100049657	0	NR_003259.1	GNAS
FRMD4A	32.32822412	180.9549634	-2.484764516	0.015879	Low	39	219	XP_005668182.1	ssc:100737056	3E-143	NM_018027.3	FRMD4A
EPB41L4B	61.34073295	9.089062089	2.754642071	0.015901	High	74	11	XP_005674634.1	ssc:100510946	0	NM_019114.4	EPB41L4B
C8H4orf29	493.21265	90.89062089	2.440006469	0.016011	High	595	110	XP_005666924.1	ssc:100516683	0	NM_025097.1	C4orf29
LOC102162607	73.7746653	11.5678972	2.672998833	0.016055	High	89	14	XR_306884.1	ssc:102162607			
LOC102161732	664.8009165	122.289199	2.442625385	0.016074	High	802	148	XR_302363.1	ssc:102161732			
LOC100621128	4.973572942	40.48764021	-3.025127019	0.016094	Low	6	49	LOC100621128				
CES3	240.3893588	43.7927537	2.45660896	0.016095	High	290	53	NM_001243625.1	ssc:100626873	0	NM_024922.5	CES3
LOC102163227	77.91930942	12.39417558	2.65231858	0.016106	High	94	15	XR_297949.1	ssc:102163227			
RSPH6A	82.06395354	13.22045395	2.633976944	0.016158	High	99	16	XP_005664680.1	ssc:102158590	2E-90	NM_030785.3	RSPH6A
SULT1C4	72.94573648	395.7873401	-2.439829903	0.016226	Low	88	479	XP_005662396.1	ssc:100623441	4E-147	NM_006588.2	SULT1C4
CDKN2B	146.7204018	799.8374638	-2.446637363	0.016237	Low	177	968	NM_214124.1	ssc:397227	4E-59	NM_078487.2	CDKN2B
LOC100518456	5.802501765	44.61903207	-2.942912256	0.016253	Low	7	54	XP_003134744.2	ssc:100518456	1E-72	NM_001195150.1	LOC100130705
NPM2	90.35324177	14.87301069	2.602879648	0.016265	High	109	18	NM_001195362.1	ssc:100154488	4E-59	NM_182795.1	NPM2
LOC102164377	23.21000706	1.652556743	3.811975246	0.016321	High	28	2	XP_005668663.1	ssc:102164377	6E-94	NM_198955.1	MGAT5B
UOX	23.21000706	1.652556743	3.811975246	0.016321	High	28	2	NM_214270.1	ssc:397510			
LOC102165355	23.21000706	1.652556743	3.811975246	0.016321	High	28	2	XR_298728.1	ssc:102165355			
XKRX	23.21000706	1.652556743	3.811975246	0.016321	High	28	2	XP_005673818.1	ssc:100518168	0	NM_212559.2	XKRX
PIWIL4	213.8636365	38.83508347	2.461258728	0.016328	High	258	47	XP_005667292.1	ssc:100517358	0	NM_152431.2	PIWIL4
CCDC170	6.631430589	48.75042393	-2.878022725	0.016369	Low	8	59	XP_005659194.1	ssc:100516653	6E-168	NM_025059.3	CCDC170
LOC100624174	145.0625441	25.61462952	2.501635126	0.016435	High	175	31	LOC100624174				
LOC100736859	639.9330518	118.1578072	2.437206025	0.016436	High	772	143	XP_005671946.1	ssc:100736859	0	NM_018086.2	FIGN
NUDT18	137.6021847	747.7819264	-2.442114226	0.016455	Low	166	905	XP_001927515.1	ssc:100156061	1E-162	NM_024815.3	NUDT18
ADK	4826.023611	838.6725473	2.524655436	0.016505	High	5822	1015	XP_005671172.1	ssc:100154626	0	NM_006721.3	ADK
LOC102160067	97.81360119	528.8181579	-2.434664721	0.016589	Low	118	640	XR_305511.1	ssc:102160067			
LOC102157897	9.11821706	61.14459951	-2.745401423	0.016601	Low	11	74	XR_298356.1	ssc:102157897			
LOC102158271	183.19327	33.05113487	2.470594789	0.016621	High	221	40	XP_005658240.1	ssc:102158271	2E-47	NM_022091.4	ASCC3
LOC100157413	1366.90363	248.7097899	2.458376331	0.016642	High	1649	301	XP_005658070.1	ssc:100157413	0	NM_015278.3	SASH1
GRPEL2	1087.554617	199.1330876	2.449282993	0.016649	High	1312	241	NM_001244870.1	ssc:100513650	2E-100	NM_152407.3	GRPEL2
MRPS27	10453.62139	1763.278045	2.567670923	0.016714	High	12611	2134	XP_003134081.1	ssc:100519229	0	NM_015084.2	MRPS27
TMEM37	123.5103947	21.48323766	2.523349127	0.016735	High	149	26	XP_003133353.1	ssc:100522166	1E-80	NM_183240.2	TMEM37
BTN2A2	1132.316773	207.3958713	2.448818539	0.016807	High	1366	251	XP_001926087.2	ssc:100156935	0	NM_181531.2	BTN2A2
KLF17	299.2433053	55.36065091	2.434386161	0.016958	High	361	67	NM_001164010.1	ssc:100302639	3E-104	NM_173484.3	KLF17
AARS2	3706.969699	653.586192	2.503790913	0.017008	High	4472	791	XP_001929628.2	ssc:100155115	0	NM_020745.3	AARS2
LOC102166211	27.35465118	2.478835115	3.464051943	0.017045	High	33	3	XR_309181.1	ssc:102166211			
LIPI	27.35465118	2.478835115	3.464051943	0.017045	High	33	3	XP_005670350.1	ssc:100154888	0	NM_198996.2	LIPI
SELRC1	1366.074701	249.5360683	2.452716112	0.017103	High	1648	302	NM_001243594.1	ssc:100627075			
TM7SF2	169.10148	917.1689926	-2.439298265	0.017137	Low	204	1110	XP_005660770.1	ssc:100514510	0	NR_102367.1	TM7SF2
LOC100624822	1068.489254	196.6542525	2.441839109	0.017202	High	1289	238	XP_003482510.1	ssc:100624822	0	NM_024751.3	GSTCD
LYRM1	102.7871741	552.7802307	-2.427045766	0.017218	Low	124	669	XP_005662153.1	ssc:100525810	2E-64	NM_020424.3	LYRM1
1-Sep	85.37966883	14.04673232	2.60365801	0.017221	High	103	17	NM_001244989.1	ssc:100514708	0	NM_052838.4	1-Sep
UMODL1	72.94573648	11.5678972	2.656697021	0.017221	High	88	14	XP_005670432.1	ssc:100620228	0	NM_173568.3	UMODL1
LOC100511417	89.52431295	14.87301069	2.589582825	0.017232	High	108	18	XP_003133982.1	ssc:100511417	0	NM_002202.2	ISL1
ANO10	218.0082806	1189.014577	-2.447311563	0.01724	Low	263	1439	XP_005669488.1	ssc:100511155	0	NM_018075.3	ANO10
TAF1B	2799.292637	499.8984149	2.485355456	0.017276	High	3377	605	XP_005662816.1	ssc:397176	0	NM_005680.2	TAF1B
LOC102164310	60.51180412	9.089062089	2.735013265	0.017286	High	73	11	XR_303047.1	ssc:102164310			
LRRN2	263.5993659	48.75042393	2.43486023	0.017316	High	318	59	XP_003130149.2	ssc:100525178	0	NM_201630.1	LRRN2
RINL	19.06536294	110.7213018	-2.53790691	0.017335	Low	23	134	XP_005664658.1	ssc:102163514	2E-129	NM_198445.3	RINL
LOC102162301	31.4992953	3.305113487	3.252547838	0.017351	High	38	4	XR_298712.1	ssc:102162301			
LOC100737267	31.4992953	3.305113487	3.252547838	0.017351	High	38	4	XP_003480491.1	ssc:100737267	0	NM_004727.2	SLC24A1
LOC100038017	31.4992953	3.305113487	3.252547838	0.017351	High	38	4	XP_005673792.1	ssc:100038017	0	NM_005296.2	LPAR4
CD72	52.22251589	7.436505346	2.811975246	0.017365	High	63	9	NM_001097493.1	ssc:100038011	3E-108	NM_001782.2	CD72
DAPK1	1016.266738	187.5651904	2.437815101	0.017387	High	1226	227	XP_003482851.1	ssc:100516103	0	NM_004938	DAPK1
FGD6	792.4559554	147.0775502	2.429753701	0.017412	High	956	178	XP_005664312.1	ssc:100517825	0	NM_018351.3	FGD6
LRRN4	43.93322765	5.783948602	2.925185857	0.017452	High	53	7	XP_003359940.1	ssc:100152719	3E-140	NM_152611.4	LRRN4
SLC2A11	97.81360119	524.686766	-2.423349408	0.017475	Low	118	635	XP_005670960.1	ssc:100125552	0	NR_104248.1	SLC2A11
LOC102167252	39.78858353	4.95767023	3.004620324	0.017478	High	48	6	XR_307630.1	ssc:102167252			
LOC100525876	11122.56696	1883.088409	2.562317143	0.017506	High	13418	2279	XP_005653411.1	ssc:100525876	0	NM_018188.3	ATAD3A
LOC102159265	43.10429883	233.8367792	-2.4395982	0.0176	Low	52	283	XR_300558.1	ssc:102159265			
TNNC2	181.5354124	33.05113487	2.457479289	0.017624	High	219	40	NM_001001862.1	ssc:414905			
LOC100738431	215.5214941	39.66136184	2.442025637	0.017684	High	260	48	XP_005662427.1	ssc:100738431	3E-123	NM_198461.3	LONRF2
LOC102158399	147.5493306	26.4409079	2.480353755	0.017736	High	178	32	XR_297559.1	ssc:102158399			
LOC100737465	419.4379847	78.49644531	2.417758291	0.017747	High	506	95	XP_003480713.1	ssc:100737465			
FOXA1	193.9693447	35.52996998	2.448720289	0.017815	High	234	43	XP_001929311.1	ssc:100156502	0	NM_004496.3	FOXA1
ERAP1	736.0887954	4171.05322	-2.502460004	0.017871	Low	888	5048	XP_005661611.1	ssc:100523953	0	NM_016442.3	ERAP1
GRIA4	26.52572236	147.9038285	-2.479195453	0.017942	Low	32	179	XP_003129869.2	ssc:100524886	0	NR_046356.1	GRIA4
TMEM9B	1008.806378	5788.079994	-2.520435558	0.017953	Low	1217	7005	XP_005667110.1	ssc:100517666	2E-82	NM_020644.2	TMEM9B
PCP4L1	528.0276606	2949.813787	-2.481938472	0.017988	Low	637	3570	XP_003125705.2	ssc:100523600	1E-23	NM_001102566.1	PCP4L1
LOC100521743	9.947145883	64.44971299	-2.695819394	0.018016	Low	12	78	XP_003357786.2	ssc:100521743	0	NM_001081.3	CUBN
LOC100738657	3.315715294	31.39857813	-3.243307189	0.018016	Low	4	38	XR_306828.1	ssc:100738657			
LOC100738884	8.289288236	56.18692928	-2.760914422	0.018068	Low	10	68	XP_003481107.2	ssc:100738884	5E-57	NM_001116.3	ADCY9
HES1	172.4171953	927.9106115	-2.42808217	0.018109	Low	208	1123	NM_001195231.1	ssc:100499567	6E-162	NM_005524.3	HES1
LOC100513627	1535.176181	281.7609248	2.445860779	0.018123	High	1852	341	NM_001244860.1	ssc:100513627	0	NM_001009899.2	KIAA2018
LOC102164820	6.631430589	47.92414556	-2.853360671	0.018139	Low	8	58	XR_299650.1	ssc:102164820			
LOC100738599	5.802501765	43.7927537	-2.915945208	0.01817	Low	7	53	XP_003481459.1	ssc:100738599	1E-69	NM_015441.1	OLFML2B
CLVS2	4.973572942	39.66136184	-2.995379676	0.018182	Low	6	48	XP_001926095.1	ssc:100155458	0	NM_001010852.3	CLVS2
LOC100737927	105.2739606	18.17812418	2.533873392	0.018199	High	127	22	XP_005666030.1	ssc:100737927	0	NM_018417.4	ADCY10
LYPD4	1027.042812	190.8703039	2.427831755	0.018269	High	1239	231	NM_001195351.1	ssc:100359350	3E-106	NM_173506	LYPD4
LOC100517015	130.1418253	23.13579441	2.491886151	0.018271	High	157	28	XP_005674311.1	ssc:100517015	2E-28	NM_181795.2	PKIB
TCEAL4	60.51180412	323.0748433	-2.416579914	0.018318	Low	73	391	NM_001243430.1	ssc:100153499	3E-73	NM_024863.4	TCEAL4

LOC102167025	138.4311135	24.78835115	2.481434021	0.018323	High	167	30	XR_297533.1	ssc:102167025			
LRRC41	668.1166318	3756.261478	-2.491125616	0.018359	Low	806	4546	XP_005665515.1	ssc:100525052	0	NM_006369.4	LRRC41
FAM54A	1276.550388	236.3156143	2.433463623	0.018514	High	1540	286	NM_001190176.1	ssc:100157026	1E-138	NM_138419.3	MTFR2
FSBP	67.97216354	10.74161883	2.661732611	0.018567	High	82	13	XP_003355069.1	ssc:100624535	1E-171	NM_001256141.1	FSBP
LOC100627376	44.76215647	240.4470062	-2.425367516	0.018578	Low	54	291	XP_005655811.1	ssc:100627376	8E-54	NM_152342.2	CDYL2
NCKIPSD	150.0361171	801.4900206	-2.417374726	0.018651	Low	181	970	XP_003132242.1	ssc:100524278	0	NM_184231.2	NCKIPSD
CSF3R	18.23643412	0.826278372	4.464051943	0.018654	High	22	1	XP_003361917.1	ssc:100622336	0	NM_172313.2	CSF3R
LOC102157528	18.23643412	0.826278372	4.464051943	0.018654	High	22	1	XP_005672489.1	ssc:102157528			
TCP11	63.82751942	9.915340461	2.686444364	0.018673	High	77	12	XP_005665956.1	ssc:100154604	0	NM_018679.5	TCP11
LOC102161733	649.8801977	122.289199	2.409876803	0.018704	High	784	148	XP_005673209.1	ssc:102161733	3E-120	NM_015135.2	NHigh205
LOC102166969	59.6828753	9.089062089	2.715113707	0.0188	High	72	11	XP_005671818.1	ssc:102166969			
ANKRD34A	872.8620513	163.6031176	2.415553425	0.018871	High	1053	198	XP_005663579.1	ssc:102165219	8E-116	NM_001039888.3	ANKRD34A
FLNC	3566.880728	21473.32232	-2.589810541	0.018878	Low	4303	25988	XP_005657826.1	ssc:100518997	0	NM_001458.4	FLNC
ATRNL1	1090.041403	203.2644794	2.422952903	0.018896	High	1315	246	XP_005671535.1	ssc:100157106	0	NR_074088.1	ATRNL1
ZNF358	910.9927771	5164.239823	-2.503044479	0.018941	Low	1099	6250	ZNF358	ssc:100511368	0	NM_018083.4	ZNF358
KCNA5	55.53823118	8.262783717	2.74878142	0.018948	High	67	10	NM_001006593.2	ssc:474166			
MGPD	6237.689398	1093.992564	2.511408784	0.019009	High	7525	1324	XP_003483700.1	ssc:397348	0	NM_001083112.2	GPD2
SNAP91	19.06536294	109.0687451	-2.516211839	0.019051	Low	23	132	XP_005659462.1	ssc:100521763	0	NR_026669.1	SNAP91
LOC100627210	15.74964765	92.54317763	-2.554807084	0.019071	Low	19	112	XR_300528.1	ssc:100627210			
NUAK1	626.6701906	3493.504956	-2.478896913	0.019116	Low	756	4228	XP_003126141.3	ssc:100523669	0	NM_014840.2	NUAK1
CHST6	2163.50423	395.7873401	2.45057257	0.01919	High	2610	479	XP_005664396.1	ssc:100522551	0	NM_021615.4	CHST6
METTL11B	0.828928824	18.17812418	-4.454811294	0.019261	Low	1	22	NM_001243649.1	ssc:100157210	5E-156	NM_001136107.1	METTL11B
LOC100512345	0.828928824	18.17812418	-4.454811294	0.019261	Low	1	22	LOC100512345				
TMPRSS7	0.828928824	18.17812418	-4.454811294	0.019261	Low	1	22	XP_003483370.1	ssc:100627225	0	NR_026734.1	TMPRSS7
LOC102157527	11.60500353	71.88621834	-2.630968249	0.019298	Low	14	87	XP_005672099.1	ssc:102157527			
LOC100624445	1396.745068	259.4514087	2.428532452	0.01931	High	1685	314	XP_005658616.1	ssc:100624445	1E-40	NR_037669.1	GGCT
PSAP	8456.731858	52725.64918	-2.640332813	0.019369	Low	10202	63811	NM_001198919.1	ssc:100153167	0	NM_002778.2	PSAP
AHNAK	7997.50529	49721.30102	-2.6362421	0.019415	Low	9648	60175	XP_005674623.1	ssc:100515251	0	NM_024060.3	AHNAK
LOC100519435	151.6939747	805.6214124	-2.408938246	0.019419	Low	183	975	XP_005666938.1	ssc:100519435	4E-164	NM_199327.2	SPRY1
AVEN	67.97216354	358.6048133	-2.399378903	0.019586	Low	82	434	XP_005653590.1	ssc:102166979	2E-28	NM_020371.2	AVEN
LOC100627730	1676.92301	310.6806678	2.432312077	0.019735	High	2023	376	XP_003357326.2	ssc:100627730	1E-42	NM_004398.3	DDX10
SNX18	138.4311135	732.0826374	-2.402838272	0.019741	Low	167	886	XP_003134014.3	ssc:100518876	0	NM_052870.2	SNX18
LOC102159180	38.13072589	204.0907578	-2.420184951	0.019896	Low	46	247	XR_306363.1	ssc:102159180			
LOC100737841	7.460359412	51.22925905	-2.779650985	0.019922	Low	9	62	XP_003481563.1	ssc:100737841	7E-136	NM_004120.4	GBP2
LOC102159430	13.26286118	0	20	0.019925	High	16	0	XR_308051.1	ssc:102159430			
LOC102168198	13.26286118	0	20	0.019925	High	16	0	XR_302580.1	ssc:102168198			
LOC102161625	13.26286118	0	20	0.019925	High	16	0	XP_005654407.1	ssc:102161625			
LOC100524527	13.26286118	0	20	0.019925	High	16	0	LOC100524527	ssc:100524527			
LOC102164330	13.26286118	0	20	0.019925	High	16	0	XR_299613.1	ssc:102164330			
LOC102164340	13.26286118	0	20	0.019925	High	16	0	XR_301043.1	ssc:102164340			
LOC100738693	13.26286118	0	20	0.019925	High	16	0	XP_005668758.1	ssc:100738693	3E-58	NM_030753.4	WNT3
LOC100736688	13.26286118	0	20	0.019925	High	16	0	LOC100736688				
ANKRD65	13.26286118	0	20	0.019925	High	16	0	XP_005653409.1	ssc:100526225	1E-120	NM_001243536.1	ANKRD65
LOC102166491	13.26286118	0	20	0.019925	High	16	0	XR_307709.1	ssc:102166491			
LOC100627297	13.26286118	0	20	0.019925	High	16	0	XP_005672595.1	ssc:100627297	0	NM_000794.3	DRD1
TEX11	13.26286118	0	20	0.019925	High	16	0	XP_003484173.1	ssc:100739555			
LOC102158197	13.26286118	0	20	0.019925	High	16	0	XR_308293.1	ssc:102158197			
LOC102162625	13.26286118	0	20	0.019925	High	16	0	XR_304256.1	ssc:102162625			
LOC102162603	13.26286118	0	20	0.019925	High	16	0	XR_306597.1	ssc:102162603			
LOC102163108	13.26286118	0	20	0.019925	High	16	0	XR_302803.1	ssc:102163108			
PRSS42	13.26286118	0	20	0.019925	High	16	0	XP_005669534.1	ssc:100739775	1E-26	XP_003960378.2	PRSS44
BIN2	13.26286118	0	20	0.019925	High	16	0	XP_005655633.1	ssc:100519772	1E-167	NM_016293.2	BIN2
LOC102167228	13.26286118	0	20	0.019925	High	16	0	XP_005654396.1	ssc:102167228			
LOC100153663	13.26286118	0	20	0.019925	High	16	0	LOC100153663				
LOC100739539	13.26286118	0	20	0.019925	High	16	0	LOC100739539		8E-119	NM_003054.4	SLC18A2
GPR151	13.26286118	0	20	0.019925	High	16	0	XP_003354388.1	ssc:100621805	0	NM_194251.2	GPR151
URB2	4864.154337	867.5922903	2.487099867	0.019955	High	5868	1050	XP_001927969.1	ssc:100157881	0	NM_014777.2	URB2
HEXA	1633.818711	9426.183665	-2.528425881	0.019957	Low	1971	11408	NM_001123221.1	ssc:100142664	0	NM_000520.4	HEXA
KIF6	22.38107824	1.652556743	3.759507826	0.019963	High	27	2	XP_003128426.3	ssc:100514850	0	NM_145027.4	KIF6
LOC100738196	22.38107824	1.652556743	3.759507826	0.019963	High	27	2	XR_305081.1	ssc:100738196			
LOC102158385	22.38107824	1.652556743	3.759507826	0.019963	High	27	2	XR_301933.1	ssc:102158385			
LOC100512828	586.0526783	111.5476802	2.393371322	0.02002	High	707	135	XP_005661574.1	ssc:100512828	1E-140	NM_001167741.1	MTX3
ST3GAL2	159.1543341	841.9776608	-2.403355511	0.020057	Low	192	1019	XP_005658555.1	ssc:100627772	0	NM_006927.3	ST3GAL2
ERCC6L	1552.583687	289.1974301	2.424544397	0.020137	High	1873	350	XP_003360412.1	ssc:100622071	0	NM_017669.2	ERCC6L
LOC102163871	30.67036647	3.305113487	3.21407369	0.02022	High	37	4	XR_298108.1	ssc:102163871			
LOC102162432	30.67036647	3.305113487	3.21407369	0.02022	High	37	4	XP_005655798.1	ssc:102162432			
LOC102159194	26.52572236	2.478835115	3.419657824	0.02028	High	32	3	XR_308182.1	ssc:102159194			
LOC102164464	14.09179	83.45411554	-2.566128317	0.020324	Low	17	101	XR_303994.1	ssc:102164464			
LOC102162673	248.6786471	47.09786719	2.400549001	0.020345	High	300	57	XP_005664190.1	ssc:102162673	5E-45	NM_020793.1	MICAL3
LOC102166090	380.47833	72.71249671	2.387539049	0.02041	High	459	88	XP_005665407.1	ssc:102166090			
LCN15	58.85394648	9.089062089	2.694935825	0.020456	High	71	11	XP_005652785.1	ssc:100514209	6E-66	NM_203347.1	LCN15
PHF2	410.3197677	2227.64649	-2.440699742	0.020469	Low	495	2696	XP_005662327.1	ssc:100514572	0	NM_024517.1	PHF2
ZNF420	193.9693447	36.35624836	2.415553425	0.02047	High	234	44	ZNF420		0	NM_144689.3	ZNF420
MMP3	0	13.22045395	-20	0.020498	Low	0	16	NM_001166308.1	ssc:396769	0	NM_002422.3	MMP3
ADAMTS16	0	13.22045395	-20	0.020498	Low	0	16	XP_003134210.3	ssc:100519115	0	NM_139056.2	ADAMTS16
IL1B	0	13.22045395	-20	0.020498	Low	0	16	NM_214055.1	ssc:397122	7E-88	NM_000576.2	IL1B
LOC102163797	0	13.22045395	-20	0.020498	Low	0	16	XP_005667561.1	ssc:102163797	1E-57	NM_002017.4	FLI1
LOC102166940	0	13.22045395	-20	0.020498	Low	0	16	LOC102166940				
LOC100738368	0	13.22045395	-20	0.020498	Low	0	16	XP_005661330.1	ssc:100738368	0	NM_032447.3	FBN3
LOC100624063	0	13.22045395	-20	0.020498	Low	0	16	XR_130524.2	ssc:100624063			
PIH1D3	0	13.22045395	-20	0.020498	Low	0	16	XP_005654349.1	ssc:100516946	2E-88	NM_173494.1	PIH1D3
LUZP2	0	13.22045395	-20	0.020498	Low	0	16	XP_003122961.4	ssc:100520176	6E-67	NM_001252010.1	LUZP2
LOC102162870	0	13.22045395	-20	0.020498	Low	0	16	XR_297598.1	ssc:102162870			
SOWAHA	0	13.22045395	-20	0.020498	Low	0	16	XP_003123975.1	ssc:100524137	0	NM_175873.4	SOWAHA
FGF7	0	13.22045395	-20	0.020498	Low	0	16	XR_300983.1	ssc:397281	6E-52	NM_002009.3	FGF7
LOC102167273	0	13.22045395	-20	0.020498	Low	0	16	XR_298579.1	ssc:102167273			
LYPD5	0	13.22045395	-20	0.020498	Low	0	16	XP_005656005.1	ssc:100626406	2E-82	NM_182573.2	LYPD5
LOC100155760	0	13.22045395	-20	0.020498	Low	0	16	NM_001130238.1	ssc:100155760	8E-61	NM_002177.1	IFNW1
IKBIP	697.9580695	3866.98278	-2.469996067	0.020543	Low	842	4680	NM_001244129.1	ssc:100512167	0	NM_201613.2	IKBIP
LOC100623790	1.657857647	22.30951604	-3.750267178	0.020652	Low	2	27	XP_005669531.1	ssc:100623790	4E-109	NR_033815.1	ALS2CL
BTC	26.52572236	144.5987151	-2.446590787	0.020698	Low	32	175	XP_005666828.1	ssc:100505411	8E-68	NM_001729.2	BTC
LOC100514211	431.0429883	82.62783717	2.383131948	0.020753	High	520	100	XP_003122517.1	ssc:100514211			
LOC102160286	78.74823824	13.22045395	2.574475933	0.020852	High	95	16	XP_005668828.1	ssc:102160286	2E-18	NM_002390.4	ADAM11
DDX18	14011.38391	2406.948897	2.541322041	0.02091	High	16903	2913	XP_005654221.1	ssc:100153560	0	NM_006773.3	DDX18
LOC100518781	272.717583	52.05553742	2.389284175	0.020981	High	329	63	XP_005663728.1	ssc:100518781	5E-152	NM_206886.3	CCDC18
ZBTB44	2704.794751	497.4195798	2.44298392	0.021082	High	3263	602	ZBTB44	ssc:100518028	0	NM_014155.4	ZBTB44
LOC100514611	151.6939747	28.09346464	2.432873321	0.021159	High	183	34	XP_005673158.1	ssc:100514611	7E-112	NM_170606.2	KMT2C
LOC100622080	238.7315012	45.44531045	2.393185612	0.02119	High	288	55	XP_005658656.1	ssc:100622080	3E-37	NM_014317.3	PDSS1
LOC100521322	259.4547218	49.5767023	2.387748576	0.021312	High	313	60	XR_304407.1	ssc:100521322			
LOC100525275	847.9941865	161.9505609	2.38850091	0.021341	High	1023	196	XP_005660916.1	ssc:100525275	1E-64	NM_148975.2	MS4A4A

SNX15	99.47145883	517.2502607	-2.378507927	0.021449	Low	120	626	XP_005660775.1	ssc:100515425	2E-119	NM_147777.3	SNX15
CELF3	46.42001412	6.610226974	2.811975246	0.02145	High	56	8	XP_005663502.1	ssc:100515828	0	NM_007185	CELF3
LOC102165737	780.8509518	149.5563853	2.384357687	0.021509	High	942	181	XP_005669144.1	ssc:102165737	5E-81	NM_016231.4	NLK
ANKLE1	518.9094436	99.97968298	2.375775934	0.021543	High	626	121	XP_005658082.1	ssc:100620785	8E-174	NR_103530.1	ANKLE1
TREX1	111.0764624	577.5685818	-2.378439131	0.021599	Low	134	699	XP_005669584.1	ssc:100155669	5E-124	NM_033629	TREX1
LOC100625556	407.0040524	78.49644531	2.37434393	0.021604	High	491	95	XP_005663202.1	ssc:100625556	9E-94	NM_199351.2	ILDR2
LOC100737019	125.9971812	656.0650272	-2.380447359	0.021639	Low	152	794	LOC100737019				
KLHL8	466.6869277	90.06434252	2.373427112	0.021695	High	563	109	XP_003129388.4	ssc:100521566	0	NM_020803.3	KLHL8
LOC102160307	86.20859765	14.87301069	2.535135041	0.021763	High	104	18	XP_005663712.1	ssc:102160307			
HSD11B2	62.16966177	9.915340461	2.648476514	0.021893	High	75	12	NM_213913.1	ssc:396948	0	NM_000196.3	HSD11B2
ATG16L2	1700.133017	319.7697299	2.410542345	0.021985	High	2051	387	XP_003129670.1	ssc:100511396	0	NM_033388.1	ATG16L2
IGDCC4	910.1638483	5046.908295	-2.471201683	0.022029	Low	1098	6108	XP_001926606.1		0	NM_020962.1	IGDCC4
WBP1L	1359.443271	7666.210733	-2.495497707	0.022099	Low	1640	9278	XP_005657509.1	ssc:100151782	3E-176	NM_017787.4	WBP1L
EDARADD	3924.149051	716.3833483	2.453576132	0.02215	High	4734	867	NM_001243663.1	ssc:100157377	5E-94	NM_145861.2	EDARADD
LOC100737752	212.2057788	40.48764021	2.38991048	0.02215	High	256	49	XP_005658717.1	ssc:100737752	0	NM_020716.2	GRAMD1B
NHigh160	5974.918961	1072.509327	2.477928952	0.022172	High	7208	1298	XP_003122865.3	ssc:100513973	0	NM_015231.1	NHigh160
MTHFD2	6672.048101	1192.31969	2.48435858	0.022189	High	8049	1443	XP_003481227.1	ssc:100737728	5E-40	NR_027405.1	MTHFD2
DBT	1815.354124	341.2529675	2.411337507	0.022238	High	2190	413	XP_003481553.1	ssc:100156530	0	NM_001918.3	DBT
ZFP36	76.26145177	394.1347833	-2.369663176	0.022256	Low	92	477	NM_001168419.1	ssc:100316849	1E-127	NM_003407.3	ZFP36
LOC100623625	81.23502471	419.7494128	-2.369354518	0.022261	Low	98	508	XP_003355955.1	ssc:100623625	1E-127	NM_003407.3	ZFP36
C9H11orf71	58.02501765	9.089062089	2.674471723	0.022269	High	70	11	XP_003129932.1	ssc:100523855	2E-32	NM_019021.3	C11orf71
RAB26	130.1418253	23.96207278	2.441260078	0.022277	High	157	29	XP_005655223.1	ssc:100516791	5E-131	NM_014353.4	RAB26
PHF11	1141.43499	6380.521586	-2.48282567	0.022278	Low	1377	7722	NM_001244327.1	ssc:100518446	2E-96	NM_016119.1	PHF11
LOC102157769	589.3683936	114.0264153	2.369801617	0.02232	High	711	138	XP_005657440.1	ssc:102157769	2E-144	NR_102264.1	DNA2
IKBKE	158.3254053	823.7995366	-2.379400542	0.022361	Low	191	997	XP_003482709.1	ssc:100519508	0	NM_014002.3	IKBKE
LOC100511078	877.0066954	168.5607878	2.379318894	0.02239	High	1058	204	XP_003126086.1	ssc:100511078	0	NM_014291.3	GCAT
LOC102161657	38.13072589	4.95767023	2.94321978	0.022408	High	46	6	LOC102161657				
TBX10	38.13072589	4.95767023	2.94321978	0.022408	High	46	6	XP_005660675.1	ssc:100524845	1E-34	NM_005995.4	TBX10
ABHD14B	294.2697324	1556.708452	-2.403287711	0.022506	Low	355	1884	XP_005669680.1	ssc:100621118	9E-103	NM_032750.2	ABHD14B
CNIH3	73.7746653	12.39417558	2.57346316	0.022534	High	89	15	XP_003130580.3	ssc:100522393	5E-90	NM_152495.1	CNIH3
LOC102160998	73.7746653	12.39417558	2.57346316	0.022534	High	89	15	XP_005670644.1	ssc:102160998			
LOC100525698	880.3224107	169.3870662	2.377708276	0.022572	High	1062	205	XP_003126081.1	ssc:100525698	0	NM_014291.3	GCAT
TMEM26	22.38107824	122.289199	-2.449945539	0.022575	Low	27	148	NM_001244582.1	ssc:100157089	6E-172	NM_178505.6	TMEM26
PUSL1	1091.699261	209.048428	2.38466638	0.022663	High	1317	253	XP_005653396.1	ssc:100524091	9E-139	NM_153339.1	PUSL1
LOC102162175	190.6536294	36.35624836	2.390678757	0.022832	High	230	44	XP_005668030.1	ssc:102162175	6E-45	NM_022366.2	TFB2M
LRRTM4	19.06536294	105.7636316	-2.47181772	0.023051	Low	23	128	XP_005662488.1	ssc:100525866	0	NM_024993.4	LRRTM4
TRIM62	121.8525371	627.9715625	-2.365562939	0.023071	Low	147	760	XP_005665260.1	ssc:100519905	0	NM_018207.2	TRIM62
PRR5	193.1404159	1004.7545	-2.379121044	0.02311	Low	233	1216	NM_001204382.1	ssc:100152201	0	NM_181333.3	PRR5
DIP2C	567.8162442	3063.013924	-2.431455898	0.023259	Low	685	3707	XP_005668325.1	ssc:100523564	0	NM_014974.2	DIP2C
LOC100623959	4.973572942	38.0088051	-2.933979131	0.023281	Low	6	46	XP_005674164.1	ssc:100623959	1E-33	NM_153343.3	ENPP6
LAD1	4.973572942	38.0088051	-2.933979131	0.023281	Low	6	46	XP_005668054.1	ssc:100622691	2E-129	NM_005558.3	LAD1
LOC100623096	242.0472165	1265.858465	-2.386755699	0.023292	Low	292	1532	XR_305640.1	ssc:100623096			
TGFBR3L	81.23502471	14.04673232	2.531867327	0.023384	High	98	17	XP_005661318.1	ssc:100520601	7E-67	NM_001195259.1	TGFBR3L
IL15RA	32.32822412	170.2133446	-2.396477984	0.023439	Low	39	206	XP_005668268.1	ssc:733692	1E-54	NR_046362.1	IL15RA
LOC100621687	1624.700494	309.028111	2.394363803	0.023506	High	1960	374	XP_005669513.1	ssc:100621687	0	NM_022405.3	SLC6A20
DEPDC1B	1133.974631	218.1374901	2.37807872	0.023543	High	1368	264	XP_005654276.1	ssc:100524052	0	NM_018369.2	DEPDC1B
LOC102164674	29.84143765	3.305113487	3.174545326	0.023601	High	36	4	LOC102164674				
LOC102166164	29.84143765	3.305113487	3.174545326	0.023601	High	36	4	XR_302445.1	ssc:102166164			
RBM47	116.8789641	21.48323766	2.443731959	0.023643	High	141	26	XP_005666667.1	ssc:100513606	0	NM_019027.3	RBM47
LOC100153192	191.4825583	992.3603244	-2.37365107	0.023658	Low	231	1201	XP_005656320.1	ssc:100153192	0	NM_014272.3	ADAMTS7
LOC100626689	72.94573648	373.477824	-2.356127019	0.023674	Low	88	452	XP_005658428.1	ssc:100626689	0	NM_001610.2	ACP2
APLP1	50.56465824	260.2776871	-2.363850356	0.023783	Low	61	315	XP_005664574.1	ssc:100524259	1E-73	NM_005166.3	APLP1
MTR	3269.29528	607.3146032	2.428463721	0.023803	High	3944	735	XP_001927093.1	ssc:397300	0	NM_000254.2	MTR
LOC100520306	498.186223	97.50084786	2.353198456	0.02382	High	601	118	XP_005663986.1	ssc:100520306	1E-135	NM_024779.4	PIP4K2C
ADAM5	17.4075053	0.826278372	4.396937747	0.02383	High	21	1	XP_005671807.1	ssc:100525189	4E-176	NM_014237.2	ADAM18
LOC100621245	1447.309726	8085.133867	-2.481897968	0.024019	Low	1746	9785	XP_005658933.1	ssc:100621245	2E-125	NM_080881.2	DBN1
STX1A	98.64253001	504.0298068	-2.353227345	0.024019	Low	119	610	XR_302112.1	ssc:100512594			
SMARCA1	363.0708257	1915.313266	-2.399257467	0.024076	Low	438	2318	XP_005673953.1	ssc:100188905	0	NM_139035.2	SMARCA1
TMEM151A	33.15715294	173.5184581	-2.387697098	0.024103	Low	40	210	XP_003122547.1	ssc:100520884	0	NM_153266.3	TMEM151A
SLC2A6	65.48537706	334.6427406	-2.353377025	0.024114	Low	79	405	XP_005654664.1	ssc:100627201	0	NM_017585.3	SLC2A6
6-Sep	13959.99032	2446.610259	2.512441735	0.024146	High	16841	2961	XP_005673925.1	ssc:100515873	0	NM_145802.3	6-Sep
LOC102159824	7.460359412	49.5767023	-2.73234527	0.024174	Low	9	60	XR_298929.1	ssc:102159824			
GPC2	116.0500353	593.2678709	-2.353936693	0.024203	Low	140	718	XP_003124388.1	ssc:100511316	0	NM_152742.1	GPC2
LOC102158126	57.19608883	9.089062089	2.653713162	0.024254	High	69	11	XP_005667853.1	ssc:102158126	5E-40	NR_073544.1	AXDND1
LOC102160482	57.19608883	9.089062089	2.653713162	0.024254	High	69	11	XP_005658654.1	ssc:102160482			
LOC102166466	43.10429883	222.268882	-2.36640232	0.02433	Low	52	269	XP_005653807.1	ssc:102166466	0	NM_031935.2	HMCN1
LOC102166035	18.23643412	100.8059614	-2.466685395	0.024399	Low	22	122	XR_306634.1	ssc:102166035			
LOC102159817	21.55214941	1.652556743	3.705060042	0.024491	High	26	2	XR_297940.1	ssc:102159817			
DMKN	21.55214941	1.652556743	3.705060042	0.024491	High	26	2	XP_005664543.1	ssc:100515381	6E-70	NR_033746.1	DMKN
LOC102164221	21.55214941	1.652556743	3.705060042	0.024491	High	26	2	LOC102164221				
LRGUK	21.55214941	1.652556743	3.705060042	0.024491	High	26	2	XP_003134708.2	ssc:100526023	0	NM_144648.1	LRGUK
LOC100739717	278.5200847	54.53437253	2.352543628	0.024502	High	336	66	XR_135312.2	ssc:100739717			
LOC100739224	0.828928824	17.35184581	-4.387697098	0.024578	Low	1	21	XR_306070.1	ssc:100739224	2E-39	NM_000231.2	SGCG
KCNT2	1298.102538	250.3623466	2.374314838	0.024617	High	1566	303	XP_003130630.3	ssc:100519680	0	NM_198503.3	KCNT2
LOC102164620	4216.760926	779.1805045	2.436105743	0.024617	High	5087	943	XP_005672589.1	ssc:102164620	2E-15	NM_022132.4	MCCC2
LOC100622285	1421.612932	7905.831461	-2.475388489	0.024636	Low	1715	9568	XP_005657028.1	ssc:100622285	1E-165	NM_005082.4	TRIM25
EPHA7	7117.182879	1287.341703	2.466911264	0.024656	High	8586	1558	XP_005659360.1	ssc:100154222	0	NM_004440.3	EPHA7
LOC100518417	9.947145883	61.14459951	-2.619870541	0.024721	Low	12	74	XP_005654939.1	ssc:100518417	0	NM_152921.1	EMR2
LOC100621699	9.947145883	61.14459951	-2.619870541	0.024721	Low	12	74	XP_005674155.1	ssc:100621699	2E-31	NM_152417.2	TMEM68
ZNF382	48.90680059	250.3623466	-2.35591061	0.024775	Low	59	303	ZNF382	ssc:100525701	0	NM_032825.4	ZNF382
LOC100157134	6.631430589	45.44531045	-2.776739389	0.024791	Low	8	55	ZNF704	ssc:100157134	0	NM_032651.1	ZNF704
MAP3K5	111.9053912	20.65695929	2.437579732	0.024937	High	135	25	XP_005659242.1	ssc:100157454	4E-124	NM_005923.3	MAP3K5
ARPC1B	1154.697851	6348.29673	-2.458854171	0.02503	Low	1393	7683	NM_001134356.1	ssc:100174964			
LOC102162967	2.486786471	25.61462952	-3.364613485	0.025033	Low	3	31	XP_005658709.1	ssc:102162967	1E-70	NM_020721.1	KIAA1210
FAM98C	300.901163	1567.450071	-2.381057901	0.025049	Low	363	1897	XP_003127161.1	ssc:100516018	2E-142	NM_174905.3	FAM98C
LOC102162766	256.9679353	50.40298068	2.350007392	0.025092	High	310	61	XP_005670534.1	ssc:102162766	5E-80	NM_173683.3	XKR6
GABRR1	64.65644824	10.74161883	2.589582825	0.0251	High	78	13	XP_001927040.1	ssc:100153555	0	NM_002042.4	GABRR1
MAP3K14	243.7050741	1260.074517	-2.370300858	0.025142	Low	294	1525	XP_003131369.1	ssc:100520402	0	NM_003954.3	MAP3K14
CPEB1	147.5493306	28.09346464	2.392890914	0.025151	High	178	34	NM_001097510.1	ssc:100048944			
TRIB3	673.9191336	3618.27299	-2.424653868	0.025158	Low	813	4379	XP_003134414.1	ssc:100518810	2E-154	NM_021158.3	TRIB3
LOC100517307	15.74964765	88.41178578	-2.488919149	0.025218	Low	19	107	XP_003131833.3	ssc:100517307	0	NM_001045.5	SLC6A4
LOC102167622	9.11821706	57.01320765	-2.644472514	0.025263	Low	11	69	XP_005658828.1	ssc:102167622	4E-67	NM_014002.3	IKBKE
RHBDL1	200.6007753	1030.36913	-2.360762188	0.025289	Low	242	1247	XP_005662298.1	ssc:102164926	0	np_003952	np_003952
C1H6orf163	1.657857647	21.48323766	-3.695819394	0.025311	Low	2	26	XP_003121330.1	ssc:100517621	5E-156	NM_001010868.2	C6orf163
TCEAL5	1.657857647	21.48323766	-3.695819394	0.025311	Low	2	26	XP_005657950.1	ssc:100523940	2E-36	NM_001012979.2	TCEAL5
LOC102163805	1.657857647	21.48323766	-3.695819394	0.025311	Low	2	26	XP_005668659.1	ssc:102163805	0	NM_173628.3	DNAH17
LOC102160840	37.30179706	4.95767023	2.91151092	0.025411	High	45	6	XR_302886.1	ssc:102160840			
IKZF1	37.30179706	4.95767023	2.91151092	0.025411	High	45	6	XP_003130482.4	ssc:100515333	1E-120	NM_006060.4	IKZF1
HSD17B13	5.802501765	41.31391859	-2.831880943	0.025521	Low	7	50	NM_001243622.1	ssc:100626886	9E-149	NM_178135.4	HSD17B13
CCDC38	119.3657506	22.30951604	2.419657824	0.025549	High	144	27	XP_005664300.1	ssc:100157054	0	NM_182496.2	CCDC38

ILK	2985.801623	17074.21627	-2.515629138	0.025552	Low	3602	20664	XP_005656654.1	ssc:100623256	0	NM_004517.3	ILK
FANCM	1198.631079	233.0105008	2.362920809	0.025553	High	1446	282	XP_005659988.1	ssc:100155917	0	NM_020937.2	FANCM
LOC102166037	824.7841795	161.9505609	2.348463196	0.025597	High	995	196	LOC102166037				
GRAMD3	263.5993659	1361.706757	-2.368997248	0.025709	Low	318	1648	NM_001244333.1	ssc:100625925			
NDUFA4L2	70.45895001	356.1259782	-2.337532799	0.025741	Low	85	431	NM_001205377.1	ssc:100156288	3E-37	NM_020142.3	NDUFA4L2
LCP1	5144.332279	949.3938491	2.437905223	0.025833	High	6206	1149	XP_005668476.1	ssc:100156254	0	NM_002298.4	LCP1
ADAMTSL1	8.289288236	52.88181579	-2.673451581	0.0259	Low	10	64	XP_005660146.1	ssc:102160697			
KLHL25	1165.473926	227.2265522	2.358713395	0.025917	High	1406	275	XP_001928010.1	ssc:100153363	0	NM_022480.3	KLHL25
TXLNG	7476.937989	1359.227921	2.459660174	0.02593	High	9020	1645	XP_003135003.3	ssc:100518815	0	NM_018360.2	TXLNG
LOC100522963	3006.524843	567.6532414	2.405015065	0.02606	High	3627	687	XP_005673315.1	ssc:100522963	0	NM_015314.2	KIAA0895
CCDC36	571.1319595	113.2001369	2.334948414	0.026085	High	689	137	XR_306780.1	ssc:100526010	1E-169	NM_178173.3	CCDC36
LOC100739113	126.82611	23.96207278	2.404027172	0.026125	High	153	29	XP_005655235.1	ssc:100739113	5E-131	NM_014353.4	RAB26
IL17RD	73.7746653	371.8252673	-2.333427436	0.026161	Low	89	450	XP_003132314.3	ssc:100513377	0	NM_017563.3	IL17RD
TMEM151B	41.44644118	211.5272632	-2.351523486	0.026215	Low	50	256	XP_001929612.1	ssc:100157535	0	NM_001137560.1	TMEM151B
LOC102165243	162.4700494	31.39857813	2.371402655	0.02623	High	196	38	LOC102165243				
SLC16A1	7334.362231	1336.918405	2.455760102	0.026279	High	8848	1618	NM_001128445.1	ssc:100127159	1E-154	NM_003051.3	SLC16A1
LOC102159568	4.973572942	37.18252673	-2.902270271	0.026384	Low	6	45	XP_005661607.1	ssc:102159568	7E-67	NM_024717.4	MCTP1
LOC102158199	106.9318182	19.83068092	2.430885079	0.026413	High	129	24	XR_302257.1	ssc:102158199			
GRIK5	1870.892355	360.2573701	2.376626703	0.026433	High	2257	436	XP_001926247.1		0	NM_175768.3	GRIK2
PDHA1	33997.68677	5805.43184	2.549961296	0.026433	High	41014	7026	XP_003484129.1	ssc:100294678	0	NM_001173456.1	PDHA1
BRI3	602.6312548	3196.87102	-2.407313135	0.026437	Low	727	3869	XP_005661908.1	ssc:100627181	1E-37	NM_015379.4	BRI3
GRIP1	675.5769912	133.8570962	2.335426571	0.026463	High	815	162	XP_005664019.1	ssc:100514286	0	NM_021150.3	GRIP1
NFE2L2	1543.46547	8523.887683	-2.465338376	0.026477	Low	1862	10316	XP_005672039.1	ssc:100516343	0	NM_006164.4	NFE2L2
LOC100624199	44.76215647	227.2265522	-2.343779982	0.026601	Low	54	275	XP_005672073.1	ssc:100624199	8E-71	NM_014585.5	SLC40A1
CDS1	254.4811488	50.40298068	2.335977832	0.026674	High	307	61	NM_001044534.1	ssc:733582	0	NM_001263.3	CDS1
FAM105A	144.2336153	728.7775239	-2.337071025	0.026721	Low	174	882	XP_003133888.1	ssc:100518874	0	NM_019018.2	FAM105A
LOC100739107	1012.122094	199.1330876	2.345578473	0.026829	High	1221	241	XP_005667610.1	ssc:100739107	0	NM_003582.2	DYRK3
TAB3	2102.163497	404.0501238	2.3792687	0.026859	High	2536	489	XP_005673583.1	ssc:100517796	0	NM_198312.1	TAB3
MYD88	533.0012336	2806.04135	-2.396325492	0.026906	Low	643	3396	NM_001099923.1	ssc:396646	5E-146	NM_002468.4	MYD88
LOC100739776	68.80109236	345.3843594	-2.327699376	0.026909	Low	83	418	XP_005670275.1	ssc:100739776	3E-55	NM_033254.2	BOC
LOC100738613	401.2015506	80.14900206	2.323570719	0.027068	High	484	97	XP_003483004.1	ssc:100738613	0	NM_172232	ABCA5
URGCP	289.2961594	1486.474791	-2.361275921	0.027169	Low	349	1799	XP_005673385.1	ssc:100525140	0	NM_017920.3	URGCP
FAM177A1	190.6536294	966.7456949	-2.342182439	0.027252	Low	230	1170	NM_001190218.1	ssc:100155589	2E-105	NM_173607.3	FAM177A1
LAMA4	1692.672658	9344.382106	-2.464796269	0.027296	Low	2042	11309	XP_003121419.3	ssc:100154581	0	NM_002290.4	LAMA4
SLC30A2	12.43393235	0	20	0.027326	High	15	0	NM_001139475.1	ssc:100170651	2E-179	NM_032513.3	SLC30A2
LOC100622164	12.43393235	0	20	0.027326	High	15	0	LOC100622164				
TDRD5	12.43393235	0	20	0.027326	High	15	0	XP_005667855.1	ssc:100524163	0	NM_173533.3	TDRD5
C6H1orf177	12.43393235	0	20	0.027326	High	15	0	XP_003356502.2	ssc:100620608	0	NM_152607.2	C1orf177
LOC102157415	12.43393235	0	20	0.027326	High	15	0	XR_304075.1	ssc:102157415			
LOC100511546	12.43393235	0	20	0.027326	High	15	0	LOC100511546				
LOC102159971	12.43393235	0	20	0.027326	High	15	0	XR_309460.1	ssc:102159971			
LOC102163596	12.43393235	0	20	0.027326	High	15	0	XR_308838.1	ssc:102163596			
LOC100624651	12.43393235	0	20	0.027326	High	15	0	XP_003360632.1	ssc:100624651	1E-82	NM_173799.3	TIGIT
IDH1	12.43393235	0	20	0.027326	High	15	0	XP_005672200.1	ssc:100170146	0	NR_073601.1	PTH2R
PROK1	12.43393235	0	20	0.027326	High	15	0	NM_001172586.1	ssc:100359362	7E-49	NM_032414.2	PROK1
QRICH2	12.43393235	0	20	0.027326	High	15	0	XP_005656983.1	ssc:100519384	0	NM_032134.1	QRICH2
LOC100154433	12.43393235	0	20	0.027326	High	15	0	LOC100154433				
DNAJB7	12.43393235	0	20	0.027326	High	15	0	XP_003481581.2	ssc:100516609	2E-91	NM_145174.1	DNAJB7
DCLK3	12.43393235	0	20	0.027326	High	15	0	XP_005652450.1	ssc:100514320	0	NM_033403.1	DCLK3
LOC100621469	12.43393235	0	20	0.027326	High	15	0	XP_003483742.1	ssc:100621469	3E-65	NR_027690.1	CERKL
LOC100623971	12.43393235	0	20	0.027326	High	15	0	XP_003356400.2	ssc:100623971	2E-70	NM_182978.3	GNAL
LOC102160857	12.43393235	0	20	0.027326	High	15	0	XP_005657197.1	ssc:102160857	0	NM_175611.2	GRIK1
LOC102167024	12.43393235	0	20	0.027326	High	15	0	XR_303272.1	ssc:102167024			
LOC102160234	12.43393235	0	20	0.027326	High	15	0	XR_299061.1	ssc:102160234			
LOC102167050	12.43393235	0	20	0.027326	High	15	0	XR_300973.1	ssc:102167050			
GP91-PHOX	12.43393235	0	20	0.027326	High	15	0	NM_214043.1	ssc:397108	0	NM_000397.3	CYBB
LOC102158719	12.43393235	0	20	0.027326	High	15	0	XR_303137.1	ssc:102158719			
KLHDC7B	12.43393235	0	20	0.027326	High	15	0	NM_001206381.1	ssc:100517826	0	NM_138433.3	KLHDC7B
LOC102162534	12.43393235	0	20	0.027326	High	15	0	XR_307789.1	ssc:102162534			
TTLL8	12.43393235	0	20	0.027326	High	15	0	XP_005653253.1	ssc:100520675			
LOC100737191	12.43393235	0	20	0.027326	High	15	0	XP_003483873.1	ssc:100737191	3E-124	NM_001190787.1	MCIDAS
LOC100158035	12.43393235	0	20	0.027326	High	15	0	LOC100158035				
LOC102163344	12.43393235	0	20	0.027326	High	15	0	XR_308835.1	ssc:102163344			
LOC100515852	12.43393235	0	20	0.027326	High	15	0	XP_005668687.1	ssc:100515852	5E-42	NM_174892.3	CD300LB
LOC100517827	12.43393235	0	20	0.027326	High	15	0	XP_005664547.1	ssc:100517827	3E-59	NM_024916.1	CD22
AVP	12.43393235	0	20	0.027326	High	15	0	NM_213952.2	ssc:396995	4E-79	NM_000490.4	AVP
LBX2	12.43393235	0	20	0.027326	High	15	0	XP_003125045.1	ssc:100514830	3E-56	NM_001282430.1	LBX2
LOC100736836	36.47286824	185.9126336	-2.349729248	0.027432	Low	44	225	XP_005664407.1	ssc:100736836	2E-72	NM_152649.2	MLKL
LOC100157427	1259.142883	247.0572331	2.349524805	0.027476	High	1519	299	XR_130251.3	ssc:100157427			
CD38	269.4018677	53.70809416	2.326548419	0.02751	High	325	65	NM_001243883.1	ssc:100511702	1E-78	NM_001775.2	CD38
CPT1B	237.0736436	47.09786719	2.331601647	0.027533	High	286	57	NM_001007191.1	ssc:399528	1E-125	NM_152247.1	CPT1B
LOC100739623	6.631430589	44.61903207	-2.750267178	0.027551	Low	8	54	XP_005668532.1	ssc:100739623	7E-63	NM_015843.1	LMO7
LOC100156127	40.61751236	5.783948602	2.811975246	0.027564	High	49	7	XP_001927974.1	ssc:100156127	1E-67	NM_138720.2	HIST1H2BD
MPC2	7146.195388	1312.956333	2.444356425	0.027573	High	8621	1589	XP_005663197.1	ssc:100154777	8E-69	NR_026550.2	MPC2
LOC102165250	29.01250883	3.305113487	3.133903341	0.027592	High	35	4	LOC102165250				
BRWD3	3281.729213	623.0138923	2.397119963	0.027718	High	3959	754	XP_003135260.2	ssc:100520060	0	NM_153252.4	BRWD3
LOC102162482	63.82751942	318.9434515	-2.321050172	0.027842	Low	77	386	XR_305617.1	ssc:102162482			
LOC100621976	59.6828753	9.915340461	2.589582825	0.027879	High	72	12	XP_005666896.1	ssc:100621976	6E-106	NM_031956.2	TTC29
HPCAL4	59.6828753	9.915340461	2.589582825	0.027879	High	72	12	NM_001123043.1	ssc:100144585	3E-110	NM_016257.3	HPCAL4
LOC100624061	1134.80356	223.9214387	2.341378014	0.027948	High	1369	271	XP_003361927.1	ssc:100624061	0	NM_005513.2	GTF2E1
ZNF394	59.6828753	298.2864922	-2.321309701	0.027982	Low	72	361	ZNF394	ssc:100522485	2E-115	NM_032164.2	ZNF394
LRFN2	129.3128965	24.78835115	2.383131948	0.027989	High	156	30	XP_001928805.1	ssc:100153249	0	NM_020737.1	LRFN2
LOC100516697	49.73572942	249.5360683	-2.326893819	0.028016	Low	60	302	LOC100516697	ssc:100516697			
LOC100518118	58.02501765	290.0237085	-2.321423879	0.028054	Low	70	351	XP_005662185.1	ssc:100518118	5E-44	NM_003498.5	SNN
SCN7A	0	12.39417558	-20	0.028072	Low	0	15	XP_005671956.1	ssc:100516881	0	NR_045628.1	SCN7A
C14H10orf90	0	12.39417558	-20	0.028072	Low	0	15	XP_005671628.1	ssc:100156286	0	NM_001004298.2	C10orf90
LOC102159171	0	12.39417558	-20	0.028072	Low	0	15	XR_299090.1	ssc:102159171			
LOC102161946	0	12.39417558	-20	0.028072	Low	0	15	XP_005673430.1	ssc:102161946	0	NM_198455.2	SSPO
LOC102164222	0	12.39417558	-20	0.028072	Low	0	15	XR_297252.1	ssc:102164222			
IL2RB	0	12.39417558	-20	0.028072	Low	0	15	XP_003355391.2	ssc:100621776	0	NM_000878.3	IL2RB
LOC102160757	0	12.39417558	-20	0.028072	Low	0	15	XR_298052.1	ssc:102160757			
SH3TC1	0	12.39417558	-20	0.028072	Low	0	15	XP_003128892.4	ssc:100522812	2E-148	NM_018986.3	SH3TC1
LOC102162948	0	12.39417558	-20	0.028072	Low	0	15	LOC102162948				
MIR199A-1	0	12.39417558	-20	0.028072	Low	0	15	NR_038596.1	ssc:100526401			
CCL4	0	12.39417558	-20	0.028072	Low	0	15	NM_213779.1	ssc:396668	9E-43	NM_002984.2	CCL4
LOC102165710	0	12.39417558	-20	0.028072	Low	0	15	XR_308934.1	ssc:102165710			
CXHXorf64	0	12.39417558	-20	0.028072	Low	0	15	XP_003484202.1	ssc:100737612	2E-95	NM_001122716.1	CXorf64
LOC100737539	0	12.39417558	-20	0.028072	Low	0	15	XR_297239.1	ssc:100737539	5E-98	NR_103766.1	MDGA2
LOC100737634	0	12.39417558	-20	0.028072	Low	0	15	XP_005667829.1	ssc:100737634	0	NM_014141.5	CNTNAP2
LOC102167920	0	12.39417558	-20	0.028072	Low	0	15	XR_301485.1	ssc:102167920			

LOC102167901	0	12.39417558	-20	0.028072	Low	0	15	XR_304632.1	ssc:102167901			
ADM5	382.1361877	76.84388857	2.314084454	0.028234	High	461	93	NM_001099939.1	ssc:100101476			
HighK3B	377.9915436	76.0176102	2.313948382	0.028257	High	456	92	XP_005661998.1	ssc:100511685	3E-107	NM_182684.1	HighK3B
LOC100738549	493.21265	99.15340461	2.314475587	0.028313	High	595	120	XP_003484366.2	ssc:100738549	0	NM_030625.2	TET1
LOC102163690	67.14323471	11.5678972	2.537115405	0.028395	High	81	14	XR_305166.1	ssc:102163690			
WNT11	67.14323471	11.5678972	2.537115405	0.028395	High	81	14	XP_005667195.1	ssc:100521912	0	NM_004626.2	WNT11
LOC102165085	276.0332983	55.36065091	2.317909501	0.02844	High	333	67	XP_005661556.1	ssc:102165085	3E-151	NM_006633.3	IQGAP2
LOC102159125	3.315715294	28.91974301	-3.124662693	0.028581	Low	4	35	XR_304469.1	ssc:102159125			
GAB3	3.315715294	28.91974301	-3.124662693	0.028581	Low	4	35	XP_005674103.1	ssc:100518409	4E-145	NR_104114.1	GAB3
11-Mar	3.315715294	28.91974301	-3.124662693	0.028581	Low	4	35	XP_003133899.3	ssc:100521230	1E-81	NM_001102562.1	11-Mar
LOC102161691	131.799683	657.7175839	-2.319121341	0.028626	Low	159	796	XP_005672700.1	ssc:102161691	8E-41	NM_020749.4	MTUS1
OAF	484.9233618	2519.322755	-2.377207296	0.028645	Low	585	3049	XP_003130002.2	ssc:100522940	1E-132	NM_178507.2	OAF
PROCR	55.53823118	276.8032545	-2.317307771	0.028711	Low	67	335	NM_001163406.1	ssc:654289	9E-94	NM_006404.4	PROCR
PHF19	62.99859059	313.1595029	-2.3135062	0.0288	Low	76	379	XP_001925612.3	ssc:100156643	0	NR_104601.1	PHF19
LRRC8C	53.88037353	268.5404708	-2.317307771	0.02883	Low	65	325	XR_303293.1	ssc:100156403	0	NM_032270.4	LRRC8C
MDFIC	293.4408036	1494.737574	-2.348750818	0.028843	Low	354	1809	XP_005673288.1	ssc:100192442	9E-117	NM_199072.4	MDFIC
C2H11orf35	36.47286824	4.95767023	2.879089442	0.028846	High	44	6	XP_005652855.1	ssc:100514751	9E-130	NM_173573.2	C11orf35
LOC102161729	24.86786471	2.478835115	3.326548419	0.028893	High	30	3	XR_301795.1	ssc:102161729			
LOC100154078	24.86786471	2.478835115	3.326548419	0.028893	High	30	3	LOC100154078				
TMEM116	24.86786471	2.478835115	3.326548419	0.028893	High	30	3	XR_299421.1	ssc:100156224	5E-151	NM_138341.2	TMEM116
GPER1	19.89429177	105.7636316	-2.410417175	0.028916	Low	24	128	XP_005655115.1	ssc:100521003	3E-169	NM_001505.2	GPER1
LOC100739280	574.4476748	115.678972	2.31204885	0.028927	High	693	140	XP_005671858.1	ssc:100739280	2E-73	NM_013964.3	NRG1
MTX3	454.2529953	91.71689926	2.308236541	0.029005	High	548	111	XP_005658838.1	ssc:100621057	5E-140	NM_001167741.1	MTX3
LOC100625581	97.81360119	485.0254042	-2.309953319	0.02908	Low	118	587	XP_005654702.1	ssc:100625581	1E-45	NM_014578.3	RHOD
POLE	1403.376498	276.8032545	2.341969297	0.029121	High	1693	335	XP_005658704.1	ssc:100620959	0	NM_006231.3	POLE
GXYLT2	79.57716707	394.1347833	-2.308262631	0.029165	Low	96	477	XP_005669783.1	ssc:100511227	0	NM_001080393.1	GXYLT2
LOC100622217	43.93322769	219.7900469	-2.322741657	0.029246	Low	53	266	XP_003357782.2	ssc:100622217	0	NM_005204.3	MAP3K8
LOC102165864	145.891473	726.2986887	-2.315667412	0.029433	Low	176	879	XP_005658088.1	ssc:102165864	2E-50	NM_033271.2	BTBD6
JHigh	2242.252468	12395.82813	-2.466834024	0.029457	Low	2705	15002	NM_214323.1	ssc:397592	0	NM_021991.2	JHigh
MDGA1	420.2669136	85.10667229	2.303961734	0.029504	High	507	103	XP_001924602.1	ssc:397529	0	NM_153487.3	MDGA1
LOC100739240	11.60500353	66.92854811	-2.527874756	0.029582	Low	14	81	XP_003481233.1	ssc:100739240	1E-67	NM_003494.3	DYSF
C18H7orf10	58.02501765	287.5448734	-2.309040155	0.029597	Low	70	348	XR_309160.1	ssc:102168155	8E-50	NM_024728.2	SUGCT
LOC100522323	2282.041051	443.7114856	2.362630941	0.029668	High	2753	537	XP_003127755.1	ssc:100522323	5E-62	NM_004102.3	FABP3
C16H5orf49	50.56465824	251.188625	-2.312569852	0.029713	Low	61	304	XP_003134195.3	ssc:100516406	2E-65	NM_001089584.2	C5orf49
SLC5A4	62.16966177	307.3755543	-2.305719796	0.02982	Low	75	372	NM_214182.1	ssc:397376	0	NM_014227.2	SLC5A4
B4GALNT1	2.486786471	24.78835115	-3.317307771	0.029883	Low	3	30	XP_005674440.1	ssc:100516440	6E-88	NM_001478.4	B4GALNT1
PKIB	281.8358	57.01320765	2.305486804	0.029898	High	340	69	XR_300705.1	ssc:100738438			
LOC102162044	51.39358706	8.262783717	2.63688854	0.029912	High	62	10	XP_005668029.1	ssc:102162044	5E-79	NM_016002.2	SCCPDH
ZACN	143.4046865	711.4256781	-2.310620875	0.030029	Low	173	861	ZACN	ssc:100623825	3E-159	NM_180990.3	ZACN
LOC102162548	96.98467236	18.17812418	2.415553425	0.030065	High	117	22	XR_297670.1	ssc:102162548			
MFI2	74.60359412	367.6938754	-2.301188105	0.030107	Low	90	445	XP_001926353.5	ssc:100156949	0	NM_033316.3	MFI2
GALR2	20.72322059	1.652556743	3.648476514	0.030132	High	25	2	XP_003484361.2	ssc:100623909	4E-167	NM_003857.3	GALR2
LOC102159553	20.72322059	1.652556743	3.648476514	0.030132	High	25	2	XR_305677.1	ssc:102159553			
LOC102157426	20.72322059	1.652556743	3.648476514	0.030132	High	25	2	XR_305811.1	ssc:102157426			
LOC102159775	20.72322059	1.652556743	3.648476514	0.030132	High	25	2	XR_304482.1	ssc:102159775			
LOC102166737	20.72322059	1.652556743	3.648476514	0.030132	High	25	2	XR_309217.1	ssc:102166737			
GRIP2	20.72322059	1.652556743	3.648476514	0.030132	High	25	2	XP_005669868.1	ssc:100513882	0	NM_001080423.2	GRIP2
LOC102157766	20.72322059	1.652556743	3.648476514	0.030132	High	25	2	XR_299150.1	ssc:102157766			
LOC102158296	20.72322059	1.652556743	3.648476514	0.030132	High	25	2	XP_005662317.1	ssc:102158296	2E-43	NM_006648.3	WNK2
SLA-DMA	20.72322059	1.652556743	3.648476514	0.030132	High	25	2	NM_001113705.1	ssc:445528	9E-105	NM_006120.3	HLA-DMA
KIAA0020	6841.97851	1276.600084	2.422106925	0.030328	High	8254	1545	XP_005660193.1	ssc:100152831	0	NM_014878.4	KIAA0020
GRIN2D	369.7022553	75.19133183	2.297725584	0.030335	High	446	91	XP_003127322.2	ssc:100521797	0	NM_000836.2	GRIN2D
PTCH1	1635.476569	323.0748433	2.339770768	0.030451	High	1973	391	XP_005668079.1	ssc:100627504	0	NM_001083607.1	PTCH1
GALNTL1	292.6118747	1477.385729	-2.335986324	0.030528	Low	353	1788	NM_001244918.1	ssc:100513992	0	NM_020692.2	GALNT16
CHST11	53.88037353	266.0616357	-2.303928741	0.030538	Low	65	322	XP_001926288.4	ssc:100157186	6E-171	NM_018413.5	CHST11
LOC102165977	16.57857647	0.826278372	4.326548419	0.030575	High	20	1	XP_005674480.1	ssc:102165977	3E-43	NM_138804.4	M1AP
TAX1BP3	1436.533651	7736.444394	-2.429078879	0.030689	Low	1733	9363	XP_003358250.1	ssc:100515576	3E-68	NM_014604.3	TAX1BP3
LOC100738903	111.9053912	21.48323766	2.380996203	0.030791	High	135	26	XP_005669932.1	ssc:100738903	0	NM_031913.3	ESYT3
KLF8	288.4672306	58.66576439	2.297816701	0.030803	High	348	71	NM_001190250.1	ssc:100154631			
LOC102159733	73.7746653	13.22045395	2.480353755	0.030815	High	89	16	XP_005662590.1	ssc:102159733			
LOC102166308	12.43393235	70.2336616	-2.497880016	0.030876	Low	15	85	XR_299392.1	ssc:102166308			
LOC102166206	181.5354124	36.35624836	2.319975765	0.030946	High	219	44	XR_301418.1	ssc:102166206			
ISCA2	373.0179706	1895.482585	-2.34524816	0.030991	Low	450	2294	XP_005656467.1	ssc:100738266	9E-68	NM_194279.3	ISCA2
LOC100625130	100.3003877	19.00440255	2.399921606	0.031004	High	121	23	XP_005653615.1	ssc:100625130	0	NM_001145292.1	PDE6B
LOC100154508	81.23502471	14.87301069	2.449405167	0.031091	High	98	18	XP_005665717.1	ssc:100154508	1E-68	NM_021065.3	HIST1H2AD
C14H10orf71	1.657857647	20.65695929	-3.639235865	0.031108	Low	2	25	XP_005671275.1	ssc:100521158	0	NM_199459.3	C10orf71
WDR78	1.657857647	20.65695929	-3.639235865	0.031108	Low	2	25	XP_003361770.1	ssc:100620493			
LOC102160082	1.657857647	20.65695929	-3.639235865	0.031108	Low	2	25	XR_301372.1	ssc:102160082			
SGCA	1.657857647	20.65695929	-3.639235865	0.031108	Low	2	25	NM_001144122.1	ssc:100240723	0	NM_001135697.1	SGCA
MAGI2	23.21000706	119.8103639	-2.367933844	0.0312	Low	28	145	XP_005667758.1	ssc:100515047	0	NM_012301.3	MAGI2
LOC102160676	47.24894295	7.436505346	2.667585337	0.031234	High	57	9	XR_303981.1	ssc:102160676			
LOC102162772	1204.433581	241.2732845	2.319614753	0.031273	High	1453	292	XR_302038.1	ssc:102162772			
IFLTD1	2048.283123	403.2238454	2.344762282	0.031403	High	2471	488	XR_297355.1	ssc:100627442	5E-150	NM_152590.3	IFLTD1
LOC102163948	54.70930236	9.089062089	2.589582825	0.031418	High	66	11	XR_308719.1	ssc:102163948			
LOC102167708	54.70930236	9.089062089	2.589582825	0.031418	High	66	11	XR_308346.1	ssc:102167708			
LOC102162885	0.828928824	16.52556743	-4.317307771	0.031496	Low	1	20	XR_299754.1	ssc:102162885			
TMEM38B	707.0762865	143.7724367	2.29807876	0.031507	High	853	174	NM_001190164.1	ssc:100153669			
BAG4	464.2001412	95.02201275	2.28841329	0.031671	High	560	115	XP_005671834.1	ssc:100738485	7E-173	NM_004874.3	BAG4
FAM101B	116.0500353	568.4795197	-2.292361413	0.031785	Low	140	688	XP_003131866.1	ssc:100524216	2E-76	NM_182705.2	FAM101B
DAPK3	1422.441861	7616.634031	-2.420783891	0.031795	Low	1716	9218	XP_005661401.1	ssc:100624415	0	NM_001348.1	DAPK3
LOC102167911	182.3643412	901.4697036	-2.305455348	0.03185	Low	220	1091	XR_299980.1	ssc:102167911			
LOC102163104	1257.485025	6692.854811	-2.412080498	0.031906	Low	1517	8100	XP_005673058.1	ssc:102163104	0	NM_198596.2	SULF2
LOC100739361	590.1973224	120.6366423	2.290529196	0.03192	High	712	146	LOC100739361				
PURA	211.37685	1048.547254	-2.310502593	0.031979	Low	255	1269	XP_005661787.1	ssc:100625926	0	NM_005859.4	PURA
LOC100625959	5.802501765	39.66136184	-2.772987254	0.032138	Low	7	48	XP_003360914.2	ssc:100625959	7E-96	NM_006558.1	KHDRBS3
HS1BP3	499.0151518	2549.895055	-2.353282345	0.03217	Low	602	3086	XP_003354961.2	ssc:100625429	3E-164	NM_022460.3	HS1BP3
PPIP5K2	5945.077523	1125.391142	2.401269109	0.032172	High	7172	1362	XP_003354281.3	ssc:100625306	0	NR_074081.1	PPIP5K2
LOC102166531	382.1361877	78.49644531	2.283387656	0.032256	High	461	95	XR_309223.1	ssc:102166531			
SARDH	28.18358	3.305113487	3.092083166	0.032306	High	34	4	NM_001253922.1	ssc:100154338	0	NM_007101.3	SARDH
LOC102162207	28.18358	3.305113487	3.092083166	0.032306	High	34	4	XR_303551.1	ssc:102162207			
RBPMS2	1786.341615	355.2996998	2.329899629	0.032553	High	2155	430	XP_005659552.1	ssc:100515517	3E-96	NM_194272.1	RBPMS2
TFAM	2058.230269	407.3552373	2.337045034	0.032596	High	2483	493	NM_001130211.1	ssc:397279			
LOC102163320	297.5854477	61.14459951	2.283006993	0.032692	High	359	74	XR_304340.1	ssc:102163320			
CDH2	179.0486259	880.8127443	-2.298483896	0.032743	Low	216	1066	HighI00028F4AC1		0	NM_001792.3	CDH2
LOC102159081	35.64393941	4.95767023	2.845922578	0.032776	High	43	6	LOC102159081				
CYP21A2	58.02501765	9.915340461	2.548940841	0.032816	High	70	12	NM_214433.1	ssc:403337	0	NM_001128590.3	CYP21A2
LHFPL3	58.02501765	9.915340461	2.548940841	0.032816	High	70	12	XP_005667775.1	ssc:100738576	5E-42	NM_199000.2	LHFPL3
LOC100623666	43.10429883	6.610226974	2.705060042	0.032838	High	52	8	XP_005667718.1	ssc:100623666	2E-132	NM_001101426.3	ISPD
FMNL1	179.0486259	879.9864659	-2.297129889	0.032939	Low	216	1065	XP_005668775.1	ssc:100520585	0	NM_052905.3	FMNL2
EPX	87.8664553	16.52556743	2.410612684	0.033148	High	106	20	XP_003131690.1	ssc:100514365	0	NM_000502.4	EPX

LOC100517259	72.11680765	349.5157512	-2.276950033	0.033456	Low	87	423	XP_005674088.1	ssc:100517259	9E-58	NM_014221.3	MTCP1
CHML	1606.46406	322.248565	2.317642866	0.033605	High	1938	390	XP_003357684.1	ssc:100521677	0	NM_001821.3	CHML
ITGA10	396.2279777	1992.157154	-2.329928803	0.033691	Low	478	2411	XP_003125838.2	ssc:100511507	0	NM_003637.3	ITGA10
ANK3	4651.119629	895.685755	2.37651348	0.033725	High	5611	1084	XP_005671069.1	ssc:100154687	0	NM_020987.3	ANK3
MIC-1	23.21000706	118.1578072	-2.34789609	0.033893	Low	28	143	MIC-1				
IRF5	4.973572942	35.52996998	-2.83668193	0.033982	Low	6	43	XP_005657821.1	ssc:100515633	0	NM_032643.3	IRF5
LOC102166028	479.12086	99.15340461	2.272655411	0.033983	High	578	120	XR_305096.1	ssc:102166028			
KIAA1211L	6.631430589	42.96647533	-2.695819394	0.034092	Low	8	52	XP_005655258.1	ssc:100526102	0	NM_207362.2	KIAA1211L
SHD	6.631430589	42.96647533	-2.695819394	0.034092	Low	8	52	XP_003123110.1	ssc:100523303	1E-162	NM_020209.3	SHD
HOPX	108.5896759	525.5130444	-2.274839629	0.03411	Low	131	636	NM_213792.2	ssc:396692	3E-35	NM_139212.3	HOPX
LOC100738308	457.5687106	2308.621771	-2.334971524	0.034155	Low	552	2794	XP_003483753.2	ssc:100738308	7E-142	NM_139266.2	STAT1
FER1L5	53.88037353	9.089062089	2.567556519	0.034277	High	65	11	XP_005659036.1	ssc:100627869	0	NM_001113382.1	FER1L5
LOC100738253	506.4755112	104.9373532	2.270964207	0.034353	High	611	127	XP_003482104.2	ssc:100738253	1E-36	NR_046177.1	MAPRE2
LOC102164987	2308.566774	458.5844963	2.331737975	0.034397	High	2785	555	XR_306325.1	ssc:102164987			
ZFPM2	56.36716001	272.6718627	-2.274239049	0.034459	Low	68	330	NM_001195374.1	ssc:100337657			
FAM133A	236.2447147	48.75042393	2.276795384	0.034574	High	285	59	XP_005673830.1	ssc:100519754	6E-46	NM_173698.2	FAM133A
NPM3	14769.85378	2715.977008	2.443112372	0.034578	High	17818	3287	XP_003359391.1	ssc:100627227	2E-75	NM_006993.2	NPM3
NUDT22	322.4533124	1602.153763	-2.312850419	0.034602	Low	389	1939	XP_005660810.1	ssc:100521244	4E-141	NM_032344.3	NUDT22
LIAS	2076.466703	414.7917426	2.323671658	0.034766	High	2505	502	NM_001243462.1	ssc:100627627	0	NM_194451.2	LIAS
LOC102167212	38.95965471	5.783948602	2.751854254	0.03481	High	47	7	XR_309191.1	ssc:102167212			
ITGB7	31.4992953	4.131391859	2.930619743	0.035033	High	38	5	XP_005652618.1	ssc:100626600	0	NR_104181.1	ITGB7
LOC102162940	31.4992953	4.131391859	2.930619743	0.035033	High	38	5	XR_302541.1	ssc:102162940			
LOC100737439	338.20296	1679.82393	-2.312348834	0.035068	Low	408	2033	XP_005663756.1	ssc:100737439	0	NM_018103.4	LRRC8D
RN5-8S	9614.745425	1805.418242	2.412915569	0.035104	High	11599	2185	NR_046250.1	ssc:100861536			
CNR1	333.2293871	69.40738323	2.263354593	0.03526	High	402	84	XP_005659346.1	ssc:100520160	0	NM_033181.3	CNR1
FAM89B	149.2071882	722.1672969	-2.275016049	0.035286	Low	180	874	XP_003360758.1	ssc:100627039	1E-56	NM_152832.2	FAM89B
LOC102165408	116.8789641	23.13579441	2.336816755	0.035297	High	141	28	XR_299918.1	ssc:102165408			
SLC16A7	532.1723048	110.7213018	2.264960621	0.035398	High	642	134	XP_005664001.1	ssc:100158241	0	NR_073056.1	SLC16A7
PRRG4	124.3393235	24.78835115	2.326548419	0.035424	High	150	30	NM_001244836.1	ssc:100513583	4E-107	NM_024081.5	PRRG4
FER1L4	124.3393235	24.78835115	2.326548419	0.035424	High	150	30	XP_005672942.1	ssc:102162661	1E-149	NM_001039112.2	FER1L6
CEL	12.43393235	68.58110485	-2.463528511	0.035435	Low	15	83	XP_003353742.1	ssc:100625953	0	NM_001807.4	CEL
SEC24D	837.2181118	4304.910317	-2.362307755	0.035582	Low	1010	5210	XP_003129278.3	ssc:100525592	0	NM_014822.2	SEC24D
LOC100620540	19.06536294	98.32712624	-2.366635483	0.035684	Low	23	119	XP_005653412.1	ssc:100620540	5E-166	NM_080601.1	PTPN11
BCL7A	3595.064308	705.6417295	2.349009793	0.035718	High	4337	854	XP_003483472.1	ssc:100736875	3E-101	NM_020993.3	BCL7A
STK36	125.9971812	606.4883248	-2.267088415	0.035763	Low	152	734	XP_005672268.1	ssc:100622459	0	NM_015690.4	STK36
C2H19orf71	378.8204724	1883.088409	-2.31351453	0.035848	Low	457	2279	XP_003123092.1	ssc:100519760	1E-88	NM_001135580.1	C19orf71
ARSK	128.4839677	618.0562221	-2.26614973	0.035985	Low	155	748	XP_005652992.1	ssc:100627205	0	NM_198150.2	ARSK
FSTL3	22.38107824	113.2001369	-2.338524256	0.035986	Low	27	137	XP_005659092.1	ssc:102164292	2E-37	NM_005860.2	FSTL3
CEP170B	588.5394648	2975.428417	-2.337886345	0.036021	Low	710	3601	XP_005656532.1	ssc:100521503	0	NM_015005.2	CEP170B
LOC102164126	49.73572942	8.262783717	2.589582825	0.036028	High	60	10	XP_005668327.1	ssc:102164126	3E-40	NM_014930.1	ZNF510
LOC100737287	211.37685	43.7927557	2.271053307	0.036172	High	255	53	XP_003480907.2	ssc:100737287	9E-174	NM_172349.2	NSD1
MMP11	545.4351659	2746.549308	-2.332140569	0.036214	Low	658	3324	XP_005670966.1	ssc:100153503	0	NM_005940.3	MMP11
TRIM3	444.3058495	2218.557428	-2.319996858	0.036243	Low	536	2685	XP_003482568.1	ssc:100739484	0	NM_033278.3	TRIM3
IPO11	577.7633901	120.6366423	2.259810611	0.036476	High	697	146	XP_003362100.1	ssc:100625727	0	NM_016338.4	IPO11
GRM2	67.97216354	12.39417558	2.455281733	0.036494	High	82	15	XP_005669707.1	ssc:100520271	0	NM_001130063.1	GRM2
TTLL6	14.92071882	79.32272369	-2.410417175	0.036531	Low	18	96	XP_005654019.1	ssc:100523818	0	NM_173623.3	TTLL6
LOC100626845	42.27537	6.610226974	2.677045666	0.036585	High	51	8	XP_005667577.1	ssc:100626845	0	np_055802	np_055802
LOC100522672	589.3683936	123.1154774	2.259157553	0.036655	High	711	149	XP_003355325.1	ssc:100522672	0	NM_000699.2	AMY2A
SLC10A4	11.60500353	64.44971299	-2.473426972	0.036773	Low	14	78	XP_003129015.2	ssc:100520624	0	NM_152679.3	SLC10A4
TAPBPL	503.1597959	2517.670199	-2.323000754	0.036927	Low	607	3047	XP_005653216.1	ssc:100520908	1E-176	NM_018009.4	TAPBPL
LOC100522668	86.20859765	16.52556743	2.383131948	0.037041	High	104	20	LOC100522668				
ENOX1	26.52572236	131.3782611	-2.308262631	0.037128	Low	32	159	XP_005668503.1	ssc:100525605	0	NM_017993.3	ENOX1
LOC100518455	2714.741897	542.0386119	2.324347511	0.037139	High	3275	656	XR_297920.1	ssc:100518455			
FRK	19.89429177	1.652556743	3.589582825	0.037172	High	24	2	XP_001925827.2	ssc:100157810	0	NM_002031.2	FRK
KCNU1	19.89429177	1.652556743	3.589582825	0.037172	High	24	2	XP_001926650.1		0	NM_001031836.2	KCNU1
GIMAP6	19.89429177	1.652556743	3.589582825	0.037172	High	24	2	XP_003360126.2	ssc:100518524	3E-89	NM_024711.5	GIMAP6
LOC100738466	847.1652577	175.9972932	2.267090185	0.03718	High	1022	213	XP_005669510.1	ssc:100738466	3E-112	NM_015340.3	LARS2
CLCN1	34.81501059	4.95767023	2.811975246	0.037274	High	42	6	XP_003134618.1	ssc:100520812	0	NR_046453.1	CLCN1
GABRA1	34.81501059	4.95767023	2.811975246	0.037274	High	42	6	XP_005672614.1	ssc:100516706	0	NM_001127648.1	GABRA1
GBE1	164.9568359	793.2272369	-2.265645651	0.037296	Low	199	960	XP_005670346.1	ssc:100156209	1E-152	NM_000158.3	GBE1
PHF16	3361.30638	665.9803676	2.335470495	0.037357	High	4055	806	XP_005673639.1	ssc:100521831	0	NM_014735.4	JADE3
CES1	33.98608177	165.2556743	-2.281683861	0.037389	Low	41	200	NM_214246.1	ssc:397478			
FAM71F1	53.05144471	9.089062089	2.545188706	0.03741	High	64	11	XP_005657828.1	ssc:100519990	2E-170	NR_104243.1	FAM71F1
LOC100037969	8.289288236	49.5767023	-2.580342176	0.037423	Low	10	60	XP_003121230.2	ssc:100037969			
WEE1	4815.247536	940.304787	2.356409597	0.037462	High	5809	1138	XR_301770.1	ssc:100521487	0	NM_003390.3	WEE1
RERG	17.4075053	90.06434252	-2.371246578	0.037504	Low	21	109	XP_003481729.1	ssc:100155884	4E-114	NM_032918.2	RERG
CERKL	384.6229742	80.97528043	2.247891475	0.037552	High	464	98	XP_005654239.1	ssc:100521824	0	NR_027690.1	CERKL
IGF2BP1	6440.776959	1242.722671	2.373730356	0.037562	High	7770	1504	XP_005654025.1	ssc:100620940	0	NM_006546.3	IGF2BP1
HLTF	7499.319067	1437.724367	2.38297249	0.037604	High	9047	1740	XR_297078.1	ssc:100520979	0	NM_139048.2	HLTF
AGO3	111.9053912	22.30951604	2.326548419	0.037606	High	135	27	XP_005665268.1	ssc:102165981	5E-101	NM_177422.2	AGO3
LOC100514826	35.64393941	172.6921797	-2.276474053	0.037608	Low	43	209	XP_003123937.3	ssc:100514826	2E-85	NM_023927.2	GRAMD3
MTBP	1103.304264	228.0528306	2.274390723	0.037753	High	1331	276	XP_005662936.1	ssc:100152418	0	NM_022045.4	MTBP
GAST	11.60500353	0	20	0.037758	High	14	0	NM_001004036.2	ssc:445524	8E-36	NM_000805.4	GAST
ANKS1B	11.60500353	0	20	0.037758	High	14	0	XP_005664274.1	ssc:100621174	3E-165	NM_181670.3	ANKS1B
IL31RA	11.60500353	0	20	0.037758	High	14	0	XP_003134027.4	ssc:100522044	0	NM_139017.5	IL31RA
LOC102159535	11.60500353	0	20	0.037758	High	14	0	XP_005652557.1	ssc:102159535			
LOC100518213	11.60500353	0	20	0.037758	High	14	0	XP_005668551.1	ssc:100518213	0	NM_005845.3	ABCC4
SLC16A14	11.60500353	0	20	0.037758	High	14	0	XP_005657647.1	ssc:100519114	9E-164	NM_152527.4	SLC16A14
LOC102159945	11.60500353	0	20	0.037758	High	14	0	XP_005668645.1	ssc:102159945			
LOC102161110	11.60500353	0	20	0.037758	High	14	0	XR_305512.1	ssc:102161110			
LOC102163833	11.60500353	0	20	0.037758	High	14	0	XP_005657359.1	ssc:102163833	1E-35	NM_133448.2	TMEM132D
LOC102166821	11.60500353	0	20	0.037758	High	14	0	XR_298230.1	ssc:102166821			
LOC102164610	11.60500353	0	20	0.037758	High	14	0	XR_307108.1	ssc:102164610			
LOC100522416	11.60500353	0	20	0.037758	High	14	0	LOC100522416	ssc:100522416			
LOC102164446	11.60500353	0	20	0.037758	High	14	0	XR_308037.1	ssc:102164446			
DQX1	11.60500353	0	20	0.037758	High	14	0	XP_005674478.1	ssc:100627998	0	NM_133637.2	DQX1
NCR3	11.60500353	0	20	0.037758	High	14	0	XP_003128360.1	ssc:100270822			
TRNAT-AGU	11.60500353	0	20	0.037758	High	14	0	TRNAT-AGU				
LOC100625090	11.60500353	0	20	0.037758	High	14	0	XR_299717.1	ssc:100625090			
LOC102164837	11.60500353	0	20	0.037758	High	14	0	XR_301688.1	ssc:102164837			
APOBEC4	11.60500353	0	20	0.037758	High	14	0	XP_003130417.1	ssc:100515680	2E-171	NM_203454.2	APOBEC4
LOC100524342	11.60500353	0	20	0.037758	High	14	0	XP_003132758.4	ssc:100524342	4E-94	NM_182909.3	FILIP1L
LOC102163249	11.60500353	0	20	0.037758	High	14	0	XR_307555.1	ssc:102163249			
LOC102165596	11.60500353	0	20	0.037758	High	14	0	XR_303605.1	ssc:102165596			
LOC102165859	11.60500353	0	20	0.037758	High	14	0	LOC102165859				
LGR6	11.60500353	0	20	0.037758	High	14	0	XP_003130662.2	ssc:100511400	0	NM_021636.2	LGR6
IFNL4	11.60500353	0	20	0.037758	High	14	0	XP_005657557.1	ssc:102161030			
PCDHGC3	1188.683933	6150.816199	-2.371412707	0.037783	Low	1434	7444	XP_003124083.1	ssc:100187729	0	NM_032403.2	PCDHGC3
LOC102157493	1736.605885	353.6471431	2.295887867	0.037827	High	2095	428	XR_302127.1	ssc:102157493			
CNTN3	987.2542289	204.9170362	2.268381712	0.037969	High	1191	248	XP_005669787.1	ssc:100511594	0	NM_014461	CNTN6

DNAJB9	376.3336859	1853.342388	-2.300045093	0.037988	Low	454	2243	XP_005673303.1	ssc:100521593	1E-121	NM_012328.2	DNAJB9
CALR3	171.5882665	35.52996998	2.271842527	0.038122	High	207	43	XP_003123521.1	ssc:100512214	0	NM_145046.4	CALR3
LOC100737528	7899.691689	1514.568255	2.382889753	0.038205	High	9530	1833	XP_005665225.1	ssc:100737528	8E-34	NM_004102.3	FABP3
LOC102160948	266.9150812	56.18692928	2.248074361	0.038231	High	322	68	XP_005659076.1	ssc:102160948	2E-43	NM_001130867.1	ERCC2
LOC100738697	1.657857647	19.83068092	-3.580342176	0.038333	Low	2	24	XP_003480533.1	ssc:100738697			
LOC100520244	87.8664553	416.4442994	-2.244739145	0.038372	Low	106	504	XP_003126314.1	ssc:100520244	3E-92	NM_002475.4	MYL6B
LOC100525452	118.5368218	563.5218495	-2.249136268	0.038391	Low	143	682	XP_005655056.1	ssc:100525452	5E-136	NM_207317.1	ZNF474
LOC100627015	441.819063	93.36945601	2.242433085	0.03857	High	533	113	XR_304759.1	ssc:100627015	1E-60	NM_001290.3	LDB2
KCNA7	74.60359412	14.04673232	2.409010579	0.038611	High	90	17	HighI0000F3BD7B		0	NM_031886.2	KCNA7
SLA-DYB	74.60359412	14.04673232	2.409010579	0.038611	High	90	17	SLA-DYB				
LOC100037944	0	11.5678972	-20	0.038731	Low	0	14	XP_003481539.1	ssc:100037944	0	NM_004000.2	CHI3L2
LOC100518202	0	11.5678972	-20	0.038731	Low	0	14	XP_003128432.1	ssc:100518202	3E-110	NM_006789.3	APOBEC2
LOC102159496	0	11.5678972	-20	0.038731	Low	0	14	XR_304236.1	ssc:102159496			
LOC102168069	0	11.5678972	-20	0.038731	Low	0	14	XP_005674259.1	ssc:102168069	1E-97	NM_001243996.1	RYR3
LOC102168116	0	11.5678972	-20	0.038731	Low	0	14	XR_309509.1	ssc:102168116			
LOC100156477	0	11.5678972	-20	0.038731	Low	0	14	XP_001928934.3	ssc:100156477	3E-110	NM_006789.3	APOBEC2
LOC100516806	0	11.5678972	-20	0.038731	Low	0	14	LOC100516806				
LOC100624214	0	11.5678972	-20	0.038731	Low	0	14	XP_005658944.1	ssc:100624214			
TLE6	0	11.5678972	-20	0.038731	Low	0	14	XP_003354048.2	ssc:100623564	7E-152	NM_024760.2	TLE6
DTX1	0	11.5678972	-20	0.038731	Low	0	14	XP_005670755.1	ssc:100524958	4E-63	NM_004416.2	DTX1
LOC100624891	0	11.5678972	-20	0.038731	Low	0	14	XP_005656569.1	ssc:100624891	9E-91	NM_021139.2	UGT2B4
LOC100623436	0	11.5678972	-20	0.038731	Low	0	14	XP_005658227.1	ssc:100623436	2E-176	NM_001201.2	BMP3
LOC102158059	0	11.5678972	-20	0.038731	Low	0	14	XP_005672690.1	ssc:102158059	2E-29	NM_182643.2	DLC1
VNN3	0	11.5678972	-20	0.038731	Low	0	14	XP_001925491.1	ssc:100152784	0	NM_004666.2	VNN1
CCM2L	0	11.5678972	-20	0.038731	Low	0	14	XP_005674628.1	ssc:100739177	0	NM_080625.3	CCM2L
LOC102162295	0	11.5678972	-20	0.038731	Low	0	14	XR_304005.1	ssc:102162295			
LOC102167618	0	11.5678972	-20	0.038731	Low	0	14	XR_300015.1	ssc:102167618			
LOC100153068	0	11.5678972	-20	0.038731	Low	0	14	XP_001925376.3	ssc:100153068	0	NM_016150.4	ASB2
LOC100155068	0	11.5678972	-20	0.038731	Low	0	14	NM_001135258.1	ssc:100155068	7E-122	NM_006287.4	TFPI
ALB	0	11.5678972	-20	0.038731	Low	0	14	NM_001005208.1	ssc:396960	0	NM_000477.5	ALB
RFC3	3309.083864	660.196419	2.325464637	0.038973	High	3992	799	NM_001190192.1	ssc:100156440	0	NM_181558.2	RFC3
WDR52	67.14323471	12.39417558	2.437579732	0.039135	High	81	15	XP_005670284.1	ssc:100739658	0	NM_018338.3	WDR52
PKCNT1	38.13072589	5.783948602	2.720827358	0.039161	High	46	7	NM_214112.1	ssc:397200	0	NM_201651.2	SLC28A1
LAMC2	25.69679353	126.4205909	-2.298571208	0.039232	Low	31	153	XP_003130413.2	ssc:100514975	0	NM_018891.2	LAMC2
HSD17B1	27.35465118	133.8570962	-2.290835559	0.039284	Low	33	162	NM_001128472.1	ssc:100147712	8E-120	NM_000413.2	HSD17B1
LOC100622805	15.74964765	0.826278372	4.252547838	0.039388	High	19	1	LOC100622805				
LOC102157488	15.74964765	0.826278372	4.252547838	0.039388	High	19	1	XP_005670512.1	ssc:102157488			
LOC100738252	15.74964765	0.826278372	4.252547838	0.039388	High	19	1	XP_003481436.1	ssc:100738252	3E-73	NM_030958.2	SLCO5A1
LOC100621278	15.74964765	0.826278372	4.252547838	0.039388	High	19	1	XP_003360826.2	ssc:100621278	1E-64	NM_173509.2	FAM163A
PLET	15.74964765	0.826278372	4.252547838	0.039388	High	19	1	NM_213744.1	ssc:396570			
FBP2	15.74964765	0.826278372	4.252547838	0.039388	High	19	1	NM_001167632.2	ssc:100134828	4E-172	NM_003837.3	FBP2
LOC102160095	15.74964765	0.826278372	4.252547838	0.039388	High	19	1	XR_297686.1	ssc:102160095			
TRNAG-UCC	15.74964765	0.826278372	4.252547838	0.039388	High	19	1	TRNAG-UCC				
LOC100627610	1264.116456	261.9302438	2.270874822	0.039443	High	1525	317	XP_003360656.3	ssc:100627610	0	NM_032730.4	RTN4IP1
TIMM8A	3140.811313	629.6241193	2.318574561	0.039671	High	3789	762	XP_003135287.1	ssc:100517443	9E-49	NM_032696.1	TIMM8A
BMP2	15.74964765	81.8015588	-2.376808782	0.039811	Low	19	99	NM_001195399.1	ssc:100157103	0	NM_001200.2	BMP2
PDK2	202.258633	967.5719733	-2.258167699	0.03988	Low	244	1171	XP_005668938.1	ssc:100516037	0	NM_002611.4	PDK2
STAT6	553.7244542	2748.201864	-2.311247836	0.0399	Low	668	3326	NM_001252101.1	ssc:100153518	0	NR_033659.1	STAT6
LOC102157784	644.9066248	136.3359313	2.241924455	0.039936	High	778	165	LOC102157784				
LOC100519177	242.0472165	51.22925905	2.240248573	0.039976	High	292	62	XP_005657769.1	ssc:100519177	6E-91	NM_130759.3	GIMAP1
ACER1	88.69538413	17.35184581	2.353769888	0.040056	High	107	21	XP_005661338.1	ssc:100622165	2E-106	NM_133492.2	ACER1
LOC100155620	772.5616636	162.7768392	2.24675465	0.040095	High	932	197	NM_001142667.1	ssc:100155620	0	NM_006996.2	SLC19A2
ULK2	875.3488377	4427.199516	-2.338464422	0.040109	Low	1056	5358	XP_005669318.1	ssc:100525780	0	NM_014683.3	ULK2
LOC102164507	489.8969347	104.1110748	2.234354721	0.040167	High	591	126	XP_005663943.1	ssc:102164507	2E-179	NM_013449.3	BAZ2A
IRAK2	14.09179	74.36505346	-2.399769931	0.040183	Low	17	90	XP_005669832.1	ssc:102161037	9E-87	NM_001570.3	IRAK2
CTSF	579.4212477	2876.275012	-2.311517129	0.040338	Low	699	3481	XP_005660701.1	ssc:100520004	0	NM_003793.3	CTSF
IFI27L2	683.8662795	3417.487345	-2.321149837	0.040432	Low	825	4136	XP_005666469.1	ssc:100152306	3E-31	NM_032036.2	IFI27L2
LOC100738381	245.3629318	52.05553742	2.236793766	0.040461	High	296	63	XP_005664214.1	ssc:100738381	9E-154	NM_006159.2	NELL2
ZNF275	368.0443977	1793.024067	-2.284443137	0.040464	Low	444	2170	ZNF275	ssc:100623368	9E-140	NM_020636.2	ZNF275
IFN-OMEGA-2	0.828928824	15.69928906	-4.243307189	0.040522	Low	1	19	NM_001166322.1	ssc:100310808	6E-47	NM_002177.1	IFNW1
KCNE3	5.802501765	38.0088051	-2.71158671	0.040588	Low	7	46	NM_214093.2	ssc:397168	3E-50	NM_005472.4	KCNE3
LOC102160792	52.22251589	9.089062089	2.522468629	0.040844	High	63	11	XR_309167.1	ssc:102160792			
ENPP5	35.64393941	170.2133446	-2.255615448	0.040972	Low	43	206	XP_005656285.1	ssc:100153376	0	NM_021572.4	ENPP5
MELK	3803.125443	760.176102	2.322779949	0.041011	High	4588	920	XP_005660305.1	ssc:100519998	0	NR_046337.1	MELK
WDR12	4278.101659	851.0667229	2.329626618	0.041062	High	5161	1030	XP_005672161.1	ssc:100512929	0	NM_018256.3	WDR12
PNOC	8.289288236	48.75042393	-2.55609463	0.041074	Low	10	59	NM_001244476.1	ssc:397257	3E-75	NM_006228.4	PNOC
KIAA1549	256.1390065	54.53437253	2.231689233	0.041078	High	309	66	XP_005657810.1	ssc:102165036	0	NM_020910.2	KIAA1549
LOC100520639	2203.292813	450.3217126	2.290633294	0.041092	High	2658	545	XR_306799.1	ssc:100520639			
LOC100514839	5784.265331	31767.09828	-2.457327678	0.041294	Low	6978	38446	XP_005663981.1	ssc:100514839	0	NM_002332.2	LRP1
LOC102161673	128.4839677	26.4409079	2.28074473	0.041317	High	155	32	XP_005666870.1	ssc:102161673	0	NM_001109977.1	FAM160A1
COL13A1	84.55074001	16.52556743	2.355117571	0.041414	High	102	20	XP_001924960.3	ssc:100157199	3E-19	NM_080815.2	COL13A1
TAF5	868.7174071	183.4397985	2.24362746	0.041434	High	1048	222	XP_001929370.2	ssc:100152210	0	NM_006951.3	TAF5
KCNK4	23.21000706	2.478835115	3.227012746	0.041482	High	28	3	XP_003122639.3	ssc:100524785	1E-165	NM_033311.1	KCNK4
DPF3	23.21000706	2.478835115	3.227012746	0.041482	High	28	3	XP_005666408.1	ssc:100156555	1E-87	NM_012074.4	DPF3
LOC102160625	23.21000706	2.478835115	3.227012746	0.041482	High	28	3	XR_302831.1	ssc:102160625			
RIPPLY3	23.21000706	2.478835115	3.227012746	0.041482	High	28	3	XP_001928108.2	ssc:100153272	9E-38	NM_018962.2	RIPPLY3
LOC102163165	23.21000706	2.478835115	3.227012746	0.041482	High	28	3	XR_306188.1	ssc:102163165			
B3GALNT1	812.3502471	171.8659013	2.240818545	0.041484	High	980	208	NM_214351.1	ssc:397634	0	NM_033169.2	B3GALNT1
ACER3	2102.992425	431.31731	2.285622132	0.041551	High	2537	522	XP_005667218.1	ssc:100524160	6E-97	NM_018367.5	ACER3
ENO3	359.7551094	1743.447364	-2.276855727	0.0416	Low	434	2110	NM_001044527.1	ssc:692156	0	NM_053013.3	ENO3
LOC100627491	699.6159271	3483.589615	-2.315939639	0.041637	Low	844	4216	XR_131410.2	ssc:100627491			
LOC102158857	17.4075053	88.41178578	-2.344529239	0.041714	Low	21	107	XR_304481.1	ssc:102158857			
LOC102158835	113.5632488	23.13579441	2.295297485	0.041768	High	137	28	XP_005661177.1	ssc:102158835			
LOC100737380	831.4156101	175.9972932	2.240016595	0.041789	High	1003	213	XP_005669553.1	ssc:100737380	0	NM_201567.1	CDC25A
ASB9	73.7746653	344.558081	-2.223549818	0.041911	Low	89	417	NM_001243703.1	ssc:100158057	2E-114	NM_024087.2	ASB9
LOC100523918	89.52431295	418.0968561	-2.223485748	0.041998	Low	108	506	XP_005668531.1	ssc:100523918	0	NM_015843.1	LMO7
PIP5K1B	55.53823118	9.915340461	2.485747014	0.042013	High	67	12	XP_003480579.2	ssc:100737867	3E-157	NM_003558.3	PIP5K1B
LOC100736638	320.7954547	1545.140555	-2.268012474	0.042081	Low	387	1870	XR_298726.1	ssc:100736638			
LOC102166535	289.2961594	61.97087788	2.22288486	0.042084	High	349	75	XP_005674340.1	ssc:102166535	1E-37	NM_022121.4	PERP
LOC100155572	187.3379141	39.66136184	2.239836781	0.042138	High	226	48	XR_304540.1	ssc:100155572			
RASGRP2	44.76215647	7.436505346	2.589582825	0.042204	High	54	9	NM_001244158.1	ssc:100626093	0	NM_153819.1	RASGRP2
TRIM8	1044.450318	5279.092517	-2.337546082	0.042363	Low	1260	6389	XP_001928939.3	ssc:100154191	0	NM_030912.2	TRIM8
SYPL2	9.11821706	52.05553742	-2.513227981	0.042411	Low	11	63	XP_003125917.1	ssc:100514960	2E-137	NM_001040709.1	SYPL2
DOK4	213.8636365	1014.66984	-2.246247266	0.042422	Low	258	1228	XP_005655844.1	ssc:100620637	0	NM_018110.3	DOK4
WNT8B	33.98608177	4.95767023	2.777209828	0.042423	High	41	6	XP_003125637.4	ssc:100513655	0	NM_003393.3	WNT8B
LOC100155923	461.7133547	99.15340461	2.219263246	0.042677	High	557	120	XR_130553.2	ssc:100155923			
TRPM6	31.4992953	150.3826637	-2.255246802	0.042795	Low	38	182	XP_005652756.1	ssc:100157775	0	NM_017662.4	TRPM6
FADD	180.7064835	851.8930013	-2.237023968	0.042812	Low	218	1031	NM_001031797.1	ssc:595129			
LOC102159328	2.486786471	23.13579441	-3.217772097	0.042818	Low	3	28	XR_299163.1	ssc:102159328			
LOC100152528	109.4186047	22.30951604	2.294126942	0.042848	High	132	27	XP_005665716.1	ssc:100152528	1E-68	NM_003524.2	HIST1H2BH

LOC102167104	127.6550388	26.4409079	2.271406865	0.042901	High	154	32	XR_301475.1	ssc:102167104			
LOC102162975	28.18358	135.509653	-2.265468839	0.042949	Low	34	164	XR_301352.1	ssc:102162975			
PDSS2	465.02907	99.97968298	2.217614048	0.042996	High	561	121	NM_001167653.1	ssc:100312977	1E-130	NM_020381.3	PDSS2
LOC100511489	1545.123327	322.248565	2.261476155	0.043024	High	1864	390	XP_003121665.1	ssc:100511489	6E-91	NM_001211.5	BUB1B
LOC102157512	58.85394648	10.74161883	2.453927726	0.043092	High	71	13	XR_299508.1	ssc:102157512			
LMF2	1760.644821	9084.104419	-2.367240389	0.043255	Low	2124	10994	NM_001206379.1	ssc:100518488	0	NM_033200.2	LMF2
ADM2	24.86786471	120.6366423	-2.278313639	0.043278	Low	30	146	NM_001206384.1	ssc:100517471	3E-47	NM_024866.5	ADM2
LOC100737748	518.9094436	111.5475802	2.217823574	0.043311	High	626	135	XP_003483718.1	ssc:100737748	0	NM_024753.4	TTC21B
LOC100620268	48.07787177	8.262783717	2.540673225	0.043468	High	58	10	XP_005658181.1	ssc:100620268	0	NM_005026.3	PIK3CD
LOC100624686	48.07787177	8.262783717	2.540673225	0.043468	High	58	10	XP_003361117.1	ssc:100624686			
HVCN1	172.4171953	809.7528043	-2.231577897	0.043478	Low	208	980	XP_005670704.1	ssc:100152115	2E-113	NM_032369.3	HVCN1
LOC100523897	9.947145883	55.36065091	-2.476506365	0.043634	Low	12	67	LOC100523897	ssc:100523897	6E-87	NM_007135.2	ZNF79
LOC100737524	9.947145883	55.36065091	-2.476506365	0.043634	Low	12	67	XP_005661782.1	ssc:100737524	0	NM_032289.2	PSD2
ALOX5AP	45.5910853	213.1798199	-2.225247218	0.043644	Low	55	258	NM_001164001.1	ssc:397392	3E-87	NM_001629.3	ALOX5AP
LOC100737905	585.2237495	2870.491063	-2.294237341	0.043644	Low	706	3474	XP_005654721.1	ssc:100737905	0	NM_003793.3	CTSF
WFIKKN2	7.460359412	44.61903207	-2.580342176	0.043773	Low	9	54	XP_005668980.1	ssc:100512012	0	NM_175575.5	WFIKKN2
RCC1	5192.410151	1034.500521	2.327469975	0.043834	High	6264	1252	XP_005665199.1	ssc:100621543	0	NR_030726.1	RCC1
LOC102160037	72.94573648	14.04673232	2.376589102	0.043853	High	88	17	XP_005669788.1	ssc:102160037	8E-52	NM_020872.1	CNTN3
DYRK1B	260.2836506	1236.112444	-2.247653387	0.043871	Low	314	1496	XP_005655892.1	ssc:100625631	0	NM_006484.1	DYRK1B
CBLB	366.38654	1761.625489	-2.265468839	0.043903	Low	442	2132	XP_003358478.2	ssc:100621690	0	NM_170662.3	CBLB
CEBPB	251.1654336	1190.667134	-2.245060312	0.044031	Low	303	1441	NM_001199889.1	ssc:397360	1E-132	NM_005194.3	CEBPB
LOC100738454	121.0236082	562.6955711	-2.217066105	0.044081	Low	146	681	XP_005654089.1	ssc:100738454	1E-75	NM_183384.2	RNF13
ASB16	37.30179706	5.783948602	2.689118499	0.044083	High	45	7	XP_005668833.1	ssc:100514598	1E-114	NM_080863.4	ASB16
LOC102166016	37.30179706	5.783948602	2.689118499	0.044083	High	45	7	XR_297893.1	ssc:102166016			
CPA5	37.30179706	5.783948602	2.689118499	0.044083	High	45	7	XP_005674244.1	ssc:100625420	0	NM_080385.4	CPA5
CLPB	3660.549685	741.1716994	2.304180606	0.04422	High	4416	897	XP_005653743.1	ssc:100524385	0	NM_030813.4	CLPB
RHBDD2	312.5061665	1491.432461	-2.254742084	0.044302	Low	377	1805	XP_005658129.1	ssc:100523781	1E-163	NM_020684.2	RHBDD2
NOL9	4545.01674	912.2113224	2.316845626	0.044325	High	5483	1104	XP_003127589.2	ssc:100518312	0	NM_024654.4	NOL9
TPP1	2199.977098	11421.64593	-2.376210157	0.044424	Low	2654	13823	XP_003357180.1	ssc:100623352	0	NM_000391.3	TPP1
NUCB1	2734.636189	14340.88742	-2.390713484	0.044449	Low	3299	17356	NM_001244488.1	ssc:100512538	0	NM_006184.5	NUCB1
PCDHA6	26.52572236	3.305113487	3.004620324	0.044467	High	32	4	XP_005674531.1	ssc:100738826	0	NM_031849.2	PCDHA6
RSPO4	26.52572236	3.305113487	3.004620324	0.044467	High	32	4	XP_003134412.2	ssc:100518279	1E-110	NM_001040007.2	RSPO4
LOC100737055	26.52572236	3.305113487	3.004620324	0.044467	High	32	4	XP_003482520.1	ssc:100737055	3E-32	NM_014395.2	DAPP1
LOC102165900	26.52572236	3.305113487	3.004620324	0.044467	High	32	4	XR_298690.1	ssc:102165900			
GPR182	26.52572236	3.305113487	3.004620324	0.044467	High	32	4	XP_003126338.1	ssc:100512467	6E-166	NM_007264.3	GPR182
ZNF518B	528.0276606	114.0264153	2.21124543	0.044615	High	637	138	ZNF518B	ssc:100622816	0	NM_053042.2	ZNF518B
LRRC7	13.26286118	69.40738323	-2.387697098	0.044647	Low	16	84	XP_005656200.1	ssc:100516204			
HAGHL	45.5910853	212.3535415	-2.219644511	0.044654	Low	55	257	XP_005662292.1	ssc:100525741	7E-141	NM_207112.1	HAGHL
IZUMO1	10.77607471	58.66576439	-2.444687077	0.044763	Low	13	71	XP_005664743.1	ssc:100524565	8E-132	NM_182575.2	IZUMO1
LOC100737169	1055.226392	5288.181579	-2.325219162	0.044881	Low	1273	6400	XP_005666111.1	ssc:100737169	7E-57	NM_003376.5	VEGFA
SASS6	916.7952789	195.8279741	2.227012746	0.045001	High	1106	237	XP_003361047.1	ssc:100622404	0	NM_194292.1	SASS6
TRMT13	130.1418253	27.26718627	2.254846954	0.045197	High	157	33	XP_005663701.1	ssc:102167932	2E-42	NM_019083.2	TRMT13
LOC100512723	140.9179	654.4124704	-2.21534536	0.045206	Low	170	792	XP_003128455.4	ssc:100512723	0	NM_019096.4	GTPBP2
LOC100739855	940.005286	4680.866976	-2.316034991	0.045217	Low	1134	5665	XP_003484031.1	ssc:100739855	2E-132	NM_005194.3	CEBPB
GLB1L	379.6494012	1818.638696	-2.260119317	0.04532	Low	458	2201	XP_001928410.1	ssc:100154356	0	NM_024506.4	GLB1L
MLKL	51.39358706	237.9681711	-2.211108367	0.045344	Low	62	288	XP_005664404.1	ssc:100736792	2E-90	NM_152649.2	MLKL
LOC100739646	23.21000706	112.3738586	-2.275487595	0.045399	Low	28	136	XP_003482943.1	ssc:100739646	3E-90	NM_017993.3	ENOX1
GALNT4	33.15715294	156.1666123	-2.235694005	0.045468	Low	40	189	XP_003481802.1	ssc:100738635	0	NM_003774.4	GALNT4
LOC100621801	33.15715294	156.1666123	-2.235694005	0.045468	Low	40	189	XP_003360781.2	ssc:100621801	0	NM_003774.4	GALNT4
LOC102157454	40.61751236	6.610226974	2.619330168	0.045486	High	49	8	XR_297367.1	ssc:102157454			
LOC100522001	79.57716707	15.69928906	2.341655312	0.045541	High	96	19	XP_005659664.1	ssc:100522001	4E-120	NM_207381.2	TNFAIP8L3
LOC100627275	440.9901342	2122.709137	-2.267088415	0.045637	Low	532	2569	XP_005655098.1	ssc:100627275	0	NM_025092.4	ATHL1
LGI2	54.70930236	9.915340461	2.464051943	0.045646	High	66	12	XP_005674273.1	ssc:100623207	2E-162	NM_018176.3	LGI2
GAS2L1	351.4658212	1676.518816	-2.254012371	0.045676	Low	424	2029	NM_001244834.1	ssc:100513569	0	NM_152237.1	GAS2L1
LOC654299	902.7034889	4478.428775	-2.310668573	0.04577	Low	1089	5420	NM_001037966.1	ssc:654299	0	NM_003214.3	TEAD3
PRDM10	285.1515153	61.97087788	2.202066389	0.045865	High	344	75	XP_003130123.3	ssc:100517360	0	NM_199439.1	PRDM10
FASN	55514.19225	10023.58293	2.469458353	0.045911	High	66971	12131	NM_001099930.1	ssc:397561	0	NM_004104.4	FASN
GPX2	19.06536294	1.652556743	3.52818228	0.045966	High	23	2	NM_001115136.1	ssc:767625	3E-107	NR_046321.1	GPX2
LOC102166864	19.06536294	1.652556743	3.52818228	0.045966	High	23	2	XR_304685.1	ssc:102166864			
CTSW	19.06536294	1.652556743	3.52818228	0.045966	High	23	2	XP_003122571.2	ssc:100525853	3E-147	NM_001335.3	CTSW
LOC100623402	19.06536294	1.652556743	3.52818228	0.045966	High	23	2	XP_005674223.1	ssc:100623402	9E-148	NM_173065.2	IFNLR1
ATOH1	19.06536294	1.652556743	3.52818228	0.045966	High	23	2	XP_003129367.1	ssc:100515497	4E-166	NM_005172.1	ATOH1
LOC102160042	19.06536294	1.652556743	3.52818228	0.045966	High	23	2	XR_301256.1	ssc:102160042			
LOC102165743	19.06536294	1.652556743	3.52818228	0.045966	High	23	2	LOC102165743				
CSMD1	19.06536294	1.652556743	3.52818228	0.045966	High	23	2	XP_005671705.1	ssc:100625335	0	NM_033225.5	CSMD1
PPP1R32	19.06536294	1.652556743	3.52818228	0.045966	High	23	2	XP_005660870.1	ssc:100521178	0	NM_145017.2	PPP1R32
CACNA2D4	19.06536294	1.652556743	3.52818228	0.045966	High	23	2	XP_003355658.2	ssc:100620898	0	NM_172364.4	CACNA2D4
ANXA1	3513.000354	18540.86038	-2.399932566	0.046025	Low	4238	22439	NM_001163998.1	ssc:396942	0	NM_000700.1	ANXA1
RPIA	3189.718113	653.586192	2.286979524	0.04607	High	3848	791	NM_001097495.1	ssc:100038014	1E-160	NM_144563.2	RPIA
IGSF1	125.9971812	26.4409079	2.252547838	0.04626	High	152	32	XP_001926045.1	ssc:100157227	0	NM_205833.3	IGSF1
TMEM141	180.7064835	841.1513824	-2.218717197	0.046277	Low	218	1018	XP_005652781.1	ssc:100512883	1E-43	NM_032928.3	TMEM141
ATAD2	3750.902927	764.3074938	2.295012846	0.046434	High	4525	925	XR_302830.1	ssc:100155780	0	NM_014109.3	ATAD2
NPAS2	313.3350953	1483.169677	-2.24290538	0.046644	Low	378	1795	XP_003124946.3	ssc:100521893	0	NM_002518.3	NPAS2
TSGA10IP	29.84143765	4.131391859	2.852617231	0.046648	High	36	5	XP_005660733.1	ssc:100516590	1E-170	NM_152762.2	TSGA10IP
ENO2	549.57981	2659.790079	-2.274911477	0.046699	Low	663	3219	XP_005652652.1	ssc:100157750	0	NM_001975.2	ENO2
HSPD1	77625.03969	13845.94668	2.487058404	0.046805	High	93645	16757	NM_001254716.1	ssc:492279	0	NM_199440.1	HSPD1
ITGA2	470.002643	102.4585181	2.197628939	0.046823	High	567	124	NM_001244272.1	ssc:397483	0	NR_073107.1	ITGA2
LOC100736775	124.3393235	572.6109116	-2.203272527	0.046842	Low	150	693	XP_005660750.1	ssc:100736775	3E-62	NM_153253.29	SIPA1
LOC100620793	96.98467256	19.83068092	2.290022543	0.046843	High	117	24	XP_003361455.1	ssc:100620793	1E-112	NM_006377.3	UNC13B
LOC102162699	321.6243836	1522.831039	-2.243307189	0.046844	Low	388	1843	XP_005672219.1	ssc:102162699			
SDCBP	2944.355181	15360.51493	-2.383202959	0.047028	Low	3552	18590	NM_001244414.2	ssc:100151838	2E-14	NM_005625.3	SDCBP
RIF1	7432.175832	1473.254337	2.334778121	0.047045	High	8966	1783	XP_003133873.1	ssc:100515692	0	NM_018151.4	RIF1
PMFBP1	86.20859765	17.35184581	2.31274262	0.047079	High	104	21	XP_005664432.1	ssc:100518840	0	NM_031293.2	PMFBP1
ENPP1	540.461593	2610.213376	-2.271903743	0.047104	Low	652	3159	XP_005652732.1	ssc:100037271	0	NM_006208.2	ENPP1
TMEM109	820.6395354	4035.543568	-2.297942446	0.047117	Low	990	4884	XP_003122718.1	ssc:100511116	3E-99	NM_024092.2	TMEM109
LOC100620578	11220.38056	2184.680015	2.360627716	0.04715	High	13536	2644	XR_298973.1	ssc:100620578	3E-30	NM_031305.2	ARHGAP24
WASF1	431.0429883	94.19573438	2.194098123	0.047274	High	520	114	XP_003121401.2	ssc:100154562	0	NM_003931.2	WASF1
LOC102161594	5228.883019	1053.504924	2.311305737	0.047333	High	6308	1275	XR_299497.1	ssc:102161594			
A3GALT2	1.657857647	19.00440255	-3.518941632	0.047346	Low	2	23	XP_005665265.1	ssc:100739829	1E-44	NM_020469.2	ABO
LOC102159557	1.657857647	19.00440255	-3.518941632	0.047346	Low	2	23	XP_005658027.1	ssc:102159557	8E-47	NM_021599.2	ADAMTS2
SLC34A3	1.657857647	19.00440255	-3.518941632	0.047346	Low	2	23	XP_005652809.1	ssc:641343	6E-59	NM_080877.2	SLC34A3
INHA	1.657857647	19.00440255	-3.518941632	0.047346	Low	2	23	NM_214189.1	ssc:397386	3E-155	NM_002191.3	INHA
LOC100626840	1.657857647	19.00440255	-3.518941632	0.047346	Low	2	23	XP_003354913.2	ssc:100626840	7E-155	NM_170672.2	RASGRP3
LOC100154914	1.657857647	19.00440255	-3.518941632	0.047346	Low	2	23	XP_005660120.1	ssc:100154914	5E-27	NM_002177.1	IFNW1
ADAMTS10	1576.622622	7979.370236	-2.339437507	0.047391	Low	1902	9657	NM_001246250.1	ssc:100624519	0	NM_030957.3	ADAMTS10
RALGAPA2	1063.515681	228.0528306	2.221401323	0.047438	High	1283	276	XP_005672795.1	ssc:100154830	0	NM_020343.3	RALGAPA2
HSPB2	68.80109236	314.8120596	-2.193987432	0.04745	Low	83	381	XP_003129916.2	ssc:100520143	1E-90	NM_001541.3	HSPB2
KLF7	74.60359412	341.2529675	-2.193524551	0.047454	Low	90	413	NM_001097487.1	ssc:100038003			
LOC102164120	161.6411206	34.70369161	2.219633215	0.047491	High	195	42	XP_005666886.1	ssc:102164120	3E-96	NM_006726.4	LRBA
UNC93B1	621.6966177	3015.089778	-2.277918326	0.047636	Low	750	3649	XP_003122484.3	ssc:100522238	0	NM_030930.2	UNC93B1

EBF4	48.90680059	224.7477171	-2.200199468	0.047746	Low	59	272	XP_005672849.1	ssc:100523648	0	NM_020833.2	EBF4
FBXL4	83.72181118	382.5668861	-2.192036576	0.047777	Low	101	463	NM_001171752.1	ssc:100156082	0	NR_103837.1	FBXL4
LGI4	19.06536294	93.36945601	-2.291996682	0.048006	Low	23	113	XP_003481877.1	ssc:100737096	0	NM_139284.2	LGI4
C4H1orf114	1352.81184	288.3711517	2.22996244	0.048051	High	1632	349	NM_001244796.1	ssc:100513278	0	NM_021179.1	CCDC181
LOC100736628	143.4046865	659.3701406	-2.200996384	0.048126	Low	173	798	XP_005670352.1	ssc:100736628	2E-152	NM_000392.3	ABCC2
NHLRC1	175.7329106	38.0088051	2.208978823	0.048281	High	212	46	NM_001258302.1	ssc:100152396			
LOC100736720	78.74823824	15.69928906	2.326548419	0.048288	High	95	19	LOC100736720				
LOC102162069	33.15715294	4.95767023	2.741585919	0.048318	High	40	6	XR_297575.1	ssc:102162069			
LOC102158566	219.6661383	47.92414556	2.196487879	0.04833	High	265	58	XR_307049.1	ssc:102158566			
LOC102160864	10.77607471	57.83948602	-2.424222974	0.048384	Low	13	70	XP_005658760.1	ssc:102160864	0	xp_003846818	xp_003846818
LOC102159188	29.01250883	136.3359313	-2.232418873	0.048413	Low	35	165	XR_300828.1	ssc:102159188			
LOC100738006	2063.203842	433.7961452	2.249797227	0.048622	High	2489	525	XP_003482423.2	ssc:100738006	0	NM_020453.3	ATP10D
MMP15	121.0236082	553.6065091	-2.193572402	0.048634	Low	146	670	XP_003127010.4	ssc:100514105	0	NM_002428.2	MMP15
LOC102157854	117.707893	24.78835115	2.247476848	0.048693	High	142	30	XR_298413.1	ssc:102157854			
LOC102161195	50.56465824	9.089062089	2.475926043	0.048733	High	61	11	XR_299408.1	ssc:102161195			
LOC100525796	50.56465824	9.089062089	2.475926043	0.048733	High	61	11	XP_005673732.1	ssc:100525796	5E-128	NM_007250.4	KLF8
ADCY9	62.16966177	283.4134815	-2.188625751	0.048749	Low	75	343	XP_005653094.1	ssc:100625423	0	NM_001116.3	ADCY9
LOC102167511	150.0361171	32.2248565	2.219063993	0.048776	High	181	39	XR_306809.1	ssc:102167511			
FGFR3	222.9818535	48.75042393	2.193439638	0.048796	High	269	59	XP_005666536.1	ssc:100514115	5E-165	NM_022965.3	FGFR3
LOC100511626	849.6520442	184.2600769	2.205128619	0.048845	High	1025	223	XP_003124122.2	ssc:100511626	0	NM_018989.1	RBM27
FKBP7	295.92759	1387.321386	-2.228985927	0.048866	Low	357	1679	XP_005657622.1	ssc:100144630	1E-118	NM_181342.2	FKBP7
LOC100624593	391.2544047	1853.342388	-2.243950531	0.048944	Low	472	2243	XP_005658463.1	ssc:100624593	4E-129	NM_001135602.1	GLB1
BMP1	2181.740664	11152.27918	-2.353787054	0.048992	Low	2632	13497	XP_003359075.1	ssc:100156461	0	NR_033404.1	BMP1
LOC100516787	82.06395354	16.52556743	2.31204885	0.049006	High	99	20	XR_115797.3	ssc:100516787			
RRAGC	1371.877203	6862.241877	-2.322528627	0.049021	Low	1655	8305	XP_005665299.1	ssc:100513798	0	NM_022157.3	RRAGC
NHE5	954.9260048	206.5695929	2.208761041	0.049102	High	1152	250	XP_005664478.1	ssc:396753	0	NM_004594.2	SLC9A5
LOC100516275	96.15574354	19.83068092	2.277638819	0.049165	High	116	24	XP_005666316.1	ssc:100516275	0	NM_033400.2	ZFHX2
LOC102158540	193.1404159	42.14019696	2.196381127	0.04954	High	233	51	XP_005664039.1	ssc:102158540	5E-57	NR_073474.1	PTPRR
1-Mar	22.38107824	2.478835115	3.174545326	0.049826	High	27	3	XP_005666735.1	ssc:100738764	2E-70	NM_017923.3	1-Mar
LOC100624694	22.38107824	2.478835115	3.174545326	0.049826	High	27	3	XP_005659018.1	ssc:100624694	2E-77	NM_001774.2	CD37
ATHL1	224.6397112	1039.458192	-2.210146841	0.04994	Low	271	1258	XP_005653042.1	ssc:100518005	0	NM_025092.4	ATHL1
