# Supplementary material for: Common microRNA–mRNA interactions exist among distinct porcine iPSC lines independent of their metastable pluripotent states
Source: Cell Death Dis. 2017 Aug 31;8(8):e3027–. doi: 10.1038/cddis.2017.426 (PMC5596602; doi:10.1038/cddis.2017.426)
Supplement: Supplementary Table 7 [file cddis2017426x8.pdf]

	piPS-LvsTarget mRNAs			piPS-FvsTarget mRNAs			piPS-LFvsTarget mRNAs	
1	PRKCZ	High		ACTL8	High		SLC6A1	High
2	DIRAS2	High		LIN28A	High		NLRP12	High
3	POU2F3	High		GABRA5	High		LOC100514082	High
4	VRTN	High		OTX2	High		VRTN	High
5	BPIFB6	High		HSD17B2	High		BPIFB6	High
6	CALB2	High		SOX21	High		NXNL2	High
7	TMEM171	High		VRTN	High		UTF1	High
8	UTF1	High		ST14	High		ACTL8	High
9	ST14	High		LOC100622870	High		LIN28A	High
10	KRT6A	High		SALL4	High		ST14	High
11	LOC102164968	High		CDH1	High		HSD17B2	High
12	HENMT1	High		LOC102159782	High		GABRR2	High
13	LOC100519925	High		TMEM171	High		INPP5D	High
14	LOC102164762	High		LOC100511106	High		GABRA5	High
15	LOC100152743	High		LOC100628205	High		OTX2	High
16	LOC102160289	High		LOC100622539	High		KRT6A	High
17	OTX2	High		LEFTY2	High		SALL4	High
18	EMSP1	High		LOC100513452	High		SOX21	High
19	CDH1	High		SLC6A1	High		HENMT1	High
20	KRT18	High		MCF2	High		CDH1	High
21	LOC102159782	High		KLHDC8A	High		LOC100511106	High
22	ERBB4	High		POU5F1	High		AIF1	High
23	LOC100511106	High		LOC100519925	High		LOC100622539	High
24	FAM43B	High		KCNJ3	High		NTRK2	High
25	SOX21	High		LOC102165113	High		LOC100519889	High
26	LOC100622539	High		NFE2L3	High		ANO9	High
27	ANO9	High		LOC100153981	High		NPTX1	High
28	KIT	High		INPP5D	High		NRARP	High
29	RAB3C	High		LOC102157759	High		FAM43B	High
30	SOX2	High		TPD52L1	High		SAMD5	High
31	PDZD4	High		AIF1	High		SOX2	High
32	LOC100512967	High		KCNK13	High		C1QL4	High
33	BATF3	High		LRAT	High		ALPL	High
34	LINGO1	High		KIAA1244	High		NHigh210	High
35	LOC100153689	High		C1QL4	High		ELMOD1	High
36	DRD2	High		LOC100738662	High		PODXL	High
37	NRARP	High		LOC100153689	High		LOC102164762	High
38	NPTX1	High		VSNL1	High		LOC100152743	High
39	LRAT	High		OCLN	High		LOC102164968	High
40	C1QL4	High		LOC102160289	High		PCNXL2	High
41	NHigh210	High		ELOVL2	High		LOC100620918	High
42	HSPA2	High		LOC100514082	High		GREB1L	High
43	CDA	High		LOC100517050	High		HSPA2	High
44	LOC102163958	High		NLRP12	High		LOC100523750	High
45	TPD52L1	High		LOC102166331	High		FAM65B	High
46	SYBU	High		ROBO2	High		CAMKV	High
47	ACTL8	High		ZSWIM5	High		LOC100513699	High
48	VSNL1	High		LOC100519889	High		LOC102160252	High
49	LOC100519889	High		LOC100621315	High		LOC100737566	High
50	LEFTY2	High		NHigh210	High		LOC100153981	High
51	KIAA1244	High		VPAC1	High		ZSWIM5	High
52	KLK15	High		LOC100517681	High		LOC102166331	High
53	LOC100511328	High		GREB1L	High		LOC100622870	High
54	LOC100737363	High		ZSCAN10	High		CHGA	High
55	FRMPD4	High		PRKCZ	High		KCNH3	High
56	FOXA3	High		EMSP1	High		DRD2	High
57	CAMSAP3	High		DNMT3B	High		TPD52L1	High
58	ALPL	High		DRD2	High		KIAA1244	High
59	NFE2L3	High		LOC100739808	High		CACNB4	High
60	LOC100738735	High		LOC100621630	High		FRMPD4	High
61	DUOX2	High		SLAIN1	High		KCNJ3	High
62	LOC102165390	High		LOC102164249	High		LRAT	High
63	OCLN	High		LOC100626670	High		NFE2L3	High
64	GABRA5	High		LYSMD2	High		MCF2	High
65	NTRK2	High		LOC100737260	High		LOC100523100	High
66	POU5F1	High		CAMKV	High		CDH8	High
67	AIF1	High		UTF1	High		TMEM171	High
68	PPP1R1A	High		SYBU	High		ATP12A	High
69	CAMKV	High		NETO2	High		LOC102157888	High
70	CHGA	High		TARSL2	High		LOC102159782	High
71	LOC100517050	High		CHGA	High		SYBU	High
72	KCNH3	High		CALB2	High		NDST3	High
73	EPCAM	High		LOC100622872	High		LOC100622674	High
74	KLHDC8A	High		LOC100512967	High		VSNL1	High
75	SALL4	High		POU2F3	High		LOC102166344	High
76	SAMD5	High		LOC100737813	High		LOC100738735	High
77	LOC102166331	High		SOWAHB	High		DIRAS2	High

78	SLC16A6	High		EHF	High		LOC100628205	High
79	ELMOD1	High		RAB3C	High		LOC100513452	High
80	SEMA5B	High		HMGCLL1	High		NMUR1	High
81	ATP2A3	High		SOX2	High		PDZD4	High
82	ROBO2	High		PDZD4	High		LOC100153689	High
83	LOC780430	High		LOC102165956	High		CRYBB1	High
84	LOC102157888	High		CCNA1	High		LOC102158081	High
85	GPR37	High		LOC102159197	High		ELAVL2	High
86	CDH8	High		RNF128	High		NPAS1	High
87	LOC102164249	High		FAM65B	High		LINGO1	High
88	INPP5D	High		LOC100739847	High		ELOVL2	High
89	ATP12A	High		LOC100623474	High		LOC100737260	High
90	LOC100739808	High		LOC100626144	High		CAMSAP3	High
91	FOXH1	High		KRT18	High		POU2F3	High
92	KCND3	High		ALPL	High		DNMT3B	High
93	LOC100517681	High		C8H4orf19	High		LOC100621315	High
94	DNMT3B	High		RNF182	High		BATF3	High
95	ETNK2	High		STOX1	High		LOC100621169	High
96	DPP10	High		FAM124A	High		POU3F2	High
97	HSD17B2	High		USP44	High		LOC100519459	High
98	LIN28A	High		PREX2	High		KLHDC8A	High
99	LOC102161608	High		LOC100514323	High		RGS4	High
100	LOC100738350	High		LOC100523750	High		KIT	High
101	NDST3	High		EPCAM	High		OCLN	High
102	LOC100523750	High		LOC100519459	High		LOC100622219	High
103	LOC100622870	High		LOC100620918	High		LOC100621385	High
104	CCDC85C	High		LOC100626715	High		BEX1	High
105	PNP	High		CACNB4	High		VPAC1	High
106	FAM65B	High		LOC102161608	High		PPP1R1A	High
107	TJP3	High		BAI3	High		SNX22	High
108	RGS4	High		LOC102167451	High		STXBP5L	High
109	EHF	High		PPP1R1A	High		PLEKHH1	High
110	STOX1	High		ETNK2	High		LOC100738686	High
111	SNX22	High		LOC100157667	High		LOC100737363	High
112	TRIM50	High		FRMPD4	High		RGS14	High
113	LOC100525793	High		LOC102157666	High		SOWAHB	High
114	CRYBB1	High		LOC102158406	High		LOC102165082	High
115	THSD7B	High		IGSF11	High		LOC100622872	High
116	CGN	High		CRIP3	High		ATP2A3	High
117	LOC102158081	High		RAB3IP	High		LOC102159197	High
118	LOC100513699	High		PODXL	High		RNF128	High
119	PREX2	High		POU3F2	High		DPP10	High
120	FGFR4	High		FAM43B	High		ADAM22	High
121	CYP2D25	High		LOC102163958	High		LOC102163958	High
122	RAPGEF4	High		SLC16A10	High		MCF2L	High
123	LOC100627431	High		AMDHD1	High		GABRB3	High
124	LOC100622872	High		SAMD5	High		LOC100625548	High
125	FAM124A	High		LOC100739780	High		RASGRF2	High
126	LOC100511417	High		LOC100622474	High		LOC102157883	High
127	LSR	High		NTRK2	High		PREX2	High
128	LOC100737139	High		LOC102159424	High		C8H4orf19	High
129	LOC100514082	High		PDE3B	High		LEFTY2	High
130	NANOG	High		LOC102159425	High		LOC100512875	High
131	CACNA2D2	High		SNX22	High		PRKCZ	High
132	ABAT	High		DIRAS2	High		FAM124A	High
133	LOC100737813	High		TSPAN33	High		FZD5	High
134	LOC100738686	High		NANOG	High		SPOCD1	High
135	ETNPPL	High		LOC100738686	High		LOC100517050	High
136	LOC102159197	High		LOC100736904	High		CRIP3	High
137	LOC100622219	High		LOC102158081	High		CDA	High
138	LOC100517809	High		LOC100521071	High		LOC100626812	High
139	LOC100739780	High		MFSD6	High		KIAA0319	High
140	POU3F2	High		ATP12A	High		TRIM50	High
141	CELF4	High		CACNA2D3	High		DSP	High
142	LOC100515261	High		LOC100525848	High		HLF	High
143	LOC100737566	High		NOR-1	High		CCDC85C	High
144	LAD1	High		LOC100513699	High		LOC102162275	High
145	HPDL	High		RHPN2	High		LOC102164249	High
146	KCNJ3	High		BPIFB6	High		LOC100739780	High
147	MCF2L	High		HS3ST5	High		HPDL	High
148	LOC102163367	High		RASGRF2	High		LOC100626144	High
149	LOC102157759	High		LOC100522330	High		LOC102167451	High
150	LOC100522330	High		LOC102161872	High		LOC102161206	High
151	GPR160	High		LOC102157888	High		FGFR4	High
152	LOC100512333	High		NXNL2	High		LOC102157759	High
153	LOC100152729	High		KIT	High		GPRIN3	High
154	PHYHD1	High		SLC25A53	High		LOC100519925	High
155	ARHGAP27	High		ELOVL3	High		GRB14	High

156	LOC100621379	High		KCNH3	High		ERBB4	High
157	LOC100738892	High		LOC100517809	High		MPP7	High
158	RGS14	High		DNAJC22	High		LOC100525496	High
159	LOC100621385	High		LOC100737490	High		LOC100737813	High
160	IQCA1	High		LOC100737780	High		LOC100157667	High
161	LOC100621315	High		FZD5	High		LOC100737780	High
162	LOC100525848	High		LOC100626812	High		LOC100515458	High
163	LOC100516621	High		NDST3	High		ABAT	High
164	LOC102165956	High		LOC100625548	High		LOC100739808	High
165	LOC102165113	High		HPRT1	High		LOC100736715	High
166	LOC100626670	High		BEX1	High		LOC100517699	High
167	LOC100514323	High		GABRB3	High		RAPGEF4	High
168	PODXL	High		SEMA3E	High		CA14	High
169	KIAA0319	High		LOC100625208	High		LOC102161872	High
170	BEX1	High		LOC100524517	High		LOC100736904	High
171	TNFAIP6	High		KRT6A	High		SLC16A6	High
172	LOC100153981	High		RAPGEF5	High		NETO2	High
173	PPP1R17	High		CCDC38	High		LOC100521376	High
174	LOC100627578	High		LOC100622219	High		RBP1	High
175	MCF2	High		LOC102164968	High		AKAP5	High
176	SOWAHB	High		LOC102161702	High		LOC100626670	High
177	TPMT	High		FOXH1	High		ZSCAN10	High
178	SEMA3E	High		CCDC85C	High		GLS2	High
179	LOC100620918	High		CAMSAP3	High		LOC100737630	High
180	PCNXL2	High		KCNJ14	High		CACNA2D2	High
181	LOC100513452	High		ADAM22	High		HMHA1	High
182	C8H4orf19	High		GUCY2C	High		ZNF311	High
183	MLXIPL	High		STXBP5L	High		LOC102165956	High
184	LOC100515339	High		PLEKHH1	High		FOXH1	High
185	LOC100515458	High		SPOCD1	High		FOXA3	High
186	GARNL3	High		PAX6	High		LOC102165113	High
187	HS3ST5	High		PROX1	High		SOX7	High
188	LOC100622674	High		FABP3	High		RAB33A	High
189	FZD3	High		SEMA5B	High		LOC100627081	High
190	LOC100525496	High		LOC102157883	High		AMDHD1	High
191	ARHGEF26	High		LOC100621385	High		STOX1	High
192	GABRA1	High		LOC100738735	High		TSPAN33	High
193	LOC100622474	High		LOC102165390	High		LOC100522330	High
194	SLC6A1	High		TPMT	High		TJP3	High
195	LOC100037976	High		GRB14	High		CALB2	High
196	MPP7	High		LOC100516787	High		EHF	High
197	LOC100511379	High		ERBB4	High		LOC100738350	High
198	LOC100737780	High		RAB33A	High		LOC100517809	High
199	LOC100518848	High		ELAVL2	High		GARNL3	High
200	LOC100626144	High		LOC102159568	High		HS3ST5	High
201	PDZK1IP1	High		LPAR3	High		LOC100157017	High
202	LOC102162598	High		LOC100737042	High		LOC780430	High
203	RHPN2	High		CRYBB1	High		LOC100511379	High
204	LOC100739139	High		FOXA1	High		LOC102159425	High
205	RASSF10	High		GNAL	High		CGN	High
206	LOC100625208	High		GPRIN3	High		LOC102164431	High
207	LOC102157883	High		1-Mar	High		PPP1R17	High
208	PDE3B	High		GARNL3	High		LOC100512967	High
209	MAOB	High		LOC100037976	High		LOC100627431	High
210	ADAM22	High		PCDH11X	High		LOC100514323	High
211	PIK3CD	High		LOC100627081	High		CACNA2D3	High
212	CDCA7	High		LOC102162275	High		FZD3	High
213	SOX7	High		SCN1A	High		KIAA1958	High
214	TSPAN33	High		GPR19	High		LOC100737139	High
215	LOC100739389	High		LOC100738350	High		LOC100622474	High
216	SLAIN1	High		SLC16A6	High		GATA-6	High
217	PPIF	High		GABRR2	High		ETNK2	High
218	VPAC1	High		LOC100737408	High		SLC25A53	High
219	LOC102161206	High		LOC102158716	High		LOC102161608	High
220	MFSD6	High		ZNF311	High		LOC100737408	High
221	SORD	High		RASSF10	High		LOC102157981	High
222	HLF	High		LOC102161206	High		KCNJ14	High
223	NETO2	High		RAB27A	High		RAB3C	High
224	LOC102167451	High		LOC100525496	High		LOC102158406	High
225	SLC1A6	High		KLK15	High		SLC16A10	High
226	PAQR5	High		SASH3	High		SOX13	High
227	ARHGAP8	High		CA14	High		LOC100625208	High
228	LOC100521071	High		ELMOD1	High		KRT18	High
229	THBD	High		LOC100622080	High		LOC100623474	High
230	PLCH1	High		LOC100523100	High		BAI3	High
231	LOC102161702	High		TIAM1	High		LOC100737042	High
232	EFHD1	High		AKAP5	High		KIF5C	High
233	SASH3	High		FZD3	High		PTCHD4	High

234	SCN9A	High		LOC396679	High		GPR4	High
235	AKAP5	High		LOC100739389	High		RAB3IP	High
236	GATA-6	High		SOX7	High		LOC100511328	High
237	NOR-1	High		HPDL	High		PAQR5	High
238	SLC16A10	High		FOXA3	High		NFASC	High
239	LOC100523100	High		KIF5C	High		GPR160	High
240	OSAP	High		LOC100627578	High		SCN1A	High
241	GPD1L	High		SCN9A	High		MSI1	High
242	MND1	High		NMUR1	High		LOC100739847	High
243	RAB3IP	High		LOC100621406	High		LOC100518848	High
244	DSP	High		LOC100156854	High		LOC102159424	High
245	TOM1L1	High		FGFR4	High		WNT8A	High
246	RNF128	High		LOC100738006	High		OPRL1	High
247	LOC396679	High		GLS2	High		RHPN2	High
248	LOC100524517	High		LOC100737630	High		CELF4	High
249	LOC100737260	High		HOMER2	High		CYP2D25	High
250	NPAS1	High		LOC100621683	High		MLXIPL	High
251	TP53I3	High		CXHXorf48	High		RNF207	High
252	KCNJ14	High		PLCH1	High		LOC102165390	High
253	KCNK13	High		XRCC2	High		LOC100512333	High
254	ELAVL2	High		TOM1L1	High		KLHL6	High
255	CRIP3	High		CDC6	High		KCNK13	High
256	LOC100737042	High		LOC100511417	High		CCNA1	High
257	CMTM8	High		BATF3	High		LOC102164131	High
258	SLC25A53	High		HSPA2	High		RNF182	High
259	EPB41L4B	High		LOC100622674	High		LOC100739389	High
260	IGSF11	High		ACN9	High		RAPGEF5	High
261	LOC102160252	High		ZNRF2	High		CNKSR2	High
262	LOC102159404	High		CGN	High		PPIF	High
263	RAB33A	High		KIAA1958	High		DNAJC22	High
264	LOC100628155	High		GPR160	High		GPR37	High
265	CDC6	High		PDSS1	High		LOC100737490	High
266	PLEKHH1	High		SEMA3G	High		LOC102157666	High
267	LOC100519459	High		LOC100628155	High		POLR3G	High
268	ZNF311	High		LOC100524058	High		LOC100627578	High
269	LOC100736904	High		LOC100737139	High		TARSL2	High
270	LOC100515686	High		SRSF12	High		LOC100738662	High
271	LOC100736869	High		POLR3G	High		LOC100525848	High
272	SEMA3G	High		LSR	High		LOC102162112	High
273	LOC100521376	High		LOC100155572	High		LOC102159402	High
274	LYSMD2	High		GPR37	High		SORD	High
275	LOC100524058	High		LOC102160718	High		ROBO2	High
276	LOC102158261	High		RBP1	High		LOC100737799	High
277	LOC100739847	High		IQCA1	High		IGSF11	High
278	LOC100737408	High		LOC102166028	High		ARHGAP27	High
279	SLC44A5	High		DSP	High		LOC102160718	High
280	KBTBD8	High		LRRN4	High		LOC102162598	High
281	SRSF12	High		SOX13	High		LPAR3	High
282	LOC102159425	High		TJP3	High		PHYHD1	High
283	1-Mar	High		LOC100522323	High		LOC100524517	High
284	LOC100737760	High		TRIM50	High		PAOX	High
285	DEPDC1B	High		LOC102166344	High		LOC100621509	High
286	LOC100739107	High		LOC102165082	High		MAOB	High
287	LOC100737799	High		WNK3	High		LOC100628155	High
288	NMUR1	High		LOC100512333	High		LOC102163494	High
289	LOC102157666	High		ANO9	High		LOC102165794	High
290	LOC100621683	High		LOC100521376	High		LOC102158232	High
291	NXNL2	High		KBTBD8	High		LOC100516621	High
292	LOC100625548	High		LOC102163367	High		PDE3B	High
293	GRB14	High		DSCC1	High		TPMT	High
294	IGFBP3	High		FAM169A	High		LOC102160475	High
295	ZNRF2	High		ARHGEF26	High		LOC100739139	High
296	LOC102159424	High		LOC100738956	High		LOC100525793	High
297	LOC102159402	High		LOC100737363	High		HMGCLL1	High
298	WNT8A	High		CDCA7	High		PAX6	High
299	LOC102165082	High		LOC102159402	High		RAB27A	High
300	DEFB123	High		ATP2A3	High		SLAIN1	High
301	GPR19	High		KIAA0319	High		LYSMD2	High
302	KIF5C	High		PCNXL2	High		REC8	High
303	EPB41L4A	High		PGM2	High		PTPRC	High
304	WDR63	High		LOC102157981	High		THBD	High
305	LOC102158578	High		TP53I3	High		PIM2	High
306	LOC102167736	High		DEPDC1B	High		HOMER2	High
307	DTL	High		LOC102160475	High		LOC102163367	High
308	OPRL1	High		LOC102162598	High		LOC100621683	High
309	ACN9	High		LOC100157017	High		SRSF12	High
310	DNAJC22	High		MCF2L	High		PGM2	High
311	PAOX	High		GPD1L	High		TIAM1	High

312	MSI1	High		MSI1	High		LOC100523701	High
313	LOC100736715	High		LOC100737566	High		POU5F1	High
314	LOC100517699	High		RGS4	High		LOC102161702	High
315	LOC100626812	High		LOC100736946	High		GCK	High
316	XRCC2	High		MND1	High		LOC102160289	High
317	SOX13	High		DTL	High		NANOG	High
318	FZD5	High		PPA2	High		MFSD6	High
319	PROX1	High		SORD	High		CDCA7	High
320	LCN2	High		CNKSR2	High		KLK15	High
321	FOXA1	High		CDA	High		DUOX2	High
322	LOC102160475	High		OSAP	High		LOC100626258	High
323	HMGCLL1	High		PTPRC	High		SEMA3G	High
324	PNAS-5	High		LOC100515458	High		LOC100737760	High
325	LOC100738613	High		LOC780430	High		DTL	High
326	GPR4	High		MAOB	High		LOC100626715	High
327	TIAM1	High		KLHL6	High		GUCY2C	High
328	LOC102165794	High		PTCHD4	High		SLC1A6	High
329	RAB27A	High		GABRA1	High		PLCH1	High
330	POLR3G	High		LOC100736869	High		CMTM8	High
331	LOC102163494	High		CNNM1	High		LOC100156854	High
332	SPOCD1	High		LINGO1	High		IQCA1	High
333	DSCC1	High		PPIF	High		LOC396679	High
334	LOC100623474	High		LOC102164131	High		LOC102158578	High
335	LOC102158406	High		WASF1	High		ELOVL3	High
336	CXHXorf48	High		DUOX2	High		LOC100515261	High
337	GLS2	High		MPP7	High		LOC100621406	High
338	LOC100737630	High		ETNPPL	High		LOC100155273	High
339	HMHA1	High		LOC102160252	High		LOC100521071	High
340	PTPRC	High		NPAS1	High		CXHXorf48	High
341	RNF182	High		HLF	High		CDC6	High
342	KIAA1958	High		LOC100516621	High		CNNM1	High
343	LOC100627892	High		REC8	High		RASSF10	High
344	CCDC38	High		ABAT	High		SEMA3E	High
345	RAPGEF5	High		LOC100518848	High		HPRT1	High
346	LOC100155273	High		EPB41L4A	High		FAM169A	High
347	CCNA1	High		SEPP1	High		LOC100524058	High
348	PDSS1	High		DEFB123	High		SCN9A	High
349	PPA2	High		LOC100515261	High		EPB41L4A	High
350	TIMM8A	High		TIMM8A	High		TP53I3	High
351	RASGRF2	High		CDH8	High		LOC100736869	High
352	LOC102158232	High		LOC102165794	High		KBTBD8	High
353	LOC102159568	High		RGS14	High		PDSS1	High
354	PIM2	High		MLXIPL	High		ETNPPL	High
355	LOC102164131	High		ARHGAP27	High		LSR	High
356	LOC102161872	High		PAQR5	High		LOC100627892	High
357	LOC100626715	High		LOC100738613	High		SASH3	High
358	PTCHD4	High		RAPGEF4	High		NOR-1	High
359	GABRR2	High		CYP2D25	High		GPR19	High
360	LOC100516787	High		LOC100739139	High		GNAL	High
361	RBP1	High		TMEM169	High		LOC100517681	High
362	HOMER2	High		RNF207	High		LOC102158261	High
363	LOC100627081	High		HENMT1	High		TOM1L1	High
364	LOC100621406	High		EPB41L4B	High		EMSP1	High
365	ELOVL2	High		LOC100512875	High		GPD1L	High
366	USP44	High		LOC100739107	High		XRCC2	High
367	LPAR3	High		CACNA2D2	High		PNAS-5	High
368	LOC100155572	High		GPR4	High		TMEM169	High
369	LOC102166028	High		LOC100511328	High		PIK3CD	High
370	KLHL6	High		LOC102164431	High		PROX1	High
371	TARSL2	High		KCND3	High		USP44	High
372	ZSCAN10	High		LOC100627892	High		ACN9	High
373	CACNB4	High		LOC100737799	High		FABP3	High
374	LOC100157667	High		LOC100621509	High		PPA2	High
375	RNF207	High		CELF4	High		WNK3	High
376	GCK	High		LOC100736715	High		EPCAM	High
377	PCDH11X	High		LOC100517699	High		MND1	High
378	LOC100157017	High		OPRL1	High		ARHGEF26	High
379	FAM169A	High		LOC102164762	High		SEMA5B	High
380	LOC100738662	High		LOC100152743	High		LOC100621630	High
381	LOC102164546	High		NFASC	High		KCND3	High
382	SCUBE2	High		PNAS-5	High		DEFB123	High
383	LOC102162275	High		LOC102158578	High		ZNRF2	High
384	LOC100621169	High		RFC3	High		OSAP	High
385	LOC100156854	High		LOC102158232	High		PCDH11X	High
386	WASF1	High		NRARP	High		DSCC1	High
387	GNAL	High		PNP	High		LOC100738956	High
388	LOC102164431	High		LOC102159404	High		LOC102158716	High
389	NLRP12	High		LOC100626258	High		LOC102159404	High

390	LOC102166344	High		LOC100525793	High		PNP	High
391	CACNA2D3	High		LOC100627431	High		EPB41L4B	High
392	LRRN4	High		PPP1R17	High		LOC100511417	High
393	LOC100621509	High		LOC100737465	High		LRRN4	High
394	ZFP36	High		LOC100514211	High		LOC100737465	High
395	LOC100737490	High		CMTM8	High		FOXA1	High
396	RFC3	High		GCK	High		FAM54A	High
397	LOC100623625	High		LOC100737760	High		LOC100514211	High
398	LOC100622080	High		HMHA1	High		LOC100622080	High
399	LOC100512875	High		WNT8A	High		DEPDC1B	High
400	BAI3	High		FAM54A	High		CCDC38	High
401	LOC100522323	High		LOC100621169	High		LOC100739107	High
402	AMDHD1	High		PIM2	High		LOC100738613	High
403	NFASC	High		LOC102158261	High		LOC100522323	High
404	GREB1L	High		POSTN	Low		LOC102166028	High
405	LOC100737465	High		COL3A1	Low		LOC100522672	High
406	PAX6	High		MGP	Low		GABRA1	High
407	FABP3	High		AEBP1	Low		RFC3	High
408	LOC102157981	High		LOC100738123	Low		TIMM8A	High
409	PGM2	High		LOC100738213	Low		LOC100155572	High
410	LOC100626258	High		COL1A1	Low		WASF1	High
411	FAM54A	High		CCL2	Low		LOC100738006	High
412	LOC100628205	High		COL4A2	Low		LOC100516787	High
413	ELOVL3	High		COL4A1	Low		1-Mar	High
414	LOC102160718	High		MFAP2	Low		COL6A2	Low
415	ZSWIM5	High		CSPG4	Low		PDGFRB	Low
416	SCN1A	High		LOC100156358	Low		MCSF	Low
417	GABRB3	High		MMP1	Low		SERPINF1	Low
418	LOC102158716	High		COL1A2	Low		LOC102159390	Low
419	LOC100514211	High		PXDN	Low		CRABP2	Low
420	LOC100738006	High		FMOD	Low		IGFBP4	Low
421	TMEM169	High		DES	Low		ITGB5	Low
422	HPRT1	High		LOC733603	Low		PDLIM4	Low
423	LOC100738956	High		LOC100623720	Low		LOC100515628	Low
424	STXBP5L	High		PRRX2	Low		DHX58	Low
425	LOC100738213	Low		COL6A3	Low		LOC100515260	Low
426	TAGLN	Low		LOC100738989	Low		NPY	Low
427	THBS1	Low		LUM	Low		GBP1	Low
428	ACTA2	Low		LOC100621401	Low		LOC100157228	Low
429	ANGPTL2	Low		BACE2	Low		IFIT3	Low
430	TNC	Low		ELN	Low		LRRN4CL	Low
431	LOC102160657	Low		LOC102160657	Low		RPS6KA2	Low
432	POSTN	Low		ENTPD3	Low		SLC6A17	Low
433	ITGA8	Low		COL8A2	Low		LOC102167439	Low
434	MGP	Low		GAS7	Low		CSF1	Low
435	ASPN	Low		LOC100157992	Low		GREM1	Low
436	LUM	Low		TNC	Low		LOC100623720	Low
437	LOC100621401	Low		LOC100152082	Low		PCDH7	Low
438	FMOD	Low		AMCF-II	Low		THBS1	Low
439	COL15A1	Low		LOC100627277	Low		COL1A1	Low
440	COL8A2	Low		SVEP1	Low		GPR124	Low
441	MXRA5	Low		PRELP	Low		BGN	Low
442	LOC733603	Low		LOC100736763	Low		ISG15	Low
443	FAM198B	Low		FAM198B	Low		NES	Low
444	LOC100620394	Low		GPR124	Low		ACTC1	Low
445	LAMA4	Low		CASP4	Low		DES	Low
446	ELN	Low		CHI3L1	Low		THY1	Low
447	LOC100738461	Low		SIX1	Low		SEMA3C	Low
448	LTBP2	Low		IRG6	Low		ITGA8	Low
449	LOC102166564	Low		LTBP2	Low		SAMD9	Low
450	MMP1	Low		C1QTNF5	Low		IGLON5	Low
451	IRG6	Low		ADM	Low		CXCL2	Low
452	COL8A1	Low		LOC100628118	Low		CNN1	Low
453	LOC102160189	Low		CMPK2	Low		CARP	Low
454	LOC100627277	Low		LOC100525680	Low		LOXL1	Low
455	LOC100156689	Low		MX2	Low		LOC100622618	Low
456	PRELP	Low		OBSL1	Low		LOC100157992	Low
457	IFIT2	Low		MCSF	Low		DKK2	Low
458	FBN1	Low		LOC100737434	Low		ARNT2	Low
459	COLL11A1	Low		LOC100737120	Low		LOC100525680	Low
460	LOC102164256	Low		LOC100156689	Low		COL4A2	Low
461	LOXL4	Low		LOC100621838	Low		MX1	Low
462	LOC100739233	Low		TIMP-3	Low		LOC100152082	Low
463	EIF2S3	Low		PDLIM4	Low		LTBP2	Low
464	LOC100628118	Low		LOC102166259	Low		LOC100736872	Low
465	MX2	Low		CXCL10	Low		SVEP1	Low
466	COL4A1	Low		LOC100153335	Low		PRRX2	Low
467	LOC102164134	Low		THY1	Low		FAP	Low

468	COL4A2	Low		PRRX1	Low		LOC100621838	Low
469	LOC102165673	Low		LOC102161109	Low		IFIT2	Low
470	COL3A1	Low		ASPN	Low		LOC100738123	Low
471	LOX	Low		ACTA2	Low		SRPX2	Low
472	LOC100157228	Low		AKR1CL1	Low		LOC102161109	Low
473	PAPP-A	Low		C3	Low		FAM198B	Low
474	LOC102161103	Low		PLA2G4A	Low		AEBP1	Low
475	NID2	Low		SERTAD4	Low		COL8A2	Low
476	COL1A2	Low		PARP12	Low		LOXL4	Low
477	LOC102166965	Low		C12H17orf67	Low		LOC100518095	Low
478	LOC100738989	Low		COL5A1	Low		KIF12	Low
479	LOC100737120	Low		AWN	Low		LOC100049650	Low
480	LOC100736872	Low		PLAC9	Low		ELN	Low
481	INHBE	Low		LOC102164776	Low		ACTA2	Low
482	LOC100153335	Low		DAAM2	Low		LOC100738989	Low
483	LOC100626687	Low		TSHZ3	Low		CCL11	Low
484	NID1	Low		LOC100514841	Low		TSHZ3	Low
485	SLC6A17	Low		LOC100620394	Low		LOC100628118	Low
486	LRRN4CL	Low		NFIX	Low		COL1A2	Low
487	CCL2	Low		LOC100739233	Low		MMP19	Low
488	LOC100511351	Low		CCL11	Low		ASPN	Low
489	LOC100153940	Low		SLC6A17	Low		IL33	Low
490	ADAM19	Low		KCNE4	Low		C12H17orf67	Low
491	SEMA3C	Low		CRABP2	Low		LOC100736763	Low
492	DAAM2	Low		KIFC3	Low		LOC100738213	Low
493	C12H17orf67	Low		LOC102159390	Low		CSPG4	Low
494	LOC100152082	Low		LOC100518132	Low		IRG6	Low
495	MMP23B	Low		PLEKHF1	Low		MGP	Low
496	LOC100621838	Low		LOC102166564	Low		PRELP	Low
497	LOC100525680	Low		CXCL2	Low		LOC100737120	Low
498	SDC2	Low		PAPP-A	Low		LOC100627277	Low
499	DDX60	Low		LOC733579	Low		LOC102165673	Low
500	AWN	Low		LOC100515546	Low		COL3A1	Low
501	LOC100738123	Low		TINAGL1	Low		LOC100621401	Low
502	LOC100157992	Low		TAGLN	Low		LOC102166564	Low
503	MFAP2	Low		TNS1	Low		POSTN	Low
504	NPY	Low		CSF1	Low		LOC102160657	Low
505	SPOCK3	Low		WFDC1	Low		COL5A1	Low
506	GREM1	Low		LOC100513671	Low		IL8	Low
507	COL1A1	Low		SLIT3	Low		CXCL10	Low
508	CCL11	Low		BOK	Low		GAS7	Low
509	FAP	Low		LOC102161219	Low		LOC100628220	Low
510	KCNE4	Low		FAP	Low		ISM1	Low
511	SRPX2	Low		LOC100049650	Low		DPT	Low
512	LOC102167439	Low		LOC100738990	Low		SERTAD4	Low
513	CHI3L1	Low		DPT	Low		LOC100622513	Low
514	LOC100737939	Low		RBP4	Low		DDX60	Low
515	CREB3L1	Low		LOC100625416	Low		MMP1	Low
516	MYLK	Low		LOC102164256	Low		ZBP1	Low
517	NT5E	Low		C1QTNF1	Low		BACE2	Low
518	CSF1	Low		LOC100628220	Low		LOC102161103	Low
519	CARP	Low		LOC100739007	Low		LUM	Low
520	COL5A3	Low		GALNTL2	Low		MX2	Low
521	CXCL10	Low		LOC100625207	Low		LOC102164776	Low
522	LOC100513671	Low		ACTC1	Low		LOC100626687	Low
523	CCN2	Low		LOC102165673	Low		KCNE4	Low
524	LOC100739307	Low		LOC100514326	Low		CHI3L1	Low
525	LOC100521530	Low		GNG11	Low		TNC	Low
526	LOC102164776	Low		LOC102164134	Low		LOC102157763	Low
527	TENM4	Low		ZNF503	Low		LOC100516442	Low
528	IFIT3	Low		IL33	Low		LOC100738461	Low
529	LOC100623720	Low		LOXL4	Low		LOC100738990	Low
530	DPT	Low		MMP23B	Low		PEG3	Low
531	ERG	Low		SAMD9	Low		LOC102166259	Low
532	GBP1	Low		LOC100739307	Low		LOC102158099	Low
533	ADAMTS2	Low		PLAU	Low		LOC100623769	Low
534	FN1	Low		WISP1	Low		FOXS1	Low
535	ITGB5	Low		CCL20	Low		AMCF-II	Low
536	FIBIN	Low		COL5A3	Low		LOC102160189	Low
537	ACTC1	Low		SLC26A7	Low		MXRA5	Low
538	ITGA11	Low		FBXO32	Low		FMOD	Low
539	LOC100628220	Low		S1PR3	Low		CCL2	Low
540	SERPINF1	Low		P4HA3	Low		EIF2S3	Low
541	LOC102159390	Low		SEMA3B	Low		LOC100737137	Low
542	LOC100622513	Low		ADAMTSL3	Low		TSPAN18	Low
543	LOC100525856	Low		PEG3	Low		LOC100153335	Low
544	VGF	Low		TNNT2	Low		LOC100513671	Low
545	TRPC6	Low		LOC102166965	Low		LOC733603	Low

546	PLAC9	Low		LOC102157763	Low		LOC733579	Low
547	PDGFRB	Low		LOC100525856	Low		LOC100625207	Low
548	DAB2	Low		COL6A2	Low		LOC100620730	Low
549	GLIS1	Low		PTGS1	Low		CCL20	Low
550	FLRT2	Low		LOC102165099	Low		AWN	Low
551	LOC100736763	Low		COL16A1	Low		COL6A3	Low
552	COL6A3	Low		LOC102158099	Low		LOC100737434	Low
553	LOC102159987	Low		IL8	Low		SDC2	Low
554	APBB1IP	Low		LOC100620730	Low		COL5A2	Low
555	LOC100739007	Low		LRRC32	Low		IFIT1	Low
556	PHACTR1	Low		SYNPO	Low		LOC100525856	Low
557	PEG3	Low		LOC100623769	Low		LOC102164335	Low
558	LOC100515546	Low		CLEC14A	Low		ZNF814	Low
559	ENTPD3	Low		GADD45B	Low		GDF10	Low
560	ISM1	Low		TRPC6	Low		ZNF134	Low
561	CNN1	Low		CLU	Low		LOC100739844	Low
562	C1QTNF5	Low		NID2	Low		EMILIN1	Low
563	PLXDC2	Low		LOC100511351	Low		SERPINE1	Low
564	CLEC14A	Low		EMILIN1	Low		PTGS2	Low
565	GAS7	Low		PODNL1	Low		VGF	Low
566	MMP19	Low		LOC102160493	Low		TENM4	Low
567	MCSF	Low		LOC100737137	Low		ERG	Low
568	EGFR	Low		ACSL5	Low		BST2	Low
569	SVEP1	Low		LOC100516442	Low		COLQ	Low
570	LOC100737434	Low		HOXC4	Low		ECEL1	Low
571	LOC102159296	Low		GDF10	Low		LOC100737503	Low
572	CSPG4	Low		GBP1	Low		SP100	Low
573	LOC100518132	Low		VASN	Low		TNNT2	Low
574	GPR124	Low		NPY	Low		COL4A1	Low
575	ARSJ	Low		CNN1	Low		VSTM4	Low
576	LOC102164335	Low		LOC102164335	Low		LOC100514326	Low
577	ACVRL1	Low		ARSI	Low		KCNK12	Low
578	LOC100515628	Low		ZNF134	Low		FIBIN	Low
579	LOC100739671	Low		MASP1	Low		EPHA3	Low
580	MYBPH	Low		ACVRL1	Low		LOC102161294	Low
581	ADM	Low		ZNF814	Low		COL14A1	Low
582	LOC100738990	Low		MYBPH	Low		WISP1	Low
583	DPP4	Low		ITGB5	Low		CLEC14A	Low
584	COLQ	Low		ECEL1	Low		KCNA6	Low
585	LOC100738803	Low		VGF	Low		MFAP2	Low
586	LOC733579	Low		LOC100738803	Low		C1QTNF1	Low
587	LOXL1	Low		TLR3	Low		LOC100515404	Low
588	CLMP	Low		CHRNG	Low		LOC100738803	Low
589	CYP7B1	Low		IGLON5	Low		ACSL5	Low
590	LOC100622713	Low		BGN	Low		LOC100739422	Low
591	THBS4	Low		SP100	Low		LOC100624693	Low
592	CCL20	Low		LOC100627133	Low		ID3	Low
593	PLA2R1	Low		ZNF606	Low		THBS4	Low
594	GBP7	Low		SRPX2	Low		ZNF606	Low
595	SLC26A7	Low		FIBIN	Low		SLC26A7	Low
596	LOC100525452	Low		HOXB8	Low		DAB2	Low
597	LOC102162332	Low		ID3	Low		TINAGL1	Low
598	VDR	Low		KCNK12	Low		LOC100739007	Low
599	MASP1	Low		COL15A1	Low		COL8A1	Low
600	AGT	Low		GLIS1	Low		LOC100513741	Low
601	LOC100514326	Low		CARP	Low		VDR	Low
602	LOC102161294	Low		LOC100515404	Low		WSCD2	Low
603	ZBP1	Low		NID1	Low		LOC102167710	Low
604	LOC102158099	Low		LOC100736977	Low		TRPC3	Low
605	IL1A	Low		ZBP1	Low		TNS1	Low
606	KCNA6	Low		IL1A	Low		LOC100625479	Low
607	AIM1	Low		OSR1	Low		NRK	Low
608	COL5A1	Low		KCNA6	Low		MYBPH	Low
609	IL33	Low		LOC100623939	Low		TRIM63	Low
610	CD14	Low		FOXL1	Low		LOC100623035	Low
611	USP18	Low		DKK2	Low		CMPK2	Low
612	ISLR2	Low		MAMLD1	Low		TPM2	Low
613	LOC100625207	Low		DPP4	Low		LOC100515181	Low
614	COL16A1	Low		LOC100737092	Low		EIF1AY	Low
615	GDNF	Low		ISLR2	Low		OSR1	Low
616	LOC102159043	Low		LOC100513632	Low		LOC100518586	Low
617	GALNTL2	Low		LOC494560	Low		MYL1	Low
618	WSCD2	Low		VDR	Low		LOC100627133	Low
619	LOC100625479	Low		FOLR2	Low		EDNRA	Low
620	NIPAL4	Low		MDFI	Low		PODNL1	Low
621	LOC100626567	Low		SPEG	Low		IGF2	Low
622	LOC100512657	Low		PDGFRB	Low		LOC102166950	Low
623	COL6A2	Low		LOC100513741	Low		GBP7	Low

624	MAP1LC3A	Low		CRISPLD2	Low		FGF10	Low
625	NRK	Low		WSCD2	Low		LOC102162332	Low
626	MYL9	Low		CLDN11	Low		HOXB5	Low
627	TRIM63	Low		LOC100626567	Low		PAX1	Low
628	LOC100515181	Low		HOXB9	Low		LOC102159296	Low
629	P4HA3	Low		LOC100623028	Low		PTGS1	Low
630	LOC100514340	Low		NRK	Low		KIAA1199	Low
631	EIF1AY	Low		TRIM63	Low		LOC100623028	Low
632	MYL1	Low		LOC100623035	Low		LOC100512657	Low
633	GDF10	Low		LOC100737413	Low		LOC100523560	Low
634	LOC100514506	Low		LOC100519278	Low		TRPC6	Low
635	LOC100156358	Low		GBP7	Low		LOC100517243	Low
636	EDNRA	Low		LOC100515181	Low		AGT	Low
637	LOC100624487	Low		LOC102161103	Low		HOXB8	Low
638	COL14A1	Low		TBX3	Low		HTRA3	Low
639	IGF2	Low		LOC100514340	Low		PLEKHF1	Low
640	LOC102166950	Low		EIF1AY	Low		IER3	Low
641	CDKN2B	Low		LRRN4CL	Low		LOC100622576	Low
642	FLT1	Low		SERPINE1	Low		ZNF211	Low
643	MYOG	Low		LOC100518586	Low		LOC100739594	Low
644	LOC100624693	Low		MYL1	Low		GPR56	Low
645	LOC100739422	Low		MXRA5	Low		UNC5B	Low
646	KIAA1199	Low		COL8A1	Low		LOC100736853	Low
647	LOC102166778	Low		PTGS2	Low		CREB3L1	Low
648	DHX58	Low		ZBTB4	Low		ZNF613	Low
649	ABI3BP	Low		THBS1	Low		LOC102160493	Low
650	LOC100622618	Low		UNC45B	Low		TNNI1	Low
651	EMILIN1	Low		NNAT	Low		LOC102157467	Low
652	LOC100737503	Low		LOC100514506	Low		ARSI	Low
653	PTGS1	Low		EDNRA	Low		LOC100737413	Low
654	LOC100513632	Low		IGF2	Low		FOXL1	Low
655	DES	Low		LOC102166950	Low		ZNF503	Low
656	LOC100516145	Low		SCARA3	Low		UNC-6	Low
657	GNG11	Low		LOC102161294	Low		LOC100626722	Low
658	FGF10	Low		MYOG	Low		COL5A3	Low
659	ASB9	Low		HOXB5	Low		ANGPTL2	Low
660	IFIT1	Low		LOC100515339	Low		PLAC9	Low
661	LOC102165099	Low		PAX1	Low		MMP23B	Low
662	ESM1	Low		SNED1	Low		DPP4	Low
663	LOC100739427	Low		LOC100523560	Low		LOC100739751	Low
664	LOC102157467	Low		LOC100738461	Low		CD14	Low
665	TNS1	Low		LOC100517243	Low		APOA1	Low
666	BGN	Low		UNC5B	Low		HOMER3	Low
667	LOC102162482	Low		LOC100622791	Low		EXO5	Low
668	DKK2	Low		COLQ	Low		MAMLD1	Low
669	RBP4	Low		LOC100620529	Low		PLAU	Low
670	LOC100736640	Low		TSPAN18	Low		OSBPL3	Low
671	ANGPTL1	Low		LOC100622576	Low		TAGLN	Low
672	GHR	Low		ZNF211	Low		LOC494560	Low
673	IFIH1	Low		LOC102162054	Low		LOC102163456	Low
674	S1PR3	Low		LOC100739594	Low		LOC100622713	Low
675	LOC100628185	Low		FOXF2	Low		KCNK2	Low
676	ZIC4	Low		LOC100523671	Low		TLR4	Low
677	KCNK2	Low		CREB3L1	Low		LOC100736658	Low
678	TLR4	Low		ZNF613	Low		ADAMTS4	Low
679	AEBP1	Low		TNNI1	Low		CD248	Low
680	LOC100736658	Low		LOC102157467	Low		BEND6	Low
681	LOC100623608	Low		FOXS1	Low		LOC102165358	Low
682	OSR1	Low		METRNL	Low		ZNF568	Low
683	PTPRU	Low		LOC100626722	Low		HOXC4	Low
684	LOC100622410	Low		DAB2	Low		SPEG	Low
685	LOC102157546	Low		LOC100626132	Low		FN1	Low
686	KIFC3	Low		LOC100739751	Low		LOC100623939	Low
687	LOC102161219	Low		C1H15orf52	Low		LOC102157546	Low
688	IL8	Low		LOC100736640	Low		LOC100626132	Low
689	LOC100620730	Low		HHAT	Low		LOC100736668	Low
690	WIF1	Low		EXO5	Low		HOXB9	Low
691	LOC100736668	Low		LOC100621379	Low		SR-PSOX	Low
692	MAMLD1	Low		LOC100152729	Low		DAAM2	Low
693	ISG15	Low		EIF2S3	Low		LOC102159043	Low
694	LOC102157763	Low		LOC100514098	Low		LOC102167727	Low
695	PAX1	Low		PRSS22	Low		LOC100738589	Low
696	PRRX2	Low		LOC102160150	Low		GGT5	Low
697	PDE1C	Low		LOC100623862	Low		TPM1	Low
698	KHDRBS3	Low		LOC102163456	Low		LOC100737912	Low
699	TMEM30B	Low		TLR4	Low		MYL4	Low
700	COL5A2	Low		LOC100739671	Low		LOC100736640	Low
701	LRRC32	Low		LOC100736658	Low		IRX2	Low

702	PCDH17	Low		LOC100153940	Low		SDK2	Low
703	BEND6	Low		HOXA3	Low		LOC100626567	Low
704	LOC102160493	Low		CD14	Low		LOC102166778	Low
705	FAT4	Low		IER3	Low		FAM180A	Low
706	LOC100517243	Low		LOC102165647	Low		LOC100737142	Low
707	MYL4	Low		LOC780415	Low		UBE2L6	Low
708	PLAUR	Low		ANPEP	Low		ZNF567	Low
709	ZBED2	Low		ZNF568	Low		LRRC32	Low
710	LOC100623668	Low		CD248	Low		LOC100620398	Low
711	OLFML2B	Low		LOC100623866	Low		LOC100516145	Low
712	OSBPL3	Low		LOC102157546	Low		VASN	Low
713	GAS1	Low		LOC100736668	Low		PARP3	Low
714	MMP14	Low		ERG	Low		LOC100522769	Low
715	ADAMTSL3	Low		CYP2C91	Low		TIMP-3	Low
716	SP100	Low		PHACTR1	Low		GULO	Low
717	LOC100736853	Low		AKR1C4	Low		LOC100514506	Low
718	FAM180A	Low		LOC102157983	Low		IFI44L	Low
719	RNASEL	Low		LOC100622976	Low		ADM	Low
720	CRYAB	Low		CRYAB	Low		MYOD1	Low
721	SLIT3	Low		LOC100738589	Low		ZNF135	Low
722	ZNF423	Low		LOC102167727	Low		MYOG	Low
723	TNN	Low		KHDRBS3	Low		MDFI	Low
724	HTR1B	Low		TMEM30B	Low		LOC100514098	Low
725	FAM78B	Low		LOXL1	Low		LOC102163961	Low
726	LOC100620398	Low		LOC100513356	Low		LOC100156358	Low
727	LOC100515404	Low		IGFBP4	Low		ACACB	Low
728	LOC100518586	Low		LOC100737912	Low		NT5E	Low
729	SULT1C4	Low		LOC102166778	Low		LOC102161219	Low
730	BACE2	Low		MYL4	Low		PLA2R1	Low
731	LOC100737466	Low		LOC100626014	Low		LOC780415	Low
732	MYOD1	Low		ZBED2	Low		PTHLH	Low
733	MYHC	Low		LOC100623668	Low		C1H15orf52	Low
734	ADRA1B	Low		SYT11	Low		ANGPTL1	Low
735	LOC100626974	Low		SLC44A5	Low		PODN	Low
736	NES	Low		LOC100625781	Low		CDH18	Low
737	PDGFRL	Low		LOC100621773	Low		ZNF583	Low
738	KCNMA1	Low		LOC100516145	Low		GNG11	Low
739	MFI2	Low		LOC102165318	Low		LOC100517161	Low
740	PHLDB2	Low		LOC100626977	Low		LOC102158748	Low
741	CD36	Low		IRX2	Low		LOC102166965	Low
742	PTHLH	Low		LOC100625479	Low		PRRX1	Low
743	TIMP-3	Low		LOC100739427	Low		LOC100625781	Low
744	ANPEP	Low		LOC102164681	Low		ISLR2	Low
745	LOC100626014	Low		TNFAIP6	Low		CLU	Low
746	LOC100514098	Low		LOC100739844	Low		PENK	Low
747	IL1RL1	Low		UNC-6	Low		COL15A1	Low
748	LOC100517161	Low		CD40	Low		TIMP2	Low
749	LOC100622715	Low		ZNF567	Low		SOX9	Low
750	LOC102158748	Low		BST2	Low		TRIM34	Low
751	C1QTNF3	Low		GAS6	Low		ADAMTSL3	Low
752	FBLN5	Low		LOC102165620	Low		IL1A	Low
753	SAMD9	Low		LOC100522769	Low		ATOH8	Low
754	CHRNG	Low		LOC100510899	Low		UNC45B	Low
755	LOC100049650	Low		NLRC5	Low		ABI3BP	Low
756	LOC100626977	Low		LOC100624693	Low		PXDN	Low
757	LOC100739844	Low		F3	Low		LOC100520029	Low
758	PENK	Low		GHR	Low		FBXO32	Low
759	PPFIA4	Low		MYOD1	Low		BATF2	Low
760	TRIM72	Low		DHX58	Low		LOC100523405	Low
761	LOC100515260	Low		ZNF135	Low		TMEM30B	Low
762	LOC102165620	Low		LOC100512171	Low		CDH15	Low
763	WDR17	Low		LOC100626974	Low		LOC100737693	Low
764	HSPB7	Low		LOC100737584	Low		RCVRN	Low
765	ADCY2	Low		LOC100739422	Low		LOC100737919	Low
766	LOC100513619	Low		KCNK2	Low		LOC100522644	Low
767	LOC102163961	Low		LOC100525036	Low		FOXF2	Low
768	IGFBP7	Low		AGT	Low		ZNF582	Low
769	BATF2	Low		ARHGEF10L	Low		CCDC69	Low
770	LOC100738589	Low		EPHA3	Low		LOC100521530	Low
771	LOC100739751	Low		ISM1	Low		SLC44A5	Low
772	IRX2	Low		CLMP	Low		SCARA3	Low
773	LOC100523405	Low		PLA2R1	Low		PTPRU	Low
774	LOC100623862	Low		TPM2	Low		MSMP	Low
775	FHL2	Low		PDGFRL	Low		GGT1	Low
776	LOC102161109	Low		SLC46A2	Low		LOC102158695	Low
777	LOC100737693	Low		CDH18	Low		LOC100626014	Low
778	SLC46A2	Low		RNASEL	Low		LOC102161288	Low
779	CRISPLD2	Low		GULO	Low		GALNTL2	Low

780	RCVRN	Low		ZNF583	Low		LOC100738804	Low
781	TCF21	Low		IL1RL1	Low		LOC100739233	Low
782	HOXB9	Low		LOC100517161	Low		SLIT3	Low
783	GPR56	Low		NR2F1	Low		ZNF350	Low
784	EPHA3	Low		LOC102158748	Low		KIFC3	Low
785	PRRX1	Low		LOC100737762	Low		ITGBL1	Low
786	LOC100516442	Low		LOC100154530	Low		IGFBP7	Low
787	LOC102165358	Low		SPARC	Low		LOC100622976	Low
788	LOC102161046	Low		LOC102160189	Low		NLRP3	Low
789	TPM2	Low		LOC100738804	Low		LOC100621009	Low
790	LOC100627808	Low		PENK	Low		LOC100620561	Low
791	ZNF503	Low		SPOCK3	Low		LOC100739307	Low
792	TLR3	Low		IFIH1	Low		LOC102164134	Low
793	LOC102159412	Low		LOC102159043	Low		LOC100623234	Low
794	ITGBL1	Low		HSPB7	Low		LOC100620394	Low
795	LOC100625270	Low		ADCY2	Low		LOC100510899	Low
796	PLA2G4A	Low		LOC100520570	Low		LOC100628185	Low
797	SERTAD4	Low		ESM1	Low		LOC102162054	Low
798	LOC102163538	Low		NTRK1	Low		IGFBP3	Low
799	LOC100737186	Low		PTPRU	Low		NID1	Low
800	LOC100622976	Low		LOC100626135	Low		INHBE	Low
801	SSC5D	Low		HTR1B	Low		LOC100626974	Low
802	NLRP3	Low		TMEM8C	Low		LOC100736977	Low
803	LOC100621009	Low		ADAMTS2	Low		SPOCK3	Low
804	LOC100620561	Low		UBE2L6	Low		LOC100520570	Low
805	PHYH	Low		LOC100621092	Low		LOC100516232	Low
806	DDX58	Low		GREM1	Low		LOC100511351	Low
807	CD44	Low		WNT2B	Low		PHACTR1	Low
808	BOK	Low		FAM180A	Low		HOXA3	Low
809	TRIM9	Low		KIAA1199	Low		SYNPO	Low
810	LOC102163288	Low		LOC100737693	Low		SNED1	Low
811	CDH18	Low		RCVRN	Low		CCN2	Low
812	OMD	Low		LOC100737919	Low		MYLPF	Low
813	PPAP2B	Low		TCF21	Low		GPNMB	Low
814	PODNL1	Low		ABI3BP	Low		LOC100739427	Low
815	LOC102163456	Low		DKK1	Low		SLC15A3	Low
816	LOC100152091	Low		ZNF582	Low		LOC100515546	Low
817	LOC100516232	Low		CCDC69	Low		LOC100627460	Low
818	KCNK12	Low		FN1	Low		LOC100622715	Low
819	PTGS2	Low		LOC100523701	Low		C1QTNF3	Low
820	ENPP6	Low		KIF12	Low		ELTD1	Low
821	FOXS1	Low		LOC100620398	Low		ELK3	Low
822	ARHGAP24	Low		SSC5D	Low		PCDH17	Low
823	KLF7	Low		ITGA8	Low		IRX4	Low
824	TRIM34	Low		TEC	Low		HHAT	Low
825	PLAU	Low		TIMP2	Low		NTNG2	Low
826	LOC102159188	Low		GNAT1	Low		LOC102164256	Low
827	LOC100520702	Low		LCN2	Low		ENTPD3	Low
828	PTER	Low		WIF1	Low		TMPRSS11F	Low
829	ZC3H12D	Low		LOC100511616	Low		IFI44	Low
830	RNF43	Low		ZNF350	Low		CRYAB	Low
831	GPNMB	Low		GGT5	Low		JAM3	Low
832	LOC100736608	Low		CCN2	Low		RNF213	Low
833	ECEL1	Low		ITGBL1	Low		LOC102165620	Low
834	CLDN11	Low		MYL9	Low		CYP7B1	Low
835	LOC102163860	Low		TNNC1	Low		LOC100737127	Low
836	TMEM140	Low		MYOM1	Low		LOC102159412	Low
837	MYOM1	Low		NLRP3	Low		LOC100154530	Low
838	FOLR2	Low		LOC100621009	Low		GLIS1	Low
839	DPEP1	Low		LOC100620561	Low		PLA2G4A	Low
840	LOC100522769	Low		SH2D3C	Low		TNNC1	Low
841	LOC100510899	Low		LOC102159968	Low		MYOM1	Low
842	NTNG2	Low		PRDM16	Low		LOC100623866	Low
843	LOC100154987	Low		LOC100157228	Low		MYHC	Low
844	ARHGEF6	Low		LOC100518095	Low		LOC102164566	Low
845	UNC45B	Low		LOC100523068	Low		KCNMA1	Low
846	TMPRSS11F	Low		ICOSLG	Low		LOC100622791	Low
847	LOC102159707	Low		TRIM9	Low		LOC100513356	Low
848	TMEM45A	Low		PODN	Low		CCDC42	Low
849	TPM1	Low		IGFBP3	Low		LOC102166489	Low
850	LOC100737919	Low		LOC100522595	Low		NFIX	Low
851	TMEM26	Low		TRPC3	Low		CHRNA1	Low
852	XIRP2	Low		C1QTNF3	Low		CD36	Low
853	ADM2	Low		TMEM45A	Low		RBP5	Low
854	LOC102164566	Low		JAM3	Low		LOC102159968	Low
855	PLEKHF1	Low		DDX58	Low		LOC102160150	Low
856	CCDC42	Low		LOC100516232	Low		TMEM200A	Low
857	LOC100514839	Low		ENPP6	Low		LOC100621905	Low

858	LOC102166489	Low		SDK2	Low		LOC100153940	Low
859	GDAP1	Low		LOC100737019	Low		SEMA3A	Low
860	LOC100737142	Low		TNFAIP3	Low		MYL9	Low
861	TNFRSF1B	Low		PARP3	Low		LOC100625694	Low
862	FAM212A	Low		FOXC2	Low		LOC100737762	Low
863	CHRNA1	Low		LOC100627460	Low		F3	Low
864	FAM65C	Low		MYLK	Low		PSP-I	Low
865	LOC102166397	Low		KCP	Low		RBP4	Low
866	LOC100621905	Low		LOC100736853	Low		LOC102167733	Low
867	MYLPF	Low		SERPINF1	Low		LOC100624137	Low
868	IL17RD	Low		NT5E	Low		CLMP	Low
869	FBXO32	Low		ELTD1	Low		LOC100623862	Low
870	LOC102164089	Low		LOC102163538	Low		CMKLR1	Low
871	SEMA3A	Low		TMEM140	Low		LOC100514841	Low
872	ARPP21	Low		IRX4	Low		LOC100620517	Low
873	LOC102158695	Low		NTNG2	Low		IFIH1	Low
874	LOC100737019	Low		TMPRSS11F	Low		CXCL11	Low
875	IL7	Low		SRPX	Low		LOC102159413	Low
876	PSP-I	Low		LOC100523405	Low		LOC102164998	Low
877	LOC102164681	Low		HOMER3	Low		LOC102164089	Low
878	GJA1	Low		MX1	Low		LOC100736608	Low
879	GVIN1	Low		CDH15	Low		POPDC3	Low
880	MSMP	Low		PLAUR	Low		MIR214	Low
881	CMKLR1	Low		LOC102164566	Low		FOLR2	Low
882	FLNC	Low		CCDC42	Low		FAT4	Low
883	LOC100737860	Low		LOC102166489	Low		TNFAIP3	Low
884	LAMA2	Low		GDAP1	Low		ZBED2	Low
885	LOC100153192	Low		COL5A2	Low		RNASEL	Low
886	GLIPR1	Low		HTRA3	Low		ZNF256	Low
887	ADAMTS4	Low		AXL	Low		NIPAL4	Low
888	CXCL11	Low		CMAH	Low		LOC100624487	Low
889	LOC102159413	Low		MYLPF	Low		LOC102157983	Low
890	LOC100738449	Low		CHRNA1	Low		LOC100621279	Low
891	LOC100738968	Low		ASB9	Low		SDK1	Low
892	ZNF211	Low		LOC100737535	Low		PTGDS	Low
893	FOXL1	Low		LOC100623234	Low		LOC102165476	Low
894	LOC102165933	Low		RBP5	Low		ZNF286A	Low
895	IER3	Low		LOC100627840	Low		TBX3	Low
896	YPEL2	Low		PCDH17	Low		NFKBIA	Low
897	ID3	Low		RELB	Low		LOC100523068	Low
898	VSTM4	Low		SEMA3A	Low		NID2	Low
899	CMPK2	Low		RXFP4	Low		LOC100522595	Low
900	TMEM8C	Low		COL14A1	Low		LOC102162312	Low
901	LOC100628148	Low		LOC100625694	Low		LOC100513982	Low
902	LOC100623513	Low		LOC100512657	Low		WIF1	Low
903	VASN	Low		LOC102158695	Low		LOC100520275	Low
904	JAM3	Low		LOC102163288	Low		DDX58	Low
905	NTRK1	Low		LOC102165358	Low		SLC41A2	Low
906	LOC100627133	Low		VSTM4	Low		LOC100152091	Low
907	LOC102161934	Low		PSP-I	Low		LOC100513632	Low
908	SYNPO2L	Low		TRIM34	Low		LOC100627840	Low
909	CD248	Low		LOC100624137	Low		ARSJ	Low
910	AFGF	Low		CMKLR1	Low		LOC100516615	Low
911	RPS6KA2	Low		MMP19	Low		LOC100626465	Low
912	TINAGL1	Low		TNN	Low		LOC102162178	Low
913	SDK1	Low		LOC100628185	Low		ZNF432	Low
914	GGT5	Low		ANKRD35	Low		METRNL	Low
915	GULO	Low		LOC100152707	Low		TLR3	Low
916	LOC102165476	Low		LOC100620517	Low		PTGIR	Low
917	LOC100621773	Low		TNFRSF1B	Low		LOC102167305	Low
918	SEMA3D	Low		PDLIM1	Low		LOC100623608	Low
919	LOC102167733	Low		CXCL11	Low		FAM212A	Low
920	CCDC69	Low		LOC100737466	Low		CASP4	Low
921	LOC102161276	Low		LOC100738968	Low		GNAT1	Low
922	LOC102162312	Low		PTER	Low		LOC100621773	Low
923	SERPINE1	Low		ANGPTL1	Low		CRISPLD2	Low
924	SPEG	Low		LEPREL2	Low		SIX1	Low
925	LOC100513982	Low		POPDC3	Low		LOC100625270	Low
926	ACSL5	Low		LOC102167736	Low		ADRA1B	Low
927	LOC102163771	Low		FLNC	Low		F5	Low
928	THY1	Low		CHD5	Low		LOC100737938	Low
929	RANBP3L	Low		LOC100622513	Low		APBB1IP	Low
930	LOC102157822	Low		LOC100628148	Low		LOC102158959	Low
931	SCG2	Low		LOC100623513	Low		KLHL41	Low
932	IRX4	Low		LOC100737503	Low		LOC100152707	Low
933	TIMP2	Low		LOC102166397	Low		SEMA3B	Low
934	LOC102161722	Low		ZNF256	Low		GLIPR1	Low
935	LOC100626465	Low		TGFB3	Low		PDGFRL	Low

936	LOC102162178	Low		ANKRD63	Low		LOC100737466	Low
937	CLU	Low		KCNMA1	Low		LOC102165647	Low
938	PTGIR	Low		LOC100621279	Low		DKK1	Low
939	LOC100514834	Low		SDK1	Low		CLDN11	Low
940	LOC102167305	Low		ZC3H12D	Low		LOC100737186	Low
941	LOC100738887	Low		LOC102163961	Low		OMD	Low
942	SLC15A3	Low		LOC102159188	Low		NKX2-2	Low
943	3-Mar	Low		APOA1	Low		LOC100737020	Low
944	ATOH8	Low		ZNF286A	Low		P4HA3	Low
945	ACACB	Low		LOC102161934	Low		LOC100739471	Low
946	ARNT2	Low		GPNMB	Low		NR2F1	Low
947	F5	Low		CD36	Low		NNAT	Low
948	GPX3	Low		BATF2	Low		LOC102163368	Low
949	LOC100525036	Low		LOC102162312	Low		TGFB3	Low
950	FOXF2	Low		LOC102159707	Low		COLL11A1	Low
951	KLHL41	Low		LOC100522644	Low		C1QTNF5	Low
952	RBP5	Low		LOC100513982	Low		LOC100511616	Low
953	LOC102159968	Low		LOC100037974	Low		SMOX	Low
954	LOC102164464	Low		LOC102167439	Low		GAS6	Low
955	ZNF613	Low		LOC100622715	Low		LOC100522650	Low
956	LOC100523068	Low		LOC100516615	Low		ZKSCAN2	Low
957	LOC102167710	Low		DPEP1	Low		LOC102160712	Low
958	LOC100737584	Low		LOC100626465	Low		GDAP1	Low
959	NKX2-2	Low		LOC102162178	Low		SH2D3C	Low
960	ANKRD35	Low		ZNF432	Low		MASP1	Low
961	HOXC4	Low		LOC100622991	Low		C3	Low
962	PODN	Low		MAFB	Low		DUSP27	Low
963	CXCL2	Low		PTGIR	Low		MAFB	Low
964	FNDC5	Low		C14H10orf54	Low		LOC100737535	Low
965	LOC102163368	Low		LOC100737468	Low		RXFP4	Low
966	C3	Low		LOC100525452	Low		WNT2B	Low
967	LOC100623939	Low		LOC102163074	Low		LOC100037976	Low
968	LOC100522644	Low		FAM212A	Low		NLRC5	Low
969	NTS	Low		MYHC	Low		LOC102166137	Low
970	LOC100737912	Low		FAM65C	Low		PARP12	Low
971	IFI44L	Low		LOC102164464	Low		LOC100624658	Low
972	HOXB8	Low		IFIT1	Low		LOC100621379	Low
973	C1QTNF7	Low		SMOX	Low		PPFIA4	Low
974	FSCN2	Low		NKX2-2	Low		MYF5	Low
975	LOC102163074	Low		OMD	Low		LOC100621670	Low
976	SSTR1	Low		GGT1	Low		LOC100739371	Low
977	LEPREL2	Low		SLCO2A1	Low		SHOX	Low
978	GAS6	Low		THBS4	Low		TNN	Low
979	TNNI1	Low		LOC102163368	Low		ACVRL1	Low
980	SRPX	Low		LOC100737142	Low		YPEL2	Low
981	MYOT	Low		ACACB	Low		KANK4	Low
982	LOC100156578	Low		SLC1A6	Low		LOC102167011	Low
983	LOC102164159	Low		PDE1C	Low		LCN2	Low
984	BICC1	Low		LOC100737020	Low		OLFML2B	Low
985	SNED1	Low		LOC100515628	Low		FOXC2	Low
986	LOC102166540	Low		NTS	Low		ARHGEF40	Low
987	SDK2	Low		PEAR1	Low		LOC102159203	Low
988	KLF6	Low		LOC100522650	Low		TMEM45A	Low
989	ELTD1	Low		LOC100516594	Low		GAS1	Low
990	MYF5	Low		ZKSCAN2	Low		LOC102166397	Low
991	SHOX	Low		LOC102160712	Low		LOC102164461	Low
992	LOC102160583	Low		USP18	Low		LOC102162793	Low
993	LOC102167011	Low		GLIPR1	Low		WFDC1	Low
994	CNNM1	Low		LOC102164998	Low		ADCY2	Low
995	SR-PSOX	Low		SSTR1	Low		LOC100625416	Low
996	ZNF568	Low		DDX60	Low		LOC102158004	Low
997	LOC102164461	Low		LOC100620105	Low		LOC100739264	Low
998	LOC100621791	Low		ZMYND15	Low		DACT3	Low
999	SEMA3B	Low		LOC100156578	Low		LOC100152729	Low
1000	LOC102162793	Low		LOC102164159	Low		RNF39	Low
1001	LOC100520029	Low		GATA-6	Low		MYLK	Low
1002	LOC100627460	Low		OAZ3	Low		LOC102165318	Low
1003	LOC100736977	Low		LOC100736608	Low		TRIM9	Low
1004	LOC100513741	Low		NFKBIA	Low		ADAMTS2	Low
1005	PDLIM3	Low		LOC100514136	Low		TMEM100	Low
1006	AKR1CL1	Low		LOC100624658	Low		ARHGEF6	Low
1007	NLRC5	Low		ITGA5	Low		LOC102159565	Low
1008	TNMD	Low		LOC100738338	Low		AFGF	Low
1009	LOC102158004	Low		PPFIA4	Low		EVA1B	Low
1010	LOC100739264	Low		MYF5	Low		F13A1	Low
1011	AMOTL2	Low		LOC100621670	Low		LOC102165879	Low
1012	CDH15	Low		LOC100739371	Low		HOGA1	Low
1013	LOC100522595	Low		SHOX	Low		USP18	Low

1014	TRIM55	Low		LOC100624487	Low		BCO2	Low
1015	LOC102162054	Low		NAALADL2	Low		LOC102161934	Low
1016	DKK1	Low		S1PR2	Low		SYNPO2	Low
1017	LOC100738804	Low		NIPAL4	Low		FNDC5	Low
1018	PRRT1	Low		LOC102159203	Low		HSPB7	Low
1019	CMAH	Low		LOC102164461	Low		LOC102163560	Low
1020	NNAT	Low		LOC100621791	Low		HTR2A	Low
1021	IGFBP4	Low		LRRC29	Low		FAM78B	Low
1022	TMEM100	Low		LOC102159265	Low		LOC100623627	Low
1023	LOC100739211	Low		C6H19orf68	Low		LOC100621447	Low
1024	LOC100521743	Low		LOC100738887	Low		GADD45B	Low
1025	F13A1	Low		PTGDS	Low		LOC100623668	Low
1026	LOC102165879	Low		DIO3	Low		LOC100738449	Low
1027	SYT11	Low		LOC102162793	Low		LOC100737092	Low
1028	BCO2	Low		LAMA4	Low		THSD7B	Low
1029	MRVI1	Low		DUSP27	Low		MYOT	Low
1030	LOC102163560	Low		UBA7	Low		ESM1	Low
1031	LOC100626132	Low		TNMD	Low		LOC102158649	Low
1032	LOC100624137	Low		LOC102158004	Low		CASP1	Low
1033	LOC100510895	Low		LOC100739264	Low		SSC5D	Low
1034	LOC100737762	Low		LOC102161276	Low		LOC102167736	Low
1035	LOC494560	Low		XIRP2	Low		PRRT1	Low
1036	TMEM200A	Low		FLT1	Low		LOC100621934	Low
1037	PCDH7	Low		LOC100513619	Low		CDRT1	Low
1038	GBP6	Low		LOC102164768	Low		GAL3ST4	Low
1039	LOC102159630	Low		SDC2	Low		NAALADL2	Low
1040	LOC100513356	Low		TRIM72	Low		MMP14	Low
1041	TTC39B	Low		NFIC	Low		IL7	Low
1042	LOC100625965	Low		GAL3ST4	Low		PPAP2B	Low
1043	LOC100620517	Low		ADRA1B	Low		FBLN5	Low
1044	LOC100621447	Low		LOC102164245	Low		LOC100621708	Low
1045	SNX18	Low		TMEM100	Low		C2H19orf38	Low
1046	ZNF814	Low		LOC100739211	Low		PLAUR	Low
1047	LOC100512714	Low		LOC100514834	Low		LOC100514136	Low
1048	PARP3	Low		RAPSN	Low		LOC100738497	Low
1049	PDLIM4	Low		SEMA3C	Low		GJA1	Low
1050	SHROOM4	Low		LOC102166137	Low		LEPREL2	Low
1051	AKR1C4	Low		LOC102157822	Low		ASPA	Low
1052	POPDC3	Low		LOC100520702	Low		TMPRSS11A	Low
1053	PRDM16	Low		LIMS2	Low		TMEM132C	Low
1054	LOC100512652	Low		F13A1	Low		TEC	Low
1055	LOC102158649	Low		LOC102165879	Low		PDLIM3	Low
1056	CASP1	Low		SLC15A3	Low		WDR17	Low
1057	LOC100620529	Low		LOC100737939	Low		LOC100520702	Low
1058	LOC100737127	Low		GNB3	Low		PID1	Low
1059	ADAMTS7	Low		LOC100738892	Low		LIMS2	Low
1060	LOC100621934	Low		LOC100622713	Low		LOC100737468	Low
1061	CDRT1	Low		SCUBE2	Low		SLCO2A1	Low
1062	LOC100523701	Low		ARHGAP8	Low		LOC100739671	Low
1063	METRNL	Low		MRVI1	Low		TNFAIP6	Low
1064	GBP5	Low		LOC102163560	Low		PHLDB2	Low
1065	LOC102167381	Low		ZIC4	Low		TRIM55	Low
1066	LOC100523671	Low		LOC102159296	Low		LOC100622410	Low
1067	TGFB3	Low		RINL	Low		GNB3	Low
1068	EPN3	Low		FAT4	Low		PRSS22	Low
1069	C2H19orf38	Low		RNF43	Low		BDKRB1	Low
1070	LOC100621708	Low		LOC102159987	Low		AZGP1	Low
1071	FRMD7	Low		ADAMTS4	Low		FLRT2	Low
1072	ANKRD63	Low		LOC102159630	Low		DDIT3	Low
1073	ZKSCAN2	Low		LOC100623627	Low		LOC102159707	Low
1074	LOC102160712	Low		LOC102158959	Low		KRT81	Low
1075	LOC100738497	Low		MSMP	Low		FSCN2	Low
1076	SEPP1	Low		LOC100626701	Low		LOC102164245	Low
1077	LOC100623028	Low		LOC102163860	Low		LOC100516594	Low
1078	ZNF134	Low		LOC100621447	Low		LOC102157611	Low
1079	SIX1	Low		LOC100512714	Low		RANBP3L	Low
1080	LOC100626722	Low		PHYH	Low		LOC100628148	Low
1081	ASPA	Low		LOC100038019	Low		ARPP21	Low
1082	TMPRSS11A	Low		C3H2orf40	Low		SSTR1	Low
1083	SGSM1	Low		SHROOM4	Low		LOC102160583	Low
1084	SPINK2	Low		LOC100622618	Low		SPARC	Low
1085	ZNF286A	Low		LOC102158649	Low		LOC102164159	Low
1086	IFI44	Low		CASP1	Low		NKX3-2	Low
1087	LOC102165318	Low		LOC100737127	Low		IFNE	Low
1088	LOC100624264	Low		LOC102166276	Low		LOC102160618	Low
1089	TRABD2A	Low		SOX9	Low		KIAA1755	Low
1090	LOC100625416	Low		LOC100739077	Low		LOC102166540	Low
1091	LOC100737535	Low		LOC100621934	Low		LOC100620269	Low

1092	AXL	Low		LOC100517234	Low		LOC102159630	Low
1093	C7H15orf59	Low		GBP5	Low		LOC100624393	Low
1094	PID1	Low		MMP14	Low		LOC100621405	Low
1095	LOC100515572	Low		LOC102160583	Low		LOC100524378	Low
1096	LRRC31	Low		EVA1B	Low		TNFRSF1B	Low
1097	LOC100737137	Low		LOC100621708	Low		LOC100626977	Low
1098	UNC5B	Low		LOC100155273	Low		GHR	Low
1099	LOC100623035	Low		AMOTL2	Low		LOC102159229	Low
1100	CRABP2	Low		PLEKHG1	Low		LOC102162209	Low
1101	LOC100739371	Low		LOC100738497	Low		FLT1	Low
1102	CASP4	Low		ARHGEF40	Low		LOC100512714	Low
1103	LOC100737841	Low		SLC41A2	Low		CHRNG	Low
1104	LOC102165647	Low		PRRT1	Low		SHROOM4	Low
1105	UNC-6	Low		ASPA	Low		ICOSLG	Low
1106	LOC102157895	Low		TMPRSS11A	Low		LOC102162300	Low
1107	LOC100155159	Low		SPINK2	Low		SCG2	Low
1108	IGLON5	Low		TMEM132C	Low		SLC46A2	Low
1109	RNF213	Low		LOC102164089	Low		LOC100622512	Low
1110	TRPC3	Low		SULT1C4	Low		ZNF660	Low
1111	C1QTNF1	Low		MYOT	Low		LOC100738887	Low
1112	LOC102158142	Low		PID1	Low		STX3	Low
1113	LOC102166308	Low		LOC100515572	Low		LOC102161046	Low
1114	KCP	Low		LOC102166540	Low		TNMD	Low
1115	FOXC2	Low		LOC102163869	Low		LOC102158142	Low
1116	TSPAN18	Low		LOC100737186	Low		PTER	Low
1117	F3	Low		LRRC31	Low		LOC102166369	Low
1118	GUCA1B	Low		LOC102162482	Low		LOC100514834	Low
1119	PNOC	Low		AZGP1	Low		LOC102159666	Low
1120	ZNF583	Low		LOC100737860	Low		NTS	Low
1121	ZBTB4	Low		LOC102157895	Low		SCN4B	Low
1122	PARP12	Low		KRT81	Low		SST	Low
1123	GNB3	Low		LOC102161722	Low		PHYH	Low
1124	LOC102164820	Low		SYNPO2	Low		PEAR1	Low
1125	IFNE	Low		PRMT8	Low		LOC100626660	Low
1126	LOC102160618	Low		LOC102157611	Low		SGSM1	Low
1127	TTNLOC100620261	Low		LOC100515686	Low		LOC102167347	Low
1128	CACNA1A	Low		IFI44L	Low		LOC100518295	Low
1129	UBA7	Low		LOC396904	Low		MYH2	Low
1130	LOC100739776	Low		LOC100624418	Low		PPP2R2B	Low
1131	RNF39	Low		LOC102167381	Low		SRPX	Low
1132	LOC100621405	Low		LOC102164820	Low		MEG3	Low
1133	LOC102158175	Low		IFNE	Low		AMOTL2	Low
1134	GNG8	Low		LOC102160618	Low		LOC100737939	Low
1135	LOC102166014	Low		KIAA1755	Low		CYP2C91	Low
1136	HOXB5	Low		TTNLOC100620261	Low		LOC102159458	Low
1137	DMPK	Low		CACNA1A	Low		SERPINB2	Low
1138	LOC102167359	Low		MAP1LC3A	Low		ZSCAN4	Low
1139	LOC102159229	Low		TRIM55	Low		LOC102163771	Low
1140	WFDC1	Low		RNF39	Low		PDE1C	Low
1141	SYNPO2	Low		LOC100624393	Low		LOC100037974	Low
1142	LOC100520275	Low		LOC100621405	Low		S1PR3	Low
1143	CCL3L1	Low		LOC100524378	Low		LOC100513619	Low
1144	SYT1	Low		B2R	Low		LOC102163860	Low
1145	EXO5	Low		ARNT2	Low		LOC100737860	Low
1146	LOC102166137	Low		GVIN1	Low		PRMT8	Low
1147	TSHZ3	Low		FSCN2	Low		LOC100621791	Low
1148	LOC102160052	Low		ADM2	Low		SYT11	Low
1149	LOC102162300	Low		LOC102167710	Low		KCNN3	Low
1150	LOC100626660	Low		COLL11A1	Low		LOC100512652	Low
1151	ZNF660	Low		FGF10	Low		LAMA2	Low
1152	CADPS	Low		LOC100520275	Low		C3H2orf40	Low
1153	NR2F1	Low		NKX3-2	Low		LOC102161426	Low
1154	RELB	Low		MEG3	Low		LOC100738788	Low
1155	KCNN3	Low		CCL3L1	Low		TTNLOC100620261	Low
1156	LOC102159666	Low		LOC100515260	Low		CACNA1A	Low
1157	AMCF-II	Low		AFGF	Low		LOC100523671	Low
1158	UBE2L6	Low		PTHLH	Low		PLEKHG1	Low
1159	SCN4B	Low		LOC102160052	Low		SLA-DQA1	Low
1160	SPMI	Low		LOC102162300	Low		LOC102166264	Low
1161	LOC100622576	Low		HOGA1	Low		LOX	Low
1162	LOC100037974	Low		SYT1	Low		FAM114A1	Low
1163	VIM	Low		BCO2	Low		KLF6	Low
1164	C6H19orf68	Low		LOC100621905	Low		GVIN1	Low
1165	LOC100152707	Low		CD44	Low		LOC100156689	Low
1166	WISP1	Low		HTR2A	Low		GBP5	Low
1167	MLKL	Low		LOC100622512	Low		ADCYAP1	Low
1168	ZNF350	Low		ZNF660	Low		LOC100627616	Low
1169	HSD17B13	Low		CADPS	Low		EFHD1	Low

1170	LOC102167347	Low		ARPP21	Low		CCL3L1	Low
1171	LOC100620105	Low		STX3	Low		TMEM8C	Low
1172	LOC102157983	Low		LOC100739537	Low		GPX3	Low
1173	LOC100517234	Low		NES	Low		SPINK2	Low
1174	ITGA10	Low		LOC102159666	Low		LOC100037951	Low
1175	STX3	Low		LOC100623608	Low		LOC102157549	Low
1176	HRC	Low		IFIT3	Low		LOC102166942	Low
1177	LOC100519278	Low		EGFR	Low		LOC100625760	Low
1178	ARSI	Low		GPR56	Low		UBA7	Low
1179	LOC100737468	Low		LOC102163771	Low		MAP1LC3A	Low
1180	LOC102160150	Low		SCN4B	Low		C7H15orf59	Low
1181	LOC100739471	Low		SST	Low		LOC100515572	Low
1182	LOC100518295	Low		SPMI	Low		C14H10orf54	Low
1183	MYH2	Low		CTSA	Low		GDNF	Low
1184	PPP2R2B	Low		LOC100510895	Low		LOC100626667	Low
1185	LOC100623769	Low		LAMA2	Low		BICC1	Low
1186	ZNF567	Low		LOC102167347	Low		XIRP2	Low
1187	MIR214	Low		LOC100152091	Low		NFIC	Low
1188	DCX	Low		LOC102166805	Low		LRRC31	Low
1189	LOC102159458	Low		LOC102167305	Low		LOC100622991	Low
1190	ZSCAN4	Low		LOC100518295	Low		DIO3	Low
1191	LOC100625959	Low		LOC102159735	Low		LOC100626135	Low
1192	NAALADL2	Low		ZNF423	Low		LOC102161276	Low
1193	KANK4	Low		LOC102167733	Low		TCF21	Low
1194	LOC100620269	Low		PIK3CD	Low		SPMI	Low
1195	LOC100736946	Low		BEND6	Low		CXCL9	Low
1196	LOC100154530	Low		DCX	Low		LOC100623513	Low
1197	LOC100627840	Low		LOC102159458	Low		CTSA	Low
1198	LOC102159203	Low		LOC100511379	Low		IL1RL1	Low
1199	ZNF256	Low		SERPINB2	Low		LOC102164680	Low
1200	STEAP4	Low		ZSCAN4	Low		LOC100626991	Low
1201	LOC100737938	Low		LOC100625959	Low		ABCC9	Low
1202	PRMT8	Low		LOC102165012	Low		LOC100625402	Low
1203	LOC102161288	Low		KANK4	Low		LOC100515686	Low
1204	LOC100623959	Low		LOC102167011	Low		ARHGDIB	Low
1205	DIO3	Low		LOC102158142	Low		LOC100738968	Low
1206	LOC100514136	Low		LOC100739471	Low		LOC102167359	Low
1207	LOC100624418	Low		FRMD7	Low		LOC100514340	Low
1208	LOC102161426	Low		STEAP4	Low		LOC100624808	Low
1209	LOC100738788	Low		LOC100154987	Low		LOC100152800	Low
1210	GUCY2C	Low		F5	Low		UNCX	Low
1211	LOC102167801	Low		FAM78B	Low		CSMD2	Low
1212	ARHGEF10L	Low		DACT3	Low		SYT1	Low
1213	DUSP27	Low		LOC102166308	Low		B2R	Low
1214	S1PR2	Low		INHBE	Low		ENPP6	Low
1215	WNT2B	Low		GNG8	Low		LOC100738892	Low
1216	SLA-DQA1	Low		LOC100736872	Low		LOC102163538	Low
1217	LOC102166264	Low		C2H19orf38	Low		FBN1	Low
1218	REC8	Low		FBLN5	Low		CCL8	Low
1219	LOC100623866	Low		IGFBP7	Low		LOC102163138	Low
1220	GAL3ST4	Low		ATF5	Low		ARHGAP8	Low
1221	TNNT2	Low		AIM1	Low		3-Mar	Low
1222	ADCYAP1	Low		FBN1	Low		LOC100624418	Low
1223	PON1	Low		LOC102161426	Low		LOC100519278	Low
1224	LOC100627616	Low		LOC102167801	Low		ZNF423	Low
1225	LOC102164998	Low		LOC102161046	Low		LOC100620105	Low
1226	LOC100518095	Low		TENM4	Low		LOC100156578	Low
1227	OBSL1	Low		DDIT3	Low		LOC102160052	Low
1228	LOC100516615	Low		ITGA10	Low		LOC100154987	Low
1229	LOC100522650	Low		SLA-DQA1	Low		SEMA3D	Low
1230	LOC100516594	Low		LOC102166264	Low		TMEM140	Low
1231	LOC102157611	Low		BDKRB1	Low		PLXDC2	Low
1232	ITGA5	Low		ADCYAP1	Low		ADAM19	Low
1233	ZNF432	Low		PON1	Low		LOC102161240	Low
1234	GGT1	Low		LOC100627616	Low		TM4SF18	Low
1235	PLEKHG1	Low		ATOH8	Low		LOC102158724	Low
1236	TBX3	Low		EPN3	Low		FAM26F	Low
1237	LOC100739594	Low		SCG2	Low		LOC100737032	Low
1238	LOC100037951	Low		LOC102159413	Low		PON1	Low
1239	LOC102157549	Low		C1QTNF7	Low		OBSL1	Low
1240	LOC102166942	Low		KLHL41	Low		LOC100627808	Low
1241	LOC100625760	Low		SNX18	Low		LOC102157895	Low
1242	CA14	Low		LOC100627808	Low		TRIM72	Low
1243	HTR2A	Low		ARHGDIB	Low		LOC100739211	Low
1244	ATF5	Low		SEMA3D	Low		PDZK1IP1	Low
1245	RXFP4	Low		LOC100037951	Low		LOC102160410	Low
1246	CYP2C91	Low		LOC102157549	Low		C6H19orf68	Low
1247	CD40	Low		LOC100625760	Low		LOC102159735	Low

1248	LOC100622016	Low		LOC100624749	Low		LOC100157320	Low
1249	LOC102167726	Low		GBP6	Low		PDLIM1	Low
1250	B2R	Low		LOC102159412	Low		LOC102157470	Low
1251	C1H15orf52	Low		IL7	Low		S1PR2	Low
1252	MX1	Low		LDB3	Low		FRMD7	Low
1253	LOC102159565	Low		LOC102158175	Low		LOC100624264	Low
1254	SH2D3C	Low		LOC100622016	Low		OAZ3	Low
1255	LOC100621670	Low		LOC100626667	Low		LOC100620529	Low
1256	CXCL9	Low		LOC102167726	Low		VIM	Low
1257	LOC100621092	Low		RNF213	Low		PRDM16	Low
1258	C3H2orf40	Low		LOC102163494	Low		LOC102158175	Low
1259	LOC102164680	Low		ZFPM2	Low		DPEP1	Low
1260	LOC100626991	Low		TTC39B	Low		LOC100736946	Low
1261	ABCC9	Low		LOC100157320	Low		HRC	Low
1262	LOC100625402	Low		LOC100155159	Low		ARHGEF10L	Low
1263	C14H10orf54	Low		DMPK	Low		BOK	Low
1264	TNNC1	Low		ARHGEF6	Low		LOC100738338	Low
1265	LOC102159735	Low		KLF6	Low		STEAP4	Low
1266	LOC100520570	Low		VIM	Low		HTR1B	Low
1267	INHBB	Low		PCDH7	Low		LOC102161722	Low
1268	MIR145	Low		LOC102164680	Low		LDB3	Low
1269	LOC100622512	Low		LOC100626991	Low		SYNPO2L	Low
1270	LOC100738599	Low		LOC100625402	Low		ITGA5	Low
1271	SPARC	Low		MIR214	Low		LOC102166014	Low
1272	SYNPO	Low		RPS6KA2	Low		LOC100155159	Low
1273	HTRA3	Low		CDRT1	Low		COL16A1	Low
1274	LOC100626135	Low		GDNF	Low		LOC102166276	Low
1275	LOC100621630	Low		SR-PSOX	Low		PAPP-A	Low
1276	LOC100624808	Low		ARSJ	Low		AIM1	Low
1277	UNCX	Low		INHBB	Low		CADPS	Low
1278	CSMD2	Low		MIR145	Low		LOC102167801	Low
1279	GNAT1	Low		PPAP2B	Low		ZBTB4	Low
1280	PDLIM1	Low		FNDC5	Low		KHDRBS3	Low
1281	RAPSN	Low		HRC	Low		LOC100525036	Low
1282	SST	Low		EFHD1	Low		SCN2B	Low
1283	LIMS2	Low		LOC102162112	Low		GUCA1B	Low
1284	TMEM132C	Low		LOC100152800	Low		GBP6	Low
1285	CCL8	Low		UNCX	Low		LOC100510895	Low
1286	LOC102163138	Low		CSMD2	Low		AKR1CL1	Low
1287	LOC100511616	Low		LOC100626354	Low		LOC100737584	Low
1288	LOC396904	Low		IFIT2	Low		ARHGAP24	Low
1289	LOC100621279	Low		LOC102161288	Low		RELB	Low
1290	LOC100737092	Low		HSD17B13	Low		C1QTNF7	Low
1291	STK36	Low		CCL8	Low		AXL	Low
1292	PTGDS	Low		LOC102163138	Low		KCP	Low
1293	LOC100738338	Low		CDKN2B	Low		LOC100520605	Low
1294	APOA1	Low		TMEM26	Low		HEPHL1	Low
1295	LOC100737413	Low		MYH2	Low		LOC102167375	Low
1296	BDKRB1	Low		FLRT2	Low		LOC100739077	Low
1297	AZGP1	Low		APBB1IP	Low		LOC102165933	Low
1298	ICOSLG	Low		FHL2	Low		LOC102159603	Low
1299	LOC102159265	Low		THBD	Low		LOC102163989	Low
1300	LOC100624658	Low		LOC100625965	Low		PTPRCAP	Low
1301	LOC100739077	Low		TRIB3	Low		LOC102167381	Low
1302	LOC102161240	Low		GPX3	Low		LOC100737008	Low
1303	TM4SF18	Low		LOC100624264	Low		CD44	Low
1304	LOC100522814	Low		ZNF793	Low		LOC100739537	Low
1305	LOC102158724	Low		LOC102161240	Low		SEPP1	Low
1306	FAM26F	Low		TM4SF18	Low		FHL2	Low
1307	LOC100737032	Low		LOC100522814	Low		LOC102163288	Low
1308	LOC100737008	Low		LOC102158724	Low		CMAH	Low
1309	LOC100622791	Low		FAM26F	Low		NTRK1	Low
1310	LOC102163869	Low		LOC100737008	Low		LOC100622016	Low
1311	SLC41A2	Low		LOC100521530	Low		LOC102167726	Low
1312	LOC102160410	Low		FGF11	Low		DMPK	Low
1313	LOC102163370	Low		LOC100623959	Low		ZNF793	Low
1314	MEG3	Low		LAD1	Low		LOC102163074	Low
1315	LOC100624749	Low		LOC100520029	Low		FAM65C	Low
1316	LOC100625694	Low		DPP10	Low		LOC102164546	Low
1317	TRIB3	Low		PDZK1IP1	Low		ADAMTS7	Low
1318	ARHGDIB	Low		LOC102160410	Low		LOC100626701	Low
1319	CNKSR2	Low		LOC102163370	Low		LRRC29	Low
1320	MAFB	Low		MYH1	Low		LOC102164768	Low
1321	LOC100622991	Low		LOC100738788	Low		ATF5	Low
1322	CHD5	Low		LOC100623625	Low		LOC100621092	Low
1323	SCARA3	Low		TPM1	Low		ANKRD35	Low
1324	MYH1	Low		LOC100625270	Low		ZMYND15	Low
1325	KIAA1755	Low		LOC102157470	Low		CD40	Low

1326	LOC102157470	Low		LOC100737228	Low		LOC396904	Low
1327	GPRIN3	Low		LOC100737938	Low		LOC100515339	Low
1328	LOC100737228	Low		LOC102159565	Low		CHD5	Low
1329	LOC100514841	Low		LOC102159229	Low		LOC100518132	Low
1330	LOC100623234	Low		LOC102162209	Low		GNG8	Low
1331	LOC100520605	Low		BICC1	Low		LOC100625965	Low
1332	LOC102166259	Low		YPEL2	Low		FGF11	Low
1333	LOC100512171	Low		GJA1	Low		LOC102163869	Low
1334	TEC	Low		PAOX	Low		EGFR	Low
1335	LOC100624393	Low		EFCAB4A	Low		INHBB	Low
1336	LOC100524378	Low		LOC100153192	Low		MIR145	Low
1337	CTSA	Low		OSBPL3	Low		EPN3	Low
1338	TLN1	Low		ZFP36	Low		LOC102164681	Low
1339	LOC102158959	Low		LOC100521743	Low		LOC100624749	Low
1340	SOX9	Low		SGSM1	Low		ANPEP	Low
1341	LOC100739537	Low		WDR63	Low		TTC39B	Low
1342	HOMER3	Low		LOC102166942	Low		DCX	Low
1343	LOC100523560	Low		C7H15orf59	Low		LOC102165012	Low
1344	EVA1B	Low		LOC100514839	Low		LOC102157822	Low
1345	LOC100038019	Low		TLN1	Low		ZC3H12D	Low
1346	LOC102162209	Low		MFI2	Low		SPTLC3	Low
1347	LOC102166276	Low		SPTLC3	Low		MUSK	Low
1348	ZNF582	Low		MUSK	Low		LOC100038019	Low
1349	LOC102166805	Low		URGCP	Low		AKR1C4	Low
1350	GADD45B	Low		LOC100739776	Low		LOC102157538	Low
1351	NFIX	Low		OLFML2B	Low		LOC100516820	Low
1352	LOC102164768	Low		IFI44	Low		RNF43	Low
1353	PEAR1	Low		GAS1	Low		LOC100626354	Low
1354	FGF11	Low		LOC100620269	Low		RAPSN	Low
1355	LOC100625781	Low		TMEM200A	Low		LOC100522814	Low
1356	SLCO2A1	Low		MLKL	Low		MYH1	Low
1357	LOC102164245	Low		ARHGAP24	Low		TLN1	Low
1358	ARHGEF40	Low		LOC100737841	Low		ANKRD63	Low
1359	HOXA3	Low		KCNN3	Low		EFCAB4A	Low
1360	KIF12	Low		CXCL9	Low		LOC100517234	Low
1361	LOC100157320	Low		SCN2B	Low		LOC102161784	Low
1362	ZFPM2	Low		LOC102157538	Low		LOC102163370	Low
1363	SPTLC3	Low		LOC100516820	Low		LOC102159987	Low
1364	MUSK	Low		SYNPO2L	Low		LOC100737228	Low
1365	LOC102166369	Low		LOC100512652	Low		TRABD2A	Low
1366	LOC102162112	Low		LOC100738449	Low		WDR63	Low
1367	LOC100626667	Low		PDLIM3	Low		LOC102166805	Low
1368	ZNF135	Low		ISG15	Low		ITGA11	Low
1369	ZNF793	Low		NPTX1	Low		LOC102165099	Low
1370	LOC100623627	Low		ABCC9	Low		LOC100512171	Low
1371	LOC100626354	Low		PPP2R2B	Low		ZIC4	Low
1372	SCN2B	Low		THSD7B	Low		SCUBE2	Low
1373	LOC102157538	Low		LOC102166369	Low		MRVI1	Low
1374	LOC100516820	Low		TCEAL5	Low		SULT1C4	Low
1375	HOGA1	Low		LOC102161784	Low		CDKN2B	Low
1376	LRRC29	Low		HEPHL1	Low		RINL	Low
1377	EFCAB4A	Low		LOC102167375	Low		LOC102159265	Low
1378	NKX3-2	Low		LOC102166014	Low		LOC100521743	Low
1379	LDB3	Low		GUCA1B	Low		LOC102164820	Low
1380	MDFI	Low		FAM114A1	Low		LOC100738599	Low
1381	OAZ3	Low		ADAMTS7	Low		FLNC	Low
1382	BST2	Low		LOX	Low		SNX18	Low
1383	KRT81	Low		IRAK2	Low		LOC100737841	Low
1384	WNK3	Low		CYP7B1	Low		LOC102164464	Low
1385	NFKBIA	Low		ADAM19	Low		LOC100737019	Low
1386	HHAT	Low		LOC100626660	Low		ZFP36	Low
1387	DDIT3	Low		LOC100624808	Low		LOC100623625	Low
1388	URGCP	Low		C14H10orf71	Low		TMEM26	Low
1389	LOC100522672	Low		LOC102159603	Low		LOC100623959	Low
1390	TCEAL5	Low		LOC102163989	Low		LAD1	Low
1391	LOC102161784	Low		PTPRCAP	Low		LOC100153192	Low
1392	HEPHL1	Low		WDR17	Low		TRIB3	Low
1393	LOC102167375	Low		ANGPTL2	Low		TCEAL5	Low
1394	LOC100626701	Low		TRABD2A	Low		HSD17B13	Low
1395	DACT3	Low		LOC100522672	Low		IL17RD	Low
1396	SERPINB2	Low		PLXDC2	Low		LOC102159568	Low
1397	LOC102165012	Low		LOC102165476	Low		LOC100739776	Low
1398	ZMYND15	Low		STK36	Low		URGCP	Low
1399	RINL	Low		PHLDB2	Low		LAMA4	Low
1400	SMOX	Low		LOC102164546	Low		LOC102162482	Low
1401	ZNF606	Low		LOC100626687	Low		MFI2	Low
1402	LOC100737020	Low		3-Mar	Low		LOC102166308	Low
1403	C14H10orf71	Low		RANBP3L	Low		C14H10orf71	Low

1404	LOC102159603	Low		PHYHD1	Low		LOC100625959	Low
1405	LOC102163989	Low		IL17RD	Low		ITGA10	Low
1406	PTPRCAP	Low		LOC102162332	Low		ZFPM2	Low
1407	IRAK2	Low		LOC100738599	Low		STK36	Low
1408	TNFAIP3	Low		LOC100737032	Low		LOC100525452	Low
1409	LOC780415	Low		ITGA11	Low		IRAK2	Low
1410	LOC102167727	Low		ELK3	Low		PNOC	Low
1411	PXDN	Low		LOC100622410	Low		LOC100514839	Low
1412	PRSS22	Low		LOC102165933	Low		ASB9	Low
1413	FAM114A1	Low		LOC102167359	Low		ADM2	Low
1414	LOC100152800	Low		PNOC	Low		MLKL	Low
1415	ELK3	Low		LOC100520605	Low		KLF7	Low
1416	NFIC	Low		KLF7	Low		LOC102159188	Low
