# Supplementary material for: Common microRNA–mRNA interactions exist among distinct porcine iPSC lines independent of their metastable pluripotent states
Source: Cell Death Dis. 2017 Aug 31;8(8):e3027–. doi: 10.1038/cddis.2017.426 (PMC5596602; doi:10.1038/cddis.2017.426)
Supplement: Supplementary Table 8 [file cddis2017426x9.pdf]

high GO	Category	GO Term	P-Value	FDR	(-log2P)
	GOTERM_BP_DIRECT	stem cell differentiation	1.93E-08	2.61985E-05	25.62954967
	GOTERM_BP_DIRECT	synaptic transmission	1.07E-05	0.007301687	16.50694547
	GOTERM_BP_DIRECT	detection of mechanical stimulus involved in sensory perception of sound	4.51E-05	0.016223	14.43660407
	GOTERM_BP_DIRECT	response to retinoic acid	5.69E-05	0.016223	14.10165202
	GOTERM_BP_DIRECT	regulation of ion transmembrane transport	6.95E-05	0.016223	13.81339315
	GOTERM_BP_DIRECT	negative regulation of oxidative phosphorylation	7.16E-05	0.016223	13.77024399
	GOTERM_BP_DIRECT	protein localization to plasma membrane	0.000212	0.037150068	12.20282684
	GOTERM_BP_DIRECT	endodermal cell fate specification	0.00029	0.037150068	11.75055164
	GOTERM_BP_DIRECT	nervous system development	0.000302	0.037150068	11.69484181
	GOTERM_BP_DIRECT	cochlea development	0.000337	0.037150068	11.53412175
**low GO**	Category	GOTerm	P-Value	FDR	(-log2P)
	GOTERM_BP_DIRECT	extracellular matrix organization	1.19E-31	3.10193E-28	102.7273335
	GOTERM_BP_DIRECT	cell adhesion	1.91E-18	2.48862E-15	58.8600877
	GOTERM_BP_DIRECT	collagen catabolic process	1.04E-16	9.01475E-14	53.09625632
	GOTERM_BP_DIRECT	extracellular matrix disassembly	2.23E-15	1.45446E-12	48.66916784
	GOTERM_BP_DIRECT	collagen fibril organization	1.03E-14	5.36917E-12	46.46302485
	GOTERM_BP_DIRECT	angiogenesis	1.19E-12	5.1783E-10	39.60835294
	GOTERM_BP_DIRECT	multicellular organismal development	4.2E-12	1.56193E-09	37.7931859
	GOTERM_BP_DIRECT	blood vessel development	7.32E-11	2.38393E-08	33.66859733
	GOTERM_BP_DIRECT	cell-matrix adhesion	2.17E-10	6.27606E-08	32.10215427
	GOTERM_BP_DIRECT	cell differentiation	3.32E-10	8.64436E-08	31.48825148
**high KEGG**	Category	PathwayTerm	P-Value	FDR	(-log2P)
	KEGG_PATHWAY	GABAergic synapse	0.000127	0.020925036	12.94494829
	KEGG_PATHWAY	Nicotine addiction	0.000977	0.080574515	9.999854804
	KEGG_PATHWAY	Morphine addiction	0.003421	0.188134287	8.191525009
	KEGG_PATHWAY	D-Glutamine and D-glutamate metabolism	0.005761	0.19983173	7.43956623
	KEGG_PATHWAY	Alanine, aspartate and glutamate metabolism	0.006056	0.19983173	7.367536533
	KEGG_PATHWAY	Maturity onset diabetes of the young	0.008074	0.222032957	6.95251397
	KEGG_PATHWAY	Nitrogen metabolism	0.010716	0.227691945	6.544144102
	KEGG_PATHWAY	Dopaminergic synapse	0.012455	0.227691945	6.327185565
	KEGG_PATHWAY	Tight junction	0.013227	0.227691945	6.240420424
	KEGG_PATHWAY	Homologous recombination	0.0138	0.227691945	6.179238963
**low KEGG**	Category	PathwayTerm	P-Value	FDR	(-log2P)
	KEGG_PATHWAY	ECM-receptor interaction	1.33E-20	2.79696E-18	66.02509387
	KEGG_PATHWAY	Focal adhesion	9.32E-17	9.78422E-15	53.25271037
	KEGG_PATHWAY	PI3K-Akt signaling pathway	7.72E-16	5.40071E-14	50.20312712
	KEGG_PATHWAY	Protein digestion and absorption	2.37E-11	1.24346E-09	35.29724258
	KEGG_PATHWAY	Amoebiasis	2.77E-10	1.16496E-08	31.74746755
	KEGG_PATHWAY	Cytokine-cytokine receptor interaction	1.86E-07	6.49866E-06	22.36070913
	KEGG_PATHWAY	Platelet activation	2.05E-06	6.13799E-05	18.89876398
	KEGG_PATHWAY	Malaria	4.01E-06	9.7433E-05	17.92952939
	KEGG_PATHWAY	Rheumatoid arthritis	4.18E-06	9.7433E-05	17.86954988
	KEGG_PATHWAY	Dilated cardiomyopathy	1.19E-05	0.000249953	16.3583709
