# Supplementary material for: Common microRNA–mRNA interactions exist among distinct porcine iPSC lines independent of their metastable pluripotent states
Source: Cell Death Dis. 2017 Aug 31;8(8):e3027–. doi: 10.1038/cddis.2017.426 (PMC5596602; doi:10.1038/cddis.2017.426)
Supplement: Supplementary Table 9 [file cddis2017426x10.pdf]

	High-expression mRNA Vs Targets	miRNAs	Low-expression mRNA Vs Targets	miRNAs
1	TARSL2	chrx_22382_mature		
2	SASH3	ssc-miR-432-5p		
3	SASH3	ssc-miR-370		
4	SASH3	NW_003613242_37866_mature		
5	SASH3	NW_003541201_39964_mature		
6	SASH3	chrx_22720_mature		
7	SASH3	chr4_9932_mature		
8	SASH3	chr4_9930_mature		
9	SASH3	chr3_8078_mature		
10	RNF207	chrx_22639_mature		
11	RGS4	chr4_10395_mature		
12	RGS14	NW_003541201_39964_mature		
13	RGS14	chr2_4023_mature		
14	RAB33A	chr6_14477_mature@@bta-miR-483		
15	OTX2	ssc-miR-206		
16	LOC100521376	ssc-miR-370		
17	LIN28A	chr15_31863_mature		
18	LIN28A	ssc-miR-370		
19	EPB41L4A	hsa-miR-4685-3p		
20	CYP2D25	ssc-miR-370		
21	CHGA	hsa-miR-4685-3p		
22	CAMSAP3	ssc-miR-370		
