# Supplementary material for: Common microRNA–mRNA interactions exist among distinct porcine iPSC lines independent of their metastable pluripotent states
Source: Cell Death Dis. 2017 Aug 31;8(8):e3027–. doi: 10.1038/cddis.2017.426 (PMC5596602; doi:10.1038/cddis.2017.426)
Supplement: Supplementary Table 10 [file cddis2017426x11.pdf]

Sus scrofa

Model ID	Model name	Score	Relative score	Start	End	Strand	predicted site sequence
MA0056.1	MZF1	8.252	0.961980175884013	1	6	-1	ggggga
MA0528.1	ZNF263	5.641	0.800867315605974	1	21	-1	ggtgcacgatttgccggggga
MA0056.1	MZF1	5.893	0.854346388995611	2	7	-1	cggggg
MA0671.1	NFIX	4.486	0.857892434580363	4	12	1	cccggcaaa
MA0498.2	MEIS1	2.576	0.835940599876542	5	11	-1	ttgccgg
MA0668.1	NEUROD2	2.193	0.801132554600654	7	16	-1	acgatttgcc
MA0877.1	Barhl1	4.014	0.824640855054073	8	17	1	gcaaatcgtg
MA0635.1	BARHL2	2.954	0.825487869022647	8	17	1	gcaaatcgtg
MA0879.1	Dlx1	3.899	0.8041976461855	8	17	-1	cacgatttgc
MA0766.1	GATA5	3.656	0.817528945555452	8	15	-1	cgatttgc
MA0711.1	OTX1	4.344	0.846336418872	9	16	1	caaatcgt
MA0712.1	OTX2	2.554	0.80176470638576	9	16	1	caaatcgt
MA1099.1	Hes1	5.218	0.802476500962731	10	19	-1	tgcacgattt
MA0067.1	Pax2	4.439	0.818310848613399	11	18	1	aatcgtgc
MA0738.1	HIC2	5.794	0.858286141820825	12	20	-1	gtgcacgat
MA0738.1	HIC2	4.756	0.835176722747661	15	23	1	gtgcaccga
MA0081.1	SPIB	4.820	0.806987596140569	19	25	1	accgaaa
MA0766.1	GATA5	5.183	0.856359867275452	21	28	-1	agatttcg
MA0714.1	PITX3	4.384	0.803693140971988	21	29	1	cgaaatctc
MA0038.1	Gfi1	8.582	0.879861377215511	22	31	1	gaaatctccc
MA0711.1	OTX1	2.336	0.80719008988965	22	29	1	gaaatctc
MA0712.1	OTX2	3.080	0.812467424081022	22	29	1	gaaatctc
MA0101.1	REL	8.151	0.852458304939653	22	31	-1	gggagatttc
MA0719.1	RHOXF1	5.125	0.904179966576141	22	29	1	gaaatctc
MA0470.1	E2F4	11.426	0.925753160925664	26	36	-1	gggcggggaga
MA0471.1	E2F6	8.243	0.882637143082899	26	36	-1	gggcggggaga
MA0057.1	MZF1(var.2)	6.671	0.832102811548913	26	35	-1	ggcggggaga
MA0738.1	HIC2	4.825	0.83671289800397	27	35	1	ctccccgcc
MA0130.1	ZNF354C	4.636	0.812679270758179	27	32	1	ctcccc
MA0039.2	Klf4	7.754	0.868248695494585	28	37	-1	cgggcggggga
MA0599.1	KLF5	6.336	0.883855677089747	28	37	1	tccccgcccg
MA0056.1	MZF1	8.510	0.973751908405084	28	33	-1	cgggga
MA0079.3	SP1	5.362	0.848600779879853	28	38	1	tccccgcccgg
MA0746.1	SP3	5.182	0.806399180101426	28	38	1	tccccgcccgg
MA0872.1	TFAP2A(var.3)	10.888	0.879689259189677	28	40	-1	tgccgggcgggga
MA0872.1	TFAP2A(var.3)	9.628	0.860338148231797	28	40	1	tccccgcccggca
MA0813.1	TFAP2B(var.3)	7.998	0.841702442005149	28	40	-1	tgccgggcgggga
MA0813.1	TFAP2B(var.3)	9.452	0.863355728679877	28	40	1	tccccgcccggca
MA0815.1	TFAP2C(var.3)	9.631	0.871179484727461	28	40	-1	tgccgggcgggga
MA0815.1	TFAP2C(var.3)	9.793	0.873424825218272	28	40	1	tccccgcccggca
MA0810.1	TFAP2A(var.2)	4.531	0.826688980401351	29	40	-1	tgccgggcgggg
MA0810.1	TFAP2A(var.2)	8.173	0.883214914898319	29	40	1	ccccgcccggca
MA0811.1	TFAP2B	4.213	0.809615632609359	29	40	-1	tgccgggcgggg
MA0811.1	TFAP2B	6.391	0.844157404744563	29	40	1	ccccgcccggca
MA0524.2	TFAP2C	4.680	0.812497570379908	29	40	-1	tgccgggcgggg
MA0524.2	TFAP2C	6.410	0.840610861515215	29	40	1	ccccgcccggca
MA0671.1	NFIX	2.726	0.816457544678349	30	38	1	cccgcccgg
MA0003.3	TFAP2A	3.233	0.806065075010476	30	40	1	cccgcccggca
MA0597.1	THAP1	7.099	0.884897527975318	31	39	1	ccgcccggc
MA0498.2	MEIS1	1.223	0.807287988489379	35	41	-1	gtgccgg
MA0503.1	Nkx2-5(var.2)	3.011	0.802290233127803	36	46	1	cggcacaccag
MA0596.1	SREBF2	6.960	0.804923364607468	37	46	-1	ctggtgtgcc
MA0801.1	MGA	3.616	0.80972597272731	38	45	-1	tggtgtgc
MA0806.1	TBX4	4.739	0.824148382744891	38	45	-1	tggtgtgc
MA0807.1	TBX5	5.231	0.808388316533211	38	45	-1	tggtgtgc
MA0808.1	TEAD3	1.561	0.822563397487036	38	45	1	gcacacca
MA0698.1	ZBTB18	5.027	0.818766636064412	38	50	-1	gagcctggtgtgc
MA0103.2	ZEB1	5.884	0.845304082167151	38	46	1	gcacaccag
MA0820.1	FIGLA	5.859	0.83385893631815	39	48	1	cacaccaggc
MA0824.1	ID4	8.312	0.884635929173185	39	48	1	cacaccaggc
MA0522.2	TCF3	5.096	0.855534751603609	39	48	1	cacaccaggc
MA0830.1	TCF4	6.152	0.881788872651408	39	48	1	cacaccaggc
MA0745.1	SNAI2	3.998	0.82779075397304	40	48	-1	gcctggtgt
MA0472.2	EGR2	6.831	0.841036140247023	45	55	1	aggctcacgca
MA0719.1	RHOXF1	7.515	0.950246508203563	45	52	-1	gtgagcct
MA0003.3	TFAP2A	5.850	0.847204212298823	45	55	1	aggctcacgca
MA0814.1	TFAP2C(var.2)	4.015	0.812480148665184	45	55	1	aggctcacgca
MA0632.1	Tcfl5	5.152	0.847925136788725	46	55	-1	tgcgtgagcc
MA0632.1	Tcfl5	5.152	0.847925136788725	46	55	1	ggctcacgca
MA0067.1	Pax2	5.005	0.84053696225247	47	54	1	gctcacgc
MA0604.1	Atf1	4.142	0.807905057177797	48	55	1	ctcacgca
MA0632.1	Tcfl5	3.786	0.804661073067487	48	57	-1	cctgcgtgag
MA0632.1	Tcfl5	3.786	0.804661073067487	48	57	1	ctcacgcagg
MA0259.1	ARNT::HIF1A	7.282	0.883145232692922	49	56	-1	ctgcgtga

MA0006.1	Ahr::Arnt	9.603	1.00000777117414	50	55	-1	tgcgtg
MA0597.1	THAP1	4.503	0.805447434907213	53	61	-1	tctccctgc
MA0599.1	KLF5	2.845	0.839445270327571	55	64	-1	ctttctccct
MA0514.1	Sox3	8.352	0.889258403359552	56	65	-1	gctttctccc
MA0515.1	Sox6	5.603	0.821311240055264	56	65	-1	gctttctccc
MA0442.1	SOX10	6.636	0.899054900725468	59	64	-1	ctttct
MA0848.1	FOXO4	3.155	0.811958958034424	60	66	1	gaaagca
MA0850.1	FOXP3	6.060	0.869508811304547	60	66	1	gaaagca
MA0820.1	FIGLA	4.229	0.801394945723089	63	72	1	agcaccagcg
MA0824.1	ID4	7.536	0.869758913535539	63	72	1	agcaccagcg
MA0499.1	Myod1	1.356	0.807643333272393	63	75	1	agcaccagcgcgc
MA0048.2	NHLH1	4.775	0.803492154676622	63	72	1	agcaccagcg
MA0506.1	NRF1	1.880	0.800204744721051	63	73	-1	gcgctggtgct
MA0522.2	TCF3	6.419	0.877777111444951	63	72	1	agcaccagcg
MA0830.1	TCF4	5.871	0.877491317712922	63	72	1	agcaccagcg
MA0646.1	GCM1	4.543	0.806201475675604	64	74	-1	cgcgctggtgc
MA0506.1	NRF1	5.461	0.84442940999577	64	74	1	gcaccagcgcg
MA0745.1	SNAI2	6.299	0.873689751356271	64	72	-1	cgctggtgc
MA0632.1	Tcfl5	4.457	0.82591303994666	67	76	-1	agcgcgctgg
MA0632.1	Tcfl5	4.457	0.82591303994666	67	76	1	ccagcgcgct
MA0632.1	Tcfl5	5.269	0.85163077035782	69	78	-1	tgagcgcgct
MA0632.1	Tcfl5	5.269	0.85163077035782	69	78	1	agcgcgctca
MA0503.1	Nkx2-5(var.2)	3.179	0.804764789771817	70	80	1	gcgcgctcagg
MA0810.1	TFAP2A(var.2)	3.216	0.806279424151073	71	82	-1	accctgagcgcg
MA0719.1	RHOXF1	0.059	0.806534318088892	72	79	-1	ctgagcgc
MA0810.1	TFAP2A(var.2)	4.353	0.823926318034393	72	83	-1	caccctgagcgc
MA0746.1	SP3	5.535	0.813350994246662	77	87	-1	ccaacaccctg
MA0148.3	FOXA1	0.929	0.80161490807119	78	92	1	agggtgttggtcatg
MA0493.1	Klf1	8.842	0.861087658492857	78	88	-1	accaacaccct
MA0039.2	Klf4	7.084	0.856865519991403	78	87	1	agggtgttgg
MA0599.1	KLF5	2.953	0.840819181507839	78	87	-1	ccaacaccct
MA0801.1	MGA	5.091	0.836557338070417	79	86	1	gggtgttg
MA0806.1	TBX4	5.946	0.848485149683486	79	86	1	gggtgttg
MA0807.1	TBX5	7.214	0.859630646652157	79	86	1	gggtgttg
MA0614.1	Foxj2	4.429	0.816226813977045	81	88	-1	accaacac
MA0476.1	FOS	2.303	0.819653792165671	82	92	1	tgttggtcatg
MA0847.1	FOXD2	5.619	0.873276008371118	82	88	-1	accaaca
MA0033.2	FOXL1	5.548	0.877157020147798	82	88	-1	accaaca
MA0848.1	FOXO4	2.982	0.808538765835091	82	88	-1	accaaca
MA0738.1	HIC2	3.761	0.813024630283501	83	91	-1	atgaccaac
MA0886.1	EMX2	3.304	0.826521735215473	86	95	-1	cgccatgacc
MA0642.1	EN2	6.125	0.860192706492977	86	95	-1	cgccatgacc
MA0661.1	MEOX1	4.656	0.822927106099434	86	95	-1	cgccatgacc
MA0661.1	MEOX1	3.805	0.80539364293863	86	95	1	ggtcatggcg
MA0067.1	Pax2	7.662	0.944874036349661	86	93	1	ggtcatgg
MA0701.1	LHX9	4.012	0.813542451469405	87	94	-1	gccatgac
MA0089.1	MAFG::NFE2L1	8.812	0.999988528354811	87	92	-1	catgac
MA0666.1	MSX1	4.062	0.810579335424453	87	94	-1	gccatgac
MA0597.1	THAP1	7.200	0.887988614030741	88	96	-1	ccgccatga
MA0748.1	YY2	11.303	0.899726955121698	88	98	-1	cgccgccatga
MA0162.2	EGR1	5.910	0.843471180376684	89	102	-1	ccctcgccgccatg
MA0089.1	MAFG::NFE2L1	4.208	0.800592934199387	89	94	1	catggc
MA0671.1	NFIX	4.185	0.850806126705076	89	97	-1	gccgccatg
MA0775.1	MEIS3	3.057	0.801506310129571	90	97	1	atggcggc
MA0161.1	NFIC	4.646	0.830809647045002	90	95	1	atggcg
MA0470.1	E2F4	5.462	0.831307045724475	91	101	1	tggcggcgagg
MA0504.1	NR2C2	7.331	0.813086269117079	91	105	1	tggcggcgaggggca
MA0599.1	KLF5	0.745	0.812730330711251	93	102	-1	ccctcgccgc
MA0079.3	SP1	5.381	0.848839821631804	94	104	-1	gcccctcgccg
MA0810.1	TFAP2A(var.2)	7.434	0.871745209902916	94	105	-1	tgcccctcgccg
MA0810.1	TFAP2A(var.2)	3.953	0.817718087996286	94	105	1	cggcgaggggca
MA0811.1	TFAP2B	6.225	0.841524744425718	94	105	-1	tgcccctcgccg
MA0811.1	TFAP2B	5.091	0.823540185380116	94	105	1	cggcgaggggca
MA0524.2	TFAP2C	5.331	0.823076618084582	94	105	-1	tgcccctcgccg
MA0524.2	TFAP2C	6.404	0.840513358771394	94	105	1	cggcgaggggca
MA0039.2	Klf4	4.658	0.815648230781376	95	104	1	ggcgaggggc
MA0599.1	KLF5	5.983	0.879365022954242	95	104	-1	gcccctcgcc
MA0673.1	NKX2-8	3.456	0.83537746320776	95	103	-1	cccctcgcc
MA0003.3	TFAP2A	2.965	0.801852125528903	95	105	-1	tgcccctcgcc
MA0057.1	MZF1(var.2)	7.546	0.859781243252386	97	106	1	cgaggggcac
MA0739.1	Hic1	6.642	0.853460157633732	98	106	-1	gtgcccctc
MA0738.1	HIC2	8.525	0.919087513197329	98	106	-1	gtgcccctc
MA0597.1	THAP1	5.256	0.828492858666952	98	106	-1	gtgcccctc
MA0258.2	ESR2	6.317	0.813220651789635	100	114	1	ggggcactgcggcag
MA0503.1	Nkx2-5(var.2)	2.876	0.800301750110292	101	111	1	gggcactgcgg
MA0099.2	FOS::JUN	5.316	0.804790114679483	107	113	-1	tgccgca

MA0671.1	NFIX	3.681	0.838940680960408	107	115	1	tgcggcaga
MA0498.2	MEIS1	1.163	0.806017362707243	108	114	-1	ctgccgc
MA0090.2	TEAD1	6.769	0.862675274553047	111	120	-1	aaaattctgc
MA0809.1	TEAD4	8.179	0.897702063040201	111	120	-1	aaaattctgc
MA0158.1	HOXA5	5.012	0.829453058691846	112	119	1	cagaattt
MA0635.1	BARHL2	2.275	0.812848135859008	113	122	-1	aaaaaattct
MA0041.1	Foxd3	6.592	0.801082580332973	114	125	1	gaattttttcct
MA0847.1	FOXD2	3.030	0.818368469042362	115	121	-1	aaaaatt
MA0087.1	Sox5	6.624	0.840728791704037	116	122	1	atttttt
MA0084.1	SRY	5.866	0.805955911773436	116	124	-1	ggaaaaaat
MA0076.2	ELK4	4.063	0.804257831874891	117	127	1	ttttttcctcc
MA0157.2	FOXO3	5.002	0.807961226849649	117	124	-1	ggaaaaaa
MA0848.1	FOXO4	2.656	0.802093779378544	117	123	-1	gaaaaaa
MA0606.1	NFAT5	5.975	0.831838029706231	117	126	1	ttttttcctc
MA0471.1	E2F6	7.885	0.877355302040208	118	128	-1	gggaggaaaaa
MA0136.2	ELF5	5.073	0.814894729498834	118	128	-1	gggaggaaaaa
MA0606.1	NFAT5	8.388	0.881025748598608	118	127	1	tttttcctcc
MA0624.1	NFATC1	8.349	0.887827337544565	118	127	1	tttttcctcc
MA0625.1	NFATC3	8.552	0.876275243382336	118	127	1	tttttcctcc
MA0152.1	NFATC2	9.490	0.931041560772187	119	125	1	ttttcct
MA0528.1	ZNF263	6.661	0.80985698272932	119	139	-1	gcagcaaagaagggaggaaaa
MA0599.1	KLF5	7.209	0.894961459130246	120	129	1	tttcctccct
MA0079.3	SP1	8.634	0.889766285794684	120	130	1	tttcctccctt
MA0516.1	SP2	5.992	0.805352993580392	120	134	1	tttcctcccttcttt
MA0081.1	SPIB	6.244	0.855637264144336	121	127	-1	ggaggaa
MA0471.1	E2F6	5.290	0.839069331353665	122	132	-1	agaagggagga
MA0056.1	MZF1	5.636	0.842620283344776	122	127	-1	ggagga
MA0597.1	THAP1	5.527	0.836786762835464	123	131	1	cctcccttc
MA0109.1	HLTF	5.436	0.872804971219847	125	134	1	tcccttcttt
MA0090.2	TEAD1	3.947	0.804530359182791	125	134	1	tcccttcttt
MA0514.1	Sox3	3.529	0.817876287026906	126	135	1	cccttctttg
MA0442.1	SOX10	4.820	0.818433532819252	127	132	1	ccttct
MA0144.2	STAT3	0.870	0.808094393174136	128	138	-1	cagcaaagaag
MA0847.1	FOXD2	4.228	0.843775665688113	130	136	-1	gcaaaga
MA0850.1	FOXP3	6.725	0.886240020070117	130	136	-1	gcaaaga
MA0442.1	SOX10	4.669	0.811729883835861	131	136	1	ctttgc
MA0019.1	Ddit3::Cebpa	7.765	0.814607077115656	134	145	1	tgctgcaatctg
MA0088.2	ZNF143	6.068	0.812056484034349	134	149	-1	cacccagattgcagca
MA0766.1	GATA5	5.562	0.865997666444987	137	144	-1	agattgca
MA0662.1	MIXL1	3.639	0.801564569663989	137	146	1	tgcaatctgg
MA0611.1	Dux	4.559	0.819596118562089	138	145	1	gcaatctg
MA0027.2	EN1	4.160	0.832563206457442	138	145	1	gcaatctg
MA0914.1	ISL2	5.090	0.82869756237402	138	145	1	gcaatctg
MA0623.1	Neurog1	5.193	0.823292792394615	138	147	-1	cccagattgc
MA0623.1	Neurog1	7.463	0.876002098882428	138	147	1	gcaatctggg
MA0827.1	OLIG3	3.682	0.807741791842136	138	147	-1	cccagattgc
MA0092.1	Hand1::Tcf3	6.144	0.804602770651927	140	149	1	aatctgggtg
MA0742.1	Klf12	6.177	0.813239853095436	140	154	-1	agccgcacccagatt
MA0079.3	SP1	1.810	0.803912553409963	143	153	-1	gccgcacccag
MA0746.1	SP3	6.691	0.836116708557974	143	153	-1	gccgcacccag
MA0006.1	Ahr::Arnt	5.297	0.817309426751426	144	149	1	tgggtg
MA0493.1	Klf1	10.485	0.888862365225811	144	154	-1	agccgcaccca
MA0039.2	Klf4	11.000	0.923397632335372	144	153	1	tgggtgcggc
MA0599.1	KLF5	6.555	0.886641663649735	144	153	-1	gccgcaccca
MA0801.1	MGA	4.037	0.817384281072698	145	152	1	gggtgcgg
MA0002.2	RUNX1	5.523	0.805784973871463	145	155	1	gggtgcggcta
MA0806.1	TBX4	4.406	0.817434096588676	145	152	1	gggtgcgg
MA0807.1	TBX5	5.247	0.80880176952459	145	152	1	gggtgcgg
MA0646.1	GCM1	4.498	0.80553596887994	146	156	1	ggtgcggctag
MA0767.1	GCM2	5.589	0.808373528677047	146	155	1	ggtgcggcta
MA0684.1	RUNX3	5.428	0.820261232673096	146	155	-1	tagccgcacc
MA0003.3	TFAP2A	3.411	0.808863228024357	150	160	1	cggctagagca
MA0814.1	TFAP2C(var.2)	3.403	0.80266488716263	150	160	1	cggctagagca
MA0102.3	CEBPA	2.401	0.826753626163121	152	162	-1	attgctctagc
MA0081.1	SPIB	5.670	0.836027074934952	155	161	1	agagcaa
MA0087.1	Sox5	6.367	0.831151705260537	156	162	-1	attgctc
MA0090.2	TEAD1	4.275	0.811288520855875	156	165	-1	caaattgctc
MA0809.1	TEAD4	6.100	0.853217246578711	156	165	-1	caaattgctc
MA0877.1	Barhl1	6.512	0.890469041803454	157	166	-1	acaaattgct
MA0635.1	BARHL2	4.139	0.847546902599839	157	166	-1	acaaattgct
MA0879.1	Dlx1	6.125	0.860528793883811	157	166	1	agcaatttgt
MA0612.1	EMX1	6.536	0.846763873641076	157	166	-1	acaaattgct
MA0644.1	ESX1	5.393	0.831579408817481	157	166	1	agcaatttgt
MA0887.1	EVX1	3.427	0.802916471085846	157	166	-1	acaaattgct
MA0888.1	EVX2	3.182	0.805189892469361	157	166	1	agcaatttgt
MA0889.1	GBX1	5.894	0.857663219524059	157	166	1	agcaatttgt

MA0890.1	GBX2	6.822	0.877410749309739	157	166	1	agcaatttgt
MA0892.1	GSX1	5.220	0.836534271459619	157	166	1	agcaatttgt
MA0893.1	GSX2	5.118	0.821684813262664	157	166	1	agcaatttgt
MA0894.1	HESX1	6.554	0.867576755334856	157	166	-1	acaaattgct
MA0900.1	HOXA2	4.185	0.825603040102218	157	166	1	agcaatttgt
MA0902.1	HOXB2	5.078	0.843890236686043	157	166	1	agcaatttgt
MA0903.1	HOXB3	5.721	0.867608939885755	157	166	1	agcaatttgt
MA0699.1	LBX2	5.256	0.818522702138256	157	166	1	agcaatttgt
MA0700.1	LHX2	3.268	0.819432189784446	157	166	-1	acaaattgct
MA0700.1	LHX2	2.222	0.801782699561217	157	166	1	agcaatttgt
MA0662.1	MIXL1	5.652	0.853953029096136	157	166	1	agcaatttgt
MA0125.1	Nobox	6.189	0.82936435549078	157	164	-1	aaattgct
MA0710.1	NOTO	5.961	0.87552776873742	157	166	-1	acaaattgct
MA0718.1	RAX	6.518	0.857018836515325	157	166	1	agcaatttgt
MA0808.1	TEAD3	0.392	0.8044664693835	157	164	-1	aaattgct
MA0724.1	VENTX	2.711	0.81336698127758	157	165	1	agcaatttg
MA0875.1	BARX1	2.778	0.814907886856419	158	165	1	gcaatttg
MA0879.1	Dlx1	4.488	0.819102877988872	158	167	-1	gacaaattgc
MA0885.1	Dlx2	6.366	0.885617774057834	158	165	1	gcaatttg
MA0880.1	Dlx3	6.283	0.871878647537569	158	165	1	gcaatttg
MA0881.1	Dlx4	6.616	0.886253166923116	158	165	1	gcaatttg
MA0882.1	DLX6	6.143	0.873066690622673	158	165	1	gcaatttg
MA0027.2	EN1	6.708	0.887928836841698	158	165	1	gcaatttg
MA0914.1	ISL2	8.022	0.904979249515833	158	165	1	gcaatttg
MA0654.1	ISX	5.545	0.859189214972164	158	165	1	gcaatttg
MA0704.1	Lhx4	4.146	0.821752104199954	158	165	1	gcaatttg
MA0705.1	Lhx8	1.328	0.805187444764903	158	165	-1	caaattgc
MA0701.1	LHX9	5.648	0.858292718108983	158	165	1	gcaatttg
MA0703.1	LMX1B	5.269	0.829806462870895	158	165	1	gcaatttg
MA0666.1	MSX1	6.097	0.860978552699148	158	165	1	gcaatttg
MA0708.1	MSX2	4.612	0.815918546385144	158	165	1	gcaatttg
MA0709.1	Msx3	6.505	0.875225062176186	158	165	1	gcaatttg
MA0668.1	NEUROD2	2.460	0.805495661054651	158	167	-1	gacaaattgc
MA0623.1	Neurog1	4.403	0.804949024938504	158	167	1	gcaatttgtc
MA0132.2	PDX1	2.464	0.813820417530302	158	165	1	gcaatttg
MA0716.1	PRRX1	5.404	0.841122223250983	158	165	1	gcaatttg
MA0075.2	Prrx2	6.054	0.854825064276216	158	165	1	gcaatttg
MA0717.1	RAX2	6.442	0.87041656062619	158	165	1	gcaatttg
MA0630.1	SHOX	6.112	0.84927225736707	158	165	-1	caaattgc
MA0720.1	Shox2	6.172	0.874955378014643	158	165	1	gcaatttg
MA0721.1	UNCX	5.790	0.84969495113426	158	165	1	gcaatttg
MA0722.1	VAX1	6.292	0.861501788132274	158	165	-1	caaattgc
MA0723.1	VAX2	6.255	0.8623391941205	158	165	-1	caaattgc
MA0725.1	VSX1	6.741	0.860940210693593	158	165	-1	caaattgc
MA0726.1	VSX2	5.803	0.829420886026564	158	165	-1	caaattgc
MA0847.1	FOXD2	3.568	0.829778378721338	160	166	-1	acaaatt
MA0158.1	HOXA5	5.527	0.847590893777919	160	167	-1	gacaaatt
MA0491.1	JUND	0.811	0.821122940649332	160	170	-1	tatgacaaatt
MA0655.1	JDP2	4.372	0.801809325909432	161	169	-1	atgacaaat
MA0489.1	JUN(var.2)	3.699	0.812533993736239	161	174	-1	attctatgacaaat
MA0490.1	JUNB	1.016	0.803013026500002	161	171	-1	ctatgacaaat
MA0670.1	NFIA	2.033	0.823366319327152	161	170	-1	tatgacaaat
MA0442.1	SOX10	4.805	0.817767607423551	161	166	1	atttgt
MA0604.1	Atf1	4.084	0.806651805176267	162	169	-1	atgacaaa
MA0774.1	MEIS2	5.535	0.826096795346533	162	169	-1	atgacaaa
MA0775.1	MEIS3	7.506	0.900026379504145	162	169	-1	atgacaaa
MA0084.1	SRY	6.786	0.836757059806185	162	170	-1	tatgacaaa
MA0605.1	Atf3	5.598	0.825698996112549	163	170	-1	tatgacaa
MA0465.1	CDX2	5.865	0.841070477628713	163	173	1	ttgtcatagaa
MA0498.2	MEIS1	9.456	0.981639022894781	163	169	-1	atgacaa
MA0161.1	NFIC	3.787	0.802038652535128	163	168	1	ttgtca
MA0519.1	Stat5a::Stat5b	0.571	0.800748973234957	163	173	1	ttgtcatagaa
MA0899.1	HOXA10	5.582	0.819861482329578	164	174	1	tgtcatagaat
MA0911.1	Hoxa11	5.546	0.80931641449105	164	175	1	tgtcatagaatc
MA0651.1	HOXC11	5.236	0.821336871832339	164	174	1	tgtcatagaat
MA0906.1	HOXC12	4.036	0.807555522594369	164	174	1	tgtcatagaat
MA0067.1	Pax2	5.776	0.870813170054101	164	171	1	tgtcatag
MA0878.1	CDX1	6.146	0.853351071376537	165	173	1	gtcatagaa
MA0905.1	HOXC10	6.760	0.872465416131608	165	174	1	gtcatagaat
MA0908.1	HOXD11	7.295	0.845452174570221	165	174	1	gtcatagaat
MA0909.1	HOXD13	5.280	0.818829739539447	165	174	1	gtcatagaat
MA0913.1	Hoxd9	5.320	0.821834160596873	165	174	1	gtcatagaat
MA0089.1	MAFG::NFE2L1	8.072	0.967939714350681	165	170	-1	tatgac
MA0090.2	TEAD1	6.543	0.85801873632708	168	177	-1	cagattctat
MA0809.1	TEAD4	6.862	0.86952192678249	168	177	-1	cagattctat
MA0766.1	GATA5	3.442	0.812087022013815	169	176	-1	agattcta

MA0623.1	Neurog1	4.321	0.803044988316477	170	179	-1	cccagattct
MA0623.1	Neurog1	6.551	0.854825496452082	170	179	1	agaatctggg
MA0827.1	OLIG3	4.221	0.816705104274573	170	179	-1	cccagattct
MA0719.1	RHOXF1	2.711	0.857650832062083	170	177	1	agaatctg
MA0607.1	Bhlha15	3.265	0.806814700906033	171	178	-1	ccagattc
MA0694.1	ZBTB7B	6.644	0.815343757748668	174	185	-1	gagccccccaga
MA0695.1	ZBTB7C	6.641	0.805737124649578	174	185	-1	gagccccccaga
MA0753.1	ZNF740	6.573	0.816022219919947	175	184	-1	agccccccag
MA0056.1	MZF1	6.469	0.880627466251957	176	181	1	tggggg
MA0478.1	FOSL2	3.321	0.819070989244286	177	187	1	ggggggctcat
MA0492.1	JUND(var.2)	0.281	0.800848679440893	177	191	-1	aaaaatgagcccccc
MA0056.1	MZF1	5.636	0.842620283344776	177	182	1	gggggg
MA0682.1	Pitx1	4.575	0.809684899547031	180	187	-1	atgagccc
MA0714.1	PITX3	4.428	0.804733272541842	180	188	-1	aatgagccc
MA0719.1	RHOXF1	8.998	0.978830893656897	180	187	-1	atgagccc
MA0793.1	POU6F2	8.119	0.860793323174769	181	190	1	ggctcatttt
MA0879.1	Dlx1	5.615	0.847622735785815	182	191	-1	aaaaatgagc
MA0879.1	Dlx1	4.758	0.825935496981928	182	191	1	gctcattttt
MA0612.1	EMX1	5.858	0.827713683546871	182	191	-1	aaaaatgagc
MA0886.1	EMX2	3.797	0.834858947857734	182	191	-1	aaaaatgagc
MA0887.1	EVX1	3.431	0.803007495034444	182	191	1	gctcattttt
MA0888.1	EVX2	3.608	0.814377198497422	182	191	1	gctcattttt
MA0892.1	GSX1	4.533	0.817877252022628	182	191	1	gctcattttt
MA0900.1	HOXA2	3.566	0.811608355961503	182	191	1	gctcattttt
MA0902.1	HOXB2	4.183	0.820640488918807	182	191	1	gctcattttt
MA0903.1	HOXB3	3.686	0.820217699086978	182	191	1	gctcattttt
MA0700.1	LHX2	2.597	0.808110193666103	182	191	1	gctcattttt
MA0706.1	MEOX2	4.298	0.803515624834874	182	191	1	gctcattttt
MA0710.1	NOTO	5.958	0.875474716819245	182	191	1	gctcattttt
MA0877.1	Barhl1	3.648	0.814995892543956	183	192	-1	gaaaaatgag
MA0635.1	BARHL2	4.319	0.850897641877387	183	192	-1	gaaaaatgag
MA0881.1	Dlx4	3.725	0.807379465169032	183	190	1	ctcatttt
MA0882.1	DLX6	3.551	0.805857283929352	183	190	1	ctcatttt
MA0701.1	LHX9	4.942	0.8389811727254	183	190	-1	aaaatgag
MA0089.1	MAFG::NFE2L1	5.264	0.846327457967442	183	188	-1	aatgag
MA0675.1	NKX6-2	4.159	0.816194916177283	183	190	1	ctcatttt
MA0722.1	VAX1	4.526	0.811370476881709	183	190	1	ctcatttt
MA0515.1	Sox6	6.759	0.843343009784452	184	193	1	tcatttttcc
MA0847.1	FOXD2	3.353	0.825218656451859	185	191	-1	aaaaatg
MA0442.1	SOX10	4.521	0.80515941993161	185	190	1	catttt
MA0076.2	ELK4	7.654	0.859926134824679	186	196	1	atttttccggc
MA0911.1	Hoxa11	5.586	0.810027441820107	186	197	-1	ggccggaaaaat
MA0136.2	ELF5	6.806	0.847198316747947	187	197	-1	ggccggaaaaa
MA0028.2	ELK1	5.613	0.837064959136889	187	196	-1	gccggaaaaa
MA0759.1	ELK3	8.226	0.859564711399539	187	196	-1	gccggaaaaa
MA0760.1	ERF	6.105	0.801493317547091	187	196	-1	gccggaaaaa
MA0474.2	ERG	7.030	0.860523215622282	187	196	-1	gccggaaaaa
MA0098.3	ETS1	8.409	0.876706719649015	187	196	-1	gccggaaaaa
MA0761.1	ETV1	8.788	0.881696920499393	187	196	-1	gccggaaaaa
MA0762.1	ETV2	5.672	0.811230384013297	187	197	-1	ggccggaaaaa
MA0764.1	ETV4	8.858	0.88521430712246	187	196	-1	gccggaaaaa
MA0765.1	ETV5	7.570	0.878239287596496	187	196	-1	gccggaaaaa
MA0645.1	ETV6	4.134	0.818832753070153	187	196	-1	gccggaaaaa
MA0156.2	FEV	8.038	0.876576834936474	187	196	-1	gccggaaaaa
MA0475.2	FLI1	7.212	0.872957871468107	187	196	-1	gccggaaaaa
MA0905.1	HOXC10	2.878	0.806879166822736	187	196	-1	gccggaaaaa
MA0906.1	HOXC12	4.187	0.809676520747457	187	197	-1	ggccggaaaaa
MA0873.1	HOXD12	5.365	0.819753463051691	187	197	-1	ggccggaaaaa
MA0913.1	Hoxd9	5.087	0.816448132332214	187	196	-1	gccggaaaaa
MA0624.1	NFATC1	9.343	0.916157540499102	187	196	1	tttttccggc
MA0625.1	NFATC3	9.898	0.910472571489069	187	196	1	tttttccggc
MA0470.1	E2F4	7.259	0.85976440136105	188	198	-1	tggccggaaaa
MA0471.1	E2F6	6.461	0.856345967836879	188	198	-1	tggccggaaaa
MA0758.1	E2F7	8.787	0.81279372198314	188	201	1	ttttccggccaatc
MA0152.1	NFATC2	8.277	0.886303295953689	188	194	1	ttttccg
MA0144.2	STAT3	2.355	0.826084506099115	188	198	-1	tggccggaaaa
MA0865.1	E2F8	13.471	0.862182886908078	189	200	1	tttccggccaat
MA0502.1	NFYB	9.667	0.868713777354735	189	203	1	tttccggccaatcgc
MA0081.1	SPIB	4.742	0.804322796910025	190	196	-1	gccggaa
MA0671.1	NFIX	6.737	0.910886717062997	192	200	1	ccggccaat
MA0161.1	NFIC	7.969	0.942108849322429	194	199	-1	ttggcc
MA0642.1	EN2	3.786	0.809505097752783	195	204	1	gccaatcgct
MA0887.1	EVX1	5.250	0.84440063565933	195	204	-1	agcgattggc
MA0888.1	EVX2	4.725	0.838466918763489	195	204	-1	agcgattggc
MA0766.1	GATA5	3.671	0.817910388794352	195	202	-1	cgattggc
MA0700.1	LHX2	2.384	0.804516177014527	195	204	1	gccaatcgct

MA0038.1	Gfi1	6.607	0.820530895030589	196	205	1	ccaatcgctt
MA0705.1	Lhx8	5.845	0.890510087379285	196	203	-1	gcgattgg
MA0705.1	Lhx8	6.738	0.907378167931564	196	203	1	ccaatcgc
MA0711.1	OTX1	2.118	0.802940139830809	196	203	1	ccaatcgc
MA0724.1	VENTX	3.756	0.830722179488503	196	204	-1	agcgattgg
MA0635.1	BARHL2	3.479	0.835260858582163	199	208	-1	caaaagcgat
MA0520.1	Stat6	10.005	0.876244398268926	201	215	1	cgcttttggagaaat
MA0520.1	Stat6	5.417	0.813305940519808	202	216	-1	catttctccaaaagc
MA0144.2	STAT3	0.440	0.802885134886095	203	213	1	cttttggagaa
MA0518.1	Stat4	4.434	0.808420743668311	204	217	1	ttttggagaaatga
MA0102.3	CEBPA	1.629	0.817504997899428	205	215	-1	atttctccaaa
MA0624.1	NFATC1	6.248	0.827946294881101	205	214	-1	tttctccaaa
MA0671.1	NFIX	4.267	0.852736615893692	205	213	-1	ttctccaaa
MA0161.1	NFIC	6.127	0.880413655041652	206	211	1	ttggag
MA0130.1	ZNF354C	4.723	0.816487152026638	206	211	-1	ctccaa
MA0056.1	MZF1	4.916	0.809768936774344	207	212	1	tggaga
MA0152.1	NFATC2	7.886	0.871882305398015	207	213	-1	ttctcca
MA0158.1	HOXA5	4.597	0.814837133331225	209	216	1	gagaaatg
MA0090.2	TEAD1	4.317	0.812153895216453	209	218	-1	ctcatttctc
MA0879.1	Dlx1	4.341	0.815382896537097	210	219	-1	gctcatttct
MA0879.1	Dlx1	4.924	0.830136292362845	210	219	1	agaaatgagc
MA0612.1	EMX1	5.858	0.827713683546871	210	219	1	agaaatgagc
MA0886.1	EMX2	4.682	0.849825343777412	210	219	1	agaaatgagc
MA0887.1	EVX1	3.695	0.809015075641904	210	219	-1	gctcatttct
MA0887.1	EVX1	3.386	0.801983475612718	210	219	1	agaaatgagc
MA0888.1	EVX2	4.004	0.822917511143225	210	219	-1	gctcatttct
MA0888.1	EVX2	3.000	0.801264799283663	210	219	1	agaaatgagc
MA0890.1	GBX2	3.146	0.805826214100892	210	219	1	agaaatgagc
MA0892.1	GSX1	5.037	0.831564497723128	210	219	-1	gctcatttct
MA0893.1	GSX2	4.527	0.805335518272402	210	219	-1	gctcatttct
MA0900.1	HOXA2	4.909	0.841971620583441	210	219	-1	gctcatttct
MA0902.1	HOXB2	4.416	0.82669321654983	210	219	-1	gctcatttct
MA0903.1	HOXB3	4.171	0.831512417410028	210	219	-1	gctcatttct
MA0492.1	JUND(var.2)	4.146	0.840267679885348	210	224	1	agaaatgagcgcatt
MA0700.1	LHX2	2.551	0.807334021055903	210	219	-1	gctcatttct
MA0706.1	MEOX2	4.684	0.813166265279946	210	219	1	agaaatgagc
MA0662.1	MIXL1	3.963	0.809996691301354	210	219	1	agaaatgagc
MA0710.1	NOTO	6.398	0.883255664818254	210	219	-1	gctcatttct
MA0881.1	Dlx4	4.026	0.815591497070789	211	218	-1	ctcatttc
MA0882.1	DLX6	3.943	0.816021669509515	211	218	-1	ctcatttc
MA0027.2	EN1	3.468	0.817526700874276	211	218	1	gaaatgag
MA0488.1	JUN	2.847	0.818726938073669	211	223	1	gaaatgagcgcat
MA0701.1	LHX9	5.019	0.841087389431542	211	218	1	gaaatgag
MA0675.1	NKX6-2	4.006	0.811983865877605	211	218	-1	ctcatttc
MA0793.1	POU6F2	6.085	0.821745129799445	211	220	-1	cgctcatttc
MA0720.1	Shox2	2.972	0.803169497439608	211	218	1	gaaatgag
MA0722.1	VAX1	4.936	0.823009116526517	211	218	-1	ctcatttc
MA0723.1	VAX2	4.524	0.816578969779566	211	218	-1	ctcatttc
MA0158.1	HOXA5	5.527	0.847590893777919	213	220	-1	cgctcatt
MA0492.1	JUND(var.2)	2.540	0.823888141796397	213	227	-1	tgcaatgcgctcatt
MA0089.1	MAFG::NFE2L1	5.264	0.846327457967442	213	218	1	aatgag
MA0488.1	JUN	1.236	0.801181379598914	214	226	-1	gcaatgcgctcat
MA0842.1	NRL	4.539	0.802073519708573	214	224	-1	aatgcgctcat
MA0719.1	RHOXF1	0.343	0.812008333914076	214	221	1	atgagcgc
MA0668.1	NEUROD2	2.693	0.809303165937728	217	226	1	agcgcattgc
MA0629.1	Rhox11	9.024	0.826235909930548	218	234	-1	attctgctgcaatgcgc
MA0019.1	Ddit3::Cebpa	8.694	0.837765756856826	219	230	-1	tgctgcaatgcg
MA0090.2	TEAD1	4.482	0.815553580204438	219	228	1	cgcattgcag
MA0809.1	TEAD4	6.357	0.858716331634316	219	228	1	cgcattgcag
MA0808.1	TEAD3	3.438	0.851620655015126	220	227	1	gcattgca
MA0833.1	ATF4	7.213	0.822477473424379	221	233	-1	ttctgctgcaatg
MA0158.1	HOXA5	4.919	0.826177682647177	221	228	-1	ctgcaatg
MA0102.3	CEBPA	4.835	0.855913161388287	222	232	1	attgcagcaga
MA0842.1	NRL	5.258	0.817542484315555	224	234	-1	attctgctgca
MA0623.1	Neurog1	4.309	0.802766348810815	227	236	-1	gcattctgct
MA0514.1	Sox3	4.790	0.83653953577156	227	236	-1	gcattctgct
MA0081.1	SPIB	6.232	0.855227295031945	227	233	1	agcagaa
MA0090.2	TEAD1	9.002	0.908684344723771	228	237	-1	cgcattctgc
MA0809.1	TEAD4	9.263	0.920896647477072	228	237	-1	cgcattctgc
MA0808.1	TEAD3	2.273	0.8335856496767	229	236	-1	gcattctg
MA0442.1	SOX10	6.352	0.886446713233527	230	235	-1	cattct
MA0117.2	Mafb	13.090	0.936541864592042	232	243	1	aatgcgctgacg
MA0842.1	NRL	11.485	0.951513614507039	232	242	1	aatgcgctgac
MA0595.1	SREBF1	7.124	0.825313643434939	233	242	-1	gtcagcgcat
MA0596.1	SREBF2	8.303	0.836287936887854	233	242	1	atgcgctgac
MA0615.1	Gmeb1	8.288	0.85715069930529	234	250	-1	tctctgacgtcagcgca

MA0492.1	JUND(var.2)	6.896	0.868314834147249	234	248	1	tgcgctgacgtcaga
MA0506.1	NRF1	3.040	0.814530522916715	234	244	-1	acgtcagcgca
MA0131.2	HINFP	7.173	0.819429957166056	235	246	-1	tgacgtcagcgc
MA0488.1	JUN	7.216	0.866310144142078	235	247	1	gcgctgacgtcag
MA0834.1	ATF7	9.122	0.871326996512933	236	249	-1	ctctgacgtcagcg
MA0834.1	ATF7	9.303	0.873328420960206	236	249	1	cgctgacgtcagag
MA0605.1	Atf3	7.288	0.86070640426761	237	244	1	gctgacgt
MA0840.1	Creb5	9.780	0.882342757048535	237	248	-1	tctgacgtcagc
MA0840.1	Creb5	9.414	0.877586748516017	237	248	1	gctgacgtcaga
MA0609.1	Crem	11.357	0.920295057833098	237	246	1	gctgacgtca
MA0639.1	DBP	5.000	0.847798127081655	237	248	-1	tctgacgtcagc
MA0639.1	DBP	5.609	0.855783556601348	237	248	1	gctgacgtcaga
MA0043.2	HLF	6.639	0.859053183395935	237	248	-1	tctgacgtcagc
MA0043.2	HLF	5.814	0.846114041758251	237	248	1	gctgacgtcaga
MA0656.1	JDP2(var.2)	10.710	0.896415839215635	237	248	-1	tctgacgtcagc
MA0656.1	JDP2(var.2)	10.848	0.897907621988041	237	248	1	gctgacgtcaga
MA0089.1	MAFG::NFE2L1	5.553	0.858843819112298	237	242	1	gctgac
MA0604.1	Atf1	8.650	0.905312988607059	238	245	1	ctgacgtc
MA0488.1	JUN	5.553	0.848198248584339	238	250	-1	tctctgacgtcag
MA0498.2	MEIS1	5.884	0.905994434664963	238	244	1	ctgacgt
MA0018.2	CREB1	11.569	1.00001610713187	239	246	-1	tgacgtca
MA0018.2	CREB1	11.569	1.00001610713187	239	246	1	tgacgtca
MA0609.1	Crem	11.768	0.928558718266003	239	248	-1	tctgacgtca
MA0862.1	GMEB2	9.330	0.916981461381433	239	246	-1	tgacgtca
MA0862.1	GMEB2	9.330	0.916981461381433	239	246	1	tgacgtca
MA0604.1	Atf1	8.650	0.905312988607059	240	247	-1	ctgacgtc
MA0605.1	Atf3	4.399	0.800862379320882	241	248	-1	tctgacgt
MA0498.2	MEIS1	5.884	0.905994434664963	241	247	-1	ctgacgt
MA0089.1	MAFG::NFE2L1	4.933	0.831992110081811	243	248	-1	tctgac

Homo sapiens

Model ID	Model name	Score	Relative score	Start	End	Strand	predicted site sequence
MA0519.1	Stat5a::Stat5b	2.069	0.819056396595807	2	12	-1	ttttccctgcg
MA0597.1	THAP1	5.850	0.846672117250332	3	11	-1	tttccctgc
MA0606.1	NFAT5	9.870	0.911235528705737	4	13	-1	tttttccctg
MA0624.1	NFATC1	7.884	0.874574274794203	4	13	-1	tttttccctg
MA0625.1	NFATC3	8.402	0.872464248422448	4	13	-1	tttttccctg
MA0606.1	NFAT5	6.009	0.832531101584532	5	14	-1	ctttttccct
MA0081.1	SPIB	4.764	0.805074406949409	5	11	1	agggaaa
MA0111.1	Spz1	9.776	0.871444493659558	5	15	1	agggaaaaagc
MA0480.1	Foxo1	4.154	0.805161938191638	6	16	-1	tgctttttccc
MA0152.1	NFATC2	9.187	0.91986621514976	6	12	-1	ttttccc
MA0514.1	Sox3	4.580	0.833431461040095	6	15	-1	gctttttccc
MA0515.1	Sox6	5.603	0.821311240055264	6	15	-1	gctttttccc
MA0442.1	SOX10	4.805	0.817767607423551	9	14	-1	cttttt
MA0850.1	FOXP3	5.205	0.847997257177385	10	16	1	aaaagca
MA0470.1	E2F4	3.641	0.802469625237995	13	23	-1	tcgcgggtgct
MA0824.1	ID4	4.464	0.810864336165889	13	22	1	agcacccgcg
MA0499.1	Myod1	2.405	0.82112860428195	13	25	1	agcacccgcgacc
MA0048.2	NHLH1	4.592	0.800396422471023	13	22	-1	cgcgggtgct
MA0048.2	NHLH1	5.160	0.810005033906981	13	22	1	agcacccgcg
MA0522.2	TCF3	6.231	0.874616443530988	13	22	1	agcacccgcg
MA0830.1	TCF4	3.785	0.845588472155333	13	22	1	agcacccgcg
MA0632.1	Tcfl5	3.922	0.808968476190539	13	22	-1	cgcgggtgct
MA0632.1	Tcfl5	3.922	0.808968476190539	13	22	1	agcacccgcg
MA0506.1	NRF1	3.483	0.820001488210404	14	24	1	gcacccgcgac
MA0745.1	SNAI2	4.276	0.833336134795525	14	22	-1	cgcgggtgc
MA0006.1	Ahr::Arnt	4.891	0.800083340398517	15	20	-1	cgggtg
MA0511.2	RUNX2	4.993	0.8182364809652	15	23	1	cacccgcga
MA0632.1	Tcfl5	4.913	0.840355509241597	15	24	-1	gtcgcgggtg
MA0632.1	Tcfl5	4.913	0.840355509241597	15	24	1	cacccgcgac
MA0810.1	TFAP2A(var.2)	5.158	0.836420380986085	15	26	1	cacccgcgacca
MA0811.1	TFAP2B	3.634	0.800433040292425	15	26	1	cacccgcgacca
MA0831.1	TFE3	5.245	0.826967361594603	15	24	1	cacccgcgac
MA0067.1	Pax2	5.591	0.86354845093179	18	25	-1	ggtcgcgg
MA0750.1	ZBTB7A	13.465	0.917364357299198	19	30	1	cgcgaccacagg
MA0734.1	GLI2	12.340	0.887698474753204	20	31	1	gcgaccacaggg
MA0694.1	ZBTB7B	6.205	0.808496749197623	20	31	1	gcgaccacaggg
MA0695.1	ZBTB7C	9.999	0.874738068433451	20	31	1	gcgaccacaggg
MA0116.1	Znf423	7.058	0.808282068135398	20	34	-1	acaccctgtggtcgc
MA0139.1	CTCF	8.961	0.806943060002373	21	39	1	cgaccacagggtgttggtc
MA0002.2	RUNX1	10.951	0.911611289680257	21	31	-1	ccctgtggtcg
MA0511.2	RUNX2	7.463	0.863765300004635	21	29	1	cgaccacag
MA0684.1	RUNX3	7.771	0.861101127602193	21	30	1	cgaccacagg

MA0751.1	ZIC4	5.389	0.80218821749146	21	35	-1	aacaccctgtggtcg
MA0116.1	Znf423	6.747	0.803084475274153	21	35	1	cgaccacagggtgtt
MA0059.1	MAX::MYC	8.372	0.810182152747926	22	32	1	gaccacagggt
MA0147.2	Myc	6.553	0.871000531884578	22	31	-1	ccctgtggtc
MA0526.1	USF2	3.163	0.821098031354332	22	32	-1	accctgtggtc
MA0696.1	ZIC1	3.856	0.808872519474453	22	35	-1	aacaccctgtggtc
MA0130.1	ZNF354C	6.331	0.886867302367815	22	27	1	gaccac
MA0104.3	Mycn	3.845	0.805111489009167	23	30	1	accacagg
MA0668.1	NEUROD2	3.488	0.822294437963676	23	32	1	accacagggt
MA0623.1	Neurog1	5.183	0.823060592806563	23	32	-1	accctgtggt
MA0871.1	TFEC	5.330	0.803477615657202	23	32	1	accacagggt
MA0746.1	SP3	5.535	0.813350994246662	27	37	-1	ccaacaccctg
MA0148.3	FOXA1	0.929	0.80161490807119	28	42	1	agggtgttggtcatg
MA0493.1	Klf1	8.842	0.861087658492857	28	38	-1	accaacaccct
MA0039.2	Klf4	7.084	0.856865519991403	28	37	1	agggtgttgg
MA0599.1	KLF5	2.953	0.840819181507839	28	37	-1	ccaacaccct
MA0801.1	MGA	5.091	0.836557338070417	29	36	1	gggtgttg
MA0806.1	TBX4	5.946	0.848485149683486	29	36	1	gggtgttg
MA0807.1	TBX5	7.214	0.859630646652157	29	36	1	gggtgttg
MA0614.1	Foxj2	4.429	0.816226813977045	31	38	-1	accaacac
MA0476.1	FOS	2.303	0.819653792165671	32	42	1	tgttggtcatg
MA0847.1	FOXD2	5.619	0.873276008371118	32	38	-1	accaaca
MA0033.2	FOXL1	5.548	0.877157020147798	32	38	-1	accaaca
MA0848.1	FOXO4	2.982	0.808538765835091	32	38	-1	accaaca
MA0738.1	HIC2	3.761	0.813024630283501	33	41	-1	atgaccaac
MA0258.2	ESR2	6.316	0.813207441192674	35	49	1	tggtcatggcggcca
MA0886.1	EMX2	3.304	0.826521735215473	36	45	-1	cgccatgacc
MA0642.1	EN2	6.125	0.860192706492977	36	45	-1	cgccatgacc
MA0661.1	MEOX1	4.656	0.822927106099434	36	45	-1	cgccatgacc
MA0661.1	MEOX1	3.805	0.80539364293863	36	45	1	ggtcatggcg
MA0067.1	Pax2	7.662	0.944874036349661	36	43	1	ggtcatgg
MA0701.1	LHX9	4.012	0.813542451469405	37	44	-1	gccatgac
MA0089.1	MAFG::NFE2L1	8.812	0.999988528354811	37	42	-1	catgac
MA0666.1	MSX1	4.062	0.810579335424453	37	44	-1	gccatgac
MA0597.1	THAP1	7.200	0.887988614030741	38	46	-1	ccgccatga
MA0748.1	YY2	12.653	0.928750251934866	38	48	-1	ggccgccatga
MA0024.3	E2F1	6.487	0.802632521673832	39	50	1	catggcggccag
MA0089.1	MAFG::NFE2L1	4.208	0.800592934199387	39	44	1	catggc
MA0671.1	NFIX	4.185	0.850806126705076	39	47	-1	gccgccatg
MA0775.1	MEIS3	3.057	0.801506310129571	40	47	1	atggcggc
MA0161.1	NFIC	4.646	0.830809647045002	40	45	1	atggcg
MA0671.1	NFIX	5.855	0.890122187009828	43	51	1	gcggccagg
MA0810.1	TFAP2A(var.2)	9.591	0.90522309038341	44	55	-1	tgcccctggccg
MA0810.1	TFAP2A(var.2)	7.526	0.87317310281168	44	55	1	cggccaggggca
MA0811.1	TFAP2B	9.132	0.887628018804524	44	55	-1	tgcccctggccg
MA0811.1	TFAP2B	8.724	0.88115738380399	44	55	1	cggccaggggca
MA0524.2	TFAP2C	8.515	0.87481807413939	44	55	-1	tgcccctggccg
MA0524.2	TFAP2C	9.566	0.891897304765522	44	55	1	cggccaggggca
MA0161.1	NFIC	6.668	0.898533687672434	45	50	-1	ctggcc
MA0003.3	TFAP2A	7.422	0.871915990601185	45	55	-1	tgcccctggcc
MA0003.3	TFAP2A	3.733	0.813925055386545	45	55	1	ggccaggggca
MA0812.1	TFAP2B(var.2)	5.587	0.834911920541139	45	55	-1	tgcccctggcc
MA0812.1	TFAP2B(var.2)	3.830	0.808274986595337	45	55	1	ggccaggggca
MA0814.1	TFAP2C(var.2)	5.221	0.831821987508451	45	55	-1	tgcccctggcc
MA0814.1	TFAP2C(var.2)	6.346	0.849864747623439	45	55	1	ggccaggggca
MA0820.1	FIGLA	4.301	0.802828937945693	46	55	-1	tgcccctggc
MA0824.1	ID4	5.863	0.837685141419841	46	55	-1	tgcccctggc
MA0522.2	TCF3	3.369	0.826500318159923	46	55	-1	tgcccctggc
MA0830.1	TCF4	3.693	0.844181443492199	46	55	-1	tgcccctggc
MA0739.1	Hic1	5.299	0.825639349391846	48	56	-1	gtgcccctg
MA0738.1	HIC2	7.745	0.901722053778188	48	56	-1	gtgcccctg
MA0597.1	THAP1	4.385	0.801836067040481	48	56	-1	gtgcccctg
MA0258.2	ESR2	6.317	0.813220651789635	50	64	1	ggggcactgcggcag
MA0503.1	Nkx2-5(var.2)	2.876	0.800301750110292	51	61	1	gggcactgcgg
MA0099.2	FOS::JUN	5.316	0.804790114679483	57	63	-1	tgccgca
MA0671.1	NFIX	3.681	0.838940680960408	57	65	1	tgcggcaga
MA0498.2	MEIS1	1.163	0.806017362707243	58	64	-1	ctgccgc
MA0090.2	TEAD1	6.769	0.862675274553047	61	70	-1	aaaattctgc
MA0809.1	TEAD4	8.179	0.897702063040201	61	70	-1	aaaattctgc
MA0158.1	HOXA5	5.012	0.829453058691846	62	69	1	cagaattt
MA0635.1	BARHL2	2.275	0.812848135859008	63	72	-1	aaaaaattct
MA0041.1	Foxd3	6.592	0.801082580332973	64	75	1	gaattttttcct
MA0847.1	FOXD2	3.030	0.818368469042362	65	71	-1	aaaaatt
MA0087.1	Sox5	6.624	0.840728791704037	66	72	1	atttttt
MA0084.1	SRY	5.866	0.805955911773436	66	74	-1	ggaaaaaat
MA0076.2	ELK4	4.063	0.804257831874891	67	77	1	ttttttcctcc

MA0157.2	FOXO3	5.002	0.807961226849649	67	74	-1	ggaaaaaa
MA0848.1	FOXO4	2.656	0.802093779378544	67	73	-1	gaaaaaa
MA0606.1	NFAT5	5.975	0.831838029706231	67	76	1	tttttcctc
MA0471.1	E2F6	7.885	0.877355302040208	68	78	-1	gggaggaaaaa
MA0136.2	ELF5	5.073	0.814894729498834	68	78	-1	gggaggaaaaa
MA0606.1	NFAT5	8.388	0.881025748598608	68	77	1	ttttcctcc
MA0624.1	NFATC1	8.349	0.887827337544565	68	77	1	ttttcctcc
MA0625.1	NFATC3	8.552	0.876275243382336	68	77	1	ttttcctcc
MA0152.1	NFATC2	9.490	0.931041560772187	69	75	1	ttttcct
MA0528.1	ZNF263	6.661	0.80985698272932	69	89	-1	gcagcaaagaagggaggaaaa
MA0599.1	KLF5	7.209	0.894961459130246	70	79	1	tttcctccct
MA0079.3	SP1	8.634	0.889766285794684	70	80	1	tttcctccctt
MA0516.1	SP2	5.992	0.805352993580392	70	84	1	tttcctcccttcttt
MA0081.1	SPIB	6.244	0.855637264144336	71	77	-1	ggaggaa
MA0471.1	E2F6	5.290	0.839069331353665	72	82	-1	agaagggagga
MA0056.1	MZF1	5.636	0.842620283344776	72	77	-1	ggagga
MA0597.1	THAP1	5.527	0.836786762835464	73	81	1	cctcccttc
MA0109.1	HLTF	5.436	0.872804971219847	75	84	1	tcccttcttt
MA0090.2	TEAD1	3.947	0.804530359182791	75	84	1	tcccttcttt
MA0514.1	Sox3	3.529	0.817876287026906	76	85	1	cccttctttg
MA0442.1	SOX10	4.820	0.818433532819252	77	82	1	ccttct
MA0144.2	STAT3	0.870	0.808094393174136	78	88	-1	cagcaaagaag
MA0847.1	FOXD2	4.228	0.843775665688113	80	86	-1	gcaaaga
MA0850.1	FOXP3	6.725	0.886240020070117	80	86	-1	gcaaaga
MA0442.1	SOX10	4.669	0.811729883835861	81	86	1	ctttgc
MA0019.1	Ddit3::Cebpa	7.765	0.814607077115656	84	95	1	tgctgcaatctg
MA0088.2	ZNF143	6.068	0.812056484034349	84	99	-1	cacccagattgcagca
MA0766.1	GATA5	5.562	0.865997666444987	87	94	-1	agattgca
MA0662.1	MIXL1	3.639	0.801564569663989	87	96	1	tgcaatctgg
MA0611.1	Dux	4.559	0.819596118562089	88	95	1	gcaatctg
MA0027.2	EN1	4.160	0.832563206457442	88	95	1	gcaatctg
MA0914.1	ISL2	5.090	0.82869756237402	88	95	1	gcaatctg
MA0623.1	Neurog1	5.193	0.823292792394615	88	97	-1	cccagattgc
MA0623.1	Neurog1	7.463	0.876002098882428	88	97	1	gcaatctggg
MA0827.1	OLIG3	3.682	0.807741791842136	88	97	-1	cccagattgc
MA0092.1	Hand1::Tcf3	6.144	0.804602770651927	90	99	1	aatctgggtg
MA0742.1	Klf12	6.177	0.813239853095436	90	104	-1	agccgcacccagatt
MA0079.3	SP1	1.810	0.803912553409963	93	103	-1	gccgcacccag
MA0746.1	SP3	6.691	0.836116708557974	93	103	-1	gccgcacccag
MA0006.1	Ahr::Arnt	5.297	0.817309426751426	94	99	1	tgggtg
MA0493.1	Klf1	10.485	0.888862365225811	94	104	-1	agccgcaccca
MA0039.2	Klf4	11.000	0.923397632335372	94	103	1	tgggtgcggc
MA0599.1	KLF5	6.555	0.886641663649735	94	103	-1	gccgcaccca
MA0801.1	MGA	4.037	0.817384281072698	95	102	1	gggtgcgg
MA0002.2	RUNX1	5.523	0.805784973871463	95	105	1	gggtgcggcta
MA0806.1	TBX4	4.406	0.817434096588676	95	102	1	gggtgcgg
MA0807.1	TBX5	5.247	0.80880176952459	95	102	1	gggtgcgg
MA0646.1	GCM1	4.498	0.80553596887994	96	106	1	ggtgcggctag
MA0767.1	GCM2	5.589	0.808373528677047	96	105	1	ggtgcggcta
MA0684.1	RUNX3	5.428	0.820261232673096	96	105	-1	tagccgcacc
MA0003.3	TFAP2A	3.411	0.808863228024357	100	110	1	cggctagagca
MA0814.1	TFAP2C(var.2)	3.403	0.80266488716263	100	110	1	cggctagagca
MA0102.3	CEBPA	2.401	0.826753626163121	102	112	-1	attgctctagc
MA0081.1	SPIB	5.670	0.836027074934952	105	111	1	agagcaa
MA0087.1	Sox5	6.367	0.831151705260537	106	112	-1	attgctc
MA0090.2	TEAD1	4.275	0.811288520855875	106	115	-1	caaattgctc
MA0809.1	TEAD4	6.100	0.853217246578711	106	115	-1	caaattgctc
MA0877.1	Barhl1	6.512	0.890469041803454	107	116	-1	acaaattgct
MA0635.1	BARHL2	4.139	0.847546902599839	107	116	-1	acaaattgct
MA0879.1	Dlx1	6.125	0.860528793883811	107	116	1	agcaatttgt
MA0612.1	EMX1	6.536	0.846763873641076	107	116	-1	acaaattgct
MA0644.1	ESX1	5.393	0.831579408817481	107	116	1	agcaatttgt
MA0887.1	EVX1	3.427	0.802916471085846	107	116	-1	acaaattgct
MA0888.1	EVX2	3.182	0.805189892469361	107	116	1	agcaatttgt
MA0889.1	GBX1	5.894	0.857663219524059	107	116	1	agcaatttgt
MA0890.1	GBX2	6.822	0.877410749309739	107	116	1	agcaatttgt
MA0892.1	GSX1	5.220	0.836534271459619	107	116	1	agcaatttgt
MA0893.1	GSX2	5.118	0.821684813262664	107	116	1	agcaatttgt
MA0894.1	HESX1	6.554	0.867576755334856	107	116	-1	acaaattgct
MA0900.1	HOXA2	4.185	0.825603040102218	107	116	1	agcaatttgt
MA0902.1	HOXB2	5.078	0.843890236686043	107	116	1	agcaatttgt
MA0903.1	HOXB3	5.721	0.867608939885755	107	116	1	agcaatttgt
MA0699.1	LBX2	5.256	0.818522702138256	107	116	1	agcaatttgt
MA0700.1	LHX2	3.268	0.819432189784446	107	116	-1	acaaattgct
MA0700.1	LHX2	2.222	0.801782699561217	107	116	1	agcaatttgt
MA0662.1	MIXL1	5.652	0.853953029096136	107	116	1	agcaatttgt

MA0125.1	Nobox	6.189	0.82936435549078	107	114	-1	aaattgct
MA0710.1	NOTO	5.961	0.87552776873742	107	116	-1	acaaattgct
MA0718.1	RAX	6.518	0.857018836515325	107	116	1	agcaatttgt
MA0808.1	TEAD3	0.392	0.8044664693835	107	114	-1	aaattgct
MA0724.1	VENTX	2.711	0.81336698127758	107	115	1	agcaatttg
MA0875.1	BARX1	2.778	0.814907886856419	108	115	1	gcaatttg
MA0879.1	Dlx1	4.488	0.819102877988872	108	117	-1	gacaaattgc
MA0885.1	Dlx2	6.366	0.885617774057834	108	115	1	gcaatttg
MA0880.1	Dlx3	6.283	0.871878647537569	108	115	1	gcaatttg
MA0881.1	Dlx4	6.616	0.886253166923116	108	115	1	gcaatttg
MA0882.1	DLX6	6.143	0.873066690622673	108	115	1	gcaatttg
MA0027.2	EN1	6.708	0.887928836841698	108	115	1	gcaatttg
MA0914.1	ISL2	8.022	0.904979249515833	108	115	1	gcaatttg
MA0654.1	ISX	5.545	0.859189214972164	108	115	1	gcaatttg
MA0704.1	Lhx4	4.146	0.821752104199954	108	115	1	gcaatttg
MA0705.1	Lhx8	1.328	0.805187444764903	108	115	-1	caaattgc
MA0701.1	LHX9	5.648	0.858292718108983	108	115	1	gcaatttg
MA0703.1	LMX1B	5.269	0.829806462870895	108	115	1	gcaatttg
MA0666.1	MSX1	6.097	0.860978552699148	108	115	1	gcaatttg
MA0708.1	MSX2	4.612	0.815918546385144	108	115	1	gcaatttg
MA0709.1	Msx3	6.505	0.875225062176186	108	115	1	gcaatttg
MA0668.1	NEUROD2	2.460	0.805495661054651	108	117	-1	gacaaattgc
MA0623.1	Neurog1	4.403	0.804949024938504	108	117	1	gcaatttgtc
MA0132.2	PDX1	2.464	0.813820417530302	108	115	1	gcaatttg
MA0716.1	PRRX1	5.404	0.841122223250983	108	115	1	gcaatttg
MA0075.2	Prrx2	6.054	0.854825064276216	108	115	1	gcaatttg
MA0717.1	RAX2	6.442	0.87041656062619	108	115	1	gcaatttg
MA0630.1	SHOX	6.112	0.84927225736707	108	115	-1	caaattgc
MA0720.1	Shox2	6.172	0.874955378014643	108	115	1	gcaatttg
MA0721.1	UNCX	5.790	0.84969495113426	108	115	1	gcaatttg
MA0722.1	VAX1	6.292	0.861501788132274	108	115	-1	caaattgc
MA0723.1	VAX2	6.255	0.8623391941205	108	115	-1	caaattgc
MA0725.1	VSX1	6.741	0.860940210693593	108	115	-1	caaattgc
MA0726.1	VSX2	5.803	0.829420886026564	108	115	-1	caaattgc
MA0847.1	FOXD2	3.568	0.829778378721338	110	116	-1	acaaatt
MA0158.1	HOXA5	5.527	0.847590893777919	110	117	-1	gacaaatt
MA0491.1	JUND	0.811	0.821122940649332	110	120	-1	tatgacaaatt
MA0655.1	JDP2	4.372	0.801809325909432	111	119	-1	atgacaaat
MA0489.1	JUN(var.2)	3.699	0.812533993736239	111	124	-1	attctatgacaaat
MA0490.1	JUNB	1.016	0.803013026500002	111	121	-1	ctatgacaaat
MA0670.1	NFIA	2.033	0.823366319327152	111	120	-1	tatgacaaat
MA0442.1	SOX10	4.805	0.817767607423551	111	116	1	atttgt
MA0604.1	Atf1	4.084	0.806651805176267	112	119	-1	atgacaaa
MA0774.1	MEIS2	5.535	0.826096795346533	112	119	-1	atgacaaa
MA0775.1	MEIS3	7.506	0.900026379504145	112	119	-1	atgacaaa
MA0084.1	SRY	6.786	0.836757059806185	112	120	-1	tatgacaaa
MA0605.1	Atf3	5.598	0.825698996112549	113	120	-1	tatgacaa
MA0465.1	CDX2	5.865	0.841070477628713	113	123	1	ttgtcatagaa
MA0498.2	MEIS1	9.456	0.981639022894781	113	119	-1	atgacaa
MA0161.1	NFIC	3.787	0.802038652535128	113	118	1	ttgtca
MA0519.1	Stat5a::Stat5b	0.571	0.800748973234957	113	123	1	ttgtcatagaa
MA0899.1	HOXA10	5.582	0.819861482329578	114	124	1	tgtcatagaat
MA0911.1	Hoxa11	5.546	0.80931641449105	114	125	1	tgtcatagaatc
MA0651.1	HOXC11	5.236	0.821336871832339	114	124	1	tgtcatagaat
MA0906.1	HOXC12	4.036	0.807555522594369	114	124	1	tgtcatagaat
MA0067.1	Pax2	5.776	0.870813170054101	114	121	1	tgtcatag
MA0878.1	CDX1	6.146	0.853351071376537	115	123	1	gtcatagaa
MA0905.1	HOXC10	6.760	0.872465416131608	115	124	1	gtcatagaat
MA0908.1	HOXD11	7.295	0.845452174570221	115	124	1	gtcatagaat
MA0909.1	HOXD13	5.280	0.818829739539447	115	124	1	gtcatagaat
MA0913.1	Hoxd9	5.320	0.821834160596873	115	124	1	gtcatagaat
MA0089.1	MAFG::NFE2L1	8.072	0.967939714350681	115	120	-1	tatgac
MA0090.2	TEAD1	6.543	0.85801873632708	118	127	-1	cagattctat
MA0809.1	TEAD4	6.862	0.86952192678249	118	127	-1	cagattctat
MA0766.1	GATA5	3.442	0.812087022013815	119	126	-1	agattcta
MA0623.1	Neurog1	4.321	0.803044988316477	120	129	-1	cccagattct
MA0623.1	Neurog1	6.551	0.854825496452082	120	129	1	agaatctggg
MA0827.1	OLIG3	4.221	0.816705104274573	120	129	-1	cccagattct
MA0719.1	RHOXF1	2.711	0.857650832062083	120	127	1	agaatctg
MA0607.1	Bhlha15	3.265	0.806814700906033	121	128	-1	ccagattc
MA0694.1	ZBTB7B	6.644	0.815343757748668	124	135	-1	gagccccccaga
MA0695.1	ZBTB7C	6.641	0.805737124649578	124	135	-1	gagccccccaga
MA0753.1	ZNF740	6.573	0.816022219919947	125	134	-1	agccccccag
MA0056.1	MZF1	6.469	0.880627466251957	126	131	1	tggggg
MA0478.1	FOSL2	3.321	0.819070989244286	127	137	1	ggggggctcat
MA0492.1	JUND(var.2)	0.281	0.800848679440893	127	141	-1	aaaaatgagcccccc

MA0056.1	MZF1	5.636	0.842620283344776	127	132	1	gggggg
MA0682.1	Pitx1	4.575	0.809684899547031	130	137	-1	atgagccc
MA0714.1	PITX3	4.428	0.804733272541842	130	138	-1	aatgagccc
MA0719.1	RHOXF1	8.998	0.978830893656897	130	137	-1	atgagccc
MA0793.1	POU6F2	8.119	0.860793323174769	131	140	1	ggctcatttt
MA0879.1	Dlx1	5.615	0.847622735785815	132	141	-1	aaaaatgagc
MA0879.1	Dlx1	4.758	0.825935496981928	132	141	1	gctcattttt
MA0612.1	EMX1	5.858	0.827713683546871	132	141	-1	aaaaatgagc
MA0886.1	EMX2	3.797	0.834858947857734	132	141	-1	aaaaatgagc
MA0887.1	EVX1	3.431	0.803007495034444	132	141	1	gctcattttt
MA0888.1	EVX2	3.608	0.814377198497422	132	141	1	gctcattttt
MA0892.1	GSX1	4.533	0.817877252022628	132	141	1	gctcattttt
MA0900.1	HOXA2	3.566	0.811608355961503	132	141	1	gctcattttt
MA0902.1	HOXB2	4.183	0.820640488918807	132	141	1	gctcattttt
MA0903.1	HOXB3	3.686	0.820217699086978	132	141	1	gctcattttt
MA0700.1	LHX2	2.597	0.808110193666103	132	141	1	gctcattttt
MA0706.1	MEOX2	4.298	0.803515624834874	132	141	1	gctcattttt
MA0710.1	NOTO	5.958	0.875474716819245	132	141	1	gctcattttt
MA0877.1	Barhl1	3.648	0.814995892543956	133	142	-1	gaaaaatgag
MA0635.1	BARHL2	4.319	0.850897641877387	133	142	-1	gaaaaatgag
MA0881.1	Dlx4	3.725	0.807379465169032	133	140	1	ctcatttt
MA0882.1	DLX6	3.551	0.805857283929352	133	140	1	ctcatttt
MA0701.1	LHX9	4.942	0.8389811727254	133	140	-1	aaaatgag
MA0089.1	MAFG::NFE2L1	5.264	0.846327457967442	133	138	-1	aatgag
MA0675.1	NKX6-2	4.159	0.816194916177283	133	140	1	ctcatttt
MA0722.1	VAX1	4.526	0.811370476881709	133	140	1	ctcatttt
MA0515.1	Sox6	6.759	0.843343009784452	134	143	1	tcatttttcc
MA0847.1	FOXD2	3.353	0.825218656451859	135	141	-1	aaaaatg
MA0442.1	SOX10	4.521	0.80515941993161	135	140	1	catttt
MA0076.2	ELK4	7.654	0.859926134824679	136	146	1	atttttccggc
MA0911.1	Hoxa11	5.586	0.810027441820107	136	147	-1	ggccggaaaaat
MA0136.2	ELF5	6.806	0.847198316747947	137	147	-1	ggccggaaaaa
MA0028.2	ELK1	5.613	0.837064959136889	137	146	-1	gccggaaaaa
MA0759.1	ELK3	8.226	0.859564711399539	137	146	-1	gccggaaaaa
MA0760.1	ERF	6.105	0.801493317547091	137	146	-1	gccggaaaaa
MA0474.2	ERG	7.030	0.860523215622282	137	146	-1	gccggaaaaa
MA0098.3	ETS1	8.409	0.876706719649015	137	146	-1	gccggaaaaa
MA0761.1	ETV1	8.788	0.881696920499393	137	146	-1	gccggaaaaa
MA0762.1	ETV2	5.672	0.811230384013297	137	147	-1	ggccggaaaaa
MA0764.1	ETV4	8.858	0.88521430712246	137	146	-1	gccggaaaaa
MA0765.1	ETV5	7.570	0.878239287596496	137	146	-1	gccggaaaaa
MA0645.1	ETV6	4.134	0.818832753070153	137	146	-1	gccggaaaaa
MA0156.2	FEV	8.038	0.876576834936474	137	146	-1	gccggaaaaa
MA0475.2	FLI1	7.212	0.872957871468107	137	146	-1	gccggaaaaa
MA0905.1	HOXC10	2.878	0.806879166822736	137	146	-1	gccggaaaaa
MA0906.1	HOXC12	4.187	0.809676520747457	137	147	-1	ggccggaaaaa
MA0873.1	HOXD12	5.365	0.819753463051691	137	147	-1	ggccggaaaaa
MA0913.1	Hoxd9	5.087	0.816448132332214	137	146	-1	gccggaaaaa
MA0624.1	NFATC1	9.343	0.916157540499102	137	146	1	tttttccggc
MA0625.1	NFATC3	9.898	0.910472571489069	137	146	1	tttttccggc
MA0470.1	E2F4	7.259	0.85976440136105	138	148	-1	tggccggaaaa
MA0471.1	E2F6	6.461	0.856345967836879	138	148	-1	tggccggaaaa
MA0758.1	E2F7	8.787	0.81279372198314	138	151	1	ttttccggccaatc
MA0152.1	NFATC2	8.277	0.886303295953689	138	144	1	ttttccg
MA0144.2	STAT3	2.355	0.826084506099115	138	148	-1	tggccggaaaa
MA0865.1	E2F8	13.471	0.862182886908078	139	150	1	tttccggccaat
MA0502.1	NFYB	11.556	0.893216558089996	139	153	1	tttccggccaatcac
MA0081.1	SPIB	4.742	0.804322796910025	140	146	-1	gccggaa
MA0671.1	NFIX	6.737	0.910886717062997	142	150	1	ccggccaat
MA0060.2	NFYA	8.221	0.81675862062606	142	159	-1	ctaaaagtgattggccgg
MA0161.1	NFIC	7.969	0.942108849322429	144	149	-1	ttggcc
MA0634.1	ALX3	4.996	0.805845853782784	145	154	1	gccaatcact
MA0879.1	Dlx1	4.235	0.812700460932415	145	154	1	gccaatcact
MA0642.1	EN2	8.686	0.915691195583886	145	154	1	gccaatcact
MA0644.1	ESX1	7.158	0.876954469510305	145	154	1	gccaatcact
MA0887.1	EVX1	6.992	0.884041565273707	145	154	-1	agtgattggc
MA0887.1	EVX1	4.414	0.825376630402373	145	154	1	gccaatcact
MA0888.1	EVX2	6.567	0.87819231243412	145	154	-1	agtgattggc
MA0888.1	EVX2	4.065	0.824233064353816	145	154	1	gccaatcact
MA0766.1	GATA5	4.436	0.837363993978242	145	152	-1	tgattggc
MA0889.1	GBX1	6.116	0.861527204000626	145	154	1	gccaatcact
MA0890.1	GBX2	5.387	0.849466296637079	145	154	1	gccaatcact
MA0892.1	GSX1	5.324	0.839358623747024	145	154	1	gccaatcact
MA0893.1	GSX2	5.059	0.820052650141809	145	154	1	gccaatcact
MA0894.1	HESX1	4.860	0.835031666297881	145	154	-1	agtgattggc
MA0900.1	HOXA2	6.465	0.877150503496126	145	154	1	gccaatcact

MA0902.1	HOXB2	5.111	0.844747489955672	145	154	1	gccaatcact
MA0903.1	HOXB3	4.637	0.842364662231763	145	154	1	gccaatcact
MA0699.1	LBX2	6.247	0.845756324236126	145	154	1	gccaatcact
MA0700.1	LHX2	4.948	0.847779363374335	145	154	1	gccaatcact
MA0658.1	LHX6	5.489	0.843589740469487	145	154	-1	agtgattggc
MA0658.1	LHX6	5.603	0.845539232583778	145	154	1	gccaatcact
MA0661.1	MEOX1	4.516	0.820042635074155	145	154	-1	agtgattggc
MA0661.1	MEOX1	5.437	0.839018333747599	145	154	1	gccaatcact
MA0706.1	MEOX2	5.424	0.831667493076198	145	154	-1	agtgattggc
MA0706.1	MEOX2	4.968	0.820266736488237	145	154	1	gccaatcact
MA0662.1	MIXL1	6.104	0.865716359281596	145	154	1	gccaatcact
MA0125.1	Nobox	6.406	0.836520913222571	145	152	-1	tgattggc
MA0718.1	RAX	5.940	0.844266616205778	145	154	1	gccaatcact
MA0876.1	BSX	2.181	0.807029409432795	146	153	1	ccaatcac
MA0885.1	Dlx2	5.528	0.865536746609707	146	153	1	ccaatcac
MA0880.1	Dlx3	6.738	0.885842257315008	146	153	1	ccaatcac
MA0881.1	Dlx4	7.033	0.897629968594321	146	153	1	ccaatcac
MA0882.1	DLX6	6.934	0.893576968668359	146	153	1	ccaatcac
MA0611.1	Dux	9.180	0.920229679511632	146	153	1	ccaatcac
MA0027.2	EN1	8.266	0.921782703458132	146	153	1	ccaatcac
MA0038.1	Gfi1	9.710	0.913747343749482	146	155	1	ccaatcactt
MA0914.1	ISL2	4.641	0.817015953394936	146	153	1	ccaatcac
MA0654.1	ISX	3.194	0.808494032961542	146	153	1	ccaatcac
MA0704.1	Lhx4	3.772	0.81315011659758	146	153	-1	gtgattgg
MA0704.1	Lhx4	5.565	0.8543890571619	146	153	1	ccaatcac
MA0705.1	Lhx8	7.188	0.915878320505501	146	153	-1	gtgattgg
MA0705.1	Lhx8	8.129	0.933653083999	146	153	1	ccaatcac
MA0701.1	LHX9	6.113	0.871012078736981	146	153	1	ccaatcac
MA0703.1	LMX1B	4.716	0.813255054757594	146	153	1	ccaatcac
MA0666.1	MSX1	7.683	0.900257746113234	146	153	1	ccaatcac
MA0708.1	MSX2	7.805	0.889408852305143	146	153	1	ccaatcac
MA0709.1	Msx3	6.321	0.871110869031688	146	153	1	ccaatcac
MA0674.1	NKX6-1	2.982	0.801894354437337	146	153	1	ccaatcac
MA0675.1	NKX6-2	5.753	0.860066904266736	146	153	1	ccaatcac
MA0716.1	PRRX1	6.772	0.876947270865098	146	153	1	ccaatcac
MA0075.2	Prrx2	7.215	0.89197272699109	146	153	1	ccaatcac
MA0717.1	RAX2	6.873	0.882964558834711	146	153	1	ccaatcac
MA0630.1	SHOX	5.914	0.842891961044758	146	153	-1	gtgattgg
MA0720.1	Shox2	5.646	0.863155573895122	146	153	1	ccaatcac
MA0721.1	UNCX	6.616	0.874005089382063	146	153	1	ccaatcac
MA0722.1	VAX1	4.843	0.820369132411963	146	153	1	ccaatcac
MA0723.1	VAX2	5.323	0.837701106838149	146	153	1	ccaatcac
MA0725.1	VSX1	5.413	0.823621234615953	146	153	1	ccaatcac
MA0726.1	VSX2	5.275	0.816042701608964	146	153	1	ccaatcac
MA0483.1	Gfi1b	6.326	0.836928685842649	147	157	1	caatcactttt
MA0067.1	Pax2	4.037	0.802524810304377	147	154	-1	agtgattg
MA0067.1	Pax2	6.410	0.895709558830021	148	155	1	aatcactt
MA0877.1	Barhl1	3.175	0.802531227879241	149	158	-1	taaaagtgat
MA0635.1	BARHL2	1.755	0.803168222390535	149	158	-1	taaaagtgat
MA0672.1	NKX2-3	2.426	0.802519019588225	149	158	1	atcactttta
MA0124.2	Nkx3-1	5.049	0.821975370215445	149	157	1	atcactttt
MA0122.2	NKX3-2	5.843	0.840028251593667	149	157	1	atcactttt
MA0676.1	Nr2e1	6.849	0.840779987501199	149	157	-1	aaaagtgat
MA0616.1	Hes2	6.643	0.802238364364073	150	162	-1	tctctaaaagtga
MA0109.1	HLTF	6.911	0.924789389609019	150	159	1	tcacttttag
MA0914.1	ISL2	5.421	0.83730917166595	150	157	1	tcactttt
MA0673.1	NKX2-8	3.019	0.827475516449228	150	158	1	tcactttta
MA0745.1	SNAI2	2.720	0.802297960120033	150	158	-1	taaaagtga
MA0877.1	Barhl1	5.635	0.867358025078391	151	160	-1	tctaaaagtg
MA0635.1	BARHL2	2.774	0.822137129745099	151	160	-1	tctaaaagtg
MA0063.1	Nkx2-5	4.772	0.81700671203629	151	157	-1	aaaagtg
MA0520.1	Stat6	12.379	0.908811071916792	151	165	1	cacttttagagaaat
MA0144.2	STAT3	4.746	0.855050405091454	154	164	1	ttttagagaaa
MA0518.1	Stat4	6.341	0.835248420310091	154	167	1	ttttagagaaatga
MA0519.1	Stat5a::Stat5b	2.030	0.818579768083742	155	165	-1	atttctctaaa
MA0606.1	NFAT5	5.036	0.812697015184912	157	166	-1	catttctcta
MA0081.1	SPIB	6.322	0.858302063374879	158	164	1	agagaaa
MA0158.1	HOXA5	4.597	0.814837133331225	159	166	1	gagaaatg
MA0090.2	TEAD1	4.317	0.812153895216453	159	168	-1	ctcatttctc
MA0879.1	Dlx1	4.341	0.815382896537097	160	169	-1	gctcatttct
MA0879.1	Dlx1	4.924	0.830136292362845	160	169	1	agaaatgagc
MA0612.1	EMX1	5.858	0.827713683546871	160	169	1	agaaatgagc
MA0886.1	EMX2	4.682	0.849825343777412	160	169	1	agaaatgagc
MA0887.1	EVX1	3.695	0.809015075641904	160	169	-1	gctcatttct
MA0887.1	EVX1	3.386	0.801983475612718	160	169	1	agaaatgagc
MA0888.1	EVX2	4.004	0.822917511143225	160	169	-1	gctcatttct

MA0888.1	EVX2	3.000	0.801264799283663	160	169	1	agaaatgagc
MA0890.1	GBX2	3.146	0.805826214100892	160	169	1	agaaatgagc
MA0892.1	GSX1	5.037	0.831564497723128	160	169	-1	gctcatttct
MA0893.1	GSX2	4.527	0.805335518272402	160	169	-1	gctcatttct
MA0900.1	HOXA2	4.909	0.841971620583441	160	169	-1	gctcatttct
MA0902.1	HOXB2	4.416	0.82669321654983	160	169	-1	gctcatttct
MA0903.1	HOXB3	4.171	0.831512417410028	160	169	-1	gctcatttct
MA0492.1	JUND(var.2)	4.146	0.840267679885348	160	174	1	agaaatgagcgcatt
MA0700.1	LHX2	2.551	0.807334021055903	160	169	-1	gctcatttct
MA0706.1	MEOX2	4.684	0.813166265279946	160	169	1	agaaatgagc
MA0662.1	MIXL1	3.963	0.809996691301354	160	169	1	agaaatgagc
MA0710.1	NOTO	6.398	0.883255664818254	160	169	-1	gctcatttct
MA0881.1	Dlx4	4.026	0.815591497070789	161	168	-1	ctcatttc
MA0882.1	DLX6	3.943	0.816021669509515	161	168	-1	ctcatttc
MA0027.2	EN1	3.468	0.817526700874276	161	168	1	gaaatgag
MA0488.1	JUN	2.847	0.818726938073669	161	173	1	gaaatgagcgcat
MA0701.1	LHX9	5.019	0.841087389431542	161	168	1	gaaatgag
MA0675.1	NKX6-2	4.006	0.811983865877605	161	168	-1	ctcatttc
MA0793.1	POU6F2	6.085	0.821745129799445	161	170	-1	cgctcatttc
MA0720.1	Shox2	2.972	0.803169497439608	161	168	1	gaaatgag
MA0722.1	VAX1	4.936	0.823009116526517	161	168	-1	ctcatttc
MA0723.1	VAX2	4.524	0.816578969779566	161	168	-1	ctcatttc
MA0158.1	HOXA5	5.527	0.847590893777919	163	170	-1	cgctcatt
MA0492.1	JUND(var.2)	2.540	0.823888141796397	163	177	-1	tgcaatgcgctcatt
MA0089.1	MAFG::NFE2L1	5.264	0.846327457967442	163	168	1	aatgag
MA0488.1	JUN	1.236	0.801181379598914	164	176	-1	gcaatgcgctcat
MA0842.1	NRL	4.539	0.802073519708573	164	174	-1	aatgcgctcat
MA0719.1	RHOXF1	0.343	0.812008333914076	164	171	1	atgagcgc
MA0668.1	NEUROD2	2.693	0.809303165937728	167	176	1	agcgcattgc
MA0629.1	Rhox11	9.024	0.826235909930548	168	184	-1	attctgctgcaatgcgc
MA0019.1	Ddit3::Cebpa	8.694	0.837765756856826	169	180	-1	tgctgcaatgcg
MA0090.2	TEAD1	4.482	0.815553580204438	169	178	1	cgcattgcag
MA0809.1	TEAD4	6.357	0.858716331634316	169	178	1	cgcattgcag
MA0808.1	TEAD3	3.438	0.851620655015126	170	177	1	gcattgca
MA0833.1	ATF4	7.213	0.822477473424379	171	183	-1	ttctgctgcaatg
MA0158.1	HOXA5	4.919	0.826177682647177	171	178	-1	ctgcaatg
MA0102.3	CEBPA	4.835	0.855913161388287	172	182	1	attgcagcaga
MA0842.1	NRL	5.258	0.817542484315555	174	184	-1	attctgctgca
MA0623.1	Neurog1	4.309	0.802766348810815	177	186	-1	gcattctgct
MA0514.1	Sox3	4.790	0.83653953577156	177	186	-1	gcattctgct
MA0081.1	SPIB	6.232	0.855227295031945	177	183	1	agcagaa
MA0090.2	TEAD1	9.002	0.908684344723771	178	187	-1	cgcattctgc
MA0809.1	TEAD4	9.263	0.920896647477072	178	187	-1	cgcattctgc
MA0808.1	TEAD3	2.273	0.8335856496767	179	186	-1	gcattctg
MA0442.1	SOX10	6.352	0.886446713233527	180	185	-1	cattct
MA0117.2	Mafb	13.090	0.936541864592042	182	193	1	aatgcgctgacg
MA0842.1	NRL	11.485	0.951513614507039	182	192	1	aatgcgctgac
MA0595.1	SREBF1	7.124	0.825313643434939	183	192	-1	gtcagcgcat
MA0596.1	SREBF2	8.303	0.836287936887854	183	192	1	atgcgctgac
MA0615.1	Gmeb1	8.288	0.85715069930529	184	200	-1	tctctgacgtcagcgca
MA0492.1	JUND(var.2)	6.896	0.868314834147249	184	198	1	tgcgctgacgtcaga
MA0506.1	NRF1	3.040	0.814530522916715	184	194	-1	acgtcagcgca
MA0615.1	Gmeb1	10.856	0.921602575054268	185	201	1	gcgctgacgtcagagac
MA0131.2	HINFP	7.173	0.819429957166056	185	196	-1	tgacgtcagcgc
MA0488.1	JUN	7.216	0.866310144142078	185	197	1	gcgctgacgtcag
MA0834.1	ATF7	9.122	0.871326996512933	186	199	-1	ctctgacgtcagcg
MA0834.1	ATF7	9.303	0.873328420960206	186	199	1	cgctgacgtcagag
MA0605.1	Atf3	7.288	0.86070640426761	187	194	1	gctgacgt
MA0840.1	Creb5	9.780	0.882342757048535	187	198	-1	tctgacgtcagc
MA0840.1	Creb5	9.414	0.877586748516017	187	198	1	gctgacgtcaga
MA0609.1	Crem	11.357	0.920295057833098	187	196	1	gctgacgtca
MA0639.1	DBP	5.000	0.847798127081655	187	198	-1	tctgacgtcagc
MA0639.1	DBP	5.609	0.855783556601348	187	198	1	gctgacgtcaga
MA0043.2	HLF	6.639	0.859053183395935	187	198	-1	tctgacgtcagc
MA0043.2	HLF	5.814	0.846114041758251	187	198	1	gctgacgtcaga
MA0656.1	JDP2(var.2)	10.710	0.896415839215635	187	198	-1	tctgacgtcagc
MA0656.1	JDP2(var.2)	10.848	0.897907621988041	187	198	1	gctgacgtcaga
MA0492.1	JUND(var.2)	3.498	0.833658750444725	187	201	-1	gtctctgacgtcagc
MA0089.1	MAFG::NFE2L1	5.553	0.858843819112298	187	192	1	gctgac
MA0604.1	Atf1	8.650	0.905312988607059	188	195	1	ctgacgtc
MA0488.1	JUN	5.553	0.848198248584339	188	200	-1	tctctgacgtcag
MA0498.2	MEIS1	5.884	0.905994434664963	188	194	1	ctgacgt
MA0018.2	CREB1	11.569	1.00001610713187	189	196	-1	tgacgtca
MA0018.2	CREB1	11.569	1.00001610713187	189	196	1	tgacgtca
MA0609.1	Crem	11.768	0.928558718266003	189	198	-1	tctgacgtca
MA0862.1	GMEB2	9.330	0.916981461381433	189	196	-1	tgacgtca

MA0862.1	GMEB2	9.330	0.916981461381433	189	196	1	tgacgtca
MA0484.1	HNF4G	7.174	0.841713851768261	189	203	1	tgacgtcagagacca
MA0504.1	NR2C2	8.246	0.824784274375956	189	203	1	tgacgtcagagacca
MA0604.1	Atf1	8.650	0.905312988607059	190	197	-1	ctgacgtc
MA0605.1	Atf3	4.399	0.800862379320882	191	198	-1	tctgacgt
MA0498.2	MEIS1	5.884	0.905994434664963	191	197	-1	ctgacgt
MA0089.1	MAFG::NFE2L1	4.933	0.831992110081811	193	198	-1	tctgac
MA0750.1	ZBTB7A	8.120	0.809167071044927	196	207	1	agagaccacccc
MA0734.1	GLI2	8.908	0.828403733395004	197	208	1	gagaccacccct
MA0160.1	NR4A2	6.947	0.835278689612702	197	204	1	gagaccac
MA0694.1	ZBTB7B	8.677	0.847052113977087	197	208	1	gagaccacccct
MA0695.1	ZBTB7C	9.725	0.869107854437983	197	208	1	gagaccacccct
MA0162.2	EGR1	4.425	0.827568074035895	198	211	1	agaccacccccttct
MA0511.2	RUNX2	4.803	0.814734264116013	198	206	1	agaccaccc
MA0684.1	RUNX3	4.525	0.804521401208745	198	207	1	agaccacccc
MA0079.3	SP1	3.295	0.822595553496615	198	208	1	agaccacccct
MA0493.1	Klf1	5.232	0.800061066097261	199	209	1	gaccaccccctt
MA0528.1	ZNF263	7.051	0.813294208394129	199	219	-1	ggaggcgcagaaggggtggtc
MA0130.1	ZNF354C	6.331	0.886867302367815	199	204	1	gaccac
MA0746.1	SP3	5.995	0.822410015512409	200	210	1	accacccccttc
MA0747.1	SP8	7.412	0.819808455065849	200	211	1	accacccccttct
MA0057.1	MZF1(var.2)	7.730	0.865601622033459	201	210	-1	gaaggggtgg
MA0803.1	TBX15	5.011	0.820720347215504	201	208	-1	aggggtgg
MA0806.1	TBX4	3.711	0.803420796653031	201	208	-1	aggggtgg
MA0807.1	TBX5	6.942	0.852601945798726	201	208	-1	aggggtgg
MA0751.1	ZIC4	5.484	0.803482544670375	201	215	1	ccacccccttctgcgc
MA0753.1	ZNF740	5.821	0.802160027179835	201	210	1	ccacccccttc
MA0528.1	ZNF263	9.330	0.83337994503541	202	222	-1	tatggaggcgcagaaggggtg
MA0672.1	NKX2-3	3.823	0.826498304758769	203	212	1	accccttctg
MA0109.1	HLTF	5.514	0.875553977751613	204	213	1	ccccttctgc
MA0673.1	NKX2-8	5.582	0.873820343776727	204	212	1	ccccttctg
MA0607.1	Bhlha15	3.265	0.806814700906033	206	213	1	ccttctgc
MA0442.1	SOX10	4.820	0.818433532819252	206	211	1	ccttct
MA0079.3	SP1	4.154	0.833402756913742	209	219	1	tctgcgcctcc
MA0057.1	MZF1(var.2)	6.639	0.8310905717609	210	219	-1	ggaggcgcag
MA0767.1	GCM2	5.260	0.801653046700999	213	222	-1	tatggaggcg
MA0108.2	TBP	9.469	0.87543183471216	213	227	-1	gtttatatggaggcg
MA0509.1	Rfx1	6.787	0.8028123617703	214	227	1	gcctccatataaac
MA0109.1	HLTF	7.464	0.944279141045773	216	225	1	ctccatataa
MA0899.1	HOXA10	5.096	0.809757625295477	216	226	1	ctccatataaa
MA0130.1	ZNF354C	4.723	0.816487152026638	216	221	1	ctccat
MA0878.1	CDX1	6.473	0.860006050772915	217	225	1	tccatataa
MA0909.1	HOXD13	4.676	0.807687632528672	217	226	1	tccatataaa
MA0913.1	Hoxd9	4.591	0.80498259576882	217	226	1	tccatataaa
MA0108.2	TBP	10.219	0.890315912299857	218	232	1	ccatataaaccccac
MA0878.1	CDX1	3.656	0.802675540193111	219	227	1	catataaac
MA0901.1	HOXB13	6.877	0.845151455545127	219	228	1	catataaacc
MA0909.1	HOXD13	4.590	0.806101173583429	219	228	1	catataaacc
MA0619.1	LIN54	3.856	0.814065950837563	219	227	-1	gtttatatg
MA0593.1	FOXP2	4.965	0.806066783213765	220	230	1	atataaacccc
MA0037.2	GATA3	3.279	0.820594050245556	220	227	1	atataaac
MA0619.1	LIN54	3.746	0.811884945303964	220	228	1	atataaacc
MA0108.2	TBP	6.048	0.807540595475476	220	234	1	atataaaccccaccc
MA0714.1	PITX3	6.326	0.849600766168741	221	229	1	tataaaccc
MA0847.1	FOXD2	3.195	0.821867790784055	222	228	1	ataaacc
MA0613.1	FOXG1	4.962	0.827900094297596	222	229	1	ataaaccc
MA0042.2	FOXI1	2.768	0.800644587675346	222	228	1	ataaacc
MA0614.1	Foxj2	4.175	0.811117272977989	222	229	1	ataaaccc
MA0033.2	FOXL1	5.844	0.882577613914127	222	228	1	ataaacc
MA0848.1	FOXO4	3.474	0.818265555211229	222	228	1	ataaacc
MA0849.1	FOXO6	2.624	0.818376184629519	222	228	1	ataaacc
MA0850.1	FOXP3	4.627	0.833454943393175	222	228	1	ataaacc
MA0711.1	OTX1	4.329	0.846043991115658	222	229	1	ataaaccc
MA0712.1	OTX2	4.816	0.847790461949794	222	229	1	ataaaccc
MA0682.1	Pitx1	5.868	0.841911904333453	222	229	1	ataaaccc
MA0719.1	RHOXF1	2.005	0.844042891313564	222	229	1	ataaaccc
MA0719.1	RHOXF1	0.057	0.806495768681673	223	230	1	taaacccc
MA0002.2	RUNX1	7.529	0.84489469927906	224	234	-1	gggtggggttt
MA0511.2	RUNX2	5.889	0.834752198317157	224	232	1	aaaccccac
MA0684.1	RUNX3	5.532	0.822074015034439	224	233	1	aaaccccacc
MA0493.1	Klf1	14.069	0.949449430917173	225	235	1	aaccccaccca
MA0742.1	Klf12	6.270	0.814475826408317	225	239	1	aaccccacccagcca
MA0741.1	KLF16	6.050	0.816813087992767	226	236	1	accccacccag
MA0039.2	Klf4	13.909	0.9728210017962	226	235	-1	tgggtggggt
MA0599.1	KLF5	12.535	0.962715634557158	226	235	1	accccaccca
MA0056.1	MZF1	4.989	0.813099698301624	226	231	-1	tggggt

MA0073.1	RREB1	12.703	0.816639148139111	226	245	1	accccacccagccagcccct
MA0079.3	SP1	8.073	0.882708263539727	226	236	1	accccacccag
MA0516.1	SP2	6.553	0.812966171312575	226	240	1	accccacccagccag
MA0746.1	SP3	9.026	0.882101088243885	226	236	1	accccacccag
MA0747.1	SP8	8.640	0.842629960785147	226	237	1	accccacccagc
MA0807.1	TBX5	5.093	0.804822284482574	227	234	-1	gggtgggg
MA0130.1	ZNF354C	6.723	0.904024652450988	227	232	1	ccccac
MA0753.1	ZNF740	7.035	0.824538620246984	227	236	1	ccccacccag
MA0810.1	TFAP2A(var.2)	3.523	0.81104424070532	228	239	1	cccacccagcca
MA0006.1	Ahr::Arnt	5.297	0.817309426751426	230	235	-1	tgggtg
MA0671.1	NFIX	6.780	0.911899046759466	233	241	1	ccagccagc
MA0092.1	Hand1::Tcf3	7.032	0.830765495507163	234	243	-1	gggctggctg
MA0498.2	MEIS1	2.079	0.825415582981183	234	240	1	cagccag
MA0597.1	THAP1	5.213	0.827176851732465	234	242	1	cagccagcc
MA0161.1	NFIC	7.219	0.916988656211363	235	240	-1	ctggct
MA0079.3	SP1	2.287	0.809913759498403	235	245	1	agccagcccct
MA0057.1	MZF1(var.2)	6.408	0.823783465791183	238	247	-1	ctaggggctg
MA0597.1	THAP1	5.793	0.844927642941826	238	246	1	cagcccta
MA0003.3	TFAP2A	6.034	0.850096685077216	239	249	1	agcccctagcg
MA0810.1	TFAP2A(var.2)	3.736	0.814350123200613	239	250	1	agcccctagcgc
MA0812.1	TFAP2B(var.2)	3.657	0.805652226451555	239	249	1	agcccctagcg
MA0814.1	TFAP2C(var.2)	4.325	0.817451931452425	239	249	-1	cgctaggggct
MA0814.1	TFAP2C(var.2)	4.531	0.820755761304592	239	249	1	agcccctagcg

Mus musculus

Model ID	Model name	Score	Relative score	Start	End	Strand	predicted site sequence
MA0147.2	Myc	3.708	0.829243067487283	4	13	-1	acatgagccc
MA0682.1	Pitx1	4.575	0.809684899547031	4	11	-1	atgagccc
MA0714.1	PITX3	4.668	0.81040671746832	4	12	-1	catgagccc
MA0719.1	RHOXF1	8.998	0.978830893656897	4	11	-1	atgagccc
MA0668.1	NEUROD2	2.576	0.807391242884853	5	14	1	ggctcatgta
MA0632.1	Tcfl5	3.675	0.801145471989114	5	14	-1	tacatgagcc
MA0632.1	Tcfl5	3.675	0.801145471989114	5	14	1	ggctcatgta
MA0710.1	NOTO	3.506	0.832113615697496	6	15	-1	ctacatgagc
MA0710.1	NOTO	2.127	0.807727417309693	6	15	1	gctcatgtag
MA0067.1	Pax2	4.036	0.802485541552365	6	13	1	gctcatgt
MA0089.1	MAFG::NFE2L1	5.823	0.870537305302994	7	12	-1	catgag
MA0673.1	NKX2-8	1.840	0.806156534233418	7	15	-1	ctacatgag
MA0144.2	STAT3	5.119	0.859569133792476	10	20	1	atgtagggaaa
MA0471.1	E2F6	4.936	0.833846505294691	11	21	1	tgtagggaaaa
MA0057.1	MZF1(var.2)	6.902	0.83940991751863	12	21	1	gtagggaaaa
MA0597.1	THAP1	5.441	0.834154748966489	12	20	-1	tttccctac
MA0056.1	MZF1	4.916	0.809768936774344	13	18	1	taggga
MA0606.1	NFAT5	9.978	0.913437051142694	13	22	-1	ttttccta
MA0624.1	NFATC1	8.121	0.881329061615355	13	22	-1	tttttcccta
MA0625.1	NFATC3	8.703	0.88011164497529	13	22	-1	tttttcccta
MA0081.1	SPIB	5.764	0.839238499648684	13	19	1	tagggaa
MA0606.1	NFAT5	6.807	0.848797906257602	14	23	-1	atttttccct
MA0081.1	SPIB	4.764	0.805074406949409	14	20	1	agggaaa
MA0111.1	Spz1	7.993	0.825087838878908	14	24	1	agggaaaaatc
MA0152.1	NFATC2	9.187	0.91986621514976	15	21	-1	ttttccc
MA0101.1	REL	6.608	0.80513913889895	16	25	-1	tgatttttcc
MA0879.1	Dlx1	5.001	0.832084854075679	18	27	1	aaaaatcacc
MA0644.1	ESX1	4.488	0.808313442853171	18	27	1	aaaaatcacc
MA0887.1	EVX1	3.604	0.806944280811302	18	27	-1	ggtgattttt
MA0887.1	EVX1	3.981	0.815523287966652	18	27	1	aaaaatcacc
MA0888.1	EVX2	3.623	0.814700695188551	18	27	-1	ggtgattttt
MA0900.1	HOXA2	4.978	0.843531609607204	18	27	1	aaaaatcacc
MA0902.1	HOXB2	4.499	0.828849338409809	18	27	1	aaaaatcacc
MA0903.1	HOXB3	3.505	0.816002556784974	18	27	1	aaaaatcacc
MA0662.1	MIXL1	3.771	0.804999878479212	18	27	1	aaaaatcacc
MA0676.1	Nr2e1	7.187	0.847631082048287	18	26	1	aaaaatcac
MA0027.2	EN1	3.326	0.814441175162124	19	26	1	aaaatcac
MA0847.1	FOXD2	2.428	0.805601246687819	19	25	1	aaaatca
MA0038.1	Gfi1	8.980	0.891817595903916	19	28	1	aaaatcacca
MA0160.1	NR4A2	6.123	0.806326298231601	19	26	1	aaaatcac
MA0620.1	Mitf	6.395	0.82890538784546	21	30	1	aatcaccagc
MA0093.2	USF1	2.684	0.808382039264973	21	31	-1	cgctggtgatt
MA0820.1	FIGLA	4.766	0.812090137716676	22	31	1	atcaccagcg
MA0033.2	FOXL1	3.427	0.838315535761363	22	28	1	atcacca
MA0522.2	TCF3	2.814	0.817169622988385	22	31	1	atcaccagcg
MA0830.1	TCF4	1.429	0.809556303347242	22	31	1	atcaccagcg
MA0056.1	MZF1	5.838	0.851836911132592	23	28	-1	tggtga
MA0745.1	SNAI2	4.378	0.835370770924638	23	31	-1	cgctggtga

MA0067.1	Pax2	4.751	0.830562699241297	27	34	-1	ggtcgctg
MA0750.1	ZBTB7A	13.264	0.91329557254015	28	39	1	agcgaccacagg
MA0734.1	GLI2	12.340	0.887698474753204	29	40	1	gcgaccacaggg
MA0694.1	ZBTB7B	6.205	0.808496749197623	29	40	1	gcgaccacaggg
MA0695.1	ZBTB7C	9.999	0.874738068433451	29	40	1	gcgaccacaggg
MA0116.1	Znf423	7.058	0.808282068135398	29	43	-1	acaccctgtggtcgc
MA0139.1	CTCF	8.961	0.806943060002373	30	48	1	cgaccacagggtgttggtc
MA0002.2	RUNX1	10.951	0.911611289680257	30	40	-1	ccctgtggtcg
MA0511.2	RUNX2	7.463	0.863765300004635	30	38	1	cgaccacag
MA0684.1	RUNX3	7.771	0.861101127602193	30	39	1	cgaccacagg
MA0751.1	ZIC4	5.389	0.80218821749146	30	44	-1	aacaccctgtggtcg
MA0116.1	Znf423	6.747	0.803084475274153	30	44	1	cgaccacagggtgtt
MA0059.1	MAX::MYC	8.372	0.810182152747926	31	41	1	gaccacagggt
MA0147.2	Myc	6.553	0.871000531884578	31	40	-1	ccctgtggtc
MA0526.1	USF2	3.163	0.821098031354332	31	41	-1	accctgtggtc
MA0696.1	ZIC1	3.856	0.808872519474453	31	44	-1	aacaccctgtggtc
MA0130.1	ZNF354C	6.331	0.886867302367815	31	36	1	gaccac
MA0104.3	Mycn	3.845	0.805111489009167	32	39	1	accacagg
MA0668.1	NEUROD2	3.488	0.822294437963676	32	41	1	accacagggt
MA0623.1	Neurog1	5.183	0.823060592806563	32	41	-1	accctgtggt
MA0871.1	TFEC	5.330	0.803477615657202	32	41	1	accacagggt
MA0746.1	SP3	5.535	0.813350994246662	36	46	-1	ccaacaccctg
MA0148.3	FOXA1	0.929	0.80161490807119	37	51	1	agggtgttggtcatg
MA0493.1	Klf1	8.842	0.861087658492857	37	47	-1	accaacaccct
MA0039.2	Klf4	7.084	0.856865519991403	37	46	1	agggtgttgg
MA0599.1	KLF5	2.953	0.840819181507839	37	46	-1	ccaacaccct
MA0801.1	MGA	5.091	0.836557338070417	38	45	1	gggtgttg
MA0806.1	TBX4	5.946	0.848485149683486	38	45	1	gggtgttg
MA0807.1	TBX5	7.214	0.859630646652157	38	45	1	gggtgttg
MA0614.1	Foxj2	4.429	0.816226813977045	40	47	-1	accaacac
MA0476.1	FOS	2.303	0.819653792165671	41	51	1	tgttggtcatg
MA0847.1	FOXD2	5.619	0.873276008371118	41	47	-1	accaaca
MA0033.2	FOXL1	5.548	0.877157020147798	41	47	-1	accaaca
MA0848.1	FOXO4	2.982	0.808538765835091	41	47	-1	accaaca
MA0738.1	HIC2	3.761	0.813024630283501	42	50	-1	atgaccaac
MA0258.2	ESR2	6.316	0.813207441192674	44	58	1	tggtcatggcggcca
MA0886.1	EMX2	3.304	0.826521735215473	45	54	-1	cgccatgacc
MA0642.1	EN2	6.125	0.860192706492977	45	54	-1	cgccatgacc
MA0661.1	MEOX1	4.656	0.822927106099434	45	54	-1	cgccatgacc
MA0661.1	MEOX1	3.805	0.80539364293863	45	54	1	ggtcatggcg
MA0067.1	Pax2	7.662	0.944874036349661	45	52	1	ggtcatgg
MA0701.1	LHX9	4.012	0.813542451469405	46	53	-1	gccatgac
MA0089.1	MAFG::NFE2L1	8.812	0.999988528354811	46	51	-1	catgac
MA0666.1	MSX1	4.062	0.810579335424453	46	53	-1	gccatgac
MA0597.1	THAP1	7.200	0.887988614030741	47	55	-1	ccgccatga
MA0748.1	YY2	12.653	0.928750251934866	47	57	-1	ggccgccatga
MA0024.3	E2F1	6.487	0.802632521673832	48	59	1	catggcggccag
MA0089.1	MAFG::NFE2L1	4.208	0.800592934199387	48	53	1	catggc
MA0671.1	NFIX	4.185	0.850806126705076	48	56	-1	gccgccatg
MA0775.1	MEIS3	3.057	0.801506310129571	49	56	1	atggcggc
MA0161.1	NFIC	4.646	0.830809647045002	49	54	1	atggcg
MA0671.1	NFIX	5.855	0.890122187009828	52	60	1	gcggccagg
MA0810.1	TFAP2A(var.2)	9.591	0.90522309038341	53	64	-1	tgcccctggccg
MA0810.1	TFAP2A(var.2)	7.526	0.87317310281168	53	64	1	cggccaggggca
MA0811.1	TFAP2B	9.132	0.887628018804524	53	64	-1	tgcccctggccg
MA0811.1	TFAP2B	8.724	0.88115738380399	53	64	1	cggccaggggca
MA0524.2	TFAP2C	8.515	0.87481807413939	53	64	-1	tgcccctggccg
MA0524.2	TFAP2C	9.566	0.891897304765522	53	64	1	cggccaggggca
MA0161.1	NFIC	6.668	0.898533687672434	54	59	-1	ctggcc
MA0003.3	TFAP2A	7.422	0.871915990601185	54	64	-1	tgcccctggcc
MA0003.3	TFAP2A	3.733	0.813925055386545	54	64	1	ggccaggggca
MA0812.1	TFAP2B(var.2)	5.587	0.834911920541139	54	64	-1	tgcccctggcc
MA0812.1	TFAP2B(var.2)	3.830	0.808274986595337	54	64	1	ggccaggggca
MA0814.1	TFAP2C(var.2)	5.221	0.831821987508451	54	64	-1	tgcccctggcc
MA0814.1	TFAP2C(var.2)	6.346	0.849864747623439	54	64	1	ggccaggggca
MA0820.1	FIGLA	4.301	0.802828937945693	55	64	-1	tgcccctggc
MA0824.1	ID4	5.863	0.837685141419841	55	64	-1	tgcccctggc
MA0522.2	TCF3	3.369	0.826500318159923	55	64	-1	tgcccctggc
MA0830.1	TCF4	3.693	0.844181443492199	55	64	-1	tgcccctggc
MA0739.1	Hic1	5.299	0.825639349391846	57	65	-1	gtgcccctg
MA0738.1	HIC2	7.745	0.901722053778188	57	65	-1	gtgcccctg
MA0597.1	THAP1	4.385	0.801836067040481	57	65	-1	gtgcccctg
MA0258.2	ESR2	6.317	0.813220651789635	59	73	1	ggggcactgcggcag
MA0503.1	Nkx2-5(var.2)	2.876	0.800301750110292	60	70	1	gggcactgcgg
MA0099.2	FOS::JUN	5.316	0.804790114679483	66	72	-1	tgccgca
MA0671.1	NFIX	3.681	0.838940680960408	66	74	1	tgcggcaga

MA0498.2	MEIS1	1.163	0.806017362707243	67	73	-1	ctgccgc
MA0461.2	Atoh1	5.813	0.832300624490779	69	78	-1	aaaatctgcc
MA0461.2	Atoh1	4.512	0.807558470065221	69	78	1	ggcagatttt
MA0038.1	Gfi1	7.364	0.843271743138937	69	78	-1	aaaatctgcc
MA0623.1	Neurog1	5.301	0.825800547945577	69	78	-1	aaaatctgcc
MA0623.1	Neurog1	4.903	0.816559004341106	69	78	1	ggcagatttt
MA0827.1	OLIG3	6.081	0.847636015451443	69	78	-1	aaaatctgcc
MA0827.1	OLIG3	3.488	0.804515664547344	69	78	1	ggcagatttt
MA0607.1	Bhlha15	3.667	0.814332968728102	70	77	1	gcagattt
MA0041.1	Foxd3	6.687	0.802977851507801	70	81	1	gcagattttttt
MA0891.1	GSC2	4.589	0.80470264752648	70	79	-1	aaaaatctgc
MA0711.1	OTX1	3.787	0.835477601519824	71	78	-1	aaaatctg
MA0712.1	OTX2	4.487	0.841096176547244	71	78	-1	aaaatctg
MA0719.1	RHOXF1	5.215	0.905914689901022	71	78	-1	aaaatctg
MA0041.1	Foxd3	6.783	0.804893072905521	72	83	1	agattttttttt
MA0041.1	Foxd3	10.579	0.880624119007061	73	84	1	gattttttttc
MA0481.1	FOXP1	8.104	0.804030638342701	74	88	-1	gaaggaaaaaaaaat
MA0913.1	Hoxd9	4.524	0.803433823692717	74	83	-1	aaaaaaaaat
MA0606.1	NFAT5	4.632	0.804461690513333	74	83	1	attttttttt
MA0087.1	Sox5	6.624	0.840728791704037	74	80	1	atttttt
MA0084.1	SRY	5.833	0.804851087985305	74	82	-1	aaaaaaaat
MA0481.1	FOXP1	12.461	0.875426717622551	75	89	-1	cgaaggaaaaaaaaa
MA0899.1	HOXA10	5.156	0.811005015052773	75	85	-1	ggaaaaaaaaa
MA0913.1	Hoxd9	6.183	0.841783269577133	75	84	-1	gaaaaaaaaa
MA0514.1	Sox3	3.745	0.821073163893556	75	84	1	tttttttttc
MA0528.1	ZNF263	9.650	0.836200232760381	75	95	-1	aaagaacgaaggaaaaaaaaa
MA0593.1	FOXP2	4.780	0.80288012719744	76	86	-1	aggaaaaaaaa
MA0913.1	Hoxd9	5.379	0.82319800466389	76	85	-1	ggaaaaaaaa
MA0514.1	Sox3	3.291	0.814353802331246	76	85	1	ttttttttcc
MA0032.2	FOXC1	3.729	0.810192806400902	77	87	-1	aaggaaaaaaa
MA0514.1	Sox3	3.212	0.813184574217981	77	86	1	tttttttcct
MA0157.2	FOXO3	5.002	0.807961226849649	78	85	-1	ggaaaaaa
MA0848.1	FOXO4	2.656	0.802093779378544	78	84	-1	gaaaaaa
MA0606.1	NFAT5	6.924	0.851182888897638	78	87	1	ttttttcctt
MA0136.2	ELF5	4.476	0.803766488536502	79	89	-1	cgaaggaaaaa
MA0606.1	NFAT5	9.303	0.899677535911714	79	88	1	tttttccttc
MA0624.1	NFATC1	8.883	0.903046983799819	79	88	1	tttttccttc
MA0625.1	NFATC3	9.353	0.896625956468141	79	88	1	tttttccttc
MA0471.1	E2F6	4.164	0.822456613437269	80	90	-1	acgaaggaaaa
MA0152.1	NFATC2	9.490	0.931041560772187	80	86	1	ttttcct
MA0768.1	LEF1	9.445	0.82072295157893	81	95	-1	aaagaacgaaggaaa
MA0109.1	HLTF	5.098	0.860892609582192	82	91	1	ttccttcgtt
MA0523.1	TCF7L2	5.894	0.823892709240715	82	95	-1	aaagaacgaaggaa
MA0143.3	Sox2	6.693	0.870981793987171	84	91	1	ccttcgtt
MA0514.1	Sox3	7.865	0.882050630053726	84	93	1	ccttcgttct
MA0515.1	Sox6	7.893	0.86495548977831	84	93	1	ccttcgttct
MA0769.1	Tcf7	7.898	0.807554805351193	84	95	-1	aaagaacgaagg
MA0442.1	SOX10	4.605	0.808888602147537	85	90	1	cttcgt
MA0442.1	SOX10	4.820	0.818433532819252	88	93	1	cgttct
MA0847.1	FOXD2	4.228	0.843775665688113	91	97	-1	gcaaaga
MA0850.1	FOXP3	6.725	0.886240020070117	91	97	-1	gcaaaga
MA0442.1	SOX10	4.669	0.811729883835861	92	97	1	ctttgc
MA0088.2	ZNF143	5.938	0.810807129435812	95	110	-1	cacccagactgcagca
MA0623.1	Neurog1	5.675	0.834484812538723	99	108	1	gcagtctggg
MA0498.2	MEIS1	2.482	0.833949952817862	100	106	-1	cagactg
MA0442.1	SOX10	4.539	0.805958530406451	100	105	1	cagtct
MA0092.1	Hand1::Tcf3	6.671	0.820129522902726	101	110	1	agtctgggtg
MA0155.1	INSM1	8.524	0.822850767294449	101	112	1	agtctgggtgcg
MA0079.3	SP1	1.810	0.803912553409963	104	114	-1	gccgcacccag
MA0746.1	SP3	6.691	0.836116708557974	104	114	-1	gccgcacccag
MA0006.1	Ahr::Arnt	5.297	0.817309426751426	105	110	1	tgggtg
MA0493.1	Klf1	10.485	0.888862365225811	105	115	-1	agccgcaccca
MA0039.2	Klf4	11.000	0.923397632335372	105	114	1	tgggtgcggc
MA0599.1	KLF5	6.555	0.886641663649735	105	114	-1	gccgcaccca
MA0801.1	MGA	4.037	0.817384281072698	106	113	1	gggtgcgg
MA0002.2	RUNX1	5.523	0.805784973871463	106	116	1	gggtgcggcta
MA0806.1	TBX4	4.406	0.817434096588676	106	113	1	gggtgcgg
MA0807.1	TBX5	5.247	0.80880176952459	106	113	1	gggtgcgg
MA0646.1	GCM1	5.964	0.827216701378698	107	117	1	ggtgcggctac
MA0767.1	GCM2	5.589	0.808373528677047	107	116	1	ggtgcggcta
MA0684.1	RUNX3	5.428	0.820261232673096	107	116	-1	tagccgcacc
MA0495.1	MAFF	6.514	0.806159135525472	110	127	1	gcggctacagcaatttgt
MA0111.1	Spz1	7.071	0.801116534499906	110	120	1	gcggctacagc
MA0719.1	RHOXF1	1.895	0.841922673916486	111	118	-1	tgtagccg
MA0003.3	TFAP2A	4.082	0.819411321689042	111	121	1	cggctacagca
MA0117.2	Mafb	6.056	0.809620705982555	114	125	-1	aaattgctgtag

MA0842.1	NRL	6.256	0.839014009597569	115	125	-1	aaattgctgta
MA0081.1	SPIB	4.704	0.803024561387453	116	122	1	acagcaa
MA0158.1	HOXA5	6.112	0.868194065671807	117	124	1	cagcaatt
MA0087.1	Sox5	6.676	0.842666567949414	117	123	-1	attgctg
MA0809.1	TEAD4	5.580	0.84209069315881	117	126	-1	caaattgctg
MA0877.1	Barhl1	6.512	0.890469041803454	118	127	-1	acaaattgct
MA0635.1	BARHL2	4.139	0.847546902599839	118	127	-1	acaaattgct
MA0879.1	Dlx1	6.125	0.860528793883811	118	127	1	agcaatttgt
MA0612.1	EMX1	6.536	0.846763873641076	118	127	-1	acaaattgct
MA0644.1	ESX1	5.393	0.831579408817481	118	127	1	agcaatttgt
MA0887.1	EVX1	3.427	0.802916471085846	118	127	-1	acaaattgct
MA0888.1	EVX2	3.182	0.805189892469361	118	127	1	agcaatttgt
MA0889.1	GBX1	5.894	0.857663219524059	118	127	1	agcaatttgt
MA0890.1	GBX2	6.822	0.877410749309739	118	127	1	agcaatttgt
MA0892.1	GSX1	5.220	0.836534271459619	118	127	1	agcaatttgt
MA0893.1	GSX2	5.118	0.821684813262664	118	127	1	agcaatttgt
MA0894.1	HESX1	6.554	0.867576755334856	118	127	-1	acaaattgct
MA0900.1	HOXA2	4.185	0.825603040102218	118	127	1	agcaatttgt
MA0902.1	HOXB2	5.078	0.843890236686043	118	127	1	agcaatttgt
MA0903.1	HOXB3	5.721	0.867608939885755	118	127	1	agcaatttgt
MA0699.1	LBX2	5.256	0.818522702138256	118	127	1	agcaatttgt
MA0700.1	LHX2	3.268	0.819432189784446	118	127	-1	acaaattgct
MA0700.1	LHX2	2.222	0.801782699561217	118	127	1	agcaatttgt
MA0662.1	MIXL1	5.652	0.853953029096136	118	127	1	agcaatttgt
MA0125.1	Nobox	6.189	0.82936435549078	118	125	-1	aaattgct
MA0710.1	NOTO	5.961	0.87552776873742	118	127	-1	acaaattgct
MA0718.1	RAX	6.518	0.857018836515325	118	127	1	agcaatttgt
MA0808.1	TEAD3	0.392	0.8044664693835	118	125	-1	aaattgct
MA0724.1	VENTX	2.711	0.81336698127758	118	126	1	agcaatttg
MA0875.1	BARX1	2.778	0.814907886856419	119	126	1	gcaatttg
MA0879.1	Dlx1	4.488	0.819102877988872	119	128	-1	gacaaattgc
MA0885.1	Dlx2	6.366	0.885617774057834	119	126	1	gcaatttg
MA0880.1	Dlx3	6.283	0.871878647537569	119	126	1	gcaatttg
MA0881.1	Dlx4	6.616	0.886253166923116	119	126	1	gcaatttg
MA0882.1	DLX6	6.143	0.873066690622673	119	126	1	gcaatttg
MA0027.2	EN1	6.708	0.887928836841698	119	126	1	gcaatttg
MA0914.1	ISL2	8.022	0.904979249515833	119	126	1	gcaatttg
MA0654.1	ISX	5.545	0.859189214972164	119	126	1	gcaatttg
MA0704.1	Lhx4	4.146	0.821752104199954	119	126	1	gcaatttg
MA0705.1	Lhx8	1.328	0.805187444764903	119	126	-1	caaattgc
MA0701.1	LHX9	5.648	0.858292718108983	119	126	1	gcaatttg
MA0703.1	LMX1B	5.269	0.829806462870895	119	126	1	gcaatttg
MA0666.1	MSX1	6.097	0.860978552699148	119	126	1	gcaatttg
MA0708.1	MSX2	4.612	0.815918546385144	119	126	1	gcaatttg
MA0709.1	Msx3	6.505	0.875225062176186	119	126	1	gcaatttg
MA0668.1	NEUROD2	2.460	0.805495661054651	119	128	-1	gacaaattgc
MA0623.1	Neurog1	4.403	0.804949024938504	119	128	1	gcaatttgtc
MA0132.2	PDX1	2.464	0.813820417530302	119	126	1	gcaatttg
MA0716.1	PRRX1	5.404	0.841122223250983	119	126	1	gcaatttg
MA0075.2	Prrx2	6.054	0.854825064276216	119	126	1	gcaatttg
MA0717.1	RAX2	6.442	0.87041656062619	119	126	1	gcaatttg
MA0630.1	SHOX	6.112	0.84927225736707	119	126	-1	caaattgc
MA0720.1	Shox2	6.172	0.874955378014643	119	126	1	gcaatttg
MA0721.1	UNCX	5.790	0.84969495113426	119	126	1	gcaatttg
MA0722.1	VAX1	6.292	0.861501788132274	119	126	-1	caaattgc
MA0723.1	VAX2	6.255	0.8623391941205	119	126	-1	caaattgc
MA0725.1	VSX1	6.741	0.860940210693593	119	126	-1	caaattgc
MA0726.1	VSX2	5.803	0.829420886026564	119	126	-1	caaattgc
MA0847.1	FOXD2	3.568	0.829778378721338	121	127	-1	acaaatt
MA0158.1	HOXA5	5.527	0.847590893777919	121	128	-1	gacaaatt
MA0491.1	JUND	0.811	0.821122940649332	121	131	-1	tatgacaaatt
MA0655.1	JDP2	4.372	0.801809325909432	122	130	-1	atgacaaat
MA0489.1	JUN(var.2)	3.699	0.812533993736239	122	135	-1	attctatgacaaat
MA0490.1	JUNB	1.016	0.803013026500002	122	132	-1	ctatgacaaat
MA0670.1	NFIA	2.033	0.823366319327152	122	131	-1	tatgacaaat
MA0442.1	SOX10	4.805	0.817767607423551	122	127	1	atttgt
MA0604.1	Atf1	4.084	0.806651805176267	123	130	-1	atgacaaa
MA0774.1	MEIS2	5.535	0.826096795346533	123	130	-1	atgacaaa
MA0775.1	MEIS3	7.506	0.900026379504145	123	130	-1	atgacaaa
MA0084.1	SRY	6.786	0.836757059806185	123	131	-1	tatgacaaa
MA0605.1	Atf3	5.598	0.825698996112549	124	131	-1	tatgacaa
MA0465.1	CDX2	5.865	0.841070477628713	124	134	1	ttgtcatagaa
MA0498.2	MEIS1	9.456	0.981639022894781	124	130	-1	atgacaa
MA0161.1	NFIC	3.787	0.802038652535128	124	129	1	ttgtca
MA0519.1	Stat5a::Stat5b	0.571	0.800748973234957	124	134	1	ttgtcatagaa
MA0899.1	HOXA10	5.582	0.819861482329578	125	135	1	tgtcatagaat

MA0911.1	Hoxa11	5.546	0.80931641449105	125	136	1	tgtcatagaatc
MA0651.1	HOXC11	5.236	0.821336871832339	125	135	1	tgtcatagaat
MA0906.1	HOXC12	4.036	0.807555522594369	125	135	1	tgtcatagaat
MA0067.1	Pax2	5.776	0.870813170054101	125	132	1	tgtcatag
MA0878.1	CDX1	6.146	0.853351071376537	126	134	1	gtcatagaa
MA0905.1	HOXC10	6.760	0.872465416131608	126	135	1	gtcatagaat
MA0908.1	HOXD11	7.295	0.845452174570221	126	135	1	gtcatagaat
MA0909.1	HOXD13	5.280	0.818829739539447	126	135	1	gtcatagaat
MA0913.1	Hoxd9	5.320	0.821834160596873	126	135	1	gtcatagaat
MA0089.1	MAFG::NFE2L1	8.072	0.967939714350681	126	131	-1	tatgac
MA0090.2	TEAD1	6.543	0.85801873632708	129	138	-1	cagattctat
MA0809.1	TEAD4	6.862	0.86952192678249	129	138	-1	cagattctat
MA0766.1	GATA5	3.442	0.812087022013815	130	137	-1	agattcta
MA0623.1	Neurog1	4.321	0.803044988316477	131	140	-1	cccagattct
MA0623.1	Neurog1	6.551	0.854825496452082	131	140	1	agaatctggg
MA0827.1	OLIG3	4.221	0.816705104274573	131	140	-1	cccagattct
MA0719.1	RHOXF1	2.711	0.857650832062083	131	138	1	agaatctg
MA0607.1	Bhlha15	3.265	0.806814700906033	132	139	-1	ccagattc
MA0694.1	ZBTB7B	6.644	0.815343757748668	135	146	-1	gagccccccaga
MA0695.1	ZBTB7C	6.641	0.805737124649578	135	146	-1	gagccccccaga
MA0753.1	ZNF740	6.573	0.816022219919947	136	145	-1	agccccccag
MA0056.1	MZF1	6.469	0.880627466251957	137	142	1	tggggg
MA0478.1	FOSL2	3.321	0.819070989244286	138	148	1	ggggggctcat
MA0492.1	JUND(var.2)	0.281	0.800848679440893	138	152	-1	aaaaatgagcccccc
MA0056.1	MZF1	5.636	0.842620283344776	138	143	1	gggggg
MA0682.1	Pitx1	4.575	0.809684899547031	141	148	-1	atgagccc
MA0714.1	PITX3	4.428	0.804733272541842	141	149	-1	aatgagccc
MA0719.1	RHOXF1	8.998	0.978830893656897	141	148	-1	atgagccc
MA0793.1	POU6F2	8.119	0.860793323174769	142	151	1	ggctcatttt
MA0879.1	Dlx1	5.615	0.847622735785815	143	152	-1	aaaaatgagc
MA0879.1	Dlx1	4.758	0.825935496981928	143	152	1	gctcattttt
MA0612.1	EMX1	5.858	0.827713683546871	143	152	-1	aaaaatgagc
MA0886.1	EMX2	3.797	0.834858947857734	143	152	-1	aaaaatgagc
MA0887.1	EVX1	3.431	0.803007495034444	143	152	1	gctcattttt
MA0888.1	EVX2	3.608	0.814377198497422	143	152	1	gctcattttt
MA0892.1	GSX1	4.533	0.817877252022628	143	152	1	gctcattttt
MA0900.1	HOXA2	3.566	0.811608355961503	143	152	1	gctcattttt
MA0902.1	HOXB2	4.183	0.820640488918807	143	152	1	gctcattttt
MA0903.1	HOXB3	3.686	0.820217699086978	143	152	1	gctcattttt
MA0700.1	LHX2	2.597	0.808110193666103	143	152	1	gctcattttt
MA0706.1	MEOX2	4.298	0.803515624834874	143	152	1	gctcattttt
MA0710.1	NOTO	5.958	0.875474716819245	143	152	1	gctcattttt
MA0877.1	Barhl1	3.648	0.814995892543956	144	153	-1	gaaaaatgag
MA0635.1	BARHL2	4.319	0.850897641877387	144	153	-1	gaaaaatgag
MA0881.1	Dlx4	3.725	0.807379465169032	144	151	1	ctcatttt
MA0882.1	DLX6	3.551	0.805857283929352	144	151	1	ctcatttt
MA0701.1	LHX9	4.942	0.8389811727254	144	151	-1	aaaatgag
MA0089.1	MAFG::NFE2L1	5.264	0.846327457967442	144	149	-1	aatgag
MA0675.1	NKX6-2	4.159	0.816194916177283	144	151	1	ctcatttt
MA0722.1	VAX1	4.526	0.811370476881709	144	151	1	ctcatttt
MA0515.1	Sox6	6.759	0.843343009784452	145	154	1	tcatttttcc
MA0847.1	FOXD2	3.353	0.825218656451859	146	152	-1	aaaaatg
MA0442.1	SOX10	4.521	0.80515941993161	146	151	1	catttt
MA0076.2	ELK4	7.654	0.859926134824679	147	157	1	atttttccggc
MA0911.1	Hoxa11	5.586	0.810027441820107	147	158	-1	ggccggaaaaat
MA0136.2	ELF5	6.806	0.847198316747947	148	158	-1	ggccggaaaaa
MA0028.2	ELK1	5.613	0.837064959136889	148	157	-1	gccggaaaaa
MA0759.1	ELK3	8.226	0.859564711399539	148	157	-1	gccggaaaaa
MA0760.1	ERF	6.105	0.801493317547091	148	157	-1	gccggaaaaa
MA0474.2	ERG	7.030	0.860523215622282	148	157	-1	gccggaaaaa
MA0098.3	ETS1	8.409	0.876706719649015	148	157	-1	gccggaaaaa
MA0761.1	ETV1	8.788	0.881696920499393	148	157	-1	gccggaaaaa
MA0762.1	ETV2	5.672	0.811230384013297	148	158	-1	ggccggaaaaa
MA0764.1	ETV4	8.858	0.88521430712246	148	157	-1	gccggaaaaa
MA0765.1	ETV5	7.570	0.878239287596496	148	157	-1	gccggaaaaa
MA0645.1	ETV6	4.134	0.818832753070153	148	157	-1	gccggaaaaa
MA0156.2	FEV	8.038	0.876576834936474	148	157	-1	gccggaaaaa
MA0475.2	FLI1	7.212	0.872957871468107	148	157	-1	gccggaaaaa
MA0905.1	HOXC10	2.878	0.806879166822736	148	157	-1	gccggaaaaa
MA0906.1	HOXC12	4.187	0.809676520747457	148	158	-1	ggccggaaaaa
MA0873.1	HOXD12	5.365	0.819753463051691	148	158	-1	ggccggaaaaa
MA0913.1	Hoxd9	5.087	0.816448132332214	148	157	-1	gccggaaaaa
MA0624.1	NFATC1	9.343	0.916157540499102	148	157	1	tttttccggc
MA0625.1	NFATC3	9.898	0.910472571489069	148	157	1	tttttccggc
MA0470.1	E2F4	7.259	0.85976440136105	149	159	-1	tggccggaaaa
MA0471.1	E2F6	6.461	0.856345967836879	149	159	-1	tggccggaaaa

MA0758.1	E2F7	8.787	0.81279372198314	149	162	1	ttttccggccaatc
MA0152.1	NFATC2	8.277	0.886303295953689	149	155	1	ttttccg
MA0144.2	STAT3	2.355	0.826084506099115	149	159	-1	tggccggaaaa
MA0865.1	E2F8	13.471	0.862182886908078	150	161	1	tttccggccaat
MA0502.1	NFYB	11.556	0.893216558089996	150	164	1	tttccggccaatcac
MA0081.1	SPIB	4.742	0.804322796910025	151	157	-1	gccggaa
MA0671.1	NFIX	6.737	0.910886717062997	153	161	1	ccggccaat
MA0060.2	NFYA	8.221	0.81675862062606	153	170	-1	ctaaaagtgattggccgg
MA0161.1	NFIC	7.969	0.942108849322429	155	160	-1	ttggcc
MA0634.1	ALX3	4.996	0.805845853782784	156	165	1	gccaatcact
MA0879.1	Dlx1	4.235	0.812700460932415	156	165	1	gccaatcact
MA0642.1	EN2	8.686	0.915691195583886	156	165	1	gccaatcact
MA0644.1	ESX1	7.158	0.876954469510305	156	165	1	gccaatcact
MA0887.1	EVX1	6.992	0.884041565273707	156	165	-1	agtgattggc
MA0887.1	EVX1	4.414	0.825376630402373	156	165	1	gccaatcact
MA0888.1	EVX2	6.567	0.87819231243412	156	165	-1	agtgattggc
MA0888.1	EVX2	4.065	0.824233064353816	156	165	1	gccaatcact
MA0766.1	GATA5	4.436	0.837363993978242	156	163	-1	tgattggc
MA0889.1	GBX1	6.116	0.861527204000626	156	165	1	gccaatcact
MA0890.1	GBX2	5.387	0.849466296637079	156	165	1	gccaatcact
MA0892.1	GSX1	5.324	0.839358623747024	156	165	1	gccaatcact
MA0893.1	GSX2	5.059	0.820052650141809	156	165	1	gccaatcact
MA0894.1	HESX1	4.860	0.835031666297881	156	165	-1	agtgattggc
MA0900.1	HOXA2	6.465	0.877150503496126	156	165	1	gccaatcact
MA0902.1	HOXB2	5.111	0.844747489955672	156	165	1	gccaatcact
MA0903.1	HOXB3	4.637	0.842364662231763	156	165	1	gccaatcact
MA0699.1	LBX2	6.247	0.845756324236126	156	165	1	gccaatcact
MA0700.1	LHX2	4.948	0.847779363374335	156	165	1	gccaatcact
MA0658.1	LHX6	5.489	0.843589740469487	156	165	-1	agtgattggc
MA0658.1	LHX6	5.603	0.845539232583778	156	165	1	gccaatcact
MA0661.1	MEOX1	4.516	0.820042635074155	156	165	-1	agtgattggc
MA0661.1	MEOX1	5.437	0.839018333747599	156	165	1	gccaatcact
MA0706.1	MEOX2	5.424	0.831667493076198	156	165	-1	agtgattggc
MA0706.1	MEOX2	4.968	0.820266736488237	156	165	1	gccaatcact
MA0662.1	MIXL1	6.104	0.865716359281596	156	165	1	gccaatcact
MA0125.1	Nobox	6.406	0.836520913222571	156	163	-1	tgattggc
MA0718.1	RAX	5.940	0.844266616205778	156	165	1	gccaatcact
MA0876.1	BSX	2.181	0.807029409432795	157	164	1	ccaatcac
MA0885.1	Dlx2	5.528	0.865536746609707	157	164	1	ccaatcac
MA0880.1	Dlx3	6.738	0.885842257315008	157	164	1	ccaatcac
MA0881.1	Dlx4	7.033	0.897629968594321	157	164	1	ccaatcac
MA0882.1	DLX6	6.934	0.893576968668359	157	164	1	ccaatcac
MA0611.1	Dux	9.180	0.920229679511632	157	164	1	ccaatcac
MA0027.2	EN1	8.266	0.921782703458132	157	164	1	ccaatcac
MA0038.1	Gfi1	9.710	0.913747343749482	157	166	1	ccaatcactt
MA0914.1	ISL2	4.641	0.817015953394936	157	164	1	ccaatcac
MA0654.1	ISX	3.194	0.808494032961542	157	164	1	ccaatcac
MA0704.1	Lhx4	3.772	0.81315011659758	157	164	-1	gtgattgg
MA0704.1	Lhx4	5.565	0.8543890571619	157	164	1	ccaatcac
MA0705.1	Lhx8	7.188	0.915878320505501	157	164	-1	gtgattgg
MA0705.1	Lhx8	8.129	0.933653083999	157	164	1	ccaatcac
MA0701.1	LHX9	6.113	0.871012078736981	157	164	1	ccaatcac
MA0703.1	LMX1B	4.716	0.813255054757594	157	164	1	ccaatcac
MA0666.1	MSX1	7.683	0.900257746113234	157	164	1	ccaatcac
MA0708.1	MSX2	7.805	0.889408852305143	157	164	1	ccaatcac
MA0709.1	Msx3	6.321	0.871110869031688	157	164	1	ccaatcac
MA0674.1	NKX6-1	2.982	0.801894354437337	157	164	1	ccaatcac
MA0675.1	NKX6-2	5.753	0.860066904266736	157	164	1	ccaatcac
MA0716.1	PRRX1	6.772	0.876947270865098	157	164	1	ccaatcac
MA0075.2	Prrx2	7.215	0.89197272699109	157	164	1	ccaatcac
MA0717.1	RAX2	6.873	0.882964558834711	157	164	1	ccaatcac
MA0630.1	SHOX	5.914	0.842891961044758	157	164	-1	gtgattgg
MA0720.1	Shox2	5.646	0.863155573895122	157	164	1	ccaatcac
MA0721.1	UNCX	6.616	0.874005089382063	157	164	1	ccaatcac
MA0722.1	VAX1	4.843	0.820369132411963	157	164	1	ccaatcac
MA0723.1	VAX2	5.323	0.837701106838149	157	164	1	ccaatcac
MA0725.1	VSX1	5.413	0.823621234615953	157	164	1	ccaatcac
MA0726.1	VSX2	5.275	0.816042701608964	157	164	1	ccaatcac
MA0483.1	Gfi1b	6.326	0.836928685842649	158	168	1	caatcactttt
MA0067.1	Pax2	4.037	0.802524810304377	158	165	-1	agtgattg
MA0067.1	Pax2	6.410	0.895709558830021	159	166	1	aatcactt
MA0877.1	Barhl1	3.175	0.802531227879241	160	169	-1	taaaagtgat
MA0635.1	BARHL2	1.755	0.803168222390535	160	169	-1	taaaagtgat
MA0672.1	NKX2-3	2.426	0.802519019588225	160	169	1	atcactttta
MA0124.2	Nkx3-1	5.049	0.821975370215445	160	168	1	atcacttttt
MA0122.2	NKX3-2	5.843	0.840028251593667	160	168	1	atcacttttt

MA0676.1	Nr2e1	6.849	0.840779987501199	160	168	-1	aaaagtgat
MA0616.1	Hes2	6.643	0.802238364364073	161	173	-1	tctctaaaagtga
MA0109.1	HLTF	6.911	0.924789389609019	161	170	1	tcacttttag
MA0914.1	ISL2	5.421	0.83730917166595	161	168	1	tcactttt
MA0673.1	NKX2-8	3.019	0.827475516449228	161	169	1	tcactttta
MA0745.1	SNAI2	2.720	0.802297960120033	161	169	-1	taaaagtga
MA0877.1	Barhl1	5.635	0.867358025078391	162	171	-1	tctaaaagtg
MA0635.1	BARHL2	2.774	0.822137129745099	162	171	-1	tctaaaagtg
MA0063.1	Nkx2-5	4.772	0.81700671203629	162	168	-1	aaaagtg
MA0520.1	Stat6	12.379	0.908811071916792	162	176	1	cacttttagagaaat
MA0144.2	STAT3	4.746	0.855050405091454	165	175	1	ttttagagaaa
MA0518.1	Stat4	6.341	0.835248420310091	165	178	1	ttttagagaaatga
MA0519.1	Stat5a::Stat5b	2.030	0.818579768083742	166	176	-1	atttctctaaa
MA0606.1	NFAT5	5.036	0.812697015184912	168	177	-1	catttctcta
MA0081.1	SPIB	6.322	0.858302063374879	169	175	1	agagaaa
MA0158.1	HOXA5	4.597	0.814837133331225	170	177	1	gagaaatg
MA0090.2	TEAD1	4.317	0.812153895216453	170	179	-1	ctcatttctc
MA0879.1	Dlx1	4.341	0.815382896537097	171	180	-1	gctcatttct
MA0879.1	Dlx1	4.924	0.830136292362845	171	180	1	agaaatgagc
MA0612.1	EMX1	5.858	0.827713683546871	171	180	1	agaaatgagc
MA0886.1	EMX2	4.682	0.849825343777412	171	180	1	agaaatgagc
MA0887.1	EVX1	3.695	0.809015075641904	171	180	-1	gctcatttct
MA0887.1	EVX1	3.386	0.801983475612718	171	180	1	agaaatgagc
MA0888.1	EVX2	4.004	0.822917511143225	171	180	-1	gctcatttct
MA0888.1	EVX2	3.000	0.801264799283663	171	180	1	agaaatgagc
MA0890.1	GBX2	3.146	0.805826214100892	171	180	1	agaaatgagc
MA0892.1	GSX1	5.037	0.831564497723128	171	180	-1	gctcatttct
MA0893.1	GSX2	4.527	0.805335518272402	171	180	-1	gctcatttct
MA0900.1	HOXA2	4.909	0.841971620583441	171	180	-1	gctcatttct
MA0902.1	HOXB2	4.416	0.82669321654983	171	180	-1	gctcatttct
MA0903.1	HOXB3	4.171	0.831512417410028	171	180	-1	gctcatttct
MA0492.1	JUND(var.2)	4.146	0.840267679885348	171	185	1	agaaatgagcgcatt
MA0700.1	LHX2	2.551	0.807334021055903	171	180	-1	gctcatttct
MA0706.1	MEOX2	4.684	0.813166265279946	171	180	1	agaaatgagc
MA0662.1	MIXL1	3.963	0.809996691301354	171	180	1	agaaatgagc
MA0710.1	NOTO	6.398	0.883255664818254	171	180	-1	gctcatttct
MA0881.1	Dlx4	4.026	0.815591497070789	172	179	-1	ctcatttc
MA0882.1	DLX6	3.943	0.816021669509515	172	179	-1	ctcatttc
MA0027.2	EN1	3.468	0.817526700874276	172	179	1	gaaatgag
MA0488.1	JUN	2.847	0.818726938073669	172	184	1	gaaatgagcgcat
MA0701.1	LHX9	5.019	0.841087389431542	172	179	1	gaaatgag
MA0675.1	NKX6-2	4.006	0.811983865877605	172	179	-1	ctcatttc
MA0793.1	POU6F2	6.085	0.821745129799445	172	181	-1	cgctcatttc
MA0720.1	Shox2	2.972	0.803169497439608	172	179	1	gaaatgag
MA0722.1	VAX1	4.936	0.823009116526517	172	179	-1	ctcatttc
MA0723.1	VAX2	4.524	0.816578969779566	172	179	-1	ctcatttc
MA0158.1	HOXA5	5.527	0.847590893777919	174	181	-1	cgctcatt
MA0492.1	JUND(var.2)	2.540	0.823888141796397	174	188	-1	tgcaatgcgctcatt
MA0089.1	MAFG::NFE2L1	5.264	0.846327457967442	174	179	1	aatgag
MA0488.1	JUN	1.236	0.801181379598914	175	187	-1	gcaatgcgctcat
MA0842.1	NRL	4.539	0.802073519708573	175	185	-1	aatgcgctcat
MA0719.1	RHOXF1	0.343	0.812008333914076	175	182	1	atgagcgc
MA0668.1	NEUROD2	2.693	0.809303165937728	178	187	1	agcgcattgc
MA0629.1	Rhox11	9.024	0.826235909930548	179	195	-1	attctgctgcaatgcgc
MA0019.1	Ddit3::Cebpa	8.694	0.837765756856826	180	191	-1	tgctgcaatgcg
MA0090.2	TEAD1	4.482	0.815553580204438	180	189	1	cgcattgcag
MA0809.1	TEAD4	6.357	0.858716331634316	180	189	1	cgcattgcag
MA0808.1	TEAD3	3.438	0.851620655015126	181	188	1	gcattgca
MA0833.1	ATF4	7.213	0.822477473424379	182	194	-1	ttctgctgcaatg
MA0158.1	HOXA5	4.919	0.826177682647177	182	189	-1	ctgcaatg
MA0102.3	CEBPA	4.835	0.855913161388287	183	193	1	attgcagcaga
MA0842.1	NRL	5.258	0.817542484315555	185	195	-1	attctgctgca
MA0623.1	Neurog1	4.309	0.802766348810815	188	197	-1	gcattctgct
MA0514.1	Sox3	4.790	0.83653953577156	188	197	-1	gcattctgct
MA0081.1	SPIB	6.232	0.855227295031945	188	194	1	agcagaa
MA0090.2	TEAD1	9.002	0.908684344723771	189	198	-1	cgcattctgc
MA0809.1	TEAD4	9.263	0.920896647477072	189	198	-1	cgcattctgc
MA0808.1	TEAD3	2.273	0.8335856496767	190	197	-1	gcattctg
MA0442.1	SOX10	6.352	0.886446713233527	191	196	-1	cattct
MA0117.2	Mafb	13.090	0.936541864592042	193	204	1	aatgcgctgacg
MA0842.1	NRL	11.485	0.951513614507039	193	203	1	aatgcgctgac
MA0595.1	SREBF1	7.124	0.825313643434939	194	203	-1	gtcagcgcat
MA0596.1	SREBF2	8.303	0.836287936887854	194	203	1	atgcgctgac
MA0615.1	Gmeb1	8.940	0.873514649067102	195	211	-1	tctttgacgtcagcgca
MA0492.1	JUND(var.2)	3.925	0.838013708579209	195	209	1	tgcgctgacgtcaaa
MA0506.1	NRF1	3.040	0.814530522916715	195	205	-1	acgtcagcgca

MA0615.1	Gmeb1	10.107	0.90280411129415	196	212	1	gcgctgacgtcaaagac
MA0131.2	HINFP	7.173	0.819429957166056	196	207	-1	tgacgtcagcgc
MA0488.1	JUN	5.079	0.843035868250984	196	208	1	gcgctgacgtcaa
MA0834.1	ATF7	6.729	0.844866174953126	197	210	-1	ctttgacgtcagcg
MA0834.1	ATF7	7.289	0.851058427386679	197	210	1	cgctgacgtcaaag
MA0638.1	CREB3	8.644	0.819644194418683	197	210	-1	ctttgacgtcagcg
MA0605.1	Atf3	7.288	0.86070640426761	198	205	1	gctgacgt
MA0840.1	Creb5	7.656	0.854742314089335	198	209	-1	tttgacgtcagc
MA0840.1	Creb5	8.226	0.862149212623583	198	209	1	gctgacgtcaaa
MA0609.1	Crem	11.357	0.920295057833098	198	207	1	gctgacgtca
MA0639.1	DBP	1.680	0.804265079453772	198	209	-1	tttgacgtcagc
MA0639.1	DBP	2.491	0.81489920644962	198	209	1	gctgacgtcaaa
MA0043.2	HLF	4.168	0.820298493715066	198	209	-1	tttgacgtcagc
MA0043.2	HLF	4.747	0.829379418573513	198	209	1	gctgacgtcaaa
MA0656.1	JDP2(var.2)	6.125	0.846851897103439	198	209	-1	tttgacgtcagc
MA0656.1	JDP2(var.2)	7.512	0.861845394968132	198	209	1	gctgacgtcaaa
MA0492.1	JUND(var.2)	1.270	0.810935456009991	198	212	-1	gtctttgacgtcagc
MA0089.1	MAFG::NFE2L1	5.553	0.858843819112298	198	203	1	gctgac
MA0844.1	XBP1	6.505	0.813638925309703	198	211	-1	tctttgacgtcagc
MA0604.1	Atf1	8.650	0.905312988607059	199	206	1	ctgacgtc
MA0488.1	JUN	1.151	0.80025563629019	199	211	-1	tctttgacgtcag
MA0498.2	MEIS1	5.884	0.905994434664963	199	205	1	ctgacgt
MA0017.2	NR2F1	2.887	0.805462539438969	199	211	1	ctgacgtcaaaga
MA0018.2	CREB1	11.569	1.00001610713187	200	207	-1	tgacgtca
MA0018.2	CREB1	11.569	1.00001610713187	200	207	1	tgacgtca
MA0608.1	Creb3l2	5.187	0.829520376612795	200	208	-1	ttgacgtca
MA0609.1	Crem	11.768	0.928558718266003	200	209	-1	tttgacgtca
MA0862.1	GMEB2	9.330	0.916981461381433	200	207	-1	tgacgtca
MA0862.1	GMEB2	9.330	0.916981461381433	200	207	1	tgacgtca
MA0484.1	HNF4G	9.568	0.872560866183796	200	214	1	tgacgtcaaagacca
MA0504.1	NR2C2	8.096	0.822866568595812	200	214	1	tgacgtcaaagacca
MA0676.1	Nr2e1	5.040	0.804112443016929	200	208	1	tgacgtcaa
MA0523.1	TCF7L2	7.182	0.841693272731515	200	213	1	tgacgtcaaagacc
MA0604.1	Atf1	8.285	0.897426144114672	201	208	-1	ttgacgtc
MA0114.3	Hnf4a	7.224	0.811228333982804	201	216	1	gacgtcaaagaccacc
MA0775.1	MEIS3	6.084	0.86853715975799	201	208	-1	ttgacgtc
MA0605.1	Atf3	4.399	0.800862379320882	202	209	-1	tttgacgt
MA0498.2	MEIS1	6.290	0.914592335790748	202	208	-1	ttgacgt
MA0442.1	SOX10	4.669	0.811729883835861	205	210	-1	ctttga
MA0160.1	NR4A2	8.132	0.876915320421688	208	215	1	aagaccac
MA0694.1	ZBTB7B	8.110	0.838208711133253	208	219	1	aagaccacccct
MA0695.1	ZBTB7C	8.977	0.853737781194881	208	219	1	aagaccacccct
MA0162.2	EGR1	4.425	0.827568074035895	209	222	1	agaccacccccttct
MA0511.2	RUNX2	4.803	0.814734264116013	209	217	1	agaccaccc
MA0684.1	RUNX3	4.525	0.804521401208745	209	218	1	agaccacccc
MA0079.3	SP1	3.295	0.822595553496615	209	219	1	agaccacccct
MA0493.1	Klf1	5.232	0.800061066097261	210	220	1	gaccaccccctt
MA0130.1	ZNF354C	6.331	0.886867302367815	210	215	1	gaccac
MA0746.1	SP3	5.995	0.822410015512409	211	221	1	accaccccttc
MA0747.1	SP8	7.412	0.819808455065849	211	222	1	accaccccttct
MA0057.1	MZF1(var.2)	7.730	0.865601622033459	212	221	-1	gaaggggtgg
MA0803.1	TBX15	5.011	0.820720347215504	212	219	-1	aggggtgg
MA0806.1	TBX4	3.711	0.803420796653031	212	219	-1	aggggtgg
MA0807.1	TBX5	6.942	0.852601945798726	212	219	-1	aggggtgg
MA0751.1	ZIC4	5.484	0.803482544670375	212	226	1	ccaccccttctgcgc
MA0753.1	ZNF740	5.821	0.802160027179835	212	221	1	ccaccccttc
MA0672.1	NKX2-3	3.823	0.826498304758769	214	223	1	accccttctg
MA0109.1	HLTF	5.514	0.875553977751613	215	224	1	ccccttctgc
MA0673.1	NKX2-8	5.582	0.873820343776727	215	223	1	ccccttctg
MA0607.1	Bhlha15	3.265	0.806814700906033	217	224	1	ccttctgc
MA0442.1	SOX10	4.820	0.818433532819252	217	222	1	ccttct
MA0108.2	TBP	10.495	0.89579325285213	220	234	-1	atataaaggcgcaga
MA0748.1	YY2	6.760	0.802058186660789	221	231	1	ctgcgcccttta
MA0108.2	TBP	11.096	0.907720360359071	222	236	-1	ttatataaaggcgca
MA0877.1	Barhl1	5.474	0.863115295668203	224	233	-1	tataaaggcg
MA0108.2	TBP	8.104	0.848342813502551	224	238	-1	gtttatataaaggcg
MA0847.1	FOXD2	2.428	0.805601246687819	226	232	-1	ataaagg
MA0850.1	FOXP3	4.521	0.830788013875655	226	232	-1	ataaagg
MA0025.1	NFIL3	7.572	0.80667359429414	226	236	-1	ttatataaagg
MA0639.1	DBP	4.090	0.835865876075217	227	238	-1	gtttatataaag
MA0639.1	DBP	3.866	0.83292870659671	227	238	1	ctttatataaac
MA0037.2	GATA3	3.921	0.830695680797781	227	234	-1	atataaag
MA0043.2	HLF	5.669	0.843839889591628	227	238	-1	gtttatataaag
MA0043.2	HLF	7.609	0.874266477200242	227	238	1	ctttatataaac
MA0619.1	LIN54	3.292	0.802883340647113	227	235	1	ctttatata
MA0792.1	POU5F1B	5.173	0.80779567472599	227	235	-1	tatataaag

MA0442.1	SOX10	4.805	0.817767607423551	227	232	1	ctttat
MA0108.2	TBP	7.462	0.835602043087482	227	241	1	ctttatataaacccc
MA0878.1	CDX1	3.940	0.808455399974674	228	236	1	tttatataa
MA0878.1	CDX1	3.940	0.808455399974674	229	237	-1	tttatataa
MA0148.3	FOXA1	1.936	0.81286975928046	229	243	-1	gtggggtttatataa
MA0025.1	NFIL3	7.731	0.810741181106468	229	239	1	ttatataaacc
MA0108.2	TBP	10.782	0.901488893209022	229	243	1	ttatataaaccccac
MA0032.2	FOXC1	3.642	0.808886847680983	230	240	1	tatataaaccc
MA0619.1	LIN54	4.444	0.825724416780798	230	238	-1	gtttatata
MA0593.1	FOXP2	4.965	0.806066783213765	231	241	1	atataaacccc
MA0037.2	GATA3	3.279	0.820594050245556	231	238	1	atataaac
MA0619.1	LIN54	3.746	0.811884945303964	231	239	1	atataaacc
MA0108.2	TBP	6.048	0.807540595475476	231	245	1	atataaaccccaccc
MA0714.1	PITX3	6.326	0.849600766168741	232	240	1	tataaaccc
MA0847.1	FOXD2	3.195	0.821867790784055	233	239	1	ataaacc
MA0613.1	FOXG1	4.962	0.827900094297596	233	240	1	ataaaccc
MA0042.2	FOXI1	2.768	0.800644587675346	233	239	1	ataaacc
MA0614.1	Foxj2	4.175	0.811117272977989	233	240	1	ataaaccc
MA0033.2	FOXL1	5.844	0.882577613914127	233	239	1	ataaacc
MA0848.1	FOXO4	3.474	0.818265555211229	233	239	1	ataaacc
MA0849.1	FOXO6	2.624	0.818376184629519	233	239	1	ataaacc
MA0850.1	FOXP3	4.627	0.833454943393175	233	239	1	ataaacc
MA0711.1	OTX1	4.329	0.846043991115658	233	240	1	ataaaccc
MA0712.1	OTX2	4.816	0.847790461949794	233	240	1	ataaaccc
MA0682.1	Pitx1	5.868	0.841911904333453	233	240	1	ataaaccc
MA0719.1	RHOXF1	2.005	0.844042891313564	233	240	1	ataaaccc
MA0719.1	RHOXF1	0.057	0.806495768681673	234	241	1	taaacccc
MA0002.2	RUNX1	7.529	0.84489469927906	235	245	-1	gggtggggttt
MA0511.2	RUNX2	5.889	0.834752198317157	235	243	1	aaaccccac
MA0684.1	RUNX3	5.532	0.822074015034439	235	244	1	aaaccccacc
MA0493.1	Klf1	14.069	0.949449430917173	236	246	1	aaccccaccca
MA0742.1	Klf12	6.270	0.814475826408317	236	250	1	aaccccacccagcca
MA0741.1	KLF16	6.050	0.816813087992767	237	247	1	accccacccag
MA0039.2	Klf4	13.909	0.9728210017962	237	246	-1	tgggtggggt
MA0599.1	KLF5	12.535	0.962715634557158	237	246	1	accccaccca
MA0056.1	MZF1	4.989	0.813099698301624	237	242	-1	tggggt
MA0079.3	SP1	8.073	0.882708263539727	237	247	1	accccacccag
MA0746.1	SP3	9.026	0.882101088243885	237	247	1	accccacccag
MA0747.1	SP8	8.640	0.842629960785147	237	248	1	accccacccagc
MA0807.1	TBX5	5.093	0.804822284482574	238	245	-1	gggtgggg
MA0130.1	ZNF354C	6.723	0.904024652450988	238	243	1	ccccac
MA0753.1	ZNF740	7.035	0.824538620246984	238	247	1	ccccacccag
MA0810.1	TFAP2A(var.2)	3.523	0.81104424070532	239	250	1	cccacccagcca
MA0006.1	Ahr::Arnt	5.297	0.817309426751426	241	246	-1	tgggtg
