# Supplementary material for: Common microRNA–mRNA interactions exist among distinct porcine iPSC lines independent of their metastable pluripotent states
Source: Cell Death Dis. 2017 Aug 31;8(8):e3027–. doi: 10.1038/cddis.2017.426 (PMC5596602; doi:10.1038/cddis.2017.426)
Supplement: Supplementary Table 11 [file cddis2017426x12.pdf]

Names	total	elements
Homo sapiens.txt Mus musculus.txt sus scrofa.txt	204	STAT3, RHOXF1, NRF1, FIGLA, HOXA10, BARX1, Nkx2-5(var.2), LBX2, TCF4, OLIG3, SP3, Sox6, Lhx4, CDX2, TCF3, EGR1, SHOX, VENTX, HLF, EN1, ZNF740, HOXD12, HOXC12, PDX1, FOXD2, GATA5, Stat4, NKX2-8, HLTF, HOXA2, E2F4, GBX2, JUND(var.2), Dlx3, JUNB, Dlx2, ELF5, TBX5, MEIS2, EVX1, TEAD4, ZBTB7B, Klf12, FOXO3, TEAD1, NRL, ETV6, Pitx1, TFAP2B, FOXA1, FOS::JUN, Gfi1, SREBF2, SOX10, JDP2(var.2), JDP2, MSX2, CEBPA, EVX2, NKX6-2, ETV5, MGA, HOXC11, Dux, ATF4, CREB1, RAX2, NOTO, THAP1, ID4, ETV1, PRRX1, GBX1, RUNX3, ETV2, YY2, Foxj2, Rhox11, Msx3, HIC2, ZNF263, Hoxd9, HESX1, Hoxa11, TFAP2C, SPIB, FOXO4, SNAI2, NFIC, Hic1, VAX2, HOXC10, E2F7, Gmeb1, GMEB2, GSX1, JUN, Atf3, FOXP3, ZNF354C, Sox5, TFAP2C(var.2), VSX2, Stat5a::Stat5b, ISL2, ELK4, Crem, MZF1, Nobox, Prrx2, ATF7, JUND, NFATC3, POU6F2, Ahr::Arnt, FOS, Klf4, Stat6, VSX1, HOXB3, PITX3, CDX1, RUNX1, EN2, Ddit3::Cebpa, OTX1, ERF, E2F8, MIXL1, KLF5, Dlx1, Shox2, UNCX, MZF1(var.2), LHX2, EMX1, MEOX2, GSX2, FLI1, NFATC2, ZNF143, NFYB, MEIS3, Hand1::Tcf3, Mafb, ETV4, HINFP, SRY, Sox3, SREBF1, Creb5, ETS1, SP1, VAX1, NFAT5, Tcfl5, RAX, ELK1, MEIS1, ELK3, FOXL1, Lhx8, JUN(var.2), OTX2, HOXB2, E2F6, NEUROD2, MEOX1, MSX1, HOXD11, Neurog1, MAFG::NFE2L1, NFIX, ERG, HOXA5, NFATC1, Atf1, Bhlha15, ZBTB7C, ESR2, TEAD3, FOSL2, GCM2, Foxd3, Klf1, GCM1, FEV, Barhl1, NR2C2, HOXD13, LHX9, NFIA, ISX, EMX2, TBX4, Pax2, Dlx4, DLX6, TFAP2A, TFAP2A(var.2), ESX1, LMX1B, DBP, BARHL2,
Homo sapiens.txt sus scrofa.txt	3	Myod1, SP2, NHLH1,
Mus musculus.txt sus scrofa.txt	1	REL,
Homo sapiens.txt Mus musculus.txt	39	Znf423, ZIC1, ALX3, NR4A2, MAX::MYC, RUNX2, FOXI1, Nkx2-5, Myc, FOXO6, NKX2-3, Spz1, NKX6-1, USF2, KLF16, E2F1, TBP, BSX, GATA3, NKX3-2, TFEC, SP8, HNF4G, Mycn, FOXP2, Hes2, NFYA, LIN54, GLI2, Nkx3-1, TBX15, TFAP2B(var.2), CTCF, ZBTB7A, FOXG1, Nr2e1, LHX6, Gfi1b, ZIC4,
sus scrofa.txt	8	ARNT，HIF1A, ZBTB18, Hes1, EGR2, TFAP2A(var.3), ZEB1, TFAP2C(var.3), TFAP2B(var.3),
Homo sapiens.txt	5	Rfx1, HOXB13, Foxo1, TFE3, RREB1,
Mus musculus.txt	19	GSC2, LEF1, Mitf, Tcf7, INSM1, POU5F1B, Creb3l2, FOXC1, NFIL3, USF1, XBP1, NR2F1, TCF7L2, Sox2, CREB3, FOXP1, Hnf4a, MAFF, Atoh1,
