# Supplementary material for: Common microRNA–mRNA interactions exist among distinct porcine iPSC lines independent of their metastable pluripotent states
Source: Cell Death Dis. 2017 Aug 31;8(8):e3027–. doi: 10.1038/cddis.2017.426 (PMC5596602; doi:10.1038/cddis.2017.426)
Supplement: Supplementary Table 12 [file cddis2017426x13.pdf]

GO	Category	GO Term	p-Value	FDR	(-log2P)
	GOTERM_BP_DIRECT	regulation of transcription from RNA polymerase II promoter	6.30E-15	4.30E-12	47.17356959
	GOTERM_BP_DIRECT	transcription, DNA-templated	1.00E-13	3.40E-11	43.18506523
	GOTERM_BP_DIRECT	positive regulation of transcription from RNA polymerase II promoter	5.30E-11	1.20E-08	34.13521668
	GOTERM_BP_DIRECT	regulation of transcription, DNA-templated	6.70E-10	1.10E-07	30.47511985
	GOTERM_BP_DIRECT	cell differentiation	1.70E-08	2.30E-06	25.80989001
	GOTERM_BP_DIRECT	anterior/posterior pattern specification	2.30E-07	2.60E-05	22.0518628
	GOTERM_BP_DIRECT	inner ear morphogenesis	5.40E-06	5.30E-04	17.49860916
	GOTERM_BP_DIRECT	proximal/distal pattern formation	5.60E-06	4.80E-04	17.44614174
	GOTERM_BP_DIRECT	multicellular organism development	1.10E-05	8.70E-04	16.47213695
	GOTERM_BP_DIRECT	negative regulation of transcription from RNA polymerase II promoter	2.70E-05	1.80E-03	15.17668107
	GOTERM_BP_DIRECT	regulation of cell differentiation	4.90E-05	3.10E-03	14.31685873
	GOTERM_BP_DIRECT	response to drug	1.00E-04	5.90E-03	13.28771238
	GOTERM_BP_DIRECT	dorsal/ventral pattern formation	1.30E-04	6.80E-03	12.90920076
	GOTERM_BP_DIRECT	positive regulation of neuron apoptotic process	6.70E-04	3.20E-02	10.54355128
	GOTERM_BP_DIRECT	odontogenesis of dentin-containing tooth	7.20E-04	3.20E-02	10.43971547
	GOTERM_BP_DIRECT	palate development	7.50E-04	3.20E-02	10.38082178
