# Supplementary material for: Common microRNA–mRNA interactions exist among distinct porcine iPSC lines independent of their metastable pluripotent states
Source: Cell Death Dis. 2017 Aug 31;8(8):e3027–. doi: 10.1038/cddis.2017.426 (PMC5596602; doi:10.1038/cddis.2017.426)
Supplement: Supplementary Table 13 [file cddis2017426x14.pdf]

**Primer for cloning PCR**	
ssc-31863-*LIN28A* WT-UTR-F	CCCTCGAGCAGAATTGAGCCGTGGTGG
ssc-31863-*LIN28A* WT-UTR-R	GAGCCTGATTGGAGTTCCTTTG
ssc-370-*LIN28A* WT-UTR-F	CCCTCGAGCCTATTCCGGGCCAATACAGT
ssc-370-*LIN28A* WT-UTR-R	GGGCAACCCAGTTAAAACCTAGCCTCC
ssc-370-*LIN28A* MUT-UTR-F	GTCTACAGTCGTCCAATACTTTTTGTTGCT
ssc-370-*LIN28A* MUT-UTR-R	TGGACGACTGTAGACCCGGAGCTGCTGCC
ssc-206-*OTX2* WT-UTR-F	CCCTCGAGAATTCCAGGTTTTGTGAAGA
ssc-206-*OTX2* WT-UTR-F	ATTTGGTTGCACATGGCTAG
pCDH-mmu-mir-370--F	GGAATTCTAGAGTTCAGAGCATAGAGCGAGTTTGA
pCDH-mmu-mir-370--R	CGGGATCCCGATCAATTGAGAAGCCTCTCTGTGCTC
pCDH-mmu-Lin28a-F	GGAATTCATGGGCTCGGTGTCCAACC
pCDH-mmu-Lin28a-R	CGGGATCCCCTCAATTCTGGGCTTCTGG
pCDH-ssc-*LIN28A* -F	GGAATTCATGGGCTCTGTGTCAAACC
pCDH-ssc-*LIN28A* -R	CGGGATCCTCAATTCTGAGCCTCTGGGA
**Primer for qRT-PCR**	
Q-ssc-*β-ACTIN* -F	GTGACAGCAGTCGGTTGGAT
Q-ssc-*β-ACTIN* -R	TTTTGGGAAGGCAGGGACTT
Q-ssc-*OCT4* -F	TGAGGCTTTGCAGCTCAGTT
Q-ssc-*OCT4* -R	TCTCCAGGTTGCCTCTCACT
Q-ssc-*SOX2* -F	CCCCGTGGTTACCTCTTCTTCC
Q-ssc-*SOX2* -R	CAGTTCACTGTCCGGCCCTCA
Q-ssc-*SALL4* -F	CCCCAACACATCAACTCGGA
Q-ssc-*SALL4* -R	ACTCGGCACAGCATTTCTCA
Q-ssc-*LIN28A* -F	TGCCGGCATCTGTAAATGGT
Q-ssc-*LIN28A* -R	CTCTCGCTCCCAATGCAGAA
Q-ssc-*NANOG* -F	AGGGCTCAGCCAGTACAGAA
Q-ssc-*NANOG* -R	CCAGCTCTGATTACCCCACA
Q-ssc-*ESRRB* -F	ATGCCTCAAAGTGGGGATGC
Q-ssc-*ESRRB* -R	TTTTAGTCAATGGCTTCTTCGCA
Q-ssc-*SOX9* -F	CATCTCTCCCAACGCCATCT
Q-ssc-*SOX9* -R	TCTCGCTTCAGGTCAGCCTT
Q-ssc-*JARID2* -F	CCCAGCACAACTCCAGTAAAGA
Q-ssc-*JARID2* -R	TCGAAGGCAGAGAAAGGTAAGA
Q-ssc-*JMJD4* -F	GCCCCTCAAAGACTACATCAG
Q-ssc-*JMJD4* -R	GAGAAGTATATGGGCAGGGTG
Q-mmu-Oct4-F	TCCCTAGGTGAGCCGTCT
Q-mmu-Oct4-R	TACCTCTGAGCCTGGTCCGAT
Q-mmu-Sox2-F	TAGAGCTAGACTCCGGGCGATGA
Q-mmu-Sox2-R	TTGCCTTAAACAAGACCACGAAA
Q-mmu-Nanog-F	TCTTCCTGGTCCCCACAGTTT
Q-mmu-Nanog-R	GCAAGAATAGTTCTCGGGATGAA
Q-mmu-Sall4-F	CCCTGGGAACTGCGATGAAG
Q-mmu-Sall4-R	TCAGAGAGACTAAAGAACTCGGC
Q-mmu-Esrrb-F	GCACCTGGGCTCTAGTTGC
Q-mmu-Esrrb-R	TACAGTCCTCGTAGCTCTTGC
Q-mmu-Gadph-F	AGGTCGGTGTGAACGGATTTG
Q-mmu-Gadph-R	TGTAGACCATGTAGTTGAGGTCA
Q-ssc-miR-370-F	CTGCTGGGGTGGAACCTGGT
Q-ssc-miR-206-F	GGCGTGGAATGTAAGGAAGTGTGTGA
Q-ssc-miR-31863-F	CTGCTCTCCTGTCTCCGCTCAG
Q-ssc-miR-R	Mir-X™ miRNA First-Strand Synthesis and SYBR® qRT-PCR
Q-U6-F	Mir-X™ miRNA First-Strand Synthesis and SYBR® qRT-PCR
Q-U6-R	Mir-X™ miRNA First-Strand Synthesis and SYBR® qRT-PCR
**oligo for pLL3.7-miR370**	
pll3.7-miR370-F	TAAGACAGAGAAGCCAGGTCACGTCTCTGCAGTTACACAGCTCACGAGTGCCTGCTGGGGTGGAACCTGGTCTGTCTGTCTTTTTTTC
pll3.7-miR370-R	TCGAGAAAAAAAGACAGACAGACCAGGTTCCACCCCAGCAGGCACTCGTGAGCTGTGTAACTGCAGAGACGTGACCTGGCTTCTCTGTCTTA
