# Supplementary material for: Common microRNA–mRNA interactions exist among distinct porcine iPSC lines independent of their metastable pluripotent states
Source: Cell Death Dis. 2017 Aug 31;8(8):e3027–. doi: 10.1038/cddis.2017.426 (PMC5596602; doi:10.1038/cddis.2017.426)
Supplement: Supplementary Figure and Table Legends [file cddis2017426x15.docx]

**Supplementary Figure and Table Legends**

**Figure S1.** Bioinformatical analysis of miR-370 promoter. (a) Venn analysis of transcription factors that bind to the miR-370 promoter of *Sus scrofa* (pig), *homo sapiens* (human) and *Mus musculus*(mouse). (b) GO analysis (biological process) of the conservative transcription factors (204) that are predicted to bind miR-370 promoter. The top 15 GO terms of biological processes are presented.

**Figure S2.** Bioinformatical analysis of the 3’UTR of mouse Lin28a mRNA. The 3’UTR of mouse Lin28a mRNA is predicted to contain a binding site for miR-370.

**Figure S3.** Effect of miR-370 on the expression of pluripotent genes in piPSCs and mESCs. (a) The expression level changes of pluripotent genes (*LIN28A*, *OCT4*, *SOX2*, *NANOG*, *SALL4* and *ESRRB*) in piPS-LF transduced with pLL3.7-basic lentivirus(vector control), miR-370 lentivirus, and miR-370+*LIN28A* lentivirus, respectively. **P<0.01, ***P<0.001. (b) The expression level changes of pluripotent genes (Lin28a, Oct4, Sox2, Nanog, Sall4 and Esrrb) in mESCs J1 transduced with pCDH-basic lentivirus(vector control), miR-370 lentivirus, and miR-370+Lin28a lentivirus, respectively. *P<0.05, **P<0.01, ***P<0.001.

**Table S1.** Differential miRNAs expressed between piPSC lines and PEFs. The data corresponds to Figure 1d.

**Table S2.** Venn analysis of differential miRNAs among the three piPSC lines. The data corresponds to Figure 1e.

**Table S3.** The predicted targets of 165 common miRNAs shared by three piPSC lines. The data corresponds to Figure 2b.

**Table S4.** The GO and KEGG analysis of 682 predicted target mRNAs among piPSC lines. The data corresponds to Figure 2c-d.

**Table S5.** The differentially expressed mRNAs between PEFs and piPSC lines. The data corresponds to Figure 3a.

**Table S6.** The Venn analysis of differential mRNAs among the three piPSC lines. The data corresponds to Figure 3b (left panel).

**Table S7.** The classification of differentially expressed mRNAs among the three piPSC lines. The data corresponds to Figure 3b (right panel).

**Table S8.** GO and KEGG analysis of the common mRNAs that differentially expressed among piPSC lines. The data corresponds to Figure 3c-d.

**Table S9.** Venn analysis of the targeted mRNAs that are predicted from common miRNAs and the common differentially expressed mRNAs from RNA-seq. The data corresponds to Figure 4b.

**Table S10.** Transcription factors potentially binding to miR-370 promoter of *Sus scrofa* (pig), *homo sapiens* (human) and *Mus musculus*(mouse).

**Table S11.** Venn analysis of the predicted transcription factors binding to miR-370 promoter. The data corresponds to Figure S1a.

**Table S12.** GO analysis of the predicted transcription factors binding to miR-370 promoter. The data corresponds to Figure S1b.

**Table S13.** Primers used in this study.
